# Supplementary material for: A high-resolution mRNA expression time course of embryonic development in zebrafish
Source: eLife. 2017 Nov 16;6:e30860. doi: 10.7554/eLife.30860 (PMC5690287; doi:10.7554/eLife.30860)
Supplement: Supplementary file 6. [file elife-30860-supp6.zip › biolayout-clusters.html]

BioLayout Clusters


## Table of BioLayout Cluster Information

Click on the cluster links for more detail on GO/ZFA enrichments.  
For the full list of genes assigned to the cluster, click on the link in the size column.

| | Cluster | Size | Number GO Terms enriched | Enriched GO Terms | Number ZFA Terms enriched | Enriched ZFA Terms | Expression Profile | | --- | --- | --- | --- | --- | --- | --- | | Cluster001 | 2360 | 83 | regulation of neurotransmitter levels (GO:0001505) neurotransmitter secretion (GO:0007269) regulation of synapse structure or activ... (GO:0050803) regulation of membrane potential (GO:0042391) signal transduction (GO:0007165) G-protein coupled receptor signaling pat... (GO:0007186) neuropeptide signaling pathway (GO:0007218) phototransduction (GO:0007602) regulation of receptor activity (GO:0010469) cytokine-mediated signaling pathway (GO:0019221) regulation of glutamate receptor signali... (GO:1900449) adenylate cyclase-inhibiting G-protein c... (GO:0007193) ion transport (GO:0006811) regulation of ion transmembrane transpor... (GO:0034765) potassium ion transport (GO:0006813) sodium ion transport (GO:0006814) regulation of ion transmembrane transpor... (GO:0032412) sodium ion transmembrane transport (GO:0035725) calcium ion transmembrane transport (GO:0070588) potassium ion transmembrane transport (GO:0071805) negative regulation of catalytic activit... (GO:0043086) negative regulation of protein kinase ac... (GO:0006469) transmission of nerve impulse (GO:0019226) neurotransmitter transport (GO:0006836) neurotransmitter secretion (GO:0007269) homophilic cell adhesion via plasma memb... (GO:0007156) cellular response to hormone stimulus (GO:0032870) synaptic transmission (GO:0007268) neuron-neuron synaptic transmission (GO:0007270) modulation of synaptic transmission (GO:0050804) neurotransmitter secretion (GO:0007269) protein homooligomerization (GO:0051260) visual perception (GO:0007601) protein-chromophore linkage (GO:0018298) regulation of exocytosis (GO:0017157) synaptic vesicle endocytosis (GO:0048488) cytosolic calcium ion homeostasis (GO:0051480) cAMP biosynthetic process (GO:0006171) extracellular region (GO:0005576) synapse (GO:0045202) presynapse (GO:0098793) presynaptic membrane (GO:0042734) postsynaptic membrane (GO:0045211) synaptic vesicle membrane (GO:0030672) cell junction (GO:0030054) plasma membrane (GO:0005886) integral component of plasma membrane (GO:0005887) presynaptic membrane (GO:0042734) postsynaptic membrane (GO:0045211) voltage-gated sodium channel complex (GO:0001518) voltage-gated potassium channel complex (GO:0008076) AMPA glutamate receptor complex (GO:0032281) integral component of membrane (GO:0016021) integral component of plasma membrane (GO:0005887) voltage-gated sodium channel complex (GO:0001518) voltage-gated potassium channel complex (GO:0008076) AMPA glutamate receptor complex (GO:0032281) neuron projection (GO:0043005) GABA-A receptor complex (GO:1902711) receptor activity (GO:0004872) photoreceptor activity (GO:0009881) G-protein coupled receptor activity (GO:0004930) glutamate receptor activity (GO:0008066) GABA-A receptor activity (GO:0004890) ionotropic glutamate receptor activity (GO:0004970) channel regulator activity (GO:0016247) cyclase activity (GO:0009975) clathrin binding (GO:0030276) phosphorus-oxygen lyase activity (GO:0016849) syntaxin binding (GO:0019905) hormone activity (GO:0005179) calcium-dependent phospholipid binding (GO:0005544) protein kinase inhibitor activity (GO:0004860) calcium ion binding (GO:0005509) anion:cation symporter activity (GO:0015296) sodium ion transmembrane transporter act... (GO:0015081) chloride channel activity (GO:0005254) extracellular ligand-gated ion channel a... (GO:0005230) ionotropic glutamate receptor activity (GO:0004970) extracellular-glutamate-gated ion channe... (GO:0005234) cGMP binding (GO:0030553) voltage-gated potassium channel activity (GO:0005249) delayed rectifier potassium channel acti... (GO:0005251) | 35 | visual system (ZFA:0001127) cranial ganglion (ZFA:0000013) pineal complex (ZFA:0001359) epiphysis (ZFA:0000019) retinal ganglion cell layer (ZFA:0000024) olfactory bulb (ZFA:0000402) retinal inner nuclear layer (ZFA:0000119) medulla oblongata (ZFA:0000545) brain nucleus (ZFA:0005575) habenula (ZFA:0000213) corpus cerebelli (ZFA:0000188) preoptic area (ZFA:0000470) diencephalic nucleus (ZFA:0001659) retinal photoreceptor layer (ZFA:0000143) ventral telencephalon (ZFA:0000304) thalamus (ZFA:0001215) trigeminal ganglion (ZFA:0000295) valvula cerebelli (ZFA:0000603) lateral line ganglion (ZFA:0000120) telencephalic nucleus (ZFA:0001660) dorsal hypothalamic zone (ZFA:0000347) neuromere (ZFA:0001328) ventro-rostral cluster (ZFA:0007002) dorsal thalamus (ZFA:0000653) spinal cord interneuron (ZFA:0000778) dorsal periventricular hypothalamus (ZFA:0000199) posterior lateral line ganglion (ZFA:0001314) ventral hypothalamic zone (ZFA:0000707) median tuberal portion (ZFA:0000392) torus longitudinalis (ZFA:0000449) rostral parvocellular preoptic nucleus (ZFA:0000426) medial zone of dorsal telencephalon (ZFA:0000391) retinal outer nuclear layer (ZFA:0001464) magnocellular preoptic nucleus (ZFA:0000248) ventral thalamus (ZFA:0000458) |  | | Cluster002 | 1931 | 13 | peroxisome organization (GO:0007031) Golgi vesicle transport (GO:0048193) intra-Golgi vesicle-mediated transport (GO:0006891) protein polyubiquitination (GO:0000209) early endosome (GO:0005769) centrosome (GO:0005813) ubiquitin-protein transferase activity (GO:0004842) ubiquitin protein ligase activity (GO:0061630) metal ion binding (GO:0046872) zinc ion binding (GO:0008270) mannosyltransferase activity (GO:0000030) acetylglucosaminyltransferase activity (GO:0008375) phosphatidylinositol binding (GO:0035091) | 0 |  |  | | Cluster003 | 910 | 6 | spermatogenesis (GO:0007283) meiosis I (GO:0007127) double-strand break repair via homologou... (GO:0000724) replication fork (GO:0005657) single-stranded DNA binding (GO:0003697) endodeoxyribonuclease activity (GO:0004520) | 0 |  |  | | Cluster004 | 407 | 2 | proteinaceous extracellular matrix (GO:0005578) actin binding (GO:0003779) | 10 | trunk musculature (ZFA:0000473) musculature system (ZFA:0000548) myotome (ZFA:0001056) embryonic structure (ZFA:0001105) cephalic musculature (ZFA:0000328) skeletal muscle (ZFA:0005277) head mesenchyme (ZFA:0000113) hyohyoideus (ZFA:0007052) head muscle (ZFA:0001652) skeletal muscle cell (ZFA:0009115) |  | | Cluster005 | 405 | 19 | oxidation-reduction process (GO:0055114) aromatic compound catabolic process (GO:0019439) organonitrogen compound catabolic proces... (GO:1901565) carbohydrate derivative catabolic proces... (GO:1901136) amino sugar metabolic process (GO:0006040) organic cyclic compound catabolic proces... (GO:1901361) proteolysis (GO:0006508) arachidonic acid metabolic process (GO:0019369) extracellular space (GO:0005615) integral component of membrane (GO:0016021) monooxygenase activity (GO:0004497) steroid hydroxylase activity (GO:0008395) oxidoreductase activity, acting on paire... (GO:0016712) oxidoreductase activity, acting on paire... (GO:0016705) oxidoreductase activity, acting on paire... (GO:0016712) heme binding (GO:0020037) serine-type endopeptidase activity (GO:0004252) iron ion binding (GO:0005506) metallocarboxypeptidase activity (GO:0004181) | 2 | digestive system (ZFA:0000339) intestinal bulb (ZFA:0001076) |  | | Cluster006 | 295 | 1 | peptidyl-serine phosphorylation (GO:0018105) | 0 |  |  | | Cluster007 | 239 | 10 | gastrulation with mouth forming second (GO:0001702) SMAD protein signal transduction (GO:0060395) mesoderm formation (GO:0001707) regulation of transcription, DNA-templat... (GO:0006355) positive regulation of pathway-restricte... (GO:0010862) nucleus (GO:0005634) nucleosome (GO:0000786) nucleic acid binding (GO:0003676) RNA polymerase II core promoter proximal... (GO:0000978) metal ion binding (GO:0046872) | 5 | hypoblast (ZFA:0000117) shield (ZFA:0000071) axial hypoblast (ZFA:0001378) prechordal plate (ZFA:0000060) axial mesoderm (ZFA:0001204) |  | | Cluster008 | 225 | 2 | nucleus (GO:0005634) nucleic acid binding (GO:0003676) | 0 |  |  | | Cluster009 | 165 | 1 | protein heterodimerization activity (GO:0046982) | 0 |  |  | | Cluster010 | 159 | 0 |  | 0 |  |  | | Cluster011 | 147 | 3 | mRNA processing (GO:0006397) nuclear pore (GO:0005643) ATP-dependent RNA helicase activity (GO:0004004) | 0 |  |  | | Cluster012 | 134 | 2 | inner ear development (GO:0048839) sequence-specific DNA binding (GO:0043565) | 6 | dorsolateral placode (ZFA:0001310) otic vesicle (ZFA:0000051) neurogenic placode (ZFA:0001309) peripheral nervous system (ZFA:0000142) neurogenic field (ZFA:0007059) otic placode (ZFA:0000138) |  | | Cluster013 | 123 | 0 |  | 1 | mesenchyme (ZFA:0000393) |  | | Cluster014 | 120 | 14 | ATP synthesis coupled proton transport (GO:0015986) mitochondrial ATP synthesis coupled elec... (GO:0042775) oxidoreductase complex (GO:1990204) respiratory chain complex (GO:0098803) respiratory chain complex IV (GO:0045277) proton-transporting ATP synthase complex (GO:0045259) proton-transporting two-sector ATPase co... (GO:0033177) proton-transporting two-sector ATPase co... (GO:0033178) mitochondrial respiratory chain (GO:0005746) inner mitochondrial membrane protein com... (GO:0098800) oxidoreductase activity, acting on NAD(P... (GO:0016651) hydrogen ion transmembrane transporter a... (GO:0015078) cytochrome-c oxidase activity (GO:0004129) ATPase activity, coupled to transmembran... (GO:0044769) | 0 |  |  | | Cluster015 | 104 | 14 | protein folding (GO:0006457) proteasome assembly (GO:0043248) regulation of translational initiation (GO:0006446) formation of translation preinitiation c... (GO:0001731) cytoplasm (GO:0005737) eukaryotic translation initiation factor... (GO:0005852) eukaryotic 43S preinitiation complex (GO:0016282) eukaryotic 48S preinitiation complex (GO:0033290) small nucleolar ribonucleoprotein comple... (GO:0005732) proteasome core complex (GO:0005839) proteasome regulatory particle (GO:0005838) unfolded protein binding (GO:0051082) translation initiation factor activity (GO:0003743) threonine-type endopeptidase activity (GO:0004298) | 0 |  |  | | Cluster016 | 104 | 6 | regulation of transcription, DNA-templat... (GO:0006355) nucleus (GO:0005634) RNA polymerase II transcription factor a... (GO:0000981) transcriptional activator activity, RNA ... (GO:0001228) sequence-specific DNA binding (GO:0043565) RNA polymerase II regulatory region sequ... (GO:0000977) | 15 | nervous system (ZFA:0000396) hindbrain (ZFA:0000029) spinal cord (ZFA:0000075) neuron (ZFA:0009248) neuronal stem cell (ZFA:0009019) forebrain (ZFA:0000109) central nervous system (ZFA:0000012) retinal ganglion cell layer (ZFA:0000024) diencephalon (ZFA:0000101) telencephalon (ZFA:0000079) midbrain (ZFA:0000128) interneuron (ZFA:0009051) spinal cord neural tube (ZFA:0007042) retina (ZFA:0000152) trigeminal ganglion (ZFA:0000295) |  | | Cluster017 | 102 | 3 | convergent extension involved in organog... (GO:0060029) cell migration to the midline involved i... (GO:0003318) heart formation (GO:0060914) | 0 |  |  | | Cluster018 | 101 | 20 | regulation of cell cycle (GO:0051726) ribosome biogenesis (GO:0042254) ribosomal large subunit biogenesis (GO:0042273) ribosomal small subunit biogenesis (GO:0042274) ribosomal large subunit assembly (GO:0000027) ribosomal small subunit assembly (GO:0000028) maturation of SSU-rRNA from tricistronic... (GO:0000462) chordate embryonic development (GO:0043009) translation (GO:0006412) cytoplasmic translation (GO:0002181) translational elongation (GO:0006414) erythrocyte differentiation (GO:0030218) ribosome (GO:0005840) small ribosomal subunit (GO:0015935) cytosolic large ribosomal subunit (GO:0022625) cytosolic small ribosomal subunit (GO:0022627) structural constituent of ribosome (GO:0003735) RNA binding (GO:0003723) rRNA binding (GO:0019843) mRNA binding (GO:0003729) | 16 | extension (ZFA:0000106) fourth ventricle (ZFA:0000110) endodermal cell (ZFA:0009139) yolk (ZFA:0000084) ball (ZFA:0000006) midbrain hindbrain boundary (ZFA:0000042) pancreas (ZFA:0000140) pancreatic bud (ZFA:0001390) pancreas primordium (ZFA:0000254) liver (ZFA:0000123) nucleate erythrocyte (ZFA:0009256) blood cell (ZFA:0009044) gut (ZFA:0000112) intestine (ZFA:0001338) post-vent region (ZFA:0001117) epithelial cell of pancreas (ZFA:0009045) |  | | Cluster019 | 75 | 10 | regulation of gene expression (GO:0010468) mRNA export from nucleus (GO:0006406) mRNA splicing, via spliceosome (GO:0000398) catalytic step 2 spliceosome (GO:0071013) U1 snRNP (GO:0005685) U2 snRNP (GO:0005686) U2-type prespliceosome (GO:0071004) nucleic acid binding (GO:0003676) RNA binding (GO:0003723) nucleotide binding (GO:0000166) | 0 |  |  | | Cluster020 | 68 | 1 | integral component of membrane (GO:0016021) | 0 |  |  | | Cluster021 | 65 | 1 | nucleus (GO:0005634) | 0 |  |  | | Cluster022 | 65 | 1 | heart contraction (GO:0060047) | 6 | trunk musculature (ZFA:0000473) myotome (ZFA:0001056) musculature system (ZFA:0000548) adaxial cell (ZFA:0000003) cardiac ventricle (ZFA:0000009) somite (ZFA:0000155) |  | | Cluster023 | 62 | 4 | regulation of transcription, DNA-templat... (GO:0006355) nucleic acid binding (GO:0003676) RNA polymerase II core promoter proximal... (GO:0000978) metal ion binding (GO:0046872) | 0 |  |  | | Cluster024 | 53 | 1 | nucleic acid binding (GO:0003676) | 0 |  |  | | Cluster025 | 52 | 11 | definitive hemopoiesis (GO:0060216) hematopoietic stem cell differentiation (GO:0060218) nuclear-transcribed mRNA catabolic proce... (GO:0000184) regulation of alternative mRNA splicing,... (GO:0000381) viral nucleocapsid (GO:0019013) precatalytic spliceosome (GO:0071011) catalytic step 2 spliceosome (GO:0071013) nucleic acid binding (GO:0003676) RNA binding (GO:0003723) mRNA binding (GO:0003729) nucleotide binding (GO:0000166) | 0 |  |  | | Cluster026 | 51 | 7 | axial mesoderm development (GO:0048318) somite development (GO:0061053) heart looping (GO:0001947) regulation of BMP signaling pathway invo... (GO:2000223) nucleic acid binding (GO:0003676) RNA polymerase II core promoter proximal... (GO:0000978) metal ion binding (GO:0046872) | 0 |  |  | | Cluster027 | 49 | 6 | multicellular organismal development (GO:0007275) regulation of transcription, DNA-templat... (GO:0006355) nucleus (GO:0005634) mitochondrial ribosome (GO:0005761) transcription factor activity, sequence-... (GO:0003700) sequence-specific DNA binding (GO:0043565) | 0 |  |  | | Cluster028 | 48 | 2 | catalytic step 2 spliceosome (GO:0071013) nucleotide binding (GO:0000166) | 0 |  |  | | Cluster029 | 44 | 0 |  | 0 |  |  | | Cluster030 | 42 | 0 |  | 0 |  |  | | Cluster031 | 42 | 0 |  | 0 |  |  | | Cluster032 | 40 | 0 |  | 0 |  |  | | Cluster033 | 38 | 0 |  | 0 |  |  | | Cluster034 | 36 | 0 |  | 0 |  |  | | Cluster035 | 35 | 2 | PcG protein complex (GO:0031519) histone methyltransferase complex (GO:0035097) | 0 |  |  | | Cluster036 | 34 | 7 | immune response (GO:0006955) blood coagulation (GO:0007596) platelet activation (GO:0030168) extracellular region (GO:0005576) extracellular space (GO:0005615) protein binding, bridging (GO:0030674) serine-type endopeptidase activity (GO:0004252) | 5 | liver (ZFA:0000123) liver primordium (ZFA:0000124) digestive system (ZFA:0000339) YSL (ZFA:0000088) yolk (ZFA:0000084) |  | | Cluster037 | 34 | 1 | secondary metabolic process (GO:0019748) | 0 |  |  | | Cluster038 | 33 | 2 | regulation of transcription, DNA-templat... (GO:0006355) sequence-specific DNA binding (GO:0043565) | 0 |  |  | | Cluster039 | 32 | 0 |  | 0 |  |  | | Cluster040 | 32 | 1 | regulation of transcription, DNA-templat... (GO:0006355) | 0 |  |  | | Cluster041 | 29 | 0 |  | 1 | epicardium (ZFA:0005057) |  | | Cluster042 | 29 | 4 | regulation of transcription, DNA-templat... (GO:0006355) nucleus (GO:0005634) protein dimerization activity (GO:0046983) sequence-specific DNA binding (GO:0043565) | 0 |  |  | | Cluster043 | 28 | 0 |  | 0 |  |  | | Cluster044 | 28 | 2 | collagen trimer (GO:0005581) extracellular matrix structural constitu... (GO:0005201) | 1 | scapulocoracoid (ZFA:0001455) |  | | Cluster045 | 27 | 1 | mitochondrial outer membrane (GO:0005741) | 0 |  |  | | Cluster046 | 27 | 0 |  | 0 |  |  | | Cluster047 | 25 | 0 |  | 0 |  |  | | Cluster048 | 25 | 0 |  | 0 |  |  | | Cluster049 | 25 | 2 | blood coagulation (GO:0007596) extracellular region (GO:0005576) | 0 |  |  | | Cluster050 | 25 | 0 |  | 4 | intestine (ZFA:0001338) liver (ZFA:0000123) intestinal bulb (ZFA:0001076) liver primordium (ZFA:0000124) |  | | Cluster051 | 24 | 0 |  | 0 |  |  | | Cluster052 | 23 | 0 |  | 0 |  |  | | Cluster053 | 23 | 5 | regulation of transcription, DNA-templat... (GO:0006355) peripheral nervous system neuron axonoge... (GO:0048936) nucleus (GO:0005634) protein dimerization activity (GO:0046983) DNA binding (GO:0003677) | 34 | rhombomere (ZFA:0001064) hindbrain (ZFA:0000029) midbrain (ZFA:0000128) telencephalon (ZFA:0000079) brainstem (ZFA:0001707) diencephalon (ZFA:0000101) tegmentum (ZFA:0000160) forebrain (ZFA:0000109) hindbrain neural keel (ZFA:0007029) hindbrain neural rod (ZFA:0007036) midbrain neural keel (ZFA:0007025) hindbrain neural plate (ZFA:0007022) midbrain neural plate (ZFA:0007019) midbrain neural rod (ZFA:0007032) optic tectum (ZFA:0000445) presumptive telencephalon (ZFA:0000571) thalamus (ZFA:0001215) basal plate midbrain region (ZFA:0000761) posterior neural plate (ZFA:0007017) brain (ZFA:0000008) presumptive diencephalon (ZFA:0000574) neuroectoderm (ZFA:0001120) spinal cord (ZFA:0000075) forebrain neural keel (ZFA:0007026) forebrain neural plate (ZFA:0007018) central nervous system (ZFA:0000012) caudal tuberculum (ZFA:0000633) spinal cord neural tube (ZFA:0007042) presumptive rhombomere 2 (ZFA:0001208) pretectum (ZFA:0000418) cerebellum (ZFA:0000100) ventricular zone (ZFA:0001083) rhombomere 1 (ZFA:0001031) rhombomere 2 (ZFA:0000822) |  | | Cluster054 | 23 | 2 | positive regulation of transcription, DN... (GO:0045893) cytokine receptor activity (GO:0004896) | 0 |  |  | | Cluster055 | 22 | 0 |  | 0 |  |  | | Cluster056 | 22 | 0 |  | 0 |  |  | | Cluster057 | 21 | 0 |  | 0 |  |  | | Cluster058 | 21 | 0 |  | 0 |  |  | | Cluster059 | 20 | 0 |  | 0 |  |  | | Cluster060 | 20 | 0 |  | 0 |  |  | | Cluster061 | 19 | 0 |  | 0 |  |  | | Cluster062 | 19 | 0 |  | 0 |  |  | | Cluster063 | 19 | 0 |  | 0 |  |  | | Cluster064 | 19 | 2 | nucleic acid binding (GO:0003676) zinc ion binding (GO:0008270) | 0 |  |  | | Cluster065 | 19 | 0 |  | 0 |  |  | | Cluster066 | 19 | 6 | rRNA processing (GO:0006364) cleavage involved in rRNA processing (GO:0000469) maturation of SSU-rRNA from tricistronic... (GO:0000462) small-subunit processome (GO:0032040) nucleolus (GO:0005730) snoRNA binding (GO:0030515) | 0 |  |  | | Cluster067 | 19 | 1 | collagen trimer (GO:0005581) | 0 |  |  | | Cluster068 | 18 | 4 | rRNA modification (GO:0000154) small nucleolar ribonucleoprotein comple... (GO:0005732) nucleolar part (GO:0044452) DNA-directed RNA polymerase II, core com... (GO:0005665) | 0 |  |  | | Cluster069 | 18 | 0 |  | 0 |  |  | | Cluster070 | 18 | 2 | nucleic acid binding (GO:0003676) metal ion binding (GO:0046872) | 0 |  |  | | Cluster071 | 18 | 0 |  | 0 |  |  | | Cluster072 | 17 | 0 |  | 0 |  |  | | Cluster073 | 17 | 0 |  | 0 |  |  | | Cluster074 | 17 | 0 |  | 0 |  |  | | Cluster075 | 17 | 0 |  | 0 |  |  | | Cluster076 | 17 | 0 |  | 0 |  |  | | Cluster077 | 16 | 0 |  | 0 |  |  | | Cluster078 | 16 | 0 |  | 0 |  |  | | Cluster079 | 16 | 0 |  | 0 |  |  | | Cluster080 | 15 | 2 | epithalamus development (GO:0021538) camera-type eye photoreceptor cell diffe... (GO:0060219) | 0 |  |  | | Cluster081 | 15 | 0 |  | 0 |  |  | | Cluster082 | 15 | 0 |  | 0 |  |  | | Cluster083 | 15 | 0 |  | 0 |  |  | | Cluster084 | 15 | 1 | neuron migration (GO:0001764) | 0 |  |  | | Cluster085 | 15 | 0 |  | 0 |  |  | | Cluster086 | 14 | 4 | proteolysis involved in cellular protein... (GO:0051603) extracellular space (GO:0005615) lysosome (GO:0005764) cysteine-type endopeptidase activity (GO:0004197) | 0 |  |  | | Cluster087 | 14 | 0 |  | 0 |  |  | | Cluster088 | 14 | 2 | regulation of transcription, DNA-templat... (GO:0006355) sequence-specific DNA binding (GO:0043565) | 1 | serotonergic neuron (ZFA:0009361) |  | | Cluster089 | 14 | 0 |  | 0 |  |  | | Cluster090 | 14 | 1 | cardiac muscle cell development (GO:0055013) | 0 |  |  | | Cluster091 | 14 | 0 |  | 0 |  |  | | Cluster092 | 14 | 0 |  | 0 |  |  | | Cluster093 | 14 | 0 |  | 0 |  |  | | Cluster094 | 14 | 3 | mitochondrial ATP synthesis coupled elec... (GO:0042775) mitochondrial respiratory chain (GO:0005746) cytochrome-c oxidase activity (GO:0004129) | 0 |  |  | | Cluster095 | 14 | 0 |  | 0 |  |  | | Cluster096 | 13 | 0 |  | 0 |  |  | | Cluster097 | 13 | 0 |  | 0 |  |  | | Cluster098 | 13 | 0 |  | 0 |  |  | | Cluster099 | 13 | 0 |  | 0 |  |  | | Cluster100 | 13 | 0 |  | 0 |  |  | | Cluster101 | 13 | 0 |  | 0 |  |  | | Cluster102 | 13 | 0 |  | 0 |  |  | | Cluster103 | 13 | 1 | serine-type endopeptidase inhibitor acti... (GO:0004867) | 1 | YSL (ZFA:0000088) |  | | Cluster104 | 12 | 0 |  | 0 |  |  | | Cluster105 | 12 | 0 |  | 0 |  |  | | Cluster106 | 12 | 0 |  | 0 |  |  | | Cluster107 | 12 | 0 |  | 0 |  |  | | Cluster108 | 12 | 0 |  | 0 |  |  | | Cluster109 | 12 | 1 | rRNA processing (GO:0006364) | 0 |  |  | | Cluster110 | 12 | 0 |  | 0 |  |  | | Cluster111 | 11 | 0 |  | 0 |  |  | | Cluster112 | 11 | 0 |  | 0 |  |  | | Cluster113 | 11 | 0 |  | 0 |  |  | | Cluster114 | 11 | 0 |  | 0 |  |  | | Cluster115 | 11 | 0 |  | 0 |  |  | | Cluster116 | 11 | 0 |  | 0 |  |  | | Cluster117 | 11 | 0 |  | 0 |  |  | | Cluster118 | 11 | 1 | potassium ion import (GO:0010107) | 0 |  |  | | Cluster119 | 11 | 0 |  | 0 |  |  | | Cluster120 | 11 | 0 |  | 0 |  |  | | Cluster121 | 11 | 0 |  | 0 |  |  | | Cluster122 | 11 | 0 |  | 0 |  |  | | Cluster123 | 10 | 0 |  | 0 |  |  | | Cluster124 | 10 | 0 |  | 0 |  |  | | Cluster125 | 10 | 0 |  | 0 |  |  | | Cluster126 | 10 | 0 |  | 0 |  |  | | Cluster127 | 10 | 2 | histone methyltransferase complex (GO:0035097) ATP-dependent DNA helicase activity (GO:0004003) | 0 |  |  | | Cluster128 | 10 | 0 |  | 0 |  |  | | Cluster129 | 10 | 0 |  | 0 |  |  | | Cluster130 | 10 | 1 | motor neuron axon guidance (GO:0008045) | 0 |  |  | | Cluster131 | 10 | 0 |  | 0 |  |  | | Cluster132 | 10 | 0 |  | 0 |  |  | | Cluster133 | 10 | 0 |  | 0 |  |  | | Cluster134 | 10 | 0 |  | 0 |  |  | | Cluster135 | 10 | 1 | RNA polymerase II core promoter proximal... (GO:0000978) | 0 |  |  | | Cluster136 | 9 | 0 |  | 0 |  |  | | Cluster137 | 9 | 0 |  | 0 |  |  | | Cluster138 | 9 | 0 |  | 0 |  |  | | Cluster139 | 9 | 1 | replication fork (GO:0005657) | 0 |  |  | | Cluster140 | 9 | 0 |  | 0 |  |  | | Cluster141 | 9 | 0 |  | 0 |  |  | | Cluster142 | 9 | 0 |  | 0 |  |  | | Cluster143 | 9 | 1 | extracellular space (GO:0005615) | 0 |  |  | | Cluster144 | 9 | 0 |  | 0 |  |  | | Cluster145 | 9 | 0 |  | 0 |  |  | | Cluster146 | 9 | 0 |  | 0 |  |  | | Cluster147 | 9 | 1 | multicellular organismal development (GO:0007275) | 0 |  |  | | Cluster148 | 9 | 0 |  | 0 |  |  | | Cluster149 | 9 | 0 |  | 0 |  |  | | Cluster150 | 9 | 0 |  | 0 |  |  | | Cluster151 | 9 | 0 |  | 0 |  |  | | Cluster152 | 9 | 0 |  | 0 |  |  | | Cluster153 | 9 | 1 | oxidation-reduction process (GO:0055114) | 0 |  |  | | Cluster154 | 9 | 0 |  | 0 |  |  | | Cluster155 | 9 | 1 | mitochondrial membrane organization (GO:0007006) | 0 |  |  | | Cluster156 | 9 | 0 |  | 0 |  |  | | Cluster157 | 9 | 0 |  | 0 |  |  | | Cluster158 | 9 | 0 |  | 0 |  |  | | Cluster159 | 9 | 0 |  | 0 |  |  | | Cluster160 | 9 | 0 |  | 0 |  |  | | Cluster161 | 9 | 1 | Notch signaling pathway (GO:0007219) | 0 |  |  | | Cluster162 | 9 | 0 |  | 0 |  |  | | Cluster163 | 8 | 1 | adenylyltransferase activity (GO:0070566) | 0 |  |  | | Cluster164 | 8 | 0 |  | 0 |  |  | | Cluster165 | 8 | 0 |  | 0 |  |  | | Cluster166 | 8 | 0 |  | 0 |  |  | | Cluster167 | 8 | 0 |  | 0 |  |  | | Cluster168 | 8 | 0 |  | 0 |  |  | | Cluster169 | 8 | 0 |  | 0 |  |  | | Cluster170 | 8 | 0 |  | 0 |  |  | | Cluster171 | 8 | 0 |  | 0 |  |  | | Cluster172 | 8 | 0 |  | 0 |  |  | | Cluster173 | 8 | 5 | DNA damage checkpoint (GO:0000077) G2/M transition of mitotic cell cycle (GO:0000086) intrinsic apoptotic signaling pathway in... (GO:0008630) negative regulation of mitotic cell cycl... (GO:1901991) mitotic DNA integrity checkpoint (GO:0044774) | 0 |  |  | | Cluster174 | 8 | 2 | tRNA modification (GO:0006400) 90S preribosome (GO:0030686) | 0 |  |  | | Cluster175 | 8 | 0 |  | 0 |  |  | | Cluster176 | 8 | 0 |  | 0 |  |  | | Cluster177 | 8 | 0 |  | 0 |  |  | | Cluster178 | 8 | 0 |  | 0 |  |  | | Cluster179 | 8 | 0 |  | 0 |  |  | | Cluster180 | 8 | 0 |  | 0 |  |  | | Cluster181 | 8 | 0 |  | 0 |  |  | | Cluster182 | 8 | 0 |  | 0 |  |  | | Cluster183 | 8 | 0 |  | 0 |  |  | | Cluster184 | 8 | 0 |  | 0 |  |  | | Cluster185 | 8 | 0 |  | 0 |  |  | | Cluster186 | 7 | 0 |  | 0 |  |  | | Cluster187 | 7 | 0 |  | 0 |  |  | | Cluster188 | 7 | 0 |  | 0 |  |  | | Cluster189 | 7 | 0 |  | 0 |  |  | | Cluster190 | 7 | 2 | dicarboxylic acid metabolic process (GO:0043648) tricarboxylic acid cycle (GO:0006099) | 0 |  |  | | Cluster191 | 7 | 0 |  | 0 |  |  | | Cluster192 | 7 | 0 |  | 0 |  |  | | Cluster193 | 7 | 0 |  | 0 |  |  | | Cluster194 | 7 | 0 |  | 0 |  |  | | Cluster195 | 7 | 0 |  | 0 |  |  | | Cluster196 | 7 | 0 |  | 0 |  |  | | Cluster197 | 7 | 0 |  | 0 |  |  | | Cluster198 | 7 | 0 |  | 0 |  |  | | Cluster199 | 7 | 0 |  | 0 |  |  | | Cluster200 | 7 | 0 |  | 0 |  |  | | Cluster201 | 7 | 0 |  | 0 |  |  | | Cluster202 | 7 | 0 |  | 0 |  |  | | Cluster203 | 7 | 0 |  | 0 |  |  | | Cluster204 | 7 | 1 | heart jogging (GO:0003146) | 0 |  |  | | Cluster205 | 7 | 0 |  | 0 |  |  | | Cluster206 | 7 | 0 |  | 0 |  |  | | Cluster207 | 7 | 0 |  | 0 |  |  | | Cluster208 | 7 | 0 |  | 0 |  |  | | Cluster209 | 7 | 0 |  | 0 |  |  | | Cluster210 | 7 | 0 |  | 0 |  |  | | Cluster211 | 7 | 0 |  | 0 |  |  | | Cluster212 | 7 | 0 |  | 0 |  |  | | Cluster213 | 7 | 0 |  | 0 |  |  | | Cluster214 | 7 | 0 |  | 1 | YSL (ZFA:0000088) |  | | Cluster215 | 7 | 0 |  | 0 |  |  | | Cluster216 | 7 | 2 | protein import into nucleus (GO:0006606) nuclear pore (GO:0005643) | 0 |  |  | | Cluster217 | 7 | 1 | cytochrome-c oxidase activity (GO:0004129) | 0 |  |  | | Cluster218 | 6 | 0 |  | 0 |  |  | | Cluster219 | 6 | 0 |  | 0 |  |  | | Cluster220 | 6 | 0 |  | 0 |  |  | | Cluster221 | 6 | 0 |  | 0 |  |  | | Cluster222 | 6 | 0 |  | 0 |  |  | | Cluster223 | 6 | 0 |  | 0 |  |  | | Cluster224 | 6 | 0 |  | 0 |  |  | | Cluster225 | 6 | 0 |  | 0 |  |  | | Cluster226 | 6 | 0 |  | 0 |  |  | | Cluster227 | 6 | 0 |  | 0 |  |  | | Cluster228 | 6 | 0 |  | 0 |  |  | | Cluster229 | 6 | 0 |  | 0 |  |  | | Cluster230 | 6 | 0 |  | 0 |  |  | | Cluster231 | 6 | 0 |  | 0 |  |  | | Cluster232 | 6 | 0 |  | 0 |  |  | | Cluster233 | 6 | 0 |  | 0 |  |  | | Cluster234 | 6 | 0 |  | 0 |  |  | | Cluster235 | 6 | 0 |  | 0 |  |  | | Cluster236 | 6 | 0 |  | 0 |  |  | | Cluster237 | 6 | 0 |  | 0 |  |  | | Cluster238 | 6 | 0 |  | 0 |  |  | | Cluster239 | 6 | 1 | calcium ion binding (GO:0005509) | 0 |  |  | | Cluster240 | 6 | 0 |  | 0 |  |  | | Cluster241 | 6 | 0 |  | 0 |  |  | | Cluster242 | 6 | 0 |  | 0 |  |  | | Cluster243 | 6 | 0 |  | 0 |  |  | | Cluster244 | 6 | 0 |  | 0 |  |  | | Cluster245 | 6 | 0 |  | 0 |  |  | | Cluster246 | 6 | 1 | nucleus (GO:0005634) | 0 |  |  | | Cluster247 | 6 | 0 |  | 0 |  |  | | Cluster248 | 6 | 0 |  | 0 |  |  | | Cluster249 | 6 | 0 |  | 0 |  |  | | Cluster250 | 6 | 0 |  | 0 |  |  | | Cluster251 | 6 | 3 | maturation of SSU-rRNA from tricistronic... (GO:0000462) nucleolus (GO:0005730) rRNA binding (GO:0019843) | 0 |  |  | | Cluster252 | 6 | 0 |  | 0 |  |  | | Cluster253 | 6 | 0 |  | 0 |  |  | | Cluster254 | 6 | 0 |  | 0 |  |  | |
