# Supplementary material for: A high-resolution mRNA expression time course of embryonic development in zebrafish
Source: eLife. 2017 Nov 16;6:e30860. doi: 10.7554/eLife.30860 (PMC5690287; doi:10.7554/eLife.30860)
Supplement: Supplementary file 6. [file elife-30860-supp6.zip › biolayout-clusters-files/Cluster001-genes.html]

Cluster001


# Cluster001: Genes

| | Ensembl ID | Gene Name | Chr | Start | End | Biotype | | --- | --- | --- | --- | --- | --- | | ENSDARG00000092071 | ABHD12B (1 of many).1 | 13 | 9556952 | 9563614 | protein\_coding | | ENSDARG00000078494 | ADAMTS14 | 12 | 47993992 | 48046495 | protein\_coding | | ENSDARG00000090624 | ADGRL3 (1 of many) | 7 | 72732962 | 72836974 | protein\_coding | | ENSDARG00000027394 | AL929252.1 | 7 | 32450958 | 32471465 | protein\_coding | | ENSDARG00000097208 | ALKBH3 (1 of many) | 25 | 29790696 | 29856669 | protein\_coding | | ENSDARG00000079569 | AMIGO3 (1 of many) | 23 | 19543784 | 19545166 | protein\_coding | | ENSDARG00000076719 | ANKRD34A | 16 | 42891959 | 42916035 | protein\_coding | | ENSDARG00000093286 | ANKRD34C | 18 | 26140154 | 26141743 | protein\_coding | | ENSDARG00000003210 | ANO2 (1 of many) | 18 | 7288019 | 7345612 | protein\_coding | | ENSDARG00000036809 | ANPEP (1 of many) | 7 | 15125125 | 15164732 | protein\_coding | | ENSDARG00000053771 | APBB3 | 21 | 40707868 | 40759542 | protein\_coding | | ENSDARG00000102424 | ARHGAP44 (1 of many) | 3 | 44485323 | 44637647 | protein\_coding | | ENSDARG00000076361 | B4GALNT2 (1 of many) | 25 | 5176782 | 5199547 | protein\_coding | | ENSDARG00000043673 | BEGAIN | 20 | 9367910 | 9433524 | protein\_coding | | ENSDARG00000098551 | BICD1 (1 of many) | 4 | 1544820 | 1564628 | protein\_coding | | ENSDARG00000075352 | BRINP3 (1 of many) | 2 | 6822833 | 6893499 | protein\_coding | | ENSDARG00000077124 | BRSK1 (1 of many) | 12 | 4610146 | 4637696 | protein\_coding | | ENSDARG00000069027 | BX005395.1 | 18 | 16809788 | 16811967 | protein\_coding | | ENSDARG00000092704 | BX072532.1 | 12 | 46424455 | 46435541 | protein\_coding | | ENSDARG00000075779 | BX323087.1 | 23 | 6397917 | 6442296 | protein\_coding | | ENSDARG00000095082 | BX571811.1 | 7 | 32880482 | 32904971 | protein\_coding | | ENSDARG00000076565 | BX640406.1 | 13 | 15044972 | 15046726 | protein\_coding | | ENSDARG00000098957 | BX908782.1 | 2 | 52197360 | 52202121 | protein\_coding | | ENSDARG00000090615 | BX957297.1 | 7 | 38424470 | 38427354 | protein\_coding | | ENSDARG00000087659 | C1QTNF4 (1 of many) | 18 | 15801949 | 15803042 | protein\_coding | | ENSDARG00000019772 | C2 | 15 | 517688 | 535747 | protein\_coding | | ENSDARG00000086931 | CABZ01029822.1 | 19 | 6937664 | 6954182 | protein\_coding | | ENSDARG00000058631 | CABZ01030107.1 | 12 | 4498909 | 4505552 | protein\_coding | | ENSDARG00000090943 | CABZ01033205.2 | 15 | 179112 | 187572 | protein\_coding | | ENSDARG00000101026 | CABZ01038709.1 | KN150709.1 | 28322 | 30415 | protein\_coding | | ENSDARG00000061481 | CABZ01041812.1 | 24 | 29770121 | 29789185 | protein\_coding | | ENSDARG00000098455 | CABZ01063757.1 | 16 | 30385064 | 30387464 | protein\_coding | | ENSDARG00000103903 | CABZ01067945.1 | 12 | 39549895 | 39581555 | protein\_coding | | ENSDARG00000013134 | CABZ01071407.1 | 17 | 39926164 | 39933597 | protein\_coding | | ENSDARG00000105274 | CABZ01074130.1 | KN150214.1 | 271 | 23344 | protein\_coding | | ENSDARG00000098269 | CABZ01079039.1 | KN149943.1 | 18756 | 25930 | protein\_coding | | ENSDARG00000100498 | CABZ01079251.1 | 3 | 13332244 | 13338732 | protein\_coding | | ENSDARG00000103822 | CABZ01081565.1 | KN150399.1 | 22 | 1756 | protein\_coding | | ENSDARG00000063158 | CABZ01081780.1 | 7 | 73115067 | 73165344 | protein\_coding | | ENSDARG00000100613 | CABZ01089777.1 | 13 | 51715298 | 51733019 | protein\_coding | | ENSDARG00000101657 | CABZ01089974.1 | KN150359.1 | 181 | 7519 | protein\_coding | | ENSDARG00000076357 | CABZ01102039.1 | 12 | 6364 | 32783 | protein\_coding | | ENSDARG00000099838 | CABZ01113374.1 | 18 | 50949408 | 50952472 | protein\_coding | | ENSDARG00000090486 | CABZ01114051.1 | 25 | 48818 | 84317 | protein\_coding | | ENSDARG00000101387 | CAMK2N1 | 11 | 3530983 | 3532680 | protein\_coding | | ENSDARG00000100166 | CASKIN1 (1 of many).1 | 1 | 7338209 | 7417384 | protein\_coding | | ENSDARG00000012204 | CDK18 | 11 | 38013303 | 38154553 | protein\_coding | | ENSDARG00000078327 | CEP170B (1 of many) | 20 | 3095757 | 3118041 | protein\_coding | | ENSDARG00000104788 | CLEC3A | 7 | 65163634 | 65171140 | protein\_coding | | ENSDARG00000060638 | CLSTN2 (1 of many) | 15 | 41598197 | 41706802 | protein\_coding | | ENSDARG00000029898 | CNGA1 (1 of many) | 1 | 43193074 | 43198258 | protein\_coding | | ENSDARG00000042107 | CNGB1 | 25 | 17411835 | 17455899 | protein\_coding | | ENSDARG00000078087 | CNNM1 | 13 | 40334634 | 40373033 | protein\_coding | | ENSDARG00000095901 | COL18A1 (1 of many) | 6 | 18619145 | 18668330 | protein\_coding | | ENSDARG00000100781 | COL28A1 (1 of many).2 | 6 | 3479561 | 3513320 | protein\_coding | | ENSDARG00000074216 | COLQ (1 of many) | 19 | 19839962 | 19859737 | protein\_coding | | ENSDARG00000011208 | COX5B (1 of many) | 8 | 52419834 | 52426519 | protein\_coding | | ENSDARG00000077736 | CPLX1 (1 of many) | 14 | 50866586 | 50892594 | protein\_coding | | ENSDARG00000102191 | CPNE8 (1 of many) | 4 | 73733122 | 73840034 | protein\_coding | | ENSDARG00000075425 | CR376723.1 | 8 | 31203380 | 31209342 | protein\_coding | | ENSDARG00000076443 | CR388231.1 | 7 | 23696175 | 23725254 | protein\_coding | | ENSDARG00000091817 | CR391998.1 | 16 | 4256425 | 4272473 | protein\_coding | | ENSDARG00000044212 | CR735126.1 | 19 | 37955768 | 37979726 | protein\_coding | | ENSDARG00000087096 | CR847847.1 | 21 | 25735371 | 25736006 | protein\_coding | | ENSDARG00000104450 | CR855996.2 | 10 | 17075904 | 17079435 | protein\_coding | | ENSDARG00000100167 | CSDC2 (1 of many) | 3 | 5098933 | 5106411 | protein\_coding | | ENSDARG00000074813 | CT030144.2 | 7 | 15946425 | 15953498 | protein\_coding | | ENSDARG00000102759 | CT583723.1 | 8 | 35129731 | 35179756 | protein\_coding | | ENSDARG00000033508 | CTXN3 | 10 | 16230687 | 16236268 | protein\_coding | | ENSDARG00000098747 | CU633479.1 | 24 | 41032106 | 41044878 | protein\_coding | | ENSDARG00000089534 | CU693369.1 | 2 | 53858767 | 53867436 | protein\_coding | | ENSDARG00000060688 | CU929225.1 | 10 | 40915175 | 40932872 | protein\_coding | | ENSDARG00000089543 | CU984579.1 | 5 | 36888191 | 36896815 | protein\_coding | | ENSDARG00000037286 | DCHS2 | 1 | 24902969 | 24959927 | protein\_coding | | ENSDARG00000063578 | DGKI | 4 | 4605698 | 4698462 | protein\_coding | | ENSDARG00000036999 | DISP3 | 8 | 51061532 | 51158521 | protein\_coding | | ENSDARG00000004187 | DNAJB5 (1 of many) | 21 | 5004589 | 5009491 | protein\_coding | | ENSDARG00000078736 | DOC2A | 3 | 15400357 | 15458428 | protein\_coding | | ENSDARG00000079216 | DPEP2 | 7 | 34996577 | 35012845 | protein\_coding | | ENSDARG00000104767 | DPP10 | 23 | 33928510 | 33975836 | protein\_coding | | ENSDARG00000103195 | DYNC1I2 (1 of many) | 6 | 3771606 | 3795958 | protein\_coding | | ENSDARG00000104210 | EFEMP1 (1 of many).1 | 22 | 35092297 | 35108032 | protein\_coding | | ENSDARG00000078141 | ELFN1 (1 of many) | 3 | 46054904 | 46057680 | protein\_coding | | ENSDARG00000053517 | EML5 | 17 | 43048924 | 43296752 | protein\_coding | | ENSDARG00000003146 | ENSDARG00000003146 | 13 | 17571723 | 17579001 | protein\_coding | | ENSDARG00000013252 | ENSDARG00000013252 | 3 | 26603291 | 26620948 | protein\_coding | | ENSDARG00000014169 | ENSDARG00000014169 | 6 | 55164916 | 55183001 | protein\_coding | | ENSDARG00000015931 | ENSDARG00000015931 | 4 | 5252371 | 5294296 | protein\_coding | | ENSDARG00000024619 | ENSDARG00000024619 | 19 | 15666928 | 15716605 | protein\_coding | | ENSDARG00000029114 | ENSDARG00000029114 | 16 | 32105755 | 32126845 | protein\_coding | | ENSDARG00000036422 | ENSDARG00000036422 | 7 | 20179096 | 20194423 | protein\_coding | | ENSDARG00000043394 | ENSDARG00000043394 | 20 | 19377349 | 19466292 | protein\_coding | | ENSDARG00000045979 | ENSDARG00000045979 | 5 | 4041699 | 4052774 | protein\_coding | | ENSDARG00000051873 | ENSDARG00000051873 | 25 | 21702849 | 21724878 | protein\_coding | | ENSDARG00000053454 | ENSDARG00000053454 | 6 | 44593202 | 44678137 | protein\_coding | | ENSDARG00000053746 | ENSDARG00000053746 | 2 | 1331990 | 1346930 | protein\_coding | | ENSDARG00000055498 | ENSDARG00000055498 | 2 | 5449942 | 5477837 | protein\_coding | | ENSDARG00000055999 | ENSDARG00000055999 | 21 | 30158052 | 30200101 | protein\_coding | | ENSDARG00000056572 | ENSDARG00000056572 | 19 | 28772530 | 28783190 | protein\_coding | | ENSDARG00000057669 | ENSDARG00000057669 | 1 | 53480127 | 53502501 | protein\_coding | | ENSDARG00000067727 | ENSDARG00000067727 | 20 | 4221954 | 4286363 | protein\_coding | | ENSDARG00000068274 | ENSDARG00000068274 | 18 | 41505649 | 41519608 | protein\_coding | | ENSDARG00000068745 | ENSDARG00000068745 | 2 | 24521355 | 24596685 | protein\_coding | | ENSDARG00000069038 | ENSDARG00000069038 | 18 | 16967882 | 16981227 | protein\_coding | | ENSDARG00000069607 | ENSDARG00000069607 | 4 | 4774151 | 4786635 | protein\_coding | | ENSDARG00000070100 | ENSDARG00000070100 | 7 | 7046534 | 7139690 | protein\_coding | | ENSDARG00000070170 | ENSDARG00000070170 | 16 | 42024406 | 42034101 | protein\_coding | | ENSDARG00000073822 | ENSDARG00000073822 | 23 | 4644933 | 4741647 | protein\_coding | | ENSDARG00000074508 | ENSDARG00000074508 | 11 | 11613451 | 11641748 | protein\_coding | | ENSDARG00000074919 | ENSDARG00000074919 | 13 | 854952 | 864083 | protein\_coding | | ENSDARG00000075043 | ENSDARG00000075043 | 1 | 50203984 | 50215255 | protein\_coding | | ENSDARG00000075706 | ENSDARG00000075706 | 2 | 31643190 | 31678391 | protein\_coding | | ENSDARG00000076070 | ENSDARG00000076070 | 20 | 14248309 | 14310718 | protein\_coding | | ENSDARG00000076127 | ENSDARG00000076127 | 13 | 12167522 | 12243118 | protein\_coding | | ENSDARG00000076268 | ENSDARG00000076268 | 5 | 5031096 | 5034674 | protein\_coding | | ENSDARG00000076873 | ENSDARG00000076873 | 8 | 31239685 | 31245136 | protein\_coding | | ENSDARG00000077725 | ENSDARG00000077725 | 17 | 1354915 | 1363561 | protein\_coding | | ENSDARG00000079741 | ENSDARG00000079741 | 9 | 50061320 | 50062804 | protein\_coding | | ENSDARG00000079873 | ENSDARG00000079873 | 1 | 7917824 | 7926453 | protein\_coding | | ENSDARG00000086665 | ENSDARG00000086665 | 10 | 23052811 | 23057077 | protein\_coding | | ENSDARG00000087663 | ENSDARG00000087663 | 24 | 38111193 | 38124696 | protein\_coding | | ENSDARG00000089920 | ENSDARG00000089920 | 13 | 2019227 | 2083922 | protein\_coding | | ENSDARG00000090126 | ENSDARG00000090126 | 15 | 15439585 | 15445857 | protein\_coding | | ENSDARG00000090381 | ENSDARG00000090381 | 17 | 2172529 | 2209370 | protein\_coding | | ENSDARG00000090526 | ENSDARG00000090526 | 12 | 30427759 | 30434120 | protein\_coding | | ENSDARG00000091099 | ENSDARG00000091099 | 13 | 9714611 | 9738069 | protein\_coding | | ENSDARG00000091135 | ENSDARG00000091135 | 20 | 20813712 | 20822946 | protein\_coding | | ENSDARG00000091408 | ENSDARG00000091408 | 2 | 54705165 | 54821647 | protein\_coding | | ENSDARG00000092260 | ENSDARG00000092260 | 23 | 45803737 | 45813729 | protein\_coding | | ENSDARG00000095819 | ENSDARG00000095819 | 2 | 16697178 | 16711693 | protein\_coding | | ENSDARG00000096701 | ENSDARG00000096701 | 12 | 34983746 | 34994351 | protein\_coding | | ENSDARG00000096908 | ENSDARG00000096908 | 6 | 34043842 | 34051541 | protein\_coding | | ENSDARG00000096920 | ENSDARG00000096920 | 5 | 3281766 | 3297378 | protein\_coding | | ENSDARG00000097285 | ENSDARG00000097285 | 25 | 10738107 | 10743999 | protein\_coding | | ENSDARG00000098090 | ENSDARG00000098090 | KN150125.1 | 15167 | 82871 | protein\_coding | | ENSDARG00000098380 | ENSDARG00000098380 | 12 | 25801639 | 25826708 | protein\_coding | | ENSDARG00000098631 | ENSDARG00000098631 | 13 | 1403988 | 1412404 | protein\_coding | | ENSDARG00000098637 | ENSDARG00000098637 | KN150600.1 | 14612 | 27709 | protein\_coding | | ENSDARG00000099488 | ENSDARG00000099488 | 20 | 51076516 | 51104490 | protein\_coding | | ENSDARG00000099648 | ENSDARG00000099648 | 21 | 45693923 | 45720749 | protein\_coding | | ENSDARG00000100032 | ENSDARG00000100032 | 21 | 42197090 | 42266680 | protein\_coding | | ENSDARG00000100145 | ENSDARG00000100145 | 2 | 18866 | 24792 | protein\_coding | | ENSDARG00000100486 | ENSDARG00000100486 | 16 | 7397798 | 7445460 | protein\_coding | | ENSDARG00000100722 | ENSDARG00000100722 | 17 | 27157852 | 27165057 | protein\_coding | | ENSDARG00000101368 | ENSDARG00000101368 | 24 | 31494211 | 31502630 | protein\_coding | | ENSDARG00000101597 | ENSDARG00000101597 | 1 | 8546133 | 8547841 | protein\_coding | | ENSDARG00000102185 | ENSDARG00000102185 | 9 | 54166770 | 54183271 | protein\_coding | | ENSDARG00000102210 | ENSDARG00000102210 | KN150265.1 | 9216 | 16968 | protein\_coding | | ENSDARG00000102288 | ENSDARG00000102288 | 17 | 47001554 | 47006177 | protein\_coding | | ENSDARG00000102364 | ENSDARG00000102364 | 15 | 12073360 | 12079822 | protein\_coding | | ENSDARG00000102406 | ENSDARG00000102406 | 13 | 1299803 | 1403357 | protein\_coding | | ENSDARG00000102436 | ENSDARG00000102436 | 3 | 13487537 | 13534551 | protein\_coding | | ENSDARG00000102443 | ENSDARG00000102443 | 18 | 51002022 | 51008559 | protein\_coding | | ENSDARG00000103993 | ENSDARG00000103993 | 15 | 43261960 | 43263570 | protein\_coding | | ENSDARG00000104084 | ENSDARG00000104084 | 13 | 21558817 | 21570873 | protein\_coding | | ENSDARG00000104264 | ENSDARG00000104264 | 2 | 28153653 | 28154336 | protein\_coding | | ENSDARG00000104339 | ENSDARG00000104339 | 6 | 65806 | 81892 | protein\_coding | | ENSDARG00000105443 | ENSDARG00000105443 | 7 | 4161881 | 4177833 | protein\_coding | | ENSDARG00000076364 | EPB41L1 | 23 | 11929240 | 12080052 | protein\_coding | | ENSDARG00000091503 | FADS6 | 12 | 46615433 | 46634873 | protein\_coding | | ENSDARG00000086300 | FAM107A | 11 | 4186180 | 4216424 | protein\_coding | | ENSDARG00000105121 | FAM135B | KN150230.1 | 49012 | 123746 | protein\_coding | | ENSDARG00000077470 | FAM163A (1 of many) | 2 | 36880016 | 36912851 | protein\_coding | | ENSDARG00000002635 | FAM184A (1 of many) | 17 | 15301499 | 15381068 | protein\_coding | | ENSDARG00000079008 | FAM189B | 16 | 23896324 | 23920890 | protein\_coding | | ENSDARG00000074317 | FAM20C (1 of many) | 3 | 61660188 | 61728620 | protein\_coding | | ENSDARG00000087709 | FAT3 (1 of many) | 10 | 25117228 | 25233341 | protein\_coding | | ENSDARG00000089519 | FBLN7 | 13 | 47461216 | 47510322 | protein\_coding | | ENSDARG00000077878 | FBXL7 (1 of many) | 24 | 1046550 | 1106484 | protein\_coding | | ENSDARG00000102508 | FLRT1 (1 of many) | 5 | 66192533 | 66194968 | protein\_coding | | ENSDARG00000097299 | FLRT2 (1 of many) | 17 | 18997558 | 19003154 | protein\_coding | | ENSDARG00000009949 | FNDC4 (1 of many) | 17 | 5740176 | 5771721 | protein\_coding | | ENSDARG00000091459 | GABRA2 (1 of many).1 | 13 | 11988189 | 12018875 | protein\_coding | | ENSDARG00000101995 | GABRG1 | 13 | 12042462 | 12070183 | protein\_coding | | ENSDARG00000060631 | GARNL3 | 5 | 32752522 | 32861217 | protein\_coding | | ENSDARG00000078258 | GGT5 (1 of many) | 10 | 41628193 | 41685320 | protein\_coding | | ENSDARG00000067999 | GJD2 (1 of many) | 17 | 50887139 | 50887999 | protein\_coding | | ENSDARG00000099844 | GNAZ | 10 | 17296339 | 17326330 | protein\_coding | | ENSDARG00000078448 | GPR137B (1 of many) | 13 | 49838527 | 49857138 | protein\_coding | | ENSDARG00000104291 | GPR151 | 14 | 51804332 | 51805766 | protein\_coding | | ENSDARG00000103736 | GRAMD2 | 7 | 53271521 | 53357072 | protein\_coding | | ENSDARG00000077715 | GRIK3 | 16 | 3564322 | 3684735 | protein\_coding | | ENSDARG00000075802 | GRIN3A | 10 | 4256154 | 4292303 | protein\_coding | | ENSDARG00000007195 | GRM2 (1 of many) | 11 | 35896293 | 35954668 | protein\_coding | | ENSDARG00000004445 | GRM5 (1 of many) | 10 | 25251786 | 25298068 | protein\_coding | | ENSDARG00000100267 | GRM7 | 22 | 33581083 | 33701899 | protein\_coding | | ENSDARG00000093185 | GUCY1A2 | 15 | 44347262 | 44382352 | protein\_coding | | ENSDARG00000056045 | GUCY2C (1 of many) | 12 | 18943610 | 18969340 | protein\_coding | | ENSDARG00000077945 | HEPACAM (1 of many) | 5 | 57734805 | 57745514 | protein\_coding | | ENSDARG00000018773 | HIVEP2 (1 of many) | 17 | 6234706 | 6242590 | protein\_coding | | ENSDARG00000069043 | HSD11B1L (1 of many) | 2 | 53729740 | 53753878 | protein\_coding | | ENSDARG00000061940 | HTR4 | 14 | 24836519 | 25011813 | protein\_coding | | ENSDARG00000010925 | HTRA3 (1 of many) | 7 | 61506635 | 61515990 | protein\_coding | | ENSDARG00000075629 | IFI30 (1 of many) | 11 | 7341087 | 7346156 | protein\_coding | | ENSDARG00000100406 | ITIH4 (1 of many) | 11 | 36933895 | 36962328 | protein\_coding | | ENSDARG00000076056 | IYD | 23 | 39882020 | 39891457 | protein\_coding | | ENSDARG00000100708 | JAKMIP3 | 12 | 41137026 | 41166962 | protein\_coding | | ENSDARG00000017108 | KCNA1 (1 of many) | 4 | 73112163 | 73113644 | protein\_coding | | ENSDARG00000079491 | KCNA3 | 6 | 49366890 | 49368727 | protein\_coding | | ENSDARG00000078650 | KCNA4 | 7 | 32325031 | 32327428 | protein\_coding | | ENSDARG00000088842 | KCNB2 | 24 | 13217853 | 13421596 | protein\_coding | | ENSDARG00000058985 | KCNJ6 | 10 | 121868 | 146686 | protein\_coding | | ENSDARG00000104868 | KCNT1 (1 of many) | 5 | 64814584 | 64917363 | protein\_coding | | ENSDARG00000070092 | KCNV1 | 16 | 41041879 | 41063292 | protein\_coding | | ENSDARG00000017162 | KIF3C (1 of many) | 20 | 47588939 | 47615219 | protein\_coding | | ENSDARG00000058332 | KRT18 (1 of many).1 | 23 | 10417951 | 10420582 | protein\_coding | | ENSDARG00000101098 | LAMP5 | KN150051.1 | 86 | 1598 | protein\_coding | | ENSDARG00000104216 | LOX (1 of many) | 8 | 235035 | 243867 | protein\_coding | | ENSDARG00000077652 | LRAT | 1 | 9434344 | 9440081 | protein\_coding | | ENSDARG00000039500 | LRFN3 (1 of many) | 18 | 49416694 | 49434039 | protein\_coding | | ENSDARG00000105059 | LRFN3 (1 of many).1 | 15 | 13158105 | 13304487 | protein\_coding | | ENSDARG00000029388 | LRG1 | 20 | 23541634 | 23545553 | protein\_coding | | ENSDARG00000087624 | LRRC2 | 8 | 507119 | 523642 | protein\_coding | | ENSDARG00000076834 | LRRC75A | 15 | 24755823 | 24801040 | protein\_coding | | ENSDARG00000091128 | LRRC75B (1 of many) | 10 | 41746654 | 41813830 | protein\_coding | | ENSDARG00000102702 | LRRN3 (1 of many) | 4 | 7201986 | 7204500 | protein\_coding | | ENSDARG00000098742 | LRTM1 | 8 | 53810398 | 53819340 | protein\_coding | | ENSDARG00000056450 | MANEAL | 19 | 17355550 | 17372846 | protein\_coding | | ENSDARG00000090326 | MAPK8IP1 (1 of many) | 7 | 72128858 | 72150596 | protein\_coding | | ENSDARG00000062959 | MARCH3 | 7 | 12546615 | 12568316 | protein\_coding | | ENSDARG00000011884 | MFN1 | 2 | 5410216 | 5445042 | protein\_coding | | ENSDARG00000009386 | MPP4 (1 of many) | 21 | 18939987 | 18956474 | protein\_coding | | ENSDARG00000076872 | MPP4 (1 of many).1 | 6 | 9631887 | 9651283 | protein\_coding | | ENSDARG00000104722 | MYL2 (1 of many) | KN150702.1 | 126256 | 146772 | protein\_coding | | ENSDARG00000099712 | MYL3 | 12 | 4801899 | 4806061 | protein\_coding | | ENSDARG00000044441 | MYO7B (1 of many) | 6 | 28061417 | 28121143 | protein\_coding | | ENSDARG00000069101 | NAPB (1 of many) | 20 | 45687726 | 45757333 | protein\_coding | | ENSDARG00000102687 | NBL1 | 22 | 20701 | 29531 | protein\_coding | | ENSDARG00000056910 | NFASC (1 of many) | 23 | 17972736 | 17991733 | protein\_coding | | ENSDARG00000077162 | NWD2 | 7 | 60746596 | 60864372 | protein\_coding | | ENSDARG00000079249 | OGDHL | 13 | 29223135 | 29273534 | protein\_coding | | ENSDARG00000003380 | PAOX | 13 | 30524722 | 30531953 | protein\_coding | | ENSDARG00000052059 | PAQR9 | 7 | 50606009 | 50607201 | protein\_coding | | ENSDARG00000036424 | PCDH20 | 11 | 42622179 | 42626846 | protein\_coding | | ENSDARG00000063264 | PCDH9 | 6 | 4712791 | 4824198 | protein\_coding | | ENSDARG00000101446 | PCP4L1 (1 of many) | 7 | 73408638 | 73434252 | protein\_coding | | ENSDARG00000100897 | PDGFRB | KN150207.1 | 85481 | 117390 | protein\_coding | | ENSDARG00000079771 | PEA15 | 7 | 73548423 | 73559834 | protein\_coding | | ENSDARG00000087452 | PELI3 | 7 | 17870119 | 17916161 | protein\_coding | | ENSDARG00000078176 | PHYHIP | 8 | 52464094 | 52470383 | protein\_coding | | ENSDARG00000095152 | PKIB (1 of many) | 16 | 34525034 | 34547872 | protein\_coding | | ENSDARG00000099344 | PLA2G10 | 3 | 43056264 | 43069959 | protein\_coding | | ENSDARG00000063519 | PLEKHB1 | 10 | 36787281 | 36794884 | protein\_coding | | ENSDARG00000104980 | PPEF2 (1 of many).1 | 5 | 52235635 | 52275043 | protein\_coding | | ENSDARG00000054931 | PPP2R5B | 7 | 24788489 | 24828990 | protein\_coding | | ENSDARG00000036446 | PRKAR2B | 25 | 3345751 | 3378002 | protein\_coding | | ENSDARG00000078102 | PSD | 13 | 22609389 | 22647309 | protein\_coding | | ENSDARG00000104937 | PTPRD | KN150000.1 | 7762 | 68639 | protein\_coding | | ENSDARG00000090292 | RALYL | 2 | 31734574 | 31758019 | protein\_coding | | ENSDARG00000075924 | RAPGEF4 (1 of many) | 6 | 6693652 | 6765121 | protein\_coding | | ENSDARG00000073824 | RASGRF1 | 18 | 26353351 | 26434780 | protein\_coding | | ENSDARG00000075054 | RASGRF2 (1 of many) | 10 | 3081322 | 3142059 | protein\_coding | | ENSDARG00000038742 | RBP1 (1 of many) | 2 | 39055643 | 39062413 | protein\_coding | | ENSDARG00000008491 | RDH13 (1 of many) | 7 | 29907103 | 29917716 | protein\_coding | | ENSDARG00000054797 | RDH13 (1 of many).2 | 19 | 42848790 | 42855162 | protein\_coding | | ENSDARG00000086756 | RGS9BP | 18 | 5166441 | 5167169 | protein\_coding | | ENSDARG00000057481 | RNF157 | 12 | 31614173 | 31650920 | protein\_coding | | ENSDARG00000059854 | RNF182 (1 of many) | 6 | 55267606 | 55268349 | protein\_coding | | ENSDARG00000078056 | RNF208 | 10 | 17144938 | 17145723 | protein\_coding | | ENSDARG00000058944 | SBK2 | 8 | 9994822 | 10009474 | protein\_coding | | ENSDARG00000104507 | SBSPON | 24 | 13176055 | 13193479 | protein\_coding | | ENSDARG00000102161 | SDK2 (1 of many) | 3 | 49990678 | 50099147 | protein\_coding | | ENSDARG00000039901 | SH3GL2 (1 of many) | 23 | 45330196 | 45355933 | protein\_coding | | ENSDARG00000098258 | SLC16A7 | KN149739.1 | 32838 | 45969 | protein\_coding | | ENSDARG00000102071 | SLC25A47 (1 of many) | 17 | 1499303 | 1504760 | protein\_coding | | ENSDARG00000063268 | SLC35G1 | 12 | 5174865 | 5189820 | protein\_coding | | ENSDARG00000056757 | SLC39A8 | 13 | 11557188 | 11581718 | protein\_coding | | ENSDARG00000055791 | SLC3A2 (1 of many) | 7 | 20283901 | 20290577 | protein\_coding | | ENSDARG00000002771 | SLC4A5 (1 of many) | 5 | 27452569 | 27502545 | protein\_coding | | ENSDARG00000004219 | SLC6A3 | 16 | 362629 | 376736 | protein\_coding | | ENSDARG00000054343 | SLC7A8 (1 of many) | 2 | 38332187 | 38354819 | protein\_coding | | ENSDARG00000068296 | SLCO3A1 (1 of many) | 7 | 52021661 | 52059973 | protein\_coding | | ENSDARG00000104443 | SLITRK1 | KN150474.1 | 160909 | 162978 | protein\_coding | | ENSDARG00000103359 | SLITRK5 (1 of many) | KN149962.1 | 65758 | 68666 | protein\_coding | | ENSDARG00000093064 | SMIM18 | 1 | 22167014 | 22170609 | protein\_coding | | ENSDARG00000077465 | SORCS2 | 7 | 39487562 | 39786833 | protein\_coding | | ENSDARG00000077349 | SORCS3 | 17 | 20301487 | 20374730 | protein\_coding | | ENSDARG00000059773 | SRCIN1 | 3 | 37094066 | 37334617 | protein\_coding | | ENSDARG00000051981 | STX3 (1 of many) | 1 | 44125013 | 44134435 | protein\_coding | | ENSDARG00000037587 | SYNPR (1 of many) | 11 | 19462148 | 19531992 | protein\_coding | | ENSDARG00000011171 | TENM2 (1 of many) | 21 | 31739541 | 31974671 | protein\_coding | | ENSDARG00000078251 | TESK1 (1 of many) | 1 | 39492767 | 39509093 | protein\_coding | | ENSDARG00000077986 | TMEM151A (1 of many) | 7 | 19630098 | 19648034 | protein\_coding | | ENSDARG00000090064 | TMEM179 (1 of many) | 17 | 45255949 | 45264641 | protein\_coding | | ENSDARG00000090876 | TMEM184B (1 of many) | 3 | 3090906 | 3114759 | protein\_coding | | ENSDARG00000104357 | TMEM229A | 4 | 4048430 | 4049281 | protein\_coding | | ENSDARG00000041644 | TMEM27 | 23 | 40979784 | 41002264 | protein\_coding | | ENSDARG00000095002 | TNNC2 (1 of many) | 23 | 20481884 | 20493900 | protein\_coding | | ENSDARG00000062013 | TRHDE (1 of many) | 4 | 23719162 | 23922297 | protein\_coding | | ENSDARG00000076044 | TRHDE (1 of many).1 | 4 | 23502555 | 23691115 | protein\_coding | | ENSDARG00000074363 | TTC9 | 13 | 36020583 | 36027740 | protein\_coding | | ENSDARG00000062902 | TULP2 | 12 | 20496462 | 20503991 | protein\_coding | | ENSDARG00000099821 | TYMP | 4 | 9539964 | 9548780 | protein\_coding | | ENSDARG00000061829 | UNC13A | 22 | 20170302 | 20216254 | protein\_coding | | ENSDARG00000097576 | VAMP1 (1 of many) | 16 | 9888778 | 9895357 | protein\_coding | | ENSDARG00000098810 | VSTM2B (1 of many).1 | 7 | 44698380 | 44821447 | protein\_coding | | ENSDARG00000098113 | WSCD1 (1 of many) | 15 | 29962216 | 29984669 | protein\_coding | | ENSDARG00000036952 | XKR7 (1 of many) | 23 | 30932555 | 30964319 | protein\_coding | | ENSDARG00000100985 | ZBTB7C | 21 | 3030380 | 3047858 | protein\_coding | | ENSDARG00000075823 | ZNF608 | 8 | 32397 | 56196 | protein\_coding | | ENSDARG00000078222 | aatka | 3 | 51644915 | 51657356 | protein\_coding | | ENSDARG00000057169 | abca4a | 24 | 28481879 | 28567829 | protein\_coding | | ENSDARG00000062661 | abca4b | 2 | 15534732 | 15626117 | protein\_coding | | ENSDARG00000056200 | abcb9 | 5 | 27418093 | 27429405 | protein\_coding | | ENSDARG00000051879 | abcc8 | 25 | 22622585 | 22751736 | protein\_coding | | ENSDARG00000077007 | abcc8b | 18 | 50044369 | 50090124 | protein\_coding | | ENSDARG00000045804 | abhd2b | 25 | 19022578 | 19041308 | protein\_coding | | ENSDARG00000018809 | abhd3 | 2 | 12064911 | 12128748 | protein\_coding | | ENSDARG00000060756 | abhd6a | 11 | 41463812 | 41487426 | protein\_coding | | ENSDARG00000103518 | abhd8a | 2 | 54075436 | 54107731 | protein\_coding | | ENSDARG00000060072 | abi3a | 3 | 33264228 | 33286112 | protein\_coding | | ENSDARG00000071095 | abi3bpb | 22 | 28496436 | 28532676 | protein\_coding | | ENSDARG00000035891 | acana | 7 | 48494529 | 48514603 | protein\_coding | | ENSDARG00000045799 | acanb | 25 | 19115921 | 19126347 | protein\_coding | | ENSDARG00000079166 | ace | 12 | 5495290 | 5547103 | protein\_coding | | ENSDARG00000031796 | ache | 7 | 25805960 | 25816614 | protein\_coding | | ENSDARG00000062549 | acmsd | 9 | 23433423 | 23443001 | protein\_coding | | ENSDARG00000018653 | acot7 | 8 | 46767791 | 46903961 | protein\_coding | | ENSDARG00000045180 | acta2 | 12 | 16966955 | 16993856 | protein\_coding | | ENSDARG00000038475 | acy1 | 22 | 36772140 | 36832541 | protein\_coding | | ENSDARG00000079204 | adam11 | 12 | 13773328 | 13848649 | protein\_coding | | ENSDARG00000062323 | adam23a | 6 | 12903067 | 12953661 | protein\_coding | | ENSDARG00000076270 | adamts13 | 8 | 45218927 | 45269491 | protein\_coding | | ENSDARG00000033544 | adamts15b | 15 | 17667466 | 17682778 | protein\_coding | | ENSDARG00000058252 | adamts15b.1 | 15 | 17687093 | 17716785 | protein\_coding | | ENSDARG00000100307 | adamts5 | 1 | 696360 | 719733 | protein\_coding | | ENSDARG00000007709 | adamts8a | 5 | 29555681 | 29576817 | protein\_coding | | ENSDARG00000088634 | adcy1b | 2 | 147297 | 167818 | protein\_coding | | ENSDARG00000058392 | adcy2a | 16 | 9381023 | 9493023 | protein\_coding | | ENSDARG00000014588 | adcy2b | 19 | 28025405 | 28192642 | protein\_coding | | ENSDARG00000004015 | adcyap1a | 7 | 71306606 | 71315593 | protein\_coding | | ENSDARG00000027740 | adcyap1b | 2 | 31313932 | 31319401 | protein\_coding | | ENSDARG00000105201 | adcyap1r1a | 2 | 51093352 | 51165708 | protein\_coding | | ENSDARG00000053724 | adcyap1r1b | 6 | 32982060 | 33039055 | protein\_coding | | ENSDARG00000075133 | adgrb1a | 16 | 37548981 | 37678464 | protein\_coding | | ENSDARG00000078529 | adgrb1b | 19 | 92480 | 107192 | protein\_coding | | ENSDARG00000091757 | adgrf6 | 20 | 35665673 | 35678453 | protein\_coding | | ENSDARG00000054137 | adgrg6 | 20 | 37480155 | 37566761 | protein\_coding | | ENSDARG00000061121 | adgrl3.1 | 1 | 22252812 | 22466844 | protein\_coding | | ENSDARG00000100086 | adipoqb | 2 | 4649979 | 4655733 | protein\_coding | | ENSDARG00000045708 | adm2a | 4 | 9879731 | 9890958 | protein\_coding | | ENSDARG00000102096 | adra2b | 8 | 40605273 | 40606853 | protein\_coding | | ENSDARG00000069669 | adra2c | 1 | 40289895 | 40291472 | protein\_coding | | ENSDARG00000099517 | adssl1 | 17 | 663357 | 695526 | protein\_coding | | ENSDARG00000052242 | aff2 | 14 | 20045308 | 20439680 | protein\_coding | | ENSDARG00000099874 | agap2 | 23 | 36552748 | 36602114 | protein\_coding | | ENSDARG00000100352 | aglb | 24 | 29796382 | 29834762 | protein\_coding | | ENSDARG00000099781 | agrp2 | 2 | 7549927 | 7560187 | protein\_coding | | ENSDARG00000016412 | agt | 13 | 23857992 | 23866391 | protein\_coding | | ENSDARG00000035552 | agtr2 | 5 | 22738570 | 22745694 | protein\_coding | | ENSDARG00000100709 | agxt2 | 21 | 11046288 | 11083035 | protein\_coding | | ENSDARG00000052099 | agxta | 6 | 27122323 | 27132766 | protein\_coding | | ENSDARG00000023537 | ahr1b | 22 | 12954436 | 13018215 | protein\_coding | | ENSDARG00000007171 | aig1 | 20 | 37758350 | 37833886 | protein\_coding | | ENSDARG00000038655 | ajap1 | 8 | 21556226 | 21639728 | protein\_coding | | ENSDARG00000012555 | ak5 | 2 | 9047835 | 9261950 | protein\_coding | | ENSDARG00000091792 | akap12a | 17 | 7606452 | 7626410 | protein\_coding | | ENSDARG00000076544 | aldh5a1 | 16 | 36977651 | 36991883 | protein\_coding | | ENSDARG00000057661 | aldoca | 15 | 16143564 | 16162509 | protein\_coding | | ENSDARG00000019702 | aldocb | 21 | 39585912 | 39616539 | protein\_coding | | ENSDARG00000095833 | alk | 17 | 47102321 | 47219486 | protein\_coding | | ENSDARG00000042562 | allc | 20 | 30452557 | 30468048 | protein\_coding | | ENSDARG00000079620 | amigo1 | 8 | 25097680 | 25101092 | protein\_coding | | ENSDARG00000074469 | amigo3 | 22 | 34454251 | 34488269 | protein\_coding | | ENSDARG00000033832 | ampd1 | 8 | 11042866 | 11074353 | protein\_coding | | ENSDARG00000007663 | amph | 2 | 31907811 | 31953867 | protein\_coding | | ENSDARG00000079302 | and2 | 2 | 31779986 | 31784434 | protein\_coding | | ENSDARG00000056873 | and3 | 10 | 22879104 | 22885306 | protein\_coding | | ENSDARG00000012071 | angptl1a | 20 | 16082899 | 16108053 | protein\_coding | | ENSDARG00000074777 | ank1b | 21 | 20990739 | 21053193 | protein\_coding | | ENSDARG00000076192 | ankrd1b | 12 | 16391898 | 16397563 | protein\_coding | | ENSDARG00000062103 | ankrd24 | 22 | 21334186 | 21368145 | protein\_coding | | ENSDARG00000055638 | ankrd33aa | 23 | 27936266 | 27948599 | protein\_coding | | ENSDARG00000058357 | ankrd33ba | 24 | 21911212 | 21914627 | protein\_coding | | ENSDARG00000028804 | ankrd9 | 17 | 29259581 | 29260768 | protein\_coding | | ENSDARG00000078901 | anks1ab | 6 | 54350421 | 54414557 | protein\_coding | | ENSDARG00000003512 | anks1b | 4 | 17895551 | 18212788 | protein\_coding | | ENSDARG00000077327 | ano11 | 23 | 24243364 | 24269172 | protein\_coding | | ENSDARG00000101363 | ano2 | 4 | 4263709 | 4350027 | protein\_coding | | ENSDARG00000015731 | ano5a | 25 | 34684597 | 34739284 | protein\_coding | | ENSDARG00000076320 | ano9a | 25 | 34650660 | 34677389 | protein\_coding | | ENSDARG00000012896 | anos1a | 1 | 31210393 | 31246942 | protein\_coding | | ENSDARG00000104118 | antxr2a | 5 | 38786315 | 38873831 | protein\_coding | | ENSDARG00000063011 | antxr2b | 10 | 9361631 | 9386104 | protein\_coding | | ENSDARG00000036456 | anxa4 | 10 | 41237313 | 41270953 | protein\_coding | | ENSDARG00000026406 | anxa5a | 14 | 47423685 | 47447115 | protein\_coding | | ENSDARG00000063007 | apc2 | 11 | 7271091 | 7310372 | protein\_coding | | ENSDARG00000098368 | aplp1 | 15 | 37204506 | 37328812 | protein\_coding | | ENSDARG00000060345 | apoda.1 | 2 | 37225342 | 37227940 | protein\_coding | | ENSDARG00000090980 | apof | 6 | 39164758 | 39170196 | protein\_coding | | ENSDARG00000007425 | apol1 | 2 | 37985657 | 37992397 | protein\_coding | | ENSDARG00000055543 | appb | 9 | 35217230 | 35260592 | protein\_coding | | ENSDARG00000045141 | aqp8a.1 | 12 | 20244235 | 20251184 | protein\_coding | | ENSDARG00000071592 | aqp8a.2 | 12 | 20233265 | 20240187 | protein\_coding | | ENSDARG00000053480 | aqp9b | 7 | 30100743 | 30118129 | protein\_coding | | ENSDARG00000070539 | arf3a | 23 | 27695084 | 27707189 | protein\_coding | | ENSDARG00000036998 | arf3b | 6 | 7283623 | 7295534 | protein\_coding | | ENSDARG00000026840 | arhgap23b | 12 | 15434804 | 15519562 | protein\_coding | | ENSDARG00000100293 | arhgef15 | 5 | 53464326 | 53514388 | protein\_coding | | ENSDARG00000061746 | arhgef9a | 5 | 21435933 | 21497253 | protein\_coding | | ENSDARG00000078624 | arhgef9b | 14 | 8836148 | 8893954 | protein\_coding | | ENSDARG00000069988 | arid5b | 21 | 11151365 | 11159187 | protein\_coding | | ENSDARG00000102393 | arl3l1 | 21 | 33138180 | 33152440 | protein\_coding | | ENSDARG00000056511 | arr3a | 10 | 26838500 | 26849684 | protein\_coding | | ENSDARG00000043241 | arrb1 | 15 | 5396814 | 5473899 | protein\_coding | | ENSDARG00000074329 | arvcfa | 10 | 465281 | 503725 | protein\_coding | | ENSDARG00000034817 | asah1b | 1 | 15961317 | 15972107 | protein\_coding | | ENSDARG00000099461 | asb10 | 24 | 34199765 | 34221443 | protein\_coding | | ENSDARG00000012948 | asb18 | 9 | 24075645 | 24083609 | protein\_coding | | ENSDARG00000008329 | asic1a | 8 | 6923560 | 6979648 | protein\_coding | | ENSDARG00000025162 | asic4a | 9 | 11063764 | 11257161 | protein\_coding | | ENSDARG00000077858 | asip1 | 6 | 49977257 | 49984676 | protein\_coding | | ENSDARG00000098249 | asmt | 9 | 56380395 | 56395051 | protein\_coding | | ENSDARG00000038297 | aspdh | 3 | 16597517 | 16611123 | protein\_coding | | ENSDARG00000075813 | asphd1 | 3 | 15122143 | 15134893 | protein\_coding | | ENSDARG00000002192 | aspn | 22 | 10450283 | 10457808 | protein\_coding | | ENSDARG00000059781 | atcayb | 2 | 55790738 | 55847125 | protein\_coding | | ENSDARG00000060481 | atl1 | 13 | 36638817 | 36658568 | protein\_coding | | ENSDARG00000040252 | atp1a1a.5 | 1 | 1690370 | 1702310 | protein\_coding | | ENSDARG00000019856 | atp1a1b | 9 | 36092369 | 36113016 | protein\_coding | | ENSDARG00000018259 | atp1a3a | 19 | 6409203 | 6466563 | protein\_coding | | ENSDARG00000104139 | atp1a3b | 16 | 10885786 | 10930007 | protein\_coding | | ENSDARG00000099203 | atp1b2a | 23 | 45020771 | 45051808 | protein\_coding | | ENSDARG00000034424 | atp1b2b | 5 | 37875378 | 37906828 | protein\_coding | | ENSDARG00000053262 | atp1b4 | 14 | 33117980 | 33129639 | protein\_coding | | ENSDARG00000063433 | atp2b2 | 11 | 1121078 | 1306091 | protein\_coding | | ENSDARG00000043474 | atp2b3a | 8 | 13964585 | 14014644 | protein\_coding | | ENSDARG00000023445 | atp2b3b | 23 | 19907872 | 20026746 | protein\_coding | | ENSDARG00000041417 | atp6ap1a | 23 | 4963653 | 4979533 | protein\_coding | | ENSDARG00000091509 | atp6ap1la | 11 | 42241902 | 42263728 | protein\_coding | | ENSDARG00000090963 | atp6ap1lb | 23 | 19632231 | 19657471 | protein\_coding | | ENSDARG00000015174 | atp6v0a1b | 24 | 7566690 | 7602868 | protein\_coding | | ENSDARG00000036577 | atp6v0cb | 24 | 39266296 | 39283763 | protein\_coding | | ENSDARG00000043465 | atp6v1b2 | 10 | 19597170 | 19609041 | protein\_coding | | ENSDARG00000059057 | atpv0e2 | 22 | 34726941 | 34732553 | protein\_coding | | ENSDARG00000077188 | atrnl1a | 13 | 19753685 | 20217313 | protein\_coding | | ENSDARG00000060862 | atxn1b | 16 | 25640277 | 25653480 | protein\_coding | | ENSDARG00000059342 | avil | 11 | 206957 | 221058 | protein\_coding | | ENSDARG00000077083 | avpr1aa | 25 | 1469193 | 1477753 | protein\_coding | | ENSDARG00000045788 | avpr1ab | 4 | 9206806 | 9213372 | protein\_coding | | ENSDARG00000007436 | avpr2aa | 23 | 25111393 | 25140580 | protein\_coding | | ENSDARG00000040547 | b3gat1a | 18 | 36123460 | 36154875 | protein\_coding | | ENSDARG00000030733 | b3gat2 | 13 | 27821238 | 27879124 | protein\_coding | | ENSDARG00000077352 | b4galnt1b | 6 | 39225062 | 39255315 | protein\_coding | | ENSDARG00000046150 | b4galnt4a | 25 | 24193608 | 24512997 | protein\_coding | | ENSDARG00000079264 | baiap3 | 3 | 47449138 | 47587519 | protein\_coding | | ENSDARG00000053358 | basp1 | 2 | 42874986 | 42899239 | protein\_coding | | ENSDARG00000099412 | bcan | 16 | 29375522 | 29400049 | protein\_coding | | ENSDARG00000045568 | bcat1 | 4 | 17290990 | 17308444 | protein\_coding | | ENSDARG00000062510 | bcl11ba | 17 | 18097522 | 18159364 | protein\_coding | | ENSDARG00000089109 | bcl2b | 2 | 13502516 | 13533483 | protein\_coding | | ENSDARG00000099128 | bcl2l16 | 8 | 52716686 | 52725743 | protein\_coding | | ENSDARG00000018817 | bdnf | 7 | 31653833 | 31670520 | protein\_coding | | ENSDARG00000087413 | bean1 | 7 | 44145220 | 44241668 | protein\_coding | | ENSDARG00000078331 | best1 | 25 | 2941205 | 2953361 | protein\_coding | | ENSDARG00000005616 | bfb | 21 | 27401172 | 27407300 | protein\_coding | | ENSDARG00000011998 | bfsp2 | 2 | 32777912 | 32785733 | protein\_coding | | ENSDARG00000017884 | bgna | 8 | 14066791 | 14088941 | protein\_coding | | ENSDARG00000037588 | bhlhe23 | 23 | 17729447 | 17731005 | protein\_coding | | ENSDARG00000070497 | bicc1b | 12 | 7337744 | 7396518 | protein\_coding | | ENSDARG00000042114 | bin1a | 9 | 7432407 | 7466371 | protein\_coding | | ENSDARG00000058082 | birc7 | 23 | 14461431 | 14474429 | protein\_coding | | ENSDARG00000041414 | bmf2 | 20 | 46447886 | 46458886 | protein\_coding | | ENSDARG00000103679 | bmp16 | 18 | 48798932 | 48814644 | protein\_coding | | ENSDARG00000011941 | bmpr2a | 6 | 12562790 | 12633543 | protein\_coding | | ENSDARG00000020057 | bmpr2b | 9 | 12988557 | 13139960 | protein\_coding | | ENSDARG00000078302 | brinp1 | 5 | 70479734 | 70688067 | protein\_coding | | ENSDARG00000014302 | brinp2 | 2 | 34397565 | 34572688 | protein\_coding | | ENSDARG00000076351 | brinp3a.1 | 6 | 35692491 | 35755453 | protein\_coding | | ENSDARG00000099069 | bsk146 | 12 | 543657 | 552260 | protein\_coding | | ENSDARG00000079161 | bsnb | 11 | 37164488 | 37322088 | protein\_coding | | ENSDARG00000099321 | btbd17a | 12 | 36398527 | 36410081 | protein\_coding | | ENSDARG00000102472 | btbd17b | 3 | 12729768 | 12739422 | protein\_coding | | ENSDARG00000087517 | btbd3b | 13 | 34915886 | 34925891 | protein\_coding | | ENSDARG00000054184 | btr06 | 7 | 15953640 | 15962916 | protein\_coding | | ENSDARG00000005179 | c17h14orf159 | 17 | 38792868 | 38831438 | protein\_coding | | ENSDARG00000028521 | c1ql3b | 3 | 31264435 | 31298775 | protein\_coding | | ENSDARG00000055317 | c1ql4b | 6 | 39814939 | 39833553 | protein\_coding | | ENSDARG00000024299 | c1qtnf4 | 7 | 38328892 | 38340983 | protein\_coding | | ENSDARG00000001818 | c3b.2 | 22 | 26109348 | 26216321 | protein\_coding | | ENSDARG00000061697 | ca14 | 16 | 9649131 | 9667201 | protein\_coding | | ENSDARG00000043589 | ca4a | 5 | 1976890 | 2014011 | protein\_coding | | ENSDARG00000042293 | ca4b | 15 | 15454860 | 15465895 | protein\_coding | | ENSDARG00000033411 | cabp1b | 10 | 41487375 | 41528487 | protein\_coding | | ENSDARG00000052016 | cabp2a | 1 | 43425989 | 43440139 | protein\_coding | | ENSDARG00000002576 | cabp5a | 12 | 3811344 | 3824994 | protein\_coding | | ENSDARG00000028485 | cabp5b | 3 | 15147024 | 15167592 | protein\_coding | | ENSDARG00000060846 | cabp7b | 5 | 31486295 | 31492703 | protein\_coding | | ENSDARG00000037905 | cacna1aa | 3 | 33637649 | 33771975 | protein\_coding | | ENSDARG00000021735 | cacna1ba | 5 | 28694465 | 28888092 | protein\_coding | | ENSDARG00000079295 | cacna1bb | 21 | 14284498 | 14534102 | protein\_coding | | ENSDARG00000008398 | cacna1c | 4 | 20597152 | 20753997 | protein\_coding | | ENSDARG00000062346 | cacna1ea | 8 | 14672837 | 14793934 | protein\_coding | | ENSDARG00000089913 | cacna1g | 12 | 27612655 | 27975985 | protein\_coding | | ENSDARG00000078169 | cacna2d2b | 22 | 9443132 | 9483379 | protein\_coding | | ENSDARG00000023886 | cacna2d4b | 25 | 18925786 | 18992528 | protein\_coding | | ENSDARG00000099045 | cacnb2a | 7 | 71600498 | 71645473 | protein\_coding | | ENSDARG00000032565 | cacng2a | 3 | 29583933 | 29714333 | protein\_coding | | ENSDARG00000102376 | cacng2b | KN149779.1 | 312 | 11581 | protein\_coding | | ENSDARG00000076401 | cacng3b | 3 | 35168570 | 35246470 | protein\_coding | | ENSDARG00000003326 | cacng5a | 6 | 19334829 | 19395601 | protein\_coding | | ENSDARG00000063006 | cacng7a | 19 | 9921846 | 9963896 | protein\_coding | | ENSDARG00000070624 | cacng7b | 16 | 13072900 | 13114046 | protein\_coding | | ENSDARG00000020450 | cacng8a | 19 | 9894362 | 9911040 | protein\_coding | | ENSDARG00000070626 | cacng8b | 16 | 12992261 | 13063619 | protein\_coding | | ENSDARG00000009930 | cadm2a | 10 | 23247027 | 23388938 | protein\_coding | | ENSDARG00000062633 | cadm2b | 15 | 8016835 | 8175414 | protein\_coding | | ENSDARG00000040291 | cadm4 | 16 | 24298046 | 24603008 | protein\_coding | | ENSDARG00000043661 | cadpsa | 11 | 19618375 | 19877812 | protein\_coding | | ENSDARG00000070567 | cadpsb | 6 | 41257949 | 41448029 | protein\_coding | | ENSDARG00000031598 | calb1 | 16 | 36812050 | 36844899 | protein\_coding | | ENSDARG00000041062 | calb2a | 18 | 21209696 | 21229785 | protein\_coding | | ENSDARG00000036344 | calb2b | 7 | 24410121 | 24428828 | protein\_coding | | ENSDARG00000056590 | calca | 7 | 27409363 | 27414828 | protein\_coding | | ENSDARG00000036695 | calcoco1a | 23 | 35882635 | 35904479 | protein\_coding | | ENSDARG00000028845 | calcr | 19 | 41129855 | 41238904 | protein\_coding | | ENSDARG00000086391 | cald1b | 25 | 20043257 | 20114024 | protein\_coding | | ENSDARG00000077021 | calhm1 | 13 | 25282224 | 25285475 | protein\_coding | | ENSDARG00000039482 | calhm2 | 13 | 25291283 | 25293836 | protein\_coding | | ENSDARG00000034187 | calm1b | 20 | 16843473 | 16875675 | protein\_coding | | ENSDARG00000075800 | calml4a | 7 | 34221298 | 34224609 | protein\_coding | | ENSDARG00000088898 | caln1 | 15 | 16628670 | 16768395 | protein\_coding | | ENSDARG00000060116 | camk1a | 6 | 46354566 | 46401593 | protein\_coding | | ENSDARG00000074905 | camk1da | 4 | 7879418 | 7974387 | protein\_coding | | ENSDARG00000044526 | camk1ga | 22 | 1032469 | 1052509 | protein\_coding | | ENSDARG00000053617 | camk2a | 21 | 43901952 | 43957792 | protein\_coding | | ENSDARG00000011065 | camk2b1 | 5 | 20427458 | 20527196 | protein\_coding | | ENSDARG00000014273 | camk2d2 | 1 | 13475960 | 13547582 | protein\_coding | | ENSDARG00000025855 | camk2n1a | 2 | 45090398 | 45093096 | protein\_coding | | ENSDARG00000090424 | camk2n2 | 24 | 25547851 | 25550105 | protein\_coding | | ENSDARG00000027963 | camkva | 6 | 42548261 | 42600475 | protein\_coding | | ENSDARG00000005141 | camkvb | 11 | 35102462 | 35179365 | protein\_coding | | ENSDARG00000007824 | camta1b | 11 | 39314639 | 39662704 | protein\_coding | | ENSDARG00000041864 | capn3a | 17 | 45191718 | 45363385 | protein\_coding | | ENSDARG00000045832 | cart2 | 25 | 19343969 | 19345470 | protein\_coding | | ENSDARG00000091836 | casp7 | 12 | 30454719 | 30468766 | protein\_coding | | ENSDARG00000057296 | cbln1 | 18 | 18115169 | 18120966 | protein\_coding | | ENSDARG00000053845 | cbln14 | 2 | 37957796 | 37978360 | protein\_coding | | ENSDARG00000087476 | cbln20 | 15 | 611392 | 615332 | protein\_coding | | ENSDARG00000077151 | cbln2b | 24 | 15501049 | 15504217 | protein\_coding | | ENSDARG00000075513 | ccdc136a | 25 | 34479375 | 34508023 | protein\_coding | | ENSDARG00000057921 | ccdc136b | 4 | 10016094 | 10048270 | protein\_coding | | ENSDARG00000039497 | ccdc85a | 13 | 26869932 | 26907957 | protein\_coding | | ENSDARG00000005343 | ccdc85al | 6 | 6579756 | 6618652 | protein\_coding | | ENSDARG00000078440 | ccdc88aa | 6 | 6144036 | 6189388 | protein\_coding | | ENSDARG00000070810 | ccka | 16 | 5875351 | 5879575 | protein\_coding | | ENSDARG00000039351 | ccl19b | 10 | 17819547 | 17822668 | protein\_coding | | ENSDARG00000070873 | ccl25b | 11 | 6264427 | 6268468 | protein\_coding | | ENSDARG00000093608 | ccl34b.1 | 24 | 27318196 | 27320879 | protein\_coding | | ENSDARG00000074772 | ccl44 | 11 | 6185425 | 6191158 | protein\_coding | | ENSDARG00000096327 | cd164l2 | 19 | 43967582 | 43976701 | protein\_coding | | ENSDARG00000022437 | cd81b | 18 | 27336211 | 27353779 | protein\_coding | | ENSDARG00000094577 | cdc42se2 | 10 | 5159171 | 5221233 | protein\_coding | | ENSDARG00000055843 | cdh10a | 2 | 28906403 | 28950751 | protein\_coding | | ENSDARG00000014215 | cdh13 | 18 | 13823978 | 14164694 | protein\_coding | | ENSDARG00000061371 | cdh18a | 2 | 29265965 | 29369407 | protein\_coding | | ENSDARG00000077996 | cdh24b | 2 | 38573867 | 38743698 | protein\_coding | | ENSDARG00000015002 | cdh4 | 11 | 20269867 | 20725817 | protein\_coding | | ENSDARG00000004643 | cdhr1a | 13 | 29350206 | 29375154 | protein\_coding | | ENSDARG00000006093 | cdk15 | 6 | 9511569 | 9556465 | protein\_coding | | ENSDARG00000045087 | cdk5r1b | 12 | 28252655 | 28255582 | protein\_coding | | ENSDARG00000071011 | cdk5r2a | 6 | 13613462 | 13615244 | protein\_coding | | ENSDARG00000078671 | cdk5r2b | 9 | 11560822 | 11563419 | protein\_coding | | ENSDARG00000015240 | cdkl5 | 11 | 29913984 | 29984949 | protein\_coding | | ENSDARG00000099719 | cdkn1d | KN149955.1 | 4149 | 15104 | protein\_coding | | ENSDARG00000058999 | cdx1b | 21 | 43984306 | 43996788 | protein\_coding | | ENSDARG00000041119 | ceacam1 | 16 | 11833328 | 11869694 | protein\_coding | | ENSDARG00000087303 | cebpd | 24 | 35676571 | 35677830 | protein\_coding | | ENSDARG00000071375 | celf5a | 22 | 19640639 | 19986626 | protein\_coding | | ENSDARG00000039881 | cemip | 7 | 10956300 | 11100971 | protein\_coding | | ENSDARG00000058892 | cep170ab | 6 | 6287896 | 6330707 | protein\_coding | | ENSDARG00000063412 | cers1 | 22 | 4791321 | 4847724 | protein\_coding | | ENSDARG00000078541 | cers3a | 7 | 9659463 | 9716563 | protein\_coding | | ENSDARG00000018619 | cers4b | 2 | 56232608 | 56265726 | protein\_coding | | ENSDARG00000101510 | cetn2 | 14 | 14356520 | 14360993 | protein\_coding | | ENSDARG00000039579 | cfd | 13 | 13550689 | 13553342 | protein\_coding | | ENSDARG00000075903 | chadlb | 12 | 18867612 | 18885960 | protein\_coding | | ENSDARG00000015854 | chata | 13 | 29278909 | 29290881 | protein\_coding | | ENSDARG00000021405 | chd3 | 7 | 20418917 | 20518928 | protein\_coding | | ENSDARG00000105083 | chd5 | 11 | 29327748 | 29403756 | protein\_coding | | ENSDARG00000008829 | chga | 20 | 27121111 | 27146901 | protein\_coding | | ENSDARG00000076500 | chgb | 20 | 45837846 | 45848296 | protein\_coding | | ENSDARG00000069254 | chrm4a | 7 | 38910410 | 38935033 | protein\_coding | | ENSDARG00000011113 | chrna10a | 15 | 38278319 | 38291798 | protein\_coding | | ENSDARG00000100991 | chrna3 | 18 | 50641553 | 50650139 | protein\_coding | | ENSDARG00000055559 | chrna6 | 1 | 21793826 | 21821324 | protein\_coding | | ENSDARG00000101702 | chrna7 | 7 | 53371605 | 53400001 | protein\_coding | | ENSDARG00000017790 | chrnb2b | 16 | 22703036 | 22760843 | protein\_coding | | ENSDARG00000052764 | chrnb3a | 1 | 21698060 | 21721303 | protein\_coding | | ENSDARG00000098245 | cidea | 24 | 2951354 | 2960385 | protein\_coding | | ENSDARG00000013687 | cilp2 | 22 | 18452334 | 18460608 | protein\_coding | | ENSDARG00000016773 | cishb | 11 | 17824872 | 17829890 | protein\_coding | | ENSDARG00000069615 | ckmt2a | 10 | 3061658 | 3068670 | protein\_coding | | ENSDARG00000039929 | ckmt2b | 5 | 50947616 | 50962166 | protein\_coding | | ENSDARG00000062084 | clcn1a | 19 | 9693238 | 9744033 | protein\_coding | | ENSDARG00000035808 | clcn4 | 11 | 43841953 | 43860928 | protein\_coding | | ENSDARG00000036463 | cldn15a | 7 | 20559126 | 20565196 | protein\_coding | | ENSDARG00000101109 | cldn15b | 5 | 53262016 | 53308625 | protein\_coding | | ENSDARG00000016081 | cldn15la | 2 | 5596660 | 5605556 | protein\_coding | | ENSDARG00000100844 | cldn15lb | 15 | 45547313 | 45574731 | protein\_coding | | ENSDARG00000044569 | cldn19 | 11 | 38737638 | 38782921 | protein\_coding | | ENSDARG00000036376 | cldn7a | 7 | 22345055 | 22355696 | protein\_coding | | ENSDARG00000042357 | cldnk | 3 | 49088239 | 49092400 | protein\_coding | | ENSDARG00000010625 | clic2 | 14 | 33542659 | 33551997 | protein\_coding | | ENSDARG00000070584 | clic5b | 20 | 44028188 | 44071781 | protein\_coding | | ENSDARG00000054456 | clip3 | 15 | 29056697 | 29076093 | protein\_coding | | ENSDARG00000031720 | clstn1 | 23 | 29654020 | 29741189 | protein\_coding | | ENSDARG00000060637 | clstn2 | 2 | 39142700 | 39590028 | protein\_coding | | ENSDARG00000073883 | clstn3 | 16 | 12211428 | 12282652 | protein\_coding | | ENSDARG00000010434 | clu | 20 | 39357596 | 39371108 | protein\_coding | | ENSDARG00000060566 | cluhb | 21 | 39320862 | 39375910 | protein\_coding | | ENSDARG00000041115 | cnfn | 16 | 11899738 | 11908092 | protein\_coding | | ENSDARG00000012125 | cnga1 | 23 | 44047661 | 44064836 | protein\_coding | | ENSDARG00000070726 | cnga3a | 6 | 38797415 | 38821046 | protein\_coding | | ENSDARG00000068242 | cngb1a | 18 | 45507408 | 45520717 | protein\_coding | | ENSDARG00000101225 | cngb3 | 24 | 31469163 | 31488588 | protein\_coding | | ENSDARG00000043662 | cnih2 | 10 | 27065160 | 27087503 | protein\_coding | | ENSDARG00000014953 | cnih3 | 20 | 36330994 | 36396486 | protein\_coding | | ENSDARG00000009020 | cnr1 | 20 | 881586 | 894360 | protein\_coding | | ENSDARG00000037337 | cnrip1b | 13 | 959651 | 966726 | protein\_coding | | ENSDARG00000045685 | cntn1b | 4 | 13734989 | 13757867 | protein\_coding | | ENSDARG00000062880 | cntn3a.1 | 23 | 11734367 | 11926423 | protein\_coding | | ENSDARG00000074524 | cntnap1 | 3 | 36762183 | 36802891 | protein\_coding | | ENSDARG00000058969 | cntnap2a | 24 | 17276384 | 17747906 | protein\_coding | | ENSDARG00000073920 | cntnap5a | 9 | 23675769 | 23817099 | protein\_coding | | ENSDARG00000075189 | cntnap5b | 22 | 13438207 | 13526348 | protein\_coding | | ENSDARG00000024032 | coch | 17 | 28726698 | 28732677 | protein\_coding | | ENSDARG00000054753 | col10a1a | 17 | 15534144 | 15538929 | protein\_coding | | ENSDARG00000012422 | col11a2 | 19 | 7260815 | 7306481 | protein\_coding | | ENSDARG00000078322 | col12a1a | 17 | 49685590 | 49879081 | protein\_coding | | ENSDARG00000069415 | col17a1a | 1 | 48923915 | 48961434 | protein\_coding | | ENSDARG00000079011 | col17a1b | 13 | 24619813 | 24649250 | protein\_coding | | ENSDARG00000076163 | col19a1 | 13 | 38865049 | 38990073 | protein\_coding | | ENSDARG00000024847 | col5a2b | 6 | 11203545 | 11260174 | protein\_coding | | ENSDARG00000074908 | col6a1 | 11 | 33733724 | 33802916 | protein\_coding | | ENSDARG00000061436 | col6a2 | 11 | 33658556 | 33697516 | protein\_coding | | ENSDARG00000077139 | col6a3 | 9 | 24103448 | 24183818 | protein\_coding | | ENSDARG00000079752 | col6a6 | 16 | 36715734 | 36765821 | protein\_coding | | ENSDARG00000003533 | col8a1b | 22 | 28696908 | 28702666 | protein\_coding | | ENSDARG00000060893 | col8a2 | 19 | 35909162 | 36009087 | protein\_coding | | ENSDARG00000073699 | col9a1a | 11 | 11291319 | 11317662 | protein\_coding | | ENSDARG00000019692 | colq | 16 | 20392245 | 20433166 | protein\_coding | | ENSDARG00000015337 | comta | 8 | 8420607 | 8441994 | protein\_coding | | ENSDARG00000023583 | coq9 | 25 | 14285005 | 14301881 | protein\_coding | | ENSDARG00000079440 | coro2ba | 7 | 33043775 | 33070771 | protein\_coding | | ENSDARG00000022509 | cox4i2 | 23 | 19018045 | 19024805 | protein\_coding | | ENSDARG00000068738 | cox5b2 | 13 | 50062547 | 50064795 | protein\_coding | | ENSDARG00000045230 | cox6b1 | 24 | 39718206 | 39722738 | protein\_coding | | ENSDARG00000097209 | cox8b | 25 | 10402836 | 10406237 | protein\_coding | | ENSDARG00000055874 | cpe | 1 | 20146545 | 20218360 | protein\_coding | | ENSDARG00000089486 | cplx3b | 18 | 5807435 | 5822219 | protein\_coding | | ENSDARG00000059978 | cplx4a | 21 | 10647218 | 10656914 | protein\_coding | | ENSDARG00000094889 | cplx4c | 2 | 55766828 | 55879954 | protein\_coding | | ENSDARG00000007753 | cpne2 | 18 | 17671336 | 17714396 | protein\_coding | | ENSDARG00000101095 | cpne4a | 16 | 41395445 | 41515510 | protein\_coding | | ENSDARG00000102584 | cpne7 | 7 | 67263455 | 67371466 | protein\_coding | | ENSDARG00000058285 | cpt1b | 18 | 8388901 | 8409547 | protein\_coding | | ENSDARG00000073716 | cpxm1a | 13 | 9243493 | 9276920 | protein\_coding | | ENSDARG00000045926 | crabp1a | 25 | 6179483 | 6209978 | protein\_coding | | ENSDARG00000060081 | crb2b | 8 | 2767882 | 2848406 | protein\_coding | | ENSDARG00000027657 | crhb | 24 | 24702509 | 24736822 | protein\_coding | | ENSDARG00000024831 | crhbp | 21 | 7217863 | 7227128 | protein\_coding | | ENSDARG00000003989 | crhr1 | 3 | 21538997 | 21759068 | protein\_coding | | ENSDARG00000077275 | crispld1a | 24 | 14709377 | 14741607 | protein\_coding | | ENSDARG00000013293 | crispld1b | 2 | 30396117 | 30412460 | protein\_coding | | ENSDARG00000056742 | crmp1 | 1 | 13661820 | 13680618 | protein\_coding | | ENSDARG00000042613 | crp3 | 24 | 38204713 | 38206185 | protein\_coding | | ENSDARG00000011989 | crx | 5 | 36332565 | 36336290 | protein\_coding | | ENSDARG00000053502 | cryaa | 1 | 28293049 | 28297091 | protein\_coding | | ENSDARG00000041141 | cryba1a | 15 | 28153818 | 28174626 | protein\_coding | | ENSDARG00000053875 | cryba1b | 21 | 39140703 | 39149628 | protein\_coding | | ENSDARG00000032929 | cryba1l1 | 14 | 47319691 | 47333038 | protein\_coding | | ENSDARG00000030349 | cryba2a | 6 | 13650768 | 13654197 | protein\_coding | | ENSDARG00000041925 | cryba2b | 9 | 11583156 | 11589229 | protein\_coding | | ENSDARG00000024548 | cryba4 | 10 | 44195430 | 44204668 | protein\_coding | | ENSDARG00000068507 | crybb1 | 10 | 44174427 | 44180594 | protein\_coding | | ENSDARG00000007576 | crybb1l1 | 14 | 47316814 | 47323257 | protein\_coding | | ENSDARG00000016793 | crybb1l2 | 1 | 43738358 | 43745614 | protein\_coding | | ENSDARG00000036140 | crybgx | 7 | 33951294 | 33953861 | protein\_coding | | ENSDARG00000087324 | crygm2d1 | 9 | 22335673 | 22528713 | protein\_coding | | ENSDARG00000087765 | crygm2d10 | 9 | 22498969 | 22500433 | protein\_coding | | ENSDARG00000069827 | crygm2d11 | 9 | 22287706 | 22288667 | protein\_coding | | ENSDARG00000069801 | crygm2d12 | 9 | 22393808 | 22394813 | protein\_coding | | ENSDARG00000057460 | crygm2d13 | 9 | 22280292 | 22281456 | protein\_coding | | ENSDARG00000087301 | crygm2d14 | 9 | 22335634 | 22336698 | protein\_coding | | ENSDARG00000069826 | crygm2d15 | 9 | 22305731 | 22306561 | protein\_coding | | ENSDARG00000076790 | crygm2d16 | 9 | 22346413 | 22347456 | protein\_coding | | ENSDARG00000088687 | crygm2d17 | 9 | 22317633 | 22318919 | protein\_coding | | ENSDARG00000086912 | crygm2d18 | 9 | 22310884 | 22311721 | protein\_coding | | ENSDARG00000076693 | crygm2d19 | 9 | 22316749 | 22377248 | protein\_coding | | ENSDARG00000086917 | crygm2d2 | 9 | 22506610 | 22507642 | protein\_coding | | ENSDARG00000091148 | crygm2d20 | 9 | 22400974 | 22461185 | protein\_coding | | ENSDARG00000086658 | crygm2d21 | 9 | 22487377 | 22488543 | protein\_coding | | ENSDARG00000088823 | crygm2d3 | 9 | 22469602 | 22470985 | protein\_coding | | ENSDARG00000087164 | crygm2d4 | 9 | 22433668 | 22470616 | protein\_coding | | ENSDARG00000069792 | crygm2d5 | 9 | 22421009 | 22422033 | protein\_coding | | ENSDARG00000073874 | crygm2d6 | 9 | 22370547 | 22371527 | protein\_coding | | ENSDARG00000076572 | crygm2d7 | 9 | 22460187 | 22461312 | protein\_coding | | ENSDARG00000069817 | crygm2d8 | 9 | 22323862 | 22507603 | protein\_coding | | ENSDARG00000073750 | crygm2d9 | 9 | 22428144 | 22429183 | protein\_coding | | ENSDARG00000041179 | crygm5 | 9 | 22534178 | 22541411 | protein\_coding | | ENSDARG00000053862 | crygmx | 12 | 30723265 | 30726777 | protein\_coding | | ENSDARG00000074001 | crygmxl2 | 12 | 30727775 | 30731153 | protein\_coding | | ENSDARG00000030411 | crygn2 | 24 | 41441956 | 41449024 | protein\_coding | | ENSDARG00000041323 | csdc2a | 12 | 18977390 | 18981616 | protein\_coding | | ENSDARG00000053624 | csf1rb | 21 | 43846478 | 43885949 | protein\_coding | | ENSDARG00000001559 | csmd2 | 19 | 36527726 | 37087903 | protein\_coding | | ENSDARG00000045150 | csnk1e | 12 | 19183582 | 19196500 | protein\_coding | | ENSDARG00000069981 | cspg5a | 16 | 42418164 | 42486683 | protein\_coding | | ENSDARG00000099793 | cspg5b | 19 | 19705129 | 19766384 | protein\_coding | | ENSDARG00000030980 | csrp1b | 6 | 54828452 | 54835861 | protein\_coding | | ENSDARG00000104292 | ctgfb | 19 | 13939305 | 13946435 | protein\_coding | | ENSDARG00000001971 | cthrc1b | 16 | 47519500 | 47548056 | protein\_coding | | ENSDARG00000062415 | ctnnd2a | 24 | 21977063 | 22395695 | protein\_coding | | ENSDARG00000068716 | cuedc1a | 10 | 37193706 | 37223693 | protein\_coding | | ENSDARG00000035553 | cx27.5 | 5 | 23118377 | 23245326 | protein\_coding | | ENSDARG00000041797 | cx28.9 | 20 | 40847644 | 40853843 | protein\_coding | | ENSDARG00000041787 | cx32.3 | 20 | 40856408 | 40858504 | protein\_coding | | ENSDARG00000070781 | cx35b | 20 | 9971266 | 9976473 | protein\_coding | | ENSDARG00000073896 | cx47.1 | 2 | 3641653 | 3667272 | protein\_coding | | ENSDARG00000075163 | cxcl20 | 5 | 42296537 | 42304152 | protein\_coding | | ENSDARG00000092283 | cxl34b.11 | 24 | 27390938 | 27394612 | protein\_coding | | ENSDARG00000051925 | cyb5r2 | 25 | 15950218 | 15961139 | protein\_coding | | ENSDARG00000070148 | cygb2 | 6 | 20537206 | 20569167 | protein\_coding | | ENSDARG00000089724 | cyldb | 11 | 25039132 | 25054093 | protein\_coding | | ENSDARG00000101195 | cyp1c1 | KN150425.1 | 103570 | 106640 | protein\_coding | | ENSDARG00000101789 | cyp1c2 | 17 | 794442 | 796628 | protein\_coding | | ENSDARG00000033802 | cyp27a7 | 9 | 38599196 | 38609345 | protein\_coding | | ENSDARG00000022631 | cyp2p9 | 20 | 25682537 | 25685779 | protein\_coding | | ENSDARG00000004262 | cyp46a1.2 | 20 | 5192739 | 5223338 | protein\_coding | | ENSDARG00000089177 | cyp46a1.3 | 20 | 5227893 | 5246623 | protein\_coding | | ENSDARG00000053068 | cyp8b1 | 2 | 3604709 | 3606683 | protein\_coding | | ENSDARG00000007807 | cyth3a | 12 | 17382685 | 17440829 | protein\_coding | | ENSDARG00000005159 | cyth3b | 3 | 56339738 | 56474271 | protein\_coding | | ENSDARG00000059939 | dab1a | 20 | 8134954 | 8431318 | protein\_coding | | ENSDARG00000003290 | dab1b | 2 | 2409917 | 2456857 | protein\_coding | | ENSDARG00000063014 | dbpa | 19 | 10258190 | 10277445 | protein\_coding | | ENSDARG00000018856 | dclk1a | 10 | 34793459 | 34927877 | protein\_coding | | ENSDARG00000056753 | dctn1b | 13 | 11449466 | 11535186 | protein\_coding | | ENSDARG00000037042 | ddhd1b | 20 | 9794692 | 9836570 | protein\_coding | | ENSDARG00000059836 | ddit3 | 6 | 58921658 | 58924855 | protein\_coding | | ENSDARG00000058695 | ddr2b | 2 | 7091690 | 7124880 | protein\_coding | | ENSDARG00000040930 | deptor | 16 | 14697920 | 14785787 | protein\_coding | | ENSDARG00000033140 | desi1a | 3 | 24842477 | 24849519 | protein\_coding | | ENSDARG00000005221 | desmb | 6 | 13742204 | 13762290 | protein\_coding | | ENSDARG00000103503 | dgat1a | 19 | 1118390 | 1141655 | protein\_coding | | ENSDARG00000054300 | dhrs1 | 2 | 38279420 | 38287347 | protein\_coding | | ENSDARG00000016348 | dip2bb | 6 | 39533599 | 39608198 | protein\_coding | | ENSDARG00000028066 | diras1a | 11 | 7314865 | 7331968 | protein\_coding | | ENSDARG00000042846 | disp2 | 17 | 31168313 | 31185317 | protein\_coding | | ENSDARG00000103591 | dkk3a | 7 | 65249346 | 65268246 | protein\_coding | | ENSDARG00000099323 | dlg2 | 10 | 29387316 | 29819169 | protein\_coding | | ENSDARG00000055459 | dlgap3 | 19 | 37871490 | 37905397 | protein\_coding | | ENSDARG00000038978 | dnajb4 | 2 | 9754451 | 9762302 | protein\_coding | | ENSDARG00000037067 | dnajc22 | 23 | 27770153 | 27776020 | protein\_coding | | ENSDARG00000023861 | dnase1l3l | 8 | 25332724 | 25338120 | protein\_coding | | ENSDARG00000061031 | dner | 18 | 46701713 | 46765152 | protein\_coding | | ENSDARG00000010042 | dnm1a | 5 | 1223652 | 1367487 | protein\_coding | | ENSDARG00000009281 | dnm1b | 5 | 62987536 | 63056535 | protein\_coding | | ENSDARG00000032238 | dnm3a | 20 | 14922901 | 15029301 | protein\_coding | | ENSDARG00000088293 | doc2b | 5 | 60249798 | 60647128 | protein\_coding | | ENSDARG00000063180 | dock3 | 22 | 31866811 | 32265768 | protein\_coding | | ENSDARG00000076826 | dpp6a | 24 | 6658375 | 6868565 | protein\_coding | | ENSDARG00000024744 | dpp6b | 2 | 12723927 | 12949975 | protein\_coding | | ENSDARG00000010267 | dpydb | 2 | 20167150 | 20462435 | protein\_coding | | ENSDARG00000079543 | dpys | 16 | 44333824 | 44360764 | protein\_coding | | ENSDARG00000059311 | dpysl5b | 4 | 785248 | 802423 | protein\_coding | | ENSDARG00000038918 | drd1b | 9 | 54917017 | 54919185 | protein\_coding | | ENSDARG00000056926 | drd2a | 15 | 22110825 | 22138583 | protein\_coding | | ENSDARG00000011091 | drd2b | 5 | 57404889 | 57503401 | protein\_coding | | ENSDARG00000038363 | drd4a | 25 | 4580646 | 4606694 | protein\_coding | | ENSDARG00000024865 | dscama | 10 | 38831870 | 39014528 | protein\_coding | | ENSDARG00000028118 | dscamb | 15 | 6150470 | 6416801 | protein\_coding | | ENSDARG00000098057 | dscaml1 | 15 | 12139421 | 12298457 | protein\_coding | | ENSDARG00000013020 | dtnbb | 17 | 37122511 | 37209081 | protein\_coding | | ENSDARG00000055206 | dtnbp1b | 19 | 26577486 | 26650996 | protein\_coding | | ENSDARG00000098108 | dusp2 | 8 | 40589566 | 40593719 | protein\_coding | | ENSDARG00000055552 | dvl1b | 11 | 24123128 | 24190703 | protein\_coding | | ENSDARG00000070734 | dyrk4 | 25 | 16689530 | 16736502 | protein\_coding | | ENSDARG00000069196 | ebf1b | 21 | 33690297 | 33961174 | protein\_coding | | ENSDARG00000056087 | ecrg4a | 9 | 6393934 | 6402668 | protein\_coding | | ENSDARG00000093413 | edil3 | 5 | 46058918 | 46382767 | protein\_coding | | ENSDARG00000060877 | edil3b | 10 | 43523820 | 43575585 | protein\_coding | | ENSDARG00000039502 | eef1a1a | 13 | 27184333 | 27194327 | protein\_coding | | ENSDARG00000069951 | eef1a1b | 1 | 30306886 | 30326291 | protein\_coding | | ENSDARG00000102291 | eef1da | 2 | 51265447 | 51297045 | protein\_coding | | ENSDARG00000035256 | eef2l2 | 5 | 40885124 | 40894678 | protein\_coding | | ENSDARG00000014675 | efna3a | 19 | 8225648 | 8420619 | protein\_coding | | ENSDARG00000033516 | efr3ba | 17 | 33277584 | 33336793 | protein\_coding | | ENSDARG00000037421 | egr1 | 14 | 21035277 | 21039140 | protein\_coding | | ENSDARG00000089156 | egr3 | 8 | 50738574 | 50744862 | protein\_coding | | ENSDARG00000045639 | elavl4 | 8 | 15968442 | 16080266 | protein\_coding | | ENSDARG00000074372 | elfn1b | 1 | 8873101 | 9028191 | protein\_coding | | ENSDARG00000063527 | elmo2 | 11 | 82235 | 99144 | protein\_coding | | ENSDARG00000062056 | elmod1 | 21 | 22062949 | 22078556 | protein\_coding | | ENSDARG00000099634 | elna | 15 | 357448 | 413825 | protein\_coding | | ENSDARG00000069017 | elnb | 21 | 40816810 | 40857306 | protein\_coding | | ENSDARG00000069632 | emp1 | 1 | 44631156 | 44641432 | protein\_coding | | ENSDARG00000022456 | eno1a | 23 | 22729926 | 22751447 | protein\_coding | | ENSDARG00000013750 | eno1b | 6 | 40664167 | 40671870 | protein\_coding | | ENSDARG00000014287 | eno2 | 19 | 5156432 | 5174246 | protein\_coding | | ENSDARG00000011257 | enpp2 | 16 | 9718819 | 9771303 | protein\_coding | | ENSDARG00000040469 | enpp6 | 14 | 3940236 | 3969501 | protein\_coding | | ENSDARG00000036944 | ensaa | 19 | 341887 | 343981 | protein\_coding | | ENSDARG00000053481 | entpd5a | 17 | 44916642 | 44927048 | protein\_coding | | ENSDARG00000076386 | epdl1 | 7 | 890713 | 902359 | protein\_coding | | ENSDARG00000104992 | epgn | 8 | 32397653 | 32403691 | protein\_coding | | ENSDARG00000022971 | epha6 | 1 | 33236468 | 33468627 | protein\_coding | | ENSDARG00000023609 | epha8 | 11 | 38449171 | 38609446 | protein\_coding | | ENSDARG00000099515 | ephx4 | 6 | 24229466 | 24258923 | protein\_coding | | ENSDARG00000005704 | eps8l3b | 8 | 25288864 | 25310828 | protein\_coding | | ENSDARG00000098618 | erap2 | 5 | 51580236 | 51598181 | protein\_coding | | ENSDARG00000063207 | erbb4a | 1 | 6133580 | 6241695 | protein\_coding | | ENSDARG00000009941 | erc1a | 25 | 21079672 | 21149032 | protein\_coding | | ENSDARG00000105178 | erc2 | 11 | 37043333 | 37158202 | protein\_coding | | ENSDARG00000004861 | esrrga | 17 | 29777835 | 29885224 | protein\_coding | | ENSDARG00000035544 | etnppl | 7 | 59773896 | 59785278 | protein\_coding | | ENSDARG00000101959 | etv1 | 15 | 34315530 | 34356315 | protein\_coding | | ENSDARG00000030782 | exoc3l2b | 15 | 20200716 | 20264320 | protein\_coding | | ENSDARG00000035649 | ext1c | 19 | 47719424 | 47744812 | protein\_coding | | ENSDARG00000020944 | ezra | 17 | 45430072 | 45659457 | protein\_coding | | ENSDARG00000008457 | faah2a | 1 | 39519203 | 39540357 | protein\_coding | | ENSDARG00000101762 | fah | 7 | 10424673 | 10447781 | protein\_coding | | ENSDARG00000020814 | faimb | 6 | 16273109 | 16279270 | protein\_coding | | ENSDARG00000070575 | fam131bb | 16 | 17481139 | 17559485 | protein\_coding | | ENSDARG00000076779 | fam13b | 14 | 24019480 | 24094236 | protein\_coding | | ENSDARG00000075858 | fam155a | 1 | 28477833 | 28587103 | protein\_coding | | ENSDARG00000102585 | fam160a1b | 23 | 45928379 | 45946941 | protein\_coding | | ENSDARG00000089742 | fam161a | 1 | 53054153 | 53067631 | protein\_coding | | ENSDARG00000014081 | fam184a | 20 | 41652090 | 41902718 | protein\_coding | | ENSDARG00000062357 | fam19a1a | 23 | 18250938 | 18360629 | protein\_coding | | ENSDARG00000088837 | fam19a1b | 11 | 17307317 | 17378997 | processed\_transcript | | ENSDARG00000062471 | fam19a4b | 11 | 17462344 | 17579352 | protein\_coding | | ENSDARG00000069160 | fam19a5a | 4 | 27262566 | 27409126 | protein\_coding | | ENSDARG00000013813 | fam219ab | 5 | 25482281 | 25493823 | protein\_coding | | ENSDARG00000043009 | fam43a | 11 | 33972924 | 33975370 | protein\_coding | | ENSDARG00000070222 | fam43b | 23 | 37221300 | 37223299 | protein\_coding | | ENSDARG00000011797 | fam46bb | 19 | 14669636 | 14684052 | protein\_coding | | ENSDARG00000062830 | fam46d | 14 | 9037846 | 9049163 | protein\_coding | | ENSDARG00000035907 | fam49a | 19 | 31046140 | 31096365 | protein\_coding | | ENSDARG00000074564 | fam57bb | 12 | 3703024 | 3719640 | protein\_coding | | ENSDARG00000101394 | fam69b | 21 | 7591526 | 7617213 | protein\_coding | | ENSDARG00000105040 | fam69c | 24 | 2893712 | 2918796 | protein\_coding | | ENSDARG00000062889 | fam78bb | 8 | 19605678 | 19617698 | protein\_coding | | ENSDARG00000019063 | fat1b | 14 | 29816215 | 29886564 | protein\_coding | | ENSDARG00000075956 | faxca | 4 | 5742284 | 5755685 | protein\_coding | | ENSDARG00000078981 | faxcb | 16 | 32676044 | 32695899 | protein\_coding | | ENSDARG00000105333 | fbn1 | 18 | 5503737 | 5528053 | protein\_coding | | ENSDARG00000060915 | fbxl16 | 3 | 28503625 | 28534449 | protein\_coding | | ENSDARG00000076952 | fer1l4 | 11 | 26165718 | 26229438 | protein\_coding | | ENSDARG00000043907 | fgf11b | 10 | 22192133 | 22279992 | protein\_coding | | ENSDARG00000027957 | fgf12a | 2 | 5675613 | 5769776 | protein\_coding | | ENSDARG00000009351 | fgf6a | 25 | 16848948 | 16855490 | protein\_coding | | ENSDARG00000059387 | fgf7 | 18 | 5405157 | 5424073 | protein\_coding | | ENSDARG00000039964 | fgfbp2a | 14 | 47461989 | 47464168 | protein\_coding | | ENSDARG00000019861 | fgl2a | 4 | 20457719 | 20460989 | protein\_coding | | ENSDARG00000042018 | fhl2a | 9 | 6600595 | 6624003 | protein\_coding | | ENSDARG00000003991 | fhl2b | 6 | 14948853 | 14974537 | protein\_coding | | ENSDARG00000014805 | fhl5 | 17 | 15597639 | 15603886 | protein\_coding | | ENSDARG00000028396 | fkbp5 | 6 | 41098522 | 41118517 | protein\_coding | | ENSDARG00000069774 | flot2b | 15 | 16451066 | 16471961 | protein\_coding | | ENSDARG00000023318 | fmn2a | 17 | 19737613 | 19814573 | protein\_coding | | ENSDARG00000010294 | fmodb | 8 | 37457061 | 37463352 | protein\_coding | | ENSDARG00000069739 | fndc5a | 13 | 45649428 | 45658998 | protein\_coding | | ENSDARG00000077096 | fndc7a | 2 | 45695892 | 45714704 | protein\_coding | | ENSDARG00000092813 | fndc7rs4 | 2 | 45765522 | 45799618 | protein\_coding | | ENSDARG00000040135 | fosaa | 17 | 50149929 | 50154935 | protein\_coding | | ENSDARG00000031683 | fosab | 20 | 46648591 | 46650720 | protein\_coding | | ENSDARG00000040623 | fosl2 | 17 | 41313752 | 41329686 | protein\_coding | | ENSDARG00000079266 | foxe1 | 1 | 26007896 | 26009278 | protein\_coding | | ENSDARG00000032705 | foxg1b | 11 | 42822027 | 42824168 | protein\_coding | | ENSDARG00000008133 | foxl1 | 18 | 17494491 | 17496353 | protein\_coding | | ENSDARG00000012833 | foxn3 | 17 | 16556988 | 16726396 | protein\_coding | | ENSDARG00000099555 | foxo1a | 15 | 3296910 | 3391535 | protein\_coding | | ENSDARG00000042904 | foxo3b | 20 | 32383886 | 32429857 | protein\_coding | | ENSDARG00000030896 | foxq1a | 2 | 848413 | 849804 | protein\_coding | | ENSDARG00000071394 | foxq2 | 22 | 20402192 | 20404396 | protein\_coding | | ENSDARG00000042984 | fpr1 | 16 | 25056685 | 25059668 | protein\_coding | | ENSDARG00000079074 | frmpd1a | 1 | 21189355 | 21216671 | protein\_coding | | ENSDARG00000074119 | frmpd2 | 12 | 2487909 | 2533727 | protein\_coding | | ENSDARG00000074865 | frmpd3 | 14 | 11456977 | 11661818 | protein\_coding | | ENSDARG00000078688 | frs3 | 6 | 40590015 | 40608737 | protein\_coding | | ENSDARG00000013921 | frya | 15 | 31948324 | 32052256 | protein\_coding | | ENSDARG00000037747 | fscn1b | 1 | 7978419 | 7986375 | protein\_coding | | ENSDARG00000025670 | fstl3 | 22 | 18859894 | 18868411 | protein\_coding | | ENSDARG00000040198 | fstl5 | 14 | 48015767 | 48327544 | protein\_coding | | ENSDARG00000007421 | ftcd | 22 | 12746100 | 12761755 | protein\_coding | | ENSDARG00000007975 | fth1b | 25 | 2933071 | 2940214 | protein\_coding | | ENSDARG00000053366 | ftr12 | 2 | 42866577 | 42869586 | protein\_coding | | ENSDARG00000016460 | fut9a | 17 | 15623686 | 15649096 | protein\_coding | | ENSDARG00000099014 | fxyd1 | 16 | 45235886 | 45258804 | protein\_coding | | ENSDARG00000100971 | fxyd6 | 15 | 12342530 | 12387717 | protein\_coding | | ENSDARG00000022810 | fzr1b | 2 | 6214756 | 6237411 | protein\_coding | | ENSDARG00000013721 | g6pca.2 | 12 | 6007978 | 6014702 | protein\_coding | | ENSDARG00000016667 | gabbr1b | 19 | 27456113 | 27676879 | protein\_coding | | ENSDARG00000068989 | gabra1 | 21 | 42037141 | 42087261 | protein\_coding | | ENSDARG00000070730 | gabra5 | 6 | 38368110 | 38429406 | protein\_coding | | ENSDARG00000079586 | gabrb2 | 14 | 21874802 | 21976602 | protein\_coding | | ENSDARG00000023771 | gabrb3 | 6 | 38384421 | 38515989 | protein\_coding | | ENSDARG00000099096 | gabrb4 | 21 | 36393096 | 36494535 | protein\_coding | | ENSDARG00000059763 | gabrd | 8 | 53303839 | 53319672 | protein\_coding | | ENSDARG00000053665 | gabrg2 | 21 | 41890252 | 41994272 | protein\_coding | | ENSDARG00000043902 | gabrr1 | 20 | 1111266 | 1130439 | protein\_coding | | ENSDARG00000052982 | gabrr2a | 17 | 15837832 | 15884690 | protein\_coding | | ENSDARG00000027153 | gabrr3a | 10 | 35423862 | 35466658 | protein\_coding | | ENSDARG00000030750 | gabrr3b | 24 | 25113730 | 25141152 | protein\_coding | | ENSDARG00000094512 | gabrz | 12 | 28940005 | 29105361 | protein\_coding | | ENSDARG00000027419 | gad1b | 6 | 3666931 | 3701293 | protein\_coding | | ENSDARG00000015537 | gad2 | 24 | 6103818 | 6129604 | protein\_coding | | ENSDARG00000104571 | gadd45ab | 6 | 23831427 | 23839345 | protein\_coding | | ENSDARG00000023448 | galnt14 | 20 | 38128560 | 38217011 | protein\_coding | | ENSDARG00000006832 | galnt9 | 5 | 16296117 | 16492486 | protein\_coding | | ENSDARG00000039914 | gapdhs | 16 | 45077926 | 45103166 | protein\_coding | | ENSDARG00000074443 | gas7a | 3 | 49275416 | 49391986 | protein\_coding | | ENSDARG00000035555 | gbgt1l3 | 5 | 23345847 | 23351647 | protein\_coding | | ENSDARG00000026820 | gc3 | 5 | 37523480 | 37560880 | protein\_coding | | ENSDARG00000079296 | gcga | 22 | 11587838 | 11596336 | protein\_coding | | ENSDARG00000070453 | gch1 | 17 | 15114044 | 15141342 | protein\_coding | | ENSDARG00000058601 | gdap1 | 24 | 14569357 | 14581344 | protein\_coding | | ENSDARG00000028628 | gdap1l1 | 23 | 35660841 | 35690970 | protein\_coding | | ENSDARG00000073891 | gdf10b | 12 | 2596978 | 2610907 | protein\_coding | | ENSDARG00000042723 | gfra2a | 8 | 42052708 | 42231756 | protein\_coding | | ENSDARG00000044015 | gfra2b | 10 | 19753385 | 19845180 | protein\_coding | | ENSDARG00000056651 | gfra4a | 13 | 14060301 | 14356532 | protein\_coding | | ENSDARG00000038185 | gh1 | 3 | 22197190 | 22202365 | protein\_coding | | ENSDARG00000025478 | gipr | 15 | 29007476 | 29041244 | protein\_coding | | ENSDARG00000021889 | gja3 | 9 | 21597118 | 21598597 | protein\_coding | | ENSDARG00000061576 | gldn | 18 | 39668649 | 39681633 | protein\_coding | | ENSDARG00000030078 | glipr1b | 18 | 7386482 | 7396290 | protein\_coding | | ENSDARG00000010958 | glmnb | 6 | 28979952 | 28990268 | protein\_coding | | ENSDARG00000012019 | glra1 | 14 | 25520001 | 25614322 | protein\_coding | | ENSDARG00000075012 | glra2 | 9 | 55334456 | 55361111 | protein\_coding | | ENSDARG00000011066 | glra3 | 1 | 37698138 | 37818921 | protein\_coding | | ENSDARG00000058103 | glra4b | 5 | 22011547 | 22040351 | protein\_coding | | ENSDARG00000052782 | glrba | 1 | 20730073 | 20790895 | protein\_coding | | ENSDARG00000052769 | glrbb | 14 | 35351575 | 35391907 | protein\_coding | | ENSDARG00000036058 | gnao1b | 7 | 28862171 | 28873396 | protein\_coding | | ENSDARG00000011487 | gnaq | 5 | 54918172 | 54930472 | protein\_coding | | ENSDARG00000044199 | gnat1 | 6 | 42921397 | 42932766 | protein\_coding | | ENSDARG00000042529 | gnat2 | 8 | 25206451 | 25228145 | protein\_coding | | ENSDARG00000035357 | gnb2 | 5 | 37663087 | 37697395 | protein\_coding | | ENSDARG00000002696 | gnb3b | 19 | 5189740 | 5218859 | protein\_coding | | ENSDARG00000099685 | gnb5a | 25 | 4711487 | 4724753 | protein\_coding | | ENSDARG00000055377 | gnb5b | 18 | 39086567 | 39124511 | protein\_coding | | ENSDARG00000024740 | gng13a | 1 | 8542970 | 8543896 | protein\_coding | | ENSDARG00000037921 | gng13b | 3 | 35459704 | 35472725 | protein\_coding | | ENSDARG00000009553 | gng3 | 21 | 26660841 | 26670357 | protein\_coding | | ENSDARG00000042970 | gng8 | 21 | 25194555 | 25199321 | protein\_coding | | ENSDARG00000035798 | gngt1 | 19 | 41269414 | 41272348 | protein\_coding | | ENSDARG00000103543 | gngt2b | 12 | 3420170 | 3423318 | protein\_coding | | ENSDARG00000105099 | golga7bb | 13 | 39893111 | 39935940 | protein\_coding | | ENSDARG00000040898 | gpa33 | 9 | 34493513 | 34500702 | protein\_coding | | ENSDARG00000019341 | gpc1a | 22 | 15480835 | 15521818 | protein\_coding | | ENSDARG00000024588 | gpc5b | 22 | 15412483 | 15475034 | protein\_coding | | ENSDARG00000100851 | gphnb | 20 | 51113428 | 51186184 | protein\_coding | | ENSDARG00000055455 | gpm6aa | 14 | 36008235 | 36038180 | protein\_coding | | ENSDARG00000004621 | gpm6ab | 1 | 37930837 | 38038600 | protein\_coding | | ENSDARG00000005739 | gpm6ba | 1 | 28157821 | 28241594 | protein\_coding | | ENSDARG00000062688 | gpnmb | 19 | 20664347 | 20685855 | protein\_coding | | ENSDARG00000039218 | gpr101 | 14 | 31549687 | 31553682 | protein\_coding | | ENSDARG00000062934 | gpr12 | 24 | 21438900 | 21443740 | protein\_coding | | ENSDARG00000077608 | gpr137bb | 11 | 44264950 | 44281050 | protein\_coding | | ENSDARG00000059610 | gpr146 | 3 | 47734932 | 47765241 | protein\_coding | | ENSDARG00000087601 | gpr153 | 8 | 46660889 | 46755417 | protein\_coding | | ENSDARG00000071086 | gpr155b | 6 | 10522754 | 10545271 | protein\_coding | | ENSDARG00000077134 | gpr158a | 24 | 6365857 | 6472080 | protein\_coding | | ENSDARG00000079665 | gpr158b | 2 | 12320790 | 12459614 | protein\_coding | | ENSDARG00000042922 | gpr173 | 8 | 23393784 | 23395123 | protein\_coding | | ENSDARG00000094860 | gpr186 | 19 | 43951868 | 43956655 | protein\_coding | | ENSDARG00000004592 | gpr22a | 4 | 2689021 | 2693376 | protein\_coding | | ENSDARG00000045383 | gpr22b | 25 | 3252391 | 3267961 | protein\_coding | | ENSDARG00000006607 | gpr27 | 6 | 43905673 | 43909785 | protein\_coding | | ENSDARG00000033296 | gpr37b | 4 | 3971334 | 3989577 | protein\_coding | | ENSDARG00000056774 | gpr37l1b | 8 | 21233178 | 21236218 | protein\_coding | | ENSDARG00000093460 | gpr52 | 22 | 17334368 | 17335480 | protein\_coding | | ENSDARG00000098526 | gpr75 | 13 | 35619740 | 35622844 | protein\_coding | | ENSDARG00000068701 | gpr85 | 4 | 6634851 | 6639035 | protein\_coding | | ENSDARG00000077080 | gprc5ba | 3 | 62130673 | 62143516 | protein\_coding | | ENSDARG00000075141 | gprc5bb | 1 | 53996195 | 54005928 | protein\_coding | | ENSDARG00000043342 | gpx3 | 14 | 50452130 | 50472652 | protein\_coding | | ENSDARG00000075383 | gramd1ba | 10 | 40283263 | 40354128 | protein\_coding | | ENSDARG00000069171 | gramd4a | 4 | 28072421 | 28169047 | protein\_coding | | ENSDARG00000022668 | grapb | 1 | 7481229 | 7508307 | protein\_coding | | ENSDARG00000019986 | grhprb | 1 | 19142866 | 19152602 | protein\_coding | | ENSDARG00000021352 | gria1a | 14 | 33785541 | 33936260 | protein\_coding | | ENSDARG00000032714 | gria1b | 21 | 26115637 | 26214049 | protein\_coding | | ENSDARG00000070173 | gria2a | 1 | 20640131 | 20716961 | protein\_coding | | ENSDARG00000052765 | gria2b | 14 | 35408071 | 35473299 | protein\_coding | | ENSDARG00000032737 | gria3a | 5 | 22848561 | 22925441 | protein\_coding | | ENSDARG00000037498 | gria3b | 14 | 12228453 | 12531038 | protein\_coding | | ENSDARG00000037496 | gria4a | 15 | 44475744 | 44790596 | protein\_coding | | ENSDARG00000074583 | grid1a | 17 | 25936612 | 26345011 | protein\_coding | | ENSDARG00000076103 | grid2ipa | 3 | 18525991 | 18560269 | protein\_coding | | ENSDARG00000033382 | grifin | 3 | 41644003 | 41649320 | protein\_coding | | ENSDARG00000040627 | grik1b | 15 | 42670892 | 42723773 | protein\_coding | | ENSDARG00000101449 | grik5 | 16 | 11592909 | 11665032 | protein\_coding | | ENSDARG00000027828 | grin1a | 21 | 11375973 | 11410343 | protein\_coding | | ENSDARG00000025728 | grin1b | 5 | 28997788 | 29043468 | protein\_coding | | ENSDARG00000034493 | grin2aa | 3 | 26940907 | 27146853 | protein\_coding | | ENSDARG00000030376 | grin2bb | 1 | 44680767 | 44861807 | protein\_coding | | ENSDARG00000078149 | grin2ca | 3 | 18854849 | 18983263 | protein\_coding | | ENSDARG00000077560 | grin2cb | 12 | 31794444 | 31898223 | protein\_coding | | ENSDARG00000058803 | grk1a | 1 | 249897 | 262959 | protein\_coding | | ENSDARG00000104685 | grk1b | 5 | 53514769 | 53550491 | protein\_coding | | ENSDARG00000020602 | grk7a | 2 | 16867863 | 16875753 | protein\_coding | | ENSDARG00000026796 | grm1a | 20 | 32144506 | 32209939 | protein\_coding | | ENSDARG00000004150 | grm2a | 6 | 42053979 | 42143600 | protein\_coding | | ENSDARG00000031712 | grm3 | 18 | 9014208 | 9088786 | protein\_coding | | ENSDARG00000017742 | grm6a | 8 | 8493822 | 8585423 | protein\_coding | | ENSDARG00000025671 | grm6b | 11 | 25396425 | 25442093 | protein\_coding | | ENSDARG00000077654 | grm8a | 4 | 3464722 | 3727719 | protein\_coding | | ENSDARG00000076508 | grm8b | 25 | 26478278 | 26794121 | protein\_coding | | ENSDARG00000037390 | gsg1l | 3 | 28587975 | 28619653 | protein\_coding | | ENSDARG00000056510 | gstk1 | 16 | 32055173 | 32059899 | protein\_coding | | ENSDARG00000042620 | gstr | 19 | 770491 | 776267 | protein\_coding | | ENSDARG00000042428 | gstt1a | 8 | 30777879 | 30782034 | protein\_coding | | ENSDARG00000027984 | gstz1 | 17 | 44992659 | 45000078 | protein\_coding | | ENSDARG00000013393 | guca1b | 23 | 19300953 | 19304284 | protein\_coding | | ENSDARG00000030758 | guca1c | 15 | 2925166 | 2940389 | protein\_coding | | ENSDARG00000101567 | guca1e | 22 | 1075740 | 1092619 | protein\_coding | | ENSDARG00000045737 | guca1g | 25 | 13996471 | 13999911 | protein\_coding | | ENSDARG00000013787 | gucy1a3 | 1 | 25092972 | 25108222 | protein\_coding | | ENSDARG00000086790 | gucy1b3 | 1 | 25110819 | 25150830 | protein\_coding | | ENSDARG00000014320 | gucy2c | 3 | 4077837 | 4112003 | protein\_coding | | ENSDARG00000025504 | gucy2f | 15 | 29429842 | 29447994 | protein\_coding | | ENSDARG00000005776 | guk1b | 8 | 18195236 | 18203786 | protein\_coding | | ENSDARG00000059391 | gyg1b | 24 | 3276070 | 3294582 | protein\_coding | | ENSDARG00000054058 | h1fx | 6 | 40771981 | 40774277 | protein\_coding | | ENSDARG00000076487 | haao | 13 | 8622644 | 8650759 | protein\_coding | | ENSDARG00000017444 | hal | 25 | 7830048 | 7850402 | protein\_coding | | ENSDARG00000045143 | hbbe2 | 12 | 20227991 | 20228755 | protein\_coding | | ENSDARG00000068220 | hbl3 | 2 | 37904820 | 37906754 | protein\_coding | | ENSDARG00000104480 | hcn1 | 21 | 10161296 | 10395898 | protein\_coding | | ENSDARG00000074419 | hcn4l | 25 | 28765744 | 28868942 | protein\_coding | | ENSDARG00000004291 | hecw1b | 2 | 50146434 | 50244094 | protein\_coding | | ENSDARG00000062447 | hecw2a | 9 | 24348856 | 24376363 | protein\_coding | | ENSDARG00000056934 | hepacama | 15 | 22376071 | 22390241 | protein\_coding | | ENSDARG00000039987 | hivep2a | 20 | 37608576 | 37726205 | protein\_coding | | ENSDARG00000075928 | hivep3a | 19 | 15402606 | 15493584 | protein\_coding | | ENSDARG00000074752 | hlfa | 3 | 11618818 | 11657952 | protein\_coding | | ENSDARG00000061011 | hlfb | 12 | 32044370 | 32050325 | protein\_coding | | ENSDARG00000070951 | hmga1b | 6 | 54134090 | 54144875 | protein\_coding | | ENSDARG00000052734 | hmgcra | 5 | 50326153 | 50346118 | protein\_coding | | ENSDARG00000102975 | hmp19 | 21 | 41396198 | 41445175 | protein\_coding | | ENSDARG00000099387 | hn1a | 6 | 22823531 | 22834268 | protein\_coding | | ENSDARG00000071565 | hnf4g | 24 | 23502612 | 23530272 | protein\_coding | | ENSDARG00000070491 | hpcal4 | 19 | 30934769 | 30975296 | protein\_coding | | ENSDARG00000100021 | hrasls | 11 | 45044195 | 45048327 | protein\_coding | | ENSDARG00000059903 | hs3st3l | 12 | 409770 | 425308 | protein\_coding | | ENSDARG00000052648 | hs3st4 | 3 | 46588988 | 46800991 | protein\_coding | | ENSDARG00000012381 | hsc70 | 15 | 37934331 | 37949384 | protein\_coding | | ENSDARG00000001975 | hsd11b2 | 7 | 34840575 | 34854897 | protein\_coding | | ENSDARG00000013251 | hsf4 | 18 | 22757440 | 22764571 | protein\_coding | | ENSDARG00000053544 | hspa4l | 17 | 43439827 | 43475779 | protein\_coding | | ENSDARG00000078411 | hspb15 | 5 | 31721158 | 31722983 | protein\_coding | | ENSDARG00000093745 | htr1aa | 8 | 31932418 | 31934203 | protein\_coding | | ENSDARG00000014907 | htra1b | 12 | 11350421 | 11419322 | protein\_coding | | ENSDARG00000091317 | hunk | 10 | 33668963 | 33678076 | protein\_coding | | ENSDARG00000051823 | hyal4 | 25 | 27092797 | 27121034 | protein\_coding | | ENSDARG00000038166 | hyal6 | 25 | 27152874 | 27189913 | protein\_coding | | ENSDARG00000062108 | iffo1b | 16 | 17207874 | 17254866 | protein\_coding | | ENSDARG00000105153 | ifitm5 | 25 | 7330274 | 7332507 | protein\_coding | | ENSDARG00000099351 | igfbp1a | 20 | 6818827 | 6822741 | protein\_coding | | ENSDARG00000039264 | igfbp5a | 6 | 18850265 | 18858473 | protein\_coding | | ENSDARG00000090833 | igfbp6b | 23 | 36479455 | 36486365 | protein\_coding | | ENSDARG00000104138 | igfbp7 | 14 | 51653568 | 51860801 | protein\_coding | | ENSDARG00000097613 | igfn1.3 | 6 | 46841851 | 46873428 | protein\_coding | | ENSDARG00000077497 | igsf10 | 15 | 1125853 | 1142706 | protein\_coding | | ENSDARG00000056084 | igsf21b | 23 | 21711707 | 21735365 | protein\_coding | | ENSDARG00000033845 | igsf9ba | 15 | 19196117 | 19314811 | protein\_coding | | ENSDARG00000058733 | ihha | 9 | 7380358 | 7388887 | protein\_coding | | ENSDARG00000052361 | il15 | 1 | 52599955 | 52625344 | protein\_coding | | ENSDARG00000102908 | il16 | 7 | 11242706 | 11342228 | protein\_coding | | ENSDARG00000104853 | il1rapl1b | 11 | 43956357 | 43965349 | protein\_coding | | ENSDARG00000077187 | impg1a | 20 | 757782 | 784426 | protein\_coding | | ENSDARG00000074839 | impg1b | 17 | 36679514 | 36734155 | protein\_coding | | ENSDARG00000019782 | impg2a | 9 | 30378018 | 30379767 | protein\_coding | | ENSDARG00000100288 | impg2b | 22 | 28370673 | 28407930 | protein\_coding | | ENSDARG00000024759 | inhbab | 2 | 21828467 | 21845680 | protein\_coding | | ENSDARG00000075201 | inpp4b | 1 | 52294447 | 52632704 | protein\_coding | | ENSDARG00000093359 | inpp5jb | 5 | 68459861 | 68499081 | protein\_coding | | ENSDARG00000035350 | ins | 5 | 37254532 | 37257543 | protein\_coding | | ENSDARG00000077709 | iqsec2 | 8 | 36770782 | 36945585 | protein\_coding | | ENSDARG00000099850 | iqsec3a | 4 | 26507291 | 26776621 | protein\_coding | | ENSDARG00000093091 | iqsec3b | 18 | 6282163 | 6337238 | protein\_coding | | ENSDARG00000040465 | irf2 | 14 | 3977141 | 4014073 | protein\_coding | | ENSDARG00000068657 | irgq2 | 15 | 38248880 | 38253031 | protein\_coding | | ENSDARG00000034420 | irx6a | 7 | 35240255 | 35244774 | protein\_coding | | ENSDARG00000074316 | itga1 | 10 | 8897429 | 9010179 | protein\_coding | | ENSDARG00000045070 | itgb3b | 12 | 26343118 | 26370384 | protein\_coding | | ENSDARG00000040985 | itgbl1 | 9 | 31941882 | 32176829 | protein\_coding | | ENSDARG00000055053 | itih1 | 11 | 3611240 | 3627406 | protein\_coding | | ENSDARG00000041505 | itm2bb | 9 | 25520036 | 25535714 | protein\_coding | | ENSDARG00000043448 | itm2ca | 15 | 40161318 | 40181831 | protein\_coding | | ENSDARG00000000161 | itsn2a | 20 | 25459819 | 25537299 | protein\_coding | | ENSDARG00000053547 | jakmip2 | 21 | 43034658 | 43083985 | protein\_coding | | ENSDARG00000086037 | jam3a | 15 | 19358111 | 19381568 | protein\_coding | | ENSDARG00000102204 | jph3 | 25 | 12543706 | 12599632 | protein\_coding | | ENSDARG00000074378 | junba | 1 | 51098615 | 51100171 | protein\_coding | | ENSDARG00000104773 | junbb | 3 | 7768815 | 7770219 | protein\_coding | | ENSDARG00000067850 | jund | 2 | 56001329 | 56007467 | protein\_coding | | ENSDARG00000059067 | jupb | 19 | 5562075 | 5676432 | protein\_coding | | ENSDARG00000059473 | kank4 | 6 | 32423934 | 32521289 | protein\_coding | | ENSDARG00000104958 | kbtbd11 | 17 | 25270831 | 25273323 | protein\_coding | | ENSDARG00000001882 | kbtbd12 | 6 | 40720280 | 40724664 | protein\_coding | | ENSDARG00000062942 | kcna1a | 25 | 19856625 | 19860327 | protein\_coding | | ENSDARG00000046014 | kcna6a | 25 | 19849145 | 19853380 | protein\_coding | | ENSDARG00000040741 | kcnab1b | 2 | 44552954 | 44657754 | protein\_coding | | ENSDARG00000062134 | kcnab2b | 23 | 21140838 | 21188181 | protein\_coding | | ENSDARG00000060095 | kcnb1 | 6 | 46505937 | 46563584 | protein\_coding | | ENSDARG00000038862 | kcnb2 | 2 | 30161446 | 30193936 | protein\_coding | | ENSDARG00000051852 | kcnc1a | 7 | 32757302 | 32815917 | protein\_coding | | ENSDARG00000032959 | kcnc1b | 25 | 7939363 | 7969988 | protein\_coding | | ENSDARG00000055855 | kcnc3a | 3 | 31821431 | 31918261 | protein\_coding | | ENSDARG00000032799 | kcnd2 | 4 | 10367008 | 10560619 | protein\_coding | | ENSDARG00000056101 | kcnd3 | 8 | 28046924 | 28213678 | protein\_coding | | ENSDARG00000027940 | kcnf1a | 20 | 33629417 | 33634364 | protein\_coding | | ENSDARG00000062565 | kcnh4a | 3 | 17397732 | 17454309 | protein\_coding | | ENSDARG00000061990 | kcnh4b | 12 | 13366778 | 13437785 | protein\_coding | | ENSDARG00000069117 | kcnh5b | 13 | 37175027 | 37258422 | protein\_coding | | ENSDARG00000062687 | kcnh7 | 6 | 10214377 | 10341428 | protein\_coding | | ENSDARG00000034808 | kcnip1b | 10 | 22034298 | 22062469 | protein\_coding | | ENSDARG00000034229 | kcnip3a | 8 | 42757447 | 42901037 | processed\_transcript | | ENSDARG00000090815 | kcnj10a | 7 | 6428836 | 6468806 | protein\_coding | | ENSDARG00000031438 | kcnj11l | 25 | 7546591 | 7552724 | protein\_coding | | ENSDARG00000062217 | kcnj3b | 6 | 13042487 | 13075278 | protein\_coding | | ENSDARG00000091306 | kcnn1a | 2 | 24754843 | 24806626 | protein\_coding | | ENSDARG00000075307 | kcnq2a | 8 | 25348895 | 25403052 | protein\_coding | | ENSDARG00000091130 | kcnq2b | 6 | 58497970 | 58505613 | protein\_coding | | ENSDARG00000060085 | kcnq3 | 2 | 43152094 | 43234337 | protein\_coding | | ENSDARG00000006891 | kcns3a | 13 | 32445327 | 32446936 | protein\_coding | | ENSDARG00000076644 | kcnv2a | 10 | 15352692 | 15356032 | protein\_coding | | ENSDARG00000004648 | kctd16a | 14 | 23514392 | 23552285 | protein\_coding | | ENSDARG00000020102 | kctd16b | 21 | 40921483 | 40936662 | protein\_coding | | ENSDARG00000068691 | kctd4 | 9 | 18868324 | 18869885 | protein\_coding | | ENSDARG00000067507 | kctd8 | 14 | 5630162 | 5697946 | protein\_coding | | ENSDARG00000069469 | khdrbs2 | 13 | 23152088 | 23258682 | protein\_coding | | ENSDARG00000101020 | khdrbs3 | KN149702.1 | 26943 | 89885 | protein\_coding | | ENSDARG00000062477 | kiaa1549la | 25 | 15257695 | 15386019 | protein\_coding | | ENSDARG00000062086 | kiaa1549lb | 18 | 28124380 | 28178016 | protein\_coding | | ENSDARG00000017338 | kidins220b | 20 | 29841008 | 29880765 | protein\_coding | | ENSDARG00000061817 | kif1aa | 6 | 27156110 | 27282466 | protein\_coding | | ENSDARG00000062024 | kif1ab | 2 | 23038666 | 23103137 | protein\_coding | | ENSDARG00000017532 | kif25 | 20 | 30999259 | 31017477 | protein\_coding | | ENSDARG00000013343 | kif26ba | 17 | 11353065 | 11564262 | protein\_coding | | ENSDARG00000024575 | kif26bb | 20 | 35110555 | 35149964 | protein\_coding | | ENSDARG00000079500 | kif3cb | 17 | 37256644 | 37266611 | protein\_coding | | ENSDARG00000098936 | kif5aa | 9 | 266338 | 296636 | protein\_coding | | ENSDARG00000059818 | kif5ab | 6 | 58774172 | 58834167 | protein\_coding | | ENSDARG00000068194 | klf9 | 5 | 25475077 | 25478012 | protein\_coding | | ENSDARG00000105322 | klhdc7a | 11 | 28386543 | 28392324 | protein\_coding | | ENSDARG00000008275 | klhl24b | 24 | 25616867 | 25643800 | protein\_coding | | ENSDARG00000003576 | klhl32 | 16 | 32283990 | 32322000 | protein\_coding | | ENSDARG00000016531 | klhl4 | 14 | 26498799 | 26545304 | protein\_coding | | ENSDARG00000086571 | knca7 | 3 | 29849761 | 29853026 | protein\_coding | | ENSDARG00000023082 | krt1-19d | 19 | 5408167 | 5416298 | protein\_coding | | ENSDARG00000026979 | krt1-c5 | 5 | 64185936 | 64201962 | protein\_coding | | ENSDARG00000036840 | krt15 | 11 | 11532157 | 11534848 | protein\_coding | | ENSDARG00000036830 | krt91 | 19 | 5462077 | 5464284 | protein\_coding | | ENSDARG00000044975 | krt94 | 11 | 11484945 | 11488236 | protein\_coding | | ENSDARG00000094526 | ksr2 | 5 | 11783402 | 12025118 | protein\_coding | | ENSDARG00000074036 | ky | 13 | 13612833 | 13623099 | protein\_coding | | ENSDARG00000099546 | kynu | 6 | 588877 | 601678 | protein\_coding | | ENSDARG00000014828 | l3mbtl1a | 23 | 4349520 | 4380119 | protein\_coding | | ENSDARG00000089063 | lactbl1a | 23 | 22037774 | 22048280 | protein\_coding | | ENSDARG00000070467 | lactbl1b | 11 | 27880662 | 27909955 | protein\_coding | | ENSDARG00000071462 | lama3 | 22 | 16356954 | 16374214 | protein\_coding | | ENSDARG00000093572 | lamc3 | 5 | 33807610 | 33942327 | protein\_coding | | ENSDARG00000036139 | lctla | 7 | 33955925 | 33964940 | protein\_coding | | ENSDARG00000016337 | lctlb | 18 | 19479145 | 19494377 | protein\_coding | | ENSDARG00000101251 | ldha | 25 | 25028201 | 25099874 | protein\_coding | | ENSDARG00000071076 | ldhbb | 25 | 3201141 | 3210843 | protein\_coding | | ENSDARG00000095553 | lenep | 16 | 22863839 | 22866056 | protein\_coding | | ENSDARG00000025903 | lgals9l1 | 15 | 31151514 | 31162442 | protein\_coding | | ENSDARG00000069701 | lgi2b | 1 | 39305567 | 39310786 | protein\_coding | | ENSDARG00000041358 | lgi3 | 8 | 51388779 | 51418277 | protein\_coding | | ENSDARG00000007715 | lgsn | 11 | 11279828 | 11283261 | protein\_coding | | ENSDARG00000021595 | lhfpl3 | 4 | 22644086 | 22698768 | protein\_coding | | ENSDARG00000078998 | lhfpl4a | 6 | 19679612 | 19717402 | protein\_coding | | ENSDARG00000076943 | lhfpl4b | 8 | 7363275 | 7372990 | protein\_coding | | ENSDARG00000041295 | lim2.5 | 16 | 49962246 | 49976886 | protein\_coding | | ENSDARG00000013414 | lin7a | 4 | 22030787 | 22130498 | protein\_coding | | ENSDARG00000037932 | lin7b | 3 | 31802179 | 31812995 | protein\_coding | | ENSDARG00000034165 | lingo1a | 25 | 7054940 | 7057060 | protein\_coding | | ENSDARG00000035899 | lingo1b | 7 | 48187463 | 48212141 | protein\_coding | | ENSDARG00000069970 | lingo2b | 13 | 12606808 | 12608683 | protein\_coding | | ENSDARG00000061970 | lingo3a | 22 | 20548884 | 20550701 | protein\_coding | | ENSDARG00000078527 | lingo4b | 16 | 29521351 | 29545712 | protein\_coding | | ENSDARG00000077261 | lmod1b | 11 | 24651554 | 24657291 | protein\_coding | | ENSDARG00000045864 | lmod2b | 4 | 4105280 | 4110678 | protein\_coding | | ENSDARG00000029177 | lnx2a | 24 | 21501141 | 21529822 | protein\_coding | | ENSDARG00000003259 | loxa | 10 | 7779840 | 7798176 | protein\_coding | | ENSDARG00000025089 | loxl4 | 13 | 40056597 | 40112923 | protein\_coding | | ENSDARG00000089824 | lpar4 | 14 | 10181976 | 10197874 | protein\_coding | | ENSDARG00000077489 | lrfn2b | 20 | 36735 | 88842 | protein\_coding | | ENSDARG00000055132 | lrfn4a | 21 | 26871217 | 26882208 | protein\_coding | | ENSDARG00000078078 | lrfn4b | 7 | 60245896 | 60293076 | protein\_coding | | ENSDARG00000019179 | lrit1a | 13 | 29331799 | 29337108 | protein\_coding | | ENSDARG00000099406 | lrit1b | 12 | 48952071 | 48955234 | protein\_coding | | ENSDARG00000030626 | lrit2 | 13 | 29339992 | 29343854 | protein\_coding | | ENSDARG00000034076 | lrp11 | 20 | 1257358 | 1270412 | protein\_coding | | ENSDARG00000097827 | lrp1aa | 11 | 1601950 | 1867375 | protein\_coding | | ENSDARG00000061517 | lrp1bb | 9 | 36546945 | 36885203 | protein\_coding | | ENSDARG00000070074 | lrp8 | 6 | 34528930 | 34828125 | protein\_coding | | ENSDARG00000040621 | lrrc32 | 10 | 252425 | 257043 | protein\_coding | | ENSDARG00000075147 | lrrc38a | 23 | 21848885 | 21870966 | protein\_coding | | ENSDARG00000087745 | lrrc38b | 11 | 28229124 | 28267804 | protein\_coding | | ENSDARG00000069402 | lrrc4.1 | 4 | 19545857 | 19550109 | protein\_coding | | ENSDARG00000003020 | lrrc4.2 | 18 | 10327404 | 10329338 | protein\_coding | | ENSDARG00000014792 | lrrc4bb | 24 | 38481281 | 38496561 | protein\_coding | | ENSDARG00000079670 | lrrc7 | 6 | 30131084 | 30323275 | protein\_coding | | ENSDARG00000063411 | lrrc73 | 13 | 3823944 | 3835659 | protein\_coding | | ENSDARG00000105302 | lrrn2 | 11 | 22616945 | 22621108 | protein\_coding | | ENSDARG00000052713 | lrrtm1 | 1 | 42075389 | 42077911 | protein\_coding | | ENSDARG00000071374 | lrrtm2 | 24 | 34720701 | 34726567 | protein\_coding | | ENSDARG00000080015 | lrrtm4l1 | 10 | 19353254 | 19382644 | protein\_coding | | ENSDARG00000045483 | lrtm2a | 4 | 20542122 | 20544751 | protein\_coding | | ENSDARG00000045811 | lrtm2b | 25 | 18943378 | 18948026 | protein\_coding | | ENSDARG00000056922 | ltbp1 | 17 | 22936591 | 23097214 | protein\_coding | | ENSDARG00000068247 | luzp2 | 18 | 37855952 | 38107091 | protein\_coding | | ENSDARG00000058558 | lzts3b | 8 | 1016357 | 1044609 | protein\_coding | | ENSDARG00000104023 | mag | 15 | 34033829 | 34075811 | protein\_coding | | ENSDARG00000077633 | magixb | 8 | 36634355 | 36753175 | protein\_coding | | ENSDARG00000024017 | mamdc1 | 17 | 28323175 | 28548667 | protein\_coding | | ENSDARG00000098345 | mamdc2a | 5 | 51862515 | 51922421 | protein\_coding | | ENSDARG00000023712 | mao | 9 | 33851779 | 33916787 | protein\_coding | | ENSDARG00000059601 | map1aa | 7 | 52555256 | 52601583 | protein\_coding | | ENSDARG00000022045 | map1ab | 25 | 19258633 | 19335293 | protein\_coding | | ENSDARG00000060326 | map1sb | 2 | 36938227 | 36949796 | protein\_coding | | ENSDARG00000103651 | map3k12 | 9 | 219491 | 235980 | protein\_coding | | ENSDARG00000013491 | map3k9 | 13 | 36034202 | 36058873 | protein\_coding | | ENSDARG00000074521 | map6a | 10 | 32617457 | 32657520 | protein\_coding | | ENSDARG00000074073 | map6b | 15 | 19979613 | 19991027 | protein\_coding | | ENSDARG00000079777 | map6d1 | 2 | 44844570 | 44867553 | protein\_coding | | ENSDARG00000078241 | map7a | 17 | 16043515 | 16061972 | protein\_coding | | ENSDARG00000068480 | map7d2a | 5 | 23457363 | 23495977 | protein\_coding | | ENSDARG00000102730 | mapk10 | 21 | 9483443 | 9620559 | protein\_coding | | ENSDARG00000102229 | mapk8ip1 | 25 | 13706663 | 13746414 | protein\_coding | | ENSDARG00000063157 | mapk8ip2 | 18 | 8297246 | 8355128 | protein\_coding | | ENSDARG00000018530 | mapkapk2b | 8 | 37432946 | 37445411 | protein\_coding | | ENSDARG00000020231 | mapre3a | 17 | 36919696 | 36948975 | protein\_coding | | ENSDARG00000089314 | mapta | 12 | 4698880 | 4767932 | protein\_coding | | ENSDARG00000037487 | march1 | 1 | 19015571 | 19063422 | protein\_coding | | ENSDARG00000056439 | march4 | 9 | 52953132 | 53000197 | protein\_coding | | ENSDARG00000055740 | march9 | 23 | 36649132 | 36676159 | protein\_coding | | ENSDARG00000069245 | matn3a | 20 | 43862981 | 43878466 | protein\_coding | | ENSDARG00000069265 | matn3b | 13 | 32496994 | 32505435 | protein\_coding | | ENSDARG00000069733 | mavs | 13 | 15051181 | 15082591 | protein\_coding | | ENSDARG00000016085 | mbnl3 | 14 | 30646440 | 30739869 | protein\_coding | | ENSDARG00000031348 | mc5ra | 19 | 12843359 | 12883880 | protein\_coding | | ENSDARG00000076480 | mcf2lb | 9 | 29775249 | 29805163 | protein\_coding | | ENSDARG00000101115 | med30 | 19 | 46779822 | 46824208 | protein\_coding | | ENSDARG00000062686 | megf11 | 18 | 19154371 | 19361726 | protein\_coding | | ENSDARG00000056726 | mettl7a | 6 | 39496231 | 39620651 | protein\_coding | | ENSDARG00000090560 | mfap5 | 16 | 12779696 | 12798837 | protein\_coding | | ENSDARG00000075159 | mfi2 | 6 | 30101250 | 30118879 | protein\_coding | | ENSDARG00000030263 | mfsd2b | 20 | 44610787 | 44659031 | protein\_coding | | ENSDARG00000041791 | mgat4c | 18 | 16398679 | 16600990 | protein\_coding | | ENSDARG00000086877 | mhc1zaa | 1 | 46433425 | 46470741 | lincRNA | | ENSDARG00000075608 | mical2a | 7 | 65309609 | 65409571 | protein\_coding | | ENSDARG00000078599 | micu3a | 1 | 15703948 | 15764187 | protein\_coding | | ENSDARG00000075450 | mkxa | 12 | 22840675 | 22888093 | protein\_coding | | ENSDARG00000063026 | mlc1 | 18 | 14958220 | 14973322 | protein\_coding | | ENSDARG00000071026 | mllt11 | 19 | 7526719 | 7531871 | protein\_coding | | ENSDARG00000026325 | mmp11a | 8 | 30788131 | 30816183 | protein\_coding | | ENSDARG00000058876 | mmp16b | 2 | 3799128 | 3846982 | protein\_coding | | ENSDARG00000018896 | mmp24 | 6 | 52362396 | 52428827 | protein\_coding | | ENSDARG00000069296 | moxd1l | 20 | 43186642 | 43214007 | protein\_coding | | ENSDARG00000010957 | mpp2b | 12 | 10161281 | 10262258 | protein\_coding | | ENSDARG00000062667 | mpp3b | 12 | 16184267 | 16227466 | protein\_coding | | ENSDARG00000058222 | mpp4a | 9 | 13714718 | 13740788 | protein\_coding | | ENSDARG00000062865 | mras | 22 | 11828358 | 11844976 | protein\_coding | | ENSDARG00000038822 | mrc1b | 2 | 31533967 | 31557620 | protein\_coding | | ENSDARG00000053561 | ms4a17a.11 | 4 | 74998757 | 75011610 | protein\_coding | | ENSDARG00000043796 | ms4a17a.7 | 4 | 75043727 | 75142798 | protein\_coding | | ENSDARG00000055876 | msmo1 | 1 | 20223614 | 20235835 | protein\_coding | | ENSDARG00000028740 | msnb | 14 | 8981669 | 9030587 | protein\_coding | | ENSDARG00000018459 | msrb2 | 24 | 16897404 | 16903499 | protein\_coding | | ENSDARG00000069133 | mstnb | 9 | 41137549 | 41141350 | protein\_coding | | ENSDARG00000102051 | mtbl | 7 | 28877564 | 28878077 | protein\_coding | | ENSDARG00000018266 | mthfd1a | 20 | 27747682 | 27784154 | protein\_coding | | ENSDARG00000056690 | mtmr1a | 7 | 25587187 | 25605773 | protein\_coding | | ENSDARG00000055852 | mtnr1ab | 14 | 29765297 | 29800848 | protein\_coding | | ENSDARG00000070331 | muc5.1 | 25 | 8279639 | 8323098 | protein\_coding | | ENSDARG00000062129 | mxra8b | 23 | 29962548 | 30173321 | protein\_coding | | ENSDARG00000045560 | mybpc1 | 4 | 17737865 | 17804176 | protein\_coding | | ENSDARG00000058799 | mybpha | 23 | 6143531 | 6192031 | protein\_coding | | ENSDARG00000009782 | myh11a | 6 | 8264326 | 8345319 | protein\_coding | | ENSDARG00000001993 | myhb | 6 | 40748131 | 40758602 | protein\_coding | | ENSDARG00000035438 | myhc4 | 5 | 31621602 | 31633709 | protein\_coding | | ENSDARG00000012944 | myhz2 | 5 | 31606225 | 31617426 | protein\_coding | | ENSDARG00000053424 | myl2b | 8 | 4277208 | 4302769 | protein\_coding | | ENSDARG00000011519 | myl4 | 3 | 22092692 | 22098054 | protein\_coding | | ENSDARG00000038123 | myl9a | 23 | 42920811 | 42930752 | protein\_coding | | ENSDARG00000055118 | mylipb | 16 | 25620961 | 25633706 | protein\_coding | | ENSDARG00000091260 | mylk4a | 2 | 381299 | 414238 | protein\_coding | | ENSDARG00000034801 | mylka | 9 | 4055593 | 4130389 | protein\_coding | | ENSDARG00000074723 | myo10l1 | 6 | 10866231 | 10991748 | protein\_coding | | ENSDARG00000006892 | myo3b | 9 | 3727829 | 3895145 | protein\_coding | | ENSDARG00000021789 | myoc | 20 | 15119577 | 15125746 | protein\_coding | | ENSDARG00000098836 | myocd | 12 | 197665 | 230365 | protein\_coding | | ENSDARG00000104836 | myom1b | 7 | 71496918 | 71566624 | protein\_coding | | ENSDARG00000056209 | myoz1a | 13 | 22134464 | 22147965 | protein\_coding | | ENSDARG00000067701 | myoz3a | 14 | 905539 | 915206 | protein\_coding | | ENSDARG00000078676 | myrf | 25 | 3772248 | 3853924 | protein\_coding | | ENSDARG00000038814 | myrip | 24 | 10984166 | 11169111 | protein\_coding | | ENSDARG00000008209 | myt1la | 20 | 30543092 | 30671150 | protein\_coding | | ENSDARG00000012347 | nacad | 16 | 27689766 | 27706372 | protein\_coding | | ENSDARG00000007149 | nadl1.2 | 23 | 28656324 | 28755234 | protein\_coding | | ENSDARG00000001835 | nalcn | 9 | 31777917 | 31936367 | protein\_coding | | ENSDARG00000013669 | napba | 17 | 4137247 | 4160303 | protein\_coding | | ENSDARG00000043012 | napgb | 2 | 54639579 | 54662991 | protein\_coding | | ENSDARG00000011334 | ncaldb | 19 | 12377572 | 12404986 | protein\_coding | | ENSDARG00000017466 | ncam2 | 9 | 35360056 | 35748212 | protein\_coding | | ENSDARG00000062087 | nceh1b | 24 | 26816589 | 26823538 | protein\_coding | | ENSDARG00000074262 | nck1a | 2 | 25308227 | 25325722 | protein\_coding | | ENSDARG00000010953 | ndel1a | 12 | 26376487 | 26396009 | protein\_coding | | ENSDARG00000076462 | ndnfl | 17 | 26551210 | 26568348 | protein\_coding | | ENSDARG00000011170 | ndrg2 | 7 | 3794730 | 3900254 | protein\_coding | | ENSDARG00000013087 | ndrg3a | 11 | 24826895 | 24901466 | protein\_coding | | ENSDARG00000103937 | ndrg4 | 25 | 13235775 | 13285349 | protein\_coding | | ENSDARG00000056108 | ndufa4 | 12 | 9842650 | 9846483 | protein\_coding | | ENSDARG00000056566 | necab1 | 19 | 31850376 | 31935364 | protein\_coding | | ENSDARG00000056745 | necab2 | 18 | 21419728 | 21631750 | protein\_coding | | ENSDARG00000074794 | necab3 | 6 | 57544912 | 57623636 | protein\_coding | | ENSDARG00000057568 | nefla | 21 | 18970961 | 18974198 | protein\_coding | | ENSDARG00000012426 | neflb | 8 | 6831204 | 6833968 | protein\_coding | | ENSDARG00000021351 | nefma | 21 | 18977214 | 18981819 | protein\_coding | | ENSDARG00000089123 | negaly6 | 16 | 24783817 | 24788571 | protein\_coding | | ENSDARG00000062797 | nell2b | 4 | 14248896 | 14330110 | protein\_coding | | ENSDARG00000071596 | neto1l | 24 | 15753298 | 15992150 | protein\_coding | | ENSDARG00000063293 | neto2b | 18 | 7541068 | 7552828 | protein\_coding | | ENSDARG00000069438 | neurl1aa | 1 | 47010692 | 47080510 | protein\_coding | | ENSDARG00000019566 | neurod1 | 9 | 44489979 | 44493329 | protein\_coding | | ENSDARG00000016854 | neurod2 | 12 | 14881785 | 14884652 | protein\_coding | | ENSDARG00000042824 | nfe2l2a | 9 | 1652136 | 1681220 | protein\_coding | | ENSDARG00000062420 | nfia | 22 | 16813197 | 17026111 | protein\_coding | | ENSDARG00000087188 | nfil3-6 | 3 | 13949850 | 13953705 | protein\_coding | | ENSDARG00000053475 | ngb | 17 | 44848187 | 44854412 | protein\_coding | | ENSDARG00000088708 | ngfra | 3 | 20007216 | 20082041 | protein\_coding | | ENSDARG00000054537 | nhsl1a | 17 | 30528932 | 30574260 | protein\_coding | | ENSDARG00000077329 | nlgn2a | 7 | 21678225 | 21866239 | protein\_coding | | ENSDARG00000079251 | nlgn2b | 10 | 22412359 | 22508875 | protein\_coding | | ENSDARG00000104786 | nlgn3a | 14 | 13919485 | 14228894 | protein\_coding | | ENSDARG00000062376 | nlgn3b | 5 | 22780088 | 22847512 | protein\_coding | | ENSDARG00000079455 | nlgn4a | 1 | 31789011 | 31960787 | protein\_coding | | ENSDARG00000028793 | nlk2 | 15 | 30276861 | 30420787 | protein\_coding | | ENSDARG00000077167 | nmbb | 7 | 29813924 | 29815958 | protein\_coding | | ENSDARG00000043820 | nme2a | 12 | 20664490 | 20674556 | protein\_coding | | ENSDARG00000099420 | nme2b.2 | 19 | 48451507 | 48454668 | protein\_coding | | ENSDARG00000004580 | nmnat2 | 2 | 36043726 | 36058363 | protein\_coding | | ENSDARG00000053528 | nog3 | 12 | 32273958 | 32275542 | protein\_coding | | ENSDARG00000058701 | nos1apb | 2 | 7054494 | 7081148 | protein\_coding | | ENSDARG00000016536 | npas2 | 5 | 22287886 | 22326924 | protein\_coding | | ENSDARG00000045016 | npffl | 11 | 256603 | 259874 | protein\_coding | | ENSDARG00000018750 | npr1b | 16 | 28968963 | 29056753 | protein\_coding | | ENSDARG00000043864 | nptnb | 18 | 1268164 | 1305037 | protein\_coding | | ENSDARG00000074671 | nptx1l | 3 | 59162016 | 59179356 | protein\_coding | | ENSDARG00000040430 | nptxra | 3 | 24352451 | 24371248 | protein\_coding | | ENSDARG00000053375 | nptxrb | 22 | 24997512 | 25005481 | protein\_coding | | ENSDARG00000036222 | npy | 19 | 20527423 | 20529826 | protein\_coding | | ENSDARG00000056541 | nr0b1 | 11 | 29435735 | 29438679 | protein\_coding | | ENSDARG00000033160 | nr1d1 | 3 | 23358104 | 23365675 | protein\_coding | | ENSDARG00000003820 | nr1d2a | 16 | 49879775 | 49907024 | protein\_coding | | ENSDARG00000009594 | nr1d2b | 19 | 17941698 | 17948550 | protein\_coding | | ENSDARG00000031161 | nr1d4a | 23 | 35696956 | 35722718 | protein\_coding | | ENSDARG00000059370 | nr1d4b | 11 | 1948136 | 1970845 | protein\_coding | | ENSDARG00000057741 | nr1h4 | 18 | 15676047 | 15698430 | protein\_coding | | ENSDARG00000017007 | nr4a2a | 9 | 5075603 | 5136894 | protein\_coding | | ENSDARG00000044532 | nr4a2b | 6 | 12227221 | 12231692 | protein\_coding | | ENSDARG00000055854 | nr4a3 | 16 | 27404450 | 27434715 | protein\_coding | | ENSDARG00000006396 | nrcama | 4 | 13651637 | 13729526 | protein\_coding | | ENSDARG00000086585 | nrg2b | 14 | 7969001 | 8131955 | protein\_coding | | ENSDARG00000089766 | nrg3b | 12 | 29235796 | 29509736 | protein\_coding | | ENSDARG00000039626 | nrgna | 5 | 57702156 | 57712647 | protein\_coding | | ENSDARG00000079985 | nrip2 | 4 | 17583519 | 17640468 | protein\_coding | | ENSDARG00000044719 | nrsn1 | 16 | 330938 | 339684 | protein\_coding | | ENSDARG00000061647 | nrxn1a | 12 | 24221098 | 24516088 | protein\_coding | | ENSDARG00000063635 | nrxn1b | 13 | 602736 | 680985 | protein\_coding | | ENSDARG00000063150 | nrxn2b | 7 | 386897 | 482462 | protein\_coding | | ENSDARG00000043746 | nrxn3a | 17 | 16898590 | 17428066 | protein\_coding | | ENSDARG00000062693 | nrxn3b | 20 | 5518645 | 5921039 | protein\_coding | | ENSDARG00000007654 | nsfa | 3 | 34637403 | 34671561 | protein\_coding | | ENSDARG00000035883 | nt5c1aa | 19 | 33179373 | 33217522 | protein\_coding | | ENSDARG00000070590 | nt5c1ab | 17 | 43547312 | 43567956 | protein\_coding | | ENSDARG00000018153 | nt5c1ba | 13 | 32315719 | 32323482 | protein\_coding | | ENSDARG00000071017 | nt5e | 20 | 805633 | 827242 | protein\_coding | | ENSDARG00000014973 | ntng1a | 24 | 29288521 | 29538493 | protein\_coding | | ENSDARG00000077367 | ntng2a | 8 | 11953547 | 12074952 | protein\_coding | | ENSDARG00000087100 | ntng2b | 21 | 3814488 | 3868031 | protein\_coding | | ENSDARG00000098511 | ntrk2b | 5 | 54956476 | 55019439 | protein\_coding | | ENSDARG00000086214 | ntrk3b | 7 | 14386931 | 14692934 | protein\_coding | | ENSDARG00000094557 | nupr1 | 3 | 15355475 | 15356358 | protein\_coding | | ENSDARG00000063224 | nxph2a | 9 | 10043444 | 10072486 | protein\_coding | | ENSDARG00000079581 | nyap2a | 15 | 35851254 | 35929443 | protein\_coding | | ENSDARG00000093357 | nyap2b | 2 | 5822988 | 5864148 | protein\_coding | | ENSDARG00000061791 | nyx | 9 | 33546265 | 33550453 | protein\_coding | | ENSDARG00000045929 | oaz2a | 25 | 6059421 | 6079041 | protein\_coding | | ENSDARG00000059815 | oaz2b | 7 | 48015986 | 48023451 | protein\_coding | | ENSDARG00000074476 | odam | 1 | 43257034 | 43262277 | protein\_coding | | ENSDARG00000061223 | ogfrl1 | 13 | 27768218 | 27786046 | protein\_coding | | ENSDARG00000018270 | olfm1a | 5 | 64050872 | 64094593 | protein\_coding | | ENSDARG00000014053 | olfm1b | 21 | 6829154 | 6891310 | protein\_coding | | ENSDARG00000071493 | olfm3a | 24 | 28607128 | 28632017 | protein\_coding | | ENSDARG00000005716 | olfml2ba | 20 | 33889277 | 33903629 | protein\_coding | | ENSDARG00000061852 | olfml3a | 23 | 24862664 | 24871045 | protein\_coding | | ENSDARG00000062171 | olfml3b | 11 | 24434060 | 24443676 | protein\_coding | | ENSDARG00000040948 | olig1 | 9 | 32917907 | 32919741 | protein\_coding | | ENSDARG00000093600 | omd | 22 | 10434208 | 10440981 | protein\_coding | | ENSDARG00000105218 | ompa | 18 | 3401904 | 3405006 | protein\_coding | | ENSDARG00000013005 | opcml | 10 | 30731486 | 30899161 | protein\_coding | | ENSDARG00000044861 | opn1lw2 | 11 | 25243430 | 25245590 | protein\_coding | | ENSDARG00000097008 | opn1mw1 | 6 | 41184333 | 41191928 | protein\_coding | | ENSDARG00000045677 | opn1sw1 | 4 | 13578816 | 13581499 | protein\_coding | | ENSDARG00000017274 | opn1sw2 | 11 | 25222081 | 25237787 | protein\_coding | | ENSDARG00000052775 | opn3 | 13 | 42119161 | 42139525 | protein\_coding | | ENSDARG00000007553 | opn4.1 | 2 | 32859915 | 32863106 | protein\_coding | | ENSDARG00000103259 | opn4xb | 5 | 7348825 | 7392782 | protein\_coding | | ENSDARG00000102430 | opn6a | 11 | 44605015 | 44618837 | protein\_coding | | ENSDARG00000037159 | oprd1b | 16 | 34206805 | 34220230 | protein\_coding | | ENSDARG00000071209 | oprl1 | 23 | 8360920 | 8449878 | protein\_coding | | ENSDARG00000053487 | osbp2 | 10 | 7958020 | 8015455 | protein\_coding | | ENSDARG00000054055 | ostn | 15 | 36369401 | 36391118 | protein\_coding | | ENSDARG00000020581 | otofb | 17 | 50234581 | 50294936 | protein\_coding | | ENSDARG00000042845 | oxt | 5 | 71303113 | 71308749 | protein\_coding | | ENSDARG00000002300 | p2rx2 | 5 | 12309138 | 12323659 | protein\_coding | | ENSDARG00000075209 | p4htm | 6 | 40736564 | 40747184 | protein\_coding | | ENSDARG00000032865 | pacsin1a | 23 | 3712122 | 3760801 | protein\_coding | | ENSDARG00000030154 | pak7 | 20 | 54907846 | 54914236 | protein\_coding | | ENSDARG00000039352 | pald1b | 13 | 30039231 | 30069276 | protein\_coding | | ENSDARG00000093480 | pamr1 | 25 | 36769471 | 36795397 | protein\_coding | | ENSDARG00000063019 | panx2 | 18 | 14934183 | 14954360 | protein\_coding | | ENSDARG00000076020 | pappa2 | 2 | 35003744 | 35120612 | protein\_coding | | ENSDARG00000074160 | paqr4b | 12 | 13126403 | 13138615 | protein\_coding | | ENSDARG00000041483 | paqr6 | 16 | 45772561 | 45815891 | protein\_coding | | ENSDARG00000034907 | paqr7b | 16 | 34568947 | 34578775 | protein\_coding | | ENSDARG00000042703 | parp6a | 18 | 994746 | 1006497 | protein\_coding | | ENSDARG00000089262 | pbx3a | 5 | 4809970 | 4902296 | protein\_coding | | ENSDARG00000013615 | pbx3b | 8 | 33920777 | 34077023 | protein\_coding | | ENSDARG00000105441 | pcdh11.1 | 14 | 26697009 | 26823749 | protein\_coding | | ENSDARG00000027041 | pcdh17 | 11 | 32313488 | 32461039 | protein\_coding | | ENSDARG00000062720 | pcdh1a | 10 | 20959780 | 21096678 | protein\_coding | | ENSDARG00000098963 | pcdh1g13 | 10 | 21765511 | 21767931 | protein\_coding | | ENSDARG00000103013 | pcdh1g22 | 10 | 21819530 | 21862679 | protein\_coding | | ENSDARG00000099931 | pcdh1g26 | 10 | 21829275 | 21862676 | protein\_coding | | ENSDARG00000104497 | pcdh1g29 | 10 | 21839895 | 21862676 | protein\_coding | | ENSDARG00000101865 | pcdh1g30 | 10 | 21843649 | 21862676 | protein\_coding | | ENSDARG00000100670 | pcdh1g31 | 10 | 21847391 | 21862676 | protein\_coding | | ENSDARG00000099035 | pcdh1g33 | 10 | 21825832 | 21862621 | protein\_coding | | ENSDARG00000057519 | pcdh1g9 | 10 | 21744170 | 21862676 | protein\_coding | | ENSDARG00000088475 | pcdh1gb9 | 10 | 21761087 | 21861202 | protein\_coding | | ENSDARG00000104826 | pcdh1gc5 | 10 | 21832573 | 21862676 | protein\_coding | | ENSDARG00000103950 | pcdh1gc6 | 10 | 21836289 | 21862678 | protein\_coding | | ENSDARG00000099783 | pcdh2ac | 14 | 2204772 | 2245103 | protein\_coding | | ENSDARG00000078898 | pcdh7a | 1 | 14451752 | 14516704 | protein\_coding | | ENSDARG00000077023 | pcdhb | 14 | 2449039 | 2451498 | protein\_coding | | ENSDARG00000063299 | pcloa | 4 | 11377065 | 11448326 | protein\_coding | | ENSDARG00000098880 | pclob | 18 | 9645957 | 9752386 | protein\_coding | | ENSDARG00000055698 | pcnxl2 | 13 | 23539211 | 23584903 | protein\_coding | | ENSDARG00000052057 | pcolceb | 7 | 50574176 | 50597691 | protein\_coding | | ENSDARG00000053130 | pcp4a | 10 | 39026022 | 39068107 | protein\_coding | | ENSDARG00000087386 | pcp4b | 15 | 6070590 | 6130803 | protein\_coding | | ENSDARG00000002600 | pcsk1 | 21 | 11592202 | 11629715 | protein\_coding | | ENSDARG00000076170 | pcsk1nl | 8 | 7340563 | 7360660 | protein\_coding | | ENSDARG00000019451 | pcsk2 | 13 | 781660 | 847559 | protein\_coding | | ENSDARG00000011886 | pdca | 2 | 21224892 | 21232367 | protein\_coding | | ENSDARG00000017634 | pdcb | 20 | 34181452 | 34187861 | protein\_coding | | ENSDARG00000032344 | pde4a | 3 | 48676551 | 48800211 | protein\_coding | | ENSDARG00000032868 | pde4ba | 6 | 30864625 | 31106880 | protein\_coding | | ENSDARG00000074233 | pde4bb | 2 | 14776378 | 14938265 | protein\_coding | | ENSDARG00000013221 | pde4ca | 2 | 25125392 | 25211331 | protein\_coding | | ENSDARG00000000380 | pde6a | 14 | 24708158 | 24736897 | protein\_coding | | ENSDARG00000100397 | pde6c | 12 | 5094288 | 5119142 | protein\_coding | | ENSDARG00000101984 | pde6g | 12 | 36286268 | 36294320 | protein\_coding | | ENSDARG00000063706 | pde8b | 21 | 7904735 | 7953492 | protein\_coding | | ENSDARG00000086778 | pdgfba | 22 | 29359751 | 29387073 | protein\_coding | | ENSDARG00000010555 | pdha1b | 24 | 24128275 | 24141018 | protein\_coding | | ENSDARG00000020876 | pdk2a | 12 | 28444506 | 28456087 | protein\_coding | | ENSDARG00000054848 | pdk4 | 19 | 41600688 | 41894937 | protein\_coding | | ENSDARG00000011023 | pdlim3a | 1 | 17028916 | 17042924 | protein\_coding | | ENSDARG00000087798 | pdyn | 23 | 41902607 | 41906920 | protein\_coding | | ENSDARG00000017127 | pdzk1ip1 | 22 | 16529669 | 16540545 | protein\_coding | | ENSDARG00000060753 | pear1 | 16 | 30480114 | 30561327 | protein\_coding | | ENSDARG00000004869 | penka | 2 | 11725092 | 11729220 | protein\_coding | | ENSDARG00000056885 | per1a | 10 | 22841347 | 22862023 | protein\_coding | | ENSDARG00000060504 | pfkla | 9 | 53087626 | 53138761 | protein\_coding | | ENSDARG00000014179 | pfkma | 23 | 27627245 | 27645126 | protein\_coding | | ENSDARG00000028000 | pfkpa | 24 | 3931994 | 3972265 | protein\_coding | | ENSDARG00000012801 | pfkpb | 2 | 48265330 | 48317277 | protein\_coding | | ENSDARG00000062998 | pglyrp2 | 6 | 8254783 | 8263369 | protein\_coding | | ENSDARG00000057439 | phf1 | 16 | 45458191 | 45477033 | protein\_coding | | ENSDARG00000077596 | phf24 | 10 | 17893570 | 17968571 | protein\_coding | | ENSDARG00000030687 | phka2 | 11 | 33607555 | 33656507 | protein\_coding | | ENSDARG00000030604 | phkg1a | 15 | 5734674 | 5754680 | protein\_coding | | ENSDARG00000086740 | phyh | 4 | 7818632 | 7827784 | protein\_coding | | ENSDARG00000003998 | phyhipla | 17 | 20943641 | 20959241 | protein\_coding | | ENSDARG00000091637 | pip4k2cb | 11 | 2387491 | 2402926 | protein\_coding | | ENSDARG00000044295 | pip5k1ba | 10 | 15923189 | 15961720 | protein\_coding | | ENSDARG00000044091 | pitpnab | 10 | 24354299 | 24401860 | protein\_coding | | ENSDARG00000091116 | pkhd1l1 | 19 | 23266334 | 23335441 | protein\_coding | | ENSDARG00000053110 | pkib | 20 | 40474787 | 40554005 | protein\_coding | | ENSDARG00000099860 | pkmb | 25 | 28716900 | 28752870 | protein\_coding | | ENSDARG00000056805 | plch2b | 11 | 25511652 | 25539702 | protein\_coding | | ENSDARG00000058200 | plcxd2 | 24 | 25028296 | 25041721 | protein\_coding | | ENSDARG00000054794 | plcxd3 | 8 | 31812164 | 31853622 | protein\_coding | | ENSDARG00000061191 | plekho1a | 19 | 42797849 | 42821953 | protein\_coding | | ENSDARG00000062756 | pllp | 18 | 18872293 | 18881566 | protein\_coding | | ENSDARG00000069404 | pln | 20 | 42014104 | 42029374 | antisense | | ENSDARG00000028552 | plppr3b | 11 | 14090327 | 14104179 | protein\_coding | | ENSDARG00000079671 | plppr4a | 2 | 20674072 | 20713308 | protein\_coding | | ENSDARG00000089101 | plrdgb | 21 | 39681913 | 39849387 | protein\_coding | | ENSDARG00000100482 | pmt | 14 | 1422194 | 1432757 | protein\_coding | | ENSDARG00000078619 | pnp5a | 7 | 6517269 | 6532726 | protein\_coding | | ENSDARG00000040942 | pnp6 | 18 | 21268195 | 21282307 | protein\_coding | | ENSDARG00000102020 | pnpla3 | 4 | 8779267 | 8794334 | protein\_coding | | ENSDARG00000043135 | pomca | 17 | 33274444 | 33281905 | protein\_coding | | ENSDARG00000036382 | ponzr6 | 7 | 22496521 | 22501978 | protein\_coding | | ENSDARG00000052558 | porcn | 8 | 21114177 | 21123879 | protein\_coding | | ENSDARG00000043806 | postna | 10 | 25800483 | 25856141 | protein\_coding | | ENSDARG00000069737 | pou4f2 | 1 | 35704909 | 35708274 | protein\_coding | | ENSDARG00000006206 | pou4f3 | 9 | 54790772 | 54792038 | protein\_coding | | ENSDARG00000099933 | ppa1a | 12 | 48146441 | 48172213 | protein\_coding | | ENSDARG00000069940 | ppap2d | 3 | 50511688 | 50576771 | protein\_coding | | ENSDARG00000013000 | ppfia2 | 4 | 22235950 | 22486898 | protein\_coding | | ENSDARG00000077053 | ppfia3 | 3 | 29913169 | 29992271 | protein\_coding | | ENSDARG00000053205 | ppfia4 | 11 | 22214185 | 22421867 | protein\_coding | | ENSDARG00000026499 | ppm1e | 10 | 32739229 | 32844972 | protein\_coding | | ENSDARG00000010231 | ppm1na | 15 | 9076988 | 9093101 | protein\_coding | | ENSDARG00000057032 | ppm1nb | 21 | 21331278 | 21336606 | protein\_coding | | ENSDARG00000071251 | ppp1r18 | 19 | 824136 | 840721 | protein\_coding | | ENSDARG00000060265 | ppp1r27a | 3 | 59561085 | 59567796 | protein\_coding | | ENSDARG00000076248 | ppp1r3db | 11 | 1383034 | 1385768 | protein\_coding | | ENSDARG00000071709 | ppp1r9bb | 12 | 9665329 | 9735645 | protein\_coding | | ENSDARG00000099221 | ppp2r2bb | 21 | 41636126 | 41764544 | protein\_coding | | ENSDARG00000056797 | ppp2r2ca | 1 | 13592828 | 13620325 | protein\_coding | | ENSDARG00000004988 | ppp3ca | 21 | 28921740 | 29039116 | protein\_coding | | ENSDARG00000025106 | ppp3cb | 14 | 7482647 | 7582339 | protein\_coding | | ENSDARG00000054683 | prdm8b | 5 | 38903983 | 38908805 | protein\_coding | | ENSDARG00000070597 | prelp | 11 | 20916304 | 20925686 | protein\_coding | | ENSDARG00000028163 | prg4b | 20 | 34126941 | 34136759 | protein\_coding | | ENSDARG00000009477 | prkar2ab | 22 | 137128 | 157672 | protein\_coding | | ENSDARG00000075949 | prkd1 | 17 | 28866014 | 28952231 | protein\_coding | | ENSDARG00000031702 | prkg1b | 12 | 6325145 | 6418106 | protein\_coding | | ENSDARG00000016570 | prlra | 21 | 11012379 | 11043314 | protein\_coding | | ENSDARG00000045760 | prmt8b | 4 | 11147769 | 11166762 | protein\_coding | | ENSDARG00000044048 | prnpb | 10 | 20671317 | 20675398 | protein\_coding | | ENSDARG00000003705 | prnprs3 | 25 | 3551980 | 3559621 | protein\_coding | | ENSDARG00000034007 | prom1b | 1 | 22022192 | 22066406 | protein\_coding | | ENSDARG00000039406 | prom2 | 13 | 28617484 | 28643897 | protein\_coding | | ENSDARG00000038018 | prph2a | 12 | 34795893 | 34800811 | protein\_coding | | ENSDARG00000014840 | prph2b | 13 | 3119600 | 3125422 | protein\_coding | | ENSDARG00000021345 | prph2l | 20 | 27485048 | 27491168 | protein\_coding | | ENSDARG00000062208 | prrt1 | 19 | 27273618 | 27284793 | protein\_coding | | ENSDARG00000103588 | prrt2 | 12 | 5046802 | 5060817 | protein\_coding | | ENSDARG00000059423 | prune2 | 5 | 54748412 | 54773128 | protein\_coding | | ENSDARG00000018066 | ptchd1 | 24 | 25204872 | 25219824 | protein\_coding | | ENSDARG00000022951 | pth2 | 17 | 33542683 | 33546512 | protein\_coding | | ENSDARG00000102340 | ptn | 4 | 1287988 | 1347047 | protein\_coding | | ENSDARG00000074866 | ptpn5 | 7 | 39355853 | 39383941 | protein\_coding | | ENSDARG00000019945 | ptprdb | 1 | 18429983 | 18544675 | protein\_coding | | ENSDARG00000079560 | ptprh | 24 | 7771167 | 7791815 | protein\_coding | | ENSDARG00000074781 | ptprt | 6 | 51180107 | 51386657 | protein\_coding | | ENSDARG00000097572 | ptprt.1 | 6 | 51010762 | 51102615 | protein\_coding | | ENSDARG00000099614 | purab | 14 | 7955303 | 7965572 | protein\_coding | | ENSDARG00000068822 | purba | 5 | 13142950 | 13145837 | protein\_coding | | ENSDARG00000037789 | pvalb1 | 3 | 60961255 | 60985163 | protein\_coding | | ENSDARG00000002768 | pvalb2 | 12 | 17588551 | 17590624 | protein\_coding | | ENSDARG00000024433 | pvalb4 | 3 | 60943484 | 60953158 | protein\_coding | | ENSDARG00000032836 | pvalb5 | 3 | 60976625 | 60985787 | protein\_coding | | ENSDARG00000009311 | pvalb6 | 3 | 24230548 | 24304357 | protein\_coding | | ENSDARG00000037790 | pvalb8 | 3 | 60921631 | 60930162 | protein\_coding | | ENSDARG00000055177 | pxdc1a | 2 | 32852413 | 32855676 | protein\_coding | | ENSDARG00000035832 | pyyb | 12 | 10266592 | 10277196 | protein\_coding | | ENSDARG00000062293 | qpctlb | 21 | 21289402 | 21299801 | protein\_coding | | ENSDARG00000069159 | rab38b | 10 | 25304778 | 25320032 | protein\_coding | | ENSDARG00000043835 | rab3ab | 11 | 6809211 | 6840754 | protein\_coding | | ENSDARG00000042803 | rab3b | 22 | 17177482 | 17182579 | protein\_coding | | ENSDARG00000034215 | rab42a | 19 | 16128021 | 16131785 | protein\_coding | | ENSDARG00000031343 | rab6bb | 2 | 23139843 | 23148014 | protein\_coding | | ENSDARG00000020795 | rac3b | 12 | 2988149 | 3007296 | protein\_coding | | ENSDARG00000035655 | rad21b | 19 | 44472677 | 44495832 | protein\_coding | | ENSDARG00000079779 | radil | 1 | 7163652 | 7210144 | protein\_coding | | ENSDARG00000068370 | ralgps1 | 8 | 32905394 | 33104970 | protein\_coding | | ENSDARG00000075271 | rapgef5a | 19 | 2834738 | 2858984 | protein\_coding | | ENSDARG00000079912 | rapgefl1 | 3 | 23204220 | 23277172 | protein\_coding | | ENSDARG00000002816 | rasgrf2b | 5 | 50796416 | 50935380 | protein\_coding | | ENSDARG00000061796 | rasgrp4 | 18 | 35761830 | 35821475 | protein\_coding | | ENSDARG00000075034 | raver2 | 6 | 31443877 | 31589483 | protein\_coding | | ENSDARG00000014746 | rbfox1 | 3 | 27853316 | 28297356 | protein\_coding | | ENSDARG00000070038 | rbp2a | 15 | 5815607 | 5827155 | protein\_coding | | ENSDARG00000093774 | rbp2b | 2 | 39050427 | 39052199 | protein\_coding | | ENSDARG00000101199 | rbp4 | 12 | 5080972 | 5085382 | protein\_coding | | ENSDARG00000044684 | rbp4l | 21 | 20302507 | 20305460 | protein\_coding | | ENSDARG00000021869 | rcan2 | 20 | 39150595 | 39285597 | protein\_coding | | ENSDARG00000019902 | rcvrn2 | 16 | 14021572 | 14031469 | protein\_coding | | ENSDARG00000009637 | rcvrn3 | 19 | 10420613 | 10433654 | protein\_coding | | ENSDARG00000052223 | rcvrna | 3 | 49406176 | 49408339 | protein\_coding | | ENSDARG00000031600 | rd3 | 20 | 14073434 | 14101855 | protein\_coding | | ENSDARG00000105060 | rdh8b | 1 | 58587428 | 58786197 | protein\_coding | | ENSDARG00000017569 | reep3a | 17 | 19587551 | 19606521 | protein\_coding | | ENSDARG00000087349 | reep6 | 11 | 7252021 | 7264539 | protein\_coding | | ENSDARG00000075670 | rereb | 6 | 40673800 | 40691506 | protein\_coding | | ENSDARG00000019396 | rergla | 4 | 12382886 | 12389686 | protein\_coding | | ENSDARG00000054324 | rerglb | 7 | 28134933 | 28141973 | protein\_coding | | ENSDARG00000054890 | rgra | 13 | 29315776 | 29325409 | protein\_coding | | ENSDARG00000089077 | rgs1 | 2 | 6599895 | 6606903 | protein\_coding | | ENSDARG00000037646 | rgs11 | 1 | 9202880 | 9230519 | protein\_coding | | ENSDARG00000040177 | rgs16 | 6 | 35460047 | 35462727 | protein\_coding | | ENSDARG00000002644 | rgs5a | 6 | 35403984 | 35456023 | protein\_coding | | ENSDARG00000017860 | rgs5b | 2 | 7014246 | 7017513 | protein\_coding | | ENSDARG00000060601 | rgs7bpa | 8 | 31892086 | 31910392 | protein\_coding | | ENSDARG00000016742 | rgs7bpb | 21 | 20896718 | 20902852 | protein\_coding | | ENSDARG00000070037 | rgs8 | 6 | 35478248 | 35504030 | protein\_coding | | ENSDARG00000037925 | rgs9a | 3 | 36236979 | 36260148 | protein\_coding | | ENSDARG00000045156 | rgs9b | 12 | 18559549 | 18595785 | protein\_coding | | ENSDARG00000009466 | rgs9bp | 7 | 41023499 | 41032124 | protein\_coding | | ENSDARG00000019253 | rhag | 20 | 14118833 | 14158103 | protein\_coding | | ENSDARG00000009018 | rhbg | 16 | 29331173 | 29342541 | protein\_coding | | ENSDARG00000003203 | rhcga | 7 | 14045860 | 14057253 | protein\_coding | | ENSDARG00000100265 | rhcgb | 25 | 8503958 | 8539319 | protein\_coding | | ENSDARG00000002193 | rho | 8 | 53668767 | 53670321 | protein\_coding | | ENSDARG00000070666 | rhol | 11 | 18933089 | 18935404 | protein\_coding | | ENSDARG00000007247 | ric8a | 25 | 10362219 | 10386420 | protein\_coding | | ENSDARG00000074660 | rilp | 15 | 28294182 | 28314707 | protein\_coding | | ENSDARG00000001154 | rimbp2 | 8 | 43946514 | 44010716 | protein\_coding | | ENSDARG00000074680 | rims1a | 13 | 27502143 | 27636880 | protein\_coding | | ENSDARG00000078902 | rims1b | 1 | 30570770 | 30772838 | protein\_coding | | ENSDARG00000101606 | rims2a | 16 | 44373881 | 44434411 | protein\_coding | | ENSDARG00000102690 | rims2b | 19 | 13181075 | 13268356 | protein\_coding | | ENSDARG00000062305 | rims3 | 19 | 25579317 | 25686932 | protein\_coding | | ENSDARG00000059523 | rims4 | 6 | 52081753 | 52156428 | protein\_coding | | ENSDARG00000012504 | rlbp1a | 7 | 13620944 | 13638474 | protein\_coding | | ENSDARG00000045808 | rlbp1b | 25 | 19007853 | 19020176 | protein\_coding | | ENSDARG00000070780 | rln3a | 3 | 19057436 | 19060897 | protein\_coding | | ENSDARG00000104458 | rnasekb | 10 | 22946661 | 22948762 | protein\_coding | | ENSDARG00000075046 | rnf151 | 12 | 18336628 | 18343101 | protein\_coding | | ENSDARG00000078817 | rnf165b | 8 | 628475 | 639917 | protein\_coding | | ENSDARG00000061049 | rnf182 | 2 | 31821364 | 31822386 | protein\_coding | | ENSDARG00000012409 | rnf207b | 23 | 21573034 | 21588631 | protein\_coding | | ENSDARG00000014891 | robo2 | 15 | 39249949 | 39384044 | protein\_coding | | ENSDARG00000078366 | robo2.1 | 15 | 38958371 | 38990931 | protein\_coding | | ENSDARG00000019752 | rom1a | 5 | 66448405 | 66467136 | protein\_coding | | ENSDARG00000026926 | rom1b | 14 | 46688014 | 46712649 | protein\_coding | | ENSDARG00000031768 | roraa | 25 | 33364220 | 33484290 | protein\_coding | | ENSDARG00000001910 | rorab | 7 | 29267488 | 29300465 | protein\_coding | | ENSDARG00000017780 | rorcb | 2 | 49683670 | 49720221 | protein\_coding | | ENSDARG00000105490 | rph3ab | 7 | 72296795 | 72338230 | protein\_coding | | ENSDARG00000053383 | rprma | 9 | 4643405 | 4644436 | protein\_coding | | ENSDARG00000056768 | rprml | 3 | 19459898 | 19461250 | protein\_coding | | ENSDARG00000079024 | rps6kl1 | 17 | 52473604 | 52493687 | protein\_coding | | ENSDARG00000057368 | rps8b | 8 | 18157404 | 18164291 | protein\_coding | | ENSDARG00000039534 | rrh | 13 | 12395568 | 12408064 | protein\_coding | | ENSDARG00000021143 | rtn1b | 20 | 20738193 | 20796280 | protein\_coding | | ENSDARG00000090035 | rtn4r | 5 | 10266180 | 10445867 | protein\_coding | | ENSDARG00000098528 | rtn4rl1b | 15 | 26432472 | 26435829 | protein\_coding | | ENSDARG00000052826 | runx3 | 13 | 44955862 | 45063957 | protein\_coding | | ENSDARG00000078125 | rusc1 | 16 | 14139163 | 14169142 | protein\_coding | | ENSDARG00000095288 | rusc2 | 5 | 18797474 | 18825541 | protein\_coding | | ENSDARG00000093628 | s100a11 | 19 | 7648838 | 7651550 | protein\_coding | | ENSDARG00000057598 | s100b | 22 | 13822359 | 13826520 | protein\_coding | | ENSDARG00000055589 | s100t | 16 | 22766428 | 22767996 | protein\_coding | | ENSDARG00000012610 | saga | 2 | 5568243 | 5593212 | protein\_coding | | ENSDARG00000038378 | sagb | 15 | 45625523 | 45647699 | protein\_coding | | ENSDARG00000054543 | samsn1a | 15 | 29631488 | 29653871 | protein\_coding | | ENSDARG00000035652 | sat1a.1 | 5 | 3999159 | 4040700 | protein\_coding | | ENSDARG00000061885 | satb2 | 9 | 32745524 | 32804842 | protein\_coding | | ENSDARG00000099819 | sb:cb1058 | 7 | 24762767 | 24769819 | protein\_coding | | ENSDARG00000057687 | sbk3 | 16 | 12820784 | 12833618 | protein\_coding | | ENSDARG00000040274 | scamp5b | 18 | 50092435 | 50102177 | protein\_coding | | ENSDARG00000008060 | scara3 | 20 | 39380970 | 39397851 | protein\_coding | | ENSDARG00000010425 | scara5 | 20 | 1333869 | 1363090 | protein\_coding | | ENSDARG00000030265 | scdb | 13 | 29879167 | 29897280 | protein\_coding | | ENSDARG00000038574 | scg2b | 2 | 47727833 | 47731033 | protein\_coding | | ENSDARG00000086288 | scg3 | 18 | 39453581 | 39492047 | protein\_coding | | ENSDARG00000032126 | scg5 | 20 | 29568927 | 29572057 | protein\_coding | | ENSDARG00000102415 | scinla | 6 | 23991141 | 24003857 | protein\_coding | | ENSDARG00000090724 | scn12aa | 2 | 52295271 | 52427287 | protein\_coding | | ENSDARG00000062744 | scn1lab | 6 | 10010985 | 10085137 | protein\_coding | | ENSDARG00000101713 | scn2b | 15 | 12589962 | 12614266 | protein\_coding | | ENSDARG00000062359 | scn3b | 15 | 22195937 | 22212128 | protein\_coding | | ENSDARG00000034588 | scn4ab | 3 | 22142529 | 22186270 | protein\_coding | | ENSDARG00000099031 | scn4ba | 15 | 12535435 | 12569521 | protein\_coding | | ENSDARG00000005775 | scn8aa | 23 | 27325085 | 27418884 | protein\_coding | | ENSDARG00000018032 | scn8ab | 6 | 39394906 | 39491654 | protein\_coding | | ENSDARG00000012194 | scp2a | 2 | 6093317 | 6127875 | protein\_coding | | ENSDARG00000078622 | scpp5 | 1 | 43208769 | 43214403 | protein\_coding | | ENSDARG00000078866 | sdk1a | 3 | 40837139 | 41150481 | protein\_coding | | ENSDARG00000017794 | sdr16c5b | 7 | 58397278 | 58416032 | protein\_coding | | ENSDARG00000074698 | sdsl | 17 | 14597861 | 14605778 | protein\_coding | | ENSDARG00000088444 | sell | 20 | 4078267 | 4089231 | processed\_transcript | | ENSDARG00000058821 | sema5a | 24 | 16249108 | 16562520 | protein\_coding | | ENSDARG00000078707 | sema7a | 25 | 26400826 | 26424580 | protein\_coding | | ENSDARG00000036031 | sept5b | 10 | 44089329 | 44117231 | protein\_coding | | ENSDARG00000014233 | sept8b | 14 | 50052433 | 50075400 | protein\_coding | | ENSDARG00000089936 | sepw2b | 3 | 19954006 | 19956493 | protein\_coding | | ENSDARG00000005924 | serpina10a | 20 | 27472304 | 27482616 | protein\_coding | | ENSDARG00000014556 | serpinb1l3 | 24 | 42226407 | 42236180 | protein\_coding | | ENSDARG00000069048 | serpinf1 | 21 | 40082895 | 40096616 | protein\_coding | | ENSDARG00000076448 | serpinf2a | 21 | 40068748 | 40081363 | protein\_coding | | ENSDARG00000089795 | sertad4 | 13 | 31676505 | 31699336 | protein\_coding | | ENSDARG00000100876 | sez6a | 10 | 36884311 | 36983836 | protein\_coding | | ENSDARG00000076052 | sez6l2 | 3 | 15079789 | 15118094 | protein\_coding | | ENSDARG00000057678 | sfrp1b | 10 | 20394032 | 20399632 | protein\_coding | | ENSDARG00000097897 | sgip1a | 6 | 30723012 | 30852849 | protein\_coding | | ENSDARG00000087176 | sgk494a | 15 | 16163436 | 16185474 | protein\_coding | | ENSDARG00000075376 | sgsm1b | 10 | 1610805 | 1647803 | protein\_coding | | ENSDARG00000036878 | sh3bgrl2 | 23 | 31478966 | 31497198 | protein\_coding | | ENSDARG00000007136 | sh3bp5lb | 19 | 27698849 | 27730839 | protein\_coding | | ENSDARG00000060539 | shank1 | 3 | 30583269 | 30730335 | protein\_coding | | ENSDARG00000062325 | shank2 | 25 | 14069047 | 14269677 | protein\_coding | | ENSDARG00000019492 | shbg | 12 | 22389796 | 22403169 | protein\_coding | | ENSDARG00000062462 | shisa7a | 16 | 13746584 | 13772394 | protein\_coding | | ENSDARG00000063144 | shisa7b | 19 | 10298664 | 10324223 | protein\_coding | | ENSDARG00000045145 | shisa9a | 12 | 19670407 | 19743711 | protein\_coding | | ENSDARG00000052642 | shisa9b | 3 | 48091072 | 48162898 | protein\_coding | | ENSDARG00000100449 | si:busm1-71b9.3 | 7 | 52900476 | 52919224 | transcribed\_unprocessed\_pseudogene | | ENSDARG00000105432 | si:cabz01029535.1 | 7 | 10111252 | 10154739 | protein\_coding | | ENSDARG00000099525 | si:ch1073-13h15.3 | 12 | 3391206 | 3408794 | protein\_coding | | ENSDARG00000095802 | si:ch1073-155h21.2 | 20 | 117846 | 124682 | protein\_coding | | ENSDARG00000100041 | si:ch1073-235m4.3 | 7 | 71786414 | 71789463 | lincRNA | | ENSDARG00000074869 | si:ch1073-291c23.2 | 13 | 212424 | 219817 | protein\_coding | | ENSDARG00000092985 | si:ch1073-309n14.1 | 21 | 43846480 | 43853754 | processed\_transcript | | ENSDARG00000105109 | si:ch1073-329i9.1 | 14 | 3464277 | 3469841 | lincRNA | | ENSDARG00000097369 | si:ch1073-358o18.3 | 17 | 27383740 | 27384391 | lincRNA | | ENSDARG00000060018 | si:ch1073-44g3.1 | 14 | 50147507 | 50149278 | protein\_coding | | ENSDARG00000101485 | si:ch1073-469d17.2 | 17 | 51112182 | 51113304 | protein\_coding | | ENSDARG00000099299 | si:ch1073-83n3.2 | 15 | 106126 | 117045 | protein\_coding | | ENSDARG00000091573 | si:ch1073-90m23.1 | 16 | 31969346 | 31978564 | protein\_coding | | ENSDARG00000088315 | si:ch211-103f14.3 | 10 | 29204382 | 29215208 | protein\_coding | | ENSDARG00000027360 | si:ch211-106h11.3 | 3 | 5665288 | 5702136 | protein\_coding | | ENSDARG00000092920 | si:ch211-106h4.12 | 2 | 31682618 | 31685893 | protein\_coding | | ENSDARG00000098164 | si:ch211-106m9.1 | 14 | 40507354 | 40535760 | protein\_coding | | ENSDARG00000052633 | si:ch211-106n13.3 | 2 | 50252858 | 50318776 | protein\_coding | | ENSDARG00000095272 | si:ch211-10p21.1 | 5 | 15992018 | 16077490 | protein\_coding | | ENSDARG00000089338 | si:ch211-110e21.3 | 3 | 59716602 | 59725613 | protein\_coding | | ENSDARG00000100139 | si:ch211-113d22.2 | 10 | 20491257 | 20496627 | protein\_coding | | ENSDARG00000070442 | si:ch211-113g11.6 | 16 | 29473043 | 29517416 | protein\_coding | | ENSDARG00000073764 | si:ch211-113j14.1 | 6 | 9235828 | 9293678 | protein\_coding | | ENSDARG00000071235 | si:ch211-117c9.5 | 23 | 6861559 | 6907273 | protein\_coding | | ENSDARG00000094286 | si:ch211-117m20.4 | 5 | 30007685 | 30012576 | protein\_coding | | ENSDARG00000092970 | si:ch211-11c15.3 | 14 | 34146315 | 34148679 | protein\_coding | | ENSDARG00000097160 | si:ch211-121j5.4 | 9 | 7262505 | 7279486 | protein\_coding | | ENSDARG00000105447 | si:ch211-129p13.1 | 18 | 43370722 | 43445013 | protein\_coding | | ENSDARG00000043141 | si:ch211-132f19.7 | 17 | 33359273 | 33386969 | protein\_coding | | ENSDARG00000093753 | si:ch211-133l5.5 | 8 | 20814172 | 20818266 | processed\_transcript | | ENSDARG00000092099 | si:ch211-133n4.6 | 19 | 3245771 | 3253014 | protein\_coding | | ENSDARG00000104775 | si:ch211-134a4.1 | 21 | 5365413 | 5374907 | protein\_coding | | ENSDARG00000092142 | si:ch211-136a13.2 | 18 | 49212355 | 49218904 | processed\_transcript | | ENSDARG00000098044 | si:ch211-136a13.3 | 18 | 49130033 | 49162174 | lincRNA | | ENSDARG00000057706 | si:ch211-137i24.10 | 5 | 23661341 | 23671156 | protein\_coding | | ENSDARG00000094561 | si:ch211-139a5.9 | 5 | 37283869 | 37285924 | protein\_coding | | ENSDARG00000093342 | si:ch211-13f8.1 | 2 | 7321360 | 7338099 | protein\_coding | | ENSDARG00000093899 | si:ch211-142k18.1 | 20 | 19879716 | 19889339 | lincRNA | | ENSDARG00000037256 | si:ch211-145b13.5 | 1 | 25317765 | 25329964 | protein\_coding | | ENSDARG00000097196 | si:ch211-145n14.3 | 21 | 42030764 | 42034017 | lincRNA | | ENSDARG00000096922 | si:ch211-147k10.6 | 25 | 29728948 | 29751060 | lincRNA | | ENSDARG00000094522 | si:ch211-14c7.2 | 18 | 46196019 | 46210563 | protein\_coding | | ENSDARG00000087426 | si:ch211-151p13.8 | 1 | 44379594 | 44393223 | protein\_coding | | ENSDARG00000081299 | si:ch211-152f6.6 | 3 | 15547317 | 15556967 | processed\_transcript | | ENSDARG00000068428 | si:ch211-153j24.3 | 20 | 46656538 | 46660748 | protein\_coding | | ENSDARG00000097187 | si:ch211-153l6.6 | 2 | 20772394 | 20786688 | protein\_coding | | ENSDARG00000076850 | si:ch211-157b11.14 | 6 | 40691821 | 40700049 | protein\_coding | | ENSDARG00000059399 | si:ch211-160o17.6 | 11 | 2621889 | 2631043 | protein\_coding | | ENSDARG00000100263 | si:ch211-161c3.5 | 6 | 53349587 | 53367449 | protein\_coding | | ENSDARG00000089919 | si:ch211-161h7.8 | 24 | 20815797 | 20819960 | protein\_coding | | ENSDARG00000097875 | si:ch211-170a17.1 | 14 | 43599395 | 43794405 | lincRNA | | ENSDARG00000096713 | si:ch211-170d12.2 | 2 | 7086907 | 7087528 | lincRNA | | ENSDARG00000046142 | si:ch211-170d8.5 | 8 | 39699504 | 39705866 | protein\_coding | | ENSDARG00000020455 | si:ch211-171h4.3 | 19 | 10496559 | 10506215 | protein\_coding | | ENSDARG00000096889 | si:ch211-173b16.4 | 5 | 68120848 | 68122344 | lincRNA | | ENSDARG00000060889 | si:ch211-173n18.4 | 6 | 39508507 | 39510839 | protein\_coding | | ENSDARG00000095831 | si:ch211-175f12.2 | 16 | 4212229 | 4227212 | protein\_coding | | ENSDARG00000098147 | si:ch211-177d9.1 | 12 | 41449760 | 41509356 | protein\_coding | | ENSDARG00000087857 | si:ch211-180a12.2 | 12 | 3961999 | 3991314 | protein\_coding | | ENSDARG00000077112 | si:ch211-180f4.1 | 8 | 8807347 | 8809581 | protein\_coding | | ENSDARG00000096655 | si:ch211-184m19.3 | 20 | 14761161 | 14765452 | antisense | | ENSDARG00000091800 | si:ch211-186e20.6 | 5 | 28212300 | 28218460 | unprocessed\_pseudogene | | ENSDARG00000077960 | si:ch211-186e20.7 | 5 | 28197642 | 28200637 | protein\_coding | | ENSDARG00000078088 | si:ch211-186j3.6 | 7 | 47143275 | 47577796 | protein\_coding | | ENSDARG00000079056 | si:ch211-194c3.5 | 13 | 13562730 | 13606702 | protein\_coding | | ENSDARG00000094836 | si:ch211-195b15.8 | 3 | 32272044 | 32275478 | protein\_coding | | ENSDARG00000089221 | si:ch211-195o20.7 | 17 | 24873355 | 24898488 | protein\_coding | | ENSDARG00000041492 | si:ch211-196f5.2 | 24 | 12594554 | 12606208 | protein\_coding | | ENSDARG00000077087 | si:ch211-196g2.4 | 8 | 46411117 | 46425876 | protein\_coding | | ENSDARG00000092574 | si:ch211-198d23.1 | 8 | 38560965 | 38583773 | protein\_coding | | ENSDARG00000091834 | si:ch211-198m17.1 | 3 | 38563567 | 38603740 | protein\_coding | | ENSDARG00000096991 | si:ch211-203d7.1 | 12 | 40032173 | 40324105 | lincRNA | | ENSDARG00000098376 | si:ch211-207l14.1 | 6 | 8940347 | 8952420 | protein\_coding | | ENSDARG00000105463 | si:ch211-210k13.6 | 11 | 37030296 | 37030892 | antisense | | ENSDARG00000071333 | si:ch211-212d10.2 | 22 | 18463723 | 18466835 | protein\_coding | | ENSDARG00000097962 | si:ch211-214g1.4 | 15 | 16978709 | 16984965 | lincRNA | | ENSDARG00000087403 | si:ch211-214p13.3 | 9 | 9273429 | 9304482 | protein\_coding | | ENSDARG00000044129 | si:ch211-214p16.1 | 17 | 25567096 | 25568467 | processed\_transcript | | ENSDARG00000094660 | si:ch211-218d20.15 | 9 | 34616543 | 34620390 | protein\_coding | | ENSDARG00000079119 | si:ch211-229d2.5 | 15 | 19862186 | 19867746 | protein\_coding | | ENSDARG00000070931 | si:ch211-232m10.6 | 19 | 10437420 | 10454705 | protein\_coding | | ENSDARG00000102650 | si:ch211-236d3.3 | 11 | 4167635 | 4175310 | lincRNA | | ENSDARG00000062059 | si:ch211-236l14.4 | 25 | 23559331 | 23648217 | protein\_coding | | ENSDARG00000105408 | si:ch211-237b12.4 | 16 | 16147507 | 16151831 | antisense | | ENSDARG00000100012 | si:ch211-239d6.4 | 19 | 25571319 | 25572968 | lincRNA | | ENSDARG00000096319 | si:ch211-239j9.1 | 6 | 52350444 | 52357146 | protein\_coding | | ENSDARG00000062618 | si:ch211-23l10.2 | 3 | 16299690 | 16308218 | protein\_coding | | ENSDARG00000068100 | si:ch211-240g9.1 | 8 | 10524193 | 10621748 | protein\_coding | | ENSDARG00000101745 | si:ch211-241a17.6 | 6 | 7296186 | 7296662 | TEC | | ENSDARG00000040703 | si:ch211-242n18.1 | 6 | 40175692 | 40184984 | processed\_transcript | | ENSDARG00000103071 | si:ch211-244c8.4 | 10 | 35594114 | 35598211 | protein\_coding | | ENSDARG00000054744 | si:ch211-244e12.7 | 15 | 31396128 | 31397307 | protein\_coding | | ENSDARG00000097693 | si:ch211-248a14.8 | 17 | 20154046 | 20169559 | protein\_coding | | ENSDARG00000092945 | si:ch211-250g4.3 | 19 | 37922518 | 37932627 | protein\_coding | | ENSDARG00000007275 | si:ch211-251b21.1 | 8 | 47644446 | 47676034 | protein\_coding | | ENSDARG00000097262 | si:ch211-255f14.2 | 8 | 43941176 | 43946758 | lincRNA | | ENSDARG00000094310 | si:ch211-255g12.6 | 8 | 6911436 | 6914625 | protein\_coding | | ENSDARG00000096616 | si:ch211-255p10.3 | 12 | 4642181 | 4648313 | protein\_coding | | ENSDARG00000055172 | si:ch211-256m1.8 | 3 | 25023530 | 25034641 | protein\_coding | | ENSDARG00000102572 | si:ch211-260e23.9 | 7 | 45731025 | 45737495 | protein\_coding | | ENSDARG00000104047 | si:ch211-262i1.3 | 11 | 14222939 | 14234303 | protein\_coding | | ENSDARG00000096508 | si:ch211-265g21.1 | 20 | 12205588 | 12209943 | lincRNA | | ENSDARG00000075491 | si:ch211-26b3.4 | 5 | 22367918 | 22448356 | protein\_coding | | ENSDARG00000097256 | si:ch211-270g19.5 | 17 | 15289465 | 15297653 | protein\_coding | | ENSDARG00000096398 | si:ch211-276a17.5 | 6 | 8103759 | 8111400 | protein\_coding | | ENSDARG00000060330 | si:ch211-284e13.4 | 5 | 37467532 | 37494337 | protein\_coding | | ENSDARG00000104200 | si:ch211-285j16.4 | 15 | 17950176 | 18000106 | lincRNA | | ENSDARG00000100703 | si:ch211-285j22.3 | 7 | 19651956 | 19655996 | protein\_coding | | ENSDARG00000011088 | si:ch211-288d18.1 | 7 | 48726988 | 48779712 | protein\_coding | | ENSDARG00000070960 | si:ch211-288g17.4 | 19 | 9425246 | 9445417 | protein\_coding | | ENSDARG00000097855 | si:ch211-3o3.9 | 9 | 41125490 | 41134147 | protein\_coding | | ENSDARG00000092534 | si:ch211-62a1.3 | 13 | 45636200 | 45638091 | protein\_coding | | ENSDARG00000091579 | si:ch211-66e2.5 | 3 | 48505116 | 48521272 | protein\_coding | | ENSDARG00000096242 | si:ch211-76l23.4 | 3 | 28692579 | 28697400 | protein\_coding | | ENSDARG00000078522 | si:ch211-80h18.1 | 19 | 3145953 | 3186392 | protein\_coding | | ENSDARG00000096558 | si:ch211-80j13.1 | 20 | 10365961 | 10435573 | lincRNA | | ENSDARG00000096505 | si:ch73-103l1.2 | 12 | 10141609 | 10155708 | protein\_coding | | ENSDARG00000003077 | si:ch73-111m19.2 | 12 | 45515616 | 45527176 | protein\_coding | | ENSDARG00000099257 | si:ch73-119p20.1 | 7 | 13122845 | 13128409 | protein\_coding | | ENSDARG00000096784 | si:ch73-123f19.1 | 12 | 2543287 | 2571787 | lincRNA | | ENSDARG00000060680 | si:ch73-127m5.1 | 12 | 35018596 | 35097265 | protein\_coding | | ENSDARG00000087364 | si:ch73-140j24.4 | 2 | 2295526 | 2313812 | protein\_coding | | ENSDARG00000097804 | si:ch73-156o22.2 | 15 | 40110757 | 40115957 | protein\_coding | | ENSDARG00000101367 | si:ch73-193i22.1 | 9 | 46891687 | 46900847 | protein\_coding | | ENSDARG00000100625 | si:ch73-197b13.1 | 22 | 38029017 | 38042730 | processed\_transcript | | ENSDARG00000086159 | si:ch73-206d17.1 | 23 | 26565454 | 26571901 | protein\_coding | | ENSDARG00000077468 | si:ch73-206p6.1 | 24 | 5176384 | 5183153 | protein\_coding | | ENSDARG00000061909 | si:ch73-233f7.1 | 14 | 2464273 | 2482033 | protein\_coding | | ENSDARG00000102095 | si:ch73-233f7.5 | 14 | 2437892 | 2440423 | protein\_coding | | ENSDARG00000099487 | si:ch73-233f7.7 | 14 | 2423089 | 2434858 | protein\_coding | | ENSDARG00000103330 | si:ch73-236c18.5 | 1 | 57720812 | 57724185 | protein\_coding | | ENSDARG00000091624 | si:ch73-240m12.1 | 5 | 8872336 | 8933732 | protein\_coding | | ENSDARG00000097420 | si:ch73-250a16.5 | 9 | 882080 | 890058 | protein\_coding | | ENSDARG00000100246 | si:ch73-264i18.3 | 3 | 5604756 | 5613091 | lincRNA | | ENSDARG00000095832 | si:ch73-269m23.4 | 23 | 45076076 | 45082118 | lincRNA | | ENSDARG00000097279 | si:ch73-274k23.3 | 5 | 63058664 | 63059938 | lincRNA | | ENSDARG00000104329 | si:ch73-281k2.5 | 8 | 19482209 | 19488256 | protein\_coding | | ENSDARG00000098392 | si:ch73-28h20.1 | 13 | 28773509 | 28783987 | protein\_coding | | ENSDARG00000076310 | si:ch73-290k24.5 | 23 | 45849028 | 45871231 | protein\_coding | | ENSDARG00000100255 | si:ch73-29l19.1 | 6 | 36817331 | 36841477 | protein\_coding | | ENSDARG00000099772 | si:ch73-302a4.4 | 14 | 844767 | 850658 | lincRNA | | ENSDARG00000101501 | si:ch73-305o9.3 | 17 | 14714238 | 14718891 | protein\_coding | | ENSDARG00000097416 | si:ch73-306e8.2 | 17 | 20596180 | 20610479 | protein\_coding | | ENSDARG00000103650 | si:ch73-329n5.1 | 12 | 44438914 | 44459486 | protein\_coding | | ENSDARG00000074285 | si:ch73-335m24.2 | 14 | 33519775 | 33531858 | protein\_coding | | ENSDARG00000036383 | si:ch73-335m24.5 | 14 | 33558662 | 33596210 | protein\_coding | | ENSDARG00000096791 | si:ch73-344o19.1 | 2 | 6269501 | 6285380 | protein\_coding | | ENSDARG00000088584 | si:ch73-352p18.4 | 22 | 1011826 | 1025858 | protein\_coding | | ENSDARG00000023940 | si:ch73-362m14.4 | 21 | 43586165 | 43611326 | protein\_coding | | ENSDARG00000098465 | si:ch73-366l1.5 | 3 | 60458961 | 60491415 | protein\_coding | | ENSDARG00000093062 | si:ch73-370c12.1 | 8 | 3630230 | 3632481 | processed\_transcript | | ENSDARG00000087224 | si:ch73-380n15.2 | 1 | 21541654 | 21679921 | protein\_coding | | ENSDARG00000091917 | si:ch73-386o14.1 | 8 | 48103919 | 48106989 | antisense | | ENSDARG00000035765 | si:ch73-388k15.2 | 15 | 6681428 | 6729989 | unprocessed\_pseudogene | | ENSDARG00000078504 | si:ch73-60h1.1 | 11 | 14618577 | 14693721 | protein\_coding | | ENSDARG00000096031 | si:ch73-6k14.2 | 1 | 48600809 | 48606070 | protein\_coding | | ENSDARG00000076998 | si:ch73-92i20.1 | 5 | 21418734 | 21428391 | protein\_coding | | ENSDARG00000094460 | si:dkey-100j22.4 | 23 | 40584316 | 40587678 | processed\_transcript | | ENSDARG00000094198 | si:dkey-100j22.7 | 23 | 40573598 | 40579184 | processed\_transcript | | ENSDARG00000062672 | si:dkey-100n10.2 | 12 | 16013750 | 16036025 | protein\_coding | | ENSDARG00000062319 | si:dkey-103g5.3 | 15 | 21926563 | 21935739 | protein\_coding | | ENSDARG00000026247 | si:dkey-105e17.1 | 18 | 50279368 | 50360255 | protein\_coding | | ENSDARG00000095421 | si:dkey-106l3.6 | 10 | 17146334 | 17174946 | processed\_transcript | | ENSDARG00000098476 | si:dkey-113d16.9 | 12 | 36253182 | 36255807 | protein\_coding | | ENSDARG00000089271 | si:dkey-114c15.7 | 5 | 22613803 | 22628595 | protein\_coding | | ENSDARG00000097706 | si:dkey-11a7.3 | 17 | 22667334 | 22669804 | antisense | | ENSDARG00000014232 | si:dkey-121j17.5 | 20 | 626267 | 654106 | protein\_coding | | ENSDARG00000045594 | si:dkey-14k9.2 | 4 | 14827375 | 14881196 | protein\_coding | | ENSDARG00000095192 | si:dkey-150k17.2 | 19 | 29204745 | 29239197 | antisense | | ENSDARG00000101125 | si:dkey-155h10.4 | 13 | 12074462 | 12075334 | lincRNA | | ENSDARG00000009568 | si:dkey-15h19.2 | 3 | 34790302 | 34852165 | protein\_coding | | ENSDARG00000089418 | si:dkey-164f24.2 | 8 | 32473501 | 32488583 | protein\_coding | | ENSDARG00000100311 | si:dkey-165e24.1 | 4 | 49692145 | 49748633 | protein\_coding | | ENSDARG00000094217 | si:dkey-17e16.15 | 21 | 25624008 | 25626846 | protein\_coding | | ENSDARG00000092673 | si:dkey-181m9.5 | 2 | 27012046 | 27017213 | processed\_transcript | | ENSDARG00000099577 | si:dkey-182g1.10 | 22 | 8742809 | 8758639 | protein\_coding | | ENSDARG00000056248 | si:dkey-183i3.5 | 21 | 25150605 | 25160498 | protein\_coding | | ENSDARG00000104326 | si:dkey-183i3.9 | 21 | 25144398 | 25146825 | protein\_coding | | ENSDARG00000053761 | si:dkey-187j14.4 | 7 | 2420214 | 2426089 | protein\_coding | | ENSDARG00000088638 | si:dkey-188p4.1 | 22 | 12908513 | 12915932 | processed\_transcript | | ENSDARG00000043770 | si:dkey-192d15.2 | 7 | 3461401 | 3475830 | protein\_coding | | ENSDARG00000097553 | si:dkey-193e13.8 | 2 | 13115580 | 13120120 | lincRNA | | ENSDARG00000094696 | si:dkey-201c13.2 | 5 | 25479025 | 25482989 | protein\_coding | | ENSDARG00000104642 | si:dkey-201i2.1 | 12 | 44467291 | 44475731 | protein\_coding | | ENSDARG00000089429 | si:dkey-205h13.2 | 23 | 26599153 | 26609334 | protein\_coding | | ENSDARG00000032838 | si:dkey-206f10.1 | 20 | 43705732 | 43747453 | protein\_coding | | ENSDARG00000089883 | si:dkey-210j14.3 | 3 | 52387074 | 52390074 | protein\_coding | | ENSDARG00000002295 | si:dkey-21p1.3 | 8 | 14816411 | 14845987 | protein\_coding | | ENSDARG00000105104 | si:dkey-223p19.1 | 21 | 45446176 | 45457555 | protein\_coding | | ENSDARG00000104930 | si:dkey-225f23.5 | 2 | 19451358 | 19460240 | protein\_coding | | ENSDARG00000092044 | si:dkey-22f5.9 | 21 | 30614369 | 30621825 | protein\_coding | | ENSDARG00000092730 | si:dkey-22i16.3 | 1 | 43200802 | 43204099 | protein\_coding | | ENSDARG00000087599 | si:dkey-237h12.3 | 14 | 36181239 | 36381114 | protein\_coding | | ENSDARG00000079446 | si:dkey-238f9.1 | 6 | 48490290 | 48690945 | protein\_coding | | ENSDARG00000097826 | si:dkey-239b22.2 | 12 | 38859724 | 38885982 | protein\_coding | | ENSDARG00000102967 | si:dkey-245f22.5 | 11 | 25237667 | 25238569 | antisense | | ENSDARG00000103167 | si:dkey-245n4.2 | 5 | 42464788 | 42470905 | protein\_coding | | ENSDARG00000013775 | si:dkey-246g23.4 | 18 | 14595573 | 14611264 | protein\_coding | | ENSDARG00000103199 | si:dkey-247k7.2 | 16 | 23424030 | 23431063 | protein\_coding | | ENSDARG00000095048 | si:dkey-250k15.7 | 16 | 11792646 | 11818857 | protein\_coding | | ENSDARG00000104231 | si:dkey-260g12.4 | 16 | 17255216 | 17280317 | protein\_coding | | ENSDARG00000098024 | si:dkey-262k9.2 | 15 | 36587381 | 36644258 | protein\_coding | | ENSDARG00000089838 | si:dkey-262k9.4 | 15 | 36648045 | 36675468 | protein\_coding | | ENSDARG00000088023 | si:dkey-26c10.5 | 16 | 12390130 | 12403561 | protein\_coding | | ENSDARG00000062606 | si:dkey-26i13.8 | 1 | 11024426 | 11046491 | protein\_coding | | ENSDARG00000068624 | si:dkey-27p18.5 | 5 | 21961087 | 21998684 | protein\_coding | | ENSDARG00000104213 | si:dkey-283b1.6 | 3 | 33257309 | 33263519 | protein\_coding | | ENSDARG00000041382 | si:dkey-283b15.2 | 16 | 5750314 | 5772236 | protein\_coding | | ENSDARG00000058492 | si:dkey-30c15.17 | 18 | 7404307 | 7415848 | protein\_coding | | ENSDARG00000057881 | si:dkey-33c12.3 | 13 | 8981754 | 8986868 | protein\_coding | | ENSDARG00000097118 | si:dkey-33c14.3 | 2 | 329611 | 331728 | lincRNA | | ENSDARG00000102555 | si:dkey-33i11.9 | 16 | 45206077 | 45211714 | protein\_coding | | ENSDARG00000055014 | si:dkey-33m11.8 | 11 | 3556576 | 3565166 | protein\_coding | | ENSDARG00000104572 | si:dkey-35h6.1 | 14 | 12730546 | 12742341 | protein\_coding | | ENSDARG00000097648 | si:dkey-35i13.1 | 15 | 4641635 | 4644672 | protein\_coding | | ENSDARG00000103639 | si:dkey-36i7.3 | 15 | 12319627 | 12339440 | protein\_coding | | ENSDARG00000098379 | si:dkey-38l22.2 | 7 | 18469280 | 18478207 | lincRNA | | ENSDARG00000092444 | si:dkey-3l5.2 | 18 | 22750666 | 22754300 | processed\_transcript | | ENSDARG00000104999 | si:dkey-45k15.3 | 13 | 7014399 | 7015543 | lincRNA | | ENSDARG00000097472 | si:dkey-45l12.1 | 3 | 46057746 | 46255409 | processed\_transcript | | ENSDARG00000086272 | si:dkey-4p15.5 | 15 | 19746108 | 19752657 | protein\_coding | | ENSDARG00000042133 | si:dkey-51e6.1 | 20 | 34079067 | 34082864 | protein\_coding | | ENSDARG00000092551 | si:dkey-56e3.2 | 1 | 40886907 | 40892282 | protein\_coding | | ENSDARG00000097110 | si:dkey-56f14.4 | 16 | 24675506 | 24683010 | protein\_coding | | ENSDARG00000055400 | si:dkey-56f14.7 | 16 | 24711416 | 24731035 | protein\_coding | | ENSDARG00000093318 | si:dkey-57a22.15 | 9 | 22193073 | 22193964 | protein\_coding | | ENSDARG00000092680 | si:dkey-58f10.12 | 5 | 38073015 | 38074574 | protein\_coding | | ENSDARG00000043436 | si:dkey-5n18.1 | 20 | 17139320 | 17141442 | protein\_coding | | ENSDARG00000099695 | si:dkey-61n16.5 | 3 | 13762104 | 13771327 | protein\_coding | | ENSDARG00000101924 | si:dkey-65b13.13 | 20 | 33610785 | 33613011 | protein\_coding | | ENSDARG00000062487 | si:dkey-6n6.1 | 8 | 14092045 | 14113506 | protein\_coding | | ENSDARG00000092039 | si:dkey-70p6.1 | 8 | 23019488 | 23060428 | protein\_coding | | ENSDARG00000080675 | si:dkey-71b5.7 | 16 | 22133134 | 22202744 | protein\_coding | | ENSDARG00000061044 | si:dkey-72l14.3 | 8 | 25954012 | 25961555 | protein\_coding | | ENSDARG00000097528 | si:dkey-7j14.5 | 16 | 43110659 | 43122515 | protein\_coding | | ENSDARG00000105420 | si:dkey-87o1.3 | 16 | 17798213 | 17799927 | lincRNA | | ENSDARG00000094990 | si:dkey-91f15.1 | 6 | 18993494 | 19005475 | protein\_coding | | ENSDARG00000057262 | si:dkey-91i10.3 | 9 | 23198739 | 23212419 | protein\_coding | | ENSDARG00000101343 | si:dkey-93h22.4 | 11 | 44721658 | 44729446 | antisense | | ENSDARG00000016923 | si:dkey-94f20.4 | 12 | 4518789 | 4541804 | protein\_coding | | ENSDARG00000040100 | si:dkey-95h12.1 | 18 | 8914254 | 8927773 | protein\_coding | | ENSDARG00000090623 | si:dkeyp-106c3.1 | 2 | 7423909 | 7540975 | protein\_coding | | ENSDARG00000100476 | si:dkeyp-110c7.4 | 5 | 36468070 | 36473663 | protein\_coding | | ENSDARG00000092632 | si:dkeyp-116h7.2 | 21 | 17947815 | 17976724 | lincRNA | | ENSDARG00000011602 | si:dkeyp-117h8.2 | 20 | 25760749 | 25810259 | protein\_coding | | ENSDARG00000075277 | si:dkeyp-14d3.1 | 8 | 35483484 | 36031303 | protein\_coding | | ENSDARG00000093936 | si:dkeyp-1h4.6 | 18 | 7632446 | 7634646 | protein\_coding | | ENSDARG00000063008 | si:dkeyp-27e10.3 | 4 | 9783385 | 9810083 | protein\_coding | | ENSDARG00000092269 | si:dkeyp-41f9.4 | 10 | 9353092 | 9358891 | protein\_coding | | ENSDARG00000087165 | si:dkeyp-69c1.7 | 21 | 22593692 | 22597554 | protein\_coding | | ENSDARG00000073704 | si:dkeyp-72g9.4 | 21 | 32442482 | 32453826 | protein\_coding | | ENSDARG00000095347 | si:dkeyp-72h1.1 | 20 | 18652074 | 18695976 | protein\_coding | | ENSDARG00000090185 | si:dkeyp-73b11.8 | 25 | 26311376 | 26315051 | protein\_coding | | ENSDARG00000076043 | si:dkeyp-73d8.9 | 2 | 32659120 | 32660520 | protein\_coding | | ENSDARG00000069292 | si:dkeyp-89c11.1 | 4 | 25662522 | 25677365 | protein\_coding | | ENSDARG00000095509 | si:dkeyp-91h9.1 | 5 | 20039192 | 20042151 | processed\_transcript | | ENSDARG00000096721 | si:rp71-1c10.11 | 12 | 28849132 | 28856100 | protein\_coding | | ENSDARG00000035861 | si:rp71-39b20.4 | 2 | 24840153 | 24849534 | protein\_coding | | ENSDARG00000058606 | sik1 | 9 | 9133879 | 9143725 | protein\_coding | | ENSDARG00000071173 | slc12a10.2 | 7 | 4194404 | 4242422 | protein\_coding | | ENSDARG00000075815 | slc12a5a | 6 | 55437979 | 55574102 | protein\_coding | | ENSDARG00000078187 | slc12a5b | 8 | 37251920 | 37358658 | protein\_coding | | ENSDARG00000045638 | slc13a1 | 25 | 27480644 | 27489521 | protein\_coding | | ENSDARG00000069478 | slc13a3 | 8 | 28658004 | 28685072 | protein\_coding | | ENSDARG00000077691 | slc13a5a | 21 | 40030116 | 40059879 | protein\_coding | | ENSDARG00000001127 | slc17a6a | 25 | 34746018 | 34766326 | protein\_coding | | ENSDARG00000041150 | slc17a6b | 7 | 32544546 | 32562465 | protein\_coding | | ENSDARG00000016480 | slc17a7a | 3 | 16462774 | 16504288 | protein\_coding | | ENSDARG00000090106 | slc17a7b | 24 | 38670118 | 38710024 | protein\_coding | | ENSDARG00000057728 | slc17a8 | 18 | 15647655 | 15666895 | protein\_coding | | ENSDARG00000020212 | slc1a1 | 7 | 69258243 | 69283431 | protein\_coding | | ENSDARG00000052138 | slc1a2a | 7 | 49592273 | 49607198 | protein\_coding | | ENSDARG00000102453 | slc1a2b | 25 | 36796114 | 36837199 | protein\_coding | | ENSDARG00000043148 | slc1a3b | 10 | 36611214 | 36648728 | protein\_coding | | ENSDARG00000010096 | slc1a6 | 11 | 6155388 | 6177802 | protein\_coding | | ENSDARG00000104204 | slc1a8a | 11 | 15010648 | 15032905 | protein\_coding | | ENSDARG00000020114 | slc20a1a | 8 | 31047865 | 31065783 | protein\_coding | | ENSDARG00000041431 | slc24a1 | 18 | 19016997 | 19050894 | protein\_coding | | ENSDARG00000042988 | slc24a2 | 1 | 11322873 | 11357590 | protein\_coding | | ENSDARG00000006760 | slc24a3 | 13 | 26943451 | 27079551 | protein\_coding | | ENSDARG00000038731 | slc25a36a | 2 | 39765645 | 39793420 | protein\_coding | | ENSDARG00000027424 | slc25a3a | 4 | 16855997 | 16864488 | protein\_coding | | ENSDARG00000027355 | slc25a4 | 1 | 16981673 | 16986152 | protein\_coding | | ENSDARG00000005853 | slc25a6 | 9 | 35057391 | 35062715 | protein\_coding | | ENSDARG00000054127 | slc26a6l | 6 | 40726018 | 40735156 | protein\_coding | | ENSDARG00000104949 | slc27a1b | 1 | 57808977 | 57826447 | protein\_coding | | ENSDARG00000053961 | slc2a11a | 6 | 40978119 | 40992937 | protein\_coding | | ENSDARG00000007412 | slc2a1b | 6 | 48069936 | 48095583 | protein\_coding | | ENSDARG00000059775 | slc32a1 | 6 | 60147713 | 60152730 | protein\_coding | | ENSDARG00000028824 | slc34a1a | 14 | 818225 | 840728 | protein\_coding | | ENSDARG00000053003 | slc35f1 | 20 | 42101392 | 42306519 | protein\_coding | | ENSDARG00000103124 | slc35f4 | 17 | 44387473 | 44471046 | protein\_coding | | ENSDARG00000056262 | slc35g2a | 2 | 25334764 | 25340807 | protein\_coding | | ENSDARG00000027065 | slc38a3a | 11 | 34562106 | 34652864 | protein\_coding | | ENSDARG00000059682 | slc43a3a | 1 | 43440003 | 43460114 | protein\_coding | | ENSDARG00000004302 | slc45a1 | 11 | 40385267 | 40409925 | protein\_coding | | ENSDARG00000060051 | slc47a2 | 21 | 39968598 | 39991977 | protein\_coding | | ENSDARG00000060303 | slc4a10b | 9 | 51758481 | 51847346 | protein\_coding | | ENSDARG00000075532 | slc4a11 | 13 | 13639988 | 13791704 | protein\_coding | | ENSDARG00000104387 | slc4a5 | 10 | 44977460 | 45054832 | protein\_coding | | ENSDARG00000015531 | slc4a8 | 6 | 40356888 | 40408398 | protein\_coding | | ENSDARG00000074860 | slc5a7a | 1 | 33510663 | 33529425 | protein\_coding | | ENSDARG00000003697 | slc5a8l | 6 | 49064636 | 49080049 | protein\_coding | | ENSDARG00000087981 | slc6a11b | 11 | 1065328 | 1104981 | protein\_coding | | ENSDARG00000062821 | slc6a15 | 18 | 16162509 | 16193409 | protein\_coding | | ENSDARG00000068787 | slc6a17 | 8 | 24842014 | 24871853 | protein\_coding | | ENSDARG00000045944 | slc6a1a | 23 | 4898862 | 4919537 | protein\_coding | | ENSDARG00000039647 | slc6a1b | 11 | 970512 | 997895 | protein\_coding | | ENSDARG00000041205 | slc6a1l | 4 | 26983691 | 27085936 | protein\_coding | | ENSDARG00000016141 | slc6a2 | 7 | 35076999 | 35108709 | protein\_coding | | ENSDARG00000061165 | slc6a4a | 15 | 28676149 | 28685626 | protein\_coding | | ENSDARG00000067964 | slc6a5 | 7 | 16977309 | 16995846 | protein\_coding | | ENSDARG00000012534 | slc6a6a | 8 | 7100518 | 7152381 | protein\_coding | | ENSDARG00000016439 | slc7a1 | 10 | 24476246 | 24524019 | protein\_coding | | ENSDARG00000008100 | slc7a10a | 7 | 38044391 | 38069416 | protein\_coding | | ENSDARG00000055154 | slc8a4a | 21 | 27010638 | 27149220 | protein\_coding | | ENSDARG00000037145 | slc8a4b | 7 | 59891923 | 59935905 | protein\_coding | | ENSDARG00000061896 | slco2a1 | 6 | 27633462 | 27674697 | protein\_coding | | ENSDARG00000054609 | slco2b1 | 21 | 40116962 | 40151124 | protein\_coding | | ENSDARG00000018726 | slco3a1 | 18 | 25087452 | 25190698 | protein\_coding | | ENSDARG00000006636 | slitrk2 | 14 | 18957255 | 18960286 | protein\_coding | | ENSDARG00000074739 | slitrk3b | 2 | 36832829 | 36837167 | protein\_coding | | ENSDARG00000079781 | slitrk4 | 14 | 18361604 | 18366918 | protein\_coding | | ENSDARG00000055754 | smc1a | 23 | 28766737 | 28787441 | protein\_coding | | ENSDARG00000015803 | smcr8b | 1 | 7850587 | 7855939 | protein\_coding | | ENSDARG00000095826 | smdt1a | 3 | 1417891 | 1421483 | protein\_coding | | ENSDARG00000036967 | smox | 1 | 41147988 | 41164822 | protein\_coding | | ENSDARG00000020609 | snap25a | 20 | 35010165 | 35053351 | protein\_coding | | ENSDARG00000058117 | snap25b | 17 | 12143918 | 12231242 | protein\_coding | | ENSDARG00000098809 | snap91 | 16 | 33051658 | 33105239 | protein\_coding | | ENSDARG00000104945 | sncb | 14 | 45619121 | 45653489 | protein\_coding | | ENSDARG00000061101 | snx19a | 5 | 29751080 | 29780440 | protein\_coding | | ENSDARG00000078057 | snx8b | 1 | 11194759 | 11213620 | protein\_coding | | ENSDARG00000054253 | sobpa | 13 | 32609691 | 32665579 | protein\_coding | | ENSDARG00000045557 | socs2 | 4 | 18443206 | 18447923 | protein\_coding | | ENSDARG00000075455 | soga3b | 23 | 40559253 | 40573494 | protein\_coding | | ENSDARG00000069361 | spa17 | 15 | 22331745 | 22351933 | protein\_coding | | ENSDARG00000074989 | sparcl1 | 1 | 36693480 | 36720533 | protein\_coding | | ENSDARG00000068782 | spg20a | 10 | 34945468 | 34963617 | protein\_coding | | ENSDARG00000017429 | sphkap | 18 | 46397891 | 46466483 | protein\_coding | | ENSDARG00000075393 | spock2 | 13 | 29924671 | 30001389 | protein\_coding | | ENSDARG00000070266 | spock3 | 1 | 19941998 | 20013359 | protein\_coding | | ENSDARG00000010029 | spon1a | 18 | 28586400 | 28901951 | protein\_coding | | ENSDARG00000002732 | spon2b | 14 | 17071287 | 17076881 | protein\_coding | | ENSDARG00000040358 | spred3 | 18 | 44603333 | 44617798 | protein\_coding | | ENSDARG00000056004 | sprn | 13 | 21552320 | 21557726 | protein\_coding | | ENSDARG00000057665 | sprn2 | 10 | 20678579 | 20679717 | protein\_coding | | ENSDARG00000097683 | sptbn5 | 17 | 29107227 | 29177256 | protein\_coding | | ENSDARG00000091658 | sptssb | 15 | 1715053 | 1722295 | protein\_coding | | ENSDARG00000043587 | srd5a2a | 1 | 50422872 | 50429329 | protein\_coding | | ENSDARG00000029368 | srpk2 | 25 | 190964 | 211940 | protein\_coding | | ENSDARG00000101057 | srsf7b | 11 | 44107327 | 44115528 | protein\_coding | | ENSDARG00000099727 | ssbp2 | 10 | 43101973 | 43200621 | protein\_coding | | ENSDARG00000040799 | sst1.1 | 15 | 36299017 | 36300882 | protein\_coding | | ENSDARG00000031649 | sst3 | 23 | 28804838 | 28805772 | protein\_coding | | ENSDARG00000104922 | sstr1a | 17 | 10091529 | 10093061 | protein\_coding | | ENSDARG00000038533 | st3gal1l2 | 19 | 4819690 | 4829438 | protein\_coding | | ENSDARG00000043814 | st6galnac1.1 | 12 | 20556313 | 20563750 | protein\_coding | | ENSDARG00000036913 | st6galnac6 | 21 | 3669162 | 3680843 | protein\_coding | | ENSDARG00000036584 | st8sia5 | 21 | 5044869 | 5076228 | protein\_coding | | ENSDARG00000006137 | star | 8 | 44709948 | 44714031 | protein\_coding | | ENSDARG00000026801 | stau2 | 24 | 12873167 | 13095638 | protein\_coding | | ENSDARG00000058476 | stc1l | 10 | 42849625 | 42856582 | protein\_coding | | ENSDARG00000102206 | stc2b | 21 | 41114832 | 41134536 | protein\_coding | | ENSDARG00000062887 | steap2 | 24 | 31895247 | 31909155 | protein\_coding | | ENSDARG00000055901 | steap4 | 16 | 27800249 | 27814549 | protein\_coding | | ENSDARG00000033655 | stmn1b | 16 | 34569485 | 34573176 | protein\_coding | | ENSDARG00000033234 | stmn2a | 16 | 16257052 | 16274342 | protein\_coding | | ENSDARG00000043932 | stmn4l | 17 | 12544466 | 12554996 | protein\_coding | | ENSDARG00000057035 | stoml3b | 10 | 24671777 | 24678779 | protein\_coding | | ENSDARG00000044605 | stx12l | 16 | 7709196 | 7724021 | protein\_coding | | ENSDARG00000000503 | stx1b | 19 | 5241820 | 5338213 | protein\_coding | | ENSDARG00000001994 | stxbp1a | 21 | 13764850 | 13824293 | protein\_coding | | ENSDARG00000056036 | stxbp1b | 5 | 28373111 | 28402105 | protein\_coding | | ENSDARG00000002656 | stxbp5a | 20 | 31843070 | 32003089 | protein\_coding | | ENSDARG00000006383 | stxbp5l | 9 | 27605008 | 27837937 | protein\_coding | | ENSDARG00000028354 | stxbp6l | 20 | 9841585 | 9861183 | protein\_coding | | ENSDARG00000013838 | sulf2b | 23 | 15447745 | 15574153 | protein\_coding | | ENSDARG00000003475 | sult1st5 | 23 | 24562536 | 24567270 | protein\_coding | | ENSDARG00000103785 | sult2st2 | 12 | 642393 | 650599 | protein\_coding | | ENSDARG00000042920 | sult3st3 | 22 | 15289272 | 15291897 | protein\_coding | | ENSDARG00000101967 | sult4a1 | 4 | 8796218 | 8809829 | protein\_coding | | ENSDARG00000026335 | susd4 | 17 | 45546201 | 45569379 | protein\_coding | | ENSDARG00000059945 | sv2a | 16 | 46137709 | 46235989 | protein\_coding | | ENSDARG00000057427 | sv2ba | 7 | 15017806 | 15057728 | protein\_coding | | ENSDARG00000060711 | sv2bb | 25 | 10941651 | 10992043 | protein\_coding | | ENSDARG00000059997 | sv2c | 5 | 45389893 | 45466405 | protein\_coding | | ENSDARG00000056833 | svopa | 5 | 19638817 | 19662085 | protein\_coding | | ENSDARG00000008186 | syk | 10 | 5533534 | 5581183 | protein\_coding | | ENSDARG00000060368 | syn1 | 8 | 31110316 | 31139087 | protein\_coding | | ENSDARG00000045945 | syn2a | 23 | 4816374 | 4839861 | protein\_coding | | ENSDARG00000101054 | syn2b | 11 | 648095 | 736223 | protein\_coding | | ENSDARG00000035944 | sync | 19 | 31403311 | 31410466 | protein\_coding | | ENSDARG00000042390 | syndig1l | 20 | 27922954 | 27965874 | protein\_coding | | ENSDARG00000063068 | syne1b | 17 | 7687722 | 8016202 | protein\_coding | | ENSDARG00000063713 | syngap1a | 19 | 231833 | 276595 | protein\_coding | | ENSDARG00000069765 | syngap1b | 16 | 45557128 | 45724504 | protein\_coding | | ENSDARG00000002564 | syngr1a | 3 | 21121079 | 21149825 | protein\_coding | | ENSDARG00000014871 | syngr3a | 3 | 40028358 | 40038343 | protein\_coding | | ENSDARG00000025034 | syngr3b | 1 | 7731872 | 7744650 | protein\_coding | | ENSDARG00000062350 | synm | 18 | 20870595 | 20880109 | protein\_coding | | ENSDARG00000042974 | sypa | 8 | 22910172 | 22929384 | protein\_coding | | ENSDARG00000002230 | sypb | 8 | 49415307 | 49443241 | protein\_coding | | ENSDARG00000045750 | syt10 | 4 | 10889906 | 10910237 | protein\_coding | | ENSDARG00000057913 | syt11a | 19 | 24398243 | 24450487 | protein\_coding | | ENSDARG00000056105 | syt11b | 16 | 28907791 | 28921489 | protein\_coding | | ENSDARG00000101776 | syt12 | 25 | 7287888 | 7315280 | protein\_coding | | ENSDARG00000030614 | syt1a | 4 | 21408781 | 21594424 | protein\_coding | | ENSDARG00000075830 | syt3 | 3 | 30384523 | 30494377 | protein\_coding | | ENSDARG00000036505 | syt4 | 7 | 23636535 | 23644961 | protein\_coding | | ENSDARG00000037941 | syt5a | 3 | 32010668 | 32019453 | protein\_coding | | ENSDARG00000011640 | syt5b | 24 | 7657261 | 7670037 | protein\_coding | | ENSDARG00000076730 | syt6a | 23 | 24876424 | 24954196 | protein\_coding | | ENSDARG00000031463 | syt6b | 11 | 24383202 | 24426415 | protein\_coding | | ENSDARG00000078060 | syt7b | 7 | 12786706 | 13085563 | protein\_coding | | ENSDARG00000003994 | syt9a | 18 | 29010482 | 29080296 | protein\_coding | | ENSDARG00000029239 | syt9b | 25 | 15745270 | 15809503 | protein\_coding | | ENSDARG00000093089 | tac3a | 23 | 28212948 | 28214878 | protein\_coding | | ENSDARG00000043475 | tagapb | 20 | 13752495 | 13777459 | protein\_coding | | ENSDARG00000045408 | tagln | 15 | 582941 | 590378 | protein\_coding | | ENSDARG00000079805 | tagln3a | 5 | 67192074 | 67200353 | protein\_coding | | ENSDARG00000094052 | taok3b | 5 | 31562531 | 31582575 | protein\_coding | | ENSDARG00000092774 | tarsl2 | 18 | 321698 | 337202 | protein\_coding | | ENSDARG00000019525 | tbcelb | 5 | 62554860 | 62592686 | protein\_coding | | ENSDARG00000061473 | tbkbp1 | 12 | 28459961 | 28563421 | protein\_coding | | ENSDARG00000004473 | tbx21 | 12 | 28634287 | 28655915 | protein\_coding | | ENSDARG00000007344 | tcap | 3 | 21110046 | 21111912 | protein\_coding | | ENSDARG00000016835 | tcirg1a | 1 | 44170905 | 44199860 | protein\_coding | | ENSDARG00000068088 | tcnl | 5 | 30017762 | 30020472 | protein\_coding | | ENSDARG00000001463 | tdh2 | 17 | 25395642 | 25406930 | protein\_coding | | ENSDARG00000071429 | tdo2a | 14 | 36397749 | 36410279 | protein\_coding | | ENSDARG00000074086 | tecpr1b | 3 | 61060347 | 61104253 | protein\_coding | | ENSDARG00000101585 | tecra | 3 | 45251917 | 45270101 | protein\_coding | | ENSDARG00000003403 | tenm1 | 5 | 20790407 | 21015713 | protein\_coding | | ENSDARG00000034718 | tfpia | 9 | 42618849 | 42682661 | protein\_coding | | ENSDARG00000099979 | tgfbr3 | 6 | 24299098 | 24498943 | protein\_coding | | ENSDARG00000030621 | th | 25 | 23957793 | 23976952 | protein\_coding | | ENSDARG00000010785 | thbs1b | 20 | 29134730 | 29148944 | protein\_coding | | ENSDARG00000095022 | thsd7ba | 9 | 10186009 | 10386265 | protein\_coding | | ENSDARG00000051749 | tigara | 25 | 16866572 | 16873522 | protein\_coding | | ENSDARG00000077257 | timd4 | 21 | 29053957 | 29063415 | protein\_coding | | ENSDARG00000060124 | timm17b | 8 | 44927217 | 44932171 | protein\_coding | | ENSDARG00000061226 | timp2a | 12 | 33889964 | 33934381 | protein\_coding | | ENSDARG00000029689 | tkta | 11 | 36722153 | 36768292 | protein\_coding | | ENSDARG00000002391 | tlcd1 | 21 | 26043520 | 26053269 | protein\_coding | | ENSDARG00000060668 | tm4sf18 | 22 | 38206663 | 38215827 | protein\_coding | | ENSDARG00000029057 | tm6sf2 | 22 | 17759579 | 17794579 | protein\_coding | | ENSDARG00000033104 | tmc2a | 5 | 54598757 | 54629158 | protein\_coding | | ENSDARG00000031757 | tmc4 | 16 | 32075060 | 32096123 | protein\_coding | | ENSDARG00000051824 | tmeff2a | 9 | 24438315 | 24602139 | protein\_coding | | ENSDARG00000058699 | tmeff2b | 6 | 8523796 | 8629707 | protein\_coding | | ENSDARG00000095259 | tmem119a | 5 | 19925038 | 19933983 | protein\_coding | | ENSDARG00000061562 | tmem121a | 17 | 33549255 | 33599852 | protein\_coding | | ENSDARG00000035163 | tmem136b | 5 | 58166645 | 58171262 | protein\_coding | | ENSDARG00000069590 | tmem150c | 10 | 5041673 | 5058519 | protein\_coding | | ENSDARG00000079908 | tmem151a | 21 | 25598165 | 25611154 | protein\_coding | | ENSDARG00000079858 | tmem163a | 9 | 23444374 | 23507251 | protein\_coding | | ENSDARG00000078659 | tmem176 | 1 | 44234139 | 44239650 | protein\_coding | | ENSDARG00000089399 | tmem176l.2 | 16 | 46673056 | 46678680 | protein\_coding | | ENSDARG00000096874 | tmem176l.3b | 7 | 66342282 | 66352874 | protein\_coding | | ENSDARG00000020758 | tmem178 | 11 | 30266472 | 30283922 | protein\_coding | | ENSDARG00000006747 | tmem178b | 4 | 12112305 | 12240311 | protein\_coding | | ENSDARG00000090140 | tmem196b | 16 | 19840059 | 19875782 | protein\_coding | | ENSDARG00000031540 | tmem200a | 20 | 2201154 | 2215338 | protein\_coding | | ENSDARG00000045273 | tmem204 | 24 | 37966105 | 37990081 | protein\_coding | | ENSDARG00000041735 | tmem237a | 9 | 13743176 | 13757734 | protein\_coding | | ENSDARG00000087916 | tmem240a | 23 | 24645271 | 24656065 | protein\_coding | | ENSDARG00000073911 | tmem255a | 14 | 33095541 | 33114350 | protein\_coding | | ENSDARG00000095937 | tmem265 | 14 | 30240367 | 30247547 | protein\_coding | | ENSDARG00000009430 | tmem30c | 9 | 30183693 | 30196731 | protein\_coding | | ENSDARG00000026771 | tmem41ab | 1 | 29618212 | 29634105 | protein\_coding | | ENSDARG00000004158 | tmem63c | 17 | 44666401 | 44809896 | protein\_coding | | ENSDARG00000038296 | tmem86b | 3 | 16612683 | 16620518 | protein\_coding | | ENSDARG00000068456 | tmem91 | 18 | 36033654 | 36065354 | protein\_coding | | ENSDARG00000100969 | tmprss13a | 15 | 12394744 | 12428992 | protein\_coding | | ENSDARG00000063149 | tmtc1 | 4 | 9393510 | 9465332 | protein\_coding | | ENSDARG00000038894 | tmx3 | 2 | 27662772 | 27674547 | protein\_coding | | ENSDARG00000001807 | tnfrsf21 | 20 | 35895119 | 35938691 | protein\_coding | | ENSDARG00000069376 | tnfsf12 | 7 | 20404974 | 20413371 | protein\_coding | | ENSDARG00000090646 | tnk2a | 24 | 25891352 | 25912744 | protein\_coding | | ENSDARG00000052615 | tnmd | 14 | 40274293 | 40355440 | protein\_coding | | ENSDARG00000073766 | tnni1d | 4 | 26368161 | 26372301 | protein\_coding | | ENSDARG00000045592 | tnni2a.1 | 25 | 30644593 | 30659073 | protein\_coding | | ENSDARG00000005841 | tnni2a.2 | 25 | 30661130 | 30671126 | protein\_coding | | ENSDARG00000013752 | tnni2a.3 | 25 | 30686679 | 30698314 | protein\_coding | | ENSDARG00000029995 | tnni2b.2 | 7 | 39115121 | 39121848 | protein\_coding | | ENSDARG00000037954 | tnnt1 | 3 | 32025426 | 32039164 | protein\_coding | | ENSDARG00000100694 | tnnt2b | 6 | 54753028 | 54787964 | protein\_coding | | ENSDARG00000045822 | tnnt2e | 4 | 7499687 | 7536058 | protein\_coding | | ENSDARG00000068457 | tnnt3b | 7 | 39173826 | 39189342 | protein\_coding | | ENSDARG00000078842 | tns1a | 6 | 18705859 | 18814425 | protein\_coding | | ENSDARG00000001760 | tnxba | 16 | 16713265 | 16741787 | protein\_coding | | ENSDARG00000042548 | tpd52l1 | 20 | 39789158 | 39833399 | protein\_coding | | ENSDARG00000057239 | tph2 | 18 | 17000698 | 17011990 | protein\_coding | | ENSDARG00000040988 | tpi1b | 16 | 12345335 | 12364092 | protein\_coding | | ENSDARG00000035338 | tppp2 | 5 | 36456665 | 36465588 | protein\_coding | | ENSDARG00000030463 | tppp3 | 7 | 34958168 | 34970794 | protein\_coding | | ENSDARG00000068240 | trim110 | 2 | 38145163 | 38159846 | protein\_coding | | ENSDARG00000101264 | trim35-12 | 3 | 44957538 | 44965103 | protein\_coding | | ENSDARG00000003970 | trim35-13 | 3 | 52352376 | 52360084 | protein\_coding | | ENSDARG00000005397 | trim3b | 10 | 26169143 | 26227490 | protein\_coding | | ENSDARG00000012367 | trim46a | 16 | 23438512 | 23464505 | protein\_coding | | ENSDARG00000058649 | trim46b | 19 | 8661756 | 8685504 | protein\_coding | | ENSDARG00000054332 | trim66 | 7 | 28151968 | 28191858 | protein\_coding | | ENSDARG00000039123 | trim9 | 13 | 36742472 | 36785295 | protein\_coding | | ENSDARG00000031875 | trpa1b | 24 | 13490968 | 13569689 | protein\_coding | | ENSDARG00000061203 | trpc1 | 24 | 5811354 | 5844423 | protein\_coding | | ENSDARG00000056625 | trpc6a | 21 | 22764652 | 22775844 | protein\_coding | | ENSDARG00000011259 | trpm1a | 7 | 29042218 | 29070214 | protein\_coding | | ENSDARG00000095696 | trpm2 | 4 | 75445046 | 75464503 | protein\_coding | | ENSDARG00000014496 | trpv6 | 16 | 12403087 | 12428961 | protein\_coding | | ENSDARG00000042189 | tspan33b | 18 | 10998743 | 11026692 | protein\_coding | | ENSDARG00000053559 | tspan3b | 7 | 29806457 | 29811781 | protein\_coding | | ENSDARG00000008407 | tspan7b | 22 | 11411023 | 11463554 | protein\_coding | | ENSDARG00000056019 | ttbk1a | 11 | 30416116 | 30480766 | protein\_coding | | ENSDARG00000056659 | ttbk1b | 17 | 22009370 | 22049638 | protein\_coding | | ENSDARG00000011693 | ttc36 | 18 | 42782718 | 42786565 | protein\_coding | | ENSDARG00000068421 | ttc9b | 18 | 36825537 | 36884378 | protein\_coding | | ENSDARG00000027584 | ttpa | 2 | 26991838 | 26997514 | protein\_coding | | ENSDARG00000076804 | ttyh1 | 16 | 13821808 | 13899788 | protein\_coding | | ENSDARG00000007678 | ttyh3b | 1 | 11094744 | 11163553 | protein\_coding | | ENSDARG00000045014 | tuba2 | 11 | 3268364 | 3275303 | protein\_coding | | ENSDARG00000078210 | tulp1b | 6 | 54530840 | 54554400 | protein\_coding | | ENSDARG00000063056 | tulp4b | 17 | 8026215 | 8053834 | protein\_coding | | ENSDARG00000090481 | tusc5a | 15 | 24909880 | 24934561 | protein\_coding | | ENSDARG00000077399 | ubash3bb | 15 | 21341003 | 21384906 | protein\_coding | | ENSDARG00000079276 | ube2ql1 | 19 | 28372786 | 28382754 | protein\_coding | | ENSDARG00000001686 | uckl1a | 8 | 22988423 | 23007297 | protein\_coding | | ENSDARG00000027799 | ucmaa | 4 | 7832998 | 7841860 | protein\_coding | | ENSDARG00000005485 | ucmab | 25 | 7858887 | 7864844 | protein\_coding | | ENSDARG00000091209 | ucp3 | 10 | 36706163 | 36729623 | protein\_coding | | ENSDARG00000005578 | ugp2a | 1 | 50353357 | 50371828 | protein\_coding | | ENSDARG00000006220 | ugt1a1 | 9 | 9998614 | 10032338 | protein\_coding | | ENSDARG00000016479 | ugt5a1 | 25 | 28964664 | 28972375 | protein\_coding | | ENSDARG00000104995 | ugt5b2.1 | 1 | 8961164 | 8968614 | protein\_coding | | ENSDARG00000099276 | ugt5b3 | 1 | 8945048 | 8968614 | protein\_coding | | ENSDARG00000091916 | ugt5b4 | 1 | 8946885 | 8968585 | protein\_coding | | ENSDARG00000058048 | ugt5e1 | 8 | 14018761 | 14022734 | protein\_coding | | ENSDARG00000097205 | ulk2 | 15 | 20907429 | 20959044 | protein\_coding | | ENSDARG00000017391 | unc13ba | 5 | 18593394 | 18735478 | protein\_coding | | ENSDARG00000098290 | unc80 | 6 | 16393850 | 16434596 | protein\_coding | | ENSDARG00000034321 | uo:ion006 | 24 | 29927996 | 29990985 | protein\_coding | | ENSDARG00000069297 | upf3a | 9 | 35093786 | 35106364 | protein\_coding | | ENSDARG00000040869 | upp1 | 16 | 16296679 | 16304638 | protein\_coding | | ENSDARG00000070394 | uroc1 | 6 | 40046665 | 40061183 | protein\_coding | | ENSDARG00000051876 | ush1c | 25 | 22546406 | 22606685 | protein\_coding | | ENSDARG00000094280 | usp21 | 23 | 20760997 | 20775520 | protein\_coding | | ENSDARG00000074120 | usp2b | 5 | 62395349 | 62415841 | protein\_coding | | ENSDARG00000021150 | valopa | 13 | 51256371 | 51284971 | protein\_coding | | ENSDARG00000031283 | vamp1 | 19 | 9541507 | 9554010 | protein\_coding | | ENSDARG00000056877 | vamp2 | 10 | 22868912 | 22876033 | protein\_coding | | ENSDARG00000004305 | vangl1 | 9 | 33198538 | 33244694 | protein\_coding | | ENSDARG00000073713 | vav3b | 2 | 16108059 | 16231597 | protein\_coding | | ENSDARG00000078247 | vip | 13 | 46698120 | 46701742 | protein\_coding | | ENSDARG00000079443 | vipb | 11 | 43109331 | 43121897 | protein\_coding | | ENSDARG00000028878 | vipr1a | 2 | 21571847 | 21642053 | protein\_coding | | ENSDARG00000012353 | vipr2 | 24 | 27176952 | 27269390 | protein\_coding | | ENSDARG00000079782 | vmhcl | 2 | 24618673 | 24633992 | protein\_coding | | ENSDARG00000023228 | vsnl1a | 20 | 35479559 | 35526858 | protein\_coding | | ENSDARG00000044053 | vsnl1b | 17 | 6558345 | 6581308 | protein\_coding | | ENSDARG00000010154 | vstm4a | 13 | 30820705 | 30837334 | protein\_coding | | ENSDARG00000055388 | vtna | 15 | 28269294 | 28276050 | protein\_coding | | ENSDARG00000075468 | vwa1 | 23 | 39739749 | 39773608 | protein\_coding | | ENSDARG00000076495 | vwc2 | 13 | 16019662 | 16060682 | protein\_coding | | ENSDARG00000077231 | vwf | 18 | 7191182 | 7269043 | protein\_coding | | ENSDARG00000059466 | wasf3a | 5 | 67329323 | 67348122 | protein\_coding | | ENSDARG00000056605 | wbp2 | 12 | 44829952 | 44847686 | protein\_coding | | ENSDARG00000042418 | wbscr17 | 15 | 16585763 | 16610450 | protein\_coding | | ENSDARG00000075098 | wdr17 | 1 | 38040141 | 38085191 | protein\_coding | | ENSDARG00000090418 | wdsub1 | 6 | 11771216 | 11798468 | protein\_coding | | ENSDARG00000089187 | wfdc2 | 23 | 12483643 | 12488808 | protein\_coding | | ENSDARG00000077882 | wisp2 | 23 | 26067179 | 26081162 | protein\_coding | | ENSDARG00000071048 | wisp3 | 20 | 288118 | 296230 | protein\_coding | | ENSDARG00000104989 | wnk2 | 11 | 36869638 | 36909302 | protein\_coding | | ENSDARG00000089361 | wu:fb59d01 | 10 | 2559635 | 2568132 | protein\_coding | | ENSDARG00000043460 | wu:fj39g12 | 16 | 17296274 | 17299777 | protein\_coding | | ENSDARG00000043410 | xkr6b | 20 | 18901942 | 19015793 | protein\_coding | | ENSDARG00000026333 | xkr7 | 23 | 9054198 | 9080056 | protein\_coding | | ENSDARG00000076820 | xkr8.2 | 19 | 25410047 | 25421386 | protein\_coding | | ENSDARG00000061692 | yjefn3 | 22 | 18364293 | 18439676 | protein\_coding | | ENSDARG00000067626 | ywhag1 | 5 | 3172127 | 3215489 | protein\_coding | | ENSDARG00000071658 | ywhag2 | 15 | 15412468 | 15421156 | protein\_coding | | ENSDARG00000042383 | zbtb25 | 20 | 27808676 | 27812880 | protein\_coding | | ENSDARG00000105255 | zbtb4 | 12 | 22407531 | 22419724 | protein\_coding | | ENSDARG00000034757 | zdhhc2 | 14 | 30086642 | 30111904 | protein\_coding | | ENSDARG00000075170 | zdhhc22 | 17 | 44653960 | 44662459 | protein\_coding | | ENSDARG00000042872 | zdhhc8a | 10 | 426624 | 452977 | protein\_coding | | ENSDARG00000078416 | zeb2b | 6 | 1149464 | 1195487 | protein\_coding | | ENSDARG00000099870 | zgc:101560 | 4 | 7382540 | 7397683 | protein\_coding | | ENSDARG00000038293 | zgc:103559 | 6 | 27136059 | 27305208 | protein\_coding | | ENSDARG00000036068 | zgc:103601 | 7 | 37922068 | 37923706 | protein\_coding | | ENSDARG00000103413 | zgc:109949 | 3 | 7727364 | 7753267 | protein\_coding | | ENSDARG00000099217 | zgc:109982 | 6 | 23977145 | 23987470 | protein\_coding | | ENSDARG00000030632 | zgc:110191 | 11 | 4129421 | 4156386 | protein\_coding | | ENSDARG00000079497 | zgc:110340 | 5 | 27266496 | 27267504 | protein\_coding | | ENSDARG00000052497 | zgc:110789 | 18 | 12066267 | 12083414 | protein\_coding | | ENSDARG00000053323 | zgc:112285 | 12 | 34950573 | 34968948 | protein\_coding | | ENSDARG00000009215 | zgc:112437 | 21 | 25736925 | 25738000 | protein\_coding | | ENSDARG00000044894 | zgc:113307 | 11 | 20889758 | 20900998 | protein\_coding | | ENSDARG00000008553 | zgc:136872 | 17 | 3048813 | 3091582 | protein\_coding | | ENSDARG00000011983 | zgc:136908 | 1 | 55109335 | 55133079 | protein\_coding | | ENSDARG00000055192 | zgc:136930 | 2 | 32796920 | 32807669 | protein\_coding | | ENSDARG00000068876 | zgc:153031 | 18 | 15163698 | 15171476 | protein\_coding | | ENSDARG00000104436 | zgc:153426 | 3 | 45413848 | 45420953 | protein\_coding | | ENSDARG00000038981 | zgc:153615 | 2 | 9831553 | 9848890 | protein\_coding | | ENSDARG00000099238 | zgc:153845 | 3 | 58992291 | 58993782 | protein\_coding | | ENSDARG00000070229 | zgc:158258 | 11 | 37416073 | 37425814 | protein\_coding | | ENSDARG00000104460 | zgc:158654 | 6 | 23652784 | 23681772 | protein\_coding | | ENSDARG00000077217 | zgc:162184 | 17 | 45665584 | 45695971 | protein\_coding | | ENSDARG00000069548 | zgc:162780 | 9 | 28882846 | 28886253 | protein\_coding | | ENSDARG00000098702 | zgc:165603 | 2 | 51866335 | 51899077 | protein\_coding | | ENSDARG00000021241 | zgc:165604 | 21 | 27521686 | 27533521 | protein\_coding | | ENSDARG00000104713 | zgc:165653 | 12 | 48643750 | 48665099 | protein\_coding | | ENSDARG00000074415 | zgc:171482 | 13 | 39530720 | 39705109 | protein\_coding | | ENSDARG00000076146 | zgc:172075 | 7 | 2436941 | 2540728 | protein\_coding | | ENSDARG00000090722 | zgc:172246 | 20 | 1391367 | 1400179 | protein\_coding | | ENSDARG00000003632 | zgc:172270 | 11 | 35981791 | 35987919 | protein\_coding | | ENSDARG00000035340 | zgc:194261 | 5 | 36374617 | 36398359 | protein\_coding | | ENSDARG00000104654 | zgc:194626 | 7 | 51479870 | 51482003 | protein\_coding | | ENSDARG00000004577 | zgc:194665 | 25 | 19901760 | 19905843 | protein\_coding | | ENSDARG00000069407 | zgc:194990 | 8 | 13123975 | 13147284 | protein\_coding | | ENSDARG00000070484 | zgc:195001 | 3 | 30072021 | 30087662 | protein\_coding | | ENSDARG00000052948 | zgc:195023 | 8 | 48614629 | 48615582 | protein\_coding | | ENSDARG00000077138 | zgc:195173 | 24 | 33365234 | 33370583 | protein\_coding | | ENSDARG00000099728 | zgc:56622 | 18 | 13213892 | 13226233 | protein\_coding | | ENSDARG00000011371 | zgc:64106 | 21 | 11892177 | 11904141 | protein\_coding | | ENSDARG00000016301 | zgc:65894 | 1 | 55548092 | 55554617 | protein\_coding | | ENSDARG00000104582 | zgc:77058 | 21 | 45566843 | 45574016 | protein\_coding | | ENSDARG00000098162 | zgc:77748 | 3 | 13319592 | 13323085 | protein\_coding | | ENSDARG00000042387 | zgc:77752 | 18 | 7635077 | 7650238 | protein\_coding | | ENSDARG00000088366 | zgc:77938 | 14 | 35552863 | 35571668 | protein\_coding | | ENSDARG00000027009 | zgc:85777 | 19 | 41274733 | 41311480 | protein\_coding | | ENSDARG00000098239 | zgc:85932 | 15 | 43452449 | 43507427 | protein\_coding | | ENSDARG00000079111 | zgc:86725 | 25 | 13990645 | 14029098 | protein\_coding | | ENSDARG00000013749 | zgc:92113 | 23 | 396785 | 406648 | protein\_coding | | ENSDARG00000034048 | zgc:92275 | 21 | 11322596 | 11341687 | protein\_coding | | ENSDARG00000041060 | zgc:92326 | 15 | 30973828 | 31186924 | protein\_coding | | ENSDARG00000004141 | zgc:92630 | 15 | 15479601 | 15501508 | protein\_coding | | ENSDARG00000006065 | znf385b | 9 | 43437937 | 43736541 | protein\_coding | | ENSDARG00000086162 | znf385d | 16 | 49504725 | 49673101 | protein\_coding | | ENSDARG00000103648 | znf536 | 7 | 45980627 | 46242630 | protein\_coding | | ENSDARG00000102025 | znf644b | 6 | 24561927 | 24598331 | protein\_coding | | ENSDARG00000020475 | znrf1 | 25 | 13309669 | 13374890 | protein\_coding | | ENSDARG00000061835 | zpld1a | 10 | 29226131 | 29233910 | protein\_coding | | ENSDARG00000101826 | zwi | 3 | 20875413 | 20881133 | processed\_transcript | |
