# Supplementary material for: A high-resolution mRNA expression time course of embryonic development in zebrafish
Source: eLife. 2017 Nov 16;6:e30860. doi: 10.7554/eLife.30860 (PMC5690287; doi:10.7554/eLife.30860)
Supplement: Supplementary file 6. [file elife-30860-supp6.zip › biolayout-clusters-files/Cluster001.html]

Cluster001


# Cluster001: Detail

### Go to ZFA detail

## GO

| | GO ID | Description | Domain | Annotated | Expected | Observed | Adjusted p-value | Genes | Ensembl IDs | | --- | --- | --- | --- | --- | --- | --- | --- | --- | | GO:0001505 | regulation of neurotransmitter levels | biological\_process | 36 | 6.0 | 25 | 2.1e-02 | stx1b lin7a comta gad2 chata snap25a mao gad1b ache lin7b nrxn3a syn2a STX3 (1 of many) snap25b cplx4a syn1 slc6a4a nrxn3b ppfia3 syt7b doc2b syn2b rims2a syt12 rims2b | ENSDARG00000000503 ENSDARG00000013414 ENSDARG00000015337 ENSDARG00000015537 ENSDARG00000015854 ENSDARG00000020609 ENSDARG00000023712 ENSDARG00000027419 ENSDARG00000031796 ENSDARG00000037932 ENSDARG00000043746 ENSDARG00000045945 ENSDARG00000051981 ENSDARG00000058117 ENSDARG00000059978 ENSDARG00000060368 ENSDARG00000061165 ENSDARG00000062693 ENSDARG00000077053 ENSDARG00000078060 ENSDARG00000088293 ENSDARG00000101054 ENSDARG00000101606 ENSDARG00000101776 ENSDARG00000102690 | | GO:0007269 | neurotransmitter secretion | biological\_process | 27 | 4.5 | 18 | 2.4e-07 | stx1b lin7a snap25a lin7b nrxn3a syn2a STX3 (1 of many) snap25b cplx4a syn1 nrxn3b ppfia3 syt7b doc2b syn2b rims2a syt12 rims2b | ENSDARG00000000503 ENSDARG00000013414 ENSDARG00000020609 ENSDARG00000037932 ENSDARG00000043746 ENSDARG00000045945 ENSDARG00000051981 ENSDARG00000058117 ENSDARG00000059978 ENSDARG00000060368 ENSDARG00000062693 ENSDARG00000077053 ENSDARG00000078060 ENSDARG00000088293 ENSDARG00000101054 ENSDARG00000101606 ENSDARG00000101776 ENSDARG00000102690 | | GO:0050803 | regulation of synapse structure or activ... | biological\_process | 17 | 2.8 | 14 | 1.1e-06 | sypb neurod2 ache sypa slc24a2 nrxn3a shisa9a shisa9b slc17a8 shisa7a nrxn3b shisa7b neto1l syt12 | ENSDARG00000002230 ENSDARG00000016854 ENSDARG00000031796 ENSDARG00000042974 ENSDARG00000042988 ENSDARG00000043746 ENSDARG00000045145 ENSDARG00000052642 ENSDARG00000057728 ENSDARG00000062462 ENSDARG00000062693 ENSDARG00000063144 ENSDARG00000071596 ENSDARG00000101776 | | GO:0042391 | regulation of membrane potential | biological\_process | 58 | 9.6 | 28 | 2.8e-02 | nalcn scn8aa cacna1c cnga1 rnf207b scn8ab cacna1ba CNGA1 (1 of many) scn4ab cacna1aa CNGB1 npffl slc26a6l kcnh4b kcnab2b cacna1ea kcnh7 scn1lab cngb1a kcnh5b cnga3a hcn4l cacna1bb cacna1g scn12aa cngb3 ENSDARG00000101368 hcn1 | ENSDARG00000001835 ENSDARG00000005775 ENSDARG00000008398 ENSDARG00000012125 ENSDARG00000012409 ENSDARG00000018032 ENSDARG00000021735 ENSDARG00000029898 ENSDARG00000034588 ENSDARG00000037905 ENSDARG00000042107 ENSDARG00000045016 ENSDARG00000054127 ENSDARG00000061990 ENSDARG00000062134 ENSDARG00000062346 ENSDARG00000062687 ENSDARG00000062744 ENSDARG00000068242 ENSDARG00000069117 ENSDARG00000070726 ENSDARG00000074419 ENSDARG00000079295 ENSDARG00000089913 ENSDARG00000090724 ENSDARG00000101225 ENSDARG00000101368 ENSDARG00000104480 | | GO:0007165 | signal transduction | biological\_process | 1596 | 264.2 | 360 | 2.2e-03 | itsn2a pde6a tnfrsf21 rorab aspn rho gnb3b rasgrf2b lrrc4.2 cacng5a tenm1 prnprs3 nr1d2a crhr1 grm2a gpr22a esrrga penka cyth3b znf385b gpr27 sh3bp5lb GRM2 (1 of many) ric8a avpr2aa opn4.1 cyth3a syk klhl24b tspan7b cnr1 fgf6a gng3 nr1d2b drd2b ndrg2 TENM2 (1 of many) enpp2 trpm1a gnaq bmpr2a glra1 vipr2 saga asb18 ndrg3a pde4ca gucy1a3 gucy2c adcy2b efna3a lrrc4bb prph2b cnih3 gabbr1b rgs7bpb cishb si:dkey-94f20.4 nr4a2a opn1sw2 kidins220b unc13ba sphkap grm6a rorcb bgna mapkapk2b bdnf dclk1a rergla rom1a bmpr2b cacng8a si:ch211-171h4.3 grk7a rac3b valopa prph2l rcan2 cd81b pth2 epha6 epha8 gng13a inhbab gipr gucy2f grm6b grm1a gc3 arhgap23b rom1b si:ch211-106h11.3 grin1a fgf12a diras1a plppr3b nlk2 calcr vipr1a pak7 nr1d4a rab6bb mc5ra grm3 roraa scg5 pde4a cacng2a si:dkey-206f10.1 pde4ba nr1d1 gpr37b lingo1a rab42a paqr7b gnb2 agtr2 gngt1 pyyb lingo1b gnao1b npy arf3b oprd1b rgs11 gng13b rgs9a prph2a drd4a sagb drd1b igfbp5a ccl19b rrh hivep2a sst1.1 deptor itgbl1 tspan33b gnat2 rab3b foxo3b gpr173 gng8 fpr1 pomca si:ch211-132f19.7 arrb1 tagapb cnih2 si:dkey-192d15.2 rab3ab nptnb gnat1 nr4a2b opn1lw2 npffl itgb3b csnk1e rgs9b gpr22b socs2 opn1sw1 avpr1ab il15 lrrtm1 glrbb opn3 glrba nog3 tspan3b csf1rb gabrg2 adgrg6 rerglb plcxd3 rgra mylipb gnb5b dvl1b nr4a3 GUCY2C (1 of many) arr3a nr0b1 gpr37l1b drd2a sfrp1b sbk3 nr1h4 birc7 glra4b adcy2a sik1 ddr2b ihha grk1a sema5a SBK2 nr1d4b fgf7 gpr146 ddit3 si:ch211-284e13.4 atl1 rgs7bpa adgrl3.1 arhgef9a nyx rasgrp4 UNC13A kcnh4b TRHDE (1 of many) kcnh4a cadm2b mras gpr12 cacng7a apc2 dock3 erbb4a pde8b syngap1a ralgps1 gpr85 gabra1 kcnh5b mstnb rab38b chrm4a lrrc4.1 adra2c mavs syngap1b ppap2d rgs8 spock3 si:ch211-113g11.6 arf3a cacng7b cacng8b rhol ccl25b wisp3 oprl1 hnf4g ppp1r9bb RASGRF1 gdf10b pde4bb itga1 zgc:171482 ccl44 ank1b sparcl1 glra2 adgrb1a gprc5bb cxcl20 rapgef5a spock2 si:ch211-26b3.4 chadlb RAPGEF4 (1 of many) hivep3a prkd1 pappa2 TRHDE (1 of many) cacng3b mcf2lb grm8b fam13b gprc5ba avpr1aa BRSK1 (1 of many) gpr158a nmbb grm8a iqsec2 asip1 wisp2 PSD vip adgrb1b arhgef9b sema7a rnf165b tns1a rps6kl1 gcga nyap2a gpr158b plppr4a radil rapgefl1 lrrtm4l1 si:ch73-206d17.1 znf385d ntrk3b nrg2b pdgfba gucy1b3 si:dkey-237h12.3 gpr153 pdyn si:ch211-180a12.2 adcy1b ngfra nrg3b rtn4r tnk2a igfbp6b hunk adgrf6 akap12a si:dkey-70p6.1 tac3a iqsec3b nyap2b gpr52 ccl34b.1 htr1aa ksr2 cdc42se2 gpr186 si:ch211-10p21.1 alk ENSDARG00000096701 opn1mw1 ntrk2b gpr75 rtn4rl1b zgc:153845 igfbp1a asb10 gnb5a agrp2 iqsec3a agap2 arhgef15 pde6c si:ch73-193i22.1 adra2b cacng2b arl3l1 ARHGAP44 (1 of many) opn6a hmp19 opn4xb gngt2b dkk3a znf536 map3k12 ndrg4 ctgfb grk1b il1rapl1b sstr1a adcyap1r1a ompa | ENSDARG00000000161 ENSDARG00000000380 ENSDARG00000001807 ENSDARG00000001910 ENSDARG00000002192 ENSDARG00000002193 ENSDARG00000002696 ENSDARG00000002816 ENSDARG00000003020 ENSDARG00000003326 ENSDARG00000003403 ENSDARG00000003705 ENSDARG00000003820 ENSDARG00000003989 ENSDARG00000004150 ENSDARG00000004592 ENSDARG00000004861 ENSDARG00000004869 ENSDARG00000005159 ENSDARG00000006065 ENSDARG00000006607 ENSDARG00000007136 ENSDARG00000007195 ENSDARG00000007247 ENSDARG00000007436 ENSDARG00000007553 ENSDARG00000007807 ENSDARG00000008186 ENSDARG00000008275 ENSDARG00000008407 ENSDARG00000009020 ENSDARG00000009351 ENSDARG00000009553 ENSDARG00000009594 ENSDARG00000011091 ENSDARG00000011170 ENSDARG00000011171 ENSDARG00000011257 ENSDARG00000011259 ENSDARG00000011487 ENSDARG00000011941 ENSDARG00000012019 ENSDARG00000012353 ENSDARG00000012610 ENSDARG00000012948 ENSDARG00000013087 ENSDARG00000013221 ENSDARG00000013787 ENSDARG00000014320 ENSDARG00000014588 ENSDARG00000014675 ENSDARG00000014792 ENSDARG00000014840 ENSDARG00000014953 ENSDARG00000016667 ENSDARG00000016742 ENSDARG00000016773 ENSDARG00000016923 ENSDARG00000017007 ENSDARG00000017274 ENSDARG00000017338 ENSDARG00000017391 ENSDARG00000017429 ENSDARG00000017742 ENSDARG00000017780 ENSDARG00000017884 ENSDARG00000018530 ENSDARG00000018817 ENSDARG00000018856 ENSDARG00000019396 ENSDARG00000019752 ENSDARG00000020057 ENSDARG00000020450 ENSDARG00000020455 ENSDARG00000020602 ENSDARG00000020795 ENSDARG00000021150 ENSDARG00000021345 ENSDARG00000021869 ENSDARG00000022437 ENSDARG00000022951 ENSDARG00000022971 ENSDARG00000023609 ENSDARG00000024740 ENSDARG00000024759 ENSDARG00000025478 ENSDARG00000025504 ENSDARG00000025671 ENSDARG00000026796 ENSDARG00000026820 ENSDARG00000026840 ENSDARG00000026926 ENSDARG00000027360 ENSDARG00000027828 ENSDARG00000027957 ENSDARG00000028066 ENSDARG00000028552 ENSDARG00000028793 ENSDARG00000028845 ENSDARG00000028878 ENSDARG00000030154 ENSDARG00000031161 ENSDARG00000031343 ENSDARG00000031348 ENSDARG00000031712 ENSDARG00000031768 ENSDARG00000032126 ENSDARG00000032344 ENSDARG00000032565 ENSDARG00000032838 ENSDARG00000032868 ENSDARG00000033160 ENSDARG00000033296 ENSDARG00000034165 ENSDARG00000034215 ENSDARG00000034907 ENSDARG00000035357 ENSDARG00000035552 ENSDARG00000035798 ENSDARG00000035832 ENSDARG00000035899 ENSDARG00000036058 ENSDARG00000036222 ENSDARG00000036998 ENSDARG00000037159 ENSDARG00000037646 ENSDARG00000037921 ENSDARG00000037925 ENSDARG00000038018 ENSDARG00000038363 ENSDARG00000038378 ENSDARG00000038918 ENSDARG00000039264 ENSDARG00000039351 ENSDARG00000039534 ENSDARG00000039987 ENSDARG00000040799 ENSDARG00000040930 ENSDARG00000040985 ENSDARG00000042189 ENSDARG00000042529 ENSDARG00000042803 ENSDARG00000042904 ENSDARG00000042922 ENSDARG00000042970 ENSDARG00000042984 ENSDARG00000043135 ENSDARG00000043141 ENSDARG00000043241 ENSDARG00000043475 ENSDARG00000043662 ENSDARG00000043770 ENSDARG00000043835 ENSDARG00000043864 ENSDARG00000044199 ENSDARG00000044532 ENSDARG00000044861 ENSDARG00000045016 ENSDARG00000045070 ENSDARG00000045150 ENSDARG00000045156 ENSDARG00000045383 ENSDARG00000045557 ENSDARG00000045677 ENSDARG00000045788 ENSDARG00000052361 ENSDARG00000052713 ENSDARG00000052769 ENSDARG00000052775 ENSDARG00000052782 ENSDARG00000053528 ENSDARG00000053559 ENSDARG00000053624 ENSDARG00000053665 ENSDARG00000054137 ENSDARG00000054324 ENSDARG00000054794 ENSDARG00000054890 ENSDARG00000055118 ENSDARG00000055377 ENSDARG00000055552 ENSDARG00000055854 ENSDARG00000056045 ENSDARG00000056511 ENSDARG00000056541 ENSDARG00000056774 ENSDARG00000056926 ENSDARG00000057678 ENSDARG00000057687 ENSDARG00000057741 ENSDARG00000058082 ENSDARG00000058103 ENSDARG00000058392 ENSDARG00000058606 ENSDARG00000058695 ENSDARG00000058733 ENSDARG00000058803 ENSDARG00000058821 ENSDARG00000058944 ENSDARG00000059370 ENSDARG00000059387 ENSDARG00000059610 ENSDARG00000059836 ENSDARG00000060330 ENSDARG00000060481 ENSDARG00000060601 ENSDARG00000061121 ENSDARG00000061746 ENSDARG00000061791 ENSDARG00000061796 ENSDARG00000061829 ENSDARG00000061990 ENSDARG00000062013 ENSDARG00000062565 ENSDARG00000062633 ENSDARG00000062865 ENSDARG00000062934 ENSDARG00000063006 ENSDARG00000063007 ENSDARG00000063180 ENSDARG00000063207 ENSDARG00000063706 ENSDARG00000063713 ENSDARG00000068370 ENSDARG00000068701 ENSDARG00000068989 ENSDARG00000069117 ENSDARG00000069133 ENSDARG00000069159 ENSDARG00000069254 ENSDARG00000069402 ENSDARG00000069669 ENSDARG00000069733 ENSDARG00000069765 ENSDARG00000069940 ENSDARG00000070037 ENSDARG00000070266 ENSDARG00000070442 ENSDARG00000070539 ENSDARG00000070624 ENSDARG00000070626 ENSDARG00000070666 ENSDARG00000070873 ENSDARG00000071048 ENSDARG00000071209 ENSDARG00000071565 ENSDARG00000071709 ENSDARG00000073824 ENSDARG00000073891 ENSDARG00000074233 ENSDARG00000074316 ENSDARG00000074415 ENSDARG00000074772 ENSDARG00000074777 ENSDARG00000074989 ENSDARG00000075012 ENSDARG00000075133 ENSDARG00000075141 ENSDARG00000075163 ENSDARG00000075271 ENSDARG00000075393 ENSDARG00000075491 ENSDARG00000075903 ENSDARG00000075924 ENSDARG00000075928 ENSDARG00000075949 ENSDARG00000076020 ENSDARG00000076044 ENSDARG00000076401 ENSDARG00000076480 ENSDARG00000076508 ENSDARG00000076779 ENSDARG00000077080 ENSDARG00000077083 ENSDARG00000077124 ENSDARG00000077134 ENSDARG00000077167 ENSDARG00000077654 ENSDARG00000077709 ENSDARG00000077858 ENSDARG00000077882 ENSDARG00000078102 ENSDARG00000078247 ENSDARG00000078529 ENSDARG00000078624 ENSDARG00000078707 ENSDARG00000078817 ENSDARG00000078842 ENSDARG00000079024 ENSDARG00000079296 ENSDARG00000079581 ENSDARG00000079665 ENSDARG00000079671 ENSDARG00000079779 ENSDARG00000079912 ENSDARG00000080015 ENSDARG00000086159 ENSDARG00000086162 ENSDARG00000086214 ENSDARG00000086585 ENSDARG00000086778 ENSDARG00000086790 ENSDARG00000087599 ENSDARG00000087601 ENSDARG00000087798 ENSDARG00000087857 ENSDARG00000088634 ENSDARG00000088708 ENSDARG00000089766 ENSDARG00000090035 ENSDARG00000090646 ENSDARG00000090833 ENSDARG00000091317 ENSDARG00000091757 ENSDARG00000091792 ENSDARG00000092039 ENSDARG00000093089 ENSDARG00000093091 ENSDARG00000093357 ENSDARG00000093460 ENSDARG00000093608 ENSDARG00000093745 ENSDARG00000094526 ENSDARG00000094577 ENSDARG00000094860 ENSDARG00000095272 ENSDARG00000095833 ENSDARG00000096701 ENSDARG00000097008 ENSDARG00000098511 ENSDARG00000098526 ENSDARG00000098528 ENSDARG00000099238 ENSDARG00000099351 ENSDARG00000099461 ENSDARG00000099685 ENSDARG00000099781 ENSDARG00000099850 ENSDARG00000099874 ENSDARG00000100293 ENSDARG00000100397 ENSDARG00000101367 ENSDARG00000102096 ENSDARG00000102376 ENSDARG00000102393 ENSDARG00000102424 ENSDARG00000102430 ENSDARG00000102975 ENSDARG00000103259 ENSDARG00000103543 ENSDARG00000103591 ENSDARG00000103648 ENSDARG00000103651 ENSDARG00000103937 ENSDARG00000104292 ENSDARG00000104685 ENSDARG00000104853 ENSDARG00000104922 ENSDARG00000105201 ENSDARG00000105218 | | GO:0007186 | G-protein coupled receptor signaling pat... | biological\_process | 234 | 38.7 | 117 | 1.9e-17 | rho crhr1 grm2a gpr22a penka gpr27 GRM2 (1 of many) ric8a avpr2aa opn4.1 cnr1 gng3 drd2b trpm1a gnaq glra1 vipr2 adcy2b gabbr1b si:dkey-94f20.4 opn1sw2 grm6a valopa pth2 gng13a gipr grm6b grm1a grin1a calcr vipr1a mc5ra grm3 scg5 si:dkey-206f10.1 gpr37b agtr2 gngt1 pyyb gnao1b npy oprd1b rgs11 gng13b rgs9a drd4a drd1b ccl19b rrh gnat2 gpr173 gng8 fpr1 pomca si:ch211-132f19.7 gnat1 opn1lw2 npffl rgs9b gpr22b opn1sw1 avpr1ab glrbb opn3 glrba gabrg2 adgrg6 rgra gpr37l1b drd2a glra4b adcy2a gpr146 adgrl3.1 gpr12 gpr85 gabra1 chrm4a adra2c rhol ccl25b oprl1 ccl44 glra2 adgrb1a gprc5bb cxcl20 grm8b gprc5ba avpr1aa gpr158a nmbb grm8a vip adgrb1b gcga gpr158b gpr153 pdyn adcy1b adgrf6 tac3a gpr52 ccl34b.1 htr1aa gpr186 si:ch211-10p21.1 opn1mw1 gpr75 si:ch73-193i22.1 adra2b opn6a hmp19 opn4xb gngt2b sstr1a adcyap1r1a | ENSDARG00000002193 ENSDARG00000003989 ENSDARG00000004150 ENSDARG00000004592 ENSDARG00000004869 ENSDARG00000006607 ENSDARG00000007195 ENSDARG00000007247 ENSDARG00000007436 ENSDARG00000007553 ENSDARG00000009020 ENSDARG00000009553 ENSDARG00000011091 ENSDARG00000011259 ENSDARG00000011487 ENSDARG00000012019 ENSDARG00000012353 ENSDARG00000014588 ENSDARG00000016667 ENSDARG00000016923 ENSDARG00000017274 ENSDARG00000017742 ENSDARG00000021150 ENSDARG00000022951 ENSDARG00000024740 ENSDARG00000025478 ENSDARG00000025671 ENSDARG00000026796 ENSDARG00000027828 ENSDARG00000028845 ENSDARG00000028878 ENSDARG00000031348 ENSDARG00000031712 ENSDARG00000032126 ENSDARG00000032838 ENSDARG00000033296 ENSDARG00000035552 ENSDARG00000035798 ENSDARG00000035832 ENSDARG00000036058 ENSDARG00000036222 ENSDARG00000037159 ENSDARG00000037646 ENSDARG00000037921 ENSDARG00000037925 ENSDARG00000038363 ENSDARG00000038918 ENSDARG00000039351 ENSDARG00000039534 ENSDARG00000042529 ENSDARG00000042922 ENSDARG00000042970 ENSDARG00000042984 ENSDARG00000043135 ENSDARG00000043141 ENSDARG00000044199 ENSDARG00000044861 ENSDARG00000045016 ENSDARG00000045156 ENSDARG00000045383 ENSDARG00000045677 ENSDARG00000045788 ENSDARG00000052769 ENSDARG00000052775 ENSDARG00000052782 ENSDARG00000053665 ENSDARG00000054137 ENSDARG00000054890 ENSDARG00000056774 ENSDARG00000056926 ENSDARG00000058103 ENSDARG00000058392 ENSDARG00000059610 ENSDARG00000061121 ENSDARG00000062934 ENSDARG00000068701 ENSDARG00000068989 ENSDARG00000069254 ENSDARG00000069669 ENSDARG00000070666 ENSDARG00000070873 ENSDARG00000071209 ENSDARG00000074772 ENSDARG00000075012 ENSDARG00000075133 ENSDARG00000075141 ENSDARG00000075163 ENSDARG00000076508 ENSDARG00000077080 ENSDARG00000077083 ENSDARG00000077134 ENSDARG00000077167 ENSDARG00000077654 ENSDARG00000078247 ENSDARG00000078529 ENSDARG00000079296 ENSDARG00000079665 ENSDARG00000087601 ENSDARG00000087798 ENSDARG00000088634 ENSDARG00000091757 ENSDARG00000093089 ENSDARG00000093460 ENSDARG00000093608 ENSDARG00000093745 ENSDARG00000094860 ENSDARG00000095272 ENSDARG00000097008 ENSDARG00000098526 ENSDARG00000101367 ENSDARG00000102096 ENSDARG00000102430 ENSDARG00000102975 ENSDARG00000103259 ENSDARG00000103543 ENSDARG00000104922 ENSDARG00000105201 | | GO:0007218 | neuropeptide signaling pathway | biological\_process | 23 | 3.8 | 16 | 5.6e-06 | penka glra1 pth2 scg5 pyyb npy oprd1b pomca npffl glrbb glrba glra4b oprl1 glra2 nmbb pdyn | ENSDARG00000004869 ENSDARG00000012019 ENSDARG00000022951 ENSDARG00000032126 ENSDARG00000035832 ENSDARG00000036222 ENSDARG00000037159 ENSDARG00000043135 ENSDARG00000045016 ENSDARG00000052769 ENSDARG00000052782 ENSDARG00000058103 ENSDARG00000071209 ENSDARG00000075012 ENSDARG00000077167 ENSDARG00000087798 | | GO:0007602 | phototransduction | biological\_process | 16 | 2.6 | 12 | 9.7e-05 | rho opn4.1 opn1sw2 grk7a valopa opn1lw2 opn1sw1 opn3 rgra rhol opn1mw1 opn4xb | ENSDARG00000002193 ENSDARG00000007553 ENSDARG00000017274 ENSDARG00000020602 ENSDARG00000021150 ENSDARG00000044861 ENSDARG00000045677 ENSDARG00000052775 ENSDARG00000054890 ENSDARG00000070666 ENSDARG00000097008 ENSDARG00000103259 | | GO:0010469 | regulation of receptor activity | biological\_process | 13 | 2.1 | 10 | 6.6e-04 | cacng5a prnprs3 klhl24b cacng8a cacng2a cacng7a cacng7b cacng8b cacng3b cacng2b | ENSDARG00000003326 ENSDARG00000003705 ENSDARG00000008275 ENSDARG00000020450 ENSDARG00000032565 ENSDARG00000063006 ENSDARG00000070624 ENSDARG00000070626 ENSDARG00000076401 ENSDARG00000102376 | | GO:0019221 | cytokine-mediated signaling pathway | biological\_process | 44 | 7.3 | 18 | 2.8e-02 | aspn lrrc4.2 lrrc4bb bgna ccl19b socs2 lrrtm1 csf1rb nyx lrrc4.1 ccl25b ccl44 cxcl20 chadlb lrrtm4l1 rtn4r ccl34b.1 rtn4rl1b | ENSDARG00000002192 ENSDARG00000003020 ENSDARG00000014792 ENSDARG00000017884 ENSDARG00000039351 ENSDARG00000045557 ENSDARG00000052713 ENSDARG00000053624 ENSDARG00000061791 ENSDARG00000069402 ENSDARG00000070873 ENSDARG00000074772 ENSDARG00000075163 ENSDARG00000075903 ENSDARG00000080015 ENSDARG00000090035 ENSDARG00000093608 ENSDARG00000098528 | | GO:1900449 | regulation of glutamate receptor signali... | biological\_process | 11 | 1.8 | 10 | 3.6e-05 | cacng5a prnprs3 klhl24b cacng8a cacng2a cacng7a cacng7b cacng8b cacng3b cacng2b | ENSDARG00000003326 ENSDARG00000003705 ENSDARG00000008275 ENSDARG00000020450 ENSDARG00000032565 ENSDARG00000063006 ENSDARG00000070624 ENSDARG00000070626 ENSDARG00000076401 ENSDARG00000102376 | | GO:0007193 | adenylate cyclase-inhibiting G-protein c... | biological\_process | 19 | 3.1 | 12 | 1.7e-03 | grm2a GRM2 (1 of many) drd2b adcy2b grm3 si:dkey-206f10.1 drd4a si:ch211-132f19.7 drd2a adcy2a chrm4a adcy1b | ENSDARG00000004150 ENSDARG00000007195 ENSDARG00000011091 ENSDARG00000014588 ENSDARG00000031712 ENSDARG00000032838 ENSDARG00000038363 ENSDARG00000043141 ENSDARG00000056926 ENSDARG00000058392 ENSDARG00000069254 ENSDARG00000088634 | | GO:0006811 | ion transport | biological\_process | 532 | 88.1 | 213 | 4.6e-22 | slc17a6a nalcn p2rx2 SLC4A5 (1 of many) rhcga cacng5a slc5a8l prnprs3 kctd16a scn8aa slc24a3 kcns3a si:ch211-251b21.1 ttyh3b fth1b slc7a10a klhl24b asic1a cacna1c rhbg slc1a6 scara5 clic2 glra3 drd2b chrna10a trpm1a glra1 cnga1 scp2a rnf207b slc6a6a si:dkey-246g23.4 trpv6 atp6v0a1b slc4a8 slc7a1 slc17a7a tcirg1a chrnb2b scn8ab atp1a3a slco3a1 rhag atp1a1b kctd16b slc20a1a slc1a1 cacng8a gria1a cacna1ba atp2b3b gabrb3 cacna2d4b asic4a grin1b slc38a3a gabrr3a grin1a kcnf1a slc34a1a CNGA1 (1 of many) grin2bb gabrr3b kcnj11l trpa1b cacng2a gria1b gria3a kcnd2 kcnc1b atp1b2b grin2aa scn4ab clcn4 si:rp71-39b20.4 atp6v0cb slc8a4b gria4a gria3b cacna1aa kcnb2 atp1a1a.5 grik1b kcnab1b slc17a6b slc6a1l atp6ap1a CNGB1 slc1a3b atp6v1b2 atp2b3a gabrr1 aqp8a.1 slc13a1 kcna6a kcnc1a abcc8 slc1a2a chrnb3a gria2b glrbb glrba gabrr2a atp1b4 gabrg2 slc26a6l slco2b1 slc8a4a chrna6 kcnc3a steap4 kcnd3 trpc6a drd2a slc17a8 glra4b atpv0e2 gabrd kcnq3 kcnb1 slc4a10b slc6a4a trpc1 slco2a1 kcnh4b clcn1a kcnab2b kcnj3b cacna1ea scn3b kcnh4a si:ch211-23l10.2 si:dkey-100n10.2 kcnh7 scn1lab slc6a15 steap2 kcna1a cacng7a atp2b2 kctd8 slc6a5 cngb1a kctd4 slc6a17 gabra1 kcnh5b slc13a3 KCNV1 ENSDARG00000070170 gria2a clic5b cacng7b cacng8b cnga3a gabra5 slc12a10.2 aqp8a.2 hcn4l grid1a glra2 mfi2 kcnq2a slc4a11 ENSDARG00000076127 cacng3b kcnv2a ttyh1 calhm1 grin2cb slc13a5a grin2ca slc12a5b best1 cacna1bb gabrb2 knca7 si:ch73-380n15.2 KCNB2 si:dkey-262k9.4 cacna1g slc17a7b scn12aa atp6ap1lb kcnq2b kcnn1a atp6ap1la gabrz trpm2 si:ch211-255p10.3 si:dkey-56f14.4 si:ch211-270g19.5 scn4ba cacnb2a gabrb4 bcl2l16 atp1b2a rhcgb fxyd6 chrna3 ano2 ENSDARG00000101368 grik5 chrna7 scn2b cacng2b slc1a2b si:dkey-36i7.3 atp1a3b slc1a8a slc4a5 hcn1 | ENSDARG00000001127 ENSDARG00000001835 ENSDARG00000002300 ENSDARG00000002771 ENSDARG00000003203 ENSDARG00000003326 ENSDARG00000003697 ENSDARG00000003705 ENSDARG00000004648 ENSDARG00000005775 ENSDARG00000006760 ENSDARG00000006891 ENSDARG00000007275 ENSDARG00000007678 ENSDARG00000007975 ENSDARG00000008100 ENSDARG00000008275 ENSDARG00000008329 ENSDARG00000008398 ENSDARG00000009018 ENSDARG00000010096 ENSDARG00000010425 ENSDARG00000010625 ENSDARG00000011066 ENSDARG00000011091 ENSDARG00000011113 ENSDARG00000011259 ENSDARG00000012019 ENSDARG00000012125 ENSDARG00000012194 ENSDARG00000012409 ENSDARG00000012534 ENSDARG00000013775 ENSDARG00000014496 ENSDARG00000015174 ENSDARG00000015531 ENSDARG00000016439 ENSDARG00000016480 ENSDARG00000016835 ENSDARG00000017790 ENSDARG00000018032 ENSDARG00000018259 ENSDARG00000018726 ENSDARG00000019253 ENSDARG00000019856 ENSDARG00000020102 ENSDARG00000020114 ENSDARG00000020212 ENSDARG00000020450 ENSDARG00000021352 ENSDARG00000021735 ENSDARG00000023445 ENSDARG00000023771 ENSDARG00000023886 ENSDARG00000025162 ENSDARG00000025728 ENSDARG00000027065 ENSDARG00000027153 ENSDARG00000027828 ENSDARG00000027940 ENSDARG00000028824 ENSDARG00000029898 ENSDARG00000030376 ENSDARG00000030750 ENSDARG00000031438 ENSDARG00000031875 ENSDARG00000032565 ENSDARG00000032714 ENSDARG00000032737 ENSDARG00000032799 ENSDARG00000032959 ENSDARG00000034424 ENSDARG00000034493 ENSDARG00000034588 ENSDARG00000035808 ENSDARG00000035861 ENSDARG00000036577 ENSDARG00000037145 ENSDARG00000037496 ENSDARG00000037498 ENSDARG00000037905 ENSDARG00000038862 ENSDARG00000040252 ENSDARG00000040627 ENSDARG00000040741 ENSDARG00000041150 ENSDARG00000041205 ENSDARG00000041417 ENSDARG00000042107 ENSDARG00000043148 ENSDARG00000043465 ENSDARG00000043474 ENSDARG00000043902 ENSDARG00000045141 ENSDARG00000045638 ENSDARG00000046014 ENSDARG00000051852 ENSDARG00000051879 ENSDARG00000052138 ENSDARG00000052764 ENSDARG00000052765 ENSDARG00000052769 ENSDARG00000052782 ENSDARG00000052982 ENSDARG00000053262 ENSDARG00000053665 ENSDARG00000054127 ENSDARG00000054609 ENSDARG00000055154 ENSDARG00000055559 ENSDARG00000055855 ENSDARG00000055901 ENSDARG00000056101 ENSDARG00000056625 ENSDARG00000056926 ENSDARG00000057728 ENSDARG00000058103 ENSDARG00000059057 ENSDARG00000059763 ENSDARG00000060085 ENSDARG00000060095 ENSDARG00000060303 ENSDARG00000061165 ENSDARG00000061203 ENSDARG00000061896 ENSDARG00000061990 ENSDARG00000062084 ENSDARG00000062134 ENSDARG00000062217 ENSDARG00000062346 ENSDARG00000062359 ENSDARG00000062565 ENSDARG00000062618 ENSDARG00000062672 ENSDARG00000062687 ENSDARG00000062744 ENSDARG00000062821 ENSDARG00000062887 ENSDARG00000062942 ENSDARG00000063006 ENSDARG00000063433 ENSDARG00000067507 ENSDARG00000067964 ENSDARG00000068242 ENSDARG00000068691 ENSDARG00000068787 ENSDARG00000068989 ENSDARG00000069117 ENSDARG00000069478 ENSDARG00000070092 ENSDARG00000070170 ENSDARG00000070173 ENSDARG00000070584 ENSDARG00000070624 ENSDARG00000070626 ENSDARG00000070726 ENSDARG00000070730 ENSDARG00000071173 ENSDARG00000071592 ENSDARG00000074419 ENSDARG00000074583 ENSDARG00000075012 ENSDARG00000075159 ENSDARG00000075307 ENSDARG00000075532 ENSDARG00000076127 ENSDARG00000076401 ENSDARG00000076644 ENSDARG00000076804 ENSDARG00000077021 ENSDARG00000077560 ENSDARG00000077691 ENSDARG00000078149 ENSDARG00000078187 ENSDARG00000078331 ENSDARG00000079295 ENSDARG00000079586 ENSDARG00000086571 ENSDARG00000087224 ENSDARG00000088842 ENSDARG00000089838 ENSDARG00000089913 ENSDARG00000090106 ENSDARG00000090724 ENSDARG00000090963 ENSDARG00000091130 ENSDARG00000091306 ENSDARG00000091509 ENSDARG00000094512 ENSDARG00000095696 ENSDARG00000096616 ENSDARG00000097110 ENSDARG00000097256 ENSDARG00000099031 ENSDARG00000099045 ENSDARG00000099096 ENSDARG00000099128 ENSDARG00000099203 ENSDARG00000100265 ENSDARG00000100971 ENSDARG00000100991 ENSDARG00000101363 ENSDARG00000101368 ENSDARG00000101449 ENSDARG00000101702 ENSDARG00000101713 ENSDARG00000102376 ENSDARG00000102453 ENSDARG00000103639 ENSDARG00000104139 ENSDARG00000104204 ENSDARG00000104387 ENSDARG00000104480 | | GO:0034765 | regulation of ion transmembrane transpor... | biological\_process | 82 | 13.6 | 48 | 1.3e-08 | nalcn cacng5a prnprs3 scn8aa kcns3a klhl24b cacna1c clic2 drd2b rnf207b scn8ab cacng8a cacna1ba kcnf1a kcnj11l cacng2a kcnd2 kcnc1b scn4ab si:rp71-39b20.4 cacna1aa kcnb2 kcna6a kcnc1a kcnc3a kcnd3 drd2a kcnb1 kcnj3b cacna1ea si:ch211-23l10.2 si:dkey-100n10.2 scn1lab kcna1a cacng7a KCNV1 clic5b cacng7b cacng8b cacng3b kcnv2a cacna1bb KCNB2 si:dkey-262k9.4 cacna1g scn12aa cacnb2a cacng2b | ENSDARG00000001835 ENSDARG00000003326 ENSDARG00000003705 ENSDARG00000005775 ENSDARG00000006891 ENSDARG00000008275 ENSDARG00000008398 ENSDARG00000010625 ENSDARG00000011091 ENSDARG00000012409 ENSDARG00000018032 ENSDARG00000020450 ENSDARG00000021735 ENSDARG00000027940 ENSDARG00000031438 ENSDARG00000032565 ENSDARG00000032799 ENSDARG00000032959 ENSDARG00000034588 ENSDARG00000035861 ENSDARG00000037905 ENSDARG00000038862 ENSDARG00000046014 ENSDARG00000051852 ENSDARG00000055855 ENSDARG00000056101 ENSDARG00000056926 ENSDARG00000060095 ENSDARG00000062217 ENSDARG00000062346 ENSDARG00000062618 ENSDARG00000062672 ENSDARG00000062744 ENSDARG00000062942 ENSDARG00000063006 ENSDARG00000070092 ENSDARG00000070584 ENSDARG00000070624 ENSDARG00000070626 ENSDARG00000076401 ENSDARG00000076644 ENSDARG00000079295 ENSDARG00000088842 ENSDARG00000089838 ENSDARG00000089913 ENSDARG00000090724 ENSDARG00000099045 ENSDARG00000102376 | | GO:0006813 | potassium ion transport | biological\_process | 74 | 12.2 | 43 | 2.8e-05 | kcns3a drd2b rnf207b atp1a3a atp1a1b kcnf1a kcnj11l kcnd2 kcnc1b atp1b2b si:rp71-39b20.4 kcnb2 atp1a1a.5 kcnab1b kcna6a kcnc1a abcc8 atp1b4 kcnc3a kcnd3 drd2a kcnq3 kcnb1 kcnh4b kcnab2b kcnj3b kcnh4a si:ch211-23l10.2 si:dkey-100n10.2 kcnh7 kcna1a kcnh5b KCNV1 hcn4l kcnq2a kcnv2a knca7 KCNB2 kcnq2b kcnn1a atp1b2a atp1a3b hcn1 | ENSDARG00000006891 ENSDARG00000011091 ENSDARG00000012409 ENSDARG00000018259 ENSDARG00000019856 ENSDARG00000027940 ENSDARG00000031438 ENSDARG00000032799 ENSDARG00000032959 ENSDARG00000034424 ENSDARG00000035861 ENSDARG00000038862 ENSDARG00000040252 ENSDARG00000040741 ENSDARG00000046014 ENSDARG00000051852 ENSDARG00000051879 ENSDARG00000053262 ENSDARG00000055855 ENSDARG00000056101 ENSDARG00000056926 ENSDARG00000060085 ENSDARG00000060095 ENSDARG00000061990 ENSDARG00000062134 ENSDARG00000062217 ENSDARG00000062565 ENSDARG00000062618 ENSDARG00000062672 ENSDARG00000062687 ENSDARG00000062942 ENSDARG00000069117 ENSDARG00000070092 ENSDARG00000074419 ENSDARG00000075307 ENSDARG00000076644 ENSDARG00000086571 ENSDARG00000088842 ENSDARG00000091130 ENSDARG00000091306 ENSDARG00000099203 ENSDARG00000104139 ENSDARG00000104480 | | GO:0006814 | sodium ion transport | biological\_process | 62 | 10.3 | 30 | 2.3e-03 | slc17a6a slc5a8l scn8aa asic1a slc17a7a scn8ab atp1a3a atp1a1b asic4a atp1b2b scn4ab atp1a1a.5 slc17a6b slc13a1 atp1b4 slc17a8 scn3b scn1lab slc13a3 ENSDARG00000070170 slc12a10.2 hcn4l slc4a11 slc13a5a si:dkey-262k9.4 slc17a7b scn12aa atp1b2a atp1a3b hcn1 | ENSDARG00000001127 ENSDARG00000003697 ENSDARG00000005775 ENSDARG00000008329 ENSDARG00000016480 ENSDARG00000018032 ENSDARG00000018259 ENSDARG00000019856 ENSDARG00000025162 ENSDARG00000034424 ENSDARG00000034588 ENSDARG00000040252 ENSDARG00000041150 ENSDARG00000045638 ENSDARG00000053262 ENSDARG00000057728 ENSDARG00000062359 ENSDARG00000062744 ENSDARG00000069478 ENSDARG00000070170 ENSDARG00000071173 ENSDARG00000074419 ENSDARG00000075532 ENSDARG00000077691 ENSDARG00000089838 ENSDARG00000090106 ENSDARG00000090724 ENSDARG00000099203 ENSDARG00000104139 ENSDARG00000104480 | | GO:0032412 | regulation of ion transmembrane transpor... | biological\_process | 21 | 3.5 | 16 | 6.6e-07 | cacng5a prnprs3 klhl24b clic2 drd2b rnf207b cacng8a cacng2a drd2a cacng7a cacng7b cacng8b cacng3b si:dkey-262k9.4 cacnb2a cacng2b | ENSDARG00000003326 ENSDARG00000003705 ENSDARG00000008275 ENSDARG00000010625 ENSDARG00000011091 ENSDARG00000012409 ENSDARG00000020450 ENSDARG00000032565 ENSDARG00000056926 ENSDARG00000063006 ENSDARG00000070624 ENSDARG00000070626 ENSDARG00000076401 ENSDARG00000089838 ENSDARG00000099045 ENSDARG00000102376 | | GO:0035725 | sodium ion transmembrane transport | biological\_process | 17 | 2.8 | 10 | 2.4e-02 | scn8aa slc17a7a atp1a3a atp1a1b atp1a1a.5 slc4a11 si:dkey-262k9.4 slc17a7b atp1a3b hcn1 | ENSDARG00000005775 ENSDARG00000016480 ENSDARG00000018259 ENSDARG00000019856 ENSDARG00000040252 ENSDARG00000075532 ENSDARG00000089838 ENSDARG00000090106 ENSDARG00000104139 ENSDARG00000104480 | | GO:0070588 | calcium ion transmembrane transport | biological\_process | 58 | 9.6 | 25 | 4.1e-04 | cacng5a cacna1c clic2 drd2b cacng8a cacna1ba atp2b3b cacna2d4b cacng2a cacna1aa atp2b3a trpc6a drd2a trpc1 cacna1ea cacng7a atp2b2 cacng7b cacng8b cacng3b cacna1bb cacna1g cacnb2a bcl2l16 cacng2b | ENSDARG00000003326 ENSDARG00000008398 ENSDARG00000010625 ENSDARG00000011091 ENSDARG00000020450 ENSDARG00000021735 ENSDARG00000023445 ENSDARG00000023886 ENSDARG00000032565 ENSDARG00000037905 ENSDARG00000043474 ENSDARG00000056625 ENSDARG00000056926 ENSDARG00000061203 ENSDARG00000062346 ENSDARG00000063006 ENSDARG00000063433 ENSDARG00000070624 ENSDARG00000070626 ENSDARG00000076401 ENSDARG00000079295 ENSDARG00000089913 ENSDARG00000099045 ENSDARG00000099128 ENSDARG00000102376 | | GO:0071805 | potassium ion transmembrane transport | biological\_process | 49 | 8.1 | 27 | 2.5e-06 | kcns3a rnf207b atp1a3a atp1a1b kcnf1a kcnj11l kcnd2 kcnc1b si:rp71-39b20.4 kcnb2 atp1a1a.5 kcna6a kcnc1a kcnc3a kcnd3 kcnq3 kcnb1 kcnj3b si:ch211-23l10.2 si:dkey-100n10.2 kcna1a KCNV1 kcnq2a kcnv2a KCNB2 atp1a3b hcn1 | ENSDARG00000006891 ENSDARG00000012409 ENSDARG00000018259 ENSDARG00000019856 ENSDARG00000027940 ENSDARG00000031438 ENSDARG00000032799 ENSDARG00000032959 ENSDARG00000035861 ENSDARG00000038862 ENSDARG00000040252 ENSDARG00000046014 ENSDARG00000051852 ENSDARG00000055855 ENSDARG00000056101 ENSDARG00000060085 ENSDARG00000060095 ENSDARG00000062217 ENSDARG00000062618 ENSDARG00000062672 ENSDARG00000062942 ENSDARG00000070092 ENSDARG00000075307 ENSDARG00000076644 ENSDARG00000088842 ENSDARG00000104139 ENSDARG00000104480 | | GO:0043086 | negative regulation of catalytic activit... | biological\_process | 84 | 13.9 | 27 | 3.0e-02 | aspn lrrc4.2 sh3bp5lb drd2b lrrc4bb bgna camk2n1a tfpia paqr7b socs2 lrrtm1 pkib drd2a birc7 oaz2b timp2a nyx lrrc4.1 adra2c chadlb lrrtm4l1 wu:fb59d01 rtn4r camk2n2 rtn4rl1b dlg2 adra2b | ENSDARG00000002192 ENSDARG00000003020 ENSDARG00000007136 ENSDARG00000011091 ENSDARG00000014792 ENSDARG00000017884 ENSDARG00000025855 ENSDARG00000034718 ENSDARG00000034907 ENSDARG00000045557 ENSDARG00000052713 ENSDARG00000053110 ENSDARG00000056926 ENSDARG00000058082 ENSDARG00000059815 ENSDARG00000061226 ENSDARG00000061791 ENSDARG00000069402 ENSDARG00000069669 ENSDARG00000075903 ENSDARG00000080015 ENSDARG00000089361 ENSDARG00000090035 ENSDARG00000090424 ENSDARG00000098528 ENSDARG00000099323 ENSDARG00000102096 | | GO:0006469 | negative regulation of protein kinase ac... | biological\_process | 37 | 6.1 | 16 | 3.0e-02 | aspn lrrc4.2 sh3bp5lb lrrc4bb bgna camk2n1a socs2 lrrtm1 pkib nyx lrrc4.1 chadlb lrrtm4l1 rtn4r camk2n2 rtn4rl1b | ENSDARG00000002192 ENSDARG00000003020 ENSDARG00000007136 ENSDARG00000014792 ENSDARG00000017884 ENSDARG00000025855 ENSDARG00000045557 ENSDARG00000052713 ENSDARG00000053110 ENSDARG00000061791 ENSDARG00000069402 ENSDARG00000075903 ENSDARG00000080015 ENSDARG00000090035 ENSDARG00000090424 ENSDARG00000098528 | | GO:0019226 | transmission of nerve impulse | biological\_process | 16 | 2.6 | 14 | 2.4e-07 | cacng5a scn8aa scn8ab cacng8a cacng2a scn4ab kcnab2b scn1lab cacng7a cacng7b cacng8b cacng3b scn12aa cacng2b | ENSDARG00000003326 ENSDARG00000005775 ENSDARG00000018032 ENSDARG00000020450 ENSDARG00000032565 ENSDARG00000034588 ENSDARG00000062134 ENSDARG00000062744 ENSDARG00000063006 ENSDARG00000070624 ENSDARG00000070626 ENSDARG00000076401 ENSDARG00000090724 ENSDARG00000102376 | | GO:0006836 | neurotransmitter transport | biological\_process | 59 | 9.8 | 33 | 1.4e-02 | stx1b slc17a6a slc6a6a lin7a slc6a2 snap25a lin7b slc6a1b slc17a6b slc6a1l nrxn3a slc6a1a syn2a STX3 (1 of many) slc17a8 snap25b cplx4a syn1 slc6a4a nrxn3b slc6a15 slc6a5 slc6a17 si:ch211-117c9.5 ppfia3 syt7b doc2b cplx3b cplx4c syn2b rims2a syt12 rims2b | ENSDARG00000000503 ENSDARG00000001127 ENSDARG00000012534 ENSDARG00000013414 ENSDARG00000016141 ENSDARG00000020609 ENSDARG00000037932 ENSDARG00000039647 ENSDARG00000041150 ENSDARG00000041205 ENSDARG00000043746 ENSDARG00000045944 ENSDARG00000045945 ENSDARG00000051981 ENSDARG00000057728 ENSDARG00000058117 ENSDARG00000059978 ENSDARG00000060368 ENSDARG00000061165 ENSDARG00000062693 ENSDARG00000062821 ENSDARG00000067964 ENSDARG00000068787 ENSDARG00000071235 ENSDARG00000077053 ENSDARG00000078060 ENSDARG00000088293 ENSDARG00000089486 ENSDARG00000094889 ENSDARG00000101054 ENSDARG00000101606 ENSDARG00000101776 ENSDARG00000102690 | | GO:0007269 | neurotransmitter secretion | biological\_process | 27 | 4.5 | 18 | 2.4e-07 | stx1b lin7a snap25a lin7b nrxn3a syn2a STX3 (1 of many) snap25b cplx4a syn1 nrxn3b ppfia3 syt7b doc2b syn2b rims2a syt12 rims2b | ENSDARG00000000503 ENSDARG00000013414 ENSDARG00000020609 ENSDARG00000037932 ENSDARG00000043746 ENSDARG00000045945 ENSDARG00000051981 ENSDARG00000058117 ENSDARG00000059978 ENSDARG00000060368 ENSDARG00000062693 ENSDARG00000077053 ENSDARG00000078060 ENSDARG00000088293 ENSDARG00000101054 ENSDARG00000101606 ENSDARG00000101776 ENSDARG00000102690 | | GO:0007156 | homophilic cell adhesion via plasma memb... | biological\_process | 78 | 12.9 | 35 | 9.4e-07 | prnprs3 cdhr1a cadm2a cdh13 cdh4 pcdh17 clstn1 DCHS2 cadm4 prnpb cdh10a pcdh1g9 crb2b clstn2 CLSTN2 (1 of many) cdh18a cadm2b pcdh1a PCDH9 clstn3 pcdhb cdh24b pcdh7a si:ch211-214p13.3 pcdh1gb9 pcdh1g33 pcdh2ac pcdh1g26 pcdh1g31 pcdh1g30 ENSDARG00000102185 pcdh1g22 pcdh1gc6 pcdh1g29 pcdh1gc5 | ENSDARG00000003705 ENSDARG00000004643 ENSDARG00000009930 ENSDARG00000014215 ENSDARG00000015002 ENSDARG00000027041 ENSDARG00000031720 ENSDARG00000037286 ENSDARG00000040291 ENSDARG00000044048 ENSDARG00000055843 ENSDARG00000057519 ENSDARG00000060081 ENSDARG00000060637 ENSDARG00000060638 ENSDARG00000061371 ENSDARG00000062633 ENSDARG00000062720 ENSDARG00000063264 ENSDARG00000073883 ENSDARG00000077023 ENSDARG00000077996 ENSDARG00000078898 ENSDARG00000087403 ENSDARG00000088475 ENSDARG00000099035 ENSDARG00000099783 ENSDARG00000099931 ENSDARG00000100670 ENSDARG00000101865 ENSDARG00000102185 ENSDARG00000103013 ENSDARG00000103950 ENSDARG00000104497 ENSDARG00000104826 | | GO:0032870 | cellular response to hormone stimulus | biological\_process | 100 | 16.6 | 31 | 8.9e-04 | rorab nr1d2a gpr22a esrrga avpr2aa nr1d2b nr4a2a rorcb nr1d4a roraa nr1d1 nfe2l2a nr4a2b gpr22b socs2 avpr1ab nr4a3 nr0b1 nr1h4 nr1d4b si:ch211-284e13.4 adgrl3.1 jund hnf4g junba avpr1aa asip1 nupr1 agrp2 junbb sstr1a | ENSDARG00000001910 ENSDARG00000003820 ENSDARG00000004592 ENSDARG00000004861 ENSDARG00000007436 ENSDARG00000009594 ENSDARG00000017007 ENSDARG00000017780 ENSDARG00000031161 ENSDARG00000031768 ENSDARG00000033160 ENSDARG00000042824 ENSDARG00000044532 ENSDARG00000045383 ENSDARG00000045557 ENSDARG00000045788 ENSDARG00000055854 ENSDARG00000056541 ENSDARG00000057741 ENSDARG00000059370 ENSDARG00000060330 ENSDARG00000061121 ENSDARG00000067850 ENSDARG00000071565 ENSDARG00000074378 ENSDARG00000077083 ENSDARG00000077858 ENSDARG00000094557 ENSDARG00000099781 ENSDARG00000104773 ENSDARG00000104922 | | GO:0007268 | synaptic transmission | biological\_process | 113 | 18.7 | 72 | 5.6e-05 | stx1b sypb drd2b chrna10a glra1 lin7a unc13ba chrnb2b snap25a oprd1b lin7b drd4a drd1b slc6a1b slc17a6b slc6a1l sypa slc24a2 nrxn3a npffl shisa9a slc6a1a syn2a STX3 (1 of many) shisa9b chrnb3a glrbb glrba chrna6 drd2a glra4b snap25b si:dkey-30c15.17 cplx4a kcnq3 syn1 slc6a4a UNC13A nlgn3b shisa7a nrxn3b shisa7b nrxn2b slc6a5 chrm4a oprl1 neto1l ppp1r9bb glra2 slc12a5a ENSDARG00000076070 ppfia3 nlgn2a syt7b slc12a5b nlgn2b nlgn4a doc2b gpr52 si:ch211-10p21.1 snap91 cacnb2a dlg2 chrna3 syn2b rims2a chrna7 syt12 rims2b hcn1 nlgn3a ompa | ENSDARG00000000503 ENSDARG00000002230 ENSDARG00000011091 ENSDARG00000011113 ENSDARG00000012019 ENSDARG00000013414 ENSDARG00000017391 ENSDARG00000017790 ENSDARG00000020609 ENSDARG00000037159 ENSDARG00000037932 ENSDARG00000038363 ENSDARG00000038918 ENSDARG00000039647 ENSDARG00000041150 ENSDARG00000041205 ENSDARG00000042974 ENSDARG00000042988 ENSDARG00000043746 ENSDARG00000045016 ENSDARG00000045145 ENSDARG00000045944 ENSDARG00000045945 ENSDARG00000051981 ENSDARG00000052642 ENSDARG00000052764 ENSDARG00000052769 ENSDARG00000052782 ENSDARG00000055559 ENSDARG00000056926 ENSDARG00000058103 ENSDARG00000058117 ENSDARG00000058492 ENSDARG00000059978 ENSDARG00000060085 ENSDARG00000060368 ENSDARG00000061165 ENSDARG00000061829 ENSDARG00000062376 ENSDARG00000062462 ENSDARG00000062693 ENSDARG00000063144 ENSDARG00000063150 ENSDARG00000067964 ENSDARG00000069254 ENSDARG00000071209 ENSDARG00000071596 ENSDARG00000071709 ENSDARG00000075012 ENSDARG00000075815 ENSDARG00000076070 ENSDARG00000077053 ENSDARG00000077329 ENSDARG00000078060 ENSDARG00000078187 ENSDARG00000079251 ENSDARG00000079455 ENSDARG00000088293 ENSDARG00000093460 ENSDARG00000095272 ENSDARG00000098809 ENSDARG00000099045 ENSDARG00000099323 ENSDARG00000100991 ENSDARG00000101054 ENSDARG00000101606 ENSDARG00000101702 ENSDARG00000101776 ENSDARG00000102690 ENSDARG00000104480 ENSDARG00000104786 ENSDARG00000105218 | | GO:0007270 | neuron-neuron synaptic transmission | biological\_process | 18 | 3.0 | 13 | 6.3e-05 | drd2b glra1 drd4a drd1b glrbb glrba drd2a glra4b slc6a5 glra2 ENSDARG00000076070 gpr52 hcn1 | ENSDARG00000011091 ENSDARG00000012019 ENSDARG00000038363 ENSDARG00000038918 ENSDARG00000052769 ENSDARG00000052782 ENSDARG00000056926 ENSDARG00000058103 ENSDARG00000067964 ENSDARG00000075012 ENSDARG00000076070 ENSDARG00000093460 ENSDARG00000104480 | | GO:0050804 | modulation of synaptic transmission | biological\_process | 26 | 4.3 | 19 | 6.6e-08 | sypb drd2b sypa slc24a2 shisa9a shisa9b drd2a si:dkey-30c15.17 cplx4a nlgn3b shisa7a shisa7b neto1l ppp1r9bb nlgn2a nlgn2b nlgn4a syt12 nlgn3a | ENSDARG00000002230 ENSDARG00000011091 ENSDARG00000042974 ENSDARG00000042988 ENSDARG00000045145 ENSDARG00000052642 ENSDARG00000056926 ENSDARG00000058492 ENSDARG00000059978 ENSDARG00000062376 ENSDARG00000062462 ENSDARG00000063144 ENSDARG00000071596 ENSDARG00000071709 ENSDARG00000077329 ENSDARG00000079251 ENSDARG00000079455 ENSDARG00000101776 ENSDARG00000104786 | | GO:0007269 | neurotransmitter secretion | biological\_process | 27 | 4.5 | 18 | 2.4e-07 | stx1b lin7a snap25a lin7b nrxn3a syn2a STX3 (1 of many) snap25b cplx4a syn1 nrxn3b ppfia3 syt7b doc2b syn2b rims2a syt12 rims2b | ENSDARG00000000503 ENSDARG00000013414 ENSDARG00000020609 ENSDARG00000037932 ENSDARG00000043746 ENSDARG00000045945 ENSDARG00000051981 ENSDARG00000058117 ENSDARG00000059978 ENSDARG00000060368 ENSDARG00000062693 ENSDARG00000077053 ENSDARG00000078060 ENSDARG00000088293 ENSDARG00000101054 ENSDARG00000101606 ENSDARG00000101776 ENSDARG00000102690 | | GO:0051260 | protein homooligomerization | biological\_process | 52 | 8.6 | 25 | 3.0e-05 | prnprs3 kctd16a kcns3a asic1a scara5 kctd16b kcnf1a kcnd2 kcnc1b si:rp71-39b20.4 kcnb2 prnpb kcna6a kcnc1a kcnc3a kcnd3 sprn2 kcnb1 kcna1a kctd8 kctd4 KCNV1 kcnv2a knca7 KCNB2 | ENSDARG00000003705 ENSDARG00000004648 ENSDARG00000006891 ENSDARG00000008329 ENSDARG00000010425 ENSDARG00000020102 ENSDARG00000027940 ENSDARG00000032799 ENSDARG00000032959 ENSDARG00000035861 ENSDARG00000038862 ENSDARG00000044048 ENSDARG00000046014 ENSDARG00000051852 ENSDARG00000055855 ENSDARG00000056101 ENSDARG00000057665 ENSDARG00000060095 ENSDARG00000062942 ENSDARG00000067507 ENSDARG00000068691 ENSDARG00000070092 ENSDARG00000076644 ENSDARG00000086571 ENSDARG00000088842 | | GO:0007601 | visual perception | biological\_process | 47 | 7.8 | 29 | 9.4e-10 | rho opn4.1 crx prph2b opn1sw2 rom1a grk7a valopa prph2l gucy2f grm6b rom1b prph2a rrh slc17a6b gnat2 opn1lw2 opn1sw1 opn3 cryaa rgra arr3a abca4a nyx abca4b rhol opn1mw1 pde6g opn4xb | ENSDARG00000002193 ENSDARG00000007553 ENSDARG00000011989 ENSDARG00000014840 ENSDARG00000017274 ENSDARG00000019752 ENSDARG00000020602 ENSDARG00000021150 ENSDARG00000021345 ENSDARG00000025504 ENSDARG00000025671 ENSDARG00000026926 ENSDARG00000038018 ENSDARG00000039534 ENSDARG00000041150 ENSDARG00000042529 ENSDARG00000044861 ENSDARG00000045677 ENSDARG00000052775 ENSDARG00000053502 ENSDARG00000054890 ENSDARG00000056511 ENSDARG00000057169 ENSDARG00000061791 ENSDARG00000062661 ENSDARG00000070666 ENSDARG00000097008 ENSDARG00000101984 ENSDARG00000103259 | | GO:0018298 | protein-chromophore linkage | biological\_process | 18 | 3.0 | 11 | 6.3e-03 | rho opn4.1 opn1sw2 valopa rrh opn1lw2 opn1sw1 opn3 rhol opn1mw1 opn4xb | ENSDARG00000002193 ENSDARG00000007553 ENSDARG00000017274 ENSDARG00000021150 ENSDARG00000039534 ENSDARG00000044861 ENSDARG00000045677 ENSDARG00000052775 ENSDARG00000070666 ENSDARG00000097008 ENSDARG00000103259 | | GO:0017157 | regulation of exocytosis | biological\_process | 20 | 3.3 | 13 | 4.1e-04 | stx1b stxbp5a syt9a stxbp5l syk syt9b sept5b syt10 RAPGEF4 (1 of many) doc2b cacna1g rims2a rims2b | ENSDARG00000000503 ENSDARG00000002656 ENSDARG00000003994 ENSDARG00000006383 ENSDARG00000008186 ENSDARG00000029239 ENSDARG00000036031 ENSDARG00000045750 ENSDARG00000075924 ENSDARG00000088293 ENSDARG00000089913 ENSDARG00000101606 ENSDARG00000102690 | | GO:0048488 | synaptic vesicle endocytosis | biological\_process | 12 | 2.0 | 8 | 3.8e-02 | syt1a si:dkey-30c15.17 nlgn3b nlgn2a nlgn2b nlgn4a syt12 nlgn3a | ENSDARG00000030614 ENSDARG00000058492 ENSDARG00000062376 ENSDARG00000077329 ENSDARG00000079251 ENSDARG00000079455 ENSDARG00000101776 ENSDARG00000104786 | | GO:0051480 | cytosolic calcium ion homeostasis | biological\_process | 21 | 3.5 | 10 | 1.4e-02 | drd2b atp2b3b calb1 calb2b calb2a slc8a4a trpc6a drd2a trpc1 atp2b2 | ENSDARG00000011091 ENSDARG00000023445 ENSDARG00000031598 ENSDARG00000036344 ENSDARG00000041062 ENSDARG00000055154 ENSDARG00000056625 ENSDARG00000056926 ENSDARG00000061203 ENSDARG00000063433 | | GO:0006171 | cAMP biosynthetic process | biological\_process | 18 | 3.0 | 12 | 7.1e-04 | drd2b adcy2b pth2 si:dkey-206f10.1 paqr7b drd1b si:ch211-132f19.7 drd2a adcy2a adra2c adcy1b adra2b | ENSDARG00000011091 ENSDARG00000014588 ENSDARG00000022951 ENSDARG00000032838 ENSDARG00000034907 ENSDARG00000038918 ENSDARG00000043141 ENSDARG00000056926 ENSDARG00000058392 ENSDARG00000069669 ENSDARG00000088634 ENSDARG00000102096 | | GO:0005576 | extracellular region | cellular\_component | 502 | 90.3 | 129 | 2.8e-03 | c3b.2 pcsk1 spon2b col8a1b adcyap1a ucmab serpina10a apol1 adamts8a fgf6a fmodb clu thbs1b col11a2 anos1a crispld1b serpinb1l3 htra1b agt gpc1a pcsk2 C2 pth2 gpc5b inhbab crhbp col5a2b si:ch211-106h11.3 crhb adcyap1b c1ql3b sst3 ache tfpia ins pyyb acana npy gh1 igfbp5a ccl19b sst1.1 oxt pomca wu:fj39g12 postna rbp4l zgc:113307 npffl adm2a acanb ENSDARG00000045979 pcolceb il15 zgc:195023 zgc:112285 nog3 col10a1a cpe ecrg4a calca sfrp1b adamts15b stc1l ihha fgf7 col8a2 timp2a olfml3a olfml3b si:dkey-6n6.1 luzp2 serpinf1 mstnb col17a1a spock3 muc5.1 si:ch211-113g11.6 prelp rln3a ccka ccl25b wisp3 cpxm1a gdf10b ccl44 sparcl1 mfi2 cxcl20 spock2 soga3b adamts13 epdl1 serpinf2a vwf crispld1a asip1 wisp2 si:ch211-186e20.7 vip col17a1b gcga scg3 nrg2b pdgfba si:ch211-180a12.2 wfdc2 wu:fb59d01 nrg3b mfap5 zgc:172246 igfbp6b cxl34b.11 omd ccl34b.1 PLA2G10 igfbp1a bcan elna agrp2 tgfbr3 adipoqb rbp4 stc2b dkk3a bmp16 igfbp7 ctgfb fbn1 | ENSDARG00000001818 ENSDARG00000002600 ENSDARG00000002732 ENSDARG00000003533 ENSDARG00000004015 ENSDARG00000005485 ENSDARG00000005924 ENSDARG00000007425 ENSDARG00000007709 ENSDARG00000009351 ENSDARG00000010294 ENSDARG00000010434 ENSDARG00000010785 ENSDARG00000012422 ENSDARG00000012896 ENSDARG00000013293 ENSDARG00000014556 ENSDARG00000014907 ENSDARG00000016412 ENSDARG00000019341 ENSDARG00000019451 ENSDARG00000019772 ENSDARG00000022951 ENSDARG00000024588 ENSDARG00000024759 ENSDARG00000024831 ENSDARG00000024847 ENSDARG00000027360 ENSDARG00000027657 ENSDARG00000027740 ENSDARG00000028521 ENSDARG00000031649 ENSDARG00000031796 ENSDARG00000034718 ENSDARG00000035350 ENSDARG00000035832 ENSDARG00000035891 ENSDARG00000036222 ENSDARG00000038185 ENSDARG00000039264 ENSDARG00000039351 ENSDARG00000040799 ENSDARG00000042845 ENSDARG00000043135 ENSDARG00000043460 ENSDARG00000043806 ENSDARG00000044684 ENSDARG00000044894 ENSDARG00000045016 ENSDARG00000045708 ENSDARG00000045799 ENSDARG00000045979 ENSDARG00000052057 ENSDARG00000052361 ENSDARG00000052948 ENSDARG00000053323 ENSDARG00000053528 ENSDARG00000054753 ENSDARG00000055874 ENSDARG00000056087 ENSDARG00000056590 ENSDARG00000057678 ENSDARG00000058252 ENSDARG00000058476 ENSDARG00000058733 ENSDARG00000059387 ENSDARG00000060893 ENSDARG00000061226 ENSDARG00000061852 ENSDARG00000062171 ENSDARG00000062487 ENSDARG00000068247 ENSDARG00000069048 ENSDARG00000069133 ENSDARG00000069415 ENSDARG00000070266 ENSDARG00000070331 ENSDARG00000070442 ENSDARG00000070597 ENSDARG00000070780 ENSDARG00000070810 ENSDARG00000070873 ENSDARG00000071048 ENSDARG00000073716 ENSDARG00000073891 ENSDARG00000074772 ENSDARG00000074989 ENSDARG00000075159 ENSDARG00000075163 ENSDARG00000075393 ENSDARG00000075455 ENSDARG00000076270 ENSDARG00000076386 ENSDARG00000076448 ENSDARG00000077231 ENSDARG00000077275 ENSDARG00000077858 ENSDARG00000077882 ENSDARG00000077960 ENSDARG00000078247 ENSDARG00000079011 ENSDARG00000079296 ENSDARG00000086288 ENSDARG00000086585 ENSDARG00000086778 ENSDARG00000087857 ENSDARG00000089187 ENSDARG00000089361 ENSDARG00000089766 ENSDARG00000090560 ENSDARG00000090722 ENSDARG00000090833 ENSDARG00000092283 ENSDARG00000093600 ENSDARG00000093608 ENSDARG00000099344 ENSDARG00000099351 ENSDARG00000099412 ENSDARG00000099634 ENSDARG00000099781 ENSDARG00000099979 ENSDARG00000100086 ENSDARG00000101199 ENSDARG00000102206 ENSDARG00000103591 ENSDARG00000103679 ENSDARG00000104138 ENSDARG00000104292 ENSDARG00000105333 | | GO:0045202 | synapse | cellular\_component | 165 | 29.7 | 109 | 1.9e-15 | stx1b slc17a6a sypb syngr1a grm2a GRM2 (1 of many) si:ch211-251b21.1 erc1a glra3 drd2b chrna10a glra1 ppfia2 lin7a syngr3a slc17a7a grm6a chrnb2b snap25a gria1a gabrb3 syngr3b grm6b grin1b grm1a gabrr3a grin1a grin2bb syt1a gabrr3b calb1 grm3 ache gria1b gria3a grin2aa sept5b calb2b atp6v0cb gsg1l gria4a gria3b lin7b grik1b calb2a slc17a6b sypa gabrr1 shisa9a syn2a STX3 (1 of many) shisa9b chrnb3a gria2b glrbb glrba gabrr2a ppfia4 gabrg2 chrna6 cpe drd2a slc17a8 snap25b si:dkey-30c15.17 gabrd cplx4a syn1 nrxn1a nlgn3b shisa7a shisa7b pcloa nrxn1b gabra1 chrm4a gria2a gabra5 ppp1r9bb grid1a rims1a glra2 ENSDARG00000076127 grm8b ppfia3 nlgn2a grin2cb grm8a grin2ca rims1b bsnb nlgn2b nlgn4a gabrb2 si:ch73-380n15.2 slc17a7b gabrz pclob gabrb4 dlg2 chrna3 syn2b grik5 rims2a chrna7 syt12 rims2b nlgn3a LRFN3 (1 of many) | ENSDARG00000000503 ENSDARG00000001127 ENSDARG00000002230 ENSDARG00000002564 ENSDARG00000004150 ENSDARG00000007195 ENSDARG00000007275 ENSDARG00000009941 ENSDARG00000011066 ENSDARG00000011091 ENSDARG00000011113 ENSDARG00000012019 ENSDARG00000013000 ENSDARG00000013414 ENSDARG00000014871 ENSDARG00000016480 ENSDARG00000017742 ENSDARG00000017790 ENSDARG00000020609 ENSDARG00000021352 ENSDARG00000023771 ENSDARG00000025034 ENSDARG00000025671 ENSDARG00000025728 ENSDARG00000026796 ENSDARG00000027153 ENSDARG00000027828 ENSDARG00000030376 ENSDARG00000030614 ENSDARG00000030750 ENSDARG00000031598 ENSDARG00000031712 ENSDARG00000031796 ENSDARG00000032714 ENSDARG00000032737 ENSDARG00000034493 ENSDARG00000036031 ENSDARG00000036344 ENSDARG00000036577 ENSDARG00000037390 ENSDARG00000037496 ENSDARG00000037498 ENSDARG00000037932 ENSDARG00000040627 ENSDARG00000041062 ENSDARG00000041150 ENSDARG00000042974 ENSDARG00000043902 ENSDARG00000045145 ENSDARG00000045945 ENSDARG00000051981 ENSDARG00000052642 ENSDARG00000052764 ENSDARG00000052765 ENSDARG00000052769 ENSDARG00000052782 ENSDARG00000052982 ENSDARG00000053205 ENSDARG00000053665 ENSDARG00000055559 ENSDARG00000055874 ENSDARG00000056926 ENSDARG00000057728 ENSDARG00000058117 ENSDARG00000058492 ENSDARG00000059763 ENSDARG00000059978 ENSDARG00000060368 ENSDARG00000061647 ENSDARG00000062376 ENSDARG00000062462 ENSDARG00000063144 ENSDARG00000063299 ENSDARG00000063635 ENSDARG00000068989 ENSDARG00000069254 ENSDARG00000070173 ENSDARG00000070730 ENSDARG00000071709 ENSDARG00000074583 ENSDARG00000074680 ENSDARG00000075012 ENSDARG00000076127 ENSDARG00000076508 ENSDARG00000077053 ENSDARG00000077329 ENSDARG00000077560 ENSDARG00000077654 ENSDARG00000078149 ENSDARG00000078902 ENSDARG00000079161 ENSDARG00000079251 ENSDARG00000079455 ENSDARG00000079586 ENSDARG00000087224 ENSDARG00000090106 ENSDARG00000094512 ENSDARG00000098880 ENSDARG00000099096 ENSDARG00000099323 ENSDARG00000100991 ENSDARG00000101054 ENSDARG00000101449 ENSDARG00000101606 ENSDARG00000101702 ENSDARG00000101776 ENSDARG00000102690 ENSDARG00000104786 ENSDARG00000105059 | | GO:0098793 | presynapse | cellular\_component | 39 | 7.0 | 29 | 2.5e-04 | stx1b slc17a6a sypb syngr1a erc1a drd2b syngr3a slc17a7a syngr3b syt1a sept5b atp6v0cb slc17a6b sypa syn2a STX3 (1 of many) drd2a slc17a8 syn1 rims1a ppfia3 rims1b bsnb slc17a7b pclob syn2b rims2a syt12 rims2b | ENSDARG00000000503 ENSDARG00000001127 ENSDARG00000002230 ENSDARG00000002564 ENSDARG00000009941 ENSDARG00000011091 ENSDARG00000014871 ENSDARG00000016480 ENSDARG00000025034 ENSDARG00000030614 ENSDARG00000036031 ENSDARG00000036577 ENSDARG00000041150 ENSDARG00000042974 ENSDARG00000045945 ENSDARG00000051981 ENSDARG00000056926 ENSDARG00000057728 ENSDARG00000060368 ENSDARG00000074680 ENSDARG00000077053 ENSDARG00000078902 ENSDARG00000079161 ENSDARG00000090106 ENSDARG00000098880 ENSDARG00000101054 ENSDARG00000101606 ENSDARG00000101776 ENSDARG00000102690 | | GO:0042734 | presynaptic membrane | cellular\_component | 13 | 2.3 | 9 | 1.8e-02 | grm2a GRM2 (1 of many) grm6a grm6b grm3 nrxn1a nrxn1b grm8b grm8a | ENSDARG00000004150 ENSDARG00000007195 ENSDARG00000017742 ENSDARG00000025671 ENSDARG00000031712 ENSDARG00000061647 ENSDARG00000063635 ENSDARG00000076508 ENSDARG00000077654 | | GO:0045211 | postsynaptic membrane | cellular\_component | 53 | 9.5 | 29 | 4.3e-07 | si:ch211-251b21.1 chrna10a glra1 chrnb2b gria1a grin1b grin1a grin2bb gria1b gria3a grin2aa gria4a gria3b grik1b chrnb3a gria2b glrbb glrba chrna6 chrm4a gria2a grid1a grin2cb grin2ca si:ch73-380n15.2 dlg2 chrna3 grik5 chrna7 | ENSDARG00000007275 ENSDARG00000011113 ENSDARG00000012019 ENSDARG00000017790 ENSDARG00000021352 ENSDARG00000025728 ENSDARG00000027828 ENSDARG00000030376 ENSDARG00000032714 ENSDARG00000032737 ENSDARG00000034493 ENSDARG00000037496 ENSDARG00000037498 ENSDARG00000040627 ENSDARG00000052764 ENSDARG00000052765 ENSDARG00000052769 ENSDARG00000052782 ENSDARG00000055559 ENSDARG00000069254 ENSDARG00000070173 ENSDARG00000074583 ENSDARG00000077560 ENSDARG00000078149 ENSDARG00000087224 ENSDARG00000099323 ENSDARG00000100991 ENSDARG00000101449 ENSDARG00000101702 | | GO:0030672 | synaptic vesicle membrane | cellular\_component | 15 | 2.7 | 12 | 7.4e-05 | slc17a6a syngr1a drd2b syngr3a slc17a7a syngr3b syt1a slc17a6b drd2a slc17a8 slc17a7b syt12 | ENSDARG00000001127 ENSDARG00000002564 ENSDARG00000011091 ENSDARG00000014871 ENSDARG00000016480 ENSDARG00000025034 ENSDARG00000030614 ENSDARG00000041150 ENSDARG00000056926 ENSDARG00000057728 ENSDARG00000090106 ENSDARG00000101776 | | GO:0030054 | cell junction | cellular\_component | 195 | 35.1 | 79 | 1.0e-13 | slc17a6a si:ch211-251b21.1 zgc:112437 cadm2a glra3 chrna10a glra1 lin7a cldn15la chrnb2b snap25a gria1a gja3 gabrb3 grin1b gabrr3a grin1a grin2bb gabrr3b ache gria1b gria3a grin2aa zgc:194261 cx27.5 cldn7a cldn15a gsg1l gria4a gria3b lin7b cadm4 grik1b gpa33 slc17a6b cx32.3 cx28.9 cldnk gabrr1 cldn19 shisa9a shisa9b chrnb3a gria2b glrbb gabrr2a gabrg2 si:ch211-244e12.7 chrna6 hepacama slc17a8 snap25b jupb gabrd cadm2b panx2 mlc1 gabra1 chrm4a gria2a gabra5 cx35b cx47.1 grid1a glra2 ENSDARG00000076127 grin2cb grin2ca gabrb2 si:ch211-214p13.3 gabrz si:dkey-91f15.1 gabrb4 cldn15lb chrna3 cldn15b grik5 chrna7 LRFN3 (1 of many) | ENSDARG00000001127 ENSDARG00000007275 ENSDARG00000009215 ENSDARG00000009930 ENSDARG00000011066 ENSDARG00000011113 ENSDARG00000012019 ENSDARG00000013414 ENSDARG00000016081 ENSDARG00000017790 ENSDARG00000020609 ENSDARG00000021352 ENSDARG00000021889 ENSDARG00000023771 ENSDARG00000025728 ENSDARG00000027153 ENSDARG00000027828 ENSDARG00000030376 ENSDARG00000030750 ENSDARG00000031796 ENSDARG00000032714 ENSDARG00000032737 ENSDARG00000034493 ENSDARG00000035340 ENSDARG00000035553 ENSDARG00000036376 ENSDARG00000036463 ENSDARG00000037390 ENSDARG00000037496 ENSDARG00000037498 ENSDARG00000037932 ENSDARG00000040291 ENSDARG00000040627 ENSDARG00000040898 ENSDARG00000041150 ENSDARG00000041787 ENSDARG00000041797 ENSDARG00000042357 ENSDARG00000043902 ENSDARG00000044569 ENSDARG00000045145 ENSDARG00000052642 ENSDARG00000052764 ENSDARG00000052765 ENSDARG00000052769 ENSDARG00000052982 ENSDARG00000053665 ENSDARG00000054744 ENSDARG00000055559 ENSDARG00000056934 ENSDARG00000057728 ENSDARG00000058117 ENSDARG00000059067 ENSDARG00000059763 ENSDARG00000062633 ENSDARG00000063019 ENSDARG00000063026 ENSDARG00000068989 ENSDARG00000069254 ENSDARG00000070173 ENSDARG00000070730 ENSDARG00000070781 ENSDARG00000073896 ENSDARG00000074583 ENSDARG00000075012 ENSDARG00000076127 ENSDARG00000077560 ENSDARG00000078149 ENSDARG00000079586 ENSDARG00000087403 ENSDARG00000094512 ENSDARG00000094990 ENSDARG00000099096 ENSDARG00000100844 ENSDARG00000100991 ENSDARG00000101109 ENSDARG00000101449 ENSDARG00000101702 ENSDARG00000105059 | | GO:0005886 | plasma membrane | cellular\_component | 961 | 172.9 | 352 | 6.3e-17 | stx1b nalcn p2rx2 rgs5a stxbp5a rasgrf2b rhcga cacng5a zgc:172270 prnprs3 crhr1 grm2a gpr22a cdhr1a scn8aa stxbp5l slc24a3 kcns3a nadl1.2 GRM2 (1 of many) ric8a si:ch211-251b21.1 avpr2aa ttyh3b syk asic1a cacna1c tspan7b rhbg zgc:112437 gng3 cadm2a scara5 glra3 drd2b chrna10a TENM2 (1 of many) glra1 vipr2 slc6a6a lin7a si:dkey-246g23.4 gucy1a3 cdh13 gucy2c adcy2b efna3a prph2b cdh4 cldn15la slc6a2 rgs7bpb si:dkey-94f20.4 grm6a chrnb2b rgs5b scn8ab atp1a3a slco3a1 mmp24 rhag gpc1a rom1a atp1a1b slc20a1a cacng8a snap25a prph2l gria1a cacna1ba gja3 cd81b epha6 atp2b3b gabrb3 cacna2d4b gpc5b gng13a dscama asic4a gucy2f grm6b grin1b grm1a gc3 rom1b pcdh17 slc38a3a gabrr3a grin1a kcnf1a stxbp6l plppr3b slc34a1a calcr vipr1a grin2bb gabrr3b mc5ra kcnj11l grm3 ache cacng2a gria1b gria3a kcnd2 si:dkey-206f10.1 kcnc1b prom1b atp1b2b grin2aa scn4ab paqr7b zgc:194261 cx27.5 gngt1 si:rp71-39b20.4 lingo1b gnao1b cldn7a si:ch73-335m24.5 cldn15a oprd1b DCHS2 gsg1l gria4a gria3b rgs11 cacna1aa gng13b rgs9a lin7b prph2a drd4a kcnb2 drd1b calhm2 slc6a1b rgs16 atp1a1a.5 cadm4 enpp6 grik1b gpa33 slc6a1l slc24a1 paqr6 cx32.3 cx28.9 tspan33b ca4b cldnk gnat2 gpr173 gng8 slc24a2 si:ch211-132f19.7 atp2b3a ca4a gabrr1 prnpb gnat1 cldn19 itgb3b shisa9a rgs9b gpr22b slc13a1 prmt8b avpr1ab slc6a1a kcna6a kcnc1a STX3 (1 of many) shisa9b chrnb3a gria2b glrbb glrba gabrr2a atp1b4 basp1 tspan3b gabrg2 slc26a6l adgrg6 clip3 slco2b1 si:ch211-244e12.7 chrna6 cdh10a kcnc3a cpe sprn kcnd3 trpc6a vamp2 drd2a abca4a pcdh1g9 glra4b snap25b adcy2a si:dkey-30c15.17 ihha jupb gabrd slc47a2 crb2b kcnq3 kcnb1 si:ch211-284e13.4 rgs7bpa slc6a4a trpc1 cdh18a nrxn1a slco2a1 TRHDE (1 of many) kcnj3b cacna1ea scn3b nlgn3b shisa7a si:ch211-23l10.2 cadm2b abca4b si:dkey-100n10.2 pcdh1a scn1lab slc6a15 kcna1a cacng7a panx2 shisa7b PCDH9 atp2b2 nrxn1b syngap1a slc6a5 ralgps1 gpr85 slc6a17 gabra1 chrm4a slc13a3 adra2c syngap1b ppap2d rgs8 KCNV1 ENSDARG00000070170 gria2a cacng7b cacng8b gabra5 cx35b slc12a10.2 oprl1 si:ch211-117c9.5 neto1l cx47.1 itga1 arvcfa grid1a glra2 kcnq2a TRHDE (1 of many) ENSDARG00000076127 cacng3b grm8b kcnv2a ttyh1 xkr8.2 calhm1 pcdhb avpr1aa gpr158a nlgn2a grin2cb grm8a slc13a5a cdh24b grin2ca best1 pcdh7a nlgn2b cacna1bb nlgn4a gabrb2 amigo1 gpr158b plppr4a si:ch73-206d17.1 ntrk3b knca7 gucy1b3 si:ch73-380n15.2 si:ch211-214p13.3 si:ch211-180a12.2 pcdh1gb9 adcy1b ngfra KCNB2 rgs1 si:dkey-262k9.4 cacna1g tnk2a scn12aa kcnq2b kcnn1a si:ch211-66e2.5 htr1aa gabrz cdc42se2 si:dkey-91f15.1 ntrk2b gpr75 erap2 snap91 pcdh1g33 cacnb2a gabrb4 atp1b2a dlg2 pcdh2ac pcdh1g26 rhcgb pcdh1g31 cldn15lb chrna3 cldn15b ano2 grik5 chrna7 scn2b pcdh1g30 adra2b ENSDARG00000102185 cacng2b pcdh1g22 gngt2b pcdh1gc6 antxr2a atp1a3b hcn1 pcdh1g29 nlgn3a pcdh1gc5 il1rapl1b sstr1a adcyap1r1a | ENSDARG00000000503 ENSDARG00000001835 ENSDARG00000002300 ENSDARG00000002644 ENSDARG00000002656 ENSDARG00000002816 ENSDARG00000003203 ENSDARG00000003326 ENSDARG00000003632 ENSDARG00000003705 ENSDARG00000003989 ENSDARG00000004150 ENSDARG00000004592 ENSDARG00000004643 ENSDARG00000005775 ENSDARG00000006383 ENSDARG00000006760 ENSDARG00000006891 ENSDARG00000007149 ENSDARG00000007195 ENSDARG00000007247 ENSDARG00000007275 ENSDARG00000007436 ENSDARG00000007678 ENSDARG00000008186 ENSDARG00000008329 ENSDARG00000008398 ENSDARG00000008407 ENSDARG00000009018 ENSDARG00000009215 ENSDARG00000009553 ENSDARG00000009930 ENSDARG00000010425 ENSDARG00000011066 ENSDARG00000011091 ENSDARG00000011113 ENSDARG00000011171 ENSDARG00000012019 ENSDARG00000012353 ENSDARG00000012534 ENSDARG00000013414 ENSDARG00000013775 ENSDARG00000013787 ENSDARG00000014215 ENSDARG00000014320 ENSDARG00000014588 ENSDARG00000014675 ENSDARG00000014840 ENSDARG00000015002 ENSDARG00000016081 ENSDARG00000016141 ENSDARG00000016742 ENSDARG00000016923 ENSDARG00000017742 ENSDARG00000017790 ENSDARG00000017860 ENSDARG00000018032 ENSDARG00000018259 ENSDARG00000018726 ENSDARG00000018896 ENSDARG00000019253 ENSDARG00000019341 ENSDARG00000019752 ENSDARG00000019856 ENSDARG00000020114 ENSDARG00000020450 ENSDARG00000020609 ENSDARG00000021345 ENSDARG00000021352 ENSDARG00000021735 ENSDARG00000021889 ENSDARG00000022437 ENSDARG00000022971 ENSDARG00000023445 ENSDARG00000023771 ENSDARG00000023886 ENSDARG00000024588 ENSDARG00000024740 ENSDARG00000024865 ENSDARG00000025162 ENSDARG00000025504 ENSDARG00000025671 ENSDARG00000025728 ENSDARG00000026796 ENSDARG00000026820 ENSDARG00000026926 ENSDARG00000027041 ENSDARG00000027065 ENSDARG00000027153 ENSDARG00000027828 ENSDARG00000027940 ENSDARG00000028354 ENSDARG00000028552 ENSDARG00000028824 ENSDARG00000028845 ENSDARG00000028878 ENSDARG00000030376 ENSDARG00000030750 ENSDARG00000031348 ENSDARG00000031438 ENSDARG00000031712 ENSDARG00000031796 ENSDARG00000032565 ENSDARG00000032714 ENSDARG00000032737 ENSDARG00000032799 ENSDARG00000032838 ENSDARG00000032959 ENSDARG00000034007 ENSDARG00000034424 ENSDARG00000034493 ENSDARG00000034588 ENSDARG00000034907 ENSDARG00000035340 ENSDARG00000035553 ENSDARG00000035798 ENSDARG00000035861 ENSDARG00000035899 ENSDARG00000036058 ENSDARG00000036376 ENSDARG00000036383 ENSDARG00000036463 ENSDARG00000037159 ENSDARG00000037286 ENSDARG00000037390 ENSDARG00000037496 ENSDARG00000037498 ENSDARG00000037646 ENSDARG00000037905 ENSDARG00000037921 ENSDARG00000037925 ENSDARG00000037932 ENSDARG00000038018 ENSDARG00000038363 ENSDARG00000038862 ENSDARG00000038918 ENSDARG00000039482 ENSDARG00000039647 ENSDARG00000040177 ENSDARG00000040252 ENSDARG00000040291 ENSDARG00000040469 ENSDARG00000040627 ENSDARG00000040898 ENSDARG00000041205 ENSDARG00000041431 ENSDARG00000041483 ENSDARG00000041787 ENSDARG00000041797 ENSDARG00000042189 ENSDARG00000042293 ENSDARG00000042357 ENSDARG00000042529 ENSDARG00000042922 ENSDARG00000042970 ENSDARG00000042988 ENSDARG00000043141 ENSDARG00000043474 ENSDARG00000043589 ENSDARG00000043902 ENSDARG00000044048 ENSDARG00000044199 ENSDARG00000044569 ENSDARG00000045070 ENSDARG00000045145 ENSDARG00000045156 ENSDARG00000045383 ENSDARG00000045638 ENSDARG00000045760 ENSDARG00000045788 ENSDARG00000045944 ENSDARG00000046014 ENSDARG00000051852 ENSDARG00000051981 ENSDARG00000052642 ENSDARG00000052764 ENSDARG00000052765 ENSDARG00000052769 ENSDARG00000052782 ENSDARG00000052982 ENSDARG00000053262 ENSDARG00000053358 ENSDARG00000053559 ENSDARG00000053665 ENSDARG00000054127 ENSDARG00000054137 ENSDARG00000054456 ENSDARG00000054609 ENSDARG00000054744 ENSDARG00000055559 ENSDARG00000055843 ENSDARG00000055855 ENSDARG00000055874 ENSDARG00000056004 ENSDARG00000056101 ENSDARG00000056625 ENSDARG00000056877 ENSDARG00000056926 ENSDARG00000057169 ENSDARG00000057519 ENSDARG00000058103 ENSDARG00000058117 ENSDARG00000058392 ENSDARG00000058492 ENSDARG00000058733 ENSDARG00000059067 ENSDARG00000059763 ENSDARG00000060051 ENSDARG00000060081 ENSDARG00000060085 ENSDARG00000060095 ENSDARG00000060330 ENSDARG00000060601 ENSDARG00000061165 ENSDARG00000061203 ENSDARG00000061371 ENSDARG00000061647 ENSDARG00000061896 ENSDARG00000062013 ENSDARG00000062217 ENSDARG00000062346 ENSDARG00000062359 ENSDARG00000062376 ENSDARG00000062462 ENSDARG00000062618 ENSDARG00000062633 ENSDARG00000062661 ENSDARG00000062672 ENSDARG00000062720 ENSDARG00000062744 ENSDARG00000062821 ENSDARG00000062942 ENSDARG00000063006 ENSDARG00000063019 ENSDARG00000063144 ENSDARG00000063264 ENSDARG00000063433 ENSDARG00000063635 ENSDARG00000063713 ENSDARG00000067964 ENSDARG00000068370 ENSDARG00000068701 ENSDARG00000068787 ENSDARG00000068989 ENSDARG00000069254 ENSDARG00000069478 ENSDARG00000069669 ENSDARG00000069765 ENSDARG00000069940 ENSDARG00000070037 ENSDARG00000070092 ENSDARG00000070170 ENSDARG00000070173 ENSDARG00000070624 ENSDARG00000070626 ENSDARG00000070730 ENSDARG00000070781 ENSDARG00000071173 ENSDARG00000071209 ENSDARG00000071235 ENSDARG00000071596 ENSDARG00000073896 ENSDARG00000074316 ENSDARG00000074329 ENSDARG00000074583 ENSDARG00000075012 ENSDARG00000075307 ENSDARG00000076044 ENSDARG00000076127 ENSDARG00000076401 ENSDARG00000076508 ENSDARG00000076644 ENSDARG00000076804 ENSDARG00000076820 ENSDARG00000077021 ENSDARG00000077023 ENSDARG00000077083 ENSDARG00000077134 ENSDARG00000077329 ENSDARG00000077560 ENSDARG00000077654 ENSDARG00000077691 ENSDARG00000077996 ENSDARG00000078149 ENSDARG00000078331 ENSDARG00000078898 ENSDARG00000079251 ENSDARG00000079295 ENSDARG00000079455 ENSDARG00000079586 ENSDARG00000079620 ENSDARG00000079665 ENSDARG00000079671 ENSDARG00000086159 ENSDARG00000086214 ENSDARG00000086571 ENSDARG00000086790 ENSDARG00000087224 ENSDARG00000087403 ENSDARG00000087857 ENSDARG00000088475 ENSDARG00000088634 ENSDARG00000088708 ENSDARG00000088842 ENSDARG00000089077 ENSDARG00000089838 ENSDARG00000089913 ENSDARG00000090646 ENSDARG00000090724 ENSDARG00000091130 ENSDARG00000091306 ENSDARG00000091579 ENSDARG00000093745 ENSDARG00000094512 ENSDARG00000094577 ENSDARG00000094990 ENSDARG00000098511 ENSDARG00000098526 ENSDARG00000098618 ENSDARG00000098809 ENSDARG00000099035 ENSDARG00000099045 ENSDARG00000099096 ENSDARG00000099203 ENSDARG00000099323 ENSDARG00000099783 ENSDARG00000099931 ENSDARG00000100265 ENSDARG00000100670 ENSDARG00000100844 ENSDARG00000100991 ENSDARG00000101109 ENSDARG00000101363 ENSDARG00000101449 ENSDARG00000101702 ENSDARG00000101713 ENSDARG00000101865 ENSDARG00000102096 ENSDARG00000102185 ENSDARG00000102376 ENSDARG00000103013 ENSDARG00000103543 ENSDARG00000103950 ENSDARG00000104118 ENSDARG00000104139 ENSDARG00000104480 ENSDARG00000104497 ENSDARG00000104786 ENSDARG00000104826 ENSDARG00000104853 ENSDARG00000104922 ENSDARG00000105201 | | GO:0005887 | integral component of plasma membrane | cellular\_component | 408 | 73.4 | 174 | 5.1e-14 | p2rx2 rhcga cacng5a grm2a gpr22a scn8aa kcns3a GRM2 (1 of many) avpr2aa asic1a tspan7b rhbg cadm2a scara5 glra3 drd2b chrna10a TENM2 (1 of many) glra1 slc6a6a si:dkey-246g23.4 prph2b slc6a2 grm6a chrnb2b scn8ab atp1a3a slco3a1 mmp24 rhag rom1a atp1a1b slc20a1a cacng8a prph2l gja3 cd81b epha6 atp2b3b dscama asic4a grm6b grin1b grm1a rom1b slc38a3a grin1a kcnf1a plppr3b grin2bb kcnj11l grm3 cacng2a gria3a kcnd2 kcnc1b atp1b2b grin2aa scn4ab si:rp71-39b20.4 si:ch73-335m24.5 oprd1b gria4a gria3b prph2a drd4a kcnb2 drd1b calhm2 slc6a1b atp1a1a.5 cadm4 slc6a1l slc24a1 tspan33b slc24a2 atp2b3a itgb3b shisa9a gpr22b slc13a1 avpr1ab slc6a1a kcna6a kcnc1a shisa9b chrnb3a gria2b atp1b4 tspan3b slc26a6l slco2b1 chrna6 kcnc3a kcnd3 trpc6a drd2a abca4a glra4b si:dkey-30c15.17 kcnq3 kcnb1 slc6a4a trpc1 slco2a1 kcnj3b scn3b nlgn3b shisa7a si:ch211-23l10.2 cadm2b abca4b si:dkey-100n10.2 scn1lab slc6a15 kcna1a cacng7a shisa7b atp2b2 slc6a5 slc6a17 chrm4a slc13a3 adra2c ppap2d KCNV1 ENSDARG00000070170 cacng7b cacng8b oprl1 si:ch211-117c9.5 neto1l itga1 glra2 kcnq2a cacng3b grm8b kcnv2a calhm1 avpr1aa nlgn2a grin2cb grm8a slc13a5a grin2ca best1 nlgn2b nlgn4a amigo1 plppr4a ntrk3b knca7 si:ch211-214p13.3 ngfra KCNB2 si:dkey-262k9.4 scn12aa kcnq2b si:ch211-66e2.5 htr1aa ntrk2b gpr75 atp1b2a dlg2 rhcgb chrna3 chrna7 scn2b adra2b cacng2b atp1a3b hcn1 nlgn3a sstr1a | ENSDARG00000002300 ENSDARG00000003203 ENSDARG00000003326 ENSDARG00000004150 ENSDARG00000004592 ENSDARG00000005775 ENSDARG00000006891 ENSDARG00000007195 ENSDARG00000007436 ENSDARG00000008329 ENSDARG00000008407 ENSDARG00000009018 ENSDARG00000009930 ENSDARG00000010425 ENSDARG00000011066 ENSDARG00000011091 ENSDARG00000011113 ENSDARG00000011171 ENSDARG00000012019 ENSDARG00000012534 ENSDARG00000013775 ENSDARG00000014840 ENSDARG00000016141 ENSDARG00000017742 ENSDARG00000017790 ENSDARG00000018032 ENSDARG00000018259 ENSDARG00000018726 ENSDARG00000018896 ENSDARG00000019253 ENSDARG00000019752 ENSDARG00000019856 ENSDARG00000020114 ENSDARG00000020450 ENSDARG00000021345 ENSDARG00000021889 ENSDARG00000022437 ENSDARG00000022971 ENSDARG00000023445 ENSDARG00000024865 ENSDARG00000025162 ENSDARG00000025671 ENSDARG00000025728 ENSDARG00000026796 ENSDARG00000026926 ENSDARG00000027065 ENSDARG00000027828 ENSDARG00000027940 ENSDARG00000028552 ENSDARG00000030376 ENSDARG00000031438 ENSDARG00000031712 ENSDARG00000032565 ENSDARG00000032737 ENSDARG00000032799 ENSDARG00000032959 ENSDARG00000034424 ENSDARG00000034493 ENSDARG00000034588 ENSDARG00000035861 ENSDARG00000036383 ENSDARG00000037159 ENSDARG00000037496 ENSDARG00000037498 ENSDARG00000038018 ENSDARG00000038363 ENSDARG00000038862 ENSDARG00000038918 ENSDARG00000039482 ENSDARG00000039647 ENSDARG00000040252 ENSDARG00000040291 ENSDARG00000041205 ENSDARG00000041431 ENSDARG00000042189 ENSDARG00000042988 ENSDARG00000043474 ENSDARG00000045070 ENSDARG00000045145 ENSDARG00000045383 ENSDARG00000045638 ENSDARG00000045788 ENSDARG00000045944 ENSDARG00000046014 ENSDARG00000051852 ENSDARG00000052642 ENSDARG00000052764 ENSDARG00000052765 ENSDARG00000053262 ENSDARG00000053559 ENSDARG00000054127 ENSDARG00000054609 ENSDARG00000055559 ENSDARG00000055855 ENSDARG00000056101 ENSDARG00000056625 ENSDARG00000056926 ENSDARG00000057169 ENSDARG00000058103 ENSDARG00000058492 ENSDARG00000060085 ENSDARG00000060095 ENSDARG00000061165 ENSDARG00000061203 ENSDARG00000061896 ENSDARG00000062217 ENSDARG00000062359 ENSDARG00000062376 ENSDARG00000062462 ENSDARG00000062618 ENSDARG00000062633 ENSDARG00000062661 ENSDARG00000062672 ENSDARG00000062744 ENSDARG00000062821 ENSDARG00000062942 ENSDARG00000063006 ENSDARG00000063144 ENSDARG00000063433 ENSDARG00000067964 ENSDARG00000068787 ENSDARG00000069254 ENSDARG00000069478 ENSDARG00000069669 ENSDARG00000069940 ENSDARG00000070092 ENSDARG00000070170 ENSDARG00000070624 ENSDARG00000070626 ENSDARG00000071209 ENSDARG00000071235 ENSDARG00000071596 ENSDARG00000074316 ENSDARG00000075012 ENSDARG00000075307 ENSDARG00000076401 ENSDARG00000076508 ENSDARG00000076644 ENSDARG00000077021 ENSDARG00000077083 ENSDARG00000077329 ENSDARG00000077560 ENSDARG00000077654 ENSDARG00000077691 ENSDARG00000078149 ENSDARG00000078331 ENSDARG00000079251 ENSDARG00000079455 ENSDARG00000079620 ENSDARG00000079671 ENSDARG00000086214 ENSDARG00000086571 ENSDARG00000087403 ENSDARG00000088708 ENSDARG00000088842 ENSDARG00000089838 ENSDARG00000090724 ENSDARG00000091130 ENSDARG00000091579 ENSDARG00000093745 ENSDARG00000098511 ENSDARG00000098526 ENSDARG00000099203 ENSDARG00000099323 ENSDARG00000100265 ENSDARG00000100991 ENSDARG00000101702 ENSDARG00000101713 ENSDARG00000102096 ENSDARG00000102376 ENSDARG00000104139 ENSDARG00000104480 ENSDARG00000104786 ENSDARG00000104922 | | GO:0042734 | presynaptic membrane | cellular\_component | 13 | 2.3 | 9 | 1.8e-02 | grm2a GRM2 (1 of many) grm6a grm6b grm3 nrxn1a nrxn1b grm8b grm8a | ENSDARG00000004150 ENSDARG00000007195 ENSDARG00000017742 ENSDARG00000025671 ENSDARG00000031712 ENSDARG00000061647 ENSDARG00000063635 ENSDARG00000076508 ENSDARG00000077654 | | GO:0045211 | postsynaptic membrane | cellular\_component | 53 | 9.5 | 29 | 4.3e-07 | si:ch211-251b21.1 chrna10a glra1 chrnb2b gria1a grin1b grin1a grin2bb gria1b gria3a grin2aa gria4a gria3b grik1b chrnb3a gria2b glrbb glrba chrna6 chrm4a gria2a grid1a grin2cb grin2ca si:ch73-380n15.2 dlg2 chrna3 grik5 chrna7 | ENSDARG00000007275 ENSDARG00000011113 ENSDARG00000012019 ENSDARG00000017790 ENSDARG00000021352 ENSDARG00000025728 ENSDARG00000027828 ENSDARG00000030376 ENSDARG00000032714 ENSDARG00000032737 ENSDARG00000034493 ENSDARG00000037496 ENSDARG00000037498 ENSDARG00000040627 ENSDARG00000052764 ENSDARG00000052765 ENSDARG00000052769 ENSDARG00000052782 ENSDARG00000055559 ENSDARG00000069254 ENSDARG00000070173 ENSDARG00000074583 ENSDARG00000077560 ENSDARG00000078149 ENSDARG00000087224 ENSDARG00000099323 ENSDARG00000100991 ENSDARG00000101449 ENSDARG00000101702 | | GO:0001518 | voltage-gated sodium channel complex | cellular\_component | 11 | 2.0 | 8 | 2.8e-02 | scn8aa scn8ab scn4ab scn3b scn1lab ENSDARG00000070170 scn12aa scn2b | ENSDARG00000005775 ENSDARG00000018032 ENSDARG00000034588 ENSDARG00000062359 ENSDARG00000062744 ENSDARG00000070170 ENSDARG00000090724 ENSDARG00000101713 | | GO:0008076 | voltage-gated potassium channel complex | cellular\_component | 25 | 4.5 | 19 | 9.4e-08 | kcns3a kcnf1a kcnd2 kcnc1b si:rp71-39b20.4 kcnb2 kcna6a kcnc1a kcnc3a kcnd3 kcnq3 kcnb1 kcna1a KCNV1 kcnq2a kcnv2a knca7 KCNB2 kcnq2b | ENSDARG00000006891 ENSDARG00000027940 ENSDARG00000032799 ENSDARG00000032959 ENSDARG00000035861 ENSDARG00000038862 ENSDARG00000046014 ENSDARG00000051852 ENSDARG00000055855 ENSDARG00000056101 ENSDARG00000060085 ENSDARG00000060095 ENSDARG00000062942 ENSDARG00000070092 ENSDARG00000075307 ENSDARG00000076644 ENSDARG00000086571 ENSDARG00000088842 ENSDARG00000091130 | | GO:0032281 | AMPA glutamate receptor complex | cellular\_component | 17 | 3.1 | 16 | 4.1e-09 | cacng5a cacng8a cacng2a gria3a gria4a gria3b shisa9a shisa9b gria2b shisa7a cacng7a shisa7b cacng7b cacng8b cacng3b cacng2b | ENSDARG00000003326 ENSDARG00000020450 ENSDARG00000032565 ENSDARG00000032737 ENSDARG00000037496 ENSDARG00000037498 ENSDARG00000045145 ENSDARG00000052642 ENSDARG00000052765 ENSDARG00000062462 ENSDARG00000063006 ENSDARG00000063144 ENSDARG00000070624 ENSDARG00000070626 ENSDARG00000076401 ENSDARG00000102376 | | GO:0016021 | integral component of membrane | cellular\_component | 2838 | 510.7 | 800 | 2.5e-28 | stx1b slc17a6a csmd2 tnfrsf21 nalcn hsd11b2 rho sypb p2rx2 tlcd1 syngr1a stxbp5a SLC4A5 (1 of many) lrrc4.2 rhcga ANO2 (1 of many) cacng5a tenm1 zgc:172270 slc5a8l crhr1 syt9a grm2a tmem63c slc45a1 vangl1 zgc:194665 gpr22a gpm6ab cdhr1a gpm6ba scn8aa slc25a6 ugt1a1 stxbp5l nrcama gpr27 slitrk2 tmem178b slc24a3 galnt9 kcns3a nadl1.2 aig1 GRM2 (1 of many) si:ch211-251b21.1 slc2a1b apol1 avpr2aa opn4.1 ttyh3b scara3 slc7a10a asic1a cacna1c tspan7b faah2a RDH13 (1 of many) rhbg cnr1 zgc:112437 tmem30c rgs9bp cadm2a slc1a6 vstm4a fmodb scara5 clic2 camk2b1 glra3 drd2b chrna10a TENM2 (1 of many) trpm1a syt5b bmpr2a glra1 cnga1 vipr2 slc6a6a ENSDARG00000013252 g6pca.2 zgc:92113 si:dkey-246g23.4 gucy1a3 gucy2c trpv6 adcy2b lrrc4bb prph2b syngr3a robo2 cnih3 cdh4 atp6v0a1b comta slc4a8 ano5a cldn15la slc6a2 lctlb slc7a1 fut9a ugt5a1 slc17a7a prlra gabbr1b tcirg1a nr4a2a pdzk1ip1 opn1sw2 kidins220b ncam2 reep3a grm6a chrnb2b scn8ab ptchd1 atp1a3a slco3a1 mmp24 lrit1a rhag gpc1a pcsk2 rom1a impg2a atp1a1b ptprdb bmpr2b slc20a1a slc1a1 cacng8a otofb tmem178 rtn1b valopa zgc:165604 prph2l gria1a lhfpl3 cacna1ba gja3 cd81b cox4i2 cyp2p9 epha6 atp2b3b galnt14 epha8 mao gabrb3 cacna2d4b dpp6b dscama syngr3b asic4a gipr gucy2f grm6b grin1b xkr7 susd4 tmem41ab grm1a gc3 rom1b pcdh17 slc38a3a gabrr3a slc25a4 slc25a3a grin1a kcnf1a dscamb stxbp6l c1ql3b plppr3b gdap1l1 slc34a1a calcr vipr1a tm6sf2 syt9b CNGA1 (1 of many) mfsd2b scdb grin2bb syt1a lrit2 b3gat2 gabrr3b vamp1 mc5ra kcnj11l tmem200a grm3 clstn1 tmc4 trpa1b cacng2a gria1b gria3a kcnd2 si:dkey-206f10.1 kcnc1b tmc2a gpr37b igsf9ba prom1b zgc:92275 lrp11 lingo1a atp1b2b grin2aa scn4ab zdhhc2 paqr7b tmem136b zgc:194261 agtr2 cx27.5 gbgt1l3 ext1c clcn4 si:rp71-39b20.4 acana lingo1b cldn7a si:ch73-335m24.5 cldn15a syt4 atp6v0cb st8sia5 st6galnac6 XKR7 (1 of many) dnajc22 slc8a4b oprd1b DCHS2 gsg1l march1 gria4a gria3b cacna1aa syt5a prph2a hyal6 tmem86b drd4a st3gal1l2 ajap1 slc25a36a mrc1b kcnb2 tmx3 drd1b prom2 calhm2 rrh slc6a1b atp1a1a.5 cadm4 enpp6 lrrc32 grik1b kcnab1b gpa33 slc17a6b slc6a1l lim2.5 atp6ap1a slc24a1 paqr6 itm2bb TMEM27 tmem237a cx32.3 mgat4c cx28.9 CNGB1 tspan33b ca4b cldnk syndig1l wbscr17 gfra2a disp2 zdhhc8a gpr173 sypa fpr1 slc24a2 si:ch211-132f19.7 slc1a3b xkr6b itm2ca atp2b3a srd5a2a ca4a cnih2 nrxn3a ms4a17a.7 st6galnac1.1 nptnb gabrr1 cldn19 stx12l nrsn1 opn1lw2 itgb3b aqp8a.1 shisa9a tmem204 gpr22b lrtm2a si:dkey-14k9.2 slc13a1 opn1sw1 syt10 avpr1ab abhd2b lrtm2b slc6a1a kcna6a si:ch211-170d8.5 tmeff2a kcnc1a abcc8 cyb5r2 STX3 (1 of many) slc1a2a zgc:110789 porcn tnmd shisa9b hs3st4 lrrtm1 hmgcra chrnb3a gria2b glrbb opn3 glrba gabrr2a slc35f1 cyp8b1 atp1b4 rprma aqp9b jakmip2 tspan3b ms4a17a.11 camk2a csf1rb gabrg2 slc2a11a slc26a6l adgrg6 slco2b1 si:ch211-244e12.7 rgra lrfn4a slc8a4a gpm6aa appb chrna6 pcnxl2 march9 SLC3A2 (1 of many) cdh10a kcnc3a msmo1 steap4 GUCY2C (1 of many) kcnd3 syt11b abcb9 slc35g2a march4 MANEAL ENSDARG00000056572 trpc6a rprml gpr37l1b svopa vamp2 drd2a hepacama stoml3b abca4a sv2ba pcdh1g9 sprn2 sfrp1b si:ch211-137i24.10 slc17a8 syt11a ccdc136b ugt5e1 glra4b cpt1b adcy2a si:dkey-30c15.17 gdap1 ddr2b tmeff2b sema5a mmp16b cntnap2a atpv0e2 gpr146 slc43a3a gabrd slc32a1 hs3st3l sv2c slc47a2 crb2b kcnq3 kcnb1 timm17b slc4a10b atl1 clstn2 CLSTN2 (1 of many) tm4sf18 sv2bb pear1 abhd6a cabp7b dner rnf182 snx19a adgrl3.1 slc6a4a trpc1 cdh18a lrp1bb tmem121a gldn nrxn1a ca14 zpld1a slco2a1 lingo3a kcnh4b TRHDE (1 of many) si:ch211-236l14.4 clcn1a kiaa1549lb nceh1b mxra8b kcnab2b prrt1 kcnj3b adam23a cacna1ea scn3b nlgn3b shisa7a kiaa1549la kcnh4a si:ch211-23l10.2 cadm2b abca4b si:dkey-100n10.2 megf11 kcnh7 gpnmb nrxn3b pcdh1a scn1lab pllp slc6a15 steap2 gpr12 kcna1a MARCH3 cacng7a si:dkeyp-27e10.3 antxr2b panx2 mlc1 syne1b shisa7b tmtc1 nrxn2b erbb4a PCDH9 SLC35G1 neto2b cers1 atp2b2 nrxn1b slc6a5 cngb1a tmem91 gpr85 slc6a17 gabra1 ENSDARG00000069038 kcnh5b fam19a5a gramd4a chrm4a moxd1l tnfsf12 lrrc4.1 col17a1a slc13a3 tmem150c ENSDARG00000069607 emp1 adra2c fndc5a ppap2d lingo2b cspg5a lrp8 KCNV1 ENSDARG00000070170 gria2a spock3 lactbl1b clic5b cacng7b cacng8b rhol cnga3a gabra5 cx35b si:ch211-232m10.6 gpr155b slc12a10.2 oprl1 si:ch211-117c9.5 lrrtm2 aqp8a.2 neto1l clstn3 cx47.1 cntnap5a tecpr1b paqr4b itga1 elfn1b hcn4l amigo3 cntnap1 fam57bb grid1a slitrk3b impg1b slc5a7a ptpn5 si:ch1073-291c23.2 glra2 adgrb1a gprc5bb lrrc38a zdhhc22 cntnap5b si:dkeyp-14d3.1 kcnq2a gramd1ba soga3b slc4a11 asphd1 syt3 fam155a TRHDE (1 of many) sez6l2 ENSDARG00000076127 ano9a brinp3a.1 cacng3b grm8b kcnv2a syt6a ttyh1 xkr8.2 dpp6a lhfpl4b calhm1 pcdhb gprc5ba avpr1aa si:ch211-196g2.4 si:ch211-180f4.1 gpr158a nmbb atrnl1a timd4 ano11 nlgn2a SORCS3 b4galnt1b FAM163A (1 of many) lrfn2b grin2cb LRAT grm8a slc13a5a cdh24b syt7b lrfn4b grin2ca slc12a5b aatka best1 robo2 lingo4b adgrb1b micu3a tmem176 myrf sdk1a pcdh7a lhfpl4a col17a1b si:ch211-229d2.5 ace adam11 nlgn2b cacna1bb nlgn4a ptprh gabrb2 amigo1 gpr158b plppr4a tmem163a ENSDARG00000079873 tmem151a lrrtm4l1 si:dkey-71b5.7 jam3a si:ch73-206d17.1 ntrk3b knca7 nrg2b RGS9BP si:ch73-380n15.2 reep6 si:ch211-214p13.3 si:dkey-237h12.3 gpr153 tmem240a si:dkey-26c10.5 si:ch211-103f14.3 pcdh1gb9 si:ch73-352p18.4 adcy1b ngfra KCNB2 caln1 bcl2b cyp46a1.3 si:ch211-110e21.3 wu:fb59d01 tmem176l.2 nrg3b si:dkey-262k9.4 cacna1g slc17a7b tmem196b si:dkeyp-73b11.8 tusc5a scn12aa TMEM184B (1 of many) atp6ap1lb pkhd1l1 kcnq2b ucp3 mylk4a kcnn1a atp6ap1la si:ch211-66e2.5 si:ch73-240m12.1 sptssb adgrf6 si:ch211-198m17.1 ugt5b4 si:dkey-70p6.1 ENSDARG00000092260 cxl34b.11 si:dkey-56e3.2 si:ch211-198d23.1 gpr52 htr1aa gabrz gpr186 si:dkey-91f15.1 thsd7ba si:dkey-250k15.7 tmem119a si:ch211-10p21.1 trpm2 alk tmem265 cd164l2 si:ch211-276a17.5 si:ch73-103l1.2 si:ch73-344o19.1 tmem176l.3b ENSDARG00000096908 opn1mw1 si:dkey-56f14.4 si:ch211-270g19.5 si:ch73-250a16.5 si:dkey-7j14.5 VAMP1 (1 of many) si:dkey-35i13.1 si:ch211-248a14.8 si:ch73-156o22.2 si:dkey-239b22.2 lrp1aa si:dkey-262k9.2 dscaml1 zgc:77748 aplp1 si:ch73-366l1.5 ntrk2b gpr75 scn4ba pcdh1g33 cacnb2a gabrb4 bcl2l16 atp1b2a ugt5b3 dlg2 lrit1b ephx4 si:ch1073-13h15.3 pcdh2ac cspg5b pcdh1g26 tgfbr3 hrasls si:ch211-161c3.5 rhcgb impg2b pcdh1g31 cldn15lb sez6a tmprss13a fxyd6 chrna3 cldn15b cyp1c1 ano2 si:ch73-193i22.1 ENSDARG00000101368 fam69b grik5 tecra chrna7 scn2b syt12 pcdh1g30 adra2b SDK2 (1 of many) ENSDARG00000102185 jph3 cacng2b ENSDARG00000102406 opn6a slc1a2b hmp19 pcdh1g22 slc35f4 opn4xb dgat1a prrt2 si:dkey-36i7.3 pcdh1gc6 mag antxr2a atp1a3b slc1a8a si:dkey-283b1.6 slc4a5 rnasekb hcn1 pcdh1g29 nlgn3a pcdh1gc5 il1rapl1b sstr1a si:dkey-225f23.5 slc27a1b ugt5b2 fam69c LRFN3 (1 of many) chd5 ifitm5 adcyap1r1a | ENSDARG00000000503 ENSDARG00000001127 ENSDARG00000001559 ENSDARG00000001807 ENSDARG00000001835 ENSDARG00000001975 ENSDARG00000002193 ENSDARG00000002230 ENSDARG00000002300 ENSDARG00000002391 ENSDARG00000002564 ENSDARG00000002656 ENSDARG00000002771 ENSDARG00000003020 ENSDARG00000003203 ENSDARG00000003210 ENSDARG00000003326 ENSDARG00000003403 ENSDARG00000003632 ENSDARG00000003697 ENSDARG00000003989 ENSDARG00000003994 ENSDARG00000004150 ENSDARG00000004158 ENSDARG00000004302 ENSDARG00000004305 ENSDARG00000004577 ENSDARG00000004592 ENSDARG00000004621 ENSDARG00000004643 ENSDARG00000005739 ENSDARG00000005775 ENSDARG00000005853 ENSDARG00000006220 ENSDARG00000006383 ENSDARG00000006396 ENSDARG00000006607 ENSDARG00000006636 ENSDARG00000006747 ENSDARG00000006760 ENSDARG00000006832 ENSDARG00000006891 ENSDARG00000007149 ENSDARG00000007171 ENSDARG00000007195 ENSDARG00000007275 ENSDARG00000007412 ENSDARG00000007425 ENSDARG00000007436 ENSDARG00000007553 ENSDARG00000007678 ENSDARG00000008060 ENSDARG00000008100 ENSDARG00000008329 ENSDARG00000008398 ENSDARG00000008407 ENSDARG00000008457 ENSDARG00000008491 ENSDARG00000009018 ENSDARG00000009020 ENSDARG00000009215 ENSDARG00000009430 ENSDARG00000009466 ENSDARG00000009930 ENSDARG00000010096 ENSDARG00000010154 ENSDARG00000010294 ENSDARG00000010425 ENSDARG00000010625 ENSDARG00000011065 ENSDARG00000011066 ENSDARG00000011091 ENSDARG00000011113 ENSDARG00000011171 ENSDARG00000011259 ENSDARG00000011640 ENSDARG00000011941 ENSDARG00000012019 ENSDARG00000012125 ENSDARG00000012353 ENSDARG00000012534 ENSDARG00000013252 ENSDARG00000013721 ENSDARG00000013749 ENSDARG00000013775 ENSDARG00000013787 ENSDARG00000014320 ENSDARG00000014496 ENSDARG00000014588 ENSDARG00000014792 ENSDARG00000014840 ENSDARG00000014871 ENSDARG00000014891 ENSDARG00000014953 ENSDARG00000015002 ENSDARG00000015174 ENSDARG00000015337 ENSDARG00000015531 ENSDARG00000015731 ENSDARG00000016081 ENSDARG00000016141 ENSDARG00000016337 ENSDARG00000016439 ENSDARG00000016460 ENSDARG00000016479 ENSDARG00000016480 ENSDARG00000016570 ENSDARG00000016667 ENSDARG00000016835 ENSDARG00000017007 ENSDARG00000017127 ENSDARG00000017274 ENSDARG00000017338 ENSDARG00000017466 ENSDARG00000017569 ENSDARG00000017742 ENSDARG00000017790 ENSDARG00000018032 ENSDARG00000018066 ENSDARG00000018259 ENSDARG00000018726 ENSDARG00000018896 ENSDARG00000019179 ENSDARG00000019253 ENSDARG00000019341 ENSDARG00000019451 ENSDARG00000019752 ENSDARG00000019782 ENSDARG00000019856 ENSDARG00000019945 ENSDARG00000020057 ENSDARG00000020114 ENSDARG00000020212 ENSDARG00000020450 ENSDARG00000020581 ENSDARG00000020758 ENSDARG00000021143 ENSDARG00000021150 ENSDARG00000021241 ENSDARG00000021345 ENSDARG00000021352 ENSDARG00000021595 ENSDARG00000021735 ENSDARG00000021889 ENSDARG00000022437 ENSDARG00000022509 ENSDARG00000022631 ENSDARG00000022971 ENSDARG00000023445 ENSDARG00000023448 ENSDARG00000023609 ENSDARG00000023712 ENSDARG00000023771 ENSDARG00000023886 ENSDARG00000024744 ENSDARG00000024865 ENSDARG00000025034 ENSDARG00000025162 ENSDARG00000025478 ENSDARG00000025504 ENSDARG00000025671 ENSDARG00000025728 ENSDARG00000026333 ENSDARG00000026335 ENSDARG00000026771 ENSDARG00000026796 ENSDARG00000026820 ENSDARG00000026926 ENSDARG00000027041 ENSDARG00000027065 ENSDARG00000027153 ENSDARG00000027355 ENSDARG00000027424 ENSDARG00000027828 ENSDARG00000027940 ENSDARG00000028118 ENSDARG00000028354 ENSDARG00000028521 ENSDARG00000028552 ENSDARG00000028628 ENSDARG00000028824 ENSDARG00000028845 ENSDARG00000028878 ENSDARG00000029057 ENSDARG00000029239 ENSDARG00000029898 ENSDARG00000030263 ENSDARG00000030265 ENSDARG00000030376 ENSDARG00000030614 ENSDARG00000030626 ENSDARG00000030733 ENSDARG00000030750 ENSDARG00000031283 ENSDARG00000031348 ENSDARG00000031438 ENSDARG00000031540 ENSDARG00000031712 ENSDARG00000031720 ENSDARG00000031757 ENSDARG00000031875 ENSDARG00000032565 ENSDARG00000032714 ENSDARG00000032737 ENSDARG00000032799 ENSDARG00000032838 ENSDARG00000032959 ENSDARG00000033104 ENSDARG00000033296 ENSDARG00000033845 ENSDARG00000034007 ENSDARG00000034048 ENSDARG00000034076 ENSDARG00000034165 ENSDARG00000034424 ENSDARG00000034493 ENSDARG00000034588 ENSDARG00000034757 ENSDARG00000034907 ENSDARG00000035163 ENSDARG00000035340 ENSDARG00000035552 ENSDARG00000035553 ENSDARG00000035555 ENSDARG00000035649 ENSDARG00000035808 ENSDARG00000035861 ENSDARG00000035891 ENSDARG00000035899 ENSDARG00000036376 ENSDARG00000036383 ENSDARG00000036463 ENSDARG00000036505 ENSDARG00000036577 ENSDARG00000036584 ENSDARG00000036913 ENSDARG00000036952 ENSDARG00000037067 ENSDARG00000037145 ENSDARG00000037159 ENSDARG00000037286 ENSDARG00000037390 ENSDARG00000037487 ENSDARG00000037496 ENSDARG00000037498 ENSDARG00000037905 ENSDARG00000037941 ENSDARG00000038018 ENSDARG00000038166 ENSDARG00000038296 ENSDARG00000038363 ENSDARG00000038533 ENSDARG00000038655 ENSDARG00000038731 ENSDARG00000038822 ENSDARG00000038862 ENSDARG00000038894 ENSDARG00000038918 ENSDARG00000039406 ENSDARG00000039482 ENSDARG00000039534 ENSDARG00000039647 ENSDARG00000040252 ENSDARG00000040291 ENSDARG00000040469 ENSDARG00000040621 ENSDARG00000040627 ENSDARG00000040741 ENSDARG00000040898 ENSDARG00000041150 ENSDARG00000041205 ENSDARG00000041295 ENSDARG00000041417 ENSDARG00000041431 ENSDARG00000041483 ENSDARG00000041505 ENSDARG00000041644 ENSDARG00000041735 ENSDARG00000041787 ENSDARG00000041791 ENSDARG00000041797 ENSDARG00000042107 ENSDARG00000042189 ENSDARG00000042293 ENSDARG00000042357 ENSDARG00000042390 ENSDARG00000042418 ENSDARG00000042723 ENSDARG00000042846 ENSDARG00000042872 ENSDARG00000042922 ENSDARG00000042974 ENSDARG00000042984 ENSDARG00000042988 ENSDARG00000043141 ENSDARG00000043148 ENSDARG00000043410 ENSDARG00000043448 ENSDARG00000043474 ENSDARG00000043587 ENSDARG00000043589 ENSDARG00000043662 ENSDARG00000043746 ENSDARG00000043796 ENSDARG00000043814 ENSDARG00000043864 ENSDARG00000043902 ENSDARG00000044569 ENSDARG00000044605 ENSDARG00000044719 ENSDARG00000044861 ENSDARG00000045070 ENSDARG00000045141 ENSDARG00000045145 ENSDARG00000045273 ENSDARG00000045383 ENSDARG00000045483 ENSDARG00000045594 ENSDARG00000045638 ENSDARG00000045677 ENSDARG00000045750 ENSDARG00000045788 ENSDARG00000045804 ENSDARG00000045811 ENSDARG00000045944 ENSDARG00000046014 ENSDARG00000046142 ENSDARG00000051824 ENSDARG00000051852 ENSDARG00000051879 ENSDARG00000051925 ENSDARG00000051981 ENSDARG00000052138 ENSDARG00000052497 ENSDARG00000052558 ENSDARG00000052615 ENSDARG00000052642 ENSDARG00000052648 ENSDARG00000052713 ENSDARG00000052734 ENSDARG00000052764 ENSDARG00000052765 ENSDARG00000052769 ENSDARG00000052775 ENSDARG00000052782 ENSDARG00000052982 ENSDARG00000053003 ENSDARG00000053068 ENSDARG00000053262 ENSDARG00000053383 ENSDARG00000053480 ENSDARG00000053547 ENSDARG00000053559 ENSDARG00000053561 ENSDARG00000053617 ENSDARG00000053624 ENSDARG00000053665 ENSDARG00000053961 ENSDARG00000054127 ENSDARG00000054137 ENSDARG00000054609 ENSDARG00000054744 ENSDARG00000054890 ENSDARG00000055132 ENSDARG00000055154 ENSDARG00000055455 ENSDARG00000055543 ENSDARG00000055559 ENSDARG00000055698 ENSDARG00000055740 ENSDARG00000055791 ENSDARG00000055843 ENSDARG00000055855 ENSDARG00000055876 ENSDARG00000055901 ENSDARG00000056045 ENSDARG00000056101 ENSDARG00000056105 ENSDARG00000056200 ENSDARG00000056262 ENSDARG00000056439 ENSDARG00000056450 ENSDARG00000056572 ENSDARG00000056625 ENSDARG00000056768 ENSDARG00000056774 ENSDARG00000056833 ENSDARG00000056877 ENSDARG00000056926 ENSDARG00000056934 ENSDARG00000057035 ENSDARG00000057169 ENSDARG00000057427 ENSDARG00000057519 ENSDARG00000057665 ENSDARG00000057678 ENSDARG00000057706 ENSDARG00000057728 ENSDARG00000057913 ENSDARG00000057921 ENSDARG00000058048 ENSDARG00000058103 ENSDARG00000058285 ENSDARG00000058392 ENSDARG00000058492 ENSDARG00000058601 ENSDARG00000058695 ENSDARG00000058699 ENSDARG00000058821 ENSDARG00000058876 ENSDARG00000058969 ENSDARG00000059057 ENSDARG00000059610 ENSDARG00000059682 ENSDARG00000059763 ENSDARG00000059775 ENSDARG00000059903 ENSDARG00000059997 ENSDARG00000060051 ENSDARG00000060081 ENSDARG00000060085 ENSDARG00000060095 ENSDARG00000060124 ENSDARG00000060303 ENSDARG00000060481 ENSDARG00000060637 ENSDARG00000060638 ENSDARG00000060668 ENSDARG00000060711 ENSDARG00000060753 ENSDARG00000060756 ENSDARG00000060846 ENSDARG00000061031 ENSDARG00000061049 ENSDARG00000061101 ENSDARG00000061121 ENSDARG00000061165 ENSDARG00000061203 ENSDARG00000061371 ENSDARG00000061517 ENSDARG00000061562 ENSDARG00000061576 ENSDARG00000061647 ENSDARG00000061697 ENSDARG00000061835 ENSDARG00000061896 ENSDARG00000061970 ENSDARG00000061990 ENSDARG00000062013 ENSDARG00000062059 ENSDARG00000062084 ENSDARG00000062086 ENSDARG00000062087 ENSDARG00000062129 ENSDARG00000062134 ENSDARG00000062208 ENSDARG00000062217 ENSDARG00000062323 ENSDARG00000062346 ENSDARG00000062359 ENSDARG00000062376 ENSDARG00000062462 ENSDARG00000062477 ENSDARG00000062565 ENSDARG00000062618 ENSDARG00000062633 ENSDARG00000062661 ENSDARG00000062672 ENSDARG00000062686 ENSDARG00000062687 ENSDARG00000062688 ENSDARG00000062693 ENSDARG00000062720 ENSDARG00000062744 ENSDARG00000062756 ENSDARG00000062821 ENSDARG00000062887 ENSDARG00000062934 ENSDARG00000062942 ENSDARG00000062959 ENSDARG00000063006 ENSDARG00000063008 ENSDARG00000063011 ENSDARG00000063019 ENSDARG00000063026 ENSDARG00000063068 ENSDARG00000063144 ENSDARG00000063149 ENSDARG00000063150 ENSDARG00000063207 ENSDARG00000063264 ENSDARG00000063268 ENSDARG00000063293 ENSDARG00000063412 ENSDARG00000063433 ENSDARG00000063635 ENSDARG00000067964 ENSDARG00000068242 ENSDARG00000068456 ENSDARG00000068701 ENSDARG00000068787 ENSDARG00000068989 ENSDARG00000069038 ENSDARG00000069117 ENSDARG00000069160 ENSDARG00000069171 ENSDARG00000069254 ENSDARG00000069296 ENSDARG00000069376 ENSDARG00000069402 ENSDARG00000069415 ENSDARG00000069478 ENSDARG00000069590 ENSDARG00000069607 ENSDARG00000069632 ENSDARG00000069669 ENSDARG00000069739 ENSDARG00000069940 ENSDARG00000069970 ENSDARG00000069981 ENSDARG00000070074 ENSDARG00000070092 ENSDARG00000070170 ENSDARG00000070173 ENSDARG00000070266 ENSDARG00000070467 ENSDARG00000070584 ENSDARG00000070624 ENSDARG00000070626 ENSDARG00000070666 ENSDARG00000070726 ENSDARG00000070730 ENSDARG00000070781 ENSDARG00000070931 ENSDARG00000071086 ENSDARG00000071173 ENSDARG00000071209 ENSDARG00000071235 ENSDARG00000071374 ENSDARG00000071592 ENSDARG00000071596 ENSDARG00000073883 ENSDARG00000073896 ENSDARG00000073920 ENSDARG00000074086 ENSDARG00000074160 ENSDARG00000074316 ENSDARG00000074372 ENSDARG00000074419 ENSDARG00000074469 ENSDARG00000074524 ENSDARG00000074564 ENSDARG00000074583 ENSDARG00000074739 ENSDARG00000074839 ENSDARG00000074860 ENSDARG00000074866 ENSDARG00000074869 ENSDARG00000075012 ENSDARG00000075133 ENSDARG00000075141 ENSDARG00000075147 ENSDARG00000075170 ENSDARG00000075189 ENSDARG00000075277 ENSDARG00000075307 ENSDARG00000075383 ENSDARG00000075455 ENSDARG00000075532 ENSDARG00000075813 ENSDARG00000075830 ENSDARG00000075858 ENSDARG00000076044 ENSDARG00000076052 ENSDARG00000076127 ENSDARG00000076320 ENSDARG00000076351 ENSDARG00000076401 ENSDARG00000076508 ENSDARG00000076644 ENSDARG00000076730 ENSDARG00000076804 ENSDARG00000076820 ENSDARG00000076826 ENSDARG00000076943 ENSDARG00000077021 ENSDARG00000077023 ENSDARG00000077080 ENSDARG00000077083 ENSDARG00000077087 ENSDARG00000077112 ENSDARG00000077134 ENSDARG00000077167 ENSDARG00000077188 ENSDARG00000077257 ENSDARG00000077327 ENSDARG00000077329 ENSDARG00000077349 ENSDARG00000077352 ENSDARG00000077470 ENSDARG00000077489 ENSDARG00000077560 ENSDARG00000077652 ENSDARG00000077654 ENSDARG00000077691 ENSDARG00000077996 ENSDARG00000078060 ENSDARG00000078078 ENSDARG00000078149 ENSDARG00000078187 ENSDARG00000078222 ENSDARG00000078331 ENSDARG00000078366 ENSDARG00000078527 ENSDARG00000078529 ENSDARG00000078599 ENSDARG00000078659 ENSDARG00000078676 ENSDARG00000078866 ENSDARG00000078898 ENSDARG00000078998 ENSDARG00000079011 ENSDARG00000079119 ENSDARG00000079166 ENSDARG00000079204 ENSDARG00000079251 ENSDARG00000079295 ENSDARG00000079455 ENSDARG00000079560 ENSDARG00000079586 ENSDARG00000079620 ENSDARG00000079665 ENSDARG00000079671 ENSDARG00000079858 ENSDARG00000079873 ENSDARG00000079908 ENSDARG00000080015 ENSDARG00000080675 ENSDARG00000086037 ENSDARG00000086159 ENSDARG00000086214 ENSDARG00000086571 ENSDARG00000086585 ENSDARG00000086756 ENSDARG00000087224 ENSDARG00000087349 ENSDARG00000087403 ENSDARG00000087599 ENSDARG00000087601 ENSDARG00000087916 ENSDARG00000088023 ENSDARG00000088315 ENSDARG00000088475 ENSDARG00000088584 ENSDARG00000088634 ENSDARG00000088708 ENSDARG00000088842 ENSDARG00000088898 ENSDARG00000089109 ENSDARG00000089177 ENSDARG00000089338 ENSDARG00000089361 ENSDARG00000089399 ENSDARG00000089766 ENSDARG00000089838 ENSDARG00000089913 ENSDARG00000090106 ENSDARG00000090140 ENSDARG00000090185 ENSDARG00000090481 ENSDARG00000090724 ENSDARG00000090876 ENSDARG00000090963 ENSDARG00000091116 ENSDARG00000091130 ENSDARG00000091209 ENSDARG00000091260 ENSDARG00000091306 ENSDARG00000091509 ENSDARG00000091579 ENSDARG00000091624 ENSDARG00000091658 ENSDARG00000091757 ENSDARG00000091834 ENSDARG00000091916 ENSDARG00000092039 ENSDARG00000092260 ENSDARG00000092283 ENSDARG00000092551 ENSDARG00000092574 ENSDARG00000093460 ENSDARG00000093745 ENSDARG00000094512 ENSDARG00000094860 ENSDARG00000094990 ENSDARG00000095022 ENSDARG00000095048 ENSDARG00000095259 ENSDARG00000095272 ENSDARG00000095696 ENSDARG00000095833 ENSDARG00000095937 ENSDARG00000096327 ENSDARG00000096398 ENSDARG00000096505 ENSDARG00000096791 ENSDARG00000096874 ENSDARG00000096908 ENSDARG00000097008 ENSDARG00000097110 ENSDARG00000097256 ENSDARG00000097420 ENSDARG00000097528 ENSDARG00000097576 ENSDARG00000097648 ENSDARG00000097693 ENSDARG00000097804 ENSDARG00000097826 ENSDARG00000097827 ENSDARG00000098024 ENSDARG00000098057 ENSDARG00000098162 ENSDARG00000098368 ENSDARG00000098465 ENSDARG00000098511 ENSDARG00000098526 ENSDARG00000099031 ENSDARG00000099035 ENSDARG00000099045 ENSDARG00000099096 ENSDARG00000099128 ENSDARG00000099203 ENSDARG00000099276 ENSDARG00000099323 ENSDARG00000099406 ENSDARG00000099515 ENSDARG00000099525 ENSDARG00000099783 ENSDARG00000099793 ENSDARG00000099931 ENSDARG00000099979 ENSDARG00000100021 ENSDARG00000100263 ENSDARG00000100265 ENSDARG00000100288 ENSDARG00000100670 ENSDARG00000100844 ENSDARG00000100876 ENSDARG00000100969 ENSDARG00000100971 ENSDARG00000100991 ENSDARG00000101109 ENSDARG00000101195 ENSDARG00000101363 ENSDARG00000101367 ENSDARG00000101368 ENSDARG00000101394 ENSDARG00000101449 ENSDARG00000101585 ENSDARG00000101702 ENSDARG00000101713 ENSDARG00000101776 ENSDARG00000101865 ENSDARG00000102096 ENSDARG00000102161 ENSDARG00000102185 ENSDARG00000102204 ENSDARG00000102376 ENSDARG00000102406 ENSDARG00000102430 ENSDARG00000102453 ENSDARG00000102975 ENSDARG00000103013 ENSDARG00000103124 ENSDARG00000103259 ENSDARG00000103503 ENSDARG00000103588 ENSDARG00000103639 ENSDARG00000103950 ENSDARG00000104023 ENSDARG00000104118 ENSDARG00000104139 ENSDARG00000104204 ENSDARG00000104213 ENSDARG00000104387 ENSDARG00000104458 ENSDARG00000104480 ENSDARG00000104497 ENSDARG00000104786 ENSDARG00000104826 ENSDARG00000104853 ENSDARG00000104922 ENSDARG00000104930 ENSDARG00000104949 ENSDARG00000104995 ENSDARG00000105040 ENSDARG00000105059 ENSDARG00000105083 ENSDARG00000105153 ENSDARG00000105201 | | GO:0005887 | integral component of plasma membrane | cellular\_component | 408 | 73.4 | 174 | 5.1e-14 | p2rx2 rhcga cacng5a grm2a gpr22a scn8aa kcns3a GRM2 (1 of many) avpr2aa asic1a tspan7b rhbg cadm2a scara5 glra3 drd2b chrna10a TENM2 (1 of many) glra1 slc6a6a si:dkey-246g23.4 prph2b slc6a2 grm6a chrnb2b scn8ab atp1a3a slco3a1 mmp24 rhag rom1a atp1a1b slc20a1a cacng8a prph2l gja3 cd81b epha6 atp2b3b dscama asic4a grm6b grin1b grm1a rom1b slc38a3a grin1a kcnf1a plppr3b grin2bb kcnj11l grm3 cacng2a gria3a kcnd2 kcnc1b atp1b2b grin2aa scn4ab si:rp71-39b20.4 si:ch73-335m24.5 oprd1b gria4a gria3b prph2a drd4a kcnb2 drd1b calhm2 slc6a1b atp1a1a.5 cadm4 slc6a1l slc24a1 tspan33b slc24a2 atp2b3a itgb3b shisa9a gpr22b slc13a1 avpr1ab slc6a1a kcna6a kcnc1a shisa9b chrnb3a gria2b atp1b4 tspan3b slc26a6l slco2b1 chrna6 kcnc3a kcnd3 trpc6a drd2a abca4a glra4b si:dkey-30c15.17 kcnq3 kcnb1 slc6a4a trpc1 slco2a1 kcnj3b scn3b nlgn3b shisa7a si:ch211-23l10.2 cadm2b abca4b si:dkey-100n10.2 scn1lab slc6a15 kcna1a cacng7a shisa7b atp2b2 slc6a5 slc6a17 chrm4a slc13a3 adra2c ppap2d KCNV1 ENSDARG00000070170 cacng7b cacng8b oprl1 si:ch211-117c9.5 neto1l itga1 glra2 kcnq2a cacng3b grm8b kcnv2a calhm1 avpr1aa nlgn2a grin2cb grm8a slc13a5a grin2ca best1 nlgn2b nlgn4a amigo1 plppr4a ntrk3b knca7 si:ch211-214p13.3 ngfra KCNB2 si:dkey-262k9.4 scn12aa kcnq2b si:ch211-66e2.5 htr1aa ntrk2b gpr75 atp1b2a dlg2 rhcgb chrna3 chrna7 scn2b adra2b cacng2b atp1a3b hcn1 nlgn3a sstr1a | ENSDARG00000002300 ENSDARG00000003203 ENSDARG00000003326 ENSDARG00000004150 ENSDARG00000004592 ENSDARG00000005775 ENSDARG00000006891 ENSDARG00000007195 ENSDARG00000007436 ENSDARG00000008329 ENSDARG00000008407 ENSDARG00000009018 ENSDARG00000009930 ENSDARG00000010425 ENSDARG00000011066 ENSDARG00000011091 ENSDARG00000011113 ENSDARG00000011171 ENSDARG00000012019 ENSDARG00000012534 ENSDARG00000013775 ENSDARG00000014840 ENSDARG00000016141 ENSDARG00000017742 ENSDARG00000017790 ENSDARG00000018032 ENSDARG00000018259 ENSDARG00000018726 ENSDARG00000018896 ENSDARG00000019253 ENSDARG00000019752 ENSDARG00000019856 ENSDARG00000020114 ENSDARG00000020450 ENSDARG00000021345 ENSDARG00000021889 ENSDARG00000022437 ENSDARG00000022971 ENSDARG00000023445 ENSDARG00000024865 ENSDARG00000025162 ENSDARG00000025671 ENSDARG00000025728 ENSDARG00000026796 ENSDARG00000026926 ENSDARG00000027065 ENSDARG00000027828 ENSDARG00000027940 ENSDARG00000028552 ENSDARG00000030376 ENSDARG00000031438 ENSDARG00000031712 ENSDARG00000032565 ENSDARG00000032737 ENSDARG00000032799 ENSDARG00000032959 ENSDARG00000034424 ENSDARG00000034493 ENSDARG00000034588 ENSDARG00000035861 ENSDARG00000036383 ENSDARG00000037159 ENSDARG00000037496 ENSDARG00000037498 ENSDARG00000038018 ENSDARG00000038363 ENSDARG00000038862 ENSDARG00000038918 ENSDARG00000039482 ENSDARG00000039647 ENSDARG00000040252 ENSDARG00000040291 ENSDARG00000041205 ENSDARG00000041431 ENSDARG00000042189 ENSDARG00000042988 ENSDARG00000043474 ENSDARG00000045070 ENSDARG00000045145 ENSDARG00000045383 ENSDARG00000045638 ENSDARG00000045788 ENSDARG00000045944 ENSDARG00000046014 ENSDARG00000051852 ENSDARG00000052642 ENSDARG00000052764 ENSDARG00000052765 ENSDARG00000053262 ENSDARG00000053559 ENSDARG00000054127 ENSDARG00000054609 ENSDARG00000055559 ENSDARG00000055855 ENSDARG00000056101 ENSDARG00000056625 ENSDARG00000056926 ENSDARG00000057169 ENSDARG00000058103 ENSDARG00000058492 ENSDARG00000060085 ENSDARG00000060095 ENSDARG00000061165 ENSDARG00000061203 ENSDARG00000061896 ENSDARG00000062217 ENSDARG00000062359 ENSDARG00000062376 ENSDARG00000062462 ENSDARG00000062618 ENSDARG00000062633 ENSDARG00000062661 ENSDARG00000062672 ENSDARG00000062744 ENSDARG00000062821 ENSDARG00000062942 ENSDARG00000063006 ENSDARG00000063144 ENSDARG00000063433 ENSDARG00000067964 ENSDARG00000068787 ENSDARG00000069254 ENSDARG00000069478 ENSDARG00000069669 ENSDARG00000069940 ENSDARG00000070092 ENSDARG00000070170 ENSDARG00000070624 ENSDARG00000070626 ENSDARG00000071209 ENSDARG00000071235 ENSDARG00000071596 ENSDARG00000074316 ENSDARG00000075012 ENSDARG00000075307 ENSDARG00000076401 ENSDARG00000076508 ENSDARG00000076644 ENSDARG00000077021 ENSDARG00000077083 ENSDARG00000077329 ENSDARG00000077560 ENSDARG00000077654 ENSDARG00000077691 ENSDARG00000078149 ENSDARG00000078331 ENSDARG00000079251 ENSDARG00000079455 ENSDARG00000079620 ENSDARG00000079671 ENSDARG00000086214 ENSDARG00000086571 ENSDARG00000087403 ENSDARG00000088708 ENSDARG00000088842 ENSDARG00000089838 ENSDARG00000090724 ENSDARG00000091130 ENSDARG00000091579 ENSDARG00000093745 ENSDARG00000098511 ENSDARG00000098526 ENSDARG00000099203 ENSDARG00000099323 ENSDARG00000100265 ENSDARG00000100991 ENSDARG00000101702 ENSDARG00000101713 ENSDARG00000102096 ENSDARG00000102376 ENSDARG00000104139 ENSDARG00000104480 ENSDARG00000104786 ENSDARG00000104922 | | GO:0001518 | voltage-gated sodium channel complex | cellular\_component | 11 | 2.0 | 8 | 2.8e-02 | scn8aa scn8ab scn4ab scn3b scn1lab ENSDARG00000070170 scn12aa scn2b | ENSDARG00000005775 ENSDARG00000018032 ENSDARG00000034588 ENSDARG00000062359 ENSDARG00000062744 ENSDARG00000070170 ENSDARG00000090724 ENSDARG00000101713 | | GO:0008076 | voltage-gated potassium channel complex | cellular\_component | 25 | 4.5 | 19 | 9.4e-08 | kcns3a kcnf1a kcnd2 kcnc1b si:rp71-39b20.4 kcnb2 kcna6a kcnc1a kcnc3a kcnd3 kcnq3 kcnb1 kcna1a KCNV1 kcnq2a kcnv2a knca7 KCNB2 kcnq2b | ENSDARG00000006891 ENSDARG00000027940 ENSDARG00000032799 ENSDARG00000032959 ENSDARG00000035861 ENSDARG00000038862 ENSDARG00000046014 ENSDARG00000051852 ENSDARG00000055855 ENSDARG00000056101 ENSDARG00000060085 ENSDARG00000060095 ENSDARG00000062942 ENSDARG00000070092 ENSDARG00000075307 ENSDARG00000076644 ENSDARG00000086571 ENSDARG00000088842 ENSDARG00000091130 | | GO:0032281 | AMPA glutamate receptor complex | cellular\_component | 17 | 3.1 | 16 | 4.1e-09 | cacng5a cacng8a cacng2a gria3a gria4a gria3b shisa9a shisa9b gria2b shisa7a cacng7a shisa7b cacng7b cacng8b cacng3b cacng2b | ENSDARG00000003326 ENSDARG00000020450 ENSDARG00000032565 ENSDARG00000032737 ENSDARG00000037496 ENSDARG00000037498 ENSDARG00000045145 ENSDARG00000052642 ENSDARG00000052765 ENSDARG00000062462 ENSDARG00000063006 ENSDARG00000063144 ENSDARG00000070624 ENSDARG00000070626 ENSDARG00000076401 ENSDARG00000102376 | | GO:0043005 | neuron projection | cellular\_component | 70 | 12.6 | 30 | 6.3e-05 | slc17a6a nadl1.2 slc6a2 snap25a calb1 stmn2a stmn1b calb2b oprd1b gria4a slc6a1b calb2a slc17a6b slc6a1l stmn4l npffl shisa9a slc6a1a shisa9b slc17a8 snap25b cplx4a slc6a4a ENSDARG00000068745 oprl1 ppp1r9bb cntnap1 il1rapl1b sstr1a ompa | ENSDARG00000001127 ENSDARG00000007149 ENSDARG00000016141 ENSDARG00000020609 ENSDARG00000031598 ENSDARG00000033234 ENSDARG00000033655 ENSDARG00000036344 ENSDARG00000037159 ENSDARG00000037496 ENSDARG00000039647 ENSDARG00000041062 ENSDARG00000041150 ENSDARG00000041205 ENSDARG00000043932 ENSDARG00000045016 ENSDARG00000045145 ENSDARG00000045944 ENSDARG00000052642 ENSDARG00000057728 ENSDARG00000058117 ENSDARG00000059978 ENSDARG00000061165 ENSDARG00000068745 ENSDARG00000071209 ENSDARG00000071709 ENSDARG00000074524 ENSDARG00000104853 ENSDARG00000104922 ENSDARG00000105218 | | GO:1902711 | GABA-A receptor complex | cellular\_component | 11 | 2.0 | 11 | 1.6e-06 | gabrb3 gabrr3a gabrr1 gabrr2a gabrg2 gabrd gabra1 gabra5 ENSDARG00000076127 gabrb2 gabrb4 | ENSDARG00000023771 ENSDARG00000027153 ENSDARG00000043902 ENSDARG00000052982 ENSDARG00000053665 ENSDARG00000059763 ENSDARG00000068989 ENSDARG00000070730 ENSDARG00000076127 ENSDARG00000079586 ENSDARG00000099096 | | GO:0004872 | receptor activity | molecular\_function | 429 | 70.0 | 170 | 4.8e-02 | rorab rho p2rx2 nr1d2a crhr1 grm2a gpr22a esrrga gpr27 GRM2 (1 of many) si:ch211-251b21.1 avpr2aa opn4.1 cnr1 nr1d2b cadm2a scara5 drd2b chrna10a enpp2 bmpr2a vipr2 prlra gabbr1b nr4a2a opn1sw2 grm6a rorcb chrnb2b ptchd1 bmpr2b grk7a valopa gria1a epha6 ahr1b epha8 gabrb3 loxl4 gipr grm6b grin1b grm1a gabrr3a grin1a prg4b calcr vipr1a grin2bb nr1d4a mc5ra grm3 roraa gria1b gria3a nr1d1 gpr37b grin2aa paqr7b agtr2 oprd1b gria4a gria3b drd4a drd1b rrh cadm4 grik1b paqr6 gfra2a disp2 gpr173 fpr1 nptnb gabrr1 gfra2b nr4a2b opn1lw2 itgb3b gpr22b opn1sw1 avpr1ab abcc8 chrnb3a gria2b opn3 gabrr2a csf1rb gabrg2 adgrg6 rgra vtna chrna6 nr4a3 nr0b1 gfra4a gpr37l1b drd2a sfrp1b nr1h4 si:dkey-30c15.17 ddr2b nr1d4b gpr146 gabrd si:ch73-127m5.1 adgrl3.1 ogfrl1 kcnh4b nlgn3b kcnh4a cadm2b gpr12 antxr2b erbb4a gpr85 gabra1 kcnh5b chrm4a adra2c gria2a rhol gabra5 oprl1 hnf4g paqr4b grid1a adgrb1a gprc5bb ENSDARG00000076127 grm8b gprc5ba avpr1aa gpr158a nlgn2a grin2cb grm8a grin2ca adgrb1b nlgn2b nlgn4a ptprh gabrb2 gpr158b ntrk3b si:ch211-214p13.3 gpr153 ngfra lpar4 adgrf6 gpr52 htr1aa gabrz gpr186 si:ch211-10p21.1 alk opn1mw1 ntrk2b gpr75 gabrb4 chrna3 grik5 chrna7 adra2b opn6a opn4xb antxr2a nlgn3a sstr1a adcyap1r1a | ENSDARG00000001910 ENSDARG00000002193 ENSDARG00000002300 ENSDARG00000003820 ENSDARG00000003989 ENSDARG00000004150 ENSDARG00000004592 ENSDARG00000004861 ENSDARG00000006607 ENSDARG00000007195 ENSDARG00000007275 ENSDARG00000007436 ENSDARG00000007553 ENSDARG00000009020 ENSDARG00000009594 ENSDARG00000009930 ENSDARG00000010425 ENSDARG00000011091 ENSDARG00000011113 ENSDARG00000011257 ENSDARG00000011941 ENSDARG00000012353 ENSDARG00000016570 ENSDARG00000016667 ENSDARG00000017007 ENSDARG00000017274 ENSDARG00000017742 ENSDARG00000017780 ENSDARG00000017790 ENSDARG00000018066 ENSDARG00000020057 ENSDARG00000020602 ENSDARG00000021150 ENSDARG00000021352 ENSDARG00000022971 ENSDARG00000023537 ENSDARG00000023609 ENSDARG00000023771 ENSDARG00000025089 ENSDARG00000025478 ENSDARG00000025671 ENSDARG00000025728 ENSDARG00000026796 ENSDARG00000027153 ENSDARG00000027828 ENSDARG00000028163 ENSDARG00000028845 ENSDARG00000028878 ENSDARG00000030376 ENSDARG00000031161 ENSDARG00000031348 ENSDARG00000031712 ENSDARG00000031768 ENSDARG00000032714 ENSDARG00000032737 ENSDARG00000033160 ENSDARG00000033296 ENSDARG00000034493 ENSDARG00000034907 ENSDARG00000035552 ENSDARG00000037159 ENSDARG00000037496 ENSDARG00000037498 ENSDARG00000038363 ENSDARG00000038918 ENSDARG00000039534 ENSDARG00000040291 ENSDARG00000040627 ENSDARG00000041483 ENSDARG00000042723 ENSDARG00000042846 ENSDARG00000042922 ENSDARG00000042984 ENSDARG00000043864 ENSDARG00000043902 ENSDARG00000044015 ENSDARG00000044532 ENSDARG00000044861 ENSDARG00000045070 ENSDARG00000045383 ENSDARG00000045677 ENSDARG00000045788 ENSDARG00000051879 ENSDARG00000052764 ENSDARG00000052765 ENSDARG00000052775 ENSDARG00000052982 ENSDARG00000053624 ENSDARG00000053665 ENSDARG00000054137 ENSDARG00000054890 ENSDARG00000055388 ENSDARG00000055559 ENSDARG00000055854 ENSDARG00000056541 ENSDARG00000056651 ENSDARG00000056774 ENSDARG00000056926 ENSDARG00000057678 ENSDARG00000057741 ENSDARG00000058492 ENSDARG00000058695 ENSDARG00000059370 ENSDARG00000059610 ENSDARG00000059763 ENSDARG00000060680 ENSDARG00000061121 ENSDARG00000061223 ENSDARG00000061990 ENSDARG00000062376 ENSDARG00000062565 ENSDARG00000062633 ENSDARG00000062934 ENSDARG00000063011 ENSDARG00000063207 ENSDARG00000068701 ENSDARG00000068989 ENSDARG00000069117 ENSDARG00000069254 ENSDARG00000069669 ENSDARG00000070173 ENSDARG00000070666 ENSDARG00000070730 ENSDARG00000071209 ENSDARG00000071565 ENSDARG00000074160 ENSDARG00000074583 ENSDARG00000075133 ENSDARG00000075141 ENSDARG00000076127 ENSDARG00000076508 ENSDARG00000077080 ENSDARG00000077083 ENSDARG00000077134 ENSDARG00000077329 ENSDARG00000077560 ENSDARG00000077654 ENSDARG00000078149 ENSDARG00000078529 ENSDARG00000079251 ENSDARG00000079455 ENSDARG00000079560 ENSDARG00000079586 ENSDARG00000079665 ENSDARG00000086214 ENSDARG00000087403 ENSDARG00000087601 ENSDARG00000088708 ENSDARG00000089824 ENSDARG00000091757 ENSDARG00000093460 ENSDARG00000093745 ENSDARG00000094512 ENSDARG00000094860 ENSDARG00000095272 ENSDARG00000095833 ENSDARG00000097008 ENSDARG00000098511 ENSDARG00000098526 ENSDARG00000099096 ENSDARG00000100991 ENSDARG00000101449 ENSDARG00000101702 ENSDARG00000102096 ENSDARG00000102430 ENSDARG00000103259 ENSDARG00000104118 ENSDARG00000104786 ENSDARG00000104922 ENSDARG00000105201 | | GO:0009881 | photoreceptor activity | molecular\_function | 18 | 2.9 | 12 | 6.1e-04 | rho opn4.1 opn1sw2 grk7a valopa rrh opn1lw2 opn1sw1 opn3 rhol opn1mw1 opn4xb | ENSDARG00000002193 ENSDARG00000007553 ENSDARG00000017274 ENSDARG00000020602 ENSDARG00000021150 ENSDARG00000039534 ENSDARG00000044861 ENSDARG00000045677 ENSDARG00000052775 ENSDARG00000070666 ENSDARG00000097008 ENSDARG00000103259 | | GO:0004930 | G-protein coupled receptor activity | molecular\_function | 153 | 25.0 | 72 | 1.5e-14 | rho crhr1 grm2a gpr22a gpr27 GRM2 (1 of many) avpr2aa opn4.1 cnr1 drd2b vipr2 gabbr1b opn1sw2 grm6a valopa gipr grm6b grm1a grin1a calcr vipr1a mc5ra grm3 gpr37b agtr2 oprd1b drd4a drd1b rrh gpr173 fpr1 opn1lw2 gpr22b opn1sw1 avpr1ab opn3 adgrg6 rgra gpr37l1b drd2a sfrp1b gpr146 adgrl3.1 gpr12 gpr85 chrm4a adra2c rhol oprl1 adgrb1a gprc5bb grm8b gprc5ba avpr1aa gpr158a grm8a adgrb1b gpr158b gpr153 lpar4 adgrf6 gpr52 htr1aa gpr186 si:ch211-10p21.1 opn1mw1 gpr75 adra2b opn6a opn4xb sstr1a adcyap1r1a | ENSDARG00000002193 ENSDARG00000003989 ENSDARG00000004150 ENSDARG00000004592 ENSDARG00000006607 ENSDARG00000007195 ENSDARG00000007436 ENSDARG00000007553 ENSDARG00000009020 ENSDARG00000011091 ENSDARG00000012353 ENSDARG00000016667 ENSDARG00000017274 ENSDARG00000017742 ENSDARG00000021150 ENSDARG00000025478 ENSDARG00000025671 ENSDARG00000026796 ENSDARG00000027828 ENSDARG00000028845 ENSDARG00000028878 ENSDARG00000031348 ENSDARG00000031712 ENSDARG00000033296 ENSDARG00000035552 ENSDARG00000037159 ENSDARG00000038363 ENSDARG00000038918 ENSDARG00000039534 ENSDARG00000042922 ENSDARG00000042984 ENSDARG00000044861 ENSDARG00000045383 ENSDARG00000045677 ENSDARG00000045788 ENSDARG00000052775 ENSDARG00000054137 ENSDARG00000054890 ENSDARG00000056774 ENSDARG00000056926 ENSDARG00000057678 ENSDARG00000059610 ENSDARG00000061121 ENSDARG00000062934 ENSDARG00000068701 ENSDARG00000069254 ENSDARG00000069669 ENSDARG00000070666 ENSDARG00000071209 ENSDARG00000075133 ENSDARG00000075141 ENSDARG00000076508 ENSDARG00000077080 ENSDARG00000077083 ENSDARG00000077134 ENSDARG00000077654 ENSDARG00000078529 ENSDARG00000079665 ENSDARG00000087601 ENSDARG00000089824 ENSDARG00000091757 ENSDARG00000093460 ENSDARG00000093745 ENSDARG00000094860 ENSDARG00000095272 ENSDARG00000097008 ENSDARG00000098526 ENSDARG00000102096 ENSDARG00000102430 ENSDARG00000103259 ENSDARG00000104922 ENSDARG00000105201 | | GO:0008066 | glutamate receptor activity | molecular\_function | 33 | 5.4 | 25 | 3.8e-03 | grm2a GRM2 (1 of many) si:ch211-251b21.1 grm6a gria1a grm6b grin1b grm1a grin1a grin2bb grm3 gria1b gria3a grin2aa gria4a gria3b grik1b gria2b gria2a grid1a grm8b grin2cb grm8a grin2ca grik5 | ENSDARG00000004150 ENSDARG00000007195 ENSDARG00000007275 ENSDARG00000017742 ENSDARG00000021352 ENSDARG00000025671 ENSDARG00000025728 ENSDARG00000026796 ENSDARG00000027828 ENSDARG00000030376 ENSDARG00000031712 ENSDARG00000032714 ENSDARG00000032737 ENSDARG00000034493 ENSDARG00000037496 ENSDARG00000037498 ENSDARG00000040627 ENSDARG00000052765 ENSDARG00000070173 ENSDARG00000074583 ENSDARG00000076508 ENSDARG00000077560 ENSDARG00000077654 ENSDARG00000078149 ENSDARG00000101449 | | GO:0004890 | GABA-A receptor activity | molecular\_function | 12 | 2.0 | 12 | 8.6e-08 | gabrb3 gabrr3a gabrr1 gabrr2a gabrg2 gabrd gabra1 gabra5 ENSDARG00000076127 gabrb2 gabrz gabrb4 | ENSDARG00000023771 ENSDARG00000027153 ENSDARG00000043902 ENSDARG00000052982 ENSDARG00000053665 ENSDARG00000059763 ENSDARG00000068989 ENSDARG00000070730 ENSDARG00000076127 ENSDARG00000079586 ENSDARG00000094512 ENSDARG00000099096 | | GO:0004970 | ionotropic glutamate receptor activity | molecular\_function | 23 | 3.8 | 17 | 3.6e-07 | si:ch211-251b21.1 gria1a grin1b grin1a grin2bb gria1b gria3a grin2aa gria4a gria3b grik1b gria2b gria2a grid1a grin2cb grin2ca grik5 | ENSDARG00000007275 ENSDARG00000021352 ENSDARG00000025728 ENSDARG00000027828 ENSDARG00000030376 ENSDARG00000032714 ENSDARG00000032737 ENSDARG00000034493 ENSDARG00000037496 ENSDARG00000037498 ENSDARG00000040627 ENSDARG00000052765 ENSDARG00000070173 ENSDARG00000074583 ENSDARG00000077560 ENSDARG00000078149 ENSDARG00000101449 | | GO:0016247 | channel regulator activity | molecular\_function | 28 | 4.6 | 16 | 9.4e-04 | cacng5a grm2a GRM2 (1 of many) cacng8a cacna2d4b cacng2a scn3b cacng7a ENSDARG00000070170 cacng7b cacng8b cacng3b si:dkey-262k9.4 si:ch211-270g19.5 scn2b cacng2b | ENSDARG00000003326 ENSDARG00000004150 ENSDARG00000007195 ENSDARG00000020450 ENSDARG00000023886 ENSDARG00000032565 ENSDARG00000062359 ENSDARG00000063006 ENSDARG00000070170 ENSDARG00000070624 ENSDARG00000070626 ENSDARG00000076401 ENSDARG00000089838 ENSDARG00000097256 ENSDARG00000101713 ENSDARG00000102376 | | GO:0009975 | cyclase activity | molecular\_function | 18 | 2.9 | 11 | 5.6e-03 | gucy1a3 gucy2c adcy2b gucy2f gc3 si:dkey-206f10.1 si:ch211-132f19.7 GUCY2C (1 of many) adcy2a gucy1b3 adcy1b | ENSDARG00000013787 ENSDARG00000014320 ENSDARG00000014588 ENSDARG00000025504 ENSDARG00000026820 ENSDARG00000032838 ENSDARG00000043141 ENSDARG00000056045 ENSDARG00000058392 ENSDARG00000086790 ENSDARG00000088634 | | GO:0030276 | clathrin binding | molecular\_function | 29 | 4.7 | 18 | 8.6e-06 | syt9a syt5b ENSDARG00000015931 syt9b syt1a syt4 syt5a syt10 syt11b syt11a syt3 syt6a syt7b DOC2A doc2b snap91 syt12 hmp19 | ENSDARG00000003994 ENSDARG00000011640 ENSDARG00000015931 ENSDARG00000029239 ENSDARG00000030614 ENSDARG00000036505 ENSDARG00000037941 ENSDARG00000045750 ENSDARG00000056105 ENSDARG00000057913 ENSDARG00000075830 ENSDARG00000076730 ENSDARG00000078060 ENSDARG00000078736 ENSDARG00000088293 ENSDARG00000098809 ENSDARG00000101776 ENSDARG00000102975 | | GO:0016849 | phosphorus-oxygen lyase activity | molecular\_function | 17 | 2.8 | 11 | 2.5e-03 | gucy1a3 gucy2c adcy2b gucy2f gc3 si:dkey-206f10.1 si:ch211-132f19.7 GUCY2C (1 of many) adcy2a gucy1b3 adcy1b | ENSDARG00000013787 ENSDARG00000014320 ENSDARG00000014588 ENSDARG00000025504 ENSDARG00000026820 ENSDARG00000032838 ENSDARG00000043141 ENSDARG00000056045 ENSDARG00000058392 ENSDARG00000086790 ENSDARG00000088634 | | GO:0019905 | syntaxin binding | molecular\_function | 46 | 7.5 | 25 | 9.1e-07 | stxbp5a syt9a stxbp5l syt5b napba snap25a syt9b syt1a syt4 syt5a napgb syt10 syt11b syt11a snap25b cplx4a NAPB (1 of many) syt3 syt6a syt7b DOC2A doc2b cplx3b cplx4c syt12 | ENSDARG00000002656 ENSDARG00000003994 ENSDARG00000006383 ENSDARG00000011640 ENSDARG00000013669 ENSDARG00000020609 ENSDARG00000029239 ENSDARG00000030614 ENSDARG00000036505 ENSDARG00000037941 ENSDARG00000043012 ENSDARG00000045750 ENSDARG00000056105 ENSDARG00000057913 ENSDARG00000058117 ENSDARG00000059978 ENSDARG00000069101 ENSDARG00000075830 ENSDARG00000076730 ENSDARG00000078060 ENSDARG00000078736 ENSDARG00000088293 ENSDARG00000089486 ENSDARG00000094889 ENSDARG00000101776 | | GO:0005179 | hormone activity | molecular\_function | 42 | 6.8 | 23 | 3.3e-06 | adcyap1a inhbab crhb adcyap1b sst3 ins pyyb npy gh1 sst1.1 oxt pomca wu:fj39g12 npffl adm2a zgc:195023 calca stc1l rln3a ccka vip gcga stc2b | ENSDARG00000004015 ENSDARG00000024759 ENSDARG00000027657 ENSDARG00000027740 ENSDARG00000031649 ENSDARG00000035350 ENSDARG00000035832 ENSDARG00000036222 ENSDARG00000038185 ENSDARG00000040799 ENSDARG00000042845 ENSDARG00000043135 ENSDARG00000043460 ENSDARG00000045016 ENSDARG00000045708 ENSDARG00000052948 ENSDARG00000056590 ENSDARG00000058476 ENSDARG00000070780 ENSDARG00000070810 ENSDARG00000078247 ENSDARG00000079296 ENSDARG00000102206 | | GO:0005544 | calcium-dependent phospholipid binding | molecular\_function | 26 | 4.2 | 14 | 3.0e-03 | syt9a syt5b anxa5a syt9b syt1a anxa4 syt5a syt10 syt3 syt6a syt7b DOC2A doc2b syt12 | ENSDARG00000003994 ENSDARG00000011640 ENSDARG00000026406 ENSDARG00000029239 ENSDARG00000030614 ENSDARG00000036456 ENSDARG00000037941 ENSDARG00000045750 ENSDARG00000075830 ENSDARG00000076730 ENSDARG00000078060 ENSDARG00000078736 ENSDARG00000088293 ENSDARG00000101776 | | GO:0004860 | protein kinase inhibitor activity | molecular\_function | 36 | 5.9 | 17 | 3.6e-03 | aspn lrrc4.2 sh3bp5lb lrrc4bb bgna camk2n1a socs2 lrrtm1 pkib nyx lrrc4.1 chadlb lrrtm4l1 rtn4r camk2n2 rtn4rl1b cdkn1d | ENSDARG00000002192 ENSDARG00000003020 ENSDARG00000007136 ENSDARG00000014792 ENSDARG00000017884 ENSDARG00000025855 ENSDARG00000045557 ENSDARG00000052713 ENSDARG00000053110 ENSDARG00000061791 ENSDARG00000069402 ENSDARG00000075903 ENSDARG00000080015 ENSDARG00000090035 ENSDARG00000090424 ENSDARG00000098528 ENSDARG00000099719 | | GO:0005509 | calcium ion binding | molecular\_function | 389 | 63.5 | 118 | 3.6e-10 | itsn2a aspn cabp5a pvalb2 syt9a cdhr1a zgc:136872 pvalb6 rcvrn3 thbs1b ncaldb myl4 syt5b guca1b sulf2b cdh13 cdh4 mmp24 rcvrn2 vsnl1a pvalb4 mmp11a anxa5a pcdh17 cabp5b syt9b syt1a guca1c calb1 clstn1 pvalb5 cabp1b calm1b kcnip1b calb2b si:ch73-335m24.5 anxa4 DCHS2 pvalb1 pvalb8 syt5a myl9a fstl5 calb2a slc24a1 capn3a slc24a2 vsnl1b guca1g syt10 cabp2a rcvrna myl2b s100t cdh10a necab1 necab2 ltbp1 pcdh1g9 s100b mmp16b crb2b clstn2 CLSTN2 (1 of many) cabp7b edil3b dner cdh18a lrp1bb rasgrp4 pcdh1a nell2b PCDH9 matn3a lrp8 spock3 hpcal4 clstn3 necab3 sparcl1 spock2 calml4a syt3 epdl1 syt6a pcdhb phf24 cdh24b syt7b micu3a DOC2A pcdh7a doc2b pcdh1gb9 caln1 edil3 s100a11 TNNC2 (1 of many) lrp1aa zgc:85932 pcdh1g33 PLA2G10 MYL3 pcdh2ac pcdh1g26 pcdh1g31 fam69b cetn2 guca1e syt12 pcdh1g30 ENSDARG00000102185 ENSDARG00000102406 pcdh1g22 pcdh1gc6 pcdh1g29 pcdh1gc5 fbn1 | ENSDARG00000000161 ENSDARG00000002192 ENSDARG00000002576 ENSDARG00000002768 ENSDARG00000003994 ENSDARG00000004643 ENSDARG00000008553 ENSDARG00000009311 ENSDARG00000009637 ENSDARG00000010785 ENSDARG00000011334 ENSDARG00000011519 ENSDARG00000011640 ENSDARG00000013393 ENSDARG00000013838 ENSDARG00000014215 ENSDARG00000015002 ENSDARG00000018896 ENSDARG00000019902 ENSDARG00000023228 ENSDARG00000024433 ENSDARG00000026325 ENSDARG00000026406 ENSDARG00000027041 ENSDARG00000028485 ENSDARG00000029239 ENSDARG00000030614 ENSDARG00000030758 ENSDARG00000031598 ENSDARG00000031720 ENSDARG00000032836 ENSDARG00000033411 ENSDARG00000034187 ENSDARG00000034808 ENSDARG00000036344 ENSDARG00000036383 ENSDARG00000036456 ENSDARG00000037286 ENSDARG00000037789 ENSDARG00000037790 ENSDARG00000037941 ENSDARG00000038123 ENSDARG00000040198 ENSDARG00000041062 ENSDARG00000041431 ENSDARG00000041864 ENSDARG00000042988 ENSDARG00000044053 ENSDARG00000045737 ENSDARG00000045750 ENSDARG00000052016 ENSDARG00000052223 ENSDARG00000053424 ENSDARG00000055589 ENSDARG00000055843 ENSDARG00000056566 ENSDARG00000056745 ENSDARG00000056922 ENSDARG00000057519 ENSDARG00000057598 ENSDARG00000058876 ENSDARG00000060081 ENSDARG00000060637 ENSDARG00000060638 ENSDARG00000060846 ENSDARG00000060877 ENSDARG00000061031 ENSDARG00000061371 ENSDARG00000061517 ENSDARG00000061796 ENSDARG00000062720 ENSDARG00000062797 ENSDARG00000063264 ENSDARG00000069245 ENSDARG00000070074 ENSDARG00000070266 ENSDARG00000070491 ENSDARG00000073883 ENSDARG00000074794 ENSDARG00000074989 ENSDARG00000075393 ENSDARG00000075800 ENSDARG00000075830 ENSDARG00000076386 ENSDARG00000076730 ENSDARG00000077023 ENSDARG00000077596 ENSDARG00000077996 ENSDARG00000078060 ENSDARG00000078599 ENSDARG00000078736 ENSDARG00000078898 ENSDARG00000088293 ENSDARG00000088475 ENSDARG00000088898 ENSDARG00000093413 ENSDARG00000093628 ENSDARG00000095002 ENSDARG00000097827 ENSDARG00000098239 ENSDARG00000099035 ENSDARG00000099344 ENSDARG00000099712 ENSDARG00000099783 ENSDARG00000099931 ENSDARG00000100670 ENSDARG00000101394 ENSDARG00000101510 ENSDARG00000101567 ENSDARG00000101776 ENSDARG00000101865 ENSDARG00000102185 ENSDARG00000102406 ENSDARG00000103013 ENSDARG00000103950 ENSDARG00000104497 ENSDARG00000104826 ENSDARG00000105333 | | GO:0015296 | anion:cation symporter activity | molecular\_function | 44 | 7.2 | 22 | 2.4e-02 | slc1a6 slc17a7a slc20a1a slc1a1 slc6a1b slc6a1l slc1a3b slc13a1 slc6a1a slc1a2a slc26a6l slc6a15 slc6a5 slc13a3 slc12a10.2 si:ch211-117c9.5 slc12a5a slc13a5a slc12a5b slc17a7b slc1a2b slc1a8a | ENSDARG00000010096 ENSDARG00000016480 ENSDARG00000020114 ENSDARG00000020212 ENSDARG00000039647 ENSDARG00000041205 ENSDARG00000043148 ENSDARG00000045638 ENSDARG00000045944 ENSDARG00000052138 ENSDARG00000054127 ENSDARG00000062821 ENSDARG00000067964 ENSDARG00000069478 ENSDARG00000071173 ENSDARG00000071235 ENSDARG00000075815 ENSDARG00000077691 ENSDARG00000078187 ENSDARG00000090106 ENSDARG00000102453 ENSDARG00000104204 | | GO:0015081 | sodium ion transmembrane transporter act... | molecular\_function | 81 | 13.2 | 40 | 4.3e-03 | scn8aa slc24a3 asic1a slc1a6 slc6a6a slc6a2 slc17a7a scn8ab atp1a3a atp1a1b slc20a1a slc1a1 asic4a scn4ab si:ch73-335m24.5 slc8a4b slc6a1b atp1a1a.5 slc6a1l slc24a1 slc24a2 slc1a3b slc13a1 slc6a1a slc1a2a slc26a6l slc8a4a slc6a4a scn1lab slc6a15 slc6a5 slc6a17 slc13a3 si:ch211-117c9.5 slc13a5a slc17a7b scn12aa slc1a2b atp1a3b slc1a8a | ENSDARG00000005775 ENSDARG00000006760 ENSDARG00000008329 ENSDARG00000010096 ENSDARG00000012534 ENSDARG00000016141 ENSDARG00000016480 ENSDARG00000018032 ENSDARG00000018259 ENSDARG00000019856 ENSDARG00000020114 ENSDARG00000020212 ENSDARG00000025162 ENSDARG00000034588 ENSDARG00000036383 ENSDARG00000037145 ENSDARG00000039647 ENSDARG00000040252 ENSDARG00000041205 ENSDARG00000041431 ENSDARG00000042988 ENSDARG00000043148 ENSDARG00000045638 ENSDARG00000045944 ENSDARG00000052138 ENSDARG00000054127 ENSDARG00000055154 ENSDARG00000061165 ENSDARG00000062744 ENSDARG00000062821 ENSDARG00000067964 ENSDARG00000068787 ENSDARG00000069478 ENSDARG00000071235 ENSDARG00000077691 ENSDARG00000090106 ENSDARG00000090724 ENSDARG00000102453 ENSDARG00000104139 ENSDARG00000104204 | | GO:0005254 | chloride channel activity | molecular\_function | 29 | 4.7 | 15 | 2.8e-03 | ttyh3b clic2 glra1 clcn4 glrbb glrba slc26a6l glra4b clcn1a clic5b glra2 ttyh1 best1 gabrz ano2 | ENSDARG00000007678 ENSDARG00000010625 ENSDARG00000012019 ENSDARG00000035808 ENSDARG00000052769 ENSDARG00000052782 ENSDARG00000054127 ENSDARG00000058103 ENSDARG00000062084 ENSDARG00000070584 ENSDARG00000075012 ENSDARG00000076804 ENSDARG00000078331 ENSDARG00000094512 ENSDARG00000101363 | | GO:0005230 | extracellular ligand-gated ion channel a... | molecular\_function | 61 | 9.9 | 44 | 1.4e-07 | p2rx2 si:ch211-251b21.1 glra3 chrna10a glra1 chrnb2b gria1a gabrb3 grin1b gabrr3a grin1a grin2bb gabrr3b gria1b gria3a grin2aa gria4a gria3b grik1b gabrr1 chrnb3a gria2b glrbb glrba gabrr2a gabrg2 chrna6 glra4b gabrd gabra1 gria2a gabra5 grid1a glra2 ENSDARG00000076127 grin2cb grin2ca gabrb2 si:ch73-380n15.2 gabrz gabrb4 chrna3 grik5 chrna7 | ENSDARG00000002300 ENSDARG00000007275 ENSDARG00000011066 ENSDARG00000011113 ENSDARG00000012019 ENSDARG00000017790 ENSDARG00000021352 ENSDARG00000023771 ENSDARG00000025728 ENSDARG00000027153 ENSDARG00000027828 ENSDARG00000030376 ENSDARG00000030750 ENSDARG00000032714 ENSDARG00000032737 ENSDARG00000034493 ENSDARG00000037496 ENSDARG00000037498 ENSDARG00000040627 ENSDARG00000043902 ENSDARG00000052764 ENSDARG00000052765 ENSDARG00000052769 ENSDARG00000052782 ENSDARG00000052982 ENSDARG00000053665 ENSDARG00000055559 ENSDARG00000058103 ENSDARG00000059763 ENSDARG00000068989 ENSDARG00000070173 ENSDARG00000070730 ENSDARG00000074583 ENSDARG00000075012 ENSDARG00000076127 ENSDARG00000077560 ENSDARG00000078149 ENSDARG00000079586 ENSDARG00000087224 ENSDARG00000094512 ENSDARG00000099096 ENSDARG00000100991 ENSDARG00000101449 ENSDARG00000101702 | | GO:0004970 | ionotropic glutamate receptor activity | molecular\_function | 23 | 3.8 | 17 | 3.6e-07 | si:ch211-251b21.1 gria1a grin1b grin1a grin2bb gria1b gria3a grin2aa gria4a gria3b grik1b gria2b gria2a grid1a grin2cb grin2ca grik5 | ENSDARG00000007275 ENSDARG00000021352 ENSDARG00000025728 ENSDARG00000027828 ENSDARG00000030376 ENSDARG00000032714 ENSDARG00000032737 ENSDARG00000034493 ENSDARG00000037496 ENSDARG00000037498 ENSDARG00000040627 ENSDARG00000052765 ENSDARG00000070173 ENSDARG00000074583 ENSDARG00000077560 ENSDARG00000078149 ENSDARG00000101449 | | GO:0005234 | extracellular-glutamate-gated ion channe... | molecular\_function | 24 | 3.9 | 18 | 7.9e-08 | si:ch211-251b21.1 gria1a grin1b grin1a grin2bb gria1b gria3a grin2aa gria4a gria3b grik1b gria2b gria2a grid1a grin2cb grin2ca gabrz grik5 | ENSDARG00000007275 ENSDARG00000021352 ENSDARG00000025728 ENSDARG00000027828 ENSDARG00000030376 ENSDARG00000032714 ENSDARG00000032737 ENSDARG00000034493 ENSDARG00000037496 ENSDARG00000037498 ENSDARG00000040627 ENSDARG00000052765 ENSDARG00000070173 ENSDARG00000074583 ENSDARG00000077560 ENSDARG00000078149 ENSDARG00000094512 ENSDARG00000101449 | | GO:0030553 | cGMP binding | molecular\_function | 13 | 2.1 | 9 | 7.9e-03 | cnga1 CNGA1 (1 of many) prkg1b CNGB1 cngb1a cnga3a cngb3 ENSDARG00000101368 pde6g | ENSDARG00000012125 ENSDARG00000029898 ENSDARG00000031702 ENSDARG00000042107 ENSDARG00000068242 ENSDARG00000070726 ENSDARG00000101225 ENSDARG00000101368 ENSDARG00000101984 | | GO:0005249 | voltage-gated potassium channel activity | molecular\_function | 57 | 9.3 | 39 | 1.4e-08 | kcns3a cnga1 kcnf1a CNGA1 (1 of many) kcnj11l kcnd2 kcnc1b si:rp71-39b20.4 kcnb2 kcnab1b CNGB1 kcna6a kcnc1a kcnc3a kcnd3 kcnq3 kcnb1 kcnh4b kcnab2b kcnj3b kcnh4a si:ch211-23l10.2 si:dkey-100n10.2 kcnh7 kcna1a cngb1a kcnh5b KCNV1 cnga3a hcn4l kcnq2a kcnv2a knca7 KCNB2 kcnj10a kcnq2b cngb3 ENSDARG00000101368 hcn1 | ENSDARG00000006891 ENSDARG00000012125 ENSDARG00000027940 ENSDARG00000029898 ENSDARG00000031438 ENSDARG00000032799 ENSDARG00000032959 ENSDARG00000035861 ENSDARG00000038862 ENSDARG00000040741 ENSDARG00000042107 ENSDARG00000046014 ENSDARG00000051852 ENSDARG00000055855 ENSDARG00000056101 ENSDARG00000060085 ENSDARG00000060095 ENSDARG00000061990 ENSDARG00000062134 ENSDARG00000062217 ENSDARG00000062565 ENSDARG00000062618 ENSDARG00000062672 ENSDARG00000062687 ENSDARG00000062942 ENSDARG00000068242 ENSDARG00000069117 ENSDARG00000070092 ENSDARG00000070726 ENSDARG00000074419 ENSDARG00000075307 ENSDARG00000076644 ENSDARG00000086571 ENSDARG00000088842 ENSDARG00000090815 ENSDARG00000091130 ENSDARG00000101225 ENSDARG00000101368 ENSDARG00000104480 | | GO:0005251 | delayed rectifier potassium channel acti... | molecular\_function | 20 | 3.3 | 15 | 2.5e-06 | kcns3a kcnf1a kcnc1b si:rp71-39b20.4 kcnb2 kcna6a kcnc1a kcnc3a kcnq3 kcnb1 kcna1a KCNV1 kcnq2a kcnv2a KCNB2 | ENSDARG00000006891 ENSDARG00000027940 ENSDARG00000032959 ENSDARG00000035861 ENSDARG00000038862 ENSDARG00000046014 ENSDARG00000051852 ENSDARG00000055855 ENSDARG00000060085 ENSDARG00000060095 ENSDARG00000062942 ENSDARG00000070092 ENSDARG00000075307 ENSDARG00000076644 ENSDARG00000088842 | |

  


### Go to GO detail

## ZFA

| | ZFA ID | Description | Annotated | Expected | Observed | Fold Enrichment | Adjusted p-value | Genes | Ensembl IDs | | --- | --- | --- | --- | --- | --- | --- | --- | --- | | ZFA:0001127 | visual system | 4 | 0.72 | 2 | 2.8 | 1.9e-26 | rlbp1a rlbp1b | ENSDARG00000012504 ENSDARG00000045808 | | ZFA:0000013 | cranial ganglion | 443 | 79.39 | 111 | 1.4 | 7.2e-22 | stx1b bdnf atp1b2a atp1a3a atp1a3b gng3 ywhag1 appb tpi1b gapdhs mllt11 atp6v0cb diras1a eno1a syt4 zgc:65894 nmnat2 vamp2 p2rx2 nsfa gria3b slc17a6b znf536 gpm6ab calm1b aldocb esrrga si:dkeyp-72g9.4 ndel1a rtn4r rtn4rl1b cnr1 aplp1 clstn1 cadm2a sncb rims2a desi1a calb2b calb2a amph gnb5b kcnip3a nptnb ndufa4 nptx1l map3k12 zgc:77058 vamp1 syt11a eno2 atp2b3a olfm1a camk2d2 itm2ca stmn4l csdc2a tmem178b cacna1aa ccdc85al rbfox1 opcml tspan7b chga stmn2a adcyap1b cadm4 gpr22a prnpb trpa1b map6a rab6bb gnb2 nhsl1a stmn1b ppp3cb atpv0e2 pacsin1a nefma gdap1 stxbp1a necab2 ngfra syn2b gpr158a st8sia5 amigo1 nlgn3b gabra1 trpm2 trpc1 kif5aa radil nlgn3a vsnl1b prkg1b lingo1a prelp si:ch211-180f4.1 syn1 nlgn2a nlgn2b nlgn4a map1aa egr1 snap25b snap25a nadl1.2 neurod1 elavl4 ldha | ENSDARG00000000503 ENSDARG00000018817 ENSDARG00000099203 ENSDARG00000018259 ENSDARG00000104139 ENSDARG00000009553 ENSDARG00000067626 ENSDARG00000055543 ENSDARG00000040988 ENSDARG00000039914 ENSDARG00000071026 ENSDARG00000036577 ENSDARG00000028066 ENSDARG00000022456 ENSDARG00000036505 ENSDARG00000016301 ENSDARG00000004580 ENSDARG00000056877 ENSDARG00000002300 ENSDARG00000007654 ENSDARG00000037498 ENSDARG00000041150 ENSDARG00000103648 ENSDARG00000004621 ENSDARG00000034187 ENSDARG00000019702 ENSDARG00000004861 ENSDARG00000073704 ENSDARG00000010953 ENSDARG00000090035 ENSDARG00000098528 ENSDARG00000009020 ENSDARG00000098368 ENSDARG00000031720 ENSDARG00000009930 ENSDARG00000104945 ENSDARG00000101606 ENSDARG00000033140 ENSDARG00000036344 ENSDARG00000041062 ENSDARG00000007663 ENSDARG00000055377 ENSDARG00000034229 ENSDARG00000043864 ENSDARG00000056108 ENSDARG00000074671 ENSDARG00000103651 ENSDARG00000104582 ENSDARG00000031283 ENSDARG00000057913 ENSDARG00000014287 ENSDARG00000043474 ENSDARG00000018270 ENSDARG00000014273 ENSDARG00000043448 ENSDARG00000043932 ENSDARG00000041323 ENSDARG00000006747 ENSDARG00000037905 ENSDARG00000005343 ENSDARG00000014746 ENSDARG00000013005 ENSDARG00000008407 ENSDARG00000008829 ENSDARG00000033234 ENSDARG00000027740 ENSDARG00000040291 ENSDARG00000004592 ENSDARG00000044048 ENSDARG00000031875 ENSDARG00000074521 ENSDARG00000031343 ENSDARG00000035357 ENSDARG00000054537 ENSDARG00000033655 ENSDARG00000025106 ENSDARG00000059057 ENSDARG00000032865 ENSDARG00000021351 ENSDARG00000058601 ENSDARG00000001994 ENSDARG00000056745 ENSDARG00000088708 ENSDARG00000101054 ENSDARG00000077134 ENSDARG00000036584 ENSDARG00000079620 ENSDARG00000062376 ENSDARG00000068989 ENSDARG00000095696 ENSDARG00000061203 ENSDARG00000098936 ENSDARG00000079779 ENSDARG00000104786 ENSDARG00000044053 ENSDARG00000031702 ENSDARG00000034165 ENSDARG00000070597 ENSDARG00000077112 ENSDARG00000060368 ENSDARG00000077329 ENSDARG00000079251 ENSDARG00000079455 ENSDARG00000059601 ENSDARG00000037421 ENSDARG00000058117 ENSDARG00000020609 ENSDARG00000007149 ENSDARG00000019566 ENSDARG00000045639 ENSDARG00000101251 | | ZFA:0001359 | pineal complex | 32 | 5.73 | 8 | 1.4 | 8.7e-22 | crx gnat2 gnat1 gngt1 gnb3b nr1d1 gngt2b neurod1 | ENSDARG00000011989 ENSDARG00000042529 ENSDARG00000044199 ENSDARG00000035798 ENSDARG00000002696 ENSDARG00000033160 ENSDARG00000103543 ENSDARG00000019566 | | ZFA:0000019 | epiphysis | 578 | 103.58 | 135 | 1.3 | 1.0e-21 | valopa atp1b2a atp1a3a atp1a3b crx atp1b2b gnat2 gnat1 per1a tpi1b gapdhs guk1b drd2a atp6v0cb fxyd6 eno1a nrsn1 rcvrn2 rbp4l gngt1 nme2a csnk1e eef2l2 vamp2 oprd1b bcat1 TULP2 opn4.1 nsfa slc17a6b gpm6ab gpm6ba penka calm1b aldocb esrrga pde6g prom1b pcp4a smox mao crispld1b saga cadm2a arl3l1 desi1a rcvrn3 rlbp1a pde6c slc25a3a scg5 ppa1a gnb5b scg3 cox4i2 pnp6 nptnb rlbp1b gnb3b igfbp7 rorab nr1d2a tph2 sh3bgrl2 nfil3-6 mpp2b arr3a syt5a opn1lw2 syt1a atp2b3a olfm1a syn2a camk2n2 faimb rorcb abhd3 ccdc85al rrh rgra opcml syngr1a stmn2a cadm4 crhb avpr1ab lrrc4.1 gpr22a nr1d1 celf5a grk1b syt5b atpv0e2 zgc:109982 pacsin1a zgc:109949 tmx3 crmp1 dpysl5b grk1a gpr146 grk7a rcvrna sagb syn2b slc6a4a dbpa crb2b lrit1b hlfa opn6a NAPB (1 of many) drd4a ppm1e ddit3 rhol opn4xb kif5aa opn3 slitrk2 cadm2b syn1 spock3 chrna6 hlfb si:dkey-260g12.4 agrp2 snap25b neurod1 nr1d2b rho opn1sw2 opn1mw1 ldha opn1sw1 | ENSDARG00000021150 ENSDARG00000099203 ENSDARG00000018259 ENSDARG00000104139 ENSDARG00000011989 ENSDARG00000034424 ENSDARG00000042529 ENSDARG00000044199 ENSDARG00000056885 ENSDARG00000040988 ENSDARG00000039914 ENSDARG00000005776 ENSDARG00000056926 ENSDARG00000036577 ENSDARG00000100971 ENSDARG00000022456 ENSDARG00000044719 ENSDARG00000019902 ENSDARG00000044684 ENSDARG00000035798 ENSDARG00000043820 ENSDARG00000045150 ENSDARG00000035256 ENSDARG00000056877 ENSDARG00000037159 ENSDARG00000045568 ENSDARG00000062902 ENSDARG00000007553 ENSDARG00000007654 ENSDARG00000041150 ENSDARG00000004621 ENSDARG00000005739 ENSDARG00000004869 ENSDARG00000034187 ENSDARG00000019702 ENSDARG00000004861 ENSDARG00000101984 ENSDARG00000034007 ENSDARG00000053130 ENSDARG00000036967 ENSDARG00000023712 ENSDARG00000013293 ENSDARG00000012610 ENSDARG00000009930 ENSDARG00000102393 ENSDARG00000033140 ENSDARG00000009637 ENSDARG00000012504 ENSDARG00000100397 ENSDARG00000027424 ENSDARG00000032126 ENSDARG00000099933 ENSDARG00000055377 ENSDARG00000086288 ENSDARG00000022509 ENSDARG00000040942 ENSDARG00000043864 ENSDARG00000045808 ENSDARG00000002696 ENSDARG00000104138 ENSDARG00000001910 ENSDARG00000003820 ENSDARG00000057239 ENSDARG00000036878 ENSDARG00000087188 ENSDARG00000010957 ENSDARG00000056511 ENSDARG00000037941 ENSDARG00000044861 ENSDARG00000030614 ENSDARG00000043474 ENSDARG00000018270 ENSDARG00000045945 ENSDARG00000090424 ENSDARG00000020814 ENSDARG00000017780 ENSDARG00000018809 ENSDARG00000005343 ENSDARG00000039534 ENSDARG00000054890 ENSDARG00000013005 ENSDARG00000002564 ENSDARG00000033234 ENSDARG00000040291 ENSDARG00000027657 ENSDARG00000045788 ENSDARG00000069402 ENSDARG00000004592 ENSDARG00000033160 ENSDARG00000071375 ENSDARG00000104685 ENSDARG00000011640 ENSDARG00000059057 ENSDARG00000099217 ENSDARG00000032865 ENSDARG00000103413 ENSDARG00000038894 ENSDARG00000056742 ENSDARG00000059311 ENSDARG00000058803 ENSDARG00000059610 ENSDARG00000020602 ENSDARG00000052223 ENSDARG00000038378 ENSDARG00000101054 ENSDARG00000061165 ENSDARG00000063014 ENSDARG00000060081 ENSDARG00000099406 ENSDARG00000074752 ENSDARG00000102430 ENSDARG00000069101 ENSDARG00000038363 ENSDARG00000026499 ENSDARG00000059836 ENSDARG00000070666 ENSDARG00000103259 ENSDARG00000098936 ENSDARG00000052775 ENSDARG00000006636 ENSDARG00000062633 ENSDARG00000060368 ENSDARG00000070266 ENSDARG00000055559 ENSDARG00000061011 ENSDARG00000104231 ENSDARG00000099781 ENSDARG00000058117 ENSDARG00000019566 ENSDARG00000009594 ENSDARG00000002193 ENSDARG00000017274 ENSDARG00000097008 ENSDARG00000101251 ENSDARG00000045677 | | ZFA:0000024 | retinal ganglion cell layer | 510 | 91.40 | 131 | 1.4 | 2.8e-18 | anos1a stx1b bdnf scn8aa robo2 robo2 ntrk2b ntrk3b gng3 ywhag1 gria1a gria1b gria1b gria2a gria2b gria3a appb glra4b grm6b sv2bb mllt11 kcnd3 diras1a nrsn1 zgc:65894 grm1a grm2a nmnat2 vamp2 nsfa gria3b slc17a6b rtn1b gpm6aa gpm6ab gpm6ba esrrga guca1c drd2b pcp4a ndel1a aplp1 camkvb cadm2a sncb rims2a desi1a calb2b calb2a scg5 gnao1b kcnip3a ndufa4 zgc:77058 rorab nr1d2a asic1a asic4a eno2 irx6a syt1a atp2b3a olfm1a cdk5r1b rgs8 itm2ca stmn4l syn2a camk2n2 gng13b rorcb cacna1aa tmem200a olfm1b lingo1b ccdc85al pvalb6 rbfox1 stmn2a adcyap1b cadm4 slc6a1b lrrc4.1 lrtm2a gpr22a nr1d1 dlg2 gnb2 nhsl1a stmn1b ppp3cb stxbp1a syn2b grm6a roraa st8sia5 amigo1 ndrg4 cdh10a scn1lab grm3 nyx oaz2b drd1b arhgef9a trpc1 kif5aa satb2 slitrk3b pappa2 vsnl1b lingo1a si:ch211-180f4.1 slitrk2 lrrtm2 nr1d4a cadm2b cacnb2a syn1 ulk2 spock3 pcdh17 pcdh1a grm8a grm8b kif5ab pcdh7a caln1 egr1 snap25b snap25a nr1d2b cdh4 | ENSDARG00000012896 ENSDARG00000000503 ENSDARG00000018817 ENSDARG00000005775 ENSDARG00000014891 ENSDARG00000078366 ENSDARG00000098511 ENSDARG00000086214 ENSDARG00000009553 ENSDARG00000067626 ENSDARG00000021352 ENSDARG00000032714 ENSDARG00000032714 ENSDARG00000070173 ENSDARG00000052765 ENSDARG00000032737 ENSDARG00000055543 ENSDARG00000058103 ENSDARG00000025671 ENSDARG00000060711 ENSDARG00000071026 ENSDARG00000056101 ENSDARG00000028066 ENSDARG00000044719 ENSDARG00000016301 ENSDARG00000026796 ENSDARG00000004150 ENSDARG00000004580 ENSDARG00000056877 ENSDARG00000007654 ENSDARG00000037498 ENSDARG00000041150 ENSDARG00000021143 ENSDARG00000055455 ENSDARG00000004621 ENSDARG00000005739 ENSDARG00000004861 ENSDARG00000030758 ENSDARG00000011091 ENSDARG00000053130 ENSDARG00000010953 ENSDARG00000098368 ENSDARG00000005141 ENSDARG00000009930 ENSDARG00000104945 ENSDARG00000101606 ENSDARG00000033140 ENSDARG00000036344 ENSDARG00000041062 ENSDARG00000032126 ENSDARG00000036058 ENSDARG00000034229 ENSDARG00000056108 ENSDARG00000104582 ENSDARG00000001910 ENSDARG00000003820 ENSDARG00000008329 ENSDARG00000025162 ENSDARG00000014287 ENSDARG00000034420 ENSDARG00000030614 ENSDARG00000043474 ENSDARG00000018270 ENSDARG00000045087 ENSDARG00000070037 ENSDARG00000043448 ENSDARG00000043932 ENSDARG00000045945 ENSDARG00000090424 ENSDARG00000037921 ENSDARG00000017780 ENSDARG00000037905 ENSDARG00000031540 ENSDARG00000014053 ENSDARG00000035899 ENSDARG00000005343 ENSDARG00000009311 ENSDARG00000014746 ENSDARG00000033234 ENSDARG00000027740 ENSDARG00000040291 ENSDARG00000039647 ENSDARG00000069402 ENSDARG00000045483 ENSDARG00000004592 ENSDARG00000033160 ENSDARG00000099323 ENSDARG00000035357 ENSDARG00000054537 ENSDARG00000033655 ENSDARG00000025106 ENSDARG00000001994 ENSDARG00000101054 ENSDARG00000017742 ENSDARG00000031768 ENSDARG00000036584 ENSDARG00000079620 ENSDARG00000103937 ENSDARG00000055843 ENSDARG00000062744 ENSDARG00000031712 ENSDARG00000061791 ENSDARG00000059815 ENSDARG00000038918 ENSDARG00000061746 ENSDARG00000061203 ENSDARG00000098936 ENSDARG00000061885 ENSDARG00000074739 ENSDARG00000076020 ENSDARG00000044053 ENSDARG00000034165 ENSDARG00000077112 ENSDARG00000006636 ENSDARG00000071374 ENSDARG00000031161 ENSDARG00000062633 ENSDARG00000099045 ENSDARG00000060368 ENSDARG00000097205 ENSDARG00000070266 ENSDARG00000027041 ENSDARG00000062720 ENSDARG00000077654 ENSDARG00000076508 ENSDARG00000059818 ENSDARG00000078898 ENSDARG00000088898 ENSDARG00000037421 ENSDARG00000058117 ENSDARG00000020609 ENSDARG00000009594 ENSDARG00000015002 | | ZFA:0000402 | olfactory bulb | 183 | 32.79 | 58 | 1.8 | 2.1e-14 | anos1a bdnf gria1a gria1b gria1b gria2a gria2b gria3a gria4a grm6b kcnd3 hsd11b2 eno1a grm1a grm2a oprd1b nsfa gria3b pvalb8 aldocb gad1b fosab ndel1a cadm2a sncb eno2 nr4a2b syt1a s100b syn2a gng13b fam49a ccdc85al stmn2a adcyap1b hivep2a cadm4 crhb kcnip1b dlg2 s100t grin1a grin1b gpr158a st8sia5 nlgn3b stxbp1b slitrk3b nlgn3a slitrk2 cadm2b syn1 il1rapl1b nlgn2a nlgn2b grm8a grm8b nr4a2a th | ENSDARG00000012896 ENSDARG00000018817 ENSDARG00000021352 ENSDARG00000032714 ENSDARG00000032714 ENSDARG00000070173 ENSDARG00000052765 ENSDARG00000032737 ENSDARG00000037496 ENSDARG00000025671 ENSDARG00000056101 ENSDARG00000001975 ENSDARG00000022456 ENSDARG00000026796 ENSDARG00000004150 ENSDARG00000037159 ENSDARG00000007654 ENSDARG00000037498 ENSDARG00000037790 ENSDARG00000019702 ENSDARG00000027419 ENSDARG00000031683 ENSDARG00000010953 ENSDARG00000009930 ENSDARG00000104945 ENSDARG00000014287 ENSDARG00000044532 ENSDARG00000030614 ENSDARG00000057598 ENSDARG00000045945 ENSDARG00000037921 ENSDARG00000035907 ENSDARG00000005343 ENSDARG00000033234 ENSDARG00000027740 ENSDARG00000039987 ENSDARG00000040291 ENSDARG00000027657 ENSDARG00000034808 ENSDARG00000099323 ENSDARG00000055589 ENSDARG00000027828 ENSDARG00000025728 ENSDARG00000077134 ENSDARG00000036584 ENSDARG00000062376 ENSDARG00000056036 ENSDARG00000074739 ENSDARG00000104786 ENSDARG00000006636 ENSDARG00000062633 ENSDARG00000060368 ENSDARG00000104853 ENSDARG00000077329 ENSDARG00000079251 ENSDARG00000077654 ENSDARG00000076508 ENSDARG00000017007 ENSDARG00000030621 | | ZFA:0000119 | retinal inner nuclear layer | 414 | 74.19 | 100 | 1.3 | 3.9e-14 | anos1a stx1b bdnf scn8aa ntrk2b crx gria1a gria1b gria1b gria2a gria2b gria3a gria4a glra4b grm6b sv2bb diras1a slc1a2b grm1a grm2a rhbg gria3b gpm6aa gpm6ab gpm6ba esrrga prom1b pcp4a ndel1a camkvb cadm2a rlbp1a pde6c lin7a calb2b calb2a gnao1b nptnb junba rorab nr1d2a asic1a nfil3-6 nr4a2b cabp1b olfm1a rgs8 syn2a camk2n2 rorcb ccdc85al opcml lin7b cadm4 crhb nr1d1 necab1 ppp3cb pdlim3a lrrtm1 cabp5b ompa bhlhe23 cabp2a syn2b grm6a roraa ush1c ndrg4 cdh10a scn1lab grm3 nyx opn6a trpm1a drd1b zgc:165604 trpc1 kif5aa slitrk3b lrrc38a lingo1a slitrk2 lrrtm2 nr1d4a nr1d4b cadm2b pcdh17 pcdh1a grm8a grm8b kif5ab pcdh7a kcnj10a si:dkey-260g12.4 egr1 snap25a neurod1 nr4a2a nr1d2b th | ENSDARG00000012896 ENSDARG00000000503 ENSDARG00000018817 ENSDARG00000005775 ENSDARG00000098511 ENSDARG00000011989 ENSDARG00000021352 ENSDARG00000032714 ENSDARG00000032714 ENSDARG00000070173 ENSDARG00000052765 ENSDARG00000032737 ENSDARG00000037496 ENSDARG00000058103 ENSDARG00000025671 ENSDARG00000060711 ENSDARG00000028066 ENSDARG00000102453 ENSDARG00000026796 ENSDARG00000004150 ENSDARG00000009018 ENSDARG00000037498 ENSDARG00000055455 ENSDARG00000004621 ENSDARG00000005739 ENSDARG00000004861 ENSDARG00000034007 ENSDARG00000053130 ENSDARG00000010953 ENSDARG00000005141 ENSDARG00000009930 ENSDARG00000012504 ENSDARG00000100397 ENSDARG00000013414 ENSDARG00000036344 ENSDARG00000041062 ENSDARG00000036058 ENSDARG00000043864 ENSDARG00000074378 ENSDARG00000001910 ENSDARG00000003820 ENSDARG00000008329 ENSDARG00000087188 ENSDARG00000044532 ENSDARG00000033411 ENSDARG00000018270 ENSDARG00000070037 ENSDARG00000045945 ENSDARG00000090424 ENSDARG00000017780 ENSDARG00000005343 ENSDARG00000013005 ENSDARG00000037932 ENSDARG00000040291 ENSDARG00000027657 ENSDARG00000033160 ENSDARG00000056566 ENSDARG00000025106 ENSDARG00000011023 ENSDARG00000052713 ENSDARG00000028485 ENSDARG00000105218 ENSDARG00000037588 ENSDARG00000052016 ENSDARG00000101054 ENSDARG00000017742 ENSDARG00000031768 ENSDARG00000051876 ENSDARG00000103937 ENSDARG00000055843 ENSDARG00000062744 ENSDARG00000031712 ENSDARG00000061791 ENSDARG00000102430 ENSDARG00000011259 ENSDARG00000038918 ENSDARG00000021241 ENSDARG00000061203 ENSDARG00000098936 ENSDARG00000074739 ENSDARG00000075147 ENSDARG00000034165 ENSDARG00000006636 ENSDARG00000071374 ENSDARG00000031161 ENSDARG00000059370 ENSDARG00000062633 ENSDARG00000027041 ENSDARG00000062720 ENSDARG00000077654 ENSDARG00000076508 ENSDARG00000059818 ENSDARG00000078898 ENSDARG00000090815 ENSDARG00000104231 ENSDARG00000037421 ENSDARG00000020609 ENSDARG00000019566 ENSDARG00000017007 ENSDARG00000009594 ENSDARG00000030621 | | ZFA:0000545 | medulla oblongata | 123 | 22.04 | 41 | 1.9 | 2.4e-12 | gria1a gria1b gria1b gria2a gria2b gria3a gria4a grm2a gria3b cnr1 ucp3 cadm2a nr4a2b syn2a crhbp cadm4 crhb cart2 stxbp1a grin1a grin1b grm6a nlgn3b stxbp1b nr4a3 slitrk3b htr1aa nlgn3a slitrk2 cadm2b syn1 pcdh17 nlgn2a nlgn2b pcdh1a nlgn4a grm8a grm8b pcdh7a slitrk4 nr4a2a th | ENSDARG00000021352 ENSDARG00000032714 ENSDARG00000032714 ENSDARG00000070173 ENSDARG00000052765 ENSDARG00000032737 ENSDARG00000037496 ENSDARG00000004150 ENSDARG00000037498 ENSDARG00000009020 ENSDARG00000091209 ENSDARG00000009930 ENSDARG00000044532 ENSDARG00000045945 ENSDARG00000024831 ENSDARG00000040291 ENSDARG00000027657 ENSDARG00000045832 ENSDARG00000001994 ENSDARG00000027828 ENSDARG00000025728 ENSDARG00000017742 ENSDARG00000062376 ENSDARG00000056036 ENSDARG00000055854 ENSDARG00000074739 ENSDARG00000093745 ENSDARG00000104786 ENSDARG00000006636 ENSDARG00000062633 ENSDARG00000060368 ENSDARG00000027041 ENSDARG00000077329 ENSDARG00000079251 ENSDARG00000062720 ENSDARG00000079455 ENSDARG00000077654 ENSDARG00000076508 ENSDARG00000078898 ENSDARG00000079781 ENSDARG00000017007 ENSDARG00000030621 | | ZFA:0005575 | brain nucleus | 6 | 1.08 | 2 | 1.9 | 8.1e-12 | ncam2 nr4a2b | ENSDARG00000017466 ENSDARG00000044532 | | ZFA:0000213 | habenula | 122 | 21.86 | 37 | 1.7 | 1.4e-10 | gria1a gria1b gria1b gria2a gria4a adcyap1a grm6b slc17a6b fosab ndel1a rtn4r mao cadm2a calb2b rgs8 syn2a cadm4 rab6bb zgc:109949 gng8 kctd8 grm6a ndrg4 stxbp1b grm3 etv1 nr4a3 trpc1 chata lrrc38a elfn1b slitrk2 cadm2b syn1 ulk2 tac3a mtbl grm8b | ENSDARG00000021352 ENSDARG00000032714 ENSDARG00000032714 ENSDARG00000070173 ENSDARG00000037496 ENSDARG00000004015 ENSDARG00000025671 ENSDARG00000041150 ENSDARG00000031683 ENSDARG00000010953 ENSDARG00000090035 ENSDARG00000023712 ENSDARG00000009930 ENSDARG00000036344 ENSDARG00000070037 ENSDARG00000045945 ENSDARG00000040291 ENSDARG00000031343 ENSDARG00000103413 ENSDARG00000042970 ENSDARG00000067507 ENSDARG00000017742 ENSDARG00000103937 ENSDARG00000056036 ENSDARG00000031712 ENSDARG00000101959 ENSDARG00000055854 ENSDARG00000061203 ENSDARG00000015854 ENSDARG00000075147 ENSDARG00000074372 ENSDARG00000006636 ENSDARG00000062633 ENSDARG00000060368 ENSDARG00000097205 ENSDARG00000093089 ENSDARG00000102051 ENSDARG00000076508 | | ZFA:0000188 | corpus cerebelli | 47 | 8.42 | 18 | 2.1 | 8.4e-09 | anos1a hsd11b2 adgrl3.1 grm1a grm2a oprd1b cnr1 mao cadm2a slc17a7a grm6a roraa grm3 fstl5 cadm2b mtbl grm8a grm8b | ENSDARG00000012896 ENSDARG00000001975 ENSDARG00000061121 ENSDARG00000026796 ENSDARG00000004150 ENSDARG00000037159 ENSDARG00000009020 ENSDARG00000023712 ENSDARG00000009930 ENSDARG00000016480 ENSDARG00000017742 ENSDARG00000031768 ENSDARG00000031712 ENSDARG00000040198 ENSDARG00000062633 ENSDARG00000102051 ENSDARG00000077654 ENSDARG00000076508 | | ZFA:0000470 | preoptic area | 121 | 21.68 | 32 | 1.5 | 3.0e-08 | bdnf gria1a gria1b gria2b gria3a gria4a sst1.1 adgrl3.1 oprd1b oxt penka gad1b prom1b pcp4a cnr1 nr4a2b crhbp crhb bsk146 nlgn3b kcnq3 htr1aa nlgn3a vip kcnq2a nlgn2a nlgn2b ccka adcyap1r1b grm8a nr4a2a th | ENSDARG00000018817 ENSDARG00000021352 ENSDARG00000032714 ENSDARG00000052765 ENSDARG00000032737 ENSDARG00000037496 ENSDARG00000040799 ENSDARG00000061121 ENSDARG00000037159 ENSDARG00000042845 ENSDARG00000004869 ENSDARG00000027419 ENSDARG00000034007 ENSDARG00000053130 ENSDARG00000009020 ENSDARG00000044532 ENSDARG00000024831 ENSDARG00000027657 ENSDARG00000099069 ENSDARG00000062376 ENSDARG00000060085 ENSDARG00000093745 ENSDARG00000104786 ENSDARG00000078247 ENSDARG00000075307 ENSDARG00000077329 ENSDARG00000079251 ENSDARG00000070810 ENSDARG00000053724 ENSDARG00000077654 ENSDARG00000017007 ENSDARG00000030621 | | ZFA:0001659 | diencephalic nucleus | 11 | 1.97 | 3 | 1.5 | 7.6e-07 | robo2 robo2 drd2a drd2b | ENSDARG00000014891 ENSDARG00000078366 ENSDARG00000056926 ENSDARG00000011091 | | ZFA:0000143 | retinal photoreceptor layer | 184 | 32.97 | 47 | 1.4 | 7.8e-07 | crx gnat2 gnat1 gc3 rbp2a guk1b rcvrn2 rbp4l gngt1 vamp2 TULP2 opn4.1 pde6a guca1c rom1b cadm2a arl3l1 rcvrn3 pde6c slc25a3a ppa1a gnb5b rdh8b cox4i2 zgc:77752 asic1a asic4a nfil3-6 arr3a ldhbb faimb rorcb grk1b zgc:109982 zgc:109949 tmx3 grk1a grk7a rcvrna crb2b lrit1b drd4a slc4a5 rho opn1sw2 opn1mw1 opn1sw1 | ENSDARG00000011989 ENSDARG00000042529 ENSDARG00000044199 ENSDARG00000026820 ENSDARG00000070038 ENSDARG00000005776 ENSDARG00000019902 ENSDARG00000044684 ENSDARG00000035798 ENSDARG00000056877 ENSDARG00000062902 ENSDARG00000007553 ENSDARG00000000380 ENSDARG00000030758 ENSDARG00000026926 ENSDARG00000009930 ENSDARG00000102393 ENSDARG00000009637 ENSDARG00000100397 ENSDARG00000027424 ENSDARG00000099933 ENSDARG00000055377 ENSDARG00000105060 ENSDARG00000022509 ENSDARG00000042387 ENSDARG00000008329 ENSDARG00000025162 ENSDARG00000087188 ENSDARG00000056511 ENSDARG00000071076 ENSDARG00000020814 ENSDARG00000017780 ENSDARG00000104685 ENSDARG00000099217 ENSDARG00000103413 ENSDARG00000038894 ENSDARG00000058803 ENSDARG00000020602 ENSDARG00000052223 ENSDARG00000060081 ENSDARG00000099406 ENSDARG00000038363 ENSDARG00000104387 ENSDARG00000002193 ENSDARG00000017274 ENSDARG00000097008 ENSDARG00000045677 | | ZFA:0000304 | ventral telencephalon | 113 | 20.25 | 29 | 1.4 | 9.0e-07 | gria1a gria1b gria2a gria2b gria4a adgrl3.1 grm1a gria3b gad1b prom1b fosab cnr1 mao cadm2a nr4a2b syn2a crhbp cadm4 crhb slc17a6a etv1 nr4a3 slitrk3b slitrk2 cadm2b syn1 pcdh17 ppp3ca nr4a2a th | ENSDARG00000021352 ENSDARG00000032714 ENSDARG00000070173 ENSDARG00000052765 ENSDARG00000037496 ENSDARG00000061121 ENSDARG00000026796 ENSDARG00000037498 ENSDARG00000027419 ENSDARG00000034007 ENSDARG00000031683 ENSDARG00000009020 ENSDARG00000023712 ENSDARG00000009930 ENSDARG00000044532 ENSDARG00000045945 ENSDARG00000024831 ENSDARG00000040291 ENSDARG00000027657 ENSDARG00000001127 ENSDARG00000101959 ENSDARG00000055854 ENSDARG00000074739 ENSDARG00000006636 ENSDARG00000062633 ENSDARG00000060368 ENSDARG00000027041 ENSDARG00000004988 ENSDARG00000017007 ENSDARG00000030621 | | ZFA:0001215 | thalamus | 102 | 18.28 | 27 | 1.5 | 1.0e-06 | anos1a valopa gria1a gria1b gria1b gria2a gria2b gria3a gria4a gria3b nr4a2b crhbp adcyap1b crhb adcyap1r1a nlgn3b cdh10a slitrk3b nlgn3a slitrk2 tac3a pcsk1nl nlgn2a nlgn2b pcdh1a adcyap1r1b pcdh7a nr4a2a | ENSDARG00000012896 ENSDARG00000021150 ENSDARG00000021352 ENSDARG00000032714 ENSDARG00000032714 ENSDARG00000070173 ENSDARG00000052765 ENSDARG00000032737 ENSDARG00000037496 ENSDARG00000037498 ENSDARG00000044532 ENSDARG00000024831 ENSDARG00000027740 ENSDARG00000027657 ENSDARG00000105201 ENSDARG00000062376 ENSDARG00000055843 ENSDARG00000074739 ENSDARG00000104786 ENSDARG00000006636 ENSDARG00000093089 ENSDARG00000076170 ENSDARG00000077329 ENSDARG00000079251 ENSDARG00000062720 ENSDARG00000053724 ENSDARG00000078898 ENSDARG00000017007 | | ZFA:0000295 | trigeminal ganglion | 201 | 36.02 | 50 | 1.4 | 1.5e-06 | runx3 scn8aa robo2 robo2 ywhag1 gria3a gria4a adcyap1a atp6v0cb eno1a grm2a nsfa gria3b gpm6ab esrrga syt9a pcp4a si:dkeyp-72g9.4 fosab ndel1a clstn1 camkvb cadm2a calb2b calca olfm1a stmn4l syn2a olfm1b stmn2a prnprs3 trpa1b cers1 rab6bb dpysl5b lgi2b st8sia5 ndrg4 scn8ab trpm2 etv1 si:ch211-11c15.3 ptprdb trpc1 kif5aa lingo1a zeb2b syn1 chrna6 neurod1 elavl4 | ENSDARG00000052826 ENSDARG00000005775 ENSDARG00000014891 ENSDARG00000078366 ENSDARG00000067626 ENSDARG00000032737 ENSDARG00000037496 ENSDARG00000004015 ENSDARG00000036577 ENSDARG00000022456 ENSDARG00000004150 ENSDARG00000007654 ENSDARG00000037498 ENSDARG00000004621 ENSDARG00000004861 ENSDARG00000003994 ENSDARG00000053130 ENSDARG00000073704 ENSDARG00000031683 ENSDARG00000010953 ENSDARG00000031720 ENSDARG00000005141 ENSDARG00000009930 ENSDARG00000036344 ENSDARG00000056590 ENSDARG00000018270 ENSDARG00000043932 ENSDARG00000045945 ENSDARG00000014053 ENSDARG00000033234 ENSDARG00000003705 ENSDARG00000031875 ENSDARG00000063412 ENSDARG00000031343 ENSDARG00000059311 ENSDARG00000069701 ENSDARG00000036584 ENSDARG00000103937 ENSDARG00000018032 ENSDARG00000095696 ENSDARG00000101959 ENSDARG00000092970 ENSDARG00000019945 ENSDARG00000061203 ENSDARG00000098936 ENSDARG00000034165 ENSDARG00000078416 ENSDARG00000060368 ENSDARG00000055559 ENSDARG00000019566 ENSDARG00000045639 | | ZFA:0000603 | valvula cerebelli | 32 | 5.73 | 12 | 2.1 | 1.6e-06 | grm1a oprd1b prom1b pcp4a ucp3 cadm2a cadm4 slc17a7a grm3 slitrk3b cadm2b slitrk4 | ENSDARG00000026796 ENSDARG00000037159 ENSDARG00000034007 ENSDARG00000053130 ENSDARG00000091209 ENSDARG00000009930 ENSDARG00000040291 ENSDARG00000016480 ENSDARG00000031712 ENSDARG00000074739 ENSDARG00000062633 ENSDARG00000079781 | | ZFA:0000120 | lateral line ganglion | 89 | 15.95 | 21 | 1.3 | 6.7e-06 | atp1a3b atp1b2b ywhag1 appb gapdhs sv2bb znf536 esrrga rtn4r clic5b gng13b dusp2 lingo1b abhd3 prnprs3 rab6bb ngfra ndrg4 tmem178 si:dkey-205h13.2 ulk2 | ENSDARG00000104139 ENSDARG00000034424 ENSDARG00000067626 ENSDARG00000055543 ENSDARG00000039914 ENSDARG00000060711 ENSDARG00000103648 ENSDARG00000004861 ENSDARG00000090035 ENSDARG00000070584 ENSDARG00000037921 ENSDARG00000098108 ENSDARG00000035899 ENSDARG00000018809 ENSDARG00000003705 ENSDARG00000031343 ENSDARG00000088708 ENSDARG00000103937 ENSDARG00000020758 ENSDARG00000089429 ENSDARG00000097205 | | ZFA:0001660 | telencephalic nucleus | 7 | 1.25 | 2 | 1.6 | 4.1e-05 | atp6v0cb drd2b | ENSDARG00000036577 ENSDARG00000011091 | | ZFA:0000347 | dorsal hypothalamic zone | 4 | 0.72 | 2 | 2.8 | 1.6e-04 | nr4a2a th | ENSDARG00000017007 ENSDARG00000030621 | | ZFA:0001328 | neuromere | 1 | 0.18 | 1 | 5.6 | 9.0e-04 | prnprs3 | ENSDARG00000003705 | | ZFA:0007002 | ventro-rostral cluster | 21 | 3.76 | 13 | 3.5 | 1.1e-03 | robo2 robo2 gria1a gria1b gria1b gria2a gria2b gria3a gria4a gria3b nlgn3b nlgn3a nlgn2a nlgn2b nlgn4a | ENSDARG00000014891 ENSDARG00000078366 ENSDARG00000021352 ENSDARG00000032714 ENSDARG00000032714 ENSDARG00000070173 ENSDARG00000052765 ENSDARG00000032737 ENSDARG00000037496 ENSDARG00000037498 ENSDARG00000062376 ENSDARG00000104786 ENSDARG00000077329 ENSDARG00000079251 ENSDARG00000079455 | | ZFA:0000653 | dorsal thalamus | 80 | 14.34 | 23 | 1.6 | 1.9e-03 | gria1a gria3a grm1a grm2a oprd1b prom1b pcp4a ndel1a cadm2a nr4a2b syn2a cadm4 cart2 cabp7b htr1aa itgb3b cadm2b syn1 pcdh17 grm8a neurod1 nr4a2a th | ENSDARG00000021352 ENSDARG00000032737 ENSDARG00000026796 ENSDARG00000004150 ENSDARG00000037159 ENSDARG00000034007 ENSDARG00000053130 ENSDARG00000010953 ENSDARG00000009930 ENSDARG00000044532 ENSDARG00000045945 ENSDARG00000040291 ENSDARG00000045832 ENSDARG00000060846 ENSDARG00000093745 ENSDARG00000045070 ENSDARG00000062633 ENSDARG00000060368 ENSDARG00000027041 ENSDARG00000077654 ENSDARG00000019566 ENSDARG00000017007 ENSDARG00000030621 | | ZFA:0000778 | spinal cord interneuron | 64 | 11.47 | 22 | 1.9 | 2.6e-03 | anos1a scn8aa gria1a gria1b gria1b gria2a gria2b gria4a gria3b slc17a6b gad1b gad2 clstn1 camkvb slc6a5 slc17a6a syn2b dab1a drd4a gphnb cadm2b syn1 nlgn4a | ENSDARG00000012896 ENSDARG00000005775 ENSDARG00000021352 ENSDARG00000032714 ENSDARG00000032714 ENSDARG00000070173 ENSDARG00000052765 ENSDARG00000037496 ENSDARG00000037498 ENSDARG00000041150 ENSDARG00000027419 ENSDARG00000015537 ENSDARG00000031720 ENSDARG00000005141 ENSDARG00000067964 ENSDARG00000001127 ENSDARG00000101054 ENSDARG00000059939 ENSDARG00000038363 ENSDARG00000100851 ENSDARG00000062633 ENSDARG00000060368 ENSDARG00000079455 | | ZFA:0000199 | dorsal periventricular hypothalamus | 35 | 6.27 | 11 | 1.8 | 3.5e-03 | anos1a ncam2 drd2a grm1a drd2b mao crhb chata tac3a mtbl th | ENSDARG00000012896 ENSDARG00000017466 ENSDARG00000056926 ENSDARG00000026796 ENSDARG00000011091 ENSDARG00000023712 ENSDARG00000027657 ENSDARG00000015854 ENSDARG00000093089 ENSDARG00000102051 ENSDARG00000030621 | | ZFA:0001314 | posterior lateral line ganglion | 72 | 12.90 | 22 | 1.7 | 6.3e-03 | scn8aa gria1b gria2a gria3a gria4a nsfa gria3b gpm6ab cadm2a chga stmn2a adgrg6 cadm4 ndrg4 cdh10a trpm2 kif5aa myo10l1 syn1 grm8b cntnap2a neurod1 cdh4 | ENSDARG00000005775 ENSDARG00000032714 ENSDARG00000070173 ENSDARG00000032737 ENSDARG00000037496 ENSDARG00000007654 ENSDARG00000037498 ENSDARG00000004621 ENSDARG00000009930 ENSDARG00000008829 ENSDARG00000033234 ENSDARG00000054137 ENSDARG00000040291 ENSDARG00000103937 ENSDARG00000055843 ENSDARG00000095696 ENSDARG00000098936 ENSDARG00000074723 ENSDARG00000060368 ENSDARG00000076508 ENSDARG00000058969 ENSDARG00000019566 ENSDARG00000015002 | | ZFA:0000707 | ventral hypothalamic zone | 8 | 1.43 | 3 | 2.1 | 9.1e-03 | crhbp crhb nlgn3a | ENSDARG00000024831 ENSDARG00000027657 ENSDARG00000104786 | | ZFA:0000392 | median tuberal portion | 1 | 0.18 | 1 | 5.6 | 9.6e-03 | th | ENSDARG00000030621 | | ZFA:0000449 | torus longitudinalis | 47 | 8.42 | 15 | 1.8 | 1.2e-02 | anos1a pclob pcloa slc17a6b cnr1 mao slc17a7a nlgn3b opn6a nlgn3a nlgn2a nlgn2b nlgn4a si:dkey-260g12.4 th | ENSDARG00000012896 ENSDARG00000098880 ENSDARG00000063299 ENSDARG00000041150 ENSDARG00000009020 ENSDARG00000023712 ENSDARG00000016480 ENSDARG00000062376 ENSDARG00000102430 ENSDARG00000104786 ENSDARG00000077329 ENSDARG00000079251 ENSDARG00000079455 ENSDARG00000104231 ENSDARG00000030621 | | ZFA:0000426 | rostral parvocellular preoptic nucleus | 32 | 5.73 | 10 | 1.7 | 1.6e-02 | anos1a oxt cadm2a calb2b crhbp crhb htr1aa cadm2b tac3a th | ENSDARG00000012896 ENSDARG00000042845 ENSDARG00000009930 ENSDARG00000036344 ENSDARG00000024831 ENSDARG00000027657 ENSDARG00000093745 ENSDARG00000062633 ENSDARG00000093089 ENSDARG00000030621 | | ZFA:0000391 | medial zone of dorsal telencephalon | 18 | 3.23 | 8 | 2.5 | 1.7e-02 | gad1b fosab mao calb2b slc17a6a nlgn3a nlgn2b mtbl | ENSDARG00000027419 ENSDARG00000031683 ENSDARG00000023712 ENSDARG00000036344 ENSDARG00000001127 ENSDARG00000104786 ENSDARG00000079251 ENSDARG00000102051 | | ZFA:0001464 | retinal outer nuclear layer | 124 | 22.22 | 29 | 1.3 | 1.8e-02 | crx gnat2 gnat1 guk1b rcvrn2 rbp4l prom1b saga cadm2a pde6c foxg1b arr3a olfm1a cadm4 pdlim3a sagb ush1c opn6a NAPB (1 of many) drd1b kif5aa cadm2b pcdh17 slc1a2a egr1 neurod1 rho opn1sw2 opn1sw1 | ENSDARG00000011989 ENSDARG00000042529 ENSDARG00000044199 ENSDARG00000005776 ENSDARG00000019902 ENSDARG00000044684 ENSDARG00000034007 ENSDARG00000012610 ENSDARG00000009930 ENSDARG00000100397 ENSDARG00000032705 ENSDARG00000056511 ENSDARG00000018270 ENSDARG00000040291 ENSDARG00000011023 ENSDARG00000038378 ENSDARG00000051876 ENSDARG00000102430 ENSDARG00000069101 ENSDARG00000038918 ENSDARG00000098936 ENSDARG00000062633 ENSDARG00000027041 ENSDARG00000052138 ENSDARG00000037421 ENSDARG00000019566 ENSDARG00000002193 ENSDARG00000017274 ENSDARG00000045677 | | ZFA:0000248 | magnocellular preoptic nucleus | 26 | 4.66 | 10 | 2.1 | 3.4e-02 | anos1a hsd11b2 oxt gad1b cadm2a cadm4 crhb cadm2b tac3a th | ENSDARG00000012896 ENSDARG00000001975 ENSDARG00000042845 ENSDARG00000027419 ENSDARG00000009930 ENSDARG00000040291 ENSDARG00000027657 ENSDARG00000062633 ENSDARG00000093089 ENSDARG00000030621 | | ZFA:0000458 | ventral thalamus | 79 | 14.16 | 21 | 1.5 | 3.8e-02 | bdnf gria1a gria1b grm1a oprd1b gad1b ndel1a cadm2a nr4a2b syn2a cadm4 igsf21b nr4a3 htr1aa cadm2b syn1 pcdh17 grm8a grm8b nr4a2a th | ENSDARG00000018817 ENSDARG00000021352 ENSDARG00000032714 ENSDARG00000026796 ENSDARG00000037159 ENSDARG00000027419 ENSDARG00000010953 ENSDARG00000009930 ENSDARG00000044532 ENSDARG00000045945 ENSDARG00000040291 ENSDARG00000056084 ENSDARG00000055854 ENSDARG00000093745 ENSDARG00000062633 ENSDARG00000060368 ENSDARG00000027041 ENSDARG00000077654 ENSDARG00000076508 ENSDARG00000017007 ENSDARG00000030621 | |
