# Supplementary material for: A high-resolution mRNA expression time course of embryonic development in zebrafish
Source: eLife. 2017 Nov 16;6:e30860. doi: 10.7554/eLife.30860 (PMC5690287; doi:10.7554/eLife.30860)
Supplement: Supplementary file 6. [file elife-30860-supp6.zip › biolayout-clusters-files/Cluster002-genes.html]

Cluster002


# Cluster002: Genes

| | Ensembl ID | Gene Name | Chr | Start | End | Biotype | | --- | --- | --- | --- | --- | --- | | ENSDARG00000079994 | AKAP13 (1 of many).1 | 25 | 11013437 | 11211798 | protein\_coding | | ENSDARG00000045666 | ANKRD50 | 14 | 444078 | 447805 | protein\_coding | | ENSDARG00000090675 | APBA3 | 11 | 5618858 | 5636887 | protein\_coding | | ENSDARG00000100359 | AREL1 | 17 | 49381028 | 49427781 | protein\_coding | | ENSDARG00000101350 | ARHGAP28 | 24 | 41886221 | 41913521 | protein\_coding | | ENSDARG00000010654 | ARHGAP42 (1 of many) | 18 | 41853404 | 41893801 | protein\_coding | | ENSDARG00000052496 | ARHGAP45 (1 of many) | 2 | 56557814 | 56632908 | protein\_coding | | ENSDARG00000062530 | ASTE1 (1 of many) | 19 | 19825871 | 19834636 | protein\_coding | | ENSDARG00000086977 | ATXN1L | 7 | 69177873 | 69192104 | protein\_coding | | ENSDARG00000077531 | BAG6 (1 of many) | 19 | 30887769 | 30923600 | protein\_coding | | ENSDARG00000062181 | BMT2 | 25 | 21673414 | 21684887 | protein\_coding | | ENSDARG00000098122 | BX005085.2 | 3 | 7170932 | 7188342 | protein\_coding | | ENSDARG00000067658 | BX470189.1 | 5 | 47324247 | 47325850 | protein\_coding | | ENSDARG00000073789 | BX537350.1 | 22 | 31074039 | 31083852 | protein\_coding | | ENSDARG00000090632 | BX601644.1 | 9 | 35026883 | 35033230 | protein\_coding | | ENSDARG00000078272 | CABP7 (1 of many) | 5 | 11002277 | 11009863 | protein\_coding | | ENSDARG00000099955 | CABZ01009880.1 | 3 | 12089188 | 12107222 | protein\_coding | | ENSDARG00000088258 | CABZ01038499.1 | 3 | 58212399 | 58237954 | protein\_coding | | ENSDARG00000100053 | CABZ01044099.1 | 24 | 41654560 | 41756952 | protein\_coding | | ENSDARG00000101896 | CABZ01048399.1 | KN149945.1 | 3164 | 7066 | protein\_coding | | ENSDARG00000104152 | CABZ01054391.1 | 4 | 71030130 | 71046860 | protein\_coding | | ENSDARG00000090942 | CABZ01054394.3 | 4 | 70966537 | 70982009 | protein\_coding | | ENSDARG00000088672 | CABZ01054962.1 | 9 | 53471604 | 53522472 | protein\_coding | | ENSDARG00000105192 | CABZ01057122.1 | 20 | 28524484 | 28531121 | protein\_coding | | ENSDARG00000100162 | CABZ01059120.1 | 20 | 47976468 | 47994412 | protein\_coding | | ENSDARG00000102282 | CABZ01063297.1 | KN150317.1 | 4727 | 5821 | protein\_coding | | ENSDARG00000087185 | CABZ01069006.1 | 5 | 60113194 | 60125240 | protein\_coding | | ENSDARG00000088367 | CABZ01069016.1 | 4 | 72300353 | 72372221 | protein\_coding | | ENSDARG00000091841 | CABZ01071909.1 | 4 | 72468478 | 72477950 | protein\_coding | | ENSDARG00000104296 | CABZ01079986.1 | 15 | 44555015 | 44561425 | protein\_coding | | ENSDARG00000098503 | CABZ01080379.1 | 24 | 41485263 | 41522933 | protein\_coding | | ENSDARG00000105295 | CABZ01084612.1 | 1 | 57938121 | 57942732 | protein\_coding | | ENSDARG00000069867 | CABZ01085857.1 | 15 | 4184849 | 4195686 | protein\_coding | | ENSDARG00000098418 | CABZ01092941.1 | KN149753.1 | 977 | 11941 | protein\_coding | | ENSDARG00000079534 | CABZ01112215.1 | 11 | 18080258 | 18081968 | protein\_coding | | ENSDARG00000102797 | CABZ01114898.1 | 24 | 36158661 | 36207564 | protein\_coding | | ENSDARG00000096307 | CACNA1I (1 of many) | 6 | 451899 | 520067 | protein\_coding | | ENSDARG00000021885 | CACUL1 | 13 | 50012247 | 50032585 | protein\_coding | | ENSDARG00000060600 | CAPN15 | 24 | 39139517 | 39197188 | protein\_coding | | ENSDARG00000036281 | CC2D1A | 1 | 54536875 | 54561631 | protein\_coding | | ENSDARG00000016464 | CDC42BPA | 20 | 35182345 | 35303401 | protein\_coding | | ENSDARG00000104831 | CEP192 | 16 | 10206217 | 10370750 | protein\_coding | | ENSDARG00000040707 | CHCHD4 (1 of many) | 6 | 40062873 | 40065928 | protein\_coding | | ENSDARG00000061357 | CHST6 | 25 | 34357267 | 34365933 | protein\_coding | | ENSDARG00000076339 | CLN5 | 9 | 16846320 | 16851655 | protein\_coding | | ENSDARG00000099568 | CNNM3 | KN150361.1 | 1369 | 16229 | protein\_coding | | ENSDARG00000074367 | CR381676.1 | 7 | 51049756 | 51081785 | protein\_coding | | ENSDARG00000060962 | CT030144.1 | 7 | 15929299 | 15943187 | protein\_coding | | ENSDARG00000102766 | DENND4C | 7 | 58766434 | 58853716 | protein\_coding | | ENSDARG00000040326 | DHX29 | 10 | 8196016 | 8238489 | protein\_coding | | ENSDARG00000077839 | DHX30 | 2 | 223147 | 245345 | protein\_coding | | ENSDARG00000025766 | DTX3 | 23 | 27114570 | 27134531 | protein\_coding | | ENSDARG00000036852 | DXO | 3 | 60389081 | 60394304 | protein\_coding | | ENSDARG00000070600 | DYRK3 | 11 | 21098788 | 21106918 | protein\_coding | | ENSDARG00000005015 | ENSDARG00000005015 | 8 | 25688928 | 25696935 | protein\_coding | | ENSDARG00000017188 | ENSDARG00000017188 | 20 | 14202081 | 14218237 | protein\_coding | | ENSDARG00000019442 | ENSDARG00000019442 | 15 | 2857286 | 2883370 | protein\_coding | | ENSDARG00000024431 | ENSDARG00000024431 | 18 | 48283858 | 48304818 | protein\_coding | | ENSDARG00000026972 | ENSDARG00000026972 | 2 | 39019850 | 39026674 | protein\_coding | | ENSDARG00000036493 | ENSDARG00000036493 | 7 | 39822780 | 39829871 | protein\_coding | | ENSDARG00000038686 | ENSDARG00000038686 | 13 | 22567765 | 22584652 | protein\_coding | | ENSDARG00000039063 | ENSDARG00000039063 | 1 | 13813268 | 13820246 | protein\_coding | | ENSDARG00000040966 | ENSDARG00000040966 | 15 | 29207250 | 29219743 | protein\_coding | | ENSDARG00000043627 | ENSDARG00000043627 | 14 | 1351494 | 1393430 | protein\_coding | | ENSDARG00000043799 | ENSDARG00000043799 | 17 | 4421283 | 4695211 | protein\_coding | | ENSDARG00000052427 | ENSDARG00000052427 | 5 | 56988681 | 57001432 | protein\_coding | | ENSDARG00000056339 | ENSDARG00000056339 | 16 | 21686384 | 21723362 | protein\_coding | | ENSDARG00000058679 | ENSDARG00000058679 | 21 | 11428535 | 11436742 | protein\_coding | | ENSDARG00000059529 | ENSDARG00000059529 | 7 | 56433785 | 56445125 | protein\_coding | | ENSDARG00000061023 | ENSDARG00000061023 | 8 | 25940801 | 25952413 | protein\_coding | | ENSDARG00000061060 | ENSDARG00000061060 | 5 | 6391512 | 6442550 | protein\_coding | | ENSDARG00000069230 | ENSDARG00000069230 | 8 | 18414340 | 18425079 | protein\_coding | | ENSDARG00000069758 | ENSDARG00000069758 | 9 | 22846107 | 22861957 | protein\_coding | | ENSDARG00000071541 | ENSDARG00000071541 | 22 | 10556614 | 10562194 | protein\_coding | | ENSDARG00000073709 | ENSDARG00000073709 | 14 | 29600952 | 29634010 | protein\_coding | | ENSDARG00000075504 | ENSDARG00000075504 | 11 | 1558159 | 1580690 | protein\_coding | | ENSDARG00000077166 | ENSDARG00000077166 | 14 | 25950443 | 25968487 | protein\_coding | | ENSDARG00000079167 | ENSDARG00000079167 | 24 | 26234400 | 26284643 | protein\_coding | | ENSDARG00000079660 | ENSDARG00000079660 | 7 | 64323604 | 64350388 | protein\_coding | | ENSDARG00000087162 | ENSDARG00000087162 | 7 | 22042363 | 22053956 | protein\_coding | | ENSDARG00000087585 | ENSDARG00000087585 | 10 | 384645 | 400401 | protein\_coding | | ENSDARG00000087867 | ENSDARG00000087867 | 16 | 2995608 | 3014378 | protein\_coding | | ENSDARG00000088354 | ENSDARG00000088354 | 19 | 12049977 | 12058555 | protein\_coding | | ENSDARG00000089567 | ENSDARG00000089567 | 12 | 13053573 | 13067914 | protein\_coding | | ENSDARG00000089893 | ENSDARG00000089893 | 22 | 39020840 | 39060036 | protein\_coding | | ENSDARG00000089922 | ENSDARG00000089922 | 3 | 52699659 | 52725759 | protein\_coding | | ENSDARG00000089954 | ENSDARG00000089954 | 17 | 6446863 | 6484305 | protein\_coding | | ENSDARG00000091489 | ENSDARG00000091489 | 24 | 31522901 | 31598788 | protein\_coding | | ENSDARG00000091872 | ENSDARG00000091872 | 5 | 26186816 | 26195285 | protein\_coding | | ENSDARG00000092290 | ENSDARG00000092290 | 1 | 22798530 | 22815795 | protein\_coding | | ENSDARG00000092464 | ENSDARG00000092464 | 5 | 22953301 | 22981515 | protein\_coding | | ENSDARG00000093999 | ENSDARG00000093999 | 5 | 62681995 | 62693747 | protein\_coding | | ENSDARG00000094249 | ENSDARG00000094249 | 13 | 404162 | 415362 | protein\_coding | | ENSDARG00000095817 | ENSDARG00000095817 | 23 | 45737526 | 45752237 | protein\_coding | | ENSDARG00000096306 | ENSDARG00000096306 | 6 | 3332856 | 3346792 | protein\_coding | | ENSDARG00000098654 | ENSDARG00000098654 | 16 | 48849208 | 48972749 | protein\_coding | | ENSDARG00000099025 | ENSDARG00000099025 | 1 | 43362583 | 43363065 | protein\_coding | | ENSDARG00000100261 | ENSDARG00000100261 | 13 | 48466885 | 48473314 | protein\_coding | | ENSDARG00000100518 | ENSDARG00000100518 | 7 | 63961241 | 64173170 | protein\_coding | | ENSDARG00000100700 | ENSDARG00000100700 | 2 | 55627925 | 55656097 | protein\_coding | | ENSDARG00000100979 | ENSDARG00000100979 | KN149713.1 | 390 | 4512 | protein\_coding | | ENSDARG00000101404 | ENSDARG00000101404 | 23 | 42307082 | 42324256 | protein\_coding | | ENSDARG00000102021 | ENSDARG00000102021 | 17 | 576808 | 582608 | protein\_coding | | ENSDARG00000102118 | ENSDARG00000102118 | 5 | 49724683 | 49790004 | protein\_coding | | ENSDARG00000102375 | ENSDARG00000102375 | 5 | 43249305 | 43258995 | protein\_coding | | ENSDARG00000102381 | ENSDARG00000102381 | KN150433.1 | 133 | 7941 | protein\_coding | | ENSDARG00000102898 | ENSDARG00000102898 | 16 | 4330179 | 4335760 | protein\_coding | | ENSDARG00000103414 | ENSDARG00000103414 | KN150088.1 | 1202 | 15437 | protein\_coding | | ENSDARG00000103608 | ENSDARG00000103608 | 11 | 43373823 | 43409713 | protein\_coding | | ENSDARG00000103613 | ENSDARG00000103613 | KN149930.1 | 60651 | 69472 | protein\_coding | | ENSDARG00000103813 | ENSDARG00000103813 | KN150046.1 | 15555 | 87808 | protein\_coding | | ENSDARG00000103854 | ENSDARG00000103854 | KN150637.1 | 439 | 12733 | protein\_coding | | ENSDARG00000104644 | ENSDARG00000104644 | KN150156.1 | 9960 | 13465 | protein\_coding | | ENSDARG00000104933 | ENSDARG00000104933 | 9 | 125940 | 137993 | protein\_coding | | ENSDARG00000105258 | ENSDARG00000105258 | 16 | 52960840 | 52979004 | protein\_coding | | ENSDARG00000090408 | ERBB4 (1 of many) | 1 | 6637459 | 6765362 | protein\_coding | | ENSDARG00000052766 | EVI5L | 1 | 44516314 | 44569954 | protein\_coding | | ENSDARG00000025518 | F8A2 | 1 | 44640835 | 44650056 | protein\_coding | | ENSDARG00000067546 | FAM109B | 5 | 9172667 | 9173449 | protein\_coding | | ENSDARG00000098860 | FAM57A | 5 | 62317947 | 62371520 | protein\_coding | | ENSDARG00000040280 | FAM84B (1 of many) | 16 | 25316931 | 25318483 | protein\_coding | | ENSDARG00000100440 | FASTKD3 | 7 | 58731516 | 58744159 | protein\_coding | | ENSDARG00000101846 | FASTKD5 | 5 | 2435438 | 2438162 | protein\_coding | | ENSDARG00000075400 | FBXO33 | 17 | 12878075 | 12884883 | protein\_coding | | ENSDARG00000095750 | FDXACB1 | 5 | 57055710 | 57069917 | protein\_coding | | ENSDARG00000022260 | FEZ2 (1 of many) | 20 | 46837341 | 46855799 | protein\_coding | | ENSDARG00000051768 | FHOD1 | 7 | 34277758 | 34345666 | protein\_coding | | ENSDARG00000035873 | FJX1 | 7 | 49526399 | 49528121 | protein\_coding | | ENSDARG00000099294 | FKBP15 | 8 | 52563928 | 52607960 | protein\_coding | | ENSDARG00000087492 | FO704810.2 | 21 | 44045723 | 44086536 | protein\_coding | | ENSDARG00000102152 | FO834898.1 | 5 | 4246450 | 4309354 | protein\_coding | | ENSDARG00000079957 | FO904944.1 | 13 | 2317441 | 2338915 | protein\_coding | | ENSDARG00000070979 | FUT9 (1 of many).1 | 3 | 51824135 | 51835523 | protein\_coding | | ENSDARG00000099919 | GABRG3 | 6 | 38134975 | 38199506 | protein\_coding | | ENSDARG00000076888 | GANAB (1 of many) | 5 | 68368160 | 68408218 | protein\_coding | | ENSDARG00000097155 | GDPGP1 | 25 | 8546641 | 8548533 | protein\_coding | | ENSDARG00000098932 | GIGYF1 (1 of many) | 7 | 20603447 | 20642521 | protein\_coding | | ENSDARG00000034941 | GLB1L2 | 18 | 44753140 | 44772061 | protein\_coding | | ENSDARG00000093240 | GMEB2 | 23 | 41909482 | 41929035 | protein\_coding | | ENSDARG00000044225 | GOLPH3 (1 of many) | 10 | 8827095 | 8839261 | protein\_coding | | ENSDARG00000101260 | GPATCH2 | 20 | 46927108 | 46963214 | protein\_coding | | ENSDARG00000076238 | GRAMD1C | 24 | 20988562 | 21005681 | protein\_coding | | ENSDARG00000102651 | GTPBP2 (1 of many) | 13 | 731469 | 743813 | protein\_coding | | ENSDARG00000025174 | HABP4 | 21 | 20360222 | 20366050 | protein\_coding | | ENSDARG00000056830 | HNRNPUL2 | 14 | 22184889 | 22198375 | protein\_coding | | ENSDARG00000037068 | IKZF4 | 23 | 27756473 | 27768632 | protein\_coding | | ENSDARG00000098655 | KIF1C | 5 | 67527171 | 67580786 | protein\_coding | | ENSDARG00000094780 | L3MBTL4 | 24 | 41939476 | 41982190 | protein\_coding | | ENSDARG00000067815 | LINGO3 (1 of many) | 2 | 57475137 | 57490614 | protein\_coding | | ENSDARG00000015563 | LPIN3 | 6 | 1615501 | 1638559 | protein\_coding | | ENSDARG00000079463 | MAN2B2 | 7 | 19257945 | 19274771 | protein\_coding | | ENSDARG00000091521 | MAP3K11 | 7 | 495901 | 598306 | protein\_coding | | ENSDARG00000104435 | MAP7 | 23 | 39961685 | 40049209 | protein\_coding | | ENSDARG00000061725 | MAST3 (1 of many) | 2 | 24982548 | 25027714 | protein\_coding | | ENSDARG00000077316 | MB21D2 | 15 | 45510394 | 45522281 | protein\_coding | | ENSDARG00000098918 | MCOLN2 | 22 | 24596312 | 24612404 | protein\_coding | | ENSDARG00000035771 | MEPCE (1 of many) | 23 | 44470357 | 44536909 | protein\_coding | | ENSDARG00000089236 | METTL15 | 7 | 31749787 | 31871336 | protein\_coding | | ENSDARG00000093253 | MFSD3 | 5 | 24936463 | 24976155 | protein\_coding | | ENSDARG00000079571 | MFSD4B | 20 | 3321747 | 3328025 | protein\_coding | | ENSDARG00000101246 | MTMR4 | 5 | 2033102 | 2134583 | protein\_coding | | ENSDARG00000074622 | MYO5A (1 of many) | 25 | 7438093 | 7475262 | protein\_coding | | ENSDARG00000068590 | NAT6 | 8 | 26352976 | 26357150 | protein\_coding | | ENSDARG00000103606 | NDST1 (1 of many) | 21 | 33216311 | 33409537 | protein\_coding | | ENSDARG00000078525 | NOCT (1 of many) | 1 | 13244183 | 13265830 | protein\_coding | | ENSDARG00000060071 | PAPD5 | 7 | 37589994 | 37623894 | protein\_coding | | ENSDARG00000061114 | PARG | 13 | 29161629 | 29217467 | protein\_coding | | ENSDARG00000104883 | PCMTD2 (1 of many) | 23 | 7809310 | 7821261 | protein\_coding | | ENSDARG00000090398 | PDE1C | 2 | 50680165 | 50852131 | protein\_coding | | ENSDARG00000075857 | PGS1 | 12 | 34097067 | 34157032 | protein\_coding | | ENSDARG00000101293 | PKN2 (1 of many) | 6 | 25324223 | 25394021 | protein\_coding | | ENSDARG00000104419 | PODXL2 | 22 | 4157534 | 4183638 | protein\_coding | | ENSDARG00000074274 | POLR2E (1 of many) | 2 | 56534383 | 56547576 | protein\_coding | | ENSDARG00000078172 | PTGFRN (1 of many).1 | 9 | 492592 | 528230 | protein\_coding | | ENSDARG00000102610 | R3HDM2 | 6 | 59647554 | 59696457 | protein\_coding | | ENSDARG00000102078 | RAB35 (1 of many) | 5 | 2486036 | 2510853 | protein\_coding | | ENSDARG00000077553 | RAP2A (1 of many) | 9 | 1163867 | 1170912 | protein\_coding | | ENSDARG00000061459 | RAPGEF2 (1 of many) | 1 | 20445467 | 20511765 | protein\_coding | | ENSDARG00000068562 | RBM33 | 7 | 40626893 | 40679726 | protein\_coding | | ENSDARG00000090904 | RNF219 | 6 | 4368873 | 4377259 | protein\_coding | | ENSDARG00000090770 | RNF31 | 2 | 28156724 | 28199141 | protein\_coding | | ENSDARG00000007813 | RNPEPL1 | 18 | 45866860 | 45888825 | protein\_coding | | ENSDARG00000091962 | RUNDC1 | 3 | 29771379 | 29779705 | protein\_coding | | ENSDARG00000071271 | SAC3D1 | 23 | 12610207 | 12677819 | protein\_coding | | ENSDARG00000032627 | SCYL2 | 4 | 26064229 | 26092014 | protein\_coding | | ENSDARG00000078574 | SENP1 | 23 | 25843860 | 25853454 | protein\_coding | | ENSDARG00000079608 | SGPP2 | 15 | 42279355 | 42299710 | protein\_coding | | ENSDARG00000028857 | SGSM1 (1 of many) | 5 | 19506558 | 19589593 | protein\_coding | | ENSDARG00000102334 | SH3RF3 | 9 | 56542461 | 56596681 | protein\_coding | | ENSDARG00000099322 | SLC37A3 | KN149790.1 | 24599 | 67602 | protein\_coding | | ENSDARG00000102387 | SLC39A14 | 5 | 15826766 | 15872411 | protein\_coding | | ENSDARG00000104981 | SLC45A4 | 19 | 947170 | 1000154 | protein\_coding | | ENSDARG00000067784 | SLC9A1 | 16 | 51598478 | 51667387 | protein\_coding | | ENSDARG00000022007 | SPEF1 | 13 | 24549224 | 24557672 | protein\_coding | | ENSDARG00000074332 | SPEN (1 of many) | 22 | 157665 | 182688 | protein\_coding | | ENSDARG00000001817 | SPIN4 (1 of many) | 2 | 55460660 | 55478306 | protein\_coding | | ENSDARG00000092855 | SRBD1 | 13 | 9564345 | 9671715 | protein\_coding | | ENSDARG00000079591 | SSH3 | 14 | 35124680 | 35156224 | protein\_coding | | ENSDARG00000087203 | STK39 | 9 | 49288784 | 49339033 | protein\_coding | | ENSDARG00000102902 | STRN4 | 15 | 11809350 | 11873756 | protein\_coding | | ENSDARG00000077440 | SYDE1 | 12 | 48615757 | 48632108 | protein\_coding | | ENSDARG00000079055 | TARBP1 | 13 | 39151181 | 39197167 | protein\_coding | | ENSDARG00000079281 | TESK1 (1 of many).1 | 7 | 24257257 | 24300299 | protein\_coding | | ENSDARG00000090696 | TMC3 | 7 | 11369514 | 11423423 | protein\_coding | | ENSDARG00000102331 | TMEM164 | KN150208.1 | 11285 | 28436 | protein\_coding | | ENSDARG00000054122 | TMEM30B | 17 | 28085524 | 28093218 | protein\_coding | | ENSDARG00000093886 | TMSB15A | 1 | 18117954 | 18118580 | protein\_coding | | ENSDARG00000062897 | TNKS (1 of many) | 5 | 8460288 | 8523305 | protein\_coding | | ENSDARG00000078751 | TPP2 | 1 | 423658 | 448580 | protein\_coding | | ENSDARG00000069331 | TRIP6 | 7 | 19727809 | 19747186 | protein\_coding | | ENSDARG00000079469 | TRPC7 (1 of many) | 14 | 1460434 | 1682860 | protein\_coding | | ENSDARG00000011897 | TSG101 (1 of many) | 7 | 49348408 | 49364539 | protein\_coding | | ENSDARG00000044812 | TTC17 | 7 | 50022709 | 50076572 | protein\_coding | | ENSDARG00000099113 | UNC13B | 10 | 16914013 | 17064759 | protein\_coding | | ENSDARG00000099133 | UNC5C | 5 | 6902191 | 6978829 | protein\_coding | | ENSDARG00000074017 | USP31 | 3 | 47242687 | 47298588 | protein\_coding | | ENSDARG00000102302 | WDR48 (1 of many) | KN150128.1 | 534 | 10800 | protein\_coding | | ENSDARG00000088799 | XXYLT1 | 11 | 34139361 | 34218723 | protein\_coding | | ENSDARG00000103525 | YTHDC2 | 5 | 929679 | 978411 | protein\_coding | | ENSDARG00000101203 | ZNF513 (1 of many) | 17 | 5776091 | 5796301 | protein\_coding | | ENSDARG00000071469 | ZNF618 | 5 | 69499365 | 69546188 | protein\_coding | | ENSDARG00000098792 | ZNRF2 (1 of many) | 16 | 53931807 | 53978315 | protein\_coding | | ENSDARG00000012468 | aacs | 5 | 18323522 | 18393690 | protein\_coding | | ENSDARG00000011855 | aak1a | 8 | 48976998 | 49055643 | protein\_coding | | ENSDARG00000077686 | aak1b | 10 | 41140467 | 41235207 | protein\_coding | | ENSDARG00000071240 | aars2 | 22 | 25534335 | 25567292 | protein\_coding | | ENSDARG00000074254 | abcb6b | 9 | 14231483 | 14302449 | protein\_coding | | ENSDARG00000077988 | abcc10 | 11 | 11927778 | 11978085 | protein\_coding | | ENSDARG00000059209 | abhd13 | 9 | 5496810 | 5502591 | protein\_coding | | ENSDARG00000058367 | abhd14a | 22 | 10668867 | 10677191 | protein\_coding | | ENSDARG00000078768 | abhd15a | 15 | 25011056 | 25025465 | protein\_coding | | ENSDARG00000043084 | abhd17ab | 8 | 19201821 | 19215925 | protein\_coding | | ENSDARG00000101961 | abhd18 | 14 | 44848397 | 44880206 | protein\_coding | | ENSDARG00000062991 | abi1b | 2 | 10070781 | 10117994 | protein\_coding | | ENSDARG00000014875 | abi2b | 6 | 9735162 | 9775735 | protein\_coding | | ENSDARG00000013841 | abl2 | 8 | 14877430 | 14946667 | protein\_coding | | ENSDARG00000060783 | acap2 | 22 | 37798012 | 37862052 | protein\_coding | | ENSDARG00000019763 | acp5a | 6 | 8119583 | 8126977 | protein\_coding | | ENSDARG00000010752 | acsl4b | 5 | 23014599 | 23070434 | protein\_coding | | ENSDARG00000037781 | acss2 | 23 | 9295576 | 9353475 | protein\_coding | | ENSDARG00000070076 | actr2b | 13 | 5818130 | 5849985 | protein\_coding | | ENSDARG00000104700 | acvr2ab | 6 | 870359 | 886002 | protein\_coding | | ENSDARG00000044422 | acvr2b | 24 | 41303914 | 41357193 | protein\_coding | | ENSDARG00000089181 | adad1 | 14 | 1226796 | 1251685 | protein\_coding | | ENSDARG00000079068 | adam12 | 17 | 32206479 | 32381596 | protein\_coding | | ENSDARG00000062561 | adck1 | 17 | 17472359 | 17739384 | protein\_coding | | ENSDARG00000073923 | adck2 | 25 | 2492098 | 2510432 | protein\_coding | | ENSDARG00000091342 | adcy5 | 9 | 38062782 | 38212202 | protein\_coding | | ENSDARG00000100869 | adcy9 | 22 | 26738364 | 26835076 | protein\_coding | | ENSDARG00000053518 | adhfe1 | 2 | 42246069 | 42260157 | protein\_coding | | ENSDARG00000002912 | adipor1a | 11 | 24562540 | 24576921 | protein\_coding | | ENSDARG00000042717 | adipor1b | 8 | 28362325 | 28374292 | protein\_coding | | ENSDARG00000060937 | adnp2a | 16 | 25458682 | 25465871 | protein\_coding | | ENSDARG00000074293 | adnpb | 23 | 20592161 | 20601373 | protein\_coding | | ENSDARG00000001857 | aff4 | 21 | 43183641 | 43251697 | protein\_coding | | ENSDARG00000071347 | aftphb | 17 | 24551587 | 24569309 | protein\_coding | | ENSDARG00000045900 | agbl5 | 4 | 724091 | 747112 | protein\_coding | | ENSDARG00000061268 | ago2 | 19 | 41894935 | 41914590 | protein\_coding | | ENSDARG00000063079 | ago3b | 19 | 10742595 | 10767788 | protein\_coding | | ENSDARG00000042821 | agps | 9 | 1587384 | 1637817 | protein\_coding | | ENSDARG00000104768 | akap17a | 9 | 56398882 | 56423275 | protein\_coding | | ENSDARG00000089802 | akap1a | 10 | 37457080 | 37471465 | protein\_coding | | ENSDARG00000074791 | aldh3a1 | 15 | 20864211 | 20870463 | protein\_coding | | ENSDARG00000054963 | alg1 | 3 | 28729441 | 28738798 | protein\_coding | | ENSDARG00000031202 | alg11 | 1 | 28947006 | 28957155 | protein\_coding | | ENSDARG00000053155 | alg3 | 2 | 45119722 | 45128346 | protein\_coding | | ENSDARG00000061235 | alg5 | 10 | 35040751 | 35058871 | protein\_coding | | ENSDARG00000076924 | als2b | 6 | 9558235 | 9625700 | protein\_coding | | ENSDARG00000008322 | ambra1a | 7 | 38984523 | 39089308 | protein\_coding | | ENSDARG00000039878 | ambra1b | 25 | 7764651 | 7794434 | protein\_coding | | ENSDARG00000079624 | amer1 | 5 | 21510627 | 21515261 | protein\_coding | | ENSDARG00000020218 | amfr | 25 | 35648014 | 35667844 | protein\_coding | | ENSDARG00000016743 | angel2 | 20 | 38017217 | 38031457 | protein\_coding | | ENSDARG00000098647 | ankdd1a | 7 | 13329536 | 13452776 | protein\_coding | | ENSDARG00000071724 | ankha | 24 | 10208761 | 10253891 | protein\_coding | | ENSDARG00000060768 | ankib1a | 19 | 44310098 | 44365180 | protein\_coding | | ENSDARG00000076829 | ankib1b | 16 | 16133666 | 16212964 | protein\_coding | | ENSDARG00000035607 | ankle2 | 5 | 18439251 | 18459002 | protein\_coding | | ENSDARG00000005948 | ankmy2a | 15 | 34715342 | 34732851 | protein\_coding | | ENSDARG00000061901 | ankrd13a | 5 | 19429524 | 19452426 | protein\_coding | | ENSDARG00000002298 | ankrd22 | 12 | 17032781 | 17036761 | protein\_coding | | ENSDARG00000036826 | ankrd52a | 23 | 32435554 | 32464211 | protein\_coding | | ENSDARG00000060917 | anln | 19 | 35846035 | 35863542 | protein\_coding | | ENSDARG00000058015 | ano3 | 25 | 34840417 | 34963616 | protein\_coding | | ENSDARG00000036147 | ano5b | 7 | 32562991 | 32624518 | protein\_coding | | ENSDARG00000102394 | ap1g1 | 7 | 68995766 | 69115102 | protein\_coding | | ENSDARG00000054337 | ap1g2 | 2 | 38306420 | 38330174 | protein\_coding | | ENSDARG00000027966 | ap1s3b | 2 | 47733074 | 47766642 | protein\_coding | | ENSDARG00000103684 | ap4e1 | 25 | 4729121 | 4759581 | protein\_coding | | ENSDARG00000031533 | ap5m1 | 17 | 44327273 | 44340257 | protein\_coding | | ENSDARG00000078111 | ap5z1 | 3 | 40734695 | 40755069 | protein\_coding | | ENSDARG00000039374 | apool | 5 | 22348057 | 22364850 | protein\_coding | | ENSDARG00000092473 | apopt1 | 13 | 15669750 | 15683784 | protein\_coding | | ENSDARG00000007566 | appbp2 | 15 | 23986999 | 24007596 | protein\_coding | | ENSDARG00000002792 | arcn1a | 5 | 29902096 | 29916493 | protein\_coding | | ENSDARG00000031214 | arcn1b | 15 | 18166258 | 18179602 | protein\_coding | | ENSDARG00000097583 | arfgap3 | 4 | 76572909 | 76580808 | protein\_coding | | ENSDARG00000074702 | arfgef2 | 23 | 19059672 | 19125589 | protein\_coding | | ENSDARG00000070055 | arfip1 | 1 | 24751870 | 24795742 | protein\_coding | | ENSDARG00000056664 | arfip2b | 10 | 26244066 | 26263965 | protein\_coding | | ENSDARG00000020488 | arhgap17a | 12 | 20290585 | 20363558 | protein\_coding | | ENSDARG00000100973 | arhgap24 | 21 | 9639181 | 9863301 | protein\_coding | | ENSDARG00000011333 | arhgap33 | 16 | 44816946 | 44911539 | protein\_coding | | ENSDARG00000060297 | arhgap35 | 5 | 37264552 | 37281192 | protein\_coding | | ENSDARG00000039265 | arhgap4a | 23 | 25062846 | 25088852 | protein\_coding | | ENSDARG00000077114 | arhgef16 | 8 | 47108567 | 47153470 | protein\_coding | | ENSDARG00000042308 | arhgef18b | 22 | 10780975 | 10861531 | protein\_coding | | ENSDARG00000055837 | arhgef1b | 16 | 26267453 | 26358969 | protein\_coding | | ENSDARG00000060415 | arhgef28 | 5 | 34228553 | 34364677 | protein\_coding | | ENSDARG00000002463 | arhgef37 | 14 | 24543424 | 24576409 | protein\_coding | | ENSDARG00000006299 | arhgef7a | 9 | 21725016 | 21756174 | protein\_coding | | ENSDARG00000101891 | arid1ab | 19 | 14490137 | 14573059 | protein\_coding | | ENSDARG00000077120 | arid5a | 8 | 52391031 | 52418117 | protein\_coding | | ENSDARG00000003616 | arih1 | 18 | 18890667 | 18911951 | protein\_coding | | ENSDARG00000036870 | arih1l | 7 | 13238732 | 13262017 | protein\_coding | | ENSDARG00000071013 | arl6ip6 | 6 | 13114313 | 13142615 | protein\_coding | | ENSDARG00000094709 | arl8 | 7 | 41435821 | 41446584 | protein\_coding | | ENSDARG00000070318 | arl8bb | 11 | 35482935 | 35494485 | protein\_coding | | ENSDARG00000013861 | armc1 | 24 | 24556606 | 24579110 | protein\_coding | | ENSDARG00000078083 | armc5 | 3 | 30910998 | 30927329 | protein\_coding | | ENSDARG00000062258 | armc8 | 9 | 28065400 | 28090860 | protein\_coding | | ENSDARG00000055676 | armt1 | 20 | 26488089 | 26492868 | protein\_coding | | ENSDARG00000074757 | arv1 | 20 | 4020975 | 4033951 | protein\_coding | | ENSDARG00000010181 | asap2a | 17 | 35149073 | 35275536 | protein\_coding | | ENSDARG00000021896 | asap3 | 17 | 27172930 | 27217406 | protein\_coding | | ENSDARG00000005246 | asb3 | 12 | 23939675 | 23955922 | protein\_coding | | ENSDARG00000006494 | asb7 | 7 | 9595830 | 9628488 | protein\_coding | | ENSDARG00000021433 | asmtl | 9 | 35063548 | 35073331 | protein\_coding | | ENSDARG00000011764 | asun | 4 | 20765066 | 20778693 | protein\_coding | | ENSDARG00000004823 | asz1 | 18 | 20653312 | 20685055 | protein\_coding | | ENSDARG00000023267 | atad1a | 5 | 16214923 | 16230064 | protein\_coding | | ENSDARG00000056609 | atad1b | 12 | 17315030 | 17334209 | protein\_coding | | ENSDARG00000101126 | atad5b | 6 | 22073459 | 22095006 | protein\_coding | | ENSDARG00000055481 | atf7b | 6 | 39615774 | 39634714 | protein\_coding | | ENSDARG00000088145 | atg4db | 3 | 6806548 | 6833464 | protein\_coding | | ENSDARG00000061039 | atp10a | 6 | 38629148 | 38728866 | protein\_coding | | ENSDARG00000003699 | atp7a | 14 | 22100128 | 22144814 | protein\_coding | | ENSDARG00000063076 | atrip | 22 | 10513611 | 10530078 | protein\_coding | | ENSDARG00000062164 | atrn | 13 | 13875126 | 14051219 | protein\_coding | | ENSDARG00000061687 | atxn1a | 19 | 32734685 | 32874674 | protein\_coding | | ENSDARG00000052897 | atxn2 | 5 | 41045326 | 41109081 | protein\_coding | | ENSDARG00000089860 | atxn7l1 | 4 | 3419757 | 3433431 | protein\_coding | | ENSDARG00000056268 | atxn7l2b | 8 | 25072268 | 25087543 | protein\_coding | | ENSDARG00000029331 | atxn7l3 | 3 | 19875969 | 19890896 | protein\_coding | | ENSDARG00000102964 | axin1 | 3 | 42878567 | 42949272 | protein\_coding | | ENSDARG00000038414 | b3galt6 | 11 | 2615758 | 2621579 | protein\_coding | | ENSDARG00000004396 | b3gnt5b | 2 | 7951389 | 7959268 | protein\_coding | | ENSDARG00000058939 | b4galnt3b | 18 | 6417973 | 6477255 | protein\_coding | | ENSDARG00000062329 | b4galt3 | 23 | 20052160 | 20084288 | protein\_coding | | ENSDARG00000021899 | b4galt7 | 14 | 26082917 | 26094041 | protein\_coding | | ENSDARG00000062553 | bach1a | 15 | 8194813 | 8215921 | protein\_coding | | ENSDARG00000036569 | bach2a | 17 | 15666119 | 15734205 | protein\_coding | | ENSDARG00000043179 | bag4 | 8 | 44605499 | 44617451 | protein\_coding | | ENSDARG00000017316 | bag5 | 13 | 15631286 | 15668299 | protein\_coding | | ENSDARG00000062799 | baiap2a | 3 | 51430277 | 51638074 | protein\_coding | | ENSDARG00000031119 | baiap2l1b | 3 | 61168723 | 61258261 | protein\_coding | | ENSDARG00000076733 | bard1 | 9 | 40832734 | 40881935 | protein\_coding | | ENSDARG00000042577 | batf3 | 20 | 37909820 | 37910984 | protein\_coding | | ENSDARG00000100611 | bcl7ba | 10 | 33300317 | 33307934 | protein\_coding | | ENSDARG00000075624 | bend5 | 8 | 15465080 | 15483202 | protein\_coding | | ENSDARG00000032037 | bet1l | 25 | 7487182 | 7546563 | protein\_coding | | ENSDARG00000061682 | bicc1a | 17 | 20963962 | 21029424 | protein\_coding | | ENSDARG00000075571 | bicd2 | 11 | 36781804 | 36821391 | protein\_coding | | ENSDARG00000010255 | bin2b | 6 | 38882630 | 38895963 | protein\_coding | | ENSDARG00000007597 | bloc1s4 | 19 | 26095315 | 26109505 | protein\_coding | | ENSDARG00000006010 | bmi1a | 24 | 17115582 | 17122816 | protein\_coding | | ENSDARG00000013076 | bmi1b | 2 | 21506868 | 21512465 | protein\_coding | | ENSDARG00000028053 | bmp1b | 10 | 40938078 | 41018117 | protein\_coding | | ENSDARG00000042688 | bora | 1 | 29919449 | 29930865 | protein\_coding | | ENSDARG00000017661 | braf | 4 | 12075927 | 12103187 | protein\_coding | | ENSDARG00000062585 | brat1 | 1 | 10984079 | 11002725 | protein\_coding | | ENSDARG00000046087 | brd2b | 16 | 48771329 | 48805770 | protein\_coding | | ENSDARG00000078904 | brd4 | 3 | 52861509 | 52969617 | protein\_coding | | ENSDARG00000074410 | brip1 | 15 | 27519442 | 27589930 | protein\_coding | | ENSDARG00000074238 | brpf3b | 8 | 10824648 | 10857568 | protein\_coding | | ENSDARG00000060089 | btaf1 | 13 | 42354792 | 42410934 | protein\_coding | | ENSDARG00000005544 | btbd3a | 1 | 50547250 | 50553802 | protein\_coding | | ENSDARG00000003069 | btbd7 | 20 | 27215777 | 27291303 | protein\_coding | | ENSDARG00000068983 | btbd9 | 13 | 44626539 | 44645563 | protein\_coding | | ENSDARG00000020298 | btg2 | 22 | 757582 | 760155 | protein\_coding | | ENSDARG00000054805 | btr09 | 7 | 24251860 | 24255873 | protein\_coding | | ENSDARG00000028850 | btr16 | 15 | 18262856 | 18273701 | protein\_coding | | ENSDARG00000103618 | btr18 | 19 | 3788106 | 3849697 | protein\_coding | | ENSDARG00000102992 | btr20 | 19 | 3821987 | 3833007 | protein\_coding | | ENSDARG00000096095 | buc | 2 | 28199466 | 28203585 | protein\_coding | | ENSDARG00000035332 | c10h21orf59 | 10 | 33629978 | 33635302 | protein\_coding | | ENSDARG00000057166 | c13h10orf11 | 13 | 16791713 | 17333662 | protein\_coding | | ENSDARG00000074137 | c2cd3 | 15 | 15322736 | 15367380 | protein\_coding | | ENSDARG00000040510 | ca15b | 12 | 4258265 | 4264718 | protein\_coding | | ENSDARG00000021374 | cab39l1 | 14 | 31136659 | 31149096 | protein\_coding | | ENSDARG00000099395 | cables1 | 2 | 4461665 | 4513167 | protein\_coding | | ENSDARG00000061590 | cachd1 | 6 | 31603837 | 31753025 | protein\_coding | | ENSDARG00000070522 | cacna1i | 3 | 28951425 | 29247588 | protein\_coding | | ENSDARG00000059347 | calml4b | 25 | 1195633 | 1243145 | protein\_coding | | ENSDARG00000035452 | camkk1a | 5 | 30683423 | 30832574 | protein\_coding | | ENSDARG00000102987 | capn7 | 16 | 36159258 | 36188051 | protein\_coding | | ENSDARG00000020749 | caprin2 | 4 | 16626050 | 16639826 | protein\_coding | | ENSDARG00000077119 | carkd | 9 | 8916458 | 8945285 | protein\_coding | | ENSDARG00000023900 | casd1 | 19 | 41419855 | 41447104 | protein\_coding | | ENSDARG00000062052 | casp2 | 16 | 17641990 | 17670462 | protein\_coding | | ENSDARG00000022718 | casp8ap2 | 20 | 24047904 | 24056914 | protein\_coding | | ENSDARG00000070272 | casp8l2 | 9 | 1336895 | 1356460 | protein\_coding | | ENSDARG00000004325 | casp9 | 23 | 25047232 | 25058731 | protein\_coding | | ENSDARG00000045601 | cax1 | 4 | 14983099 | 14992171 | protein\_coding | | ENSDARG00000009958 | cbl | 15 | 23272777 | 23330140 | protein\_coding | | ENSDARG00000036587 | cbr1 | 1 | 46474610 | 46482000 | protein\_coding | | ENSDARG00000087181 | cbx7b | 12 | 18794411 | 18801102 | protein\_coding | | ENSDARG00000098771 | cbx8b | 6 | 21887985 | 21891310 | protein\_coding | | ENSDARG00000058578 | ccdc106b | 19 | 10406435 | 10411901 | protein\_coding | | ENSDARG00000062695 | ccdc126 | 19 | 20579503 | 20592679 | protein\_coding | | ENSDARG00000098734 | ccdc14 | 6 | 9033318 | 9046541 | protein\_coding | | ENSDARG00000039937 | ccdc172 | 13 | 20393939 | 20403966 | protein\_coding | | ENSDARG00000027851 | ccdc191 | 24 | 20947737 | 21135124 | protein\_coding | | ENSDARG00000052379 | ccdc22 | 8 | 23593893 | 23616792 | protein\_coding | | ENSDARG00000043334 | ccdc6a | 17 | 20654127 | 20676162 | protein\_coding | | ENSDARG00000017595 | ccdc6b | 12 | 7575660 | 7605029 | protein\_coding | | ENSDARG00000014966 | ccdc82 | 21 | 40662848 | 40686192 | protein\_coding | | ENSDARG00000045683 | ccdc87 | 4 | 13616882 | 13625445 | protein\_coding | | ENSDARG00000040678 | ccdc93 | 9 | 38543657 | 38558343 | protein\_coding | | ENSDARG00000035963 | ccdc96 | 7 | 39460311 | 39467443 | protein\_coding | | ENSDARG00000013705 | ccm2 | 20 | 18874546 | 18895206 | protein\_coding | | ENSDARG00000007657 | ccnh | 5 | 47312429 | 47323929 | protein\_coding | | ENSDARG00000059917 | ccnj | 13 | 50135391 | 50150769 | protein\_coding | | ENSDARG00000075919 | ccser1 | 8 | 28715314 | 28863567 | protein\_coding | | ENSDARG00000087749 | ccser2a | 17 | 26459274 | 26519537 | protein\_coding | | ENSDARG00000091535 | ccser2b | 12 | 12916501 | 13042926 | protein\_coding | | ENSDARG00000021483 | cdc14b | 8 | 1156389 | 1201504 | protein\_coding | | ENSDARG00000055470 | cdc16 | 9 | 35110816 | 35128071 | protein\_coding | | ENSDARG00000010792 | cdc25b | 13 | 14784342 | 14798244 | protein\_coding | | ENSDARG00000056258 | cdc27 | 3 | 21905565 | 21924945 | protein\_coding | | ENSDARG00000044265 | cdc37l1 | 10 | 11014557 | 11040261 | protein\_coding | | ENSDARG00000019383 | cdc42bpb | 20 | 18492277 | 18635919 | protein\_coding | | ENSDARG00000053959 | cdc42ep2 | 5 | 37120624 | 37130036 | protein\_coding | | ENSDARG00000023724 | cdc42se1 | 16 | 38361855 | 38383944 | protein\_coding | | ENSDARG00000102750 | cdh1 | 7 | 52788342 | 52847585 | protein\_coding | | ENSDARG00000056683 | cdk5 | 24 | 33686326 | 33894218 | protein\_coding | | ENSDARG00000035853 | cdkn2aip | 7 | 52678789 | 52689009 | protein\_coding | | ENSDARG00000035952 | cdr2a | 3 | 58375897 | 58400508 | protein\_coding | | ENSDARG00000038985 | cdyl | 2 | 23967137 | 24021771 | protein\_coding | | ENSDARG00000036073 | cebpg | 7 | 37818413 | 37823927 | protein\_coding | | ENSDARG00000060361 | cep104 | 8 | 22276376 | 22305724 | protein\_coding | | ENSDARG00000058000 | cep350 | 8 | 14521673 | 14571579 | protein\_coding | | ENSDARG00000102349 | cep55l | 12 | 5067713 | 5354827 | protein\_coding | | ENSDARG00000039229 | cep78 | 8 | 51948490 | 51968033 | protein\_coding | | ENSDARG00000101236 | cep83 | 4 | 20404271 | 20414431 | protein\_coding | | ENSDARG00000058716 | cept1a | 8 | 12913454 | 12932116 | protein\_coding | | ENSDARG00000013704 | cers2a | 19 | 8856132 | 8894031 | protein\_coding | | ENSDARG00000018281 | cfap161 | 18 | 7131958 | 7138441 | protein\_coding | | ENSDARG00000056381 | cfap97 | 1 | 16988107 | 17000336 | protein\_coding | | ENSDARG00000055607 | cgna | 16 | 22609741 | 22669813 | protein\_coding | | ENSDARG00000015471 | chd1l | 6 | 36866233 | 36899693 | protein\_coding | | ENSDARG00000075543 | chd8 | 2 | 38105186 | 38133405 | protein\_coding | | ENSDARG00000025820 | chek2 | 5 | 9551342 | 9577606 | protein\_coding | | ENSDARG00000043553 | ches1 | 20 | 16577987 | 16649329 | protein\_coding | | ENSDARG00000075347 | chfr | 5 | 17276479 | 17309303 | protein\_coding | | ENSDARG00000008541 | chia.4 | 23 | 17999936 | 18006800 | protein\_coding | | ENSDARG00000053166 | chic1 | 14 | 32611528 | 32619537 | protein\_coding | | ENSDARG00000019588 | chico | 2 | 52812843 | 52861811 | protein\_coding | | ENSDARG00000003845 | chm | 21 | 34652274 | 34759419 | protein\_coding | | ENSDARG00000038064 | chmp3 | 17 | 43478194 | 43485742 | protein\_coding | | ENSDARG00000007418 | chmp4c | 24 | 10929743 | 10936033 | protein\_coding | | ENSDARG00000025788 | chp2 | 15 | 20470411 | 20476554 | protein\_coding | | ENSDARG00000078392 | chpfa | 9 | 11274759 | 11292025 | protein\_coding | | ENSDARG00000028786 | chst12a | 3 | 41584502 | 41591248 | protein\_coding | | ENSDARG00000079027 | chsy1 | 7 | 9080747 | 9265811 | protein\_coding | | ENSDARG00000070430 | chtopb | 16 | 28946612 | 28952539 | protein\_coding | | ENSDARG00000011662 | chuk | 13 | 25234303 | 25249190 | protein\_coding | | ENSDARG00000089781 | ciz1a | 5 | 1184374 | 1220371 | protein\_coding | | ENSDARG00000089461 | ciz1b | 5 | 62966965 | 62982612 | protein\_coding | | ENSDARG00000022466 | clcn5b | 7 | 22582464 | 22626285 | protein\_coding | | ENSDARG00000078722 | clip1a | 5 | 66509734 | 66571931 | protein\_coding | | ENSDARG00000030830 | cmtr1 | 23 | 35263861 | 35297957 | protein\_coding | | ENSDARG00000032932 | cnksr1 | 17 | 26972140 | 27030146 | protein\_coding | | ENSDARG00000061195 | cnnm2a | 13 | 28487636 | 28512810 | protein\_coding | | ENSDARG00000074309 | cnnm4b | 5 | 12971842 | 13040711 | protein\_coding | | ENSDARG00000058528 | cnot10 | 16 | 7786188 | 7813490 | protein\_coding | | ENSDARG00000019842 | cnot3b | 19 | 10336298 | 10376248 | protein\_coding | | ENSDARG00000007639 | cnot4b | 4 | 12244849 | 12287218 | protein\_coding | | ENSDARG00000070822 | cnp | 3 | 17783392 | 17794555 | protein\_coding | | ENSDARG00000027078 | cnppd1 | 1 | 5464152 | 5472039 | protein\_coding | | ENSDARG00000086283 | cnsta | 17 | 11921739 | 11981404 | protein\_coding | | ENSDARG00000105131 | cog1 | 3 | 36260520 | 36277849 | protein\_coding | | ENSDARG00000004037 | cog2 | 13 | 23865620 | 23888466 | protein\_coding | | ENSDARG00000101999 | cog3 | 9 | 54303110 | 54359511 | protein\_coding | | ENSDARG00000103149 | cog6 | 15 | 3468297 | 3547119 | protein\_coding | | ENSDARG00000002798 | cog8 | 18 | 5236073 | 5244747 | protein\_coding | | ENSDARG00000069857 | coq3 | 16 | 32698320 | 32708917 | protein\_coding | | ENSDARG00000102463 | cox18 | 14 | 51641203 | 51651268 | protein\_coding | | ENSDARG00000053877 | cpdb | 21 | 39152340 | 39181275 | protein\_coding | | ENSDARG00000056691 | cpeb4 | 14 | 23799328 | 23813594 | protein\_coding | | ENSDARG00000105114 | crebrf | 21 | 41160802 | 41191154 | protein\_coding | | ENSDARG00000056462 | crp2 | 24 | 37962545 | 38209408 | protein\_coding | | ENSDARG00000105102 | crsp7 | 2 | 24902476 | 24909586 | processed\_transcript | | ENSDARG00000002396 | cry-dash | 24 | 20049603 | 20064055 | protein\_coding | | ENSDARG00000091131 | cry1bb | 22 | 748818 | 792003 | protein\_coding | | ENSDARG00000102403 | cry2 | 25 | 13750483 | 13774721 | protein\_coding | | ENSDARG00000067996 | csk | 25 | 20503999 | 20569100 | protein\_coding | | ENSDARG00000100236 | cspp1a | 24 | 18545431 | 18587976 | protein\_coding | | ENSDARG00000038429 | csrnp1b | 24 | 19563190 | 19574821 | protein\_coding | | ENSDARG00000016519 | ctdnep1a | 5 | 23543492 | 23552996 | protein\_coding | | ENSDARG00000035781 | ctdnep1b | 7 | 69211764 | 69229740 | protein\_coding | | ENSDARG00000061587 | ctdspl2a | 25 | 32085766 | 32108458 | protein\_coding | | ENSDARG00000060586 | ctdspl2b | 7 | 31103233 | 31122429 | protein\_coding | | ENSDARG00000089646 | ctdspl3 | 11 | 5819947 | 5839031 | protein\_coding | | ENSDARG00000018162 | ctnnal1 | 19 | 35164567 | 35286446 | protein\_coding | | ENSDARG00000051851 | ctu2 | 7 | 54991548 | 55024301 | protein\_coding | | ENSDARG00000098284 | ctxn1 | 22 | 4035606 | 4061809 | protein\_coding | | ENSDARG00000054748 | cuedc1b | 15 | 31307275 | 31356654 | protein\_coding | | ENSDARG00000039365 | cuedc2 | 13 | 29794947 | 29805809 | protein\_coding | | ENSDARG00000013965 | cul2 | 24 | 2287915 | 2316842 | protein\_coding | | ENSDARG00000043134 | cux1b | 15 | 2556747 | 2580262 | protein\_coding | | ENSDARG00000086345 | cux2b | 8 | 3987872 | 4270767 | protein\_coding | | ENSDARG00000025718 | cxxc1b | 11 | 25302082 | 25313229 | protein\_coding | | ENSDARG00000055295 | cyb561d1 | 11 | 36147474 | 36153717 | protein\_coding | | ENSDARG00000020898 | cyb5r4 | 16 | 3171760 | 3200431 | protein\_coding | | ENSDARG00000060058 | cylda | 7 | 37244389 | 37283950 | protein\_coding | | ENSDARG00000033566 | cyp17a1 | 13 | 28470745 | 28480515 | protein\_coding | | ENSDARG00000053966 | cyp17a2 | 23 | 45825339 | 45862659 | protein\_coding | | ENSDARG00000044002 | cyp2x7 | 25 | 17275890 | 17282481 | protein\_coding | | ENSDARG00000060210 | d2hgdh | 2 | 41696862 | 41713542 | protein\_coding | | ENSDARG00000015059 | daam1a | 17 | 14459424 | 14551104 | protein\_coding | | ENSDARG00000031761 | dab2 | 5 | 31723622 | 31738713 | protein\_coding | | ENSDARG00000074447 | dapk3 | 2 | 37487119 | 37495961 | protein\_coding | | ENSDARG00000070846 | dazap1 | 11 | 5883307 | 5926489 | protein\_coding | | ENSDARG00000086550 | dbf4b | 12 | 13850367 | 13867004 | protein\_coding | | ENSDARG00000054334 | dctpp1 | 1 | 23866585 | 23867920 | protein\_coding | | ENSDARG00000076074 | dda1 | 11 | 5947931 | 5956393 | protein\_coding | | ENSDARG00000037618 | ddit4 | 12 | 48356811 | 48358937 | protein\_coding | | ENSDARG00000060411 | ddx28 | 7 | 34692278 | 34694177 | protein\_coding | | ENSDARG00000020573 | ddx3a | 9 | 33523731 | 33543992 | protein\_coding | | ENSDARG00000061338 | ddx6 | 18 | 43873729 | 43890590 | protein\_coding | | ENSDARG00000029783 | dedd | 8 | 14024890 | 14042829 | protein\_coding | | ENSDARG00000101868 | dennd1b | 22 | 23259483 | 23453425 | protein\_coding | | ENSDARG00000073738 | dennd2c | 8 | 10981346 | 11039525 | protein\_coding | | ENSDARG00000075362 | dfnb31a | 21 | 13884282 | 14079009 | protein\_coding | | ENSDARG00000104793 | dgke | 6 | 21896067 | 21910379 | protein\_coding | | ENSDARG00000039851 | dhdds | 16 | 51290775 | 51314187 | protein\_coding | | ENSDARG00000003270 | dhps | 3 | 13273823 | 13287705 | protein\_coding | | ENSDARG00000012816 | dia1a | 2 | 8463104 | 8514194 | protein\_coding | | ENSDARG00000090785 | diaph3 | 11 | 31417517 | 31802038 | protein\_coding | | ENSDARG00000005350 | dip2ba | 23 | 27432181 | 27516020 | protein\_coding | | ENSDARG00000060559 | dis3 | 1 | 29932518 | 29958333 | protein\_coding | | ENSDARG00000053200 | dis3l | 7 | 33969013 | 33985167 | protein\_coding | | ENSDARG00000033259 | dis3l2 | 2 | 5929226 | 5972522 | protein\_coding | | ENSDARG00000044417 | disp1 | 20 | 51676029 | 51829201 | protein\_coding | | ENSDARG00000075825 | dlec1 | 24 | 20186198 | 20225192 | protein\_coding | | ENSDARG00000009677 | dlg1 | 6 | 29870215 | 30092361 | protein\_coding | | ENSDARG00000076796 | dlg3 | 14 | 32729970 | 32837683 | protein\_coding | | ENSDARG00000025824 | dmtf1 | 4 | 9652367 | 9666494 | protein\_coding | | ENSDARG00000100285 | dnai2a | 12 | 36242666 | 36250292 | protein\_coding | | ENSDARG00000058494 | dnaja3a | 3 | 11985830 | 12007472 | protein\_coding | | ENSDARG00000015088 | dnajb11 | 9 | 32830280 | 32837979 | protein\_coding | | ENSDARG00000099383 | dnajb1a | 3 | 45005411 | 45018310 | protein\_coding | | ENSDARG00000001940 | dnajc1 | 2 | 21516936 | 21524499 | protein\_coding | | ENSDARG00000023927 | dnajc24 | 25 | 14773625 | 14834945 | protein\_coding | | ENSDARG00000078675 | dock7 | 6 | 32339160 | 32417722 | protein\_coding | | ENSDARG00000099177 | dok1b | 7 | 58910236 | 58943520 | protein\_coding | | ENSDARG00000061992 | dot1l | 22 | 20757739 | 20813388 | protein\_coding | | ENSDARG00000012219 | dpf2 | 5 | 37144290 | 37157665 | protein\_coding | | ENSDARG00000057973 | dph1 | 15 | 25975236 | 26153799 | protein\_coding | | ENSDARG00000043360 | dph3 | 6 | 15000567 | 15004256 | protein\_coding | | ENSDARG00000023002 | dtl | 20 | 13245428 | 13278185 | protein\_coding | | ENSDARG00000032933 | dtx2 | 5 | 58883970 | 58925888 | protein\_coding | | ENSDARG00000089260 | dus1l | 3 | 36093201 | 36103055 | protein\_coding | | ENSDARG00000099528 | dus3l | 19 | 420100 | 431790 | protein\_coding | | ENSDARG00000056396 | dusp22a | 18 | 30532976 | 30586434 | protein\_coding | | ENSDARG00000015707 | dvl3a | 2 | 9904233 | 9946261 | protein\_coding | | ENSDARG00000022944 | ecd | 17 | 20168868 | 20182889 | protein\_coding | | ENSDARG00000061737 | ece1 | 11 | 27672518 | 27803385 | protein\_coding | | ENSDARG00000069703 | efhb | 16 | 49405893 | 49422632 | protein\_coding | | ENSDARG00000069318 | efr3bb | 20 | 42993795 | 43062827 | protein\_coding | | ENSDARG00000004632 | egln1b | 11 | 31294946 | 31340632 | protein\_coding | | ENSDARG00000070029 | ehhadh | 9 | 12457023 | 12472523 | protein\_coding | | ENSDARG00000068157 | ehmt1a | 5 | 28890031 | 28914576 | protein\_coding | | ENSDARG00000026634 | ehmt1b | 21 | 14547518 | 14595390 | protein\_coding | | ENSDARG00000093182 | eif2ak1 | 12 | 17469912 | 17481084 | protein\_coding | | ENSDARG00000002549 | eif3eb | 19 | 23039945 | 23079965 | protein\_coding | | ENSDARG00000077215 | eif4g3a | 11 | 27581616 | 27670556 | protein\_coding | | ENSDARG00000034060 | elac2 | 3 | 10569414 | 10615841 | protein\_coding | | ENSDARG00000074742 | elmod3 | 8 | 44719229 | 44744650 | protein\_coding | | ENSDARG00000017199 | elp2 | 19 | 35104525 | 35155320 | protein\_coding | | ENSDARG00000057255 | emc1 | 23 | 21334749 | 21350971 | protein\_coding | | ENSDARG00000035282 | emc6 | 5 | 41522657 | 41523641 | protein\_coding | | ENSDARG00000076913 | eme1 | 12 | 21550856 | 21563036 | protein\_coding | | ENSDARG00000032049 | enah | 20 | 36517068 | 36714434 | protein\_coding | | ENSDARG00000006877 | enpp4 | 17 | 5441918 | 5453418 | protein\_coding | | ENSDARG00000038422 | entpd4 | 8 | 50162188 | 50191580 | protein\_coding | | ENSDARG00000032324 | epb41l5 | 9 | 29059952 | 29128435 | protein\_coding | | ENSDARG00000007485 | epc2 | 9 | 25536945 | 25555672 | protein\_coding | | ENSDARG00000055163 | epo | 7 | 21624135 | 21652055 | protein\_coding | | ENSDARG00000021859 | erap1b | 5 | 25633964 | 25647560 | protein\_coding | | ENSDARG00000044281 | erbb2ip | 10 | 15496737 | 15641030 | protein\_coding | | ENSDARG00000012403 | ercc6l2 | 8 | 29889483 | 29921589 | protein\_coding | | ENSDARG00000044692 | eri1 | 21 | 19871123 | 19879385 | protein\_coding | | ENSDARG00000086309 | erlec1 | 13 | 35624858 | 35639196 | protein\_coding | | ENSDARG00000015228 | ero1a | 17 | 15010286 | 15021706 | protein\_coding | | ENSDARG00000077057 | esco1 | 24 | 36289316 | 36308005 | protein\_coding | | ENSDARG00000074915 | etaa1 | 1 | 50893451 | 50902118 | protein\_coding | | ENSDARG00000069763 | etv5a | 9 | 22820918 | 22838182 | protein\_coding | | ENSDARG00000070839 | evi5b | 6 | 29019766 | 29095912 | protein\_coding | | ENSDARG00000098669 | exd1 | 17 | 53236875 | 53240912 | protein\_coding | | ENSDARG00000016117 | exd2 | 17 | 51707633 | 51729640 | protein\_coding | | ENSDARG00000014582 | exoc3 | 2 | 50370581 | 50392107 | protein\_coding | | ENSDARG00000058180 | exoc3l4 | 13 | 7055620 | 7081894 | protein\_coding | | ENSDARG00000060323 | exoc5 | 17 | 44300005 | 44327067 | protein\_coding | | ENSDARG00000103155 | ext1a | 16 | 48518855 | 48631492 | protein\_coding | | ENSDARG00000101019 | ext1b | 19 | 46829094 | 46865351 | protein\_coding | | ENSDARG00000026811 | extl3 | 20 | 50137614 | 50211732 | protein\_coding | | ENSDARG00000005673 | f3b | 2 | 15432152 | 15438378 | protein\_coding | | ENSDARG00000022418 | faf1 | 8 | 16239066 | 16370728 | protein\_coding | | ENSDARG00000016866 | fam102ab | 5 | 31123251 | 31173056 | protein\_coding | | ENSDARG00000015293 | fam110a | 8 | 49217089 | 49218714 | protein\_coding | | ENSDARG00000079252 | fam117ab | 12 | 9811633 | 9838233 | protein\_coding | | ENSDARG00000030804 | fam120b | 13 | 5958270 | 5996874 | protein\_coding | | ENSDARG00000036500 | fam122b | 14 | 31189644 | 31206263 | protein\_coding | | ENSDARG00000037320 | fam131c | 23 | 24373973 | 24416822 | protein\_coding | | ENSDARG00000059843 | fam135a | 13 | 39056209 | 39102492 | protein\_coding | | ENSDARG00000061021 | fam160a1a | 1 | 23778552 | 23823844 | protein\_coding | | ENSDARG00000078188 | fam160a2 | 9 | 33084257 | 33101892 | protein\_coding | | ENSDARG00000033451 | fam173b | 2 | 30497374 | 30499676 | protein\_coding | | ENSDARG00000098907 | fam175b | 17 | 31788812 | 32068137 | protein\_coding | | ENSDARG00000102787 | fam193a | 1 | 36593251 | 36664585 | protein\_coding | | ENSDARG00000102831 | fam193b | 14 | 44800142 | 44844177 | protein\_coding | | ENSDARG00000016396 | fam219aa | 21 | 12996148 | 13026197 | protein\_coding | | ENSDARG00000078578 | fam222bb | 15 | 16441241 | 16448162 | protein\_coding | | ENSDARG00000012726 | fam26e.2 | 16 | 32251630 | 32254698 | protein\_coding | | ENSDARG00000010437 | fam46c | 9 | 20969944 | 20973178 | protein\_coding | | ENSDARG00000045417 | fam49bb | 24 | 10652425 | 10757164 | protein\_coding | | ENSDARG00000037537 | fam58a | 23 | 20042613 | 20050871 | protein\_coding | | ENSDARG00000004866 | fam63a | 19 | 7535071 | 7559672 | protein\_coding | | ENSDARG00000062955 | fam69aa | 2 | 10858448 | 10903631 | protein\_coding | | ENSDARG00000089357 | fam73b | 21 | 3699022 | 3718224 | protein\_coding | | ENSDARG00000075296 | fam83c | 6 | 52468140 | 52484567 | protein\_coding | | ENSDARG00000038012 | fam83fa | 3 | 29338441 | 29353049 | protein\_coding | | ENSDARG00000032859 | fam84b | 19 | 33552044 | 33553283 | protein\_coding | | ENSDARG00000079979 | fam89a | 20 | 4000439 | 4005399 | protein\_coding | | ENSDARG00000062269 | fan1 | 18 | 21699197 | 21736572 | protein\_coding | | ENSDARG00000067596 | fancb | 9 | 55367242 | 55391868 | protein\_coding | | ENSDARG00000055232 | fancc | 8 | 30103392 | 30146885 | protein\_coding | | ENSDARG00000077075 | fastk | 2 | 32595799 | 32611559 | protein\_coding | | ENSDARG00000007443 | fbxl18 | 1 | 7954051 | 7962830 | protein\_coding | | ENSDARG00000012135 | fbxl2 | 19 | 43090721 | 43113510 | protein\_coding | | ENSDARG00000075567 | fbxl20 | 19 | 4911765 | 4934925 | protein\_coding | | ENSDARG00000043046 | fbxl5 | 23 | 46024219 | 46040986 | protein\_coding | | ENSDARG00000061936 | fbxo10 | 1 | 18909330 | 18939412 | protein\_coding | | ENSDARG00000016897 | fbxo15 | 24 | 15510810 | 15524835 | protein\_coding | | ENSDARG00000058561 | fbxo30a | 17 | 7463042 | 7471280 | protein\_coding | | ENSDARG00000019311 | fbxo30b | 23 | 1552824 | 1562575 | protein\_coding | | ENSDARG00000071259 | fbxo34 | 17 | 10627565 | 10657851 | protein\_coding | | ENSDARG00000086484 | fbxw10 | 12 | 35851343 | 35861735 | protein\_coding | | ENSDARG00000017230 | fbxw11b | 10 | 21487273 | 21513212 | protein\_coding | | ENSDARG00000009745 | fbxw2 | 5 | 31779873 | 31796776 | protein\_coding | | ENSDARG00000099139 | fbxw5 | 5 | 53811288 | 53835099 | protein\_coding | | ENSDARG00000060994 | fbxw7 | 1 | 24178200 | 24453610 | protein\_coding | | ENSDARG00000035389 | fcho2 | 5 | 34523506 | 34600621 | protein\_coding | | ENSDARG00000003462 | fech | 21 | 1622166 | 1644440 | protein\_coding | | ENSDARG00000102147 | fem1c | 8 | 423393 | 425620 | protein\_coding | | ENSDARG00000012196 | fer | 5 | 56573351 | 56640936 | protein\_coding | | ENSDARG00000087666 | fibpb | 21 | 25568763 | 25576644 | protein\_coding | | ENSDARG00000035595 | ficd | 5 | 19981824 | 19987472 | protein\_coding | | ENSDARG00000043328 | fip1l1a | 20 | 22840119 | 22899121 | protein\_coding | | ENSDARG00000001734 | fkbp8 | 22 | 20900871 | 20925470 | protein\_coding | | ENSDARG00000095322 | fkbpl | 23 | 19179713 | 19193100 | protein\_coding | | ENSDARG00000037433 | fmr1 | 14 | 19859232 | 19894036 | protein\_coding | | ENSDARG00000027916 | fntb | 20 | 28901098 | 28924881 | protein\_coding | | ENSDARG00000037872 | foxk1 | 3 | 40759885 | 40791557 | protein\_coding | | ENSDARG00000045105 | foxn2b | 12 | 24676736 | 24711078 | protein\_coding | | ENSDARG00000061549 | foxo1b | 10 | 31865366 | 31917614 | protein\_coding | | ENSDARG00000044809 | fpgs | 21 | 6008797 | 6029062 | protein\_coding | | ENSDARG00000074599 | frmd4ba | 6 | 43253429 | 43390384 | protein\_coding | | ENSDARG00000055436 | ftr97 | 20 | 27021944 | 27038363 | protein\_coding | | ENSDARG00000074356 | fxn | 8 | 11287605 | 11301902 | protein\_coding | | ENSDARG00000104500 | fyco1a | 23 | 40071028 | 40150499 | protein\_coding | | ENSDARG00000054344 | gabpb2b | 16 | 38387751 | 38397524 | protein\_coding | | ENSDARG00000090654 | gak | 5 | 8936560 | 9036019 | protein\_coding | | ENSDARG00000073728 | gal3st3 | 7 | 6918453 | 7016410 | protein\_coding | | ENSDARG00000002401 | gale | 17 | 24861921 | 24872527 | protein\_coding | | ENSDARG00000028088 | galk1 | 12 | 33689138 | 33704383 | protein\_coding | | ENSDARG00000004059 | galk2 | 25 | 31979837 | 31989252 | protein\_coding | | ENSDARG00000055490 | galnt12 | 19 | 34642988 | 34672546 | protein\_coding | | ENSDARG00000100889 | galnt18a | 25 | 17593165 | 17729114 | protein\_coding | | ENSDARG00000014386 | galnt6 | 23 | 26836797 | 26858230 | protein\_coding | | ENSDARG00000078959 | gb:eh507706 | 11 | 30400352 | 30414570 | protein\_coding | | ENSDARG00000003244 | gbp3 | 2 | 44041370 | 44102997 | protein\_coding | | ENSDARG00000045541 | gcc1 | 4 | 20074303 | 20080517 | protein\_coding | | ENSDARG00000013095 | gclc | 13 | 2261361 | 2296388 | protein\_coding | | ENSDARG00000021569 | gdap2 | 9 | 20995458 | 21042615 | protein\_coding | | ENSDARG00000003229 | gdf9 | 14 | 22159985 | 22166938 | protein\_coding | | ENSDARG00000061145 | gfod2 | 7 | 28773738 | 28781318 | protein\_coding | | ENSDARG00000055569 | ghdc | 12 | 13489119 | 13511235 | protein\_coding | | ENSDARG00000037589 | gid8b | 23 | 17583451 | 17594432 | protein\_coding | | ENSDARG00000089088 | gig2n | 21 | 22598799 | 22600804 | processed\_transcript | | ENSDARG00000088260 | gig2p | 5 | 57052046 | 57053712 | protein\_coding | | ENSDARG00000038847 | gins3 | 25 | 13303567 | 13312360 | protein\_coding | | ENSDARG00000039489 | git1 | 15 | 28498787 | 28547670 | protein\_coding | | ENSDARG00000042791 | git2b | 10 | 40362817 | 40396293 | protein\_coding | | ENSDARG00000036155 | gla | 14 | 38592127 | 38606494 | protein\_coding | | ENSDARG00000016837 | glipr2l | 16 | 27680366 | 27687560 | protein\_coding | | ENSDARG00000020948 | gmcl1 | 10 | 7714746 | 7743329 | protein\_coding | | ENSDARG00000035957 | gmnn | 19 | 31998028 | 32140417 | protein\_coding | | ENSDARG00000010002 | gna11a | 22 | 21825890 | 21872423 | protein\_coding | | ENSDARG00000053326 | gna11b | 2 | 52876118 | 52956422 | protein\_coding | | ENSDARG00000043094 | gnas | 6 | 49813178 | 49874367 | protein\_coding | | ENSDARG00000036293 | gnb1l | 5 | 14721794 | 14783292 | protein\_coding | | ENSDARG00000074401 | gnpat | 20 | 3844866 | 3873735 | protein\_coding | | ENSDARG00000008979 | golga1 | 8 | 41460289 | 41511431 | protein\_coding | | ENSDARG00000052851 | golph3 | 5 | 40482381 | 40503491 | protein\_coding | | ENSDARG00000023117 | gopc | 20 | 42349838 | 42384324 | protein\_coding | | ENSDARG00000053995 | gpank1 | 1 | 25342433 | 25351851 | protein\_coding | | ENSDARG00000053575 | gpat2 | 8 | 40927594 | 41139397 | protein\_coding | | ENSDARG00000052747 | gpatch3 | 16 | 53703577 | 53714784 | protein\_coding | | ENSDARG00000032199 | gpc3 | 14 | 30836638 | 31125591 | protein\_coding | | ENSDARG00000045289 | gpr137c | 17 | 14957637 | 15004194 | protein\_coding | | ENSDARG00000090804 | gpr155a | 9 | 2472649 | 2505223 | protein\_coding | | ENSDARG00000074540 | gpr63 | 20 | 417176 | 422500 | protein\_coding | | ENSDARG00000035558 | gps2 | 5 | 23559246 | 23570738 | protein\_coding | | ENSDARG00000017311 | gpsm2 | 2 | 45638034 | 45657706 | protein\_coding | | ENSDARG00000102026 | gpsm2l | 23 | 40345648 | 40363886 | protein\_coding | | ENSDARG00000000588 | grasp | 23 | 32273057 | 32309591 | protein\_coding | | ENSDARG00000105276 | grb2a | 6 | 22873428 | 22910672 | protein\_coding | | ENSDARG00000078815 | grk5l | 8 | 44629634 | 44701354 | protein\_coding | | ENSDARG00000025081 | grnb | 24 | 36451170 | 36476519 | protein\_coding | | ENSDARG00000036494 | gstcd | 1 | 49307881 | 49421777 | protein\_coding | | ENSDARG00000020642 | gtdc1 | 9 | 26029851 | 26102091 | protein\_coding | | ENSDARG00000026701 | gtf2h1 | 25 | 3071111 | 3091371 | protein\_coding | | ENSDARG00000071583 | gtf3ab | 24 | 21495461 | 21497684 | protein\_coding | | ENSDARG00000004574 | gtf3c2 | 20 | 38640435 | 38681282 | protein\_coding | | ENSDARG00000069538 | gtf3c4 | 21 | 17376977 | 17388021 | protein\_coding | | ENSDARG00000053467 | gtpbp1 | 3 | 33308901 | 33328050 | protein\_coding | | ENSDARG00000042900 | gtpbp1l | 22 | 14811716 | 14827120 | protein\_coding | | ENSDARG00000061091 | gtpbp6 | 6 | 37463895 | 37477016 | protein\_coding | | ENSDARG00000076776 | gucy2g | 12 | 31345975 | 31361212 | protein\_coding | | ENSDARG00000018542 | hapln4 | 22 | 17803289 | 17806275 | protein\_coding | | ENSDARG00000019156 | haus5 | 8 | 20865764 | 20882744 | protein\_coding | | ENSDARG00000087573 | hdac11 | 11 | 26896058 | 26950302 | protein\_coding | | ENSDARG00000098349 | hdac4 | 9 | 46083552 | 46441023 | protein\_coding | | ENSDARG00000053934 | hdhd3 | 6 | 41021029 | 41024329 | protein\_coding | | ENSDARG00000023999 | heatr5a | 17 | 28607396 | 28652951 | protein\_coding | | ENSDARG00000042877 | heca | 20 | 36918025 | 36941108 | protein\_coding | | ENSDARG00000079818 | helq | 21 | 19294138 | 19310888 | protein\_coding | | ENSDARG00000018871 | henmt1 | 20 | 33841186 | 33846295 | protein\_coding | | ENSDARG00000075887 | herc3 | 1 | 49995590 | 50047712 | protein\_coding | | ENSDARG00000024314 | herpud1 | 18 | 17611573 | 17619512 | protein\_coding | | ENSDARG00000061216 | hexdc | 12 | 26520066 | 26529368 | protein\_coding | | ENSDARG00000074428 | hhat | 17 | 25758939 | 25813178 | protein\_coding | | ENSDARG00000013117 | hiat1a | 2 | 37673621 | 37702184 | protein\_coding | | ENSDARG00000100497 | hic2 | 10 | 3166057 | 3176307 | protein\_coding | | ENSDARG00000100583 | hid1b | 12 | 46242580 | 46278940 | protein\_coding | | ENSDARG00000031915 | hif1an | 13 | 29822183 | 29849765 | protein\_coding | | ENSDARG00000004851 | hinfp | 5 | 29996324 | 30006650 | protein\_coding | | ENSDARG00000077445 | hip1ra | 10 | 44619730 | 44634003 | protein\_coding | | ENSDARG00000037154 | hivep3b | 16 | 34367364 | 34387992 | protein\_coding | | ENSDARG00000101482 | hk2 | 5 | 13370084 | 13503972 | protein\_coding | | ENSDARG00000027082 | hmbox1a | 17 | 16290189 | 16316632 | protein\_coding | | ENSDARG00000018913 | hmbox1b | 20 | 49975324 | 49999099 | protein\_coding | | ENSDARG00000103025 | hmgcs1 | 10 | 6009365 | 6033188 | protein\_coding | | ENSDARG00000077823 | hmgxb3 | 14 | 2819993 | 2853762 | protein\_coding | | ENSDARG00000078452 | hmox2b | 3 | 12018193 | 12032316 | protein\_coding | | ENSDARG00000101010 | hpdl | 12 | 46853086 | 46864817 | protein\_coding | | ENSDARG00000026170 | hps1 | 13 | 40560243 | 40590812 | protein\_coding | | ENSDARG00000013795 | hps4 | 8 | 39734141 | 39758581 | protein\_coding | | ENSDARG00000071062 | hps5 | 25 | 3022687 | 3066700 | protein\_coding | | ENSDARG00000078163 | hpse2 | 13 | 40377595 | 40542385 | protein\_coding | | ENSDARG00000098107 | hs3st1l1 | KN150267.1 | 45328 | 46447 | protein\_coding | | ENSDARG00000103846 | hspa5 | 5 | 2440676 | 2448586 | protein\_coding | | ENSDARG00000028386 | htatip2 | 7 | 16844308 | 16851210 | protein\_coding | | ENSDARG00000068557 | htr5al | 7 | 40801781 | 40822425 | protein\_coding | | ENSDARG00000052866 | htt | 1 | 40591177 | 40671330 | protein\_coding | | ENSDARG00000040338 | hvcn1 | 10 | 8443229 | 8454264 | protein\_coding | | ENSDARG00000056764 | hydin | 18 | 21284683 | 21412335 | protein\_coding | | ENSDARG00000013670 | hyou1 | 10 | 29883780 | 29905126 | protein\_coding | | ENSDARG00000057688 | ical1 | 6 | 12635220 | 12665318 | protein\_coding | | ENSDARG00000058461 | ice1 | 16 | 9509759 | 9529456 | protein\_coding | | ENSDARG00000086670 | ice2 | 25 | 33486726 | 33523098 | protein\_coding | | ENSDARG00000002184 | igbp1 | 5 | 35930683 | 35938955 | protein\_coding | | ENSDARG00000075158 | igdcc3 | 7 | 31230388 | 31347061 | protein\_coding | | ENSDARG00000027423 | igf1ra | 18 | 20892396 | 21058514 | protein\_coding | | ENSDARG00000056491 | ikzf5 | 17 | 21797166 | 21802362 | protein\_coding | | ENSDARG00000007743 | il15l | 15 | 20416391 | 20427799 | protein\_coding | | ENSDARG00000101756 | im:7141269 | 4 | 68816117 | 68823793 | protein\_coding | | ENSDARG00000089139 | im:7145024 | 25 | 8936274 | 8963844 | protein\_coding | | ENSDARG00000086626 | im:7147486 | 16 | 25151848 | 25157798 | protein\_coding | | ENSDARG00000024964 | inadl | 22 | 16542853 | 16732703 | protein\_coding | | ENSDARG00000104907 | ing2 | 1 | 39263378 | 39265657 | protein\_coding | | ENSDARG00000104727 | ino80da | 9 | 1029976 | 1046606 | protein\_coding | | ENSDARG00000061437 | inpp5f | 13 | 25375130 | 25402235 | protein\_coding | | ENSDARG00000071524 | insrb | 22 | 10897688 | 11024562 | protein\_coding | | ENSDARG00000091678 | ints12 | 1 | 25744883 | 25750361 | protein\_coding | | ENSDARG00000029291 | ipmkb | 12 | 7160276 | 7201600 | protein\_coding | | ENSDARG00000003446 | ippk | 22 | 10486650 | 10509498 | protein\_coding | | ENSDARG00000092483 | iqcc | 13 | 33167888 | 33171536 | protein\_coding | | ENSDARG00000005468 | irf2bp1 | 18 | 46363526 | 46371498 | protein\_coding | | ENSDARG00000054087 | irs1 | 15 | 36030580 | 36072101 | protein\_coding | | ENSDARG00000012942 | itgb5 | 9 | 22866634 | 22937146 | protein\_coding | | ENSDARG00000017565 | itk | 14 | 33704970 | 33734196 | protein\_coding | | ENSDARG00000002994 | itpkca | 21 | 21242523 | 21262359 | protein\_coding | | ENSDARG00000067741 | itpkcb | 18 | 35454487 | 35485150 | protein\_coding | | ENSDARG00000061741 | itpr3 | 8 | 21222107 | 21320997 | protein\_coding | | ENSDARG00000013946 | ivns1abpb | 2 | 23723750 | 23735157 | protein\_coding | | ENSDARG00000033707 | jade1 | 17 | 26809636 | 26829627 | protein\_coding | | ENSDARG00000028581 | jkamp | 17 | 14435473 | 14443785 | protein\_coding | | ENSDARG00000102896 | jmjd6 | 3 | 60497710 | 60509790 | protein\_coding | | ENSDARG00000071548 | josd1 | 12 | 18784533 | 18791520 | protein\_coding | | ENSDARG00000070150 | jtb | 19 | 42657963 | 42666748 | protein\_coding | | ENSDARG00000045951 | kat5b | 5 | 66054184 | 66070430 | protein\_coding | | ENSDARG00000076923 | kazna | 8 | 26695393 | 26784601 | protein\_coding | | ENSDARG00000057468 | kcnc2 | 4 | 2211211 | 2347876 | protein\_coding | | ENSDARG00000061622 | kcng3 | 12 | 25118009 | 25125857 | protein\_coding | | ENSDARG00000029881 | kcnh2a | 2 | 24425043 | 24513019 | protein\_coding | | ENSDARG00000002666 | kcnj1b | 10 | 39382935 | 39386332 | protein\_coding | | ENSDARG00000104563 | kcnk9 | 19 | 4546893 | 4614553 | protein\_coding | | ENSDARG00000017115 | kctd10 | 5 | 18932683 | 18941149 | protein\_coding | | ENSDARG00000078133 | kdm2ab | 14 | 21457276 | 21484982 | protein\_coding | | ENSDARG00000019103 | kdm4ab | 2 | 17779059 | 17820911 | protein\_coding | | ENSDARG00000061504 | kdm4c | 1 | 21244264 | 21278976 | protein\_coding | | ENSDARG00000062187 | kif14 | 22 | 22412915 | 22451238 | protein\_coding | | ENSDARG00000008022 | kif18a | 7 | 31705614 | 31750703 | protein\_coding | | ENSDARG00000009733 | kif21b | 11 | 2786076 | 2904713 | protein\_coding | | ENSDARG00000079862 | kl | 10 | 34057584 | 34077996 | protein\_coding | | ENSDARG00000045130 | klhl11 | 12 | 13922965 | 13927881 | protein\_coding | | ENSDARG00000001930 | klhl15 | 24 | 16820786 | 16835309 | protein\_coding | | ENSDARG00000004306 | klhl18 | 2 | 32760589 | 32773488 | protein\_coding | | ENSDARG00000021739 | klhl24a | 2 | 44718202 | 44751457 | protein\_coding | | ENSDARG00000053876 | klhl26 | 11 | 6871162 | 6886142 | protein\_coding | | ENSDARG00000044980 | kpna6 | 19 | 30274546 | 30300316 | protein\_coding | | ENSDARG00000068714 | ksr1a | 10 | 37229271 | 37313231 | protein\_coding | | ENSDARG00000054301 | kti12 | 18 | 26789367 | 26799238 | protein\_coding | | ENSDARG00000051934 | kxd1 | 25 | 20596523 | 20604270 | protein\_coding | | ENSDARG00000060500 | l2hgdh | 13 | 36540830 | 36554699 | protein\_coding | | ENSDARG00000025983 | l3mbtl3 | 20 | 2112730 | 2179604 | protein\_coding | | ENSDARG00000042902 | lace1b | 20 | 32434643 | 32502572 | protein\_coding | | ENSDARG00000014914 | lamp2 | 14 | 33133457 | 33141276 | protein\_coding | | ENSDARG00000005126 | large | 4 | 73574647 | 73618127 | protein\_coding | | ENSDARG00000005355 | larp6 | 18 | 284467 | 305708 | protein\_coding | | ENSDARG00000076153 | lca5 | 23 | 31453984 | 31474299 | protein\_coding | | ENSDARG00000103320 | lclat1 | 13 | 51747141 | 51754203 | protein\_coding | | ENSDARG00000100908 | lcor | 22 | 34809193 | 34843257 | protein\_coding | | ENSDARG00000039750 | ldlrap1b | 13 | 45458995 | 45491957 | protein\_coding | | ENSDARG00000062374 | leng9 | 16 | 13916149 | 13927951 | protein\_coding | | ENSDARG00000030641 | letmd1 | 23 | 32152515 | 32173575 | protein\_coding | | ENSDARG00000055525 | lgalslb | 13 | 24421804 | 24423578 | protein\_coding | | ENSDARG00000002330 | lhx8a | 2 | 11422196 | 11426569 | protein\_coding | | ENSDARG00000078092 | limd2 | 3 | 31719137 | 31743424 | protein\_coding | | ENSDARG00000028475 | lin9 | 20 | 43794130 | 43818164 | protein\_coding | | ENSDARG00000086879 | lins1 | 7 | 9628586 | 9650785 | protein\_coding | | ENSDARG00000069980 | lman1 | 21 | 10609403 | 10638624 | protein\_coding | | ENSDARG00000061265 | lmln | 9 | 38692751 | 38707129 | protein\_coding | | ENSDARG00000006639 | lnpb | 6 | 10566887 | 10628224 | protein\_coding | | ENSDARG00000100394 | lnpep | 5 | 51545534 | 51569577 | protein\_coding | | ENSDARG00000013542 | lpgat1 | 20 | 13885547 | 13978360 | protein\_coding | | ENSDARG00000100679 | lrch3 | 18 | 3482744 | 3548800 | protein\_coding | | ENSDARG00000045574 | lrmp | 4 | 17263620 | 17289952 | protein\_coding | | ENSDARG00000023479 | lrp13 | 5 | 8674940 | 8712120 | protein\_coding | | ENSDARG00000076425 | lrrc29 | 25 | 17382864 | 17397964 | protein\_coding | | ENSDARG00000078415 | lrrc3 | 9 | 42355791 | 42362957 | protein\_coding | | ENSDARG00000017708 | lrrc40 | 6 | 30123189 | 30131993 | protein\_coding | | ENSDARG00000030591 | lrrc42 | 2 | 26949139 | 26955399 | protein\_coding | | ENSDARG00000078535 | lrrcc1 | 2 | 31704104 | 31728151 | protein\_coding | | ENSDARG00000043177 | lsm1 | 8 | 44619229 | 44624307 | protein\_coding | | ENSDARG00000090854 | lsm11 | 14 | 35052422 | 35065409 | protein\_coding | | ENSDARG00000025386 | lsm14b | 23 | 540603 | 554390 | protein\_coding | | ENSDARG00000024693 | lysmd3 | 5 | 48021042 | 48029069 | protein\_coding | | ENSDARG00000015905 | lztr1 | 5 | 12223829 | 12242995 | protein\_coding | | ENSDARG00000077207 | lzts2b | 12 | 47827857 | 47864688 | protein\_coding | | ENSDARG00000018432 | m6pr | 16 | 17671938 | 17678906 | protein\_coding | | ENSDARG00000053691 | maea | 14 | 20826622 | 20843823 | protein\_coding | | ENSDARG00000101869 | magi3a | 23 | 26305445 | 26562860 | protein\_coding | | ENSDARG00000079552 | maml3 | 1 | 39615806 | 39801465 | protein\_coding | | ENSDARG00000076592 | man1b1b | 5 | 28579921 | 28595276 | protein\_coding | | ENSDARG00000102200 | man2a1 | 5 | 56335621 | 56534135 | protein\_coding | | ENSDARG00000063177 | manf | 22 | 32274258 | 32280927 | protein\_coding | | ENSDARG00000006609 | map2k2a | 22 | 20363643 | 20378216 | protein\_coding | | ENSDARG00000008279 | map2k7 | 1 | 45148132 | 45164383 | protein\_coding | | ENSDARG00000062884 | map3k2 | 6 | 9306909 | 9329987 | protein\_coding | | ENSDARG00000020469 | map3k7 | 20 | 24251896 | 24284282 | protein\_coding | | ENSDARG00000023110 | mapk7 | 12 | 4183588 | 4202062 | protein\_coding | | ENSDARG00000077364 | mapk9 | 21 | 32315932 | 32341398 | protein\_coding | | ENSDARG00000076066 | march6 | 2 | 30506313 | 30543188 | protein\_coding | | ENSDARG00000062489 | march8 | 13 | 17620874 | 17880216 | protein\_coding | | ENSDARG00000032458 | mark2b | 7 | 24651510 | 24704179 | protein\_coding | | ENSDARG00000019345 | mark3a | 13 | 15449766 | 15537751 | protein\_coding | | ENSDARG00000026630 | mark3b | 20 | 43895788 | 44020611 | protein\_coding | | ENSDARG00000009218 | mars2 | 14 | 23381804 | 23387569 | protein\_coding | | ENSDARG00000086505 | mast3b | 11 | 13222576 | 13283824 | protein\_coding | | ENSDARG00000012932 | mat2b | 21 | 32031966 | 32048891 | protein\_coding | | ENSDARG00000087330 | maza | 3 | 20946287 | 20988622 | protein\_coding | | ENSDARG00000025699 | mbd1b | 11 | 25322456 | 25338583 | protein\_coding | | ENSDARG00000059689 | mblac2 | 5 | 48008236 | 48013011 | protein\_coding | | ENSDARG00000042551 | mboat2b | 20 | 29788022 | 29840281 | protein\_coding | | ENSDARG00000060868 | mbtd1 | 12 | 33437692 | 33457896 | protein\_coding | | ENSDARG00000037532 | mcm3l | 20 | 6526576 | 6542515 | protein\_coding | | ENSDARG00000002285 | mcoln1a | 1 | 44979892 | 45008779 | protein\_coding | | ENSDARG00000058372 | mcph1 | 13 | 6158134 | 6195551 | protein\_coding | | ENSDARG00000073970 | mctp2b | 7 | 15488195 | 15540009 | protein\_coding | | ENSDARG00000101175 | mcu | 13 | 4593614 | 4720303 | protein\_coding | | ENSDARG00000014218 | mecp2 | 8 | 7597842 | 7618909 | protein\_coding | | ENSDARG00000053884 | med13a | 10 | 28143413 | 28231115 | protein\_coding | | ENSDARG00000009953 | med14 | 9 | 34588625 | 34614872 | protein\_coding | | ENSDARG00000037410 | med28 | 1 | 20616449 | 20619659 | protein\_coding | | ENSDARG00000043912 | mei4 | 20 | 791336 | 802400 | protein\_coding | | ENSDARG00000059461 | mepce | 5 | 67995789 | 68011974 | protein\_coding | | ENSDARG00000101914 | metrnl | 3 | 44328048 | 44344245 | protein\_coding | | ENSDARG00000102828 | mfsd11 | 3 | 60524143 | 60544463 | protein\_coding | | ENSDARG00000030630 | mfsd2aa | 13 | 35766544 | 35828850 | protein\_coding | | ENSDARG00000023768 | mfsd4a | 11 | 38162114 | 38191201 | protein\_coding | | ENSDARG00000015997 | mfsd5 | 23 | 35688139 | 35691457 | protein\_coding | | ENSDARG00000054583 | mfsd6b | 9 | 41681044 | 41705925 | protein\_coding | | ENSDARG00000014761 | mfsd6l | 3 | 25719012 | 25722031 | protein\_coding | | ENSDARG00000012407 | mgat1a | 15 | 35009549 | 35020419 | protein\_coding | | ENSDARG00000052408 | mgat2 | 20 | 54277595 | 54285690 | protein\_coding | | ENSDARG00000038069 | mgat3a | 3 | 25106851 | 25138203 | protein\_coding | | ENSDARG00000063330 | mgat4a | 9 | 7114716 | 7235341 | protein\_coding | | ENSDARG00000100072 | mgrn1a | 12 | 20000597 | 20025890 | protein\_coding | | ENSDARG00000074765 | mhc1zja | 3 | 615199 | 634091 | protein\_coding | | ENSDARG00000008184 | mia3 | 20 | 51946487 | 52019921 | protein\_coding | | ENSDARG00000011809 | mical1 | 23 | 39569295 | 39635413 | protein\_coding | | ENSDARG00000079811 | micall1a | 6 | 19476205 | 19510755 | protein\_coding | | ENSDARG00000102366 | micall2a | 3 | 42395995 | 42429513 | protein\_coding | | ENSDARG00000060991 | mief1 | 3 | 25005605 | 25017499 | protein\_coding | | ENSDARG00000002377 | mief2 | 1 | 7837212 | 7847898 | protein\_coding | | ENSDARG00000071413 | mier2 | 22 | 21491753 | 21520262 | protein\_coding | | ENSDARG00000021288 | mier3b | 8 | 17107789 | 17121472 | protein\_coding | | ENSDARG00000045172 | minpp1b | 12 | 17360733 | 17371011 | protein\_coding | | ENSDARG00000075867 | mkl1a | 3 | 24564301 | 24634579 | protein\_coding | | ENSDARG00000088307 | mkl2a | 12 | 19854526 | 19923227 | protein\_coding | | ENSDARG00000007630 | mkrn2 | 11 | 516867 | 547980 | protein\_coding | | ENSDARG00000028295 | mkrn4 | 8 | 23704718 | 23709974 | protein\_coding | | ENSDARG00000059657 | mks1 | 5 | 55420634 | 55448811 | protein\_coding | | ENSDARG00000056334 | mlh3 | 23 | 24662639 | 24672415 | protein\_coding | | ENSDARG00000059474 | mlxip | 5 | 66637447 | 66671547 | protein\_coding | | ENSDARG00000104732 | mlycd | 18 | 14265041 | 14306291 | protein\_coding | | ENSDARG00000079067 | mms19 | 12 | 2769772 | 2813555 | protein\_coding | | ENSDARG00000002077 | mnat1 | 13 | 31517821 | 31551501 | protein\_coding | | ENSDARG00000012953 | mob1bb | 21 | 5854779 | 5867155 | protein\_coding | | ENSDARG00000019113 | mob3a | 8 | 20383752 | 20395384 | protein\_coding | | ENSDARG00000078585 | mon1a | 11 | 34909423 | 34923074 | protein\_coding | | ENSDARG00000077434 | mon1bb | 11 | 25455779 | 25471695 | protein\_coding | | ENSDARG00000006272 | mpp5a | 17 | 34238686 | 34291568 | protein\_coding | | ENSDARG00000102470 | mpp7a | 12 | 23054743 | 23244518 | protein\_coding | | ENSDARG00000101913 | mpzl3 | KN150212.1 | 7721 | 14605 | protein\_coding | | ENSDARG00000075754 | mri1 | 1 | 54513499 | 54532968 | protein\_coding | | ENSDARG00000059658 | mrm1 | 5 | 55448879 | 55455611 | protein\_coding | | ENSDARG00000018040 | msantd4 | 15 | 44442396 | 44453240 | protein\_coding | | ENSDARG00000063276 | msh3 | 5 | 50602191 | 50792853 | protein\_coding | | ENSDARG00000039430 | msl2b | 24 | 26862038 | 26866231 | protein\_coding | | ENSDARG00000029587 | msra | 20 | 39007335 | 39143302 | protein\_coding | | ENSDARG00000005448 | mtfmt | 7 | 52478003 | 52486502 | protein\_coding | | ENSDARG00000060394 | mtfr2 | 23 | 31902101 | 31986758 | protein\_coding | | ENSDARG00000063213 | mtif2 | 6 | 6118232 | 6138344 | protein\_coding | | ENSDARG00000010601 | mtmr10 | 7 | 30454323 | 30497395 | protein\_coding | | ENSDARG00000022378 | mtmr1b | 21 | 34053577 | 34083716 | protein\_coding | | ENSDARG00000098201 | mtmr3 | 8 | 3876368 | 3974415 | protein\_coding | | ENSDARG00000016794 | mtmr6 | 24 | 24887166 | 24914365 | protein\_coding | | ENSDARG00000076155 | mtpap | 12 | 23543116 | 23553402 | protein\_coding | | ENSDARG00000104906 | mtr | 12 | 47134909 | 47162786 | protein\_coding | | ENSDARG00000059604 | mtss1 | 6 | 50581721 | 50686643 | protein\_coding | | ENSDARG00000071562 | mtus1a | 14 | 29945299 | 30017327 | protein\_coding | | ENSDARG00000052527 | mul1b | 23 | 37184880 | 37189154 | protein\_coding | | ENSDARG00000076309 | mxra5b | 9 | 56182786 | 56204193 | protein\_coding | | ENSDARG00000095241 | mybl2a | 23 | 4409364 | 4418867 | protein\_coding | | ENSDARG00000006003 | mycla | 13 | 35778503 | 35782443 | protein\_coding | | ENSDARG00000104235 | myo5c | 25 | 4661003 | 4707196 | protein\_coding | | ENSDARG00000077410 | myo9b | 2 | 37702464 | 37762526 | protein\_coding | | ENSDARG00000006112 | myof | 12 | 10479630 | 10529345 | protein\_coding | | ENSDARG00000034693 | mysm1 | 20 | 9108457 | 9135313 | protein\_coding | | ENSDARG00000057491 | n4bp1 | 18 | 18416926 | 18433477 | protein\_coding | | ENSDARG00000030368 | naa15b | 1 | 39895219 | 39923643 | protein\_coding | | ENSDARG00000075446 | naa25 | 10 | 3364282 | 3394005 | protein\_coding | | ENSDARG00000078238 | nacc1 | 11 | 30955896 | 30971524 | protein\_coding | | ENSDARG00000062753 | naif1 | 8 | 2583841 | 2591383 | protein\_coding | | ENSDARG00000045620 | nansa | 1 | 11649016 | 11657992 | protein\_coding | | ENSDARG00000008593 | nbas | 20 | 32933033 | 33272020 | protein\_coding | | ENSDARG00000099547 | nbeal1 | 6 | 4091161 | 4208732 | protein\_coding | | ENSDARG00000040303 | nbn | 16 | 23922886 | 23960443 | protein\_coding | | ENSDARG00000089586 | ncam3 | 16 | 44953296 | 44978508 | protein\_coding | | ENSDARG00000103902 | nde1 | 3 | 36529727 | 36542530 | protein\_coding | | ENSDARG00000104225 | ndel1b | 3 | 44439270 | 44466666 | protein\_coding | | ENSDARG00000060678 | ndst2a | 12 | 35102028 | 35239828 | protein\_coding | | ENSDARG00000022350 | nek1 | 20 | 23769161 | 23814917 | protein\_coding | | ENSDARG00000087564 | nek11 | 16 | 41221917 | 41393370 | protein\_coding | | ENSDARG00000061411 | nelfa | 7 | 24220756 | 24234261 | protein\_coding | | ENSDARG00000096382 | neu3.1 | 21 | 21754707 | 21758135 | protein\_coding | | ENSDARG00000020872 | nfat5a | 25 | 36523587 | 36550446 | protein\_coding | | ENSDARG00000105261 | nfkb1 | 14 | 50609994 | 50657925 | protein\_coding | | ENSDARG00000070127 | nfxl1 | 23 | 44069414 | 44127523 | protein\_coding | | ENSDARG00000001621 | nfyal | 8 | 24995061 | 25015299 | protein\_coding | | ENSDARG00000098414 | ninl.1 | 13 | 9859594 | 9885119 | protein\_coding | | ENSDARG00000043077 | nisch | 22 | 10331488 | 10367998 | protein\_coding | | ENSDARG00000099527 | nkiras1 | 19 | 17918519 | 17926983 | protein\_coding | | ENSDARG00000087133 | nlgn1.1 | 11 | 9823657 | 10001798 | protein\_coding | | ENSDARG00000077761 | nlgn4b | 9 | 55792844 | 55828632 | protein\_coding | | ENSDARG00000019233 | nln | 10 | 15450471 | 15489467 | protein\_coding | | ENSDARG00000055548 | npat | 15 | 25593025 | 25607327 | protein\_coding | | ENSDARG00000012871 | npepl1 | 6 | 49743511 | 49762223 | protein\_coding | | ENSDARG00000010712 | npffr1l3 | 10 | 516901 | 530983 | protein\_coding | | ENSDARG00000009046 | nphp1 | 17 | 22052501 | 22071305 | protein\_coding | | ENSDARG00000069014 | nphp4 | 8 | 21956658 | 22253010 | protein\_coding | | ENSDARG00000076391 | npm2a | 8 | 37716829 | 37722476 | protein\_coding | | ENSDARG00000056089 | nrbp1 | 20 | 26103444 | 26125834 | protein\_coding | | ENSDARG00000077818 | nrg2a | 21 | 28517347 | 28700626 | protein\_coding | | ENSDARG00000099315 | nsdhl | 14 | 14341154 | 14353564 | protein\_coding | | ENSDARG00000021324 | nsun4 | 2 | 10362664 | 10371613 | protein\_coding | | ENSDARG00000103478 | nsun6 | 7 | 71585169 | 71621170 | protein\_coding | | ENSDARG00000062949 | nt5dc3 | 25 | 18830238 | 18850865 | protein\_coding | | ENSDARG00000036158 | nudcd1 | 19 | 23174793 | 23259157 | protein\_coding | | ENSDARG00000062335 | nudt14 | 20 | 21369122 | 21442698 | protein\_coding | | ENSDARG00000008014 | nudt17 | 16 | 43097070 | 43107743 | protein\_coding | | ENSDARG00000016256 | nudt3a | 23 | 3768043 | 3778541 | protein\_coding | | ENSDARG00000103523 | nudt6 | 14 | 533602 | 542518 | protein\_coding | | ENSDARG00000007722 | nudt8 | 15 | 539034 | 545207 | protein\_coding | | ENSDARG00000069066 | nufip2 | 15 | 30246230 | 30257039 | protein\_coding | | ENSDARG00000027279 | numb | 17 | 51732871 | 51804088 | protein\_coding | | ENSDARG00000061690 | nxpe3 | 9 | 34420882 | 34434782 | protein\_coding | | ENSDARG00000057426 | oard1 | 23 | 19294038 | 19299366 | protein\_coding | | ENSDARG00000024815 | ogfrl2 | 11 | 19928913 | 19935436 | protein\_coding | | ENSDARG00000061761 | opa3 | 18 | 35857744 | 35861546 | protein\_coding | | ENSDARG00000011515 | orai1a | 8 | 40236764 | 40248638 | protein\_coding | | ENSDARG00000025555 | ormdl3 | 12 | 22109193 | 22116785 | protein\_coding | | ENSDARG00000004634 | osbp | 1 | 52027465 | 52064523 | protein\_coding | | ENSDARG00000076328 | osbpl11 | 9 | 38715086 | 38746533 | protein\_coding | | ENSDARG00000033251 | osbpl3a | 19 | 20404546 | 20448724 | protein\_coding | | ENSDARG00000076002 | osbpl5 | 25 | 23261376 | 23353414 | protein\_coding | | ENSDARG00000012981 | osbpl7 | 12 | 28658687 | 28680055 | protein\_coding | | ENSDARG00000022648 | otud3 | 23 | 39718777 | 39733770 | protein\_coding | | ENSDARG00000017220 | otud7b | 16 | 45964246 | 46015941 | protein\_coding | | ENSDARG00000044752 | p2rx4a | 21 | 15627092 | 15636424 | protein\_coding | | ENSDARG00000105116 | p4hb | 6 | 21732143 | 21757077 | protein\_coding | | ENSDARG00000087996 | pabpn1l | 7 | 55335968 | 55347048 | protein\_coding | | ENSDARG00000032013 | pafah1b1a | 15 | 25337920 | 25384398 | protein\_coding | | ENSDARG00000018110 | pak4 | 15 | 25516827 | 25550745 | protein\_coding | | ENSDARG00000074675 | pan2 | 23 | 32053071 | 32081287 | protein\_coding | | ENSDARG00000098453 | pank4 | 11 | 15743708 | 15781153 | protein\_coding | | ENSDARG00000012141 | papolg | 13 | 25693895 | 25711624 | protein\_coding | | ENSDARG00000059858 | pappab | 5 | 63232111 | 63422813 | protein\_coding | | ENSDARG00000041145 | paqr5a | 18 | 20470822 | 20476995 | protein\_coding | | ENSDARG00000044672 | parapinopsinb | 11 | 36515630 | 36526814 | protein\_coding | | ENSDARG00000101719 | pargl | 10 | 45358795 | 45383265 | protein\_coding | | ENSDARG00000102634 | parn | 1 | 58608230 | 58618094 | protein\_coding | | ENSDARG00000042496 | parp12a | 18 | 12870408 | 12889298 | protein\_coding | | ENSDARG00000044404 | pars2 | 6 | 30697782 | 30702212 | protein\_coding | | ENSDARG00000076584 | patz1 | 6 | 40953691 | 40970784 | protein\_coding | | ENSDARG00000052388 | pcgf5b | 12 | 16356788 | 16386340 | protein\_coding | | ENSDARG00000019501 | pcnxl3 | 21 | 27539063 | 27583006 | protein\_coding | | ENSDARG00000069003 | pcyox1l | 21 | 41587918 | 41611487 | protein\_coding | | ENSDARG00000004492 | pcyt1ab | 18 | 46011156 | 46022120 | protein\_coding | | ENSDARG00000041022 | pdcd4b | 22 | 29709788 | 29740786 | protein\_coding | | ENSDARG00000038976 | pdcd7 | 7 | 13400637 | 13416937 | protein\_coding | | ENSDARG00000030964 | pde12 | 11 | 42215490 | 42234888 | protein\_coding | | ENSDARG00000018491 | pdia4 | 24 | 17190259 | 17197039 | protein\_coding | | ENSDARG00000056509 | pdik1l | 19 | 15581194 | 15613029 | protein\_coding | | ENSDARG00000076308 | pdp1 | 16 | 26943311 | 26947192 | protein\_coding | | ENSDARG00000058571 | pdp2 | 18 | 7493874 | 7497443 | protein\_coding | | ENSDARG00000012563 | pdss2 | 13 | 32530094 | 32595728 | protein\_coding | | ENSDARG00000101886 | pdxdc1 | 3 | 36474855 | 36502918 | protein\_coding | | ENSDARG00000079756 | peak1 | 25 | 7172760 | 7253783 | protein\_coding | | ENSDARG00000091592 | peli1a | 1 | 54370677 | 54391290 | protein\_coding | | ENSDARG00000044083 | peli1b | 17 | 24461479 | 24502981 | protein\_coding | | ENSDARG00000098904 | pex1 | 19 | 34263585 | 34289125 | protein\_coding | | ENSDARG00000041511 | pex10 | 8 | 47329338 | 47339289 | protein\_coding | | ENSDARG00000060707 | pex11a | 18 | 25559695 | 25564428 | protein\_coding | | ENSDARG00000069147 | pex11b | 19 | 24484000 | 24490874 | protein\_coding | | ENSDARG00000035149 | pex12 | 5 | 60898934 | 60904277 | protein\_coding | | ENSDARG00000071037 | pex13 | 17 | 23908395 | 23917049 | protein\_coding | | ENSDARG00000062421 | pex2 | 24 | 23057371 | 23069084 | protein\_coding | | ENSDARG00000070654 | pex5 | 16 | 12173674 | 12206539 | protein\_coding | | ENSDARG00000062465 | pgap1 | 9 | 24315242 | 24334979 | protein\_coding | | ENSDARG00000014704 | pgm2l1 | 15 | 5125032 | 5157646 | protein\_coding | | ENSDARG00000056695 | phc2a | 23 | 22136932 | 22204265 | protein\_coding | | ENSDARG00000074604 | phc3 | 2 | 37441804 | 37467477 | protein\_coding | | ENSDARG00000075509 | phf12b | 15 | 24613789 | 24627777 | protein\_coding | | ENSDARG00000061458 | phf14 | 19 | 38580875 | 38740613 | protein\_coding | | ENSDARG00000030887 | phf23a | 5 | 23736518 | 23741418 | protein\_coding | | ENSDARG00000036305 | phf23b | 7 | 26345049 | 26356060 | protein\_coding | | ENSDARG00000004046 | phf6 | 14 | 31148265 | 31167430 | protein\_coding | | ENSDARG00000074843 | phldb2b | 24 | 24948125 | 25021546 | protein\_coding | | ENSDARG00000045378 | pi15a | 24 | 14657425 | 14666215 | protein\_coding | | ENSDARG00000076724 | pi4kaa | 10 | 3185947 | 3260848 | protein\_coding | | ENSDARG00000062445 | pias1b | 18 | 20001339 | 20039045 | protein\_coding | | ENSDARG00000013006 | pibf1 | 1 | 33882690 | 33952784 | protein\_coding | | ENSDARG00000012866 | picalma | 10 | 29251323 | 29338540 | protein\_coding | | ENSDARG00000098984 | pid1 | 18 | 46636360 | 46686334 | protein\_coding | | ENSDARG00000041040 | piga | 9 | 30564143 | 30574313 | protein\_coding | | ENSDARG00000011743 | pigo | 10 | 17790446 | 17802444 | protein\_coding | | ENSDARG00000017813 | pigs | 21 | 39537136 | 39581058 | protein\_coding | | ENSDARG00000078285 | pik3ap1 | 13 | 22963293 | 22998528 | protein\_coding | | ENSDARG00000060841 | pik3c2a | 18 | 27018731 | 27090761 | protein\_coding | | ENSDARG00000003250 | pik3cd | 23 | 29830058 | 29886287 | protein\_coding | | ENSDARG00000060469 | pik3r4 | 16 | 36767346 | 36794550 | protein\_coding | | ENSDARG00000102762 | pik3r5 | 6 | 23462442 | 23506668 | protein\_coding | | ENSDARG00000055129 | pim3 | 25 | 29219404 | 29223895 | protein\_coding | | ENSDARG00000001929 | pink1 | 23 | 37364148 | 37376349 | protein\_coding | | ENSDARG00000098600 | pithd1 | 19 | 44282201 | 44285110 | protein\_coding | | ENSDARG00000062601 | piwil2 | 5 | 15927850 | 15971391 | protein\_coding | | ENSDARG00000100296 | pja2 | 5 | 56540020 | 56563901 | protein\_coding | | ENSDARG00000086471 | pkig | 23 | 25952779 | 26016318 | protein\_coding | | ENSDARG00000062748 | pkn1b | 3 | 5868579 | 5919580 | protein\_coding | | ENSDARG00000101983 | pkn2 | 2 | 22761235 | 22824459 | protein\_coding | | ENSDARG00000079585 | pkn3 | 5 | 31230323 | 31257192 | protein\_coding | | ENSDARG00000070953 | pla2g4f.2 | 17 | 21458056 | 21474459 | protein\_coding | | ENSDARG00000056601 | plekha3 | 11 | 29509686 | 29520880 | protein\_coding | | ENSDARG00000013928 | plekhb2 | 22 | 16406134 | 16416929 | protein\_coding | | ENSDARG00000021141 | plekhf2 | 16 | 40475841 | 40509072 | protein\_coding | | ENSDARG00000058459 | plekhg2 | 15 | 20345573 | 20414948 | protein\_coding | | ENSDARG00000040851 | plekhh2 | 13 | 8515398 | 8560491 | protein\_coding | | ENSDARG00000099954 | plekhm1 | 12 | 4885494 | 4928196 | protein\_coding | | ENSDARG00000030633 | plekhm3 | 9 | 28329439 | 28393622 | protein\_coding | | ENSDARG00000028896 | plpp2b | 22 | 21524441 | 21559692 | protein\_coding | | ENSDARG00000043527 | plpp6 | 20 | 15252219 | 15262800 | protein\_coding | | ENSDARG00000003811 | plxnb2a | 4 | 14450478 | 14532812 | protein\_coding | | ENSDARG00000000476 | pms1 | 9 | 41164422 | 41223275 | protein\_coding | | ENSDARG00000070798 | pmvk | 16 | 6996508 | 7004249 | protein\_coding | | ENSDARG00000062986 | pnpla7a | 21 | 13517511 | 13565556 | protein\_coding | | ENSDARG00000021110 | poc1b | 25 | 18270578 | 18339081 | protein\_coding | | ENSDARG00000092305 | poldip2 | 15 | 24741640 | 24754995 | protein\_coding | | ENSDARG00000070231 | polh | 13 | 683288 | 694273 | protein\_coding | | ENSDARG00000103470 | polm | 10 | 45150418 | 45182439 | protein\_coding | | ENSDARG00000052025 | pomgnt1 | 6 | 33092149 | 33111076 | protein\_coding | | ENSDARG00000010941 | pomgnt2 | 16 | 8225786 | 8248651 | protein\_coding | | ENSDARG00000067670 | pomt1 | 5 | 50464750 | 50481438 | protein\_coding | | ENSDARG00000055027 | pomt2 | 17 | 31679694 | 31695078 | protein\_coding | | ENSDARG00000020191 | ppef1 | 11 | 30006784 | 30033774 | protein\_coding | | ENSDARG00000029168 | ppfibp2b | 25 | 15842875 | 15944685 | protein\_coding | | ENSDARG00000002016 | ppil2 | 5 | 12408564 | 12479769 | protein\_coding | | ENSDARG00000021380 | ppm1db | 5 | 56244674 | 56254530 | protein\_coding | | ENSDARG00000005786 | ppm1f | 5 | 12680812 | 12706661 | protein\_coding | | ENSDARG00000063218 | ppm1la | 2 | 6353886 | 6380628 | protein\_coding | | ENSDARG00000102167 | ppox | 16 | 42339044 | 42402839 | protein\_coding | | ENSDARG00000010784 | ppp1r12a | 4 | 21768015 | 21863183 | protein\_coding | | ENSDARG00000009142 | ppp1r13bb | 20 | 20831379 | 20913953 | protein\_coding | | ENSDARG00000068128 | ppp1r15b | 18 | 27095475 | 27103450 | protein\_coding | | ENSDARG00000076980 | ppp1r16a | 12 | 13704511 | 13751670 | protein\_coding | | ENSDARG00000068916 | ppp1r35 | 20 | 54802141 | 54812753 | protein\_coding | | ENSDARG00000078458 | ppp1r37 | 18 | 48869580 | 48941214 | protein\_coding | | ENSDARG00000044691 | ppp1r3b | 21 | 19879820 | 19883298 | protein\_coding | | ENSDARG00000077513 | ppp1r3da | 23 | 12225930 | 12227784 | protein\_coding | | ENSDARG00000057632 | ppp1r42 | 24 | 23584887 | 23595725 | protein\_coding | | ENSDARG00000014428 | ppp2r5d | 13 | 4104072 | 4175145 | protein\_coding | | ENSDARG00000070570 | ppp4ca | 3 | 26105408 | 26113385 | protein\_coding | | ENSDARG00000026540 | ppp4r2a | 23 | 10870218 | 10892449 | protein\_coding | | ENSDARG00000010407 | ppp4r4 | 20 | 27431670 | 27473891 | protein\_coding | | ENSDARG00000045540 | ppp6r2a | 4 | 20093499 | 20109782 | protein\_coding | | ENSDARG00000043624 | pqlc2 | 8 | 10899818 | 10915138 | protein\_coding | | ENSDARG00000104251 | prdm10 | 18 | 44327466 | 44366272 | protein\_coding | | ENSDARG00000078161 | prdm1c | 20 | 48631729 | 48657296 | protein\_coding | | ENSDARG00000005382 | prdm9 | 12 | 9743681 | 9758394 | protein\_coding | | ENSDARG00000009505 | prelid3b | 6 | 60117277 | 60127158 | protein\_coding | | ENSDARG00000075793 | prex1 | 8 | 25404869 | 25550781 | protein\_coding | | ENSDARG00000020982 | prickle2a | 8 | 53674394 | 53697273 | protein\_coding | | ENSDARG00000060596 | prkaa1 | 5 | 32638392 | 32655606 | protein\_coding | | ENSDARG00000009208 | prkcda | 6 | 40525834 | 40546671 | protein\_coding | | ENSDARG00000018382 | prkcha | 13 | 31586370 | 31618520 | protein\_coding | | ENSDARG00000079967 | prkd3 | 17 | 42335576 | 42523698 | protein\_coding | | ENSDARG00000054741 | prkg2 | 5 | 39079614 | 39098071 | protein\_coding | | ENSDARG00000020964 | prkrirb | 15 | 19964465 | 19973177 | protein\_coding | | ENSDARG00000069706 | prmt6 | 24 | 29278178 | 29279660 | protein\_coding | | ENSDARG00000036755 | prmt9 | 1 | 36014013 | 36040120 | protein\_coding | | ENSDARG00000019326 | prpsap2 | 3 | 39458704 | 39471368 | protein\_coding | | ENSDARG00000076246 | prune | 16 | 38289995 | 38316853 | protein\_coding | | ENSDARG00000102114 | ptdss1b | 19 | 46476269 | 46500877 | protein\_coding | | ENSDARG00000056623 | ptenb | 12 | 17277978 | 17302300 | protein\_coding | | ENSDARG00000068415 | ptgesl | 8 | 30999055 | 31004432 | protein\_coding | | ENSDARG00000039997 | ptp4a3 | 19 | 25504987 | 25564811 | protein\_coding | | ENSDARG00000062248 | ptpn21 | 17 | 2430311 | 2513697 | protein\_coding | | ENSDARG00000098689 | ptpn23b | 19 | 19668419 | 19697802 | protein\_coding | | ENSDARG00000035986 | ptpn2b | 19 | 12443259 | 12473291 | protein\_coding | | ENSDARG00000000631 | ptpn4a | 9 | 29129939 | 29219036 | protein\_coding | | ENSDARG00000000183 | ptpn4b | 22 | 11750166 | 11803650 | protein\_coding | | ENSDARG00000077495 | ptpn9a | 25 | 6276434 | 6304724 | protein\_coding | | ENSDARG00000073902 | ptpn9b | 1 | 18401611 | 18419997 | protein\_coding | | ENSDARG00000028976 | pus3 | 24 | 36429472 | 36441728 | protein\_coding | | ENSDARG00000069780 | pus7l | 4 | 14177585 | 14193405 | protein\_coding | | ENSDARG00000006604 | pvrl3b | 21 | 21412360 | 21478830 | protein\_coding | | ENSDARG00000103148 | pvrl4 | 2 | 57911535 | 57945244 | protein\_coding | | ENSDARG00000003144 | pxmp2 | 5 | 19611974 | 19619202 | protein\_coding | | ENSDARG00000098639 | pycr1b | 11 | 44909333 | 45103774 | protein\_coding | | ENSDARG00000098687 | pygo1 | 18 | 2867437 | 2874719 | protein\_coding | | ENSDARG00000039459 | qsox1 | 8 | 14465905 | 14517080 | protein\_coding | | ENSDARG00000101432 | rab11fip2 | 13 | 19479381 | 19511257 | protein\_coding | | ENSDARG00000103207 | rab11fip4b | 6 | 21947330 | 21968869 | protein\_coding | | ENSDARG00000104179 | rab11fip5a | 13 | 14960272 | 15011949 | protein\_coding | | ENSDARG00000004151 | rab23 | 13 | 1251662 | 1264711 | protein\_coding | | ENSDARG00000054032 | rab24 | 21 | 38685239 | 38692022 | protein\_coding | | ENSDARG00000029036 | rab32a | 20 | 32106392 | 32142303 | protein\_coding | | ENSDARG00000058425 | rab35b | 8 | 3590287 | 3616166 | protein\_coding | | ENSDARG00000044136 | rab3gap2 | 17 | 25591435 | 25612457 | protein\_coding | | ENSDARG00000061208 | rab40c | 3 | 26620990 | 26656559 | protein\_coding | | ENSDARG00000101170 | rab42b | 16 | 35448668 | 35453012 | protein\_coding | | ENSDARG00000016059 | rab5b | 9 | 27939771 | 27965887 | protein\_coding | | ENSDARG00000067920 | rab8a | 2 | 55539492 | 55565208 | protein\_coding | | ENSDARG00000059600 | rabep1 | 5 | 58360470 | 58409978 | protein\_coding | | ENSDARG00000061213 | rabep2 | 3 | 26026202 | 26040797 | protein\_coding | | ENSDARG00000014430 | rabgef1 | 10 | 32913056 | 32936440 | protein\_coding | | ENSDARG00000068587 | rabggtb | 2 | 26807322 | 26813459 | protein\_coding | | ENSDARG00000027938 | rad18 | 6 | 41811137 | 41889862 | protein\_coding | | ENSDARG00000022493 | rad21l1 | 8 | 49128737 | 49151876 | protein\_coding | | ENSDARG00000068919 | rad51c | 10 | 32719320 | 32728648 | protein\_coding | | ENSDARG00000062138 | ranbp10 | 18 | 21962485 | 22031443 | protein\_coding | | ENSDARG00000008727 | ranbp3a | 11 | 6298115 | 6319463 | protein\_coding | | ENSDARG00000043593 | rapgef1a | 8 | 11741842 | 11806637 | protein\_coding | | ENSDARG00000015971 | rapgef1b | 21 | 3951798 | 4059004 | protein\_coding | | ENSDARG00000074307 | rapgef5b | 16 | 19223658 | 19346161 | protein\_coding | | ENSDARG00000035535 | rasa1a | 5 | 47211125 | 47317603 | protein\_coding | | ENSDARG00000036257 | rasal2 | 2 | 35750371 | 35869204 | protein\_coding | | ENSDARG00000059064 | rassf3 | 4 | 4529260 | 4590682 | protein\_coding | | ENSDARG00000074721 | rassf9 | 18 | 16325326 | 16337792 | protein\_coding | | ENSDARG00000043480 | rbbp8 | 24 | 36315956 | 36335871 | protein\_coding | | ENSDARG00000073705 | rbfa | 16 | 25445401 | 25453025 | protein\_coding | | ENSDARG00000060521 | rbm27 | 9 | 54736805 | 54780270 | protein\_coding | | ENSDARG00000079452 | rbm46 | 1 | 9443052 | 9453341 | protein\_coding | | ENSDARG00000020841 | rbm7 | 18 | 47305694 | 47311455 | protein\_coding | | ENSDARG00000003398 | rbpja | 1 | 13768150 | 13777507 | protein\_coding | | ENSDARG00000052091 | rbpjb | 7 | 61770294 | 61896771 | protein\_coding | | ENSDARG00000079578 | rbpms2b | 7 | 47979862 | 47990857 | protein\_coding | | ENSDARG00000090687 | rcbtb2 | 15 | 3136843 | 3171822 | protein\_coding | | ENSDARG00000077909 | rce1a | 7 | 17923980 | 17935449 | protein\_coding | | ENSDARG00000031434 | rcor1 | 17 | 29234699 | 29254396 | protein\_coding | | ENSDARG00000002467 | rdh14b | 20 | 9433709 | 9440282 | protein\_coding | | ENSDARG00000059556 | recql5 | 3 | 59695019 | 59736207 | protein\_coding | | ENSDARG00000086173 | relb | 15 | 17164390 | 17189195 | protein\_coding | | ENSDARG00000018312 | rell1 | 7 | 60920495 | 60969616 | protein\_coding | | ENSDARG00000002877 | reps1 | 20 | 39320173 | 39357001 | protein\_coding | | ENSDARG00000034989 | retsatl | 9 | 45193262 | 45197770 | protein\_coding | | ENSDARG00000006545 | rgp1 | 7 | 24360357 | 24374383 | protein\_coding | | ENSDARG00000021521 | rgs14a | 14 | 853381 | 880928 | protein\_coding | | ENSDARG00000079816 | rhbdd3 | 5 | 24378833 | 24382797 | protein\_coding | | ENSDARG00000016868 | rhobtb2a | 5 | 42857792 | 42902590 | protein\_coding | | ENSDARG00000028401 | rhogc | 5 | 35994996 | 35997483 | protein\_coding | | ENSDARG00000025953 | rhoq | 12 | 25018268 | 25058919 | protein\_coding | | ENSDARG00000100867 | rictora | 5 | 8029291 | 8109579 | protein\_coding | | ENSDARG00000016399 | riok1 | 2 | 21405598 | 21418508 | protein\_coding | | ENSDARG00000104290 | ripk2 | 2 | 7739609 | 7772985 | protein\_coding | | ENSDARG00000063553 | rlf | 17 | 24571776 | 24584111 | protein\_coding | | ENSDARG00000078802 | rnf111 | 7 | 30290443 | 30353324 | protein\_coding | | ENSDARG00000040635 | rnf11b | 8 | 16389010 | 16428741 | protein\_coding | | ENSDARG00000041802 | rnf141 | 18 | 16760025 | 16767078 | protein\_coding | | ENSDARG00000089981 | rnf146 | 23 | 40633081 | 40643185 | protein\_coding | | ENSDARG00000009524 | rnf150b | 23 | 43968631 | 43985629 | protein\_coding | | ENSDARG00000087782 | rnf152 | 2 | 28214104 | 28215418 | protein\_coding | | ENSDARG00000017636 | rnf167 | 5 | 67358554 | 67378503 | protein\_coding | | ENSDARG00000105172 | rnf168 | 15 | 3902595 | 3921710 | protein\_coding | | ENSDARG00000056387 | rnf17 | 9 | 29737884 | 29760485 | protein\_coding | | ENSDARG00000090319 | rnf212 | 14 | 17091882 | 17111063 | protein\_coding | | ENSDARG00000087893 | rnf216 | 3 | 40463261 | 40523010 | protein\_coding | | ENSDARG00000060944 | rnf217 | 20 | 39846813 | 39886404 | protein\_coding | | ENSDARG00000074074 | rnf26 | 15 | 23421335 | 23423542 | protein\_coding | | ENSDARG00000062055 | rnf38 | 1 | 18170123 | 18240734 | protein\_coding | | ENSDARG00000069082 | rnmtl1b | 21 | 39649608 | 39661211 | protein\_coding | | ENSDARG00000058993 | rock1 | 2 | 2976734 | 3059937 | protein\_coding | | ENSDARG00000015176 | ror1 | 6 | 31785133 | 32001026 | protein\_coding | | ENSDARG00000044339 | rp2 | 6 | 37536705 | 37547985 | protein\_coding | | ENSDARG00000051754 | rpgrip1l | 25 | 35540133 | 35577507 | protein\_coding | | ENSDARG00000070131 | rpp25l | 16 | 41582584 | 41585681 | protein\_coding | | ENSDARG00000027428 | rpp30 | 12 | 16397418 | 16403390 | protein\_coding | | ENSDARG00000035556 | rps6ka3a | 5 | 23505868 | 23525437 | protein\_coding | | ENSDARG00000060192 | rpusd1 | 3 | 35412559 | 35419970 | protein\_coding | | ENSDARG00000069829 | rragca | 16 | 33209828 | 33225089 | protein\_coding | | ENSDARG00000099049 | rragcb | 19 | 4196079 | 4206261 | protein\_coding | | ENSDARG00000063701 | rreb1a | 24 | 2350529 | 2454270 | protein\_coding | | ENSDARG00000089033 | rrnad1 | 16 | 29442128 | 29450312 | protein\_coding | | ENSDARG00000102589 | rsf1a | 18 | 2967706 | 2989096 | protein\_coding | | ENSDARG00000052903 | rtkn2b | 21 | 11163797 | 11193855 | protein\_coding | | ENSDARG00000098261 | rtkn2b.1 | 21 | 11218425 | 11235248 | protein\_coding | | ENSDARG00000078752 | rubcn | 22 | 35299457 | 35337996 | protein\_coding | | ENSDARG00000078685 | rufy1 | 14 | 6710207 | 6738282 | protein\_coding | | ENSDARG00000009961 | rundc3b | 16 | 43174151 | 43214337 | protein\_coding | | ENSDARG00000032820 | rxfp2a | 10 | 33790782 | 33923566 | protein\_coding | | ENSDARG00000037773 | rybpa | 23 | 10827714 | 10851430 | protein\_coding | | ENSDARG00000062977 | samd13 | 11 | 8143033 | 8151310 | protein\_coding | | ENSDARG00000056924 | sap130a | 6 | 28027829 | 28059256 | protein\_coding | | ENSDARG00000070452 | saraf | 1 | 11362429 | 11371639 | protein\_coding | | ENSDARG00000007179 | sash1a | 20 | 31525940 | 31840938 | protein\_coding | | ENSDARG00000062968 | sbf1 | 4 | 9910595 | 9999080 | protein\_coding | | ENSDARG00000060865 | scai | 5 | 31395530 | 31446144 | protein\_coding | | ENSDARG00000101557 | scarb1 | 10 | 44853122 | 44899915 | protein\_coding | | ENSDARG00000086819 | scn1a | 9 | 50208646 | 50304215 | protein\_coding | | ENSDARG00000003027 | scnm1 | 16 | 29750934 | 29755589 | protein\_coding | | ENSDARG00000079854 | scyl1 | 14 | 46587743 | 46616649 | protein\_coding | | ENSDARG00000024026 | sdf2 | 21 | 25984801 | 25991510 | protein\_coding | | ENSDARG00000061413 | sec23ip | 13 | 25418974 | 25435858 | protein\_coding | | ENSDARG00000071906 | sec24b | 14 | 1106160 | 1142311 | protein\_coding | | ENSDARG00000002916 | sec31b | 12 | 48435280 | 48484427 | protein\_coding | | ENSDARG00000073892 | secisbp2 | 5 | 65568866 | 65592370 | protein\_coding | | ENSDARG00000004581 | sel1l | 20 | 27088326 | 27104208 | protein\_coding | | ENSDARG00000036571 | sema3e | 18 | 9524530 | 9607588 | protein\_coding | | ENSDARG00000062352 | sema4ab | 19 | 24741296 | 24807944 | protein\_coding | | ENSDARG00000073837 | senp2 | 6 | 11161842 | 11180208 | protein\_coding | | ENSDARG00000079992 | senp6b | 17 | 36902137 | 36913403 | protein\_coding | | ENSDARG00000087421 | serbp1b | 2 | 2788236 | 2800194 | protein\_coding | | ENSDARG00000053425 | serinc2l | 16 | 34505036 | 34523775 | protein\_coding | | ENSDARG00000062244 | setd2 | 16 | 16345733 | 16395474 | protein\_coding | | ENSDARG00000036592 | setd4 | 1 | 46415036 | 46423087 | protein\_coding | | ENSDARG00000022996 | setx | 21 | 3764328 | 3808546 | protein\_coding | | ENSDARG00000062460 | sgk3 | 24 | 23613336 | 23639527 | protein\_coding | | ENSDARG00000019038 | sgsm3 | 3 | 33329361 | 33362449 | protein\_coding | | ENSDARG00000069958 | sh2b3 | 5 | 9050688 | 9120857 | protein\_coding | | ENSDARG00000067958 | sh3gl1a | 2 | 49723396 | 49777228 | protein\_coding | | ENSDARG00000007302 | sh3gl3b | 25 | 19425003 | 19438380 | protein\_coding | | ENSDARG00000008983 | sh3glb2a | 8 | 2699273 | 2731240 | protein\_coding | | ENSDARG00000070470 | sh3rf1 | 20 | 23843474 | 23937867 | protein\_coding | | ENSDARG00000040853 | shoc2 | 22 | 29647451 | 29690986 | protein\_coding | | ENSDARG00000094181 | shroom1 | 21 | 43258377 | 43315507 | protein\_coding | | ENSDARG00000095081 | si:ch1073-111a5.3 | 10 | 8660149 | 8662573 | lincRNA | | ENSDARG00000087674 | si:ch1073-127d16.1 | 19 | 6985389 | 6989757 | protein\_coding | | ENSDARG00000095640 | si:ch1073-15f19.2 | 10 | 24808480 | 24825245 | protein\_coding | | ENSDARG00000089107 | si:ch1073-189o9.1 | 5 | 3494115 | 3516117 | protein\_coding | | ENSDARG00000104485 | si:ch1073-224n8.1 | 5 | 36904156 | 36910733 | protein\_coding | | ENSDARG00000093773 | si:ch1073-296i8.2 | 25 | 2921276 | 2932834 | protein\_coding | | ENSDARG00000055934 | si:ch1073-416j23.1 | 9 | 10810977 | 10833593 | protein\_coding | | ENSDARG00000097692 | si:ch211-116o3.5 | 15 | 14149230 | 14162565 | protein\_coding | | ENSDARG00000099779 | si:ch211-11n16.2 | 15 | 14442872 | 14484607 | protein\_coding | | ENSDARG00000100646 | si:ch211-127l15.7 | 14 | 31564921 | 31717382 | antisense | | ENSDARG00000104860 | si:ch211-128m15.3 | 13 | 3743819 | 3748727 | protein\_coding | | ENSDARG00000055250 | si:ch211-132b12.8 | 18 | 40708358 | 40718345 | protein\_coding | | ENSDARG00000095920 | si:ch211-132p20.5 | 4 | 1727576 | 1743814 | lincRNA | | ENSDARG00000027676 | si:ch211-133l5.8 | 8 | 20829273 | 20830358 | protein\_coding | | ENSDARG00000098940 | si:ch211-134o20.4 | 7 | 38044406 | 38065155 | antisense | | ENSDARG00000068926 | si:ch211-137a8.2 | 15 | 29092019 | 29138994 | protein\_coding | | ENSDARG00000042063 | si:ch211-145c1.1 | 9 | 8311533 | 8335698 | protein\_coding | | ENSDARG00000069009 | si:ch211-147a11.3 | 8 | 22262325 | 22267022 | protein\_coding | | ENSDARG00000091941 | si:ch211-147m20.1 | 18 | 24490994 | 24496753 | transcribed\_unprocessed\_pseudogene | | ENSDARG00000094469 | si:ch211-148l7.4 | 23 | 34007697 | 34026575 | protein\_coding | | ENSDARG00000054313 | si:ch211-14a17.6 | 2 | 38303086 | 38305696 | protein\_coding | | ENSDARG00000061221 | si:ch211-157j23.2 | 24 | 5771294 | 5780374 | protein\_coding | | ENSDARG00000095333 | si:ch211-157j23.3.1 | 24 | 5782479 | 5789236 | protein\_coding | | ENSDARG00000096774 | si:ch211-157l15.4 | 20 | 37592278 | 37702246 | antisense | | ENSDARG00000020611 | si:ch211-160d20.3 | 18 | 36801922 | 36819033 | protein\_coding | | ENSDARG00000079290 | si:ch211-162k9.5 | 15 | 32529084 | 32535245 | protein\_coding | | ENSDARG00000097877 | si:ch211-167b20.8 | 8 | 25714733 | 25729713 | protein\_coding | | ENSDARG00000094509 | si:ch211-16k18.1 | 2 | 39961322 | 40060983 | processed\_transcript | | ENSDARG00000077288 | si:ch211-188c16.1 | 2 | 2357554 | 2373654 | protein\_coding | | ENSDARG00000095566 | si:ch211-188m17.3 | 1 | 40158903 | 40198970 | processed\_transcript | | ENSDARG00000086679 | si:ch211-197l9.5 | 23 | 31114527 | 31133819 | protein\_coding | | ENSDARG00000093192 | si:ch211-198m1.1 | 3 | 18786266 | 18817913 | protein\_coding | | ENSDARG00000029170 | si:ch211-200p22.4 | 7 | 23485534 | 23577053 | protein\_coding | | ENSDARG00000099986 | si:ch211-207i20.2 | 12 | 9445032 | 9460217 | protein\_coding | | ENSDARG00000094075 | si:ch211-208c9.1 | 20 | 39699221 | 39783434 | processed\_transcript | | ENSDARG00000092856 | si:ch211-210c8.7 | 23 | 33804661 | 33812414 | protein\_coding | | ENSDARG00000058254 | si:ch211-213d14.1 | 15 | 17792158 | 17812297 | protein\_coding | | ENSDARG00000092556 | si:ch211-214j8.12 | 5 | 25531629 | 25536570 | protein\_coding | | ENSDARG00000056165 | si:ch211-215a10.4 | 19 | 30862812 | 30880929 | protein\_coding | | ENSDARG00000103484 | si:ch211-215k15.5 | 16 | 42567697 | 42569229 | protein\_coding | | ENSDARG00000096962 | si:ch211-225o7.1 | 5 | 37650835 | 37655591 | processed\_transcript | | ENSDARG00000070178 | si:ch211-225p5.3 | 16 | 42098330 | 42106105 | protein\_coding | | ENSDARG00000101271 | si:ch211-227n13.3 | 11 | 33598375 | 33606106 | protein\_coding | | ENSDARG00000100586 | si:ch211-233e16.4 | 16 | 40571706 | 40634018 | antisense | | ENSDARG00000097665 | si:ch211-235m3.10 | 15 | 2635574 | 2647598 | protein\_coding | | ENSDARG00000008906 | si:ch211-241e1.5 | 2 | 1065544 | 1072160 | protein\_coding | | ENSDARG00000100015 | si:ch211-248k15.2 | 4 | 75739446 | 75744332 | protein\_coding | | ENSDARG00000078457 | si:ch211-250e5.16 | 22 | 25734129 | 25735394 | protein\_coding | | ENSDARG00000097345 | si:ch211-250m6.10 | 4 | 76205358 | 76301005 | antisense | | ENSDARG00000103477 | si:ch211-255f4.5 | 22 | 1500420 | 1507777 | protein\_coding | | ENSDARG00000030945 | si:ch211-259g3.4 | 15 | 16187125 | 16241581 | protein\_coding | | ENSDARG00000104641 | si:ch211-261d7.6 | 16 | 25201795 | 25209919 | protein\_coding | | ENSDARG00000099896 | si:ch211-261o3.3 | 7 | 67506796 | 67508534 | lincRNA | | ENSDARG00000092965 | si:ch211-266i10.1 | 24 | 32295135 | 32338889 | processed\_transcript | | ENSDARG00000104955 | si:ch211-271c18.3 | 7 | 35163781 | 35332489 | antisense | | ENSDARG00000010524 | si:ch211-282j22.3 | 21 | 13665680 | 13688130 | protein\_coding | | ENSDARG00000098768 | si:ch211-284a16.4 | 20 | 49087295 | 49153203 | lincRNA | | ENSDARG00000094725 | si:ch211-284e13.5 | 5 | 37496576 | 37502078 | protein\_coding | | ENSDARG00000097707 | si:ch211-285i13.1 | 2 | 32883369 | 33042827 | lincRNA | | ENSDARG00000092912 | si:ch211-3o3.7 | 9 | 41141332 | 41151786 | processed\_transcript | | ENSDARG00000099086 | si:ch211-69b22.4 | 14 | 31734227 | 31807887 | antisense | | ENSDARG00000030129 | si:ch211-87m7.2 | 8 | 532918 | 556463 | protein\_coding | | ENSDARG00000093040 | si:ch211-89b14.1 | 9 | 11851176 | 11884102 | processed\_transcript | | ENSDARG00000094290 | si:ch211-8o18.1 | 5 | 59095531 | 59106890 | processed\_transcript | | ENSDARG00000089227 | si:ch211-91p5.3 | 25 | 27185804 | 27222776 | protein\_coding | | ENSDARG00000096530 | si:ch211-93g23.3 | 1 | 5967046 | 5970181 | lincRNA | | ENSDARG00000103871 | si:ch73-109d9.2 | 1 | 43339047 | 43347634 | protein\_coding | | ENSDARG00000097902 | si:ch73-204p21.2 | 17 | 16038199 | 16076645 | protein\_coding | | ENSDARG00000095974 | si:ch73-211l13.2 | 3 | 1364259 | 1377848 | protein\_coding | | ENSDARG00000104904 | si:ch73-221f6.1 | 1 | 57865998 | 57870808 | protein\_coding | | ENSDARG00000000423 | si:ch73-314g15.3 | 7 | 32358524 | 32374050 | protein\_coding | | ENSDARG00000102462 | si:ch73-315i10.1 | 14 | 28261677 | 28354703 | antisense | | ENSDARG00000091640 | si:ch73-352p4.5 | 4 | 5823741 | 5826078 | protein\_coding | | ENSDARG00000089503 | si:ch73-367f21.5 | 19 | 6999210 | 7004765 | protein\_coding | | ENSDARG00000101358 | si:ch73-378g22.1 | 24 | 1631413 | 1661232 | protein\_coding | | ENSDARG00000093833 | si:ch73-382f3.1 | 2 | 27730983 | 27735924 | protein\_coding | | ENSDARG00000091909 | si:ch73-59c19.1 | 16 | 46383904 | 46426438 | protein\_coding | | ENSDARG00000095463 | si:ch73-90k17.1 | 1 | 44624268 | 44628331 | protein\_coding | | ENSDARG00000068846 | si:ch73-95l15.3 | 15 | 35075583 | 35080222 | protein\_coding | | ENSDARG00000098021 | si:dkey-111k8.2 | 3 | 61138846 | 61144539 | protein\_coding | | ENSDARG00000058719 | si:dkey-119f1.1 | 2 | 6409741 | 6545459 | protein\_coding | | ENSDARG00000096452 | si:dkey-11p10.11 | 24 | 24999159 | 25039370 | antisense | | ENSDARG00000094542 | si:dkey-121h17.7 | 21 | 5027451 | 5033741 | protein\_coding | | ENSDARG00000069328 | si:dkey-12e7.1 | 18 | 21112769 | 21116902 | protein\_coding | | ENSDARG00000097701 | si:dkey-12h9.17 | 20 | 26160620 | 26166854 | antisense | | ENSDARG00000093306 | si:dkey-149i8.3 | 23 | 28464566 | 28492966 | processed\_transcript | | ENSDARG00000097810 | si:dkey-151m6.6 | 2 | 28319918 | 28382989 | lincRNA | | ENSDARG00000105043 | si:dkey-152b24.6 | 16 | 46610102 | 46613265 | protein\_coding | | ENSDARG00000101668 | si:dkey-161l11.5 | 2 | 36110496 | 36111862 | processed\_transcript | | ENSDARG00000092980 | si:dkey-166k9.2 | 20 | 39145235 | 39149820 | lincRNA | | ENSDARG00000089862 | si:dkey-16j16.4 | 17 | 40956756 | 41000720 | protein\_coding | | ENSDARG00000098326 | si:dkey-172h23.1 | 7 | 14747562 | 14836088 | lincRNA | | ENSDARG00000052680 | si:dkey-182g1.2 | 22 | 8592836 | 8740803 | protein\_coding | | ENSDARG00000087424 | si:dkey-187j14.6 | 7 | 2406286 | 2477306 | protein\_coding | | ENSDARG00000103328 | si:dkey-188e21.1 | 8 | 21798053 | 21891717 | lincRNA | | ENSDARG00000102843 | si:dkey-204g14.5 | 5 | 68896091 | 68901105 | processed\_transcript | | ENSDARG00000096971 | si:dkey-206p8.1 | 17 | 46403834 | 46456000 | protein\_coding | | ENSDARG00000104170 | si:dkey-208k4.2 | 19 | 18847533 | 18850528 | protein\_coding | | ENSDARG00000022905 | si:dkey-20i20.12 | 22 | 2699549 | 2702647 | protein\_coding | | ENSDARG00000089951 | si:dkey-210j14.5 | 3 | 52421914 | 52428578 | protein\_coding | | ENSDARG00000096428 | si:dkey-217d24.6 | 6 | 8217859 | 8231210 | protein\_coding | | ENSDARG00000062199 | si:dkey-217l24.1 | 25 | 21919634 | 21998289 | protein\_coding | | ENSDARG00000075805 | si:dkey-219c3.2 | 25 | 20174491 | 20257829 | protein\_coding | | ENSDARG00000102926 | si:dkey-223p19.2 | 21 | 45460284 | 45467420 | protein\_coding | | ENSDARG00000093255 | si:dkey-229d11.3 | 21 | 43665048 | 43671244 | protein\_coding | | ENSDARG00000094392 | si:dkey-229d11.5 | 21 | 43650636 | 43654783 | protein\_coding | | ENSDARG00000097217 | si:dkey-229e3.2 | 17 | 39794341 | 39804007 | protein\_coding | | ENSDARG00000086259 | si:dkey-234l24.9 | 4 | 14107662 | 14109961 | unprocessed\_pseudogene | | ENSDARG00000023036 | si:dkey-23a23.2 | 4 | 29079208 | 29095592 | protein\_coding | | ENSDARG00000041248 | si:dkey-241l7.6 | 20 | 54223766 | 54245097 | protein\_coding | | ENSDARG00000034227 | si:dkey-243i1.1 | 15 | 26916033 | 26954152 | protein\_coding | | ENSDARG00000090013 | si:dkey-248g15.2 | 17 | 2302917 | 2359296 | protein\_coding | | ENSDARG00000092169 | si:dkey-250k15.4 | 16 | 11888632 | 11894384 | protein\_coding | | ENSDARG00000075974 | si:dkey-258f14.3 | 8 | 13605281 | 13709925 | protein\_coding | | ENSDARG00000096229 | si:dkey-260c8.8 | 3 | 37648493 | 37664425 | protein\_coding | | ENSDARG00000101942 | si:dkey-260j18.2 | 15 | 14694733 | 14706229 | protein\_coding | | ENSDARG00000097882 | si:dkey-27e18.3 | 5 | 14544090 | 14675811 | lincRNA | | ENSDARG00000089780 | si:dkey-288a3.2 | 17 | 18791068 | 18852314 | protein\_coding | | ENSDARG00000097263 | si:dkey-28g23.3 | 17 | 28927618 | 29005538 | antisense | | ENSDARG00000057238 | si:dkey-30k6.5 | 21 | 20350229 | 20359775 | protein\_coding | | ENSDARG00000096761 | si:dkey-33g13.1 | 12 | 32316801 | 32380311 | lincRNA | | ENSDARG00000094587 | si:dkey-35m8.1 | 5 | 61389645 | 61468213 | protein\_coding | | ENSDARG00000094273 | si:dkey-3k20.4 | 22 | 20685456 | 20687676 | protein\_coding | | ENSDARG00000096565 | si:dkey-45e15.9 | 16 | 21125766 | 21129688 | antisense | | ENSDARG00000095191 | si:dkey-57k2.7 | 2 | 37478183 | 37480005 | protein\_coding | | ENSDARG00000068865 | si:dkey-61p9.7 | 4 | 76212365 | 76220612 | protein\_coding | | ENSDARG00000088084 | si:dkey-66i24.8 | 3 | 30956667 | 30959719 | protein\_coding | | ENSDARG00000096869 | si:dkey-69p21.1 | 2 | 40768781 | 40857985 | lincRNA | | ENSDARG00000079897 | si:dkey-7k24.5 | 6 | 46479142 | 46492202 | protein\_coding | | ENSDARG00000097406 | si:dkey-81e3.2 | 25 | 21420003 | 21426395 | protein\_coding | | ENSDARG00000097096 | si:dkey-81j5.4 | 9 | 51660712 | 51745410 | antisense | | ENSDARG00000091366 | si:dkey-84k17.2 | 17 | 33735709 | 33755078 | protein\_coding | | ENSDARG00000086529 | si:dkey-90l23.2 | 9 | 8384283 | 8390070 | protein\_coding | | ENSDARG00000104658 | si:dkey-95j14.1 | 19 | 19806680 | 19810575 | protein\_coding | | ENSDARG00000093997 | si:dkey-9i23.15 | 1 | 44224119 | 44227684 | protein\_coding | | ENSDARG00000094214 | si:dkeyp-117b8.4 | 5 | 61278873 | 61288302 | protein\_coding | | ENSDARG00000042025 | si:dkeyp-118h3.6 | 9 | 6629529 | 6640082 | protein\_coding | | ENSDARG00000099628 | si:dkeyp-121d2.4 | 14 | 50011055 | 50018302 | lincRNA | | ENSDARG00000086245 | si:dkeyp-121d2.7 | 14 | 49956563 | 49973299 | protein\_coding | | ENSDARG00000079263 | si:dkeyp-2e4.2 | 13 | 44711439 | 44715420 | protein\_coding | | ENSDARG00000093704 | si:dkeyp-46h3.8 | 19 | 27964236 | 27966545 | protein\_coding | | ENSDARG00000098664 | si:dkeyp-53d3.3 | 22 | 1423804 | 1429952 | protein\_coding | | ENSDARG00000079930 | si:dkeyp-68b7.5 | 2 | 57810755 | 57827100 | protein\_coding | | ENSDARG00000038476 | si:dkeyp-68b7.7 | 2 | 57777903 | 57785674 | protein\_coding | | ENSDARG00000092766 | si:dkeyp-68b7.7.1 | 2 | 57798727 | 57803551 | protein\_coding | | ENSDARG00000086712 | si:dkeyp-97b10.3 | 15 | 1487925 | 1519886 | protein\_coding | | ENSDARG00000096017 | si:dkeyp-98a7.2 | 22 | 25662015 | 25663251 | transcribed\_unprocessed\_pseudogene | | ENSDARG00000087584 | si:dkeyp-98a7.9 | 22 | 25714283 | 25715510 | protein\_coding | | ENSDARG00000089828 | si:rp71-19m20.1 | 12 | 11314327 | 11332988 | protein\_coding | | ENSDARG00000053792 | si:rp71-1g18.1 | 2 | 38073163 | 38083848 | protein\_coding | | ENSDARG00000077252 | si:rp71-36a1.5 | 22 | 5796401 | 5809877 | protein\_coding | | ENSDARG00000068982 | si:rp71-56i13.6 | 10 | 29968873 | 30001524 | protein\_coding | | ENSDARG00000011418 | sigmar1 | 10 | 14530506 | 14536120 | protein\_coding | | ENSDARG00000059812 | sin3ab | 7 | 48074496 | 48123446 | protein\_coding | | ENSDARG00000061699 | sipa1l3 | 18 | 37139088 | 37220542 | protein\_coding | | ENSDARG00000060645 | sirt7 | 22 | 38948315 | 38960226 | protein\_coding | | ENSDARG00000012947 | ska2 | 10 | 33425740 | 33435784 | protein\_coding | | ENSDARG00000032114 | slain2 | 20 | 23547143 | 23573219 | protein\_coding | | ENSDARG00000104508 | slc10a7 | 23 | 43746618 | 43760648 | protein\_coding | | ENSDARG00000074384 | slc12a8 | 9 | 38832735 | 38872444 | protein\_coding | | ENSDARG00000045051 | slc16a3 | 12 | 33249273 | 33258671 | protein\_coding | | ENSDARG00000011049 | slc17a9b | 23 | 17596524 | 17622802 | protein\_coding | | ENSDARG00000059712 | slc19a2 | 6 | 16309632 | 16329153 | protein\_coding | | ENSDARG00000017365 | slc23a2 | 8 | 42910728 | 42979902 | protein\_coding | | ENSDARG00000102362 | slc25a12 | 6 | 3800499 | 3821963 | protein\_coding | | ENSDARG00000060564 | slc25a21 | 17 | 38082742 | 38285436 | protein\_coding | | ENSDARG00000010572 | slc25a25a | 8 | 2547525 | 2557783 | protein\_coding | | ENSDARG00000035468 | slc25a25b | 5 | 31179758 | 31206460 | protein\_coding | | ENSDARG00000074533 | slc25a38b | 6 | 7564123 | 7577984 | protein\_coding | | ENSDARG00000035905 | slc25a44b | 7 | 48025066 | 48030641 | protein\_coding | | ENSDARG00000056163 | slc25a51a | 1 | 18118742 | 18123869 | protein\_coding | | ENSDARG00000043021 | slc26a11 | 22 | 11470038 | 11490723 | protein\_coding | | ENSDARG00000006240 | slc27a1a | 3 | 52290351 | 52349020 | protein\_coding | | ENSDARG00000046053 | slc27a6 | 10 | 16543580 | 16582484 | protein\_coding | | ENSDARG00000076899 | slc2a13b | 25 | 35158994 | 35176589 | protein\_coding | | ENSDARG00000013222 | slc35a3a | 8 | 19589540 | 19606475 | protein\_coding | | ENSDARG00000020981 | slc35a3b | 2 | 10192671 | 10242444 | protein\_coding | | ENSDARG00000000001 | slc35a5 | 9 | 34302882 | 34312656 | protein\_coding | | ENSDARG00000059850 | slc35f3b | 13 | 39220486 | 39274077 | protein\_coding | | ENSDARG00000000442 | slc39a13 | 7 | 32422740 | 32443388 | protein\_coding | | ENSDARG00000073952 | slc4a7 | 16 | 42789489 | 42866306 | protein\_coding | | ENSDARG00000014599 | slc5a6b | 17 | 6378427 | 6398913 | protein\_coding | | ENSDARG00000102377 | slc6a16b | 24 | 38730739 | 38757174 | protein\_coding | | ENSDARG00000009209 | slc9a6a | 14 | 31278668 | 31307586 | protein\_coding | | ENSDARG00000003328 | slf2 | 13 | 9280372 | 9325928 | protein\_coding | | ENSDARG00000074153 | slitrk5b | 9 | 53995822 | 53998392 | protein\_coding | | ENSDARG00000098605 | slx1b | 7 | 59202658 | 59213225 | protein\_coding | | ENSDARG00000061446 | smap2 | 19 | 38800135 | 38832455 | protein\_coding | | ENSDARG00000098919 | smarcc1b | 19 | 19769223 | 19806274 | protein\_coding | | ENSDARG00000104374 | smchd1 | 7 | 71345104 | 71469295 | protein\_coding | | ENSDARG00000052818 | smcr8a | 3 | 40113550 | 40117092 | protein\_coding | | ENSDARG00000061378 | smg8 | 10 | 35294628 | 35304642 | protein\_coding | | ENSDARG00000070358 | smim12 | 19 | 37548580 | 37552332 | protein\_coding | | ENSDARG00000052701 | smpd2b | 6 | 54672622 | 54696942 | protein\_coding | | ENSDARG00000020730 | smpd4 | 21 | 18870508 | 18890264 | protein\_coding | | ENSDARG00000053119 | smpdl3a | 20 | 40440313 | 40470383 | protein\_coding | | ENSDARG00000016086 | smurf1 | 3 | 40548354 | 40626690 | protein\_coding | | ENSDARG00000038067 | smurf2 | 3 | 25151399 | 25239009 | protein\_coding | | ENSDARG00000058050 | smyd3 | 17 | 11574854 | 11904105 | protein\_coding | | ENSDARG00000044433 | sned1 | 6 | 27461318 | 27520064 | protein\_coding | | ENSDARG00000004405 | snx10a | 19 | 18655502 | 18667599 | protein\_coding | | ENSDARG00000052522 | snx11 | 12 | 20483317 | 20494286 | protein\_coding | | ENSDARG00000013828 | snx13 | 19 | 2097105 | 2147996 | protein\_coding | | ENSDARG00000020397 | snx15 | 7 | 19702542 | 19714401 | protein\_coding | | ENSDARG00000091418 | snx17 | 20 | 19536583 | 19612117 | protein\_coding | | ENSDARG00000067713 | snx18a | 5 | 39672695 | 39692146 | protein\_coding | | ENSDARG00000062770 | snx21 | 23 | 12493755 | 12518730 | protein\_coding | | ENSDARG00000053527 | snx24 | 10 | 7768581 | 7776239 | protein\_coding | | ENSDARG00000033804 | snx27a | 19 | 8693914 | 8728969 | protein\_coding | | ENSDARG00000020442 | snx5 | 13 | 33535868 | 33545386 | protein\_coding | | ENSDARG00000069302 | snx9b | 20 | 43119308 | 43182422 | protein\_coding | | ENSDARG00000017386 | sobpb | 4 | 805480 | 824452 | protein\_coding | | ENSDARG00000088187 | sowah1 | 23 | 37807266 | 37857985 | processed\_transcript | | ENSDARG00000040266 | sox19b | 7 | 26223390 | 26226754 | protein\_coding | | ENSDARG00000076763 | sp2 | 11 | 11999844 | 12024222 | protein\_coding | | ENSDARG00000001549 | sp3a | 9 | 2756196 | 2771266 | protein\_coding | | ENSDARG00000024933 | spast | 1 | 50377480 | 50395086 | protein\_coding | | ENSDARG00000062837 | spata13 | 24 | 21719544 | 21758186 | protein\_coding | | ENSDARG00000059131 | spdya | 17 | 24790820 | 24796819 | protein\_coding | | ENSDARG00000029480 | specc1 | 5 | 62122699 | 62303733 | protein\_coding | | ENSDARG00000042232 | specc1lb | 21 | 13136389 | 13187672 | protein\_coding | | ENSDARG00000069893 | sphk2 | 16 | 32182142 | 32191984 | protein\_coding | | ENSDARG00000003084 | spire2 | 25 | 34383041 | 34406648 | protein\_coding | | ENSDARG00000013837 | spo11 | 23 | 6610481 | 6640151 | protein\_coding | | ENSDARG00000100519 | spop | 3 | 20508463 | 20643916 | protein\_coding | | ENSDARG00000069485 | spryd7b | 9 | 29825874 | 29832882 | protein\_coding | | ENSDARG00000033889 | spty2d1 | 25 | 24139901 | 24150452 | protein\_coding | | ENSDARG00000053619 | sra1 | 21 | 43887098 | 43896243 | protein\_coding | | ENSDARG00000008107 | src | 23 | 39454019 | 39557789 | protein\_coding | | ENSDARG00000056780 | ssh1a | 5 | 19737763 | 19786809 | protein\_coding | | ENSDARG00000054827 | ssuh2.4 | 22 | 31055540 | 31071495 | protein\_coding | | ENSDARG00000021973 | st13 | 3 | 24957057 | 24970178 | protein\_coding | | ENSDARG00000079654 | st3gal1 | 19 | 4857224 | 4876847 | protein\_coding | | ENSDARG00000007494 | st3gal8 | 11 | 26330876 | 26352813 | protein\_coding | | ENSDARG00000088515 | st6gal2b | 6 | 15310192 | 15365090 | protein\_coding | | ENSDARG00000018788 | st8sia2 | 18 | 25040963 | 25065314 | protein\_coding | | ENSDARG00000011783 | stag2a | 5 | 22984255 | 23013947 | protein\_coding | | ENSDARG00000086906 | stambpb | 8 | 52544596 | 52561664 | protein\_coding | | ENSDARG00000014003 | stard8 | 5 | 35186081 | 35266020 | protein\_coding | | ENSDARG00000022712 | stat3 | 3 | 16880865 | 16957645 | protein\_coding | | ENSDARG00000060723 | stim1a | 15 | 38319611 | 38396148 | protein\_coding | | ENSDARG00000101894 | stk10 | 21 | 42789139 | 42872424 | protein\_coding | | ENSDARG00000023878 | stk24b | 6 | 12247741 | 12316624 | protein\_coding | | ENSDARG00000061095 | stk36 | 5 | 29791995 | 29811012 | protein\_coding | | ENSDARG00000018516 | stk38b | 22 | 866780 | 881747 | protein\_coding | | ENSDARG00000045482 | stk38l | 4 | 20503124 | 20514929 | protein\_coding | | ENSDARG00000062467 | stra6l | 1 | 11485522 | 11504143 | protein\_coding | | ENSDARG00000045228 | stub1 | 24 | 39768933 | 39780340 | protein\_coding | | ENSDARG00000025033 | stx5a | 14 | 8288253 | 8310549 | protein\_coding | | ENSDARG00000103173 | stx8 | 6 | 23218143 | 23331259 | protein\_coding | | ENSDARG00000029234 | stxbp5b | 17 | 7075373 | 7194240 | protein\_coding | | ENSDARG00000013461 | supt7l | 17 | 40902972 | 40923793 | protein\_coding | | ENSDARG00000077728 | supv3l1 | 13 | 22907135 | 22921316 | protein\_coding | | ENSDARG00000075461 | suv420h2 | 3 | 30737484 | 30757573 | protein\_coding | | ENSDARG00000093423 | swsap1 | 3 | 14415961 | 14421714 | protein\_coding | | ENSDARG00000103187 | syce2 | 3 | 13780014 | 13787605 | protein\_coding | | ENSDARG00000003904 | sycp1 | 6 | 49054088 | 49062433 | protein\_coding | | ENSDARG00000079190 | sycp2l | 24 | 8702790 | 8716682 | protein\_coding | | ENSDARG00000017606 | sys1 | 6 | 54430247 | 54436821 | protein\_coding | | ENSDARG00000102433 | sytl4 | 14 | 35084225 | 35101111 | protein\_coding | | ENSDARG00000021509 | tab2 | 20 | 1308683 | 1329470 | protein\_coding | | ENSDARG00000062063 | tab3 | 8 | 18509827 | 18527267 | protein\_coding | | ENSDARG00000003769 | tada2b | 7 | 39467488 | 39471335 | protein\_coding | | ENSDARG00000028937 | taf1b | 20 | 29609480 | 29630131 | protein\_coding | | ENSDARG00000068192 | taf4a | 11 | 20787195 | 20827670 | protein\_coding | | ENSDARG00000075732 | taf4b | 20 | 18360830 | 18396654 | protein\_coding | | ENSDARG00000079684 | taf6l | 7 | 17701668 | 17718950 | protein\_coding | | ENSDARG00000034001 | tango6 | 18 | 17054452 | 17085998 | protein\_coding | | ENSDARG00000067723 | tank | 9 | 52019180 | 52034070 | protein\_coding | | ENSDARG00000103688 | tank.1 | 9 | 51916345 | 51923414 | protein\_coding | | ENSDARG00000074899 | taok2a | 3 | 21007992 | 21054468 | protein\_coding | | ENSDARG00000100620 | taok2b | 12 | 3752151 | 3804001 | protein\_coding | | ENSDARG00000079402 | tapbp.1 | 19 | 7119769 | 7143239 | protein\_coding | | ENSDARG00000098288 | tax1bp1a | 19 | 19978260 | 20026421 | protein\_coding | | ENSDARG00000102070 | tbc1d10aa | 8 | 40386580 | 40412444 | protein\_coding | | ENSDARG00000003520 | tbc1d14 | 7 | 39408927 | 39431215 | protein\_coding | | ENSDARG00000026988 | tbc1d22b | 8 | 10302140 | 10412156 | protein\_coding | | ENSDARG00000061986 | tbc1d2b | 25 | 25494804 | 25551674 | protein\_coding | | ENSDARG00000041734 | tbc1d32 | 20 | 40869535 | 40943883 | protein\_coding | | ENSDARG00000036212 | tbc1d5 | 19 | 20819964 | 20862433 | protein\_coding | | ENSDARG00000059900 | tbc1d9 | 1 | 52687140 | 52726370 | protein\_coding | | ENSDARG00000022918 | tbcc | 12 | 34790466 | 34791760 | protein\_coding | | ENSDARG00000013667 | tbck | 1 | 49554781 | 49650122 | protein\_coding | | ENSDARG00000014994 | tbp | 13 | 24253719 | 24265553 | protein\_coding | | ENSDARG00000074996 | tbrg1 | 18 | 35254008 | 35279178 | protein\_coding | | ENSDARG00000040036 | tcf19l | 19 | 6912424 | 6925175 | protein\_coding | | ENSDARG00000078348 | tcf20 | 3 | 1490887 | 1513801 | protein\_coding | | ENSDARG00000105142 | tcirg1b | 14 | 16471655 | 16504836 | protein\_coding | | ENSDARG00000031890 | tcp11l1 | 25 | 15176970 | 15189190 | protein\_coding | | ENSDARG00000035954 | tdp2b | 19 | 31973181 | 31989008 | protein\_coding | | ENSDARG00000007465 | tdrd1 | 12 | 30230934 | 30244596 | protein\_coding | | ENSDARG00000025421 | tdrd3 | 11 | 42473942 | 42523317 | protein\_coding | | ENSDARG00000071450 | tdrd5 | 22 | 17235608 | 17254453 | protein\_coding | | ENSDARG00000070052 | tdrd6 | 20 | 35535421 | 35561392 | protein\_coding | | ENSDARG00000058710 | terf1 | 24 | 13192794 | 13205053 | protein\_coding | | ENSDARG00000060306 | tfcp2 | 23 | 33730474 | 33753734 | protein\_coding | | ENSDARG00000098903 | tfe3a | 8 | 7739969 | 7770650 | protein\_coding | | ENSDARG00000053939 | tgfa | 6 | 40994873 | 41011845 | protein\_coding | | ENSDARG00000061508 | tgfbrap1 | 6 | 14909888 | 14938436 | protein\_coding | | ENSDARG00000053082 | thada | 13 | 8560761 | 8621443 | protein\_coding | | ENSDARG00000059020 | thap1 | 5 | 424738 | 434194 | protein\_coding | | ENSDARG00000077323 | thap4 | 2 | 22997472 | 23003799 | protein\_coding | | ENSDARG00000000151 | thraa | 3 | 34558514 | 34624097 | protein\_coding | | ENSDARG00000042467 | tlk1a | 9 | 3577000 | 3637687 | protein\_coding | | ENSDARG00000100715 | tlk1b | 6 | 3716847 | 3763903 | protein\_coding | | ENSDARG00000010779 | tlk2 | 3 | 19536133 | 19568194 | protein\_coding | | ENSDARG00000022970 | tm9sf1 | 24 | 11749610 | 11767971 | protein\_coding | | ENSDARG00000075108 | tmco3 | 1 | 219451 | 230344 | protein\_coding | | ENSDARG00000088560 | tmem104 | 3 | 18691131 | 18774175 | protein\_coding | | ENSDARG00000045075 | tmem106a | 12 | 27139852 | 27145672 | protein\_coding | | ENSDARG00000070866 | tmem11 | 3 | 15678626 | 15686340 | protein\_coding | | ENSDARG00000055763 | tmem115 | 8 | 25873419 | 25879165 | protein\_coding | | ENSDARG00000088356 | tmem117 | 25 | 686657 | 696053 | protein\_coding | | ENSDARG00000056259 | tmem131 | 6 | 29322659 | 29386531 | protein\_coding | | ENSDARG00000068705 | tmem168a | 4 | 6726483 | 6740842 | protein\_coding | | ENSDARG00000062168 | tmem168b | 25 | 21652838 | 21666202 | protein\_coding | | ENSDARG00000076376 | tmem175 | 5 | 39394130 | 39403553 | protein\_coding | | ENSDARG00000099491 | tmem176l.1 | 16 | 46551398 | 46558439 | protein\_coding | | ENSDARG00000045147 | tmem184ba | 12 | 19198783 | 19218346 | protein\_coding | | ENSDARG00000034685 | tmem198b | 6 | 13650420 | 13740721 | protein\_coding | | ENSDARG00000016968 | tmem214 | 4 | 750652 | 766348 | protein\_coding | | ENSDARG00000104097 | tmem222b | 19 | 14132368 | 14173697 | protein\_coding | | ENSDARG00000074248 | tmem237b | 6 | 9663144 | 9680995 | protein\_coding | | ENSDARG00000061553 | tmem246 | 11 | 30389167 | 30395462 | protein\_coding | | ENSDARG00000060442 | tmem259 | 11 | 14015123 | 14044472 | protein\_coding | | ENSDARG00000101297 | tmem266 | 25 | 32362888 | 32375587 | protein\_coding | | ENSDARG00000070028 | tmem41aa | 9 | 12473291 | 12484318 | protein\_coding | | ENSDARG00000080006 | tmem41b | 7 | 64794132 | 64890039 | protein\_coding | | ENSDARG00000063414 | tmem5 | 18 | 13094492 | 13153265 | protein\_coding | | ENSDARG00000061916 | tmem51b | 11 | 25595603 | 25615596 | protein\_coding | | ENSDARG00000005625 | tmem57a | 19 | 29716624 | 29746644 | protein\_coding | | ENSDARG00000012741 | tmem57b | 13 | 45387466 | 45434859 | protein\_coding | | ENSDARG00000053890 | tmem62 | 17 | 37306121 | 37333728 | protein\_coding | | ENSDARG00000031956 | tmem63a | 4 | 5656612 | 5682687 | protein\_coding | | ENSDARG00000061723 | tmem64 | 19 | 31944730 | 31966205 | protein\_coding | | ENSDARG00000042716 | tmem68 | 2 | 28020128 | 28023915 | protein\_coding | | ENSDARG00000086737 | tmem68.1 | 2 | 27996357 | 28000672 | protein\_coding | | ENSDARG00000078773 | tmem70 | 2 | 30266444 | 30268814 | protein\_coding | | ENSDARG00000076861 | tmem79b | 16 | 30396152 | 30404580 | protein\_coding | | ENSDARG00000057997 | tmf1 | 11 | 17622707 | 17648295 | protein\_coding | | ENSDARG00000035273 | tmtc2b | 18 | 11747031 | 11940951 | protein\_coding | | ENSDARG00000041565 | tnfaip1 | 15 | 20593465 | 20605453 | protein\_coding | | ENSDARG00000070165 | tnfrsf1b | 11 | 40167484 | 40193180 | protein\_coding | | ENSDARG00000035390 | tnpo1 | 5 | 34602388 | 34652803 | protein\_coding | | ENSDARG00000070512 | tnrc5 | 1 | 9778581 | 9789519 | protein\_coding | | ENSDARG00000102795 | tob2 | 3 | 5125187 | 5132323 | lincRNA | | ENSDARG00000104581 | tom1 | 3 | 25776754 | 25808388 | protein\_coding | | ENSDARG00000029639 | tomm70a | 6 | 28955480 | 28971099 | protein\_coding | | ENSDARG00000100163 | tox4b | 7 | 1393944 | 1407868 | protein\_coding | | ENSDARG00000079000 | tp53bp1 | 7 | 52612631 | 52662501 | protein\_coding | | ENSDARG00000069430 | tp53i11a | 7 | 26378125 | 26434621 | protein\_coding | | ENSDARG00000100420 | tpcn2 | 7 | 54699112 | 54752352 | protein\_coding | | ENSDARG00000029432 | tph1a | 25 | 8015128 | 8035938 | protein\_coding | | ENSDARG00000025012 | tpi1a | 19 | 5177732 | 5186827 | protein\_coding | | ENSDARG00000036057 | tradd | 7 | 28844749 | 28853428 | protein\_coding | | ENSDARG00000069482 | traf1 | 8 | 12371135 | 12394980 | protein\_coding | | ENSDARG00000022000 | traf3 | 17 | 29210539 | 29232295 | protein\_coding | | ENSDARG00000010300 | traf3ip1 | 9 | 45804931 | 45867008 | protein\_coding | | ENSDARG00000038964 | traf4b | 21 | 38960953 | 38979802 | protein\_coding | | ENSDARG00000028058 | traf6 | 7 | 48450062 | 48465942 | protein\_coding | | ENSDARG00000060207 | traf7 | 3 | 35478965 | 35505219 | protein\_coding | | ENSDARG00000041304 | trak1 | 16 | 5887144 | 5974240 | protein\_coding | | ENSDARG00000033171 | trappc6bl | 15 | 14140047 | 14147183 | protein\_coding | | ENSDARG00000077911 | treh | 18 | 43853482 | 43872547 | protein\_coding | | ENSDARG00000076546 | trhra | 16 | 38870454 | 38875047 | protein\_coding | | ENSDARG00000069420 | trim23 | 10 | 11832643 | 11882115 | protein\_coding | | ENSDARG00000031817 | trim2a | 1 | 24631616 | 24679452 | protein\_coding | | ENSDARG00000102505 | trim32 | KN149782.1 | 29665 | 32060 | protein\_coding | | ENSDARG00000035009 | trim35-27 | 14 | 635163 | 745362 | protein\_coding | | ENSDARG00000079238 | trim59 | 15 | 1609447 | 1619228 | protein\_coding | | ENSDARG00000060901 | trim62 | 11 | 40067646 | 40154830 | protein\_coding | | ENSDARG00000005679 | trip10a | 3 | 54179534 | 54245686 | protein\_coding | | ENSDARG00000078381 | trip11 | 13 | 33043768 | 33077359 | protein\_coding | | ENSDARG00000037245 | trmo | 1 | 25977114 | 25982677 | protein\_coding | | ENSDARG00000040033 | trmt11 | 16 | 40093413 | 40124113 | protein\_coding | | ENSDARG00000069278 | trmt5 | 13 | 31551538 | 31557475 | protein\_coding | | ENSDARG00000008480 | trmt61a | 13 | 15570857 | 15620302 | protein\_coding | | ENSDARG00000043734 | trmu | 4 | 28349354 | 28364250 | protein\_coding | | ENSDARG00000070504 | trpc5a | 1 | 9802771 | 9936348 | protein\_coding | | ENSDARG00000061941 | trpv4 | 5 | 19291229 | 19342661 | protein\_coding | | ENSDARG00000026048 | tsc1a | 5 | 29052360 | 29067268 | protein\_coding | | ENSDARG00000057918 | tsc1b | 21 | 17265154 | 17303196 | protein\_coding | | ENSDARG00000041839 | tsc22d2 | 22 | 35229268 | 35287631 | protein\_coding | | ENSDARG00000036844 | tsen54 | 3 | 60299983 | 60334005 | protein\_coding | | ENSDARG00000040854 | tsg101a | 25 | 25037519 | 25047203 | protein\_coding | | ENSDARG00000036144 | tssc4 | 7 | 32626573 | 32628693 | protein\_coding | | ENSDARG00000063242 | ttc13 | 20 | 4034078 | 4077871 | protein\_coding | | ENSDARG00000102541 | ttc21a | 24 | 41134511 | 41160107 | protein\_coding | | ENSDARG00000069241 | ttc32 | 20 | 43849445 | 43853693 | protein\_coding | | ENSDARG00000035406 | ttc33 | 5 | 34821613 | 34856116 | protein\_coding | | ENSDARG00000013329 | ttc5 | 7 | 19276297 | 19286438 | protein\_coding | | ENSDARG00000091967 | ttf1 | 5 | 62666251 | 62680443 | protein\_coding | | ENSDARG00000104105 | ttf2 | 9 | 9956251 | 9978827 | protein\_coding | | ENSDARG00000062418 | ttl | 13 | 15787457 | 15798410 | protein\_coding | | ENSDARG00000013079 | tubgcp2 | 12 | 9145783 | 9180415 | protein\_coding | | ENSDARG00000029133 | tubgcp3 | 1 | 45714062 | 45759038 | protein\_coding | | ENSDARG00000077442 | tubgcp5 | 6 | 37645799 | 37674049 | protein\_coding | | ENSDARG00000077037 | txlna | 19 | 30249145 | 30266089 | protein\_coding | | ENSDARG00000074895 | tysnd1 | 13 | 30005225 | 30012677 | protein\_coding | | ENSDARG00000038222 | tyw3 | 2 | 11335716 | 11342622 | protein\_coding | | ENSDARG00000057987 | uba3 | 11 | 17650547 | 17668502 | protein\_coding | | ENSDARG00000100107 | ubald2 | 3 | 60605103 | 60631323 | protein\_coding | | ENSDARG00000058746 | ubap1 | 21 | 11773344 | 11791889 | protein\_coding | | ENSDARG00000015292 | ube2g1a | 5 | 30386359 | 30394955 | protein\_coding | | ENSDARG00000033489 | ube2j1 | 20 | 1156925 | 1171084 | protein\_coding | | ENSDARG00000061161 | ube2j2 | 23 | 36747893 | 36761829 | protein\_coding | | ENSDARG00000058740 | ube2r2 | 21 | 11752613 | 11763515 | protein\_coding | | ENSDARG00000029215 | ube2z | 3 | 23453364 | 23466292 | protein\_coding | | ENSDARG00000055737 | ube3a | 6 | 38775840 | 38792528 | protein\_coding | | ENSDARG00000035978 | ube3c | 7 | 40312871 | 40359648 | protein\_coding | | ENSDARG00000044492 | ublcp1 | 21 | 35270484 | 35291051 | protein\_coding | | ENSDARG00000063169 | ubn1 | 1 | 8574820 | 8593926 | protein\_coding | | ENSDARG00000079623 | ubtd1b | 12 | 2745457 | 2766126 | protein\_coding | | ENSDARG00000087180 | ubxn2a | 20 | 44668933 | 44678943 | protein\_coding | | ENSDARG00000026871 | uchl1 | 1 | 21957017 | 21961685 | protein\_coding | | ENSDARG00000006074 | uck2a | 8 | 21082253 | 21101034 | protein\_coding | | ENSDARG00000009266 | uevld | 25 | 24083519 | 24105028 | protein\_coding | | ENSDARG00000058221 | ugcg | 10 | 11079954 | 11135889 | protein\_coding | | ENSDARG00000008200 | ugp2b | 17 | 24427417 | 24452188 | protein\_coding | | ENSDARG00000074481 | ulk1b | 21 | 14742702 | 14781523 | protein\_coding | | ENSDARG00000040455 | unc50 | 6 | 3824021 | 3837379 | protein\_coding | | ENSDARG00000099034 | unc5a | KN150681.1 | 20136 | 278214 | protein\_coding | | ENSDARG00000061541 | unc5db | 5 | 26667102 | 26797459 | protein\_coding | | ENSDARG00000000935 | unk | 12 | 35754332 | 35799148 | protein\_coding | | ENSDARG00000003217 | urb2 | 13 | 24103882 | 24130159 | protein\_coding | | ENSDARG00000007250 | use1 | 22 | 11041160 | 11049386 | protein\_coding | | ENSDARG00000077431 | usf3 | 24 | 21042757 | 21058370 | protein\_coding | | ENSDARG00000076079 | ush1gb | 12 | 46281735 | 46295062 | protein\_coding | | ENSDARG00000056414 | usp1 | 6 | 32407588 | 32424813 | protein\_coding | | ENSDARG00000056842 | usp30 | 5 | 19621286 | 19631888 | protein\_coding | | ENSDARG00000016163 | usp33 | 2 | 8919150 | 8979491 | protein\_coding | | ENSDARG00000075798 | usp38 | 1 | 34741279 | 34756707 | protein\_coding | | ENSDARG00000077300 | usp42 | 12 | 17448801 | 17470341 | protein\_coding | | ENSDARG00000075013 | usp45 | 16 | 32722487 | 32773169 | protein\_coding | | ENSDARG00000045343 | usp46 | 20 | 23184700 | 23212627 | protein\_coding | | ENSDARG00000102645 | usp47 | 7 | 65174438 | 65239640 | protein\_coding | | ENSDARG00000034825 | uspl1 | 10 | 36495535 | 36512227 | protein\_coding | | ENSDARG00000006044 | ust | 20 | 31406485 | 31524511 | protein\_coding | | ENSDARG00000023815 | uvrag | 10 | 32320997 | 32550444 | protein\_coding | | ENSDARG00000014303 | vac14 | 25 | 17493670 | 17514904 | protein\_coding | | ENSDARG00000046048 | vapal | 2 | 58448983 | 58499222 | protein\_coding | | ENSDARG00000056717 | vars2 | 19 | 27814945 | 27864383 | protein\_coding | | ENSDARG00000099483 | vdra | 23 | 45976378 | 45980632 | protein\_coding | | ENSDARG00000075215 | vetz | 4 | 25923231 | 25958213 | protein\_coding | | ENSDARG00000061030 | vezf1b | 15 | 31363284 | 31384634 | protein\_coding | | ENSDARG00000103690 | vipas39 | 20 | 18454826 | 18483162 | protein\_coding | | ENSDARG00000043644 | vkorc1l1 | 5 | 1737479 | 1782476 | protein\_coding | | ENSDARG00000006257 | vldlr | 10 | 15296893 | 15347025 | protein\_coding | | ENSDARG00000036338 | vps11 | 10 | 29864689 | 29882216 | protein\_coding | | ENSDARG00000070433 | vps18 | 20 | 28455179 | 28462484 | protein\_coding | | ENSDARG00000015823 | vps26b | 15 | 19388956 | 19395691 | protein\_coding | | ENSDARG00000039319 | vps37b | 5 | 27399733 | 27416652 | protein\_coding | | ENSDARG00000062016 | vps51 | 10 | 27000252 | 27010717 | protein\_coding | | ENSDARG00000019195 | vps9d1 | 18 | 14665462 | 14699886 | protein\_coding | | ENSDARG00000098289 | vstm2l | 6 | 1991490 | 2000078 | protein\_coding | | ENSDARG00000039270 | vti1b | 13 | 32909986 | 32924406 | protein\_coding | | ENSDARG00000044144 | wapla | 17 | 25838280 | 25867081 | protein\_coding | | ENSDARG00000024209 | wasf2 | 19 | 43928093 | 43947531 | protein\_coding | | ENSDARG00000063457 | wash1 | 18 | 6339598 | 6363077 | protein\_coding | | ENSDARG00000014113 | wasla | 25 | 27301091 | 27369608 | protein\_coding | | ENSDARG00000013245 | wbp1la | 13 | 28449907 | 28466315 | protein\_coding | | ENSDARG00000069507 | wbscr27 | 21 | 25759270 | 25765381 | protein\_coding | | ENSDARG00000077009 | wdfy4 | 13 | 30850627 | 30938417 | protein\_coding | | ENSDARG00000041239 | wdr20b | 20 | 54247692 | 54261750 | protein\_coding | | ENSDARG00000003197 | wdr21 | 16 | 3284951 | 3288548 | protein\_coding | | ENSDARG00000053386 | wdr27 | 13 | 35402773 | 35484286 | protein\_coding | | ENSDARG00000078136 | wdr47a | 2 | 45529435 | 45566688 | protein\_coding | | ENSDARG00000102734 | wdr48a | 24 | 41176453 | 41201526 | protein\_coding | | ENSDARG00000023152 | wdr73 | 21 | 21783242 | 21787649 | protein\_coding | | ENSDARG00000079702 | wdr81 | 15 | 26620072 | 26638072 | protein\_coding | | ENSDARG00000059760 | wdtc1 | 16 | 55239743 | 55270956 | protein\_coding | | ENSDARG00000037871 | wipi2 | 3 | 40679799 | 40695360 | protein\_coding | | ENSDARG00000098211 | wrn | 10 | 7522683 | 7596960 | protein\_coding | | ENSDARG00000005218 | wrnip1 | 2 | 361498 | 377000 | protein\_coding | | ENSDARG00000058608 | wsb2 | 5 | 12049334 | 12060352 | protein\_coding | | ENSDARG00000087530 | wu:fe05a04 | 16 | 25256800 | 25266510 | protein\_coding | | ENSDARG00000076041 | wwc1 | 21 | 36780561 | 36886559 | protein\_coding | | ENSDARG00000061654 | wwc3 | 6 | 30504182 | 30606230 | protein\_coding | | ENSDARG00000068939 | xaf1 | 15 | 29221671 | 29229385 | protein\_coding | | ENSDARG00000039996 | xkr8.3 | 19 | 25487830 | 25497488 | protein\_coding | | ENSDARG00000010281 | xpo4 | 9 | 21496031 | 21546972 | protein\_coding | | ENSDARG00000029671 | xpr1b | 2 | 7127763 | 7185724 | protein\_coding | | ENSDARG00000010732 | xrcc4 | 10 | 43289281 | 43355590 | protein\_coding | | ENSDARG00000059557 | xylt2 | 3 | 59886578 | 59937322 | protein\_coding | | ENSDARG00000062655 | ydjc | 5 | 12826548 | 12835407 | protein\_coding | | ENSDARG00000014474 | yipf1 | 2 | 26943167 | 26948684 | protein\_coding | | ENSDARG00000030957 | yipf4 | 17 | 22557636 | 22565760 | protein\_coding | | ENSDARG00000007279 | yipf5 | 21 | 40907130 | 40915335 | protein\_coding | | ENSDARG00000075192 | yme1l1a | 24 | 32610503 | 32634677 | protein\_coding | | ENSDARG00000104401 | yme1l1b | 2 | 4205053 | 4229880 | protein\_coding | | ENSDARG00000057939 | ythdf3 | 24 | 24140577 | 24155176 | protein\_coding | | ENSDARG00000006978 | zak | 9 | 2890883 | 2930145 | protein\_coding | | ENSDARG00000019706 | zar1 | 20 | 23524714 | 23527239 | protein\_coding | | ENSDARG00000023040 | zbtb1 | 20 | 27813624 | 27821008 | protein\_coding | | ENSDARG00000074548 | zbtb17 | 23 | 24311497 | 24326681 | protein\_coding | | ENSDARG00000043285 | zbtb21 | 10 | 32702542 | 32707828 | protein\_coding | | ENSDARG00000003251 | zbtb22b | 19 | 7082245 | 7088229 | protein\_coding | | ENSDARG00000075533 | zbtb24 | 17 | 6778050 | 6797852 | protein\_coding | | ENSDARG00000036235 | zbtb3 | 14 | 35065204 | 35068457 | protein\_coding | | ENSDARG00000045983 | zbtb43 | 5 | 4497505 | 4507679 | protein\_coding | | ENSDARG00000061110 | zbtb44 | 5 | 29530934 | 29551128 | protein\_coding | | ENSDARG00000079547 | zbtb47b | 24 | 20444840 | 20496371 | protein\_coding | | ENSDARG00000102111 | zbtb49 | 14 | 223940 | 227457 | protein\_coding | | ENSDARG00000079250 | zbtb5 | 1 | 19115504 | 19128258 | processed\_transcript | | ENSDARG00000059226 | zbtb8a | 17 | 24850970 | 24860602 | protein\_coding | | ENSDARG00000005271 | zc3h12a | 16 | 4183537 | 4202690 | protein\_coding | | ENSDARG00000015889 | zc3h15 | 9 | 11693594 | 11705288 | protein\_coding | | ENSDARG00000090751 | zc3h3 | 6 | 32732748 | 32849695 | protein\_coding | | ENSDARG00000070271 | zcchc11 | 23 | 42789238 | 42811271 | protein\_coding | | ENSDARG00000055443 | zcchc7 | 1 | 20917534 | 21021088 | protein\_coding | | ENSDARG00000101144 | zdhhc13 | 7 | 16257219 | 16294158 | protein\_coding | | ENSDARG00000015989 | zdhhc16b | 12 | 2814851 | 2834851 | protein\_coding | | ENSDARG00000069807 | zdhhc18a | 16 | 34038289 | 34052305 | protein\_coding | | ENSDARG00000055066 | zdhhc20a | 10 | 35610192 | 35636226 | protein\_coding | | ENSDARG00000058178 | zdhhc20b | 24 | 24842341 | 24859490 | protein\_coding | | ENSDARG00000016263 | zdhhc5a | 1 | 43822213 | 43865130 | protein\_coding | | ENSDARG00000075721 | zdhhc6 | 12 | 31307655 | 31323680 | protein\_coding | | ENSDARG00000002271 | zfand5b | 10 | 15644963 | 15655183 | protein\_coding | | ENSDARG00000061147 | zfp64 | 23 | 38875850 | 38886683 | protein\_coding | | ENSDARG00000103453 | zfpl1 | 5 | 52290988 | 52318196 | protein\_coding | | ENSDARG00000060430 | zfyve28 | 17 | 43575090 | 43604326 | protein\_coding | | ENSDARG00000101903 | zfyve9a | 2 | 19631765 | 19705558 | protein\_coding | | ENSDARG00000040179 | zgc:101562 | 20 | 2625692 | 2630403 | protein\_coding | | ENSDARG00000037852 | zgc:101663 | 23 | 358019 | 381744 | protein\_coding | | ENSDARG00000054934 | zgc:101765 | 7 | 24855472 | 24858424 | protein\_coding | | ENSDARG00000029307 | zgc:101800 | 24 | 33548390 | 33566264 | protein\_coding | | ENSDARG00000045785 | zgc:103499 | 25 | 17829024 | 17838275 | protein\_coding | | ENSDARG00000035532 | zgc:110329 | 5 | 27534535 | 27549614 | protein\_coding | | ENSDARG00000077085 | zgc:110821 | 22 | 2566614 | 2643228 | protein\_coding | | ENSDARG00000099060 | zgc:111868 | 12 | 42208868 | 42213007 | protein\_coding | | ENSDARG00000037739 | zgc:112980 | 1 | 7855560 | 7869187 | protein\_coding | | ENSDARG00000053483 | zgc:113054 | 6 | 43452685 | 43477221 | protein\_coding | | ENSDARG00000089142 | zgc:113119 | 4 | 74873981 | 74883735 | protein\_coding | | ENSDARG00000045842 | zgc:113263 | 4 | 5333022 | 5359588 | protein\_coding | | ENSDARG00000037813 | zgc:113278 | 23 | 4287440 | 4304652 | protein\_coding | | ENSDARG00000053263 | zgc:113372 | 17 | 49537352 | 49542461 | protein\_coding | | ENSDARG00000038133 | zgc:113411 | 3 | 24510398 | 24519345 | protein\_coding | | ENSDARG00000035680 | zgc:113424 | 19 | 35410909 | 35412259 | protein\_coding | | ENSDARG00000051793 | zgc:113426 | 25 | 19572322 | 19579024 | protein\_coding | | ENSDARG00000094616 | zgc:113984.1 | 9 | 8412495 | 8415105 | protein\_coding | | ENSDARG00000040725 | zgc:114130 | 15 | 14098297 | 14102270 | protein\_coding | | ENSDARG00000039082 | zgc:123010 | 13 | 40121489 | 40156916 | protein\_coding | | ENSDARG00000055613 | zgc:123238 | 16 | 22430123 | 22445829 | protein\_coding | | ENSDARG00000055970 | zgc:136971 | 8 | 26413500 | 26430692 | protein\_coding | | ENSDARG00000095281 | zgc:152652 | 24 | 9841893 | 9850767 | protein\_coding | | ENSDARG00000099969 | zgc:152863 | 15 | 25554141 | 25564253 | protein\_coding | | ENSDARG00000102586 | zgc:152938 | 4 | 71393012 | 71405841 | protein\_coding | | ENSDARG00000037002 | zgc:152968 | 1 | 35253866 | 35266370 | protein\_coding | | ENSDARG00000070458 | zgc:153292 | 3 | 30949838 | 30955928 | protein\_coding | | ENSDARG00000092445 | zgc:153352 | 5 | 8156776 | 8177952 | protein\_coding | | ENSDARG00000070166 | zgc:153499 | 9 | 8393930 | 8433744 | protein\_coding | | ENSDARG00000053257 | zgc:153733 | 3 | 32693595 | 32700257 | protein\_coding | | ENSDARG00000015922 | zgc:153952 | 3 | 16692142 | 16723110 | protein\_coding | | ENSDARG00000099373 | zgc:158320 | 9 | 355235 | 356868 | protein\_coding | | ENSDARG00000062757 | zgc:158403 | 3 | 14492162 | 14538310 | protein\_coding | | ENSDARG00000060259 | zgc:162160 | 16 | 42243131 | 42288088 | protein\_coding | | ENSDARG00000079934 | zgc:162200 | 8 | 37027317 | 37072792 | protein\_coding | | ENSDARG00000070416 | zgc:162816 | 11 | 31346421 | 31358614 | protein\_coding | | ENSDARG00000095034 | zgc:162928 | 24 | 19065729 | 19244165 | protein\_coding | | ENSDARG00000053695 | zgc:162948 | 4 | 75676777 | 75689678 | protein\_coding | | ENSDARG00000003968 | zgc:162969 | 11 | 6436396 | 6442653 | protein\_coding | | ENSDARG00000076134 | zgc:162972 | 19 | 6977259 | 6982426 | protein\_coding | | ENSDARG00000076437 | zgc:163143 | 3 | 34472344 | 34483593 | protein\_coding | | ENSDARG00000078212 | zgc:165515 | 4 | 72608312 | 72627706 | protein\_coding | | ENSDARG00000104715 | zgc:171422 | KN149790.1 | 76923 | 94226 | protein\_coding | | ENSDARG00000105118 | zgc:171566 | 22 | 4145054 | 4157103 | protein\_coding | | ENSDARG00000102473 | zgc:171673 | 4 | 74755263 | 74767304 | protein\_coding | | ENSDARG00000016908 | zgc:171779 | 3 | 31745615 | 31747505 | protein\_coding | | ENSDARG00000092026 | zgc:173545 | 11 | 18076373 | 18090251 | protein\_coding | | ENSDARG00000070048 | zgc:173548 | 11 | 6976750 | 6979759 | protein\_coding | | ENSDARG00000077078 | zgc:173726 | 22 | 2341126 | 2505590 | protein\_coding | | ENSDARG00000076442 | zgc:174310 | 4 | 75673766 | 75732453 | protein\_coding | | ENSDARG00000075314 | zgc:174906 | 23 | 36344219 | 36350308 | protein\_coding | | ENSDARG00000077741 | zgc:175135 | 11 | 17903094 | 17920427 | protein\_coding | | ENSDARG00000105499 | zgc:175135.1 | 11 | 17918698 | 17920499 | protein\_coding | | ENSDARG00000044325 | zgc:193690 | 23 | 36992752 | 37010768 | protein\_coding | | ENSDARG00000101901 | zgc:193711 | 5 | 56951195 | 56953707 | protein\_coding | | ENSDARG00000076552 | zgc:193801 | 21 | 15993409 | 16006524 | protein\_coding | | ENSDARG00000078917 | zgc:195245 | 8 | 23755331 | 23759163 | protein\_coding | | ENSDARG00000003017 | zgc:55512 | 9 | 8990372 | 8994433 | protein\_coding | | ENSDARG00000057184 | zgc:55781 | 2 | 21962115 | 21967431 | protein\_coding | | ENSDARG00000011723 | zgc:56556 | 2 | 28302006 | 28319894 | protein\_coding | | ENSDARG00000027461 | zgc:56719 | 3 | 17897036 | 17912043 | protein\_coding | | ENSDARG00000068840 | zgc:66024 | 15 | 35076599 | 35086098 | protein\_coding | | ENSDARG00000040081 | zgc:66432 | 16 | 42107966 | 42116585 | protein\_coding | | ENSDARG00000057707 | zgc:66443 | 3 | 15520152 | 15529307 | protein\_coding | | ENSDARG00000022952 | zgc:66448 | 16 | 25192312 | 25200440 | protein\_coding | | ENSDARG00000051959 | zgc:66449 | 25 | 17484993 | 17491460 | protein\_coding | | ENSDARG00000026453 | zgc:66474 | 3 | 15496392 | 15517913 | protein\_coding | | ENSDARG00000099612 | zgc:66483 | 21 | 2287906 | 2296278 | protein\_coding | | ENSDARG00000017337 | zgc:77929 | 11 | 11349031 | 11353365 | protein\_coding | | ENSDARG00000055162 | zhx2 | 16 | 25693746 | 25728194 | protein\_coding | | ENSDARG00000035434 | zmat5 | 5 | 31475956 | 31480039 | protein\_coding | | ENSDARG00000035823 | zmym4 | 19 | 40482404 | 40526815 | protein\_coding | | ENSDARG00000100773 | zmynd19 | 5 | 53690305 | 53748895 | protein\_coding | | ENSDARG00000101562 | znf1014 | 2 | 195958 | 204848 | protein\_coding | | ENSDARG00000088331 | znf1028 | 3 | 61105601 | 61135046 | protein\_coding | | ENSDARG00000068400 | znf131 | 8 | 31397505 | 31407907 | protein\_coding | | ENSDARG00000061373 | znf142 | 9 | 38580047 | 38589637 | protein\_coding | | ENSDARG00000095890 | znf236 | 16 | 6809639 | 6859968 | protein\_coding | | ENSDARG00000077013 | znf280d | 7 | 52437770 | 52476566 | protein\_coding | | ENSDARG00000043973 | znf292b | 20 | 207906 | 223285 | protein\_coding | | ENSDARG00000098348 | znf326 | 6 | 25057899 | 25065884 | protein\_coding | | ENSDARG00000087985 | znf365 | 17 | 43633060 | 43648155 | protein\_coding | | ENSDARG00000105056 | znf45l | 22 | 1335294 | 1344250 | protein\_coding | | ENSDARG00000052164 | znf507 | 7 | 46970267 | 46993072 | protein\_coding | | ENSDARG00000000760 | znf511 | 13 | 24534884 | 24538808 | protein\_coding | | ENSDARG00000019961 | znf513 | 20 | 19524240 | 19533183 | protein\_coding | | ENSDARG00000077143 | znf526 | 16 | 11336318 | 11350522 | protein\_coding | | ENSDARG00000087074 | znf574 | 16 | 11406801 | 11453825 | protein\_coding | | ENSDARG00000014945 | znf598 | 3 | 40043395 | 40060749 | protein\_coding | | ENSDARG00000103388 | znf609a | 25 | 734878 | 915906 | protein\_coding | | ENSDARG00000101361 | znf644a | 2 | 22850713 | 22875318 | protein\_coding | | ENSDARG00000093469 | znf653 | 3 | 14422013 | 14456318 | protein\_coding | | ENSDARG00000025226 | znf668 | 12 | 28840864 | 28845731 | protein\_coding | | ENSDARG00000102774 | znf687a | 16 | 22114220 | 22135362 | protein\_coding | | ENSDARG00000078434 | znf692 | 8 | 19945627 | 19968980 | protein\_coding | | ENSDARG00000071868 | znf711 | 14 | 11669330 | 11714639 | protein\_coding | | ENSDARG00000013279 | znf76 | 22 | 699488 | 715669 | protein\_coding | | ENSDARG00000036698 | znf865 | 19 | 10282245 | 10295340 | protein\_coding | | ENSDARG00000086494 | znf970 | 15 | 820733 | 828548 | protein\_coding | | ENSDARG00000058562 | znf990 | 22 | 9861692 | 9870696 | protein\_coding | | ENSDARG00000055415 | zp2l1 | 9 | 35822255 | 35824642 | protein\_coding | | ENSDARG00000090768 | zp3.2 | 17 | 2393135 | 2419350 | protein\_coding | | ENSDARG00000039828 | zp3b | 2 | 6341364 | 6346914 | protein\_coding | | ENSDARG00000092919 | zp3c | 1 | 48869805 | 48876991 | protein\_coding | | ENSDARG00000075700 | zswim2 | 6 | 11525322 | 11528911 | protein\_coding | | ENSDARG00000018738 | zw10 | 21 | 22999709 | 23010001 | protein\_coding | | ENSDARG00000075733 | zyx | 16 | 17394475 | 17439750 | protein\_coding | | ENSDARG00000094380 | zzef1 | 5 | 30436741 | 30509829 | protein\_coding | |
