# Supplementary material for: A high-resolution mRNA expression time course of embryonic development in zebrafish
Source: eLife. 2017 Nov 16;6:e30860. doi: 10.7554/eLife.30860 (PMC5690287; doi:10.7554/eLife.30860)
Supplement: Supplementary file 6. [file elife-30860-supp6.zip › biolayout-clusters-files/Cluster002.html]

Cluster002


# Cluster002: Detail

### Go to ZFA detail

## GO

| | GO ID | Description | Domain | Annotated | Expected | Observed | Adjusted p-value | Genes | Ensembl IDs | | --- | --- | --- | --- | --- | --- | --- | --- | --- | | GO:0007031 | peroxisome organization | biological\_process | 13 | 1.8 | 8 | 0.02515 | pex12 pex10 pik3r4 pex11a pex11b pex5 pex13 pex1 | ENSDARG00000035149 ENSDARG00000041511 ENSDARG00000060469 ENSDARG00000060707 ENSDARG00000069147 ENSDARG00000070654 ENSDARG00000071037 ENSDARG00000098904 | | GO:0048193 | Golgi vesicle transport | biological\_process | 53 | 7.4 | 24 | 0.02362 | arcn1a cog8 cog2 nbas golga1 sys1 zw10 spast stx5a arcn1b cux1b GOLPH3 (1 of many) gcc1 golph3 tmem115 rab35b vps51 rab8a sec24b trip11 cnsta cog3 cog6 cog1 | ENSDARG00000002792 ENSDARG00000002798 ENSDARG00000004037 ENSDARG00000008593 ENSDARG00000008979 ENSDARG00000017606 ENSDARG00000018738 ENSDARG00000024933 ENSDARG00000025033 ENSDARG00000031214 ENSDARG00000043134 ENSDARG00000044225 ENSDARG00000045541 ENSDARG00000052851 ENSDARG00000055763 ENSDARG00000058425 ENSDARG00000062016 ENSDARG00000067920 ENSDARG00000071906 ENSDARG00000078381 ENSDARG00000086283 ENSDARG00000101999 ENSDARG00000103149 ENSDARG00000105131 | | GO:0006891 | intra-Golgi vesicle-mediated transport | biological\_process | 17 | 2.4 | 10 | 0.00559 | cog8 cog2 golga1 sys1 cux1b gcc1 trip11 cog3 cog6 cog1 | ENSDARG00000002798 ENSDARG00000004037 ENSDARG00000008979 ENSDARG00000017606 ENSDARG00000043134 ENSDARG00000045541 ENSDARG00000078381 ENSDARG00000101999 ENSDARG00000103149 ENSDARG00000105131 | | GO:0000209 | protein polyubiquitination | biological\_process | 47 | 6.6 | 17 | 0.03048 | ppil2 zgc:55512 arih1 ube2g1a amfr dtl traf6 arih1l stub1 ankib1a rnf217 ube2j2 chfr ankib1b rnf152 rnf146 rnf168 | ENSDARG00000002016 ENSDARG00000003017 ENSDARG00000003616 ENSDARG00000015292 ENSDARG00000020218 ENSDARG00000023002 ENSDARG00000028058 ENSDARG00000036870 ENSDARG00000045228 ENSDARG00000060768 ENSDARG00000060944 ENSDARG00000061161 ENSDARG00000075347 ENSDARG00000076829 ENSDARG00000087782 ENSDARG00000089981 ENSDARG00000105172 | | GO:0005769 | early endosome | cellular\_component | 21 | 2.8 | 10 | 0.04064 | rab5b snx5 plekhf2 snx27a zfyve28 tgfbrap1 wash1 snx17 vipas39 rab11fip5a | ENSDARG00000016059 ENSDARG00000020442 ENSDARG00000021141 ENSDARG00000033804 ENSDARG00000060430 ENSDARG00000061508 ENSDARG00000063457 ENSDARG00000091418 ENSDARG00000103690 ENSDARG00000104179 | | GO:0005813 | centrosome | cellular\_component | 71 | 9.6 | 21 | 0.02540 | ippk snx10a traf3ip1 tubgcp2 usp33 poc1b dtl spast tubgcp3 pafah1b1a cep78 lrmp ppp1r42 cep350 lzts2b tubgcp5 wdr81 cspp1a cep83 nde1 ndel1b | ENSDARG00000003446 ENSDARG00000004405 ENSDARG00000010300 ENSDARG00000013079 ENSDARG00000016163 ENSDARG00000021110 ENSDARG00000023002 ENSDARG00000024933 ENSDARG00000029133 ENSDARG00000032013 ENSDARG00000039229 ENSDARG00000045574 ENSDARG00000057632 ENSDARG00000058000 ENSDARG00000077207 ENSDARG00000077442 ENSDARG00000079702 ENSDARG00000100236 ENSDARG00000101236 ENSDARG00000103902 ENSDARG00000104225 | | GO:0004842 | ubiquitin-protein transferase activity | molecular\_function | 156 | 22.4 | 49 | 0.04572 | klhl15 ppil2 zgc:55512 arih1 klhl18 cnot4b cbl cul2 ube2g1a smurf1 fbxo30b amfr klhl24a traf3 dtl rad18 traf6 ube2z ube2j1 pex12 ube3c bach2a arih1l smurf2 traf4b msl2b rnf141 stub1 mul1b klhl26 ube3a fbxo30a ube2r2 traf7 ankib1a rnf217 ube2j2 bach1a traf1 chfr herc3 ankib1b rnf152 rnf146 pja2 si:dkey-260j18.2 btr20 btr18 rnf168 | ENSDARG00000001930 ENSDARG00000002016 ENSDARG00000003017 ENSDARG00000003616 ENSDARG00000004306 ENSDARG00000007639 ENSDARG00000009958 ENSDARG00000013965 ENSDARG00000015292 ENSDARG00000016086 ENSDARG00000019311 ENSDARG00000020218 ENSDARG00000021739 ENSDARG00000022000 ENSDARG00000023002 ENSDARG00000027938 ENSDARG00000028058 ENSDARG00000029215 ENSDARG00000033489 ENSDARG00000035149 ENSDARG00000035978 ENSDARG00000036569 ENSDARG00000036870 ENSDARG00000038067 ENSDARG00000038964 ENSDARG00000039430 ENSDARG00000041802 ENSDARG00000045228 ENSDARG00000052527 ENSDARG00000053876 ENSDARG00000055737 ENSDARG00000058561 ENSDARG00000058740 ENSDARG00000060207 ENSDARG00000060768 ENSDARG00000060944 ENSDARG00000061161 ENSDARG00000062553 ENSDARG00000069482 ENSDARG00000075347 ENSDARG00000075887 ENSDARG00000076829 ENSDARG00000087782 ENSDARG00000089981 ENSDARG00000100296 ENSDARG00000101942 ENSDARG00000102992 ENSDARG00000103618 ENSDARG00000105172 | | GO:0061630 | ubiquitin protein ligase activity | molecular\_function | 72 | 10.4 | 24 | 0.00965 | ppil2 zgc:55512 arih1 cul2 ube2g1a smurf1 fbxo30b amfr rad18 ube2z ube2j1 ube3c arih1l smurf2 msl2b ube3a fbxo30a ube2r2 ankib1a rnf217 ube2j2 ankib1b pja2 btr20 | ENSDARG00000002016 ENSDARG00000003017 ENSDARG00000003616 ENSDARG00000013965 ENSDARG00000015292 ENSDARG00000016086 ENSDARG00000019311 ENSDARG00000020218 ENSDARG00000027938 ENSDARG00000029215 ENSDARG00000033489 ENSDARG00000035978 ENSDARG00000036870 ENSDARG00000038067 ENSDARG00000039430 ENSDARG00000055737 ENSDARG00000058561 ENSDARG00000058740 ENSDARG00000060768 ENSDARG00000060944 ENSDARG00000061161 ENSDARG00000076829 ENSDARG00000100296 ENSDARG00000102992 | | GO:0046872 | metal ion binding | molecular\_function | 2036 | 292.8 | 338 | 0.00018 | thraa si:ch73-314g15.3 znf511 unk sp3a mnat1 zfand5b lhx8a reps1 scnm1 zbtb22b arih1 atp7a tada2b phf6 egln1b hinfp large zc3h12a prdm9 ppm1f ankmy2a bmi1a vldlr lnpb enpp4 tdrd1 mkrn2 cnot4b nudt17 si:ch211-241e1.5 prkcda rnf150b cbl si:ch211-282j22.3 slc25a25a mical1 dpf2 npepl1 bmi1b gclc znf76 armc1 rabgef1 znf598 zc3h15 zdhhc16b usp33 zdhhc5a CDC42BPA otud7b sobpb rnf167 braf prkcha henmt1 kdm4ab nln fbxo30b prpsap2 cdc42bpb znf513 ppef1 amfr map3k7 smpd4 cyb5r4 prickle2a plekhf2 ppm1db tab2 erap1b traf3 si:dkey-20i20.12 zgc:66448 zbtb1 lrp13 znf668 mbd1b cxxc1b DTX3 chp2 l3mbtl3 zgc:66474 ehmt1b ENSDARG00000026972 rad18 bmp1b traf6 mkrn4 btr16 taf1b atxn7l3 tph1a phf23a trim2a hif1an dtx2 dis3l2 cyp17a1 jade1 mysm1 trim35-27 pex12 zmat5 slc25a25b rps6ka3a zmym4 tdp2b zbtb3 phf23b vps11 znf865 arih1l IKZF4 hivep3b rybpa zgc:101663 zgc:113411 si:dkeyp-68b7.7 traf4b zgc:123010 msl2b zgc:101562 ca15b rnf11b zgc:114130 pex10 rnf141 arhgef18b parp12a fbxl5 znf292b cyp2x7 acvr2b sned1 agbl5 zbtb43 znf507 pcgf5b mul1b EVI5L zgc:113372 adhfe1 zgc:162948 si:rp71-1g18.1 cpdb cyp17a2 btr09 zdhhc20a zhx2 ftr97 zcchc7 atf7b si:ch1073-416j23.1 rnf17 ikzf5 ptenb phc2a si:dkey-30k6.5 zgc:66443 zdhhc20b dnaja3a fbxo30a znf990 pdp2 ENSDARG00000058679 rock1 thap1 zbtb8a calml4b tbc1d9 traf7 arhgef28 zfyve28 rbm27 ankib1a trim62 adnp2a rnf217 vezf1b atp10a zbtb44 zfp64 znf142 sec23ip phf14 kdm4c rnf38 tab3 fan1 nudt14 leng9 pex2 pias1b march8 nt5dc3 ppm1la rlf rreb1a ehmt1a znf131 xaf1 trim23 traf1 efhb zdhhc18a zgc:173548 nfxl1 vps18 sh3rf1 ppp4ca pla2g4f.2 gtf3ab znf711 sec24b rnf26 brpf3b adnpb fxn zbtb17 phc3 usp45 chfr ENSDARG00000075504 phf12b zbtb24 zswim2 zdhhc6 zyx si:dkey-258f14.3 march6 zgc:162972 pdp1 zgc:174310 zgc:193801 patz1 man1b1b sp2 ankib1b znf280d zgc:173726 zgc:110821 znf526 myo9b usf3 limd2 prdm1c zgc:165515 tcf20 znf692 rnf111 adam12 trim59 si:dkeyp-2e4.2 zbtb47b ENSDARG00000079660 micall1a si:dkeyp-68b7.5 prkd3 znf970 dbf4b im:7147486 znf574 maza wu:fe05a04 si:ch1073-127d16.1 rnf152 si:dkey-66i24.8 znf1028 ciz1b si:ch73-367f21.5 ciz1a si:dkey-210j14.5 rnf146 zc3h3 ints12 znf653 zzef1 si:ch211-148l7.4 si:ch211-284e13.5 FDXACB1 znf236 si:dkey-217d24.6 si:dkey-111k8.2 mtmr3 tax1bp1a znf326 hdac4 ninl si:dkeyp-53d3.3 pygo1 vdra dus3l zgc:66483 si:ch211-11n16.2 mgrn1a pja2 lnpep hic2 zmynd19 hpdl ext1b zdhhc13 znf644a znf1014 im:7141269 zfyve9a zbtb49 man2a1 slc25a12 micall2a sytl4 zgc:171673 trim32 rsf1a parn cdh1 znf687a jmjd6 btr20 ext1a znf609a zfpl1 polm btr18 ttf2 si:dkey-208k4.2 prdm10 fyco1a acvr2ab zgc:171422 dgke si:ch73-221f6.1 mtr ing2 znf45l rnf168 | ENSDARG00000000151 ENSDARG00000000423 ENSDARG00000000760 ENSDARG00000000935 ENSDARG00000001549 ENSDARG00000002077 ENSDARG00000002271 ENSDARG00000002330 ENSDARG00000002877 ENSDARG00000003027 ENSDARG00000003251 ENSDARG00000003616 ENSDARG00000003699 ENSDARG00000003769 ENSDARG00000004046 ENSDARG00000004632 ENSDARG00000004851 ENSDARG00000005126 ENSDARG00000005271 ENSDARG00000005382 ENSDARG00000005786 ENSDARG00000005948 ENSDARG00000006010 ENSDARG00000006257 ENSDARG00000006639 ENSDARG00000006877 ENSDARG00000007465 ENSDARG00000007630 ENSDARG00000007639 ENSDARG00000008014 ENSDARG00000008906 ENSDARG00000009208 ENSDARG00000009524 ENSDARG00000009958 ENSDARG00000010524 ENSDARG00000010572 ENSDARG00000011809 ENSDARG00000012219 ENSDARG00000012871 ENSDARG00000013076 ENSDARG00000013095 ENSDARG00000013279 ENSDARG00000013861 ENSDARG00000014430 ENSDARG00000014945 ENSDARG00000015889 ENSDARG00000015989 ENSDARG00000016163 ENSDARG00000016263 ENSDARG00000016464 ENSDARG00000017220 ENSDARG00000017386 ENSDARG00000017636 ENSDARG00000017661 ENSDARG00000018382 ENSDARG00000018871 ENSDARG00000019103 ENSDARG00000019233 ENSDARG00000019311 ENSDARG00000019326 ENSDARG00000019383 ENSDARG00000019961 ENSDARG00000020191 ENSDARG00000020218 ENSDARG00000020469 ENSDARG00000020730 ENSDARG00000020898 ENSDARG00000020982 ENSDARG00000021141 ENSDARG00000021380 ENSDARG00000021509 ENSDARG00000021859 ENSDARG00000022000 ENSDARG00000022905 ENSDARG00000022952 ENSDARG00000023040 ENSDARG00000023479 ENSDARG00000025226 ENSDARG00000025699 ENSDARG00000025718 ENSDARG00000025766 ENSDARG00000025788 ENSDARG00000025983 ENSDARG00000026453 ENSDARG00000026634 ENSDARG00000026972 ENSDARG00000027938 ENSDARG00000028053 ENSDARG00000028058 ENSDARG00000028295 ENSDARG00000028850 ENSDARG00000028937 ENSDARG00000029331 ENSDARG00000029432 ENSDARG00000030887 ENSDARG00000031817 ENSDARG00000031915 ENSDARG00000032933 ENSDARG00000033259 ENSDARG00000033566 ENSDARG00000033707 ENSDARG00000034693 ENSDARG00000035009 ENSDARG00000035149 ENSDARG00000035434 ENSDARG00000035468 ENSDARG00000035556 ENSDARG00000035823 ENSDARG00000035954 ENSDARG00000036235 ENSDARG00000036305 ENSDARG00000036338 ENSDARG00000036698 ENSDARG00000036870 ENSDARG00000037068 ENSDARG00000037154 ENSDARG00000037773 ENSDARG00000037852 ENSDARG00000038133 ENSDARG00000038476 ENSDARG00000038964 ENSDARG00000039082 ENSDARG00000039430 ENSDARG00000040179 ENSDARG00000040510 ENSDARG00000040635 ENSDARG00000040725 ENSDARG00000041511 ENSDARG00000041802 ENSDARG00000042308 ENSDARG00000042496 ENSDARG00000043046 ENSDARG00000043973 ENSDARG00000044002 ENSDARG00000044422 ENSDARG00000044433 ENSDARG00000045900 ENSDARG00000045983 ENSDARG00000052164 ENSDARG00000052388 ENSDARG00000052527 ENSDARG00000052766 ENSDARG00000053263 ENSDARG00000053518 ENSDARG00000053695 ENSDARG00000053792 ENSDARG00000053877 ENSDARG00000053966 ENSDARG00000054805 ENSDARG00000055066 ENSDARG00000055162 ENSDARG00000055436 ENSDARG00000055443 ENSDARG00000055481 ENSDARG00000055934 ENSDARG00000056387 ENSDARG00000056491 ENSDARG00000056623 ENSDARG00000056695 ENSDARG00000057238 ENSDARG00000057707 ENSDARG00000058178 ENSDARG00000058494 ENSDARG00000058561 ENSDARG00000058562 ENSDARG00000058571 ENSDARG00000058679 ENSDARG00000058993 ENSDARG00000059020 ENSDARG00000059226 ENSDARG00000059347 ENSDARG00000059900 ENSDARG00000060207 ENSDARG00000060415 ENSDARG00000060430 ENSDARG00000060521 ENSDARG00000060768 ENSDARG00000060901 ENSDARG00000060937 ENSDARG00000060944 ENSDARG00000061030 ENSDARG00000061039 ENSDARG00000061110 ENSDARG00000061147 ENSDARG00000061373 ENSDARG00000061413 ENSDARG00000061458 ENSDARG00000061504 ENSDARG00000062055 ENSDARG00000062063 ENSDARG00000062269 ENSDARG00000062335 ENSDARG00000062374 ENSDARG00000062421 ENSDARG00000062445 ENSDARG00000062489 ENSDARG00000062949 ENSDARG00000063218 ENSDARG00000063553 ENSDARG00000063701 ENSDARG00000068157 ENSDARG00000068400 ENSDARG00000068939 ENSDARG00000069420 ENSDARG00000069482 ENSDARG00000069703 ENSDARG00000069807 ENSDARG00000070048 ENSDARG00000070127 ENSDARG00000070433 ENSDARG00000070470 ENSDARG00000070570 ENSDARG00000070953 ENSDARG00000071583 ENSDARG00000071868 ENSDARG00000071906 ENSDARG00000074074 ENSDARG00000074238 ENSDARG00000074293 ENSDARG00000074356 ENSDARG00000074548 ENSDARG00000074604 ENSDARG00000075013 ENSDARG00000075347 ENSDARG00000075504 ENSDARG00000075509 ENSDARG00000075533 ENSDARG00000075700 ENSDARG00000075721 ENSDARG00000075733 ENSDARG00000075974 ENSDARG00000076066 ENSDARG00000076134 ENSDARG00000076308 ENSDARG00000076442 ENSDARG00000076552 ENSDARG00000076584 ENSDARG00000076592 ENSDARG00000076763 ENSDARG00000076829 ENSDARG00000077013 ENSDARG00000077078 ENSDARG00000077085 ENSDARG00000077143 ENSDARG00000077410 ENSDARG00000077431 ENSDARG00000078092 ENSDARG00000078161 ENSDARG00000078212 ENSDARG00000078348 ENSDARG00000078434 ENSDARG00000078802 ENSDARG00000079068 ENSDARG00000079238 ENSDARG00000079263 ENSDARG00000079547 ENSDARG00000079660 ENSDARG00000079811 ENSDARG00000079930 ENSDARG00000079967 ENSDARG00000086494 ENSDARG00000086550 ENSDARG00000086626 ENSDARG00000087074 ENSDARG00000087330 ENSDARG00000087530 ENSDARG00000087674 ENSDARG00000087782 ENSDARG00000088084 ENSDARG00000088331 ENSDARG00000089461 ENSDARG00000089503 ENSDARG00000089781 ENSDARG00000089951 ENSDARG00000089981 ENSDARG00000090751 ENSDARG00000091678 ENSDARG00000093469 ENSDARG00000094380 ENSDARG00000094469 ENSDARG00000094725 ENSDARG00000095750 ENSDARG00000095890 ENSDARG00000096428 ENSDARG00000098021 ENSDARG00000098201 ENSDARG00000098288 ENSDARG00000098348 ENSDARG00000098349 ENSDARG00000098414 ENSDARG00000098664 ENSDARG00000098687 ENSDARG00000099483 ENSDARG00000099528 ENSDARG00000099612 ENSDARG00000099779 ENSDARG00000100072 ENSDARG00000100296 ENSDARG00000100394 ENSDARG00000100497 ENSDARG00000100773 ENSDARG00000101010 ENSDARG00000101019 ENSDARG00000101144 ENSDARG00000101361 ENSDARG00000101562 ENSDARG00000101756 ENSDARG00000101903 ENSDARG00000102111 ENSDARG00000102200 ENSDARG00000102362 ENSDARG00000102366 ENSDARG00000102433 ENSDARG00000102473 ENSDARG00000102505 ENSDARG00000102589 ENSDARG00000102634 ENSDARG00000102750 ENSDARG00000102774 ENSDARG00000102896 ENSDARG00000102992 ENSDARG00000103155 ENSDARG00000103388 ENSDARG00000103453 ENSDARG00000103470 ENSDARG00000103618 ENSDARG00000104105 ENSDARG00000104170 ENSDARG00000104251 ENSDARG00000104500 ENSDARG00000104700 ENSDARG00000104715 ENSDARG00000104793 ENSDARG00000104904 ENSDARG00000104906 ENSDARG00000104907 ENSDARG00000105056 ENSDARG00000105172 | | GO:0008270 | zinc ion binding | molecular\_function | 677 | 97.4 | 138 | 0.00152 | thraa unk mnat1 zfand5b lhx8a arih1 tada2b phf6 bmi1a mkrn2 cnot4b rnf150b cbl mical1 dpf2 bmi1b rabgef1 znf598 zdhhc16b usp33 zdhhc5a otud7b rnf167 kdm4ab fbxo30b amfr prickle2a tab2 erap1b traf3 mbd1b cxxc1b DTX3 l3mbtl3 ehmt1b ENSDARG00000026972 rad18 bmp1b traf6 mkrn4 btr16 atxn7l3 phf23a trim2a dtx2 jade1 trim35-27 pex12 zmat5 zmym4 phf23b vps11 arih1l rybpa zgc:113411 traf4b msl2b ca15b rnf11b pex10 rnf141 agbl5 pcgf5b mul1b EVI5L cpdb btr09 zdhhc20a ftr97 zcchc7 rnf17 phc2a zdhhc20b fbxo30a ENSDARG00000058679 traf7 ankib1a trim62 rnf217 phf14 kdm4c rnf38 tab3 pex2 pias1b march8 ehmt1a xaf1 trim23 traf1 zdhhc18a nfxl1 vps18 sh3rf1 sec24b rnf26 brpf3b phc3 usp45 chfr ENSDARG00000075504 phf12b zswim2 zdhhc6 zyx march6 ankib1b limd2 tcf20 rnf111 adam12 trim59 ENSDARG00000079660 micall1a dbf4b rnf152 ciz1b ciz1a rnf146 ints12 zzef1 pygo1 vdra mgrn1a pja2 lnpep zdhhc13 man2a1 micall2a trim32 rsf1a btr20 zfpl1 btr18 ttf2 mtr ing2 rnf168 | ENSDARG00000000151 ENSDARG00000000935 ENSDARG00000002077 ENSDARG00000002271 ENSDARG00000002330 ENSDARG00000003616 ENSDARG00000003769 ENSDARG00000004046 ENSDARG00000006010 ENSDARG00000007630 ENSDARG00000007639 ENSDARG00000009524 ENSDARG00000009958 ENSDARG00000011809 ENSDARG00000012219 ENSDARG00000013076 ENSDARG00000014430 ENSDARG00000014945 ENSDARG00000015989 ENSDARG00000016163 ENSDARG00000016263 ENSDARG00000017220 ENSDARG00000017636 ENSDARG00000019103 ENSDARG00000019311 ENSDARG00000020218 ENSDARG00000020982 ENSDARG00000021509 ENSDARG00000021859 ENSDARG00000022000 ENSDARG00000025699 ENSDARG00000025718 ENSDARG00000025766 ENSDARG00000025983 ENSDARG00000026634 ENSDARG00000026972 ENSDARG00000027938 ENSDARG00000028053 ENSDARG00000028058 ENSDARG00000028295 ENSDARG00000028850 ENSDARG00000029331 ENSDARG00000030887 ENSDARG00000031817 ENSDARG00000032933 ENSDARG00000033707 ENSDARG00000035009 ENSDARG00000035149 ENSDARG00000035434 ENSDARG00000035823 ENSDARG00000036305 ENSDARG00000036338 ENSDARG00000036870 ENSDARG00000037773 ENSDARG00000038133 ENSDARG00000038964 ENSDARG00000039430 ENSDARG00000040510 ENSDARG00000040635 ENSDARG00000041511 ENSDARG00000041802 ENSDARG00000045900 ENSDARG00000052388 ENSDARG00000052527 ENSDARG00000052766 ENSDARG00000053877 ENSDARG00000054805 ENSDARG00000055066 ENSDARG00000055436 ENSDARG00000055443 ENSDARG00000056387 ENSDARG00000056695 ENSDARG00000058178 ENSDARG00000058561 ENSDARG00000058679 ENSDARG00000060207 ENSDARG00000060768 ENSDARG00000060901 ENSDARG00000060944 ENSDARG00000061458 ENSDARG00000061504 ENSDARG00000062055 ENSDARG00000062063 ENSDARG00000062421 ENSDARG00000062445 ENSDARG00000062489 ENSDARG00000068157 ENSDARG00000068939 ENSDARG00000069420 ENSDARG00000069482 ENSDARG00000069807 ENSDARG00000070127 ENSDARG00000070433 ENSDARG00000070470 ENSDARG00000071906 ENSDARG00000074074 ENSDARG00000074238 ENSDARG00000074604 ENSDARG00000075013 ENSDARG00000075347 ENSDARG00000075504 ENSDARG00000075509 ENSDARG00000075700 ENSDARG00000075721 ENSDARG00000075733 ENSDARG00000076066 ENSDARG00000076829 ENSDARG00000078092 ENSDARG00000078348 ENSDARG00000078802 ENSDARG00000079068 ENSDARG00000079238 ENSDARG00000079660 ENSDARG00000079811 ENSDARG00000086550 ENSDARG00000087782 ENSDARG00000089461 ENSDARG00000089781 ENSDARG00000089981 ENSDARG00000091678 ENSDARG00000094380 ENSDARG00000098687 ENSDARG00000099483 ENSDARG00000100072 ENSDARG00000100296 ENSDARG00000100394 ENSDARG00000101144 ENSDARG00000102200 ENSDARG00000102366 ENSDARG00000102505 ENSDARG00000102589 ENSDARG00000102992 ENSDARG00000103453 ENSDARG00000103618 ENSDARG00000104105 ENSDARG00000104906 ENSDARG00000104907 ENSDARG00000105172 | | GO:0000030 | mannosyltransferase activity | molecular\_function | 29 | 4.2 | 13 | 0.00864 | large pomgnt2 galnt6 sdf2 alg11 alg3 alg1 pomt2 galnt12 alg5 pomt1 zdhhc18a galnt18a | ENSDARG00000005126 ENSDARG00000010941 ENSDARG00000014386 ENSDARG00000024026 ENSDARG00000031202 ENSDARG00000053155 ENSDARG00000054963 ENSDARG00000055027 ENSDARG00000055490 ENSDARG00000061235 ENSDARG00000067670 ENSDARG00000069807 ENSDARG00000100889 | | GO:0008375 | acetylglucosaminyltransferase activity | molecular\_function | 38 | 5.5 | 17 | 0.00157 | b3gnt5b large pomgnt2 mgat1a galnt6 extl3 zgc:101663 mgat3a pomgnt1 mgat2 galnt12 xylt2 ccdc126 mgat4a galnt18a ext1b ext1a | ENSDARG00000004396 ENSDARG00000005126 ENSDARG00000010941 ENSDARG00000012407 ENSDARG00000014386 ENSDARG00000026811 ENSDARG00000037852 ENSDARG00000038069 ENSDARG00000052025 ENSDARG00000052408 ENSDARG00000055490 ENSDARG00000059557 ENSDARG00000062695 ENSDARG00000063330 ENSDARG00000100889 ENSDARG00000101019 ENSDARG00000103155 | | GO:0035091 | phosphatidylinositol binding | molecular\_function | 66 | 9.5 | 24 | 0.04064 | snx10a arhgap33 picalma snx13 snx15 snx5 si:ch211-200p22.4 snx27a fcho2 wipi2 nisch GOLPH3 (1 of many) snx11 golph3 snx24 zfyve28 pik3c2a sgk3 snx21 snx18a snx9b arfip1 amer1 snx17 | ENSDARG00000004405 ENSDARG00000011333 ENSDARG00000012866 ENSDARG00000013828 ENSDARG00000020397 ENSDARG00000020442 ENSDARG00000029170 ENSDARG00000033804 ENSDARG00000035389 ENSDARG00000037871 ENSDARG00000043077 ENSDARG00000044225 ENSDARG00000052522 ENSDARG00000052851 ENSDARG00000053527 ENSDARG00000060430 ENSDARG00000060841 ENSDARG00000062460 ENSDARG00000062770 ENSDARG00000067713 ENSDARG00000069302 ENSDARG00000070055 ENSDARG00000079624 ENSDARG00000091418 | |
