# Supplementary material for: A high-resolution mRNA expression time course of embryonic development in zebrafish
Source: eLife. 2017 Nov 16;6:e30860. doi: 10.7554/eLife.30860 (PMC5690287; doi:10.7554/eLife.30860)
Supplement: Supplementary file 6. [file elife-30860-supp6.zip › biolayout-clusters-files/Cluster003-genes.html]

Cluster003


# Cluster003: Genes

| | Ensembl ID | Gene Name | Chr | Start | End | Biotype | | --- | --- | --- | --- | --- | --- | | ENSDARG00000059503 | ACSF3 | 7 | 55651329 | 55736488 | protein\_coding | | ENSDARG00000002670 | ATG14 | 17 | 10592332 | 10610643 | protein\_coding | | ENSDARG00000101094 | ATP11B | 22 | 37006588 | 37064215 | protein\_coding | | ENSDARG00000079546 | BX548000.1 | 11 | 29295038 | 29304002 | protein\_coding | | ENSDARG00000078394 | CABZ01059627.1 | 10 | 19611604 | 19627894 | protein\_coding | | ENSDARG00000100177 | CABZ01066957.1 | KN149968.1 | 3248 | 13337 | protein\_coding | | ENSDARG00000090998 | CABZ01071911.1 | 4 | 72538760 | 72554520 | protein\_coding | | ENSDARG00000099654 | CABZ01072765.1 | 7 | 68897233 | 68901516 | protein\_coding | | ENSDARG00000100626 | CABZ01080599.1 | 18 | 5066176 | 5078495 | protein\_coding | | ENSDARG00000098959 | CABZ01113771.1 | KN149914.1 | 21 | 16939 | protein\_coding | | ENSDARG00000045199 | CAPN1 (1 of many) | 7 | 6548425 | 6618314 | protein\_coding | | ENSDARG00000105136 | CCDC50 | 22 | 24291683 | 24335635 | protein\_coding | | ENSDARG00000091326 | CEP120 | 8 | 59241 | 108563 | protein\_coding | | ENSDARG00000091754 | CHCHD5 | 3 | 52640493 | 52644341 | protein\_coding | | ENSDARG00000100186 | CLCC1 | 22 | 24576375 | 24594828 | protein\_coding | | ENSDARG00000088956 | CNTD1 | 12 | 3453137 | 3461097 | protein\_coding | | ENSDARG00000068231 | CRACR2A (1 of many) | 18 | 11588074 | 11626753 | protein\_coding | | ENSDARG00000101340 | CST7 | KN150663.1 | 111 | 632 | protein\_coding | | ENSDARG00000098327 | CT573860.1 | 3 | 54551940 | 54563492 | protein\_coding | | ENSDARG00000091234 | CU019646.2 | 7 | 19987960 | 19989382 | protein\_coding | | ENSDARG00000095486 | CU467646.2 | 4 | 75536652 | 75537791 | protein\_coding | | ENSDARG00000101763 | CU467646.5 | 4 | 75560210 | 75560911 | protein\_coding | | ENSDARG00000058946 | CU856539.2 | 10 | 2573044 | 2641613 | protein\_coding | | ENSDARG00000100088 | CU915738.1 | 15 | 37122103 | 37202477 | protein\_coding | | ENSDARG00000088039 | CU929391.1 | 15 | 44180421 | 44200742 | protein\_coding | | ENSDARG00000073961 | CYSTM1 (1 of many) | 21 | 32018822 | 32027735 | protein\_coding | | ENSDARG00000011493 | DCUN1D3 | 24 | 39630927 | 39647408 | protein\_coding | | ENSDARG00000053246 | DEPDC7 (1 of many) | 25 | 5845288 | 5855100 | protein\_coding | | ENSDARG00000102325 | EHMT2 | 19 | 1334979 | 1356617 | protein\_coding | | ENSDARG00000037528 | ENSDARG00000037528 | 20 | 6543313 | 6550986 | protein\_coding | | ENSDARG00000043963 | ENSDARG00000043963 | 20 | 170642 | 194206 | protein\_coding | | ENSDARG00000045254 | ENSDARG00000045254 | 24 | 37810920 | 37820708 | protein\_coding | | ENSDARG00000075385 | ENSDARG00000075385 | 16 | 1200492 | 1212591 | protein\_coding | | ENSDARG00000082979 | ENSDARG00000082979 | 9 | 52367104 | 52419620 | protein\_coding | | ENSDARG00000087461 | ENSDARG00000087461 | 12 | 34753499 | 34769026 | protein\_coding | | ENSDARG00000092508 | ENSDARG00000092508 | 5 | 62609269 | 62626111 | protein\_coding | | ENSDARG00000094043 | ENSDARG00000094043 | 5 | 62628507 | 62645290 | protein\_coding | | ENSDARG00000096554 | ENSDARG00000096554 | 1 | 25983044 | 26006725 | protein\_coding | | ENSDARG00000098194 | ENSDARG00000098194 | 12 | 1671374 | 1735160 | protein\_coding | | ENSDARG00000098209 | ENSDARG00000098209 | 14 | 49779239 | 49798951 | protein\_coding | | ENSDARG00000098474 | ENSDARG00000098474 | KN150447.1 | 650 | 5694 | protein\_coding | | ENSDARG00000098623 | ENSDARG00000098623 | 11 | 11319837 | 11336232 | protein\_coding | | ENSDARG00000098674 | ENSDARG00000098674 | 18 | 50863799 | 50876414 | protein\_coding | | ENSDARG00000098784 | ENSDARG00000098784 | KN150307.1 | 6472 | 7270 | protein\_coding | | ENSDARG00000099385 | ENSDARG00000099385 | 7 | 39139162 | 39169054 | protein\_coding | | ENSDARG00000101309 | ENSDARG00000101309 | 11 | 44962340 | 44974258 | protein\_coding | | ENSDARG00000102050 | ENSDARG00000102050 | 23 | 43770357 | 43785552 | protein\_coding | | ENSDARG00000102419 | ENSDARG00000102419 | 16 | 55370283 | 55381910 | protein\_coding | | ENSDARG00000104448 | ENSDARG00000104448 | 17 | 132565 | 133308 | protein\_coding | | ENSDARG00000077696 | FAM188B | 8 | 18470429 | 18480673 | protein\_coding | | ENSDARG00000099605 | FO834829.1 | 9 | 173138 | 174127 | protein\_coding | | ENSDARG00000004864 | FOXR2 | 5 | 58009362 | 58014746 | protein\_coding | | ENSDARG00000075685 | FRMPD4 | 9 | 54510852 | 54561158 | protein\_coding | | ENSDARG00000102426 | FUT9 (1 of many).4 | 8 | 3330419 | 3354399 | protein\_coding | | ENSDARG00000076111 | GXYLT1 (1 of many) | 25 | 242637 | 246814 | protein\_coding | | ENSDARG00000057114 | HPF1 | 1 | 13333896 | 13340263 | protein\_coding | | ENSDARG00000075416 | HSCB | 5 | 9579462 | 9588793 | protein\_coding | | ENSDARG00000098650 | IQGAP3 | 16 | 54970569 | 55003510 | protein\_coding | | ENSDARG00000054539 | LRP10 | 7 | 23881897 | 23891771 | protein\_coding | | ENSDARG00000088999 | METTL4 | 7 | 71335010 | 71343798 | protein\_coding | | ENSDARG00000094300 | NUPR2 | 5 | 1732440 | 1734163 | protein\_coding | | ENSDARG00000100872 | OTUD1 | 24 | 9157812 | 9159092 | protein\_coding | | ENSDARG00000088768 | PQLC1 | 19 | 11488961 | 11574452 | protein\_coding | | ENSDARG00000078775 | RASA2 | 18 | 41102555 | 41199405 | protein\_coding | | ENSDARG00000038186 | RDM1 | 3 | 22204482 | 22210558 | protein\_coding | | ENSDARG00000077516 | RECQL4 | 19 | 2897387 | 2928587 | protein\_coding | | ENSDARG00000098968 | RSBN1 | 23 | 26257231 | 26301554 | protein\_coding | | ENSDARG00000104128 | SNX2 | 8 | 293181 | 324522 | protein\_coding | | ENSDARG00000098537 | SPATA22 | 5 | 61626050 | 61644616 | protein\_coding | | ENSDARG00000071594 | TIMM21 | 24 | 15995957 | 16005340 | protein\_coding | | ENSDARG00000076934 | WDR60 | 7 | 39851919 | 39871425 | protein\_coding | | ENSDARG00000098540 | ZC3H7B (1 of many) | 3 | 4548100 | 4590736 | protein\_coding | | ENSDARG00000077546 | ZDHHC1 | 7 | 34919753 | 34941339 | protein\_coding | | ENSDARG00000074041 | abca5 | 12 | 38633103 | 38681547 | protein\_coding | | ENSDARG00000062795 | abcb7 | 14 | 10675689 | 10764142 | protein\_coding | | ENSDARG00000035571 | abhd17b | 5 | 25133621 | 25153049 | protein\_coding | | ENSDARG00000025797 | abhd2a | 7 | 13573155 | 13611473 | protein\_coding | | ENSDARG00000099240 | acap3b | 11 | 3316944 | 3475577 | protein\_coding | | ENSDARG00000052124 | accs | 25 | 8844357 | 8872571 | protein\_coding | | ENSDARG00000101060 | acrc | 14 | 14361705 | 14381441 | protein\_coding | | ENSDARG00000061201 | acsf2 | 12 | 26405807 | 26447463 | protein\_coding | | ENSDARG00000093003 | acy3.1 | 1 | 51864674 | 51870541 | protein\_coding | | ENSDARG00000001452 | adam8a | 13 | 24541031 | 24549186 | protein\_coding | | ENSDARG00000003544 | adarb1b | 9 | 45692862 | 45740888 | protein\_coding | | ENSDARG00000004889 | aga | 14 | 36088127 | 36096935 | protein\_coding | | ENSDARG00000100854 | ago4 | 19 | 48104155 | 48186562 | protein\_coding | | ENSDARG00000063187 | agpat5 | 13 | 6130932 | 6151224 | protein\_coding | | ENSDARG00000058088 | aifm1 | 5 | 22046814 | 22069974 | protein\_coding | | ENSDARG00000069037 | akip1 | 18 | 16961241 | 16964912 | protein\_coding | | ENSDARG00000026862 | aktip | 7 | 36195838 | 36212201 | protein\_coding | | ENSDARG00000052247 | alkbh4 | 5 | 60782897 | 60788045 | protein\_coding | | ENSDARG00000060256 | alkbh8 | 16 | 42206032 | 42225585 | protein\_coding | | ENSDARG00000074779 | alms1 | 13 | 9036596 | 9074136 | protein\_coding | | ENSDARG00000100376 | amdhd2 | 3 | 13369691 | 13396326 | protein\_coding | | ENSDARG00000078435 | anapc13 | 21 | 24499628 | 24500456 | protein\_coding | | ENSDARG00000101285 | anapc15 | 15 | 46895634 | 46900156 | protein\_coding | | ENSDARG00000103831 | ankrd13c | 11 | 45024959 | 45042768 | protein\_coding | | ENSDARG00000009023 | ankrd28b | 16 | 20250042 | 20346917 | protein\_coding | | ENSDARG00000023508 | ankrd49 | 15 | 2887692 | 2894167 | protein\_coding | | ENSDARG00000102335 | ano1 | 7 | 54052996 | 54160853 | protein\_coding | | ENSDARG00000096454 | ap1m2 | 6 | 94528 | 436629 | protein\_coding | | ENSDARG00000078805 | ap5b1 | 11 | 38850394 | 38856975 | protein\_coding | | ENSDARG00000008472 | apex2 | 8 | 22489568 | 22493679 | protein\_coding | | ENSDARG00000005612 | aph1b | 7 | 28905774 | 28915786 | protein\_coding | | ENSDARG00000101626 | arf6a | 20 | 54525232 | 54527311 | protein\_coding | | ENSDARG00000078326 | arhgap10 | 1 | 36039911 | 36180662 | protein\_coding | | ENSDARG00000083189 | arhgap19 | 22 | 34984597 | 35003428 | protein\_coding | | ENSDARG00000001210 | arl14ep | 7 | 32353534 | 32358308 | protein\_coding | | ENSDARG00000006791 | arntl1a | 25 | 17797384 | 17822410 | protein\_coding | | ENSDARG00000061081 | arpp21 | 19 | 43316976 | 43347789 | protein\_coding | | ENSDARG00000068148 | arrdc1a | 5 | 28916691 | 28931795 | protein\_coding | | ENSDARG00000013058 | ascc2 | 5 | 17508158 | 17531257 | protein\_coding | | ENSDARG00000101037 | asf1ba | 3 | 45359507 | 45369581 | protein\_coding | | ENSDARG00000060392 | ash2l | 10 | 2732055 | 2761621 | protein\_coding | | ENSDARG00000033361 | asl | 21 | 31216455 | 31237784 | protein\_coding | | ENSDARG00000043287 | atg101 | 10 | 32713636 | 32719899 | protein\_coding | | ENSDARG00000052104 | atg4b | 6 | 27100239 | 27114452 | protein\_coding | | ENSDARG00000057719 | atl2 | 13 | 7949200 | 7972585 | protein\_coding | | ENSDARG00000004270 | atl3 | 14 | 30433718 | 30454510 | protein\_coding | | ENSDARG00000061890 | atp13a2 | 23 | 24584876 | 24616411 | protein\_coding | | ENSDARG00000060978 | atp2a3 | 5 | 30567703 | 30669038 | protein\_coding | | ENSDARG00000060980 | atp8b1 | 21 | 3467098 | 3548981 | protein\_coding | | ENSDARG00000087961 | avd | 10 | 21401734 | 21404746 | protein\_coding | | ENSDARG00000054937 | badb | 7 | 24858363 | 24862696 | protein\_coding | | ENSDARG00000029305 | baiap2l1a | 12 | 17694071 | 17741458 | protein\_coding | | ENSDARG00000011818 | batf | 20 | 46668830 | 46742836 | protein\_coding | | ENSDARG00000099745 | baz1a | 17 | 9881300 | 9903962 | protein\_coding | | ENSDARG00000071046 | bbip1 | 22 | 29699659 | 29703161 | protein\_coding | | ENSDARG00000014676 | bckdhb | 16 | 5166061 | 5205719 | protein\_coding | | ENSDARG00000007783 | blk | 20 | 19275448 | 19297273 | protein\_coding | | ENSDARG00000096603 | bmb | 25 | 35742338 | 35760296 | protein\_coding | | ENSDARG00000090234 | bmf1 | 23 | 39339698 | 39348017 | protein\_coding | | ENSDARG00000020009 | bmp2k | 5 | 38499019 | 38552614 | protein\_coding | | ENSDARG00000008807 | bokb | 2 | 22984564 | 22994784 | protein\_coding | | ENSDARG00000102802 | brdt | 6 | 24264869 | 24293106 | protein\_coding | | ENSDARG00000017835 | brf1a | 13 | 33238012 | 33353891 | protein\_coding | | ENSDARG00000002060 | brf2 | 10 | 41357723 | 41381002 | protein\_coding | | ENSDARG00000098112 | btc | 21 | 19332139 | 19342654 | protein\_coding | | ENSDARG00000069065 | btg3 | 10 | 28484191 | 28494303 | protein\_coding | | ENSDARG00000035171 | btg4 | 5 | 57254394 | 57256476 | protein\_coding | | ENSDARG00000045139 | ca7 | 7 | 44463128 | 44474538 | protein\_coding | | ENSDARG00000036658 | cab39l | 1 | 45907270 | 45926051 | protein\_coding | | ENSDARG00000076964 | cables2a | 23 | 30971482 | 30992776 | protein\_coding | | ENSDARG00000019208 | camsap1a | 21 | 4547569 | 4599559 | protein\_coding | | ENSDARG00000102865 | carnmt1 | KN150339.1 | 41271 | 49830 | protein\_coding | | ENSDARG00000104702 | cat | 25 | 7318706 | 7328383 | protein\_coding | | ENSDARG00000067517 | ccdc15 | 5 | 57751528 | 57780641 | protein\_coding | | ENSDARG00000102145 | ccdc77 | 25 | 3423667 | 3437048 | protein\_coding | | ENSDARG00000043236 | ccna1 | 10 | 34964476 | 34972124 | protein\_coding | | ENSDARG00000051923 | ccnb1 | 5 | 54088191 | 54091953 | protein\_coding | | ENSDARG00000036180 | ccnb2 | 7 | 30355228 | 30359966 | protein\_coding | | ENSDARG00000009607 | ccnc | 4 | 5755916 | 5766937 | protein\_coding | | ENSDARG00000098622 | ccne1 | 7 | 45747466 | 45757513 | protein\_coding | | ENSDARG00000055124 | ccnl1a | 18 | 41521910 | 41536296 | protein\_coding | | ENSDARG00000099692 | ccnyl1 | 9 | 28426632 | 28444283 | protein\_coding | | ENSDARG00000100741 | cdc20 | 2 | 19568522 | 19585305 | protein\_coding | | ENSDARG00000069708 | cdc34a | 22 | 22115917 | 22139405 | protein\_coding | | ENSDARG00000043720 | cdc45 | 8 | 4677738 | 4704456 | protein\_coding | | ENSDARG00000023584 | cdc7 | 2 | 11080343 | 11094166 | protein\_coding | | ENSDARG00000088639 | cdca9 | 12 | 22551104 | 22624564 | protein\_coding | | ENSDARG00000070686 | cdipt | 3 | 20994745 | 21006814 | protein\_coding | | ENSDARG00000043640 | cenpn | 18 | 29920701 | 29927756 | protein\_coding | | ENSDARG00000079679 | cep112 | 3 | 55433153 | 55774217 | protein\_coding | | ENSDARG00000002991 | cep135 | 20 | 25812628 | 25836225 | protein\_coding | | ENSDARG00000075725 | cep152 | 25 | 31904362 | 31952384 | protein\_coding | | ENSDARG00000074636 | cep170b | 17 | 1799244 | 1863912 | protein\_coding | | ENSDARG00000002826 | cep57l1 | 20 | 32238683 | 32246141 | protein\_coding | | ENSDARG00000060215 | cep85 | 16 | 41734969 | 41764956 | protein\_coding | | ENSDARG00000102407 | cep97 | 1 | 11822 | 16373 | protein\_coding | | ENSDARG00000002365 | cers5 | 22 | 5728244 | 5792301 | protein\_coding | | ENSDARG00000041215 | cetn4 | 14 | 1334847 | 1342544 | protein\_coding | | ENSDARG00000016255 | chmp4ba | 6 | 49902812 | 49921667 | protein\_coding | | ENSDARG00000041362 | chmp7 | 8 | 51364448 | 51380769 | protein\_coding | | ENSDARG00000101167 | chst3b | 12 | 48984866 | 48989308 | protein\_coding | | ENSDARG00000102674 | ckap5 | 25 | 13649151 | 13693664 | protein\_coding | | ENSDARG00000104550 | clcn2b | KN150171.1 | 31423 | 90706 | protein\_coding | | ENSDARG00000099950 | cldn17 | 15 | 42604215 | 42637647 | protein\_coding | | ENSDARG00000014047 | cldn7b | 10 | 22175316 | 22250266 | protein\_coding | | ENSDARG00000006580 | cldnd | 21 | 25740820 | 25741596 | protein\_coding | | ENSDARG00000003701 | cldng | 1 | 32912476 | 32914601 | protein\_coding | | ENSDARG00000077584 | cln6a | 7 | 34216421 | 34221131 | protein\_coding | | ENSDARG00000036064 | cnep1r1 | 7 | 37987491 | 37993122 | protein\_coding | | ENSDARG00000032116 | cnot7 | 14 | 30073444 | 30084540 | protein\_coding | | ENSDARG00000020043 | cnot8 | 21 | 36756905 | 36768305 | protein\_coding | | ENSDARG00000003757 | cnpy1 | 2 | 30002689 | 30012503 | protein\_coding | | ENSDARG00000074662 | cntf | 1 | 39377050 | 39384763 | protein\_coding | | ENSDARG00000042747 | coasy | 24 | 36387588 | 36413000 | protein\_coding | | ENSDARG00000089858 | cobll1a | 6 | 10098381 | 10130064 | protein\_coding | | ENSDARG00000040070 | coil | 3 | 35990184 | 35997636 | protein\_coding | | ENSDARG00000060380 | coq6 | 17 | 44896103 | 44915162 | protein\_coding | | ENSDARG00000075933 | cox15 | 13 | 583650 | 594094 | protein\_coding | | ENSDARG00000069920 | cox17 | 9 | 21424820 | 21427376 | protein\_coding | | ENSDARG00000008454 | cpeb1b | 7 | 47967931 | 47978459 | protein\_coding | | ENSDARG00000062025 | cpox | 8 | 18566030 | 18582931 | protein\_coding | | ENSDARG00000056480 | cpvl | 16 | 20690911 | 20709101 | protein\_coding | | ENSDARG00000011583 | cry1ab | 18 | 15138006 | 15161184 | protein\_coding | | ENSDARG00000006963 | cse1l | 11 | 5522383 | 5543878 | protein\_coding | | ENSDARG00000006125 | csnk1db | 12 | 33219521 | 33235804 | protein\_coding | | ENSDARG00000017522 | ctdp1 | 19 | 11712659 | 11928658 | protein\_coding | | ENSDARG00000062116 | ctdsplb | 2 | 21698180 | 21725335 | protein\_coding | | ENSDARG00000057328 | cth1 | 7 | 17695244 | 17698337 | protein\_coding | | ENSDARG00000011777 | cttn | 18 | 50921184 | 50928863 | protein\_coding | | ENSDARG00000020986 | ctu1 | 8 | 3364403 | 3380735 | protein\_coding | | ENSDARG00000091760 | cwf19l2 | 15 | 44260260 | 44325065 | protein\_coding | | ENSDARG00000077765 | cx44.2 | 6 | 18941182 | 18949592 | protein\_coding | | ENSDARG00000000069 | dap | 24 | 21925116 | 21958411 | protein\_coding | | ENSDARG00000060093 | dapk1 | 5 | 44054986 | 44195753 | protein\_coding | | ENSDARG00000103666 | dapk2b | 14 | 44566727 | 44730307 | protein\_coding | | ENSDARG00000036214 | dazl | 19 | 20804781 | 20819548 | protein\_coding | | ENSDARG00000077790 | dcaf15 | 1 | 53409707 | 53424922 | protein\_coding | | ENSDARG00000099642 | dcun1d1 | 2 | 7900441 | 7913517 | protein\_coding | | ENSDARG00000074431 | ddb1 | 18 | 50803251 | 50808967 | protein\_coding | | ENSDARG00000103083 | ddb1.1 | 18 | 50963148 | 50967512 | protein\_coding | | ENSDARG00000011072 | ddx11 | 18 | 6362076 | 6394151 | protein\_coding | | ENSDARG00000030250 | dennd2db | 22 | 818830 | 839988 | protein\_coding | | ENSDARG00000011605 | dennd6b | 4 | 20081781 | 20092654 | protein\_coding | | ENSDARG00000018165 | depdc1a | 8 | 16677104 | 16689861 | protein\_coding | | ENSDARG00000042401 | derl2 | 5 | 58327448 | 58339456 | protein\_coding | | ENSDARG00000004460 | desi2 | 13 | 11417979 | 11438093 | protein\_coding | | ENSDARG00000098247 | det1 | 25 | 12302541 | 12316199 | protein\_coding | | ENSDARG00000035564 | dgcr8 | 5 | 24016637 | 24028586 | protein\_coding | | ENSDARG00000098175 | dgkab | 11 | 2311706 | 2321026 | protein\_coding | | ENSDARG00000098364 | dgkab.1 | 11 | 2421914 | 2435399 | protein\_coding | | ENSDARG00000075395 | dguok | 5 | 68930050 | 68935242 | protein\_coding | | ENSDARG00000014956 | diablob | 21 | 21486446 | 21574221 | protein\_coding | | ENSDARG00000025513 | dip2cb | 2 | 42646290 | 42729836 | protein\_coding | | ENSDARG00000009783 | dirc2 | 9 | 37557178 | 37643333 | protein\_coding | | ENSDARG00000078759 | dna2 | 13 | 22691258 | 22709317 | protein\_coding | | ENSDARG00000087473 | dnajb12b | 12 | 48363371 | 48391431 | protein\_coding | | ENSDARG00000041896 | dnajc5ga | 20 | 38861074 | 38884462 | protein\_coding | | ENSDARG00000031116 | dnal4a | 3 | 24381411 | 24387948 | protein\_coding | | ENSDARG00000022813 | dnd1 | 14 | 7091395 | 7102955 | protein\_coding | | ENSDARG00000030756 | dnmt1 | 3 | 54336611 | 54352679 | protein\_coding | | ENSDARG00000036915 | dolpp1 | 21 | 3733037 | 3754042 | protein\_coding | | ENSDARG00000052606 | dpp9 | 8 | 47688216 | 47733073 | protein\_coding | | ENSDARG00000000853 | dstyk | 22 | 435537 | 469215 | protein\_coding | | ENSDARG00000059386 | dtwd1 | 18 | 5390216 | 5393084 | protein\_coding | | ENSDARG00000052103 | dtymk | 6 | 27112930 | 27118297 | protein\_coding | | ENSDARG00000077607 | dus2 | 25 | 35699956 | 35713406 | protein\_coding | | ENSDARG00000076330 | dusp12 | 2 | 21002892 | 21057122 | protein\_coding | | ENSDARG00000039850 | dusp22b | 2 | 1011546 | 1028491 | protein\_coding | | ENSDARG00000042555 | dym | 21 | 3324764 | 3462281 | protein\_coding | | ENSDARG00000007278 | ect2 | 11 | 10404945 | 10472696 | protein\_coding | | ENSDARG00000025094 | edem1 | 6 | 42935221 | 42951648 | protein\_coding | | ENSDARG00000020279 | efcab7 | 6 | 32041730 | 32059069 | protein\_coding | | ENSDARG00000005163 | efr3a | 2 | 43023190 | 43090855 | protein\_coding | | ENSDARG00000052115 | ehf | 25 | 10313814 | 10340100 | protein\_coding | | ENSDARG00000005078 | eif4e3 | 23 | 10789072 | 10810349 | protein\_coding | | ENSDARG00000045095 | elac1 | 5 | 6291778 | 6301560 | protein\_coding | | ENSDARG00000040732 | elavl2 | 22 | 38299579 | 38321088 | protein\_coding | | ENSDARG00000042005 | elp3 | 20 | 34768507 | 34797775 | protein\_coding | | ENSDARG00000017093 | emc8 | 18 | 30522436 | 30526474 | protein\_coding | | ENSDARG00000008808 | eml2 | 15 | 28948262 | 28974935 | protein\_coding | | ENSDARG00000006640 | eomesa | 19 | 861017 | 875079 | protein\_coding | | ENSDARG00000040534 | epcam | 13 | 8508749 | 8511340 | protein\_coding | | ENSDARG00000014161 | ercc4 | 12 | 19837163 | 19852658 | protein\_coding | | ENSDARG00000077304 | erg | 10 | 160347 | 167782 | protein\_coding | | ENSDARG00000021991 | erlin1 | 13 | 24571301 | 24586946 | protein\_coding | | ENSDARG00000075354 | espl1 | 6 | 39671314 | 39703429 | protein\_coding | | ENSDARG00000018814 | esrp2 | 7 | 34523352 | 34550359 | protein\_coding | | ENSDARG00000056832 | exo1 | 17 | 22545867 | 22553535 | protein\_coding | | ENSDARG00000063191 | extl2 | 2 | 7251631 | 7260085 | protein\_coding | | ENSDARG00000068856 | f2rl1.1 | 21 | 7329352 | 7334884 | protein\_coding | | ENSDARG00000052374 | faf2 | 14 | 45842972 | 45863060 | protein\_coding | | ENSDARG00000031657 | fahd1 | 3 | 18645830 | 18646819 | protein\_coding | | ENSDARG00000102566 | fam102aa | 8 | 2597651 | 2680757 | protein\_coding | | ENSDARG00000002685 | fam113 | 7 | 24559419 | 24567270 | protein\_coding | | ENSDARG00000079508 | fam114a2 | 21 | 37738208 | 37750421 | protein\_coding | | ENSDARG00000062970 | fam129ba | 21 | 13702719 | 13759960 | protein\_coding | | ENSDARG00000008026 | fam129bb | 5 | 28316728 | 28369009 | protein\_coding | | ENSDARG00000063270 | fam134a | 1 | 5492171 | 5512940 | protein\_coding | | ENSDARG00000079788 | fam171a1 | 16 | 28612534 | 28640166 | protein\_coding | | ENSDARG00000043339 | fam175a | 21 | 19283168 | 19292440 | protein\_coding | | ENSDARG00000078383 | fam195b | 3 | 59573808 | 59585761 | protein\_coding | | ENSDARG00000052638 | fam210b | 6 | 58922776 | 59361811 | protein\_coding | | ENSDARG00000020929 | fam49ba | 2 | 32232054 | 32279069 | protein\_coding | | ENSDARG00000020979 | fam65c | 6 | 51676228 | 51763504 | protein\_coding | | ENSDARG00000017111 | fam8a1a | 19 | 32614678 | 32619506 | protein\_coding | | ENSDARG00000053574 | fancd2 | 6 | 42293772 | 42339461 | protein\_coding | | ENSDARG00000019250 | fancf | 25 | 34789712 | 34791207 | protein\_coding | | ENSDARG00000024967 | fancg | 5 | 40896337 | 40909186 | protein\_coding | | ENSDARG00000007885 | fancl | 13 | 26573447 | 26650347 | protein\_coding | | ENSDARG00000104186 | fbxo43 | 16 | 54681445 | 54850016 | protein\_coding | | ENSDARG00000022623 | fbxo44 | 23 | 16822463 | 16831412 | protein\_coding | | ENSDARG00000007477 | fbxo8 | 1 | 37626865 | 37642318 | protein\_coding | | ENSDARG00000104806 | fbxw11a | KN150273.1 | 28692 | 68022 | protein\_coding | | ENSDARG00000062636 | fem1b | 25 | 1254885 | 1259917 | protein\_coding | | ENSDARG00000069129 | fhdc3 | 10 | 26229094 | 26241053 | protein\_coding | | ENSDARG00000087166 | figla | 5 | 13339099 | 13348079 | protein\_coding | | ENSDARG00000016427 | fignl1 | 13 | 15802176 | 15812945 | protein\_coding | | ENSDARG00000062385 | flcn | 16 | 13928380 | 13955877 | protein\_coding | | ENSDARG00000059701 | flii | 3 | 40064173 | 40090757 | protein\_coding | | ENSDARG00000097351 | flnbl | 11 | 41515653 | 41529899 | protein\_coding | | ENSDARG00000061778 | fmn2b | 12 | 47448300 | 47543432 | protein\_coding | | ENSDARG00000020131 | fnbp1l | 8 | 14949301 | 15043927 | protein\_coding | | ENSDARG00000071198 | fopnl | 6 | 8356046 | 8362196 | protein\_coding | | ENSDARG00000057089 | ftsj1 | 21 | 21133976 | 21142115 | protein\_coding | | ENSDARG00000076811 | ganab | 14 | 46527170 | 46571349 | protein\_coding | | ENSDARG00000045880 | gas2l3 | 4 | 1839999 | 1871335 | protein\_coding | | ENSDARG00000027016 | gbf1 | 13 | 7243361 | 7417020 | protein\_coding | | ENSDARG00000018953 | gclm | 8 | 15231718 | 15239547 | protein\_coding | | ENSDARG00000037995 | gdf3 | 17 | 4081036 | 4093820 | protein\_coding | | ENSDARG00000105394 | gemin6 | 11 | 45091164 | 45092804 | protein\_coding | | ENSDARG00000103667 | gga3 | 6 | 22844394 | 22862650 | protein\_coding | | ENSDARG00000055086 | ggnbp2 | 5 | 3682348 | 3719311 | protein\_coding | | ENSDARG00000002304 | gins2 | 18 | 30502776 | 30521235 | protein\_coding | | ENSDARG00000068123 | gkap1 | 8 | 49717618 | 49739985 | protein\_coding | | ENSDARG00000069409 | gkup | 15 | 21327185 | 21339951 | protein\_coding | | ENSDARG00000043559 | gle1 | 8 | 2484982 | 2516238 | protein\_coding | | ENSDARG00000088439 | gm2a | 14 | 25927110 | 25930546 | protein\_coding | | ENSDARG00000018174 | gnai2a | 11 | 34659499 | 34780201 | protein\_coding | | ENSDARG00000017294 | gnai2b | 6 | 53051837 | 53146007 | protein\_coding | | ENSDARG00000099771 | gne | 1 | 11608342 | 11761059 | protein\_coding | | ENSDARG00000037307 | gnpda1 | 14 | 23411967 | 23416458 | protein\_coding | | ENSDARG00000063197 | golga2 | 5 | 1375388 | 1432828 | protein\_coding | | ENSDARG00000075331 | golga4 | 13 | 47772087 | 47870501 | protein\_coding | | ENSDARG00000069525 | gpn3 | 21 | 16979701 | 16986018 | protein\_coding | | ENSDARG00000096651 | gpr108 | 1 | 58365635 | 58373910 | protein\_coding | | ENSDARG00000034975 | gpr157 | 23 | 22671073 | 22679364 | protein\_coding | | ENSDARG00000030038 | grcc10 | 20 | 14562950 | 14567032 | protein\_coding | | ENSDARG00000069139 | grik1a | 10 | 25441299 | 25608934 | protein\_coding | | ENSDARG00000087187 | grip2a | 8 | 7161969 | 7289642 | protein\_coding | | ENSDARG00000069865 | grxcr1 | 13 | 9108303 | 9110919 | protein\_coding | | ENSDARG00000029501 | gsk3aa | 19 | 6131879 | 6164753 | protein\_coding | | ENSDARG00000015681 | gsk3ab | 16 | 11351519 | 11401246 | protein\_coding | | ENSDARG00000011000 | gtf2a1 | 20 | 16538158 | 16554029 | protein\_coding | | ENSDARG00000053685 | gtf2a2 | 7 | 29080350 | 29084934 | protein\_coding | | ENSDARG00000018650 | gtpbp3 | 11 | 6012922 | 6040504 | protein\_coding | | ENSDARG00000068438 | gucd1 | 8 | 30721396 | 30733001 | protein\_coding | | ENSDARG00000102892 | h1m | KN150456.1 | 14113 | 19625 | protein\_coding | | ENSDARG00000062280 | hace1 | 16 | 2844733 | 2880152 | protein\_coding | | ENSDARG00000036038 | harbi1 | 7 | 38536769 | 38539358 | protein\_coding | | ENSDARG00000101652 | haus6 | 7 | 58853983 | 58863210 | protein\_coding | | ENSDARG00000037432 | haus7 | 23 | 19896091 | 19905396 | protein\_coding | | ENSDARG00000031246 | hbegfb | 21 | 30124600 | 30132252 | protein\_coding | | ENSDARG00000034957 | hddc2 | 20 | 39782937 | 39787156 | protein\_coding | | ENSDARG00000061864 | hdlbpb | 12 | 22282889 | 22323842 | protein\_coding | | ENSDARG00000004392 | hdr | 5 | 26804793 | 26814928 | protein\_coding | | ENSDARG00000073686 | heatr6 | 21 | 38697388 | 38747431 | protein\_coding | | ENSDARG00000043055 | hiat1b | 22 | 10684031 | 10705566 | protein\_coding | | ENSDARG00000027749 | hirip3 | 3 | 15043705 | 15060691 | protein\_coding | | ENSDARG00000053810 | hnrnpc | 2 | 38042847 | 38051917 | protein\_coding | | ENSDARG00000024598 | homezb | 7 | 23736009 | 23742696 | protein\_coding | | ENSDARG00000099235 | hook2 | 3 | 7877216 | 7935490 | protein\_coding | | ENSDARG00000008884 | hprt1 | 14 | 31169608 | 31181229 | protein\_coding | | ENSDARG00000012249 | hs3st1 | 14 | 270424 | 271305 | protein\_coding | | ENSDARG00000075984 | hsbp1l1 | 19 | 11484117 | 11486132 | protein\_coding | | ENSDARG00000020241 | icmt | 23 | 21565600 | 21572423 | protein\_coding | | ENSDARG00000071699 | ids | 14 | 20572373 | 20594524 | protein\_coding | | ENSDARG00000043934 | il17a/f2 | 17 | 12575959 | 12579792 | protein\_coding | | ENSDARG00000001442 | inppl1b | 14 | 32452958 | 32484094 | protein\_coding | | ENSDARG00000078888 | iqgap1 | 7 | 50370776 | 50489379 | protein\_coding | | ENSDARG00000045681 | irf5 | 4 | 13605424 | 13615972 | protein\_coding | | ENSDARG00000101986 | irf6 | 22 | 1153396 | 1165267 | protein\_coding | | ENSDARG00000051888 | ist1 | 7 | 56171157 | 56183387 | protein\_coding | | ENSDARG00000099199 | itchb | 23 | 43639607 | 43703501 | protein\_coding | | ENSDARG00000039650 | itm2cb | 2 | 48430300 | 48444821 | protein\_coding | | ENSDARG00000054973 | itsn2b | 17 | 30456544 | 30504080 | protein\_coding | | ENSDARG00000058996 | jam2a | 1 | 599405 | 613068 | protein\_coding | | ENSDARG00000060102 | kank1 | 5 | 44246213 | 44340737 | protein\_coding | | ENSDARG00000021827 | katna1 | 20 | 23961758 | 23977191 | protein\_coding | | ENSDARG00000042522 | katnbl1 | 20 | 29306736 | 29313357 | protein\_coding | | ENSDARG00000087226 | kbtbd2 | 12 | 14111383 | 14124478 | protein\_coding | | ENSDARG00000012390 | kcnk5b | 20 | 9235775 | 9251298 | protein\_coding | | ENSDARG00000059798 | kcnq1 | 7 | 48282850 | 48384453 | protein\_coding | | ENSDARG00000073859 | kif13bb | 20 | 50012476 | 50089891 | protein\_coding | | ENSDARG00000054978 | kifc3 | 18 | 45529529 | 45595459 | protein\_coding | | ENSDARG00000007764 | klhdc10 | 4 | 14992161 | 15005163 | protein\_coding | | ENSDARG00000061786 | klhl2 | 1 | 20239115 | 20277329 | protein\_coding | | ENSDARG00000063577 | klhl35 | 10 | 233694 | 244906 | protein\_coding | | ENSDARG00000101270 | klhl8 | 21 | 30655 | 39713 | protein\_coding | | ENSDARG00000027169 | kpna7 | 10 | 25243704 | 25246787 | protein\_coding | | ENSDARG00000040224 | krcp | 7 | 24610515 | 24617477 | protein\_coding | | ENSDARG00000030933 | ksr1b | 15 | 31189862 | 31266560 | protein\_coding | | ENSDARG00000040803 | lactb | 25 | 32770665 | 32780947 | protein\_coding | | ENSDARG00000092610 | lamp1 | 9 | 34571114 | 34580690 | protein\_coding | | ENSDARG00000045140 | lcmt1 | 12 | 20256502 | 20287920 | protein\_coding | | ENSDARG00000020493 | lgi1a | 13 | 290957 | 299852 | protein\_coding | | ENSDARG00000031044 | lipg | 8 | 32367704 | 32376810 | protein\_coding | | ENSDARG00000007108 | lipia | 21 | 34910543 | 34938382 | protein\_coding | | ENSDARG00000009693 | llgl1 | 3 | 39828496 | 39912909 | protein\_coding | | ENSDARG00000007751 | lmnl3 | 23 | 46000368 | 46012740 | protein\_coding | | ENSDARG00000053535 | lmo7b | 1 | 33633277 | 33718216 | protein\_coding | | ENSDARG00000020354 | lmx1a | 20 | 34018898 | 34037381 | protein\_coding | | ENSDARG00000063646 | lnpa | 9 | 2020028 | 2044714 | protein\_coding | | ENSDARG00000071478 | lnx2b | 14 | 32578170 | 32605615 | protein\_coding | | ENSDARG00000102765 | lonp1 | 22 | 3143174 | 3165692 | protein\_coding | | ENSDARG00000011506 | lpcat1 | 19 | 28706989 | 28749941 | protein\_coding | | ENSDARG00000035028 | lpcat4 | 7 | 1337849 | 1359886 | protein\_coding | | ENSDARG00000102684 | lrrc1 | 13 | 2122952 | 2151885 | protein\_coding | | ENSDARG00000014005 | lrrc45 | 12 | 2958790 | 2985114 | protein\_coding | | ENSDARG00000099291 | lsr | 15 | 34080865 | 34106943 | protein\_coding | | ENSDARG00000075528 | lyrm9 | 15 | 30430102 | 30431212 | protein\_coding | | ENSDARG00000100528 | map1lc3c | 11 | 44536220 | 44539854 | protein\_coding | | ENSDARG00000060805 | map1sa | 22 | 36853851 | 36888457 | protein\_coding | | ENSDARG00000098405 | map3k1 | 10 | 5903143 | 6005333 | protein\_coding | | ENSDARG00000039125 | map4k5 | 13 | 36579753 | 36655744 | protein\_coding | | ENSDARG00000037276 | map9 | 1 | 25046938 | 25059059 | protein\_coding | | ENSDARG00000028721 | mapk14b | 11 | 2641176 | 2691019 | protein\_coding | | ENSDARG00000042927 | mapre1a | 8 | 23360710 | 23367406 | protein\_coding | | ENSDARG00000100300 | marveld3 | 7 | 68964816 | 68965461 | protein\_coding | | ENSDARG00000019364 | mbip | 17 | 38348262 | 38360794 | protein\_coding | | ENSDARG00000045428 | mcee | 7 | 30508635 | 30511893 | protein\_coding | | ENSDARG00000009779 | mcl1a | 19 | 348522 | 353024 | protein\_coding | | ENSDARG00000045815 | mcm10 | 4 | 7842500 | 7861102 | protein\_coding | | ENSDARG00000021402 | mcm3ap | 9 | 38647919 | 38671686 | protein\_coding | | ENSDARG00000002305 | me3 | 9 | 34437199 | 34451029 | protein\_coding | | ENSDARG00000070623 | med10 | 19 | 28386069 | 28389928 | protein\_coding | | ENSDARG00000009472 | med19b | 1 | 43881066 | 43890186 | protein\_coding | | ENSDARG00000010823 | memo1 | 13 | 35385061 | 35401865 | protein\_coding | | ENSDARG00000019715 | metap1d | 1 | 30142300 | 30170234 | protein\_coding | | ENSDARG00000068893 | mettl5 | 9 | 48425607 | 48434240 | protein\_coding | | ENSDARG00000070085 | mettl6 | 16 | 40390491 | 40394179 | protein\_coding | | ENSDARG00000060001 | mettl9 | 12 | 956855 | 964213 | protein\_coding | | ENSDARG00000061908 | mfsd12a | 2 | 23425596 | 23443993 | protein\_coding | | ENSDARG00000103235 | mfsd8 | 12 | 22507485 | 22523996 | protein\_coding | | ENSDARG00000017834 | micall2b | 1 | 8775649 | 8802103 | protein\_coding | | ENSDARG00000104845 | mipol1 | 17 | 10169598 | 10342313 | protein\_coding | | ENSDARG00000044766 | mitd1 | 9 | 7296794 | 7308984 | protein\_coding | | ENSDARG00000018411 | mknk1 | 11 | 13118909 | 13132092 | protein\_coding | | ENSDARG00000041665 | mkrn1 | 4 | 12293425 | 12308985 | protein\_coding | | ENSDARG00000059630 | mlec | 8 | 39836940 | 39850585 | protein\_coding | | ENSDARG00000034616 | mlf2 | 16 | 31871914 | 31881151 | protein\_coding | | ENSDARG00000025948 | mlh1 | 13 | 42476346 | 42491579 | protein\_coding | | ENSDARG00000060383 | mmaa | 1 | 35224408 | 35238232 | protein\_coding | | ENSDARG00000069744 | mos | 15 | 17162329 | 17163538 | protein\_coding | | ENSDARG00000045416 | mppe1 | 24 | 8839066 | 8861714 | protein\_coding | | ENSDARG00000105014 | mre11a | 15 | 2894267 | 2924135 | protein\_coding | | ENSDARG00000011885 | mrpl19 | 20 | 25541424 | 25546156 | protein\_coding | | ENSDARG00000057805 | mrpl28 | 15 | 17192260 | 17202618 | protein\_coding | | ENSDARG00000039667 | mrpl38 | 12 | 44867469 | 44886420 | protein\_coding | | ENSDARG00000103198 | mrps2 | 21 | 7560451 | 7566573 | protein\_coding | | ENSDARG00000018073 | mrps22 | 15 | 6944386 | 6969449 | protein\_coding | | ENSDARG00000104111 | mrps30 | 5 | 52324832 | 52357459 | protein\_coding | | ENSDARG00000019732 | mtch2 | 7 | 38395808 | 38406832 | protein\_coding | | ENSDARG00000053087 | mthfr | 8 | 47228641 | 47262086 | protein\_coding | | ENSDARG00000099741 | mvb12a | 1 | 58530502 | 58536716 | protein\_coding | | ENSDARG00000004130 | mvk | 5 | 18990398 | 19005895 | protein\_coding | | ENSDARG00000098350 | mxd3 | 14 | 15762755 | 15777243 | protein\_coding | | ENSDARG00000008494 | myl6 | 6 | 39876641 | 39895990 | protein\_coding | | ENSDARG00000008859 | mylipa | 19 | 26756513 | 26794432 | protein\_coding | | ENSDARG00000027825 | naa50 | 24 | 21030432 | 21039194 | protein\_coding | | ENSDARG00000060362 | nadkb | 2 | 37284784 | 37299291 | protein\_coding | | ENSDARG00000075113 | nanog | 24 | 12689785 | 12694862 | protein\_coding | | ENSDARG00000068255 | nanos3 | 1 | 54569719 | 54570845 | protein\_coding | | ENSDARG00000039208 | nasp | 6 | 33926464 | 33932098 | protein\_coding | | ENSDARG00000061185 | nat15 | 3 | 27655759 | 27665509 | protein\_coding | | ENSDARG00000005058 | ncapd2 | 2 | 44896905 | 44924594 | protein\_coding | | ENSDARG00000021439 | ncoa4 | 13 | 3784664 | 3805959 | protein\_coding | | ENSDARG00000010052 | ndrg3b | 23 | 15076417 | 15234844 | protein\_coding | | ENSDARG00000018061 | neil1 | 25 | 26383938 | 26389898 | protein\_coding | | ENSDARG00000005619 | nek2 | 20 | 13987151 | 13997930 | protein\_coding | | ENSDARG00000073899 | nemp1 | 23 | 32015509 | 32027112 | protein\_coding | | ENSDARG00000020204 | nf2a | 5 | 11227616 | 11309187 | protein\_coding | | ENSDARG00000054162 | nfatc4 | 2 | 37793198 | 37821157 | protein\_coding | | ENSDARG00000038687 | nfkb2 | 13 | 22586916 | 22605097 | protein\_coding | | ENSDARG00000062237 | nfs1 | 11 | 24110809 | 24118477 | protein\_coding | | ENSDARG00000058893 | nhej1 | 6 | 6928720 | 6951894 | protein\_coding | | ENSDARG00000060298 | nin | 13 | 36674296 | 36719113 | protein\_coding | | ENSDARG00000055912 | nipa2 | 6 | 37755714 | 37767614 | protein\_coding | | ENSDARG00000070116 | nit1 | 7 | 7261665 | 7274119 | protein\_coding | | ENSDARG00000026767 | nol11 | 6 | 17860353 | 17904326 | protein\_coding | | ENSDARG00000090912 | npc2 | 17 | 43031045 | 43041345 | protein\_coding | | ENSDARG00000032951 | nrbp2a | 2 | 32690146 | 32755340 | protein\_coding | | ENSDARG00000062372 | nrd1b | 22 | 17188133 | 17209814 | protein\_coding | | ENSDARG00000074726 | nrde2 | 17 | 26700257 | 26732281 | protein\_coding | | ENSDARG00000098780 | nsmce1 | 3 | 43361659 | 43376773 | protein\_coding | | ENSDARG00000056665 | nsun2 | 19 | 28274643 | 28318286 | protein\_coding | | ENSDARG00000105182 | ntan1 | 6 | 762975 | 929541 | protein\_coding | | ENSDARG00000042881 | nthl1 | 1 | 53465895 | 53478130 | protein\_coding | | ENSDARG00000058728 | nudt2 | 21 | 11742252 | 11747464 | protein\_coding | | ENSDARG00000012222 | nup35 | 9 | 12288266 | 12298777 | protein\_coding | | ENSDARG00000052334 | ociad1 | 20 | 23346520 | 23355776 | protein\_coding | | ENSDARG00000003091 | oclnb | 5 | 25612468 | 25620718 | protein\_coding | | ENSDARG00000000529 | ofd1 | 9 | 55126923 | 55155199 | protein\_coding | | ENSDARG00000099455 | ogt.2 | 14 | 14382163 | 14400187 | protein\_coding | | ENSDARG00000004154 | orai1b | 10 | 42085704 | 42093037 | protein\_coding | | ENSDARG00000039217 | orc1 | 6 | 33947047 | 33963193 | protein\_coding | | ENSDARG00000076474 | org | 22 | 20664980 | 20670259 | protein\_coding | | ENSDARG00000058022 | ormdl2 | 23 | 32035852 | 32043255 | protein\_coding | | ENSDARG00000013830 | osbpl2b | 23 | 9573568 | 9616997 | protein\_coding | | ENSDARG00000069298 | osbpl9 | 8 | 16580086 | 16638872 | protein\_coding | | ENSDARG00000077810 | otud4 | 23 | 43868175 | 43890603 | protein\_coding | | ENSDARG00000071082 | p4ha1b | 17 | 20127567 | 20147370 | protein\_coding | | ENSDARG00000002597 | pabpc1l | 8 | 45326436 | 45344091 | protein\_coding | | ENSDARG00000042892 | paip1 | 5 | 8180014 | 8206519 | protein\_coding | | ENSDARG00000031637 | pan3 | 24 | 21575130 | 21603526 | protein\_coding | | ENSDARG00000017703 | paqr3a | 5 | 38554464 | 38571149 | protein\_coding | | ENSDARG00000029944 | parpbp | 4 | 17349754 | 17364289 | protein\_coding | | ENSDARG00000001249 | pcgf1 | 14 | 5087796 | 5100721 | protein\_coding | | ENSDARG00000062198 | pcm1 | 1 | 15928379 | 15961224 | protein\_coding | | ENSDARG00000033012 | pcnt | 6 | 11036839 | 11127460 | protein\_coding | | ENSDARG00000069968 | pcsk7 | 15 | 557335 | 583010 | protein\_coding | | ENSDARG00000018285 | pdpk1b | 24 | 37414132 | 37438516 | protein\_coding | | ENSDARG00000088959 | pdxka | 9 | 19812494 | 19846653 | protein\_coding | | ENSDARG00000088699 | pdzd8 | 17 | 23800426 | 23875002 | protein\_coding | | ENSDARG00000099900 | peo1 | 12 | 33705369 | 33716131 | protein\_coding | | ENSDARG00000055540 | pfkfb4a | 11 | 34295203 | 34314935 | protein\_coding | | ENSDARG00000044596 | pgap2 | 21 | 30937002 | 30957882 | protein\_coding | | ENSDARG00000027172 | phactr2 | 13 | 23782128 | 23825822 | protein\_coding | | ENSDARG00000032221 | phactr4b | 16 | 34243999 | 34331140 | protein\_coding | | ENSDARG00000098739 | phc2b.1 | KN150699.1 | 14153 | 15112 | protein\_coding | | ENSDARG00000054208 | phkg2 | 12 | 28774071 | 28786288 | protein\_coding | | ENSDARG00000042215 | pias4b | 2 | 57312951 | 57339837 | protein\_coding | | ENSDARG00000020289 | pif1 | 2 | 17425681 | 17443683 | protein\_coding | | ENSDARG00000105011 | pigb | 18 | 1329209 | 1347365 | protein\_coding | | ENSDARG00000009113 | pigc | 20 | 14914906 | 14919512 | protein\_coding | | ENSDARG00000067752 | pigl | 5 | 41396736 | 41453917 | protein\_coding | | ENSDARG00000101952 | pigv | 16 | 34033830 | 34037986 | protein\_coding | | ENSDARG00000103038 | pik3r3a | 2 | 3115626 | 3146824 | protein\_coding | | ENSDARG00000023532 | pinx1 | 20 | 19107120 | 19159657 | protein\_coding | | ENSDARG00000006508 | pip5k1bb | 8 | 11196463 | 11286438 | protein\_coding | | ENSDARG00000034080 | plcd1b | 2 | 21729842 | 21780493 | protein\_coding | | ENSDARG00000087921 | plce1 | 12 | 5216481 | 5365326 | protein\_coding | | ENSDARG00000038442 | plcg1 | 23 | 2847746 | 2929123 | protein\_coding | | ENSDARG00000059951 | pld6 | 1 | 51568884 | 51576825 | protein\_coding | | ENSDARG00000030064 | plekha1a | 17 | 21867987 | 21893492 | protein\_coding | | ENSDARG00000045870 | plekha5 | 4 | 3017952 | 3215254 | protein\_coding | | ENSDARG00000078850 | plekhm2 | 23 | 24276008 | 24299982 | protein\_coding | | ENSDARG00000058471 | plk1 | 1 | 8469030 | 8477732 | protein\_coding | | ENSDARG00000057672 | plpp5 | 10 | 20570046 | 20579681 | protein\_coding | | ENSDARG00000075672 | pms2 | 12 | 17481654 | 17497650 | protein\_coding | | ENSDARG00000100028 | pole2 | 13 | 116839 | 124711 | protein\_coding | | ENSDARG00000039613 | poll | 1 | 30925274 | 30939775 | protein\_coding | | ENSDARG00000012572 | polr3f | 22 | 25007116 | 25015479 | protein\_coding | | ENSDARG00000052455 | porcnl | 8 | 22523051 | 22537534 | protein\_coding | | ENSDARG00000045749 | ppfibp1a | 4 | 10814412 | 10836771 | protein\_coding | | ENSDARG00000052423 | ppp1r12c | 6 | 19527180 | 19556466 | protein\_coding | | ENSDARG00000011239 | ppp1r14aa | 5 | 36130573 | 36139356 | protein\_coding | | ENSDARG00000102009 | ppp2r2d | 12 | 41182815 | 41217604 | protein\_coding | | ENSDARG00000076439 | ppp4cb | 12 | 3927586 | 3941577 | protein\_coding | | ENSDARG00000053447 | ppp4r2b | 6 | 44199781 | 44210135 | protein\_coding | | ENSDARG00000039980 | ppt1 | 19 | 44145087 | 44161971 | protein\_coding | | ENSDARG00000060288 | prosc | 23 | 34037088 | 34049605 | protein\_coding | | ENSDARG00000097591 | proser3 | 15 | 37660997 | 37688015 | protein\_coding | | ENSDARG00000087193 | prrg2 | 3 | 32285234 | 32292584 | protein\_coding | | ENSDARG00000030753 | prrg4 | 18 | 45669608 | 45678106 | protein\_coding | | ENSDARG00000018742 | psme4b | 12 | 23870057 | 23931860 | protein\_coding | | ENSDARG00000022652 | psmf1 | 6 | 51796297 | 51811096 | protein\_coding | | ENSDARG00000102338 | ptar1 | 5 | 51950257 | 51975361 | protein\_coding | | ENSDARG00000103699 | ptpn13 | 21 | 9294531 | 9469539 | protein\_coding | | ENSDARG00000016481 | ptpn2a | 16 | 10164590 | 10180985 | protein\_coding | | ENSDARG00000075421 | pttg1 | 14 | 34218166 | 34220968 | protein\_coding | | ENSDARG00000040039 | pttg1ipb | 9 | 12684075 | 12687937 | protein\_coding | | ENSDARG00000045562 | pus1 | 21 | 14729854 | 14735640 | protein\_coding | | ENSDARG00000088590 | pxna | 5 | 6110303 | 6155553 | protein\_coding | | ENSDARG00000043105 | qtrt1 | 3 | 50303638 | 50317508 | protein\_coding | | ENSDARG00000099593 | r3hcc1 | 10 | 20435275 | 20444780 | protein\_coding | | ENSDARG00000046124 | rab11fip1a | 8 | 37965422 | 37989350 | protein\_coding | | ENSDARG00000036462 | rab11fip1b | 10 | 41395008 | 41430728 | protein\_coding | | ENSDARG00000101534 | rab3db | 3 | 14362870 | 14387776 | protein\_coding | | ENSDARG00000037046 | rad51b | 20 | 9487232 | 9579619 | protein\_coding | | ENSDARG00000026400 | rad51d | 5 | 60188326 | 60201189 | protein\_coding | | ENSDARG00000096415 | raf1a | 23 | 4773818 | 4811692 | protein\_coding | | ENSDARG00000059406 | raf1b | 11 | 479967 | 513754 | protein\_coding | | ENSDARG00000098786 | rangrf | 5 | 53633542 | 53647493 | protein\_coding | | ENSDARG00000015649 | rap2c | 14 | 30634849 | 30642697 | protein\_coding | | ENSDARG00000036645 | rcbtb1 | 1 | 45944004 | 45958809 | protein\_coding | | ENSDARG00000019746 | rfng | 12 | 3019707 | 3042662 | protein\_coding | | ENSDARG00000056078 | rftn2 | 9 | 32502174 | 32532927 | protein\_coding | | ENSDARG00000079329 | rfwd2 | 2 | 35134170 | 35154760 | protein\_coding | | ENSDARG00000087752 | rfwd3 | 25 | 36679404 | 36691723 | protein\_coding | | ENSDARG00000071213 | rgl3a | 6 | 7974576 | 8008935 | protein\_coding | | ENSDARG00000056328 | rhot2 | 24 | 37646008 | 37671776 | protein\_coding | | ENSDARG00000062650 | rif1 | 9 | 22970150 | 22991303 | protein\_coding | | ENSDARG00000043359 | rmnd5b | 14 | 50232269 | 50258062 | protein\_coding | | ENSDARG00000042571 | rnaseh1 | 20 | 30475924 | 30482691 | protein\_coding | | ENSDARG00000055563 | rnasen | 12 | 13306593 | 13351481 | protein\_coding | | ENSDARG00000104076 | rnf126 | 2 | 55112154 | 55430033 | protein\_coding | | ENSDARG00000016867 | rnf128a | 5 | 23173620 | 23193178 | protein\_coding | | ENSDARG00000057101 | rnf180 | 21 | 20903386 | 20909022 | protein\_coding | | ENSDARG00000036831 | rnf41 | 23 | 32382283 | 32407677 | protein\_coding | | ENSDARG00000044949 | rnf8 | 20 | 51550554 | 51573432 | protein\_coding | | ENSDARG00000015917 | rnft1 | 10 | 28273640 | 28282544 | protein\_coding | | ENSDARG00000104413 | rogdi | 3 | 36286130 | 36298847 | protein\_coding | | ENSDARG00000063021 | rpain | 5 | 3568032 | 3585396 | protein\_coding | | ENSDARG00000040350 | rpp38 | 16 | 28657872 | 28659558 | protein\_coding | | ENSDARG00000006553 | rras | 3 | 32279032 | 32284407 | protein\_coding | | ENSDARG00000035074 | rtel1 | 11 | 12754247 | 12801608 | protein\_coding | | ENSDARG00000102810 | samd1a | 3 | 45280380 | 45302483 | protein\_coding | | ENSDARG00000102736 | sars2 | 15 | 14706025 | 14731172 | protein\_coding | | ENSDARG00000006196 | sav1 | 13 | 36661245 | 36672372 | protein\_coding | | ENSDARG00000079645 | sc:d217 | 22 | 7866226 | 7976496 | protein\_coding | | ENSDARG00000017010 | scamp2l | 7 | 56787509 | 56805311 | protein\_coding | | ENSDARG00000067859 | scospondin | 24 | 40129042 | 40283614 | protein\_coding | | ENSDARG00000078947 | sdccag8 | 13 | 10940807 | 11034256 | protein\_coding | | ENSDARG00000103516 | sec24c | 12 | 35442097 | 35481458 | protein\_coding | | ENSDARG00000041951 | selo | 18 | 14894895 | 14910623 | protein\_coding | | ENSDARG00000061138 | senp7b | 22 | 28286616 | 28358870 | protein\_coding | | ENSDARG00000033861 | sepsecs | 7 | 71196416 | 71214765 | protein\_coding | | ENSDARG00000052673 | sept7b | 16 | 47455184 | 47491472 | protein\_coding | | ENSDARG00000020235 | sept9a | 3 | 34325814 | 34456895 | protein\_coding | | ENSDARG00000056795 | serpine1 | 7 | 25994391 | 25998821 | protein\_coding | | ENSDARG00000070012 | sesn2 | 19 | 43745518 | 43761860 | protein\_coding | | ENSDARG00000041243 | setdb1a | 19 | 9192623 | 9225467 | protein\_coding | | ENSDARG00000053992 | sfi1 | 6 | 40902515 | 40925435 | protein\_coding | | ENSDARG00000052520 | sgms2 | 1 | 49289826 | 49306817 | protein\_coding | | ENSDARG00000100083 | sgut1 | 1 | 27283617 | 27325511 | protein\_coding | | ENSDARG00000014324 | sh2d5 | 11 | 37371775 | 37383761 | protein\_coding | | ENSDARG00000019160 | sh3bp5la | 15 | 34996056 | 35008250 | protein\_coding | | ENSDARG00000035470 | sh3glb2b | 5 | 31044307 | 31089758 | protein\_coding | | ENSDARG00000074393 | sh3yl1 | 17 | 30587856 | 30635775 | protein\_coding | | ENSDARG00000102068 | shcbp1 | 7 | 45556360 | 45579994 | protein\_coding | | ENSDARG00000058522 | shq1 | 6 | 44091653 | 44163726 | protein\_coding | | ENSDARG00000095799 | si:ch1073-228j22.1 | 23 | 44644973 | 44646007 | protein\_coding | | ENSDARG00000105279 | si:ch211-108c17.2 | 4 | 68824615 | 68857695 | protein\_coding | | ENSDARG00000087869 | si:ch211-11k18.4 | 3 | 26044237 | 26052949 | protein\_coding | | ENSDARG00000045790 | si:ch211-125a15.1 | 4 | 9337903 | 9349669 | protein\_coding | | ENSDARG00000076534 | si:ch211-14a17.10 | 2 | 38249367 | 38278944 | protein\_coding | | ENSDARG00000104543 | si:ch211-152f22.2 | 12 | 37039776 | 37096670 | lincRNA | | ENSDARG00000086336 | si:ch211-157b11.12 | 6 | 40709626 | 40715668 | protein\_coding | | ENSDARG00000097765 | si:ch211-198p11.6 | 3 | 39413904 | 39422641 | lincRNA | | ENSDARG00000011498 | si:ch211-212o1.2 | 18 | 18011562 | 18071478 | protein\_coding | | ENSDARG00000096676 | si:ch211-213i16.4 | 20 | 46004044 | 46079720 | processed\_transcript | | ENSDARG00000044061 | si:ch211-233f11.5 | 6 | 54488700 | 54496368 | protein\_coding | | ENSDARG00000042213 | si:ch211-233h19.2 | 18 | 8959747 | 8997554 | protein\_coding | | ENSDARG00000076798 | si:ch211-261p9.4 | 9 | 16304390 | 16310832 | protein\_coding | | ENSDARG00000092538 | si:ch211-262i10.2 | 21 | 19595615 | 19649043 | antisense | | ENSDARG00000086840 | si:ch211-266k8.4 | 25 | 16371460 | 16455735 | protein\_coding | | ENSDARG00000053387 | si:ch211-276k2.2 | 22 | 35455330 | 35467696 | processed\_transcript | | ENSDARG00000093983 | si:ch211-285d14.3 | 4 | 16020460 | 16023902 | processed\_transcript | | ENSDARG00000104472 | si:ch211-39i2.2 | 14 | 7418467 | 7437744 | antisense | | ENSDARG00000070656 | si:ch211-69g19.2 | 16 | 12161404 | 12169880 | protein\_coding | | ENSDARG00000075022 | si:ch211-93e11.8 | 12 | 4775851 | 4800421 | protein\_coding | | ENSDARG00000105209 | si:ch73-103b11.2 | 24 | 39339528 | 39447371 | protein\_coding | | ENSDARG00000097848 | si:ch73-111k20.3 | 15 | 510812 | 516506 | lincRNA | | ENSDARG00000096628 | si:ch73-119o17.1 | 12 | 14100037 | 14105041 | protein\_coding | | ENSDARG00000075349 | si:ch73-138n13.1 | 8 | 42635025 | 42705804 | protein\_coding | | ENSDARG00000103066 | si:ch73-14h10.2 | 6 | 28218412 | 28219711 | protein\_coding | | ENSDARG00000079467 | si:ch73-15b2.5 | 6 | 40566312 | 40575014 | protein\_coding | | ENSDARG00000087392 | si:ch73-160p18.3 | 23 | 44750690 | 44757539 | protein\_coding | | ENSDARG00000099607 | si:ch73-160p18.4 | 23 | 44759149 | 44761365 | protein\_coding | | ENSDARG00000089075 | si:ch73-173p19.1 | 24 | 33478288 | 33514547 | protein\_coding | | ENSDARG00000095174 | si:ch73-243b8.4 | 22 | 17607843 | 17627936 | protein\_coding | | ENSDARG00000073787 | si:ch73-303b9.1 | 11 | 1520638 | 1524121 | protein\_coding | | ENSDARG00000103224 | si:ch73-350k19.1 | 19 | 8841870 | 8849639 | protein\_coding | | ENSDARG00000087596 | si:ch73-95l15.5 | 15 | 35105347 | 35128147 | protein\_coding | | ENSDARG00000062893 | si:dkey-103i16.6 | 18 | 15303481 | 15321485 | protein\_coding | | ENSDARG00000057743 | si:dkey-10f21.4 | 2 | 15366500 | 15373258 | protein\_coding | | ENSDARG00000093201 | si:dkey-112e17.1 | 5 | 17318219 | 17372713 | protein\_coding | | ENSDARG00000093058 | si:dkey-13i19.8 | 4 | 16795577 | 16797268 | protein\_coding | | ENSDARG00000103386 | si:dkey-160o24.4 | 14 | 8077435 | 8077809 | unprocessed\_pseudogene | | ENSDARG00000100936 | si:dkey-163f14.5 | 11 | 30005771 | 30006373 | processed\_transcript | | ENSDARG00000037967 | si:dkey-16l2.16 | 3 | 32530051 | 32537505 | protein\_coding | | ENSDARG00000078389 | si:dkey-188i13.7 | 13 | 37497386 | 37505202 | protein\_coding | | ENSDARG00000103211 | si:dkey-19b23.8 | 7 | 20348706 | 20359071 | protein\_coding | | ENSDARG00000055833 | si:dkey-216e24.3 | 9 | 33426098 | 33427503 | protein\_coding | | ENSDARG00000091813 | si:dkey-21a6.5 | 17 | 43528752 | 43542557 | protein\_coding | | ENSDARG00000060584 | si:dkey-226m8.9 | 5 | 32621971 | 32636484 | protein\_coding | | ENSDARG00000028784 | si:dkey-24p1.6 | 15 | 38406836 | 38428290 | protein\_coding | | ENSDARG00000097212 | si:dkey-24p1.6.1 | 15 | 38397800 | 38401297 | protein\_coding | | ENSDARG00000021849 | si:dkey-256h2.1 | 16 | 47428510 | 47446580 | protein\_coding | | ENSDARG00000067718 | si:dkey-3h3.3 | 5 | 40235448 | 40239708 | protein\_coding | | ENSDARG00000092651 | si:dkey-57a22.11 | 9 | 22166514 | 22172790 | protein\_coding | | ENSDARG00000099247 | si:dkey-68o6.5 | 3 | 24465374 | 24473157 | protein\_coding | | ENSDARG00000087331 | si:dkey-77f5.3 | 15 | 1072440 | 1077408 | protein\_coding | | ENSDARG00000094602 | si:dkey-7n6.2 | 24 | 22614277 | 22624822 | protein\_coding | | ENSDARG00000094055 | si:dkey-88l16.3 | 10 | 15024858 | 15058920 | protein\_coding | | ENSDARG00000092525 | si:dkey-88l16.4 | 10 | 14988884 | 14991248 | protein\_coding | | ENSDARG00000094850 | si:dkey-88l16.5 | 10 | 15066653 | 15073301 | protein\_coding | | ENSDARG00000042120 | si:dkey-97o5.1 | 20 | 34091701 | 34107356 | protein\_coding | | ENSDARG00000090986 | si:dkeyp-115e12.6 | 14 | 8332194 | 8395765 | protein\_coding | | ENSDARG00000043004 | si:dkeyp-117h8.4 | 20 | 25740380 | 25746060 | protein\_coding | | ENSDARG00000071083 | si:dkeyp-34c12.1 | 22 | 28790541 | 28828432 | protein\_coding | | ENSDARG00000102751 | si:dkeyp-50f5.4 | 20 | 48907670 | 48957464 | antisense | | ENSDARG00000096954 | si:dkeyp-67f1.1 | 6 | 46307913 | 46318645 | protein\_coding | | ENSDARG00000088923 | si:dkeyp-98a7.10 | 22 | 25591517 | 25592783 | protein\_coding | | ENSDARG00000105424 | si:rp71-46j2.7 | 14 | 32526434 | 32535966 | protein\_coding | | ENSDARG00000017163 | sinup | 4 | 20455168 | 20456893 | protein\_coding | | ENSDARG00000103598 | siva1 | 17 | 699767 | 709078 | protein\_coding | | ENSDARG00000013457 | skiv2l2 | 10 | 8239127 | 8307394 | protein\_coding | | ENSDARG00000069428 | slbp2 | 21 | 30251875 | 30256891 | protein\_coding | | ENSDARG00000104573 | slc12a2.1 | 10 | 16347708 | 16385688 | protein\_coding | | ENSDARG00000015425 | slc24a4a | 17 | 38836777 | 38939839 | protein\_coding | | ENSDARG00000057110 | slc25a1a | 10 | 23090939 | 23099871 | protein\_coding | | ENSDARG00000022424 | slc26a5 | 4 | 14933782 | 14955274 | protein\_coding | | ENSDARG00000001767 | slc29a2 | 21 | 27240990 | 27376599 | protein\_coding | | ENSDARG00000007180 | slc30a4 | 18 | 5657371 | 5674422 | protein\_coding | | ENSDARG00000007886 | slc35b2 | 20 | 51295464 | 51454060 | protein\_coding | | ENSDARG00000054312 | slc38a6 | 13 | 31557523 | 31577420 | protein\_coding | | ENSDARG00000032769 | slc38a9 | 10 | 6949031 | 6989143 | protein\_coding | | ENSDARG00000026109 | slc48a1b | 6 | 38898622 | 38904962 | protein\_coding | | ENSDARG00000052330 | slc4a2b | 24 | 33386709 | 33451857 | protein\_coding | | ENSDARG00000005392 | slc5a5 | 8 | 13327245 | 13344646 | protein\_coding | | ENSDARG00000042859 | slc5a6a | 20 | 36743882 | 36768781 | protein\_coding | | ENSDARG00000000730 | slc6a13 | 6 | 41121374 | 41141395 | protein\_coding | | ENSDARG00000068286 | slc7a4 | 8 | 36467874 | 36483473 | protein\_coding | | ENSDARG00000020699 | slc9a8 | 23 | 4008749 | 4080798 | protein\_coding | | ENSDARG00000020764 | slmapb | 23 | 19663663 | 19678252 | protein\_coding | | ENSDARG00000014041 | smarcad1a | 8 | 29626736 | 29643183 | protein\_coding | | ENSDARG00000033647 | smarcb1a | 8 | 30920472 | 30935259 | protein\_coding | | ENSDARG00000005799 | smim14 | 1 | 22606884 | 22617569 | protein\_coding | | ENSDARG00000012874 | snap23.1 | 17 | 33462808 | 33478182 | protein\_coding | | ENSDARG00000029569 | snapc1a | 20 | 20414901 | 20425119 | protein\_coding | | ENSDARG00000008395 | snupn | 25 | 6308297 | 6320433 | protein\_coding | | ENSDARG00000077317 | snx29 | 12 | 19286499 | 19537382 | protein\_coding | | ENSDARG00000004647 | spice1 | 24 | 21185626 | 21199022 | protein\_coding | | ENSDARG00000012467 | spint1b | 20 | 3201392 | 3220609 | protein\_coding | | ENSDARG00000011925 | spns1 | 3 | 15305519 | 15325280 | protein\_coding | | ENSDARG00000056515 | spsb1 | 23 | 22569763 | 22596752 | protein\_coding | | ENSDARG00000042995 | sptlc1 | 10 | 5233976 | 5264942 | protein\_coding | | ENSDARG00000030743 | sptlc3 | 13 | 34678237 | 34736446 | protein\_coding | | ENSDARG00000045789 | srgap1b | 4 | 9278868 | 9334119 | protein\_coding | | ENSDARG00000073997 | srpk1a | 8 | 23840222 | 23858156 | protein\_coding | | ENSDARG00000061124 | srpr | 18 | 44832719 | 44854401 | protein\_coding | | ENSDARG00000058237 | ssbp3a | 2 | 11150880 | 11243811 | protein\_coding | | ENSDARG00000038938 | ssfa2 | 9 | 44503238 | 44618638 | protein\_coding | | ENSDARG00000088440 | ssh2a | 15 | 28615722 | 28654271 | protein\_coding | | ENSDARG00000101300 | ssna1 | 5 | 53898839 | 53908012 | protein\_coding | | ENSDARG00000039521 | ssx2ipa | 2 | 1645230 | 1665657 | protein\_coding | | ENSDARG00000061173 | st14a | 18 | 44538745 | 44571298 | protein\_coding | | ENSDARG00000043816 | st6galnac1.2 | 12 | 20569436 | 20578998 | protein\_coding | | ENSDARG00000054211 | st8sia7.1 | 23 | 45085609 | 45107044 | protein\_coding | | ENSDARG00000062137 | stag1b | 24 | 26869553 | 26916005 | protein\_coding | | ENSDARG00000002127 | stam | 2 | 31454329 | 31469388 | protein\_coding | | ENSDARG00000024904 | stil | 22 | 15933735 | 15946255 | protein\_coding | | ENSDARG00000070122 | stk11ip | 6 | 18894179 | 18937547 | protein\_coding | | ENSDARG00000060390 | stk26 | 21 | 43425128 | 43462378 | protein\_coding | | ENSDARG00000011312 | stk3 | 19 | 33220116 | 33232987 | protein\_coding | | ENSDARG00000057497 | stk35l | 6 | 38848140 | 38863813 | protein\_coding | | ENSDARG00000062142 | stoml1 | 25 | 22183893 | 22195353 | protein\_coding | | ENSDARG00000103135 | stoml2 | 10 | 16899545 | 16910357 | protein\_coding | | ENSDARG00000044039 | stx11a | 17 | 7570286 | 7575859 | protein\_coding | | ENSDARG00000052518 | stx4 | 12 | 20505631 | 20518294 | protein\_coding | | ENSDARG00000007603 | stxbp2 | 3 | 52985899 | 53019622 | protein\_coding | | ENSDARG00000057699 | styx | 17 | 15212109 | 15221854 | protein\_coding | | ENSDARG00000016532 | suco | 20 | 14788407 | 14885924 | protein\_coding | | ENSDARG00000056801 | sufu | 13 | 11696580 | 11737328 | protein\_coding | | ENSDARG00000104390 | sumo2a | 6 | 22836193 | 22842247 | protein\_coding | | ENSDARG00000026799 | suv39h1a | 8 | 23374230 | 23383866 | protein\_coding | | ENSDARG00000094010 | swt1 | 20 | 34440770 | 34485857 | protein\_coding | | ENSDARG00000026723 | syncripl | 17 | 50182483 | 50206348 | protein\_coding | | ENSDARG00000005454 | tacc3 | 13 | 35754732 | 35766411 | protein\_coding | | ENSDARG00000060751 | taok3a | 5 | 14859978 | 14993966 | protein\_coding | | ENSDARG00000062081 | tbc1d1 | 1 | 17952411 | 18079304 | protein\_coding | | ENSDARG00000036868 | tbpl1 | 23 | 31669910 | 31678776 | protein\_coding | | ENSDARG00000097302 | tbpl2 | 17 | 10582092 | 10591519 | protein\_coding | | ENSDARG00000035605 | tchp | 5 | 19363994 | 19375452 | protein\_coding | | ENSDARG00000078447 | tctn1 | 10 | 8457417 | 8476116 | protein\_coding | | ENSDARG00000075217 | tdrd12 | 25 | 36590670 | 36610080 | protein\_coding | | ENSDARG00000032808 | tdrd7a | 1 | 11508900 | 11541317 | protein\_coding | | ENSDARG00000053474 | tdrkh | 12 | 33460435 | 33481399 | protein\_coding | | ENSDARG00000044584 | tex261 | 7 | 8448599 | 8466190 | protein\_coding | | ENSDARG00000030636 | tex30 | 9 | 27582722 | 27585681 | protein\_coding | | ENSDARG00000067916 | tgs1 | 2 | 27958137 | 27996176 | protein\_coding | | ENSDARG00000035018 | thy1 | 5 | 29193905 | 29203140 | protein\_coding | | ENSDARG00000045605 | ticrr | 25 | 29873832 | 29894982 | unprocessed\_pseudogene | | ENSDARG00000043898 | timm10b | 10 | 24792764 | 24796536 | protein\_coding | | ENSDARG00000053466 | timmdc1 | 1 | 28032152 | 28057874 | protein\_coding | | ENSDARG00000032353 | tiprl | 10 | 30830 | 37425 | protein\_coding | | ENSDARG00000023443 | tjp2b | 8 | 11287586 | 11505136 | protein\_coding | | ENSDARG00000046127 | tk2 | 7 | 44260088 | 44265831 | protein\_coding | | ENSDARG00000057042 | tm2d1 | 22 | 16732740 | 16749249 | protein\_coding | | ENSDARG00000076618 | tm2d3 | 18 | 315472 | 321342 | protein\_coding | | ENSDARG00000093658 | tm7sf3 | 4 | 1883512 | 1908147 | protein\_coding | | ENSDARG00000004875 | tmc6b | 11 | 44805164 | 44830247 | protein\_coding | | ENSDARG00000060954 | tmcc1b | 11 | 18029346 | 18071715 | protein\_coding | | ENSDARG00000069099 | tmco1 | 8 | 21072422 | 21078161 | protein\_coding | | ENSDARG00000008765 | tmed5 | 20 | 14893325 | 14910090 | protein\_coding | | ENSDARG00000046022 | tmed7 | 8 | 150872 | 155813 | protein\_coding | | ENSDARG00000098105 | tmem106c | 3 | 1603611 | 1619318 | protein\_coding | | ENSDARG00000013694 | tmem110 | 11 | 18097026 | 18112323 | protein\_coding | | ENSDARG00000042929 | tmem120b | 10 | 42111605 | 42131554 | protein\_coding | | ENSDARG00000036247 | tmem144b | 14 | 35544539 | 35552453 | protein\_coding | | ENSDARG00000055989 | tmem161b | 5 | 47376470 | 47419268 | protein\_coding | | ENSDARG00000037484 | tmem192 | 1 | 18956608 | 18972837 | protein\_coding | | ENSDARG00000104996 | tmem203 | 5 | 53850882 | 53852524 | protein\_coding | | ENSDARG00000039340 | tmem209 | 4 | 5085437 | 5100371 | protein\_coding | | ENSDARG00000078333 | tmem234 | 13 | 33184223 | 33186948 | protein\_coding | | ENSDARG00000090478 | tmem238b | 16 | 17762264 | 17763224 | protein\_coding | | ENSDARG00000042214 | tmem243a | 18 | 8954094 | 8957981 | protein\_coding | | ENSDARG00000042341 | tmem251 | 20 | 27188449 | 27191763 | protein\_coding | | ENSDARG00000043555 | tmem30ab | 20 | 16740265 | 16761268 | protein\_coding | | ENSDARG00000041332 | tmem33 | 14 | 47369016 | 47378854 | protein\_coding | | ENSDARG00000018956 | tmem39b | 13 | 33138207 | 33150860 | protein\_coding | | ENSDARG00000068193 | tmem44 | 11 | 33938751 | 33956779 | protein\_coding | | ENSDARG00000068575 | tmem51a | 8 | 26681899 | 26690820 | protein\_coding | | ENSDARG00000038789 | tmem53 | 2 | 32863384 | 32867403 | protein\_coding | | ENSDARG00000071250 | tmem79a | 19 | 791729 | 797027 | protein\_coding | | ENSDARG00000103117 | tmprss4a | 15 | 12497789 | 12527918 | protein\_coding | | ENSDARG00000012860 | tmprss4b | 5 | 37290368 | 37312921 | protein\_coding | | ENSDARG00000038787 | tmub1 | 2 | 32577965 | 32591726 | protein\_coding | | ENSDARG00000058068 | tnfsf10l4 | 5 | 22076175 | 22079742 | protein\_coding | | ENSDARG00000068760 | tnks1bp1 | 5 | 5953586 | 6004893 | protein\_coding | | ENSDARG00000098486 | tollip | 7 | 72027220 | 72041305 | protein\_coding | | ENSDARG00000052827 | top3a | 3 | 40093430 | 40112776 | protein\_coding | | ENSDARG00000099801 | topaz1 | 19 | 48729905 | 48748712 | protein\_coding | | ENSDARG00000037260 | toporsa | 1 | 25411427 | 25416247 | protein\_coding | | ENSDARG00000006025 | tp53i11b | 18 | 27457720 | 27591078 | protein\_coding | | ENSDARG00000042793 | tpp1 | 17 | 30826918 | 30838829 | protein\_coding | | ENSDARG00000011262 | traip | 11 | 34900842 | 34909179 | protein\_coding | | ENSDARG00000033768 | trappc13 | 10 | 15382649 | 15424023 | protein\_coding | | ENSDARG00000026068 | trappc5 | 1 | 44971932 | 44977649 | protein\_coding | | ENSDARG00000016463 | trappc6b | 23 | 32095272 | 32100667 | protein\_coding | | ENSDARG00000034518 | trdmt1 | 24 | 32236986 | 32281769 | protein\_coding | | ENSDARG00000079884 | trim107 | 6 | 1604030 | 1613964 | protein\_coding | | ENSDARG00000062794 | trim36 | 8 | 389149 | 410770 | protein\_coding | | ENSDARG00000076473 | trim37 | 10 | 32865760 | 32907987 | protein\_coding | | ENSDARG00000006385 | triobpb | 3 | 1398377 | 1411821 | protein\_coding | | ENSDARG00000098074 | trip4 | 7 | 52984582 | 53129633 | protein\_coding | | ENSDARG00000032876 | trit1 | 19 | 31135454 | 31212769 | protein\_coding | | ENSDARG00000060338 | trmt12 | 14 | 35074318 | 35082463 | protein\_coding | | ENSDARG00000033957 | trmt44 | 7 | 59372036 | 59414674 | protein\_coding | | ENSDARG00000036232 | trpm7 | 18 | 113928 | 127873 | protein\_coding | | ENSDARG00000074554 | tspan31 | 23 | 36608252 | 36627840 | protein\_coding | | ENSDARG00000007025 | ttc9c | 14 | 46362716 | 46376443 | protein\_coding | | ENSDARG00000073773 | tti2 | 8 | 45373419 | 45387436 | protein\_coding | | ENSDARG00000103537 | ttll12 | 4 | 8742609 | 8778419 | protein\_coding | | ENSDARG00000010727 | ttyh2l | 3 | 51309032 | 51403059 | protein\_coding | | ENSDARG00000074289 | tuba4l | 13 | 6118796 | 6124054 | protein\_coding | | ENSDARG00000001969 | txlng | 11 | 12724637 | 12744464 | protein\_coding | | ENSDARG00000101291 | txndc11 | 1 | 58597651 | 58607041 | protein\_coding | | ENSDARG00000058962 | txnl4b | 1 | 518807 | 522897 | protein\_coding | | ENSDARG00000100374 | txnrd1 | 6 | 661726 | 688111 | protein\_coding | | ENSDARG00000042894 | tyms | 7 | 71293904 | 71300188 | protein\_coding | | ENSDARG00000062987 | tyw1 | 15 | 16803138 | 16936578 | protein\_coding | | ENSDARG00000060238 | uacab | 7 | 33529484 | 33558930 | protein\_coding | | ENSDARG00000013082 | uap1l1 | 5 | 31211509 | 31217242 | protein\_coding | | ENSDARG00000099749 | ube2d2l | 14 | 50663183 | 50694896 | protein\_coding | | ENSDARG00000102827 | ubfd1 | 3 | 43240590 | 43254054 | protein\_coding | | ENSDARG00000070164 | ubr2 | 13 | 3130121 | 3191423 | protein\_coding | | ENSDARG00000005536 | ubr7 | 20 | 27193952 | 27208619 | protein\_coding | | ENSDARG00000078973 | uckl1b | 23 | 13772473 | 13843661 | protein\_coding | | ENSDARG00000042527 | unga | 5 | 66672850 | 66687381 | protein\_coding | | ENSDARG00000068176 | uqcc1 | 6 | 52521473 | 52567507 | protein\_coding | | ENSDARG00000093382 | uqcc3 | 10 | 27044553 | 27047871 | protein\_coding | | ENSDARG00000027491 | uros | 12 | 48750693 | 48759117 | protein\_coding | | ENSDARG00000076303 | usp11 | 8 | 8605283 | 8644348 | protein\_coding | | ENSDARG00000027501 | usp20 | 5 | 32698488 | 32730325 | protein\_coding | | ENSDARG00000040990 | usp37 | 9 | 715590 | 746553 | protein\_coding | | ENSDARG00000043510 | vamp4 | 20 | 15072051 | 15109131 | protein\_coding | | ENSDARG00000102960 | vps54 | 1 | 54332940 | 54366628 | protein\_coding | | ENSDARG00000021547 | vrk2 | 13 | 26649554 | 26668794 | protein\_coding | | ENSDARG00000029768 | waplb | 12 | 11611843 | 11725355 | protein\_coding | | ENSDARG00000098583 | wbp1 | 5 | 71599384 | 71605753 | protein\_coding | | ENSDARG00000074590 | wdpcp | 17 | 24312560 | 24421271 | protein\_coding | | ENSDARG00000100664 | wdr1 | 14 | 300359 | 304526 | protein\_coding | | ENSDARG00000006812 | wdr26b | 20 | 46795301 | 46830331 | protein\_coding | | ENSDARG00000009851 | wdr41 | 21 | 7868135 | 7892423 | protein\_coding | | ENSDARG00000038543 | wdr48b | 2 | 50071662 | 50099822 | protein\_coding | | ENSDARG00000053554 | wdr76 | 7 | 29930461 | 29938640 | protein\_coding | | ENSDARG00000041600 | wdr83 | 22 | 26244646 | 26254280 | protein\_coding | | ENSDARG00000079241 | wdr90 | 24 | 37596941 | 37704914 | protein\_coding | | ENSDARG00000012718 | wee2 | 18 | 20571376 | 20576916 | protein\_coding | | ENSDARG00000045298 | wipf2a | 11 | 11861980 | 11906380 | protein\_coding | | ENSDARG00000009557 | wrap73 | 8 | 48827313 | 48859003 | protein\_coding | | ENSDARG00000017928 | xrcc3 | 20 | 20996949 | 21001885 | protein\_coding | | ENSDARG00000043260 | xylb | 24 | 41370410 | 41403848 | protein\_coding | | ENSDARG00000011999 | ybey | 9 | 712147 | 715571 | protein\_coding | | ENSDARG00000038308 | ykt6 | 10 | 2814034 | 2823765 | protein\_coding | | ENSDARG00000055973 | zar1l | 10 | 34056317 | 34058363 | protein\_coding | | ENSDARG00000098273 | zbtb14 | 24 | 42048194 | 42054026 | protein\_coding | | ENSDARG00000044038 | zbtb2a | 17 | 7629068 | 7635198 | protein\_coding | | ENSDARG00000102195 | zc3h7a | 3 | 43073452 | 43107405 | protein\_coding | | ENSDARG00000034982 | zcchc8 | 5 | 66575518 | 66589498 | protein\_coding | | ENSDARG00000071872 | zdhhc15b | 14 | 10648331 | 10668636 | protein\_coding | | ENSDARG00000105034 | zdhhc18b | 19 | 14592639 | 14609723 | protein\_coding | | ENSDARG00000105134 | zdhhc24 | 14 | 33600405 | 33605378 | protein\_coding | | ENSDARG00000103511 | zfand2a | 3 | 42667852 | 42687238 | protein\_coding | | ENSDARG00000098443 | zfand6 | 7 | 10320011 | 10341044 | protein\_coding | | ENSDARG00000038694 | zgc:101744 | 2 | 37106905 | 37121165 | protein\_coding | | ENSDARG00000037199 | zgc:101851 | 9 | 900883 | 904718 | protein\_coding | | ENSDARG00000077664 | zgc:110239 | 19 | 42921499 | 42933706 | protein\_coding | | ENSDARG00000059412 | zgc:111976 | 24 | 1027795 | 1040340 | protein\_coding | | ENSDARG00000037405 | zgc:112083 | 15 | 20054336 | 20068140 | protein\_coding | | ENSDARG00000038271 | zgc:113293 | 2 | 216852 | 222153 | protein\_coding | | ENSDARG00000026616 | zgc:113425 | 14 | 47203515 | 47211081 | protein\_coding | | ENSDARG00000052109 | zgc:114041 | 5 | 65453275 | 65467687 | protein\_coding | | ENSDARG00000044956 | zgc:114123 | 23 | 2786419 | 2804310 | protein\_coding | | ENSDARG00000077178 | zgc:152977 | 12 | 10440823 | 10475068 | protein\_coding | | ENSDARG00000070611 | zgc:152986 | 6 | 40470026 | 40472985 | protein\_coding | | ENSDARG00000055622 | zgc:153345 | 10 | 33449969 | 33459662 | protein\_coding | | ENSDARG00000071555 | zgc:153675 | 22 | 795950 | 800581 | protein\_coding | | ENSDARG00000086017 | zgc:153681 | 14 | 8587515 | 8634485 | protein\_coding | | ENSDARG00000070461 | zgc:154061 | 17 | 52854307 | 52927043 | protein\_coding | | ENSDARG00000068484 | zgc:158270 | 11 | 44187930 | 44198491 | processed\_transcript | | ENSDARG00000102076 | zgc:158852 | 14 | 14850121 | 14851161 | protein\_coding | | ENSDARG00000046141 | zgc:162025 | 8 | 39782749 | 39789143 | protein\_coding | | ENSDARG00000016724 | zgc:162339 | 15 | 21772051 | 21781435 | protein\_coding | | ENSDARG00000070040 | zgc:162344 | 21 | 11943610 | 11981582 | protein\_coding | | ENSDARG00000069846 | zgc:162944 | 9 | 22015855 | 22020719 | protein\_coding | | ENSDARG00000074100 | zgc:163014 | 17 | 45000249 | 45011772 | protein\_coding | | ENSDARG00000076214 | zgc:165514 | 15 | 44161036 | 44165591 | protein\_coding | | ENSDARG00000092924 | zgc:171474 | 24 | 9871275 | 9874126 | protein\_coding | | ENSDARG00000078638 | zgc:171750 | 24 | 9863040 | 9865772 | protein\_coding | | ENSDARG00000092885 | zgc:171977 | 24 | 9836242 | 9838956 | protein\_coding | | ENSDARG00000004529 | zgc:173742 | 18 | 14297589 | 14363548 | protein\_coding | | ENSDARG00000078807 | zgc:193538 | 5 | 24580585 | 24583492 | protein\_coding | | ENSDARG00000078130 | zgc:193593 | 17 | 24898949 | 24920444 | protein\_coding | | ENSDARG00000014208 | zgc:55413 | 15 | 35244487 | 35254971 | protein\_coding | | ENSDARG00000010782 | zgc:55621 | 15 | 35082153 | 35102957 | protein\_coding | | ENSDARG00000103845 | zgc:55733 | 7 | 3944123 | 3966557 | protein\_coding | | ENSDARG00000029292 | zgc:55943 | 2 | 10160261 | 10173908 | protein\_coding | | ENSDARG00000010332 | zgc:56231 | 7 | 58449051 | 58455392 | protein\_coding | | ENSDARG00000043562 | zgc:65997 | 17 | 23529093 | 23532044 | protein\_coding | | ENSDARG00000030357 | zgc:66313 | 17 | 43749943 | 43773770 | protein\_coding | | ENSDARG00000099519 | zgc:66475 | 2 | 12547210 | 12579888 | protein\_coding | | ENSDARG00000102636 | zgc:73340 | 15 | 33844939 | 33876171 | protein\_coding | | ENSDARG00000015657 | zgc:77112 | 8 | 52455806 | 52459439 | protein\_coding | | ENSDARG00000093176 | zgc:77118 | 15 | 35257363 | 35268363 | protein\_coding | | ENSDARG00000017985 | zgc:77739 | 1 | 11707405 | 11711086 | protein\_coding | | ENSDARG00000030563 | zgc:77880 | 1 | 9621500 | 9631095 | protein\_coding | | ENSDARG00000005098 | zgc:86764 | 21 | 20273580 | 20286180 | protein\_coding | | ENSDARG00000098592 | zgc:92140 | 8 | 19370669 | 19437155 | protein\_coding | | ENSDARG00000040161 | zgc:92287 | 23 | 18106226 | 18131615 | protein\_coding | | ENSDARG00000040513 | zgc:92313 | 12 | 4204693 | 4212232 | protein\_coding | | ENSDARG00000076939 | znf451 | 13 | 1279495 | 1296949 | protein\_coding | | ENSDARG00000056307 | znf706 | 19 | 12286630 | 12293268 | protein\_coding | | ENSDARG00000091538 | zranb3 | 22 | 12189247 | 12305165 | protein\_coding | | ENSDARG00000059983 | zufsp | 16 | 32230455 | 32242741 | protein\_coding | |
