# Supplementary material for: A high-resolution mRNA expression time course of embryonic development in zebrafish
Source: eLife. 2017 Nov 16;6:e30860. doi: 10.7554/eLife.30860 (PMC5690287; doi:10.7554/eLife.30860)
Supplement: Supplementary file 6. [file elife-30860-supp6.zip › biolayout-clusters-files/Cluster003.html]

Cluster003


# Cluster003: Detail

### Go to ZFA detail

## GO

| | GO ID | Description | Domain | Annotated | Expected | Observed | Adjusted p-value | Genes | Ensembl IDs | | --- | --- | --- | --- | --- | --- | --- | --- | --- | | GO:0007283 | spermatogenesis | biological\_process | 24 | 1.68 | 8 | 3.8e-02 | psme4b mlh1 tdrd7a dazl rnf8 styx pld6 brdt | ENSDARG00000018742 ENSDARG00000025948 ENSDARG00000032808 ENSDARG00000036214 ENSDARG00000044949 ENSDARG00000057699 ENSDARG00000059951 ENSDARG00000102802 | | GO:0007127 | meiosis I | biological\_process | 13 | 0.91 | 7 | 2.4e-03 | ercc4 xrcc3 mlh1 rad51d rad51b pttg1 brdt | ENSDARG00000014161 ENSDARG00000017928 ENSDARG00000025948 ENSDARG00000026400 ENSDARG00000037046 ENSDARG00000075421 ENSDARG00000102802 | | GO:0000724 | double-strand break repair via homologou... | biological\_process | 26 | 1.82 | 11 | 1.3e-04 | gins2 ercc4 fignl1 xrcc3 cdc7 rad51d parpbp rtel1 rad51b cdc45 nsmce1 | ENSDARG00000002304 ENSDARG00000014161 ENSDARG00000016427 ENSDARG00000017928 ENSDARG00000023584 ENSDARG00000026400 ENSDARG00000029944 ENSDARG00000035074 ENSDARG00000037046 ENSDARG00000043720 ENSDARG00000098780 | | GO:0005657 | replication fork | cellular\_component | 16 | 1.15 | 9 | 8.6e-05 | gins2 zgc:173742 xrcc3 pif1 rad51d rad51b cdc45 mcm10 pole2 | ENSDARG00000002304 ENSDARG00000004529 ENSDARG00000017928 ENSDARG00000020289 ENSDARG00000026400 ENSDARG00000037046 ENSDARG00000043720 ENSDARG00000045815 ENSDARG00000100028 | | GO:0003697 | single-stranded DNA binding | molecular\_function | 27 | 1.83 | 11 | 1.5e-04 | nup35 ercc4 xrcc3 mlh1 rad51d rad51b cdc45 mcm10 ssbp3a pms2 lonp1 | ENSDARG00000012222 ENSDARG00000014161 ENSDARG00000017928 ENSDARG00000025948 ENSDARG00000026400 ENSDARG00000037046 ENSDARG00000043720 ENSDARG00000045815 ENSDARG00000058237 ENSDARG00000075672 ENSDARG00000102765 | | GO:0004520 | endodeoxyribonuclease activity | molecular\_function | 34 | 2.30 | 12 | 4.1e-03 | apex2 ercc4 xrcc3 neil1 rad51d harbi1 rad51b nthl1 mppe1 exo1 pld6 dna2 | ENSDARG00000008472 ENSDARG00000014161 ENSDARG00000017928 ENSDARG00000018061 ENSDARG00000026400 ENSDARG00000036038 ENSDARG00000037046 ENSDARG00000042881 ENSDARG00000045416 ENSDARG00000056832 ENSDARG00000059951 ENSDARG00000078759 | |
