# Supplementary material for: A high-resolution mRNA expression time course of embryonic development in zebrafish
Source: eLife. 2017 Nov 16;6:e30860. doi: 10.7554/eLife.30860 (PMC5690287; doi:10.7554/eLife.30860)
Supplement: Supplementary file 6. [file elife-30860-supp6.zip › biolayout-clusters-files/Cluster004-genes.html]

Cluster004


# Cluster004: Genes

| | Ensembl ID | Gene Name | Chr | Start | End | Biotype | | --- | --- | --- | --- | --- | --- | | ENSDARG00000056478 | ACAP2 (1 of many) | 2 | 5975744 | 6012419 | protein\_coding | | ENSDARG00000105296 | AP3B2 | 25 | 5573708 | 5613876 | protein\_coding | | ENSDARG00000076434 | ARHGAP22 (1 of many) | 12 | 2412104 | 2457737 | protein\_coding | | ENSDARG00000077545 | BRSK1 (1 of many).1 | 3 | 32162061 | 32189027 | protein\_coding | | ENSDARG00000086724 | CABZ01020207.1 | 6 | 47485066 | 47507826 | protein\_coding | | ENSDARG00000031618 | CABZ01038517.1 | 3 | 58450693 | 58488954 | protein\_coding | | ENSDARG00000099804 | CABZ01059119.1 | 20 | 47937555 | 47971653 | protein\_coding | | ENSDARG00000100753 | CABZ01076275.1 | KN150405.1 | 1085 | 65848 | protein\_coding | | ENSDARG00000101809 | CABZ01078594.1 | KN150637.1 | 22430 | 37942 | protein\_coding | | ENSDARG00000016545 | CRAT (1 of many) | 8 | 44932723 | 44982549 | protein\_coding | | ENSDARG00000033201 | CRIP2 | 20 | 9441226 | 9474710 | protein\_coding | | ENSDARG00000101858 | DST | 13 | 1441977 | 1589054 | protein\_coding | | ENSDARG00000039232 | DUSP8 (1 of many) | 18 | 49945735 | 49959998 | protein\_coding | | ENSDARG00000102755 | EML6 | 11 | 42640693 | 42688166 | protein\_coding | | ENSDARG00000044767 | ENSDARG00000044767 | 3 | 15027422 | 15037193 | protein\_coding | | ENSDARG00000045947 | ENSDARG00000045947 | 12 | 4340966 | 4444705 | protein\_coding | | ENSDARG00000057353 | ENSDARG00000057353 | 7 | 17804264 | 17822251 | protein\_coding | | ENSDARG00000062790 | ENSDARG00000062790 | 13 | 28689778 | 28771841 | protein\_coding | | ENSDARG00000071460 | ENSDARG00000071460 | 24 | 38267933 | 38304065 | protein\_coding | | ENSDARG00000076623 | ENSDARG00000076623 | 19 | 40466 | 61377 | protein\_coding | | ENSDARG00000095339 | ENSDARG00000095339 | 14 | 6322136 | 6420990 | protein\_coding | | ENSDARG00000099362 | ENSDARG00000099362 | 25 | 36888884 | 36897948 | protein\_coding | | ENSDARG00000100596 | ENSDARG00000100596 | 14 | 6814211 | 6822571 | protein\_coding | | ENSDARG00000101716 | ENSDARG00000101716 | 15 | 18560 | 22866 | protein\_coding | | ENSDARG00000079656 | FAM171A2 (1 of many) | 24 | 36925010 | 36938874 | protein\_coding | | ENSDARG00000103940 | FRRS1L | 16 | 26076986 | 26083790 | protein\_coding | | ENSDARG00000103744 | HACD1 | 7 | 58718618 | 58728640 | protein\_coding | | ENSDARG00000059534 | JPH3 (1 of many) | 7 | 56476257 | 56492328 | protein\_coding | | ENSDARG00000078508 | KCP | 18 | 6939348 | 7016213 | protein\_coding | | ENSDARG00000028618 | KRT18 (1 of many) | 6 | 39362841 | 39367931 | protein\_coding | | ENSDARG00000060434 | MAP1B | 5 | 34674156 | 34701756 | protein\_coding | | ENSDARG00000043079 | MMP23B | 8 | 53595810 | 53614159 | protein\_coding | | ENSDARG00000038709 | MYADM (1 of many) | 2 | 37767559 | 37772671 | protein\_coding | | ENSDARG00000075433 | MYOM2 | 11 | 11217475 | 11277249 | protein\_coding | | ENSDARG00000090564 | PDZD4 (1 of many) | 23 | 222886 | 236307 | protein\_coding | | ENSDARG00000098823 | PDZD4 (1 of many).1 | 8 | 22582640 | 22592579 | protein\_coding | | ENSDARG00000052033 | PLPP7 (1 of many) | 5 | 71213814 | 71222233 | protein\_coding | | ENSDARG00000056704 | RAMP1 | 9 | 29007243 | 29056816 | protein\_coding | | ENSDARG00000098969 | RELN | 18 | 50929817 | 50945792 | protein\_coding | | ENSDARG00000020284 | RNF19B (1 of many) | 20 | 19473845 | 19522913 | protein\_coding | | ENSDARG00000009567 | SPEG (1 of many) | 9 | 41810270 | 41879284 | protein\_coding | | ENSDARG00000061956 | SYTL2 | 10 | 29351639 | 29377662 | protein\_coding | | ENSDARG00000103775 | THBS1 (1 of many) | KN150104.1 | 737 | 17802 | protein\_coding | | ENSDARG00000078362 | TNC (1 of many) | 5 | 5215683 | 5338943 | protein\_coding | | ENSDARG00000039726 | YIPF7 | 13 | 9100478 | 9104516 | protein\_coding | | ENSDARG00000015985 | abcc9 | 4 | 14658198 | 14715780 | protein\_coding | | ENSDARG00000033854 | abrab | 19 | 47180422 | 47186367 | protein\_coding | | ENSDARG00000055652 | aclyb | 12 | 13935340 | 14039767 | protein\_coding | | ENSDARG00000099197 | actc1b | 17 | 117124 | 124703 | protein\_coding | | ENSDARG00000013755 | actn3a | 21 | 27346198 | 27368283 | protein\_coding | | ENSDARG00000001431 | actn3b | 7 | 6814866 | 6898486 | protein\_coding | | ENSDARG00000060532 | adam22 | 16 | 43248969 | 43381181 | protein\_coding | | ENSDARG00000074581 | add2 | 5 | 13281751 | 13335244 | protein\_coding | | ENSDARG00000076994 | adgra2 | 8 | 37990927 | 38126777 | protein\_coding | | ENSDARG00000089292 | adgrl1a | 3 | 50686602 | 50834993 | protein\_coding | | ENSDARG00000015263 | adma | 7 | 66651122 | 66653410 | protein\_coding | | ENSDARG00000002071 | adss | 13 | 11357478 | 11404476 | protein\_coding | | ENSDARG00000103811 | agla | 2 | 20814178 | 20868861 | protein\_coding | | ENSDARG00000093453 | ahdc1 | 19 | 43843804 | 43904702 | protein\_coding | | ENSDARG00000001950 | ak1 | 5 | 70925989 | 70948273 | protein\_coding | | ENSDARG00000077295 | akap6 | 17 | 8831194 | 9109995 | protein\_coding | | ENSDARG00000088332 | alx4a | 7 | 26622211 | 26653567 | protein\_coding | | ENSDARG00000088514 | and1 | 24 | 26134462 | 26138318 | protein\_coding | | ENSDARG00000092143 | ank1a | 5 | 68129839 | 68157061 | protein\_coding | | ENSDARG00000043313 | ank2b | 7 | 57583734 | 57796505 | protein\_coding | | ENSDARG00000061736 | ank3a | 17 | 20680225 | 20877630 | protein\_coding | | ENSDARG00000077582 | ank3b | 12 | 7612388 | 7902807 | protein\_coding | | ENSDARG00000014652 | ankrd13b | 15 | 28549986 | 28606774 | protein\_coding | | ENSDARG00000015780 | ankrd46b | 7 | 50635889 | 50642178 | protein\_coding | | ENSDARG00000074328 | apba1b | 10 | 15819093 | 15855160 | protein\_coding | | ENSDARG00000060639 | apba2b | 7 | 30552697 | 30644815 | protein\_coding | | ENSDARG00000102004 | apoea | 19 | 10936233 | 10940769 | protein\_coding | | ENSDARG00000023713 | aqp1a.1 | 2 | 2895633 | 2907365 | protein\_coding | | ENSDARG00000059472 | arhgap31 | 5 | 66992838 | 67042999 | protein\_coding | | ENSDARG00000027063 | arpc1b | 3 | 40667153 | 40678754 | protein\_coding | | ENSDARG00000087741 | asb14b | 23 | 19564040 | 19577187 | protein\_coding | | ENSDARG00000003797 | asb2a.1 | 20 | 27397942 | 27412585 | protein\_coding | | ENSDARG00000053222 | asb5b | 1 | 38086657 | 38098381 | protein\_coding | | ENSDARG00000061352 | bcl11aa | 13 | 25716078 | 25794774 | protein\_coding | | ENSDARG00000079286 | bcr | 21 | 17073962 | 17222352 | protein\_coding | | ENSDARG00000042172 | c7a | 8 | 31863760 | 31890097 | protein\_coding | | ENSDARG00000014488 | ca2 | 24 | 32735385 | 32750019 | protein\_coding | | ENSDARG00000042552 | cacna1sb | 8 | 25748471 | 25789264 | protein\_coding | | ENSDARG00000014804 | cacna2d1a | 4 | 19779504 | 19895170 | protein\_coding | | ENSDARG00000062174 | cacnb4b | 6 | 13956935 | 14017542 | protein\_coding | | ENSDARG00000046079 | cacng6b | 16 | 12946023 | 12976431 | protein\_coding | | ENSDARG00000031075 | cadm1a | 21 | 23916576 | 24375822 | protein\_coding | | ENSDARG00000057013 | cadm3 | 2 | 44195990 | 44330615 | protein\_coding | | ENSDARG00000010758 | capn12 | 18 | 36683646 | 36748629 | protein\_coding | | ENSDARG00000052748 | capn1b | 22 | 26278244 | 26303997 | protein\_coding | | ENSDARG00000008165 | caspa | 16 | 42043519 | 42054512 | protein\_coding | | ENSDARG00000052039 | caspb | 1 | 57315223 | 57329056 | protein\_coding | | ENSDARG00000038716 | casq1a | 2 | 38057524 | 38071766 | protein\_coding | | ENSDARG00000018105 | casq1b | 7 | 73495849 | 73530654 | protein\_coding | | ENSDARG00000100052 | cckb | KN150698.1 | 1656 | 8433 | protein\_coding | | ENSDARG00000068214 | ccni | 21 | 5673971 | 5692282 | protein\_coding | | ENSDARG00000103713 | cd248a | 7 | 18111923 | 18115006 | protein\_coding | | ENSDARG00000002131 | celf2 | 4 | 24165751 | 24310950 | protein\_coding | | ENSDARG00000070045 | celf4 | 21 | 11987250 | 12179915 | protein\_coding | | ENSDARG00000053583 | cers6 | 9 | 49172637 | 49239755 | protein\_coding | | ENSDARG00000014106 | cfl2 | 17 | 9851027 | 9869190 | protein\_coding | | ENSDARG00000063631 | ch1073-291c23.1 | 13 | 222884 | 247011 | protein\_coding | | ENSDARG00000045071 | chad | 12 | 26421209 | 26434339 | protein\_coding | | ENSDARG00000074498 | chd9 | 7 | 36281135 | 36347613 | protein\_coding | | ENSDARG00000103109 | chl1b | 6 | 44817141 | 45074233 | protein\_coding | | ENSDARG00000057025 | chrna2b | 20 | 19894037 | 19924932 | protein\_coding | | ENSDARG00000012269 | clcn1b | 16 | 17569175 | 17633913 | protein\_coding | | ENSDARG00000020031 | cldn11a | 2 | 26341792 | 26345539 | protein\_coding | | ENSDARG00000075993 | clic5a | 17 | 5409042 | 5426249 | protein\_coding | | ENSDARG00000053122 | clvs2 | 20 | 40372781 | 40422780 | protein\_coding | | ENSDARG00000091683 | cnrip1a | 1 | 50950085 | 50962753 | protein\_coding | | ENSDARG00000009014 | col11a1b | 2 | 15720603 | 15894604 | protein\_coding | | ENSDARG00000019601 | col12a1b | 20 | 49647131 | 49880731 | protein\_coding | | ENSDARG00000009194 | col16a1 | 19 | 38886452 | 39048413 | protein\_coding | | ENSDARG00000012405 | col1a1a | 3 | 23090499 | 23108910 | protein\_coding | | ENSDARG00000035809 | col1a1b | 12 | 3060966 | 3098750 | protein\_coding | | ENSDARG00000020007 | col1a2 | 19 | 41396101 | 41415860 | protein\_coding | | ENSDARG00000077084 | col28a1 | 19 | 25893980 | 25927516 | protein\_coding | | ENSDARG00000024492 | col9a2 | 19 | 38834939 | 38880434 | protein\_coding | | ENSDARG00000037845 | col9a3 | 23 | 482721 | 511987 | protein\_coding | | ENSDARG00000025679 | comtb | 11 | 25382276 | 25393826 | protein\_coding | | ENSDARG00000061918 | cplx2 | 14 | 25237359 | 25279849 | protein\_coding | | ENSDARG00000018997 | cplx2l | 1 | 11576180 | 11591781 | protein\_coding | | ENSDARG00000062054 | cpt1ab | 25 | 23494442 | 23546729 | protein\_coding | | ENSDARG00000104049 | crebl2 | 4 | 144009 | 147740 | protein\_coding | | ENSDARG00000014803 | cryba1l2 | 1 | 43720617 | 43741477 | protein\_coding | | ENSDARG00000007655 | crybb1l3 | 15 | 28163276 | 28171527 | protein\_coding | | ENSDARG00000102986 | csf1ra | 14 | 3170816 | 3202049 | protein\_coding | | ENSDARG00000078227 | cspg4 | 25 | 6379739 | 6501879 | protein\_coding | | ENSDARG00000011961 | csrp2 | 4 | 2581518 | 2595823 | protein\_coding | | ENSDARG00000040251 | ctsk | 16 | 29558314 | 29567926 | protein\_coding | | ENSDARG00000069451 | cx50.5 | 9 | 30619442 | 30622032 | protein\_coding | | ENSDARG00000003142 | dachc | 1 | 29781209 | 29885073 | protein\_coding | | ENSDARG00000069440 | dachd | 9 | 31009544 | 31251958 | protein\_coding | | ENSDARG00000012066 | dcn | 4 | 16379786 | 16417761 | protein\_coding | | ENSDARG00000071877 | dhrs7cb | 12 | 449322 | 461216 | protein\_coding | | ENSDARG00000025302 | dixdc1a | 15 | 18194087 | 18219969 | protein\_coding | | ENSDARG00000070425 | dll4 | 20 | 28346074 | 28354934 | protein\_coding | | ENSDARG00000013110 | dmtn | 10 | 20150231 | 20246676 | protein\_coding | | ENSDARG00000102696 | dnajc11b | 11 | 41777627 | 41798906 | protein\_coding | | ENSDARG00000078108 | dok1a | 1 | 40775558 | 40782074 | protein\_coding | | ENSDARG00000071549 | ecm2 | 22 | 10461255 | 10473098 | protein\_coding | | ENSDARG00000089334 | ednrba | 1 | 27820834 | 27826119 | protein\_coding | | ENSDARG00000003032 | eif4a1b | 5 | 23704613 | 23715544 | protein\_coding | | ENSDARG00000004402 | elovl6 | 14 | 35890812 | 35928396 | protein\_coding | | ENSDARG00000042840 | eml1 | 17 | 30962000 | 31042018 | protein\_coding | | ENSDARG00000039007 | eno3 | 23 | 44795344 | 44807255 | protein\_coding | | ENSDARG00000060243 | enox1 | 9 | 18301974 | 18561215 | protein\_coding | | ENSDARG00000037373 | ephb2a | 23 | 21876859 | 22020056 | protein\_coding | | ENSDARG00000056950 | epyc | 4 | 16329909 | 16345101 | protein\_coding | | ENSDARG00000099720 | evlb | 20 | 54646858 | 54661063 | protein\_coding | | ENSDARG00000074921 | fam117ba | 6 | 12618797 | 12630969 | protein\_coding | | ENSDARG00000070952 | fam131ba | 19 | 9614083 | 9685190 | protein\_coding | | ENSDARG00000042815 | fam150bb | 17 | 30644385 | 30649074 | protein\_coding | | ENSDARG00000055099 | fam184b | 1 | 22815786 | 22867138 | protein\_coding | | ENSDARG00000076862 | fam198a | 16 | 8275415 | 8338224 | protein\_coding | | ENSDARG00000077054 | fam198b | 14 | 35536562 | 35542705 | protein\_coding | | ENSDARG00000063018 | fam78ba | 2 | 10258280 | 10264755 | protein\_coding | | ENSDARG00000078468 | fap | 9 | 51693922 | 51729916 | protein\_coding | | ENSDARG00000069775 | fbxo40.1 | 21 | 22690154 | 22694227 | protein\_coding | | ENSDARG00000091119 | fbxo40.2 | 21 | 22694685 | 22700623 | protein\_coding | | ENSDARG00000009242 | fev | 9 | 11577030 | 11580405 | protein\_coding | | ENSDARG00000023174 | fez1 | 10 | 31794630 | 31838756 | protein\_coding | | ENSDARG00000088801 | fgd5a | 11 | 18234215 | 18306228 | protein\_coding | | ENSDARG00000056633 | fgf13b | 10 | 26754898 | 26782418 | protein\_coding | | ENSDARG00000052625 | fkbp1b | 20 | 44566339 | 44598239 | protein\_coding | | ENSDARG00000075597 | flrt1b | 14 | 46896395 | 46945107 | protein\_coding | | ENSDARG00000044895 | fmoda | 11 | 20865327 | 20871205 | protein\_coding | | ENSDARG00000015399 | foxf1 | 18 | 30868983 | 30872111 | protein\_coding | | ENSDARG00000070053 | foxg1d | 13 | 47154 | 49910 | protein\_coding | | ENSDARG00000102626 | frem2b | 15 | 32578491 | 32738123 | protein\_coding | | ENSDARG00000076496 | frmd4bb | 23 | 791326 | 831319 | protein\_coding | | ENSDARG00000043457 | gapdh | 16 | 17284687 | 17292143 | protein\_coding | | ENSDARG00000095378 | gdf10a | 13 | 31155210 | 31159175 | protein\_coding | | ENSDARG00000016160 | gmpr | 19 | 32885876 | 32900349 | protein\_coding | | ENSDARG00000043701 | gpd1a | 8 | 6989501 | 7008945 | protein\_coding | | ENSDARG00000092517 | gpsm1a | 5 | 63522678 | 63531753 | protein\_coding | | ENSDARG00000088116 | gstm.3 | 8 | 25283033 | 25287875 | protein\_coding | | ENSDARG00000038559 | h1f0 | 24 | 37679309 | 37680639 | protein\_coding | | ENSDARG00000039051 | hhatla | 2 | 21679743 | 21694039 | protein\_coding | | ENSDARG00000005139 | hhatlb | 24 | 20414590 | 20427751 | protein\_coding | | ENSDARG00000009134 | hlx1 | 20 | 52383620 | 52387406 | protein\_coding | | ENSDARG00000101759 | homer1b | 5 | 51128390 | 51184404 | protein\_coding | | ENSDARG00000056015 | hoxb13a | 3 | 23523685 | 23525286 | protein\_coding | | ENSDARG00000069425 | hsbp1a | 7 | 26357955 | 26362866 | protein\_coding | | ENSDARG00000052450 | hspb2 | 5 | 56958990 | 56971040 | protein\_coding | | ENSDARG00000009978 | icn | 16 | 23516157 | 23517520 | protein\_coding | | ENSDARG00000005526 | igfn1.1 | 6 | 46877007 | 46939362 | protein\_coding | | ENSDARG00000053248 | inab | 1 | 31131836 | 31135149 | protein\_coding | | ENSDARG00000051875 | islr2 | 25 | 22523768 | 22541638 | protein\_coding | | ENSDARG00000013371 | isoc2 | 16 | 12903791 | 12919766 | protein\_coding | | ENSDARG00000078717 | itga8 | 16 | 28449135 | 28579384 | protein\_coding | | ENSDARG00000022689 | itgb1b.2 | 2 | 43719748 | 43733981 | protein\_coding | | ENSDARG00000038826 | jph1b | 2 | 30307186 | 30340764 | protein\_coding | | ENSDARG00000028625 | jph2 | 23 | 35622804 | 35657312 | protein\_coding | | ENSDARG00000077165 | kcnj3a | 9 | 4508684 | 4543063 | protein\_coding | | ENSDARG00000056929 | kdm6bb | 10 | 22754607 | 22791207 | protein\_coding | | ENSDARG00000076027 | kif5c | 9 | 23370534 | 23406327 | protein\_coding | | ENSDARG00000053803 | klhl43 | 19 | 44195272 | 44223752 | protein\_coding | | ENSDARG00000020785 | lama4 | 20 | 230251 | 271330 | protein\_coding | | ENSDARG00000045524 | lamb1b | 4 | 25715280 | 25762109 | protein\_coding | | ENSDARG00000099974 | ldb3b | 12 | 25933693 | 25973150 | protein\_coding | | ENSDARG00000100133 | lect1 | 9 | 54185533 | 54206826 | protein\_coding | | ENSDARG00000033227 | lect2l | 14 | 36545210 | 36546749 | protein\_coding | | ENSDARG00000088711 | lgals1l1 | 3 | 28808917 | 28815915 | protein\_coding | | ENSDARG00000054942 | lgals2a | 3 | 28808783 | 28829415 | protein\_coding | | ENSDARG00000006896 | lhx6 | 10 | 9539143 | 9561661 | protein\_coding | | ENSDARG00000015445 | lim2.4 | 19 | 10622847 | 10635427 | protein\_coding | | ENSDARG00000029710 | lrrc30a | 24 | 41778204 | 41779303 | protein\_coding | | ENSDARG00000045580 | lum | 4 | 16362632 | 16365316 | protein\_coding | | ENSDARG00000103849 | mdh1ab | 17 | 24421131 | 24426001 | polymorphic\_pseudogene | | ENSDARG00000042033 | mettl11b | 20 | 34552766 | 34563604 | protein\_coding | | ENSDARG00000042925 | mfng | 22 | 15225144 | 15254255 | protein\_coding | | ENSDARG00000037285 | mipa | 23 | 27141684 | 27146233 | protein\_coding | | ENSDARG00000013963 | mipb | 23 | 6703639 | 6707251 | protein\_coding | | ENSDARG00000070991 | mlpha | 6 | 15479051 | 15514746 | protein\_coding | | ENSDARG00000009825 | mmp23bb | 18 | 45993010 | 46007507 | protein\_coding | | ENSDARG00000019521 | mpx | 10 | 7815556 | 7827254 | protein\_coding | | ENSDARG00000043802 | ms4a17a.9 | 4 | 75032057 | 75055899 | protein\_coding | | ENSDARG00000024789 | mxc | 9 | 32994776 | 33007893 | protein\_coding | | ENSDARG00000011615 | mybpc3 | 7 | 31567170 | 31628058 | protein\_coding | | ENSDARG00000003081 | mybphb | 6 | 55022626 | 55070995 | protein\_coding | | ENSDARG00000067990 | myhz1.1 | 5 | 31698473 | 31708976 | protein\_coding | | ENSDARG00000067995 | myhz1.2 | 5 | 31681664 | 31692849 | protein\_coding | | ENSDARG00000053254 | mylpfa | 3 | 32681334 | 32686893 | protein\_coding | | ENSDARG00000002589 | mylpfb | 12 | 14964454 | 14972693 | protein\_coding | | ENSDARG00000017441 | mylz3 | 1 | 6855476 | 6871030 | protein\_coding | | ENSDARG00000061249 | myom1a | 2 | 30932655 | 30976380 | protein\_coding | | ENSDARG00000071445 | myoz1b | 12 | 26293886 | 26317516 | protein\_coding | | ENSDARG00000012311 | myoz2a | 7 | 69588478 | 69616337 | protein\_coding | | ENSDARG00000015025 | nadl1.1 | 23 | 584289 | 624554 | protein\_coding | | ENSDARG00000062397 | ndst1a | 14 | 2736184 | 2780693 | protein\_coding | | ENSDARG00000032630 | neb | 9 | 22994268 | 23081969 | protein\_coding | | ENSDARG00000043697 | nefmb | 8 | 6819914 | 6823342 | protein\_coding | | ENSDARG00000090690 | nell2a | 25 | 558243 | 668986 | protein\_coding | | ENSDARG00000036168 | nfatc1 | 19 | 22632455 | 22720211 | protein\_coding | | ENSDARG00000043237 | nfil3-2 | 3 | 13933319 | 13935340 | protein\_coding | | ENSDARG00000068710 | nid1a | 13 | 49753659 | 49826548 | protein\_coding | | ENSDARG00000067848 | nmrk2 | 2 | 55859095 | 55866484 | protein\_coding | | ENSDARG00000010423 | npsn | 7 | 38382223 | 38388219 | protein\_coding | | ENSDARG00000009341 | nrap | 12 | 30471697 | 30503538 | protein\_coding | | ENSDARG00000071860 | nrn1a | 24 | 2523717 | 2528911 | protein\_coding | | ENSDARG00000061303 | oca2 | 6 | 37881002 | 38060286 | protein\_coding | | ENSDARG00000034270 | ogdha | 8 | 45754605 | 45830602 | protein\_coding | | ENSDARG00000079045 | opn8b | 20 | 44413442 | 44449616 | protein\_coding | | ENSDARG00000043483 | otx5 | 15 | 47316335 | 47321752 | protein\_coding | | ENSDARG00000059259 | pabpc4 | 17 | 25287168 | 25308294 | protein\_coding | | ENSDARG00000014910 | panx1b | 5 | 37240761 | 37252355 | protein\_coding | | ENSDARG00000027867 | paplna | 17 | 51034383 | 51110523 | protein\_coding | | ENSDARG00000053829 | pax9 | 17 | 38288884 | 38343480 | protein\_coding | | ENSDARG00000094974 | pbld1 | 13 | 22713380 | 22717034 | transcribed\_unprocessed\_pseudogene | | ENSDARG00000026359 | pbld2 | 13 | 22715966 | 22731983 | protein\_coding | | ENSDARG00000100494 | pbx1a | 2 | 19049120 | 19181730 | protein\_coding | | ENSDARG00000101131 | pbx1b | 6 | 2170329 | 2294610 | protein\_coding | | ENSDARG00000006456 | pdgfrl | 14 | 30018810 | 30025882 | protein\_coding | | ENSDARG00000014527 | pdk3a | 24 | 24799178 | 24814433 | protein\_coding | | ENSDARG00000014248 | pdlim3b | 14 | 29442194 | 29459793 | protein\_coding | | ENSDARG00000105357 | pdlim7 | 14 | 45893100 | 45905468 | protein\_coding | | ENSDARG00000060263 | pecam1 | 3 | 59491910 | 59525948 | protein\_coding | | ENSDARG00000036045 | penkb | 7 | 58384420 | 58393646 | protein\_coding | | ENSDARG00000069498 | phkg1b | 21 | 25639492 | 25649163 | protein\_coding | | ENSDARG00000070498 | phyhiplb | 12 | 7412092 | 7480413 | protein\_coding | | ENSDARG00000052462 | pisd | 5 | 56819036 | 56858726 | protein\_coding | | ENSDARG00000036542 | pknox1.2 | 1 | 46268999 | 46293309 | protein\_coding | | ENSDARG00000060813 | plekha7a | 18 | 27118774 | 27323729 | protein\_coding | | ENSDARG00000090752 | plk2a | 10 | 4476569 | 4485777 | protein\_coding | | ENSDARG00000019328 | plxna4 | 4 | 15219174 | 15442963 | protein\_coding | | ENSDARG00000091298 | pmela | 11 | 3186364 | 3203860 | protein\_coding | | ENSDARG00000033760 | pmelb | 23 | 35573739 | 35591533 | protein\_coding | | ENSDARG00000025024 | pnoca | 17 | 16102352 | 16125316 | protein\_coding | | ENSDARG00000058551 | popdc3 | 16 | 7736559 | 7741361 | protein\_coding | | ENSDARG00000104267 | postnb | 15 | 32852115 | 32882686 | protein\_coding | | ENSDARG00000061977 | ppfibp2a | 18 | 29166932 | 29213794 | protein\_coding | | ENSDARG00000090468 | ppp1r3aa | 4 | 6563794 | 6572040 | protein\_coding | | ENSDARG00000100093 | ppp2r2ba | 14 | 51807023 | 51837181 | protein\_coding | | ENSDARG00000062277 | ppp2r2cb | 14 | 20906838 | 20922414 | protein\_coding | | ENSDARG00000077722 | ppp2r3a | 2 | 25517837 | 25635547 | protein\_coding | | ENSDARG00000001933 | prkag2b | 2 | 13374016 | 13465667 | protein\_coding | | ENSDARG00000034173 | prkcq | 4 | 25402043 | 25454181 | protein\_coding | | ENSDARG00000086705 | prob1 | 21 | 30048128 | 30054247 | protein\_coding | | ENSDARG00000055158 | prox1a | 17 | 32870009 | 32913277 | protein\_coding | | ENSDARG00000028306 | prph | 11 | 3191072 | 3266090 | protein\_coding | | ENSDARG00000097224 | prr33 | 25 | 30811423 | 30819003 | protein\_coding | | ENSDARG00000100691 | prss35 | 16 | 33041852 | 33050822 | protein\_coding | | ENSDARG00000017246 | prx | 18 | 46224211 | 46243758 | protein\_coding | | ENSDARG00000013317 | pygmb | 7 | 22552671 | 22581015 | protein\_coding | | ENSDARG00000053449 | pyya | 3 | 33209225 | 33213066 | protein\_coding | | ENSDARG00000040190 | qdpra | 14 | 47886148 | 47909986 | protein\_coding | | ENSDARG00000002026 | qkib | 13 | 3533388 | 3713096 | protein\_coding | | ENSDARG00000090086 | rab11bb | 2 | 56339322 | 56383670 | protein\_coding | | ENSDARG00000019312 | rab39ba | 21 | 30503770 | 30508426 | protein\_coding | | ENSDARG00000036501 | rab39bb | 7 | 23601384 | 23613507 | protein\_coding | | ENSDARG00000014462 | rab3c | 8 | 17019580 | 17031652 | protein\_coding | | ENSDARG00000020497 | rab7 | 11 | 16044454 | 16080508 | protein\_coding | | ENSDARG00000010083 | rbfox3 | 12 | 32936247 | 33150482 | protein\_coding | | ENSDARG00000056639 | rltpr | 18 | 22313569 | 22407305 | protein\_coding | | ENSDARG00000091161 | rpz | 16 | 46434860 | 46457126 | protein\_coding | | ENSDARG00000075718 | rpz5 | 16 | 46526040 | 46529577 | protein\_coding | | ENSDARG00000006497 | rtn1a | 13 | 31294137 | 31339989 | protein\_coding | | ENSDARG00000057027 | rtn2b | 21 | 21337641 | 21349484 | protein\_coding | | ENSDARG00000003680 | runx1t1 | 19 | 34114430 | 34199596 | protein\_coding | | ENSDARG00000023797 | ryr1b | 18 | 35516112 | 35755583 | protein\_coding | | ENSDARG00000071331 | ryr3 | 20 | 29365565 | 29517834 | protein\_coding | | ENSDARG00000060222 | scn1ba | 16 | 41923676 | 41947713 | protein\_coding | | ENSDARG00000035695 | scxa | 19 | 3060575 | 3068672 | protein\_coding | | ENSDARG00000034300 | sema3c | 4 | 22892650 | 22931969 | protein\_coding | | ENSDARG00000030656 | sept3 | 1 | 44924847 | 44942160 | protein\_coding | | ENSDARG00000052341 | sgcb | 20 | 23274610 | 23282662 | protein\_coding | | ENSDARG00000038107 | sgcg | 15 | 9351181 | 9377625 | protein\_coding | | ENSDARG00000006598 | sgk2b | 16 | 26656984 | 26663672 | protein\_coding | | ENSDARG00000021633 | sh3bgr | 15 | 5936000 | 5980821 | protein\_coding | | ENSDARG00000061758 | sh3pxd2ab | 17 | 20502245 | 20539125 | protein\_coding | | ENSDARG00000025891 | shox | 9 | 34832052 | 34838510 | protein\_coding | | ENSDARG00000100353 | si:ch1073-398f15.1 | 21 | 175761 | 185138 | protein\_coding | | ENSDARG00000060656 | si:ch211-10a23.2 | 13 | 32987078 | 32992790 | protein\_coding | | ENSDARG00000029296 | si:ch211-134n22.1 | 14 | 10797704 | 10962361 | protein\_coding | | ENSDARG00000076332 | si:ch211-159i8.4 | 14 | 17295732 | 17316427 | protein\_coding | | ENSDARG00000086027 | si:ch211-168d23.3 | 17 | 51675275 | 51700713 | protein\_coding | | ENSDARG00000097617 | si:ch211-178n15.1 | 2 | 55936555 | 55971430 | protein\_coding | | ENSDARG00000007912 | si:ch211-196i2.1 | 21 | 14156675 | 14213430 | protein\_coding | | ENSDARG00000103231 | si:ch211-199i15.5 | 20 | 40340331 | 40356572 | protein\_coding | | ENSDARG00000098011 | si:ch211-209m20.7 | 15 | 31042185 | 31043291 | antisense | | ENSDARG00000076547 | si:ch211-221f10.2 | 14 | 23220865 | 23295599 | protein\_coding | | ENSDARG00000100270 | si:ch211-235e15.1 | 24 | 30343717 | 30348120 | antisense | | ENSDARG00000071353 | si:ch211-235e9.8 | 14 | 47087003 | 47126112 | protein\_coding | | ENSDARG00000092240 | si:ch211-243a20.3 | 1 | 44660095 | 44674627 | protein\_coding | | ENSDARG00000057903 | si:ch211-266g18.10 | 17 | 15441468 | 15490342 | protein\_coding | | ENSDARG00000093244 | si:ch211-32p8.1 | 24 | 38508701 | 38509617 | processed\_transcript | | ENSDARG00000087508 | si:ch211-39i22.1 | 9 | 56247362 | 56292510 | protein\_coding | | ENSDARG00000076996 | si:ch73-182a11.2 | 2 | 6238973 | 6270216 | protein\_coding | | ENSDARG00000103358 | si:ch73-187p21.1 | 6 | 39527388 | 39527969 | TEC | | ENSDARG00000061672 | si:ch73-334d15.1 | 24 | 36833557 | 36840166 | protein\_coding | | ENSDARG00000096257 | si:ch73-367p23.2 | 3 | 32394549 | 32399290 | protein\_coding | | ENSDARG00000101586 | si:ch73-389b16.1 | 6 | 24040948 | 24044114 | protein\_coding | | ENSDARG00000098941 | si:ch73-57a19.4 | 13 | 4583835 | 4585520 | lincRNA | | ENSDARG00000071458 | si:dkey-121a11.3 | 22 | 16419782 | 16451752 | protein\_coding | | ENSDARG00000004386 | si:dkey-153k10.9 | 4 | 9668801 | 9689503 | protein\_coding | | ENSDARG00000057504 | si:dkey-202g17.3 | 15 | 9096750 | 9113436 | protein\_coding | | ENSDARG00000101128 | si:dkey-211f22.5 | 22 | 20695830 | 20722084 | protein\_coding | | ENSDARG00000091513 | si:dkey-22o12.2 | 1 | 29539783 | 29562893 | protein\_coding | | ENSDARG00000090973 | si:dkey-264d12.1 | 11 | 39768645 | 39809282 | protein\_coding | | ENSDARG00000103315 | si:dkey-30d10.1 | 7 | 36277575 | 36283633 | lincRNA | | ENSDARG00000097738 | si:dkey-31n5.4 | 3 | 23089722 | 23107761 | antisense | | ENSDARG00000105188 | si:dkey-76i15.1 | 14 | 33448983 | 33483081 | protein\_coding | | ENSDARG00000017036 | si:dkeyp-57f11.2 | 3 | 38971021 | 39031346 | protein\_coding | | ENSDARG00000095615 | si:dkeyp-86h10.3 | 16 | 20503552 | 20506327 | protein\_coding | | ENSDARG00000058004 | six2a | 13 | 10100220 | 10103090 | protein\_coding | | ENSDARG00000069030 | skor1a | 7 | 33683537 | 33690401 | protein\_coding | | ENSDARG00000006356 | slc18a3a | 13 | 29291599 | 29294004 | protein\_coding | | ENSDARG00000059690 | slc29a4 | 3 | 40173552 | 40207875 | protein\_coding | | ENSDARG00000045447 | slc35g2b | 24 | 3382067 | 3388211 | protein\_coding | | ENSDARG00000071685 | slco5a1 | 24 | 19447474 | 19553027 | protein\_coding | | ENSDARG00000077946 | smarcc2 | 6 | 39838938 | 39869134 | protein\_coding | | ENSDARG00000038786 | smarcd3a | 2 | 32531413 | 32575577 | protein\_coding | | ENSDARG00000098226 | smpd3 | 25 | 12967714 | 13058023 | protein\_coding | | ENSDARG00000009280 | smyd1a | 5 | 71326683 | 71341070 | protein\_coding | | ENSDARG00000034423 | sncga | 13 | 22528703 | 22542716 | protein\_coding | | ENSDARG00000061259 | sost | 12 | 27239964 | 27244292 | protein\_coding | | ENSDARG00000098834 | sox4b | 16 | 68069 | 69930 | protein\_coding | | ENSDARG00000077069 | srgn | 13 | 22940131 | 22944004 | protein\_coding | | ENSDARG00000086327 | srrm4 | 5 | 15167657 | 15316987 | protein\_coding | | ENSDARG00000041747 | sspn | 18 | 16122119 | 16134156 | protein\_coding | | ENSDARG00000033161 | sst1.2 | 15 | 36257986 | 36262308 | protein\_coding | | ENSDARG00000014190 | sst2 | 2 | 5814471 | 5816961 | protein\_coding | | ENSDARG00000009499 | syne1a | 20 | 26196440 | 26460352 | protein\_coding | | ENSDARG00000077157 | synpo2b | 1 | 25267662 | 25272368 | protein\_coding | | ENSDARG00000077356 | sypl1 | 4 | 3376539 | 3385058 | protein\_coding | | ENSDARG00000014490 | tac1 | 19 | 26065403 | 26068962 | protein\_coding | | ENSDARG00000071304 | tbc1d2 | 14 | 5981037 | 6023097 | protein\_coding | | ENSDARG00000004415 | tcf7l2 | 12 | 30864834 | 30988414 | protein\_coding | | ENSDARG00000102106 | tgm1l1 | 2 | 52076466 | 52126677 | protein\_coding | | ENSDARG00000073810 | thbs2b | 12 | 30538145 | 30567025 | protein\_coding | | ENSDARG00000046007 | tmcc2 | 22 | 554086 | 562293 | protein\_coding | | ENSDARG00000075980 | tmem125b | 2 | 6022020 | 6030473 | protein\_coding | | ENSDARG00000060569 | tmem229b | 17 | 37677119 | 37702243 | protein\_coding | | ENSDARG00000069423 | tmie | 2 | 1881031 | 1936505 | protein\_coding | | ENSDARG00000077547 | tmlhe | 21 | 43674767 | 43700640 | protein\_coding | | ENSDARG00000020890 | tmod4 | 16 | 29716075 | 29749570 | protein\_coding | | ENSDARG00000070835 | tnnc2 | 23 | 20496318 | 20500956 | protein\_coding | | ENSDARG00000036671 | tnni1al | 25 | 19992332 | 19999199 | protein\_coding | | ENSDARG00000002988 | tnnt2d | 25 | 20118310 | 20128930 | protein\_coding | | ENSDARG00000076847 | tnrc6c1 | 3 | 34129247 | 34208461 | protein\_coding | | ENSDARG00000100055 | tox2 | 6 | 57903518 | 58152418 | protein\_coding | | ENSDARG00000104682 | tpm2 | 10 | 6317005 | 6373961 | protein\_coding | | ENSDARG00000033683 | tpma | 7 | 29680931 | 29689316 | protein\_coding | | ENSDARG00000018264 | trim101 | 23 | 17528348 | 17543803 | protein\_coding | | ENSDARG00000070507 | trpc4a | 10 | 25867549 | 25898389 | protein\_coding | | ENSDARG00000032389 | tspan12 | 4 | 10575973 | 10600213 | protein\_coding | | ENSDARG00000086033 | tspan5a | 7 | 69254125 | 69399342 | protein\_coding | | ENSDARG00000028213 | ttna | 9 | 42931207 | 43272173 | protein\_coding | | ENSDARG00000039522 | tubb2 | 2 | 1667471 | 1677238 | protein\_coding | | ENSDARG00000104801 | tubb6 | 24 | 2942256 | 2948662 | protein\_coding | | ENSDARG00000094792 | twf2a | 6 | 41455468 | 41504230 | protein\_coding | | ENSDARG00000056151 | tyrp1b | 1 | 18639900 | 18653111 | protein\_coding | | ENSDARG00000002607 | unm\_sa1614 | 11 | 26238537 | 26324730 | protein\_coding | | ENSDARG00000034700 | vegfab | 4 | 5560118 | 5589254 | protein\_coding | | ENSDARG00000045003 | vsg1 | 11 | 6046487 | 6051235 | protein\_coding | | ENSDARG00000058003 | wfdc1 | 18 | 14626840 | 14649200 | protein\_coding | | ENSDARG00000041117 | wnt2 | 18 | 20624175 | 20647483 | protein\_coding | | ENSDARG00000037889 | wnt9b | 3 | 37433027 | 37439762 | protein\_coding | | ENSDARG00000071113 | xirp2a | 6 | 9934702 | 9998111 | protein\_coding | | ENSDARG00000091090 | xirp2b | 9 | 49751277 | 49795830 | protein\_coding | | ENSDARG00000033599 | zgc:100906 | 19 | 27133851 | 27146027 | protein\_coding | | ENSDARG00000056650 | zgc:113276 | 11 | 29689569 | 29699753 | protein\_coding | | ENSDARG00000063614 | zgc:153395 | 10 | 14589795 | 14599941 | protein\_coding | | ENSDARG00000079699 | zgc:162612 | 3 | 25977162 | 25978451 | protein\_coding | | ENSDARG00000105001 | zgc:91999 | 24 | 38382414 | 38401016 | protein\_coding | |
