# Supplementary material for: A high-resolution mRNA expression time course of embryonic development in zebrafish
Source: eLife. 2017 Nov 16;6:e30860. doi: 10.7554/eLife.30860 (PMC5690287; doi:10.7554/eLife.30860)
Supplement: Supplementary file 6. [file elife-30860-supp6.zip › biolayout-clusters-files/Cluster004.html]

Cluster004


# Cluster004: Detail

### Go to ZFA detail

## GO

| | GO ID | Description | Domain | Annotated | Expected | Observed | Adjusted p-value | Genes | Ensembl IDs | | --- | --- | --- | --- | --- | --- | --- | --- | --- | | GO:0005578 | proteinaceous extracellular matrix | cellular\_component | 111 | 3.5 | 15 | 0.00041 | col11a1b col1a1a col1a2 paplna col1a1b col9a3 wnt9b wnt2 fmoda lum epyc ch1073-291c23.1 ecm2 lect1 postnb | ENSDARG00000009014 ENSDARG00000012405 ENSDARG00000020007 ENSDARG00000027867 ENSDARG00000035809 ENSDARG00000037845 ENSDARG00000037889 ENSDARG00000041117 ENSDARG00000044895 ENSDARG00000045580 ENSDARG00000056950 ENSDARG00000063631 ENSDARG00000071549 ENSDARG00000100133 ENSDARG00000104267 | | GO:0003779 | actin binding | molecular\_function | 171 | 5.2 | 19 | 0.00160 | actn3b nrap syne1a dmtn actn3a cfl2 si:dkeyp-57f11.2 tmod4 arpc1b ttna neb tpma abrab myhz1.1 myhz1.2 xirp2a xirp2b twf2a evlb | ENSDARG00000001431 ENSDARG00000009341 ENSDARG00000009499 ENSDARG00000013110 ENSDARG00000013755 ENSDARG00000014106 ENSDARG00000017036 ENSDARG00000020890 ENSDARG00000027063 ENSDARG00000028213 ENSDARG00000032630 ENSDARG00000033683 ENSDARG00000033854 ENSDARG00000067990 ENSDARG00000067995 ENSDARG00000071113 ENSDARG00000091090 ENSDARG00000094792 ENSDARG00000099720 | |

  


### Go to GO detail

## ZFA

| | ZFA ID | Description | Annotated | Expected | Observed | Fold Enrichment | Adjusted p-value | Genes | Ensembl IDs | | --- | --- | --- | --- | --- | --- | --- | --- | --- | | ZFA:0000473 | trunk musculature | 162 | 5.02 | 12 | 2.4 | 2.0e-11 | actn3a actn3b sgcb eno3 pdlim7 tnni1al lama4 homer1b shox ryr1b tpma mylpfa | ENSDARG00000013755 ENSDARG00000001431 ENSDARG00000052341 ENSDARG00000039007 ENSDARG00000105357 ENSDARG00000036671 ENSDARG00000020785 ENSDARG00000101759 ENSDARG00000025891 ENSDARG00000023797 ENSDARG00000033683 ENSDARG00000053254 | | ZFA:0000548 | musculature system | 273 | 8.46 | 23 | 2.7 | 1.2e-10 | actc1b myhz1.1 mylz3 ttna gapdh myom1a ldb3b mdh1ab postnb pabpc4 col9a2 mybphb smyd1a jph2 tpm2 mylpfb neb lgals2a asb5b pdlim3b scn1ba gdf10a mylpfa | ENSDARG00000099197 ENSDARG00000067990 ENSDARG00000017441 ENSDARG00000028213 ENSDARG00000043457 ENSDARG00000061249 ENSDARG00000099974 ENSDARG00000103849 ENSDARG00000104267 ENSDARG00000059259 ENSDARG00000024492 ENSDARG00000003081 ENSDARG00000009280 ENSDARG00000028625 ENSDARG00000104682 ENSDARG00000002589 ENSDARG00000032630 ENSDARG00000054942 ENSDARG00000053222 ENSDARG00000014248 ENSDARG00000060222 ENSDARG00000095378 ENSDARG00000053254 | | ZFA:0001056 | myotome | 825 | 25.56 | 55 | 2.2 | 1.2e-10 | actc1b myhz1.1 actn3a ctsk ttna gapdh ogdha myom1a ldb3b twf2a mdh1ab col1a1a postnb pabpc4 mybphb smyd1a jph2 xirp2b sgcg celf2 eno3 ca2 xirp2a fkbp1b cfl2 hhatla tpm2 myoz1b lama4 trim101 casq1a zgc:91999 homer1b ak1 mylpfb ryr3 neb ENSDARG00000100596 igfn1.1 asb5b pbld2 hhatlb zgc:113276 tnnt2d pdlim3b capn1b mybpc3 cers6 ryr1b gdf10a synpo2b pax9 tpma mylpfa tmod4 | ENSDARG00000099197 ENSDARG00000067990 ENSDARG00000013755 ENSDARG00000040251 ENSDARG00000028213 ENSDARG00000043457 ENSDARG00000034270 ENSDARG00000061249 ENSDARG00000099974 ENSDARG00000094792 ENSDARG00000103849 ENSDARG00000012405 ENSDARG00000104267 ENSDARG00000059259 ENSDARG00000003081 ENSDARG00000009280 ENSDARG00000028625 ENSDARG00000091090 ENSDARG00000038107 ENSDARG00000002131 ENSDARG00000039007 ENSDARG00000014488 ENSDARG00000071113 ENSDARG00000052625 ENSDARG00000014106 ENSDARG00000039051 ENSDARG00000104682 ENSDARG00000071445 ENSDARG00000020785 ENSDARG00000018264 ENSDARG00000038716 ENSDARG00000105001 ENSDARG00000101759 ENSDARG00000001950 ENSDARG00000002589 ENSDARG00000071331 ENSDARG00000032630 ENSDARG00000100596 ENSDARG00000005526 ENSDARG00000053222 ENSDARG00000026359 ENSDARG00000005139 ENSDARG00000056650 ENSDARG00000002988 ENSDARG00000014248 ENSDARG00000052748 ENSDARG00000011615 ENSDARG00000053583 ENSDARG00000023797 ENSDARG00000095378 ENSDARG00000077157 ENSDARG00000053829 ENSDARG00000033683 ENSDARG00000053254 ENSDARG00000020890 | | ZFA:0001105 | embryonic structure | 15 | 0.46 | 1 | 2.2 | 8.4e-09 | capn1b | ENSDARG00000052748 | | ZFA:0000328 | cephalic musculature | 93 | 2.88 | 14 | 4.9 | 2.8e-08 | actc1b myhz1.1 myom1a sgcg hhatla pdlim7 tpm2 tnni1al trim101 zgc:91999 ak1 neb mybpc3 tpma | ENSDARG00000099197 ENSDARG00000067990 ENSDARG00000061249 ENSDARG00000038107 ENSDARG00000039051 ENSDARG00000105357 ENSDARG00000104682 ENSDARG00000036671 ENSDARG00000018264 ENSDARG00000105001 ENSDARG00000001950 ENSDARG00000032630 ENSDARG00000011615 ENSDARG00000033683 | | ZFA:0005277 | skeletal muscle | 170 | 5.27 | 19 | 3.6 | 4.6e-06 | actc1b tnnc2 pbx1a ttna smyd1a xirp2b xirp2a pdlim7 lum trim101 casq1a neb rtn2b casq1b mybpc3 ryr1b slco5a1 mylpfa tmod4 | ENSDARG00000099197 ENSDARG00000070835 ENSDARG00000100494 ENSDARG00000028213 ENSDARG00000009280 ENSDARG00000091090 ENSDARG00000071113 ENSDARG00000105357 ENSDARG00000045580 ENSDARG00000018264 ENSDARG00000038716 ENSDARG00000032630 ENSDARG00000057027 ENSDARG00000018105 ENSDARG00000011615 ENSDARG00000023797 ENSDARG00000071685 ENSDARG00000053254 ENSDARG00000020890 | | ZFA:0000113 | head mesenchyme | 160 | 4.96 | 11 | 2.2 | 2.3e-03 | ctsk dcn six2a lect1 col9a3 xirp2a pdlim7 pdgfrl lum lhx6 pax9 | ENSDARG00000040251 ENSDARG00000012066 ENSDARG00000058004 ENSDARG00000100133 ENSDARG00000037845 ENSDARG00000071113 ENSDARG00000105357 ENSDARG00000006456 ENSDARG00000045580 ENSDARG00000006896 ENSDARG00000053829 | | ZFA:0007052 | hyohyoideus | 19 | 0.59 | 5 | 8.5 | 1.2e-02 | ttna actn3b tnni1al mylpfa tmod4 | ENSDARG00000028213 ENSDARG00000001431 ENSDARG00000036671 ENSDARG00000053254 ENSDARG00000020890 | | ZFA:0001652 | head muscle | 24 | 0.74 | 1 | 1.4 | 1.2e-02 | mylpfa | ENSDARG00000053254 | | ZFA:0009115 | skeletal muscle cell | 89 | 2.76 | 6 | 2.2 | 4.0e-02 | actc1b tnnc2 ttna neb scn1ba tpma | ENSDARG00000099197 ENSDARG00000070835 ENSDARG00000028213 ENSDARG00000032630 ENSDARG00000060222 ENSDARG00000033683 | |
