# Supplementary material for: A high-resolution mRNA expression time course of embryonic development in zebrafish
Source: eLife. 2017 Nov 16;6:e30860. doi: 10.7554/eLife.30860 (PMC5690287; doi:10.7554/eLife.30860)
Supplement: Supplementary file 6. [file elife-30860-supp6.zip › biolayout-clusters-files/Cluster005-genes.html]

Cluster005


# Cluster005: Genes

| | Ensembl ID | Gene Name | Chr | Start | End | Biotype | | --- | --- | --- | --- | --- | --- | | ENSDARG00000030778 | BX323854.2 | 24 | 40102848 | 40104407 | protein\_coding | | ENSDARG00000078349 | BX324215.3 | 22 | 30709100 | 30713494 | protein\_coding | | ENSDARG00000094120 | BX548047.2 | 2 | 48126682 | 48129830 | protein\_coding | | ENSDARG00000100184 | BX908782.2 | 2 | 52180833 | 52189501 | protein\_coding | | ENSDARG00000101633 | CABZ01050166.1 | 16 | 18422909 | 18453939 | protein\_coding | | ENSDARG00000102894 | CABZ01057159.1 | 21 | 726765 | 732096 | protein\_coding | | ENSDARG00000057408 | CABZ01067232.1 | 15 | 45653529 | 45658748 | protein\_coding | | ENSDARG00000102907 | CABZ01072043.1 | 21 | 49418 | 65146 | protein\_coding | | ENSDARG00000104672 | CABZ01074397.1 | KN149880.1 | 6192 | 50193 | protein\_coding | | ENSDARG00000090707 | CABZ01079981.1 | 16 | 26039777 | 26040958 | protein\_coding | | ENSDARG00000098538 | CABZ01112049.1 | KN150189.1 | 3 | 10065 | protein\_coding | | ENSDARG00000103342 | CABZ01113193.1 | KN149797.1 | 55564 | 70688 | protein\_coding | | ENSDARG00000017314 | CELA1 (1 of many) | 22 | 7409630 | 7414668 | protein\_coding | | ENSDARG00000043168 | CELA1 (1 of many).1 | 22 | 7467473 | 7479048 | protein\_coding | | ENSDARG00000043171 | CELA1 (1 of many).2 | 22 | 7457021 | 7462702 | protein\_coding | | ENSDARG00000043173 | CELA1 (1 of many).3 | 22 | 7450619 | 7453909 | protein\_coding | | ENSDARG00000077058 | CELA1 (1 of many).5 | 22 | 7712365 | 7724545 | protein\_coding | | ENSDARG00000095462 | CELA1 (1 of many).6 | 22 | 7433161 | 7446506 | protein\_coding | | ENSDARG00000100465 | CLRN3 | 12 | 42897462 | 42908694 | protein\_coding | | ENSDARG00000101535 | COL10A1 (1 of many) | 20 | 494770 | 498370 | protein\_coding | | ENSDARG00000091431 | CR352285.1 | 11 | 36971967 | 36972962 | protein\_coding | | ENSDARG00000103034 | CRISP3 | 16 | 50157425 | 50176281 | protein\_coding | | ENSDARG00000031534 | ENSDARG00000031534 | 3 | 19319037 | 19327733 | protein\_coding | | ENSDARG00000041685 | ENSDARG00000041685 | 15 | 21178414 | 21303684 | protein\_coding | | ENSDARG00000056791 | ENSDARG00000056791 | 3 | 19311251 | 19316277 | protein\_coding | | ENSDARG00000068586 | ENSDARG00000068586 | 14 | 45706669 | 45738362 | protein\_coding | | ENSDARG00000077799 | ENSDARG00000077799 | 23 | 45844302 | 45846431 | protein\_coding | | ENSDARG00000087633 | ENSDARG00000087633 | 21 | 14687592 | 14706825 | protein\_coding | | ENSDARG00000087805 | ENSDARG00000087805 | 6 | 30469874 | 30477653 | protein\_coding | | ENSDARG00000088044 | ENSDARG00000088044 | 4 | 9523889 | 9532429 | protein\_coding | | ENSDARG00000089853 | ENSDARG00000089853 | 1 | 46293820 | 46298613 | protein\_coding | | ENSDARG00000095217 | ENSDARG00000095217 | 22 | 237708 | 250084 | protein\_coding | | ENSDARG00000096445 | ENSDARG00000096445 | 17 | 25631548 | 25719061 | protein\_coding | | ENSDARG00000098278 | ENSDARG00000098278 | 15 | 36517368 | 36561825 | protein\_coding | | ENSDARG00000098791 | ENSDARG00000098791 | 25 | 4634061 | 4654207 | protein\_coding | | ENSDARG00000104569 | ENSDARG00000104569 | 18 | 2211054 | 2233382 | protein\_coding | | ENSDARG00000086512 | FP102464.1 | 5 | 10742928 | 10770835 | protein\_coding | | ENSDARG00000093043 | FP236810.1 | 5 | 8855219 | 8865030 | protein\_coding | | ENSDARG00000093303 | IFITM3 | 5 | 32658136 | 32658926 | protein\_coding | | ENSDARG00000102442 | IZUMO1R | 15 | 46903511 | 46910452 | protein\_coding | | ENSDARG00000040683 | MEP1B (1 of many) | 2 | 270897 | 280926 | protein\_coding | | ENSDARG00000101629 | MGAM | 18 | 2028736 | 2117027 | protein\_coding | | ENSDARG00000102414 | MYH13 (1 of many) | 22 | 281041 | 295380 | protein\_coding | | ENSDARG00000020952 | NAALADL1 | 5 | 25648227 | 25660972 | protein\_coding | | ENSDARG00000099558 | NPC1L1 | 8 | 40485278 | 40513758 | protein\_coding | | ENSDARG00000041960 | OLFM4 (1 of many) | 17 | 52494712 | 52501298 | protein\_coding | | ENSDARG00000087625 | OLFM4 (1 of many).1 | 17 | 52503650 | 52509632 | protein\_coding | | ENSDARG00000074457 | PFKFB1 (1 of many) | 23 | 12926691 | 13032155 | protein\_coding | | ENSDARG00000090899 | SYCN (1 of many) | 21 | 25182290 | 25182797 | protein\_coding | | ENSDARG00000005913 | TGM1 (1 of many) | 23 | 40277439 | 40297528 | protein\_coding | | ENSDARG00000102692 | TGM1 (1 of many).1 | 23 | 40305254 | 40318053 | protein\_coding | | ENSDARG00000073768 | TMEM125 (1 of many) | 6 | 34098323 | 34098991 | protein\_coding | | ENSDARG00000056781 | ZAN | 7 | 26055316 | 26094149 | protein\_coding | | ENSDARG00000070078 | abcb11b | 11 | 13165966 | 13211184 | protein\_coding | | ENSDARG00000010936 | abcb4 | 16 | 28694619 | 28723718 | protein\_coding | | ENSDARG00000063475 | abcg1 | 15 | 5037461 | 5083836 | protein\_coding | | ENSDARG00000100075 | abcg2a | 23 | 46061346 | 46091925 | protein\_coding | | ENSDARG00000079361 | abcg2b | 1 | 7988950 | 8007858 | protein\_coding | | ENSDARG00000063078 | abcg5 | 13 | 10812862 | 10836132 | protein\_coding | | ENSDARG00000062156 | abi3bpa | 9 | 30321752 | 30350158 | protein\_coding | | ENSDARG00000019207 | abo | 5 | 24521253 | 24533658 | protein\_coding | | ENSDARG00000016918 | ace2 | 11 | 29734494 | 29749173 | protein\_coding | | ENSDARG00000075931 | acsl5 | 12 | 31323677 | 31346317 | protein\_coding | | ENSDARG00000003113 | ada | 23 | 26019907 | 26050115 | protein\_coding | | ENSDARG00000105416 | adgrg4a | 14 | 31380857 | 31410840 | protein\_coding | | ENSDARG00000025595 | agmo | 15 | 34476753 | 34560556 | protein\_coding | | ENSDARG00000018478 | agxtb | 2 | 23021092 | 23033000 | protein\_coding | | ENSDARG00000026764 | ahsg1 | 22 | 37920796 | 37927972 | protein\_coding | | ENSDARG00000069293 | ahsg2 | 2 | 10336525 | 10343921 | protein\_coding | | ENSDARG00000098063 | alp3 | 15 | 44598837 | 44617951 | protein\_coding | | ENSDARG00000053774 | alpi.2 | 22 | 38791904 | 38806070 | protein\_coding | | ENSDARG00000032469 | ampd3b | 18 | 16773233 | 16806734 | protein\_coding | | ENSDARG00000013856 | amy2a | 2 | 15944165 | 15958382 | protein\_coding | | ENSDARG00000041083 | anpepa | 18 | 21088462 | 21108597 | protein\_coding | | ENSDARG00000103878 | anpepb | 25 | 10714755 | 10730975 | protein\_coding | | ENSDARG00000074075 | antxr1b | 10 | 41052466 | 41125589 | protein\_coding | | ENSDARG00000061355 | aoc1 | 24 | 40113443 | 40121599 | protein\_coding | | ENSDARG00000075614 | apoc4 | 16 | 24060898 | 24062096 | protein\_coding | | ENSDARG00000060350 | apoda.2 | 2 | 37225004 | 37233532 | protein\_coding | | ENSDARG00000007086 | aqp10a | 16 | 22373353 | 22378875 | protein\_coding | | ENSDARG00000057429 | arg1 | 12 | 9559308 | 9579927 | protein\_coding | | ENSDARG00000012829 | asah2 | 12 | 6084098 | 6109089 | protein\_coding | | ENSDARG00000001870 | atp1a1a.4 | 1 | 1541981 | 1558365 | protein\_coding | | ENSDARG00000035458 | atp2a1l | 12 | 13205881 | 13223442 | protein\_coding | | ENSDARG00000099216 | atp8b2 | 16 | 22321647 | 22336024 | protein\_coding | | ENSDARG00000015887 | b2ml | 8 | 50953262 | 50961527 | protein\_coding | | ENSDARG00000052376 | b3gnt2a | 1 | 52836283 | 52844567 | protein\_coding | | ENSDARG00000103659 | bco1l | 7 | 65016199 | 65028910 | protein\_coding | | ENSDARG00000099961 | bnip3 | 12 | 40534029 | 40549950 | protein\_coding | | ENSDARG00000024503 | c6ast3 | 7 | 38370856 | 38373302 | protein\_coding | | ENSDARG00000052578 | c6ast4 | 7 | 38364283 | 38367551 | protein\_coding | | ENSDARG00000016391 | calcoco1b | 11 | 2166340 | 2186038 | protein\_coding | | ENSDARG00000052515 | calcoco2 | 12 | 20519228 | 20531049 | protein\_coding | | ENSDARG00000008788 | camk1gb | 23 | 39661200 | 39704990 | protein\_coding | | ENSDARG00000015134 | camkk1b | 15 | 15378107 | 15395479 | protein\_coding | | ENSDARG00000068232 | cbln12 | 2 | 38139087 | 38143329 | protein\_coding | | ENSDARG00000090969 | cbln18 | 15 | 596153 | 607842 | protein\_coding | | ENSDARG00000019294 | cbln8 | 7 | 73231373 | 73233805 | protein\_coding | | ENSDARG00000038025 | cbx7a | 3 | 24855597 | 24864069 | protein\_coding | | ENSDARG00000032639 | cd36 | 4 | 22937497 | 22950494 | protein\_coding | | ENSDARG00000038199 | cda | 11 | 3566547 | 3573731 | protein\_coding | | ENSDARG00000017490 | cel.1 | 21 | 11308619 | 11318737 | protein\_coding | | ENSDARG00000029822 | cel.2 | 21 | 11292403 | 11301351 | protein\_coding | | ENSDARG00000100635 | chia.1 | 11 | 23755508 | 23761368 | protein\_coding | | ENSDARG00000099185 | chia.2 | 11 | 23764646 | 23770131 | protein\_coding | | ENSDARG00000009612 | chia.3 | 11 | 23776004 | 23778941 | protein\_coding | | ENSDARG00000093193 | chia.6 | 23 | 18013268 | 18067324 | protein\_coding | | ENSDARG00000041078 | chka | 18 | 21133752 | 21156308 | protein\_coding | | ENSDARG00000068515 | chs1 | 13 | 155651 | 171568 | protein\_coding | | ENSDARG00000016290 | clca1 | 15 | 14173695 | 14184474 | protein\_coding | | ENSDARG00000055595 | clul1 | 2 | 31380068 | 31393389 | protein\_coding | | ENSDARG00000017193 | cnn1a | 1 | 51580770 | 51588864 | protein\_coding | | ENSDARG00000054588 | cox6a2 | 3 | 30909081 | 30911260 | protein\_coding | | ENSDARG00000069464 | cox7a1 | 15 | 20467352 | 20470064 | protein\_coding | | ENSDARG00000030915 | cpa1 | 25 | 18351786 | 18357616 | protein\_coding | | ENSDARG00000043722 | cpa4 | 25 | 16485772 | 16493061 | protein\_coding | | ENSDARG00000021339 | cpa5 | 25 | 18363524 | 18374295 | protein\_coding | | ENSDARG00000045442 | cpb1 | 24 | 4723602 | 4733953 | protein\_coding | | ENSDARG00000077688 | cpo | 1 | 4878346 | 4889770 | protein\_coding | | ENSDARG00000078876 | crygm2b | 9 | 22257543 | 22258495 | protein\_coding | | ENSDARG00000044875 | crygm2e | 9 | 22269253 | 22270120 | protein\_coding | | ENSDARG00000078134 | crygm2f | 9 | 22533726 | 22534631 | protein\_coding | | ENSDARG00000032206 | cthl | 6 | 37364437 | 37425603 | protein\_coding | | ENSDARG00000090428 | ctrb1 | 7 | 34804498 | 34807090 | protein\_coding | | ENSDARG00000068680 | ctrl | 15 | 37918673 | 37927575 | protein\_coding | | ENSDARG00000101051 | ctsbb | 20 | 53081289 | 53097303 | protein\_coding | | ENSDARG00000003902 | ctsl.1 | 17 | 27439841 | 27444267 | protein\_coding | | ENSDARG00000013771 | ctss2.2 | 16 | 29579850 | 29585354 | protein\_coding | | ENSDARG00000087013 | cubn | 24 | 32120983 | 32235633 | protein\_coding | | ENSDARG00000025311 | cuzd1.2 | 17 | 21813883 | 21842446 | protein\_coding | | ENSDARG00000099169 | cxcl8b.3 | 7 | 7585787 | 7590293 | protein\_coding | | ENSDARG00000098315 | cyp1a | 18 | 5738730 | 5749620 | protein\_coding | | ENSDARG00000103277 | cyp24a1 | 6 | 55869049 | 55881711 | protein\_coding | | ENSDARG00000102805 | cyp2aa12 | 23 | 42327252 | 42340826 | protein\_coding | | ENSDARG00000021172 | cyp2ad2 | 20 | 25635579 | 25643093 | protein\_coding | | ENSDARG00000022650 | cyp2ad3 | 20 | 25649335 | 25652586 | protein\_coding | | ENSDARG00000042956 | cyp2ad6 | 20 | 25626712 | 25634649 | protein\_coding | | ENSDARG00000104593 | cyp2k18 | 3 | 12558901 | 12563983 | protein\_coding | | ENSDARG00000101861 | cyp2k19 | 3 | 12580933 | 12588861 | protein\_coding | | ENSDARG00000042953 | cyp2n13 | 20 | 25616169 | 25623821 | protein\_coding | | ENSDARG00000042978 | cyp2p6 | 20 | 25664015 | 25669351 | protein\_coding | | ENSDARG00000042982 | cyp2p8 | 20 | 25676301 | 25680912 | protein\_coding | | ENSDARG00000068290 | cyp2x12 | 7 | 51860721 | 51870025 | protein\_coding | | ENSDARG00000070775 | cyp2x9 | 25 | 17300336 | 17307177 | protein\_coding | | ENSDARG00000103295 | cyp3a65 | 1 | 58631257 | 58641843 | protein\_coding | | ENSDARG00000037873 | cyp3c3 | 3 | 40806103 | 40813924 | protein\_coding | | ENSDARG00000070021 | cyp3c4 | 3 | 40794236 | 40803866 | protein\_coding | | ENSDARG00000061585 | cyp4v7 | 14 | 29634954 | 29751221 | protein\_coding | | ENSDARG00000062132 | cyp4v8 | 1 | 17207369 | 17219708 | protein\_coding | | ENSDARG00000069018 | cyp7a1 | 2 | 22492072 | 22494735 | protein\_coding | | ENSDARG00000016494 | ddc | 16 | 8384813 | 8429020 | protein\_coding | | ENSDARG00000002758 | dedd1 | 16 | 10950261 | 10972139 | protein\_coding | | ENSDARG00000018846 | dgat2 | 10 | 32557416 | 32580360 | protein\_coding | | ENSDARG00000042112 | dio1 | 8 | 18003858 | 18006377 | protein\_coding | | ENSDARG00000068181 | dpep1 | 7 | 56314147 | 56335995 | protein\_coding | | ENSDARG00000053620 | ebi3 | 2 | 42087185 | 42093248 | protein\_coding | | ENSDARG00000056744 | ela2 | 8 | 21344204 | 21349649 | protein\_coding | | ENSDARG00000056765 | ela2l | 8 | 21334686 | 21340400 | protein\_coding | | ENSDARG00000007276 | ela3l | 5 | 25511443 | 25518702 | protein\_coding | | ENSDARG00000044204 | endou | 6 | 39907485 | 39914904 | protein\_coding | | ENSDARG00000053526 | enpp7.1 | 12 | 32298622 | 32306144 | protein\_coding | | ENSDARG00000005565 | entpd8 | 6 | 41041221 | 41081499 | protein\_coding | | ENSDARG00000103498 | epd | 5 | 37237092 | 37239157 | protein\_coding | | ENSDARG00000055539 | epdl2 | 21 | 28476362 | 28486959 | protein\_coding | | ENSDARG00000040255 | ephx2 | 17 | 5859013 | 5918854 | protein\_coding | | ENSDARG00000101979 | eps8l3a | 22 | 1169968 | 1186507 | protein\_coding | | ENSDARG00000037894 | ezh1 | 3 | 36717712 | 36750793 | protein\_coding | | ENSDARG00000038439 | fabp10a | 16 | 52626257 | 52631239 | protein\_coding | | ENSDARG00000044566 | fabp6 | 21 | 30006359 | 30010658 | protein\_coding | | ENSDARG00000019532 | fads2 | 25 | 4007596 | 4023060 | protein\_coding | | ENSDARG00000058218 | fam151a | 2 | 11285449 | 11299496 | protein\_coding | | ENSDARG00000054641 | fam46ab | 23 | 40382465 | 40390138 | protein\_coding | | ENSDARG00000023820 | faxdc2 | 21 | 45802099 | 45813782 | protein\_coding | | ENSDARG00000019686 | fgl2b | 25 | 21169925 | 21176221 | protein\_coding | | ENSDARG00000010764 | flj13639 | 15 | 43285404 | 43315792 | protein\_coding | | ENSDARG00000075676 | fndc7b | 23 | 1569761 | 1582076 | protein\_coding | | ENSDARG00000003909 | ftr01 | 2 | 11886671 | 11894106 | protein\_coding | | ENSDARG00000012355 | galnt8a.1 | 25 | 16636936 | 16646047 | protein\_coding | | ENSDARG00000089310 | gc | 5 | 45077668 | 45124680 | protein\_coding | | ENSDARG00000002986 | gda | 5 | 25108216 | 25122968 | protein\_coding | | ENSDARG00000007671 | ghrb | 21 | 20706240 | 20855258 | protein\_coding | | ENSDARG00000071306 | gip | 12 | 20540762 | 20543290 | protein\_coding | | ENSDARG00000002917 | gls2b | 22 | 10068764 | 10081306 | protein\_coding | | ENSDARG00000101074 | glud1b | 12 | 25754134 | 25790584 | protein\_coding | | ENSDARG00000102888 | gpr39 | 9 | 56808818 | 56877232 | protein\_coding | | ENSDARG00000098320 | grm1b | 17 | 7223372 | 7283219 | protein\_coding | | ENSDARG00000089362 | grn1 | 19 | 40599557 | 40605129 | protein\_coding | | ENSDARG00000088641 | grn2 | 19 | 40609342 | 40611978 | protein\_coding | | ENSDARG00000022183 | gsto1 | 13 | 25249982 | 25258601 | protein\_coding | | ENSDARG00000057498 | habp2 | 12 | 31598337 | 31605164 | protein\_coding | | ENSDARG00000058005 | hgd | 24 | 23814313 | 23825872 | protein\_coding | | ENSDARG00000018351 | hpda | 5 | 2544815 | 2582079 | protein\_coding | | ENSDARG00000044935 | hpdb | 8 | 34797182 | 34807434 | protein\_coding | | ENSDARG00000033594 | ido1 | 8 | 50474521 | 50494149 | protein\_coding | | ENSDARG00000037836 | igfals | 3 | 47892673 | 47895923 | protein\_coding | | ENSDARG00000038666 | igfbp1b | 2 | 135384 | 138963 | protein\_coding | | ENSDARG00000022413 | ing5a | 22 | 2932874 | 3074863 | protein\_coding | | ENSDARG00000059163 | irbp | 12 | 2630348 | 2635423 | protein\_coding | | ENSDARG00000056018 | itga6l | 14 | 41163533 | 41189466 | protein\_coding | | ENSDARG00000102722 | itih3b | 11 | 36910728 | 36932669 | protein\_coding | | ENSDARG00000075914 | kcnj14 | 16 | 16916701 | 16931520 | protein\_coding | | ENSDARG00000062906 | kcnv2b | 5 | 8573560 | 8585540 | protein\_coding | | ENSDARG00000091127 | klf15 | 23 | 35088769 | 35096994 | protein\_coding | | ENSDARG00000040278 | klhl38b | 16 | 25330825 | 25335382 | protein\_coding | | ENSDARG00000044976 | krt93 | 11 | 11470924 | 11474587 | protein\_coding | | ENSDARG00000014356 | krt95 | 11 | 12206950 | 12215293 | protein\_coding | | ENSDARG00000060671 | lct | 9 | 46569453 | 46582158 | protein\_coding | | ENSDARG00000029476 | ldlra | 3 | 19149569 | 19173949 | protein\_coding | | ENSDARG00000038153 | lgals2b | 6 | 579652 | 586261 | protein\_coding | | ENSDARG00000020239 | lpin1 | 17 | 30425555 | 30453489 | protein\_coding | | ENSDARG00000007906 | lrp2b | 12 | 16083802 | 16180592 | protein\_coding | | ENSDARG00000043102 | lxn | 15 | 1220620 | 1238131 | protein\_coding | | ENSDARG00000090618 | malrd1 | 7 | 41355442 | 41412931 | protein\_coding | | ENSDARG00000036186 | mbpa | 19 | 22229724 | 22248307 | protein\_coding | | ENSDARG00000029747 | mep1a.1 | 20 | 35570409 | 35578705 | protein\_coding | | ENSDARG00000008029 | mep1a.2 | 20 | 35581191 | 35593580 | protein\_coding | | ENSDARG00000037533 | mep1b | 20 | 6503701 | 6526592 | protein\_coding | | ENSDARG00000015164 | mknk2b | 8 | 20406799 | 20420076 | protein\_coding | | ENSDARG00000054898 | ms4a17a.17 | 4 | 74912105 | 74933261 | protein\_coding | | ENSDARG00000014024 | ms4a17a.4 | 4 | 75175989 | 75185694 | protein\_coding | | ENSDARG00000098949 | mslna | 3 | 43134641 | 43171972 | protein\_coding | | ENSDARG00000079647 | muc13b | 12 | 16057649 | 16073704 | protein\_coding | | ENSDARG00000099470 | muc5.3 | 7 | 72041890 | 72088651 | protein\_coding | | ENSDARG00000074852 | myo15b | 12 | 33560798 | 33605743 | protein\_coding | | ENSDARG00000077201 | myo7bb | 2 | 23224954 | 23272307 | protein\_coding | | ENSDARG00000058327 | neu3.3 | 21 | 21766727 | 21771823 | protein\_coding | | ENSDARG00000030616 | nfe2l1a | 3 | 23942172 | 23962596 | protein\_coding | | ENSDARG00000055752 | npas4a | 14 | 30296641 | 30302505 | protein\_coding | | ENSDARG00000087753 | npas4b | 21 | 25532208 | 25536849 | protein\_coding | | ENSDARG00000102082 | nr3c2 | 1 | 36203197 | 36356009 | protein\_coding | | ENSDARG00000039116 | nr5a5 | 3 | 53202529 | 53210586 | protein\_coding | | ENSDARG00000052035 | nxnl1 | 1 | 57269540 | 57273534 | protein\_coding | | ENSDARG00000044280 | opn1mw2 | 6 | 41188784 | 41191924 | protein\_coding | | ENSDARG00000098051 | opn6b | 17 | 28743623 | 28753837 | protein\_coding | | ENSDARG00000006522 | otop2 | 3 | 56834314 | 56850916 | protein\_coding | | ENSDARG00000021555 | park2 | 13 | 3219977 | 3383257 | protein\_coding | | ENSDARG00000013522 | pck1 | 6 | 60060245 | 60067174 | protein\_coding | | ENSDARG00000102558 | pde6h | 6 | 326189 | 372673 | protein\_coding | | ENSDARG00000018263 | pdia2 | 1 | 9270378 | 9280654 | protein\_coding | | ENSDARG00000059054 | pdk2b | 19 | 5756655 | 5769491 | protein\_coding | | ENSDARG00000062304 | pdpk1a | 3 | 47331057 | 47356060 | protein\_coding | | ENSDARG00000055656 | pdzd3b | 5 | 30080651 | 30097755 | protein\_coding | | ENSDARG00000022261 | pdzk1 | 9 | 5884055 | 5914090 | protein\_coding | | ENSDARG00000055591 | pipox | 15 | 24214825 | 24243628 | protein\_coding | | ENSDARG00000009153 | pla2g1b | 8 | 39690364 | 39699893 | protein\_coding | | ENSDARG00000043729 | plac8.1 | 10 | 11424127 | 11426917 | protein\_coding | | ENSDARG00000011929 | plp1b | 5 | 22764321 | 22776298 | protein\_coding | | ENSDARG00000093078 | plscr3a | 5 | 23730666 | 23734933 | protein\_coding | | ENSDARG00000029230 | pnp4b | 16 | 36854040 | 36880749 | protein\_coding | | ENSDARG00000100504 | ppp1r27b | 6 | 21725640 | 21727741 | protein\_coding | | ENSDARG00000026229 | prnpa | 8 | 43046732 | 43050188 | protein\_coding | | ENSDARG00000079274 | prss59.1 | 16 | 26139127 | 26140409 | protein\_coding | | ENSDARG00000073742 | prss59.2 | 16 | 26143918 | 26145252 | protein\_coding | | ENSDARG00000087911 | psme4a | 13 | 35563917 | 35619133 | protein\_coding | | ENSDARG00000034705 | pvalb7 | 22 | 29219499 | 29293152 | protein\_coding | | ENSDARG00000089933 | rasal3 | 6 | 8235059 | 8254575 | protein\_coding | | ENSDARG00000095512 | rca2.2 | 23 | 26195265 | 26203013 | protein\_coding | | ENSDARG00000017882 | rdh1 | 9 | 48982037 | 49003321 | protein\_coding | | ENSDARG00000017653 | rgs13 | 2 | 6563685 | 6570358 | protein\_coding | | ENSDARG00000041848 | rh50 | 8 | 37226964 | 37231090 | protein\_coding | | ENSDARG00000007080 | rhcgl1 | 6 | 36403815 | 36413793 | protein\_coding | | ENSDARG00000087197 | ros1 | 17 | 15239153 | 15266715 | protein\_coding | | ENSDARG00000088377 | rp1l1b | 20 | 19220217 | 19249094 | protein\_coding | | ENSDARG00000052012 | rtn4rl2a | 1 | 43789774 | 43794335 | protein\_coding | | ENSDARG00000037495 | rtn4rl2b | 14 | 16891479 | 16895295 | protein\_coding | | ENSDARG00000037425 | s100a10a | 19 | 8687958 | 8689348 | protein\_coding | | ENSDARG00000090416 | scpp1 | 1 | 36682488 | 36685878 | protein\_coding | | ENSDARG00000020693 | sesn1 | 20 | 32249476 | 32328278 | protein\_coding | | ENSDARG00000092858 | si:ch1073-126c3.2 | 13 | 20404177 | 20409808 | protein\_coding | | ENSDARG00000105351 | si:ch211-113d11.6 | 14 | 47246412 | 47250894 | protein\_coding | | ENSDARG00000092976 | si:ch211-127i16.2 | 10 | 19470201 | 19566494 | protein\_coding | | ENSDARG00000068374 | si:ch211-132b12.7 | 18 | 40678423 | 40694600 | protein\_coding | | ENSDARG00000095633 | si:ch211-133l5.7 | 8 | 20800499 | 20806257 | protein\_coding | | ENSDARG00000076011 | si:ch211-149b19.3 | 23 | 44007366 | 44027316 | protein\_coding | | ENSDARG00000076122 | si:ch211-160b11.4 | 9 | 35169682 | 35177642 | protein\_coding | | ENSDARG00000096809 | si:ch211-168b3.2 | 2 | 6015373 | 6021469 | protein\_coding | | ENSDARG00000028478 | si:ch211-173n18.3 | 6 | 39502087 | 39505845 | protein\_coding | | ENSDARG00000096269 | si:ch211-173n18.5 | 6 | 39494310 | 39509050 | antisense | | ENSDARG00000091847 | si:ch211-181d7.1.1 | 15 | 42766916 | 42780368 | protein\_coding | | ENSDARG00000068621 | si:ch211-181d7.3 | 15 | 42785207 | 42804045 | protein\_coding | | ENSDARG00000079980 | si:ch211-202h22.9 | 1 | 53948840 | 53959727 | protein\_coding | | ENSDARG00000093780 | si:ch211-212c13.6 | 15 | 21024337 | 21042516 | unprocessed\_pseudogene | | ENSDARG00000094975 | si:ch211-214p16.2 | 17 | 25569234 | 25570039 | processed\_transcript | | ENSDARG00000039423 | si:ch211-217g15.3 | 13 | 24454774 | 24457770 | antisense | | ENSDARG00000056915 | si:ch211-237l4.6 | 10 | 22813070 | 22816998 | protein\_coding | | ENSDARG00000039393 | si:ch211-240l19.5 | 4 | 8034176 | 8035335 | protein\_coding | | ENSDARG00000074613 | si:ch211-240l19.6 | 4 | 8031162 | 8031807 | protein\_coding | | ENSDARG00000077880 | si:ch211-255i20.3 | 14 | 17077150 | 17087942 | protein\_coding | | ENSDARG00000101473 | si:ch211-25g7.5 | 21 | 45551864 | 45554058 | protein\_coding | | ENSDARG00000087532 | si:ch211-278p9.1 | 17 | 7286301 | 7300520 | protein\_coding | | ENSDARG00000105450 | si:ch211-63p21.1 | 7 | 4024968 | 4048813 | protein\_coding | | ENSDARG00000095362 | si:ch211-77g15.32 | 2 | 48132498 | 48134378 | processed\_transcript | | ENSDARG00000070972 | si:ch211-81a5.8 | 19 | 9255241 | 9260933 | protein\_coding | | ENSDARG00000101726 | si:ch211-93f2.1 | 25 | 29942634 | 29986550 | protein\_coding | | ENSDARG00000104818 | si:ch211-93f2.1.1 | 25 | 29919797 | 29941612 | protein\_coding | | ENSDARG00000070558 | si:ch211-93g23.2 | 1 | 5907562 | 5948371 | protein\_coding | | ENSDARG00000079901 | si:ch73-15n24.1 | 19 | 9376319 | 9421210 | protein\_coding | | ENSDARG00000031278 | si:ch73-193i2.2 | 13 | 3194067 | 3218358 | protein\_coding | | ENSDARG00000091584 | si:ch73-217n20.1 | 23 | 42844195 | 42859783 | protein\_coding | | ENSDARG00000038009 | si:ch73-233k15.1 | 3 | 29533515 | 29540811 | antisense | | ENSDARG00000097503 | si:ch73-288o11.5 | 17 | 20585177 | 20591476 | protein\_coding | | ENSDARG00000095963 | si:ch73-361h17.1 | 23 | 45168964 | 45182069 | protein\_coding | | ENSDARG00000090164 | si:ch73-362m14.2 | 21 | 43541002 | 43554944 | protein\_coding | | ENSDARG00000092890 | si:ch73-44m9.5 | 22 | 7709120 | 7709703 | protein\_coding | | ENSDARG00000002219 | si:dkey-103j14.5 | 13 | 23103309 | 23140126 | protein\_coding | | ENSDARG00000045835 | si:dkey-14d8.6 | 4 | 5522222 | 5525253 | protein\_coding | | ENSDARG00000045834 | si:dkey-14d8.7 | 4 | 5528531 | 5531272 | protein\_coding | | ENSDARG00000091715 | si:dkey-162h11.2 | 7 | 781251 | 816015 | protein\_coding | | ENSDARG00000088432 | si:dkey-162h11.3 | 7 | 825581 | 844337 | protein\_coding | | ENSDARG00000096722 | si:dkey-16i5.8 | 12 | 19279980 | 19281076 | protein\_coding | | ENSDARG00000070511 | si:dkey-183j2.10 | 11 | 25497862 | 25512444 | protein\_coding | | ENSDARG00000099633 | si:dkey-186o21.1 | 10 | 45078110 | 45092189 | protein\_coding | | ENSDARG00000092725 | si:dkey-188i13.8 | 13 | 37508014 | 37510257 | protein\_coding | | ENSDARG00000095294 | si:dkey-200l5.4 | 10 | 17773859 | 17787940 | protein\_coding | | ENSDARG00000098736 | si:dkey-201i2.4 | 12 | 44312132 | 44328649 | protein\_coding | | ENSDARG00000104721 | si:dkey-203a12.9 | 14 | 15317254 | 15319909 | protein\_coding | | ENSDARG00000079307 | si:dkey-205h13.1 | 23 | 26577335 | 26596485 | protein\_coding | | ENSDARG00000076972 | si:dkey-208k22.3 | 2 | 24709801 | 24713436 | protein\_coding | | ENSDARG00000096739 | si:dkey-219e21.2 | 12 | 22486542 | 22496861 | protein\_coding | | ENSDARG00000094840 | si:dkey-21e2.13 | 22 | 19252450 | 19253793 | protein\_coding | | ENSDARG00000092788 | si:dkey-21e2.15 | 22 | 19239414 | 19266283 | protein\_coding | | ENSDARG00000094077 | si:dkey-21e2.16 | 22 | 19265008 | 19288760 | protein\_coding | | ENSDARG00000092532 | si:dkey-21e2.4 | 22 | 19163884 | 19165169 | protein\_coding | | ENSDARG00000094910 | si:dkey-22i16.7 | 1 | 43244465 | 43247662 | protein\_coding | | ENSDARG00000011581 | si:dkey-23k10.5 | 15 | 36492316 | 36500339 | protein\_coding | | ENSDARG00000075958 | si:dkey-266f7.9 | 19 | 8167566 | 8178059 | protein\_coding | | ENSDARG00000095402 | si:dkey-273o13.3 | 5 | 66483073 | 66486050 | processed\_transcript | | ENSDARG00000026383 | si:dkey-27j5.5 | 1 | 56562858 | 56565498 | protein\_coding | | ENSDARG00000097533 | si:dkey-29l4.4 | 15 | 36283700 | 36288038 | lincRNA | | ENSDARG00000097762 | si:dkey-33c14.2 | 2 | 323082 | 325591 | lincRNA | | ENSDARG00000092361 | si:dkey-79f11.7 | 1 | 7282469 | 7291658 | protein\_coding | | ENSDARG00000095649 | si:dkey-97a13.12 | 2 | 42239038 | 42242508 | protein\_coding | | ENSDARG00000063376 | si:dkeyp-74b6.2 | 7 | 1152309 | 1163758 | protein\_coding | | ENSDARG00000029890 | si:dkeyp-86f7.4 | 14 | 9126613 | 9141101 | protein\_coding | | ENSDARG00000068493 | si:zfos-411a11.2 | 15 | 88262 | 92917 | protein\_coding | | ENSDARG00000014916 | slc10a2 | 9 | 27528759 | 27533937 | protein\_coding | | ENSDARG00000055253 | slc12a10.3 | 7 | 4246837 | 4282175 | protein\_coding | | ENSDARG00000053853 | slc13a2 | 21 | 39055231 | 39073444 | protein\_coding | | ENSDARG00000100315 | slc15a1a | 9 | 981940 | 1003279 | protein\_coding | | ENSDARG00000044528 | slc15a1b | 6 | 12321569 | 12353186 | protein\_coding | | ENSDARG00000013926 | slc16a9a | 17 | 20619234 | 20646780 | protein\_coding | | ENSDARG00000006447 | slc19a3a | 15 | 35281488 | 35298822 | protein\_coding | | ENSDARG00000061103 | slc19a3b | 18 | 46372367 | 46382519 | protein\_coding | | ENSDARG00000032465 | slc1a8b | 2 | 37128047 | 37139894 | protein\_coding | | ENSDARG00000005335 | slc22a4 | 21 | 45859467 | 45865189 | protein\_coding | | ENSDARG00000062182 | slc22a7b.3 | 17 | 21962781 | 21973871 | protein\_coding | | ENSDARG00000059923 | slc25a47a | 20 | 53839078 | 53852407 | protein\_coding | | ENSDARG00000003615 | slc26a3.2 | 4 | 18815341 | 18834056 | protein\_coding | | ENSDARG00000060879 | slc28a1 | 18 | 26846708 | 26867693 | protein\_coding | | ENSDARG00000012903 | slc34a2a | 1 | 13745921 | 13756389 | protein\_coding | | ENSDARG00000013871 | slc5a1 | 10 | 17177667 | 17201797 | protein\_coding | | ENSDARG00000019932 | slc5a11 | 3 | 18505933 | 18525948 | protein\_coding | | ENSDARG00000068387 | slc6a18 | 19 | 555789 | 657630 | protein\_coding | | ENSDARG00000018621 | slc6a19a.1 | 19 | 663987 | 677904 | protein\_coding | | ENSDARG00000091560 | slc6a19a.2 | 19 | 684693 | 704191 | protein\_coding | | ENSDARG00000056719 | slc6a19b | 16 | 19168425 | 19192910 | protein\_coding | | ENSDARG00000005894 | slc7a9 | 7 | 38239906 | 38415134 | protein\_coding | | ENSDARG00000038095 | socs1a | 3 | 26682187 | 26684082 | protein\_coding | | ENSDARG00000075015 | soul5 | 3 | 53221493 | 53230596 | protein\_coding | | ENSDARG00000091609 | spink4 | 1 | 11448329 | 11451812 | protein\_coding | | ENSDARG00000044276 | spp1 | 10 | 15162592 | 15170652 | protein\_coding | | ENSDARG00000031647 | stat2 | 6 | 39126008 | 39162886 | protein\_coding | | ENSDARG00000100955 | stk24a | 9 | 1004584 | 1020324 | protein\_coding | | ENSDARG00000041540 | sult1st2 | 8 | 46505864 | 46517213 | protein\_coding | | ENSDARG00000018361 | sult1st3 | 8 | 46505998 | 46573569 | protein\_coding | | ENSDARG00000006811 | sult1st6 | 12 | 15159554 | 15166784 | protein\_coding | | ENSDARG00000093619 | sult3st2 | 22 | 15310369 | 15315782 | protein\_coding | | ENSDARG00000103687 | sycn.2 | 21 | 25189953 | 25190586 | protein\_coding | | ENSDARG00000002249 | tbxas1 | 18 | 12611854 | 12904635 | protein\_coding | | ENSDARG00000023176 | tdo2b | 11 | 33821730 | 33834881 | protein\_coding | | ENSDARG00000101595 | tgm1l2 | 23 | 40337189 | 40342145 | protein\_coding | | ENSDARG00000100795 | timp4a | 11 | 687888 | 692043 | protein\_coding | | ENSDARG00000040747 | tm4sf4 | 22 | 38174320 | 38184761 | protein\_coding | | ENSDARG00000055185 | tm4sf5 | 7 | 21574699 | 21581867 | protein\_coding | | ENSDARG00000088079 | tmed6 | 25 | 36715178 | 36717625 | protein\_coding | | ENSDARG00000059305 | tmem37 | 9 | 917562 | 923562 | protein\_coding | | ENSDARG00000042993 | try | 16 | 25165732 | 25181539 | protein\_coding | | ENSDARG00000037191 | ttr | 20 | 6098575 | 6114084 | protein\_coding | | ENSDARG00000070000 | txnipb | 16 | 42965296 | 42968883 | protein\_coding | | ENSDARG00000023151 | ucp1 | 1 | 52739716 | 52754176 | protein\_coding | | ENSDARG00000036833 | upp2 | 9 | 16160546 | 16171735 | protein\_coding | | ENSDARG00000040466 | vil1 | 9 | 45109816 | 45152397 | protein\_coding | | ENSDARG00000098805 | vwa11 | 1 | 43368763 | 43386303 | protein\_coding | | ENSDARG00000055240 | xdh | 17 | 33388199 | 33421929 | protein\_coding | | ENSDARG00000026017 | xpnpep2 | 14 | 33251312 | 33273480 | protein\_coding | | ENSDARG00000006588 | zgc:111983 | 1 | 44472118 | 44476098 | protein\_coding | | ENSDARG00000039730 | zgc:112160 | 7 | 34790652 | 34794980 | protein\_coding | | ENSDARG00000096110 | zgc:123278 | 25 | 25286470 | 25288967 | protein\_coding | | ENSDARG00000102720 | zgc:123284 | 3 | 44952350 | 44955045 | protein\_coding | | ENSDARG00000093844 | zgc:136461 | 7 | 34796559 | 34798330 | protein\_coding | | ENSDARG00000040738 | zgc:153846 | 9 | 22206499 | 22207435 | protein\_coding | | ENSDARG00000061858 | zgc:153968 | 3 | 21095202 | 21100120 | protein\_coding | | ENSDARG00000100792 | zgc:154142 | 1 | 50150870 | 50194361 | protein\_coding | | ENSDARG00000067806 | zgc:158432 | 5 | 35938382 | 35948871 | protein\_coding | | ENSDARG00000058462 | zgc:158846 | 6 | 39339195 | 39346723 | protein\_coding | | ENSDARG00000052905 | zgc:165423 | 3 | 39398156 | 39408231 | protein\_coding | | ENSDARG00000052176 | zgc:171711 | 3 | 53233694 | 53253911 | protein\_coding | | ENSDARG00000056762 | zgc:172079 | 7 | 26178547 | 26186619 | protein\_coding | | ENSDARG00000091136 | zgc:174259 | 5 | 28230255 | 28251254 | protein\_coding | | ENSDARG00000088263 | zgc:174356 | 17 | 39294852 | 39368659 | protein\_coding | | ENSDARG00000099111 | zgc:175280 | 5 | 67134351 | 67156546 | protein\_coding | | ENSDARG00000079532 | zgc:194242 | 23 | 5178849 | 5185000 | protein\_coding | | ENSDARG00000023759 | zgc:73226 | 5 | 5859173 | 5886756 | protein\_coding | | ENSDARG00000101749 | zgc:92161 | 3 | 44893986 | 44912921 | protein\_coding | | ENSDARG00000040282 | zgc:92590 | 16 | 25211903 | 25215043 | protein\_coding | |
