# Supplementary material for: A high-resolution mRNA expression time course of embryonic development in zebrafish
Source: eLife. 2017 Nov 16;6:e30860. doi: 10.7554/eLife.30860 (PMC5690287; doi:10.7554/eLife.30860)
Supplement: Supplementary file 6. [file elife-30860-supp6.zip › biolayout-clusters-files/Cluster005.html]

Cluster005


# Cluster005: Detail

### Go to ZFA detail

## GO

| | GO ID | Description | Domain | Annotated | Expected | Observed | Adjusted p-value | Genes | Ensembl IDs | | --- | --- | --- | --- | --- | --- | --- | --- | --- | | GO:0055114 | oxidation-reduction process | biological\_process | 343 | 10.44 | 35 | 2.5e-09 | tbxas1 hpda fads2 cyp2ad2 park2 cyp2ad3 tdo2b faxdc2 agmo ido1 cyp3c3 dio1 cyp2n13 cyp2ad6 cyp2p6 cyp2p8 hpdb xdh pipox hgd aoc1 cyp4v7 cyp4v8 si:zfos-411a11.2 cyp7a1 cyp3c4 cyp2x9 si:ch211-127i16.2 cyp1a glud1b cyp2k19 cyp2aa12 cyp24a1 cyp3a65 cyp2k18 | ENSDARG00000002249 ENSDARG00000018351 ENSDARG00000019532 ENSDARG00000021172 ENSDARG00000021555 ENSDARG00000022650 ENSDARG00000023176 ENSDARG00000023820 ENSDARG00000025595 ENSDARG00000033594 ENSDARG00000037873 ENSDARG00000042112 ENSDARG00000042953 ENSDARG00000042956 ENSDARG00000042978 ENSDARG00000042982 ENSDARG00000044935 ENSDARG00000055240 ENSDARG00000055591 ENSDARG00000058005 ENSDARG00000061355 ENSDARG00000061585 ENSDARG00000062132 ENSDARG00000068493 ENSDARG00000069018 ENSDARG00000070021 ENSDARG00000070775 ENSDARG00000092976 ENSDARG00000098315 ENSDARG00000101074 ENSDARG00000101861 ENSDARG00000102805 ENSDARG00000103277 ENSDARG00000103295 ENSDARG00000104593 | | GO:0019439 | aromatic compound catabolic process | biological\_process | 81 | 2.47 | 8 | 2.8e-02 | gda ada hpda tdo2b upp2 cda xdh hgd | ENSDARG00000002986 ENSDARG00000003113 ENSDARG00000018351 ENSDARG00000023176 ENSDARG00000036833 ENSDARG00000038199 ENSDARG00000055240 ENSDARG00000058005 | | GO:1901565 | organonitrogen compound catabolic proces... | biological\_process | 63 | 1.92 | 15 | 4.6e-06 | gda ada chia.3 hpda tdo2b cda anpepa enpp7.1 xdh hgd neu3.3 chia.6 chia.2 chia.1 anpepb | ENSDARG00000002986 ENSDARG00000003113 ENSDARG00000009612 ENSDARG00000018351 ENSDARG00000023176 ENSDARG00000038199 ENSDARG00000041083 ENSDARG00000053526 ENSDARG00000055240 ENSDARG00000058005 ENSDARG00000058327 ENSDARG00000093193 ENSDARG00000099185 ENSDARG00000100635 ENSDARG00000103878 | | GO:1901136 | carbohydrate derivative catabolic proces... | biological\_process | 28 | 0.85 | 7 | 3.8e-03 | ada chia.3 cda neu3.3 chia.6 chia.2 chia.1 | ENSDARG00000003113 ENSDARG00000009612 ENSDARG00000038199 ENSDARG00000058327 ENSDARG00000093193 ENSDARG00000099185 ENSDARG00000100635 | | GO:0006040 | amino sugar metabolic process | biological\_process | 15 | 0.46 | 5 | 1.5e-02 | chia.3 chs1 chia.6 chia.2 chia.1 | ENSDARG00000009612 ENSDARG00000068515 ENSDARG00000093193 ENSDARG00000099185 ENSDARG00000100635 | | GO:1901361 | organic cyclic compound catabolic proces... | biological\_process | 88 | 2.68 | 9 | 1.6e-02 | gda ada hpda tdo2b upp2 cda xdh hgd cyp7a1 | ENSDARG00000002986 ENSDARG00000003113 ENSDARG00000018351 ENSDARG00000023176 ENSDARG00000036833 ENSDARG00000038199 ENSDARG00000055240 ENSDARG00000058005 ENSDARG00000069018 | | GO:0006508 | proteolysis | biological\_process | 489 | 14.88 | 46 | 6.6e-17 | ctsl.1 ela3l mep1a.2 ctss2.2 ace2 CELA1 (1 of many) cpa5 park2 c6ast3 mep1a.1 cpa1 mep1b zgc:112160 zgc:92590 MEP1B (1 of many) anpepa try CELA1 (1 of many) CELA1 (1 of many) CELA1 (1 of many) cpa4 cpb1 c6ast4 zgc:165423 ela2 ela2l habp2 irbp zgc:153968 dpep1 ctrl prss59.2 cpo prss59.1 psme4a ctrb1 si:dkey-21e2.4 si:dkey-21e2.15 si:ch73-44m9.5 zgc:136461 si:dkey-21e2.16 si:dkey-21e2.13 CELA1 (1 of many) zgc:154142 ctsbb anpepb | ENSDARG00000003902 ENSDARG00000007276 ENSDARG00000008029 ENSDARG00000013771 ENSDARG00000016918 ENSDARG00000017314 ENSDARG00000021339 ENSDARG00000021555 ENSDARG00000024503 ENSDARG00000029747 ENSDARG00000030915 ENSDARG00000037533 ENSDARG00000039730 ENSDARG00000040282 ENSDARG00000040683 ENSDARG00000041083 ENSDARG00000042993 ENSDARG00000043168 ENSDARG00000043171 ENSDARG00000043173 ENSDARG00000043722 ENSDARG00000045442 ENSDARG00000052578 ENSDARG00000052905 ENSDARG00000056744 ENSDARG00000056765 ENSDARG00000057498 ENSDARG00000059163 ENSDARG00000061858 ENSDARG00000068181 ENSDARG00000068680 ENSDARG00000073742 ENSDARG00000077688 ENSDARG00000079274 ENSDARG00000087911 ENSDARG00000090428 ENSDARG00000092532 ENSDARG00000092788 ENSDARG00000092890 ENSDARG00000093844 ENSDARG00000094077 ENSDARG00000094840 ENSDARG00000095462 ENSDARG00000100792 ENSDARG00000101051 ENSDARG00000103878 | | GO:0019369 | arachidonic acid metabolic process | biological\_process | 11 | 0.33 | 8 | 2.5e-08 | cyp2ad2 cyp2ad3 cyp2n13 cyp2ad6 cyp2p6 cyp2p8 cyp2k19 cyp2k18 | ENSDARG00000021172 ENSDARG00000022650 ENSDARG00000042953 ENSDARG00000042956 ENSDARG00000042978 ENSDARG00000042982 ENSDARG00000101861 ENSDARG00000104593 | | GO:0005615 | extracellular space | cellular\_component | 241 | 7.42 | 25 | 3.0e-05 | ctsl.1 ela3l ctss2.2 CELA1 (1 of many) cpa5 cpa1 igfbp1b zgc:92590 ENSDARG00000041685 CELA1 (1 of many) CELA1 (1 of many) cpa4 cpb1 ahsg2 prss59.2 cpo prss59.1 gc zgc:174259 si:ch73-44m9.5 CELA1 (1 of many) zgc:154142 ctsbb si:ch211-93f2.1 si:ch211-93f2.1 | ENSDARG00000003902 ENSDARG00000007276 ENSDARG00000013771 ENSDARG00000017314 ENSDARG00000021339 ENSDARG00000030915 ENSDARG00000038666 ENSDARG00000040282 ENSDARG00000041685 ENSDARG00000043171 ENSDARG00000043173 ENSDARG00000043722 ENSDARG00000045442 ENSDARG00000069293 ENSDARG00000073742 ENSDARG00000077688 ENSDARG00000079274 ENSDARG00000089310 ENSDARG00000091136 ENSDARG00000092890 ENSDARG00000095462 ENSDARG00000100792 ENSDARG00000101051 ENSDARG00000101726 ENSDARG00000104818 | | GO:0016021 | integral component of membrane | cellular\_component | 2838 | 87.35 | 122 | 2.8e-02 | atp1a1a.4 tbxas1 slc26a3.2 slc22a4 entpd8 slc7a9 slc19a3a otop2 zgc:111983 rhcgl1 aqp10a ghrb lrp2b mep1a.2 abcb4 plp1b galnt8a.1 asah2 slc34a2a slc5a1 slc16a9a ms4a17a.4 slc10a2 clca1 ace2 cel.1 rdh1 slc6a19a.1 dgat2 fads2 slc5a11 NAALADL1 cyp2ad2 ucp1 zgc:73226 faxdc2 agmo xpnpep2 prnpa ldlra cel.2 si:dkeyp-86f7.4 slc1a8b cd36 atp2a1l mep1b cyp3c3 igfbp1b MEP1B (1 of many) tm4sf4 anpepa rh50 dio1 cyp2p6 cyp2p8 opn1mw2 slc15a1b b3gnt2a enpp7.1 slc13a2 ms4a17a.17 tm4sf5 slc12a10.3 itga6l slc6a19b zgc:172079 habp2 tmem37 slc25a47a lct slc28a1 slc19a3b cyp4v8 slc22a7b.3 kcnv2b abcg5 abcg1 zgc:158432 slc6a18 chs1 cyp7a1 ahsg2 cox7a1 cyp3c4 abcb11b antxr1b soul5 kcnj14 acsl5 si:dkey-208k22.3 si:dkey-205h13.1 abcg2b muc13b si:ch211-202h22.9 ros1 tmed6 zgc:174356 malrd1 slc6a19a.2 si:dkey-188i13.8 si:dkey-21e2.15 si:ch211-127i16.2 IFITM3 si:ch211-133l5.7 si:ch73-361h17.1 ENSDARG00000096445 si:dkey-16i5.8 si:ch211-168b3.2 opn6b grm1b ENSDARG00000098791 zgc:175280 atp8b2 bnip3 abcg2a slc15a1a zgc:154142 si:ch211-25g7.5 cyp2aa12 gpr39 cyp3a65 anpepb | ENSDARG00000001870 ENSDARG00000002249 ENSDARG00000003615 ENSDARG00000005335 ENSDARG00000005565 ENSDARG00000005894 ENSDARG00000006447 ENSDARG00000006522 ENSDARG00000006588 ENSDARG00000007080 ENSDARG00000007086 ENSDARG00000007671 ENSDARG00000007906 ENSDARG00000008029 ENSDARG00000010936 ENSDARG00000011929 ENSDARG00000012355 ENSDARG00000012829 ENSDARG00000012903 ENSDARG00000013871 ENSDARG00000013926 ENSDARG00000014024 ENSDARG00000014916 ENSDARG00000016290 ENSDARG00000016918 ENSDARG00000017490 ENSDARG00000017882 ENSDARG00000018621 ENSDARG00000018846 ENSDARG00000019532 ENSDARG00000019932 ENSDARG00000020952 ENSDARG00000021172 ENSDARG00000023151 ENSDARG00000023759 ENSDARG00000023820 ENSDARG00000025595 ENSDARG00000026017 ENSDARG00000026229 ENSDARG00000029476 ENSDARG00000029822 ENSDARG00000029890 ENSDARG00000032465 ENSDARG00000032639 ENSDARG00000035458 ENSDARG00000037533 ENSDARG00000037873 ENSDARG00000038666 ENSDARG00000040683 ENSDARG00000040747 ENSDARG00000041083 ENSDARG00000041848 ENSDARG00000042112 ENSDARG00000042978 ENSDARG00000042982 ENSDARG00000044280 ENSDARG00000044528 ENSDARG00000052376 ENSDARG00000053526 ENSDARG00000053853 ENSDARG00000054898 ENSDARG00000055185 ENSDARG00000055253 ENSDARG00000056018 ENSDARG00000056719 ENSDARG00000056762 ENSDARG00000057498 ENSDARG00000059305 ENSDARG00000059923 ENSDARG00000060671 ENSDARG00000060879 ENSDARG00000061103 ENSDARG00000062132 ENSDARG00000062182 ENSDARG00000062906 ENSDARG00000063078 ENSDARG00000063475 ENSDARG00000067806 ENSDARG00000068387 ENSDARG00000068515 ENSDARG00000069018 ENSDARG00000069293 ENSDARG00000069464 ENSDARG00000070021 ENSDARG00000070078 ENSDARG00000074075 ENSDARG00000075015 ENSDARG00000075914 ENSDARG00000075931 ENSDARG00000076972 ENSDARG00000079307 ENSDARG00000079361 ENSDARG00000079647 ENSDARG00000079980 ENSDARG00000087197 ENSDARG00000088079 ENSDARG00000088263 ENSDARG00000090618 ENSDARG00000091560 ENSDARG00000092725 ENSDARG00000092788 ENSDARG00000092976 ENSDARG00000093303 ENSDARG00000095633 ENSDARG00000095963 ENSDARG00000096445 ENSDARG00000096722 ENSDARG00000096809 ENSDARG00000098051 ENSDARG00000098320 ENSDARG00000098791 ENSDARG00000099111 ENSDARG00000099216 ENSDARG00000099961 ENSDARG00000100075 ENSDARG00000100315 ENSDARG00000100792 ENSDARG00000101473 ENSDARG00000102805 ENSDARG00000102888 ENSDARG00000103295 ENSDARG00000103878 | | GO:0004497 | monooxygenase activity | molecular\_function | 72 | 2.20 | 21 | 8.6e-03 | tbxas1 cyp2ad2 cyp2ad3 agmo cyp3c3 cyp2n13 cyp2ad6 cyp2p6 cyp2p8 cyp4v7 cyp4v8 si:zfos-411a11.2 cyp7a1 cyp3c4 cyp2x9 cyp1a cyp2k19 cyp2aa12 cyp24a1 cyp3a65 cyp2k18 | ENSDARG00000002249 ENSDARG00000021172 ENSDARG00000022650 ENSDARG00000025595 ENSDARG00000037873 ENSDARG00000042953 ENSDARG00000042956 ENSDARG00000042978 ENSDARG00000042982 ENSDARG00000061585 ENSDARG00000062132 ENSDARG00000068493 ENSDARG00000069018 ENSDARG00000070021 ENSDARG00000070775 ENSDARG00000098315 ENSDARG00000101861 ENSDARG00000102805 ENSDARG00000103277 ENSDARG00000103295 ENSDARG00000104593 | | GO:0008395 | steroid hydroxylase activity | molecular\_function | 26 | 0.80 | 12 | 8.9e-10 | cyp2ad2 cyp2ad3 cyp2n13 cyp2ad6 cyp2p6 cyp2p8 si:zfos-411a11.2 cyp7a1 cyp2x9 cyp2k19 cyp2aa12 cyp2k18 | ENSDARG00000021172 ENSDARG00000022650 ENSDARG00000042953 ENSDARG00000042956 ENSDARG00000042978 ENSDARG00000042982 ENSDARG00000068493 ENSDARG00000069018 ENSDARG00000070775 ENSDARG00000101861 ENSDARG00000102805 ENSDARG00000104593 | | GO:0016712 | oxidoreductase activity, acting on paire... | molecular\_function | 15 | 0.46 | 10 | 4.1e-10 | cyp2ad2 cyp2ad3 cyp2ad6 cyp2p6 cyp2p8 si:zfos-411a11.2 cyp1a cyp2k19 cyp2aa12 cyp2k18 | ENSDARG00000021172 ENSDARG00000022650 ENSDARG00000042956 ENSDARG00000042978 ENSDARG00000042982 ENSDARG00000068493 ENSDARG00000098315 ENSDARG00000101861 ENSDARG00000102805 ENSDARG00000104593 | | GO:0016705 | oxidoreductase activity, acting on paire... | molecular\_function | 97 | 2.97 | 23 | 1.0e-04 | tbxas1 pdia2 fads2 cyp2ad2 cyp2ad3 agmo cyp3c3 cyp2n13 cyp2ad6 cyp2p6 cyp2p8 cyp4v7 cyp4v8 si:zfos-411a11.2 cyp7a1 cyp3c4 cyp2x9 cyp1a cyp2k19 cyp2aa12 cyp24a1 cyp3a65 cyp2k18 | ENSDARG00000002249 ENSDARG00000018263 ENSDARG00000019532 ENSDARG00000021172 ENSDARG00000022650 ENSDARG00000025595 ENSDARG00000037873 ENSDARG00000042953 ENSDARG00000042956 ENSDARG00000042978 ENSDARG00000042982 ENSDARG00000061585 ENSDARG00000062132 ENSDARG00000068493 ENSDARG00000069018 ENSDARG00000070021 ENSDARG00000070775 ENSDARG00000098315 ENSDARG00000101861 ENSDARG00000102805 ENSDARG00000103277 ENSDARG00000103295 ENSDARG00000104593 | | GO:0016712 | oxidoreductase activity, acting on paire... | molecular\_function | 15 | 0.46 | 10 | 4.1e-10 | cyp2ad2 cyp2ad3 cyp2ad6 cyp2p6 cyp2p8 si:zfos-411a11.2 cyp1a cyp2k19 cyp2aa12 cyp2k18 | ENSDARG00000021172 ENSDARG00000022650 ENSDARG00000042956 ENSDARG00000042978 ENSDARG00000042982 ENSDARG00000068493 ENSDARG00000098315 ENSDARG00000101861 ENSDARG00000102805 ENSDARG00000104593 | | GO:0020037 | heme binding | molecular\_function | 70 | 2.14 | 22 | 1.2e-14 | tbxas1 cyp2ad2 cyp2ad3 tdo2b ido1 cyp3c3 cyp2n13 cyp2ad6 cyp2p6 cyp2p8 cyp4v7 cyp4v8 si:zfos-411a11.2 cyp7a1 cyp3c4 cyp2x9 cyp1a cyp2k19 cyp2aa12 cyp24a1 cyp3a65 cyp2k18 | ENSDARG00000002249 ENSDARG00000021172 ENSDARG00000022650 ENSDARG00000023176 ENSDARG00000033594 ENSDARG00000037873 ENSDARG00000042953 ENSDARG00000042956 ENSDARG00000042978 ENSDARG00000042982 ENSDARG00000061585 ENSDARG00000062132 ENSDARG00000068493 ENSDARG00000069018 ENSDARG00000070021 ENSDARG00000070775 ENSDARG00000098315 ENSDARG00000101861 ENSDARG00000102805 ENSDARG00000103277 ENSDARG00000103295 ENSDARG00000104593 | | GO:0004252 | serine-type endopeptidase activity | molecular\_function | 79 | 2.42 | 25 | 6.9e-17 | ela3l CELA1 (1 of many) zgc:112160 zgc:92590 try CELA1 (1 of many) CELA1 (1 of many) CELA1 (1 of many) zgc:165423 ela2 ela2l habp2 zgc:153968 ctrl prss59.2 prss59.1 ctrb1 si:dkey-21e2.4 si:dkey-21e2.15 si:ch73-44m9.5 zgc:136461 si:dkey-21e2.16 si:dkey-21e2.13 CELA1 (1 of many) zgc:154142 | ENSDARG00000007276 ENSDARG00000017314 ENSDARG00000039730 ENSDARG00000040282 ENSDARG00000042993 ENSDARG00000043168 ENSDARG00000043171 ENSDARG00000043173 ENSDARG00000052905 ENSDARG00000056744 ENSDARG00000056765 ENSDARG00000057498 ENSDARG00000061858 ENSDARG00000068680 ENSDARG00000073742 ENSDARG00000079274 ENSDARG00000090428 ENSDARG00000092532 ENSDARG00000092788 ENSDARG00000092890 ENSDARG00000093844 ENSDARG00000094077 ENSDARG00000094840 ENSDARG00000095462 ENSDARG00000100792 | | GO:0005506 | iron ion binding | molecular\_function | 92 | 2.81 | 23 | 6.4e-13 | tbxas1 cyp2ad2 cyp2ad3 faxdc2 agmo cyp3c3 cyp2n13 cyp2ad6 cyp2p6 cyp2p8 xdh cyp4v7 cyp4v8 si:zfos-411a11.2 cyp7a1 cyp3c4 cyp2x9 cyp1a cyp2k19 cyp2aa12 cyp24a1 cyp3a65 cyp2k18 | ENSDARG00000002249 ENSDARG00000021172 ENSDARG00000022650 ENSDARG00000023820 ENSDARG00000025595 ENSDARG00000037873 ENSDARG00000042953 ENSDARG00000042956 ENSDARG00000042978 ENSDARG00000042982 ENSDARG00000055240 ENSDARG00000061585 ENSDARG00000062132 ENSDARG00000068493 ENSDARG00000069018 ENSDARG00000070021 ENSDARG00000070775 ENSDARG00000098315 ENSDARG00000101861 ENSDARG00000102805 ENSDARG00000103277 ENSDARG00000103295 ENSDARG00000104593 | | GO:0004181 | metallocarboxypeptidase activity | molecular\_function | 14 | 0.43 | 5 | 1.0e-02 | cpa5 cpa1 cpa4 cpb1 cpo | ENSDARG00000021339 ENSDARG00000030915 ENSDARG00000043722 ENSDARG00000045442 ENSDARG00000077688 | |

  


### Go to GO detail

## ZFA

| | ZFA ID | Description | Annotated | Expected | Observed | Fold Enrichment | Adjusted p-value | Genes | Ensembl IDs | | --- | --- | --- | --- | --- | --- | --- | --- | --- | | ZFA:0000339 | digestive system | 78 | 2.4 | 4 | 1.7 | 1.1e-09 | slc15a1b ddc CELA1 (1 of many) ela3l | ENSDARG00000044528 ENSDARG00000016494 ENSDARG00000043173 ENSDARG00000007276 | | ZFA:0001076 | intestinal bulb | 276 | 8.4 | 25 | 3.0 | 4.0e-08 | ucp1 nr5a5 slc15a1b sult1st2 glud1b rdh1 pdzk1 gpr39 slc5a1 pnp4b chia.1 chia.3 ada aqp10a cd36 tm4sf4 cyp2n13 s100a10a si:ch211-93f2.1 si:ch211-93f2.1 cyp3c3 alpi.2 grn1 neu3.3 cyp4v8 abcb4 | ENSDARG00000023151 ENSDARG00000039116 ENSDARG00000044528 ENSDARG00000041540 ENSDARG00000101074 ENSDARG00000017882 ENSDARG00000022261 ENSDARG00000102888 ENSDARG00000013871 ENSDARG00000029230 ENSDARG00000100635 ENSDARG00000009612 ENSDARG00000003113 ENSDARG00000007086 ENSDARG00000032639 ENSDARG00000040747 ENSDARG00000042953 ENSDARG00000037425 ENSDARG00000101726 ENSDARG00000104818 ENSDARG00000037873 ENSDARG00000053774 ENSDARG00000089362 ENSDARG00000058327 ENSDARG00000062132 ENSDARG00000010936 | |
