# Supplementary material for: A high-resolution mRNA expression time course of embryonic development in zebrafish
Source: eLife. 2017 Nov 16;6:e30860. doi: 10.7554/eLife.30860 (PMC5690287; doi:10.7554/eLife.30860)
Supplement: Supplementary file 6. [file elife-30860-supp6.zip › biolayout-clusters-files/Cluster006-genes.html]

Cluster006


# Cluster006: Genes

| | Ensembl ID | Gene Name | Chr | Start | End | Biotype | | --- | --- | --- | --- | --- | --- | | ENSDARG00000059888 | AGO3 (1 of many) | 16 | 51137729 | 51208263 | protein\_coding | | ENSDARG00000068593 | CU326366.2 | 10 | 43059021 | 43069864 | protein\_coding | | ENSDARG00000098286 | CU695232.1 | 13 | 46514197 | 46528961 | protein\_coding | | ENSDARG00000016538 | ENSDARG00000016538 | 4 | 9466133 | 9475502 | protein\_coding | | ENSDARG00000016674 | ENSDARG00000016674 | 3 | 1054435 | 1060784 | protein\_coding | | ENSDARG00000045127 | ENSDARG00000045127 | 21 | 30223450 | 30247709 | protein\_coding | | ENSDARG00000058630 | ENSDARG00000058630 | 21 | 11237606 | 11274643 | protein\_coding | | ENSDARG00000060395 | ENSDARG00000060395 | 2 | 37464786 | 37477740 | protein\_coding | | ENSDARG00000078822 | ENSDARG00000078822 | 2 | 10684763 | 10780420 | protein\_coding | | ENSDARG00000086112 | ENSDARG00000086112 | 10 | 32107046 | 32114218 | protein\_coding | | ENSDARG00000086579 | ENSDARG00000086579 | 1 | 50998008 | 51016982 | protein\_coding | | ENSDARG00000096566 | ENSDARG00000096566 | 1 | 50971707 | 50992837 | protein\_coding | | ENSDARG00000099419 | ENSDARG00000099419 | KN150241.1 | 21743 | 38113 | protein\_coding | | ENSDARG00000100633 | ENSDARG00000100633 | 10 | 6382061 | 6408572 | protein\_coding | | ENSDARG00000102717 | ENSDARG00000102717 | 16 | 39287736 | 39291769 | protein\_coding | | ENSDARG00000102965 | ENSDARG00000102965 | 20 | 54762163 | 54778132 | protein\_coding | | ENSDARG00000103823 | ENSDARG00000103823 | 9 | 52126813 | 52225580 | protein\_coding | | ENSDARG00000099814 | FBXO46 | 15 | 14656667 | 14664339 | protein\_coding | | ENSDARG00000060248 | FGD4 (1 of many) | 4 | 3308063 | 3325117 | protein\_coding | | ENSDARG00000079482 | GID4 | 3 | 61903399 | 61913334 | protein\_coding | | ENSDARG00000098123 | GNG12 (1 of many) | 6 | 23855565 | 23953807 | protein\_coding | | ENSDARG00000042608 | ICA1 | 16 | 47283329 | 47309126 | protein\_coding | | ENSDARG00000041602 | MMADHC (1 of many) | 22 | 12570367 | 12579927 | protein\_coding | | ENSDARG00000073761 | MYO19 | 5 | 3619704 | 3650110 | protein\_coding | | ENSDARG00000078313 | MYO9B (1 of many) | 22 | 4947319 | 5043866 | protein\_coding | | ENSDARG00000006848 | PARP9 | 9 | 23947593 | 23954611 | protein\_coding | | ENSDARG00000023916 | PHACTR1 | 20 | 52969202 | 53002470 | protein\_coding | | ENSDARG00000101080 | PWWP2B | 13 | 51485145 | 51503843 | protein\_coding | | ENSDARG00000104709 | SPRTN | 20 | 3981495 | 3996252 | protein\_coding | | ENSDARG00000078615 | USP53 (1 of many) | 7 | 69491080 | 69554133 | protein\_coding | | ENSDARG00000016528 | WDCP | 20 | 44600031 | 44613102 | protein\_coding | | ENSDARG00000019265 | ZBTB39 | 23 | 36538493 | 36547531 | protein\_coding | | ENSDARG00000056672 | abcb8 | 24 | 33895310 | 33915493 | protein\_coding | | ENSDARG00000058452 | abhd17c | 7 | 10669756 | 10764279 | protein\_coding | | ENSDARG00000058352 | acot9.1 | 24 | 21814820 | 21828284 | protein\_coding | | ENSDARG00000005640 | adat3 | 9 | 41234138 | 41238705 | protein\_coding | | ENSDARG00000093008 | adgrf3b | 20 | 35592808 | 35612359 | protein\_coding | | ENSDARG00000089162 | afap1l1a | 14 | 34222112 | 34293646 | protein\_coding | | ENSDARG00000092644 | ago1 | 16 | 51220329 | 51287850 | protein\_coding | | ENSDARG00000014532 | aida | 20 | 51918753 | 51941980 | protein\_coding | | ENSDARG00000037935 | aldh16a1 | 3 | 31948689 | 31966801 | protein\_coding | | ENSDARG00000028259 | aldh3a2a | 15 | 20894936 | 20904031 | protein\_coding | | ENSDARG00000059856 | alkbh2 | 5 | 41199743 | 41204245 | protein\_coding | | ENSDARG00000057435 | amacr | 21 | 19381961 | 19404981 | protein\_coding | | ENSDARG00000078335 | amot | 21 | 37791846 | 37865664 | protein\_coding | | ENSDARG00000077567 | angel1 | 17 | 52526597 | 52543831 | protein\_coding | | ENSDARG00000099918 | arl4aa | 15 | 34211767 | 34217000 | protein\_coding | | ENSDARG00000041381 | arntl2 | 18 | 15601115 | 15642344 | protein\_coding | | ENSDARG00000023396 | atg5 | 16 | 7463117 | 7499186 | protein\_coding | | ENSDARG00000060197 | atp2c1 | 16 | 41635613 | 41717069 | protein\_coding | | ENSDARG00000035565 | atp6v0a2b | 5 | 24040062 | 24064069 | protein\_coding | | ENSDARG00000063001 | atp8a1 | 14 | 45981387 | 46014354 | protein\_coding | | ENSDARG00000070605 | b4galnt2.2 | 3 | 22914666 | 22928740 | protein\_coding | | ENSDARG00000044781 | bace2 | 15 | 6462514 | 6501957 | protein\_coding | | ENSDARG00000077912 | bcorl1 | 14 | 41039067 | 41070900 | protein\_coding | | ENSDARG00000061409 | bivm | 6 | 29304749 | 29314571 | protein\_coding | | ENSDARG00000037491 | bmp15 | 7 | 51493273 | 51500618 | protein\_coding | | ENSDARG00000005115 | calub | 18 | 7056314 | 7072501 | protein\_coding | | ENSDARG00000062307 | ccdc61 | 21 | 21227352 | 21241197 | protein\_coding | | ENSDARG00000033046 | ccni2 | 21 | 43371628 | 43402999 | protein\_coding | | ENSDARG00000026070 | cd82b | 18 | 27593208 | 27648151 | protein\_coding | | ENSDARG00000026577 | cdk2 | 11 | 3233117 | 3240409 | protein\_coding | | ENSDARG00000038500 | cep41 | 25 | 18339901 | 18348879 | protein\_coding | | ENSDARG00000039411 | cep68 | 13 | 24283967 | 24298656 | protein\_coding | | ENSDARG00000101735 | chn1 | 9 | 2530715 | 2562211 | protein\_coding | | ENSDARG00000003973 | chordc1a | 15 | 43938371 | 43954698 | protein\_coding | | ENSDARG00000052859 | chp1 | 13 | 44898852 | 44926782 | protein\_coding | | ENSDARG00000032577 | clcn6 | 8 | 48640726 | 48680358 | protein\_coding | | ENSDARG00000037865 | cln3 | 3 | 39437535 | 39457481 | protein\_coding | | ENSDARG00000075525 | cln8 | 17 | 25163616 | 25176480 | protein\_coding | | ENSDARG00000021048 | clptm1l | 19 | 1906395 | 1921560 | protein\_coding | | ENSDARG00000036345 | cmtr2 | 7 | 24443683 | 24448498 | protein\_coding | | ENSDARG00000101072 | cnot6b | 14 | 49747037 | 49777505 | protein\_coding | | ENSDARG00000033345 | cntln | 1 | 26133295 | 26321174 | protein\_coding | | ENSDARG00000104342 | csnk1g1 | 7 | 53831696 | 53947896 | protein\_coding | | ENSDARG00000005458 | csnk1g2a | 2 | 23383390 | 23423097 | protein\_coding | | ENSDARG00000012818 | csnk2a2a | 7 | 56450335 | 56465562 | protein\_coding | | ENSDARG00000014439 | dgkza | 7 | 38740129 | 38884365 | protein\_coding | | ENSDARG00000055629 | dia1b | 24 | 5623833 | 5684470 | protein\_coding | | ENSDARG00000060849 | dlgap4a | 23 | 42943521 | 42990918 | protein\_coding | | ENSDARG00000102295 | dnaja3b | 22 | 25911885 | 25923370 | protein\_coding | | ENSDARG00000039363 | dnajb12a | 13 | 29904914 | 29913945 | protein\_coding | | ENSDARG00000020953 | dnajb6b | 7 | 40289124 | 40307716 | protein\_coding | | ENSDARG00000056005 | dnajc18 | 21 | 30202854 | 30217490 | protein\_coding | | ENSDARG00000069937 | dnm2a | 3 | 50320272 | 50402475 | protein\_coding | | ENSDARG00000063032 | dok6 | 24 | 14792774 | 14962954 | protein\_coding | | ENSDARG00000038812 | e2f5 | 2 | 31693572 | 31703185 | protein\_coding | | ENSDARG00000043643 | ehbp1 | 17 | 24090611 | 24296914 | protein\_coding | | ENSDARG00000062139 | eif2ak3 | 13 | 13814226 | 13873554 | protein\_coding | | ENSDARG00000000568 | ell | 22 | 20822471 | 20899593 | protein\_coding | | ENSDARG00000098874 | exoc6b | 7 | 24864576 | 25022889 | protein\_coding | | ENSDARG00000063344 | fam162a | 24 | 20782716 | 20790247 | protein\_coding | | ENSDARG00000052697 | fam172a | 5 | 49114518 | 49431837 | protein\_coding | | ENSDARG00000028715 | fam188a | 16 | 28335414 | 28436707 | protein\_coding | | ENSDARG00000078391 | fam98a | 17 | 23192001 | 23201529 | protein\_coding | | ENSDARG00000033949 | fbxo16 | 20 | 35305141 | 35351762 | protein\_coding | | ENSDARG00000071492 | fbxo45 | 24 | 28346741 | 28358674 | protein\_coding | | ENSDARG00000069662 | fgf11a | 7 | 22128454 | 22256066 | protein\_coding | | ENSDARG00000035339 | ftr99 | 5 | 36440501 | 36447015 | protein\_coding | | ENSDARG00000090183 | gapvd1 | 8 | 34423823 | 34509309 | protein\_coding | | ENSDARG00000079588 | gatsl3 | 5 | 11485079 | 11530957 | protein\_coding | | ENSDARG00000029051 | gbe1a | 10 | 24079431 | 24327137 | protein\_coding | | ENSDARG00000025275 | gbgt1l1 | 5 | 23198495 | 23212061 | protein\_coding | | ENSDARG00000079020 | gcfc2 | 17 | 32579343 | 32598745 | protein\_coding | | ENSDARG00000023627 | ggps1 | 13 | 49436655 | 49478694 | protein\_coding | | ENSDARG00000086903 | gig2o | 5 | 29781783 | 29782772 | protein\_coding | | ENSDARG00000009488 | gipc2 | 2 | 9762735 | 9792845 | protein\_coding | | ENSDARG00000068981 | glceb | 7 | 33136067 | 33172854 | protein\_coding | | ENSDARG00000075850 | gnal2 | 24 | 36130092 | 36139575 | protein\_coding | | ENSDARG00000043006 | gnav1 | 22 | 11508710 | 11564732 | protein\_coding | | ENSDARG00000039279 | golga5 | 13 | 33080564 | 33096961 | protein\_coding | | ENSDARG00000026654 | gosr1 | 15 | 24860208 | 24902390 | protein\_coding | | ENSDARG00000103200 | gpr107 | 5 | 31860300 | 31889643 | protein\_coding | | ENSDARG00000063069 | gripap1 | 8 | 9660526 | 9753685 | protein\_coding | | ENSDARG00000032157 | grk6 | 21 | 36938286 | 37052289 | protein\_coding | | ENSDARG00000061385 | haus3 | 7 | 24119782 | 24127272 | protein\_coding | | ENSDARG00000014866 | hprt1l | 25 | 36502300 | 36513143 | protein\_coding | | ENSDARG00000070606 | ikbke | 11 | 21204002 | 21243528 | protein\_coding | | ENSDARG00000040910 | ildr1b | 9 | 34451419 | 34459881 | protein\_coding | | ENSDARG00000021466 | ireb2 | 25 | 6100373 | 6134291 | protein\_coding | | ENSDARG00000011909 | itpr2 | 18 | 15968902 | 16074064 | protein\_coding | | ENSDARG00000015576 | itprip | 13 | 25272963 | 25277937 | protein\_coding | | ENSDARG00000071009 | kif20ba | 17 | 23450950 | 23489528 | protein\_coding | | ENSDARG00000101120 | kif3b | 23 | 7776695 | 7802528 | protein\_coding | | ENSDARG00000055965 | klc3 | 15 | 23721786 | 23742181 | protein\_coding | | ENSDARG00000038801 | klhl20 | 2 | 34128882 | 34143661 | protein\_coding | | ENSDARG00000062962 | kmt2ba | 19 | 9313751 | 9356315 | protein\_coding | | ENSDARG00000003751 | lats1 | 20 | 1219635 | 1248043 | protein\_coding | | ENSDARG00000070098 | lcorl | 1 | 22882485 | 22905182 | protein\_coding | | ENSDARG00000054941 | ldlrad4 | 16 | 30663537 | 30745696 | protein\_coding | | ENSDARG00000078094 | lmf2a | 18 | 6747178 | 6768952 | protein\_coding | | ENSDARG00000030012 | lrrfip1a | 9 | 24255063 | 24304755 | protein\_coding | | ENSDARG00000028848 | lsm12a | 3 | 22853550 | 22863885 | protein\_coding | | ENSDARG00000075556 | luzp1 | 17 | 27988800 | 28054982 | protein\_coding | | ENSDARG00000053113 | ly75 | 11 | 11136993 | 11209439 | protein\_coding | | ENSDARG00000054789 | lysmd1 | 16 | 29756085 | 29762166 | protein\_coding | | ENSDARG00000060771 | map7d3 | 14 | 31328189 | 31375098 | protein\_coding | | ENSDARG00000028082 | mapkapk5 | 5 | 68781104 | 68799535 | protein\_coding | | ENSDARG00000025076 | marveld2a | 10 | 2919871 | 2944236 | protein\_coding | | ENSDARG00000055566 | mastl | 24 | 5863797 | 5878951 | protein\_coding | | ENSDARG00000074326 | mcm8 | 17 | 4167568 | 4207427 | protein\_coding | | ENSDARG00000003910 | med13b | 15 | 20124911 | 20189599 | protein\_coding | | ENSDARG00000055838 | mettl16 | 15 | 25278906 | 25333763 | protein\_coding | | ENSDARG00000077863 | mettl22 | 3 | 27751592 | 27784709 | protein\_coding | | ENSDARG00000045754 | mettl25 | 4 | 11031872 | 11054826 | protein\_coding | | ENSDARG00000018145 | mid1ip1l | 14 | 41099114 | 41101782 | protein\_coding | | ENSDARG00000025576 | mkln1 | 4 | 11692074 | 11764375 | protein\_coding | | ENSDARG00000011373 | mknk2a | 2 | 23351141 | 23369301 | protein\_coding | | ENSDARG00000043185 | mllt1a | 8 | 20162070 | 20198717 | protein\_coding | | ENSDARG00000043877 | mmachc | 20 | 1364848 | 1372194 | protein\_coding | | ENSDARG00000062872 | mrs2 | 16 | 10373384 | 10402035 | protein\_coding | | ENSDARG00000090447 | mtbp | 16 | 15207623 | 15257125 | protein\_coding | | ENSDARG00000045304 | mtfr1 | 24 | 24581782 | 24588081 | protein\_coding | | ENSDARG00000059642 | mtmr14 | 6 | 19639601 | 19666863 | protein\_coding | | ENSDARG00000008592 | mtmr8 | 5 | 21523788 | 21549052 | protein\_coding | | ENSDARG00000025500 | mtx1a | 16 | 43035101 | 43061434 | protein\_coding | | ENSDARG00000079686 | naa30 | 17 | 44349465 | 44362947 | protein\_coding | | ENSDARG00000020079 | neil3 | 14 | 36074542 | 36088123 | protein\_coding | | ENSDARG00000040152 | nek4 | 11 | 3975360 | 4004601 | protein\_coding | | ENSDARG00000076179 | ngef | 15 | 40228027 | 40284882 | protein\_coding | | ENSDARG00000060758 | nphs1 | 15 | 36698534 | 36869503 | protein\_coding | | ENSDARG00000038991 | nsfb | 12 | 22189146 | 22234233 | protein\_coding | | ENSDARG00000071671 | nudt22 | 14 | 22170427 | 22182235 | protein\_coding | | ENSDARG00000075851 | oafa | 15 | 22458807 | 22496595 | protein\_coding | | ENSDARG00000007734 | obfc1 | 1 | 46808656 | 46822252 | protein\_coding | | ENSDARG00000068124 | opn7d | 8 | 49489257 | 49506828 | protein\_coding | | ENSDARG00000075682 | orc6 | 7 | 41532117 | 41541288 | protein\_coding | | ENSDARG00000079886 | otulinb | 2 | 31959137 | 31970284 | protein\_coding | | ENSDARG00000078185 | pacs2 | 13 | 36320156 | 36400412 | protein\_coding | | ENSDARG00000062114 | papd7 | 19 | 28223192 | 28246595 | protein\_coding | | ENSDARG00000075008 | pask | 2 | 44659326 | 44692871 | protein\_coding | | ENSDARG00000045486 | pawr | 4 | 21602008 | 21745618 | protein\_coding | | ENSDARG00000002967 | pdap1b | 3 | 40135846 | 40142886 | protein\_coding | | ENSDARG00000044298 | phax | 10 | 16071141 | 16077294 | protein\_coding | | ENSDARG00000013881 | pi4k2b | 1 | 14572167 | 14598717 | protein\_coding | | ENSDARG00000038097 | pigq | 3 | 26661675 | 26675161 | protein\_coding | | ENSDARG00000036005 | pitpnb | 10 | 4046072 | 4086400 | protein\_coding | | ENSDARG00000059982 | poc5 | 5 | 45338163 | 45358685 | protein\_coding | | ENSDARG00000037358 | polr3e | 12 | 978827 | 998873 | protein\_coding | | ENSDARG00000101472 | pop4 | 7 | 45691401 | 45699275 | protein\_coding | | ENSDARG00000007409 | ppifa | 13 | 30441722 | 30445349 | protein\_coding | | ENSDARG00000031985 | ppp2r4 | 5 | 32197737 | 32213839 | protein\_coding | | ENSDARG00000099379 | ppp4r1l | 6 | 49542625 | 49549027 | protein\_coding | | ENSDARG00000022254 | prkcbb | 3 | 34876222 | 35121017 | protein\_coding | | ENSDARG00000016093 | qtrtd1 | 24 | 20915267 | 20946098 | protein\_coding | | ENSDARG00000098344 | rab18b | 2 | 4178475 | 4191669 | protein\_coding | | ENSDARG00000005049 | rab20 | 9 | 8909198 | 8916913 | protein\_coding | | ENSDARG00000007711 | rab3il1 | 25 | 2973440 | 2992370 | protein\_coding | | ENSDARG00000092134 | rad54b | 16 | 26858644 | 26884207 | protein\_coding | | ENSDARG00000094896 | rbm33b | 2 | 30012057 | 30064104 | protein\_coding | | ENSDARG00000031382 | reep2 | 14 | 21299471 | 21320760 | protein\_coding | | ENSDARG00000021046 | rhbdd1 | 15 | 36075125 | 36086933 | protein\_coding | | ENSDARG00000099996 | rhot1a | 3 | 26158092 | 26188899 | protein\_coding | | ENSDARG00000100850 | rnf103 | 14 | 15778255 | 15786299 | protein\_coding | | ENSDARG00000037574 | rps6kal | 14 | 8634312 | 8696391 | protein\_coding | | ENSDARG00000006279 | rragd | 17 | 15780167 | 15896378 | protein\_coding | | ENSDARG00000053558 | rtkn2a | 17 | 43620356 | 43632832 | protein\_coding | | ENSDARG00000004131 | rttn | 24 | 14987704 | 15118657 | protein\_coding | | ENSDARG00000018096 | scap | 16 | 41110129 | 41153750 | protein\_coding | | ENSDARG00000027046 | scyl3 | 20 | 34109692 | 34126090 | protein\_coding | | ENSDARG00000056532 | serinc2 | 19 | 15504001 | 15516356 | protein\_coding | | ENSDARG00000076995 | sharpin | 2 | 341557 | 351972 | protein\_coding | | ENSDARG00000062109 | shdb | 22 | 21373530 | 21440830 | protein\_coding | | ENSDARG00000087145 | si:ch1073-296d18.1 | 19 | 12036993 | 12047673 | protein\_coding | | ENSDARG00000075173 | si:ch1073-322p19.1 | 3 | 1328182 | 1337664 | protein\_coding | | ENSDARG00000079888 | si:ch1073-357b18.3 | 12 | 290966 | 292459 | processed\_pseudogene | | ENSDARG00000091930 | si:ch211-12e13.1 | 5 | 8713680 | 8716786 | protein\_coding | | ENSDARG00000045423 | si:ch211-146l10.8 | 24 | 9887515 | 9890430 | protein\_coding | | ENSDARG00000086459 | si:ch211-165g14.1 | 3 | 58417524 | 58433067 | protein\_coding | | ENSDARG00000096789 | si:ch211-169j21.4 | 20 | 34231035 | 34244717 | antisense | | ENSDARG00000097772 | si:ch211-185a18.2 | 17 | 10919172 | 11214407 | protein\_coding | | ENSDARG00000088276 | si:ch211-190p8.2 | 24 | 34183500 | 34185913 | protein\_coding | | ENSDARG00000092310 | si:ch211-199g17.2 | 19 | 43984189 | 44008335 | protein\_coding | | ENSDARG00000095852 | si:ch211-205p4.1 | 16 | 7634738 | 7703768 | lincRNA | | ENSDARG00000087046 | si:ch211-222k6.2 | 22 | 10024512 | 10026611 | protein\_coding | | ENSDARG00000101341 | si:ch211-262i1.6 | 11 | 14285786 | 14288453 | protein\_coding | | ENSDARG00000093353 | si:ch211-263m18.4 | 21 | 43656859 | 43660949 | protein\_coding | | ENSDARG00000090473 | si:ch211-269k10.5 | 16 | 53366124 | 53385514 | protein\_coding | | ENSDARG00000103186 | si:ch73-389k6.1 | 4 | 74723830 | 74754136 | protein\_coding | | ENSDARG00000100357 | si:ch73-44m9.3.1 | 22 | 8126523 | 8144595 | protein\_coding | | ENSDARG00000069661 | si:dkey-121j17.6 | 20 | 570958 | 576730 | protein\_coding | | ENSDARG00000079530 | si:dkey-17m8.1 | 16 | 17763838 | 17793093 | protein\_coding | | ENSDARG00000095912 | si:dkey-229b18.3 | 9 | 30679758 | 30691264 | protein\_coding | | ENSDARG00000017173 | si:dkey-29p10.4 | 18 | 27355616 | 27376493 | protein\_coding | | ENSDARG00000104199 | si:dkey-43p13.5 | 3 | 42380533 | 42386247 | protein\_coding | | ENSDARG00000069542 | si:dkey-8e10.2 | 8 | 9963220 | 9974967 | protein\_coding | | ENSDARG00000060366 | slc12a9 | 5 | 37615771 | 37648309 | protein\_coding | | ENSDARG00000073743 | slc25a37 | 8 | 50202096 | 50229269 | protein\_coding | | ENSDARG00000069745 | slc35f2 | 21 | 22079170 | 22085676 | protein\_coding | | ENSDARG00000026149 | slc46a1 | 15 | 28242762 | 28253653 | protein\_coding | | ENSDARG00000061525 | slka | 13 | 24650729 | 24695992 | protein\_coding | | ENSDARG00000006389 | smad2 | 10 | 14913017 | 14985162 | protein\_coding | | ENSDARG00000016977 | snx27b | 16 | 23580568 | 23609342 | protein\_coding | | ENSDARG00000022659 | snx4 | 9 | 38778286 | 38811407 | protein\_coding | | ENSDARG00000068814 | spata2l | 18 | 14715603 | 14719926 | protein\_coding | | ENSDARG00000035868 | spire1a | 19 | 33392162 | 33485037 | protein\_coding | | ENSDARG00000018976 | sptlc2a | 17 | 17784891 | 17836282 | protein\_coding | | ENSDARG00000009764 | srek1 | 5 | 54537404 | 54578336 | protein\_coding | | ENSDARG00000015374 | st3gal3a | 6 | 3174085 | 3245200 | protein\_coding | | ENSDARG00000037556 | st3gal5 | 14 | 9175425 | 9193206 | protein\_coding | | ENSDARG00000100804 | st6galnac4 | 21 | 3689534 | 3692901 | protein\_coding | | ENSDARG00000014348 | stk17b | 9 | 24381501 | 24390290 | protein\_coding | | ENSDARG00000101543 | strumpellin | 16 | 43387951 | 43414169 | protein\_coding | | ENSDARG00000026338 | styxl1 | 10 | 35313791 | 35318508 | protein\_coding | | ENSDARG00000057286 | swap70b | 18 | 16887767 | 16927774 | protein\_coding | | ENSDARG00000017842 | syvn1 | 14 | 26154635 | 26167624 | protein\_coding | | ENSDARG00000070618 | tatdn2 | 6 | 40442386 | 40451863 | protein\_coding | | ENSDARG00000036994 | tbc1d16 | 3 | 18287657 | 18348934 | protein\_coding | | ENSDARG00000075333 | tbc1d25 | 8 | 23637387 | 23649255 | protein\_coding | | ENSDARG00000008966 | tbl1xr1b | 2 | 7966666 | 8002697 | protein\_coding | | ENSDARG00000061450 | tesk2 | 2 | 27674827 | 27722744 | protein\_coding | | ENSDARG00000070704 | thap7 | 19 | 24543322 | 24552010 | protein\_coding | | ENSDARG00000010681 | tjap1 | 13 | 3851433 | 4005645 | protein\_coding | | ENSDARG00000035515 | tm2d2 | 5 | 26320837 | 26326404 | protein\_coding | | ENSDARG00000104538 | tmem184a | 3 | 42217260 | 42251594 | protein\_coding | | ENSDARG00000086457 | tnfaip8l1 | 22 | 4769086 | 4787454 | protein\_coding | | ENSDARG00000077052 | tnrc18 | 3 | 40265494 | 40346993 | protein\_coding | | ENSDARG00000100559 | tor2a | 5 | 64423484 | 64432750 | protein\_coding | | ENSDARG00000062362 | tpcn1 | 5 | 22697570 | 22727424 | protein\_coding | | ENSDARG00000099885 | trim105 | 14 | 15666561 | 15684798 | protein\_coding | | ENSDARG00000038306 | tsc22d1 | 9 | 18724793 | 18807341 | protein\_coding | | ENSDARG00000007918 | ttc27 | 17 | 22740290 | 22927281 | protein\_coding | | ENSDARG00000063062 | ubn2a | 6 | 7502407 | 7529425 | protein\_coding | | ENSDARG00000098902 | ubxn8 | 10 | 7664998 | 7677640 | protein\_coding | | ENSDARG00000040407 | usp22 | 12 | 1464323 | 1497733 | protein\_coding | | ENSDARG00000055433 | vps26bl | 10 | 31856352 | 31862063 | protein\_coding | | ENSDARG00000079220 | wdr20a | 17 | 1511928 | 1525001 | protein\_coding | | ENSDARG00000099757 | wdr47b | 22 | 24532577 | 24563954 | protein\_coding | | ENSDARG00000060312 | wdr62 | 15 | 5578335 | 5636605 | protein\_coding | | ENSDARG00000105278 | wu:fa19b12 | KN150339.1 | 36068 | 39511 | protein\_coding | | ENSDARG00000041217 | xpo6 | 3 | 14935494 | 14981638 | protein\_coding | | ENSDARG00000041428 | yrdc | 16 | 4353570 | 4361437 | protein\_coding | | ENSDARG00000005586 | zbtb20 | 5 | 66854811 | 66982733 | protein\_coding | | ENSDARG00000013492 | zbtb34 | 8 | 33123270 | 33145445 | protein\_coding | | ENSDARG00000067950 | zcchc2 | 2 | 50327706 | 50365752 | protein\_coding | | ENSDARG00000076703 | zfand4 | 13 | 17888717 | 17938429 | protein\_coding | | ENSDARG00000026682 | zgc:101583 | 14 | 38542338 | 38549526 | protein\_coding | | ENSDARG00000030803 | zgc:110006 | 10 | 35115996 | 35164823 | protein\_coding | | ENSDARG00000069102 | zgc:112962 | 8 | 21020388 | 21039669 | protein\_coding | | ENSDARG00000052923 | zgc:113162 | 21 | 13286684 | 13335815 | protein\_coding | | ENSDARG00000057006 | zgc:113201 | 13 | 12532391 | 12534745 | protein\_coding | | ENSDARG00000038898 | zgc:113691 | 2 | 26936334 | 26941160 | protein\_coding | | ENSDARG00000068663 | zgc:152951 | 9 | 19332504 | 19358826 | protein\_coding | | ENSDARG00000070604 | zgc:162509 | 16 | 14075803 | 14086815 | protein\_coding | | ENSDARG00000101787 | zgc:162592 | 7 | 67475826 | 67483397 | protein\_coding | | ENSDARG00000074163 | zgc:162879 | 18 | 25768306 | 25785021 | protein\_coding | | ENSDARG00000071643 | zgc:171490 | 22 | 6516851 | 6532772 | protein\_coding | | ENSDARG00000042054 | zgc:171971 | 12 | 7234887 | 7247048 | protein\_coding | | ENSDARG00000037645 | zgc:173570 | 23 | 16904720 | 16923172 | protein\_coding | | ENSDARG00000045424 | zgc:173856 | 24 | 9878267 | 9880955 | protein\_coding | | ENSDARG00000103024 | zgc:175136 | 19 | 14247153 | 14250772 | protein\_coding | | ENSDARG00000067669 | zglp1 | 6 | 135095 | 139337 | protein\_coding | | ENSDARG00000057249 | zmynd11 | 24 | 27145584 | 27177186 | protein\_coding | | ENSDARG00000091947 | znf106b | 20 | 26037280 | 26062287 | protein\_coding | | ENSDARG00000021086 | znf367 | 8 | 1144725 | 1154072 | protein\_coding | |
