# Supplementary material for: A high-resolution mRNA expression time course of embryonic development in zebrafish
Source: eLife. 2017 Nov 16;6:e30860. doi: 10.7554/eLife.30860 (PMC5690287; doi:10.7554/eLife.30860)
Supplement: Supplementary file 6. [file elife-30860-supp6.zip › biolayout-clusters-files/Cluster006.html]

Cluster006


# Cluster006: Detail

### Go to ZFA detail

## GO

| | GO ID | Description | Domain | Annotated | Expected | Observed | Adjusted p-value | Genes | Ensembl IDs | | --- | --- | --- | --- | --- | --- | --- | --- | --- | | GO:0018105 | peptidyl-serine phosphorylation | biological\_process | 50 | 1.1 | 7 | 0.025 | lats1 csnk1g2a mknk2a prkcbb mapkapk5 mastl csnk1g1 | ENSDARG00000003751 ENSDARG00000005458 ENSDARG00000011373 ENSDARG00000022254 ENSDARG00000028082 ENSDARG00000055566 ENSDARG00000104342 | |
