# Supplementary material for: A high-resolution mRNA expression time course of embryonic development in zebrafish
Source: eLife. 2017 Nov 16;6:e30860. doi: 10.7554/eLife.30860 (PMC5690287; doi:10.7554/eLife.30860)
Supplement: Supplementary file 6. [file elife-30860-supp6.zip › biolayout-clusters-files/Cluster007-genes.html]

Cluster007


# Cluster007: Genes

| | Ensembl ID | Gene Name | Chr | Start | End | Biotype | | --- | --- | --- | --- | --- | --- | | ENSDARG00000024602 | ACBD3 (1 of many) | 17 | 33422958 | 33434079 | protein\_coding | | ENSDARG00000098111 | BX005448.1 | KN149682.1 | 48874 | 53897 | protein\_coding | | ENSDARG00000098447 | BX321873.1 | 4 | 36350259 | 36465216 | protein\_coding | | ENSDARG00000104750 | BX323023.2 | 4 | 29817305 | 29862193 | protein\_coding | | ENSDARG00000103008 | BX324179.3 | 4 | 34172073 | 34179372 | protein\_coding | | ENSDARG00000086839 | BX324216.1 | 22 | 9998189 | 10005581 | protein\_coding | | ENSDARG00000102016 | BX469910.2 | 4 | 56988719 | 56994019 | protein\_coding | | ENSDARG00000098877 | BX510909.1 | KN149695.1 | 52920 | 60511 | protein\_coding | | ENSDARG00000104294 | BX510909.3 | KN149695.1 | 99691 | 111469 | protein\_coding | | ENSDARG00000087544 | BX510934.1 | 4 | 55439551 | 55441277 | protein\_coding | | ENSDARG00000104762 | BX530097.1 | 4 | 29775758 | 29835564 | protein\_coding | | ENSDARG00000098986 | BX548067.1 | 4 | 38939438 | 38949041 | protein\_coding | | ENSDARG00000102311 | BX572103.3 | 4 | 65078832 | 65090234 | protein\_coding | | ENSDARG00000099193 | BX640461.2 | 4 | 52586627 | 52602725 | protein\_coding | | ENSDARG00000100076 | BX640547.1 | 4 | 50924288 | 50933098 | protein\_coding | | ENSDARG00000104407 | BX649453.3 | 4 | 36164175 | 36180953 | protein\_coding | | ENSDARG00000103741 | BX649620.1 | 4 | 31108576 | 31118245 | protein\_coding | | ENSDARG00000079652 | CAAP1 | 7 | 73615539 | 73620533 | protein\_coding | | ENSDARG00000102897 | CABZ01002345.1 | 4 | 70443121 | 70457107 | protein\_coding | | ENSDARG00000104652 | CABZ01017723.1 | KN150666.1 | 729 | 13375 | protein\_coding | | ENSDARG00000100023 | CABZ01019474.1 | KN150080.1 | 8916 | 21154 | protein\_coding | | ENSDARG00000100390 | CABZ01021450.2 | 4 | 71354577 | 71367468 | protein\_coding | | ENSDARG00000102749 | CABZ01021450.3 | 4 | 71289786 | 71390457 | protein\_coding | | ENSDARG00000102090 | CABZ01021453.1 | 4 | 71273520 | 71279036 | protein\_coding | | ENSDARG00000101974 | CABZ01021454.1 | 4 | 71239374 | 71251160 | protein\_coding | | ENSDARG00000101451 | CABZ01033540.1 | 4 | 68454152 | 68461979 | protein\_coding | | ENSDARG00000076082 | CABZ01041962.1 | 5 | 6393351 | 6397953 | protein\_coding | | ENSDARG00000093254 | CABZ01051641.1 | 4 | 70143228 | 70150606 | protein\_coding | | ENSDARG00000098960 | CABZ01051641.2 | 4 | 70151515 | 70160994 | protein\_coding | | ENSDARG00000095569 | CABZ01059309.1 | 4 | 42250204 | 42251451 | protein\_coding | | ENSDARG00000098464 | CABZ01060490.1 | KN149696.1 | 57065 | 67873 | protein\_coding | | ENSDARG00000099731 | CABZ01066719.1 | KN150616.1 | 60067 | 61320 | protein\_coding | | ENSDARG00000099884 | CABZ01074982.1 | KN149696.1 | 361611 | 368613 | protein\_coding | | ENSDARG00000099245 | CABZ01081752.1 | KN150703.1 | 23158 | 47525 | protein\_coding | | ENSDARG00000101105 | CR382372.1 | 4 | 29340387 | 29350323 | protein\_coding | | ENSDARG00000104041 | CR382372.2 | 4 | 29315506 | 29333427 | protein\_coding | | ENSDARG00000099486 | CR387997.1 | 4 | 29050408 | 29059067 | protein\_coding | | ENSDARG00000104496 | CR388042.3 | 4 | 51979572 | 51991944 | protein\_coding | | ENSDARG00000101395 | CR388132.1 | 4 | 29209957 | 29225170 | protein\_coding | | ENSDARG00000104136 | CR450780.5 | 4 | 60131649 | 60320535 | protein\_coding | | ENSDARG00000101797 | CT737127.2 | 4 | 66074040 | 66106973 | protein\_coding | | ENSDARG00000104385 | CT978957.2 | 5 | 68922755 | 68923819 | pseudogene | | ENSDARG00000101278 | CU207245.2 | 4 | 30789852 | 30795470 | protein\_coding | | ENSDARG00000003313 | ENSDARG00000003313 | 24 | 38770546 | 38807189 | protein\_coding | | ENSDARG00000028096 | ENSDARG00000028096 | 10 | 15088718 | 15090662 | protein\_coding | | ENSDARG00000089094 | ENSDARG00000089094 | 14 | 8136134 | 8143582 | protein\_coding | | ENSDARG00000104501 | ENSDARG00000104501 | 7 | 73595914 | 73597184 | protein\_coding | | ENSDARG00000105418 | ENSDARG00000105418 | 7 | 6279684 | 6280174 | protein\_coding | | ENSDARG00000103701 | FO393424.1 | 22 | 8939730 | 8943408 | protein\_coding | | ENSDARG00000100350 | FO681350.1 | 4 | 34015923 | 34020403 | protein\_coding | | ENSDARG00000090740 | FO704783.1 | 3 | 7608191 | 7609507 | protein\_coding | | ENSDARG00000087837 | FO834877.1 | 4 | 42228513 | 42240454 | protein\_coding | | ENSDARG00000096631 | MED9 | 12 | 1375488 | 1378162 | protein\_coding | | ENSDARG00000076638 | MEX3A | 16 | 46793710 | 46799742 | protein\_coding | | ENSDARG00000076104 | SEMA4B (1 of many) | 7 | 29969778 | 29995889 | protein\_coding | | ENSDARG00000075229 | SLC46A2 | 10 | 4923885 | 4934671 | protein\_coding | | ENSDARG00000105028 | TOPORS (1 of many) | KN149959.1 | 21059 | 34349 | protein\_coding | | ENSDARG00000094901 | abcc6b.2 | 3 | 36604025 | 36682332 | protein\_coding | | ENSDARG00000055678 | akap12b | 20 | 26509747 | 26568217 | protein\_coding | | ENSDARG00000078941 | ano9a.1 | 25 | 34650718 | 34651914 | protein\_coding | | ENSDARG00000094729 | apela | 1 | 18975010 | 18980963 | protein\_coding | | ENSDARG00000002172 | aplnra | 8 | 40286105 | 40287812 | protein\_coding | | ENSDARG00000036670 | aplnrb | 10 | 8670575 | 8672661 | protein\_coding | | ENSDARG00000055381 | bambia | 12 | 23302889 | 23309137 | protein\_coding | | ENSDARG00000018260 | bmp7a | 11 | 7097959 | 7137701 | protein\_coding | | ENSDARG00000034855 | ccnb3 | 7 | 22710746 | 22733535 | protein\_coding | | ENSDARG00000098529 | ccne2 | 16 | 40625710 | 40635748 | protein\_coding | | ENSDARG00000104903 | cdkn1cb | 25 | 7345633 | 7346897 | protein\_coding | | ENSDARG00000045561 | dram1 | 4 | 17673437 | 17680905 | protein\_coding | | ENSDARG00000058454 | dynll1 | 8 | 2428707 | 2432928 | protein\_coding | | ENSDARG00000011245 | esrp1 | 16 | 26769430 | 26803277 | protein\_coding | | ENSDARG00000018303 | etv4 | 12 | 27444735 | 27477334 | protein\_coding | | ENSDARG00000044511 | etv5b | 6 | 13902582 | 13910699 | protein\_coding | | ENSDARG00000012788 | foxa3 | 18 | 46352556 | 46356251 | protein\_coding | | ENSDARG00000042485 | foxd5 | 8 | 30443713 | 30444864 | protein\_coding | | ENSDARG00000059680 | fscn1a | 3 | 40434589 | 40447631 | protein\_coding | | ENSDARG00000025403 | ftr83 | 5 | 30141574 | 30149340 | protein\_coding | | ENSDARG00000067524 | fut9b | 8 | 3249016 | 3354488 | protein\_coding | | ENSDARG00000099213 | glyr1 | 3 | 36299448 | 36316307 | protein\_coding | | ENSDARG00000016364 | gna15.1 | 22 | 21996958 | 22011329 | protein\_coding | | ENSDARG00000015472 | gpc4 | 14 | 30749837 | 30810834 | protein\_coding | | ENSDARG00000037244 | gpr137 | 14 | 26141122 | 26152596 | protein\_coding | | ENSDARG00000020504 | h3f3a | 3 | 47824036 | 47825789 | protein\_coding | | ENSDARG00000017917 | her7 | 5 | 68036503 | 68037818 | protein\_coding | | ENSDARG00000094154 | hist2h3c | 25 | 34546698 | 34547197 | protein\_coding | | ENSDARG00000036162 | hnrnpa0b | 14 | 38505360 | 38507852 | protein\_coding | | ENSDARG00000051890 | hp | 7 | 56276111 | 56282910 | protein\_coding | | ENSDARG00000012025 | hs6st2 | 14 | 30741548 | 30747516 | protein\_coding | | ENSDARG00000096948 | im:7143333 | 15 | 780191 | 784869 | protein\_coding | | ENSDARG00000097825 | im:7154473 | 17 | 43892123 | 43897088 | lincRNA | | ENSDARG00000020541 | ism1 | 13 | 34639135 | 34667481 | protein\_coding | | ENSDARG00000078518 | kazald2 | 14 | 5498094 | 5510205 | protein\_coding | | ENSDARG00000045067 | kcnk1a | 12 | 30590953 | 30597209 | protein\_coding | | ENSDARG00000070475 | khdrbs1b | 19 | 30761363 | 30771746 | protein\_coding | | ENSDARG00000038792 | klf17 | 2 | 33343304 | 33345470 | protein\_coding | | ENSDARG00000019920 | lft1 | 20 | 35171102 | 35173508 | protein\_coding | | ENSDARG00000044059 | lft2 | 17 | 8151004 | 8155745 | protein\_coding | | ENSDARG00000070792 | lrrc15 | 6 | 36482003 | 36485801 | protein\_coding | | ENSDARG00000099911 | lrrc8c | 6 | 25090028 | 25102001 | protein\_coding | | ENSDARG00000100836 | lrrc8c.1 | 6 | 25088214 | 25088974 | TEC | | ENSDARG00000035147 | lrwd1 | 5 | 60790561 | 60820153 | protein\_coding | | ENSDARG00000006409 | mapk12b | 4 | 5515120 | 5521871 | protein\_coding | | ENSDARG00000035715 | marcksl1b | 19 | 30210751 | 30213499 | protein\_coding | | ENSDARG00000017078 | mespaa | 7 | 15081532 | 15084042 | protein\_coding | | ENSDARG00000041051 | mid1ip1a | 9 | 30452442 | 30453669 | protein\_coding | | ENSDARG00000101279 | ndr2 | 12 | 48295205 | 48302968 | protein\_coding | | ENSDARG00000042004 | nfya | 11 | 35992288 | 36017681 | protein\_coding | | ENSDARG00000031126 | notum1a | 3 | 57878417 | 57908864 | protein\_coding | | ENSDARG00000063661 | nuak2 | 22 | 470567 | 497731 | protein\_coding | | ENSDARG00000014091 | osr1 | 13 | 32013920 | 32019993 | protein\_coding | | ENSDARG00000044625 | pcf11 | 21 | 22842229 | 22851770 | protein\_coding | | ENSDARG00000012591 | pdcd10b | 2 | 36738900 | 36749506 | protein\_coding | | ENSDARG00000059001 | pim2 | 8 | 8889285 | 8895484 | protein\_coding | | ENSDARG00000068190 | pimr188 | 8 | 46049742 | 46052806 | protein\_coding | | ENSDARG00000012044 | polr3gla | 19 | 24961790 | 24971712 | protein\_coding | | ENSDARG00000071005 | ppp1r3ca | 17 | 23291353 | 23294154 | protein\_coding | | ENSDARG00000103546 | purbb | 10 | 45132924 | 45134683 | protein\_coding | | ENSDARG00000100636 | rbm12 | 11 | 26138384 | 26145791 | protein\_coding | | ENSDARG00000011613 | rbm39a | 11 | 24101746 | 24107807 | protein\_coding | | ENSDARG00000077060 | rbm6 | 6 | 42669158 | 42693200 | protein\_coding | | ENSDARG00000070434 | rhov | 20 | 28465652 | 28469047 | protein\_coding | | ENSDARG00000098942 | rp9 | 19 | 34486757 | 34502185 | protein\_coding | | ENSDARG00000090673 | si:ch1073-104i17.1 | 22 | 4563533 | 4576667 | protein\_coding | | ENSDARG00000105340 | si:ch1073-153i20.3 | 7 | 6292762 | 6293214 | protein\_coding | | ENSDARG00000096602 | si:ch1073-357b18.4 | 12 | 279193 | 281061 | protein\_coding | | ENSDARG00000096794 | si:ch211-10j20.7 | 20 | 39555717 | 39570401 | antisense | | ENSDARG00000096216 | si:ch211-162i8.7 | 4 | 44507344 | 45106942 | protein\_coding | | ENSDARG00000094887 | si:ch211-170d8.2 | 8 | 39762144 | 39764368 | protein\_coding | | ENSDARG00000095328 | si:ch211-191i18.4 | 19 | 42666302 | 42669003 | protein\_coding | | ENSDARG00000101935 | si:ch211-195b11.4 | 14 | 38505190 | 38507061 | antisense | | ENSDARG00000093317 | si:ch211-209j10.6 | 23 | 18978331 | 18986729 | protein\_coding | | ENSDARG00000103581 | si:ch211-212k5.1 | 4 | 56021464 | 56309088 | protein\_coding | | ENSDARG00000098892 | si:ch211-223a21.1 | 4 | 62629808 | 62641336 | protein\_coding | | ENSDARG00000100842 | si:ch211-223a21.4 | 4 | 62868354 | 62874985 | protein\_coding | | ENSDARG00000089875 | si:ch211-226o13.2 | 4 | 43151470 | 43162693 | protein\_coding | | ENSDARG00000096222 | si:ch211-245n8.4 | 4 | 48939903 | 48944071 | protein\_coding | | ENSDARG00000093354 | si:ch211-57i17.2 | 20 | 46832962 | 46836207 | protein\_coding | | ENSDARG00000095745 | si:ch73-138e16.3 | 22 | 1268284 | 1273241 | protein\_coding | | ENSDARG00000098681 | si:ch73-138e16.7 | 22 | 1306616 | 1312248 | processed\_transcript | | ENSDARG00000086223 | si:ch73-144d13.4 | 15 | 616517 | 623024 | protein\_coding | | ENSDARG00000103636 | si:dkey-122c11.4 | 4 | 55451358 | 55465325 | protein\_coding | | ENSDARG00000096152 | si:dkey-149m13.4 | 4 | 57611745 | 57619388 | protein\_coding | | ENSDARG00000094653 | si:dkey-149m13.5 | 4 | 57601473 | 57609592 | protein\_coding | | ENSDARG00000092329 | si:dkey-156j15.1 | 4 | 49155195 | 49160476 | protein\_coding | | ENSDARG00000090160 | si:dkey-156k2.3 | 4 | 48010069 | 48138012 | protein\_coding | | ENSDARG00000086236 | si:dkey-163m14.7 | 21 | 7393309 | 7398561 | protein\_coding | | ENSDARG00000101829 | si:dkey-16b10.2 | 4 | 50303954 | 50315604 | protein\_coding | | ENSDARG00000098265 | si:dkey-16p19.1.1 | 4 | 60494922 | 60560691 | protein\_coding | | ENSDARG00000100961 | si:dkey-176f19.6 | 4 | 35715983 | 35766868 | protein\_coding | | ENSDARG00000103471 | si:dkey-199m13.4 | 4 | 30408999 | 30506241 | protein\_coding | | ENSDARG00000104479 | si:dkey-1b17.3 | 22 | 1633230 | 1637040 | processed\_transcript | | ENSDARG00000092617 | si:dkey-22h13.2 | 4 | 39815650 | 39823026 | protein\_coding | | ENSDARG00000101460 | si:dkey-233e3.3 | 4 | 30305991 | 30339599 | protein\_coding | | ENSDARG00000102316 | si:dkey-237m9.2 | 4 | 41247628 | 41257018 | protein\_coding | | ENSDARG00000101197 | si:dkey-238d18.15 | 15 | 38026364 | 38027550 | protein\_coding | | ENSDARG00000076252 | si:dkey-247i3.1 | 4 | 33566413 | 33575978 | protein\_coding | | ENSDARG00000093713 | si:dkey-256i11.2 | 4 | 44666428 | 44743608 | protein\_coding | | ENSDARG00000103417 | si:dkey-269o24.6 | 4 | 54959337 | 55070633 | protein\_coding | | ENSDARG00000102673 | si:dkey-26i24.1 | 4 | 59721238 | 59727962 | protein\_coding | | ENSDARG00000098293 | si:dkey-27i16.2 | 14 | 14255369 | 14260854 | protein\_coding | | ENSDARG00000098087 | si:dkey-30f3.2 | 4 | 62081475 | 62116419 | protein\_coding | | ENSDARG00000100479 | si:dkey-43f9.4 | 4 | 31281914 | 31524416 | protein\_coding | | ENSDARG00000096026 | si:dkey-4e4.1 | 4 | 57489608 | 57495604 | protein\_coding | | ENSDARG00000098170 | si:dkey-4e4.1.1 | 4 | 41745855 | 41752647 | protein\_coding | | ENSDARG00000096189 | si:dkey-54j5.2 | 4 | 42521012 | 42529112 | protein\_coding | | ENSDARG00000103213 | si:dkey-56m15.8 | 4 | 52963960 | 52992446 | protein\_coding | | ENSDARG00000104697 | si:dkey-57k17.1 | 4 | 55990877 | 56002805 | protein\_coding | | ENSDARG00000102008 | si:dkey-72l17.6 | 4 | 32181085 | 32214101 | protein\_coding | | ENSDARG00000104489 | si:dkey-74i1.5 | 15 | 32775311 | 32777798 | lincRNA | | ENSDARG00000096901 | si:dkey-7i4.11 | 15 | 903073 | 919005 | protein\_coding | | ENSDARG00000098032 | si:dkey-7i4.13 | 15 | 847124 | 902937 | protein\_coding | | ENSDARG00000097180 | si:dkey-7i4.21 | 15 | 762638 | 794205 | protein\_coding | | ENSDARG00000079010 | si:dkey-7i4.5 | 15 | 871401 | 889864 | protein\_coding | | ENSDARG00000086668 | si:dkey-7j22.4 | 4 | 43898252 | 44020391 | protein\_coding | | ENSDARG00000095126 | si:dkey-82i20.2 | 4 | 59016295 | 59020474 | protein\_coding | | ENSDARG00000093612 | si:dkey-92i17.2 | 14 | 10301432 | 10311979 | protein\_coding | | ENSDARG00000092141 | si:dkeyp-100a1.6 | 8 | 47197688 | 47208002 | protein\_coding | | ENSDARG00000089158 | si:dkeyp-104f11.6 | 20 | 47240179 | 47248145 | protein\_coding | | ENSDARG00000103250 | si:dkeyp-35e5.10 | 4 | 60913281 | 60920604 | protein\_coding | | ENSDARG00000102581 | si:dkeyp-44b5.5 | 4 | 50684378 | 50754643 | protein\_coding | | ENSDARG00000096011 | si:dkeyp-4f2.3 | 4 | 33467752 | 33629011 | protein\_coding | | ENSDARG00000096456 | si:dkeyp-82h4.3 | 4 | 40515829 | 40522631 | protein\_coding | | ENSDARG00000051914 | slc14a2 | 5 | 51521297 | 51531221 | protein\_coding | | ENSDARG00000042954 | slc17a9a | 8 | 23131005 | 23142964 | protein\_coding | | ENSDARG00000012946 | syap1 | 23 | 46015885 | 46023057 | protein\_coding | | ENSDARG00000101576 | ta | 19 | 14325345 | 14329397 | protein\_coding | | ENSDARG00000007329 | tbx16 | 8 | 51747378 | 51767075 | protein\_coding | | ENSDARG00000089572 | tfpt | 16 | 4702441 | 4711625 | protein\_coding | | ENSDARG00000024828 | tmem134 | 1 | 39164468 | 39177845 | protein\_coding | | ENSDARG00000074776 | tmem154 | 1 | 24463247 | 24486257 | protein\_coding | | ENSDARG00000099609 | tpbga | 19 | 14197154 | 14199792 | protein\_coding | | ENSDARG00000036082 | tph1b | 7 | 32835182 | 32843734 | protein\_coding | | ENSDARG00000051761 | trim44 | 25 | 36762077 | 36767489 | protein\_coding | | ENSDARG00000100483 | vent | 13 | 50293013 | 50299989 | protein\_coding | | ENSDARG00000099761 | vox | 13 | 50307003 | 50309275 | protein\_coding | | ENSDARG00000093864 | wee1 | 18 | 16929360 | 16935155 | protein\_coding | | ENSDARG00000042796 | yy1a | 17 | 30840045 | 30846289 | protein\_coding | | ENSDARG00000040487 | zgc:113176 | 17 | 49198622 | 49223711 | protein\_coding | | ENSDARG00000100294 | zgc:113209 | 4 | 74835205 | 74842847 | protein\_coding | | ENSDARG00000068941 | zgc:113983 | 25 | 34557395 | 34558700 | protein\_coding | | ENSDARG00000077587 | zgc:113984 | 25 | 34645565 | 34645975 | protein\_coding | | ENSDARG00000069505 | zgc:136892 | 21 | 25757365 | 25759363 | protein\_coding | | ENSDARG00000068784 | zgc:153073 | 13 | 43009117 | 43027327 | protein\_coding | | ENSDARG00000041359 | zgc:161969 | 6 | 16341836 | 16348939 | protein\_coding | | ENSDARG00000105345 | zgc:173552.8 | 7 | 6293815 | 6294284 | protein\_coding | | ENSDARG00000103723 | zgc:173702 | 4 | 67023063 | 67063035 | protein\_coding | | ENSDARG00000105137 | zgc:173705 | 4 | 50303962 | 50436607 | protein\_coding | | ENSDARG00000097244 | zgc:174263 | 2 | 314214 | 321548 | protein\_coding | | ENSDARG00000100257 | zgc:174314 | 4 | 55789088 | 55803146 | protein\_coding | | ENSDARG00000079222 | zgc:174315 | 4 | 28996915 | 29003958 | protein\_coding | | ENSDARG00000088375 | zgc:174704 | 4 | 48723853 | 48729079 | protein\_coding | | ENSDARG00000037178 | zic2b | 1 | 29027828 | 29056964 | protein\_coding | | ENSDARG00000103777 | znf1001.1 | 3 | 7623483 | 7650198 | protein\_coding | | ENSDARG00000099917 | znf1005 | 5 | 68904574 | 68928635 | protein\_coding | | ENSDARG00000098582 | znf1040 | 4 | 45987326 | 46105351 | protein\_coding | | ENSDARG00000076272 | znf1041 | 4 | 28429541 | 28434905 | protein\_coding | | ENSDARG00000098071 | znf1049 | 4 | 41228841 | 41238471 | protein\_coding | | ENSDARG00000096210 | znf1050 | 4 | 57263220 | 57271530 | protein\_coding | | ENSDARG00000098270 | znf1053 | 4 | 42005580 | 42013350 | protein\_coding | | ENSDARG00000103310 | znf1053.1 | 4 | 51229598 | 51238471 | protein\_coding | | ENSDARG00000086449 | znf1055 | 4 | 33439391 | 33447071 | protein\_coding | | ENSDARG00000100329 | znf1056 | 4 | 59980299 | 59996767 | protein\_coding | | ENSDARG00000088000 | znf1057 | 4 | 47841075 | 47905558 | protein\_coding | | ENSDARG00000074009 | znf1059 | 4 | 28860207 | 29154519 | protein\_coding | | ENSDARG00000103441 | znf1060 | 4 | 41536956 | 41549373 | protein\_coding | | ENSDARG00000096007 | znf1067 | 4 | 28429500 | 28463053 | protein\_coding | | ENSDARG00000104561 | znf1081 | 4 | 67756436 | 67761721 | protein\_coding | | ENSDARG00000104006 | znf1082 | 4 | 59792599 | 59797711 | protein\_coding | | ENSDARG00000076255 | znf1084 | 4 | 57135167 | 57336533 | protein\_coding | | ENSDARG00000073915 | znf1085 | 4 | 32572471 | 32576051 | protein\_coding | | ENSDARG00000088847 | znf1089 | 4 | 62438189 | 62450854 | protein\_coding | | ENSDARG00000104964 | znf1093 | 4 | 37854536 | 37859835 | protein\_coding | | ENSDARG00000098991 | znf1095 | 4 | 63587614 | 63593741 | protein\_coding | | ENSDARG00000104887 | znf1105 | 4 | 62762890 | 62773362 | protein\_coding | | ENSDARG00000076054 | znf1140 | 4 | 29353763 | 29598881 | protein\_coding | | ENSDARG00000103689 | znf1148 | 4 | 41610767 | 41629634 | protein\_coding | | ENSDARG00000099641 | znf569l | 4 | 53520406 | 53528844 | protein\_coding | | ENSDARG00000101623 | znf992 | 4 | 35994749 | 36120033 | protein\_coding | | ENSDARG00000008333 | znfl2a | 2 | 129176 | 133115 | protein\_coding | |
