# Supplementary material for: A high-resolution mRNA expression time course of embryonic development in zebrafish
Source: eLife. 2017 Nov 16;6:e30860. doi: 10.7554/eLife.30860 (PMC5690287; doi:10.7554/eLife.30860)
Supplement: Supplementary file 6. [file elife-30860-supp6.zip › biolayout-clusters-files/Cluster007.html]

Cluster007


# Cluster007: Detail

### Go to ZFA detail

## GO

| | GO ID | Description | Domain | Annotated | Expected | Observed | Adjusted p-value | Genes | Ensembl IDs | | --- | --- | --- | --- | --- | --- | --- | --- | --- | | GO:0001702 | gastrulation with mouth forming second | biological\_process | 34 | 0.42 | 5 | 3.6e-02 | aplnra gpc4 mespaa aplnrb rhov | ENSDARG00000002172 ENSDARG00000015472 ENSDARG00000017078 ENSDARG00000036670 ENSDARG00000070434 | | GO:0060395 | SMAD protein signal transduction | biological\_process | 22 | 0.27 | 4 | 3.3e-02 | bmp7a lft1 lft2 ndr2 | ENSDARG00000018260 ENSDARG00000019920 ENSDARG00000044059 ENSDARG00000101279 | | GO:0001707 | mesoderm formation | biological\_process | 29 | 0.36 | 5 | 4.8e-02 | mespaa vox vent ndr2 ta | ENSDARG00000017078 ENSDARG00000099761 ENSDARG00000100483 ENSDARG00000101279 ENSDARG00000101576 | | GO:0006355 | regulation of transcription, DNA-templat... | biological\_process | 1072 | 13.20 | 38 | 3.0e-07 | tbx16 polr3gla foxa3 osr1 her7 bmp7a etv4 klf17 nfya foxd5 etv5b bambia znf1085 znf1084 znf1089 si:dkey-156j15.1 wee1 si:dkey-149m13.4 si:ch211-162i8.7 si:dkeyp-82h4.3 MED9 si:ch211-223a21.1 znf1095 si:dkey-43f9.4 vent ndr2 ta si:dkey-237m9.2 si:dkey-56m15.8 si:dkey-269o24.6 purbb si:ch211-212k5.1 znf1148 znf1082 znf1081 si:dkey-57k17.1 znf1105 znf1093 | ENSDARG00000007329 ENSDARG00000012044 ENSDARG00000012788 ENSDARG00000014091 ENSDARG00000017917 ENSDARG00000018260 ENSDARG00000018303 ENSDARG00000038792 ENSDARG00000042004 ENSDARG00000042485 ENSDARG00000044511 ENSDARG00000055381 ENSDARG00000073915 ENSDARG00000076255 ENSDARG00000088847 ENSDARG00000092329 ENSDARG00000093864 ENSDARG00000096152 ENSDARG00000096216 ENSDARG00000096456 ENSDARG00000096631 ENSDARG00000098892 ENSDARG00000098991 ENSDARG00000100479 ENSDARG00000100483 ENSDARG00000101279 ENSDARG00000101576 ENSDARG00000102316 ENSDARG00000103213 ENSDARG00000103417 ENSDARG00000103546 ENSDARG00000103581 ENSDARG00000103689 ENSDARG00000104006 ENSDARG00000104561 ENSDARG00000104697 ENSDARG00000104887 ENSDARG00000104964 | | GO:0010862 | positive regulation of pathway-restricte... | biological\_process | 22 | 0.27 | 4 | 3.3e-02 | bmp7a lft1 lft2 ndr2 | ENSDARG00000018260 ENSDARG00000019920 ENSDARG00000044059 ENSDARG00000101279 | | GO:0005634 | nucleus | cellular\_component | 1915 | 22.67 | 50 | 1.5e-09 | tbx16 esrp1 rbm39a polr3gla foxa3 osr1 her7 etv4 h3f3a ccnb3 lrwd1 klf17 zgc:113176 mid1ip1a nfya foxd5 etv5b pcf11 zgc:113983 znf1085 znf1084 zgc:113984 ano9a znf1089 si:dkey-156j15.1 wee1 si:dkey-149m13.4 si:ch211-162i8.7 si:dkeyp-82h4.3 MED9 ccne2 si:ch211-223a21.1 znf1095 glyr1 vox si:dkey-43f9.4 vent ta si:dkey-237m9.2 si:dkey-56m15.8 si:dkey-269o24.6 purbb si:ch211-212k5.1 znf1148 znf1082 znf1081 si:dkey-57k17.1 znf1105 cdkn1cb znf1093 | ENSDARG00000007329 ENSDARG00000011245 ENSDARG00000011613 ENSDARG00000012044 ENSDARG00000012788 ENSDARG00000014091 ENSDARG00000017917 ENSDARG00000018303 ENSDARG00000020504 ENSDARG00000034855 ENSDARG00000035147 ENSDARG00000038792 ENSDARG00000040487 ENSDARG00000041051 ENSDARG00000042004 ENSDARG00000042485 ENSDARG00000044511 ENSDARG00000044625 ENSDARG00000068941 ENSDARG00000073915 ENSDARG00000076255 ENSDARG00000077587 ENSDARG00000078941 ENSDARG00000088847 ENSDARG00000092329 ENSDARG00000093864 ENSDARG00000096152 ENSDARG00000096216 ENSDARG00000096456 ENSDARG00000096631 ENSDARG00000098529 ENSDARG00000098892 ENSDARG00000098991 ENSDARG00000099213 ENSDARG00000099761 ENSDARG00000100479 ENSDARG00000100483 ENSDARG00000101576 ENSDARG00000102316 ENSDARG00000103213 ENSDARG00000103417 ENSDARG00000103546 ENSDARG00000103581 ENSDARG00000103689 ENSDARG00000104006 ENSDARG00000104561 ENSDARG00000104697 ENSDARG00000104887 ENSDARG00000104903 ENSDARG00000104964 | | GO:0000786 | nucleosome | cellular\_component | 25 | 0.30 | 4 | 4.8e-02 | h3f3a zgc:113983 zgc:113984 ano9a | ENSDARG00000020504 ENSDARG00000068941 ENSDARG00000077587 ENSDARG00000078941 | | GO:0003676 | nucleic acid binding | molecular\_function | 1873 | 30.63 | 102 | 2.5e-28 | tbx16 znfl2a esrp1 rbm39a foxa3 osr1 her7 etv4 h3f3a lrwd1 hnrnpa0b zic2b klf17 zgc:113176 zgc:161969 nfya foxd5 yy1a etv5b pcf11 zgc:113983 khdrbs1b znf1085 znf1059 znf1140 si:dkey-247i3.1 znf1084 znf1041 rbm6 zgc:113984 ano9a si:dkey-7i4.5 zgc:174315 si:ch73-144d13.4 znf1055 si:dkey-7j22.4 znf1057 zgc:174704 znf1089 si:dkeyp-104f11.6 si:ch211-226o13.2 si:dkey-156k2.3 si:dkey-156j15.1 si:dkey-22h13.2 si:dkey-256i11.2 wee1 si:dkey-149m13.5 si:ch73-138e16.3 znf1067 si:dkey-4e4.1 si:dkey-149m13.4 si:dkey-54j5.2 znf1050 si:ch211-162i8.7 si:ch211-245n8.4 si:dkeyp-82h4.3 si:dkey-7i4.11 im:7143333 si:dkey-7i4.21 zgc:174263 si:dkey-7i4.13 znf1049 si:dkey-30f3.2 znf1040 si:ch211-223a21.1 rp9 znf1095 znf569l vox znf1005 zgc:174314 zgc:113209 znf1056 si:dkey-43f9.4 vent rbm12 si:ch211-223a21.4 si:dkey-233e3.3 ta znf992 si:dkey-16b10.2 si:dkey-72l17.6 si:dkey-237m9.2 si:dkey-26i24.1 si:dkey-56m15.8 si:dkeyp-35e5.10 znf1053 si:dkey-269o24.6 znf1060 si:dkey-199m13.4 purbb si:ch211-212k5.1 si:dkey-122c11.4 znf1148 zgc:173702 znf1001 znf1082 znf1081 si:dkey-57k17.1 znf1105 znf1093 zgc:173705 | ENSDARG00000007329 ENSDARG00000008333 ENSDARG00000011245 ENSDARG00000011613 ENSDARG00000012788 ENSDARG00000014091 ENSDARG00000017917 ENSDARG00000018303 ENSDARG00000020504 ENSDARG00000035147 ENSDARG00000036162 ENSDARG00000037178 ENSDARG00000038792 ENSDARG00000040487 ENSDARG00000041359 ENSDARG00000042004 ENSDARG00000042485 ENSDARG00000042796 ENSDARG00000044511 ENSDARG00000044625 ENSDARG00000068941 ENSDARG00000070475 ENSDARG00000073915 ENSDARG00000074009 ENSDARG00000076054 ENSDARG00000076252 ENSDARG00000076255 ENSDARG00000076272 ENSDARG00000077060 ENSDARG00000077587 ENSDARG00000078941 ENSDARG00000079010 ENSDARG00000079222 ENSDARG00000086223 ENSDARG00000086449 ENSDARG00000086668 ENSDARG00000088000 ENSDARG00000088375 ENSDARG00000088847 ENSDARG00000089158 ENSDARG00000089875 ENSDARG00000090160 ENSDARG00000092329 ENSDARG00000092617 ENSDARG00000093713 ENSDARG00000093864 ENSDARG00000094653 ENSDARG00000095745 ENSDARG00000096007 ENSDARG00000096026 ENSDARG00000096152 ENSDARG00000096189 ENSDARG00000096210 ENSDARG00000096216 ENSDARG00000096222 ENSDARG00000096456 ENSDARG00000096901 ENSDARG00000096948 ENSDARG00000097180 ENSDARG00000097244 ENSDARG00000098032 ENSDARG00000098071 ENSDARG00000098087 ENSDARG00000098582 ENSDARG00000098892 ENSDARG00000098942 ENSDARG00000098991 ENSDARG00000099641 ENSDARG00000099761 ENSDARG00000099917 ENSDARG00000100257 ENSDARG00000100294 ENSDARG00000100329 ENSDARG00000100479 ENSDARG00000100483 ENSDARG00000100636 ENSDARG00000100842 ENSDARG00000101460 ENSDARG00000101576 ENSDARG00000101623 ENSDARG00000101829 ENSDARG00000102008 ENSDARG00000102316 ENSDARG00000102673 ENSDARG00000103213 ENSDARG00000103250 ENSDARG00000103310 ENSDARG00000103417 ENSDARG00000103441 ENSDARG00000103471 ENSDARG00000103546 ENSDARG00000103581 ENSDARG00000103636 ENSDARG00000103689 ENSDARG00000103723 ENSDARG00000103777 ENSDARG00000104006 ENSDARG00000104561 ENSDARG00000104697 ENSDARG00000104887 ENSDARG00000104964 ENSDARG00000105137 | | GO:0000978 | RNA polymerase II core promoter proximal... | molecular\_function | 68 | 1.11 | 20 | 5.3e-18 | znf1085 znf1084 znf1089 si:dkey-156j15.1 si:dkey-149m13.4 si:ch211-162i8.7 si:dkeyp-82h4.3 si:ch211-223a21.1 znf1095 si:dkey-43f9.4 si:dkey-237m9.2 si:dkey-56m15.8 si:dkey-269o24.6 si:ch211-212k5.1 znf1148 znf1082 znf1081 si:dkey-57k17.1 znf1105 znf1093 | ENSDARG00000073915 ENSDARG00000076255 ENSDARG00000088847 ENSDARG00000092329 ENSDARG00000096152 ENSDARG00000096216 ENSDARG00000096456 ENSDARG00000098892 ENSDARG00000098991 ENSDARG00000100479 ENSDARG00000102316 ENSDARG00000103213 ENSDARG00000103417 ENSDARG00000103581 ENSDARG00000103689 ENSDARG00000104006 ENSDARG00000104561 ENSDARG00000104697 ENSDARG00000104887 ENSDARG00000104964 | | GO:0046872 | metal ion binding | molecular\_function | 2036 | 33.29 | 81 | 6.1e-26 | znfl2a osr1 ftr83 tph1b zic2b klf17 yy1a trim44 znf1085 znf1059 znf1140 si:dkey-247i3.1 znf1084 znf1041 rbm6 si:dkey-7i4.5 zgc:174315 si:ch73-144d13.4 znf1055 si:dkey-7j22.4 znf1057 zgc:174704 znf1089 si:dkeyp-104f11.6 si:ch211-226o13.2 si:dkey-156k2.3 si:dkey-156j15.1 si:dkey-22h13.2 si:dkey-256i11.2 wee1 si:dkey-149m13.5 si:ch73-138e16.3 znf1067 si:dkey-4e4.1 si:dkey-149m13.4 si:dkey-54j5.2 znf1050 si:ch211-162i8.7 si:ch211-245n8.4 si:dkeyp-82h4.3 si:dkey-7i4.11 im:7143333 si:dkey-7i4.21 zgc:174263 si:dkey-7i4.13 znf1049 si:dkey-30f3.2 znf1040 si:ch211-223a21.1 rp9 znf1095 znf569l znf1005 zgc:174314 zgc:113209 znf1056 si:dkey-43f9.4 si:ch211-223a21.4 si:dkey-233e3.3 znf992 si:dkey-16b10.2 si:dkey-72l17.6 si:dkey-237m9.2 si:dkey-26i24.1 si:dkey-56m15.8 si:dkeyp-35e5.10 znf1053 si:dkey-269o24.6 znf1060 si:dkey-199m13.4 si:ch211-212k5.1 si:dkey-122c11.4 znf1148 zgc:173702 znf1001 znf1082 znf1081 si:dkey-57k17.1 znf1105 znf1093 zgc:173705 | ENSDARG00000008333 ENSDARG00000014091 ENSDARG00000025403 ENSDARG00000036082 ENSDARG00000037178 ENSDARG00000038792 ENSDARG00000042796 ENSDARG00000051761 ENSDARG00000073915 ENSDARG00000074009 ENSDARG00000076054 ENSDARG00000076252 ENSDARG00000076255 ENSDARG00000076272 ENSDARG00000077060 ENSDARG00000079010 ENSDARG00000079222 ENSDARG00000086223 ENSDARG00000086449 ENSDARG00000086668 ENSDARG00000088000 ENSDARG00000088375 ENSDARG00000088847 ENSDARG00000089158 ENSDARG00000089875 ENSDARG00000090160 ENSDARG00000092329 ENSDARG00000092617 ENSDARG00000093713 ENSDARG00000093864 ENSDARG00000094653 ENSDARG00000095745 ENSDARG00000096007 ENSDARG00000096026 ENSDARG00000096152 ENSDARG00000096189 ENSDARG00000096210 ENSDARG00000096216 ENSDARG00000096222 ENSDARG00000096456 ENSDARG00000096901 ENSDARG00000096948 ENSDARG00000097180 ENSDARG00000097244 ENSDARG00000098032 ENSDARG00000098071 ENSDARG00000098087 ENSDARG00000098582 ENSDARG00000098892 ENSDARG00000098942 ENSDARG00000098991 ENSDARG00000099641 ENSDARG00000099917 ENSDARG00000100257 ENSDARG00000100294 ENSDARG00000100329 ENSDARG00000100479 ENSDARG00000100842 ENSDARG00000101460 ENSDARG00000101623 ENSDARG00000101829 ENSDARG00000102008 ENSDARG00000102316 ENSDARG00000102673 ENSDARG00000103213 ENSDARG00000103250 ENSDARG00000103310 ENSDARG00000103417 ENSDARG00000103441 ENSDARG00000103471 ENSDARG00000103581 ENSDARG00000103636 ENSDARG00000103689 ENSDARG00000103723 ENSDARG00000103777 ENSDARG00000104006 ENSDARG00000104561 ENSDARG00000104697 ENSDARG00000104887 ENSDARG00000104964 ENSDARG00000105137 | |

  


### Go to GO detail

## ZFA

| | ZFA ID | Description | Annotated | Expected | Observed | Fold Enrichment | Adjusted p-value | Genes | Ensembl IDs | | --- | --- | --- | --- | --- | --- | --- | --- | --- | | ZFA:0000117 | hypoblast | 112 | 1.74 | 12 | 6.9 | 7.8e-05 | mespaa klf17 akap12b zic2b aplnrb aplnra osr1 apela foxa3 ta ndr2 tbx16 | ENSDARG00000017078 ENSDARG00000038792 ENSDARG00000055678 ENSDARG00000037178 ENSDARG00000036670 ENSDARG00000002172 ENSDARG00000014091 ENSDARG00000094729 ENSDARG00000012788 ENSDARG00000101576 ENSDARG00000101279 ENSDARG00000007329 | | ZFA:0000071 | shield | 118 | 1.83 | 8 | 4.4 | 1.3e-03 | bambia ism1 osr1 foxa3 ta foxd5 etv4 lft1 | ENSDARG00000055381 ENSDARG00000020541 ENSDARG00000014091 ENSDARG00000012788 ENSDARG00000101576 ENSDARG00000042485 ENSDARG00000018303 ENSDARG00000019920 | | ZFA:0001378 | axial hypoblast | 22 | 0.34 | 4 | 11.8 | 2.1e-03 | klf17 ta ndr2 lft1 | ENSDARG00000038792 ENSDARG00000101576 ENSDARG00000101279 ENSDARG00000019920 | | ZFA:0000060 | prechordal plate | 96 | 1.49 | 9 | 6.0 | 1.2e-02 | bmp7a gpc4 aplnra foxa3 ta ndr2 tbx16 lft1 lft2 | ENSDARG00000018260 ENSDARG00000015472 ENSDARG00000002172 ENSDARG00000012788 ENSDARG00000101576 ENSDARG00000101279 ENSDARG00000007329 ENSDARG00000019920 ENSDARG00000044059 | | ZFA:0001204 | axial mesoderm | 89 | 1.38 | 6 | 4.3 | 1.9e-02 | tph1b foxa3 ta ndr2 etv4 lft1 | ENSDARG00000036082 ENSDARG00000012788 ENSDARG00000101576 ENSDARG00000101279 ENSDARG00000018303 ENSDARG00000019920 | |
