# Supplementary material for: A high-resolution mRNA expression time course of embryonic development in zebrafish
Source: eLife. 2017 Nov 16;6:e30860. doi: 10.7554/eLife.30860 (PMC5690287; doi:10.7554/eLife.30860)
Supplement: Supplementary file 6. [file elife-30860-supp6.zip › biolayout-clusters-files/Cluster008-genes.html]

Cluster008


# Cluster008: Genes

| | Ensembl ID | Gene Name | Chr | Start | End | Biotype | | --- | --- | --- | --- | --- | --- | | ENSDARG00000073976 | CABZ01051901.1 | 1 | 44604598 | 44613781 | protein\_coding | | ENSDARG00000102550 | CABZ01080370.1 | 15 | 49205 | 61507 | protein\_coding | | ENSDARG00000102800 | CABZ01081752.2 | KN150703.1 | 36969 | 40261 | protein\_coding | | ENSDARG00000101782 | CR388132.2 | 4 | 29157891 | 29166800 | protein\_coding | | ENSDARG00000011609 | ENSDARG00000011609 | 3 | 11813072 | 11861589 | protein\_coding | | ENSDARG00000073673 | ENSDARG00000073673 | 7 | 69816793 | 69833076 | protein\_coding | | ENSDARG00000074645 | ENSDARG00000074645 | 5 | 68686816 | 68713084 | protein\_coding | | ENSDARG00000075737 | ENSDARG00000075737 | 3 | 129460 | 141984 | protein\_coding | | ENSDARG00000078166 | ENSDARG00000078166 | 13 | 5114581 | 5128717 | protein\_coding | | ENSDARG00000080016 | ENSDARG00000080016 | 4 | 72206750 | 72211616 | protein\_coding | | ENSDARG00000087529 | ENSDARG00000087529 | 19 | 48400187 | 48409501 | protein\_coding | | ENSDARG00000092846 | ENSDARG00000092846 | 25 | 34592204 | 34596715 | protein\_coding | | ENSDARG00000104943 | ENSDARG00000104943 | 13 | 33342711 | 33348402 | protein\_coding | | ENSDARG00000077095 | HIPK1 (1 of many) | 11 | 2580537 | 2614625 | protein\_coding | | ENSDARG00000043571 | KIF2A | 8 | 12887647 | 12911230 | protein\_coding | | ENSDARG00000086107 | MTERF1 | 19 | 432296 | 436291 | protein\_coding | | ENSDARG00000102963 | SIRT6 | 2 | 53469010 | 53506514 | protein\_coding | | ENSDARG00000075226 | SMAD4 (1 of many).1 | 10 | 572970 | 730884 | protein\_coding | | ENSDARG00000100031 | TADA1 | 2 | 116698 | 120305 | protein\_coding | | ENSDARG00000030665 | TCERG1 (1 of many) | 21 | 41768899 | 41805011 | protein\_coding | | ENSDARG00000025467 | aatf | 5 | 55607653 | 55636125 | protein\_coding | | ENSDARG00000091433 | abhd17aa | 2 | 49304929 | 49329681 | protein\_coding | | ENSDARG00000043666 | abt1 | 8 | 8445079 | 8451140 | protein\_coding | | ENSDARG00000042623 | acbd3 | 20 | 30799646 | 30819003 | protein\_coding | | ENSDARG00000098235 | actr5 | 9 | 331792 | 340661 | protein\_coding | | ENSDARG00000060109 | aggf1 | 21 | 8004117 | 8031230 | protein\_coding | | ENSDARG00000100317 | ahsa1b | 17 | 43736751 | 43749935 | protein\_coding | | ENSDARG00000006062 | akap1b | 15 | 30959652 | 30976609 | protein\_coding | | ENSDARG00000067601 | arfgap2 | 18 | 44856355 | 44880880 | protein\_coding | | ENSDARG00000012848 | arih2 | 8 | 26015484 | 26038262 | protein\_coding | | ENSDARG00000105036 | arl6ip4 | 5 | 68960544 | 68973730 | protein\_coding | | ENSDARG00000039215 | arrb2a | 10 | 22921674 | 22937966 | protein\_coding | | ENSDARG00000011597 | atxn2l | 3 | 15244628 | 15275891 | protein\_coding | | ENSDARG00000055300 | atxn7l2a | 11 | 36117310 | 36145884 | protein\_coding | | ENSDARG00000001939 | b3gat3 | 7 | 24744009 | 24756079 | protein\_coding | | ENSDARG00000104404 | b4galt1l | 14 | 51724467 | 51751466 | protein\_coding | | ENSDARG00000021112 | c1d | 13 | 4528754 | 4535741 | protein\_coding | | ENSDARG00000042671 | c2h1orf27 | 2 | 21208829 | 21221772 | protein\_coding | | ENSDARG00000043451 | cab39 | 15 | 40130409 | 40155878 | protein\_coding | | ENSDARG00000023858 | ccdc174 | 6 | 40432237 | 40437489 | protein\_coding | | ENSDARG00000026185 | ccdc94 | 2 | 52456477 | 52470678 | protein\_coding | | ENSDARG00000017525 | ccnt1 | 23 | 35551968 | 35565702 | protein\_coding | | ENSDARG00000036510 | ccnt2b | 22 | 12132767 | 12147026 | protein\_coding | | ENSDARG00000057016 | cdc14ab | 22 | 16165001 | 16248966 | protein\_coding | | ENSDARG00000043797 | cdc5l | 17 | 4903020 | 4947809 | protein\_coding | | ENSDARG00000020201 | cdc73 | 2 | 12162955 | 12219576 | protein\_coding | | ENSDARG00000058943 | cdcp1a | 16 | 2609899 | 2632856 | protein\_coding | | ENSDARG00000044811 | cdk9 | 21 | 6031473 | 6045060 | protein\_coding | | ENSDARG00000045857 | cebpz | 20 | 3272730 | 3292516 | protein\_coding | | ENSDARG00000056473 | chaf1b | 9 | 30732393 | 30744979 | protein\_coding | | ENSDARG00000058480 | chtf18 | 1 | 8514920 | 8543093 | protein\_coding | | ENSDARG00000063663 | clp1 | 14 | 49994479 | 50010874 | protein\_coding | | ENSDARG00000061802 | cnot2 | 25 | 28235649 | 28270308 | protein\_coding | | ENSDARG00000008255 | cnot6a | 21 | 32724343 | 32748354 | protein\_coding | | ENSDARG00000008235 | cog5 | 4 | 2618672 | 2707599 | protein\_coding | | ENSDARG00000068182 | crb3b | 1 | 55511120 | 55515632 | protein\_coding | | ENSDARG00000104148 | crebbpb | 3 | 9671381 | 9755772 | protein\_coding | | ENSDARG00000100108 | cul3b | 15 | 1801694 | 1829467 | protein\_coding | | ENSDARG00000014008 | cwc22 | 9 | 43750728 | 43842474 | protein\_coding | | ENSDARG00000005774 | ddx3b | 6 | 59900573 | 59918191 | protein\_coding | | ENSDARG00000009748 | dffb | 8 | 22255959 | 22261873 | protein\_coding | | ENSDARG00000105398 | dnajc14 | 11 | 2531258 | 2540470 | protein\_coding | | ENSDARG00000067613 | dnajc25 | 5 | 69178330 | 69187449 | protein\_coding | | ENSDARG00000038243 | e4f1 | 3 | 18564403 | 18587386 | protein\_coding | | ENSDARG00000019834 | edrf1 | 17 | 8596840 | 8634892 | protein\_coding | | ENSDARG00000017439 | eif4ba | 23 | 10461918 | 10514486 | protein\_coding | | ENSDARG00000020377 | eif4g2a | 7 | 66410585 | 66430956 | protein\_coding | | ENSDARG00000100965 | ep400 | 8 | 43673372 | 43733984 | protein\_coding | | ENSDARG00000021985 | ercc2 | 15 | 23744753 | 23757094 | protein\_coding | | ENSDARG00000063417 | erf | 19 | 6222882 | 6274417 | protein\_coding | | ENSDARG00000014685 | esco2 | 20 | 39442010 | 39456275 | protein\_coding | | ENSDARG00000037708 | exosc10 | 23 | 15015902 | 15047724 | protein\_coding | | ENSDARG00000060168 | fam160b1 | 13 | 43476525 | 43503546 | protein\_coding | | ENSDARG00000074168 | fam208b | 8 | 11136325 | 11164425 | protein\_coding | | ENSDARG00000077883 | fam83d | 11 | 25090542 | 25099305 | protein\_coding | | ENSDARG00000043663 | faub | 10 | 27028382 | 27030290 | protein\_coding | | ENSDARG00000034497 | fbxl14a | 25 | 20966365 | 20968482 | protein\_coding | | ENSDARG00000076839 | ftr86 | 15 | 545650 | 555950 | protein\_coding | | ENSDARG00000007943 | gabpb2a | 19 | 7513785 | 7523023 | protein\_coding | | ENSDARG00000104483 | gipc1 | 3 | 44980692 | 44992541 | protein\_coding | | ENSDARG00000004806 | grwd1 | 16 | 13970179 | 13980779 | protein\_coding | | ENSDARG00000037706 | gss | 23 | 14987010 | 15013446 | protein\_coding | | ENSDARG00000000542 | gtf2e1 | 9 | 27880915 | 27909327 | protein\_coding | | ENSDARG00000012672 | gtf2e2 | 1 | 22161006 | 22181535 | protein\_coding | | ENSDARG00000053127 | helb | 4 | 12771102 | 12782333 | protein\_coding | | ENSDARG00000045660 | hic1l | 25 | 25469480 | 25480639 | protein\_coding | | ENSDARG00000054304 | homeza | 2 | 38289064 | 38293330 | protein\_coding | | ENSDARG00000034326 | ing3 | 4 | 10605261 | 10616506 | protein\_coding | | ENSDARG00000016811 | ints3 | 19 | 27055873 | 27089636 | protein\_coding | | ENSDARG00000067913 | ints9 | 20 | 50092005 | 50125386 | protein\_coding | | ENSDARG00000019614 | jade3 | 6 | 37500871 | 37535239 | protein\_coding | | ENSDARG00000062268 | jarid2b | 19 | 26386749 | 26572618 | protein\_coding | | ENSDARG00000029556 | kansl3 | 8 | 51321800 | 51354281 | protein\_coding | | ENSDARG00000031770 | kat7b | 12 | 5947749 | 5965354 | protein\_coding | | ENSDARG00000056050 | kctd17 | 12 | 18914506 | 18943424 | protein\_coding | | ENSDARG00000074131 | kif5ba | 2 | 43831052 | 43886561 | protein\_coding | | ENSDARG00000033099 | kif7 | 7 | 14147454 | 14201049 | protein\_coding | | ENSDARG00000060661 | klf8 | 21 | 37985520 | 38063512 | protein\_coding | | ENSDARG00000100631 | klhl12 | 8 | 28377079 | 28392045 | protein\_coding | | ENSDARG00000074346 | l3mbtl1b | 16 | 26635911 | 26654971 | protein\_coding | | ENSDARG00000008490 | ltv1 | 1 | 53205934 | 53220896 | protein\_coding | | ENSDARG00000000857 | mapk14a | 8 | 23806332 | 23839480 | protein\_coding | | ENSDARG00000032103 | mapk6 | 18 | 39191489 | 39207433 | protein\_coding | | ENSDARG00000034396 | mars | 6 | 58927009 | 58980788 | protein\_coding | | ENSDARG00000070278 | mettl14 | 1 | 19370565 | 19375218 | protein\_coding | | ENSDARG00000102587 | mios | 16 | 47345188 | 47366438 | protein\_coding | | ENSDARG00000026454 | mis12 | 5 | 58322714 | 58326524 | protein\_coding | | ENSDARG00000041155 | morf4l1 | 18 | 20044957 | 20056241 | protein\_coding | | ENSDARG00000028894 | mrgbp | 23 | 16143616 | 16160314 | protein\_coding | | ENSDARG00000007198 | mta1 | 17 | 37563223 | 37671306 | protein\_coding | | ENSDARG00000091619 | mvb12ba | 5 | 4679082 | 4750939 | protein\_coding | | ENSDARG00000020405 | napab | 18 | 44680536 | 44703361 | protein\_coding | | ENSDARG00000044597 | ncbp3 | 21 | 30959381 | 30977122 | protein\_coding | | ENSDARG00000013472 | nedd1 | 25 | 19443068 | 19459035 | protein\_coding | | ENSDARG00000103472 | nedd8l | 2 | 38305958 | 38308028 | protein\_coding | | ENSDARG00000100536 | nkrf | 14 | 32512074 | 32518659 | protein\_coding | | ENSDARG00000044143 | nol8 | 22 | 10395313 | 10411006 | protein\_coding | | ENSDARG00000033965 | nup58 | 24 | 24863033 | 24885078 | protein\_coding | | ENSDARG00000024669 | oser1 | 6 | 52717379 | 52725572 | protein\_coding | | ENSDARG00000098946 | pald1a | 12 | 48222912 | 48289031 | protein\_coding | | ENSDARG00000037140 | pfkfb1 | 23 | 26090686 | 26111428 | protein\_coding | | ENSDARG00000075252 | phrf1 | 25 | 25413417 | 25451290 | protein\_coding | | ENSDARG00000092979 | pias2 | 5 | 40876290 | 40885056 | protein\_coding | | ENSDARG00000024642 | pip5k1ab | 16 | 9835138 | 9867668 | protein\_coding | | ENSDARG00000054778 | pogza | 19 | 42863188 | 42886564 | protein\_coding | | ENSDARG00000044622 | polq | 21 | 22813046 | 22835296 | protein\_coding | | ENSDARG00000055113 | pom121 | 10 | 35225784 | 35242450 | protein\_coding | | ENSDARG00000103144 | ppp1r21 | 13 | 81454 | 127754 | protein\_coding | | ENSDARG00000090337 | pprc1 | 13 | 11875429 | 11889091 | protein\_coding | | ENSDARG00000063252 | prcc | 19 | 7588404 | 7597372 | protein\_coding | | ENSDARG00000021225 | prkci | 2 | 37383094 | 37419143 | protein\_coding | | ENSDARG00000042489 | prkrira | 10 | 32114832 | 32120545 | protein\_coding | | ENSDARG00000086425 | prpf3 | 16 | 9541721 | 9562689 | protein\_coding | | ENSDARG00000061040 | pum2 | 20 | 6045273 | 6087828 | protein\_coding | | ENSDARG00000075526 | pwwp2a | 21 | 29980662 | 29996011 | protein\_coding | | ENSDARG00000061024 | rabggta | 23 | 1693767 | 1717703 | protein\_coding | | ENSDARG00000069056 | ranbp3b | 2 | 53891161 | 53947296 | protein\_coding | | ENSDARG00000044255 | rbm18 | 10 | 9512824 | 9537385 | protein\_coding | | ENSDARG00000018077 | rbp1 | 2 | 39062698 | 39070965 | protein\_coding | | ENSDARG00000103046 | rest | 14 | 51702553 | 51717598 | protein\_coding | | ENSDARG00000013575 | rfx2 | 8 | 20040902 | 20106166 | protein\_coding | | ENSDARG00000079306 | rlim | 14 | 10983837 | 10998043 | protein\_coding | | ENSDARG00000074676 | rprd2b | 16 | 1174845 | 1187226 | protein\_coding | | ENSDARG00000058230 | rps6kb1b | 15 | 17470898 | 17488568 | protein\_coding | | ENSDARG00000071288 | samhd1 | 23 | 513709 | 533149 | protein\_coding | | ENSDARG00000079352 | sap130b | 2 | 23276704 | 23310446 | protein\_coding | | ENSDARG00000008032 | sart3 | 5 | 19944829 | 19977589 | protein\_coding | | ENSDARG00000057248 | sclt1 | 1 | 11677655 | 11710458 | protein\_coding | | ENSDARG00000099439 | serp1 | 18 | 3475229 | 3479349 | protein\_coding | | ENSDARG00000040614 | sestd1 | 9 | 43348067 | 43411442 | protein\_coding | | ENSDARG00000045294 | sh3glb1a | 24 | 28304119 | 28318743 | protein\_coding | | ENSDARG00000075884 | shprh | 17 | 7377187 | 7432128 | protein\_coding | | ENSDARG00000105479 | si:ch211-126c2.4 | 14 | 30558568 | 30565080 | protein\_coding | | ENSDARG00000045342 | si:ch211-140b10.6 | 24 | 21531747 | 21536250 | protein\_coding | | ENSDARG00000092746 | si:ch211-150o23.2 | 8 | 17751595 | 17755226 | protein\_coding | | ENSDARG00000101750 | si:ch211-196p9.1 | 7 | 19428284 | 19441794 | protein\_coding | | ENSDARG00000057681 | si:ch211-266o15.1 | 17 | 14723371 | 14772645 | protein\_coding | | ENSDARG00000010331 | si:ch211-26b3.2 | 5 | 22332556 | 22345937 | protein\_coding | | ENSDARG00000074282 | si:ch73-12o23.1 | 1 | 8815534 | 8843089 | protein\_coding | | ENSDARG00000104783 | si:ch73-221f6.4 | 1 | 57885271 | 57889928 | protein\_coding | | ENSDARG00000095332 | si:dkey-14d8.1 | 4 | 5354339 | 5363069 | protein\_coding | | ENSDARG00000078814 | si:dkey-34m19.3 | 6 | 10465969 | 10479689 | protein\_coding | | ENSDARG00000093672 | si:dkey-4c15.10 | 22 | 2374278 | 2429060 | protein\_coding | | ENSDARG00000094410 | si:dkey-4c15.14 | 22 | 2173125 | 2373297 | processed\_transcript | | ENSDARG00000068064 | sirt1 | 13 | 50035737 | 50053580 | protein\_coding | | ENSDARG00000061414 | slx4 | 22 | 26936448 | 26956680 | protein\_coding | | ENSDARG00000003827 | snapc1b | 13 | 37460601 | 37474710 | protein\_coding | | ENSDARG00000087528 | snip1 | 16 | 33976834 | 33983894 | protein\_coding | | ENSDARG00000015863 | socs6b | 2 | 30439895 | 30446812 | protein\_coding | | ENSDARG00000010563 | spopla | 9 | 10079517 | 10096631 | protein\_coding | | ENSDARG00000025220 | sppl3 | 10 | 17277406 | 17476444 | protein\_coding | | ENSDARG00000020820 | srp68 | 6 | 17928753 | 17955319 | protein\_coding | | ENSDARG00000037929 | strada | 3 | 31765315 | 31785676 | protein\_coding | | ENSDARG00000055753 | suv39h1b | 8 | 25731824 | 25742405 | protein\_coding | | ENSDARG00000028321 | synj2bp | 13 | 36431373 | 36440428 | protein\_coding | | ENSDARG00000102307 | taf2 | 16 | 9689797 | 9715382 | protein\_coding | | ENSDARG00000018325 | taf5 | 1 | 48770861 | 48787727 | protein\_coding | | ENSDARG00000025808 | taf5l | 1 | 53249352 | 53259096 | protein\_coding | | ENSDARG00000077870 | taf9 | 21 | 38676340 | 38682935 | protein\_coding | | ENSDARG00000056886 | tbccd1 | 9 | 27930222 | 27939190 | protein\_coding | | ENSDARG00000102365 | tceb3 | 19 | 44227121 | 44238148 | protein\_coding | | ENSDARG00000013453 | tdrd9 | 13 | 31271617 | 31292896 | protein\_coding | | ENSDARG00000036055 | thap11 | 7 | 28770315 | 28771925 | protein\_coding | | ENSDARG00000056517 | thoc3 | 14 | 25208223 | 25213710 | protein\_coding | | ENSDARG00000077950 | tor1 | 21 | 3624130 | 3635904 | protein\_coding | | ENSDARG00000002031 | tox4a | 2 | 38085361 | 38098331 | protein\_coding | | ENSDARG00000090821 | tp53rk | 11 | 2338433 | 2343404 | protein\_coding | | ENSDARG00000019137 | tram1 | 24 | 14070412 | 14090405 | protein\_coding | | ENSDARG00000021021 | trim35-28 | 16 | 26566076 | 26572879 | protein\_coding | | ENSDARG00000102846 | trmt13 | 22 | 16285470 | 16291616 | protein\_coding | | ENSDARG00000100623 | trrap | 12 | 17805997 | 17983242 | protein\_coding | | ENSDARG00000041361 | ttk | 16 | 5210829 | 5243550 | protein\_coding | | ENSDARG00000063588 | uba5 | 24 | 9487823 | 9549544 | protein\_coding | | ENSDARG00000013990 | ube2q2 | 25 | 28984846 | 29020944 | protein\_coding | | ENSDARG00000037017 | ube4b | 23 | 29580748 | 29627157 | protein\_coding | | ENSDARG00000100508 | ubn2b | 3 | 7954040 | 8011465 | protein\_coding | | ENSDARG00000103422 | usp10 | 7 | 68919986 | 68961689 | protein\_coding | | ENSDARG00000060633 | usp16 | 15 | 41333552 | 41353899 | protein\_coding | | ENSDARG00000098433 | utp6 | 6 | 22053123 | 22060439 | protein\_coding | | ENSDARG00000058605 | vsig10 | 5 | 12062665 | 12086671 | protein\_coding | | ENSDARG00000012577 | waca | 12 | 23250323 | 23481699 | protein\_coding | | ENSDARG00000041113 | wdr18 | 11 | 13584498 | 13731792 | protein\_coding | | ENSDARG00000090581 | wdr4 | 9 | 19666818 | 19678395 | protein\_coding | | ENSDARG00000096411 | wu:fc32g12 | 6 | 6512200 | 6516644 | protein\_coding | | ENSDARG00000073811 | wu:fi34b01 | 2 | 9849468 | 9854239 | protein\_coding | | ENSDARG00000077299 | yars2 | 25 | 2172621 | 2185896 | protein\_coding | | ENSDARG00000005941 | yes1 | 2 | 31287864 | 31380189 | protein\_coding | | ENSDARG00000027978 | yy1b | 20 | 54633023 | 54646040 | protein\_coding | | ENSDARG00000039263 | zbtb48 | 23 | 25014758 | 25028614 | protein\_coding | | ENSDARG00000041402 | zc3h14 | 20 | 46538860 | 46619357 | protein\_coding | | ENSDARG00000019484 | zdhhc7 | 18 | 5199243 | 5215664 | protein\_coding | | ENSDARG00000035570 | zgc:101016 | 5 | 25126326 | 25133592 | protein\_coding | | ENSDARG00000078075 | zgc:101577 | 6 | 54316900 | 54340460 | protein\_coding | | ENSDARG00000035221 | zgc:110249 | 3 | 68122 | 210085 | protein\_coding | | ENSDARG00000055889 | zgc:153215 | 16 | 27626491 | 27631929 | protein\_coding | | ENSDARG00000104611 | zgc:173517 | 3 | 7492049 | 7601598 | protein\_coding | | ENSDARG00000096528 | zgc:174928.1 | 1 | 57873296 | 57877805 | protein\_coding | | ENSDARG00000102033 | zgc:193790 | 21 | 2310465 | 2316113 | protein\_coding | | ENSDARG00000104285 | zgc:63733 | 22 | 35037694 | 35087777 | protein\_coding | | ENSDARG00000054307 | zgc:77151 | 21 | 37300022 | 37342059 | protein\_coding | | ENSDARG00000046071 | zgc:77838 | 21 | 4858484 | 4901897 | protein\_coding | | ENSDARG00000019774 | zhx3 | 23 | 2929159 | 2957326 | protein\_coding | | ENSDARG00000035910 | znf281b | 12 | 16038267 | 16053612 | protein\_coding | | ENSDARG00000105370 | znf628 | 16 | 12730821 | 12738889 | protein\_coding | | ENSDARG00000070939 | znf740a | 11 | 2229561 | 2274410 | protein\_coding | |
