# Supplementary material for: A high-resolution mRNA expression time course of embryonic development in zebrafish
Source: eLife. 2017 Nov 16;6:e30860. doi: 10.7554/eLife.30860 (PMC5690287; doi:10.7554/eLife.30860)
Supplement: Supplementary file 6. [file elife-30860-supp6.zip › biolayout-clusters-files/Cluster008.html]

Cluster008


# Cluster008: Detail

### Go to ZFA detail

## GO

| | GO ID | Description | Domain | Annotated | Expected | Observed | Adjusted p-value | Genes | Ensembl IDs | | --- | --- | --- | --- | --- | --- | --- | --- | --- | | GO:0005634 | nucleus | cellular\_component | 1915 | 32 | 69 | 2.5e-04 | mapk14a grwd1 mta1 sart3 dffb spopla ENSDARG00000011609 waca gtf2e2 arih2 tdrd9 rfx2 cwc22 esco2 ints3 ccnt1 taf5 zhx3 cdc73 c1d prkci ercc2 aatf taf5l mis12 mrgbp kansl3 kat7b nup58 ing3 thap11 ccnt2b exosc10 e4f1 morf4l1 zc3h14 abt1 ncbp3 helb homeza zgc:77151 suv39h1b chaf1b thoc3 cdc14ab slx4 cnot2 jarid2b erf ints9 mettl14 samhd1 l3mbtl1b SMAD4 (1 of many) shprh tor1 prpf3 pprc1 wdr4 tp53rk actr5 utp6 pald1a ubn2b taf2 tceb3 rest crebbpb arl6ip4 | ENSDARG00000000857 ENSDARG00000004806 ENSDARG00000007198 ENSDARG00000008032 ENSDARG00000009748 ENSDARG00000010563 ENSDARG00000011609 ENSDARG00000012577 ENSDARG00000012672 ENSDARG00000012848 ENSDARG00000013453 ENSDARG00000013575 ENSDARG00000014008 ENSDARG00000014685 ENSDARG00000016811 ENSDARG00000017525 ENSDARG00000018325 ENSDARG00000019774 ENSDARG00000020201 ENSDARG00000021112 ENSDARG00000021225 ENSDARG00000021985 ENSDARG00000025467 ENSDARG00000025808 ENSDARG00000026454 ENSDARG00000028894 ENSDARG00000029556 ENSDARG00000031770 ENSDARG00000033965 ENSDARG00000034326 ENSDARG00000036055 ENSDARG00000036510 ENSDARG00000037708 ENSDARG00000038243 ENSDARG00000041155 ENSDARG00000041402 ENSDARG00000043666 ENSDARG00000044597 ENSDARG00000053127 ENSDARG00000054304 ENSDARG00000054307 ENSDARG00000055753 ENSDARG00000056473 ENSDARG00000056517 ENSDARG00000057016 ENSDARG00000061414 ENSDARG00000061802 ENSDARG00000062268 ENSDARG00000063417 ENSDARG00000067913 ENSDARG00000070278 ENSDARG00000071288 ENSDARG00000074346 ENSDARG00000075226 ENSDARG00000075884 ENSDARG00000077950 ENSDARG00000086425 ENSDARG00000090337 ENSDARG00000090581 ENSDARG00000090821 ENSDARG00000098235 ENSDARG00000098433 ENSDARG00000098946 ENSDARG00000100508 ENSDARG00000102307 ENSDARG00000102365 ENSDARG00000103046 ENSDARG00000104148 ENSDARG00000105036 | | GO:0003676 | nucleic acid binding | molecular\_function | 1873 | 32 | 63 | 8.6e-06 | gtf2e1 ddx3b akap1b mta1 sart3 atxn2l ENSDARG00000011609 gtf2e2 arih2 tdrd9 rfx2 cwc22 eif4ba zhx3 eif4g2a srp68 c1d ercc2 yy1b zgc:110249 znf281b thap11 exosc10 e4f1 zbtb48 zc3h14 prkrira abt1 cdc5l nol8 rbm18 ncbp3 polq hic1l helb homeza zgc:77151 pogza zgc:153215 aggf1 klf8 pum2 jarid2b erf mettl14 znf740a samhd1 SMAD4 (1 of many) shprh yars2 si:dkey-34m19.3 pprc1 si:dkey-4c15.10 si:dkey-14d8.1 zgc:174928 utp6 nkrf zgc:193790 taf2 tceb3 rest zgc:173517 si:ch73-221f6.4 | ENSDARG00000000542 ENSDARG00000005774 ENSDARG00000006062 ENSDARG00000007198 ENSDARG00000008032 ENSDARG00000011597 ENSDARG00000011609 ENSDARG00000012672 ENSDARG00000012848 ENSDARG00000013453 ENSDARG00000013575 ENSDARG00000014008 ENSDARG00000017439 ENSDARG00000019774 ENSDARG00000020377 ENSDARG00000020820 ENSDARG00000021112 ENSDARG00000021985 ENSDARG00000027978 ENSDARG00000035221 ENSDARG00000035910 ENSDARG00000036055 ENSDARG00000037708 ENSDARG00000038243 ENSDARG00000039263 ENSDARG00000041402 ENSDARG00000042489 ENSDARG00000043666 ENSDARG00000043797 ENSDARG00000044143 ENSDARG00000044255 ENSDARG00000044597 ENSDARG00000044622 ENSDARG00000045660 ENSDARG00000053127 ENSDARG00000054304 ENSDARG00000054307 ENSDARG00000054778 ENSDARG00000055889 ENSDARG00000060109 ENSDARG00000060661 ENSDARG00000061040 ENSDARG00000062268 ENSDARG00000063417 ENSDARG00000070278 ENSDARG00000070939 ENSDARG00000071288 ENSDARG00000075226 ENSDARG00000075884 ENSDARG00000077299 ENSDARG00000078814 ENSDARG00000090337 ENSDARG00000093672 ENSDARG00000095332 ENSDARG00000096528 ENSDARG00000098433 ENSDARG00000100536 ENSDARG00000102033 ENSDARG00000102307 ENSDARG00000102365 ENSDARG00000103046 ENSDARG00000104611 ENSDARG00000104783 | |
