# Supplementary material for: A high-resolution mRNA expression time course of embryonic development in zebrafish
Source: eLife. 2017 Nov 16;6:e30860. doi: 10.7554/eLife.30860 (PMC5690287; doi:10.7554/eLife.30860)
Supplement: Supplementary file 6. [file elife-30860-supp6.zip › biolayout-clusters-files/Cluster009-genes.html]

Cluster009


# Cluster009: Genes

| | Ensembl ID | Gene Name | Chr | Start | End | Biotype | | --- | --- | --- | --- | --- | --- | | ENSDARG00000080337 | AC024175.4 | MT | 2043 | 3725 | Mt\_rRNA | | ENSDARG00000101316 | CABZ01104045.1 | KN149734.1 | 1077 | 3890 | protein\_coding | | ENSDARG00000030508 | CCDC134 (1 of many) | 3 | 662601 | 671629 | protein\_coding | | ENSDARG00000057890 | ENSDARG00000057890 | 13 | 9007896 | 9036512 | protein\_coding | | ENSDARG00000069251 | ENSDARG00000069251 | 20 | 43839944 | 43846622 | protein\_coding | | ENSDARG00000074764 | ENSDARG00000074764 | 3 | 3687347 | 3699510 | protein\_coding | | ENSDARG00000077405 | ENSDARG00000077405 | 23 | 45487012 | 45605143 | protein\_coding | | ENSDARG00000079034 | ENSDARG00000079034 | 7 | 20325726 | 20330953 | protein\_coding | | ENSDARG00000098911 | ENSDARG00000098911 | 25 | 3381634 | 3392855 | protein\_coding | | ENSDARG00000100660 | ENSDARG00000100660 | 4 | 16217 | 28312 | protein\_coding | | ENSDARG00000093374 | EPB41L2 | 20 | 2260592 | 2391775 | protein\_coding | | ENSDARG00000077605 | FRRS1 (1 of many) | 24 | 29902374 | 29925048 | protein\_coding | | ENSDARG00000000887 | GTSF1 (1 of many) | 23 | 32174671 | 32181077 | protein\_coding | | ENSDARG00000090072 | KRTCAP3 | 17 | 8421432 | 8435352 | protein\_coding | | ENSDARG00000045542 | LAMTOR4 | 4 | 20062502 | 20065607 | protein\_coding | | ENSDARG00000092671 | PARD3B (1 of many) | 1 | 5100815 | 5387972 | protein\_coding | | ENSDARG00000099396 | RBPMS | 7 | 73887220 | 73890702 | protein\_coding | | ENSDARG00000053468 | adam10a | 7 | 30137782 | 30220896 | protein\_coding | | ENSDARG00000095947 | adkb | 12 | 46826210 | 46851821 | protein\_coding | | ENSDARG00000074667 | akt1s1 | 3 | 30059577 | 30069715 | protein\_coding | | ENSDARG00000044301 | atf1 | 6 | 39511898 | 39524296 | protein\_coding | | ENSDARG00000052073 | blcap | 6 | 51769761 | 51771635 | protein\_coding | | ENSDARG00000011299 | blzf1 | 1 | 496822 | 510441 | protein\_coding | | ENSDARG00000025071 | brd8 | 14 | 7391285 | 7433649 | protein\_coding | | ENSDARG00000035540 | brwd3 | 5 | 22470183 | 22517004 | protein\_coding | | ENSDARG00000019881 | bsg | 22 | 18904449 | 18921486 | protein\_coding | | ENSDARG00000094590 | c2cd5 | 18 | 6498662 | 6744560 | protein\_coding | | ENSDARG00000013312 | cadps2 | 25 | 27494383 | 27826751 | protein\_coding | | ENSDARG00000044938 | cbx2 | 3 | 10182781 | 10195448 | protein\_coding | | ENSDARG00000011094 | ccna2 | 14 | 47357470 | 47387584 | protein\_coding | | ENSDARG00000027357 | cd2bp2 | 3 | 32700328 | 32710669 | protein\_coding | | ENSDARG00000012627 | cdc34b | 2 | 49273362 | 49297073 | protein\_coding | | ENSDARG00000009942 | cdc6 | 11 | 11799843 | 11809145 | protein\_coding | | ENSDARG00000077620 | cdca7a | 9 | 2873743 | 2886230 | protein\_coding | | ENSDARG00000038802 | cenpl | 2 | 34145520 | 34155182 | protein\_coding | | ENSDARG00000002190 | chmp2bb | 1 | 32825987 | 32836677 | protein\_coding | | ENSDARG00000029856 | cnn3b | 2 | 15379820 | 15406705 | protein\_coding | | ENSDARG00000070261 | crlf3 | 3 | 35682532 | 35717606 | protein\_coding | | ENSDARG00000044820 | cstf1 | 11 | 27570901 | 27580388 | protein\_coding | | ENSDARG00000102441 | ctnna1 | 24 | 34602051 | 34794691 | protein\_coding | | ENSDARG00000023472 | ctnnb2 | 19 | 48326047 | 48346964 | protein\_coding | | ENSDARG00000044345 | cyfip1 | 6 | 37677184 | 37753013 | protein\_coding | | ENSDARG00000007349 | dmrt1 | 5 | 44344664 | 44391191 | protein\_coding | | ENSDARG00000004680 | dnajb6a | 2 | 30119752 | 30132934 | protein\_coding | | ENSDARG00000104959 | dnajc17 | 17 | 626928 | 971042 | protein\_coding | | ENSDARG00000069995 | dpy19l1l | 16 | 42763802 | 42779743 | protein\_coding | | ENSDARG00000105098 | dr1 | 6 | 23955612 | 23966867 | protein\_coding | | ENSDARG00000041203 | drap1 | 7 | 605715 | 616429 | protein\_coding | | ENSDARG00000070795 | eaf1 | 16 | 7003633 | 7014867 | protein\_coding | | ENSDARG00000010432 | eaf2 | 9 | 23192339 | 23197072 | protein\_coding | | ENSDARG00000063140 | exd3 | 10 | 10921262 | 11011219 | protein\_coding | | ENSDARG00000039020 | fbxo5 | 13 | 46652095 | 46665352 | protein\_coding | | ENSDARG00000056410 | fdx1 | 9 | 29709950 | 29724707 | protein\_coding | | ENSDARG00000022968 | fxr1 | 22 | 37607823 | 37630492 | protein\_coding | | ENSDARG00000040059 | gbp | 16 | 41004449 | 41005807 | protein\_coding | | ENSDARG00000076962 | gdpd5b | 15 | 29455034 | 29551636 | protein\_coding | | ENSDARG00000057676 | golga7 | 10 | 20406774 | 20414761 | protein\_coding | | ENSDARG00000091931 | gpatch4 | 16 | 29090669 | 29096051 | protein\_coding | | ENSDARG00000099358 | hccsb | 24 | 31144688 | 31155500 | protein\_coding | | ENSDARG00000086458 | hdac10 | 25 | 4888591 | 4906636 | protein\_coding | | ENSDARG00000003021 | hdac8 | 7 | 51367586 | 51434924 | protein\_coding | | ENSDARG00000022303 | higd1a | 2 | 1825741 | 1830943 | protein\_coding | | ENSDARG00000031198 | hmg20a | 25 | 7147635 | 7162306 | protein\_coding | | ENSDARG00000014569 | hnrnpr | 19 | 74225 | 82426 | protein\_coding | | ENSDARG00000075570 | ide | 13 | 42183628 | 42224100 | protein\_coding | | ENSDARG00000092181 | im:7140055 | 20 | 51248475 | 51264716 | protein\_coding | | ENSDARG00000026489 | khsrp | 1 | 54282538 | 54294585 | protein\_coding | | ENSDARG00000102624 | kif22 | 12 | 5013087 | 5036879 | protein\_coding | | ENSDARG00000076464 | lamtor1 | 15 | 44046695 | 44054447 | protein\_coding | | ENSDARG00000017315 | larp7 | 7 | 59267786 | 59284453 | protein\_coding | | ENSDARG00000042561 | lpar2b | 1 | 58415318 | 58439198 | protein\_coding | | ENSDARG00000033852 | mad1l1 | 1 | 9055259 | 9174410 | protein\_coding | | ENSDARG00000033231 | mcm6l | 2 | 50465508 | 50491283 | protein\_coding | | ENSDARG00000099431 | med24 | 12 | 10605398 | 10636606 | protein\_coding | | ENSDARG00000038005 | med25 | 3 | 30323507 | 30357273 | protein\_coding | | ENSDARG00000010553 | mmgt1 | 14 | 31275263 | 31279094 | protein\_coding | | ENSDARG00000043685 | nabp1b | 6 | 8463138 | 8469163 | protein\_coding | | ENSDARG00000070109 | ncapg | 1 | 22868739 | 22882491 | protein\_coding | | ENSDARG00000014898 | ncbp2 | 22 | 38943980 | 38948244 | protein\_coding | | ENSDARG00000061062 | nemp2 | 9 | 41657972 | 41679863 | protein\_coding | | ENSDARG00000070151 | nfyc | 19 | 42486675 | 42549522 | protein\_coding | | ENSDARG00000033450 | nkap | 14 | 33078186 | 33084919 | protein\_coding | | ENSDARG00000005108 | oclna | 10 | 2897604 | 2915729 | protein\_coding | | ENSDARG00000103959 | pak1 | 18 | 3268382 | 3312641 | protein\_coding | | ENSDARG00000024681 | pane1 | 1 | 54483432 | 54488960 | protein\_coding | | ENSDARG00000030237 | pgrmc2 | 17 | 26785079 | 26795239 | protein\_coding | | ENSDARG00000035133 | pho | 5 | 61656480 | 61672957 | protein\_coding | | ENSDARG00000031020 | pip4k2ca | 23 | 36494070 | 36522695 | protein\_coding | | ENSDARG00000038754 | plk3 | 2 | 35621180 | 35629332 | protein\_coding | | ENSDARG00000033126 | prkrip1 | 5 | 58564712 | 58578565 | protein\_coding | | ENSDARG00000068261 | pros1 | 1 | 348113 | 358987 | protein\_coding | | ENSDARG00000053101 | prpf38b | 2 | 45802296 | 45810947 | protein\_coding | | ENSDARG00000011683 | prtfdc1 | 2 | 12471976 | 12496870 | protein\_coding | | ENSDARG00000012234 | psme3 | 12 | 27115368 | 27122688 | protein\_coding | | ENSDARG00000012588 | ptdss1a | 16 | 53391579 | 53415626 | protein\_coding | | ENSDARG00000035676 | ptp4a2b | 19 | 35871702 | 35905616 | protein\_coding | | ENSDARG00000061048 | ranbp9 | 2 | 31823384 | 31844742 | protein\_coding | | ENSDARG00000037919 | rbbp6 | 3 | 35276597 | 35291688 | protein\_coding | | ENSDARG00000006395 | rbm25b | 20 | 51318713 | 51373345 | protein\_coding | | ENSDARG00000037158 | rcc1 | 16 | 34231583 | 34240972 | protein\_coding | | ENSDARG00000012747 | rer1 | 8 | 48953701 | 48970839 | protein\_coding | | ENSDARG00000099709 | rhoac | 23 | 893900 | 903470 | protein\_coding | | ENSDARG00000014577 | rhpn2 | 7 | 38078407 | 38120872 | protein\_coding | | ENSDARG00000016830 | rimkla | 8 | 26848966 | 26868169 | protein\_coding | | ENSDARG00000032997 | rnf185 | 5 | 1110042 | 1125794 | protein\_coding | | ENSDARG00000020711 | rrm2 | 20 | 29589590 | 29596547 | protein\_coding | | ENSDARG00000078069 | rrm2.1 | 19 | 48003903 | 48010821 | protein\_coding | | ENSDARG00000015543 | s100a1 | 16 | 29450942 | 29452592 | protein\_coding | | ENSDARG00000027595 | selt1b | 18 | 41537685 | 41549064 | protein\_coding | | ENSDARG00000010721 | sept6 | 14 | 32957467 | 32987717 | protein\_coding | | ENSDARG00000032606 | sept8a | 21 | 43333509 | 43379717 | protein\_coding | | ENSDARG00000029353 | serpine2 | 15 | 43360809 | 43403043 | protein\_coding | | ENSDARG00000105231 | setd8a | 10 | 44446445 | 44455688 | protein\_coding | | ENSDARG00000019963 | sfxn1 | 14 | 50090849 | 50122523 | protein\_coding | | ENSDARG00000097973 | si:ch1073-190k2.1 | 15 | 46098135 | 46100286 | protein\_coding | | ENSDARG00000093216 | si:ch211-146l10.7 | 24 | 9854691 | 9857562 | protein\_coding | | ENSDARG00000097385 | si:ch211-15d5.12 | 17 | 24682670 | 24685377 | protein\_coding | | ENSDARG00000007204 | si:ch211-195b21.5 | 17 | 23202324 | 23212998 | protein\_coding | | ENSDARG00000099044 | si:ch211-199m8.2 | 11 | 29319572 | 29324354 | processed\_transcript | | ENSDARG00000045847 | si:ch211-214j24.10 | 4 | 5308913 | 5316766 | protein\_coding | | ENSDARG00000088713 | si:ch211-226h8.14 | 22 | 25307696 | 25308923 | protein\_coding | | ENSDARG00000086221 | si:ch211-226h8.4 | 22 | 25113298 | 25210211 | protein\_coding | | ENSDARG00000089765 | si:ch211-286b5.4 | 1 | 54572456 | 54586089 | protein\_coding | | ENSDARG00000100697 | si:ch73-52f24.4 | 16 | 51328512 | 51349886 | processed\_transcript | | ENSDARG00000104636 | si:dkey-112a7.4 | 7 | 22358745 | 22361781 | protein\_coding | | ENSDARG00000097948 | si:dkey-151m15.7 | 3 | 29704429 | 29732738 | antisense | | ENSDARG00000008049 | si:dkey-42i9.4 | 22 | 10510764 | 10512030 | protein\_coding | | ENSDARG00000069401 | si:dkeyp-114g9.1 | 20 | 42070202 | 42095768 | protein\_coding | | ENSDARG00000012002 | slc38a7 | 7 | 51520671 | 51539701 | protein\_coding | | ENSDARG00000037238 | smad5 | 14 | 26169095 | 26200091 | protein\_coding | | ENSDARG00000038882 | smc4 | 15 | 1569735 | 1608220 | protein\_coding | | ENSDARG00000099913 | snrnp35 | 10 | 44456590 | 44793486 | protein\_coding | | ENSDARG00000013800 | snrpd3 | 8 | 30733666 | 30736366 | protein\_coding | | ENSDARG00000069601 | snx30 | 10 | 4940461 | 4961625 | protein\_coding | | ENSDARG00000011555 | spag7 | 5 | 67380962 | 67395630 | protein\_coding | | ENSDARG00000069476 | spint2 | 15 | 20303409 | 20314784 | protein\_coding | | ENSDARG00000017809 | stard3 | 19 | 5052461 | 5069510 | protein\_coding | | ENSDARG00000075030 | stx10 | 11 | 30927095 | 30939791 | protein\_coding | | ENSDARG00000007720 | sub1b | 5 | 40543410 | 40551177 | protein\_coding | | ENSDARG00000098141 | supt3h | 17 | 5052359 | 5267079 | protein\_coding | | ENSDARG00000070834 | taf13 | 23 | 20502496 | 20503398 | protein\_coding | | ENSDARG00000070471 | tarbp2 | 6 | 46723658 | 46733498 | protein\_coding | | ENSDARG00000037503 | thoc2 | 14 | 12085892 | 12180608 | protein\_coding | | ENSDARG00000038290 | thoc5 | 25 | 3634016 | 3659907 | protein\_coding | | ENSDARG00000002909 | tjp3 | 22 | 17632632 | 17663895 | protein\_coding | | ENSDARG00000026908 | tmed2 | 10 | 3419528 | 3427326 | protein\_coding | | ENSDARG00000102746 | tmem39a | 1 | 39313 | 44565 | protein\_coding | | ENSDARG00000069433 | tnk1 | 7 | 26274297 | 26293591 | protein\_coding | | ENSDARG00000043484 | ube2d2 | 14 | 7585612 | 7595079 | protein\_coding | | ENSDARG00000040286 | ubl7b | 18 | 50527305 | 50535988 | protein\_coding | | ENSDARG00000103409 | uhrf1 | 22 | 3955795 | 3986463 | protein\_coding | | ENSDARG00000032327 | usp36 | 3 | 57513132 | 57560267 | protein\_coding | | ENSDARG00000012450 | vmp1 | 15 | 17407297 | 17437330 | protein\_coding | | ENSDARG00000037334 | wdr92 | 13 | 1001457 | 1010880 | protein\_coding | | ENSDARG00000070709 | wu:fi42e03 | 11 | 29290370 | 29292977 | protein\_coding | | ENSDARG00000091652 | xrn1 | 2 | 16928421 | 16978669 | protein\_coding | | ENSDARG00000055510 | ypel3 | 3 | 26055282 | 26061089 | protein\_coding | | ENSDARG00000069671 | zgc:123096 | 9 | 24310167 | 24313062 | protein\_coding | | ENSDARG00000032156 | zgc:171776 | 9 | 8418425 | 8420789 | protein\_coding | | ENSDARG00000076476 | zgc:173770 | 4 | 75550814 | 75557022 | protein\_coding | | ENSDARG00000100254 | zgc:85789 | 5 | 61645947 | 61654645 | protein\_coding | | ENSDARG00000060113 | znf395a | 17 | 16082227 | 16100451 | protein\_coding | | ENSDARG00000105346 | zp2.2 | 20 | 54420785 | 54422602 | protein\_coding | | ENSDARG00000042130 | zp3a.2 | 20 | 34084586 | 34086548 | protein\_coding | | ENSDARG00000059252 | zpcx | 17 | 25309960 | 25313056 | protein\_coding | |
