# Supplementary material for: A high-resolution mRNA expression time course of embryonic development in zebrafish
Source: eLife. 2017 Nov 16;6:e30860. doi: 10.7554/eLife.30860 (PMC5690287; doi:10.7554/eLife.30860)
Supplement: Supplementary file 6. [file elife-30860-supp6.zip › biolayout-clusters-files/Cluster009.html]

Cluster009


# Cluster009: Detail

### Go to ZFA detail

## GO

| | GO ID | Description | Domain | Annotated | Expected | Observed | Adjusted p-value | Genes | Ensembl IDs | | --- | --- | --- | --- | --- | --- | --- | --- | --- | | GO:0046982 | protein heterodimerization activity | molecular\_function | 72 | 0.89 | 7 | 0.0071 | smad5 drap1 atf1 nfyc taf13 supt3h dr1 | ENSDARG00000037238 ENSDARG00000041203 ENSDARG00000044301 ENSDARG00000070151 ENSDARG00000070834 ENSDARG00000098141 ENSDARG00000105098 | |
