# Supplementary material for: A high-resolution mRNA expression time course of embryonic development in zebrafish
Source: eLife. 2017 Nov 16;6:e30860. doi: 10.7554/eLife.30860 (PMC5690287; doi:10.7554/eLife.30860)
Supplement: Supplementary file 6. [file elife-30860-supp6.zip › biolayout-clusters-files/Cluster010-genes.html]

Cluster010


# Cluster010: Genes

| | Ensembl ID | Gene Name | Chr | Start | End | Biotype | | --- | --- | --- | --- | --- | --- | | ENSDARG00000087822 | AGAP3 | 24 | 34252773 | 34449300 | protein\_coding | | ENSDARG00000079681 | CABZ01059627.2 | 10 | 19616546 | 19618372 | protein\_coding | | ENSDARG00000103158 | CABZ01078098.1 | 11 | 44014334 | 44026831 | protein\_coding | | ENSDARG00000090617 | CTIF | 21 | 3090902 | 3189840 | protein\_coding | | ENSDARG00000008835 | ENSDARG00000008835 | 3 | 3619979 | 3665840 | protein\_coding | | ENSDARG00000068971 | ENSDARG00000068971 | 5 | 6375795 | 6385002 | protein\_coding | | ENSDARG00000076771 | ENSDARG00000076771 | 3 | 3633668 | 3646691 | protein\_coding | | ENSDARG00000088862 | ENSDARG00000088862 | 17 | 50719992 | 50751012 | protein\_coding | | ENSDARG00000090625 | ENSDARG00000090625 | 3 | 32440215 | 32451660 | protein\_coding | | ENSDARG00000103296 | ENSDARG00000103296 | 21 | 196699 | 204634 | protein\_coding | | ENSDARG00000036489 | MAF1 | 7 | 39812625 | 39820999 | protein\_coding | | ENSDARG00000104777 | MOV10L1 | 2 | 168264 | 179584 | protein\_coding | | ENSDARG00000079723 | TNRC6B (1 of many) | 3 | 3552914 | 3590006 | protein\_coding | | ENSDARG00000063297 | abcb6a | 1 | 5437398 | 5460831 | protein\_coding | | ENSDARG00000075627 | abhd10b | 24 | 21782013 | 21789056 | protein\_coding | | ENSDARG00000018968 | acvr1ba | 23 | 27873978 | 27896379 | protein\_coding | | ENSDARG00000052142 | acvr1bb | 6 | 2040703 | 2065438 | protein\_coding | | ENSDARG00000062785 | adpgk2 | 17 | 11291605 | 11312634 | protein\_coding | | ENSDARG00000099579 | aldh18a1 | 12 | 30674010 | 30694031 | protein\_coding | | ENSDARG00000017617 | alg6 | 6 | 32078180 | 32100197 | protein\_coding | | ENSDARG00000013476 | arhgef39 | 4 | 12414356 | 12478379 | protein\_coding | | ENSDARG00000012684 | atp2b1a | 4 | 16077964 | 16125664 | protein\_coding | | ENSDARG00000010301 | b4galt6 | 20 | 6120074 | 6153511 | protein\_coding | | ENSDARG00000102974 | baz2a | 23 | 6739474 | 6793703 | protein\_coding | | ENSDARG00000099148 | bzw1b | 9 | 426759 | 434282 | protein\_coding | | ENSDARG00000103317 | capn5a | 18 | 3378658 | 3430601 | protein\_coding | | ENSDARG00000038302 | ccar2 | 10 | 2848825 | 2871231 | protein\_coding | | ENSDARG00000004636 | ccdc9 | 15 | 26971759 | 26998665 | protein\_coding | | ENSDARG00000063726 | cdk12 | 19 | 4939865 | 4988856 | protein\_coding | | ENSDARG00000051854 | cdt1 | 7 | 55217108 | 55229201 | protein\_coding | | ENSDARG00000052703 | cisd2 | 1 | 42985087 | 42990977 | protein\_coding | | ENSDARG00000097598 | clocka.1 | 20 | 22168487 | 22170421 | antisense | | ENSDARG00000019498 | cry5 | 10 | 39347036 | 39362396 | protein\_coding | | ENSDARG00000003382 | cwc25 | 2 | 5797542 | 5811904 | protein\_coding | | ENSDARG00000000729 | daxx | 19 | 7095394 | 7114745 | protein\_coding | | ENSDARG00000056923 | dbr1 | 9 | 28049586 | 28057521 | protein\_coding | | ENSDARG00000019743 | dctn1a | 7 | 903916 | 954058 | protein\_coding | | ENSDARG00000006477 | ddi2 | 6 | 40557015 | 40565973 | protein\_coding | | ENSDARG00000014373 | ddx4 | 10 | 6925423 | 6946874 | protein\_coding | | ENSDARG00000022614 | ddx43 | 13 | 27202206 | 27223583 | protein\_coding | | ENSDARG00000041394 | dnajb1b | 1 | 55068227 | 55072557 | protein\_coding | | ENSDARG00000017272 | eapp | 17 | 9795574 | 9806534 | protein\_coding | | ENSDARG00000040021 | ebag9 | 16 | 39235071 | 39245286 | protein\_coding | | ENSDARG00000053840 | ei24 | 10 | 39272734 | 39287976 | protein\_coding | | ENSDARG00000014565 | eif4e1b | 5 | 67110293 | 67121906 | protein\_coding | | ENSDARG00000038639 | elovl6l | 13 | 21536071 | 21541681 | protein\_coding | | ENSDARG00000056152 | fam3c | 4 | 20113016 | 20119857 | protein\_coding | | ENSDARG00000062178 | fam65a | 18 | 22185564 | 22294858 | protein\_coding | | ENSDARG00000012432 | fam76b | 5 | 23081415 | 23092539 | protein\_coding | | ENSDARG00000087545 | fbxo42 | 22 | 218902 | 220374 | protein\_coding | | ENSDARG00000102402 | fut11 | 14 | 14302536 | 14331148 | protein\_coding | | ENSDARG00000061282 | glg1a | 25 | 36648751 | 36678336 | protein\_coding | | ENSDARG00000037924 | gna13b | 3 | 36202981 | 36235305 | protein\_coding | | ENSDARG00000029612 | gpkow | 8 | 36559357 | 36585639 | protein\_coding | | ENSDARG00000012199 | gpt2 | 7 | 41607356 | 41619953 | protein\_coding | | ENSDARG00000078754 | gra | 16 | 16314417 | 16318203 | protein\_coding | | ENSDARG00000063670 | gtf2a1l | 13 | 70245 | 75621 | protein\_coding | | ENSDARG00000103578 | gtf2b | 2 | 22824423 | 22836638 | protein\_coding | | ENSDARG00000011934 | gyg1a | 2 | 25281132 | 25289185 | protein\_coding | | ENSDARG00000015206 | hnrnpua | 17 | 11316506 | 11341400 | protein\_coding | | ENSDARG00000070603 | hspa12a | 17 | 21201098 | 21260386 | protein\_coding | | ENSDARG00000104734 | kat2a | 3 | 17503969 | 17531176 | protein\_coding | | ENSDARG00000010603 | kctd9b | 10 | 42714137 | 42727442 | protein\_coding | | ENSDARG00000079834 | kdf1a | 16 | 55183625 | 55211235 | protein\_coding | | ENSDARG00000062267 | kdm3b | 14 | 21325632 | 21363352 | protein\_coding | | ENSDARG00000014943 | kif23 | 18 | 20447226 | 20469346 | protein\_coding | | ENSDARG00000056079 | l3mbtl2 | 12 | 18848857 | 18866622 | protein\_coding | | ENSDARG00000015824 | lemd3 | 4 | 12917379 | 12931237 | protein\_coding | | ENSDARG00000023920 | llgl2 | 12 | 33637378 | 33688023 | protein\_coding | | ENSDARG00000032188 | lrrc8aa | 21 | 4091726 | 4140380 | protein\_coding | | ENSDARG00000006600 | lsm14aa | 25 | 35384163 | 35404687 | protein\_coding | | ENSDARG00000029609 | macrod1 | 14 | 46802162 | 46980420 | protein\_coding | | ENSDARG00000009681 | med27 | 8 | 11846966 | 11950360 | protein\_coding | | ENSDARG00000008105 | mettl2a | 3 | 19515579 | 19533079 | protein\_coding | | ENSDARG00000061451 | n4bp2 | 1 | 22681755 | 22703076 | protein\_coding | | ENSDARG00000024109 | naa40 | 7 | 24728903 | 24740440 | protein\_coding | | ENSDARG00000021120 | ndc1 | 2 | 26921219 | 26934994 | protein\_coding | | ENSDARG00000035505 | nelfb | 5 | 28948830 | 28959774 | protein\_coding | | ENSDARG00000053963 | npm2b | 10 | 44706310 | 44713501 | protein\_coding | | ENSDARG00000038225 | nras | 8 | 11077049 | 11093990 | protein\_coding | | ENSDARG00000023591 | nrbf2b | 12 | 8531842 | 8541884 | protein\_coding | | ENSDARG00000051920 | nsmaf | 7 | 57841673 | 57876412 | protein\_coding | | ENSDARG00000024311 | nsmce4a | 13 | 28655364 | 28666619 | protein\_coding | | ENSDARG00000078073 | nudt5 | 4 | 7861755 | 7867376 | protein\_coding | | ENSDARG00000074955 | nup98 | 21 | 40331228 | 40375086 | protein\_coding | | ENSDARG00000102421 | papl | KN150131.1 | 536 | 14150 | protein\_coding | | ENSDARG00000010583 | pard3 | 2 | 43355555 | 43539830 | protein\_coding | | ENSDARG00000017423 | patl1 | 1 | 52129959 | 52155986 | protein\_coding | | ENSDARG00000053152 | pcmtd1 | 24 | 35297157 | 35331815 | protein\_coding | | ENSDARG00000058202 | pex16 | 18 | 44919968 | 44941720 | protein\_coding | | ENSDARG00000034768 | phf3 | 13 | 37527961 | 37547686 | protein\_coding | | ENSDARG00000033666 | pi4k2a | 1 | 54059702 | 54077905 | protein\_coding | | ENSDARG00000007081 | pias4a | 22 | 20381432 | 20394682 | protein\_coding | | ENSDARG00000041699 | piwil1 | 8 | 44231274 | 44305828 | protein\_coding | | ENSDARG00000008953 | pofut1 | 23 | 7758070 | 7775552 | protein\_coding | | ENSDARG00000076169 | pold3 | 21 | 21706963 | 21717994 | protein\_coding | | ENSDARG00000032155 | ppm1aa | 13 | 31415323 | 31475900 | protein\_coding | | ENSDARG00000099226 | ppp1cc | KN150702.1 | 75503 | 111816 | protein\_coding | | ENSDARG00000054007 | ppp1r2 | 22 | 37869773 | 37899304 | protein\_coding | | ENSDARG00000009740 | ppp1r7 | 2 | 17027462 | 17039216 | protein\_coding | | ENSDARG00000021996 | ppp2r2aa | 8 | 5174537 | 5208902 | protein\_coding | | ENSDARG00000067639 | prpf4 | 5 | 56772054 | 56783574 | protein\_coding | | ENSDARG00000037506 | prps1b | 14 | 11770751 | 11800632 | protein\_coding | | ENSDARG00000020334 | ptpn11a | 10 | 3427890 | 3452371 | protein\_coding | | ENSDARG00000001769 | ptpra | 21 | 15660572 | 15720340 | protein\_coding | | ENSDARG00000086859 | qdprb2 | 1 | 23691822 | 23695150 | protein\_coding | | ENSDARG00000074849 | rac1a | 12 | 10427054 | 10438605 | protein\_coding | | ENSDARG00000032373 | rnf145b | 21 | 33967222 | 33998160 | protein\_coding | | ENSDARG00000076227 | ror2 | 10 | 5267750 | 5407205 | protein\_coding | | ENSDARG00000043404 | rpp21 | 22 | 15402716 | 15410859 | protein\_coding | | ENSDARG00000028442 | rsg1 | 6 | 41449005 | 41454308 | protein\_coding | | ENSDARG00000019951 | sec62 | 2 | 37262925 | 37283446 | protein\_coding | | ENSDARG00000056741 | senp3b | 7 | 26247672 | 26260872 | protein\_coding | | ENSDARG00000105310 | si:cabz01039271.2 | 11 | 44163417 | 44165632 | protein\_coding | | ENSDARG00000090870 | si:ch1073-263o8.2 | 21 | 43635262 | 43641455 | protein\_coding | | ENSDARG00000063300 | si:ch1073-385i9.2 | 16 | 52039524 | 52090962 | processed\_transcript | | ENSDARG00000094478 | si:ch1073-75o15.4 | 9 | 8402398 | 8407566 | protein\_coding | | ENSDARG00000095991 | si:ch211-199c19.4 | 1 | 52729772 | 52733296 | processed\_transcript | | ENSDARG00000104936 | si:ch211-225h24.2 | 23 | 35303783 | 35370945 | protein\_coding | | ENSDARG00000099967 | si:ch73-160p18.5 | 23 | 44737127 | 44745320 | lincRNA | | ENSDARG00000062831 | si:ch73-22o12.1 | 16 | 10666602 | 10747118 | protein\_coding | | ENSDARG00000096447 | si:dkey-192l18.10 | 24 | 1110226 | 1111863 | lincRNA | | ENSDARG00000092057 | si:dkey-90l23.1 | 9 | 8393906 | 8396412 | protein\_coding | | ENSDARG00000045927 | slc25a44a | 25 | 6138607 | 6147805 | protein\_coding | | ENSDARG00000023394 | slc37a2 | 5 | 57785050 | 57812213 | protein\_coding | | ENSDARG00000038870 | slu7 | 14 | 47016681 | 47031436 | protein\_coding | | ENSDARG00000042187 | smek1 | 20 | 33564282 | 33584850 | protein\_coding | | ENSDARG00000060767 | smg7 | 2 | 36063721 | 36096068 | protein\_coding | | ENSDARG00000037423 | smim19 | 14 | 20941437 | 20948528 | protein\_coding | | ENSDARG00000088347 | sp1 | 11 | 19668 | 28747 | protein\_coding | | ENSDARG00000019973 | stk38a | 8 | 25571702 | 25586398 | protein\_coding | | ENSDARG00000100011 | strn | 11 | 44543693 | 44595962 | protein\_coding | | ENSDARG00000062919 | tceanc2 | 2 | 11094807 | 11096950 | protein\_coding | | ENSDARG00000002536 | tm9sf4 | 23 | 7614825 | 7644920 | protein\_coding | | ENSDARG00000058323 | tmbim1 | 6 | 12761180 | 12787308 | protein\_coding | | ENSDARG00000076362 | tmem260 | 17 | 44001719 | 44078179 | protein\_coding | | ENSDARG00000009031 | tnikb | 24 | 26346863 | 26487155 | protein\_coding | | ENSDARG00000045680 | tnpo3 | 4 | 13587840 | 13606309 | protein\_coding | | ENSDARG00000018840 | trpc4apa | 11 | 24919758 | 24942714 | protein\_coding | | ENSDARG00000036190 | txnl4a | 19 | 21208914 | 21212587 | protein\_coding | | ENSDARG00000022213 | uck2b | 2 | 6140034 | 6153534 | protein\_coding | | ENSDARG00000009629 | unc119.1 | 8 | 39814730 | 39825914 | protein\_coding | | ENSDARG00000004132 | usp19 | 23 | 20218771 | 20251211 | protein\_coding | | ENSDARG00000012314 | usp25 | 10 | 38226704 | 38299877 | protein\_coding | | ENSDARG00000036056 | vrk3 | 7 | 28794765 | 28809405 | protein\_coding | | ENSDARG00000096562 | wu:fj29h11 | 12 | 554562 | 622546 | protein\_coding | | ENSDARG00000039754 | xpc | 8 | 25988851 | 26011811 | protein\_coding | | ENSDARG00000101900 | xrn2 | 20 | 48979684 | 49067343 | protein\_coding | | ENSDARG00000037177 | zc3h13 | 1 | 28962768 | 28993976 | protein\_coding | | ENSDARG00000062651 | zc3hc1 | 4 | 15007462 | 15012136 | protein\_coding | | ENSDARG00000091092 | zcchc10 | 21 | 43175837 | 43182063 | protein\_coding | | ENSDARG00000045691 | zcrb1 | 4 | 13926429 | 13932659 | protein\_coding | | ENSDARG00000045159 | zdhhc4 | 12 | 18449581 | 18456558 | protein\_coding | | ENSDARG00000027639 | zgc:165539 | 10 | 21584544 | 21587736 | protein\_coding | | ENSDARG00000076949 | zgc:173837 | 10 | 19637947 | 19640520 | protein\_coding | | ENSDARG00000013161 | zgc:55558 | 3 | 16513573 | 16530171 | protein\_coding | | ENSDARG00000102630 | zgc:56628 | 2 | 22753644 | 22757287 | protein\_coding | | ENSDARG00000075916 | zgc:66472 | 1 | 43287551 | 43299847 | protein\_coding | | ENSDARG00000071414 | zgc:91940 | 12 | 33351932 | 33358596 | protein\_coding | |
