# Supplementary material for: A high-resolution mRNA expression time course of embryonic development in zebrafish
Source: eLife. 2017 Nov 16;6:e30860. doi: 10.7554/eLife.30860 (PMC5690287; doi:10.7554/eLife.30860)
Supplement: Supplementary file 6. [file elife-30860-supp6.zip › biolayout-clusters-files/Cluster011-genes.html]

Cluster011


# Cluster011: Genes

| | Ensembl ID | Gene Name | Chr | Start | End | Biotype | | --- | --- | --- | --- | --- | --- | | ENSDARG00000105074 | BX000701.4 | 3 | 7154523 | 7155920 | protein\_coding | | ENSDARG00000091595 | CABZ01079490.1 | 20 | 51834473 | 51843328 | protein\_coding | | ENSDARG00000090020 | CABZ01084613.1 | 1 | 57944278 | 57951994 | protein\_coding | | ENSDARG00000102276 | CR391944.1 | 3 | 149395 | 174093 | protein\_coding | | ENSDARG00000029445 | EIF1B | 24 | 32603272 | 32608012 | protein\_coding | | ENSDARG00000042887 | ENSDARG00000042887 | 1 | 53510235 | 53519707 | protein\_coding | | ENSDARG00000089303 | ENSDARG00000089303 | 25 | 10681255 | 10694595 | protein\_coding | | ENSDARG00000100562 | ENSDARG00000100562 | KN149710.1 | 4356 | 26370 | protein\_coding | | ENSDARG00000104316 | ENSDARG00000104316 | KN149997.1 | 4401 | 6296 | protein\_coding | | ENSDARG00000103579 | FO704584.2 | 22 | 1958383 | 1965824 | protein\_coding | | ENSDARG00000086896 | FUBP3 | 5 | 853581 | 881489 | protein\_coding | | ENSDARG00000099164 | NACC1 (1 of many) | 3 | 33613372 | 33627128 | protein\_coding | | ENSDARG00000035697 | SPIN4 (1 of many).1 | 8 | 556547 | 566334 | protein\_coding | | ENSDARG00000098135 | STK40 | 16 | 36465290 | 36487812 | protein\_coding | | ENSDARG00000014986 | acvr1l | 2 | 41674898 | 41695081 | protein\_coding | | ENSDARG00000009336 | aif1l | 5 | 33949212 | 33975349 | protein\_coding | | ENSDARG00000077732 | alyref | 3 | 59589107 | 59599428 | protein\_coding | | ENSDARG00000012892 | ammecr1 | 21 | 44685034 | 44707619 | protein\_coding | | ENSDARG00000005122 | atp2a2b | 21 | 17014356 | 17071851 | protein\_coding | | ENSDARG00000016904 | bckdk | 5 | 60823697 | 60869289 | protein\_coding | | ENSDARG00000089505 | bod1l1 | 14 | 13022 | 31613 | protein\_coding | | ENSDARG00000006527 | brd3a | 21 | 18229683 | 18238701 | protein\_coding | | ENSDARG00000074927 | bub1bb | 17 | 1950175 | 1969881 | protein\_coding | | ENSDARG00000029911 | casc3 | 3 | 23298405 | 23331406 | protein\_coding | | ENSDARG00000004189 | cbx1a | 3 | 23919495 | 23941034 | protein\_coding | | ENSDARG00000073962 | ccdc32 | 17 | 31204798 | 31212008 | protein\_coding | | ENSDARG00000034146 | ccnk | 20 | 5031205 | 5051510 | protein\_coding | | ENSDARG00000012140 | ccnl1b | 2 | 36681716 | 36691548 | protein\_coding | | ENSDARG00000092136 | cenpw | 16 | 40181441 | 40203650 | protein\_coding | | ENSDARG00000102643 | chmp1a | 25 | 36864777 | 36871561 | protein\_coding | | ENSDARG00000057234 | chtopa | 19 | 24313443 | 24333913 | protein\_coding | | ENSDARG00000099631 | cmtm6 | 16 | 41518497 | 41537991 | protein\_coding | | ENSDARG00000055797 | cnpy4 | 7 | 20207703 | 20212500 | protein\_coding | | ENSDARG00000024325 | col4a3bpa | 5 | 50266441 | 50321383 | protein\_coding | | ENSDARG00000028971 | cpsf2 | 20 | 50249890 | 50283091 | protein\_coding | | ENSDARG00000018904 | cstf3 | 18 | 45703162 | 45765046 | protein\_coding | | ENSDARG00000002267 | dcaf13 | 16 | 44686550 | 44707135 | protein\_coding | | ENSDARG00000009862 | dcps | 10 | 39362498 | 39373238 | protein\_coding | | ENSDARG00000005699 | ddx19 | 11 | 3503971 | 3516194 | protein\_coding | | ENSDARG00000037928 | ddx42 | 3 | 31747728 | 31761294 | protein\_coding | | ENSDARG00000045257 | decr2 | 24 | 37821294 | 37833842 | protein\_coding | | ENSDARG00000051785 | dhx33 | 5 | 1699971 | 1726309 | protein\_coding | | ENSDARG00000100013 | dhx38 | 7 | 67525899 | 67574013 | protein\_coding | | ENSDARG00000054707 | dhx8 | 12 | 27479093 | 27496981 | protein\_coding | | ENSDARG00000045167 | dlgap5 | 12 | 17511480 | 17533809 | protein\_coding | | ENSDARG00000057648 | dnttip2 | 8 | 15240169 | 15248604 | protein\_coding | | ENSDARG00000056184 | dvl2 | 7 | 20008123 | 20043565 | protein\_coding | | ENSDARG00000020759 | ef1 | 14 | 40378579 | 40483227 | protein\_coding | | ENSDARG00000057167 | eif4g2b | 18 | 16726798 | 16737142 | protein\_coding | | ENSDARG00000006251 | ell2 | 21 | 11656469 | 11682336 | protein\_coding | | ENSDARG00000100185 | elovl7b | KN150589.1 | 3100 | 5397 | protein\_coding | | ENSDARG00000002479 | ercc6l | 5 | 23409785 | 23422719 | protein\_coding | | ENSDARG00000104392 | eri2 | 1 | 58475217 | 58476755 | protein\_coding | | ENSDARG00000036100 | eya3 | 19 | 25307524 | 25332674 | protein\_coding | | ENSDARG00000055630 | foxh1 | 12 | 13685437 | 13692198 | protein\_coding | | ENSDARG00000008396 | ftr24 | 2 | 47990797 | 48069643 | protein\_coding | | ENSDARG00000044760 | gnaia | 25 | 20900017 | 20933353 | protein\_coding | | ENSDARG00000040024 | gpd1l | 16 | 39298612 | 39317195 | protein\_coding | | ENSDARG00000068820 | h2afva | 5 | 13147071 | 13152470 | protein\_coding | | ENSDARG00000008818 | hsf1 | 19 | 3115552 | 3141370 | protein\_coding | | ENSDARG00000068175 | ing5b | 2 | 36623562 | 36626305 | protein\_coding | | ENSDARG00000100419 | ints1 | 3 | 42293262 | 42354956 | protein\_coding | | ENSDARG00000070698 | kbtbd8 | 11 | 16439990 | 16453409 | protein\_coding | | ENSDARG00000007383 | kcnk6 | 18 | 46173086 | 46185444 | protein\_coding | | ENSDARG00000001558 | kifc1 | 19 | 7072045 | 7081678 | protein\_coding | | ENSDARG00000037060 | kmt2d | 23 | 27830443 | 27869341 | protein\_coding | | ENSDARG00000023190 | kpna4 | 15 | 1631599 | 1657576 | protein\_coding | | ENSDARG00000004745 | lmbr1l | 23 | 27777208 | 27800971 | protein\_coding | | ENSDARG00000076324 | lmtk2 | 3 | 60988583 | 61056186 | protein\_coding | | ENSDARG00000001162 | matr3l1.1.1 | 14 | 24766073 | 24781335 | protein\_coding | | ENSDARG00000024844 | max | 20 | 28929869 | 28939725 | protein\_coding | | ENSDARG00000074695 | mertka | 13 | 47334335 | 47400025 | protein\_coding | | ENSDARG00000003213 | naa15a | 14 | 47131542 | 47167785 | protein\_coding | | ENSDARG00000021097 | nelfcd | 6 | 49783231 | 49802520 | protein\_coding | | ENSDARG00000024561 | nolc1 | 13 | 4844625 | 4863813 | protein\_coding | | ENSDARG00000020482 | nono | 5 | 22089476 | 22099210 | protein\_coding | | ENSDARG00000017454 | nup50 | 4 | 73504434 | 73532058 | protein\_coding | | ENSDARG00000020527 | nup62l | 14 | 10318897 | 10350197 | protein\_coding | | ENSDARG00000099039 | pcbp2 | 9 | 252945 | 264178 | protein\_coding | | ENSDARG00000055022 | pds5a | 1 | 22632263 | 22679790 | protein\_coding | | ENSDARG00000057531 | pgap3 | 12 | 9411822 | 9430775 | protein\_coding | | ENSDARG00000017427 | phf20l1 | 16 | 31642875 | 31665054 | protein\_coding | | ENSDARG00000100396 | pip5k1aa | 19 | 10973540 | 10996973 | protein\_coding | | ENSDARG00000037283 | plrg1 | 1 | 24960088 | 24975775 | protein\_coding | | ENSDARG00000100455 | pnkp | 15 | 14242762 | 14258018 | protein\_coding | | ENSDARG00000056127 | polr2gl | 14 | 26417521 | 26420948 | protein\_coding | | ENSDARG00000044774 | pou5f3 | 21 | 13589124 | 13593983 | protein\_coding | | ENSDARG00000001888 | ppm1ba | 13 | 10549952 | 10598850 | protein\_coding | | ENSDARG00000022430 | ppp1r8b | 16 | 4684308 | 4702368 | protein\_coding | | ENSDARG00000095904 | prpf31 | 16 | 32128329 | 32136642 | protein\_coding | | ENSDARG00000039213 | prpf38a | 6 | 33942139 | 33947006 | protein\_coding | | ENSDARG00000008784 | prpf4ba | 2 | 32829184 | 32842890 | protein\_coding | | ENSDARG00000040310 | prpf4bb | 24 | 11332699 | 11365687 | protein\_coding | | ENSDARG00000044304 | prrc1 | 10 | 16207414 | 16228572 | protein\_coding | | ENSDARG00000011863 | ptk7a | 22 | 38408216 | 38473168 | protein\_coding | | ENSDARG00000046024 | pym1 | 8 | 387611 | 465291 | protein\_coding | | ENSDARG00000019797 | qrich1 | 11 | 17862768 | 17885623 | protein\_coding | | ENSDARG00000089243 | r3hcc1l | 1 | 54270408 | 54280812 | protein\_coding | | ENSDARG00000015460 | racgap1 | 23 | 33831745 | 33848713 | protein\_coding | | ENSDARG00000063031 | rad54l2 | 22 | 10577181 | 10609340 | protein\_coding | | ENSDARG00000006782 | rb1 | 21 | 22924130 | 22980873 | protein\_coding | | ENSDARG00000079717 | rbm12b | 16 | 16324644 | 16329883 | protein\_coding | | ENSDARG00000031346 | rbm17 | 4 | 25507906 | 25526393 | protein\_coding | | ENSDARG00000038113 | rprd1b | 23 | 43514585 | 43561371 | protein\_coding | | ENSDARG00000008947 | rtf1 | 13 | 33098996 | 33115704 | protein\_coding | | ENSDARG00000040607 | rtfdc1 | 6 | 56092622 | 56155108 | protein\_coding | | ENSDARG00000078954 | rxrba | 19 | 7330116 | 7355919 | protein\_coding | | ENSDARG00000018681 | saal1 | 25 | 8056447 | 8077891 | protein\_coding | | ENSDARG00000031261 | sap30bp | 8 | 13027045 | 13060487 | protein\_coding | | ENSDARG00000054010 | scaf1 | 3 | 32346068 | 32358907 | protein\_coding | | ENSDARG00000045885 | scaf11 | 4 | 1770241 | 1791066 | protein\_coding | | ENSDARG00000077093 | scaf4a | 10 | 25738998 | 25755170 | protein\_coding | | ENSDARG00000024124 | sde2 | 22 | 26257721 | 26269652 | protein\_coding | | ENSDARG00000005675 | sec61a1l | 6 | 42380375 | 42391072 | protein\_coding | | ENSDARG00000100114 | sf3a3 | 19 | 17388152 | 17400633 | protein\_coding | | ENSDARG00000070284 | sft2d3 | 2 | 5494354 | 5495990 | protein\_coding | | ENSDARG00000087238 | si:ch211-161h7.4 | 24 | 20748195 | 20763830 | protein\_coding | | ENSDARG00000074624 | si:ch211-198a12.6 | 13 | 18376600 | 18381844 | protein\_coding | | ENSDARG00000042000 | si:ch211-63o20.7 | 20 | 34799531 | 34808649 | protein\_coding | | ENSDARG00000074098 | si:ch211-86h15.1 | 11 | 22462716 | 22469821 | protein\_coding | | ENSDARG00000004937 | skp2 | 21 | 22310484 | 22322700 | protein\_coding | | ENSDARG00000041481 | smg5 | 16 | 45726697 | 45767497 | protein\_coding | | ENSDARG00000062476 | snapc5 | 18 | 19498636 | 19502096 | protein\_coding | | ENSDARG00000035625 | snrnp27 | 5 | 13267526 | 13278509 | protein\_coding | | ENSDARG00000018890 | snrpa | 18 | 35428179 | 35432944 | protein\_coding | | ENSDARG00000039424 | snrpb2 | 1 | 51067723 | 51075976 | protein\_coding | | ENSDARG00000091563 | snw1 | 20 | 5336408 | 5355538 | protein\_coding | | ENSDARG00000004874 | spata6l | 7 | 69240864 | 69256454 | protein\_coding | | ENSDARG00000017659 | sps2 | 23 | 18634523 | 18642179 | protein\_coding | | ENSDARG00000074084 | strip1 | 22 | 9493356 | 9508264 | protein\_coding | | ENSDARG00000006524 | supt6h | 21 | 25992252 | 26021852 | protein\_coding | | ENSDARG00000056338 | szrd1 | 23 | 24575377 | 24582280 | protein\_coding | | ENSDARG00000003531 | tcea1 | 2 | 30756147 | 30764477 | protein\_coding | | ENSDARG00000015757 | tmem50a | 13 | 45348279 | 45358660 | protein\_coding | | ENSDARG00000038239 | tnpo2 | 3 | 18657784 | 18689492 | protein\_coding | | ENSDARG00000016630 | tprb | 20 | 34136402 | 34167077 | protein\_coding | | ENSDARG00000086126 | trim33l | 16 | 28829394 | 28855794 | protein\_coding | | ENSDARG00000104825 | trim35-7 | 3 | 5510485 | 5523471 | protein\_coding | | ENSDARG00000069527 | ube2g1b | 21 | 16994999 | 17001271 | protein\_coding | | ENSDARG00000062506 | zc3h18 | 18 | 17090121 | 17158534 | protein\_coding | | ENSDARG00000099273 | zgc:103508 | 10 | 22948886 | 22956589 | protein\_coding | | ENSDARG00000070800 | zgc:109744 | 19 | 22757488 | 22762280 | protein\_coding | | ENSDARG00000103134 | zgc:65873 | KN149934.1 | 8687 | 12326 | protein\_coding | | ENSDARG00000060695 | znf346 | 21 | 37405593 | 37423314 | protein\_coding | | ENSDARG00000087536 | znf407 | 16 | 6786517 | 6806893 | protein\_coding | | ENSDARG00000078164 | znf576.2 | 16 | 25248411 | 25254813 | protein\_coding | | ENSDARG00000004594 | znf800a | 25 | 26457947 | 26469000 | protein\_coding | |
