# Supplementary material for: A high-resolution mRNA expression time course of embryonic development in zebrafish
Source: eLife. 2017 Nov 16;6:e30860. doi: 10.7554/eLife.30860 (PMC5690287; doi:10.7554/eLife.30860)
Supplement: Supplementary file 6. [file elife-30860-supp6.zip › biolayout-clusters-files/Cluster011.html]

Cluster011


# Cluster011: Detail

### Go to ZFA detail

## GO

| | GO ID | Description | Domain | Annotated | Expected | Observed | Adjusted p-value | Genes | Ensembl IDs | | --- | --- | --- | --- | --- | --- | --- | --- | --- | | GO:0006397 | mRNA processing | biological\_process | 164 | 1.90 | 14 | 0.043 | snrpa cstf3 nono cpsf2 casc3 snrnp27 prpf38a snrpb2 dhx8 polr2gl snw1 prpf31 dhx38 sf3a3 | ENSDARG00000018890 ENSDARG00000018904 ENSDARG00000020482 ENSDARG00000028971 ENSDARG00000029911 ENSDARG00000035625 ENSDARG00000039213 ENSDARG00000039424 ENSDARG00000054707 ENSDARG00000056127 ENSDARG00000091563 ENSDARG00000095904 ENSDARG00000100013 ENSDARG00000100114 | | GO:0005643 | nuclear pore | cellular\_component | 23 | 0.26 | 4 | 0.030 | tprb nup50 nup62l kpna4 | ENSDARG00000016630 ENSDARG00000017454 ENSDARG00000020527 ENSDARG00000023190 | | GO:0004004 | ATP-dependent RNA helicase activity | molecular\_function | 42 | 0.47 | 5 | 0.025 | ddx19 ddx42 dhx33 dhx8 dhx38 | ENSDARG00000005699 ENSDARG00000037928 ENSDARG00000051785 ENSDARG00000054707 ENSDARG00000100013 | |
