# Supplementary material for: A high-resolution mRNA expression time course of embryonic development in zebrafish
Source: eLife. 2017 Nov 16;6:e30860. doi: 10.7554/eLife.30860 (PMC5690287; doi:10.7554/eLife.30860)
Supplement: Supplementary file 6. [file elife-30860-supp6.zip › biolayout-clusters-files/Cluster012-genes.html]

Cluster012


# Cluster012: Genes

| | Ensembl ID | Gene Name | Chr | Start | End | Biotype | | --- | --- | --- | --- | --- | --- | | ENSDARG00000052118 | ADAMTSL5 | 7 | 50201789 | 50244236 | protein\_coding | | ENSDARG00000077590 | ARSJ | 7 | 57374730 | 57388691 | protein\_coding | | ENSDARG00000074745 | CABZ01044297.1 | 13 | 1596016 | 1650722 | protein\_coding | | ENSDARG00000006990 | CRELD1 | 8 | 54089727 | 54097634 | protein\_coding | | ENSDARG00000014717 | DYNC1H1 | 17 | 1527965 | 1599371 | protein\_coding | | ENSDARG00000004658 | ENSDARG00000004658 | 7 | 7145551 | 7164981 | protein\_coding | | ENSDARG00000010556 | ENSDARG00000010556 | 12 | 4223765 | 4243723 | protein\_coding | | ENSDARG00000040162 | ENSDARG00000040162 | 17 | 23278904 | 23282297 | protein\_coding | | ENSDARG00000105285 | ENSDARG00000105285 | 14 | 344941 | 373782 | protein\_coding | | ENSDARG00000104393 | NPR2 | 5 | 53938900 | 54016006 | protein\_coding | | ENSDARG00000079632 | POLD4 | 1 | 44005751 | 44009634 | protein\_coding | | ENSDARG00000092716 | VASH1 | 17 | 53163671 | 53172586 | protein\_coding | | ENSDARG00000067545 | adam19b | 14 | 6239789 | 6307375 | protein\_coding | | ENSDARG00000079388 | agrn | 23 | 23559210 | 23988136 | protein\_coding | | ENSDARG00000004932 | anos1b | 22 | 38837166 | 38927144 | protein\_coding | | ENSDARG00000099995 | apbb2b | 1 | 19237583 | 19296647 | protein\_coding | | ENSDARG00000004034 | arhgdig | 3 | 27582768 | 27638390 | protein\_coding | | ENSDARG00000088630 | arhgef10la | 11 | 29083968 | 29316263 | protein\_coding | | ENSDARG00000074244 | arhgef10lb | 23 | 21610776 | 21708782 | protein\_coding | | ENSDARG00000019062 | arpc5b | 20 | 34248791 | 34252729 | protein\_coding | | ENSDARG00000037555 | atoh8 | 14 | 9201277 | 9216350 | protein\_coding | | ENSDARG00000056427 | auts2a | 10 | 27220977 | 27237406 | protein\_coding | | ENSDARG00000035005 | bag2 | 13 | 1274633 | 1278484 | protein\_coding | | ENSDARG00000090190 | bcam | 16 | 24113376 | 24220541 | protein\_coding | | ENSDARG00000063230 | bmp7b | 23 | 6487442 | 6588127 | protein\_coding | | ENSDARG00000032369 | btbd6b | 20 | 21028154 | 21033109 | protein\_coding | | ENSDARG00000100252 | capzb | 22 | 97120 | 111800 | protein\_coding | | ENSDARG00000040917 | cbfb | 18 | 23005047 | 23083599 | protein\_coding | | ENSDARG00000004318 | cbwd | 8 | 30416468 | 30440840 | protein\_coding | | ENSDARG00000051975 | cd99 | 1 | 32622830 | 32647456 | protein\_coding | | ENSDARG00000068156 | chchd7 | 7 | 58418161 | 58427454 | protein\_coding | | ENSDARG00000052063 | col4a5 | 7 | 50828152 | 50919360 | protein\_coding | | ENSDARG00000052061 | col4a6 | 7 | 50645637 | 50827420 | protein\_coding | | ENSDARG00000078865 | cxxc5a | 21 | 28826426 | 28883550 | protein\_coding | | ENSDARG00000077121 | cyp26b1 | 7 | 25052585 | 25077440 | protein\_coding | | ENSDARG00000010132 | dacha | 21 | 34329966 | 34623776 | protein\_coding | | ENSDARG00000042296 | dlx5a | 19 | 41885161 | 41887173 | protein\_coding | | ENSDARG00000044420 | dnajc19 | 22 | 37599176 | 37606102 | protein\_coding | | ENSDARG00000011141 | dpysl5a | 17 | 36951984 | 36989064 | protein\_coding | | ENSDARG00000044521 | eef1b2 | 6 | 12967027 | 12972720 | protein\_coding | | ENSDARG00000030053 | eef1db | 20 | 52740570 | 52777029 | protein\_coding | | ENSDARG00000056119 | eef1g | 14 | 26421586 | 26431184 | protein\_coding | | ENSDARG00000039373 | epha3 | 9 | 16437405 | 16619849 | protein\_coding | | ENSDARG00000009250 | etfb | 16 | 50010488 | 50024954 | protein\_coding | | ENSDARG00000102701 | eva1ba | 16 | 35991347 | 36022379 | protein\_coding | | ENSDARG00000018984 | eya2 | 6 | 55925603 | 56013158 | protein\_coding | | ENSDARG00000098832 | fam168a | 18 | 6551969 | 6650101 | protein\_coding | | ENSDARG00000058115 | fgfr2 | 13 | 46162095 | 46292968 | protein\_coding | | ENSDARG00000054632 | fli1a | 18 | 48429318 | 48493178 | protein\_coding | | ENSDARG00000004830 | flot2a | 5 | 42233881 | 42272564 | protein\_coding | | ENSDARG00000058133 | foxd2 | 8 | 19642284 | 19644555 | protein\_coding | | ENSDARG00000076120 | foxp4 | 11 | 21749775 | 21933824 | protein\_coding | | ENSDARG00000089009 | frmpd1b | 14 | 4084157 | 4166417 | protein\_coding | | ENSDARG00000039576 | fstl1b | 9 | 9757456 | 9864112 | protein\_coding | | ENSDARG00000061335 | galnt1 | 16 | 26989972 | 27227033 | protein\_coding | | ENSDARG00000025826 | gna12a | 3 | 41505779 | 41564380 | protein\_coding | | ENSDARG00000011459 | gsna | 21 | 17535785 | 17566546 | protein\_coding | | ENSDARG00000043182 | hhla2a.1 | 10 | 35473239 | 35493764 | protein\_coding | | ENSDARG00000095651 | hmx1 | 1 | 40209826 | 40211965 | protein\_coding | | ENSDARG00000007941 | hmx4 | 1 | 40206963 | 40208545 | protein\_coding | | ENSDARG00000054030 | hoxb5b | 12 | 27038299 | 27040375 | protein\_coding | | ENSDARG00000088140 | hsd17b7 | 6 | 34959968 | 34974891 | protein\_coding | | ENSDARG00000030278 | idh3a | 25 | 25670955 | 25734031 | protein\_coding | | ENSDARG00000034043 | irx5a | 7 | 35435288 | 35438786 | protein\_coding | | ENSDARG00000013168 | jag1b | 13 | 35213350 | 35264263 | protein\_coding | | ENSDARG00000044001 | lgals3l | 17 | 10668625 | 10682400 | protein\_coding | | ENSDARG00000003022 | limk1b | 21 | 40782090 | 40811425 | protein\_coding | | ENSDARG00000105018 | lrrc20 | 12 | 48306862 | 48330377 | protein\_coding | | ENSDARG00000004307 | lypd6 | 9 | 23260280 | 23307481 | protein\_coding | | ENSDARG00000028533 | macf1a | 19 | 35452782 | 35752907 | protein\_coding | | ENSDARG00000042021 | mapk12a | 18 | 14876157 | 14891923 | protein\_coding | | ENSDARG00000077690 | mb21d2 | 2 | 5651195 | 5669890 | protein\_coding | | ENSDARG00000005368 | mcamb | 15 | 23336597 | 23407487 | protein\_coding | | ENSDARG00000024478 | mpc2 | 9 | 34412812 | 34418287 | protein\_coding | | ENSDARG00000045658 | msrb3 | 4 | 12893619 | 12915356 | protein\_coding | | ENSDARG00000101023 | msx2b | 21 | 41283835 | 41286879 | protein\_coding | | ENSDARG00000053196 | mtor | 8 | 46069730 | 46314039 | protein\_coding | | ENSDARG00000078976 | nanos1 | 14 | 4810207 | 4811015 | protein\_coding | | ENSDARG00000052695 | nr2f1a | 5 | 49093202 | 49100038 | protein\_coding | | ENSDARG00000096546 | nrp2a | 1 | 4908655 | 5048297 | protein\_coding | | ENSDARG00000022531 | ntn1b | 3 | 25578166 | 25683274 | protein\_coding | | ENSDARG00000001913 | palmda | 2 | 20748567 | 20769972 | protein\_coding | | ENSDARG00000009585 | papola | 20 | 4669863 | 4695220 | protein\_coding | | ENSDARG00000024276 | pcbp4 | 22 | 32334907 | 32553561 | protein\_coding | | ENSDARG00000018691 | phf2 | 11 | 27397327 | 27455694 | protein\_coding | | ENSDARG00000062590 | pleca | 19 | 22823496 | 23037488 | protein\_coding | | ENSDARG00000103712 | plpp7 | 5 | 63687581 | 63697617 | protein\_coding | | ENSDARG00000031228 | podxl | 4 | 11733307 | 11752188 | protein\_coding | | ENSDARG00000103994 | ppiab | 10 | 45499085 | 45505655 | protein\_coding | | ENSDARG00000069013 | prdx4 | 5 | 3965601 | 3985654 | protein\_coding | | ENSDARG00000033971 | prrx1a | 2 | 23509485 | 23517057 | protein\_coding | | ENSDARG00000016404 | ptch1 | 8 | 29953792 | 30056947 | protein\_coding | | ENSDARG00000073684 | quo | 6 | 1895787 | 1958852 | protein\_coding | | ENSDARG00000090039 | reck | 24 | 336750 | 373416 | protein\_coding | | ENSDARG00000070735 | rnd2 | 3 | 19701197 | 19750174 | protein\_coding | | ENSDARG00000101047 | rtcb | 4 | 663262 | 671985 | protein\_coding | | ENSDARG00000019235 | sema3aa | 4 | 11312466 | 11369641 | protein\_coding | | ENSDARG00000007362 | sft2d1 | 13 | 4239996 | 4272320 | protein\_coding | | ENSDARG00000013360 | sh3gl3a | 7 | 12587595 | 12656391 | protein\_coding | | ENSDARG00000095843 | si:ch1073-95o12.1 | 23 | 45759494 | 45769796 | protein\_coding | | ENSDARG00000100582 | si:ch211-195b11.3 | 14 | 38445944 | 38451570 | protein\_coding | | ENSDARG00000097072 | si:ch211-229g11.6 | 15 | 27423949 | 27431261 | lincRNA | | ENSDARG00000044261 | si:ch211-243g18.2 | 10 | 9716739 | 9737507 | protein\_coding | | ENSDARG00000054645 | si:ch211-51c14.1 | 3 | 30534353 | 30554559 | protein\_coding | | ENSDARG00000096770 | si:ch73-21k16.6 | 20 | 47053371 | 47056048 | lincRNA | | ENSDARG00000032482 | si:dkey-40c11.2 | 5 | 42930270 | 43008148 | protein\_coding | | ENSDARG00000039304 | six1a | 13 | 31489511 | 31491745 | protein\_coding | | ENSDARG00000026473 | six1b | 20 | 20600196 | 20606245 | protein\_coding | | ENSDARG00000044954 | slit1a | 13 | 425030 | 508194 | protein\_coding | | ENSDARG00000034268 | slit3 | 14 | 24194573 | 24463916 | protein\_coding | | ENSDARG00000098852 | smdt1b | 1 | 53402767 | 53403777 | protein\_coding | | ENSDARG00000061603 | sorbs2b | 14 | 29466783 | 29565866 | protein\_coding | | ENSDARG00000069866 | sox1a | 9 | 21911864 | 21913792 | protein\_coding | | ENSDARG00000015536 | sox6 | 7 | 26980282 | 27181168 | protein\_coding | | ENSDARG00000063292 | ssuh2rs1 | 8 | 7292664 | 7330474 | protein\_coding | | ENSDARG00000004714 | tcf12 | 7 | 52241602 | 52434814 | protein\_coding | | ENSDARG00000005479 | tenm3 | 1 | 38669803 | 39061840 | protein\_coding | | ENSDARG00000076928 | tet2 | 1 | 25662646 | 25693594 | protein\_coding | | ENSDARG00000062646 | tet3 | 5 | 13809699 | 13826742 | protein\_coding | | ENSDARG00000019367 | tgfb3 | 17 | 51987801 | 52020693 | protein\_coding | | ENSDARG00000011273 | tlx2 | 14 | 5349845 | 5383529 | protein\_coding | | ENSDARG00000024954 | tmed4 | 23 | 34051193 | 34060231 | protein\_coding | | ENSDARG00000077777 | tmsb4x | 9 | 54612562 | 54614146 | protein\_coding | | ENSDARG00000032317 | tox | 2 | 22324851 | 22447193 | protein\_coding | | ENSDARG00000078854 | tusc3 | 1 | 15441312 | 15527298 | protein\_coding | | ENSDARG00000068262 | vamp5 | 5 | 27697097 | 27707317 | protein\_coding | | ENSDARG00000060477 | vps8 | 1 | 29279647 | 29506758 | protein\_coding | | ENSDARG00000005541 | wif1 | 4 | 12932914 | 12957869 | protein\_coding | | ENSDARG00000040657 | wipi1 | 3 | 36155388 | 36176805 | protein\_coding | | ENSDARG00000044827 | wnt7aa | 11 | 27239410 | 27253853 | protein\_coding | | ENSDARG00000088449 | wu:fb55g09 | 22 | 10372875 | 10373770 | protein\_coding | | ENSDARG00000094133 | wu:fc21g02 | 1 | 44205214 | 44224710 | protein\_coding | | ENSDARG00000038151 | zgc:92360 | 6 | 561006 | 574539 | protein\_coding | | ENSDARG00000060900 | znf362a | 11 | 39911016 | 39940476 | protein\_coding | |
