# Supplementary material for: A high-resolution mRNA expression time course of embryonic development in zebrafish
Source: eLife. 2017 Nov 16;6:e30860. doi: 10.7554/eLife.30860 (PMC5690287; doi:10.7554/eLife.30860)
Supplement: Supplementary file 6. [file elife-30860-supp6.zip › biolayout-clusters-files/Cluster012.html]

Cluster012


# Cluster012: Detail

### Go to ZFA detail

## GO

| | GO ID | Description | Domain | Annotated | Expected | Observed | Adjusted p-value | Genes | Ensembl IDs | | --- | --- | --- | --- | --- | --- | --- | --- | --- | | GO:0048839 | inner ear development | biological\_process | 94 | 1.1 | 6 | 0.0175 | dacha tlx2 jag1b eya2 six1b six1a | ENSDARG00000010132 ENSDARG00000011273 ENSDARG00000013168 ENSDARG00000018984 ENSDARG00000026473 ENSDARG00000039304 | | GO:0043565 | sequence-specific DNA binding | molecular\_function | 494 | 5.1 | 15 | 0.0038 | tcf12 hmx4 tlx2 six1b prrx1a irx5a six1a dlx5a nr2f1a hoxb5b fli1a foxd2 foxp4 hmx1 msx2b | ENSDARG00000004714 ENSDARG00000007941 ENSDARG00000011273 ENSDARG00000026473 ENSDARG00000033971 ENSDARG00000034043 ENSDARG00000039304 ENSDARG00000042296 ENSDARG00000052695 ENSDARG00000054030 ENSDARG00000054632 ENSDARG00000058133 ENSDARG00000076120 ENSDARG00000095651 ENSDARG00000101023 | |

  


### Go to GO detail

## ZFA

| | ZFA ID | Description | Annotated | Expected | Observed | Fold Enrichment | Adjusted p-value | Genes | Ensembl IDs | | --- | --- | --- | --- | --- | --- | --- | --- | --- | | ZFA:0001310 | dorsolateral placode | 9 | 0.09 | 1 | 11.1 | 0.00087 | six1b | ENSDARG00000026473 | | ZFA:0000051 | otic vesicle | 1008 | 10.19 | 28 | 2.7 | 0.00087 | anos1b hoxb5b irx5a jag1b dacha flot2a agrn col4a5 cyp26b1 fgfr2 tgfb3 btbd6b six1b six1a lypd6 eya2 wnt7aa hmx4 bmp7b bcam hmx1 col4a6 nr2f1a ptch1 fli1a msx2b mapk12a dlx5a | ENSDARG00000004932 ENSDARG00000054030 ENSDARG00000034043 ENSDARG00000013168 ENSDARG00000010132 ENSDARG00000004830 ENSDARG00000079388 ENSDARG00000052063 ENSDARG00000077121 ENSDARG00000058115 ENSDARG00000019367 ENSDARG00000032369 ENSDARG00000026473 ENSDARG00000039304 ENSDARG00000004307 ENSDARG00000018984 ENSDARG00000044827 ENSDARG00000007941 ENSDARG00000063230 ENSDARG00000090190 ENSDARG00000095651 ENSDARG00000052061 ENSDARG00000052695 ENSDARG00000016404 ENSDARG00000054632 ENSDARG00000101023 ENSDARG00000042021 ENSDARG00000042296 | | ZFA:0001309 | neurogenic placode | 16 | 0.16 | 1 | 6.2 | 0.00090 | six1b | ENSDARG00000026473 | | ZFA:0000142 | peripheral nervous system | 47 | 0.48 | 1 | 2.1 | 0.03435 | reck | ENSDARG00000090039 | | ZFA:0007059 | neurogenic field | 14 | 0.14 | 1 | 7.1 | 0.03435 | six1b | ENSDARG00000026473 | | ZFA:0000138 | otic placode | 384 | 3.88 | 6 | 1.5 | 0.03435 | fgfr2 tgfb3 btbd6b six1b lypd6 hmx1 | ENSDARG00000058115 ENSDARG00000019367 ENSDARG00000032369 ENSDARG00000026473 ENSDARG00000004307 ENSDARG00000095651 | |
