# Supplementary material for: A high-resolution mRNA expression time course of embryonic development in zebrafish
Source: eLife. 2017 Nov 16;6:e30860. doi: 10.7554/eLife.30860 (PMC5690287; doi:10.7554/eLife.30860)
Supplement: Supplementary file 6. [file elife-30860-supp6.zip › biolayout-clusters-files/Cluster013-genes.html]

Cluster013


# Cluster013: Genes

| | Ensembl ID | Gene Name | Chr | Start | End | Biotype | | --- | --- | --- | --- | --- | --- | | ENSDARG00000088137 | ADGRG2 (1 of many) | 24 | 25139784 | 25156591 | protein\_coding | | ENSDARG00000100606 | ARHGEF25 (1 of many) | 23 | 27985254 | 28088628 | protein\_coding | | ENSDARG00000037196 | ARID5B | 12 | 8134180 | 8347970 | protein\_coding | | ENSDARG00000089549 | BAALC (1 of many).1 | 20 | 19680252 | 19690795 | protein\_coding | | ENSDARG00000100981 | CCDC39 | 2 | 7870827 | 7898610 | protein\_coding | | ENSDARG00000099902 | ENSDARG00000099902 | 11 | 36983634 | 37008024 | protein\_coding | | ENSDARG00000101951 | ENSDARG00000101951 | 19 | 47924915 | 47940788 | protein\_coding | | ENSDARG00000099115 | FO904953.1 | 19 | 890369 | 910641 | protein\_coding | | ENSDARG00000014939 | KCNN2 | 10 | 17634143 | 17720921 | protein\_coding | | ENSDARG00000099137 | LTBP4 | 18 | 49007999 | 49083558 | protein\_coding | | ENSDARG00000036619 | MGAT3 (1 of many) | 6 | 184257 | 187438 | protein\_coding | | ENSDARG00000098826 | PLCL2 | 16 | 50189141 | 50321462 | protein\_coding | | ENSDARG00000061233 | abcc5 | 18 | 45795209 | 45856643 | protein\_coding | | ENSDARG00000088168 | ablim3 | 14 | 34293831 | 34431592 | protein\_coding | | ENSDARG00000029124 | adamts15a | 5 | 29578857 | 29608915 | protein\_coding | | ENSDARG00000056331 | ahcyl1 | 8 | 24952751 | 24992018 | protein\_coding | | ENSDARG00000013976 | anxa13 | 24 | 6331094 | 6346445 | protein\_coding | | ENSDARG00000053279 | apln | 14 | 33382636 | 33417401 | protein\_coding | | ENSDARG00000057437 | apodb | 24 | 26161321 | 26183680 | protein\_coding | | ENSDARG00000057606 | arl4ca | 22 | 13952995 | 13954616 | protein\_coding | | ENSDARG00000008413 | atp11a | 1 | 45572241 | 45710153 | protein\_coding | | ENSDARG00000077492 | atp8a2 | 24 | 21226105 | 21353825 | protein\_coding | | ENSDARG00000044092 | atpif1b | 17 | 24594854 | 24596323 | protein\_coding | | ENSDARG00000043388 | atraid | 20 | 19613144 | 19617057 | protein\_coding | | ENSDARG00000063255 | btbd11a | 4 | 11814929 | 12008555 | protein\_coding | | ENSDARG00000036457 | cacng6a | 19 | 9832092 | 9879945 | protein\_coding | | ENSDARG00000099441 | cbx4 | 3 | 18406159 | 18414290 | protein\_coding | | ENSDARG00000000002 | ccdc80 | 9 | 34279971 | 34304024 | protein\_coding | | ENSDARG00000036337 | cers3b | 18 | 7350120 | 7367033 | protein\_coding | | ENSDARG00000098158 | cpxm1b | 14 | 51607628 | 51638769 | protein\_coding | | ENSDARG00000063095 | ctsf | 14 | 6834588 | 6858237 | protein\_coding | | ENSDARG00000041799 | cx43 | 20 | 40818053 | 40823420 | protein\_coding | | ENSDARG00000062154 | dip2ca | 24 | 26944115 | 27143882 | protein\_coding | | ENSDARG00000015566 | dnmt3ab | 17 | 36991995 | 37107374 | protein\_coding | | ENSDARG00000076119 | emid1 | 5 | 24383419 | 24508628 | protein\_coding | | ENSDARG00000077039 | esama | 10 | 31701907 | 31782410 | protein\_coding | | ENSDARG00000099124 | f3a | 24 | 31340149 | 31353495 | protein\_coding | | ENSDARG00000090338 | fam20cb | 12 | 18112683 | 18221779 | protein\_coding | | ENSDARG00000104315 | fhl3b | 19 | 4158668 | 4187549 | protein\_coding | | ENSDARG00000019371 | flt1 | 24 | 21605636 | 21663645 | protein\_coding | | ENSDARG00000055926 | foxi3a | 21 | 30314561 | 30316256 | protein\_coding | | ENSDARG00000059432 | fsd1l | 5 | 69036164 | 69079691 | protein\_coding | | ENSDARG00000059438 | galnt18b | 7 | 66035398 | 66229179 | protein\_coding | | ENSDARG00000045413 | gcm2 | 24 | 8687217 | 8691526 | protein\_coding | | ENSDARG00000039959 | gdnfa | 10 | 1384194 | 1401415 | protein\_coding | | ENSDARG00000005085 | ggctb | 16 | 54000890 | 54023177 | protein\_coding | | ENSDARG00000015053 | grip1 | 4 | 13034769 | 13256964 | protein\_coding | | ENSDARG00000071059 | gtpbp2 | 22 | 28909098 | 28922660 | protein\_coding | | ENSDARG00000061194 | hectd2 | 12 | 16279407 | 16332151 | protein\_coding | | ENSDARG00000060008 | hhipl2 | 17 | 45518126 | 45542684 | protein\_coding | | ENSDARG00000059351 | hnrnpa3 | 9 | 1690531 | 1701653 | protein\_coding | | ENSDARG00000030307 | hspa12b | 7 | 59806101 | 59839781 | protein\_coding | | ENSDARG00000002204 | hspb11 | 21 | 20347201 | 20348242 | protein\_coding | | ENSDARG00000029544 | id2b | 20 | 29882048 | 29884331 | protein\_coding | | ENSDARG00000033307 | igf2b | 25 | 23941816 | 23949322 | protein\_coding | | ENSDARG00000025348 | igfbp5b | 9 | 47020157 | 47041700 | protein\_coding | | ENSDARG00000016457 | irf9 | 12 | 13244494 | 13268580 | protein\_coding | | ENSDARG00000078475 | klhl23 | 9 | 48433983 | 48487088 | protein\_coding | | ENSDARG00000019579 | ldb2a | 14 | 47691630 | 47845744 | protein\_coding | | ENSDARG00000059060 | lgalsla | 1 | 54403146 | 54412336 | protein\_coding | | ENSDARG00000054451 | loxl1 | 18 | 50128943 | 50156230 | protein\_coding | | ENSDARG00000069441 | lpar6b | 21 | 22948470 | 22949672 | protein\_coding | | ENSDARG00000021590 | magi2a | 4 | 23037943 | 23362010 | protein\_coding | | ENSDARG00000087616 | maptb | 3 | 22027456 | 22085894 | protein\_coding | | ENSDARG00000018460 | mbnl2 | 1 | 1953747 | 2070067 | protein\_coding | | ENSDARG00000009418 | mef2cb | 5 | 47529487 | 47634295 | protein\_coding | | ENSDARG00000032319 | mest | 4 | 15997479 | 16002460 | protein\_coding | | ENSDARG00000002235 | mmp14a | 7 | 23853134 | 23878513 | protein\_coding | | ENSDARG00000017676 | mmp2 | 7 | 35138573 | 35161424 | protein\_coding | | ENSDARG00000076135 | mmrn2a | 13 | 22489234 | 22515684 | protein\_coding | | ENSDARG00000035322 | myh7bb | 23 | 18796372 | 18830994 | protein\_coding | | ENSDARG00000020924 | myo1ca | 5 | 37184999 | 37216987 | protein\_coding | | ENSDARG00000099871 | myo7aa | 18 | 3096447 | 3184792 | protein\_coding | | ENSDARG00000099843 | nedd4a | 18 | 618400 | 721582 | protein\_coding | | ENSDARG00000061099 | nfasca | 11 | 23466553 | 23646948 | protein\_coding | | ENSDARG00000018721 | npnt | 1 | 49441614 | 49547182 | protein\_coding | | ENSDARG00000100940 | nr5a2 | 22 | 22607310 | 22694452 | protein\_coding | | ENSDARG00000044132 | ogn | 22 | 10424921 | 10430198 | protein\_coding | | ENSDARG00000029718 | p2rx3b | 1 | 44044851 | 44057087 | protein\_coding | | ENSDARG00000021140 | pabpc1b | 19 | 12202416 | 12226237 | protein\_coding | | ENSDARG00000069608 | palm2 | 10 | 4874958 | 4918318 | protein\_coding | | ENSDARG00000099729 | pcdh10a | 1 | 12076661 | 12110610 | protein\_coding | | ENSDARG00000060610 | pcdh7b | 7 | 63014726 | 63261279 | protein\_coding | | ENSDARG00000019304 | phactr3b | 11 | 1396149 | 1483185 | protein\_coding | | ENSDARG00000019130 | plk2b | 8 | 17042980 | 17050483 | protein\_coding | | ENSDARG00000067829 | ppargc1a | 7 | 70128523 | 70219243 | protein\_coding | | ENSDARG00000101569 | ppargc1b | 14 | 24637886 | 24704339 | protein\_coding | | ENSDARG00000007682 | ppdpfa | 8 | 23192081 | 23194796 | protein\_coding | | ENSDARG00000061830 | prss12 | 1 | 19376401 | 19449424 | protein\_coding | | ENSDARG00000068275 | ptx3a | 18 | 41374245 | 41384928 | protein\_coding | | ENSDARG00000089717 | qpct | 17 | 42567226 | 42598288 | protein\_coding | | ENSDARG00000090062 | rac3a | 3 | 36119253 | 36130504 | protein\_coding | | ENSDARG00000032623 | rcan3 | 17 | 26861334 | 26908186 | protein\_coding | | ENSDARG00000045519 | sfmbt2 | 4 | 25311776 | 25373594 | protein\_coding | | ENSDARG00000060148 | sh3pxd2aa | 1 | 46894468 | 46995656 | protein\_coding | | ENSDARG00000063054 | shank3b | 4 | 9552315 | 9585797 | protein\_coding | | ENSDARG00000089645 | si:ch1073-406l10.2 | 24 | 33374035 | 33377364 | protein\_coding | | ENSDARG00000094488 | si:ch211-214j8.15 | 5 | 25500635 | 25509210 | lincRNA | | ENSDARG00000059567 | si:ch73-281f12.4 | 3 | 58655434 | 58739508 | protein\_coding | | ENSDARG00000076843 | si:dkey-174i8.1 | 13 | 21973848 | 21984345 | protein\_coding | | ENSDARG00000033089 | si:dkeyp-8h9.1 | 21 | 38409577 | 38415440 | processed\_transcript | | ENSDARG00000013295 | slc2a3a | 19 | 9766283 | 9793605 | protein\_coding | | ENSDARG00000009901 | slc38a5a | 8 | 25590878 | 25606671 | protein\_coding | | ENSDARG00000098769 | slitrk6 | 1 | 3723907 | 3726666 | protein\_coding | | ENSDARG00000103435 | sorbs1 | 13 | 23002216 | 23096010 | protein\_coding | | ENSDARG00000039256 | speg | 6 | 19035640 | 19094973 | protein\_coding | | ENSDARG00000101655 | sstr5 | 3 | 11134947 | 11156436 | protein\_coding | | ENSDARG00000079542 | stard13a | 10 | 34371859 | 34460817 | protein\_coding | | ENSDARG00000070162 | stox2a | 1 | 39180254 | 39259478 | protein\_coding | | ENSDARG00000074390 | tmem176l.4 | 16 | 46684939 | 46697749 | protein\_coding | | ENSDARG00000036059 | tmppe | 7 | 28881907 | 28890773 | protein\_coding | | ENSDARG00000043416 | tnfaip2a | 20 | 18762041 | 18785769 | protein\_coding | | ENSDARG00000088709 | tnfaip8l3 | 18 | 39541460 | 39602593 | protein\_coding | | ENSDARG00000018569 | tnfrsf1a | 16 | 17154114 | 17164463 | protein\_coding | | ENSDARG00000076292 | tns2a | 23 | 10534985 | 10652287 | protein\_coding | | ENSDARG00000006868 | trh | 8 | 54107707 | 54110479 | protein\_coding | | ENSDARG00000103476 | trpm1b | 25 | 34135366 | 34175192 | protein\_coding | | ENSDARG00000070479 | tspan13b | 19 | 30800669 | 30811898 | protein\_coding | | ENSDARG00000020107 | usp2a | 15 | 20964895 | 21003847 | protein\_coding | | ENSDARG00000079564 | vmhc | 2 | 24600869 | 24614260 | protein\_coding | | ENSDARG00000038794 | zgc:113531 | 2 | 33474092 | 33483153 | protein\_coding | | ENSDARG00000055897 | zgc:154093 | 15 | 23758249 | 23764472 | protein\_coding | | ENSDARG00000040290 | zgc:174637 | 16 | 24741930 | 24753588 | protein\_coding | |
