# Supplementary material for: A high-resolution mRNA expression time course of embryonic development in zebrafish
Source: eLife. 2017 Nov 16;6:e30860. doi: 10.7554/eLife.30860 (PMC5690287; doi:10.7554/eLife.30860)
Supplement: Supplementary file 6. [file elife-30860-supp6.zip › biolayout-clusters-files/Cluster013.html]

Cluster013


## Cluster013: Detail

### Go to GO detail

## ZFA

| | ZFA ID | Description | Annotated | Expected | Observed | Fold Enrichment | Adjusted p-value | Genes | Ensembl IDs | | --- | --- | --- | --- | --- | --- | --- | --- | --- | | ZFA:0000393 | mesenchyme | 50 | 0.47 | 1 | 2.1 | 0.024 | cx43 | ENSDARG00000041799 | |
