# Supplementary material for: A high-resolution mRNA expression time course of embryonic development in zebrafish
Source: eLife. 2017 Nov 16;6:e30860. doi: 10.7554/eLife.30860 (PMC5690287; doi:10.7554/eLife.30860)
Supplement: Supplementary file 6. [file elife-30860-supp6.zip › biolayout-clusters-files/Cluster014-genes.html]

Cluster014


# Cluster014: Genes

| | Ensembl ID | Gene Name | Chr | Start | End | Biotype | | --- | --- | --- | --- | --- | --- | | ENSDARG00000068460 | BX842701.1 | 7 | 39192029 | 39216728 | protein\_coding | | ENSDARG00000098488 | CABZ01118154.1 | 14 | 1716212 | 1717790 | protein\_coding | | ENSDARG00000078114 | CFP (1 of many) | 8 | 23668753 | 23680641 | protein\_coding | | ENSDARG00000031678 | COL5A2 (1 of many) | 9 | 42851401 | 42894621 | protein\_coding | | ENSDARG00000053091 | DAB2 | 5 | 43261196 | 43334966 | protein\_coding | | ENSDARG00000087456 | ENSDARG00000087456 | 20 | 50246506 | 50249425 | protein\_coding | | ENSDARG00000089066 | ENSDARG00000089066 | 7 | 51195924 | 51253587 | protein\_coding | | ENSDARG00000079712 | GAL3ST4 | 7 | 26122555 | 26137319 | protein\_coding | | ENSDARG00000003795 | IDH2 | 18 | 26112296 | 26130964 | protein\_coding | | ENSDARG00000077964 | LIMD1 | 16 | 3090229 | 3134617 | protein\_coding | | ENSDARG00000055339 | MIPEP (1 of many) | 10 | 39148073 | 39179933 | protein\_coding | | ENSDARG00000090036 | RHOBTB3 | 21 | 11688855 | 11727751 | protein\_coding | | ENSDARG00000100308 | SEMA4F | 7 | 59032057 | 59167909 | protein\_coding | | ENSDARG00000033993 | SLC19A1 | 6 | 18590575 | 18612767 | protein\_coding | | ENSDARG00000096003 | USMG5 | 1 | 48787717 | 48790472 | protein\_coding | | ENSDARG00000024101 | adra1d | 1 | 41256046 | 41281418 | protein\_coding | | ENSDARG00000035859 | angptl4 | 2 | 24881111 | 24886867 | protein\_coding | | ENSDARG00000052950 | arhgap23a | 3 | 39171184 | 39313570 | protein\_coding | | ENSDARG00000070083 | atp5b | 11 | 13150239 | 13155400 | protein\_coding | | ENSDARG00000045514 | atp5c1 | 4 | 25184101 | 25192354 | protein\_coding | | ENSDARG00000019404 | atp5d | 22 | 18741083 | 18754010 | protein\_coding | | ENSDARG00000095897 | atp5e | 6 | 49894835 | 49903487 | protein\_coding | | ENSDARG00000011553 | atp5f1 | 8 | 25310354 | 25319570 | protein\_coding | | ENSDARG00000098355 | atp5h | 3 | 44405174 | 44414425 | protein\_coding | | ENSDARG00000068940 | atp5ib | 5 | 8565744 | 8568795 | protein\_coding | | ENSDARG00000014313 | atp5j | 1 | 614903 | 619459 | protein\_coding | | ENSDARG00000011841 | atp5l | 5 | 30099107 | 30101738 | protein\_coding | | ENSDARG00000079496 | bicd1a | 18 | 12089392 | 12156609 | protein\_coding | | ENSDARG00000070314 | cald1l2 | 4 | 7406749 | 7494862 | processed\_transcript | | ENSDARG00000056206 | camk2g2 | 13 | 21989239 | 22112865 | protein\_coding | | ENSDARG00000100119 | caskb | 6 | 59808618 | 59892880 | protein\_coding | | ENSDARG00000005232 | ccdc85ca | 17 | 17894533 | 18005855 | protein\_coding | | ENSDARG00000063677 | ccny | 24 | 4341215 | 4392623 | protein\_coding | | ENSDARG00000052783 | cdc42ep3 | 13 | 41998506 | 42017577 | protein\_coding | | ENSDARG00000092677 | coa6 | 4 | 4823574 | 4826047 | protein\_coding | | ENSDARG00000032970 | cox4i1 | 18 | 30529585 | 30531987 | protein\_coding | | ENSDARG00000088383 | cox5aa | 25 | 28631943 | 28636923 | protein\_coding | | ENSDARG00000099663 | cox5ab | 18 | 892085 | 895920 | protein\_coding | | ENSDARG00000022438 | cox6a1 | 8 | 39726484 | 39732222 | protein\_coding | | ENSDARG00000038577 | cox6c | 6 | 52235442 | 52241530 | protein\_coding | | ENSDARG00000053217 | cox7a2a | 17 | 49883252 | 49895461 | protein\_coding | | ENSDARG00000098250 | cox7b | 21 | 37757816 | 37758999 | protein\_coding | | ENSDARG00000044241 | dram2b | 22 | 839971 | 849298 | protein\_coding | | ENSDARG00000020521 | exoc6 | 12 | 8930432 | 9049723 | protein\_coding | | ENSDARG00000053104 | fam102ba | 2 | 45812495 | 45838130 | protein\_coding | | ENSDARG00000052556 | fgfrl1b | 14 | 41233086 | 41311495 | protein\_coding | | ENSDARG00000014181 | foxp1b | 6 | 43600209 | 43892926 | protein\_coding | | ENSDARG00000015449 | fut8a | 17 | 33766904 | 33953977 | protein\_coding | | ENSDARG00000014806 | hacd2 | 9 | 38219680 | 38233144 | protein\_coding | | ENSDARG00000013057 | hoxb5a | 3 | 23577143 | 23579547 | protein\_coding | | ENSDARG00000043531 | jun | 20 | 15653154 | 15655103 | protein\_coding | | ENSDARG00000053542 | kctd12.2 | 1 | 33563357 | 33564835 | protein\_coding | | ENSDARG00000029072 | klf6a | 24 | 4109697 | 4117128 | protein\_coding | | ENSDARG00000039255 | klhl21 | 23 | 24999866 | 25012607 | protein\_coding | | ENSDARG00000071518 | krt222 | 22 | 11689098 | 11705539 | protein\_coding | | ENSDARG00000104701 | map7d1b | 19 | 3969059 | 4079437 | protein\_coding | | ENSDARG00000102744 | mgst3 | 20 | 48757462 | 48773436 | protein\_coding | | ENSDARG00000008388 | mmp14b | 2 | 38178990 | 38201313 | protein\_coding | | ENSDARG00000093448 | mpc1 | 13 | 151013 | 154950 | protein\_coding | | ENSDARG00000079823 | mrps36 | 8 | 17127782 | 17132118 | protein\_coding | | ENSDARG00000069755 | mtmr11 | 16 | 46034240 | 46124884 | protein\_coding | | ENSDARG00000030598 | nampta | 4 | 3345056 | 3372125 | protein\_coding | | ENSDARG00000077256 | nat8l | 7 | 24186039 | 24201850 | protein\_coding | | ENSDARG00000036329 | ndufa1 | 14 | 33073666 | 33075125 | protein\_coding | | ENSDARG00000098584 | ndufa13 | KN149883.1 | 2235 | 5251 | protein\_coding | | ENSDARG00000021984 | ndufa2 | 21 | 44010330 | 44014059 | protein\_coding | | ENSDARG00000041400 | ndufa3 | 16 | 5818134 | 5821760 | protein\_coding | | ENSDARG00000038028 | ndufa6 | 3 | 1413666 | 1417451 | protein\_coding | | ENSDARG00000028889 | ndufb10 | 3 | 18255396 | 18261228 | protein\_coding | | ENSDARG00000043467 | ndufb11 | 8 | 14142359 | 14146718 | protein\_coding | | ENSDARG00000045490 | ndufb2 | 4 | 22759194 | 22762156 | protein\_coding | | ENSDARG00000075709 | ndufb3 | 9 | 708005 | 711306 | protein\_coding | | ENSDARG00000019332 | ndufb4 | 24 | 23825882 | 23840744 | protein\_coding | | ENSDARG00000033789 | ndufb7 | 1 | 55102477 | 55108071 | protein\_coding | | ENSDARG00000010113 | ndufb8 | 12 | 48486420 | 48493711 | protein\_coding | | ENSDARG00000007526 | ndufs2 | 7 | 18791483 | 18816272 | protein\_coding | | ENSDARG00000015385 | ndufs3 | 7 | 38317608 | 38323338 | protein\_coding | | ENSDARG00000052840 | ndufs4 | 5 | 39885350 | 39901579 | protein\_coding | | ENSDARG00000074552 | ndufs7 | 11 | 5845377 | 5855460 | protein\_coding | | ENSDARG00000013044 | ndufv2 | 2 | 54911027 | 54960533 | protein\_coding | | ENSDARG00000043066 | nog2 | 24 | 38926971 | 38928962 | protein\_coding | | ENSDARG00000043130 | notch2 | 8 | 19516900 | 19587707 | protein\_coding | | ENSDARG00000102153 | nrp1a | 24 | 1163433 | 1307962 | protein\_coding | | ENSDARG00000070494 | pdgfra | 20 | 22535507 | 22576582 | protein\_coding | | ENSDARG00000042874 | phlda2 | 25 | 23427248 | 23428538 | protein\_coding | | ENSDARG00000059933 | plpp3 | 20 | 7977476 | 8036607 | protein\_coding | | ENSDARG00000076280 | ppp1r1b | 19 | 4996331 | 5045916 | protein\_coding | | ENSDARG00000037895 | ramp2 | 3 | 36694853 | 36704638 | protein\_coding | | ENSDARG00000044179 | rbms2a | 11 | 1874658 | 1923924 | protein\_coding | | ENSDARG00000055305 | ret | 13 | 25454106 | 25502306 | protein\_coding | | ENSDARG00000015627 | rgs6 | 20 | 28605504 | 28739151 | protein\_coding | | ENSDARG00000004301 | rhogb | 21 | 30900995 | 30915214 | protein\_coding | | ENSDARG00000089767 | rnf130 | 14 | 36425298 | 36523151 | protein\_coding | | ENSDARG00000026784 | robo1 | 15 | 39431053 | 39755758 | protein\_coding | | ENSDARG00000101406 | rplp2 | 6 | 49510152 | 49511900 | protein\_coding | | ENSDARG00000035175 | schip1 | 15 | 1407434 | 1471798 | protein\_coding | | ENSDARG00000011672 | sema3b | 8 | 26564692 | 26623305 | protein\_coding | | ENSDARG00000011163 | sema3fa | 6 | 42821801 | 42918238 | protein\_coding | | ENSDARG00000035136 | sepw1 | 5 | 61335866 | 61342425 | protein\_coding | | ENSDARG00000088816 | si:ch211-120j21.1 | 8 | 19769780 | 19872039 | protein\_coding | | ENSDARG00000088975 | si:ch211-14k19.8 | 1 | 8415729 | 8430910 | protein\_coding | | ENSDARG00000037867 | si:ch211-194i10.5 | 3 | 40154927 | 40159609 | protein\_coding | | ENSDARG00000096887 | si:ch211-220p4.1 | 25 | 30472410 | 30597957 | lincRNA | | ENSDARG00000094466 | si:ch73-199e17.1 | 9 | 7697593 | 7705611 | protein\_coding | | ENSDARG00000104181 | si:dkey-152p16.6 | 14 | 11760681 | 11765665 | protein\_coding | | ENSDARG00000094695 | si:dkey-204f11.64 | 3 | 34010999 | 34017592 | protein\_coding | | ENSDARG00000037059 | slc44a2 | 6 | 8029275 | 8075505 | protein\_coding | | ENSDARG00000004246 | slit2 | 1 | 23092610 | 23219297 | protein\_coding | | ENSDARG00000011582 | sox5 | 4 | 16992394 | 17085808 | protein\_coding | | ENSDARG00000023694 | spon1b | 25 | 15551302 | 15704492 | protein\_coding | | ENSDARG00000010318 | srpx | 9 | 30483327 | 30506044 | protein\_coding | | ENSDARG00000018025 | tbx2a | 5 | 55834500 | 55843608 | protein\_coding | | ENSDARG00000012060 | thbs3b | 19 | 24831911 | 24859397 | protein\_coding | | ENSDARG00000089354 | tspan4a | 18 | 50811126 | 50834473 | protein\_coding | | ENSDARG00000052304 | uqcrc1 | 6 | 20276833 | 20291828 | protein\_coding | | ENSDARG00000014794 | uqcrc2a | 3 | 58096746 | 58133544 | protein\_coding | | ENSDARG00000044813 | vps33b | 7 | 50103321 | 50122619 | protein\_coding | | ENSDARG00000037267 | zgc:158263 | 23 | 26806691 | 26824737 | protein\_coding | | ENSDARG00000022891 | zgc:175214 | 1 | 48836783 | 48853696 | protein\_coding | | ENSDARG00000092873 | zgc:193541 | 20 | 20826558 | 20830541 | protein\_coding | |
