# Supplementary material for: A high-resolution mRNA expression time course of embryonic development in zebrafish
Source: eLife. 2017 Nov 16;6:e30860. doi: 10.7554/eLife.30860 (PMC5690287; doi:10.7554/eLife.30860)
Supplement: Supplementary file 6. [file elife-30860-supp6.zip › biolayout-clusters-files/Cluster014.html]

Cluster014


# Cluster014: Detail

### Go to ZFA detail

## GO

| | GO ID | Description | Domain | Annotated | Expected | Observed | Adjusted p-value | Genes | Ensembl IDs | | --- | --- | --- | --- | --- | --- | --- | --- | --- | | GO:0015986 | ATP synthesis coupled proton transport | biological\_process | 16 | 0.15 | 9 | 1.3e-12 | atp5f1 atp5l atp5j atp5d atp5c1 atp5ib atp5b atp5e atp5h | ENSDARG00000011553 ENSDARG00000011841 ENSDARG00000014313 ENSDARG00000019404 ENSDARG00000045514 ENSDARG00000068940 ENSDARG00000070083 ENSDARG00000095897 ENSDARG00000098355 | | GO:0042775 | mitochondrial ATP synthesis coupled elec... | biological\_process | 14 | 0.13 | 7 | 4.8e-09 | ndufb8 ndufv2 uqcrc2a cox4i1 uqcrc1 cox5aa cox5ab | ENSDARG00000010113 ENSDARG00000013044 ENSDARG00000014794 ENSDARG00000032970 ENSDARG00000052304 ENSDARG00000088383 ENSDARG00000099663 | | GO:1990204 | oxidoreductase complex | cellular\_component | 20 | 0.21 | 7 | 1.7e-07 | ndufb8 ndufv2 uqcrc2a ndufa3 ndufb2 uqcrc1 mrps36 | ENSDARG00000010113 ENSDARG00000013044 ENSDARG00000014794 ENSDARG00000041400 ENSDARG00000045490 ENSDARG00000052304 ENSDARG00000079823 | | GO:0098803 | respiratory chain complex | cellular\_component | 19 | 0.20 | 10 | 1.6e-08 | ndufb8 ndufv2 uqcrc2a cox6a1 cox4i1 ndufa3 ndufb2 uqcrc1 cox5aa cox5ab | ENSDARG00000010113 ENSDARG00000013044 ENSDARG00000014794 ENSDARG00000022438 ENSDARG00000032970 ENSDARG00000041400 ENSDARG00000045490 ENSDARG00000052304 ENSDARG00000088383 ENSDARG00000099663 | | GO:0045277 | respiratory chain complex IV | cellular\_component | 10 | 0.10 | 4 | 5.3e-04 | cox6a1 cox4i1 cox5aa cox5ab | ENSDARG00000022438 ENSDARG00000032970 ENSDARG00000088383 ENSDARG00000099663 | | GO:0045259 | proton-transporting ATP synthase complex | cellular\_component | 12 | 0.12 | 9 | 4.6e-14 | atp5f1 atp5l atp5j atp5d atp5c1 atp5ib atp5b atp5e atp5h | ENSDARG00000011553 ENSDARG00000011841 ENSDARG00000014313 ENSDARG00000019404 ENSDARG00000045514 ENSDARG00000068940 ENSDARG00000070083 ENSDARG00000095897 ENSDARG00000098355 | | GO:0033177 | proton-transporting two-sector ATPase co... | cellular\_component | 17 | 0.18 | 5 | 1.5e-04 | atp5f1 atp5l atp5j atp5ib atp5h | ENSDARG00000011553 ENSDARG00000011841 ENSDARG00000014313 ENSDARG00000068940 ENSDARG00000098355 | | GO:0033178 | proton-transporting two-sector ATPase co... | cellular\_component | 12 | 0.12 | 4 | 1.2e-03 | atp5d atp5c1 atp5b atp5e | ENSDARG00000019404 ENSDARG00000045514 ENSDARG00000070083 ENSDARG00000095897 | | GO:0005746 | mitochondrial respiratory chain | cellular\_component | 21 | 0.22 | 12 | 4.6e-17 | ndufb8 ndufv2 uqcrc2a cox6a1 cox4i1 ndufa3 ndufb2 uqcrc1 cox7a2a cox5aa cox7b cox5ab | ENSDARG00000010113 ENSDARG00000013044 ENSDARG00000014794 ENSDARG00000022438 ENSDARG00000032970 ENSDARG00000041400 ENSDARG00000045490 ENSDARG00000052304 ENSDARG00000053217 ENSDARG00000088383 ENSDARG00000098250 ENSDARG00000099663 | | GO:0098800 | inner mitochondrial membrane protein com... | cellular\_component | 30 | 0.31 | 17 | 8.6e-25 | ndufb8 atp5f1 atp5l ndufv2 atp5j uqcrc2a atp5d cox6a1 cox4i1 ndufa3 ndufb2 uqcrc1 atp5ib cox5aa atp5e atp5h cox5ab | ENSDARG00000010113 ENSDARG00000011553 ENSDARG00000011841 ENSDARG00000013044 ENSDARG00000014313 ENSDARG00000014794 ENSDARG00000019404 ENSDARG00000022438 ENSDARG00000032970 ENSDARG00000041400 ENSDARG00000045490 ENSDARG00000052304 ENSDARG00000068940 ENSDARG00000088383 ENSDARG00000095897 ENSDARG00000098355 ENSDARG00000099663 | | GO:0016651 | oxidoreductase activity, acting on NAD(P... | molecular\_function | 26 | 0.26 | 8 | 2.8e-08 | ndufs2 ndufb8 ndufv2 ndufs3 ndufb4 ndufb7 ndufs4 ndufs7 | ENSDARG00000007526 ENSDARG00000010113 ENSDARG00000013044 ENSDARG00000015385 ENSDARG00000019332 ENSDARG00000033789 ENSDARG00000052840 ENSDARG00000074552 | | GO:0015078 | hydrogen ion transmembrane transporter a... | molecular\_function | 58 | 0.59 | 17 | 1.8e-06 | atp5f1 atp5l atp5j atp5d cox6a1 cox4i1 cox6c atp5c1 cox7a2a atp5ib atp5b cox5aa coa6 atp5e cox7b atp5h cox5ab | ENSDARG00000011553 ENSDARG00000011841 ENSDARG00000014313 ENSDARG00000019404 ENSDARG00000022438 ENSDARG00000032970 ENSDARG00000038577 ENSDARG00000045514 ENSDARG00000053217 ENSDARG00000068940 ENSDARG00000070083 ENSDARG00000088383 ENSDARG00000092677 ENSDARG00000095897 ENSDARG00000098250 ENSDARG00000098355 ENSDARG00000099663 | | GO:0004129 | cytochrome-c oxidase activity | molecular\_function | 22 | 0.22 | 8 | 5.8e-09 | cox6a1 cox4i1 cox6c cox7a2a cox5aa coa6 cox7b cox5ab | ENSDARG00000022438 ENSDARG00000032970 ENSDARG00000038577 ENSDARG00000053217 ENSDARG00000088383 ENSDARG00000092677 ENSDARG00000098250 ENSDARG00000099663 | | GO:0044769 | ATPase activity, coupled to transmembran... | molecular\_function | 20 | 0.20 | 5 | 2.4e-02 | atp5f1 atp5d atp5c1 atp5b atp5e | ENSDARG00000011553 ENSDARG00000019404 ENSDARG00000045514 ENSDARG00000070083 ENSDARG00000095897 | |
