# Supplementary material for: A high-resolution mRNA expression time course of embryonic development in zebrafish
Source: eLife. 2017 Nov 16;6:e30860. doi: 10.7554/eLife.30860 (PMC5690287; doi:10.7554/eLife.30860)
Supplement: Supplementary file 6. [file elife-30860-supp6.zip › biolayout-clusters-files/Cluster015-genes.html]

Cluster015


# Cluster015: Genes

| | Ensembl ID | Gene Name | Chr | Start | End | Biotype | | --- | --- | --- | --- | --- | --- | | ENSDARG00000079317 | DLC1 | 1 | 14604453 | 14642737 | protein\_coding | | ENSDARG00000100954 | WARS | 17 | 1507534 | 1509311 | protein\_coding | | ENSDARG00000003132 | apip | 7 | 49628690 | 49635865 | protein\_coding | | ENSDARG00000055945 | asph | 2 | 27000649 | 27026548 | protein\_coding | | ENSDARG00000020946 | auts2b | 15 | 20069034 | 20088473 | protein\_coding | | ENSDARG00000046133 | b3galnt2 | 13 | 49484119 | 49517183 | protein\_coding | | ENSDARG00000021753 | ccdc25 | 20 | 39435821 | 39441251 | protein\_coding | | ENSDARG00000016173 | cct3 | 16 | 30450770 | 30463348 | protein\_coding | | ENSDARG00000013475 | cct4 | 1 | 53068959 | 53082043 | protein\_coding | | ENSDARG00000045399 | cct5 | 24 | 16760769 | 16768478 | protein\_coding | | ENSDARG00000021252 | cct6a | 21 | 41807149 | 41826474 | protein\_coding | | ENSDARG00000077815 | cdc26 | 5 | 56689325 | 56691377 | protein\_coding | | ENSDARG00000018693 | cdh2 | 20 | 17840340 | 17919655 | protein\_coding | | ENSDARG00000079049 | cercam | 10 | 10393447 | 10421893 | protein\_coding | | ENSDARG00000030723 | cldn11b | 24 | 26287500 | 26296277 | protein\_coding | | ENSDARG00000018010 | crtap | 19 | 43039695 | 43048286 | protein\_coding | | ENSDARG00000070119 | ctc1 | 23 | 45270774 | 45325842 | protein\_coding | | ENSDARG00000055100 | cxcl12b | 22 | 27067102 | 27083041 | protein\_coding | | ENSDARG00000100371 | eef2b | 2 | 56935780 | 56961732 | protein\_coding | | ENSDARG00000091402 | eif2b1 | 5 | 68866490 | 68876492 | protein\_coding | | ENSDARG00000059654 | eif3ba | 3 | 41573108 | 41583074 | protein\_coding | | ENSDARG00000016443 | eif3c | 12 | 3907441 | 3925632 | protein\_coding | | ENSDARG00000021257 | eif3d | 3 | 29509995 | 29524083 | protein\_coding | | ENSDARG00000090697 | eif3ea | 16 | 38606804 | 38681937 | protein\_coding | | ENSDARG00000016889 | eif3g | 3 | 54327385 | 54335221 | protein\_coding | | ENSDARG00000102452 | eif3ha | 16 | 48340303 | 48458621 | protein\_coding | | ENSDARG00000068289 | eif3k | 15 | 47257741 | 47260916 | protein\_coding | | ENSDARG00000013931 | eif3m | 18 | 45648403 | 45661122 | protein\_coding | | ENSDARG00000101082 | eif3s6ip | 13 | 43101718 | 43116871 | protein\_coding | | ENSDARG00000098401 | fam136a | 10 | 7712560 | 7714261 | protein\_coding | | ENSDARG00000021882 | fbxl3b | 6 | 60003136 | 60012393 | protein\_coding | | ENSDARG00000101244 | gli1 | 6 | 59430937 | 59510495 | protein\_coding | | ENSDARG00000025641 | gli2a | 9 | 37332041 | 37487113 | protein\_coding | | ENSDARG00000022550 | gxylt1b | 4 | 13893924 | 13903339 | protein\_coding | | ENSDARG00000006514 | her6 | 6 | 36573189 | 36574950 | protein\_coding | | ENSDARG00000056438 | her9 | 23 | 23472969 | 23474754 | protein\_coding | | ENSDARG00000029150 | hsp90ab1 | 20 | 51384538 | 51398620 | protein\_coding | | ENSDARG00000023495 | ift74 | 5 | 446758 | 472748 | protein\_coding | | ENSDARG00000061478 | igf2bp1 | 3 | 23387589 | 23444567 | protein\_coding | | ENSDARG00000015722 | kctd5a | 3 | 47309053 | 47329016 | protein\_coding | | ENSDARG00000058328 | lsm7 | 22 | 10722829 | 10726365 | protein\_coding | | ENSDARG00000057683 | mcm6 | 6 | 12733207 | 12755502 | protein\_coding | | ENSDARG00000063030 | mesdc2 | 7 | 11134595 | 11142549 | protein\_coding | | ENSDARG00000090462 | mrpl20 | 22 | 19300 | 62124 | protein\_coding | | ENSDARG00000062916 | mrpl37 | 2 | 11130424 | 11148171 | protein\_coding | | ENSDARG00000079391 | mrpl42 | 4 | 1972324 | 1975523 | protein\_coding | | ENSDARG00000041340 | mrpl51 | 16 | 4711692 | 4717682 | protein\_coding | | ENSDARG00000007285 | mrpl57 | 17 | 26590119 | 26592471 | protein\_coding | | ENSDARG00000071679 | mydgf | 22 | 4750818 | 4761094 | protein\_coding | | ENSDARG00000020053 | nkd1 | 7 | 37314603 | 37351032 | protein\_coding | | ENSDARG00000100990 | nme3 | 3 | 15840747 | 15850368 | protein\_coding | | ENSDARG00000044565 | ola1 | 6 | 10505839 | 10518080 | protein\_coding | | ENSDARG00000045846 | osgep | 4 | 5319062 | 5325401 | protein\_coding | | ENSDARG00000071212 | p3h1 | 11 | 38813659 | 38843299 | protein\_coding | | ENSDARG00000057299 | pbdc1 | 10 | 20145686 | 20148631 | protein\_coding | | ENSDARG00000061228 | pfdn4 | 23 | 38219956 | 38228587 | protein\_coding | | ENSDARG00000037108 | pfdn6 | 14 | 30211007 | 30216813 | protein\_coding | | ENSDARG00000032780 | pigf | 12 | 25006394 | 25019614 | protein\_coding | | ENSDARG00000074933 | polg2 | 6 | 17912466 | 17926678 | protein\_coding | | ENSDARG00000013734 | polr3glb | 16 | 42925929 | 42939674 | protein\_coding | | ENSDARG00000032296 | pomp | 21 | 40689710 | 40694631 | protein\_coding | | ENSDARG00000030161 | ppp1r14bb | 7 | 60054110 | 60091341 | protein\_coding | | ENSDARG00000101560 | psma1 | 25 | 36881720 | 36886703 | protein\_coding | | ENSDARG00000045928 | psma4 | 25 | 6084275 | 6096165 | protein\_coding | | ENSDARG00000013966 | psma6b | 20 | 16984745 | 17007040 | protein\_coding | | ENSDARG00000010965 | psma8 | 20 | 18406539 | 18414281 | protein\_coding | | ENSDARG00000009640 | psmb1 | 13 | 1012433 | 1021119 | protein\_coding | | ENSDARG00000031511 | psmb2 | 19 | 47835136 | 47864494 | protein\_coding | | ENSDARG00000002240 | psmb6 | 23 | 44720406 | 44729632 | protein\_coding | | ENSDARG00000043561 | psmc1b | 20 | 16822350 | 16841374 | protein\_coding | | ENSDARG00000020101 | psmc2 | 4 | 14928193 | 14933604 | protein\_coding | | ENSDARG00000003189 | psmd1 | 22 | 37696218 | 37781619 | protein\_coding | | ENSDARG00000016239 | psmd13 | 25 | 10388297 | 10399973 | protein\_coding | | ENSDARG00000023279 | psmd4b | 16 | 9871359 | 9887026 | protein\_coding | | ENSDARG00000035987 | psmg2 | 19 | 12430541 | 12440593 | protein\_coding | | ENSDARG00000101766 | ptmab | KN150442.1 | 34777 | 37978 | protein\_coding | | ENSDARG00000041317 | rangap1a | 3 | 4591776 | 4619905 | protein\_coding | | ENSDARG00000070006 | rcn1 | 7 | 15570892 | 15582365 | protein\_coding | | ENSDARG00000074242 | serbp1a | 6 | 34948214 | 34955363 | protein\_coding | | ENSDARG00000087093 | si:ch211-157c3.4 | 3 | 27511544 | 27517003 | protein\_coding | | ENSDARG00000076532 | si:ch211-222l21.1 | 11 | 40413471 | 40431991 | protein\_coding | | ENSDARG00000059109 | si:dkeyp-113d7.1 | 19 | 5399501 | 5403844 | protein\_coding | | ENSDARG00000087636 | si:dkeyp-86f7.3 | 14 | 9115496 | 9122234 | protein\_coding | | ENSDARG00000031983 | six4b | 20 | 20585154 | 20592488 | protein\_coding | | ENSDARG00000102793 | slc16a6a | KN150708.1 | 18003 | 31610 | protein\_coding | | ENSDARG00000104687 | slc16a9b | 12 | 7555068 | 7573326 | protein\_coding | | ENSDARG00000033175 | snrpe | 11 | 37453347 | 37458996 | protein\_coding | | ENSDARG00000105037 | snrpf | 7 | 19122711 | 19126053 | protein\_coding | | ENSDARG00000099667 | snrpg | 23 | 34030819 | 34035458 | protein\_coding | | ENSDARG00000052435 | spred2a | 1 | 50800750 | 50831175 | protein\_coding | | ENSDARG00000014165 | ssr3 | 18 | 34566501 | 34573734 | protein\_coding | | ENSDARG00000069109 | sssca1 | 10 | 26865400 | 26870331 | protein\_coding | | ENSDARG00000069497 | sumf2 | 21 | 25633329 | 25639791 | protein\_coding | | ENSDARG00000044194 | tcta | 6 | 43018380 | 43021686 | protein\_coding | | ENSDARG00000013004 | tdg.1 | 25 | 18866831 | 18880132 | protein\_coding | | ENSDARG00000071670 | tma7 | 22 | 5633683 | 5638292 | protein\_coding | | ENSDARG00000035914 | tmem167a | 10 | 43282408 | 43289264 | protein\_coding | | ENSDARG00000097797 | tomm6 | 6 | 40575009 | 40575734 | protein\_coding | | ENSDARG00000092693 | tpt1 | 1 | 33974974 | 33981243 | protein\_coding | | ENSDARG00000069184 | ubtd2 | 21 | 35181320 | 35247119 | protein\_coding | | ENSDARG00000062948 | wasf3b | 24 | 21395668 | 21433091 | protein\_coding | | ENSDARG00000013207 | zeb1b | 12 | 26658914 | 26760366 | protein\_coding | | ENSDARG00000012035 | zgc:100832 | 23 | 16888988 | 16896529 | protein\_coding | | ENSDARG00000062326 | zgc:158409 | 5 | 22465835 | 22468975 | protein\_coding | |
