# Supplementary material for: A high-resolution mRNA expression time course of embryonic development in zebrafish
Source: eLife. 2017 Nov 16;6:e30860. doi: 10.7554/eLife.30860 (PMC5690287; doi:10.7554/eLife.30860)
Supplement: Supplementary file 6. [file elife-30860-supp6.zip › biolayout-clusters-files/Cluster015.html]

Cluster015


# Cluster015: Detail

### Go to ZFA detail

## GO

| | GO ID | Description | Domain | Annotated | Expected | Observed | Adjusted p-value | Genes | Ensembl IDs | | --- | --- | --- | --- | --- | --- | --- | --- | --- | | GO:0006457 | protein folding | biological\_process | 64 | 0.62 | 7 | 6.6e-04 | cct4 cct3 cct6a hsp90ab1 pfdn6 cct5 pfdn4 | ENSDARG00000013475 ENSDARG00000016173 ENSDARG00000021252 ENSDARG00000029150 ENSDARG00000037108 ENSDARG00000045399 ENSDARG00000061228 | | GO:0043248 | proteasome assembly | biological\_process | 11 | 0.11 | 3 | 3.6e-02 | psmd4b pomp psmg2 | ENSDARG00000023279 ENSDARG00000032296 ENSDARG00000035987 | | GO:0006446 | regulation of translational initiation | biological\_process | 21 | 0.20 | 9 | 3.6e-11 | eif3m eif3c eif3g eif3d eif3ba eif3k eif3ea eif2b1 eif3s6ip | ENSDARG00000013931 ENSDARG00000016443 ENSDARG00000016889 ENSDARG00000021257 ENSDARG00000059654 ENSDARG00000068289 ENSDARG00000090697 ENSDARG00000091402 ENSDARG00000101082 | | GO:0001731 | formation of translation preinitiation c... | biological\_process | 13 | 0.13 | 9 | 9.1e-14 | eif3m eif3c eif3g eif3d eif3ba eif3k eif3ea eif3s6ip eif3ha | ENSDARG00000013931 ENSDARG00000016443 ENSDARG00000016889 ENSDARG00000021257 ENSDARG00000059654 ENSDARG00000068289 ENSDARG00000090697 ENSDARG00000101082 ENSDARG00000102452 | | GO:0005737 | cytoplasm | cellular\_component | 2387 | 25.43 | 49 | 2.8e-03 | psmb6 apip psmd1 mrpl57 psmb1 psma8 cct4 eif3m psma6b cct3 eif3c eif3g cdh2 nkd1 psmc2 cct6a eif3d psmd4b hsp90ab1 ppp1r14bb psmb2 pomp pigf snrpe tmem167a pfdn6 mrpl51 psmc1b ola1 cct5 psma4 b3galnt2 eif3ba pfdn4 igf2bp1 mrpl37 mesdc2 eif3k ubtd2 polg2 mrpl20 eif3ea eif2b1 tpt1 tomm6 snrpg eif3s6ip psma1 eif3ha | ENSDARG00000002240 ENSDARG00000003132 ENSDARG00000003189 ENSDARG00000007285 ENSDARG00000009640 ENSDARG00000010965 ENSDARG00000013475 ENSDARG00000013931 ENSDARG00000013966 ENSDARG00000016173 ENSDARG00000016443 ENSDARG00000016889 ENSDARG00000018693 ENSDARG00000020053 ENSDARG00000020101 ENSDARG00000021252 ENSDARG00000021257 ENSDARG00000023279 ENSDARG00000029150 ENSDARG00000030161 ENSDARG00000031511 ENSDARG00000032296 ENSDARG00000032780 ENSDARG00000033175 ENSDARG00000035914 ENSDARG00000037108 ENSDARG00000041340 ENSDARG00000043561 ENSDARG00000044565 ENSDARG00000045399 ENSDARG00000045928 ENSDARG00000046133 ENSDARG00000059654 ENSDARG00000061228 ENSDARG00000061478 ENSDARG00000062916 ENSDARG00000063030 ENSDARG00000068289 ENSDARG00000069184 ENSDARG00000074933 ENSDARG00000090462 ENSDARG00000090697 ENSDARG00000091402 ENSDARG00000092693 ENSDARG00000097797 ENSDARG00000099667 ENSDARG00000101082 ENSDARG00000101560 ENSDARG00000102452 | | GO:0005852 | eukaryotic translation initiation factor... | cellular\_component | 14 | 0.15 | 9 | 5.8e-13 | eif3m eif3c eif3g eif3d eif3ba eif3k eif3ea eif3s6ip eif3ha | ENSDARG00000013931 ENSDARG00000016443 ENSDARG00000016889 ENSDARG00000021257 ENSDARG00000059654 ENSDARG00000068289 ENSDARG00000090697 ENSDARG00000101082 ENSDARG00000102452 | | GO:0016282 | eukaryotic 43S preinitiation complex | cellular\_component | 12 | 0.13 | 9 | 6.3e-14 | eif3m eif3c eif3g eif3d eif3ba eif3k eif3ea eif3s6ip eif3ha | ENSDARG00000013931 ENSDARG00000016443 ENSDARG00000016889 ENSDARG00000021257 ENSDARG00000059654 ENSDARG00000068289 ENSDARG00000090697 ENSDARG00000101082 ENSDARG00000102452 | | GO:0033290 | eukaryotic 48S preinitiation complex | cellular\_component | 14 | 0.15 | 9 | 5.8e-13 | eif3m eif3c eif3g eif3d eif3ba eif3k eif3ea eif3s6ip eif3ha | ENSDARG00000013931 ENSDARG00000016443 ENSDARG00000016889 ENSDARG00000021257 ENSDARG00000059654 ENSDARG00000068289 ENSDARG00000090697 ENSDARG00000101082 ENSDARG00000102452 | | GO:0005732 | small nucleolar ribonucleoprotein comple... | cellular\_component | 11 | 0.12 | 3 | 4.6e-02 | lsm7 snrpg snrpf | ENSDARG00000058328 ENSDARG00000099667 ENSDARG00000105037 | | GO:0005839 | proteasome core complex | cellular\_component | 14 | 0.15 | 7 | 1.0e-08 | psmb6 psmb1 psma8 psma6b psmb2 psma4 psma1 | ENSDARG00000002240 ENSDARG00000009640 ENSDARG00000010965 ENSDARG00000013966 ENSDARG00000031511 ENSDARG00000045928 ENSDARG00000101560 | | GO:0005838 | proteasome regulatory particle | cellular\_component | 11 | 0.12 | 4 | 9.7e-04 | psmd1 psmc2 psmd4b psmc1b | ENSDARG00000003189 ENSDARG00000020101 ENSDARG00000023279 ENSDARG00000043561 | | GO:0051082 | unfolded protein binding | molecular\_function | 27 | 0.25 | 7 | 7.9e-07 | cct4 cct3 cct6a hsp90ab1 pfdn6 cct5 pfdn4 | ENSDARG00000013475 ENSDARG00000016173 ENSDARG00000021252 ENSDARG00000029150 ENSDARG00000037108 ENSDARG00000045399 ENSDARG00000061228 | | GO:0003743 | translation initiation factor activity | molecular\_function | 38 | 0.35 | 10 | 2.2e-10 | eif3m eif3c eif3g eif3d eif3ba eif3k eif3ea eif2b1 eif3s6ip eif3ha | ENSDARG00000013931 ENSDARG00000016443 ENSDARG00000016889 ENSDARG00000021257 ENSDARG00000059654 ENSDARG00000068289 ENSDARG00000090697 ENSDARG00000091402 ENSDARG00000101082 ENSDARG00000102452 | | GO:0004298 | threonine-type endopeptidase activity | molecular\_function | 14 | 0.13 | 7 | 3.3e-09 | psmb6 psmb1 psma8 psma6b psmb2 psma4 psma1 | ENSDARG00000002240 ENSDARG00000009640 ENSDARG00000010965 ENSDARG00000013966 ENSDARG00000031511 ENSDARG00000045928 ENSDARG00000101560 | |
