# Supplementary material for: A high-resolution mRNA expression time course of embryonic development in zebrafish
Source: eLife. 2017 Nov 16;6:e30860. doi: 10.7554/eLife.30860 (PMC5690287; doi:10.7554/eLife.30860)
Supplement: Supplementary file 6. [file elife-30860-supp6.zip › biolayout-clusters-files/Cluster016-genes.html]

Cluster016


# Cluster016: Genes

| | Ensembl ID | Gene Name | Chr | Start | End | Biotype | | --- | --- | --- | --- | --- | --- | | ENSDARG00000006901 | AEBP1 (1 of many) | 8 | 44765667 | 44806787 | protein\_coding | | ENSDARG00000079012 | CBFA2T3 | 7 | 55354018 | 55406429 | protein\_coding | | ENSDARG00000022615 | ENSDARG00000022615 | 2 | 1073933 | 1115009 | protein\_coding | | ENSDARG00000041998 | ENSDARG00000041998 | 9 | 9344387 | 9370021 | protein\_coding | | ENSDARG00000098817 | ENSDARG00000098817 | 24 | 37230175 | 37243208 | protein\_coding | | ENSDARG00000100128 | ENSDARG00000100128 | 2 | 59444057 | 59531877 | protein\_coding | | ENSDARG00000102165 | ENSDARG00000102165 | KN150518.1 | 16340 | 40355 | protein\_coding | | ENSDARG00000003803 | LHX3 | 5 | 71028034 | 71046618 | protein\_coding | | ENSDARG00000092624 | SPTBN4 | 18 | 46031942 | 46137909 | protein\_coding | | ENSDARG00000101282 | ZSWIM6 | 10 | 44416135 | 44542315 | protein\_coding | | ENSDARG00000060149 | ablim1a | 13 | 43373558 | 43466114 | protein\_coding | | ENSDARG00000075222 | amer2 | 24 | 24924435 | 24927858 | protein\_coding | | ENSDARG00000097964 | arhgef33 | 17 | 24650127 | 24662564 | protein\_coding | | ENSDARG00000004472 | atat1 | 19 | 27895340 | 27922145 | protein\_coding | | ENSDARG00000071678 | atcaya | 22 | 4719361 | 4732282 | protein\_coding | | ENSDARG00000004074 | bach2b | 20 | 24073848 | 24223781 | protein\_coding | | ENSDARG00000035508 | barhl1a | 5 | 29146202 | 29150224 | protein\_coding | | ENSDARG00000019013 | barhl1b | 21 | 17441857 | 17445829 | protein\_coding | | ENSDARG00000104361 | barhl2 | 6 | 24718043 | 24721872 | protein\_coding | | ENSDARG00000058039 | bhlhe22 | 24 | 24316167 | 24318172 | protein\_coding | | ENSDARG00000075501 | cdnf | 4 | 9587052 | 9591486 | protein\_coding | | ENSDARG00000034668 | celf3a | 19 | 8897732 | 8961763 | protein\_coding | | ENSDARG00000006983 | celf3b | 16 | 1306497 | 1336651 | protein\_coding | | ENSDARG00000055825 | celsr3 | 8 | 26106079 | 26193581 | protein\_coding | | ENSDARG00000035327 | ckma | 5 | 36231926 | 36237693 | protein\_coding | | ENSDARG00000040565 | ckmb | 15 | 23708453 | 23711813 | protein\_coding | | ENSDARG00000074558 | cntnap2b | 2 | 50542525 | 50638237 | protein\_coding | | ENSDARG00000008660 | coro1b | 7 | 18365720 | 18403802 | protein\_coding | | ENSDARG00000077817 | cxxc4 | 1 | 25557997 | 25601033 | protein\_coding | | ENSDARG00000079850 | dchs1b | 10 | 26307359 | 26468302 | protein\_coding | | ENSDARG00000104664 | dclk1b | 15 | 32963413 | 33033289 | protein\_coding | | ENSDARG00000102845 | dclk2b | 23 | 45876454 | 45907313 | protein\_coding | | ENSDARG00000025309 | dpf3 | 20 | 28531696 | 28598602 | protein\_coding | | ENSDARG00000002587 | dpysl3 | 21 | 42975807 | 43026872 | protein\_coding | | ENSDARG00000103490 | dpysl4 | 12 | 41086234 | 41124405 | protein\_coding | | ENSDARG00000060948 | dync1i1 | 16 | 25401941 | 25422802 | protein\_coding | | ENSDARG00000099849 | ebf1a | 14 | 34673342 | 34910320 | protein\_coding | | ENSDARG00000100244 | ebf3a | 12 | 42281039 | 42424191 | protein\_coding | | ENSDARG00000039701 | emx2 | 13 | 19191704 | 19196675 | protein\_coding | | ENSDARG00000020122 | endou2 | 21 | 41850330 | 41859887 | protein\_coding | | ENSDARG00000086892 | erfl1 | 16 | 26360737 | 26382435 | protein\_coding | | ENSDARG00000007697 | fabp7a | 17 | 15425585 | 15427954 | protein\_coding | | ENSDARG00000017195 | foxf2a | 2 | 840714 | 844060 | protein\_coding | | ENSDARG00000070389 | foxf2b | 20 | 26790946 | 26793854 | protein\_coding | | ENSDARG00000099744 | gap43 | KN150239.1 | 1 | 14235 | protein\_coding | | ENSDARG00000056831 | gng2 | 13 | 11913000 | 11956360 | protein\_coding | | ENSDARG00000103826 | gpib | 18 | 512830 | 1011132 | protein\_coding | | ENSDARG00000044550 | hif1al2 | 21 | 27290747 | 27303205 | protein\_coding | | ENSDARG00000070954 | hmx2 | 17 | 21762207 | 21764316 | protein\_coding | | ENSDARG00000070955 | hmx3a | 17 | 21496581 | 21773277 | protein\_coding | | ENSDARG00000070340 | hoxc5a | 23 | 36019935 | 36021708 | protein\_coding | | ENSDARG00000067714 | hspb3 | 5 | 39696398 | 39696850 | protein\_coding | | ENSDARG00000001785 | irx2a | 16 | 539456 | 544003 | protein\_coding | | ENSDARG00000035648 | irx4a | 16 | 287438 | 292336 | protein\_coding | | ENSDARG00000004023 | isl1 | 5 | 40128460 | 40133892 | protein\_coding | | ENSDARG00000041071 | jagn1a | 9 | 30297895 | 30300359 | protein\_coding | | ENSDARG00000044769 | kctd13 | 3 | 15064180 | 15077866 | protein\_coding | | ENSDARG00000039458 | lhx4 | 8 | 14416228 | 14446894 | protein\_coding | | ENSDARG00000056979 | lhx9 | 22 | 23228025 | 23238701 | protein\_coding | | ENSDARG00000068974 | lim2.1 | 10 | 30009935 | 30016825 | protein\_coding | | ENSDARG00000037402 | lim2.3 | 15 | 19717075 | 19723846 | protein\_coding | | ENSDARG00000102047 | mab21l1 | 15 | 33212960 | 33215221 | protein\_coding | | ENSDARG00000015266 | mab21l2 | 1 | 23385414 | 23387270 | protein\_coding | | ENSDARG00000096375 | march7.2 | 6 | 11529914 | 11576641 | protein\_coding | | ENSDARG00000098240 | meis2a | 17 | 52736268 | 52835080 | protein\_coding | | ENSDARG00000015184 | mpp3a | 3 | 38506129 | 38551062 | protein\_coding | | ENSDARG00000037639 | nkx3.2 | 14 | 6196 | 7908 | protein\_coding | | ENSDARG00000088411 | notum2 | 6 | 10708590 | 10728460 | protein\_coding | | ENSDARG00000052960 | nppa | 8 | 48624271 | 48628895 | protein\_coding | | ENSDARG00000045904 | nr2e3 | 25 | 22177094 | 22181107 | protein\_coding | | ENSDARG00000007406 | phox2a | 15 | 46976198 | 46984969 | protein\_coding | | ENSDARG00000005559 | pou4f1 | 6 | 4379877 | 4382908 | protein\_coding | | ENSDARG00000044541 | ppp1r14ba | 21 | 26959581 | 26974142 | protein\_coding | | ENSDARG00000078701 | prdm13 | 16 | 32795561 | 32801870 | protein\_coding | | ENSDARG00000063036 | psd2 | 14 | 7821643 | 7943223 | protein\_coding | | ENSDARG00000021113 | ptmaa | 6 | 29763580 | 29804468 | protein\_coding | | ENSDARG00000051814 | ptprz1a | 25 | 27896969 | 28000405 | protein\_coding | | ENSDARG00000075183 | rnf220b | 8 | 18206793 | 18280323 | protein\_coding | | ENSDARG00000040214 | scrt1b | 16 | 31228156 | 31230788 | protein\_coding | | ENSDARG00000056092 | si:dkey-12h9.6 | 20 | 26127428 | 26142980 | protein\_coding | | ENSDARG00000087979 | si:dkey-245g22.3 | 2 | 12192662 | 12197706 | antisense | | ENSDARG00000056519 | si:dkey-280e21.3 | 14 | 25168086 | 25205292 | protein\_coding | | ENSDARG00000031316 | six6b | 20 | 20631329 | 20634192 | protein\_coding | | ENSDARG00000062448 | skor1b | 18 | 19985519 | 19992953 | protein\_coding | | ENSDARG00000070929 | sox14 | 6 | 26567418 | 26569360 | protein\_coding | | ENSDARG00000004588 | sox4a | 19 | 29201926 | 29205186 | protein\_coding | | ENSDARG00000070537 | stmn2b | 19 | 32183957 | 32203238 | protein\_coding | | ENSDARG00000042041 | tal2 | 5 | 8843646 | 8845563 | protein\_coding | | ENSDARG00000012667 | tfap2b | 20 | 48211715 | 48230662 | protein\_coding | | ENSDARG00000008861 | tfap2e | 19 | 47871836 | 47896188 | protein\_coding | | ENSDARG00000061479 | thsd7aa | 19 | 38033293 | 38256283 | protein\_coding | | ENSDARG00000029069 | tnni2a.4 | 25 | 30699939 | 30711182 | protein\_coding | | ENSDARG00000055216 | tuba1c | 23 | 35591854 | 35595371 | protein\_coding | | ENSDARG00000102976 | uncx | 3 | 42646541 | 42650067 | protein\_coding | | ENSDARG00000062479 | vcam1 | 22 | 16144331 | 16154579 | protein\_coding | | ENSDARG00000076373 | vopp1 | 24 | 464125 | 475199 | protein\_coding | | ENSDARG00000040159 | wnt4b | 16 | 31846615 | 31860152 | protein\_coding | | ENSDARG00000103057 | zfhx3 | 7 | 67980241 | 68138801 | protein\_coding | | ENSDARG00000075542 | zfhx4 | 24 | 23097140 | 23175049 | protein\_coding | | ENSDARG00000057141 | zgc:110045 | 13 | 16087891 | 16126854 | protein\_coding | | ENSDARG00000044375 | zgc:158291 | 21 | 38264843 | 38267535 | protein\_coding | | ENSDARG00000060597 | zgc:158659 | 23 | 44424928 | 44467486 | protein\_coding | | ENSDARG00000079738 | znf219 | 7 | 73371701 | 73398250 | protein\_coding | | ENSDARG00000076171 | znf827 | 1 | 35285840 | 35419350 | protein\_coding | |
