# Supplementary material for: A high-resolution mRNA expression time course of embryonic development in zebrafish
Source: eLife. 2017 Nov 16;6:e30860. doi: 10.7554/eLife.30860 (PMC5690287; doi:10.7554/eLife.30860)
Supplement: Supplementary file 6. [file elife-30860-supp6.zip › biolayout-clusters-files/Cluster016.html]

Cluster016


# Cluster016: Detail

### Go to ZFA detail

## GO

| | GO ID | Description | Domain | Annotated | Expected | Observed | Adjusted p-value | Genes | Ensembl IDs | | --- | --- | --- | --- | --- | --- | --- | --- | --- | | GO:0006355 | regulation of transcription, DNA-templat... | biological\_process | 1072 | 10.34 | 34 | 2.0e-10 | irx2a isl1 bach2b pou4f1 phox2a tfap2e tfap2b foxf2a barhl1b dpf3 barhl1a irx4a nkx3.2 lhx4 emx2 zgc:158291 hif1al2 nr2e3 lhx9 bhlhe22 skor1b hoxc5a foxf2b sox14 hmx2 hmx3a zfhx4 erfl1 meis2a ebf1a ebf3a uncx zfhx3 barhl2 | ENSDARG00000001785 ENSDARG00000004023 ENSDARG00000004074 ENSDARG00000005559 ENSDARG00000007406 ENSDARG00000008861 ENSDARG00000012667 ENSDARG00000017195 ENSDARG00000019013 ENSDARG00000025309 ENSDARG00000035508 ENSDARG00000035648 ENSDARG00000037639 ENSDARG00000039458 ENSDARG00000039701 ENSDARG00000044375 ENSDARG00000044550 ENSDARG00000045904 ENSDARG00000056979 ENSDARG00000058039 ENSDARG00000062448 ENSDARG00000070340 ENSDARG00000070389 ENSDARG00000070929 ENSDARG00000070954 ENSDARG00000070955 ENSDARG00000075542 ENSDARG00000086892 ENSDARG00000098240 ENSDARG00000099849 ENSDARG00000100244 ENSDARG00000102976 ENSDARG00000103057 ENSDARG00000104361 | | GO:0005634 | nucleus | cellular\_component | 1915 | 16.55 | 39 | 1.0e-09 | irx2a isl1 bach2b pou4f1 phox2a tfap2e tfap2b mab21l2 foxf2a barhl1b ptmaa dpf3 six6b celf3a barhl1a irx4a nkx3.2 lhx4 emx2 tal2 zgc:158291 hif1al2 nr2e3 lhx9 skor1b hoxc5a foxf2b sox14 hmx2 hmx3a zfhx4 erfl1 meis2a ebf1a ebf3a mab21l1 uncx zfhx3 barhl2 | ENSDARG00000001785 ENSDARG00000004023 ENSDARG00000004074 ENSDARG00000005559 ENSDARG00000007406 ENSDARG00000008861 ENSDARG00000012667 ENSDARG00000015266 ENSDARG00000017195 ENSDARG00000019013 ENSDARG00000021113 ENSDARG00000025309 ENSDARG00000031316 ENSDARG00000034668 ENSDARG00000035508 ENSDARG00000035648 ENSDARG00000037639 ENSDARG00000039458 ENSDARG00000039701 ENSDARG00000042041 ENSDARG00000044375 ENSDARG00000044550 ENSDARG00000045904 ENSDARG00000056979 ENSDARG00000062448 ENSDARG00000070340 ENSDARG00000070389 ENSDARG00000070929 ENSDARG00000070954 ENSDARG00000070955 ENSDARG00000075542 ENSDARG00000086892 ENSDARG00000098240 ENSDARG00000099849 ENSDARG00000100244 ENSDARG00000102047 ENSDARG00000102976 ENSDARG00000103057 ENSDARG00000104361 | | GO:0000981 | RNA polymerase II transcription factor a... | molecular\_function | 192 | 1.77 | 14 | 1.7e-03 | tfap2e tfap2b foxf2a barhl1b barhl1a tal2 hif1al2 nr2e3 bhlhe22 foxf2b erfl1 ebf1a ebf3a barhl2 | ENSDARG00000008861 ENSDARG00000012667 ENSDARG00000017195 ENSDARG00000019013 ENSDARG00000035508 ENSDARG00000042041 ENSDARG00000044550 ENSDARG00000045904 ENSDARG00000058039 ENSDARG00000070389 ENSDARG00000086892 ENSDARG00000099849 ENSDARG00000100244 ENSDARG00000104361 | | GO:0001228 | transcriptional activator activity, RNA ... | molecular\_function | 23 | 0.21 | 5 | 4.6e-04 | barhl1b barhl1a ebf1a ebf3a barhl2 | ENSDARG00000019013 ENSDARG00000035508 ENSDARG00000099849 ENSDARG00000100244 ENSDARG00000104361 | | GO:0043565 | sequence-specific DNA binding | molecular\_function | 494 | 4.56 | 27 | 2.8e-09 | irx2a isl1 bach2b pou4f1 phox2a tfap2e tfap2b foxf2a barhl1b barhl1a irx4a nkx3.2 lhx4 emx2 tal2 zgc:158291 nr2e3 lhx9 hoxc5a foxf2b hmx2 hmx3a zfhx4 erfl1 uncx zfhx3 barhl2 | ENSDARG00000001785 ENSDARG00000004023 ENSDARG00000004074 ENSDARG00000005559 ENSDARG00000007406 ENSDARG00000008861 ENSDARG00000012667 ENSDARG00000017195 ENSDARG00000019013 ENSDARG00000035508 ENSDARG00000035648 ENSDARG00000037639 ENSDARG00000039458 ENSDARG00000039701 ENSDARG00000042041 ENSDARG00000044375 ENSDARG00000045904 ENSDARG00000056979 ENSDARG00000070340 ENSDARG00000070389 ENSDARG00000070954 ENSDARG00000070955 ENSDARG00000075542 ENSDARG00000086892 ENSDARG00000102976 ENSDARG00000103057 ENSDARG00000104361 | | GO:0000977 | RNA polymerase II regulatory region sequ... | molecular\_function | 109 | 1.01 | 7 | 1.6e-02 | bach2b tfap2e tfap2b barhl1b barhl1a tal2 barhl2 | ENSDARG00000004074 ENSDARG00000008861 ENSDARG00000012667 ENSDARG00000019013 ENSDARG00000035508 ENSDARG00000042041 ENSDARG00000104361 | |

  


### Go to GO detail

## ZFA

| | ZFA ID | Description | Annotated | Expected | Observed | Fold Enrichment | Adjusted p-value | Genes | Ensembl IDs | | --- | --- | --- | --- | --- | --- | --- | --- | --- | | ZFA:0000396 | nervous system | 430 | 3.41 | 6 | 1.8 | 0.00022 | phox2a fabp7a ptmaa bhlhe22 tuba1c isl1 | ENSDARG00000007406 ENSDARG00000007697 ENSDARG00000021113 ENSDARG00000058039 ENSDARG00000055216 ENSDARG00000004023 | | ZFA:0000029 | hindbrain | 1756 | 13.92 | 34 | 2.4 | 0.00022 | phox2a wnt4b fabp7a hmx3a mab21l2 meis2a mab21l1 zfhx3 ptmaa sox4a coro1b bhlhe22 irx2a tfap2e irx4a scrt1b lhx9 barhl1a tfap2b stmn2b dpysl3 dpysl4 barhl2 barhl1b thsd7aa kctd13 ebf3a vcam1 zfhx4 prdm13 ebf1a znf219 isl1 gap43 | ENSDARG00000007406 ENSDARG00000040159 ENSDARG00000007697 ENSDARG00000070955 ENSDARG00000015266 ENSDARG00000098240 ENSDARG00000102047 ENSDARG00000103057 ENSDARG00000021113 ENSDARG00000004588 ENSDARG00000008660 ENSDARG00000058039 ENSDARG00000001785 ENSDARG00000008861 ENSDARG00000035648 ENSDARG00000040214 ENSDARG00000056979 ENSDARG00000035508 ENSDARG00000012667 ENSDARG00000070537 ENSDARG00000002587 ENSDARG00000103490 ENSDARG00000104361 ENSDARG00000019013 ENSDARG00000061479 ENSDARG00000044769 ENSDARG00000100244 ENSDARG00000062479 ENSDARG00000075542 ENSDARG00000078701 ENSDARG00000099849 ENSDARG00000079738 ENSDARG00000004023 ENSDARG00000099744 | | ZFA:0000075 | spinal cord | 1319 | 10.46 | 27 | 2.6 | 0.00022 | fabp7a hmx3a mab21l2 meis2a mab21l1 psd2 ptmaa sox4a tal2 coro1b bhlhe22 irx2a irx4a dpf3 scrt1b tfap2b gng2 stmn2b dpysl3 dpysl4 barhl2 thsd7aa atcaya prdm13 ebf1a isl1 gap43 | ENSDARG00000007697 ENSDARG00000070955 ENSDARG00000015266 ENSDARG00000098240 ENSDARG00000102047 ENSDARG00000063036 ENSDARG00000021113 ENSDARG00000004588 ENSDARG00000042041 ENSDARG00000008660 ENSDARG00000058039 ENSDARG00000001785 ENSDARG00000035648 ENSDARG00000025309 ENSDARG00000040214 ENSDARG00000012667 ENSDARG00000056831 ENSDARG00000070537 ENSDARG00000002587 ENSDARG00000103490 ENSDARG00000104361 ENSDARG00000061479 ENSDARG00000071678 ENSDARG00000078701 ENSDARG00000099849 ENSDARG00000004023 ENSDARG00000099744 | | ZFA:0009248 | neuron | 504 | 4.00 | 15 | 3.8 | 0.00028 | phox2a fabp7a hmx3a mab21l2 mab21l1 sox4a bhlhe22 irx4a tfap2b stmn2b barhl2 tuba1c atcaya isl1 gap43 | ENSDARG00000007406 ENSDARG00000007697 ENSDARG00000070955 ENSDARG00000015266 ENSDARG00000102047 ENSDARG00000004588 ENSDARG00000058039 ENSDARG00000035648 ENSDARG00000012667 ENSDARG00000070537 ENSDARG00000104361 ENSDARG00000055216 ENSDARG00000071678 ENSDARG00000004023 ENSDARG00000099744 | | ZFA:0009019 | neuronal stem cell | 13 | 0.10 | 1 | 10.0 | 0.00031 | isl1 | ENSDARG00000004023 | | ZFA:0000109 | forebrain | 883 | 7.00 | 16 | 2.3 | 0.00062 | fabp7a mab21l1 zfhx3 coro1b bhlhe22 scrt1b lhx9 tfap2b stmn2b barhl2 kctd13 tuba1c vcam1 zfhx4 isl1 emx2 | ENSDARG00000007697 ENSDARG00000102047 ENSDARG00000103057 ENSDARG00000008660 ENSDARG00000058039 ENSDARG00000040214 ENSDARG00000056979 ENSDARG00000012667 ENSDARG00000070537 ENSDARG00000104361 ENSDARG00000044769 ENSDARG00000055216 ENSDARG00000062479 ENSDARG00000075542 ENSDARG00000004023 ENSDARG00000039701 | | ZFA:0000012 | central nervous system | 1346 | 10.67 | 18 | 1.7 | 0.00070 | phox2a fabp7a hmx3a ptmaa sox4a coro1b bhlhe22 irx4a dchs1b scrt1b tfap2b gng2 stmn2b thsd7aa ppp1r14ba tuba1c atcaya isl1 | ENSDARG00000007406 ENSDARG00000007697 ENSDARG00000070955 ENSDARG00000021113 ENSDARG00000004588 ENSDARG00000008660 ENSDARG00000058039 ENSDARG00000035648 ENSDARG00000079850 ENSDARG00000040214 ENSDARG00000012667 ENSDARG00000056831 ENSDARG00000070537 ENSDARG00000061479 ENSDARG00000044541 ENSDARG00000055216 ENSDARG00000071678 ENSDARG00000004023 | | ZFA:0000024 | retinal ganglion cell layer | 510 | 4.04 | 16 | 4.0 | 0.00242 | mab21l2 meis2a zfhx3 sox4a bhlhe22 irx2a irx4a dchs1b barhl1a gng2 barhl2 celsr3 zfhx4 ebf1a isl1 gap43 | ENSDARG00000015266 ENSDARG00000098240 ENSDARG00000103057 ENSDARG00000004588 ENSDARG00000058039 ENSDARG00000001785 ENSDARG00000035648 ENSDARG00000079850 ENSDARG00000035508 ENSDARG00000056831 ENSDARG00000104361 ENSDARG00000055825 ENSDARG00000075542 ENSDARG00000099849 ENSDARG00000004023 ENSDARG00000099744 | | ZFA:0000101 | diencephalon | 1085 | 8.60 | 25 | 2.9 | 0.00336 | fabp7a hmx3a meis2a ptmaa sox4a coro1b bhlhe22 irx2a irx4a nr2e3 scrt1b lhx9 barhl1a gng2 stmn2b dpysl3 dpysl4 barhl2 barhl1b kctd13 vcam1 zfhx4 isl1 emx2 gap43 | ENSDARG00000007697 ENSDARG00000070955 ENSDARG00000098240 ENSDARG00000021113 ENSDARG00000004588 ENSDARG00000008660 ENSDARG00000058039 ENSDARG00000001785 ENSDARG00000035648 ENSDARG00000045904 ENSDARG00000040214 ENSDARG00000056979 ENSDARG00000035508 ENSDARG00000056831 ENSDARG00000070537 ENSDARG00000002587 ENSDARG00000103490 ENSDARG00000104361 ENSDARG00000019013 ENSDARG00000044769 ENSDARG00000062479 ENSDARG00000075542 ENSDARG00000004023 ENSDARG00000039701 ENSDARG00000099744 | | ZFA:0000079 | telencephalon | 1004 | 7.96 | 22 | 2.8 | 0.00336 | fabp7a hmx3a meis2a mab21l1 zfhx3 ptmaa sox4a coro1b tfap2e scrt1b lhx9 stmn2b dpysl3 dpysl4 barhl2 thsd7aa kctd13 zfhx4 cntnap2b isl1 emx2 gap43 | ENSDARG00000007697 ENSDARG00000070955 ENSDARG00000098240 ENSDARG00000102047 ENSDARG00000103057 ENSDARG00000021113 ENSDARG00000004588 ENSDARG00000008660 ENSDARG00000008861 ENSDARG00000040214 ENSDARG00000056979 ENSDARG00000070537 ENSDARG00000002587 ENSDARG00000103490 ENSDARG00000104361 ENSDARG00000061479 ENSDARG00000044769 ENSDARG00000075542 ENSDARG00000074558 ENSDARG00000004023 ENSDARG00000039701 ENSDARG00000099744 | | ZFA:0000128 | midbrain | 1312 | 10.40 | 20 | 1.9 | 0.00336 | pou4f1 fabp7a mab21l2 meis2a mab21l1 zfhx3 sox4a bhlhe22 irx2a irx4a tfap2b gng2 dpysl3 dpysl4 barhl2 thsd7aa kctd13 zfhx4 ebf1a gap43 | ENSDARG00000005559 ENSDARG00000007697 ENSDARG00000015266 ENSDARG00000098240 ENSDARG00000102047 ENSDARG00000103057 ENSDARG00000004588 ENSDARG00000058039 ENSDARG00000001785 ENSDARG00000035648 ENSDARG00000012667 ENSDARG00000056831 ENSDARG00000002587 ENSDARG00000103490 ENSDARG00000104361 ENSDARG00000061479 ENSDARG00000044769 ENSDARG00000075542 ENSDARG00000099849 ENSDARG00000099744 | | ZFA:0009051 | interneuron | 16 | 0.13 | 1 | 7.7 | 0.02227 | isl1 | ENSDARG00000004023 | | ZFA:0007042 | spinal cord neural tube | 91 | 0.72 | 3 | 4.2 | 0.02280 | scrt1b dpysl3 dpysl4 | ENSDARG00000040214 ENSDARG00000002587 ENSDARG00000103490 | | ZFA:0000152 | retina | 1650 | 13.08 | 27 | 2.1 | 0.02703 | pou4f1 fabp7a mab21l2 meis2a mab21l1 zfhx3 ptmaa sox4a coro1b bhlhe22 irx2a tfap2e irx4a nr2e3 dchs1b barhl1a tfap2b stmn2b six6b barhl2 barhl1b thsd7aa kctd13 prdm13 ebf1a isl1 gap43 | ENSDARG00000005559 ENSDARG00000007697 ENSDARG00000015266 ENSDARG00000098240 ENSDARG00000102047 ENSDARG00000103057 ENSDARG00000021113 ENSDARG00000004588 ENSDARG00000008660 ENSDARG00000058039 ENSDARG00000001785 ENSDARG00000008861 ENSDARG00000035648 ENSDARG00000045904 ENSDARG00000079850 ENSDARG00000035508 ENSDARG00000012667 ENSDARG00000070537 ENSDARG00000031316 ENSDARG00000104361 ENSDARG00000019013 ENSDARG00000061479 ENSDARG00000044769 ENSDARG00000078701 ENSDARG00000099849 ENSDARG00000004023 ENSDARG00000099744 | | ZFA:0000295 | trigeminal ganglion | 201 | 1.59 | 9 | 5.7 | 0.02942 | fabp7a ptmaa irx2a irx4a stmn2b dpysl3 dpysl4 isl1 gap43 | ENSDARG00000007697 ENSDARG00000021113 ENSDARG00000001785 ENSDARG00000035648 ENSDARG00000070537 ENSDARG00000002587 ENSDARG00000103490 ENSDARG00000004023 ENSDARG00000099744 | |
