# Supplementary material for: A high-resolution mRNA expression time course of embryonic development in zebrafish
Source: eLife. 2017 Nov 16;6:e30860. doi: 10.7554/eLife.30860 (PMC5690287; doi:10.7554/eLife.30860)
Supplement: Supplementary file 6. [file elife-30860-supp6.zip › biolayout-clusters-files/Cluster017-genes.html]

Cluster017


# Cluster017: Genes

| | Ensembl ID | Gene Name | Chr | Start | End | Biotype | | --- | --- | --- | --- | --- | --- | | ENSDARG00000059751 | ABTB2 (1 of many) | 7 | 48999885 | 49046965 | protein\_coding | | ENSDARG00000099678 | BX569789.3 | 3 | 24544481 | 24550856 | protein\_coding | | ENSDARG00000098640 | CU984600.3 | 9 | 535602 | 544884 | protein\_coding | | ENSDARG00000021566 | ENSDARG00000021566 | 14 | 196090 | 204764 | protein\_coding | | ENSDARG00000022807 | ENSDARG00000022807 | 16 | 13534374 | 13563698 | protein\_coding | | ENSDARG00000088835 | ENSDARG00000088835 | 7 | 23778698 | 23795704 | protein\_coding | | ENSDARG00000060627 | HIP1R (1 of many) | 10 | 44583879 | 44619301 | protein\_coding | | ENSDARG00000078801 | MSANTD1 | 7 | 18297426 | 18302301 | protein\_coding | | ENSDARG00000079790 | PLPP1 (1 of many).1 | 8 | 584616 | 601889 | protein\_coding | | ENSDARG00000098867 | PRDM15 | 10 | 33325511 | 33357665 | protein\_coding | | ENSDARG00000079340 | SLC47A1 (1 of many).1 | 15 | 26683128 | 26703950 | protein\_coding | | ENSDARG00000030006 | SLC6A7 | 21 | 30757656 | 30794206 | protein\_coding | | ENSDARG00000103580 | TWSG1 (1 of many) | KN150650.1 | 1929 | 3012 | protein\_coding | | ENSDARG00000010347 | acer1 | 8 | 20200336 | 20213807 | protein\_coding | | ENSDARG00000056250 | add3b | 17 | 20228341 | 20267694 | protein\_coding | | ENSDARG00000026090 | adprm | 12 | 90491 | 93198 | protein\_coding | | ENSDARG00000058226 | ak3 | 10 | 11042656 | 11053762 | protein\_coding | | ENSDARG00000018971 | b3gnt5a | 11 | 10557566 | 10566685 | protein\_coding | | ENSDARG00000062173 | camsap2a | 22 | 22317347 | 22391772 | protein\_coding | | ENSDARG00000070142 | cart4 | 19 | 42672093 | 42674921 | protein\_coding | | ENSDARG00000087863 | cd44a | 7 | 49609974 | 49620215 | protein\_coding | | ENSDARG00000043128 | cldne | 15 | 2675312 | 2677394 | protein\_coding | | ENSDARG00000043133 | cldnf | 15 | 2686210 | 2689068 | protein\_coding | | ENSDARG00000011701 | ctsll | 5 | 23802077 | 23805867 | protein\_coding | | ENSDARG00000092660 | cyp27c1 | 6 | 9332610 | 9346410 | protein\_coding | | ENSDARG00000079653 | cyp2x6 | 25 | 17262737 | 17271179 | protein\_coding | | ENSDARG00000062632 | duox | 25 | 31547311 | 31608285 | protein\_coding | | ENSDARG00000056012 | eve1 | 3 | 23511387 | 23513282 | protein\_coding | | ENSDARG00000090063 | fa2h | 25 | 13462592 | 13518463 | protein\_coding | | ENSDARG00000060830 | fam83hb | 19 | 44016582 | 44043734 | protein\_coding | | ENSDARG00000037677 | fgf24 | 14 | 5200149 | 5239663 | protein\_coding | | ENSDARG00000055713 | fmnl1a | 3 | 37055828 | 37093442 | protein\_coding | | ENSDARG00000027807 | fynrk | 20 | 451800 | 475349 | protein\_coding | | ENSDARG00000017821 | gata5 | 23 | 7445756 | 7457056 | protein\_coding | | ENSDARG00000103589 | gata6 | 2 | 4366260 | 4376938 | protein\_coding | | ENSDARG00000099329 | h2afy | 21 | 45538226 | 45550257 | protein\_coding | | ENSDARG00000021494 | hnf4a | 23 | 25906148 | 25939315 | protein\_coding | | ENSDARG00000054842 | hsd17b14 | 3 | 29838301 | 29846653 | protein\_coding | | ENSDARG00000023188 | lcp1 | 9 | 56663226 | 56705090 | protein\_coding | | ENSDARG00000089016 | mapkapk3 | 11 | 34322176 | 34367076 | protein\_coding | | ENSDARG00000068761 | mespab | 25 | 10918621 | 10919879 | protein\_coding | | ENSDARG00000060005 | mtrf1l | 13 | 46637515 | 46647222 | protein\_coding | | ENSDARG00000021688 | mxa | 1 | 6878883 | 6898529 | protein\_coding | | ENSDARG00000038281 | natd1 | 3 | 15667844 | 15674300 | protein\_coding | | ENSDARG00000005320 | nipsnap1 | 5 | 11532585 | 11563164 | protein\_coding | | ENSDARG00000077726 | nocta | 14 | 47040711 | 47054844 | protein\_coding | | ENSDARG00000044685 | nr0b2a | 16 | 55167505 | 55172525 | protein\_coding | | ENSDARG00000012777 | nucks1b | 23 | 18192185 | 18203936 | protein\_coding | | ENSDARG00000036175 | pcdh1b | 14 | 37205325 | 37447047 | protein\_coding | | ENSDARG00000029075 | pfkfb4b | 6 | 39996069 | 40031887 | protein\_coding | | ENSDARG00000055046 | ponzr5 | 7 | 22386409 | 22392814 | protein\_coding | | ENSDARG00000045371 | prdm14 | 24 | 14302034 | 14312440 | protein\_coding | | ENSDARG00000035415 | ptger4b | 5 | 34798592 | 34801415 | protein\_coding | | ENSDARG00000060315 | rabgap1l2 | 2 | 36916525 | 36922738 | protein\_coding | | ENSDARG00000015611 | rasl11b | 20 | 23123885 | 23127123 | protein\_coding | | ENSDARG00000024940 | rnf144b | 16 | 43464814 | 43488697 | protein\_coding | | ENSDARG00000015822 | sesn3 | 15 | 35185038 | 35234766 | protein\_coding | | ENSDARG00000011564 | sfpq | 19 | 40438808 | 40468271 | protein\_coding | | ENSDARG00000103000 | si:ch211-107p11.3 | 7 | 26569943 | 26572871 | protein\_coding | | ENSDARG00000056836 | si:ch211-125o16.4 | 17 | 22524812 | 22532842 | protein\_coding | | ENSDARG00000068250 | si:ch211-151h10.2 | 18 | 44309699 | 44323466 | protein\_coding | | ENSDARG00000105127 | si:ch211-155e24.3 | 2 | 57894234 | 57901774 | protein\_coding | | ENSDARG00000093214 | si:ch211-284e13.9 | 5 | 37593300 | 37596927 | protein\_coding | | ENSDARG00000093131 | si:ch211-57f7.7 | 13 | 15722685 | 15734343 | protein\_coding | | ENSDARG00000102146 | si:ch211-69b7.6 | 11 | 11907959 | 11926604 | protein\_coding | | ENSDARG00000098999 | si:ch73-311h14.2 | 15 | 6971834 | 6980124 | protein\_coding | | ENSDARG00000097123 | si:ch73-70c5.2 | 24 | 38777319 | 38782952 | antisense | | ENSDARG00000060340 | si:dkey-157l19.2 | 13 | 35864445 | 35908807 | protein\_coding | | ENSDARG00000096689 | si:dkey-66i24.9 | 3 | 30962613 | 30966688 | protein\_coding | | ENSDARG00000101135 | si:dkey-85k7.7 | 7 | 21592999 | 21608008 | protein\_coding | | ENSDARG00000092225 | si:dkeyp-13a3.10 | 2 | 16916959 | 16927006 | protein\_coding | | ENSDARG00000095283 | si:dkeyp-2e4.3.1 | 13 | 44703792 | 44706915 | protein\_coding | | ENSDARG00000090099 | si:dkeyp-67a8.4 | 7 | 5420100 | 5427391 | protein\_coding | | ENSDARG00000104963 | si:rp71-84b10.3 | 13 | 15444145 | 15449160 | lincRNA | | ENSDARG00000068572 | slc16a1b | 8 | 26904474 | 26942640 | protein\_coding | | ENSDARG00000029832 | slc26a1 | 21 | 20287926 | 20295912 | protein\_coding | | ENSDARG00000036865 | slc2a12 | 23 | 31681722 | 31707085 | protein\_coding | | ENSDARG00000100917 | slc30a8 | 19 | 46751130 | 46769977 | protein\_coding | | ENSDARG00000091061 | slc38a3b | 6 | 53149117 | 53227563 | protein\_coding | | ENSDARG00000075831 | slc7a8a | 7 | 23937454 | 23965207 | protein\_coding | | ENSDARG00000074842 | snx8a | 3 | 41948594 | 41978969 | protein\_coding | | ENSDARG00000100591 | sox32 | 7 | 58522099 | 58523763 | protein\_coding | | ENSDARG00000004017 | spag1a | 19 | 12065986 | 12098914 | protein\_coding | | ENSDARG00000016783 | srsf6b | 11 | 1495539 | 1504277 | protein\_coding | | ENSDARG00000045421 | stard3nl | 2 | 31855224 | 31870130 | protein\_coding | | ENSDARG00000035694 | stm | 19 | 3196620 | 3226980 | protein\_coding | | ENSDARG00000042231 | stx11b.2 | 20 | 26636252 | 26638081 | protein\_coding | | ENSDARG00000041947 | styk1 | 16 | 32014343 | 32022239 | protein\_coding | | ENSDARG00000086826 | sult6b1 | 11 | 43083065 | 43095802 | protein\_coding | | ENSDARG00000028346 | tesca | 5 | 11340688 | 11404952 | protein\_coding | | ENSDARG00000098745 | tfec | 4 | 5988658 | 6039484 | protein\_coding | | ENSDARG00000046148 | tnfaip8l2b | 16 | 29763942 | 29779166 | protein\_coding | | ENSDARG00000013598 | tnfb | 15 | 34841616 | 34844692 | protein\_coding | | ENSDARG00000059792 | trpm5 | 7 | 48402571 | 48429274 | protein\_coding | | ENSDARG00000039899 | zbtb7a | 22 | 20347386 | 20360278 | protein\_coding | | ENSDARG00000040628 | zgc:110333 | 15 | 42604012 | 42618861 | protein\_coding | | ENSDARG00000017474 | zgc:110699 | 7 | 38540542 | 38551129 | protein\_coding | | ENSDARG00000026296 | zgc:112994 | 23 | 38228278 | 38251320 | protein\_coding | | ENSDARG00000060977 | zgc:153018 | 14 | 46352694 | 46359266 | protein\_coding | | ENSDARG00000069379 | zgc:158868 | 18 | 22120313 | 22127312 | protein\_coding | | ENSDARG00000104716 | zgc:91849 | 9 | 445838 | 456908 | protein\_coding | | ENSDARG00000103917 | znf185 | 14 | 14317356 | 14337627 | protein\_coding | |
