# Supplementary material for: A high-resolution mRNA expression time course of embryonic development in zebrafish
Source: eLife. 2017 Nov 16;6:e30860. doi: 10.7554/eLife.30860 (PMC5690287; doi:10.7554/eLife.30860)
Supplement: Supplementary file 6. [file elife-30860-supp6.zip › biolayout-clusters-files/Cluster017.html]

Cluster017


# Cluster017: Detail

### Go to ZFA detail

## GO

| | GO ID | Description | Domain | Annotated | Expected | Observed | Adjusted p-value | Genes | Ensembl IDs | | --- | --- | --- | --- | --- | --- | --- | --- | --- | | GO:0060029 | convergent extension involved in organog... | biological\_process | 15 | 0.11 | 3 | 0.038 | gata5 sox32 gata6 | ENSDARG00000017821 ENSDARG00000100591 ENSDARG00000103589 | | GO:0003318 | cell migration to the midline involved i... | biological\_process | 10 | 0.07 | 3 | 0.010 | gata5 sox32 gata6 | ENSDARG00000017821 ENSDARG00000100591 ENSDARG00000103589 | | GO:0060914 | heart formation | biological\_process | 11 | 0.08 | 3 | 0.014 | gata5 sox32 gata6 | ENSDARG00000017821 ENSDARG00000100591 ENSDARG00000103589 | |
