# Supplementary material for: A high-resolution mRNA expression time course of embryonic development in zebrafish
Source: eLife. 2017 Nov 16;6:e30860. doi: 10.7554/eLife.30860 (PMC5690287; doi:10.7554/eLife.30860)
Supplement: Supplementary file 6. [file elife-30860-supp6.zip › biolayout-clusters-files/Cluster018-genes.html]

Cluster018


# Cluster018: Genes

| | Ensembl ID | Gene Name | Chr | Start | End | Biotype | | --- | --- | --- | --- | --- | --- | | ENSDARG00000077717 | ENSDARG00000077717 | 11 | 1369582 | 1374046 | protein\_coding | | ENSDARG00000092787 | ENSDARG00000092787 | 10 | 27036701 | 27037769 | protein\_coding | | ENSDARG00000090568 | MRPL52 | 7 | 23843537 | 23846005 | protein\_coding | | ENSDARG00000089836 | MRPS12 | 21 | 25524679 | 25528787 | protein\_coding | | ENSDARG00000098458 | RPL37A | 11 | 44862932 | 44868144 | protein\_coding | | ENSDARG00000092807 | RPL41 | 23 | 25378890 | 25380062 | protein\_coding | | ENSDARG00000093606 | RPS11 (1 of many) | 8 | 26683540 | 26684096 | protein\_coding | | ENSDARG00000046157 | RPS17 | 25 | 180028 | 186921 | protein\_coding | | ENSDARG00000070922 | cnbpb | 11 | 465089 | 471356 | protein\_coding | | ENSDARG00000058666 | dennd2da | 8 | 12951667 | 12991592 | protein\_coding | | ENSDARG00000075650 | dscr3 | 10 | 76864 | 81705 | protein\_coding | | ENSDARG00000041397 | eif2b2 | 20 | 46619358 | 46630459 | protein\_coding | | ENSDARG00000017235 | eif5a | 24 | 26336259 | 26340304 | protein\_coding | | ENSDARG00000099022 | faua | 20 | 54512620 | 54522396 | protein\_coding | | ENSDARG00000022684 | fkbp1aa | 8 | 49180999 | 49183731 | protein\_coding | | ENSDARG00000041619 | gnb2l1 | 14 | 6639895 | 6647038 | protein\_coding | | ENSDARG00000068434 | h3f3b.1.2 | 15 | 47272417 | 47277435 | protein\_coding | | ENSDARG00000059367 | mfap2 | 22 | 71558 | 75573 | protein\_coding | | ENSDARG00000008920 | mrpl53 | 19 | 32570697 | 32577219 | protein\_coding | | ENSDARG00000005513 | naca | 23 | 17939610 | 17947998 | protein\_coding | | ENSDARG00000025581 | rpl10 | 23 | 19786214 | 19789516 | protein\_coding | | ENSDARG00000042905 | rpl10a | 8 | 23716883 | 23721746 | protein\_coding | | ENSDARG00000043509 | rpl11 | 19 | 44185328 | 44191343 | protein\_coding | | ENSDARG00000099380 | rpl13 | 7 | 67244350 | 67253785 | protein\_coding | | ENSDARG00000044093 | rpl13a | 17 | 24596690 | 24599878 | protein\_coding | | ENSDARG00000103433 | rpl14 | 19 | 48704918 | 48711698 | protein\_coding | | ENSDARG00000009285 | rpl15 | 19 | 17899379 | 17934819 | protein\_coding | | ENSDARG00000057556 | rpl17 | 21 | 18960867 | 19030881 | protein\_coding | | ENSDARG00000029533 | rpl18 | 16 | 17069809 | 17083831 | protein\_coding | | ENSDARG00000025073 | rpl18a | 4 | 377849 | 385003 | protein\_coding | | ENSDARG00000013307 | rpl19 | 3 | 16080111 | 16082642 | protein\_coding | | ENSDARG00000010516 | rpl21 | 24 | 21476480 | 21480351 | protein\_coding | | ENSDARG00000070437 | rpl22 | 11 | 29405282 | 29410773 | protein\_coding | | ENSDARG00000010244 | rpl22l1 | 24 | 26330141 | 26334800 | protein\_coding | | ENSDARG00000053457 | rpl23 | 3 | 33292272 | 33296089 | protein\_coding | | ENSDARG00000006316 | rpl23a | 21 | 26035179 | 26037945 | protein\_coding | | ENSDARG00000099104 | rpl24 | 1 | 6408 | 12027 | protein\_coding | | ENSDARG00000102317 | rpl26 | 14 | 51848100 | 51852677 | protein\_coding | | ENSDARG00000015128 | rpl27 | 3 | 29763124 | 29768388 | protein\_coding | | ENSDARG00000005791 | rpl28 | 16 | 52026723 | 52031084 | protein\_coding | | ENSDARG00000003599 | rpl3 | 3 | 29736371 | 29740522 | protein\_coding | | ENSDARG00000035871 | rpl30 | 19 | 33268019 | 33273660 | protein\_coding | | ENSDARG00000053365 | rpl31 | 9 | 56637734 | 56644520 | protein\_coding | | ENSDARG00000054818 | rpl32 | 22 | 31089896 | 31094871 | protein\_coding | | ENSDARG00000029500 | rpl34 | 7 | 59344162 | 59359437 | protein\_coding | | ENSDARG00000018334 | rpl35 | 21 | 5766296 | 5774259 | protein\_coding | | ENSDARG00000088030 | rpl35a | 18 | 45784345 | 45792199 | protein\_coding | | ENSDARG00000100588 | rpl36 | 22 | 3136603 | 3142183 | protein\_coding | | ENSDARG00000058105 | rpl36a | 5 | 22006839 | 22011386 | protein\_coding | | ENSDARG00000034291 | rpl37 | 21 | 5030037 | 5041088 | protein\_coding | | ENSDARG00000006413 | rpl38 | 12 | 38382041 | 38387421 | protein\_coding | | ENSDARG00000036316 | rpl39 | 14 | 32991459 | 32993751 | protein\_coding | | ENSDARG00000041182 | rpl4 | 18 | 19462004 | 19467203 | protein\_coding | | ENSDARG00000020197 | rpl5a | 2 | 10903677 | 10920022 | protein\_coding | | ENSDARG00000015862 | rpl5b | 6 | 29096033 | 29106832 | protein\_coding | | ENSDARG00000058451 | rpl6 | 8 | 2435244 | 2447841 | protein\_coding | | ENSDARG00000007320 | rpl7 | 2 | 30193943 | 30198820 | protein\_coding | | ENSDARG00000019230 | rpl7a | 5 | 32683991 | 32687538 | protein\_coding | | ENSDARG00000014867 | rpl8 | 9 | 33512349 | 33519041 | protein\_coding | | ENSDARG00000037350 | rpl9 | 1 | 22578764 | 22583765 | protein\_coding | | ENSDARG00000051783 | rplp0 | 5 | 1685335 | 1698173 | protein\_coding | | ENSDARG00000021864 | rplp1 | 19 | 1902223 | 1905513 | protein\_coding | | ENSDARG00000011201 | rplp2l | 7 | 12707126 | 12715981 | protein\_coding | | ENSDARG00000034897 | rps10 | 6 | 54166588 | 54172591 | protein\_coding | | ENSDARG00000053058 | rps11 | 3 | 37645371 | 37647386 | protein\_coding | | ENSDARG00000036875 | rps12 | 23 | 31505425 | 31509391 | protein\_coding | | ENSDARG00000036298 | rps13 | 7 | 26770122 | 26772810 | protein\_coding | | ENSDARG00000036629 | rps14 | 21 | 33418568 | 33422984 | protein\_coding | | ENSDARG00000070849 | rps15 | 11 | 5929595 | 5934367 | protein\_coding | | ENSDARG00000010160 | rps15a | 3 | 26214861 | 26220785 | protein\_coding | | ENSDARG00000045487 | rps16 | 4 | 21928035 | 21931668 | protein\_coding | | ENSDARG00000104011 | rps17 | 7 | 53822639 | 53828979 | protein\_coding | | ENSDARG00000100392 | rps18 | 19 | 19154755 | 19164172 | protein\_coding | | ENSDARG00000030602 | rps19 | 16 | 24274933 | 24280294 | protein\_coding | | ENSDARG00000077291 | rps2 | 3 | 18249136 | 18253443 | protein\_coding | | ENSDARG00000036044 | rps20 | 7 | 58443547 | 58447960 | processed\_transcript | | ENSDARG00000025850 | rps21 | 23 | 9285466 | 9293608 | protein\_coding | | ENSDARG00000021838 | rps23 | 11 | 42265833 | 42270653 | protein\_coding | | ENSDARG00000039347 | rps24 | 13 | 30094812 | 30104770 | protein\_coding | | ENSDARG00000041811 | rps25 | 10 | 39182357 | 39187699 | protein\_coding | | ENSDARG00000037071 | rps26 | 23 | 27748995 | 27754481 | protein\_coding | | ENSDARG00000030408 | rps26l | 6 | 49522053 | 49527658 | protein\_coding | | ENSDARG00000023298 | rps27.1 | 19 | 8824335 | 8829646 | protein\_coding | | ENSDARG00000055475 | rps27.2 | 16 | 23873584 | 23885452 | protein\_coding | | ENSDARG00000032725 | rps27a | 6 | 6113517 | 6116350 | protein\_coding | | ENSDARG00000035860 | rps28 | 2 | 24851946 | 24854047 | protein\_coding | | ENSDARG00000041232 | rps29 | 20 | 54289895 | 54296503 | protein\_coding | | ENSDARG00000103007 | rps3 | 18 | 3045142 | 3056786 | protein\_coding | | ENSDARG00000035692 | rps3a | 1 | 23664686 | 23668824 | protein\_coding | | ENSDARG00000014690 | rps4x | 7 | 51358745 | 51364629 | protein\_coding | | ENSDARG00000043453 | rps5 | 15 | 40117891 | 40124704 | protein\_coding | | ENSDARG00000019778 | rps6 | 1 | 44342464 | 44348300 | protein\_coding | | ENSDARG00000042566 | rps7 | 20 | 30469473 | 30474342 | protein\_coding | | ENSDARG00000055996 | rps8a | 2 | 26842812 | 26846234 | protein\_coding | | ENSDARG00000011405 | rps9 | 16 | 31966476 | 31969065 | protein\_coding | | ENSDARG00000019181 | rpsa | 6 | 7554889 | 7563163 | protein\_coding | | ENSDARG00000068567 | shha | 7 | 40603939 | 40611263 | protein\_coding | | ENSDARG00000097753 | slirp | 17 | 17743331 | 17744965 | protein\_coding | | ENSDARG00000097824 | smco4 | 15 | 41280851 | 41288566 | protein\_coding | | ENSDARG00000041435 | uba52 | 2 | 56215213 | 56224882 | protein\_coding | | ENSDARG00000039008 | zgc:85858 | 23 | 44828391 | 44837148 | protein\_coding | |
