# Supplementary material for: A high-resolution mRNA expression time course of embryonic development in zebrafish
Source: eLife. 2017 Nov 16;6:e30860. doi: 10.7554/eLife.30860 (PMC5690287; doi:10.7554/eLife.30860)
Supplement: Supplementary file 6. [file elife-30860-supp6.zip › biolayout-clusters-files/Cluster018.html]

Cluster018


# Cluster018: Detail

### Go to ZFA detail

## GO

| | GO ID | Description | Domain | Annotated | Expected | Observed | Adjusted p-value | Genes | Ensembl IDs | | --- | --- | --- | --- | --- | --- | --- | --- | --- | | GO:0051726 | regulation of cell cycle | biological\_process | 194 | 2.05 | 11 | 1.4e-03 | rpl23a rpl7 rps15a rpl35 rps29 rps7 rps8a rpl36a rpl13 rps18 rpl36 | ENSDARG00000006316 ENSDARG00000007320 ENSDARG00000010160 ENSDARG00000018334 ENSDARG00000041232 ENSDARG00000042566 ENSDARG00000055996 ENSDARG00000058105 ENSDARG00000099380 ENSDARG00000100392 ENSDARG00000100588 | | GO:0042254 | ribosome biogenesis | biological\_process | 128 | 1.35 | 31 | 2.5e-03 | rpl3 rpl23a rpl7 rpl35 rpsa rpl7a rps6 rpl5a rps27.1 rpl10 rps21 rpl34 rps19 rps10 rps28 rps14 rps24 rps7 rps5 rpl11 rps16 RPS17 rplp0 rps27.2 rps8a rpl6 rpl35a rpl24 rpl26 rpl14 rps17 | ENSDARG00000003599 ENSDARG00000006316 ENSDARG00000007320 ENSDARG00000018334 ENSDARG00000019181 ENSDARG00000019230 ENSDARG00000019778 ENSDARG00000020197 ENSDARG00000023298 ENSDARG00000025581 ENSDARG00000025850 ENSDARG00000029500 ENSDARG00000030602 ENSDARG00000034897 ENSDARG00000035860 ENSDARG00000036629 ENSDARG00000039347 ENSDARG00000042566 ENSDARG00000043453 ENSDARG00000043509 ENSDARG00000045487 ENSDARG00000046157 ENSDARG00000051783 ENSDARG00000055475 ENSDARG00000055996 ENSDARG00000058451 ENSDARG00000088030 ENSDARG00000099104 ENSDARG00000102317 ENSDARG00000103433 ENSDARG00000104011 | | GO:0042273 | ribosomal large subunit biogenesis | biological\_process | 28 | 0.30 | 12 | 1.1e-02 | rpl3 rpl23a rpl7 rpl35 rpl5a rpl10 rpl11 rpl6 rpl35a rpl24 rpl26 rpl14 | ENSDARG00000003599 ENSDARG00000006316 ENSDARG00000007320 ENSDARG00000018334 ENSDARG00000020197 ENSDARG00000025581 ENSDARG00000043509 ENSDARG00000058451 ENSDARG00000088030 ENSDARG00000099104 ENSDARG00000102317 ENSDARG00000103433 | | GO:0042274 | ribosomal small subunit biogenesis | biological\_process | 35 | 0.37 | 15 | 2.1e-02 | rpsa rps6 rps27.1 rps21 rps19 rps10 rps14 rps24 rps7 rps5 rps16 RPS17 rps27.2 rps8a rps17 | ENSDARG00000019181 ENSDARG00000019778 ENSDARG00000023298 ENSDARG00000025850 ENSDARG00000030602 ENSDARG00000034897 ENSDARG00000036629 ENSDARG00000039347 ENSDARG00000042566 ENSDARG00000043453 ENSDARG00000045487 ENSDARG00000046157 ENSDARG00000055475 ENSDARG00000055996 ENSDARG00000104011 | | GO:0000027 | ribosomal large subunit assembly | biological\_process | 11 | 0.12 | 7 | 9.4e-10 | rpl3 rpl23a rpl5a rpl10 rpl11 rpl6 rpl24 | ENSDARG00000003599 ENSDARG00000006316 ENSDARG00000020197 ENSDARG00000025581 ENSDARG00000043509 ENSDARG00000058451 ENSDARG00000099104 | | GO:0000028 | ribosomal small subunit assembly | biological\_process | 13 | 0.14 | 9 | 1.9e-13 | rpsa rps27.1 rps19 rps10 rps14 rps5 RPS17 rps27.2 rps17 | ENSDARG00000019181 ENSDARG00000023298 ENSDARG00000030602 ENSDARG00000034897 ENSDARG00000036629 ENSDARG00000043453 ENSDARG00000046157 ENSDARG00000055475 ENSDARG00000104011 | | GO:0000462 | maturation of SSU-rRNA from tricistronic... | biological\_process | 21 | 0.22 | 6 | 1.4e-05 | rpsa rps21 rps14 rps24 rps16 rps8a | ENSDARG00000019181 ENSDARG00000025850 ENSDARG00000036629 ENSDARG00000039347 ENSDARG00000045487 ENSDARG00000055996 | | GO:0043009 | chordate embryonic development | biological\_process | 303 | 3.20 | 22 | 1.8e-19 | rpl28 rpl38 rps15a rplp2l rps4x rpl35 rpl5a rps19 rps3a rps14 rps29 rps7 rpl11 rplp0 rps8a rpl36a rpl6 shha rps15 rpl35a rpl24 rps3 | ENSDARG00000005791 ENSDARG00000006413 ENSDARG00000010160 ENSDARG00000011201 ENSDARG00000014690 ENSDARG00000018334 ENSDARG00000020197 ENSDARG00000030602 ENSDARG00000035692 ENSDARG00000036629 ENSDARG00000041232 ENSDARG00000042566 ENSDARG00000043509 ENSDARG00000051783 ENSDARG00000055996 ENSDARG00000058105 ENSDARG00000058451 ENSDARG00000068567 ENSDARG00000070849 ENSDARG00000088030 ENSDARG00000099104 ENSDARG00000103007 | | GO:0006412 | translation | biological\_process | 264 | 2.79 | 80 | 2.5e-28 | rpl3 rpl28 rpl23a rpl38 rpl7 rpl15 rps15a rpl22l1 rpl21 rplp2l rps9 rpl19 rps4x rpl8 rpl27 rpl5b eif5a rpl35 rpsa rps6 rpl5a rps23 rplp1 rps27.1 rpl18a rpl10 rps21 rpl34 rpl18 rps26l rps19 rps27a rpl37 rps3a rps28 rpl30 rps13 rpl39 rps14 rps12 rps26 rpl9 rps24 rpl4 rps29 eif2b2 uba52 rps7 rpl10a rps5 rpl11 rpl13a rps16 RPS17 rps11 rpl31 rpl23 rpl32 rps27.2 rps8a rpl17 rpl36a rpl6 rpl22 rps15 rps2 ENSDARG00000077717 rpl35a MRPS12 RPS11 (1 of many) RPL37A faua rpl24 rpl13 rps18 rpl36 rpl26 rps3 rpl14 rps17 | ENSDARG00000003599 ENSDARG00000005791 ENSDARG00000006316 ENSDARG00000006413 ENSDARG00000007320 ENSDARG00000009285 ENSDARG00000010160 ENSDARG00000010244 ENSDARG00000010516 ENSDARG00000011201 ENSDARG00000011405 ENSDARG00000013307 ENSDARG00000014690 ENSDARG00000014867 ENSDARG00000015128 ENSDARG00000015862 ENSDARG00000017235 ENSDARG00000018334 ENSDARG00000019181 ENSDARG00000019778 ENSDARG00000020197 ENSDARG00000021838 ENSDARG00000021864 ENSDARG00000023298 ENSDARG00000025073 ENSDARG00000025581 ENSDARG00000025850 ENSDARG00000029500 ENSDARG00000029533 ENSDARG00000030408 ENSDARG00000030602 ENSDARG00000032725 ENSDARG00000034291 ENSDARG00000035692 ENSDARG00000035860 ENSDARG00000035871 ENSDARG00000036298 ENSDARG00000036316 ENSDARG00000036629 ENSDARG00000036875 ENSDARG00000037071 ENSDARG00000037350 ENSDARG00000039347 ENSDARG00000041182 ENSDARG00000041232 ENSDARG00000041397 ENSDARG00000041435 ENSDARG00000042566 ENSDARG00000042905 ENSDARG00000043453 ENSDARG00000043509 ENSDARG00000044093 ENSDARG00000045487 ENSDARG00000046157 ENSDARG00000053058 ENSDARG00000053365 ENSDARG00000053457 ENSDARG00000054818 ENSDARG00000055475 ENSDARG00000055996 ENSDARG00000057556 ENSDARG00000058105 ENSDARG00000058451 ENSDARG00000070437 ENSDARG00000070849 ENSDARG00000077291 ENSDARG00000077717 ENSDARG00000088030 ENSDARG00000089836 ENSDARG00000093606 ENSDARG00000098458 ENSDARG00000099022 ENSDARG00000099104 ENSDARG00000099380 ENSDARG00000100392 ENSDARG00000100588 ENSDARG00000102317 ENSDARG00000103007 ENSDARG00000103433 ENSDARG00000104011 | | GO:0002181 | cytoplasmic translation | biological\_process | 20 | 0.21 | 11 | 3.8e-15 | rpl7 rpl15 rpl8 rpl9 rpl31 rpl6 rpl22 ENSDARG00000077717 rpl35a rpl36 rpl26 | ENSDARG00000007320 ENSDARG00000009285 ENSDARG00000014867 ENSDARG00000037350 ENSDARG00000053365 ENSDARG00000058451 ENSDARG00000070437 ENSDARG00000077717 ENSDARG00000088030 ENSDARG00000100588 ENSDARG00000102317 | | GO:0006414 | translational elongation | biological\_process | 25 | 0.26 | 4 | 3.0e-02 | rplp2l rps9 eif5a rplp1 | ENSDARG00000011201 ENSDARG00000011405 ENSDARG00000017235 ENSDARG00000021864 | | GO:0030218 | erythrocyte differentiation | biological\_process | 52 | 0.55 | 10 | 7.1e-07 | rpl27 rpl35 rplp1 rps27.1 rps19 rps14 rps29 rpl11 rpl35a rps3 | ENSDARG00000015128 ENSDARG00000018334 ENSDARG00000021864 ENSDARG00000023298 ENSDARG00000030602 ENSDARG00000036629 ENSDARG00000041232 ENSDARG00000043509 ENSDARG00000088030 ENSDARG00000103007 | | GO:0005840 | ribosome | cellular\_component | 119 | 1.31 | 82 | 3.3e-27 | rpl3 rpl28 rpl23a rpl38 rpl7 rpl15 rps15a rpl22l1 rpl21 rplp2l rps9 rpl19 rps4x rpl8 rpl27 rpl5b rpl35 rpsa rpl7a rps6 rpl5a rps23 rplp1 rps27.1 rpl18a rpl10 rps21 rpl34 rpl18 rps26l rps19 rps27a rpl37 rps10 rps3a rps28 rpl30 rps13 rpl39 rps14 rps12 rps26 rpl9 rps24 rpl4 rps29 uba52 rps25 rps7 rpl10a rps5 rpl11 rpl13a rps16 RPS17 rplp0 rps11 rpl31 rpl23 rpl32 rps27.2 rps8a rpl17 rpl36a rpl6 rpl22 rps15 rps2 ENSDARG00000077717 rpl35a MRPS12 RPS11 (1 of many) RPL37A faua rpl24 rpl13 rps18 rpl36 rpl26 rps3 rpl14 rps17 | ENSDARG00000003599 ENSDARG00000005791 ENSDARG00000006316 ENSDARG00000006413 ENSDARG00000007320 ENSDARG00000009285 ENSDARG00000010160 ENSDARG00000010244 ENSDARG00000010516 ENSDARG00000011201 ENSDARG00000011405 ENSDARG00000013307 ENSDARG00000014690 ENSDARG00000014867 ENSDARG00000015128 ENSDARG00000015862 ENSDARG00000018334 ENSDARG00000019181 ENSDARG00000019230 ENSDARG00000019778 ENSDARG00000020197 ENSDARG00000021838 ENSDARG00000021864 ENSDARG00000023298 ENSDARG00000025073 ENSDARG00000025581 ENSDARG00000025850 ENSDARG00000029500 ENSDARG00000029533 ENSDARG00000030408 ENSDARG00000030602 ENSDARG00000032725 ENSDARG00000034291 ENSDARG00000034897 ENSDARG00000035692 ENSDARG00000035860 ENSDARG00000035871 ENSDARG00000036298 ENSDARG00000036316 ENSDARG00000036629 ENSDARG00000036875 ENSDARG00000037071 ENSDARG00000037350 ENSDARG00000039347 ENSDARG00000041182 ENSDARG00000041232 ENSDARG00000041435 ENSDARG00000041811 ENSDARG00000042566 ENSDARG00000042905 ENSDARG00000043453 ENSDARG00000043509 ENSDARG00000044093 ENSDARG00000045487 ENSDARG00000046157 ENSDARG00000051783 ENSDARG00000053058 ENSDARG00000053365 ENSDARG00000053457 ENSDARG00000054818 ENSDARG00000055475 ENSDARG00000055996 ENSDARG00000057556 ENSDARG00000058105 ENSDARG00000058451 ENSDARG00000070437 ENSDARG00000070849 ENSDARG00000077291 ENSDARG00000077717 ENSDARG00000088030 ENSDARG00000089836 ENSDARG00000093606 ENSDARG00000098458 ENSDARG00000099022 ENSDARG00000099104 ENSDARG00000099380 ENSDARG00000100392 ENSDARG00000100588 ENSDARG00000102317 ENSDARG00000103007 ENSDARG00000103433 ENSDARG00000104011 | | GO:0015935 | small ribosomal subunit | cellular\_component | 36 | 0.40 | 30 | 4.1e-03 | rps9 rps4x rpsa rps6 rps23 rps27.1 rps21 rps26l rps19 rps10 rps3a rps28 rps13 rps14 rps12 rps26 rps24 rps29 rps7 rps5 rps16 RPS17 rps27.2 rps8a rps15 rps2 MRPS12 faua rps3 rps17 | ENSDARG00000011405 ENSDARG00000014690 ENSDARG00000019181 ENSDARG00000019778 ENSDARG00000021838 ENSDARG00000023298 ENSDARG00000025850 ENSDARG00000030408 ENSDARG00000030602 ENSDARG00000034897 ENSDARG00000035692 ENSDARG00000035860 ENSDARG00000036298 ENSDARG00000036629 ENSDARG00000036875 ENSDARG00000037071 ENSDARG00000039347 ENSDARG00000041232 ENSDARG00000042566 ENSDARG00000043453 ENSDARG00000045487 ENSDARG00000046157 ENSDARG00000055475 ENSDARG00000055996 ENSDARG00000070849 ENSDARG00000077291 ENSDARG00000089836 ENSDARG00000099022 ENSDARG00000103007 ENSDARG00000104011 | | GO:0022625 | cytosolic large ribosomal subunit | cellular\_component | 38 | 0.42 | 36 | 2.5e-28 | rpl3 rpl28 rpl23a rpl38 rpl7 rpl15 rpl21 rpl19 rpl8 rpl27 rpl35 rpl5a rpl18a rpl10 rpl34 rpl18 rpl37 rpl30 rpl39 rpl9 rpl4 rpl11 rpl13a rpl31 rpl23 rpl32 rpl36a rpl6 rpl22 ENSDARG00000077717 rpl35a rpl24 rpl13 rpl36 rpl26 rpl14 | ENSDARG00000003599 ENSDARG00000005791 ENSDARG00000006316 ENSDARG00000006413 ENSDARG00000007320 ENSDARG00000009285 ENSDARG00000010516 ENSDARG00000013307 ENSDARG00000014867 ENSDARG00000015128 ENSDARG00000018334 ENSDARG00000020197 ENSDARG00000025073 ENSDARG00000025581 ENSDARG00000029500 ENSDARG00000029533 ENSDARG00000034291 ENSDARG00000035871 ENSDARG00000036316 ENSDARG00000037350 ENSDARG00000041182 ENSDARG00000043509 ENSDARG00000044093 ENSDARG00000053365 ENSDARG00000053457 ENSDARG00000054818 ENSDARG00000058105 ENSDARG00000058451 ENSDARG00000070437 ENSDARG00000077717 ENSDARG00000088030 ENSDARG00000099104 ENSDARG00000099380 ENSDARG00000100588 ENSDARG00000102317 ENSDARG00000103433 | | GO:0022627 | cytosolic small ribosomal subunit | cellular\_component | 29 | 0.32 | 27 | 2.5e-28 | rps9 rps4x rpsa rps6 rps23 rps27.1 rps21 rps26l rps19 rps10 rps3a rps28 rps13 rps14 rps12 rps26 rps24 rps29 rps7 rps5 rps16 RPS17 rps27.2 rps8a rps15 faua rps17 | ENSDARG00000011405 ENSDARG00000014690 ENSDARG00000019181 ENSDARG00000019778 ENSDARG00000021838 ENSDARG00000023298 ENSDARG00000025850 ENSDARG00000030408 ENSDARG00000030602 ENSDARG00000034897 ENSDARG00000035692 ENSDARG00000035860 ENSDARG00000036298 ENSDARG00000036629 ENSDARG00000036875 ENSDARG00000037071 ENSDARG00000039347 ENSDARG00000041232 ENSDARG00000042566 ENSDARG00000043453 ENSDARG00000045487 ENSDARG00000046157 ENSDARG00000055475 ENSDARG00000055996 ENSDARG00000070849 ENSDARG00000099022 ENSDARG00000104011 | | GO:0003735 | structural constituent of ribosome | molecular\_function | 132 | 1.32 | 79 | 2.5e-28 | rpl3 rpl28 rpl23a rpl38 rpl7 rpl15 rps15a rpl22l1 rpl21 rplp2l rps9 rpl19 rps4x rpl8 rpl27 rpl5b rpl35 rpsa rps6 rpl5a rps23 rplp1 rps27.1 rpl18a rpl10 rps21 rpl34 rpl18 rps26l rps19 rps27a rpl37 rps10 rps3a rps28 rpl30 rps13 rpl39 rps14 rps12 rps26 rpl9 rps24 rpl4 rps29 uba52 rps7 rpl10a rps5 rpl11 rpl13a rps16 RPS17 rps11 rpl31 rpl23 rpl32 rps27.2 rps8a rpl17 rpl36a rpl6 rpl22 rps15 rps2 ENSDARG00000077717 rpl35a MRPS12 RPS11 (1 of many) RPL37A faua rpl24 rpl13 rps18 rpl36 rpl26 rps3 rpl14 rps17 | ENSDARG00000003599 ENSDARG00000005791 ENSDARG00000006316 ENSDARG00000006413 ENSDARG00000007320 ENSDARG00000009285 ENSDARG00000010160 ENSDARG00000010244 ENSDARG00000010516 ENSDARG00000011201 ENSDARG00000011405 ENSDARG00000013307 ENSDARG00000014690 ENSDARG00000014867 ENSDARG00000015128 ENSDARG00000015862 ENSDARG00000018334 ENSDARG00000019181 ENSDARG00000019778 ENSDARG00000020197 ENSDARG00000021838 ENSDARG00000021864 ENSDARG00000023298 ENSDARG00000025073 ENSDARG00000025581 ENSDARG00000025850 ENSDARG00000029500 ENSDARG00000029533 ENSDARG00000030408 ENSDARG00000030602 ENSDARG00000032725 ENSDARG00000034291 ENSDARG00000034897 ENSDARG00000035692 ENSDARG00000035860 ENSDARG00000035871 ENSDARG00000036298 ENSDARG00000036316 ENSDARG00000036629 ENSDARG00000036875 ENSDARG00000037071 ENSDARG00000037350 ENSDARG00000039347 ENSDARG00000041182 ENSDARG00000041232 ENSDARG00000041435 ENSDARG00000042566 ENSDARG00000042905 ENSDARG00000043453 ENSDARG00000043509 ENSDARG00000044093 ENSDARG00000045487 ENSDARG00000046157 ENSDARG00000053058 ENSDARG00000053365 ENSDARG00000053457 ENSDARG00000054818 ENSDARG00000055475 ENSDARG00000055996 ENSDARG00000057556 ENSDARG00000058105 ENSDARG00000058451 ENSDARG00000070437 ENSDARG00000070849 ENSDARG00000077291 ENSDARG00000077717 ENSDARG00000088030 ENSDARG00000089836 ENSDARG00000093606 ENSDARG00000098458 ENSDARG00000099022 ENSDARG00000099104 ENSDARG00000099380 ENSDARG00000100392 ENSDARG00000100588 ENSDARG00000102317 ENSDARG00000103007 ENSDARG00000103433 ENSDARG00000104011 | | GO:0003723 | RNA binding | molecular\_function | 423 | 4.23 | 32 | 1.4e-04 | rpl38 rpl7 rpl22l1 rps9 rps4x rpl8 rpl5b eif5a rpl35 rpl5a rpl34 rpl18 rps26l rpl37 rpl30 rps13 rpl39 rps14 rps26 rpl9 eif2b2 rpl10a rps5 rpl13a rpl23 rpl22 rps15 rps2 rpl35a rpl13 rps18 rps3 | ENSDARG00000006413 ENSDARG00000007320 ENSDARG00000010244 ENSDARG00000011405 ENSDARG00000014690 ENSDARG00000014867 ENSDARG00000015862 ENSDARG00000017235 ENSDARG00000018334 ENSDARG00000020197 ENSDARG00000029500 ENSDARG00000029533 ENSDARG00000030408 ENSDARG00000034291 ENSDARG00000035871 ENSDARG00000036298 ENSDARG00000036316 ENSDARG00000036629 ENSDARG00000037071 ENSDARG00000037350 ENSDARG00000041397 ENSDARG00000042905 ENSDARG00000043453 ENSDARG00000044093 ENSDARG00000053457 ENSDARG00000070437 ENSDARG00000070849 ENSDARG00000077291 ENSDARG00000088030 ENSDARG00000099380 ENSDARG00000100392 ENSDARG00000103007 | | GO:0019843 | rRNA binding | molecular\_function | 26 | 0.26 | 12 | 1.0e-15 | rps9 rps4x rpl8 rpl5b rpl5a rpl37 rps13 rps14 rpl9 rps5 rpl23 rps18 | ENSDARG00000011405 ENSDARG00000014690 ENSDARG00000014867 ENSDARG00000015862 ENSDARG00000020197 ENSDARG00000034291 ENSDARG00000036298 ENSDARG00000036629 ENSDARG00000037350 ENSDARG00000043453 ENSDARG00000053457 ENSDARG00000100392 | | GO:0003729 | mRNA binding | molecular\_function | 54 | 0.54 | 6 | 3.8e-03 | rpl35 rps26l rps14 rps26 rps5 rpl13a | ENSDARG00000018334 ENSDARG00000030408 ENSDARG00000036629 ENSDARG00000037071 ENSDARG00000043453 ENSDARG00000044093 | |

  


### Go to GO detail

## ZFA

| | ZFA ID | Description | Annotated | Expected | Observed | Fold Enrichment | Adjusted p-value | Genes | Ensembl IDs | | --- | --- | --- | --- | --- | --- | --- | --- | --- | | ZFA:0000106 | extension | 222 | 1.81 | 31 | 17.1 | 1.7e-27 | rplp0 rps5 rpl35 rps18 rpl24 rpl36a rpl14 rpl27 rps3 rps8a rps14 rpl7 rplp1 rpl6 rps15a rpl38 rps12 rps15 rpl13 rps19 rps7 rpl19 rps11 rpsa rps29 rpl11 rpl35a rps4x rpl28 rps27.1 rplp2l | ENSDARG00000051783 ENSDARG00000043453 ENSDARG00000018334 ENSDARG00000100392 ENSDARG00000099104 ENSDARG00000058105 ENSDARG00000103433 ENSDARG00000015128 ENSDARG00000103007 ENSDARG00000055996 ENSDARG00000036629 ENSDARG00000007320 ENSDARG00000021864 ENSDARG00000058451 ENSDARG00000010160 ENSDARG00000006413 ENSDARG00000036875 ENSDARG00000070849 ENSDARG00000099380 ENSDARG00000030602 ENSDARG00000042566 ENSDARG00000013307 ENSDARG00000053058 ENSDARG00000019181 ENSDARG00000041232 ENSDARG00000043509 ENSDARG00000088030 ENSDARG00000014690 ENSDARG00000005791 ENSDARG00000023298 ENSDARG00000011201 | | ZFA:0000110 | fourth ventricle | 167 | 1.36 | 28 | 20.6 | 3.3e-25 | rplp0 rps5 rpl35 rps18 rpl24 rpl36a rpl14 rpl5a rps3 rps8a rpl7 rpl6 rps15a rpl38 rps12 rps15 rps3a rpl13 rps19 rps7 rpl19 rps11 rps29 rpl11 rpl35a rpl28 rplp2l gnb2l1 | ENSDARG00000051783 ENSDARG00000043453 ENSDARG00000018334 ENSDARG00000100392 ENSDARG00000099104 ENSDARG00000058105 ENSDARG00000103433 ENSDARG00000020197 ENSDARG00000103007 ENSDARG00000055996 ENSDARG00000007320 ENSDARG00000058451 ENSDARG00000010160 ENSDARG00000006413 ENSDARG00000036875 ENSDARG00000070849 ENSDARG00000035692 ENSDARG00000099380 ENSDARG00000030602 ENSDARG00000042566 ENSDARG00000013307 ENSDARG00000053058 ENSDARG00000041232 ENSDARG00000043509 ENSDARG00000088030 ENSDARG00000005791 ENSDARG00000011201 ENSDARG00000041619 | | ZFA:0009139 | endodermal cell | 51 | 0.42 | 19 | 45.2 | 4.8e-25 | rpl24 rpl27 rpl23a rpl21 rpl9 rpl30 rpl6 rpl23 rpl4 rpl34 rpl18a rpl8 rpl19 rpl36 rpl11 rpl37 rpl35a rpl15 rpl28 | ENSDARG00000099104 ENSDARG00000015128 ENSDARG00000006316 ENSDARG00000010516 ENSDARG00000037350 ENSDARG00000035871 ENSDARG00000058451 ENSDARG00000053457 ENSDARG00000041182 ENSDARG00000029500 ENSDARG00000025073 ENSDARG00000014867 ENSDARG00000013307 ENSDARG00000100588 ENSDARG00000043509 ENSDARG00000034291 ENSDARG00000088030 ENSDARG00000009285 ENSDARG00000005791 | | ZFA:0000084 | yolk | 295 | 2.41 | 25 | 10.4 | 2.3e-13 | rps5 rpl35 rps18 rpl24 rpl36a naca rpl14 rps14 rpl9 rpl7 rplp1 rpl6 rps15a rps12 rps15 rpl13 rps7 rpl19 rps11 rpsa rpl36 rps29 rpl11 rpl28 gnb2l1 | ENSDARG00000043453 ENSDARG00000018334 ENSDARG00000100392 ENSDARG00000099104 ENSDARG00000058105 ENSDARG00000005513 ENSDARG00000103433 ENSDARG00000036629 ENSDARG00000037350 ENSDARG00000007320 ENSDARG00000021864 ENSDARG00000058451 ENSDARG00000010160 ENSDARG00000036875 ENSDARG00000070849 ENSDARG00000099380 ENSDARG00000042566 ENSDARG00000013307 ENSDARG00000053058 ENSDARG00000019181 ENSDARG00000100588 ENSDARG00000041232 ENSDARG00000043509 ENSDARG00000005791 ENSDARG00000041619 | | ZFA:0000006 | ball | 58 | 0.47 | 14 | 29.8 | 4.8e-13 | rpl35 rpl24 rpl36a rps3 rps8a rpl6 rps15a rps3a rps19 rps29 rpl35a rps4x rpl28 rplp2l | ENSDARG00000018334 ENSDARG00000099104 ENSDARG00000058105 ENSDARG00000103007 ENSDARG00000055996 ENSDARG00000058451 ENSDARG00000010160 ENSDARG00000035692 ENSDARG00000030602 ENSDARG00000041232 ENSDARG00000088030 ENSDARG00000014690 ENSDARG00000005791 ENSDARG00000011201 | | ZFA:0000042 | midbrain hindbrain boundary | 522 | 4.27 | 27 | 6.3 | 6.2e-12 | rplp0 rpl35 rpl36a rpl3 rpl14 rpl5a rps3 rps8a rpl9 rpl10 rplp1 rpl6 rps15a rpl38 rps15 rps3a rpl13 rps19 rps7 rpl19 rps11 rpsa rps29 rpl11 rpl35a rpl28 rplp2l | ENSDARG00000051783 ENSDARG00000018334 ENSDARG00000058105 ENSDARG00000003599 ENSDARG00000103433 ENSDARG00000020197 ENSDARG00000103007 ENSDARG00000055996 ENSDARG00000037350 ENSDARG00000025581 ENSDARG00000021864 ENSDARG00000058451 ENSDARG00000010160 ENSDARG00000006413 ENSDARG00000070849 ENSDARG00000035692 ENSDARG00000099380 ENSDARG00000030602 ENSDARG00000042566 ENSDARG00000013307 ENSDARG00000053058 ENSDARG00000019181 ENSDARG00000041232 ENSDARG00000043509 ENSDARG00000088030 ENSDARG00000005791 ENSDARG00000011201 | | ZFA:0000140 | pancreas | 354 | 2.89 | 23 | 8.0 | 2.9e-11 | rpl24 rpl36a rpl3 rpl27 rpl5a rpl23a rpl21 rpl9 rpl30 rpl6 rpl23 rpl4 rpl34 rpl18a rpl8 rpl19 rpl36 rpl11 rpl37 rpl35a rpl15 rpl28 shha | ENSDARG00000099104 ENSDARG00000058105 ENSDARG00000003599 ENSDARG00000015128 ENSDARG00000020197 ENSDARG00000006316 ENSDARG00000010516 ENSDARG00000037350 ENSDARG00000035871 ENSDARG00000058451 ENSDARG00000053457 ENSDARG00000041182 ENSDARG00000029500 ENSDARG00000025073 ENSDARG00000014867 ENSDARG00000013307 ENSDARG00000100588 ENSDARG00000043509 ENSDARG00000034291 ENSDARG00000088030 ENSDARG00000009285 ENSDARG00000005791 ENSDARG00000068567 | | ZFA:0001390 | pancreatic bud | 145 | 1.18 | 2 | 1.7 | 1.6e-10 | rpl36a rpl23a | ENSDARG00000058105 ENSDARG00000006316 | | ZFA:0000254 | pancreas primordium | 91 | 0.74 | 1 | 1.4 | 3.8e-10 | shha | ENSDARG00000068567 | | ZFA:0000123 | liver | 1962 | 16.03 | 39 | 2.4 | 5.2e-08 | rplp0 rps5 rps18 rpl24 rpl36a rpl3 rpl13a rpl27 rpl5a rpl23a rpl21 rps8a rpl9 rpl7 rpl30 rplp1 rpl6 rpl23 rps12 rpl4 slirp rpl13 rpl34 rpl18a rpl8 rps7 rps17 rpl19 rps11 rpsa rpl36 rps29 rpl11 rpl37 rpl35a rpl15 rps6 rpl28 shha | ENSDARG00000051783 ENSDARG00000043453 ENSDARG00000100392 ENSDARG00000099104 ENSDARG00000058105 ENSDARG00000003599 ENSDARG00000044093 ENSDARG00000015128 ENSDARG00000020197 ENSDARG00000006316 ENSDARG00000010516 ENSDARG00000055996 ENSDARG00000037350 ENSDARG00000007320 ENSDARG00000035871 ENSDARG00000021864 ENSDARG00000058451 ENSDARG00000053457 ENSDARG00000036875 ENSDARG00000041182 ENSDARG00000097753 ENSDARG00000099380 ENSDARG00000029500 ENSDARG00000025073 ENSDARG00000014867 ENSDARG00000042566 ENSDARG00000104011 ENSDARG00000013307 ENSDARG00000053058 ENSDARG00000019181 ENSDARG00000100588 ENSDARG00000041232 ENSDARG00000043509 ENSDARG00000034291 ENSDARG00000088030 ENSDARG00000009285 ENSDARG00000019778 ENSDARG00000005791 ENSDARG00000068567 | | ZFA:0009256 | nucleate erythrocyte | 157 | 1.28 | 13 | 10.2 | 4.3e-07 | rpl35 rpl36a rpl27 rpl5a rps14 rps24 rps19 rps7 rps29 rpl11 rpl35a rps27.1 rplp2l | ENSDARG00000018334 ENSDARG00000058105 ENSDARG00000015128 ENSDARG00000020197 ENSDARG00000036629 ENSDARG00000039347 ENSDARG00000030602 ENSDARG00000042566 ENSDARG00000041232 ENSDARG00000043509 ENSDARG00000088030 ENSDARG00000023298 ENSDARG00000011201 | | ZFA:0009044 | blood cell | 86 | 0.70 | 1 | 1.4 | 2.3e-04 | rps7 | ENSDARG00000042566 | | ZFA:0000112 | gut | 1099 | 8.98 | 22 | 2.5 | 6.6e-04 | rps5 rps18 rpl24 rpl3 rps8a rpl9 rpl7 rplp1 rpl6 rps12 rps15 rpl13 rps7 rps17 rpl19 rps11 rpsa rpl36 rps29 rpl11 rpl28 shha | ENSDARG00000043453 ENSDARG00000100392 ENSDARG00000099104 ENSDARG00000003599 ENSDARG00000055996 ENSDARG00000037350 ENSDARG00000007320 ENSDARG00000021864 ENSDARG00000058451 ENSDARG00000036875 ENSDARG00000070849 ENSDARG00000099380 ENSDARG00000042566 ENSDARG00000104011 ENSDARG00000013307 ENSDARG00000053058 ENSDARG00000019181 ENSDARG00000100588 ENSDARG00000041232 ENSDARG00000043509 ENSDARG00000005791 ENSDARG00000068567 | | ZFA:0001338 | intestine | 863 | 7.05 | 22 | 3.1 | 2.9e-03 | rpl24 rpl36a rpl13a rpl27 rpl5a rpl23a rpl21 rpl9 rpl30 rpl6 rpl23 rpl4 rpl34 rpl18a rpl8 rpl19 rpl36 rpl11 rpl37 rpl35a rpl15 rpl28 | ENSDARG00000099104 ENSDARG00000058105 ENSDARG00000044093 ENSDARG00000015128 ENSDARG00000020197 ENSDARG00000006316 ENSDARG00000010516 ENSDARG00000037350 ENSDARG00000035871 ENSDARG00000058451 ENSDARG00000053457 ENSDARG00000041182 ENSDARG00000029500 ENSDARG00000025073 ENSDARG00000014867 ENSDARG00000013307 ENSDARG00000100588 ENSDARG00000043509 ENSDARG00000034291 ENSDARG00000088030 ENSDARG00000009285 ENSDARG00000005791 | | ZFA:0001117 | post-vent region | 811 | 6.63 | 23 | 3.5 | 8.4e-03 | rplp0 rpl35 rpl24 rpl27 rpl5a rps3 rps8a rplp1 rpl6 rps15a rpl38 rps15 rps3a rps19 rps7 rps29 rpl11 rpl35a rps4x rpl28 rps27.1 rplp2l shha | ENSDARG00000051783 ENSDARG00000018334 ENSDARG00000099104 ENSDARG00000015128 ENSDARG00000020197 ENSDARG00000103007 ENSDARG00000055996 ENSDARG00000021864 ENSDARG00000058451 ENSDARG00000010160 ENSDARG00000006413 ENSDARG00000070849 ENSDARG00000035692 ENSDARG00000030602 ENSDARG00000042566 ENSDARG00000041232 ENSDARG00000043509 ENSDARG00000088030 ENSDARG00000014690 ENSDARG00000005791 ENSDARG00000023298 ENSDARG00000011201 ENSDARG00000068567 | | ZFA:0009045 | epithelial cell of pancreas | 5 | 0.04 | 3 | 75.0 | 2.1e-02 | rpl36a rpl23a rpl6 | ENSDARG00000058105 ENSDARG00000006316 ENSDARG00000058451 | |
