# Supplementary material for: A high-resolution mRNA expression time course of embryonic development in zebrafish
Source: eLife. 2017 Nov 16;6:e30860. doi: 10.7554/eLife.30860 (PMC5690287; doi:10.7554/eLife.30860)
Supplement: Supplementary file 6. [file elife-30860-supp6.zip › biolayout-clusters-files/Cluster019-genes.html]

Cluster019


# Cluster019: Genes

| | Ensembl ID | Gene Name | Chr | Start | End | Biotype | | --- | --- | --- | --- | --- | --- | | ENSDARG00000039969 | akirin1 | 16 | 33167187 | 33186550 | protein\_coding | | ENSDARG00000051886 | ankrd11 | 7 | 55883482 | 56108603 | protein\_coding | | ENSDARG00000008380 | brd7 | 7 | 37471041 | 37484085 | protein\_coding | | ENSDARG00000017084 | bud31 | 3 | 40142740 | 40145983 | protein\_coding | | ENSDARG00000010481 | bzw1a | 22 | 14092301 | 14101288 | protein\_coding | | ENSDARG00000059866 | cactin | 2 | 49874566 | 49892873 | protein\_coding | | ENSDARG00000054272 | caprin1b | 18 | 38266789 | 38290290 | protein\_coding | | ENSDARG00000102546 | cdk11b | 8 | 53615204 | 53627383 | protein\_coding | | ENSDARG00000103145 | cdk2ap2 | 1 | 39291817 | 39297764 | protein\_coding | | ENSDARG00000074758 | csde1 | 8 | 22500490 | 22521306 | protein\_coding | | ENSDARG00000098114 | ctsa | 6 | 55386994 | 55411507 | protein\_coding | | ENSDARG00000006225 | ddx39aa | 1 | 44504617 | 44514040 | protein\_coding | | ENSDARG00000015111 | ddx39ab | 3 | 5709137 | 5734107 | protein\_coding | | ENSDARG00000099458 | ddx46 | 21 | 45575350 | 45590015 | protein\_coding | | ENSDARG00000037291 | dpf2l | 14 | 23970647 | 23980560 | protein\_coding | | ENSDARG00000006567 | dus4l | 4 | 2707836 | 2712424 | protein\_coding | | ENSDARG00000038156 | dusp11 | 10 | 42305876 | 42318938 | protein\_coding | | ENSDARG00000043446 | efhd1 | 15 | 40341024 | 40365152 | protein\_coding | | ENSDARG00000070046 | eny2 | 16 | 38990726 | 38993653 | protein\_coding | | ENSDARG00000039830 | gng5 | 2 | 6331262 | 6337281 | protein\_coding | | ENSDARG00000099865 | hnrnpabb | 21 | 3013302 | 3020566 | protein\_coding | | ENSDARG00000058660 | ilf3a | 6 | 8130186 | 8156576 | protein\_coding | | ENSDARG00000097691 | kiaa0101 | 25 | 176216 | 179949 | protein\_coding | | ENSDARG00000061774 | mbd3a | 2 | 57026751 | 57051112 | protein\_coding | | ENSDARG00000101216 | meaf6 | 19 | 4093617 | 4102205 | protein\_coding | | ENSDARG00000028539 | morc2 | 25 | 3582428 | 3619734 | protein\_coding | | ENSDARG00000005576 | mphosph8 | 9 | 21651594 | 21678107 | protein\_coding | | ENSDARG00000013031 | mta2 | 7 | 17656063 | 17691942 | protein\_coding | | ENSDARG00000004343 | nelfe | 19 | 27338887 | 27347771 | protein\_coding | | ENSDARG00000037958 | nosip | 3 | 32400830 | 32404664 | protein\_coding | | ENSDARG00000099679 | papd4 | 5 | 51186539 | 51201303 | protein\_coding | | ENSDARG00000015851 | pnn | 17 | 13089027 | 13099693 | protein\_coding | | ENSDARG00000099375 | poldip3 | 3 | 1440357 | 1449751 | protein\_coding | | ENSDARG00000044431 | ppig | 6 | 3532621 | 3547566 | protein\_coding | | ENSDARG00000075559 | ppm1g | 13 | 6060299 | 6077117 | protein\_coding | | ENSDARG00000003486 | ppp1caa | 3 | 30757284 | 30778645 | protein\_coding | | ENSDARG00000071566 | ppp1cab | 12 | 13167652 | 13185146 | protein\_coding | | ENSDARG00000015239 | prp19 | 14 | 15982315 | 15995749 | protein\_coding | | ENSDARG00000058467 | prpf40a | 9 | 4708852 | 4755652 | protein\_coding | | ENSDARG00000091367 | prpf6 | 23 | 7066181 | 7118425 | protein\_coding | | ENSDARG00000039910 | rbm42 | 16 | 44915113 | 44933590 | protein\_coding | | ENSDARG00000015853 | rnps1 | 3 | 9492965 | 9505071 | protein\_coding | | ENSDARG00000059357 | sarnp | 11 | 3241006 | 3250721 | protein\_coding | | ENSDARG00000099664 | sep15 | 2 | 22697769 | 22707799 | protein\_coding | | ENSDARG00000102889 | sept15 | 25 | 12756606 | 12810396 | protein\_coding | | ENSDARG00000041887 | sf3a1 | 8 | 36522382 | 36537802 | protein\_coding | | ENSDARG00000021107 | sf3a2 | 2 | 57710669 | 57723075 | protein\_coding | | ENSDARG00000018574 | sf3b4 | 19 | 7505422 | 7511933 | protein\_coding | | ENSDARG00000016855 | sf3b5 | 13 | 23767525 | 23771282 | protein\_coding | | ENSDARG00000091631 | si:ch211-197h24.6 | 16 | 27608685 | 27624273 | protein\_coding | | ENSDARG00000019004 | smarcd1 | 22 | 6979216 | 6995547 | protein\_coding | | ENSDARG00000002538 | smu1a | 14 | 5922162 | 5938914 | protein\_coding | | ENSDARG00000023160 | snrnp40 | 19 | 43434971 | 43446958 | protein\_coding | | ENSDARG00000024651 | snrpa1 | 7 | 9045442 | 9060094 | protein\_coding | | ENSDARG00000011125 | snrpb | 6 | 57481473 | 57488547 | protein\_coding | | ENSDARG00000009871 | snrpc | 6 | 54422305 | 54425887 | protein\_coding | | ENSDARG00000101066 | srp14 | 17 | 1660955 | 1682500 | protein\_coding | | ENSDARG00000098367 | srp54 | 17 | 9918326 | 9933992 | protein\_coding | | ENSDARG00000017762 | srrt | 5 | 3920894 | 3964446 | protein\_coding | | ENSDARG00000013729 | srsf6a | 23 | 4321557 | 4333083 | protein\_coding | | ENSDARG00000035325 | srsf7a | 5 | 36295392 | 36298991 | protein\_coding | | ENSDARG00000008097 | srsf9 | 10 | 1659229 | 1669328 | protein\_coding | | ENSDARG00000029252 | ssb | 6 | 3556227 | 3567809 | protein\_coding | | ENSDARG00000035993 | sumo3b | 9 | 12677502 | 12681781 | protein\_coding | | ENSDARG00000070019 | taf15 | 15 | 1861600 | 1880476 | protein\_coding | | ENSDARG00000052536 | tia1 | 5 | 56280644 | 56294330 | protein\_coding | | ENSDARG00000092112 | tomm40 | 16 | 25758075 | 25765716 | protein\_coding | | ENSDARG00000002168 | tra2b | 9 | 12607874 | 12623574 | protein\_coding | | ENSDARG00000041830 | tsn | 9 | 23118806 | 23128641 | protein\_coding | | ENSDARG00000070161 | vamp3 | 11 | 39664683 | 39690467 | protein\_coding | | ENSDARG00000042642 | wtap | 20 | 31028535 | 31035367 | protein\_coding | | ENSDARG00000006399 | ywhae1 | 15 | 24811179 | 24819630 | protein\_coding | | ENSDARG00000022564 | zgc:56106 | 18 | 27327026 | 27334221 | protein\_coding | | ENSDARG00000004525 | zgc:63882 | 2 | 3203013 | 3220814 | protein\_coding | | ENSDARG00000015538 | znf207a | 3 | 34104499 | 34115517 | protein\_coding | |
