# Supplementary material for: A high-resolution mRNA expression time course of embryonic development in zebrafish
Source: eLife. 2017 Nov 16;6:e30860. doi: 10.7554/eLife.30860 (PMC5690287; doi:10.7554/eLife.30860)
Supplement: Supplementary file 6. [file elife-30860-supp6.zip › biolayout-clusters-files/Cluster019.html]

Cluster019


# Cluster019: Detail

### Go to ZFA detail

## GO

| | GO ID | Description | Domain | Annotated | Expected | Observed | Adjusted p-value | Genes | Ensembl IDs | | --- | --- | --- | --- | --- | --- | --- | --- | --- | | GO:0010468 | regulation of gene expression | biological\_process | 1192 | 7.75 | 12 | 2.1e-03 | ddx39aa snrpc bzw1a mta2 ddx39ab srrt wtap mbd3a eny2 csde1 ddx46 meaf6 | ENSDARG00000006225 ENSDARG00000009871 ENSDARG00000010481 ENSDARG00000013031 ENSDARG00000015111 ENSDARG00000017762 ENSDARG00000042642 ENSDARG00000061774 ENSDARG00000070046 ENSDARG00000074758 ENSDARG00000099458 ENSDARG00000101216 | | GO:0006406 | mRNA export from nucleus | biological\_process | 13 | 0.08 | 3 | 1.8e-02 | ddx39aa ddx39ab eny2 | ENSDARG00000006225 ENSDARG00000015111 ENSDARG00000070046 | | GO:0000398 | mRNA splicing, via spliceosome | biological\_process | 99 | 0.64 | 13 | 1.7e-07 | ddx39aa snrpc snrpb ddx39ab sf3b5 bud31 sf3a2 snrpa1 sf3a1 wtap prpf40a prpf6 ddx46 | ENSDARG00000006225 ENSDARG00000009871 ENSDARG00000011125 ENSDARG00000015111 ENSDARG00000016855 ENSDARG00000017084 ENSDARG00000021107 ENSDARG00000024651 ENSDARG00000041887 ENSDARG00000042642 ENSDARG00000058467 ENSDARG00000091367 ENSDARG00000099458 | | GO:0071013 | catalytic step 2 spliceosome | cellular\_component | 28 | 0.17 | 4 | 5.8e-03 | snrpb sf3a2 sf3a1 prpf6 | ENSDARG00000011125 ENSDARG00000021107 ENSDARG00000041887 ENSDARG00000091367 | | GO:0005685 | U1 snRNP | cellular\_component | 15 | 0.09 | 3 | 2.4e-02 | snrpc snrpb prpf40a | ENSDARG00000009871 ENSDARG00000011125 ENSDARG00000058467 | | GO:0005686 | U2 snRNP | cellular\_component | 15 | 0.09 | 5 | 5.3e-06 | snrpb sf3b5 sf3a2 snrpa1 sf3a1 | ENSDARG00000011125 ENSDARG00000016855 ENSDARG00000021107 ENSDARG00000024651 ENSDARG00000041887 | | GO:0071004 | U2-type prespliceosome | cellular\_component | 14 | 0.09 | 5 | 3.6e-06 | snrpc snrpb sf3a2 sf3a1 prpf40a | ENSDARG00000009871 ENSDARG00000011125 ENSDARG00000021107 ENSDARG00000041887 ENSDARG00000058467 | | GO:0003676 | nucleic acid binding | molecular\_function | 1873 | 12.50 | 30 | 5.8e-04 | tra2b nelfe ddx39aa srsf9 snrpc snrpb mta2 srsf6a ddx39ab rnps1 sf3b4 sf3a2 snrpa1 ssb srsf7a gng5 rbm42 tsn sf3a1 tia1 prpf40a ilf3a mbd3a taf15 csde1 srp54 poldip3 ddx46 hnrnpabb srp14 | ENSDARG00000002168 ENSDARG00000004343 ENSDARG00000006225 ENSDARG00000008097 ENSDARG00000009871 ENSDARG00000011125 ENSDARG00000013031 ENSDARG00000013729 ENSDARG00000015111 ENSDARG00000015853 ENSDARG00000018574 ENSDARG00000021107 ENSDARG00000024651 ENSDARG00000029252 ENSDARG00000035325 ENSDARG00000039830 ENSDARG00000039910 ENSDARG00000041830 ENSDARG00000041887 ENSDARG00000052536 ENSDARG00000058467 ENSDARG00000058660 ENSDARG00000061774 ENSDARG00000070019 ENSDARG00000074758 ENSDARG00000098367 ENSDARG00000099375 ENSDARG00000099458 ENSDARG00000099865 ENSDARG00000101066 | | GO:0003723 | RNA binding | molecular\_function | 423 | 2.82 | 12 | 1.4e-02 | snrpc snrpb rnps1 snrpa1 ssb rbm42 sf3a1 prpf40a ilf3a srp54 ddx46 srp14 | ENSDARG00000009871 ENSDARG00000011125 ENSDARG00000015853 ENSDARG00000024651 ENSDARG00000029252 ENSDARG00000039910 ENSDARG00000041887 ENSDARG00000058467 ENSDARG00000058660 ENSDARG00000098367 ENSDARG00000099458 ENSDARG00000101066 | | GO:0000166 | nucleotide binding | molecular\_function | 1424 | 9.50 | 22 | 2.5e-07 | tra2b nelfe ddx39aa dus4l srsf9 srsf6a ddx39ab rnps1 sf3b4 ssb srsf7a rbm42 tia1 mbd3a taf15 srp54 poldip3 ddx46 papd4 hnrnpabb cdk11b sept15 | ENSDARG00000002168 ENSDARG00000004343 ENSDARG00000006225 ENSDARG00000006567 ENSDARG00000008097 ENSDARG00000013729 ENSDARG00000015111 ENSDARG00000015853 ENSDARG00000018574 ENSDARG00000029252 ENSDARG00000035325 ENSDARG00000039910 ENSDARG00000052536 ENSDARG00000061774 ENSDARG00000070019 ENSDARG00000098367 ENSDARG00000099375 ENSDARG00000099458 ENSDARG00000099679 ENSDARG00000099865 ENSDARG00000102546 ENSDARG00000102889 | |
