# Supplementary material for: A high-resolution mRNA expression time course of embryonic development in zebrafish
Source: eLife. 2017 Nov 16;6:e30860. doi: 10.7554/eLife.30860 (PMC5690287; doi:10.7554/eLife.30860)
Supplement: Supplementary file 6. [file elife-30860-supp6.zip › biolayout-clusters-files/Cluster020-genes.html]

Cluster020


# Cluster020: Genes

| | Ensembl ID | Gene Name | Chr | Start | End | Biotype | | --- | --- | --- | --- | --- | --- | | ENSDARG00000089562 | BX957322.1 | 10 | 21565262 | 21575024 | protein\_coding | | ENSDARG00000104358 | CLASP1 (1 of many) | 11 | 43710172 | 43812283 | protein\_coding | | ENSDARG00000067824 | CNTNAP3 | 10 | 2147869 | 2366584 | protein\_coding | | ENSDARG00000076679 | ENSDARG00000076679 | 3 | 61765145 | 61816674 | protein\_coding | | ENSDARG00000079784 | ENSDARG00000079784 | 2 | 42363530 | 42375285 | protein\_coding | | ENSDARG00000098751 | FQ323163.2 | 5 | 2400604 | 2420331 | protein\_coding | | ENSDARG00000016739 | LRRC4C | 7 | 49836463 | 49838358 | protein\_coding | | ENSDARG00000039203 | MFF (1 of many) | 2 | 11867577 | 11879324 | protein\_coding | | ENSDARG00000089665 | OXR1 (1 of many) | 16 | 43513678 | 43587773 | protein\_coding | | ENSDARG00000044600 | PLPPR2 (1 of many) | 6 | 7632303 | 7731505 | protein\_coding | | ENSDARG00000062165 | TUB | 7 | 27705559 | 27769983 | protein\_coding | | ENSDARG00000063354 | abtb1 | 8 | 7054192 | 7080332 | protein\_coding | | ENSDARG00000025667 | adgrb2 | 19 | 39179493 | 39285530 | protein\_coding | | ENSDARG00000059832 | adgrb3 | 13 | 38176775 | 38468550 | protein\_coding | | ENSDARG00000005800 | ampd3a | 7 | 66612828 | 66644895 | protein\_coding | | ENSDARG00000068323 | astn1 | 2 | 34589765 | 34999969 | protein\_coding | | ENSDARG00000076701 | b4galnt4b | 7 | 48806077 | 48998411 | protein\_coding | | ENSDARG00000002945 | bgnb | 23 | 20087687 | 20125026 | protein\_coding | | ENSDARG00000006923 | cacna1ab | 11 | 30570759 | 30779151 | protein\_coding | | ENSDARG00000023683 | cacna1fb | 8 | 22838479 | 22905944 | protein\_coding | | ENSDARG00000077428 | camta1a | 23 | 30134833 | 30504802 | protein\_coding | | ENSDARG00000067841 | ccdc177 | 17 | 48135832 | 48138547 | protein\_coding | | ENSDARG00000025325 | ccsapa | 1 | 53294487 | 53304190 | protein\_coding | | ENSDARG00000005842 | cd9a | 18 | 7163615 | 7184958 | protein\_coding | | ENSDARG00000073802 | cntnap5l | 11 | 33163708 | 33291989 | protein\_coding | | ENSDARG00000079962 | doc2d | 8 | 13812294 | 13918167 | protein\_coding | | ENSDARG00000061255 | dusp3a | 12 | 27194634 | 27214463 | protein\_coding | | ENSDARG00000070625 | enpp5 | 20 | 18821728 | 18836698 | protein\_coding | | ENSDARG00000057671 | epas1b | 13 | 8043589 | 8114318 | protein\_coding | | ENSDARG00000061042 | gabbr2 | 19 | 34724254 | 35092618 | protein\_coding | | ENSDARG00000012987 | gpia | 25 | 35408675 | 35434057 | protein\_coding | | ENSDARG00000059368 | gria4b | 21 | 21857431 | 22045874 | protein\_coding | | ENSDARG00000055302 | grid2 | 8 | 28891457 | 29578471 | protein\_coding | | ENSDARG00000040156 | grm4 | 6 | 46980636 | 47386724 | protein\_coding | | ENSDARG00000031049 | igsf21a | 11 | 28395714 | 28835255 | protein\_coding | | ENSDARG00000037020 | kif1b | 23 | 29469645 | 29579132 | protein\_coding | | ENSDARG00000009160 | kmo | 13 | 41967911 | 42180452 | protein\_coding | | ENSDARG00000027602 | lrfn1 | 15 | 45778772 | 45917030 | protein\_coding | | ENSDARG00000094171 | lrp1ba.1 | 22 | 14336629 | 14451352 | protein\_coding | | ENSDARG00000057527 | mef2ab | 7 | 15411076 | 15471453 | protein\_coding | | ENSDARG00000074255 | micu3b | 14 | 30113177 | 30150277 | protein\_coding | | ENSDARG00000088181 | mpped1 | 4 | 8851094 | 8902324 | protein\_coding | | ENSDARG00000062222 | mxra8a | 11 | 24192307 | 24221170 | protein\_coding | | ENSDARG00000061836 | nfixb | 3 | 14618564 | 14908664 | protein\_coding | | ENSDARG00000056156 | npdc1b | 5 | 28270215 | 28315065 | protein\_coding | | ENSDARG00000077228 | ntrk3a | 25 | 11699178 | 12107456 | protein\_coding | | ENSDARG00000030832 | otofa | 20 | 31173609 | 31309677 | protein\_coding | | ENSDARG00000101325 | oxr1a | 16 | 42209076 | 42393073 | protein\_coding | | ENSDARG00000054378 | pcbp3 | 9 | 42238770 | 42324299 | protein\_coding | | ENSDARG00000069767 | pvrl1a | 21 | 22552972 | 22598657 | protein\_coding | | ENSDARG00000043497 | scrn2 | 20 | 14739445 | 14784917 | protein\_coding | | ENSDARG00000009106 | serinc1 | 20 | 40560489 | 40575409 | protein\_coding | | ENSDARG00000079414 | sez6b | 15 | 24277582 | 24609415 | protein\_coding | | ENSDARG00000023600 | sh3gl2 | 1 | 26047004 | 26088859 | protein\_coding | | ENSDARG00000074425 | si:busm1-57f23.1 | 10 | 13277981 | 13281248 | protein\_coding | | ENSDARG00000038974 | si:ch73-112n4.1 | 8 | 5256337 | 5323075 | processed\_transcript | | ENSDARG00000093851 | si:ch73-332k15.1 | 8 | 12413746 | 12631408 | processed\_transcript | | ENSDARG00000073747 | si:dkey-1j5.4 | 20 | 36477387 | 36490676 | protein\_coding | | ENSDARG00000058248 | si:dkeyp-77h1.4 | 16 | 10455375 | 10478396 | protein\_coding | | ENSDARG00000016994 | ssrp1b | 1 | 44009414 | 44036628 | protein\_coding | | ENSDARG00000030106 | stmn4 | 20 | 34870767 | 34965935 | protein\_coding | | ENSDARG00000052654 | thrab | 12 | 21677911 | 21918131 | protein\_coding | | ENSDARG00000090145 | tmem240b | 22 | 344474 | 349945 | protein\_coding | | ENSDARG00000020771 | tnr | 2 | 35258307 | 35491658 | protein\_coding | | ENSDARG00000092722 | unc5da | 21 | 16177024 | 16303833 | protein\_coding | | ENSDARG00000062449 | xpr1a | 8 | 14206771 | 14338185 | protein\_coding | | ENSDARG00000052787 | zdhhc12b | 5 | 31220111 | 31226420 | protein\_coding | | ENSDARG00000073845 | zgc:110843 | 14 | 7596260 | 7611747 | protein\_coding | |
