# Supplementary material for: A high-resolution mRNA expression time course of embryonic development in zebrafish
Source: eLife. 2017 Nov 16;6:e30860. doi: 10.7554/eLife.30860 (PMC5690287; doi:10.7554/eLife.30860)
Supplement: Supplementary file 6. [file elife-30860-supp6.zip › biolayout-clusters-files/Cluster020.html]

Cluster020


# Cluster020: Detail

### Go to ZFA detail

## GO

| | GO ID | Description | Domain | Annotated | Expected | Observed | Adjusted p-value | Genes | Ensembl IDs | | --- | --- | --- | --- | --- | --- | --- | --- | --- | | GO:0016021 | integral component of membrane | cellular\_component | 2838 | 14 | 31 | 0.0015 | cd9a cacna1ab serinc1 kmo LRRC4C cacna1fb adgrb2 lrfn1 otofa igsf21a MFF (1 of many) grm4 zdhhc12b grid2 si:dkeyp-77h1.4 gria4b adgrb3 gabbr2 mxra8a xpr1a CNTNAP3 astn1 pvrl1a enpp5 zgc:110843 ntrk3a sez6b tmem240b unc5da lrp1ba oxr1a | ENSDARG00000005842 ENSDARG00000006923 ENSDARG00000009106 ENSDARG00000009160 ENSDARG00000016739 ENSDARG00000023683 ENSDARG00000025667 ENSDARG00000027602 ENSDARG00000030832 ENSDARG00000031049 ENSDARG00000039203 ENSDARG00000040156 ENSDARG00000052787 ENSDARG00000055302 ENSDARG00000058248 ENSDARG00000059368 ENSDARG00000059832 ENSDARG00000061042 ENSDARG00000062222 ENSDARG00000062449 ENSDARG00000067824 ENSDARG00000068323 ENSDARG00000069767 ENSDARG00000070625 ENSDARG00000073845 ENSDARG00000077228 ENSDARG00000079414 ENSDARG00000090145 ENSDARG00000092722 ENSDARG00000094171 ENSDARG00000101325 | |
