# Supplementary material for: A high-resolution mRNA expression time course of embryonic development in zebrafish
Source: eLife. 2017 Nov 16;6:e30860. doi: 10.7554/eLife.30860 (PMC5690287; doi:10.7554/eLife.30860)
Supplement: Supplementary file 6. [file elife-30860-supp6.zip › biolayout-clusters-files/Cluster021-genes.html]

Cluster021


# Cluster021: Genes

| | Ensembl ID | Gene Name | Chr | Start | End | Biotype | | --- | --- | --- | --- | --- | --- | | ENSDARG00000104169 | ENSDARG00000104169 | 7 | 19373654 | 19390449 | protein\_coding | | ENSDARG00000042520 | UTP14C | 5 | 66795750 | 66820910 | protein\_coding | | ENSDARG00000062000 | abtb2b | 25 | 24519169 | 24626325 | protein\_coding | | ENSDARG00000001999 | adamts18 | 7 | 42124583 | 42212627 | protein\_coding | | ENSDARG00000077253 | alkbh6 | 5 | 36049911 | 36054545 | protein\_coding | | ENSDARG00000040295 | apoeb | 16 | 24045712 | 24048429 | protein\_coding | | ENSDARG00000101680 | arl1 | 4 | 5901688 | 5904842 | protein\_coding | | ENSDARG00000052907 | bcl7a | 5 | 41192848 | 41199729 | protein\_coding | | ENSDARG00000105046 | ccnf | 3 | 12401858 | 12433670 | protein\_coding | | ENSDARG00000014571 | ctnnb1 | 16 | 6281340 | 6304825 | protein\_coding | | ENSDARG00000005057 | dimt1l | 8 | 17148848 | 17157329 | protein\_coding | | ENSDARG00000054950 | eef2kmt | 3 | 28738768 | 28741914 | protein\_coding | | ENSDARG00000074050 | efnb2b | 1 | 6948686 | 6976437 | protein\_coding | | ENSDARG00000045873 | frs2a | 4 | 2215469 | 2250418 | protein\_coding | | ENSDARG00000029248 | fubp1 | 2 | 8851288 | 8865099 | protein\_coding | | ENSDARG00000014722 | her1 | 5 | 68017991 | 68024396 | protein\_coding | | ENSDARG00000019335 | hes6 | 2 | 48434297 | 48450532 | protein\_coding | | ENSDARG00000028335 | hmga1a | 23 | 3808555 | 3816789 | protein\_coding | | ENSDARG00000035324 | hnrpl | 5 | 36299538 | 36311750 | protein\_coding | | ENSDARG00000102937 | hspbp1 | 3 | 13450868 | 13468536 | protein\_coding | | ENSDARG00000101032 | iqcb1 | 6 | 8990055 | 9000776 | protein\_coding | | ENSDARG00000040110 | kif20bb | 12 | 16403765 | 16430481 | protein\_coding | | ENSDARG00000058358 | krt8 | 23 | 10314656 | 10319318 | protein\_coding | | ENSDARG00000052553 | lig3 | 5 | 60163092 | 60188361 | protein\_coding | | ENSDARG00000101624 | lmnb2 | 22 | 3103813 | 3135084 | protein\_coding | | ENSDARG00000039543 | mrps6 | 13 | 4866847 | 4872153 | protein\_coding | | ENSDARG00000098313 | nadka.1 | 8 | 53174521 | 53190961 | protein\_coding | | ENSDARG00000098361 | nudcd3 | 10 | 45213090 | 45223570 | protein\_coding | | ENSDARG00000007221 | pbk | 20 | 39456850 | 39465127 | protein\_coding | | ENSDARG00000033596 | polr2c | 25 | 14273093 | 14282435 | protein\_coding | | ENSDARG00000036625 | polr2f | 3 | 2032082 | 2041540 | protein\_coding | | ENSDARG00000063161 | ppwd1 | 10 | 11809553 | 11830327 | protein\_coding | | ENSDARG00000100209 | prpf39 | 20 | 54478484 | 54508839 | protein\_coding | | ENSDARG00000045628 | rab34a | 15 | 15523952 | 15533203 | protein\_coding | | ENSDARG00000008141 | rbl1 | 6 | 51543723 | 51574006 | protein\_coding | | ENSDARG00000019191 | sept12 | 3 | 28371577 | 28496605 | protein\_coding | | ENSDARG00000031495 | seta | 8 | 2526713 | 2533111 | protein\_coding | | ENSDARG00000056138 | sf3b1 | 9 | 32350877 | 32366371 | protein\_coding | | ENSDARG00000018425 | si:ch211-175g6.7 | 16 | 25767545 | 25784522 | protein\_coding | | ENSDARG00000097333 | si:ch211-213d14.3 | 15 | 17815128 | 17818666 | antisense | | ENSDARG00000103322 | si:ch73-347e22.8 | 19 | 11019007 | 11031914 | protein\_coding | | ENSDARG00000097134 | si:dkey-7i4.7 | 15 | 862634 | 866659 | protein\_coding | | ENSDARG00000076419 | si:dkeyp-117b11.2 | 8 | 2157165 | 2171585 | protein\_coding | | ENSDARG00000054570 | smg1 | 3 | 30987184 | 31027595 | protein\_coding | | ENSDARG00000077536 | snrnp200 | 8 | 31021381 | 31044640 | protein\_coding | | ENSDARG00000042851 | srp9 | 20 | 36720928 | 36723690 | protein\_coding | | ENSDARG00000040031 | tardbp | 6 | 45935895 | 45947239 | protein\_coding | | ENSDARG00000039806 | tb | 13 | 4312373 | 4325167 | protein\_coding | | ENSDARG00000059337 | tgif1 | 24 | 9147023 | 9159774 | protein\_coding | | ENSDARG00000045525 | tmcc3 | 4 | 25765527 | 25823131 | protein\_coding | | ENSDARG00000056475 | trnau1apb | 2 | 24654984 | 24661785 | protein\_coding | | ENSDARG00000077836 | ttc22 | 6 | 30681184 | 30689160 | protein\_coding | | ENSDARG00000028198 | ube2v2 | 24 | 35634201 | 35646176 | protein\_coding | | ENSDARG00000052975 | ubqln4 | 16 | 46699358 | 46714454 | protein\_coding | | ENSDARG00000077948 | usp54a | 13 | 22150533 | 22243586 | protein\_coding | | ENSDARG00000079659 | wdr53 | 15 | 2534924 | 2544416 | protein\_coding | | ENSDARG00000063229 | xpo1b | 17 | 23945731 | 23969186 | protein\_coding | | ENSDARG00000045874 | yeats4 | 4 | 2210379 | 2213122 | protein\_coding | | ENSDARG00000070658 | zbtb12.2 | 19 | 27372135 | 27380125 | protein\_coding | | ENSDARG00000015123 | zgc:101000 | 22 | 5637518 | 5652648 | protein\_coding | | ENSDARG00000025577 | zgc:66447 | 14 | 11714922 | 11745640 | protein\_coding | | ENSDARG00000071497 | zic3 | 14 | 31671375 | 31676301 | protein\_coding | | ENSDARG00000100142 | znf1083 | 4 | 30399529 | 30408316 | protein\_coding | | ENSDARG00000090054 | znf318 | 13 | 4179087 | 4204685 | protein\_coding | | ENSDARG00000071714 | znf983 | 22 | 2814437 | 2822968 | protein\_coding | |
