# Supplementary material for: A high-resolution mRNA expression time course of embryonic development in zebrafish
Source: eLife. 2017 Nov 16;6:e30860. doi: 10.7554/eLife.30860 (PMC5690287; doi:10.7554/eLife.30860)
Supplement: Supplementary file 6. [file elife-30860-supp6.zip › biolayout-clusters-files/Cluster021.html]

Cluster021


# Cluster021: Detail

### Go to ZFA detail

## GO

| | GO ID | Description | Domain | Annotated | Expected | Observed | Adjusted p-value | Genes | Ensembl IDs | | --- | --- | --- | --- | --- | --- | --- | --- | --- | | GO:0005634 | nucleus | cellular\_component | 1915 | 8.8 | 21 | 0.033 | rbl1 ctnnb1 her1 hes6 ube2v2 hmga1a seta polr2c hnrpl polr2f tb UTP14C lig3 trnau1apb krt8 tgif1 alkbh6 znf1083 prpf39 lmnb2 ccnf | ENSDARG00000008141 ENSDARG00000014571 ENSDARG00000014722 ENSDARG00000019335 ENSDARG00000028198 ENSDARG00000028335 ENSDARG00000031495 ENSDARG00000033596 ENSDARG00000035324 ENSDARG00000036625 ENSDARG00000039806 ENSDARG00000042520 ENSDARG00000052553 ENSDARG00000056475 ENSDARG00000058358 ENSDARG00000059337 ENSDARG00000077253 ENSDARG00000100142 ENSDARG00000100209 ENSDARG00000101624 ENSDARG00000105046 | |
