# Supplementary material for: A high-resolution mRNA expression time course of embryonic development in zebrafish
Source: eLife. 2017 Nov 16;6:e30860. doi: 10.7554/eLife.30860 (PMC5690287; doi:10.7554/eLife.30860)
Supplement: Supplementary file 6. [file elife-30860-supp6.zip › biolayout-clusters-files/Cluster022-genes.html]

Cluster022


# Cluster022: Genes

| | Ensembl ID | Gene Name | Chr | Start | End | Biotype | | --- | --- | --- | --- | --- | --- | | ENSDARG00000068589 | CABZ01079764.1 | 21 | 333325 | 337610 | protein\_coding | | ENSDARG00000036426 | CDA | 7 | 20266250 | 20269640 | protein\_coding | | ENSDARG00000061272 | SLC41A3 | 23 | 34928263 | 34970891 | protein\_coding | | ENSDARG00000063621 | aadac | 15 | 1084571 | 1105466 | protein\_coding | | ENSDARG00000070480 | agr2 | 19 | 30814138 | 30816783 | protein\_coding | | ENSDARG00000034470 | aldoab | 12 | 4015415 | 4033293 | protein\_coding | | ENSDARG00000016128 | ap3m2 | 5 | 13874891 | 13890228 | protein\_coding | | ENSDARG00000010472 | atp1a2a | 2 | 44350077 | 44402539 | protein\_coding | | ENSDARG00000058548 | bves | 16 | 7745315 | 7762634 | protein\_coding | | ENSDARG00000056499 | ca6 | 23 | 22708356 | 22724128 | protein\_coding | | ENSDARG00000034307 | chrne | 5 | 37839946 | 37850929 | protein\_coding | | ENSDARG00000061379 | cmya5 | 5 | 51202163 | 51237459 | protein\_coding | | ENSDARG00000017174 | dlx2b | 1 | 30174005 | 30175776 | protein\_coding | | ENSDARG00000035835 | eef2k | 12 | 10344114 | 10372127 | protein\_coding | | ENSDARG00000012397 | eya4 | 23 | 31513113 | 31586036 | protein\_coding | | ENSDARG00000035056 | fgf13a | 14 | 31843664 | 32063455 | protein\_coding | | ENSDARG00000001676 | gpm6bb | 9 | 55161688 | 55250696 | protein\_coding | | ENSDARG00000051880 | kcnj11 | 25 | 22756170 | 22757315 | protein\_coding | | ENSDARG00000019753 | kcnn3 | 16 | 23683876 | 23804617 | protein\_coding | | ENSDARG00000056938 | kera | 4 | 16348180 | 16356251 | protein\_coding | | ENSDARG00000002084 | lamb2 | 23 | 20253816 | 20332147 | protein\_coding | | ENSDARG00000039133 | lamb4 | 25 | 31221722 | 31292009 | protein\_coding | | ENSDARG00000056322 | ldb3a | 13 | 22346315 | 22422237 | protein\_coding | | ENSDARG00000014976 | lims2 | 9 | 116315 | 122190 | protein\_coding | | ENSDARG00000015947 | matn4 | 6 | 52791720 | 52847865 | protein\_coding | | ENSDARG00000062481 | mlphb | 9 | 24198144 | 24212500 | protein\_coding | | ENSDARG00000076348 | mylk3 | 7 | 41541238 | 41578440 | protein\_coding | | ENSDARG00000059838 | myom3 | 16 | 52500890 | 52622526 | protein\_coding | | ENSDARG00000076312 | myot | 14 | 11916529 | 12001566 | protein\_coding | | ENSDARG00000054321 | ngs | 4 | 5767777 | 5776325 | protein\_coding | | ENSDARG00000068126 | nppc | 22 | 38628601 | 38635152 | protein\_coding | | ENSDARG00000043309 | obscna | 8 | 18333465 | 18363075 | protein\_coding | | ENSDARG00000052405 | pak6b | 20 | 53716231 | 53763432 | protein\_coding | | ENSDARG00000026882 | palm1a | 11 | 14142143 | 14210196 | protein\_coding | | ENSDARG00000019117 | parvb | 25 | 4908915 | 4937095 | protein\_coding | | ENSDARG00000105344 | pkd1l2a | 7 | 64953104 | 64967909 | protein\_coding | | ENSDARG00000022503 | pkd2l1 | 13 | 25324869 | 25340926 | protein\_coding | | ENSDARG00000060457 | pmp22b | 12 | 37100594 | 37124929 | protein\_coding | | ENSDARG00000041133 | rapsn | 18 | 20505347 | 20521949 | protein\_coding | | ENSDARG00000077618 | rin3 | 17 | 33501178 | 33533519 | protein\_coding | | ENSDARG00000074156 | sgca | 12 | 3043488 | 3058291 | protein\_coding | | ENSDARG00000098573 | sgcd | 21 | 35617273 | 35806729 | protein\_coding | | ENSDARG00000092035 | si:ch211-156j16.1 | 8 | 26799307 | 26819706 | protein\_coding | | ENSDARG00000096896 | si:ch211-222f23.7 | 9 | 1573132 | 1577756 | lincRNA | | ENSDARG00000093000 | si:dkeyp-77c8.2 | 13 | 37060413 | 37063670 | protein\_coding | | ENSDARG00000002593 | slc45a2 | 21 | 19409306 | 19447653 | protein\_coding | | ENSDARG00000045302 | smpx | 24 | 25326245 | 25352979 | protein\_coding | | ENSDARG00000030490 | sptb | 17 | 38619132 | 38651437 | protein\_coding | | ENSDARG00000076484 | stab1 | 22 | 10251738 | 10324699 | protein\_coding | | ENSDARG00000098883 | stac3 | 9 | 308230 | 319880 | protein\_coding | | ENSDARG00000028275 | sult1st1 | 8 | 46438659 | 46501315 | protein\_coding | | ENSDARG00000002582 | tbx15 | 9 | 21224016 | 21257108 | protein\_coding | | ENSDARG00000074094 | tgm2b | 6 | 1959701 | 1988145 | protein\_coding | | ENSDARG00000040374 | tmem182a | 9 | 6937220 | 6949196 | protein\_coding | | ENSDARG00000024047 | tmem38a | 11 | 7148884 | 7169677 | protein\_coding | | ENSDARG00000037539 | tnnc1b | 23 | 20183743 | 20186253 | protein\_coding | | ENSDARG00000042559 | tnni1c | 18 | 6484983 | 6494059 | protein\_coding | | ENSDARG00000041779 | trdn | 20 | 40247976 | 40328743 | protein\_coding | | ENSDARG00000094675 | tusc5b | 21 | 39195839 | 39208589 | protein\_coding | | ENSDARG00000019646 | twist3 | 23 | 2669 | 9354 | protein\_coding | | ENSDARG00000039077 | tyr | 15 | 43776250 | 43795256 | protein\_coding | | ENSDARG00000003695 | vdac3 | 8 | 2354040 | 2375744 | protein\_coding | | ENSDARG00000044695 | wu:fb92b05 | 21 | 19797436 | 19805234 | protein\_coding | | ENSDARG00000040118 | zgc:113232 | 16 | 38409970 | 38439851 | protein\_coding | | ENSDARG00000104823 | znf648 | 6 | 23787505 | 23790067 | protein\_coding | |
