# Supplementary material for: A high-resolution mRNA expression time course of embryonic development in zebrafish
Source: eLife. 2017 Nov 16;6:e30860. doi: 10.7554/eLife.30860 (PMC5690287; doi:10.7554/eLife.30860)
Supplement: Supplementary file 6. [file elife-30860-supp6.zip › biolayout-clusters-files/Cluster022.html]

Cluster022


# Cluster022: Detail

### Go to ZFA detail

## GO

| | GO ID | Description | Domain | Annotated | Expected | Observed | Adjusted p-value | Genes | Ensembl IDs | | --- | --- | --- | --- | --- | --- | --- | --- | --- | | GO:0060047 | heart contraction | biological\_process | 78 | 0.46 | 7 | 0.0048 | atp1a2a lims2 parvb tnnc1b tnni1c myot sgcd | ENSDARG00000010472 ENSDARG00000014976 ENSDARG00000019117 ENSDARG00000037539 ENSDARG00000042559 ENSDARG00000076312 ENSDARG00000098573 | |

  


### Go to GO detail

## ZFA

| | ZFA ID | Description | Annotated | Expected | Observed | Fold Enrichment | Adjusted p-value | Genes | Ensembl IDs | | --- | --- | --- | --- | --- | --- | --- | --- | --- | | ZFA:0000473 | trunk musculature | 162 | 0.83 | 7 | 8.4 | 1.4e-05 | lamb4 parvb rapsn ldb3a bves tnnc1b lamb2 | ENSDARG00000039133 ENSDARG00000019117 ENSDARG00000041133 ENSDARG00000056322 ENSDARG00000058548 ENSDARG00000037539 ENSDARG00000002084 | | ZFA:0001056 | myotome | 825 | 4.21 | 17 | 4.0 | 1.3e-04 | atp1a2a eef2k sgcd rapsn ldb3a tmem38a aldoab vdac3 tnnc1b stac3 trdn sgca fgf13a CDA palm1a lims2 lamb2 | ENSDARG00000010472 ENSDARG00000035835 ENSDARG00000098573 ENSDARG00000041133 ENSDARG00000056322 ENSDARG00000024047 ENSDARG00000034470 ENSDARG00000003695 ENSDARG00000037539 ENSDARG00000098883 ENSDARG00000041779 ENSDARG00000074156 ENSDARG00000035056 ENSDARG00000036426 ENSDARG00000026882 ENSDARG00000014976 ENSDARG00000002084 | | ZFA:0000548 | musculature system | 273 | 1.39 | 7 | 5.0 | 1.3e-03 | eef2k rapsn tmem38a aldoab tnnc1b chrne sgca | ENSDARG00000035835 ENSDARG00000041133 ENSDARG00000024047 ENSDARG00000034470 ENSDARG00000037539 ENSDARG00000034307 ENSDARG00000074156 | | ZFA:0000003 | adaxial cell | 399 | 2.03 | 12 | 5.9 | 2.1e-03 | ldb3a tmem38a aldoab vdac3 tnnc1b stac3 chrne trdn sgca palm1a lims2 lamb2 | ENSDARG00000056322 ENSDARG00000024047 ENSDARG00000034470 ENSDARG00000003695 ENSDARG00000037539 ENSDARG00000098883 ENSDARG00000034307 ENSDARG00000041779 ENSDARG00000074156 ENSDARG00000026882 ENSDARG00000014976 ENSDARG00000002084 | | ZFA:0000009 | cardiac ventricle | 216 | 1.10 | 7 | 6.4 | 8.5e-03 | sptb mylk3 parvb tnnc1b eya4 lims2 myot | ENSDARG00000030490 ENSDARG00000076348 ENSDARG00000019117 ENSDARG00000037539 ENSDARG00000012397 ENSDARG00000014976 ENSDARG00000076312 | | ZFA:0000155 | somite | 1639 | 8.35 | 19 | 2.3 | 1.2e-02 | twist3 atp1a2a mylk3 sgcd parvb ldb3a tmem38a aldoab vdac3 bves stac3 chrne CDA matn4 eya4 palm1a lims2 pmp22b lamb2 | ENSDARG00000019646 ENSDARG00000010472 ENSDARG00000076348 ENSDARG00000098573 ENSDARG00000019117 ENSDARG00000056322 ENSDARG00000024047 ENSDARG00000034470 ENSDARG00000003695 ENSDARG00000058548 ENSDARG00000098883 ENSDARG00000034307 ENSDARG00000036426 ENSDARG00000015947 ENSDARG00000012397 ENSDARG00000026882 ENSDARG00000014976 ENSDARG00000060457 ENSDARG00000002084 | |
