# Supplementary material for: A high-resolution mRNA expression time course of embryonic development in zebrafish
Source: eLife. 2017 Nov 16;6:e30860. doi: 10.7554/eLife.30860 (PMC5690287; doi:10.7554/eLife.30860)
Supplement: Supplementary file 6. [file elife-30860-supp6.zip › biolayout-clusters-files/Cluster023-genes.html]

Cluster023


# Cluster023: Genes

| | Ensembl ID | Gene Name | Chr | Start | End | Biotype | | --- | --- | --- | --- | --- | --- | | ENSDARG00000101349 | BX005448.2 | KN149682.1 | 60997 | 66835 | protein\_coding | | ENSDARG00000104638 | BX324155.1 | 4 | 46447572 | 46456087 | protein\_coding | | ENSDARG00000101418 | BX324179.1 | 4 | 34103310 | 34166767 | protein\_coding | | ENSDARG00000098062 | BX511215.1 | 4 | 65419237 | 65546219 | protein\_coding | | ENSDARG00000093220 | BX571664.1 | 4 | 40439384 | 40454597 | protein\_coding | | ENSDARG00000098872 | BX649453.1 | 4 | 36238097 | 36238477 | protein\_coding | | ENSDARG00000100673 | BX927193.1 | 4 | 44999378 | 45003832 | protein\_coding | | ENSDARG00000100502 | CABZ01038979.1 | KN150372.1 | 16021 | 21360 | protein\_coding | | ENSDARG00000067599 | CABZ01076985.1 | KN150530.1 | 2388 | 4392 | protein\_coding | | ENSDARG00000100124 | CR361547.1 | 4 | 38741916 | 38851751 | protein\_coding | | ENSDARG00000098190 | CR383668.1 | 4 | 30539280 | 30540146 | protein\_coding | | ENSDARG00000068464 | CR388046.1 | 7 | 29521616 | 29523113 | protein\_coding | | ENSDARG00000104160 | CR628395.2 | 4 | 49753455 | 49757633 | protein\_coding | | ENSDARG00000100543 | CT573234.3 | 4 | 65799260 | 65808705 | protein\_coding | | ENSDARG00000104586 | CU302413.1 | 4 | 30684985 | 30690221 | protein\_coding | | ENSDARG00000043126 | blf | 22 | 9887325 | 9894010 | protein\_coding | | ENSDARG00000030905 | cited2 | 20 | 37012264 | 37014791 | protein\_coding | | ENSDARG00000070360 | fam212aa | 11 | 34786649 | 34794614 | protein\_coding | | ENSDARG00000039943 | fam46ba | 16 | 34092703 | 34102413 | protein\_coding | | ENSDARG00000008796 | her5 | 14 | 30202694 | 30204352 | protein\_coding | | ENSDARG00000055374 | irf4b | 20 | 26657084 | 26666203 | protein\_coding | | ENSDARG00000015906 | mxtx2 | 12 | 29121372 | 29122913 | protein\_coding | | ENSDARG00000097873 | si:ch211-195b13.9 | 19 | 25338308 | 25351043 | lincRNA | | ENSDARG00000104745 | si:ch211-207e19.15 | 4 | 54217709 | 54338630 | protein\_coding | | ENSDARG00000103700 | si:ch211-239d6.2 | 19 | 25569843 | 25571035 | protein\_coding | | ENSDARG00000093761 | si:ch211-250k18.7 | 22 | 9256807 | 9285442 | protein\_coding | | ENSDARG00000104932 | si:ch211-261d9.1 | 4 | 45735928 | 45737111 | protein\_coding | | ENSDARG00000096571 | si:ch211-281p14.2 | 7 | 278539 | 280047 | protein\_coding | | ENSDARG00000099350 | si:ch211-283l16.1 | 4 | 63321676 | 63328191 | protein\_coding | | ENSDARG00000102731 | si:ch73-299h12.1.1 | 21 | 2233876 | 2235896 | protein\_coding | | ENSDARG00000098851 | si:dkey-11d20.1 | 4 | 68530330 | 68541229 | protein\_coding | | ENSDARG00000094859 | si:dkey-11o15.10 | 21 | 15339969 | 15341635 | protein\_coding | | ENSDARG00000094101 | si:dkey-122c11.8 | 4 | 38643083 | 38649649 | protein\_coding | | ENSDARG00000098094 | si:dkey-146c18.5 | 4 | 59889283 | 60020041 | protein\_coding | | ENSDARG00000091062 | si:dkey-199k11.6 | 4 | 39556477 | 39559533 | protein\_coding | | ENSDARG00000101731 | si:dkey-199k11.8 | 4 | 39560310 | 39562003 | lincRNA | | ENSDARG00000097347 | si:dkey-204l2.4 | 4 | 59021583 | 59024284 | lincRNA | | ENSDARG00000103283 | si:dkey-238o14.7 | 4 | 67775150 | 67778999 | protein\_coding | | ENSDARG00000098536 | si:dkey-25i10.1 | 4 | 56629223 | 56637027 | protein\_coding | | ENSDARG00000095635 | si:dkey-261j4.5 | 5 | 60916569 | 60924727 | protein\_coding | | ENSDARG00000076160 | si:dkey-285e18.2 | 4 | 40293530 | 40297358 | protein\_coding | | ENSDARG00000088523 | si:dkey-5i16.5 | 4 | 47686564 | 47703891 | protein\_coding | | ENSDARG00000100632 | si:dkey-72l17.1 | 4 | 31983618 | 31987973 | processed\_transcript | | ENSDARG00000100509 | si:dkey-82i20.2.1 | 4 | 61452714 | 61469697 | protein\_coding | | ENSDARG00000104681 | si:dkeyp-87d1.1 | 4 | 36446874 | 36451395 | protein\_coding | | ENSDARG00000093129 | si:rp71-45k5.2 | 19 | 35407313 | 35408509 | protein\_coding | | ENSDARG00000068409 | vgll4l | 8 | 31090032 | 31098305 | protein\_coding | | ENSDARG00000077712 | zgc:113886 | 17 | 49932200 | 49992665 | protein\_coding | | ENSDARG00000070705 | zgc:64022 | 19 | 24539186 | 24541234 | protein\_coding | | ENSDARG00000092000 | znf1020 | 4 | 46954149 | 46970174 | protein\_coding | | ENSDARG00000105067 | znf1043 | 4 | 66064982 | 66116330 | protein\_coding | | ENSDARG00000102791 | znf1045.1 | 4 | 51799273 | 51805283 | protein\_coding | | ENSDARG00000104074 | znf1052 | 4 | 30567126 | 30573715 | protein\_coding | | ENSDARG00000093994 | znf1058 | 4 | 48911465 | 48924864 | protein\_coding | | ENSDARG00000099670 | znf1074 | 4 | 53701265 | 53711220 | protein\_coding | | ENSDARG00000099872 | znf1092 | 4 | 37970003 | 37983154 | protein\_coding | | ENSDARG00000100723 | znf1097 | 4 | 55917550 | 55928600 | protein\_coding | | ENSDARG00000092475 | znf1109 | 4 | 57902138 | 57987536 | protein\_coding | | ENSDARG00000098758 | znf1125 | 4 | 35848811 | 35856282 | protein\_coding | | ENSDARG00000091502 | znf1127 | 4 | 56962059 | 56970101 | protein\_coding | | ENSDARG00000091176 | znf995 | 4 | 39601670 | 39764287 | protein\_coding | | ENSDARG00000101137 | znf999 | 4 | 55044050 | 55088179 | protein\_coding | |
