# Supplementary material for: A high-resolution mRNA expression time course of embryonic development in zebrafish
Source: eLife. 2017 Nov 16;6:e30860. doi: 10.7554/eLife.30860 (PMC5690287; doi:10.7554/eLife.30860)
Supplement: Supplementary file 6. [file elife-30860-supp6.zip › biolayout-clusters-files/Cluster023.html]

Cluster023


# Cluster023: Detail

### Go to ZFA detail

## GO

| | GO ID | Description | Domain | Annotated | Expected | Observed | Adjusted p-value | Genes | Ensembl IDs | | --- | --- | --- | --- | --- | --- | --- | --- | --- | | GO:0006355 | regulation of transcription, DNA-templat... | biological\_process | 1072 | 2.24 | 10 | 3.8e-03 | her5 cited2 irf4b vgll4l znf1109 si:rp71-45k5.2 znf1125 znf1092 znf1097 si:ch211-207e19.15 | ENSDARG00000008796 ENSDARG00000030905 ENSDARG00000055374 ENSDARG00000068409 ENSDARG00000092475 ENSDARG00000093129 ENSDARG00000098758 ENSDARG00000099872 ENSDARG00000100723 ENSDARG00000104745 | | GO:0003676 | nucleic acid binding | molecular\_function | 1873 | 6.04 | 24 | 1.3e-08 | her5 mxtx2 blf irf4b zgc:113886 znf995 znf1020 znf1109 si:rp71-45k5.2 znf1058 si:dkey-261j4.5 si:dkey-146c18.5 si:dkey-25i10.1 znf1125 si:ch211-283l16.1 znf1074 znf1092 si:dkey-82i20.2 znf1097 znf999 si:ch73-299h12.1 znf1052 si:ch211-207e19.15 znf1043 | ENSDARG00000008796 ENSDARG00000015906 ENSDARG00000043126 ENSDARG00000055374 ENSDARG00000077712 ENSDARG00000091176 ENSDARG00000092000 ENSDARG00000092475 ENSDARG00000093129 ENSDARG00000093994 ENSDARG00000095635 ENSDARG00000098094 ENSDARG00000098536 ENSDARG00000098758 ENSDARG00000099350 ENSDARG00000099670 ENSDARG00000099872 ENSDARG00000100509 ENSDARG00000100723 ENSDARG00000101137 ENSDARG00000102731 ENSDARG00000104074 ENSDARG00000104745 ENSDARG00000105067 | | GO:0000978 | RNA polymerase II core promoter proximal... | molecular\_function | 68 | 0.22 | 5 | 5.6e-04 | znf1109 znf1125 znf1092 znf1097 si:ch211-207e19.15 | ENSDARG00000092475 ENSDARG00000098758 ENSDARG00000099872 ENSDARG00000100723 ENSDARG00000104745 | | GO:0046872 | metal ion binding | molecular\_function | 2036 | 6.57 | 20 | 3.6e-05 | blf zgc:113886 znf995 znf1020 znf1109 znf1058 si:dkey-261j4.5 si:dkey-146c18.5 si:dkey-25i10.1 znf1125 si:ch211-283l16.1 znf1074 znf1092 si:dkey-82i20.2 znf1097 znf999 si:ch73-299h12.1 znf1052 si:ch211-207e19.15 znf1043 | ENSDARG00000043126 ENSDARG00000077712 ENSDARG00000091176 ENSDARG00000092000 ENSDARG00000092475 ENSDARG00000093994 ENSDARG00000095635 ENSDARG00000098094 ENSDARG00000098536 ENSDARG00000098758 ENSDARG00000099350 ENSDARG00000099670 ENSDARG00000099872 ENSDARG00000100509 ENSDARG00000100723 ENSDARG00000101137 ENSDARG00000102731 ENSDARG00000104074 ENSDARG00000104745 ENSDARG00000105067 | |
