# Supplementary material for: A high-resolution mRNA expression time course of embryonic development in zebrafish
Source: eLife. 2017 Nov 16;6:e30860. doi: 10.7554/eLife.30860 (PMC5690287; doi:10.7554/eLife.30860)
Supplement: Supplementary file 6. [file elife-30860-supp6.zip › biolayout-clusters-files/Cluster024-genes.html]

Cluster024


# Cluster024: Genes

| | Ensembl ID | Gene Name | Chr | Start | End | Biotype | | --- | --- | --- | --- | --- | --- | | ENSDARG00000089604 | CABZ01054405.1 | 4 | 70717551 | 70726459 | protein\_coding | | ENSDARG00000036043 | ZNF408 | 7 | 38473657 | 38479132 | protein\_coding | | ENSDARG00000103610 | actr8 | 8 | 53536644 | 53553949 | protein\_coding | | ENSDARG00000036687 | bcl9 | 1 | 46640412 | 46717831 | protein\_coding | | ENSDARG00000079015 | brca2 | 15 | 32054010 | 32070540 | protein\_coding | | ENSDARG00000074747 | brwd1 | 15 | 5841142 | 5904882 | protein\_coding | | ENSDARG00000041533 | ccdc130 | 22 | 26219527 | 26231706 | protein\_coding | | ENSDARG00000060510 | cul4b | 14 | 33145837 | 33165395 | protein\_coding | | ENSDARG00000041586 | dhx40 | 15 | 17322221 | 17349166 | protein\_coding | | ENSDARG00000013997 | ern1 | 3 | 55163897 | 55222546 | protein\_coding | | ENSDARG00000095007 | fam60al.1 | 7 | 28782089 | 28790495 | protein\_coding | | ENSDARG00000045528 | fgd6 | 4 | 25873378 | 25919673 | protein\_coding | | ENSDARG00000003200 | foxm1 | 4 | 5799394 | 5822952 | protein\_coding | | ENSDARG00000037018 | gab1 | 1 | 34762810 | 34877632 | protein\_coding | | ENSDARG00000041252 | gemin4 | 15 | 24648417 | 24653384 | protein\_coding | | ENSDARG00000009735 | gigfy2 | 2 | 48349114 | 48401679 | protein\_coding | | ENSDARG00000061159 | gltscr1 | 18 | 44636963 | 44653514 | protein\_coding | | ENSDARG00000105206 | hmgcrb | 21 | 2480189 | 2507425 | protein\_coding | | ENSDARG00000010266 | igf2bp3 | 19 | 20616730 | 20661173 | protein\_coding | | ENSDARG00000017037 | ikbkg | 23 | 18647878 | 18671366 | protein\_coding | | ENSDARG00000008238 | manba | 13 | 11584563 | 11611602 | protein\_coding | | ENSDARG00000063574 | mtg2 | 23 | 534314 | 539746 | protein\_coding | | ENSDARG00000060150 | psmd11a | 3 | 34672491 | 34687393 | protein\_coding | | ENSDARG00000036772 | pygo2 | 19 | 7708145 | 7714137 | protein\_coding | | ENSDARG00000056984 | rad9a | 14 | 21626496 | 21635158 | protein\_coding | | ENSDARG00000102997 | rrn3 | 3 | 36447619 | 36460642 | protein\_coding | | ENSDARG00000007231 | ryk | 6 | 27523904 | 27625140 | protein\_coding | | ENSDARG00000060646 | senp5 | 22 | 38928238 | 38942626 | protein\_coding | | ENSDARG00000060030 | setd1a | 3 | 32582443 | 32610864 | protein\_coding | | ENSDARG00000002339 | sgf29 | 3 | 15339925 | 15346810 | protein\_coding | | ENSDARG00000035821 | si:ch211-173p18.3 | 19 | 40528857 | 40578679 | protein\_coding | | ENSDARG00000057907 | si:ch73-181d5.4 | 23 | 444776 | 465408 | protein\_coding | | ENSDARG00000097086 | si:dkey-172k15.4 | 4 | 75644408 | 75652002 | protein\_coding | | ENSDARG00000071589 | si:dkey-253d23.2 | 22 | 9801947 | 9838713 | protein\_coding | | ENSDARG00000074125 | si:dkey-4c15.5 | 22 | 2074051 | 2218994 | protein\_coding | | ENSDARG00000073801 | si:dkeyp-104h9.5 | 8 | 47840369 | 47855464 | protein\_coding | | ENSDARG00000092327 | si:dkeyp-2e4.3 | 13 | 44698870 | 44699823 | protein\_coding | | ENSDARG00000062472 | sin3b | 8 | 14174041 | 14201318 | protein\_coding | | ENSDARG00000034160 | slc4a1ap | 17 | 40924016 | 40948050 | protein\_coding | | ENSDARG00000078658 | supt20 | 10 | 35067354 | 35108347 | protein\_coding | | ENSDARG00000063649 | tead3b | 6 | 54568412 | 54663588 | protein\_coding | | ENSDARG00000010445 | trabd | 18 | 14917033 | 14932989 | protein\_coding | | ENSDARG00000009436 | ubxn7 | 2 | 17042293 | 17068773 | protein\_coding | | ENSDARG00000041429 | vwa9 | 18 | 19005614 | 19016853 | protein\_coding | | ENSDARG00000074146 | zgc:113452 | 2 | 294798 | 303872 | protein\_coding | | ENSDARG00000069957 | zgc:162936 | 15 | 965722 | 971012 | protein\_coding | | ENSDARG00000078281 | zgc:173575 | 3 | 61967830 | 62093437 | protein\_coding | | ENSDARG00000092605 | znf1003 | 22 | 2374918 | 2513717 | protein\_coding | | ENSDARG00000054957 | znf1015 | 4 | 71932052 | 71945671 | protein\_coding | | ENSDARG00000042969 | znf1035 | 16 | 25096962 | 25123823 | protein\_coding | | ENSDARG00000096851 | znf1143 | 3 | 61149602 | 61154685 | protein\_coding | | ENSDARG00000070786 | znf770 | 20 | 10070510 | 10077890 | protein\_coding | | ENSDARG00000075470 | znf989 | 4 | 72009519 | 72016929 | protein\_coding | |
