# Supplementary material for: A high-resolution mRNA expression time course of embryonic development in zebrafish
Source: eLife. 2017 Nov 16;6:e30860. doi: 10.7554/eLife.30860 (PMC5690287; doi:10.7554/eLife.30860)
Supplement: Supplementary file 6. [file elife-30860-supp6.zip › biolayout-clusters-files/Cluster024.html]

Cluster024


# Cluster024: Detail

### Go to ZFA detail

## GO

| | GO ID | Description | Domain | Annotated | Expected | Observed | Adjusted p-value | Genes | Ensembl IDs | | --- | --- | --- | --- | --- | --- | --- | --- | --- | | GO:0003676 | nucleic acid binding | molecular\_function | 1873 | 8.8 | 18 | 0.00046 | foxm1 igf2bp3 si:ch211-173p18.3 dhx40 znf1035 znf1015 setd1a zgc:162936 znf770 si:dkey-253d23.2 si:dkey-4c15.5 zgc:113452 znf989 zgc:173575 brca2 fam60al znf1143 si:dkey-172k15.4 | ENSDARG00000003200 ENSDARG00000010266 ENSDARG00000035821 ENSDARG00000041586 ENSDARG00000042969 ENSDARG00000054957 ENSDARG00000060030 ENSDARG00000069957 ENSDARG00000070786 ENSDARG00000071589 ENSDARG00000074125 ENSDARG00000074146 ENSDARG00000075470 ENSDARG00000078281 ENSDARG00000079015 ENSDARG00000095007 ENSDARG00000096851 ENSDARG00000097086 | |
