# Supplementary material for: A high-resolution mRNA expression time course of embryonic development in zebrafish
Source: eLife. 2017 Nov 16;6:e30860. doi: 10.7554/eLife.30860 (PMC5690287; doi:10.7554/eLife.30860)
Supplement: Supplementary file 6. [file elife-30860-supp6.zip › biolayout-clusters-files/Cluster025-genes.html]

Cluster025


# Cluster025: Genes

| | Ensembl ID | Gene Name | Chr | Start | End | Biotype | | --- | --- | --- | --- | --- | --- | | ENSDARG00000089355 | ENSDARG00000089355 | 13 | 4210386 | 4238587 | protein\_coding | | ENSDARG00000023330 | anp32b | 1 | 25966643 | 25971141 | protein\_coding | | ENSDARG00000003860 | cbx3a | 19 | 18669752 | 18672871 | protein\_coding | | ENSDARG00000040158 | cdc42l | 16 | 31859656 | 31868937 | protein\_coding | | ENSDARG00000007901 | crnkl1 | 20 | 49274034 | 49293895 | protein\_coding | | ENSDARG00000090788 | cstf2 | 14 | 40994592 | 41020938 | protein\_coding | | ENSDARG00000007099 | cx43.4 | 9 | 46605108 | 46615368 | protein\_coding | | ENSDARG00000016484 | dkc1 | 21 | 43706834 | 43721295 | protein\_coding | | ENSDARG00000102978 | eif4a3 | 11 | 44841827 | 44859344 | protein\_coding | | ENSDARG00000015835 | eif4e2rs1 | 23 | 44622538 | 44839570 | protein\_coding | | ENSDARG00000038074 | ergic3 | 6 | 50393828 | 50414978 | protein\_coding | | ENSDARG00000032866 | erh | 20 | 33591045 | 33594249 | protein\_coding | | ENSDARG00000020465 | ewsr1b | 5 | 24369396 | 24378762 | protein\_coding | | ENSDARG00000069289 | gabpa | 9 | 35205328 | 35216332 | protein\_coding | | ENSDARG00000019897 | gpat4 | 10 | 41440500 | 41478169 | protein\_coding | | ENSDARG00000045248 | h3f3b.1.1 | 24 | 37800832 | 37805131 | protein\_coding | | ENSDARG00000015427 | hdac1 | 19 | 30221386 | 30231335 | protein\_coding | | ENSDARG00000036161 | hnrnpa0l | 14 | 38509118 | 38510619 | protein\_coding | | ENSDARG00000011020 | hnrnpa1a | 11 | 2062910 | 2227366 | protein\_coding | | ENSDARG00000036675 | hnrnpa1b | 23 | 36207736 | 36211615 | protein\_coding | | ENSDARG00000018914 | hnrnpk | 8 | 49756899 | 49777472 | protein\_coding | | ENSDARG00000059303 | hnrnpl2 | 15 | 5796361 | 5811333 | protein\_coding | | ENSDARG00000061735 | hnrnpm | 22 | 17506023 | 17519073 | protein\_coding | | ENSDARG00000014591 | ilf2 | 19 | 37914532 | 37921451 | protein\_coding | | ENSDARG00000105177 | ilf3b | 3 | 13692708 | 13727858 | protein\_coding | | ENSDARG00000052856 | khdrbs1a | 13 | 44873005 | 44885471 | protein\_coding | | ENSDARG00000014366 | luc7l3 | 3 | 58746483 | 58764456 | protein\_coding | | ENSDARG00000038635 | magoh | 8 | 21101222 | 21103647 | protein\_coding | | ENSDARG00000102571 | metap2b | 25 | 3106068 | 3121599 | protein\_coding | | ENSDARG00000059398 | myef2 | 18 | 5552073 | 5567123 | protein\_coding | | ENSDARG00000006092 | rad21a | 16 | 48467048 | 48488254 | protein\_coding | | ENSDARG00000012553 | rap1aa | 8 | 28248407 | 28255413 | protein\_coding | | ENSDARG00000022129 | rbm4.3 | 7 | 22520975 | 22523944 | protein\_coding | | ENSDARG00000016516 | rbm8a | 16 | 45955459 | 45963361 | protein\_coding | | ENSDARG00000005945 | sart1 | 21 | 27266057 | 27288076 | protein\_coding | | ENSDARG00000058292 | sephs1 | 18 | 8409583 | 8422101 | protein\_coding | | ENSDARG00000009753 | sf3b6 | 20 | 35530313 | 35535061 | protein\_coding | | ENSDARG00000045914 | si:ch211-51e12.7 | 4 | 295240 | 304523 | protein\_coding | | ENSDARG00000100558 | slbp | 14 | 14536122 | 14540603 | protein\_coding | | ENSDARG00000017397 | smarcc1a | 16 | 42519772 | 42557505 | protein\_coding | | ENSDARG00000016871 | smarce1 | 3 | 34856362 | 34865480 | protein\_coding | | ENSDARG00000077126 | snrnp70 | 3 | 31793353 | 31802060 | protein\_coding | | ENSDARG00000098574 | srsf11 | 11 | 45010560 | 45022580 | protein\_coding | | ENSDARG00000057691 | srsf1a | 15 | 16136058 | 16140496 | protein\_coding | | ENSDARG00000057484 | srsf2a | 12 | 31605771 | 31611846 | protein\_coding | | ENSDARG00000059360 | srsf3b | 22 | 575530 | 585244 | protein\_coding | | ENSDARG00000053668 | stag2b | 14 | 28180255 | 28221464 | protein\_coding | | ENSDARG00000019572 | taf7 | 14 | 40485243 | 40506848 | protein\_coding | | ENSDARG00000103004 | tpra | 2 | 21153615 | 21208696 | protein\_coding | | ENSDARG00000015325 | u2af1 | 9 | 9252409 | 9264740 | protein\_coding | | ENSDARG00000061490 | u2surp | 2 | 26967743 | 26987310 | protein\_coding | | ENSDARG00000102632 | ubc | 10 | 44845660 | 44848120 | protein\_coding | |
