# Supplementary material for: A high-resolution mRNA expression time course of embryonic development in zebrafish
Source: eLife. 2017 Nov 16;6:e30860. doi: 10.7554/eLife.30860 (PMC5690287; doi:10.7554/eLife.30860)
Supplement: Supplementary file 6. [file elife-30860-supp6.zip › biolayout-clusters-files/Cluster025.html]

Cluster025


# Cluster025: Detail

### Go to ZFA detail

## GO

| | GO ID | Description | Domain | Annotated | Expected | Observed | Adjusted p-value | Genes | Ensembl IDs | | --- | --- | --- | --- | --- | --- | --- | --- | --- | | GO:0060216 | definitive hemopoiesis | biological\_process | 23 | 0.10 | 3 | 3.0e-02 | dkc1 myef2 metap2b | ENSDARG00000016484 ENSDARG00000059398 ENSDARG00000102571 | | GO:0060218 | hematopoietic stem cell differentiation | biological\_process | 23 | 0.10 | 3 | 3.0e-02 | hdac1 snrnp70 metap2b | ENSDARG00000015427 ENSDARG00000077126 ENSDARG00000102571 | | GO:0000184 | nuclear-transcribed mRNA catabolic proce... | biological\_process | 14 | 0.06 | 3 | 6.6e-03 | rbm8a magoh eif4a3 | ENSDARG00000016516 ENSDARG00000038635 ENSDARG00000102978 | | GO:0000381 | regulation of alternative mRNA splicing,... | biological\_process | 11 | 0.05 | 3 | 3.0e-03 | rbm8a magoh eif4a3 | ENSDARG00000016516 ENSDARG00000038635 ENSDARG00000102978 | | GO:0019013 | viral nucleocapsid | cellular\_component | 29 | 0.14 | 4 | 2.4e-03 | hnrnpk hnrnpa1b hnrnpl2 snrnp70 | ENSDARG00000018914 ENSDARG00000036675 ENSDARG00000059303 ENSDARG00000077126 | | GO:0071011 | precatalytic spliceosome | cellular\_component | 17 | 0.08 | 3 | 1.6e-02 | crnkl1 sf3b6 snrnp70 | ENSDARG00000007901 ENSDARG00000009753 ENSDARG00000077126 | | GO:0071013 | catalytic step 2 spliceosome | cellular\_component | 28 | 0.13 | 4 | 2.1e-03 | crnkl1 sf3b6 magoh eif4a3 | ENSDARG00000007901 ENSDARG00000009753 ENSDARG00000038635 ENSDARG00000102978 | | GO:0003676 | nucleic acid binding | molecular\_function | 1873 | 9.17 | 33 | 6.9e-05 | rad21a sf3b6 hnrnpa1a luc7l3 ilf2 u2af1 eif4e2rs1 dkc1 rbm8a smarcc1a hnrnpk taf7 ewsr1b rbm4.3 hnrnpa0l hnrnpa1b magoh h3f3b.1 khdrbs1a srsf2a srsf1a hnrnpl2 srsf3b myef2 u2surp hnrnpm gabpa snrnp70 cstf2 srsf11 slbp eif4a3 ilf3b | ENSDARG00000006092 ENSDARG00000009753 ENSDARG00000011020 ENSDARG00000014366 ENSDARG00000014591 ENSDARG00000015325 ENSDARG00000015835 ENSDARG00000016484 ENSDARG00000016516 ENSDARG00000017397 ENSDARG00000018914 ENSDARG00000019572 ENSDARG00000020465 ENSDARG00000022129 ENSDARG00000036161 ENSDARG00000036675 ENSDARG00000038635 ENSDARG00000045248 ENSDARG00000052856 ENSDARG00000057484 ENSDARG00000057691 ENSDARG00000059303 ENSDARG00000059360 ENSDARG00000059398 ENSDARG00000061490 ENSDARG00000061735 ENSDARG00000069289 ENSDARG00000077126 ENSDARG00000090788 ENSDARG00000098574 ENSDARG00000100558 ENSDARG00000102978 ENSDARG00000105177 | | GO:0003723 | RNA binding | molecular\_function | 423 | 2.07 | 18 | 2.2e-07 | luc7l3 ilf2 u2af1 eif4e2rs1 dkc1 rbm8a hnrnpk magoh khdrbs1a srsf1a hnrnpl2 u2surp hnrnpm snrnp70 cstf2 slbp eif4a3 ilf3b | ENSDARG00000014366 ENSDARG00000014591 ENSDARG00000015325 ENSDARG00000015835 ENSDARG00000016484 ENSDARG00000016516 ENSDARG00000018914 ENSDARG00000038635 ENSDARG00000052856 ENSDARG00000057691 ENSDARG00000059303 ENSDARG00000061490 ENSDARG00000061735 ENSDARG00000077126 ENSDARG00000090788 ENSDARG00000100558 ENSDARG00000102978 ENSDARG00000105177 | | GO:0003729 | mRNA binding | molecular\_function | 54 | 0.26 | 5 | 1.5e-03 | luc7l3 rbm8a snrnp70 cstf2 slbp | ENSDARG00000014366 ENSDARG00000016516 ENSDARG00000077126 ENSDARG00000090788 ENSDARG00000100558 | | GO:0000166 | nucleotide binding | molecular\_function | 1424 | 6.97 | 23 | 1.5e-12 | sf3b6 hnrnpa1a rap1aa ilf2 u2af1 rbm8a ewsr1b rbm4.3 hnrnpa0l hnrnpa1b cdc42l srsf2a srsf1a sephs1 hnrnpl2 srsf3b myef2 u2surp hnrnpm snrnp70 cstf2 srsf11 eif4a3 | ENSDARG00000009753 ENSDARG00000011020 ENSDARG00000012553 ENSDARG00000014591 ENSDARG00000015325 ENSDARG00000016516 ENSDARG00000020465 ENSDARG00000022129 ENSDARG00000036161 ENSDARG00000036675 ENSDARG00000040158 ENSDARG00000057484 ENSDARG00000057691 ENSDARG00000058292 ENSDARG00000059303 ENSDARG00000059360 ENSDARG00000059398 ENSDARG00000061490 ENSDARG00000061735 ENSDARG00000077126 ENSDARG00000090788 ENSDARG00000098574 ENSDARG00000102978 | |
