# Supplementary material for: A high-resolution mRNA expression time course of embryonic development in zebrafish
Source: eLife. 2017 Nov 16;6:e30860. doi: 10.7554/eLife.30860 (PMC5690287; doi:10.7554/eLife.30860)
Supplement: Supplementary file 6. [file elife-30860-supp6.zip › biolayout-clusters-files/Cluster026-genes.html]

Cluster026


# Cluster026: Genes

| | Ensembl ID | Gene Name | Chr | Start | End | Biotype | | --- | --- | --- | --- | --- | --- | | ENSDARG00000099693 | AL935065.2 | 5 | 37217717 | 37220725 | protein\_coding | | ENSDARG00000098501 | BX248128.1 | 4 | 60483415 | 60484212 | protein\_coding | | ENSDARG00000090220 | BX324003.1 | 4 | 32764185 | 32865433 | protein\_coding | | ENSDARG00000090452 | BX470115.1 | 4 | 47146892 | 47148050 | protein\_coding | | ENSDARG00000102347 | BX640584.4 | 4 | 50465753 | 50474941 | protein\_coding | | ENSDARG00000098302 | BX664622.2 | KN149689.1 | 35308 | 42104 | protein\_coding | | ENSDARG00000102600 | CABZ01003344.2 | KN149847.1 | 66755 | 67936 | protein\_coding | | ENSDARG00000104013 | CABZ01024856.2 | KN149992.1 | 51454 | 56497 | protein\_coding | | ENSDARG00000088927 | CR450793.1 | 10 | 26973395 | 26977091 | protein\_coding | | ENSDARG00000102371 | CU570769.1 | 22 | 1235691 | 1243852 | protein\_coding | | ENSDARG00000087212 | CU655961.2 | 15 | 44095364 | 44101436 | protein\_coding | | ENSDARG00000104576 | ENSDARG00000104576 | 24 | 2827466 | 2830305 | protein\_coding | | ENSDARG00000104944 | HIST2H3A (1 of many).3 | 25 | 34615323 | 34615733 | protein\_coding | | ENSDARG00000059081 | PRSS35 (1 of many) | 23 | 2507402 | 2509033 | protein\_coding | | ENSDARG00000055043 | depdc7 | 18 | 45708660 | 45735762 | protein\_coding | | ENSDARG00000040955 | dharma | 15 | 29078414 | 29079617 | protein\_coding | | ENSDARG00000102778 | fgf17 | 8 | 52745814 | 52758875 | protein\_coding | | ENSDARG00000013576 | gadd45bb | 2 | 36957292 | 36959531 | protein\_coding | | ENSDARG00000036987 | has2 | 16 | 15479514 | 15497339 | protein\_coding | | ENSDARG00000086374 | isg15 | 5 | 19608232 | 19609484 | protein\_coding | | ENSDARG00000031698 | mylk2 | 23 | 32112855 | 32134352 | protein\_coding | | ENSDARG00000061773 | neurl1ab | 17 | 20480393 | 20498790 | protein\_coding | | ENSDARG00000058917 | nnr | 15 | 1624904 | 1625949 | protein\_coding | | ENSDARG00000021201 | noto | 13 | 14845116 | 14847112 | protein\_coding | | ENSDARG00000102694 | si:ch211-103a14.1 | 22 | 1233341 | 1234762 | processed\_transcript | | ENSDARG00000093284 | si:ch211-173m16.2 | 9 | 3029108 | 3031295 | protein\_coding | | ENSDARG00000086296 | si:ch211-209p16.1 | 4 | 44084822 | 44105274 | protein\_coding | | ENSDARG00000040141 | si:ch73-138e16.5 | 22 | 1283689 | 1290245 | protein\_coding | | ENSDARG00000101790 | si:ch73-299h12.3 | 21 | 2243522 | 2247420 | protein\_coding | | ENSDARG00000093787 | si:dkey-182g1.8 | 22 | 8761089 | 8767672 | lincRNA | | ENSDARG00000099837 | si:dkey-199m13.7 | 4 | 30362395 | 30419839 | protein\_coding | | ENSDARG00000094118 | si:dkey-21o22.2 | 4 | 19138997 | 19142141 | protein\_coding | | ENSDARG00000099284 | si:dkey-228b2.6 | 21 | 13050027 | 13052465 | protein\_coding | | ENSDARG00000097023 | si:dkey-234l24.10.1 | 4 | 14175390 | 14187880 | lincRNA | | ENSDARG00000035151 | si:dkey-261j4.3 | 5 | 60945975 | 60953404 | protein\_coding | | ENSDARG00000101476 | si:dkey-65j4.2 | 10 | 37541645 | 37543920 | protein\_coding | | ENSDARG00000097098 | si:zfos-44a5.1 | 3 | 61285679 | 61287356 | protein\_coding | | ENSDARG00000002353 | tagapa | 17 | 45421506 | 45427401 | protein\_coding | | ENSDARG00000101593 | zgc:163077 | 21 | 2144974 | 2147476 | protein\_coding | | ENSDARG00000091994 | znf1046 | 4 | 44360251 | 44369333 | protein\_coding | | ENSDARG00000100750 | znf1047 | 4 | 54780018 | 54792719 | protein\_coding | | ENSDARG00000094888 | znf1061 | 4 | 48610827 | 48619113 | protein\_coding | | ENSDARG00000095149 | znf1062 | 4 | 40615413 | 40624403 | protein\_coding | | ENSDARG00000101245 | znf1063 | 4 | 46813156 | 46821473 | protein\_coding | | ENSDARG00000087645 | znf1064 | 4 | 47114891 | 47141723 | protein\_coding | | ENSDARG00000090366 | znf1065 | 4 | 33440720 | 33533611 | protein\_coding | | ENSDARG00000091477 | znf1066 | 4 | 48490171 | 48501412 | protein\_coding | | ENSDARG00000098384 | znf1101 | 4 | 67518275 | 67594925 | protein\_coding | | ENSDARG00000103405 | znf1102.2 | 4 | 37161731 | 37168614 | protein\_coding | | ENSDARG00000096029 | znf1138 | 4 | 44506972 | 44514308 | protein\_coding | | ENSDARG00000103047 | znf995.1 | 4 | 61892724 | 61897139 | protein\_coding | |
