# Supplementary material for: A high-resolution mRNA expression time course of embryonic development in zebrafish
Source: eLife. 2017 Nov 16;6:e30860. doi: 10.7554/eLife.30860 (PMC5690287; doi:10.7554/eLife.30860)
Supplement: Supplementary file 6. [file elife-30860-supp6.zip › biolayout-clusters-files/Cluster026.html]

Cluster026


# Cluster026: Detail

### Go to ZFA detail

## GO

| | GO ID | Description | Domain | Annotated | Expected | Observed | Adjusted p-value | Genes | Ensembl IDs | | --- | --- | --- | --- | --- | --- | --- | --- | --- | | GO:0048318 | axial mesoderm development | biological\_process | 10 | 0.02 | 2 | 3.3e-02 | noto dharma | ENSDARG00000021201 ENSDARG00000040955 | | GO:0061053 | somite development | biological\_process | 83 | 0.14 | 4 | 4.3e-02 | gadd45bb noto has2 dharma | ENSDARG00000013576 ENSDARG00000021201 ENSDARG00000036987 ENSDARG00000040955 | | GO:0001947 | heart looping | biological\_process | 66 | 0.11 | 3 | 4.6e-02 | noto has2 dharma | ENSDARG00000021201 ENSDARG00000036987 ENSDARG00000040955 | | GO:2000223 | regulation of BMP signaling pathway invo... | biological\_process | 11 | 0.02 | 3 | 1.8e-04 | noto has2 dharma | ENSDARG00000021201 ENSDARG00000036987 ENSDARG00000040955 | | GO:0003676 | nucleic acid binding | molecular\_function | 1873 | 5.00 | 18 | 9.9e-06 | noto si:dkey-261j4.3 dharma si:ch211-209p16.1 znf1064 znf1065 znf1066 znf1046 znf1061 znf1062 znf1138 si:zfos-44a5.1 znf1101 si:dkey-199m13.7 znf1047 znf1063 zgc:163077 si:ch73-299h12.3 | ENSDARG00000021201 ENSDARG00000035151 ENSDARG00000040955 ENSDARG00000086296 ENSDARG00000087645 ENSDARG00000090366 ENSDARG00000091477 ENSDARG00000091994 ENSDARG00000094888 ENSDARG00000095149 ENSDARG00000096029 ENSDARG00000097098 ENSDARG00000098384 ENSDARG00000099837 ENSDARG00000100750 ENSDARG00000101245 ENSDARG00000101593 ENSDARG00000101790 | | GO:0000978 | RNA polymerase II core promoter proximal... | molecular\_function | 68 | 0.18 | 4 | 7.1e-03 | si:ch211-209p16.1 znf1138 znf1101 si:dkey-199m13.7 | ENSDARG00000086296 ENSDARG00000096029 ENSDARG00000098384 ENSDARG00000099837 | | GO:0046872 | metal ion binding | molecular\_function | 2036 | 5.44 | 17 | 1.3e-06 | si:dkey-261j4.3 neurl1ab si:ch211-209p16.1 znf1064 znf1065 znf1066 znf1046 znf1061 znf1062 znf1138 si:zfos-44a5.1 znf1101 si:dkey-199m13.7 znf1047 znf1063 zgc:163077 si:ch73-299h12.3 | ENSDARG00000035151 ENSDARG00000061773 ENSDARG00000086296 ENSDARG00000087645 ENSDARG00000090366 ENSDARG00000091477 ENSDARG00000091994 ENSDARG00000094888 ENSDARG00000095149 ENSDARG00000096029 ENSDARG00000097098 ENSDARG00000098384 ENSDARG00000099837 ENSDARG00000100750 ENSDARG00000101245 ENSDARG00000101593 ENSDARG00000101790 | |
