# Supplementary material for: A high-resolution mRNA expression time course of embryonic development in zebrafish
Source: eLife. 2017 Nov 16;6:e30860. doi: 10.7554/eLife.30860 (PMC5690287; doi:10.7554/eLife.30860)
Supplement: Supplementary file 6. [file elife-30860-supp6.zip › biolayout-clusters-files/Cluster027-genes.html]

Cluster027


# Cluster027: Genes

| | Ensembl ID | Gene Name | Chr | Start | End | Biotype | | --- | --- | --- | --- | --- | --- | | ENSDARG00000022085 | NOS1AP | 5 | 32933749 | 33006646 | protein\_coding | | ENSDARG00000088157 | SMOC2 | 13 | 43130259 | 43191116 | protein\_coding | | ENSDARG00000021137 | adgrv1 | 5 | 48031936 | 48288616 | protein\_coding | | ENSDARG00000036637 | arl11 | 1 | 45961383 | 45963296 | protein\_coding | | ENSDARG00000005002 | brf1b | 20 | 21002118 | 21117226 | protein\_coding | | ENSDARG00000071051 | cbx6a | 22 | 29125907 | 29133881 | protein\_coding | | ENSDARG00000003061 | cd276 | 18 | 50446908 | 50527477 | protein\_coding | | ENSDARG00000023542 | cdh7 | 2 | 27354781 | 27439019 | protein\_coding | | ENSDARG00000061328 | cdon | 18 | 44059598 | 44135697 | protein\_coding | | ENSDARG00000071410 | cks2 | 22 | 21288341 | 21321375 | protein\_coding | | ENSDARG00000060106 | crb2a | 21 | 8386235 | 8420653 | protein\_coding | | ENSDARG00000003779 | ctnnd2b | 2 | 30570723 | 30710209 | protein\_coding | | ENSDARG00000017988 | dse | 20 | 545611 | 567877 | protein\_coding | | ENSDARG00000098295 | efna3b | 16 | 23172294 | 23274851 | protein\_coding | | ENSDARG00000099650 | fgd | KN149731.1 | 730 | 6892 | protein\_coding | | ENSDARG00000070769 | foxg1a | 17 | 29100064 | 29102399 | protein\_coding | | ENSDARG00000052131 | gli3 | 24 | 11387903 | 11681138 | protein\_coding | | ENSDARG00000068436 | h3f3b.1.3 | 15 | 47263247 | 47270078 | protein\_coding | | ENSDARG00000103824 | hint1 | 10 | 7678111 | 7682097 | protein\_coding | | ENSDARG00000103862 | hoxa3a | 19 | 20181999 | 20207294 | protein\_coding | | ENSDARG00000000175 | hoxb2a | 3 | 23621602 | 23624283 | protein\_coding | | ENSDARG00000029263 | hoxb3a | 3 | 23567486 | 23618858 | protein\_coding | | ENSDARG00000013533 | hoxb4a | 3 | 23580291 | 23593434 | protein\_coding | | ENSDARG00000010630 | hoxb6a | 3 | 23573156 | 23575198 | protein\_coding | | ENSDARG00000054025 | hoxb8b | 12 | 27026249 | 27028811 | protein\_coding | | ENSDARG00000092809 | hoxc9a | 23 | 35996457 | 35998685 | protein\_coding | | ENSDARG00000057859 | hoxd10a | 9 | 1966910 | 1969274 | protein\_coding | | ENSDARG00000059280 | hoxd3a | 9 | 1936252 | 1958755 | protein\_coding | | ENSDARG00000101076 | irx3a | 7 | 35770017 | 35773362 | protein\_coding | | ENSDARG00000002937 | meis1a | 1 | 50831295 | 50881287 | protein\_coding | | ENSDARG00000045091 | mrpl10 | 12 | 28681720 | 28684740 | protein\_coding | | ENSDARG00000013075 | mrpl11 | 14 | 30217047 | 30280036 | protein\_coding | | ENSDARG00000045696 | mrpl23 | 25 | 23679895 | 23718401 | protein\_coding | | ENSDARG00000010710 | msi1 | 8 | 39573169 | 39613442 | protein\_coding | | ENSDARG00000019835 | nkx2.1 | 17 | 38314823 | 38317040 | protein\_coding | | ENSDARG00000105112 | ntn1a | 6 | 23332727 | 23455336 | protein\_coding | | ENSDARG00000070069 | pitx3 | 13 | 7224110 | 7243014 | protein\_coding | | ENSDARG00000030537 | psmc1a | 17 | 8741836 | 8784241 | protein\_coding | | ENSDARG00000018124 | psmd3 | 19 | 48388925 | 48495346 | protein\_coding | | ENSDARG00000055026 | ptch2 | 2 | 33985967 | 34010363 | protein\_coding | | ENSDARG00000100422 | ptpro | 4 | 57288 | 85726 | protein\_coding | | ENSDARG00000070427 | s100v1 | 16 | 28819780 | 28825383 | protein\_coding | | ENSDARG00000098798 | si:dkey-25e11.10 | 19 | 20209462 | 20210673 | transcribed\_unprocessed\_pseudogene | | ENSDARG00000008131 | sox1b | 1 | 45503101 | 45504875 | protein\_coding | | ENSDARG00000008540 | sox21b | 9 | 53736849 | 53738215 | protein\_coding | | ENSDARG00000021195 | st8sia6 | 3 | 16116124 | 16135459 | protein\_coding | | ENSDARG00000021916 | vax1 | 17 | 21412857 | 21422488 | protein\_coding | | ENSDARG00000105129 | zgc:158345 | KN150169.1 | 895 | 6392 | protein\_coding | | ENSDARG00000016022 | zic5 | 9 | 31463362 | 31503578 | protein\_coding | |
