# Supplementary material for: A high-resolution mRNA expression time course of embryonic development in zebrafish
Source: eLife. 2017 Nov 16;6:e30860. doi: 10.7554/eLife.30860 (PMC5690287; doi:10.7554/eLife.30860)
Supplement: Supplementary file 6. [file elife-30860-supp6.zip › biolayout-clusters-files/Cluster027.html]

Cluster027


# Cluster027: Detail

### Go to ZFA detail

## GO

| | GO ID | Description | Domain | Annotated | Expected | Observed | Adjusted p-value | Genes | Ensembl IDs | | --- | --- | --- | --- | --- | --- | --- | --- | --- | | GO:0007275 | multicellular organismal development | biological\_process | 1969 | 9.15 | 31 | 7.1e-08 | hoxb2a meis1a ctnnd2b brf1b sox1b sox21b hoxb6a msi1 hoxb4a zic5 psmd3 nkx2.1 adgrv1 st8sia6 vax1 cdh7 hoxb3a gli3 hoxb8b ptch2 hoxd10a hoxd3a crb2a pitx3 foxg1a hoxc9a efna3b ptpro irx3a hoxa3a ntn1a | ENSDARG00000000175 ENSDARG00000002937 ENSDARG00000003779 ENSDARG00000005002 ENSDARG00000008131 ENSDARG00000008540 ENSDARG00000010630 ENSDARG00000010710 ENSDARG00000013533 ENSDARG00000016022 ENSDARG00000018124 ENSDARG00000019835 ENSDARG00000021137 ENSDARG00000021195 ENSDARG00000021916 ENSDARG00000023542 ENSDARG00000029263 ENSDARG00000052131 ENSDARG00000054025 ENSDARG00000055026 ENSDARG00000057859 ENSDARG00000059280 ENSDARG00000060106 ENSDARG00000070069 ENSDARG00000070769 ENSDARG00000092809 ENSDARG00000098295 ENSDARG00000100422 ENSDARG00000101076 ENSDARG00000103862 ENSDARG00000105112 | | GO:0006355 | regulation of transcription, DNA-templat... | biological\_process | 1072 | 4.98 | 20 | 5.3e-07 | hoxb2a meis1a brf1b sox1b sox21b hoxb6a hoxb4a nkx2.1 vax1 hoxb3a psmc1a gli3 hoxb8b hoxd10a hoxd3a pitx3 foxg1a hoxc9a irx3a hoxa3a | ENSDARG00000000175 ENSDARG00000002937 ENSDARG00000005002 ENSDARG00000008131 ENSDARG00000008540 ENSDARG00000010630 ENSDARG00000013533 ENSDARG00000019835 ENSDARG00000021916 ENSDARG00000029263 ENSDARG00000030537 ENSDARG00000052131 ENSDARG00000054025 ENSDARG00000057859 ENSDARG00000059280 ENSDARG00000070069 ENSDARG00000070769 ENSDARG00000092809 ENSDARG00000101076 ENSDARG00000103862 | | GO:0005634 | nucleus | cellular\_component | 1915 | 9.07 | 21 | 7.6e-04 | hoxb2a meis1a brf1b sox1b sox21b hoxb6a hoxb4a nkx2.1 vax1 hoxb3a psmc1a hoxb8b hoxd10a hoxd3a h3f3b.1 pitx3 foxg1a cbx6a hoxc9a irx3a hoxa3a | ENSDARG00000000175 ENSDARG00000002937 ENSDARG00000005002 ENSDARG00000008131 ENSDARG00000008540 ENSDARG00000010630 ENSDARG00000013533 ENSDARG00000019835 ENSDARG00000021916 ENSDARG00000029263 ENSDARG00000030537 ENSDARG00000054025 ENSDARG00000057859 ENSDARG00000059280 ENSDARG00000068436 ENSDARG00000070069 ENSDARG00000070769 ENSDARG00000071051 ENSDARG00000092809 ENSDARG00000101076 ENSDARG00000103862 | | GO:0005761 | mitochondrial ribosome | cellular\_component | 16 | 0.08 | 3 | 1.3e-02 | mrpl11 mrpl10 mrpl23 | ENSDARG00000013075 ENSDARG00000045091 ENSDARG00000045696 | | GO:0003700 | transcription factor activity, sequence-... | molecular\_function | 434 | 2.03 | 10 | 3.3e-04 | hoxb2a sox1b hoxb6a hoxb4a hoxb3a hoxb8b hoxd3a pitx3 foxg1a hoxa3a | ENSDARG00000000175 ENSDARG00000008131 ENSDARG00000010630 ENSDARG00000013533 ENSDARG00000029263 ENSDARG00000054025 ENSDARG00000059280 ENSDARG00000070069 ENSDARG00000070769 ENSDARG00000103862 | | GO:0043565 | sequence-specific DNA binding | molecular\_function | 494 | 2.31 | 15 | 6.3e-07 | hoxb2a sox21b hoxb6a hoxb4a nkx2.1 vax1 hoxb3a hoxb8b hoxd10a hoxd3a pitx3 foxg1a hoxc9a irx3a hoxa3a | ENSDARG00000000175 ENSDARG00000008540 ENSDARG00000010630 ENSDARG00000013533 ENSDARG00000019835 ENSDARG00000021916 ENSDARG00000029263 ENSDARG00000054025 ENSDARG00000057859 ENSDARG00000059280 ENSDARG00000070069 ENSDARG00000070769 ENSDARG00000092809 ENSDARG00000101076 ENSDARG00000103862 | |
