# Supplementary material for: A high-resolution mRNA expression time course of embryonic development in zebrafish
Source: eLife. 2017 Nov 16;6:e30860. doi: 10.7554/eLife.30860 (PMC5690287; doi:10.7554/eLife.30860)
Supplement: Supplementary file 6. [file elife-30860-supp6.zip › biolayout-clusters-files/Cluster028-genes.html]

Cluster028


# Cluster028: Genes

| | Ensembl ID | Gene Name | Chr | Start | End | Biotype | | --- | --- | --- | --- | --- | --- | | ENSDARG00000025858 | ENSDARG00000025858 | 8 | 49103270 | 49123267 | protein\_coding | | ENSDARG00000101720 | HIST2H3A (1 of many).2 | 7 | 5843964 | 5844461 | protein\_coding | | ENSDARG00000070828 | actl6a | 6 | 29396167 | 29405143 | protein\_coding | | ENSDARG00000033597 | api5 | 25 | 29688048 | 29709032 | protein\_coding | | ENSDARG00000100019 | arhgap11a | 17 | 92794 | 114131 | protein\_coding | | ENSDARG00000096877 | cab39.1 | 15 | 40157680 | 40159211 | lincRNA | | ENSDARG00000052674 | csnk1a1 | 14 | 38422663 | 38443305 | protein\_coding | | ENSDARG00000038954 | ctnnbip1 | 23 | 29785028 | 29825189 | protein\_coding | | ENSDARG00000057940 | dido1 | 11 | 18372168 | 18395070 | protein\_coding | | ENSDARG00000005827 | eif2s1a | 17 | 34296399 | 34306287 | protein\_coding | | ENSDARG00000016429 | farp2 | 6 | 26957532 | 27048068 | protein\_coding | | ENSDARG00000009657 | fgfr1op2 | 4 | 1873586 | 1881607 | protein\_coding | | ENSDARG00000017741 | g3bp1 | 14 | 25626128 | 25637922 | protein\_coding | | ENSDARG00000058127 | hcfc2 | 4 | 9182743 | 9195378 | protein\_coding | | ENSDARG00000004735 | hnrnpub | 20 | 35155755 | 35167597 | protein\_coding | | ENSDARG00000063466 | isy1 | 11 | 306674 | 460034 | protein\_coding | | ENSDARG00000034734 | med6 | 13 | 36009290 | 36015654 | protein\_coding | | ENSDARG00000018241 | mfap1 | 7 | 50126890 | 50134953 | protein\_coding | | ENSDARG00000098432 | paf1 | 15 | 46784544 | 46791459 | protein\_coding | | ENSDARG00000008706 | paip2b | 1 | 40762984 | 40769300 | protein\_coding | | ENSDARG00000040111 | pi4kb | 16 | 38209700 | 38249789 | protein\_coding | | ENSDARG00000099966 | polr2a | 12 | 22438848 | 22452078 | protein\_coding | | ENSDARG00000009273 | ppm1da | 15 | 27000320 | 27006528 | protein\_coding | | ENSDARG00000101947 | ptbp2a | 24 | 30898551 | 30924968 | protein\_coding | | ENSDARG00000103032 | rap2b | 18 | 50710614 | 50713150 | protein\_coding | | ENSDARG00000012723 | rbm14a | 21 | 27333899 | 27344918 | protein\_coding | | ENSDARG00000010238 | rbm22 | 21 | 33177332 | 33196792 | protein\_coding | | ENSDARG00000041853 | rbm39b | 8 | 37199321 | 37217386 | protein\_coding | | ENSDARG00000055080 | rbm4.2 | 7 | 22524926 | 22529708 | protein\_coding | | ENSDARG00000018854 | scaf4b | 15 | 42329291 | 42390857 | protein\_coding | | ENSDARG00000018049 | sf3b2 | 7 | 6620508 | 6664991 | protein\_coding | | ENSDARG00000095135 | si:ch73-266f23.1.1 | 4 | 71829275 | 71856445 | protein\_coding | | ENSDARG00000077926 | si:dkey-48p11.3 | 4 | 9050672 | 9054031 | protein\_coding | | ENSDARG00000055389 | si:dkey-67c22.2 | 9 | 36023109 | 36033190 | protein\_coding | | ENSDARG00000092816 | si:dkey-93n13.2 | 3 | 15477468 | 15485513 | protein\_coding | | ENSDARG00000037393 | slc43a1a | 14 | 21779351 | 21800766 | protein\_coding | | ENSDARG00000052728 | sltm | 7 | 30267362 | 30289300 | protein\_coding | | ENSDARG00000030155 | ssbp3b | 8 | 17975266 | 18000927 | protein\_coding | | ENSDARG00000026476 | tia1l | 5 | 24006819 | 24013968 | protein\_coding | | ENSDARG00000070545 | top1l | 11 | 25058750 | 25074061 | protein\_coding | | ENSDARG00000012505 | u2af2a | 16 | 12922352 | 12931648 | protein\_coding | | ENSDARG00000019188 | ube2l3a | 10 | 3052927 | 3058851 | protein\_coding | | ENSDARG00000008748 | ube2na | 18 | 15809598 | 15824651 | protein\_coding | | ENSDARG00000016302 | upf1 | 2 | 56045567 | 56111198 | protein\_coding | | ENSDARG00000014498 | ythdf2 | 17 | 27390955 | 27402356 | protein\_coding | | ENSDARG00000035700 | zgc:101664 | 8 | 450430 | 456021 | protein\_coding | | ENSDARG00000035678 | zgc:91910 | 19 | 35426009 | 35429927 | protein\_coding | | ENSDARG00000004956 | zmat2 | 21 | 30256869 | 30263354 | protein\_coding | |
