# Supplementary material for: A high-resolution mRNA expression time course of embryonic development in zebrafish
Source: eLife. 2017 Nov 16;6:e30860. doi: 10.7554/eLife.30860 (PMC5690287; doi:10.7554/eLife.30860)
Supplement: Supplementary file 6. [file elife-30860-supp6.zip › biolayout-clusters-files/Cluster028.html]

Cluster028


# Cluster028: Detail

### Go to ZFA detail

## GO

| | GO ID | Description | Domain | Annotated | Expected | Observed | Adjusted p-value | Genes | Ensembl IDs | | --- | --- | --- | --- | --- | --- | --- | --- | --- | | GO:0071013 | catalytic step 2 spliceosome | cellular\_component | 28 | 0.11 | 3 | 4.3e-02 | rbm22 sf3b2 isy1 | ENSDARG00000010238 ENSDARG00000018049 ENSDARG00000063466 | | GO:0000166 | nucleotide binding | molecular\_function | 1424 | 5.54 | 16 | 1.4e-05 | ube2na rbm22 u2af2a rbm14a upf1 g3bp1 scaf4b ube2l3a tia1l pi4kb rbm39b csnk1a1 sltm rbm4.2 ptbp2a rap2b | ENSDARG00000008748 ENSDARG00000010238 ENSDARG00000012505 ENSDARG00000012723 ENSDARG00000016302 ENSDARG00000017741 ENSDARG00000018854 ENSDARG00000019188 ENSDARG00000026476 ENSDARG00000040111 ENSDARG00000041853 ENSDARG00000052674 ENSDARG00000052728 ENSDARG00000055080 ENSDARG00000101947 ENSDARG00000103032 | |
