# Supplementary material for: A high-resolution mRNA expression time course of embryonic development in zebrafish
Source: eLife. 2017 Nov 16;6:e30860. doi: 10.7554/eLife.30860 (PMC5690287; doi:10.7554/eLife.30860)
Supplement: Supplementary file 6. [file elife-30860-supp6.zip › biolayout-clusters-files/Cluster029-genes.html]

Cluster029


# Cluster029: Genes

| | Ensembl ID | Gene Name | Chr | Start | End | Biotype | | --- | --- | --- | --- | --- | --- | | ENSDARG00000060941 | ANKFN1 | 12 | 32177662 | 32259449 | protein\_coding | | ENSDARG00000036575 | CU424462.1 | 7 | 19154615 | 19168102 | protein\_coding | | ENSDARG00000099566 | ENSDARG00000099566 | 5 | 7691642 | 7734513 | protein\_coding | | ENSDARG00000098374 | FLNB (1 of many).1 | 11 | 41904792 | 41984388 | protein\_coding | | ENSDARG00000105159 | PHKA1 (1 of many) | 7 | 51437416 | 51474604 | protein\_coding | | ENSDARG00000099962 | PHLPP2 | 7 | 69029537 | 69060650 | protein\_coding | | ENSDARG00000062192 | TBC1D8B | 5 | 23138033 | 23171422 | protein\_coding | | ENSDARG00000102471 | TRAK2 | 6 | 4024088 | 4067699 | protein\_coding | | ENSDARG00000034883 | acbd5a | 24 | 5886566 | 5903753 | protein\_coding | | ENSDARG00000010070 | adam9 | 8 | 45842260 | 45931812 | protein\_coding | | ENSDARG00000003383 | asb8 | 23 | 27571416 | 27579620 | protein\_coding | | ENSDARG00000029439 | atp2a2a | 8 | 11649527 | 11707308 | protein\_coding | | ENSDARG00000003631 | clockb | 1 | 18740067 | 18773516 | protein\_coding | | ENSDARG00000096073 | col19a1.1 | 13 | 38688631 | 38730266 | protein\_coding | | ENSDARG00000056587 | cyp2r1 | 7 | 27184128 | 27426041 | protein\_coding | | ENSDARG00000000324 | dcaf8 | 2 | 38019347 | 38039234 | protein\_coding | | ENSDARG00000086808 | ddhd1a | 17 | 50577108 | 50612329 | protein\_coding | | ENSDARG00000019205 | fam120c | 23 | 18460646 | 18489529 | protein\_coding | | ENSDARG00000077284 | gdpd5a | 10 | 38488410 | 38554053 | protein\_coding | | ENSDARG00000045415 | gnal | 24 | 8764353 | 8833331 | protein\_coding | | ENSDARG00000036764 | hax1 | 19 | 7880093 | 7886207 | protein\_coding | | ENSDARG00000063594 | hipk1a | 23 | 36693826 | 36743183 | protein\_coding | | ENSDARG00000021209 | kctd9a | 8 | 51600038 | 51612629 | protein\_coding | | ENSDARG00000053026 | kif19 | 12 | 38541303 | 38630908 | protein\_coding | | ENSDARG00000006889 | mpped2a | 7 | 16096912 | 16209238 | protein\_coding | | ENSDARG00000037101 | mtmr7a | 14 | 30058232 | 30071827 | protein\_coding | | ENSDARG00000074902 | nol4la | 8 | 23231611 | 23307025 | protein\_coding | | ENSDARG00000102066 | ntn4 | 7 | 19082043 | 19117052 | protein\_coding | | ENSDARG00000003934 | nxnl2 | 10 | 2554655 | 2558026 | protein\_coding | | ENSDARG00000003776 | pip4k2aa | 24 | 16925001 | 16982578 | protein\_coding | | ENSDARG00000068246 | plcb3 | 7 | 59951153 | 60046639 | protein\_coding | | ENSDARG00000057374 | sfxn3 | 13 | 18546436 | 18560049 | protein\_coding | | ENSDARG00000089019 | si:ch211-175m2.5 | 14 | 20935341 | 20940801 | protein\_coding | | ENSDARG00000023933 | skila | 2 | 37324670 | 37371986 | protein\_coding | | ENSDARG00000070832 | snrka | 16 | 8135109 | 8219070 | protein\_coding | | ENSDARG00000067607 | srebf1 | 3 | 61824619 | 61850706 | protein\_coding | | ENSDARG00000056218 | tnika | 2 | 26002462 | 26160855 | protein\_coding | | ENSDARG00000104409 | tnk2b | 2 | 4888417 | 4956992 | protein\_coding | | ENSDARG00000020326 | tyk2 | 3 | 48527579 | 48599773 | protein\_coding | | ENSDARG00000024229 | ubl7a | 25 | 31332690 | 31352936 | protein\_coding | | ENSDARG00000062518 | ulk1a | 8 | 43741528 | 43782435 | protein\_coding | | ENSDARG00000070441 | zdhhc17 | 4 | 2551935 | 2578466 | protein\_coding | | ENSDARG00000087417 | zdhhc5b | 14 | 50019744 | 50044225 | protein\_coding | | ENSDARG00000071683 | zfr2 | 22 | 4671913 | 4715449 | protein\_coding | |
