# Supplementary material for: A high-resolution mRNA expression time course of embryonic development in zebrafish
Source: eLife. 2017 Nov 16;6:e30860. doi: 10.7554/eLife.30860 (PMC5690287; doi:10.7554/eLife.30860)
Supplement: Supplementary file 6. [file elife-30860-supp6.zip › biolayout-clusters-files/Cluster030-genes.html]

Cluster030


# Cluster030: Genes

| | Ensembl ID | Gene Name | Chr | Start | End | Biotype | | --- | --- | --- | --- | --- | --- | | ENSDARG00000088389 | CABZ01092285.1 | 2 | 29111 | 31213 | protein\_coding | | ENSDARG00000104871 | LEPROTL1 | KN149978.1 | 12205 | 15439 | protein\_coding | | ENSDARG00000078256 | OVOL1 (1 of many) | 21 | 25588421 | 25594777 | protein\_coding | | ENSDARG00000036245 | WIPF3 | 19 | 19862782 | 19873772 | protein\_coding | | ENSDARG00000025372 | admp | 2 | 53756683 | 53772703 | protein\_coding | | ENSDARG00000077044 | arl6ip5a | 6 | 43236677 | 43244095 | protein\_coding | | ENSDARG00000102300 | ca9 | 10 | 6413482 | 6453356 | protein\_coding | | ENSDARG00000006110 | chd | 15 | 46557484 | 46594812 | protein\_coding | | ENSDARG00000078254 | ftr14l | 13 | 18402013 | 18408928 | protein\_coding | | ENSDARG00000038569 | fzd8b | 2 | 48100996 | 48103509 | protein\_coding | | ENSDARG00000059327 | gata2a | 11 | 3828494 | 3846289 | protein\_coding | | ENSDARG00000038703 | hkdc1 | 13 | 22887048 | 22901353 | protein\_coding | | ENSDARG00000019747 | hsd3b2 | 20 | 1251700 | 1255349 | protein\_coding | | ENSDARG00000041065 | hspb1 | 5 | 3238284 | 3255083 | protein\_coding | | ENSDARG00000052158 | il23r | 6 | 34884773 | 34911792 | protein\_coding | | ENSDARG00000039070 | im:7138239 | 2 | 53773527 | 53797673 | protein\_coding | | ENSDARG00000040432 | klf2b | 19 | 17832518 | 17838270 | protein\_coding | | ENSDARG00000001414 | mctp2a | 18 | 23375175 | 23467233 | protein\_coding | | ENSDARG00000055479 | mmp20b | 15 | 25621657 | 25636600 | protein\_coding | | ENSDARG00000030703 | otx1a | 1 | 50277467 | 50286158 | protein\_coding | | ENSDARG00000008403 | phospho1 | 3 | 15656117 | 15661237 | protein\_coding | | ENSDARG00000038235 | pkdccb | 17 | 31134252 | 31147256 | protein\_coding | | ENSDARG00000070713 | prss60.1 | 6 | 39088433 | 39093794 | protein\_coding | | ENSDARG00000088143 | sema4gb | 12 | 33730532 | 33792398 | protein\_coding | | ENSDARG00000045898 | si:ch211-152c2.3 | 4 | 828562 | 831585 | protein\_coding | | ENSDARG00000073912 | si:ch211-202h22.7 | 1 | 53972397 | 53982272 | protein\_coding | | ENSDARG00000078138 | si:ch211-202h22.8 | 1 | 53960475 | 53971115 | protein\_coding | | ENSDARG00000093494 | si:ch211-217k17.9 | 1 | 51757707 | 51765926 | protein\_coding | | ENSDARG00000062502 | si:ch73-196l6.5 | 19 | 22733677 | 22743831 | protein\_coding | | ENSDARG00000087793 | si:dkey-54n8.2 | 15 | 25641544 | 25649623 | protein\_coding | | ENSDARG00000097088 | si:dkey-60d5.3 | 10 | 5409204 | 5410681 | protein\_coding | | ENSDARG00000101021 | slc22a5 | 21 | 45634836 | 45646180 | protein\_coding | | ENSDARG00000089837 | slc35d2 | 8 | 1122185 | 1141217 | protein\_coding | | ENSDARG00000079525 | slc39a5 | 23 | 32409340 | 32425648 | protein\_coding | | ENSDARG00000010124 | sp5l | 23 | 28096555 | 28099402 | protein\_coding | | ENSDARG00000098837 | tgm5l | 6 | 2012019 | 2028822 | protein\_coding | | ENSDARG00000025920 | tiam1b | 15 | 42440773 | 42595002 | protein\_coding | | ENSDARG00000061841 | tiparp | 18 | 34623291 | 34666062 | protein\_coding | | ENSDARG00000089756 | traf3ip2b | 11 | 43823544 | 43835810 | protein\_coding | | ENSDARG00000102389 | ved | 10 | 45150450 | 45155584 | protein\_coding | | ENSDARG00000039443 | zgc:110353 | 8 | 24225420 | 24231694 | protein\_coding | | ENSDARG00000077855 | znrf2a | 19 | 1981133 | 1997548 | protein\_coding | |
