# Supplementary material for: A high-resolution mRNA expression time course of embryonic development in zebrafish
Source: eLife. 2017 Nov 16;6:e30860. doi: 10.7554/eLife.30860 (PMC5690287; doi:10.7554/eLife.30860)
Supplement: Supplementary file 6. [file elife-30860-supp6.zip › biolayout-clusters-files/Cluster031-genes.html]

Cluster031


# Cluster031: Genes

| | Ensembl ID | Gene Name | Chr | Start | End | Biotype | | --- | --- | --- | --- | --- | --- | | ENSDARG00000091457 | ENSDARG00000091457 | 20 | 53438776 | 53441527 | protein\_coding | | ENSDARG00000105002 | FP103009.1 | 13 | 36409584 | 36419624 | protein\_coding | | ENSDARG00000089138 | LONRF2 | 1 | 27697917 | 27779773 | protein\_coding | | ENSDARG00000036094 | PIAS1 (1 of many) | 7 | 33611440 | 33677985 | protein\_coding | | ENSDARG00000102503 | SLC9A3R1 (1 of many) | KN149968.1 | 15773 | 67125 | protein\_coding | | ENSDARG00000018179 | acvrl1 | 23 | 27909106 | 27930563 | protein\_coding | | ENSDARG00000104279 | appa | 1 | 622441 | 661520 | protein\_coding | | ENSDARG00000092067 | atn1 | 16 | 31890786 | 31913397 | protein\_coding | | ENSDARG00000009447 | atp5g3b | 6 | 10595417 | 10600495 | protein\_coding | | ENSDARG00000088593 | chst1 | 25 | 13921209 | 13924711 | protein\_coding | | ENSDARG00000036375 | cyfip2 | 14 | 33643035 | 33704055 | protein\_coding | | ENSDARG00000069113 | dbn1 | 21 | 37116243 | 37287062 | protein\_coding | | ENSDARG00000013847 | egfra | 2 | 3864859 | 3989336 | protein\_coding | | ENSDARG00000062542 | fgf12b | 15 | 45391445 | 45492160 | protein\_coding | | ENSDARG00000068708 | ifrd1 | 4 | 6791224 | 6800753 | protein\_coding | | ENSDARG00000099758 | igf2bp2b | 1 | 29639262 | 29706908 | protein\_coding | | ENSDARG00000071863 | itgb1a | 24 | 1116468 | 1155290 | protein\_coding | | ENSDARG00000060925 | jarid2a | 16 | 25492047 | 25585697 | protein\_coding | | ENSDARG00000073857 | klf7a | 1 | 5705564 | 5796397 | protein\_coding | | ENSDARG00000045275 | klhl14 | 24 | 35993027 | 36019174 | protein\_coding | | ENSDARG00000034896 | ldb2b | 1 | 22114328 | 22144085 | protein\_coding | | ENSDARG00000008720 | lmo3 | 4 | 12613874 | 12680444 | protein\_coding | | ENSDARG00000033138 | lyrm2 | 20 | 1175560 | 1178183 | protein\_coding | | ENSDARG00000099970 | malat1 | 14 | 46643944 | 46651420 | lincRNA | | ENSDARG00000045316 | map7d2b | 24 | 23975598 | 24018027 | protein\_coding | | ENSDARG00000028676 | nuak1b | 25 | 5808536 | 5836133 | protein\_coding | | ENSDARG00000035352 | pafah1b2 | 5 | 37363067 | 37373400 | protein\_coding | | ENSDARG00000051756 | pdhx | 25 | 36857607 | 36864690 | protein\_coding | | ENSDARG00000042785 | pitx1 | 21 | 45776822 | 45785097 | protein\_coding | | ENSDARG00000005883 | rfx1a | 3 | 19001063 | 19050911 | protein\_coding | | ENSDARG00000036965 | rnf24 | 1 | 41066429 | 41116690 | protein\_coding | | ENSDARG00000055530 | sertad2b | 13 | 24370513 | 24394095 | protein\_coding | | ENSDARG00000012138 | sgce | 19 | 41451548 | 41482453 | protein\_coding | | ENSDARG00000096632 | si:dkey-22o12.7 | 1 | 29635491 | 29637129 | lincRNA | | ENSDARG00000102445 | si:dkey-22o22.2 | 16 | 40641012 | 40777423 | protein\_coding | | ENSDARG00000058008 | six3a | 13 | 10125532 | 10128908 | protein\_coding | | ENSDARG00000019185 | slain1a | 9 | 17340937 | 17379010 | protein\_coding | | ENSDARG00000003293 | sox9a | 12 | 1927596 | 1931236 | protein\_coding | | ENSDARG00000041449 | spred1 | 17 | 52553192 | 52572819 | protein\_coding | | ENSDARG00000013623 | vdac2 | 13 | 17583304 | 17598482 | protein\_coding | | ENSDARG00000060349 | wasf1 | 20 | 53501325 | 53560720 | protein\_coding | | ENSDARG00000034086 | zgc:65895 | 6 | 59762784 | 59767333 | protein\_coding | |
