# Supplementary material for: A high-resolution mRNA expression time course of embryonic development in zebrafish
Source: eLife. 2017 Nov 16;6:e30860. doi: 10.7554/eLife.30860 (PMC5690287; doi:10.7554/eLife.30860)
Supplement: Supplementary file 6. [file elife-30860-supp6.zip › biolayout-clusters-files/Cluster032-genes.html]

Cluster032


# Cluster032: Genes

| | Ensembl ID | Gene Name | Chr | Start | End | Biotype | | --- | --- | --- | --- | --- | --- | | ENSDARG00000023868 | AKAP13 (1 of many) | 7 | 14838906 | 15008833 | protein\_coding | | ENSDARG00000079483 | ENSDARG00000079483 | 10 | 44665103 | 44693011 | protein\_coding | | ENSDARG00000087688 | ENSDARG00000087688 | 20 | 44892698 | 45417496 | protein\_coding | | ENSDARG00000009493 | MAP3K13 | 9 | 53151081 | 53274697 | protein\_coding | | ENSDARG00000055839 | MARCH4 (1 of many) | 1 | 29150759 | 29235401 | protein\_coding | | ENSDARG00000002231 | PLPP2 (1 of many) | 2 | 52639034 | 52713278 | protein\_coding | | ENSDARG00000102887 | abhd4 | 2 | 52052402 | 52064345 | protein\_coding | | ENSDARG00000020123 | adck3 | 20 | 38555205 | 38616247 | protein\_coding | | ENSDARG00000075111 | als2a | 9 | 13657452 | 13705602 | protein\_coding | | ENSDARG00000079470 | cdk17 | 4 | 7710225 | 7803296 | protein\_coding | | ENSDARG00000026834 | cdr2l | 3 | 59659895 | 59690351 | protein\_coding | | ENSDARG00000003206 | chchd6a | 23 | 34120882 | 34258488 | protein\_coding | | ENSDARG00000068602 | dalrd3 | 8 | 26064669 | 26078936 | protein\_coding | | ENSDARG00000021895 | disc1 | 13 | 48766403 | 48854203 | protein\_coding | | ENSDARG00000057729 | ephb6 | 16 | 12444372 | 12579132 | protein\_coding | | ENSDARG00000100402 | fgd1 | 8 | 7655608 | 7733716 | protein\_coding | | ENSDARG00000070543 | grin2ab | 1 | 8081513 | 8233791 | protein\_coding | | ENSDARG00000005651 | hrasb | 7 | 49365841 | 49378572 | protein\_coding | | ENSDARG00000087247 | kcnab2a | 11 | 40864061 | 41087770 | protein\_coding | | ENSDARG00000061000 | klhdc8a | 11 | 37765910 | 37816192 | protein\_coding | | ENSDARG00000076297 | nfatc3a | 7 | 34555995 | 34656380 | protein\_coding | | ENSDARG00000008192 | pank1a | 17 | 23534678 | 23557656 | protein\_coding | | ENSDARG00000076143 | ppip5k1a | 7 | 52486771 | 52545500 | protein\_coding | | ENSDARG00000005861 | ralgapa2 | 17 | 41486332 | 41809982 | protein\_coding | | ENSDARG00000076891 | rfxap | 10 | 35014723 | 35017898 | protein\_coding | | ENSDARG00000009886 | rnf114 | 23 | 3560693 | 3568727 | protein\_coding | | ENSDARG00000062019 | rufy2 | 13 | 22746017 | 22776810 | protein\_coding | | ENSDARG00000103978 | sh3bp5b | 16 | 36118244 | 36154631 | protein\_coding | | ENSDARG00000062575 | si:ch211-210g13.5 | 3 | 17196732 | 17373423 | protein\_coding | | ENSDARG00000062538 | si:dkey-215k6.1 | 5 | 17927410 | 18269512 | protein\_coding | | ENSDARG00000063159 | si:dkey-32e23.4 | 8 | 50989996 | 51038519 | protein\_coding | | ENSDARG00000033446 | si:dkey-57h18.2 | 12 | 26451088 | 26468304 | protein\_coding | | ENSDARG00000076754 | slc9a7 | 6 | 37551489 | 37622754 | protein\_coding | | ENSDARG00000000837 | snx9a | 17 | 33205783 | 33249329 | protein\_coding | | ENSDARG00000039022 | stk25b | 2 | 22962024 | 22981070 | protein\_coding | | ENSDARG00000060835 | tecpr2 | 17 | 29265900 | 29295543 | protein\_coding | | ENSDARG00000074760 | ttc7a | 13 | 8361387 | 8424031 | protein\_coding | | ENSDARG00000074471 | vps39 | 17 | 49995401 | 50034417 | protein\_coding | | ENSDARG00000069600 | zgc:109889 | 7 | 23224846 | 23268544 | protein\_coding | | ENSDARG00000099753 | zgc:63863 | 19 | 1148491 | 1181375 | protein\_coding | |
