# Supplementary material for: A high-resolution mRNA expression time course of embryonic development in zebrafish
Source: eLife. 2017 Nov 16;6:e30860. doi: 10.7554/eLife.30860 (PMC5690287; doi:10.7554/eLife.30860)
Supplement: Supplementary file 6. [file elife-30860-supp6.zip › biolayout-clusters-files/Cluster033-genes.html]

Cluster033


# Cluster033: Genes

| | Ensembl ID | Gene Name | Chr | Start | End | Biotype | | --- | --- | --- | --- | --- | --- | | ENSDARG00000009026 | ANK2 | 1 | 13386954 | 13473738 | protein\_coding | | ENSDARG00000086945 | ENSDARG00000086945 | 12 | 2345031 | 2349371 | protein\_coding | | ENSDARG00000101631 | ETFA | 25 | 32348404 | 32393802 | protein\_coding | | ENSDARG00000017128 | MYOF (1 of many) | 13 | 29108284 | 29158764 | protein\_coding | | ENSDARG00000101452 | SUSD2 | 5 | 15881082 | 15899043 | protein\_coding | | ENSDARG00000061764 | ahnak | 14 | 26461227 | 26498229 | protein\_coding | | ENSDARG00000078989 | alpk3a | 18 | 26737409 | 26770761 | protein\_coding | | ENSDARG00000037030 | casz1 | 23 | 29029709 | 29138989 | protein\_coding | | ENSDARG00000075549 | cdh5 | 7 | 44106370 | 44180006 | protein\_coding | | ENSDARG00000053362 | cilp | 7 | 31160992 | 31170276 | protein\_coding | | ENSDARG00000040362 | ehd2b | 18 | 44656350 | 44669790 | protein\_coding | | ENSDARG00000063682 | fhod3a | 19 | 712092 | 769179 | protein\_coding | | ENSDARG00000105215 | kdrl | 14 | 32643455 | 32704641 | protein\_coding | | ENSDARG00000070961 | lepr | 6 | 31197144 | 31246782 | protein\_coding | | ENSDARG00000062662 | lmod3 | 23 | 14058901 | 14066565 | protein\_coding | | ENSDARG00000008263 | mfsd4b | 23 | 18176737 | 18188547 | protein\_coding | | ENSDARG00000077301 | mlf1 | 15 | 1187271 | 1201855 | protein\_coding | | ENSDARG00000054031 | mxd4 | 1 | 25378155 | 25402858 | protein\_coding | | ENSDARG00000075752 | myo18aa | 10 | 37006999 | 37131603 | protein\_coding | | ENSDARG00000055875 | pag1 | 19 | 32463776 | 32562798 | protein\_coding | | ENSDARG00000021987 | plecb | 16 | 5352142 | 5597848 | protein\_coding | | ENSDARG00000055786 | prss23 | 14 | 30462151 | 30467860 | protein\_coding | | ENSDARG00000058646 | ptprna | 9 | 7570141 | 7641240 | protein\_coding | | ENSDARG00000011422 | ryr1a | 10 | 33462959 | 33596476 | protein\_coding | | ENSDARG00000101181 | s100w | 16 | 23488570 | 23490344 | protein\_coding | | ENSDARG00000075759 | samd4a | 17 | 15025889 | 15109575 | protein\_coding | | ENSDARG00000089917 | sh3tc2 | 14 | 34440639 | 34458023 | protein\_coding | | ENSDARG00000042857 | si:ch211-246m6.4 | 22 | 14102695 | 14103939 | protein\_coding | | ENSDARG00000096654 | si:dkey-119m7.8 | 20 | 331780 | 333984 | protein\_coding | | ENSDARG00000100712 | si:dkey-19b23.12 | 7 | 20251376 | 20256803 | protein\_coding | | ENSDARG00000000241 | slc40a1 | 9 | 41301340 | 41312091 | protein\_coding | | ENSDARG00000043646 | slc6a8 | 8 | 9013538 | 9080373 | protein\_coding | | ENSDARG00000103969 | smyhc2 | 24 | 40956716 | 41025918 | protein\_coding | | ENSDARG00000041006 | st3gal4 | 18 | 48701315 | 48750631 | protein\_coding | | ENSDARG00000075436 | sytl2b | 15 | 19761514 | 19789200 | protein\_coding | | ENSDARG00000004105 | tie1 | 6 | 33998984 | 34024189 | protein\_coding | | ENSDARG00000008880 | usp28 | 21 | 23014306 | 23074309 | protein\_coding | | ENSDARG00000069529 | zgc:153981 | 13 | 24703749 | 24707527 | protein\_coding | |
