# Supplementary material for: A high-resolution mRNA expression time course of embryonic development in zebrafish
Source: eLife. 2017 Nov 16;6:e30860. doi: 10.7554/eLife.30860 (PMC5690287; doi:10.7554/eLife.30860)
Supplement: Supplementary file 6. [file elife-30860-supp6.zip › biolayout-clusters-files/Cluster034-genes.html]

Cluster034


# Cluster034: Genes

| | Ensembl ID | Gene Name | Chr | Start | End | Biotype | | --- | --- | --- | --- | --- | --- | | ENSDARG00000100757 | BNC1 | KN150342.1 | 51991 | 60094 | protein\_coding | | ENSDARG00000088805 | ENSDARG00000088805 | 16 | 29109044 | 29119290 | protein\_coding | | ENSDARG00000042535 | actc1a | 20 | 29574265 | 29580635 | protein\_coding | | ENSDARG00000060127 | adamts3 | 5 | 44722592 | 44936510 | protein\_coding | | ENSDARG00000089749 | aqp8b | 3 | 18422539 | 18426128 | protein\_coding | | ENSDARG00000057113 | c6 | 21 | 20758940 | 20896022 | protein\_coding | | ENSDARG00000021720 | col7a1 | 6 | 40186699 | 40314609 | protein\_coding | | ENSDARG00000079964 | dlx2a | 9 | 3425857 | 3429216 | protein\_coding | | ENSDARG00000023290 | fabp3 | 19 | 43385843 | 43423869 | protein\_coding | | ENSDARG00000052045 | ggt5a | 8 | 39964715 | 39995260 | protein\_coding | | ENSDARG00000091511 | gpx7 | 23 | 42763354 | 42775848 | protein\_coding | | ENSDARG00000043322 | gsx2 | 20 | 22583375 | 22584948 | protein\_coding | | ENSDARG00000089769 | hapln1a | 5 | 45890469 | 45905545 | protein\_coding | | ENSDARG00000056027 | hoxb8a | 3 | 23557361 | 23559933 | protein\_coding | | ENSDARG00000058365 | hspb8 | 5 | 15319431 | 15340268 | protein\_coding | | ENSDARG00000019125 | klhl40b | 24 | 20430840 | 20437253 | protein\_coding | | ENSDARG00000027930 | naprt | 20 | 52461541 | 52502394 | protein\_coding | | ENSDARG00000058597 | nt5c3a | 16 | 7299460 | 7322162 | protein\_coding | | ENSDARG00000003684 | obsl1a | 6 | 19096973 | 19129073 | protein\_coding | | ENSDARG00000095896 | pou3f3b | 6 | 14823242 | 14826420 | protein\_coding | | ENSDARG00000046013 | rasl11a | 7 | 51039664 | 51046024 | protein\_coding | | ENSDARG00000021184 | rbfox1l | 16 | 31944539 | 31963980 | protein\_coding | | ENSDARG00000094752 | rpe65b | 8 | 16652658 | 16662200 | protein\_coding | | ENSDARG00000060927 | si:ch211-136a13.1 | 18 | 49230766 | 49250620 | protein\_coding | | ENSDARG00000099621 | si:ch211-69b22.5 | 14 | 31731461 | 31748803 | protein\_coding | | ENSDARG00000104297 | si:ch73-380l10.2 | 7 | 50491024 | 50498396 | protein\_coding | | ENSDARG00000098045 | si:dkey-172f14.2 | 5 | 49103746 | 49122419 | antisense | | ENSDARG00000104028 | si:dkey-74k8.3 | 14 | 20755259 | 20766954 | protein\_coding | | ENSDARG00000087784 | si:dkeyp-110a12.4 | 17 | 7438178 | 7460380 | protein\_coding | | ENSDARG00000028173 | slc4a2a | 2 | 32408679 | 32474705 | protein\_coding | | ENSDARG00000005629 | smyd2b | 20 | 582190 | 600163 | protein\_coding | | ENSDARG00000070155 | tuba8l3 | 9 | 7684247 | 7695465 | protein\_coding | | ENSDARG00000005574 | vsx2 | 17 | 31635677 | 31642707 | protein\_coding | | ENSDARG00000016343 | zgc:92518 | 1 | 471601 | 476746 | protein\_coding | | ENSDARG00000031307 | zic4 | 24 | 4946075 | 4950664 | protein\_coding | | ENSDARG00000071496 | zic6 | 14 | 31681958 | 31685785 | protein\_coding | |
