# Supplementary material for: A high-resolution mRNA expression time course of embryonic development in zebrafish
Source: eLife. 2017 Nov 16;6:e30860. doi: 10.7554/eLife.30860 (PMC5690287; doi:10.7554/eLife.30860)
Supplement: Supplementary file 6. [file elife-30860-supp6.zip › biolayout-clusters-files/Cluster035-genes.html]

Cluster035


# Cluster035: Genes

| | Ensembl ID | Gene Name | Chr | Start | End | Biotype | | --- | --- | --- | --- | --- | --- | | ENSDARG00000099449 | CR762398.1 | 5 | 22541102 | 22580102 | protein\_coding | | ENSDARG00000062148 | ENSDARG00000062148 | 9 | 30407082 | 30437215 | protein\_coding | | ENSDARG00000071570 | ENSDARG00000071570 | 22 | 10202845 | 10232759 | protein\_coding | | ENSDARG00000077648 | FNIP2 | 1 | 19072058 | 19109665 | protein\_coding | | ENSDARG00000038785 | abcf2a | 2 | 32522336 | 32530327 | protein\_coding | | ENSDARG00000062002 | adnpa | 11 | 25165945 | 25171020 | protein\_coding | | ENSDARG00000006038 | aebp2 | 4 | 2993088 | 3015195 | protein\_coding | | ENSDARG00000069619 | atf7ip | 1 | 44878260 | 44924675 | protein\_coding | | ENSDARG00000102832 | baz1b | 18 | 41570630 | 41601492 | protein\_coding | | ENSDARG00000015222 | cbll1 | 4 | 18847819 | 18852095 | protein\_coding | | ENSDARG00000059870 | clk2b | 16 | 54655228 | 54679400 | protein\_coding | | ENSDARG00000042087 | dmap1 | 8 | 18320390 | 18332024 | protein\_coding | | ENSDARG00000053505 | fbxo22 | 7 | 29691428 | 29697724 | protein\_coding | | ENSDARG00000037353 | frs2b | 25 | 34222970 | 34235669 | protein\_coding | | ENSDARG00000059308 | golt1bb | 25 | 3216030 | 3221674 | protein\_coding | | ENSDARG00000098359 | il17rd | 11 | 42352132 | 42429271 | protein\_coding | | ENSDARG00000077943 | ints5 | 14 | 46564672 | 46571198 | protein\_coding | | ENSDARG00000002552 | mapkapk2a | 11 | 20994110 | 21057604 | protein\_coding | | ENSDARG00000042563 | mis18bp1 | 17 | 28594783 | 28606397 | protein\_coding | | ENSDARG00000061468 | ncaph | 5 | 27659502 | 27670792 | protein\_coding | | ENSDARG00000058212 | ndnl2 | 23 | 13144339 | 13157054 | protein\_coding | | ENSDARG00000005606 | paxip1 | 2 | 29919143 | 29939875 | protein\_coding | | ENSDARG00000029045 | peli2 | 17 | 43916927 | 43966968 | protein\_coding | | ENSDARG00000013379 | ppp6r3 | 25 | 10505387 | 10533690 | protein\_coding | | ENSDARG00000010381 | rnf2 | 2 | 23695803 | 23706282 | protein\_coding | | ENSDARG00000075194 | si:dkey-56e3.3 | 1 | 40894935 | 40904945 | protein\_coding | | ENSDARG00000039354 | ska1 | 10 | 17585842 | 17593497 | protein\_coding | | ENSDARG00000070256 | suz12a | 3 | 35719884 | 35735532 | protein\_coding | | ENSDARG00000060438 | tbl1x | 9 | 55503475 | 55539236 | protein\_coding | | ENSDARG00000042344 | trmt6 | 20 | 45850603 | 45868586 | protein\_coding | | ENSDARG00000073710 | usp7 | 3 | 27475663 | 27509981 | protein\_coding | | ENSDARG00000086150 | wrap53 | 7 | 20651628 | 20674073 | protein\_coding | | ENSDARG00000027353 | zmym2 | 9 | 21619442 | 21649295 | protein\_coding | | ENSDARG00000061174 | znf740b | 23 | 36302769 | 36319905 | protein\_coding | | ENSDARG00000042129 | zp3a.1 | 20 | 34088922 | 34090819 | protein\_coding | |
