# Supplementary material for: A high-resolution mRNA expression time course of embryonic development in zebrafish
Source: eLife. 2017 Nov 16;6:e30860. doi: 10.7554/eLife.30860 (PMC5690287; doi:10.7554/eLife.30860)
Supplement: Supplementary file 6. [file elife-30860-supp6.zip › biolayout-clusters-files/Cluster035.html]

Cluster035


# Cluster035: Detail

### Go to ZFA detail

## GO

| | GO ID | Description | Domain | Annotated | Expected | Observed | Adjusted p-value | Genes | Ensembl IDs | | --- | --- | --- | --- | --- | --- | --- | --- | --- | | GO:0031519 | PcG protein complex | cellular\_component | 13 | 0.03 | 3 | 0.00069 | aebp2 rnf2 suz12a | ENSDARG00000006038 ENSDARG00000010381 ENSDARG00000070256 | | GO:0035097 | histone methyltransferase complex | cellular\_component | 17 | 0.04 | 3 | 0.00163 | aebp2 rnf2 suz12a | ENSDARG00000006038 ENSDARG00000010381 ENSDARG00000070256 | |
