# Supplementary material for: A high-resolution mRNA expression time course of embryonic development in zebrafish
Source: eLife. 2017 Nov 16;6:e30860. doi: 10.7554/eLife.30860 (PMC5690287; doi:10.7554/eLife.30860)
Supplement: Supplementary file 6. [file elife-30860-supp6.zip › biolayout-clusters-files/Cluster036-genes.html]

Cluster036


# Cluster036: Genes

| | Ensembl ID | Gene Name | Chr | Start | End | Biotype | | --- | --- | --- | --- | --- | --- | | ENSDARG00000038424 | ENSDARG00000038424 | 16 | 16757226 | 16793741 | protein\_coding | | ENSDARG00000021154 | PRODH2 | 12 | 3889403 | 3904003 | protein\_coding | | ENSDARG00000101324 | apoa1b | 15 | 31389 | 33853 | protein\_coding | | ENSDARG00000015866 | apoa2 | 16 | 24063946 | 24065322 | protein\_coding | | ENSDARG00000040298 | apoa4b.1 | 16 | 23999038 | 24000948 | protein\_coding | | ENSDARG00000092155 | apoc2 | 16 | 24069723 | 24076129 | protein\_coding | | ENSDARG00000058318 | c1qtnf9 | 24 | 21762695 | 21768260 | protein\_coding | | ENSDARG00000039516 | c8a | 2 | 2374646 | 2390764 | protein\_coding | | ENSDARG00000039517 | c8b | 2 | 2387407 | 2401596 | protein\_coding | | ENSDARG00000016319 | c9 | 5 | 31745034 | 31755136 | protein\_coding | | ENSDARG00000041569 | ces2 | 18 | 17419404 | 17426543 | protein\_coding | | ENSDARG00000055278 | cfb | 21 | 27379589 | 27394702 | protein\_coding | | ENSDARG00000100442 | cfh | 22 | 23615379 | 23721721 | protein\_coding | | ENSDARG00000102456 | cfhl4 | 22 | 23587103 | 23598808 | protein\_coding | | ENSDARG00000037144 | cpb2 | 1 | 28955747 | 28960129 | protein\_coding | | ENSDARG00000036041 | f2 | 7 | 38479613 | 38488900 | protein\_coding | | ENSDARG00000020741 | fga | 1 | 9379390 | 9387428 | protein\_coding | | ENSDARG00000008969 | fgb | 1 | 9367652 | 9375385 | protein\_coding | | ENSDARG00000037281 | fgg | 1 | 24978970 | 24988554 | protein\_coding | | ENSDARG00000070844 | gamt | 11 | 5858668 | 5868489 | protein\_coding | | ENSDARG00000027992 | hao2 | 9 | 21335993 | 21353748 | protein\_coding | | ENSDARG00000095807 | hp.1 | 8 | 50154548 | 50159302 | protein\_coding | | ENSDARG00000053232 | itgb1b.1 | 2 | 43719683 | 43753656 | protein\_coding | | ENSDARG00000018529 | lipf | 12 | 17036768 | 17079154 | protein\_coding | | ENSDARG00000007988 | masp2 | 23 | 28843684 | 28869616 | protein\_coding | | ENSDARG00000023111 | plg | 20 | 42791378 | 42805774 | protein\_coding | | ENSDARG00000037883 | prcp | 15 | 11295851 | 11381296 | protein\_coding | | ENSDARG00000061383 | serpinf2b | 15 | 26608143 | 26619776 | protein\_coding | | ENSDARG00000058053 | serping1 | 8 | 13922950 | 13934921 | protein\_coding | | ENSDARG00000097746 | si:rp71-77l1.1 | 16 | 45121094 | 45127883 | protein\_coding | | ENSDARG00000054447 | slc29a1b | 11 | 43127548 | 43166351 | protein\_coding | | ENSDARG00000036848 | slc43a2a | 15 | 28435947 | 28475836 | protein\_coding | | ENSDARG00000053831 | vtnb | 21 | 39022454 | 39036049 | protein\_coding | | ENSDARG00000100952 | wu:fj16a03 | 13 | 2774426 | 2779553 | protein\_coding | |
