# Supplementary material for: A high-resolution mRNA expression time course of embryonic development in zebrafish
Source: eLife. 2017 Nov 16;6:e30860. doi: 10.7554/eLife.30860 (PMC5690287; doi:10.7554/eLife.30860)
Supplement: Supplementary file 6. [file elife-30860-supp6.zip › biolayout-clusters-files/Cluster036.html]

Cluster036


# Cluster036: Detail

### Go to ZFA detail

## GO

| | GO ID | Description | Domain | Annotated | Expected | Observed | Adjusted p-value | Genes | Ensembl IDs | | --- | --- | --- | --- | --- | --- | --- | --- | --- | | GO:0006955 | immune response | biological\_process | 122 | 0.35 | 6 | 0.02794 | masp2 c9 c8a c8b vtnb cfb | ENSDARG00000007988 ENSDARG00000016319 ENSDARG00000039516 ENSDARG00000039517 ENSDARG00000053831 ENSDARG00000055278 | | GO:0007596 | blood coagulation | biological\_process | 36 | 0.10 | 6 | 0.00559 | fgb fga plg f2 cpb2 fgg | ENSDARG00000008969 ENSDARG00000020741 ENSDARG00000023111 ENSDARG00000036041 ENSDARG00000037144 ENSDARG00000037281 | | GO:0030168 | platelet activation | biological\_process | 14 | 0.04 | 3 | 0.00196 | fgb fga fgg | ENSDARG00000008969 ENSDARG00000020741 ENSDARG00000037281 | | GO:0005576 | extracellular region | cellular\_component | 502 | 1.55 | 16 | 0.00025 | masp2 fgb fga plg f2 cpb2 fgg ENSDARG00000038424 apoa4b.1 ces2 cfb serping1 c1qtnf9 serpinf2b apoc2 apoa1b | ENSDARG00000007988 ENSDARG00000008969 ENSDARG00000020741 ENSDARG00000023111 ENSDARG00000036041 ENSDARG00000037144 ENSDARG00000037281 ENSDARG00000038424 ENSDARG00000040298 ENSDARG00000041569 ENSDARG00000055278 ENSDARG00000058053 ENSDARG00000058318 ENSDARG00000061383 ENSDARG00000092155 ENSDARG00000101324 | | GO:0005615 | extracellular space | cellular\_component | 241 | 0.74 | 10 | 0.00003 | fgb fga cpb2 fgg ENSDARG00000038424 ces2 serping1 serpinf2b apoc2 apoa1b | ENSDARG00000008969 ENSDARG00000020741 ENSDARG00000037144 ENSDARG00000037281 ENSDARG00000038424 ENSDARG00000041569 ENSDARG00000058053 ENSDARG00000061383 ENSDARG00000092155 ENSDARG00000101324 | | GO:0030674 | protein binding, bridging | molecular\_function | 14 | 0.04 | 3 | 0.00218 | fgb fga fgg | ENSDARG00000008969 ENSDARG00000020741 ENSDARG00000037281 | | GO:0004252 | serine-type endopeptidase activity | molecular\_function | 79 | 0.24 | 4 | 0.02108 | masp2 plg f2 cfb | ENSDARG00000007988 ENSDARG00000023111 ENSDARG00000036041 ENSDARG00000055278 | |

  


### Go to GO detail

## ZFA

| | ZFA ID | Description | Annotated | Expected | Observed | Fold Enrichment | Adjusted p-value | Genes | Ensembl IDs | | --- | --- | --- | --- | --- | --- | --- | --- | --- | | ZFA:0000123 | liver | 1962 | 5.24 | 23 | 4.4 | 9.9e-12 | apoa2 hp serping1 apoa4b.1 plg apoc2 cfhl4 f2 fgb fga hao2 fgg PRODH2 c8a vtnb cfh apoa1b c9 gamt masp2 itgb1b.1 ces2 cfb | ENSDARG00000015866 ENSDARG00000095807 ENSDARG00000058053 ENSDARG00000040298 ENSDARG00000023111 ENSDARG00000092155 ENSDARG00000102456 ENSDARG00000036041 ENSDARG00000008969 ENSDARG00000020741 ENSDARG00000027992 ENSDARG00000037281 ENSDARG00000021154 ENSDARG00000039516 ENSDARG00000053831 ENSDARG00000100442 ENSDARG00000101324 ENSDARG00000016319 ENSDARG00000070844 ENSDARG00000007988 ENSDARG00000053232 ENSDARG00000041569 ENSDARG00000055278 | | ZFA:0000124 | liver primordium | 32 | 0.09 | 1 | 11.1 | 2.2e-10 | vtnb | ENSDARG00000053831 | | ZFA:0000339 | digestive system | 78 | 0.21 | 1 | 4.8 | 9.7e-07 | gamt | ENSDARG00000070844 | | ZFA:0000088 | YSL | 581 | 1.55 | 13 | 8.4 | 7.0e-06 | apoa2 apoa4b.1 f2 fgb fga hao2 fgg PRODH2 vtnb apoa1b c9 gamt ces2 | ENSDARG00000015866 ENSDARG00000040298 ENSDARG00000036041 ENSDARG00000008969 ENSDARG00000020741 ENSDARG00000027992 ENSDARG00000037281 ENSDARG00000021154 ENSDARG00000053831 ENSDARG00000101324 ENSDARG00000016319 ENSDARG00000070844 ENSDARG00000041569 | | ZFA:0000084 | yolk | 295 | 0.79 | 3 | 3.8 | 1.9e-05 | apoc2 gamt ces2 | ENSDARG00000092155 ENSDARG00000070844 ENSDARG00000041569 | |
