# Supplementary material for: A high-resolution mRNA expression time course of embryonic development in zebrafish
Source: eLife. 2017 Nov 16;6:e30860. doi: 10.7554/eLife.30860 (PMC5690287; doi:10.7554/eLife.30860)
Supplement: Supplementary file 6. [file elife-30860-supp6.zip › biolayout-clusters-files/Cluster037-genes.html]

Cluster037


# Cluster037: Genes

| | Ensembl ID | Gene Name | Chr | Start | End | Biotype | | --- | --- | --- | --- | --- | --- | | ENSDARG00000011076 | MREG | 19 | 5073824 | 5082746 | protein\_coding | | ENSDARG00000055618 | acta1b | 13 | 24148571 | 24153992 | protein\_coding | | ENSDARG00000045633 | asb15a | 25 | 27380908 | 27399476 | protein\_coding | | ENSDARG00000020574 | atp2a1 | 3 | 25950472 | 26026195 | protein\_coding | | ENSDARG00000007407 | barx1 | 11 | 27295919 | 27298545 | protein\_coding | | ENSDARG00000025912 | bscl2l | 14 | 5852328 | 5869113 | protein\_coding | | ENSDARG00000029457 | cacna1sa | 22 | 895552 | 951586 | protein\_coding | | ENSDARG00000008982 | casq2 | 9 | 33286543 | 33296491 | protein\_coding | | ENSDARG00000014059 | cldn5b | 10 | 44147674 | 44149320 | protein\_coding | | ENSDARG00000101816 | col5a3b | 1 | 58545607 | 58581062 | protein\_coding | | ENSDARG00000006008 | dct | 9 | 53788137 | 53813085 | protein\_coding | | ENSDARG00000006865 | glra4a | 14 | 38661802 | 38734225 | protein\_coding | | ENSDARG00000043074 | grp | 21 | 10673258 | 10680716 | protein\_coding | | ENSDARG00000038658 | murca | 2 | 42341698 | 42349182 | protein\_coding | | ENSDARG00000021265 | mybpc2b | 24 | 38371736 | 38460977 | protein\_coding | | ENSDARG00000014196 | myl1 | 9 | 39184437 | 39195522 | protein\_coding | | ENSDARG00000060797 | pfkmb | 6 | 39742181 | 39767459 | protein\_coding | | ENSDARG00000057571 | pgam2 | 21 | 18965041 | 18969995 | protein\_coding | | ENSDARG00000042027 | prrx1b | 20 | 34608799 | 34618162 | protein\_coding | | ENSDARG00000014479 | ptf1a | 2 | 29727324 | 29728228 | protein\_coding | | ENSDARG00000022817 | pvalb3 | 12 | 17583344 | 17585643 | protein\_coding | | ENSDARG00000002194 | rhd | 13 | 45357468 | 45387357 | protein\_coding | | ENSDARG00000097289 | rnf183.1 | 15 | 35023044 | 35027655 | protein\_coding | | ENSDARG00000103278 | si:ch211-136k14.3 | 14 | 16781696 | 16791489 | lincRNA | | ENSDARG00000096997 | si:dkey-53i3.1 | 23 | 7237681 | 7283507 | lincRNA | | ENSDARG00000056028 | slc22a7a | 11 | 30482561 | 30495782 | protein\_coding | | ENSDARG00000024771 | slc24a5 | 18 | 5532057 | 5546139 | protein\_coding | | ENSDARG00000029894 | slc2a15a | 13 | 28286847 | 28311636 | protein\_coding | | ENSDARG00000077293 | synpo2la | 13 | 22119255 | 22133029 | protein\_coding | | ENSDARG00000054911 | tmsb | 21 | 26352696 | 26356038 | protein\_coding | | ENSDARG00000030270 | tnnt3a | 25 | 30827790 | 30840981 | protein\_coding | | ENSDARG00000024829 | tnw | 2 | 35526961 | 35584559 | protein\_coding | | ENSDARG00000029204 | tyrp1a | 7 | 73941331 | 73947361 | protein\_coding | | ENSDARG00000104814 | wu:fc46h12 | 2 | 7684712 | 7693267 | protein\_coding | |
