# Supplementary material for: A high-resolution mRNA expression time course of embryonic development in zebrafish
Source: eLife. 2017 Nov 16;6:e30860. doi: 10.7554/eLife.30860 (PMC5690287; doi:10.7554/eLife.30860)
Supplement: Supplementary file 6. [file elife-30860-supp6.zip › biolayout-clusters-files/Cluster037.html]

Cluster037


# Cluster037: Detail

### Go to ZFA detail

## GO

| | GO ID | Description | Domain | Annotated | Expected | Observed | Adjusted p-value | Genes | Ensembl IDs | | --- | --- | --- | --- | --- | --- | --- | --- | --- | | GO:0019748 | secondary metabolic process | biological\_process | 11 | 0.03 | 3 | 0.00089 | slc24a5 tyrp1a pgam2 | ENSDARG00000024771 ENSDARG00000029204 ENSDARG00000057571 | |
