# Supplementary material for: A high-resolution mRNA expression time course of embryonic development in zebrafish
Source: eLife. 2017 Nov 16;6:e30860. doi: 10.7554/eLife.30860 (PMC5690287; doi:10.7554/eLife.30860)
Supplement: Supplementary file 6. [file elife-30860-supp6.zip › biolayout-clusters-files/Cluster038-genes.html]

Cluster038


# Cluster038: Genes

| | Ensembl ID | Gene Name | Chr | Start | End | Biotype | | --- | --- | --- | --- | --- | --- | | ENSDARG00000020265 | angptl6 | 3 | 54269625 | 54296045 | protein\_coding | | ENSDARG00000103456 | cyp2aa11 | 23 | 42381303 | 42392857 | protein\_coding | | ENSDARG00000021488 | cyth1b | 12 | 33950865 | 34091972 | protein\_coding | | ENSDARG00000071560 | dlx4b | 12 | 5684659 | 5693798 | protein\_coding | | ENSDARG00000039412 | dmrta2 | 8 | 16221508 | 16223351 | protein\_coding | | ENSDARG00000020417 | emx3 | 14 | 26065432 | 26078939 | protein\_coding | | ENSDARG00000038868 | eng2b | 2 | 29992886 | 29996423 | protein\_coding | | ENSDARG00000023952 | fam167ab | 20 | 18840935 | 18857670 | protein\_coding | | ENSDARG00000074812 | fhdc1 | 1 | 24708685 | 24747977 | protein\_coding | | ENSDARG00000005023 | fkbp9 | 19 | 43021713 | 43038014 | protein\_coding | | ENSDARG00000101919 | foxj1a | 3 | 60637106 | 60838792 | protein\_coding | | ENSDARG00000068213 | fzd10 | 8 | 44302564 | 44305545 | protein\_coding | | ENSDARG00000005510 | gdf6b | 19 | 23414858 | 23418560 | protein\_coding | | ENSDARG00000043928 | gpn1 | 17 | 12504345 | 12521201 | protein\_coding | | ENSDARG00000059231 | hephl1a | 15 | 2458157 | 2509361 | protein\_coding | | ENSDARG00000038205 | her2 | 11 | 41354000 | 41357664 | protein\_coding | | ENSDARG00000059276 | hoxd4a | 9 | 1947668 | 1950347 | protein\_coding | | ENSDARG00000075864 | igsf9a | 10 | 19069384 | 19135185 | protein\_coding | | ENSDARG00000090297 | ldlrad2 | 11 | 36215498 | 36227833 | protein\_coding | | ENSDARG00000020332 | nkx2.9 | 17 | 38307520 | 38308805 | protein\_coding | | ENSDARG00000033172 | nr2f5 | 16 | 46327621 | 46360585 | protein\_coding | | ENSDARG00000038139 | pdgfbb | 3 | 24145203 | 24165966 | protein\_coding | | ENSDARG00000033029 | pkd1b | 12 | 21576321 | 21626795 | protein\_coding | | ENSDARG00000005128 | rnaseh2b | 9 | 29860807 | 29868426 | protein\_coding | | ENSDARG00000067606 | rsph4a | 5 | 61256480 | 61268123 | protein\_coding | | ENSDARG00000088195 | si:ch211-168f7.5 | 14 | 46377865 | 46407547 | protein\_coding | | ENSDARG00000075487 | si:ch211-267e7.3 | 2 | 20897422 | 20941343 | protein\_coding | | ENSDARG00000097445 | si:dkey-195m11.8 | 6 | 39373051 | 39381197 | protein\_coding | | ENSDARG00000031664 | sox21a | 6 | 7257046 | 7258174 | protein\_coding | | ENSDARG00000056666 | sp8b | 16 | 19730795 | 19732953 | protein\_coding | | ENSDARG00000040925 | wnt10b | 23 | 27714930 | 27721228 | protein\_coding | | ENSDARG00000071208 | wnt4a | 11 | 38905892 | 38940961 | protein\_coding | | ENSDARG00000074526 | zbtb16b | 15 | 18317520 | 18425453 | protein\_coding | |
