# Supplementary material for: A high-resolution mRNA expression time course of embryonic development in zebrafish
Source: eLife. 2017 Nov 16;6:e30860. doi: 10.7554/eLife.30860 (PMC5690287; doi:10.7554/eLife.30860)
Supplement: Supplementary file 6. [file elife-30860-supp6.zip › biolayout-clusters-files/Cluster038.html]

Cluster038


# Cluster038: Detail

### Go to ZFA detail

## GO

| | GO ID | Description | Domain | Annotated | Expected | Observed | Adjusted p-value | Genes | Ensembl IDs | | --- | --- | --- | --- | --- | --- | --- | --- | --- | | GO:0006355 | regulation of transcription, DNA-templat... | biological\_process | 1072 | 2.9 | 13 | 0.0010 | gdf6b nkx2.9 emx3 sox21a nr2f5 her2 eng2b dmrta2 sp8b hoxd4a wnt4a dlx4b foxj1a | ENSDARG00000005510 ENSDARG00000020332 ENSDARG00000020417 ENSDARG00000031664 ENSDARG00000033172 ENSDARG00000038205 ENSDARG00000038868 ENSDARG00000039412 ENSDARG00000056666 ENSDARG00000059276 ENSDARG00000071208 ENSDARG00000071560 ENSDARG00000101919 | | GO:0043565 | sequence-specific DNA binding | molecular\_function | 494 | 1.5 | 9 | 0.0013 | nkx2.9 emx3 sox21a nr2f5 eng2b dmrta2 hoxd4a dlx4b foxj1a | ENSDARG00000020332 ENSDARG00000020417 ENSDARG00000031664 ENSDARG00000033172 ENSDARG00000038868 ENSDARG00000039412 ENSDARG00000059276 ENSDARG00000071560 ENSDARG00000101919 | |
