# Supplementary material for: A high-resolution mRNA expression time course of embryonic development in zebrafish
Source: eLife. 2017 Nov 16;6:e30860. doi: 10.7554/eLife.30860 (PMC5690287; doi:10.7554/eLife.30860)
Supplement: Supplementary file 6. [file elife-30860-supp6.zip › biolayout-clusters-files/Cluster039-genes.html]

Cluster039


# Cluster039: Genes

| | Ensembl ID | Gene Name | Chr | Start | End | Biotype | | --- | --- | --- | --- | --- | --- | | ENSDARG00000044437 | cdca5 | 5 | 66496914 | 66502431 | protein\_coding | | ENSDARG00000091831 | ddx27 | 6 | 57439450 | 57479601 | protein\_coding | | ENSDARG00000100505 | ddx52 | 21 | 38808077 | 38822076 | protein\_coding | | ENSDARG00000020913 | ddx56 | 10 | 2829206 | 2848564 | protein\_coding | | ENSDARG00000104066 | dnaja2 | 7 | 41623474 | 41635240 | protein\_coding | | ENSDARG00000070463 | e2f3 | 19 | 29687162 | 29707234 | protein\_coding | | ENSDARG00000012274 | eif4e1c | 13 | 18180418 | 18193477 | protein\_coding | | ENSDARG00000043976 | etf1b | 10 | 21477336 | 21486503 | protein\_coding | | ENSDARG00000071500 | fam207a | 22 | 12674344 | 12701779 | protein\_coding | | ENSDARG00000058287 | gpalpp1 | 1 | 33952837 | 33961797 | protein\_coding | | ENSDARG00000053021 | grsf1 | 5 | 42343819 | 42350810 | protein\_coding | | ENSDARG00000099742 | heatr1 | 12 | 9582066 | 9662762 | protein\_coding | | ENSDARG00000058030 | hspa14 | 4 | 9591570 | 9600775 | protein\_coding | | ENSDARG00000040245 | kpnb3 | 1 | 2112613 | 2151759 | protein\_coding | | ENSDARG00000012495 | mphosph10 | 7 | 30501841 | 30508425 | protein\_coding | | ENSDARG00000075795 | nol7 | 2 | 31846965 | 31850129 | protein\_coding | | ENSDARG00000030022 | nup188 | 21 | 4375506 | 4442080 | protein\_coding | | ENSDARG00000002971 | phc1 | 16 | 31988515 | 32006319 | protein\_coding | | ENSDARG00000099222 | prpsap1 | 3 | 59011021 | 59197155 | protein\_coding | | ENSDARG00000007196 | rae1 | 6 | 20266405 | 20273840 | protein\_coding | | ENSDARG00000043960 | rpf2 | 20 | 162255 | 166354 | protein\_coding | | ENSDARG00000103337 | rrp1 | 9 | 188606 | 197253 | protein\_coding | | ENSDARG00000105117 | sdad1 | 21 | 9274234 | 9291641 | protein\_coding | | ENSDARG00000103553 | sf3b3 | 18 | 18561352 | 18595773 | protein\_coding | | ENSDARG00000055760 | srm | 23 | 28792322 | 28800565 | protein\_coding | | ENSDARG00000098822 | tcerg1a | 14 | 51765594 | 51802874 | protein\_coding | | ENSDARG00000034600 | tmem165 | 20 | 22286624 | 22293517 | protein\_coding | | ENSDARG00000075593 | trim71 | 16 | 7825312 | 7876111 | protein\_coding | | ENSDARG00000005772 | tsr2 | 8 | 8302493 | 8311092 | protein\_coding | | ENSDARG00000057321 | tut1 | 12 | 10299831 | 10314158 | protein\_coding | | ENSDARG00000101332 | uba2 | 25 | 12715119 | 12728170 | protein\_coding | | ENSDARG00000041908 | usp39 | 5 | 68055847 | 68072444 | protein\_coding | |
