# Supplementary material for: A high-resolution mRNA expression time course of embryonic development in zebrafish
Source: eLife. 2017 Nov 16;6:e30860. doi: 10.7554/eLife.30860 (PMC5690287; doi:10.7554/eLife.30860)
Supplement: Supplementary file 6. [file elife-30860-supp6.zip › biolayout-clusters-files/Cluster040-genes.html]

Cluster040


# Cluster040: Genes

| | Ensembl ID | Gene Name | Chr | Start | End | Biotype | | --- | --- | --- | --- | --- | --- | | ENSDARG00000103162 | CABZ01046954.1 | 25 | 32784669 | 32790239 | protein\_coding | | ENSDARG00000089563 | CU633855.1 | 15 | 47227238 | 47234869 | protein\_coding | | ENSDARG00000096081 | ENSDARG00000096081 | 25 | 25343089 | 25352721 | protein\_coding | | ENSDARG00000053133 | b3gnt7l | 8 | 22268081 | 22269647 | protein\_coding | | ENSDARG00000015072 | dmrt2a | 5 | 44407809 | 44410963 | protein\_coding | | ENSDARG00000042826 | egr2b | 12 | 8445995 | 8466435 | protein\_coding | | ENSDARG00000078052 | emilin3a | 8 | 22941252 | 22944677 | protein\_coding | | ENSDARG00000100475 | fgf10b | 5 | 8265223 | 8312820 | protein\_coding | | ENSDARG00000043962 | fgf18b | 10 | 21513935 | 21526684 | protein\_coding | | ENSDARG00000020746 | gfi1aa | 2 | 10983145 | 11002050 | protein\_coding | | ENSDARG00000105013 | hoxa9a | 19 | 20174479 | 20179194 | protein\_coding | | ENSDARG00000056030 | hoxb7a | 3 | 23561299 | 23565660 | protein\_coding | | ENSDARG00000096956 | hoxc10a.1 | 23 | 35982622 | 35989431 | antisense | | ENSDARG00000101954 | hoxc6b | 11 | 2099938 | 2107059 | protein\_coding | | ENSDARG00000045553 | hsd17b2 | 25 | 35437928 | 35449387 | protein\_coding | | ENSDARG00000095019 | lmo2 | 18 | 38307869 | 38315657 | protein\_coding | | ENSDARG00000007891 | meox1 | 12 | 27370865 | 27377907 | protein\_coding | | ENSDARG00000097947 | mespbb | 25 | 10929107 | 10930111 | protein\_coding | | ENSDARG00000007277 | myf5 | 4 | 22020253 | 22024132 | protein\_coding | | ENSDARG00000011821 | plod2 | 24 | 5205922 | 5282920 | protein\_coding | | ENSDARG00000052148 | ptgs1 | 5 | 64226204 | 64265272 | protein\_coding | | ENSDARG00000075177 | ribc2 | 25 | 16651075 | 16658940 | protein\_coding | | ENSDARG00000097849 | si:ch211-72a16.11 | 3 | 23555650 | 23556152 | lincRNA | | ENSDARG00000097523 | si:ch73-364h19.1 | 3 | 59855208 | 59884369 | protein\_coding | | ENSDARG00000096750 | si:dkey-11c5.14 | 12 | 27028757 | 27032217 | antisense | | ENSDARG00000096563 | si:dkey-269i1.2 | 12 | 16621178 | 16624673 | transcribed\_unprocessed\_pseudogene | | ENSDARG00000096421 | si:dkey-45d16.4 | 6 | 20402888 | 20459122 | protein\_coding | | ENSDARG00000097150 | si:dkey-81p22.14 | 23 | 35993543 | 35996565 | antisense | | ENSDARG00000027734 | srsf5b | 20 | 28967090 | 28981950 | protein\_coding | | ENSDARG00000011785 | tbx6 | 12 | 3875779 | 3886305 | protein\_coding | | ENSDARG00000067544 | tcf15 | 8 | 28584475 | 28586259 | protein\_coding | | ENSDARG00000076745 | zgc:193811 | 3 | 31813890 | 31819581 | protein\_coding | |
