# Supplementary material for: A high-resolution mRNA expression time course of embryonic development in zebrafish
Source: eLife. 2017 Nov 16;6:e30860. doi: 10.7554/eLife.30860 (PMC5690287; doi:10.7554/eLife.30860)
Supplement: Supplementary file 6. [file elife-30860-supp6.zip › biolayout-clusters-files/Cluster040.html]

Cluster040


# Cluster040: Detail

### Go to ZFA detail

## GO

| | GO ID | Description | Domain | Annotated | Expected | Observed | Adjusted p-value | Genes | Ensembl IDs | | --- | --- | --- | --- | --- | --- | --- | --- | --- | | GO:0006355 | regulation of transcription, DNA-templat... | biological\_process | 1072 | 2.5 | 9 | 0.018 | myf5 meox1 tbx6 dmrt2a gfi1aa egr2b hoxb7a hoxc6b hoxa9a | ENSDARG00000007277 ENSDARG00000007891 ENSDARG00000011785 ENSDARG00000015072 ENSDARG00000020746 ENSDARG00000042826 ENSDARG00000056030 ENSDARG00000101954 ENSDARG00000105013 | |
