# Supplementary material for: A high-resolution mRNA expression time course of embryonic development in zebrafish
Source: eLife. 2017 Nov 16;6:e30860. doi: 10.7554/eLife.30860 (PMC5690287; doi:10.7554/eLife.30860)
Supplement: Supplementary file 6. [file elife-30860-supp6.zip › biolayout-clusters-files/Cluster041-genes.html]

Cluster041


# Cluster041: Genes

| | Ensembl ID | Gene Name | Chr | Start | End | Biotype | | --- | --- | --- | --- | --- | --- | | ENSDARG00000103605 | BX322657.1 | 22 | 1394855 | 1398780 | protein\_coding | | ENSDARG00000056204 | ENSDARG00000056204 | 5 | 27432296 | 27441562 | protein\_coding | | ENSDARG00000069696 | ENSDARG00000069696 | 8 | 22478123 | 22486816 | protein\_coding | | ENSDARG00000086815 | MYADM (1 of many).1 | 7 | 5372918 | 5399165 | protein\_coding | | ENSDARG00000074867 | STIM2 (1 of many) | 1 | 13781848 | 13798179 | protein\_coding | | ENSDARG00000016981 | bcar3 | 8 | 15045876 | 15192353 | protein\_coding | | ENSDARG00000019995 | bmp4 | 17 | 50485001 | 50507066 | protein\_coding | | ENSDARG00000059606 | ccdc84 | 5 | 58017841 | 58026555 | protein\_coding | | ENSDARG00000070914 | dusp6 | 25 | 18229898 | 18234103 | protein\_coding | | ENSDARG00000008377 | epn2 | 3 | 39514391 | 39548360 | protein\_coding | | ENSDARG00000019808 | evpla | 6 | 17959266 | 17983562 | protein\_coding | | ENSDARG00000069105 | fgfr4 | 21 | 37498712 | 37509505 | protein\_coding | | ENSDARG00000027612 | gatad1 | 19 | 44373790 | 44380949 | protein\_coding | | ENSDARG00000010137 | ldb1a | 13 | 28574621 | 28615304 | protein\_coding | | ENSDARG00000063572 | perp | 13 | 1025245 | 1040404 | protein\_coding | | ENSDARG00000058800 | rab25a | 19 | 7495017 | 7501942 | protein\_coding | | ENSDARG00000094673 | rhoab | 8 | 25880910 | 25886887 | protein\_coding | | ENSDARG00000011533 | sema6dl | 18 | 40364806 | 40397670 | protein\_coding | | ENSDARG00000071733 | si:ch211-207i20.3 | 12 | 9472634 | 9479138 | protein\_coding | | ENSDARG00000088298 | si:ch211-235i11.4 | 17 | 50345338 | 50351748 | protein\_coding | | ENSDARG00000102950 | si:ch211-241b2.1 | 21 | 2276332 | 2284240 | protein\_coding | | ENSDARG00000053800 | si:ch211-276c2.4 | 15 | 41736239 | 41751261 | protein\_coding | | ENSDARG00000093188 | si:dkey-75a21.2 | 8 | 46319471 | 46340711 | protein\_coding | | ENSDARG00000103893 | srsf2b | 3 | 60514329 | 60522590 | protein\_coding | | ENSDARG00000003866 | tm9sf2 | 1 | 28994209 | 29020444 | protein\_coding | | ENSDARG00000035889 | zbtb8b | 19 | 32918627 | 32931436 | protein\_coding | | ENSDARG00000093406 | zgc:111986 | 19 | 31992078 | 31997131 | protein\_coding | | ENSDARG00000070571 | zgc:153953 | 11 | 24479109 | 24519200 | protein\_coding | | ENSDARG00000102267 | znf750 | 3 | 44109468 | 44113103 | protein\_coding | |
