# Supplementary material for: A high-resolution mRNA expression time course of embryonic development in zebrafish
Source: eLife. 2017 Nov 16;6:e30860. doi: 10.7554/eLife.30860 (PMC5690287; doi:10.7554/eLife.30860)
Supplement: Supplementary file 6. [file elife-30860-supp6.zip › biolayout-clusters-files/Cluster041.html]

Cluster041


## Cluster041: Detail

### Go to GO detail

## ZFA

| | ZFA ID | Description | Annotated | Expected | Observed | Fold Enrichment | Adjusted p-value | Genes | Ensembl IDs | | --- | --- | --- | --- | --- | --- | --- | --- | --- | | ZFA:0005057 | epicardium | 14 | 0.03 | 3 | 100 | 0.015 | dusp6 fgfr4 bmp4 | ENSDARG00000070914 ENSDARG00000069105 ENSDARG00000019995 | |
