# Supplementary material for: A high-resolution mRNA expression time course of embryonic development in zebrafish
Source: eLife. 2017 Nov 16;6:e30860. doi: 10.7554/eLife.30860 (PMC5690287; doi:10.7554/eLife.30860)
Supplement: Supplementary file 6. [file elife-30860-supp6.zip › biolayout-clusters-files/Cluster042-genes.html]

Cluster042


# Cluster042: Genes

| | Ensembl ID | Gene Name | Chr | Start | End | Biotype | | --- | --- | --- | --- | --- | --- | | ENSDARG00000020655 | INSM2 | 17 | 12770864 | 12772390 | protein\_coding | | ENSDARG00000070555 | LDLRAD4 (1 of many) | 19 | 12525415 | 12703327 | protein\_coding | | ENSDARG00000069552 | atoh7 | 13 | 25319231 | 25320275 | protein\_coding | | ENSDARG00000063153 | bcl11ab | 6 | 6333844 | 6384350 | protein\_coding | | ENSDARG00000054150 | cx23 | 20 | 37391233 | 37395395 | protein\_coding | | ENSDARG00000059255 | evx2 | 9 | 2001904 | 2006309 | protein\_coding | | ENSDARG00000010591 | foxn4 | 5 | 18881685 | 18896698 | protein\_coding | | ENSDARG00000074897 | hes2.1 | 8 | 46930867 | 46935793 | protein\_coding | | ENSDARG00000068168 | hes2.2 | 8 | 46905979 | 46907268 | protein\_coding | | ENSDARG00000097082 | im:7152348 | 6 | 27893811 | 27901400 | protein\_coding | | ENSDARG00000091756 | insm1a | 20 | 48675359 | 48677782 | protein\_coding | | ENSDARG00000058421 | lgi1b | 12 | 5154819 | 5172314 | protein\_coding | | ENSDARG00000102879 | myt1b | 23 | 7826444 | 7892877 | protein\_coding | | ENSDARG00000003469 | neurod4 | 23 | 28417447 | 28420498 | protein\_coding | | ENSDARG00000025495 | nhlh2 | 9 | 33167556 | 33170914 | protein\_coding | | ENSDARG00000099427 | nkx1.2lb | 14 | 14759307 | 14762999 | protein\_coding | | ENSDARG00000014201 | otpa | 21 | 7844209 | 7848873 | protein\_coding | | ENSDARG00000058379 | otpb | 5 | 50969399 | 50972728 | protein\_coding | | ENSDARG00000019658 | pou2f2a | 16 | 11027350 | 11100338 | protein\_coding | | ENSDARG00000100821 | scrt1a | 19 | 1626607 | 1629639 | protein\_coding | | ENSDARG00000056175 | scrt2 | 8 | 28493793 | 28500237 | protein\_coding | | ENSDARG00000101584 | si:ch211-132d3.4 | 7 | 14108459 | 14135906 | lincRNA | | ENSDARG00000095715 | si:ch211-202e12.3 | 5 | 47458644 | 47524110 | lincRNA | | ENSDARG00000098395 | si:ch73-166c6.2 | 25 | 8448372 | 8474177 | lincRNA | | ENSDARG00000098512 | si:ch73-215f7.1 | 10 | 43671441 | 43711816 | protein\_coding | | ENSDARG00000095557 | si:dkey-11n6.3 | 22 | 18222314 | 18255462 | processed\_transcript | | ENSDARG00000025847 | sox12 | 11 | 23949994 | 23954312 | protein\_coding | | ENSDARG00000058598 | sox18 | 23 | 8862173 | 8864910 | protein\_coding | | ENSDARG00000013460 | wfikkn2a | 3 | 17947960 | 17954918 | protein\_coding | |
