# Supplementary material for: A high-resolution mRNA expression time course of embryonic development in zebrafish
Source: eLife. 2017 Nov 16;6:e30860. doi: 10.7554/eLife.30860 (PMC5690287; doi:10.7554/eLife.30860)
Supplement: Supplementary file 6. [file elife-30860-supp6.zip › biolayout-clusters-files/Cluster042.html]

Cluster042


# Cluster042: Detail

### Go to ZFA detail

## GO

| | GO ID | Description | Domain | Annotated | Expected | Observed | Adjusted p-value | Genes | Ensembl IDs | | --- | --- | --- | --- | --- | --- | --- | --- | --- | | GO:0006355 | regulation of transcription, DNA-templat... | biological\_process | 1072 | 1.99 | 11 | 0.00028 | neurod4 foxn4 otpa pou2f2a otpb evx2 bcl11ab atoh7 insm1a nkx1.2lb myt1b | ENSDARG00000003469 ENSDARG00000010591 ENSDARG00000014201 ENSDARG00000019658 ENSDARG00000058379 ENSDARG00000059255 ENSDARG00000063153 ENSDARG00000069552 ENSDARG00000091756 ENSDARG00000099427 ENSDARG00000102879 | | GO:0005634 | nucleus | cellular\_component | 1915 | 3.63 | 12 | 0.00330 | neurod4 foxn4 otpa pou2f2a nhlh2 otpb evx2 bcl11ab atoh7 insm1a nkx1.2lb myt1b | ENSDARG00000003469 ENSDARG00000010591 ENSDARG00000014201 ENSDARG00000019658 ENSDARG00000025495 ENSDARG00000058379 ENSDARG00000059255 ENSDARG00000063153 ENSDARG00000069552 ENSDARG00000091756 ENSDARG00000099427 ENSDARG00000102879 | | GO:0046983 | protein dimerization activity | molecular\_function | 226 | 0.48 | 5 | 0.02134 | neurod4 nhlh2 hes2.2 atoh7 hes2.1 | ENSDARG00000003469 ENSDARG00000025495 ENSDARG00000068168 ENSDARG00000069552 ENSDARG00000074897 | | GO:0043565 | sequence-specific DNA binding | molecular\_function | 494 | 1.04 | 9 | 0.00056 | foxn4 otpa pou2f2a nhlh2 otpb evx2 bcl11ab insm1a nkx1.2lb | ENSDARG00000010591 ENSDARG00000014201 ENSDARG00000019658 ENSDARG00000025495 ENSDARG00000058379 ENSDARG00000059255 ENSDARG00000063153 ENSDARG00000091756 ENSDARG00000099427 | |
