# Supplementary material for: A high-resolution mRNA expression time course of embryonic development in zebrafish
Source: eLife. 2017 Nov 16;6:e30860. doi: 10.7554/eLife.30860 (PMC5690287; doi:10.7554/eLife.30860)
Supplement: Supplementary file 6. [file elife-30860-supp6.zip › biolayout-clusters-files/Cluster043-genes.html]

Cluster043


# Cluster043: Genes

| | Ensembl ID | Gene Name | Chr | Start | End | Biotype | | --- | --- | --- | --- | --- | --- | | ENSDARG00000101281 | CORIN | 23 | 44137901 | 44188769 | protein\_coding | | ENSDARG00000075557 | apcdd1l | 6 | 49652223 | 49674823 | protein\_coding | | ENSDARG00000058011 | arxa | 24 | 23791655 | 23797548 | protein\_coding | | ENSDARG00000102490 | asb2b | 17 | 53224112 | 53231022 | protein\_coding | | ENSDARG00000101701 | bmp5 | 13 | 1741459 | 1775441 | protein\_coding | | ENSDARG00000052000 | cav2 | 25 | 18460188 | 18465395 | protein\_coding | | ENSDARG00000038288 | cd151 | 25 | 3684074 | 3704613 | protein\_coding | | ENSDARG00000036080 | cd81a | 7 | 32712290 | 32752250 | protein\_coding | | ENSDARG00000056627 | cxcl14 | 14 | 24103554 | 24113428 | protein\_coding | | ENSDARG00000100657 | egfl7 | 21 | 7666975 | 7703748 | protein\_coding | | ENSDARG00000014259 | eya1 | 24 | 13780647 | 13871040 | protein\_coding | | ENSDARG00000040080 | fli1b | 16 | 42067953 | 42096849 | protein\_coding | | ENSDARG00000102138 | foxa1 | 17 | 10162037 | 10164898 | protein\_coding | | ENSDARG00000052154 | lix1 | 5 | 64133318 | 64150043 | protein\_coding | | ENSDARG00000053666 | myb | 23 | 31888892 | 31903512 | protein\_coding | | ENSDARG00000045695 | myca | 24 | 10273098 | 10275662 | protein\_coding | | ENSDARG00000007220 | ncam1b | 15 | 18431302 | 18638742 | protein\_coding | | ENSDARG00000098578 | pdgfab | 3 | 12773481 | 12818979 | protein\_coding | | ENSDARG00000029865 | rassf2a | 8 | 42992157 | 43042282 | protein\_coding | | ENSDARG00000071684 | rx1 | 22 | 4614881 | 4622426 | protein\_coding | | ENSDARG00000094128 | si:dkeyp-10a3.2 | 5 | 49082727 | 49091584 | lincRNA | | ENSDARG00000068397 | tns2b | 6 | 39016341 | 39053783 | protein\_coding | | ENSDARG00000061462 | vwa7 | 18 | 40535216 | 40560282 | protein\_coding | | ENSDARG00000071107 | wnt7bb | 25 | 1911947 | 1990135 | protein\_coding | | ENSDARG00000030722 | xirp1 | 2 | 24093963 | 24113167 | protein\_coding | | ENSDARG00000004378 | yrk | 19 | 43763478 | 43836818 | protein\_coding | | ENSDARG00000098181 | zgc:113184 | 11 | 2485460 | 2502451 | protein\_coding | | ENSDARG00000015567 | zic1 | 24 | 4938793 | 4941978 | protein\_coding | |
