# Supplementary material for: A high-resolution mRNA expression time course of embryonic development in zebrafish
Source: eLife. 2017 Nov 16;6:e30860. doi: 10.7554/eLife.30860 (PMC5690287; doi:10.7554/eLife.30860)
Supplement: Supplementary file 6. [file elife-30860-supp6.zip › biolayout-clusters-files/Cluster044-genes.html]

Cluster044


# Cluster044: Genes

| | Ensembl ID | Gene Name | Chr | Start | End | Biotype | | --- | --- | --- | --- | --- | --- | | ENSDARG00000089986 | TP53INP2 | 23 | 2597815 | 2610837 | protein\_coding | | ENSDARG00000011175 | atp6v1d | 20 | 51275386 | 51287449 | protein\_coding | | ENSDARG00000022315 | atp6v1g1 | 5 | 56649896 | 56655340 | protein\_coding | | ENSDARG00000069989 | bnc2 | 1 | 26330354 | 26515375 | protein\_coding | | ENSDARG00000026165 | col11a1a | 24 | 28684862 | 28814144 | protein\_coding | | ENSDARG00000069093 | col2a1a | 8 | 21163335 | 21187388 | protein\_coding | | ENSDARG00000011407 | col2a1b | 11 | 32007 | 110423 | protein\_coding | | ENSDARG00000012593 | col5a1 | 21 | 6634384 | 6803529 | protein\_coding | | ENSDARG00000092124 | cox14 | 22 | 5722411 | 5726538 | protein\_coding | | ENSDARG00000070681 | fam132a | 23 | 24778883 | 24824394 | protein\_coding | | ENSDARG00000056122 | gdi1 | 23 | 26136608 | 26150497 | protein\_coding | | ENSDARG00000053499 | isl2b | 7 | 29721739 | 29725744 | protein\_coding | | ENSDARG00000069946 | itga6b | 1 | 30215219 | 30260789 | protein\_coding | | ENSDARG00000028507 | itgb4 | 8 | 13069045 | 13121882 | protein\_coding | | ENSDARG00000076566 | kank3 | 8 | 47769629 | 47834326 | protein\_coding | | ENSDARG00000053509 | kazald3 | 8 | 42602990 | 42617667 | protein\_coding | | ENSDARG00000101619 | minos1 | 23 | 39851458 | 39858323 | protein\_coding | | ENSDARG00000018178 | pgm2 | 1 | 18086231 | 18110982 | protein\_coding | | ENSDARG00000010728 | scin | 19 | 31760362 | 31785583 | protein\_coding | | ENSDARG00000038608 | sdhc | 2 | 44339654 | 44346842 | protein\_coding | | ENSDARG00000070050 | sfrp2 | 1 | 24800495 | 24805304 | protein\_coding | | ENSDARG00000096917 | si:ch211-227m13.1 | 22 | 13251916 | 13325579 | protein\_coding | | ENSDARG00000019353 | sparc | 14 | 25641650 | 25659615 | protein\_coding | | ENSDARG00000052712 | suclg1 | 1 | 42951316 | 42983420 | protein\_coding | | ENSDARG00000104647 | surf4 | KN149679.1 | 9850 | 32544 | protein\_coding | | ENSDARG00000004497 | tspan33a | 4 | 14958605 | 14971812 | protein\_coding | | ENSDARG00000070391 | tspan4b | 3 | 32455296 | 32464680 | protein\_coding | | ENSDARG00000090369 | zgc:86896 | 3 | 36146118 | 36154684 | protein\_coding | |
