# Supplementary material for: A high-resolution mRNA expression time course of embryonic development in zebrafish
Source: eLife. 2017 Nov 16;6:e30860. doi: 10.7554/eLife.30860 (PMC5690287; doi:10.7554/eLife.30860)
Supplement: Supplementary file 6. [file elife-30860-supp6.zip › biolayout-clusters-files/Cluster044.html]

Cluster044


# Cluster044: Detail

### Go to ZFA detail

## GO

| | GO ID | Description | Domain | Annotated | Expected | Observed | Adjusted p-value | Genes | Ensembl IDs | | --- | --- | --- | --- | --- | --- | --- | --- | --- | | GO:0005581 | collagen trimer | cellular\_component | 41 | 0.11 | 4 | 1.0e-03 | col2a1b col5a1 col11a1a col2a1a | ENSDARG00000011407 ENSDARG00000012593 ENSDARG00000026165 ENSDARG00000069093 | | GO:0005201 | extracellular matrix structural constitu... | molecular\_function | 26 | 0.06 | 4 | 9.9e-05 | col2a1b col5a1 col11a1a col2a1a | ENSDARG00000011407 ENSDARG00000012593 ENSDARG00000026165 ENSDARG00000069093 | |

  


### Go to GO detail

## ZFA

| | ZFA ID | Description | Annotated | Expected | Observed | Fold Enrichment | Adjusted p-value | Genes | Ensembl IDs | | --- | --- | --- | --- | --- | --- | --- | --- | --- | | ZFA:0001455 | scapulocoracoid | 9 | 0.02 | 3 | 150 | 0.047 | sparc col2a1b col2a1a | ENSDARG00000019353 ENSDARG00000011407 ENSDARG00000069093 | |
