# Supplementary material for: A high-resolution mRNA expression time course of embryonic development in zebrafish
Source: eLife. 2017 Nov 16;6:e30860. doi: 10.7554/eLife.30860 (PMC5690287; doi:10.7554/eLife.30860)
Supplement: Supplementary file 6. [file elife-30860-supp6.zip › biolayout-clusters-files/Cluster045-genes.html]

Cluster045


# Cluster045: Genes

| | Ensembl ID | Gene Name | Chr | Start | End | Biotype | | --- | --- | --- | --- | --- | --- | | ENSDARG00000035985 | FAM210A (1 of many) | 19 | 12710886 | 12728880 | protein\_coding | | ENSDARG00000098304 | TAOK1 (1 of many) | 21 | 40505741 | 40556760 | protein\_coding | | ENSDARG00000021564 | VDAC3 (1 of many) | 10 | 3299525 | 3317901 | protein\_coding | | ENSDARG00000104719 | abcc1 | 3 | 36571624 | 36602104 | protein\_coding | | ENSDARG00000075435 | akap11 | 9 | 17964223 | 17998920 | protein\_coding | | ENSDARG00000026028 | ankrd44 | 9 | 32295582 | 32347740 | protein\_coding | | ENSDARG00000090656 | arid4b | 13 | 49306381 | 49375937 | protein\_coding | | ENSDARG00000062521 | atp9b | 19 | 22490145 | 22593063 | protein\_coding | | ENSDARG00000102061 | b3galt2 | 22 | 24131243 | 24142525 | protein\_coding | | ENSDARG00000070513 | brpf3a | 11 | 25475156 | 25496294 | protein\_coding | | ENSDARG00000020857 | ccdc149b | 1 | 39315988 | 39334939 | protein\_coding | | ENSDARG00000011703 | clocka | 20 | 22167664 | 22276330 | protein\_coding | | ENSDARG00000076068 | crtc1a | 2 | 24922400 | 24947674 | protein\_coding | | ENSDARG00000070213 | etnk2 | 11 | 37581500 | 37613287 | protein\_coding | | ENSDARG00000088505 | fam217b | 23 | 12217089 | 12223715 | protein\_coding | | ENSDARG00000057680 | foxj2 | 16 | 12868175 | 12897070 | protein\_coding | | ENSDARG00000077249 | gmip | 3 | 52482902 | 52547793 | protein\_coding | | ENSDARG00000079504 | mfn2 | 8 | 47908742 | 47959911 | protein\_coding | | ENSDARG00000058771 | nav1b | 6 | 54871403 | 55012866 | protein\_coding | | ENSDARG00000069654 | ppp6r2b | 18 | 6856782 | 6897029 | protein\_coding | | ENSDARG00000071772 | si:ch211-253p2.2 | 12 | 6509286 | 6518178 | protein\_coding | | ENSDARG00000057272 | slc30a9 | 14 | 44343014 | 44363315 | protein\_coding | | ENSDARG00000102308 | ttc39c | 22 | 16324285 | 16351690 | protein\_coding | | ENSDARG00000061243 | ubash3ba | 5 | 29251280 | 29296615 | protein\_coding | | ENSDARG00000061180 | vps45 | 19 | 42827850 | 42847259 | protein\_coding | | ENSDARG00000040131 | zfyve26 | 13 | 32934657 | 32984483 | protein\_coding | | ENSDARG00000056245 | zgc:162952 | 5 | 27925056 | 27953754 | processed\_transcript | |
