# Supplementary material for: A high-resolution mRNA expression time course of embryonic development in zebrafish
Source: eLife. 2017 Nov 16;6:e30860. doi: 10.7554/eLife.30860 (PMC5690287; doi:10.7554/eLife.30860)
Supplement: Supplementary file 6. [file elife-30860-supp6.zip › biolayout-clusters-files/Cluster045.html]

Cluster045


# Cluster045: Detail

### Go to ZFA detail

## GO

| | GO ID | Description | Domain | Annotated | Expected | Observed | Adjusted p-value | Genes | Ensembl IDs | | --- | --- | --- | --- | --- | --- | --- | --- | --- | | GO:0005741 | mitochondrial outer membrane | cellular\_component | 38 | 0.07 | 3 | 0.011 | VDAC3 (1 of many) mfn2 arid4b | ENSDARG00000021564 ENSDARG00000079504 ENSDARG00000090656 | |
