# Supplementary material for: A high-resolution mRNA expression time course of embryonic development in zebrafish
Source: eLife. 2017 Nov 16;6:e30860. doi: 10.7554/eLife.30860 (PMC5690287; doi:10.7554/eLife.30860)
Supplement: Supplementary file 6. [file elife-30860-supp6.zip › biolayout-clusters-files/Cluster046-genes.html]

Cluster046


# Cluster046: Genes

| | Ensembl ID | Gene Name | Chr | Start | End | Biotype | | --- | --- | --- | --- | --- | --- | | ENSDARG00000103792 | CABZ01076737.1 | 5 | 2855023 | 2879613 | protein\_coding | | ENSDARG00000089538 | RNF166 | 7 | 54922348 | 54990874 | protein\_coding | | ENSDARG00000043026 | TTBK2 (1 of many) | 20 | 26012252 | 26029265 | protein\_coding | | ENSDARG00000069249 | aftpha | 1 | 54417663 | 54432263 | protein\_coding | | ENSDARG00000016350 | cap1 | 19 | 44120314 | 44141474 | protein\_coding | | ENSDARG00000076757 | ephb1 | 2 | 46706335 | 46876773 | protein\_coding | | ENSDARG00000058692 | fzd3b | 17 | 8341511 | 8413187 | protein\_coding | | ENSDARG00000008503 | glcci1 | 19 | 25768066 | 25843032 | protein\_coding | | ENSDARG00000090585 | gpc1b | 2 | 46179680 | 46331769 | protein\_coding | | ENSDARG00000053389 | jade2 | 21 | 45345222 | 45370257 | protein\_coding | | ENSDARG00000015937 | kptn | 5 | 36311472 | 36324359 | protein\_coding | | ENSDARG00000063583 | map2k4a | 3 | 10626054 | 10667989 | protein\_coding | | ENSDARG00000023914 | mark4a | 5 | 36168521 | 36222265 | protein\_coding | | ENSDARG00000010047 | neu3.2 | 21 | 21760027 | 21762656 | protein\_coding | | ENSDARG00000060705 | nsd1b | 21 | 37668363 | 37713145 | protein\_coding | | ENSDARG00000045422 | otulina | 24 | 10162685 | 10169548 | protein\_coding | | ENSDARG00000024827 | rnf150a | 1 | 52645123 | 52664286 | protein\_coding | | ENSDARG00000042545 | sema3ga | 8 | 25631090 | 25678140 | protein\_coding | | ENSDARG00000105288 | si:ch211-79l17.1 | 18 | 443278 | 510253 | protein\_coding | | ENSDARG00000078267 | si:dkey-181m9.8 | 2 | 27034227 | 27065326 | protein\_coding | | ENSDARG00000044381 | siah2l | 21 | 38611596 | 38670148 | protein\_coding | | ENSDARG00000039661 | slc36a4 | 15 | 41215158 | 41277631 | protein\_coding | | ENSDARG00000091821 | smc6 | 17 | 6510240 | 6588816 | protein\_coding | | ENSDARG00000054501 | stambpa | 10 | 42282908 | 42303028 | protein\_coding | | ENSDARG00000013842 | tbc1d19 | 7 | 61938352 | 62002626 | protein\_coding | | ENSDARG00000069157 | tmem135 | 10 | 25351336 | 25366627 | protein\_coding | | ENSDARG00000007737 | zyg11 | 20 | 16274098 | 16302296 | protein\_coding | |
