# Supplementary material for: A high-resolution mRNA expression time course of embryonic development in zebrafish
Source: eLife. 2017 Nov 16;6:e30860. doi: 10.7554/eLife.30860 (PMC5690287; doi:10.7554/eLife.30860)
Supplement: Supplementary file 6. [file elife-30860-supp6.zip › biolayout-clusters-files/Cluster047-genes.html]

Cluster047


# Cluster047: Genes

| | Ensembl ID | Gene Name | Chr | Start | End | Biotype | | --- | --- | --- | --- | --- | --- | | ENSDARG00000090997 | ENSDARG00000090997 | 14 | 8198296 | 8267365 | protein\_coding | | ENSDARG00000105442 | ENSDARG00000105442 | 11 | 44200326 | 44210756 | protein\_coding | | ENSDARG00000105141 | FO704635.1 | 2 | 43805217 | 43845109 | protein\_coding | | ENSDARG00000055223 | ablim2 | 12 | 22558896 | 22694628 | protein\_coding | | ENSDARG00000038583 | abraa | 6 | 52064305 | 52072830 | protein\_coding | | ENSDARG00000104256 | bco1 | 7 | 65037326 | 65066446 | protein\_coding | | ENSDARG00000004060 | bhlhe40 | 11 | 35497669 | 35501340 | protein\_coding | | ENSDARG00000011473 | calcrla | 9 | 42558892 | 42616683 | protein\_coding | | ENSDARG00000091006 | cobl | 16 | 8616892 | 8749764 | protein\_coding | | ENSDARG00000037910 | filip1l | 11 | 43976547 | 44029701 | protein\_coding | | ENSDARG00000102447 | gdpd4a | 18 | 3195885 | 3244649 | protein\_coding | | ENSDARG00000075282 | irs2b | 9 | 8450909 | 8475730 | protein\_coding | | ENSDARG00000016939 | itgb2 | 9 | 45160186 | 45182714 | protein\_coding | | ENSDARG00000002764 | miox | 18 | 6773233 | 6783325 | protein\_coding | | ENSDARG00000094809 | ms4a17a.14 | 4 | 74942686 | 74994344 | protein\_coding | | ENSDARG00000029766 | nr1i2 | 9 | 9524490 | 9584607 | protein\_coding | | ENSDARG00000025615 | prr15la | 3 | 24059256 | 24066722 | protein\_coding | | ENSDARG00000071437 | ptprc | 22 | 22962427 | 23053079 | protein\_coding | | ENSDARG00000055416 | serpinb1 | 20 | 26981578 | 26987759 | protein\_coding | | ENSDARG00000057826 | si:ch73-61d6.3 | 8 | 13150004 | 13183024 | protein\_coding | | ENSDARG00000099026 | si:dkey-185m8.2 | 7 | 8078548 | 8095430 | protein\_coding | | ENSDARG00000092759 | si:dkey-61p9.9 | 4 | 76245352 | 76361603 | protein\_coding | | ENSDARG00000021250 | slc25a48 | 14 | 25293358 | 25301757 | protein\_coding | | ENSDARG00000098686 | tmprss2 | 10 | 38764432 | 38786259 | protein\_coding | | ENSDARG00000086685 | trim63b | 9 | 24089834 | 24091346 | protein\_coding | |
