# Supplementary material for: A high-resolution mRNA expression time course of embryonic development in zebrafish
Source: eLife. 2017 Nov 16;6:e30860. doi: 10.7554/eLife.30860 (PMC5690287; doi:10.7554/eLife.30860)
Supplement: Supplementary file 6. [file elife-30860-supp6.zip › biolayout-clusters-files/Cluster048-genes.html]

Cluster048


# Cluster048: Genes

| | Ensembl ID | Gene Name | Chr | Start | End | Biotype | | --- | --- | --- | --- | --- | --- | | ENSDARG00000034836 | KRT84 | 8 | 7041426 | 7049959 | protein\_coding | | ENSDARG00000068369 | angptl2b | 8 | 33010683 | 33042174 | protein\_coding | | ENSDARG00000099677 | bambib | 2 | 4562140 | 4568204 | protein\_coding | | ENSDARG00000073978 | crabp2a | 16 | 14493359 | 14506883 | protein\_coding | | ENSDARG00000058256 | draxin | 11 | 15796553 | 15878479 | protein\_coding | | ENSDARG00000075249 | fam171a2 | 3 | 21150110 | 21217930 | protein\_coding | | ENSDARG00000091660 | fgfbp1b.1 | 1 | 21986690 | 21987732 | protein\_coding | | ENSDARG00000077298 | gas1a | 5 | 43864327 | 43896936 | protein\_coding | | ENSDARG00000056023 | hoxb9a | 3 | 23546803 | 23551888 | protein\_coding | | ENSDARG00000010408 | igsf9b | 8 | 4797695 | 4904043 | protein\_coding | | ENSDARG00000014180 | ildr2 | 9 | 34465307 | 34491527 | protein\_coding | | ENSDARG00000061794 | jam3b | 21 | 24948365 | 25010386 | protein\_coding | | ENSDARG00000015674 | msx3 | 13 | 24531788 | 24534218 | protein\_coding | | ENSDARG00000104735 | nkx6.2 | 13 | 51154848 | 51158678 | protein\_coding | | ENSDARG00000055505 | pdgfaa | 22 | 25971126 | 26080385 | protein\_coding | | ENSDARG00000036630 | plp2 | 8 | 49322016 | 49356619 | protein\_coding | | ENSDARG00000105190 | rhbdl3 | 6 | 22152171 | 22227174 | protein\_coding | | ENSDARG00000023220 | selt2 | 14 | 17274918 | 17282721 | protein\_coding | | ENSDARG00000088885 | si:ch1073-340i21.3 | 15 | 46114202 | 46121452 | protein\_coding | | ENSDARG00000094004 | si:ch73-108h4.3 | 9 | 1370658 | 1374172 | processed\_transcript | | ENSDARG00000075057 | si:dkeyp-33b5.4 | 17 | 12817238 | 12853418 | protein\_coding | | ENSDARG00000027199 | smad1 | 1 | 35165017 | 35196915 | protein\_coding | | ENSDARG00000036721 | tomm40l | 19 | 10912437 | 10922990 | protein\_coding | | ENSDARG00000035129 | zgc:101858 | 5 | 64175502 | 64180136 | protein\_coding | | ENSDARG00000104367 | zgc:194469 | 6 | 12870808 | 12883741 | protein\_coding | |
