# Supplementary material for: A high-resolution mRNA expression time course of embryonic development in zebrafish
Source: eLife. 2017 Nov 16;6:e30860. doi: 10.7554/eLife.30860 (PMC5690287; doi:10.7554/eLife.30860)
Supplement: Supplementary file 6. [file elife-30860-supp6.zip › biolayout-clusters-files/Cluster049-genes.html]

Cluster049


# Cluster049: Genes

| | Ensembl ID | Gene Name | Chr | Start | End | Biotype | | --- | --- | --- | --- | --- | --- | | ENSDARG00000070918 | ENSDARG00000070918 | 22 | 38276174 | 38287021 | protein\_coding | | ENSDARG00000100782 | F7 (1 of many) | 1 | 109798 | 119146 | protein\_coding | | ENSDARG00000016704 | PBLD (1 of many) | 13 | 22709371 | 22713355 | protein\_coding | | ENSDARG00000098724 | RGR (1 of many) | 12 | 48943520 | 48948929 | protein\_coding | | ENSDARG00000004296 | ambp | 10 | 10843326 | 10865634 | protein\_coding | | ENSDARG00000003808 | aqp3a | 5 | 42406269 | 42411661 | protein\_coding | | ENSDARG00000030694 | atp6v1e1b | 4 | 5231556 | 5238765 | protein\_coding | | ENSDARG00000015654 | ca15a | 12 | 4283383 | 4309398 | protein\_coding | | ENSDARG00000010312 | cp | 22 | 38220813 | 38246841 | protein\_coding | | ENSDARG00000093671 | crp4 | 24 | 38210999 | 38211778 | unprocessed\_pseudogene | | ENSDARG00000088581 | f10 | 1 | 135903 | 142469 | protein\_coding | | ENSDARG00000055705 | f5 | 9 | 34317821 | 34338811 | protein\_coding | | ENSDARG00000034862 | f7 | 1 | 119960 | 125246 | protein\_coding | | ENSDARG00000068849 | fam83ha | 13 | 42438606 | 42453377 | protein\_coding | | ENSDARG00000052470 | igfbp2a | 6 | 18861319 | 18891956 | protein\_coding | | ENSDARG00000102956 | mmp17b | 14 | 49824438 | 49859874 | protein\_coding | | ENSDARG00000033742 | nt5c1bb | 20 | 9289560 | 9313469 | protein\_coding | | ENSDARG00000073757 | pdzrn3a | 23 | 10893547 | 10979461 | protein\_coding | | ENSDARG00000093549 | sepp1a | 8 | 31426220 | 31434102 | protein\_coding | | ENSDARG00000105411 | si:ch211-113d11.5 | 14 | 47233712 | 47240802 | protein\_coding | | ENSDARG00000096857 | si:ch73-126m5.4 | 6 | 48381486 | 48382464 | processed\_transcript | | ENSDARG00000104380 | si:dkey-238k10.2 | 4 | 76377194 | 76528321 | protein\_coding | | ENSDARG00000090352 | si:dkey-97i18.5 | 16 | 31668075 | 31670257 | processed\_transcript | | ENSDARG00000055523 | slc22a6l | 21 | 28441968 | 28457627 | protein\_coding | | ENSDARG00000036481 | tcn2 | 5 | 26195312 | 26205947 | protein\_coding | |
