# Supplementary material for: A high-resolution mRNA expression time course of embryonic development in zebrafish
Source: eLife. 2017 Nov 16;6:e30860. doi: 10.7554/eLife.30860 (PMC5690287; doi:10.7554/eLife.30860)
Supplement: Supplementary file 6. [file elife-30860-supp6.zip › biolayout-clusters-files/Cluster049.html]

Cluster049


# Cluster049: Detail

### Go to ZFA detail

## GO

| | GO ID | Description | Domain | Annotated | Expected | Observed | Adjusted p-value | Genes | Ensembl IDs | | --- | --- | --- | --- | --- | --- | --- | --- | --- | | GO:0007596 | blood coagulation | biological\_process | 36 | 0.06 | 4 | 0.00132 | f7 f5 f10 F7 (1 of many) | ENSDARG00000034862 ENSDARG00000055705 ENSDARG00000088581 ENSDARG00000100782 | | GO:0005576 | extracellular region | cellular\_component | 502 | 0.95 | 7 | 0.00016 | ambp f7 igfbp2a ENSDARG00000070918 f10 sepp1a F7 (1 of many) | ENSDARG00000004296 ENSDARG00000034862 ENSDARG00000052470 ENSDARG00000070918 ENSDARG00000088581 ENSDARG00000093549 ENSDARG00000100782 | |
