# Supplementary material for: A high-resolution mRNA expression time course of embryonic development in zebrafish
Source: eLife. 2017 Nov 16;6:e30860. doi: 10.7554/eLife.30860 (PMC5690287; doi:10.7554/eLife.30860)
Supplement: Supplementary file 6. [file elife-30860-supp6.zip › biolayout-clusters-files/Cluster050-genes.html]

Cluster050


# Cluster050: Genes

| | Ensembl ID | Gene Name | Chr | Start | End | Biotype | | --- | --- | --- | --- | --- | --- | | ENSDARG00000076900 | PROZ | 1 | 144284 | 151998 | protein\_coding | | ENSDARG00000031782 | aim1a | 17 | 25463075 | 25531858 | protein\_coding | | ENSDARG00000044365 | angptl3 | 6 | 32357283 | 32362239 | protein\_coding | | ENSDARG00000043279 | aqp12 | 22 | 2857785 | 2870671 | protein\_coding | | ENSDARG00000043719 | c3a.6 | 1 | 55360638 | 55410849 | protein\_coding | | ENSDARG00000093068 | c3b.1 | 22 | 26160287 | 26216378 | protein\_coding | | ENSDARG00000099389 | cdo1 | KN149707.1 | 3861 | 5958 | protein\_coding | | ENSDARG00000016598 | ckmt1 | 25 | 21732229 | 21750005 | protein\_coding | | ENSDARG00000028336 | dhdhl | 18 | 1077870 | 1084699 | protein\_coding | | ENSDARG00000059227 | fabp1b.1 | 8 | 933678 | 940431 | protein\_coding | | ENSDARG00000009550 | foxi3b | 14 | 34174022 | 34176273 | protein\_coding | | ENSDARG00000057992 | fstb | 10 | 9134644 | 9143264 | protein\_coding | | ENSDARG00000036942 | gpd1c | 19 | 24933 | 325377 | protein\_coding | | ENSDARG00000039832 | gsta.2 | 13 | 699294 | 710792 | protein\_coding | | ENSDARG00000025338 | hagh | 3 | 18624771 | 18642794 | protein\_coding | | ENSDARG00000051939 | pcxb | 7 | 60151878 | 60525938 | protein\_coding | | ENSDARG00000038258 | proca | 2 | 5544659 | 5564039 | protein\_coding | | ENSDARG00000098645 | rgn | 6 | 8895450 | 8904447 | protein\_coding | | ENSDARG00000021208 | serpind1 | 8 | 1761000 | 1776527 | protein\_coding | | ENSDARG00000096712 | si:dkey-193p11.2 | 12 | 25794550 | 25796504 | protein\_coding | | ENSDARG00000058032 | si:dkey-6n6.2 | 8 | 14120316 | 14132871 | protein\_coding | | ENSDARG00000034273 | sid4 | 1 | 57971018 | 57981003 | protein\_coding | | ENSDARG00000056196 | slc2a2 | 2 | 26161209 | 26489761 | protein\_coding | | ENSDARG00000038106 | slc37a4a | 15 | 9318549 | 9339495 | protein\_coding | | ENSDARG00000097606 | tmem82 | 23 | 24559116 | 24562155 | protein\_coding | |
