# Supplementary material for: A high-resolution mRNA expression time course of embryonic development in zebrafish
Source: eLife. 2017 Nov 16;6:e30860. doi: 10.7554/eLife.30860 (PMC5690287; doi:10.7554/eLife.30860)
Supplement: Supplementary file 6. [file elife-30860-supp6.zip › biolayout-clusters-files/Cluster050.html]

Cluster050


## Cluster050: Detail

### Go to GO detail

## ZFA

| | ZFA ID | Description | Annotated | Expected | Observed | Fold Enrichment | Adjusted p-value | Genes | Ensembl IDs | | --- | --- | --- | --- | --- | --- | --- | --- | --- | | ZFA:0001338 | intestine | 863 | 1.82 | 8 | 4.4 | 0.00017 | angptl3 slc2a2 c3b.1 rgn c3a.6 fabp1b.1 gsta.2 gsta.2 | ENSDARG00000044365 ENSDARG00000056196 ENSDARG00000093068 ENSDARG00000098645 ENSDARG00000043719 ENSDARG00000059227 ENSDARG00000039832 ENSDARG00000039832 | | ZFA:0000123 | liver | 1962 | 4.13 | 14 | 3.4 | 0.00669 | pcxb angptl3 slc2a2 c3b.1 hagh serpind1 cdo1 slc37a4a aqp12 rgn c3a.6 fabp1b.1 sid4 proca | ENSDARG00000051939 ENSDARG00000044365 ENSDARG00000056196 ENSDARG00000093068 ENSDARG00000025338 ENSDARG00000021208 ENSDARG00000099389 ENSDARG00000038106 ENSDARG00000043279 ENSDARG00000098645 ENSDARG00000043719 ENSDARG00000059227 ENSDARG00000034273 ENSDARG00000038258 | | ZFA:0001076 | intestinal bulb | 276 | 0.58 | 7 | 12.1 | 0.01035 | slc2a2 ckmt1 hagh cdo1 slc37a4a aqp12 fabp1b.1 | ENSDARG00000056196 ENSDARG00000016598 ENSDARG00000025338 ENSDARG00000099389 ENSDARG00000038106 ENSDARG00000043279 ENSDARG00000059227 | | ZFA:0000124 | liver primordium | 32 | 0.07 | 1 | 14.3 | 0.03518 | angptl3 | ENSDARG00000044365 | |
