# Supplementary material for: A high-resolution mRNA expression time course of embryonic development in zebrafish
Source: eLife. 2017 Nov 16;6:e30860. doi: 10.7554/eLife.30860 (PMC5690287; doi:10.7554/eLife.30860)
Supplement: Supplementary file 6. [file elife-30860-supp6.zip › biolayout-clusters-files/Cluster051-genes.html]

Cluster051


# Cluster051: Genes

| | Ensembl ID | Gene Name | Chr | Start | End | Biotype | | --- | --- | --- | --- | --- | --- | | ENSDARG00000063407 | ENSDARG00000063407 | 16 | 1275707 | 1294474 | protein\_coding | | ENSDARG00000060542 | LGR4 | 7 | 32097876 | 32189070 | protein\_coding | | ENSDARG00000090976 | NABP2 | 11 | 2437223 | 2446570 | protein\_coding | | ENSDARG00000030417 | PKD1 | 1 | 53597305 | 53684756 | protein\_coding | | ENSDARG00000077778 | adamts9 | 11 | 18995537 | 19065460 | protein\_coding | | ENSDARG00000091726 | aebp1 | 10 | 2779932 | 2811020 | protein\_coding | | ENSDARG00000028661 | cntfr | 10 | 13502011 | 13814728 | protein\_coding | | ENSDARG00000074059 | dlg5a | 13 | 16211735 | 16366066 | protein\_coding | | ENSDARG00000032617 | fgfrl1a | 14 | 17137260 | 17258120 | protein\_coding | | ENSDARG00000059768 | gpatch8 | 3 | 37520586 | 37558134 | protein\_coding | | ENSDARG00000001634 | kirrela | 7 | 18818202 | 18894659 | protein\_coding | | ENSDARG00000056647 | klhl3 | 14 | 23986670 | 24007727 | protein\_coding | | ENSDARG00000102506 | lrp2a | 9 | 48717448 | 48974769 | protein\_coding | | ENSDARG00000013310 | map3k15 | 24 | 25768121 | 25843795 | protein\_coding | | ENSDARG00000006617 | napga | 24 | 742695 | 986399 | protein\_coding | | ENSDARG00000012499 | per1b | 7 | 50547395 | 50571221 | protein\_coding | | ENSDARG00000054060 | pof1b | 21 | 38463379 | 38534328 | protein\_coding | | ENSDARG00000071692 | prex2 | 24 | 18804246 | 19062637 | protein\_coding | | ENSDARG00000040321 | rx2 | 2 | 55709127 | 55737506 | protein\_coding | | ENSDARG00000095580 | si:ch211-67e16.11 | 9 | 14181008 | 14226301 | protein\_coding | | ENSDARG00000079175 | si:ch211-79k12.1 | 16 | 24607792 | 24634997 | protein\_coding | | ENSDARG00000040920 | si:dkey-49n23.1 | 22 | 30862501 | 30986315 | protein\_coding | | ENSDARG00000077326 | slc45a4 | 16 | 30950355 | 30974606 | protein\_coding | | ENSDARG00000101512 | tmx4 | 17 | 3842400 | 3867696 | protein\_coding | |
