# Supplementary material for: A high-resolution mRNA expression time course of embryonic development in zebrafish
Source: eLife. 2017 Nov 16;6:e30860. doi: 10.7554/eLife.30860 (PMC5690287; doi:10.7554/eLife.30860)
Supplement: Supplementary file 6. [file elife-30860-supp6.zip › biolayout-clusters-files/Cluster052-genes.html]

Cluster052


# Cluster052: Genes

| | Ensembl ID | Gene Name | Chr | Start | End | Biotype | | --- | --- | --- | --- | --- | --- | | ENSDARG00000098142 | CABZ01079303.1 | KN149850.1 | 11648 | 12280 | protein\_coding | | ENSDARG00000053716 | ENSDARG00000053716 | 5 | 36066562 | 36074870 | protein\_coding | | ENSDARG00000067668 | ENSDARG00000067668 | 15 | 47637147 | 47643338 | protein\_coding | | ENSDARG00000076366 | ENSDARG00000076366 | 3 | 3603985 | 3631427 | protein\_coding | | ENSDARG00000042456 | MAD2L2 | 8 | 22324043 | 22327152 | protein\_coding | | ENSDARG00000070568 | atad5a | 3 | 26113482 | 26135593 | protein\_coding | | ENSDARG00000077116 | ccnjl | 21 | 30018077 | 30045826 | protein\_coding | | ENSDARG00000103787 | chd1 | 10 | 6520167 | 6587619 | protein\_coding | | ENSDARG00000102294 | fbxo11a.1 | 13 | 48090005 | 48140757 | protein\_coding | | ENSDARG00000042031 | gorab | 20 | 34598273 | 34604616 | protein\_coding | | ENSDARG00000034427 | hn1l | 3 | 15743239 | 15752429 | protein\_coding | | ENSDARG00000087377 | lbh | 13 | 51610360 | 51617488 | protein\_coding | | ENSDARG00000037560 | mtm1 | 7 | 25554418 | 25584611 | protein\_coding | | ENSDARG00000007271 | mynn | 24 | 26183746 | 26198361 | protein\_coding | | ENSDARG00000079244 | pigw | 5 | 3673802 | 3678014 | protein\_coding | | ENSDARG00000102891 | ppp1r26 | 21 | 7540435 | 7551433 | protein\_coding | | ENSDARG00000058306 | prpf18 | 18 | 8444101 | 8450499 | protein\_coding | | ENSDARG00000034893 | rarab | 3 | 32933804 | 33127907 | protein\_coding | | ENSDARG00000089967 | si:ch211-59o9.10 | 25 | 19462696 | 19476195 | protein\_coding | | ENSDARG00000089861 | usp44 | 4 | 26032542 | 26046634 | protein\_coding | | ENSDARG00000009754 | zc3h11a | 11 | 23796488 | 23806729 | protein\_coding | | ENSDARG00000037652 | zgc:56409 | 1 | 9189184 | 9201215 | protein\_coding | | ENSDARG00000074544 | znf1007 | 15 | 1022938 | 1032630 | protein\_coding | |
