# Supplementary material for: A high-resolution mRNA expression time course of embryonic development in zebrafish
Source: eLife. 2017 Nov 16;6:e30860. doi: 10.7554/eLife.30860 (PMC5690287; doi:10.7554/eLife.30860)
Supplement: Supplementary file 6. [file elife-30860-supp6.zip › biolayout-clusters-files/Cluster053-genes.html]

Cluster053


# Cluster053: Genes

| | Ensembl ID | Gene Name | Chr | Start | End | Biotype | | --- | --- | --- | --- | --- | --- | | ENSDARG00000044676 | GADD45G (1 of many) | 21 | 20673941 | 20674948 | protein\_coding | | ENSDARG00000038386 | ascl1a | 4 | 17428135 | 17430217 | protein\_coding | | ENSDARG00000101628 | ascl1b.1 | 7 | 49442974 | 49444419 | protein\_coding | | ENSDARG00000099564 | atoh1b | KN150642.1 | 9441 | 10244 | protein\_coding | | ENSDARG00000079029 | dhx32b | 17 | 32390610 | 32407978 | protein\_coding | | ENSDARG00000010791 | dla | 1 | 53353714 | 53366437 | protein\_coding | | ENSDARG00000004232 | dlb | 5 | 36093706 | 36101607 | protein\_coding | | ENSDARG00000042525 | ebf2 | 5 | 66712276 | 66761462 | protein\_coding | | ENSDARG00000016725 | gadd45gb.1 | 21 | 20678384 | 20679925 | protein\_coding | | ENSDARG00000044924 | gdf11 | 11 | 15478583 | 15622280 | protein\_coding | | ENSDARG00000035735 | gsx1 | 5 | 67231933 | 67233402 | protein\_coding | | ENSDARG00000056400 | helt | 1 | 16900455 | 16902108 | protein\_coding | | ENSDARG00000007097 | her13 | 15 | 7190431 | 7193386 | protein\_coding | | ENSDARG00000056732 | her4.1 | 23 | 21526006 | 21527063 | protein\_coding | | ENSDARG00000056729 | her4.2 | 23 | 21542652 | 21544471 | protein\_coding | | ENSDARG00000094426 | her4.2.1 | 23 | 21528601 | 21534583 | protein\_coding | | ENSDARG00000009822 | her4.4 | 23 | 21535470 | 21544507 | protein\_coding | | ENSDARG00000053301 | insm1b | 17 | 41475068 | 41477271 | protein\_coding | | ENSDARG00000060115 | lrrn1 | 6 | 43078980 | 43094639 | protein\_coding | | ENSDARG00000103732 | plp1a | 14 | 14271683 | 14291136 | protein\_coding | | ENSDARG00000009823 | pou3f1 | 16 | 33639086 | 33641955 | protein\_coding | | ENSDARG00000103982 | si:ch211-193l2.6 | 11 | 41401886 | 41406010 | processed\_transcript | | ENSDARG00000093156 | si:ch73-21g5.7 | 23 | 21546552 | 21547925 | protein\_coding | |
