# Supplementary material for: A high-resolution mRNA expression time course of embryonic development in zebrafish
Source: eLife. 2017 Nov 16;6:e30860. doi: 10.7554/eLife.30860 (PMC5690287; doi:10.7554/eLife.30860)
Supplement: Supplementary file 6. [file elife-30860-supp6.zip › biolayout-clusters-files/Cluster053.html]

Cluster053


# Cluster053: Detail

### Go to ZFA detail

## GO

| | GO ID | Description | Domain | Annotated | Expected | Observed | Adjusted p-value | Genes | Ensembl IDs | | --- | --- | --- | --- | --- | --- | --- | --- | --- | | GO:0006355 | regulation of transcription, DNA-templat... | biological\_process | 1072 | 2.49 | 12 | 3.0e-04 | her13 her4.4 pou3f1 gsx1 ascl1a ebf2 insm1b helt her4.2 her4.1 her4.2 ascl1b | ENSDARG00000007097 ENSDARG00000009822 ENSDARG00000009823 ENSDARG00000035735 ENSDARG00000038386 ENSDARG00000042525 ENSDARG00000053301 ENSDARG00000056400 ENSDARG00000056729 ENSDARG00000056732 ENSDARG00000094426 ENSDARG00000101628 | | GO:0048936 | peripheral nervous system neuron axonoge... | biological\_process | 12 | 0.03 | 4 | 2.5e-06 | her4.4 her4.2 her4.1 her4.2 | ENSDARG00000009822 ENSDARG00000056729 ENSDARG00000056732 ENSDARG00000094426 | | GO:0005634 | nucleus | cellular\_component | 1915 | 4.31 | 14 | 9.1e-05 | her13 her4.4 pou3f1 gadd45gb.1 gsx1 ascl1a ebf2 insm1b helt her4.2 her4.1 dhx32b her4.2 ascl1b | ENSDARG00000007097 ENSDARG00000009822 ENSDARG00000009823 ENSDARG00000016725 ENSDARG00000035735 ENSDARG00000038386 ENSDARG00000042525 ENSDARG00000053301 ENSDARG00000056400 ENSDARG00000056729 ENSDARG00000056732 ENSDARG00000079029 ENSDARG00000094426 ENSDARG00000101628 | | GO:0046983 | protein dimerization activity | molecular\_function | 226 | 0.50 | 10 | 3.0e-09 | her13 her4.4 ascl1a ebf2 helt her4.2 her4.1 her4.2 atoh1b ascl1b | ENSDARG00000007097 ENSDARG00000009822 ENSDARG00000038386 ENSDARG00000042525 ENSDARG00000056400 ENSDARG00000056729 ENSDARG00000056732 ENSDARG00000094426 ENSDARG00000099564 ENSDARG00000101628 | | GO:0003677 | DNA binding | molecular\_function | 966 | 2.15 | 12 | 6.6e-04 | her13 her4.4 pou3f1 gsx1 ascl1a ebf2 insm1b helt her4.2 her4.1 her4.2 ascl1b | ENSDARG00000007097 ENSDARG00000009822 ENSDARG00000009823 ENSDARG00000035735 ENSDARG00000038386 ENSDARG00000042525 ENSDARG00000053301 ENSDARG00000056400 ENSDARG00000056729 ENSDARG00000056732 ENSDARG00000094426 ENSDARG00000101628 | |

  


### Go to GO detail

## ZFA

| | ZFA ID | Description | Annotated | Expected | Observed | Fold Enrichment | Adjusted p-value | Genes | Ensembl IDs | | --- | --- | --- | --- | --- | --- | --- | --- | --- | | ZFA:0001064 | rhombomere | 265 | 0.45 | 10 | 22.2 | 8.1e-07 | insm1b gsx1 lrrn1 dlb ascl1b dla pou3f1 her4.1 ascl1a ebf2 | ENSDARG00000053301 ENSDARG00000035735 ENSDARG00000060115 ENSDARG00000004232 ENSDARG00000101628 ENSDARG00000010791 ENSDARG00000009823 ENSDARG00000056732 ENSDARG00000038386 ENSDARG00000042525 | | ZFA:0000029 | hindbrain | 1756 | 2.98 | 15 | 5.0 | 1.6e-06 | plp1a insm1b gadd45gb.1 gsx1 atoh1b her13 her4.2 her4.2 lrrn1 dlb ascl1b dla pou3f1 her4.1 ascl1a ebf2 | ENSDARG00000103732 ENSDARG00000053301 ENSDARG00000016725 ENSDARG00000035735 ENSDARG00000099564 ENSDARG00000007097 ENSDARG00000094426 ENSDARG00000056729 ENSDARG00000060115 ENSDARG00000004232 ENSDARG00000101628 ENSDARG00000010791 ENSDARG00000009823 ENSDARG00000056732 ENSDARG00000038386 ENSDARG00000042525 | | ZFA:0000128 | midbrain | 1312 | 2.23 | 14 | 6.3 | 1.7e-06 | plp1a insm1b gadd45gb.1 helt gsx1 her13 her4.2 her4.2 lrrn1 ascl1b dla pou3f1 her4.1 ascl1a ebf2 | ENSDARG00000103732 ENSDARG00000053301 ENSDARG00000016725 ENSDARG00000056400 ENSDARG00000035735 ENSDARG00000007097 ENSDARG00000094426 ENSDARG00000056729 ENSDARG00000060115 ENSDARG00000101628 ENSDARG00000010791 ENSDARG00000009823 ENSDARG00000056732 ENSDARG00000038386 ENSDARG00000042525 | | ZFA:0000079 | telencephalon | 1004 | 1.71 | 12 | 7.0 | 9.3e-06 | plp1a insm1b gadd45gb.1 her13 her4.2 her4.2 lrrn1 dlb ascl1b dla pou3f1 ascl1a ebf2 | ENSDARG00000103732 ENSDARG00000053301 ENSDARG00000016725 ENSDARG00000007097 ENSDARG00000094426 ENSDARG00000056729 ENSDARG00000060115 ENSDARG00000004232 ENSDARG00000101628 ENSDARG00000010791 ENSDARG00000009823 ENSDARG00000038386 ENSDARG00000042525 | | ZFA:0001707 | brainstem | 9 | 0.02 | 1 | 50.0 | 5.6e-05 | atoh1b | ENSDARG00000099564 | | ZFA:0000101 | diencephalon | 1085 | 1.84 | 10 | 5.4 | 6.9e-05 | plp1a insm1b her4.2 her4.2 dlb ascl1b dla pou3f1 her4.1 ascl1a ebf2 | ENSDARG00000103732 ENSDARG00000053301 ENSDARG00000094426 ENSDARG00000056729 ENSDARG00000004232 ENSDARG00000101628 ENSDARG00000010791 ENSDARG00000009823 ENSDARG00000056732 ENSDARG00000038386 ENSDARG00000042525 | | ZFA:0000160 | tegmentum | 611 | 1.04 | 10 | 9.6 | 7.9e-05 | plp1a insm1b helt her4.2 her4.2 lrrn1 dlb ascl1b dla pou3f1 ascl1a | ENSDARG00000103732 ENSDARG00000053301 ENSDARG00000056400 ENSDARG00000094426 ENSDARG00000056729 ENSDARG00000060115 ENSDARG00000004232 ENSDARG00000101628 ENSDARG00000010791 ENSDARG00000009823 ENSDARG00000038386 | | ZFA:0000109 | forebrain | 883 | 1.50 | 8 | 5.3 | 1.0e-04 | plp1a insm1b gsx1 ascl1b dla pou3f1 her4.1 ascl1a | ENSDARG00000103732 ENSDARG00000053301 ENSDARG00000035735 ENSDARG00000101628 ENSDARG00000010791 ENSDARG00000009823 ENSDARG00000056732 ENSDARG00000038386 | | ZFA:0007029 | hindbrain neural keel | 90 | 0.15 | 2 | 13.3 | 1.0e-04 | lrrn1 her4.1 | ENSDARG00000060115 ENSDARG00000056732 | | ZFA:0007036 | hindbrain neural rod | 41 | 0.07 | 2 | 28.6 | 1.0e-04 | her4.1 ebf2 | ENSDARG00000056732 ENSDARG00000042525 | | ZFA:0007025 | midbrain neural keel | 149 | 0.25 | 4 | 16.0 | 1.0e-04 | insm1b her4.2 her4.2 dla ebf2 | ENSDARG00000053301 ENSDARG00000094426 ENSDARG00000056729 ENSDARG00000010791 ENSDARG00000042525 | | ZFA:0007022 | hindbrain neural plate | 180 | 0.31 | 3 | 9.7 | 1.0e-04 | her4.2 her4.2 ascl1b dla | ENSDARG00000094426 ENSDARG00000056729 ENSDARG00000101628 ENSDARG00000010791 | | ZFA:0007019 | midbrain neural plate | 10 | 0.02 | 1 | 50.0 | 1.0e-04 | ebf2 | ENSDARG00000042525 | | ZFA:0007032 | midbrain neural rod | 76 | 0.13 | 2 | 15.4 | 1.1e-04 | her4.1 ebf2 | ENSDARG00000056732 ENSDARG00000042525 | | ZFA:0000445 | optic tectum | 1276 | 2.17 | 12 | 5.5 | 1.2e-04 | plp1a gadd45gb.1 gdf11 gsx1 her4.2 her4.2 lrrn1 dlb ascl1b dla pou3f1 her4.1 ascl1a | ENSDARG00000103732 ENSDARG00000016725 ENSDARG00000044924 ENSDARG00000035735 ENSDARG00000094426 ENSDARG00000056729 ENSDARG00000060115 ENSDARG00000004232 ENSDARG00000101628 ENSDARG00000010791 ENSDARG00000009823 ENSDARG00000056732 ENSDARG00000038386 | | ZFA:0000571 | presumptive telencephalon | 90 | 0.15 | 4 | 26.7 | 1.7e-04 | her4.2 her4.2 ascl1b dla her4.1 | ENSDARG00000094426 ENSDARG00000056729 ENSDARG00000101628 ENSDARG00000010791 ENSDARG00000056732 | | ZFA:0001215 | thalamus | 102 | 0.17 | 4 | 23.5 | 2.1e-04 | dlb dla pou3f1 her4.1 | ENSDARG00000004232 ENSDARG00000010791 ENSDARG00000009823 ENSDARG00000056732 | | ZFA:0000761 | basal plate midbrain region | 292 | 0.50 | 6 | 12.0 | 8.1e-04 | her4.2 her4.2 lrrn1 dlb ascl1b dla ascl1a | ENSDARG00000094426 ENSDARG00000056729 ENSDARG00000060115 ENSDARG00000004232 ENSDARG00000101628 ENSDARG00000010791 ENSDARG00000038386 | | ZFA:0007017 | posterior neural plate | 8 | 0.01 | 1 | 100.0 | 1.0e-03 | dla | ENSDARG00000010791 | | ZFA:0000008 | brain | 2929 | 4.98 | 7 | 1.4 | 1.0e-03 | gdf11 dlb ascl1b dla pou3f1 her4.1 ascl1a | ENSDARG00000044924 ENSDARG00000004232 ENSDARG00000101628 ENSDARG00000010791 ENSDARG00000009823 ENSDARG00000056732 ENSDARG00000038386 | | ZFA:0000574 | presumptive diencephalon | 118 | 0.20 | 4 | 20.0 | 1.6e-03 | dla pou3f1 her4.1 ascl1a | ENSDARG00000010791 ENSDARG00000009823 ENSDARG00000056732 ENSDARG00000038386 | | ZFA:0001120 | neuroectoderm | 96 | 0.16 | 3 | 18.8 | 2.2e-03 | dlb dla her4.1 | ENSDARG00000004232 ENSDARG00000010791 ENSDARG00000056732 | | ZFA:0000075 | spinal cord | 1319 | 2.24 | 11 | 4.9 | 2.7e-03 | plp1a gsx1 her13 her4.2 her4.2 lrrn1 dlb ascl1b dla pou3f1 her4.1 ascl1a | ENSDARG00000103732 ENSDARG00000035735 ENSDARG00000007097 ENSDARG00000094426 ENSDARG00000056729 ENSDARG00000060115 ENSDARG00000004232 ENSDARG00000101628 ENSDARG00000010791 ENSDARG00000009823 ENSDARG00000056732 ENSDARG00000038386 | | ZFA:0007026 | forebrain neural keel | 110 | 0.19 | 2 | 10.5 | 4.1e-03 | dla her4.1 | ENSDARG00000010791 ENSDARG00000056732 | | ZFA:0007018 | forebrain neural plate | 24 | 0.04 | 1 | 25.0 | 4.2e-03 | pou3f1 | ENSDARG00000009823 | | ZFA:0000012 | central nervous system | 1346 | 2.29 | 8 | 3.5 | 4.2e-03 | insm1b gadd45gb.1 lrrn1 ascl1b dla pou3f1 her4.1 ascl1a | ENSDARG00000053301 ENSDARG00000016725 ENSDARG00000060115 ENSDARG00000101628 ENSDARG00000010791 ENSDARG00000009823 ENSDARG00000056732 ENSDARG00000038386 | | ZFA:0000633 | caudal tuberculum | 94 | 0.16 | 3 | 18.8 | 5.2e-03 | dlb dla ebf2 | ENSDARG00000004232 ENSDARG00000010791 ENSDARG00000042525 | | ZFA:0007042 | spinal cord neural tube | 91 | 0.15 | 3 | 20.0 | 8.5e-03 | ascl1b dla ebf2 | ENSDARG00000101628 ENSDARG00000010791 ENSDARG00000042525 | | ZFA:0001208 | presumptive rhombomere 2 | 24 | 0.04 | 1 | 25.0 | 1.1e-02 | ebf2 | ENSDARG00000042525 | | ZFA:0000418 | pretectum | 75 | 0.13 | 4 | 30.8 | 2.1e-02 | helt gsx1 ascl1b ascl1a | ENSDARG00000056400 ENSDARG00000035735 ENSDARG00000101628 ENSDARG00000038386 | | ZFA:0000100 | cerebellum | 506 | 0.86 | 7 | 8.1 | 2.2e-02 | insm1b gdf11 atoh1b her4.2 her4.2 dla pou3f1 ebf2 | ENSDARG00000053301 ENSDARG00000044924 ENSDARG00000099564 ENSDARG00000094426 ENSDARG00000056729 ENSDARG00000010791 ENSDARG00000009823 ENSDARG00000042525 | | ZFA:0001083 | ventricular zone | 386 | 0.66 | 6 | 9.1 | 3.0e-02 | plp1a dlb ascl1b dla her4.1 ascl1a | ENSDARG00000103732 ENSDARG00000004232 ENSDARG00000101628 ENSDARG00000010791 ENSDARG00000056732 ENSDARG00000038386 | | ZFA:0001031 | rhombomere 1 | 57 | 0.10 | 2 | 20.0 | 3.3e-02 | atoh1b pou3f1 | ENSDARG00000099564 ENSDARG00000009823 | | ZFA:0000822 | rhombomere 2 | 88 | 0.15 | 4 | 26.7 | 3.4e-02 | insm1b gsx1 ascl1b ascl1a | ENSDARG00000053301 ENSDARG00000035735 ENSDARG00000101628 ENSDARG00000038386 | |
