# Supplementary material for: A high-resolution mRNA expression time course of embryonic development in zebrafish
Source: eLife. 2017 Nov 16;6:e30860. doi: 10.7554/eLife.30860 (PMC5690287; doi:10.7554/eLife.30860)
Supplement: Supplementary file 6. [file elife-30860-supp6.zip › biolayout-clusters-files/Cluster054-genes.html]

Cluster054


# Cluster054: Genes

| | Ensembl ID | Gene Name | Chr | Start | End | Biotype | | --- | --- | --- | --- | --- | --- | | ENSDARG00000103703 | CLDN23 | 10 | 15092350 | 15095495 | protein\_coding | | ENSDARG00000078962 | DUOXA2 | 25 | 31533283 | 31547255 | protein\_coding | | ENSDARG00000094160 | ENSDARG00000094160 | 1 | 46139577 | 46141648 | protein\_coding | | ENSDARG00000076747 | TMCC1 (1 of many) | 8 | 54148793 | 54169089 | protein\_coding | | ENSDARG00000056346 | acap1 | 7 | 19806885 | 19906412 | protein\_coding | | ENSDARG00000056561 | asb11 | 9 | 30553921 | 30560501 | protein\_coding | | ENSDARG00000042725 | cebpb | 8 | 28414259 | 28415774 | protein\_coding | | ENSDARG00000057633 | cxcr4a | 6 | 12687144 | 12688869 | protein\_coding | | ENSDARG00000041959 | cxcr4b | 9 | 10721532 | 10723557 | protein\_coding | | ENSDARG00000038068 | ddx5 | 3 | 25139313 | 25144893 | protein\_coding | | ENSDARG00000069790 | dynll2a | 15 | 16130069 | 16134709 | protein\_coding | | ENSDARG00000055647 | ftr82 | 5 | 30106980 | 30115072 | protein\_coding | | ENSDARG00000059073 | gsc | 17 | 19323179 | 19325685 | protein\_coding | | ENSDARG00000078986 | mplkip | 2 | 21947485 | 21949722 | protein\_coding | | ENSDARG00000069382 | mxtx1 | 13 | 21526263 | 21529753 | protein\_coding | | ENSDARG00000102435 | plekhf1 | 7 | 45703171 | 45704590 | protein\_coding | | ENSDARG00000087844 | plekhn1 | 23 | 23305585 | 23326117 | protein\_coding | | ENSDARG00000003193 | rassf7b | 7 | 49381812 | 49417900 | protein\_coding | | ENSDARG00000097776 | si:ch1073-80i24.3 | 17 | 48907077 | 48908338 | protein\_coding | | ENSDARG00000097122 | si:ch211-194e18.1 | 6 | 6219226 | 6229155 | lincRNA | | ENSDARG00000010145 | tor4aa | 10 | 3323409 | 3332058 | protein\_coding | | ENSDARG00000004256 | wnt11 | 5 | 35913452 | 35919964 | protein\_coding | | ENSDARG00000102536 | zgc:174646 | 4 | 36669995 | 36680862 | protein\_coding | |
