# Supplementary material for: A high-resolution mRNA expression time course of embryonic development in zebrafish
Source: eLife. 2017 Nov 16;6:e30860. doi: 10.7554/eLife.30860 (PMC5690287; doi:10.7554/eLife.30860)
Supplement: Supplementary file 6. [file elife-30860-supp6.zip › biolayout-clusters-files/Cluster054.html]

Cluster054


# Cluster054: Detail

### Go to ZFA detail

## GO

| | GO ID | Description | Domain | Annotated | Expected | Observed | Adjusted p-value | Genes | Ensembl IDs | | --- | --- | --- | --- | --- | --- | --- | --- | --- | | GO:0045893 | positive regulation of transcription, DN... | biological\_process | 157 | 0.20 | 4 | 0.012 | wnt11 ddx5 asb11 mxtx1 | ENSDARG00000004256 ENSDARG00000038068 ENSDARG00000056561 ENSDARG00000069382 | | GO:0004896 | cytokine receptor activity | molecular\_function | 13 | 0.02 | 2 | 0.043 | cxcr4b cxcr4a | ENSDARG00000041959 ENSDARG00000057633 | |
