# Supplementary material for: A high-resolution mRNA expression time course of embryonic development in zebrafish
Source: eLife. 2017 Nov 16;6:e30860. doi: 10.7554/eLife.30860 (PMC5690287; doi:10.7554/eLife.30860)
Supplement: Supplementary file 6. [file elife-30860-supp6.zip › biolayout-clusters-files/Cluster055-genes.html]

Cluster055


# Cluster055: Genes

| | Ensembl ID | Gene Name | Chr | Start | End | Biotype | | --- | --- | --- | --- | --- | --- | | ENSDARG00000052351 | ENSDARG00000052351 | 6 | 19965584 | 19982979 | protein\_coding | | ENSDARG00000100056 | SCAF8 | 17 | 48844292 | 48862655 | protein\_coding | | ENSDARG00000063005 | anapc7 | 8 | 11708778 | 11732387 | protein\_coding | | ENSDARG00000051798 | brd1b | 25 | 29245664 | 29267880 | protein\_coding | | ENSDARG00000052565 | cenpi | 14 | 40877623 | 40904868 | protein\_coding | | ENSDARG00000102024 | chmp6b | 12 | 35486908 | 35490394 | protein\_coding | | ENSDARG00000038967 | cul3a | 2 | 47789877 | 47827290 | protein\_coding | | ENSDARG00000010571 | ezh2 | 24 | 17201102 | 17220037 | protein\_coding | | ENSDARG00000015638 | gemin2 | 17 | 13078332 | 13086886 | protein\_coding | | ENSDARG00000104372 | gnb1b | 6 | 53304598 | 53333880 | protein\_coding | | ENSDARG00000056099 | gtf2h5 | 20 | 26146163 | 26148697 | protein\_coding | | ENSDARG00000012519 | hcfc1b | 8 | 7488096 | 7549084 | protein\_coding | | ENSDARG00000023648 | idh3g | 23 | 25195454 | 25208566 | protein\_coding | | ENSDARG00000016318 | med7 | 14 | 33723935 | 33725623 | protein\_coding | | ENSDARG00000099080 | sbno1 | 10 | 44421795 | 44441266 | protein\_coding | | ENSDARG00000009136 | tp53bp2a | 13 | 516409 | 534181 | protein\_coding | | ENSDARG00000060065 | ubap2b | 8 | 45285864 | 45320773 | protein\_coding | | ENSDARG00000013732 | vta1 | 20 | 37397820 | 37473781 | protein\_coding | | ENSDARG00000016447 | ythdf1 | 8 | 23083878 | 23099013 | protein\_coding | | ENSDARG00000045824 | zgc:101783 | 25 | 19635753 | 19649892 | protein\_coding | | ENSDARG00000055383 | zgc:66160 | 3 | 27464195 | 27470970 | protein\_coding | | ENSDARG00000061424 | znf646p | 12 | 28831806 | 28842019 | protein\_coding | |
