# Supplementary material for: A high-resolution mRNA expression time course of embryonic development in zebrafish
Source: eLife. 2017 Nov 16;6:e30860. doi: 10.7554/eLife.30860 (PMC5690287; doi:10.7554/eLife.30860)
Supplement: Supplementary file 6. [file elife-30860-supp6.zip › biolayout-clusters-files/Cluster056-genes.html]

Cluster056


# Cluster056: Genes

| | Ensembl ID | Gene Name | Chr | Start | End | Biotype | | --- | --- | --- | --- | --- | --- | | ENSDARG00000091086 | ALX3 | 8 | 24777393 | 24788908 | protein\_coding | | ENSDARG00000041464 | CABZ01047424.1 | 16 | 53894398 | 53896034 | pseudogene | | ENSDARG00000035538 | atp6v0a2a | 10 | 8030046 | 8074768 | protein\_coding | | ENSDARG00000006128 | cep170aa | 13 | 10849304 | 10940489 | protein\_coding | | ENSDARG00000101677 | chrnb4 | 18 | 50630376 | 50638871 | protein\_coding | | ENSDARG00000013125 | dlx1a | 9 | 3416570 | 3418577 | protein\_coding | | ENSDARG00000032083 | dpysl2b | 10 | 18994837 | 19035037 | protein\_coding | | ENSDARG00000014420 | elavl3 | 3 | 48848938 | 48865522 | protein\_coding | | ENSDARG00000006773 | elovl4a | 16 | 5256794 | 5267654 | protein\_coding | | ENSDARG00000098730 | eng1b | 1 | 6995895 | 7000026 | protein\_coding | | ENSDARG00000062017 | fsd1 | 8 | 18593664 | 18617163 | protein\_coding | | ENSDARG00000087704 | gfra3 | 14 | 7145893 | 7215035 | protein\_coding | | ENSDARG00000070338 | hoxc4a | 23 | 36032080 | 36033760 | protein\_coding | | ENSDARG00000031222 | lhx2b | 8 | 3026371 | 3042643 | protein\_coding | | ENSDARG00000056181 | ncam1a | 21 | 23297510 | 23710342 | protein\_coding | | ENSDARG00000062147 | otc | 9 | 30457294 | 30463666 | protein\_coding | | ENSDARG00000032578 | pax2b | 12 | 45637903 | 45714171 | protein\_coding | | ENSDARG00000091029 | phox2bb | 14 | 16759662 | 16763059 | protein\_coding | | ENSDARG00000092354 | si:ch211-284e13.6 | 5 | 37510231 | 37521973 | protein\_coding | | ENSDARG00000099446 | slit1b | 22 | 34867222 | 34961840 | protein\_coding | | ENSDARG00000037997 | tubb5 | 16 | 10427991 | 10436718 | protein\_coding | | ENSDARG00000070809 | znf516 | 19 | 22113826 | 22182138 | protein\_coding | |
