# Supplementary material for: A high-resolution mRNA expression time course of embryonic development in zebrafish
Source: eLife. 2017 Nov 16;6:e30860. doi: 10.7554/eLife.30860 (PMC5690287; doi:10.7554/eLife.30860)
Supplement: Supplementary file 6. [file elife-30860-supp6.zip › biolayout-clusters-files/Cluster057-genes.html]

Cluster057


# Cluster057: Genes

| | Ensembl ID | Gene Name | Chr | Start | End | Biotype | | --- | --- | --- | --- | --- | --- | | ENSDARG00000075990 | acap3a | 23 | 36765033 | 36812314 | protein\_coding | | ENSDARG00000039456 | acbd6 | 8 | 14343567 | 14404107 | protein\_coding | | ENSDARG00000011727 | b9d1 | 3 | 39549667 | 39554208 | protein\_coding | | ENSDARG00000005092 | cep89 | 7 | 38123939 | 38228826 | protein\_coding | | ENSDARG00000017591 | fat1a | 1 | 16683985 | 16831997 | protein\_coding | | ENSDARG00000004372 | fmnl3 | 23 | 25428411 | 25522331 | protein\_coding | | ENSDARG00000040157 | glt8d1 | 11 | 3951118 | 3968498 | protein\_coding | | ENSDARG00000007034 | hnrpkl | 5 | 54933638 | 54955030 | protein\_coding | | ENSDARG00000070343 | hoxc6a | 23 | 36016455 | 36018761 | protein\_coding | | ENSDARG00000070346 | hoxc8a | 23 | 36002382 | 36004832 | protein\_coding | | ENSDARG00000086645 | hs3st3b1b | 12 | 36797210 | 36831559 | protein\_coding | | ENSDARG00000004363 | lhfp | 10 | 32007478 | 32094403 | protein\_coding | | ENSDARG00000089878 | nedd9 | 19 | 3713078 | 3766896 | protein\_coding | | ENSDARG00000038446 | nrp2b | 9 | 14396367 | 14533631 | protein\_coding | | ENSDARG00000059746 | plod1a | 8 | 47861081 | 47906825 | protein\_coding | | ENSDARG00000074750 | qser1 | 18 | 45680536 | 45701025 | protein\_coding | | ENSDARG00000056150 | rbms2b | 23 | 25782246 | 25842224 | protein\_coding | | ENSDARG00000095268 | si:dkey-261h17.1 | 23 | 32573385 | 32606081 | protein\_coding | | ENSDARG00000054879 | six3b | 12 | 25509325 | 25511981 | protein\_coding | | ENSDARG00000103730 | vgll4b | 11 | 743125 | 937615 | protein\_coding | | ENSDARG00000014796 | wnt11r | 10 | 32160445 | 32179344 | protein\_coding | |
