# Supplementary material for: A high-resolution mRNA expression time course of embryonic development in zebrafish
Source: eLife. 2017 Nov 16;6:e30860. doi: 10.7554/eLife.30860 (PMC5690287; doi:10.7554/eLife.30860)
Supplement: Supplementary file 6. [file elife-30860-supp6.zip › biolayout-clusters-files/Cluster058-genes.html]

Cluster058


# Cluster058: Genes

| | Ensembl ID | Gene Name | Chr | Start | End | Biotype | | --- | --- | --- | --- | --- | --- | | ENSDARG00000101347 | ENSDARG00000101347 | KN149909.1 | 22449 | 27319 | protein\_coding | | ENSDARG00000052928 | arf6b | 17 | 45748613 | 45751207 | protein\_coding | | ENSDARG00000087937 | cdk4 | 11 | 170968 | 179157 | protein\_coding | | ENSDARG00000103672 | cirbpa | 2 | 58654657 | 58662360 | protein\_coding | | ENSDARG00000045776 | cnbpa | 23 | 4763271 | 4769380 | protein\_coding | | ENSDARG00000077776 | csnk2b | 19 | 27146075 | 27152123 | protein\_coding | | ENSDARG00000003564 | dohh | 8 | 20820910 | 20826591 | protein\_coding | | ENSDARG00000006200 | eif4g1a | 2 | 17130062 | 17170539 | protein\_coding | | ENSDARG00000103799 | kars | 7 | 67206105 | 67225532 | protein\_coding | | ENSDARG00000055360 | llph | 4 | 22576864 | 22579351 | protein\_coding | | ENSDARG00000089626 | ptges3b | 23 | 6802144 | 6815848 | protein\_coding | | ENSDARG00000057026 | ran | 14 | 21388292 | 21396669 | protein\_coding | | ENSDARG00000101877 | rbm34 | 11 | 44354989 | 44373137 | protein\_coding | | ENSDARG00000098934 | rrp7a | 3 | 1450695 | 1453600 | protein\_coding | | ENSDARG00000052344 | tbl3 | 3 | 61936766 | 61958022 | protein\_coding | | ENSDARG00000100264 | thumpd1 | 1 | 58479921 | 58483848 | protein\_coding | | ENSDARG00000041391 | tmed10 | 20 | 46632878 | 46638114 | protein\_coding | | ENSDARG00000101323 | trnau1apa | 19 | 14056883 | 14071017 | protein\_coding | | ENSDARG00000090309 | ubxn1 | 14 | 46484350 | 46495313 | protein\_coding | | ENSDARG00000105012 | utp23 | 19 | 46371396 | 46377098 | protein\_coding | | ENSDARG00000015161 | zgc:92664 | 7 | 25801921 | 25805385 | protein\_coding | |
