# Supplementary material for: A high-resolution mRNA expression time course of embryonic development in zebrafish
Source: eLife. 2017 Nov 16;6:e30860. doi: 10.7554/eLife.30860 (PMC5690287; doi:10.7554/eLife.30860)
Supplement: Supplementary file 6. [file elife-30860-supp6.zip › biolayout-clusters-files/Cluster059-genes.html]

Cluster059


# Cluster059: Genes

| | Ensembl ID | Gene Name | Chr | Start | End | Biotype | | --- | --- | --- | --- | --- | --- | | ENSDARG00000036767 | ENSDARG00000036767 | 19 | 7763184 | 7772050 | protein\_coding | | ENSDARG00000045089 | ENSDARG00000045089 | 13 | 20248410 | 20250503 | protein\_coding | | ENSDARG00000074642 | FO704620.1 | 12 | 45932328 | 45952111 | protein\_coding | | ENSDARG00000062947 | amn | 3 | 56808754 | 56810392 | protein\_coding | | ENSDARG00000087359 | c3a.2 | 1 | 55560378 | 55597435 | protein\_coding | | ENSDARG00000052207 | c3a.3 | 1 | 55560378 | 55651273 | protein\_coding | | ENSDARG00000094511 | ccl20b | 24 | 25861689 | 25864571 | protein\_coding | | ENSDARG00000004954 | grna | 3 | 29783585 | 29810515 | protein\_coding | | ENSDARG00000043093 | mpeg1.2 | 8 | 19325655 | 19329417 | protein\_coding | | ENSDARG00000052336 | ociad2 | 20 | 23336353 | 23345740 | protein\_coding | | ENSDARG00000079227 | plekhs1 | 12 | 30402484 | 30408327 | protein\_coding | | ENSDARG00000086947 | si:ch211-147m6.1 | 25 | 13087219 | 13092173 | protein\_coding | | ENSDARG00000074322 | si:ch211-194m7.3 | 25 | 13094886 | 13097591 | protein\_coding | | ENSDARG00000097157 | si:ch211-207n23.2 | 15 | 29660671 | 29665803 | protein\_coding | | ENSDARG00000031588 | si:dkey-239b22.1 | 12 | 38755162 | 38800368 | protein\_coding | | ENSDARG00000102895 | si:dkey-31i7.1 | 12 | 44286967 | 44297682 | protein\_coding | | ENSDARG00000097725 | si:dkey-96g2.1 | 15 | 42085706 | 42090626 | protein\_coding | | ENSDARG00000007769 | sult5a1 | 7 | 56128616 | 56305341 | protein\_coding | | ENSDARG00000104829 | zgc:112146 | 3 | 44928328 | 44933917 | protein\_coding | | ENSDARG00000052779 | zgc:153932 | 12 | 45959768 | 45985231 | protein\_coding | |
