# Supplementary material for: A high-resolution mRNA expression time course of embryonic development in zebrafish
Source: eLife. 2017 Nov 16;6:e30860. doi: 10.7554/eLife.30860 (PMC5690287; doi:10.7554/eLife.30860)
Supplement: Supplementary file 6. [file elife-30860-supp6.zip › biolayout-clusters-files/Cluster060-genes.html]

Cluster060


# Cluster060: Genes

| | Ensembl ID | Gene Name | Chr | Start | End | Biotype | | --- | --- | --- | --- | --- | --- | | ENSDARG00000099301 | ENSDARG00000099301 | KN149781.1 | 4959 | 8644 | protein\_coding | | ENSDARG00000102015 | MSI1 (1 of many) | 5 | 5907326 | 5942427 | protein\_coding | | ENSDARG00000038557 | anks3 | 24 | 37677118 | 37798123 | protein\_coding | | ENSDARG00000006487 | anp32a | 7 | 33074474 | 33080382 | protein\_coding | | ENSDARG00000025535 | clint1a | 14 | 35029665 | 35048201 | protein\_coding | | ENSDARG00000103783 | cyyr1 | 1 | 665125 | 674564 | protein\_coding | | ENSDARG00000020850 | eef1a1l1 | 19 | 43523334 | 43539373 | protein\_coding | | ENSDARG00000075612 | ercc6 | 13 | 30743721 | 30779522 | protein\_coding | | ENSDARG00000020258 | ewsr1a | 10 | 8016885 | 8029717 | protein\_coding | | ENSDARG00000089302 | hnrnpa0a | 14 | 23980311 | 23983166 | protein\_coding | | ENSDARG00000042029 | mbd3b | 22 | 20267550 | 20284447 | protein\_coding | | ENSDARG00000102184 | mib1 | 2 | 4233463 | 4320343 | protein\_coding | | ENSDARG00000045527 | nr2c1 | 4 | 25840714 | 25869810 | protein\_coding | | ENSDARG00000076542 | pogzb | 16 | 38168972 | 38186638 | protein\_coding | | ENSDARG00000006242 | ptp4a1 | 11 | 11284208 | 11291298 | protein\_coding | | ENSDARG00000068432 | si:dkey-56m19.5 | 7 | 44373571 | 44378272 | protein\_coding | | ENSDARG00000101085 | ss18l2 | 18 | 439471 | 441719 | protein\_coding | | ENSDARG00000058696 | tbl1xr1a | 11 | 8557912 | 8674362 | protein\_coding | | ENSDARG00000015966 | yaf2 | 4 | 13906673 | 13922357 | protein\_coding | | ENSDARG00000004757 | ybx1 | 8 | 47153363 | 47161620 | protein\_coding | |
