# Supplementary material for: A high-resolution mRNA expression time course of embryonic development in zebrafish
Source: eLife. 2017 Nov 16;6:e30860. doi: 10.7554/eLife.30860 (PMC5690287; doi:10.7554/eLife.30860)
Supplement: Supplementary file 6. [file elife-30860-supp6.zip › biolayout-clusters-files/Cluster061-genes.html]

Cluster061


# Cluster061: Genes

| | Ensembl ID | Gene Name | Chr | Start | End | Biotype | | --- | --- | --- | --- | --- | --- | | ENSDARG00000043631 | bcas2 | 8 | 10971058 | 10979061 | protein\_coding | | ENSDARG00000016691 | cd9b | 4 | 4226081 | 4252760 | protein\_coding | | ENSDARG00000020311 | cnih1 | 13 | 36466362 | 36481109 | protein\_coding | | ENSDARG00000008370 | csnk1da | 3 | 36043605 | 36080746 | protein\_coding | | ENSDARG00000055120 | ctsba | 17 | 32670420 | 32679003 | protein\_coding | | ENSDARG00000006924 | fbxo38 | 14 | 38316533 | 38349449 | protein\_coding | | ENSDARG00000052190 | fdx1l | 6 | 130711 | 134907 | protein\_coding | | ENSDARG00000032129 | gtf2f1 | 3 | 33792036 | 33811811 | protein\_coding | | ENSDARG00000104889 | kpnb1 | 19 | 48364180 | 48377425 | protein\_coding | | ENSDARG00000071426 | lrrc59 | 12 | 31668164 | 31672959 | protein\_coding | | ENSDARG00000042056 | mau2 | 2 | 58790298 | 58805176 | protein\_coding | | ENSDARG00000054903 | mta3 | 12 | 25132439 | 25193150 | protein\_coding | | ENSDARG00000007377 | odc1 | 17 | 51654873 | 51668516 | protein\_coding | | ENSDARG00000101018 | ptdss2 | 25 | 7358771 | 7389283 | protein\_coding | | ENSDARG00000044380 | rbmx2 | 21 | 38591273 | 38600037 | protein\_coding | | ENSDARG00000030949 | srprb | 15 | 2547189 | 2556068 | protein\_coding | | ENSDARG00000037777 | stk16 | 1 | 4724634 | 4736456 | protein\_coding | | ENSDARG00000024416 | twistnb | 19 | 2213210 | 2222343 | protein\_coding | | ENSDARG00000023303 | zgc:66427 | 2 | 37884423 | 37892190 | protein\_coding | |
