# Supplementary material for: A high-resolution mRNA expression time course of embryonic development in zebrafish
Source: eLife. 2017 Nov 16;6:e30860. doi: 10.7554/eLife.30860 (PMC5690287; doi:10.7554/eLife.30860)
Supplement: Supplementary file 6. [file elife-30860-supp6.zip › biolayout-clusters-files/Cluster062-genes.html]

Cluster062


# Cluster062: Genes

| | Ensembl ID | Gene Name | Chr | Start | End | Biotype | | --- | --- | --- | --- | --- | --- | | ENSDARG00000056862 | cep63 | 6 | 27906072 | 27934909 | protein\_coding | | ENSDARG00000054264 | cog4 | 7 | 28752976 | 28768127 | protein\_coding | | ENSDARG00000061372 | cog7 | 24 | 37149730 | 37178223 | protein\_coding | | ENSDARG00000062834 | ecsit | 7 | 17553356 | 17564948 | protein\_coding | | ENSDARG00000053070 | gosr2 | 3 | 37507578 | 37519002 | protein\_coding | | ENSDARG00000024184 | narf | 12 | 33359811 | 33373046 | protein\_coding | | ENSDARG00000068894 | nrip1b | 10 | 38312798 | 38372376 | protein\_coding | | ENSDARG00000060785 | pcnxl4 | 13 | 31390087 | 31405058 | protein\_coding | | ENSDARG00000039490 | pitpnaa | 15 | 28477788 | 28492135 | protein\_coding | | ENSDARG00000042728 | plaa | 7 | 61666888 | 61693676 | protein\_coding | | ENSDARG00000032704 | qrsl1 | 17 | 25545588 | 25557606 | protein\_coding | | ENSDARG00000040959 | rabl3 | 9 | 32838330 | 32841673 | protein\_coding | | ENSDARG00000042833 | rffl | 15 | 4558093 | 4589616 | protein\_coding | | ENSDARG00000005547 | rint1 | 4 | 18511036 | 18523574 | protein\_coding | | ENSDARG00000097712 | si:ch1073-443n13.2 | 16 | 16689562 | 16695838 | lincRNA | | ENSDARG00000007976 | si:ch211-220f16.2 | 18 | 29991478 | 30043225 | protein\_coding | | ENSDARG00000040527 | siae | 18 | 36601807 | 36617685 | protein\_coding | | ENSDARG00000069301 | tmem177 | 9 | 29220153 | 29228760 | protein\_coding | | ENSDARG00000077650 | tnks | 21 | 19889405 | 20072213 | protein\_coding | |
