# Supplementary material for: A high-resolution mRNA expression time course of embryonic development in zebrafish
Source: eLife. 2017 Nov 16;6:e30860. doi: 10.7554/eLife.30860 (PMC5690287; doi:10.7554/eLife.30860)
Supplement: Supplementary file 6. [file elife-30860-supp6.zip › biolayout-clusters-files/Cluster063-genes.html]

Cluster063


# Cluster063: Genes

| | Ensembl ID | Gene Name | Chr | Start | End | Biotype | | --- | --- | --- | --- | --- | --- | | ENSDARG00000069929 | CABZ01053976.1 | 16 | 53238144 | 53303526 | protein\_coding | | ENSDARG00000058775 | SLC22A3 | 17 | 6173587 | 6191620 | protein\_coding | | ENSDARG00000016375 | asns | 19 | 26070140 | 26093779 | protein\_coding | | ENSDARG00000043716 | cldn5a | 8 | 4641497 | 4643053 | protein\_coding | | ENSDARG00000012013 | cpa6 | 24 | 18739210 | 18775152 | protein\_coding | | ENSDARG00000056029 | cyp26c1 | 17 | 19503020 | 19515492 | protein\_coding | | ENSDARG00000054454 | epha4a | 2 | 40102182 | 40170399 | protein\_coding | | ENSDARG00000053868 | etv2 | 16 | 42058781 | 42063826 | protein\_coding | | ENSDARG00000020884 | gli2b | 11 | 43577108 | 43631693 | protein\_coding | | ENSDARG00000031138 | irx3b | 25 | 35901116 | 36105780 | protein\_coding | | ENSDARG00000042282 | itga6a | 9 | 3458133 | 3514959 | protein\_coding | | ENSDARG00000040944 | ntd5 | 16 | 13678577 | 13705265 | protein\_coding | | ENSDARG00000010192 | pax3a | 2 | 47517819 | 47578737 | protein\_coding | | ENSDARG00000026395 | rfx4 | 18 | 15365605 | 15405108 | protein\_coding | | ENSDARG00000036252 | rras2 | 7 | 27562937 | 27616642 | protein\_coding | | ENSDARG00000086950 | si:ch211-269k10.2 | 16 | 53306767 | 53360547 | protein\_coding | | ENSDARG00000061845 | si:dkeyp-110e4.6 | 14 | 26128889 | 26139062 | protein\_coding | | ENSDARG00000055554 | wnt1 | 23 | 27725129 | 27731964 | protein\_coding | | ENSDARG00000021959 | zgc:101100 | 8 | 20456237 | 20459694 | protein\_coding | |
