# Supplementary material for: A high-resolution mRNA expression time course of embryonic development in zebrafish
Source: eLife. 2017 Nov 16;6:e30860. doi: 10.7554/eLife.30860 (PMC5690287; doi:10.7554/eLife.30860)
Supplement: Supplementary file 6. [file elife-30860-supp6.zip › biolayout-clusters-files/Cluster064-genes.html]

Cluster064


# Cluster064: Genes

| | Ensembl ID | Gene Name | Chr | Start | End | Biotype | | --- | --- | --- | --- | --- | --- | | ENSDARG00000019365 | KRT23 | 11 | 11582375 | 11591449 | protein\_coding | | ENSDARG00000058538 | alcamb | 15 | 19902726 | 19943255 | protein\_coding | | ENSDARG00000052731 | ankrd31 | 5 | 50355422 | 50364494 | protein\_coding | | ENSDARG00000036292 | cdx4 | 14 | 32621404 | 32624855 | protein\_coding | | ENSDARG00000089042 | foxb1a | 25 | 33668744 | 33670725 | protein\_coding | | ENSDARG00000070546 | msgn1 | 4 | 292588 | 294558 | protein\_coding | | ENSDARG00000006350 | nkx1.2la | 13 | 40644738 | 40649640 | protein\_coding | | ENSDARG00000055868 | rsl1d1 | 12 | 19262741 | 19272094 | protein\_coding | | ENSDARG00000094456 | si:ch211-105d18.8 | 20 | 51355692 | 51360284 | antisense | | ENSDARG00000074024 | si:ch211-152n14.4 | 6 | 19444281 | 19448656 | protein\_coding | | ENSDARG00000074359 | si:ch211-155k24.1 | 19 | 20559527 | 20564146 | protein\_coding | | ENSDARG00000101498 | si:ch211-155k24.1.1 | KN150451.1 | 13411 | 14916 | protein\_coding | | ENSDARG00000094197 | si:ch211-196c10.11 | 8 | 23224357 | 23228957 | protein\_coding | | ENSDARG00000102552 | si:dkey-103d23.5.1 | 18 | 5625861 | 5627378 | protein\_coding | | ENSDARG00000077877 | si:dkey-14o18.1 | 19 | 9852492 | 9857150 | protein\_coding | | ENSDARG00000079126 | si:dkey-250i3.3 | 9 | 31947924 | 31952506 | protein\_coding | | ENSDARG00000079036 | si:dkeyp-11g8.3 | 20 | 34471856 | 34476777 | protein\_coding | | ENSDARG00000006939 | tbx6l | 5 | 41680219 | 41697180 | protein\_coding | | ENSDARG00000026236 | zgc:56585 | 21 | 34801321 | 34809569 | protein\_coding | |
