# Supplementary material for: A high-resolution mRNA expression time course of embryonic development in zebrafish
Source: eLife. 2017 Nov 16;6:e30860. doi: 10.7554/eLife.30860 (PMC5690287; doi:10.7554/eLife.30860)
Supplement: Supplementary file 6. [file elife-30860-supp6.zip › biolayout-clusters-files/Cluster064.html]

Cluster064


# Cluster064: Detail

### Go to ZFA detail

## GO

| | GO ID | Description | Domain | Annotated | Expected | Observed | Adjusted p-value | Genes | Ensembl IDs | | --- | --- | --- | --- | --- | --- | --- | --- | --- | | GO:0003676 | nucleic acid binding | molecular\_function | 1873 | 3.8 | 14 | 3.6e-05 | nkx1.2la tbx6l cdx4 rsl1d1 msgn1 si:ch211-152n14.4 si:ch211-155k24.1 si:dkey-14o18.1 si:dkeyp-11g8.3 si:dkey-250i3.3 foxb1a si:ch211-196c10.11 si:ch211-155k24.1 si:dkey-103d23.5 | ENSDARG00000006350 ENSDARG00000006939 ENSDARG00000036292 ENSDARG00000055868 ENSDARG00000070546 ENSDARG00000074024 ENSDARG00000074359 ENSDARG00000077877 ENSDARG00000079036 ENSDARG00000079126 ENSDARG00000089042 ENSDARG00000094197 ENSDARG00000101498 ENSDARG00000102552 | | GO:0008270 | zinc ion binding | molecular\_function | 677 | 1.4 | 8 | 5.6e-03 | si:ch211-152n14.4 si:ch211-155k24.1 si:dkey-14o18.1 si:dkeyp-11g8.3 si:dkey-250i3.3 si:ch211-196c10.11 si:ch211-155k24.1 si:dkey-103d23.5 | ENSDARG00000074024 ENSDARG00000074359 ENSDARG00000077877 ENSDARG00000079036 ENSDARG00000079126 ENSDARG00000094197 ENSDARG00000101498 ENSDARG00000102552 | |
