# Supplementary material for: A high-resolution mRNA expression time course of embryonic development in zebrafish
Source: eLife. 2017 Nov 16;6:e30860. doi: 10.7554/eLife.30860 (PMC5690287; doi:10.7554/eLife.30860)
Supplement: Supplementary file 6. [file elife-30860-supp6.zip › biolayout-clusters-files/Cluster065-genes.html]

Cluster065


# Cluster065: Genes

| | Ensembl ID | Gene Name | Chr | Start | End | Biotype | | --- | --- | --- | --- | --- | --- | | ENSDARG00000076673 | DSP (1 of many) | 20 | 53044041 | 53077408 | protein\_coding | | ENSDARG00000036371 | acta1a | 1 | 53286191 | 53293789 | protein\_coding | | ENSDARG00000077082 | agtrap | 11 | 15905882 | 16105164 | protein\_coding | | ENSDARG00000055592 | capn2b | 22 | 26308166 | 26334019 | protein\_coding | | ENSDARG00000034211 | capn2l | 22 | 26423303 | 26646980 | protein\_coding | | ENSDARG00000030975 | ccdc80l1 | 6 | 16279783 | 16296383 | protein\_coding | | ENSDARG00000054616 | cldni | 3 | 30790404 | 30795479 | protein\_coding | | ENSDARG00000018688 | elk3 | 4 | 7644184 | 7705036 | protein\_coding | | ENSDARG00000095774 | emd | 23 | 19887334 | 19895880 | protein\_coding | | ENSDARG00000069912 | hmga2 | 4 | 12820423 | 12863238 | protein\_coding | | ENSDARG00000055679 | mto1 | 12 | 14172527 | 14271811 | protein\_coding | | ENSDARG00000098788 | pcdh2g16 | 14 | 1976550 | 2166806 | protein\_coding | | ENSDARG00000056913 | pcolcea | 10 | 22819881 | 22834379 | protein\_coding | | ENSDARG00000037655 | pls3 | 14 | 13147113 | 13219444 | protein\_coding | | ENSDARG00000039577 | ptk2bb | 20 | 19927796 | 19964548 | protein\_coding | | ENSDARG00000000804 | rassf6 | 10 | 13251461 | 13277893 | protein\_coding | | ENSDARG00000101481 | rbp5 | 16 | 31755126 | 31763217 | protein\_coding | | ENSDARG00000027611 | sdpra | 9 | 24412199 | 24431723 | protein\_coding | | ENSDARG00000087956 | she | 16 | 22672336 | 22691334 | protein\_coding | |
