# Supplementary material for: A high-resolution mRNA expression time course of embryonic development in zebrafish
Source: eLife. 2017 Nov 16;6:e30860. doi: 10.7554/eLife.30860 (PMC5690287; doi:10.7554/eLife.30860)
Supplement: Supplementary file 6. [file elife-30860-supp6.zip › biolayout-clusters-files/Cluster066-genes.html]

Cluster066


# Cluster066: Genes

| | Ensembl ID | Gene Name | Chr | Start | End | Biotype | | --- | --- | --- | --- | --- | --- | | ENSDARG00000099280 | UTP20 | 4 | 5859348 | 5901925 | protein\_coding | | ENSDARG00000054154 | bms1 | 12 | 29125222 | 29155460 | protein\_coding | | ENSDARG00000039887 | c1qbp | 5 | 3590950 | 3604310 | protein\_coding | | ENSDARG00000104024 | ccdc137 | 3 | 12741692 | 12752160 | protein\_coding | | ENSDARG00000105286 | ddx54 | 5 | 71409979 | 71624237 | protein\_coding | | ENSDARG00000098080 | gnl2 | 16 | 33947976 | 33971847 | protein\_coding | | ENSDARG00000054540 | imp4 | 5 | 26247037 | 26251887 | protein\_coding | | ENSDARG00000045372 | ngdn | 24 | 14396594 | 14412739 | protein\_coding | | ENSDARG00000059075 | nip7 | 18 | 5229548 | 5236026 | protein\_coding | | ENSDARG00000016080 | nob1 | 18 | 5245562 | 5260451 | protein\_coding | | ENSDARG00000060027 | nom1 | 7 | 40373853 | 40386814 | protein\_coding | | ENSDARG00000104227 | nop10 | 7 | 1369427 | 1372832 | protein\_coding | | ENSDARG00000052480 | pdcd11 | 1 | 48790597 | 48836753 | protein\_coding | | ENSDARG00000040649 | prickle1a | 25 | 291701 | 325877 | protein\_coding | | ENSDARG00000014817 | ranbp1 | 5 | 18502909 | 18510797 | protein\_coding | | ENSDARG00000077544 | toe1 | 2 | 27724227 | 27730300 | protein\_coding | | ENSDARG00000056720 | utp3 | 11 | 26132765 | 26137959 | protein\_coding | | ENSDARG00000095879 | wdr46 | 8 | 53201936 | 53218175 | protein\_coding | | ENSDARG00000007217 | wdr55 | 21 | 34780324 | 34805752 | protein\_coding | |
