# Supplementary material for: A high-resolution mRNA expression time course of embryonic development in zebrafish
Source: eLife. 2017 Nov 16;6:e30860. doi: 10.7554/eLife.30860 (PMC5690287; doi:10.7554/eLife.30860)
Supplement: Supplementary file 6. [file elife-30860-supp6.zip › biolayout-clusters-files/Cluster066.html]

Cluster066


# Cluster066: Detail

### Go to ZFA detail

## GO

| | GO ID | Description | Domain | Annotated | Expected | Observed | Adjusted p-value | Genes | Ensembl IDs | | --- | --- | --- | --- | --- | --- | --- | --- | --- | | GO:0006364 | rRNA processing | biological\_process | 85 | 0.16 | 9 | 5.8e-05 | wdr55 nob1 ngdn bms1 imp4 utp3 wdr46 gnl2 nop10 | ENSDARG00000007217 ENSDARG00000016080 ENSDARG00000045372 ENSDARG00000054154 ENSDARG00000054540 ENSDARG00000056720 ENSDARG00000095879 ENSDARG00000098080 ENSDARG00000104227 | | GO:0000469 | cleavage involved in rRNA processing | biological\_process | 10 | 0.02 | 2 | 3.6e-02 | nob1 gnl2 | ENSDARG00000016080 ENSDARG00000098080 | | GO:0000462 | maturation of SSU-rRNA from tricistronic... | biological\_process | 21 | 0.04 | 3 | 1.8e-03 | ngdn utp3 wdr46 | ENSDARG00000045372 ENSDARG00000056720 ENSDARG00000095879 | | GO:0032040 | small-subunit processome | cellular\_component | 29 | 0.05 | 5 | 3.6e-07 | ngdn pdcd11 imp4 utp3 wdr46 | ENSDARG00000045372 ENSDARG00000052480 ENSDARG00000054540 ENSDARG00000056720 ENSDARG00000095879 | | GO:0005730 | nucleolus | cellular\_component | 111 | 0.21 | 9 | 7.1e-11 | wdr55 ngdn pdcd11 imp4 utp3 nip7 wdr46 gnl2 nop10 | ENSDARG00000007217 ENSDARG00000045372 ENSDARG00000052480 ENSDARG00000054540 ENSDARG00000056720 ENSDARG00000059075 ENSDARG00000095879 ENSDARG00000098080 ENSDARG00000104227 | | GO:0030515 | snoRNA binding | molecular\_function | 14 | 0.02 | 2 | 4.3e-02 | imp4 nop10 | ENSDARG00000054540 ENSDARG00000104227 | |
