# Supplementary material for: A high-resolution mRNA expression time course of embryonic development in zebrafish
Source: eLife. 2017 Nov 16;6:e30860. doi: 10.7554/eLife.30860 (PMC5690287; doi:10.7554/eLife.30860)
Supplement: Supplementary file 6. [file elife-30860-supp6.zip › biolayout-clusters-files/Cluster067-genes.html]

Cluster067


# Cluster067: Genes

| | Ensembl ID | Gene Name | Chr | Start | End | Biotype | | --- | --- | --- | --- | --- | --- | | ENSDARG00000105053 | ENSDARG00000105053 | KN150365.1 | 3552 | 6428 | protein\_coding | | ENSDARG00000026052 | ccdc3 | 4 | 7993245 | 8005834 | protein\_coding | | ENSDARG00000070868 | cfap126 | 23 | 20050777 | 20055470 | protein\_coding | | ENSDARG00000098294 | col5a3a | 3 | 53518593 | 53664800 | protein\_coding | | ENSDARG00000069692 | col7a1l | 4 | 15890682 | 15967505 | protein\_coding | | ENSDARG00000077403 | col8a1a | 9 | 30279910 | 30292032 | protein\_coding | | ENSDARG00000062262 | ednrab | 23 | 45958860 | 45970337 | protein\_coding | | ENSDARG00000094324 | efemp2a | 14 | 30329348 | 30363761 | protein\_coding | | ENSDARG00000062892 | foxe3 | 8 | 19636569 | 19638220 | protein\_coding | | ENSDARG00000030494 | hfe2 | 16 | 42980041 | 42990870 | protein\_coding | | ENSDARG00000078280 | nkx3-1 | 8 | 50268284 | 50270802 | protein\_coding | | ENSDARG00000001721 | oc90 | 2 | 43093134 | 43110935 | protein\_coding | | ENSDARG00000014358 | optc | 11 | 22952865 | 22966343 | protein\_coding | | ENSDARG00000037383 | pax5 | 1 | 21032884 | 21122617 | protein\_coding | | ENSDARG00000097268 | si:ch73-171a6.2 | 24 | 39225512 | 39226377 | lincRNA | | ENSDARG00000093135 | si:dkey-117n7.2 | 4 | 15943320 | 15945092 | protein\_coding | | ENSDARG00000092064 | si:dkey-117n7.4 | 4 | 15955538 | 15965275 | protein\_coding | | ENSDARG00000094973 | wnt16 | 4 | 10707906 | 10743490 | protein\_coding | | ENSDARG00000007184 | zbtb16a | 21 | 23124769 | 23271216 | protein\_coding | |
