# Supplementary material for: A high-resolution mRNA expression time course of embryonic development in zebrafish
Source: eLife. 2017 Nov 16;6:e30860. doi: 10.7554/eLife.30860 (PMC5690287; doi:10.7554/eLife.30860)
Supplement: Supplementary file 6. [file elife-30860-supp6.zip › biolayout-clusters-files/Cluster067.html]

Cluster067


# Cluster067: Detail

### Go to ZFA detail

## GO

| | GO ID | Description | Domain | Annotated | Expected | Observed | Adjusted p-value | Genes | Ensembl IDs | | --- | --- | --- | --- | --- | --- | --- | --- | --- | | GO:0005581 | collagen trimer | cellular\_component | 41 | 0.06 | 3 | 0.0074 | col7a1l col8a1a col5a3a | ENSDARG00000069692 ENSDARG00000077403 ENSDARG00000098294 | |
