# Supplementary material for: A high-resolution mRNA expression time course of embryonic development in zebrafish
Source: eLife. 2017 Nov 16;6:e30860. doi: 10.7554/eLife.30860 (PMC5690287; doi:10.7554/eLife.30860)
Supplement: Supplementary file 6. [file elife-30860-supp6.zip › biolayout-clusters-files/Cluster068-genes.html]

Cluster068


# Cluster068: Genes

| | Ensembl ID | Gene Name | Chr | Start | End | Biotype | | --- | --- | --- | --- | --- | --- | | ENSDARG00000087950 | IWS1 | 15 | 46398784 | 46439142 | protein\_coding | | ENSDARG00000009484 | arf1 | 2 | 3220758 | 3235048 | protein\_coding | | ENSDARG00000037009 | banf1 | 21 | 26673106 | 26678575 | protein\_coding | | ENSDARG00000053912 | fbl | 16 | 42227627 | 42236061 | protein\_coding | | ENSDARG00000005551 | hnrnph1l | 21 | 30132915 | 30145069 | protein\_coding | | ENSDARG00000098507 | lyar | 14 | 219860 | 221783 | protein\_coding | | ENSDARG00000012820 | nop56 | 21 | 11866130 | 11877571 | protein\_coding | | ENSDARG00000014329 | npm1a | 10 | 22065025 | 22075667 | protein\_coding | | ENSDARG00000078473 | nucks1a | 11 | 38272933 | 38287189 | protein\_coding | | ENSDARG00000098783 | polr2b | 14 | 51664725 | 51693123 | protein\_coding | | ENSDARG00000038505 | polr2h | 22 | 36554983 | 36561348 | protein\_coding | | ENSDARG00000043854 | ppil4 | 20 | 2956250 | 2991286 | protein\_coding | | ENSDARG00000097478 | qkia | 17 | 27706527 | 27797172 | protein\_coding | | ENSDARG00000042590 | rbm19 | 6 | 45842625 | 45867245 | protein\_coding | | ENSDARG00000010487 | sae1 | 15 | 23787355 | 23803839 | protein\_coding | | ENSDARG00000003920 | setb | 21 | 13130056 | 13133937 | protein\_coding | | ENSDARG00000105293 | srsf4 | 19 | 13492437 | 13502024 | protein\_coding | | ENSDARG00000105412 | zgc:194285.1 | 14 | 46621284 | 46627586 | protein\_coding | |
