# Supplementary material for: A high-resolution mRNA expression time course of embryonic development in zebrafish
Source: eLife. 2017 Nov 16;6:e30860. doi: 10.7554/eLife.30860 (PMC5690287; doi:10.7554/eLife.30860)
Supplement: Supplementary file 6. [file elife-30860-supp6.zip › biolayout-clusters-files/Cluster068.html]

Cluster068


# Cluster068: Detail

### Go to ZFA detail

## GO

| | GO ID | Description | Domain | Annotated | Expected | Observed | Adjusted p-value | Genes | Ensembl IDs | | --- | --- | --- | --- | --- | --- | --- | --- | --- | | GO:0000154 | rRNA modification | biological\_process | 13 | 0.02 | 2 | 0.036 | nop56 fbl | ENSDARG00000012820 ENSDARG00000053912 | | GO:0005732 | small nucleolar ribonucleoprotein comple... | cellular\_component | 11 | 0.02 | 2 | 0.030 | nop56 fbl | ENSDARG00000012820 ENSDARG00000053912 | | GO:0044452 | nucleolar part | cellular\_component | 31 | 0.05 | 3 | 0.003 | nop56 polr2h fbl | ENSDARG00000012820 ENSDARG00000038505 ENSDARG00000053912 | | GO:0005665 | DNA-directed RNA polymerase II, core com... | cellular\_component | 11 | 0.02 | 2 | 0.030 | polr2h polr2b | ENSDARG00000038505 ENSDARG00000098783 | |
