# Supplementary material for: A high-resolution mRNA expression time course of embryonic development in zebrafish
Source: eLife. 2017 Nov 16;6:e30860. doi: 10.7554/eLife.30860 (PMC5690287; doi:10.7554/eLife.30860)
Supplement: Supplementary file 6. [file elife-30860-supp6.zip › biolayout-clusters-files/Cluster069-genes.html]

Cluster069


# Cluster069: Genes

| | Ensembl ID | Gene Name | Chr | Start | End | Biotype | | --- | --- | --- | --- | --- | --- | | ENSDARG00000074223 | MDFIC | 4 | 6331002 | 6407585 | protein\_coding | | ENSDARG00000088357 | acadl | 18 | 39506478 | 39524839 | protein\_coding | | ENSDARG00000061634 | aifm4 | 15 | 25503449 | 25513529 | protein\_coding | | ENSDARG00000031387 | cers4a | 22 | 4323288 | 4348731 | protein\_coding | | ENSDARG00000060316 | cish | 6 | 41506318 | 41509213 | protein\_coding | | ENSDARG00000043081 | ctsz | 6 | 49772719 | 49782709 | protein\_coding | | ENSDARG00000018966 | cyb5r1 | 11 | 24548762 | 24562059 | protein\_coding | | ENSDARG00000026875 | fam57ba | 3 | 20892773 | 20913038 | protein\_coding | | ENSDARG00000061472 | gba2 | 7 | 24374029 | 24402239 | protein\_coding | | ENSDARG00000035872 | hsd17b12b | 7 | 49948761 | 50000097 | protein\_coding | | ENSDARG00000057626 | josd2 | 16 | 13793687 | 13816650 | protein\_coding | | ENSDARG00000101234 | nsmfb | 5 | 27833062 | 27890461 | protein\_coding | | ENSDARG00000056121 | serac1 | 20 | 26149214 | 26161156 | protein\_coding | | ENSDARG00000062515 | tecpr1a | 12 | 17638446 | 17688665 | protein\_coding | | ENSDARG00000061621 | them4 | 16 | 17857144 | 17891708 | protein\_coding | | ENSDARG00000037409 | tmem144a | 1 | 20620228 | 20628545 | protein\_coding | | ENSDARG00000062973 | ttll7 | 11 | 7971404 | 8116424 | protein\_coding | | ENSDARG00000035630 | ypel1 | 5 | 12536889 | 12586399 | protein\_coding | |
