# Supplementary material for: A high-resolution mRNA expression time course of embryonic development in zebrafish
Source: eLife. 2017 Nov 16;6:e30860. doi: 10.7554/eLife.30860 (PMC5690287; doi:10.7554/eLife.30860)
Supplement: Supplementary file 6. [file elife-30860-supp6.zip › biolayout-clusters-files/Cluster070-genes.html]

Cluster070


# Cluster070: Genes

| | Ensembl ID | Gene Name | Chr | Start | End | Biotype | | --- | --- | --- | --- | --- | --- | | ENSDARG00000089598 | CABZ01054396.1 | 4 | 70897882 | 70912559 | protein\_coding | | ENSDARG00000075545 | ENSDARG00000075545 | 19 | 5218138 | 5235125 | protein\_coding | | ENSDARG00000055561 | c1galt1b | 6 | 39192866 | 39201534 | protein\_coding | | ENSDARG00000007045 | cnot4a | 25 | 19906078 | 19927200 | protein\_coding | | ENSDARG00000074688 | fbrs | 12 | 26943400 | 26969976 | protein\_coding | | ENSDARG00000062511 | golga3 | 5 | 18460426 | 18502505 | protein\_coding | | ENSDARG00000090895 | kansl1a | 12 | 4651133 | 4690648 | protein\_coding | | ENSDARG00000018559 | kdm7ab | 25 | 17148132 | 17213901 | protein\_coding | | ENSDARG00000075639 | si:ch211-89o9.6 | 1 | 40784552 | 40789989 | protein\_coding | | ENSDARG00000056847 | si:dkey-27c15.3 | 19 | 27094690 | 27139146 | protein\_coding | | ENSDARG00000037073 | si:dkey-60a16.1 | 20 | 5022355 | 5027411 | protein\_coding | | ENSDARG00000041081 | suv420h1 | 18 | 21124150 | 21131152 | protein\_coding | | ENSDARG00000052170 | uap1 | 6 | 35052811 | 35068789 | protein\_coding | | ENSDARG00000017886 | zbtb11 | 6 | 29154215 | 29169327 | protein\_coding | | ENSDARG00000014775 | zgc:113220 | 22 | 2392909 | 2404827 | protein\_coding | | ENSDARG00000032285 | zgc:113294 | 15 | 855753 | 861668 | protein\_coding | | ENSDARG00000091869 | zgc:171727 | 4 | 71652635 | 71770329 | protein\_coding | | ENSDARG00000076988 | znf839 | 17 | 31606393 | 31622882 | protein\_coding | |
