# Supplementary material for: A high-resolution mRNA expression time course of embryonic development in zebrafish
Source: eLife. 2017 Nov 16;6:e30860. doi: 10.7554/eLife.30860 (PMC5690287; doi:10.7554/eLife.30860)
Supplement: Supplementary file 6. [file elife-30860-supp6.zip › biolayout-clusters-files/Cluster070.html]

Cluster070


# Cluster070: Detail

### Go to ZFA detail

## GO

| | GO ID | Description | Domain | Annotated | Expected | Observed | Adjusted p-value | Genes | Ensembl IDs | | --- | --- | --- | --- | --- | --- | --- | --- | --- | | GO:0003676 | nucleic acid binding | molecular\_function | 1873 | 2.9 | 9 | 0.0071 | cnot4a zgc:113220 zbtb11 zgc:113294 si:dkey-60a16.1 si:dkey-27c15.3 ENSDARG00000075545 si:ch211-89o9.6 zgc:171727 | ENSDARG00000007045 ENSDARG00000014775 ENSDARG00000017886 ENSDARG00000032285 ENSDARG00000037073 ENSDARG00000056847 ENSDARG00000075545 ENSDARG00000075639 ENSDARG00000091869 | | GO:0046872 | metal ion binding | molecular\_function | 2036 | 3.2 | 10 | 0.0180 | cnot4a zgc:113220 zbtb11 kdm7ab zgc:113294 c1galt1b ENSDARG00000075545 si:ch211-89o9.6 znf839 zgc:171727 | ENSDARG00000007045 ENSDARG00000014775 ENSDARG00000017886 ENSDARG00000018559 ENSDARG00000032285 ENSDARG00000055561 ENSDARG00000075545 ENSDARG00000075639 ENSDARG00000076988 ENSDARG00000091869 | |
