# Supplementary material for: A high-resolution mRNA expression time course of embryonic development in zebrafish
Source: eLife. 2017 Nov 16;6:e30860. doi: 10.7554/eLife.30860 (PMC5690287; doi:10.7554/eLife.30860)
Supplement: Supplementary file 6. [file elife-30860-supp6.zip › biolayout-clusters-files/Cluster071-genes.html]

Cluster071


# Cluster071: Genes

| | Ensembl ID | Gene Name | Chr | Start | End | Biotype | | --- | --- | --- | --- | --- | --- | | ENSDARG00000036894 | AIMP1 (1 of many) | 23 | 45640856 | 45657895 | protein\_coding | | ENSDARG00000104539 | OLFML2A (1 of many) | 21 | 8050462 | 8103947 | protein\_coding | | ENSDARG00000030514 | acsl1a | 1 | 16844514 | 16894615 | protein\_coding | | ENSDARG00000055045 | casp3b | 14 | 4018384 | 4038615 | protein\_coding | | ENSDARG00000009021 | chrna1 | 6 | 10576910 | 10593496 | protein\_coding | | ENSDARG00000040045 | cldn1 | 2 | 45475 | 48529 | protein\_coding | | ENSDARG00000031483 | col9a1b | 13 | 38993570 | 39046663 | protein\_coding | | ENSDARG00000042707 | cx30.3 | 9 | 21590320 | 21594197 | protein\_coding | | ENSDARG00000045958 | egfl6 | 9 | 54688229 | 54723923 | protein\_coding | | ENSDARG00000017299 | fabp11a | 19 | 32579389 | 32581848 | protein\_coding | | ENSDARG00000062592 | myl10 | 10 | 33227953 | 33236059 | protein\_coding | | ENSDARG00000042245 | myl13 | 2 | 2628812 | 2639928 | protein\_coding | | ENSDARG00000037266 | myoz2b | 1 | 25226762 | 25245850 | protein\_coding | | ENSDARG00000070818 | pax7b | 23 | 20936394 | 21012544 | protein\_coding | | ENSDARG00000105223 | pmp22a | 3 | 49821187 | 49825090 | protein\_coding | | ENSDARG00000069823 | proca1 | 15 | 15509091 | 15513907 | protein\_coding | | ENSDARG00000100443 | si:ch211-71k24.8 | 13 | 2301030 | 2301736 | lincRNA | | ENSDARG00000036876 | zgc:153284 | 23 | 31499962 | 31509993 | protein\_coding | |
