# Supplementary material for: A high-resolution mRNA expression time course of embryonic development in zebrafish
Source: eLife. 2017 Nov 16;6:e30860. doi: 10.7554/eLife.30860 (PMC5690287; doi:10.7554/eLife.30860)
Supplement: Supplementary file 6. [file elife-30860-supp6.zip › biolayout-clusters-files/Cluster072-genes.html]

Cluster072


# Cluster072: Genes

| | Ensembl ID | Gene Name | Chr | Start | End | Biotype | | --- | --- | --- | --- | --- | --- | | ENSDARG00000056294 | SBK1 | 24 | 37473369 | 37487542 | protein\_coding | | ENSDARG00000052419 | ankrd12 | 2 | 54966494 | 55053809 | protein\_coding | | ENSDARG00000052644 | ca10a | 12 | 21105913 | 21428583 | protein\_coding | | ENSDARG00000055857 | dopey2 | 9 | 33343982 | 33384864 | protein\_coding | | ENSDARG00000058038 | eml3 | 7 | 17564352 | 17653511 | protein\_coding | | ENSDARG00000026762 | fam126a | 19 | 2672019 | 2734038 | protein\_coding | | ENSDARG00000039436 | il13ra2 | 5 | 36501224 | 36517625 | protein\_coding | | ENSDARG00000062053 | kif1bp | 13 | 22953408 | 22964556 | protein\_coding | | ENSDARG00000001898 | manea | 17 | 15650934 | 15659275 | protein\_coding | | ENSDARG00000074892 | pde6d | 6 | 29702931 | 29715434 | protein\_coding | | ENSDARG00000017757 | pik3cg | 4 | 3329844 | 3340268 | protein\_coding | | ENSDARG00000009771 | ppme1 | 21 | 25344070 | 25358625 | protein\_coding | | ENSDARG00000061656 | ptpdc1a | 11 | 27264103 | 27290487 | protein\_coding | | ENSDARG00000038859 | rgs20 | 2 | 30773096 | 30800969 | protein\_coding | | ENSDARG00000089204 | smim13 | 19 | 3777039 | 3785889 | protein\_coding | | ENSDARG00000078604 | tbc1d10b | 3 | 32657864 | 32679650 | protein\_coding | | ENSDARG00000086342 | zgc:101566 | 25 | 13936445 | 13990977 | protein\_coding | |
