# Supplementary material for: A high-resolution mRNA expression time course of embryonic development in zebrafish
Source: eLife. 2017 Nov 16;6:e30860. doi: 10.7554/eLife.30860 (PMC5690287; doi:10.7554/eLife.30860)
Supplement: Supplementary file 6. [file elife-30860-supp6.zip › biolayout-clusters-files/Cluster073-genes.html]

Cluster073


# Cluster073: Genes

| | Ensembl ID | Gene Name | Chr | Start | End | Biotype | | --- | --- | --- | --- | --- | --- | | ENSDARG00000094107 | ENSDARG00000094107 | 5 | 62595208 | 62601552 | protein\_coding | | ENSDARG00000079639 | PRRC2B | 5 | 71232141 | 71274138 | protein\_coding | | ENSDARG00000058729 | akirin2 | 20 | 2699210 | 2709191 | protein\_coding | | ENSDARG00000036956 | asxl1 | 23 | 30803590 | 30826116 | protein\_coding | | ENSDARG00000012485 | aurka | 6 | 60130110 | 60144746 | protein\_coding | | ENSDARG00000041226 | cdc40 | 20 | 53563194 | 53596582 | protein\_coding | | ENSDARG00000087759 | cnih4 | 16 | 2232276 | 2235182 | protein\_coding | | ENSDARG00000016177 | eif4enif1 | 6 | 40925036 | 40950135 | protein\_coding | | ENSDARG00000044491 | kif20a | 21 | 34845295 | 34892129 | protein\_coding | | ENSDARG00000045515 | kin | 4 | 25192374 | 25199231 | protein\_coding | | ENSDARG00000099972 | nup153 | 19 | 46428693 | 46468343 | protein\_coding | | ENSDARG00000055291 | rab18a | 12 | 22800258 | 22812305 | protein\_coding | | ENSDARG00000097414 | si:ch211-106e7.2 | 25 | 28419458 | 28449680 | protein\_coding | | ENSDARG00000017744 | smc2 | 1 | 18842183 | 18868864 | protein\_coding | | ENSDARG00000074623 | tbc1d31 | 16 | 25381357 | 25400104 | protein\_coding | | ENSDARG00000088318 | ubap2a | 21 | 11824160 | 11862685 | protein\_coding | | ENSDARG00000078355 | zc3h4 | 15 | 23774064 | 23786353 | protein\_coding | |
