# Supplementary material for: A high-resolution mRNA expression time course of embryonic development in zebrafish
Source: eLife. 2017 Nov 16;6:e30860. doi: 10.7554/eLife.30860 (PMC5690287; doi:10.7554/eLife.30860)
Supplement: Supplementary file 6. [file elife-30860-supp6.zip › biolayout-clusters-files/Cluster074-genes.html]

Cluster074


# Cluster074: Genes

| | Ensembl ID | Gene Name | Chr | Start | End | Biotype | | --- | --- | --- | --- | --- | --- | | ENSDARG00000098431 | COMP | 11 | 6934461 | 6964183 | protein\_coding | | ENSDARG00000045438 | ENSDARG00000045438 | 24 | 4402486 | 4408464 | protein\_coding | | ENSDARG00000030176 | ITGB1BP2 | 7 | 23456612 | 23474827 | protein\_coding | | ENSDARG00000079462 | PTGES3L | 12 | 5969131 | 5973281 | protein\_coding | | ENSDARG00000037558 | asb12b | 14 | 8959899 | 8975218 | protein\_coding | | ENSDARG00000086647 | chrng | 2 | 45228491 | 45244745 | protein\_coding | | ENSDARG00000005762 | col14a1a | 16 | 14794796 | 15160722 | protein\_coding | | ENSDARG00000001859 | dbx1b | 25 | 14411167 | 14414082 | protein\_coding | | ENSDARG00000044588 | emp2 | 3 | 26896939 | 26925338 | protein\_coding | | ENSDARG00000018820 | flncb | 4 | 10065903 | 10131044 | protein\_coding | | ENSDARG00000010478 | hsp90aa1.1 | 20 | 54192871 | 54222597 | protein\_coding | | ENSDARG00000075463 | mss51 | 24 | 39213356 | 39222136 | protein\_coding | | ENSDARG00000070486 | rbp7b | 6 | 45930359 | 45931486 | protein\_coding | | ENSDARG00000104431 | slc1a3a | 5 | 7606712 | 7666984 | protein\_coding | | ENSDARG00000074002 | slc6a11a | 6 | 40631530 | 40648667 | protein\_coding | | ENSDARG00000069388 | tmem88b | 7 | 21521169 | 21540116 | protein\_coding | | ENSDARG00000089235 | tspearb | 9 | 12625610 | 12653528 | protein\_coding | |
