# Supplementary material for: A high-resolution mRNA expression time course of embryonic development in zebrafish
Source: eLife. 2017 Nov 16;6:e30860. doi: 10.7554/eLife.30860 (PMC5690287; doi:10.7554/eLife.30860)
Supplement: Supplementary file 6. [file elife-30860-supp6.zip › biolayout-clusters-files/Cluster075-genes.html]

Cluster075


# Cluster075: Genes

| | Ensembl ID | Gene Name | Chr | Start | End | Biotype | | --- | --- | --- | --- | --- | --- | | ENSDARG00000056379 | ENSDARG00000056379 | 16 | 21975668 | 21981009 | protein\_coding | | ENSDARG00000074930 | ENSDARG00000074930 | 19 | 11061985 | 11068663 | protein\_coding | | ENSDARG00000078878 | METTL21C (1 of many).1 | 1 | 458829 | 466986 | protein\_coding | | ENSDARG00000089924 | aldh2.1 | 5 | 68802115 | 68848178 | protein\_coding | | ENSDARG00000009196 | anxa3a | 5 | 38487211 | 38495470 | protein\_coding | | ENSDARG00000024141 | cav3 | 6 | 41998530 | 42006244 | protein\_coding | | ENSDARG00000055160 | chadla | 3 | 24978420 | 24989291 | protein\_coding | | ENSDARG00000002831 | col4a4 | 15 | 36093919 | 36197432 | protein\_coding | | ENSDARG00000058656 | desma | 9 | 7537473 | 7560935 | protein\_coding | | ENSDARG00000014321 | eng1a | 9 | 781413 | 785636 | protein\_coding | | ENSDARG00000056464 | fitm1 | 2 | 24649203 | 24653031 | protein\_coding | | ENSDARG00000045142 | hbz | 12 | 20220577 | 20231667 | protein\_coding | | ENSDARG00000003732 | mitfa | 6 | 43392851 | 43435741 | protein\_coding | | ENSDARG00000096419 | si:ch211-114n24.7 | 6 | 6838125 | 6840177 | lincRNA | | ENSDARG00000096905 | si:ch73-23l24.1 | 6 | 15123732 | 15132479 | protein\_coding | | ENSDARG00000086957 | si:dkeyp-118a3.2 | 1 | 18610304 | 18638595 | protein\_coding | | ENSDARG00000100719 | urah | 18 | 31038125 | 31045193 | protein\_coding | |
