# Supplementary material for: A high-resolution mRNA expression time course of embryonic development in zebrafish
Source: eLife. 2017 Nov 16;6:e30860. doi: 10.7554/eLife.30860 (PMC5690287; doi:10.7554/eLife.30860)
Supplement: Supplementary file 6. [file elife-30860-supp6.zip › biolayout-clusters-files/Cluster076-genes.html]

Cluster076


# Cluster076: Genes

| | Ensembl ID | Gene Name | Chr | Start | End | Biotype | | --- | --- | --- | --- | --- | --- | | ENSDARG00000017049 | adsl | 3 | 24076695 | 24088079 | protein\_coding | | ENSDARG00000021193 | coro1cb | 5 | 31282956 | 31304409 | protein\_coding | | ENSDARG00000034309 | cox10 | 12 | 36716741 | 36785783 | protein\_coding | | ENSDARG00000015793 | creb3l1 | 7 | 38626663 | 38683616 | protein\_coding | | ENSDARG00000021664 | fzd3a | 20 | 35305976 | 35343281 | protein\_coding | | ENSDARG00000040556 | ift20 | 15 | 23849583 | 23854907 | protein\_coding | | ENSDARG00000023703 | kctd6b | 23 | 19743742 | 19747426 | protein\_coding | | ENSDARG00000063244 | lix1l | 19 | 7608184 | 7621902 | protein\_coding | | ENSDARG00000045836 | mapk11 | 4 | 5498376 | 5513867 | protein\_coding | | ENSDARG00000104031 | mrpl33 | 17 | 41034650 | 41044519 | protein\_coding | | ENSDARG00000045936 | pax6b | 7 | 15619410 | 15648344 | protein\_coding | | ENSDARG00000006878 | phf21aa | 7 | 38552758 | 38590524 | protein\_coding | | ENSDARG00000005645 | robo3 | 10 | 31414255 | 31619799 | protein\_coding | | ENSDARG00000079062 | samd1b | 1 | 55140002 | 55162454 | protein\_coding | | ENSDARG00000092467 | si:ch73-46j18.5 | 10 | 1680371 | 1690649 | protein\_coding | | ENSDARG00000034195 | top2b | 19 | 18229785 | 18316319 | protein\_coding | | ENSDARG00000042708 | tuba8l | 8 | 28326213 | 28330720 | protein\_coding | |
