# Supplementary material for: A high-resolution mRNA expression time course of embryonic development in zebrafish
Source: eLife. 2017 Nov 16;6:e30860. doi: 10.7554/eLife.30860 (PMC5690287; doi:10.7554/eLife.30860)
Supplement: Supplementary file 6. [file elife-30860-supp6.zip › biolayout-clusters-files/Cluster077-genes.html]

Cluster077


# Cluster077: Genes

| | Ensembl ID | Gene Name | Chr | Start | End | Biotype | | --- | --- | --- | --- | --- | --- | | ENSDARG00000044694 | ENSDARG00000044694 | 21 | 19807558 | 19829715 | protein\_coding | | ENSDARG00000006215 | akr1b1 | 4 | 14901287 | 14907761 | protein\_coding | | ENSDARG00000104491 | ampd2a | 22 | 1186157 | 1228328 | protein\_coding | | ENSDARG00000005749 | cand2 | 22 | 31096349 | 31110475 | protein\_coding | | ENSDARG00000087457 | ecscr | 21 | 29040814 | 29054735 | protein\_coding | | ENSDARG00000008414 | exoc3l2a | 5 | 36143402 | 36163241 | protein\_coding | | ENSDARG00000074283 | inpp5d | 6 | 28150255 | 28201941 | protein\_coding | | ENSDARG00000099390 | lama2 | 20 | 1883458 | 1959629 | protein\_coding | | ENSDARG00000063358 | micu1 | 13 | 4743300 | 4841101 | protein\_coding | | ENSDARG00000020143 | pah | 4 | 17402704 | 17420557 | protein\_coding | | ENSDARG00000004227 | pde3a | 4 | 2822543 | 2938001 | protein\_coding | | ENSDARG00000061304 | ppp1r9a | 19 | 41488368 | 41573823 | protein\_coding | | ENSDARG00000044808 | slc4a4b | 21 | 5887439 | 6002027 | protein\_coding | | ENSDARG00000038465 | stmn3 | 6 | 58549105 | 58569351 | protein\_coding | | ENSDARG00000000563 | ttnb | 9 | 42931205 | 43071913 | protein\_coding | | ENSDARG00000044615 | zak.1 | 6 | 6767468 | 6810419 | protein\_coding | |
