# Supplementary figures and images for: A high-resolution mRNA expression time course of embryonic development in zebrafish
Source: eLife. 2017 Nov 16;6:e30860. doi: 10.7554/eLife.30860 (PMC5690287; doi:10.7554/eLife.30860)

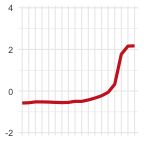

Supplement: Supplementary file 6. [file elife-30860-supp6.zip › biolayout-clusters-files/Cluster001.png]

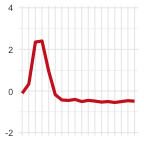

Supplement: Supplementary file 6. [file elife-30860-supp6.zip › biolayout-clusters-files/Cluster002.png]

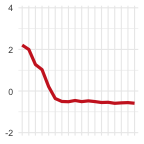

Supplement: Supplementary file 6. [file elife-30860-supp6.zip › biolayout-clusters-files/Cluster003.png]

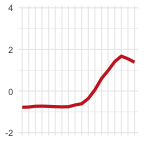

Supplement: Supplementary file 6. [file elife-30860-supp6.zip › biolayout-clusters-files/Cluster004.png]

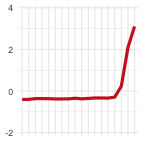

Supplement: Supplementary file 6. [file elife-30860-supp6.zip › biolayout-clusters-files/Cluster005.png]

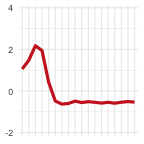

Supplement: Supplementary file 6. [file elife-30860-supp6.zip › biolayout-clusters-files/Cluster006.png]

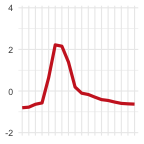

Supplement: Supplementary file 6. [file elife-30860-supp6.zip › biolayout-clusters-files/Cluster007.png]

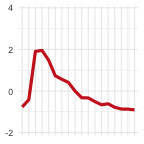

Supplement: Supplementary file 6. [file elife-30860-supp6.zip › biolayout-clusters-files/Cluster008.png]

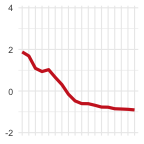

Supplement: Supplementary file 6. [file elife-30860-supp6.zip › biolayout-clusters-files/Cluster009.png]

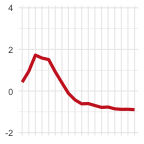

Supplement: Supplementary file 6. [file elife-30860-supp6.zip › biolayout-clusters-files/Cluster010.png]

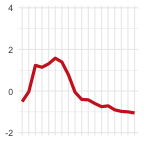

Supplement: Supplementary file 6. [file elife-30860-supp6.zip › biolayout-clusters-files/Cluster011.png]

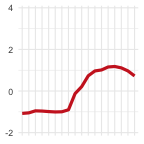

Supplement: Supplementary file 6. [file elife-30860-supp6.zip › biolayout-clusters-files/Cluster012.png]

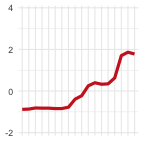

Supplement: Supplementary file 6. [file elife-30860-supp6.zip › biolayout-clusters-files/Cluster013.png]

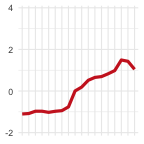

Supplement: Supplementary file 6. [file elife-30860-supp6.zip › biolayout-clusters-files/Cluster014.png]

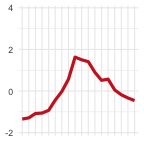

Supplement: Supplementary file 6. [file elife-30860-supp6.zip › biolayout-clusters-files/Cluster015.png]

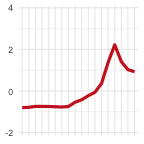

Supplement: Supplementary file 6. [file elife-30860-supp6.zip › biolayout-clusters-files/Cluster016.png]

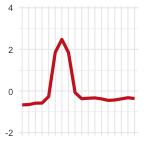

Supplement: Supplementary file 6. [file elife-30860-supp6.zip › biolayout-clusters-files/Cluster017.png]

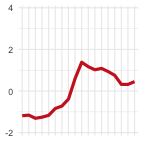

Supplement: Supplementary file 6. [file elife-30860-supp6.zip › biolayout-clusters-files/Cluster018.png]

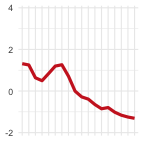

Supplement: Supplementary file 6. [file elife-30860-supp6.zip › biolayout-clusters-files/Cluster019.png]

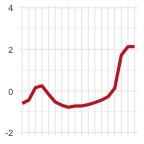

Supplement: Supplementary file 6. [file elife-30860-supp6.zip › biolayout-clusters-files/Cluster020.png]

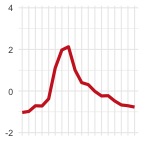

Supplement: Supplementary file 6. [file elife-30860-supp6.zip › biolayout-clusters-files/Cluster021.png]

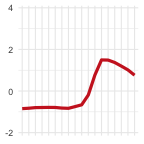

Supplement: Supplementary file 6. [file elife-30860-supp6.zip › biolayout-clusters-files/Cluster022.png]

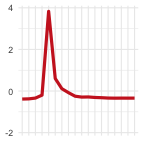

Supplement: Supplementary file 6. [file elife-30860-supp6.zip › biolayout-clusters-files/Cluster023.png]

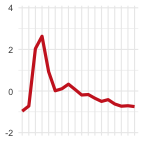

Supplement: Supplementary file 6. [file elife-30860-supp6.zip › biolayout-clusters-files/Cluster024.png]

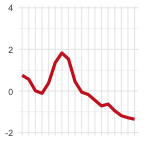

Supplement: Supplementary file 6. [file elife-30860-supp6.zip › biolayout-clusters-files/Cluster025.png]

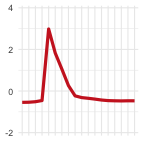

Supplement: Supplementary file 6. [file elife-30860-supp6.zip › biolayout-clusters-files/Cluster026.png]

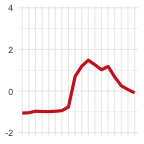

Supplement: Supplementary file 6. [file elife-30860-supp6.zip › biolayout-clusters-files/Cluster027.png]

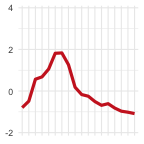

Supplement: Supplementary file 6. [file elife-30860-supp6.zip › biolayout-clusters-files/Cluster028.png]

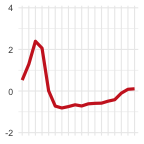

Supplement: Supplementary file 6. [file elife-30860-supp6.zip › biolayout-clusters-files/Cluster029.png]

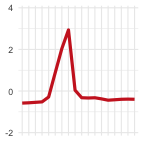

Supplement: Supplementary file 6. [file elife-30860-supp6.zip › biolayout-clusters-files/Cluster030.png]

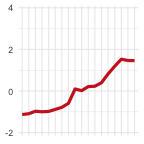

Supplement: Supplementary file 6. [file elife-30860-supp6.zip › biolayout-clusters-files/Cluster031.png]

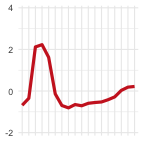

Supplement: Supplementary file 6. [file elife-30860-supp6.zip › biolayout-clusters-files/Cluster032.png]

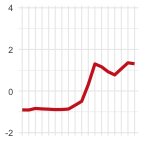

Supplement: Supplementary file 6. [file elife-30860-supp6.zip › biolayout-clusters-files/Cluster033.png]

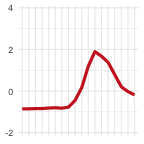

Supplement: Supplementary file 6. [file elife-30860-supp6.zip › biolayout-clusters-files/Cluster034.png]

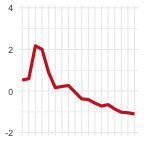

Supplement: Supplementary file 6. [file elife-30860-supp6.zip › biolayout-clusters-files/Cluster035.png]

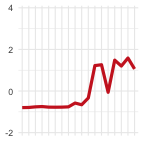

Supplement: Supplementary file 6. [file elife-30860-supp6.zip › biolayout-clusters-files/Cluster036.png]

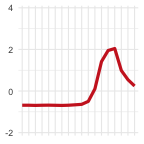

Supplement: Supplementary file 6. [file elife-30860-supp6.zip › biolayout-clusters-files/Cluster037.png]

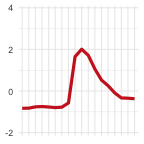

Supplement: Supplementary file 6. [file elife-30860-supp6.zip › biolayout-clusters-files/Cluster038.png]

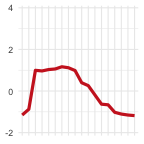

Supplement: Supplementary file 6. [file elife-30860-supp6.zip › biolayout-clusters-files/Cluster039.png]

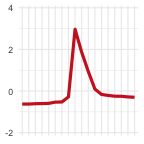

Supplement: Supplementary file 6. [file elife-30860-supp6.zip › biolayout-clusters-files/Cluster040.png]

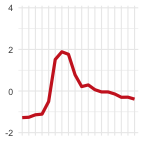

Supplement: Supplementary file 6. [file elife-30860-supp6.zip › biolayout-clusters-files/Cluster041.png]

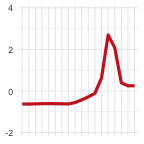

Supplement: Supplementary file 6. [file elife-30860-supp6.zip › biolayout-clusters-files/Cluster042.png]

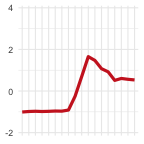

Supplement: Supplementary file 6. [file elife-30860-supp6.zip › biolayout-clusters-files/Cluster043.png]

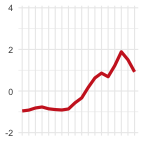

Supplement: Supplementary file 6. [file elife-30860-supp6.zip › biolayout-clusters-files/Cluster044.png]

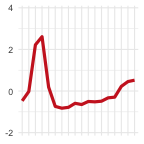

Supplement: Supplementary file 6. [file elife-30860-supp6.zip › biolayout-clusters-files/Cluster045.png]

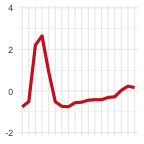

Supplement: Supplementary file 6. [file elife-30860-supp6.zip › biolayout-clusters-files/Cluster046.png]

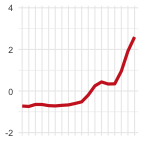

Supplement: Supplementary file 6. [file elife-30860-supp6.zip › biolayout-clusters-files/Cluster047.png]

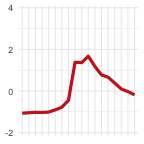

Supplement: Supplementary file 6. [file elife-30860-supp6.zip › biolayout-clusters-files/Cluster048.png]

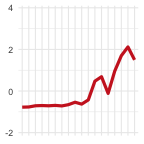

Supplement: Supplementary file 6. [file elife-30860-supp6.zip › biolayout-clusters-files/Cluster049.png]

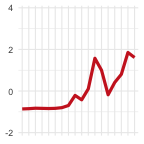

Supplement: Supplementary file 6. [file elife-30860-supp6.zip › biolayout-clusters-files/Cluster050.png]

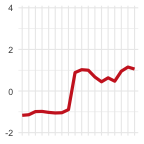

Supplement: Supplementary file 6. [file elife-30860-supp6.zip › biolayout-clusters-files/Cluster051.png]

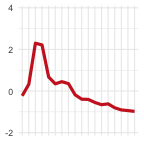

Supplement: Supplementary file 6. [file elife-30860-supp6.zip › biolayout-clusters-files/Cluster052.png]

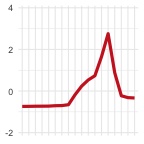

Supplement: Supplementary file 6. [file elife-30860-supp6.zip › biolayout-clusters-files/Cluster053.png]

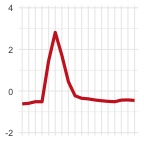

Supplement: Supplementary file 6. [file elife-30860-supp6.zip › biolayout-clusters-files/Cluster054.png]

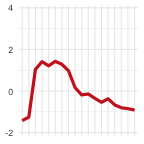

Supplement: Supplementary file 6. [file elife-30860-supp6.zip › biolayout-clusters-files/Cluster055.png]

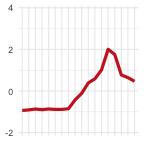

Supplement: Supplementary file 6. [file elife-30860-supp6.zip › biolayout-clusters-files/Cluster056.png]

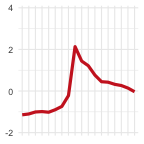

Supplement: Supplementary file 6. [file elife-30860-supp6.zip › biolayout-clusters-files/Cluster057.png]

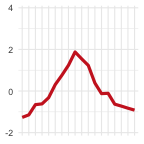

Supplement: Supplementary file 6. [file elife-30860-supp6.zip › biolayout-clusters-files/Cluster058.png]

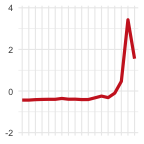

Supplement: Supplementary file 6. [file elife-30860-supp6.zip › biolayout-clusters-files/Cluster059.png]

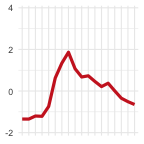

Supplement: Supplementary file 6. [file elife-30860-supp6.zip › biolayout-clusters-files/Cluster060.png]

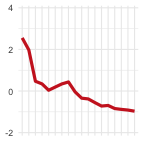

Supplement: Supplementary file 6. [file elife-30860-supp6.zip › biolayout-clusters-files/Cluster061.png]

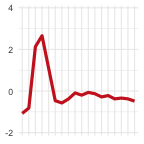

Supplement: Supplementary file 6. [file elife-30860-supp6.zip › biolayout-clusters-files/Cluster062.png]

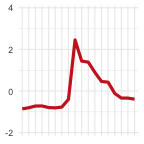

Supplement: Supplementary file 6. [file elife-30860-supp6.zip › biolayout-clusters-files/Cluster063.png]

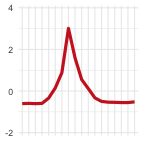

Supplement: Supplementary file 6. [file elife-30860-supp6.zip › biolayout-clusters-files/Cluster064.png]

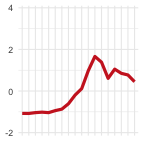

Supplement: Supplementary file 6. [file elife-30860-supp6.zip › biolayout-clusters-files/Cluster065.png]

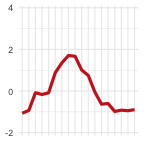

Supplement: Supplementary file 6. [file elife-30860-supp6.zip › biolayout-clusters-files/Cluster066.png]

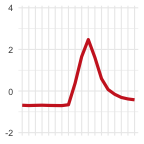

Supplement: Supplementary file 6. [file elife-30860-supp6.zip › biolayout-clusters-files/Cluster067.png]

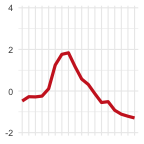

Supplement: Supplementary file 6. [file elife-30860-supp6.zip › biolayout-clusters-files/Cluster068.png]

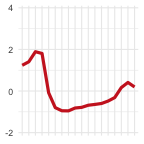

Supplement: Supplementary file 6. [file elife-30860-supp6.zip › biolayout-clusters-files/Cluster069.png]

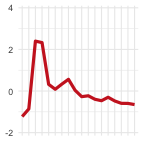

Supplement: Supplementary file 6. [file elife-30860-supp6.zip › biolayout-clusters-files/Cluster070.png]

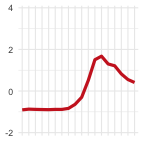

Supplement: Supplementary file 6. [file elife-30860-supp6.zip › biolayout-clusters-files/Cluster071.png]

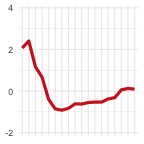

Supplement: Supplementary file 6. [file elife-30860-supp6.zip › biolayout-clusters-files/Cluster072.png]

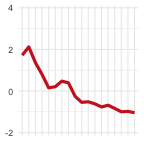

Supplement: Supplementary file 6. [file elife-30860-supp6.zip › biolayout-clusters-files/Cluster073.png]

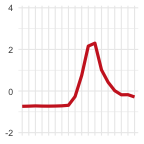

Supplement: Supplementary file 6. [file elife-30860-supp6.zip › biolayout-clusters-files/Cluster074.png]

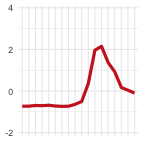

Supplement: Supplementary file 6. [file elife-30860-supp6.zip › biolayout-clusters-files/Cluster075.png]

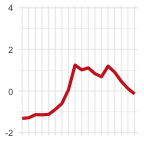

Supplement: Supplementary file 6. [file elife-30860-supp6.zip › biolayout-clusters-files/Cluster076.png]
