# Supplementary material for: A high-resolution mRNA expression time course of embryonic development in zebrafish
Source: eLife. 2017 Nov 16;6:e30860. doi: 10.7554/eLife.30860 (PMC5690287; doi:10.7554/eLife.30860)
Supplement: Supplementary file 9. [file elife-30860-supp9.zip › chr_expression_domains.pdf]

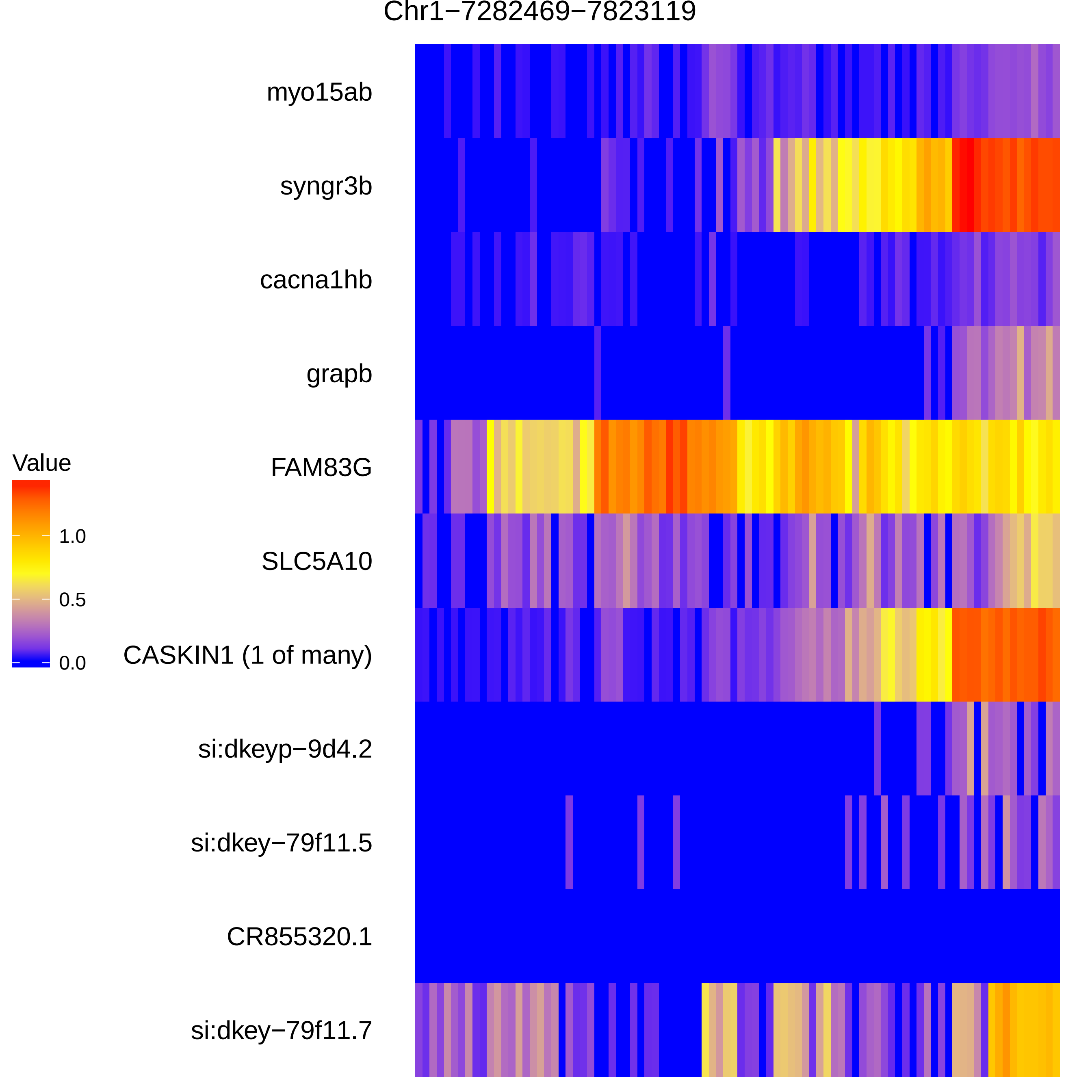
Chr1−7282469−7823119
myo15ab
syngr3b
cacna1hb
grapb
FAM83G
SLC5A10
CASKIN1 (1 of many)
si:dkeyp−9d4.2
si:dkey−79f11.5
CR855320.1
si:dkey−79f11.7
Value
1.0
0.5
0.0

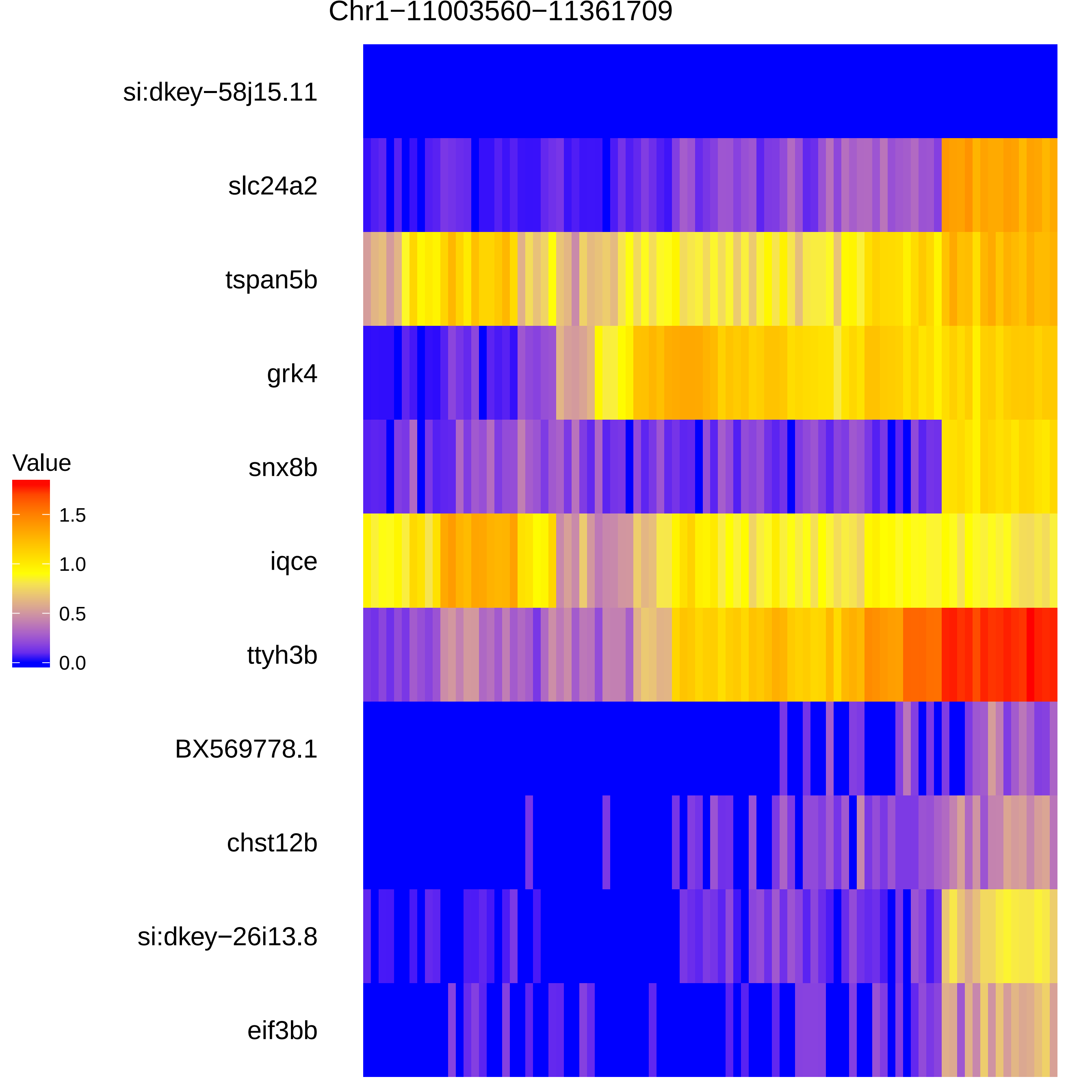
Chr1−11003560−11361709
si:dkey−58j15.11
slc24a2
tspan5b
grk4
snx8b
iqce
ttyh3b
BX569778.1
chst12b
si:dkey−26i13.8
eif3bb
Value
1.5
1.0
0.5
0.0

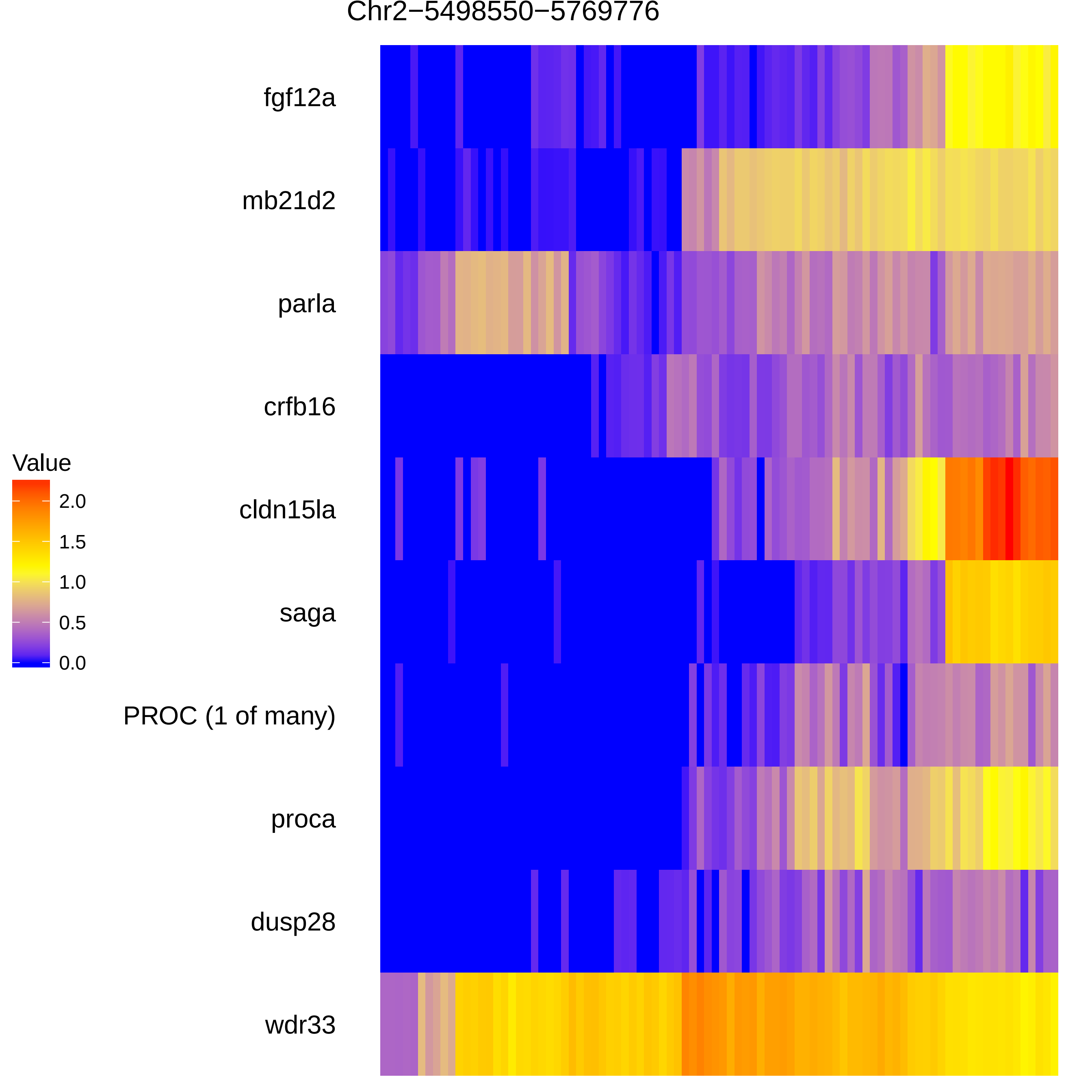
Chr2−5498550−5769776
fgf12a
mb21d2
parla
crfb16
cldn15la
saga
PROC (1 of many)
proca
dusp28
wdr33
Value
2.0
1.5
1.0
0.5
0.0

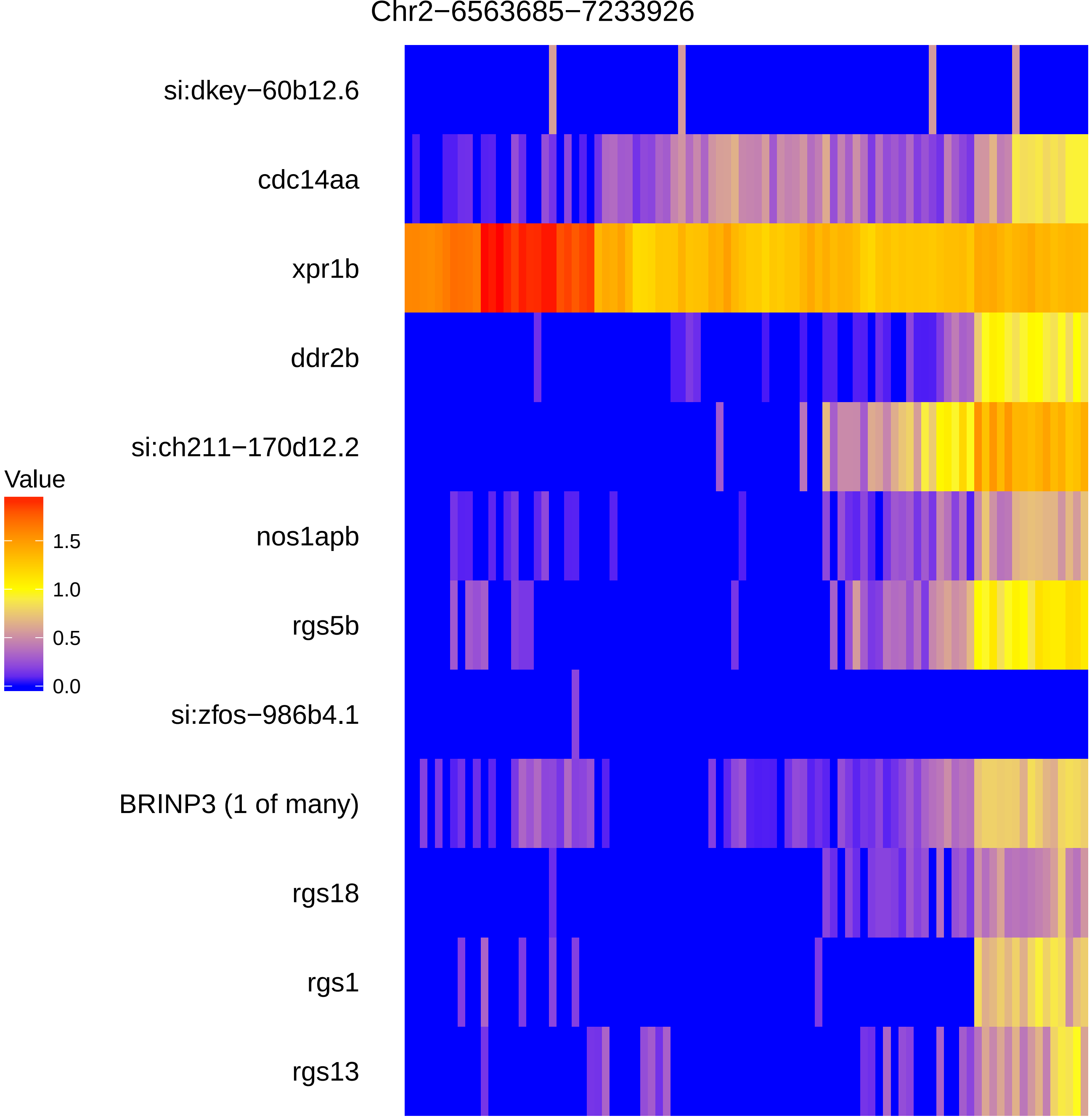
Chr2−6563685−7233926
si:dkey−60b12.6
cdc14aa
xpr1b
ddr2b
si:ch211−170d12.2
nos1apb
rgs5b
si:zfos−986b4.1
BRINP3 (1 of many)
rgs18
rgs1
rgs13
Value
1.5
1.0
0.5
0.0

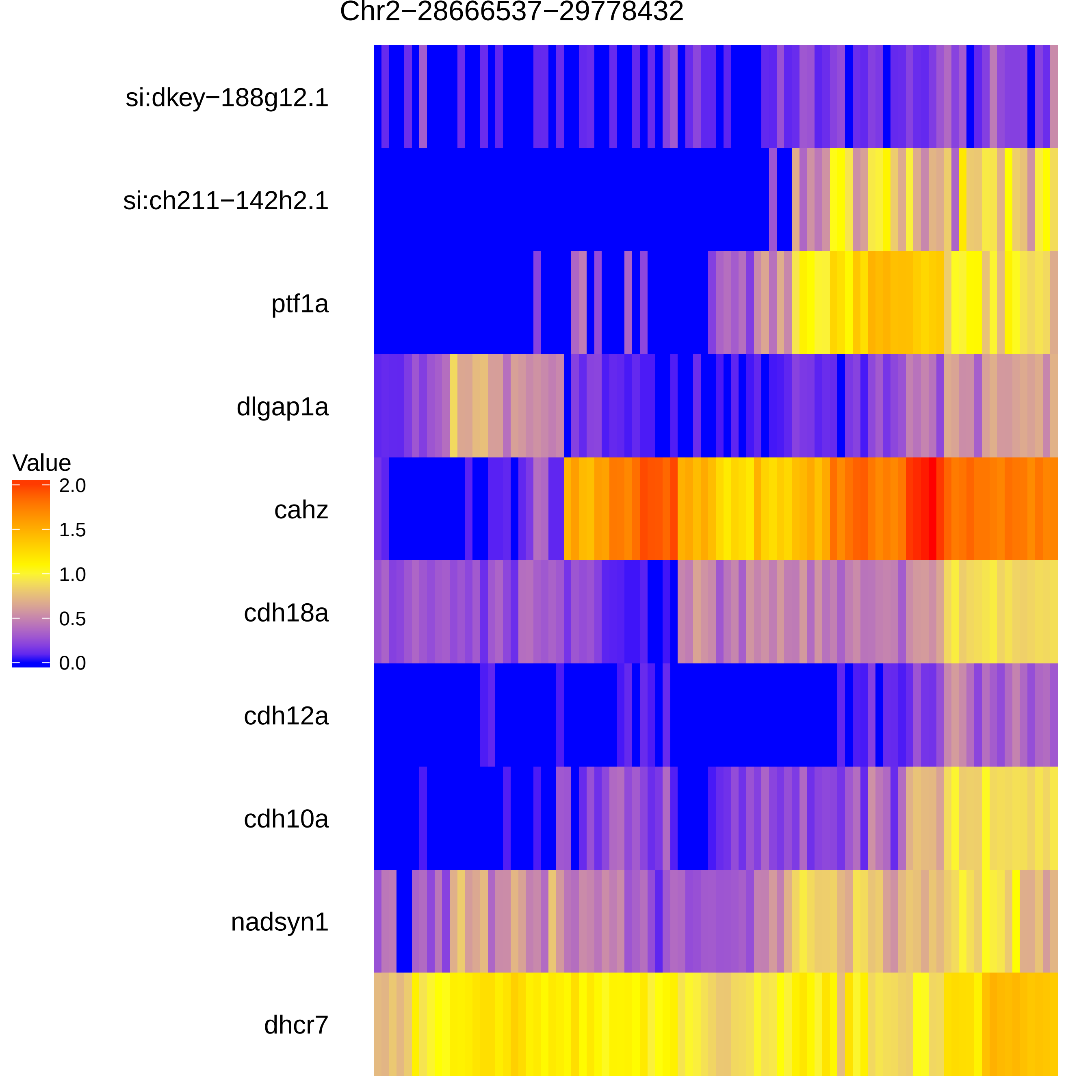
Chr2−28666537−29778432
si:dkey−188g12.1
si:ch211−142h2.1
ptf1a
dlgap1a
cahz
cdh18a
cdh12a
cdh10a
nadsyn1
dhcr7
Value
2.0
1.5
1.0
0.5
0.0

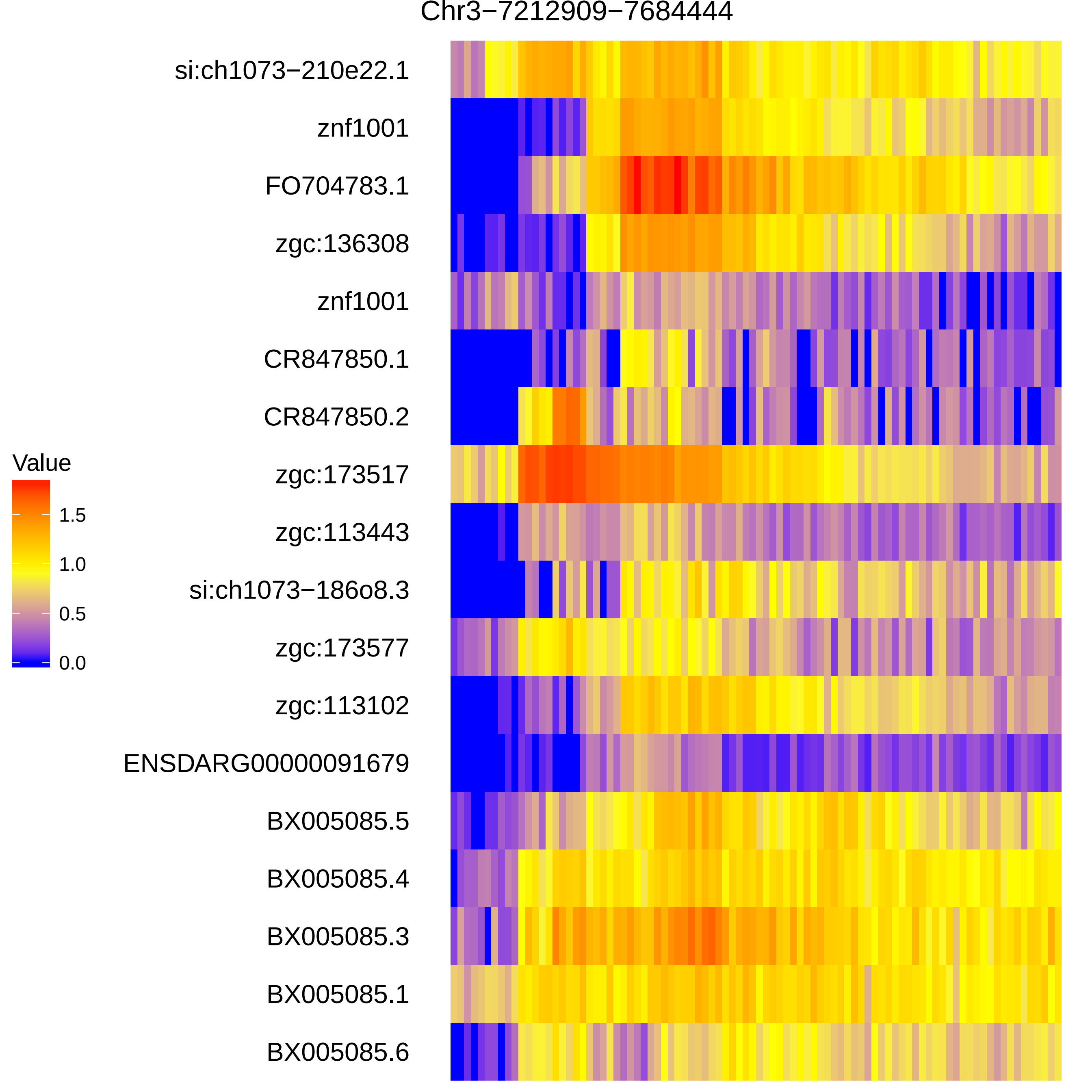
Chr3−7212909−7684444
si:ch1073−210e22.1
znf1001
FO704783.1
zgc:136308
znf1001
CR847850.1
CR847850.2
zgc:173517
zgc:113443
si:ch1073−186o8.3
zgc:173577
zgc:113102
ENSDARG00000091679
BX005085.5
BX005085.4
BX005085.3
BX005085.1
BX005085.6
Value
1.5
1.0
0.5
0.0

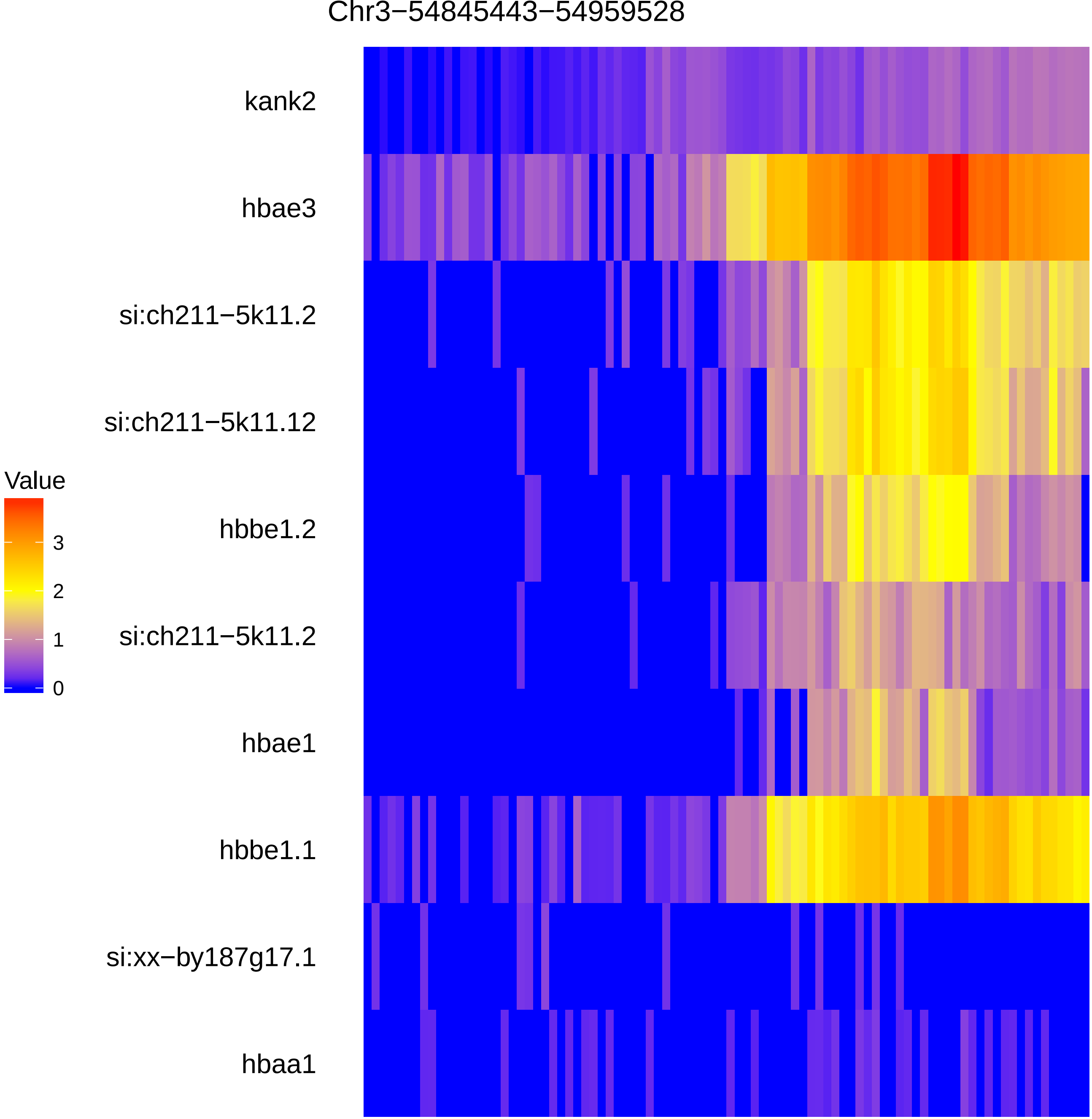
Chr3−54845443−54959528
kank2
hbae3
si:ch211−5k11.2
si:ch211−5k11.12
hbbe1.2
si:ch211−5k11.2
hbae1
hbbe1.1
si:xx−by187g17.1
hbaa1
Value
3
2
1
0

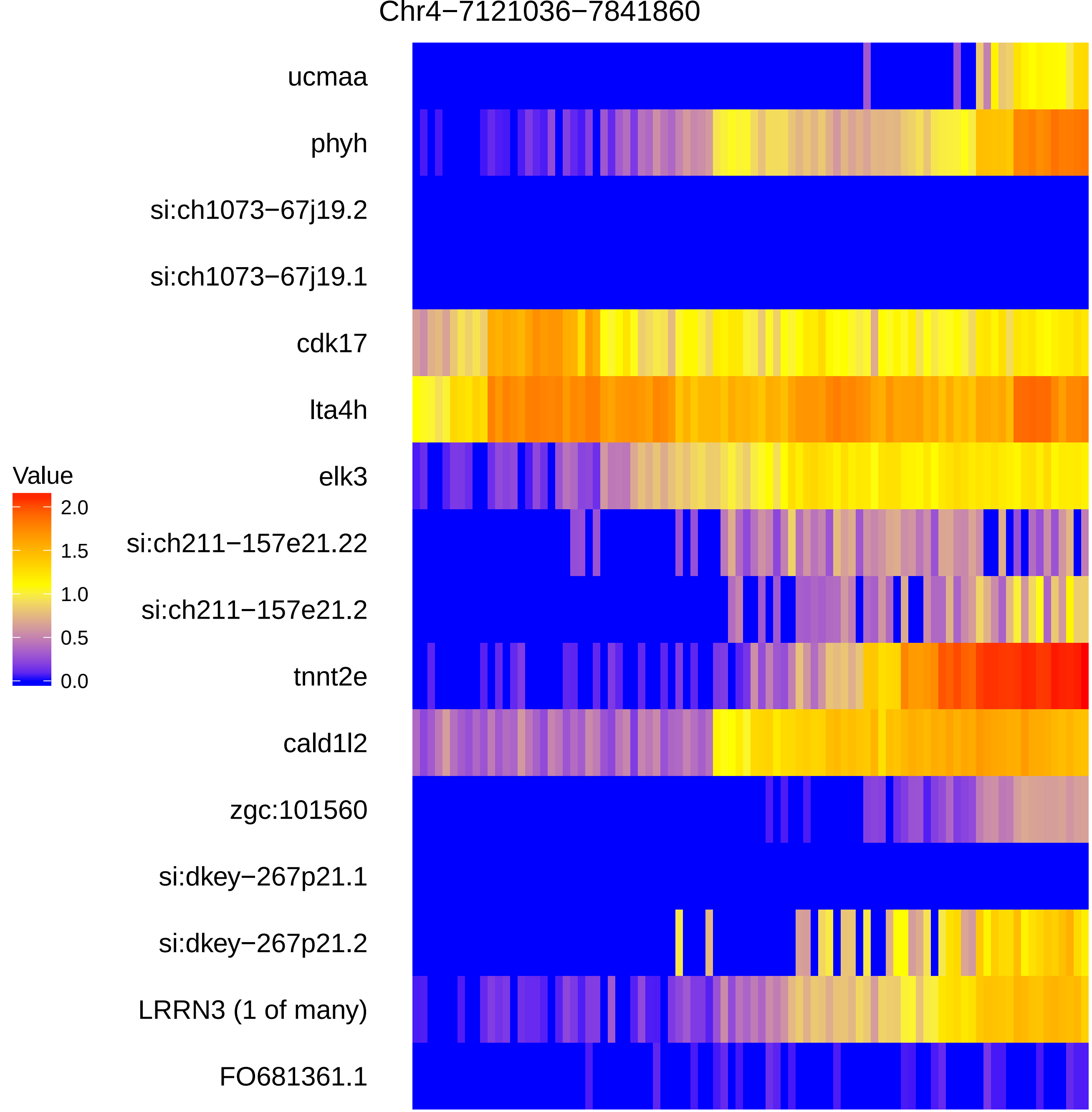
Chr4−7121036−7841860
ucmaa
phyh
si:ch1073−67j19.2
si:ch1073−67j19.1
cdk17
lta4h
elk3
si:ch211−157e21.22
si:ch211−157e21.2
tnnt2e
cald1l2
zgc:101560
si:dkey−267p21.1
si:dkey−267p21.2
LRRN3 (1 of many)
FO681361.1
Value
2.0
1.5
1.0
0.5
0.0

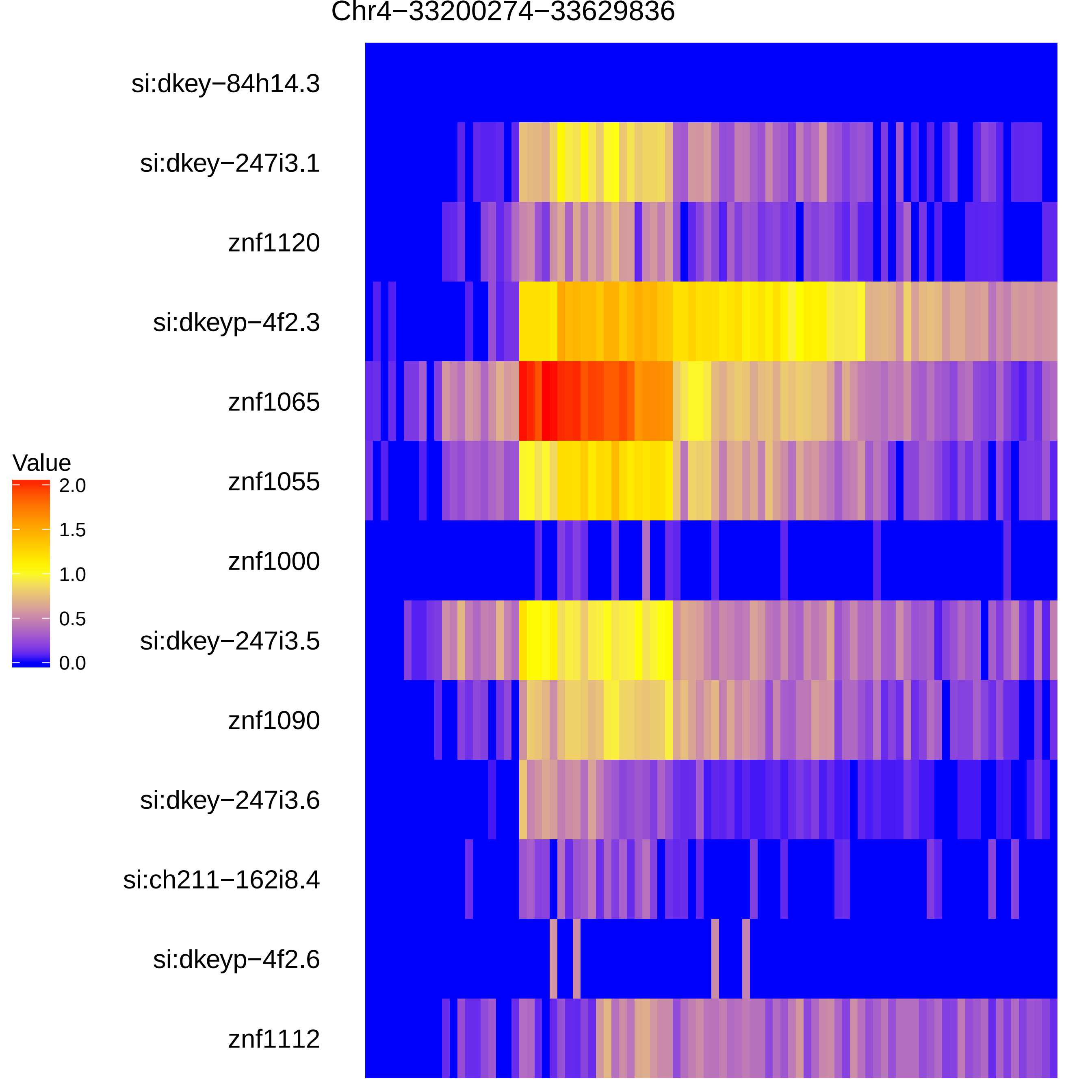

Chr4−33200274−33629836
si:dkey−84h14.3
si:dkey−247i3.1
znf1120
si:dkeyp−4f2.3
znf1065
znf1055
znf1000
si:dkey−247i3.5
znf1090
si:dkey−247i3.6
si:ch211−162i8.4
si:dkeyp−4f2.6
znf1112
Value
2.0
1.5
1.0
0.5
0.0

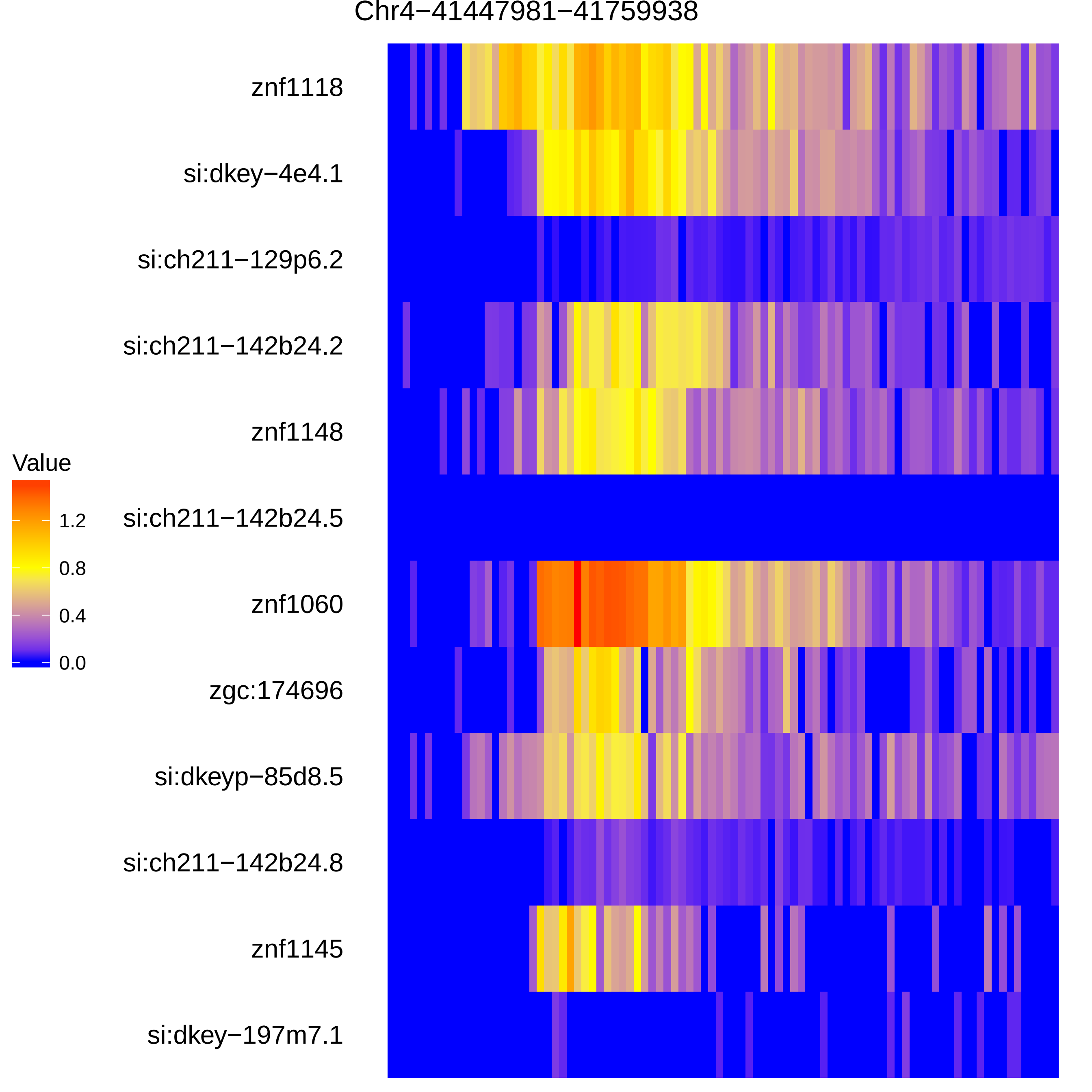
Chr4−41447981−41759938
znf1118
si:dkey−4e4.1
si:ch211−129p6.2
si:ch211−142b24.2
znf1148
si:ch211−142b24.5
znf1060
zgc:174696
si:dkeyp−85d8.5
si:ch211−142b24.8
znf1145
si:dkey−197m7.1
Value
1.2
0.8
0.4
0.0

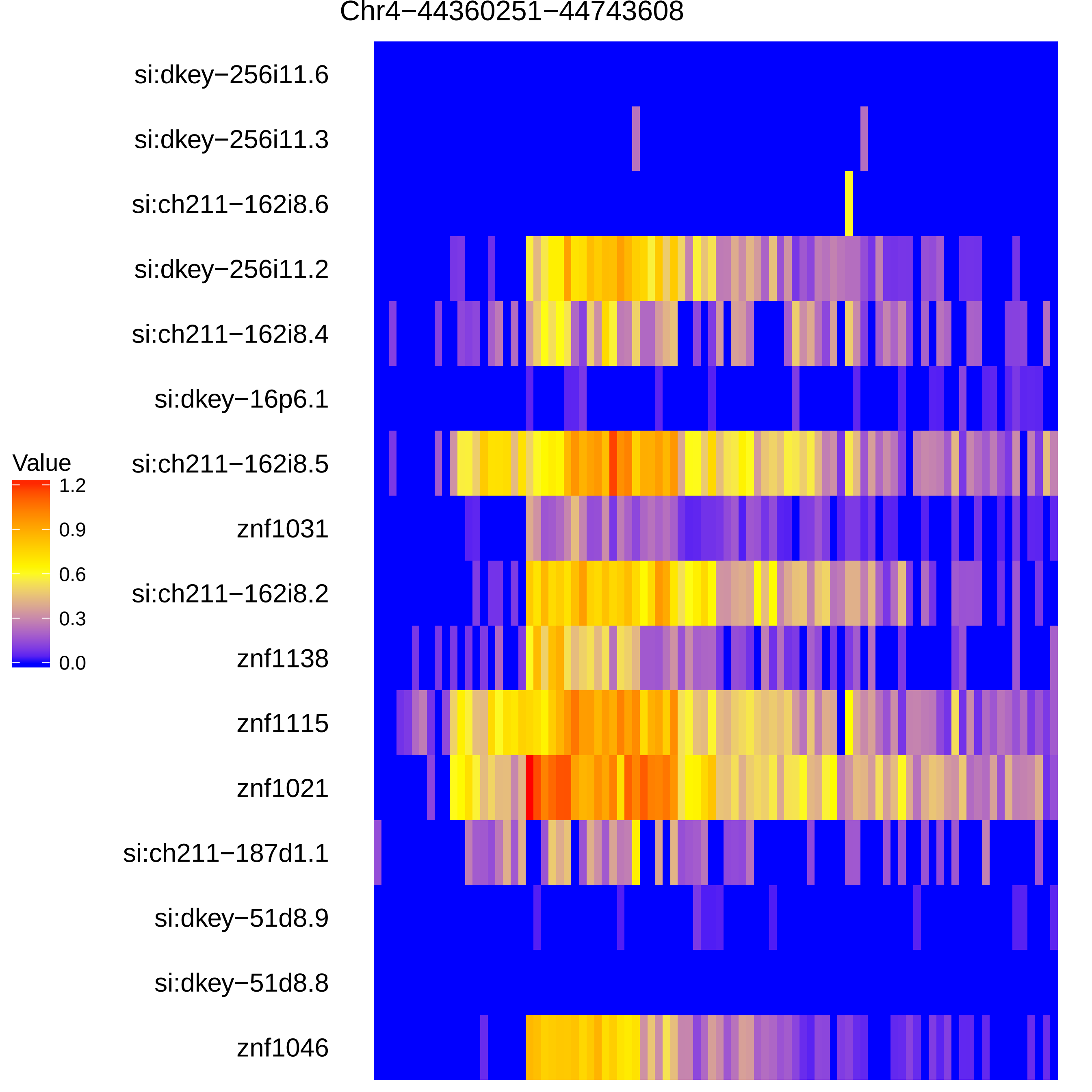
Chr4−44360251−44743608
si:dkey−256i11.6
si:dkey−256i11.3
si:ch211−162i8.6
si:dkey−256i11.2
si:ch211−162i8.4
si:dkey−16p6.1
si:ch211−162i8.5
znf1031
si:ch211−162i8.2
znf1138
znf1115
znf1021
si:ch211−187d1.1
si:dkey−51d8.9
si:dkey−51d8.8
znf1046
Value
1.2
0.9
0.6
0.3
0.0

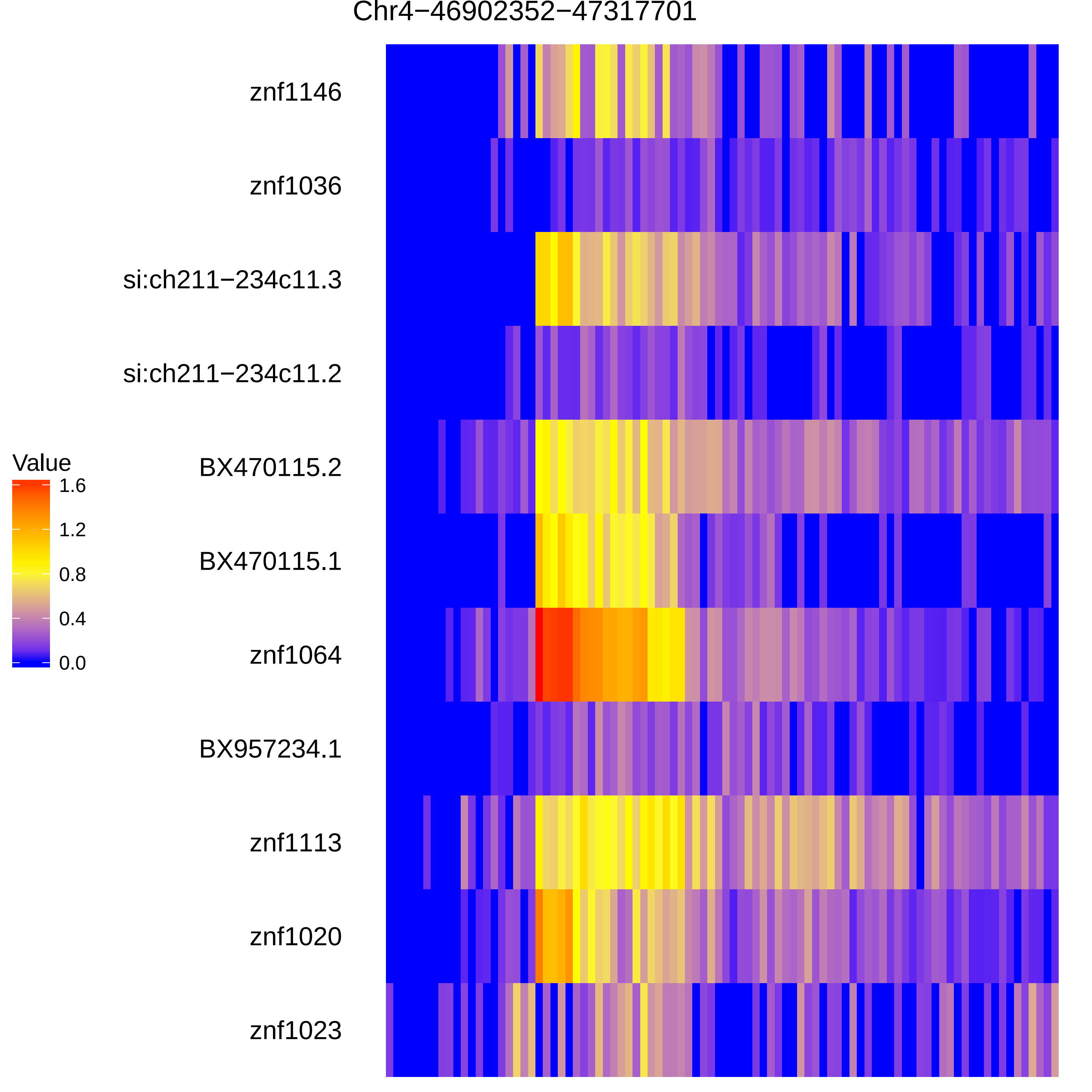
Chr4−46902352−47317701
znf1146
znf1036
si:ch211−234c11.3
si:ch211−234c11.2
BX470115.2
BX470115.1
znf1064
BX957234.1
znf1113
znf1020
znf1023
Value
1.6
1.2
0.8
0.4
0.0

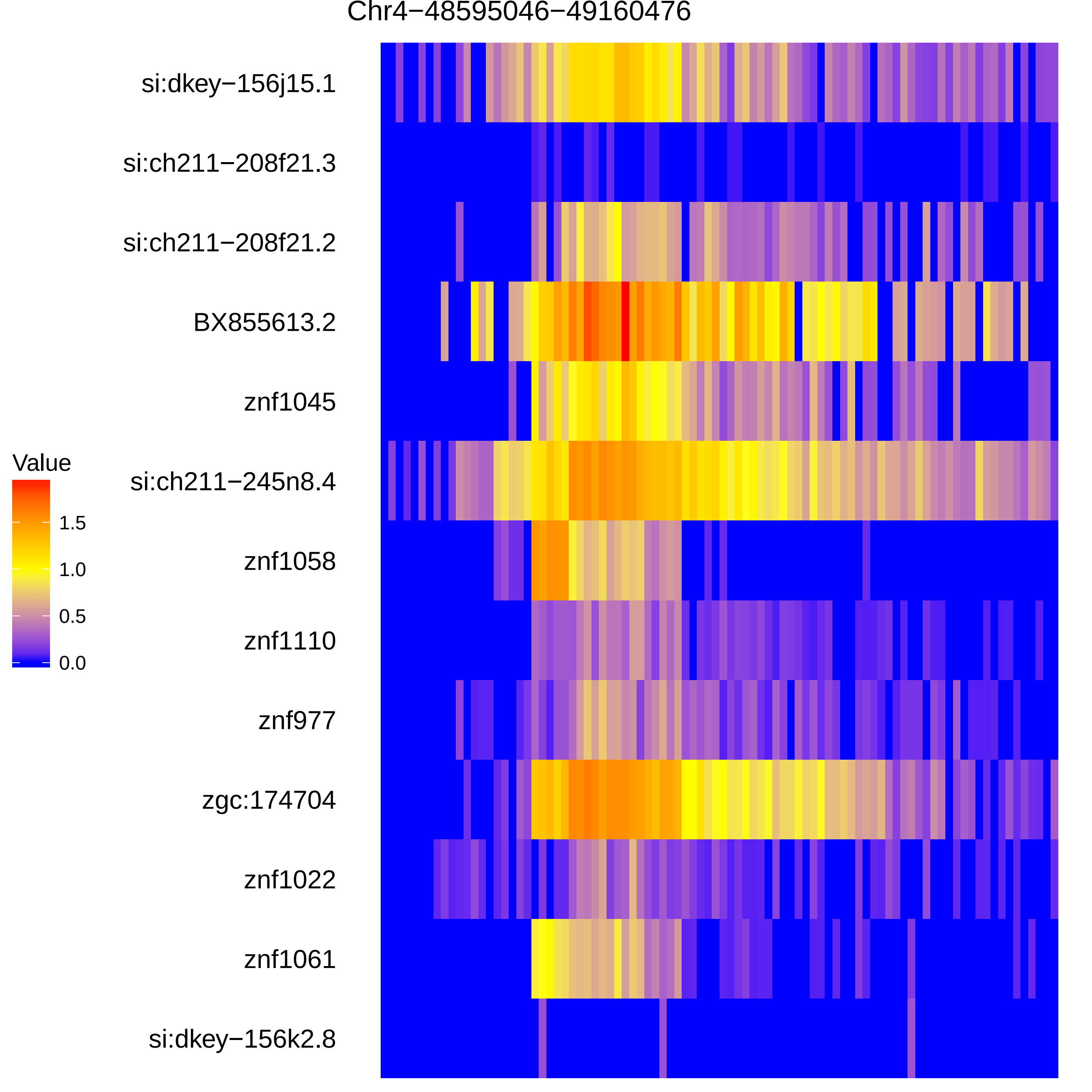

Chr4−48595046−49160476
si:dkey−156j15.1
si:ch211−208f21.3
si:ch211−208f21.2
BX855613.2
znf1045
si:ch211−245n8.4
znf1058
znf1110
znf977
zgc:174704
znf1022
znf1061
si:dkey−156k2.8
Value
1.5
1.0
0.5
0.0

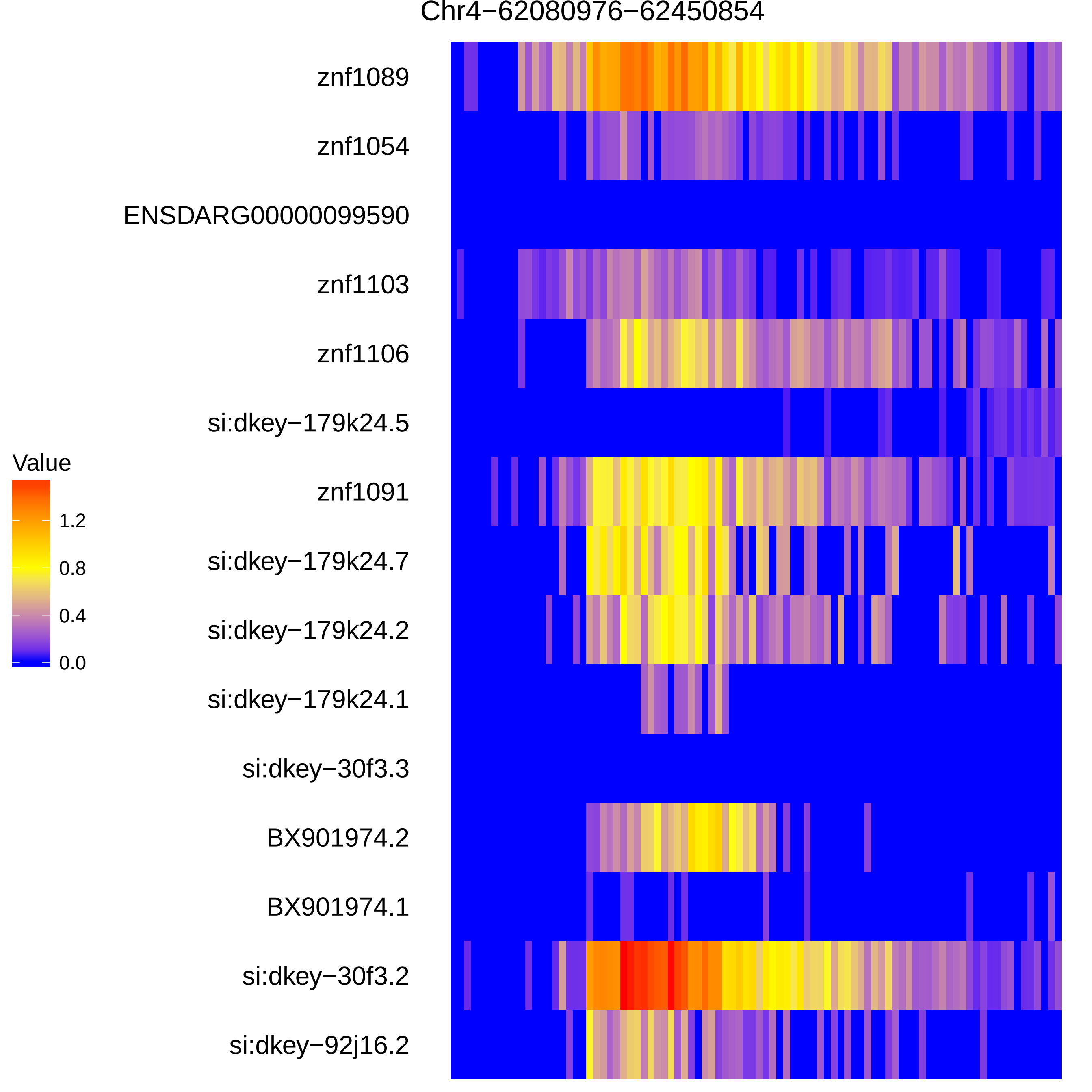
Chr4−62080976−62450854
znf1089
znf1054
ENSDARG00000099590
znf1103
znf1106
si:dkey−179k24.5
znf1091
si:dkey−179k24.7
si:dkey−179k24.2
si:dkey−179k24.1
si:dkey−30f3.3
BX901974.2
BX901974.1
si:dkey−30f3.2
si:dkey−92j16.2
Value
1.2
0.8
0.4
0.0

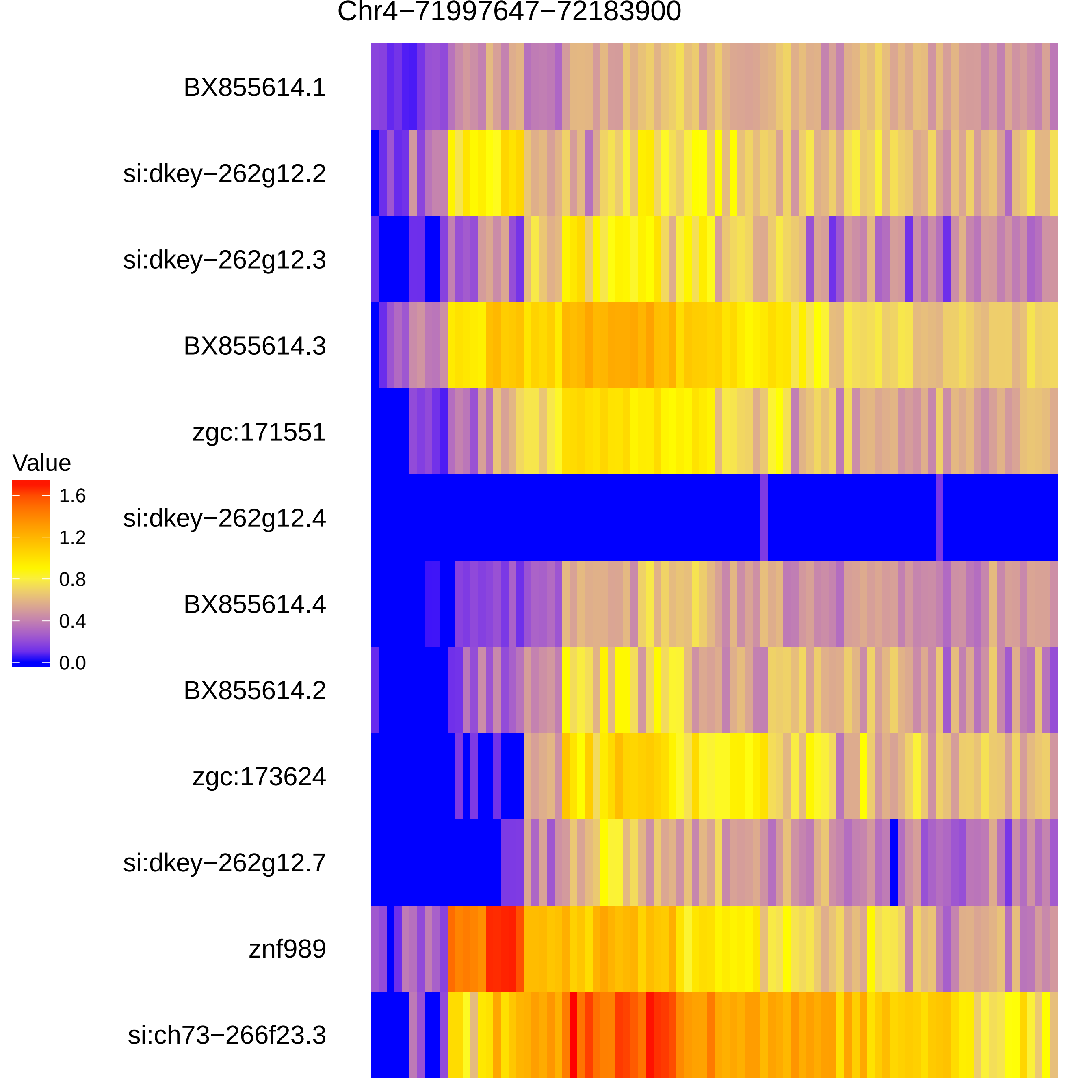
Chr4−71997647−72183900
BX855614.1
si:dkey−262g12.2
si:dkey−262g12.3
BX855614.3
zgc:171551
si:dkey−262g12.4
BX855614.4
BX855614.2
zgc:173624
si:dkey−262g12.7
znf989
si:ch73−266f23.3
Value
1.6
1.2
0.8
0.4
0.0

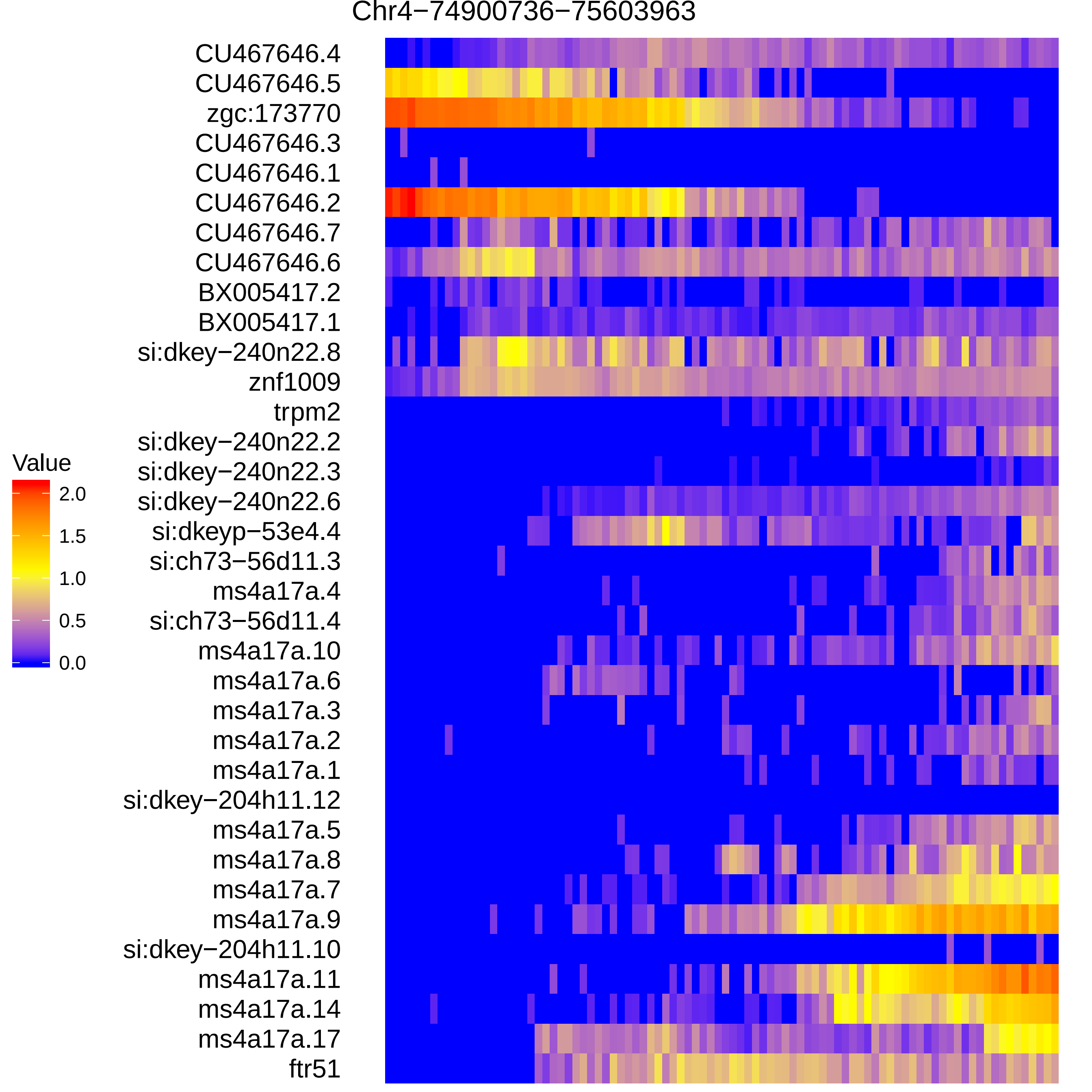

Chr4−74900736−75603963
CU467646.4
CU467646.5
zgc:173770
CU467646.3
CU467646.1
CU467646.2
CU467646.7
CU467646.6
BX005417.2
BX005417.1
si:dkey−240n22.8
znf1009
trpm2
si:dkey−240n22.2
si:dkey−240n22.3
si:dkey−240n22.6
si:dkeyp−53e4.4
si:ch73−56d11.3
ms4a17a.4
si:ch73−56d11.4
ms4a17a.10
ms4a17a.6
ms4a17a.3
ms4a17a.2
ms4a17a.1
si:dkey−204h11.12
ms4a17a.5
ms4a17a.8
ms4a17a.7
ms4a17a.9
si:dkey−204h11.10
ms4a17a.11
ms4a17a.14
ms4a17a.17
ftr51
Value
2.0
1.5
1.0
0.5
0.0

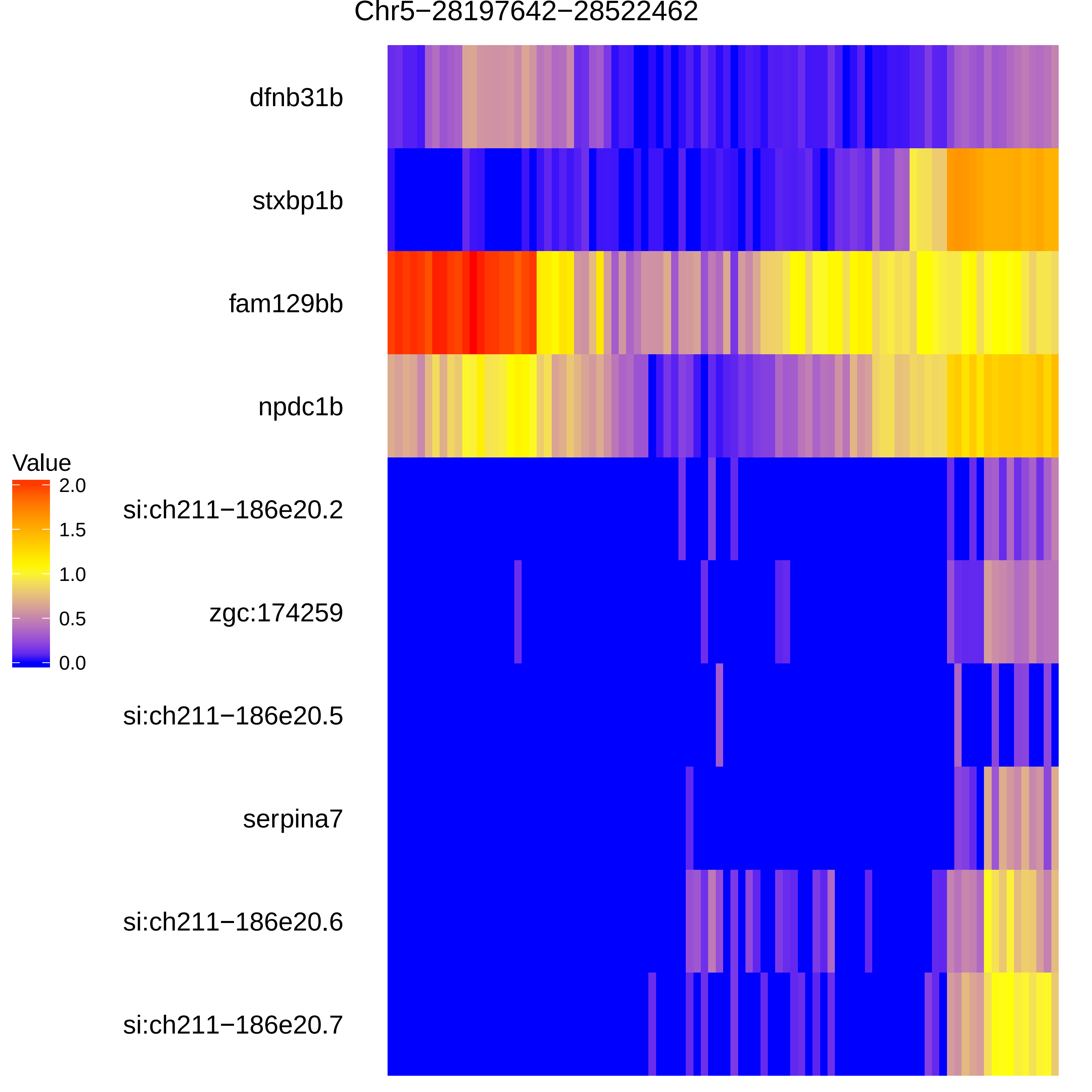
Chr5−28197642−28522462
dfnb31b
stxbp1b
fam129bb
npdc1b
si:ch211−186e20.2
zgc:174259
si:ch211−186e20.5
serpina7
si:ch211−186e20.6
si:ch211−186e20.7
Value
2.0
1.5
1.0
0.5
0.0

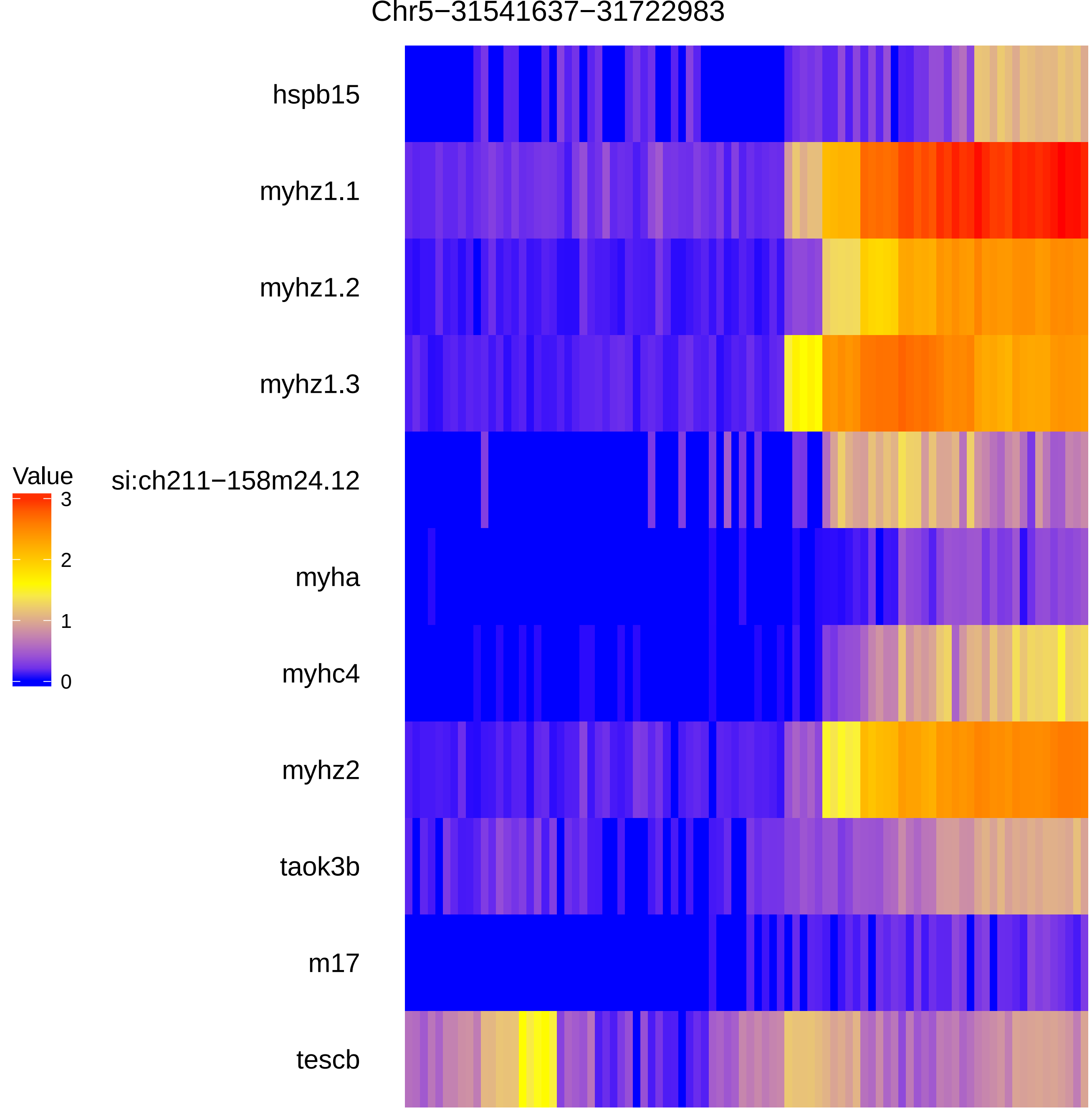
Chr5−31541637−31722983
hspb15
myhz1.1
myhz1.2
myhz1.3
si:ch211−158m24.12
myha
myhc4
myhz2
taok3b
m17
tescb
Value
3
2
1
0

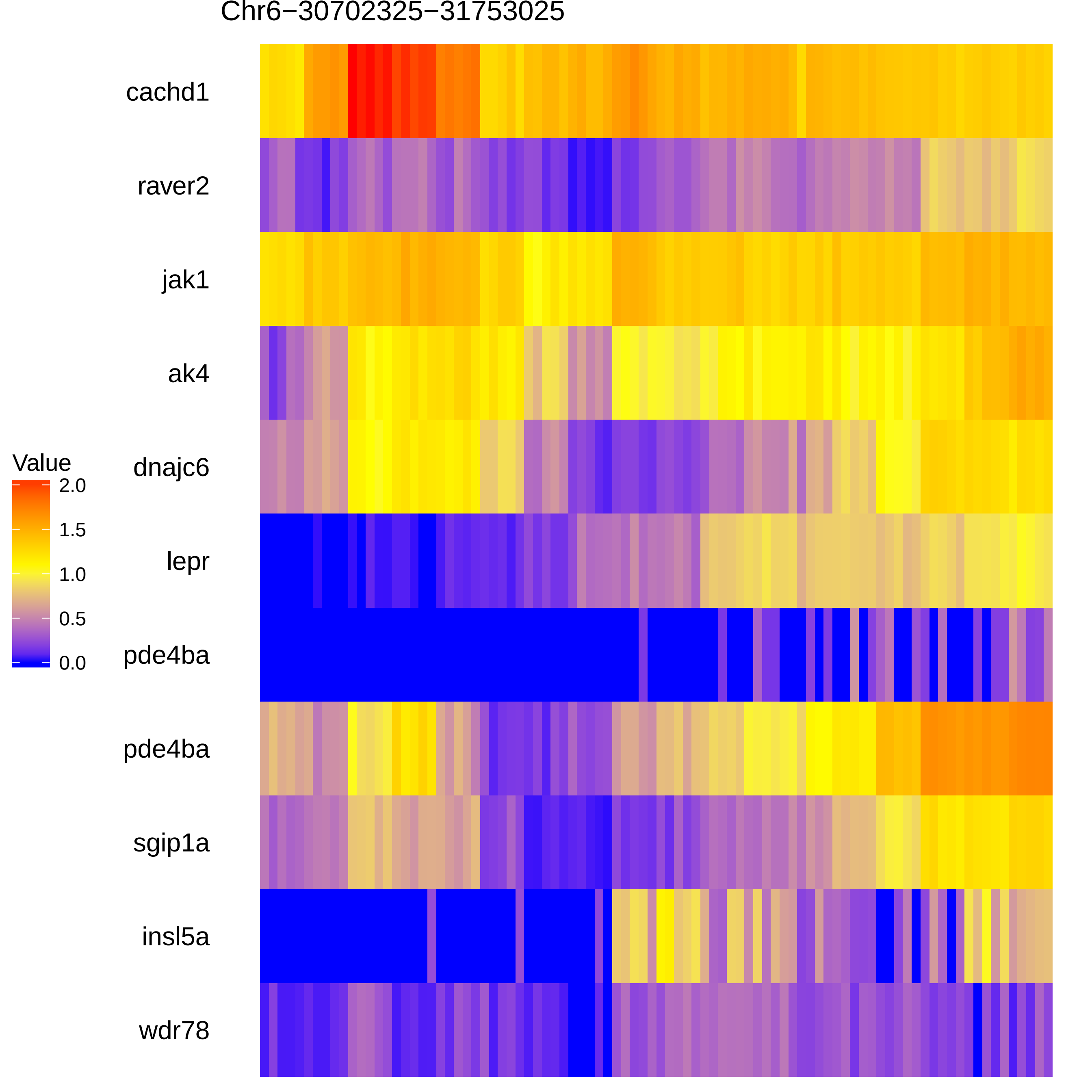

Chr6−30702325−31753025
cachd1
raver2
jak1
ak4
dnajc6
lepr
pde4ba
pde4ba
sgip1a
insl5a
wdr78
Value
2.0
1.5
1.0
0.5
0.0

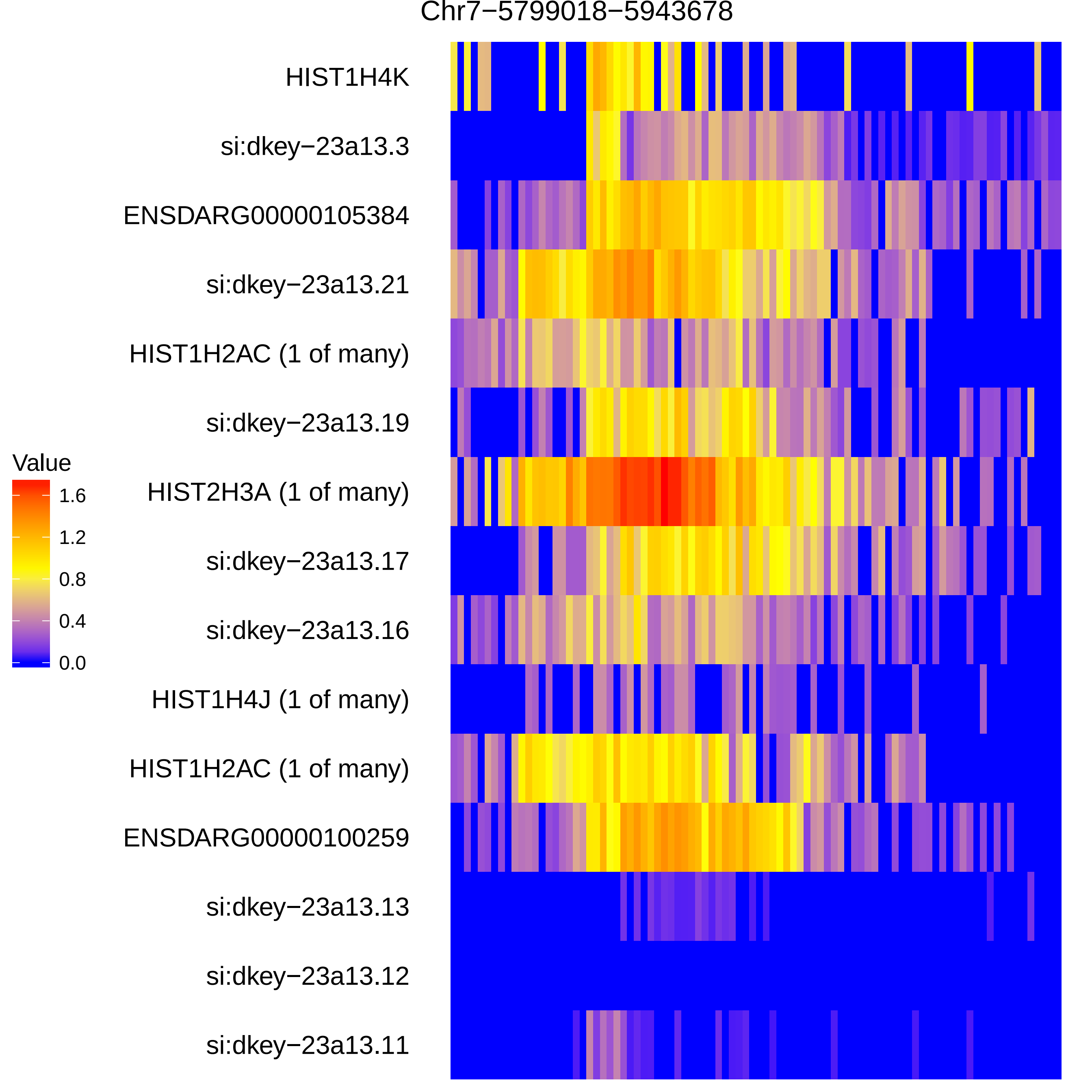
Chr7−5799018−5943678
HIST1H4K
si:dkey−23a13.3
ENSDARG00000105384
si:dkey−23a13.21
HIST1H2AC (1 of many)
si:dkey−23a13.19
HIST2H3A (1 of many)
si:dkey−23a13.17
si:dkey−23a13.16
HIST1H4J (1 of many)
HIST1H2AC (1 of many)
ENSDARG00000100259
si:dkey−23a13.13
si:dkey−23a13.12
si:dkey−23a13.11
Value
1.6
1.2
0.8
0.4
0.0

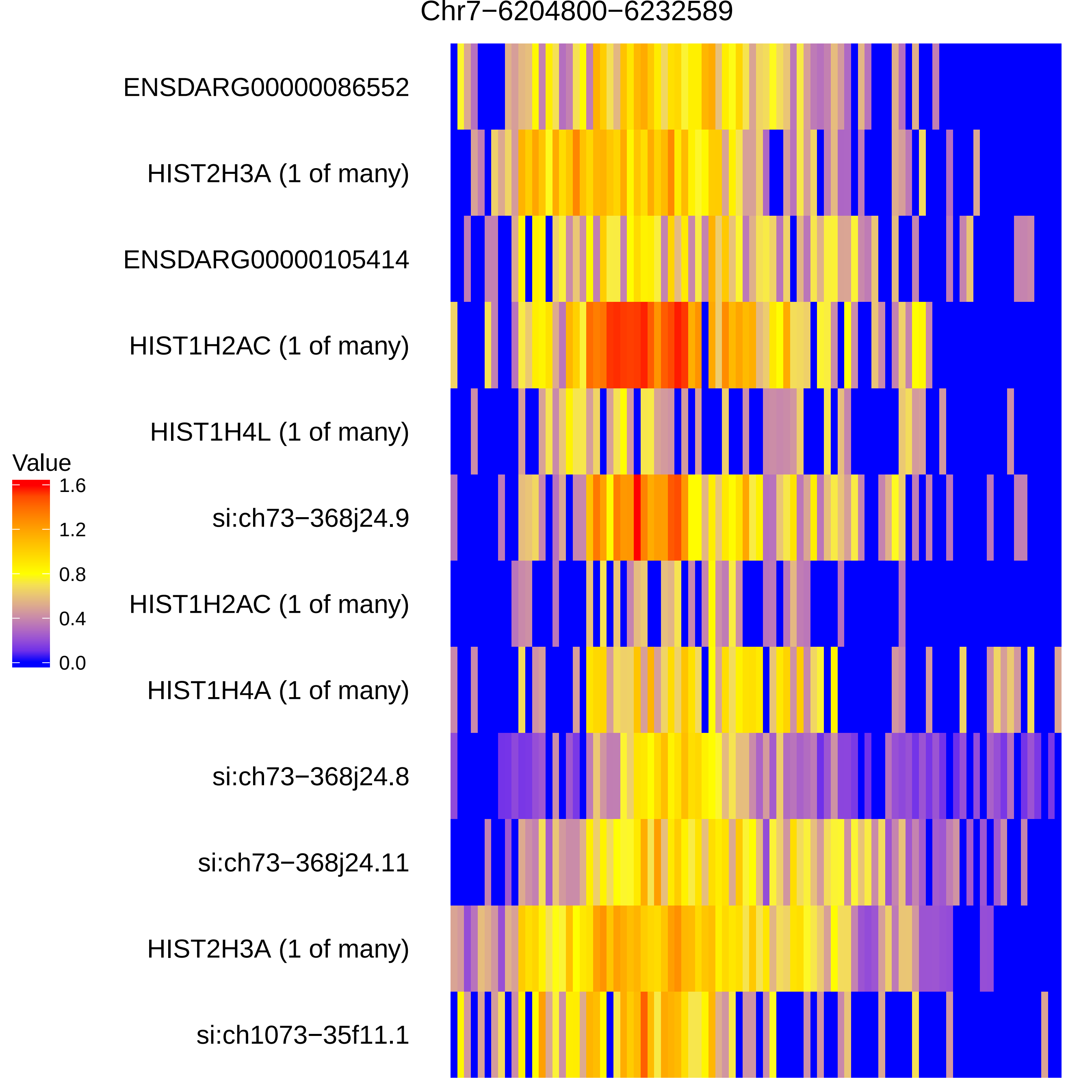

Chr7−6204800−6232589
ENSDARG00000086552
HIST2H3A (1 of many)
ENSDARG00000105414
HIST1H2AC (1 of many)
HIST1H4L (1 of many)
si:ch73−368j24.9
HIST1H2AC (1 of many)
HIST1H4A (1 of many)
si:ch73−368j24.8
si:ch73−368j24.11
HIST2H3A (1 of many)
si:ch1073−35f11.1
Value
1.6
1.2
0.8
0.4
0.0

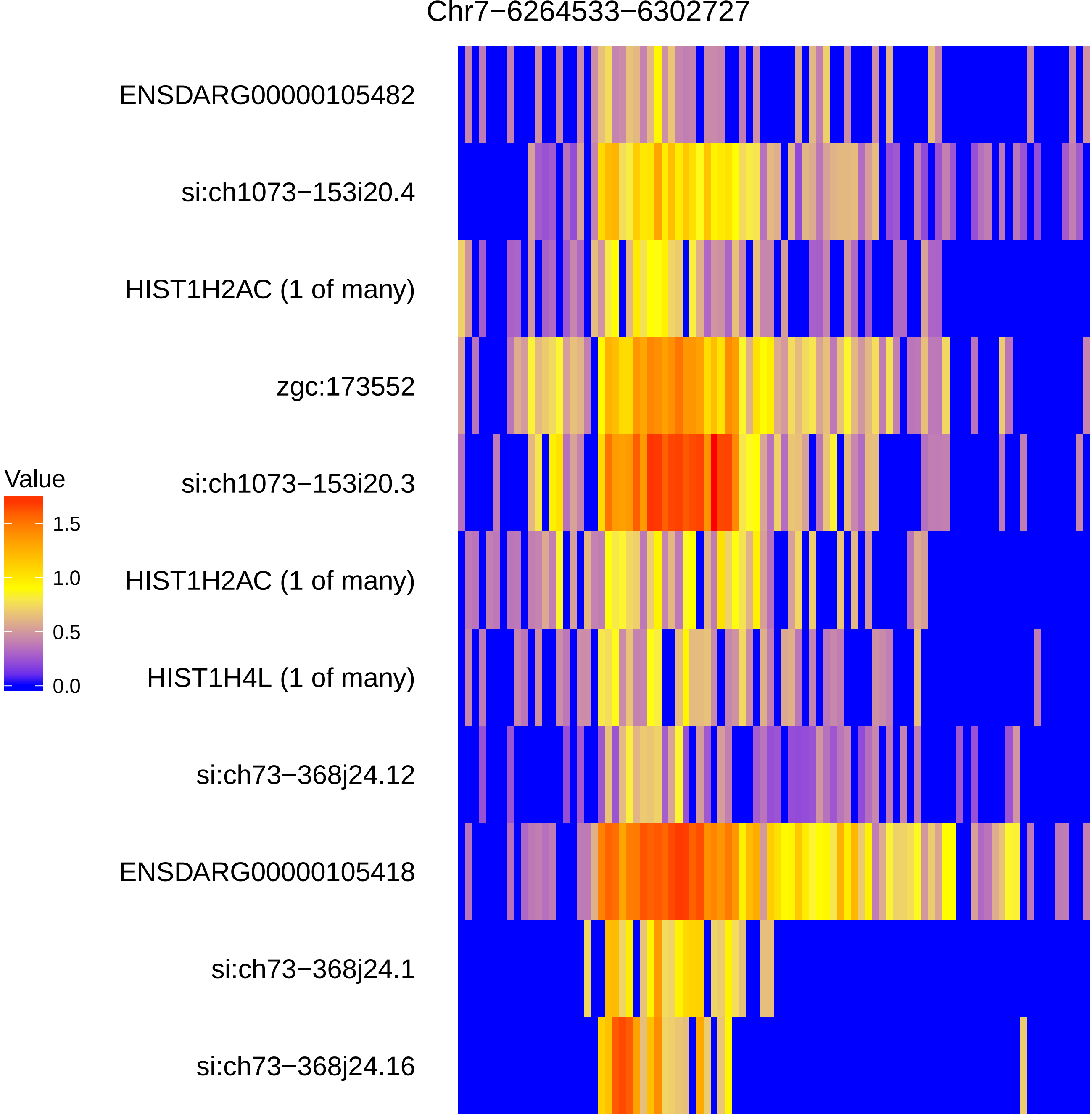
Chr7−6264533−6302727
ENSDARG00000105482
si:ch1073−153i20.4
HIST1H2AC (1 of many)
zgc:173552
si:ch1073−153i20.3
HIST1H2AC (1 of many)
HIST1H4L (1 of many)
si:ch73−368j24.12
ENSDARG00000105418
si:ch73−368j24.1
si:ch73−368j24.16
Value
1.5
1.0
0.5
0.0

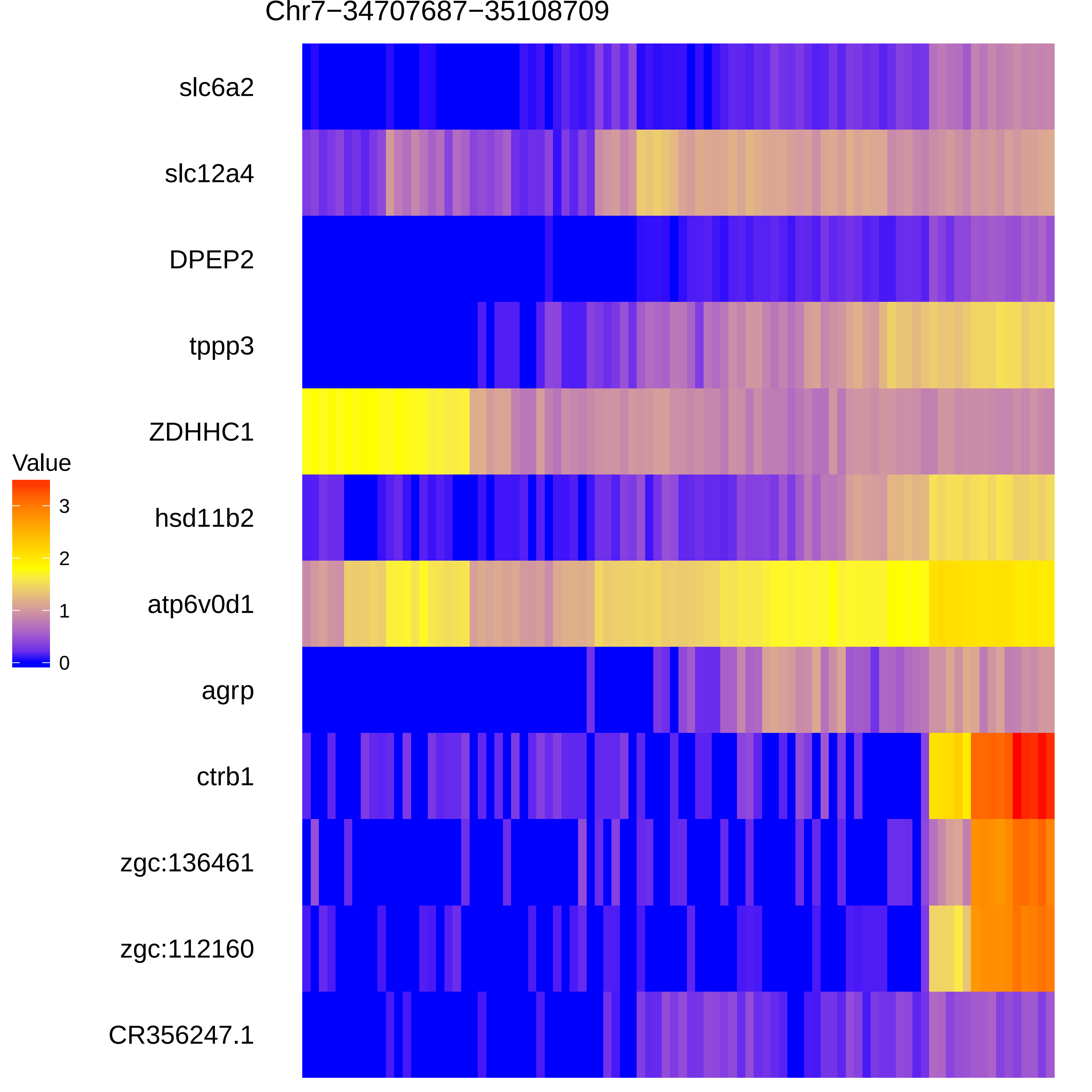
Chr7−34707687−35108709
slc6a2
slc12a4
DPEP2
tppp3
ZDHHC1
hsd11b2
atp6v0d1
agrp
ctrb1
zgc:136461
zgc:112160
CR356247.1
Value
3
2
1
0

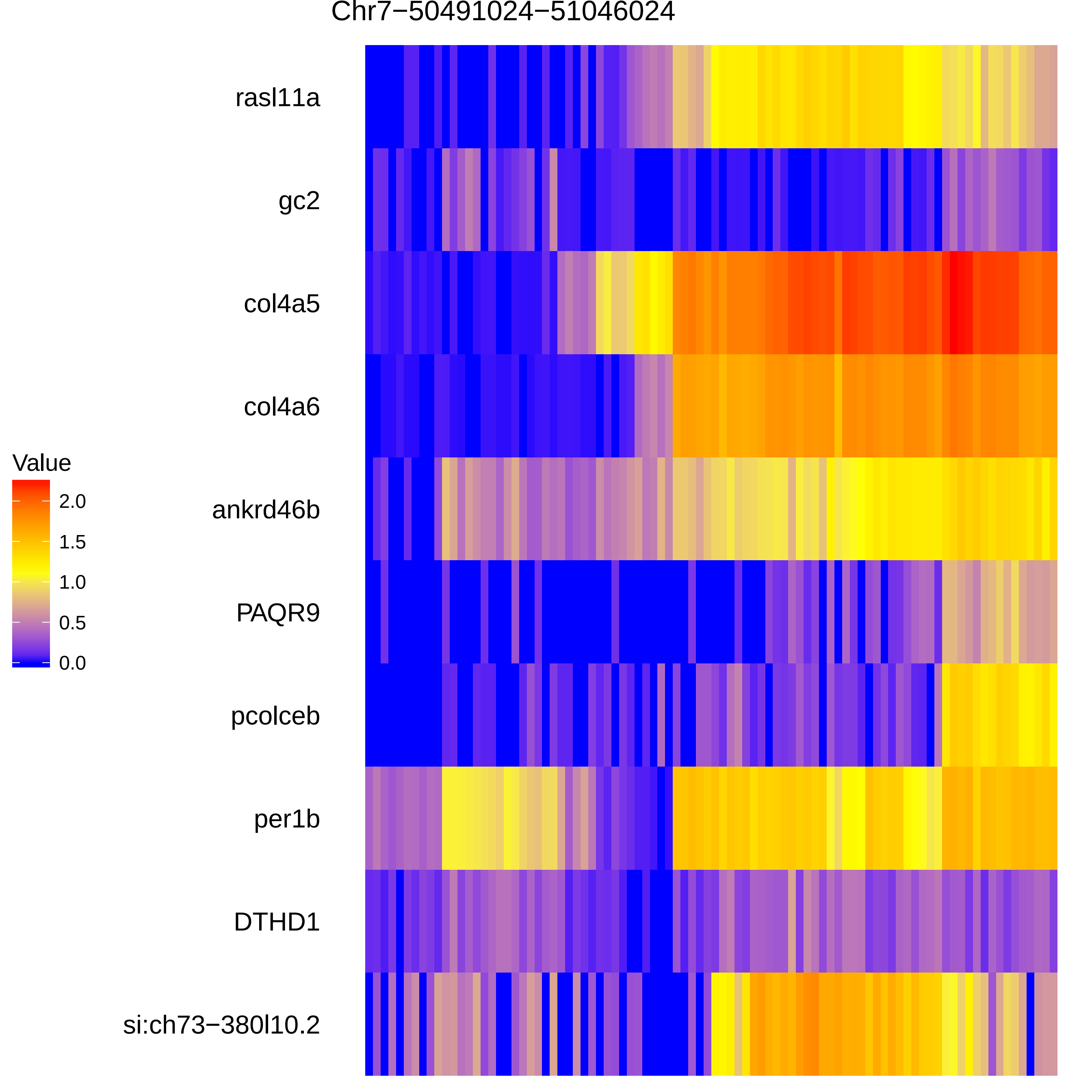
Chr7−50491024−51046024
rasl11a
gc2
col4a5
col4a6
ankrd46b
PAQR9
pcolceb
per1b
DTHD1
si:ch73−380l10.2
Value
2.0
1.5
1.0
0.5
0.0

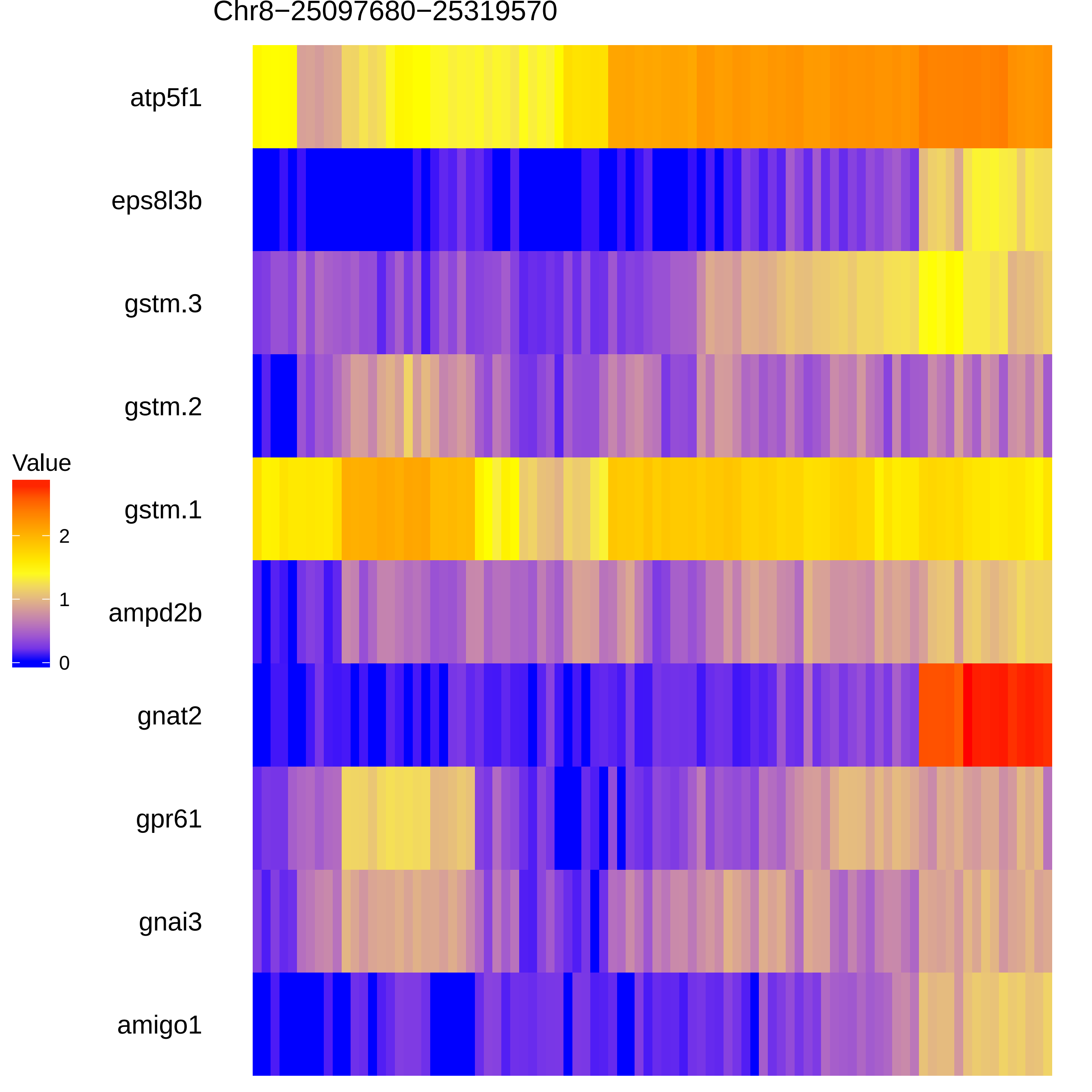
Chr8−25097680−25319570
atp5f1
eps8l3b
gstm.3
gstm.2
gstm.1
ampd2b
gnat2
gpr61
gnai3
amigo1
Value
2
1
0

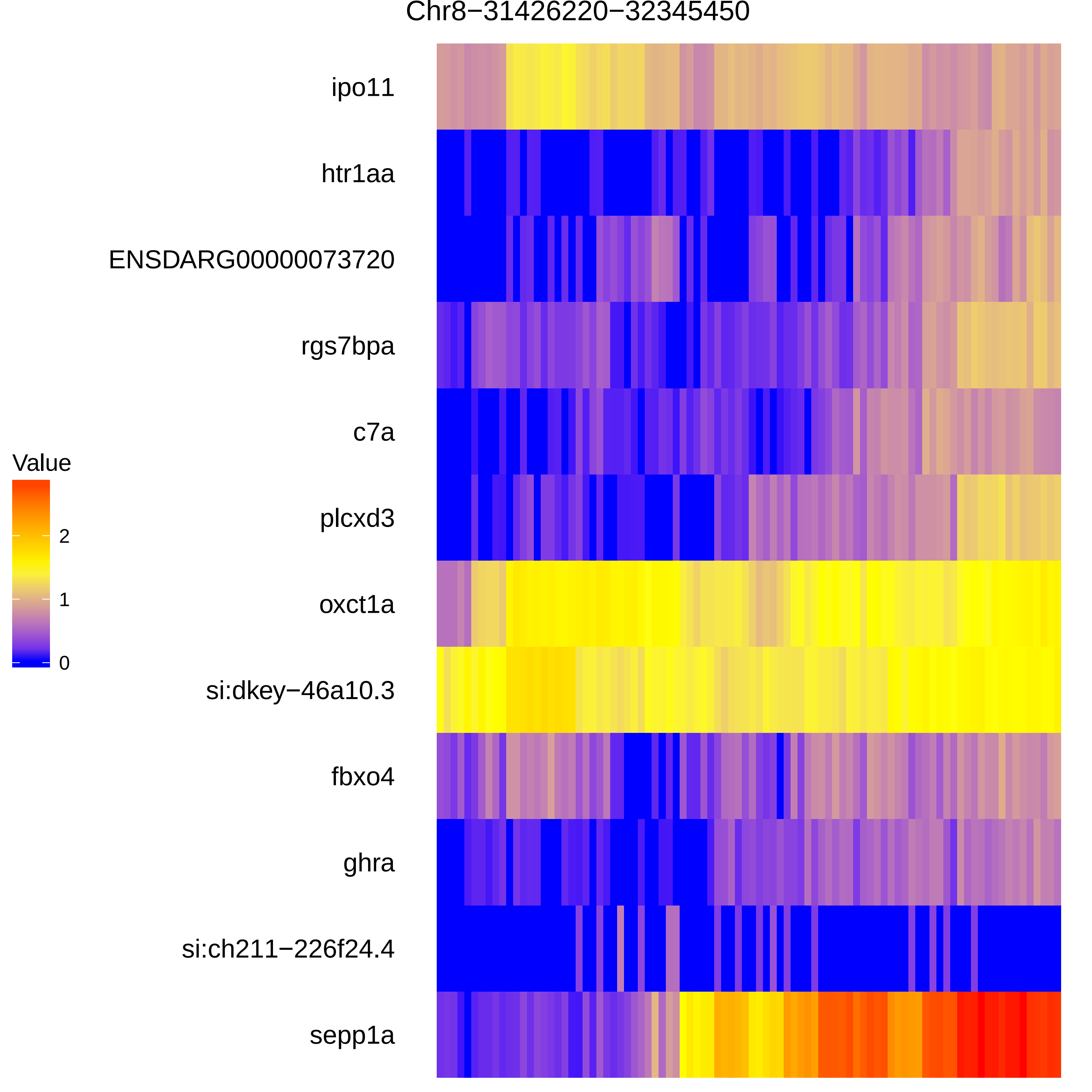
Chr8−31426220−32345450
ipo11
htr1aa
ENSDARG00000073720
rgs7bpa
c7a
plcxd3
oxct1a
si:dkey−46a10.3
fbxo4
ghra
si:ch211−226f24.4
sepp1a
Value
2
1
0

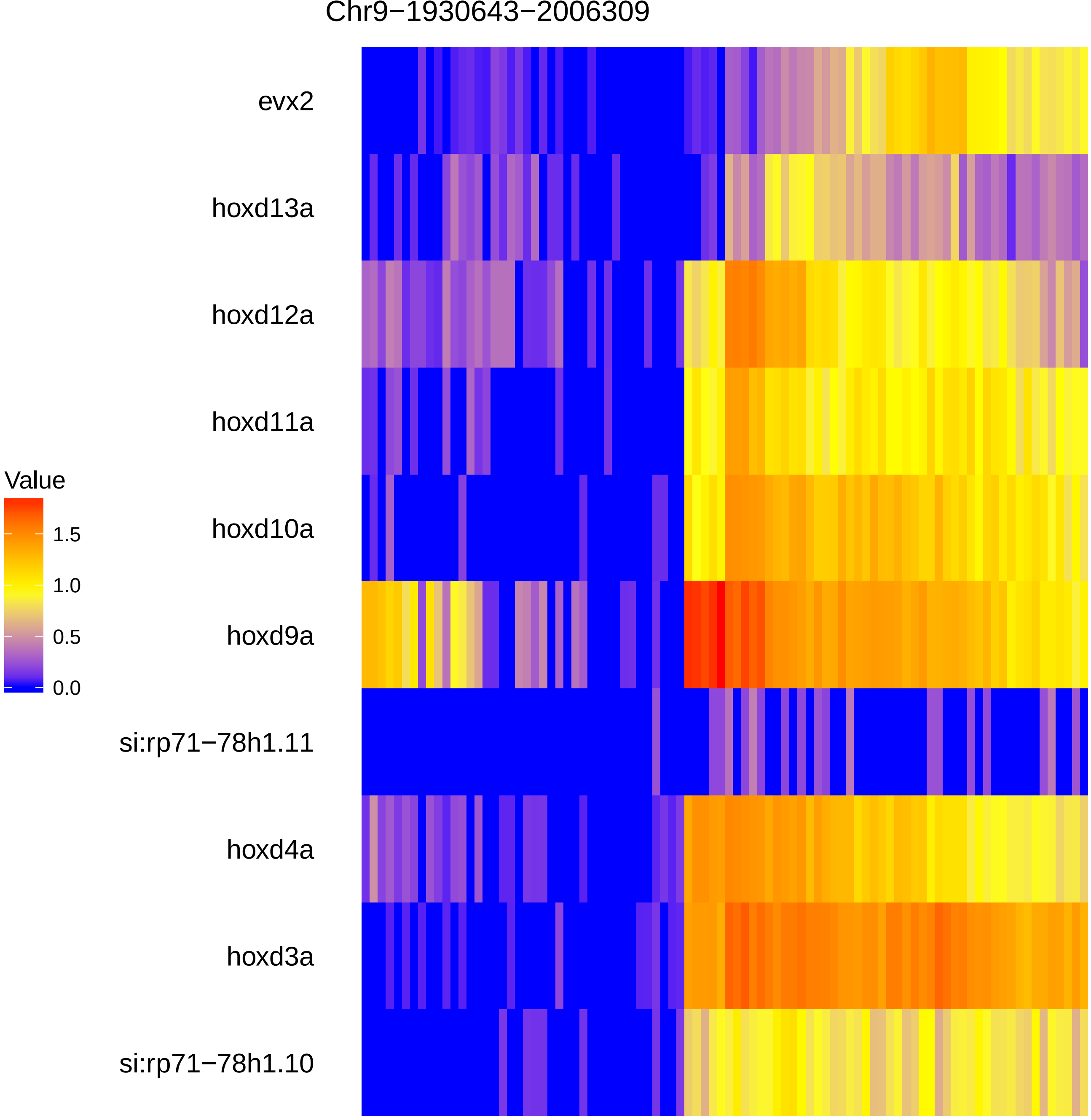
Chr9−1930643−2006309
evx2
hoxd13a
hoxd12a
hoxd11a
hoxd10a
hoxd9a
si:rp71−78h1.11
hoxd4a
hoxd3a
si:rp71−78h1.10
Value
1.5
1.0
0.5
0.0

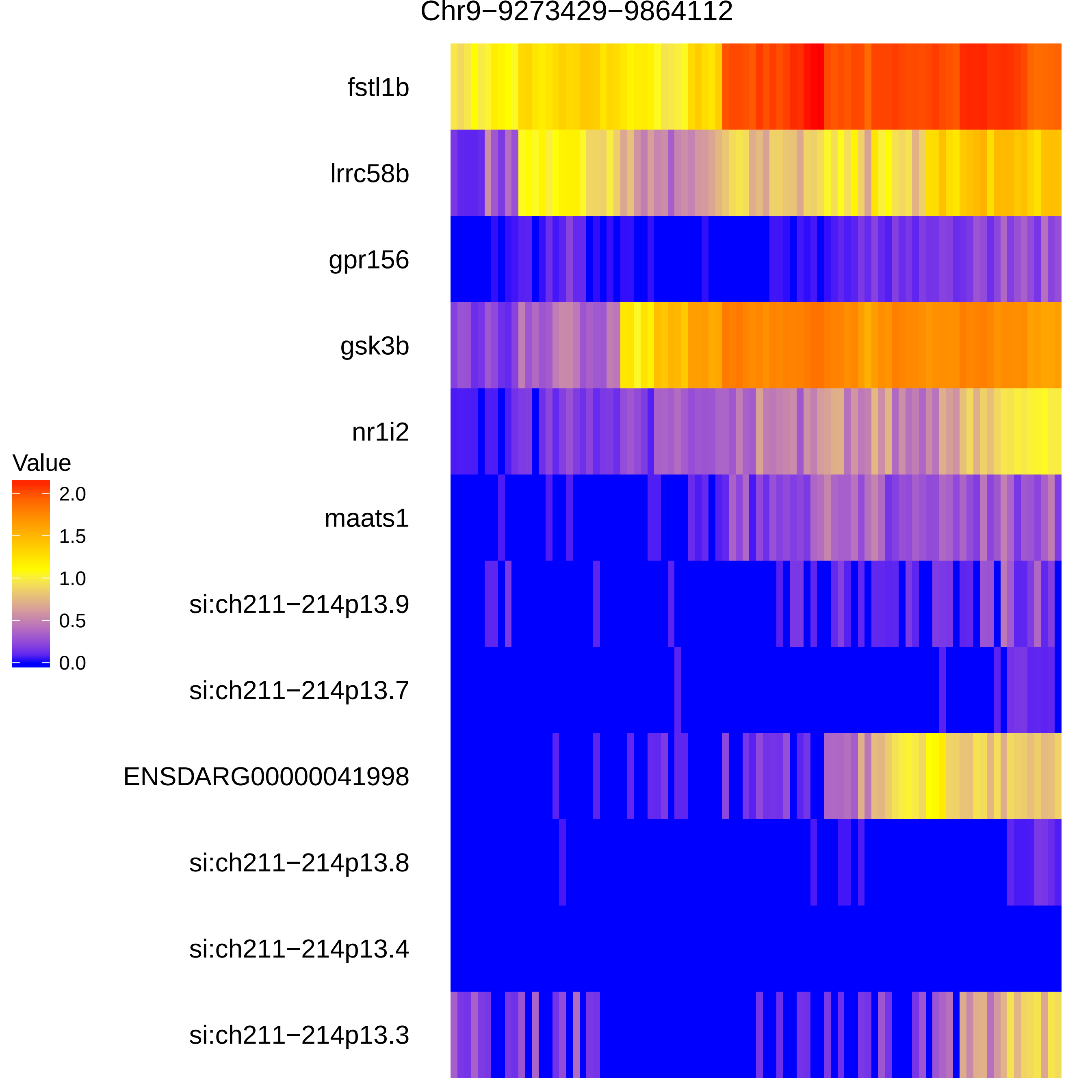
Chr9−9273429−9864112
fstl1b
lrrc58b
gpr156
gsk3b
nr1i2
maats1
si:ch211−214p13.9
si:ch211−214p13.7
ENSDARG00000041998
si:ch211−214p13.8
si:ch211−214p13.4
si:ch211−214p13.3
Value
2.0
1.5
1.0
0.5
0.0

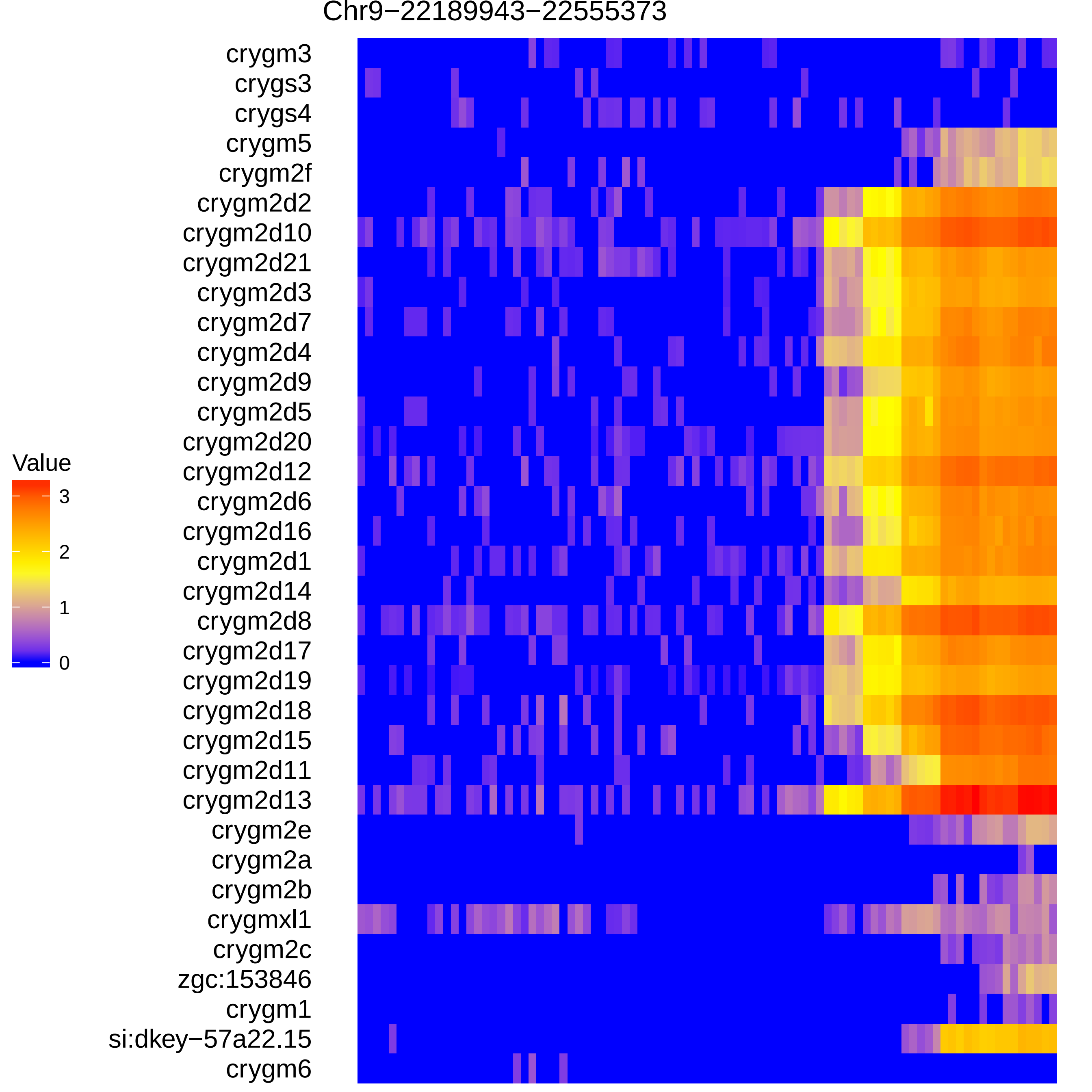

Chr9−22189943−22555373
crygm3
crygs3
crygs4
crygm5
crygm2f
crygm2d2
crygm2d10
crygm2d21
crygm2d3
crygm2d7
crygm2d4
crygm2d9
crygm2d5
crygm2d20
crygm2d12
crygm2d6
crygm2d16
crygm2d1
crygm2d14
crygm2d8
crygm2d17
crygm2d19
crygm2d18
crygm2d15
crygm2d11
crygm2d13
crygm2e
crygm2a
crygm2b
crygmxl1
crygm2c
zgc:153846
crygm1
si:dkey−57a22.15
crygm6
Value
3
2
1
0

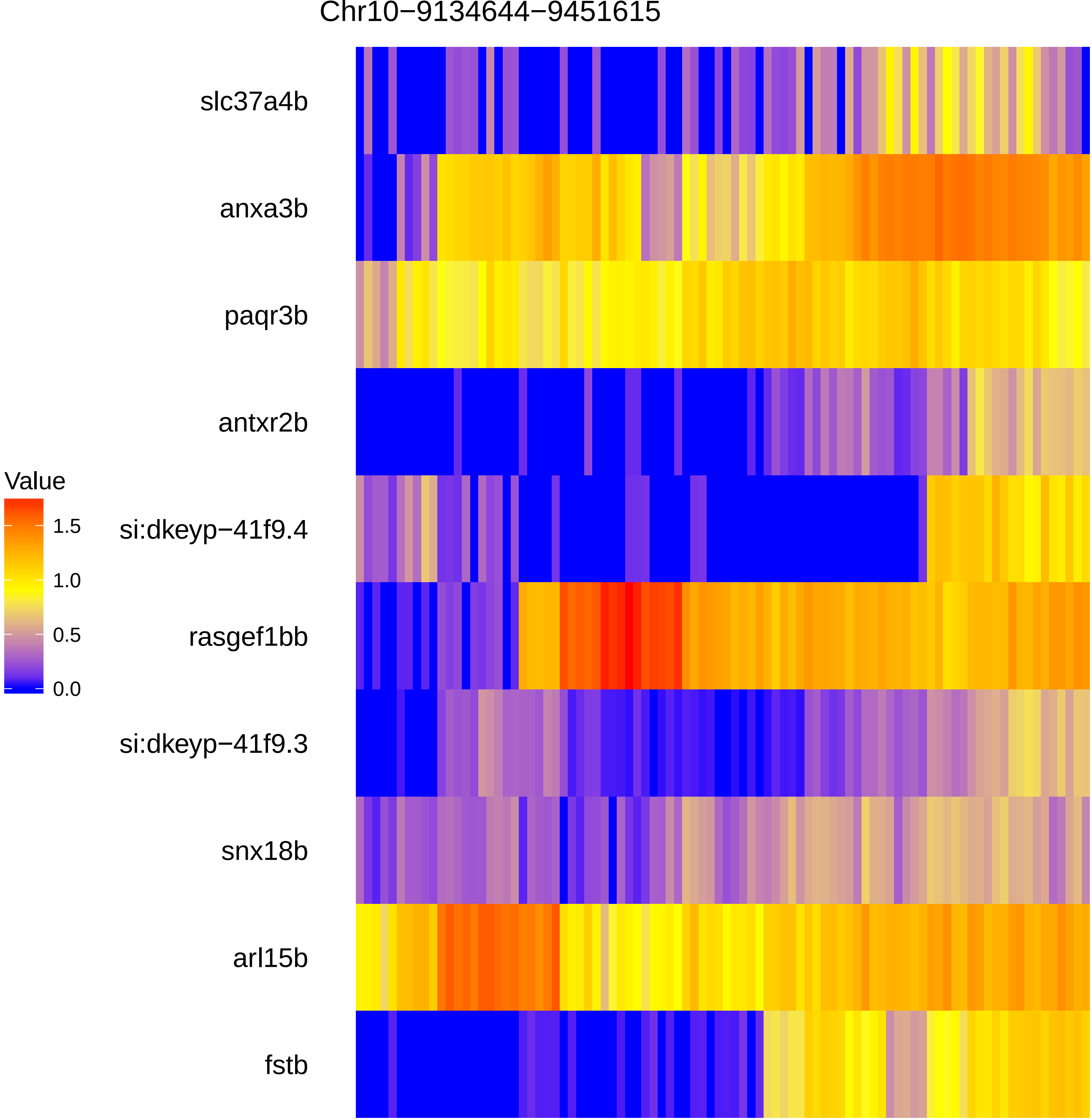
Chr10−9134644−9451615
slc37a4b
anxa3b
paqr3b
antxr2b
si:dkeyp−41f9.4
rasgef1bb
si:dkeyp−41f9.3
snx18b
arl15b
fstb
Value
1.5
1.0
0.5
0.0

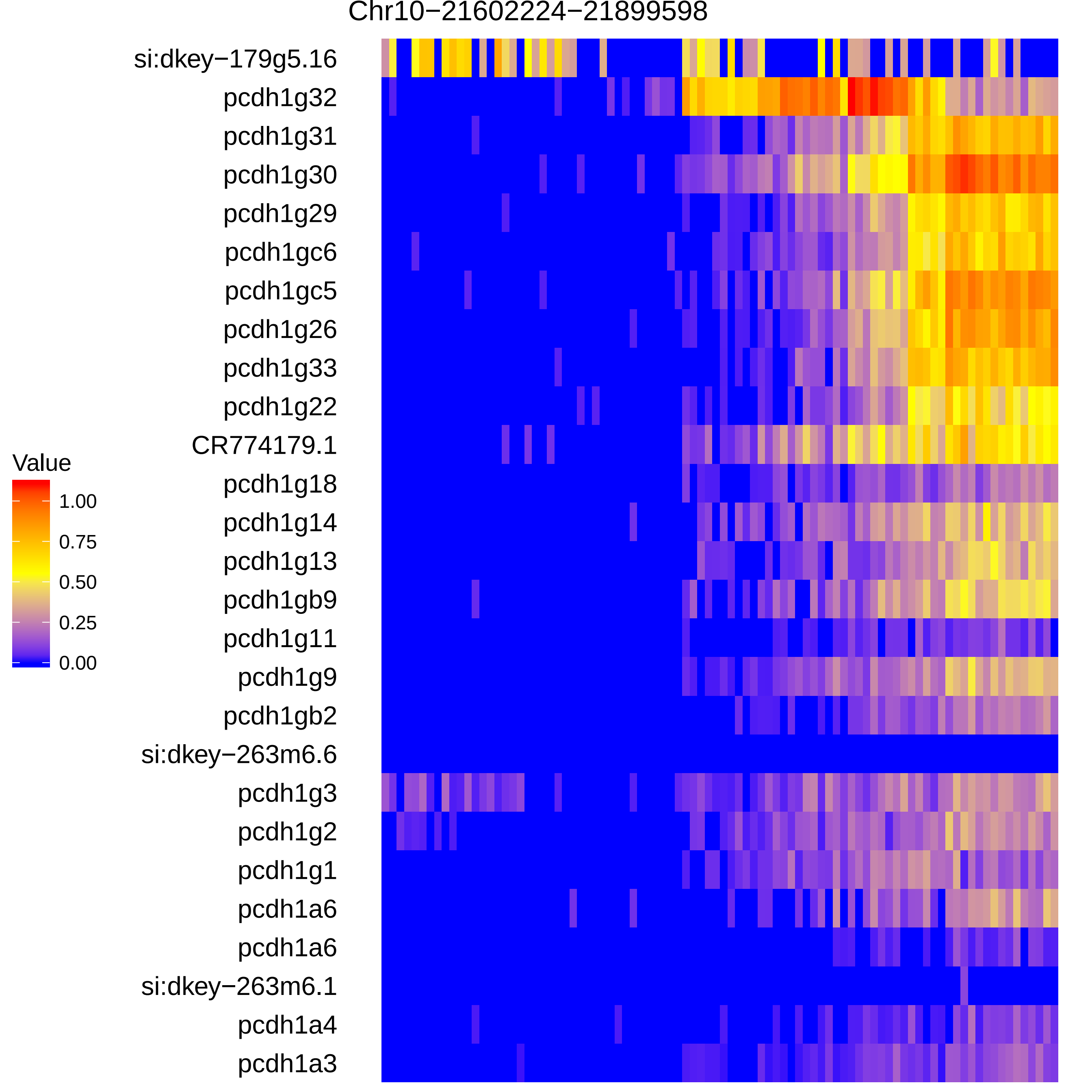

Chr10−21602224−21899598
si:dkey−179g5.16
pcdh1g32
pcdh1g31
pcdh1g30
pcdh1g29
pcdh1gc6
pcdh1gc5
pcdh1g26
pcdh1g33
pcdh1g22
CR774179.1
pcdh1g18
pcdh1g14
pcdh1g13
pcdh1gb9
pcdh1g11
pcdh1g9
pcdh1gb2
si:dkey−263m6.6
pcdh1g3
pcdh1g2
pcdh1g1
pcdh1a6
pcdh1a6
si:dkey−263m6.1
pcdh1a4
pcdh1a3
Value
1.00
0.75
0.50
0.25
0.00

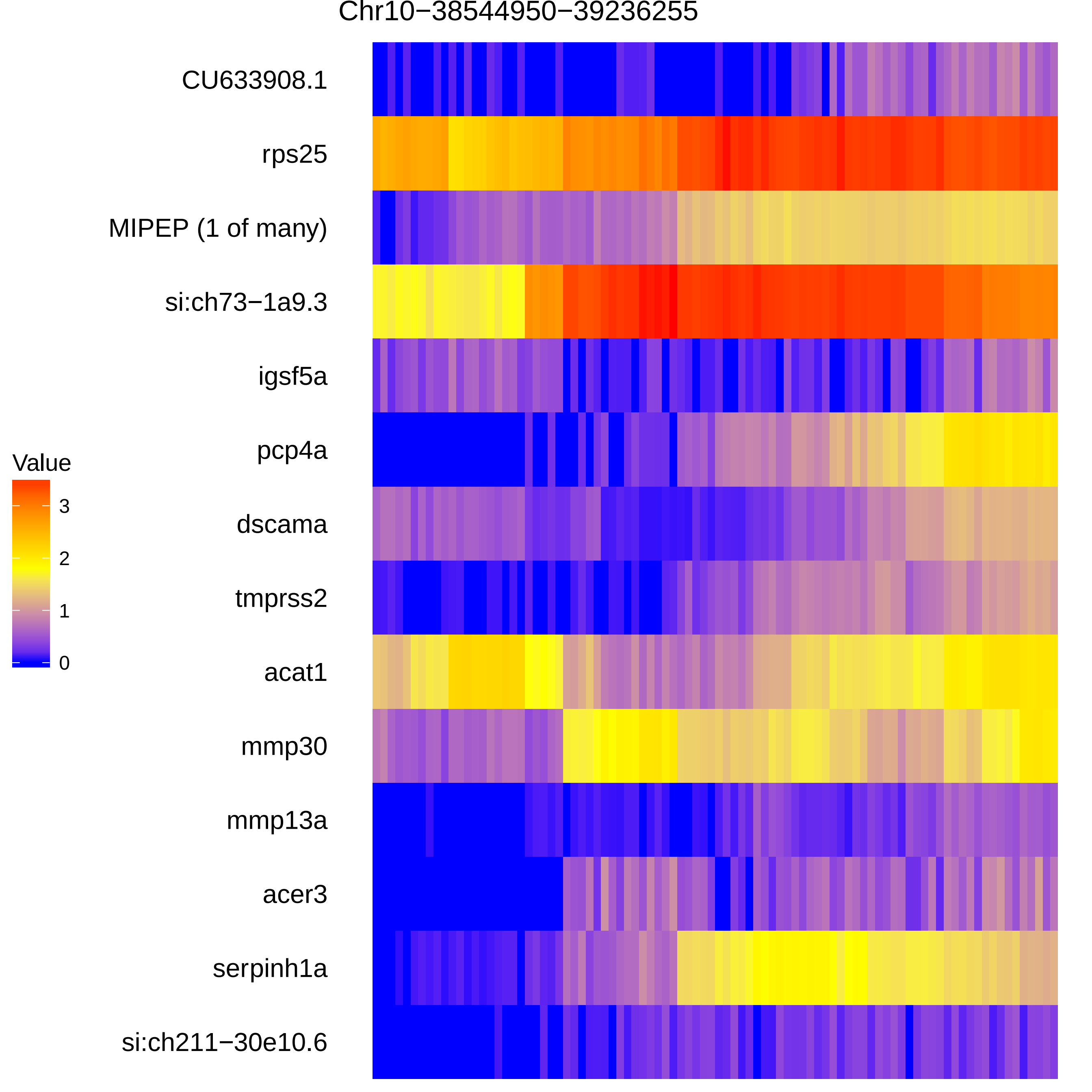
Chr10−38544950−39236255
CU633908.1
rps25
MIPEP (1 of many)
si:ch73−1a9.3
igsf5a
pcp4a
dscama
tmprss2
acat1
mmp30
mmp13a
acer3
serpinh1a
si:ch211−30e10.6
Value
3
2
1
0

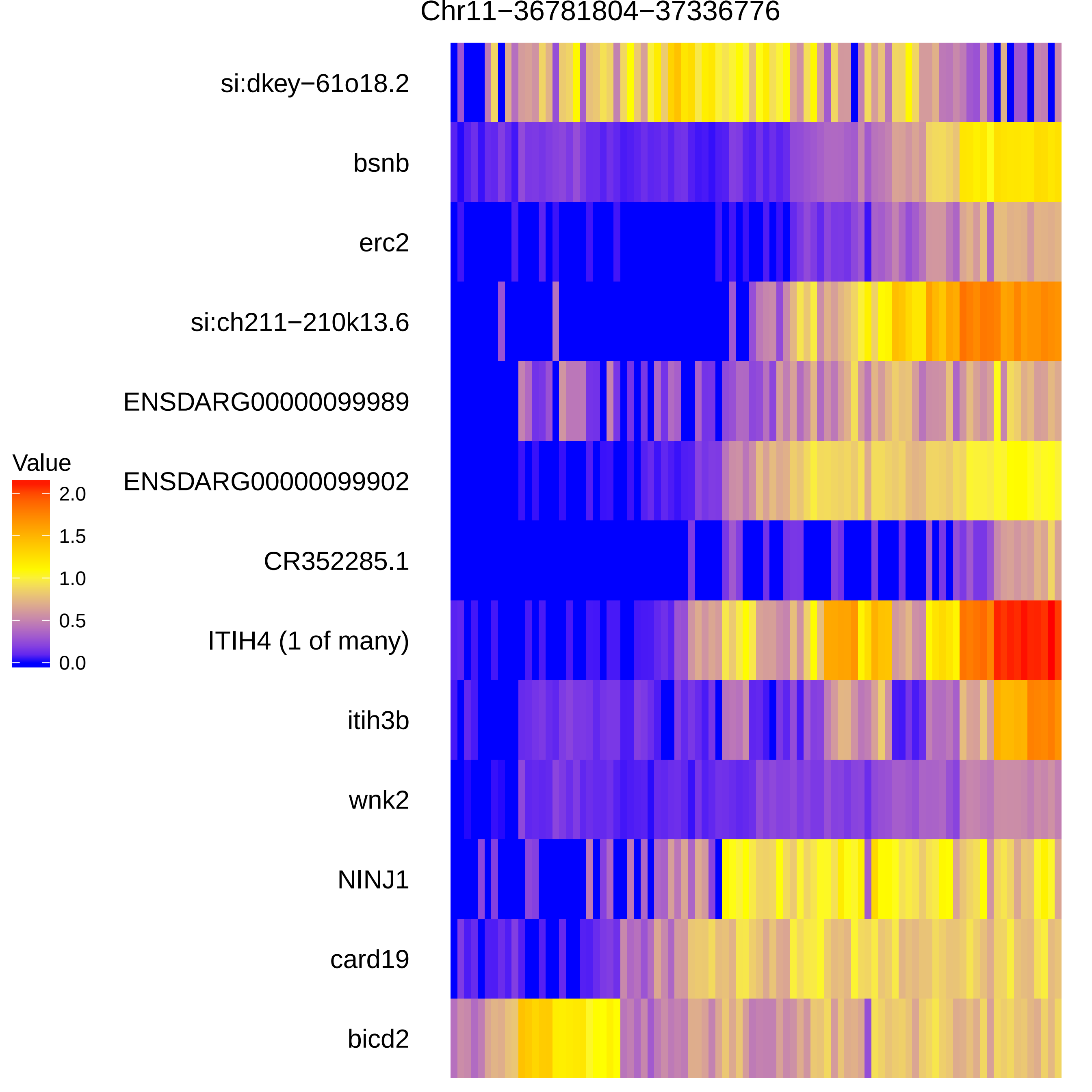
Chr11−36781804−37336776
si:dkey−61o18.2
bsnb
erc2
si:ch211−210k13.6
ENSDARG00000099989
ENSDARG00000099902
CR352285.1
ITIH4 (1 of many)
itih3b
wnk2
NINJ1
card19
bicd2
Value
2.0
1.5
1.0
0.5
0.0

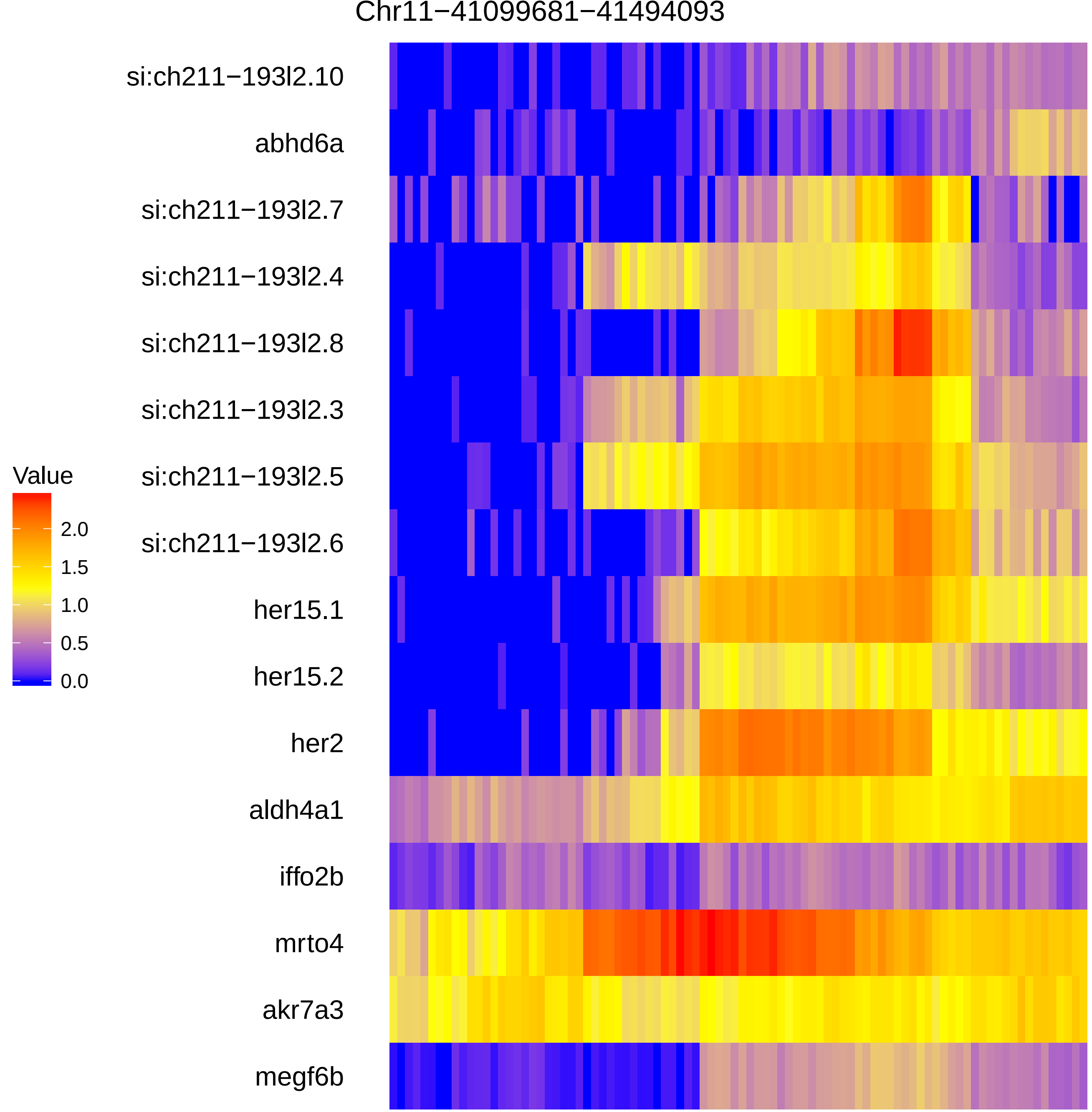
Chr11−41099681−41494093
si:ch211−193l2.10
abhd6a
si:ch211−193l2.7
si:ch211−193l2.4
si:ch211−193l2.8
si:ch211−193l2.3
si:ch211−193l2.5
si:ch211−193l2.6
her15.1
her15.2
her2
aldh4a1
iffo2b
mrto4
akr7a3
megf6b
Value
2.0
1.5
1.0
0.5
0.0

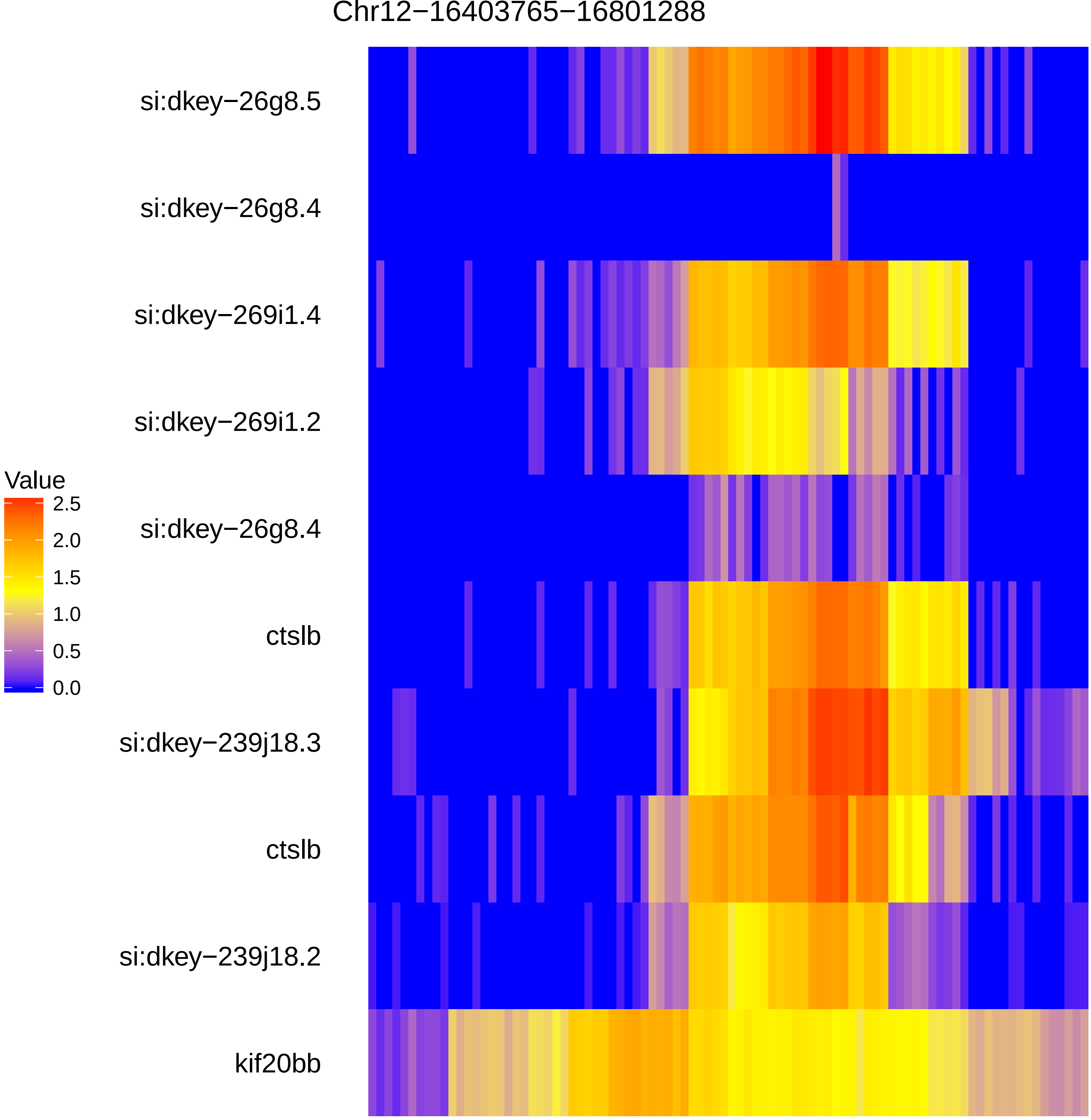
Chr12−16403765−16801288
si:dkey−26g8.5
si:dkey−26g8.4
si:dkey−269i1.4
si:dkey−269i1.2
si:dkey−26g8.4
ctslb
si:dkey−239j18.3
ctslb
si:dkey−239j18.2
kif20bb
Value
2.5
2.0
1.5
1.0
0.5
0.0

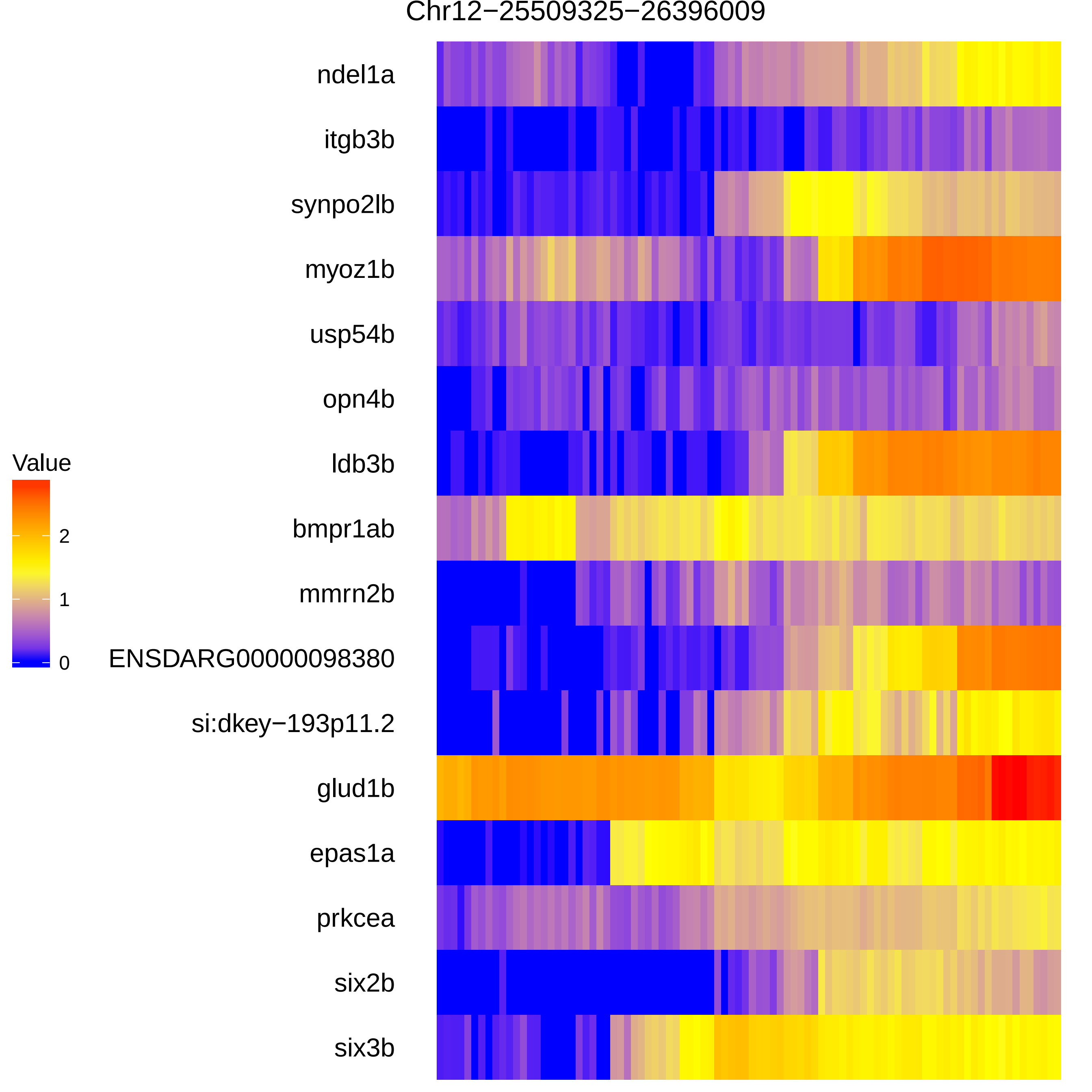
Chr12−25509325−26396009
ndel1a
itgb3b
synpo2lb
myoz1b
usp54b
opn4b
ldb3b
bmpr1ab
mmrn2b
ENSDARG00000098380
si:dkey−193p11.2
glud1b
epas1a
prkcea
six2b
six3b
Value
2
1
0

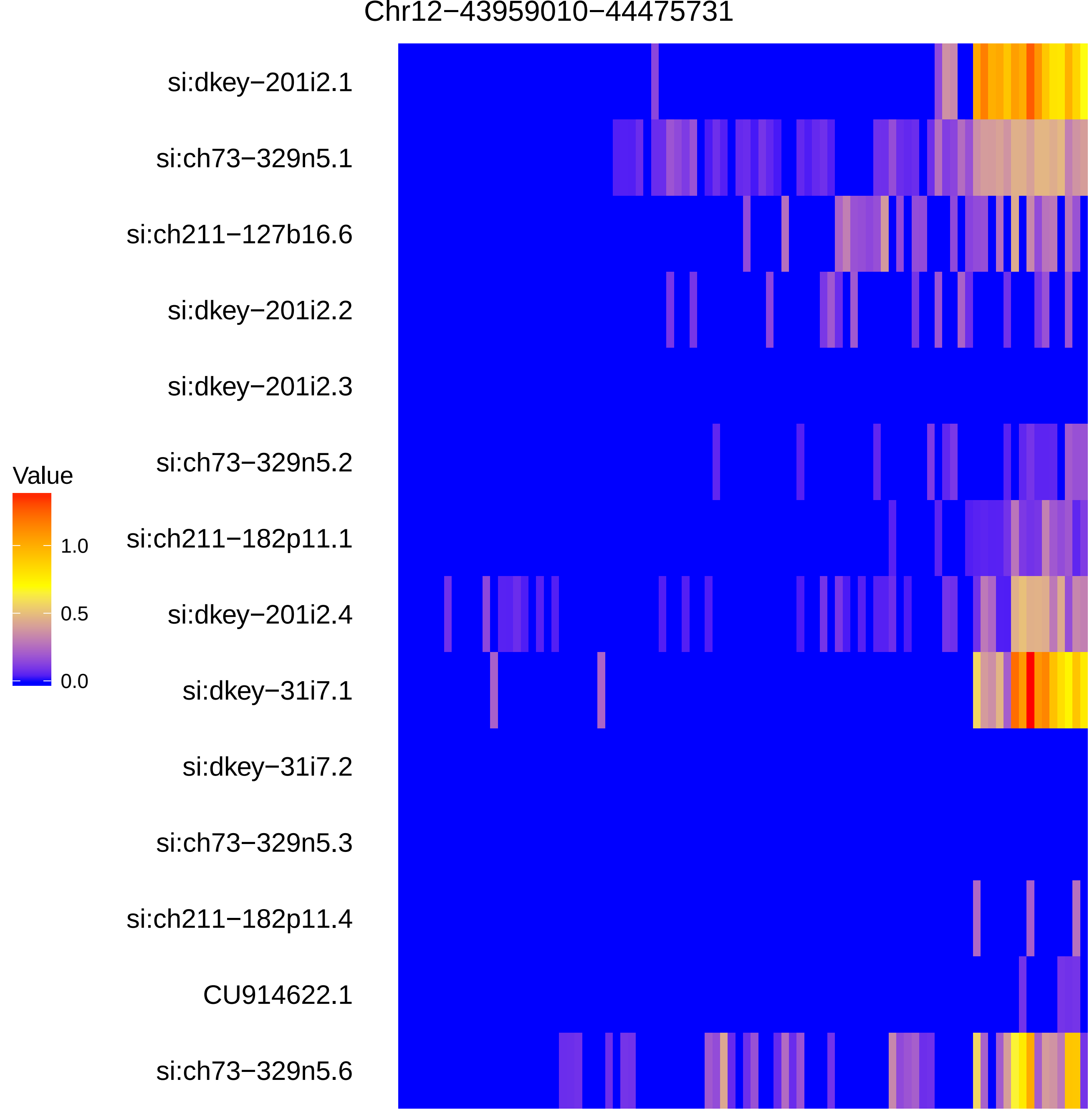
Chr12−43959010−44475731
si:dkey−201i2.1
si:ch73−329n5.1
si:ch211−127b16.6
si:dkey−201i2.2
si:dkey−201i2.3
si:ch73−329n5.2
si:ch211−182p11.1
si:dkey−201i2.4
si:dkey−31i7.1
si:dkey−31i7.2
si:ch73−329n5.3
si:ch211−182p11.4
CU914622.1
si:ch73−329n5.6
Value
1.0
0.5
0.0

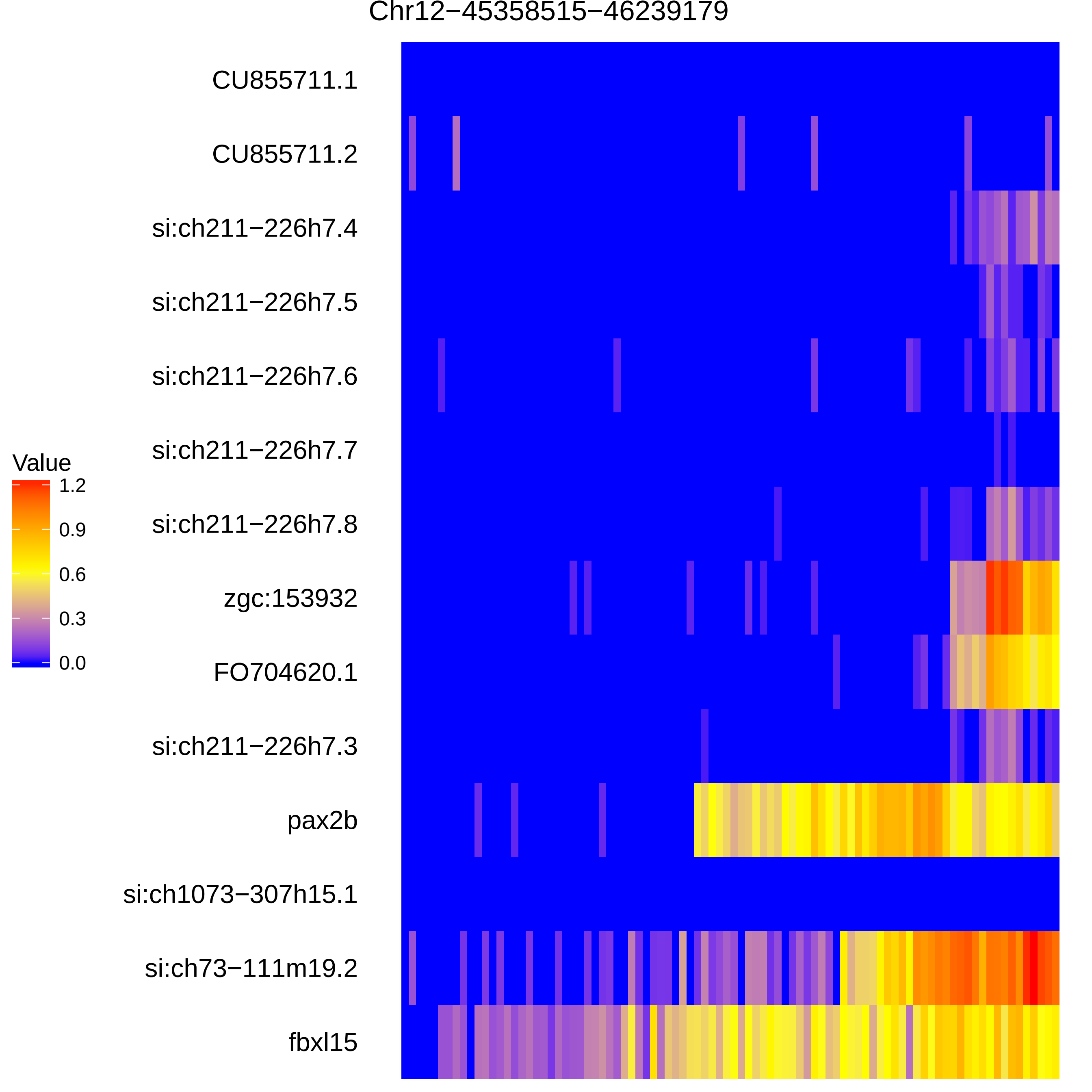
Chr12−45358515−46239179
CU855711.1
CU855711.2
si:ch211−226h7.4
si:ch211−226h7.5
si:ch211−226h7.6
si:ch211−226h7.7
si:ch211−226h7.8
zgc:153932
FO704620.1
si:ch211−226h7.3
pax2b
si:ch1073−307h15.1
si:ch73−111m19.2
fbxl15
Value
1.2
0.9
0.6
0.3
0.0

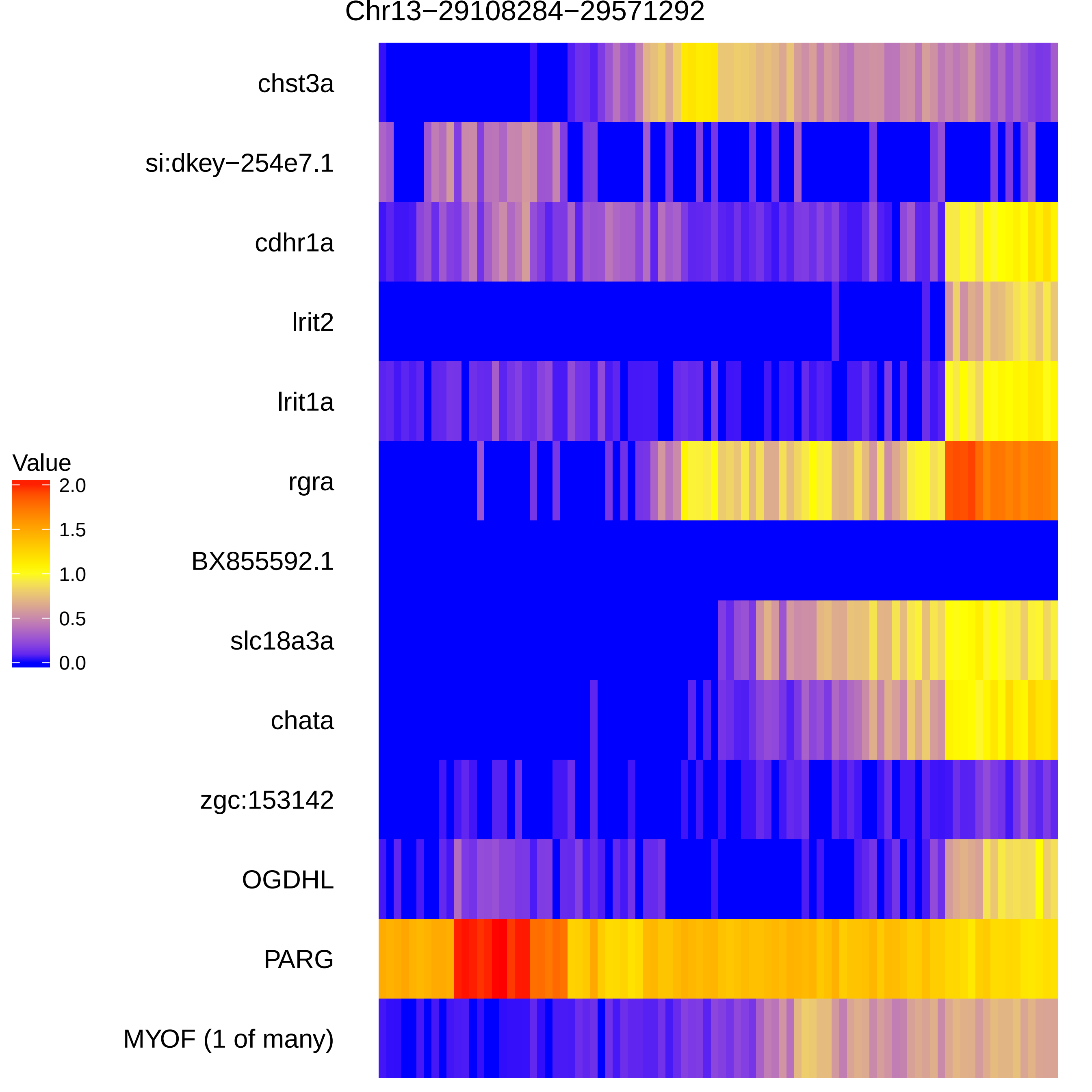
Chr13−29108284−29571292
chst3a
si:dkey−254e7.1
cdhr1a
lrit2
lrit1a
rgra
BX855592.1
slc18a3a
chata
zgc:153142
OGDHL
PARG
MYOF (1 of many)
Value
2.0
1.5
1.0
0.5
0.0

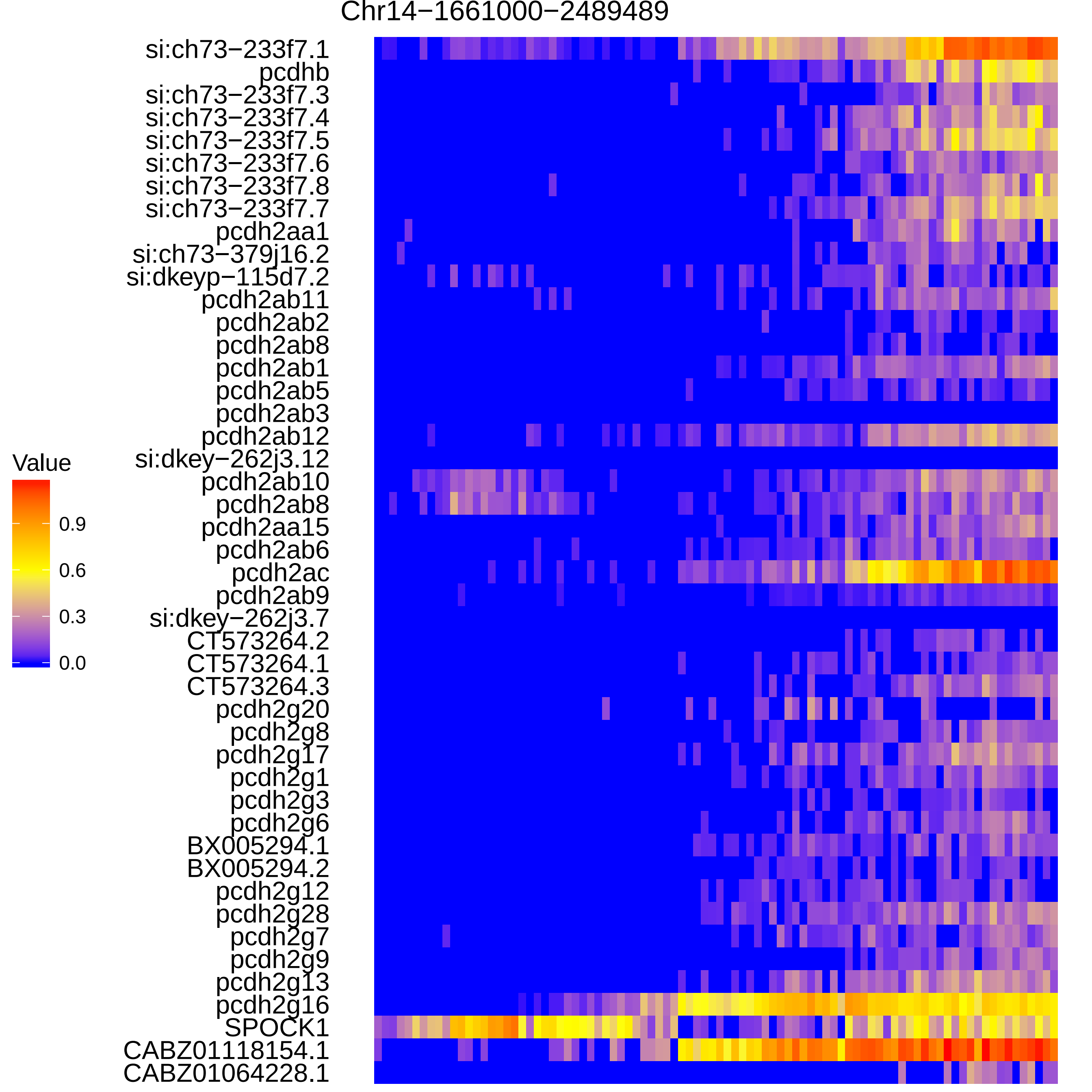
Chr14−1661000−2489489
si:ch73−233f7.1
pcdhb
si:ch73−233f7.3
si:ch73−233f7.4
si:ch73−233f7.5
si:ch73−233f7.6
si:ch73−233f7.8
si:ch73−233f7.7
pcdh2aa1
si:ch73−379j16.2
si:dkeyp−115d7.2
pcdh2ab11
pcdh2ab2
pcdh2ab8
pcdh2ab1
pcdh2ab5
pcdh2ab3
pcdh2ab12
si:dkey−262j3.12
pcdh2ab10
pcdh2ab8
pcdh2aa15
pcdh2ab6
pcdh2ac
pcdh2ab9
si:dkey−262j3.7
CT573264.2
CT573264.1
CT573264.3
pcdh2g20
pcdh2g8
pcdh2g17
pcdh2g1
pcdh2g3
pcdh2g6
BX005294.1
BX005294.2
pcdh2g12
pcdh2g28
pcdh2g7
pcdh2g9
pcdh2g13
pcdh2g16
SPOCK1
CABZ01118154.1
CABZ01064228.1
Value
0.9
0.6
0.3
0.0

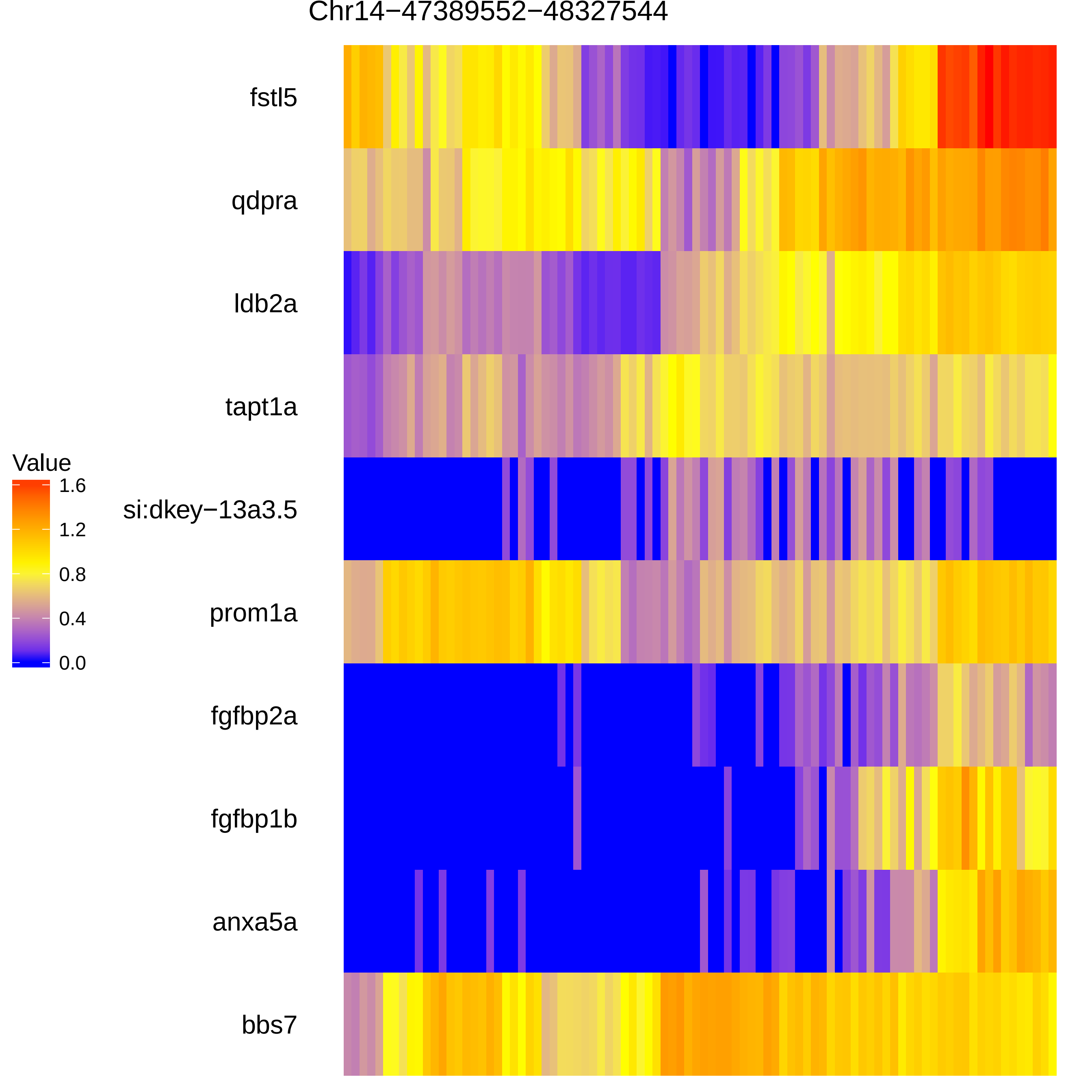
Chr14−47389552−48327544
fstl5
qdpra
ldb2a
tapt1a
si:dkey−13a3.5
prom1a
fgfbp2a
fgfbp1b
anxa5a
bbs7
Value
1.6
1.2
0.8
0.4
0.0

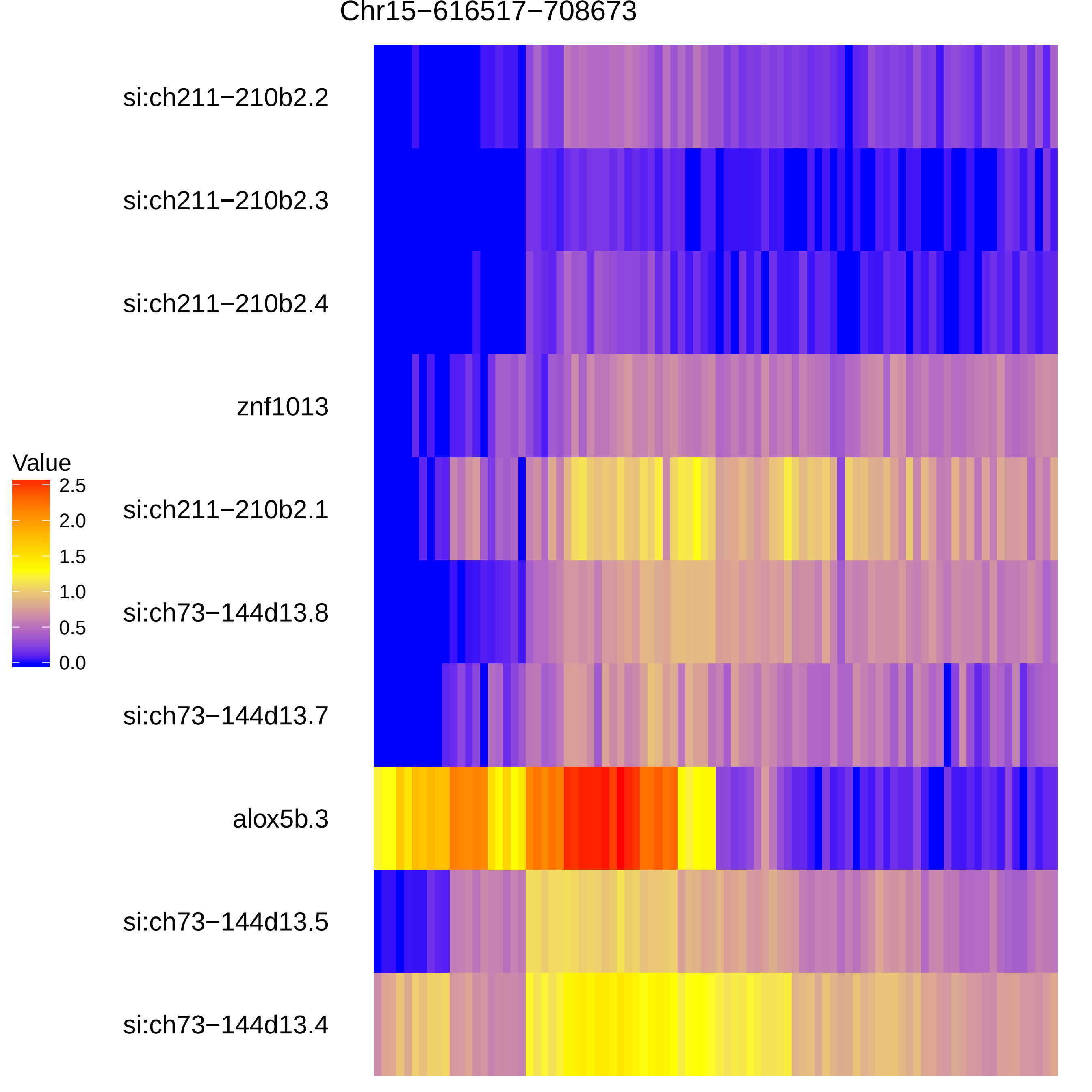
Chr15−616517−708673
si:ch211−210b2.2
si:ch211−210b2.3
si:ch211−210b2.4
znf1013
si:ch211−210b2.1
si:ch73−144d13.8
si:ch73−144d13.7
alox5b.3
si:ch73−144d13.5
si:ch73−144d13.4
Value
2.5
2.0
1.5
1.0
0.5
0.0

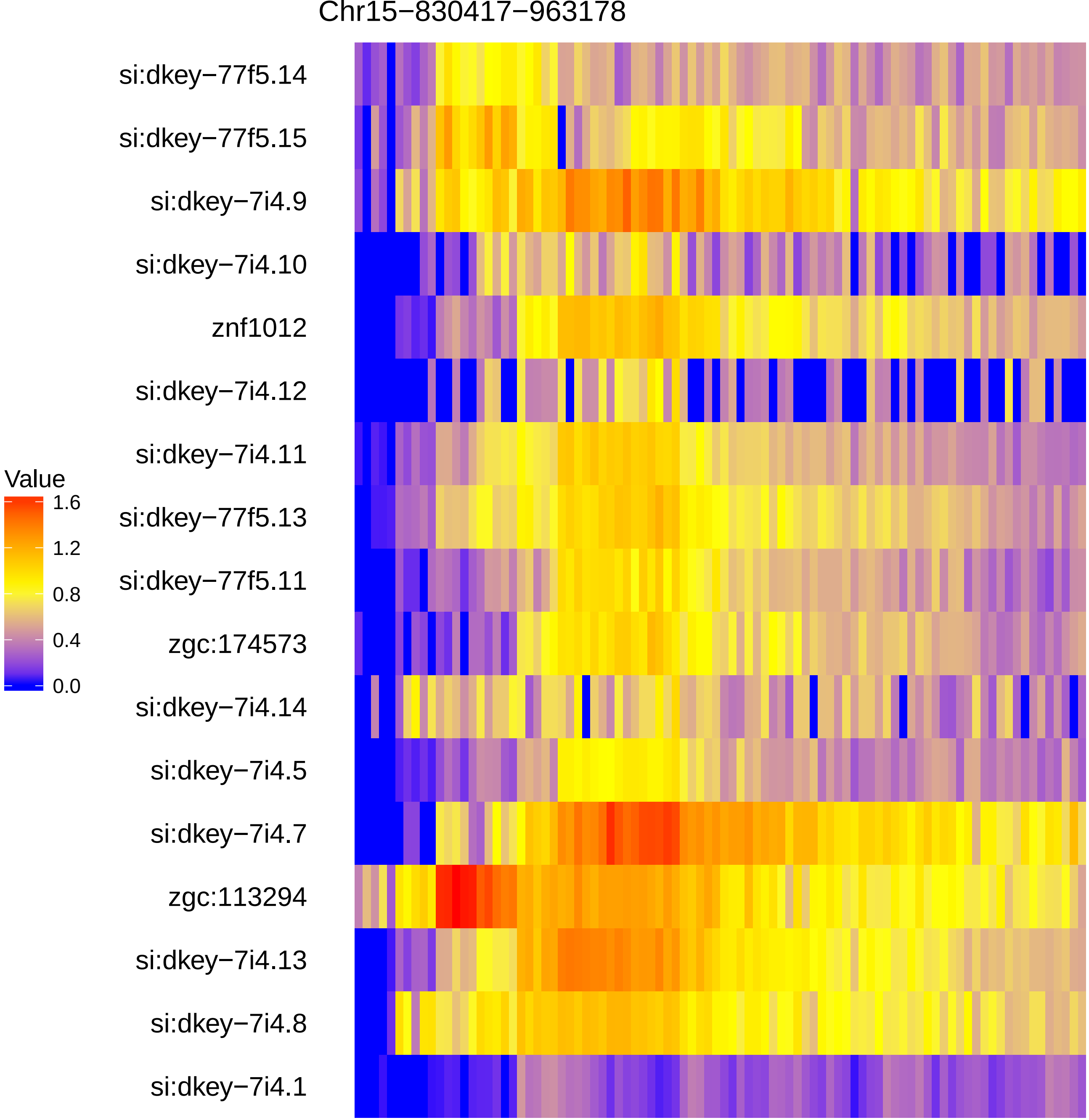
Chr15−830417−963178
si:dkey−77f5.14
si:dkey−77f5.15
si:dkey−7i4.9
si:dkey−7i4.10
znf1012
si:dkey−7i4.12
si:dkey−7i4.11
si:dkey−77f5.13
si:dkey−77f5.11
zgc:174573
si:dkey−7i4.14
si:dkey−7i4.5
si:dkey−7i4.7
zgc:113294
si:dkey−7i4.13
si:dkey−7i4.8
si:dkey−7i4.1
Value
1.6
1.2
0.8
0.4
0.0

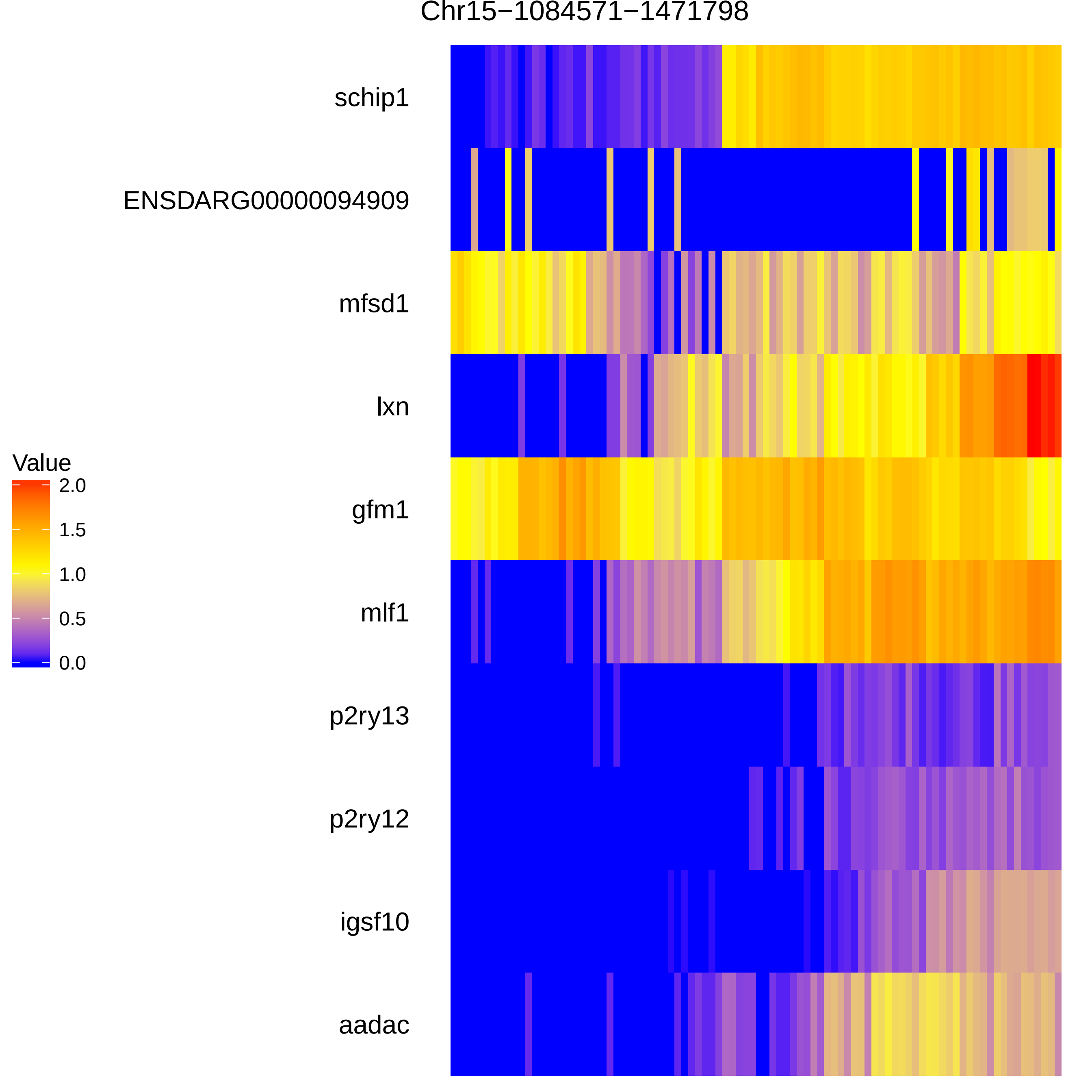

Chr15−1084571−1471798
schip1
ENSDARG00000094909
mfsd1
lxn
gfm1
mlf1
p2ry13
p2ry12
igsf10
aadac
Value
2.0
1.5
1.0
0.5
0.0

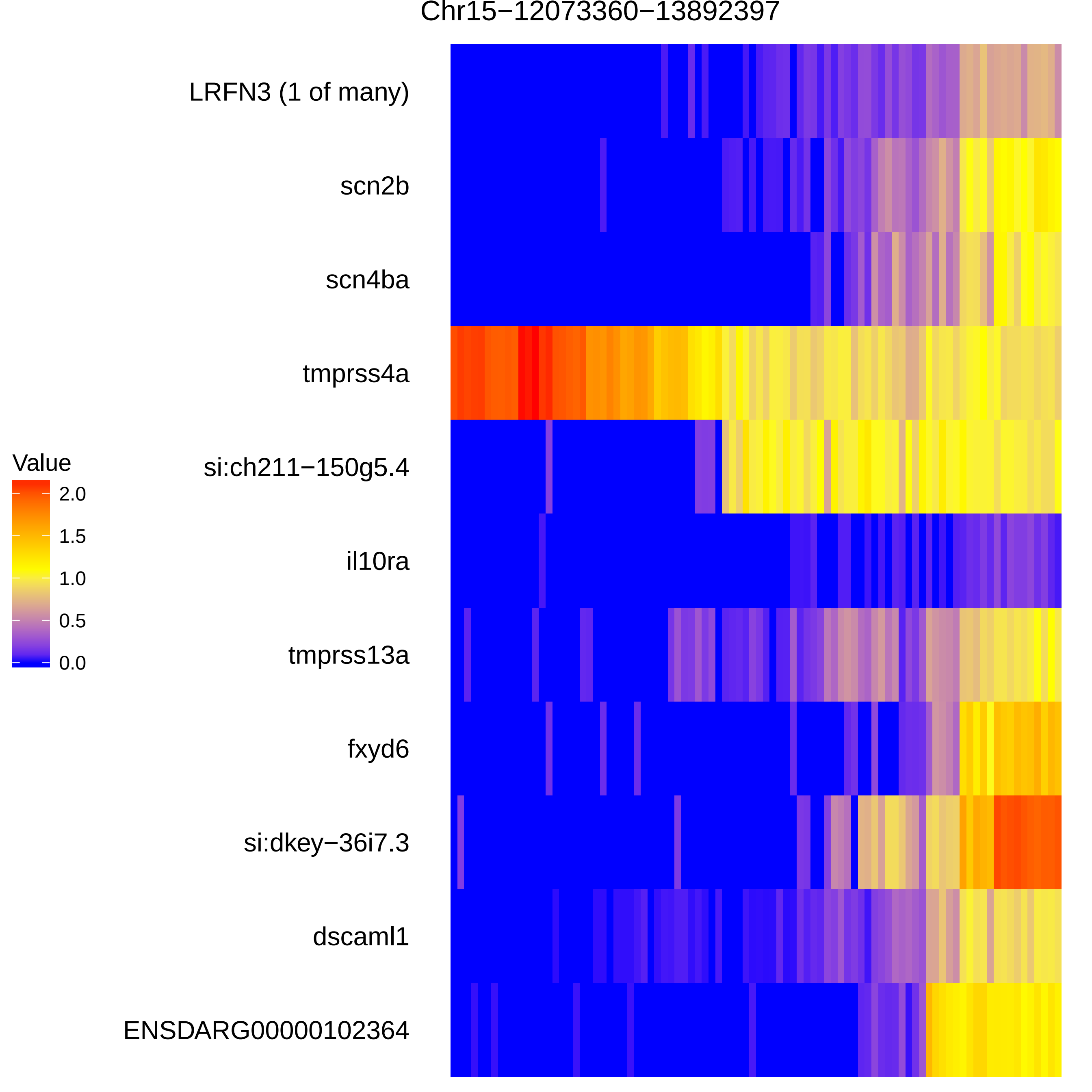
Chr15−12073360−13892397
LRFN3 (1 of many)
scn2b
scn4ba
tmprss4a
si:ch211−150g5.4
il10ra
tmprss13a
fxyd6
si:dkey−36i7.3
dscaml1
ENSDARG00000102364
Value
2.0
1.5
1.0
0.5
0.0

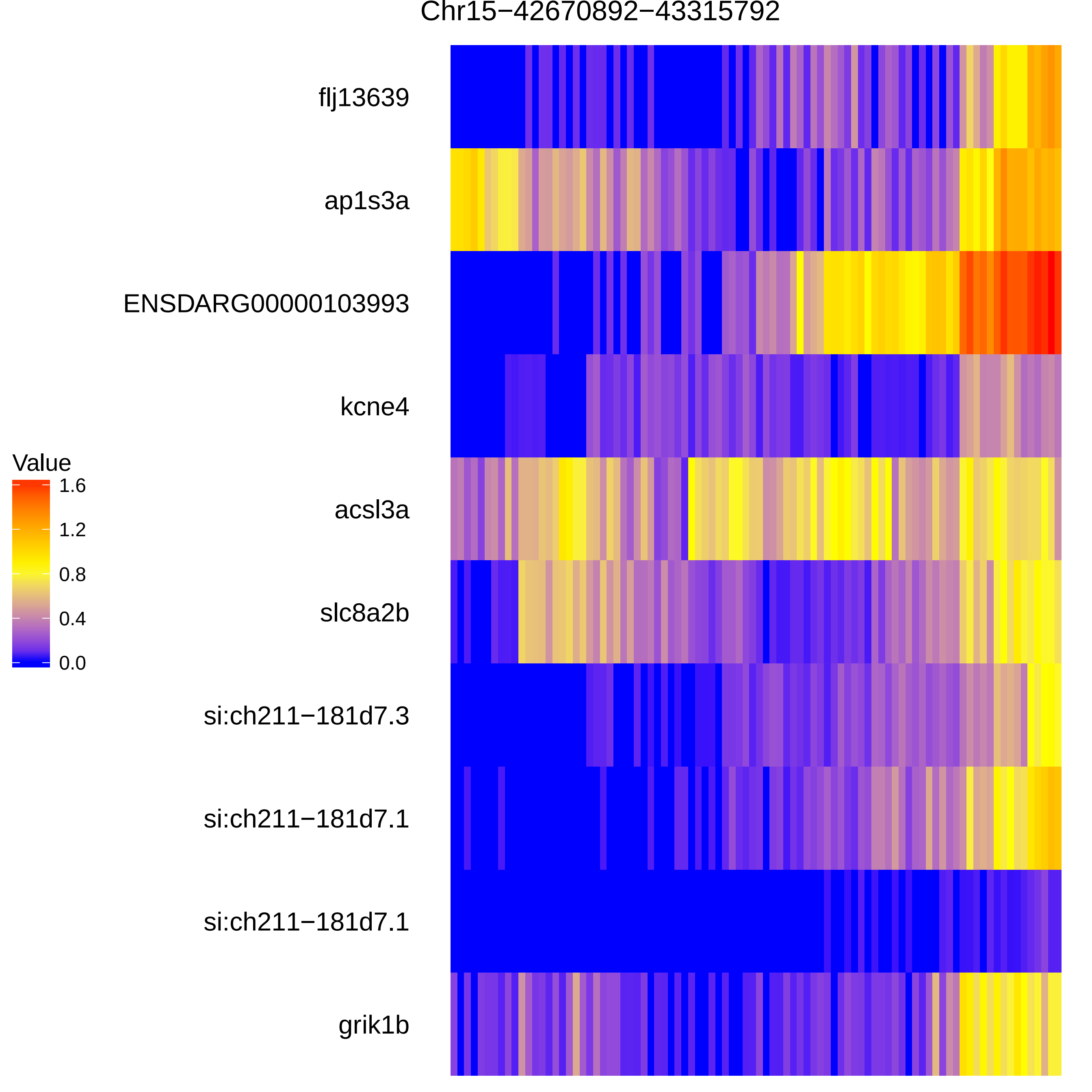
Chr15−42670892−43315792
flj13639
ap1s3a
ENSDARG00000103993
kcne4
acsl3a
slc8a2b
si:ch211−181d7.3
si:ch211−181d7.1
si:ch211−181d7.1
grik1b
Value
1.6
1.2
0.8
0.4
0.0

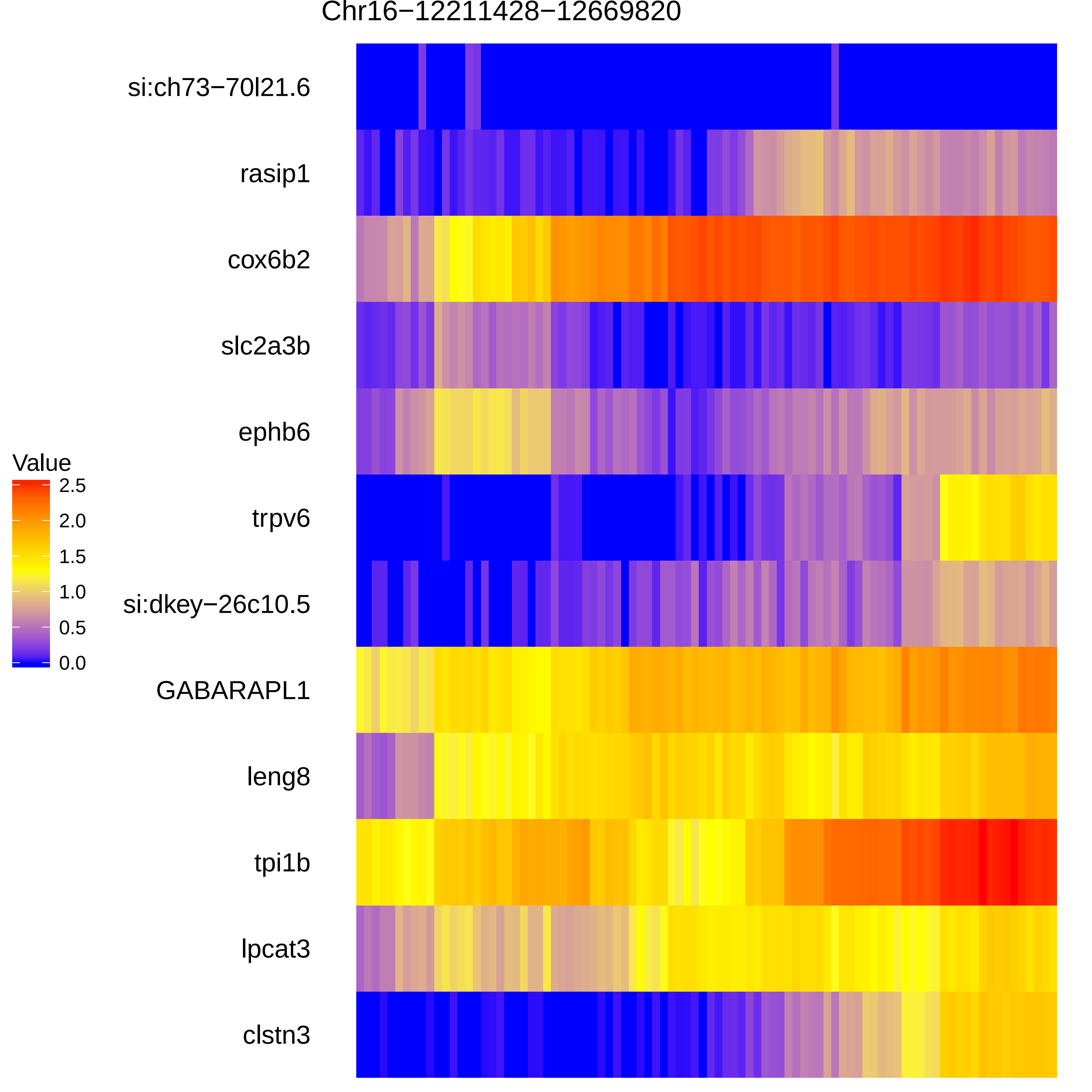
Chr16−12211428−12669820
si:ch73−70l21.6
rasip1
cox6b2
slc2a3b
ephb6
trpv6
si:dkey−26c10.5
GABARAPL1
leng8
tpi1b
lpcat3
clstn3
Value
2.5
2.0
1.5
1.0
0.5
0.0

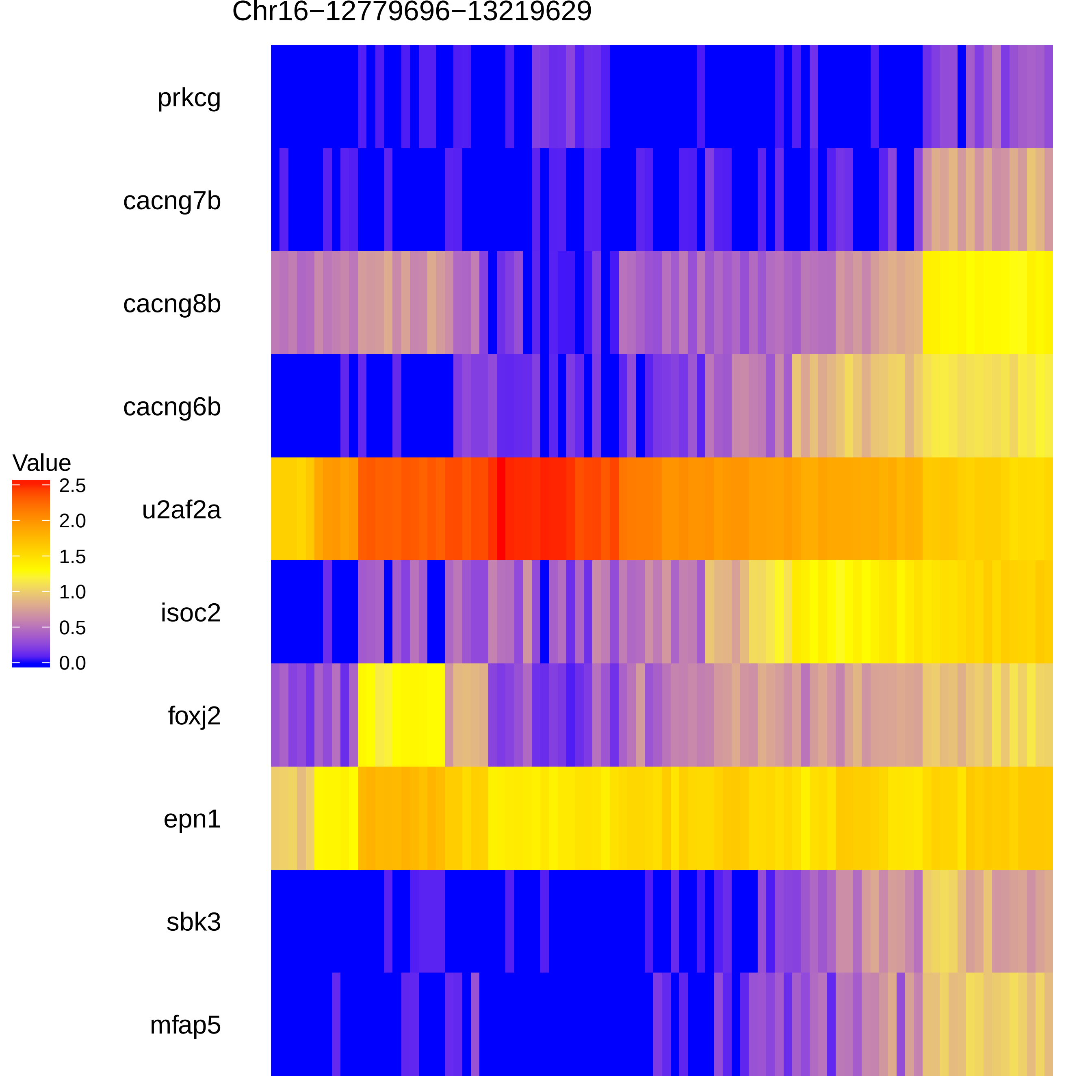

Chr16−12779696−13219629
prkcg
cacng7b
cacng8b
cacng6b
u2af2a
isoc2
foxj2
epn1
sbk3
mfap5
Value
2.5
2.0
1.5
1.0
0.5
0.0

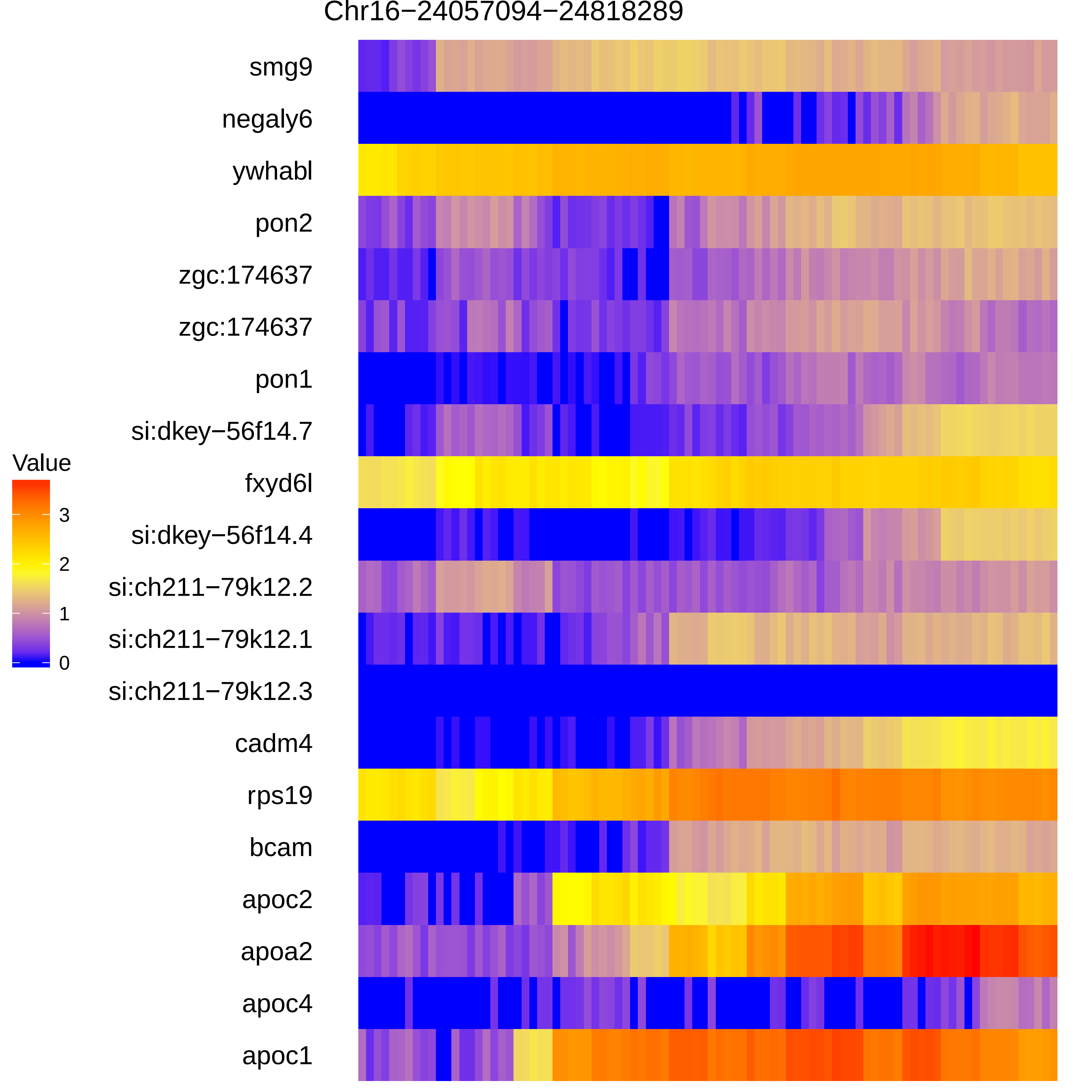
Chr16−24057094−24818289
smg9
negaly6
ywhabl
pon2
zgc:174637
zgc:174637
pon1
si:dkey−56f14.7
fxyd6l
si:dkey−56f14.4
si:ch211−79k12.2
si:ch211−79k12.1
si:ch211−79k12.3
cadm4
rps19
bcam
apoc2
apoa2
apoc4
apoc1
Value
3
2
1
0

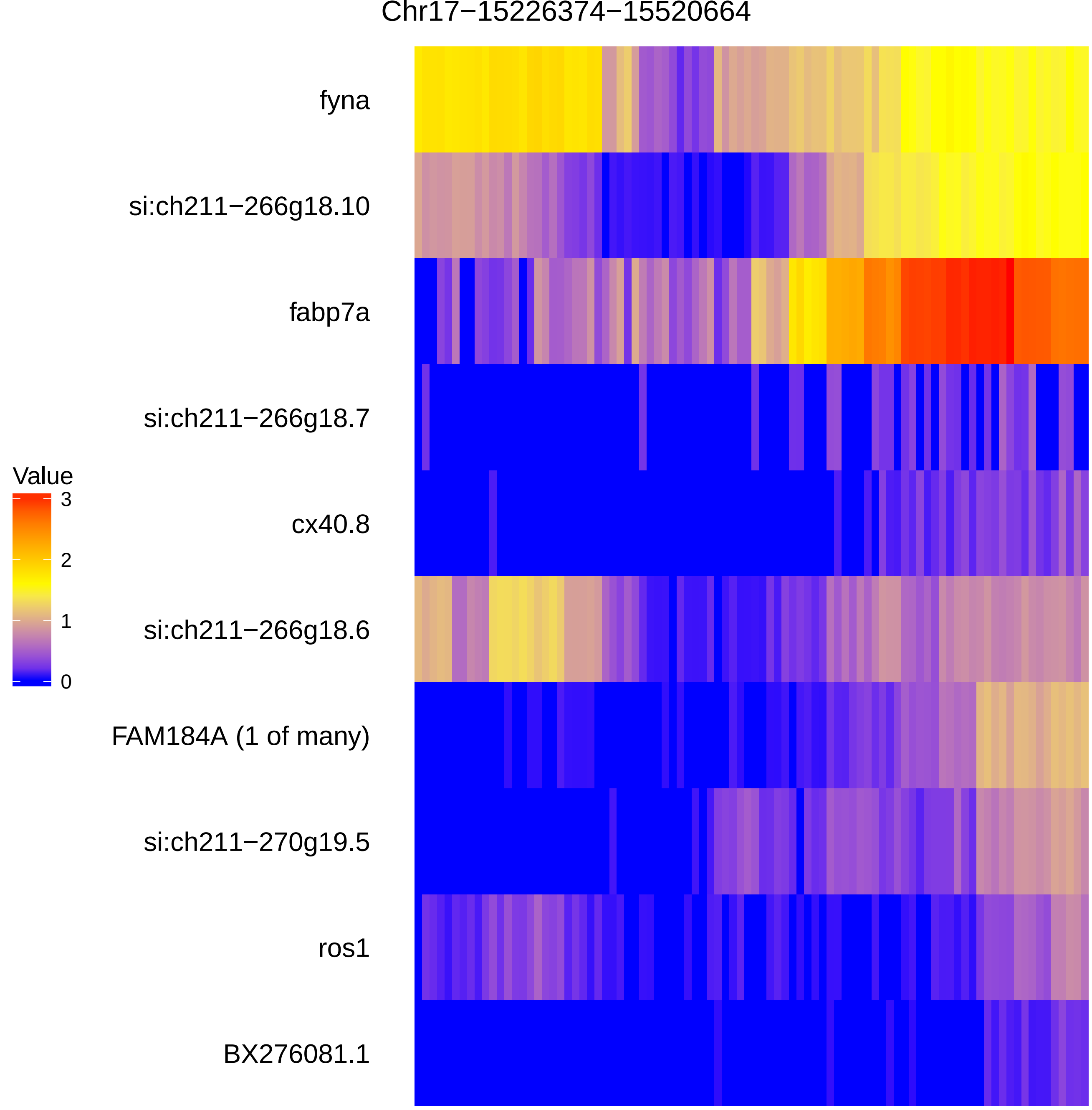

Chr17−15226374−15520664
fyna
si:ch211−266g18.10
fabp7a
si:ch211−266g18.7
cx40.8
si:ch211−266g18.6
FAM184A (1 of many)
si:ch211−270g19.5
ros1
BX276081.1
Value
3
2
1
0

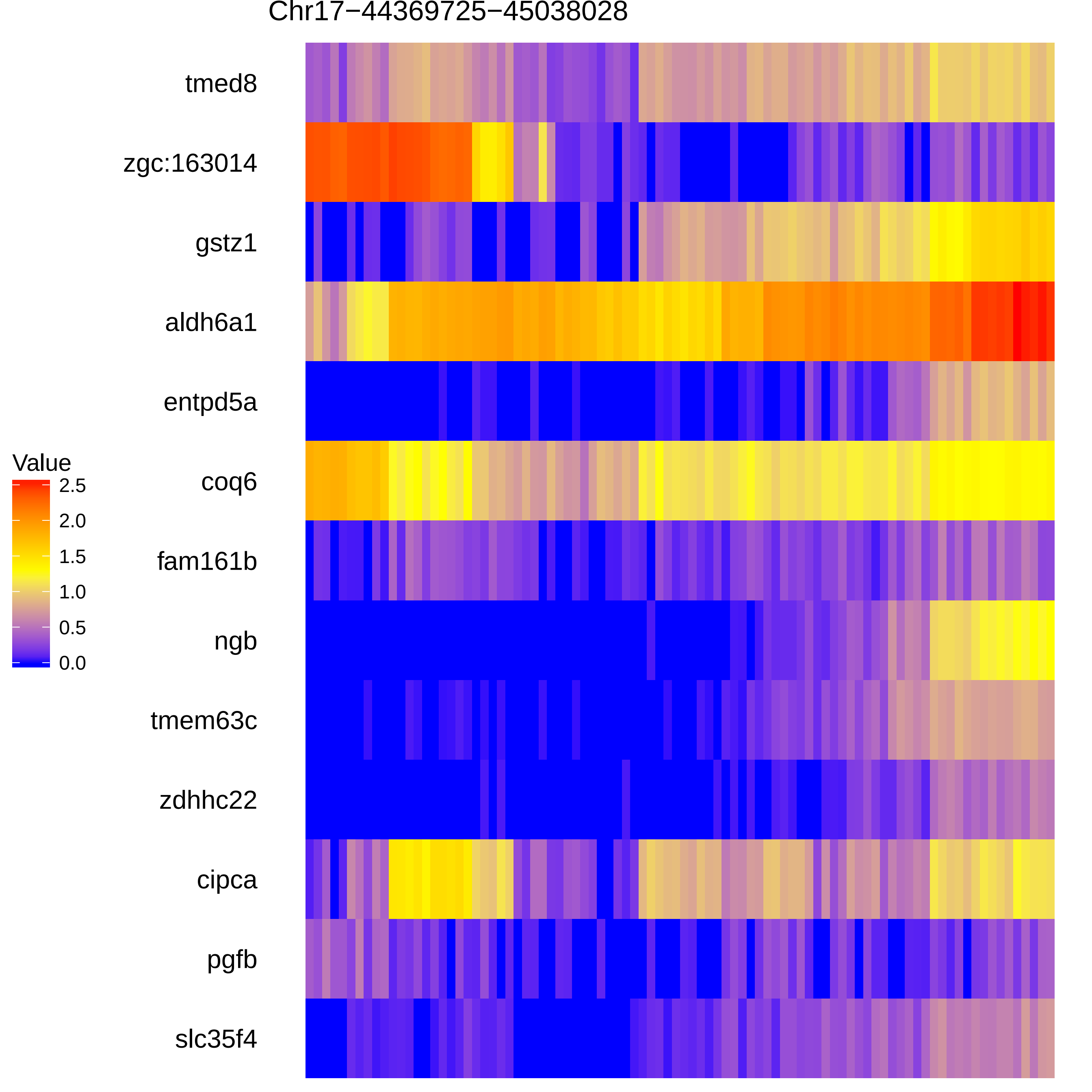
Chr17−44369725−45038028
tmed8
zgc:163014
gstz1
aldh6a1
entpd5a
coq6
fam161b
ngb
tmem63c
zdhhc22
cipca
pgfb
slc35f4
Value
2.5
2.0
1.5
1.0
0.5
0.0

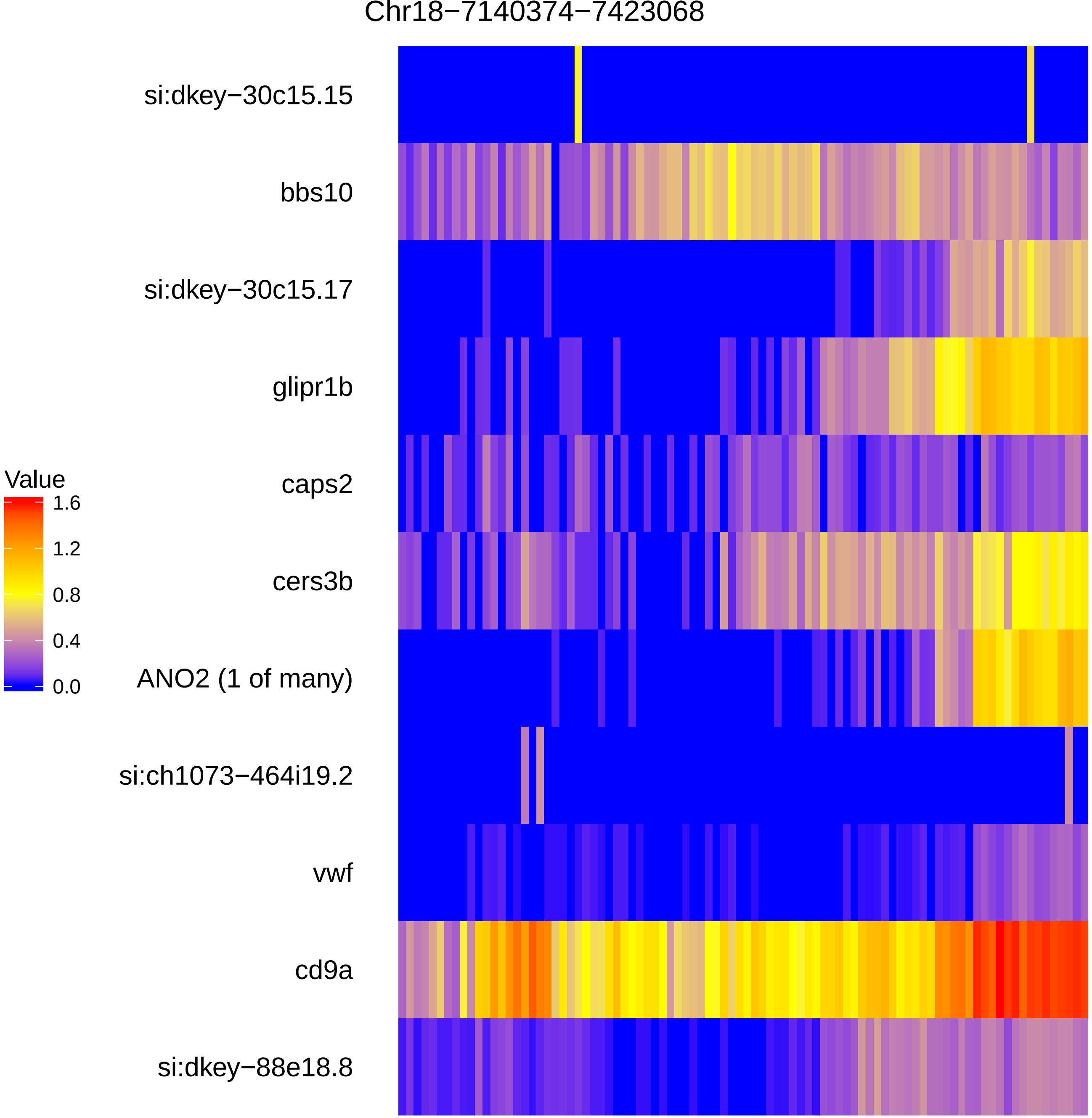
Chr18−7140374−7423068
si:dkey−30c15.15
bbs10
si:dkey−30c15.17
glipr1b
caps2
cers3b
ANO2 (1 of many)
si:ch1073−464i19.2
vwf
cd9a
si:dkey−88e18.8
Value
1.6
1.2
0.8
0.4
0.0

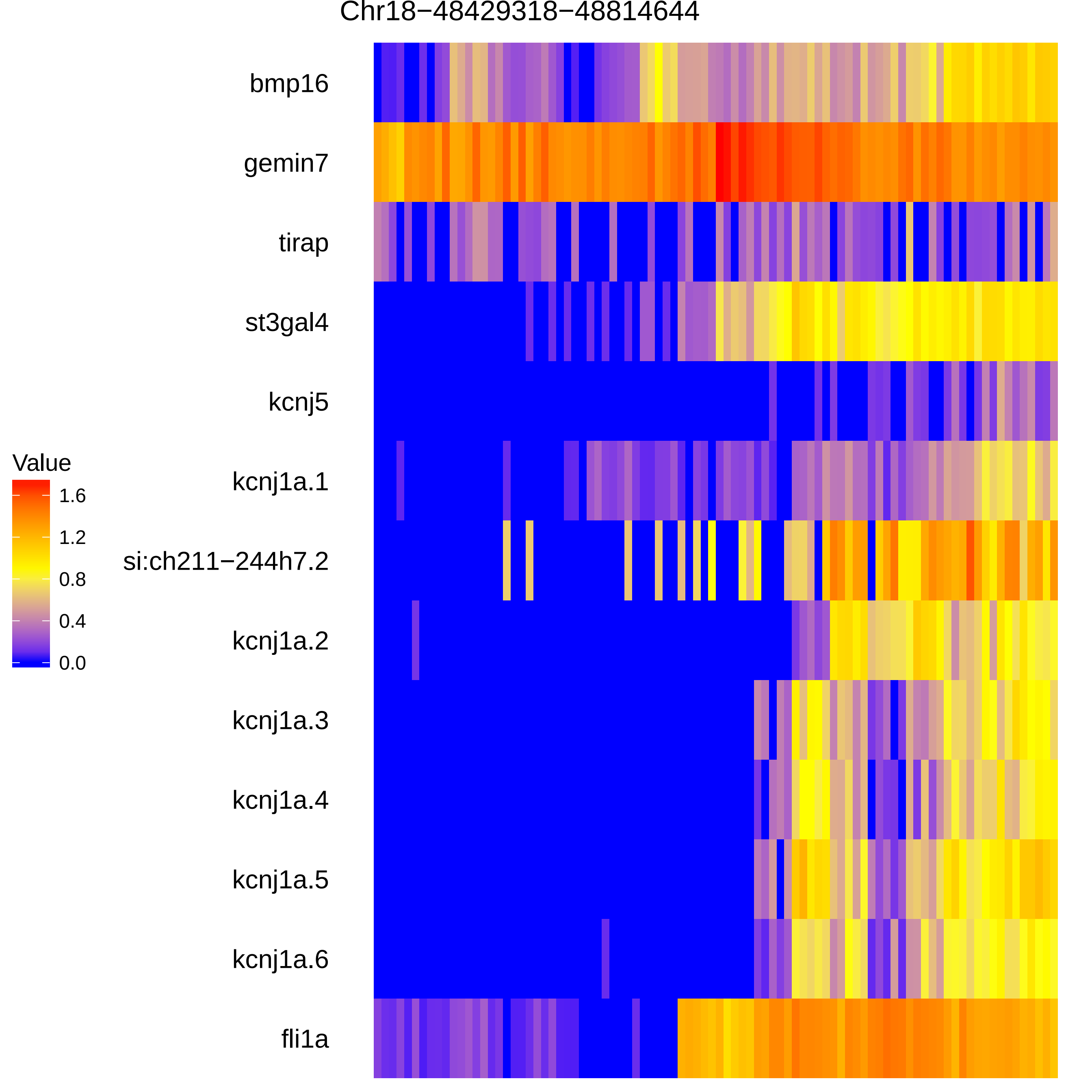

Chr18−48429318−48814644
bmp16
gemin7
tirap
st3gal4
kcnj5
kcnj1a.1
si:ch211−244h7.2
kcnj1a.2
kcnj1a.3
kcnj1a.4
kcnj1a.5
kcnj1a.6
fli1a
Value
1.6
1.2
0.8
0.4
0.0

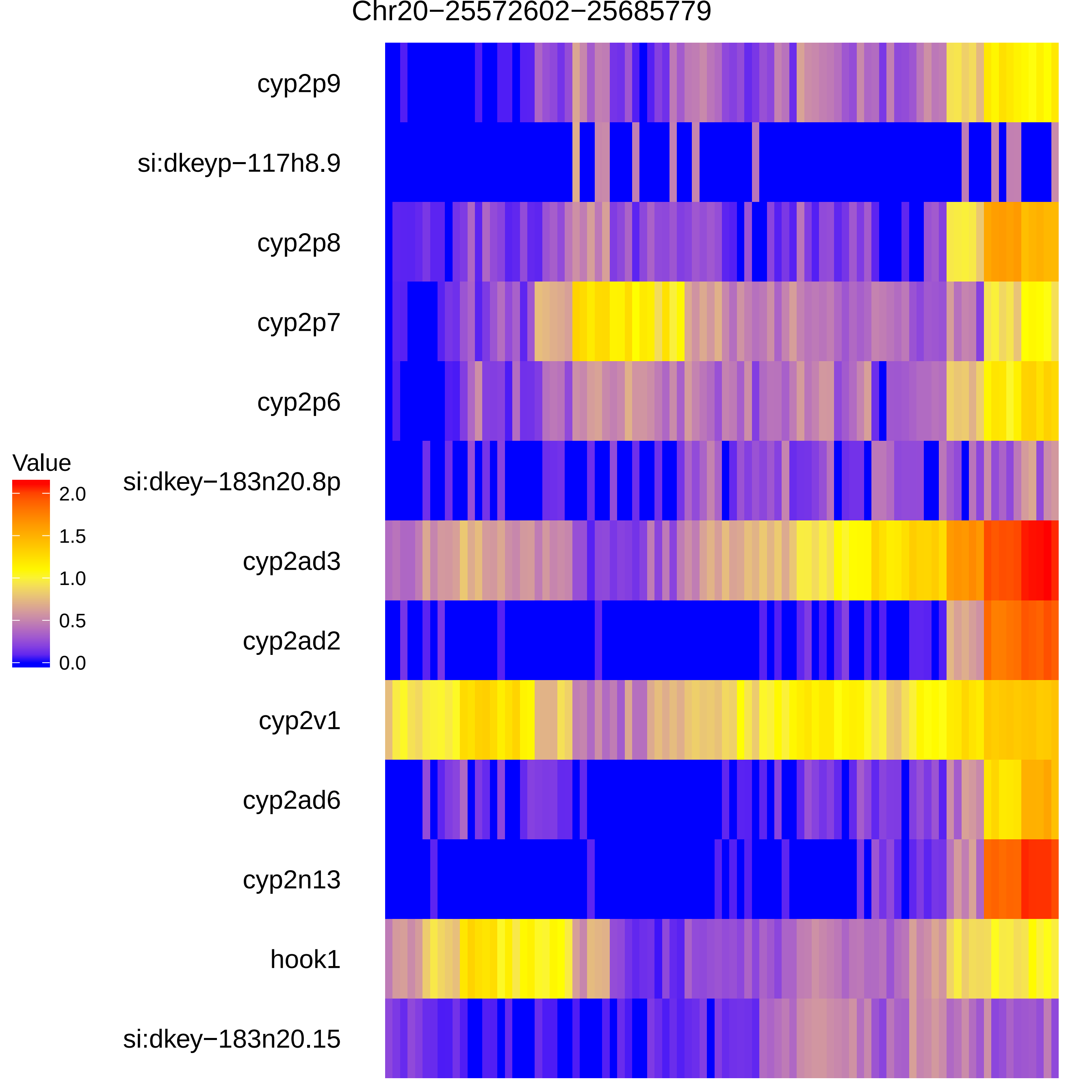
Chr20−25572602−25685779
cyp2p9
si:dkeyp−117h8.9
cyp2p8
cyp2p7
cyp2p6
si:dkey−183n20.8p
cyp2ad3
cyp2ad2
cyp2v1
cyp2ad6
cyp2n13
hook1
si:dkey−183n20.15
Value
2.0
1.5
1.0
0.5
0.0

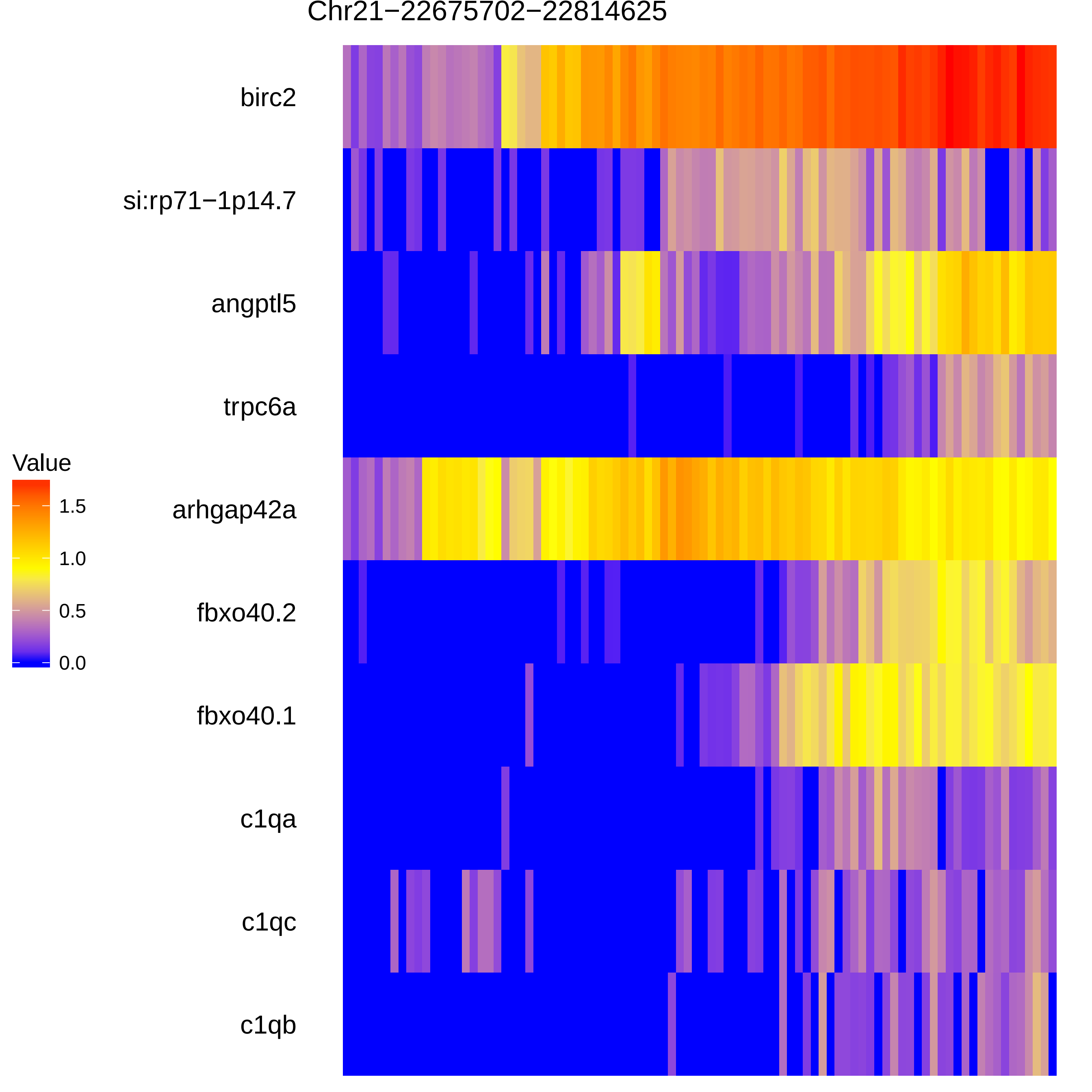
Chr21−22675702−22814625
birc2
si:rp71−1p14.7
angptl5
trpc6a
arhgap42a
fbxo40.2
fbxo40.1
c1qa
c1qc
c1qb
Value
1.5
1.0
0.5
0.0

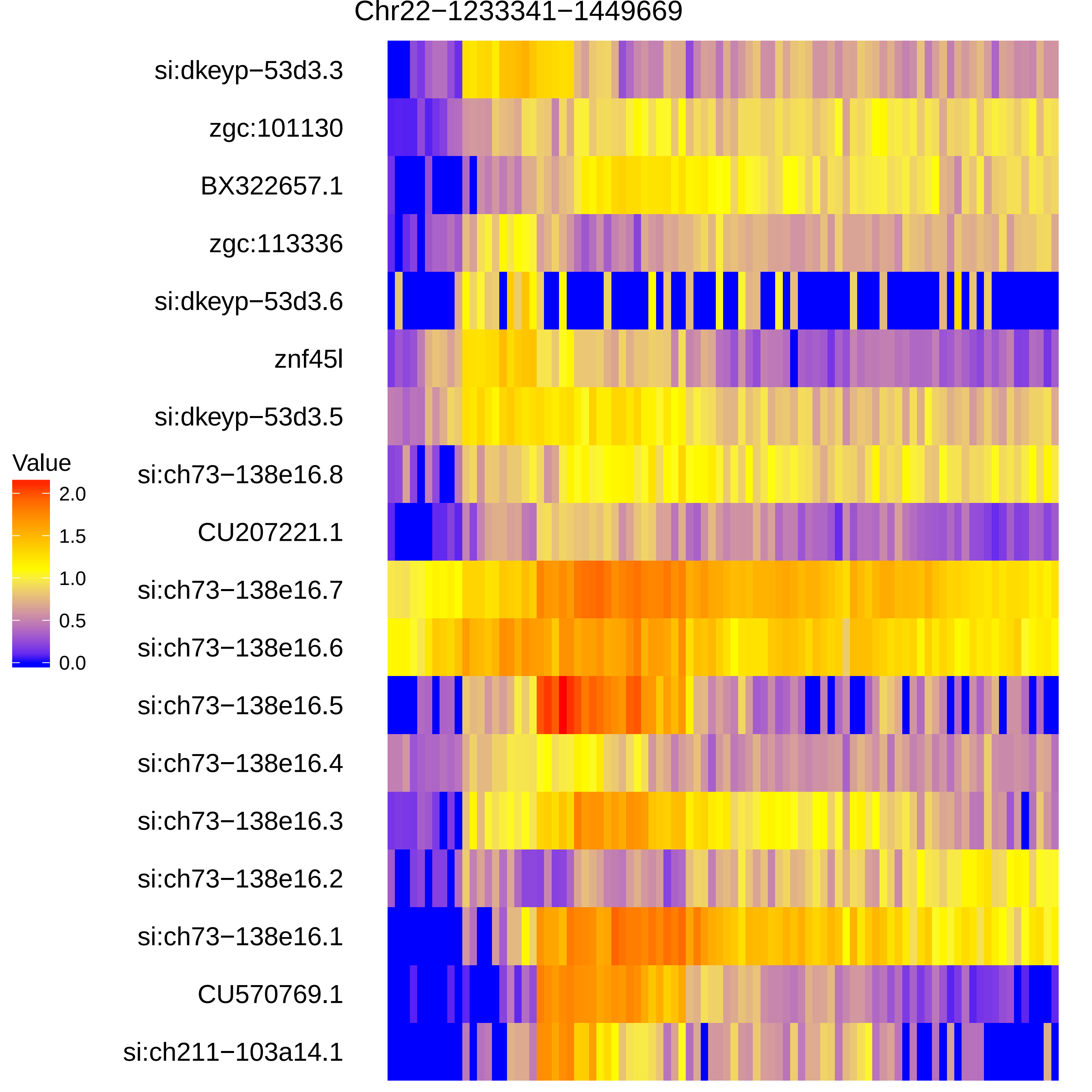
Chr22−1233341−1449669
si:dkeyp−53d3.3
zgc:101130
BX322657.1
zgc:113336
si:dkeyp−53d3.6
znf45l
si:dkeyp−53d3.5
si:ch73−138e16.8
CU207221.1
si:ch73−138e16.7
si:ch73−138e16.6
si:ch73−138e16.5
si:ch73−138e16.4
si:ch73−138e16.3
si:ch73−138e16.2
si:ch73−138e16.1
CU570769.1
si:ch211−103a14.1
Value
2.0
1.5
1.0
0.5
0.0

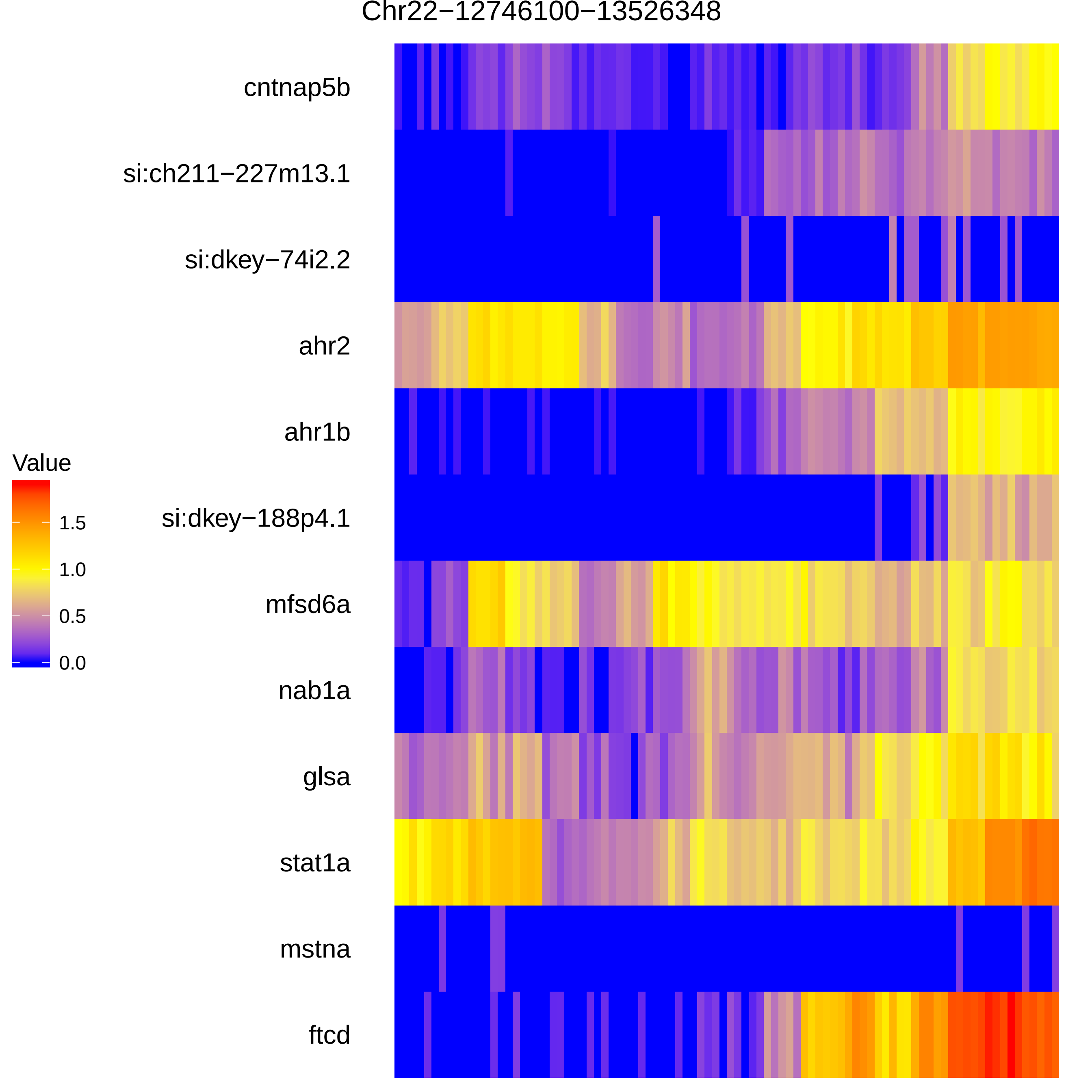
Chr22−12746100−13526348
cntnap5b
si:ch211−227m13.1
si:dkey−74i2.2
ahr2
ahr1b
si:dkey−188p4.1
mfsd6a
nab1a
glsa
stat1a
mstna
ftcd
Value
1.5
1.0
0.5
0.0

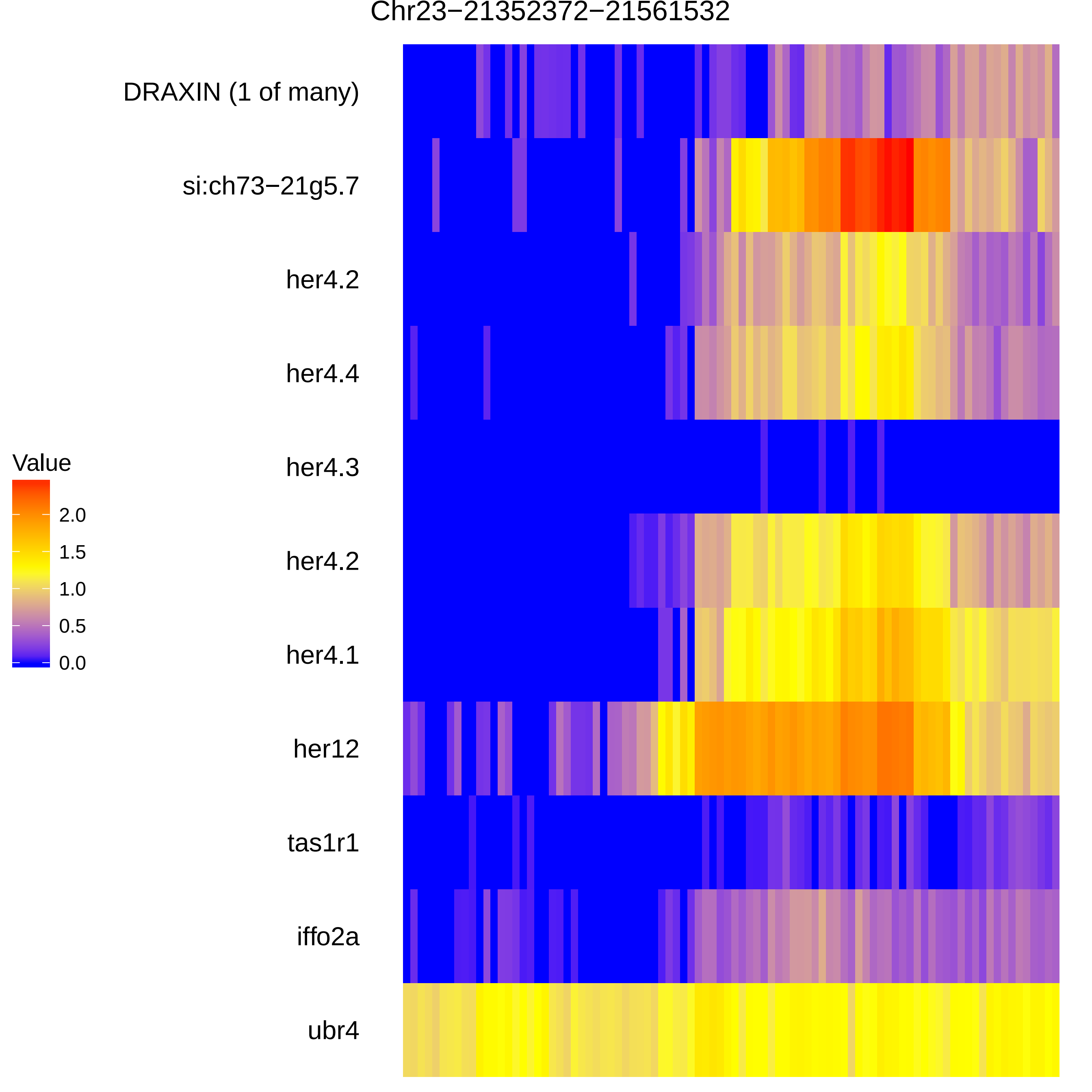

Chr23−21352372−21561532
DRAXIN (1 of many)
si:ch73−21g5.7
her4.2
her4.4
her4.3
her4.2
her4.1
her12
tas1r1
iffo2a
ubr4
Value
2.0
1.5
1.0
0.5
0.0

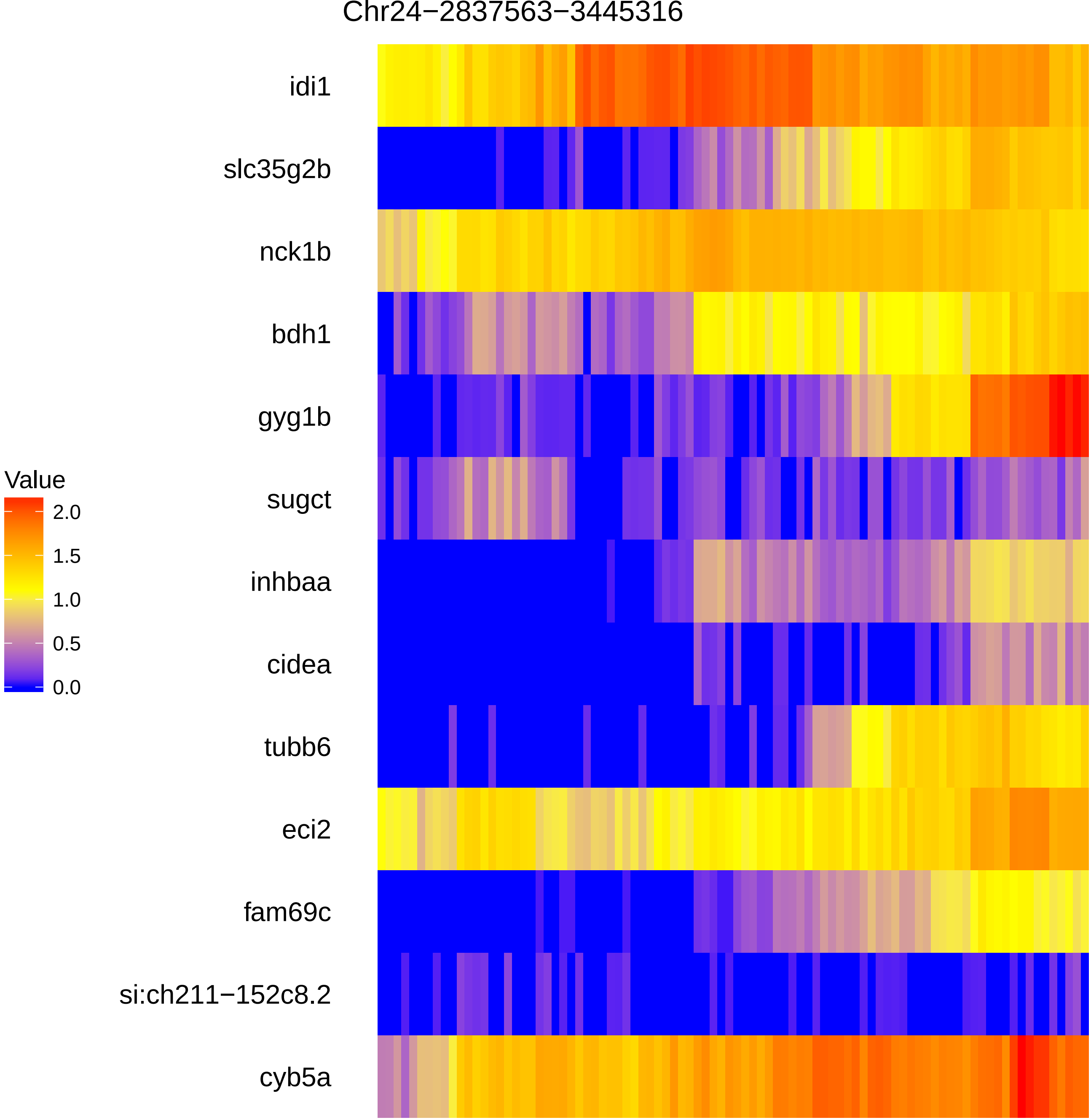
Chr24−2837563−3445316
idi1
slc35g2b
nck1b
bdh1
gyg1b
sugct
inhbaa
cidea
tubb6
eci2
fam69c
si:ch211−152c8.2
cyb5a
Value
2.0
1.5
1.0
0.5
0.0

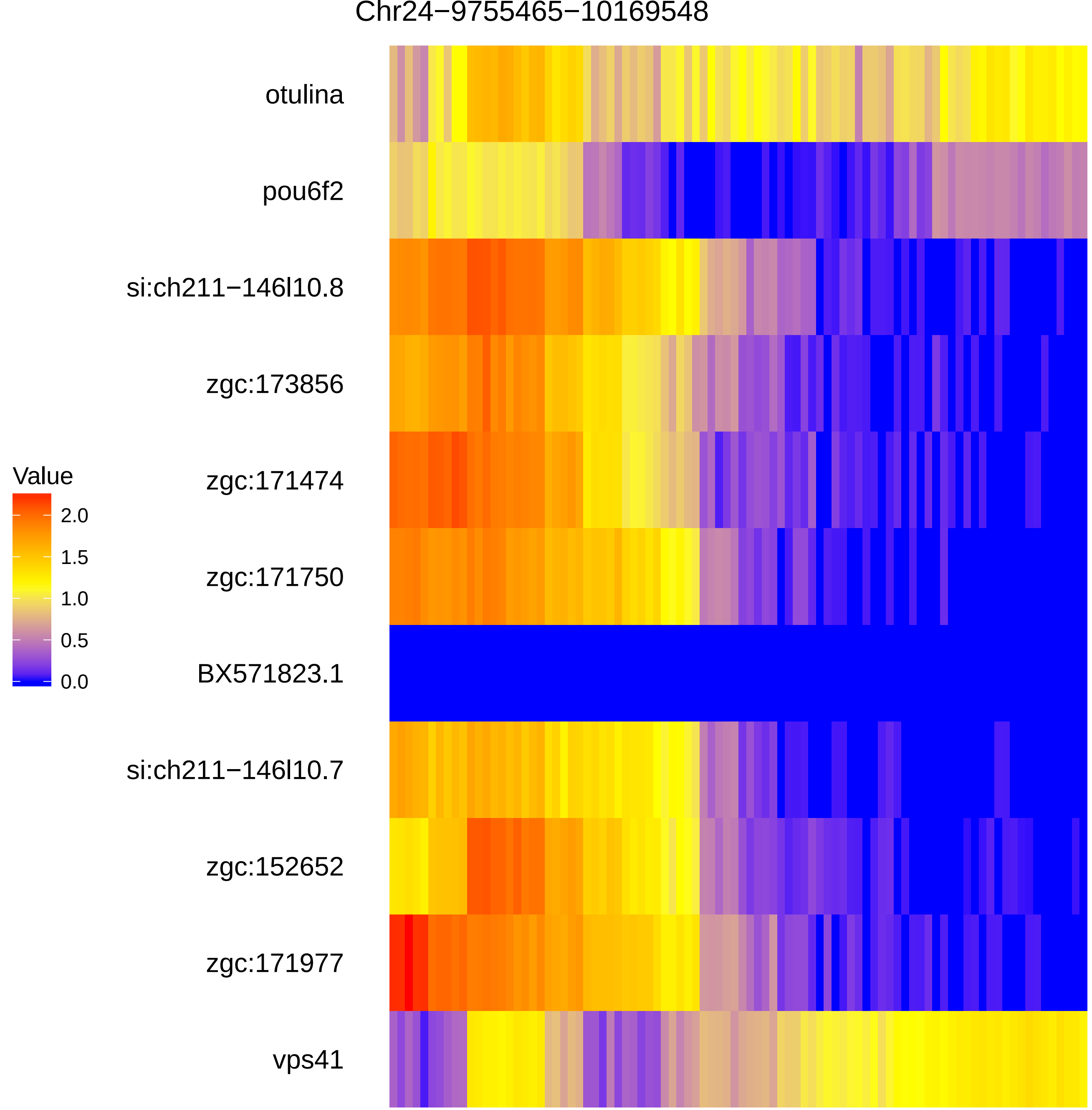
Chr24−9755465−10169548
otulina
pou6f2
si:ch211−146l10.8
zgc:173856
zgc:171474
zgc:171750
BX571823.1
si:ch211−146l10.7
zgc:152652
zgc:171977
vps41
Value
2.0
1.5
1.0
0.5
0.0

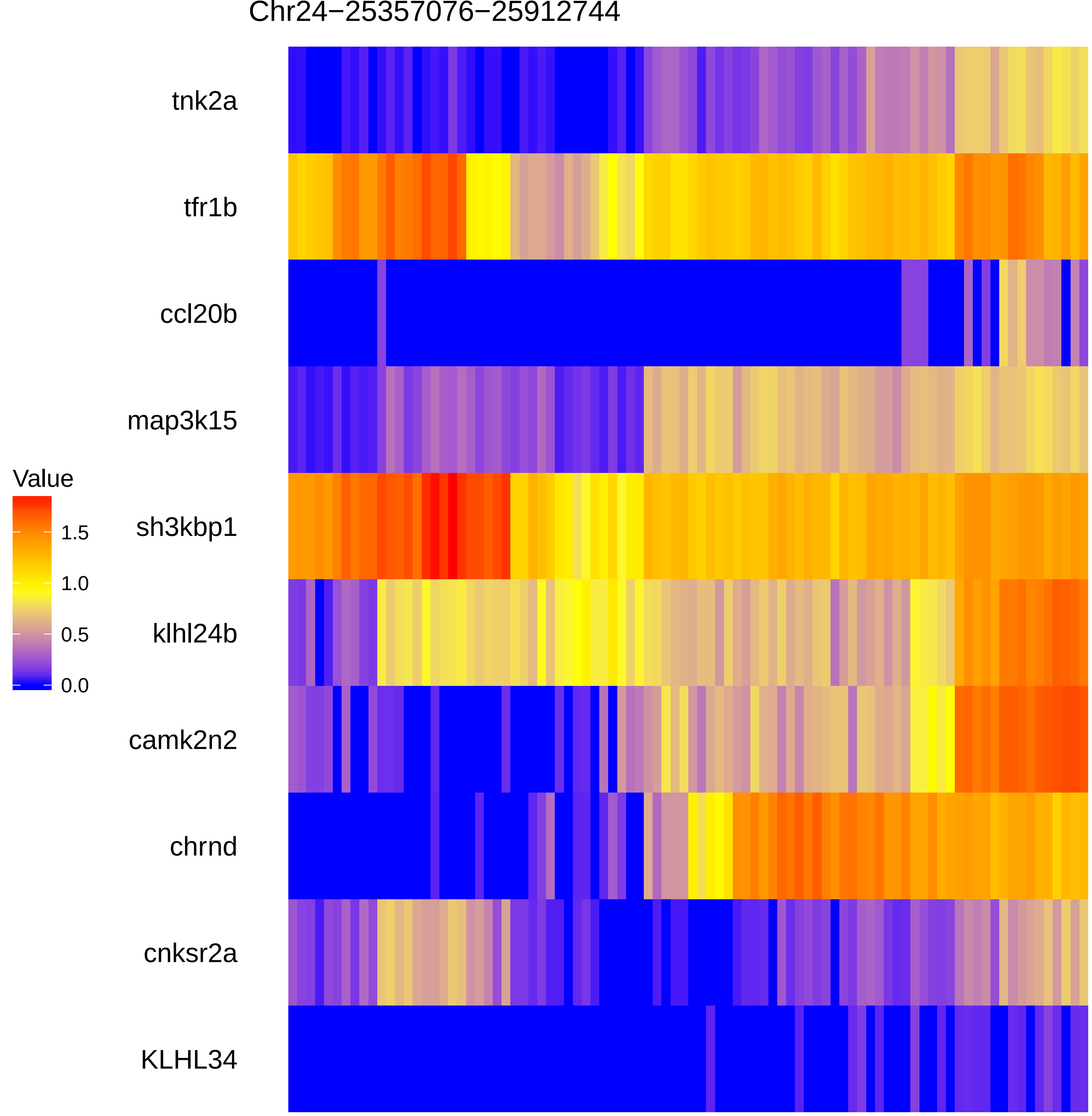
Chr24−25357076−25912744
tnk2a
tfr1b
ccl20b
map3k15
sh3kbp1
klhl24b
camk2n2
chrnd
cnksr2a
KLHL34
Value
1.5
1.0
0.5
0.0

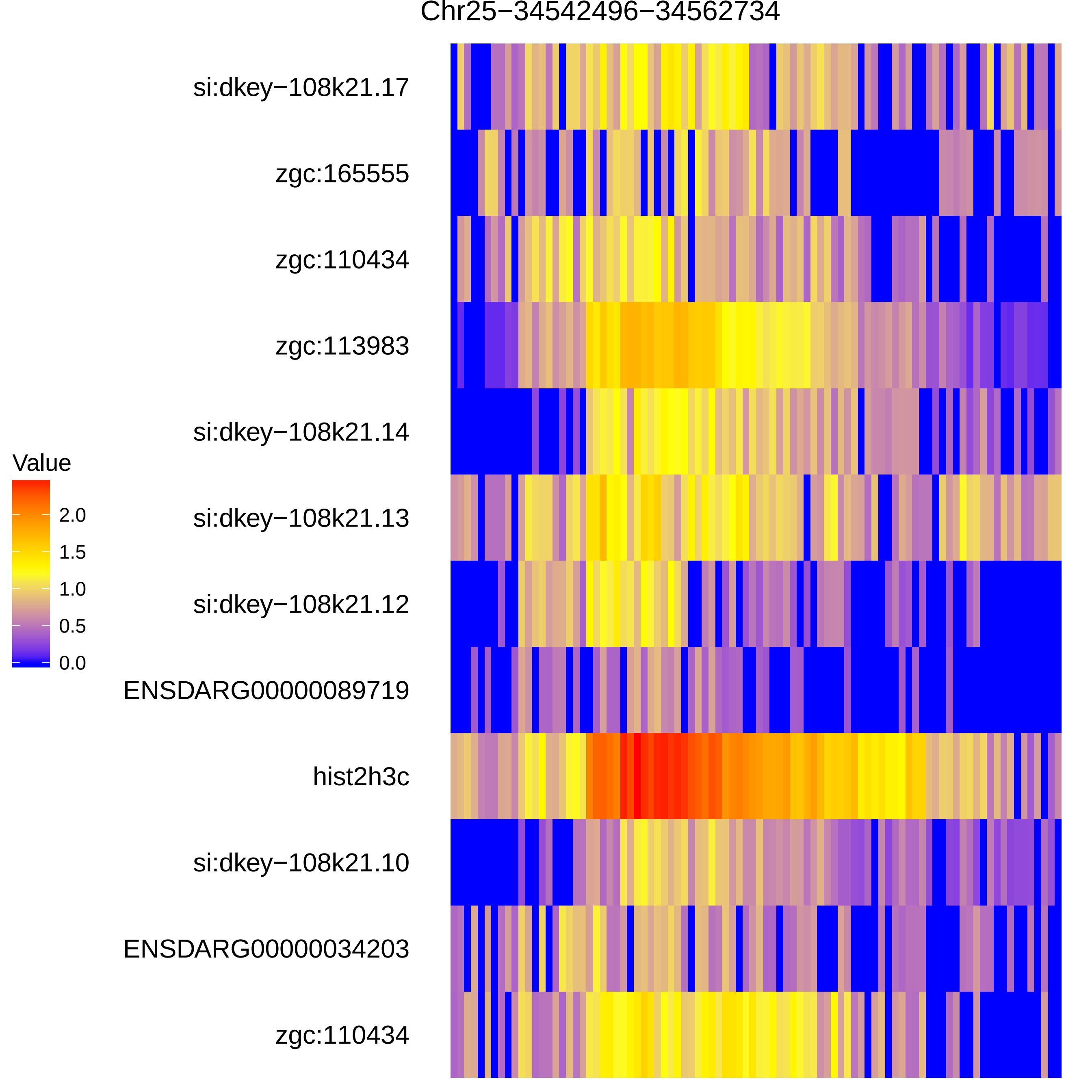

Chr25−34542496−34562734
si:dkey−108k21.17
zgc:165555
zgc:110434
zgc:113983
si:dkey−108k21.14
si:dkey−108k21.13
si:dkey−108k21.12
ENSDARG00000089719
hist2h3c
si:dkey−108k21.10
ENSDARG00000034203
zgc:110434
Value
2.0
1.5
1.0
0.5
0.0
