# Supplementary material for: A high-resolution mRNA expression time course of embryonic development in zebrafish
Source: eLife. 2017 Nov 16;6:e30860. doi: 10.7554/eLife.30860 (PMC5690287; doi:10.7554/eLife.30860)
Supplement: Supplementary file 14. [file elife-30860-supp14.pdf]

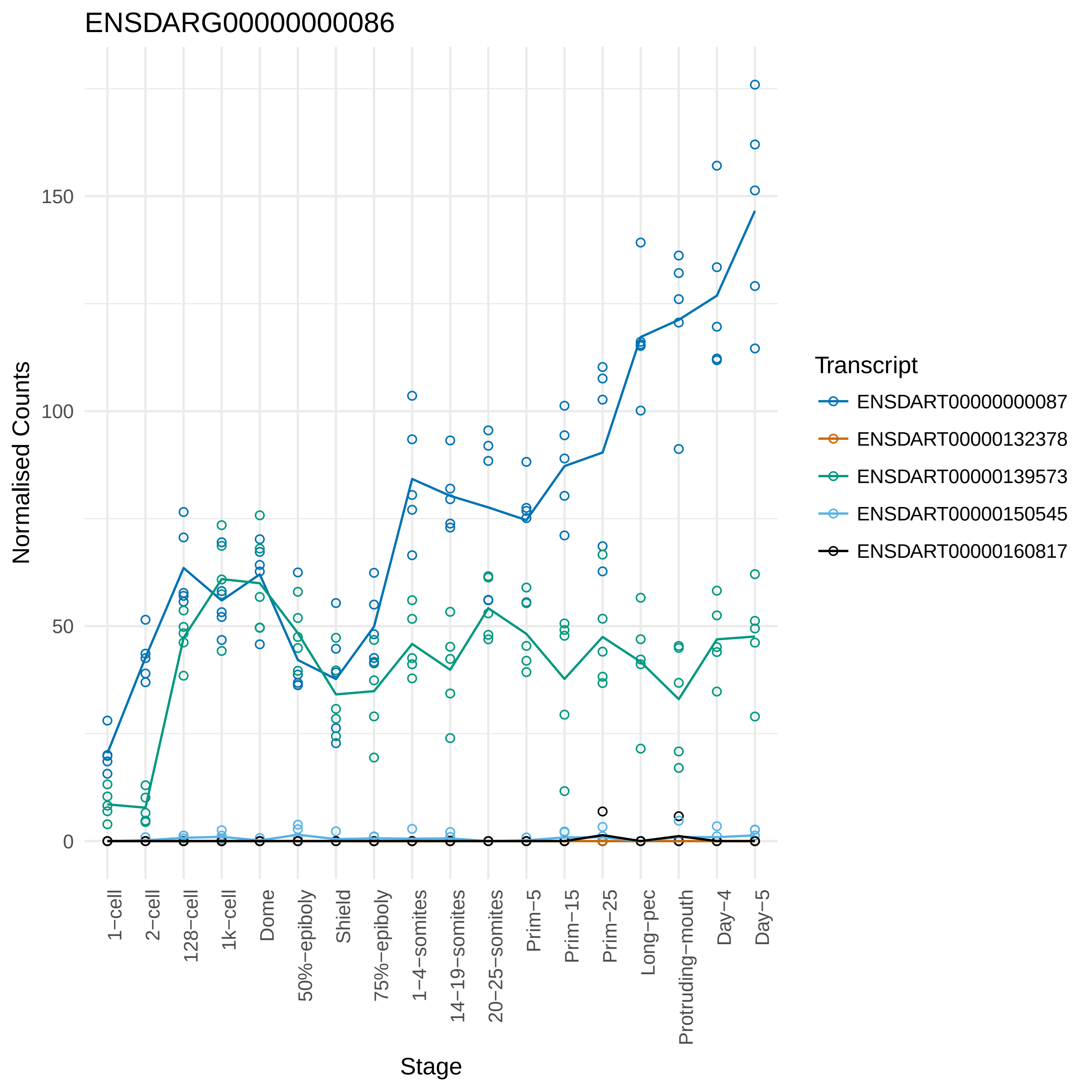

ENSDARG00000000086
Normalised Counts
150
100
50
0
1−cell
2−cell
128−cell
1k−cell
Dome
50%−epiboly
Shield
75%−epiboly
1−4−somites
14−19−somites
20−25−somites
Prim−5
Prim−15
Prim−25
Long−pec
Protruding−mouth
Day−4
Day−5
Stage
Transcript
ENSDART00000000087
ENSDART00000132378
ENSDART00000139573
ENSDART00000150545
ENSDART00000160817

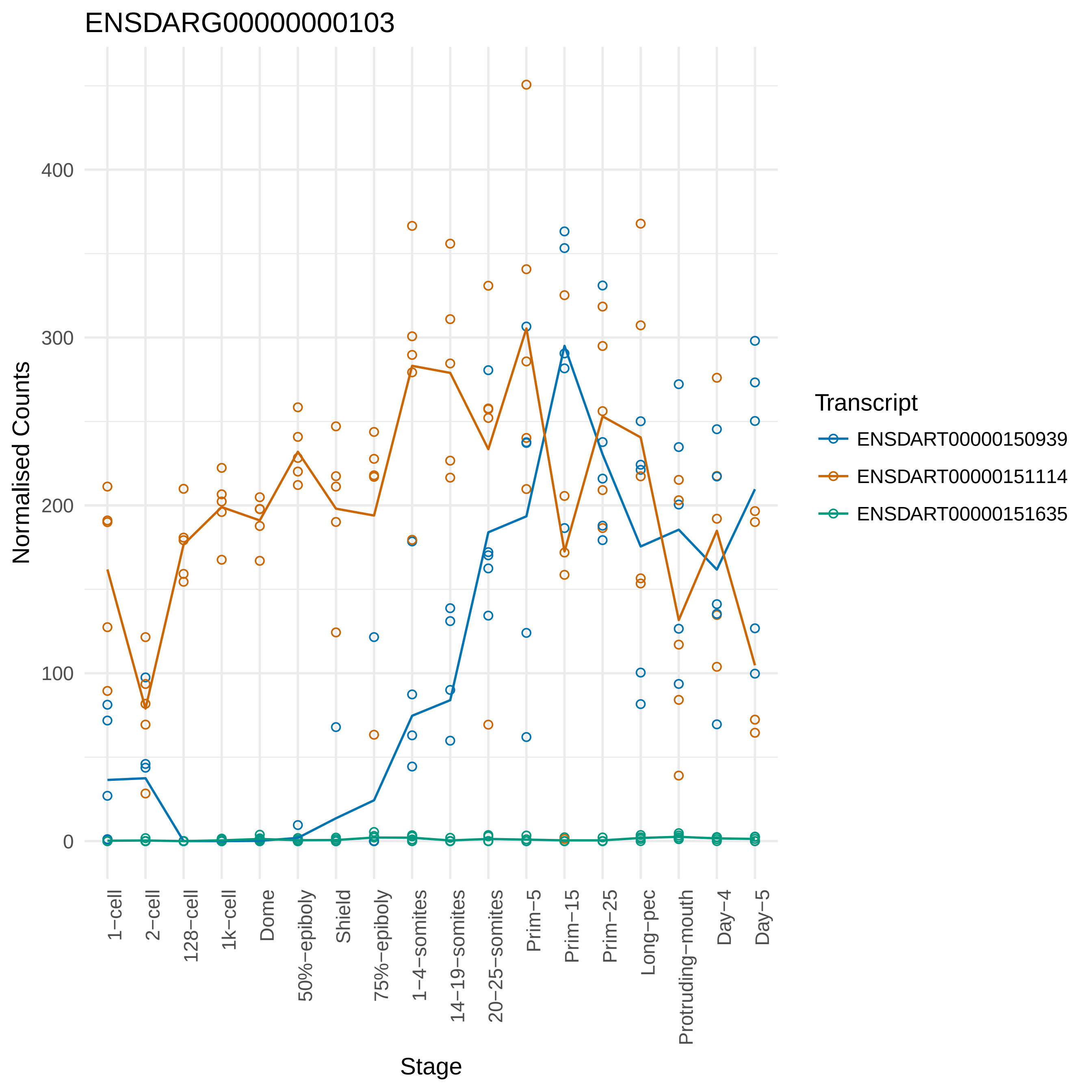
ENSDARG00000000103
Normalised Counts
400
300
200
100
0
1−cell
2−cell
128−cell
1k−cell
Dome
50%−epiboly
Shield
75%−epiboly
1−4−somites
14−19−somites
20−25−somites
Prim−5
Prim−15
Prim−25
Long−pec
Protruding−mouth
Day−4
Day−5
Stage
Transcript
ENSDART00000150939
ENSDART00000151114
ENSDART00000151635

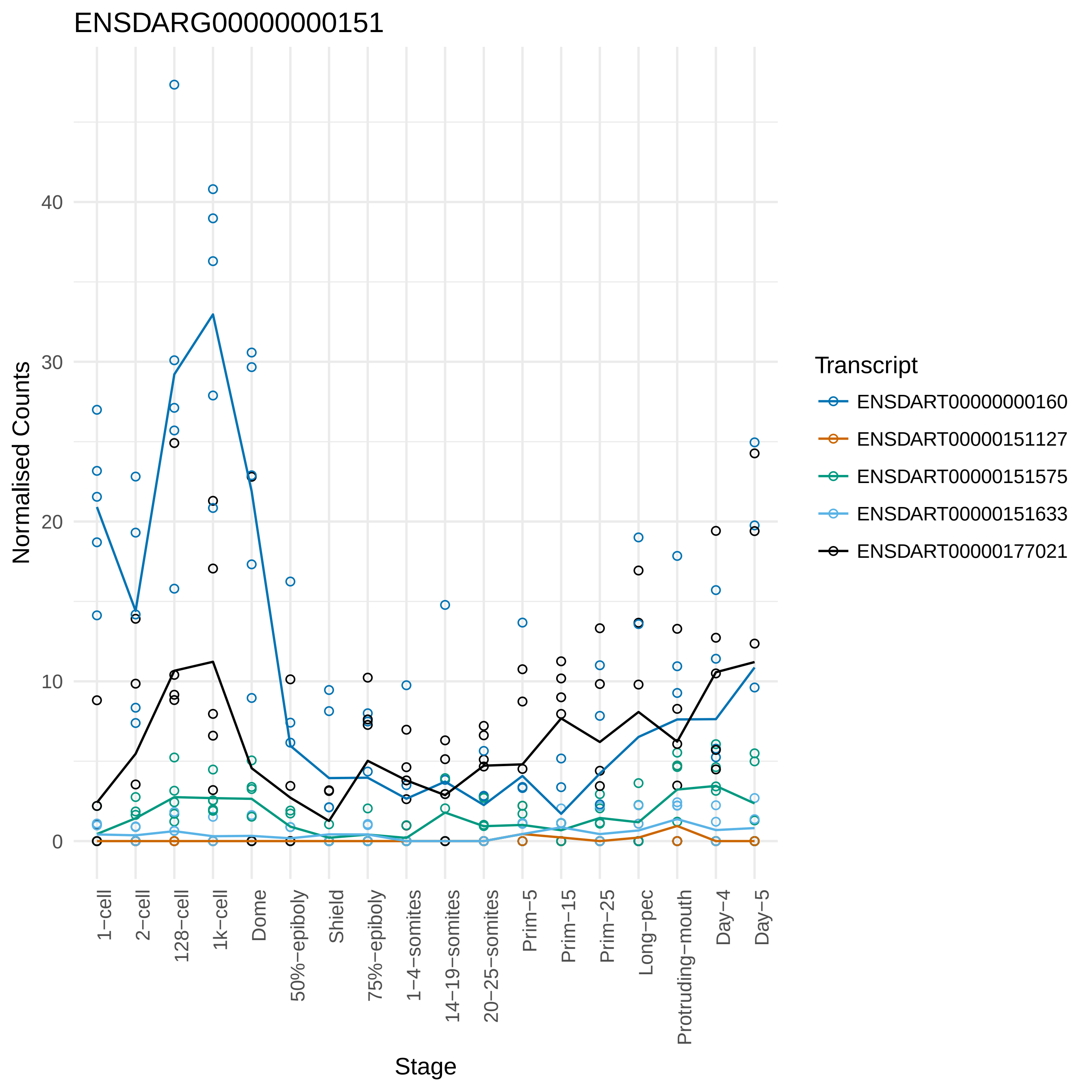
ENSDARG00000000151
Normalised Counts
0
10
20
30
40
Stage
1−cell
2−cell
128−cell
1k−cell
Dome
50%−epiboly
Shield
75%−epiboly
1−4−somites
14−19−somites
20−25−somites
Prim−5
Prim−15
Prim−25
Long−pec
Protruding−mouth
Day−4
Day−5
Transcript
ENSDART00000000160
ENSDART00000151127
ENSDART00000151575
ENSDART00000151633
ENSDART00000177021

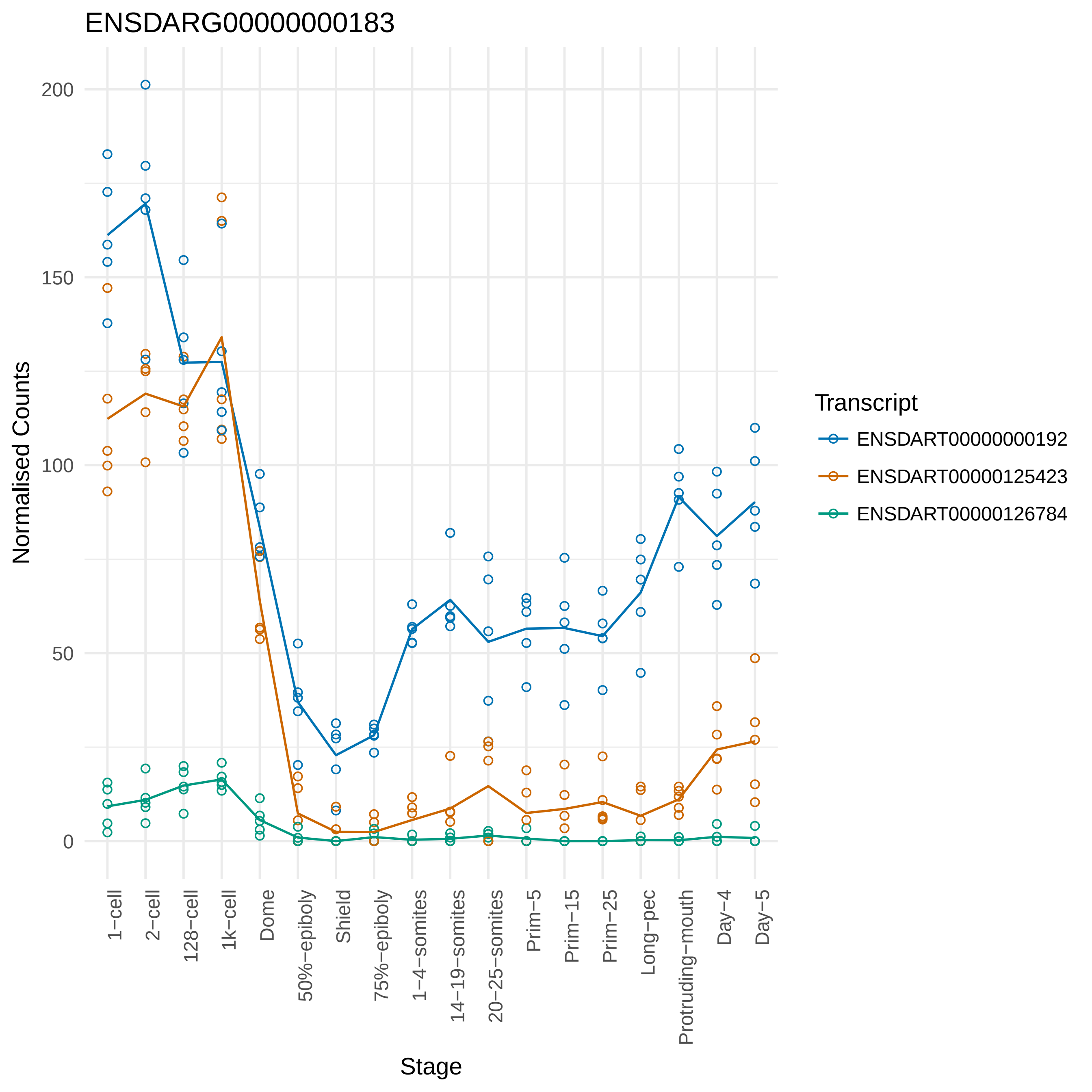
ENSDARG00000000183
Normalised Counts
0
50
100
150
200
1−cell
2−cell
128−cell
1k−cell
Dome
50%−epiboly
Shield
75%−epiboly
1−4−somites
14−19−somites
20−25−somites
Prim−5
Prim−15
Prim−25
Long−pec
Protruding−mouth
Day−4
Day−5
Stage
Transcript
ENSDART00000000192
ENSDART00000125423
ENSDART00000126784

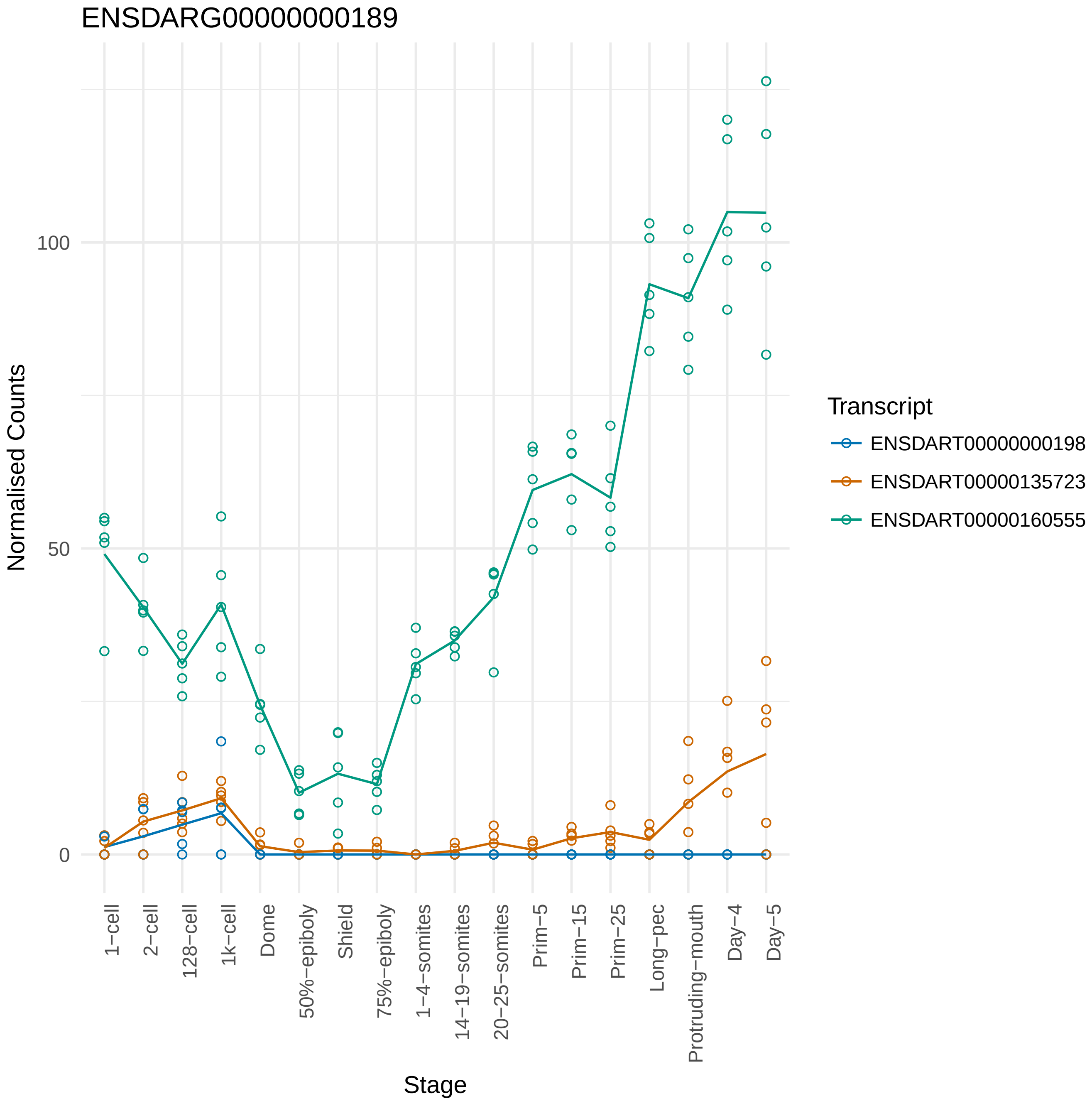

ENSDARG00000000189
Normalised Counts
100
50
0
1−cell
2−cell
128−cell
1k−cell
Dome
50%−epiboly
Shield
75%−epiboly
1−4−somites
14−19−somites
20−25−somites
Prim−5
Prim−15
Prim−25
Long−pec
Protruding−mouth
Day−4
Day−5
Stage
Transcript
ENSDART00000000198
ENSDART00000135723
ENSDART00000160555

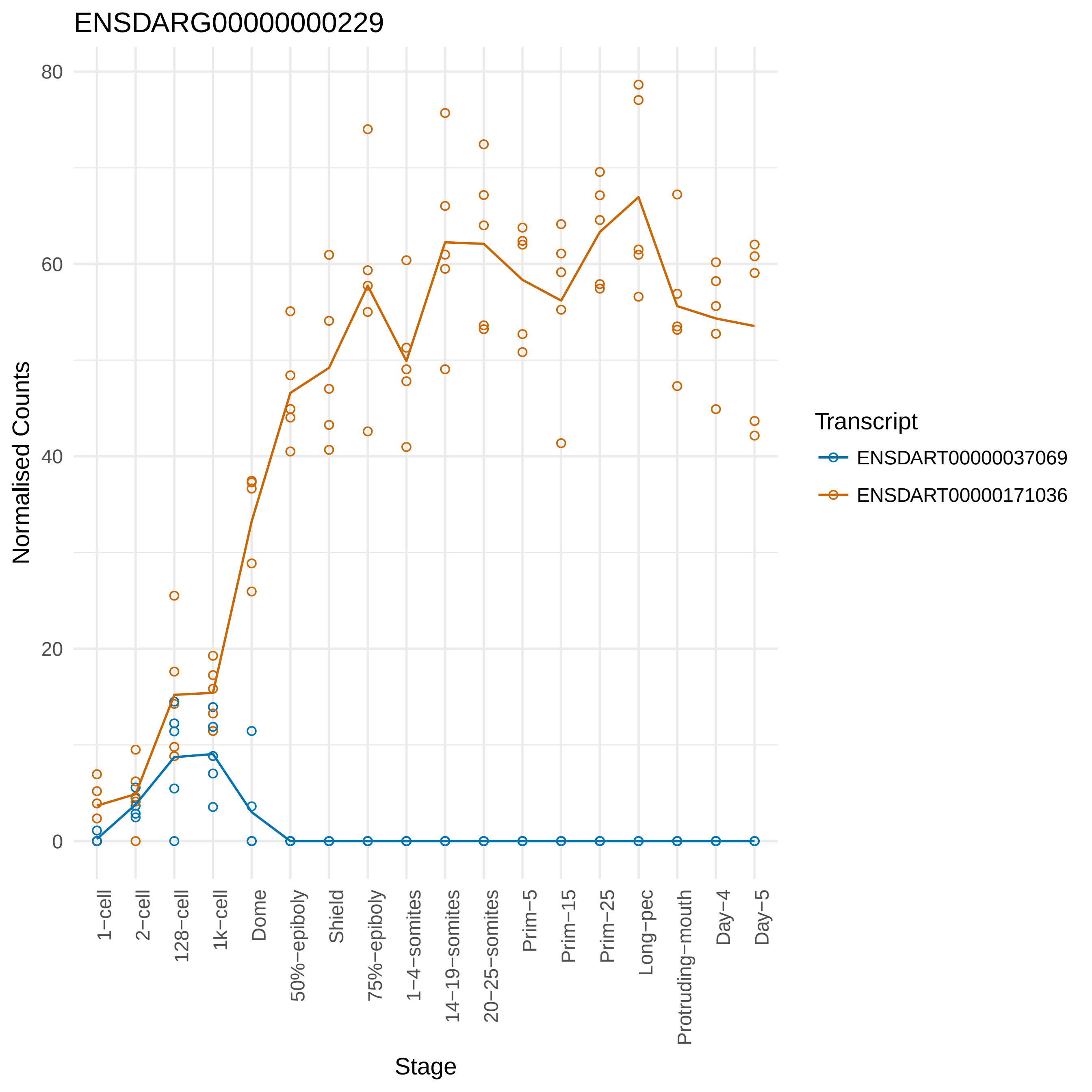
ENSDARG00000000229
Normalised Counts
80
60
40
20
0
1−cell
2−cell
128−cell
1k−cell
Dome
50%−epiboly
Shield
75%−epiboly
1−4−somites
14−19−somites
20−25−somites
Prim−5
Prim−15
Prim−25
Long−pec
Protruding−mouth
Day−4
Day−5
Stage
Transcript
ENSDART00000037069
ENSDART00000171036

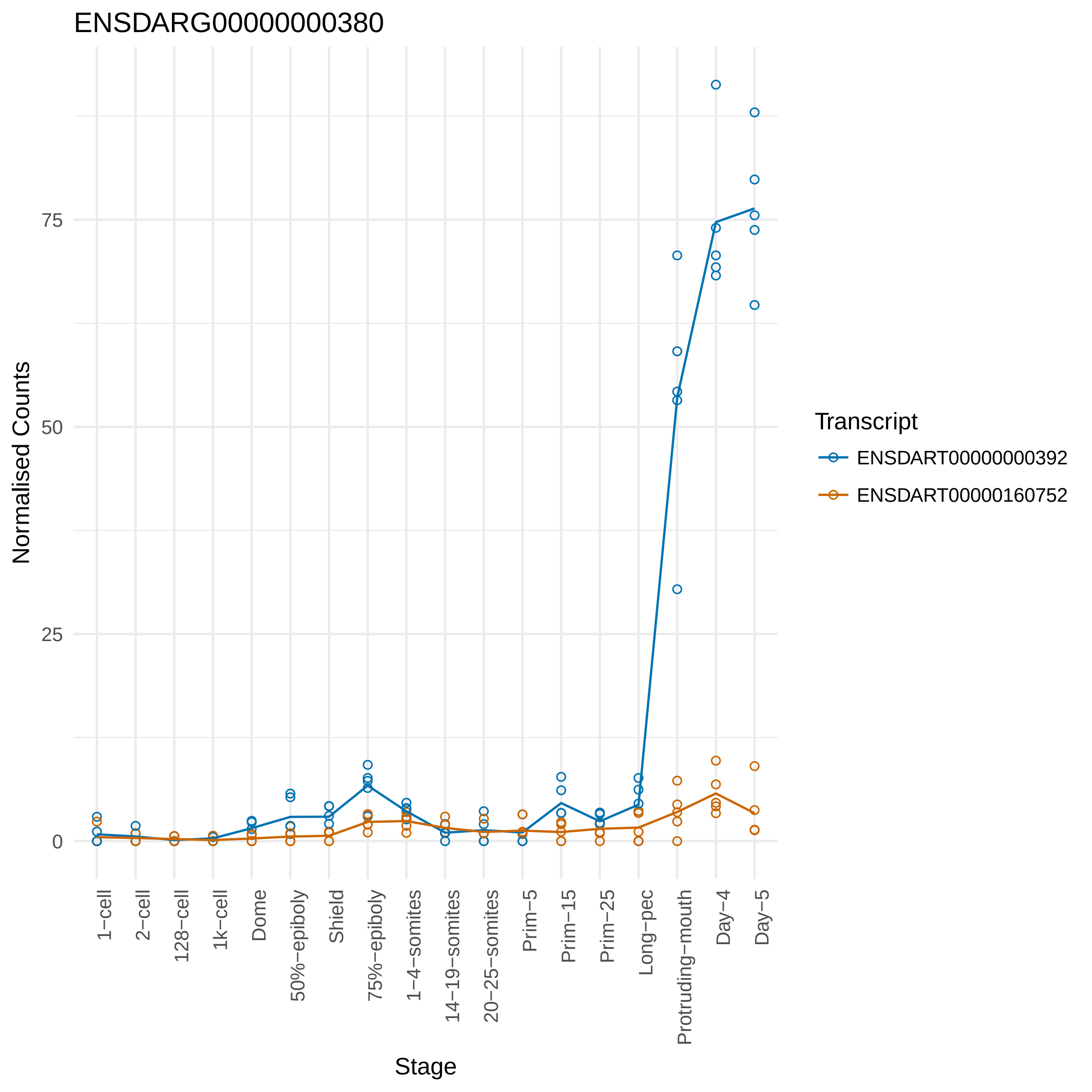

ENSDARG00000000380
Normalised Counts
0
25
50
75
Stage
1−cell
2−cell
128−cell
1k−cell
Dome
50%−epiboly
Shield
75%−epiboly
1−4−somites
14−19−somites
20−25−somites
Prim−5
Prim−15
Prim−25
Long−pec
Protruding−mouth
Day−4
Day−5
Transcript
ENSDART00000000392
ENSDART00000160752

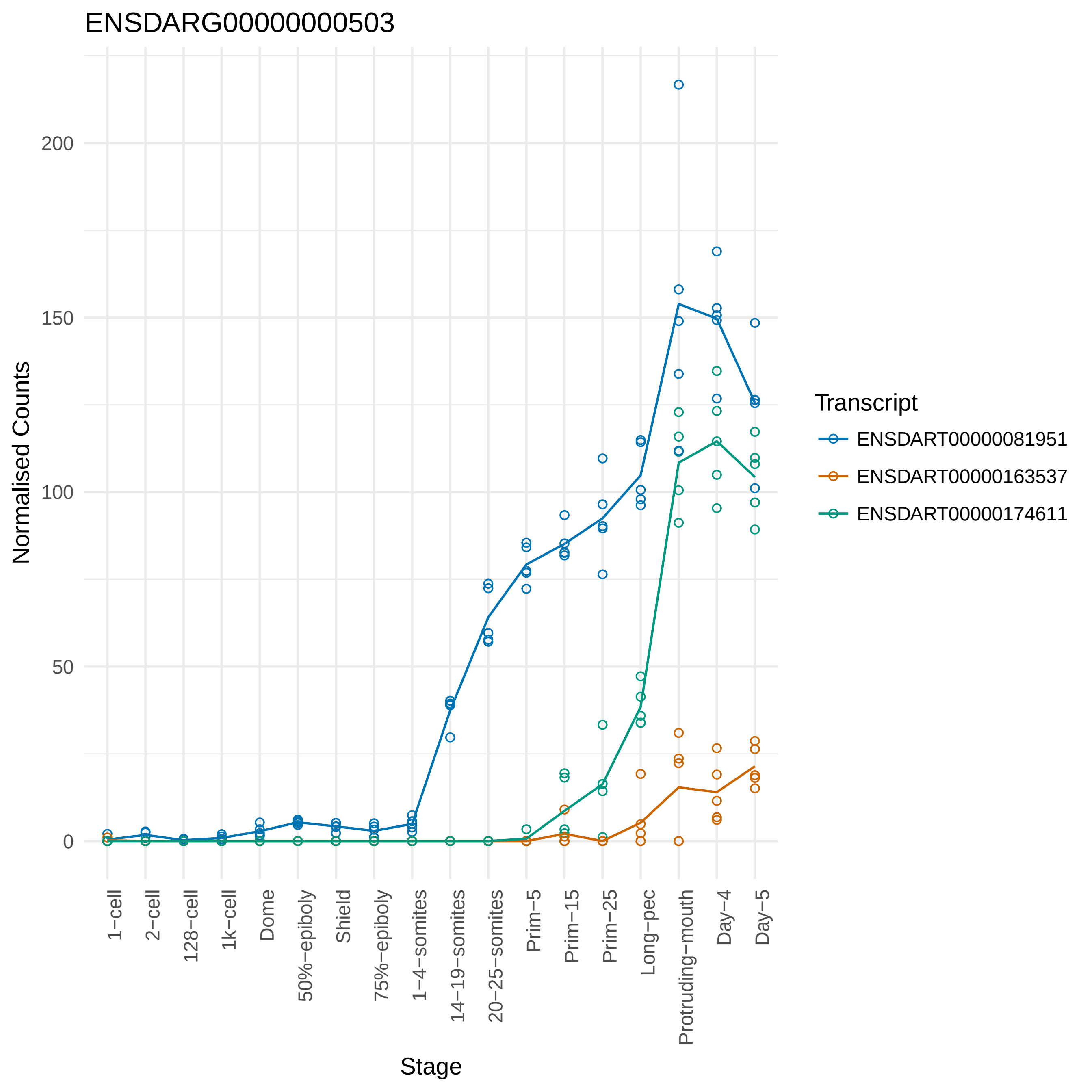

ENSDARG00000000503
Normalised Counts
0
50
100
150
200
Stage
1−cell
2−cell
128−cell
1k−cell
Dome
50%−epiboly
Shield
75%−epiboly
1−4−somites
14−19−somites
20−25−somites
Prim−5
Prim−15
Prim−25
Long−pec
Protruding−mouth
Day−4
Day−5
Transcript
ENSDART00000081951
ENSDART00000163537
ENSDART00000174611

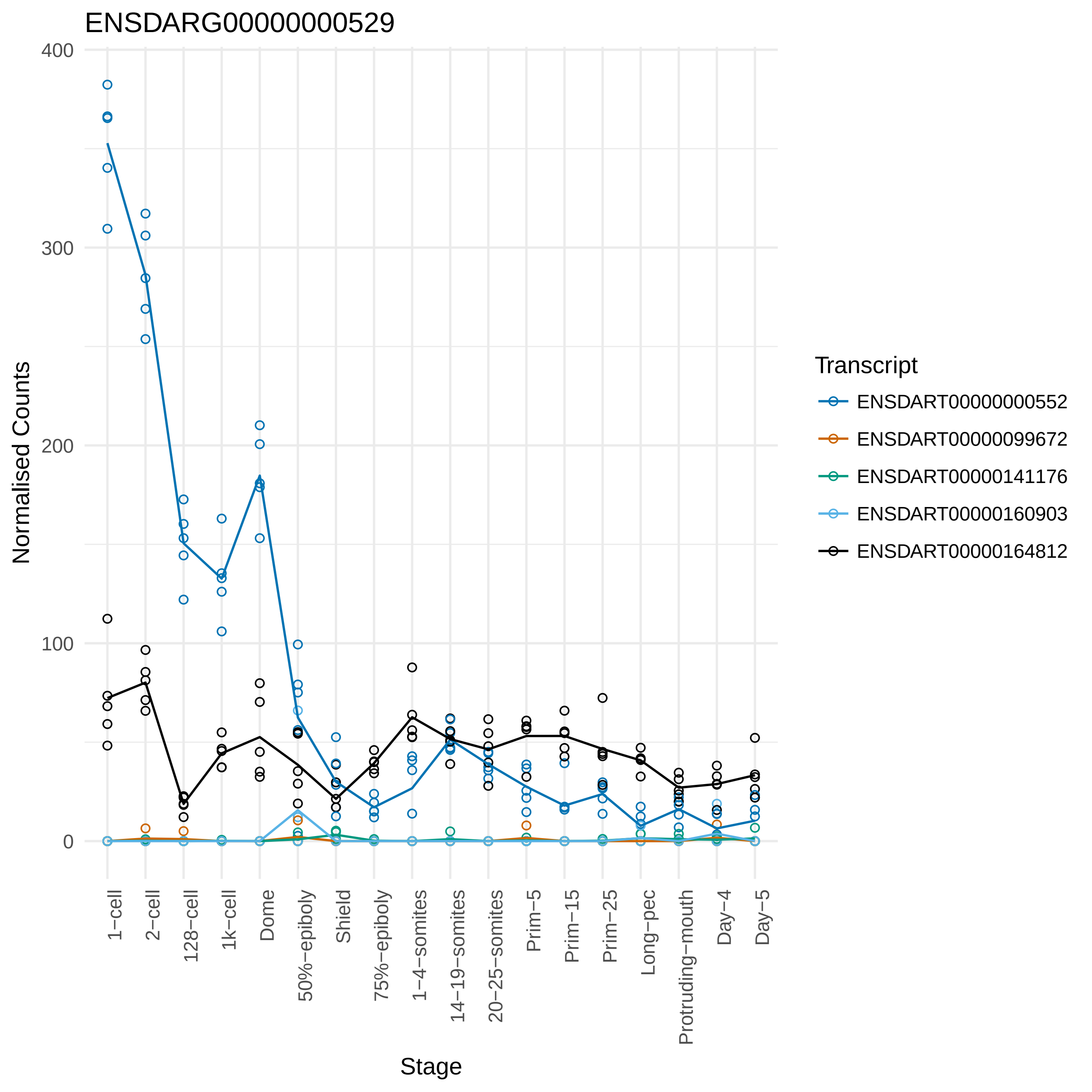
ENSDARG00000000529
Normalised Counts
400
300
200
100
0
1−cell
2−cell
128−cell
1k−cell
Dome
50%−epiboly
Shield
75%−epiboly
1−4−somites
14−19−somites
20−25−somites
Prim−5
Prim−15
Prim−25
Long−pec
Protruding−mouth
Day−4
Day−5
Stage
Transcript
ENSDART00000000552
ENSDART00000099672
ENSDART00000141176
ENSDART00000160903
ENSDART00000164812

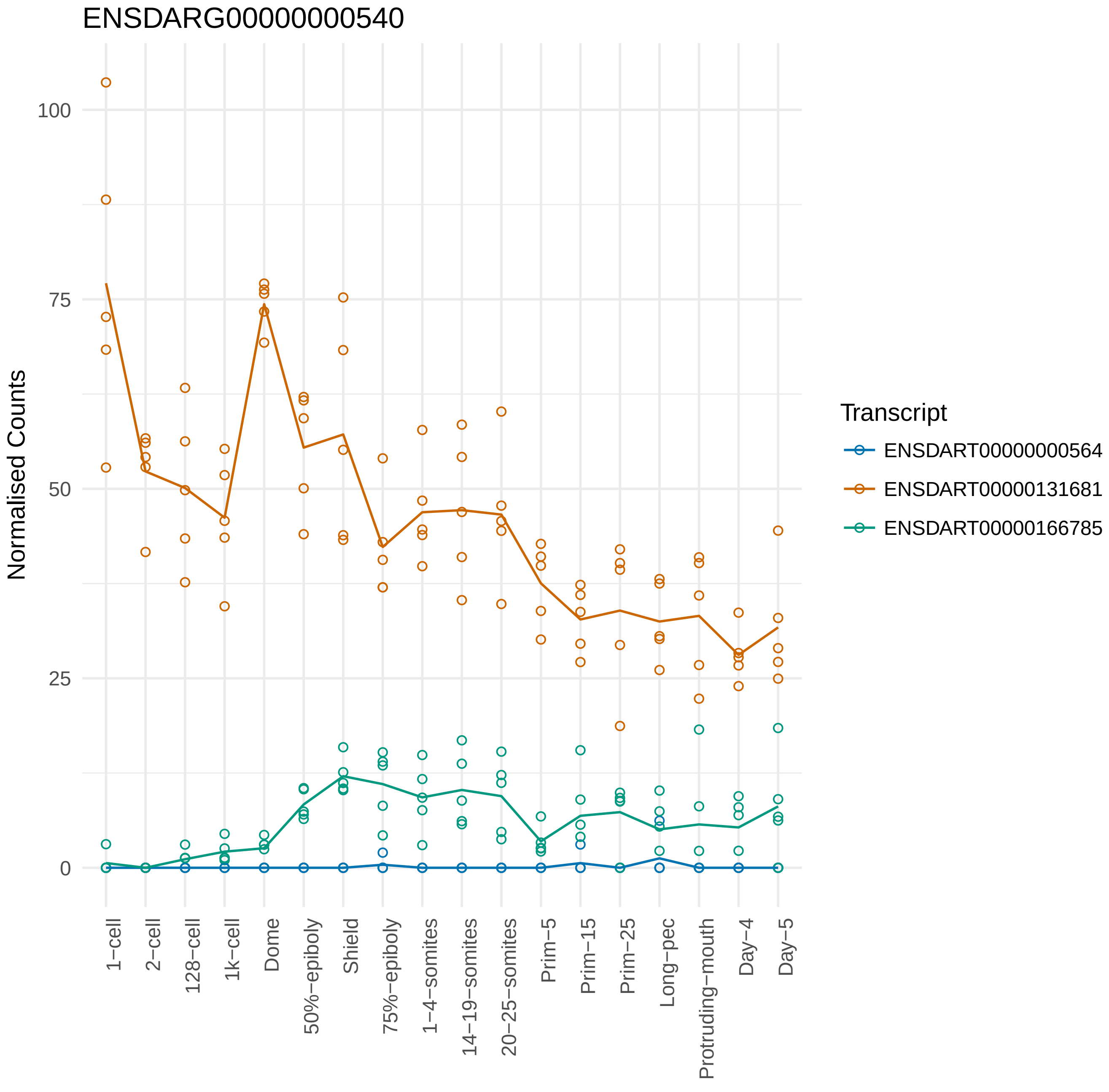
ENSDARG00000000540
Normalised Counts
100
75
50
25
0
1−cell
2−cell
128−cell
1k−cell
Dome
50%−epiboly
Shield
75%−epiboly
1−4−somites
14−19−somites
20−25−somites
Prim−5
Prim−15
Prim−25
Long−pec
Protruding−mouth
Day−4
Day−5
Stage
Transcript
ENSDART00000000564
ENSDART00000131681
ENSDART00000166785

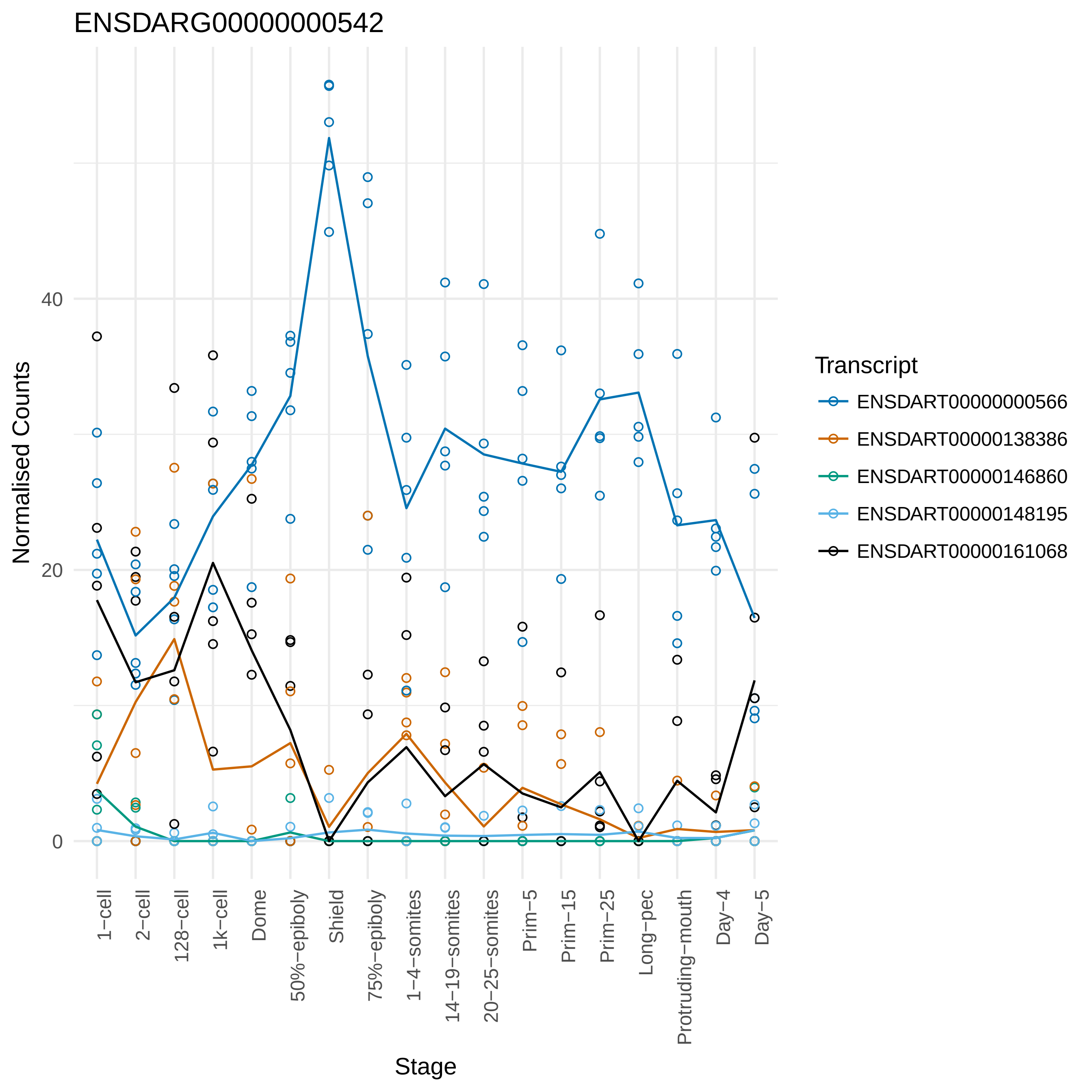

ENSDARG00000000542
Normalised Counts
0
20
40
Stage
1−cell
2−cell
128−cell
1k−cell
Dome
50%−epiboly
Shield
75%−epiboly
1−4−somites
14−19−somites
20−25−somites
Prim−5
Prim−15
Prim−25
Long−pec
Protruding−mouth
Day−4
Day−5
Transcript
ENSDART00000000566
ENSDART00000138386
ENSDART00000146860
ENSDART00000148195
ENSDART00000161068

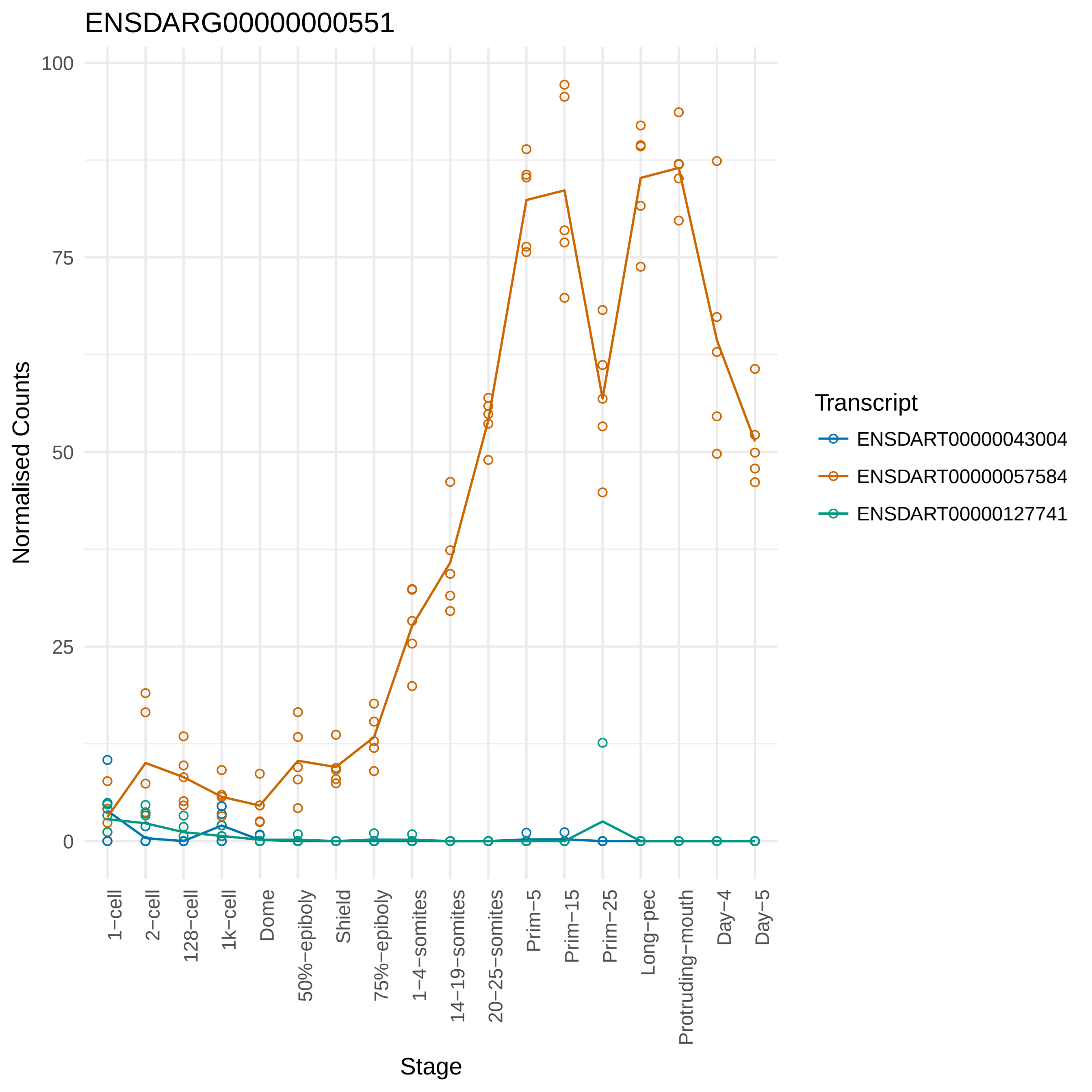

ENSDARG00000000551
Normalised Counts
100
75
50
25
0
1−cell
2−cell
128−cell
1k−cell
Dome
50%−epiboly
Shield
75%−epiboly
1−4−somites
14−19−somites
20−25−somites
Prim−5
Prim−15
Prim−25
Long−pec
Protruding−mouth
Day−4
Day−5
Stage
Transcript
ENSDART00000043004
ENSDART00000057584
ENSDART00000127741

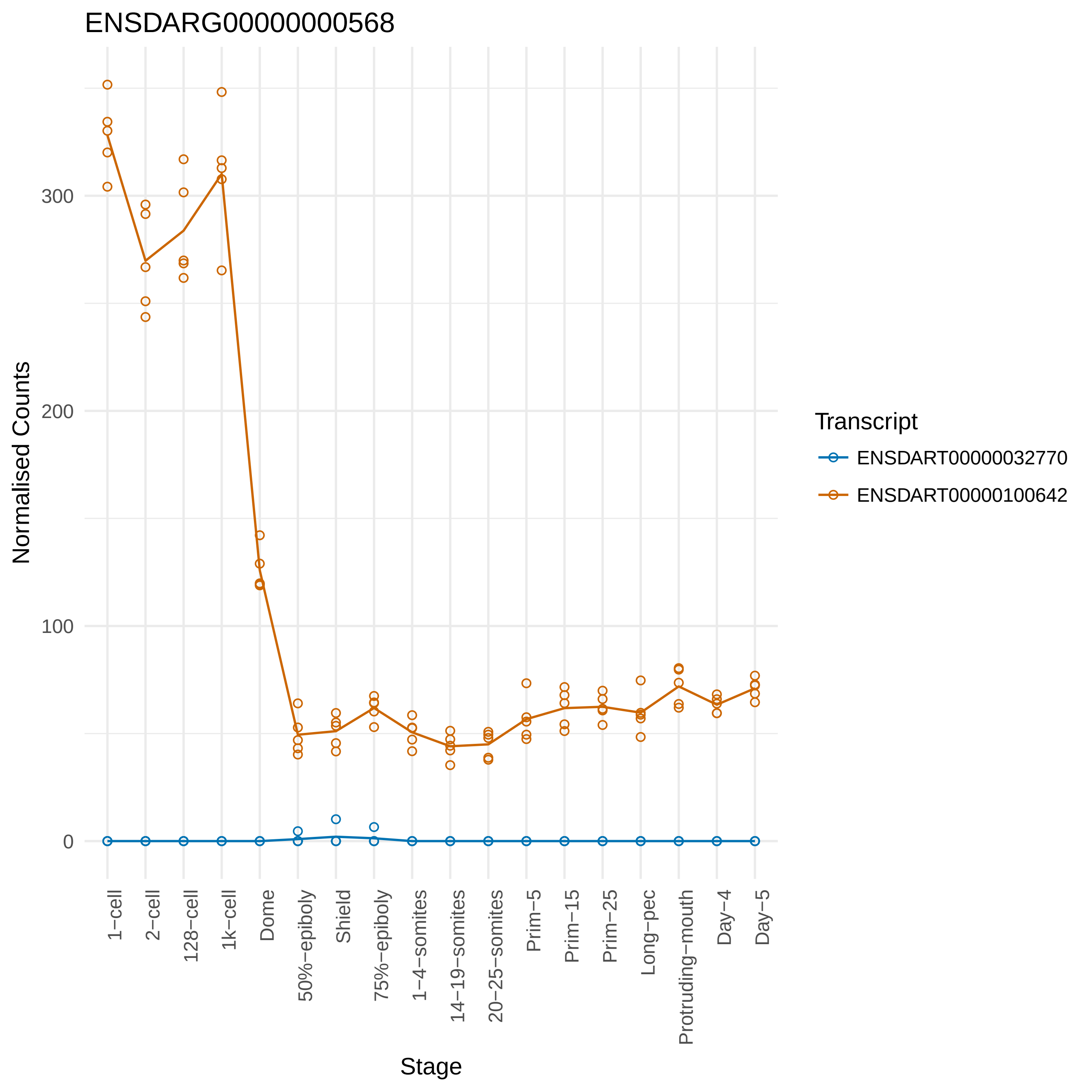

ENSDARG00000000568
Normalised Counts
300
200
100
0
Stage
1−cell
2−cell
128−cell
1k−cell
Dome
50%−epiboly
Shield
75%−epiboly
1−4−somites
14−19−somites
20−25−somites
Prim−5
Prim−15
Prim−25
Long−pec
Protruding−mouth
Day−4
Day−5
Transcript
ENSDART00000032770
ENSDART00000100642

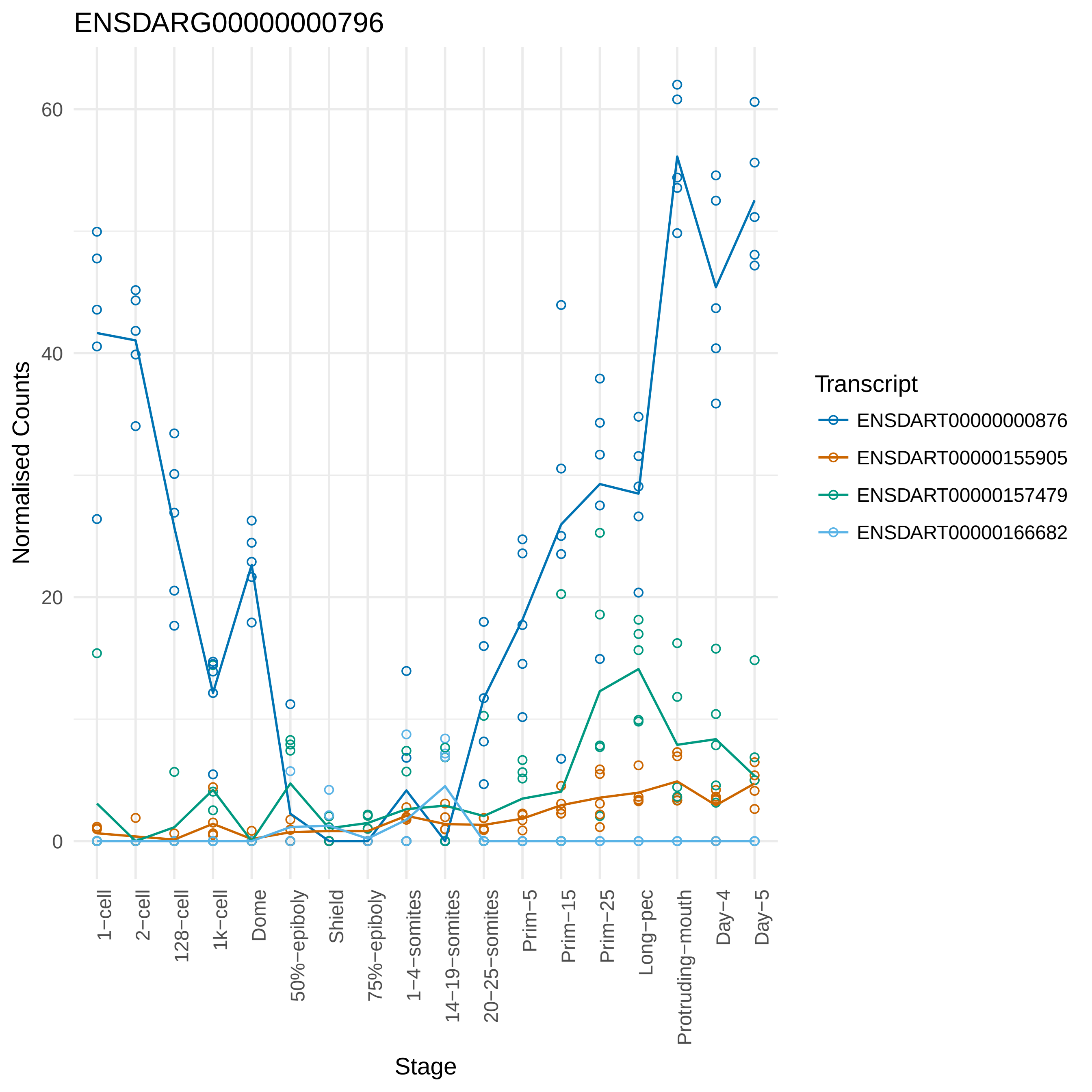

ENSDARG00000000796
Normalised Counts
0
20
40
60
1−cell
2−cell
128−cell
1k−cell
Dome
50%−epiboly
Shield
75%−epiboly
1−4−somites
14−19−somites
20−25−somites
Prim−5
Prim−15
Prim−25
Long−pec
Protruding−mouth
Day−4
Day−5
Stage
Transcript
ENSDART00000000876
ENSDART00000155905
ENSDART00000157479
ENSDART00000166682

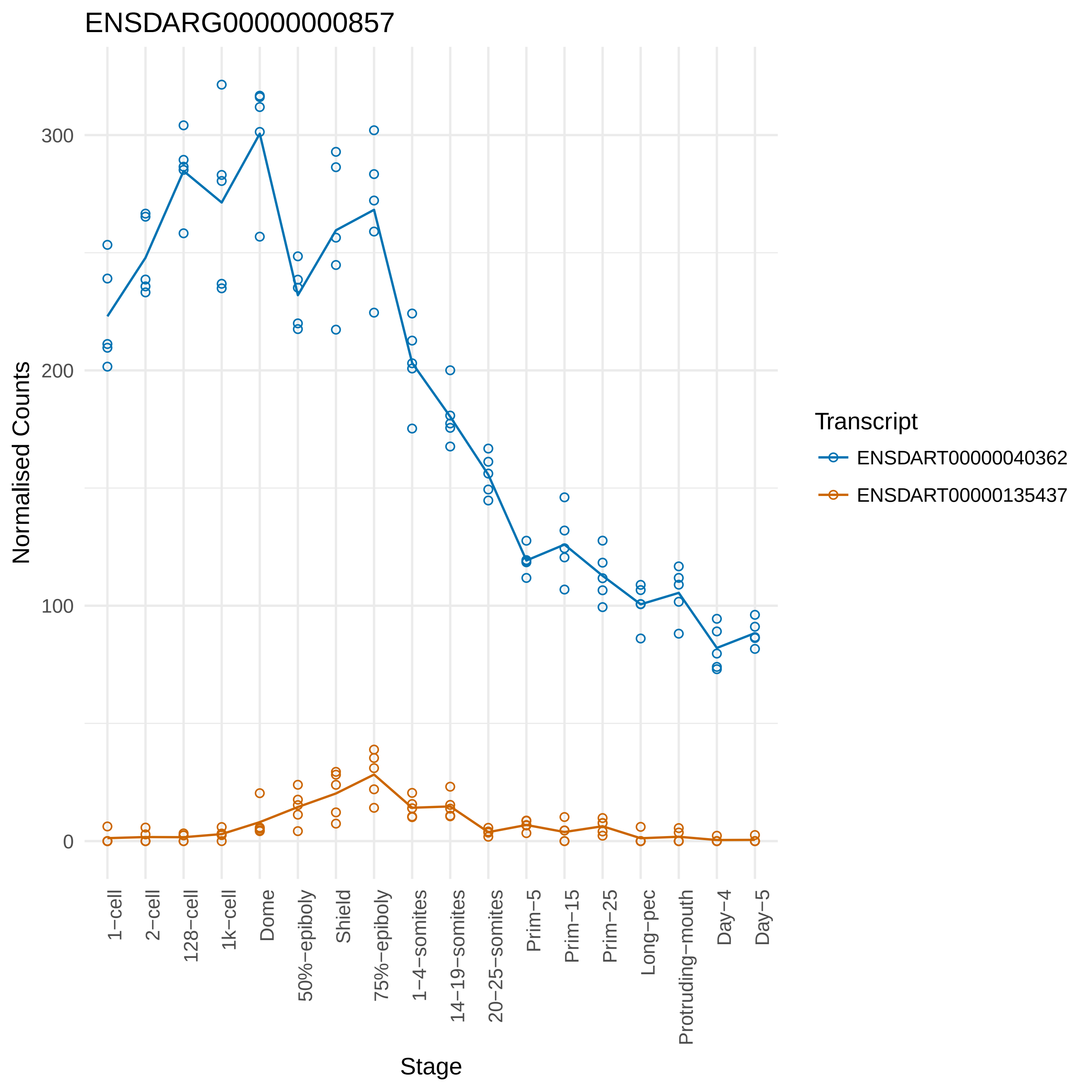

ENSDARG00000000857
Normalised Counts
300
200
100
0
Stage
1−cell
2−cell
128−cell
1k−cell
Dome
50%−epiboly
Shield
75%−epiboly
1−4−somites
14−19−somites
20−25−somites
Prim−5
Prim−15
Prim−25
Long−pec
Protruding−mouth
Day−4
Day−5
Transcript
ENSDART00000040362
ENSDART00000135437

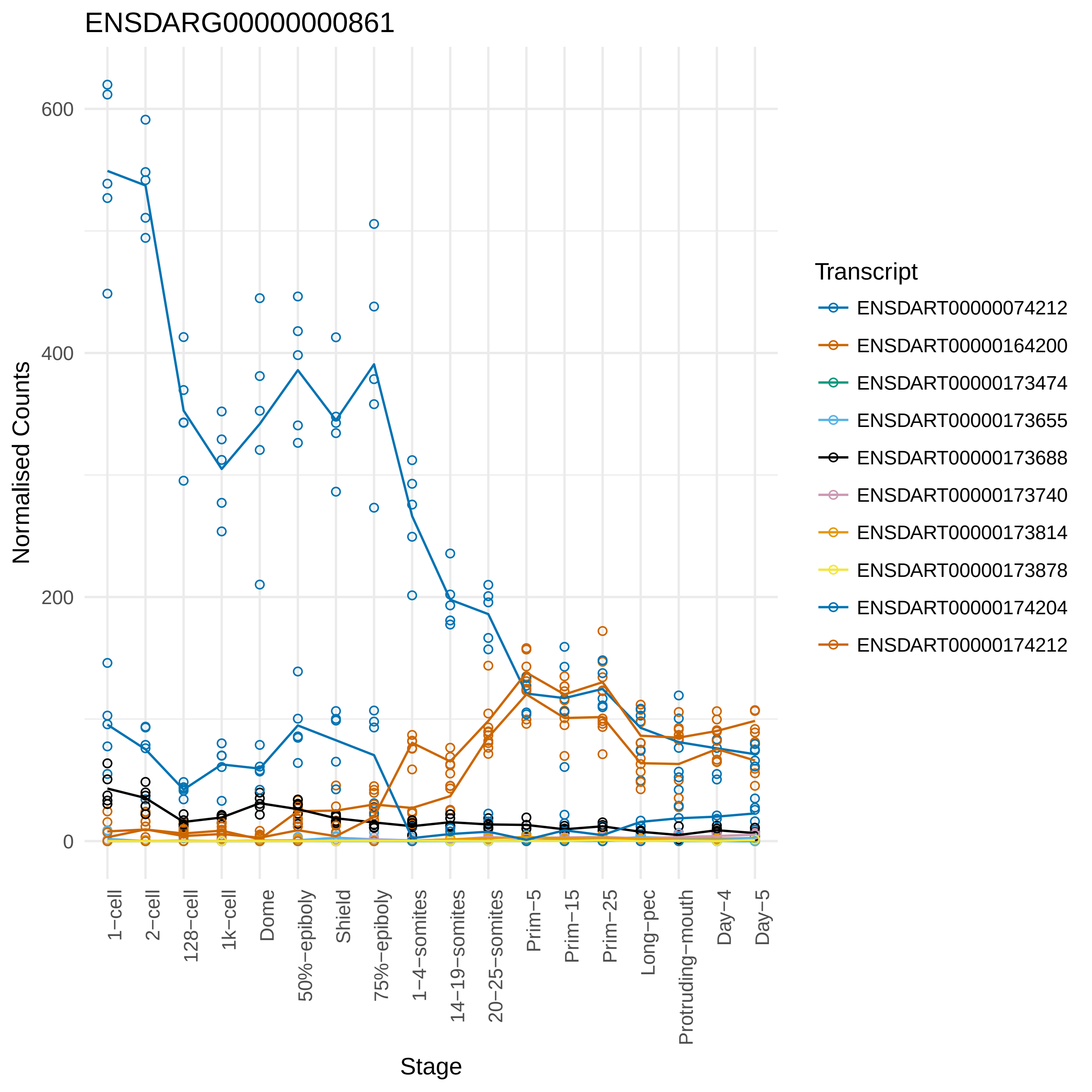

ENSDARG00000000861
Normalised Counts
600
400
200
0
Stage
1−cell
2−cell
128−cell
1k−cell
Dome
50%−epiboly
Shield
75%−epiboly
1−4−somites
14−19−somites
20−25−somites
Prim−5
Prim−15
Prim−25
Long−pec
Protruding−mouth
Day−4
Day−5
Transcript
ENSDART00000074212
ENSDART00000164200
ENSDART00000173474
ENSDART00000173655
ENSDART00000173688
ENSDART00000173740
ENSDART00000173814
ENSDART00000173878
ENSDART00000174204
ENSDART00000174212

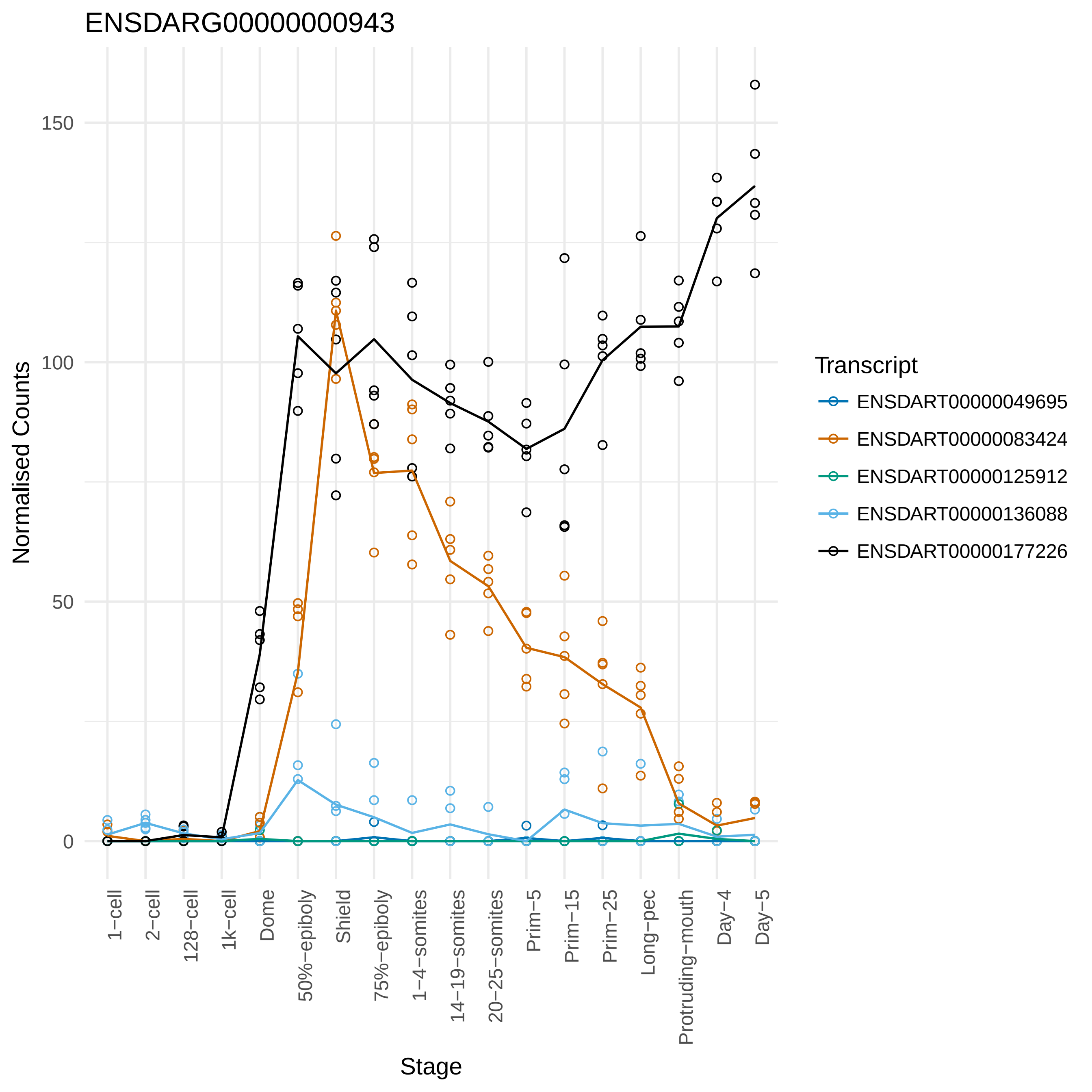
ENSDARG00000000943
Normalised Counts
150
100
50
0
1−cell
2−cell
128−cell
1k−cell
Dome
50%−epiboly
Shield
75%−epiboly
1−4−somites
14−19−somites
20−25−somites
Prim−5
Prim−15
Prim−25
Long−pec
Protruding−mouth
Day−4
Day−5
Stage
Transcript
ENSDART00000049695
ENSDART00000083424
ENSDART00000125912
ENSDART00000136088
ENSDART00000177226

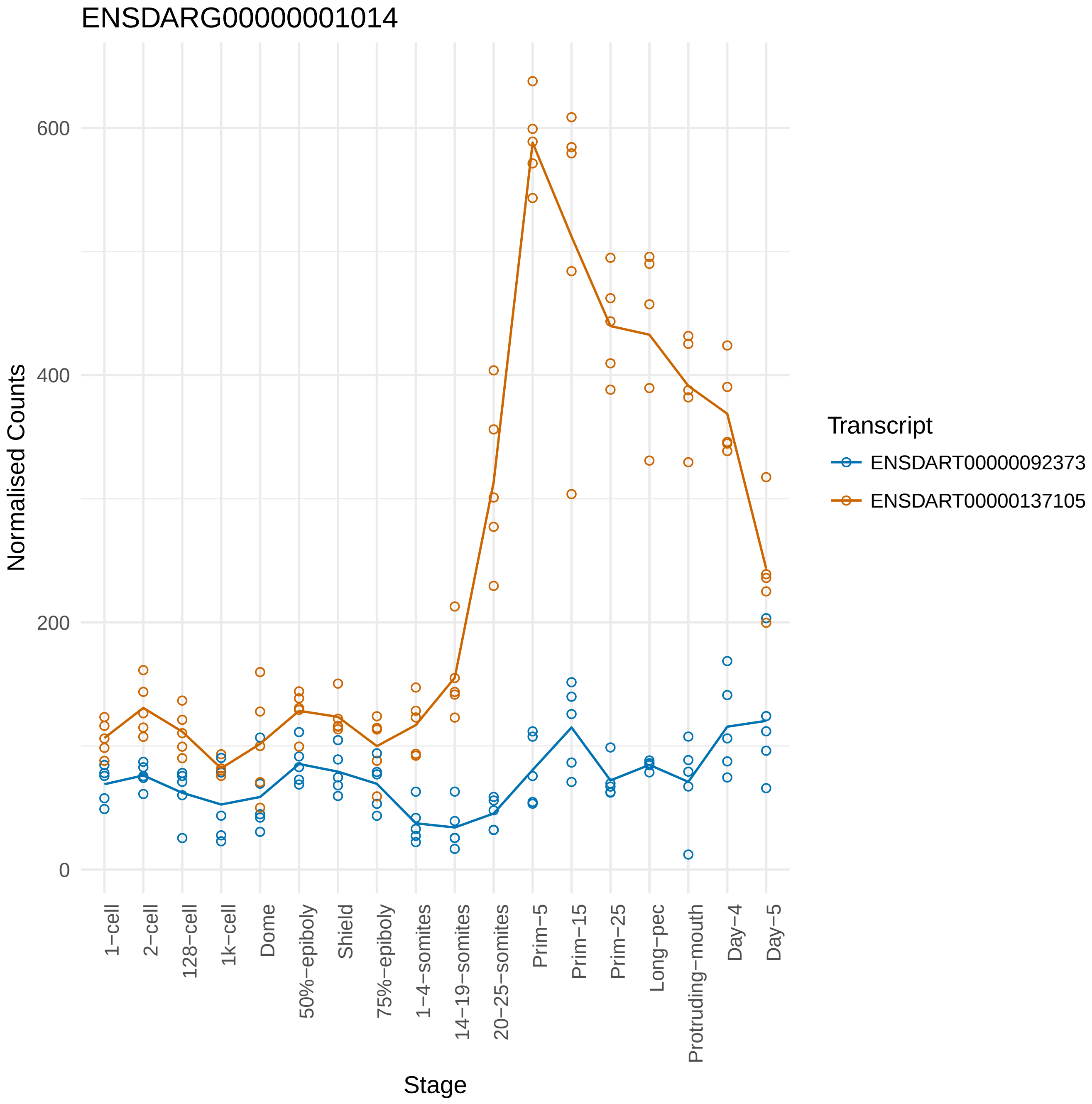

ENSDARG00000001014
Normalised Counts
0
200
400
600
Stage
1−cell
2−cell
128−cell
1k−cell
Dome
50%−epiboly
Shield
75%−epiboly
1−4−somites
14−19−somites
20−25−somites
Prim−5
Prim−15
Prim−25
Long−pec
Protruding−mouth
Day−4
Day−5
Transcript
ENSDART00000092373
ENSDART00000137105

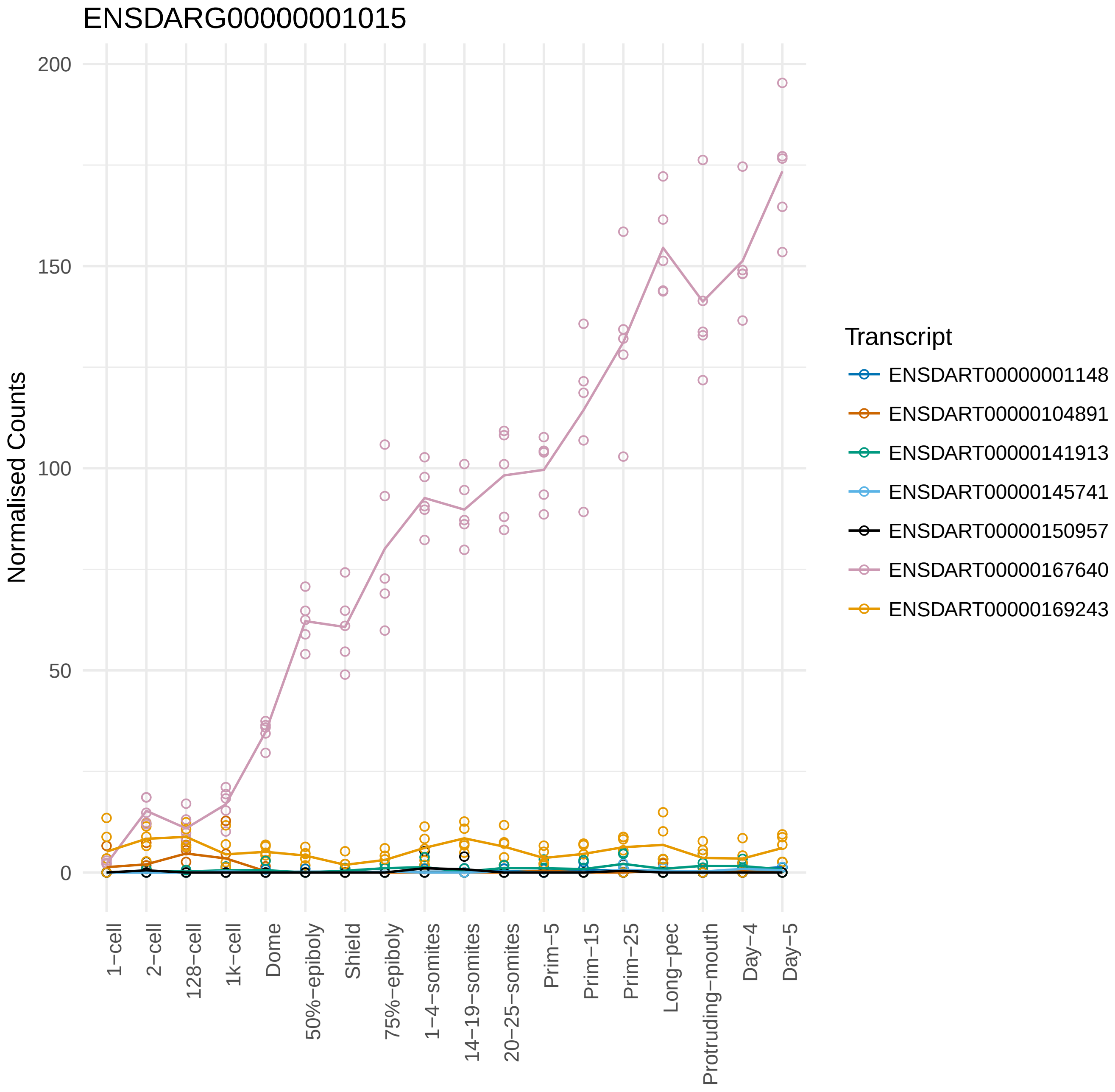

ENSDARG00000001015
Normalised Counts
0
50
100
150
200
1−cell
2−cell
128−cell
1k−cell
Dome
50%−epiboly
Shield
75%−epiboly
1−4−somites
14−19−somites
20−25−somites
Prim−5
Prim−15
Prim−25
Long−pec
Protruding−mouth
Day−4
Day−5
Stage
Transcript
ENSDART00000001148
ENSDART00000104891
ENSDART00000141913
ENSDART00000145741
ENSDART00000150957
ENSDART00000167640
ENSDART00000169243

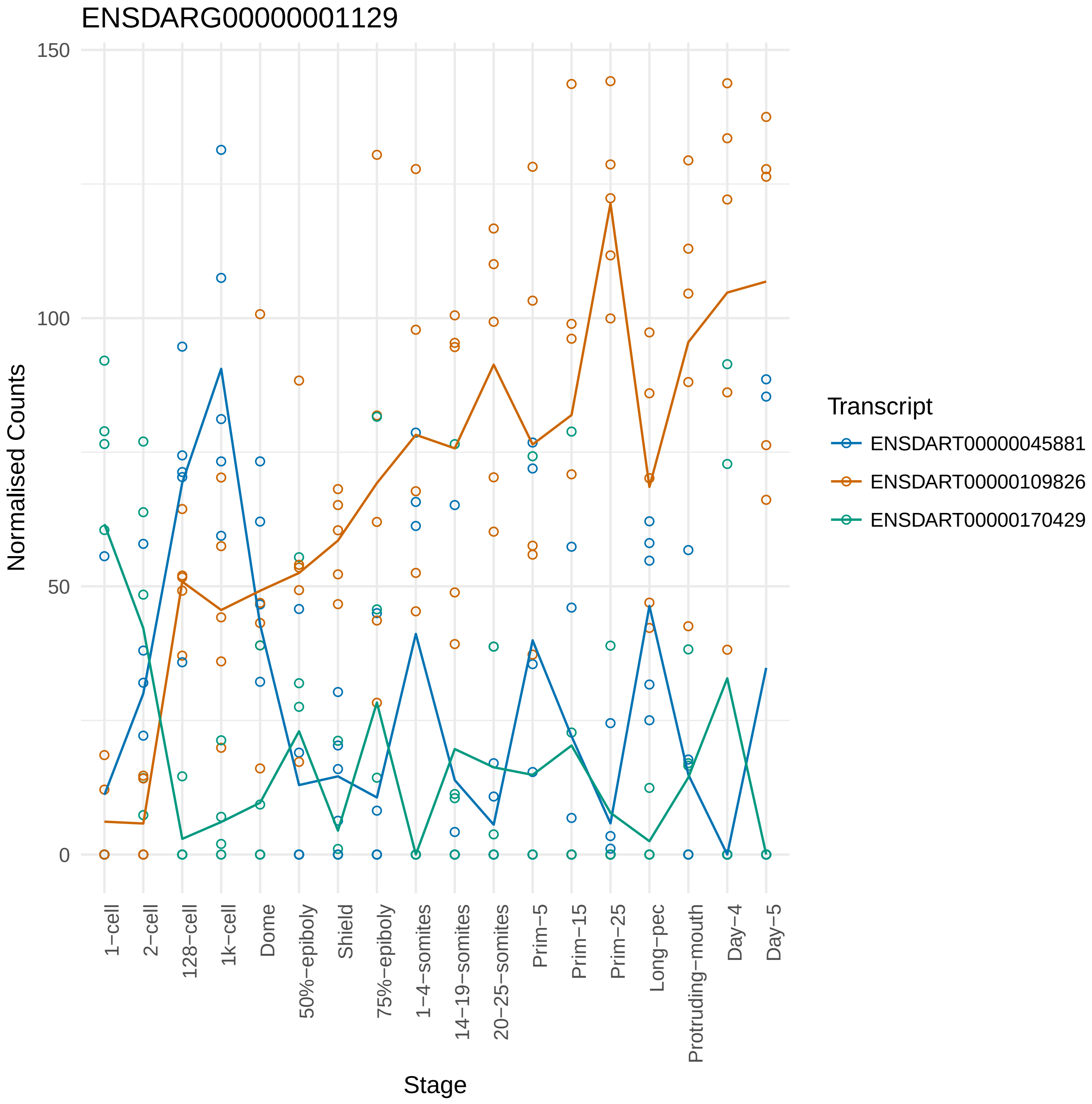
ENSDARG00000001129
Normalised Counts
150
100
50
0
1−cell
2−cell
128−cell
1k−cell
Dome
50%−epiboly
Shield
75%−epiboly
1−4−somites
14−19−somites
20−25−somites
Prim−5
Prim−15
Prim−25
Long−pec
Protruding−mouth
Day−4
Day−5
Stage
Transcript
ENSDART00000045881
ENSDART00000109826
ENSDART00000170429

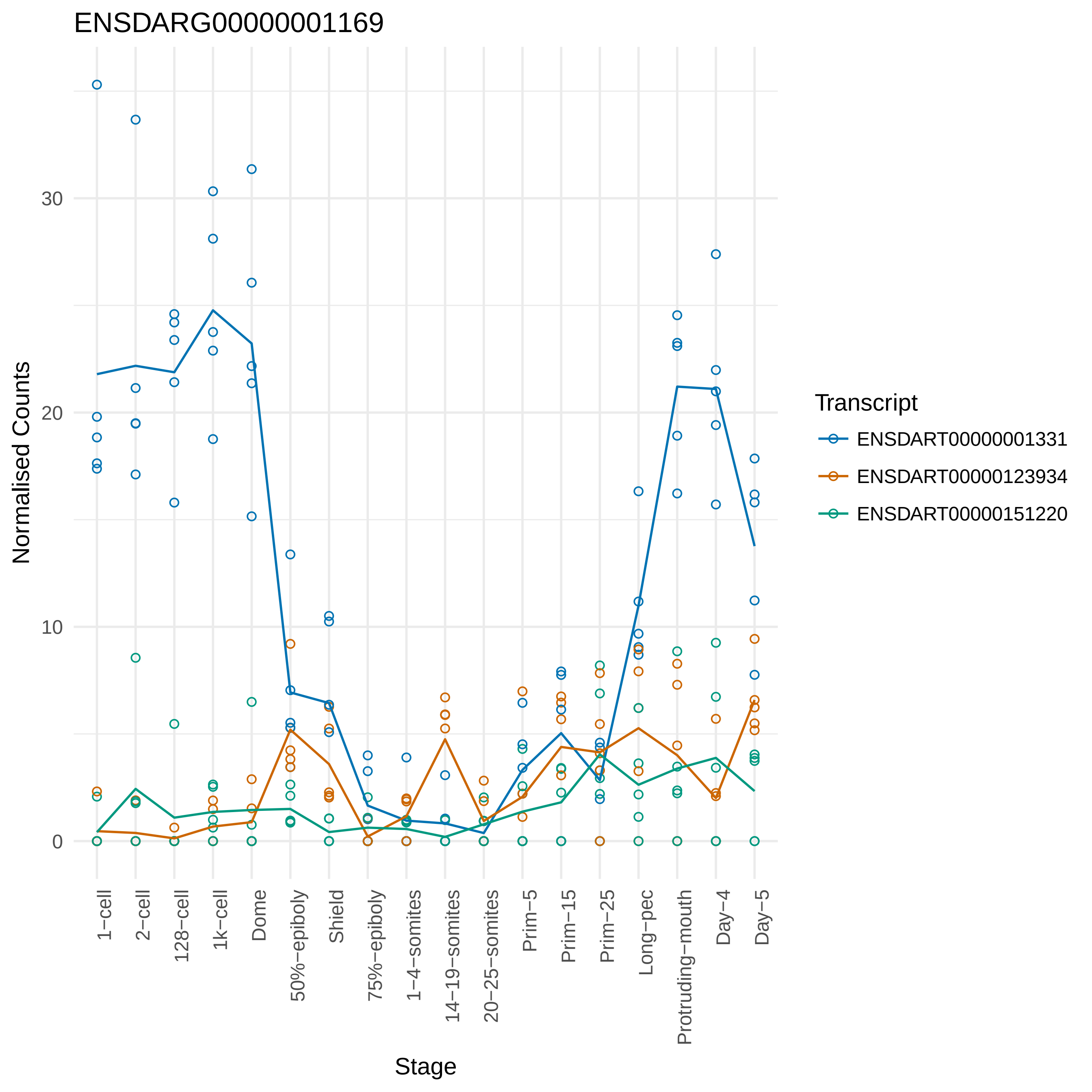
ENSDARG00000001169
Normalised Counts
30
20
10
0
1−cell
2−cell
128−cell
1k−cell
Dome
50%−epiboly
Shield
75%−epiboly
1−4−somites
14−19−somites
20−25−somites
Prim−5
Prim−15
Prim−25
Long−pec
Protruding−mouth
Day−4
Day−5
Stage
Transcript
ENSDART00000001331
ENSDART00000123934
ENSDART00000151220

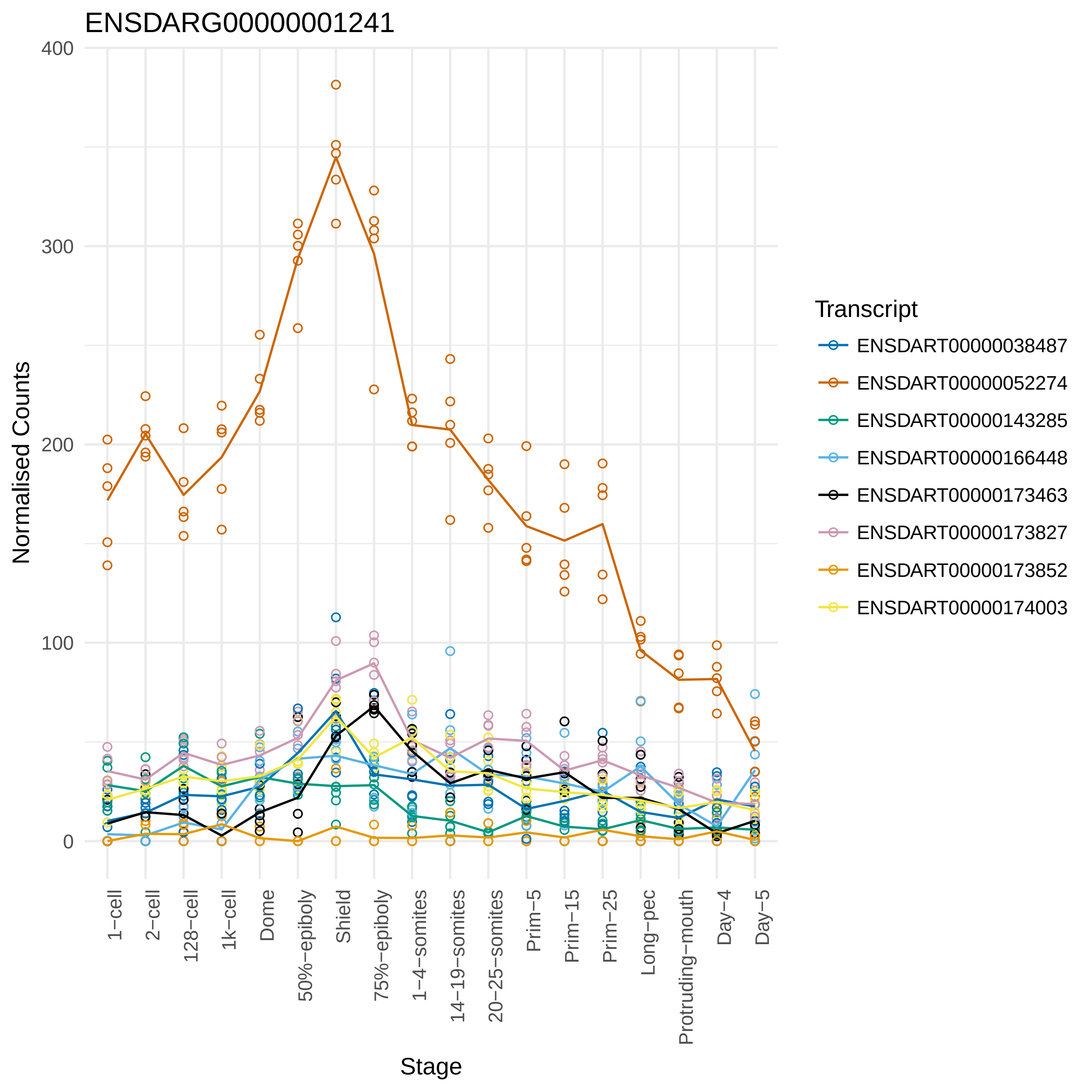

ENSDARG00000001241
Normalised Counts
400
300
200
100
0
1−cell
2−cell
128−cell
1k−cell
Dome
50%−epiboly
Shield
75%−epiboly
1−4−somites
14−19−somites
20−25−somites
Prim−5
Prim−15
Prim−25
Long−pec
Protruding−mouth
Day−4
Day−5
Stage
Transcript
ENSDART00000038487
ENSDART00000052274
ENSDART00000143285
ENSDART00000166448
ENSDART00000173463
ENSDART00000173827
ENSDART00000173852
ENSDART00000174003

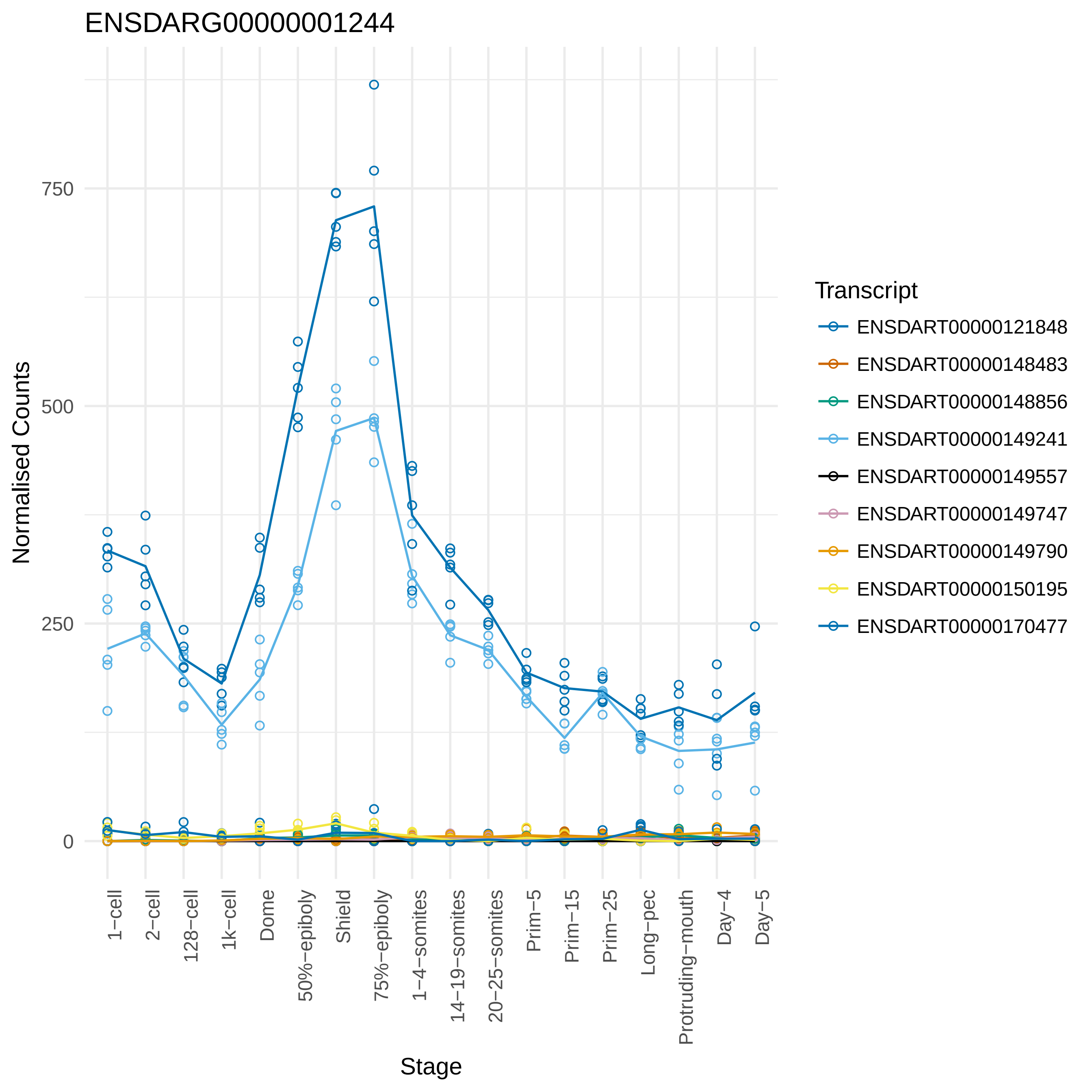
ENSDARG00000001244
Normalised Counts
750
500
250
0
1−cell
2−cell
128−cell
1k−cell
Dome
50%−epiboly
Shield
75%−epiboly
1−4−somites
14−19−somites
20−25−somites
Prim−5
Prim−15
Prim−25
Long−pec
Protruding−mouth
Day−4
Day−5
Stage
Transcript
ENSDART00000121848
ENSDART00000148483
ENSDART00000148856
ENSDART00000149241
ENSDART00000149557
ENSDART00000149747
ENSDART00000149790
ENSDART00000150195
ENSDART00000170477

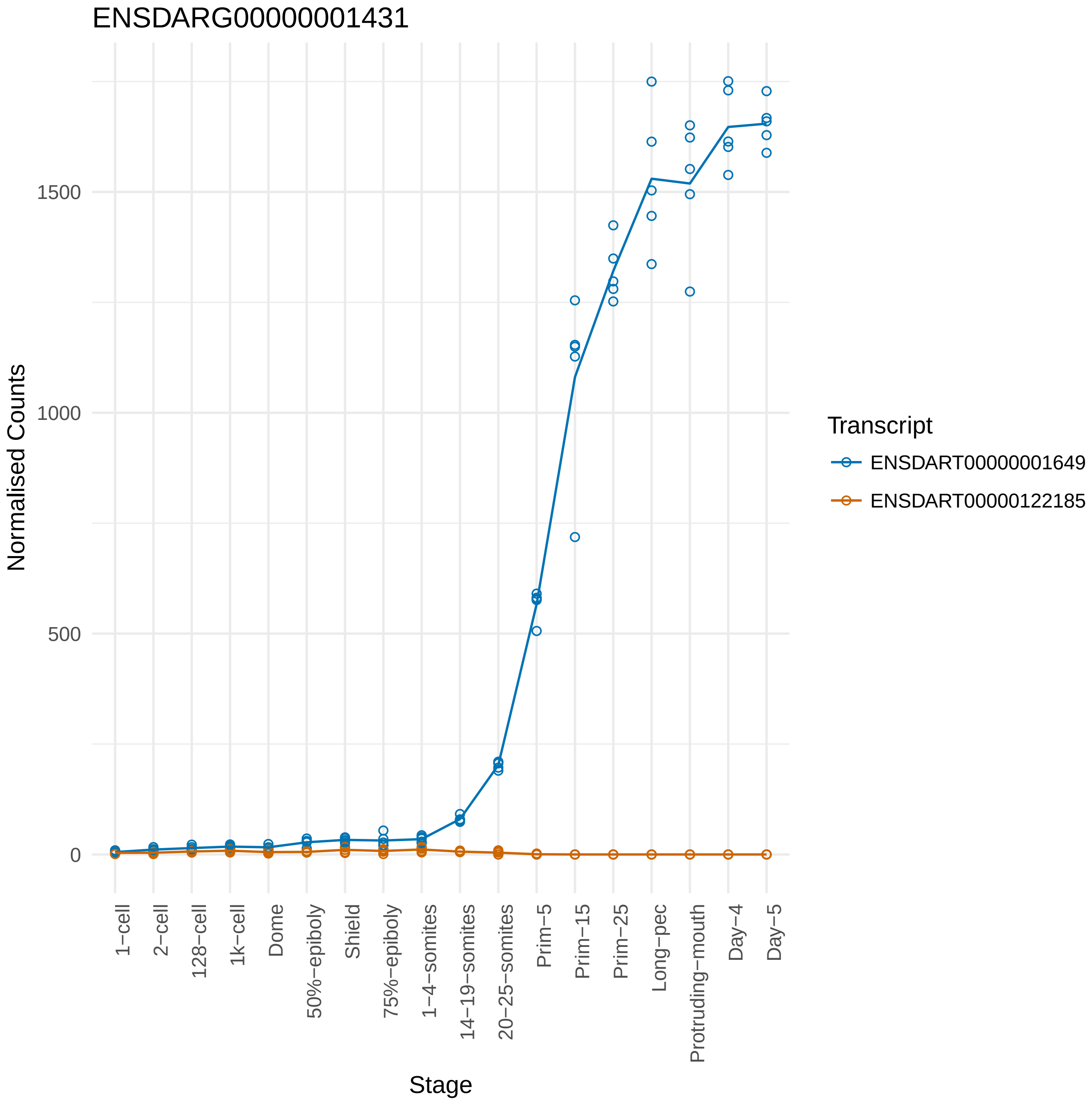
ENSDARG00000001431
Normalised Counts
0
500
1000
1500
Stage
1−cell
2−cell
128−cell
1k−cell
Dome
50%−epiboly
Shield
75%−epiboly
1−4−somites
14−19−somites
20−25−somites
Prim−5
Prim−15
Prim−25
Long−pec
Protruding−mouth
Day−4
Day−5
Transcript
ENSDART00000001649
ENSDART00000122185

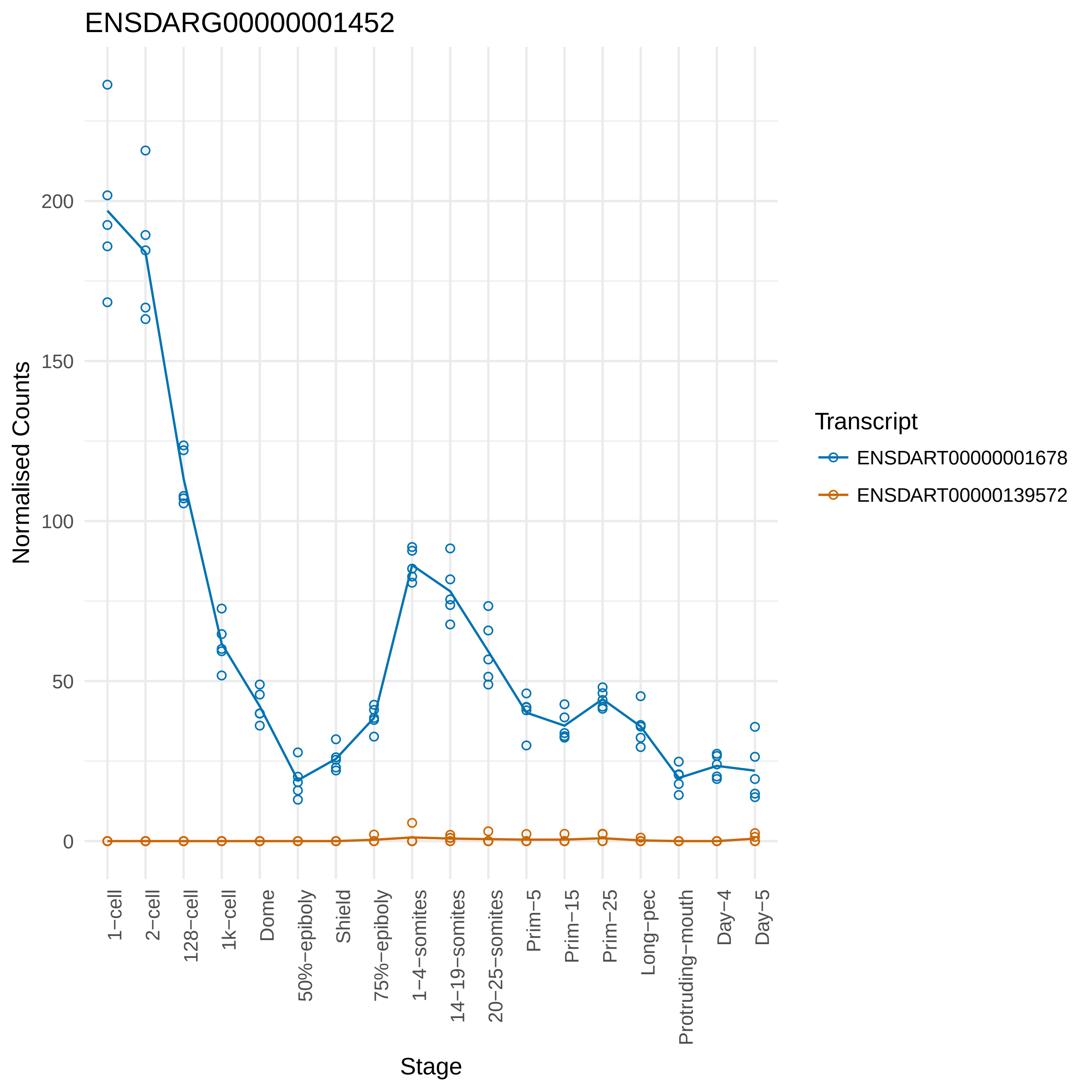
ENSDARG00000001452
Normalised Counts
200
150
100
50
0
1−cell
2−cell
128−cell
1k−cell
Dome
50%−epiboly
Shield
75%−epiboly
1−4−somites
14−19−somites
20−25−somites
Prim−5
Prim−15
Prim−25
Long−pec
Protruding−mouth
Day−4
Day−5
Stage
Transcript
ENSDART00000001678
ENSDART00000139572

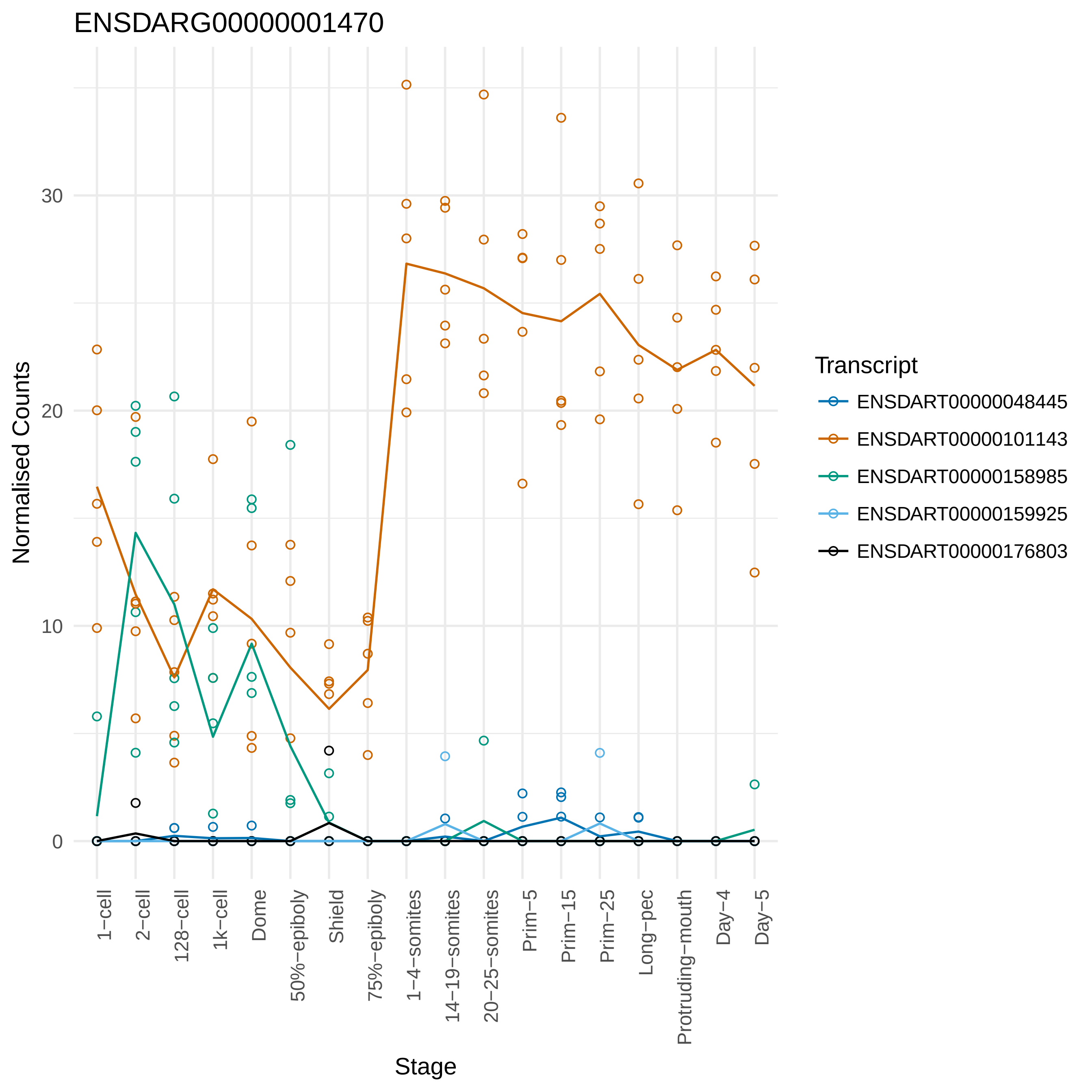
ENSDARG00000001470
Normalised Counts
30
20
10
0
Stage
1−cell
2−cell
128−cell
1k−cell
Dome
50%−epiboly
Shield
75%−epiboly
1−4−somites
14−19−somites
20−25−somites
Prim−5
Prim−15
Prim−25
Long−pec
Protruding−mouth
Day−4
Day−5
Transcript
ENSDART00000048445
ENSDART00000101143
ENSDART00000158985
ENSDART00000159925
ENSDART00000176803

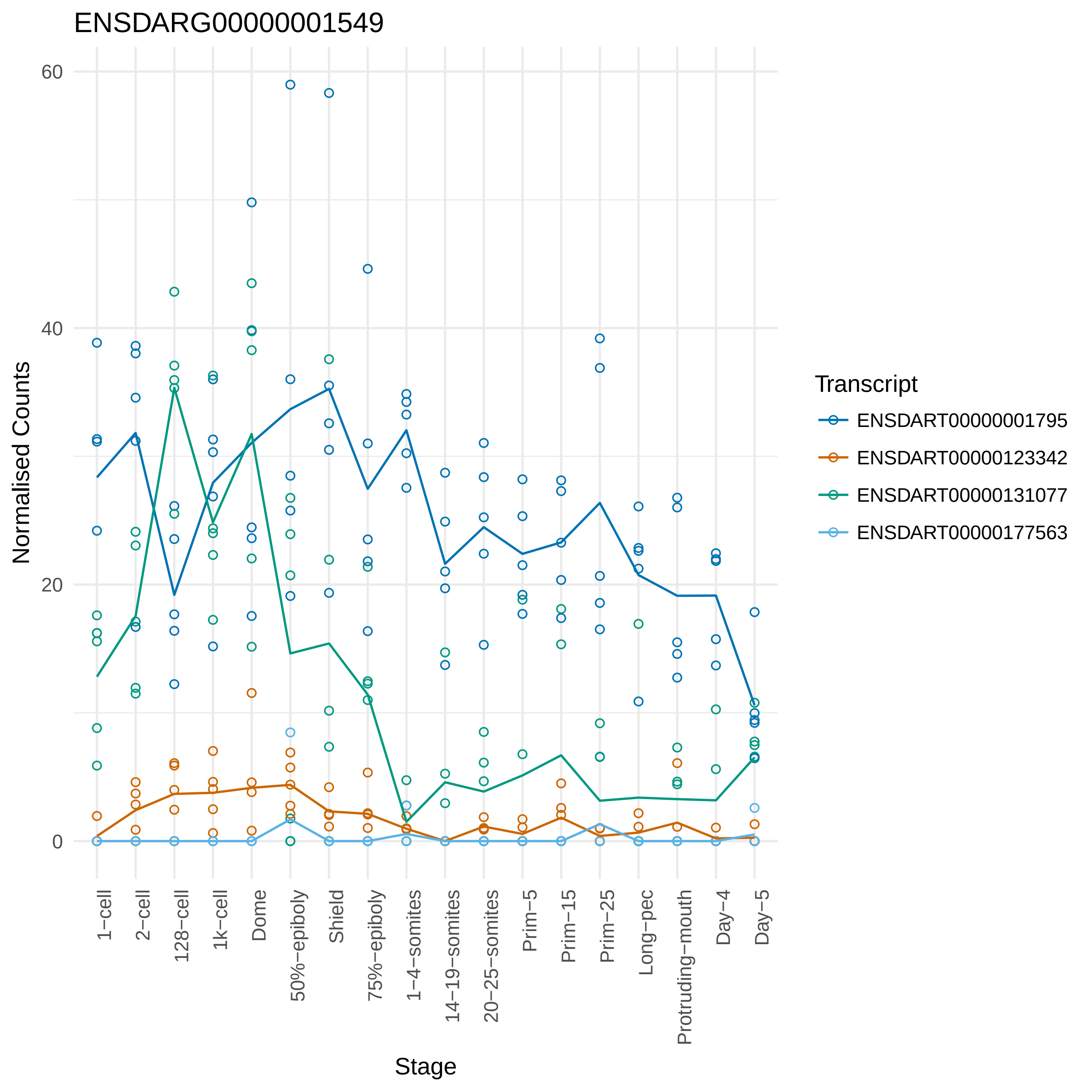
ENSDARG00000001549
Normalised Counts
0
20
40
60
1−cell
2−cell
128−cell
1k−cell
Dome
50%−epiboly
Shield
75%−epiboly
1−4−somites
14−19−somites
20−25−somites
Prim−5
Prim−15
Prim−25
Long−pec
Protruding−mouth
Day−4
Day−5
Stage
Transcript
ENSDART00000001795
ENSDART00000123342
ENSDART00000131077
ENSDART00000177563

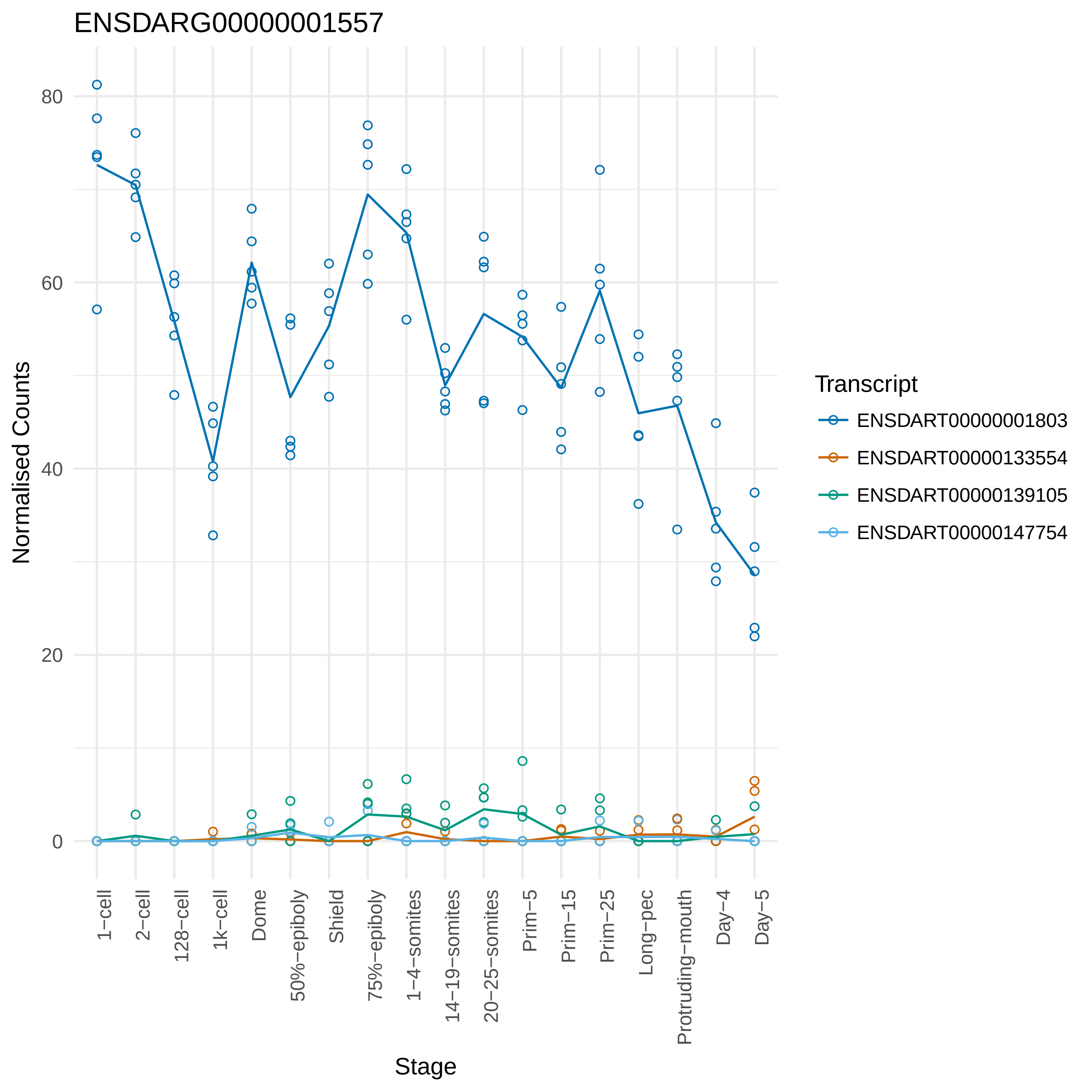

ENSDARG00000001557
Normalised Counts
80
60
40
20
0
1−cell
2−cell
128−cell
1k−cell
Dome
50%−epiboly
Shield
75%−epiboly
1−4−somites
14−19−somites
20−25−somites
Prim−5
Prim−15
Prim−25
Long−pec
Protruding−mouth
Day−4
Day−5
Stage
Transcript
ENSDART00000001803
ENSDART00000133554
ENSDART00000139105
ENSDART00000147754

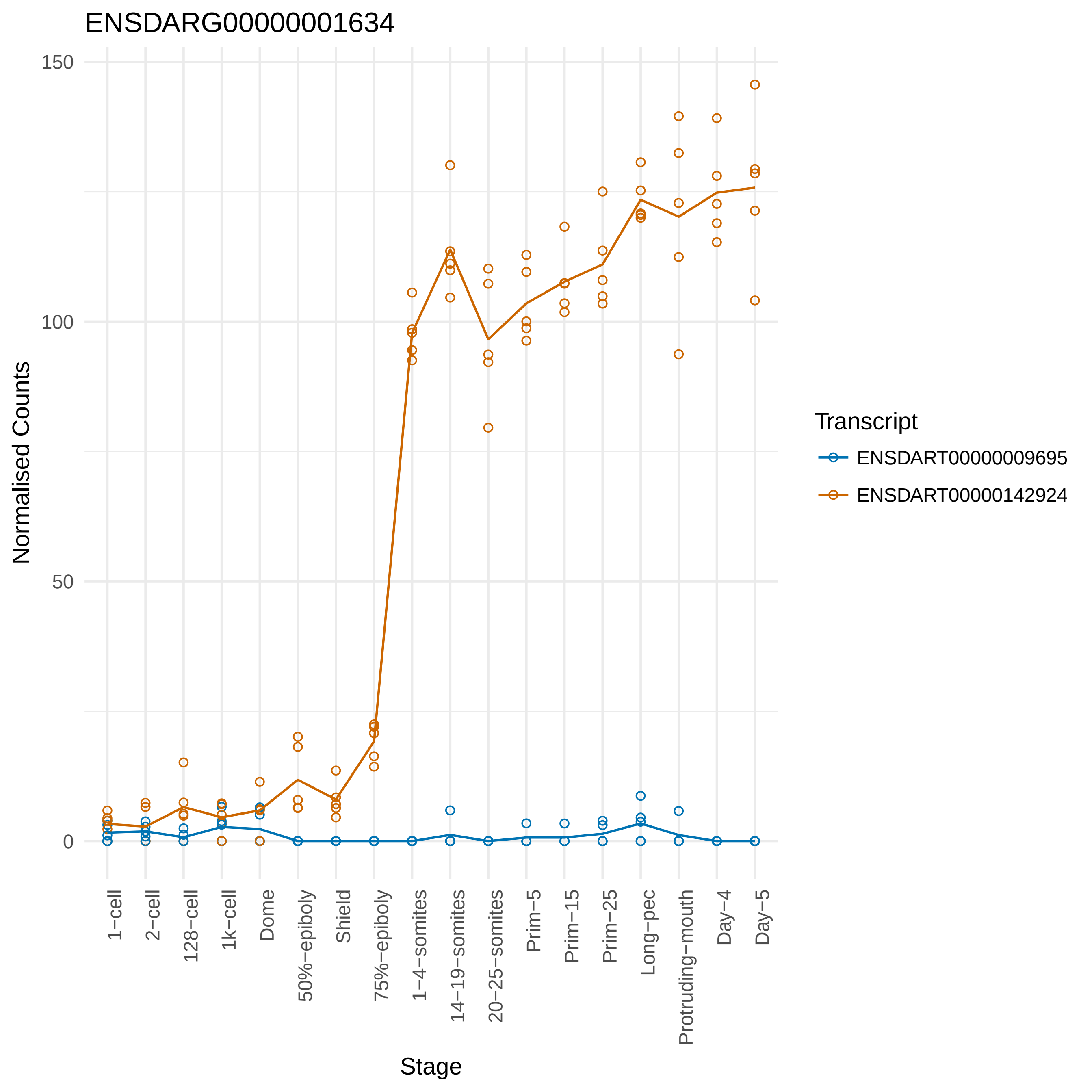
ENSDARG00000001634
Normalised Counts
150
100
50
0
1−cell
2−cell
128−cell
1k−cell
Dome
50%−epiboly
Shield
75%−epiboly
1−4−somites
14−19−somites
20−25−somites
Prim−5
Prim−15
Prim−25
Long−pec
Protruding−mouth
Day−4
Day−5
Stage
Transcript
ENSDART00000009695
ENSDART00000142924

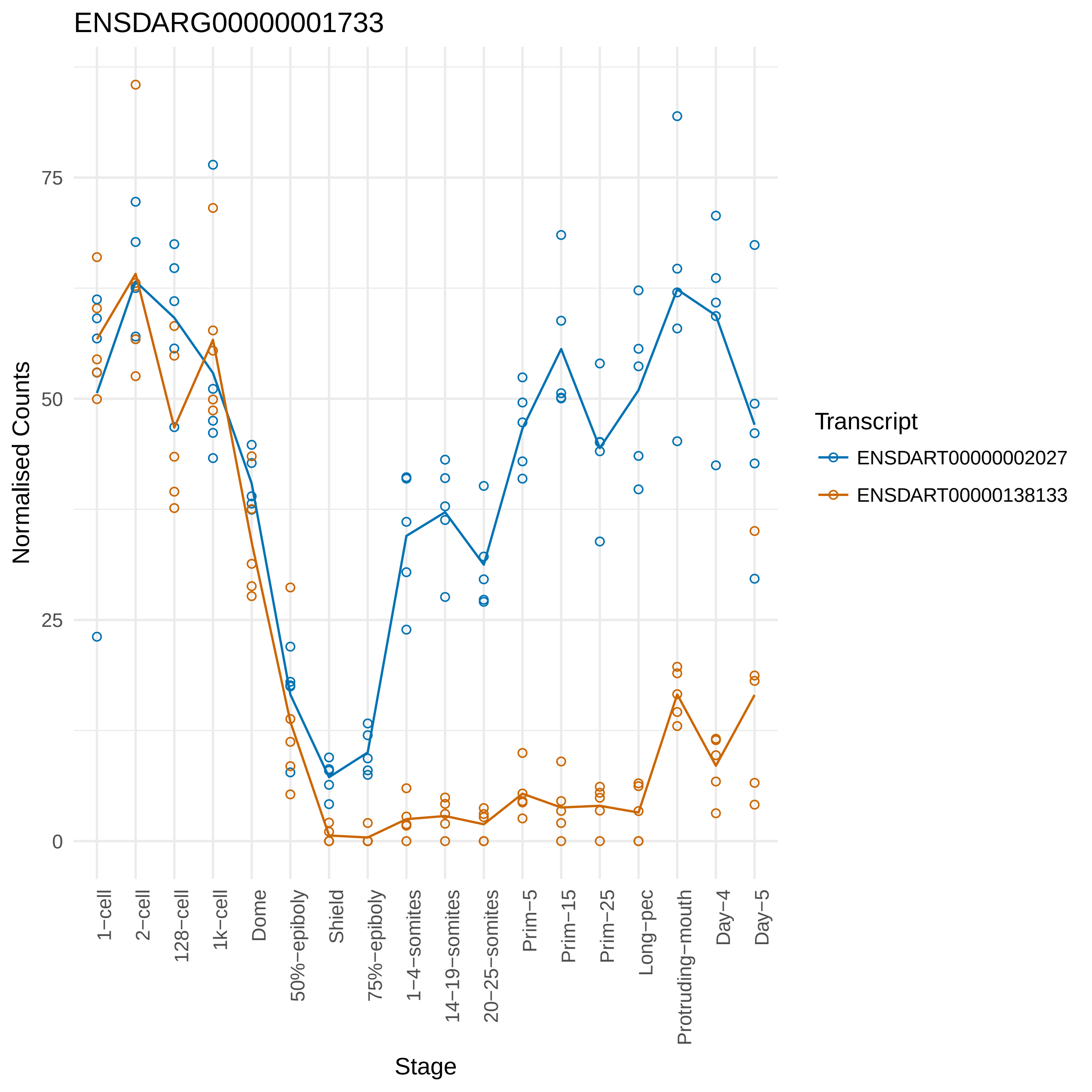

ENSDARG00000001733
Normalised Counts
75
50
25
0
1−cell
2−cell
128−cell
1k−cell
Dome
50%−epiboly
Shield
75%−epiboly
1−4−somites
14−19−somites
20−25−somites
Prim−5
Prim−15
Prim−25
Long−pec
Protruding−mouth
Day−4
Day−5
Stage
Transcript
ENSDART00000002027
ENSDART00000138133

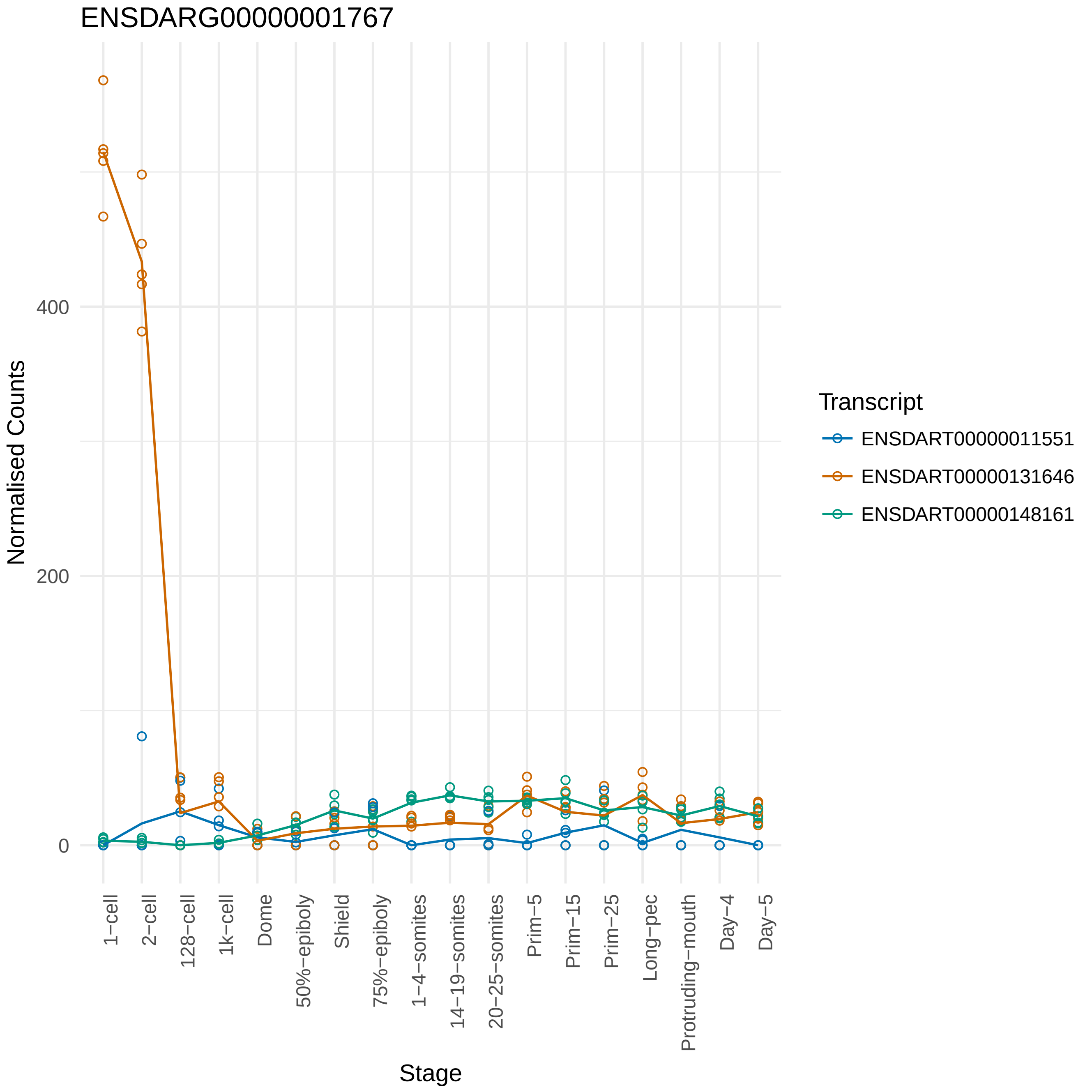

ENSDARG00000001767
Normalised Counts
400
200
0
Stage
1−cell
2−cell
128−cell
1k−cell
Dome
50%−epiboly
Shield
75%−epiboly
1−4−somites
14−19−somites
20−25−somites
Prim−5
Prim−15
Prim−25
Long−pec
Protruding−mouth
Day−4
Day−5
Transcript
ENSDART00000011551
ENSDART00000131646
ENSDART00000148161

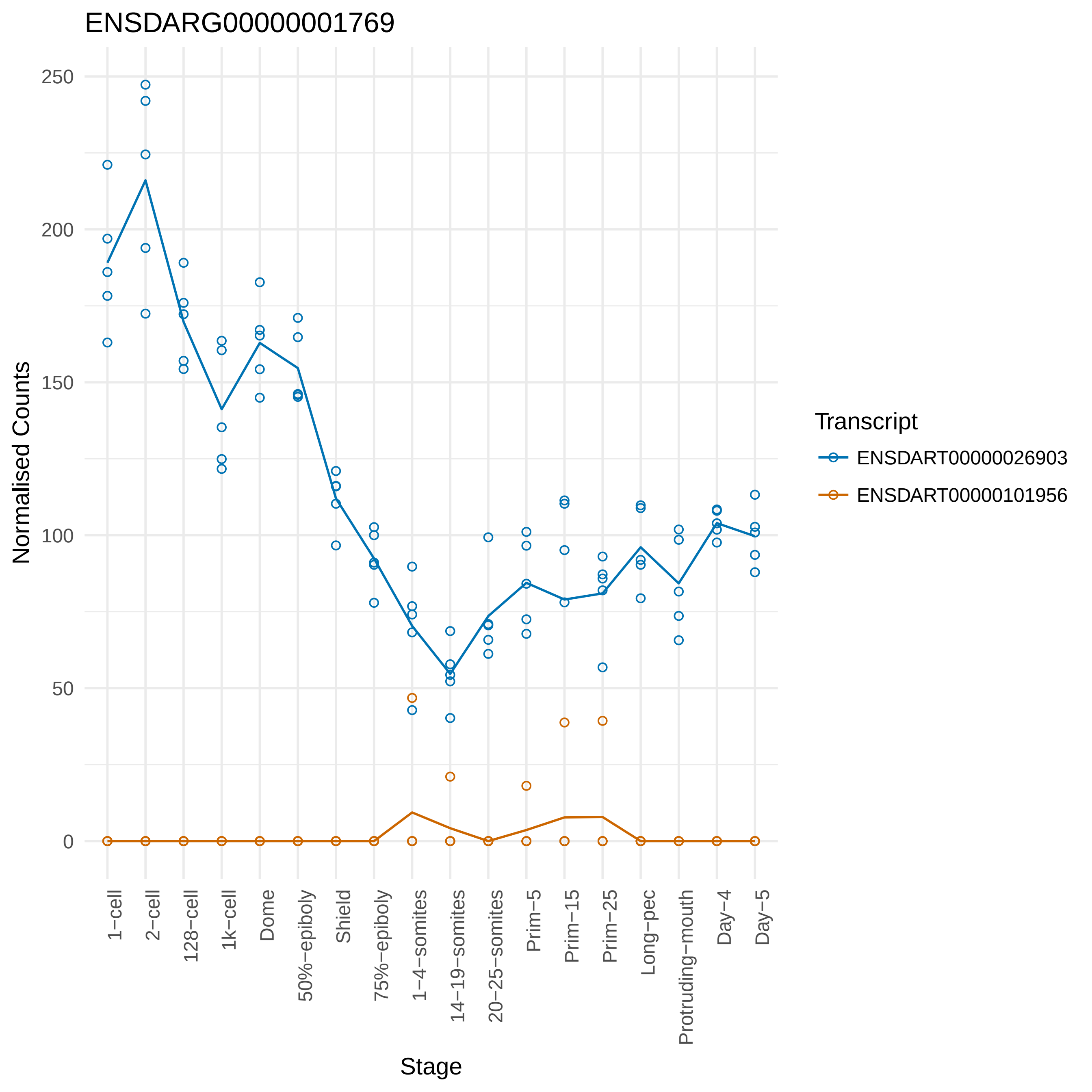

ENSDARG00000001769
Normalised Counts
0
50
100
150
200
250
Stage
1−cell
2−cell
128−cell
1k−cell
Dome
50%−epiboly
Shield
75%−epiboly
1−4−somites
14−19−somites
20−25−somites
Prim−5
Prim−15
Prim−25
Long−pec
Protruding−mouth
Day−4
Day−5
Transcript
ENSDART00000026903
ENSDART00000101956

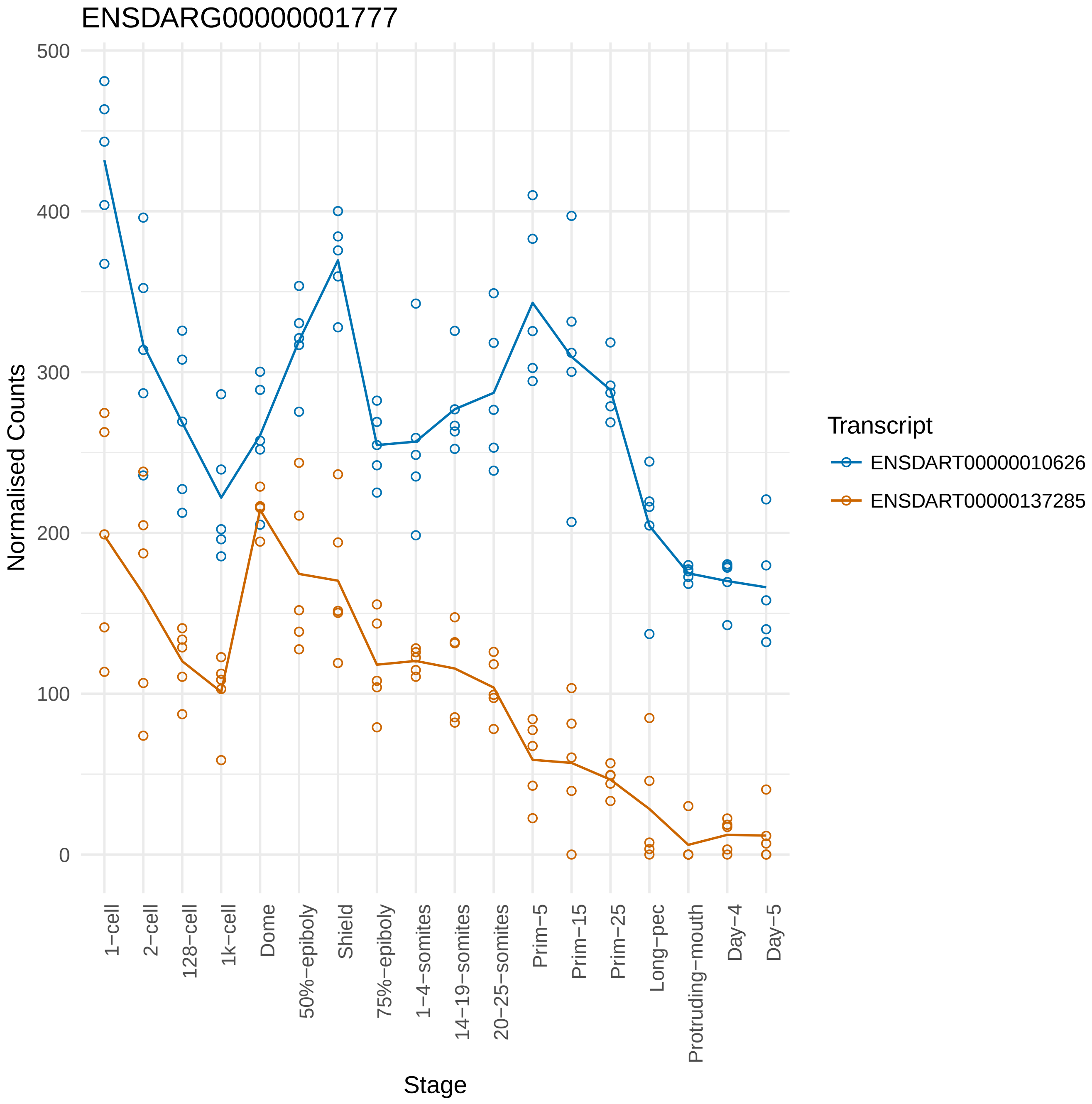
ENSDARG00000001777
Normalised Counts
0
100
200
300
400
500
1−cell
2−cell
128−cell
1k−cell
Dome
50%−epiboly
Shield
75%−epiboly
1−4−somites
14−19−somites
20−25−somites
Prim−5
Prim−15
Prim−25
Long−pec
Protruding−mouth
Day−4
Day−5
Stage
Transcript
ENSDART00000010626
ENSDART00000137285

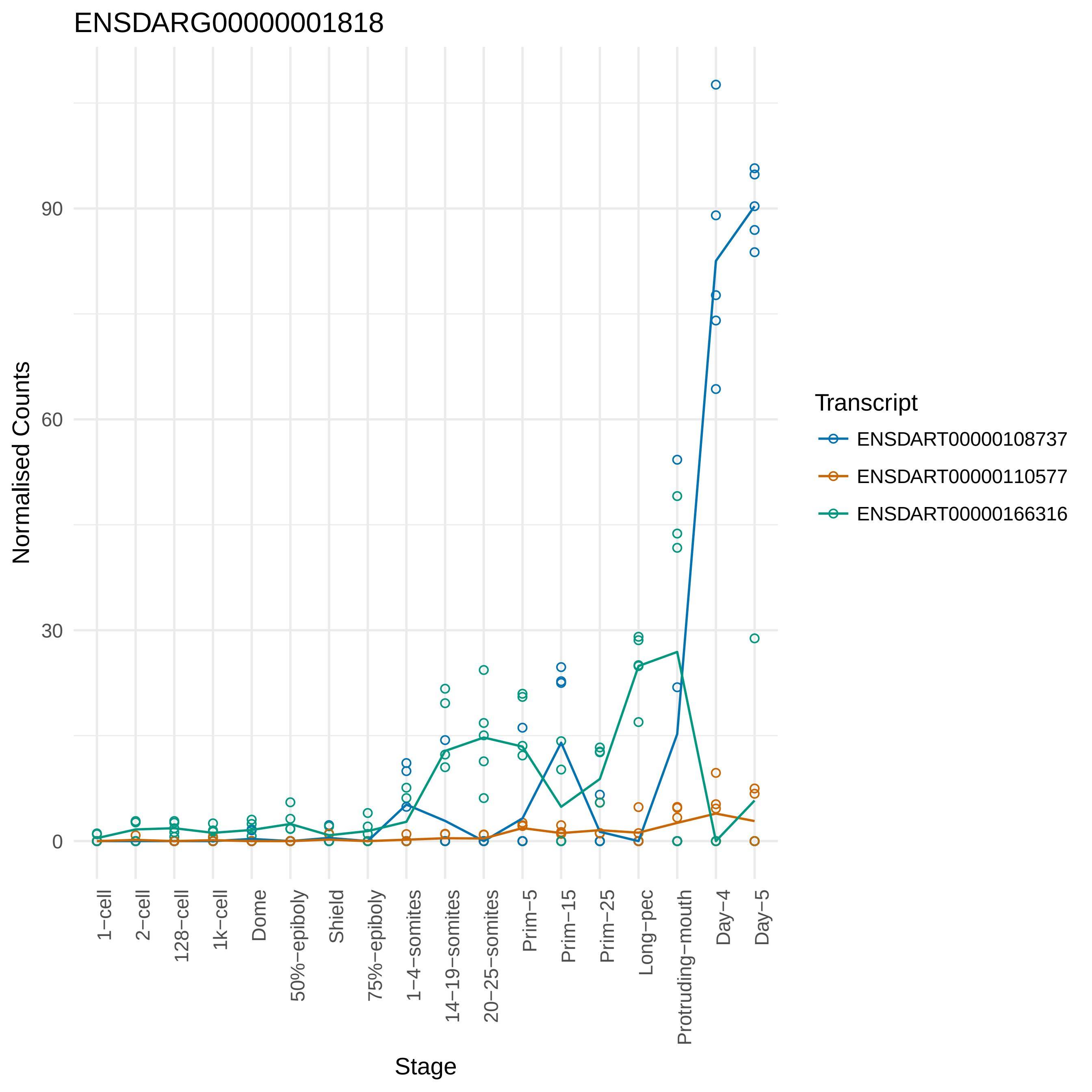
ENSDARG00000001818
Normalised Counts
0
30
60
90
Stage
1−cell
2−cell
128−cell
1k−cell
Dome
50%−epiboly
Shield
75%−epiboly
1−4−somites
14−19−somites
20−25−somites
Prim−5
Prim−15
Prim−25
Long−pec
Protruding−mouth
Day−4
Day−5
Transcript
ENSDART00000108737
ENSDART00000110577
ENSDART00000166316

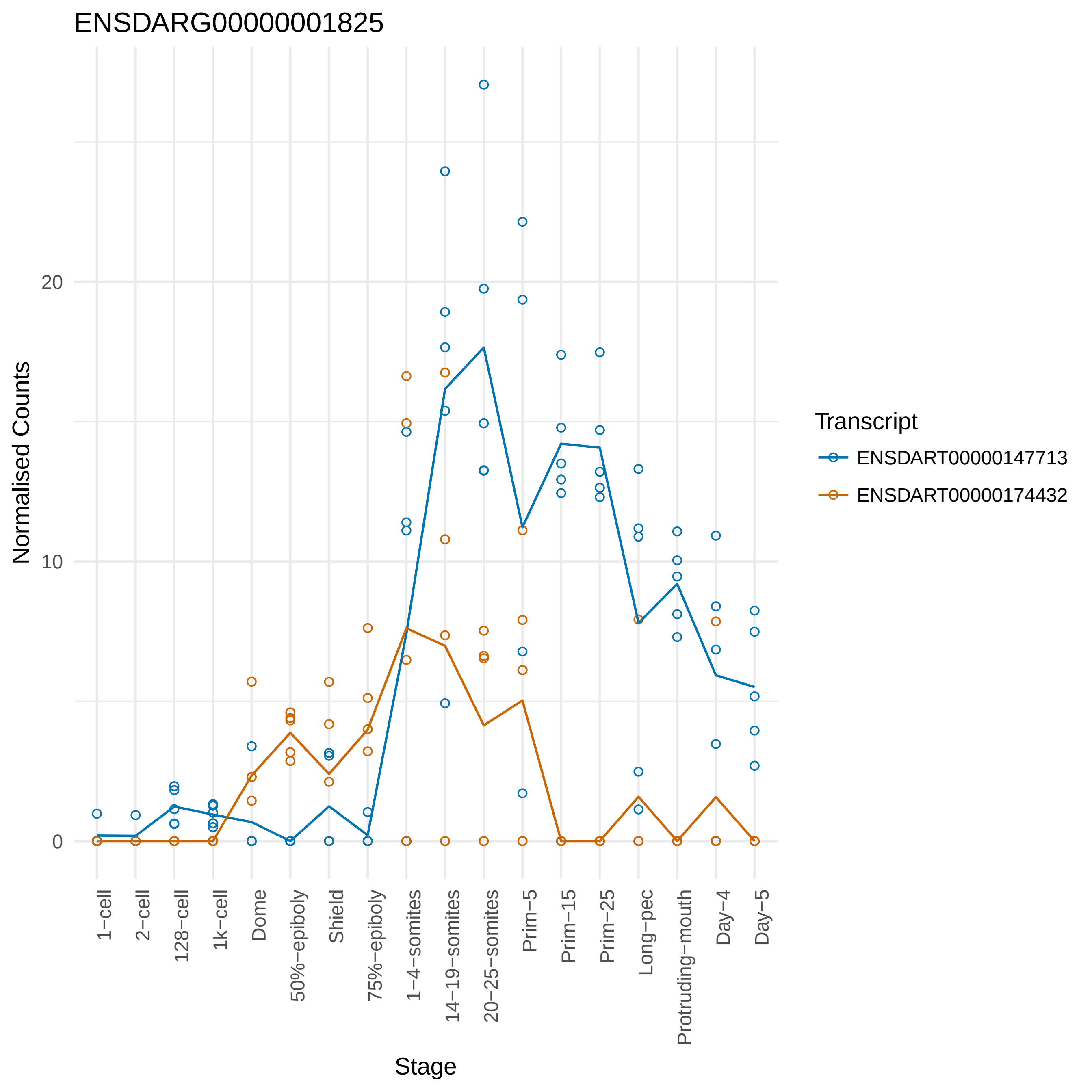
ENSDARG00000001825
Normalised Counts
20
10
0
1−cell
2−cell
128−cell
1k−cell
Dome
50%−epiboly
Shield
75%−epiboly
1−4−somites
14−19−somites
20−25−somites
Prim−5
Prim−15
Prim−25
Long−pec
Protruding−mouth
Day−4
Day−5
Stage
Transcript
ENSDART00000147713
ENSDART00000174432

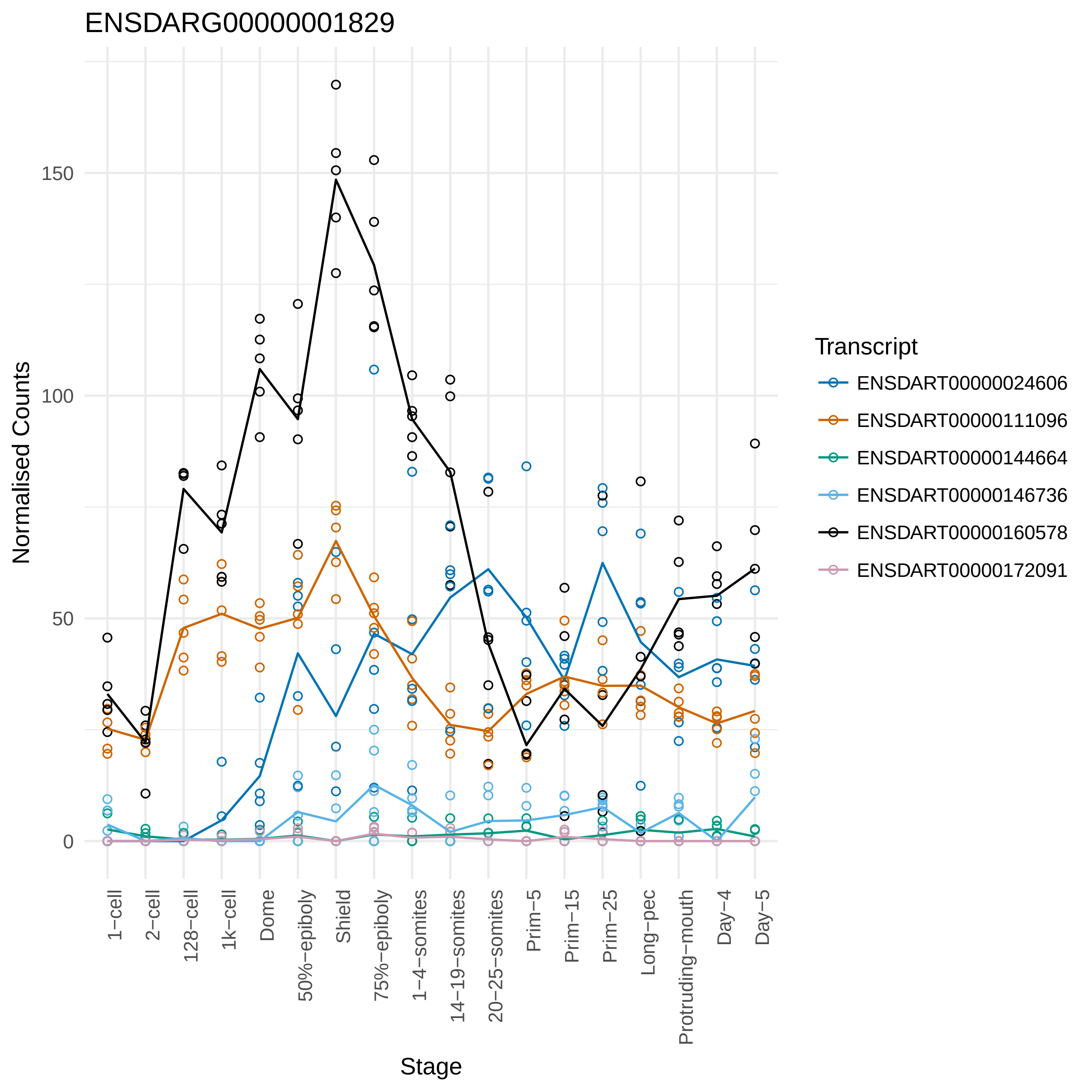

ENSDARG00000001829
Normalised Counts
0
50
100
150
Stage
1−cell
2−cell
128−cell
1k−cell
Dome
50%−epiboly
Shield
75%−epiboly
1−4−somites
14−19−somites
20−25−somites
Prim−5
Prim−15
Prim−25
Long−pec
Protruding−mouth
Day−4
Day−5
Transcript
ENSDART00000024606
ENSDART00000111096
ENSDART00000144664
ENSDART00000146736
ENSDART00000160578
ENSDART00000172091

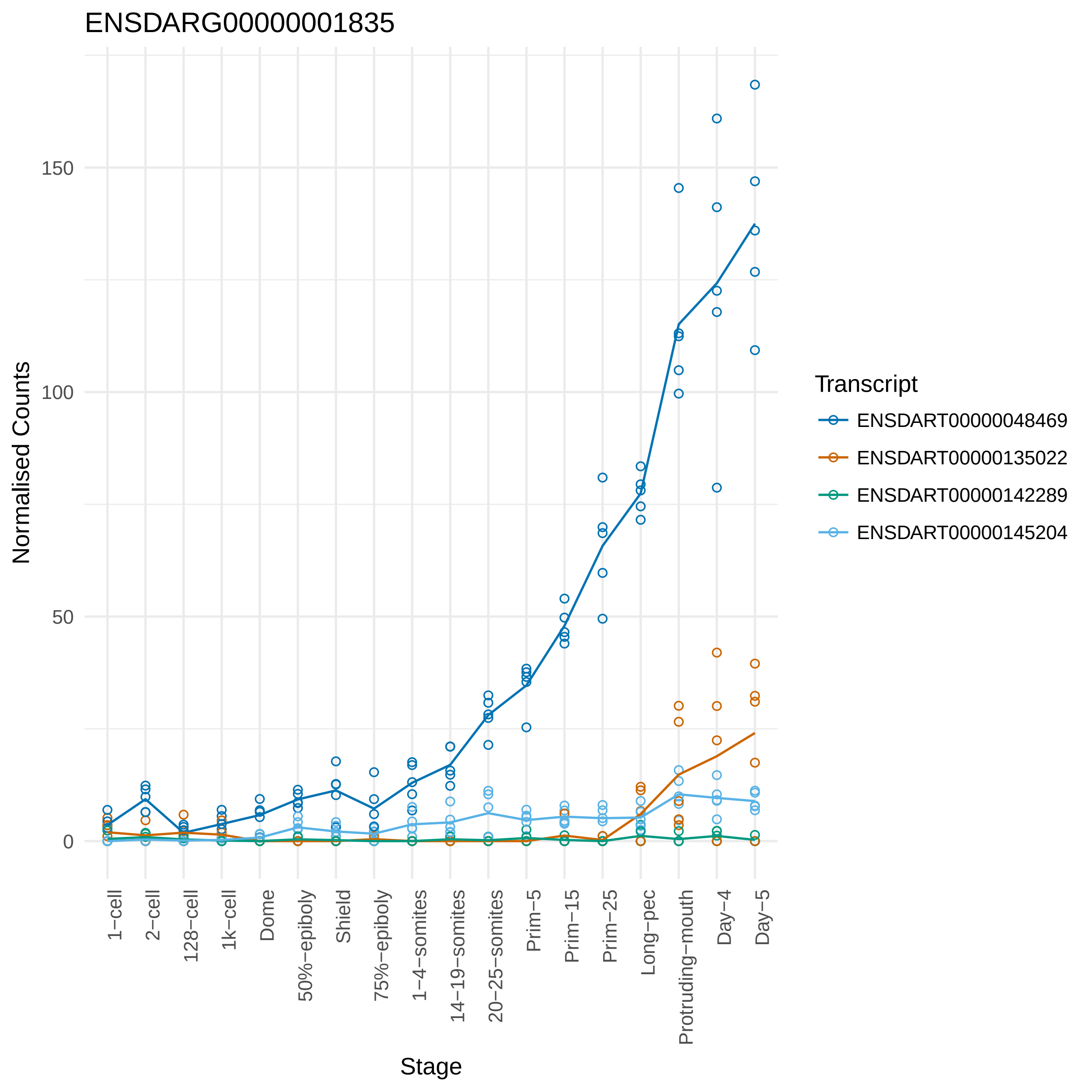

ENSDARG00000001835
Normalised Counts
150
100
50
0
1−cell
2−cell
128−cell
1k−cell
Dome
50%−epiboly
Shield
75%−epiboly
1−4−somites
14−19−somites
20−25−somites
Prim−5
Prim−15
Prim−25
Long−pec
Protruding−mouth
Day−4
Day−5
Stage
Transcript
ENSDART00000048469
ENSDART00000135022
ENSDART00000142289
ENSDART00000145204

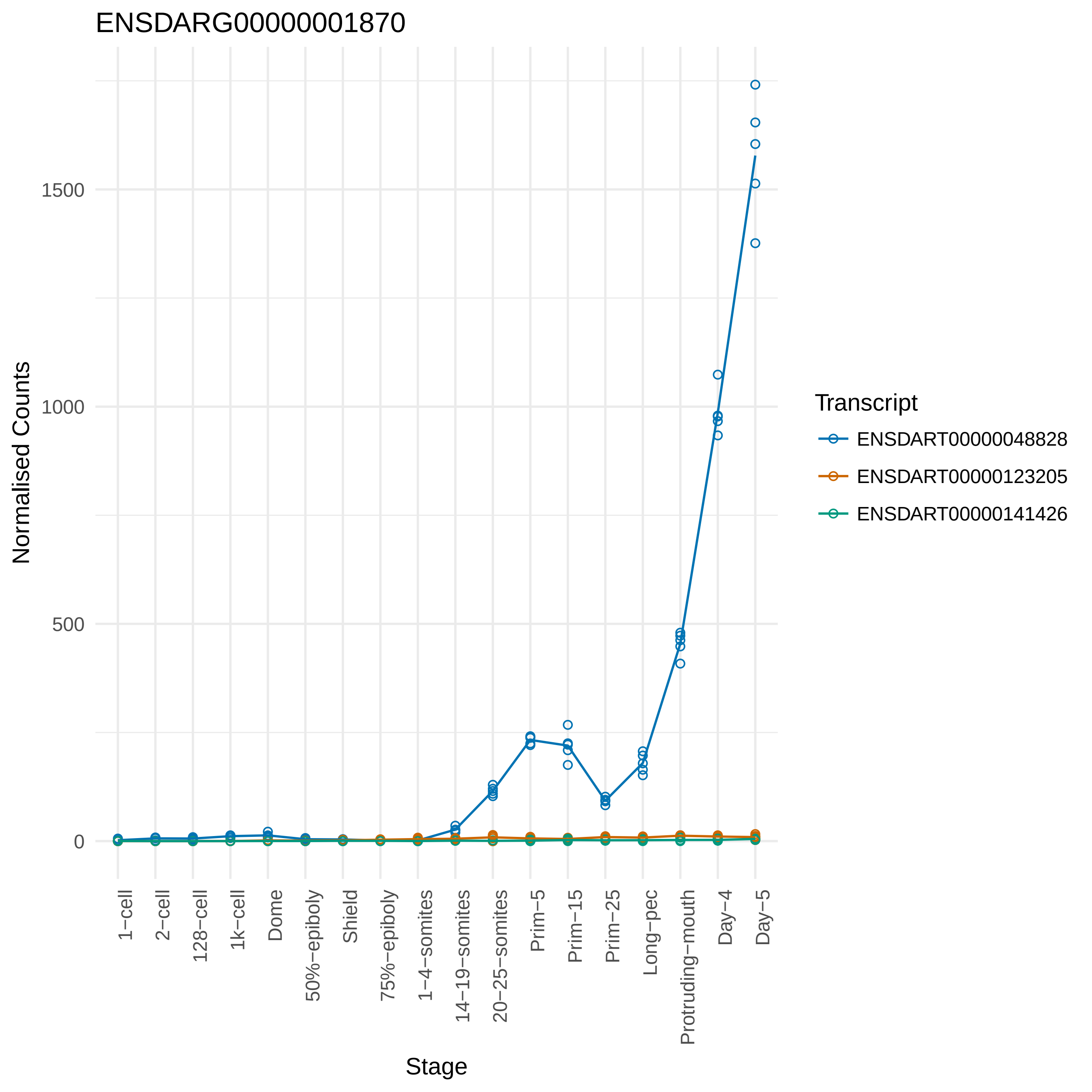

ENSDARG00000001870
Normalised Counts
1500
1000
500
0
1−cell
2−cell
128−cell
1k−cell
Dome
50%−epiboly
Shield
75%−epiboly
1−4−somites
14−19−somites
20−25−somites
Prim−5
Prim−15
Prim−25
Long−pec
Protruding−mouth
Day−4
Day−5
Stage
Transcript
ENSDART00000048828
ENSDART00000123205
ENSDART00000141426

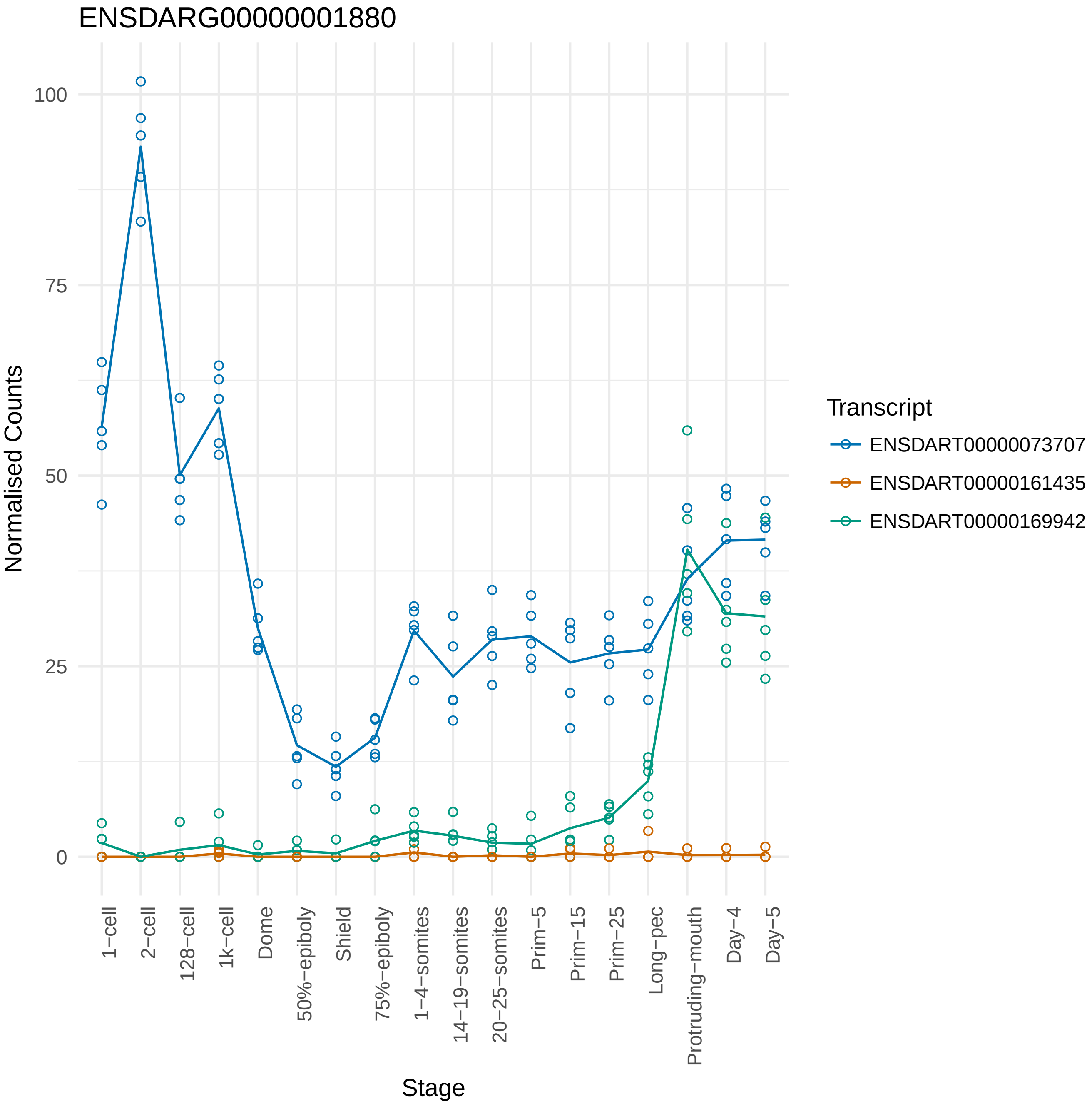

ENSDARG00000001880
Normalised Counts
100
75
50
25
0
1−cell
2−cell
128−cell
1k−cell
Dome
50%−epiboly
Shield
75%−epiboly
1−4−somites
14−19−somites
20−25−somites
Prim−5
Prim−15
Prim−25
Long−pec
Protruding−mouth
Day−4
Day−5
Stage
Transcript
ENSDART00000073707
ENSDART00000161435
ENSDART00000169942

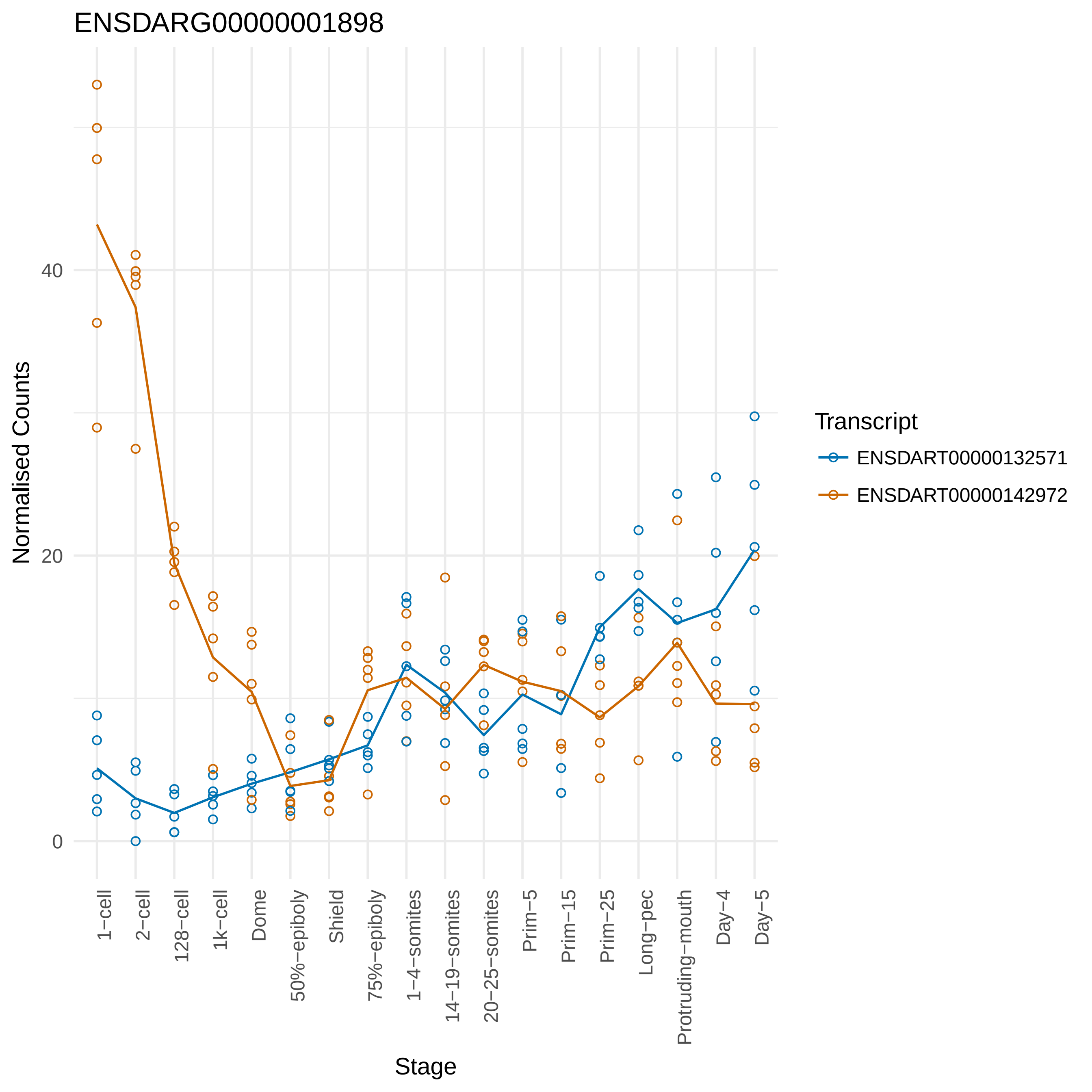
ENSDARG00000001898
Normalised Counts
0
20
40
Stage
1−cell
2−cell
128−cell
1k−cell
Dome
50%−epiboly
Shield
75%−epiboly
1−4−somites
14−19−somites
20−25−somites
Prim−5
Prim−15
Prim−25
Long−pec
Protruding−mouth
Day−4
Day−5
Transcript
ENSDART00000132571
ENSDART00000142972

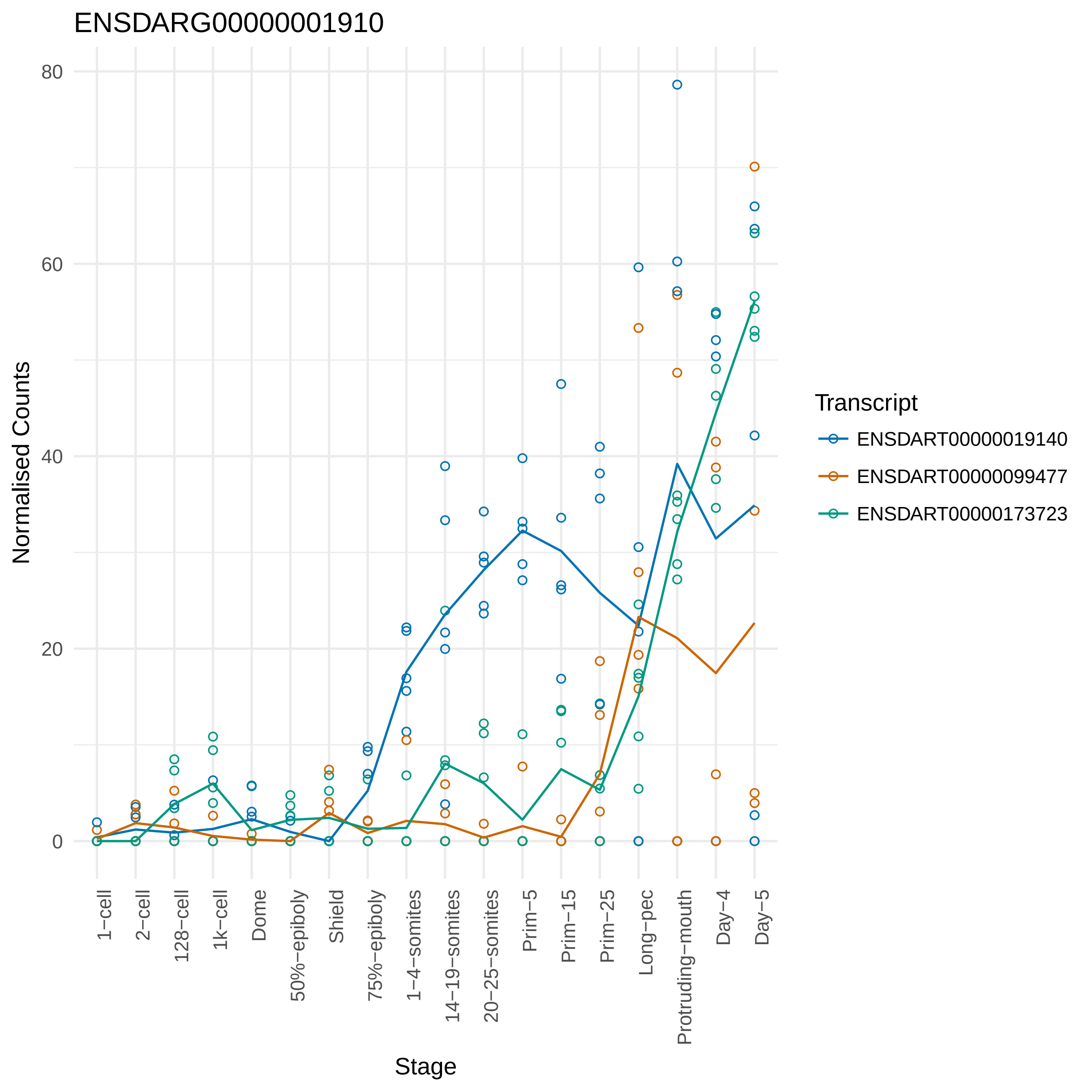

ENSDARG00000001910
Normalised Counts
80
60
40
20
0
1−cell
2−cell
128−cell
1k−cell
Dome
50%−epiboly
Shield
75%−epiboly
1−4−somites
14−19−somites
20−25−somites
Prim−5
Prim−15
Prim−25
Long−pec
Protruding−mouth
Day−4
Day−5
Stage
Transcript
ENSDART00000019140
ENSDART00000099477
ENSDART00000173723

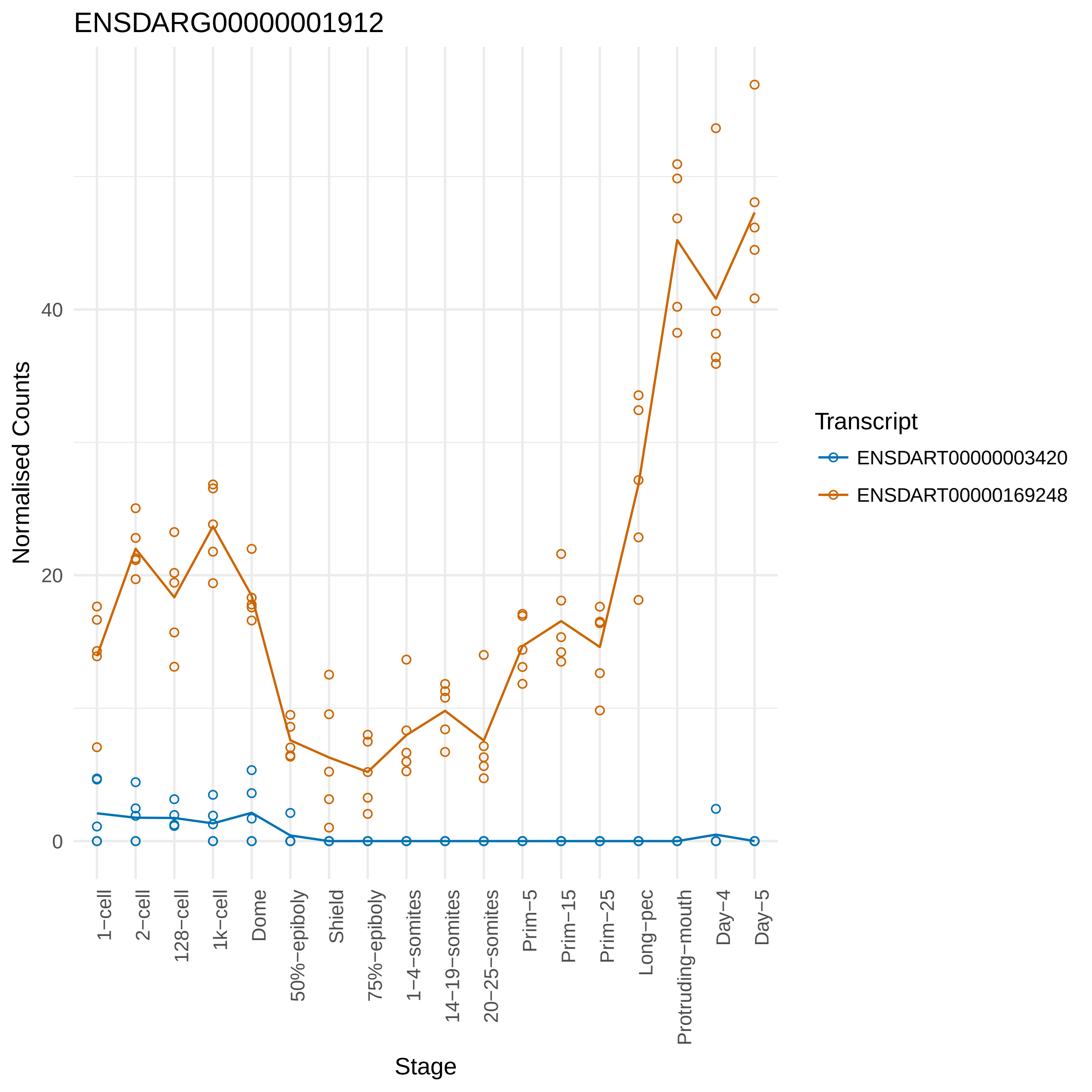
ENSDARG00000001912
Normalised Counts
0
20
40
Stage
1−cell
2−cell
128−cell
1k−cell
Dome
50%−epiboly
Shield
75%−epiboly
1−4−somites
14−19−somites
20−25−somites
Prim−5
Prim−15
Prim−25
Long−pec
Protruding−mouth
Day−4
Day−5
Transcript
ENSDART00000003420
ENSDART00000169248

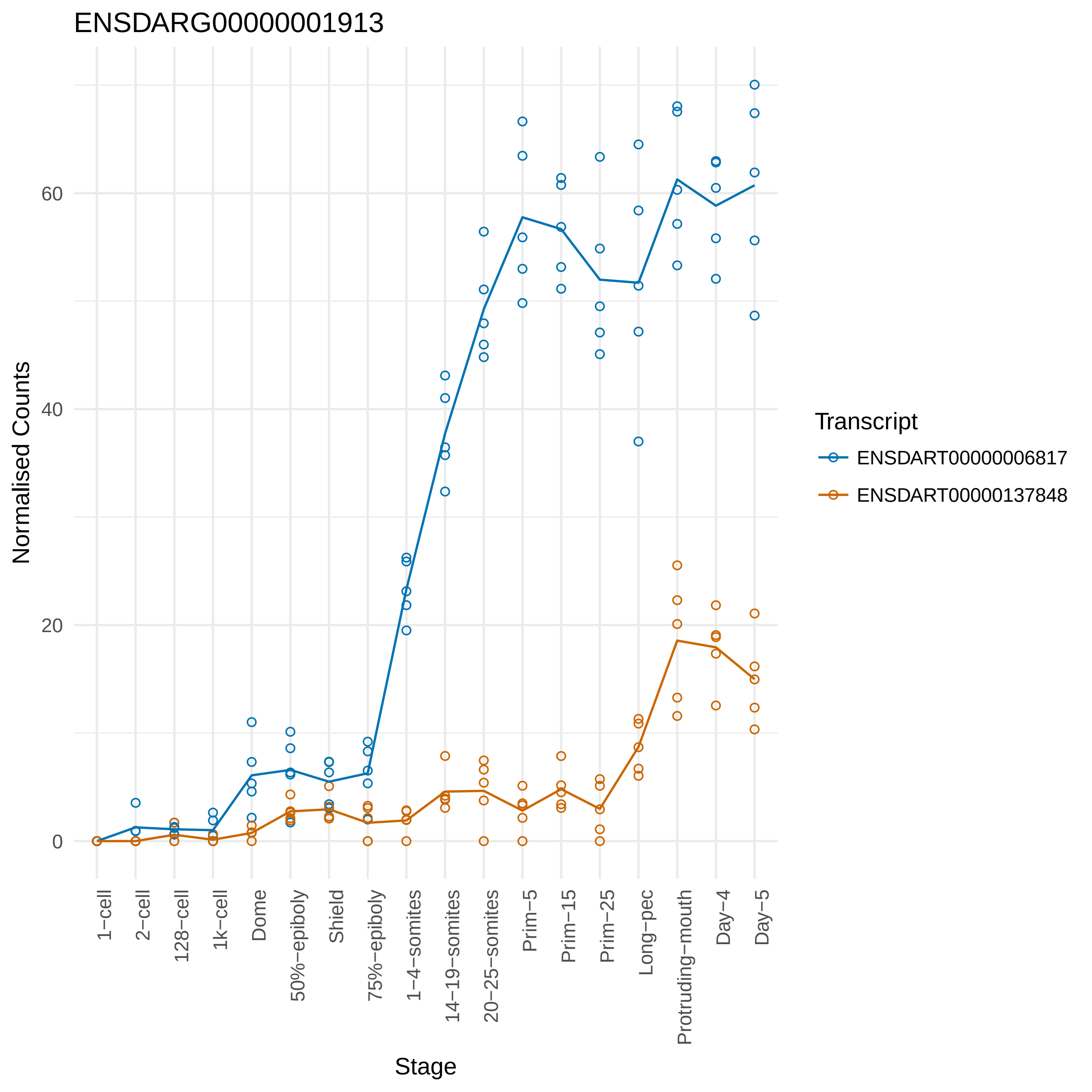

ENSDARG00000001913
Normalised Counts
60
40
20
0
1−cell
2−cell
128−cell
1k−cell
Dome
50%−epiboly
Shield
75%−epiboly
1−4−somites
14−19−somites
20−25−somites
Prim−5
Prim−15
Prim−25
Long−pec
Protruding−mouth
Day−4
Day−5
Stage
Transcript
ENSDART00000006817
ENSDART00000137848

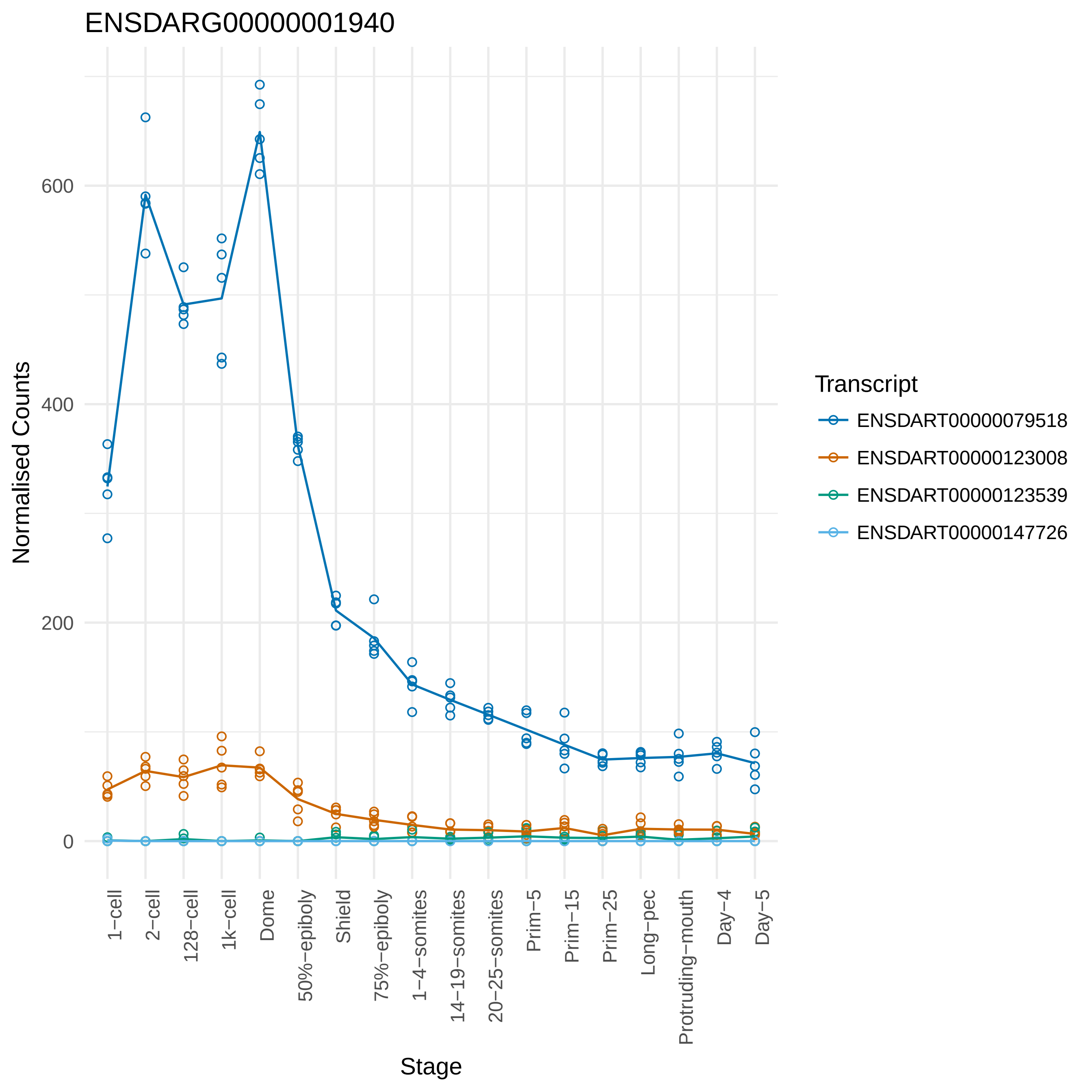

ENSDARG00000001940
Normalised Counts
600
400
200
0
1−cell
2−cell
128−cell
1k−cell
Dome
50%−epiboly
Shield
75%−epiboly
1−4−somites
14−19−somites
20−25−somites
Prim−5
Prim−15
Prim−25
Long−pec
Protruding−mouth
Day−4
Day−5
Stage
Transcript
ENSDART00000079518
ENSDART00000123008
ENSDART00000123539
ENSDART00000147726

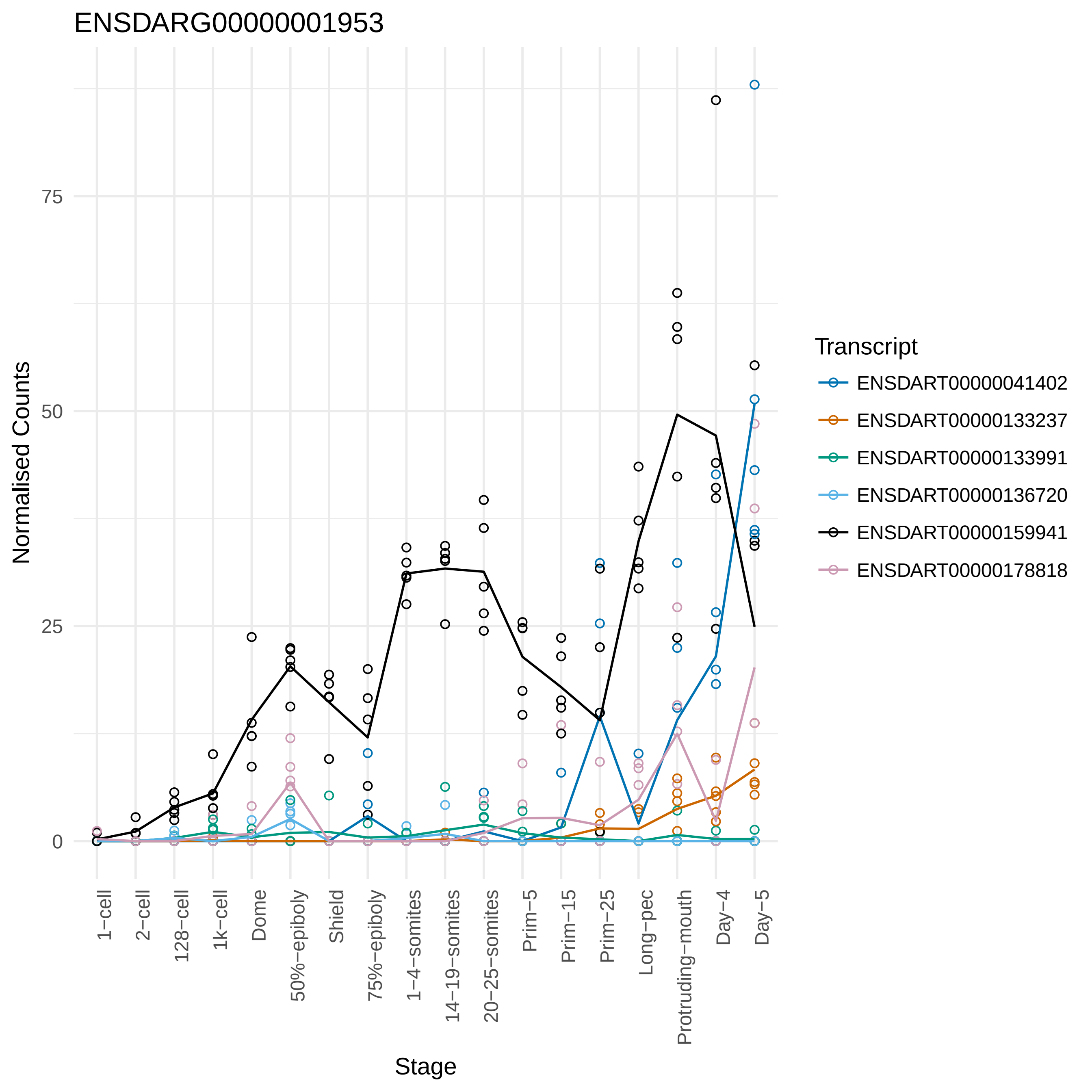
ENSDARG00000001953
Normalised Counts
0
25
50
75
Stage
1−cell
2−cell
128−cell
1k−cell
Dome
50%−epiboly
Shield
75%−epiboly
1−4−somites
14−19−somites
20−25−somites
Prim−5
Prim−15
Prim−25
Long−pec
Protruding−mouth
Day−4
Day−5
Transcript
ENSDART00000041402
ENSDART00000133237
ENSDART00000133991
ENSDART00000136720
ENSDART00000159941
ENSDART00000178818

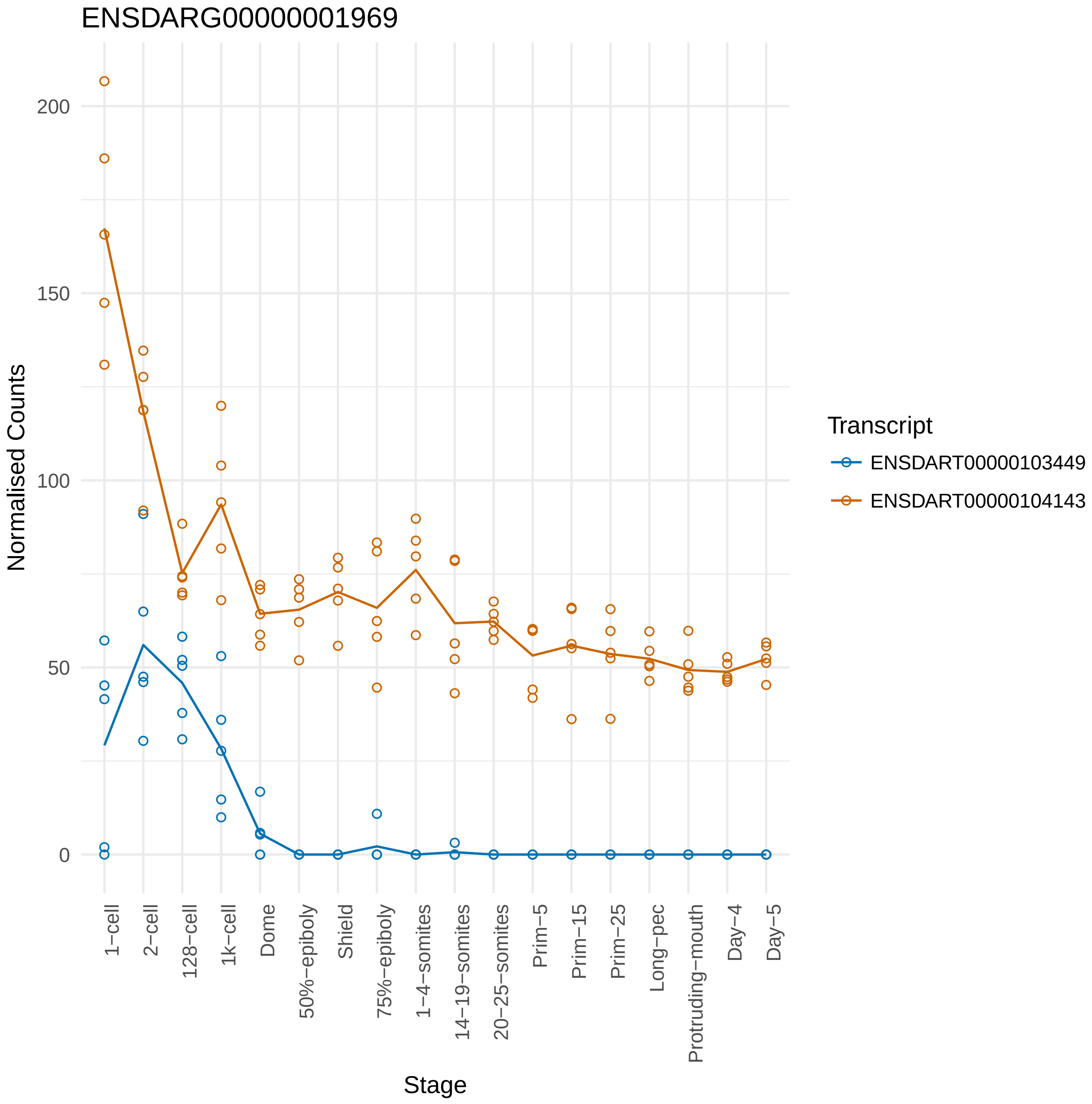
ENSDARG00000001969
Normalised Counts
0
50
100
150
200
1−cell
2−cell
128−cell
1k−cell
Dome
50%−epiboly
Shield
75%−epiboly
1−4−somites
14−19−somites
20−25−somites
Prim−5
Prim−15
Prim−25
Long−pec
Protruding−mouth
Day−4
Day−5
Stage
Transcript
ENSDART00000103449
ENSDART00000104143

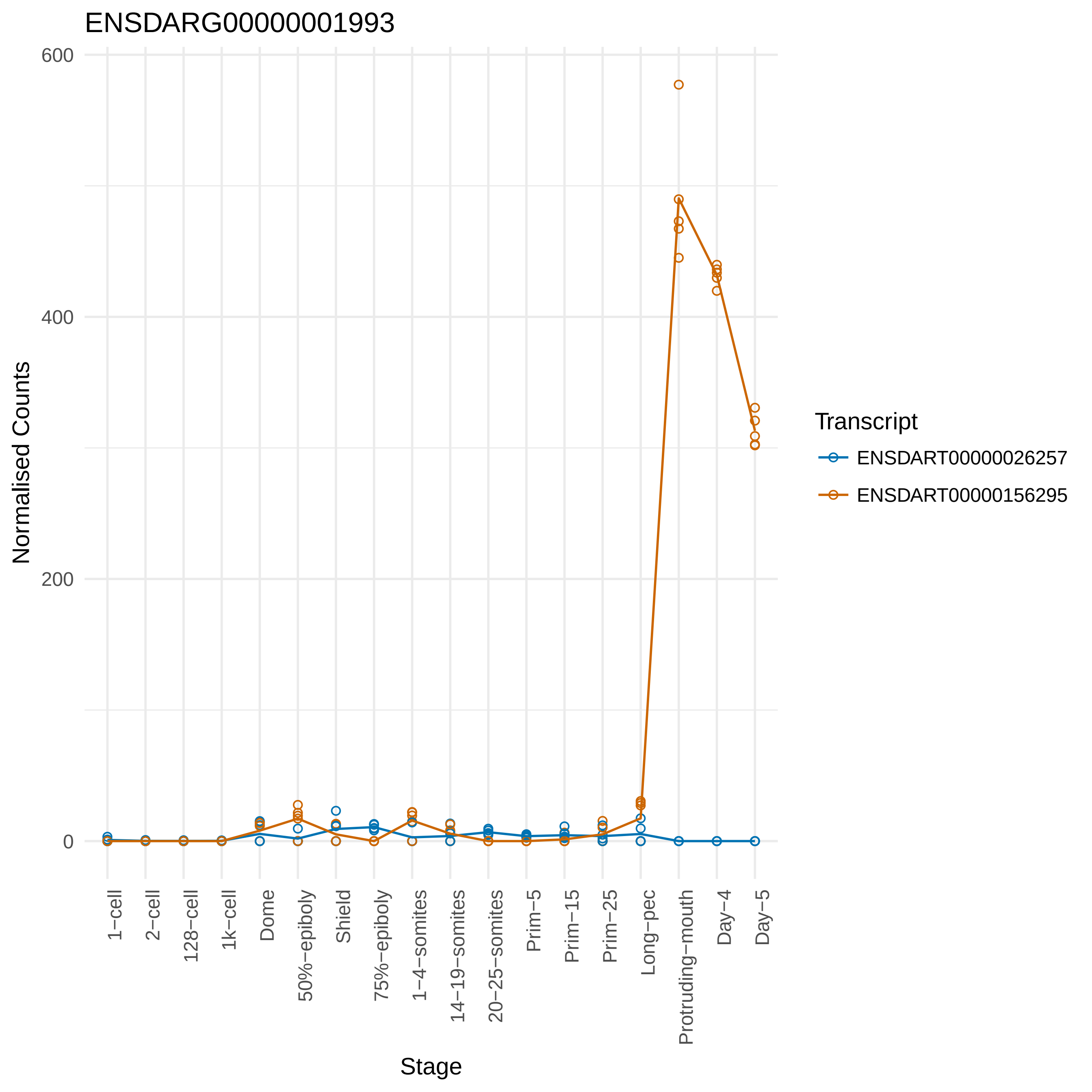

ENSDARG00000001993
Normalised Counts
600
400
200
0
1−cell
2−cell
128−cell
1k−cell
Dome
50%−epiboly
Shield
75%−epiboly
1−4−somites
14−19−somites
20−25−somites
Prim−5
Prim−15
Prim−25
Long−pec
Protruding−mouth
Day−4
Day−5
Stage
Transcript
ENSDART00000026257
ENSDART00000156295

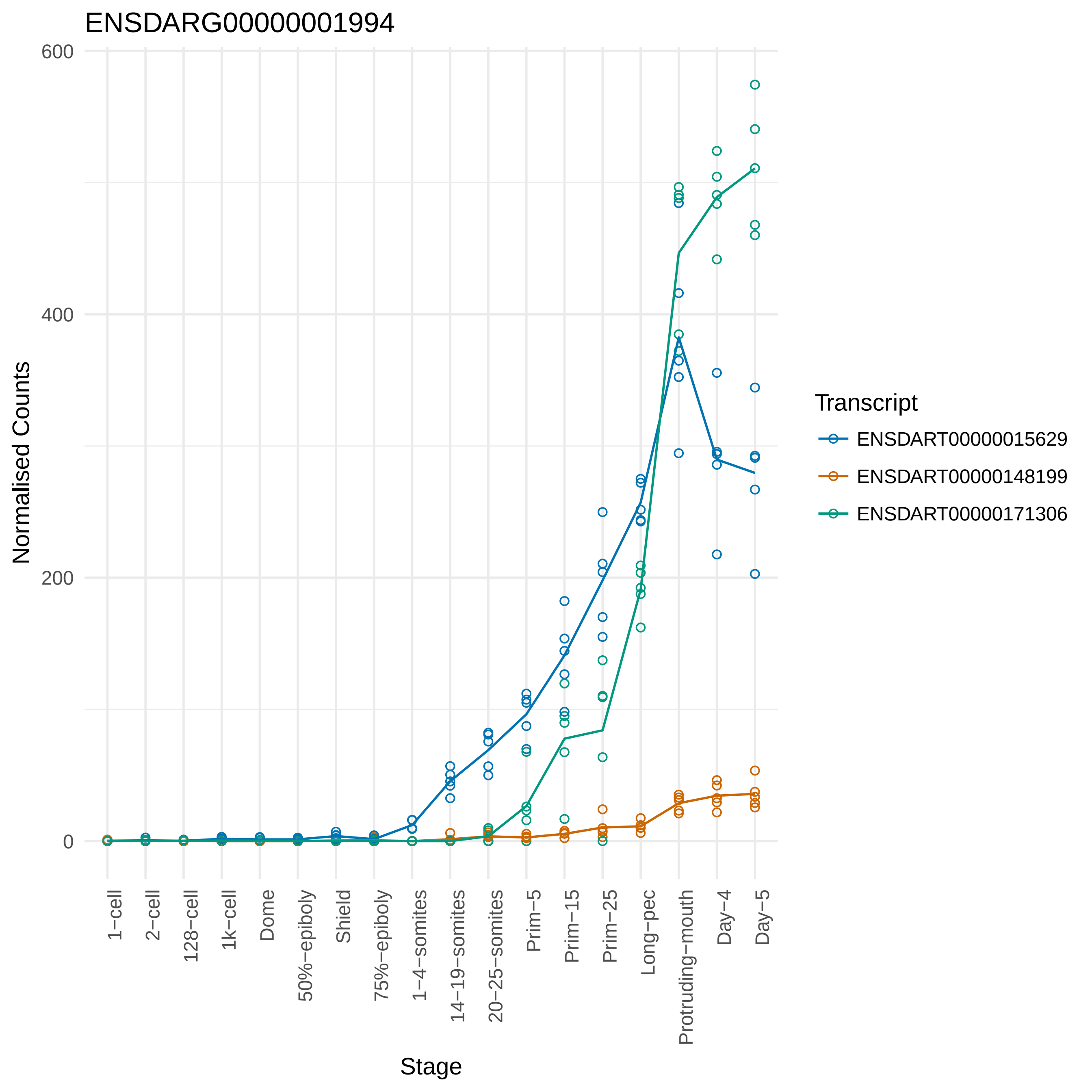
ENSDARG00000001994
Normalised Counts
600
400
200
0
1−cell
2−cell
128−cell
1k−cell
Dome
50%−epiboly
Shield
75%−epiboly
1−4−somites
14−19−somites
20−25−somites
Prim−5
Prim−15
Prim−25
Long−pec
Protruding−mouth
Day−4
Day−5
Stage
Transcript
ENSDART00000015629
ENSDART00000148199
ENSDART00000171306

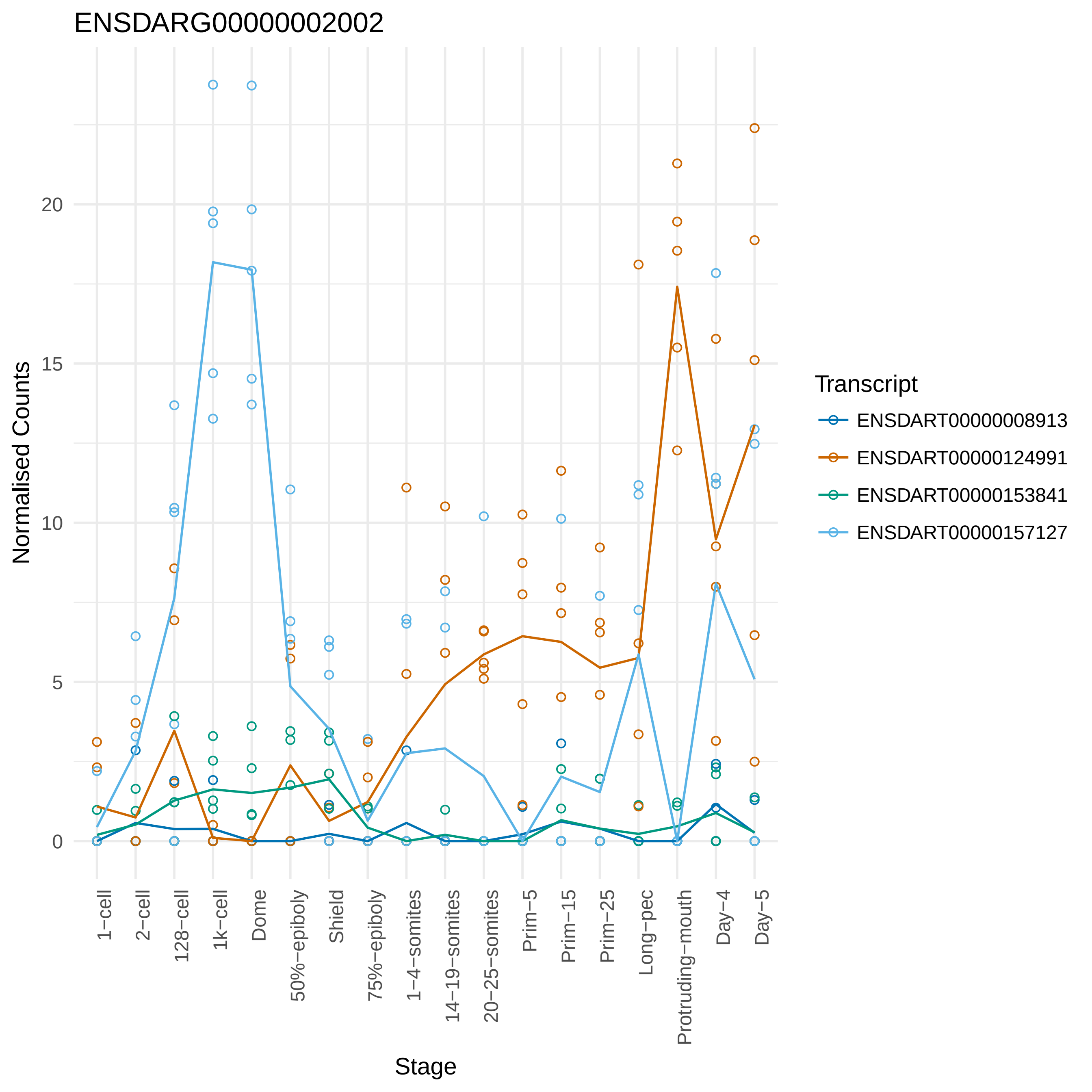

ENSDARG00000002002
Normalised Counts
0
5
10
15
20
Stage
1−cell
2−cell
128−cell
1k−cell
Dome
50%−epiboly
Shield
75%−epiboly
1−4−somites
14−19−somites
20−25−somites
Prim−5
Prim−15
Prim−25
Long−pec
Protruding−mouth
Day−4
Day−5
Transcript
ENSDART00000008913
ENSDART00000124991
ENSDART00000153841
ENSDART00000157127

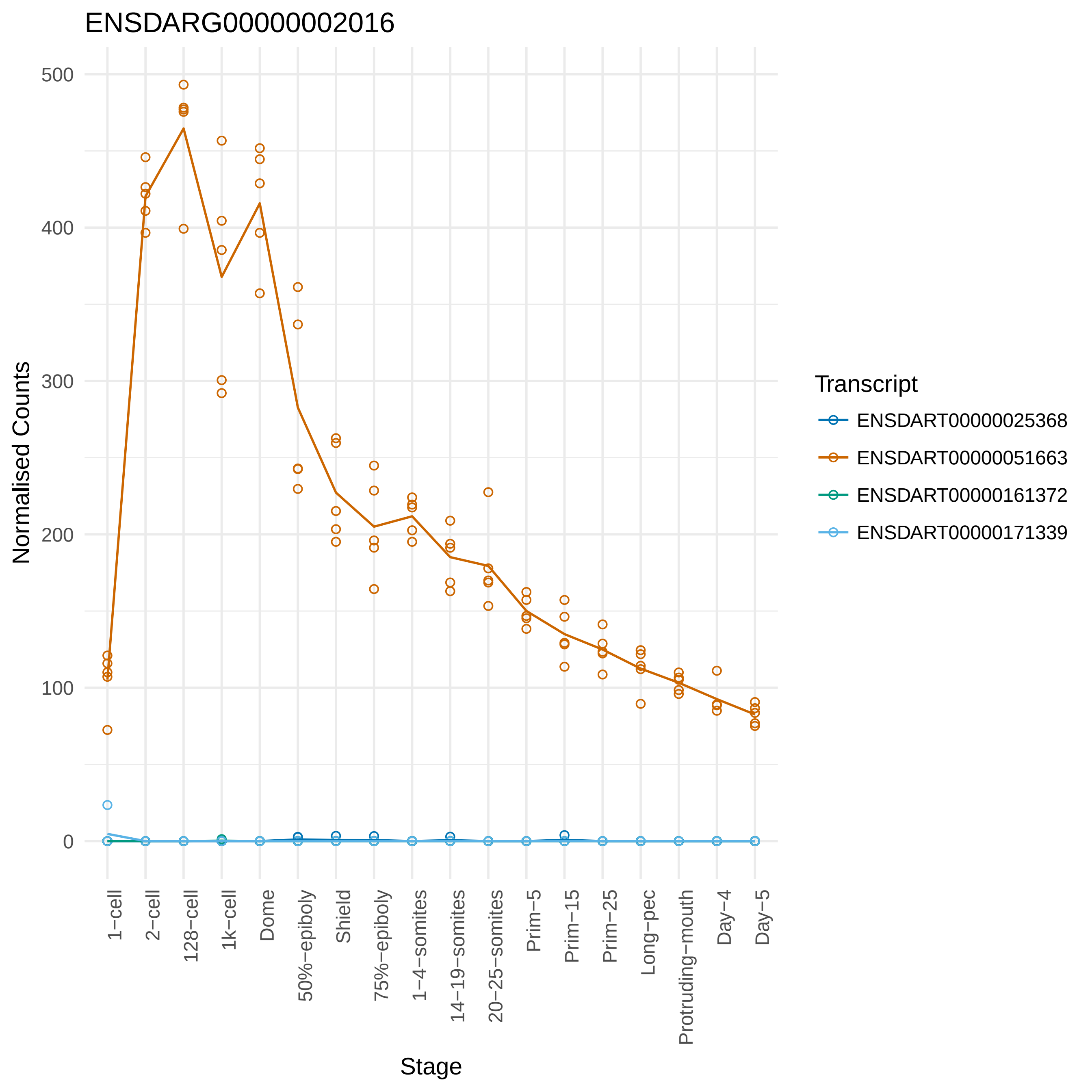
ENSDARG00000002016
Normalised Counts
500
400
300
200
100
0
1−cell
2−cell
128−cell
1k−cell
Dome
50%−epiboly
Shield
75%−epiboly
1−4−somites
14−19−somites
20−25−somites
Prim−5
Prim−15
Prim−25
Long−pec
Protruding−mouth
Day−4
Day−5
Stage
Transcript
ENSDART00000025368
ENSDART00000051663
ENSDART00000161372
ENSDART00000171339

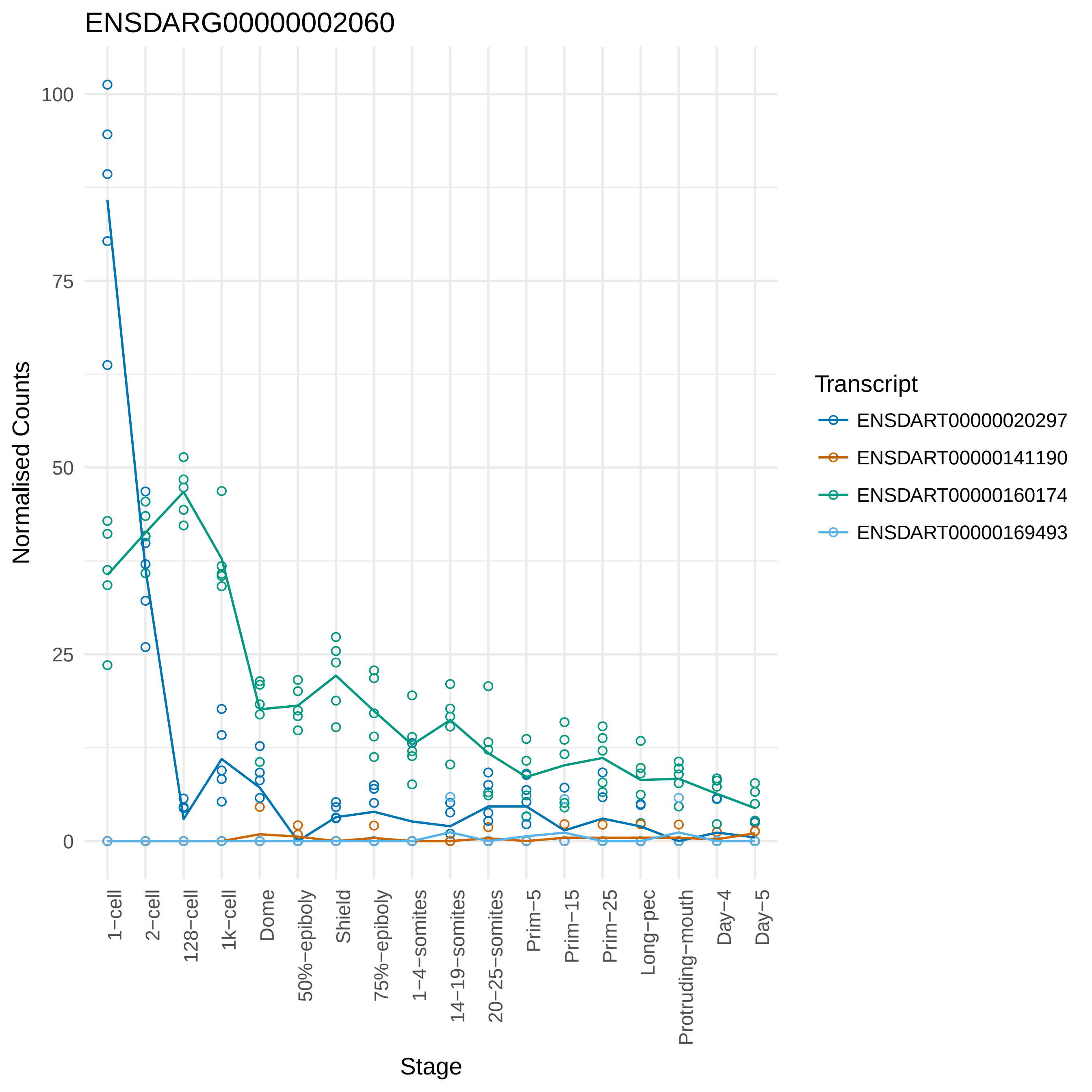
ENSDARG00000002060
Normalised Counts
100
75
50
25
0
1−cell
2−cell
128−cell
1k−cell
Dome
50%−epiboly
Shield
75%−epiboly
1−4−somites
14−19−somites
20−25−somites
Prim−5
Prim−15
Prim−25
Long−pec
Protruding−mouth
Day−4
Day−5
Stage
Transcript
ENSDART00000020297
ENSDART00000141190
ENSDART00000160174
ENSDART00000169493

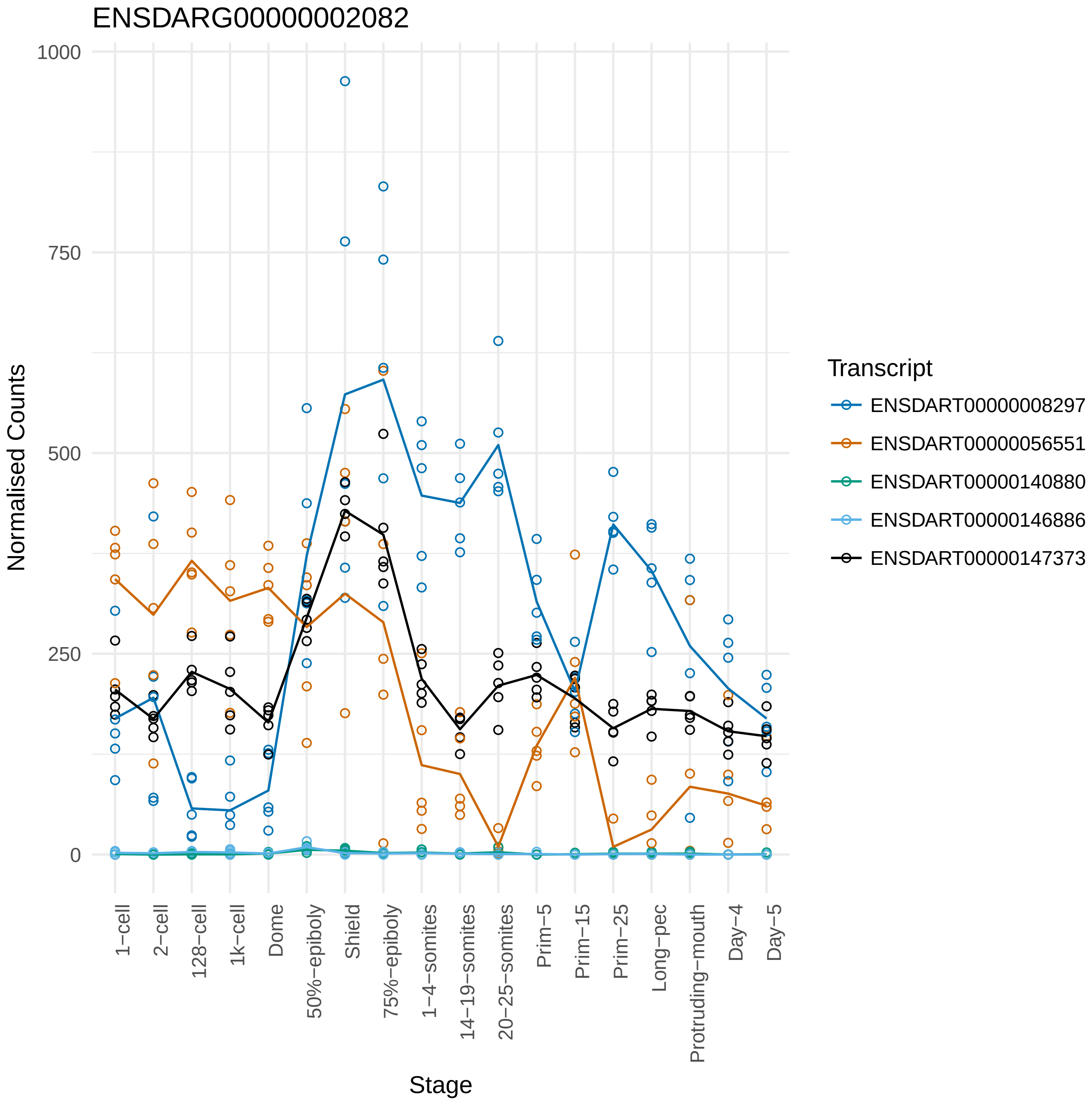

ENSDARG00000002082
Normalised Counts
1000
750
500
250
0
1−cell
2−cell
128−cell
1k−cell
Dome
50%−epiboly
Shield
75%−epiboly
1−4−somites
14−19−somites
20−25−somites
Prim−5
Prim−15
Prim−25
Long−pec
Protruding−mouth
Day−4
Day−5
Stage
Transcript
ENSDART00000008297
ENSDART00000056551
ENSDART00000140880
ENSDART00000146886
ENSDART00000147373

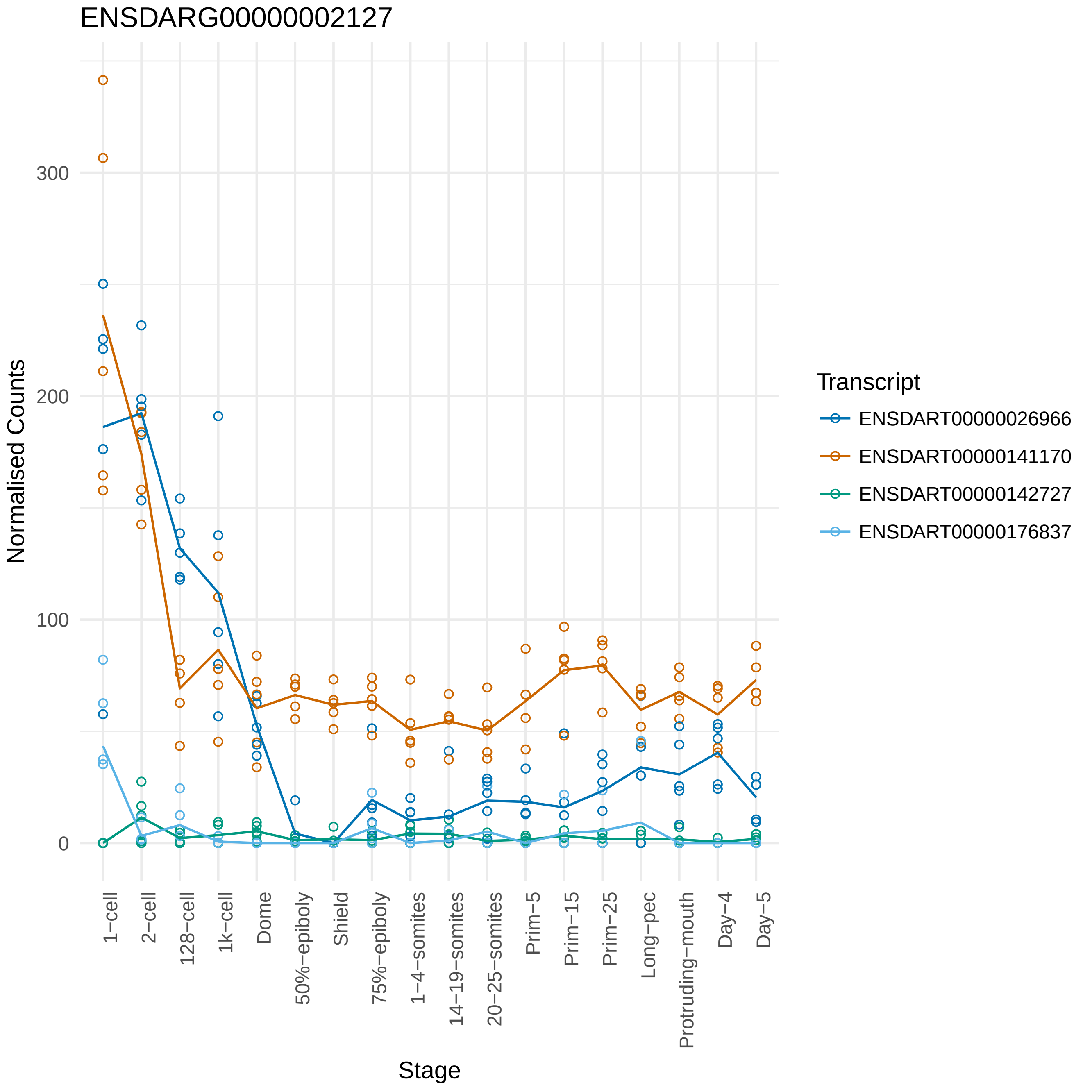

ENSDARG00000002127
Normalised Counts
300
200
100
0
Stage
1−cell
2−cell
128−cell
1k−cell
Dome
50%−epiboly
Shield
75%−epiboly
1−4−somites
14−19−somites
20−25−somites
Prim−5
Prim−15
Prim−25
Long−pec
Protruding−mouth
Day−4
Day−5
Transcript
ENSDART00000026966
ENSDART00000141170
ENSDART00000142727
ENSDART00000176837

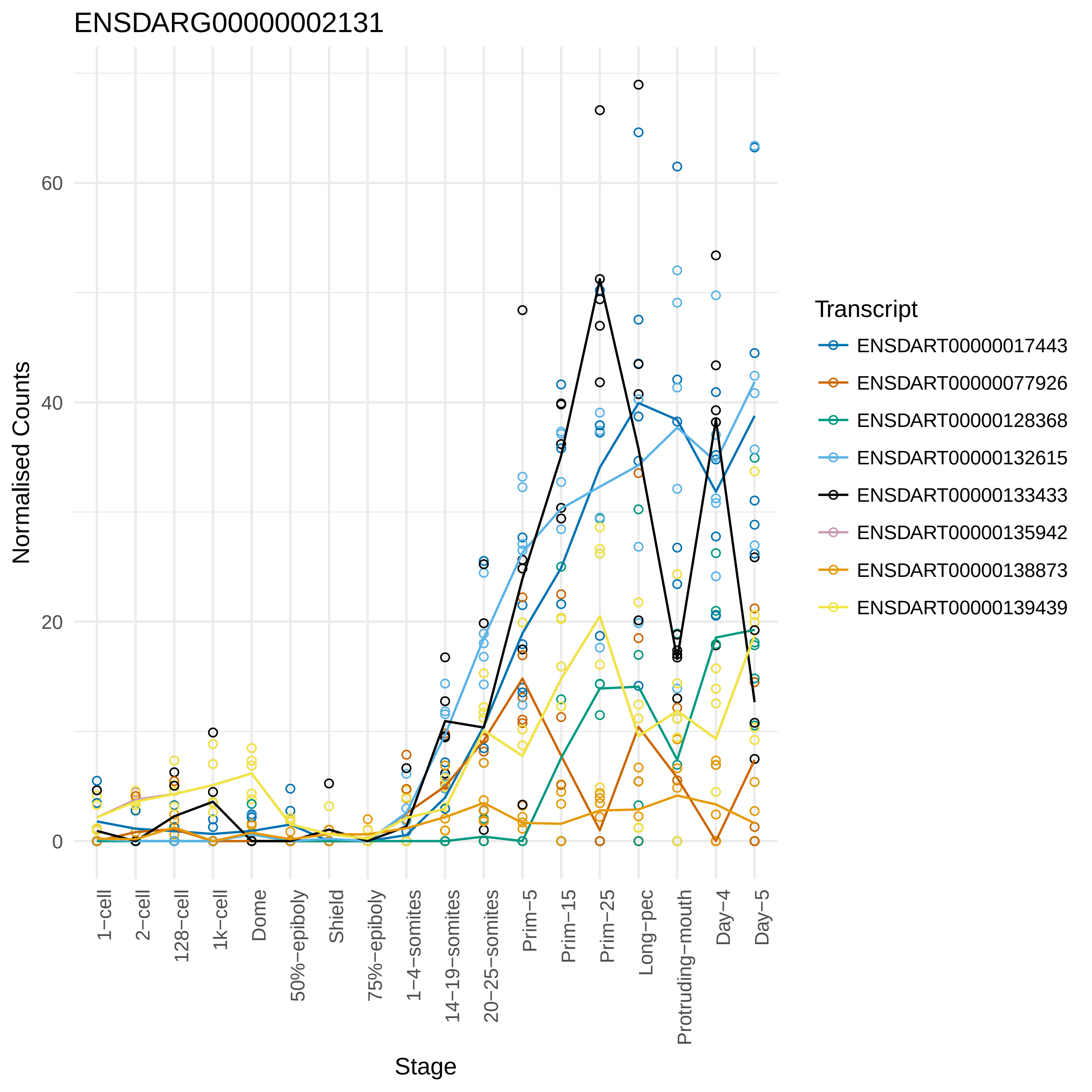

ENSDARG00000002131
Normalised Counts
0
20
40
60
Stage
1−cell
2−cell
128−cell
1k−cell
Dome
50%−epiboly
Shield
75%−epiboly
1−4−somites
14−19−somites
20−25−somites
Prim−5
Prim−15
Prim−25
Long−pec
Protruding−mouth
Day−4
Day−5
Transcript
ENSDART00000017443
ENSDART00000077926
ENSDART00000128368
ENSDART00000132615
ENSDART00000133433
ENSDART00000135942
ENSDART00000138873
ENSDART00000139439

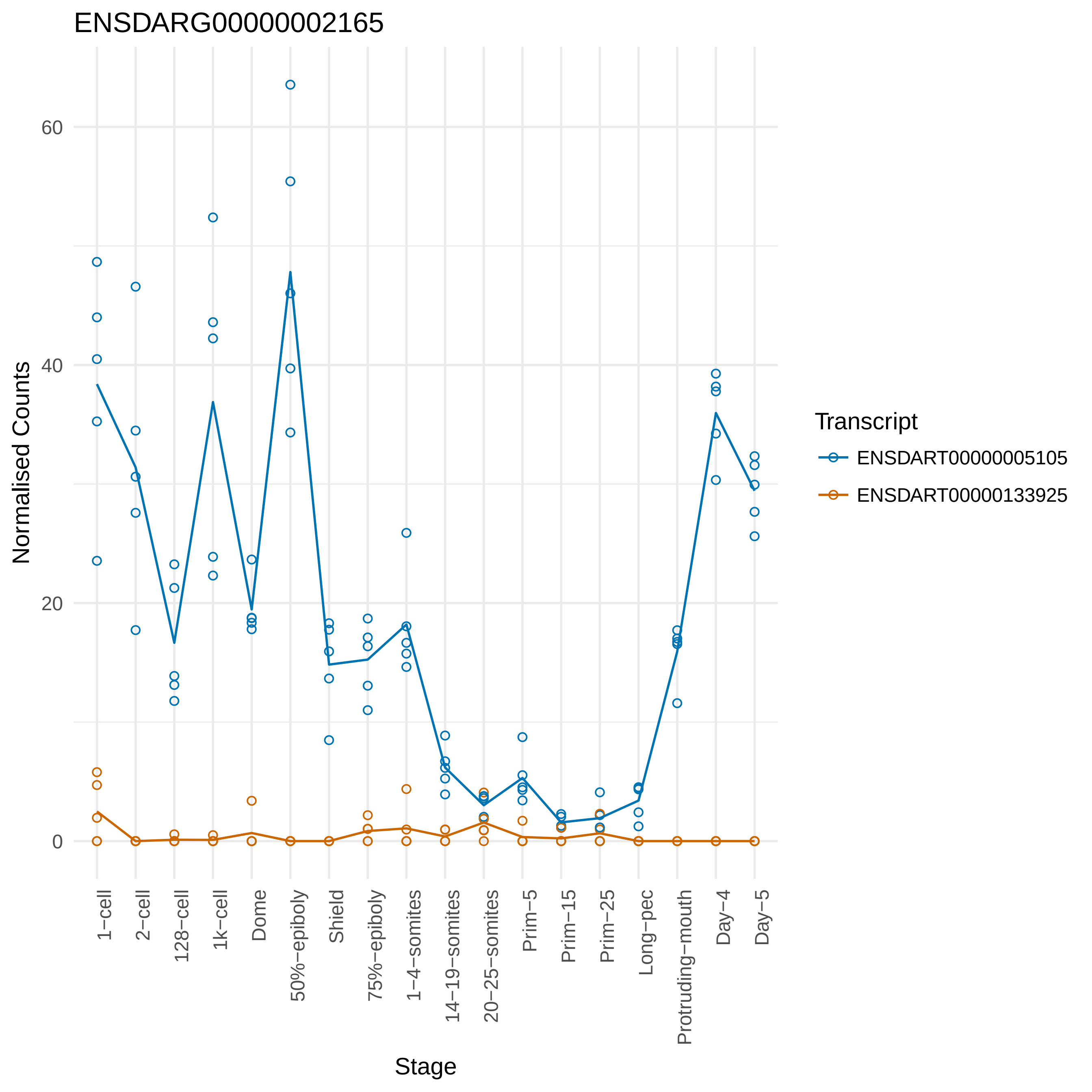
ENSDARG00000002165
Normalised Counts
60
40
20
0
1−cell
2−cell
128−cell
1k−cell
Dome
50%−epiboly
Shield
75%−epiboly
1−4−somites
14−19−somites
20−25−somites
Prim−5
Prim−15
Prim−25
Long−pec
Protruding−mouth
Day−4
Day−5
Stage
Transcript
ENSDART00000005105
ENSDART00000133925

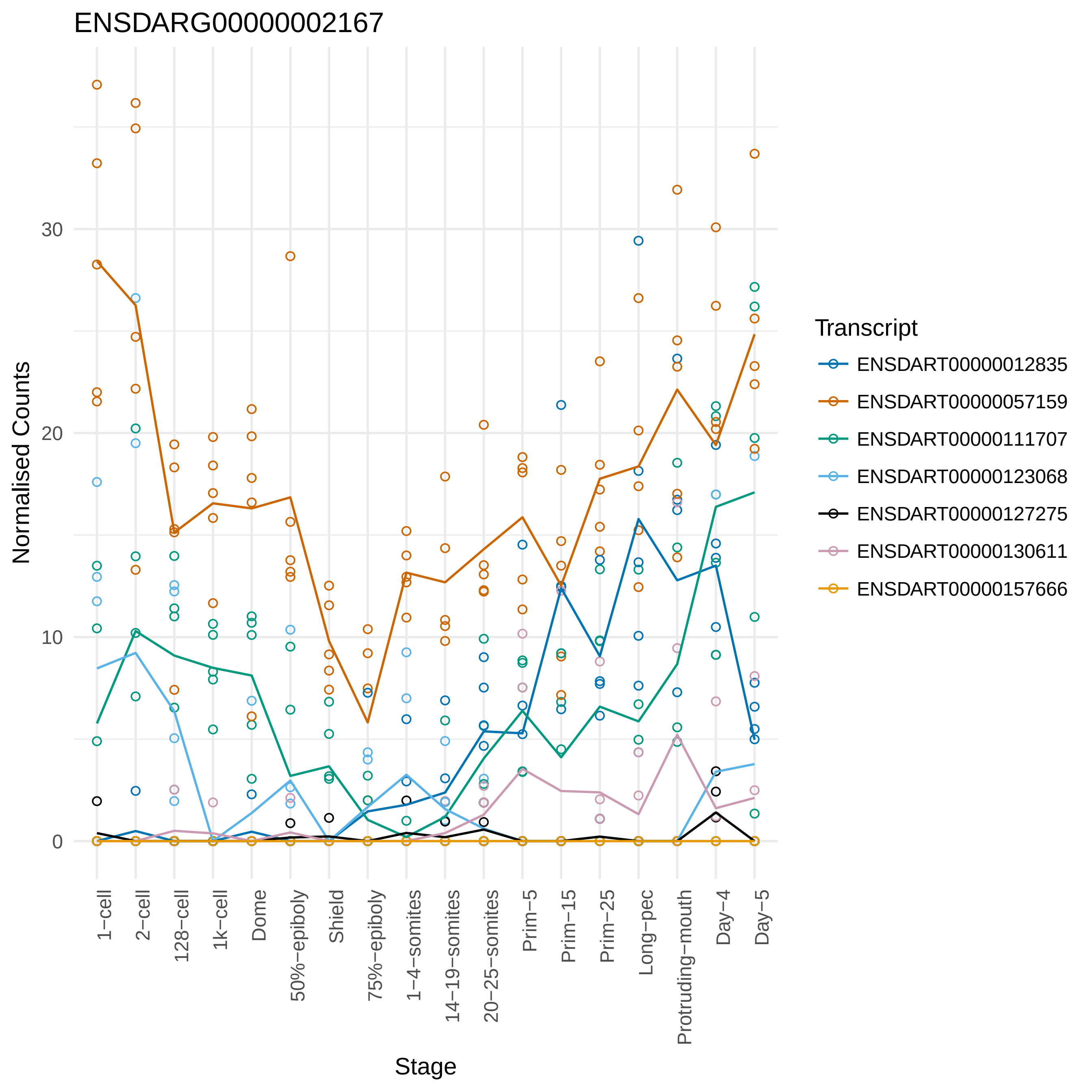
ENSDARG00000002167
Normalised Counts
30
20
10
0
Stage
1−cell
2−cell
128−cell
1k−cell
Dome
50%−epiboly
Shield
75%−epiboly
1−4−somites
14−19−somites
20−25−somites
Prim−5
Prim−15
Prim−25
Long−pec
Protruding−mouth
Day−4
Day−5
Transcript
ENSDART00000012835
ENSDART00000057159
ENSDART00000111707
ENSDART00000123068
ENSDART00000127275
ENSDART00000130611
ENSDART00000157666

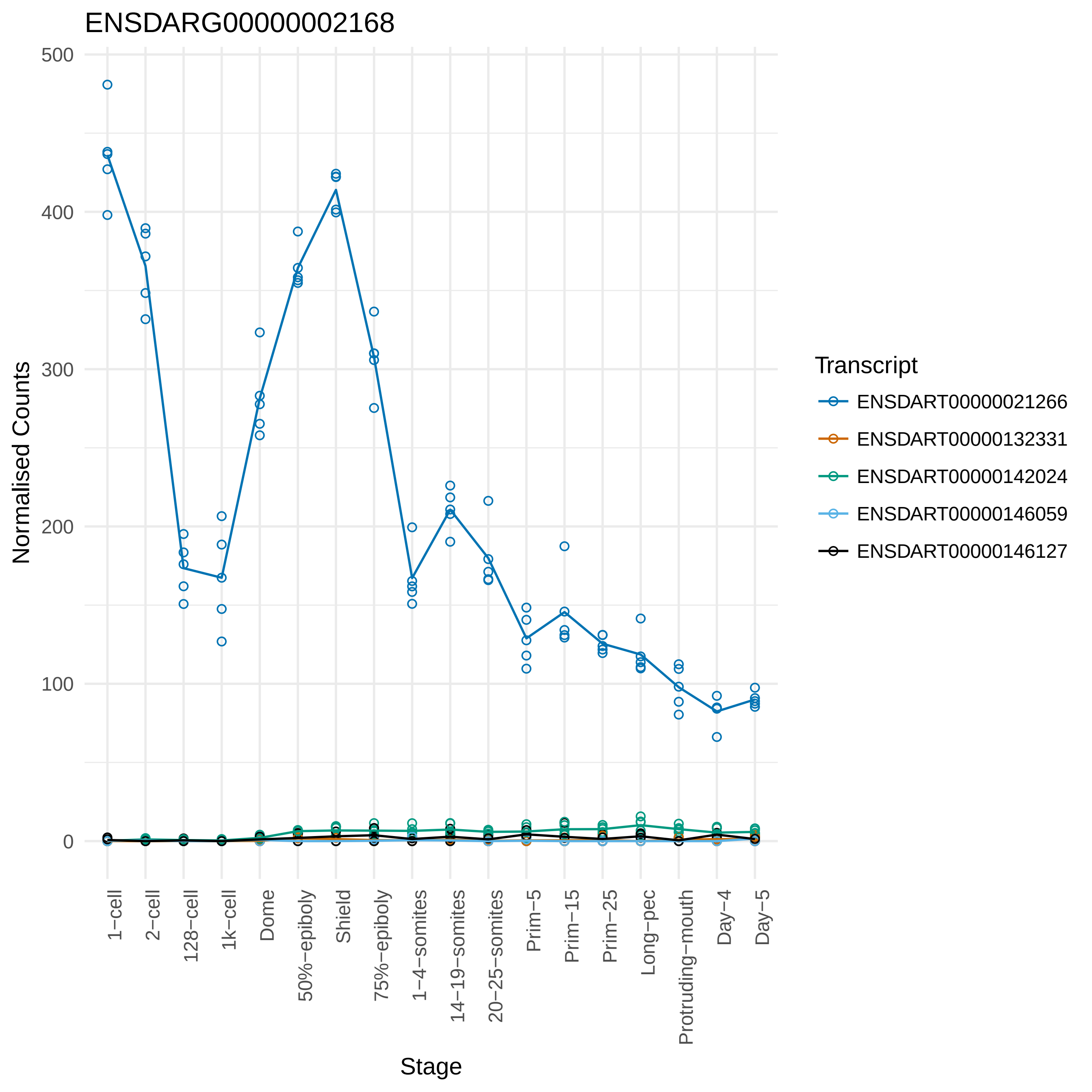

ENSDARG00000002168
Normalised Counts
500
400
300
200
100
0
1−cell
2−cell
128−cell
1k−cell
Dome
50%−epiboly
Shield
75%−epiboly
1−4−somites
14−19−somites
20−25−somites
Prim−5
Prim−15
Prim−25
Long−pec
Protruding−mouth
Day−4
Day−5
Stage
Transcript
ENSDART00000021266
ENSDART00000132331
ENSDART00000142024
ENSDART00000146059
ENSDART00000146127

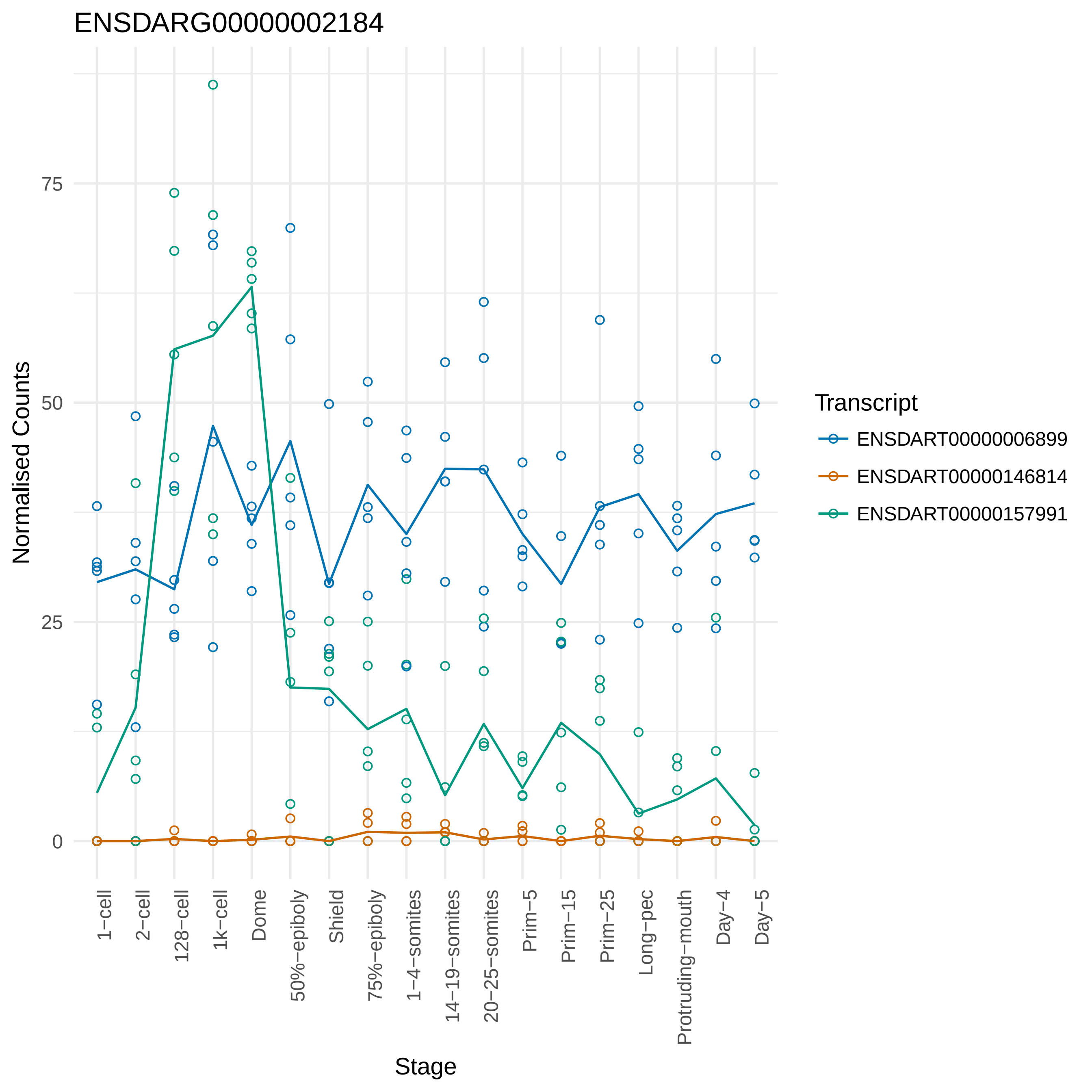

ENSDARG00000002184
Normalised Counts
75
50
25
0
1−cell
2−cell
128−cell
1k−cell
Dome
50%−epiboly
Shield
75%−epiboly
1−4−somites
14−19−somites
20−25−somites
Prim−5
Prim−15
Prim−25
Long−pec
Protruding−mouth
Day−4
Day−5
Stage
Transcript
ENSDART00000006899
ENSDART00000146814
ENSDART00000157991

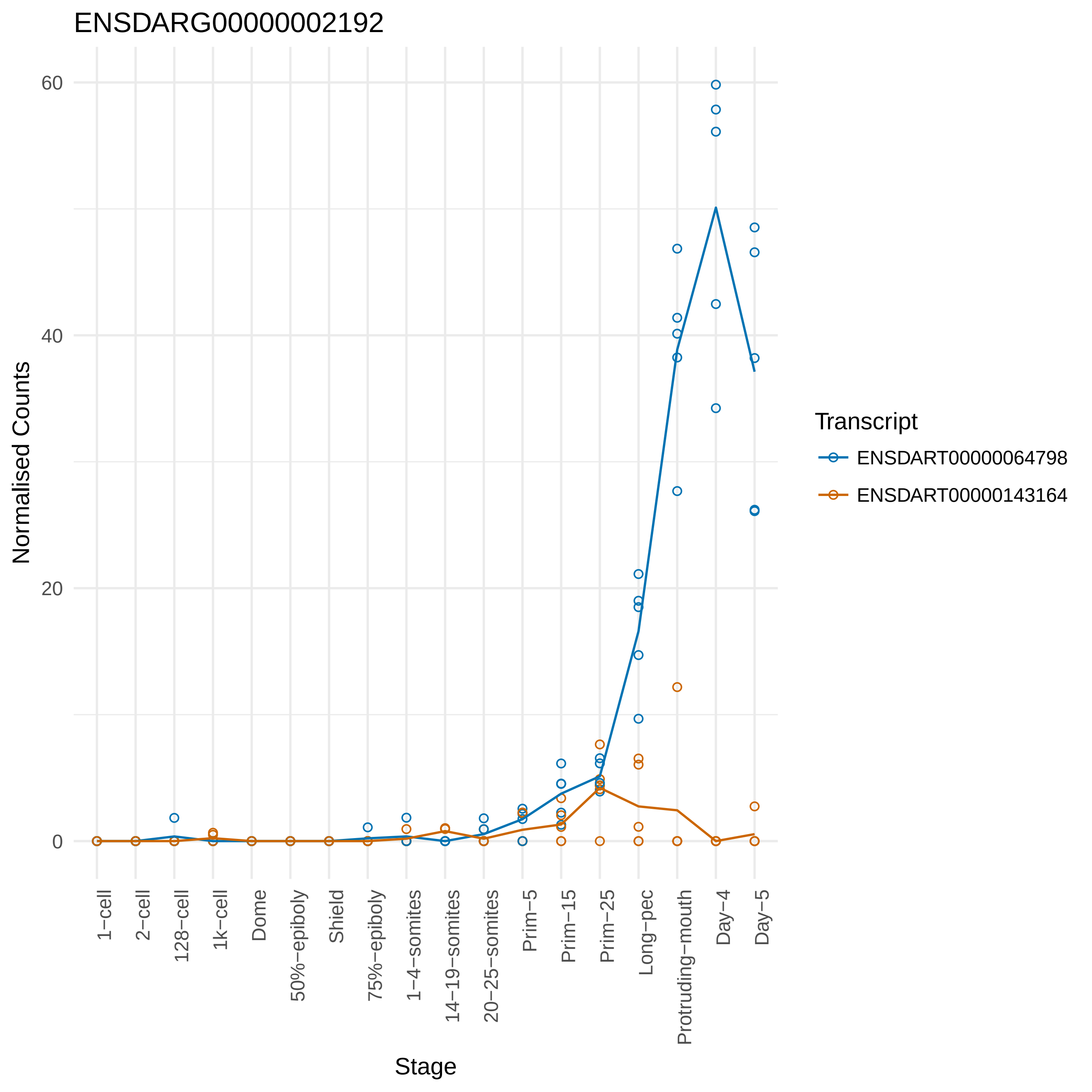

ENSDARG00000002192
Normalised Counts
60
40
20
0
1−cell
2−cell
128−cell
1k−cell
Dome
50%−epiboly
Shield
75%−epiboly
1−4−somites
14−19−somites
20−25−somites
Prim−5
Prim−15
Prim−25
Long−pec
Protruding−mouth
Day−4
Day−5
Stage
Transcript
ENSDART00000064798
ENSDART00000143164

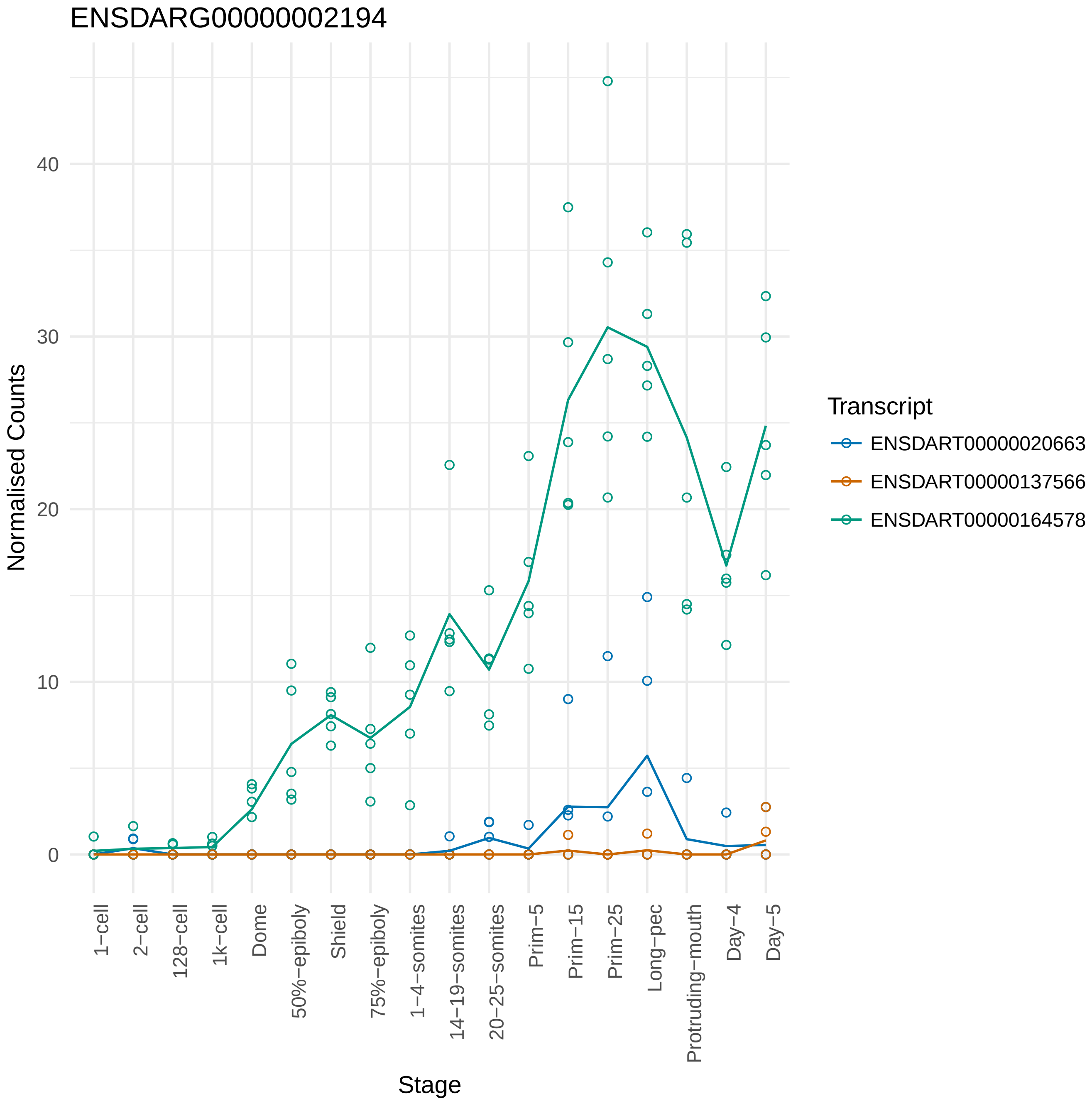
ENSDARG00000002194
Normalised Counts
Stage
Transcript
ENSDART00000020663
ENSDART00000137566
ENSDART00000164578
0
10
20
30
40
1−cell
2−cell
128−cell
1k−cell
Dome
50%−epiboly
Shield
75%−epiboly
1−4−somites
14−19−somites
20−25−somites
Prim−5
Prim−15
Prim−25
Long−pec
Protruding−mouth
Day−4
Day−5

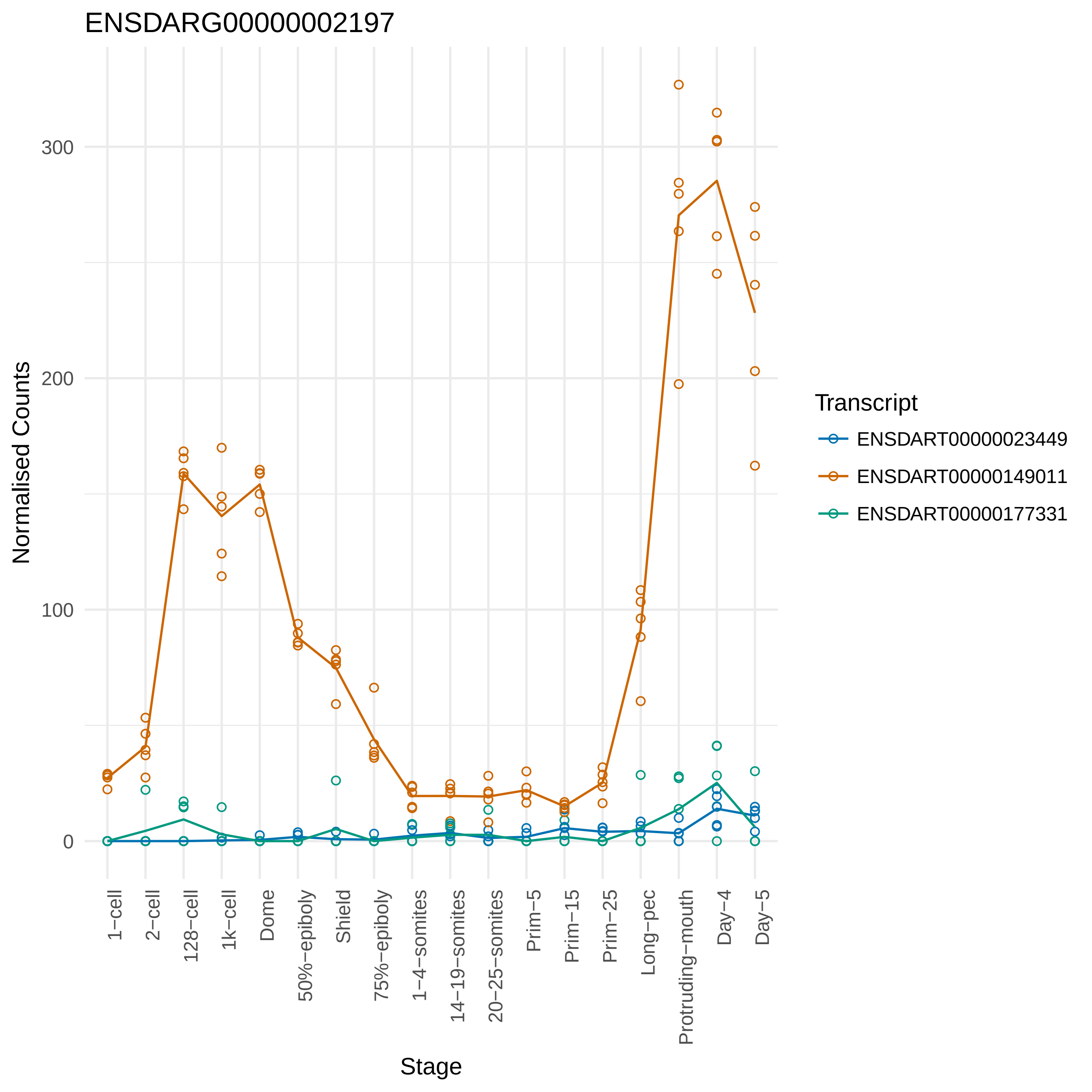

ENSDARG00000002197
Normalised Counts
300
200
100
0
Stage
1−cell
2−cell
128−cell
1k−cell
Dome
50%−epiboly
Shield
75%−epiboly
1−4−somites
14−19−somites
20−25−somites
Prim−5
Prim−15
Prim−25
Long−pec
Protruding−mouth
Day−4
Day−5
Transcript
ENSDART00000023449
ENSDART00000149011
ENSDART00000177331

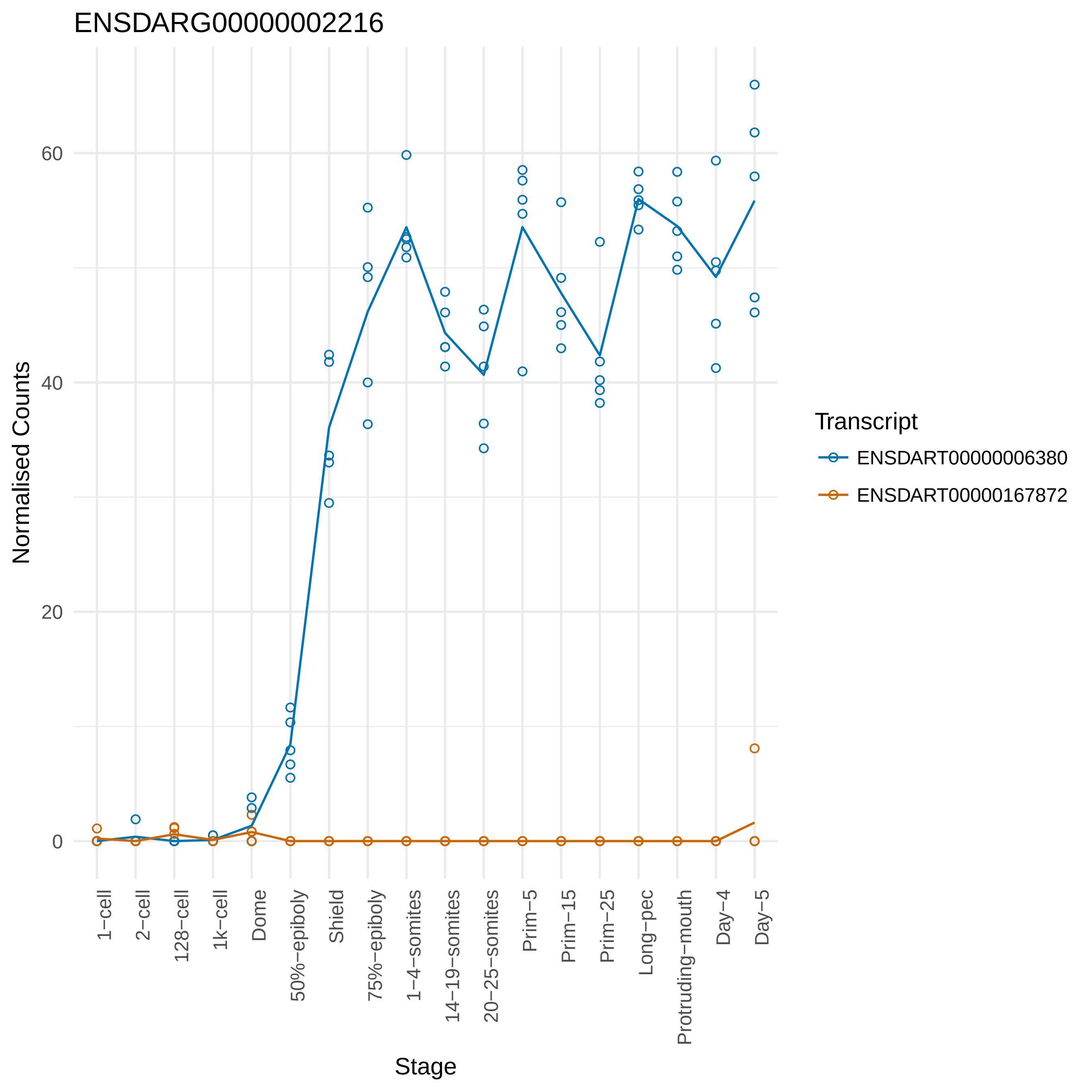

ENSDARG00000002216
Normalised Counts
60
40
20
0
1−cell
2−cell
128−cell
1k−cell
Dome
50%−epiboly
Shield
75%−epiboly
1−4−somites
14−19−somites
20−25−somites
Prim−5
Prim−15
Prim−25
Long−pec
Protruding−mouth
Day−4
Day−5
Stage
Transcript
ENSDART00000006380
ENSDART00000167872

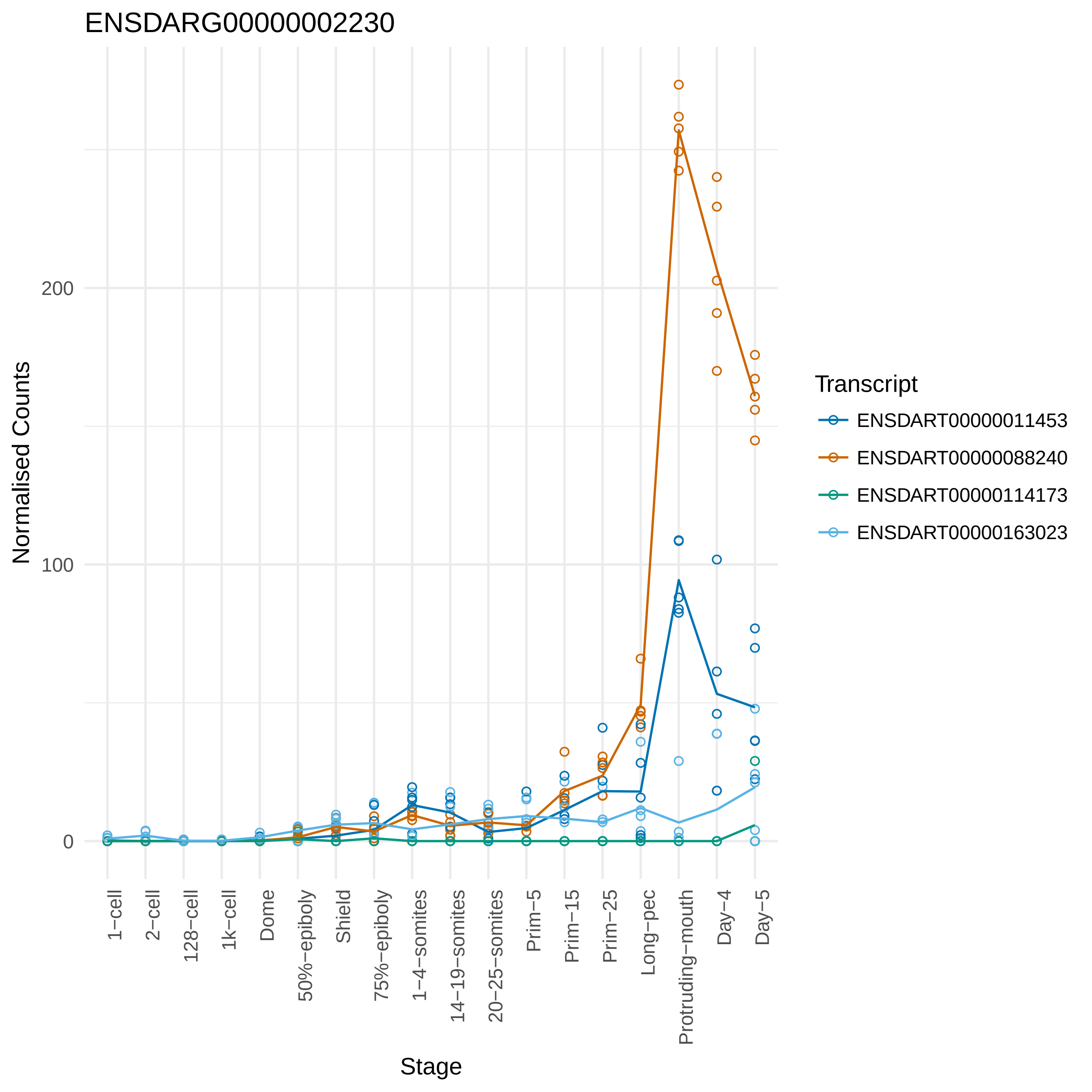

ENSDARG00000002230
Normalised Counts
200
100
0
1−cell
2−cell
128−cell
1k−cell
Dome
50%−epiboly
Shield
75%−epiboly
1−4−somites
14−19−somites
20−25−somites
Prim−5
Prim−15
Prim−25
Long−pec
Protruding−mouth
Day−4
Day−5
Stage
Transcript
ENSDART00000011453
ENSDART00000088240
ENSDART00000114173
ENSDART00000163023

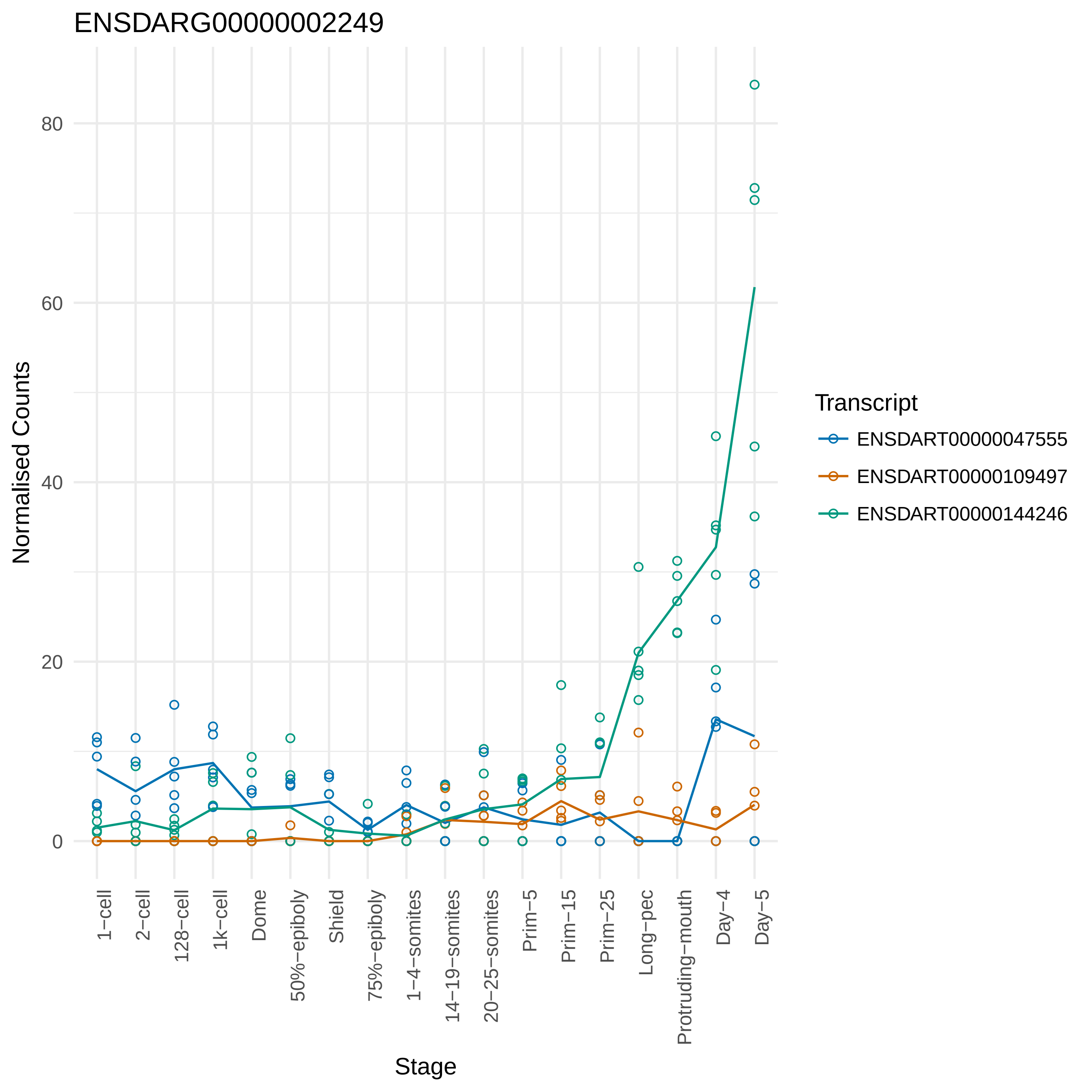

ENSDARG00000002249
Normalised Counts
0
20
40
60
80
1−cell
2−cell
128−cell
1k−cell
Dome
50%−epiboly
Shield
75%−epiboly
1−4−somites
14−19−somites
20−25−somites
Prim−5
Prim−15
Prim−25
Long−pec
Protruding−mouth
Day−4
Day−5
Stage
Transcript
ENSDART00000047555
ENSDART00000109497
ENSDART00000144246

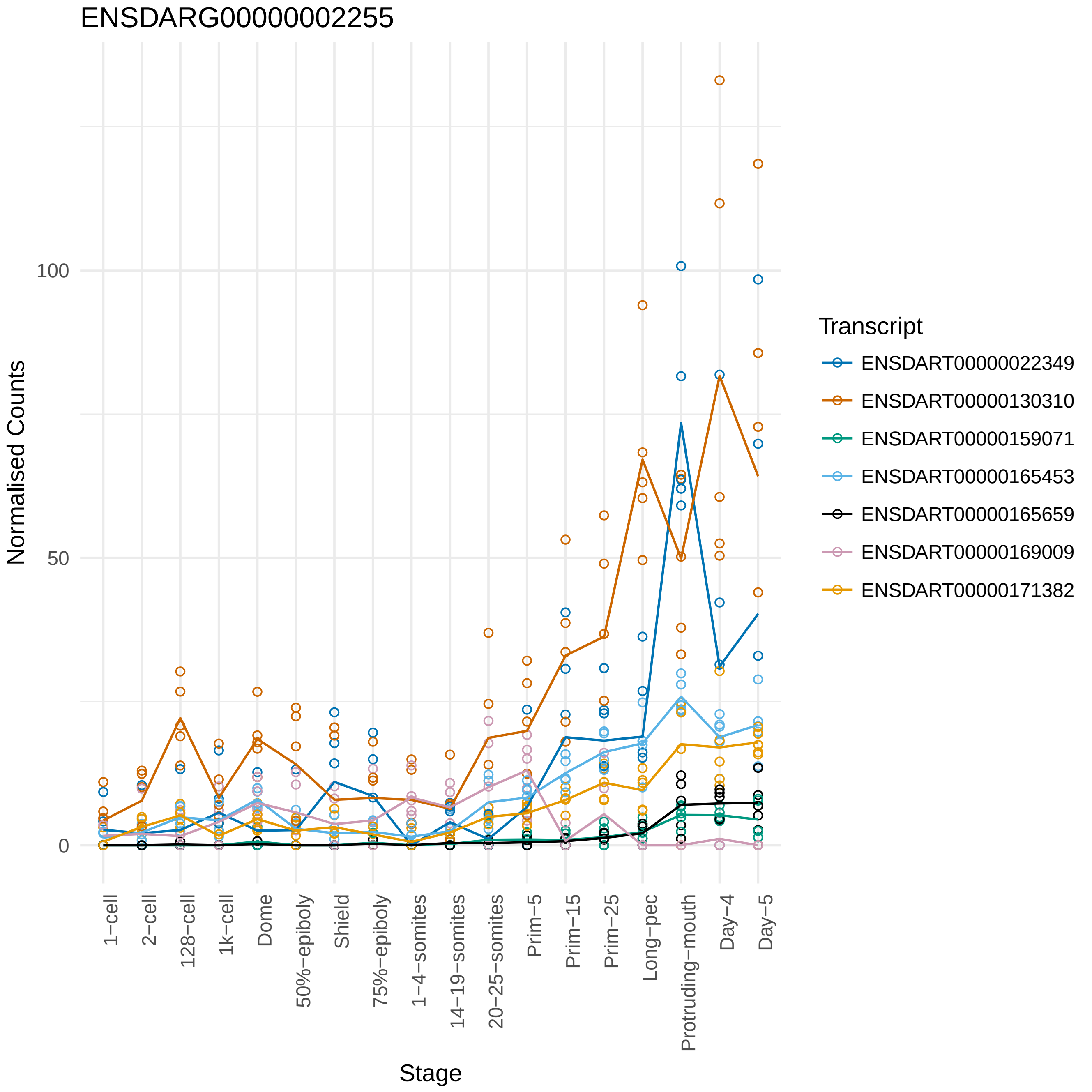

ENSDARG00000002255
Normalised Counts
100
50
0
1−cell
2−cell
128−cell
1k−cell
Dome
50%−epiboly
Shield
75%−epiboly
1−4−somites
14−19−somites
20−25−somites
Prim−5
Prim−15
Prim−25
Long−pec
Protruding−mouth
Day−4
Day−5
Stage
Transcript
ENSDART00000022349
ENSDART00000130310
ENSDART00000159071
ENSDART00000165453
ENSDART00000165659
ENSDART00000169009
ENSDART00000171382

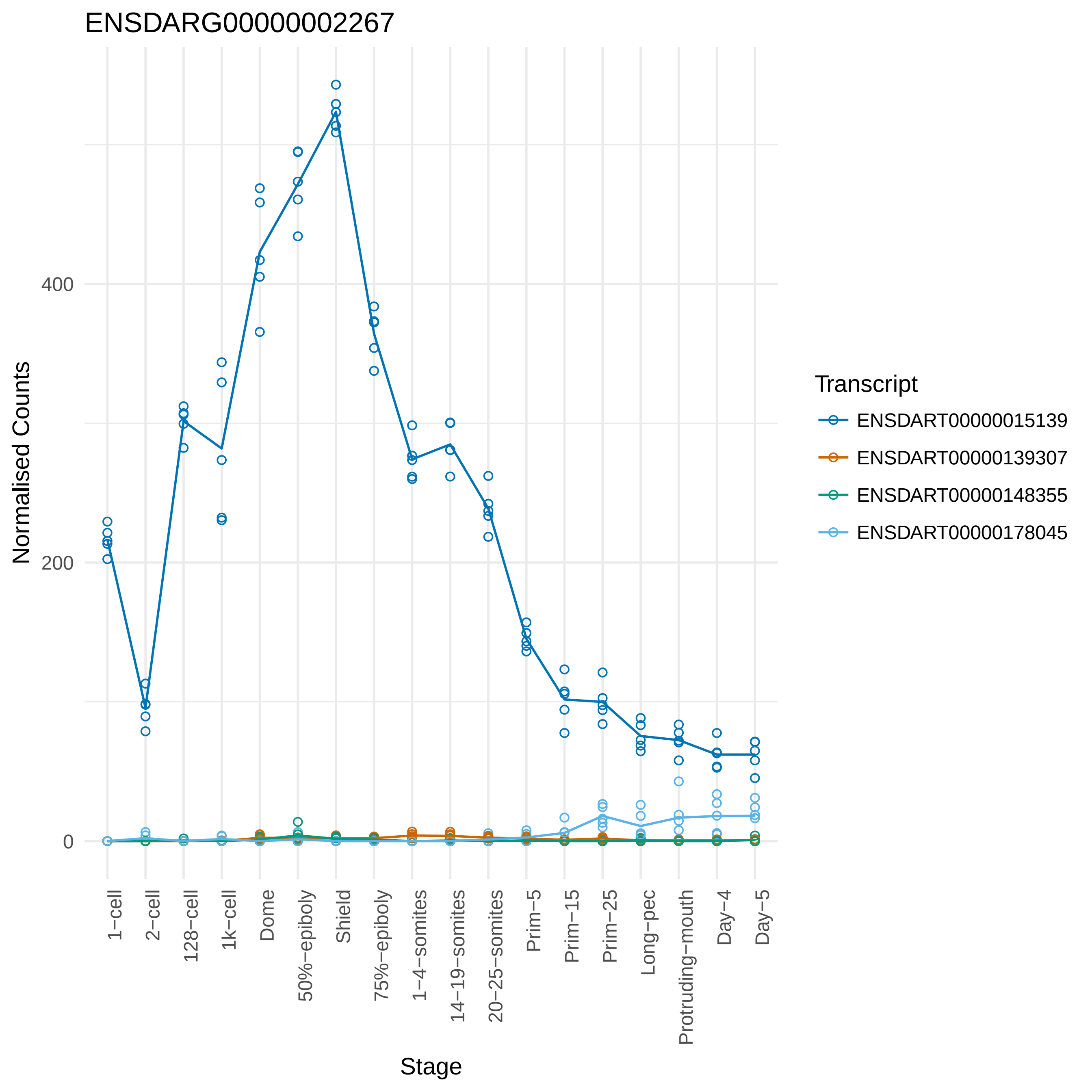
ENSDARG00000002267
Normalised Counts
400
200
0
1−cell
2−cell
128−cell
1k−cell
Dome
50%−epiboly
Shield
75%−epiboly
1−4−somites
14−19−somites
20−25−somites
Prim−5
Prim−15
Prim−25
Long−pec
Protruding−mouth
Day−4
Day−5
Stage
Transcript
ENSDART00000015139
ENSDART00000139307
ENSDART00000148355
ENSDART00000178045

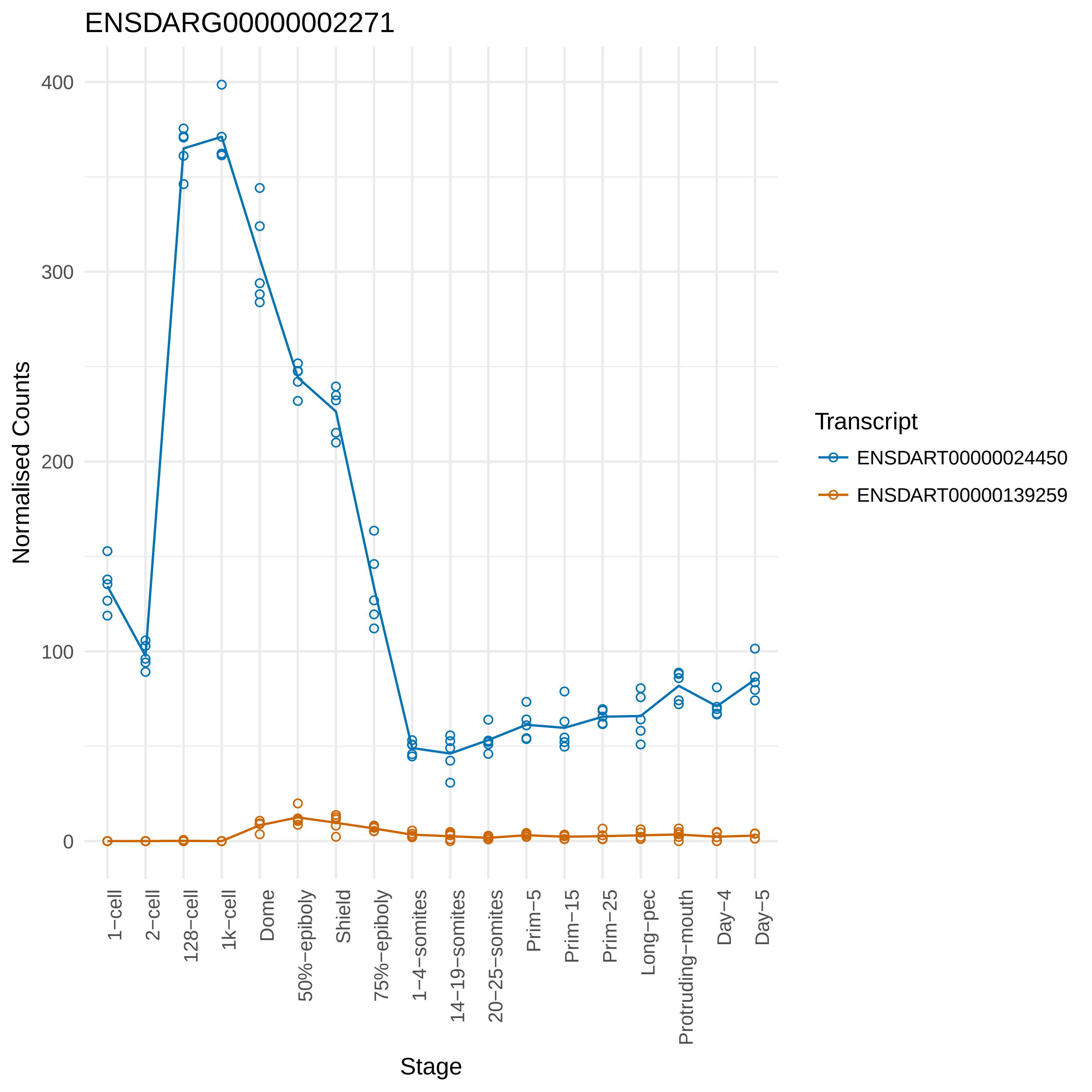
ENSDARG00000002271
Normalised Counts
400
300
200
100
0
1−cell
2−cell
128−cell
1k−cell
Dome
50%−epiboly
Shield
75%−epiboly
1−4−somites
14−19−somites
20−25−somites
Prim−5
Prim−15
Prim−25
Long−pec
Protruding−mouth
Day−4
Day−5
Stage
Transcript
ENSDART00000024450
ENSDART00000139259

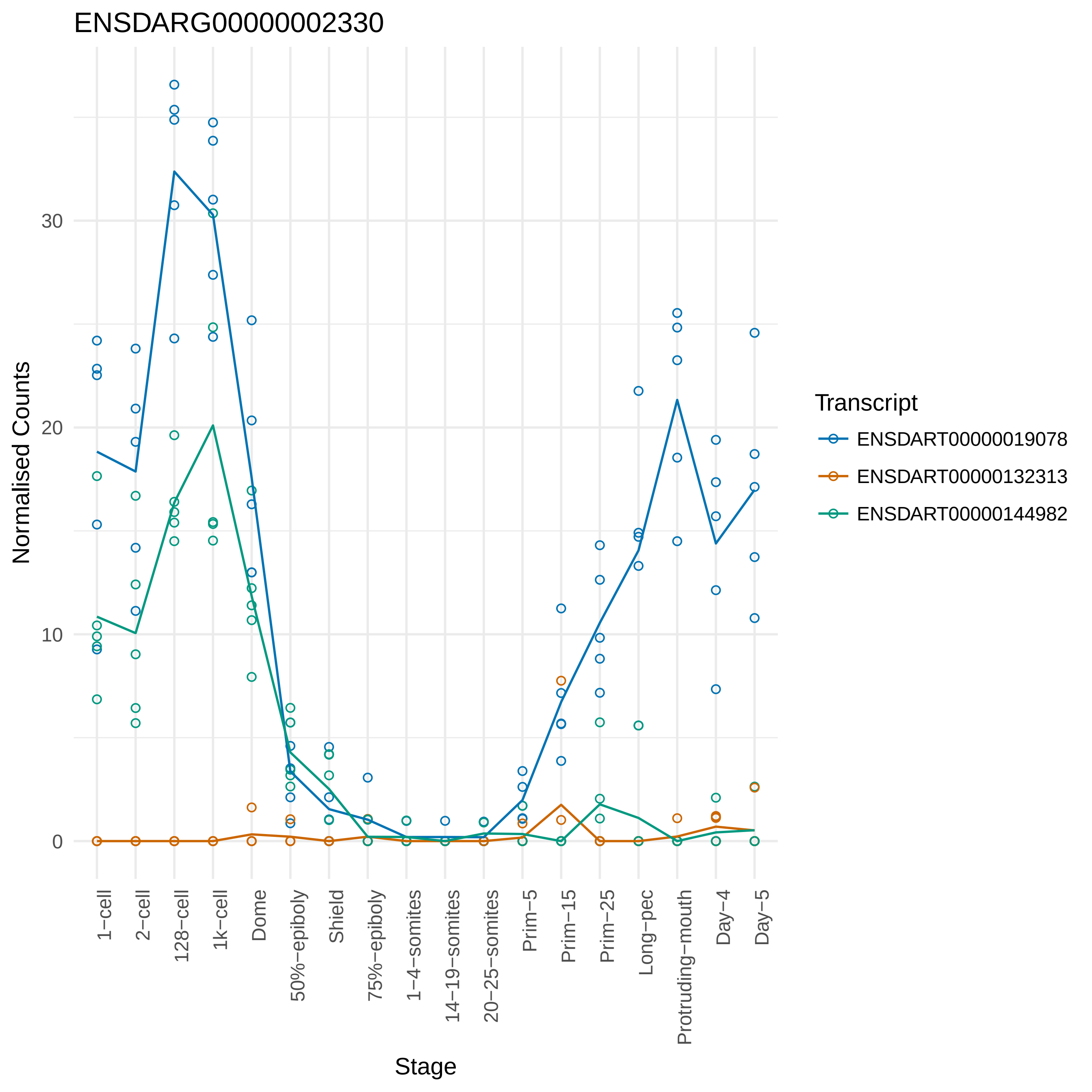
ENSDARG00000002330
Normalised Counts
0
10
20
30
1−cell
2−cell
128−cell
1k−cell
Dome
50%−epiboly
Shield
75%−epiboly
1−4−somites
14−19−somites
20−25−somites
Prim−5
Prim−15
Prim−25
Long−pec
Protruding−mouth
Day−4
Day−5
Stage
Transcript
ENSDART00000019078
ENSDART00000132313
ENSDART00000144982

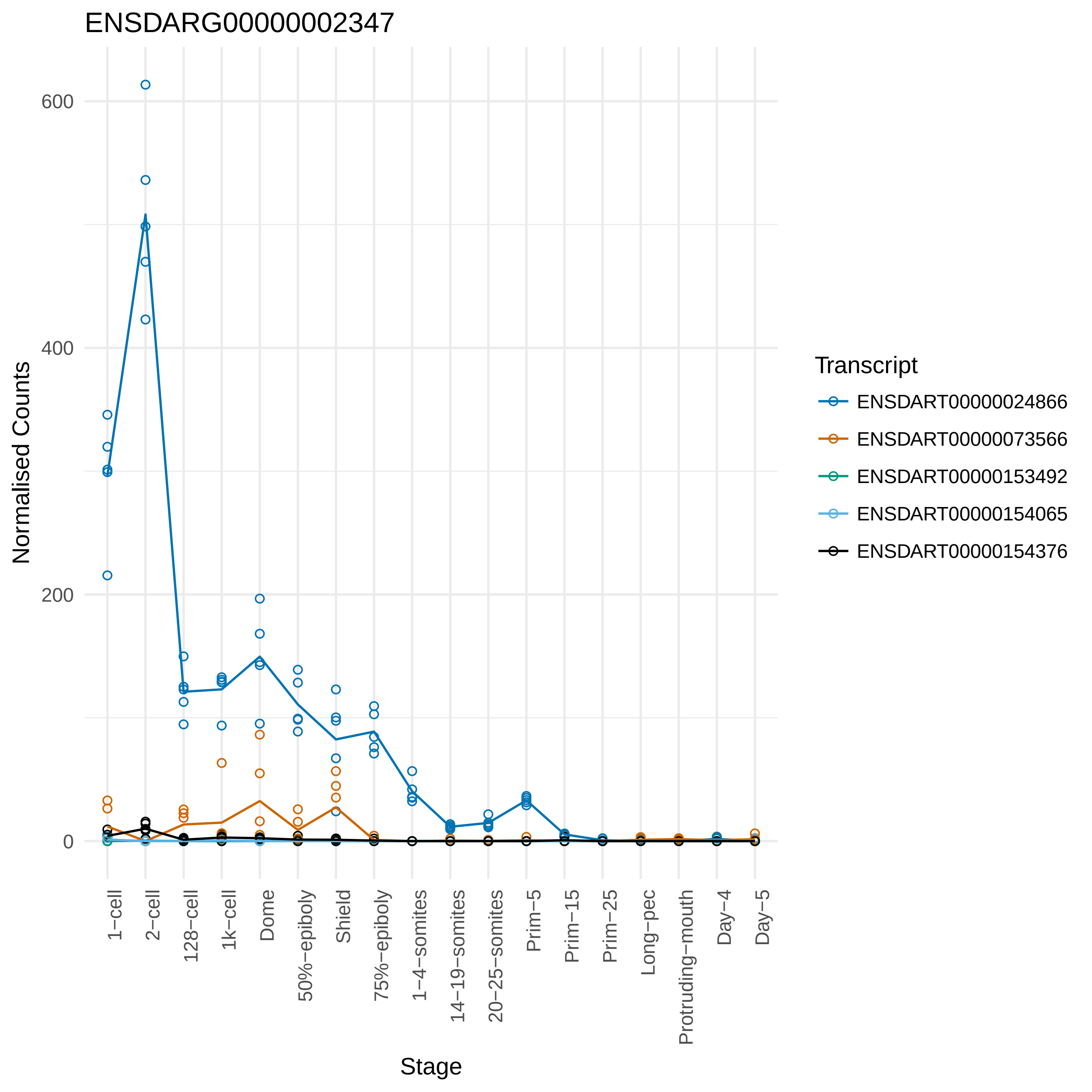

ENSDARG00000002347
Normalised Counts
600
400
200
0
1−cell
2−cell
128−cell
1k−cell
Dome
50%−epiboly
Shield
75%−epiboly
1−4−somites
14−19−somites
20−25−somites
Prim−5
Prim−15
Prim−25
Long−pec
Protruding−mouth
Day−4
Day−5
Stage
Transcript
ENSDART00000024866
ENSDART00000073566
ENSDART00000153492
ENSDART00000154065
ENSDART00000154376

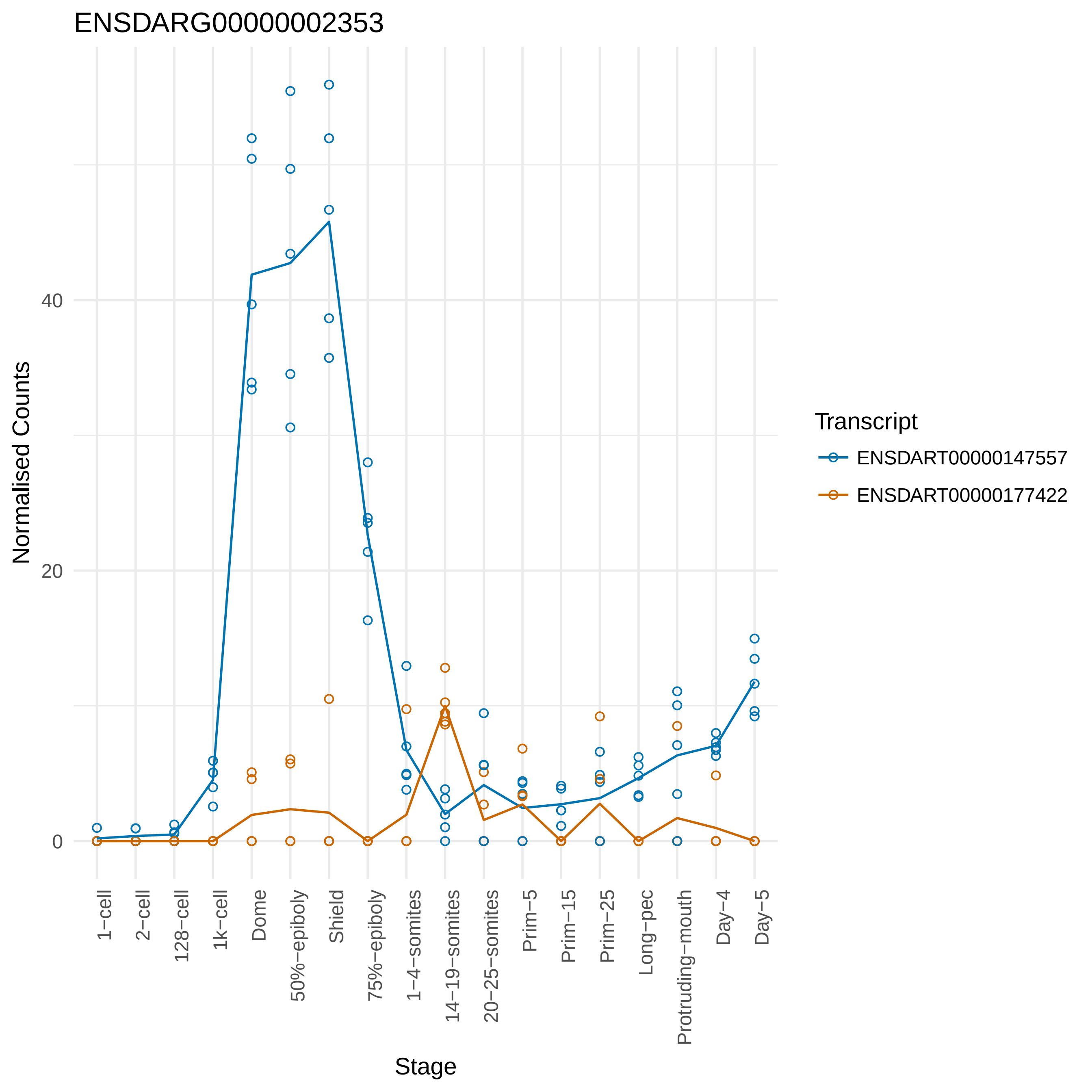

ENSDARG00000002353
Normalised Counts
0
20
40
1−cell
2−cell
128−cell
1k−cell
Dome
50%−epiboly
Shield
75%−epiboly
1−4−somites
14−19−somites
20−25−somites
Prim−5
Prim−15
Prim−25
Long−pec
Protruding−mouth
Day−4
Day−5
Stage
Transcript
ENSDART00000147557
ENSDART00000177422

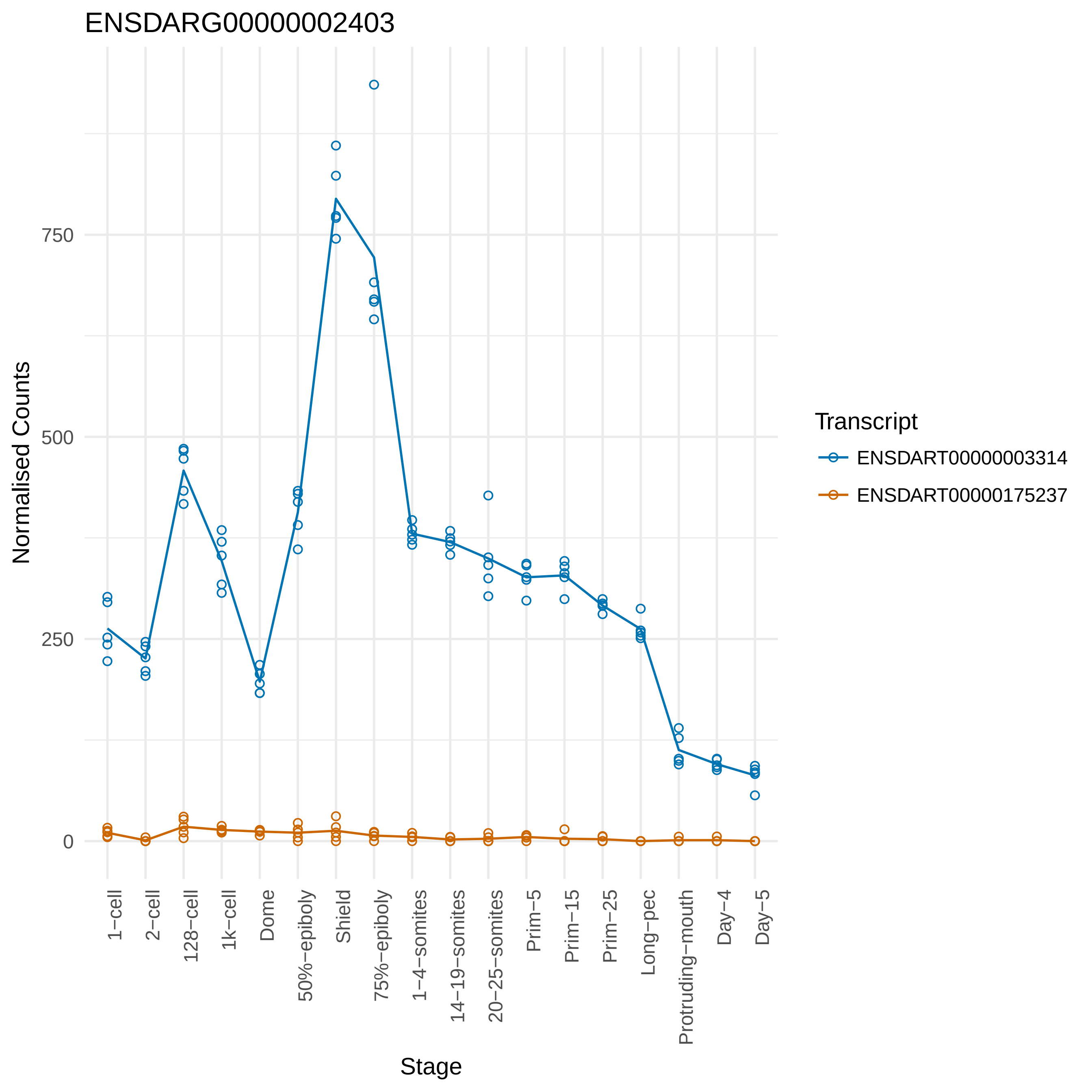

ENSDARG00000002403
Normalised Counts
750
500
250
0
1−cell
2−cell
128−cell
1k−cell
Dome
50%−epiboly
Shield
75%−epiboly
1−4−somites
14−19−somites
20−25−somites
Prim−5
Prim−15
Prim−25
Long−pec
Protruding−mouth
Day−4
Day−5
Stage
Transcript
ENSDART00000003314
ENSDART00000175237

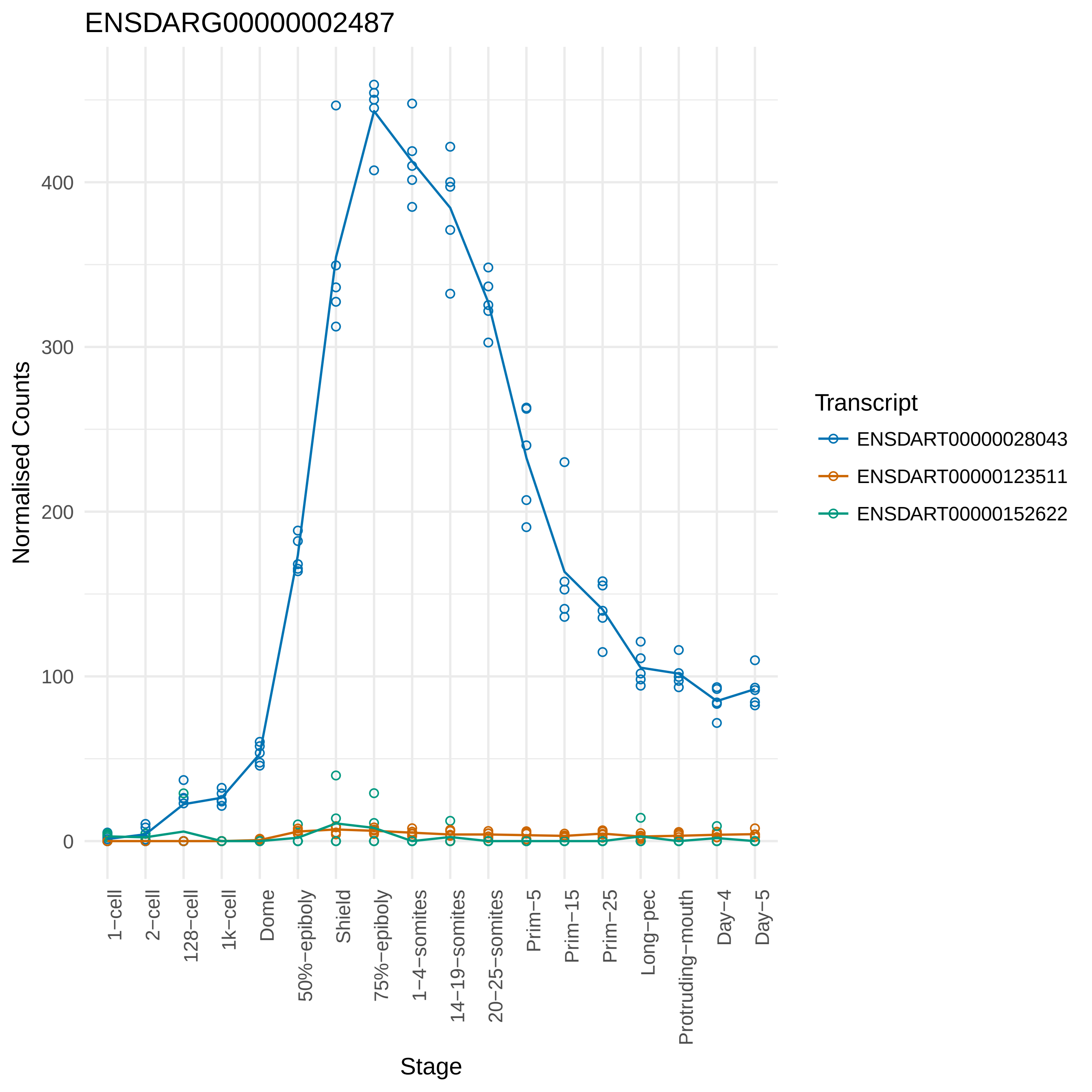
ENSDARG00000002487
Normalised Counts
0
100
200
300
400
Stage
1−cell
2−cell
128−cell
1k−cell
Dome
50%−epiboly
Shield
75%−epiboly
1−4−somites
14−19−somites
20−25−somites
Prim−5
Prim−15
Prim−25
Long−pec
Protruding−mouth
Day−4
Day−5
Transcript
ENSDART00000028043
ENSDART00000123511
ENSDART00000152622

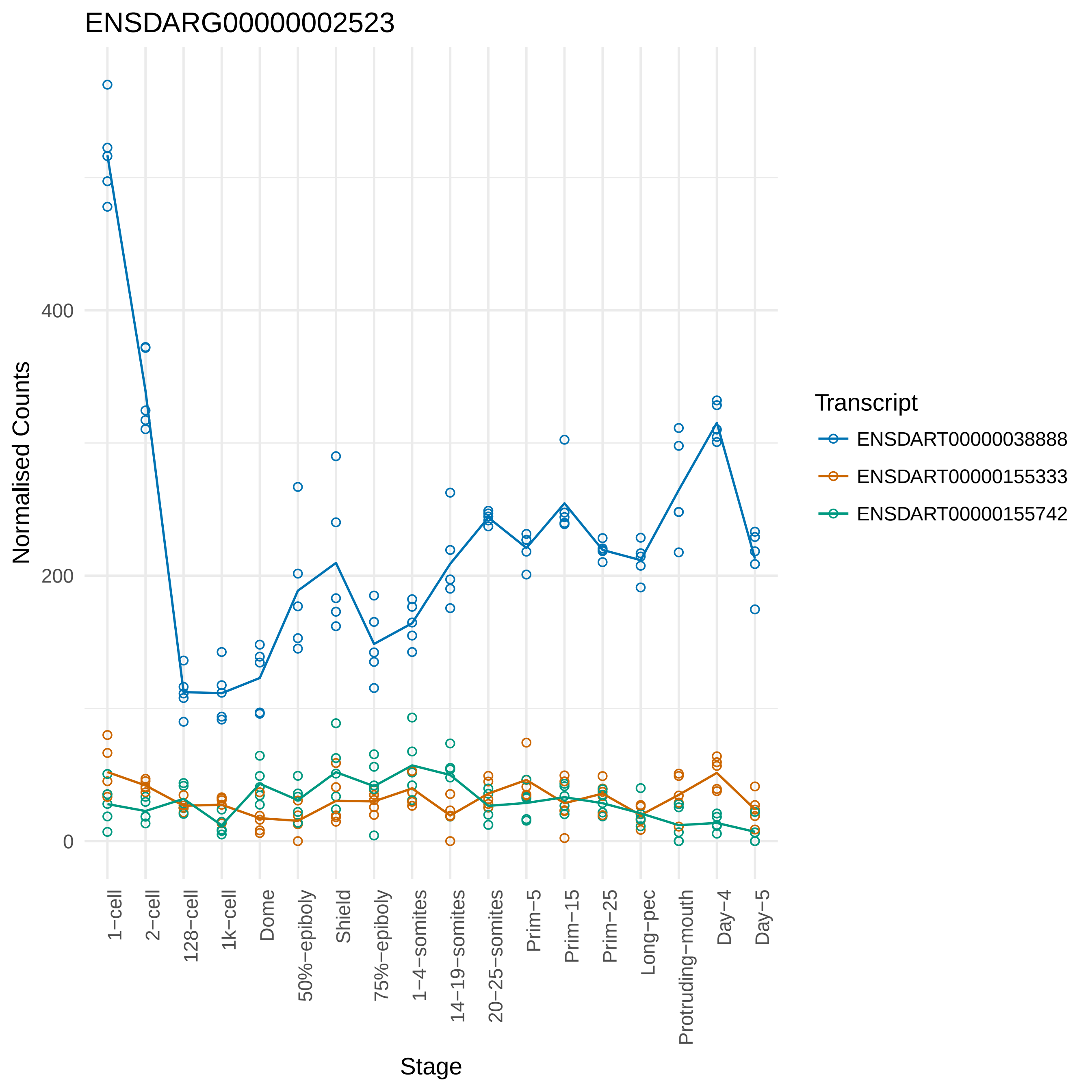

ENSDARG00000002523
Normalised Counts
400
200
0
Stage
1−cell
2−cell
128−cell
1k−cell
Dome
50%−epiboly
Shield
75%−epiboly
1−4−somites
14−19−somites
20−25−somites
Prim−5
Prim−15
Prim−25
Long−pec
Protruding−mouth
Day−4
Day−5
Transcript
ENSDART00000038888
ENSDART00000155333
ENSDART00000155742

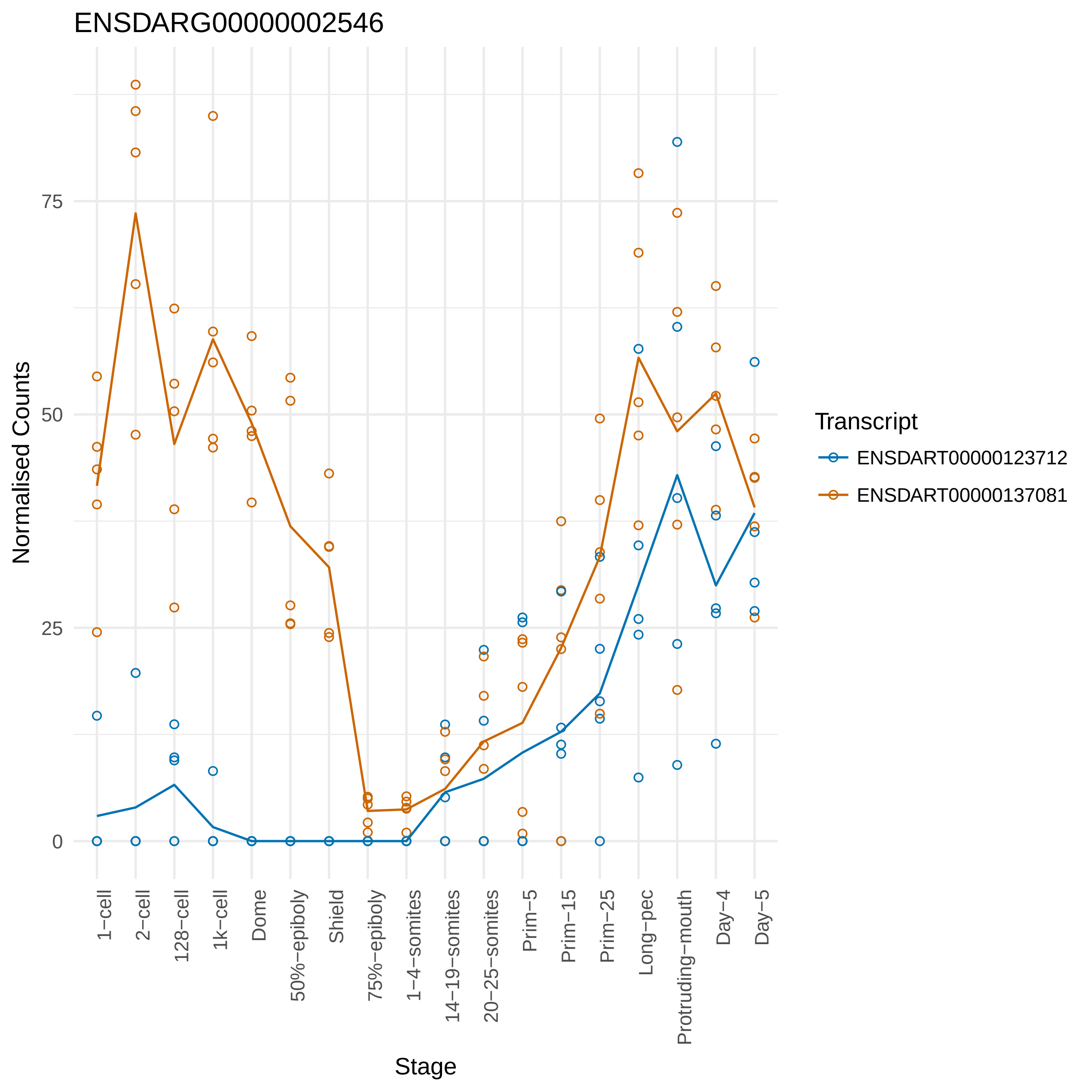
ENSDARG00000002546
Normalised Counts
0
25
50
75
1−cell
2−cell
128−cell
1k−cell
Dome
50%−epiboly
Shield
75%−epiboly
1−4−somites
14−19−somites
20−25−somites
Prim−5
Prim−15
Prim−25
Long−pec
Protruding−mouth
Day−4
Day−5
Stage
Transcript
ENSDART00000123712
ENSDART00000137081

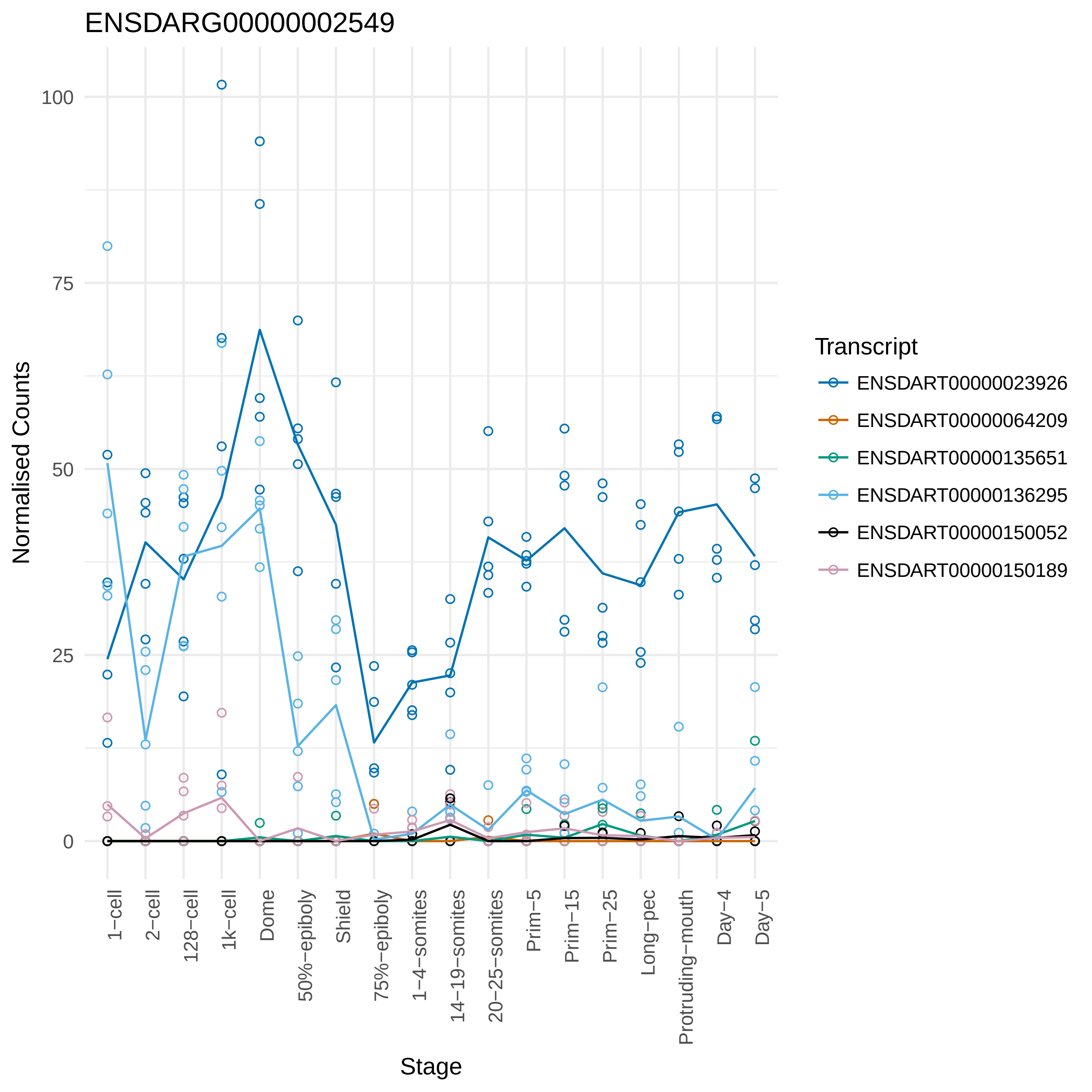

ENSDARG00000002549
Normalised Counts
100
75
50
25
0
Stage
1−cell
2−cell
128−cell
1k−cell
Dome
50%−epiboly
Shield
75%−epiboly
1−4−somites
14−19−somites
20−25−somites
Prim−5
Prim−15
Prim−25
Long−pec
Protruding−mouth
Day−4
Day−5
Transcript
ENSDART00000023926
ENSDART00000064209
ENSDART00000135651
ENSDART00000136295
ENSDART00000150052
ENSDART00000150189

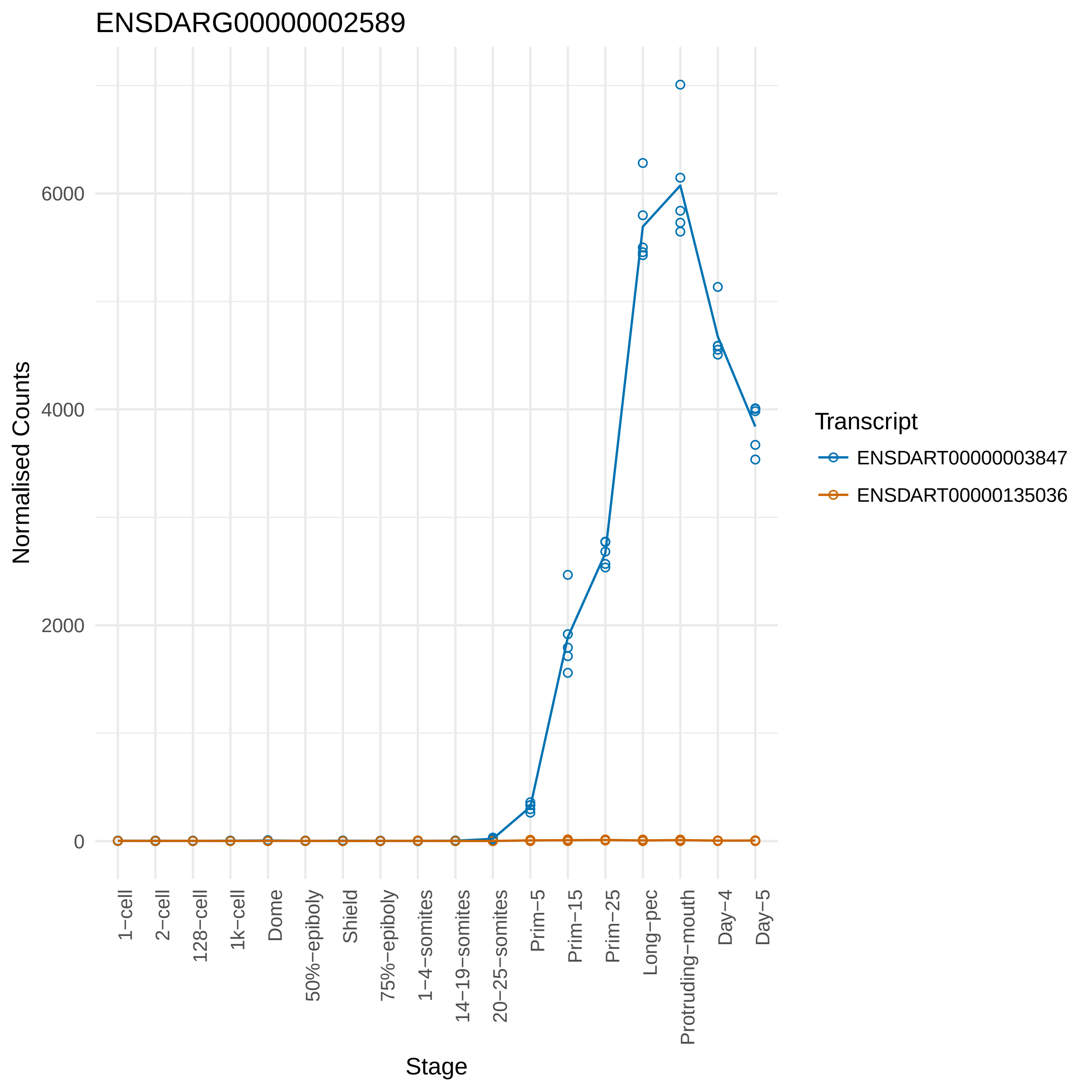

ENSDARG00000002589
Normalised Counts
6000
4000
2000
0
1−cell
2−cell
128−cell
1k−cell
Dome
50%−epiboly
Shield
75%−epiboly
1−4−somites
14−19−somites
20−25−somites
Prim−5
Prim−15
Prim−25
Long−pec
Protruding−mouth
Day−4
Day−5
Stage
Transcript
ENSDART00000003847
ENSDART00000135036

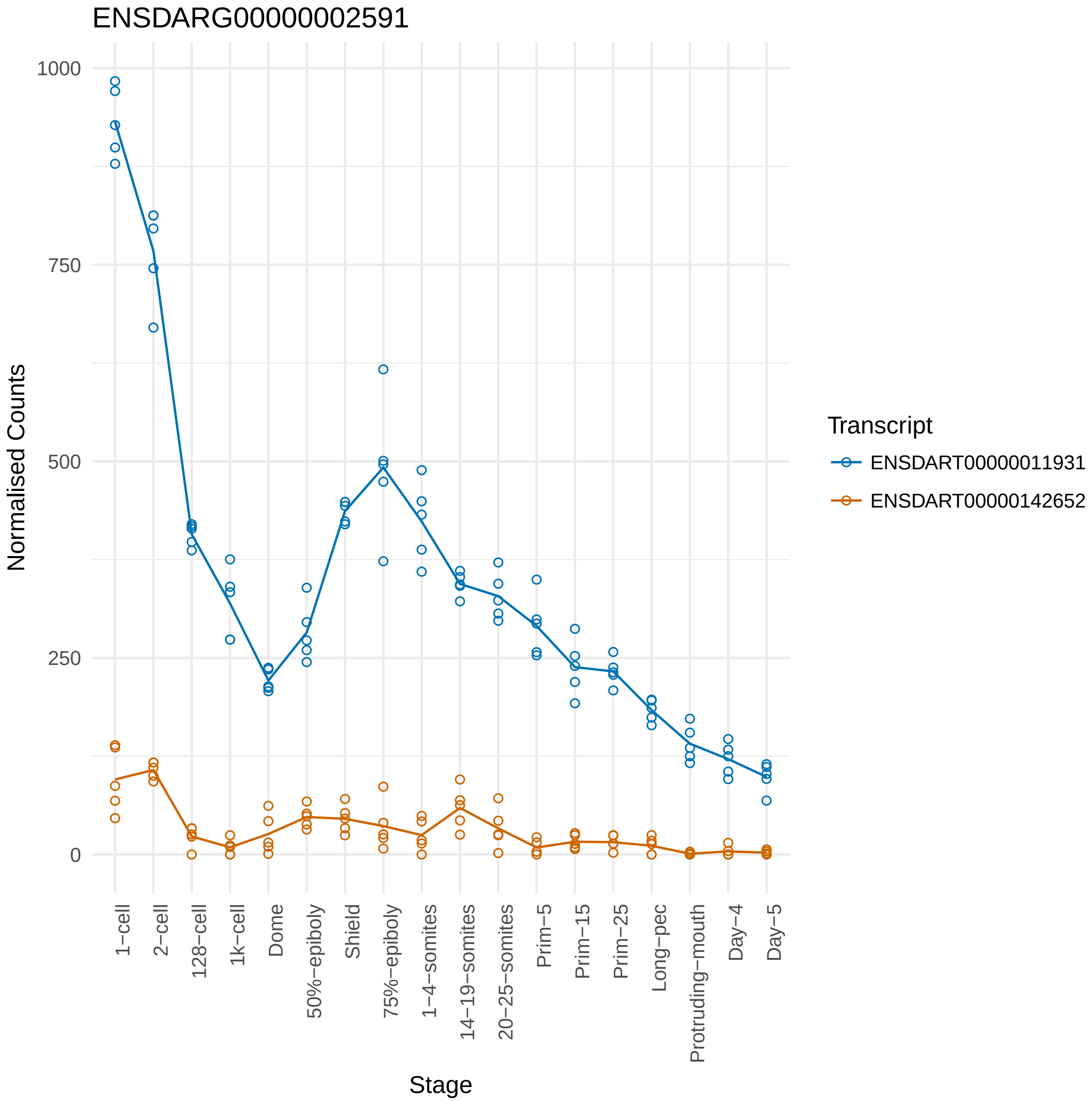

ENSDARG00000002591
Normalised Counts
1000
750
500
250
0
1−cell
2−cell
128−cell
1k−cell
Dome
50%−epiboly
Shield
75%−epiboly
1−4−somites
14−19−somites
20−25−somites
Prim−5
Prim−15
Prim−25
Long−pec
Protruding−mouth
Day−4
Day−5
Stage
Transcript
ENSDART00000011931
ENSDART00000142652

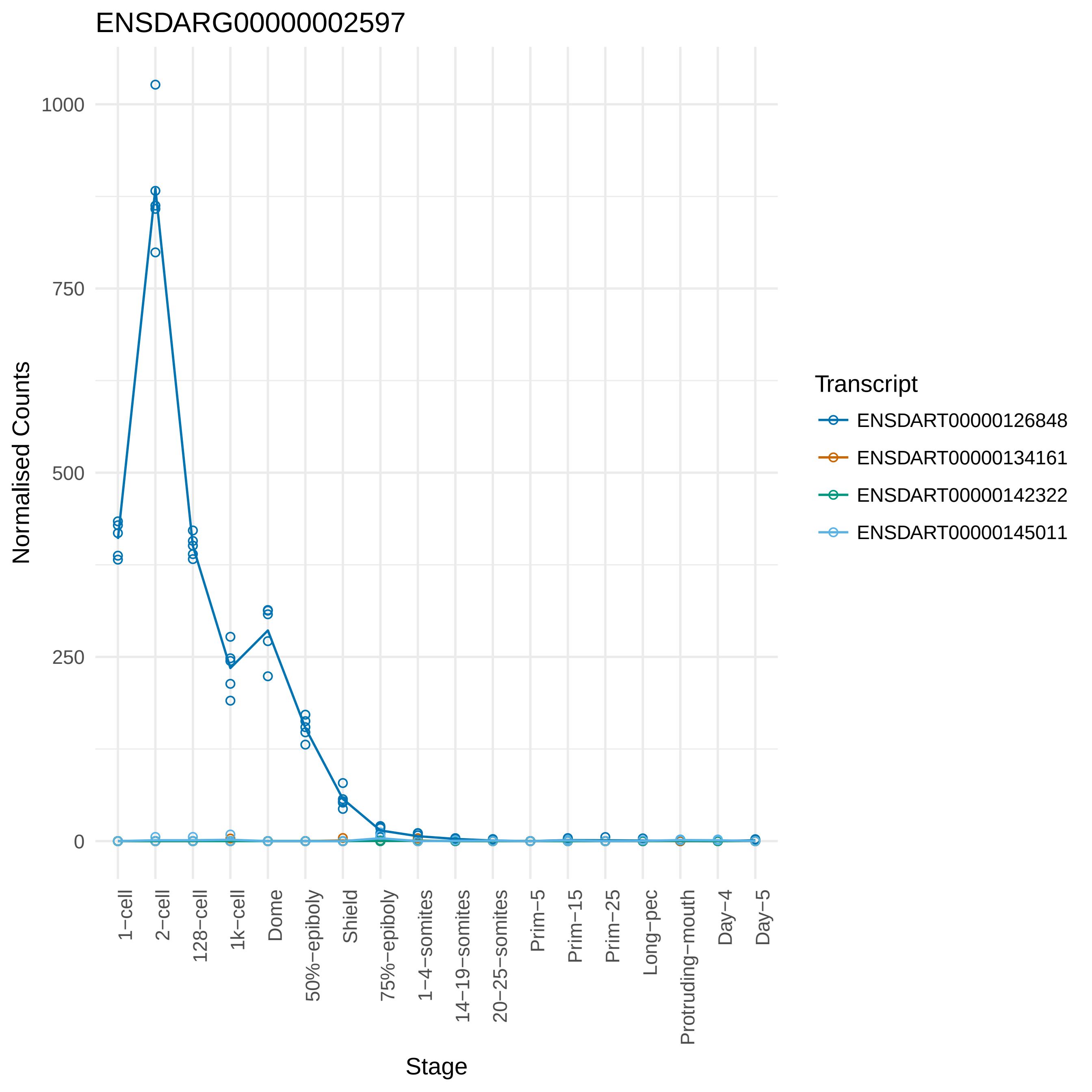
ENSDARG00000002597
Normalised Counts
1000
750
500
250
0
1−cell
2−cell
128−cell
1k−cell
Dome
50%−epiboly
Shield
75%−epiboly
1−4−somites
14−19−somites
20−25−somites
Prim−5
Prim−15
Prim−25
Long−pec
Protruding−mouth
Day−4
Day−5
Stage
Transcript
ENSDART00000126848
ENSDART00000134161
ENSDART00000142322
ENSDART00000145011

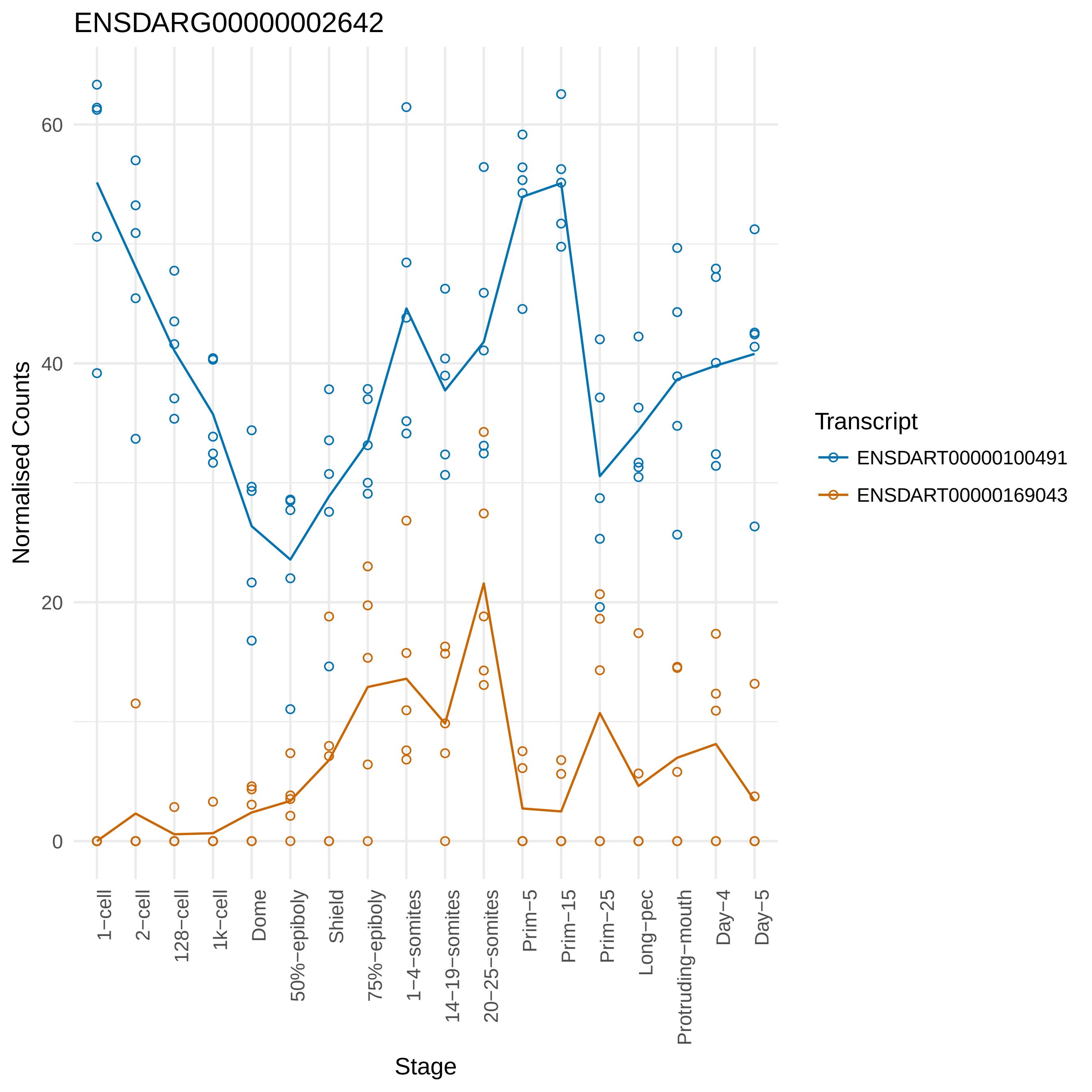
ENSDARG00000002642
Normalised Counts
0
20
40
60
Stage
1−cell
2−cell
128−cell
1k−cell
Dome
50%−epiboly
Shield
75%−epiboly
1−4−somites
14−19−somites
20−25−somites
Prim−5
Prim−15
Prim−25
Long−pec
Protruding−mouth
Day−4
Day−5
Transcript
ENSDART00000100491
ENSDART00000169043

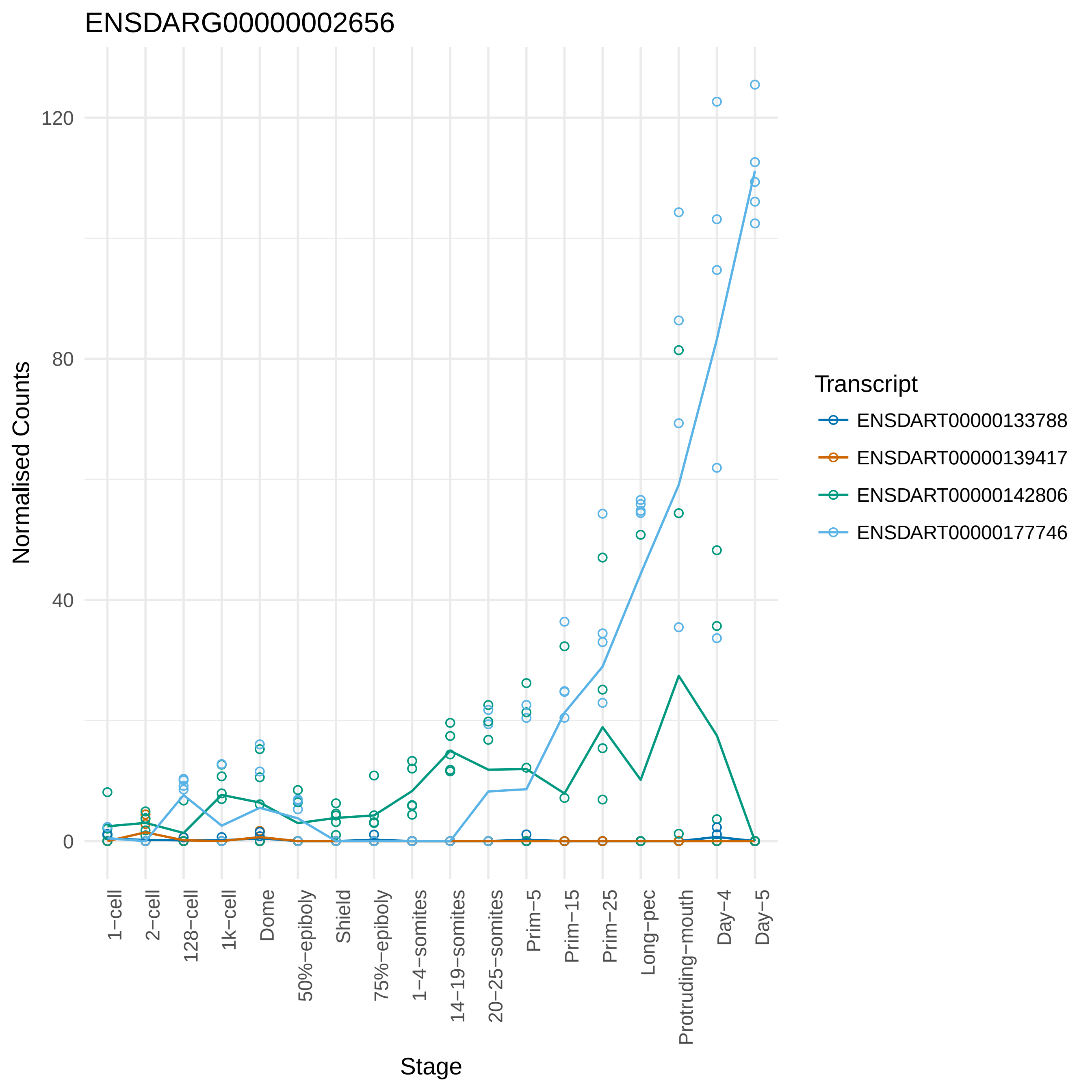

ENSDARG00000002656
Normalised Counts
0
40
80
120
Stage
1−cell
2−cell
128−cell
1k−cell
Dome
50%−epiboly
Shield
75%−epiboly
1−4−somites
14−19−somites
20−25−somites
Prim−5
Prim−15
Prim−25
Long−pec
Protruding−mouth
Day−4
Day−5
Transcript
ENSDART00000133788
ENSDART00000139417
ENSDART00000142806
ENSDART00000177746

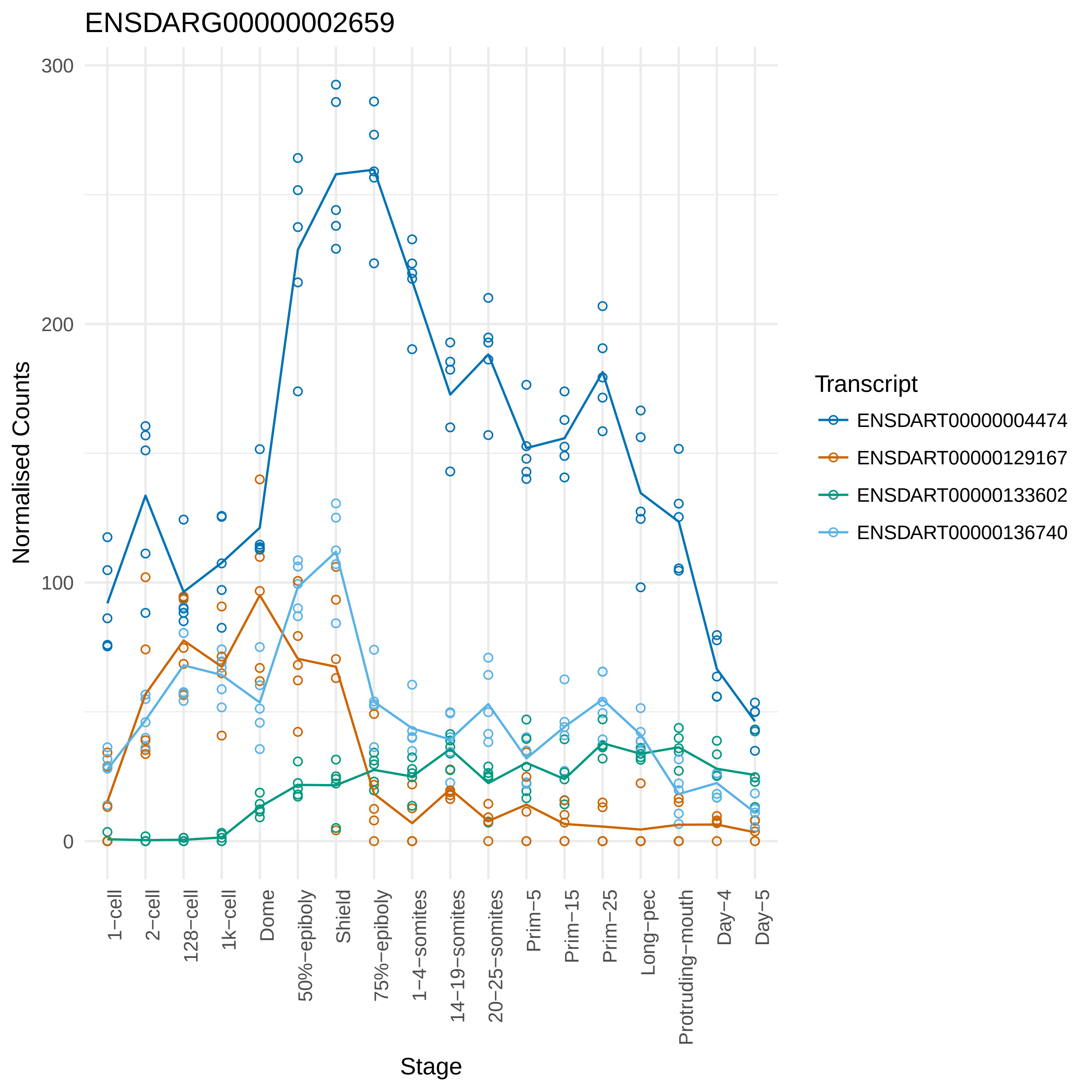
ENSDARG00000002659
Normalised Counts
300
200
100
0
1−cell
2−cell
128−cell
1k−cell
Dome
50%−epiboly
Shield
75%−epiboly
1−4−somites
14−19−somites
20−25−somites
Prim−5
Prim−15
Prim−25
Long−pec
Protruding−mouth
Day−4
Day−5
Stage
Transcript
ENSDART00000004474
ENSDART00000129167
ENSDART00000133602
ENSDART00000136740

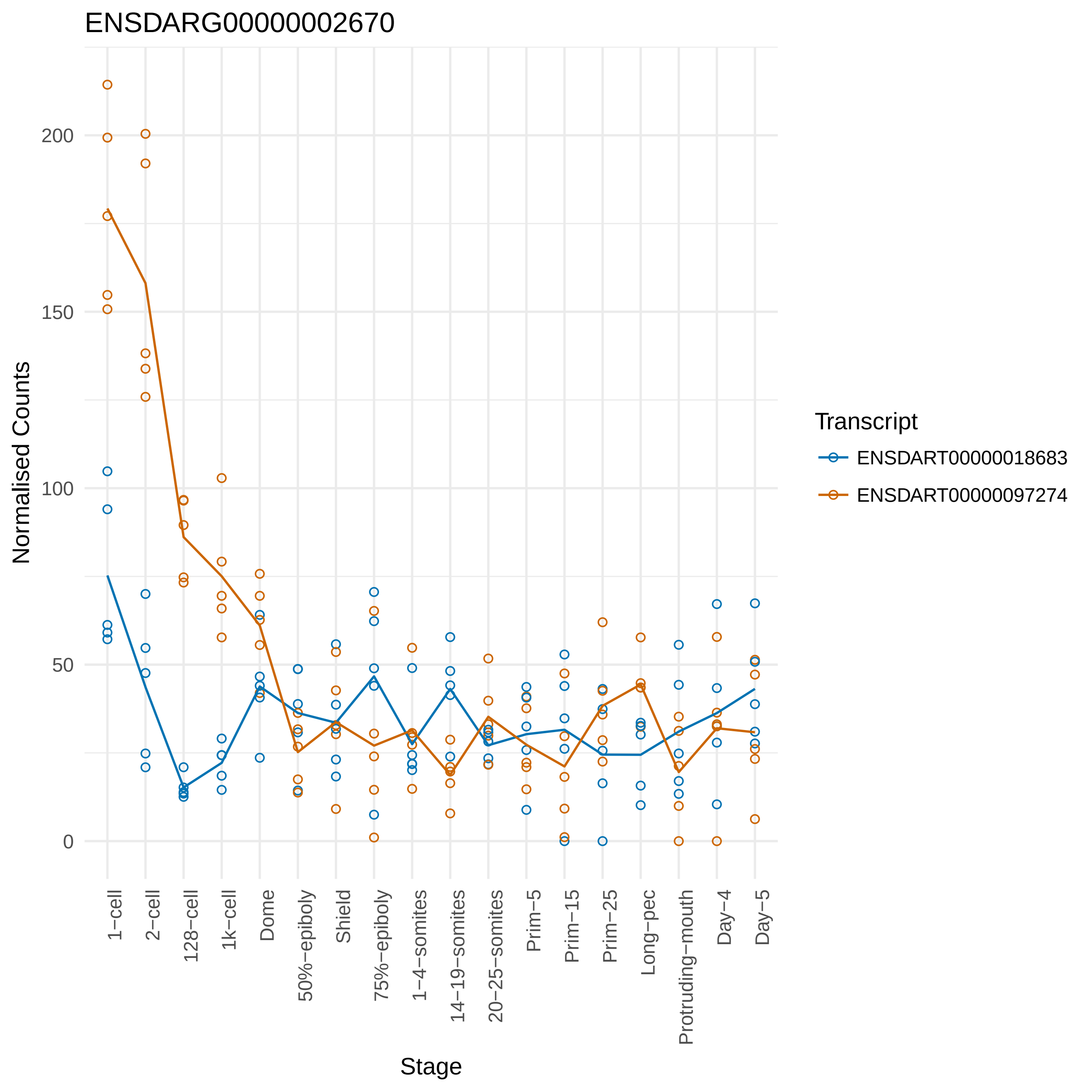

ENSDARG00000002670
Normalised Counts
200
150
100
50
0
1−cell
2−cell
128−cell
1k−cell
Dome
50%−epiboly
Shield
75%−epiboly
1−4−somites
14−19−somites
20−25−somites
Prim−5
Prim−15
Prim−25
Long−pec
Protruding−mouth
Day−4
Day−5
Stage
Transcript
ENSDART00000018683
ENSDART00000097274

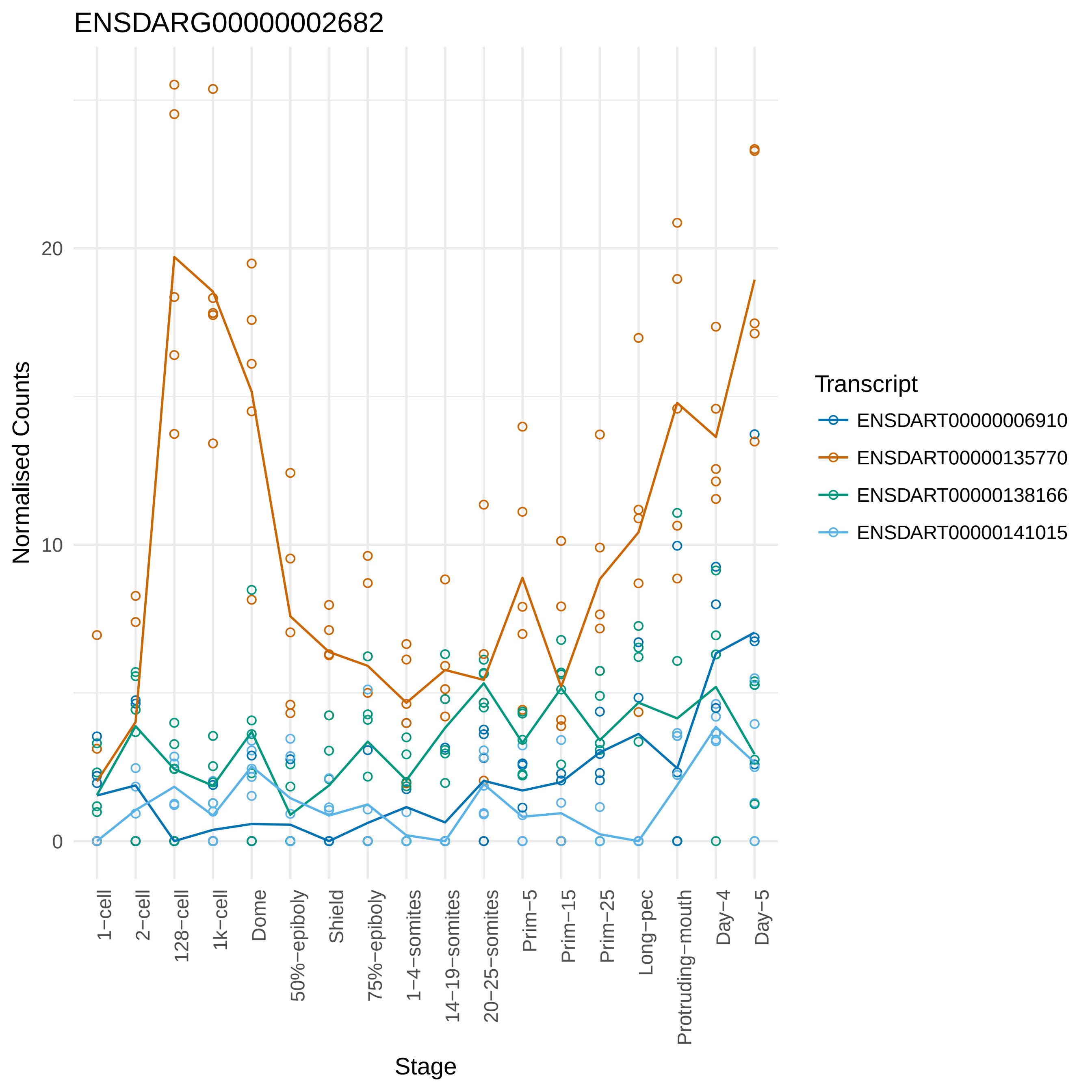

ENSDARG00000002682
Normalised Counts
20
10
0
1−cell
2−cell
128−cell
1k−cell
Dome
50%−epiboly
Shield
75%−epiboly
1−4−somites
14−19−somites
20−25−somites
Prim−5
Prim−15
Prim−25
Long−pec
Protruding−mouth
Day−4
Day−5
Stage
Transcript
ENSDART00000006910
ENSDART00000135770
ENSDART00000138166
ENSDART00000141015

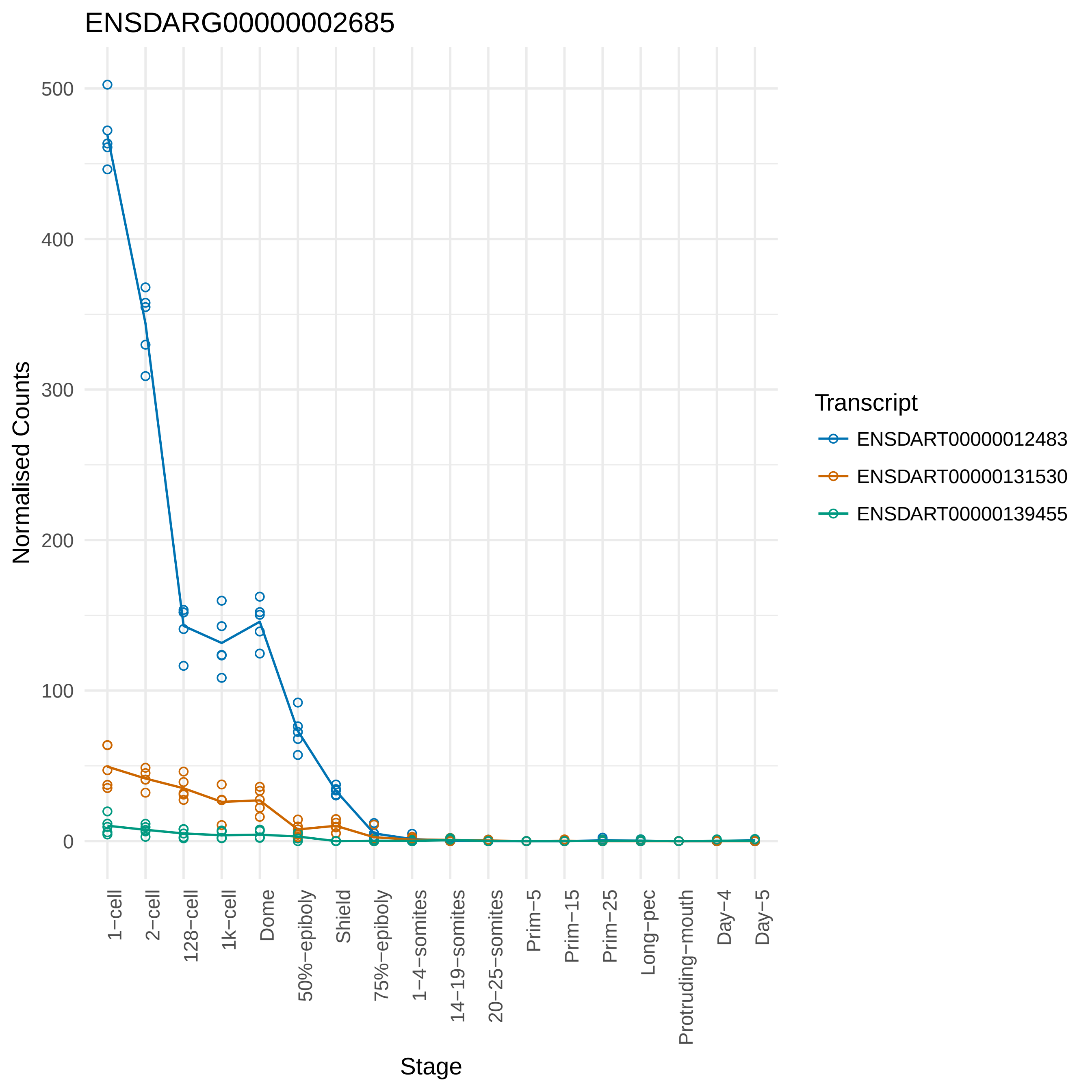
ENSDARG00000002685
Normalised Counts
0
100
200
300
400
500
1−cell
2−cell
128−cell
1k−cell
Dome
50%−epiboly
Shield
75%−epiboly
1−4−somites
14−19−somites
20−25−somites
Prim−5
Prim−15
Prim−25
Long−pec
Protruding−mouth
Day−4
Day−5
Stage
Transcript
ENSDART00000012483
ENSDART00000131530
ENSDART00000139455

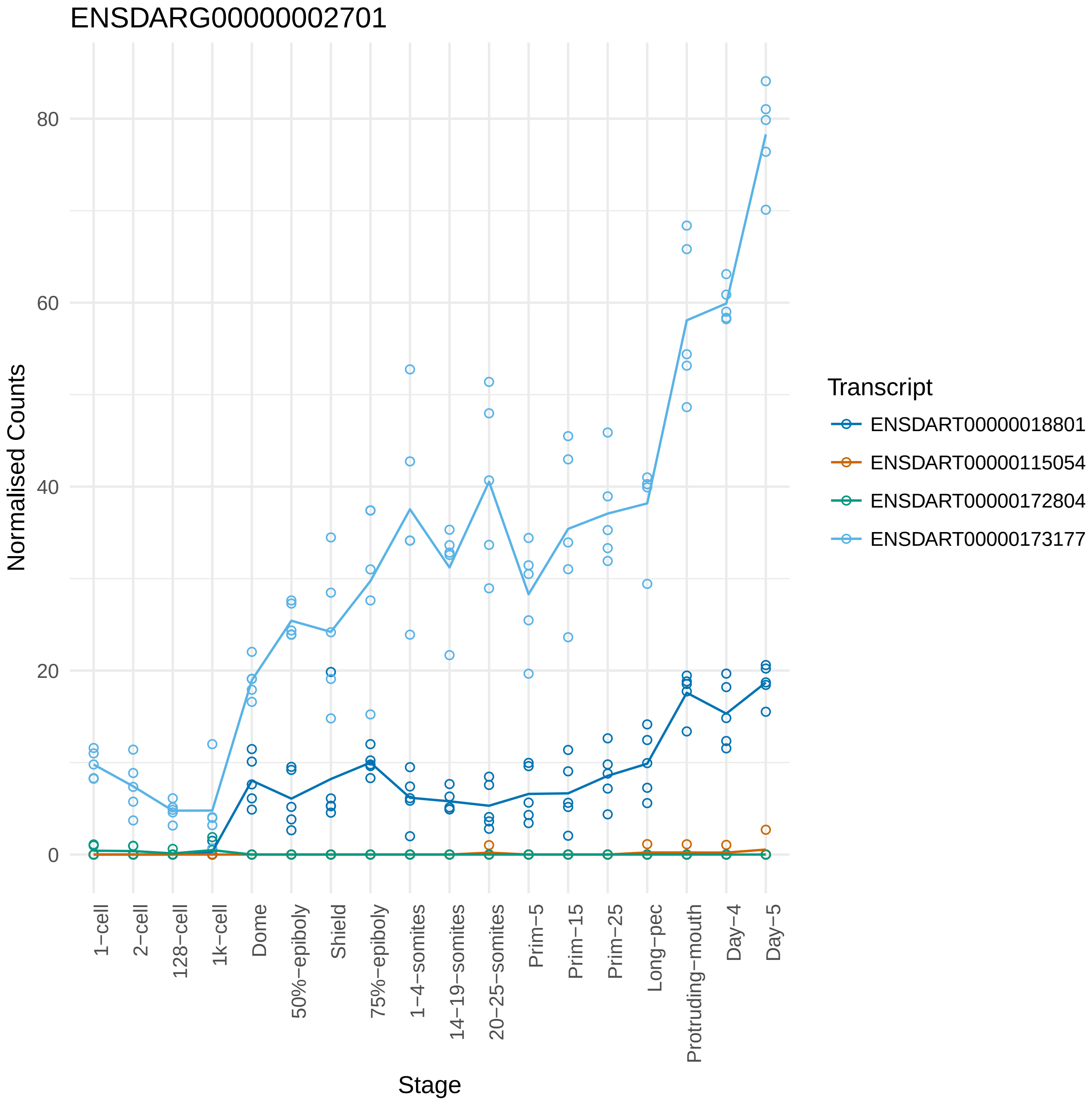
ENSDARG00000002701
Normalised Counts
0
20
40
60
80
1−cell
2−cell
128−cell
1k−cell
Dome
50%−epiboly
Shield
75%−epiboly
1−4−somites
14−19−somites
20−25−somites
Prim−5
Prim−15
Prim−25
Long−pec
Protruding−mouth
Day−4
Day−5
Stage
Transcript
ENSDART00000018801
ENSDART00000115054
ENSDART00000172804
ENSDART00000173177

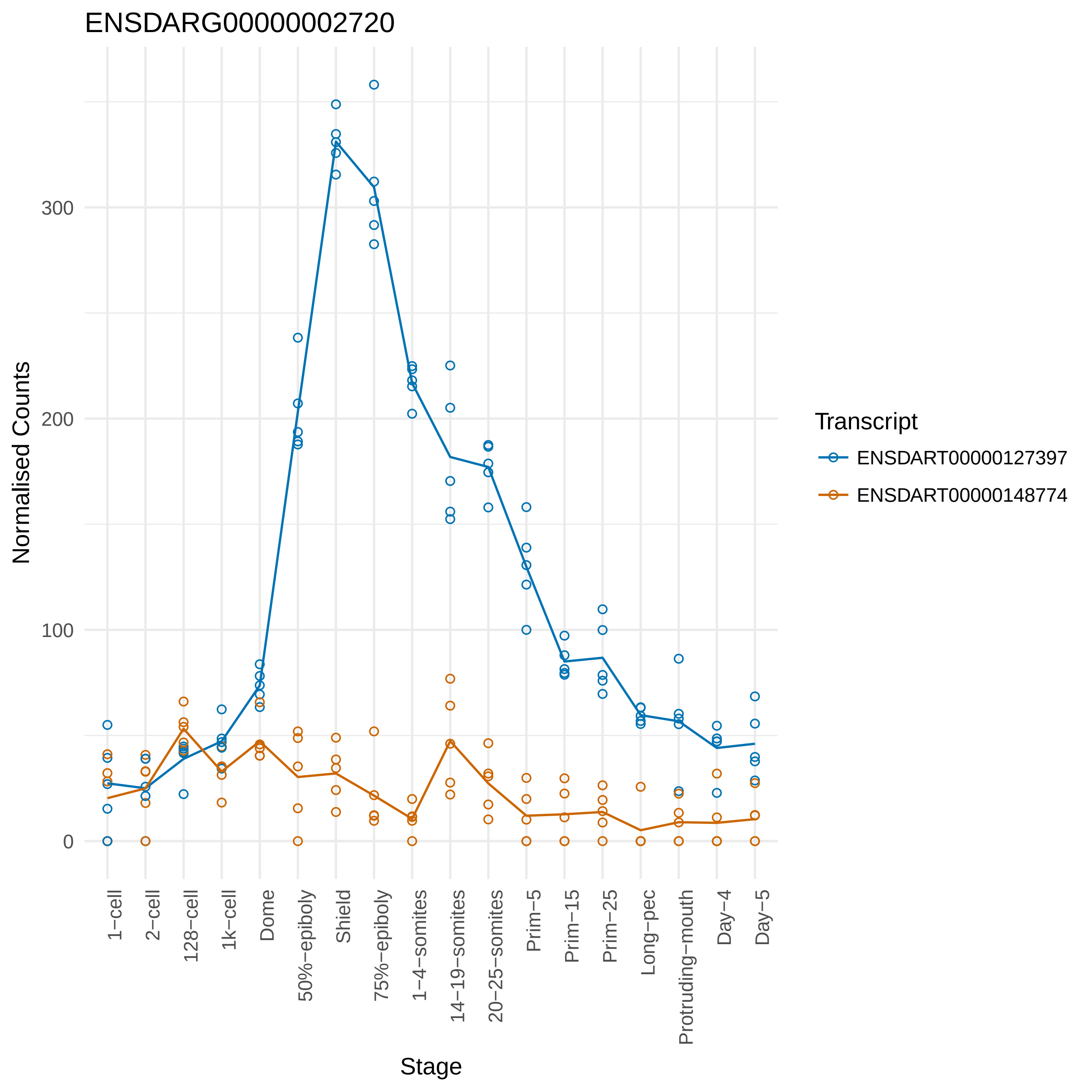
ENSDARG00000002720
Normalised Counts
300
200
100
0
Stage
1−cell
2−cell
128−cell
1k−cell
Dome
50%−epiboly
Shield
75%−epiboly
1−4−somites
14−19−somites
20−25−somites
Prim−5
Prim−15
Prim−25
Long−pec
Protruding−mouth
Day−4
Day−5
Transcript
ENSDART00000127397
ENSDART00000148774

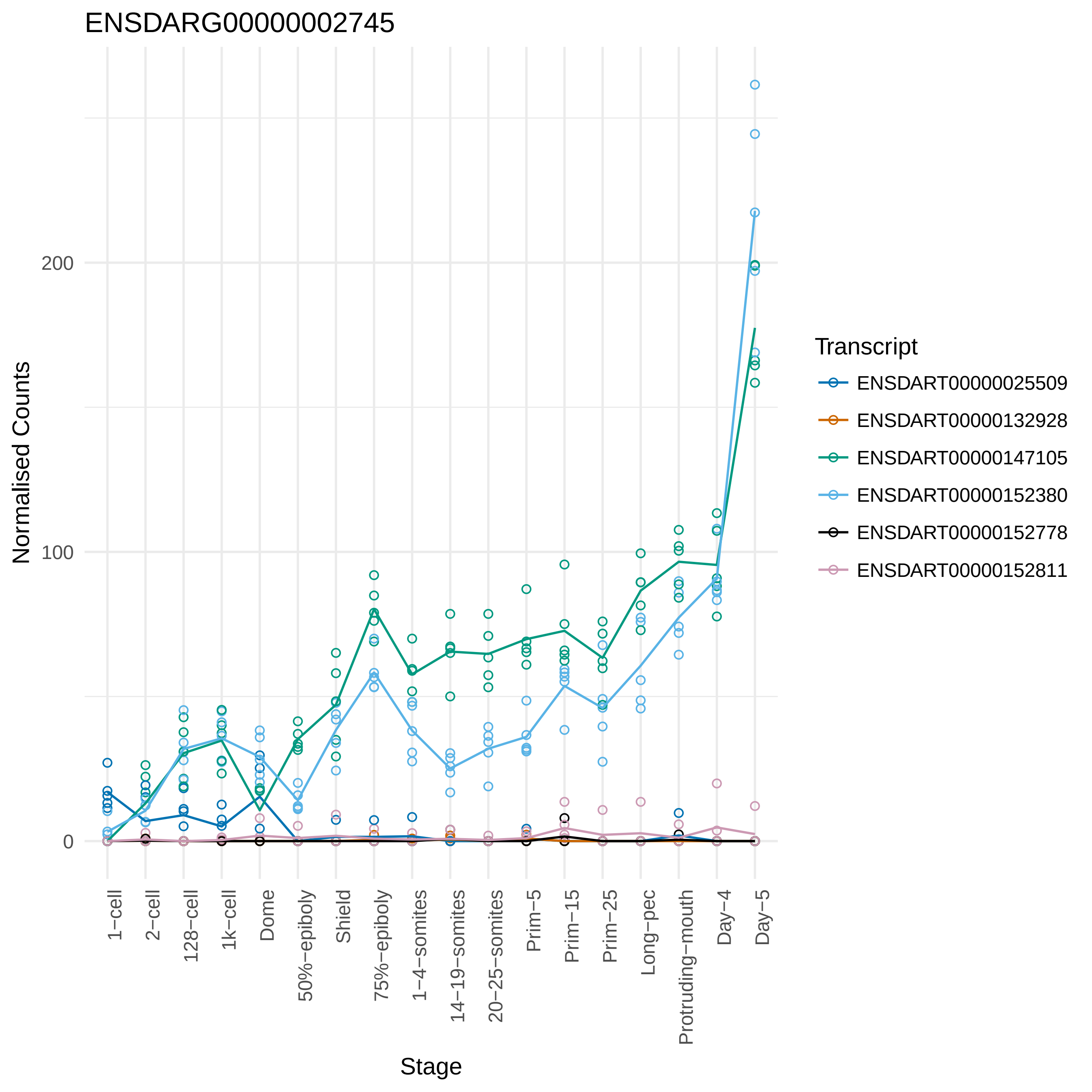

ENSDARG00000002745
Normalised Counts
0
100
200
Stage
1−cell
2−cell
128−cell
1k−cell
Dome
50%−epiboly
Shield
75%−epiboly
1−4−somites
14−19−somites
20−25−somites
Prim−5
Prim−15
Prim−25
Long−pec
Protruding−mouth
Day−4
Day−5
Transcript
ENSDART00000025509
ENSDART00000132928
ENSDART00000147105
ENSDART00000152380
ENSDART00000152778
ENSDART00000152811

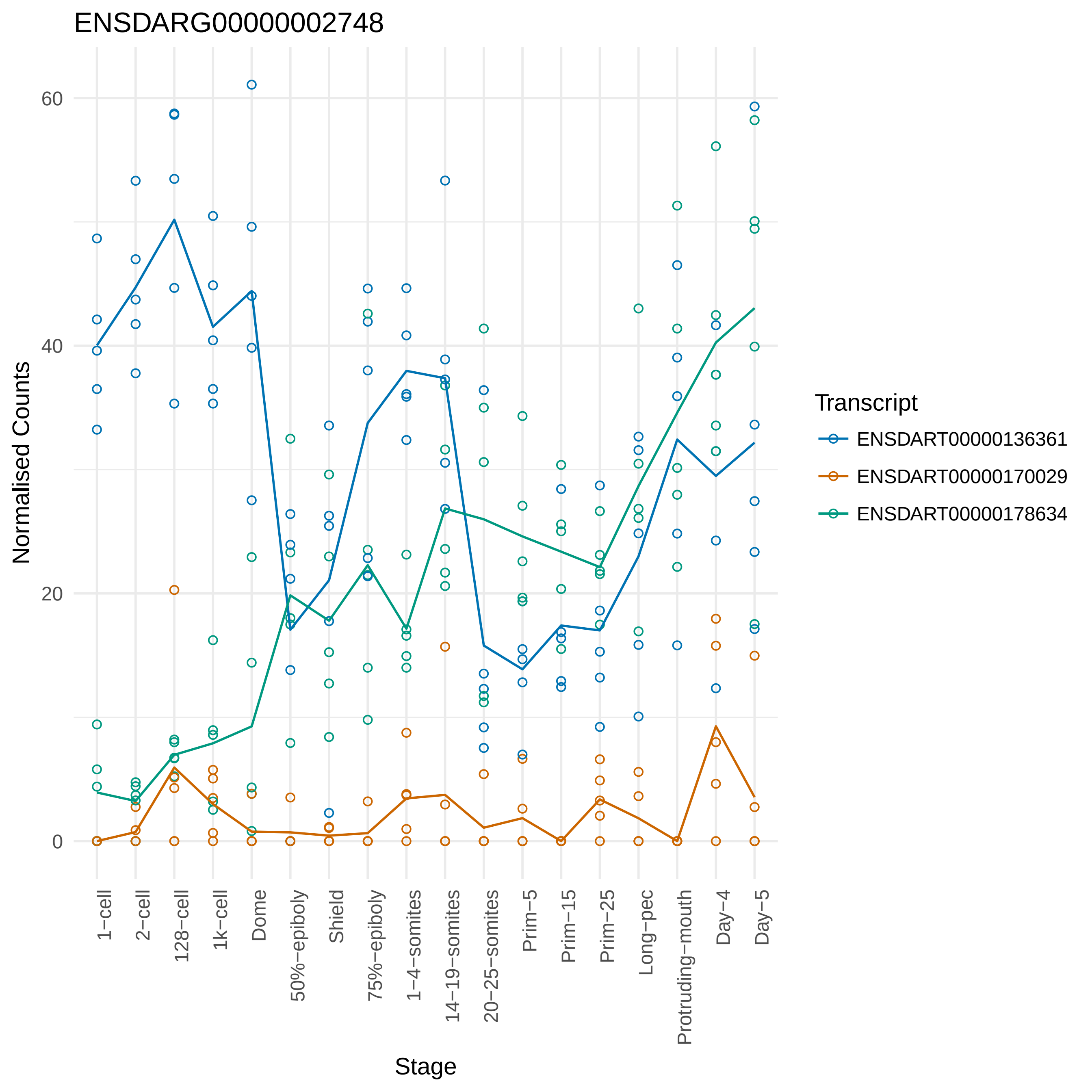
ENSDARG00000002748
Normalised Counts
0
20
40
60
1−cell
2−cell
128−cell
1k−cell
Dome
50%−epiboly
Shield
75%−epiboly
1−4−somites
14−19−somites
20−25−somites
Prim−5
Prim−15
Prim−25
Long−pec
Protruding−mouth
Day−4
Day−5
Stage
Transcript
ENSDART00000136361
ENSDART00000170029
ENSDART00000178634

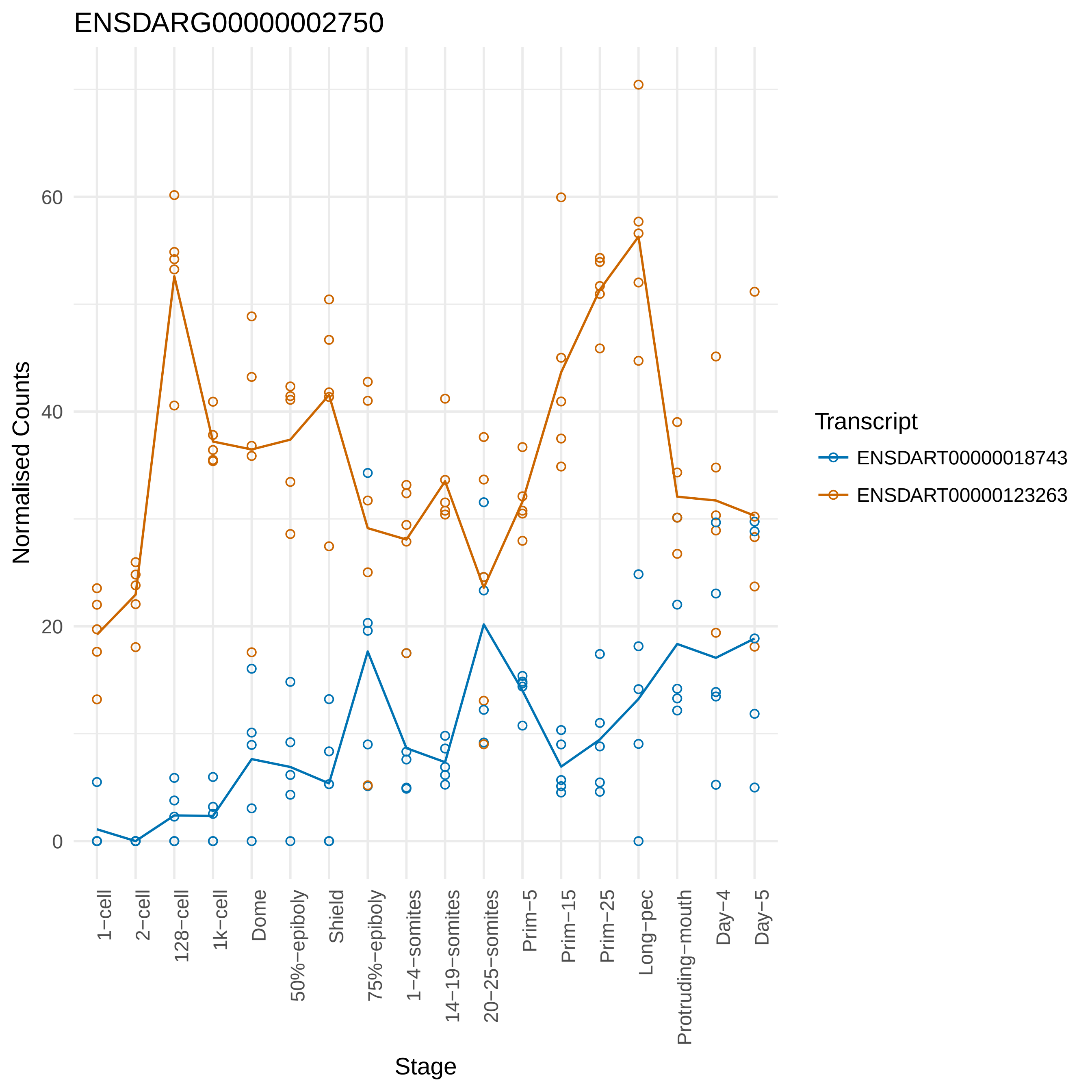

ENSDARG00000002750
Normalised Counts
0
20
40
60
Stage
1−cell
2−cell
128−cell
1k−cell
Dome
50%−epiboly
Shield
75%−epiboly
1−4−somites
14−19−somites
20−25−somites
Prim−5
Prim−15
Prim−25
Long−pec
Protruding−mouth
Day−4
Day−5
Transcript
ENSDART00000018743
ENSDART00000123263

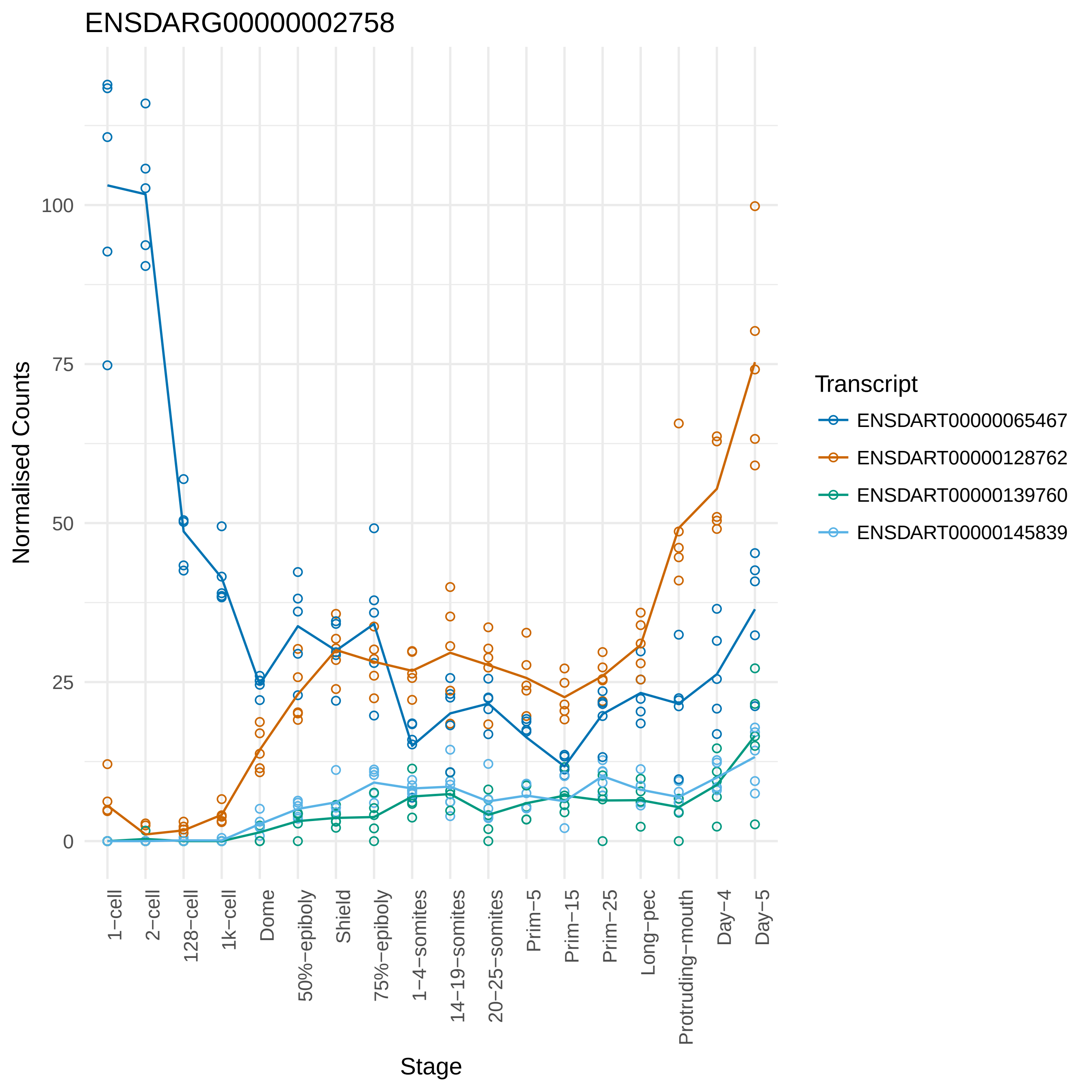
ENSDARG00000002758
Normalised Counts
100
75
50
25
0
1−cell
2−cell
128−cell
1k−cell
Dome
50%−epiboly
Shield
75%−epiboly
1−4−somites
14−19−somites
20−25−somites
Prim−5
Prim−15
Prim−25
Long−pec
Protruding−mouth
Day−4
Day−5
Stage
Transcript
ENSDART00000065467
ENSDART00000128762
ENSDART00000139760
ENSDART00000145839

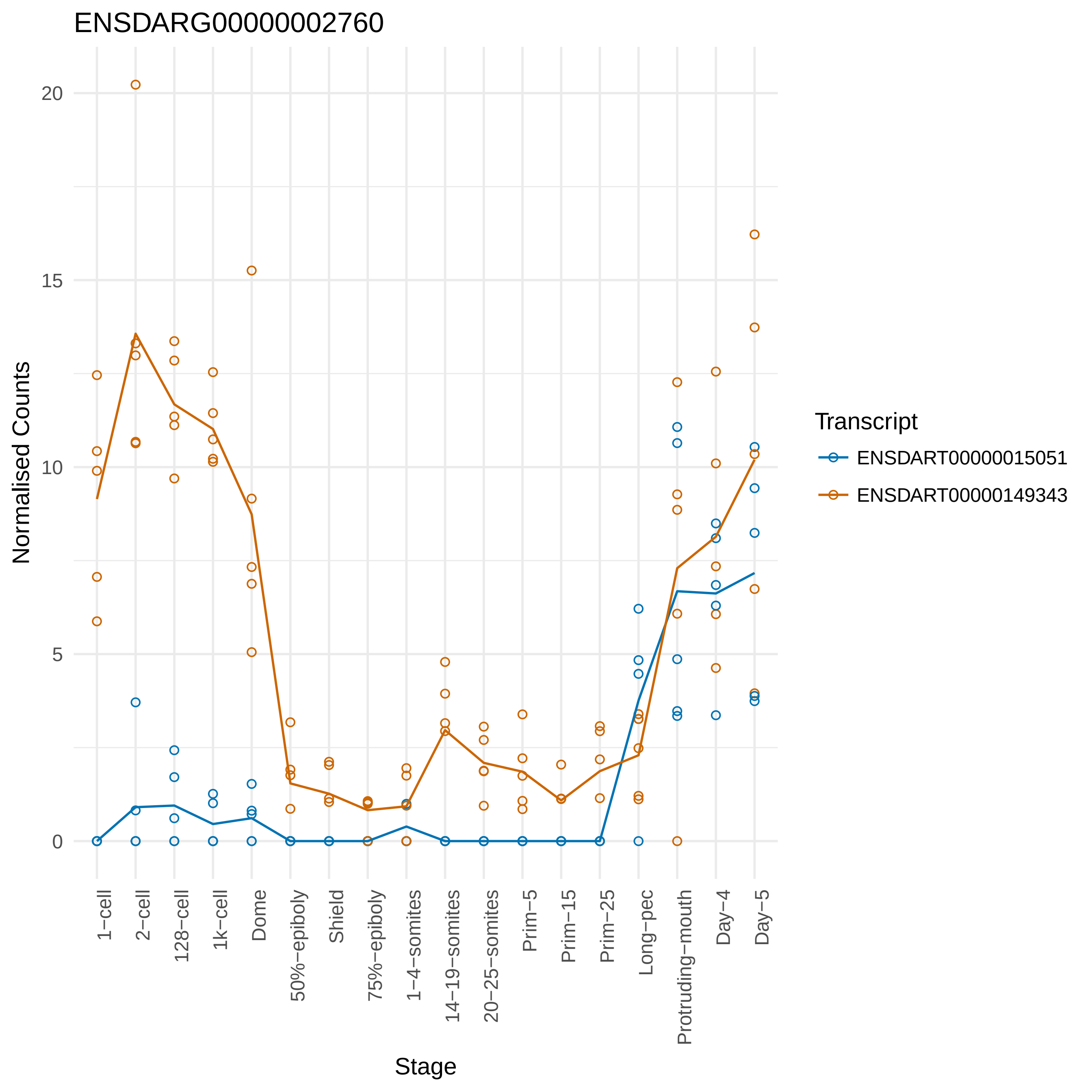
ENSDARG00000002760
Normalised Counts
0
5
10
15
20
Stage
1−cell
2−cell
128−cell
1k−cell
Dome
50%−epiboly
Shield
75%−epiboly
1−4−somites
14−19−somites
20−25−somites
Prim−5
Prim−15
Prim−25
Long−pec
Protruding−mouth
Day−4
Day−5
Transcript
ENSDART00000015051
ENSDART00000149343

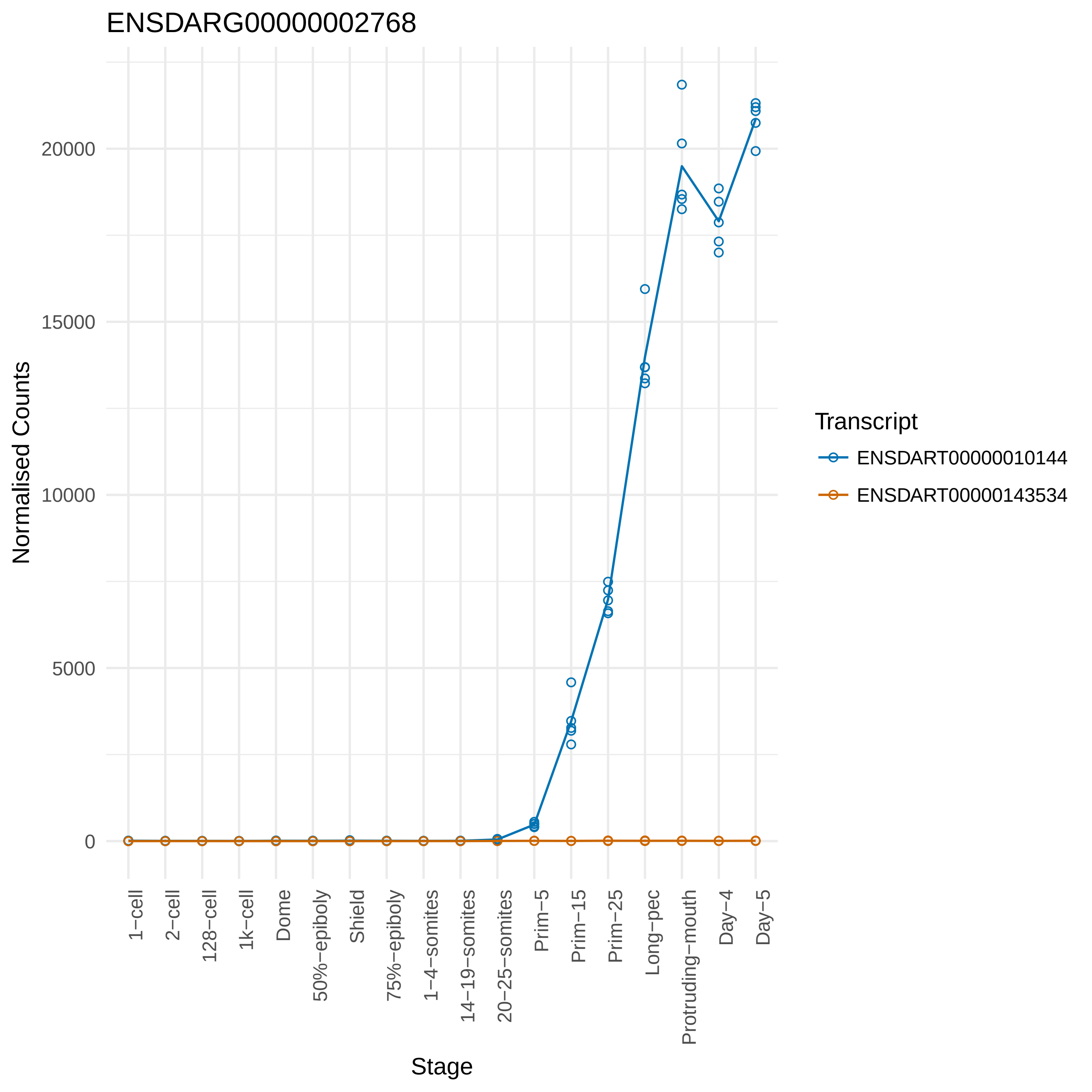

ENSDARG00000002768
Normalised Counts
0
5000
10000
15000
20000
Stage
1−cell
2−cell
128−cell
1k−cell
Dome
50%−epiboly
Shield
75%−epiboly
1−4−somites
14−19−somites
20−25−somites
Prim−5
Prim−15
Prim−25
Long−pec
Protruding−mouth
Day−4
Day−5
Transcript
ENSDART00000010144
ENSDART00000143534

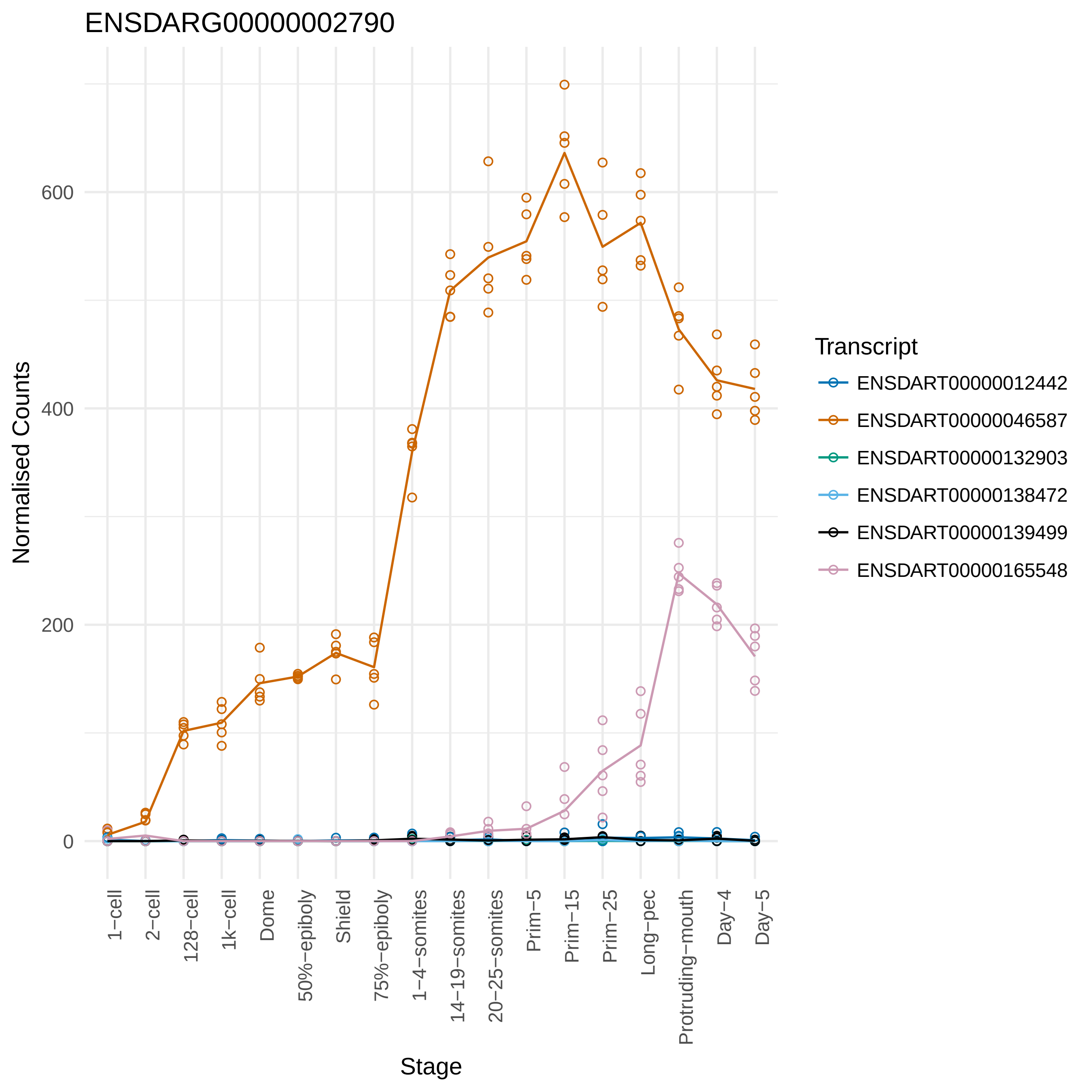

ENSDARG00000002790
Normalised Counts
0
200
400
600
1−cell
2−cell
128−cell
1k−cell
Dome
50%−epiboly
Shield
75%−epiboly
1−4−somites
14−19−somites
20−25−somites
Prim−5
Prim−15
Prim−25
Long−pec
Protruding−mouth
Day−4
Day−5
Stage
Transcript
ENSDART00000012442
ENSDART00000046587
ENSDART00000132903
ENSDART00000138472
ENSDART00000139499
ENSDART00000165548

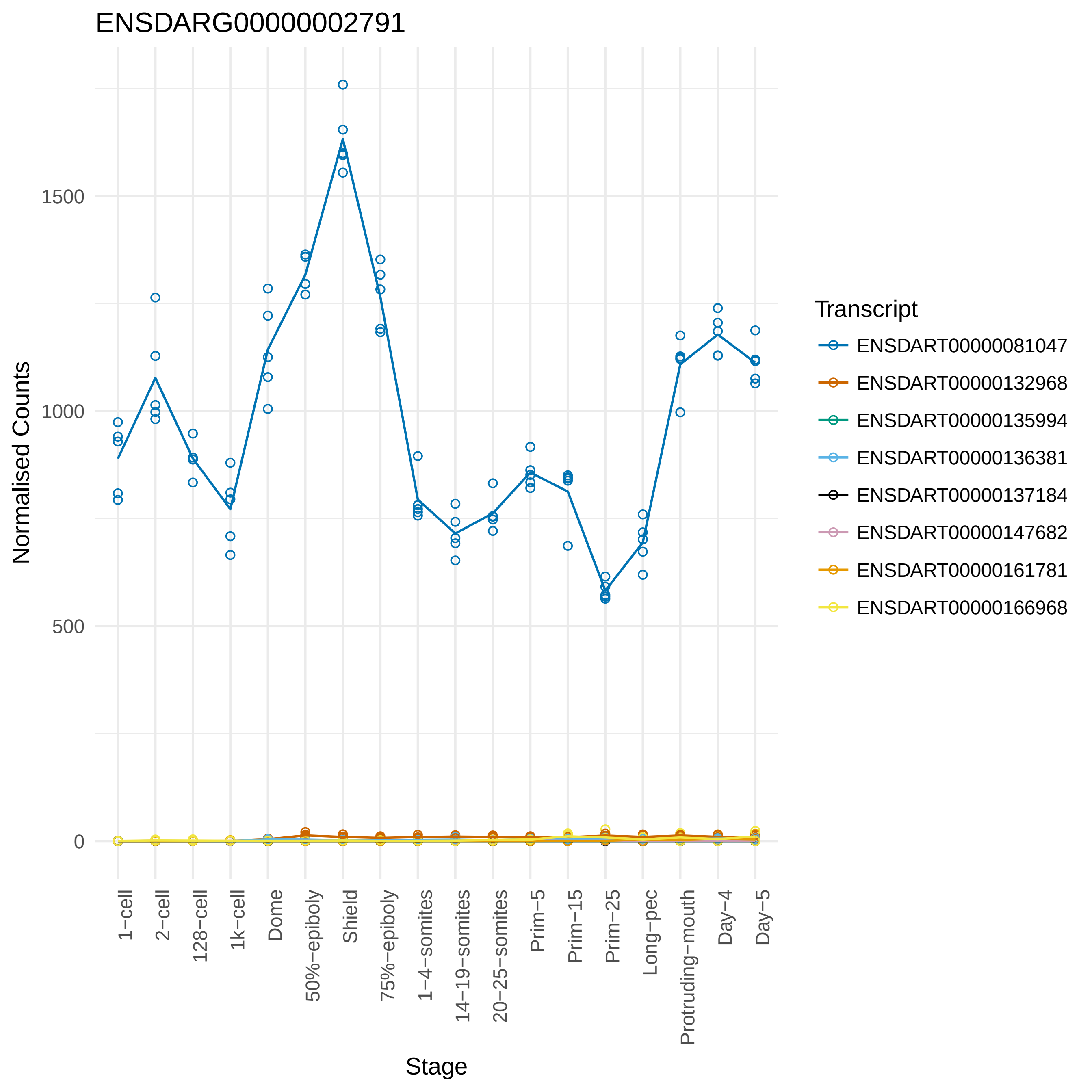
ENSDARG00000002791
Normalised Counts
0
500
1000
1500
Stage
1−cell
2−cell
128−cell
1k−cell
Dome
50%−epiboly
Shield
75%−epiboly
1−4−somites
14−19−somites
20−25−somites
Prim−5
Prim−15
Prim−25
Long−pec
Protruding−mouth
Day−4
Day−5
Transcript
ENSDART00000081047
ENSDART00000132968
ENSDART00000135994
ENSDART00000136381
ENSDART00000137184
ENSDART00000147682
ENSDART00000161781
ENSDART00000166968

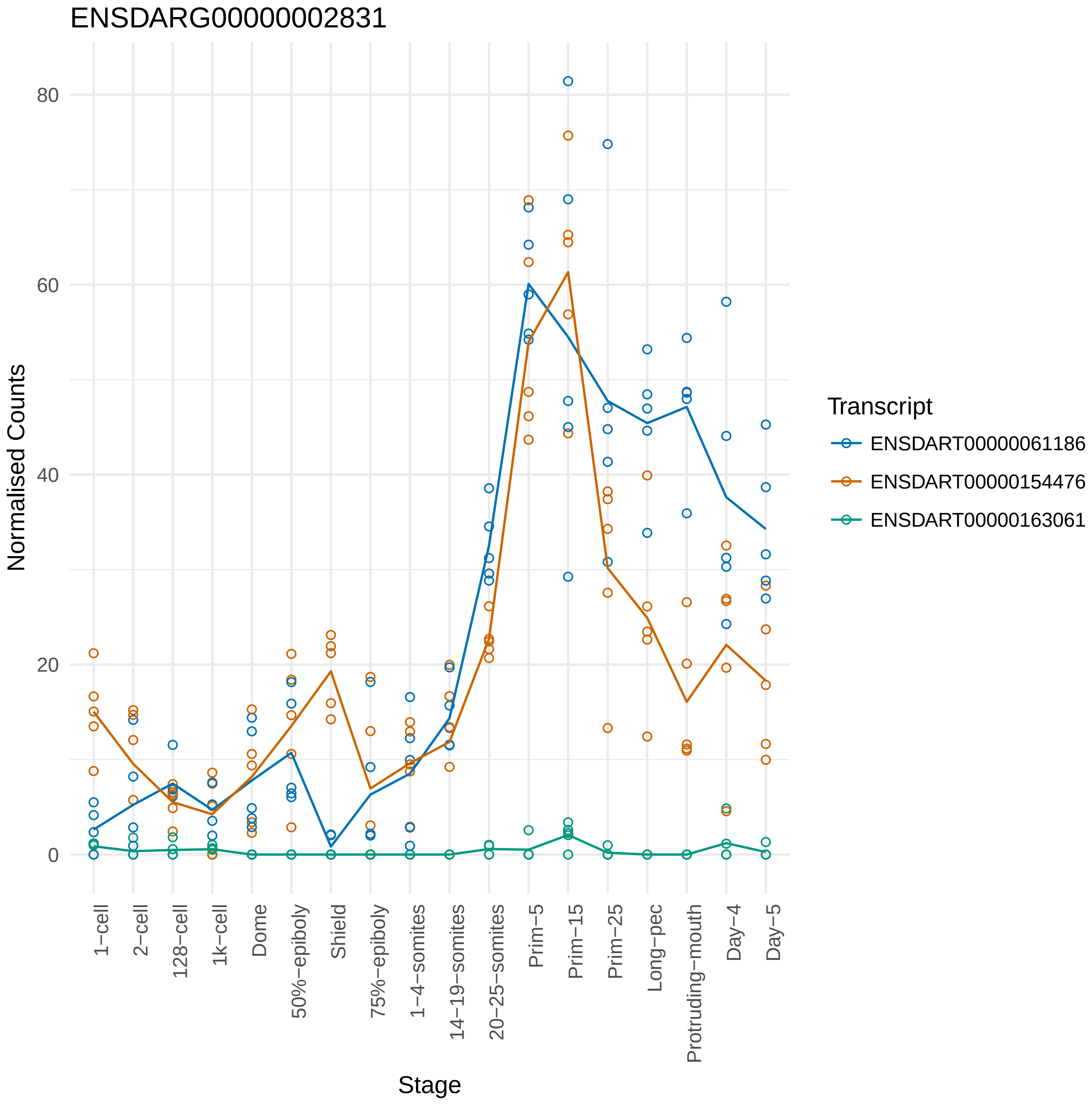

ENSDARG00000002831
Normalised Counts
0
20
40
60
80
1−cell
2−cell
128−cell
1k−cell
Dome
50%−epiboly
Shield
75%−epiboly
1−4−somites
14−19−somites
20−25−somites
Prim−5
Prim−15
Prim−25
Long−pec
Protruding−mouth
Day−4
Day−5
Stage
Transcript
ENSDART00000061186
ENSDART00000154476
ENSDART00000163061

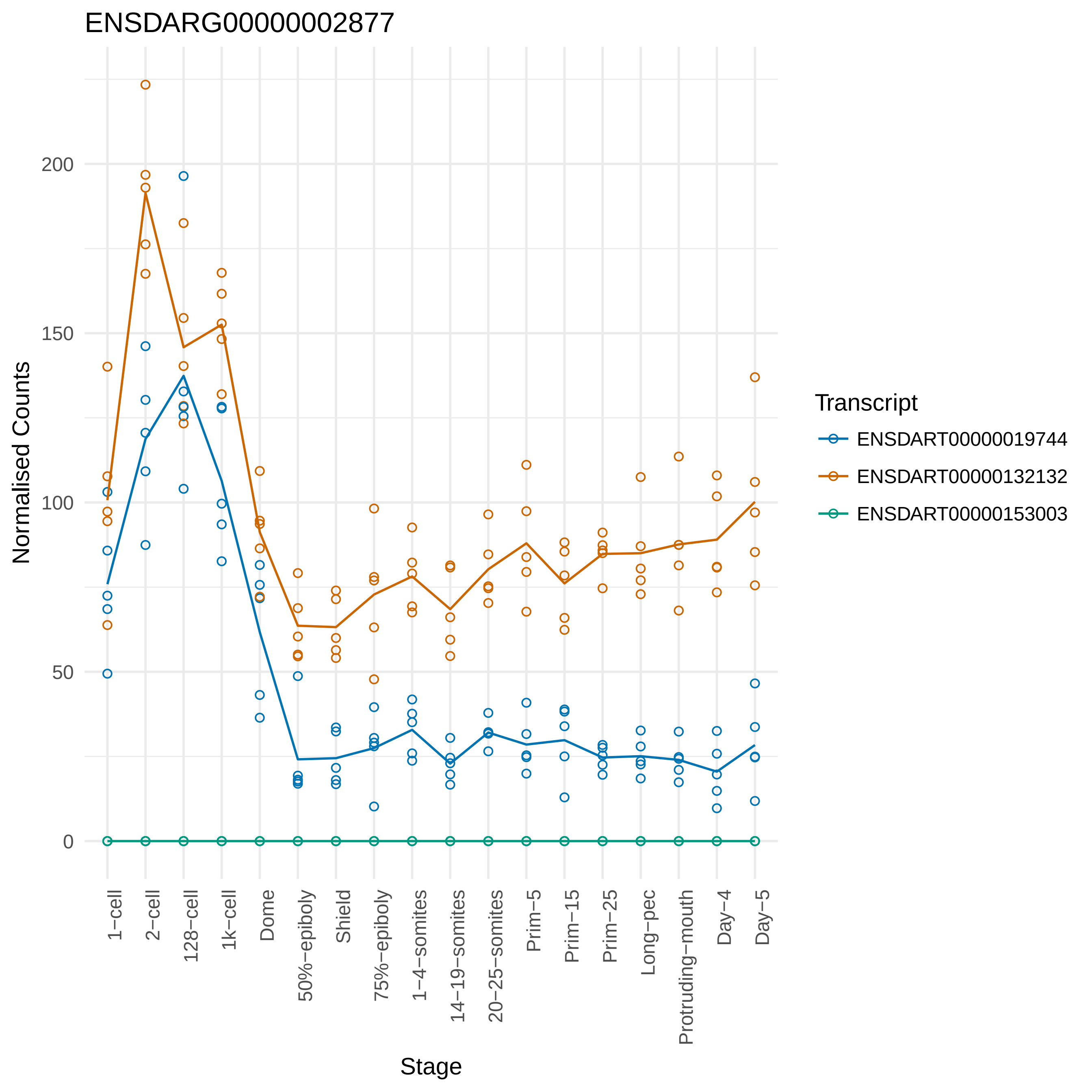
ENSDARG00000002877
Normalised Counts
200
150
100
50
0
1−cell
2−cell
128−cell
1k−cell
Dome
50%−epiboly
Shield
75%−epiboly
1−4−somites
14−19−somites
20−25−somites
Prim−5
Prim−15
Prim−25
Long−pec
Protruding−mouth
Day−4
Day−5
Stage
Transcript
ENSDART00000019744
ENSDART00000132132
ENSDART00000153003

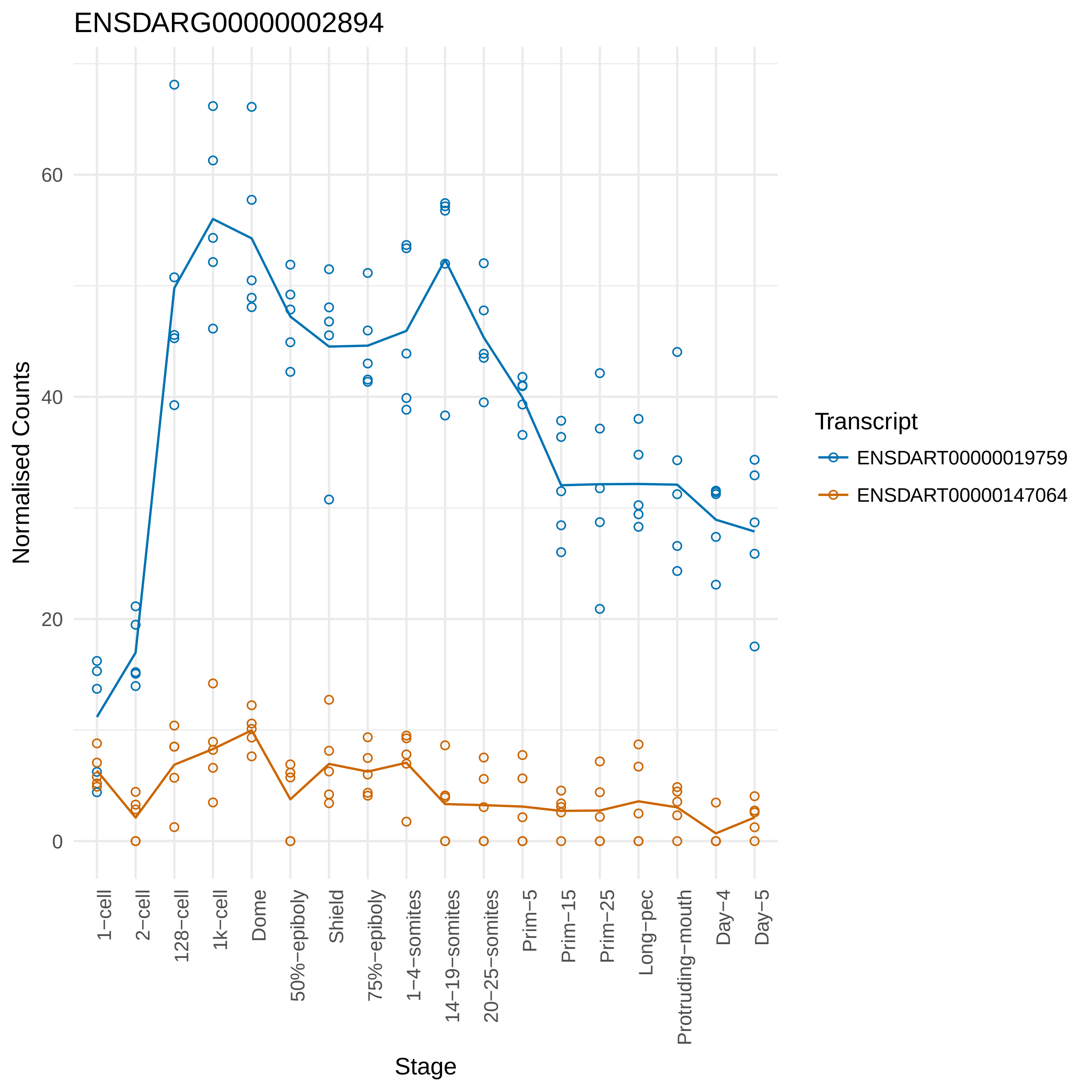
ENSDARG00000002894
Normalised Counts
0
20
40
60
Stage
1−cell
2−cell
128−cell
1k−cell
Dome
50%−epiboly
Shield
75%−epiboly
1−4−somites
14−19−somites
20−25−somites
Prim−5
Prim−15
Prim−25
Long−pec
Protruding−mouth
Day−4
Day−5
Transcript
ENSDART00000019759
ENSDART00000147064

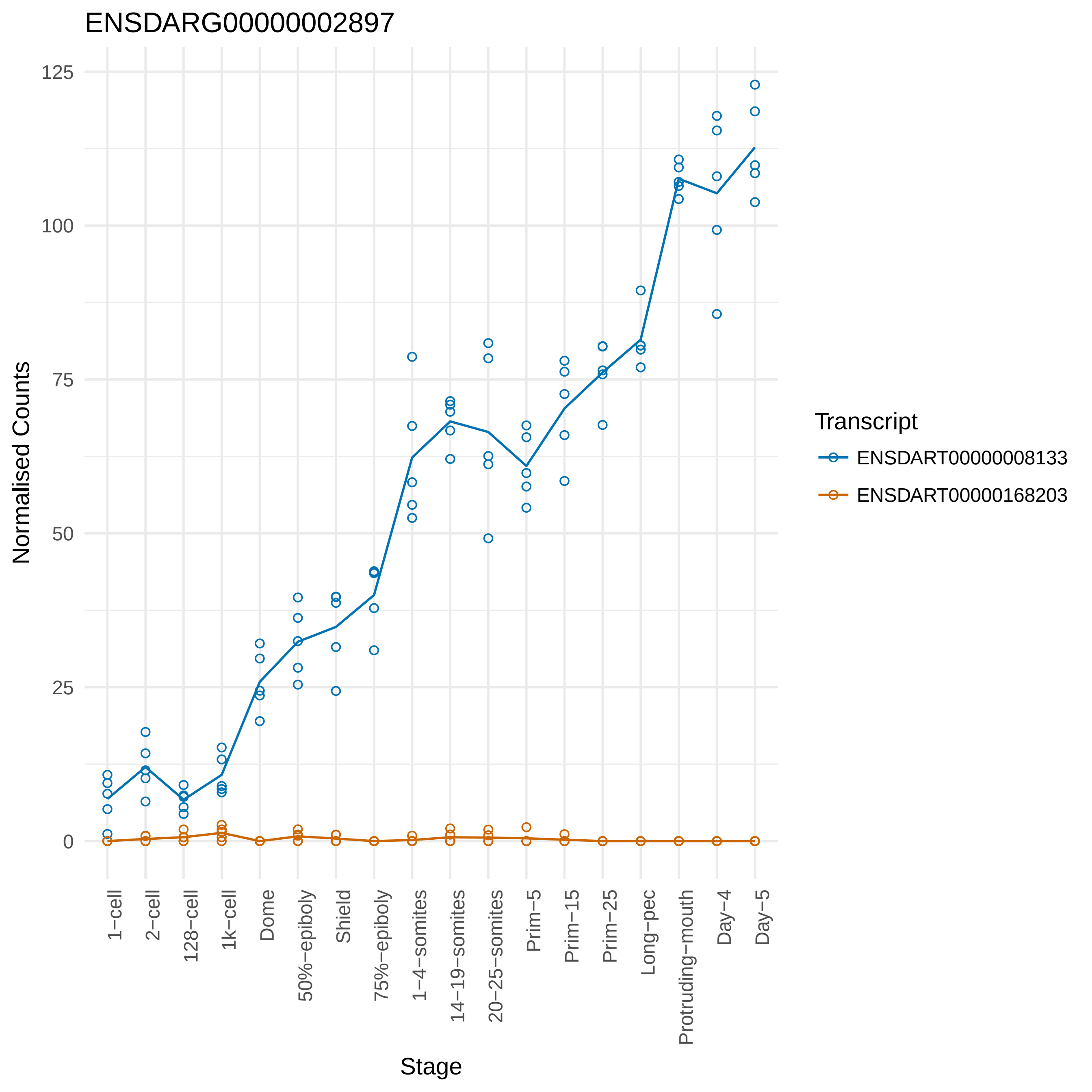

ENSDARG00000002897
Normalised Counts
125
100
75
50
25
0
1−cell
2−cell
128−cell
1k−cell
Dome
50%−epiboly
Shield
75%−epiboly
1−4−somites
14−19−somites
20−25−somites
Prim−5
Prim−15
Prim−25
Long−pec
Protruding−mouth
Day−4
Day−5
Stage
Transcript
ENSDART00000008133
ENSDART00000168203

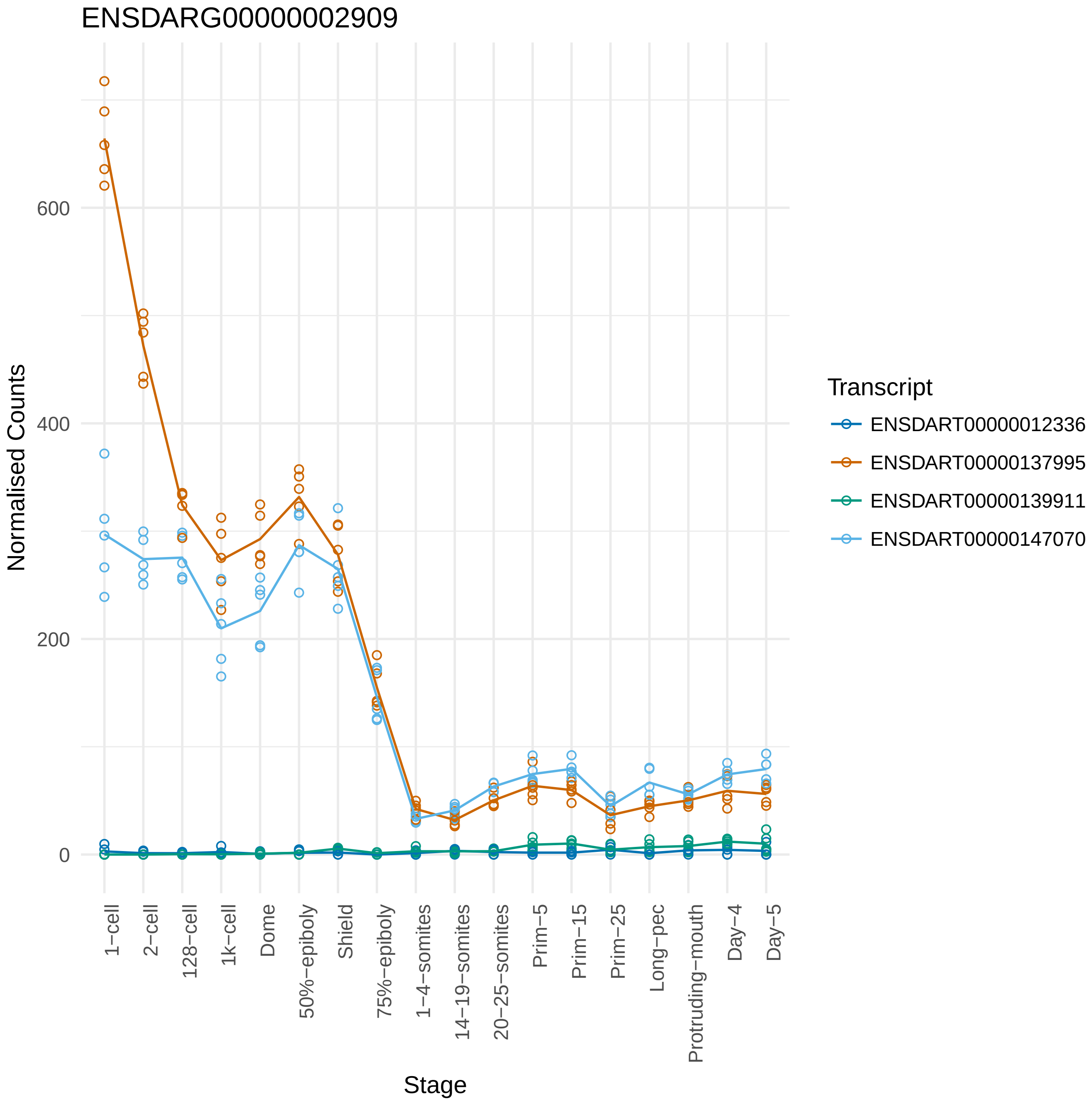
ENSDARG00000002909
Normalised Counts
600
400
200
0
Stage
1−cell
2−cell
128−cell
1k−cell
Dome
50%−epiboly
Shield
75%−epiboly
1−4−somites
14−19−somites
20−25−somites
Prim−5
Prim−15
Prim−25
Long−pec
Protruding−mouth
Day−4
Day−5
Transcript
ENSDART00000012336
ENSDART00000137995
ENSDART00000139911
ENSDART00000147070

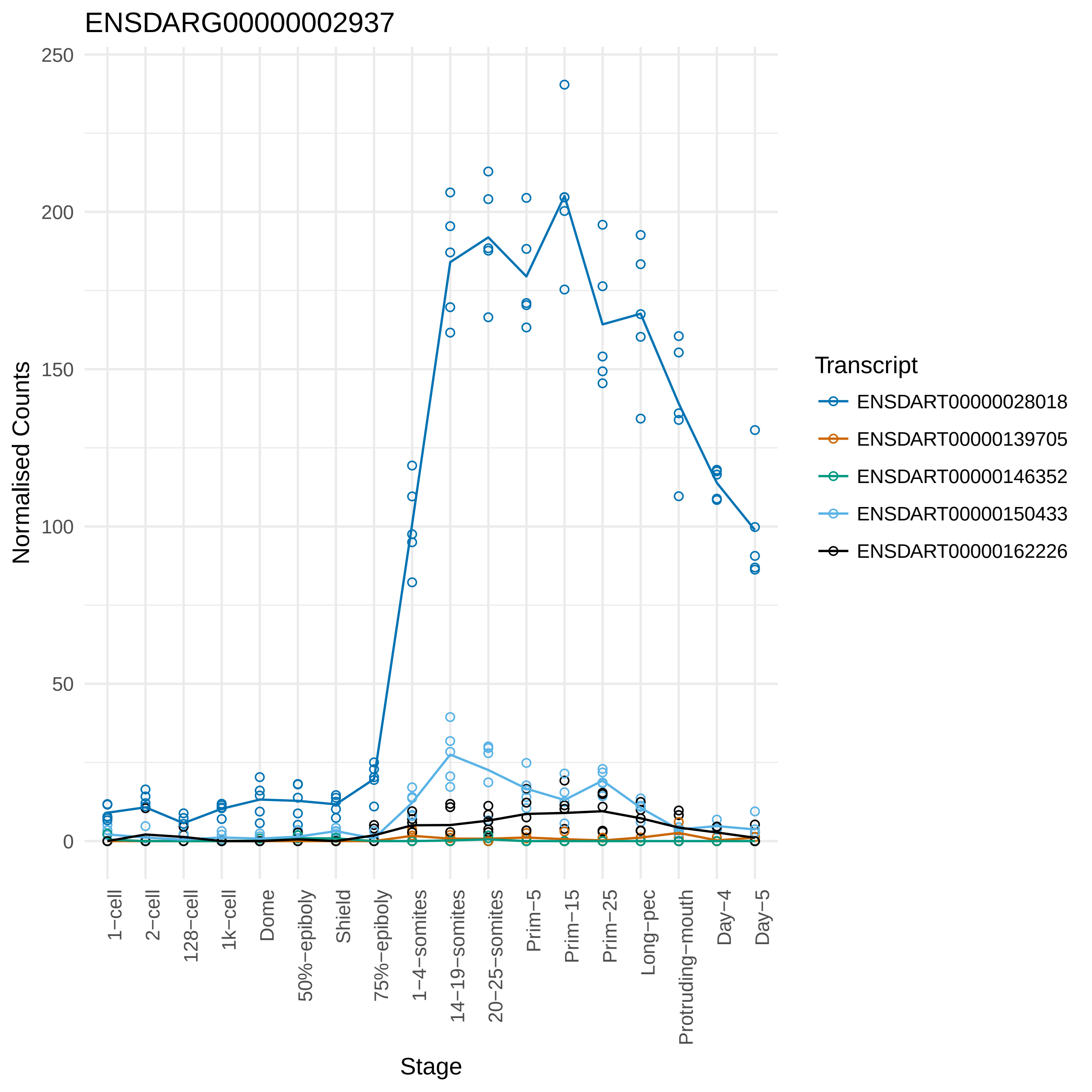

ENSDARG00000002937
Normalised Counts
0
50
100
150
200
250
1−cell
2−cell
128−cell
1k−cell
Dome
50%−epiboly
Shield
75%−epiboly
1−4−somites
14−19−somites
20−25−somites
Prim−5
Prim−15
Prim−25
Long−pec
Protruding−mouth
Day−4
Day−5
Stage
Transcript
ENSDART00000028018
ENSDART00000139705
ENSDART00000146352
ENSDART00000150433
ENSDART00000162226

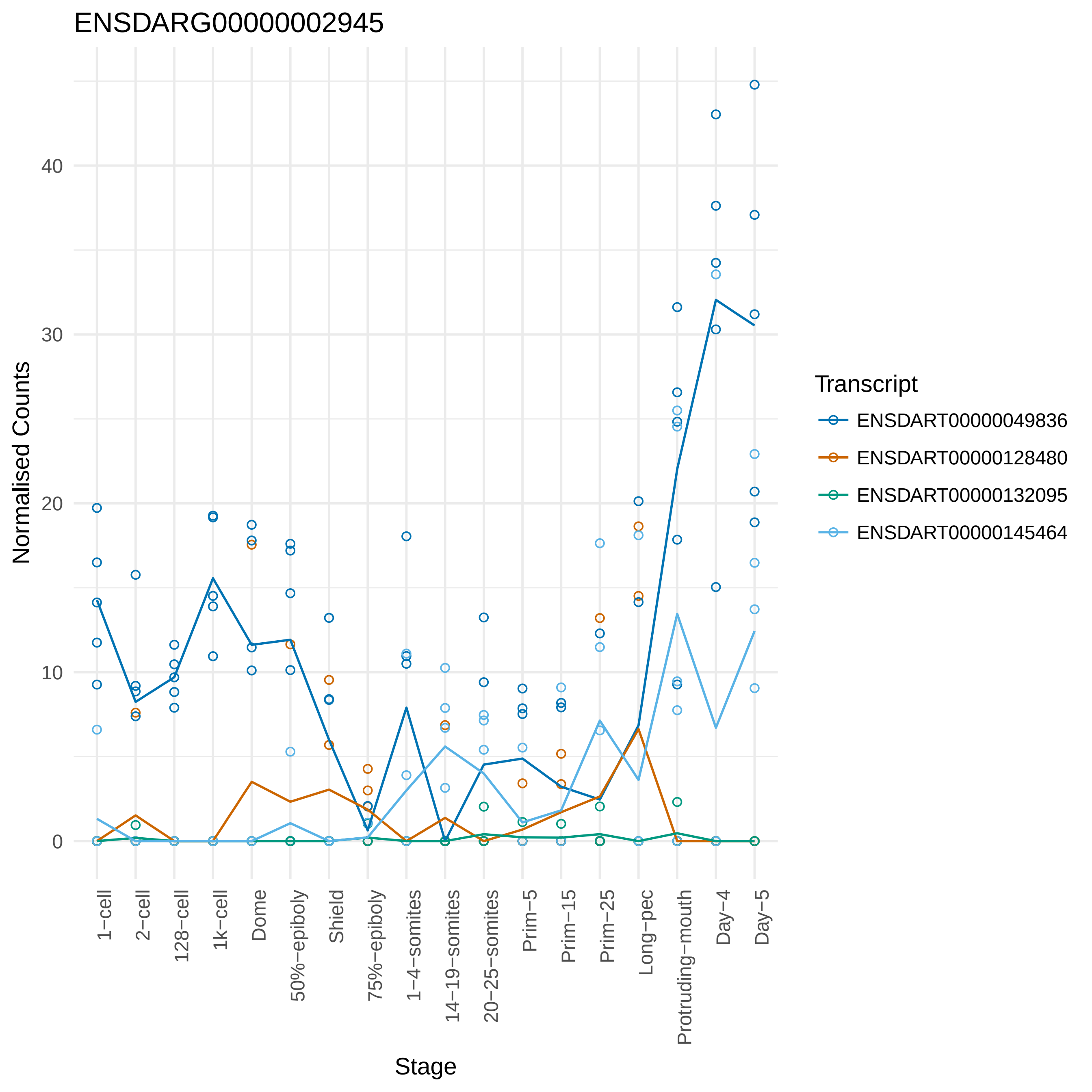
ENSDARG00000002945
Normalised Counts
0
10
20
30
40
1−cell
2−cell
128−cell
1k−cell
Dome
50%−epiboly
Shield
75%−epiboly
1−4−somites
14−19−somites
20−25−somites
Prim−5
Prim−15
Prim−25
Long−pec
Protruding−mouth
Day−4
Day−5
Stage
Transcript
ENSDART00000049836
ENSDART00000128480
ENSDART00000132095
ENSDART00000145464

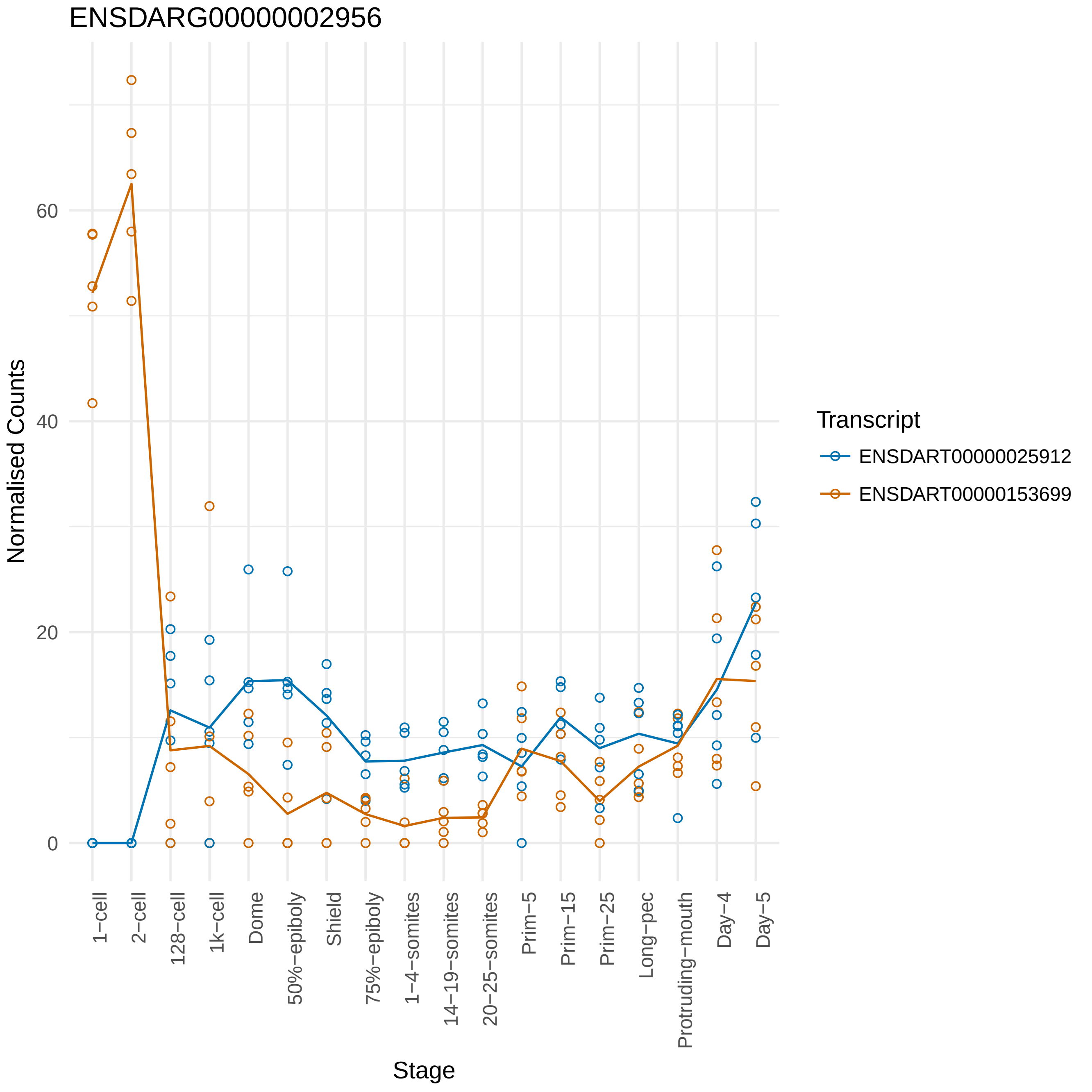

ENSDARG00000002956
Normalised Counts
0
20
40
60
1−cell
2−cell
128−cell
1k−cell
Dome
50%−epiboly
Shield
75%−epiboly
1−4−somites
14−19−somites
20−25−somites
Prim−5
Prim−15
Prim−25
Long−pec
Protruding−mouth
Day−4
Day−5
Stage
Transcript
ENSDART00000025912
ENSDART00000153699

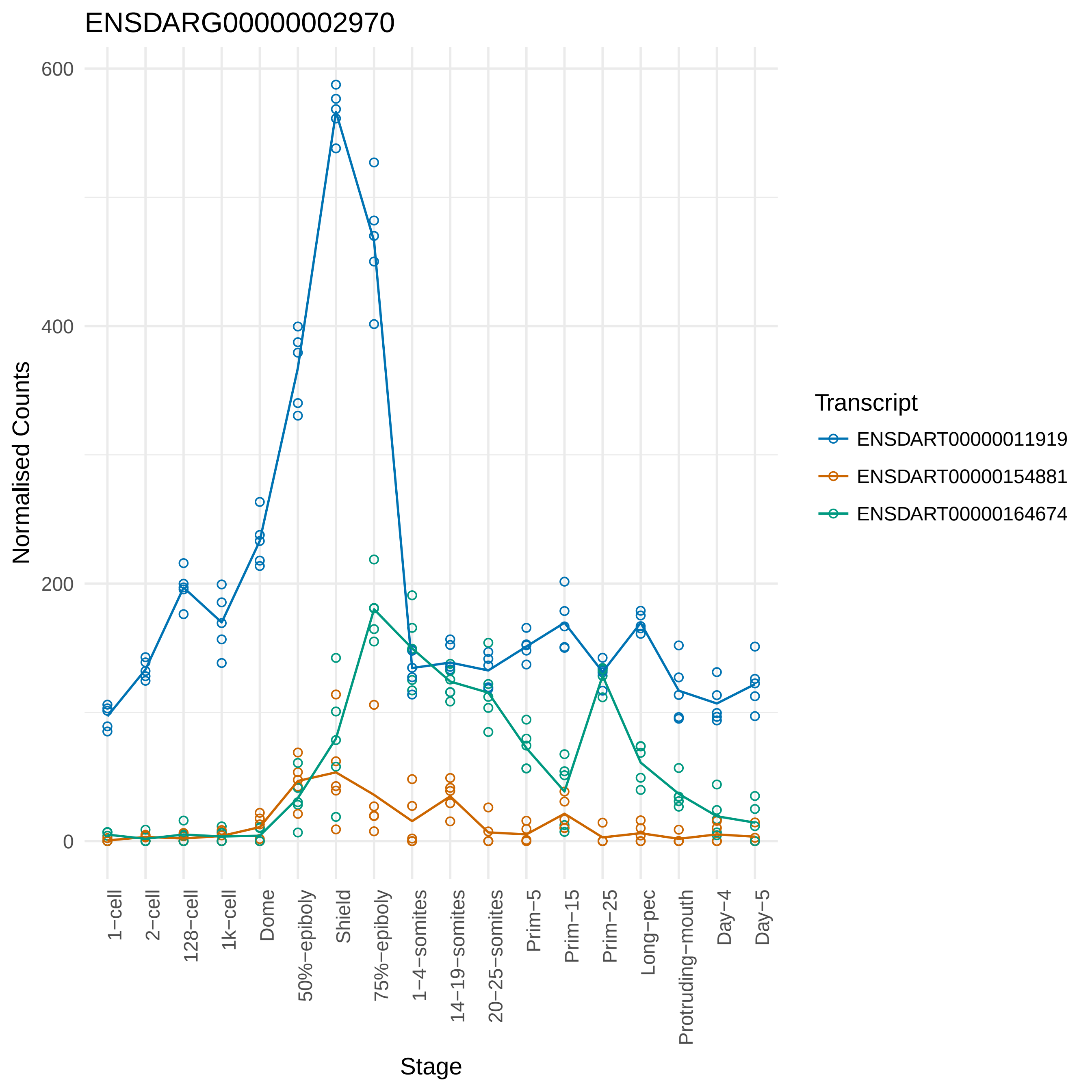

ENSDARG00000002970
Normalised Counts
600
400
200
0
1−cell
2−cell
128−cell
1k−cell
Dome
50%−epiboly
Shield
75%−epiboly
1−4−somites
14−19−somites
20−25−somites
Prim−5
Prim−15
Prim−25
Long−pec
Protruding−mouth
Day−4
Day−5
Stage
Transcript
ENSDART00000011919
ENSDART00000154881
ENSDART00000164674

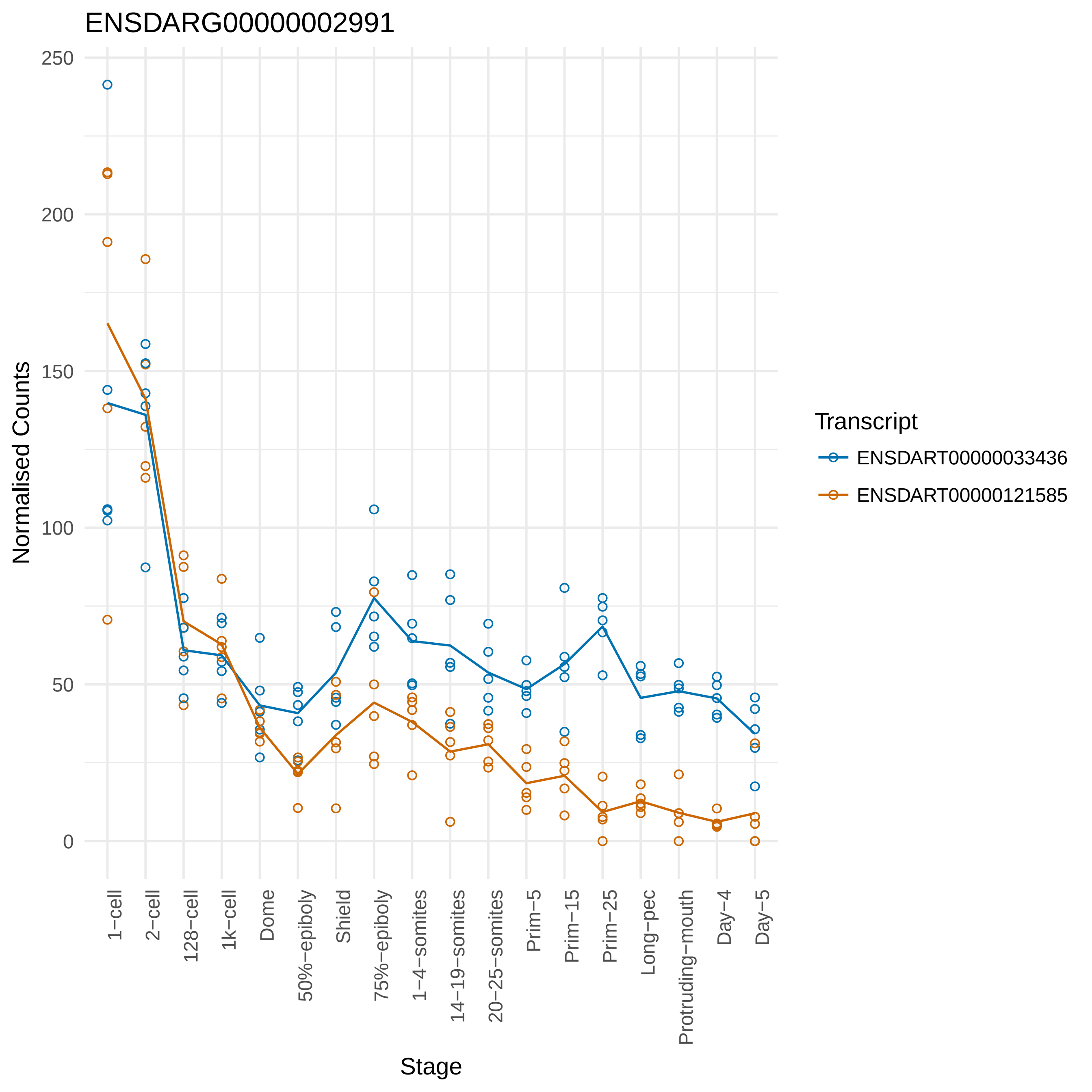
ENSDARG00000002991
Normalised Counts
250
200
150
100
50
0
1−cell
2−cell
128−cell
1k−cell
Dome
50%−epiboly
Shield
75%−epiboly
1−4−somites
14−19−somites
20−25−somites
Prim−5
Prim−15
Prim−25
Long−pec
Protruding−mouth
Day−4
Day−5
Stage
Transcript
ENSDART00000033436
ENSDART00000121585

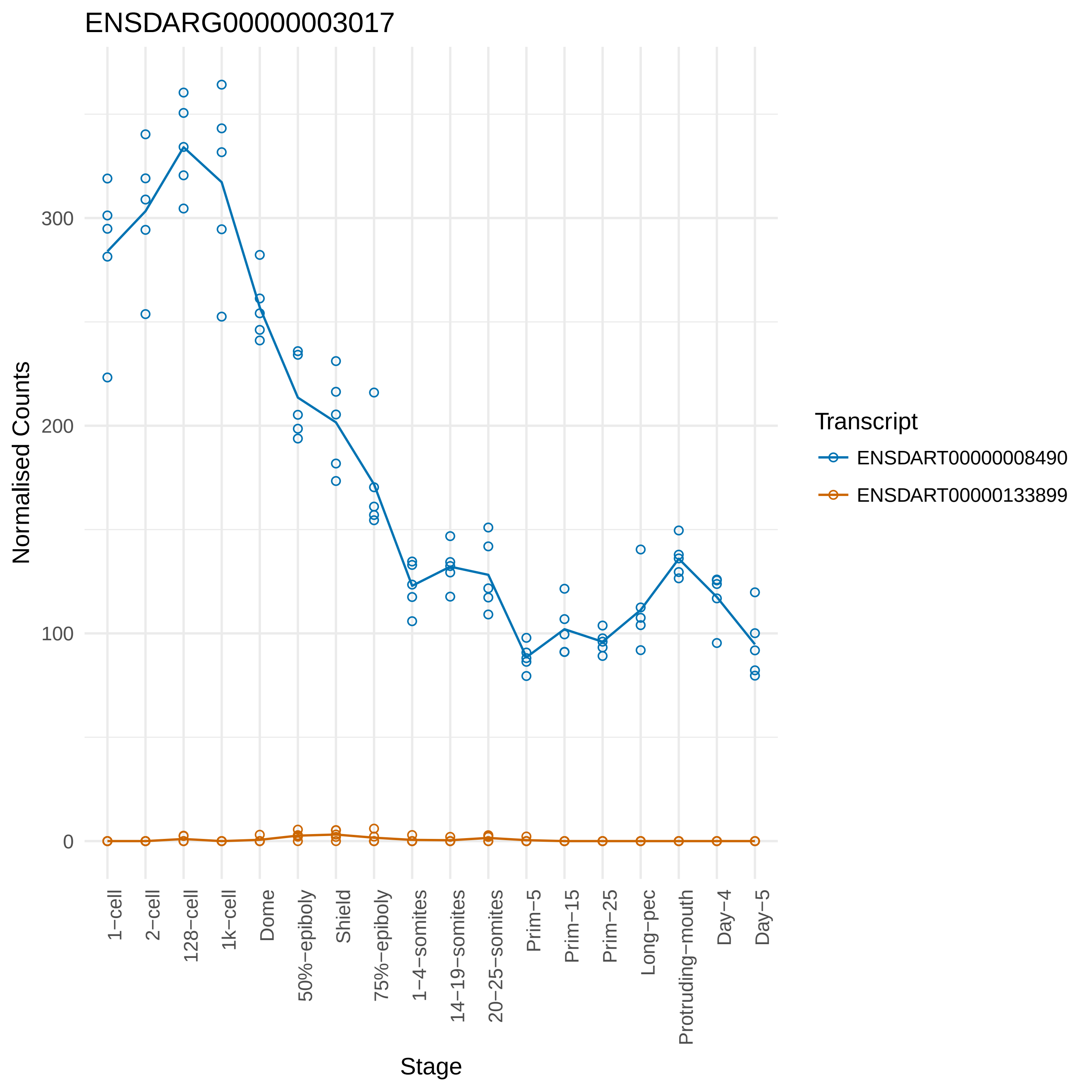

ENSDARG00000003017
Normalised Counts
300
200
100
0
1−cell
2−cell
128−cell
1k−cell
Dome
50%−epiboly
Shield
75%−epiboly
1−4−somites
14−19−somites
20−25−somites
Prim−5
Prim−15
Prim−25
Long−pec
Protruding−mouth
Day−4
Day−5
Stage
Transcript
ENSDART00000008490
ENSDART00000133899

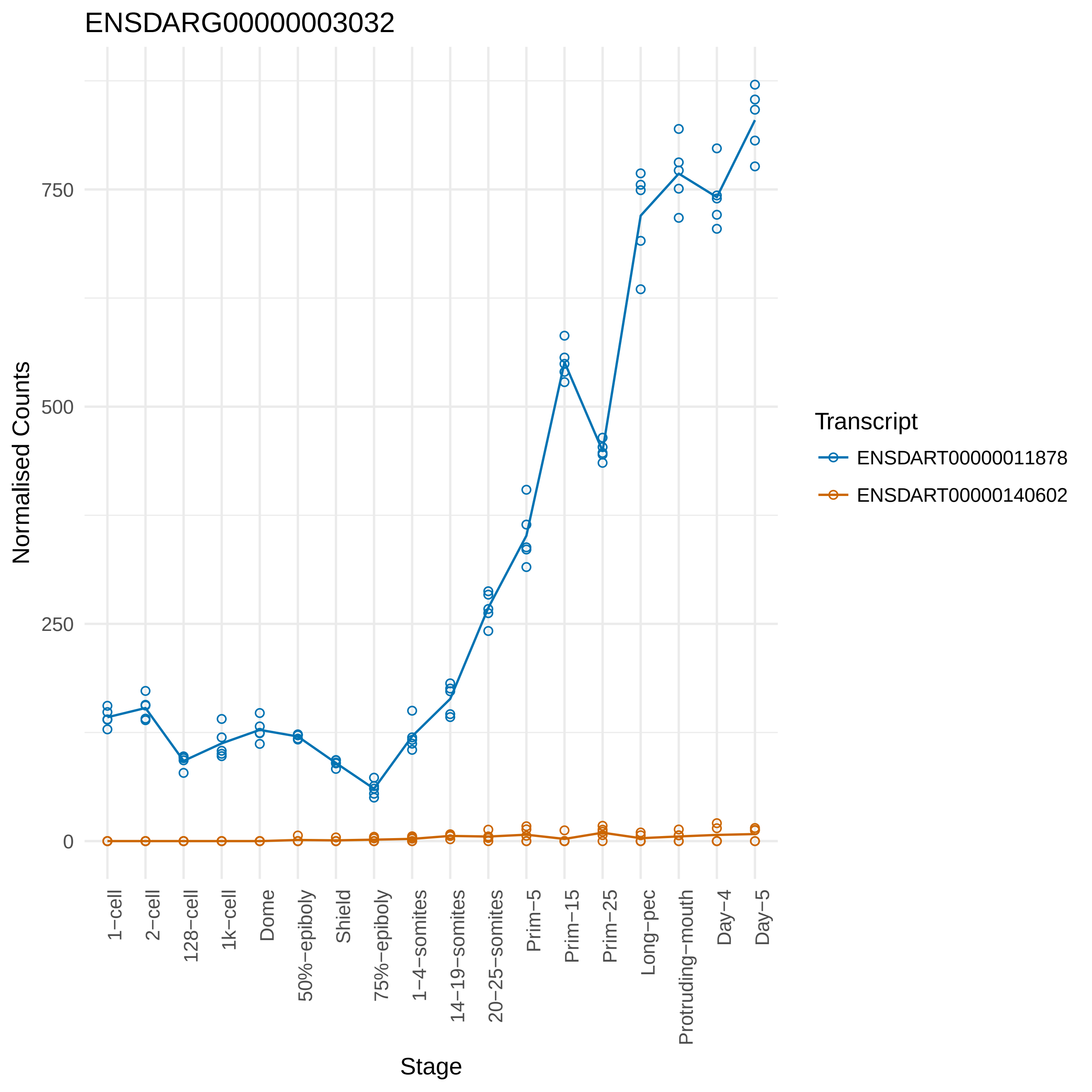

ENSDARG00000003032
Normalised Counts
750
500
250
0
1−cell
2−cell
128−cell
1k−cell
Dome
50%−epiboly
Shield
75%−epiboly
1−4−somites
14−19−somites
20−25−somites
Prim−5
Prim−15
Prim−25
Long−pec
Protruding−mouth
Day−4
Day−5
Stage
Transcript
ENSDART00000011878
ENSDART00000140602

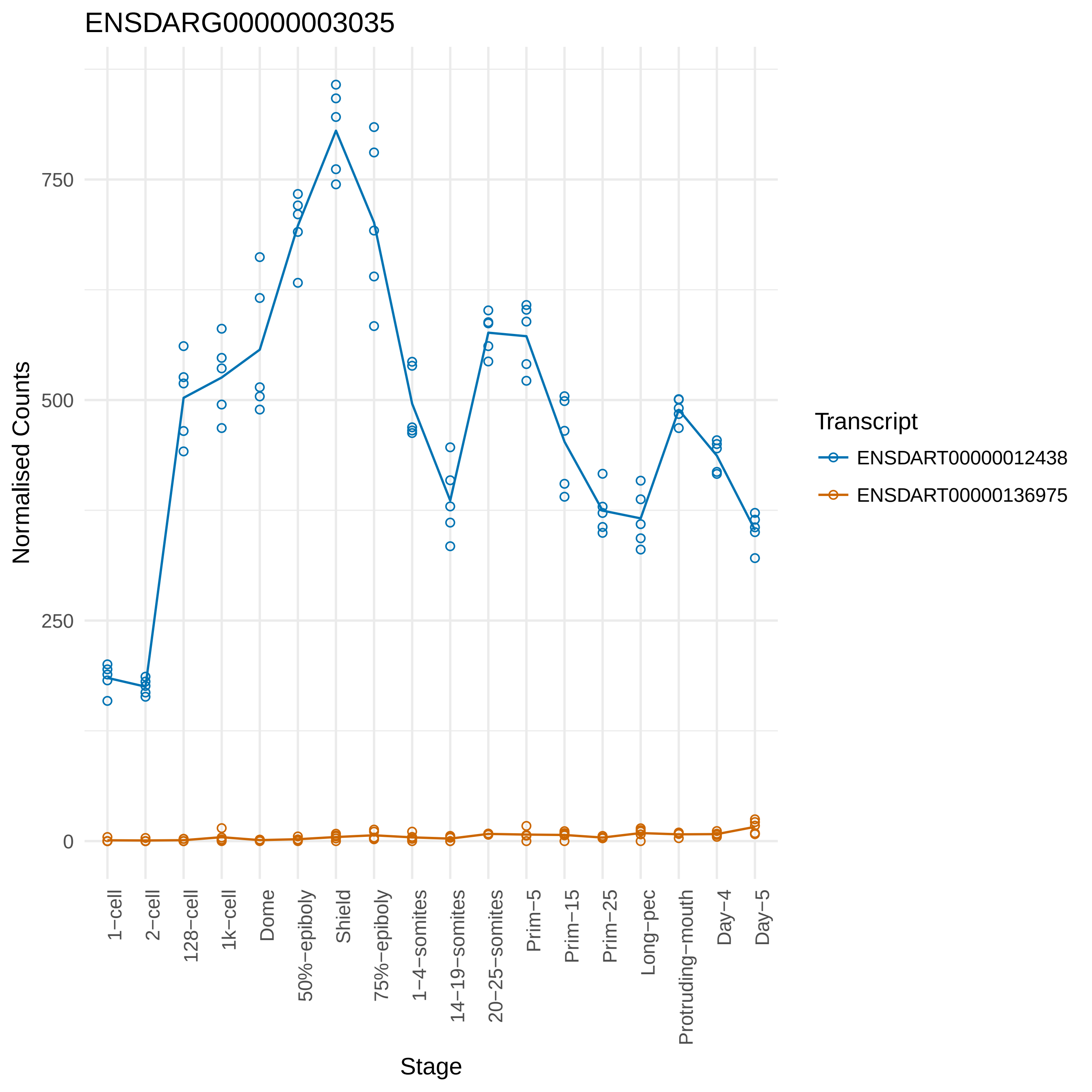

ENSDARG00000003035
Normalised Counts
750
500
250
0
1−cell
2−cell
128−cell
1k−cell
Dome
50%−epiboly
Shield
75%−epiboly
1−4−somites
14−19−somites
20−25−somites
Prim−5
Prim−15
Prim−25
Long−pec
Protruding−mouth
Day−4
Day−5
Stage
Transcript
ENSDART00000012438
ENSDART00000136975

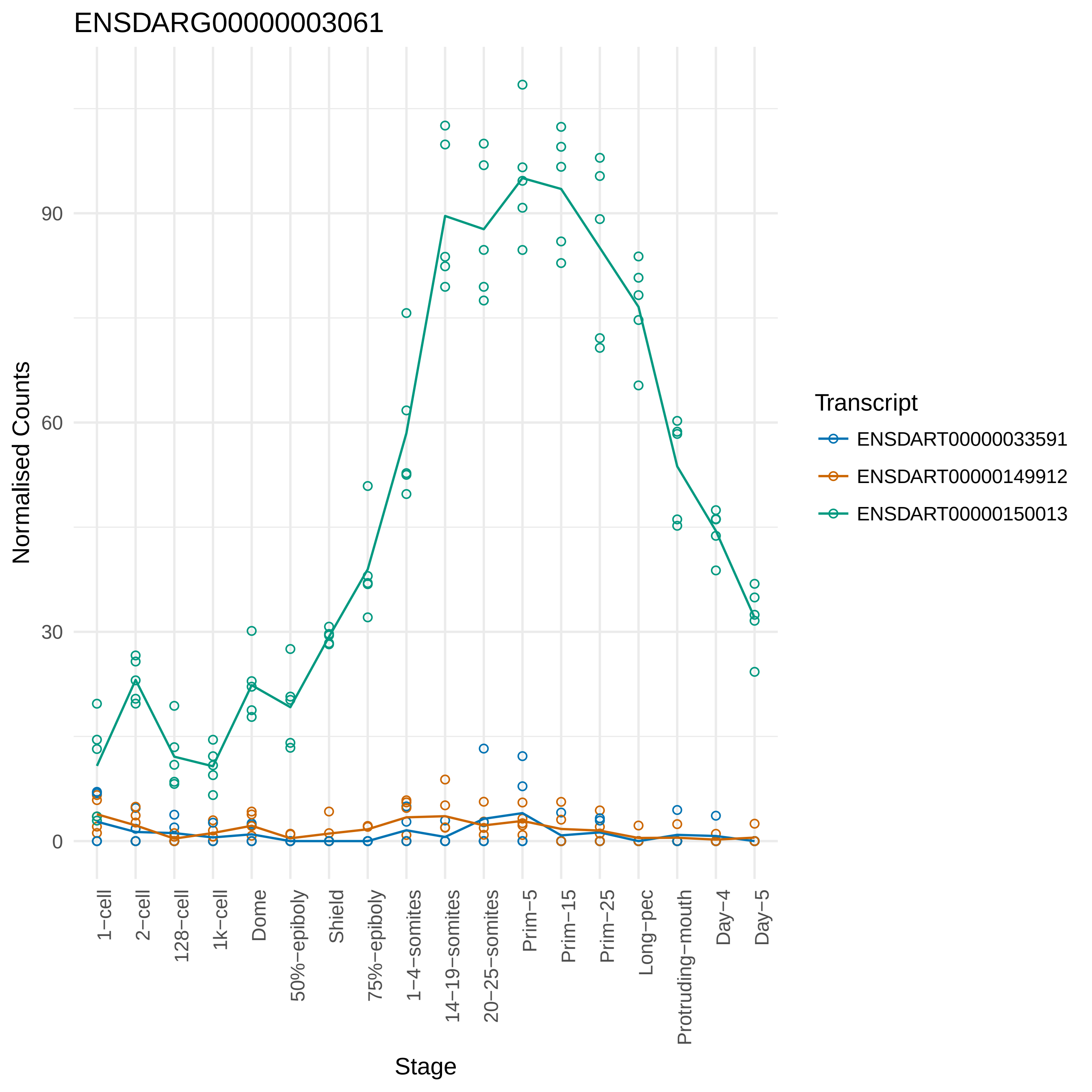

ENSDARG00000003061
Normalised Counts
0
30
60
90
Stage
1−cell
2−cell
128−cell
1k−cell
Dome
50%−epiboly
Shield
75%−epiboly
1−4−somites
14−19−somites
20−25−somites
Prim−5
Prim−15
Prim−25
Long−pec
Protruding−mouth
Day−4
Day−5
Transcript
ENSDART00000033591
ENSDART00000149912
ENSDART00000150013

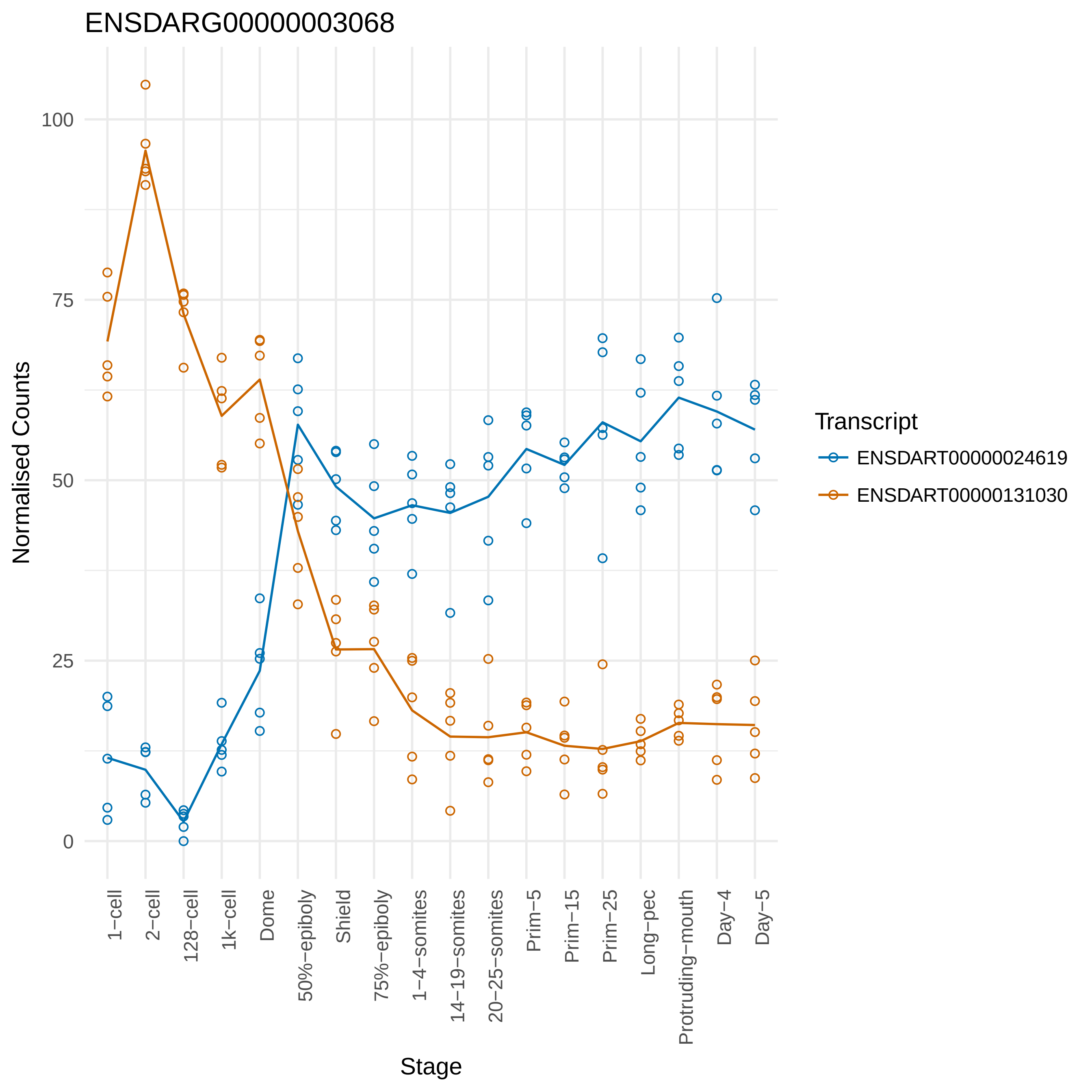

ENSDARG00000003068
Normalised Counts
0
25
50
75
100
1−cell
2−cell
128−cell
1k−cell
Dome
50%−epiboly
Shield
75%−epiboly
1−4−somites
14−19−somites
20−25−somites
Prim−5
Prim−15
Prim−25
Long−pec
Protruding−mouth
Day−4
Day−5
Stage
Transcript
ENSDART00000024619
ENSDART00000131030

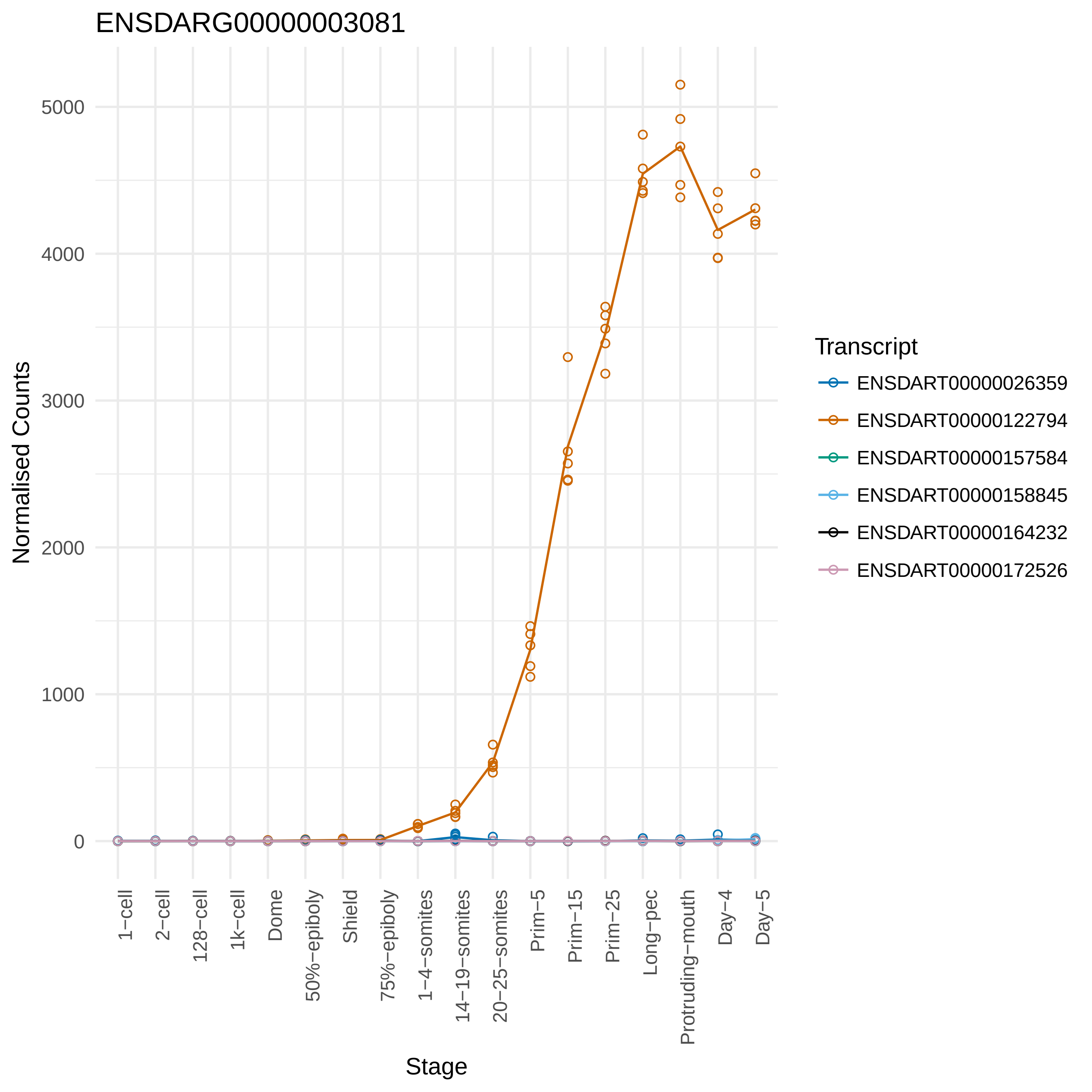

ENSDARG00000003081
Normalised Counts
0
1000
2000
3000
4000
5000
1−cell
2−cell
128−cell
1k−cell
Dome
50%−epiboly
Shield
75%−epiboly
1−4−somites
14−19−somites
20−25−somites
Prim−5
Prim−15
Prim−25
Long−pec
Protruding−mouth
Day−4
Day−5
Stage
Transcript
ENSDART00000026359
ENSDART00000122794
ENSDART00000157584
ENSDART00000158845
ENSDART00000164232
ENSDART00000172526

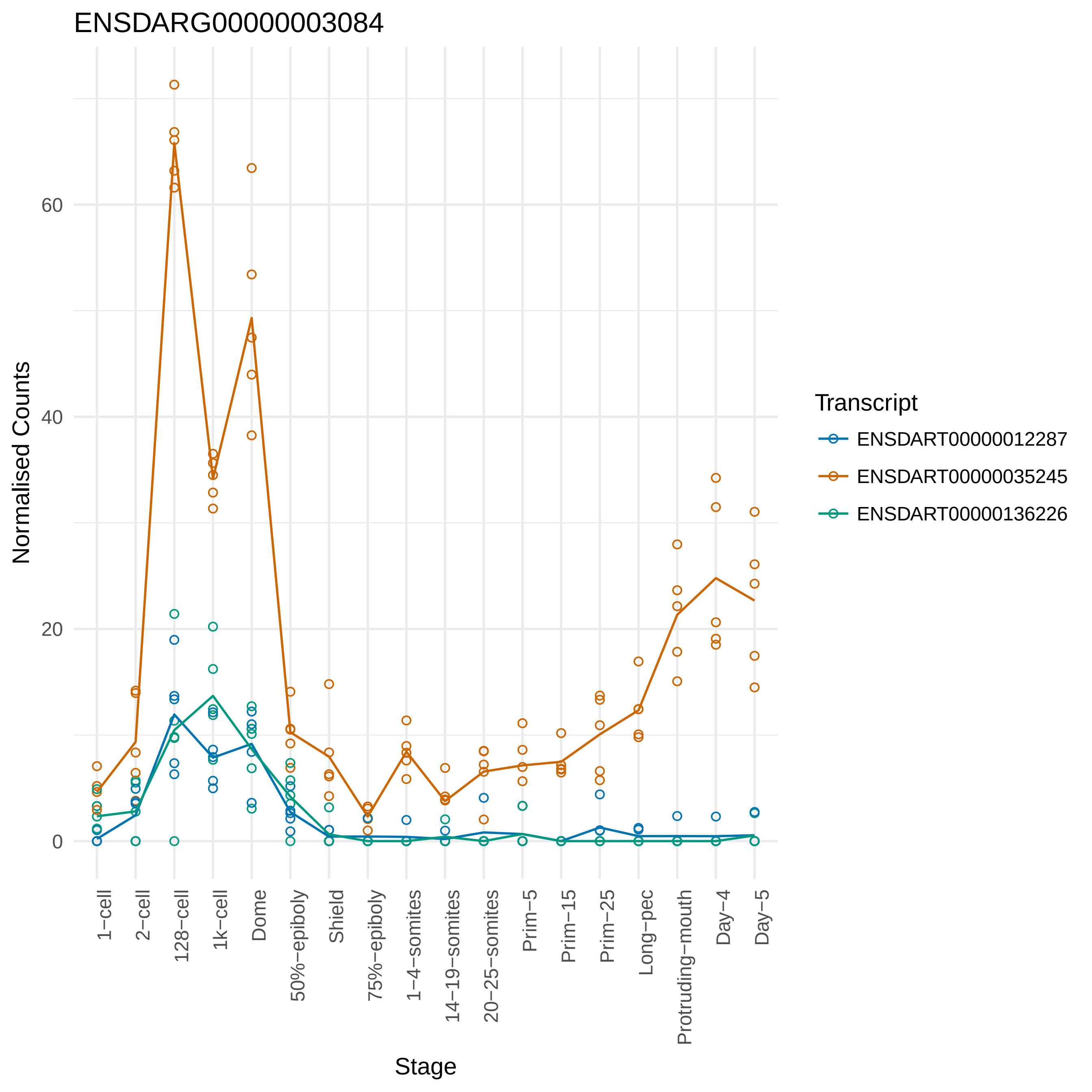
ENSDARG00000003084
Normalised Counts
0
20
40
60
Stage
1−cell
2−cell
128−cell
1k−cell
Dome
50%−epiboly
Shield
75%−epiboly
1−4−somites
14−19−somites
20−25−somites
Prim−5
Prim−15
Prim−25
Long−pec
Protruding−mouth
Day−4
Day−5
Transcript
ENSDART00000012287
ENSDART00000035245
ENSDART00000136226

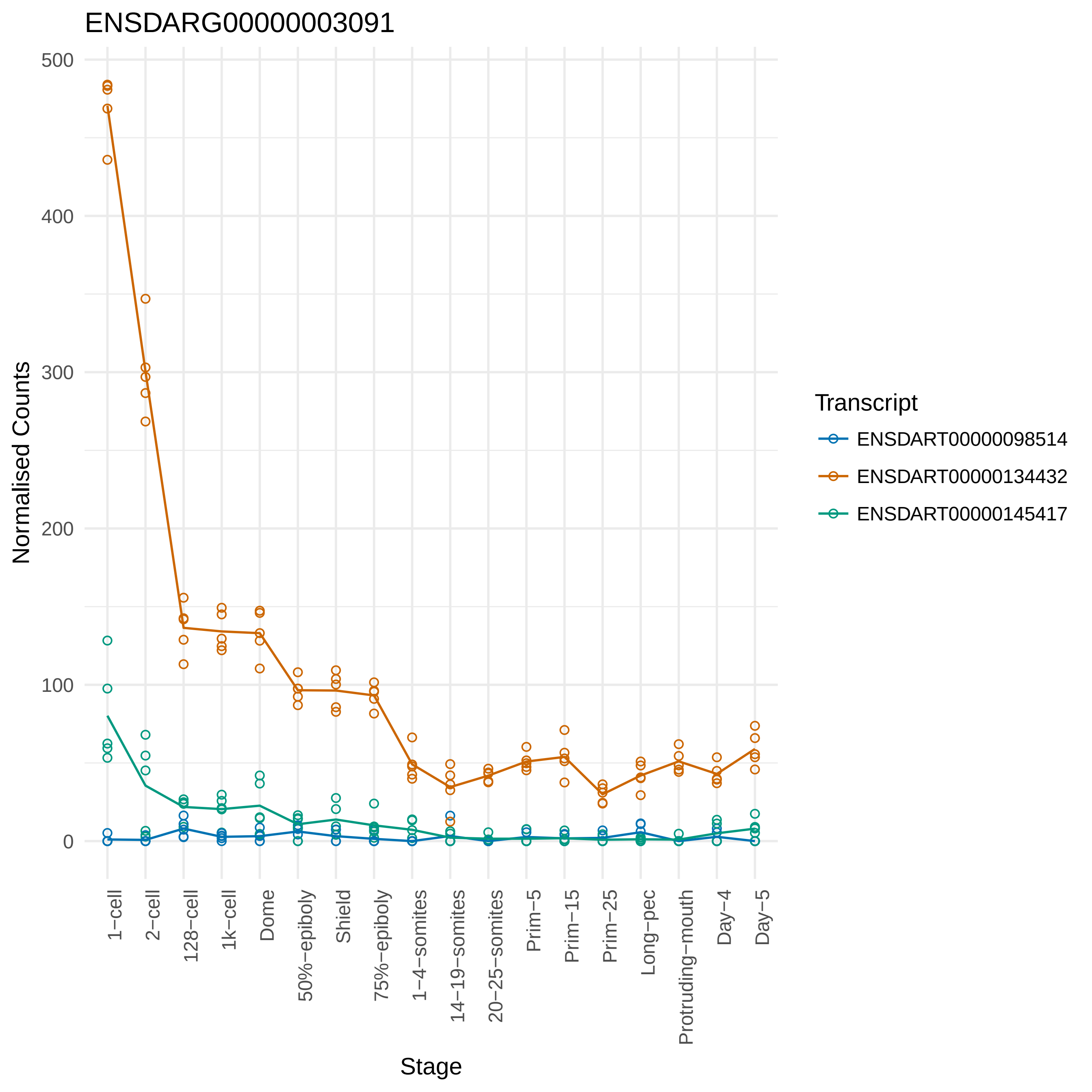
ENSDARG00000003091
Normalised Counts
0
100
200
300
400
500
Stage
1−cell
2−cell
128−cell
1k−cell
Dome
50%−epiboly
Shield
75%−epiboly
1−4−somites
14−19−somites
20−25−somites
Prim−5
Prim−15
Prim−25
Long−pec
Protruding−mouth
Day−4
Day−5
Transcript
ENSDART00000098514
ENSDART00000134432
ENSDART00000145417

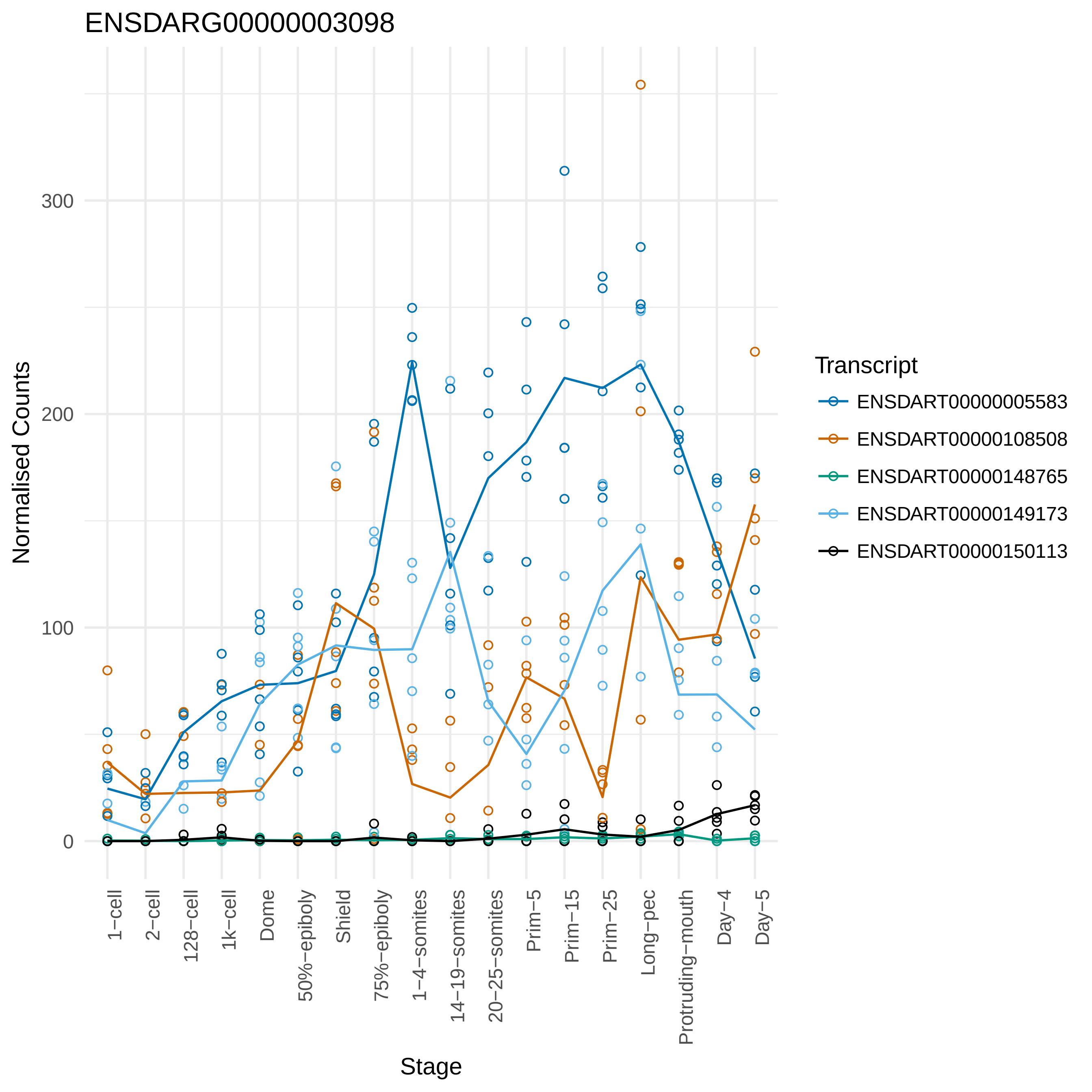

ENSDARG00000003098
Normalised Counts
300
200
100
0
1−cell
2−cell
128−cell
1k−cell
Dome
50%−epiboly
Shield
75%−epiboly
1−4−somites
14−19−somites
20−25−somites
Prim−5
Prim−15
Prim−25
Long−pec
Protruding−mouth
Day−4
Day−5
Stage
Transcript
ENSDART00000005583
ENSDART00000108508
ENSDART00000148765
ENSDART00000149173
ENSDART00000150113

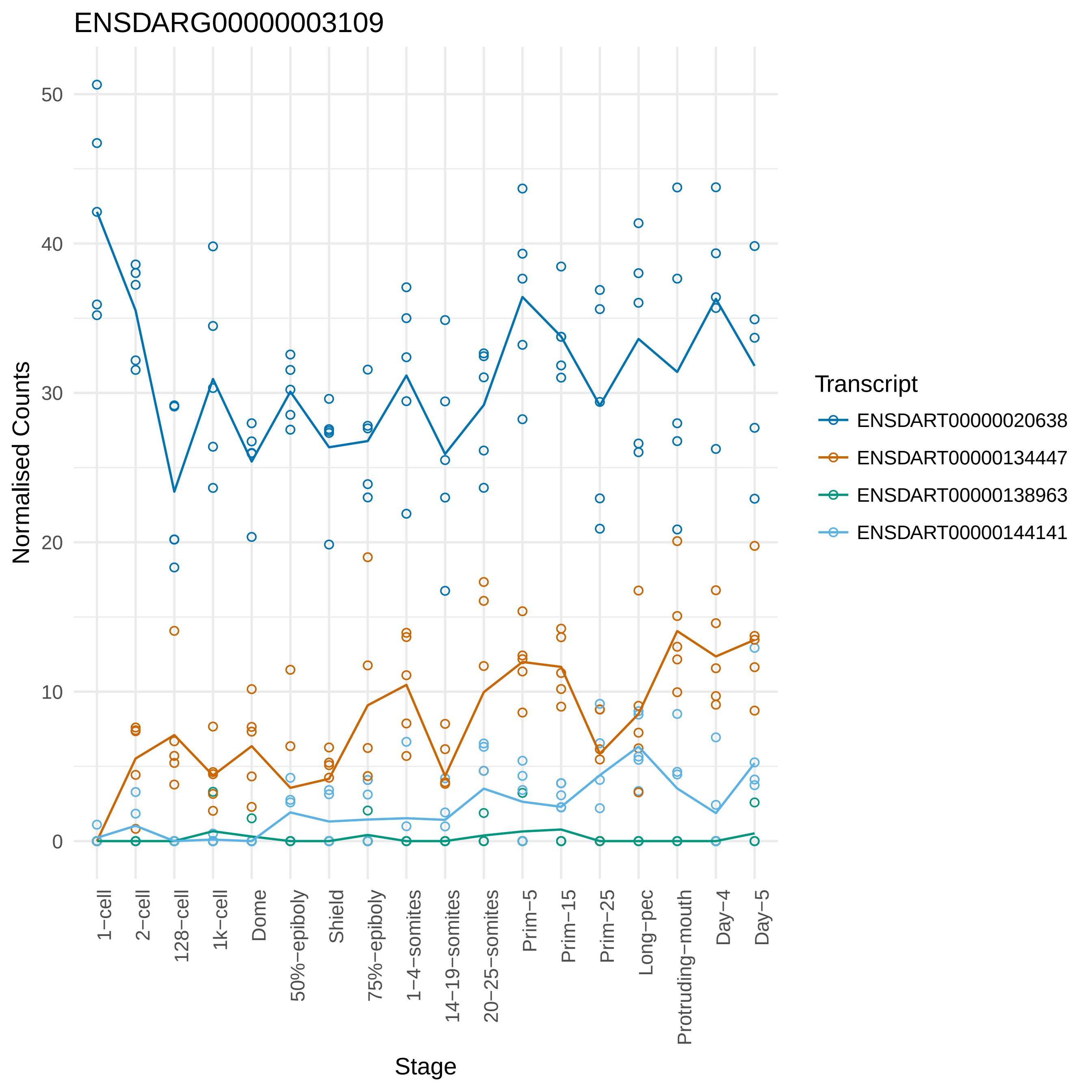
ENSDARG00000003109
Normalised Counts
50
40
30
20
10
0
1−cell
2−cell
128−cell
1k−cell
Dome
50%−epiboly
Shield
75%−epiboly
1−4−somites
14−19−somites
20−25−somites
Prim−5
Prim−15
Prim−25
Long−pec
Protruding−mouth
Day−4
Day−5
Stage
Transcript
ENSDART00000020638
ENSDART00000134447
ENSDART00000138963
ENSDART00000144141

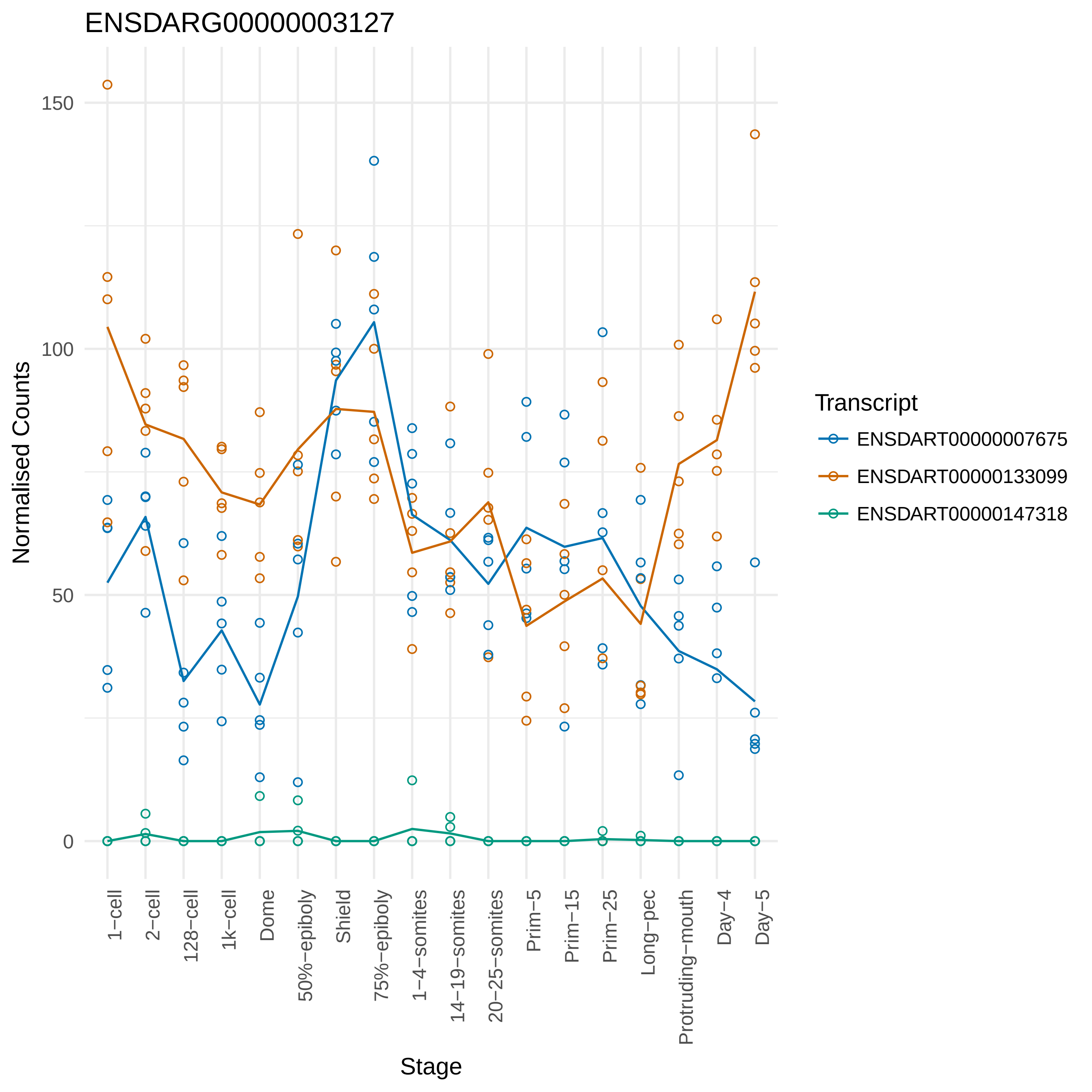

ENSDARG00000003127
Normalised Counts
150
100
50
0
1−cell
2−cell
128−cell
1k−cell
Dome
50%−epiboly
Shield
75%−epiboly
1−4−somites
14−19−somites
20−25−somites
Prim−5
Prim−15
Prim−25
Long−pec
Protruding−mouth
Day−4
Day−5
Stage
Transcript
ENSDART00000007675
ENSDART00000133099
ENSDART00000147318

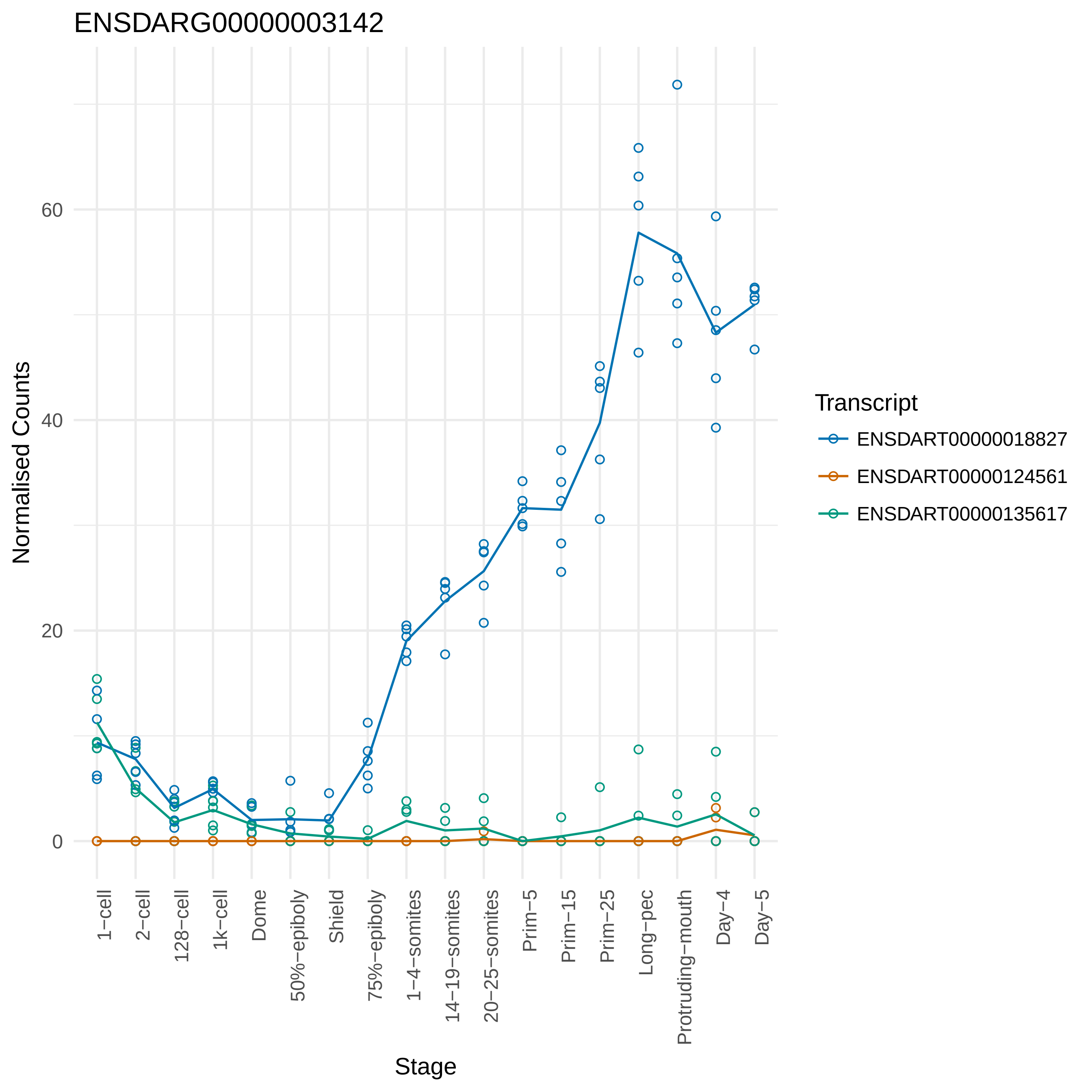

ENSDARG00000003142
Normalised Counts
0
20
40
60
Stage
1−cell
2−cell
128−cell
1k−cell
Dome
50%−epiboly
Shield
75%−epiboly
1−4−somites
14−19−somites
20−25−somites
Prim−5
Prim−15
Prim−25
Long−pec
Protruding−mouth
Day−4
Day−5
Transcript
ENSDART00000018827
ENSDART00000124561
ENSDART00000135617

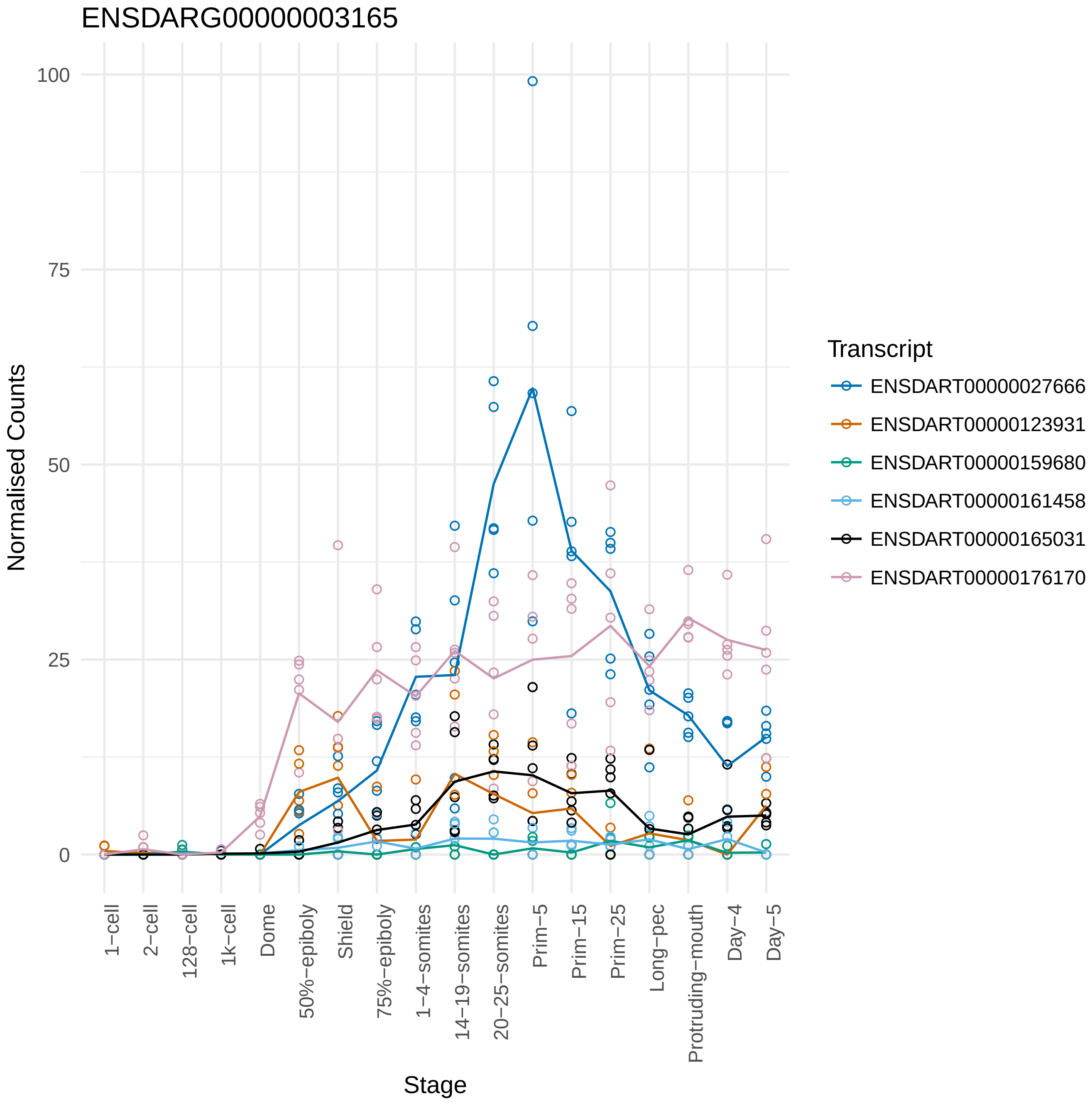

ENSDARG00000003165
Normalised Counts
100
75
50
25
0
1−cell
2−cell
128−cell
1k−cell
Dome
50%−epiboly
Shield
75%−epiboly
1−4−somites
14−19−somites
20−25−somites
Prim−5
Prim−15
Prim−25
Long−pec
Protruding−mouth
Day−4
Day−5
Stage
Transcript
ENSDART00000027666
ENSDART00000123931
ENSDART00000159680
ENSDART00000161458
ENSDART00000165031
ENSDART00000176170

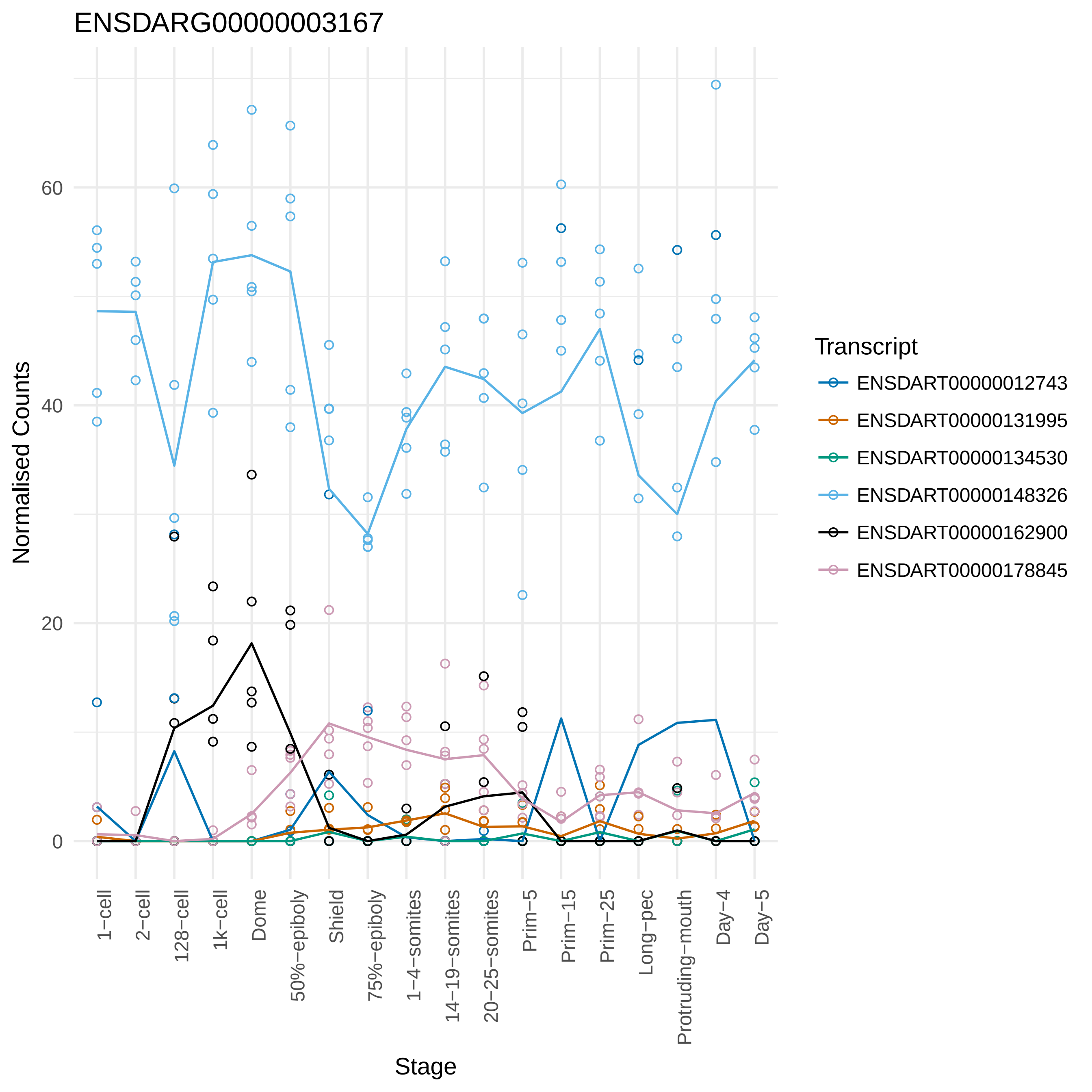

ENSDARG00000003167
Normalised Counts
0
20
40
60
1−cell
2−cell
128−cell
1k−cell
Dome
50%−epiboly
Shield
75%−epiboly
1−4−somites
14−19−somites
20−25−somites
Prim−5
Prim−15
Prim−25
Long−pec
Protruding−mouth
Day−4
Day−5
Stage
Transcript
ENSDART00000012743
ENSDART00000131995
ENSDART00000134530
ENSDART00000148326
ENSDART00000162900
ENSDART00000178845

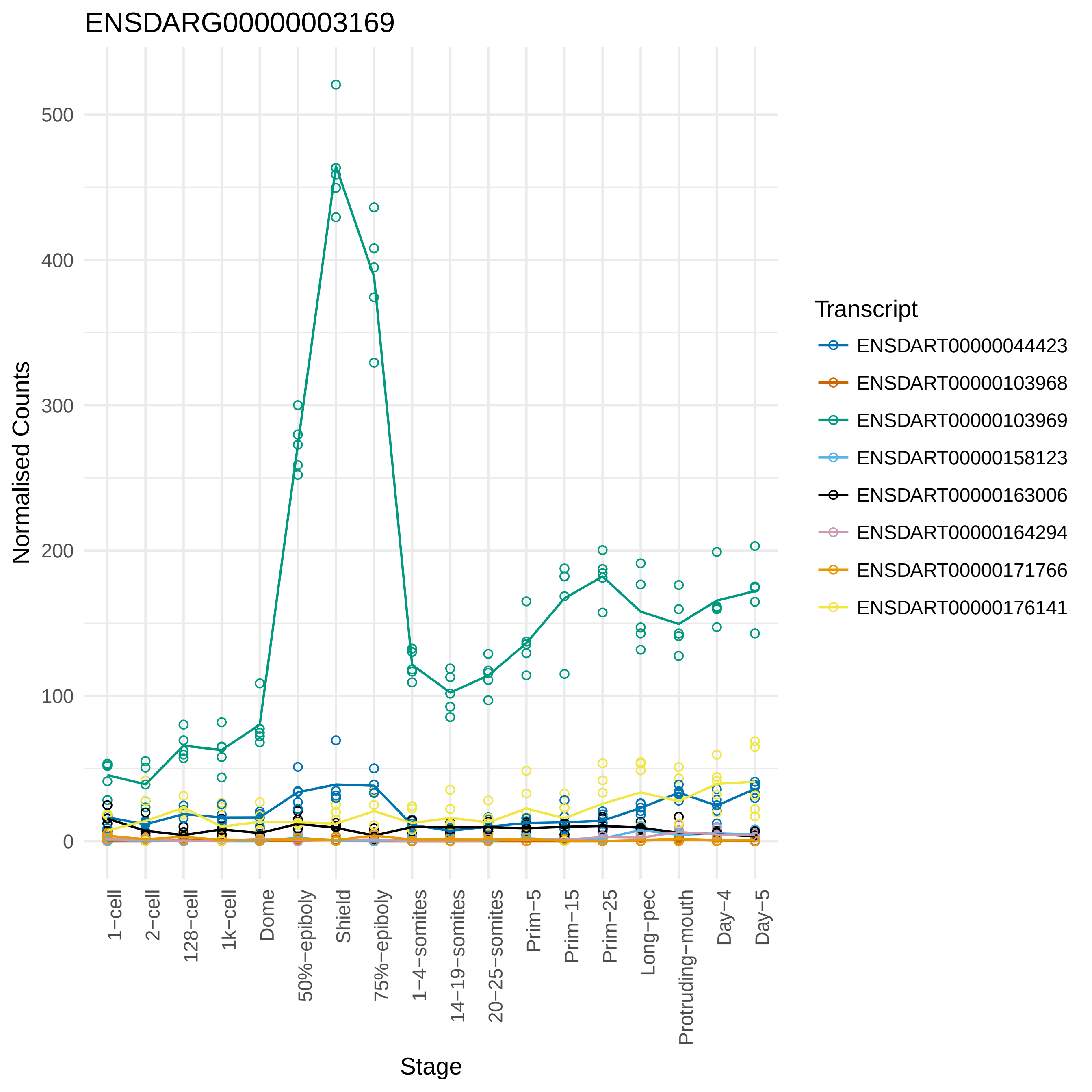

ENSDARG00000003169
Normalised Counts
500
400
300
200
100
0
Stage
1−cell
2−cell
128−cell
1k−cell
Dome
50%−epiboly
Shield
75%−epiboly
1−4−somites
14−19−somites
20−25−somites
Prim−5
Prim−15
Prim−25
Long−pec
Protruding−mouth
Day−4
Day−5
Transcript
ENSDART00000044423
ENSDART00000103968
ENSDART00000103969
ENSDART00000158123
ENSDART00000163006
ENSDART00000164294
ENSDART00000171766
ENSDART00000176141

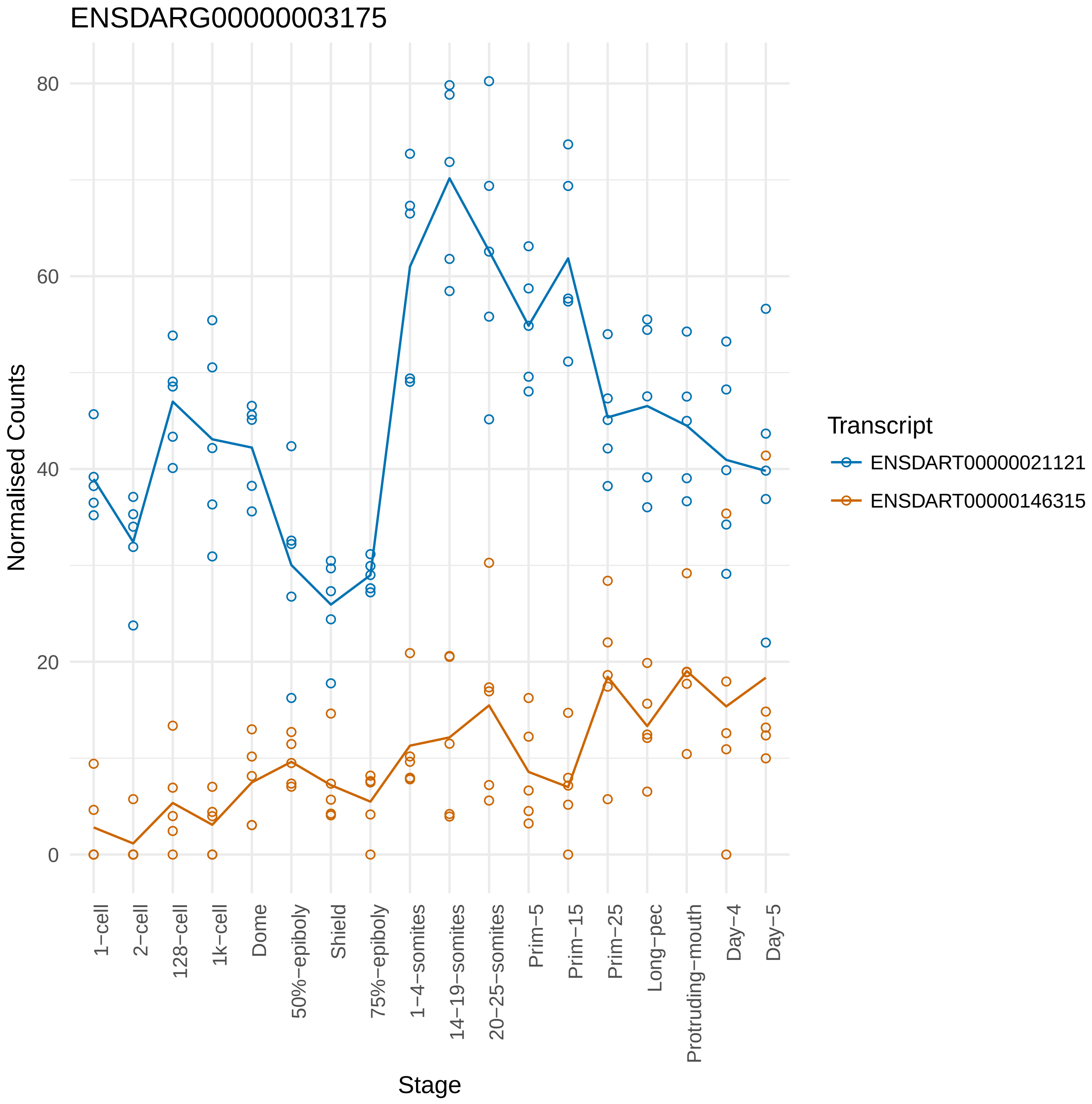

ENSDARG00000003175
Normalised Counts
0
20
40
60
80
1−cell
2−cell
128−cell
1k−cell
Dome
50%−epiboly
Shield
75%−epiboly
1−4−somites
14−19−somites
20−25−somites
Prim−5
Prim−15
Prim−25
Long−pec
Protruding−mouth
Day−4
Day−5
Stage
Transcript
ENSDART00000021121
ENSDART00000146315

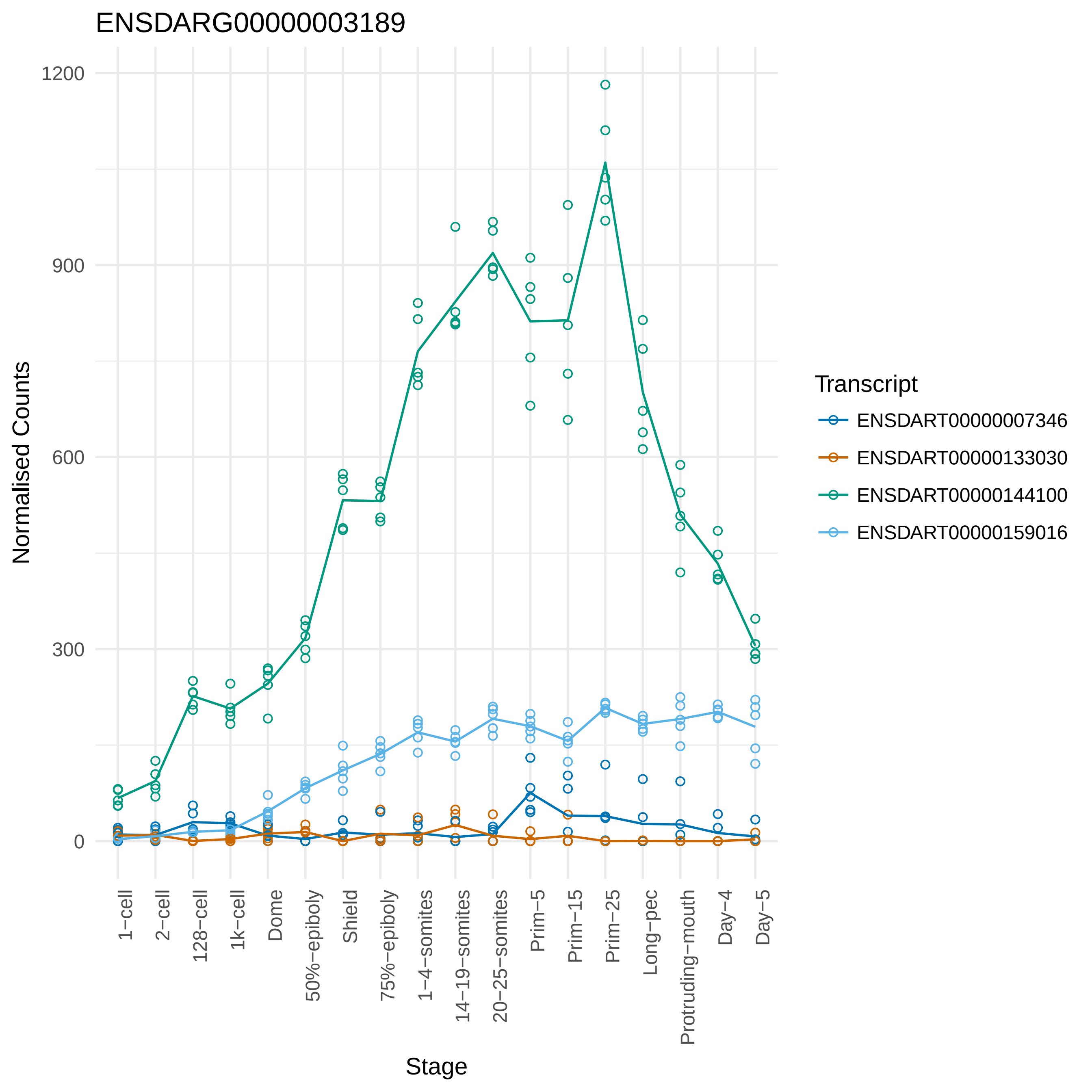

ENSDARG00000003189
Normalised Counts
1200
900
600
300
0
1−cell
2−cell
128−cell
1k−cell
Dome
50%−epiboly
Shield
75%−epiboly
1−4−somites
14−19−somites
20−25−somites
Prim−5
Prim−15
Prim−25
Long−pec
Protruding−mouth
Day−4
Day−5
Stage
Transcript
ENSDART00000007346
ENSDART00000133030
ENSDART00000144100
ENSDART00000159016

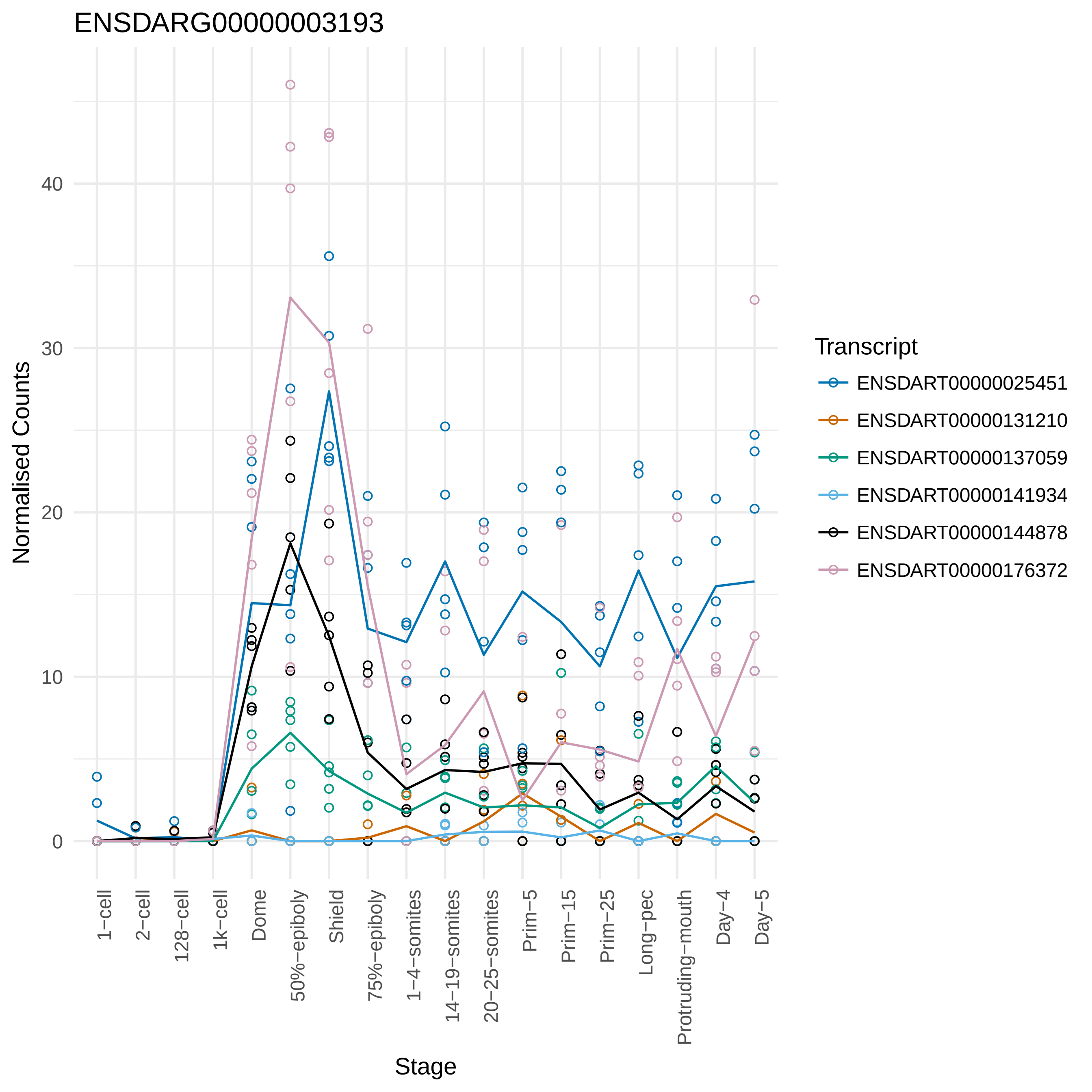
ENSDARG00000003193
Normalised Counts
0
10
20
30
40
1−cell
2−cell
128−cell
1k−cell
Dome
50%−epiboly
Shield
75%−epiboly
1−4−somites
14−19−somites
20−25−somites
Prim−5
Prim−15
Prim−25
Long−pec
Protruding−mouth
Day−4
Day−5
Stage
Transcript
ENSDART00000025451
ENSDART00000131210
ENSDART00000137059
ENSDART00000141934
ENSDART00000144878
ENSDART00000176372

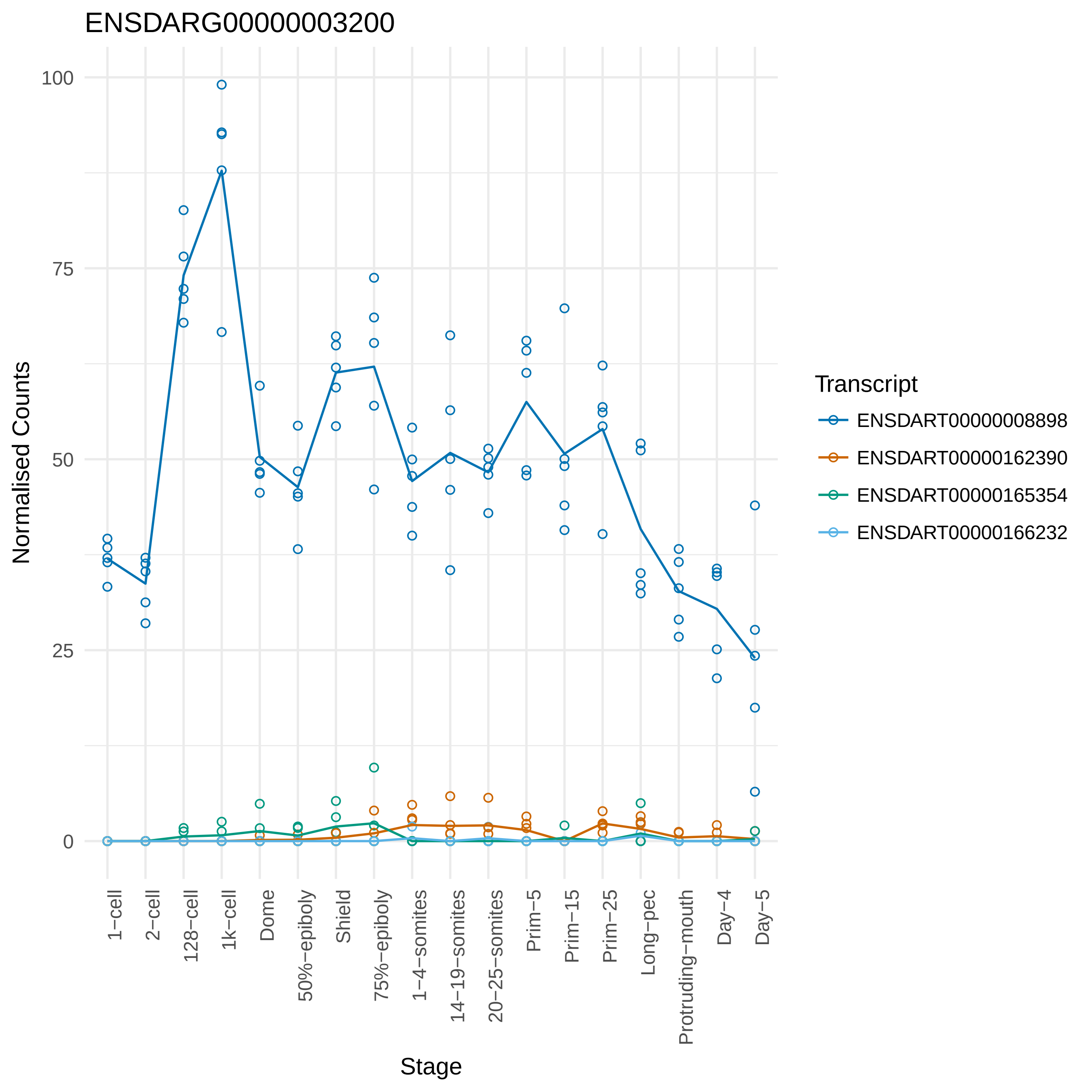

ENSDARG00000003200
Normalised Counts
100
75
50
25
0
1−cell
2−cell
128−cell
1k−cell
Dome
50%−epiboly
Shield
75%−epiboly
1−4−somites
14−19−somites
20−25−somites
Prim−5
Prim−15
Prim−25
Long−pec
Protruding−mouth
Day−4
Day−5
Stage
Transcript
ENSDART00000008898
ENSDART00000162390
ENSDART00000165354
ENSDART00000166232

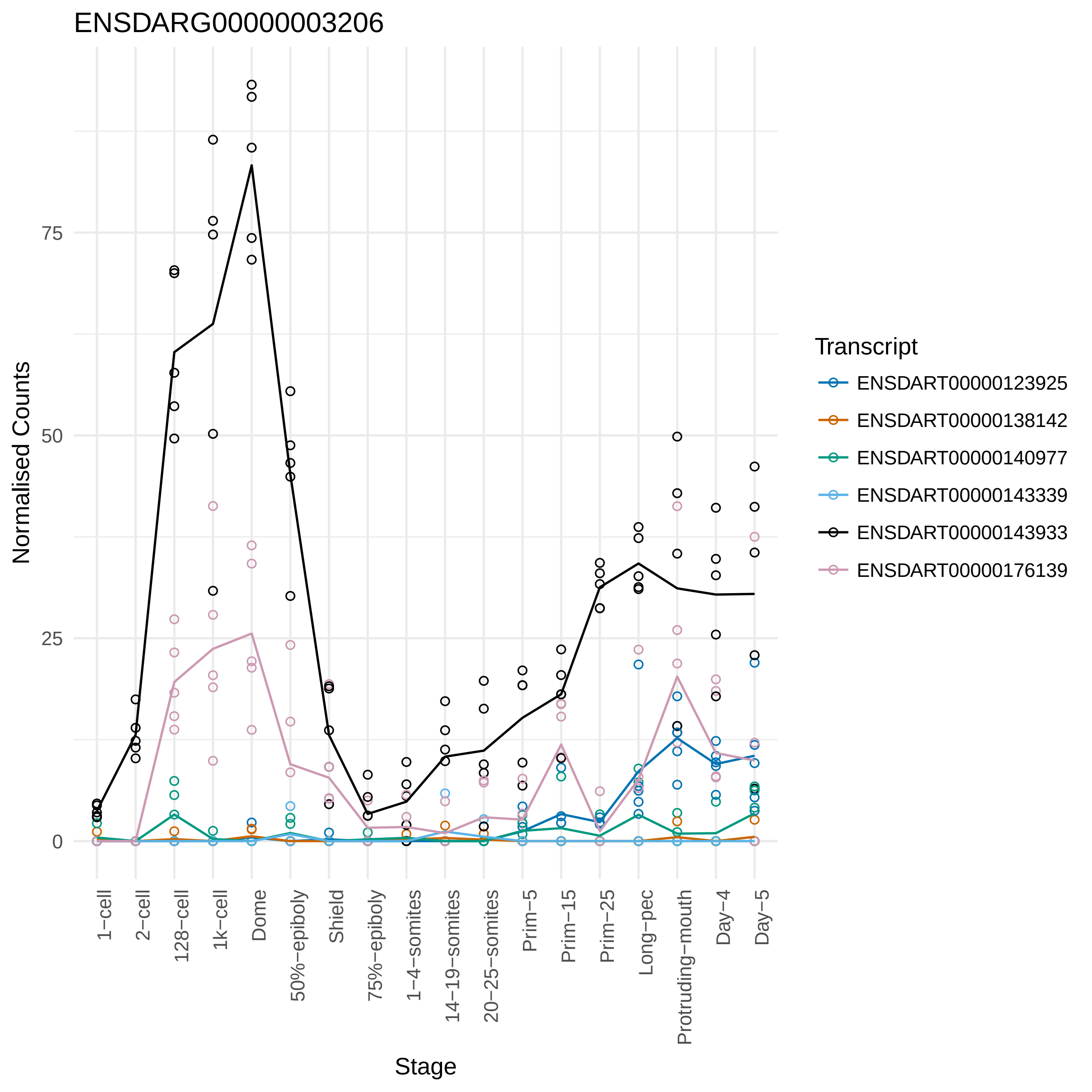
ENSDARG00000003206
Normalised Counts
0
25
50
75
Stage
1−cell
2−cell
128−cell
1k−cell
Dome
50%−epiboly
Shield
75%−epiboly
1−4−somites
14−19−somites
20−25−somites
Prim−5
Prim−15
Prim−25
Long−pec
Protruding−mouth
Day−4
Day−5
Transcript
ENSDART00000123925
ENSDART00000138142
ENSDART00000140977
ENSDART00000143339
ENSDART00000143933
ENSDART00000176139

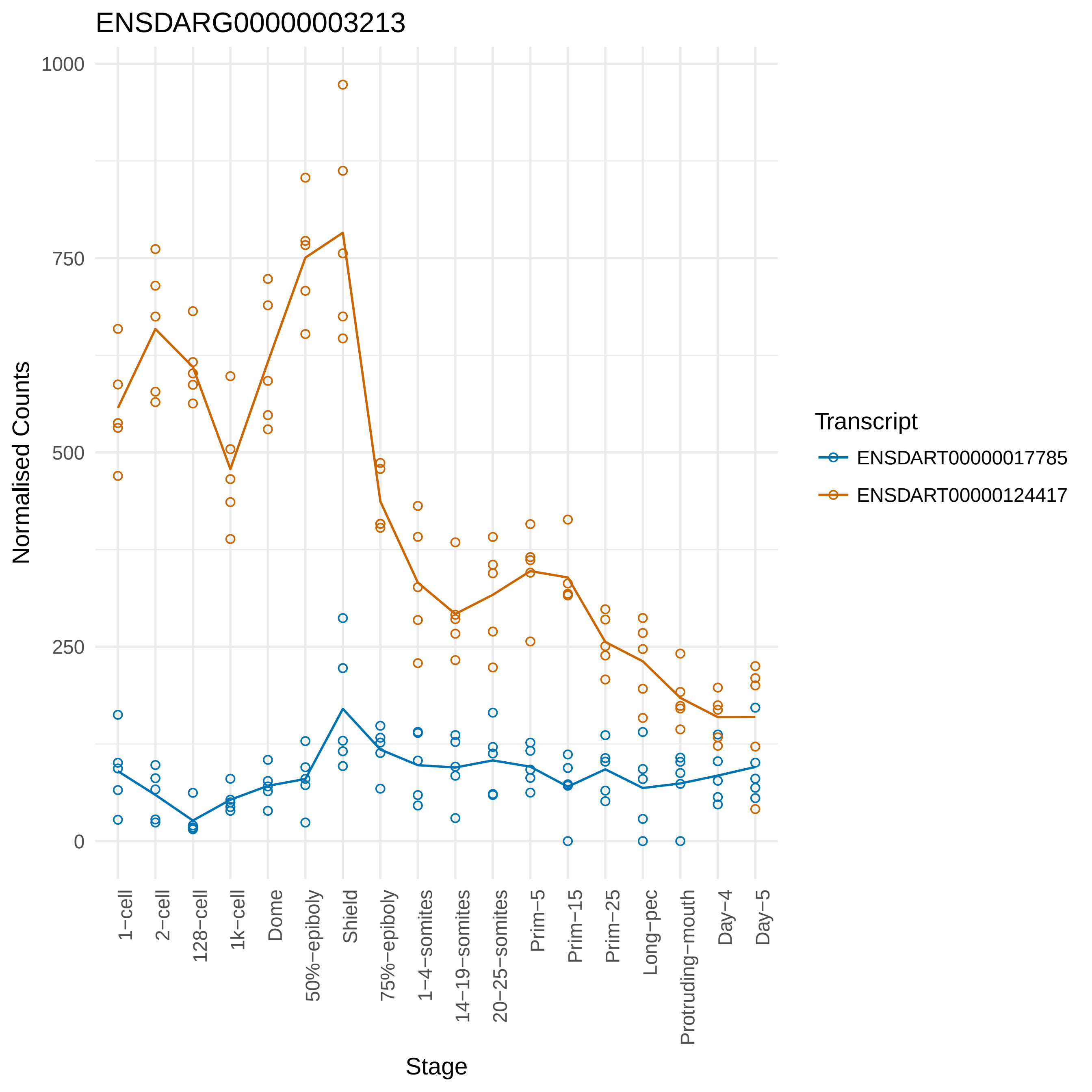

ENSDARG00000003213
Normalised Counts
1000
750
500
250
0
1−cell
2−cell
128−cell
1k−cell
Dome
50%−epiboly
Shield
75%−epiboly
1−4−somites
14−19−somites
20−25−somites
Prim−5
Prim−15
Prim−25
Long−pec
Protruding−mouth
Day−4
Day−5
Stage
Transcript
ENSDART00000017785
ENSDART00000124417

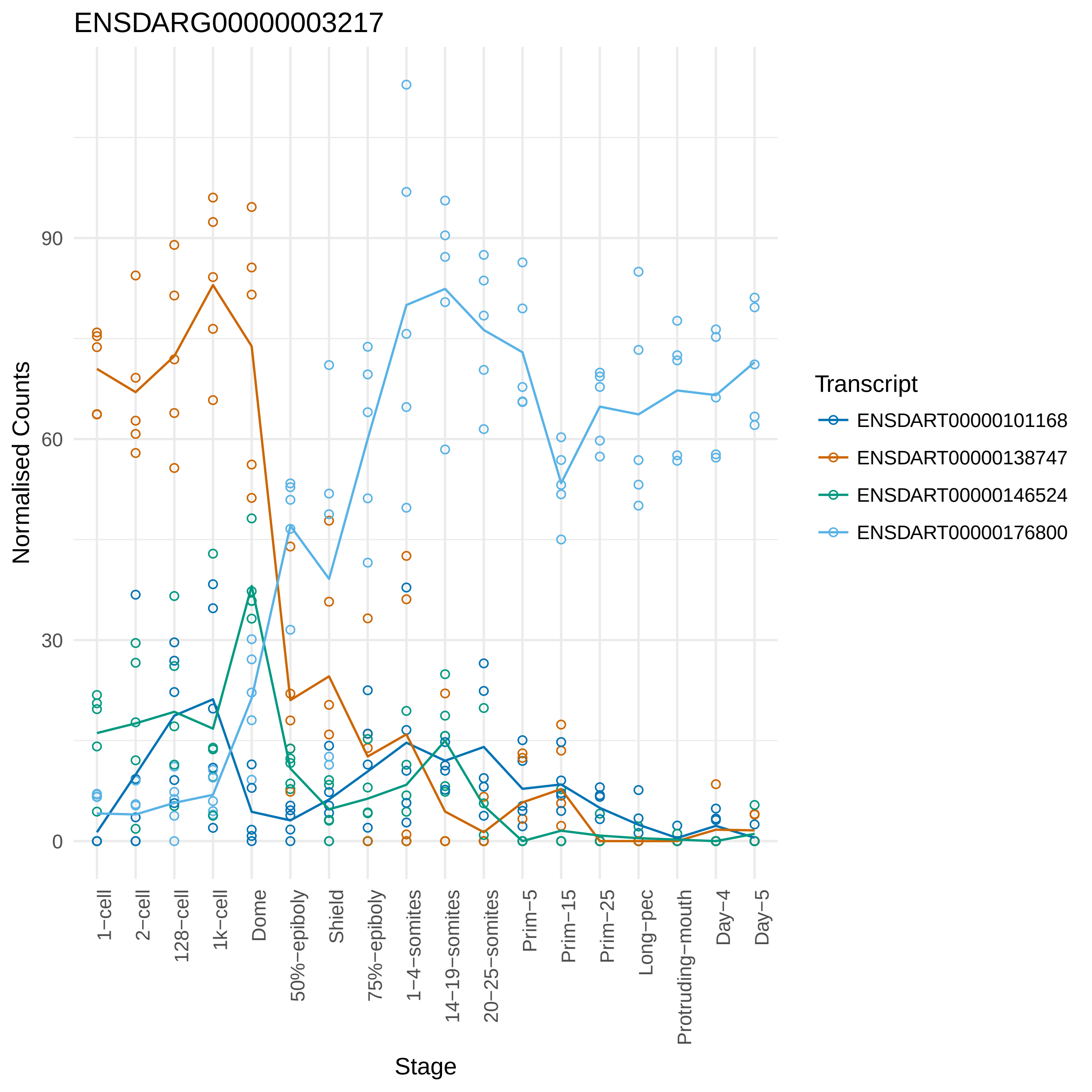
ENSDARG00000003217
Normalised Counts
90
60
30
0
1−cell
2−cell
128−cell
1k−cell
Dome
50%−epiboly
Shield
75%−epiboly
1−4−somites
14−19−somites
20−25−somites
Prim−5
Prim−15
Prim−25
Long−pec
Protruding−mouth
Day−4
Day−5
Stage
Transcript
ENSDART00000101168
ENSDART00000138747
ENSDART00000146524
ENSDART00000176800

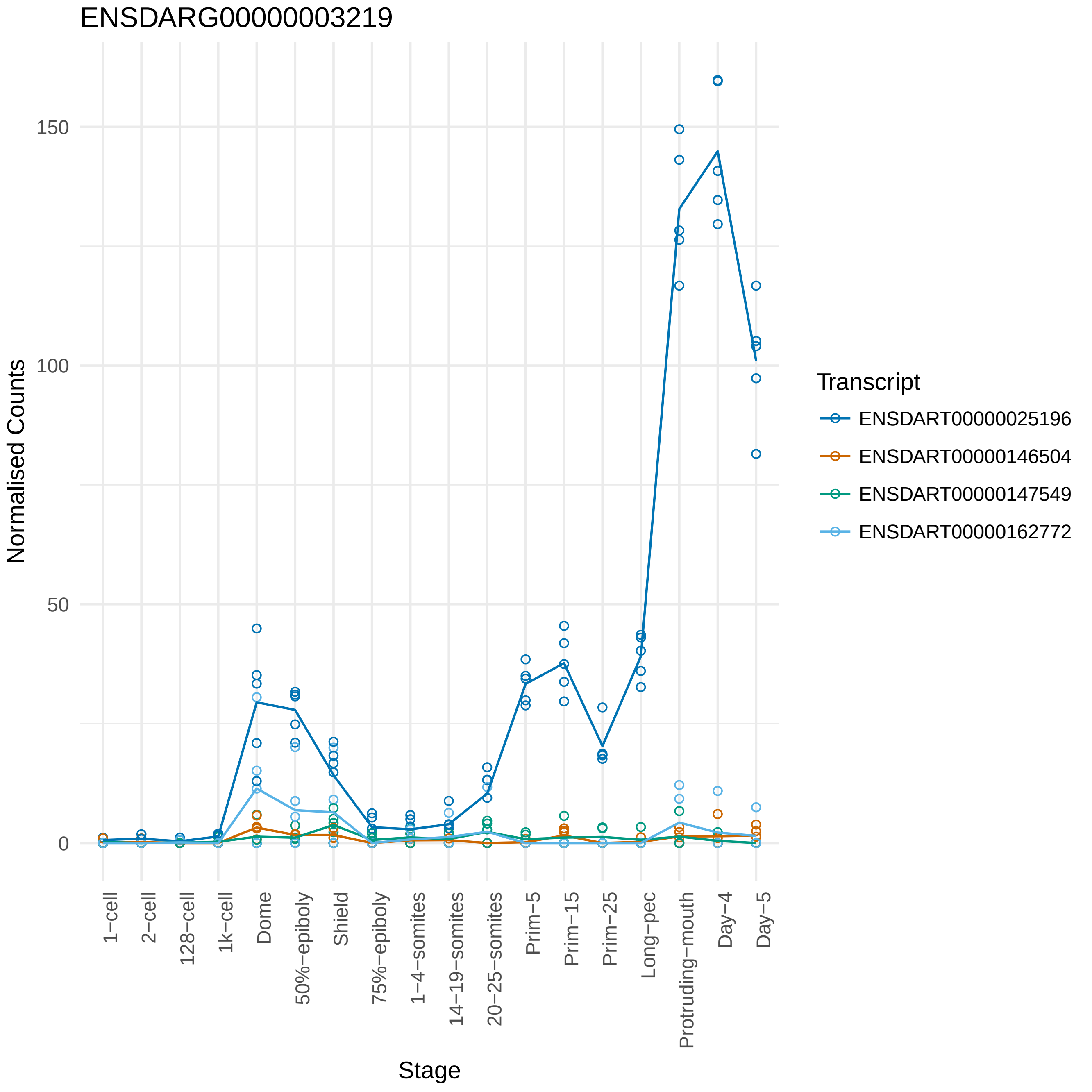

ENSDARG00000003219
Normalised Counts
0
50
100
150
Stage
1−cell
2−cell
128−cell
1k−cell
Dome
50%−epiboly
Shield
75%−epiboly
1−4−somites
14−19−somites
20−25−somites
Prim−5
Prim−15
Prim−25
Long−pec
Protruding−mouth
Day−4
Day−5
Transcript
ENSDART00000025196
ENSDART00000146504
ENSDART00000147549
ENSDART00000162772

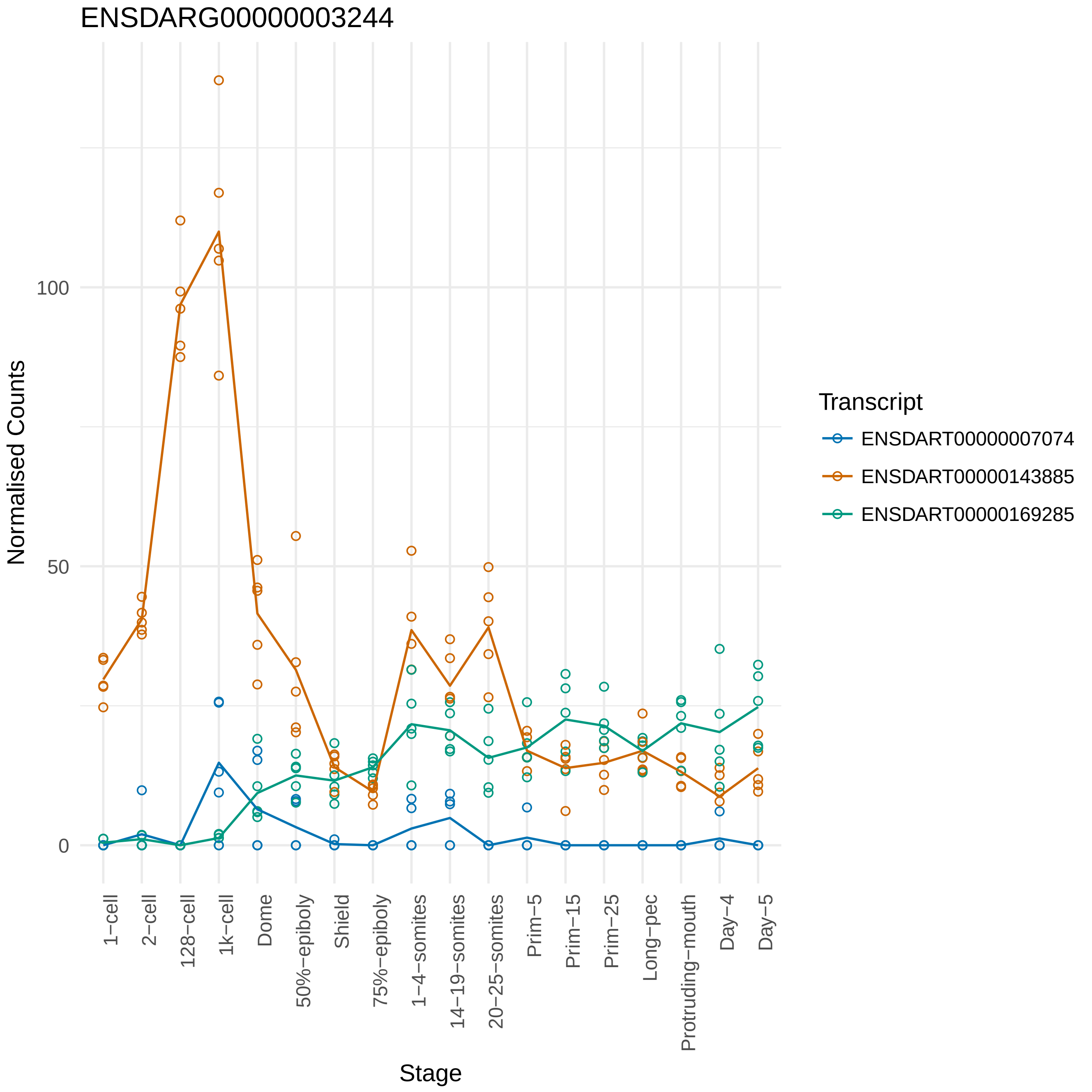
ENSDARG00000003244
Normalised Counts
100
50
0
1−cell
2−cell
128−cell
1k−cell
Dome
50%−epiboly
Shield
75%−epiboly
1−4−somites
14−19−somites
20−25−somites
Prim−5
Prim−15
Prim−25
Long−pec
Protruding−mouth
Day−4
Day−5
Stage
Transcript
ENSDART00000007074
ENSDART00000143885
ENSDART00000169285

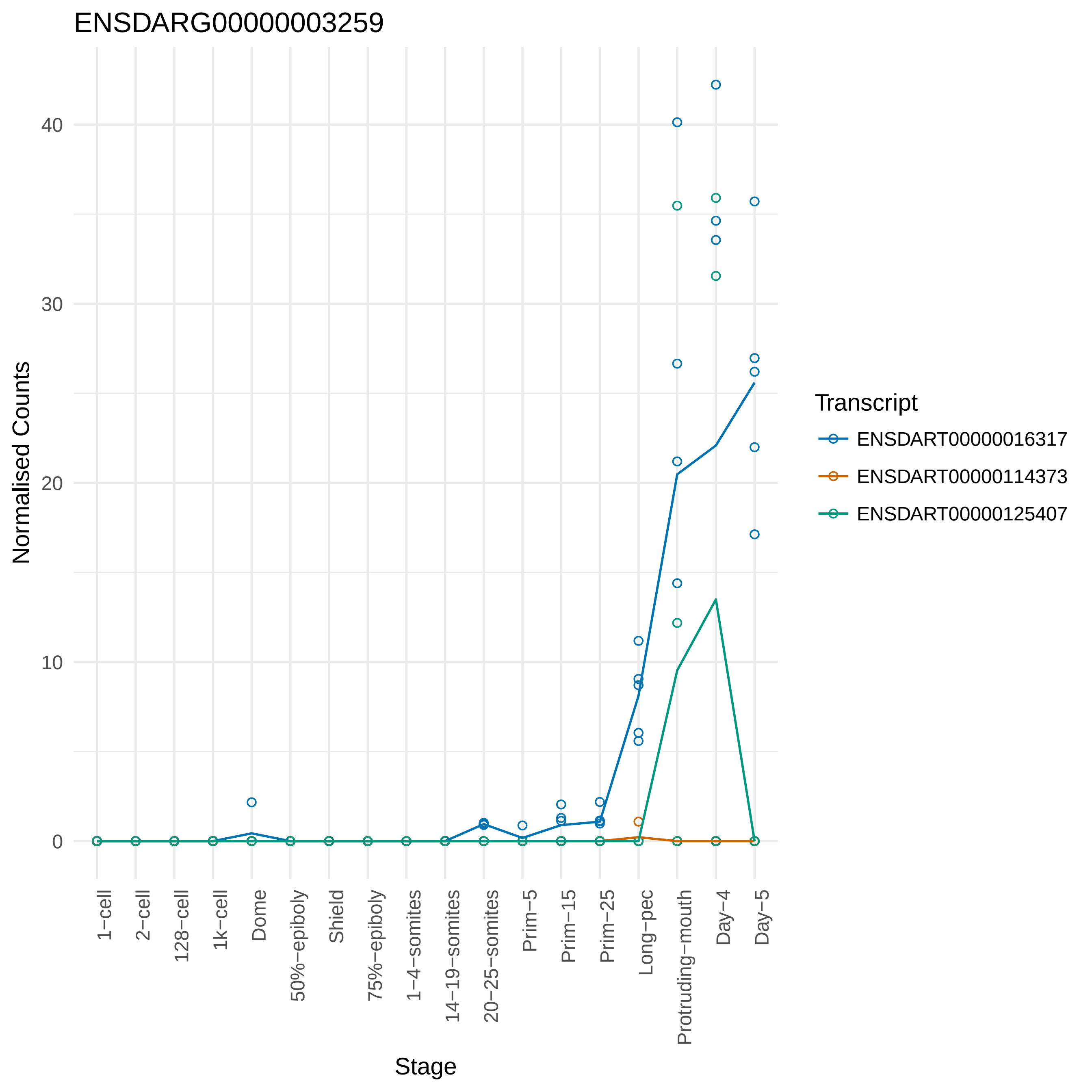

ENSDARG00000003259
Normalised Counts
0
10
20
30
40
1−cell
2−cell
128−cell
1k−cell
Dome
50%−epiboly
Shield
75%−epiboly
1−4−somites
14−19−somites
20−25−somites
Prim−5
Prim−15
Prim−25
Long−pec
Protruding−mouth
Day−4
Day−5
Stage
Transcript
ENSDART00000016317
ENSDART00000114373
ENSDART00000125407

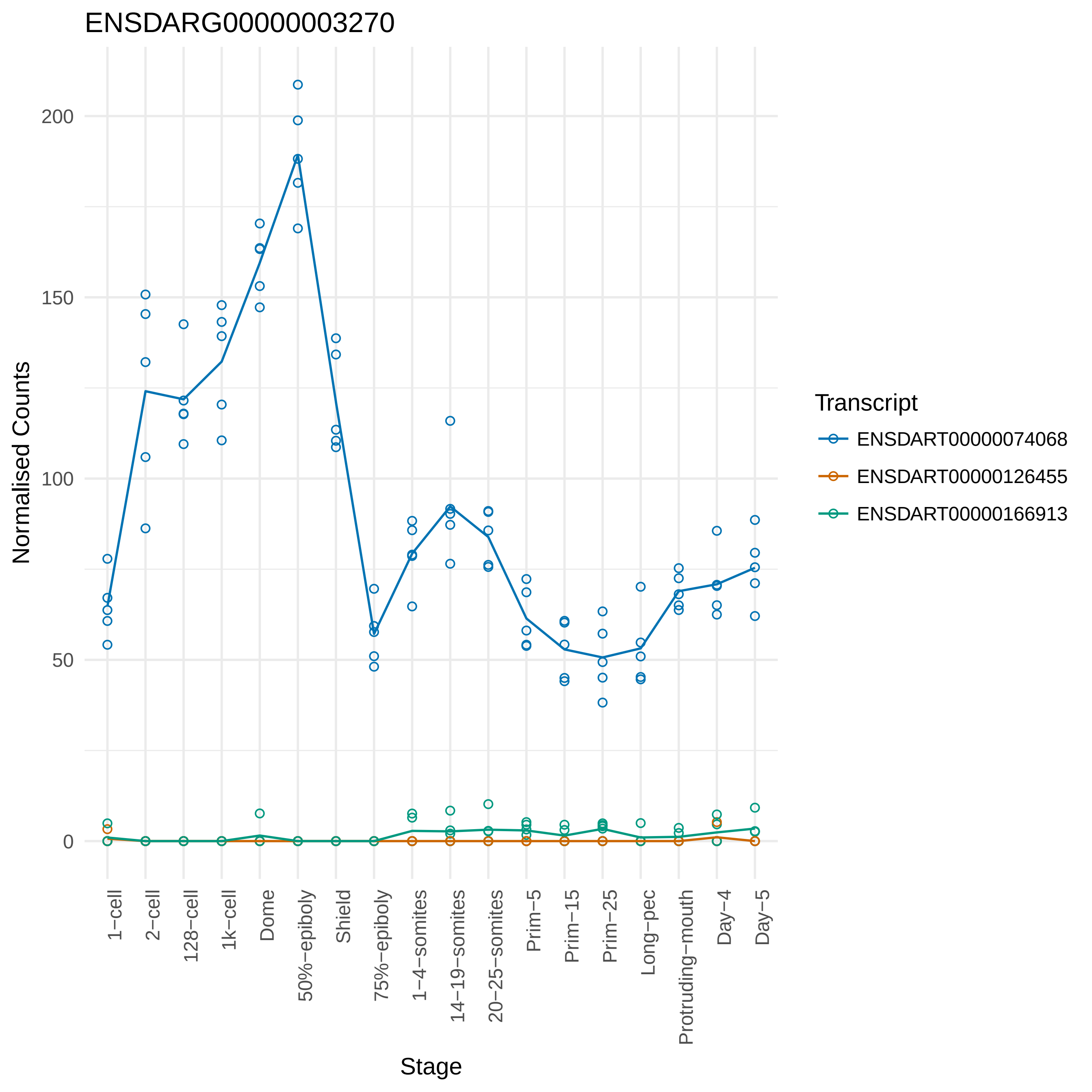

ENSDARG00000003270
Normalised Counts
200
150
100
50
0
1−cell
2−cell
128−cell
1k−cell
Dome
50%−epiboly
Shield
75%−epiboly
1−4−somites
14−19−somites
20−25−somites
Prim−5
Prim−15
Prim−25
Long−pec
Protruding−mouth
Day−4
Day−5
Stage
Transcript
ENSDART00000074068
ENSDART00000126455
ENSDART00000166913

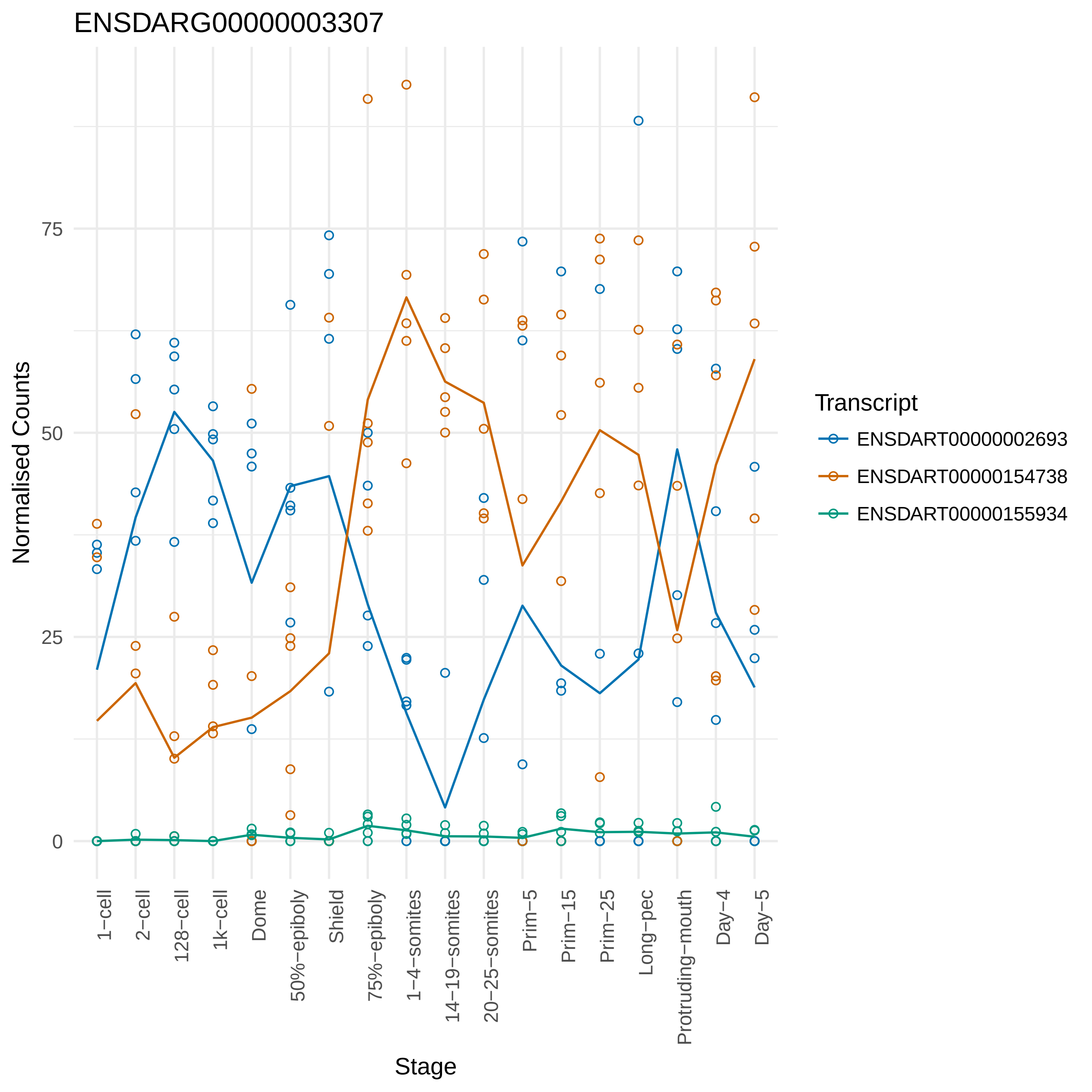
ENSDARG00000003307
Normalised Counts
75
50
25
0
1−cell
2−cell
128−cell
1k−cell
Dome
50%−epiboly
Shield
75%−epiboly
1−4−somites
14−19−somites
20−25−somites
Prim−5
Prim−15
Prim−25
Long−pec
Protruding−mouth
Day−4
Day−5
Stage
Transcript
ENSDART00000002693
ENSDART00000154738
ENSDART00000155934

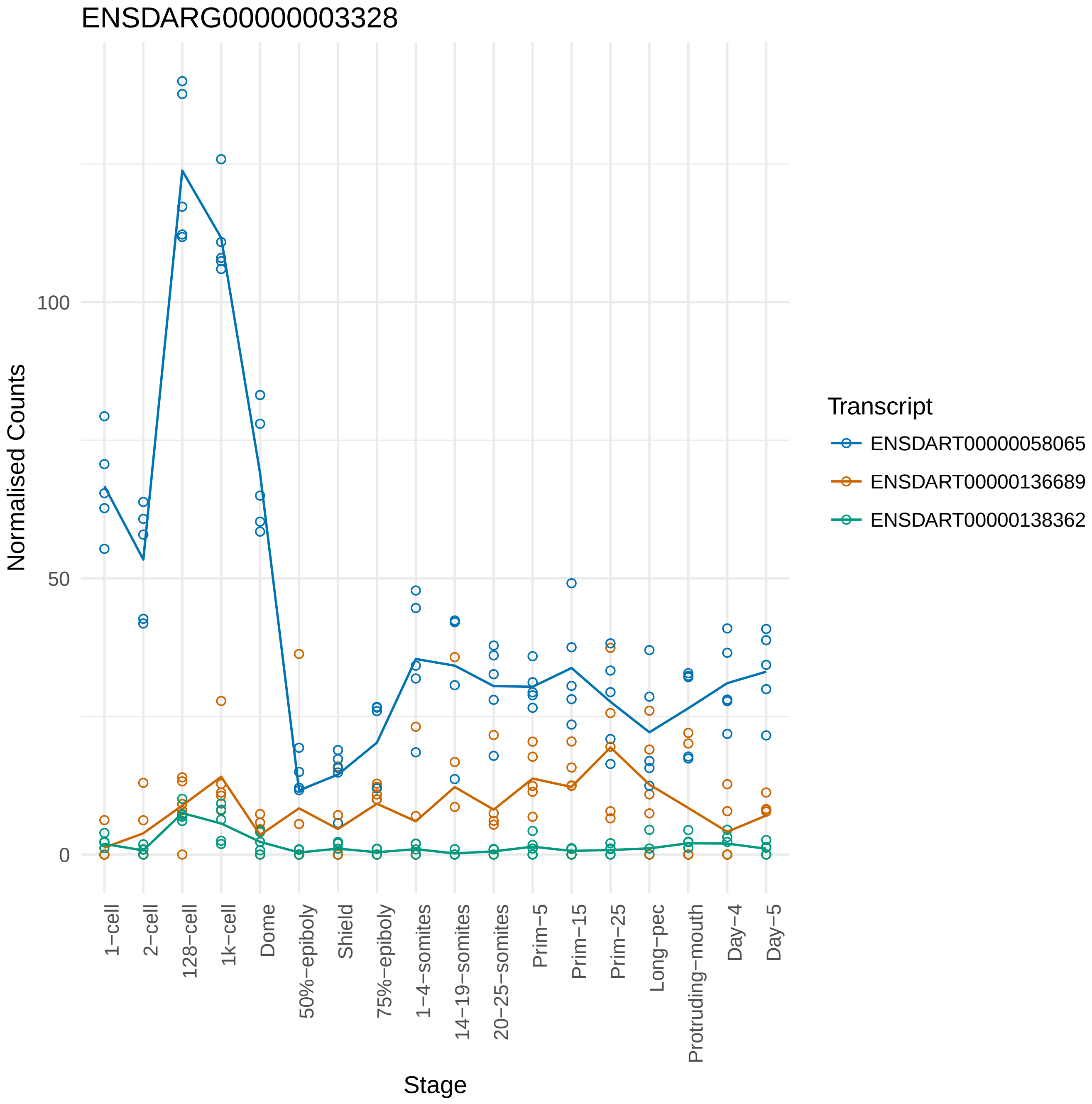

ENSDARG00000003328
Normalised Counts
0
50
100
Stage
1−cell
2−cell
128−cell
1k−cell
Dome
50%−epiboly
Shield
75%−epiboly
1−4−somites
14−19−somites
20−25−somites
Prim−5
Prim−15
Prim−25
Long−pec
Protruding−mouth
Day−4
Day−5
Transcript
ENSDART00000058065
ENSDART00000136689
ENSDART00000138362

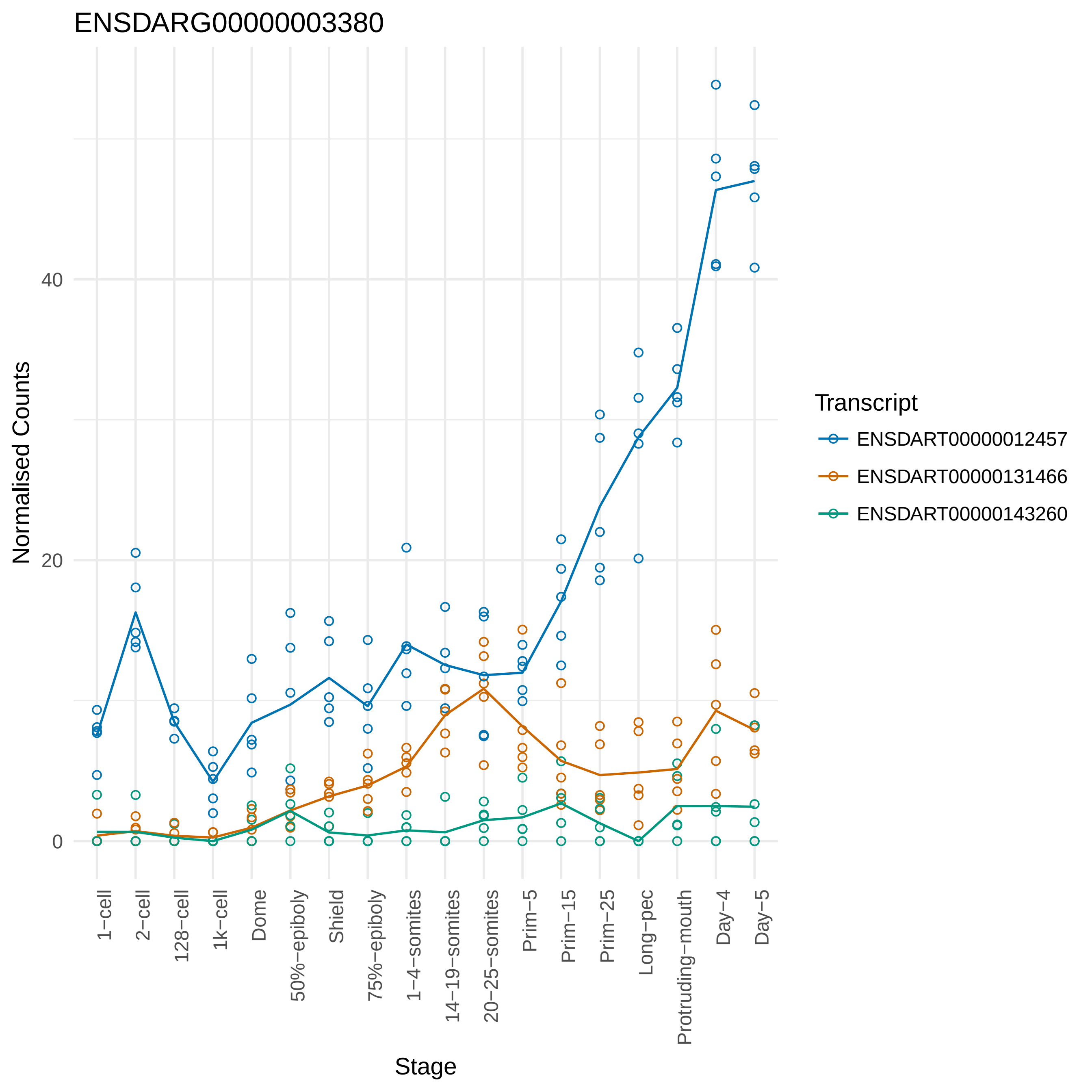
ENSDARG00000003380
Normalised Counts
0
20
40
1−cell
2−cell
128−cell
1k−cell
Dome
50%−epiboly
Shield
75%−epiboly
1−4−somites
14−19−somites
20−25−somites
Prim−5
Prim−15
Prim−25
Long−pec
Protruding−mouth
Day−4
Day−5
Stage
Transcript
ENSDART00000012457
ENSDART00000131466
ENSDART00000143260

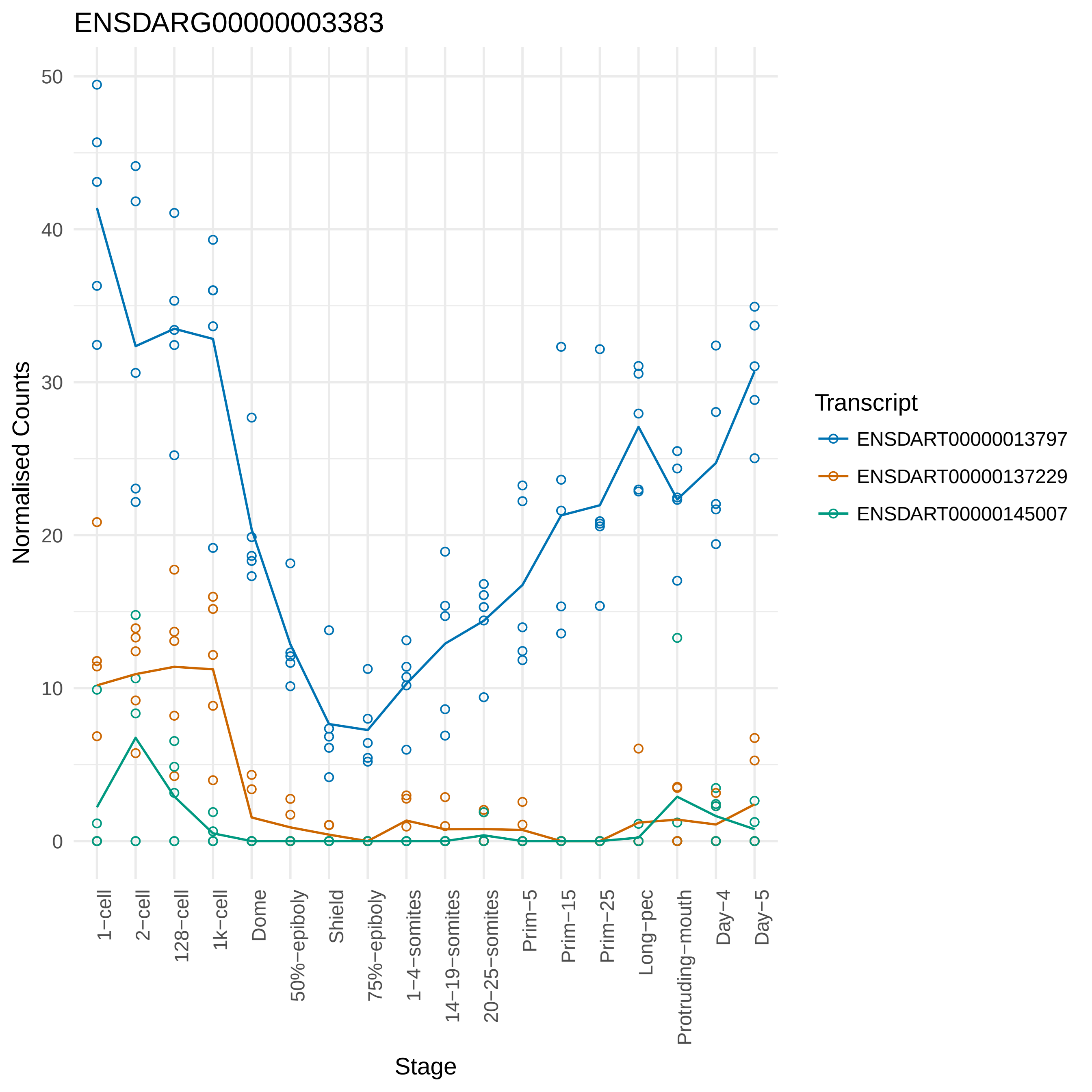

ENSDARG00000003383
Normalised Counts
50
40
30
20
10
0
1−cell
2−cell
128−cell
1k−cell
Dome
50%−epiboly
Shield
75%−epiboly
1−4−somites
14−19−somites
20−25−somites
Prim−5
Prim−15
Prim−25
Long−pec
Protruding−mouth
Day−4
Day−5
Stage
Transcript
ENSDART00000013797
ENSDART00000137229
ENSDART00000145007

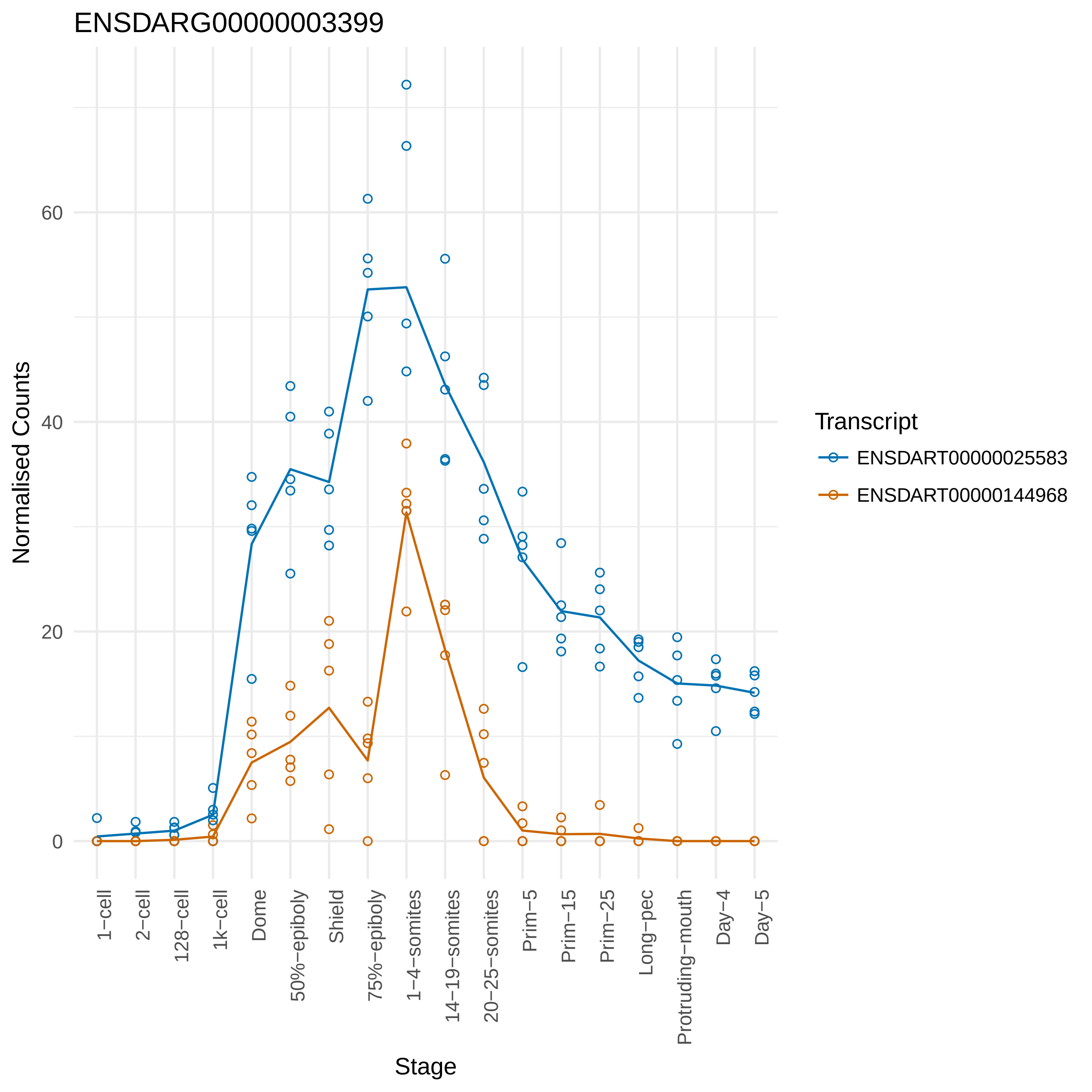

ENSDARG00000003399
Normalised Counts
0
20
40
60
Stage
1−cell
2−cell
128−cell
1k−cell
Dome
50%−epiboly
Shield
75%−epiboly
1−4−somites
14−19−somites
20−25−somites
Prim−5
Prim−15
Prim−25
Long−pec
Protruding−mouth
Day−4
Day−5
Transcript
ENSDART00000025583
ENSDART00000144968

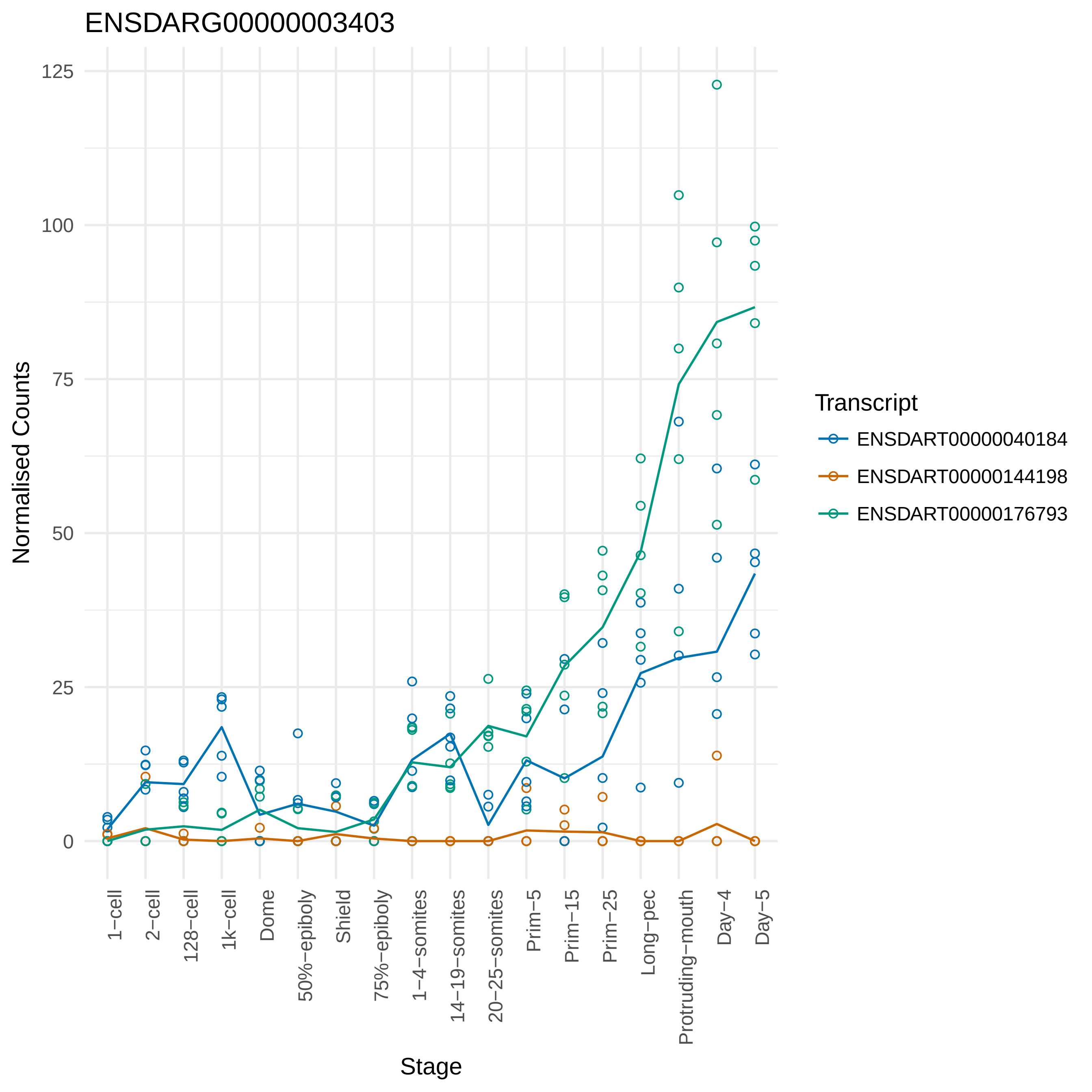

ENSDARG00000003403
Normalised Counts
125
100
75
50
25
0
1−cell
2−cell
128−cell
1k−cell
Dome
50%−epiboly
Shield
75%−epiboly
1−4−somites
14−19−somites
20−25−somites
Prim−5
Prim−15
Prim−25
Long−pec
Protruding−mouth
Day−4
Day−5
Stage
Transcript
ENSDART00000040184
ENSDART00000144198
ENSDART00000176793

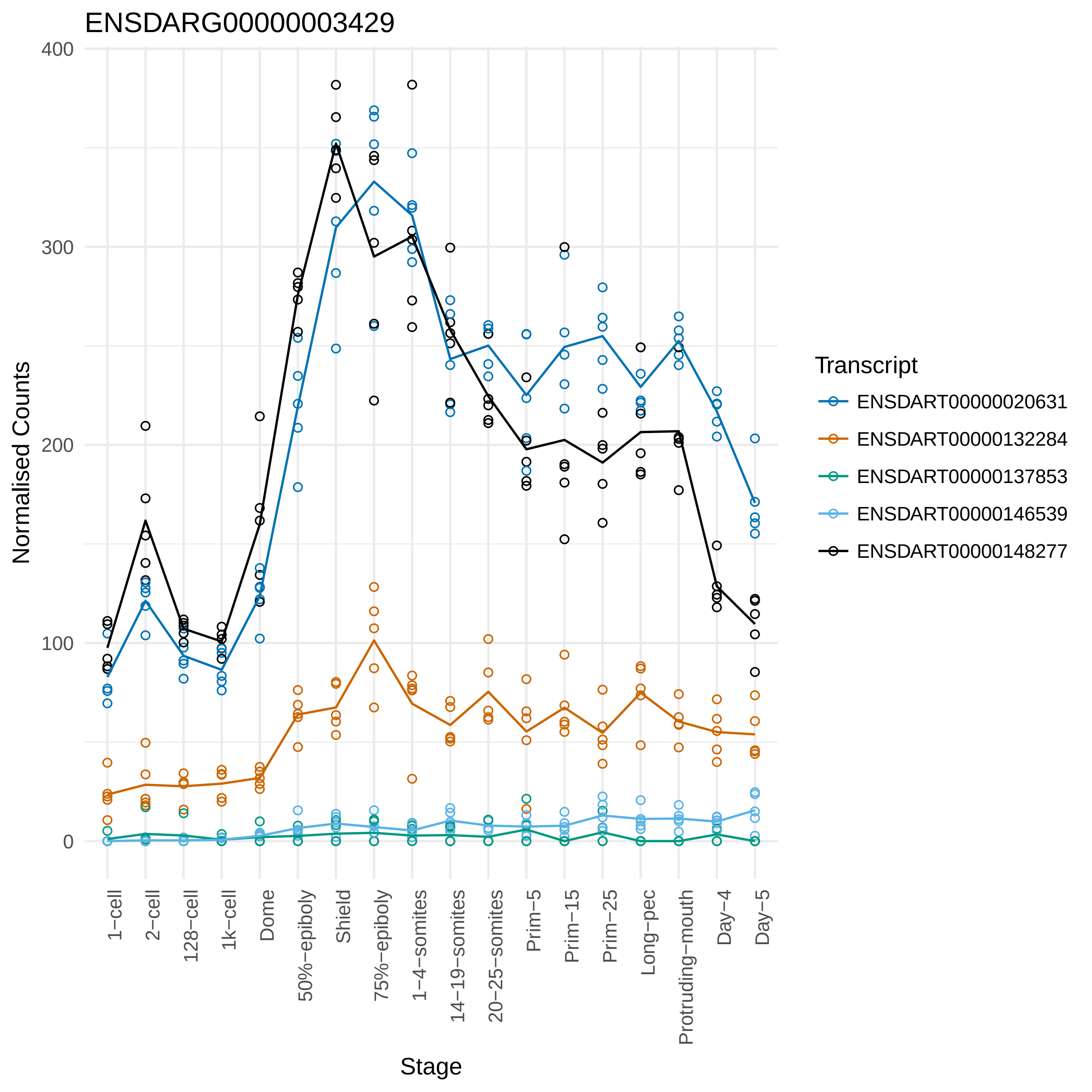

ENSDARG00000003429
Normalised Counts
400
300
200
100
0
1−cell
2−cell
128−cell
1k−cell
Dome
50%−epiboly
Shield
75%−epiboly
1−4−somites
14−19−somites
20−25−somites
Prim−5
Prim−15
Prim−25
Long−pec
Protruding−mouth
Day−4
Day−5
Stage
Transcript
ENSDART00000020631
ENSDART00000132284
ENSDART00000137853
ENSDART00000146539
ENSDART00000148277

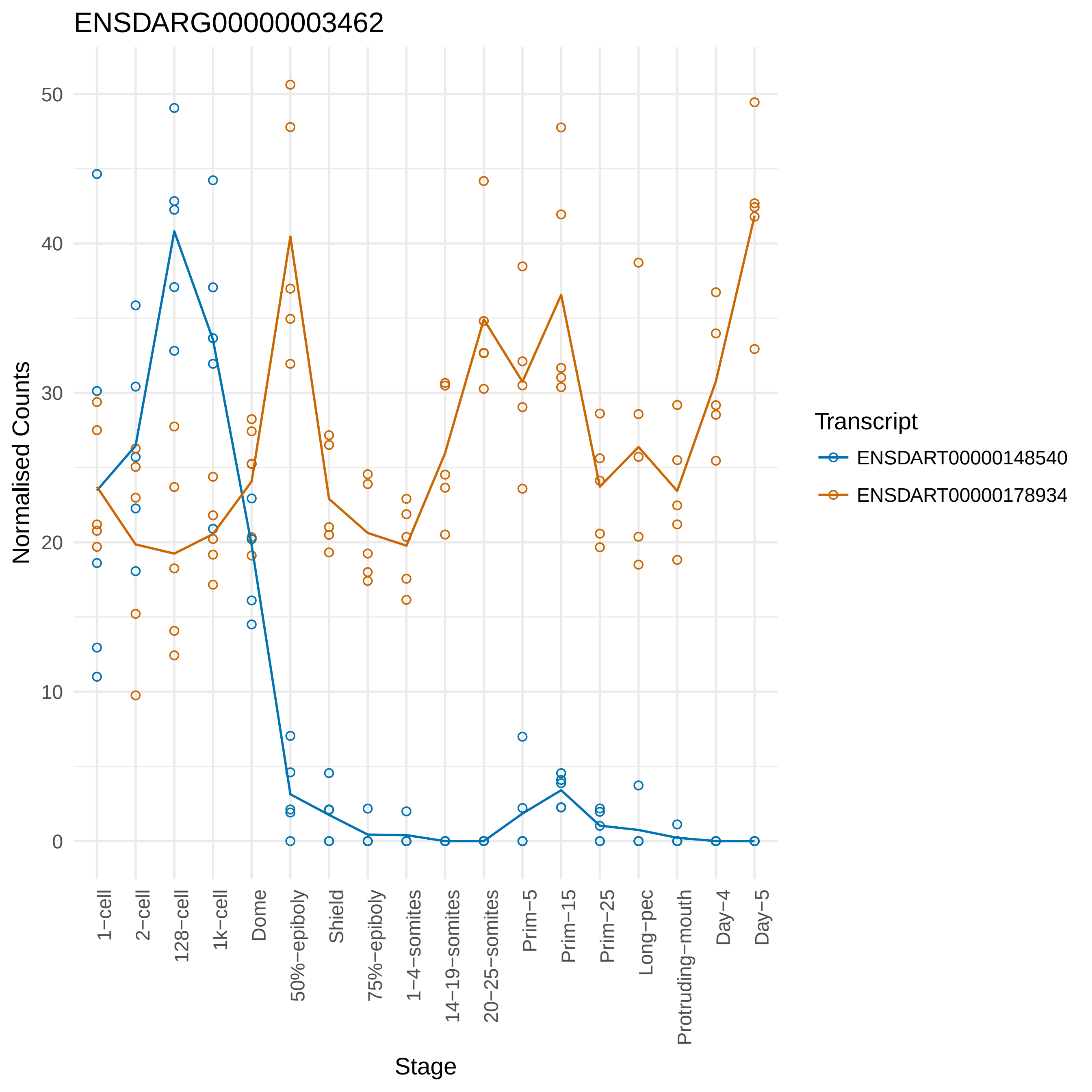

ENSDARG00000003462
Normalised Counts
0
10
20
30
40
50
1−cell
2−cell
128−cell
1k−cell
Dome
50%−epiboly
Shield
75%−epiboly
1−4−somites
14−19−somites
20−25−somites
Prim−5
Prim−15
Prim−25
Long−pec
Protruding−mouth
Day−4
Day−5
Stage
Transcript
ENSDART00000148540
ENSDART00000178934

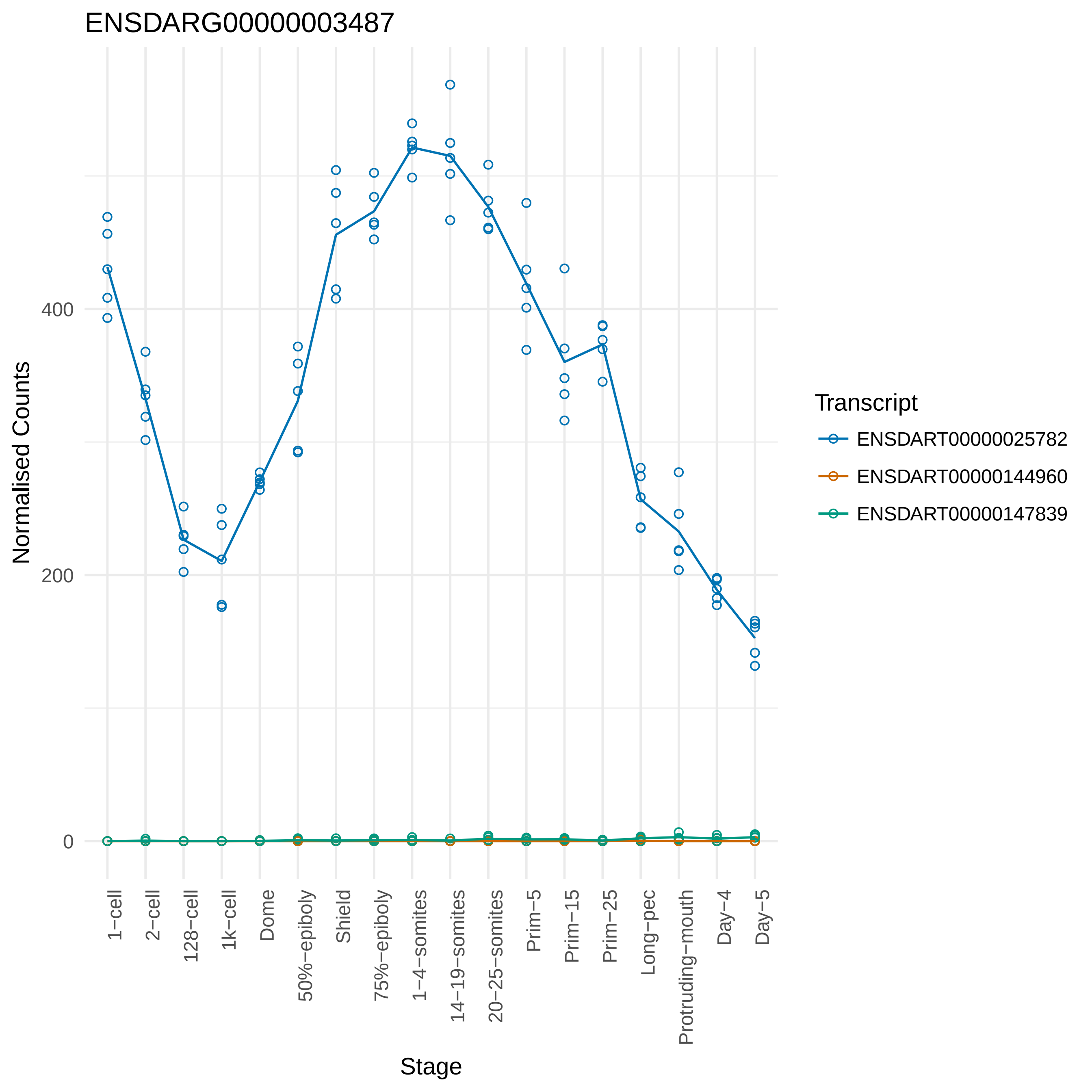

ENSDARG00000003487
Normalised Counts
400
200
0
1−cell
2−cell
128−cell
1k−cell
Dome
50%−epiboly
Shield
75%−epiboly
1−4−somites
14−19−somites
20−25−somites
Prim−5
Prim−15
Prim−25
Long−pec
Protruding−mouth
Day−4
Day−5
Stage
Transcript
ENSDART00000025782
ENSDART00000144960
ENSDART00000147839

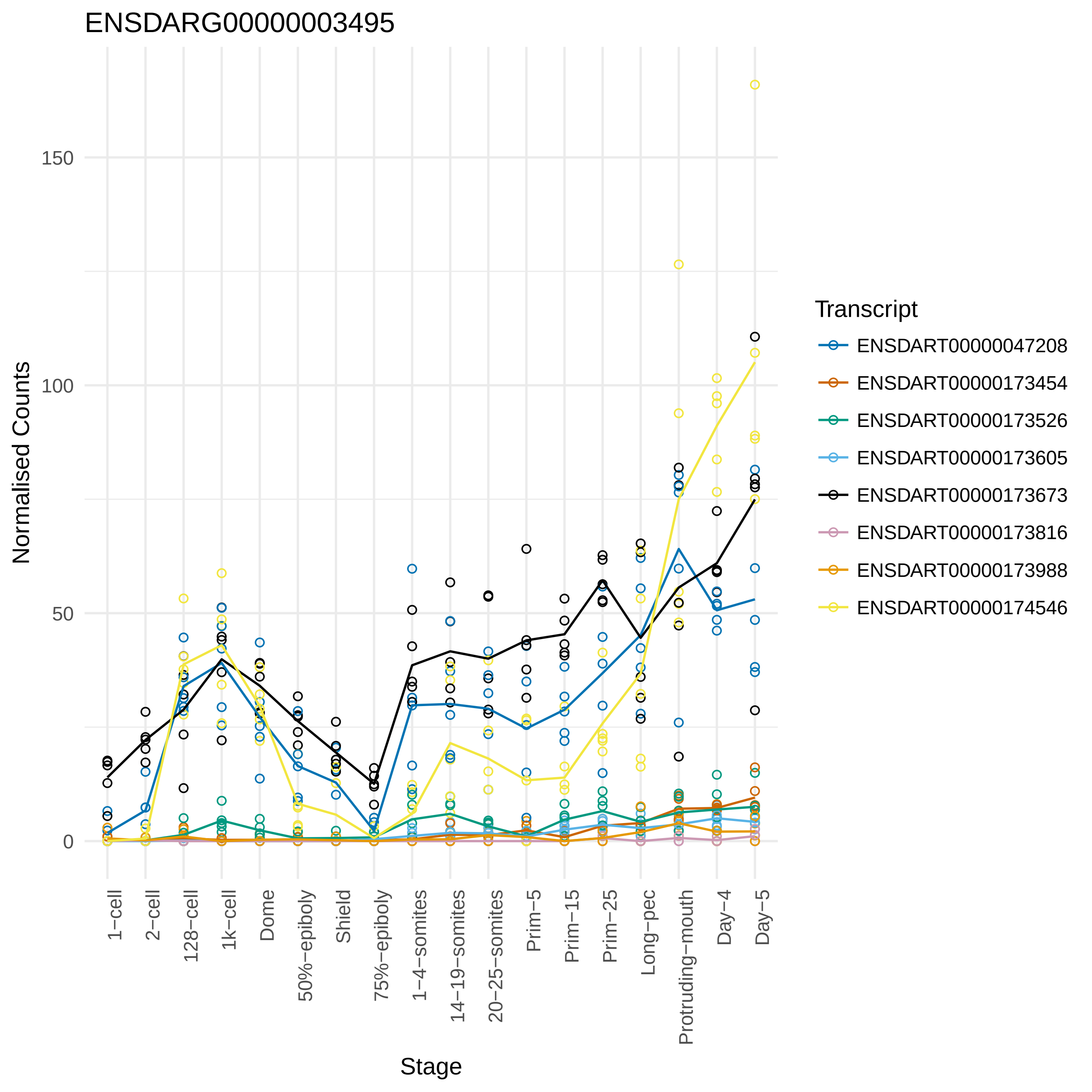

ENSDARG00000003495
Normalised Counts
0
50
100
150
Stage
1−cell
2−cell
128−cell
1k−cell
Dome
50%−epiboly
Shield
75%−epiboly
1−4−somites
14−19−somites
20−25−somites
Prim−5
Prim−15
Prim−25
Long−pec
Protruding−mouth
Day−4
Day−5
Transcript
ENSDART00000047208
ENSDART00000173454
ENSDART00000173526
ENSDART00000173605
ENSDART00000173673
ENSDART00000173816
ENSDART00000173988
ENSDART00000174546

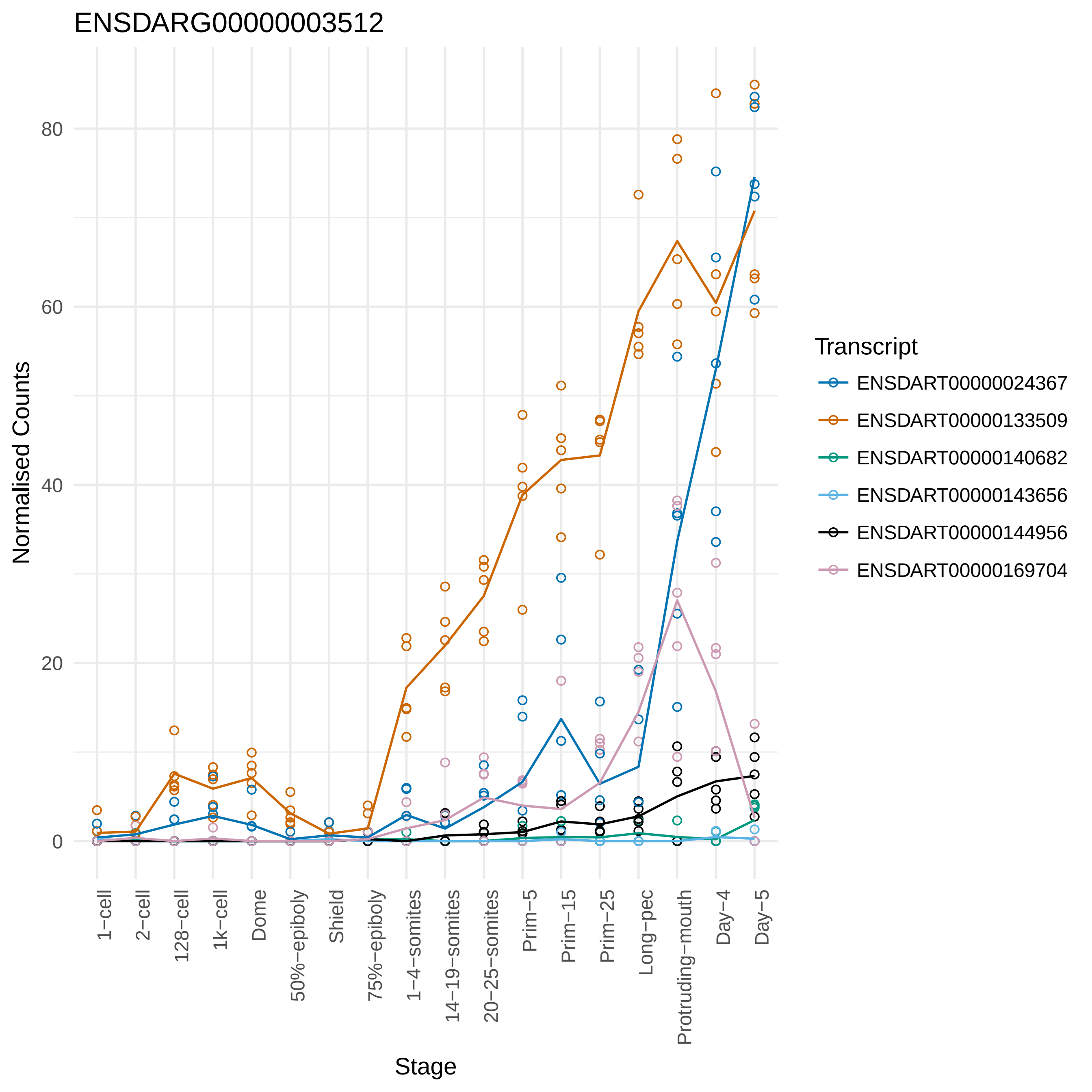
ENSDARG00000003512
Normalised Counts
0
20
40
60
80
1−cell
2−cell
128−cell
1k−cell
Dome
50%−epiboly
Shield
75%−epiboly
1−4−somites
14−19−somites
20−25−somites
Prim−5
Prim−15
Prim−25
Long−pec
Protruding−mouth
Day−4
Day−5
Stage
Transcript
ENSDART00000024367
ENSDART00000133509
ENSDART00000140682
ENSDART00000143656
ENSDART00000144956
ENSDART00000169704

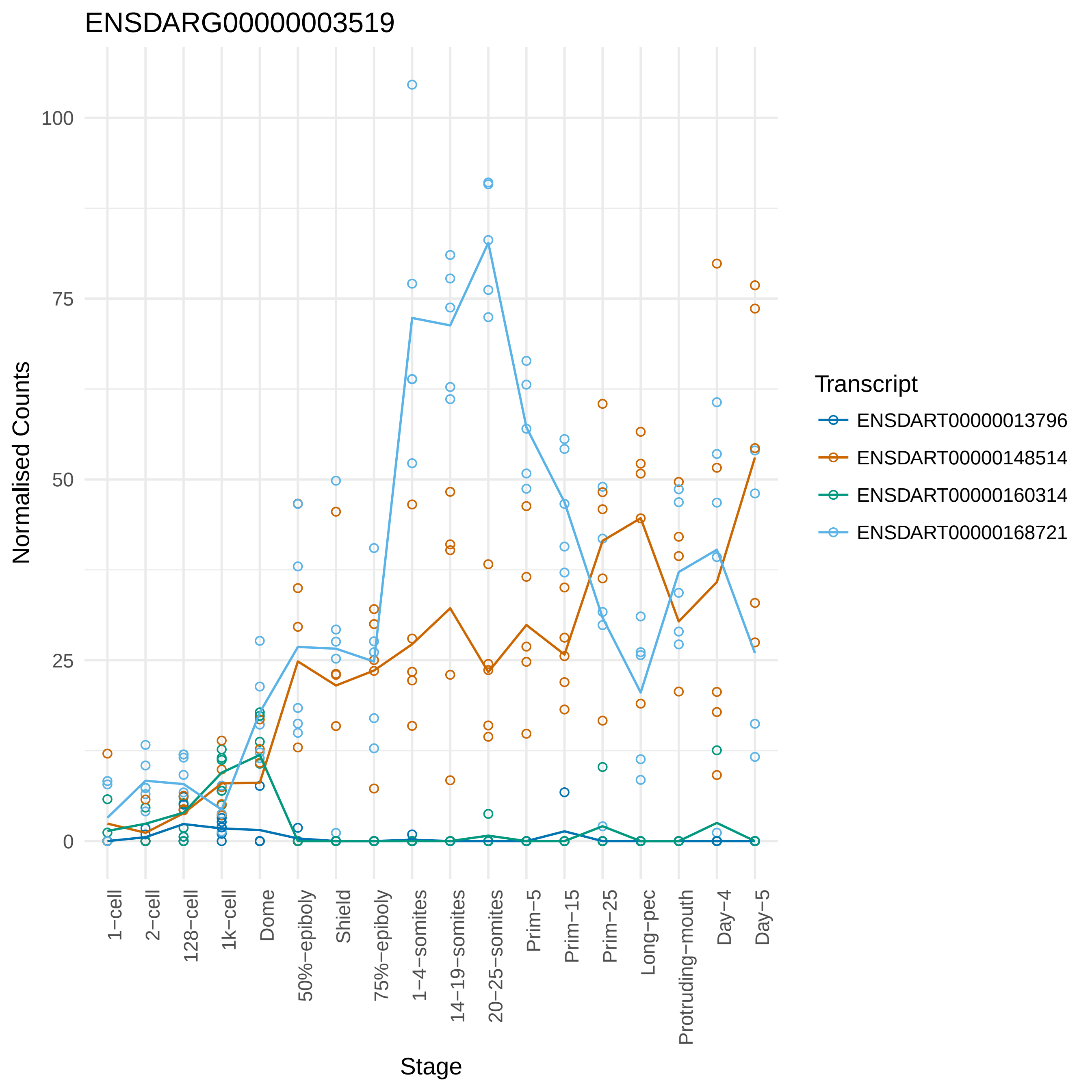

ENSDARG00000003519
Normalised Counts
100
75
50
25
0
1−cell
2−cell
128−cell
1k−cell
Dome
50%−epiboly
Shield
75%−epiboly
1−4−somites
14−19−somites
20−25−somites
Prim−5
Prim−15
Prim−25
Long−pec
Protruding−mouth
Day−4
Day−5
Stage
Transcript
ENSDART00000013796
ENSDART00000148514
ENSDART00000160314
ENSDART00000168721

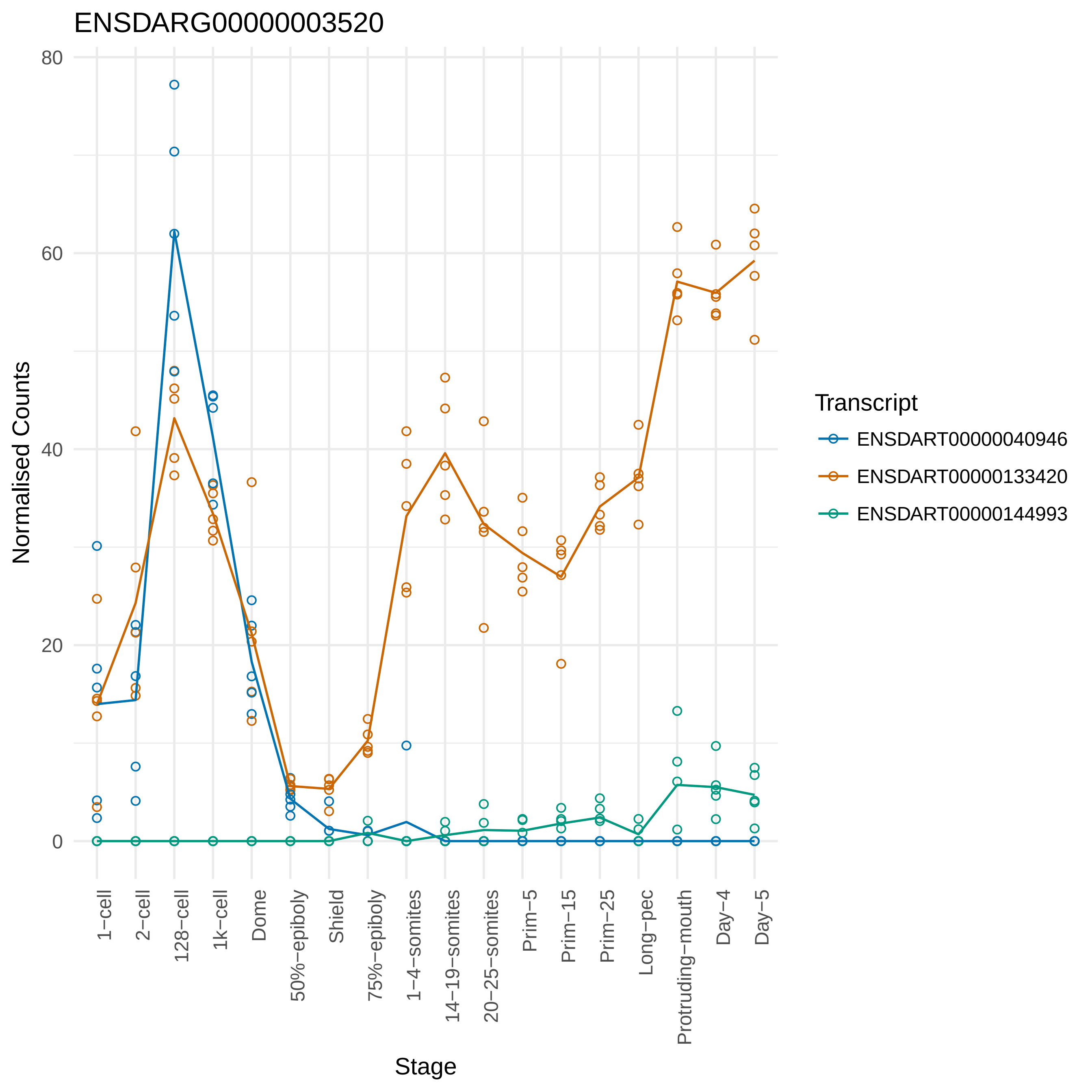

ENSDARG00000003520
Normalised Counts
0
20
40
60
80
Stage
1−cell
2−cell
128−cell
1k−cell
Dome
50%−epiboly
Shield
75%−epiboly
1−4−somites
14−19−somites
20−25−somites
Prim−5
Prim−15
Prim−25
Long−pec
Protruding−mouth
Day−4
Day−5
Transcript
ENSDART00000040946
ENSDART00000133420
ENSDART00000144993

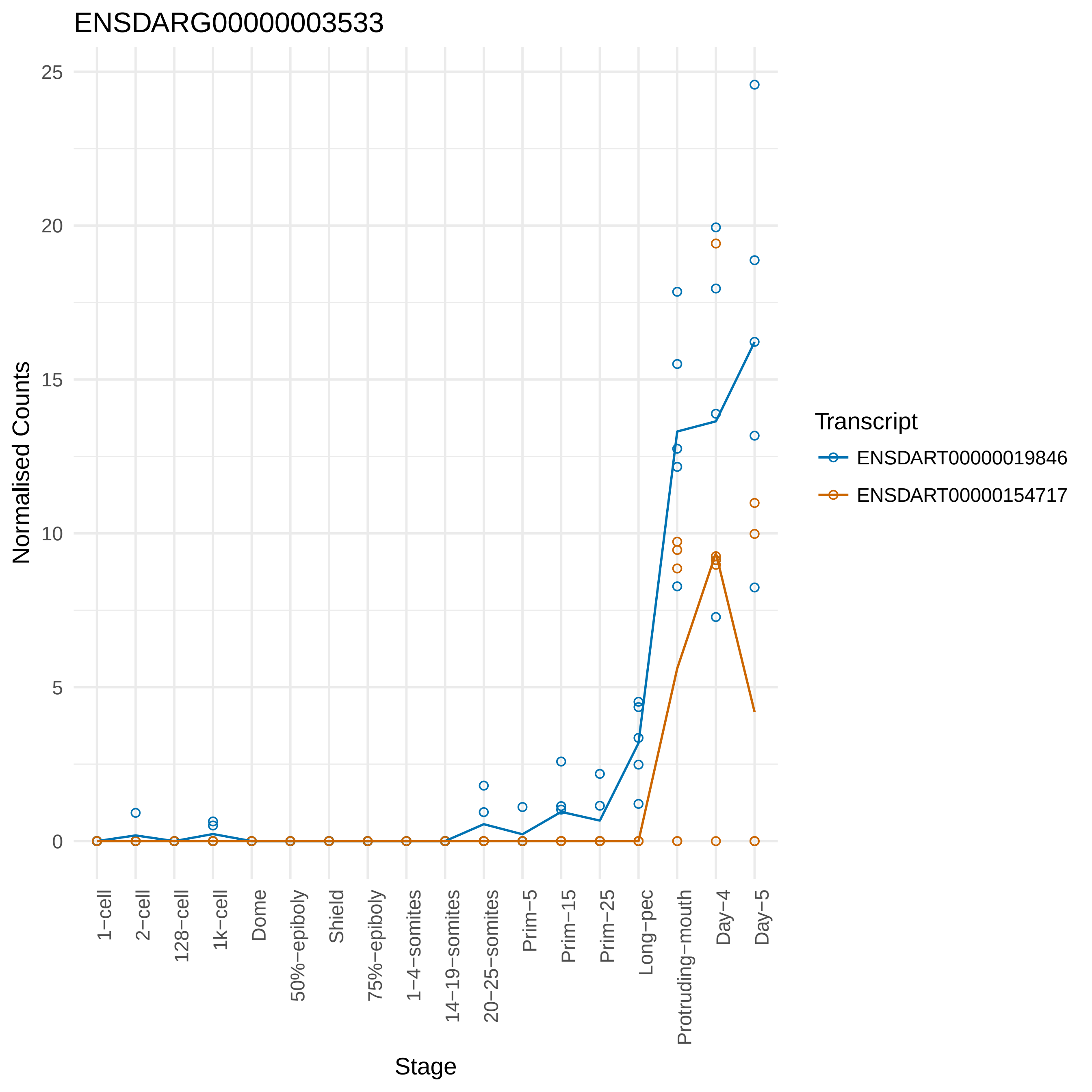

ENSDARG00000003533
Normalised Counts
0
5
10
15
20
25
1−cell
2−cell
128−cell
1k−cell
Dome
50%−epiboly
Shield
75%−epiboly
1−4−somites
14−19−somites
20−25−somites
Prim−5
Prim−15
Prim−25
Long−pec
Protruding−mouth
Day−4
Day−5
Stage
Transcript
ENSDART00000019846
ENSDART00000154717

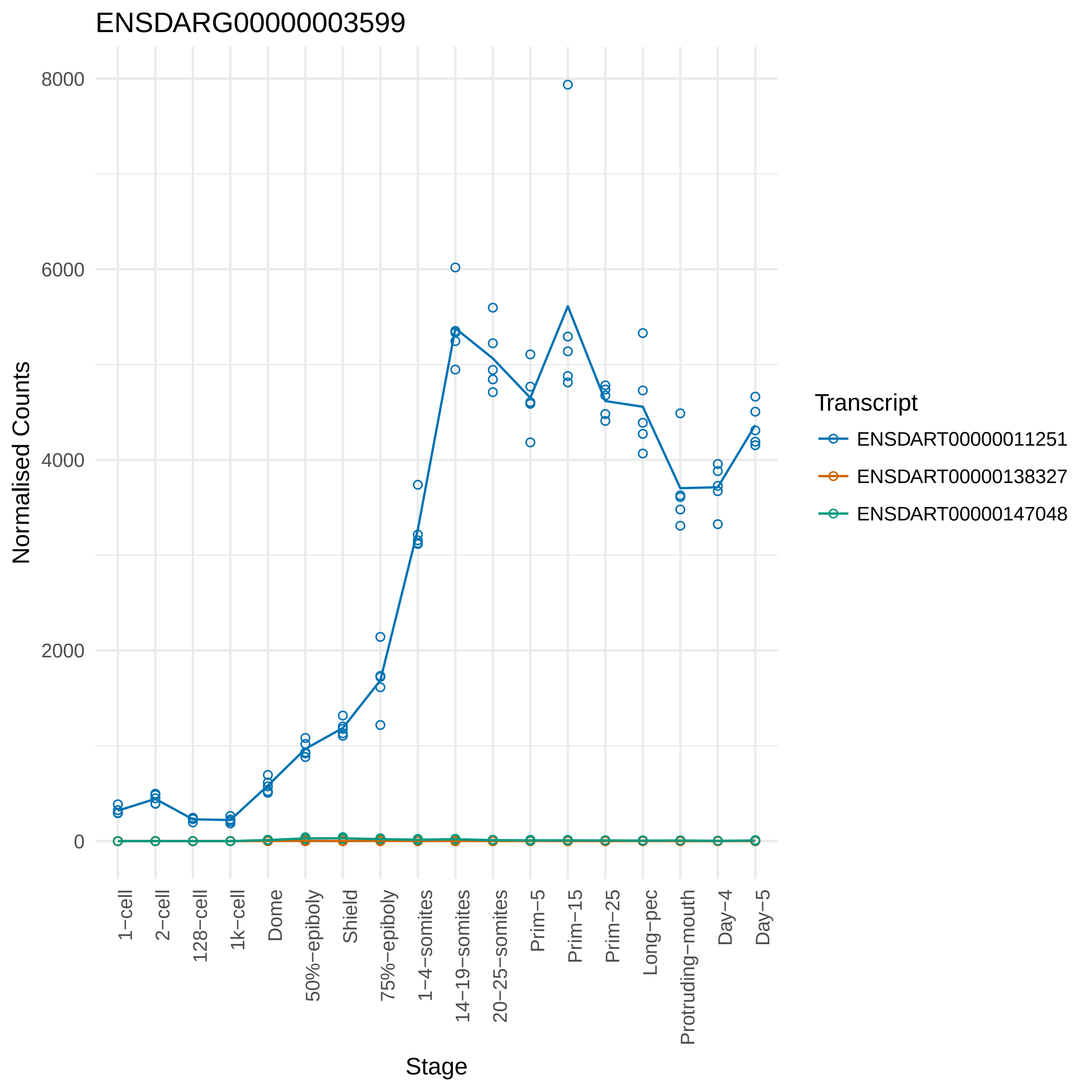

ENSDARG00000003599
Normalised Counts
8000
6000
4000
2000
0
Stage
1−cell
2−cell
128−cell
1k−cell
Dome
50%−epiboly
Shield
75%−epiboly
1−4−somites
14−19−somites
20−25−somites
Prim−5
Prim−15
Prim−25
Long−pec
Protruding−mouth
Day−4
Day−5
Transcript
ENSDART00000011251
ENSDART00000138327
ENSDART00000147048

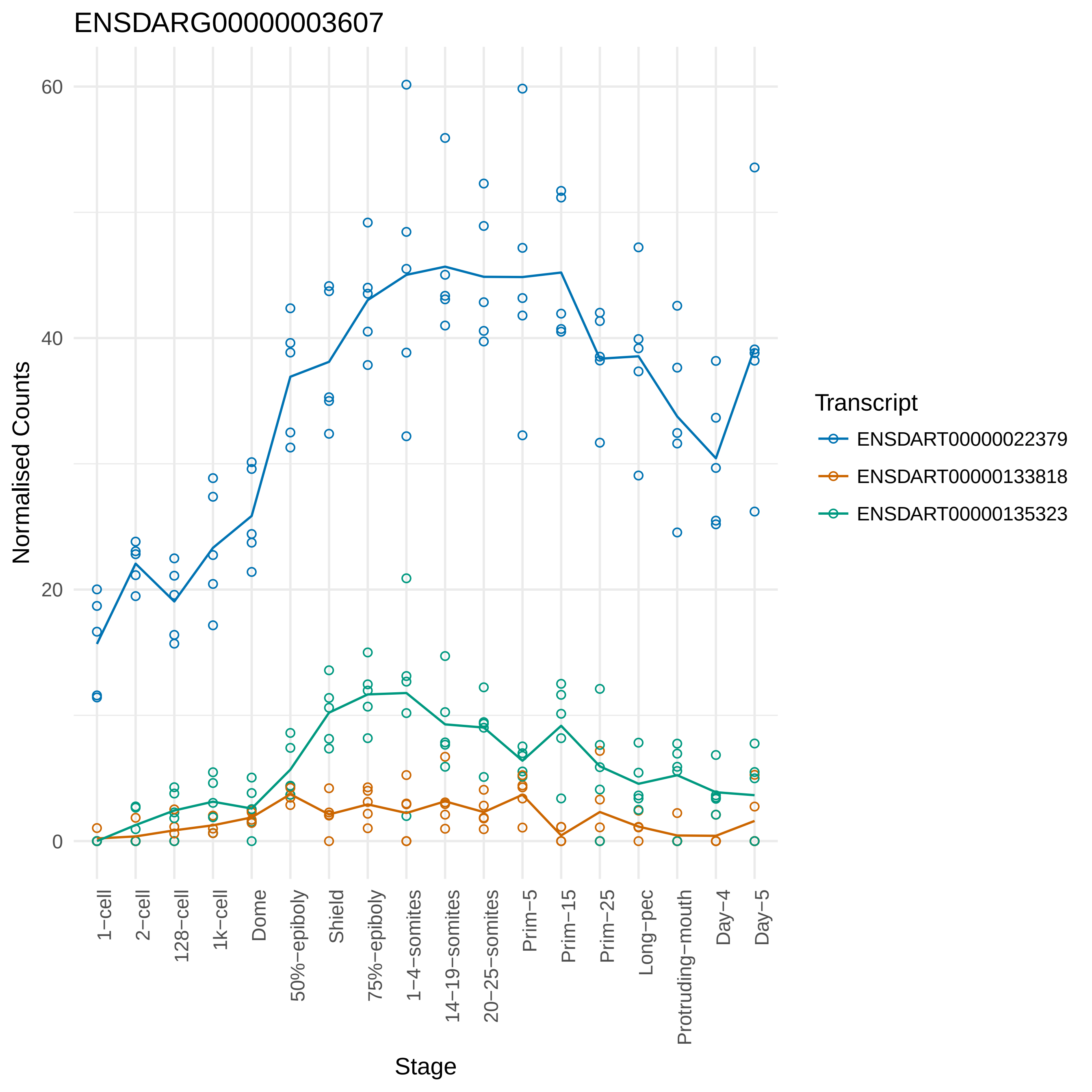
ENSDARG00000003607
Normalised Counts
60
40
20
0
1−cell
2−cell
128−cell
1k−cell
Dome
50%−epiboly
Shield
75%−epiboly
1−4−somites
14−19−somites
20−25−somites
Prim−5
Prim−15
Prim−25
Long−pec
Protruding−mouth
Day−4
Day−5
Stage
Transcript
ENSDART00000022379
ENSDART00000133818
ENSDART00000135323

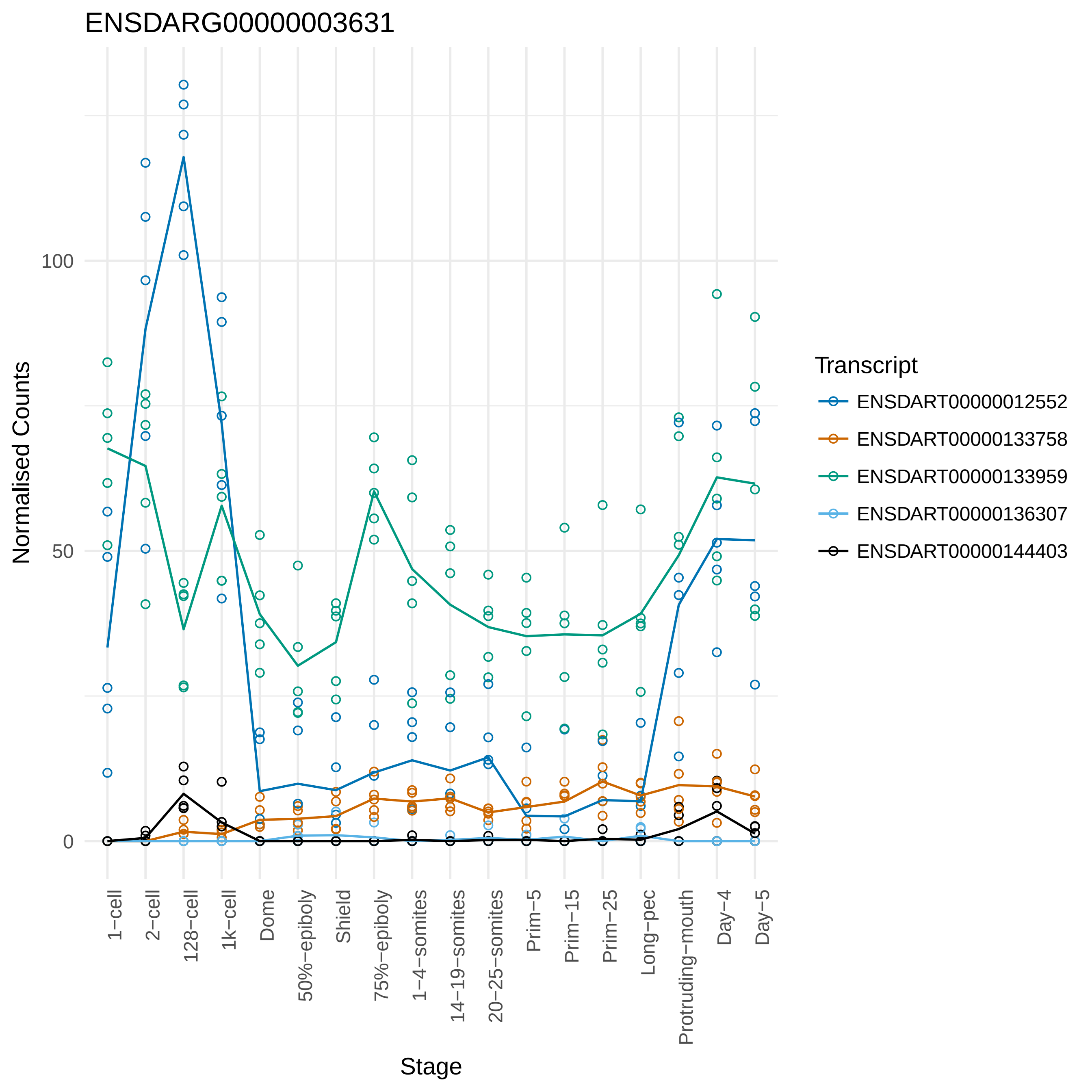

ENSDARG00000003631
Normalised Counts
100
50
0
1−cell
2−cell
128−cell
1k−cell
Dome
50%−epiboly
Shield
75%−epiboly
1−4−somites
14−19−somites
20−25−somites
Prim−5
Prim−15
Prim−25
Long−pec
Protruding−mouth
Day−4
Day−5
Stage
Transcript
ENSDART00000012552
ENSDART00000133758
ENSDART00000133959
ENSDART00000136307
ENSDART00000144403

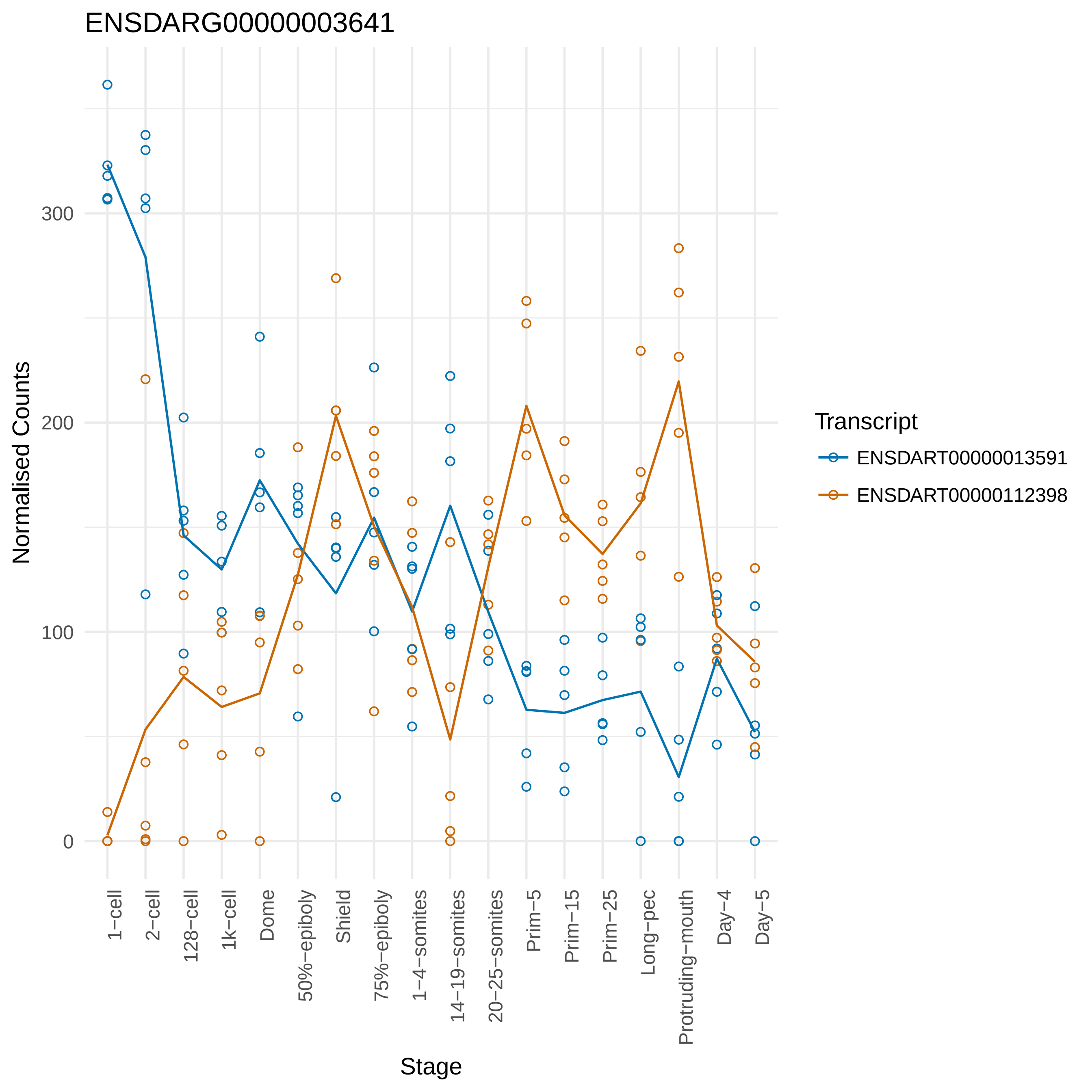

ENSDARG00000003641
Normalised Counts
0
100
200
300
Stage
1−cell
2−cell
128−cell
1k−cell
Dome
50%−epiboly
Shield
75%−epiboly
1−4−somites
14−19−somites
20−25−somites
Prim−5
Prim−15
Prim−25
Long−pec
Protruding−mouth
Day−4
Day−5
Transcript
ENSDART00000013591
ENSDART00000112398

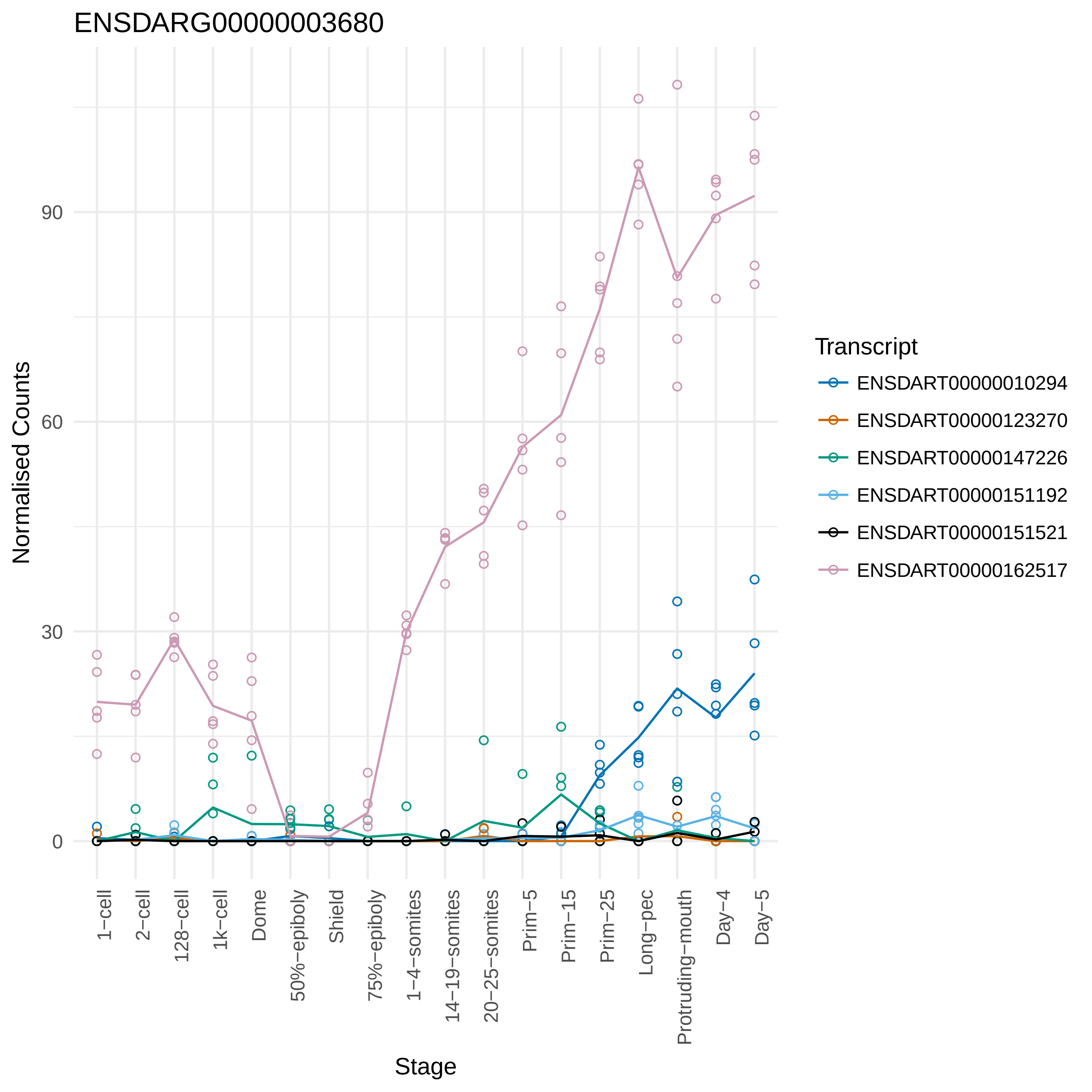

ENSDARG00000003680
Normalised Counts
0
30
60
90
Stage
1−cell
2−cell
128−cell
1k−cell
Dome
50%−epiboly
Shield
75%−epiboly
1−4−somites
14−19−somites
20−25−somites
Prim−5
Prim−15
Prim−25
Long−pec
Protruding−mouth
Day−4
Day−5
Transcript
ENSDART00000010294
ENSDART00000123270
ENSDART00000147226
ENSDART00000151192
ENSDART00000151521
ENSDART00000162517

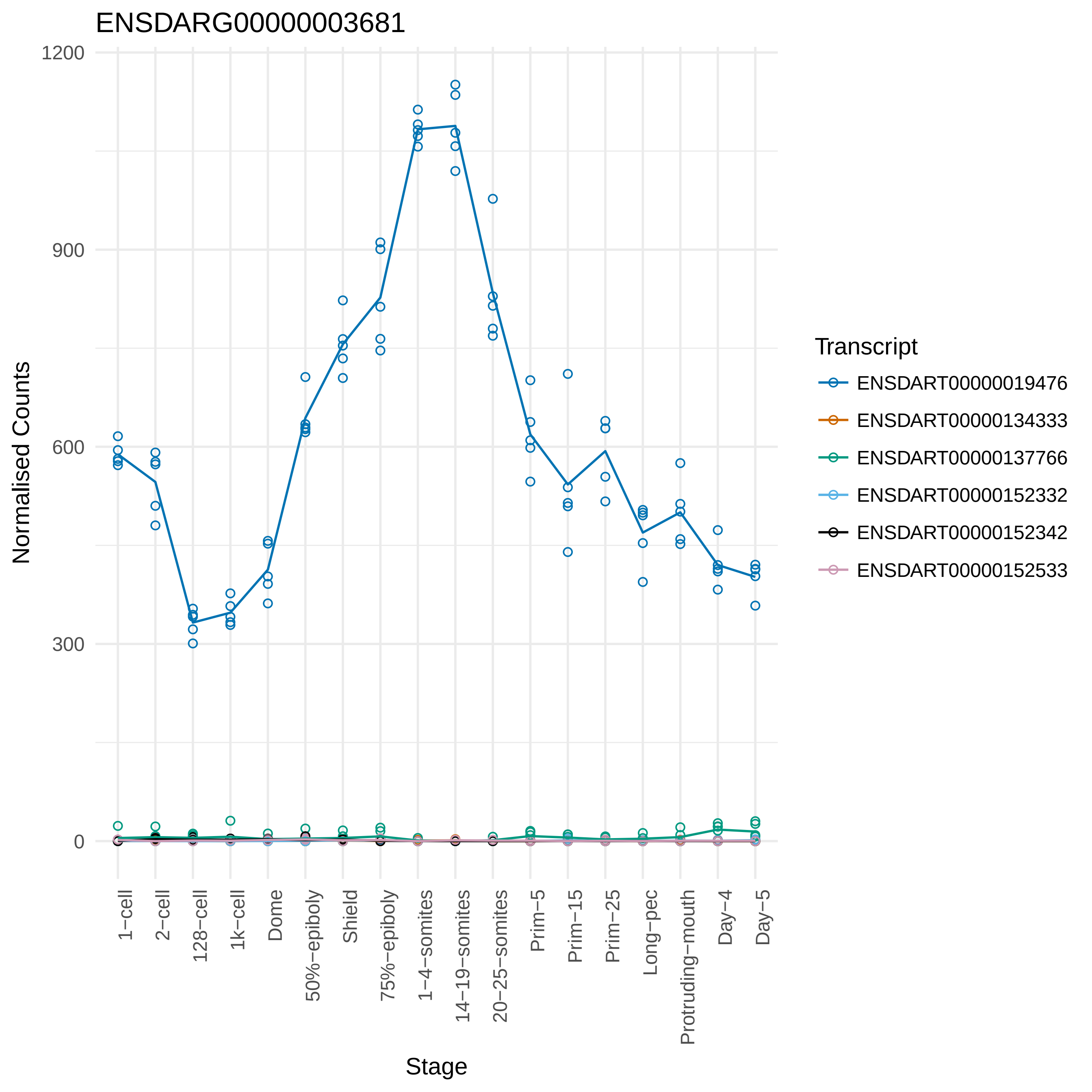

ENSDARG00000003681
Normalised Counts
1200
900
600
300
0
Stage
1−cell
2−cell
128−cell
1k−cell
Dome
50%−epiboly
Shield
75%−epiboly
1−4−somites
14−19−somites
20−25−somites
Prim−5
Prim−15
Prim−25
Long−pec
Protruding−mouth
Day−4
Day−5
Transcript
ENSDART00000019476
ENSDART00000134333
ENSDART00000137766
ENSDART00000152332
ENSDART00000152342
ENSDART00000152533

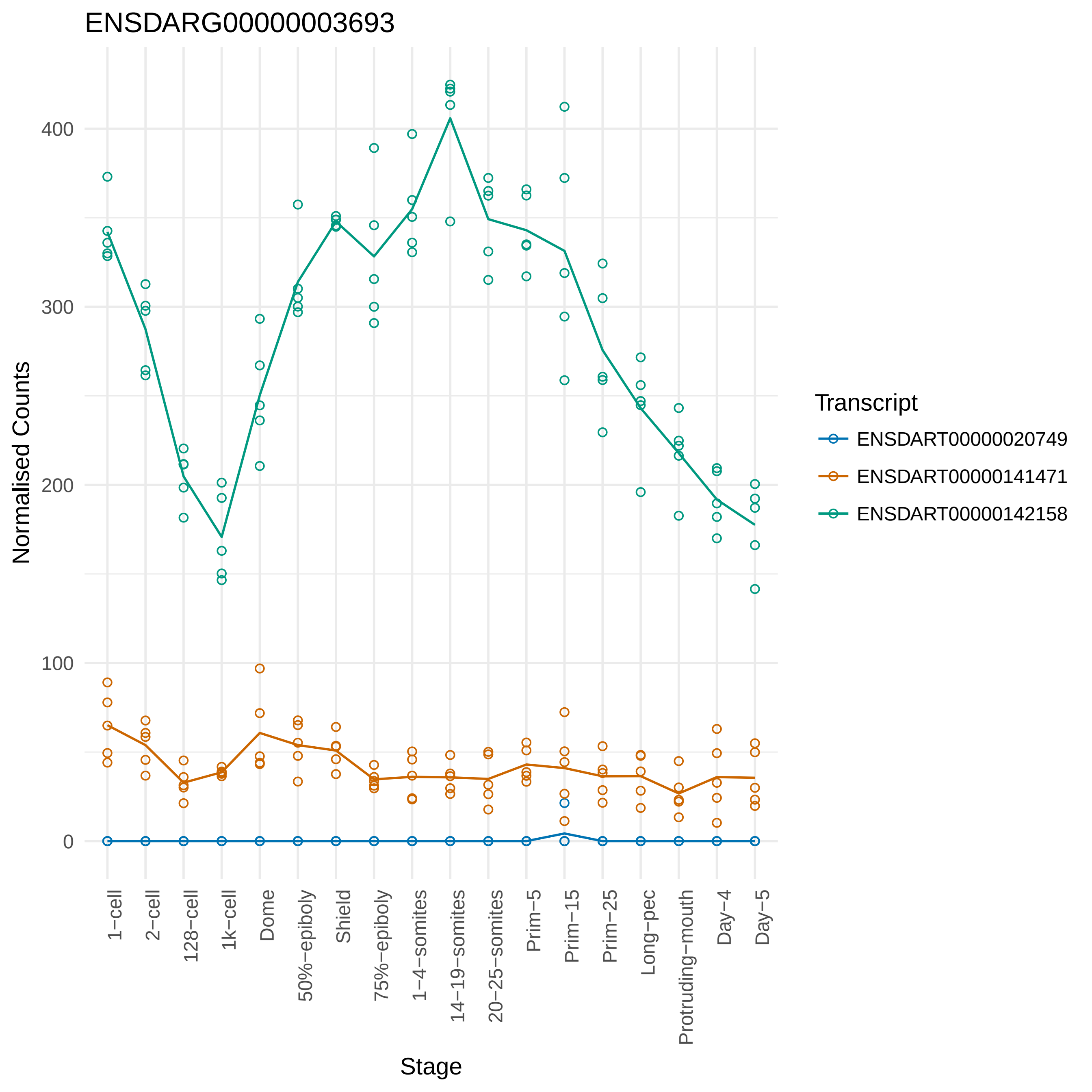
ENSDARG00000003693
Normalised Counts
0
100
200
300
400
Stage
1−cell
2−cell
128−cell
1k−cell
Dome
50%−epiboly
Shield
75%−epiboly
1−4−somites
14−19−somites
20−25−somites
Prim−5
Prim−15
Prim−25
Long−pec
Protruding−mouth
Day−4
Day−5
Transcript
ENSDART00000020749
ENSDART00000141471
ENSDART00000142158

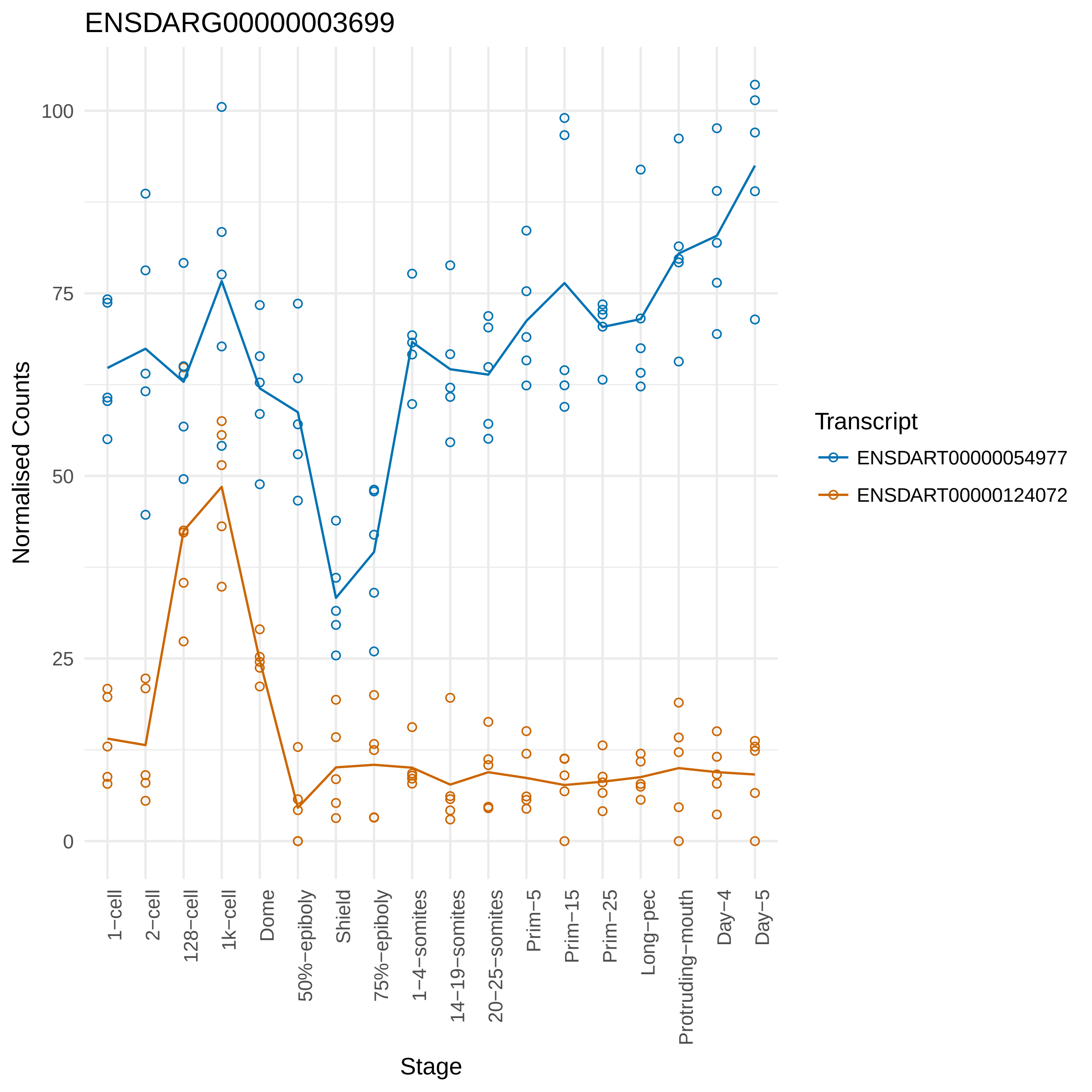

ENSDARG00000003699
Normalised Counts
100
75
50
25
0
1−cell
2−cell
128−cell
1k−cell
Dome
50%−epiboly
Shield
75%−epiboly
1−4−somites
14−19−somites
20−25−somites
Prim−5
Prim−15
Prim−25
Long−pec
Protruding−mouth
Day−4
Day−5
Stage
Transcript
ENSDART00000054977
ENSDART00000124072

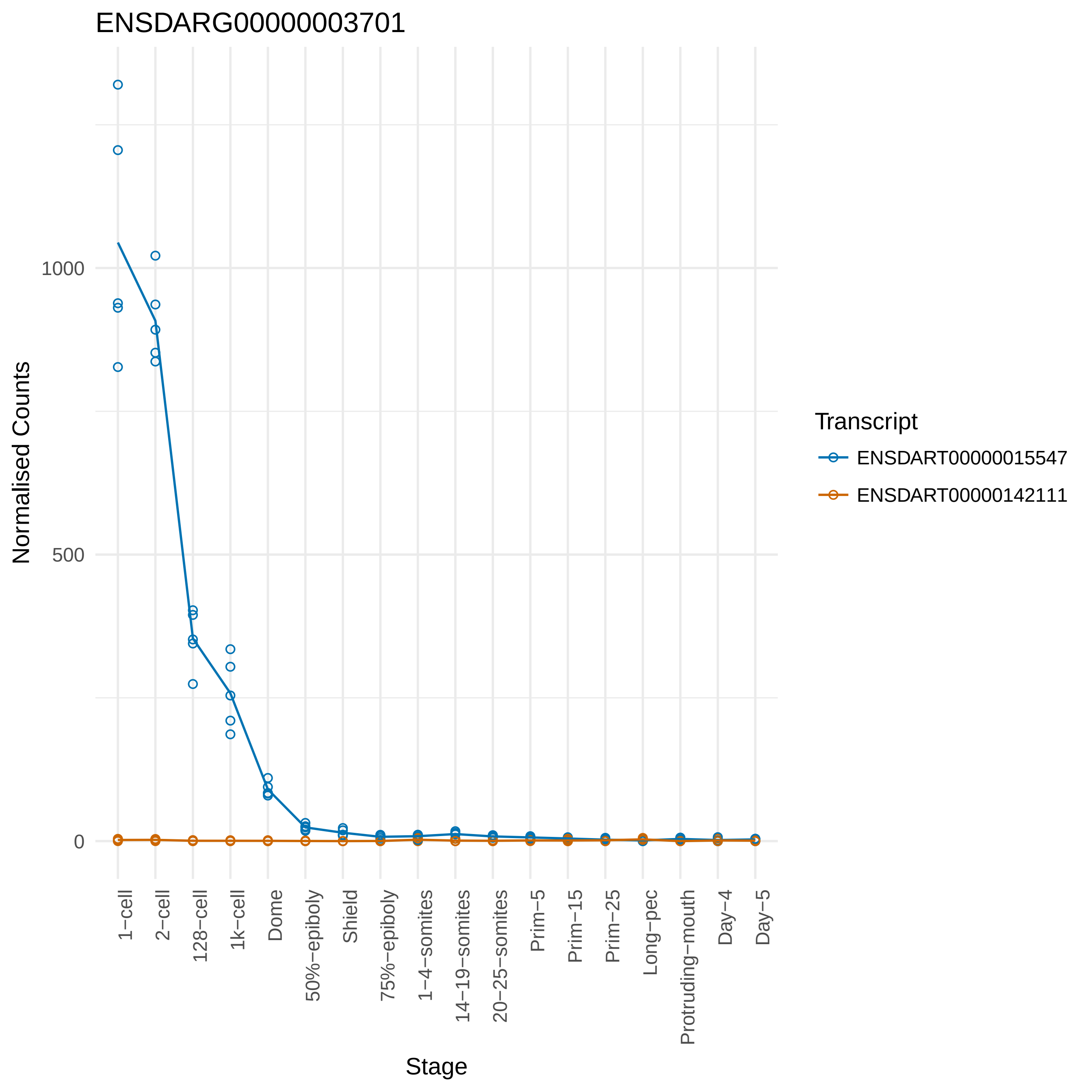
ENSDARG00000003701
Normalised Counts
1000
500
0
1−cell
2−cell
128−cell
1k−cell
Dome
50%−epiboly
Shield
75%−epiboly
1−4−somites
14−19−somites
20−25−somites
Prim−5
Prim−15
Prim−25
Long−pec
Protruding−mouth
Day−4
Day−5
Stage
Transcript
ENSDART00000015547
ENSDART00000142111

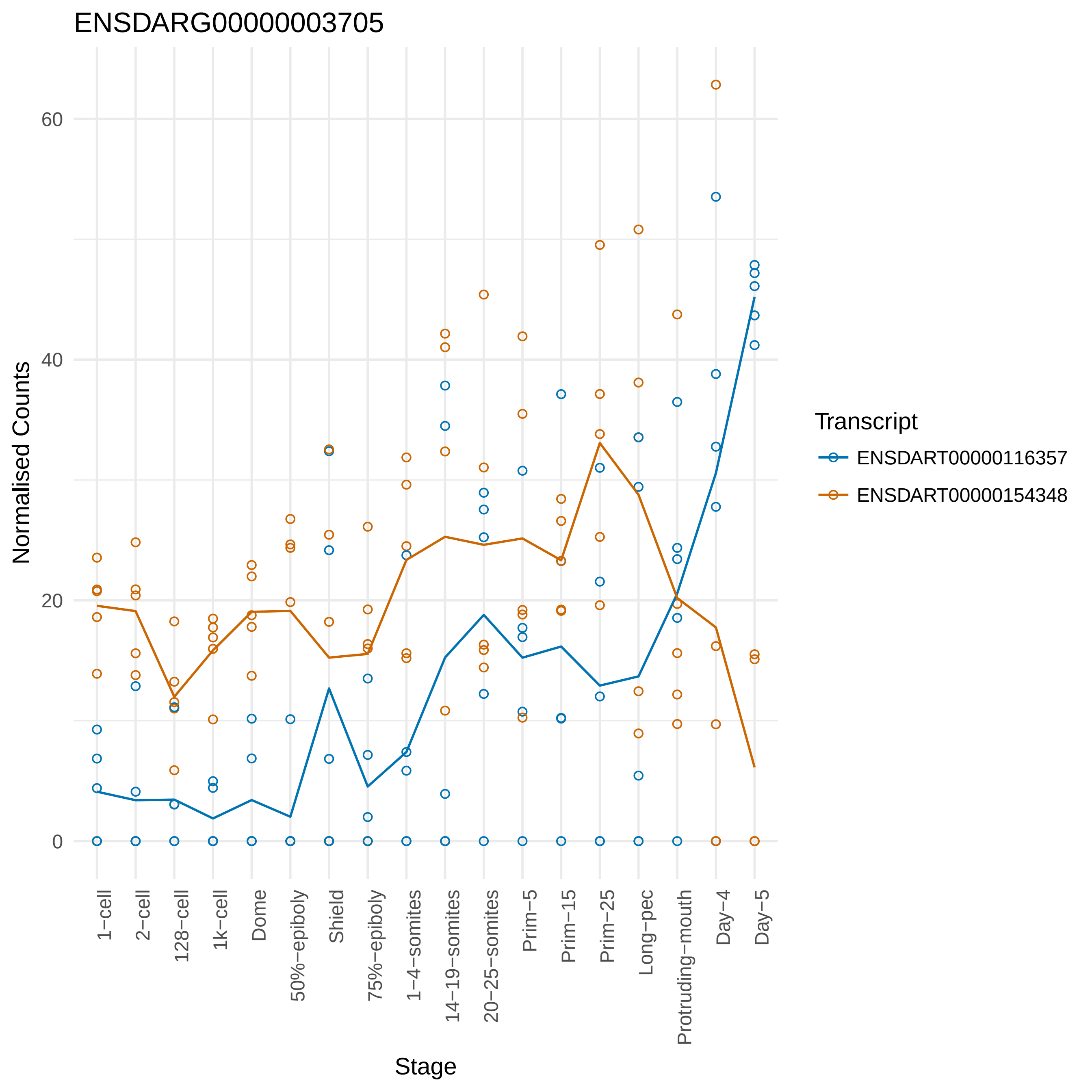

ENSDARG00000003705
Normalised Counts
0
20
40
60
1−cell
2−cell
128−cell
1k−cell
Dome
50%−epiboly
Shield
75%−epiboly
1−4−somites
14−19−somites
20−25−somites
Prim−5
Prim−15
Prim−25
Long−pec
Protruding−mouth
Day−4
Day−5
Stage
Transcript
ENSDART00000116357
ENSDART00000154348

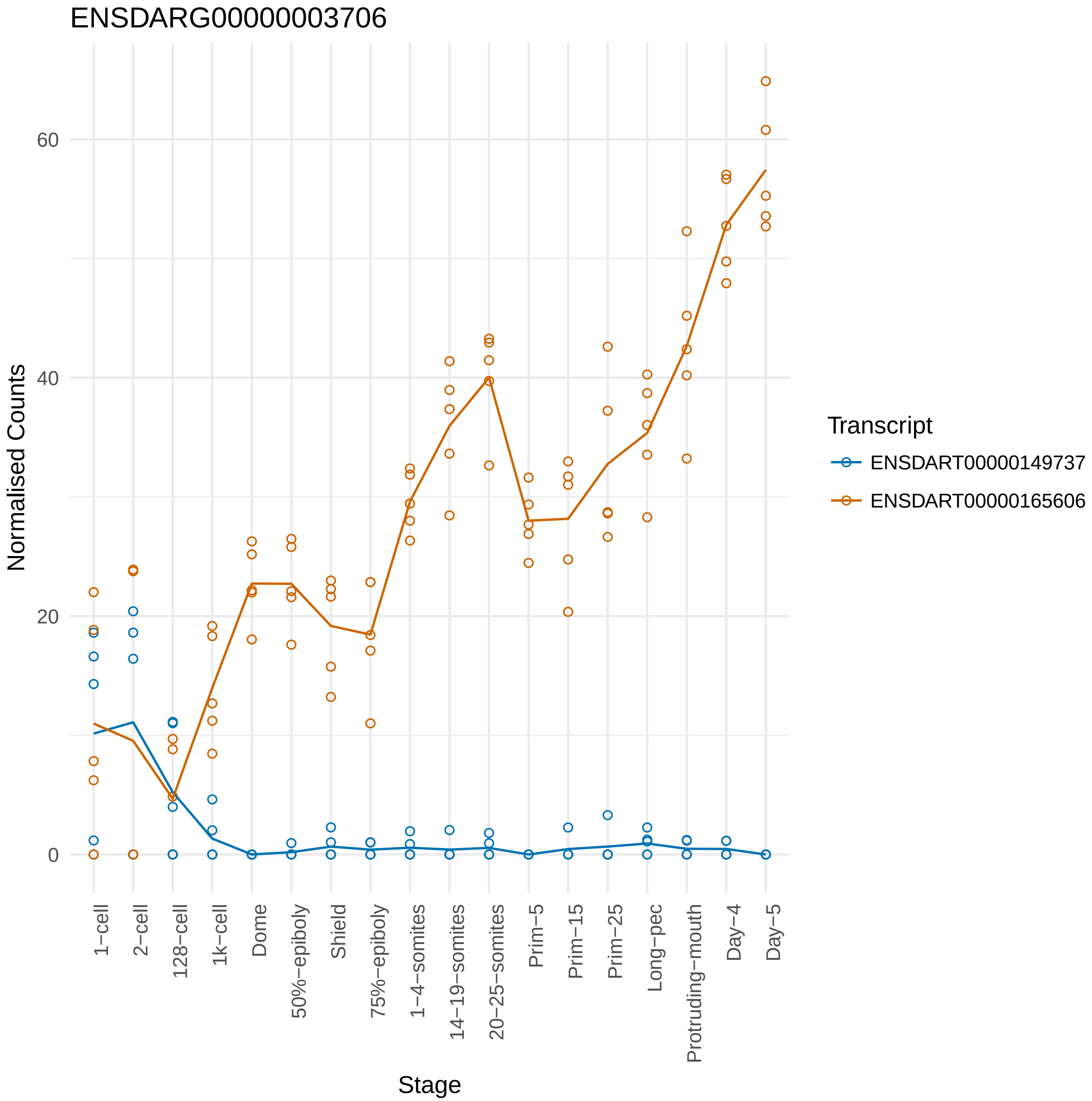
ENSDARG00000003706
Normalised Counts
60
40
20
0
1−cell
2−cell
128−cell
1k−cell
Dome
50%−epiboly
Shield
75%−epiboly
1−4−somites
14−19−somites
20−25−somites
Prim−5
Prim−15
Prim−25
Long−pec
Protruding−mouth
Day−4
Day−5
Stage
Transcript
ENSDART00000149737
ENSDART00000165606

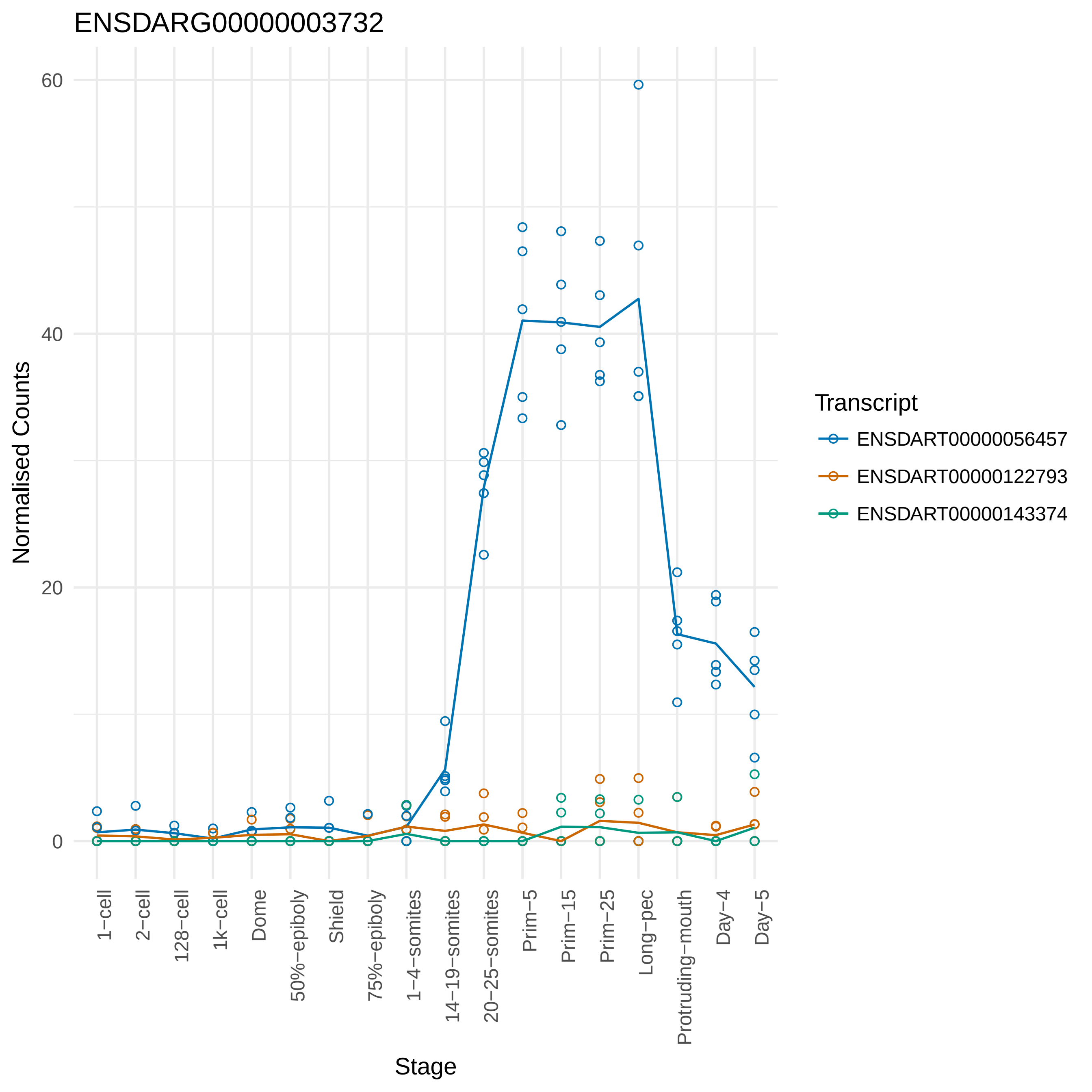

ENSDARG00000003732
Normalised Counts
60
40
20
0
Stage
1−cell
2−cell
128−cell
1k−cell
Dome
50%−epiboly
Shield
75%−epiboly
1−4−somites
14−19−somites
20−25−somites
Prim−5
Prim−15
Prim−25
Long−pec
Protruding−mouth
Day−4
Day−5
Transcript
ENSDART00000056457
ENSDART00000122793
ENSDART00000143374

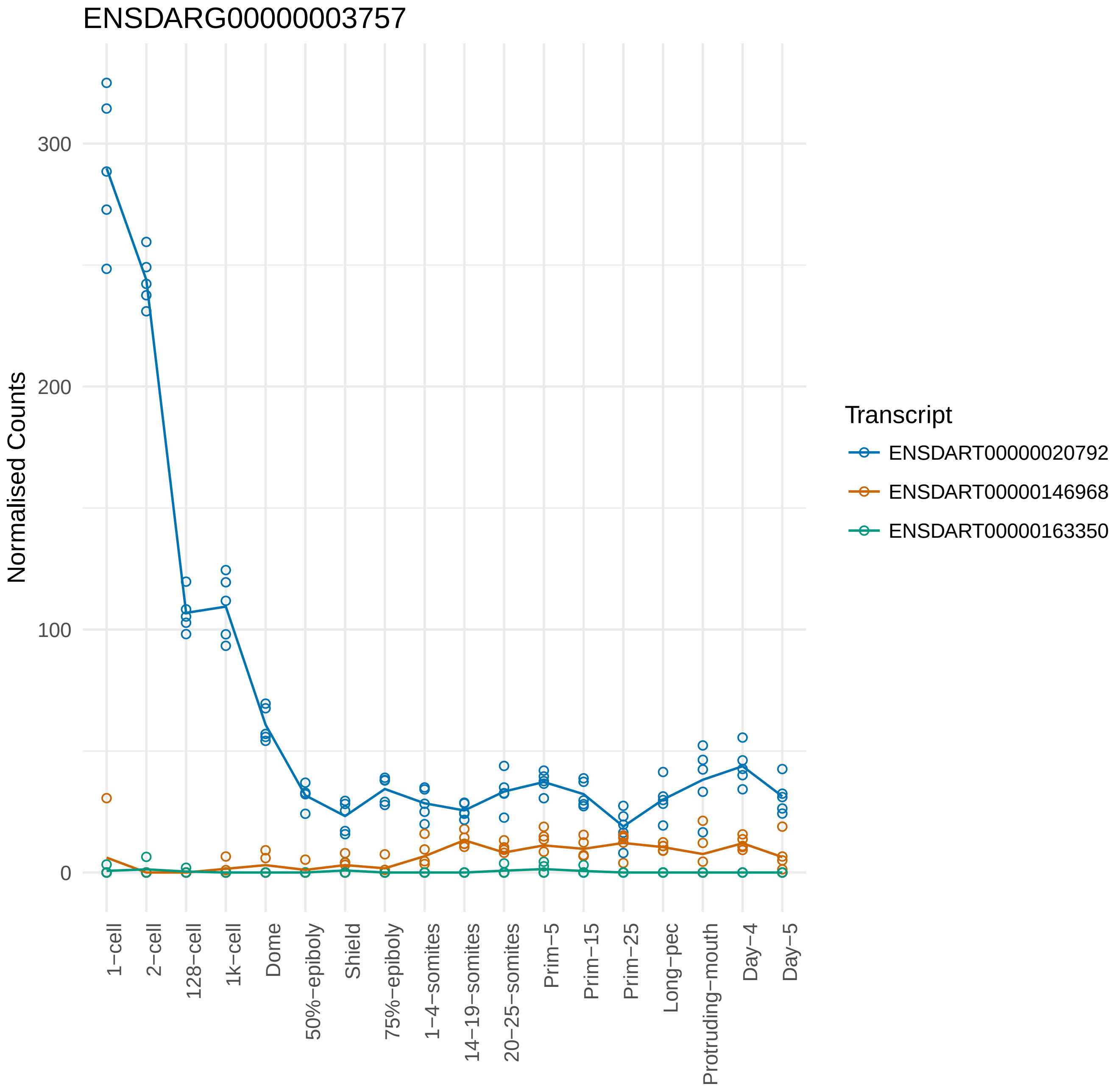
ENSDARG00000003757
Normalised Counts
300
200
100
0
1−cell
2−cell
128−cell
1k−cell
Dome
50%−epiboly
Shield
75%−epiboly
1−4−somites
14−19−somites
20−25−somites
Prim−5
Prim−15
Prim−25
Long−pec
Protruding−mouth
Day−4
Day−5
Stage
Transcript
ENSDART00000020792
ENSDART00000146968
ENSDART00000163350

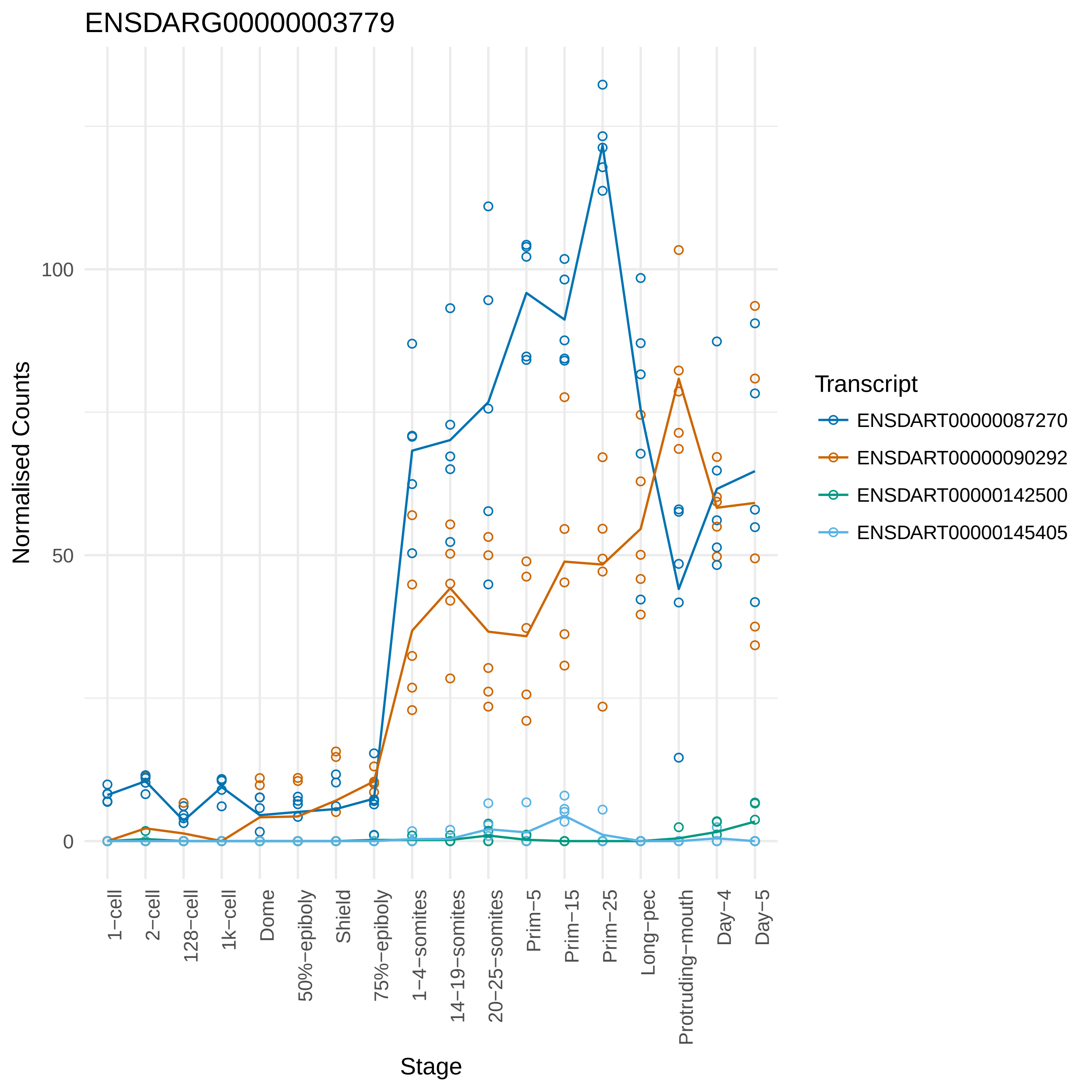
ENSDARG00000003779
Normalised Counts
0
50
100
Stage
1−cell
2−cell
128−cell
1k−cell
Dome
50%−epiboly
Shield
75%−epiboly
1−4−somites
14−19−somites
20−25−somites
Prim−5
Prim−15
Prim−25
Long−pec
Protruding−mouth
Day−4
Day−5
Transcript
ENSDART00000087270
ENSDART00000090292
ENSDART00000142500
ENSDART00000145405

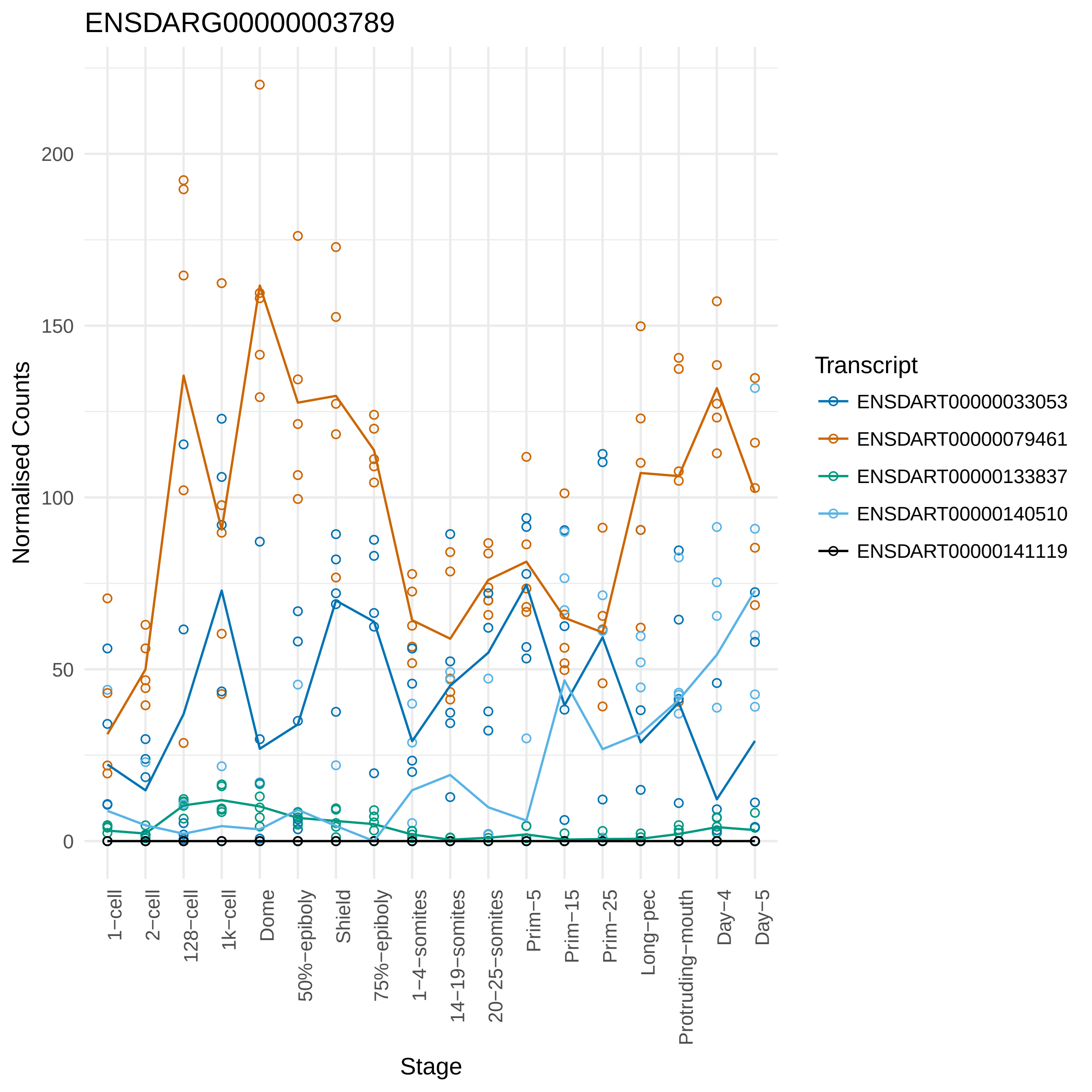

ENSDARG00000003789
Normalised Counts
200
150
100
50
0
1−cell
2−cell
128−cell
1k−cell
Dome
50%−epiboly
Shield
75%−epiboly
1−4−somites
14−19−somites
20−25−somites
Prim−5
Prim−15
Prim−25
Long−pec
Protruding−mouth
Day−4
Day−5
Stage
Transcript
ENSDART00000033053
ENSDART00000079461
ENSDART00000133837
ENSDART00000140510
ENSDART00000141119

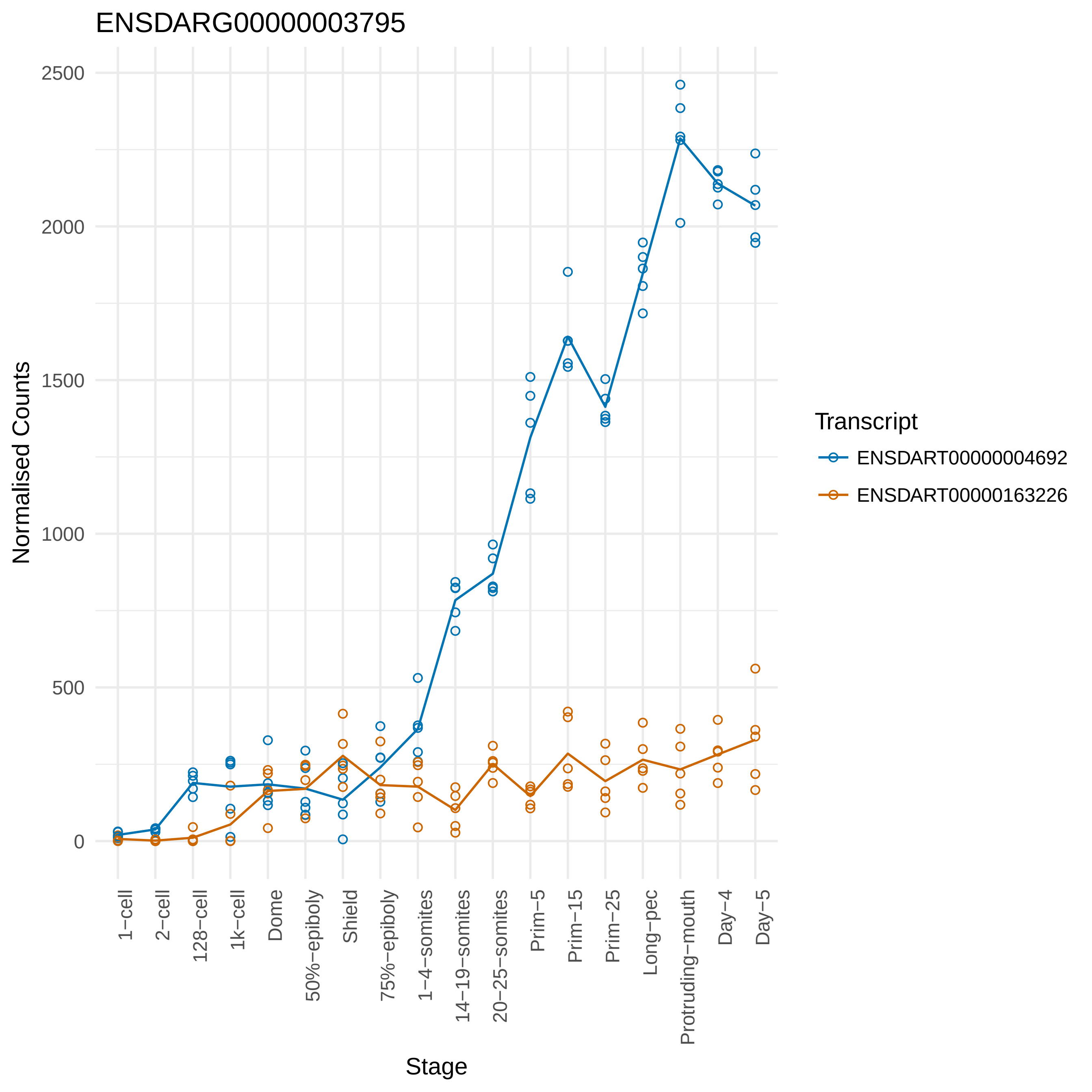

ENSDARG00000003795
Normalised Counts
2500
2000
1500
1000
500
0
Stage
1−cell
2−cell
128−cell
1k−cell
Dome
50%−epiboly
Shield
75%−epiboly
1−4−somites
14−19−somites
20−25−somites
Prim−5
Prim−15
Prim−25
Long−pec
Protruding−mouth
Day−4
Day−5
Transcript
ENSDART00000004692
ENSDART00000163226

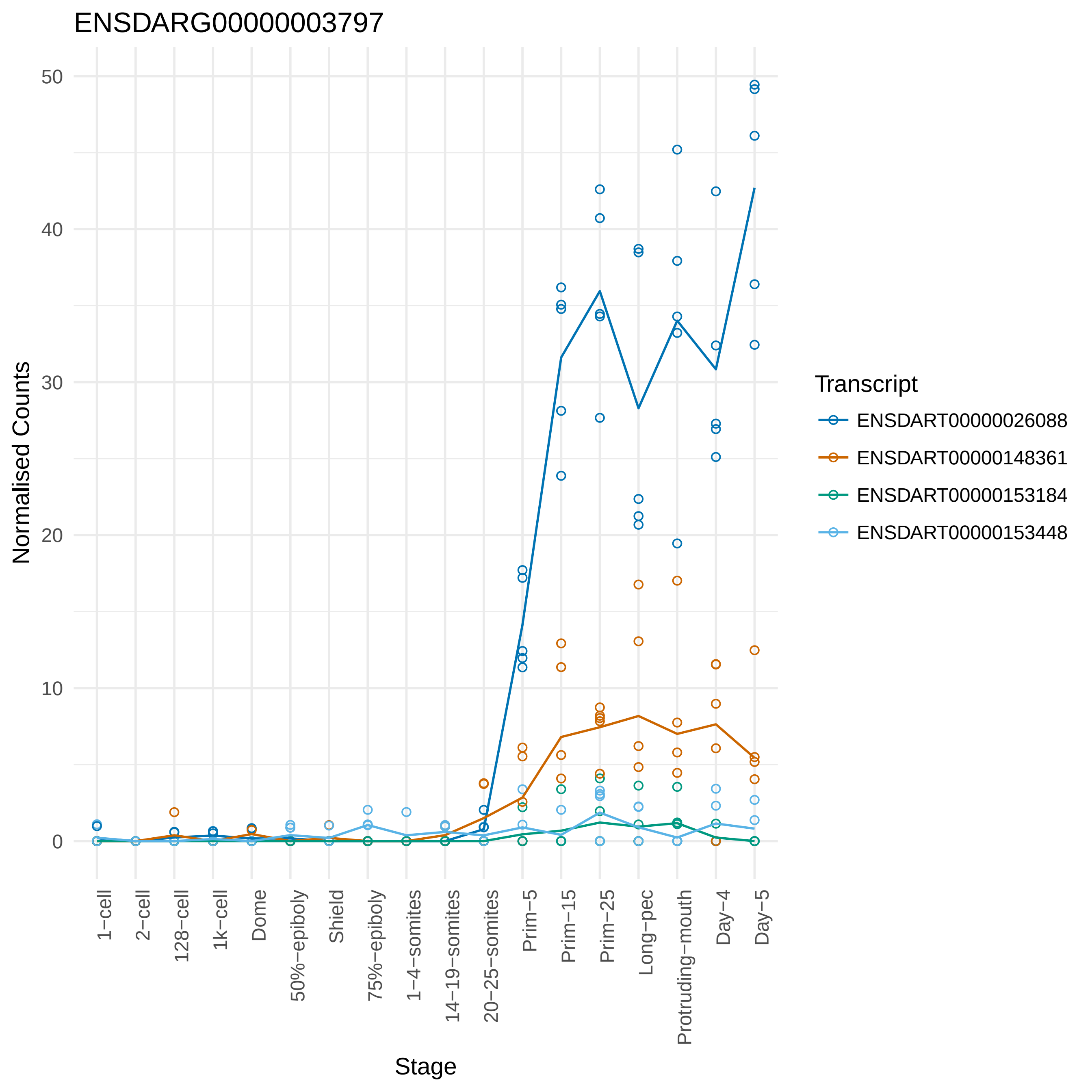

ENSDARG00000003797
Normalised Counts
50
40
30
20
10
0
1−cell
2−cell
128−cell
1k−cell
Dome
50%−epiboly
Shield
75%−epiboly
1−4−somites
14−19−somites
20−25−somites
Prim−5
Prim−15
Prim−25
Long−pec
Protruding−mouth
Day−4
Day−5
Stage
Transcript
ENSDART00000026088
ENSDART00000148361
ENSDART00000153184
ENSDART00000153448

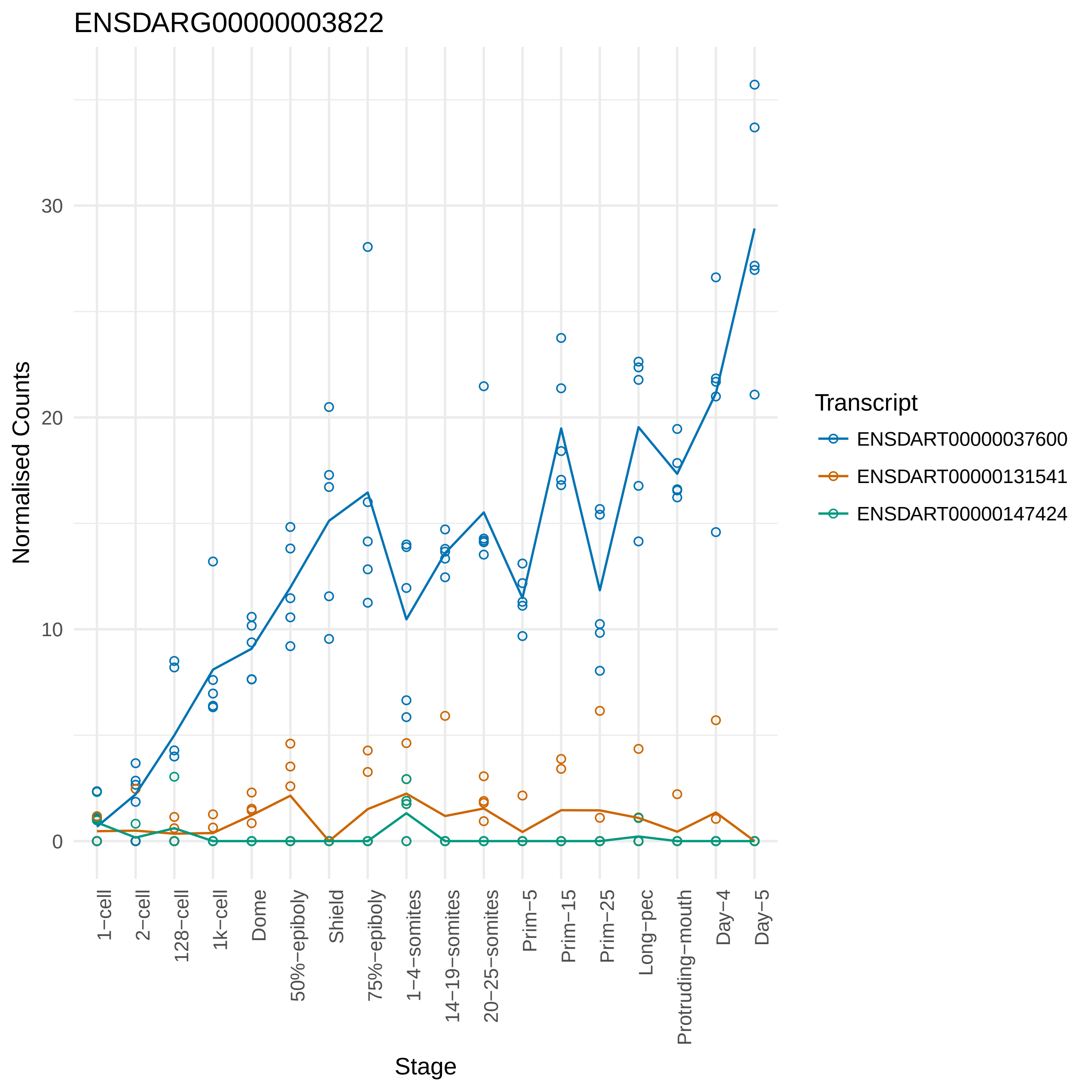
ENSDARG00000003822
Normalised Counts
0
10
20
30
1−cell
2−cell
128−cell
1k−cell
Dome
50%−epiboly
Shield
75%−epiboly
1−4−somites
14−19−somites
20−25−somites
Prim−5
Prim−15
Prim−25
Long−pec
Protruding−mouth
Day−4
Day−5
Stage
Transcript
ENSDART00000037600
ENSDART00000131541
ENSDART00000147424

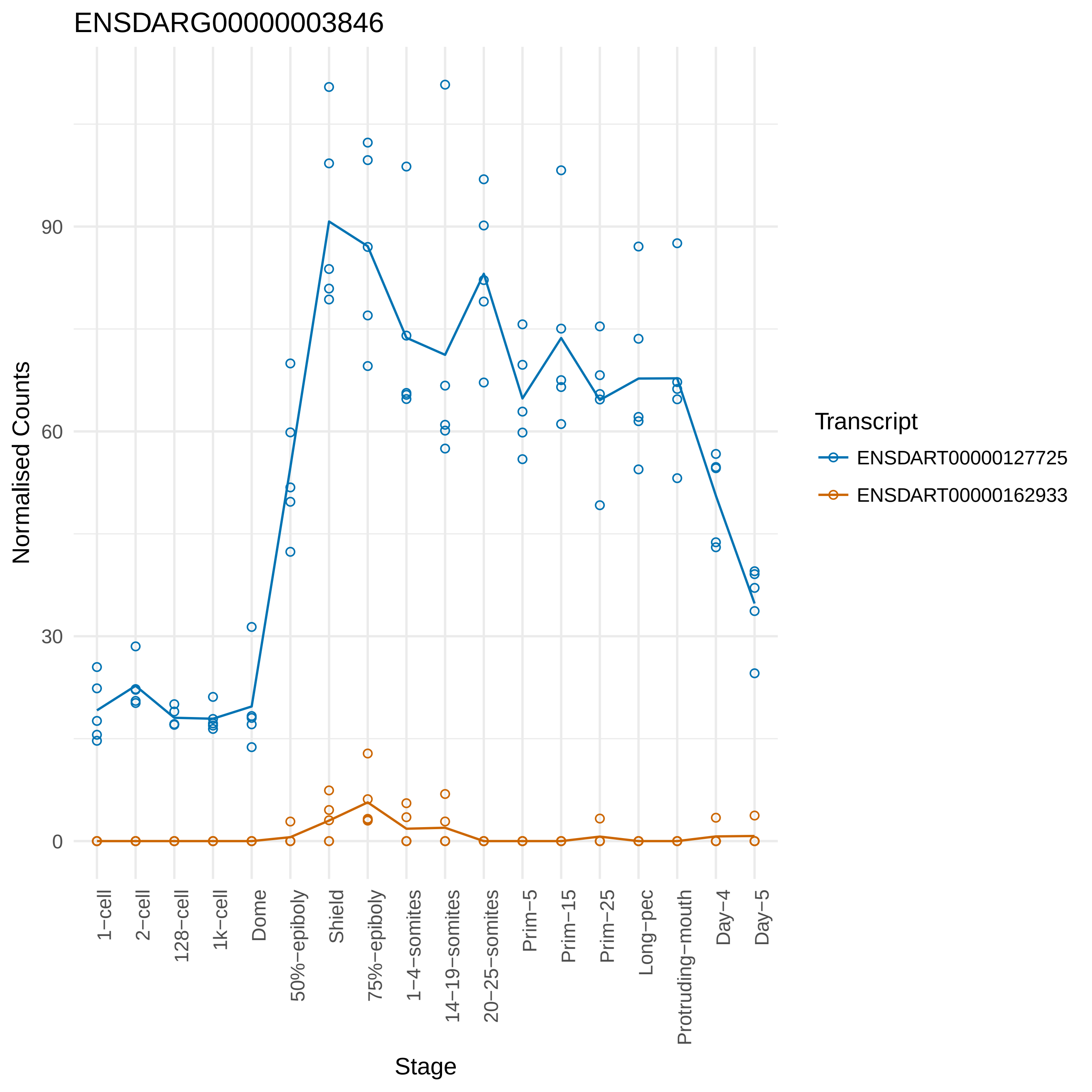

ENSDARG00000003846
Normalised Counts
0
30
60
90
1−cell
2−cell
128−cell
1k−cell
Dome
50%−epiboly
Shield
75%−epiboly
1−4−somites
14−19−somites
20−25−somites
Prim−5
Prim−15
Prim−25
Long−pec
Protruding−mouth
Day−4
Day−5
Stage
Transcript
ENSDART00000127725
ENSDART00000162933

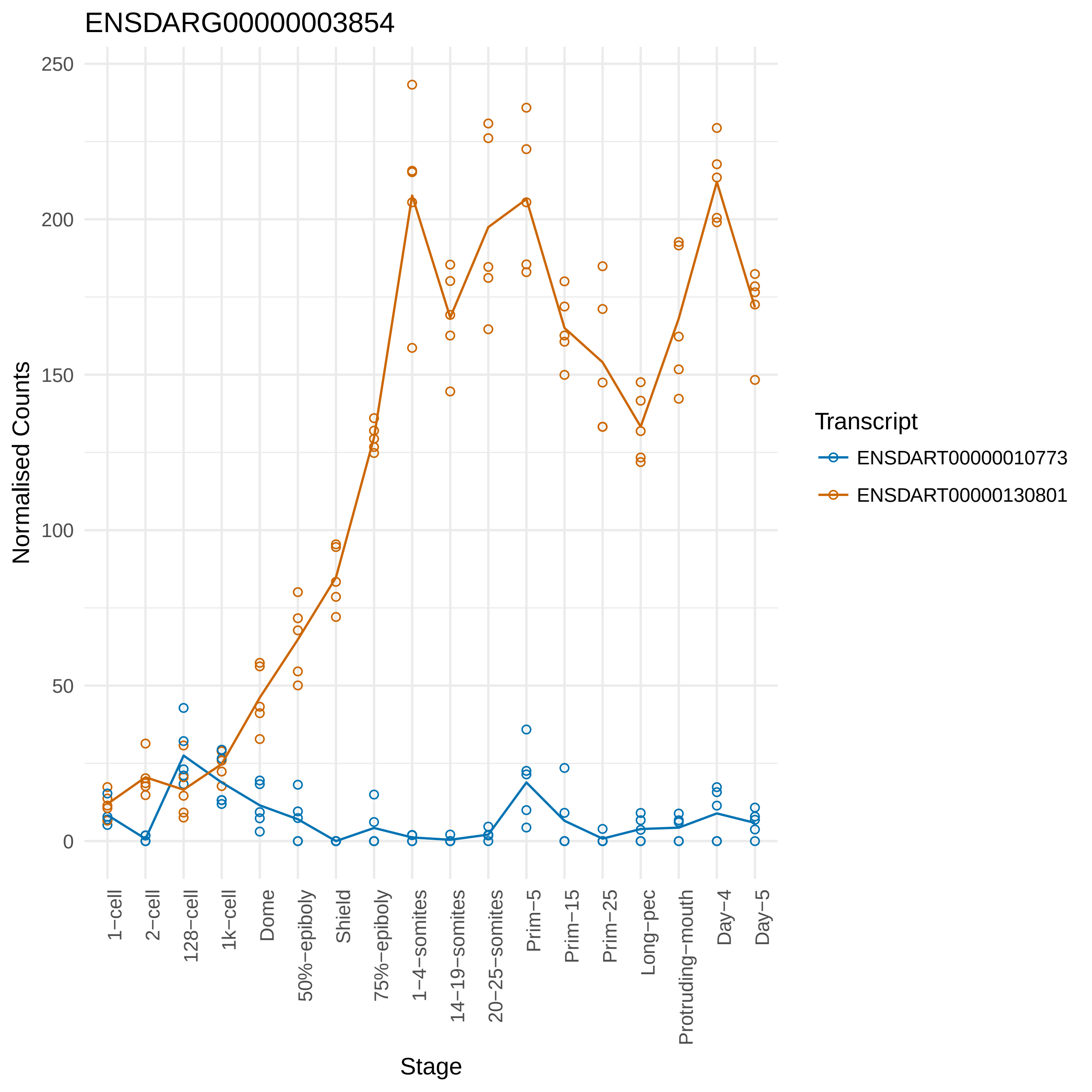

ENSDARG00000003854
Normalised Counts
250
200
150
100
50
0
1−cell
2−cell
128−cell
1k−cell
Dome
50%−epiboly
Shield
75%−epiboly
1−4−somites
14−19−somites
20−25−somites
Prim−5
Prim−15
Prim−25
Long−pec
Protruding−mouth
Day−4
Day−5
Stage
Transcript
ENSDART00000010773
ENSDART00000130801

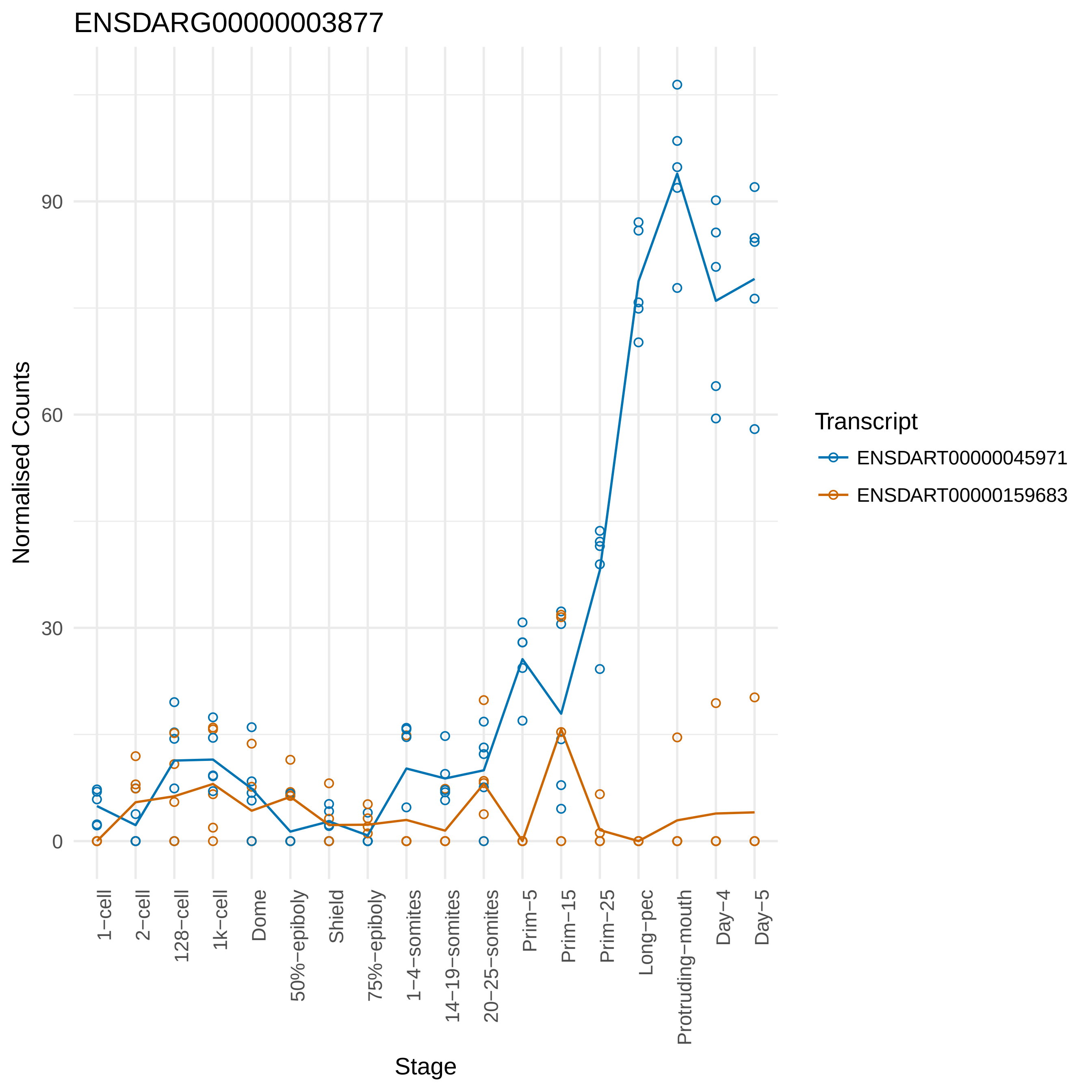

ENSDARG00000003877
Normalised Counts
0
30
60
90
1−cell
2−cell
128−cell
1k−cell
Dome
50%−epiboly
Shield
75%−epiboly
1−4−somites
14−19−somites
20−25−somites
Prim−5
Prim−15
Prim−25
Long−pec
Protruding−mouth
Day−4
Day−5
Stage
Transcript
ENSDART00000045971
ENSDART00000159683

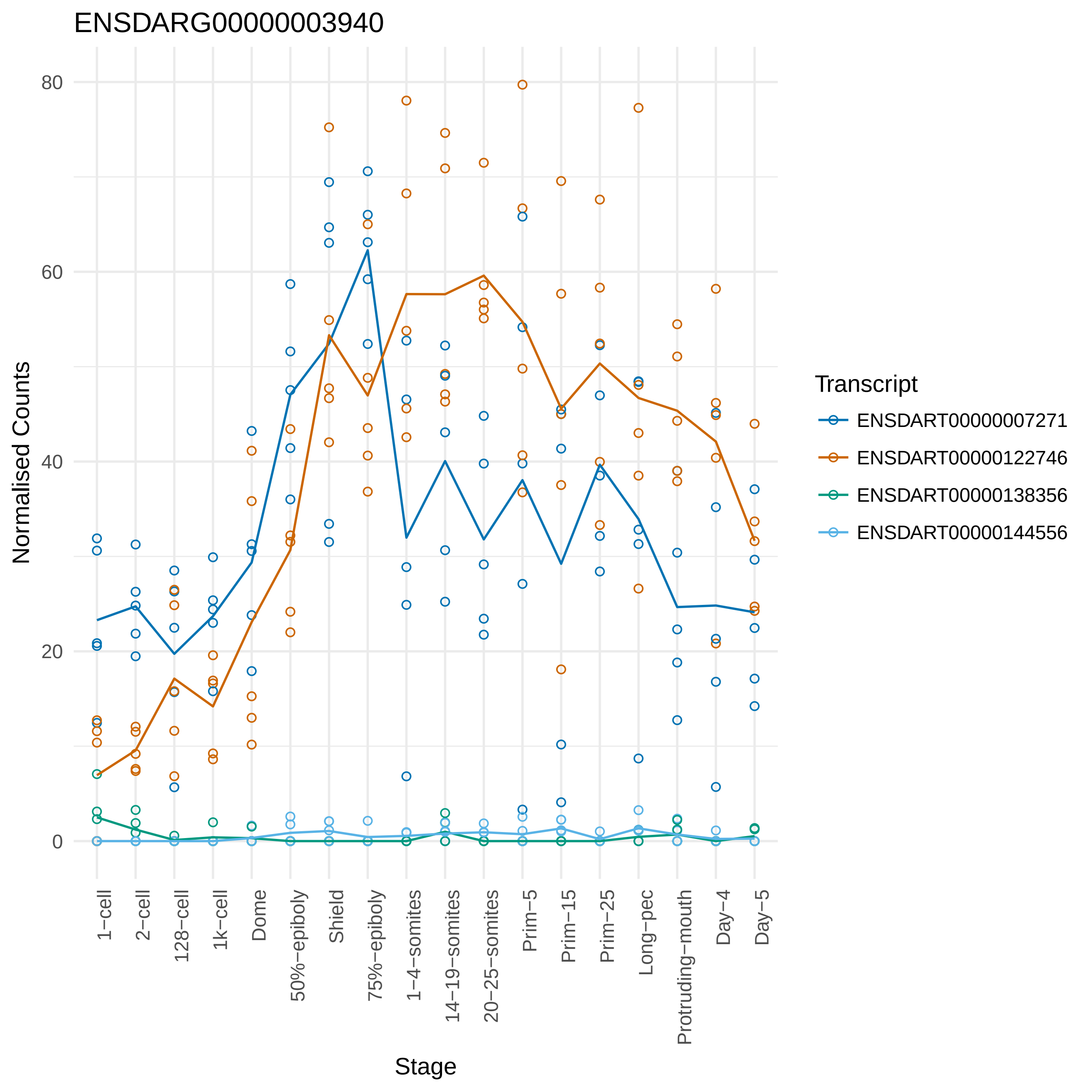

ENSDARG00000003940
Normalised Counts
0
20
40
60
80
Stage
1−cell
2−cell
128−cell
1k−cell
Dome
50%−epiboly
Shield
75%−epiboly
1−4−somites
14−19−somites
20−25−somites
Prim−5
Prim−15
Prim−25
Long−pec
Protruding−mouth
Day−4
Day−5
Transcript
ENSDART00000007271
ENSDART00000122746
ENSDART00000138356
ENSDART00000144556

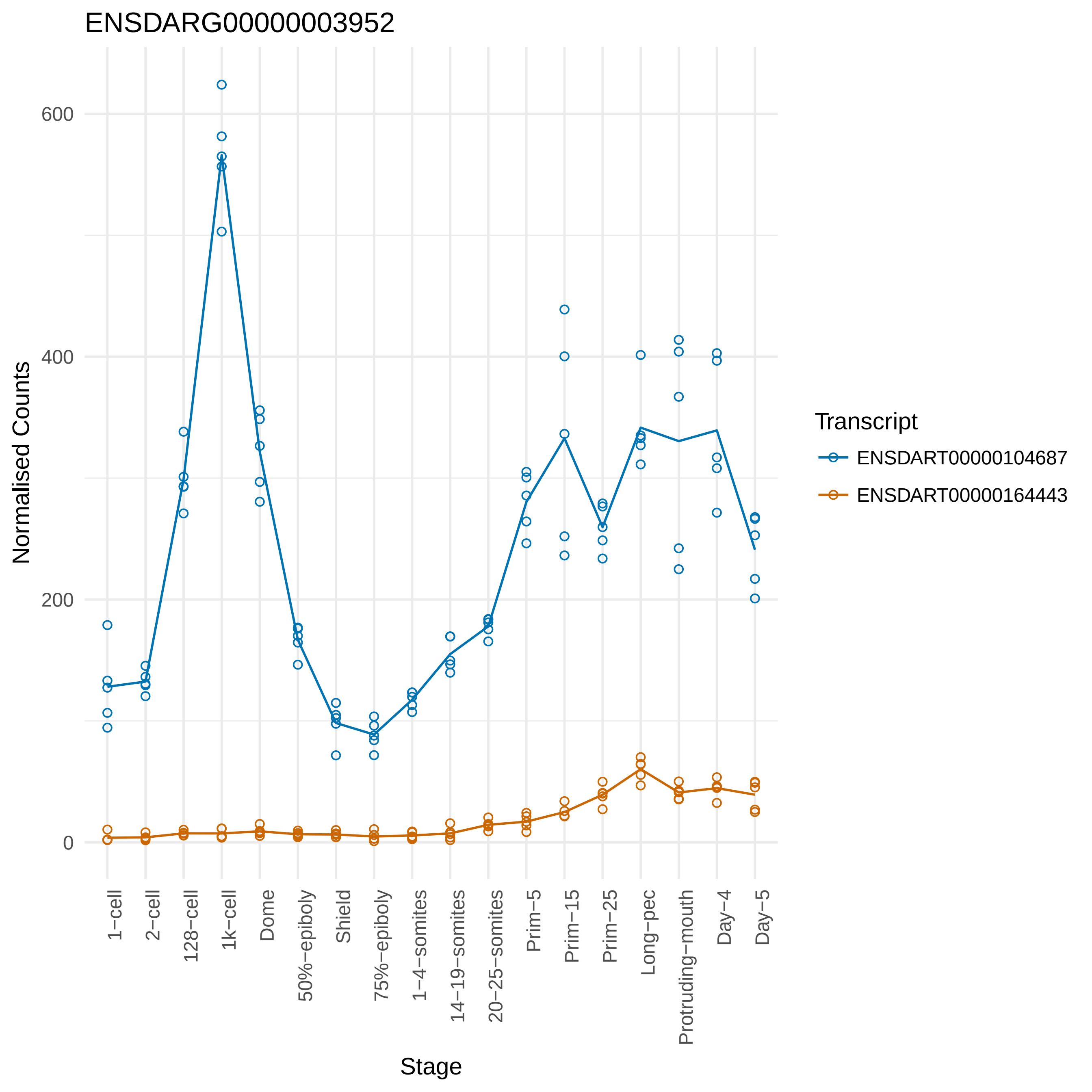

ENSDARG00000003952
Normalised Counts
600
400
200
0
1−cell
2−cell
128−cell
1k−cell
Dome
50%−epiboly
Shield
75%−epiboly
1−4−somites
14−19−somites
20−25−somites
Prim−5
Prim−15
Prim−25
Long−pec
Protruding−mouth
Day−4
Day−5
Stage
Transcript
ENSDART00000104687
ENSDART00000164443

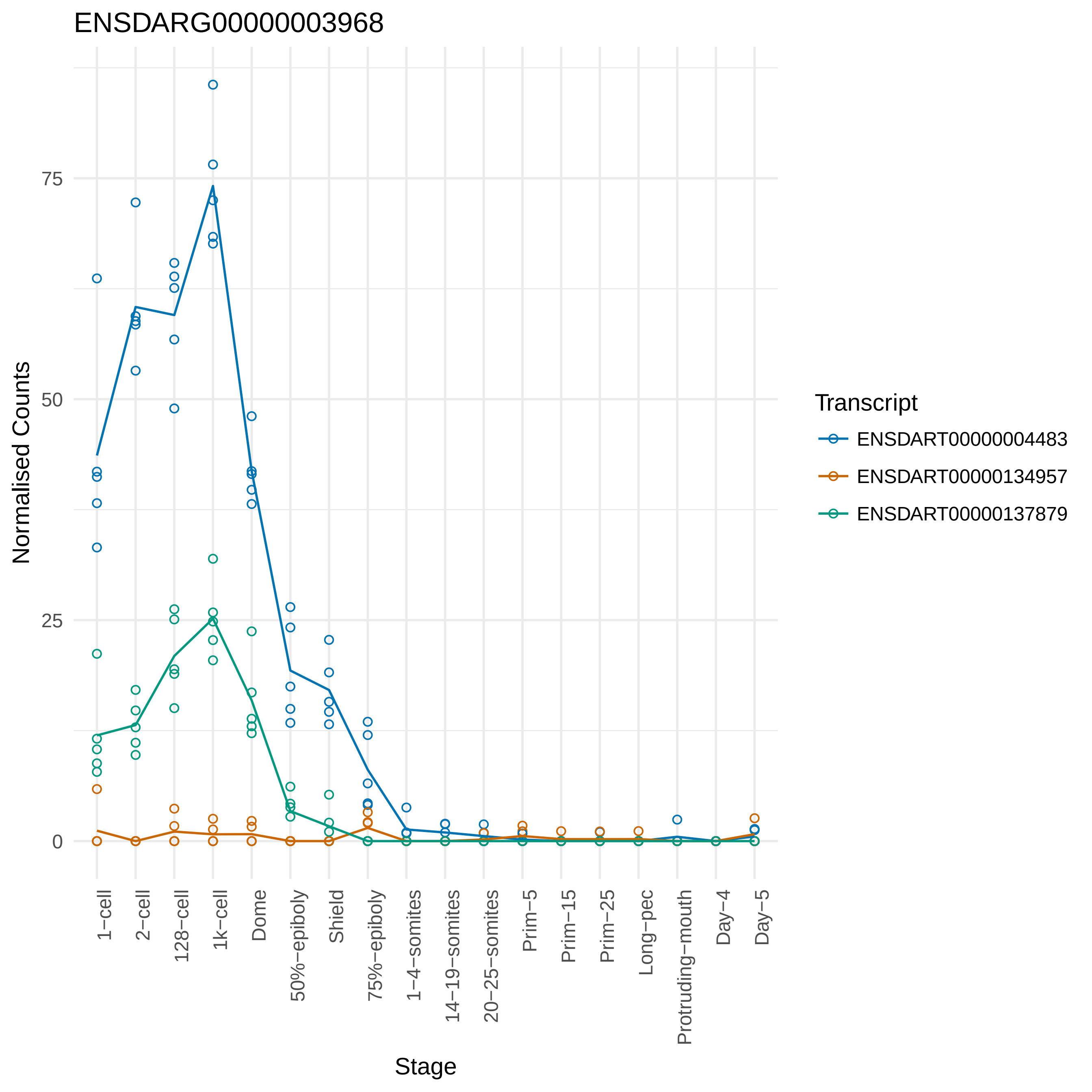

ENSDARG00000003968
Normalised Counts
75
50
25
0
1−cell
2−cell
128−cell
1k−cell
Dome
50%−epiboly
Shield
75%−epiboly
1−4−somites
14−19−somites
20−25−somites
Prim−5
Prim−15
Prim−25
Long−pec
Protruding−mouth
Day−4
Day−5
Stage
Transcript
ENSDART00000004483
ENSDART00000134957
ENSDART00000137879

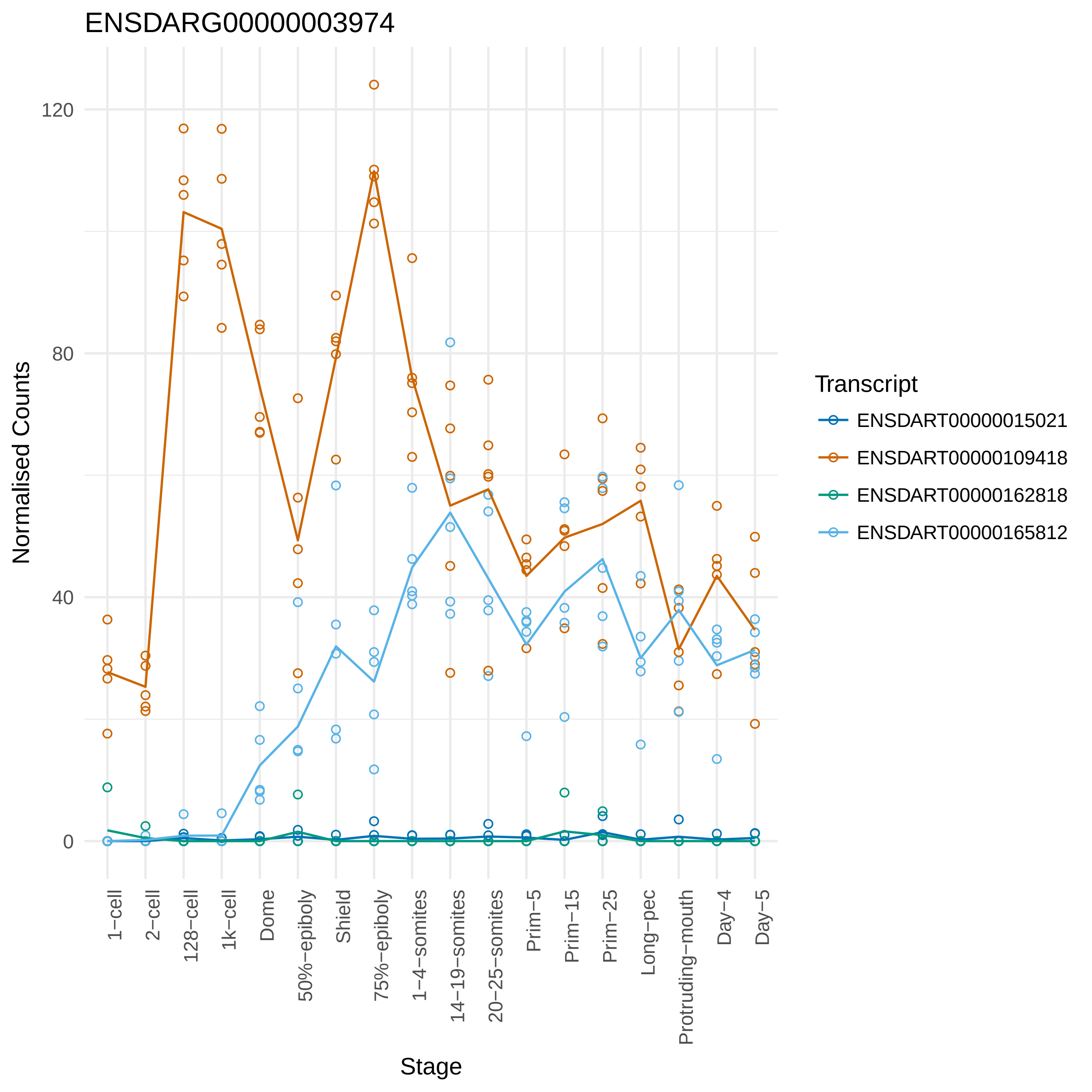

ENSDARG00000003974
Normalised Counts
0
40
80
120
Stage
1−cell
2−cell
128−cell
1k−cell
Dome
50%−epiboly
Shield
75%−epiboly
1−4−somites
14−19−somites
20−25−somites
Prim−5
Prim−15
Prim−25
Long−pec
Protruding−mouth
Day−4
Day−5
Transcript
ENSDART00000015021
ENSDART00000109418
ENSDART00000162818
ENSDART00000165812

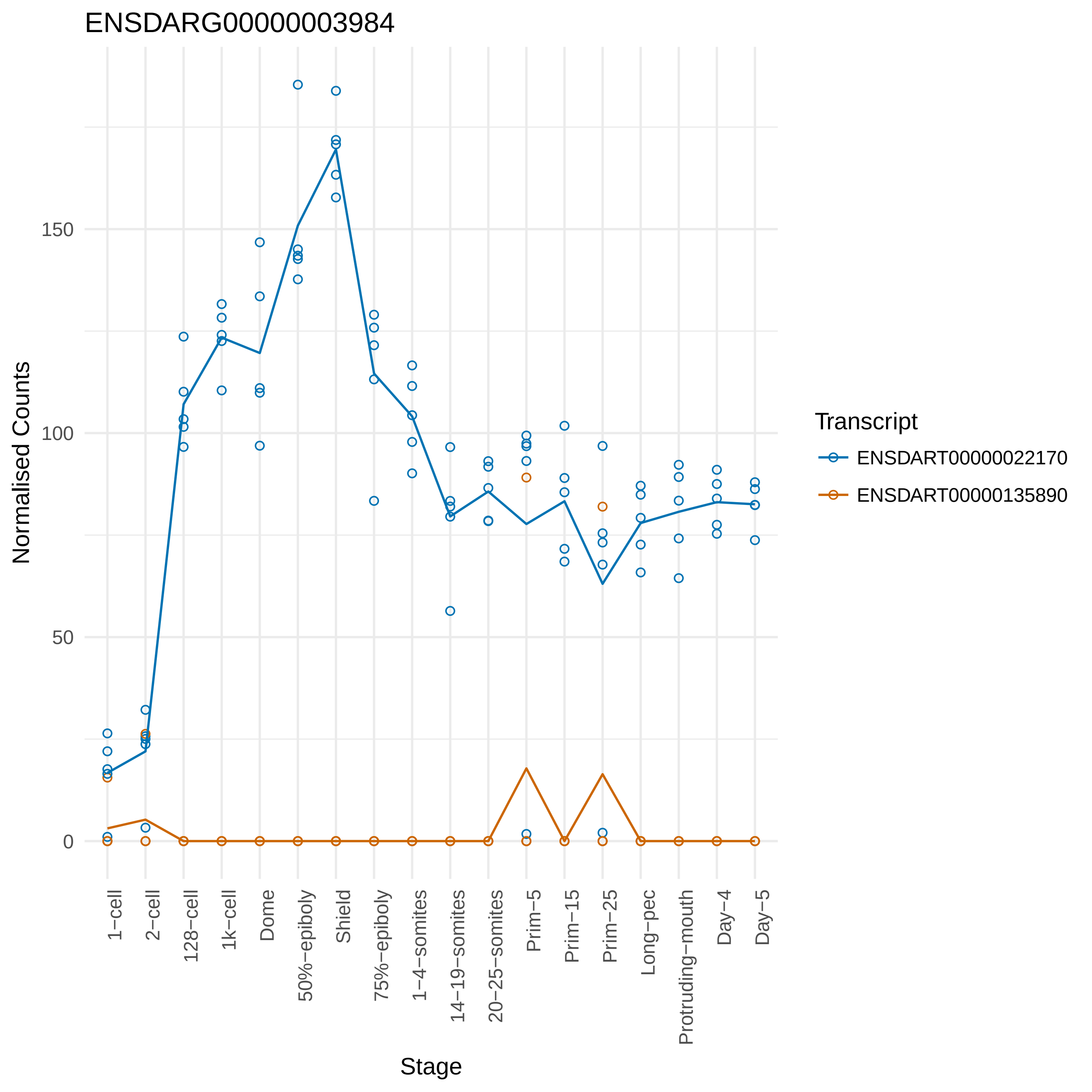
ENSDARG00000003984
Normalised Counts
150
100
50
0
1−cell
2−cell
128−cell
1k−cell
Dome
50%−epiboly
Shield
75%−epiboly
1−4−somites
14−19−somites
20−25−somites
Prim−5
Prim−15
Prim−25
Long−pec
Protruding−mouth
Day−4
Day−5
Stage
Transcript
ENSDART00000022170
ENSDART00000135890

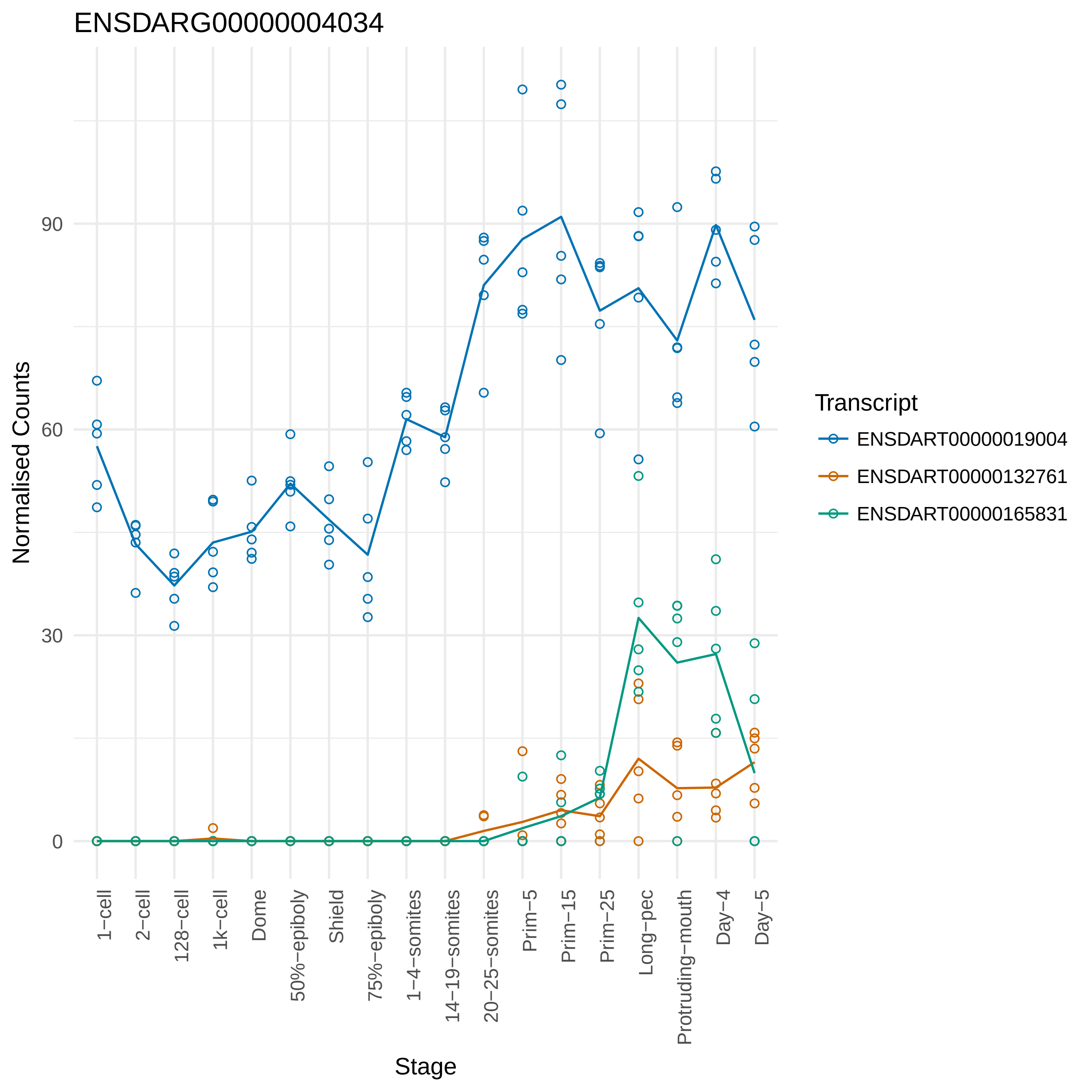
ENSDARG00000004034
Normalised Counts
0
30
60
90
Stage
1−cell
2−cell
128−cell
1k−cell
Dome
50%−epiboly
Shield
75%−epiboly
1−4−somites
14−19−somites
20−25−somites
Prim−5
Prim−15
Prim−25
Long−pec
Protruding−mouth
Day−4
Day−5
Transcript
ENSDART00000019004
ENSDART00000132761
ENSDART00000165831

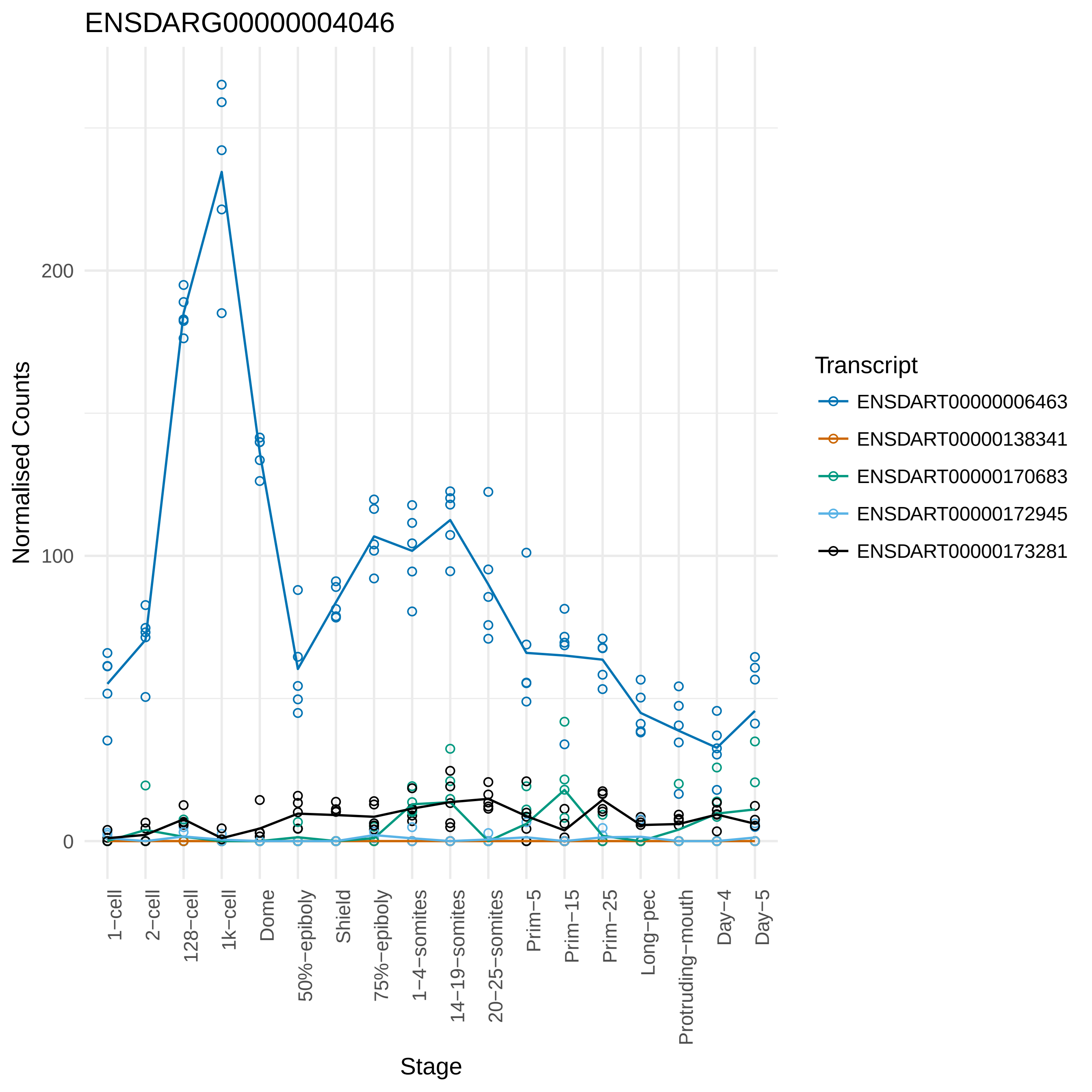
ENSDARG00000004046
Normalised Counts
0
100
200
1−cell
2−cell
128−cell
1k−cell
Dome
50%−epiboly
Shield
75%−epiboly
1−4−somites
14−19−somites
20−25−somites
Prim−5
Prim−15
Prim−25
Long−pec
Protruding−mouth
Day−4
Day−5
Stage
Transcript
ENSDART00000006463
ENSDART00000138341
ENSDART00000170683
ENSDART00000172945
ENSDART00000173281

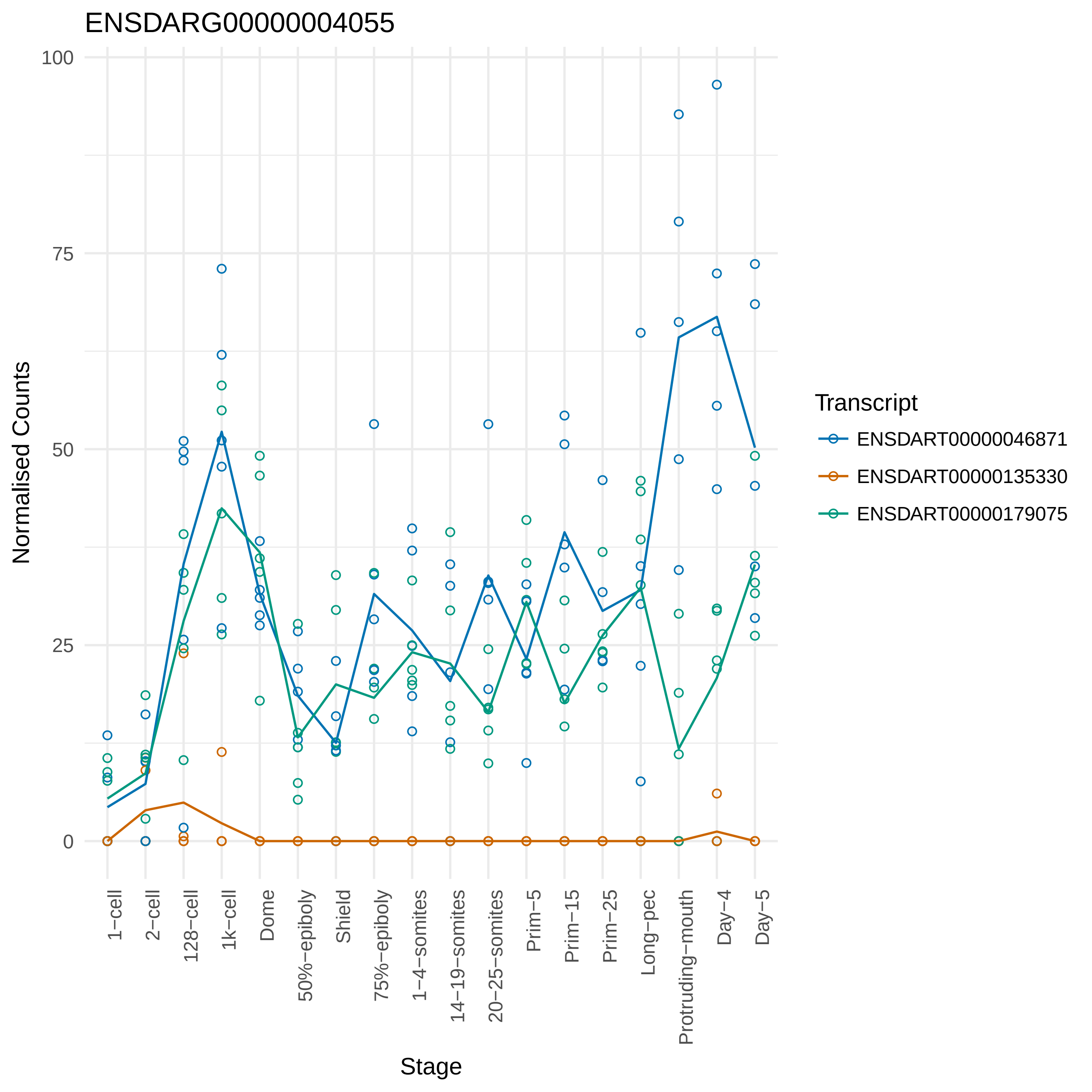

ENSDARG00000004055
Normalised Counts
100
75
50
25
0
1−cell
2−cell
128−cell
1k−cell
Dome
50%−epiboly
Shield
75%−epiboly
1−4−somites
14−19−somites
20−25−somites
Prim−5
Prim−15
Prim−25
Long−pec
Protruding−mouth
Day−4
Day−5
Stage
Transcript
ENSDART00000046871
ENSDART00000135330
ENSDART00000179075

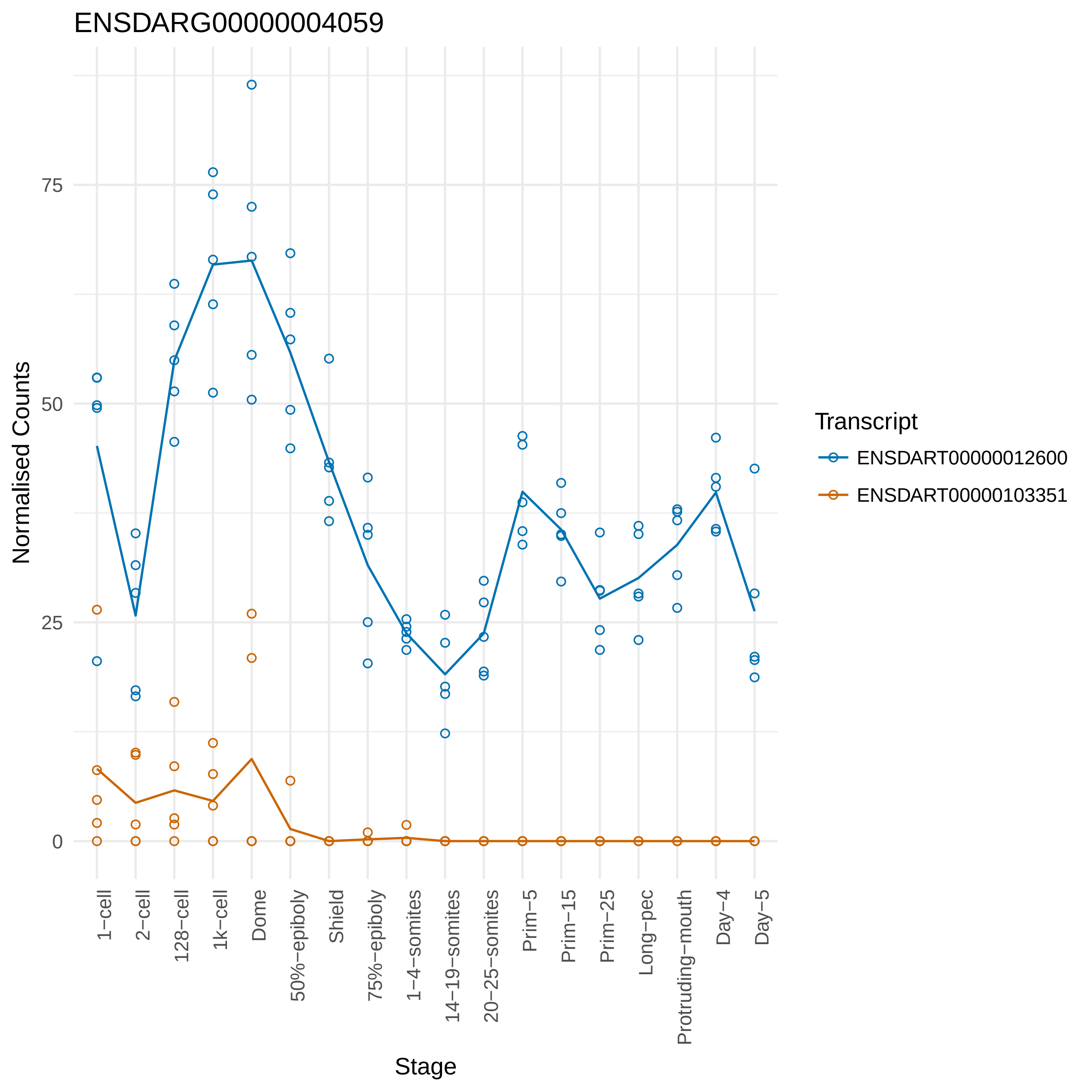

ENSDARG00000004059
Normalised Counts
75
50
25
0
1−cell
2−cell
128−cell
1k−cell
Dome
50%−epiboly
Shield
75%−epiboly
1−4−somites
14−19−somites
20−25−somites
Prim−5
Prim−15
Prim−25
Long−pec
Protruding−mouth
Day−4
Day−5
Stage
Transcript
ENSDART00000012600
ENSDART00000103351

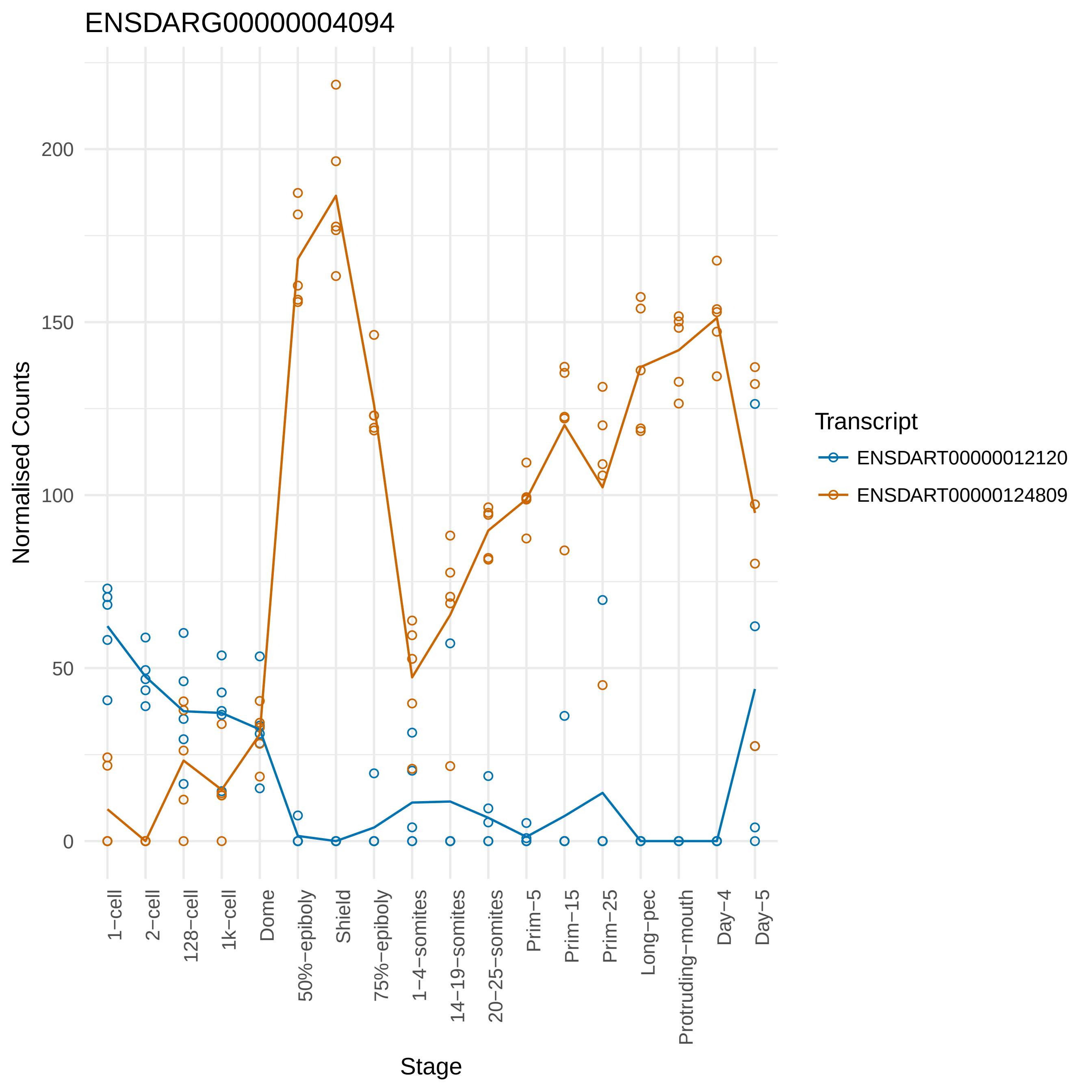

ENSDARG00000004094
Normalised Counts
0
50
100
150
200
1−cell
2−cell
128−cell
1k−cell
Dome
50%−epiboly
Shield
75%−epiboly
1−4−somites
14−19−somites
20−25−somites
Prim−5
Prim−15
Prim−25
Long−pec
Protruding−mouth
Day−4
Day−5
Stage
Transcript
ENSDART00000012120
ENSDART00000124809

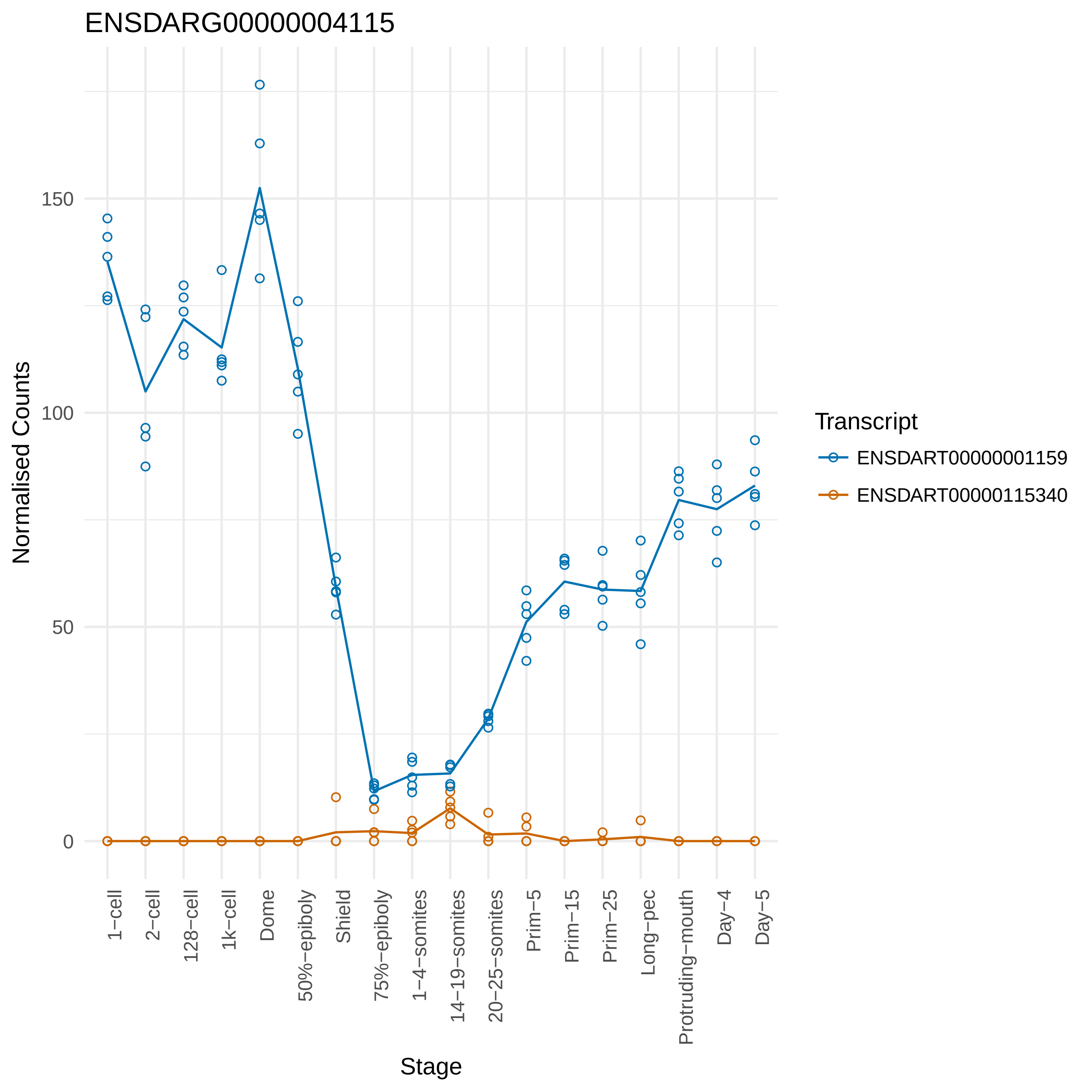

ENSDARG00000004115
Normalised Counts
150
100
50
0
1−cell
2−cell
128−cell
1k−cell
Dome
50%−epiboly
Shield
75%−epiboly
1−4−somites
14−19−somites
20−25−somites
Prim−5
Prim−15
Prim−25
Long−pec
Protruding−mouth
Day−4
Day−5
Stage
Transcript
ENSDART00000001159
ENSDART00000115340

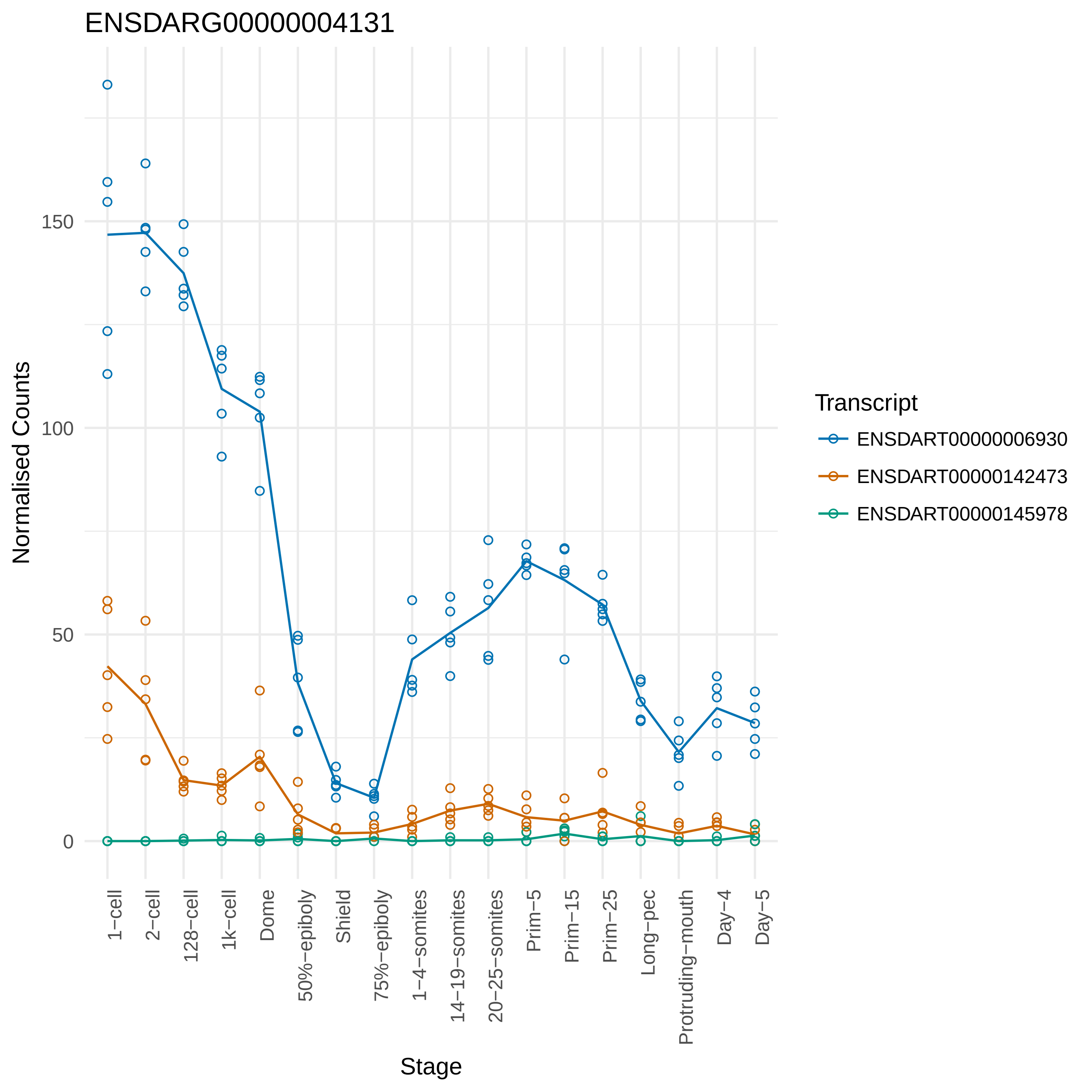

ENSDARG00000004131
Normalised Counts
150
100
50
0
1−cell
2−cell
128−cell
1k−cell
Dome
50%−epiboly
Shield
75%−epiboly
1−4−somites
14−19−somites
20−25−somites
Prim−5
Prim−15
Prim−25
Long−pec
Protruding−mouth
Day−4
Day−5
Stage
Transcript
ENSDART00000006930
ENSDART00000142473
ENSDART00000145978

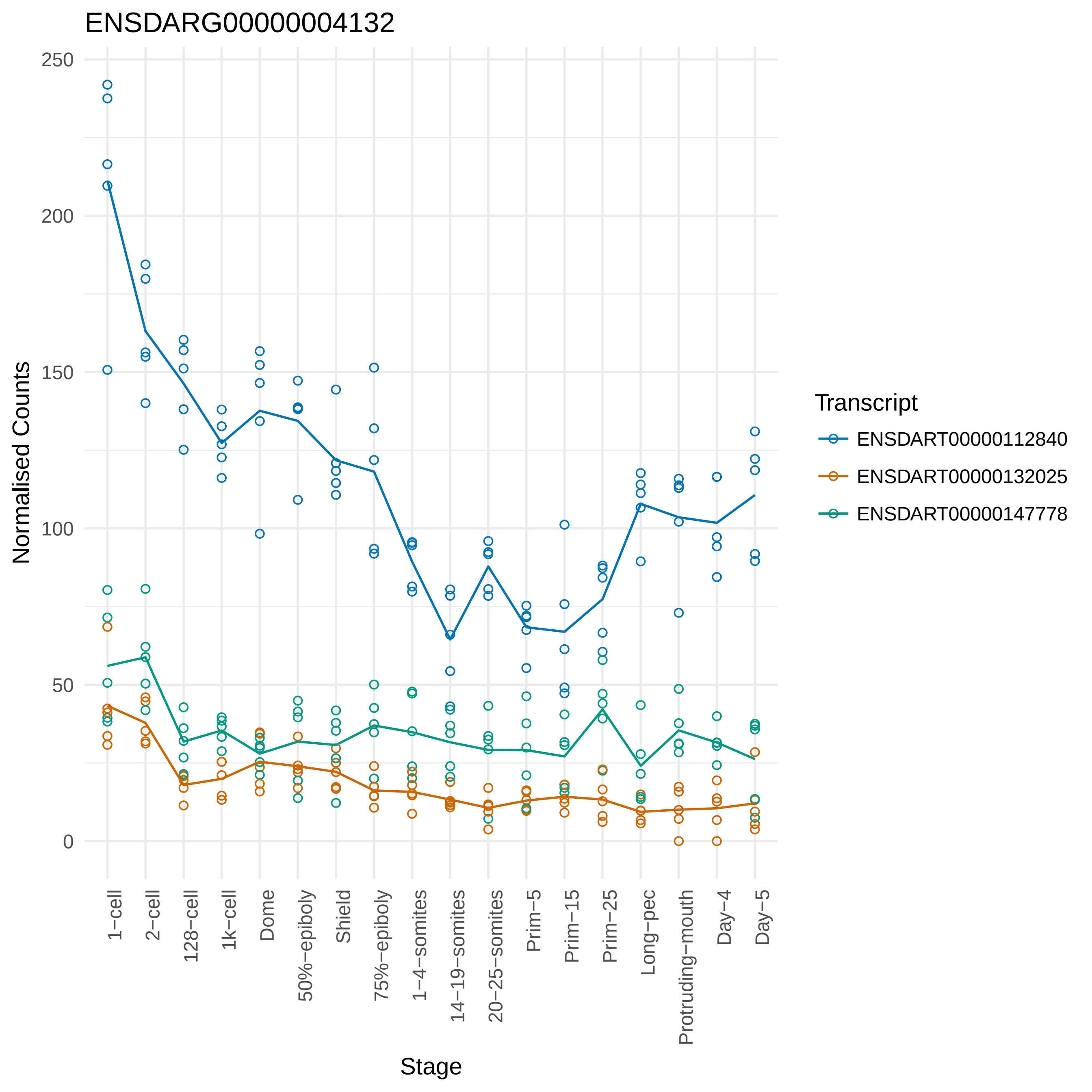

ENSDARG00000004132
Normalised Counts
250
200
150
100
50
0
1−cell
2−cell
128−cell
1k−cell
Dome
50%−epiboly
Shield
75%−epiboly
1−4−somites
14−19−somites
20−25−somites
Prim−5
Prim−15
Prim−25
Long−pec
Protruding−mouth
Day−4
Day−5
Stage
Transcript
ENSDART00000112840
ENSDART00000132025
ENSDART00000147778

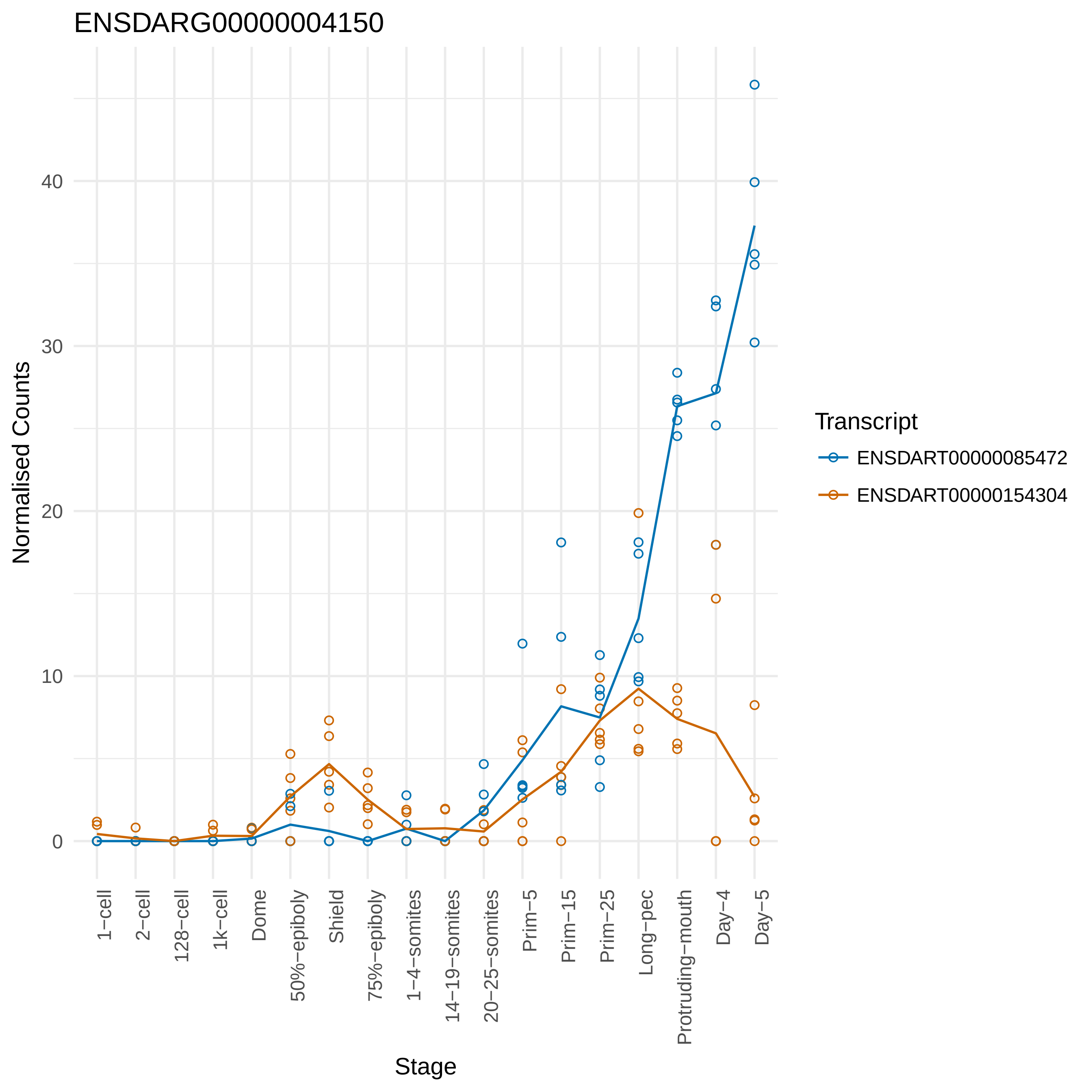

ENSDARG00000004150
Normalised Counts
0
10
20
30
40
Stage
1−cell
2−cell
128−cell
1k−cell
Dome
50%−epiboly
Shield
75%−epiboly
1−4−somites
14−19−somites
20−25−somites
Prim−5
Prim−15
Prim−25
Long−pec
Protruding−mouth
Day−4
Day−5
Transcript
ENSDART00000085472
ENSDART00000154304

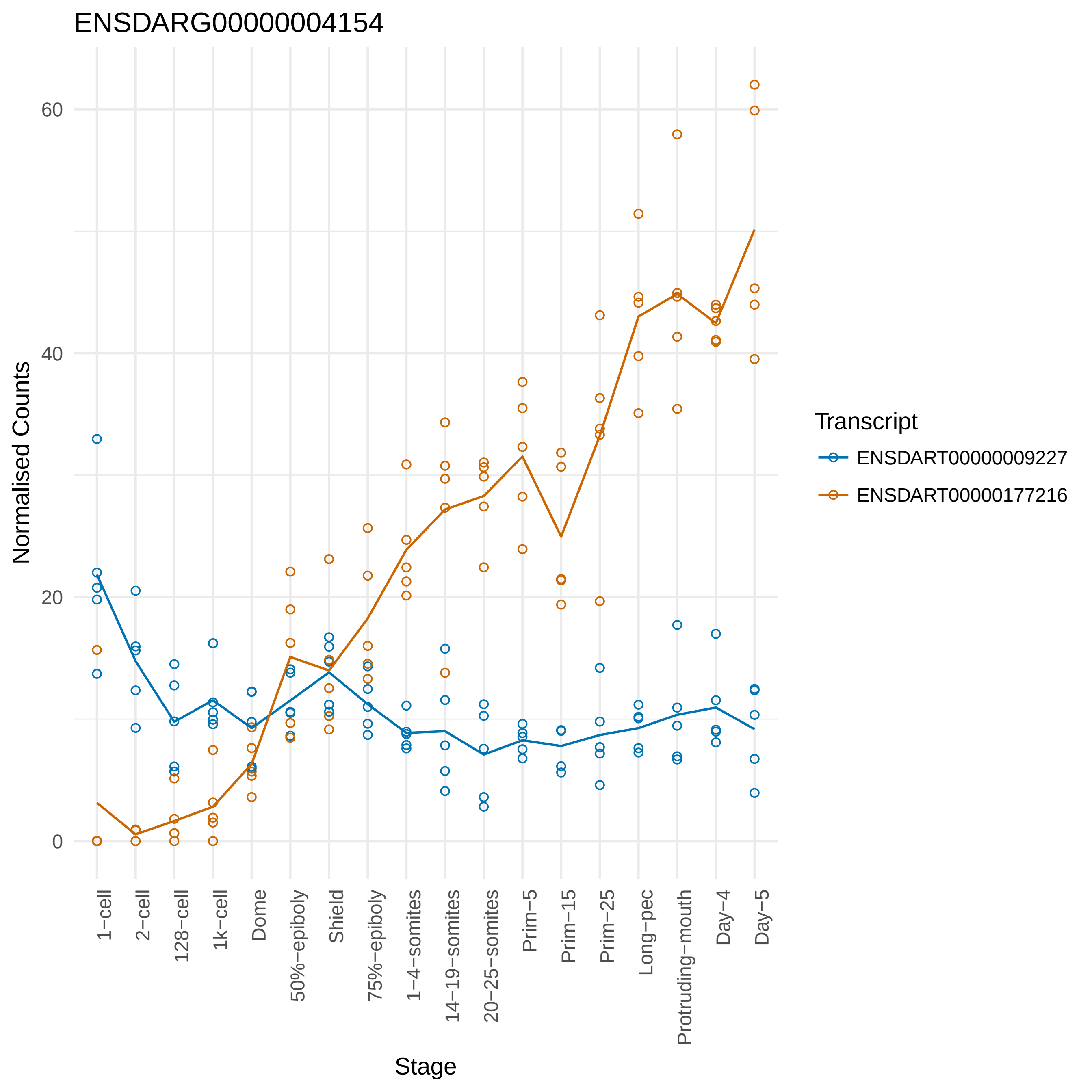
ENSDARG00000004154
Normalised Counts
60
40
20
0
1−cell
2−cell
128−cell
1k−cell
Dome
50%−epiboly
Shield
75%−epiboly
1−4−somites
14−19−somites
20−25−somites
Prim−5
Prim−15
Prim−25
Long−pec
Protruding−mouth
Day−4
Day−5
Stage
Transcript
ENSDART00000009227
ENSDART00000177216

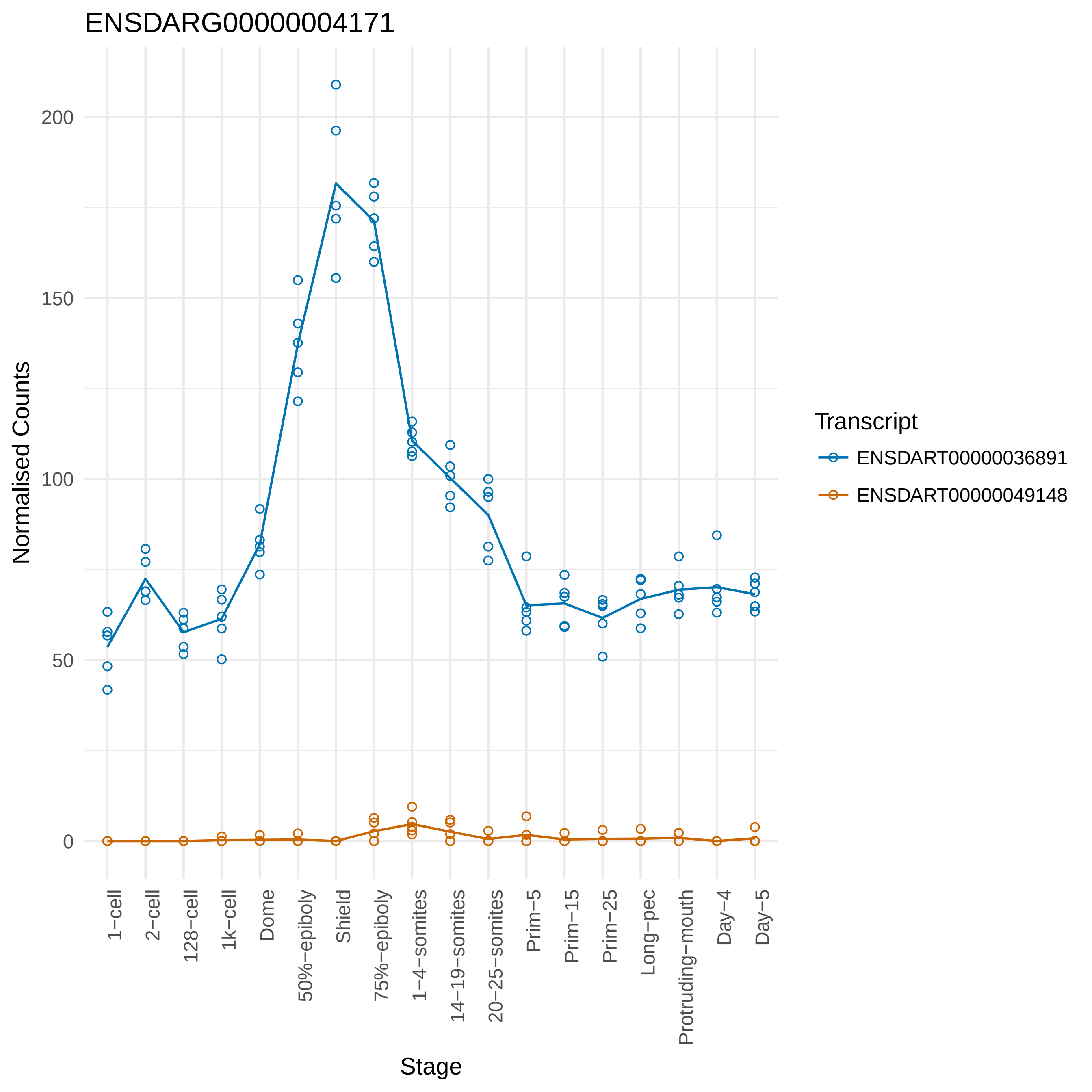

ENSDARG00000004171
Normalised Counts
200
150
100
50
0
1−cell
2−cell
128−cell
1k−cell
Dome
50%−epiboly
Shield
75%−epiboly
1−4−somites
14−19−somites
20−25−somites
Prim−5
Prim−15
Prim−25
Long−pec
Protruding−mouth
Day−4
Day−5
Stage
Transcript
ENSDART00000036891
ENSDART00000049148

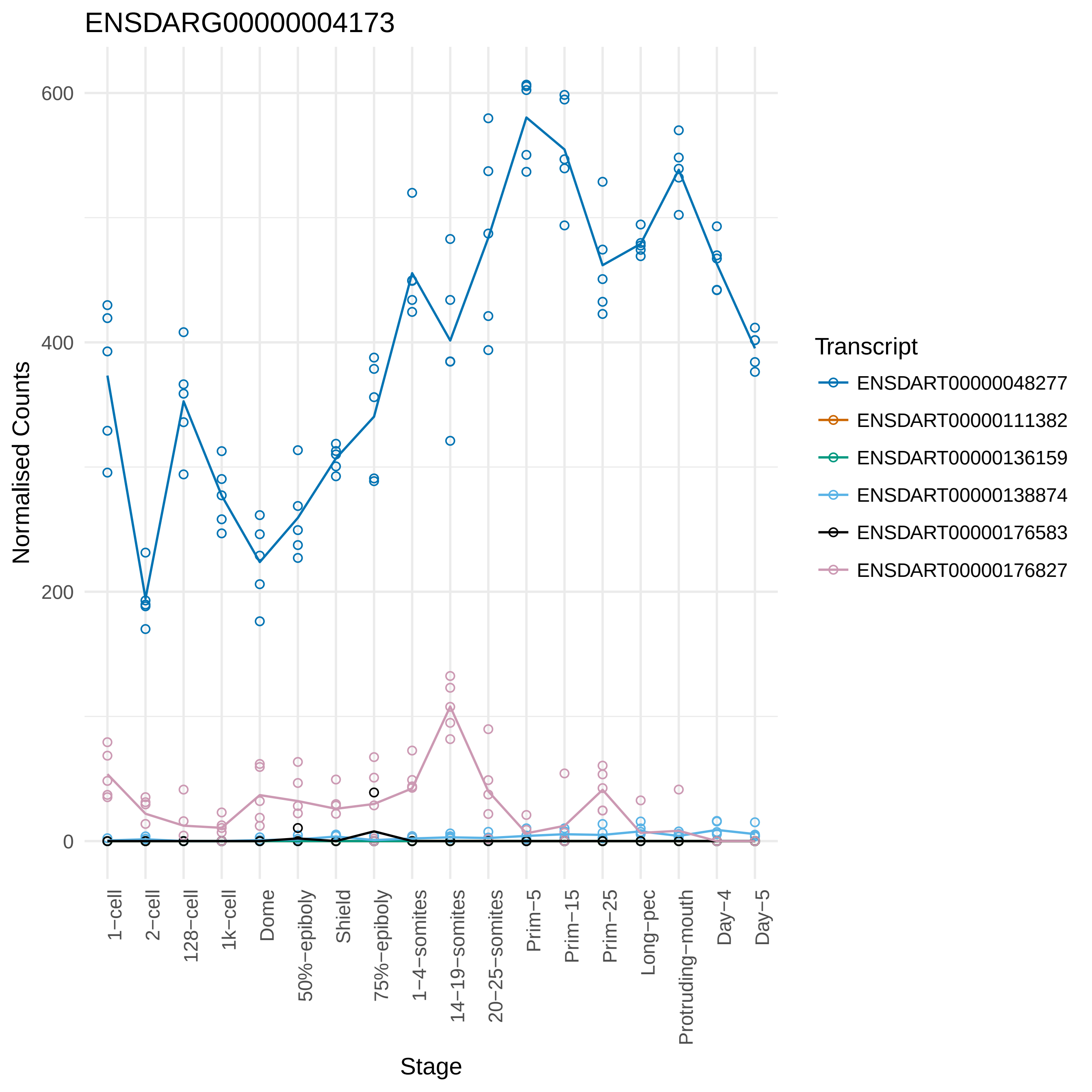
ENSDARG00000004173
Normalised Counts
600
400
200
0
Stage
1−cell
2−cell
128−cell
1k−cell
Dome
50%−epiboly
Shield
75%−epiboly
1−4−somites
14−19−somites
20−25−somites
Prim−5
Prim−15
Prim−25
Long−pec
Protruding−mouth
Day−4
Day−5
Transcript
ENSDART00000048277
ENSDART00000111382
ENSDART00000136159
ENSDART00000138874
ENSDART00000176583
ENSDART00000176827

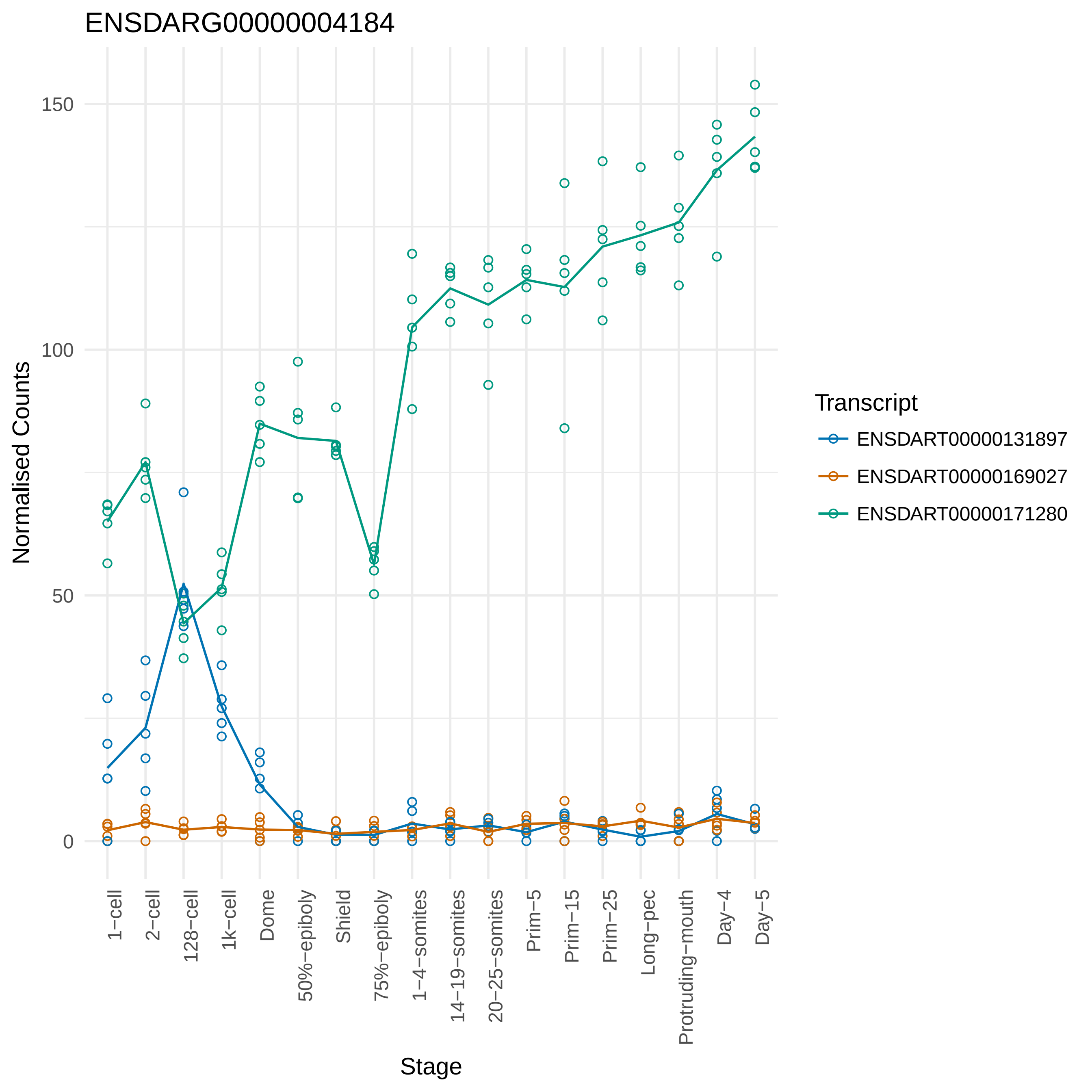

ENSDARG00000004184
Normalised Counts
150
100
50
0
1−cell
2−cell
128−cell
1k−cell
Dome
50%−epiboly
Shield
75%−epiboly
1−4−somites
14−19−somites
20−25−somites
Prim−5
Prim−15
Prim−25
Long−pec
Protruding−mouth
Day−4
Day−5
Stage
Transcript
ENSDART00000131897
ENSDART00000169027
ENSDART00000171280

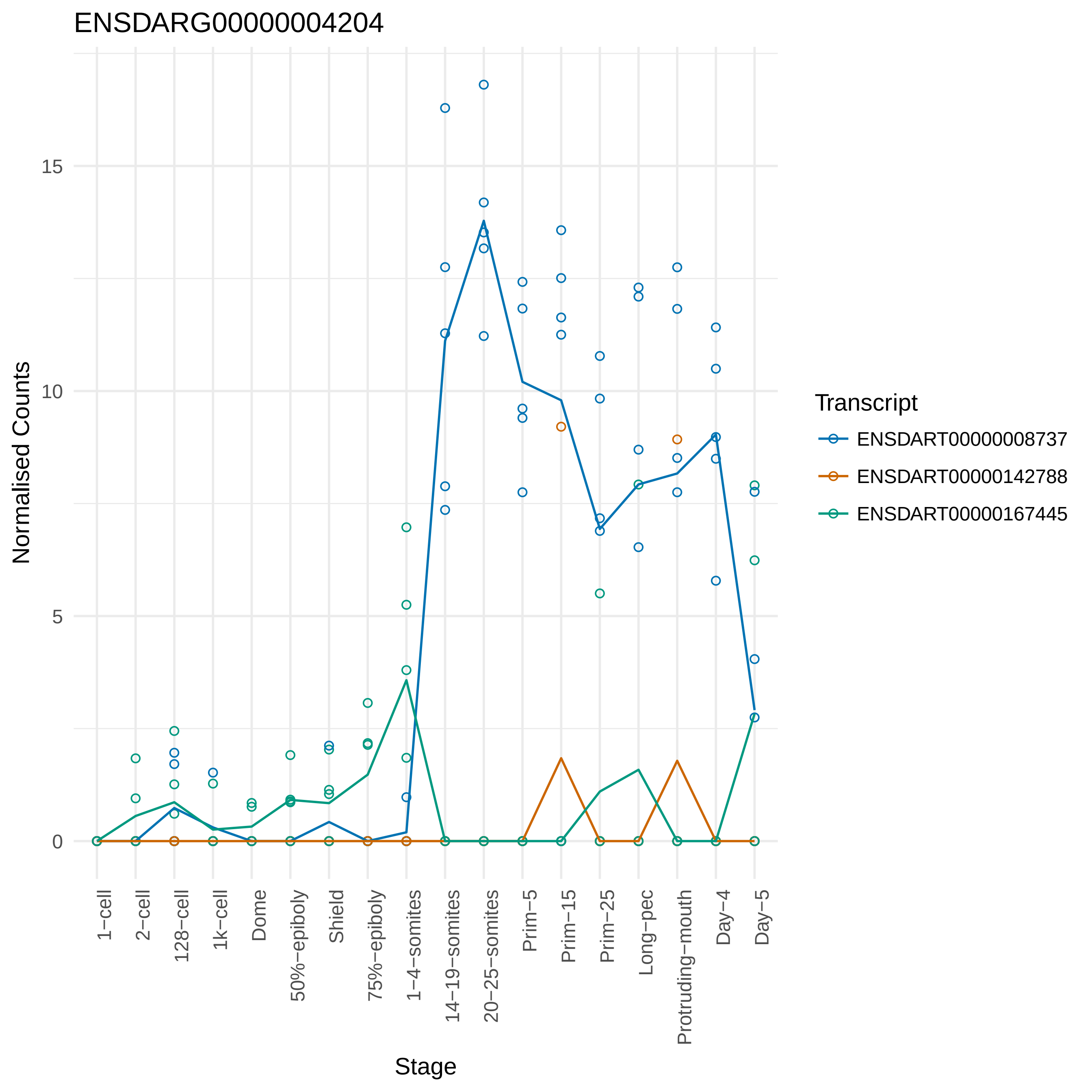

ENSDARG00000004204
Normalised Counts
15
10
5
0
1−cell
2−cell
128−cell
1k−cell
Dome
50%−epiboly
Shield
75%−epiboly
1−4−somites
14−19−somites
20−25−somites
Prim−5
Prim−15
Prim−25
Long−pec
Protruding−mouth
Day−4
Day−5
Stage
Transcript
ENSDART00000008737
ENSDART00000142788
ENSDART00000167445

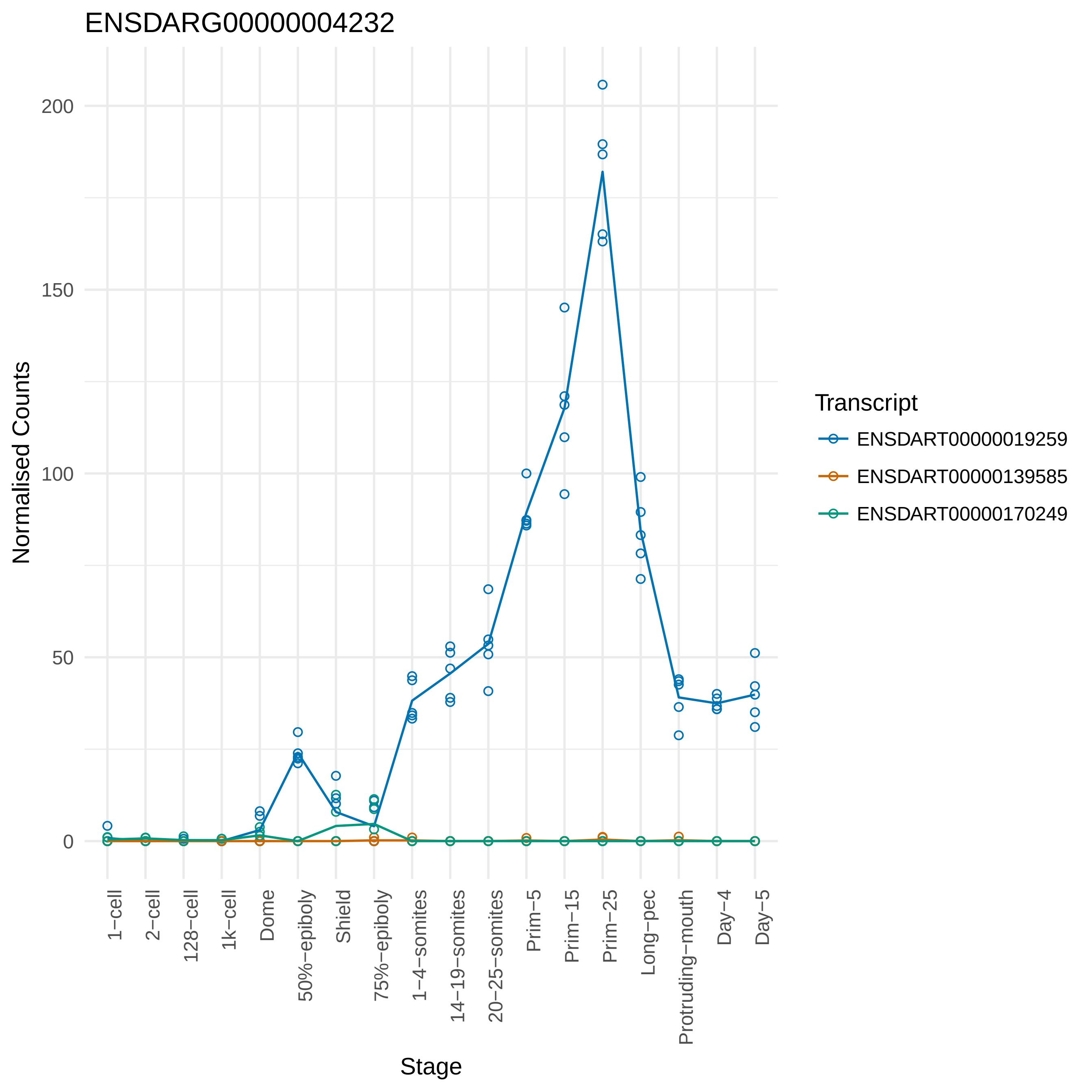

ENSDARG00000004232
Normalised Counts
200
150
100
50
0
1−cell
2−cell
128−cell
1k−cell
Dome
50%−epiboly
Shield
75%−epiboly
1−4−somites
14−19−somites
20−25−somites
Prim−5
Prim−15
Prim−25
Long−pec
Protruding−mouth
Day−4
Day−5
Stage
Transcript
ENSDART00000019259
ENSDART00000139585
ENSDART00000170249

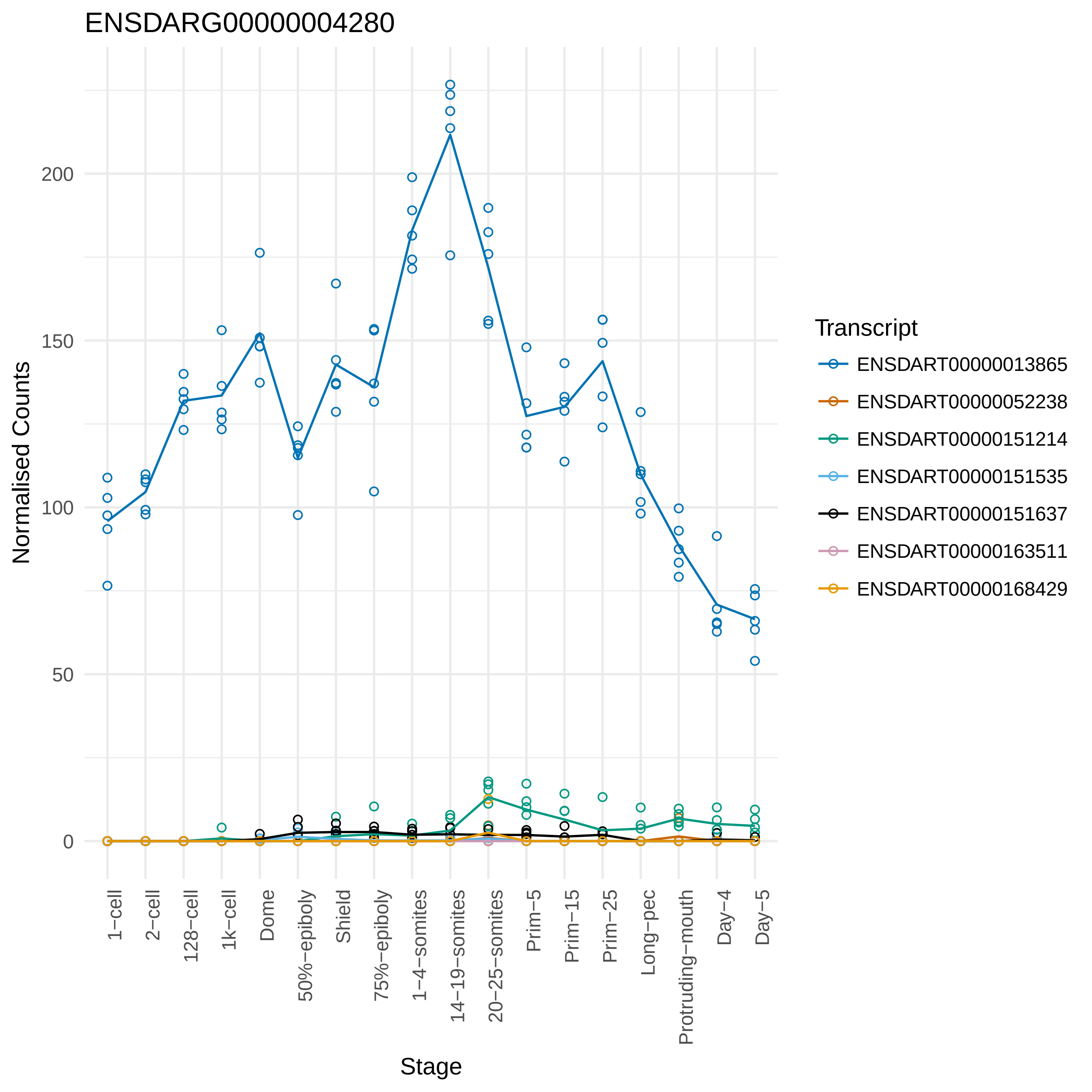

ENSDARG00000004280
Normalised Counts
200
150
100
50
0
1−cell
2−cell
128−cell
1k−cell
Dome
50%−epiboly
Shield
75%−epiboly
1−4−somites
14−19−somites
20−25−somites
Prim−5
Prim−15
Prim−25
Long−pec
Protruding−mouth
Day−4
Day−5
Stage
Transcript
ENSDART00000013865
ENSDART00000052238
ENSDART00000151214
ENSDART00000151535
ENSDART00000151637
ENSDART00000163511
ENSDART00000168429

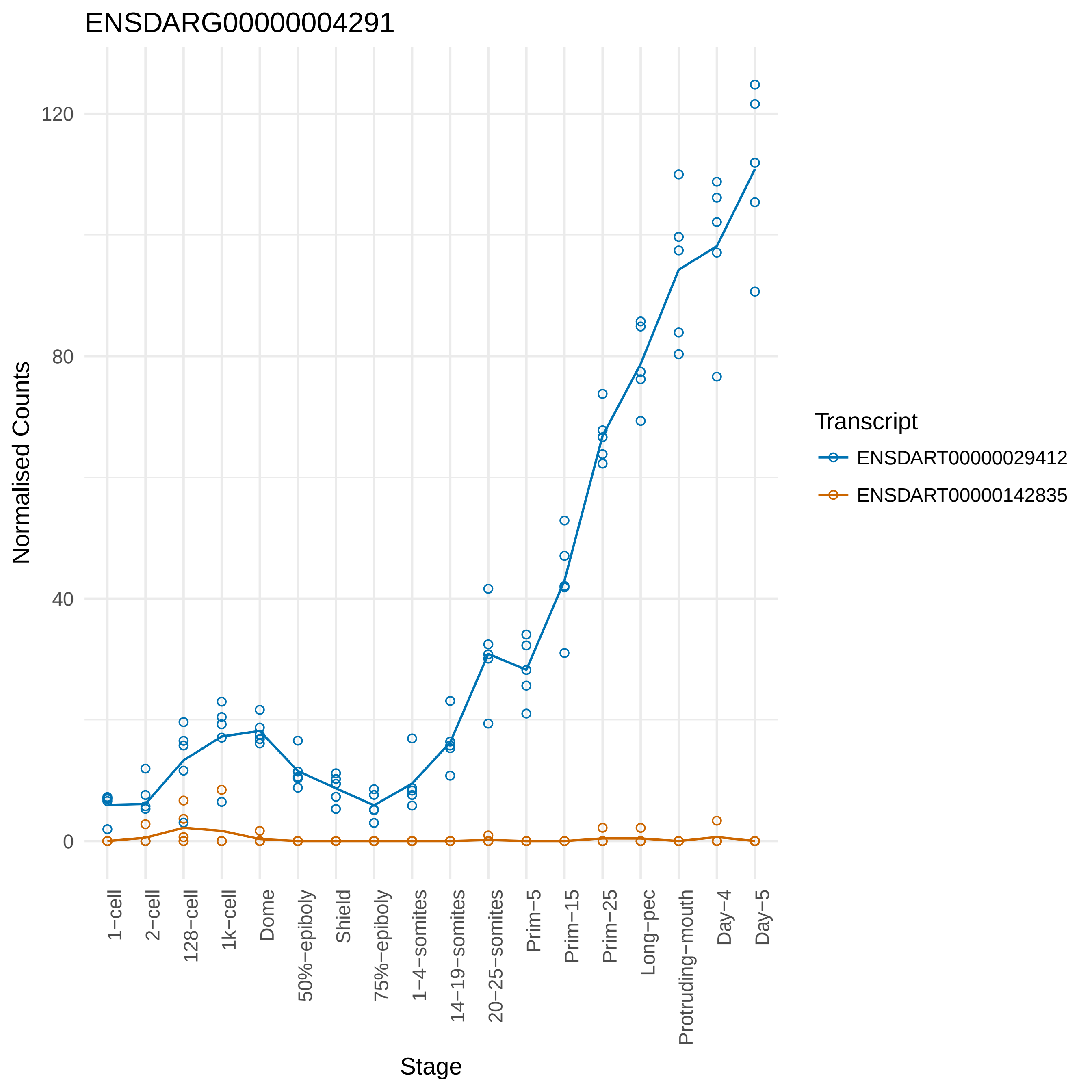

ENSDARG00000004291
Normalised Counts
120
80
40
0
1−cell
2−cell
128−cell
1k−cell
Dome
50%−epiboly
Shield
75%−epiboly
1−4−somites
14−19−somites
20−25−somites
Prim−5
Prim−15
Prim−25
Long−pec
Protruding−mouth
Day−4
Day−5
Stage
Transcript
ENSDART00000029412
ENSDART00000142835

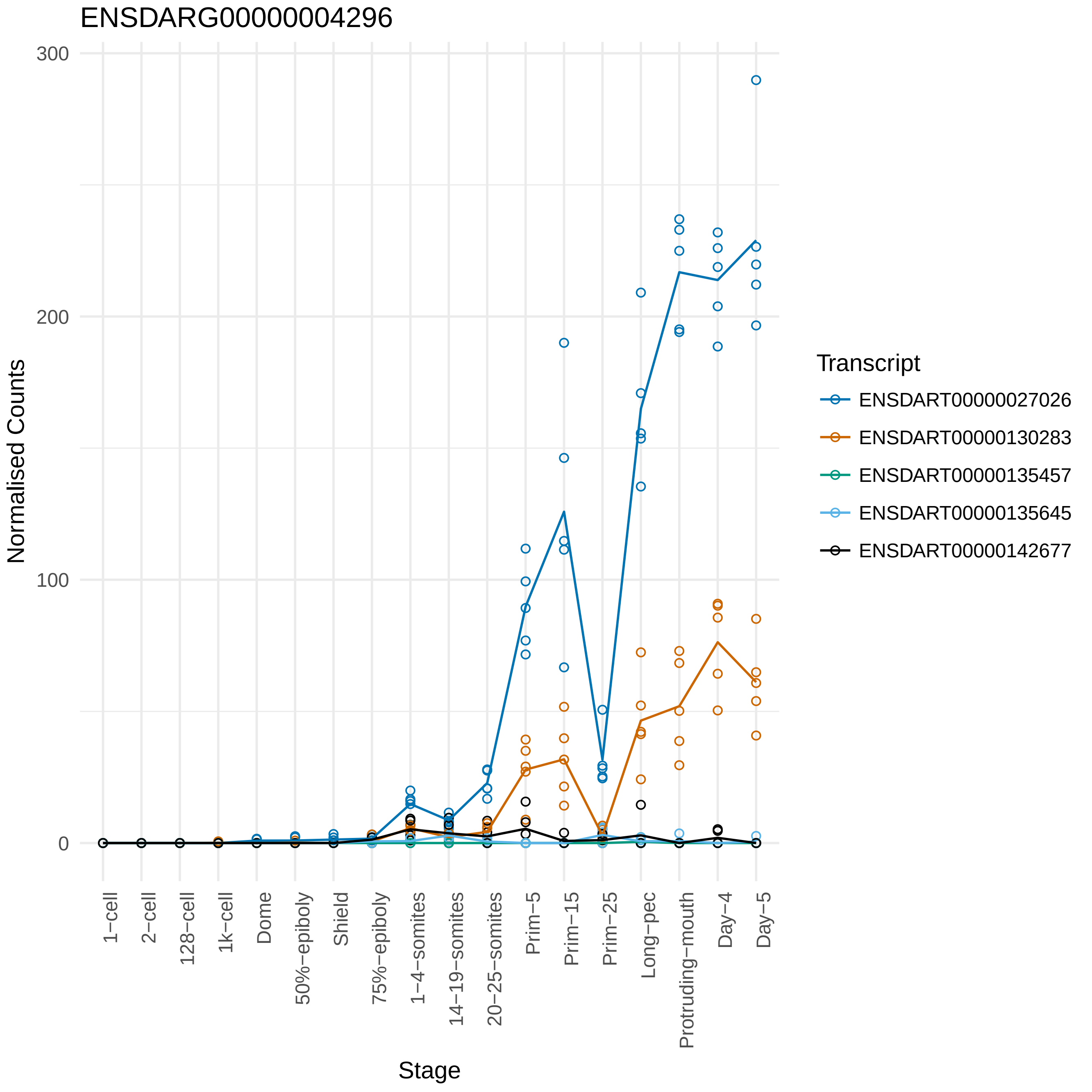

ENSDARG00000004296
Normalised Counts
300
200
100
0
1−cell
2−cell
128−cell
1k−cell
Dome
50%−epiboly
Shield
75%−epiboly
1−4−somites
14−19−somites
20−25−somites
Prim−5
Prim−15
Prim−25
Long−pec
Protruding−mouth
Day−4
Day−5
Stage
Transcript
ENSDART00000027026
ENSDART00000130283
ENSDART00000135457
ENSDART00000135645
ENSDART00000142677

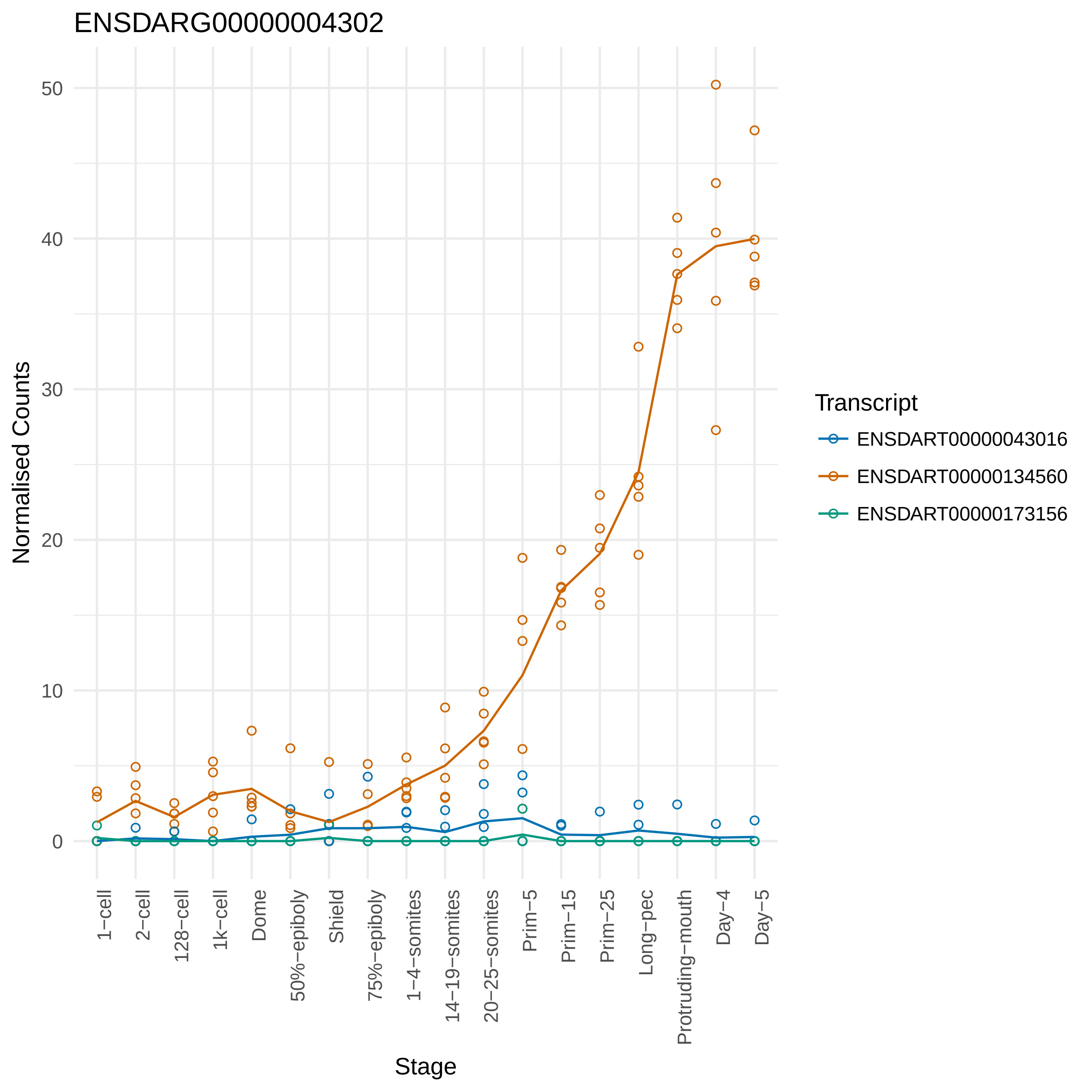

ENSDARG00000004302
Normalised Counts
50
40
30
20
10
0
1−cell
2−cell
128−cell
1k−cell
Dome
50%−epiboly
Shield
75%−epiboly
1−4−somites
14−19−somites
20−25−somites
Prim−5
Prim−15
Prim−25
Long−pec
Protruding−mouth
Day−4
Day−5
Stage
Transcript
ENSDART00000043016
ENSDART00000134560
ENSDART00000173156

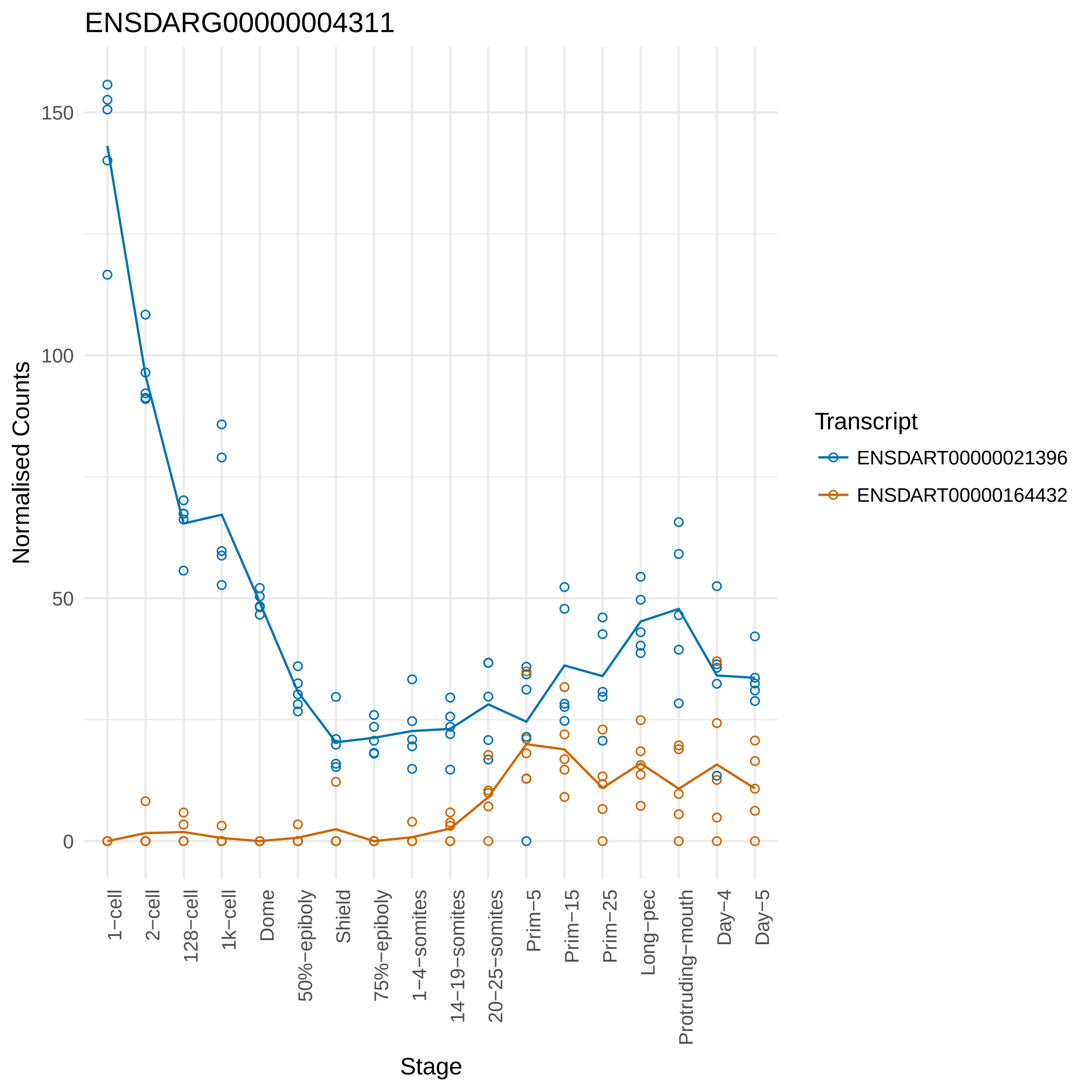

ENSDARG00000004311
Normalised Counts
150
100
50
0
1−cell
2−cell
128−cell
1k−cell
Dome
50%−epiboly
Shield
75%−epiboly
1−4−somites
14−19−somites
20−25−somites
Prim−5
Prim−15
Prim−25
Long−pec
Protruding−mouth
Day−4
Day−5
Stage
Transcript
ENSDART00000021396
ENSDART00000164432

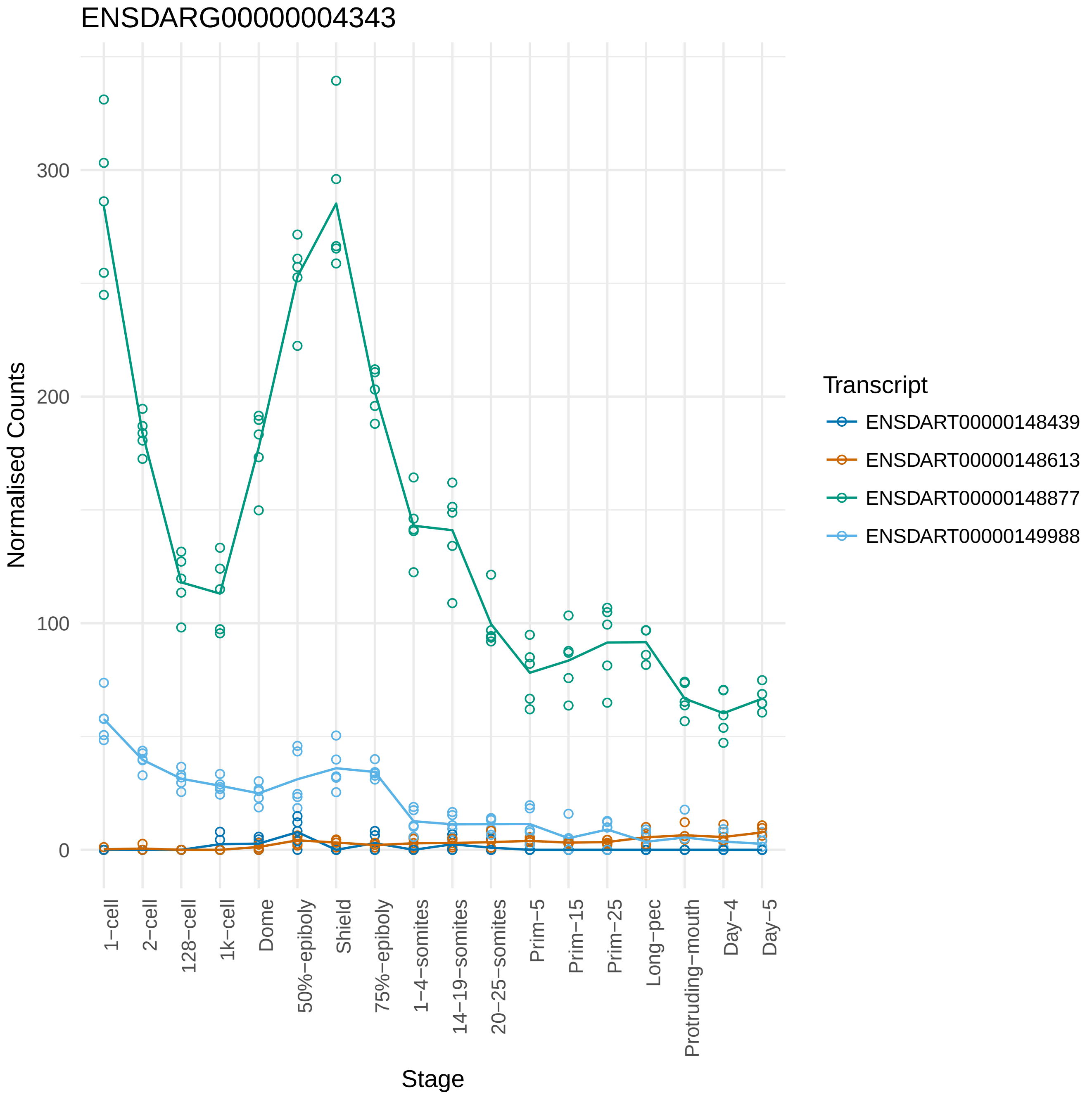

ENSDARG00000004343
Normalised Counts
300
200
100
0
1−cell
2−cell
128−cell
1k−cell
Dome
50%−epiboly
Shield
75%−epiboly
1−4−somites
14−19−somites
20−25−somites
Prim−5
Prim−15
Prim−25
Long−pec
Protruding−mouth
Day−4
Day−5
Stage
Transcript
ENSDART00000148439
ENSDART00000148613
ENSDART00000148877
ENSDART00000149988

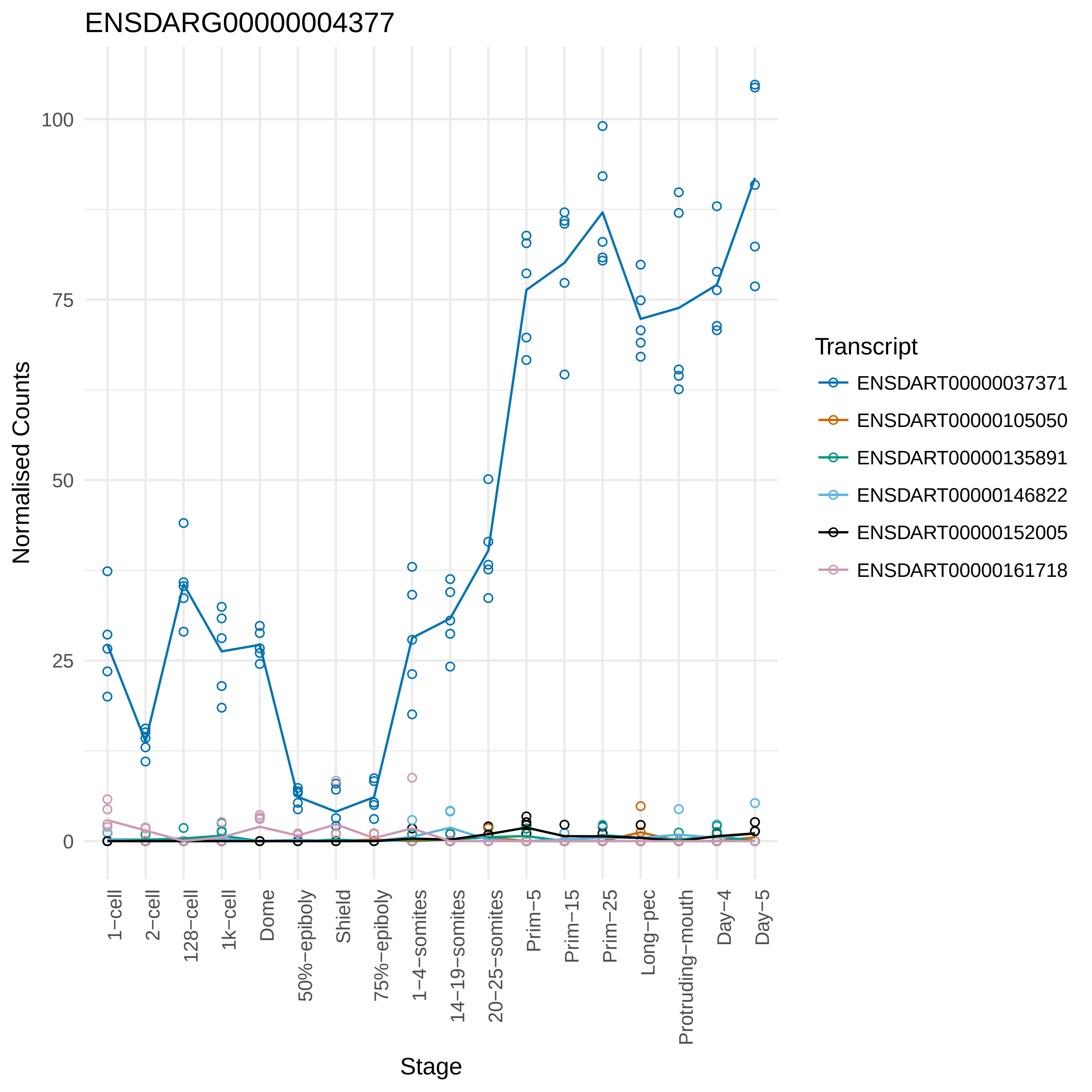

ENSDARG00000004377
Normalised Counts
100
75
50
25
0
1−cell
2−cell
128−cell
1k−cell
Dome
50%−epiboly
Shield
75%−epiboly
1−4−somites
14−19−somites
20−25−somites
Prim−5
Prim−15
Prim−25
Long−pec
Protruding−mouth
Day−4
Day−5
Stage
Transcript
ENSDART00000037371
ENSDART00000105050
ENSDART00000135891
ENSDART00000146822
ENSDART00000152005
ENSDART00000161718

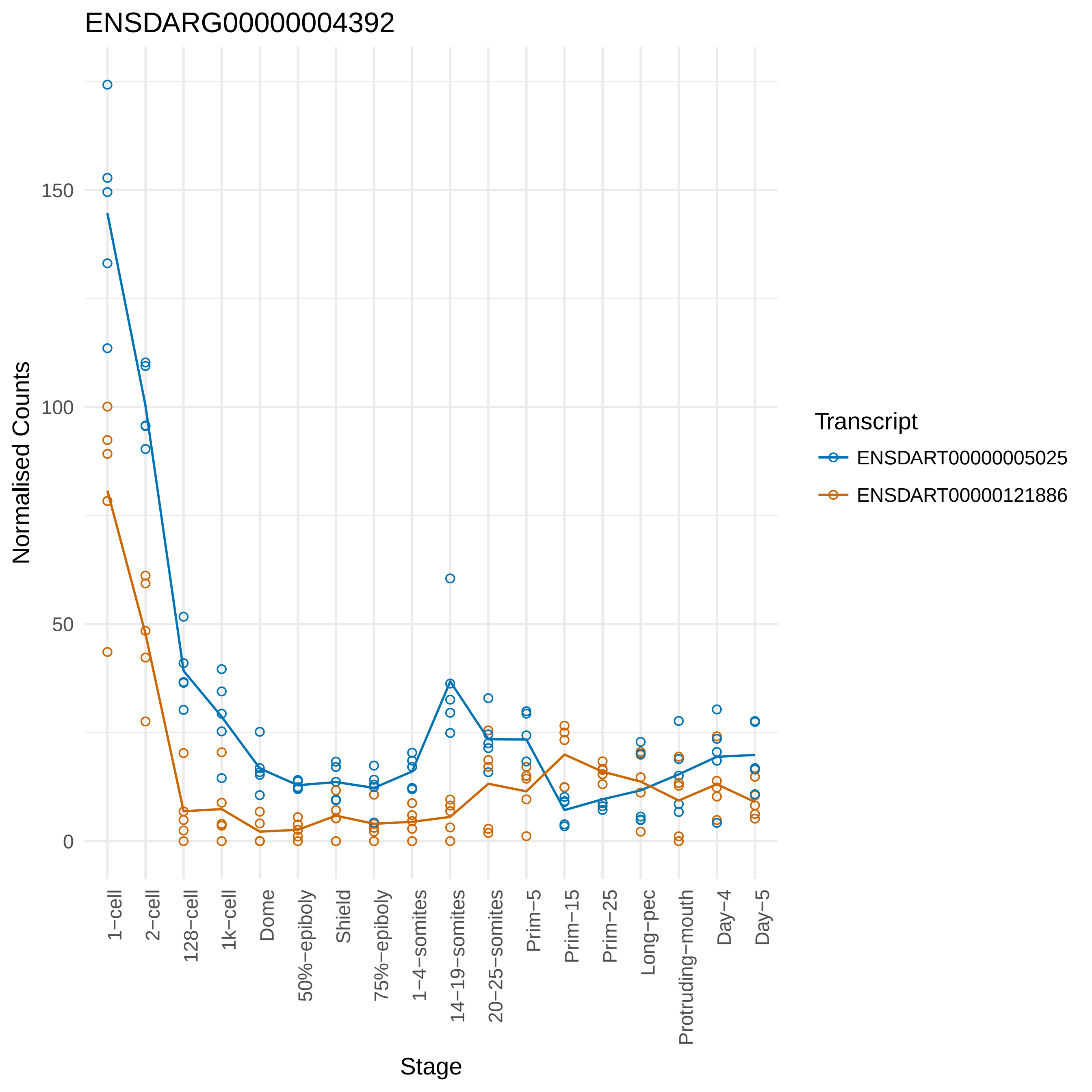
ENSDARG00000004392
Normalised Counts
150
100
50
0
1−cell
2−cell
128−cell
1k−cell
Dome
50%−epiboly
Shield
75%−epiboly
1−4−somites
14−19−somites
20−25−somites
Prim−5
Prim−15
Prim−25
Long−pec
Protruding−mouth
Day−4
Day−5
Stage
Transcript
ENSDART00000005025
ENSDART00000121886

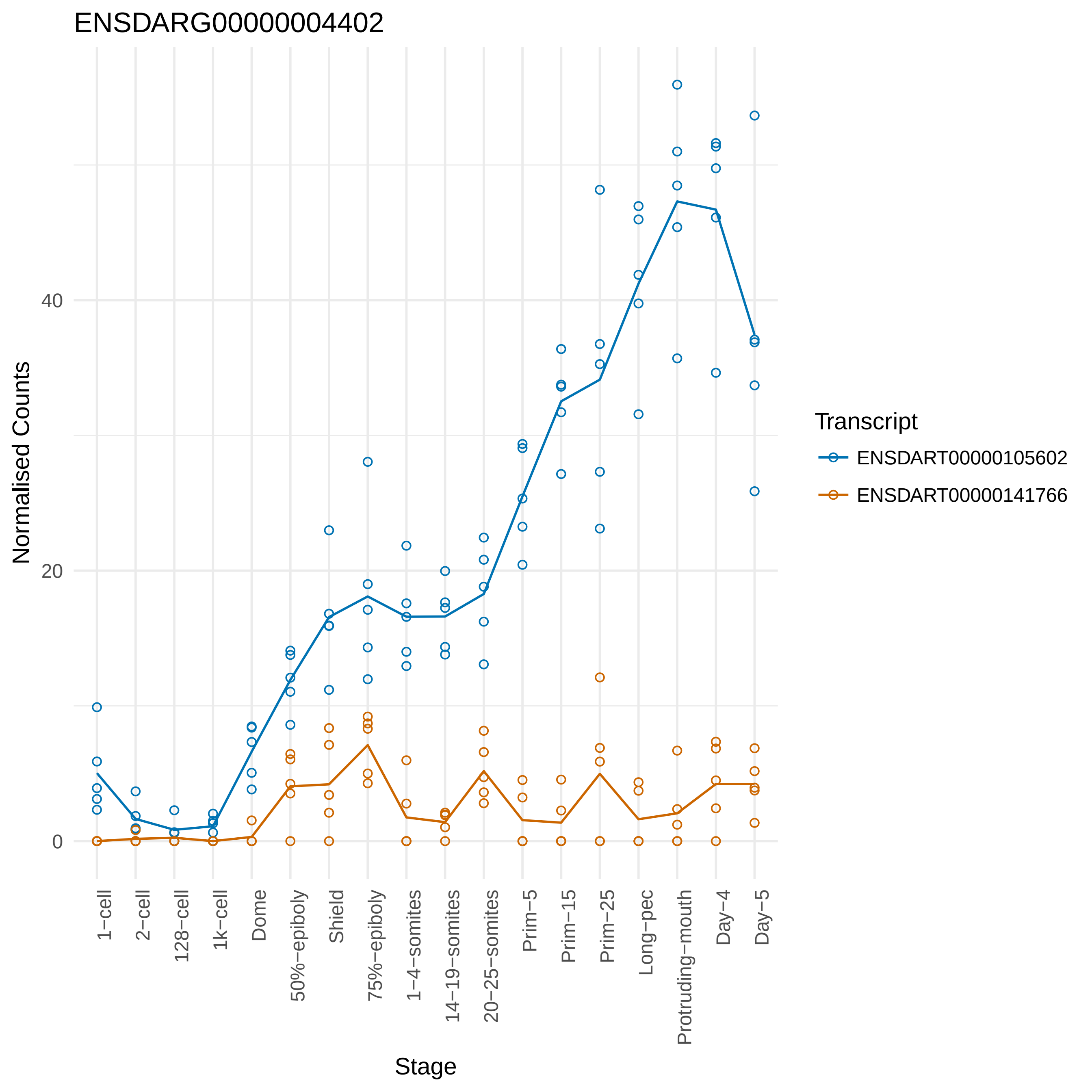

ENSDARG00000004402
Normalised Counts
0
20
40
Stage
1−cell
2−cell
128−cell
1k−cell
Dome
50%−epiboly
Shield
75%−epiboly
1−4−somites
14−19−somites
20−25−somites
Prim−5
Prim−15
Prim−25
Long−pec
Protruding−mouth
Day−4
Day−5
Transcript
ENSDART00000105602
ENSDART00000141766

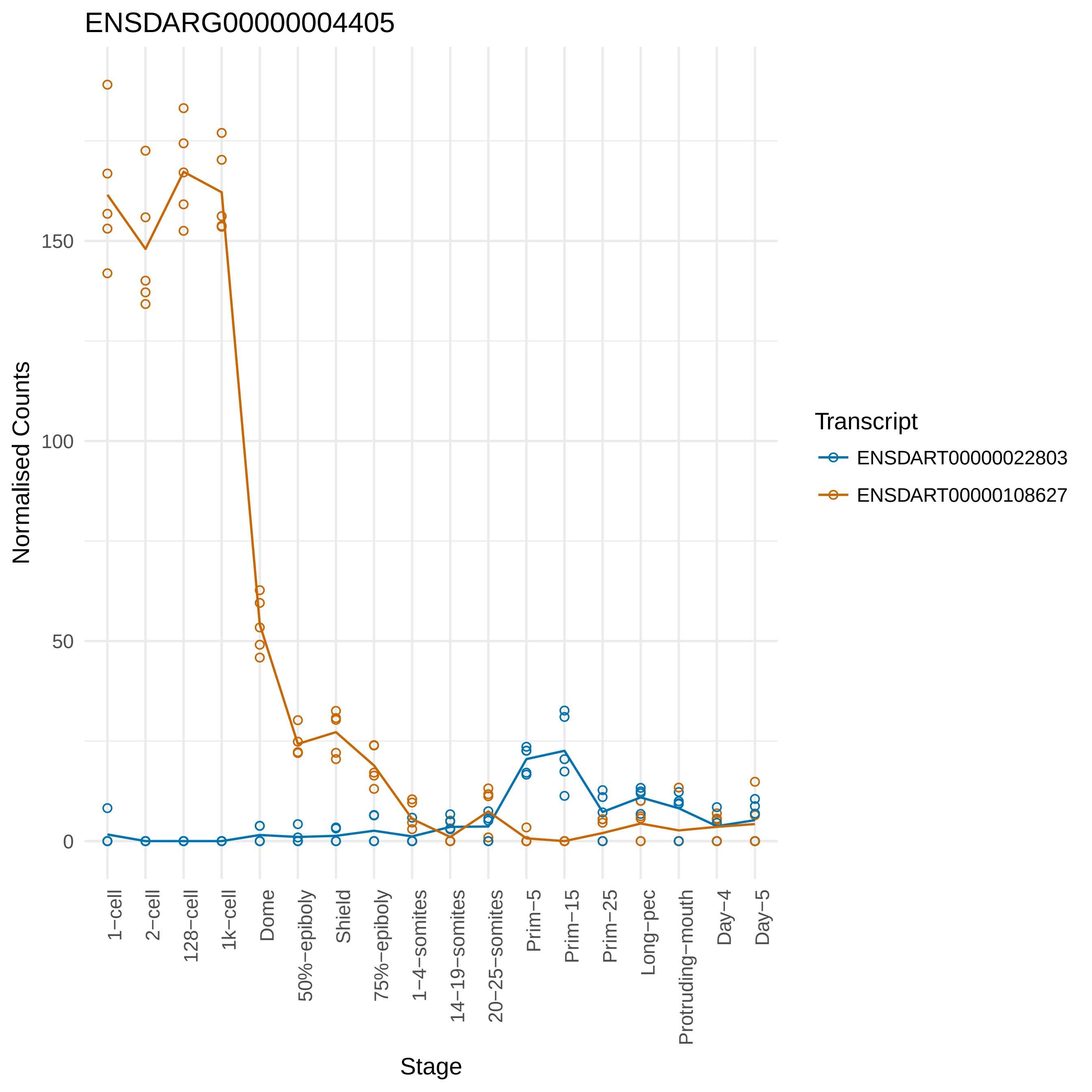
ENSDARG00000004405
Normalised Counts
150
100
50
0
1−cell
2−cell
128−cell
1k−cell
Dome
50%−epiboly
Shield
75%−epiboly
1−4−somites
14−19−somites
20−25−somites
Prim−5
Prim−15
Prim−25
Long−pec
Protruding−mouth
Day−4
Day−5
Stage
Transcript
ENSDART00000022803
ENSDART00000108627

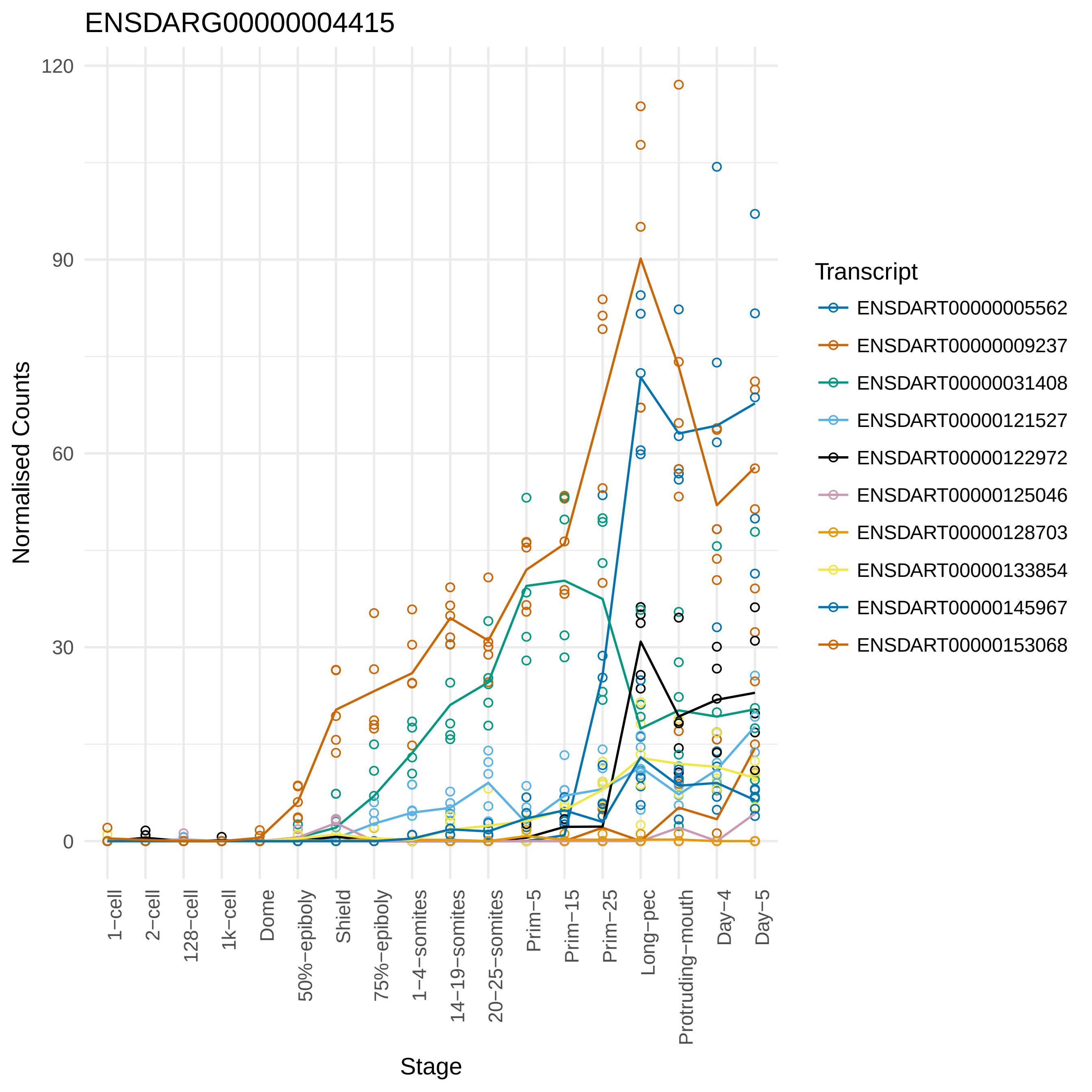
ENSDARG00000004415
Normalised Counts
120
90
60
30
0
1−cell
2−cell
128−cell
1k−cell
Dome
50%−epiboly
Shield
75%−epiboly
1−4−somites
14−19−somites
20−25−somites
Prim−5
Prim−15
Prim−25
Long−pec
Protruding−mouth
Day−4
Day−5
Stage
Transcript
ENSDART00000005562
ENSDART00000009237
ENSDART00000031408
ENSDART00000121527
ENSDART00000122972
ENSDART00000125046
ENSDART00000128703
ENSDART00000133854
ENSDART00000145967
ENSDART00000153068

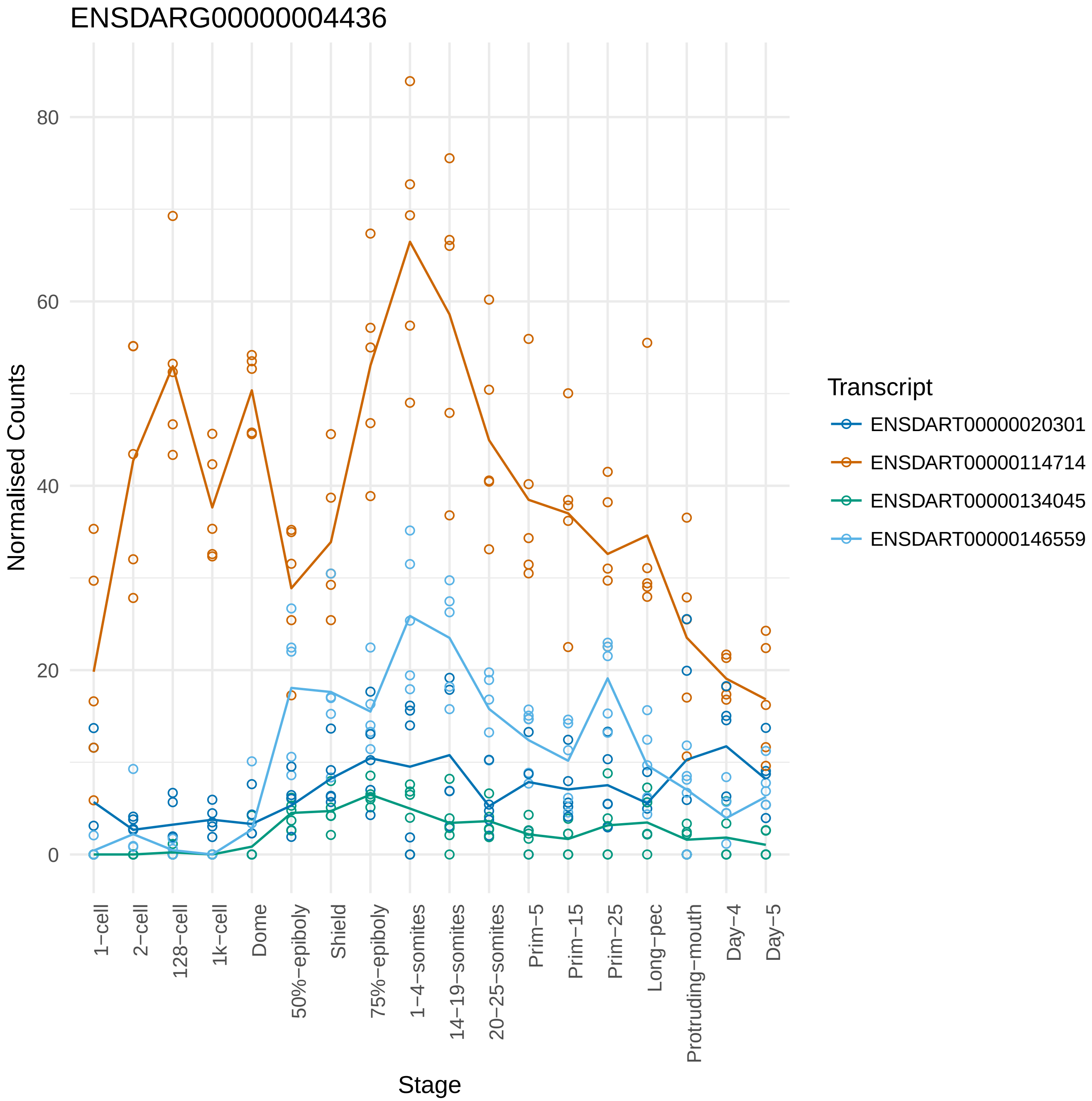

ENSDARG00000004436
Normalised Counts
0
20
40
60
80
Stage
1−cell
2−cell
128−cell
1k−cell
Dome
50%−epiboly
Shield
75%−epiboly
1−4−somites
14−19−somites
20−25−somites
Prim−5
Prim−15
Prim−25
Long−pec
Protruding−mouth
Day−4
Day−5
Transcript
ENSDART00000020301
ENSDART00000114714
ENSDART00000134045
ENSDART00000146559

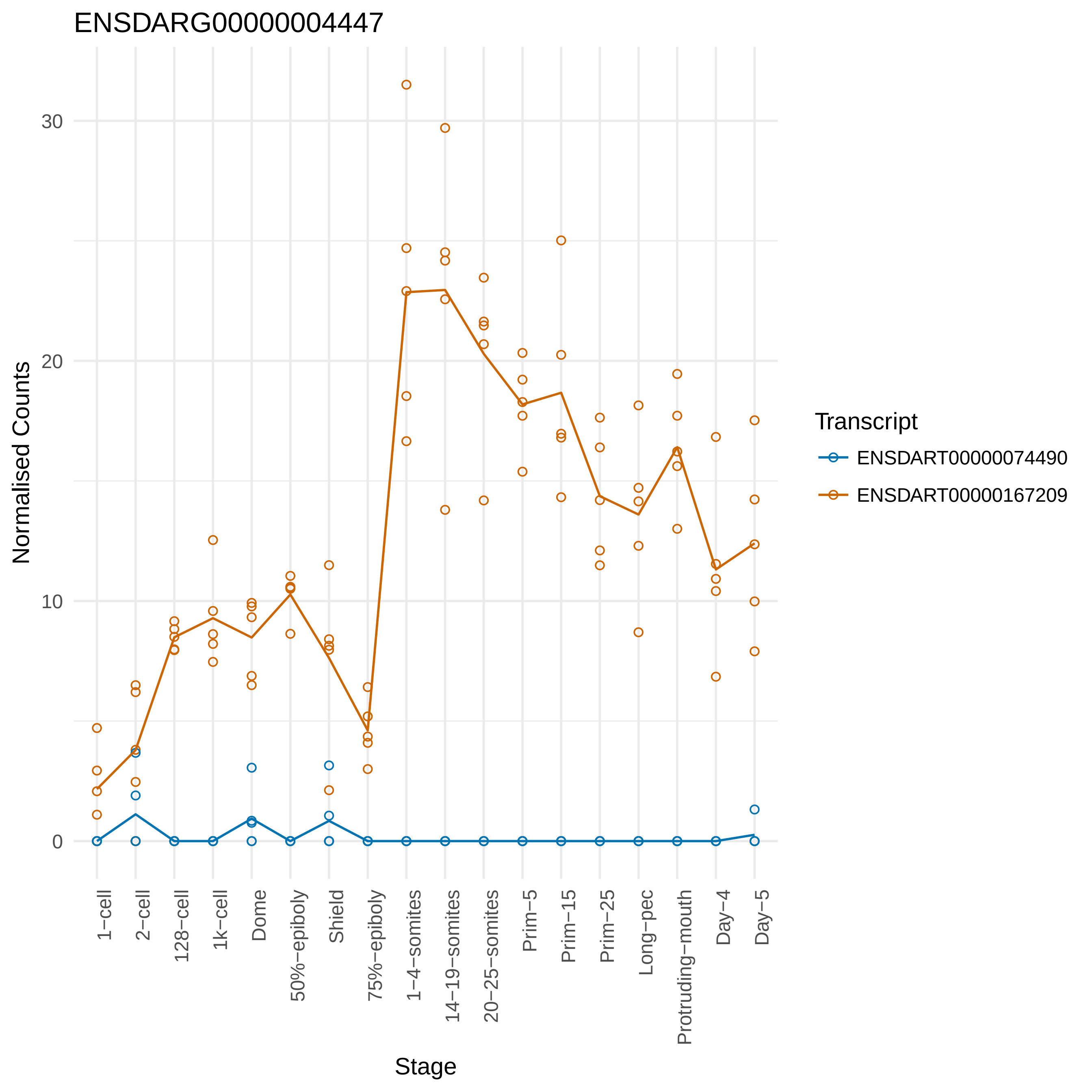

ENSDARG00000004447
Normalised Counts
30
20
10
0
1−cell
2−cell
128−cell
1k−cell
Dome
50%−epiboly
Shield
75%−epiboly
1−4−somites
14−19−somites
20−25−somites
Prim−5
Prim−15
Prim−25
Long−pec
Protruding−mouth
Day−4
Day−5
Stage
Transcript
ENSDART00000074490
ENSDART00000167209

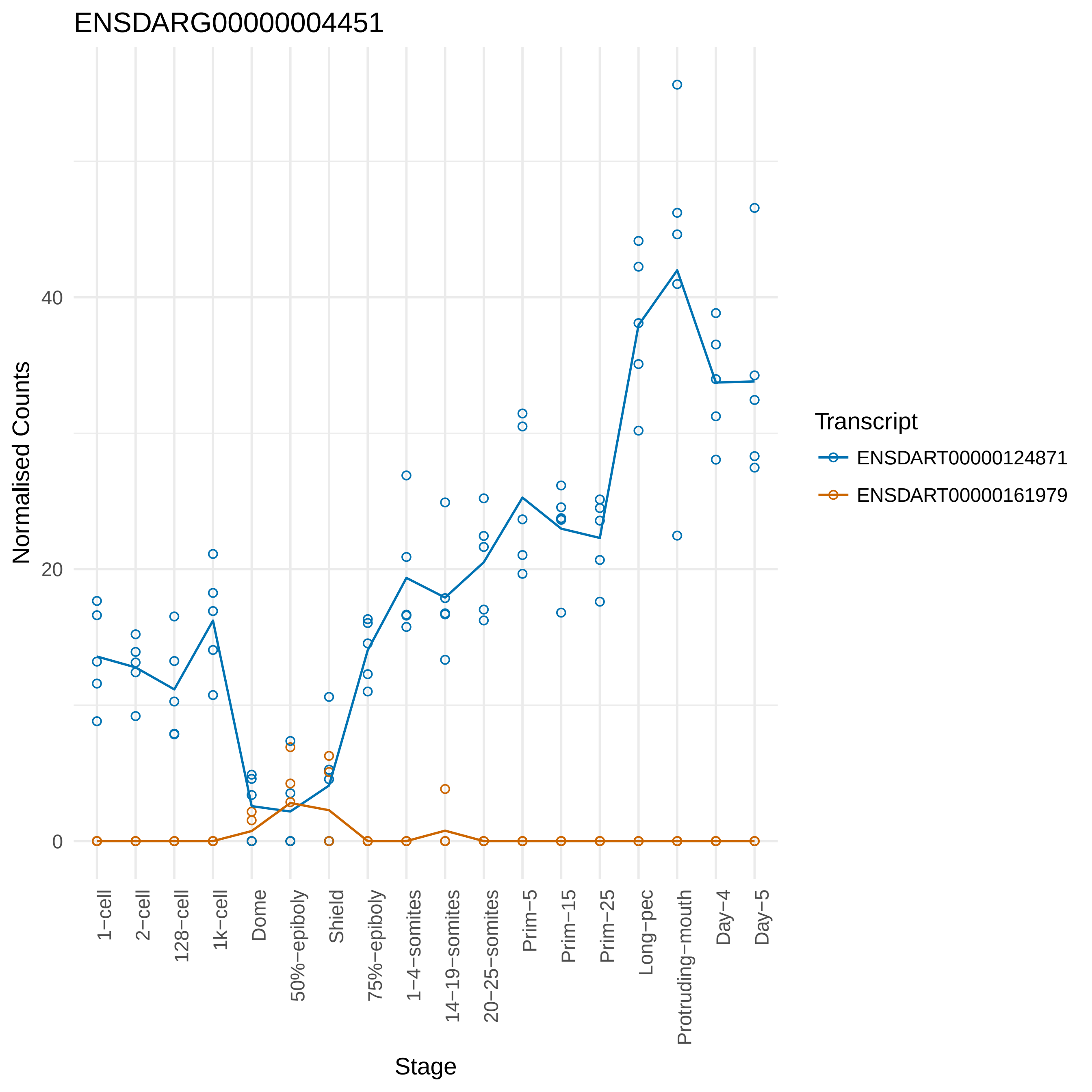
ENSDARG00000004451
Normalised Counts
0
20
40
Stage
1−cell
2−cell
128−cell
1k−cell
Dome
50%−epiboly
Shield
75%−epiboly
1−4−somites
14−19−somites
20−25−somites
Prim−5
Prim−15
Prim−25
Long−pec
Protruding−mouth
Day−4
Day−5
Transcript
ENSDART00000124871
ENSDART00000161979

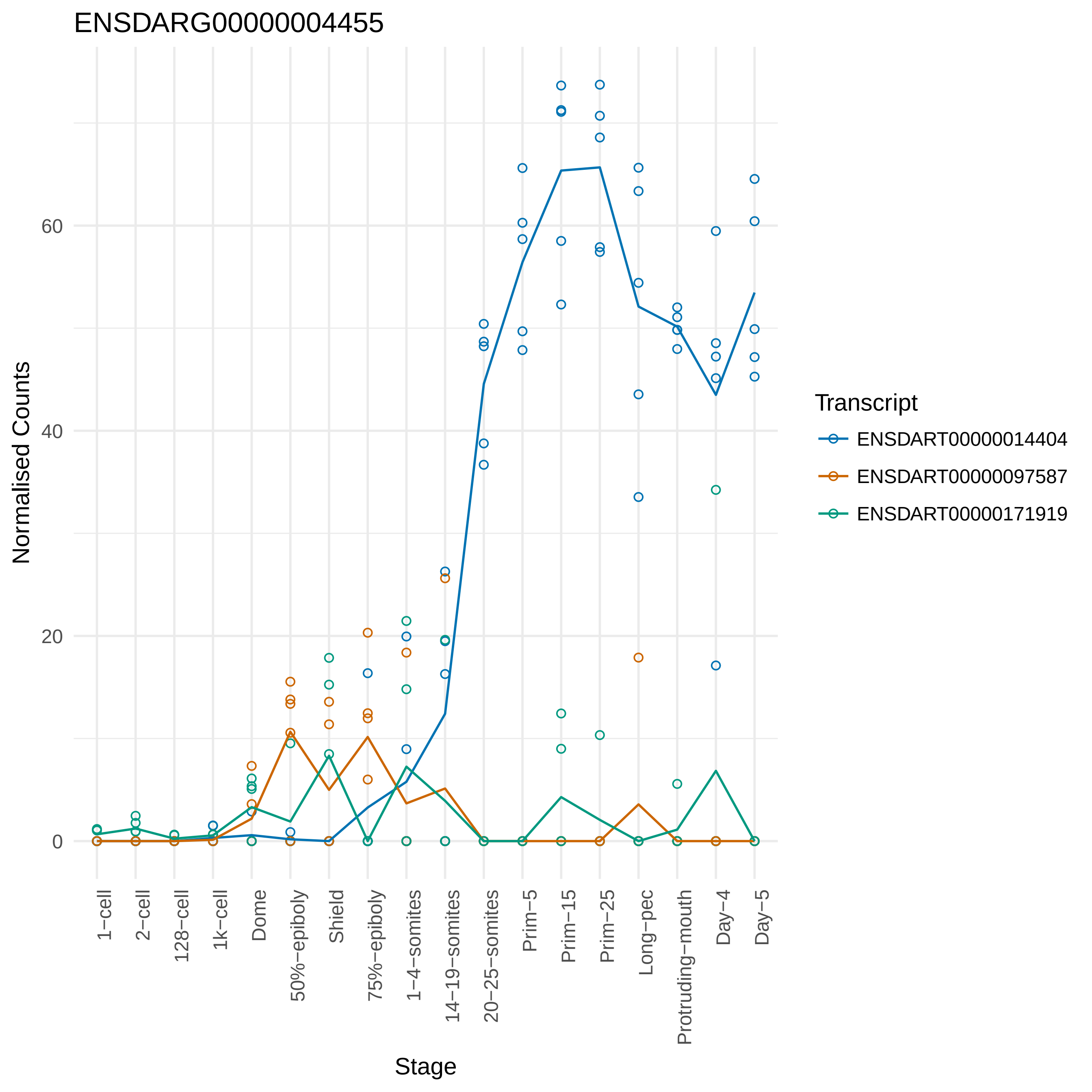

ENSDARG00000004455
Normalised Counts
0
20
40
60
Stage
1−cell
2−cell
128−cell
1k−cell
Dome
50%−epiboly
Shield
75%−epiboly
1−4−somites
14−19−somites
20−25−somites
Prim−5
Prim−15
Prim−25
Long−pec
Protruding−mouth
Day−4
Day−5
Transcript
ENSDART00000014404
ENSDART00000097587
ENSDART00000171919

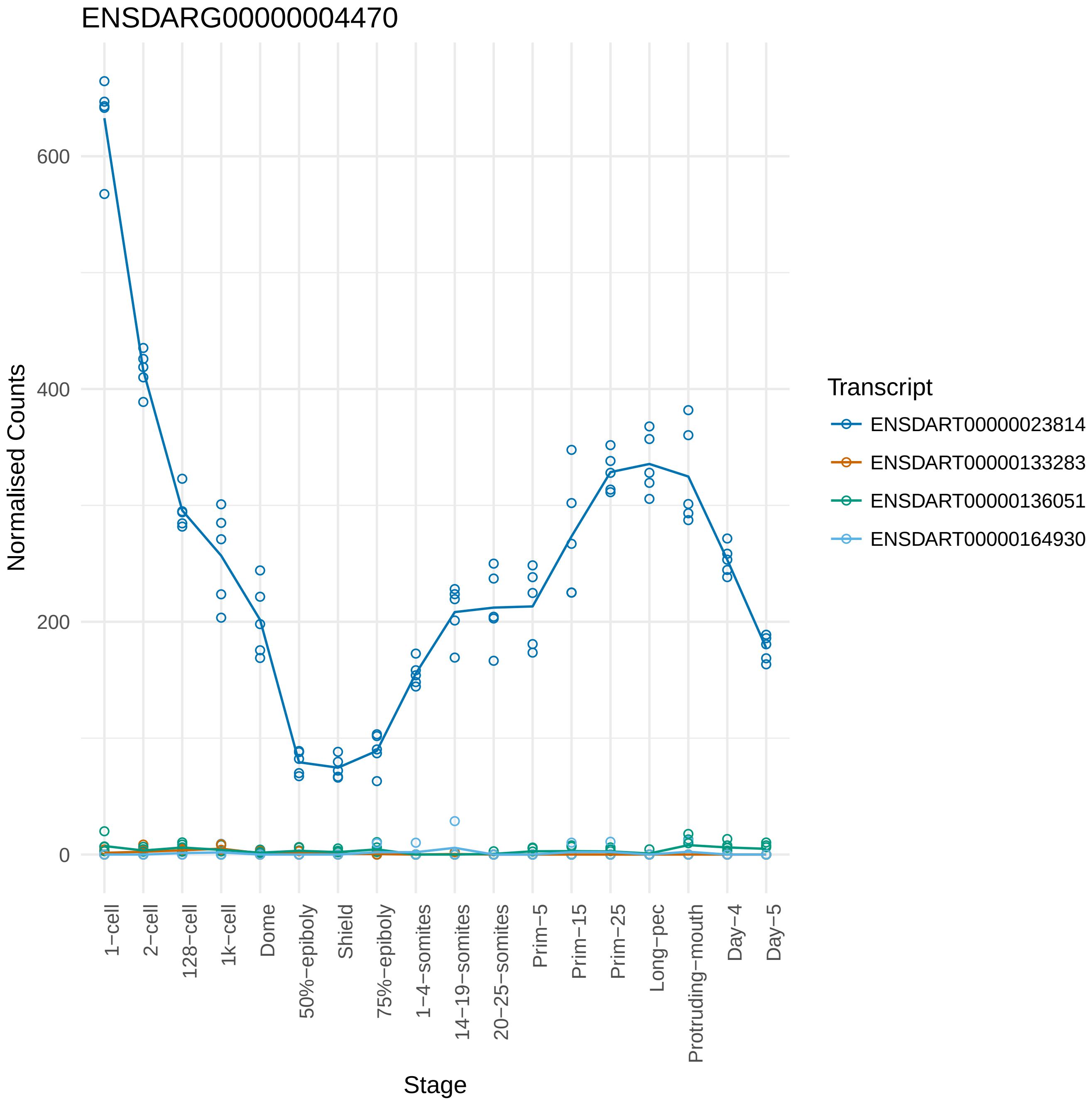

ENSDARG00000004470
Normalised Counts
600
400
200
0
1−cell
2−cell
128−cell
1k−cell
Dome
50%−epiboly
Shield
75%−epiboly
1−4−somites
14−19−somites
20−25−somites
Prim−5
Prim−15
Prim−25
Long−pec
Protruding−mouth
Day−4
Day−5
Stage
Transcript
ENSDART00000023814
ENSDART00000133283
ENSDART00000136051
ENSDART00000164930

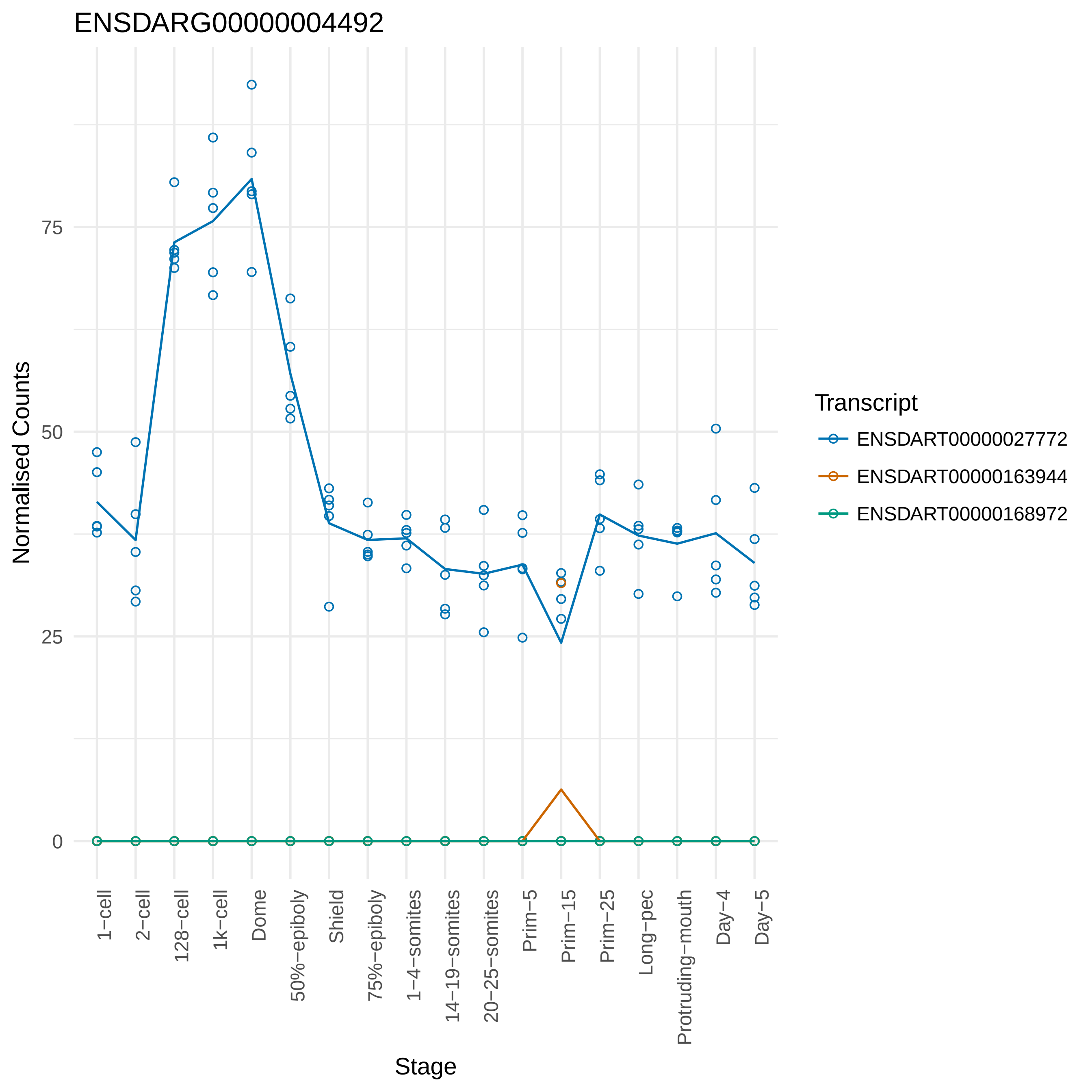

ENSDARG00000004492
Normalised Counts
75
50
25
0
1−cell
2−cell
128−cell
1k−cell
Dome
50%−epiboly
Shield
75%−epiboly
1−4−somites
14−19−somites
20−25−somites
Prim−5
Prim−15
Prim−25
Long−pec
Protruding−mouth
Day−4
Day−5
Stage
Transcript
ENSDART00000027772
ENSDART00000163944
ENSDART00000168972

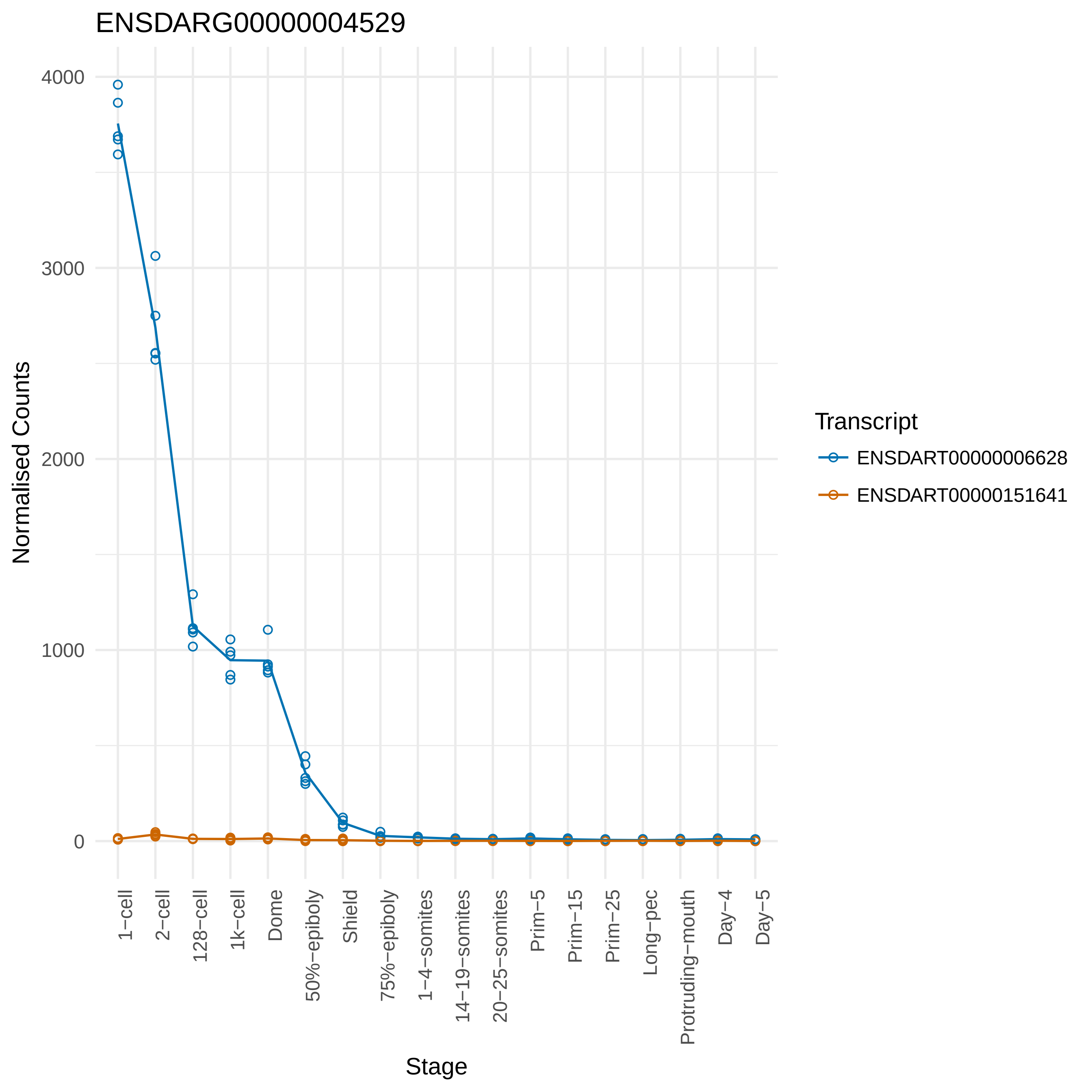

ENSDARG00000004529
Normalised Counts
0
1000
2000
3000
4000
Stage
1−cell
2−cell
128−cell
1k−cell
Dome
50%−epiboly
Shield
75%−epiboly
1−4−somites
14−19−somites
20−25−somites
Prim−5
Prim−15
Prim−25
Long−pec
Protruding−mouth
Day−4
Day−5
Transcript
ENSDART00000006628
ENSDART00000151641

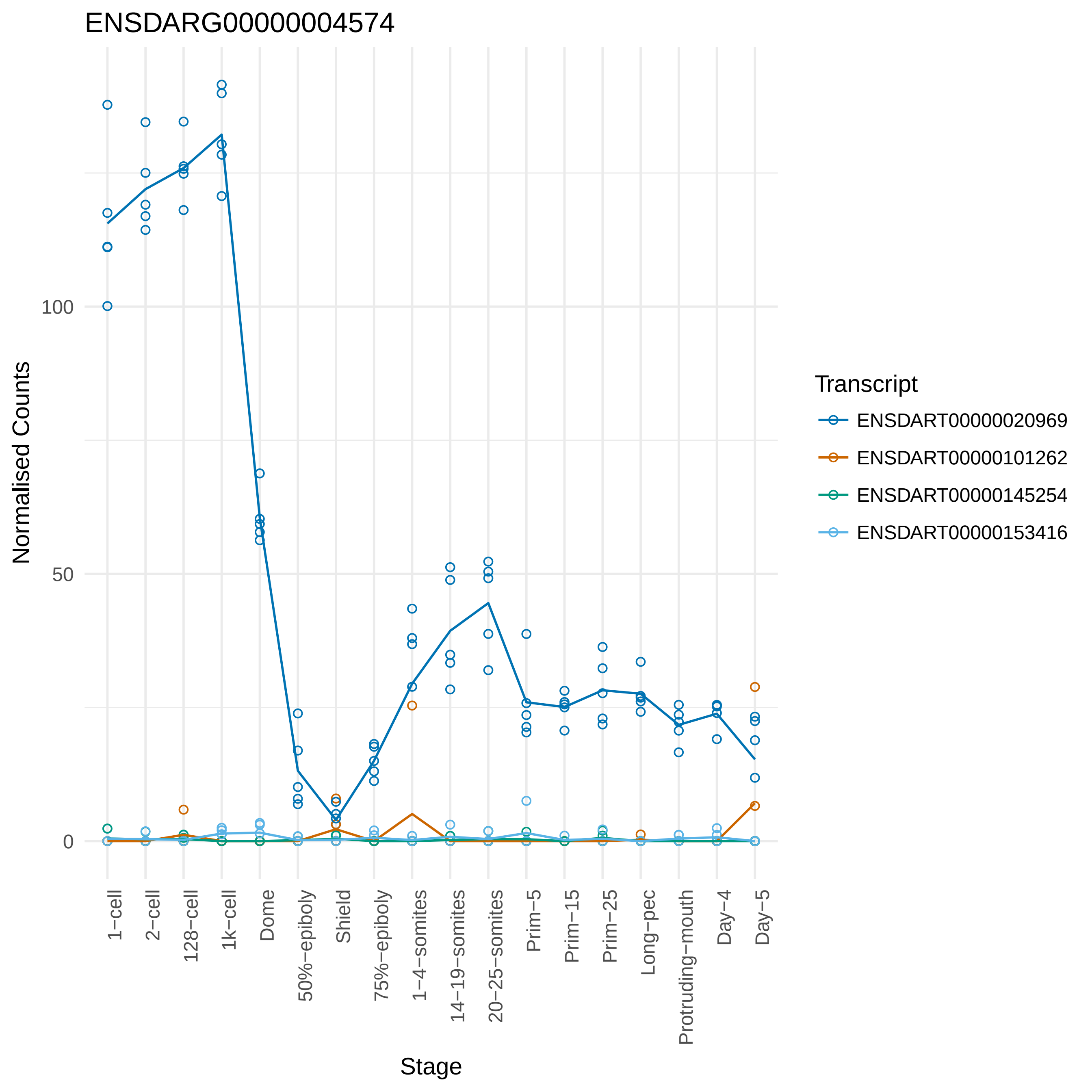

ENSDARG00000004574
Normalised Counts
100
50
0
Stage
1−cell
2−cell
128−cell
1k−cell
Dome
50%−epiboly
Shield
75%−epiboly
1−4−somites
14−19−somites
20−25−somites
Prim−5
Prim−15
Prim−25
Long−pec
Protruding−mouth
Day−4
Day−5
Transcript
ENSDART00000020969
ENSDART00000101262
ENSDART00000145254
ENSDART00000153416

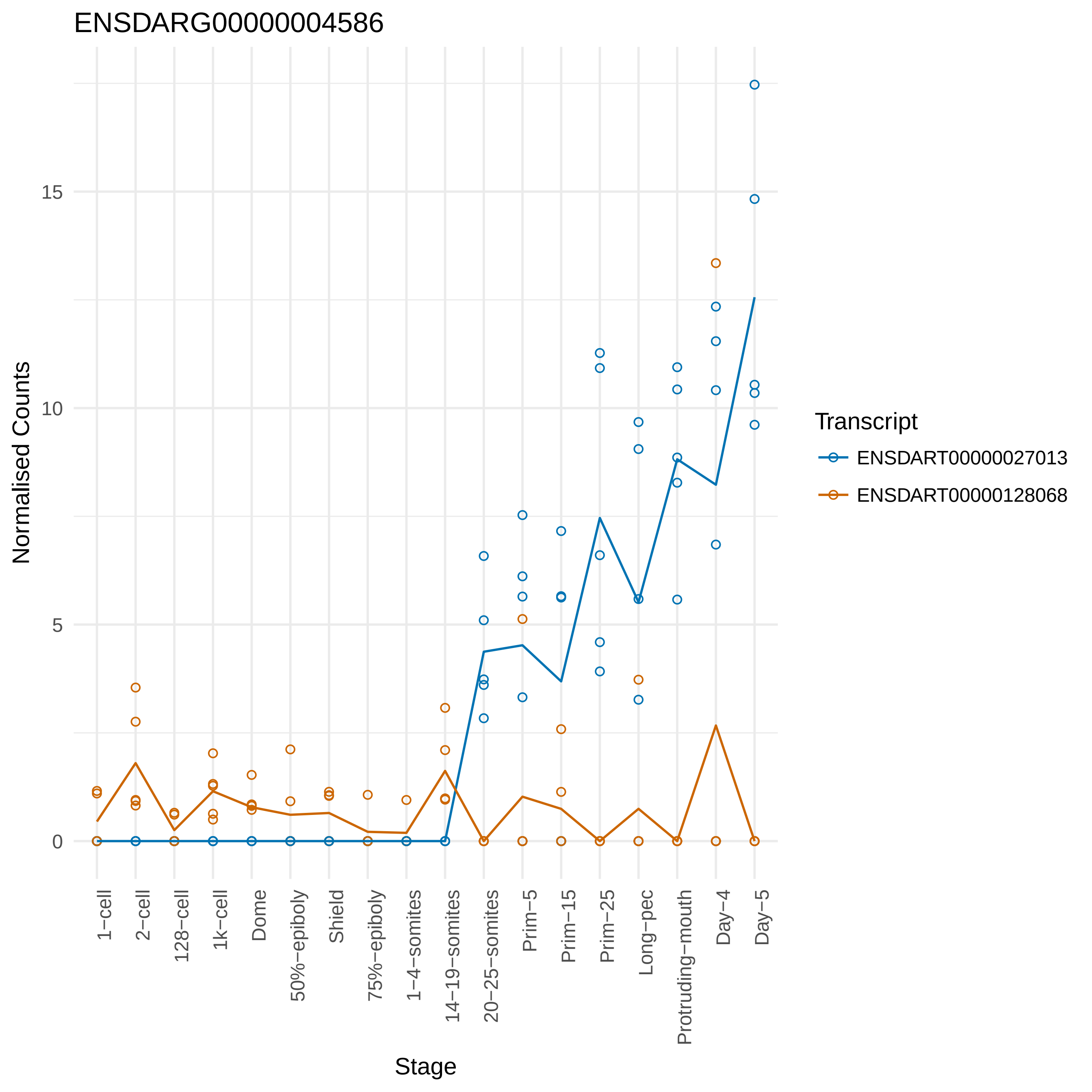
ENSDARG00000004586
Normalised Counts
0
5
10
15
1−cell
2−cell
128−cell
1k−cell
Dome
50%−epiboly
Shield
75%−epiboly
1−4−somites
14−19−somites
20−25−somites
Prim−5
Prim−15
Prim−25
Long−pec
Protruding−mouth
Day−4
Day−5
Stage
Transcript
ENSDART00000027013
ENSDART00000128068

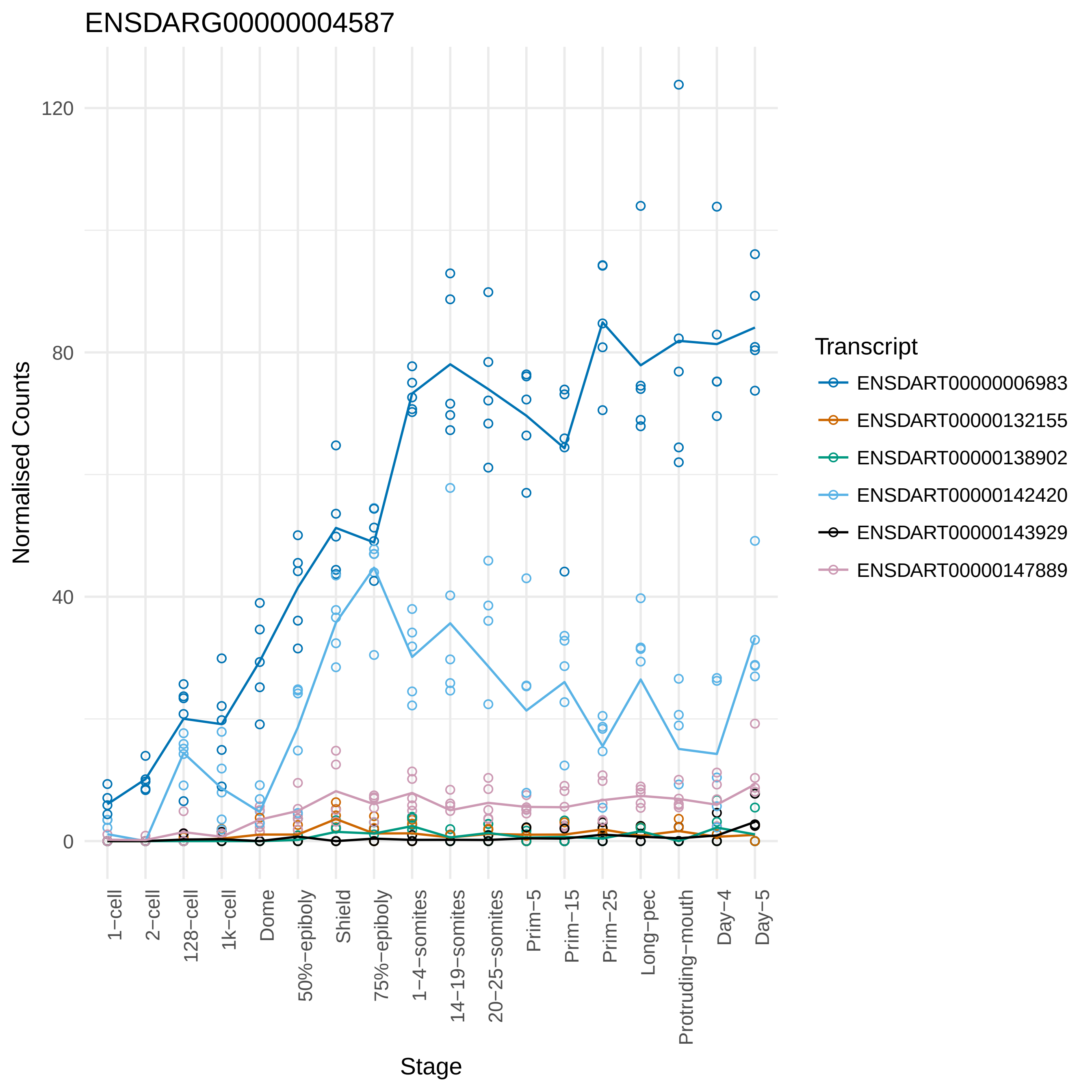

ENSDARG00000004587
Normalised Counts
120
80
40
0
1−cell
2−cell
128−cell
1k−cell
Dome
50%−epiboly
Shield
75%−epiboly
1−4−somites
14−19−somites
20−25−somites
Prim−5
Prim−15
Prim−25
Long−pec
Protruding−mouth
Day−4
Day−5
Stage
Transcript
ENSDART00000006983
ENSDART00000132155
ENSDART00000138902
ENSDART00000142420
ENSDART00000143929
ENSDART00000147889

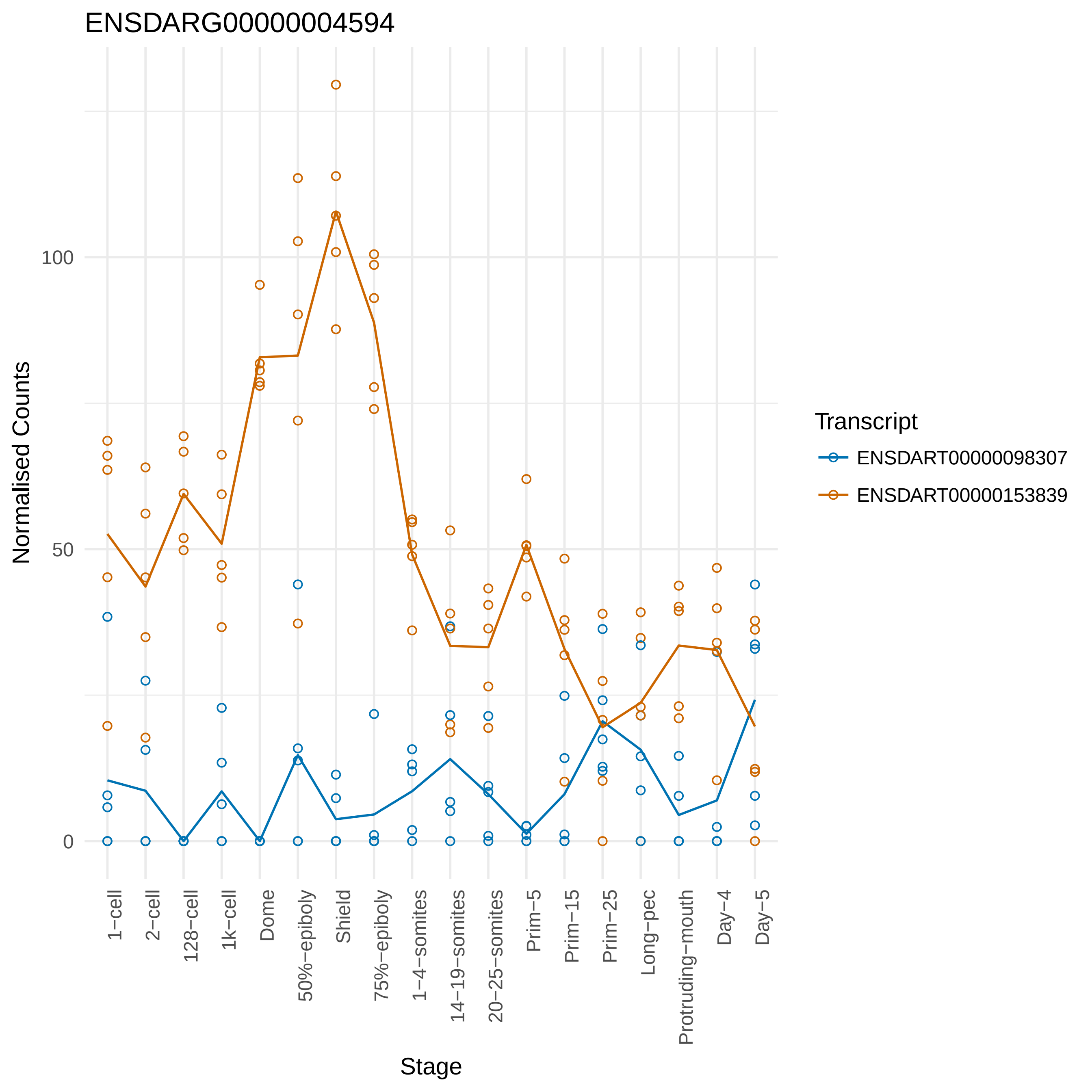

ENSDARG00000004594
Normalised Counts
100
50
0
1−cell
2−cell
128−cell
1k−cell
Dome
50%−epiboly
Shield
75%−epiboly
1−4−somites
14−19−somites
20−25−somites
Prim−5
Prim−15
Prim−25
Long−pec
Protruding−mouth
Day−4
Day−5
Stage
Transcript
ENSDART00000098307
ENSDART00000153839

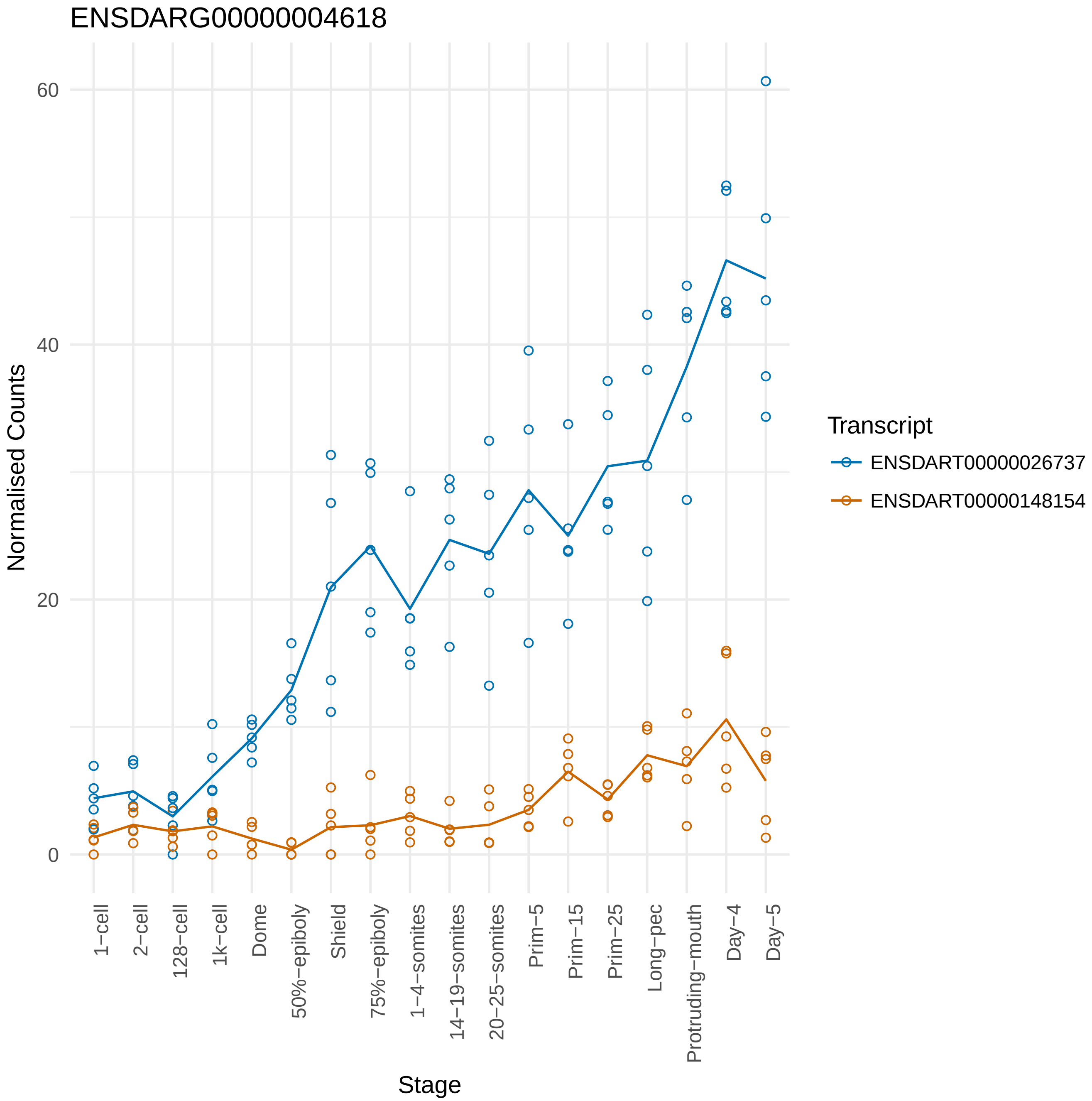
ENSDARG00000004618
Normalised Counts
60
40
20
0
1−cell
2−cell
128−cell
1k−cell
Dome
50%−epiboly
Shield
75%−epiboly
1−4−somites
14−19−somites
20−25−somites
Prim−5
Prim−15
Prim−25
Long−pec
Protruding−mouth
Day−4
Day−5
Stage
Transcript
ENSDART00000026737
ENSDART00000148154

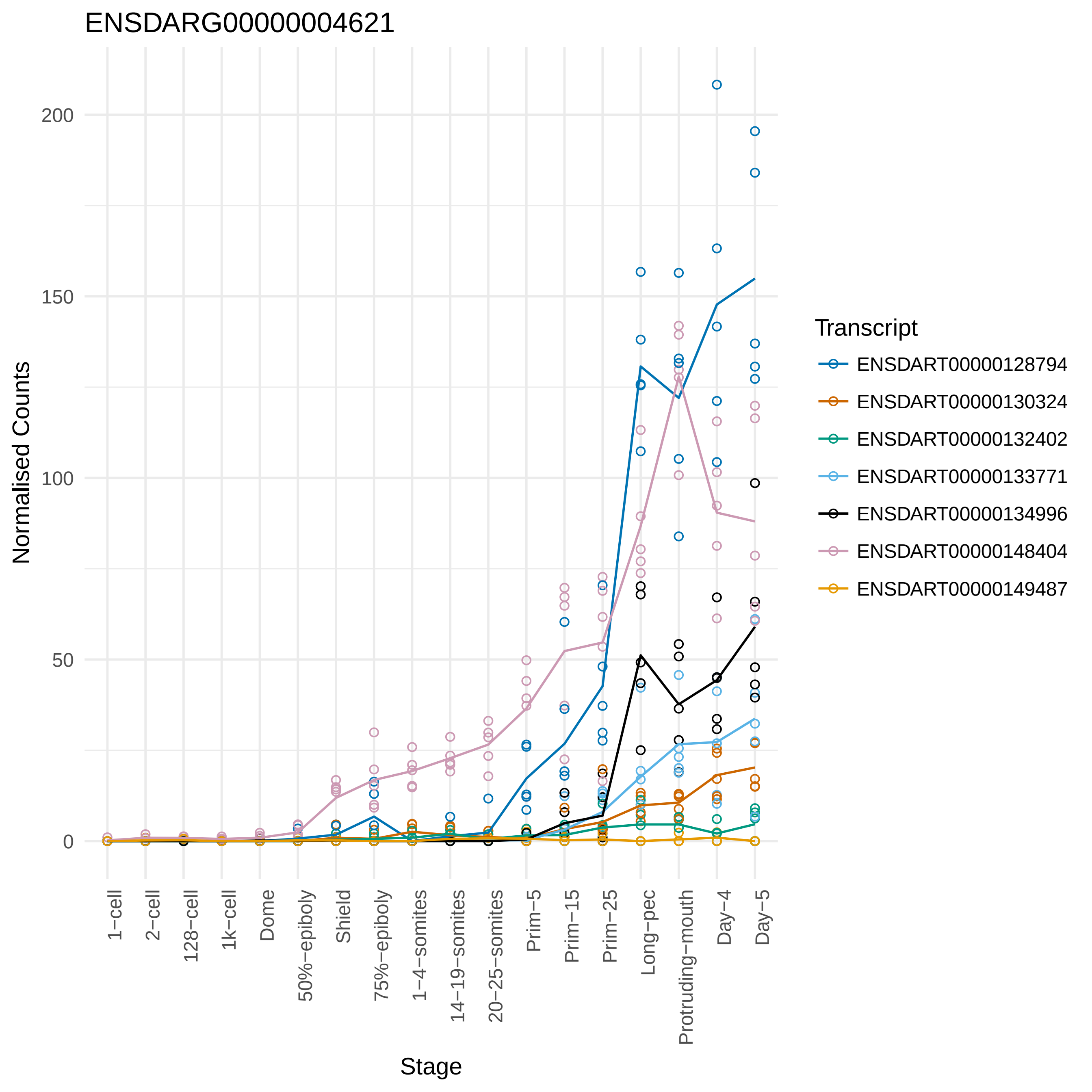

ENSDARG00000004621
Normalised Counts
200
150
100
50
0
1−cell
2−cell
128−cell
1k−cell
Dome
50%−epiboly
Shield
75%−epiboly
1−4−somites
14−19−somites
20−25−somites
Prim−5
Prim−15
Prim−25
Long−pec
Protruding−mouth
Day−4
Day−5
Stage
Transcript
ENSDART00000128794
ENSDART00000130324
ENSDART00000132402
ENSDART00000133771
ENSDART00000134996
ENSDART00000148404
ENSDART00000149487

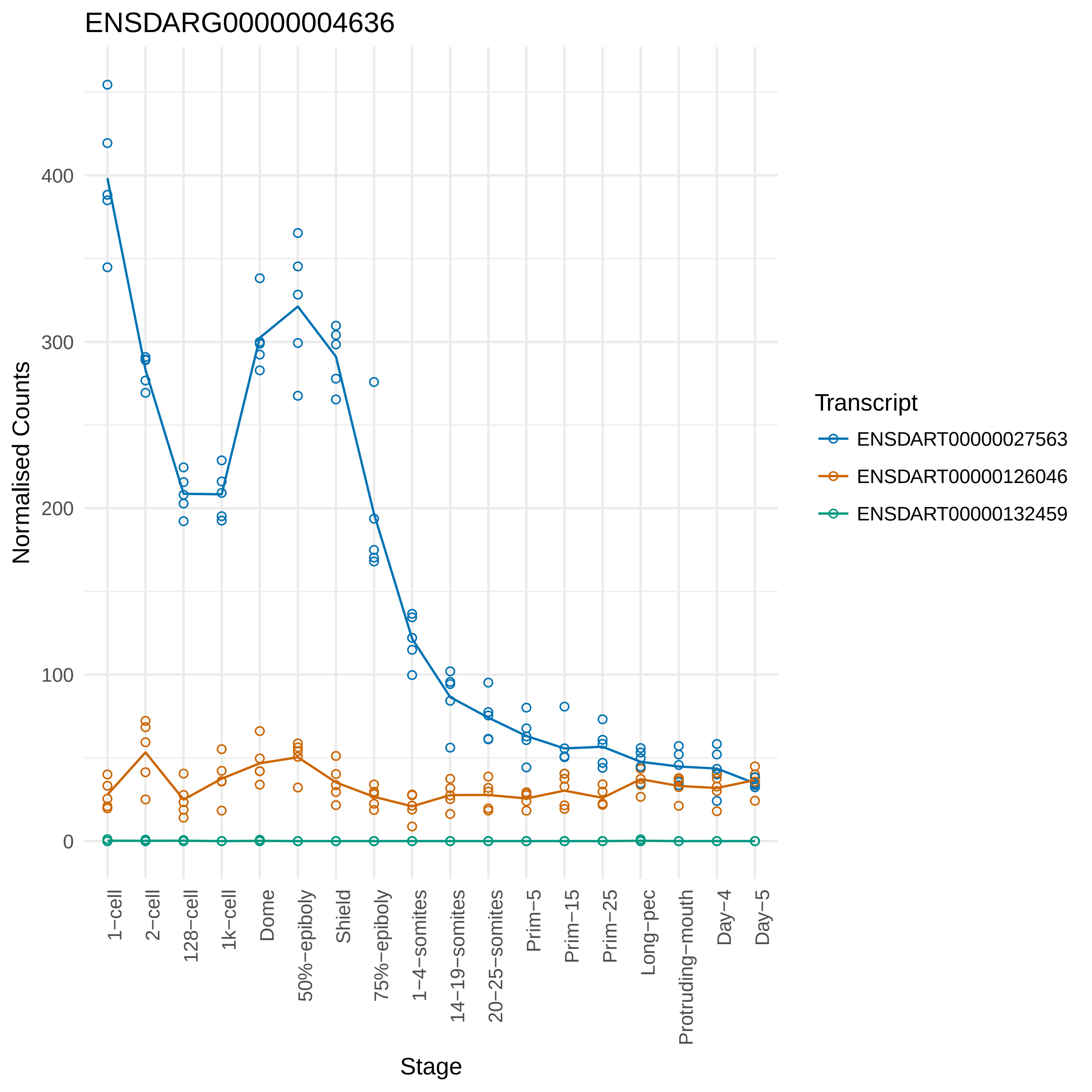

ENSDARG00000004636
Normalised Counts
0
100
200
300
400
Stage
1−cell
2−cell
128−cell
1k−cell
Dome
50%−epiboly
Shield
75%−epiboly
1−4−somites
14−19−somites
20−25−somites
Prim−5
Prim−15
Prim−25
Long−pec
Protruding−mouth
Day−4
Day−5
Transcript
ENSDART00000027563
ENSDART00000126046
ENSDART00000132459

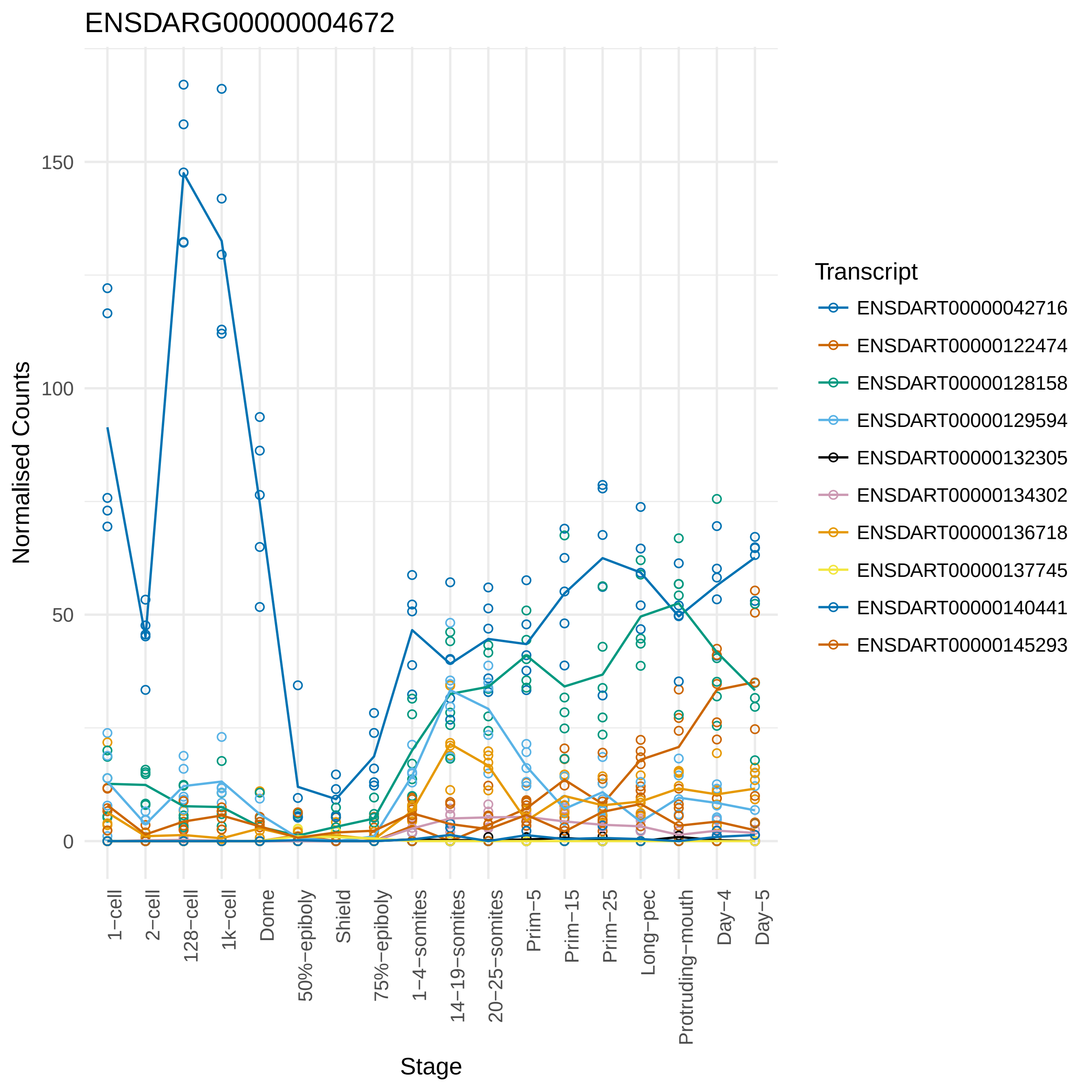

ENSDARG00000004672
Normalised Counts
150
100
50
0
1−cell
2−cell
128−cell
1k−cell
Dome
50%−epiboly
Shield
75%−epiboly
1−4−somites
14−19−somites
20−25−somites
Prim−5
Prim−15
Prim−25
Long−pec
Protruding−mouth
Day−4
Day−5
Stage
Transcript
ENSDART00000042716
ENSDART00000122474
ENSDART00000128158
ENSDART00000129594
ENSDART00000132305
ENSDART00000134302
ENSDART00000136718
ENSDART00000137745
ENSDART00000140441
ENSDART00000145293

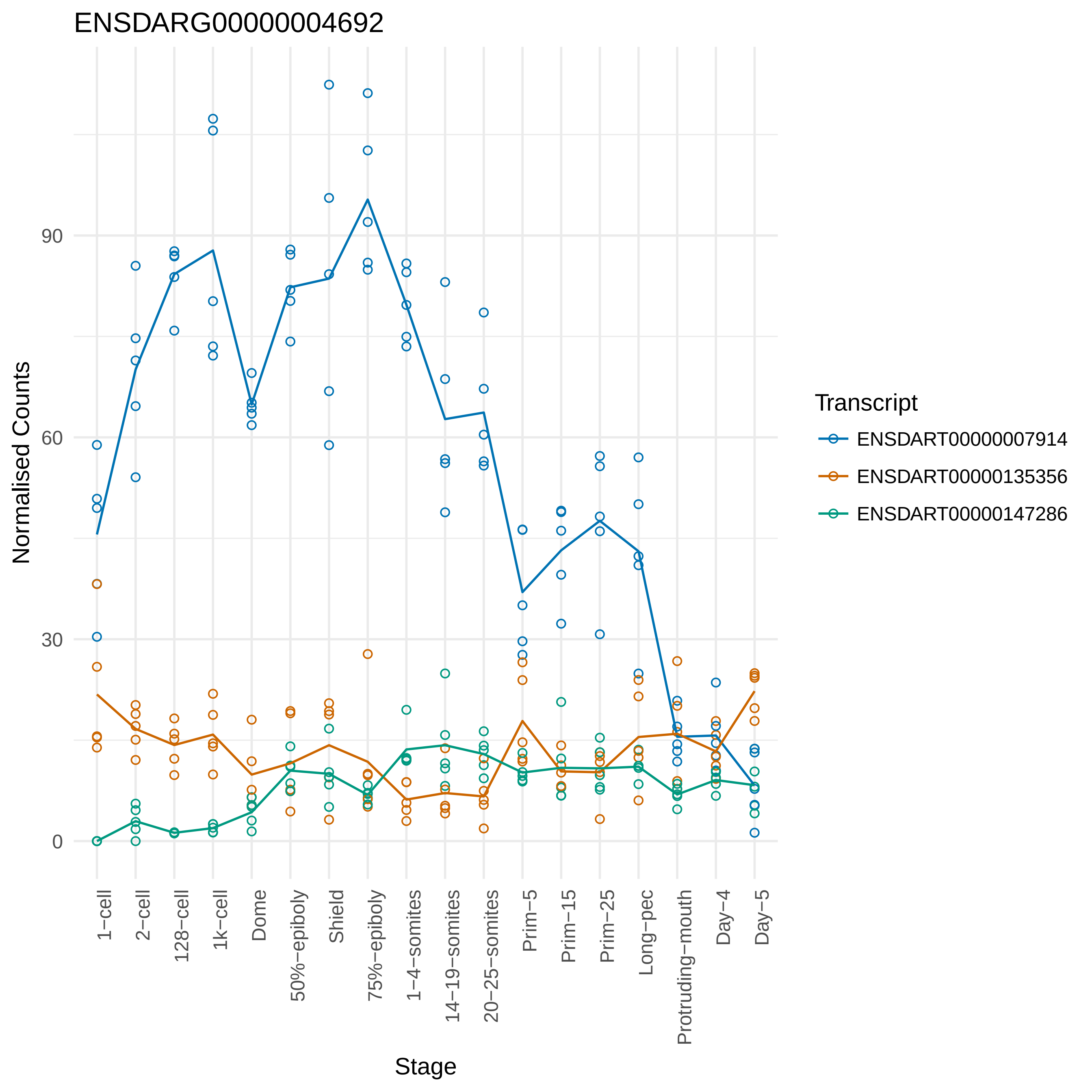

ENSDARG00000004692
Normalised Counts
90
60
30
0
Stage
1−cell
2−cell
128−cell
1k−cell
Dome
50%−epiboly
Shield
75%−epiboly
1−4−somites
14−19−somites
20−25−somites
Prim−5
Prim−15
Prim−25
Long−pec
Protruding−mouth
Day−4
Day−5
Transcript
ENSDART00000007914
ENSDART00000135356
ENSDART00000147286

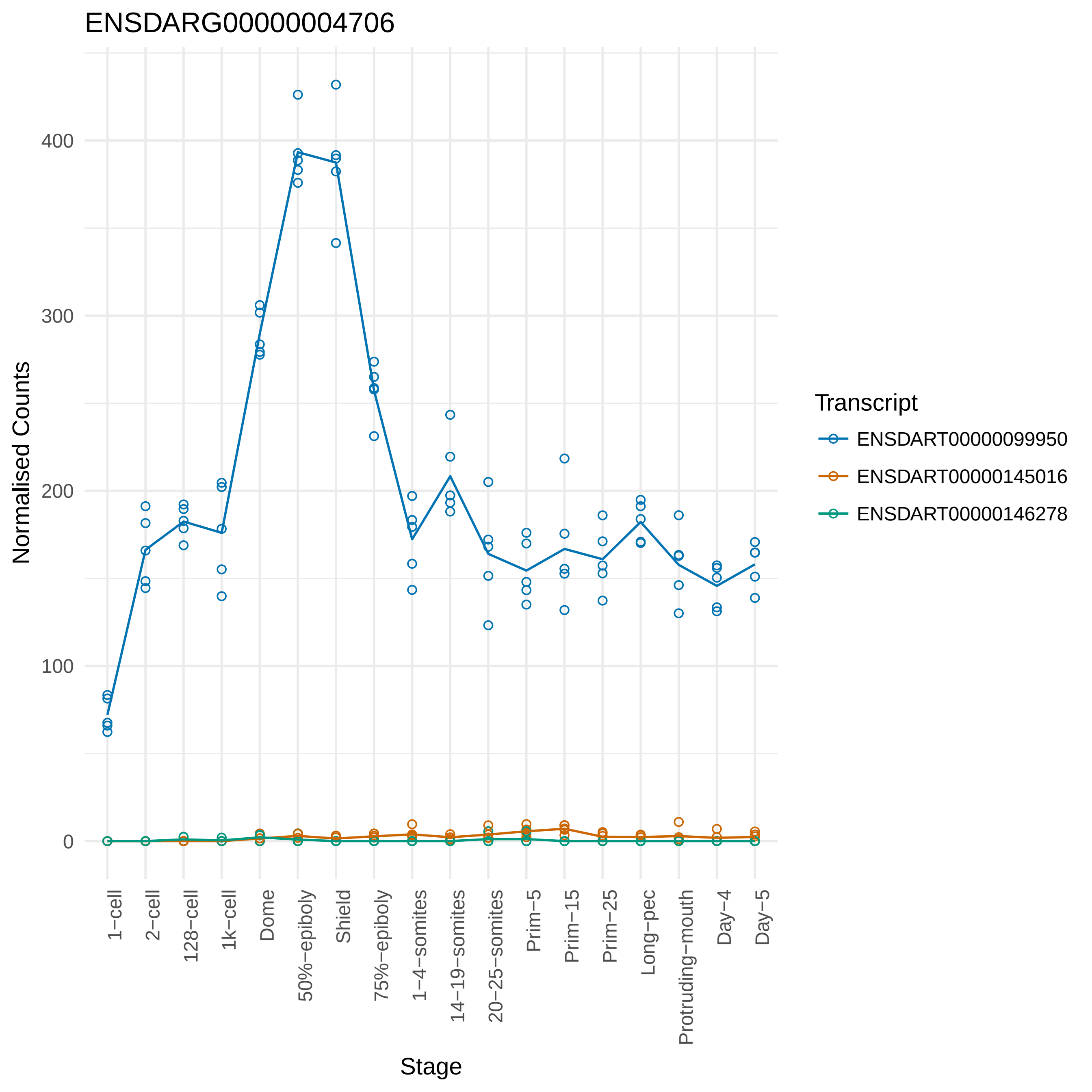

ENSDARG00000004706
Normalised Counts
400
300
200
100
0
1−cell
2−cell
128−cell
1k−cell
Dome
50%−epiboly
Shield
75%−epiboly
1−4−somites
14−19−somites
20−25−somites
Prim−5
Prim−15
Prim−25
Long−pec
Protruding−mouth
Day−4
Day−5
Stage
Transcript
ENSDART00000099950
ENSDART00000145016
ENSDART00000146278

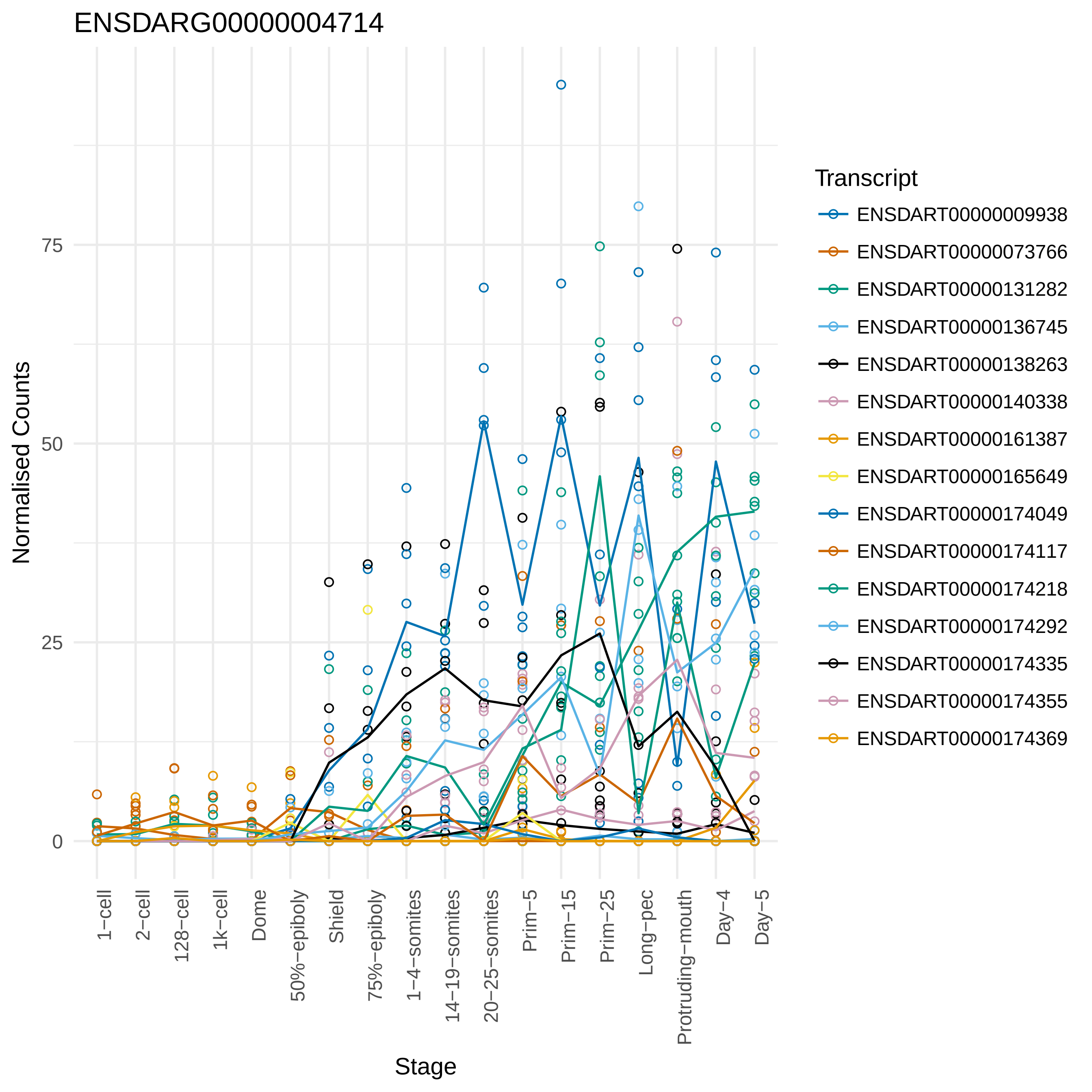
ENSDARG00000004714
Normalised Counts
Stage
0
25
50
75
1−cell
2−cell
128−cell
1k−cell
Dome
50%−epiboly
Shield
75%−epiboly
1−4−somites
14−19−somites
20−25−somites
Prim−5
Prim−15
Prim−25
Long−pec
Protruding−mouth
Day−4
Day−5
Transcript
ENSDART00000009938
ENSDART00000073766
ENSDART00000131282
ENSDART00000136745
ENSDART00000138263
ENSDART00000140338
ENSDART00000161387
ENSDART00000165649
ENSDART00000174049
ENSDART00000174117
ENSDART00000174218
ENSDART00000174292
ENSDART00000174335
ENSDART00000174355
ENSDART00000174369

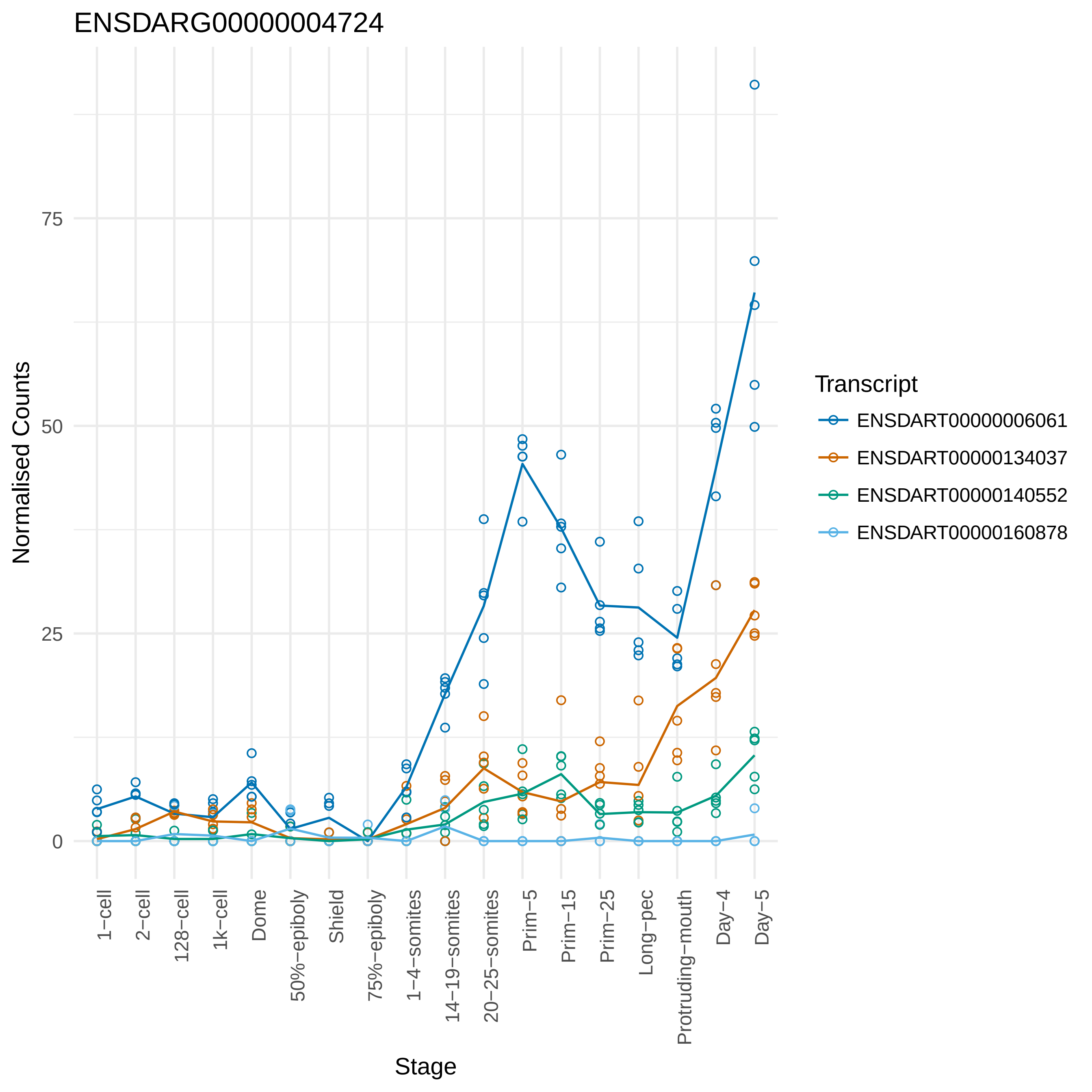

ENSDARG00000004724
Normalised Counts
0
25
50
75
Stage
1−cell
2−cell
128−cell
1k−cell
Dome
50%−epiboly
Shield
75%−epiboly
1−4−somites
14−19−somites
20−25−somites
Prim−5
Prim−15
Prim−25
Long−pec
Protruding−mouth
Day−4
Day−5
Transcript
ENSDART00000006061
ENSDART00000134037
ENSDART00000140552
ENSDART00000160878

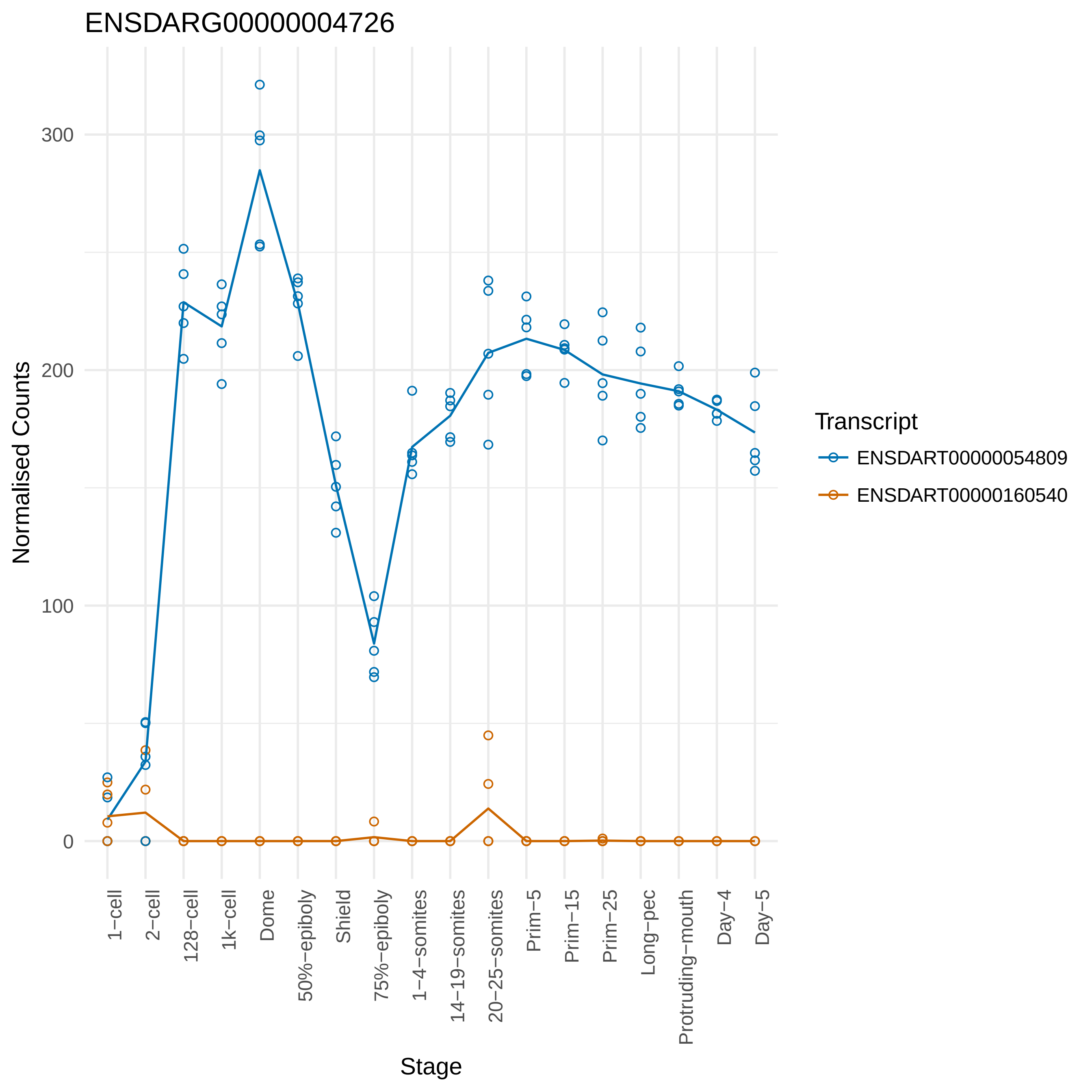

ENSDARG00000004726
Normalised Counts
300
200
100
0
1−cell
2−cell
128−cell
1k−cell
Dome
50%−epiboly
Shield
75%−epiboly
1−4−somites
14−19−somites
20−25−somites
Prim−5
Prim−15
Prim−25
Long−pec
Protruding−mouth
Day−4
Day−5
Stage
Transcript
ENSDART00000054809
ENSDART00000160540

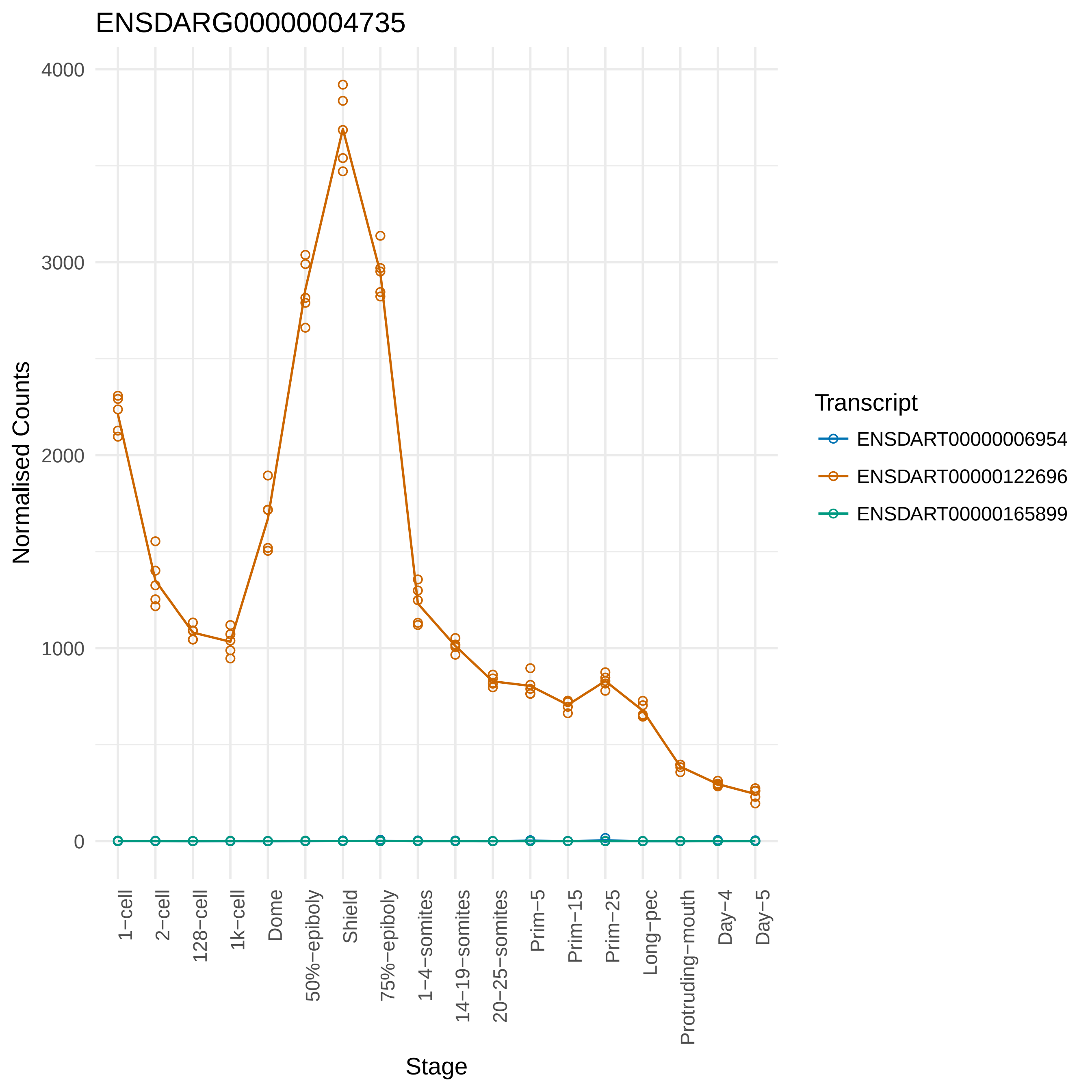

ENSDARG00000004735
Normalised Counts
4000
3000
2000
1000
0
1−cell
2−cell
128−cell
1k−cell
Dome
50%−epiboly
Shield
75%−epiboly
1−4−somites
14−19−somites
20−25−somites
Prim−5
Prim−15
Prim−25
Long−pec
Protruding−mouth
Day−4
Day−5
Stage
Transcript
ENSDART00000006954
ENSDART00000122696
ENSDART00000165899

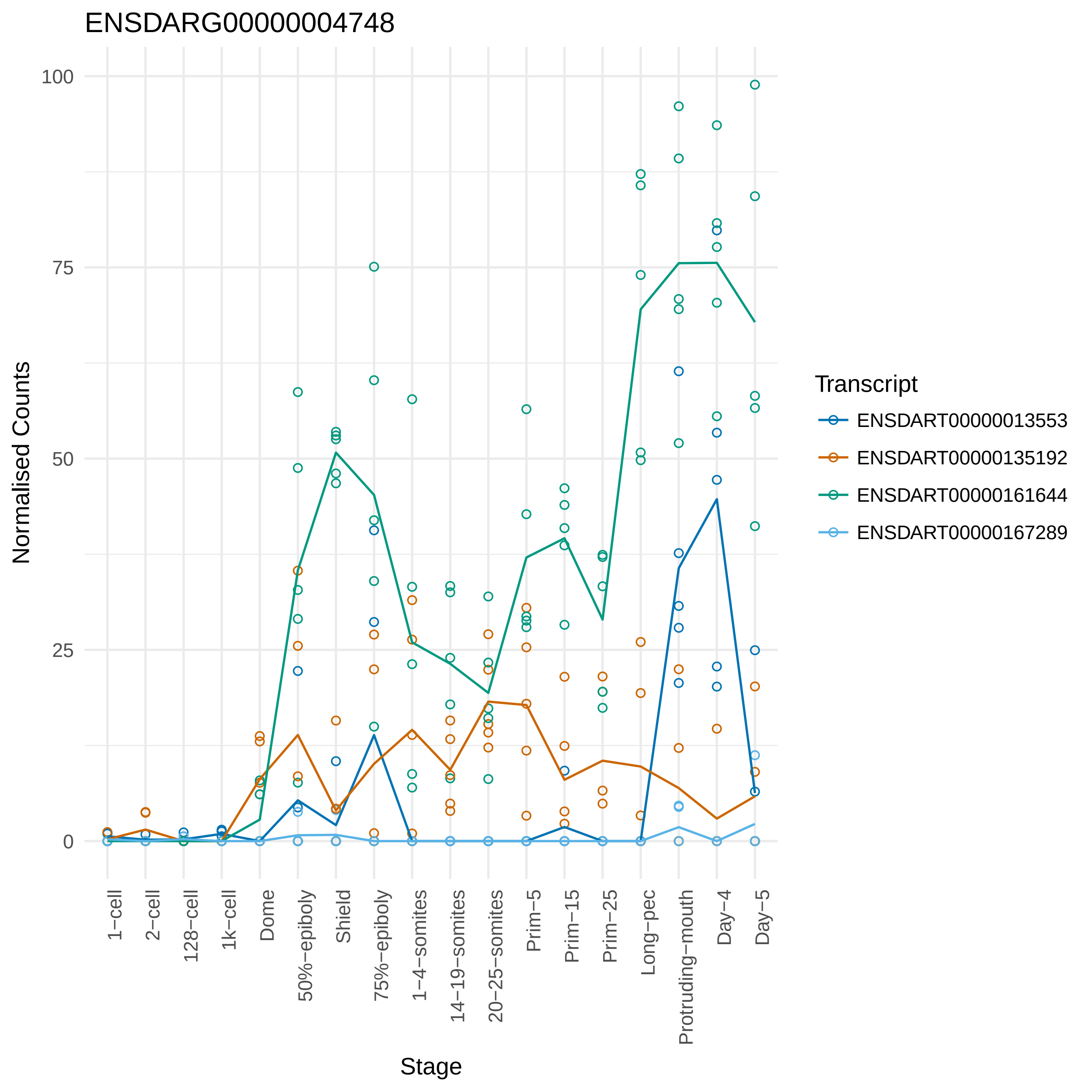

ENSDARG00000004748
Normalised Counts
100
75
50
25
0
Stage
1−cell
2−cell
128−cell
1k−cell
Dome
50%−epiboly
Shield
75%−epiboly
1−4−somites
14−19−somites
20−25−somites
Prim−5
Prim−15
Prim−25
Long−pec
Protruding−mouth
Day−4
Day−5
Transcript
ENSDART00000013553
ENSDART00000135192
ENSDART00000161644
ENSDART00000167289

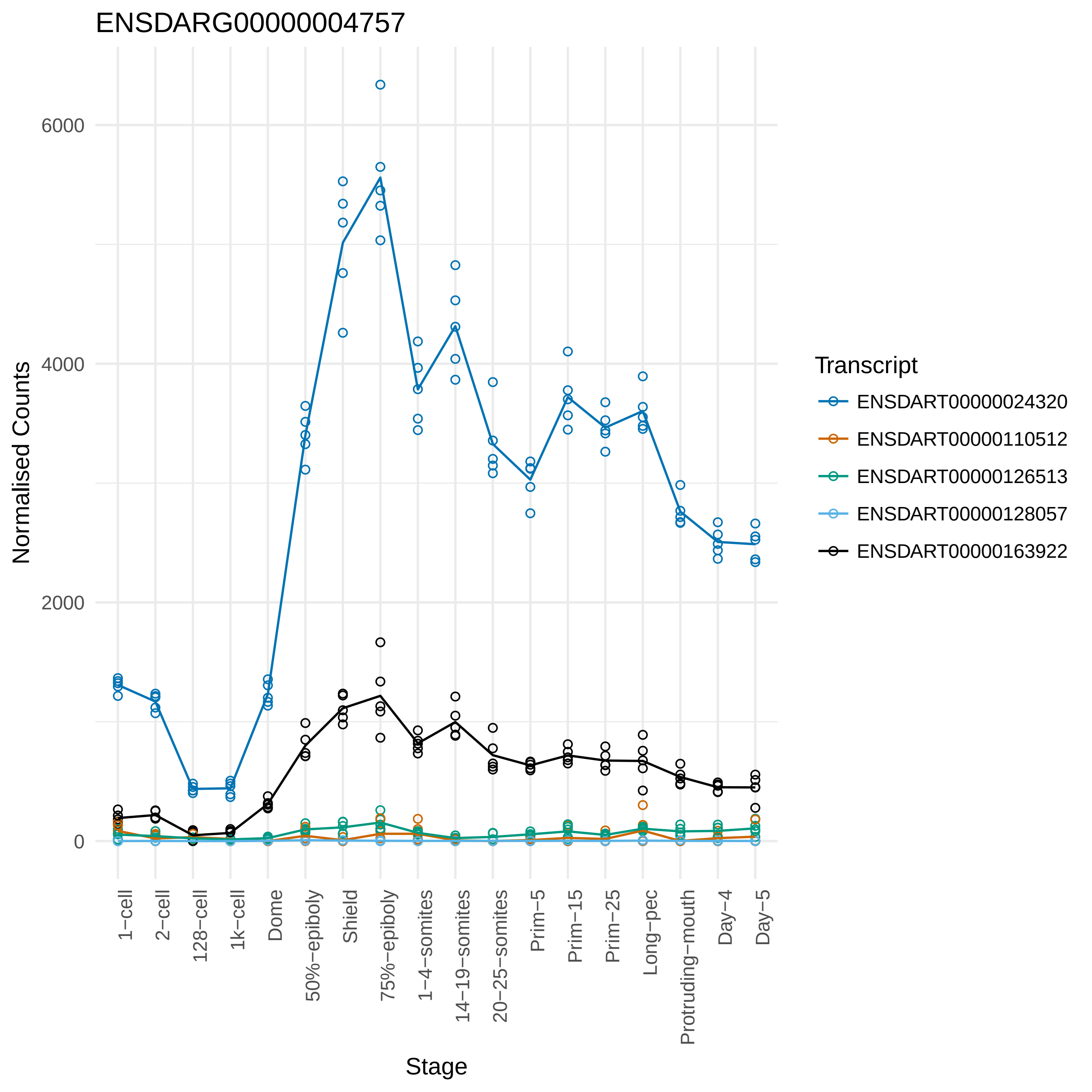

ENSDARG00000004757
Normalised Counts
6000
4000
2000
0
1−cell
2−cell
128−cell
1k−cell
Dome
50%−epiboly
Shield
75%−epiboly
1−4−somites
14−19−somites
20−25−somites
Prim−5
Prim−15
Prim−25
Long−pec
Protruding−mouth
Day−4
Day−5
Stage
Transcript
ENSDART00000024320
ENSDART00000110512
ENSDART00000126513
ENSDART00000128057
ENSDART00000163922

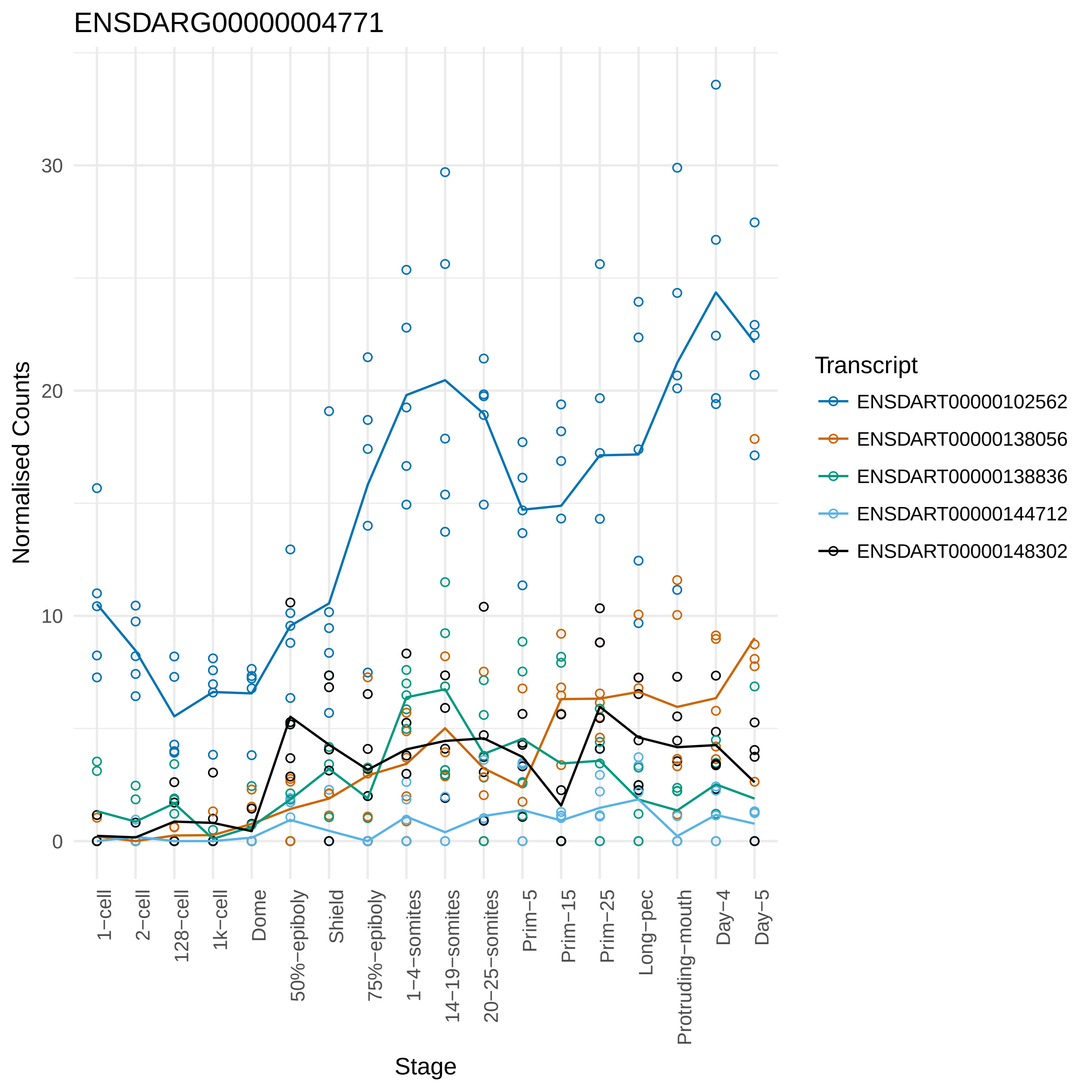
ENSDARG00000004771
Normalised Counts
0
10
20
30
1−cell
2−cell
128−cell
1k−cell
Dome
50%−epiboly
Shield
75%−epiboly
1−4−somites
14−19−somites
20−25−somites
Prim−5
Prim−15
Prim−25
Long−pec
Protruding−mouth
Day−4
Day−5
Stage
Transcript
ENSDART00000102562
ENSDART00000138056
ENSDART00000138836
ENSDART00000144712
ENSDART00000148302

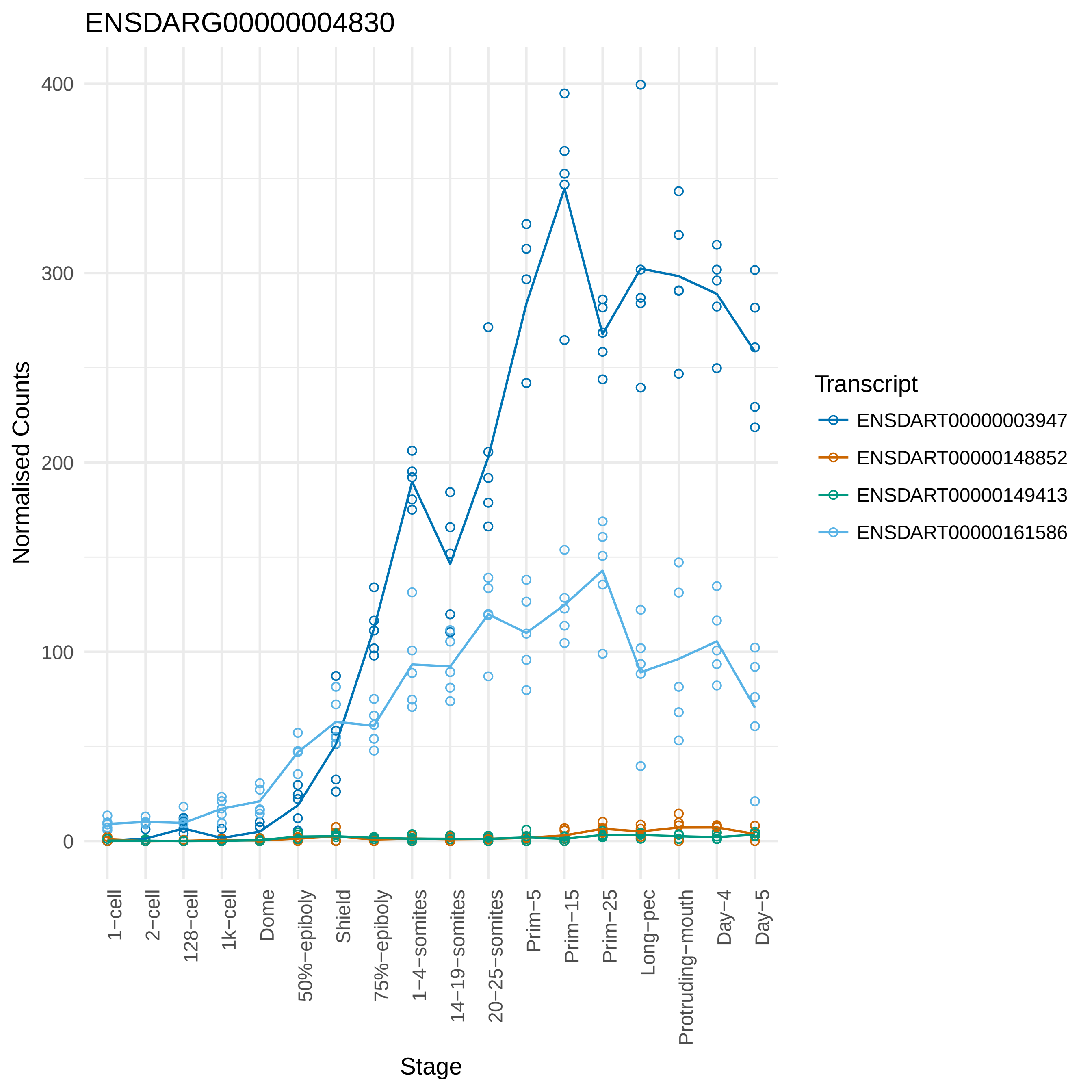

ENSDARG00000004830
Normalised Counts
0
100
200
300
400
1−cell
2−cell
128−cell
1k−cell
Dome
50%−epiboly
Shield
75%−epiboly
1−4−somites
14−19−somites
20−25−somites
Prim−5
Prim−15
Prim−25
Long−pec
Protruding−mouth
Day−4
Day−5
Stage
Transcript
ENSDART00000003947
ENSDART00000148852
ENSDART00000149413
ENSDART00000161586

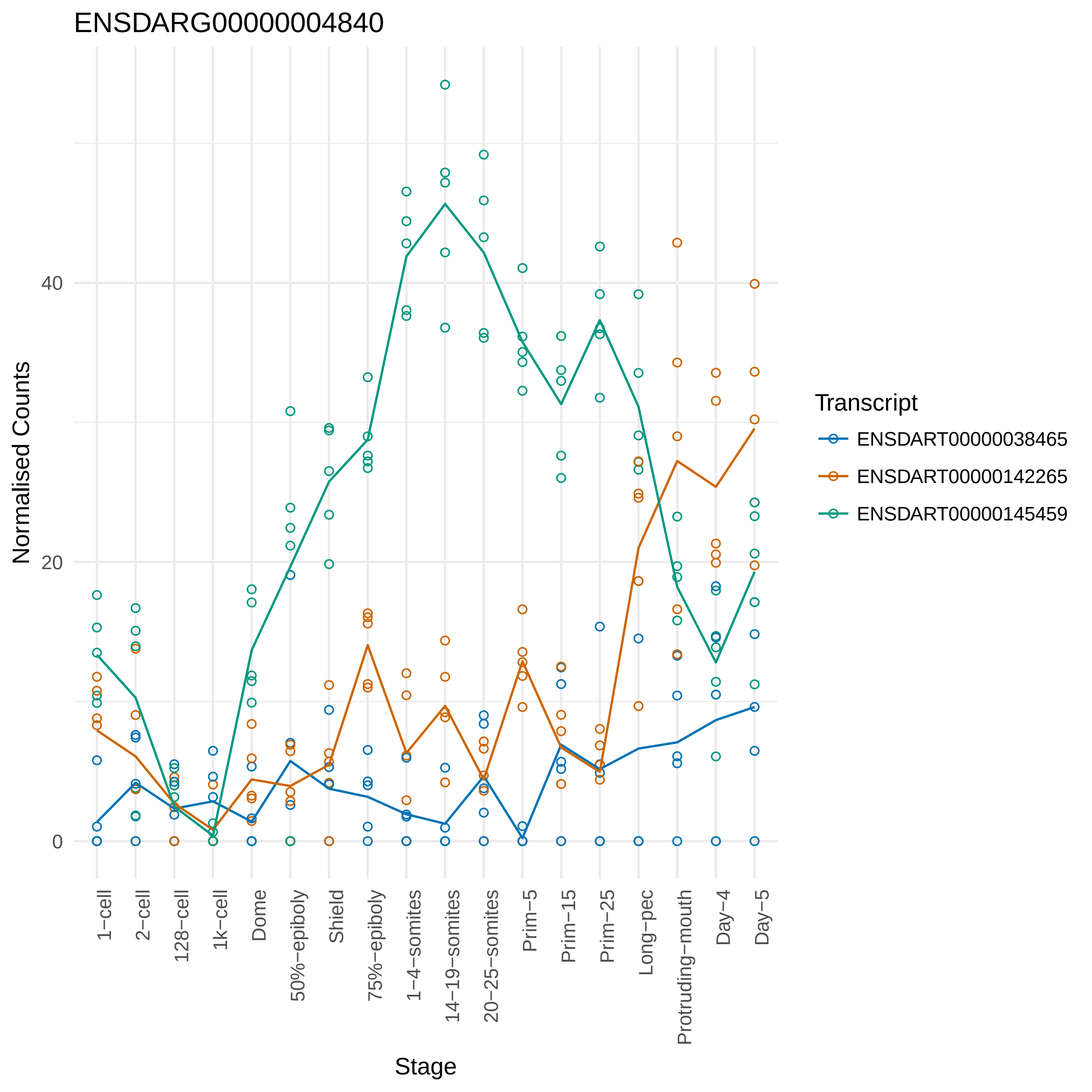
ENSDARG00000004840
Normalised Counts
0
20
40
1−cell
2−cell
128−cell
1k−cell
Dome
50%−epiboly
Shield
75%−epiboly
1−4−somites
14−19−somites
20−25−somites
Prim−5
Prim−15
Prim−25
Long−pec
Protruding−mouth
Day−4
Day−5
Stage
Transcript
ENSDART00000038465
ENSDART00000142265
ENSDART00000145459

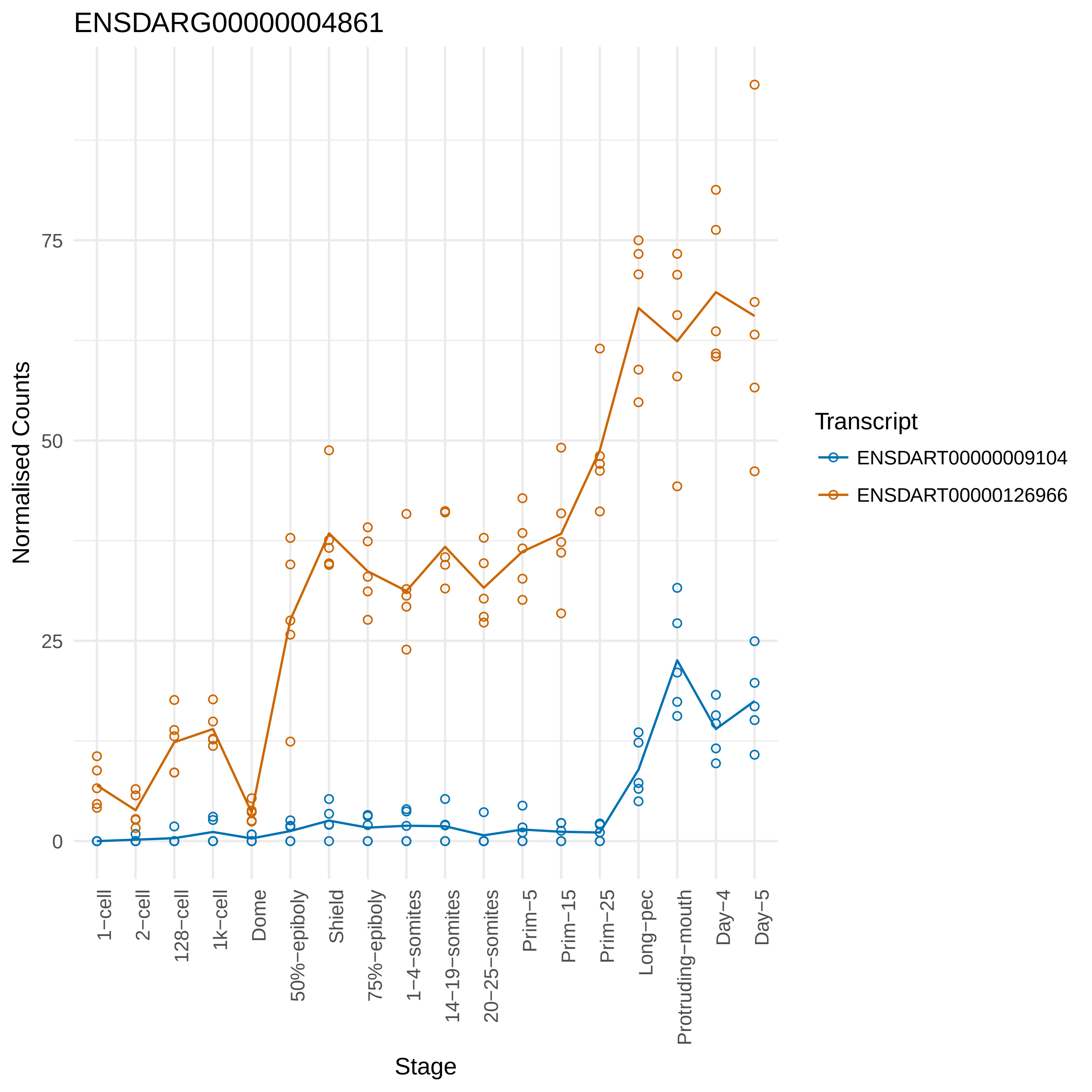
ENSDARG00000004861
Normalised Counts
0
25
50
75
Stage
1−cell
2−cell
128−cell
1k−cell
Dome
50%−epiboly
Shield
75%−epiboly
1−4−somites
14−19−somites
20−25−somites
Prim−5
Prim−15
Prim−25
Long−pec
Protruding−mouth
Day−4
Day−5
Transcript
ENSDART00000009104
ENSDART00000126966

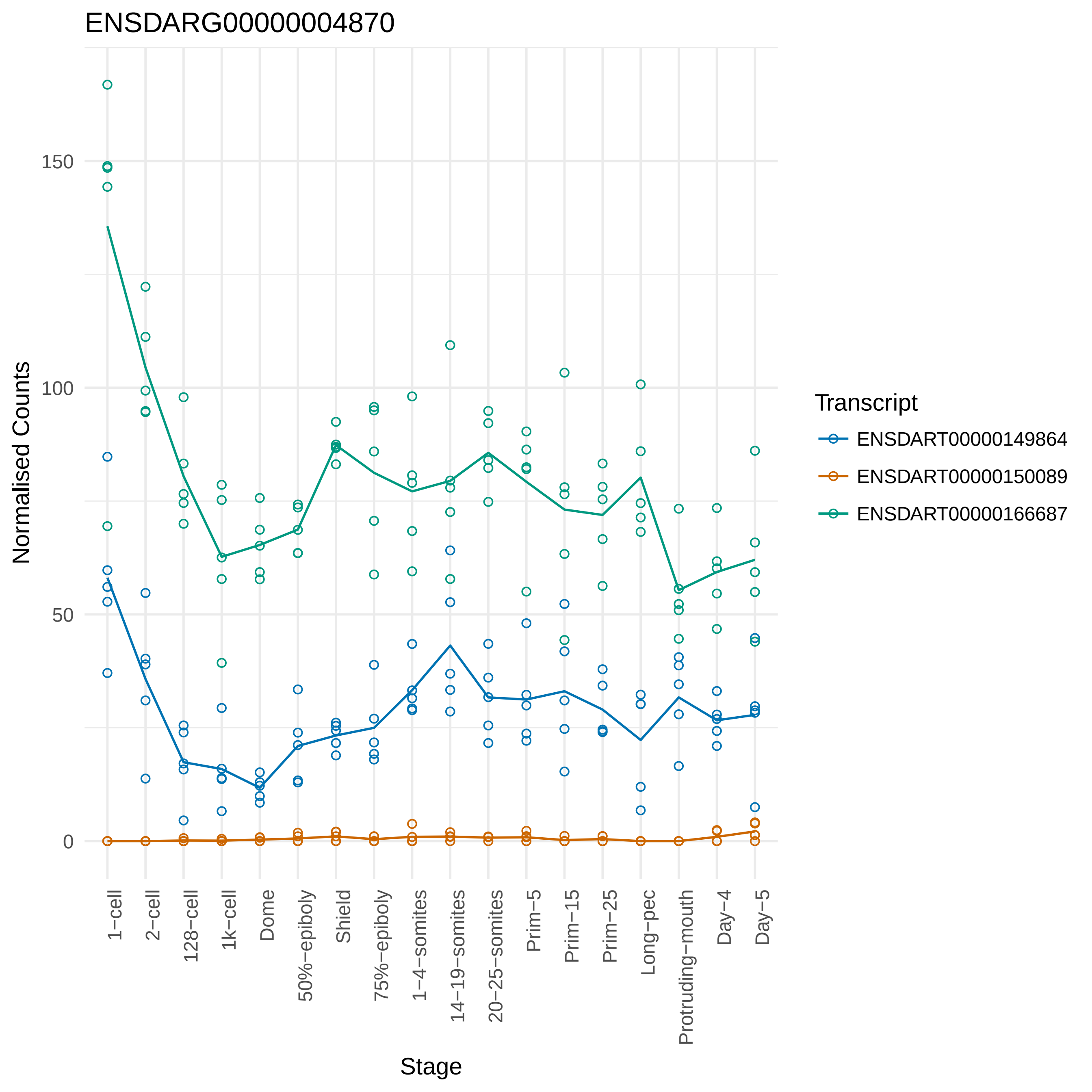

ENSDARG00000004870
Normalised Counts
150
100
50
0
1−cell
2−cell
128−cell
1k−cell
Dome
50%−epiboly
Shield
75%−epiboly
1−4−somites
14−19−somites
20−25−somites
Prim−5
Prim−15
Prim−25
Long−pec
Protruding−mouth
Day−4
Day−5
Stage
Transcript
ENSDART00000149864
ENSDART00000150089
ENSDART00000166687

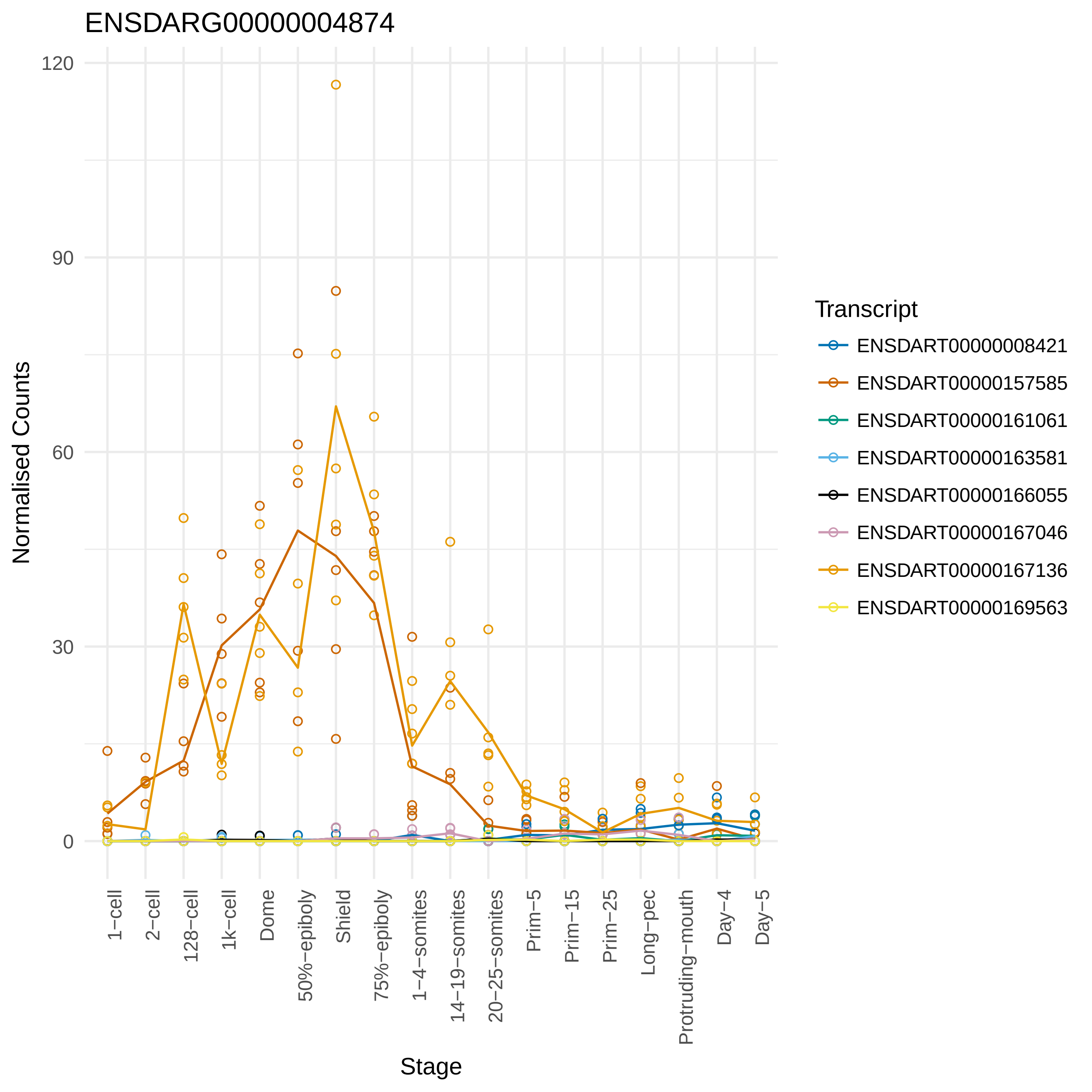
ENSDARG00000004874
Normalised Counts
120
90
60
30
0
1−cell
2−cell
128−cell
1k−cell
Dome
50%−epiboly
Shield
75%−epiboly
1−4−somites
14−19−somites
20−25−somites
Prim−5
Prim−15
Prim−25
Long−pec
Protruding−mouth
Day−4
Day−5
Stage
Transcript
ENSDART00000008421
ENSDART00000157585
ENSDART00000161061
ENSDART00000163581
ENSDART00000166055
ENSDART00000167046
ENSDART00000167136
ENSDART00000169563

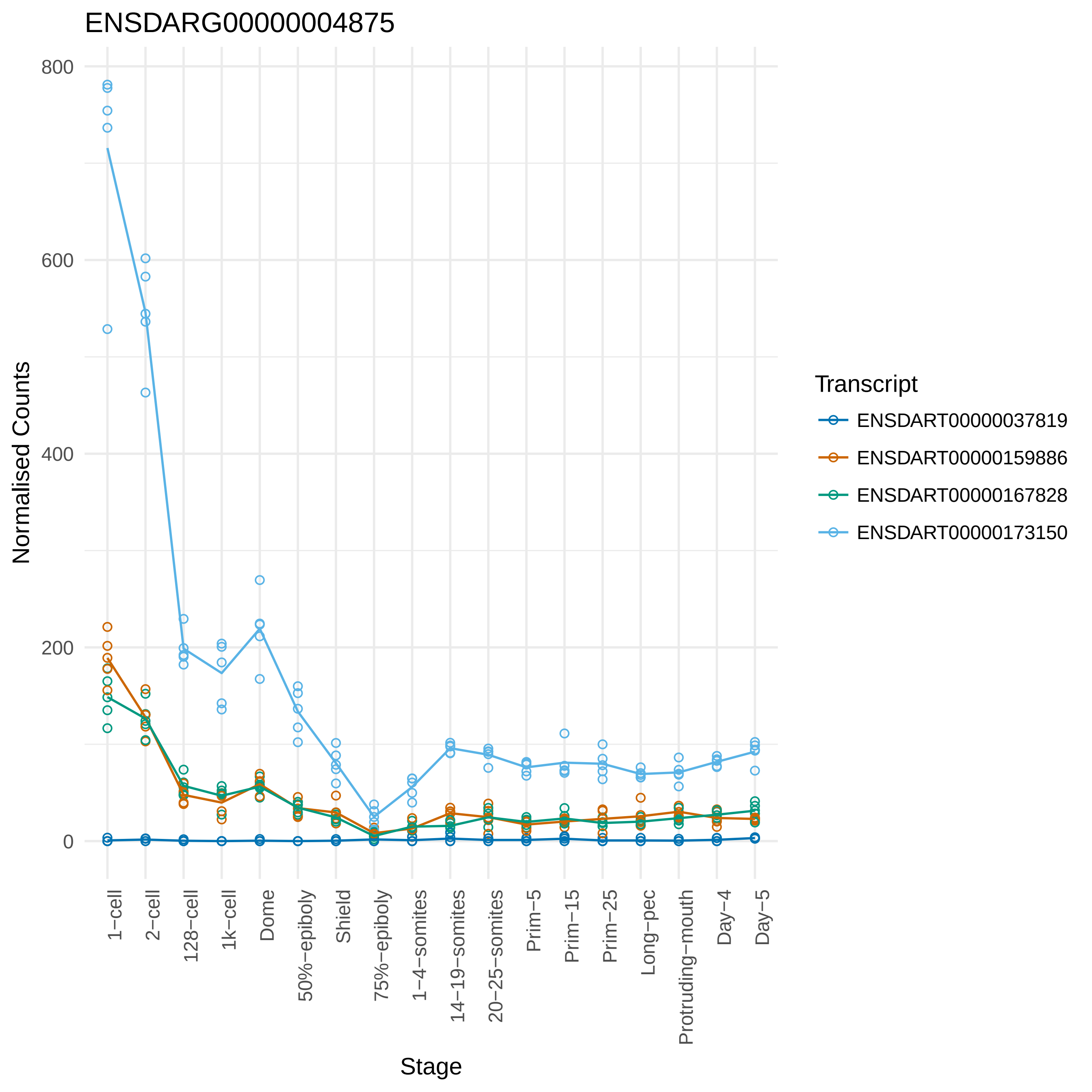

ENSDARG00000004875
Normalised Counts
800
600
400
200
0
Stage
1−cell
2−cell
128−cell
1k−cell
Dome
50%−epiboly
Shield
75%−epiboly
1−4−somites
14−19−somites
20−25−somites
Prim−5
Prim−15
Prim−25
Long−pec
Protruding−mouth
Day−4
Day−5
Transcript
ENSDART00000037819
ENSDART00000159886
ENSDART00000167828
ENSDART00000173150

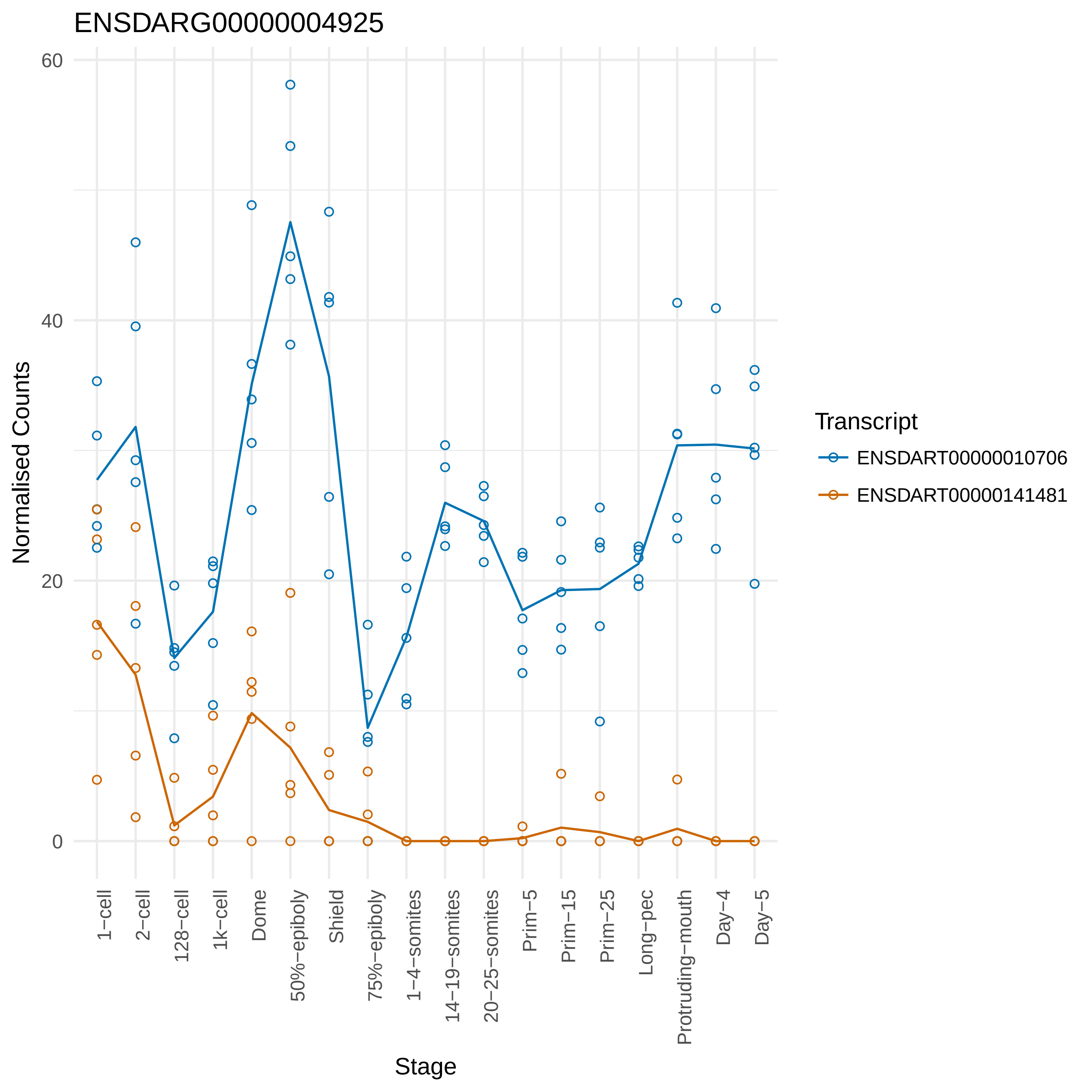
ENSDARG00000004925
Normalised Counts
60
40
20
0
1−cell
2−cell
128−cell
1k−cell
Dome
50%−epiboly
Shield
75%−epiboly
1−4−somites
14−19−somites
20−25−somites
Prim−5
Prim−15
Prim−25
Long−pec
Protruding−mouth
Day−4
Day−5
Stage
Transcript
ENSDART00000010706
ENSDART00000141481

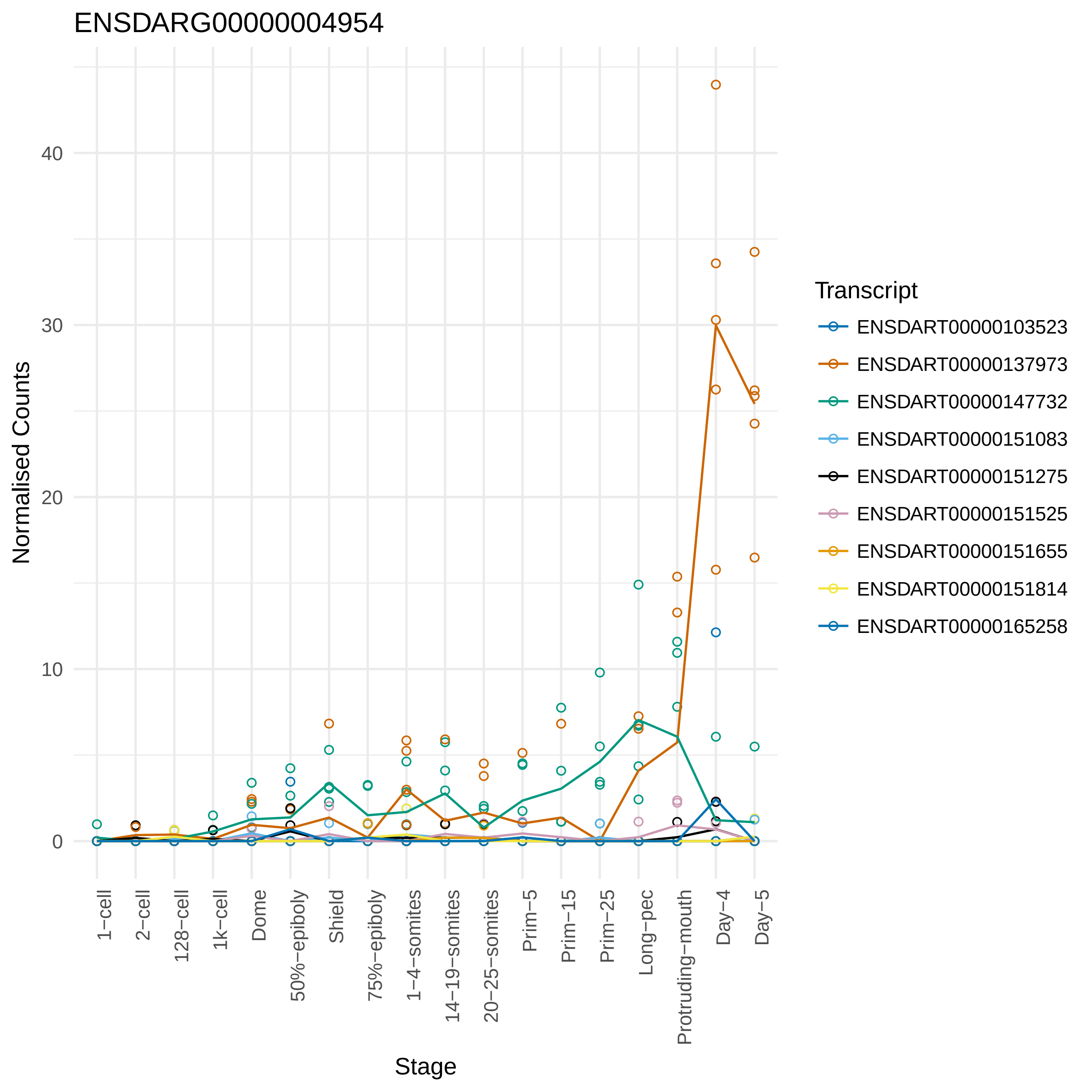
ENSDARG00000004954
Normalised Counts
0
10
20
30
40
1−cell
2−cell
128−cell
1k−cell
Dome
50%−epiboly
Shield
75%−epiboly
1−4−somites
14−19−somites
20−25−somites
Prim−5
Prim−15
Prim−25
Long−pec
Protruding−mouth
Day−4
Day−5
Stage
Transcript
ENSDART00000103523
ENSDART00000137973
ENSDART00000147732
ENSDART00000151083
ENSDART00000151275
ENSDART00000151525
ENSDART00000151655
ENSDART00000151814
ENSDART00000165258

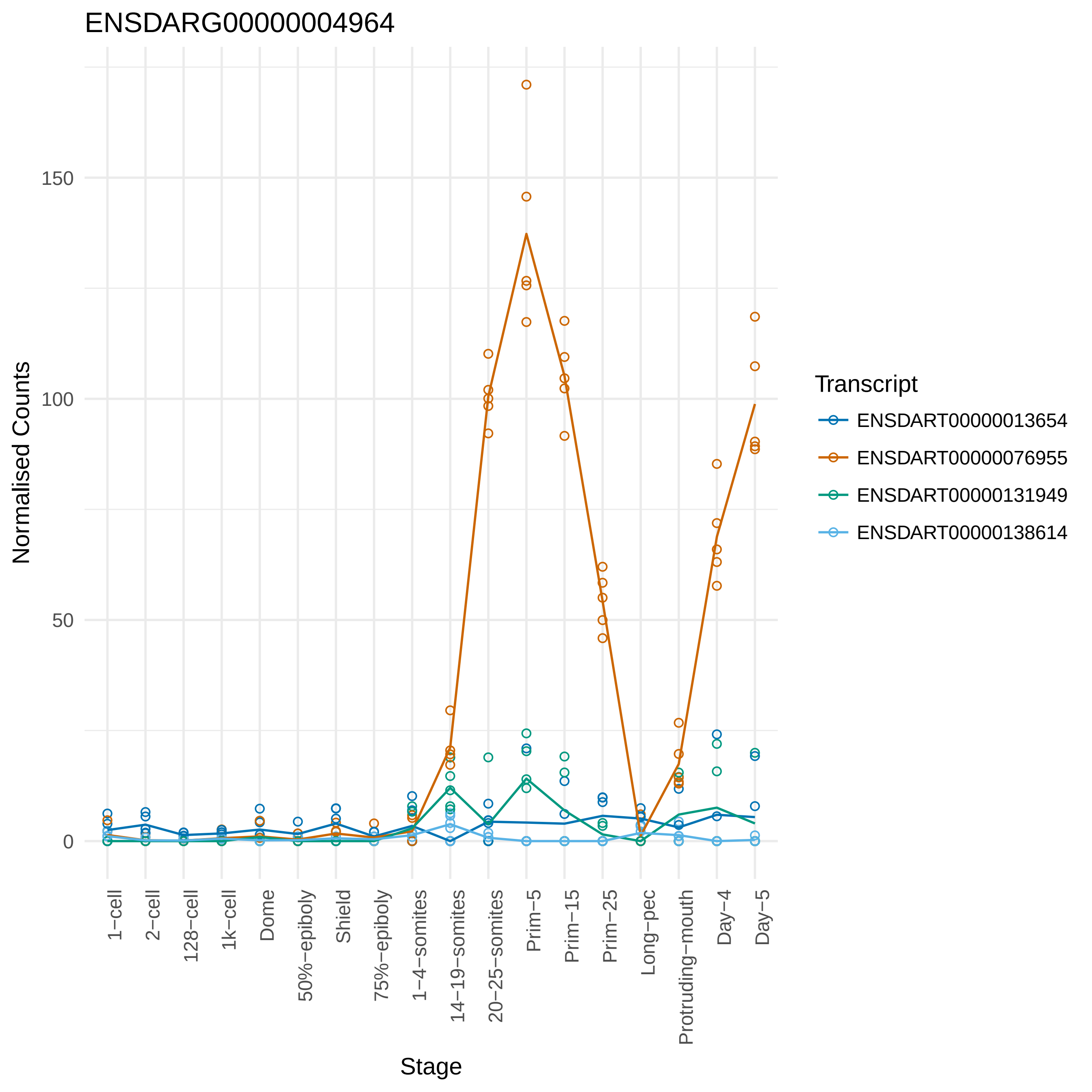

ENSDARG00000004964
Normalised Counts
150
100
50
0
1−cell
2−cell
128−cell
1k−cell
Dome
50%−epiboly
Shield
75%−epiboly
1−4−somites
14−19−somites
20−25−somites
Prim−5
Prim−15
Prim−25
Long−pec
Protruding−mouth
Day−4
Day−5
Stage
Transcript
ENSDART00000013654
ENSDART00000076955
ENSDART00000131949
ENSDART00000138614

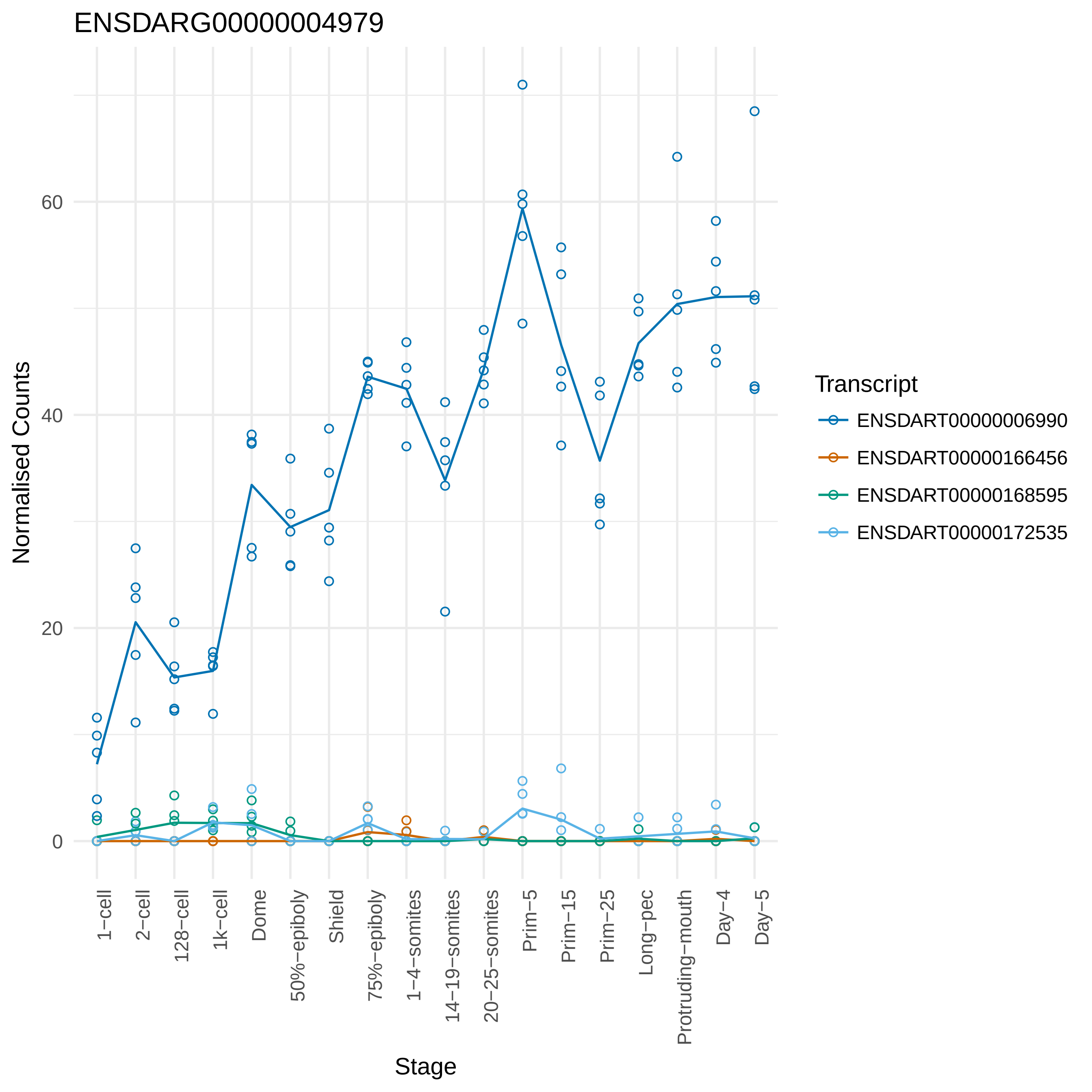

ENSDARG00000004979
Normalised Counts
0
20
40
60
Stage
1−cell
2−cell
128−cell
1k−cell
Dome
50%−epiboly
Shield
75%−epiboly
1−4−somites
14−19−somites
20−25−somites
Prim−5
Prim−15
Prim−25
Long−pec
Protruding−mouth
Day−4
Day−5
Transcript
ENSDART00000006990
ENSDART00000166456
ENSDART00000168595
ENSDART00000172535

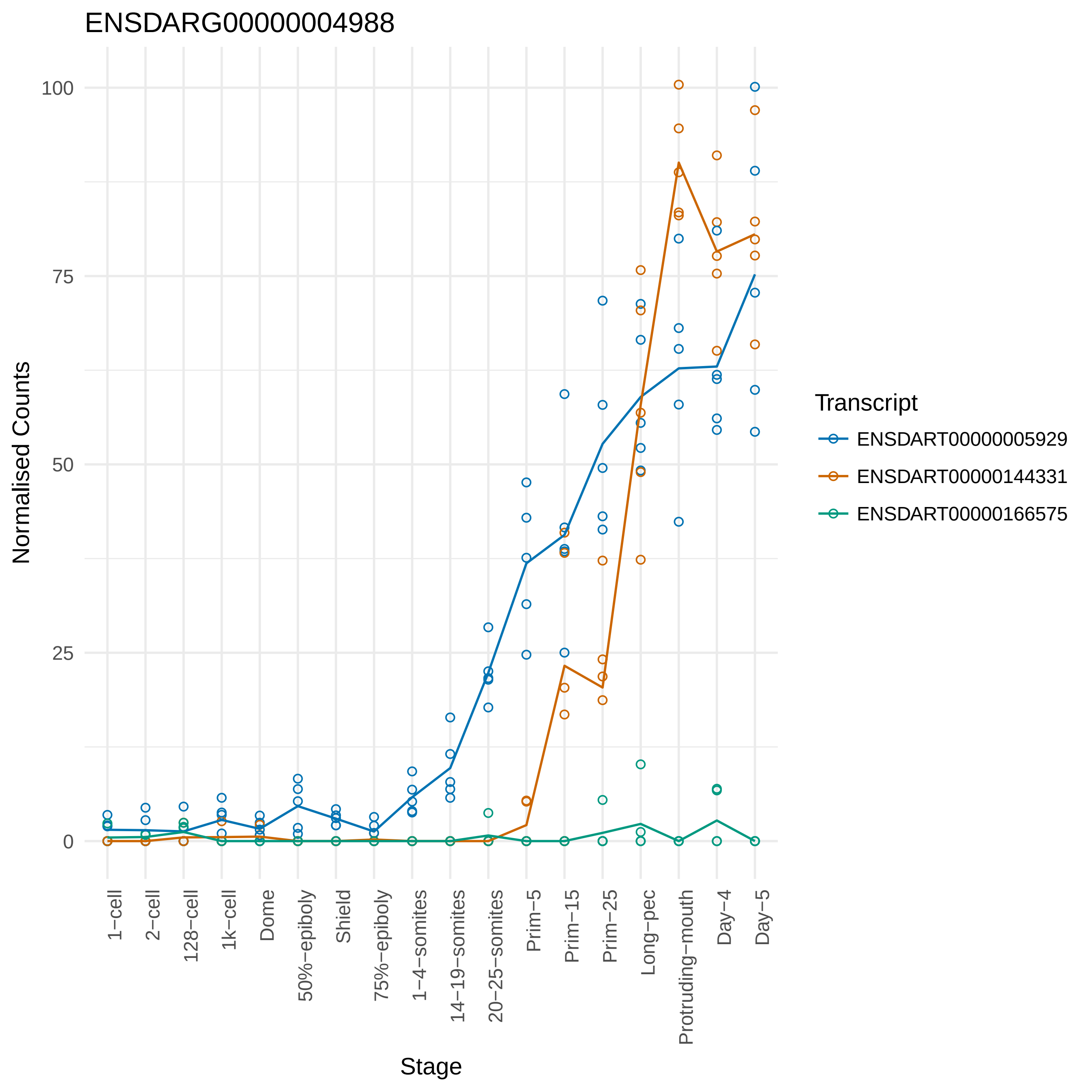
ENSDARG00000004988
Normalised Counts
100
75
50
25
0
1−cell
2−cell
128−cell
1k−cell
Dome
50%−epiboly
Shield
75%−epiboly
1−4−somites
14−19−somites
20−25−somites
Prim−5
Prim−15
Prim−25
Long−pec
Protruding−mouth
Day−4
Day−5
Stage
Transcript
ENSDART00000005929
ENSDART00000144331
ENSDART00000166575

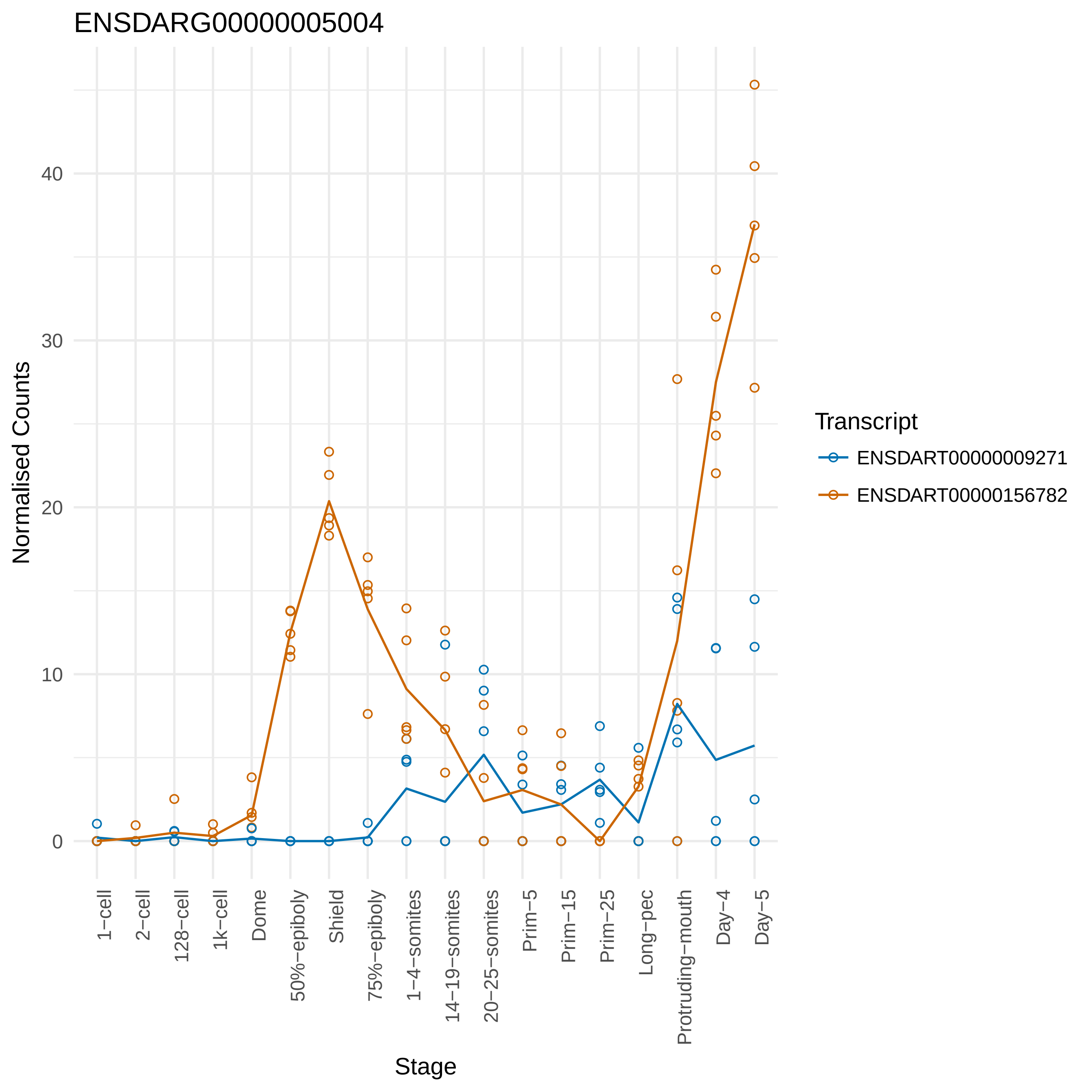

ENSDARG00000005004
Normalised Counts
0
10
20
30
40
1−cell
2−cell
128−cell
1k−cell
Dome
50%−epiboly
Shield
75%−epiboly
1−4−somites
14−19−somites
20−25−somites
Prim−5
Prim−15
Prim−25
Long−pec
Protruding−mouth
Day−4
Day−5
Stage
Transcript
ENSDART00000009271
ENSDART00000156782

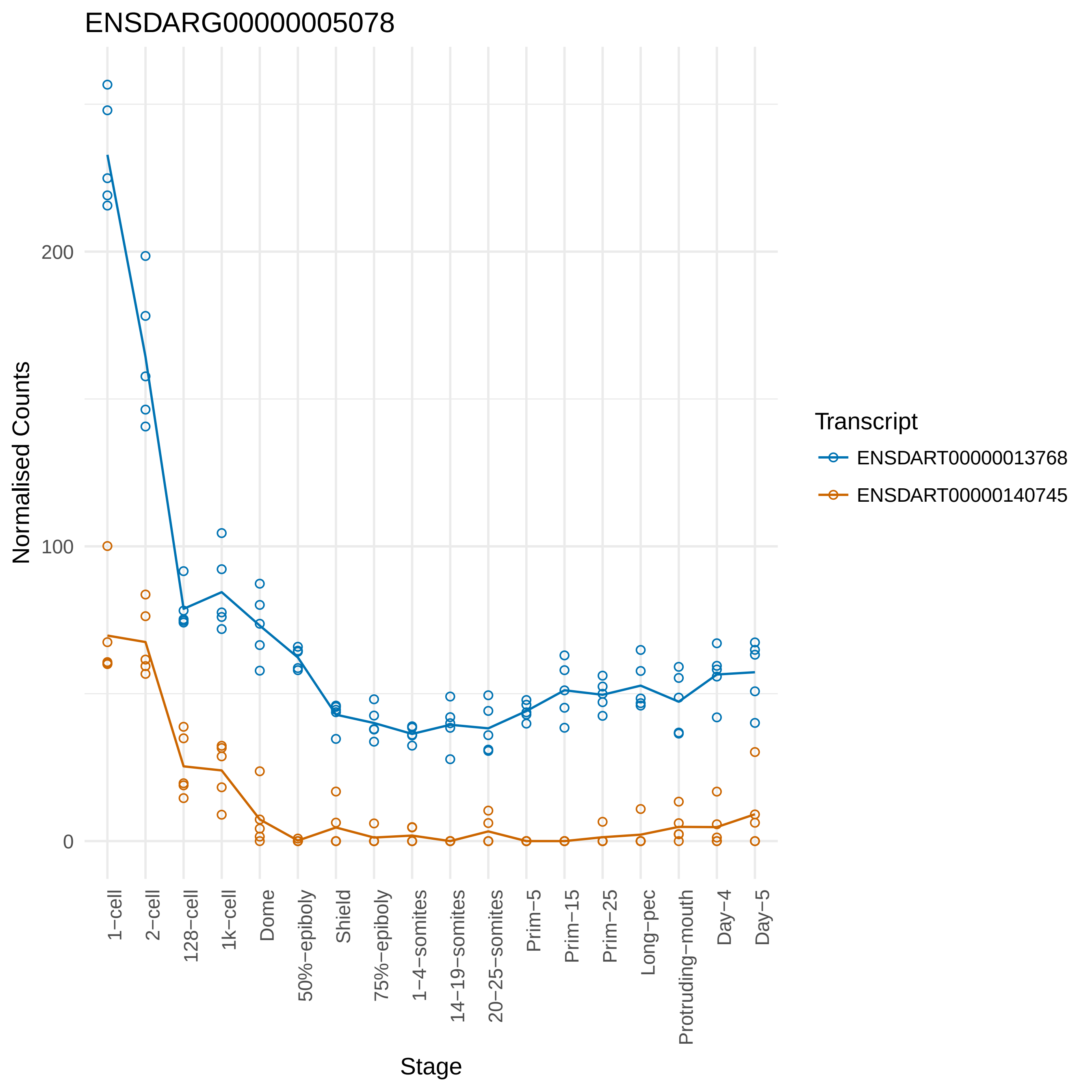
ENSDARG00000005078
Normalised Counts
200
100
0
1−cell
2−cell
128−cell
1k−cell
Dome
50%−epiboly
Shield
75%−epiboly
1−4−somites
14−19−somites
20−25−somites
Prim−5
Prim−15
Prim−25
Long−pec
Protruding−mouth
Day−4
Day−5
Stage
Transcript
ENSDART00000013768
ENSDART00000140745

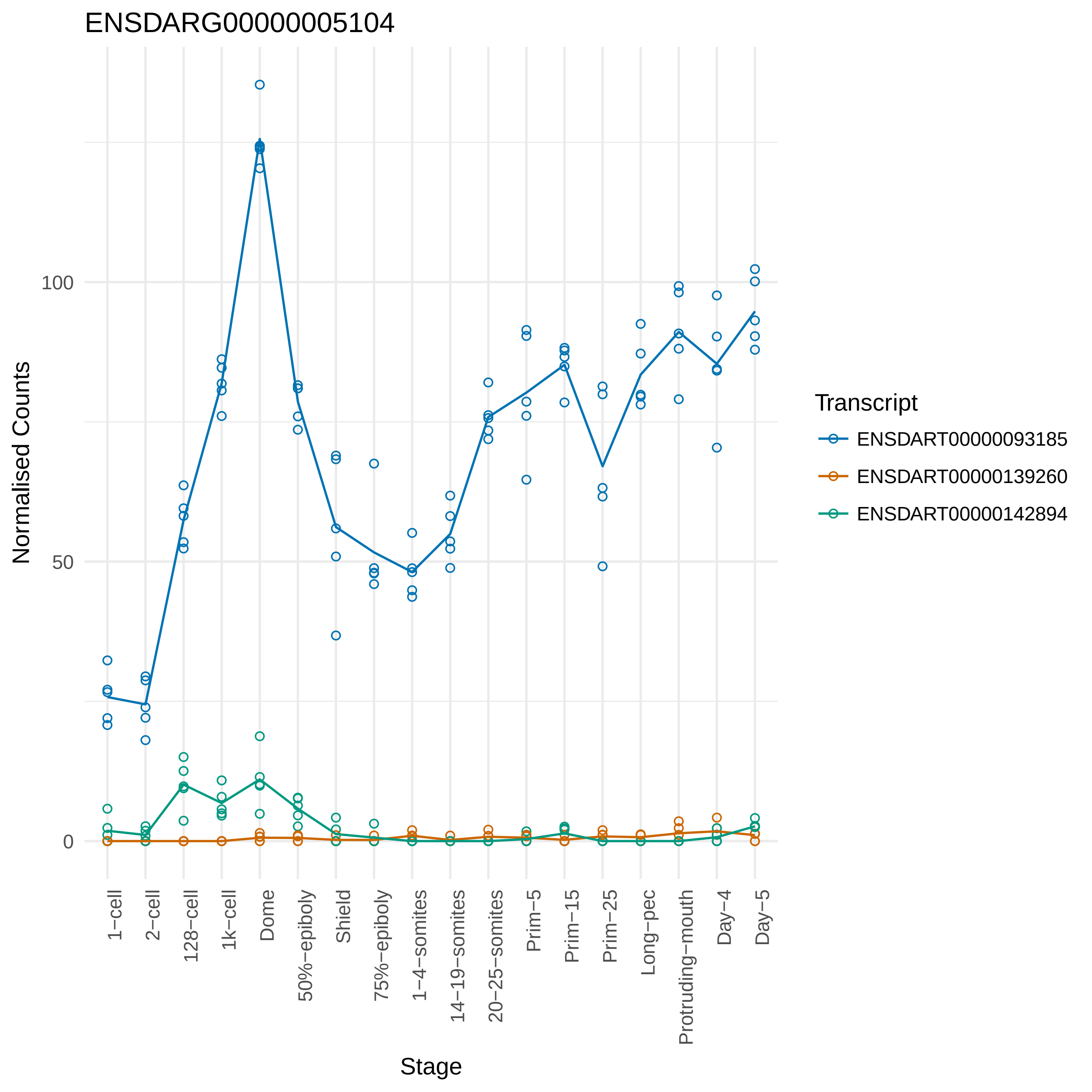

ENSDARG00000005104
Normalised Counts
100
50
0
1−cell
2−cell
128−cell
1k−cell
Dome
50%−epiboly
Shield
75%−epiboly
1−4−somites
14−19−somites
20−25−somites
Prim−5
Prim−15
Prim−25
Long−pec
Protruding−mouth
Day−4
Day−5
Stage
Transcript
ENSDART00000093185
ENSDART00000139260
ENSDART00000142894

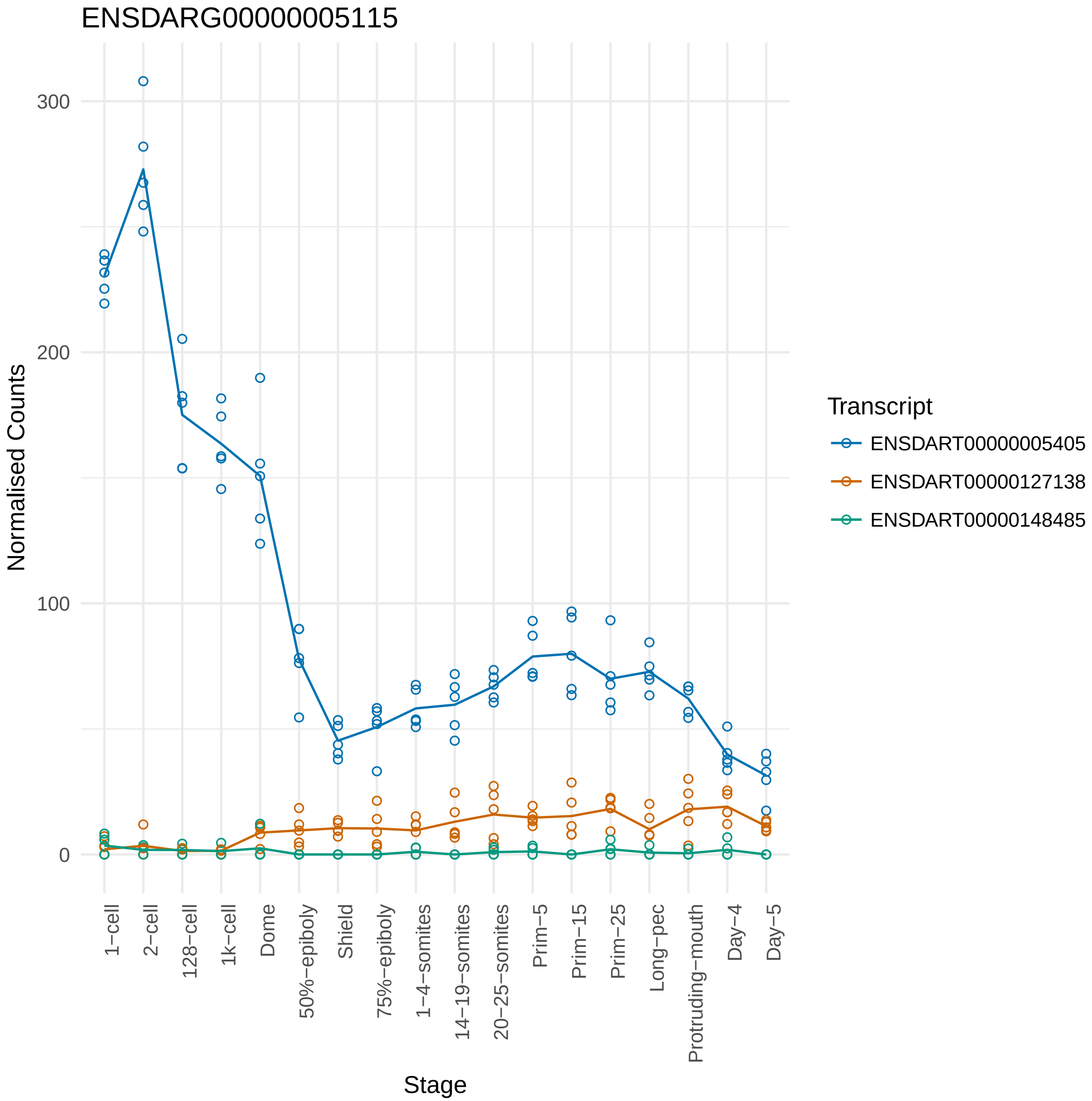

ENSDARG00000005115
Normalised Counts
300
200
100
0
1−cell
2−cell
128−cell
1k−cell
Dome
50%−epiboly
Shield
75%−epiboly
1−4−somites
14−19−somites
20−25−somites
Prim−5
Prim−15
Prim−25
Long−pec
Protruding−mouth
Day−4
Day−5
Stage
Transcript
ENSDART00000005405
ENSDART00000127138
ENSDART00000148485

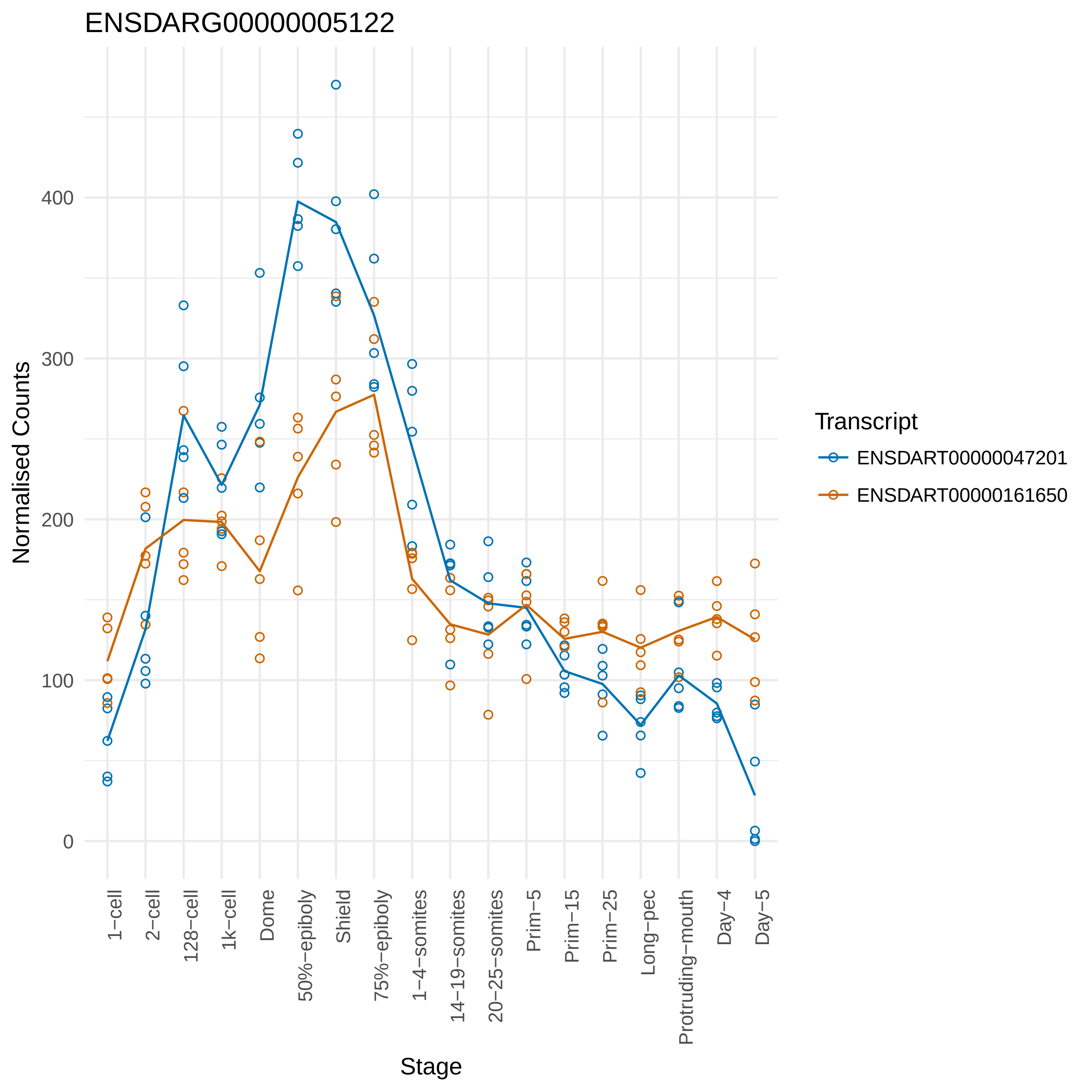
ENSDARG00000005122
Normalised Counts
400
300
200
100
0
1−cell
2−cell
128−cell
1k−cell
Dome
50%−epiboly
Shield
75%−epiboly
1−4−somites
14−19−somites
20−25−somites
Prim−5
Prim−15
Prim−25
Long−pec
Protruding−mouth
Day−4
Day−5
Stage
Transcript
ENSDART00000047201
ENSDART00000161650

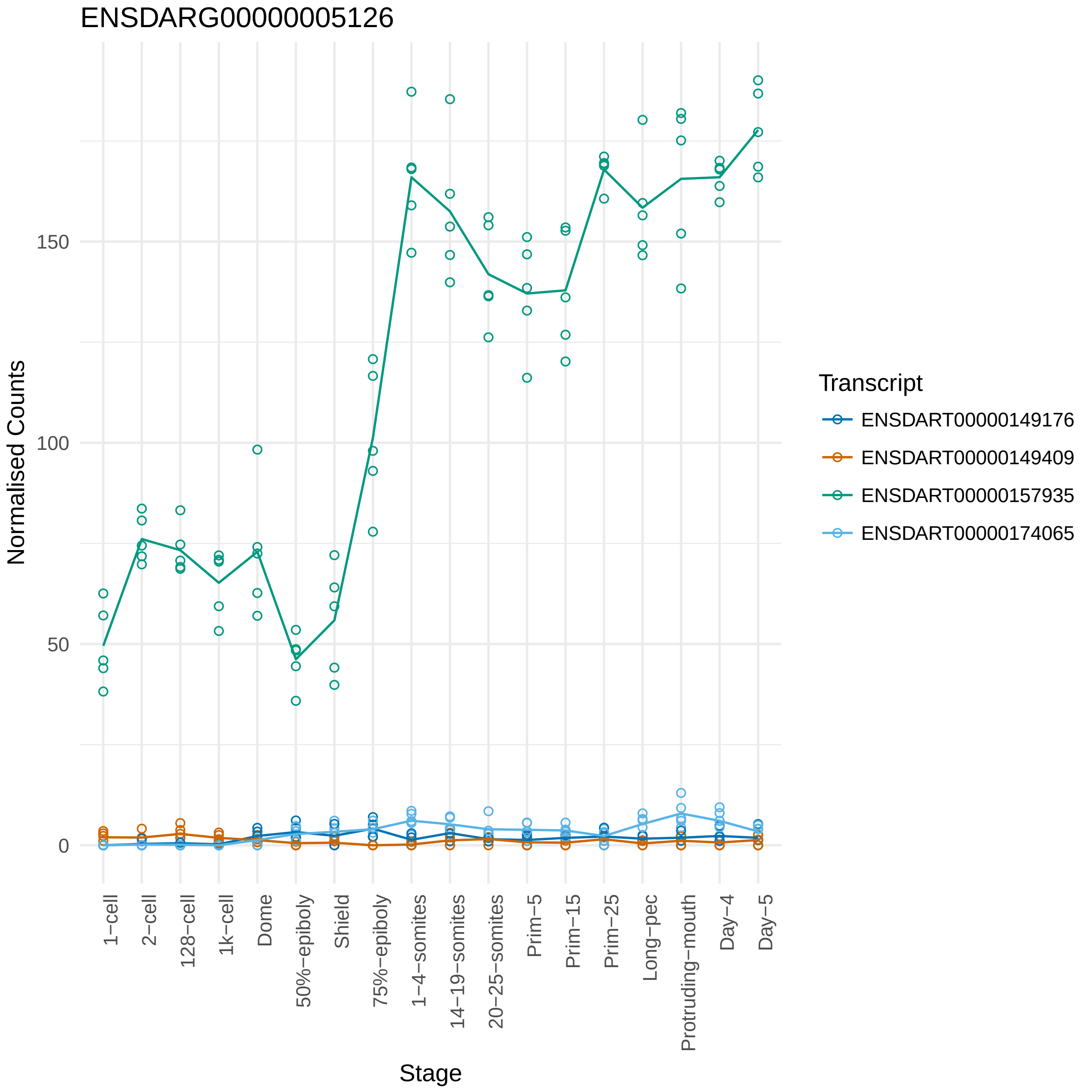

ENSDARG00000005126
Normalised Counts
150
100
50
0
Stage
1−cell
2−cell
128−cell
1k−cell
Dome
50%−epiboly
Shield
75%−epiboly
1−4−somites
14−19−somites
20−25−somites
Prim−5
Prim−15
Prim−25
Long−pec
Protruding−mouth
Day−4
Day−5
Transcript
ENSDART00000149176
ENSDART00000149409
ENSDART00000157935
ENSDART00000174065

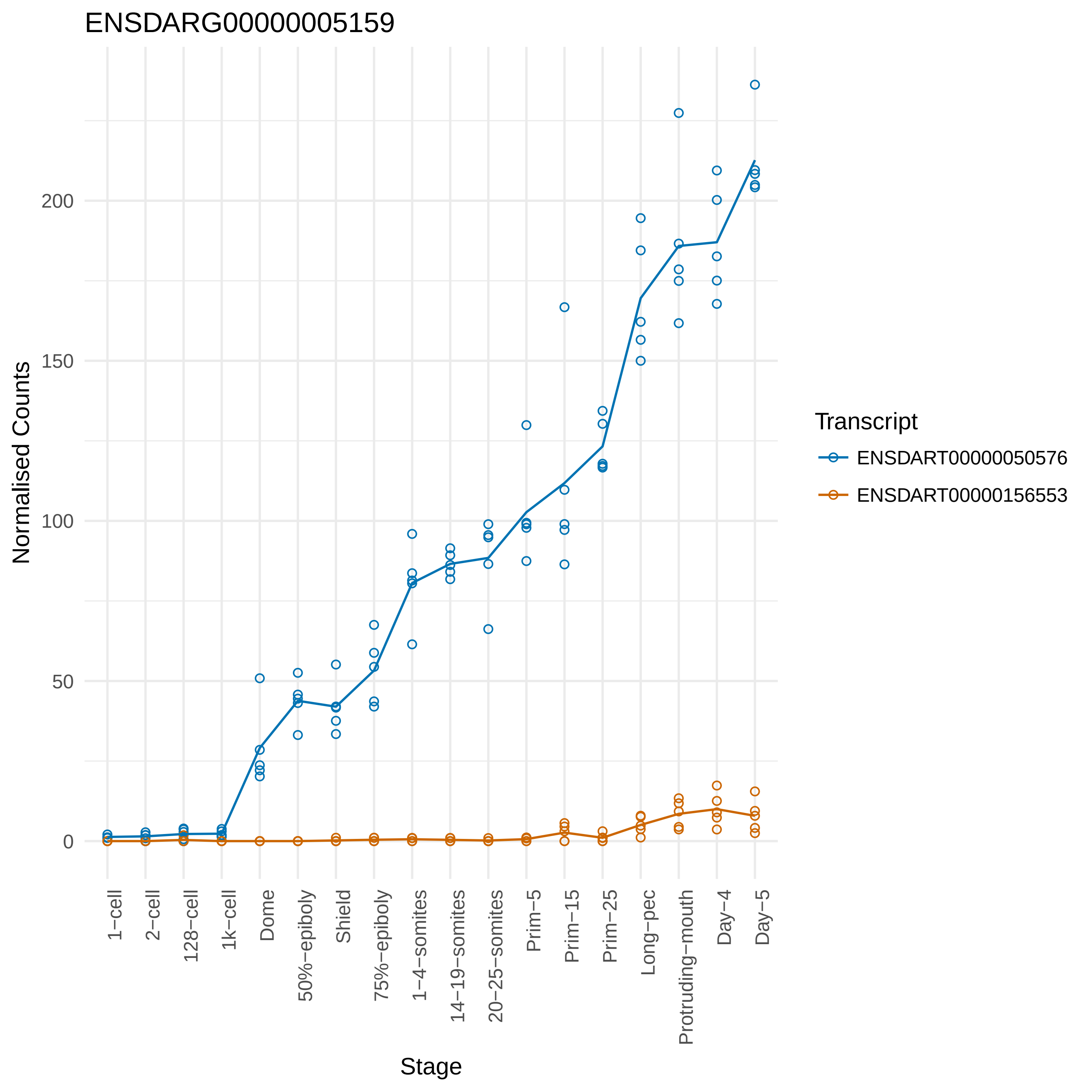
ENSDARG00000005159
Normalised Counts
0
50
100
150
200
Stage
1−cell
2−cell
128−cell
1k−cell
Dome
50%−epiboly
Shield
75%−epiboly
1−4−somites
14−19−somites
20−25−somites
Prim−5
Prim−15
Prim−25
Long−pec
Protruding−mouth
Day−4
Day−5
Transcript
ENSDART00000050576
ENSDART00000156553

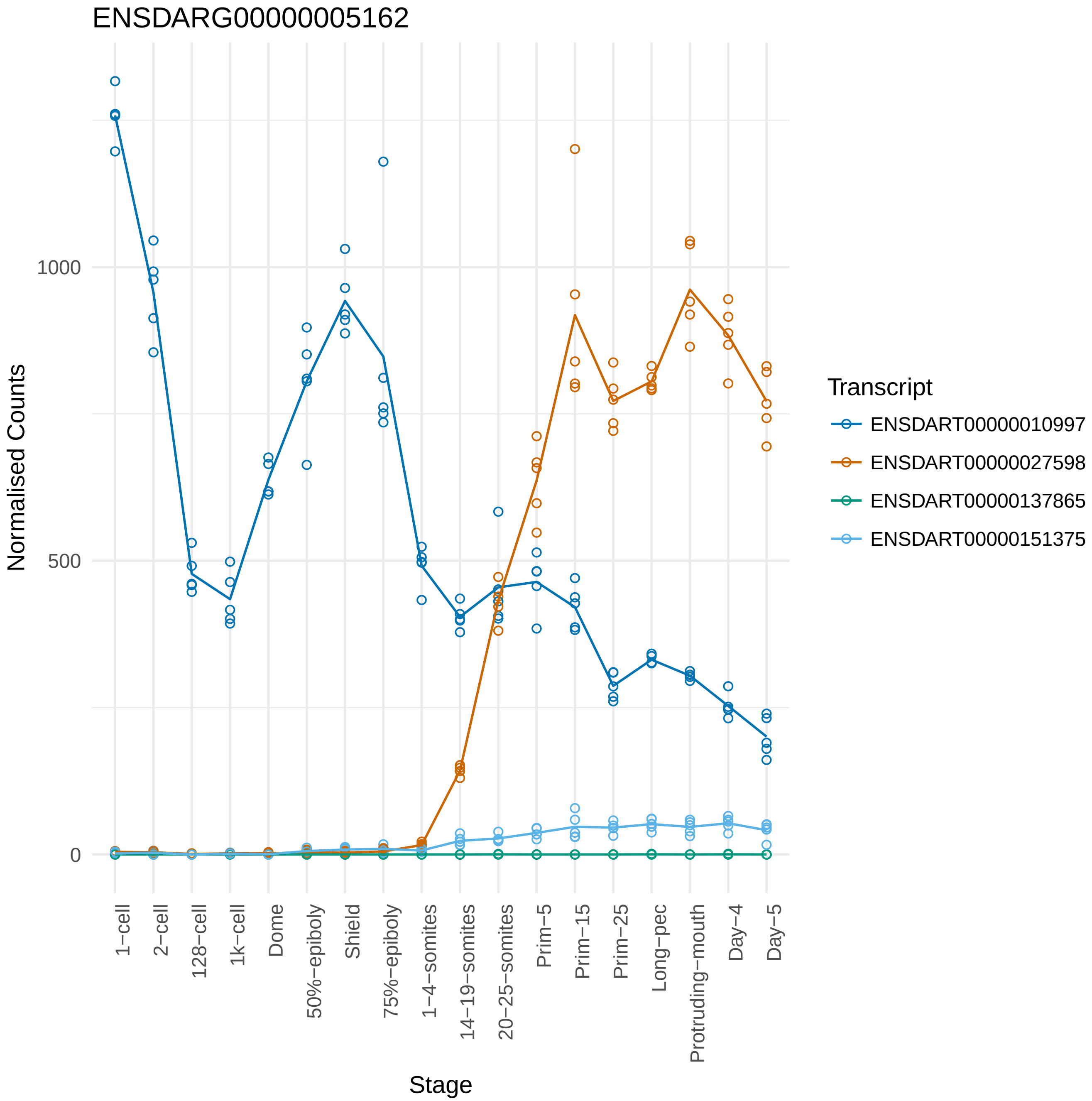
ENSDARG00000005162
Normalised Counts
1000
500
0
Stage
1−cell
2−cell
128−cell
1k−cell
Dome
50%−epiboly
Shield
75%−epiboly
1−4−somites
14−19−somites
20−25−somites
Prim−5
Prim−15
Prim−25
Long−pec
Protruding−mouth
Day−4
Day−5
Transcript
ENSDART00000010997
ENSDART00000027598
ENSDART00000137865
ENSDART00000151375

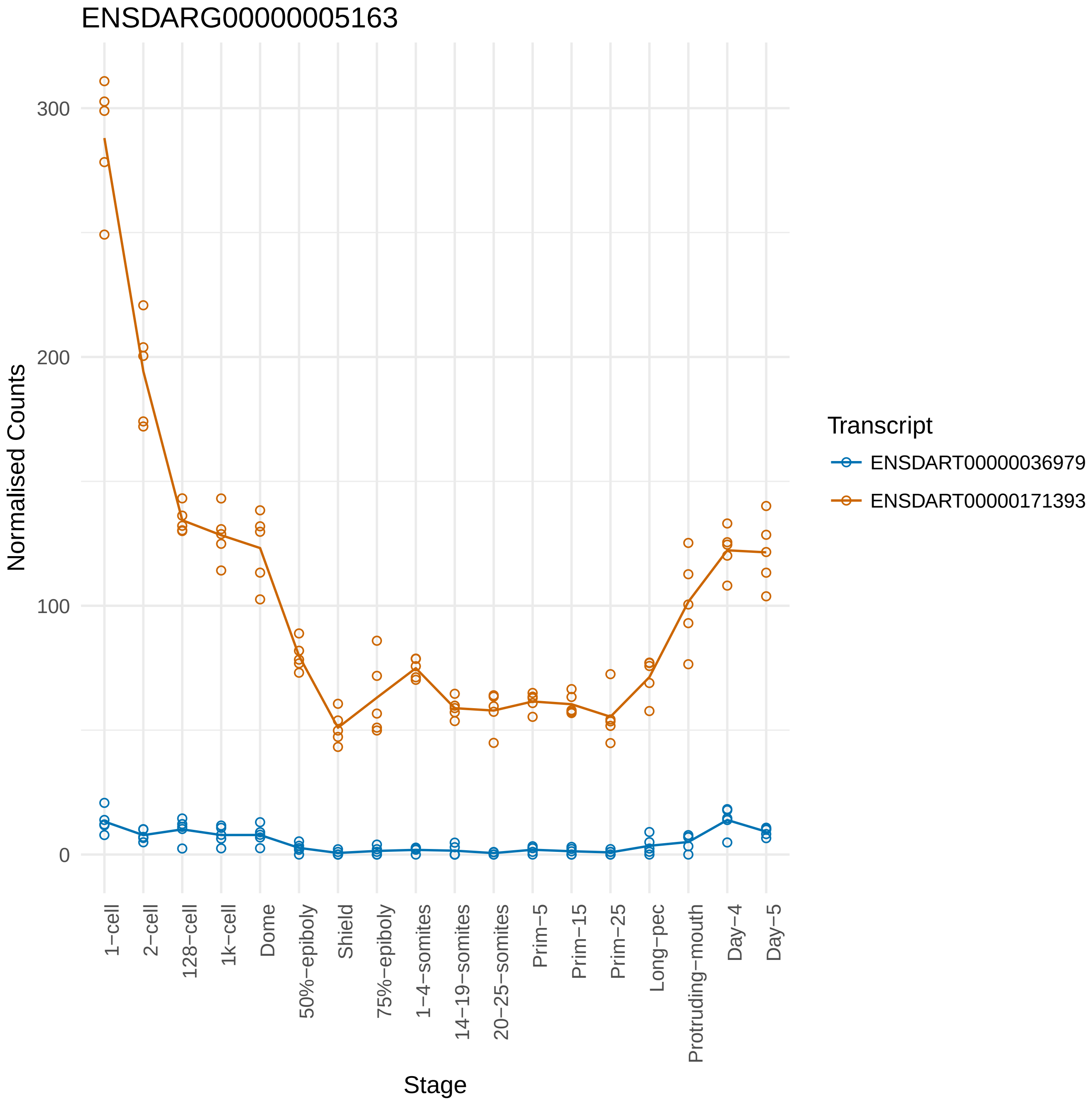

ENSDARG00000005163
Normalised Counts
300
200
100
0
1−cell
2−cell
128−cell
1k−cell
Dome
50%−epiboly
Shield
75%−epiboly
1−4−somites
14−19−somites
20−25−somites
Prim−5
Prim−15
Prim−25
Long−pec
Protruding−mouth
Day−4
Day−5
Stage
Transcript
ENSDART00000036979
ENSDART00000171393

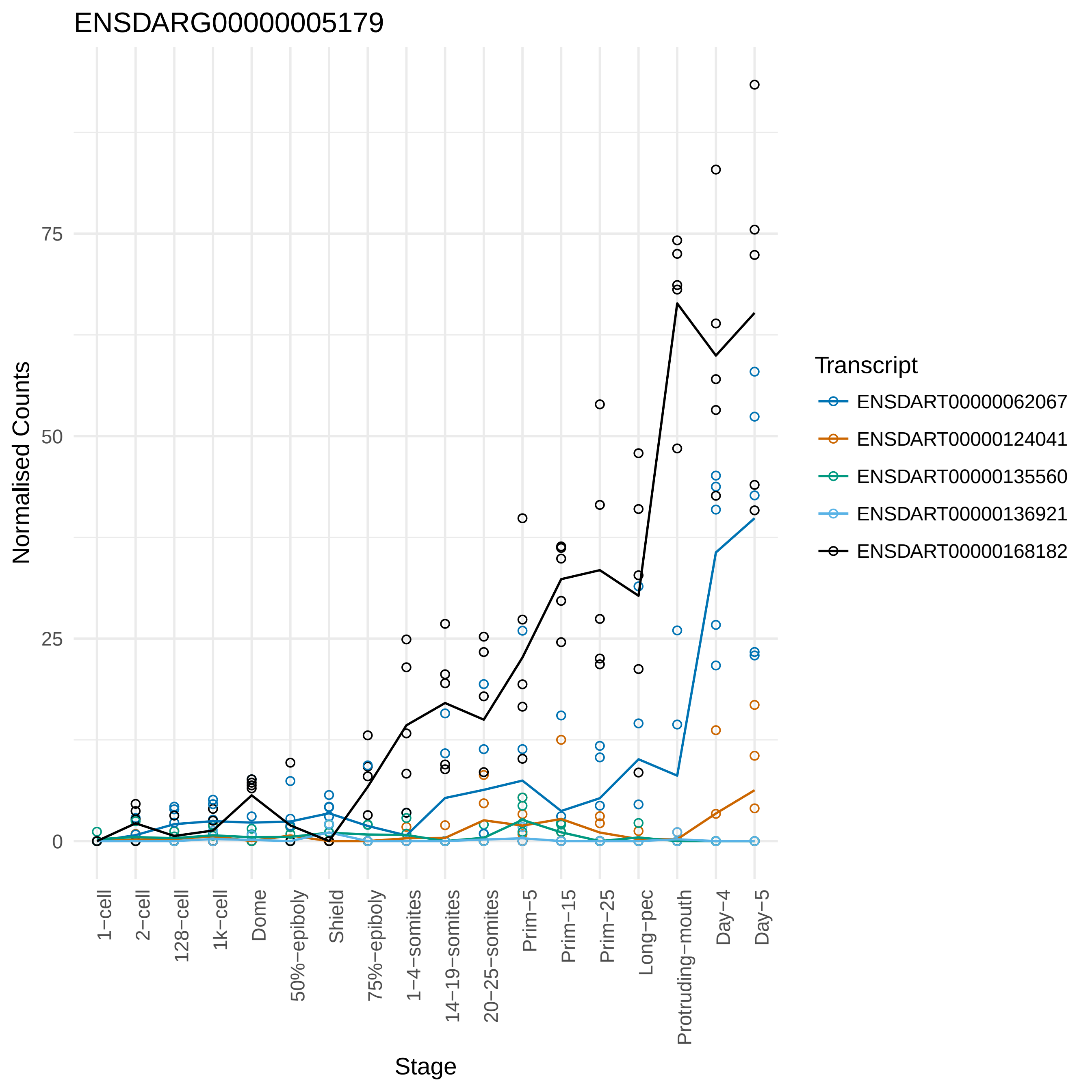

ENSDARG00000005179
Normalised Counts
75
50
25
0
1−cell
2−cell
128−cell
1k−cell
Dome
50%−epiboly
Shield
75%−epiboly
1−4−somites
14−19−somites
20−25−somites
Prim−5
Prim−15
Prim−25
Long−pec
Protruding−mouth
Day−4
Day−5
Stage
Transcript
ENSDART00000062067
ENSDART00000124041
ENSDART00000135560
ENSDART00000136921
ENSDART00000168182

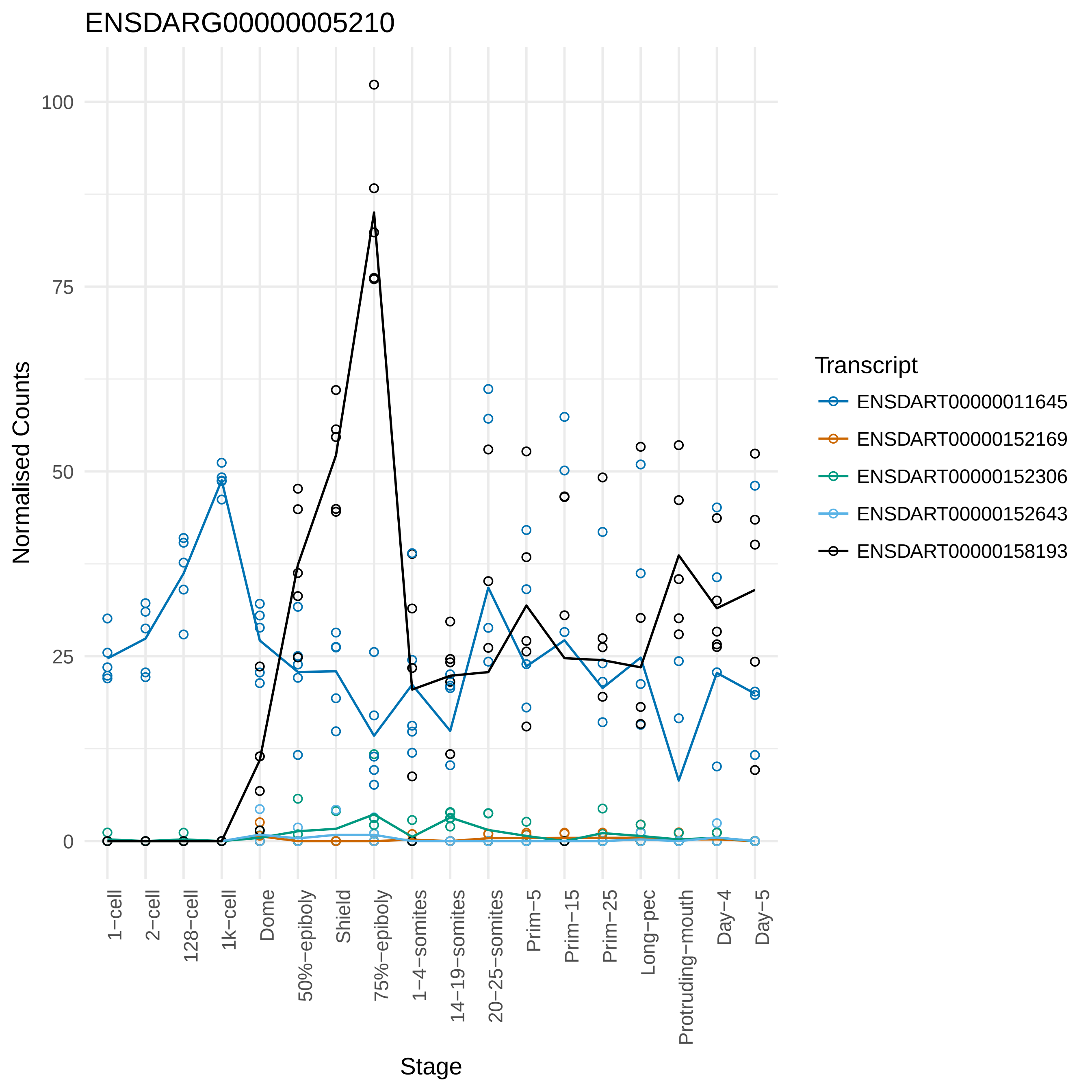

ENSDARG00000005210
Normalised Counts
100
75
50
25
0
1−cell
2−cell
128−cell
1k−cell
Dome
50%−epiboly
Shield
75%−epiboly
1−4−somites
14−19−somites
20−25−somites
Prim−5
Prim−15
Prim−25
Long−pec
Protruding−mouth
Day−4
Day−5
Stage
Transcript
ENSDART00000011645
ENSDART00000152169
ENSDART00000152306
ENSDART00000152643
ENSDART00000158193

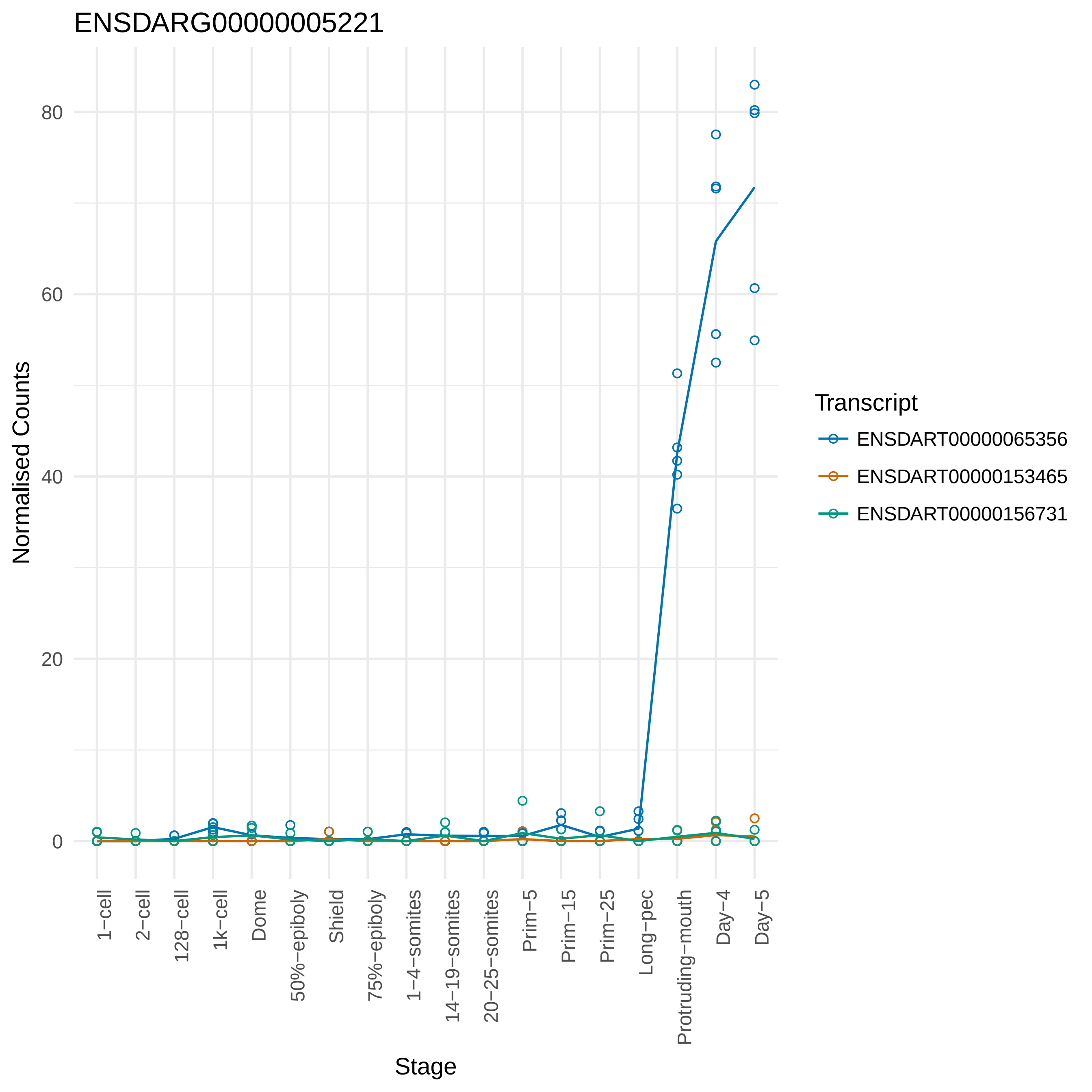

ENSDARG00000005221
Normalised Counts
0
20
40
60
80
1−cell
2−cell
128−cell
1k−cell
Dome
50%−epiboly
Shield
75%−epiboly
1−4−somites
14−19−somites
20−25−somites
Prim−5
Prim−15
Prim−25
Long−pec
Protruding−mouth
Day−4
Day−5
Stage
Transcript
ENSDART00000065356
ENSDART00000153465
ENSDART00000156731

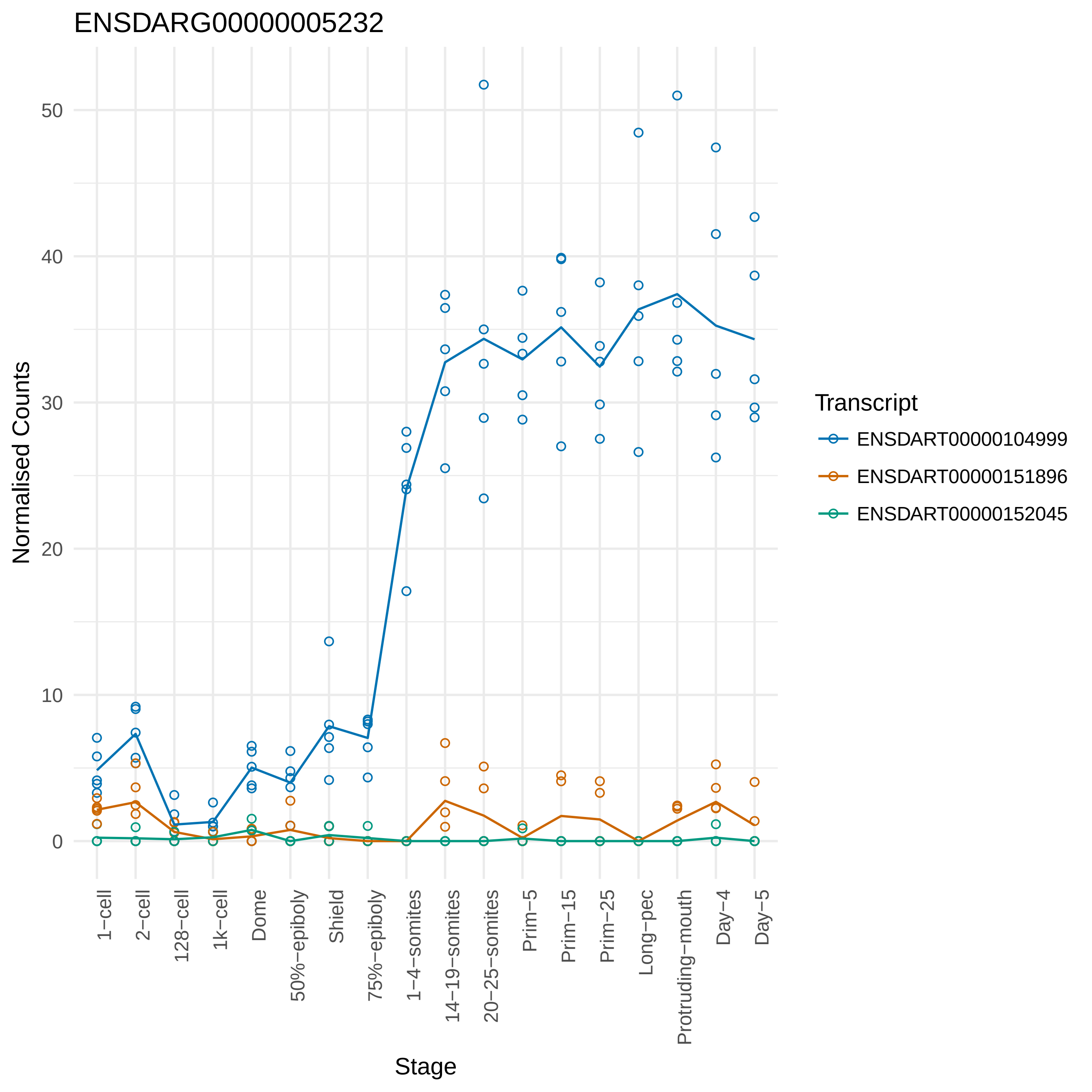
ENSDARG00000005232
Normalised Counts
0
10
20
30
40
50
1−cell
2−cell
128−cell
1k−cell
Dome
50%−epiboly
Shield
75%−epiboly
1−4−somites
14−19−somites
20−25−somites
Prim−5
Prim−15
Prim−25
Long−pec
Protruding−mouth
Day−4
Day−5
Stage
Transcript
ENSDART00000104999
ENSDART00000151896
ENSDART00000152045

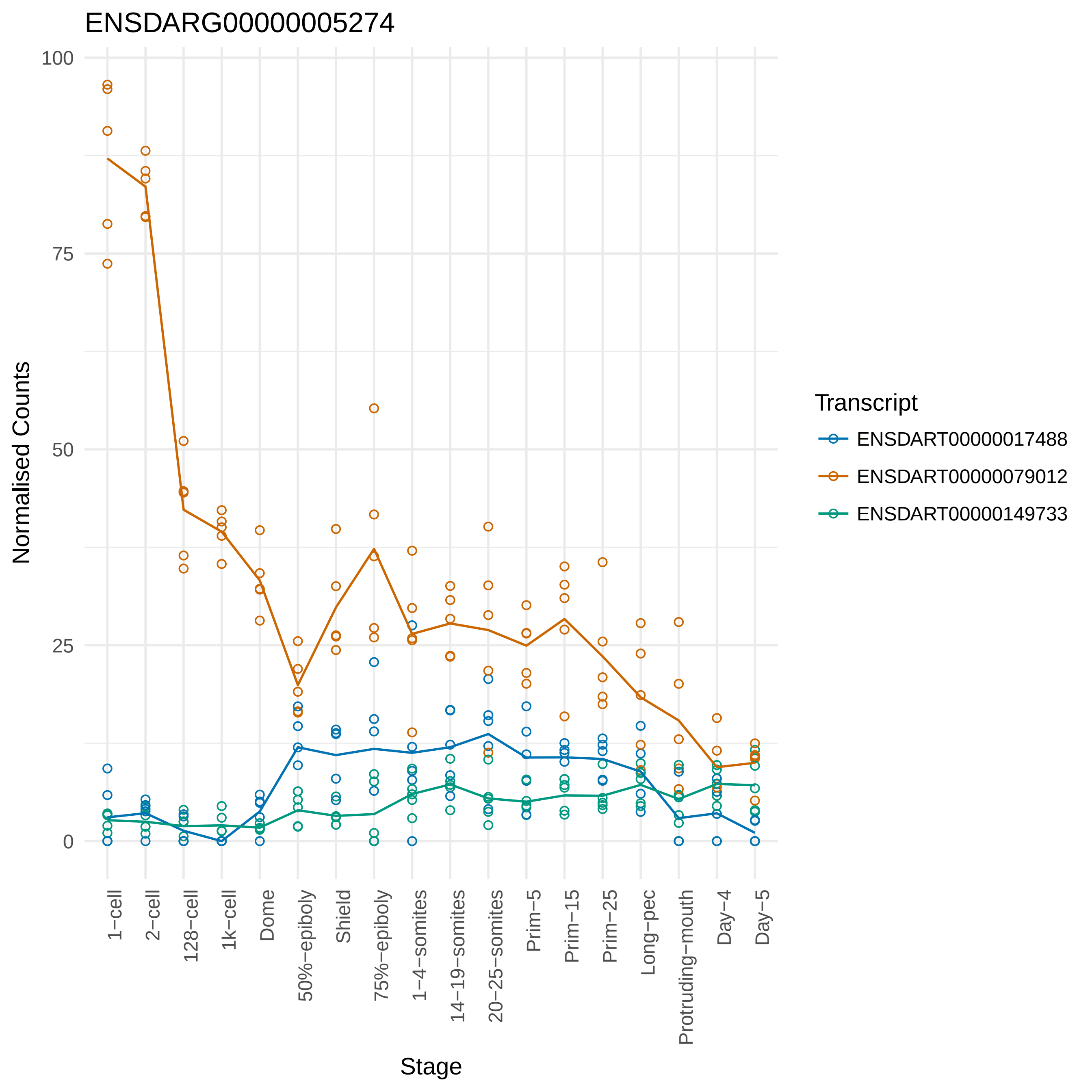

ENSDARG00000005274
Normalised Counts
100
75
50
25
0
1−cell
2−cell
128−cell
1k−cell
Dome
50%−epiboly
Shield
75%−epiboly
1−4−somites
14−19−somites
20−25−somites
Prim−5
Prim−15
Prim−25
Long−pec
Protruding−mouth
Day−4
Day−5
Stage
Transcript
ENSDART00000017488
ENSDART00000079012
ENSDART00000149733

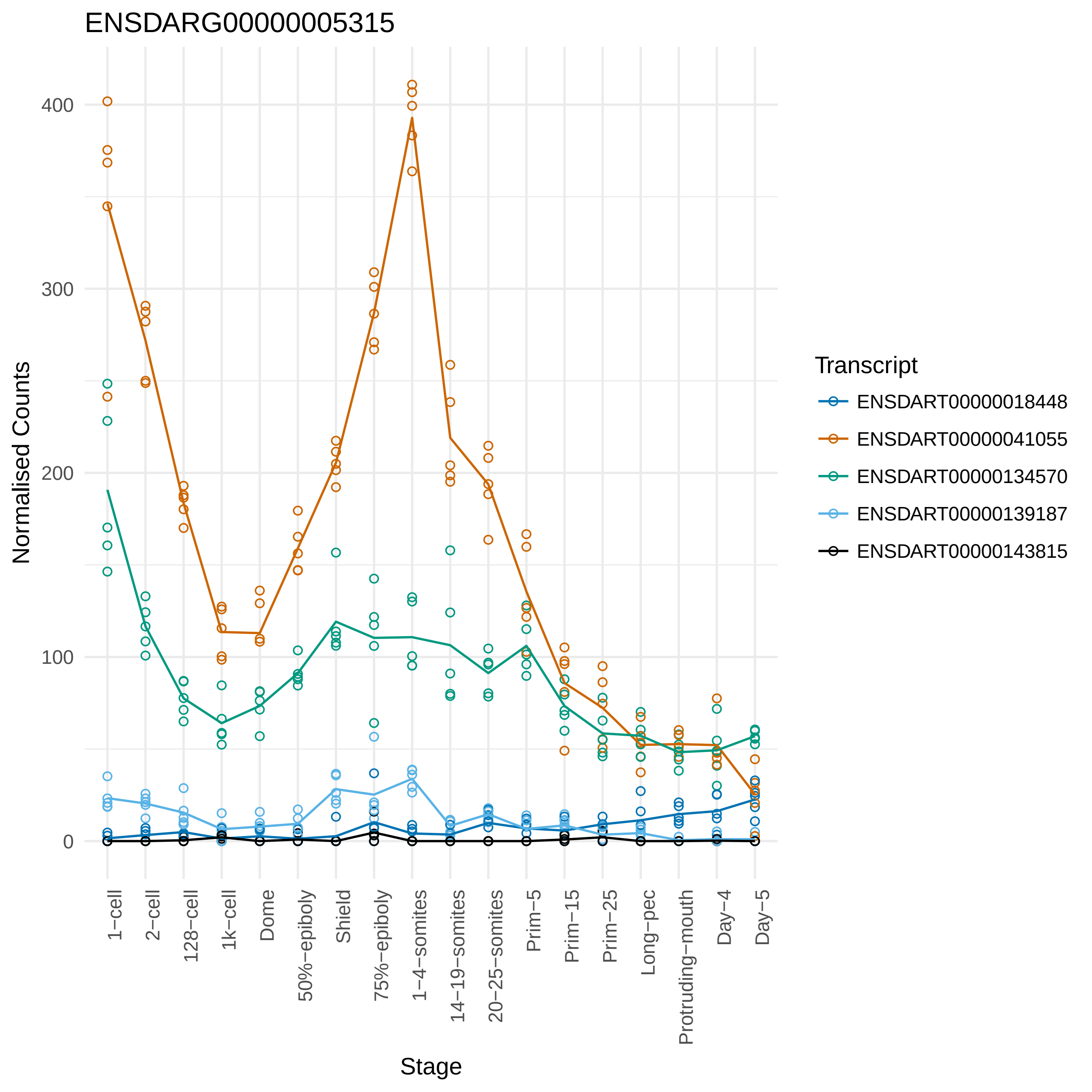

ENSDARG00000005315
Normalised Counts
400
300
200
100
0
1−cell
2−cell
128−cell
1k−cell
Dome
50%−epiboly
Shield
75%−epiboly
1−4−somites
14−19−somites
20−25−somites
Prim−5
Prim−15
Prim−25
Long−pec
Protruding−mouth
Day−4
Day−5
Stage
Transcript
ENSDART00000018448
ENSDART00000041055
ENSDART00000134570
ENSDART00000139187
ENSDART00000143815

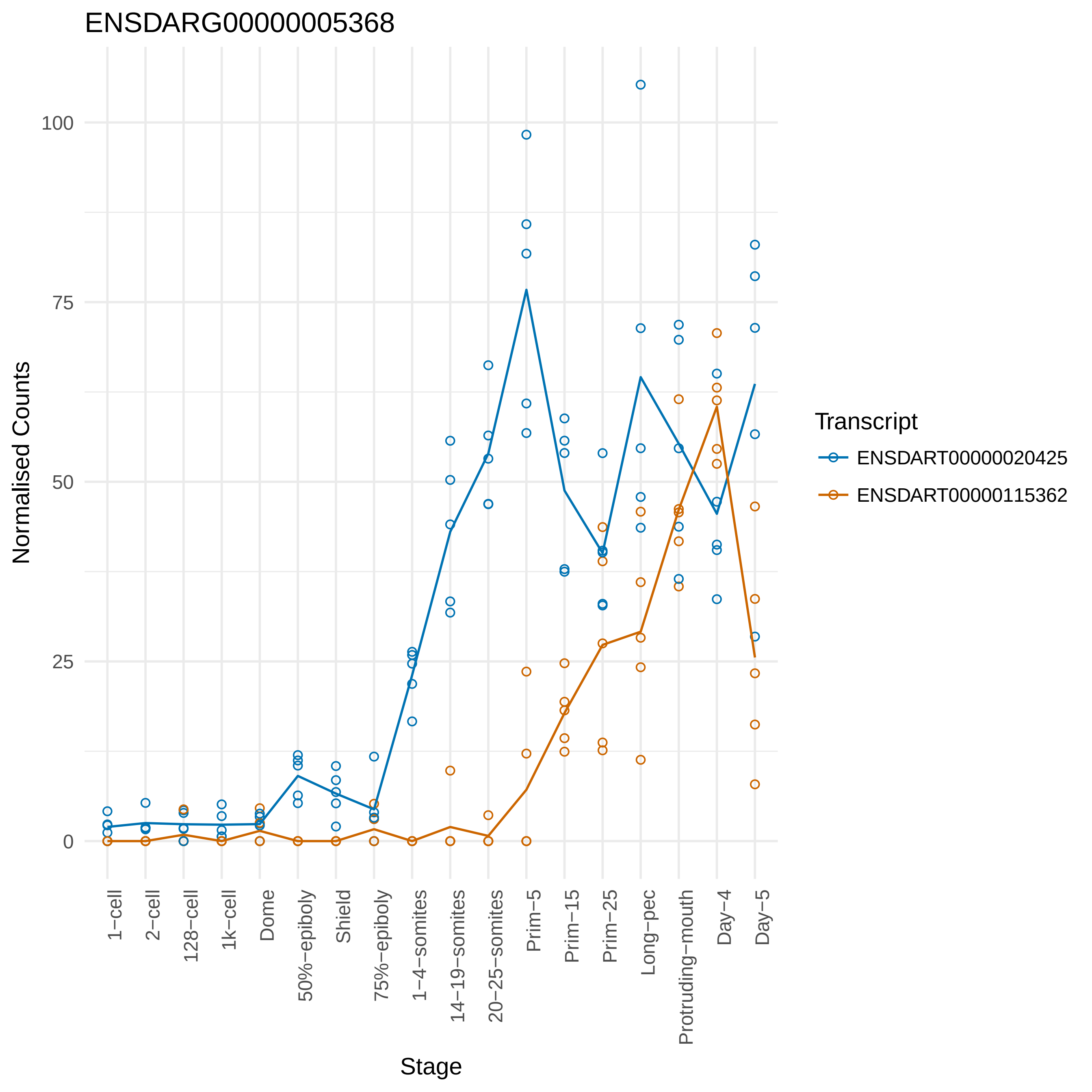

ENSDARG00000005368
Normalised Counts
100
75
50
25
0
1−cell
2−cell
128−cell
1k−cell
Dome
50%−epiboly
Shield
75%−epiboly
1−4−somites
14−19−somites
20−25−somites
Prim−5
Prim−15
Prim−25
Long−pec
Protruding−mouth
Day−4
Day−5
Stage
Transcript
ENSDART00000020425
ENSDART00000115362

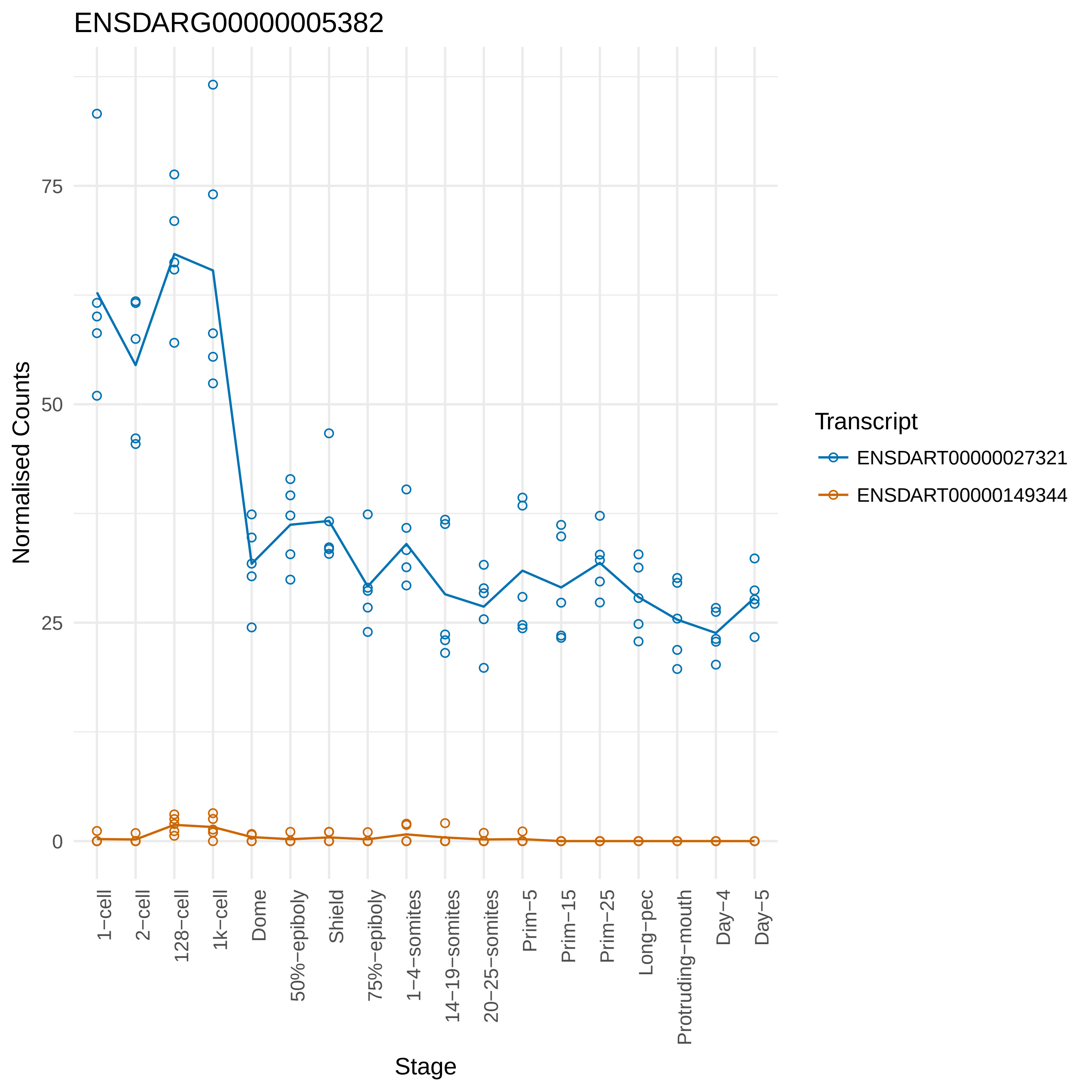

ENSDARG00000005382
Normalised Counts
75
50
25
0
1−cell
2−cell
128−cell
1k−cell
Dome
50%−epiboly
Shield
75%−epiboly
1−4−somites
14−19−somites
20−25−somites
Prim−5
Prim−15
Prim−25
Long−pec
Protruding−mouth
Day−4
Day−5
Stage
Transcript
ENSDART00000027321
ENSDART00000149344

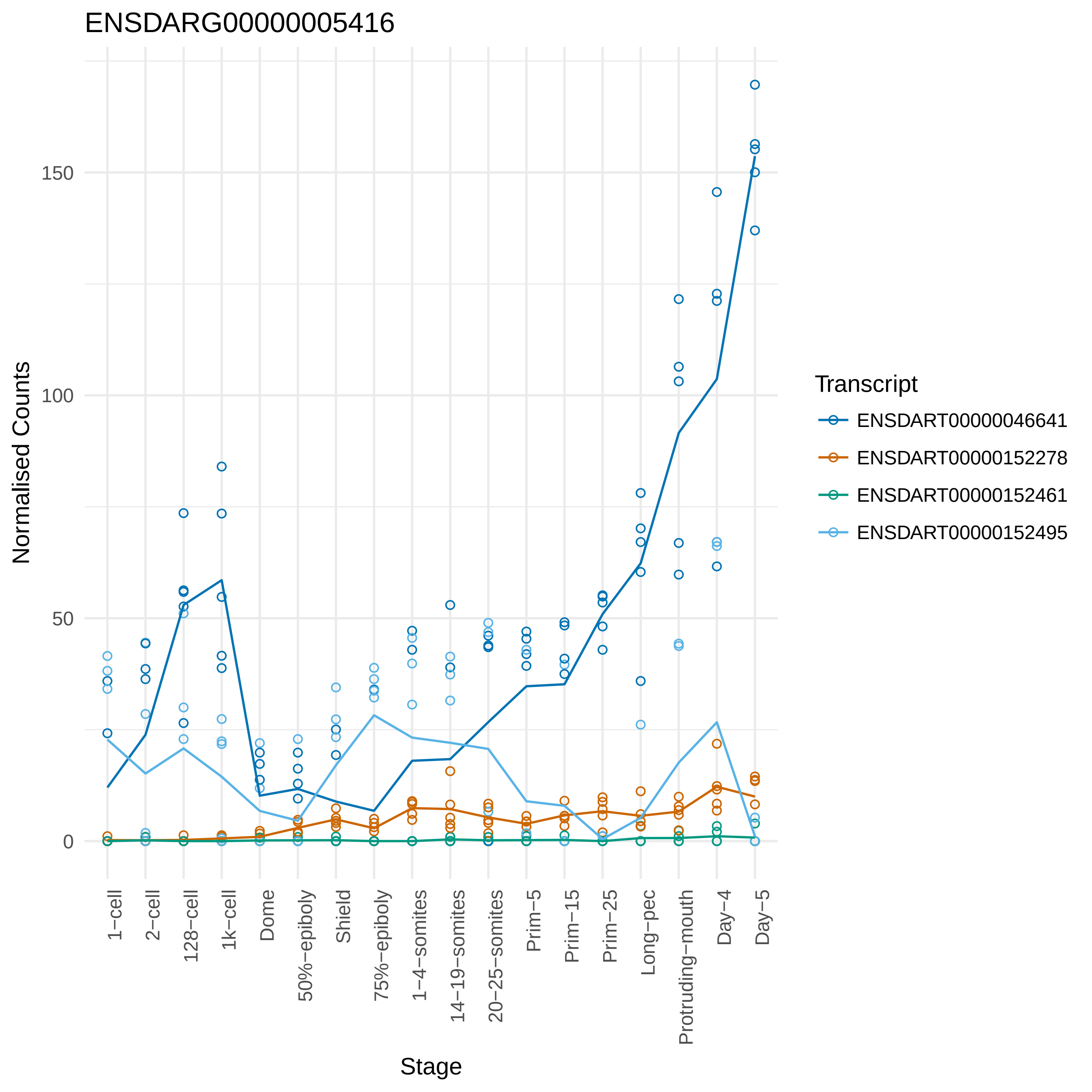

ENSDARG00000005416
Normalised Counts
0
50
100
150
1−cell
2−cell
128−cell
1k−cell
Dome
50%−epiboly
Shield
75%−epiboly
1−4−somites
14−19−somites
20−25−somites
Prim−5
Prim−15
Prim−25
Long−pec
Protruding−mouth
Day−4
Day−5
Stage
Transcript
ENSDART00000046641
ENSDART00000152278
ENSDART00000152461
ENSDART00000152495

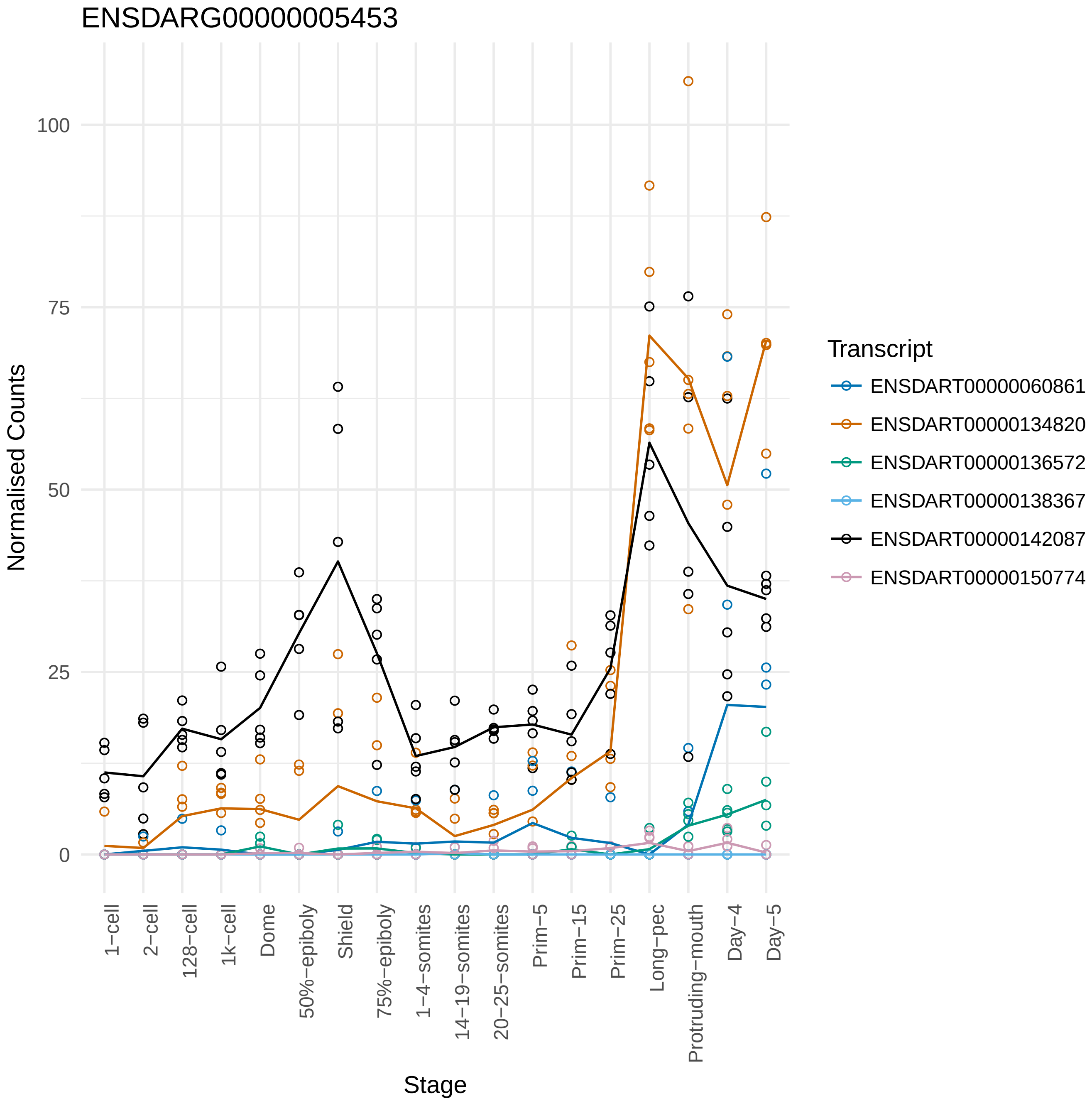

ENSDARG00000005453
Normalised Counts
0
25
50
75
100
1−cell
2−cell
128−cell
1k−cell
Dome
50%−epiboly
Shield
75%−epiboly
1−4−somites
14−19−somites
20−25−somites
Prim−5
Prim−15
Prim−25
Long−pec
Protruding−mouth
Day−4
Day−5
Stage
Transcript
ENSDART00000060861
ENSDART00000134820
ENSDART00000136572
ENSDART00000138367
ENSDART00000142087
ENSDART00000150774

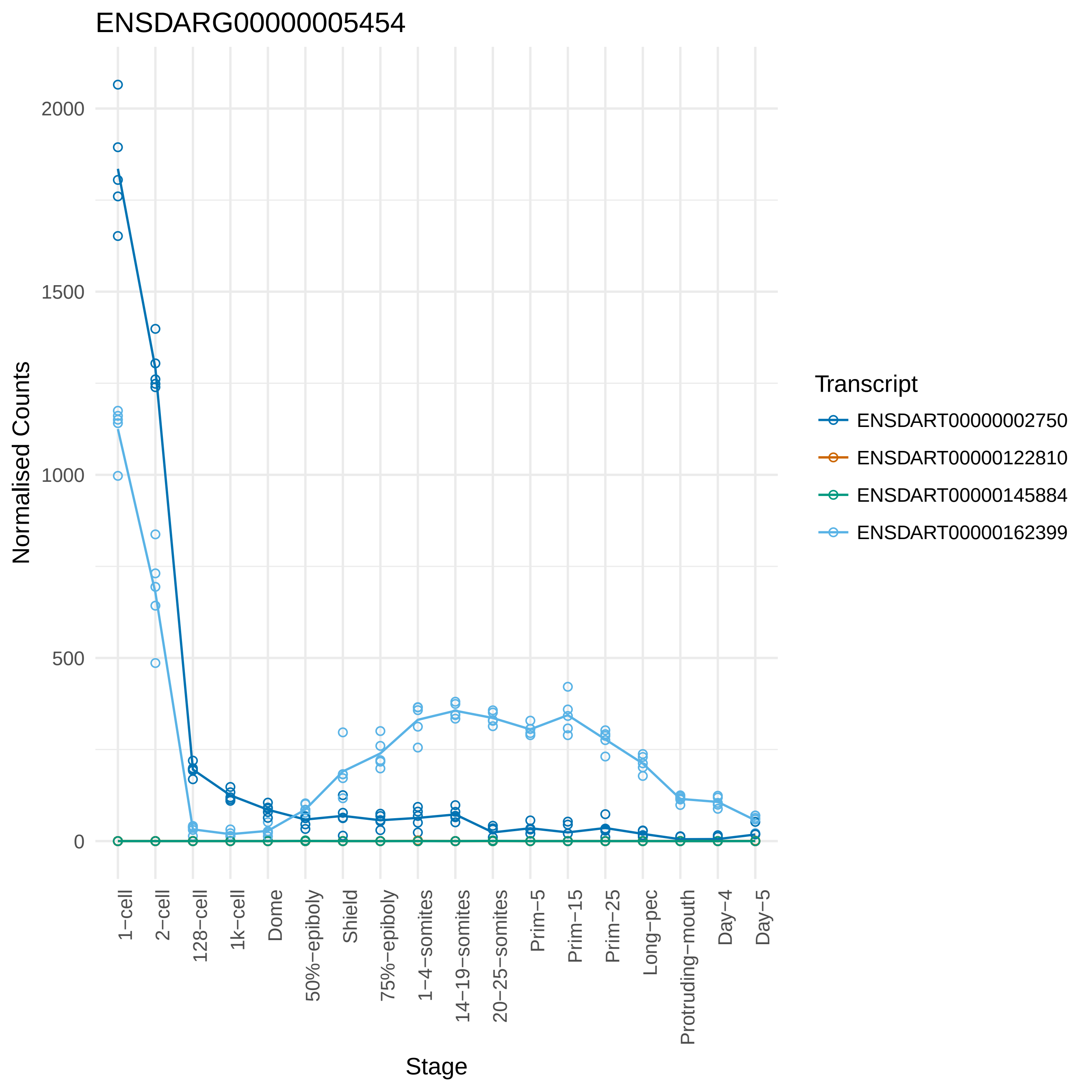

ENSDARG00000005454
Normalised Counts
0
500
1000
1500
2000
Stage
1−cell
2−cell
128−cell
1k−cell
Dome
50%−epiboly
Shield
75%−epiboly
1−4−somites
14−19−somites
20−25−somites
Prim−5
Prim−15
Prim−25
Long−pec
Protruding−mouth
Day−4
Day−5
Transcript
ENSDART00000002750
ENSDART00000122810
ENSDART00000145884
ENSDART00000162399

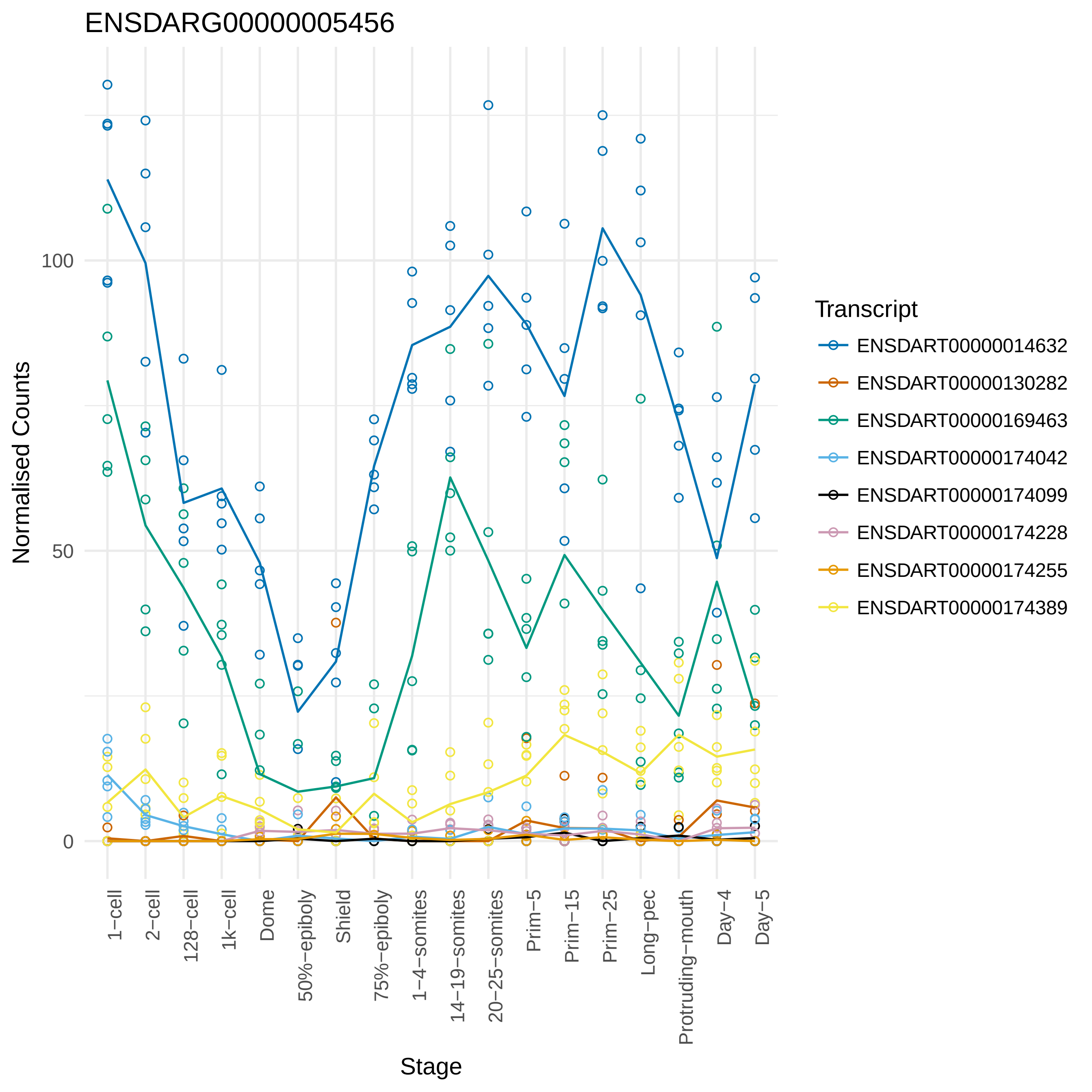

ENSDARG00000005456
Normalised Counts
100
50
0
1−cell
2−cell
128−cell
1k−cell
Dome
50%−epiboly
Shield
75%−epiboly
1−4−somites
14−19−somites
20−25−somites
Prim−5
Prim−15
Prim−25
Long−pec
Protruding−mouth
Day−4
Day−5
Stage
Transcript
ENSDART00000014632
ENSDART00000130282
ENSDART00000169463
ENSDART00000174042
ENSDART00000174099
ENSDART00000174228
ENSDART00000174255
ENSDART00000174389

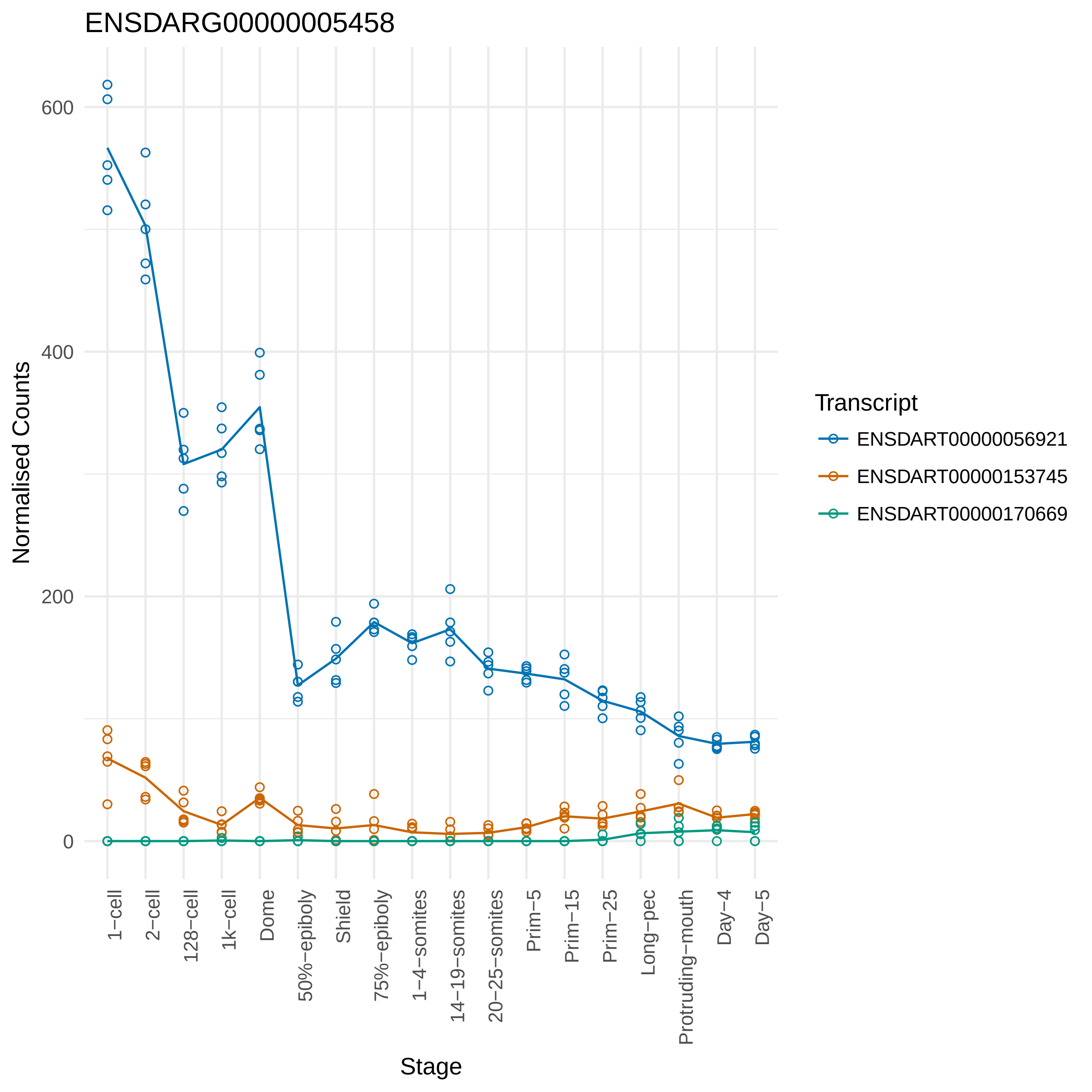

ENSDARG00000005458
Normalised Counts
600
400
200
0
Stage
1−cell
2−cell
128−cell
1k−cell
Dome
50%−epiboly
Shield
75%−epiboly
1−4−somites
14−19−somites
20−25−somites
Prim−5
Prim−15
Prim−25
Long−pec
Protruding−mouth
Day−4
Day−5
Transcript
ENSDART00000056921
ENSDART00000153745
ENSDART00000170669

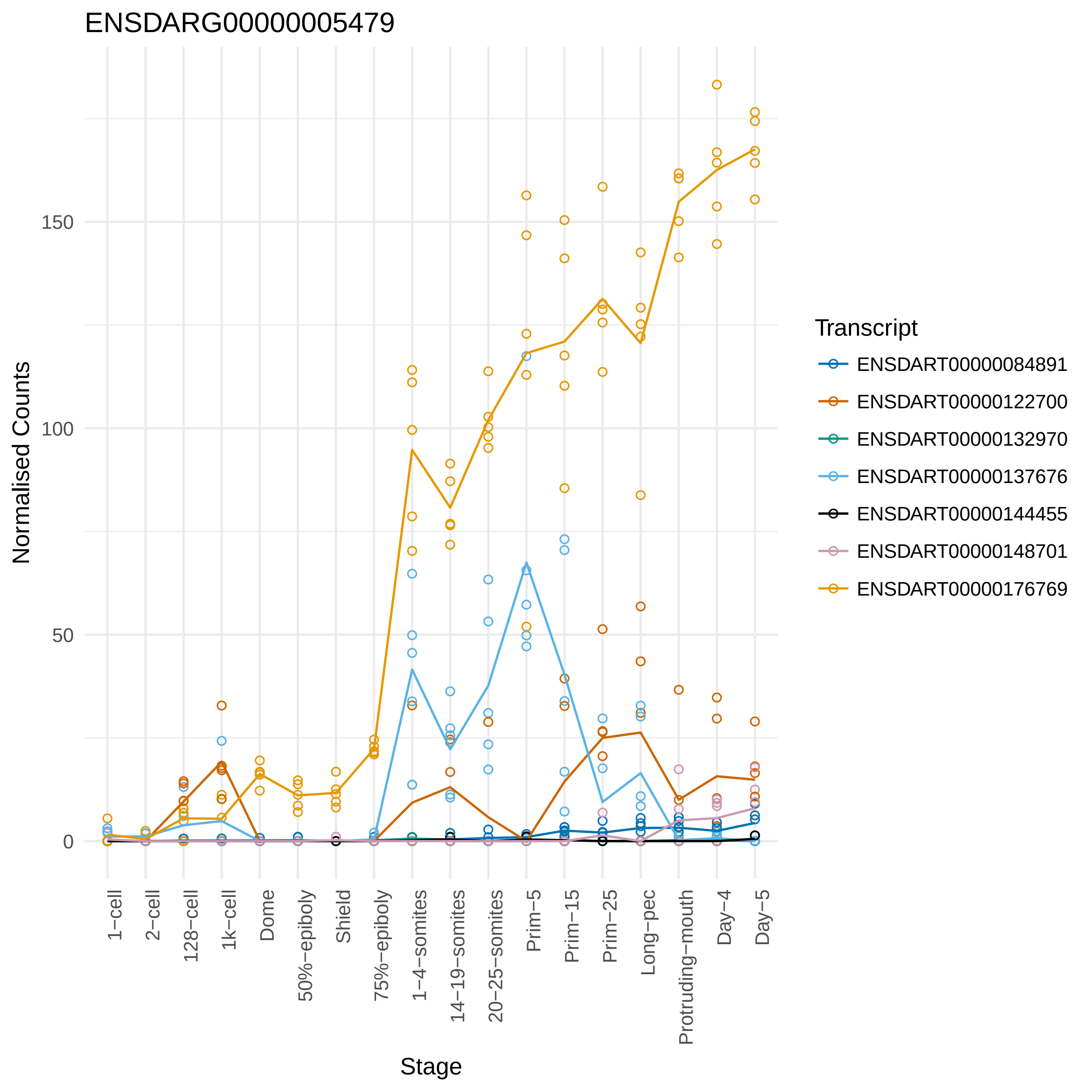

ENSDARG00000005479
Normalised Counts
150
100
50
0
1−cell
2−cell
128−cell
1k−cell
Dome
50%−epiboly
Shield
75%−epiboly
1−4−somites
14−19−somites
20−25−somites
Prim−5
Prim−15
Prim−25
Long−pec
Protruding−mouth
Day−4
Day−5
Stage
Transcript
ENSDART00000084891
ENSDART00000122700
ENSDART00000132970
ENSDART00000137676
ENSDART00000144455
ENSDART00000148701
ENSDART00000176769

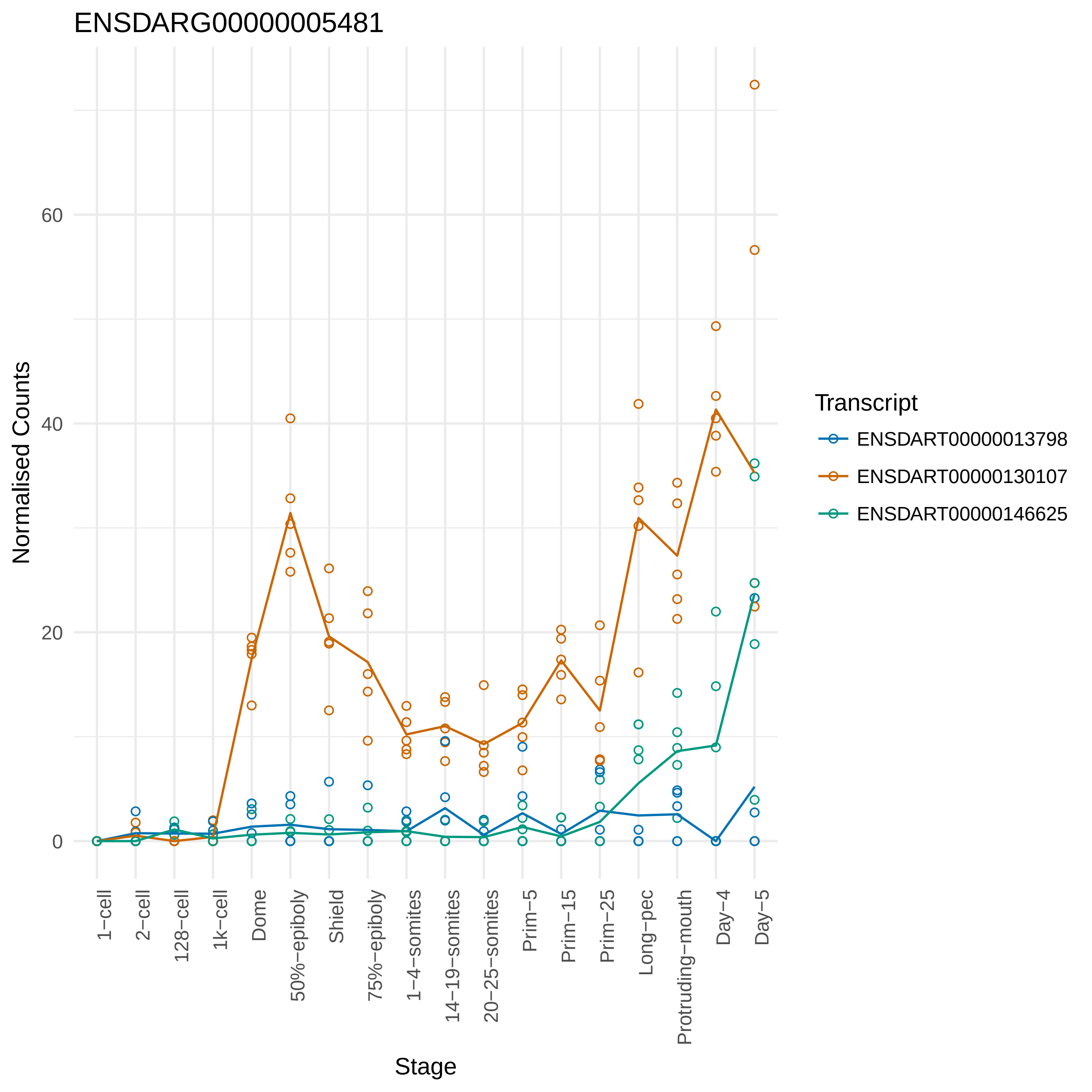

ENSDARG00000005481
Normalised Counts
0
20
40
60
Stage
1−cell
2−cell
128−cell
1k−cell
Dome
50%−epiboly
Shield
75%−epiboly
1−4−somites
14−19−somites
20−25−somites
Prim−5
Prim−15
Prim−25
Long−pec
Protruding−mouth
Day−4
Day−5
Transcript
ENSDART00000013798
ENSDART00000130107
ENSDART00000146625

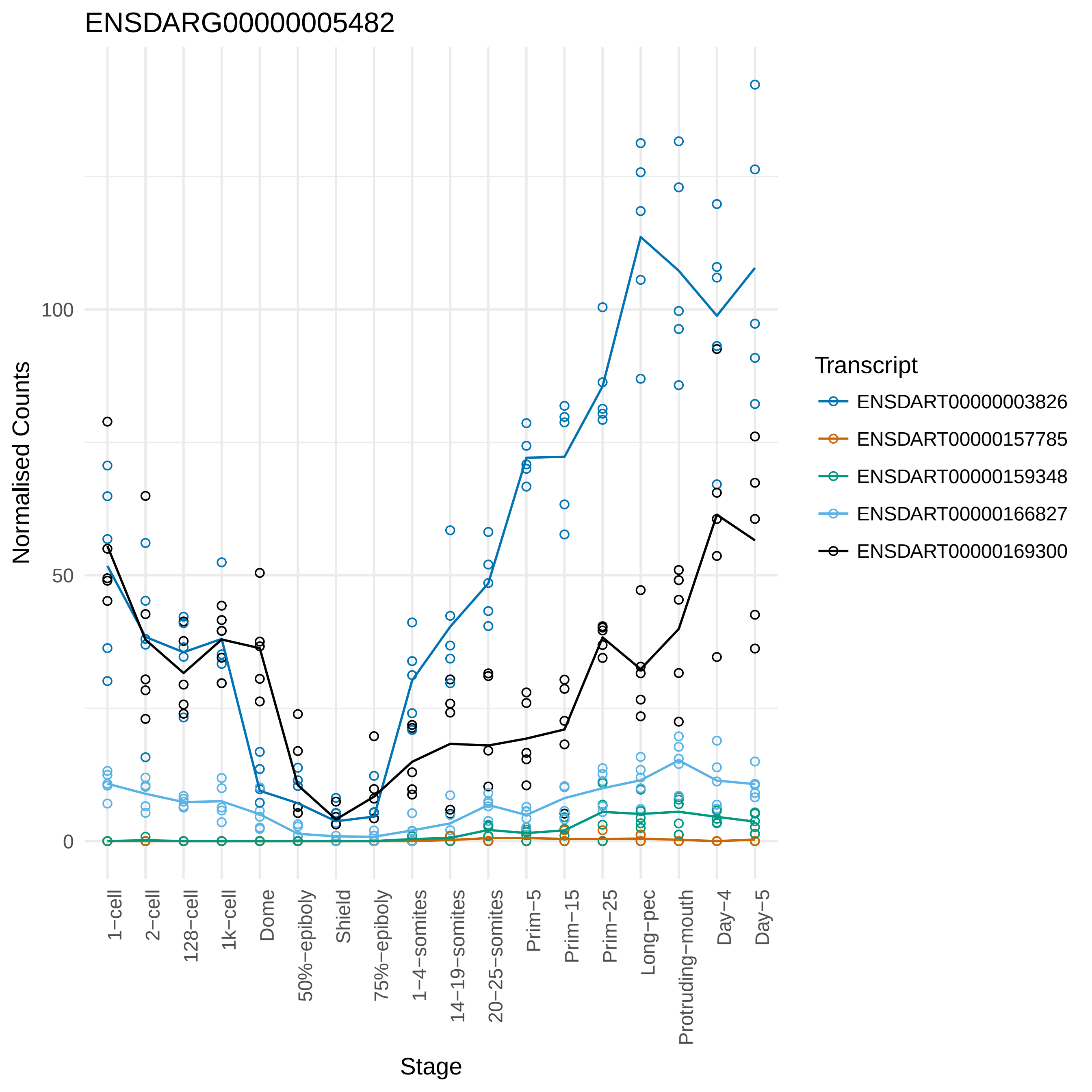
ENSDARG00000005482
Normalised Counts
100
50
0
1−cell
2−cell
128−cell
1k−cell
Dome
50%−epiboly
Shield
75%−epiboly
1−4−somites
14−19−somites
20−25−somites
Prim−5
Prim−15
Prim−25
Long−pec
Protruding−mouth
Day−4
Day−5
Stage
Transcript
ENSDART00000003826
ENSDART00000157785
ENSDART00000159348
ENSDART00000166827
ENSDART00000169300

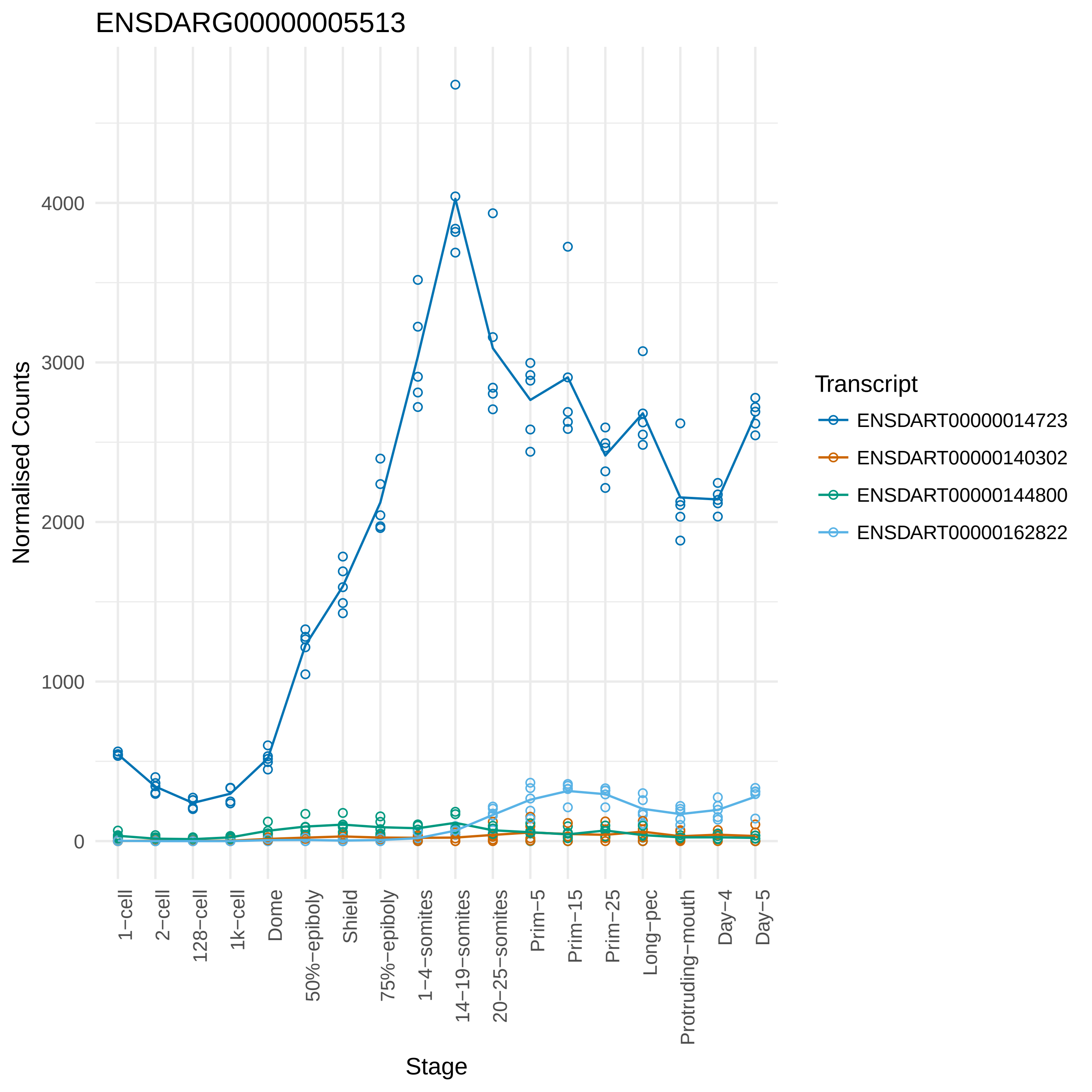
ENSDARG00000005513
Normalised Counts
0
1000
2000
3000
4000
Stage
1−cell
2−cell
128−cell
1k−cell
Dome
50%−epiboly
Shield
75%−epiboly
1−4−somites
14−19−somites
20−25−somites
Prim−5
Prim−15
Prim−25
Long−pec
Protruding−mouth
Day−4
Day−5
Transcript
ENSDART00000014723
ENSDART00000140302
ENSDART00000144800
ENSDART00000162822

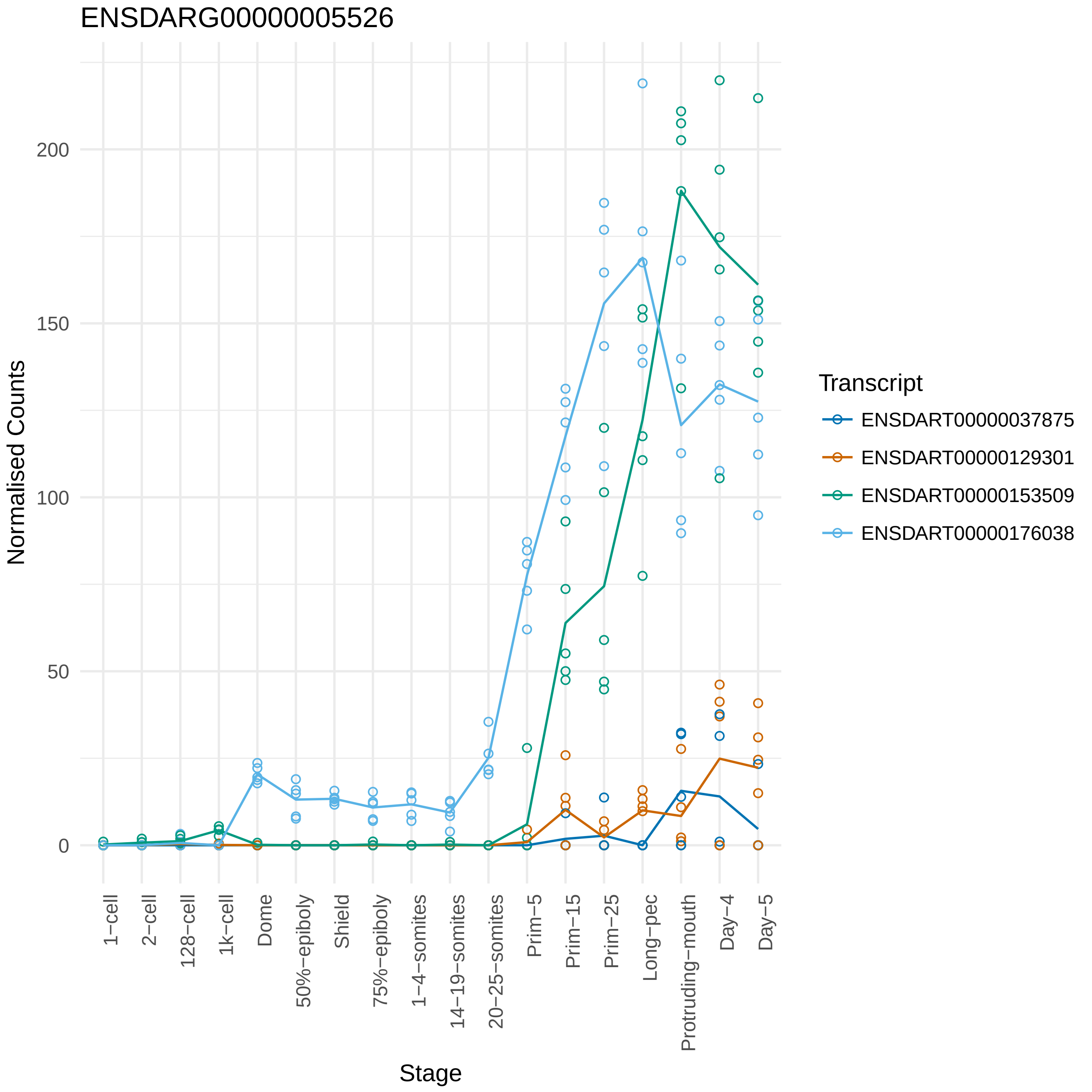

ENSDARG00000005526
Normalised Counts
0
50
100
150
200
Stage
1−cell
2−cell
128−cell
1k−cell
Dome
50%−epiboly
Shield
75%−epiboly
1−4−somites
14−19−somites
20−25−somites
Prim−5
Prim−15
Prim−25
Long−pec
Protruding−mouth
Day−4
Day−5
Transcript
ENSDART00000037875
ENSDART00000129301
ENSDART00000153509
ENSDART00000176038

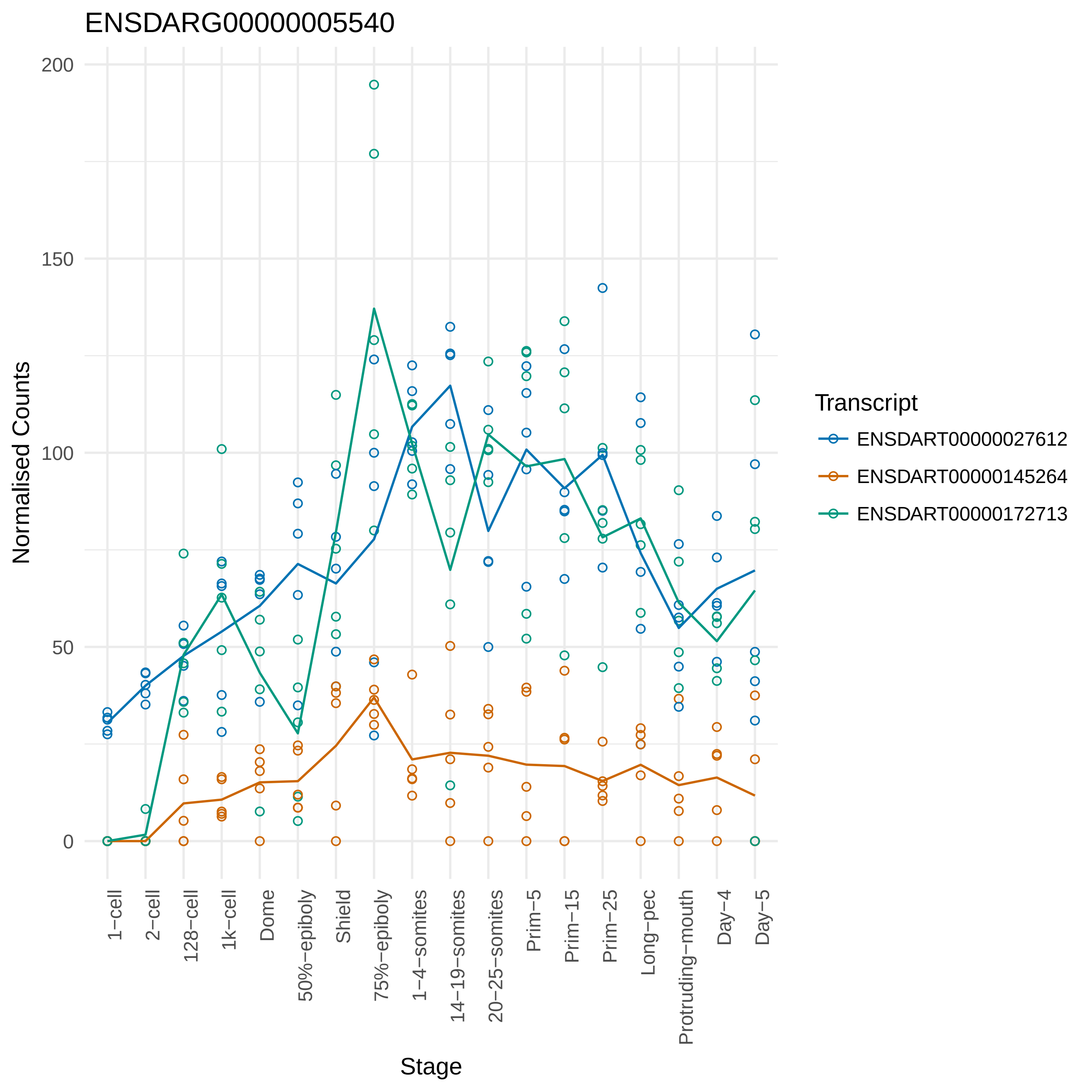

ENSDARG00000005540
Normalised Counts
200
150
100
50
0
1−cell
2−cell
128−cell
1k−cell
Dome
50%−epiboly
Shield
75%−epiboly
1−4−somites
14−19−somites
20−25−somites
Prim−5
Prim−15
Prim−25
Long−pec
Protruding−mouth
Day−4
Day−5
Stage
Transcript
ENSDART00000027612
ENSDART00000145264
ENSDART00000172713

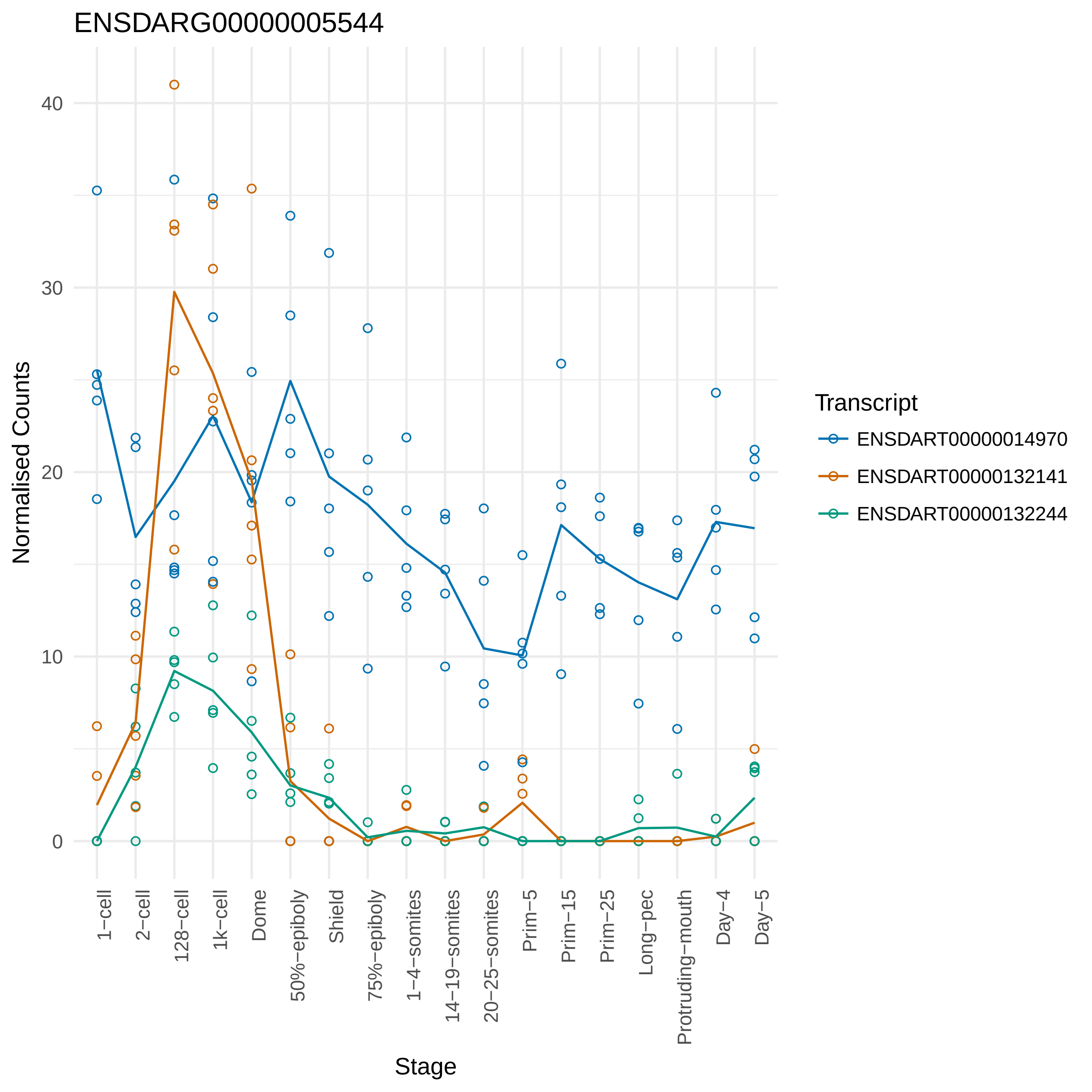

ENSDARG00000005544
Normalised Counts
0
10
20
30
40
Stage
1−cell
2−cell
128−cell
1k−cell
Dome
50%−epiboly
Shield
75%−epiboly
1−4−somites
14−19−somites
20−25−somites
Prim−5
Prim−15
Prim−25
Long−pec
Protruding−mouth
Day−4
Day−5
Transcript
ENSDART00000014970
ENSDART00000132141
ENSDART00000132244

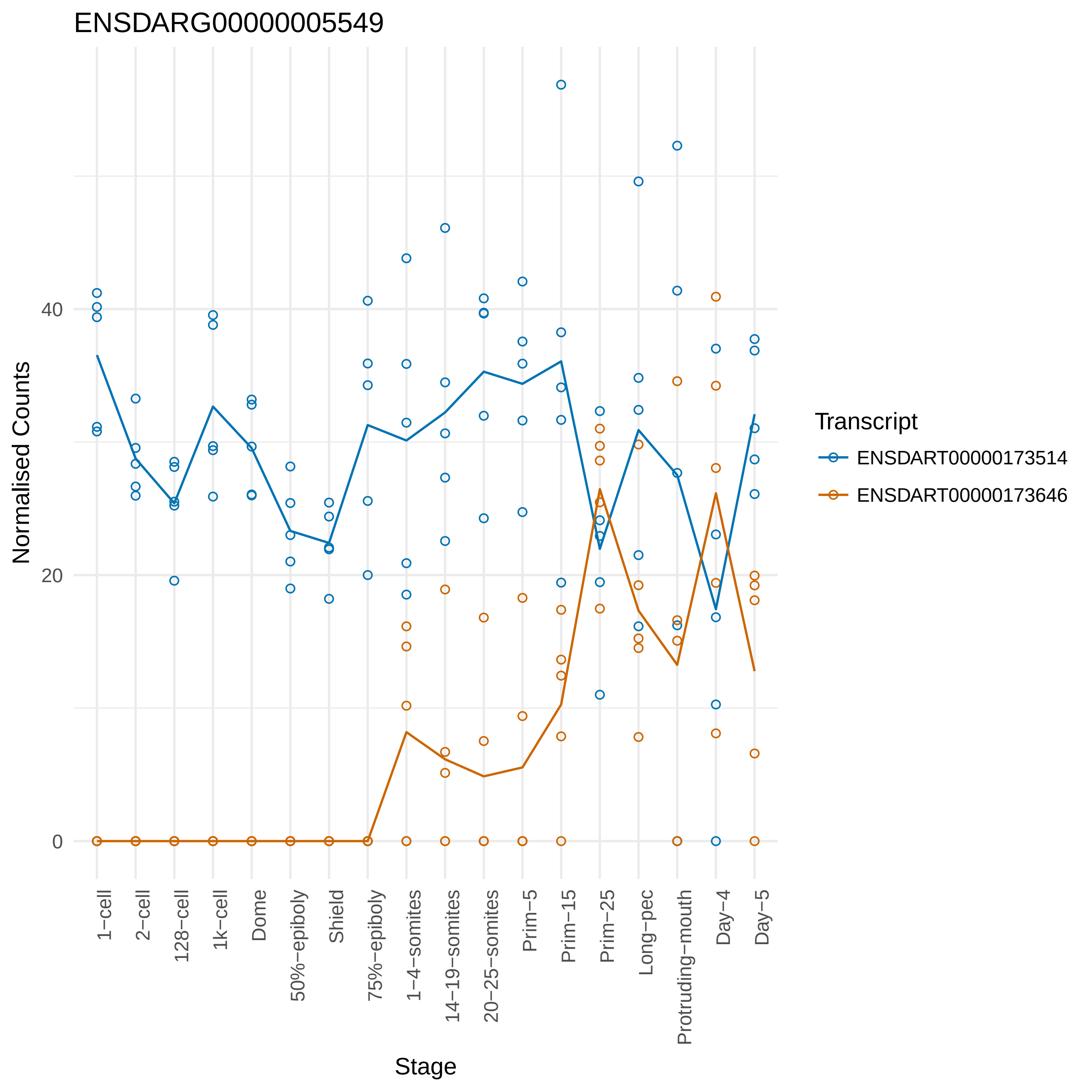

ENSDARG00000005549
Normalised Counts
0
20
40
Stage
1−cell
2−cell
128−cell
1k−cell
Dome
50%−epiboly
Shield
75%−epiboly
1−4−somites
14−19−somites
20−25−somites
Prim−5
Prim−15
Prim−25
Long−pec
Protruding−mouth
Day−4
Day−5
Transcript
ENSDART00000173514
ENSDART00000173646

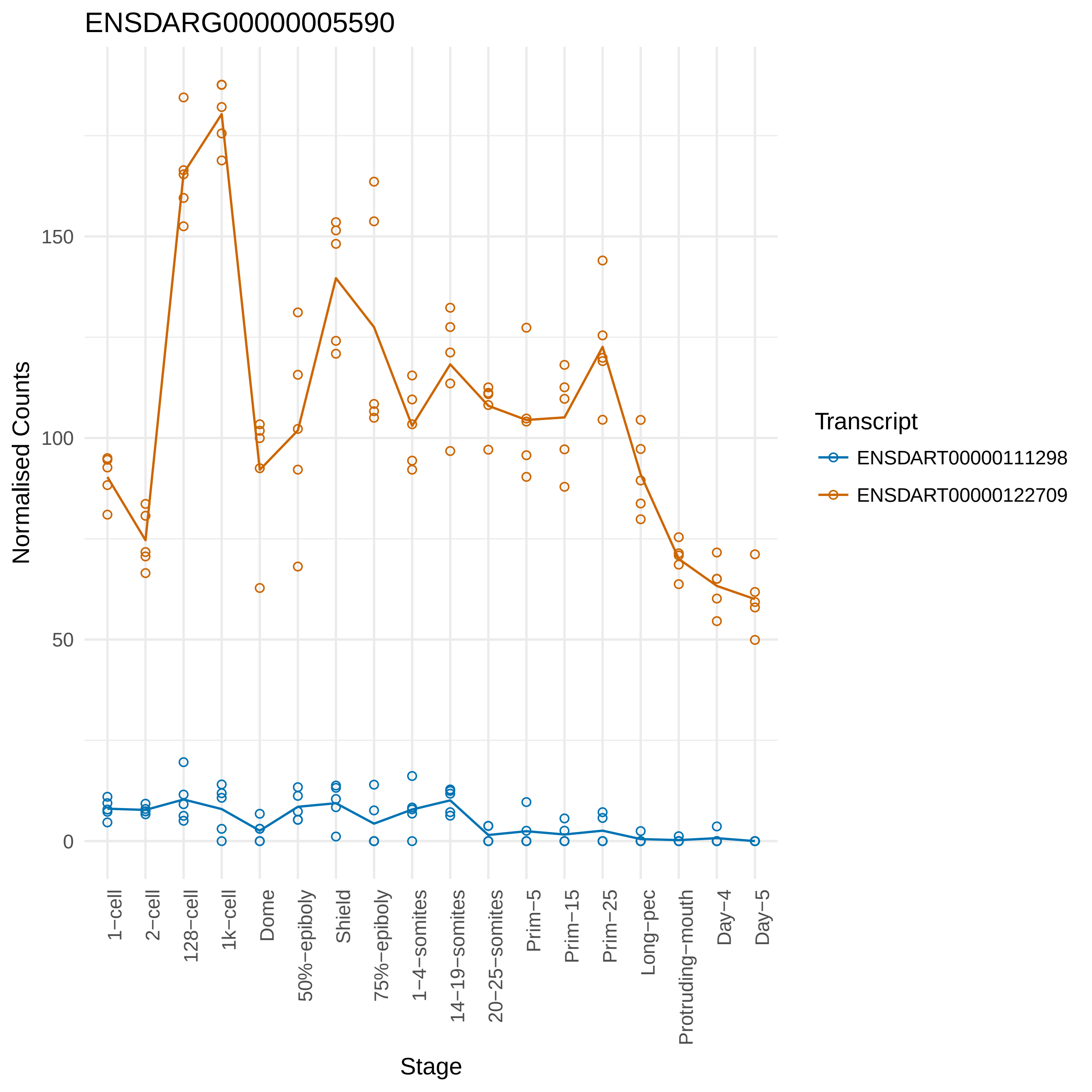

ENSDARG00000005590
Normalised Counts
150
100
50
0
1−cell
2−cell
128−cell
1k−cell
Dome
50%−epiboly
Shield
75%−epiboly
1−4−somites
14−19−somites
20−25−somites
Prim−5
Prim−15
Prim−25
Long−pec
Protruding−mouth
Day−4
Day−5
Stage
Transcript
ENSDART00000111298
ENSDART00000122709

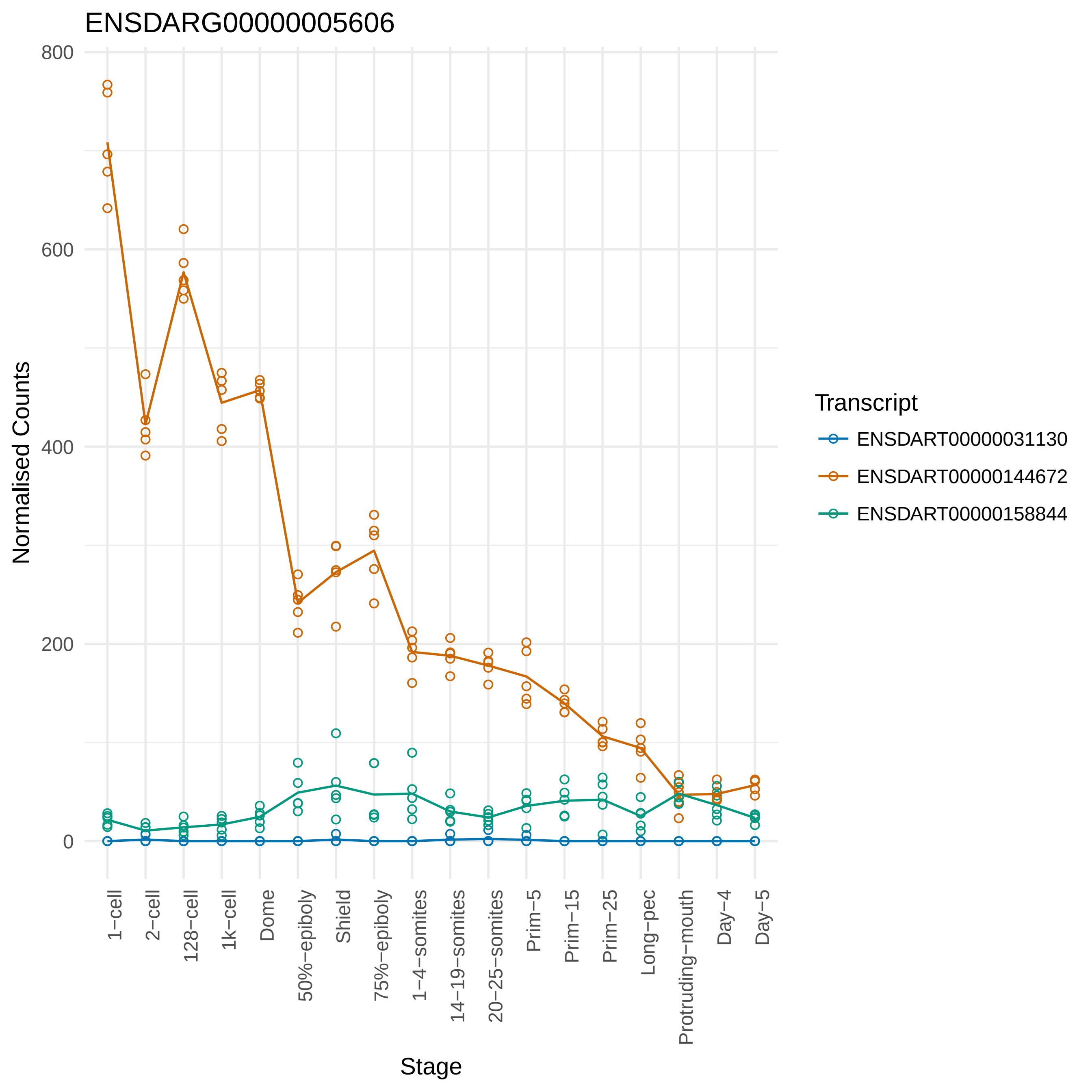
ENSDARG00000005606
Normalised Counts
800
600
400
200
0
1−cell
2−cell
128−cell
1k−cell
Dome
50%−epiboly
Shield
75%−epiboly
1−4−somites
14−19−somites
20−25−somites
Prim−5
Prim−15
Prim−25
Long−pec
Protruding−mouth
Day−4
Day−5
Stage
Transcript
ENSDART00000031130
ENSDART00000144672
ENSDART00000158844

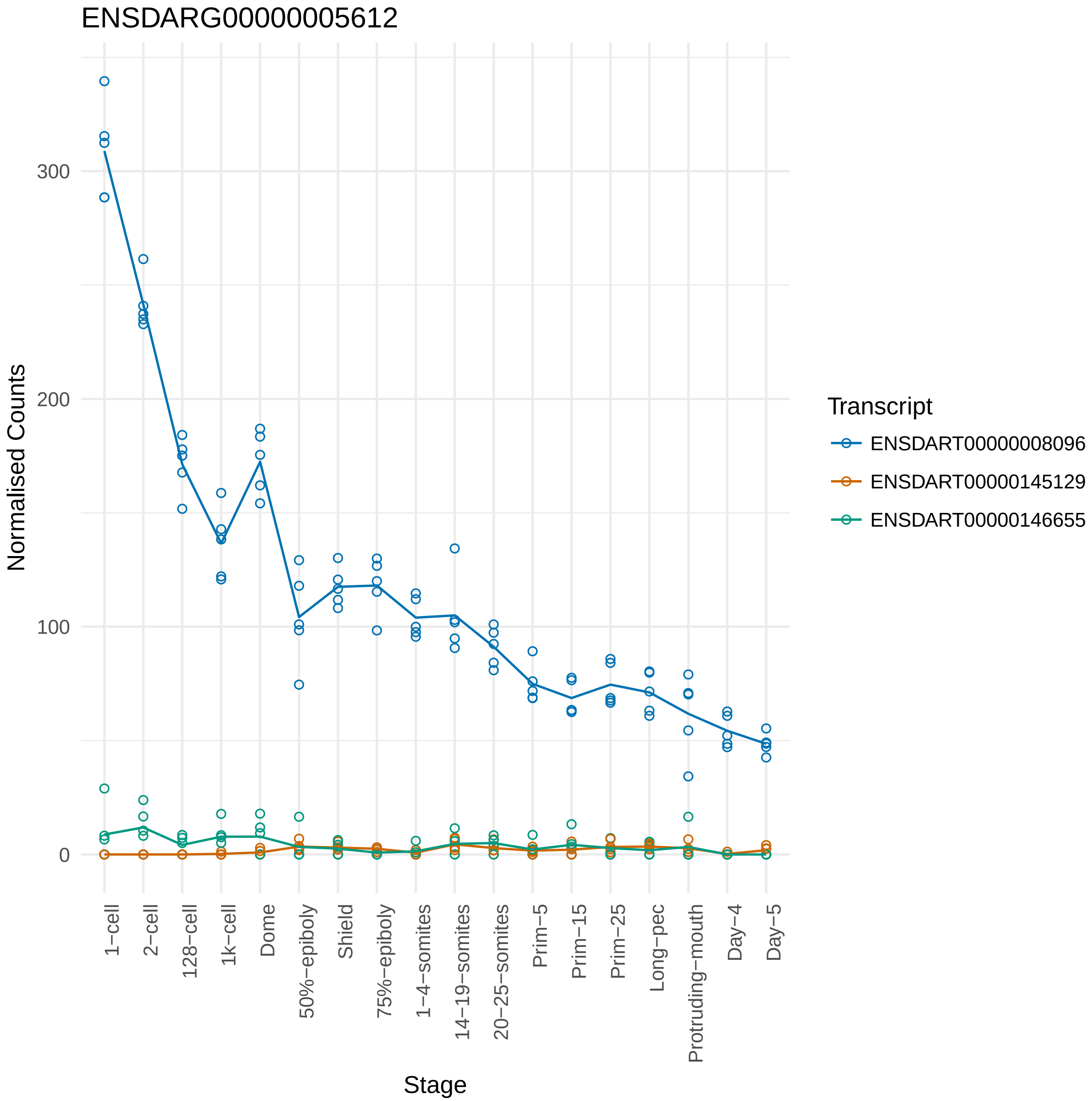

ENSDARG00000005612
Normalised Counts
0
100
200
300
Stage
1−cell
2−cell
128−cell
1k−cell
Dome
50%−epiboly
Shield
75%−epiboly
1−4−somites
14−19−somites
20−25−somites
Prim−5
Prim−15
Prim−25
Long−pec
Protruding−mouth
Day−4
Day−5
Transcript
ENSDART00000008096
ENSDART00000145129
ENSDART00000146655

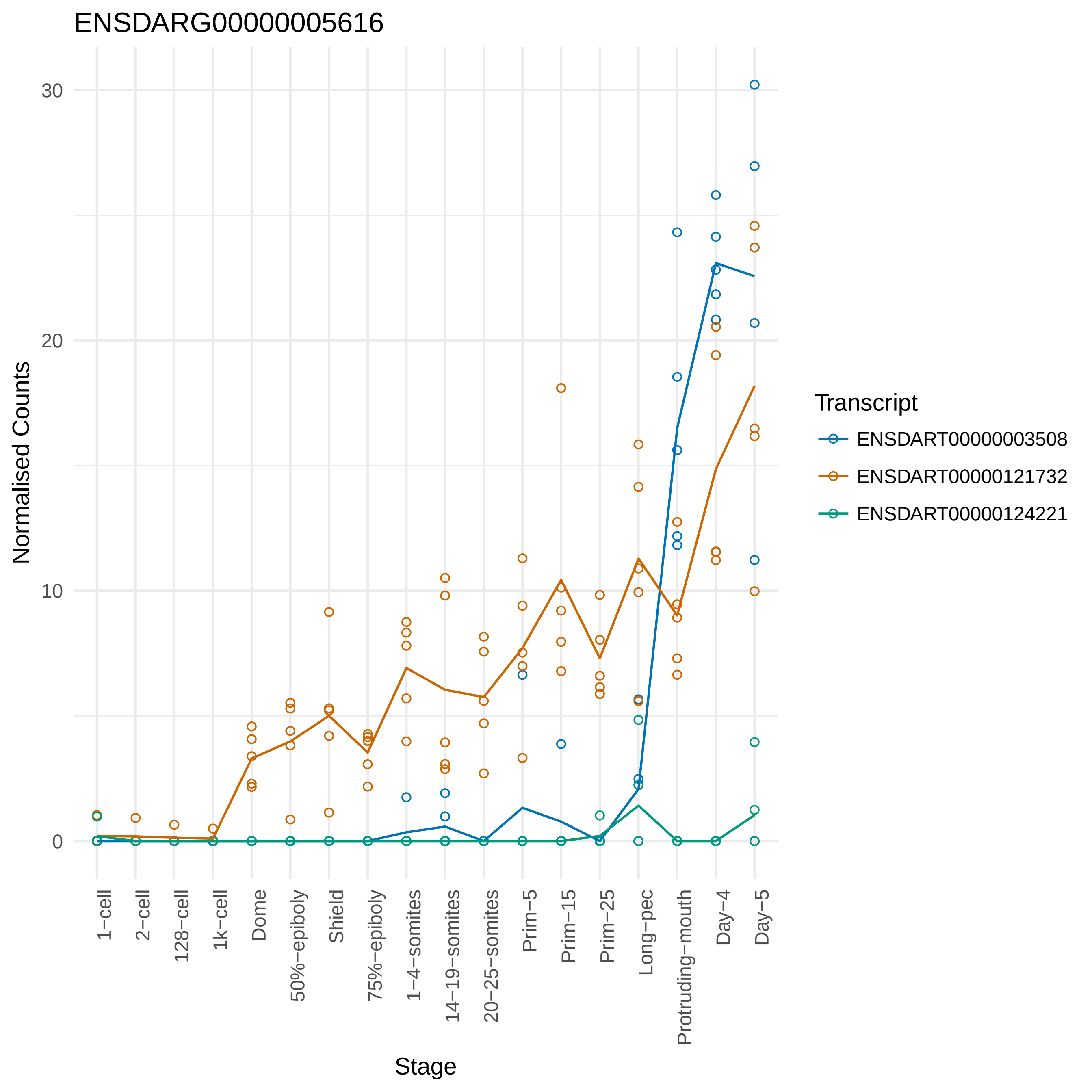

ENSDARG00000005616
Normalised Counts
0
10
20
30
1−cell
2−cell
128−cell
1k−cell
Dome
50%−epiboly
Shield
75%−epiboly
1−4−somites
14−19−somites
20−25−somites
Prim−5
Prim−15
Prim−25
Long−pec
Protruding−mouth
Day−4
Day−5
Stage
Transcript
ENSDART00000003508
ENSDART00000121732
ENSDART00000124221

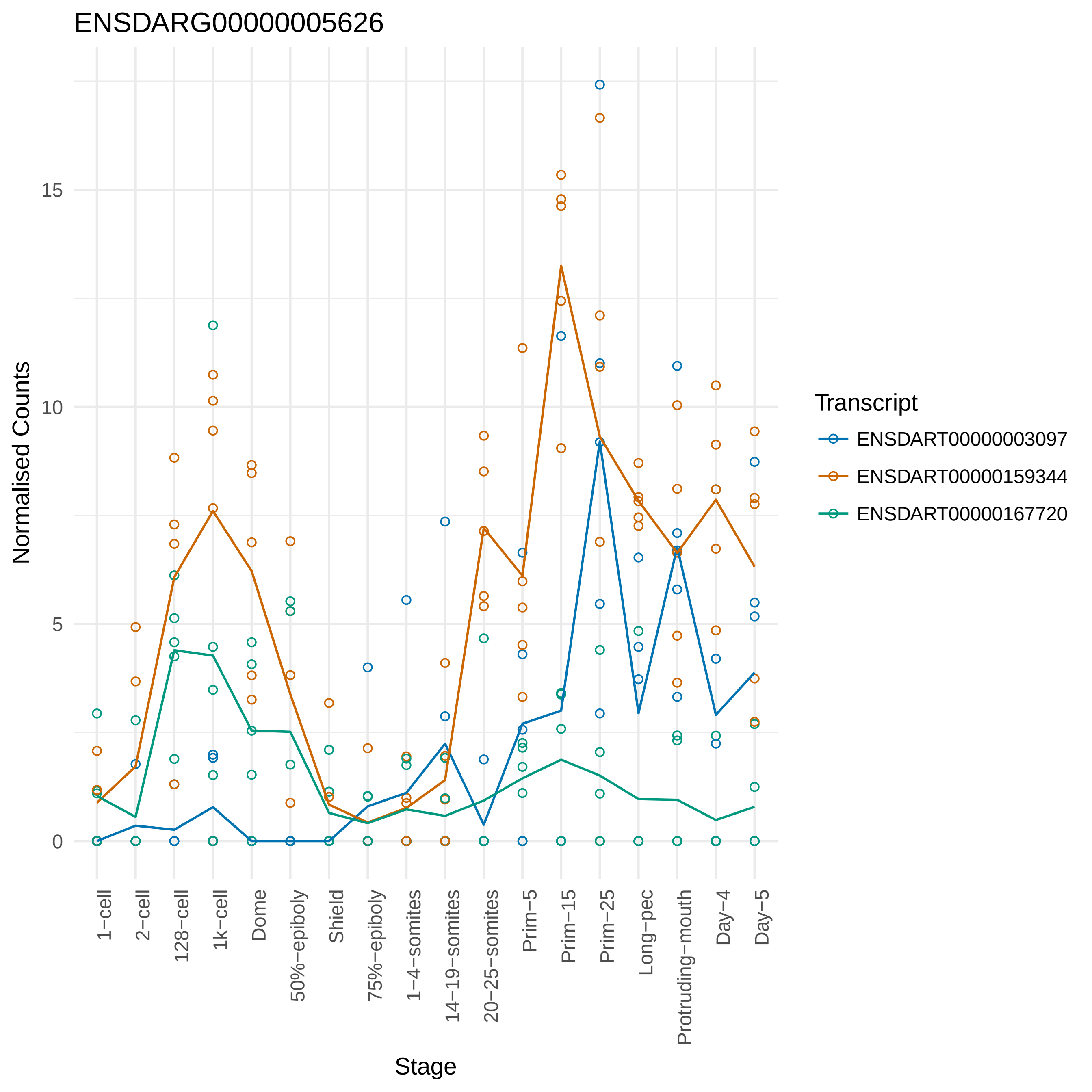
ENSDARG00000005626
Normalised Counts
0
5
10
15
Stage
1−cell
2−cell
128−cell
1k−cell
Dome
50%−epiboly
Shield
75%−epiboly
1−4−somites
14−19−somites
20−25−somites
Prim−5
Prim−15
Prim−25
Long−pec
Protruding−mouth
Day−4
Day−5
Transcript
ENSDART00000003097
ENSDART00000159344
ENSDART00000167720

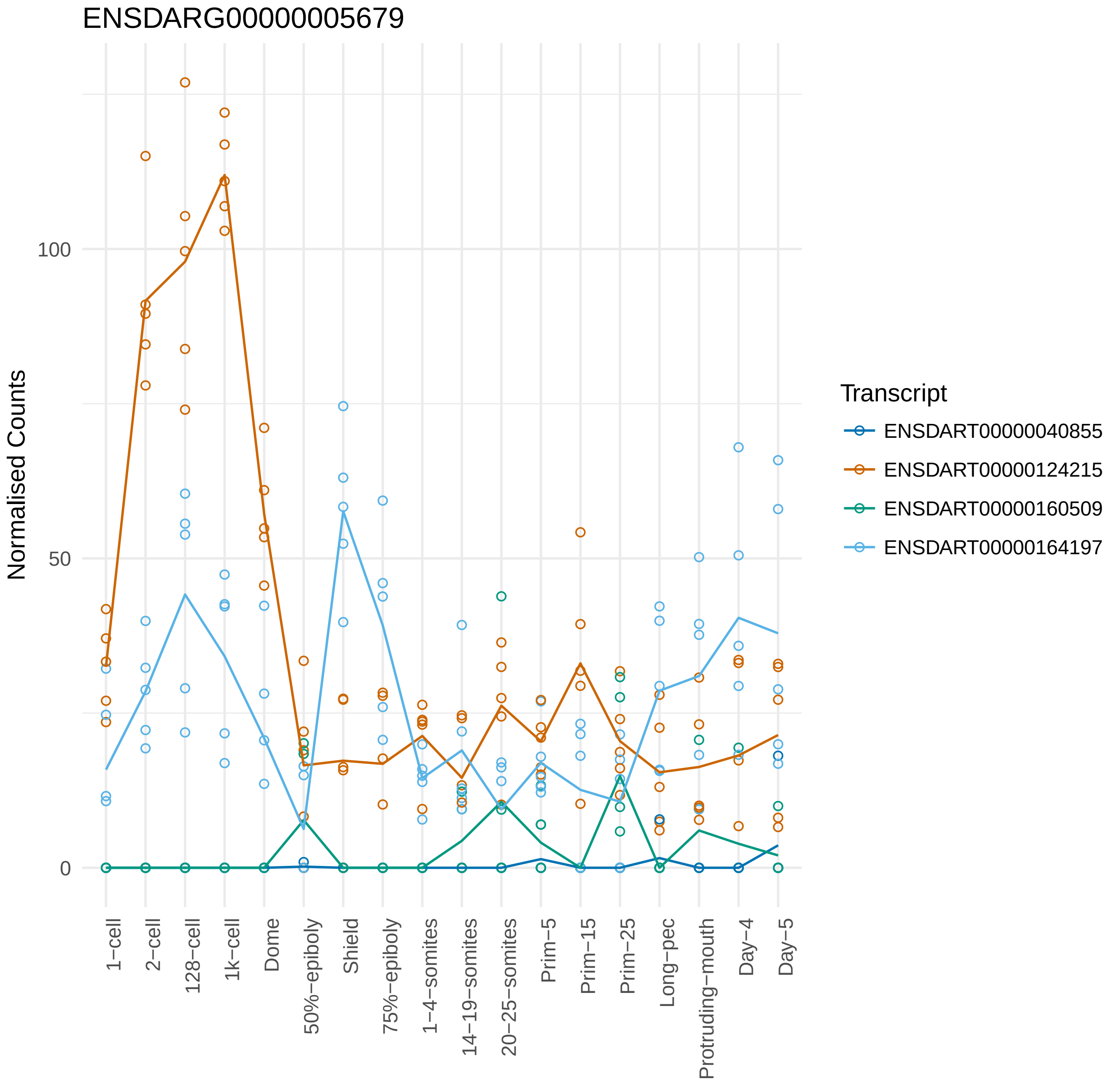

ENSDARG00000005679
Normalised Counts
100
50
0
Stage
1−cell
2−cell
128−cell
1k−cell
Dome
50%−epiboly
Shield
75%−epiboly
1−4−somites
14−19−somites
20−25−somites
Prim−5
Prim−15
Prim−25
Long−pec
Protruding−mouth
Day−4
Day−5
Transcript
ENSDART00000040855
ENSDART00000124215
ENSDART00000160509
ENSDART00000164197

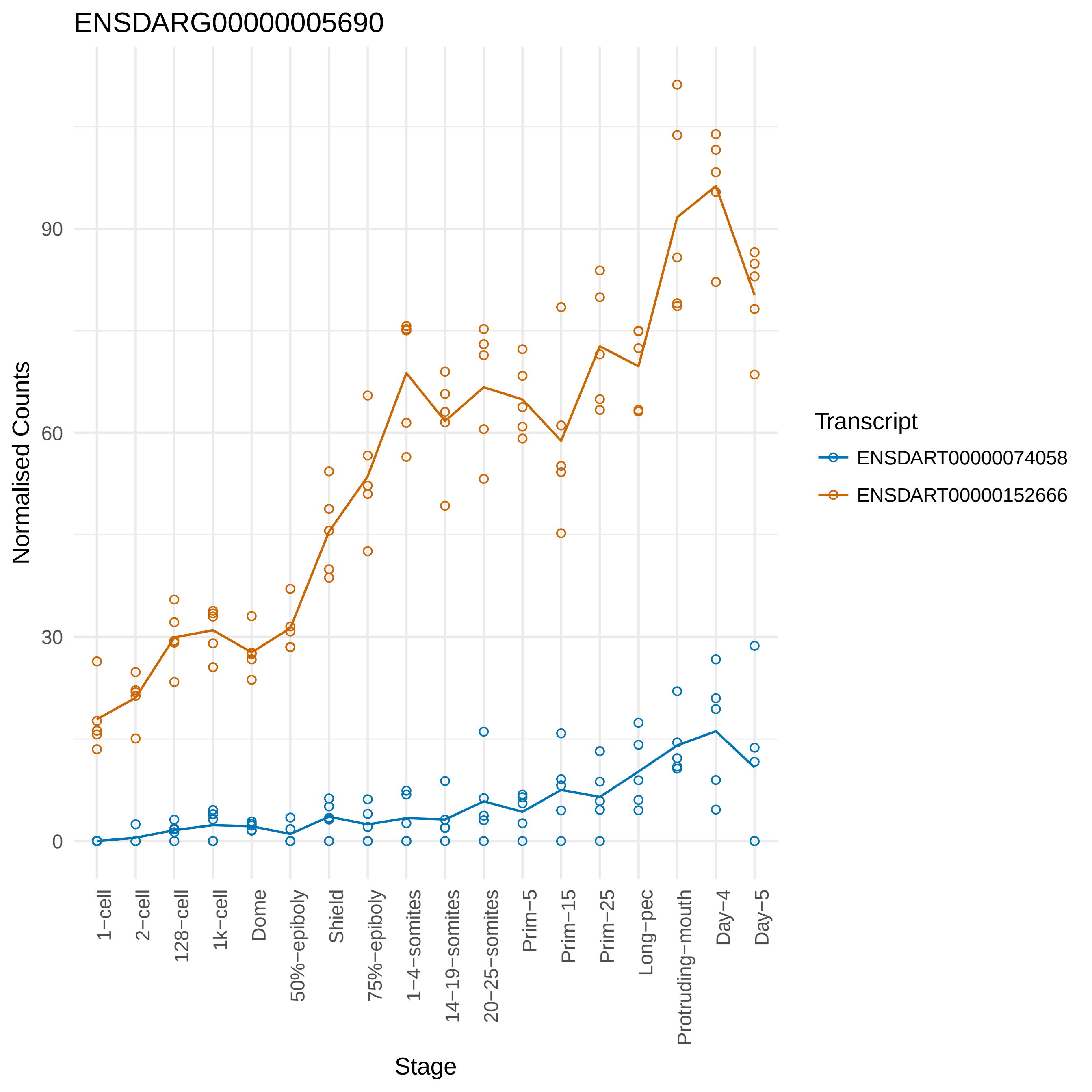

ENSDARG00000005690
Normalised Counts
0
30
60
90
1−cell
2−cell
128−cell
1k−cell
Dome
50%−epiboly
Shield
75%−epiboly
1−4−somites
14−19−somites
20−25−somites
Prim−5
Prim−15
Prim−25
Long−pec
Protruding−mouth
Day−4
Day−5
Stage
Transcript
ENSDART00000074058
ENSDART00000152666

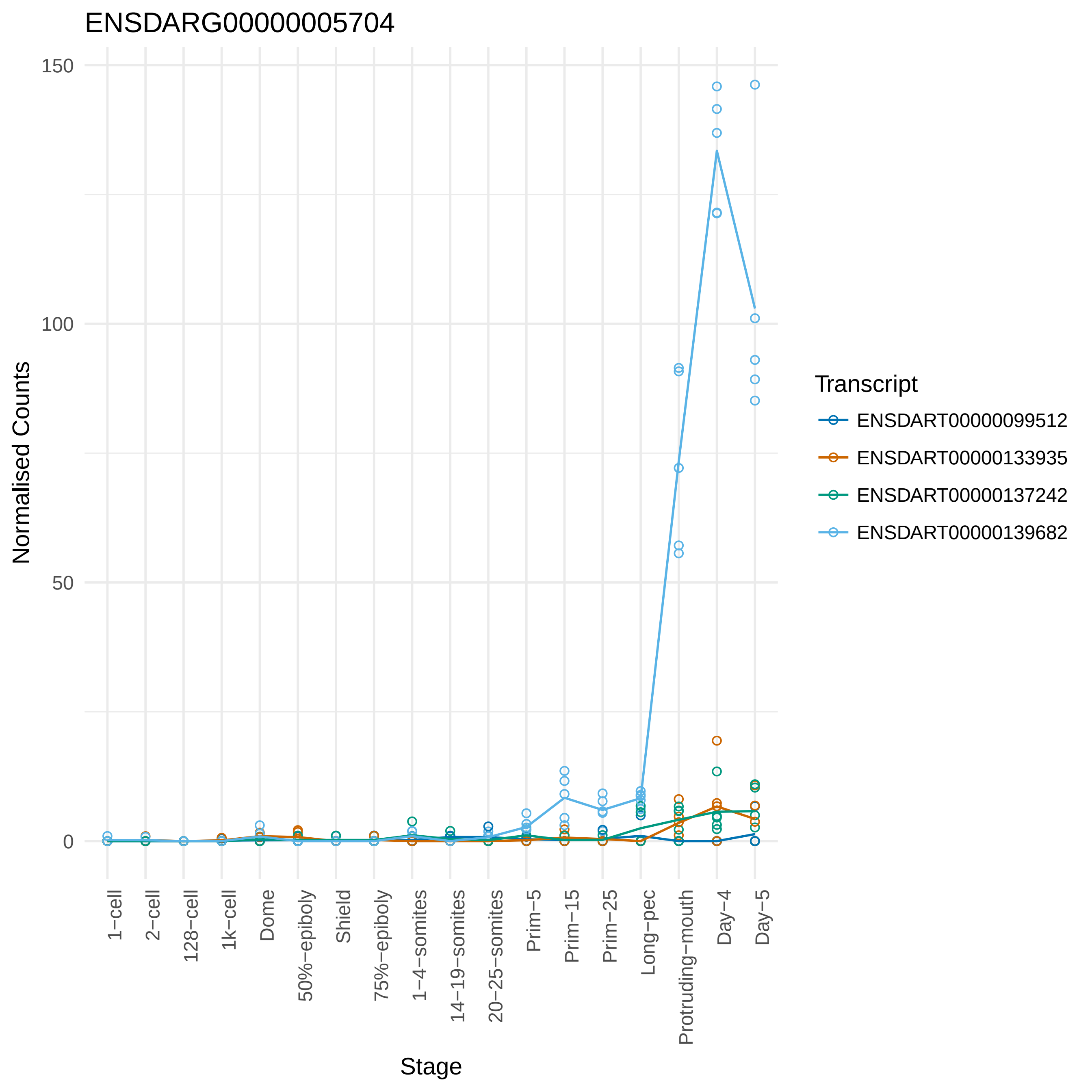

ENSDARG00000005704
Normalised Counts
150
100
50
0
1−cell
2−cell
128−cell
1k−cell
Dome
50%−epiboly
Shield
75%−epiboly
1−4−somites
14−19−somites
20−25−somites
Prim−5
Prim−15
Prim−25
Long−pec
Protruding−mouth
Day−4
Day−5
Stage
Transcript
ENSDART00000099512
ENSDART00000133935
ENSDART00000137242
ENSDART00000139682

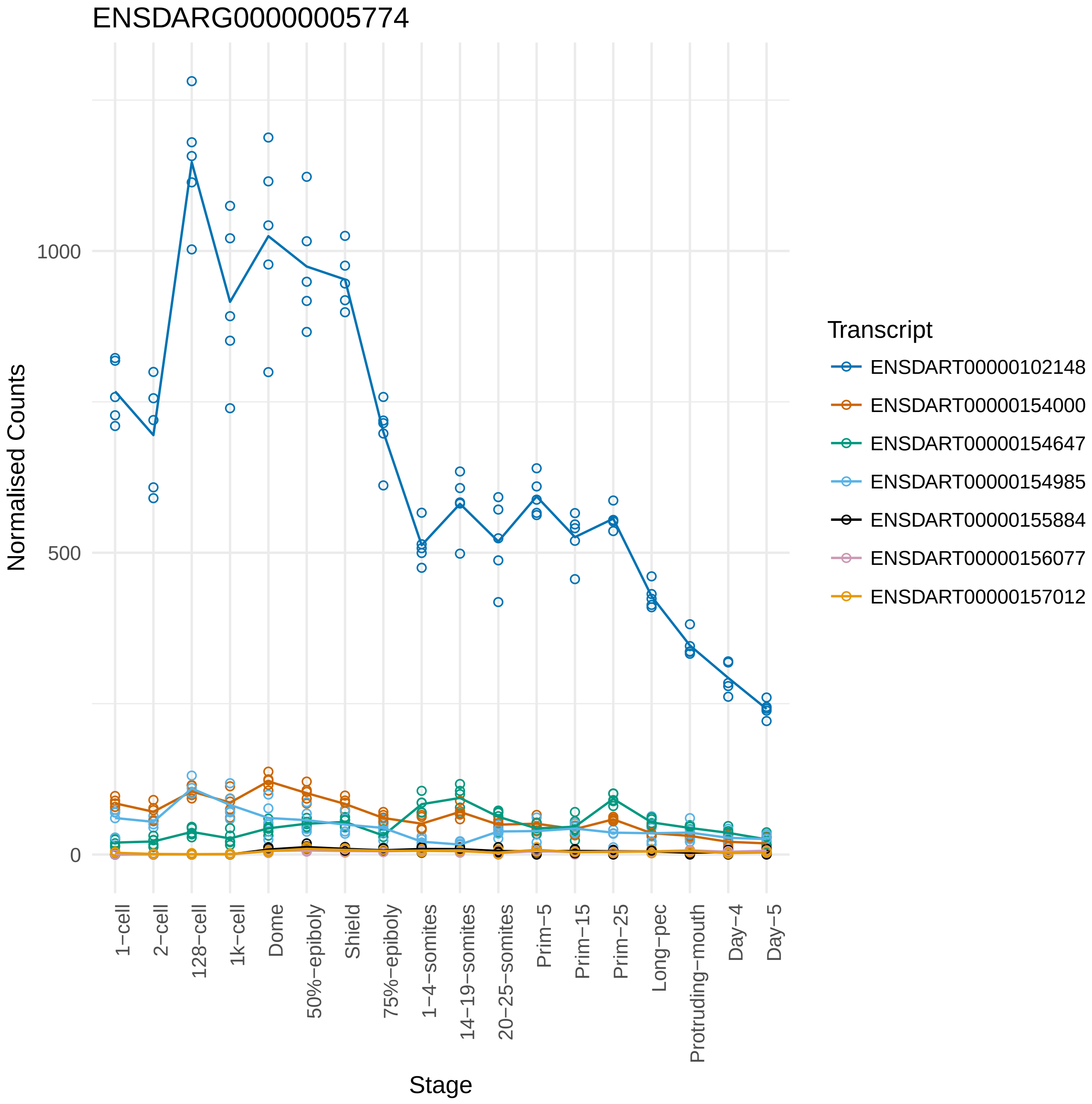

ENSDARG00000005774
Normalised Counts
1000
500
0
1−cell
2−cell
128−cell
1k−cell
Dome
50%−epiboly
Shield
75%−epiboly
1−4−somites
14−19−somites
20−25−somites
Prim−5
Prim−15
Prim−25
Long−pec
Protruding−mouth
Day−4
Day−5
Stage
Transcript
ENSDART00000102148
ENSDART00000154000
ENSDART00000154647
ENSDART00000154985
ENSDART00000155884
ENSDART00000156077
ENSDART00000157012

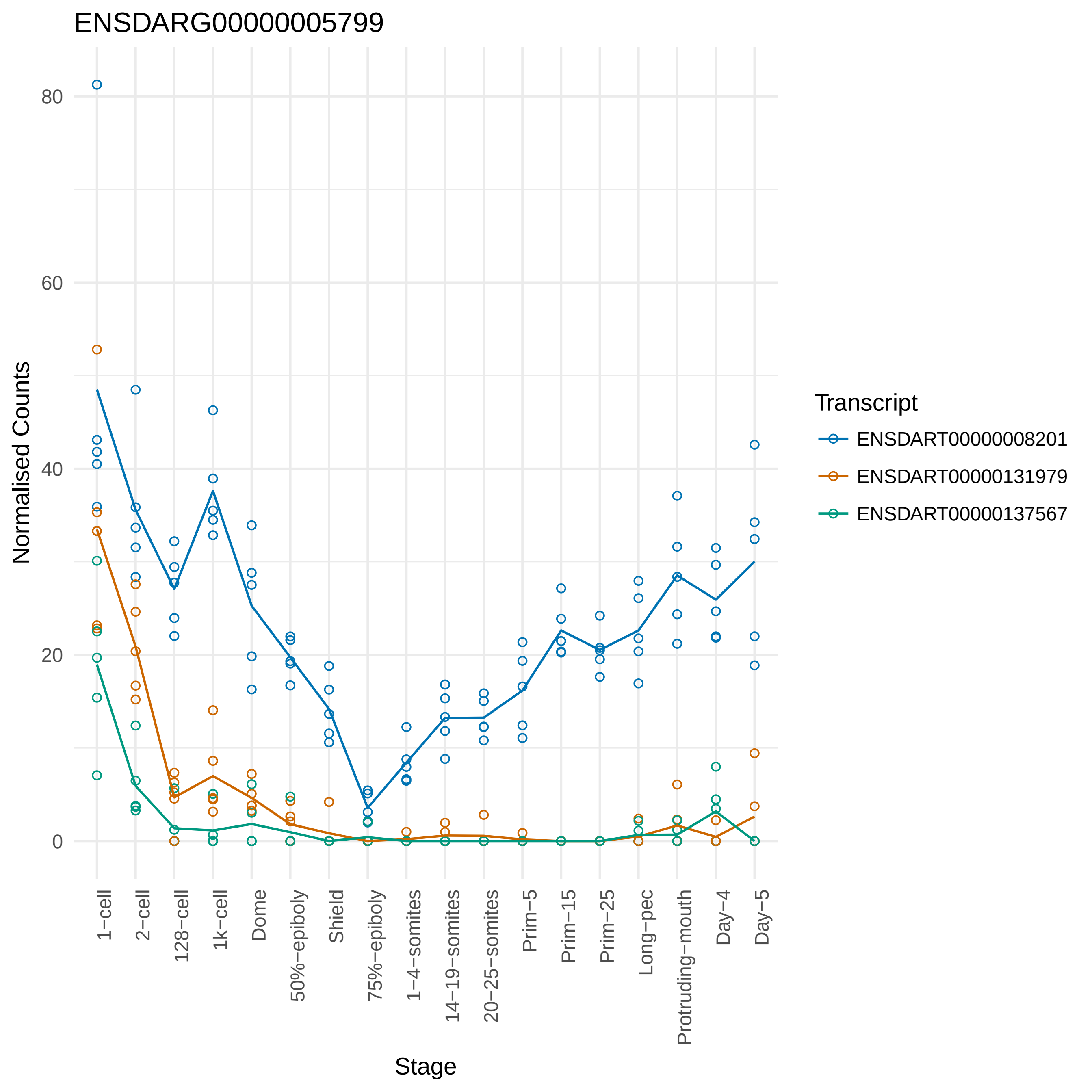
ENSDARG00000005799
Normalised Counts
0
20
40
60
80
1−cell
2−cell
128−cell
1k−cell
Dome
50%−epiboly
Shield
75%−epiboly
1−4−somites
14−19−somites
20−25−somites
Prim−5
Prim−15
Prim−25
Long−pec
Protruding−mouth
Day−4
Day−5
Stage
Transcript
ENSDART00000008201
ENSDART00000131979
ENSDART00000137567

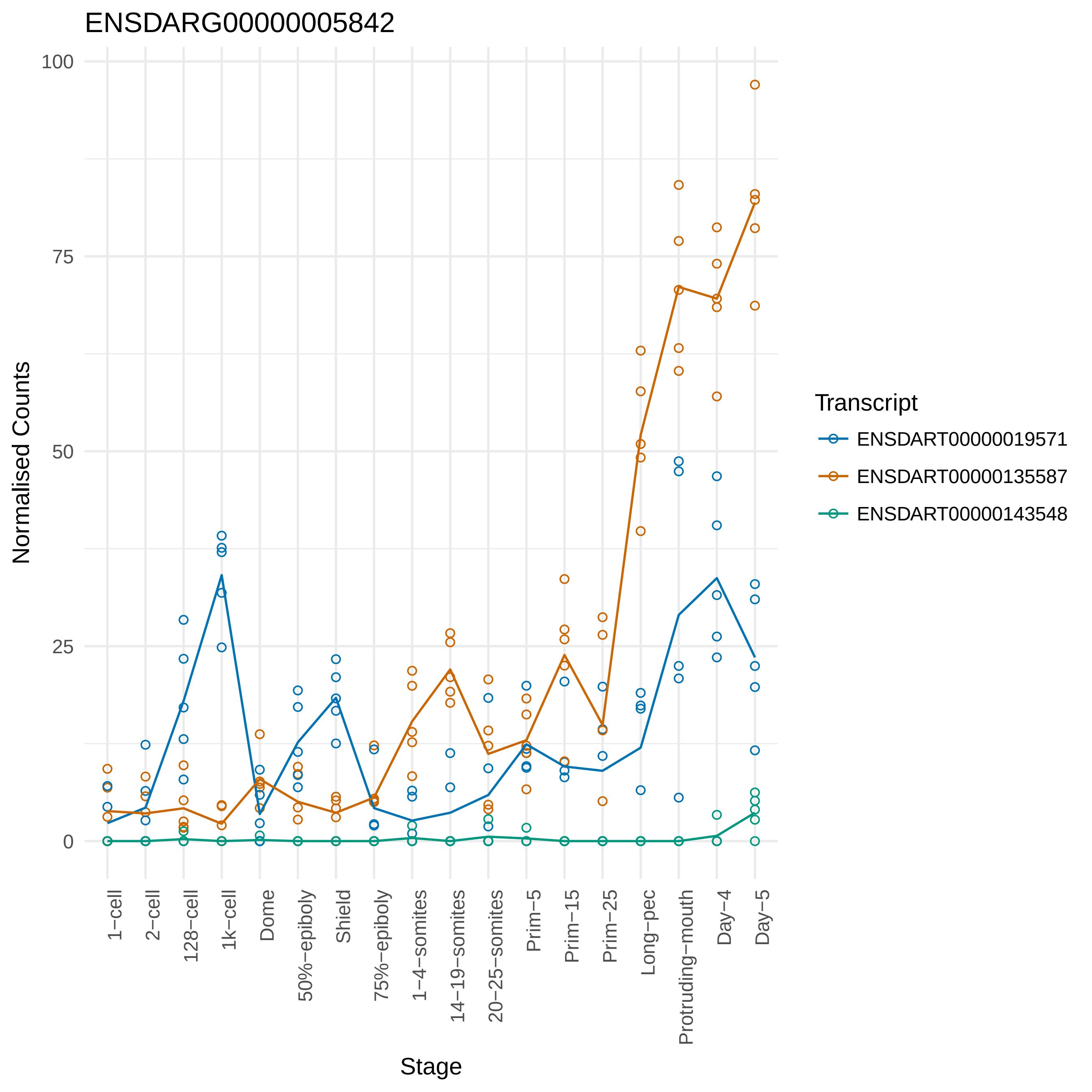
ENSDARG00000005842
Normalised Counts
100
75
50
25
0
Stage
1−cell
2−cell
128−cell
1k−cell
Dome
50%−epiboly
Shield
75%−epiboly
1−4−somites
14−19−somites
20−25−somites
Prim−5
Prim−15
Prim−25
Long−pec
Protruding−mouth
Day−4
Day−5
Transcript
ENSDART00000019571
ENSDART00000135587
ENSDART00000143548

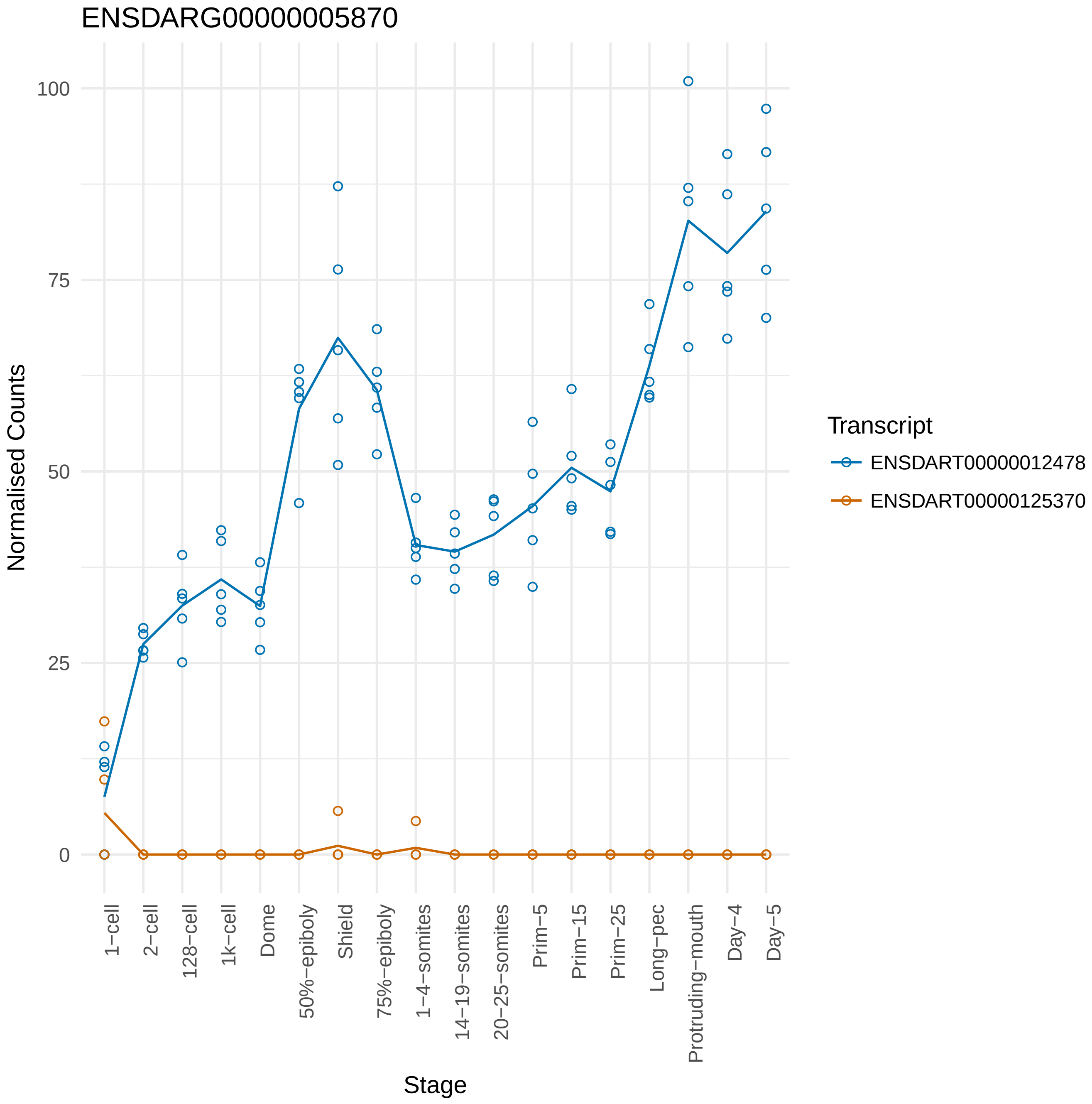

ENSDARG00000005870
Normalised Counts
100
75
50
25
0
1−cell
2−cell
128−cell
1k−cell
Dome
50%−epiboly
Shield
75%−epiboly
1−4−somites
14−19−somites
20−25−somites
Prim−5
Prim−15
Prim−25
Long−pec
Protruding−mouth
Day−4
Day−5
Stage
Transcript
ENSDART00000012478
ENSDART00000125370

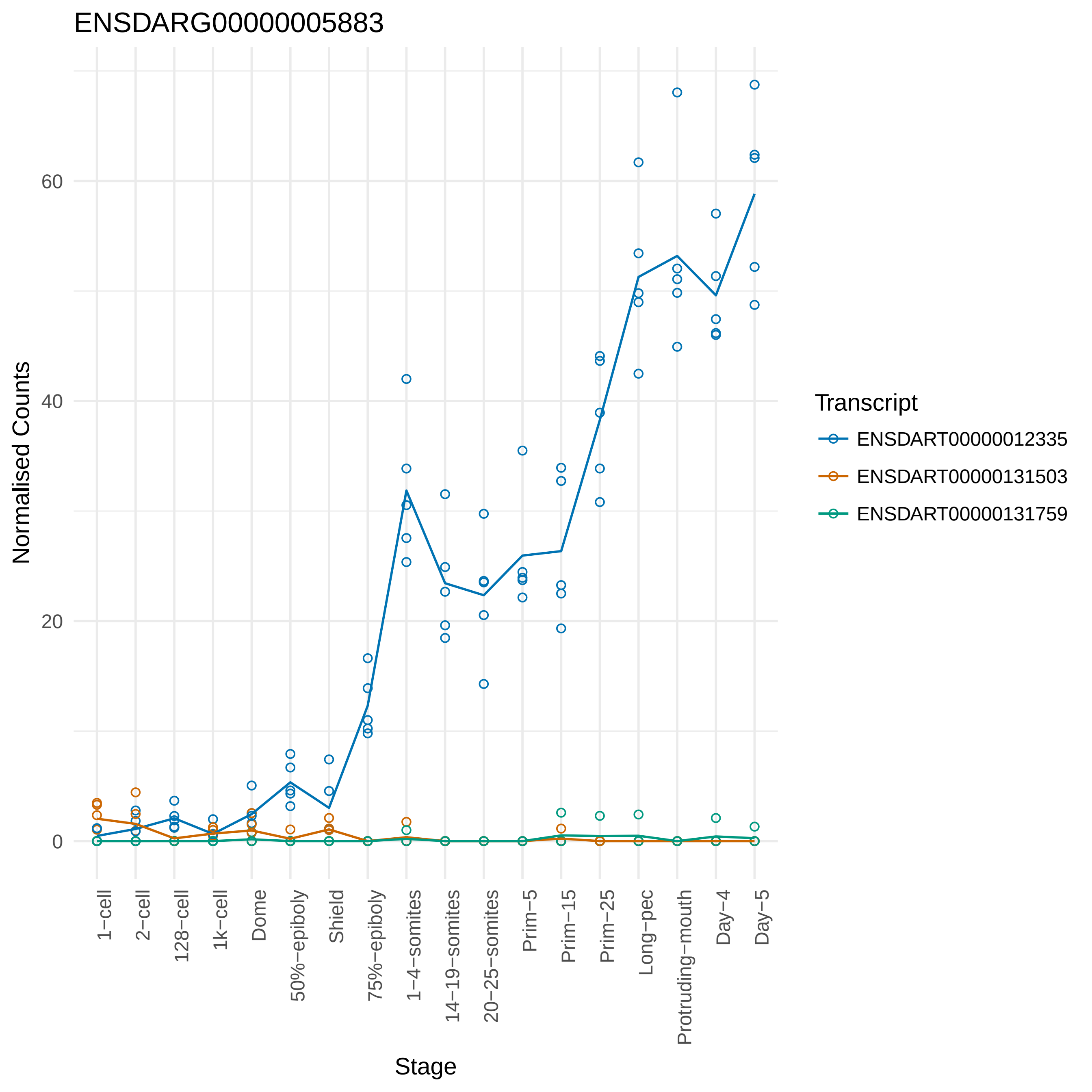
ENSDARG00000005883
Normalised Counts
0
20
40
60
Stage
1−cell
2−cell
128−cell
1k−cell
Dome
50%−epiboly
Shield
75%−epiboly
1−4−somites
14−19−somites
20−25−somites
Prim−5
Prim−15
Prim−25
Long−pec
Protruding−mouth
Day−4
Day−5
Transcript
ENSDART00000012335
ENSDART00000131503
ENSDART00000131759

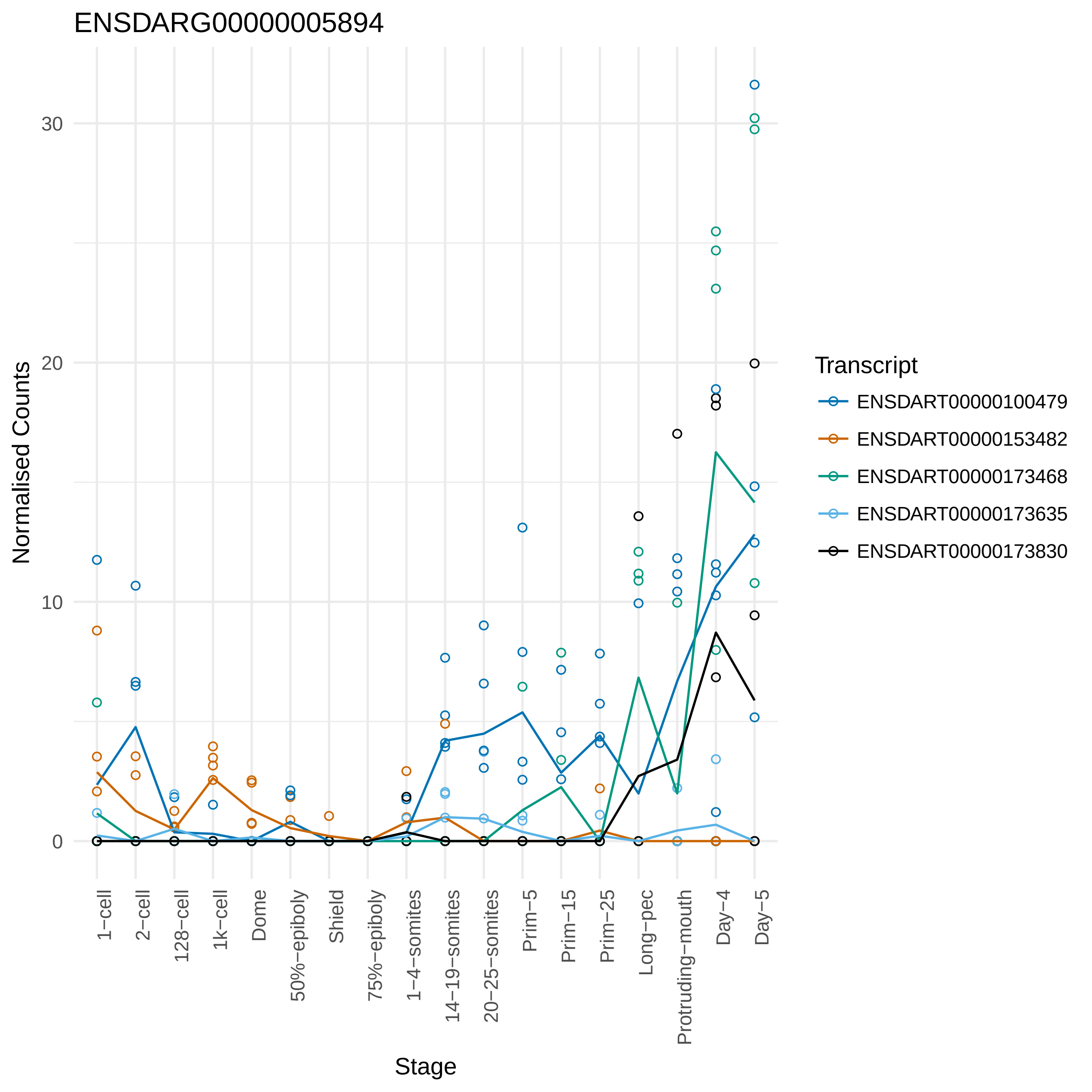
ENSDARG00000005894
Normalised Counts
30
20
10
0
Stage
1−cell
2−cell
128−cell
1k−cell
Dome
50%−epiboly
Shield
75%−epiboly
1−4−somites
14−19−somites
20−25−somites
Prim−5
Prim−15
Prim−25
Long−pec
Protruding−mouth
Day−4
Day−5
Transcript
ENSDART00000100479
ENSDART00000153482
ENSDART00000173468
ENSDART00000173635
ENSDART00000173830

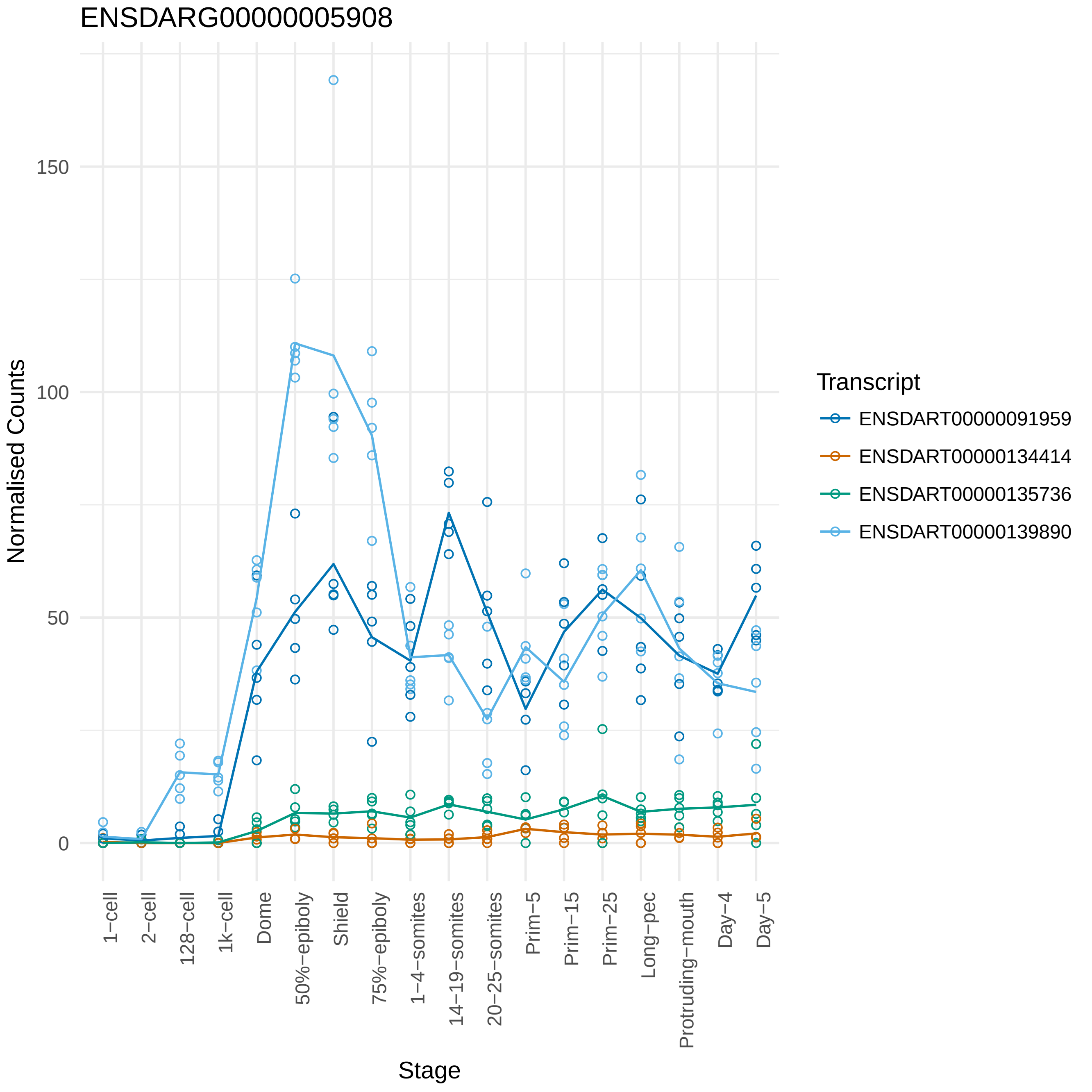
ENSDARG00000005908
Normalised Counts
150
100
50
0
1−cell
2−cell
128−cell
1k−cell
Dome
50%−epiboly
Shield
75%−epiboly
1−4−somites
14−19−somites
20−25−somites
Prim−5
Prim−15
Prim−25
Long−pec
Protruding−mouth
Day−4
Day−5
Stage
Transcript
ENSDART00000091959
ENSDART00000134414
ENSDART00000135736
ENSDART00000139890

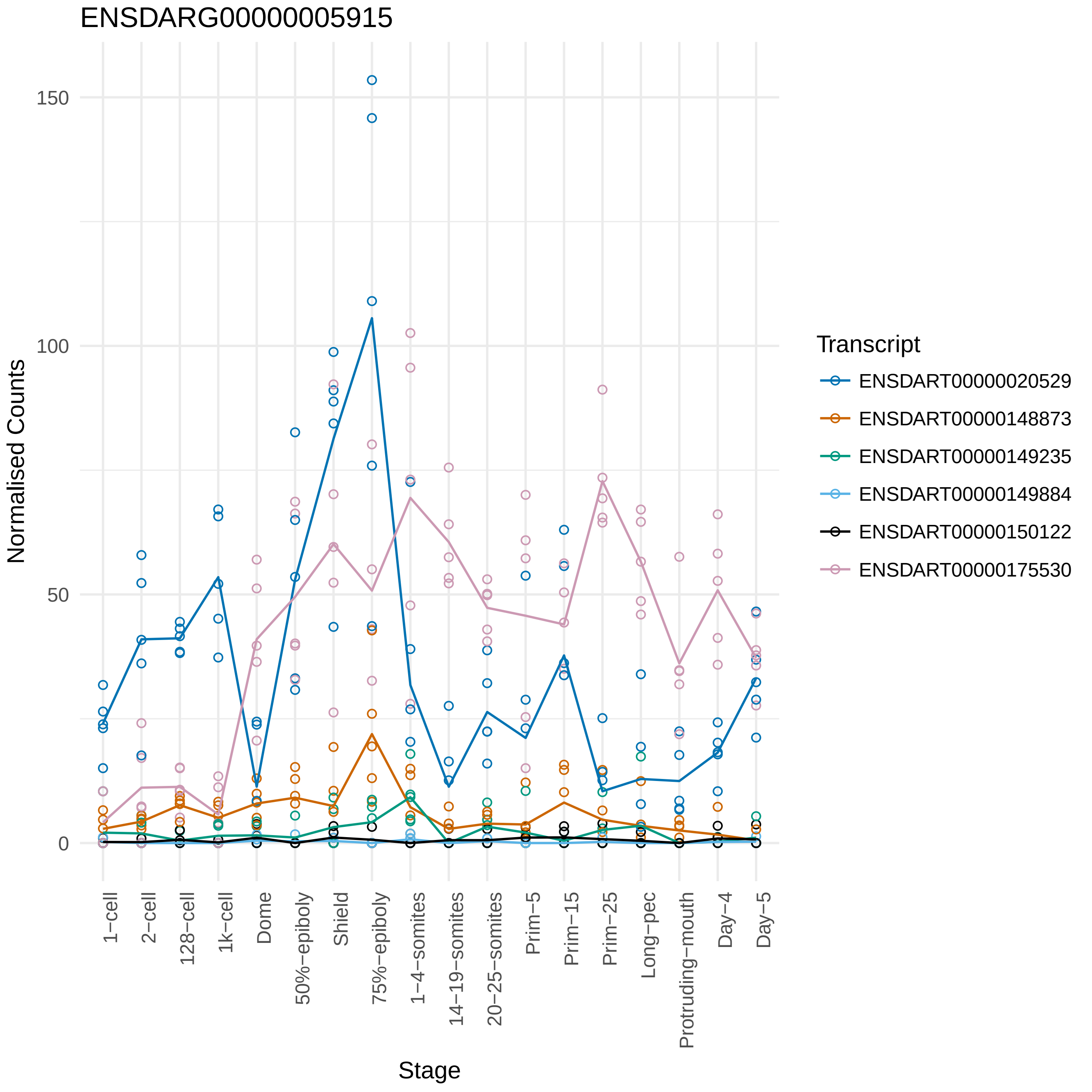
ENSDARG00000005915
Normalised Counts
150
100
50
0
1−cell
2−cell
128−cell
1k−cell
Dome
50%−epiboly
Shield
75%−epiboly
1−4−somites
14−19−somites
20−25−somites
Prim−5
Prim−15
Prim−25
Long−pec
Protruding−mouth
Day−4
Day−5
Stage
Transcript
ENSDART00000020529
ENSDART00000148873
ENSDART00000149235
ENSDART00000149884
ENSDART00000150122
ENSDART00000175530

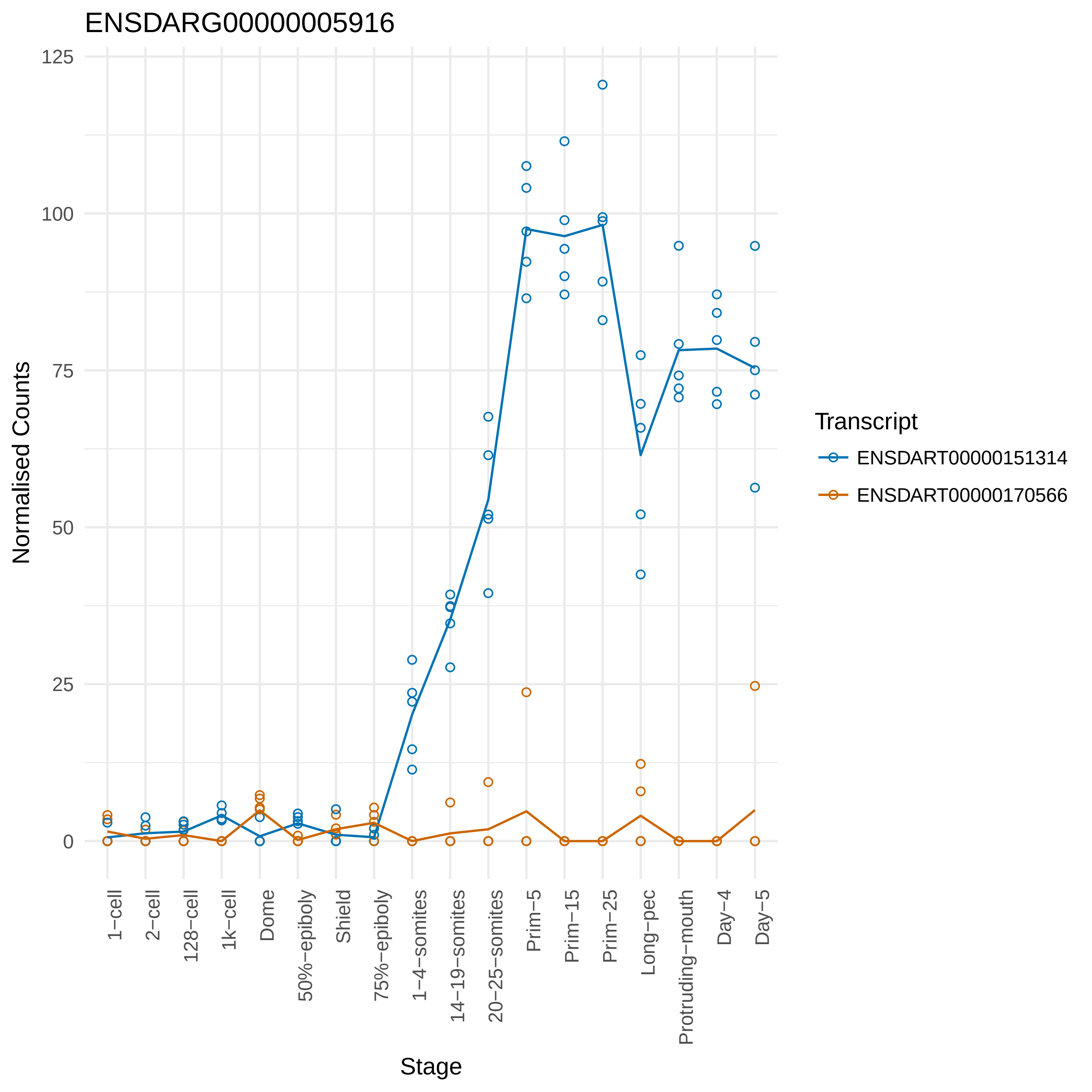

ENSDARG00000005916
Normalised Counts
125
100
75
50
25
0
1−cell
2−cell
128−cell
1k−cell
Dome
50%−epiboly
Shield
75%−epiboly
1−4−somites
14−19−somites
20−25−somites
Prim−5
Prim−15
Prim−25
Long−pec
Protruding−mouth
Day−4
Day−5
Stage
Transcript
ENSDART00000151314
ENSDART00000170566

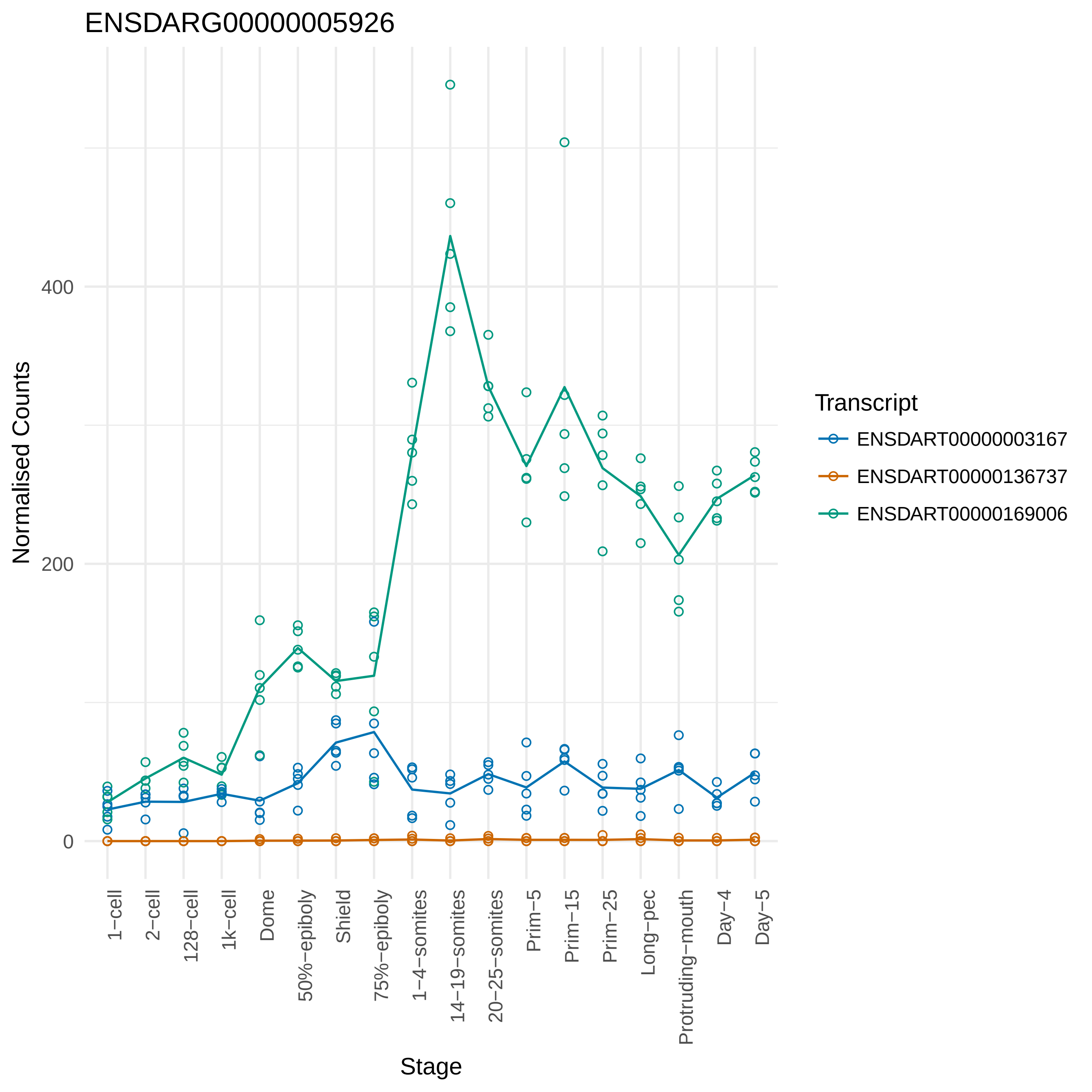

ENSDARG00000005926
Normalised Counts
400
200
0
1−cell
2−cell
128−cell
1k−cell
Dome
50%−epiboly
Shield
75%−epiboly
1−4−somites
14−19−somites
20−25−somites
Prim−5
Prim−15
Prim−25
Long−pec
Protruding−mouth
Day−4
Day−5
Stage
Transcript
ENSDART00000003167
ENSDART00000136737
ENSDART00000169006

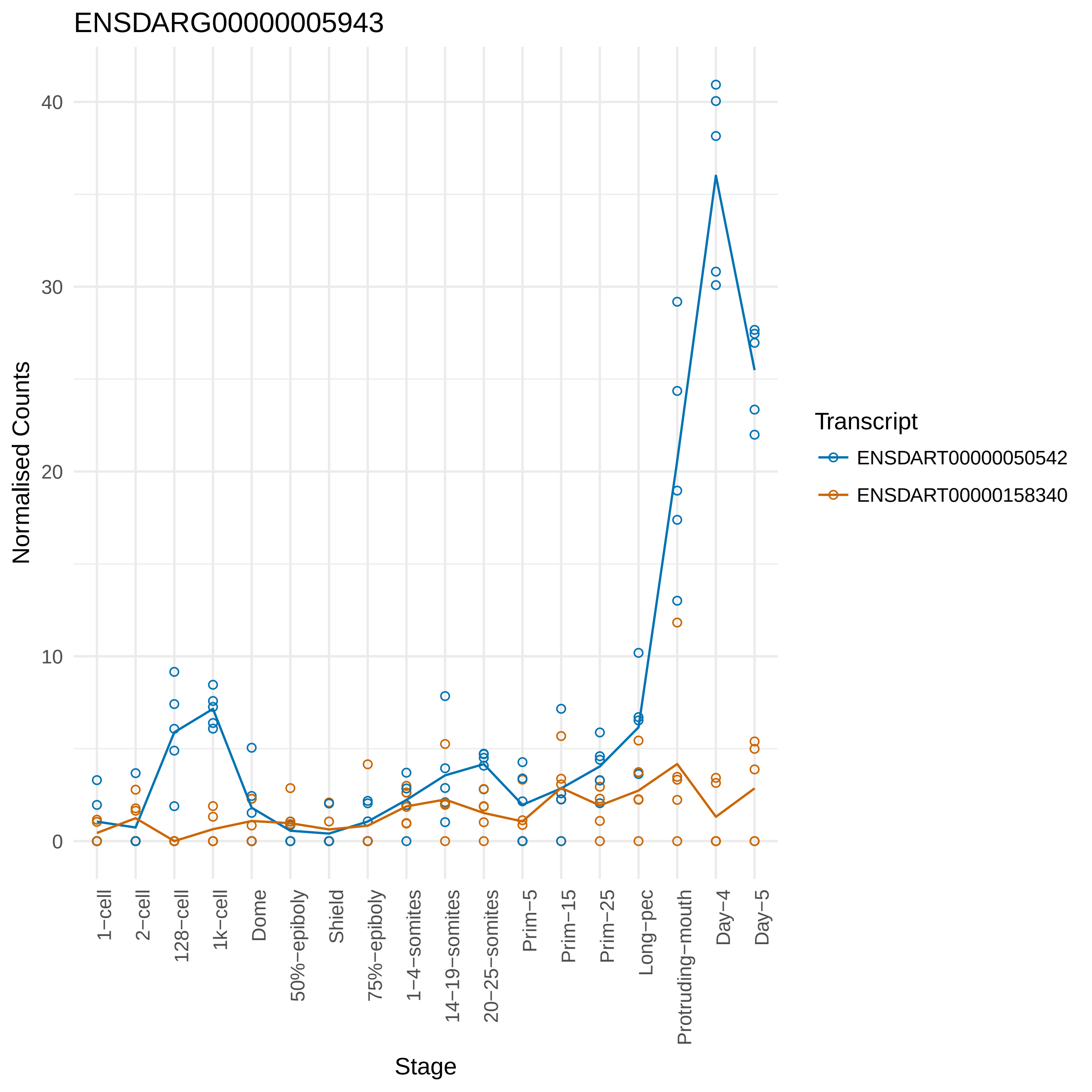

ENSDARG00000005943
Normalised Counts
0
10
20
30
40
1−cell
2−cell
128−cell
1k−cell
Dome
50%−epiboly
Shield
75%−epiboly
1−4−somites
14−19−somites
20−25−somites
Prim−5
Prim−15
Prim−25
Long−pec
Protruding−mouth
Day−4
Day−5
Stage
Transcript
ENSDART00000050542
ENSDART00000158340

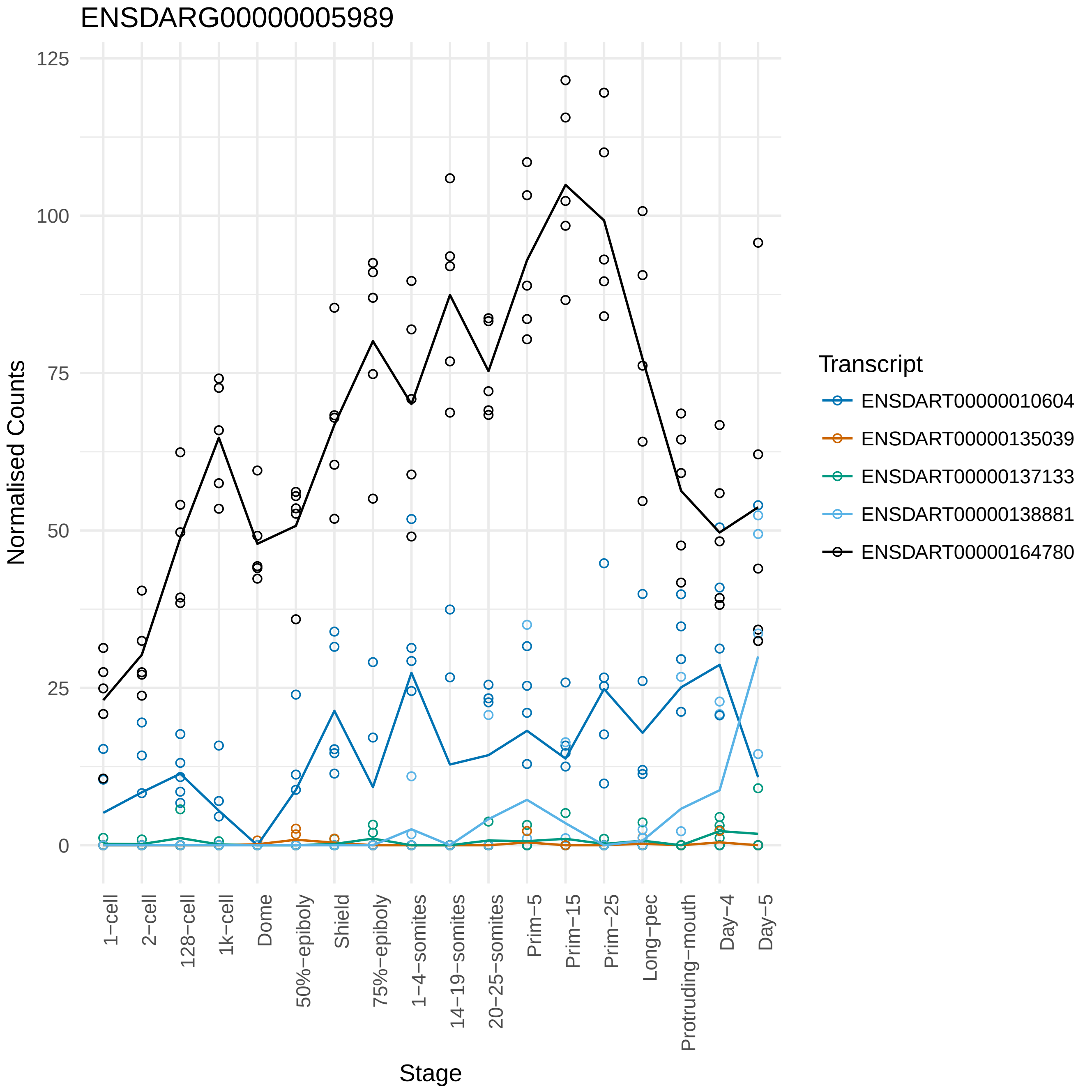

ENSDARG00000005989
Normalised Counts
125
100
75
50
25
0
Stage
1−cell
2−cell
128−cell
1k−cell
Dome
50%−epiboly
Shield
75%−epiboly
1−4−somites
14−19−somites
20−25−somites
Prim−5
Prim−15
Prim−25
Long−pec
Protruding−mouth
Day−4
Day−5
Transcript
ENSDART00000010604
ENSDART00000135039
ENSDART00000137133
ENSDART00000138881
ENSDART00000164780

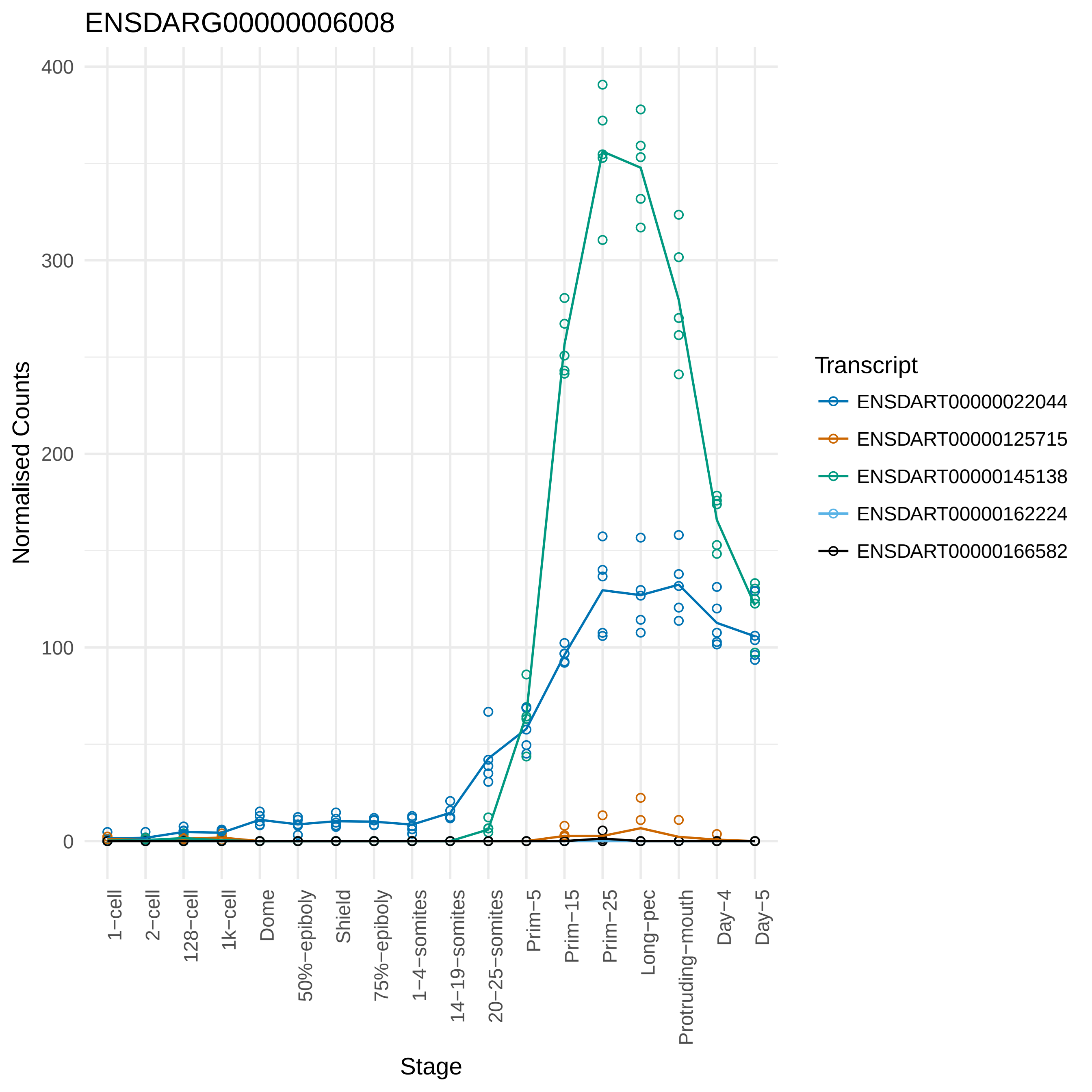
ENSDARG00000006008
Normalised Counts
400
300
200
100
0
1−cell
2−cell
128−cell
1k−cell
Dome
50%−epiboly
Shield
75%−epiboly
1−4−somites
14−19−somites
20−25−somites
Prim−5
Prim−15
Prim−25
Long−pec
Protruding−mouth
Day−4
Day−5
Stage
Transcript
ENSDART00000022044
ENSDART00000125715
ENSDART00000145138
ENSDART00000162224
ENSDART00000166582

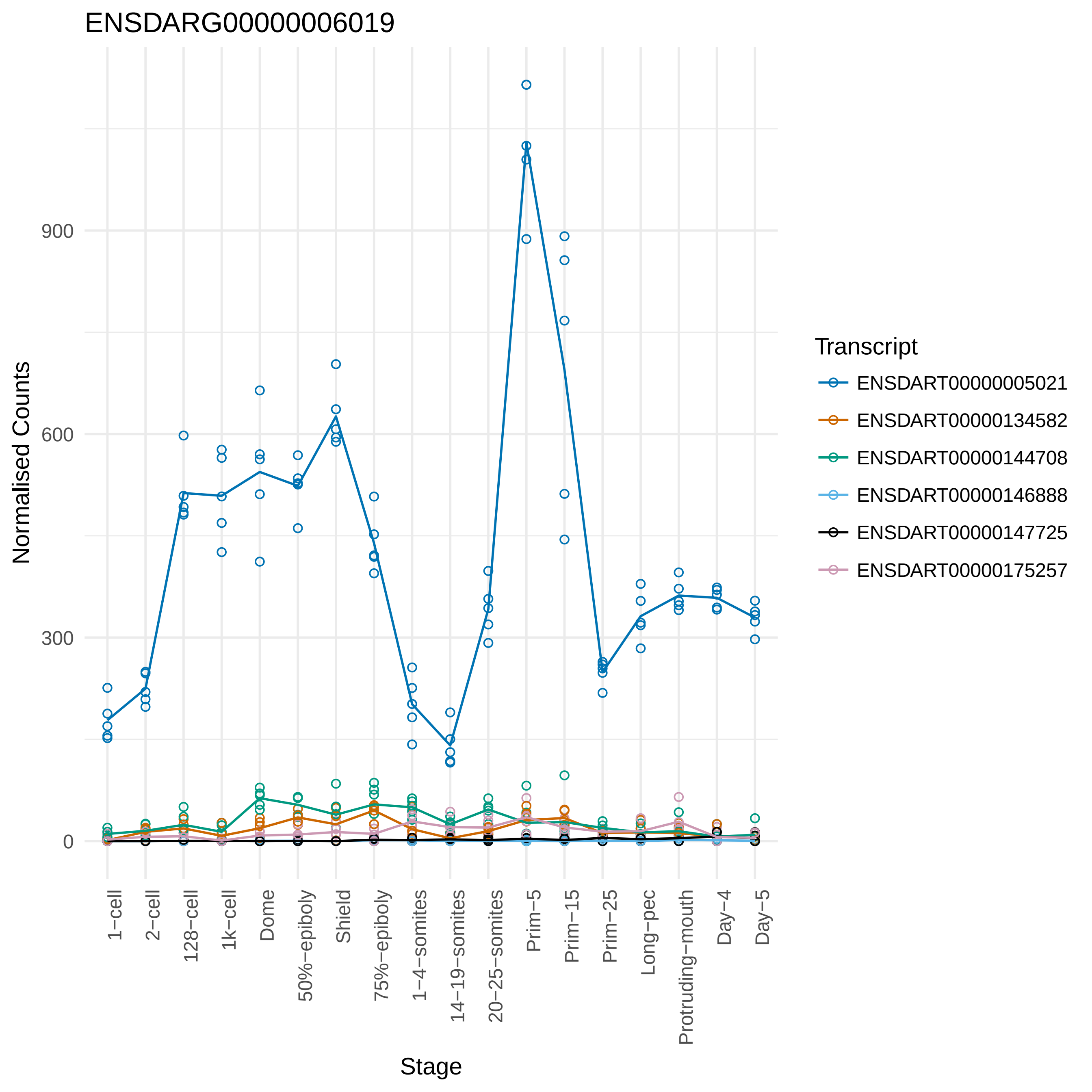
ENSDARG00000006019
Normalised Counts
0
300
600
900
1−cell
2−cell
128−cell
1k−cell
Dome
50%−epiboly
Shield
75%−epiboly
1−4−somites
14−19−somites
20−25−somites
Prim−5
Prim−15
Prim−25
Long−pec
Protruding−mouth
Day−4
Day−5
Stage
Transcript
ENSDART00000005021
ENSDART00000134582
ENSDART00000144708
ENSDART00000146888
ENSDART00000147725
ENSDART00000175257

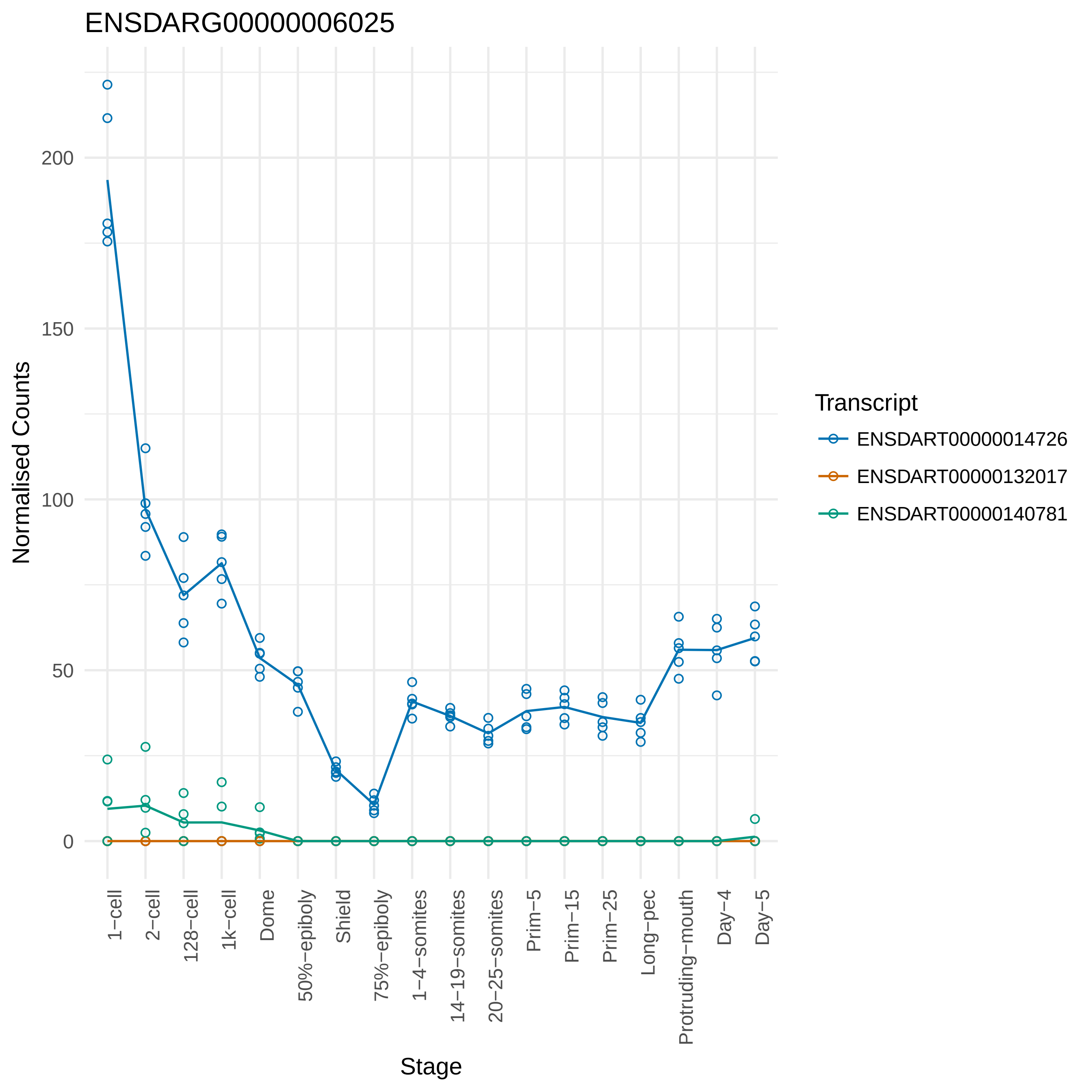

ENSDARG00000006025
Normalised Counts
200
150
100
50
0
1−cell
2−cell
128−cell
1k−cell
Dome
50%−epiboly
Shield
75%−epiboly
1−4−somites
14−19−somites
20−25−somites
Prim−5
Prim−15
Prim−25
Long−pec
Protruding−mouth
Day−4
Day−5
Stage
Transcript
ENSDART00000014726
ENSDART00000132017
ENSDART00000140781

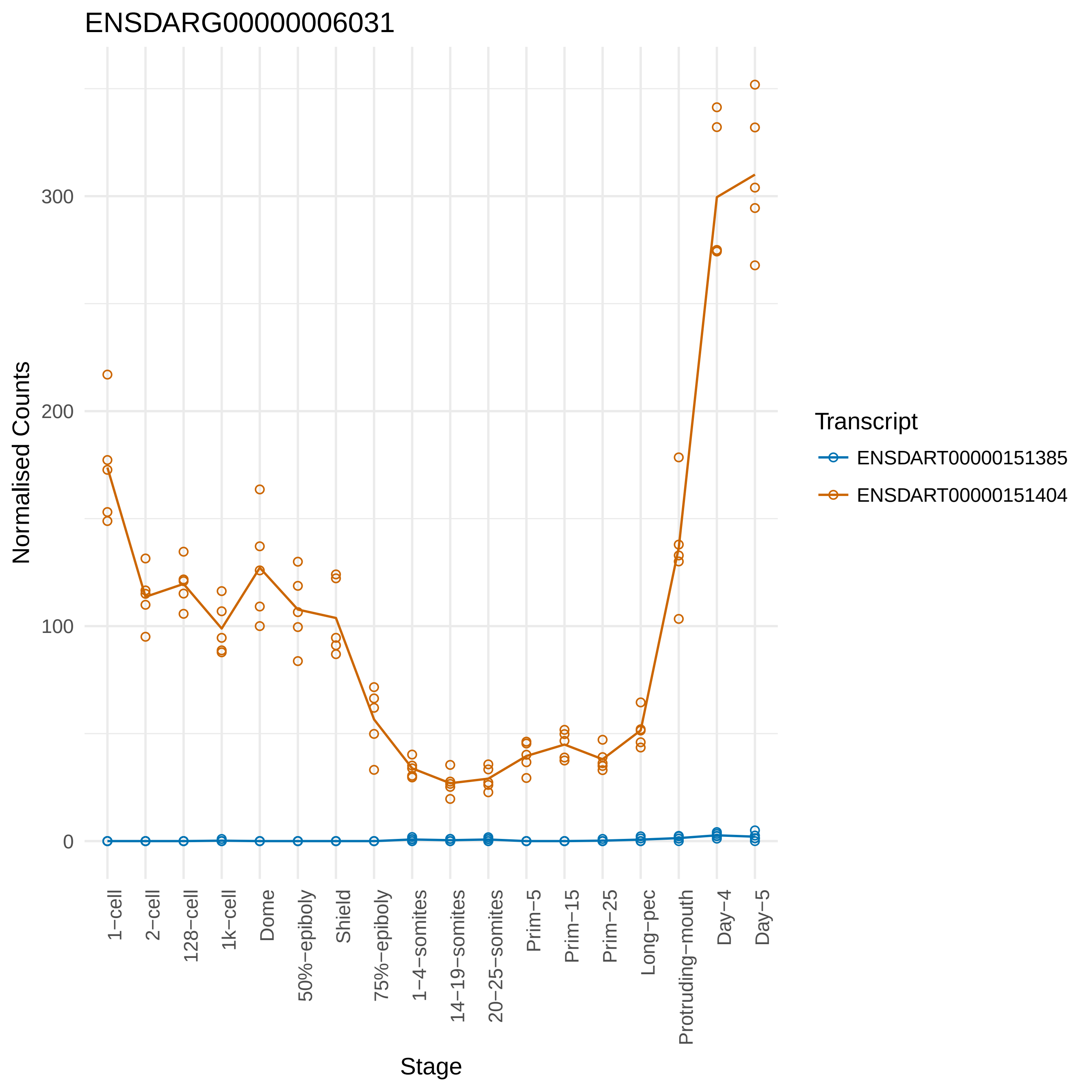

ENSDARG00000006031
Normalised Counts
300
200
100
0
1−cell
2−cell
128−cell
1k−cell
Dome
50%−epiboly
Shield
75%−epiboly
1−4−somites
14−19−somites
20−25−somites
Prim−5
Prim−15
Prim−25
Long−pec
Protruding−mouth
Day−4
Day−5
Stage
Transcript
ENSDART00000151385
ENSDART00000151404

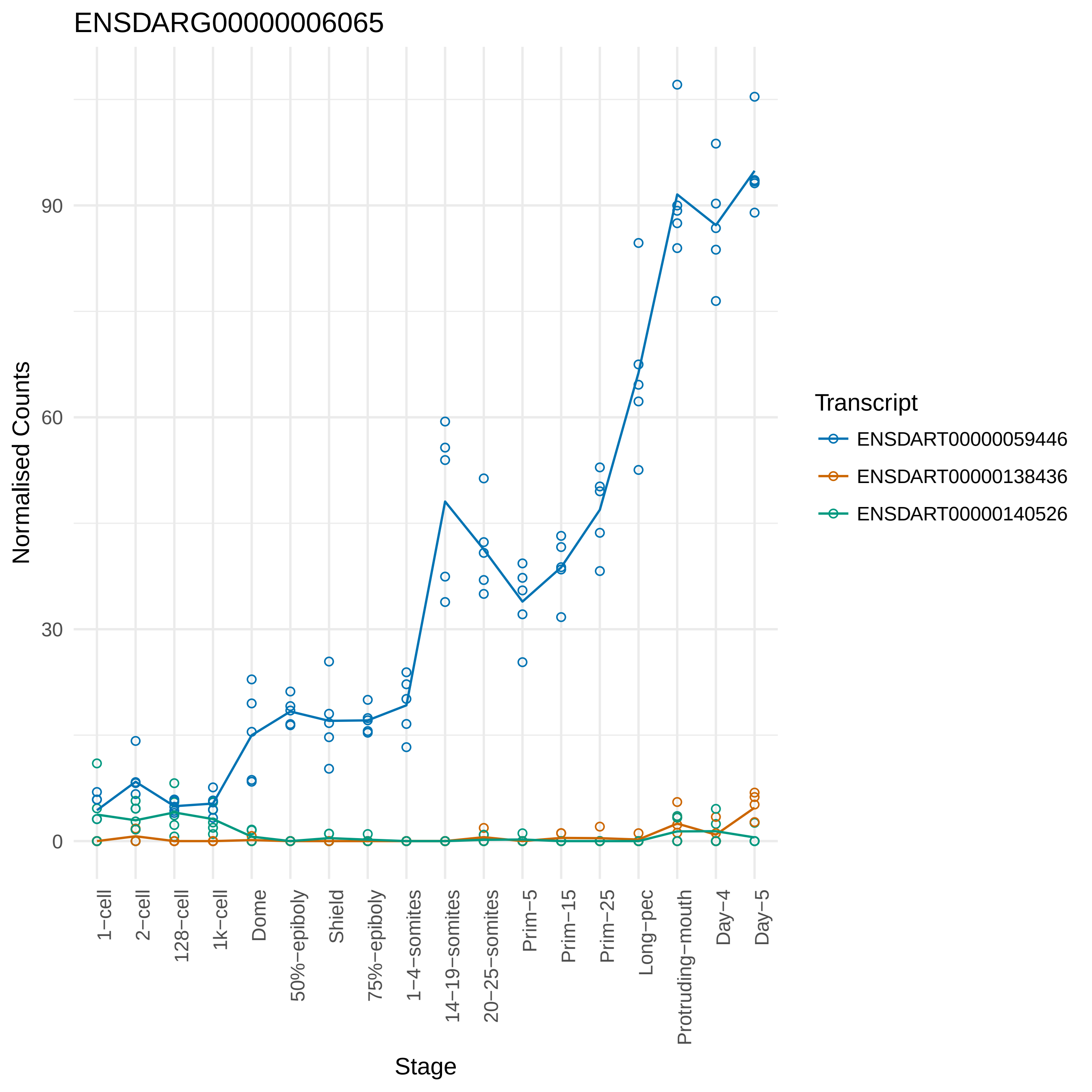

ENSDARG00000006065
Normalised Counts
0
30
60
90
Stage
1−cell
2−cell
128−cell
1k−cell
Dome
50%−epiboly
Shield
75%−epiboly
1−4−somites
14−19−somites
20−25−somites
Prim−5
Prim−15
Prim−25
Long−pec
Protruding−mouth
Day−4
Day−5
Transcript
ENSDART00000059446
ENSDART00000138436
ENSDART00000140526

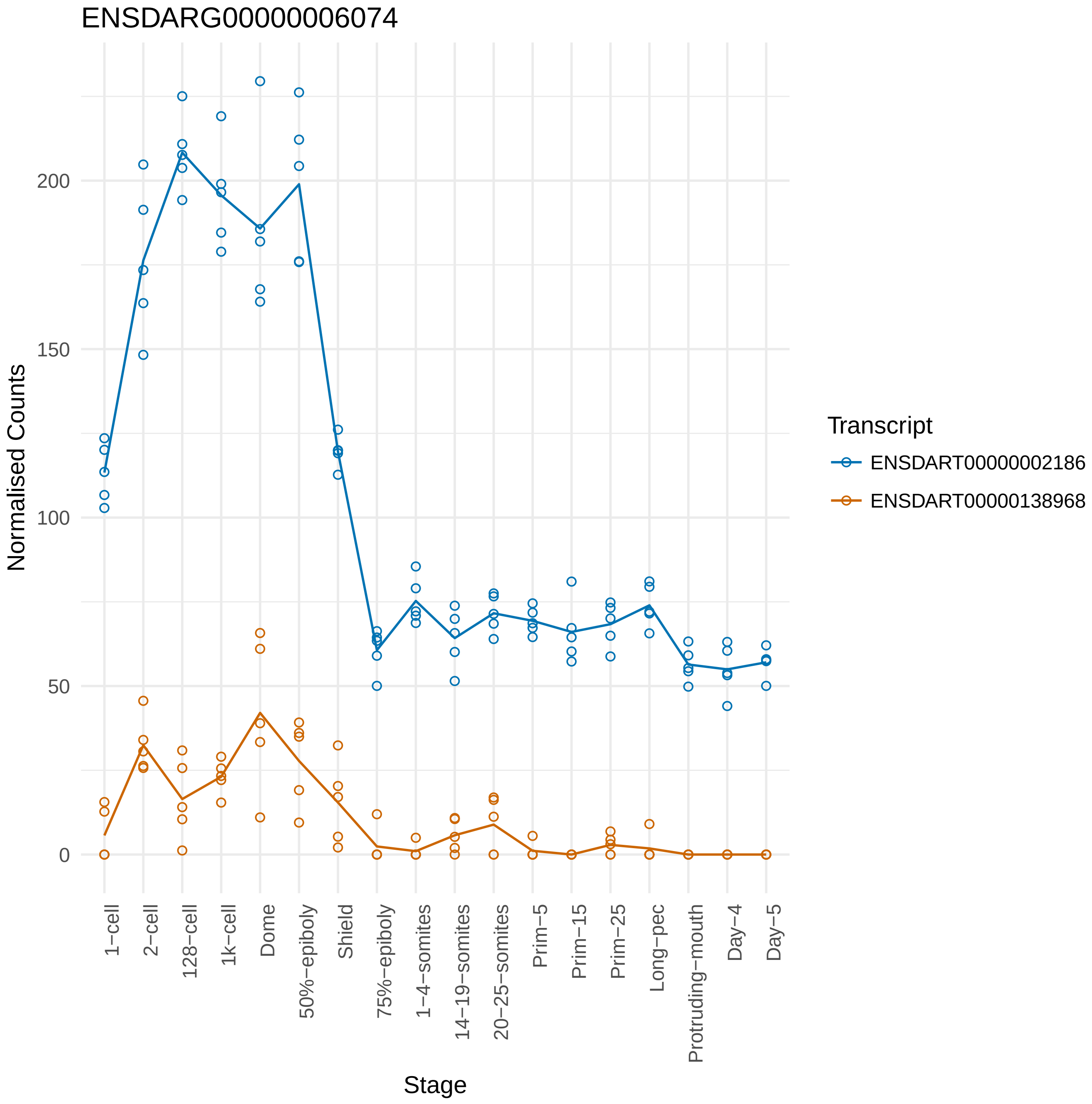
ENSDARG00000006074
Normalised Counts
0
50
100
150
200
Stage
1−cell
2−cell
128−cell
1k−cell
Dome
50%−epiboly
Shield
75%−epiboly
1−4−somites
14−19−somites
20−25−somites
Prim−5
Prim−15
Prim−25
Long−pec
Protruding−mouth
Day−4
Day−5
Transcript
ENSDART00000002186
ENSDART00000138968

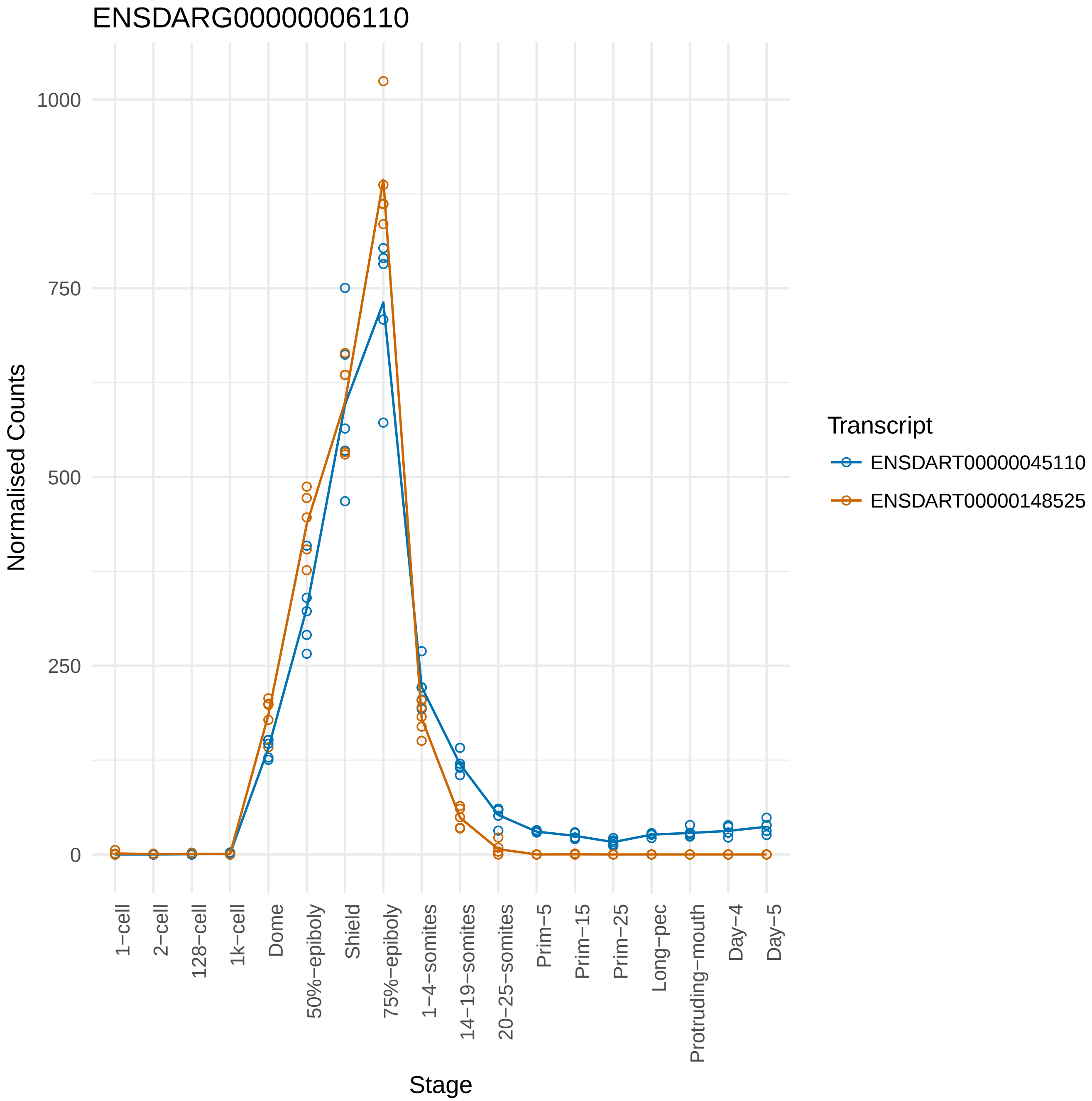

ENSDARG00000006110
Normalised Counts
1000
750
500
250
0
1−cell
2−cell
128−cell
1k−cell
Dome
50%−epiboly
Shield
75%−epiboly
1−4−somites
14−19−somites
20−25−somites
Prim−5
Prim−15
Prim−25
Long−pec
Protruding−mouth
Day−4
Day−5
Stage
Transcript
ENSDART00000045110
ENSDART00000148525

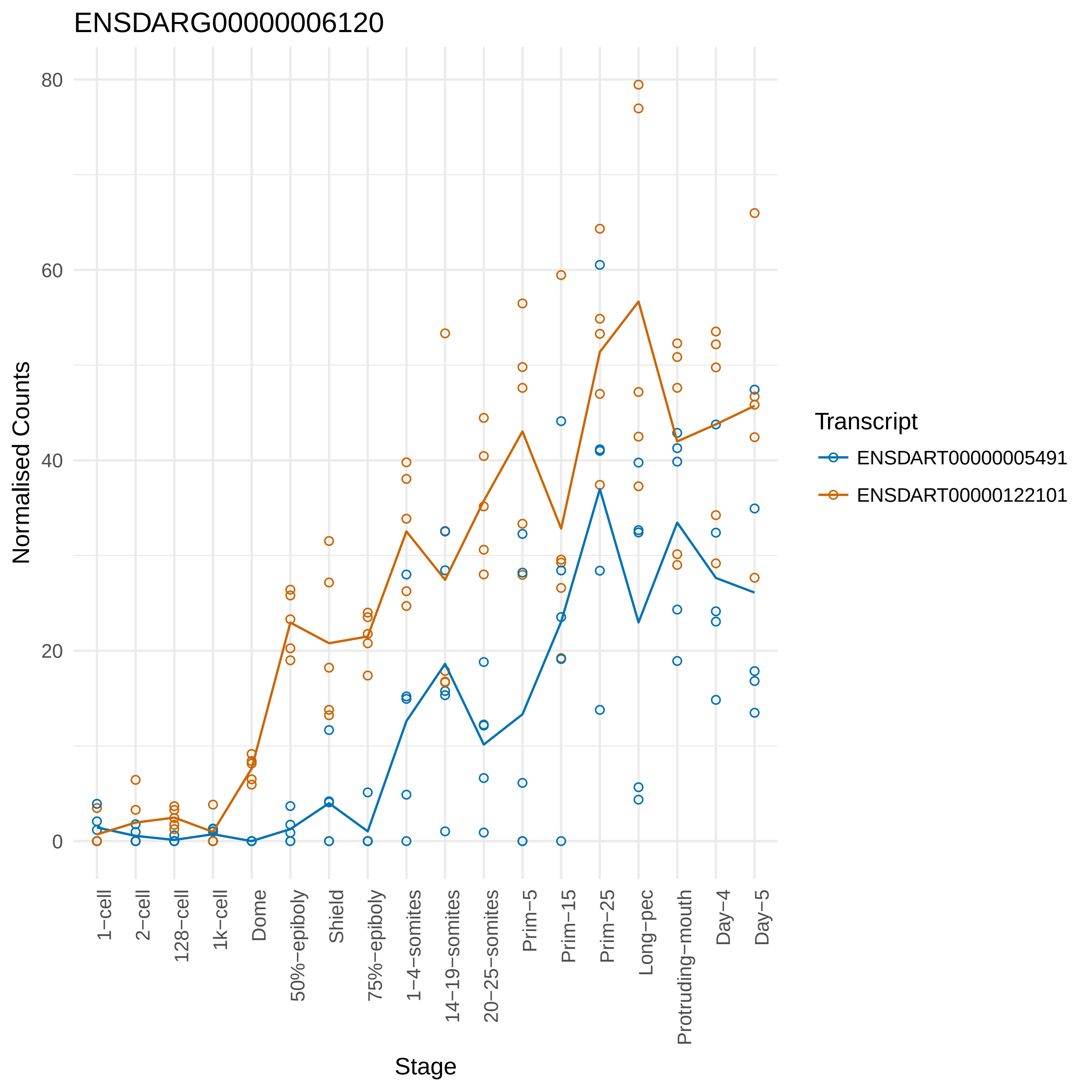

ENSDARG00000006120
Normalised Counts
0
20
40
60
80
Stage
1−cell
2−cell
128−cell
1k−cell
Dome
50%−epiboly
Shield
75%−epiboly
1−4−somites
14−19−somites
20−25−somites
Prim−5
Prim−15
Prim−25
Long−pec
Protruding−mouth
Day−4
Day−5
Transcript
ENSDART00000005491
ENSDART00000122101

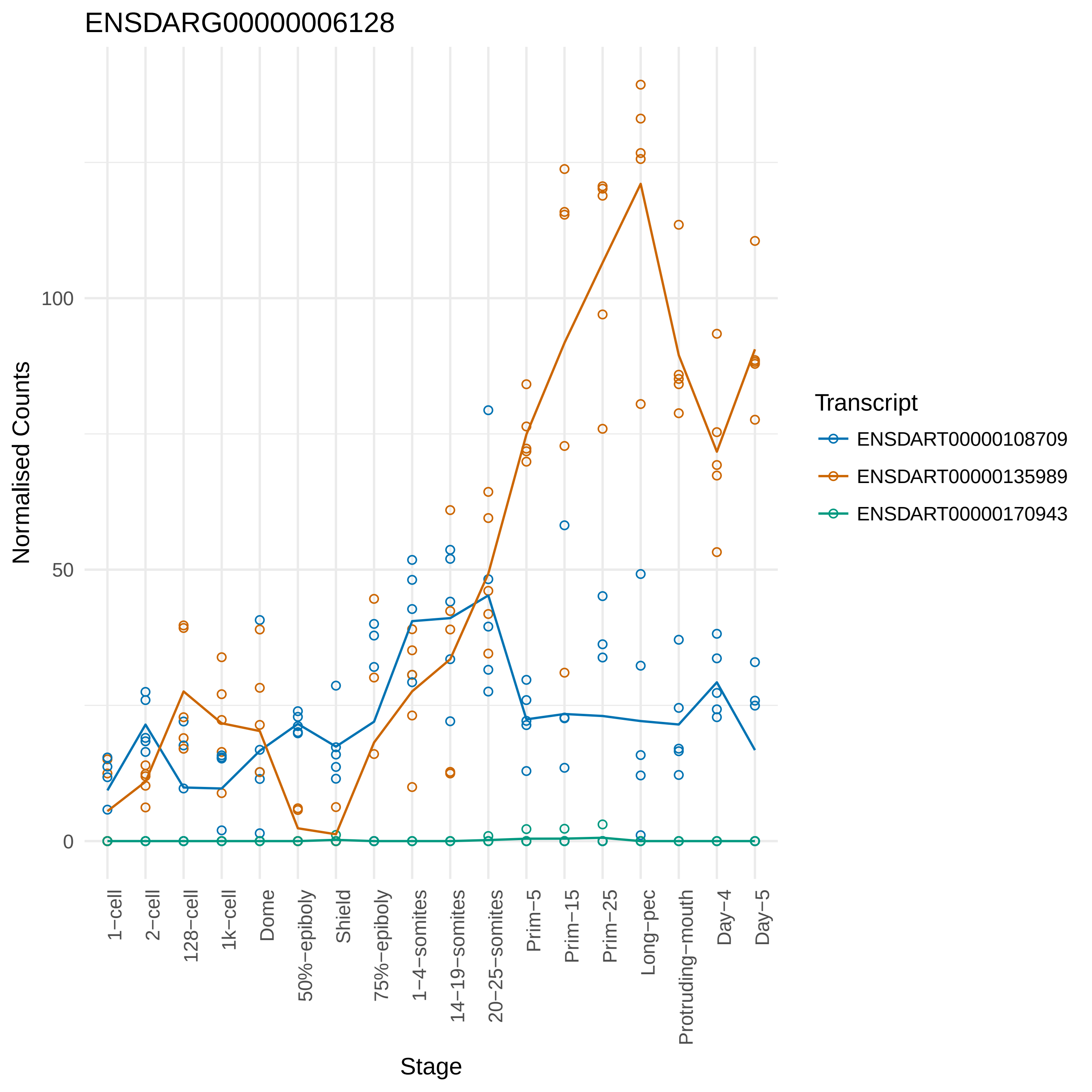
ENSDARG00000006128
Normalised Counts
100
50
0
1−cell
2−cell
128−cell
1k−cell
Dome
50%−epiboly
Shield
75%−epiboly
1−4−somites
14−19−somites
20−25−somites
Prim−5
Prim−15
Prim−25
Long−pec
Protruding−mouth
Day−4
Day−5
Stage
Transcript
ENSDART00000108709
ENSDART00000135989
ENSDART00000170943

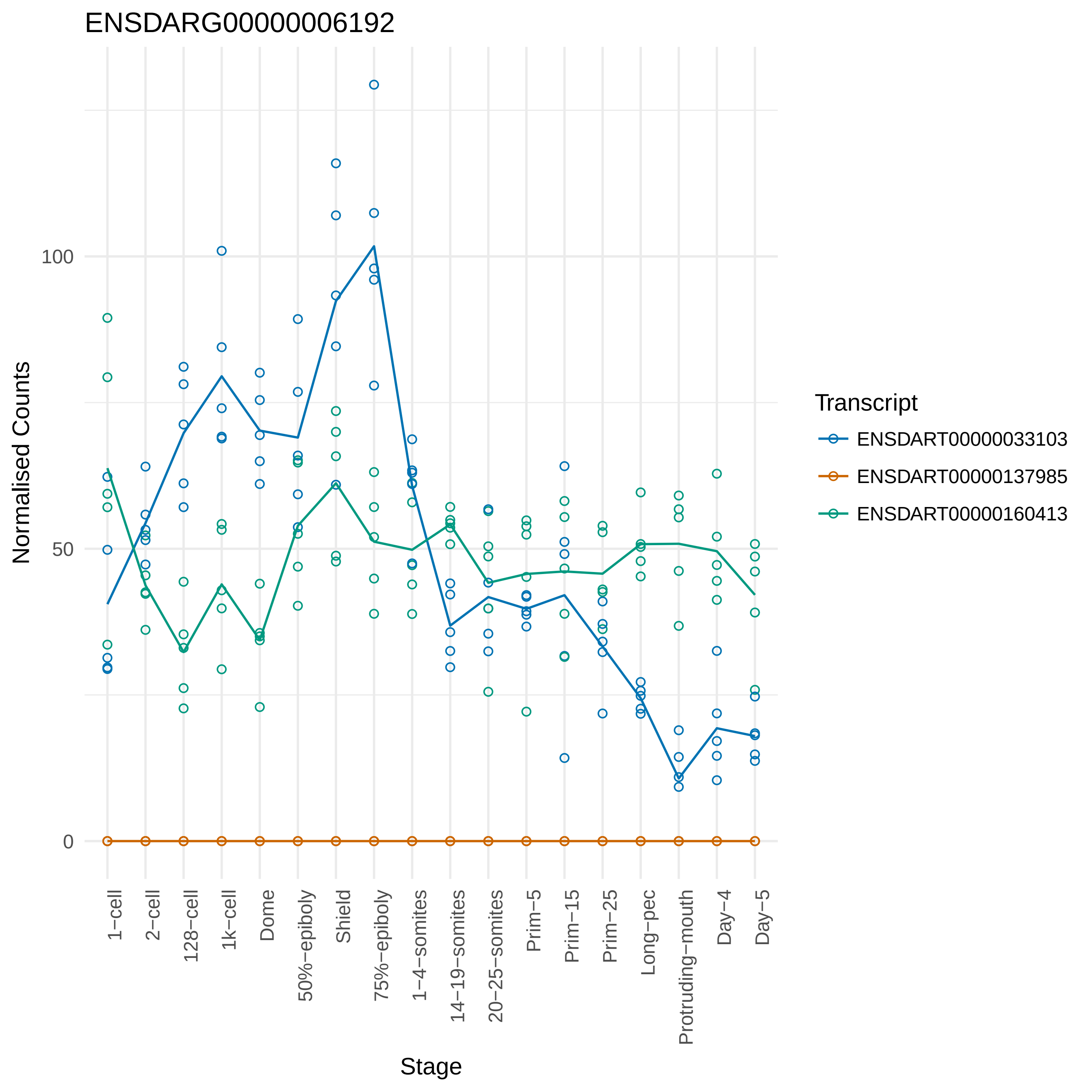

ENSDARG00000006192
Normalised Counts
100
50
0
1−cell
2−cell
128−cell
1k−cell
Dome
50%−epiboly
Shield
75%−epiboly
1−4−somites
14−19−somites
20−25−somites
Prim−5
Prim−15
Prim−25
Long−pec
Protruding−mouth
Day−4
Day−5
Stage
Transcript
ENSDART00000033103
ENSDART00000137985
ENSDART00000160413

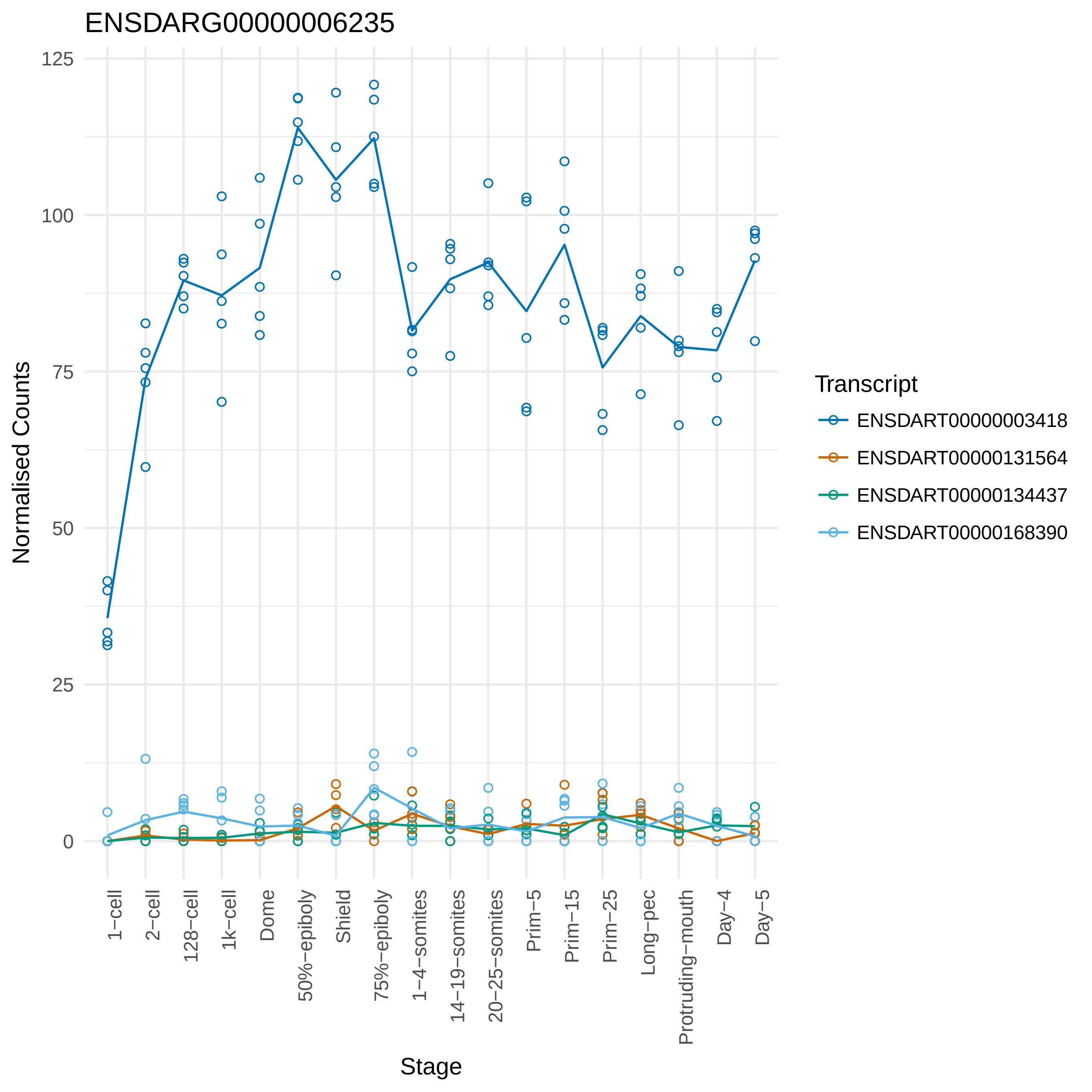

ENSDARG00000006235
Normalised Counts
125
100
75
50
25
0
Stage
1−cell
2−cell
128−cell
1k−cell
Dome
50%−epiboly
Shield
75%−epiboly
1−4−somites
14−19−somites
20−25−somites
Prim−5
Prim−15
Prim−25
Long−pec
Protruding−mouth
Day−4
Day−5
Transcript
ENSDART00000003418
ENSDART00000131564
ENSDART00000134437
ENSDART00000168390

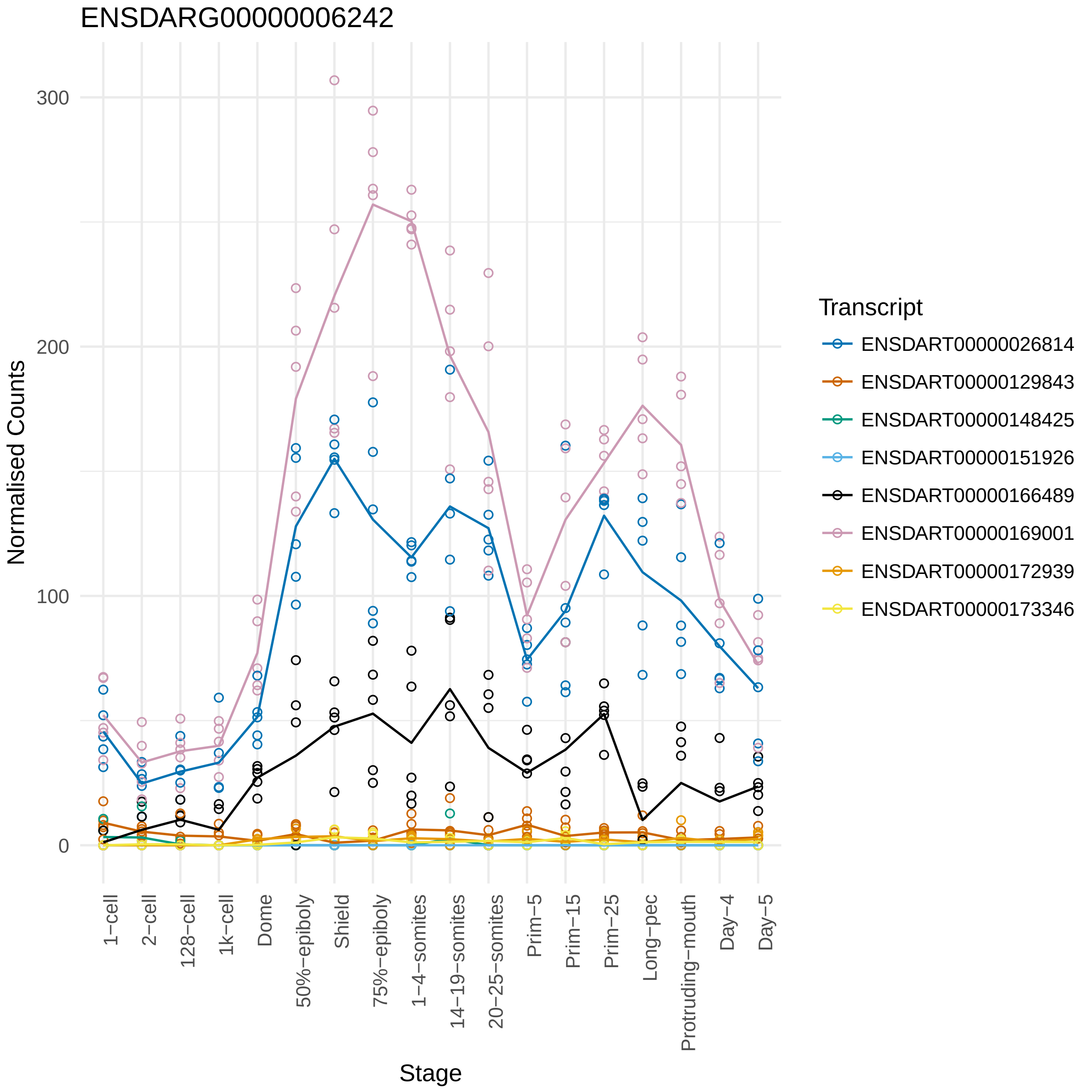

ENSDARG00000006242
Normalised Counts
300
200
100
0
1−cell
2−cell
128−cell
1k−cell
Dome
50%−epiboly
Shield
75%−epiboly
1−4−somites
14−19−somites
20−25−somites
Prim−5
Prim−15
Prim−25
Long−pec
Protruding−mouth
Day−4
Day−5
Stage
Transcript
ENSDART00000026814
ENSDART00000129843
ENSDART00000148425
ENSDART00000151926
ENSDART00000166489
ENSDART00000169001
ENSDART00000172939
ENSDART00000173346

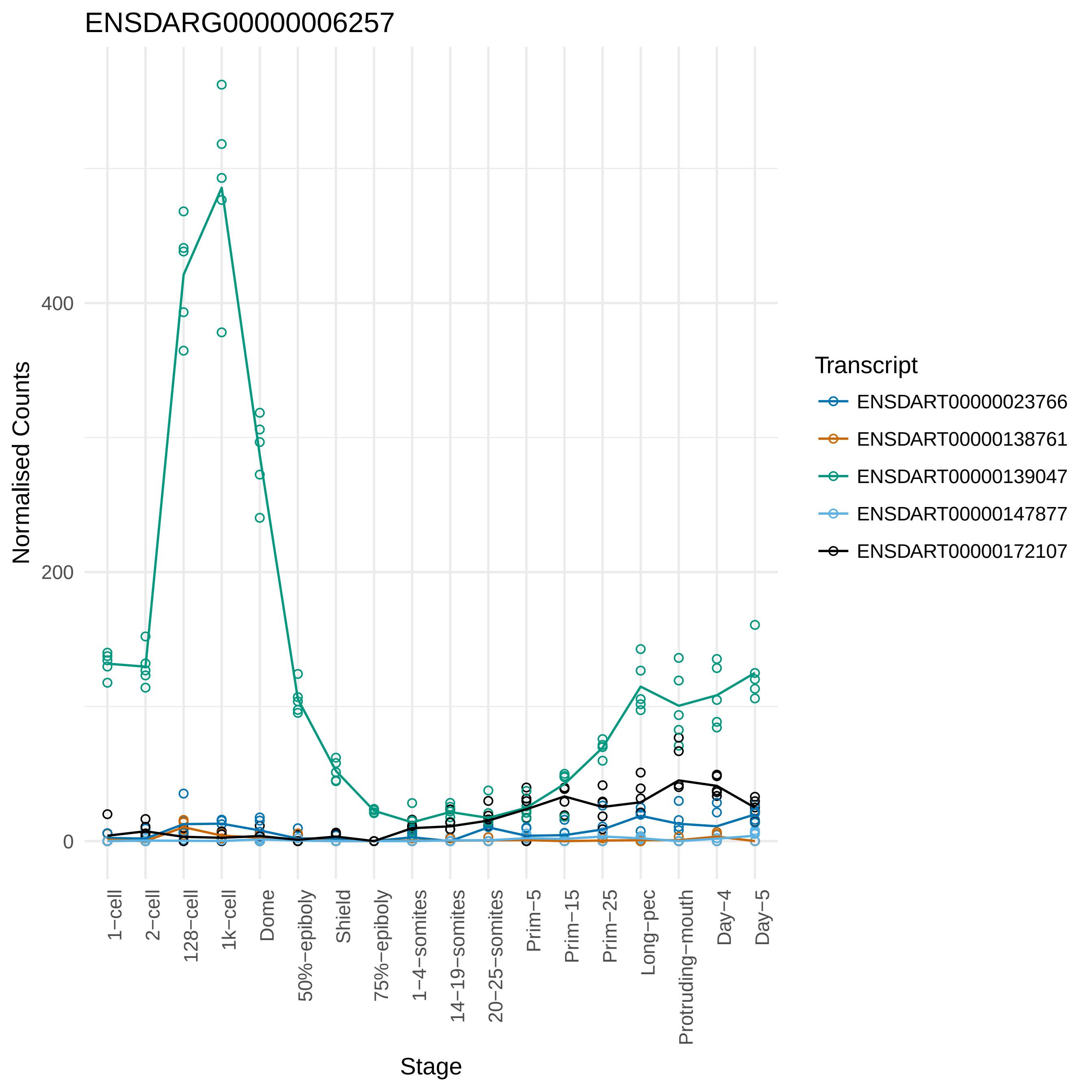

ENSDARG00000006257
Normalised Counts
400
200
0
1−cell
2−cell
128−cell
1k−cell
Dome
50%−epiboly
Shield
75%−epiboly
1−4−somites
14−19−somites
20−25−somites
Prim−5
Prim−15
Prim−25
Long−pec
Protruding−mouth
Day−4
Day−5
Stage
Transcript
ENSDART00000023766
ENSDART00000138761
ENSDART00000139047
ENSDART00000147877
ENSDART00000172107

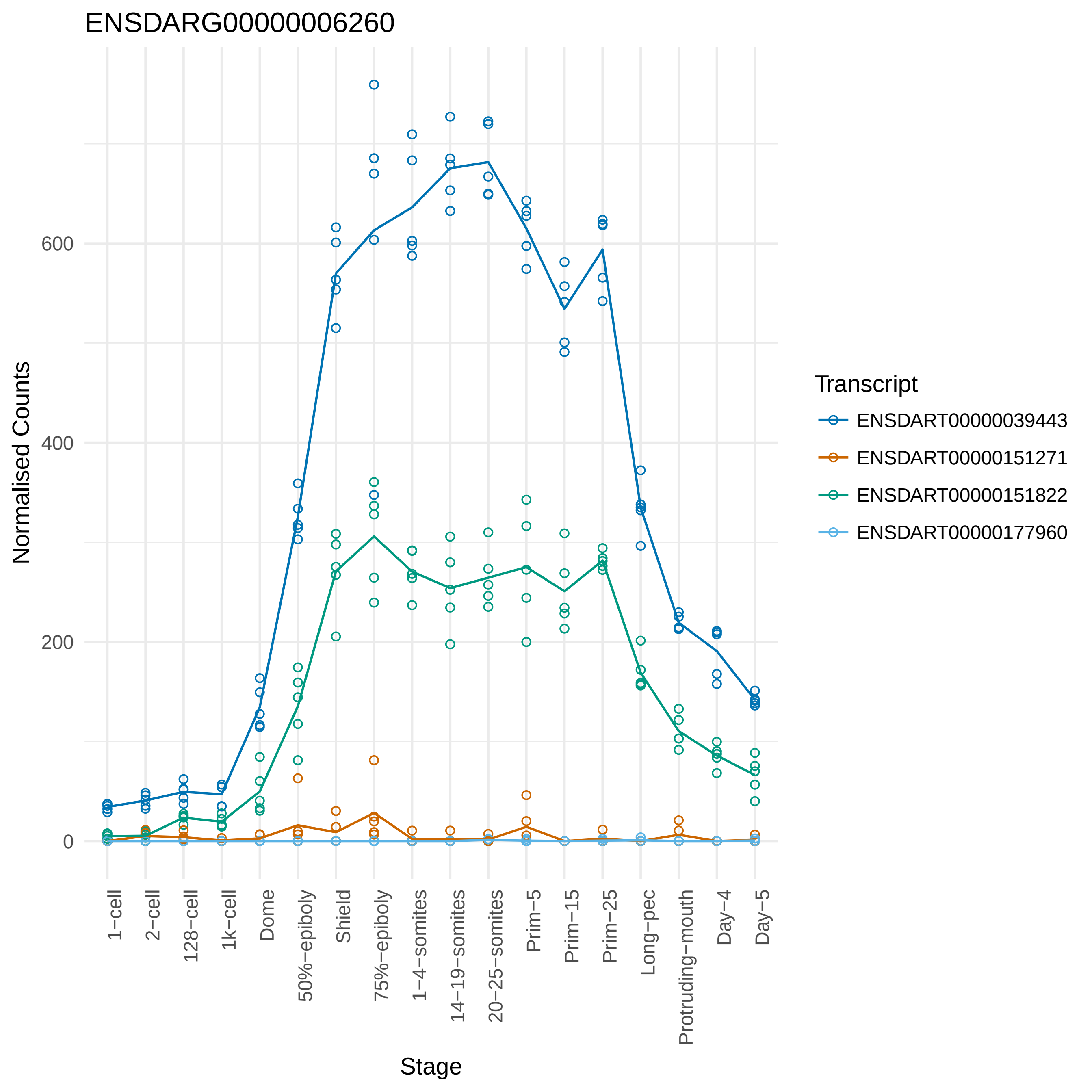

ENSDARG00000006260
Normalised Counts
0
200
400
600
1−cell
2−cell
128−cell
1k−cell
Dome
50%−epiboly
Shield
75%−epiboly
1−4−somites
14−19−somites
20−25−somites
Prim−5
Prim−15
Prim−25
Long−pec
Protruding−mouth
Day−4
Day−5
Stage
Transcript
ENSDART00000039443
ENSDART00000151271
ENSDART00000151822
ENSDART00000177960

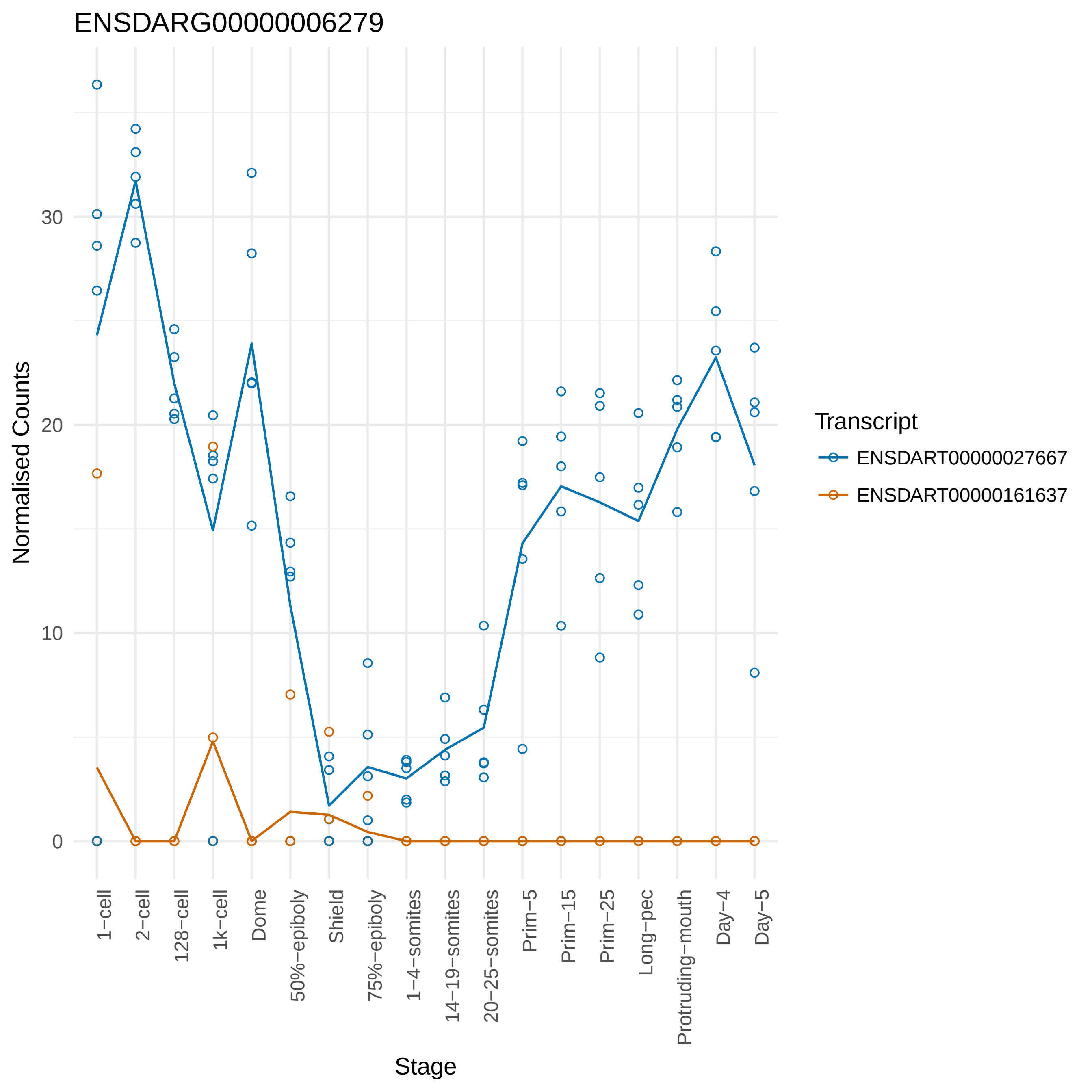
ENSDARG00000006279
Normalised Counts
0
10
20
30
Stage
1−cell
2−cell
128−cell
1k−cell
Dome
50%−epiboly
Shield
75%−epiboly
1−4−somites
14−19−somites
20−25−somites
Prim−5
Prim−15
Prim−25
Long−pec
Protruding−mouth
Day−4
Day−5
Transcript
ENSDART00000027667
ENSDART00000161637

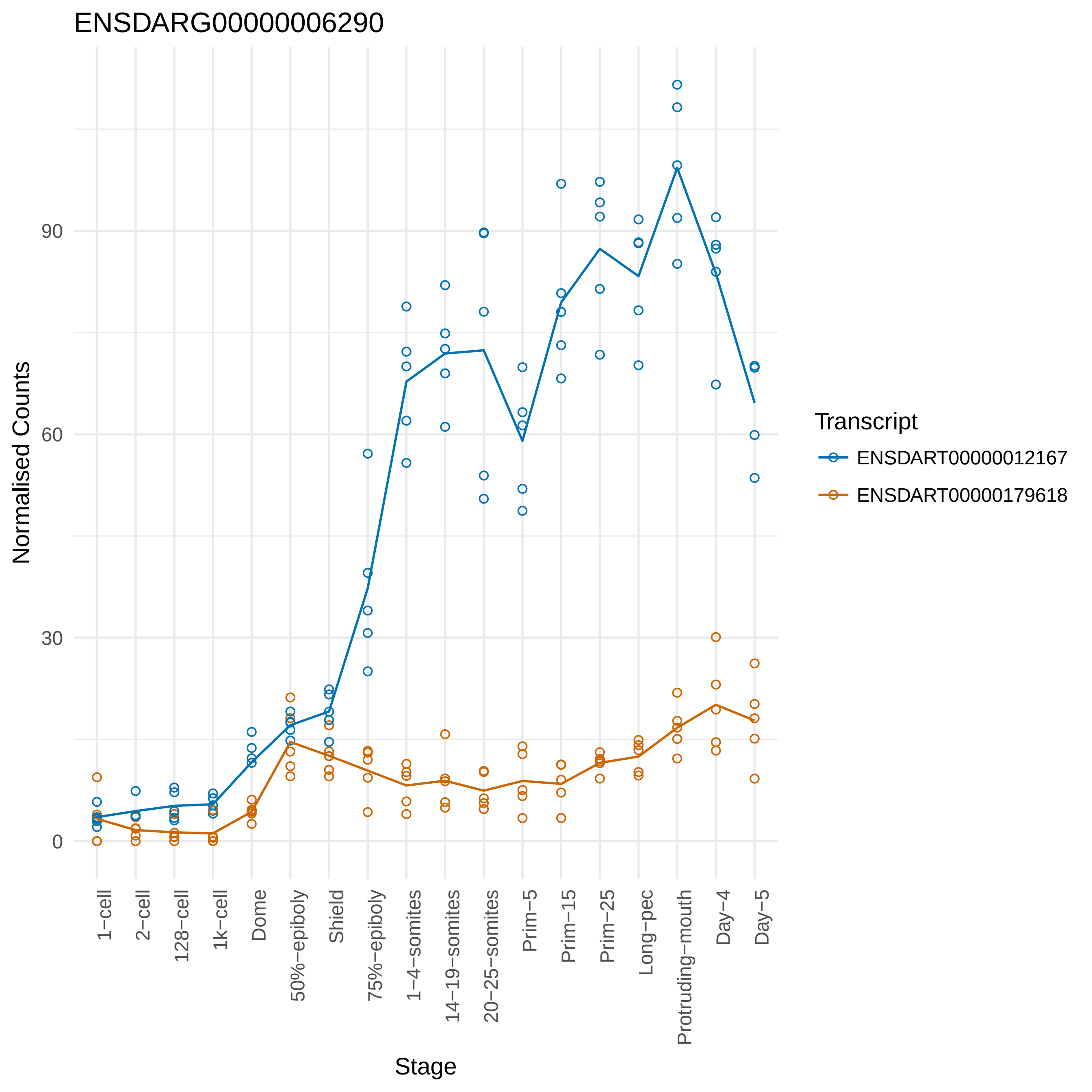

ENSDARG00000006290
Normalised Counts
0
30
60
90
1−cell
2−cell
128−cell
1k−cell
Dome
50%−epiboly
Shield
75%−epiboly
1−4−somites
14−19−somites
20−25−somites
Prim−5
Prim−15
Prim−25
Long−pec
Protruding−mouth
Day−4
Day−5
Stage
Transcript
ENSDART00000012167
ENSDART00000179618

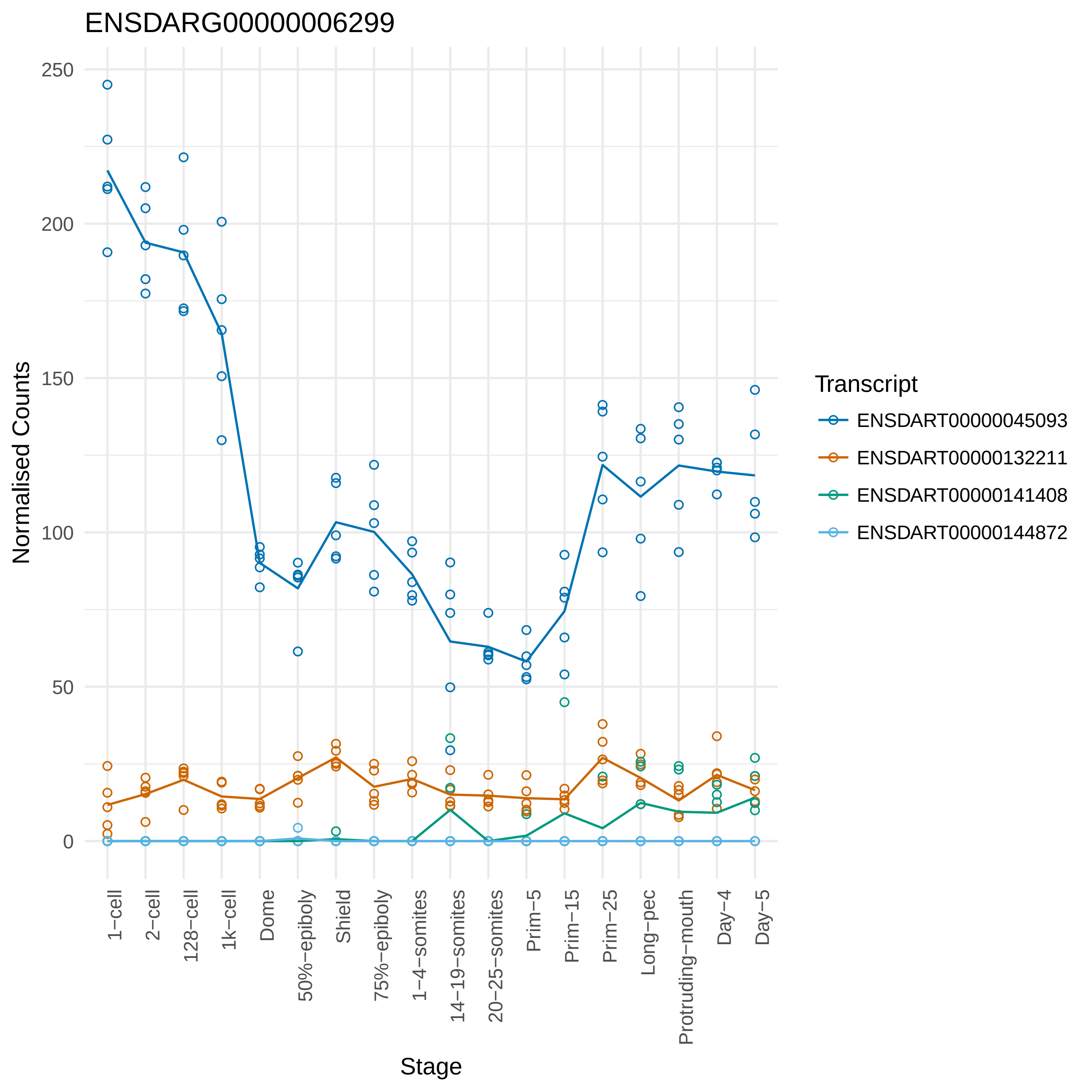

ENSDARG00000006299
Normalised Counts
250
200
150
100
50
0
1−cell
2−cell
128−cell
1k−cell
Dome
50%−epiboly
Shield
75%−epiboly
1−4−somites
14−19−somites
20−25−somites
Prim−5
Prim−15
Prim−25
Long−pec
Protruding−mouth
Day−4
Day−5
Stage
Transcript
ENSDART00000045093
ENSDART00000132211
ENSDART00000141408
ENSDART00000144872

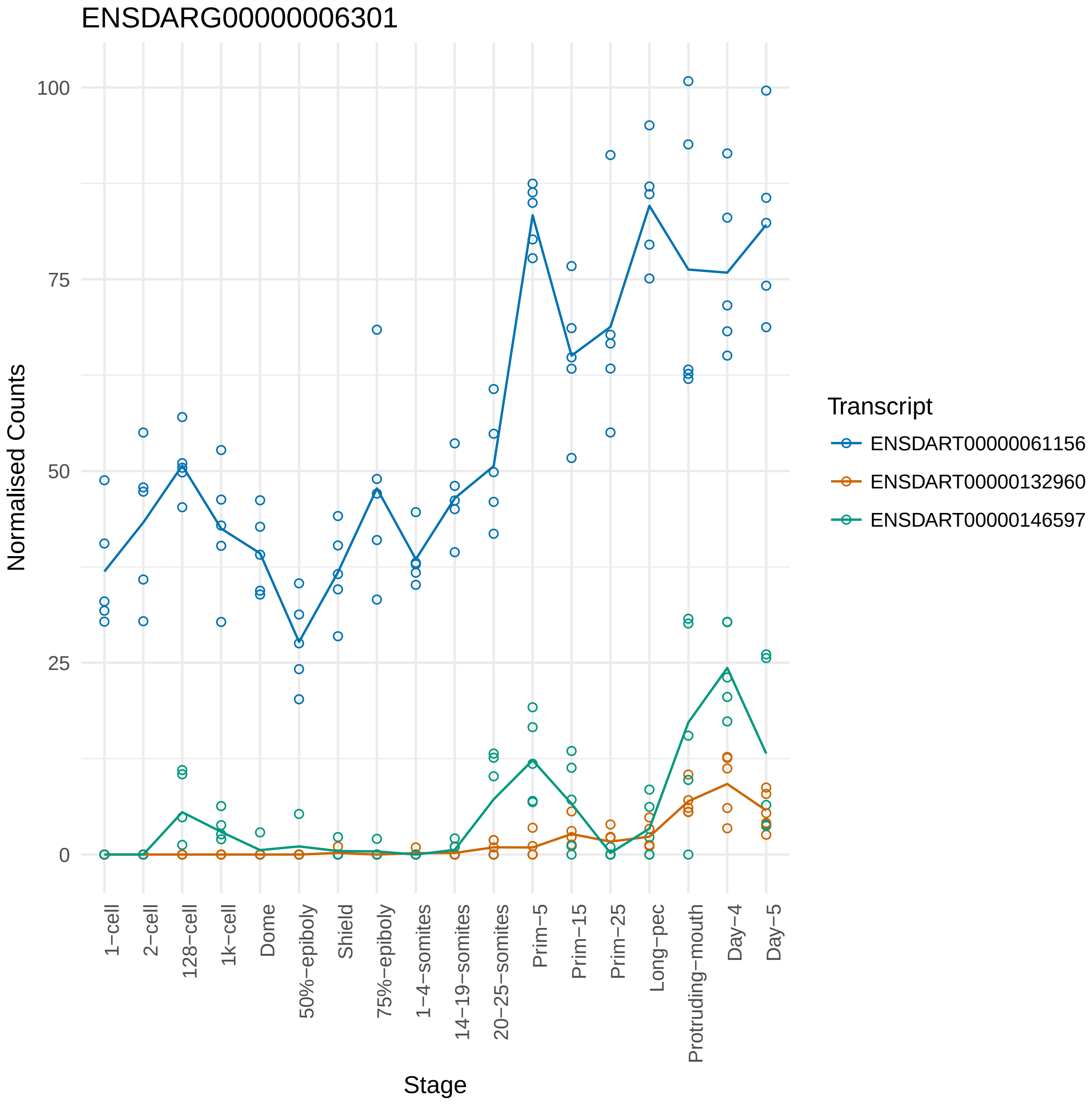

ENSDARG00000006301
Normalised Counts
100
75
50
25
0
1−cell
2−cell
128−cell
1k−cell
Dome
50%−epiboly
Shield
75%−epiboly
1−4−somites
14−19−somites
20−25−somites
Prim−5
Prim−15
Prim−25
Long−pec
Protruding−mouth
Day−4
Day−5
Stage
Transcript
ENSDART00000061156
ENSDART00000132960
ENSDART00000146597

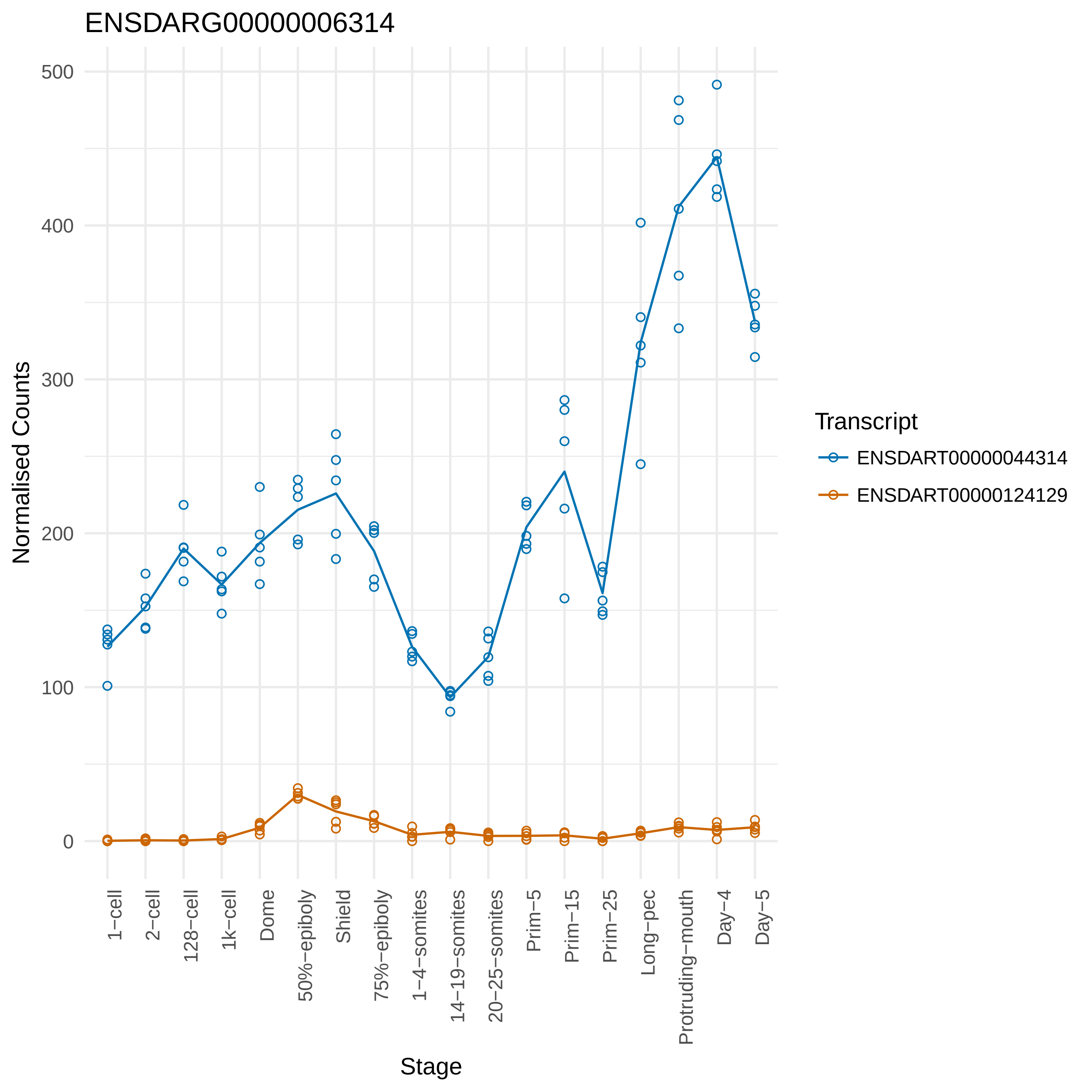
ENSDARG00000006314
Normalised Counts
500
400
300
200
100
0
1−cell
2−cell
128−cell
1k−cell
Dome
50%−epiboly
Shield
75%−epiboly
1−4−somites
14−19−somites
20−25−somites
Prim−5
Prim−15
Prim−25
Long−pec
Protruding−mouth
Day−4
Day−5
Stage
Transcript
ENSDART00000044314
ENSDART00000124129

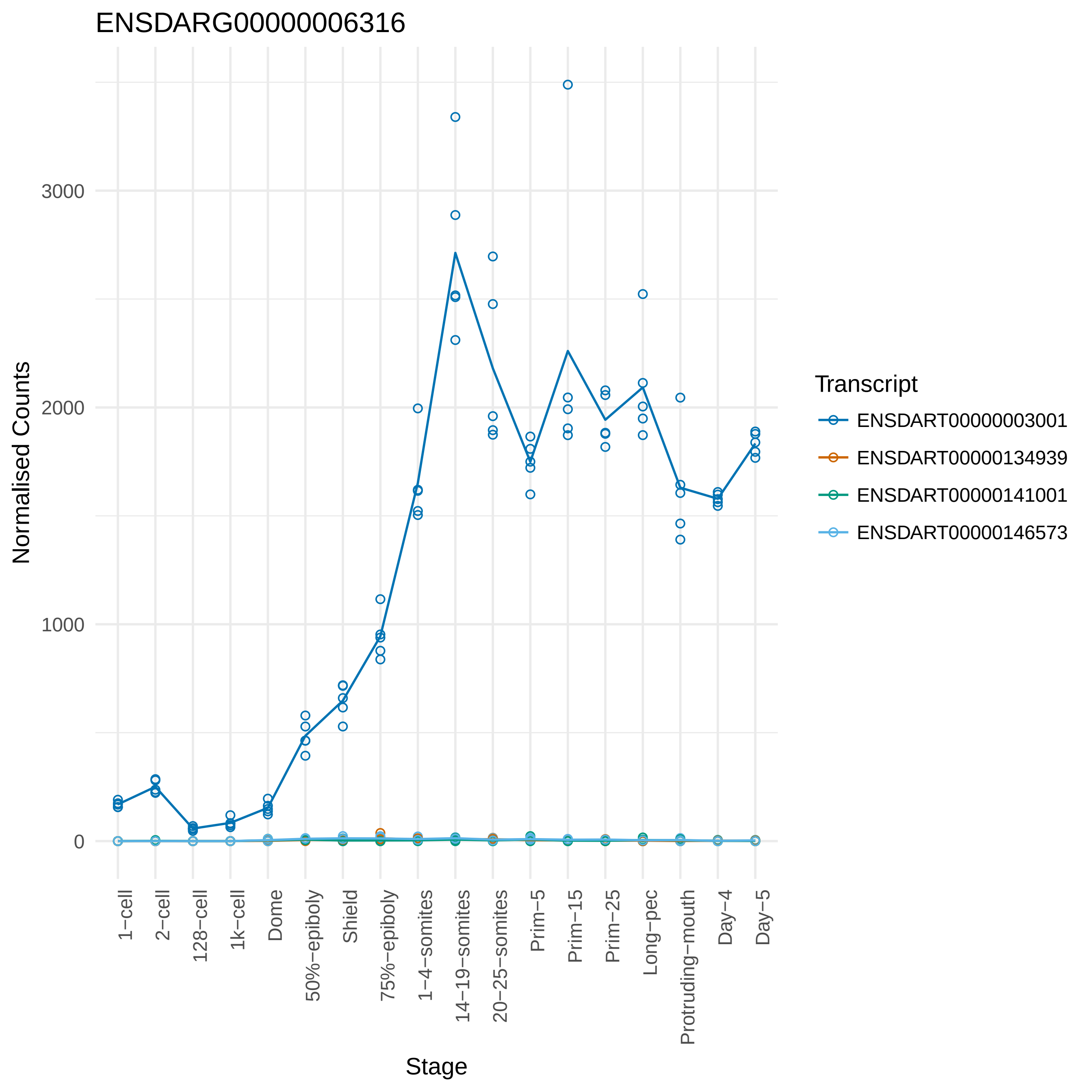
ENSDARG00000006316
Normalised Counts
0
1000
2000
3000
Stage
1−cell
2−cell
128−cell
1k−cell
Dome
50%−epiboly
Shield
75%−epiboly
1−4−somites
14−19−somites
20−25−somites
Prim−5
Prim−15
Prim−25
Long−pec
Protruding−mouth
Day−4
Day−5
Transcript
ENSDART00000003001
ENSDART00000134939
ENSDART00000141001
ENSDART00000146573

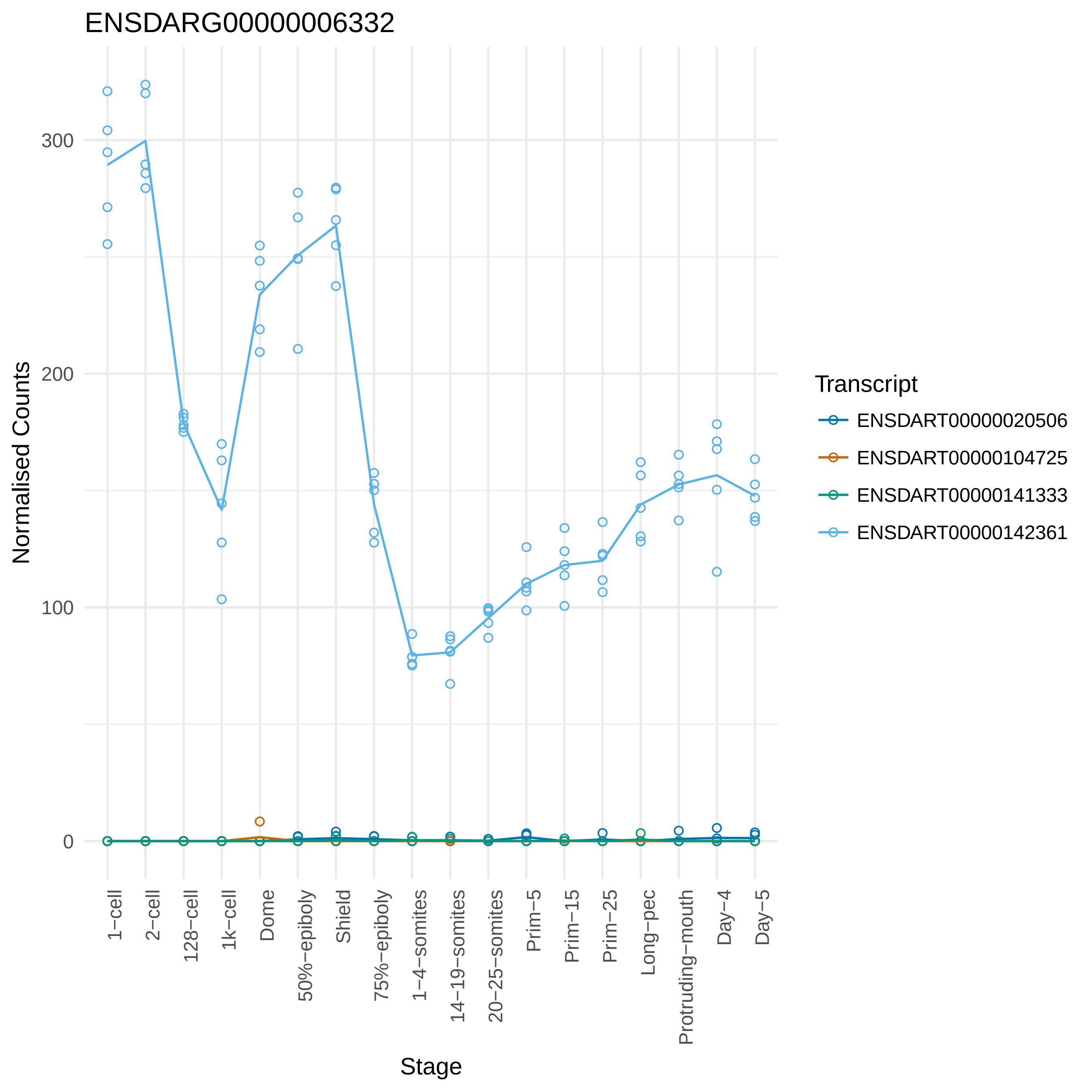
ENSDARG00000006332
Normalised Counts
300
200
100
0
1−cell
2−cell
128−cell
1k−cell
Dome
50%−epiboly
Shield
75%−epiboly
1−4−somites
14−19−somites
20−25−somites
Prim−5
Prim−15
Prim−25
Long−pec
Protruding−mouth
Day−4
Day−5
Stage
Transcript
ENSDART00000020506
ENSDART00000104725
ENSDART00000141333
ENSDART00000142361

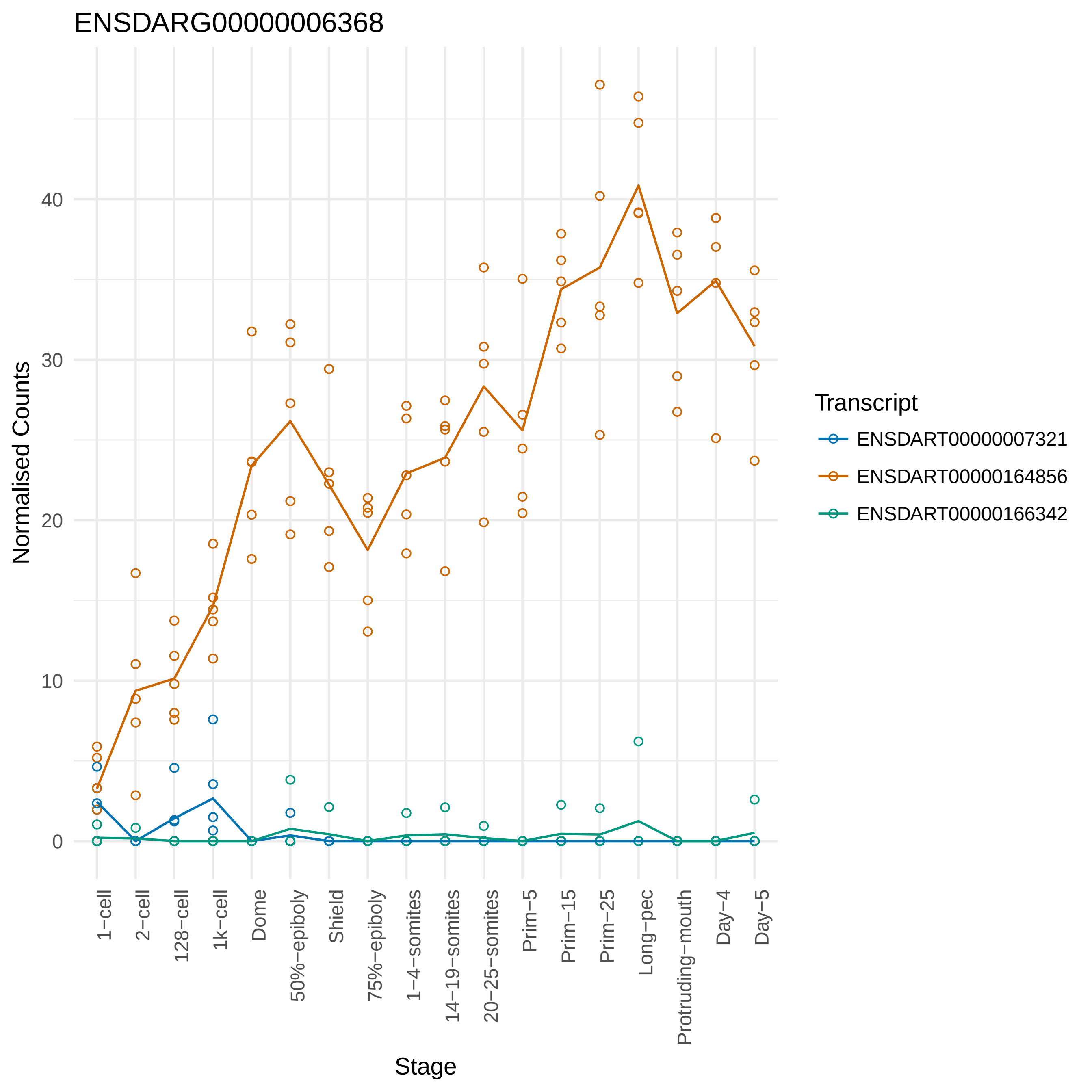

ENSDARG00000006368
Normalised Counts
0
10
20
30
40
Stage
1−cell
2−cell
128−cell
1k−cell
Dome
50%−epiboly
Shield
75%−epiboly
1−4−somites
14−19−somites
20−25−somites
Prim−5
Prim−15
Prim−25
Long−pec
Protruding−mouth
Day−4
Day−5
Transcript
ENSDART00000007321
ENSDART00000164856
ENSDART00000166342

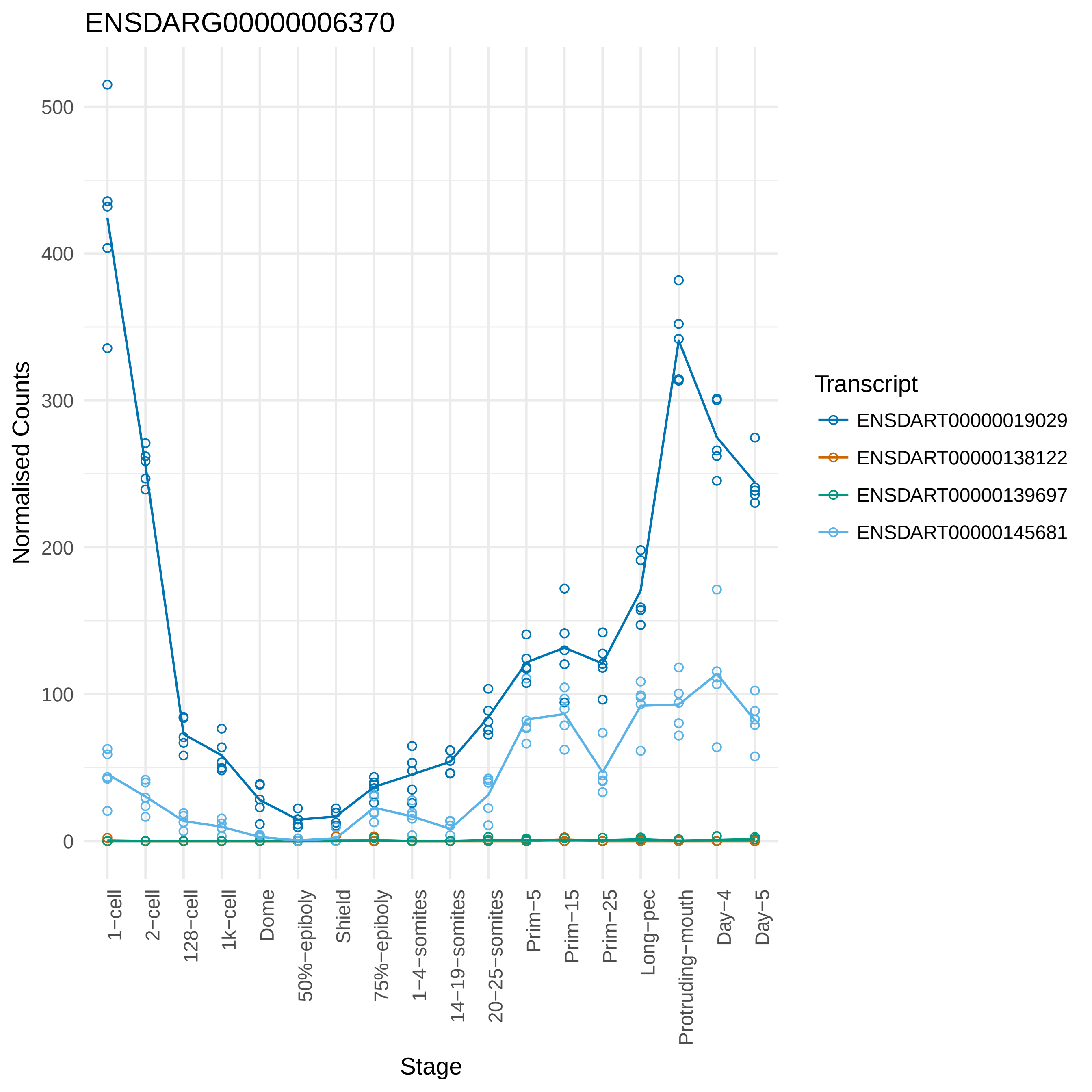

ENSDARG00000006370
Normalised Counts
500
400
300
200
100
0
1−cell
2−cell
128−cell
1k−cell
Dome
50%−epiboly
Shield
75%−epiboly
1−4−somites
14−19−somites
20−25−somites
Prim−5
Prim−15
Prim−25
Long−pec
Protruding−mouth
Day−4
Day−5
Stage
Transcript
ENSDART00000019029
ENSDART00000138122
ENSDART00000139697
ENSDART00000145681

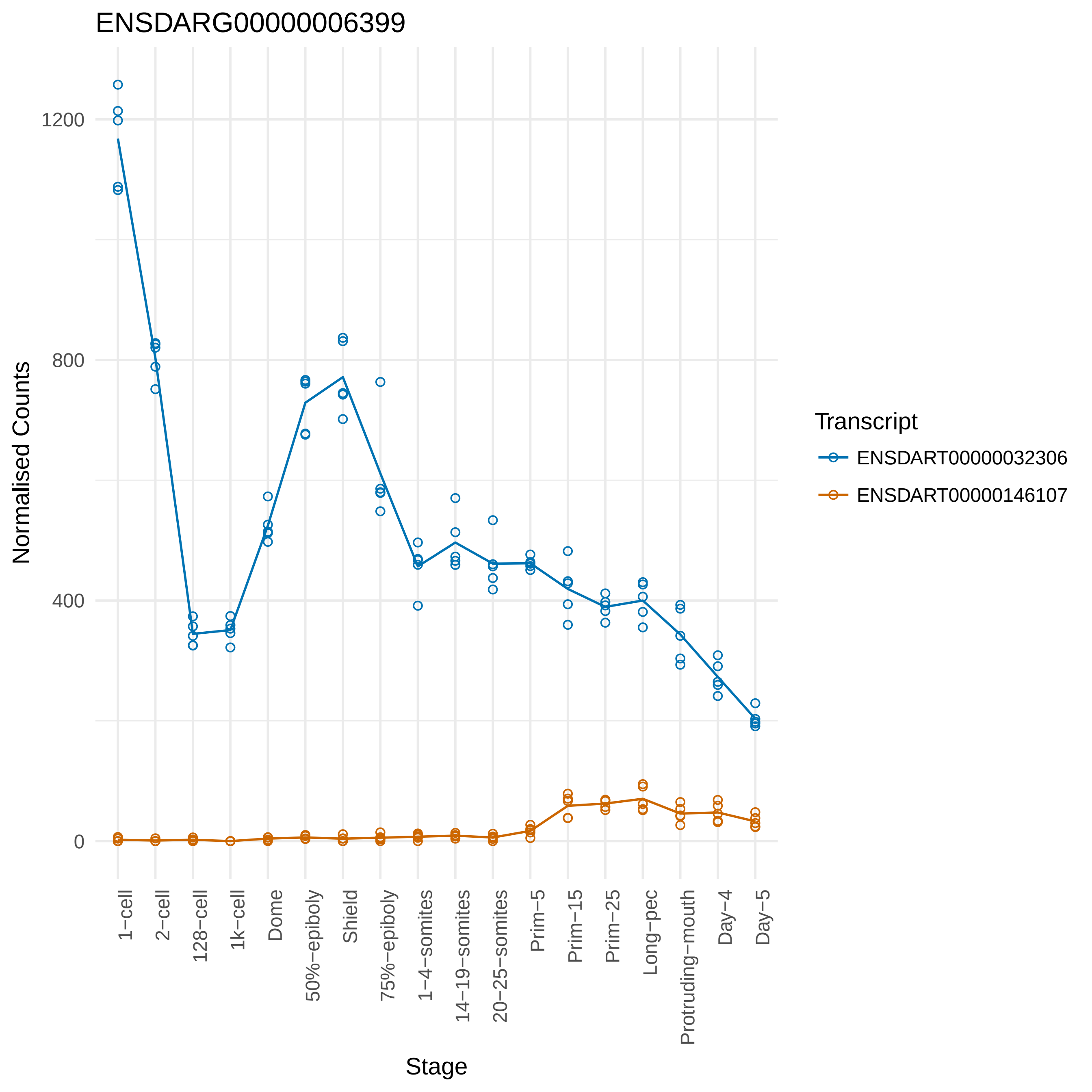

ENSDARG00000006399
Normalised Counts
1200
800
400
0
Stage
1−cell
2−cell
128−cell
1k−cell
Dome
50%−epiboly
Shield
75%−epiboly
1−4−somites
14−19−somites
20−25−somites
Prim−5
Prim−15
Prim−25
Long−pec
Protruding−mouth
Day−4
Day−5
Transcript
ENSDART00000032306
ENSDART00000146107

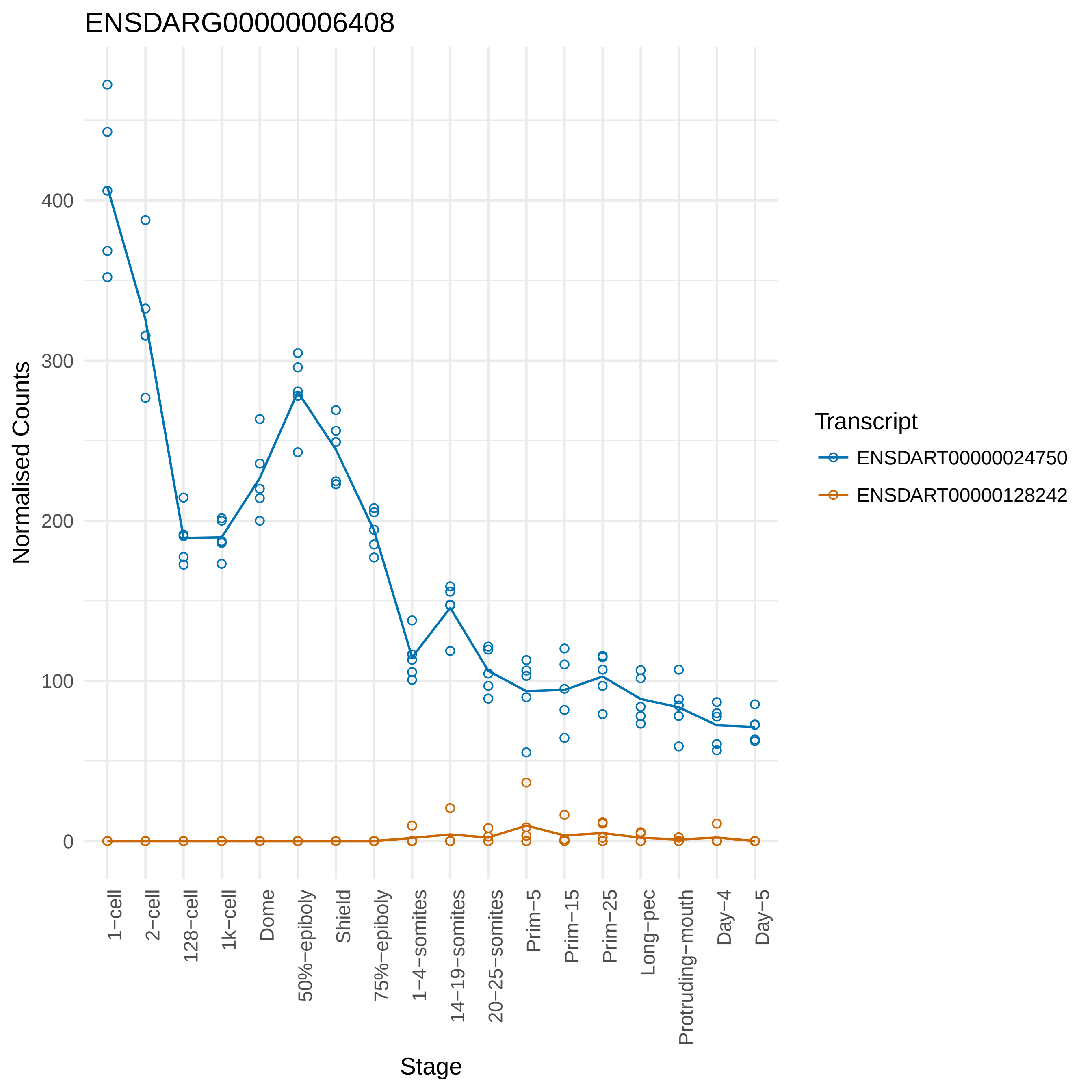

ENSDARG00000006408
Normalised Counts
0
100
200
300
400
Stage
1−cell
2−cell
128−cell
1k−cell
Dome
50%−epiboly
Shield
75%−epiboly
1−4−somites
14−19−somites
20−25−somites
Prim−5
Prim−15
Prim−25
Long−pec
Protruding−mouth
Day−4
Day−5
Transcript
ENSDART00000024750
ENSDART00000128242

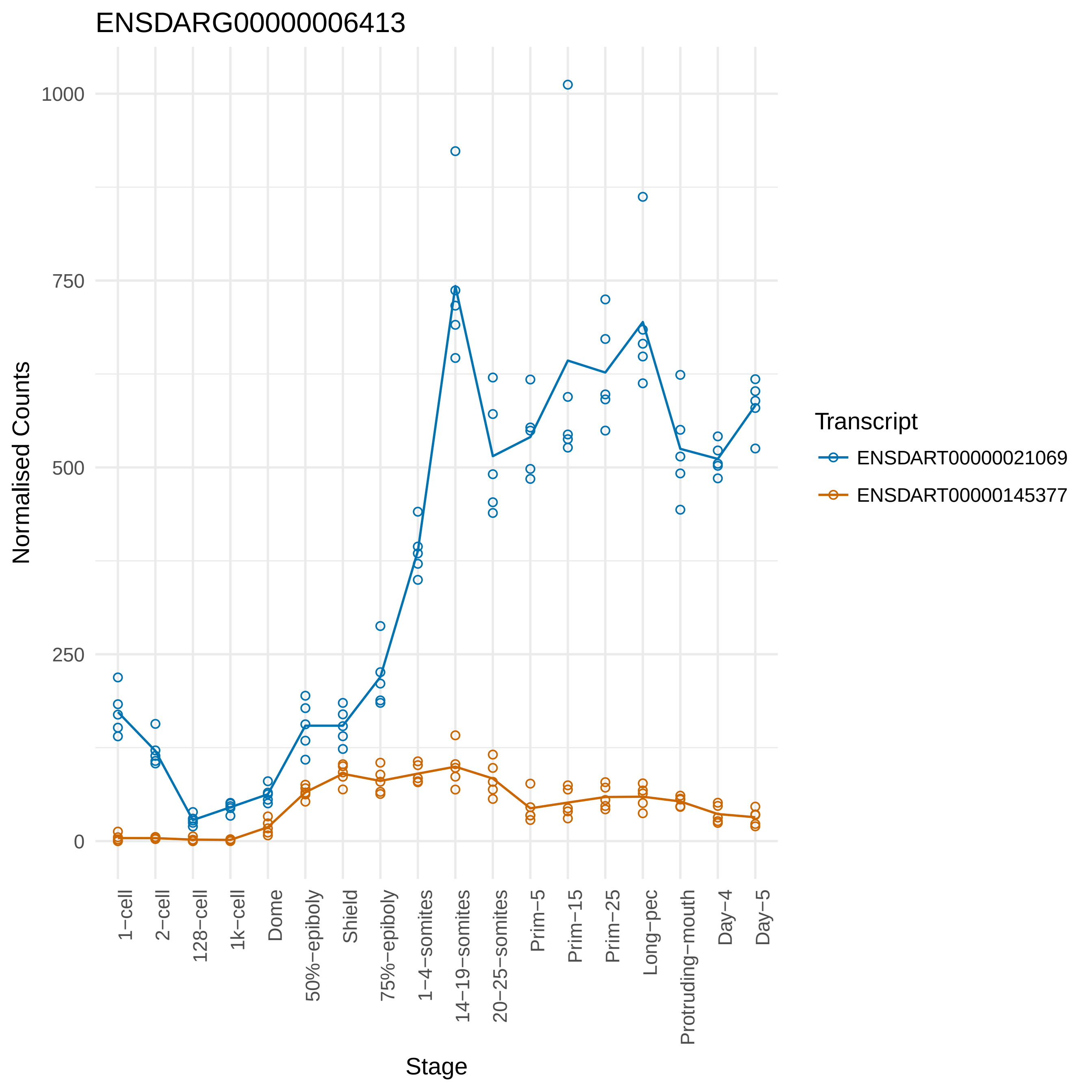

ENSDARG00000006413
Normalised Counts
1000
750
500
250
0
Stage
1−cell
2−cell
128−cell
1k−cell
Dome
50%−epiboly
Shield
75%−epiboly
1−4−somites
14−19−somites
20−25−somites
Prim−5
Prim−15
Prim−25
Long−pec
Protruding−mouth
Day−4
Day−5
Transcript
ENSDART00000021069
ENSDART00000145377

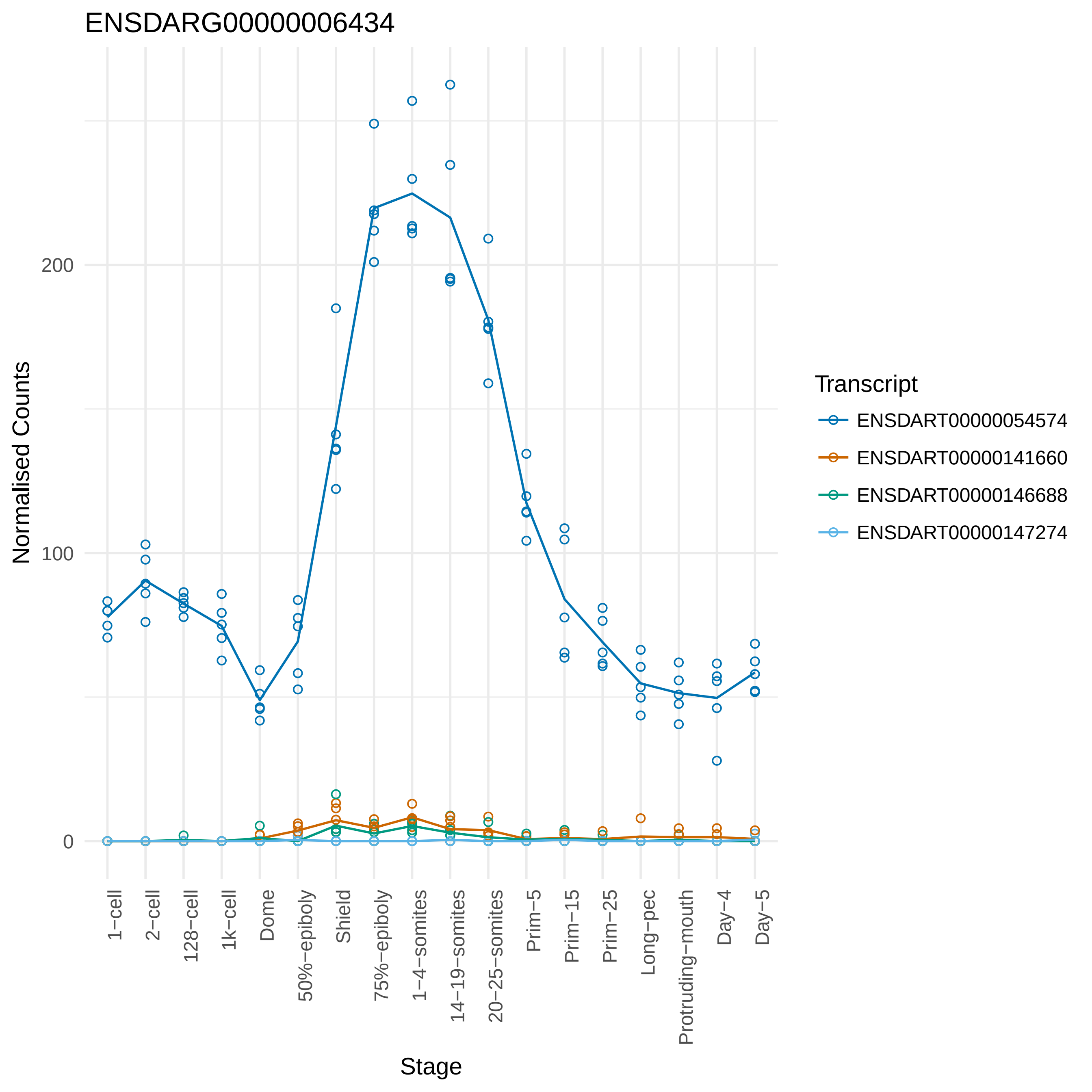

ENSDARG00000006434
Normalised Counts
200
100
0
1−cell
2−cell
128−cell
1k−cell
Dome
50%−epiboly
Shield
75%−epiboly
1−4−somites
14−19−somites
20−25−somites
Prim−5
Prim−15
Prim−25
Long−pec
Protruding−mouth
Day−4
Day−5
Stage
Transcript
ENSDART00000054574
ENSDART00000141660
ENSDART00000146688
ENSDART00000147274

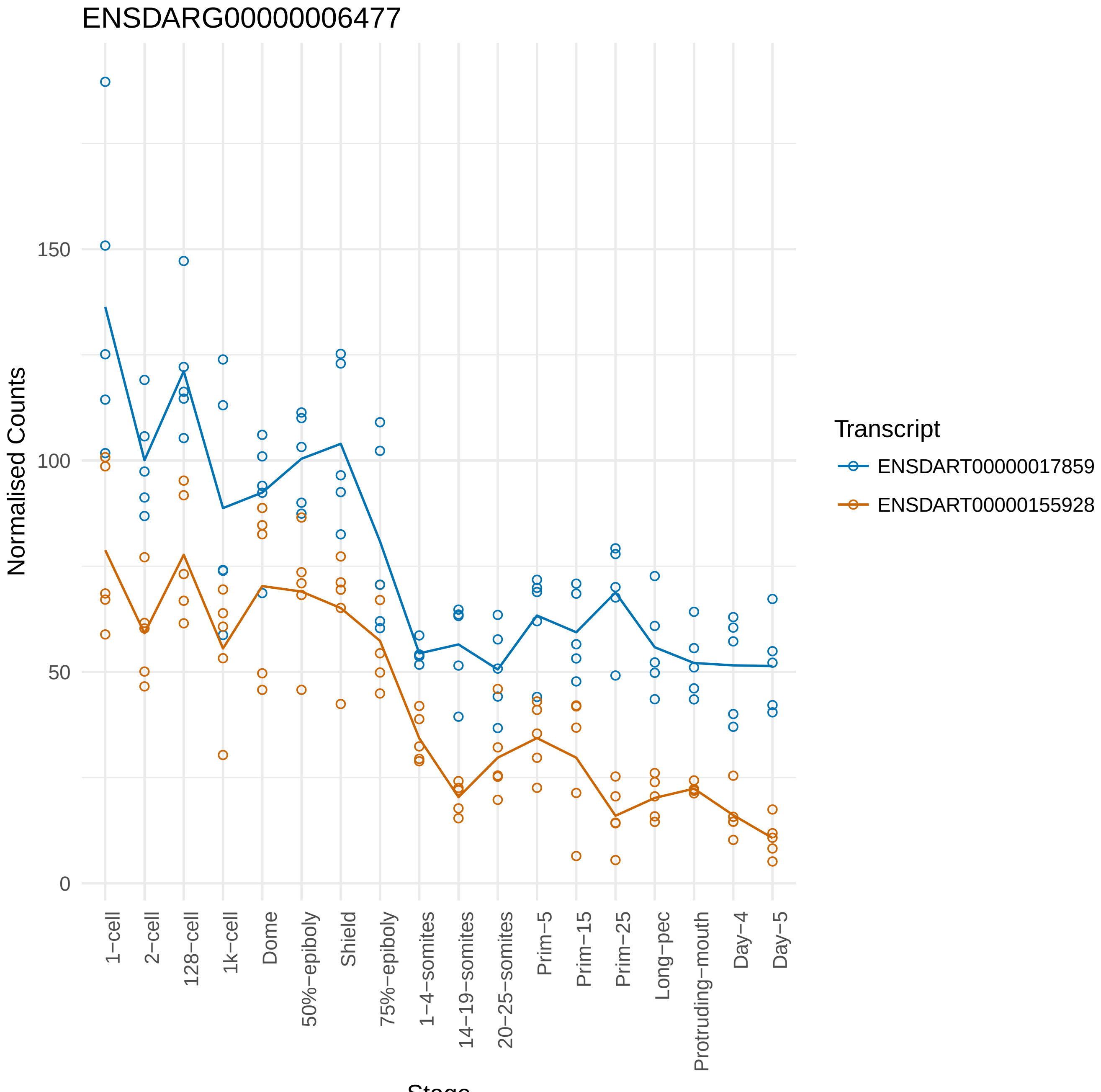

ENSDARG00000006477
Normalised Counts
150
100
50
0
1−cell
2−cell
128−cell
1k−cell
Dome
50%−epiboly
Shield
75%−epiboly
1−4−somites
14−19−somites
20−25−somites
Prim−5
Prim−15
Prim−25
Long−pec
Protruding−mouth
Day−4
Day−5
Stage
Transcript
ENSDART00000017859
ENSDART00000155928

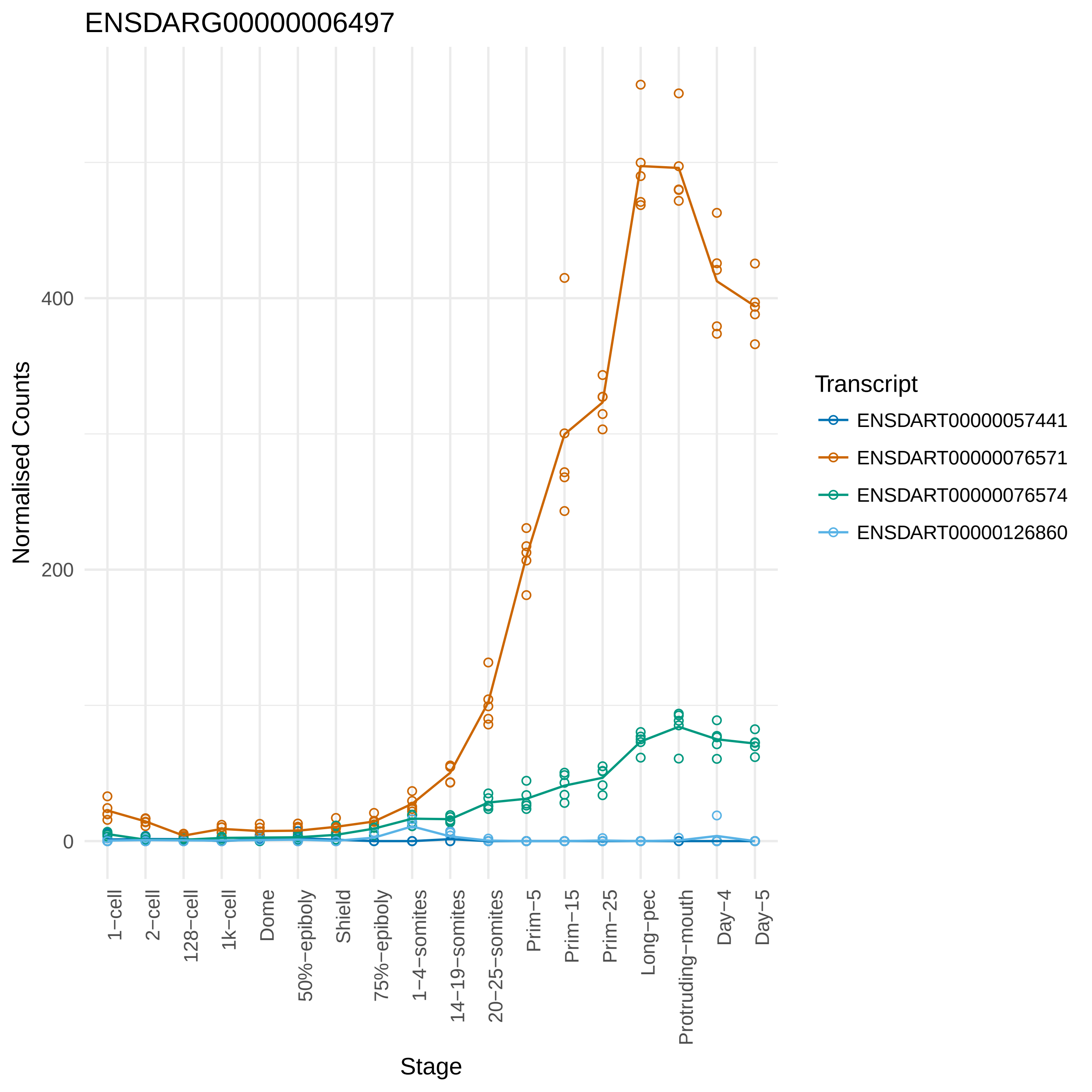

ENSDARG00000006497
Normalised Counts
400
200
0
1−cell
2−cell
128−cell
1k−cell
Dome
50%−epiboly
Shield
75%−epiboly
1−4−somites
14−19−somites
20−25−somites
Prim−5
Prim−15
Prim−25
Long−pec
Protruding−mouth
Day−4
Day−5
Stage
Transcript
ENSDART00000057441
ENSDART00000076571
ENSDART00000076574
ENSDART00000126860

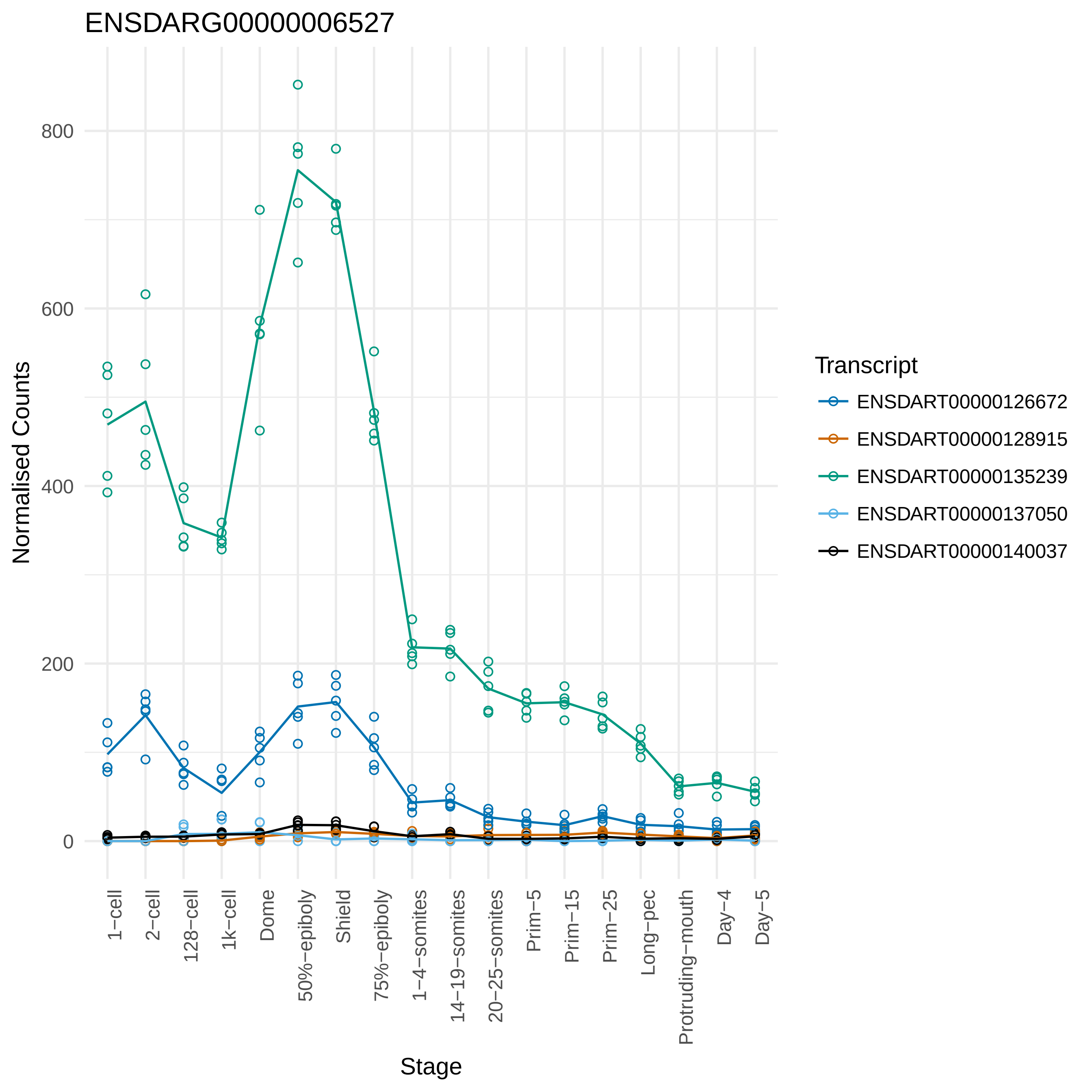

ENSDARG00000006527
Normalised Counts
800
600
400
200
0
1−cell
2−cell
128−cell
1k−cell
Dome
50%−epiboly
Shield
75%−epiboly
1−4−somites
14−19−somites
20−25−somites
Prim−5
Prim−15
Prim−25
Long−pec
Protruding−mouth
Day−4
Day−5
Stage
Transcript
ENSDART00000126672
ENSDART00000128915
ENSDART00000135239
ENSDART00000137050
ENSDART00000140037

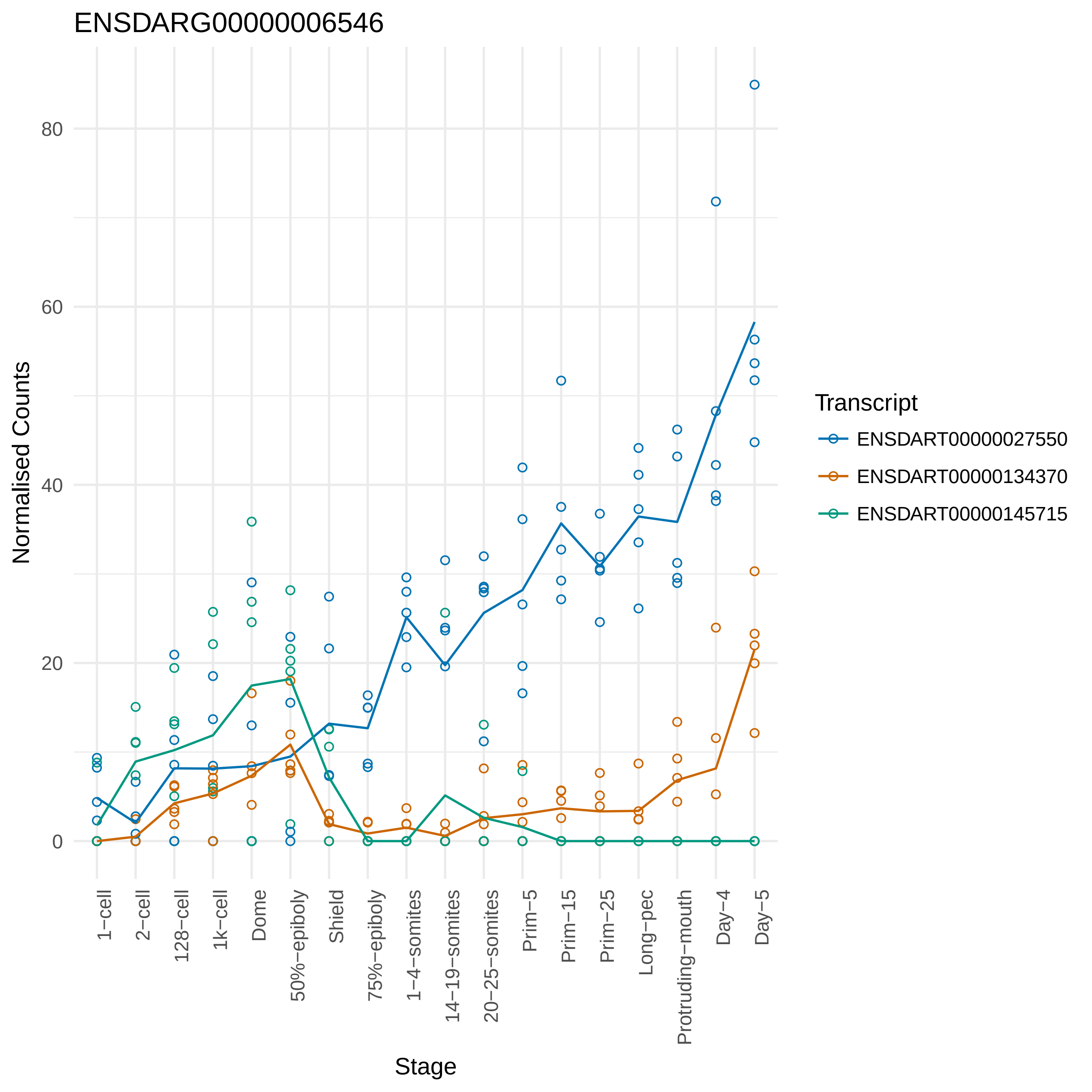

ENSDARG00000006546
Normalised Counts
0
20
40
60
80
Stage
1−cell
2−cell
128−cell
1k−cell
Dome
50%−epiboly
Shield
75%−epiboly
1−4−somites
14−19−somites
20−25−somites
Prim−5
Prim−15
Prim−25
Long−pec
Protruding−mouth
Day−4
Day−5
Transcript
ENSDART00000027550
ENSDART00000134370
ENSDART00000145715

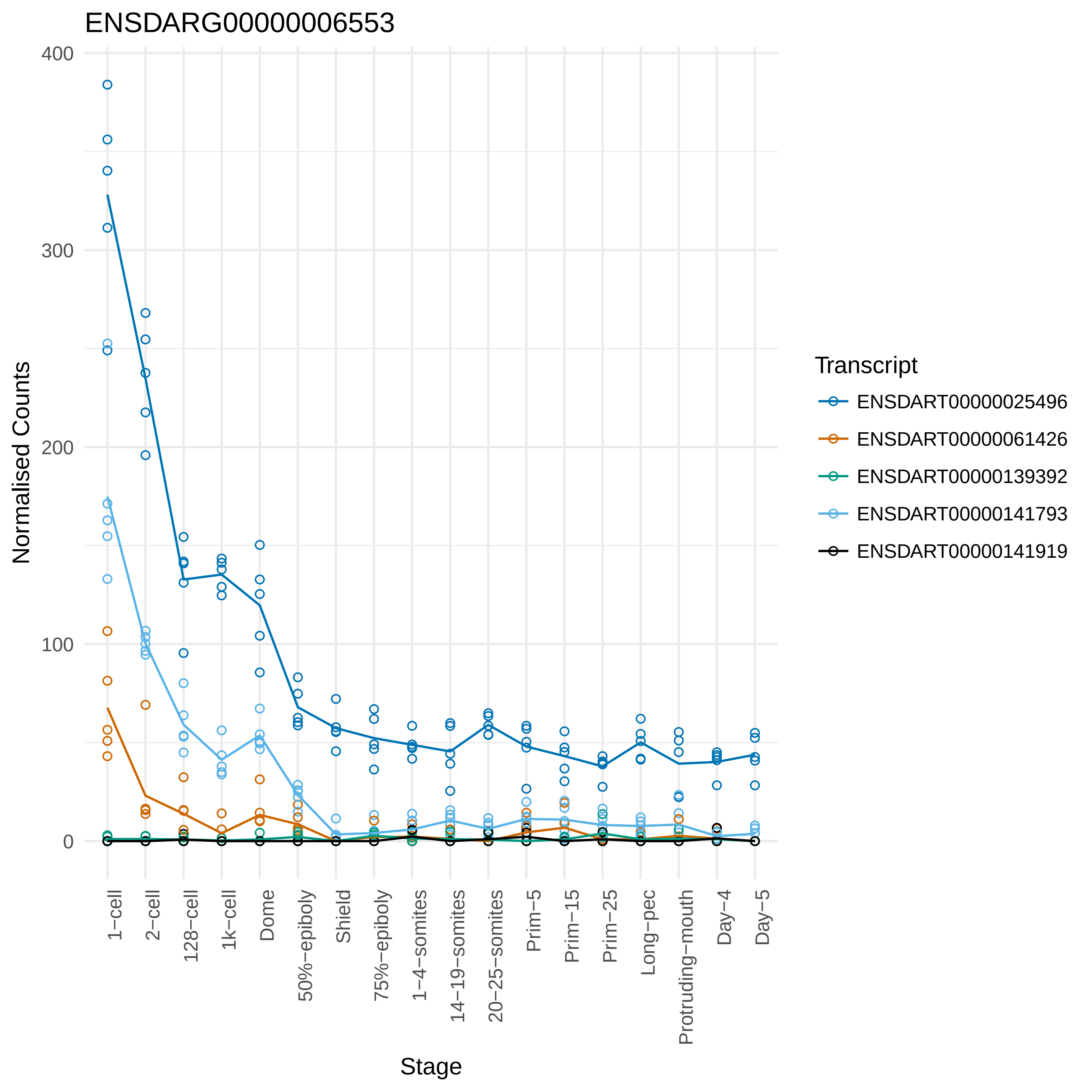
ENSDARG00000006553
Normalised Counts
400
300
200
100
0
1−cell
2−cell
128−cell
1k−cell
Dome
50%−epiboly
Shield
75%−epiboly
1−4−somites
14−19−somites
20−25−somites
Prim−5
Prim−15
Prim−25
Long−pec
Protruding−mouth
Day−4
Day−5
Stage
Transcript
ENSDART00000025496
ENSDART00000061426
ENSDART00000139392
ENSDART00000141793
ENSDART00000141919

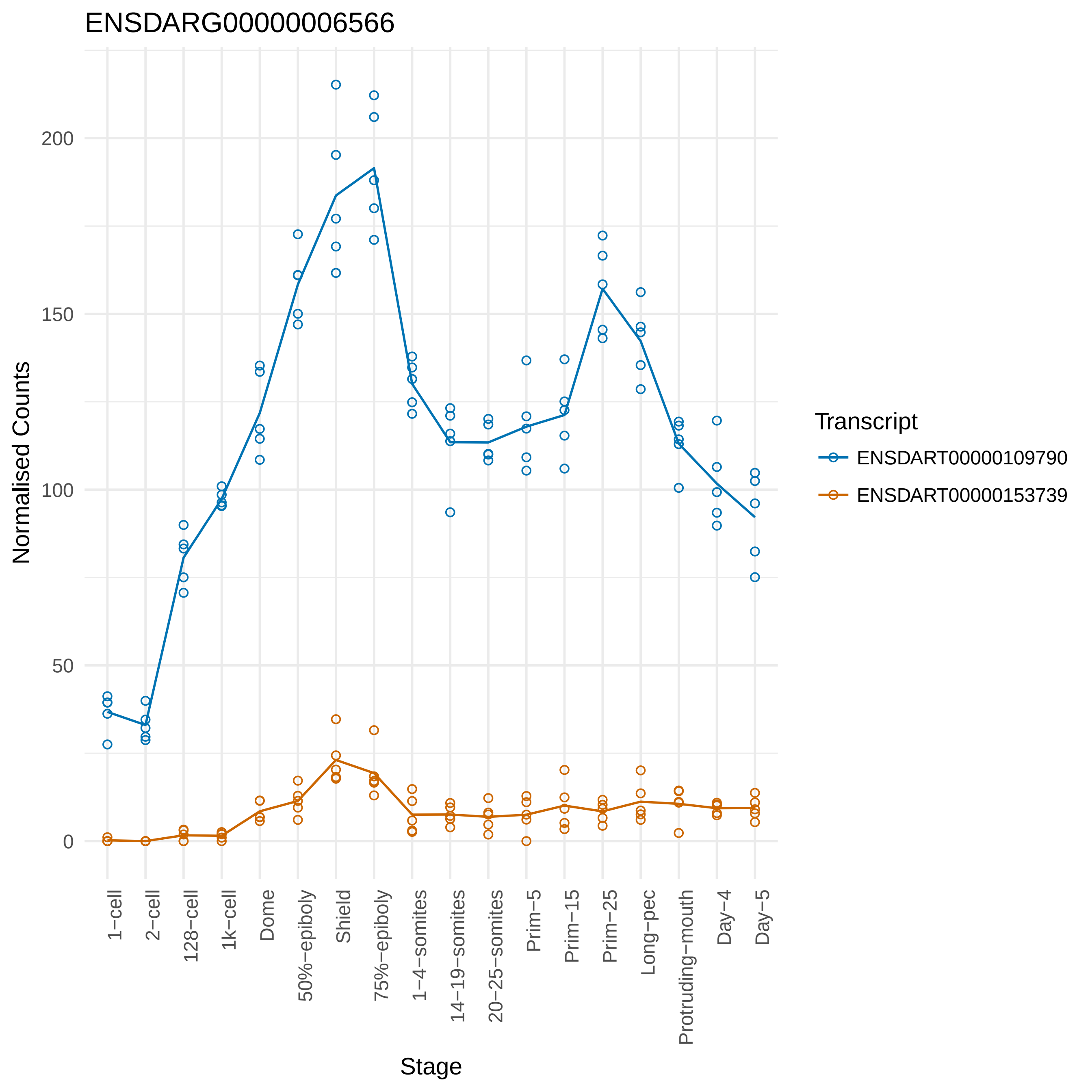

ENSDARG00000006566
Normalised Counts
0
50
100
150
200
Stage
1−cell
2−cell
128−cell
1k−cell
Dome
50%−epiboly
Shield
75%−epiboly
1−4−somites
14−19−somites
20−25−somites
Prim−5
Prim−15
Prim−25
Long−pec
Protruding−mouth
Day−4
Day−5
Transcript
ENSDART00000109790
ENSDART00000153739

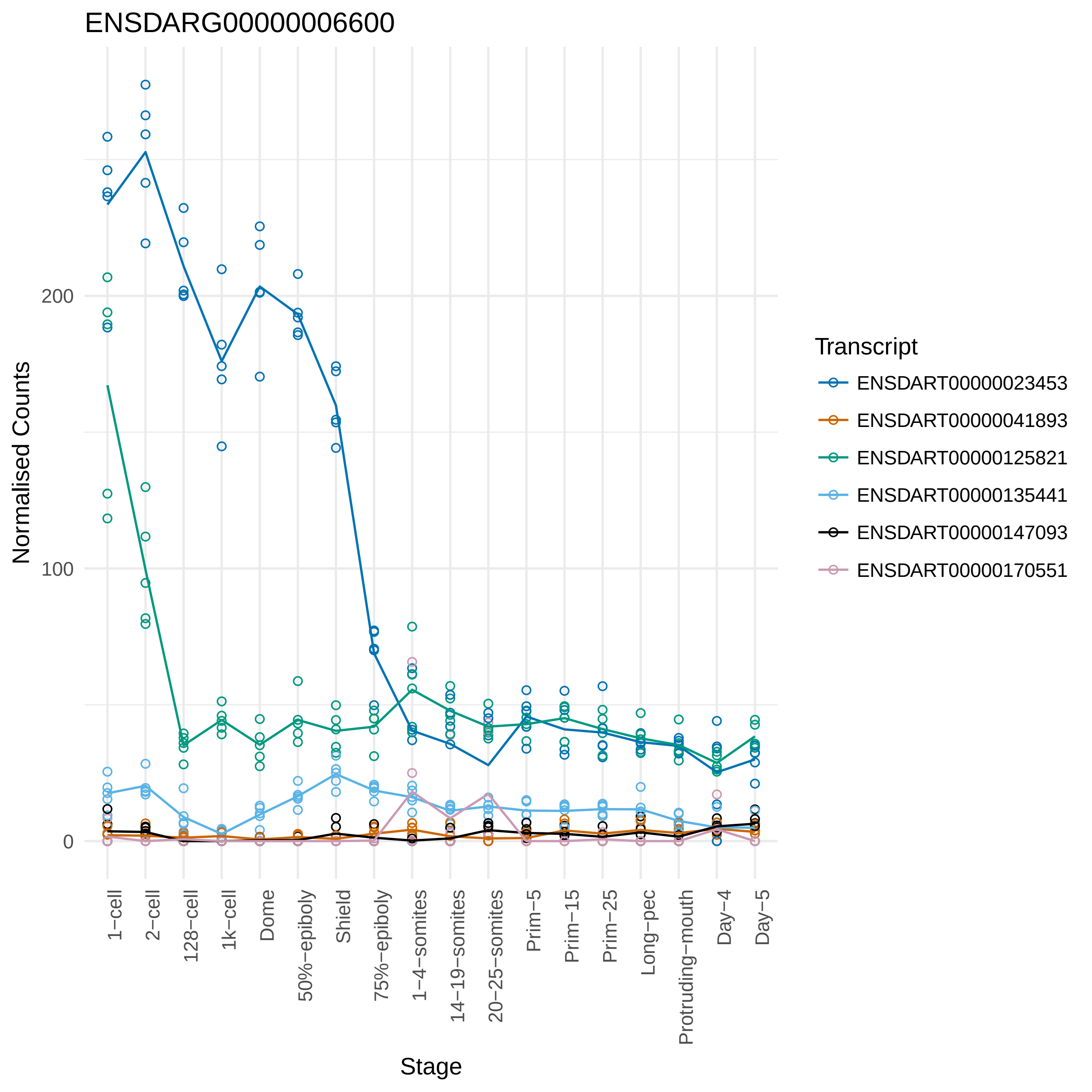
ENSDARG00000006600
Normalised Counts
200
100
0
Stage
1−cell
2−cell
128−cell
1k−cell
Dome
50%−epiboly
Shield
75%−epiboly
1−4−somites
14−19−somites
20−25−somites
Prim−5
Prim−15
Prim−25
Long−pec
Protruding−mouth
Day−4
Day−5
Transcript
ENSDART00000023453
ENSDART00000041893
ENSDART00000125821
ENSDART00000135441
ENSDART00000147093
ENSDART00000170551

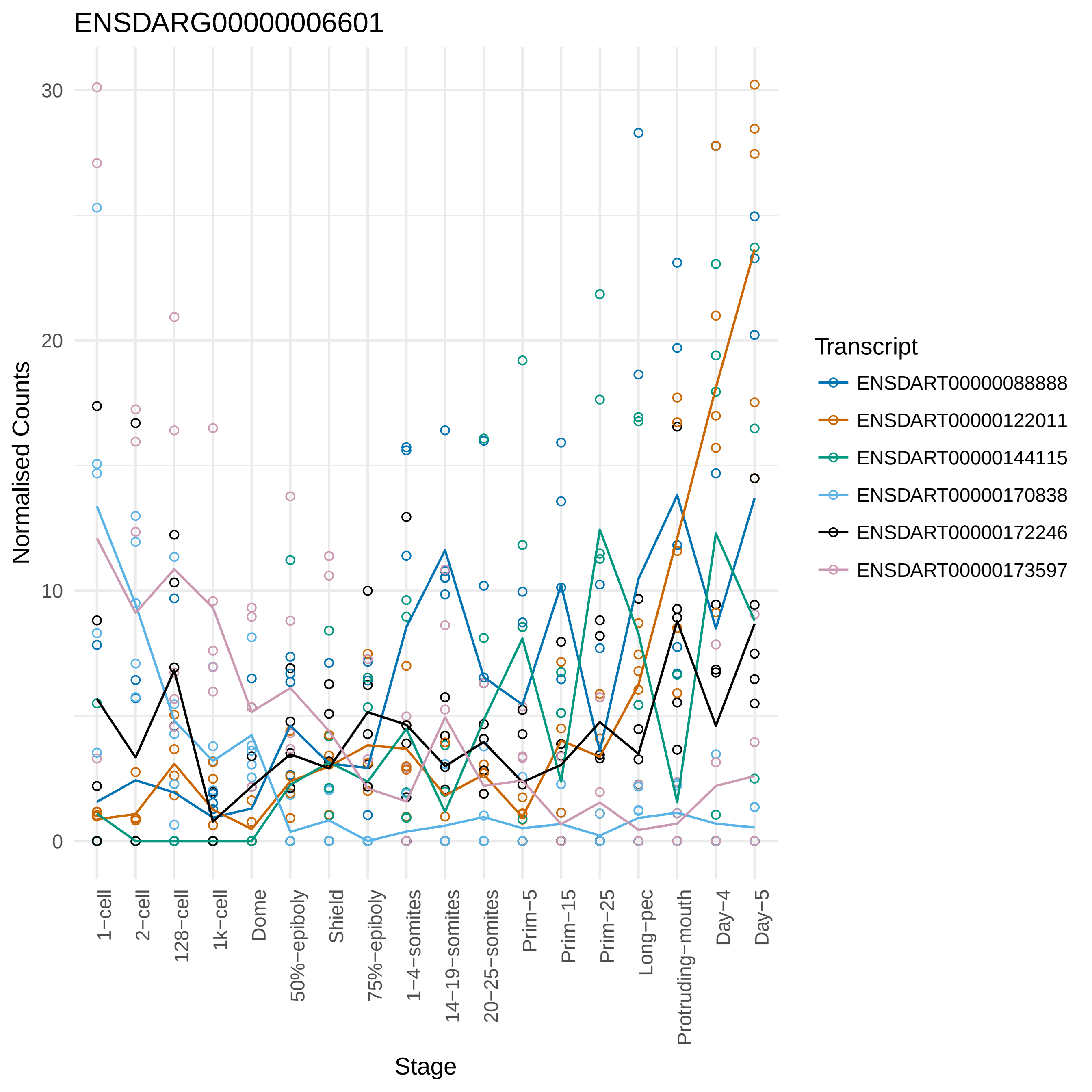
ENSDARG00000006601
Normalised Counts
0
10
20
30
Stage
1−cell
2−cell
128−cell
1k−cell
Dome
50%−epiboly
Shield
75%−epiboly
1−4−somites
14−19−somites
20−25−somites
Prim−5
Prim−15
Prim−25
Long−pec
Protruding−mouth
Day−4
Day−5
Transcript
ENSDART00000088888
ENSDART00000122011
ENSDART00000144115
ENSDART00000170838
ENSDART00000172246
ENSDART00000173597

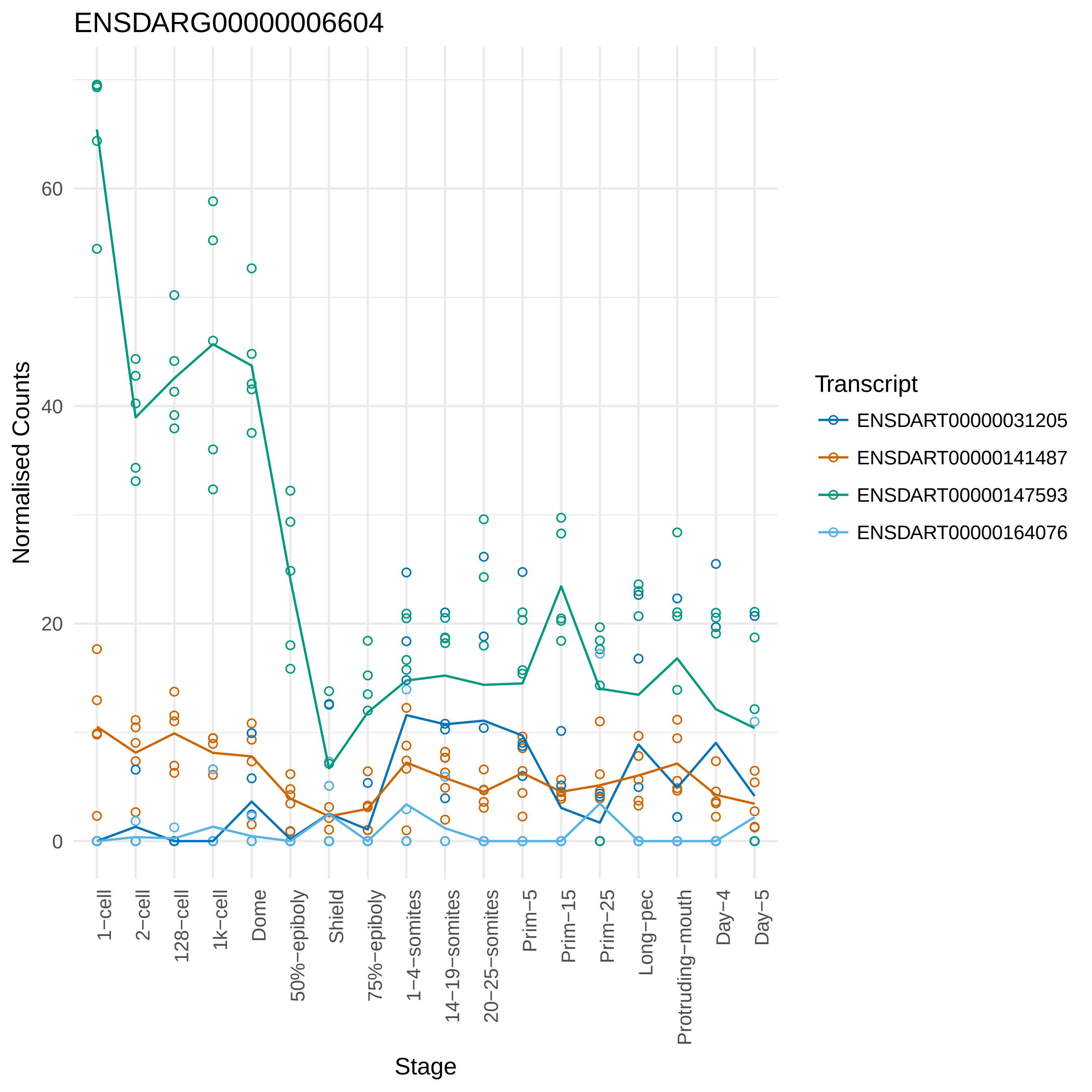
ENSDARG00000006604
Normalised Counts
0
20
40
60
Stage
1−cell
2−cell
128−cell
1k−cell
Dome
50%−epiboly
Shield
75%−epiboly
1−4−somites
14−19−somites
20−25−somites
Prim−5
Prim−15
Prim−25
Long−pec
Protruding−mouth
Day−4
Day−5
Transcript
ENSDART00000031205
ENSDART00000141487
ENSDART00000147593
ENSDART00000164076

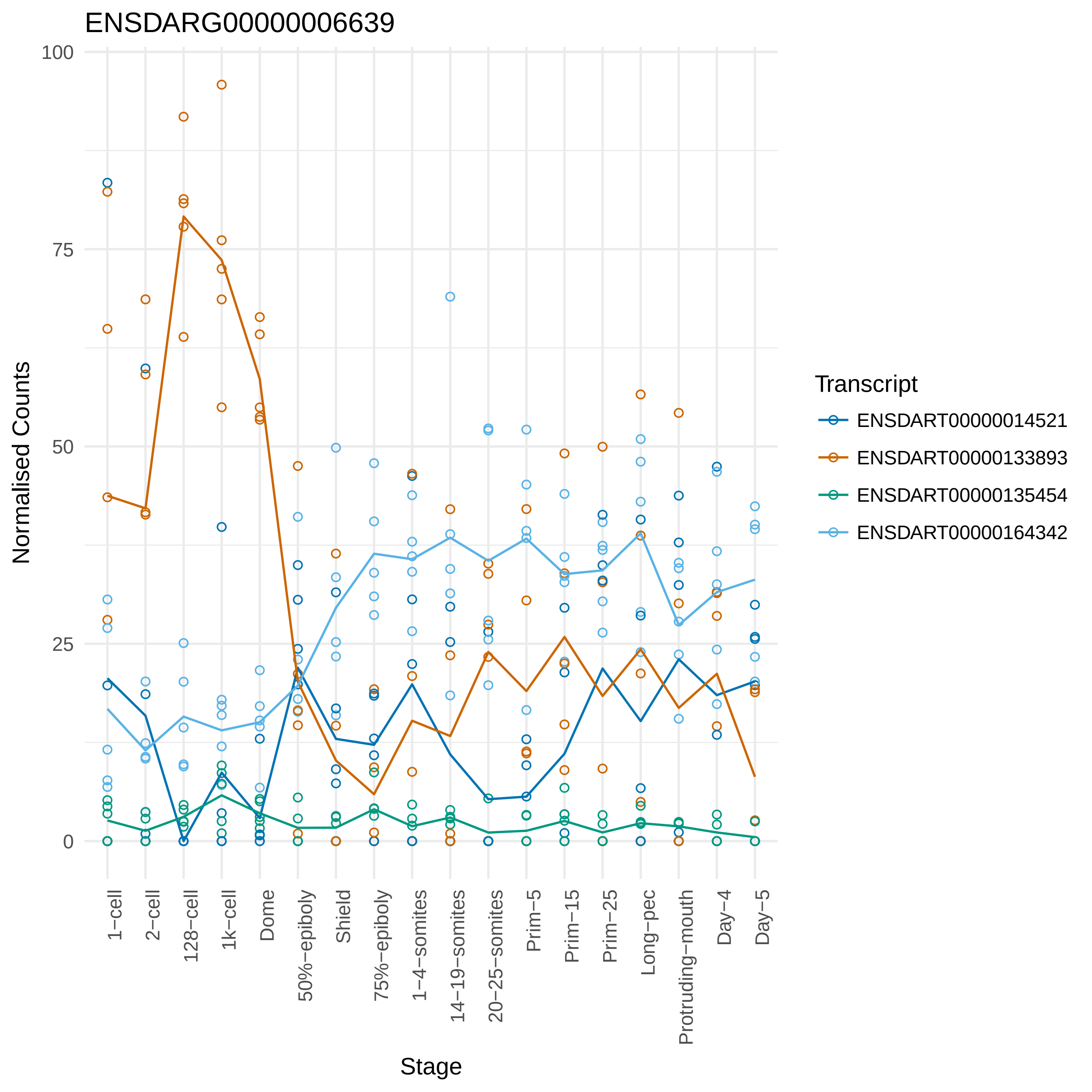

ENSDARG00000006639
Normalised Counts
100
75
50
25
0
1−cell
2−cell
128−cell
1k−cell
Dome
50%−epiboly
Shield
75%−epiboly
1−4−somites
14−19−somites
20−25−somites
Prim−5
Prim−15
Prim−25
Long−pec
Protruding−mouth
Day−4
Day−5
Stage
Transcript
ENSDART00000014521
ENSDART00000133893
ENSDART00000135454
ENSDART00000164342

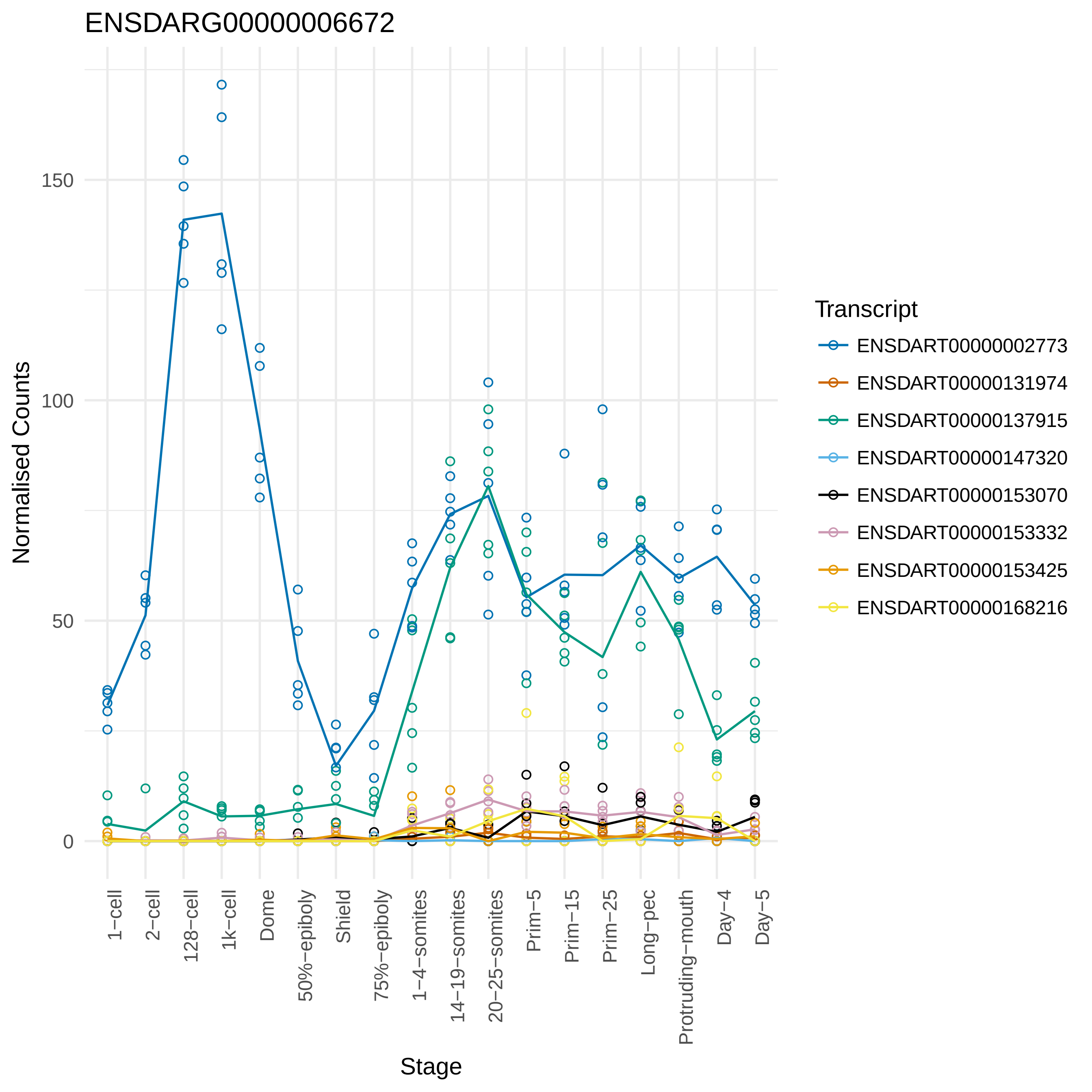

ENSDARG00000006672
Normalised Counts
150
100
50
0
1−cell
2−cell
128−cell
1k−cell
Dome
50%−epiboly
Shield
75%−epiboly
1−4−somites
14−19−somites
20−25−somites
Prim−5
Prim−15
Prim−25
Long−pec
Protruding−mouth
Day−4
Day−5
Stage
Transcript
ENSDART00000002773
ENSDART00000131974
ENSDART00000137915
ENSDART00000147320
ENSDART00000153070
ENSDART00000153332
ENSDART00000153425
ENSDART00000168216

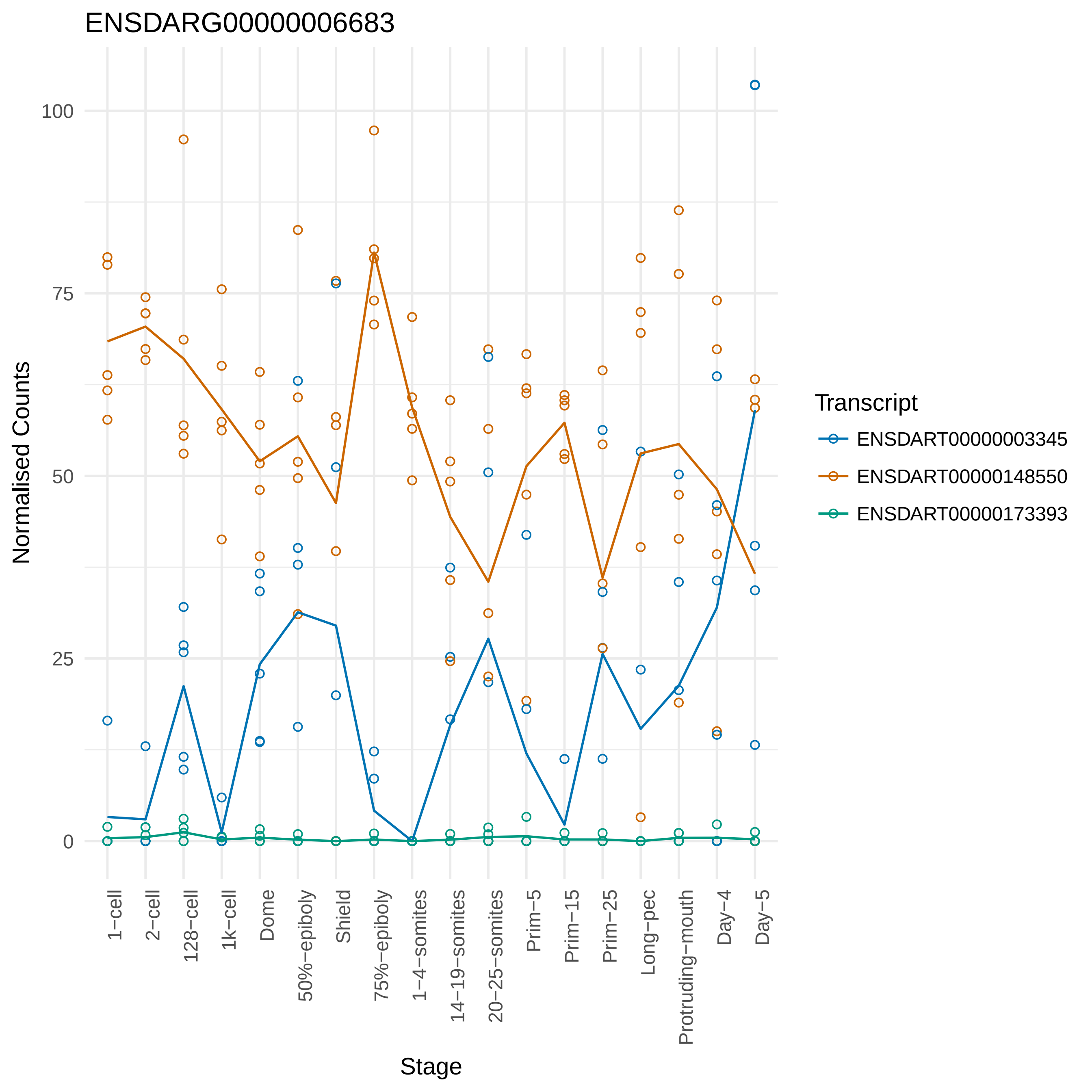
ENSDARG00000006683
Normalised Counts
100
75
50
25
0
1−cell
2−cell
128−cell
1k−cell
Dome
50%−epiboly
Shield
75%−epiboly
1−4−somites
14−19−somites
20−25−somites
Prim−5
Prim−15
Prim−25
Long−pec
Protruding−mouth
Day−4
Day−5
Stage
Transcript
ENSDART00000003345
ENSDART00000148550
ENSDART00000173393

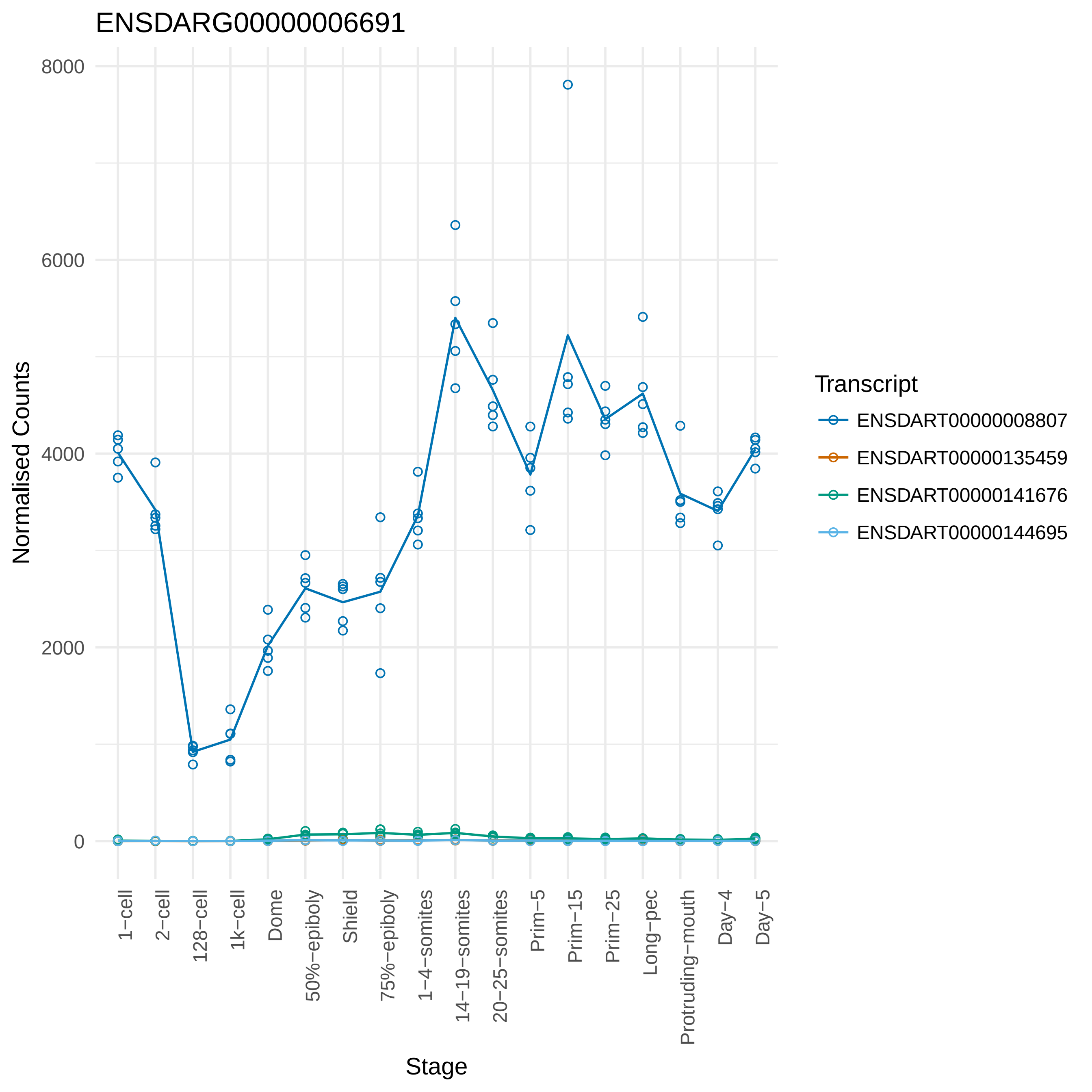

ENSDARG00000006691
Normalised Counts
8000
6000
4000
2000
0
1−cell
2−cell
128−cell
1k−cell
Dome
50%−epiboly
Shield
75%−epiboly
1−4−somites
14−19−somites
20−25−somites
Prim−5
Prim−15
Prim−25
Long−pec
Protruding−mouth
Day−4
Day−5
Stage
Transcript
ENSDART00000008807
ENSDART00000135459
ENSDART00000141676
ENSDART00000144695

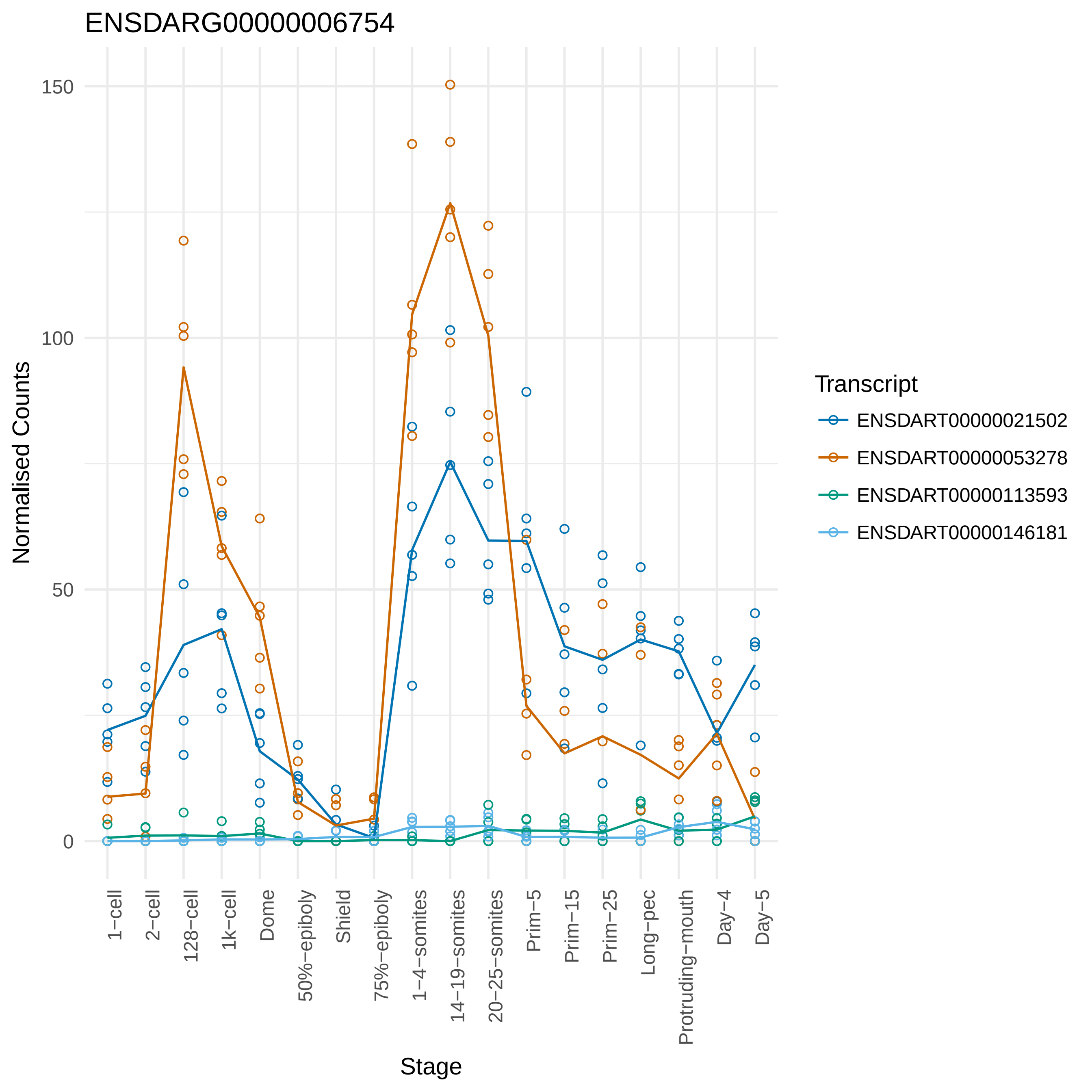
ENSDARG00000006754
Normalised Counts
150
100
50
0
1−cell
2−cell
128−cell
1k−cell
Dome
50%−epiboly
Shield
75%−epiboly
1−4−somites
14−19−somites
20−25−somites
Prim−5
Prim−15
Prim−25
Long−pec
Protruding−mouth
Day−4
Day−5
Stage
Transcript
ENSDART00000021502
ENSDART00000053278
ENSDART00000113593
ENSDART00000146181

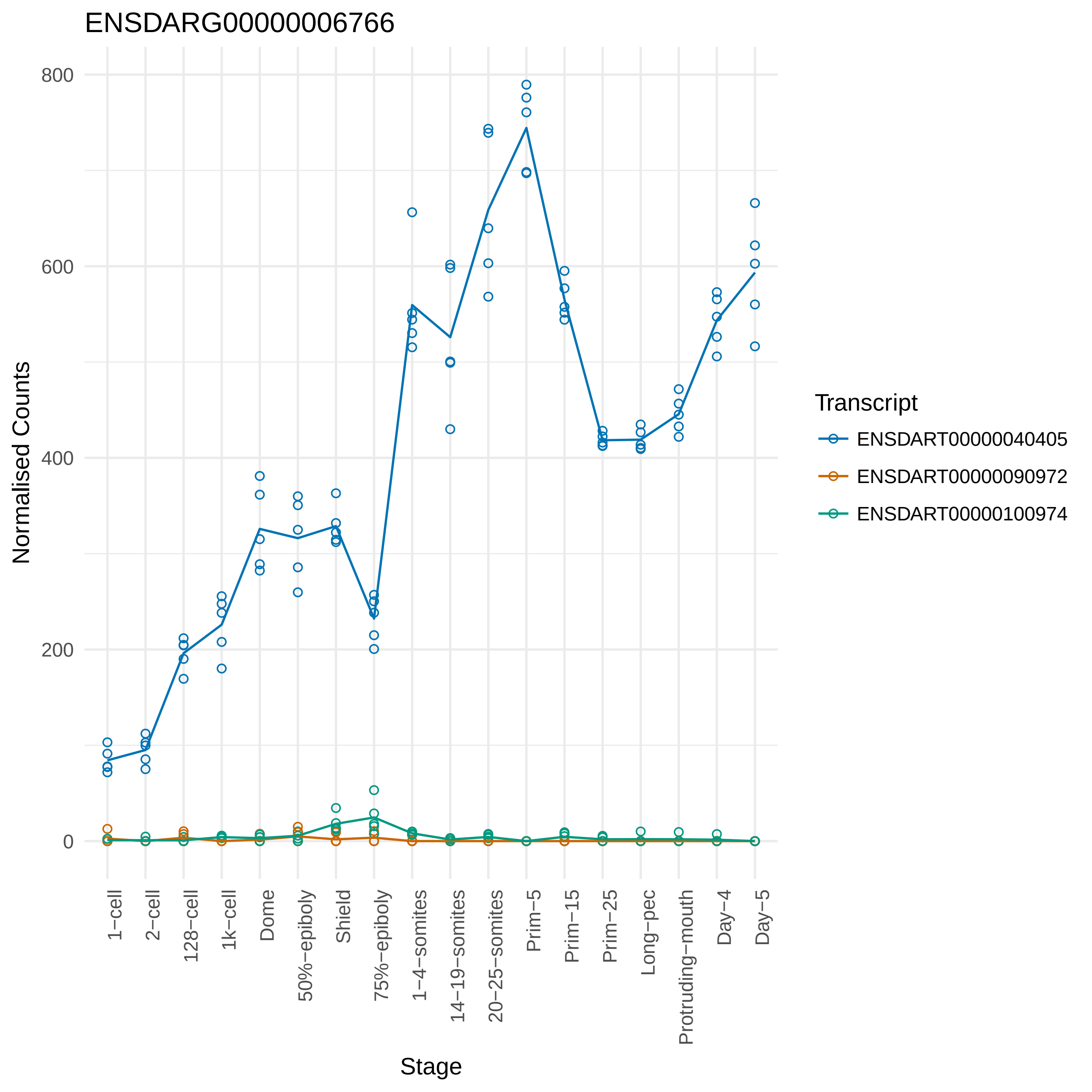

ENSDARG00000006766
Normalised Counts
800
600
400
200
0
1−cell
2−cell
128−cell
1k−cell
Dome
50%−epiboly
Shield
75%−epiboly
1−4−somites
14−19−somites
20−25−somites
Prim−5
Prim−15
Prim−25
Long−pec
Protruding−mouth
Day−4
Day−5
Stage
Transcript
ENSDART00000040405
ENSDART00000090972
ENSDART00000100974

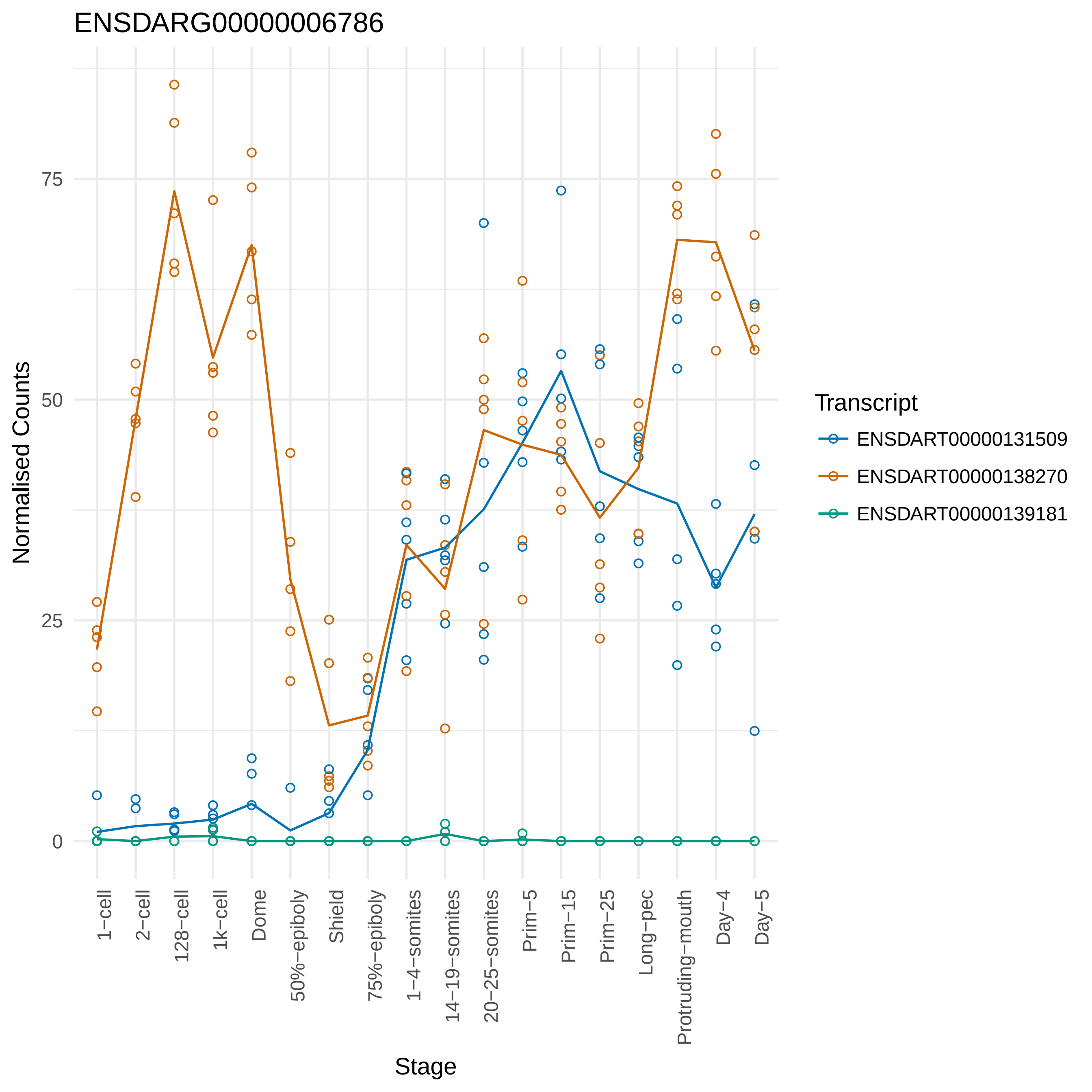

ENSDARG00000006786
Normalised Counts
0
25
50
75
Stage
1−cell
2−cell
128−cell
1k−cell
Dome
50%−epiboly
Shield
75%−epiboly
1−4−somites
14−19−somites
20−25−somites
Prim−5
Prim−15
Prim−25
Long−pec
Protruding−mouth
Day−4
Day−5
Transcript
ENSDART00000131509
ENSDART00000138270
ENSDART00000139181

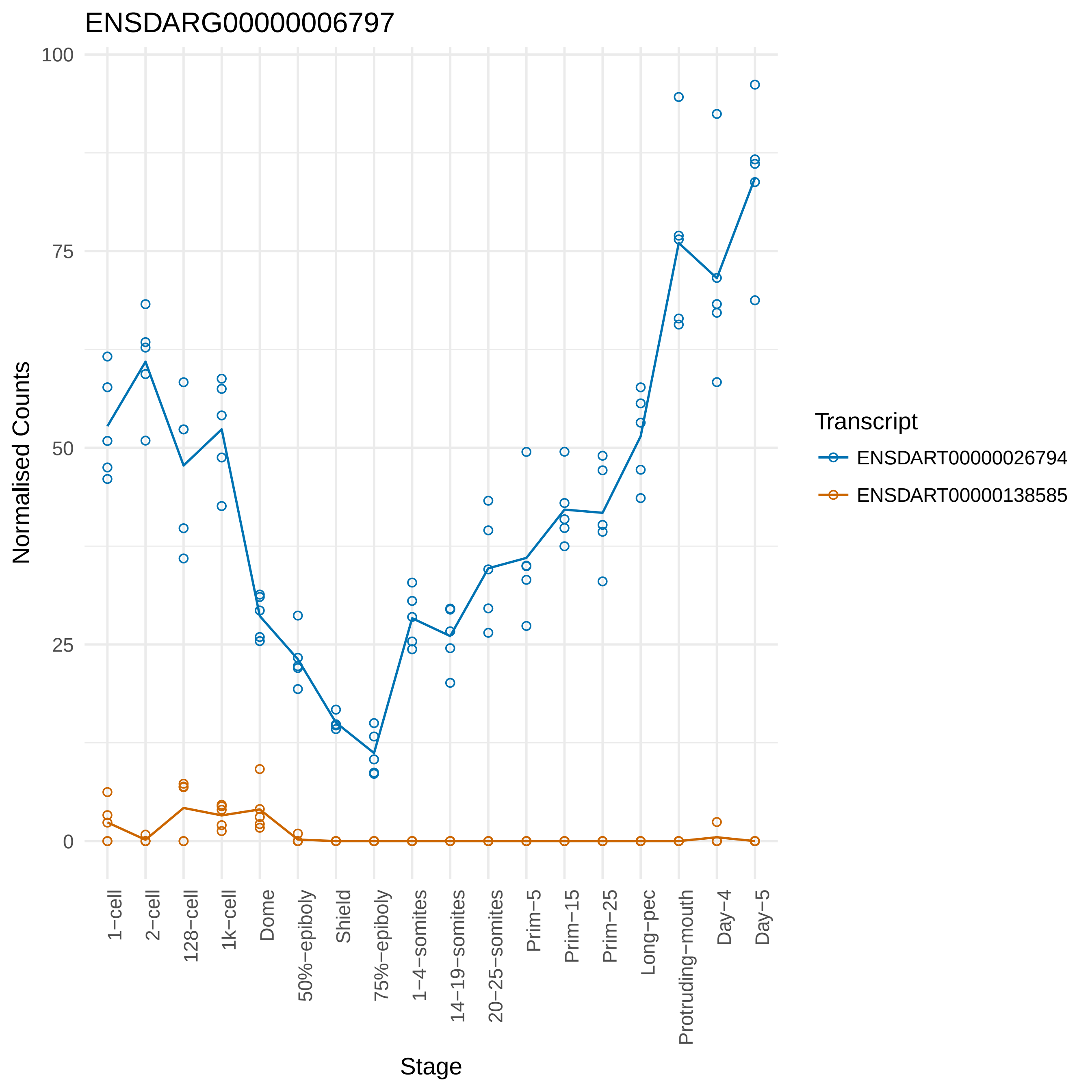
ENSDARG00000006797
Normalised Counts
100
75
50
25
0
1−cell
2−cell
128−cell
1k−cell
Dome
50%−epiboly
Shield
75%−epiboly
1−4−somites
14−19−somites
20−25−somites
Prim−5
Prim−15
Prim−25
Long−pec
Protruding−mouth
Day−4
Day−5
Stage
Transcript
ENSDART00000026794
ENSDART00000138585

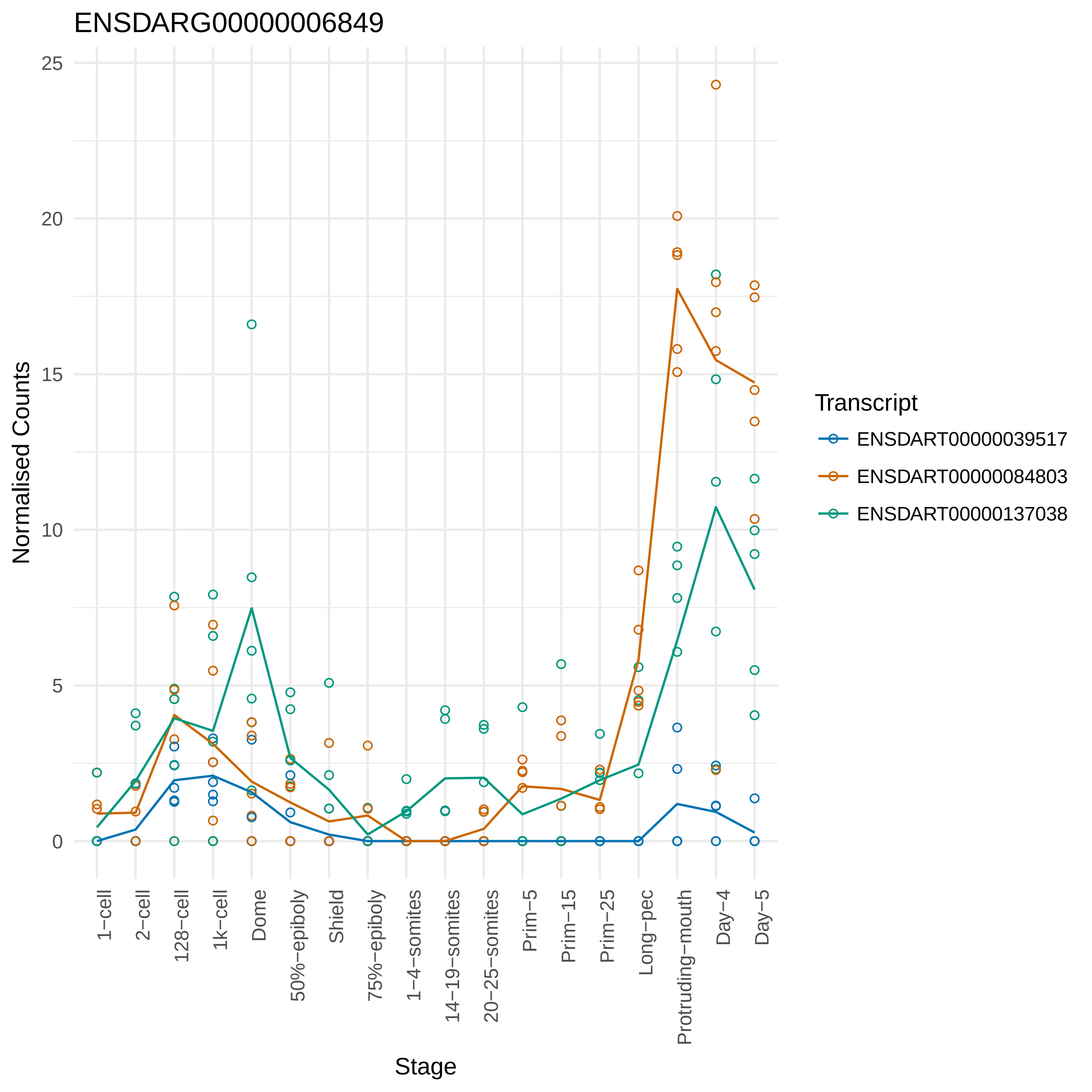

ENSDARG00000006849
Normalised Counts
0
5
10
15
20
25
1−cell
2−cell
128−cell
1k−cell
Dome
50%−epiboly
Shield
75%−epiboly
1−4−somites
14−19−somites
20−25−somites
Prim−5
Prim−15
Prim−25
Long−pec
Protruding−mouth
Day−4
Day−5
Stage
Transcript
ENSDART00000039517
ENSDART00000084803
ENSDART00000137038

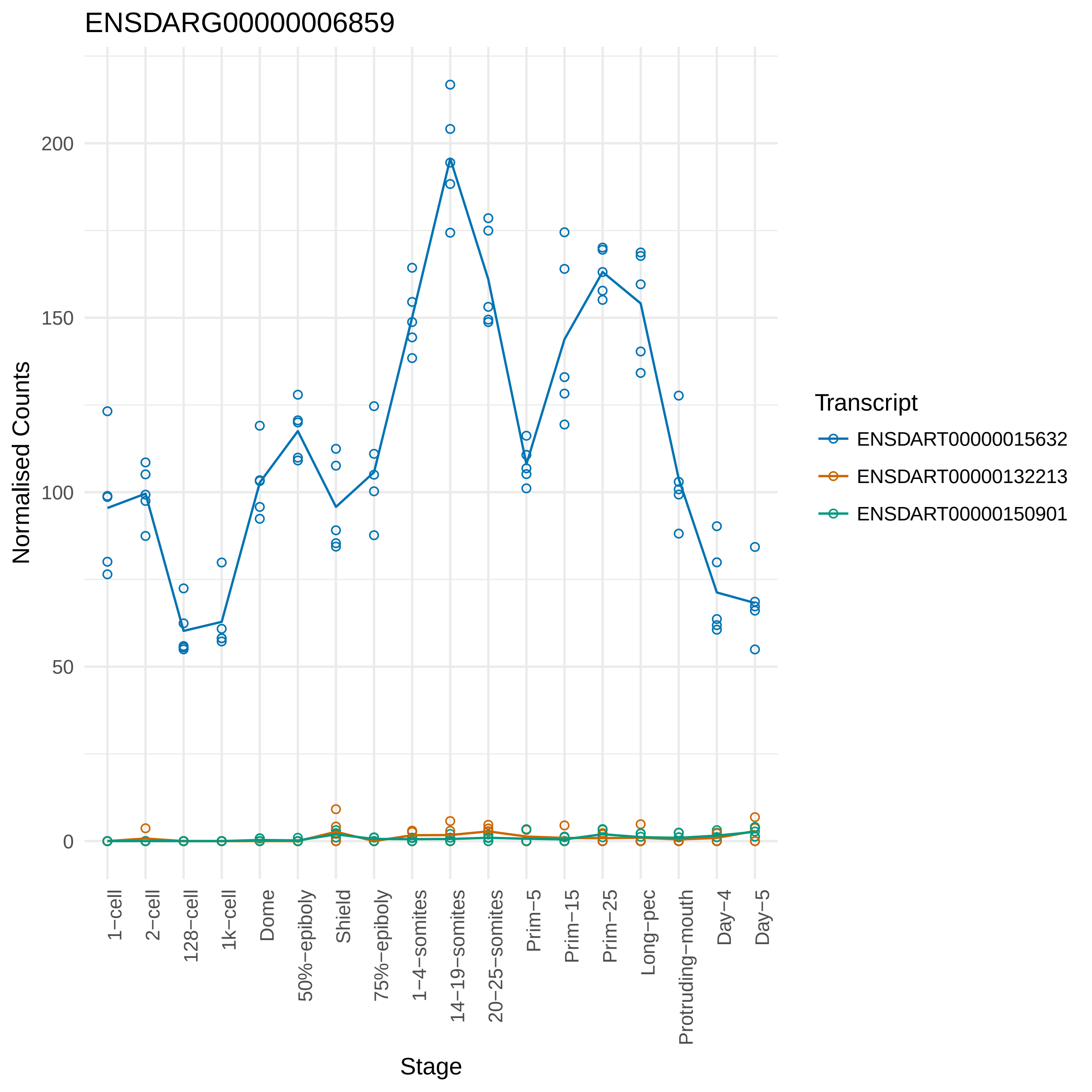

ENSDARG00000006859
Normalised Counts
200
150
100
50
0
1−cell
2−cell
128−cell
1k−cell
Dome
50%−epiboly
Shield
75%−epiboly
1−4−somites
14−19−somites
20−25−somites
Prim−5
Prim−15
Prim−25
Long−pec
Protruding−mouth
Day−4
Day−5
Stage
Transcript
ENSDART00000015632
ENSDART00000132213
ENSDART00000150901

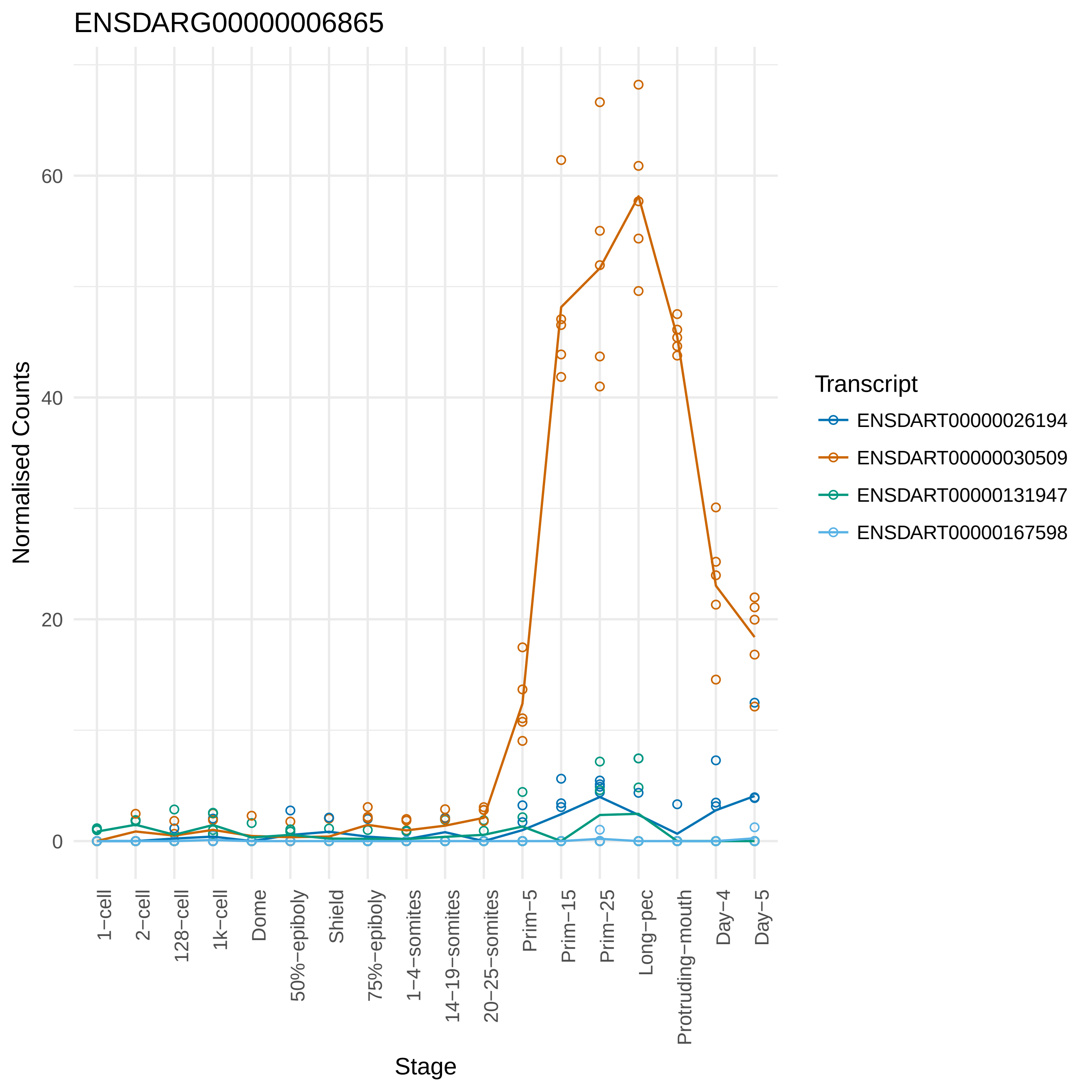

ENSDARG00000006865
Normalised Counts
0
20
40
60
1−cell
2−cell
128−cell
1k−cell
Dome
50%−epiboly
Shield
75%−epiboly
1−4−somites
14−19−somites
20−25−somites
Prim−5
Prim−15
Prim−25
Long−pec
Protruding−mouth
Day−4
Day−5
Stage
Transcript
ENSDART00000026194
ENSDART00000030509
ENSDART00000131947
ENSDART00000167598

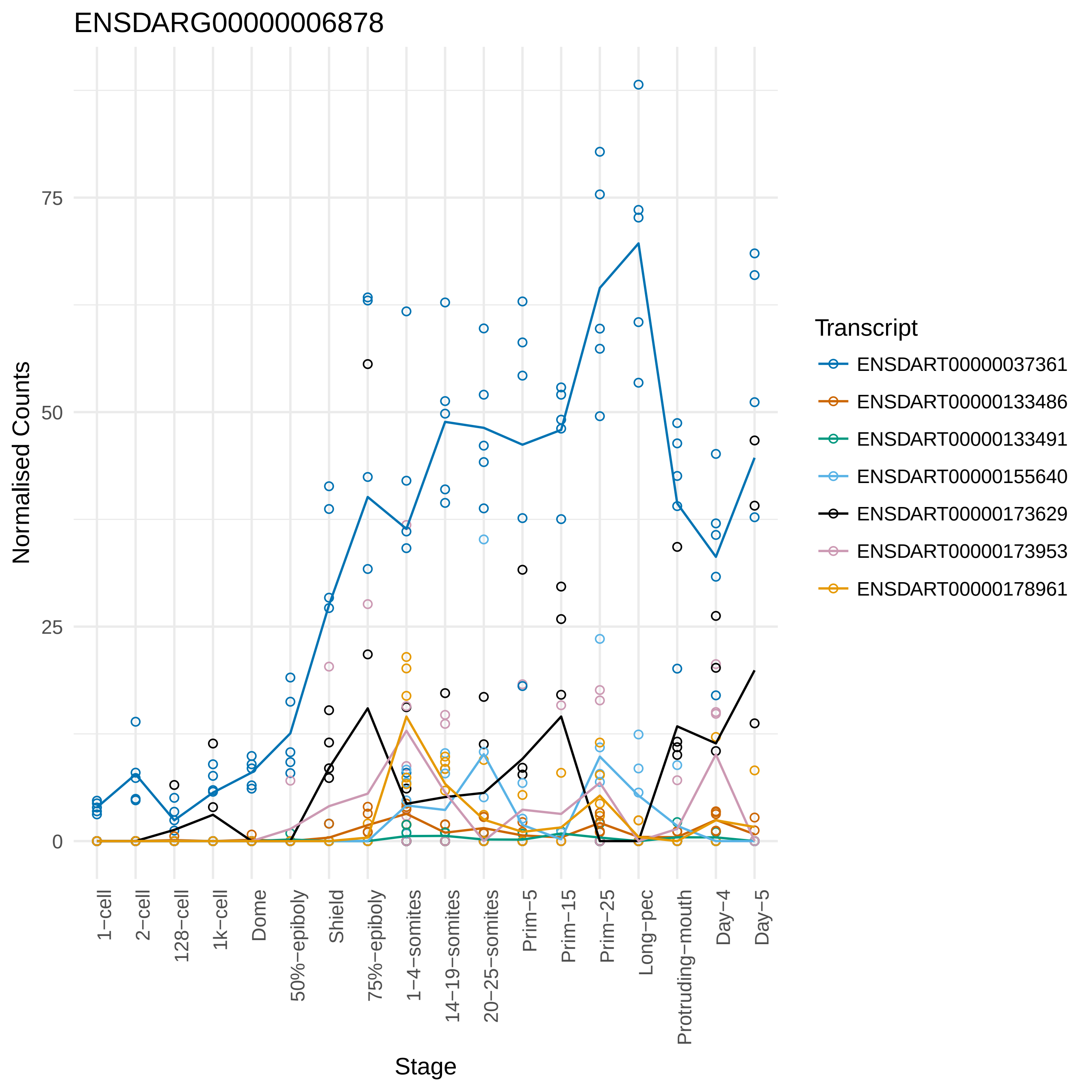
ENSDARG00000006878
Normalised Counts
0
25
50
75
Stage
1−cell
2−cell
128−cell
1k−cell
Dome
50%−epiboly
Shield
75%−epiboly
1−4−somites
14−19−somites
20−25−somites
Prim−5
Prim−15
Prim−25
Long−pec
Protruding−mouth
Day−4
Day−5
Transcript
ENSDART00000037361
ENSDART00000133486
ENSDART00000133491
ENSDART00000155640
ENSDART00000173629
ENSDART00000173953
ENSDART00000178961

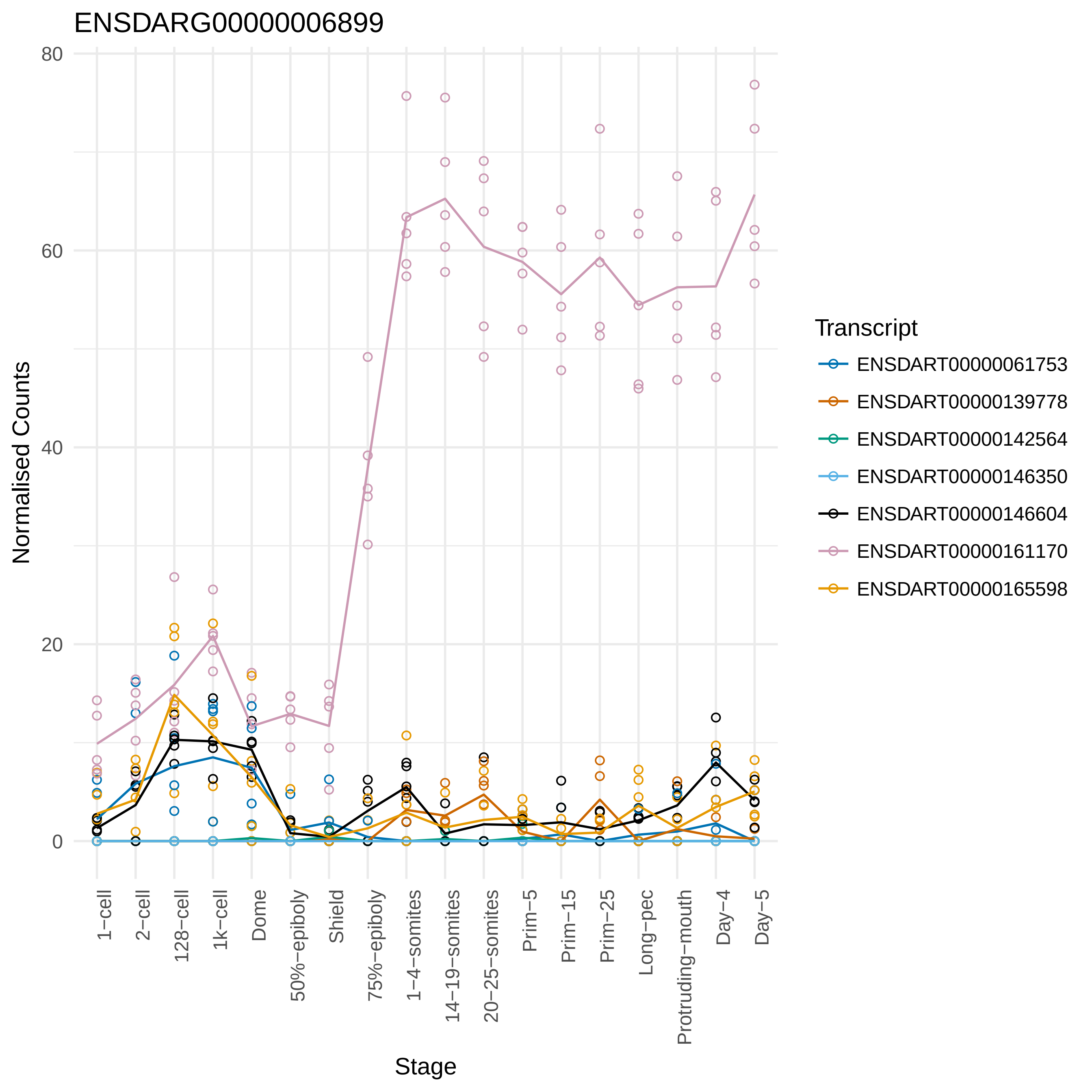

ENSDARG00000006899
Normalised Counts
0
20
40
60
80
1−cell
2−cell
128−cell
1k−cell
Dome
50%−epiboly
Shield
75%−epiboly
1−4−somites
14−19−somites
20−25−somites
Prim−5
Prim−15
Prim−25
Long−pec
Protruding−mouth
Day−4
Day−5
Stage
Transcript
ENSDART00000061753
ENSDART00000139778
ENSDART00000142564
ENSDART00000146350
ENSDART00000146604
ENSDART00000161170
ENSDART00000165598

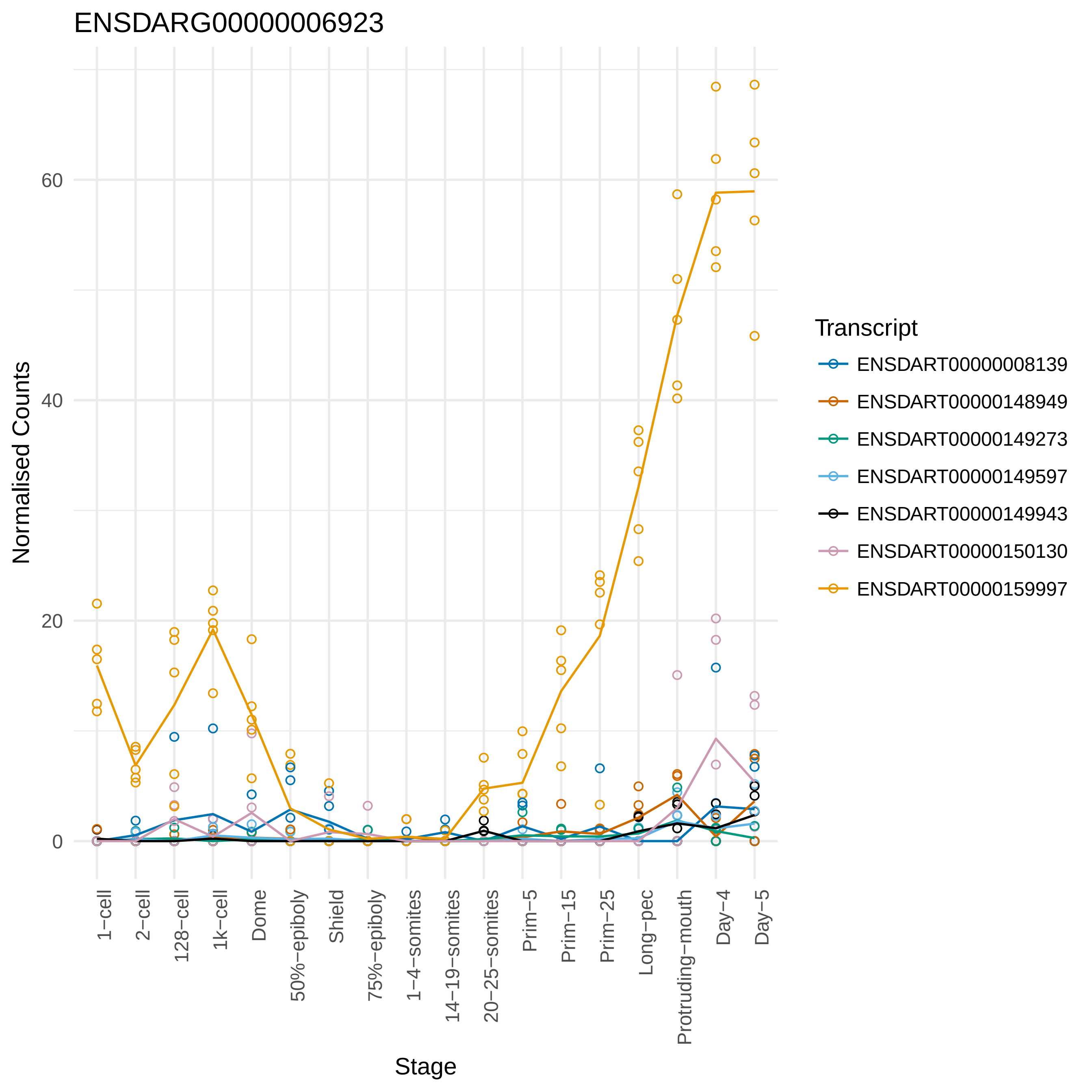
ENSDARG00000006923
Normalised Counts
0
20
40
60
Stage
1−cell
2−cell
128−cell
1k−cell
Dome
50%−epiboly
Shield
75%−epiboly
1−4−somites
14−19−somites
20−25−somites
Prim−5
Prim−15
Prim−25
Long−pec
Protruding−mouth
Day−4
Day−5
Transcript
ENSDART00000008139
ENSDART00000148949
ENSDART00000149273
ENSDART00000149597
ENSDART00000149943
ENSDART00000150130
ENSDART00000159997

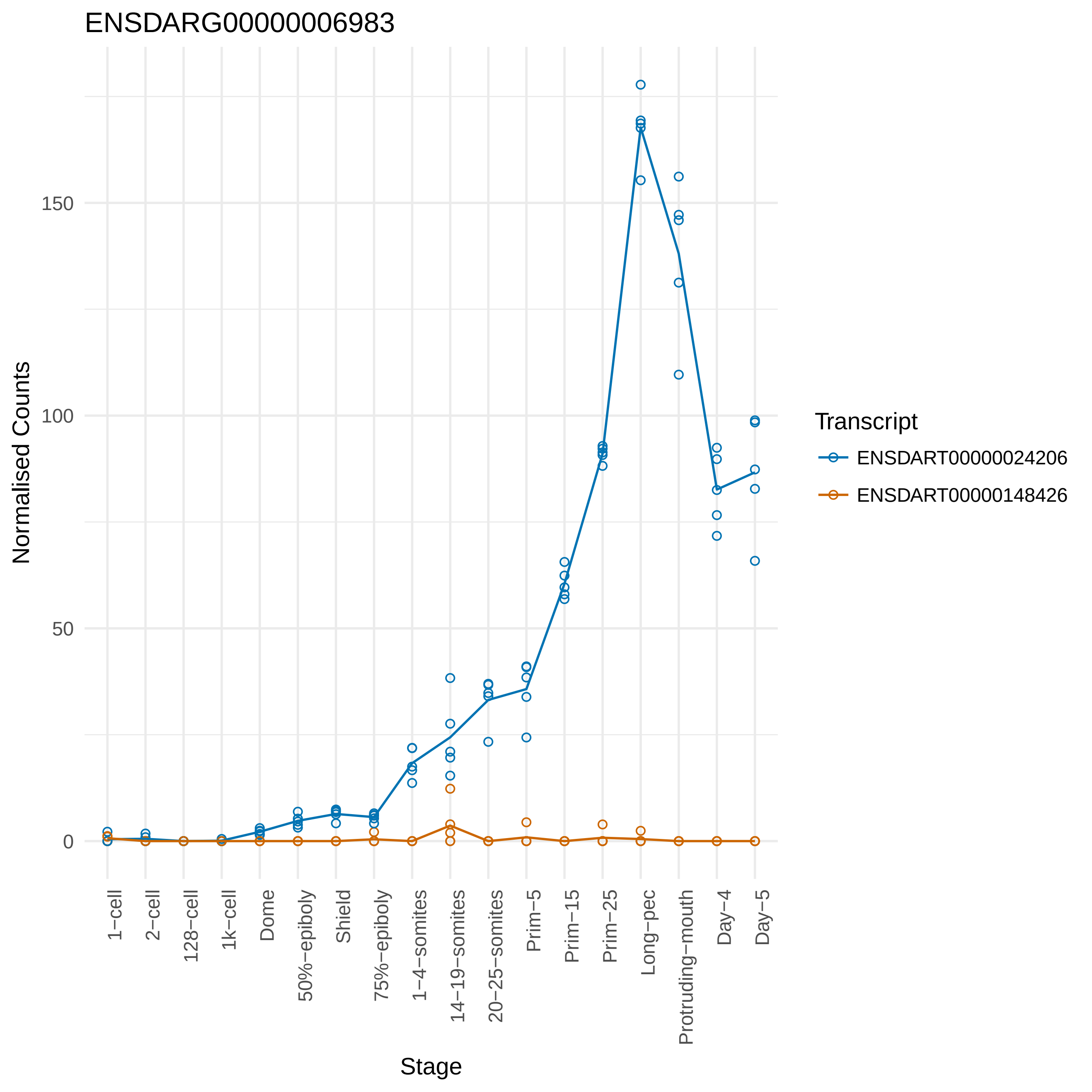

ENSDARG00000006983
Normalised Counts
150
100
50
0
1−cell
2−cell
128−cell
1k−cell
Dome
50%−epiboly
Shield
75%−epiboly
1−4−somites
14−19−somites
20−25−somites
Prim−5
Prim−15
Prim−25
Long−pec
Protruding−mouth
Day−4
Day−5
Stage
Transcript
ENSDART00000024206
ENSDART00000148426

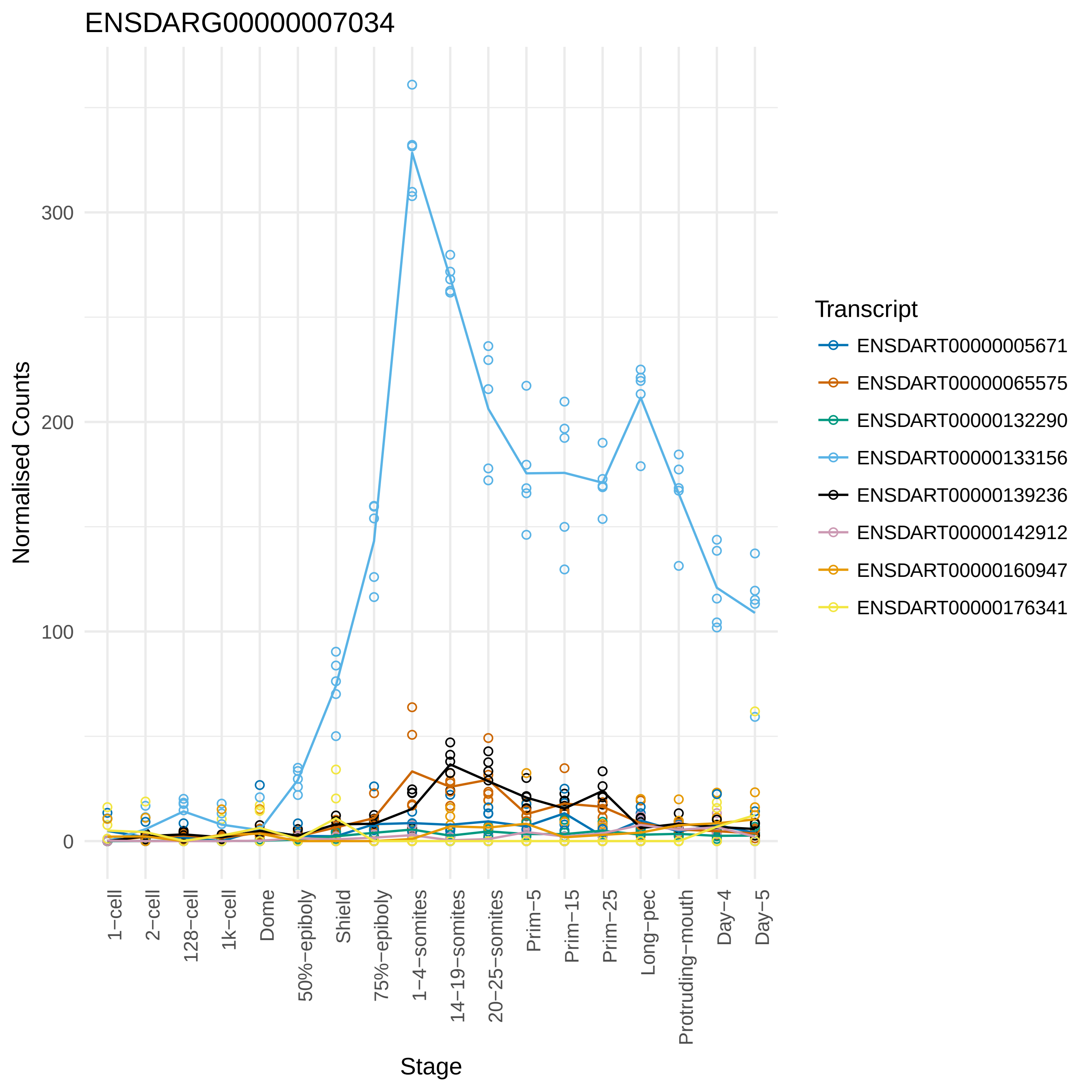
ENSDARG00000007034
Normalised Counts
0
100
200
300
Stage
1−cell
2−cell
128−cell
1k−cell
Dome
50%−epiboly
Shield
75%−epiboly
1−4−somites
14−19−somites
20−25−somites
Prim−5
Prim−15
Prim−25
Long−pec
Protruding−mouth
Day−4
Day−5
Transcript
ENSDART00000005671
ENSDART00000065575
ENSDART00000132290
ENSDART00000133156
ENSDART00000139236
ENSDART00000142912
ENSDART00000160947
ENSDART00000176341

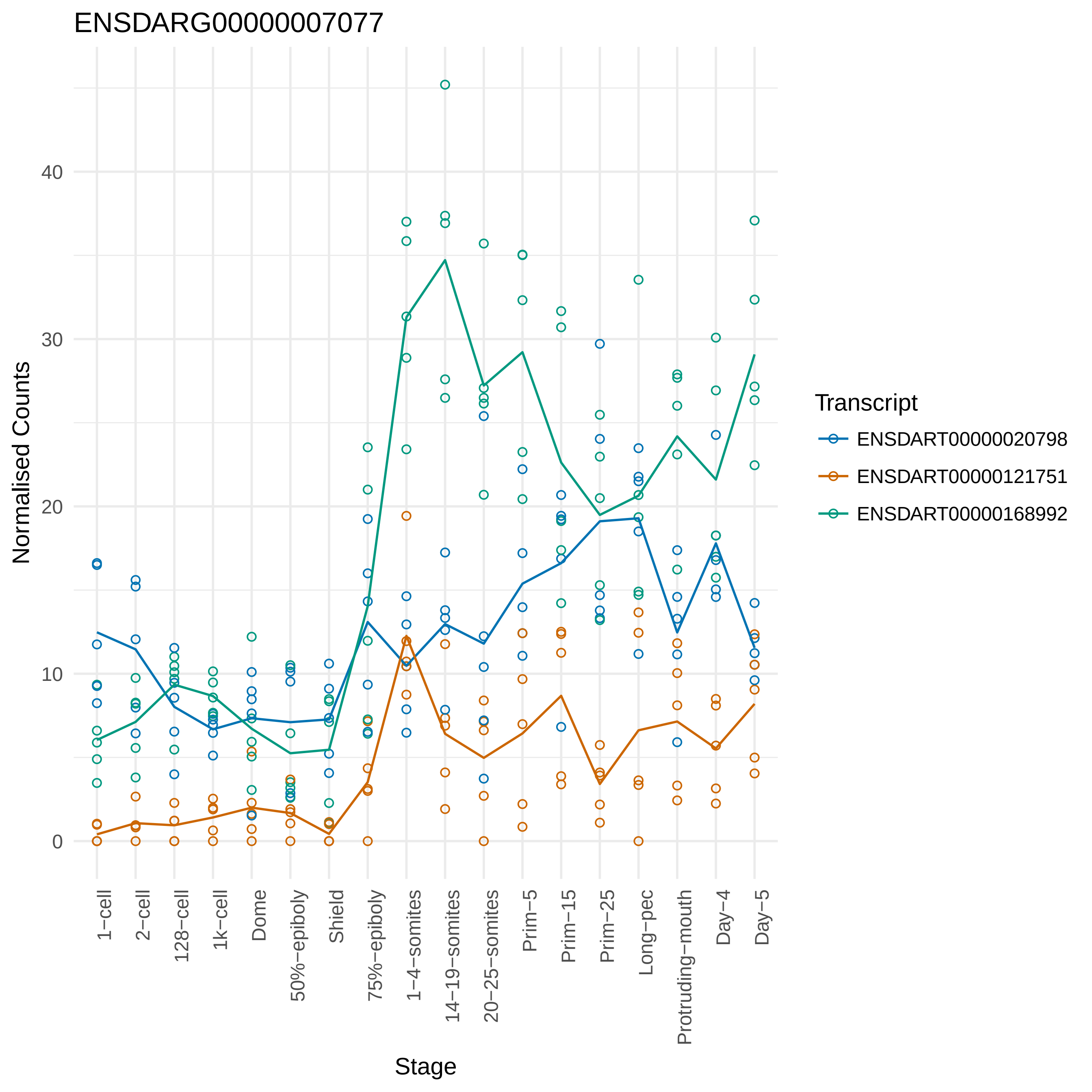

ENSDARG00000007077
Normalised Counts
0
10
20
30
40
Stage
1−cell
2−cell
128−cell
1k−cell
Dome
50%−epiboly
Shield
75%−epiboly
1−4−somites
14−19−somites
20−25−somites
Prim−5
Prim−15
Prim−25
Long−pec
Protruding−mouth
Day−4
Day−5
Transcript
ENSDART00000020798
ENSDART00000121751
ENSDART00000168992

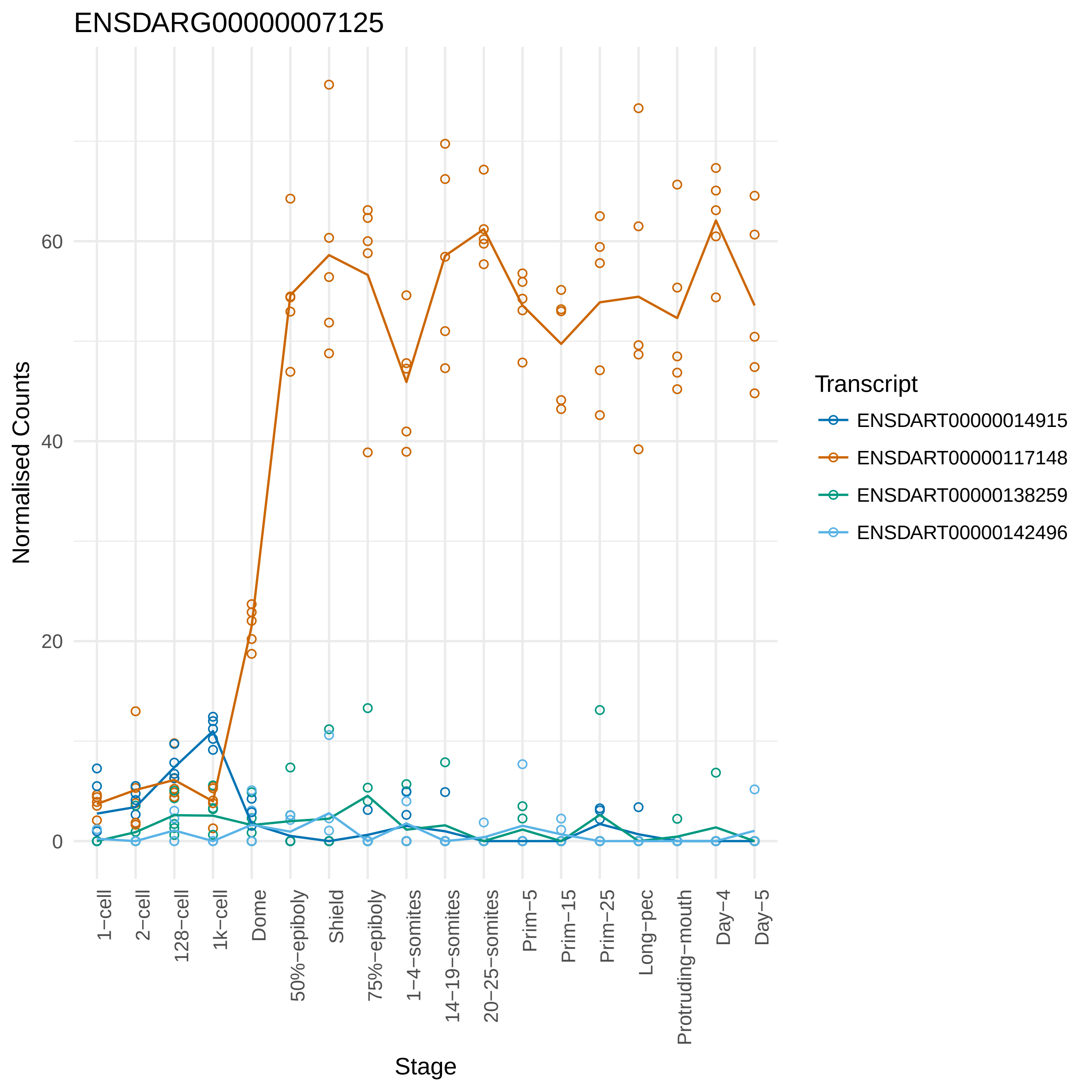

ENSDARG00000007125
Normalised Counts
0
20
40
60
Stage
1−cell
2−cell
128−cell
1k−cell
Dome
50%−epiboly
Shield
75%−epiboly
1−4−somites
14−19−somites
20−25−somites
Prim−5
Prim−15
Prim−25
Long−pec
Protruding−mouth
Day−4
Day−5
Transcript
ENSDART00000014915
ENSDART00000117148
ENSDART00000138259
ENSDART00000142496

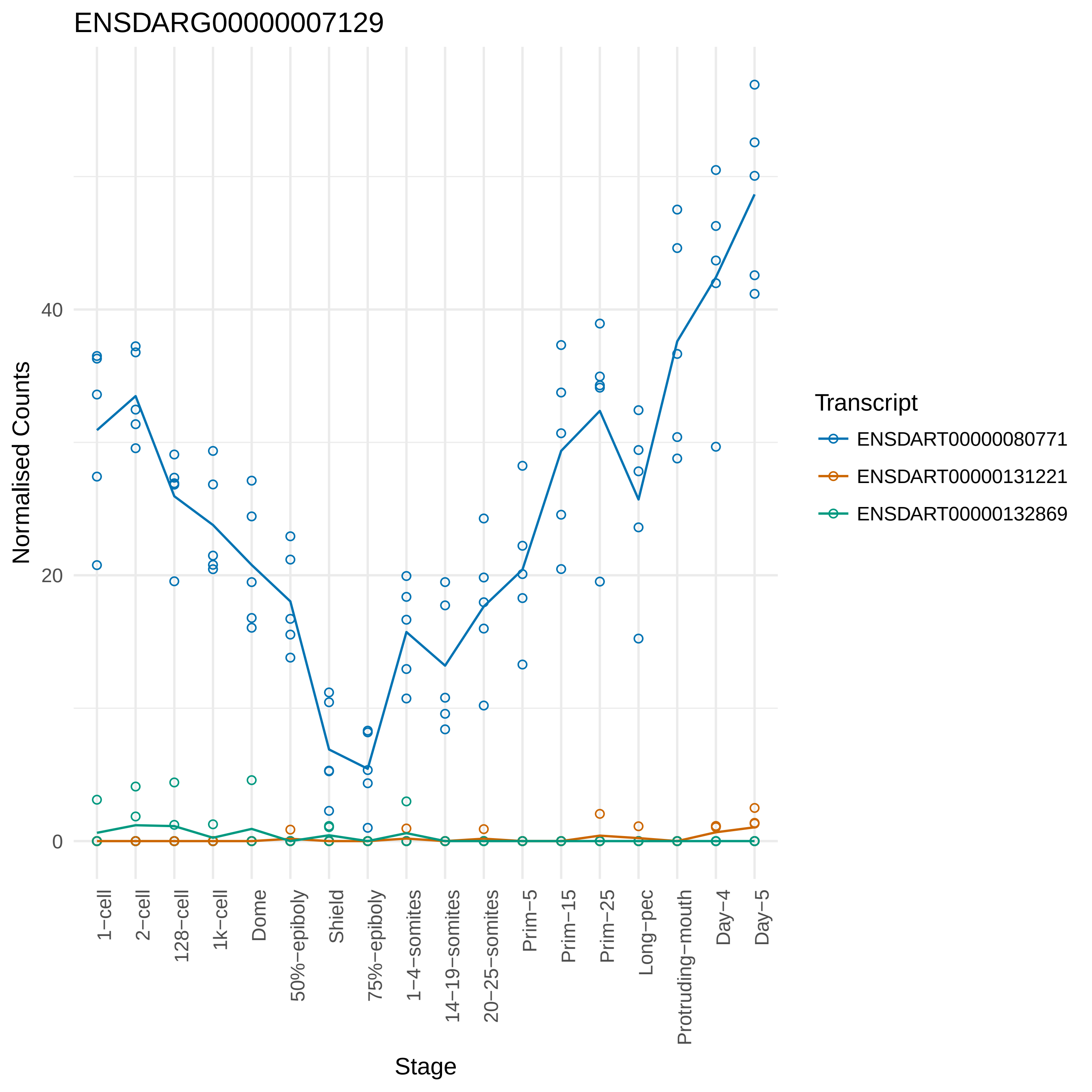

ENSDARG00000007129
Normalised Counts
0
20
40
1−cell
2−cell
128−cell
1k−cell
Dome
50%−epiboly
Shield
75%−epiboly
1−4−somites
14−19−somites
20−25−somites
Prim−5
Prim−15
Prim−25
Long−pec
Protruding−mouth
Day−4
Day−5
Stage
Transcript
ENSDART00000080771
ENSDART00000131221
ENSDART00000132869

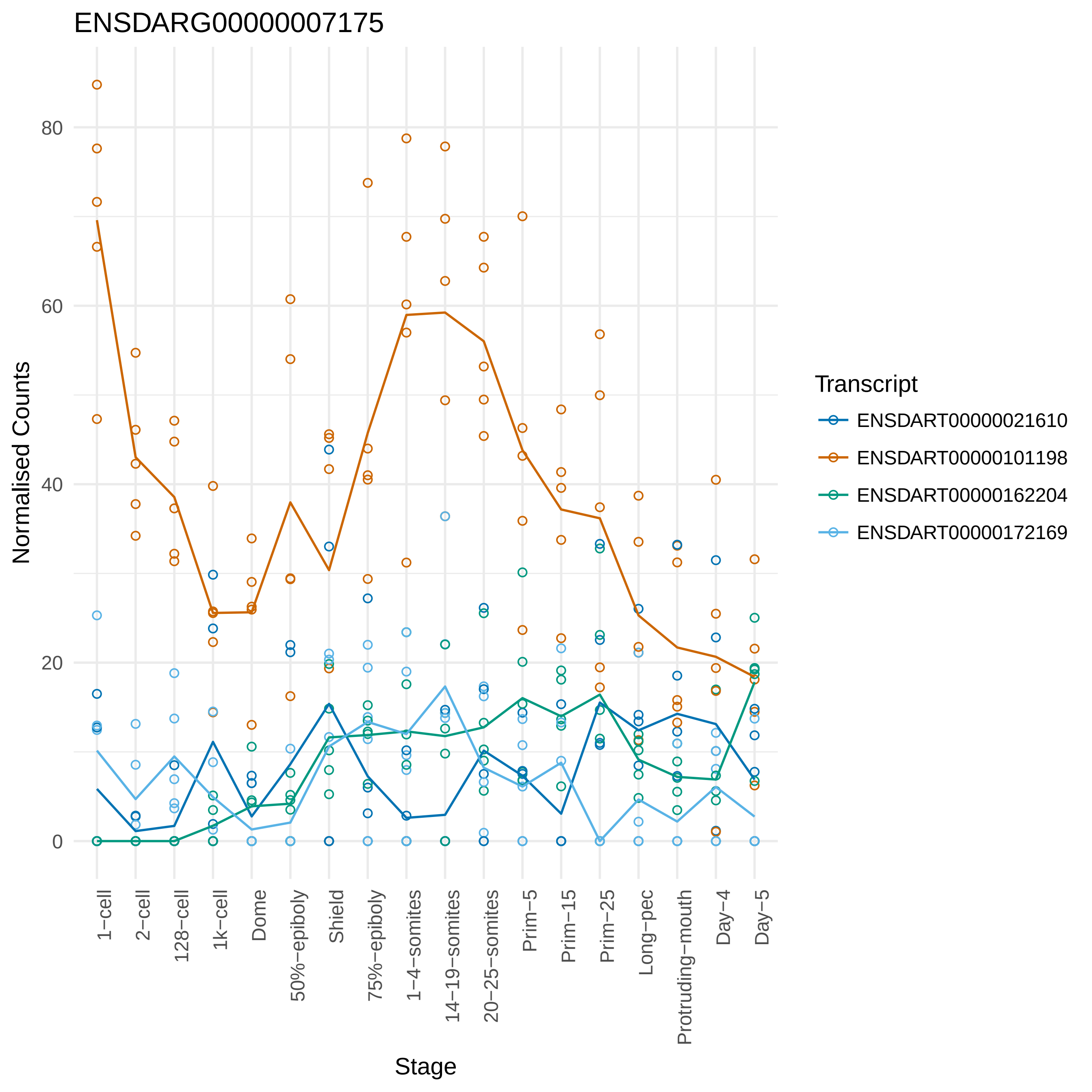
ENSDARG00000007175
Normalised Counts
0
20
40
60
80
1−cell
2−cell
128−cell
1k−cell
Dome
50%−epiboly
Shield
75%−epiboly
1−4−somites
14−19−somites
20−25−somites
Prim−5
Prim−15
Prim−25
Long−pec
Protruding−mouth
Day−4
Day−5
Stage
Transcript
ENSDART00000021610
ENSDART00000101198
ENSDART00000162204
ENSDART00000172169

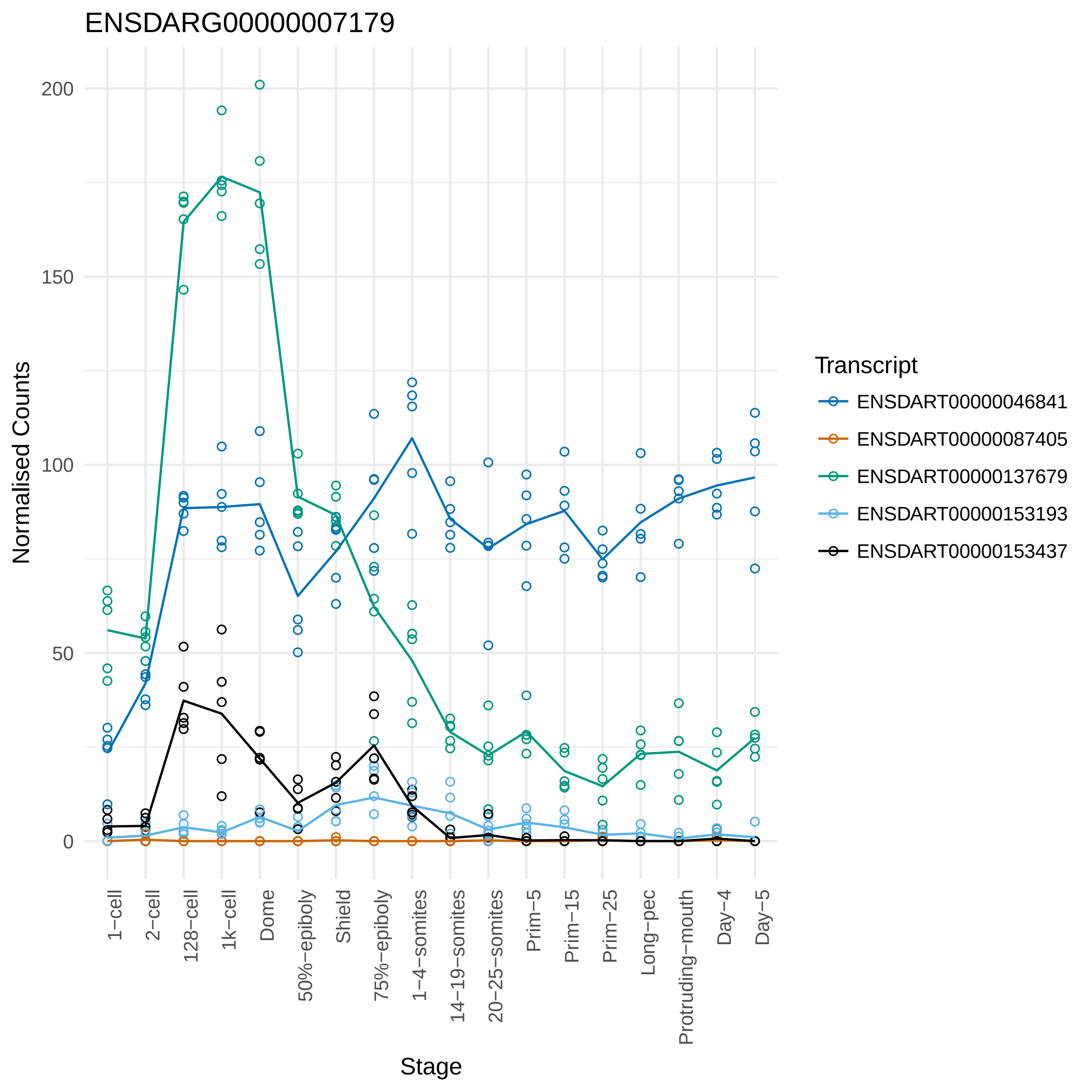

ENSDARG00000007179
Normalised Counts
200
150
100
50
0
1−cell
2−cell
128−cell
1k−cell
Dome
50%−epiboly
Shield
75%−epiboly
1−4−somites
14−19−somites
20−25−somites
Prim−5
Prim−15
Prim−25
Long−pec
Protruding−mouth
Day−4
Day−5
Stage
Transcript
ENSDART00000046841
ENSDART00000087405
ENSDART00000137679
ENSDART00000153193
ENSDART00000153437

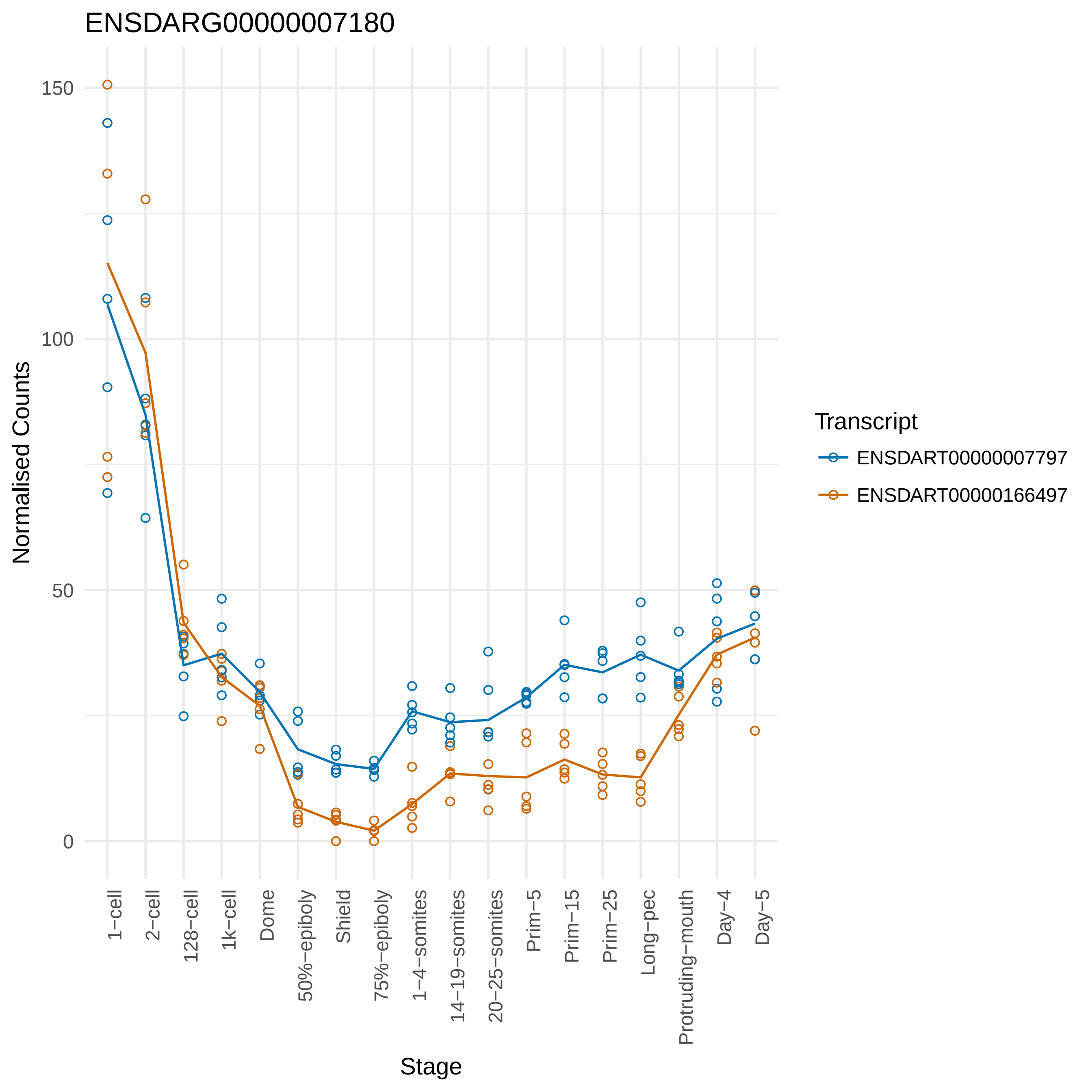

ENSDARG00000007180
Normalised Counts
150
100
50
0
1−cell
2−cell
128−cell
1k−cell
Dome
50%−epiboly
Shield
75%−epiboly
1−4−somites
14−19−somites
20−25−somites
Prim−5
Prim−15
Prim−25
Long−pec
Protruding−mouth
Day−4
Day−5
Stage
Transcript
ENSDART00000007797
ENSDART00000166497

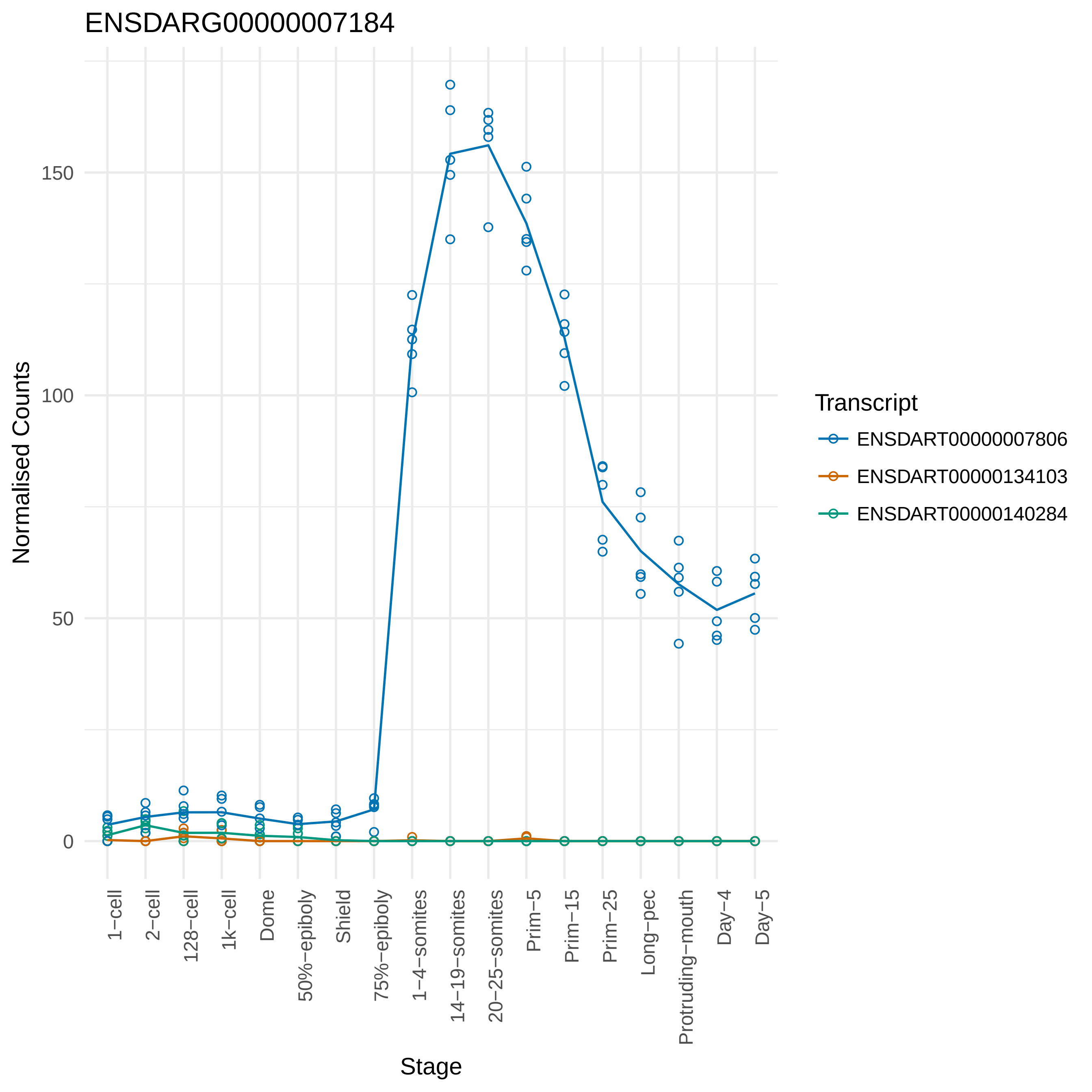
ENSDARG00000007184
Normalised Counts
0
50
100
150
Stage
1−cell
2−cell
128−cell
1k−cell
Dome
50%−epiboly
Shield
75%−epiboly
1−4−somites
14−19−somites
20−25−somites
Prim−5
Prim−15
Prim−25
Long−pec
Protruding−mouth
Day−4
Day−5
Transcript
ENSDART00000007806
ENSDART00000134103
ENSDART00000140284

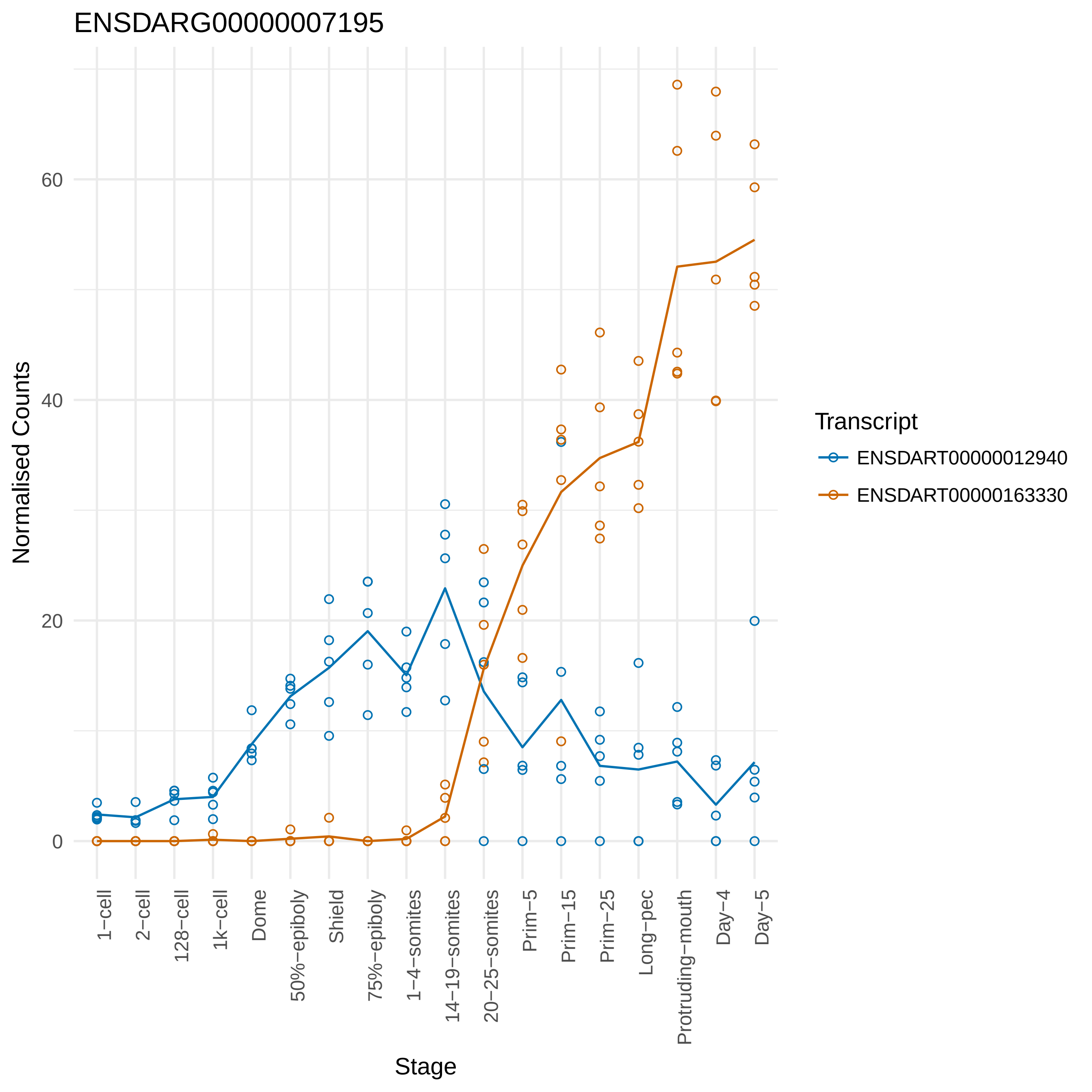

ENSDARG00000007195
Normalised Counts
0
20
40
60
1−cell
2−cell
128−cell
1k−cell
Dome
50%−epiboly
Shield
75%−epiboly
1−4−somites
14−19−somites
20−25−somites
Prim−5
Prim−15
Prim−25
Long−pec
Protruding−mouth
Day−4
Day−5
Stage
Transcript
ENSDART00000012940
ENSDART00000163330

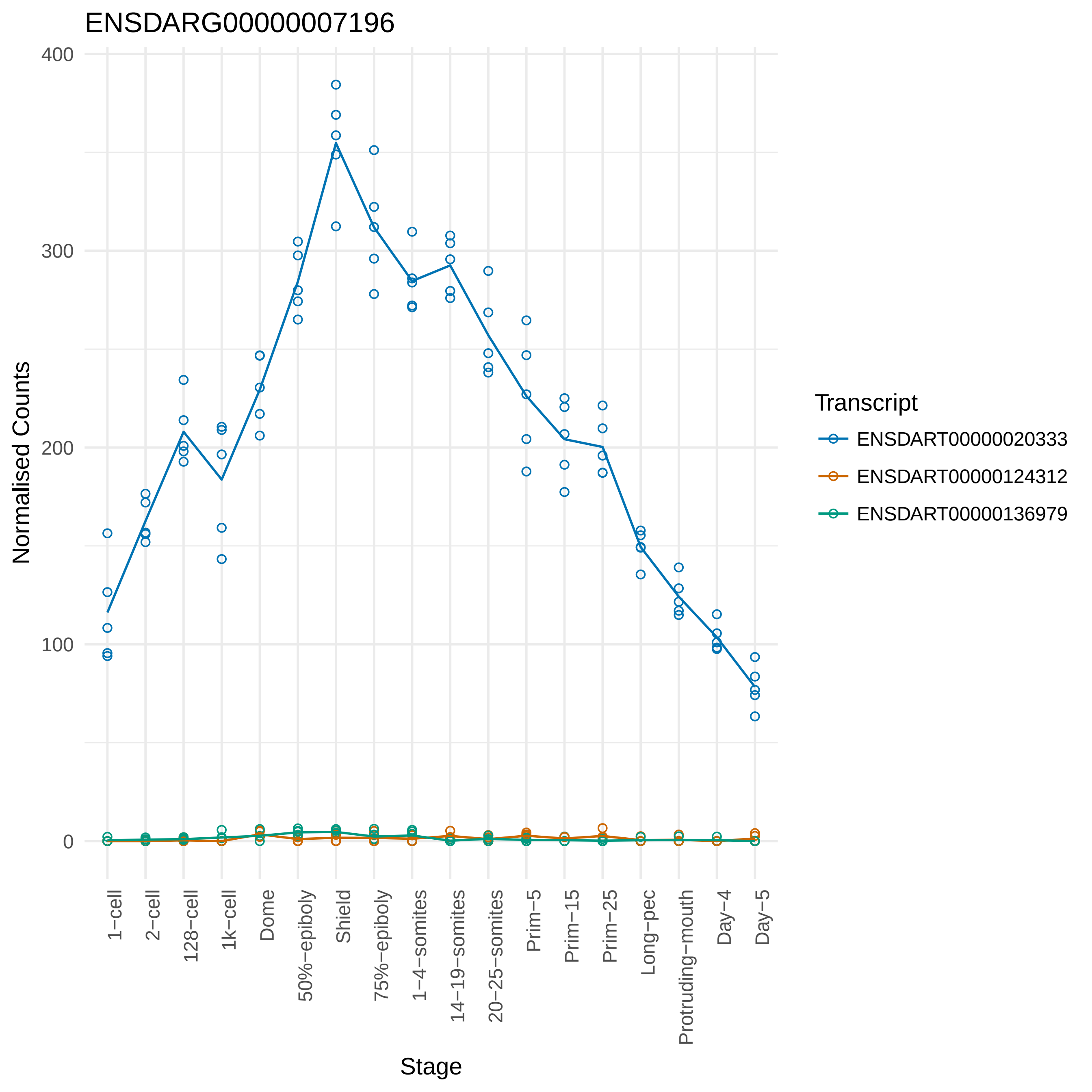

ENSDARG00000007196
Normalised Counts
400
300
200
100
0
1−cell
2−cell
128−cell
1k−cell
Dome
50%−epiboly
Shield
75%−epiboly
1−4−somites
14−19−somites
20−25−somites
Prim−5
Prim−15
Prim−25
Long−pec
Protruding−mouth
Day−4
Day−5
Stage
Transcript
ENSDART00000020333
ENSDART00000124312
ENSDART00000136979

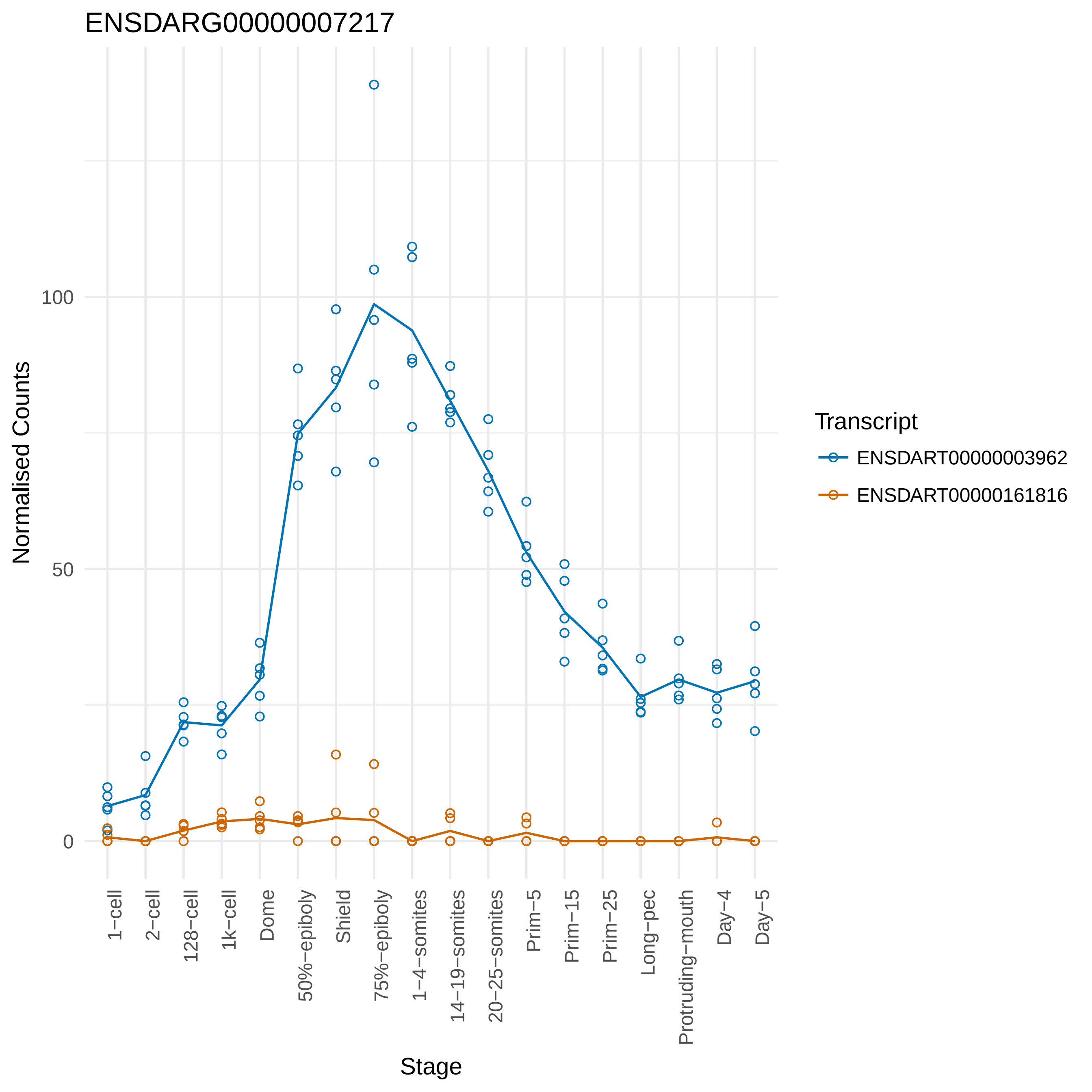

ENSDARG00000007217
Normalised Counts
100
50
0
1−cell
2−cell
128−cell
1k−cell
Dome
50%−epiboly
Shield
75%−epiboly
1−4−somites
14−19−somites
20−25−somites
Prim−5
Prim−15
Prim−25
Long−pec
Protruding−mouth
Day−4
Day−5
Stage
Transcript
ENSDART00000003962
ENSDART00000161816

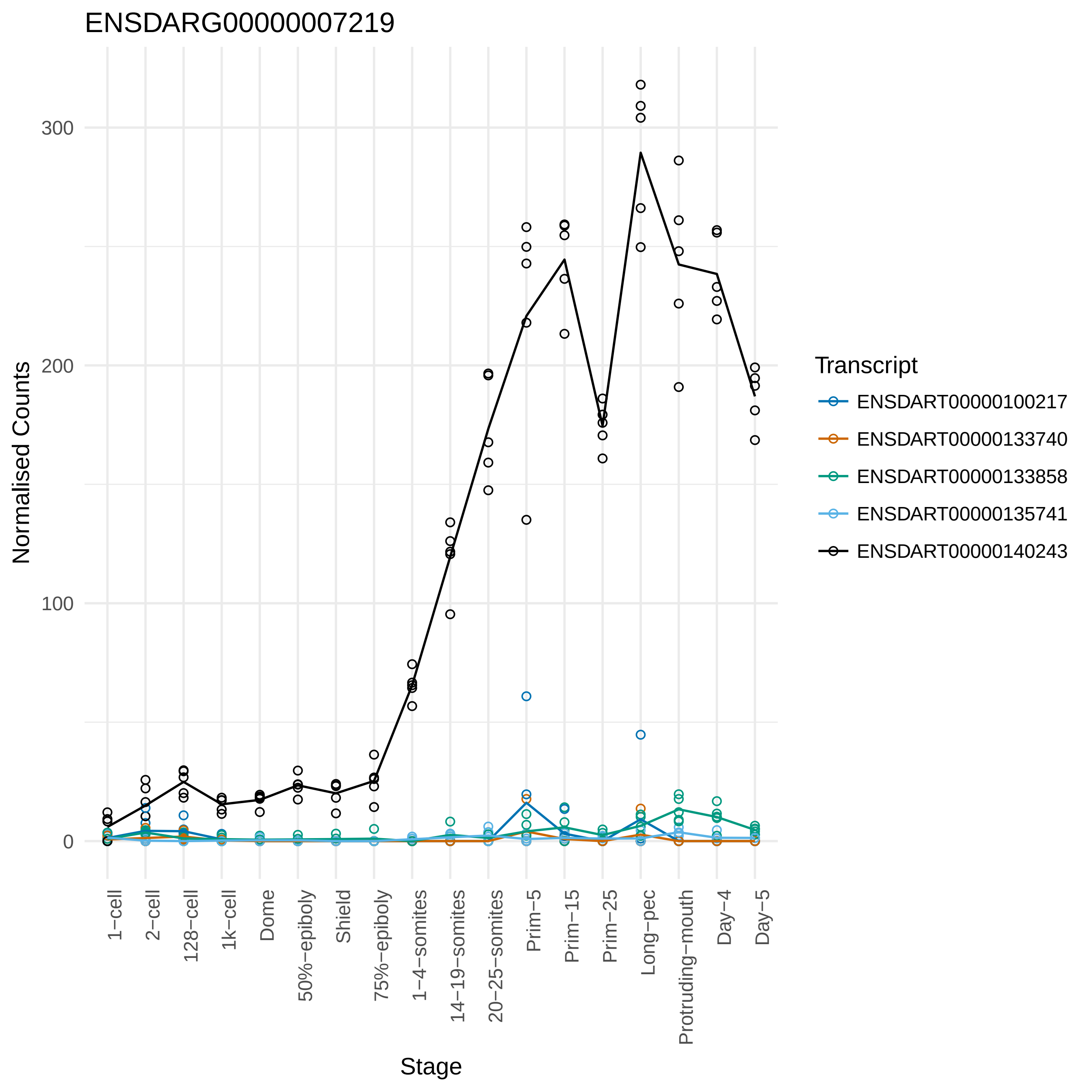

ENSDARG00000007219
Normalised Counts
300
200
100
0
Stage
1−cell
2−cell
128−cell
1k−cell
Dome
50%−epiboly
Shield
75%−epiboly
1−4−somites
14−19−somites
20−25−somites
Prim−5
Prim−15
Prim−25
Long−pec
Protruding−mouth
Day−4
Day−5
Transcript
ENSDART00000100217
ENSDART00000133740
ENSDART00000133858
ENSDART00000135741
ENSDART00000140243

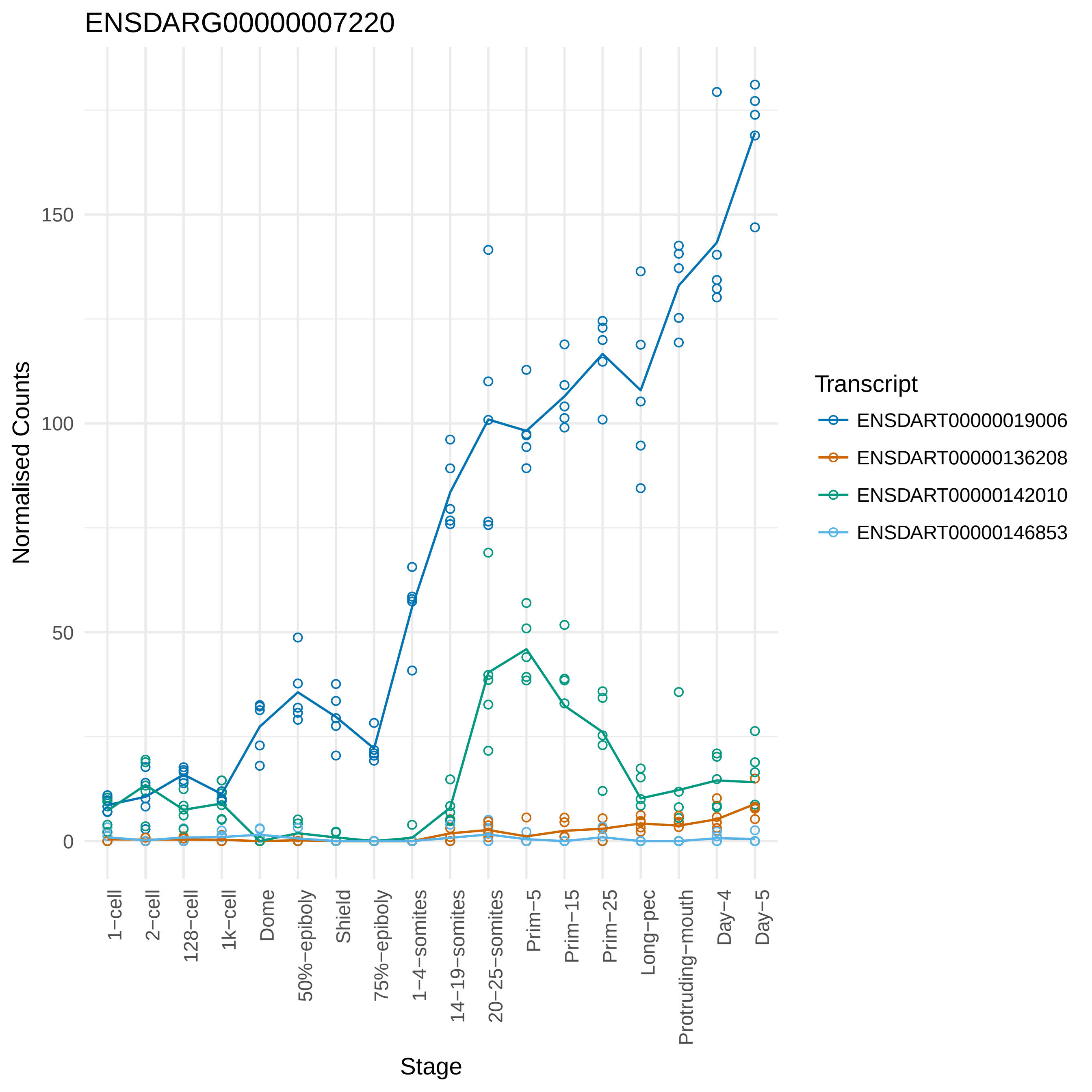

ENSDARG00000007220
Normalised Counts
150
100
50
0
1−cell
2−cell
128−cell
1k−cell
Dome
50%−epiboly
Shield
75%−epiboly
1−4−somites
14−19−somites
20−25−somites
Prim−5
Prim−15
Prim−25
Long−pec
Protruding−mouth
Day−4
Day−5
Stage
Transcript
ENSDART00000019006
ENSDART00000136208
ENSDART00000142010
ENSDART00000146853

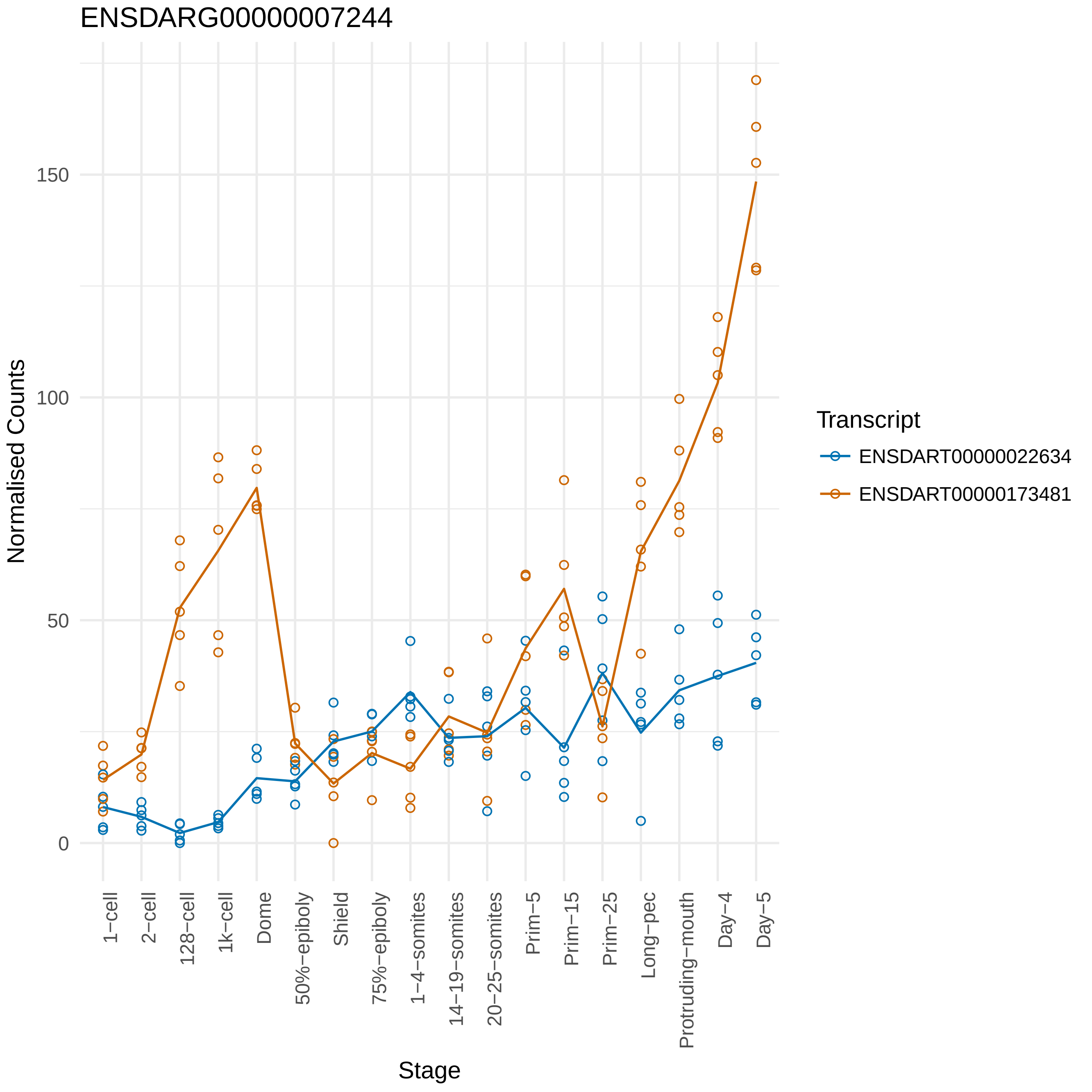

ENSDARG00000007244
Normalised Counts
150
100
50
0
1−cell
2−cell
128−cell
1k−cell
Dome
50%−epiboly
Shield
75%−epiboly
1−4−somites
14−19−somites
20−25−somites
Prim−5
Prim−15
Prim−25
Long−pec
Protruding−mouth
Day−4
Day−5
Stage
Transcript
ENSDART00000022634
ENSDART00000173481

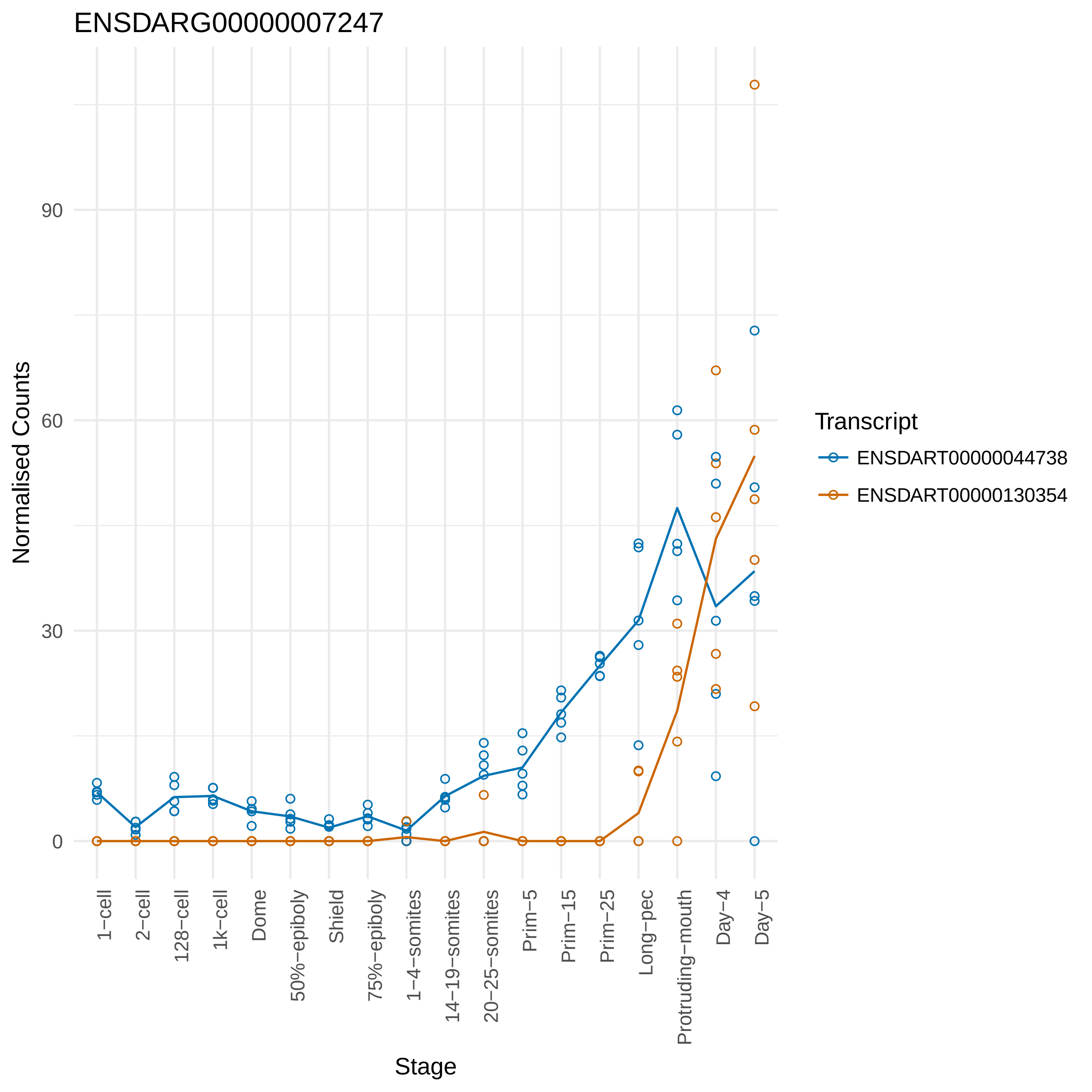

ENSDARG00000007247
Normalised Counts
0
30
60
90
1−cell
2−cell
128−cell
1k−cell
Dome
50%−epiboly
Shield
75%−epiboly
1−4−somites
14−19−somites
20−25−somites
Prim−5
Prim−15
Prim−25
Long−pec
Protruding−mouth
Day−4
Day−5
Stage
Transcript
ENSDART00000044738
ENSDART00000130354

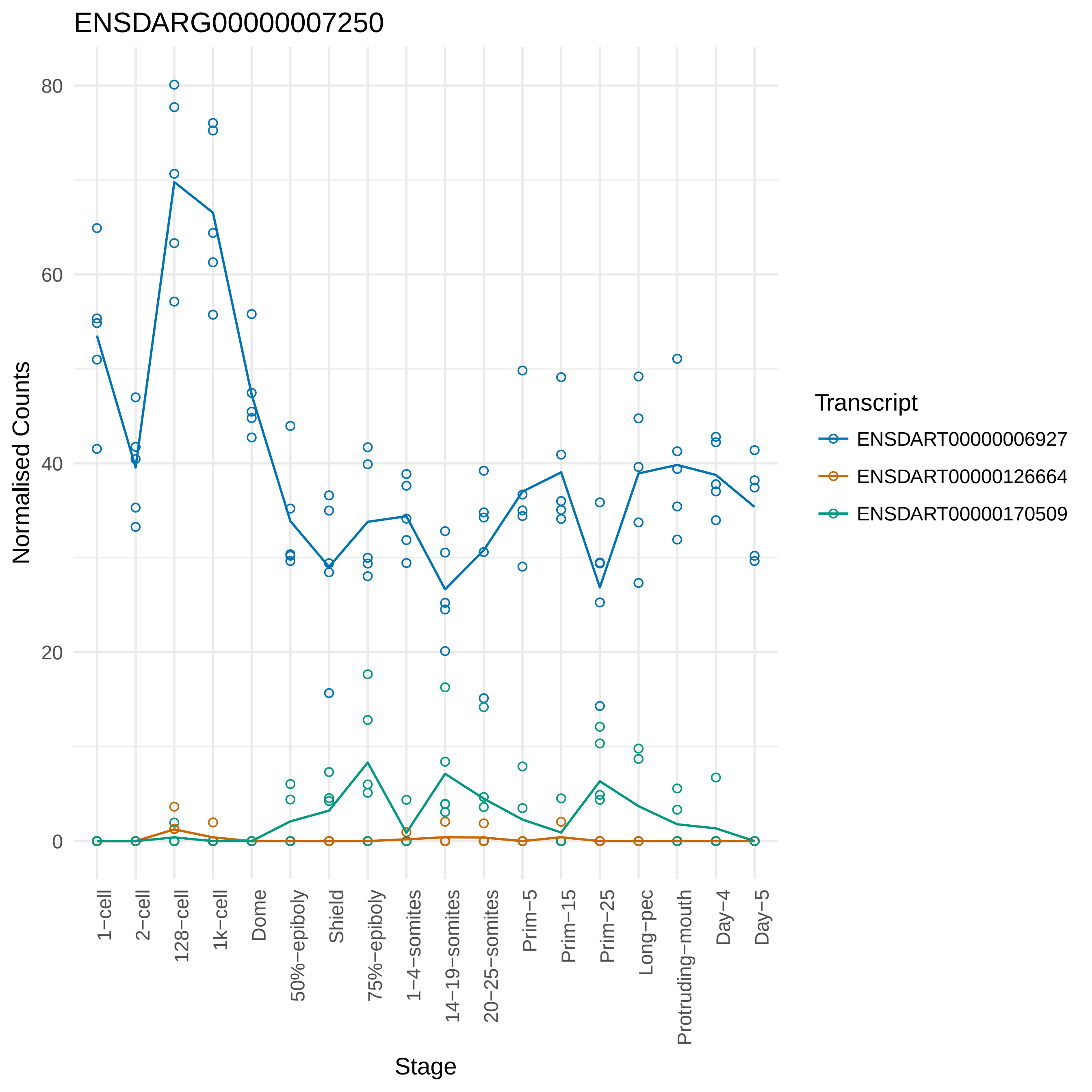
ENSDARG00000007250
Normalised Counts
0
20
40
60
80
1−cell
2−cell
128−cell
1k−cell
Dome
50%−epiboly
Shield
75%−epiboly
1−4−somites
14−19−somites
20−25−somites
Prim−5
Prim−15
Prim−25
Long−pec
Protruding−mouth
Day−4
Day−5
Stage
Transcript
ENSDART00000006927
ENSDART00000126664
ENSDART00000170509

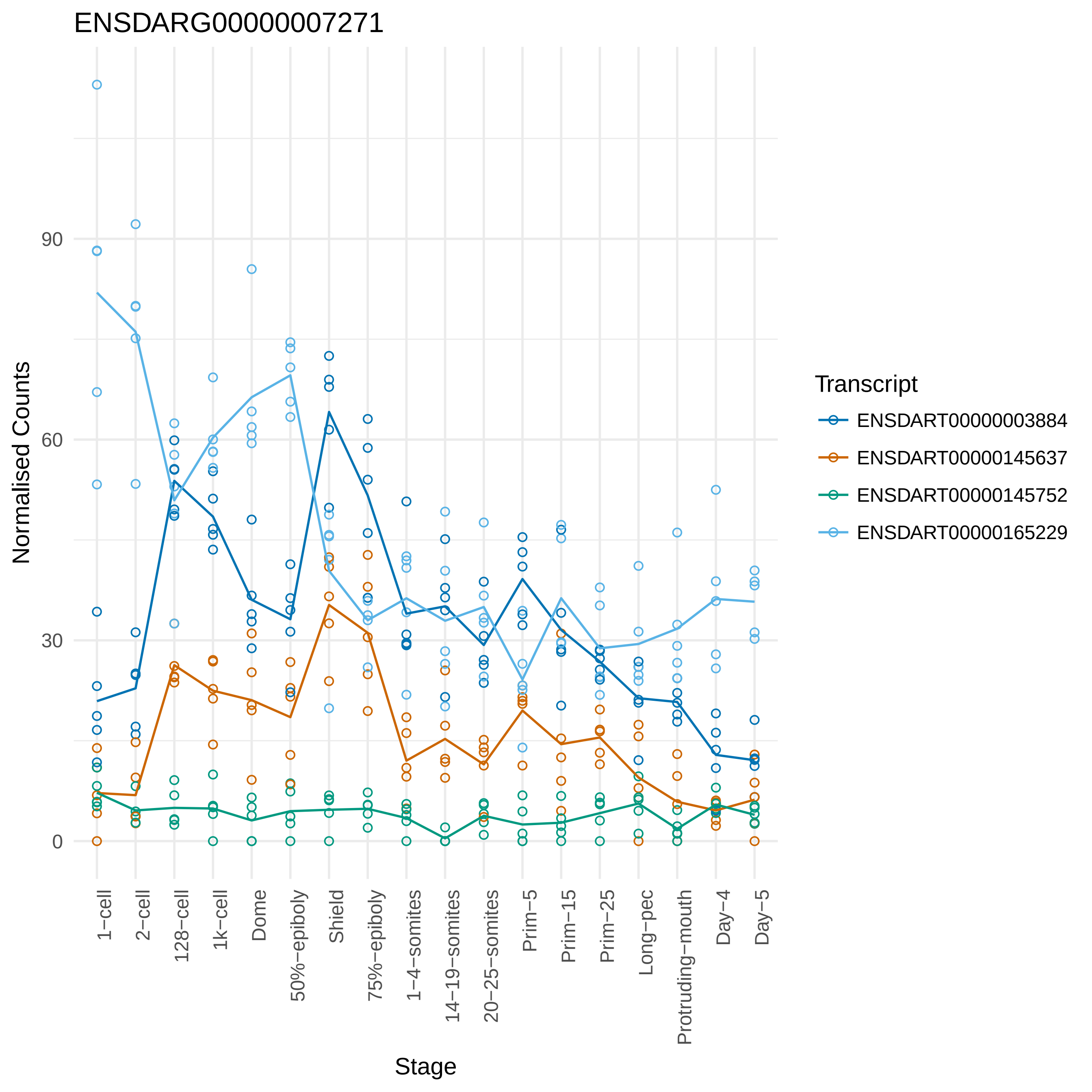

ENSDARG00000007271
Normalised Counts
0
30
60
90
1−cell
2−cell
128−cell
1k−cell
Dome
50%−epiboly
Shield
75%−epiboly
1−4−somites
14−19−somites
20−25−somites
Prim−5
Prim−15
Prim−25
Long−pec
Protruding−mouth
Day−4
Day−5
Stage
Transcript
ENSDART00000003884
ENSDART00000145637
ENSDART00000145752
ENSDART00000165229

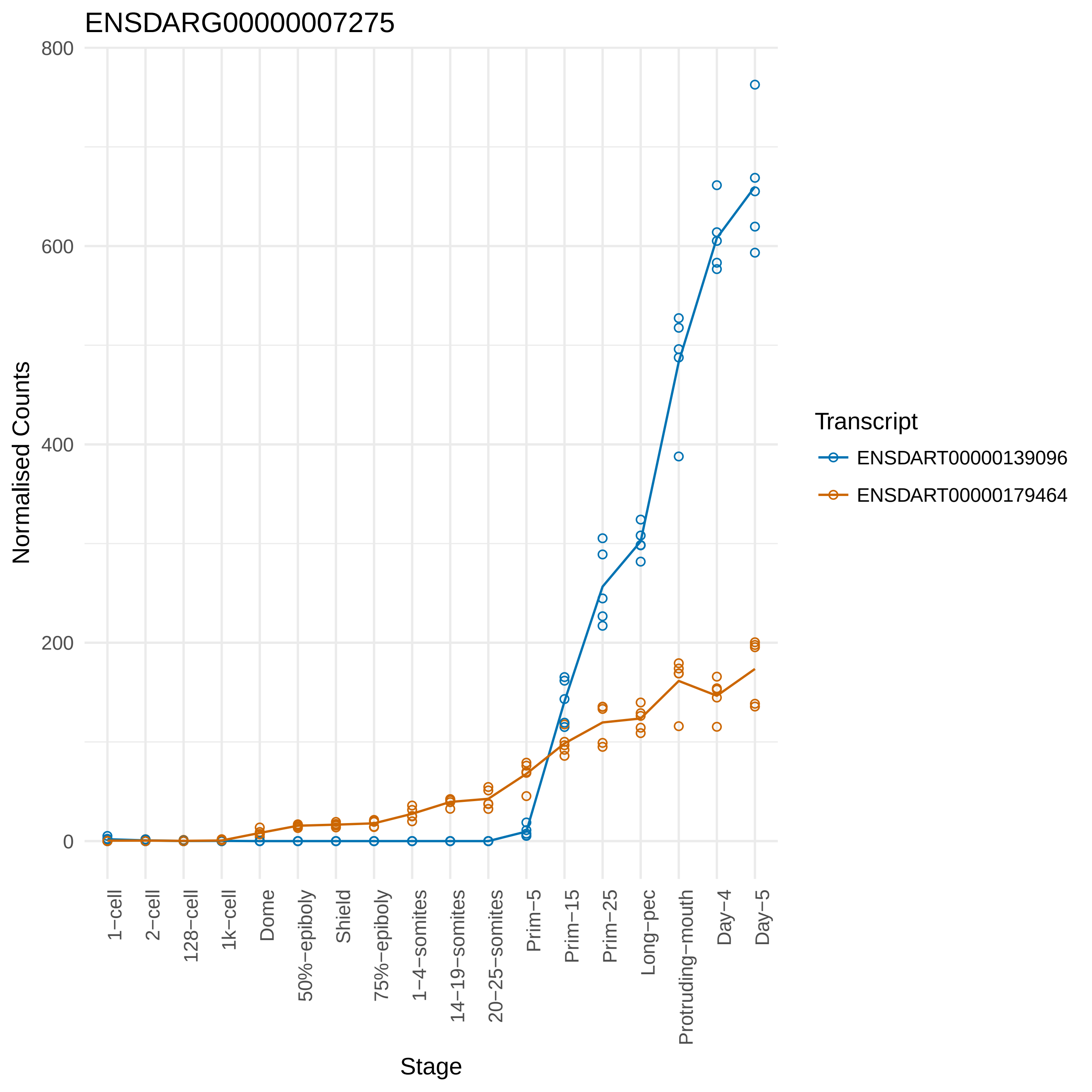
ENSDARG00000007275
Normalised Counts
0
200
400
600
800
Stage
1−cell
2−cell
128−cell
1k−cell
Dome
50%−epiboly
Shield
75%−epiboly
1−4−somites
14−19−somites
20−25−somites
Prim−5
Prim−15
Prim−25
Long−pec
Protruding−mouth
Day−4
Day−5
Transcript
ENSDART00000139096
ENSDART00000179464

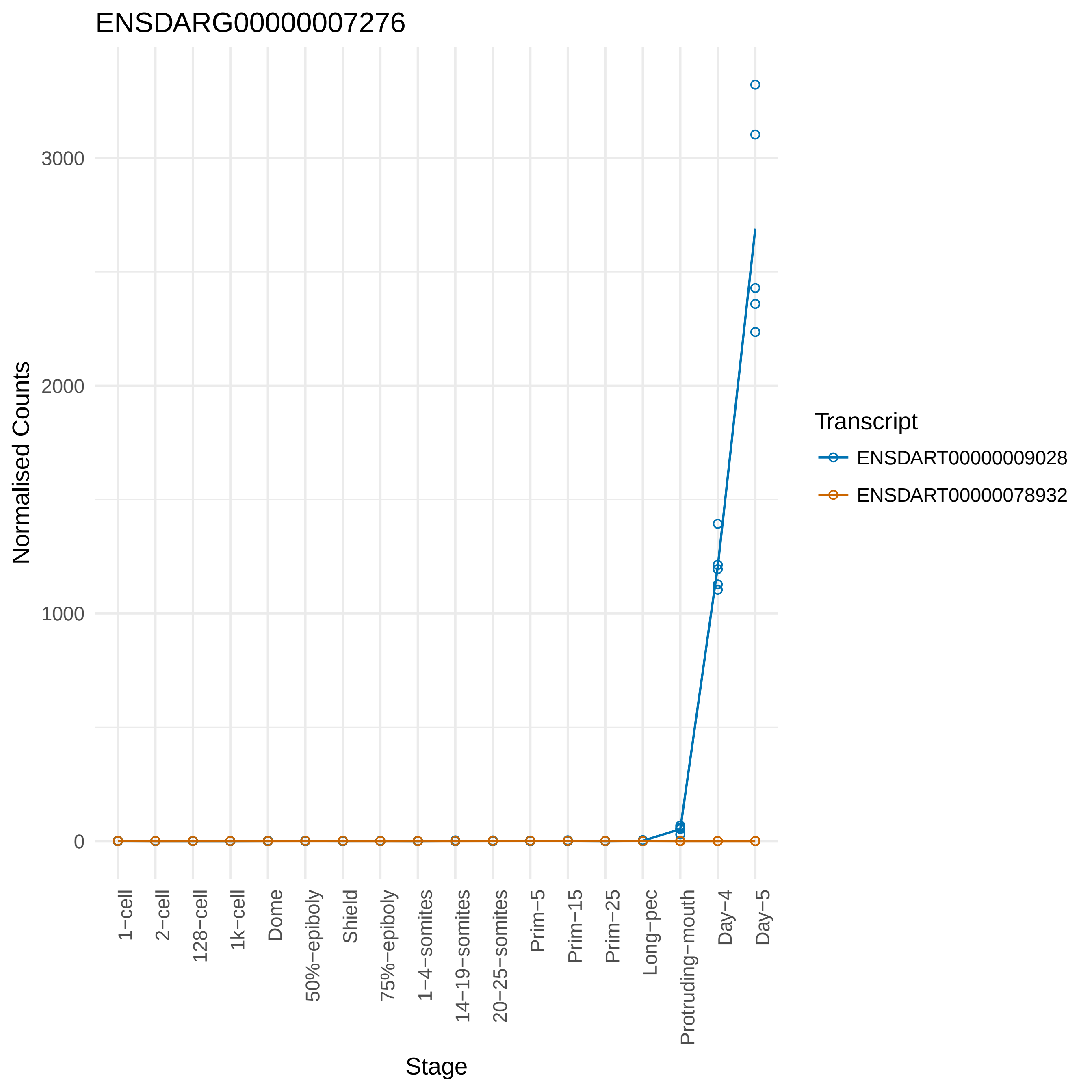
ENSDARG00000007276
Normalised Counts
3000
2000
1000
0
1−cell
2−cell
128−cell
1k−cell
Dome
50%−epiboly
Shield
75%−epiboly
1−4−somites
14−19−somites
20−25−somites
Prim−5
Prim−15
Prim−25
Long−pec
Protruding−mouth
Day−4
Day−5
Stage
Transcript
ENSDART00000009028
ENSDART00000078932

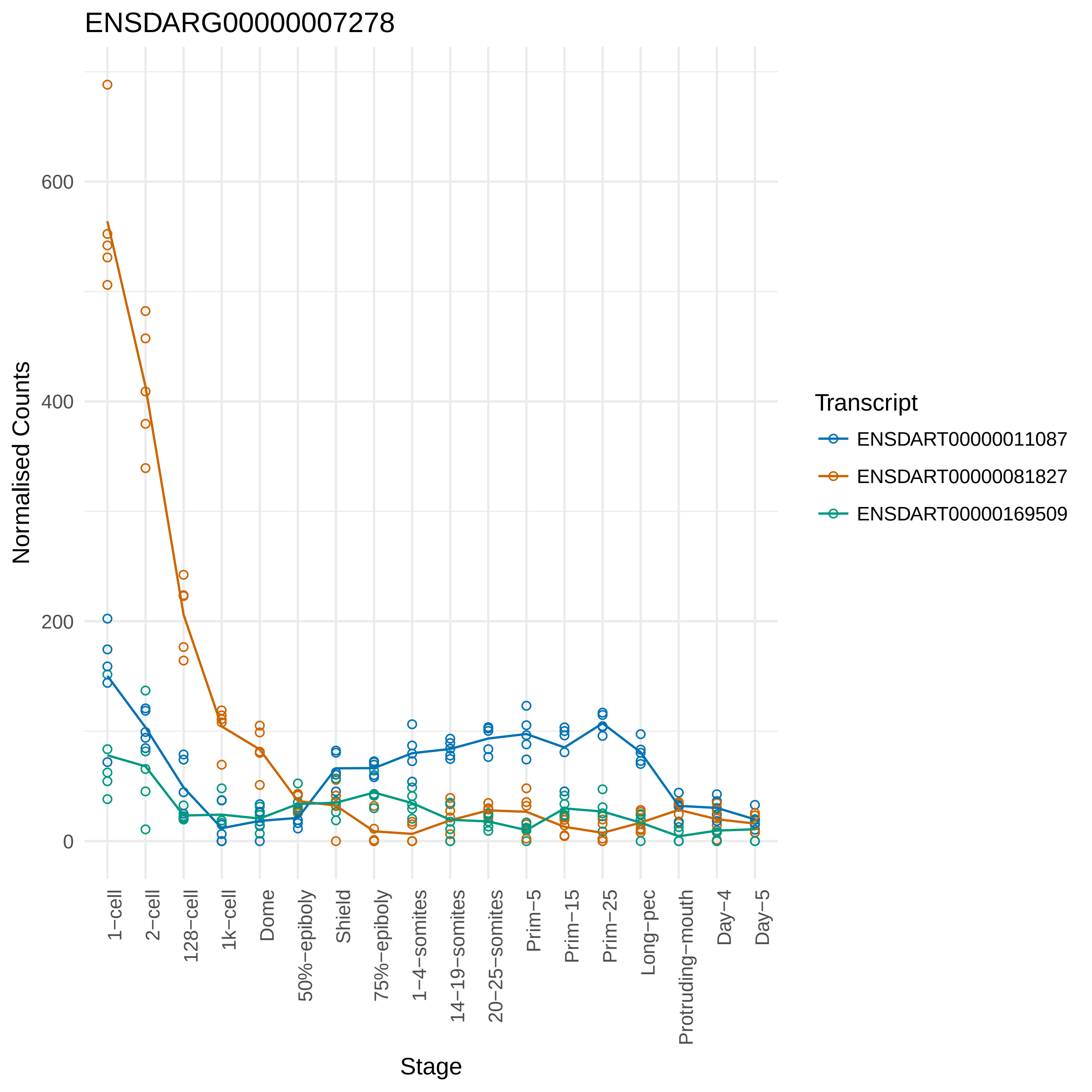
ENSDARG00000007278
Normalised Counts
600
400
200
0
1−cell
2−cell
128−cell
1k−cell
Dome
50%−epiboly
Shield
75%−epiboly
1−4−somites
14−19−somites
20−25−somites
Prim−5
Prim−15
Prim−25
Long−pec
Protruding−mouth
Day−4
Day−5
Stage
Transcript
ENSDART00000011087
ENSDART00000081827
ENSDART00000169509

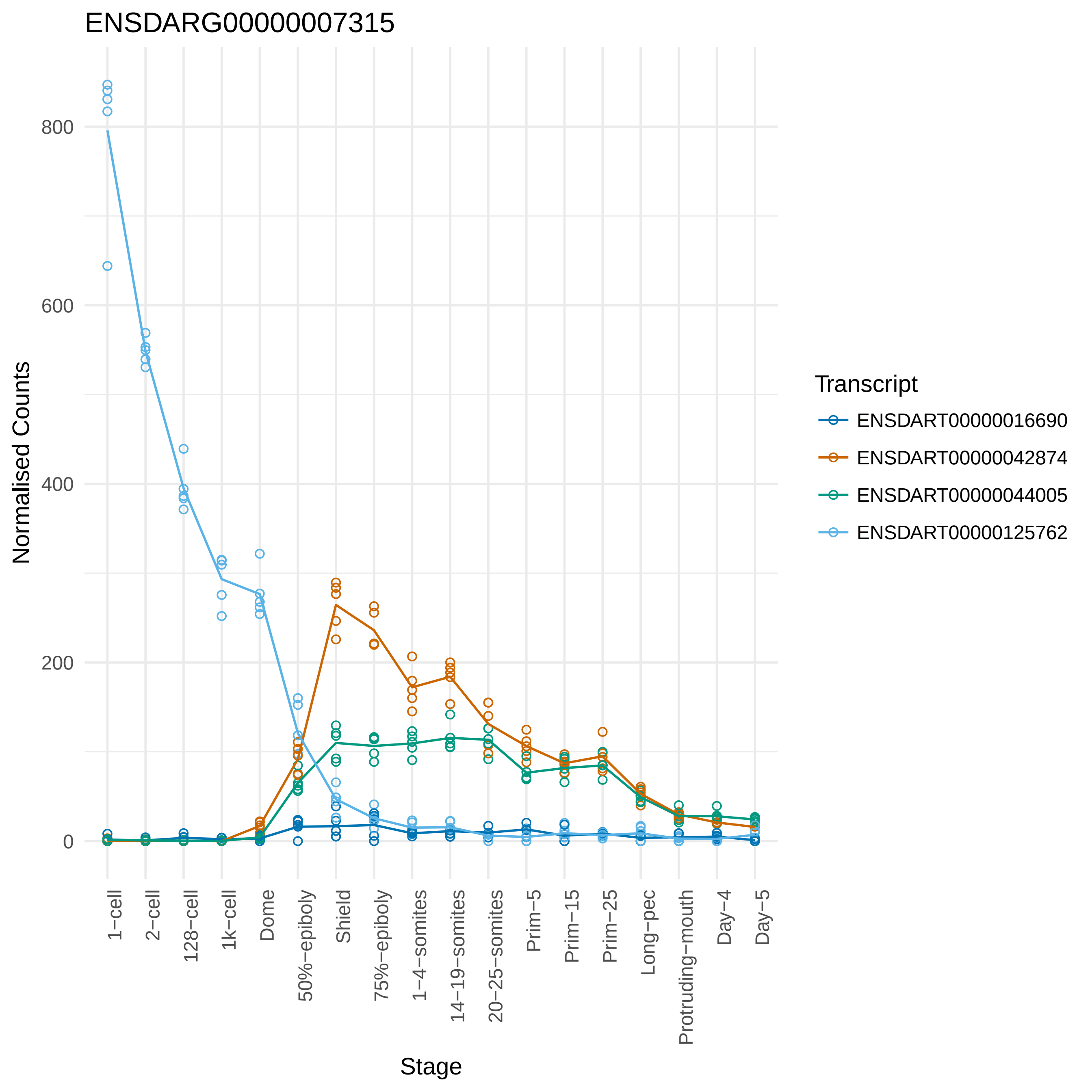

ENSDARG00000007315
Normalised Counts
0
200
400
600
800
Stage
1−cell
2−cell
128−cell
1k−cell
Dome
50%−epiboly
Shield
75%−epiboly
1−4−somites
14−19−somites
20−25−somites
Prim−5
Prim−15
Prim−25
Long−pec
Protruding−mouth
Day−4
Day−5
Transcript
ENSDART00000016690
ENSDART00000042874
ENSDART00000044005
ENSDART00000125762

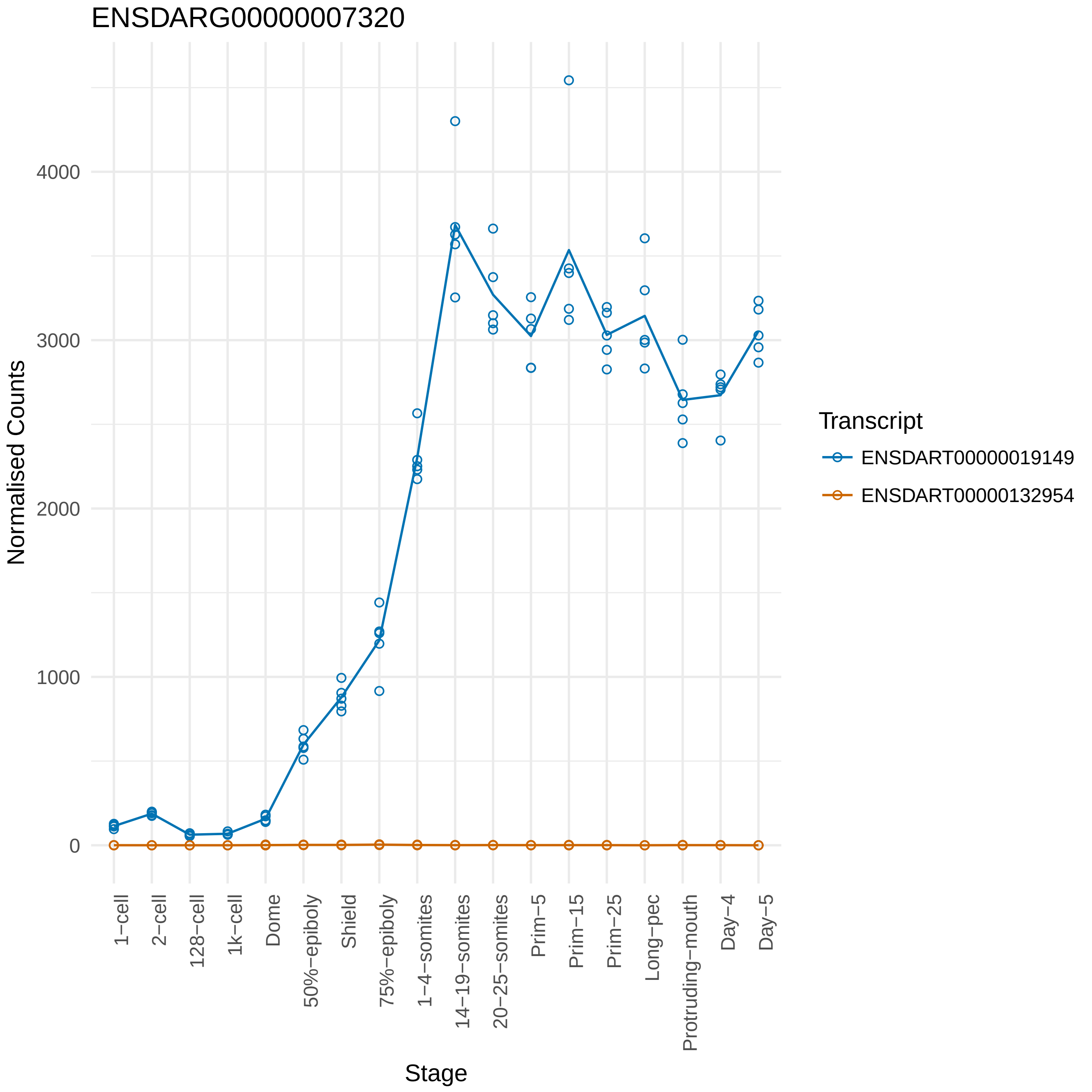

ENSDARG00000007320
Normalised Counts
0
1000
2000
3000
4000
Stage
1−cell
2−cell
128−cell
1k−cell
Dome
50%−epiboly
Shield
75%−epiboly
1−4−somites
14−19−somites
20−25−somites
Prim−5
Prim−15
Prim−25
Long−pec
Protruding−mouth
Day−4
Day−5
Transcript
ENSDART00000019149
ENSDART00000132954

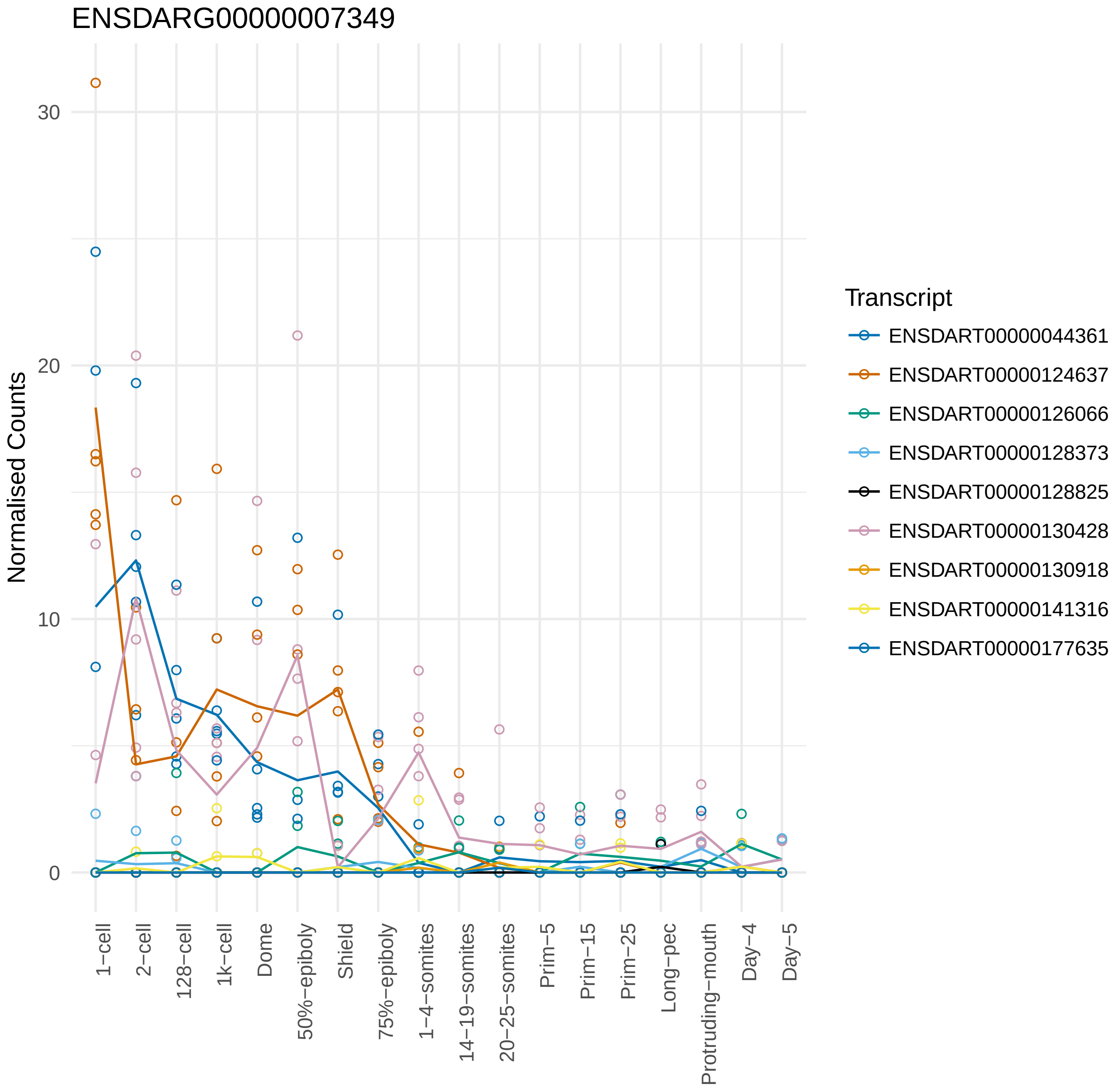

ENSDARG00000007349
Normalised Counts
30
20
10
0
1−cell
2−cell
128−cell
1k−cell
Dome
50%−epiboly
Shield
75%−epiboly
1−4−somites
14−19−somites
20−25−somites
Prim−5
Prim−15
Prim−25
Long−pec
Protruding−mouth
Day−4
Day−5
Stage
Transcript
ENSDART00000044361
ENSDART00000124637
ENSDART00000126066
ENSDART00000128373
ENSDART00000128825
ENSDART00000130428
ENSDART00000130918
ENSDART00000141316
ENSDART00000177635

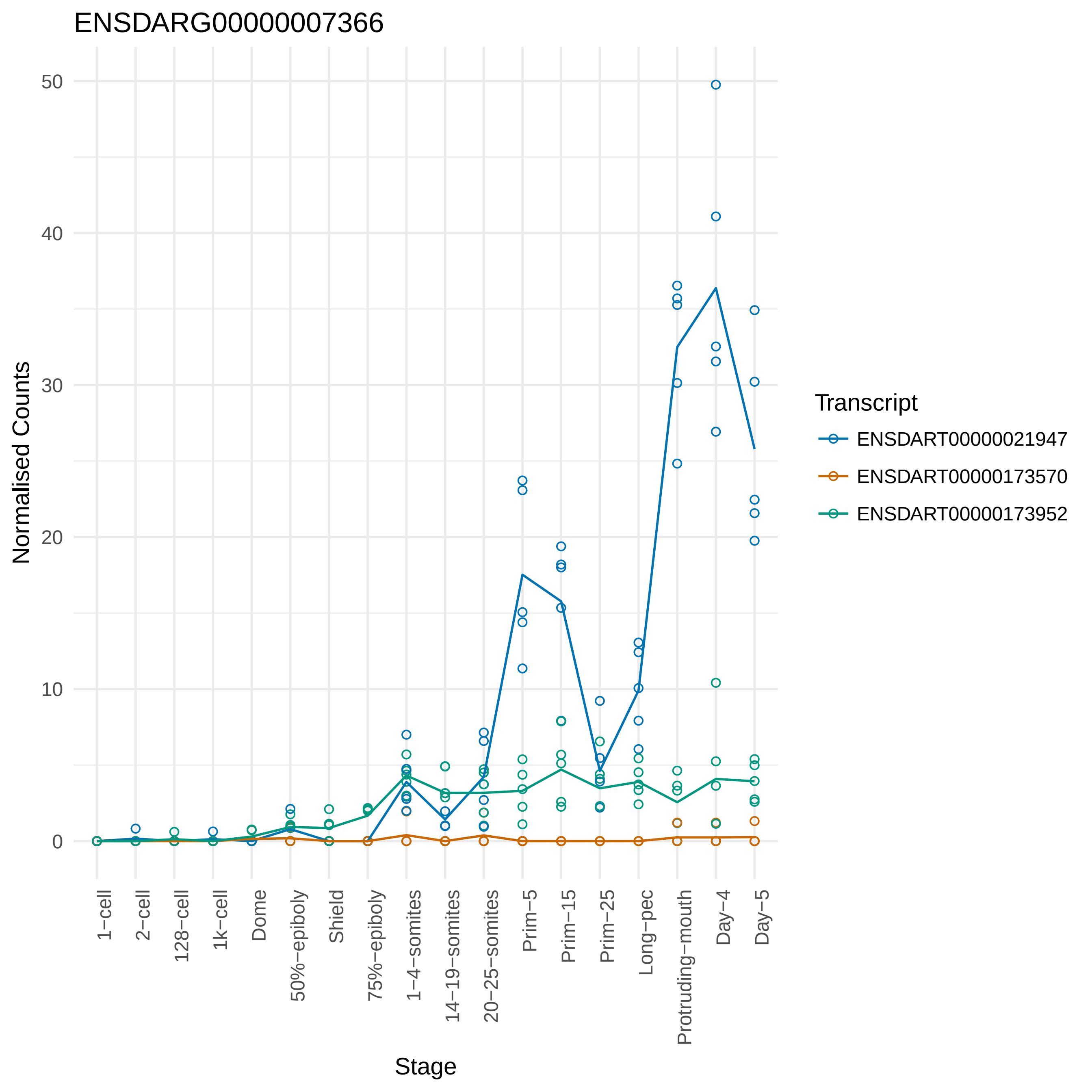

ENSDARG00000007366
Normalised Counts
0
10
20
30
40
50
1−cell
2−cell
128−cell
1k−cell
Dome
50%−epiboly
Shield
75%−epiboly
1−4−somites
14−19−somites
20−25−somites
Prim−5
Prim−15
Prim−25
Long−pec
Protruding−mouth
Day−4
Day−5
Stage
Transcript
ENSDART00000021947
ENSDART00000173570
ENSDART00000173952

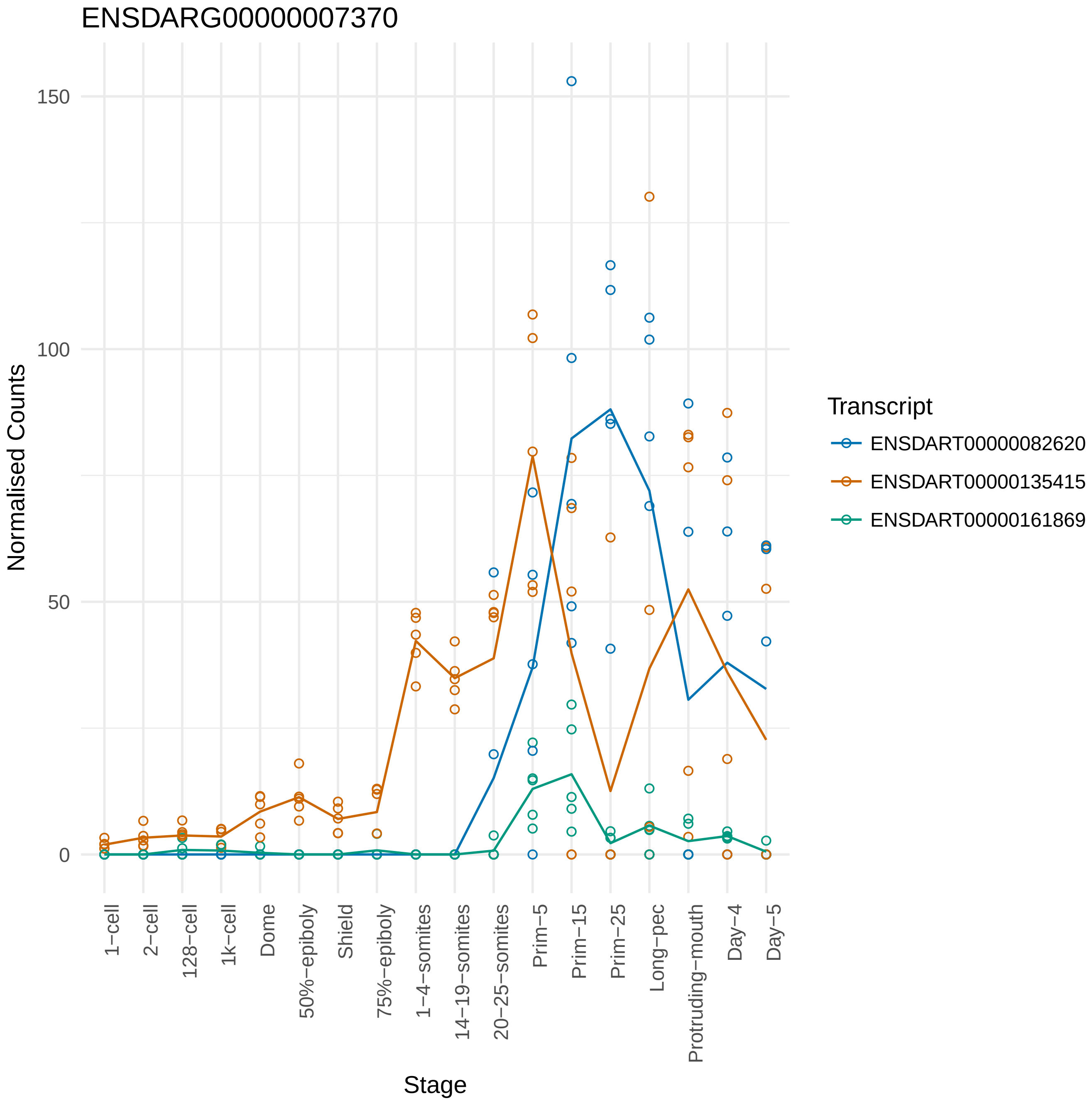

ENSDARG00000007370
Normalised Counts
150
100
50
0
1−cell
2−cell
128−cell
1k−cell
Dome
50%−epiboly
Shield
75%−epiboly
1−4−somites
14−19−somites
20−25−somites
Prim−5
Prim−15
Prim−25
Long−pec
Protruding−mouth
Day−4
Day−5
Stage
Transcript
ENSDART00000082620
ENSDART00000135415
ENSDART00000161869

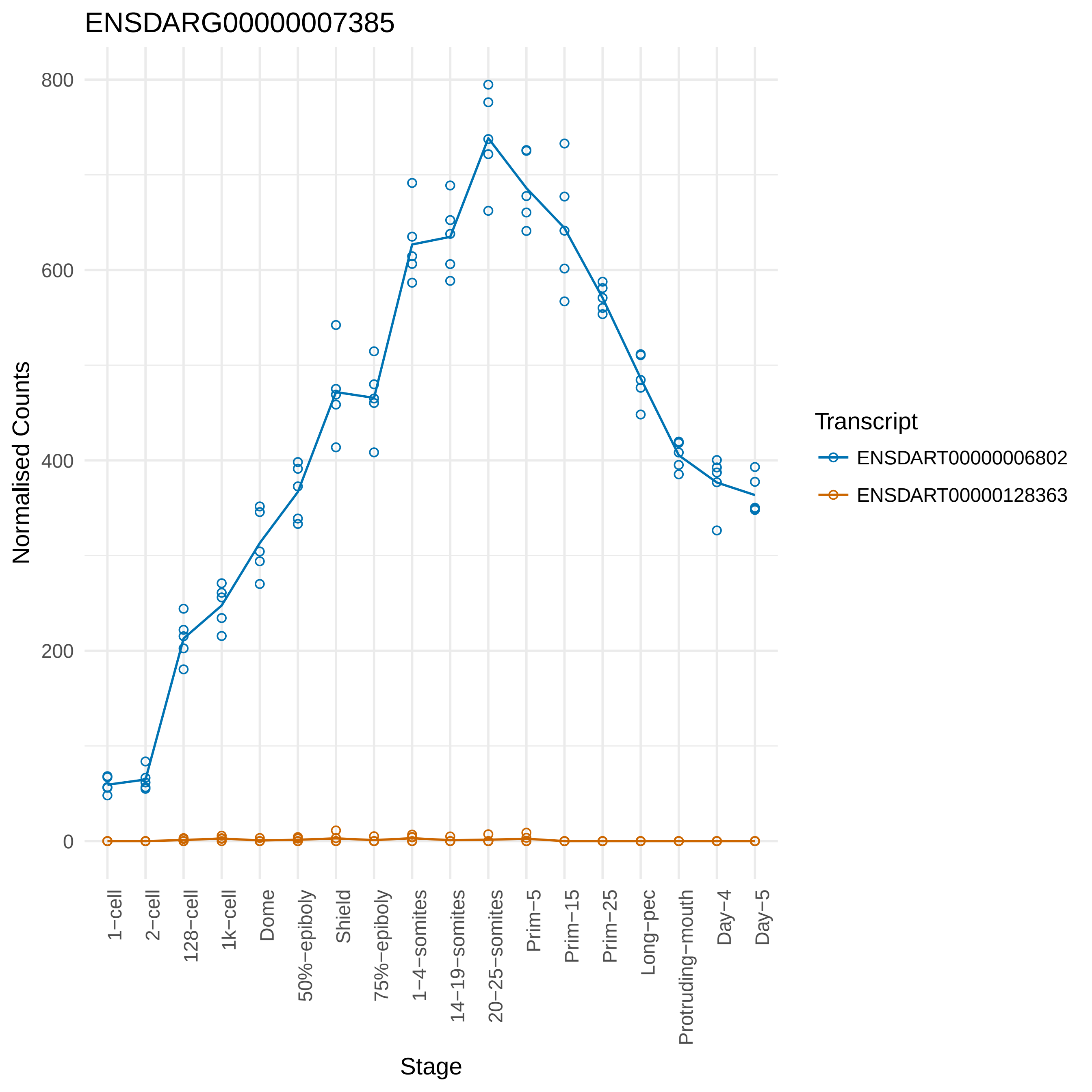

ENSDARG00000007385
Normalised Counts
800
600
400
200
0
Stage
1−cell
2−cell
128−cell
1k−cell
Dome
50%−epiboly
Shield
75%−epiboly
1−4−somites
14−19−somites
20−25−somites
Prim−5
Prim−15
Prim−25
Long−pec
Protruding−mouth
Day−4
Day−5
Transcript
ENSDART00000006802
ENSDART00000128363

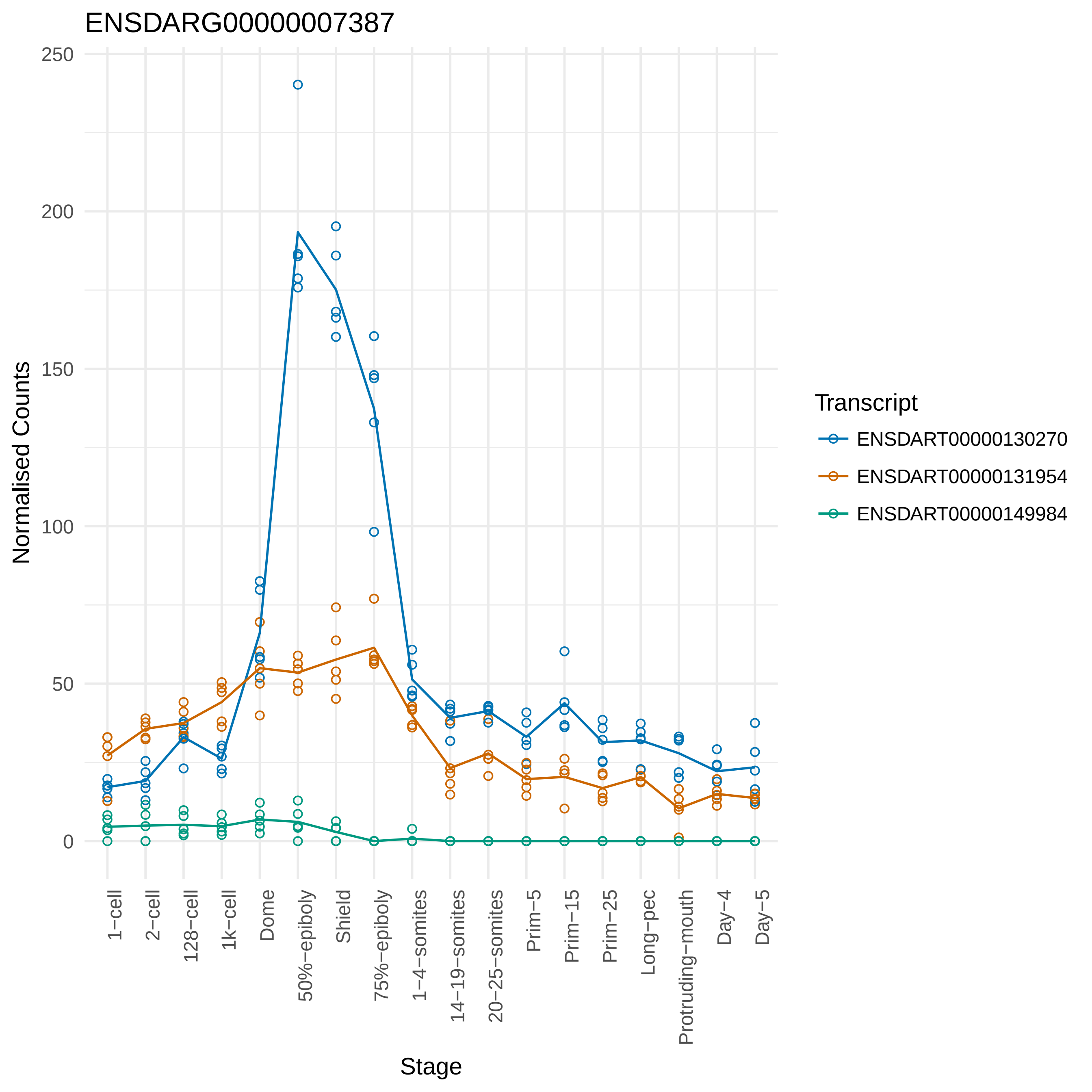
ENSDARG00000007387
Normalised Counts
0
50
100
150
200
250
Stage
1−cell
2−cell
128−cell
1k−cell
Dome
50%−epiboly
Shield
75%−epiboly
1−4−somites
14−19−somites
20−25−somites
Prim−5
Prim−15
Prim−25
Long−pec
Protruding−mouth
Day−4
Day−5
Transcript
ENSDART00000130270
ENSDART00000131954
ENSDART00000149984

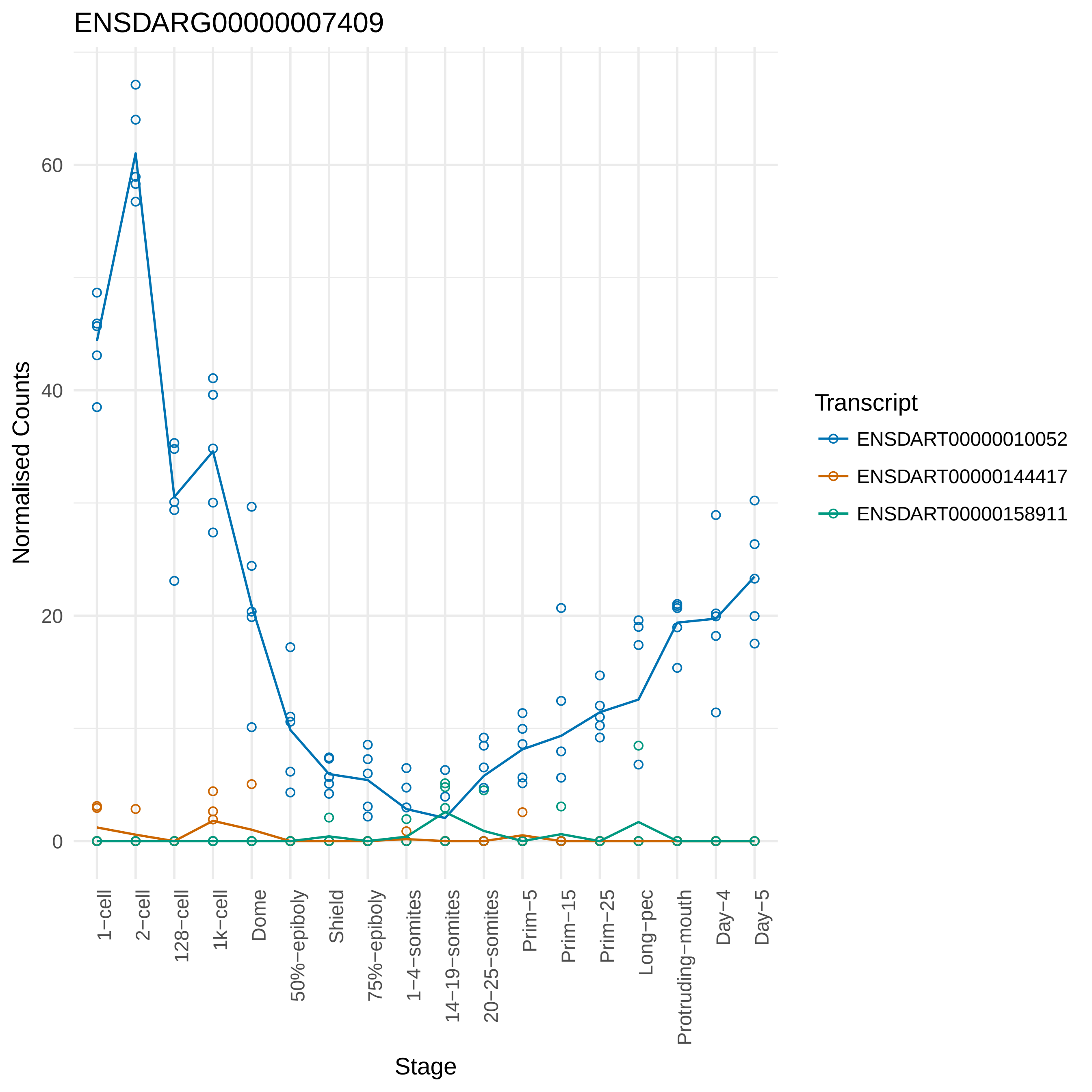
ENSDARG00000007409
Normalised Counts
0
20
40
60
Stage
1−cell
2−cell
128−cell
1k−cell
Dome
50%−epiboly
Shield
75%−epiboly
1−4−somites
14−19−somites
20−25−somites
Prim−5
Prim−15
Prim−25
Long−pec
Protruding−mouth
Day−4
Day−5
Transcript
ENSDART00000010052
ENSDART00000144417
ENSDART00000158911

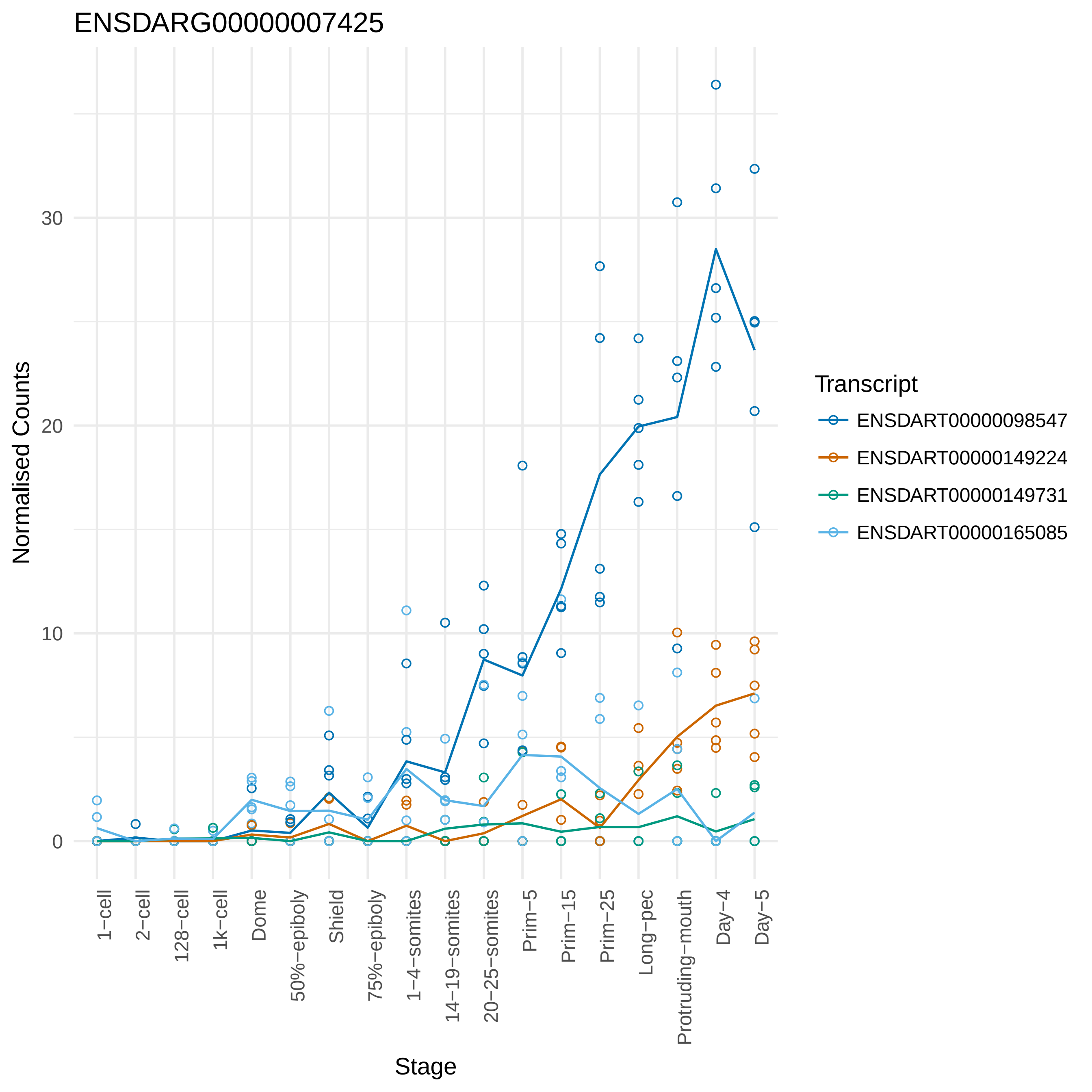
ENSDARG00000007425
Normalised Counts
0
10
20
30
Stage
1−cell
2−cell
128−cell
1k−cell
Dome
50%−epiboly
Shield
75%−epiboly
1−4−somites
14−19−somites
20−25−somites
Prim−5
Prim−15
Prim−25
Long−pec
Protruding−mouth
Day−4
Day−5
Transcript
ENSDART00000098547
ENSDART00000149224
ENSDART00000149731
ENSDART00000165085

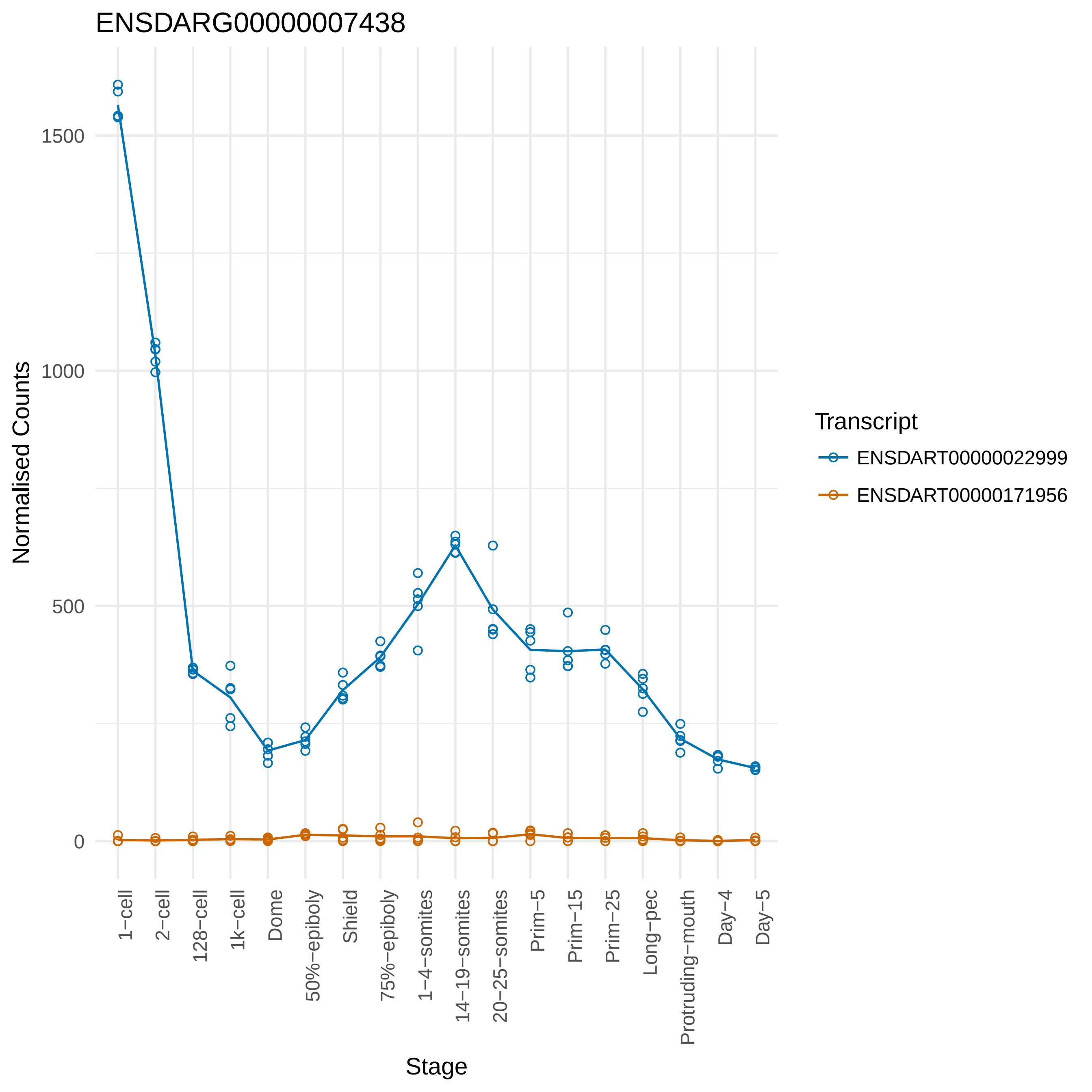

ENSDARG00000007438
Normalised Counts
1500
1000
500
0
1−cell
2−cell
128−cell
1k−cell
Dome
50%−epiboly
Shield
75%−epiboly
1−4−somites
14−19−somites
20−25−somites
Prim−5
Prim−15
Prim−25
Long−pec
Protruding−mouth
Day−4
Day−5
Stage
Transcript
ENSDART00000022999
ENSDART00000171956

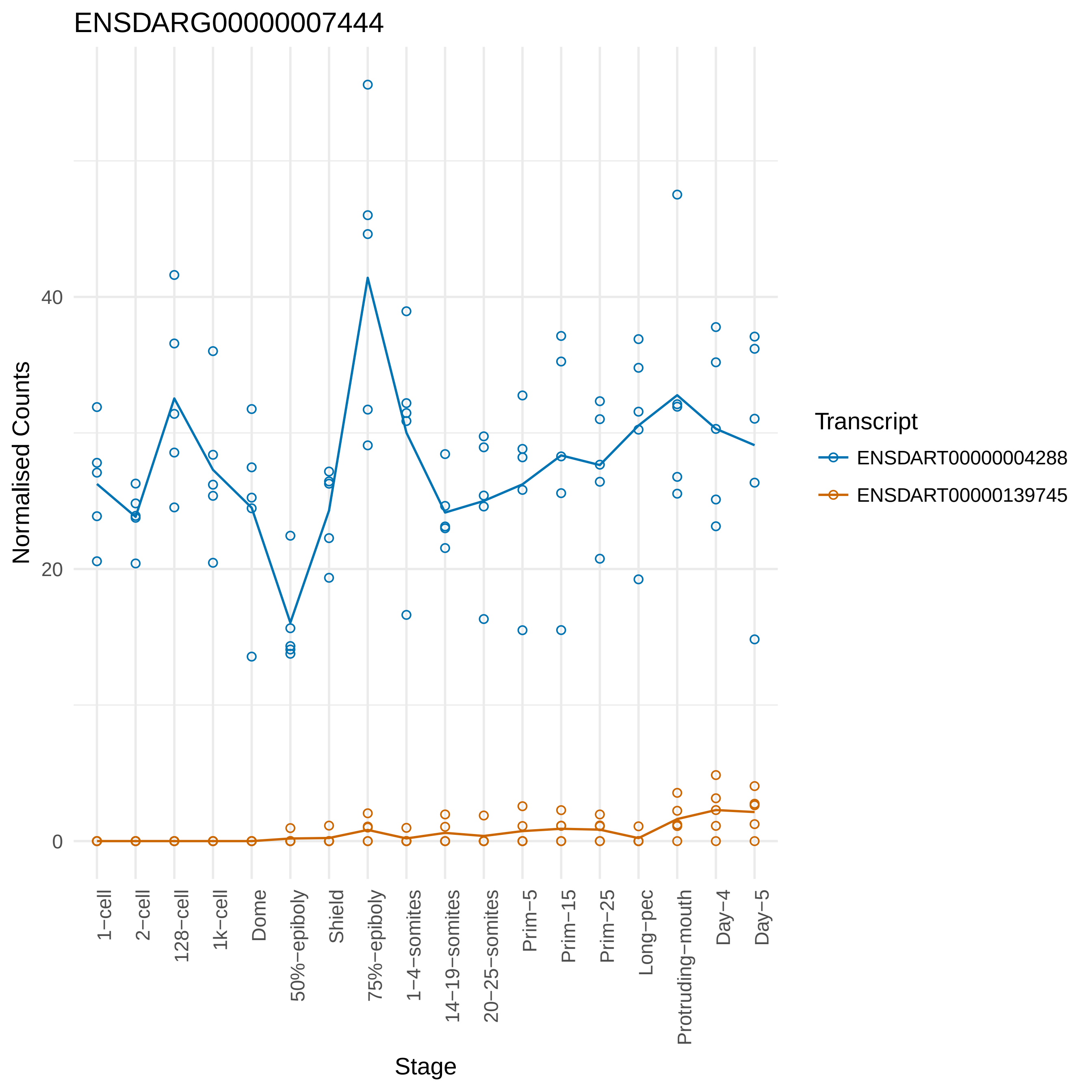
ENSDARG00000007444
Normalised Counts
0
20
40
Stage
1−cell
2−cell
128−cell
1k−cell
Dome
50%−epiboly
Shield
75%−epiboly
1−4−somites
14−19−somites
20−25−somites
Prim−5
Prim−15
Prim−25
Long−pec
Protruding−mouth
Day−4
Day−5
Transcript
ENSDART00000004288
ENSDART00000139745

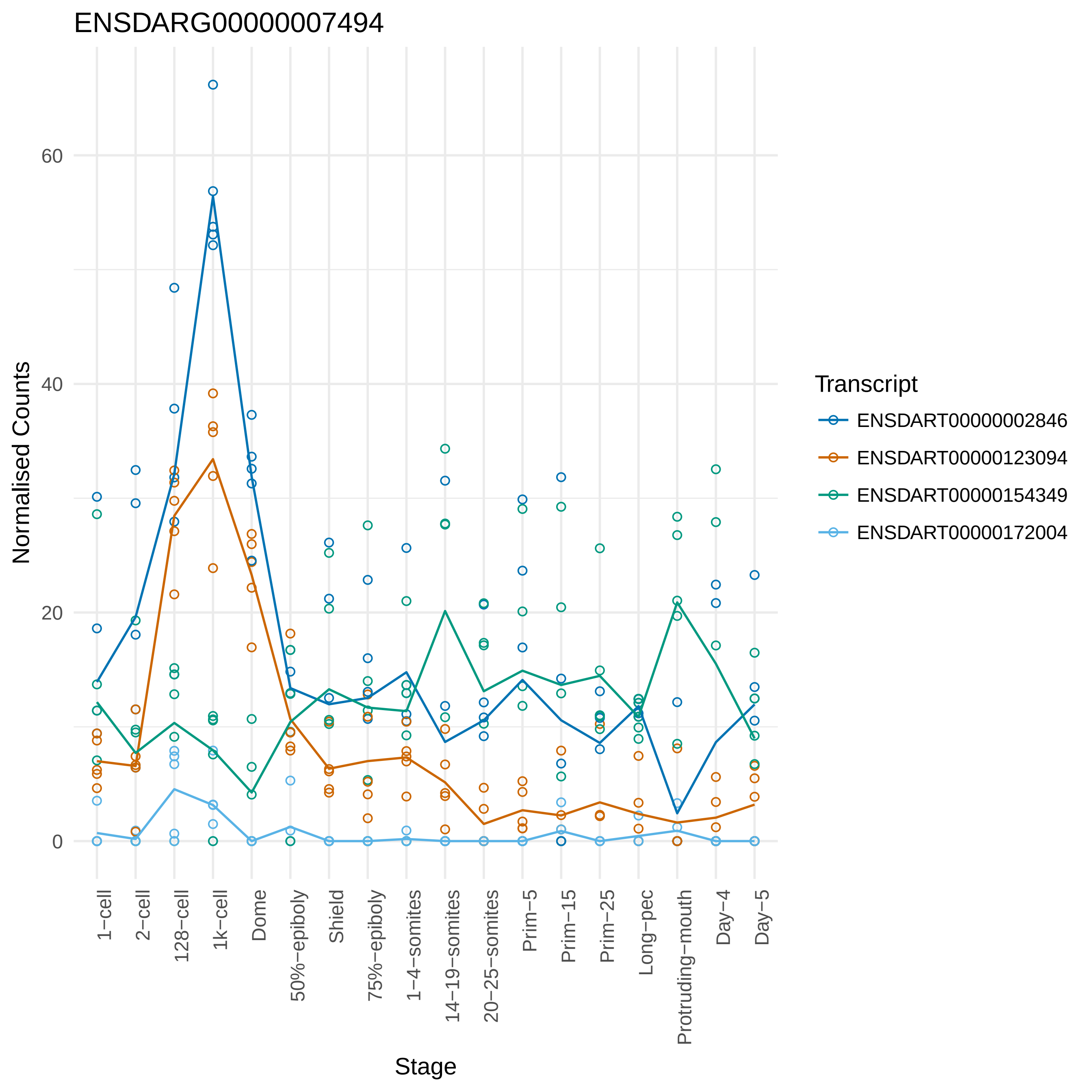

ENSDARG00000007494
Normalised Counts
0
20
40
60
Stage
1−cell
2−cell
128−cell
1k−cell
Dome
50%−epiboly
Shield
75%−epiboly
1−4−somites
14−19−somites
20−25−somites
Prim−5
Prim−15
Prim−25
Long−pec
Protruding−mouth
Day−4
Day−5
Transcript
ENSDART00000002846
ENSDART00000123094
ENSDART00000154349
ENSDART00000172004

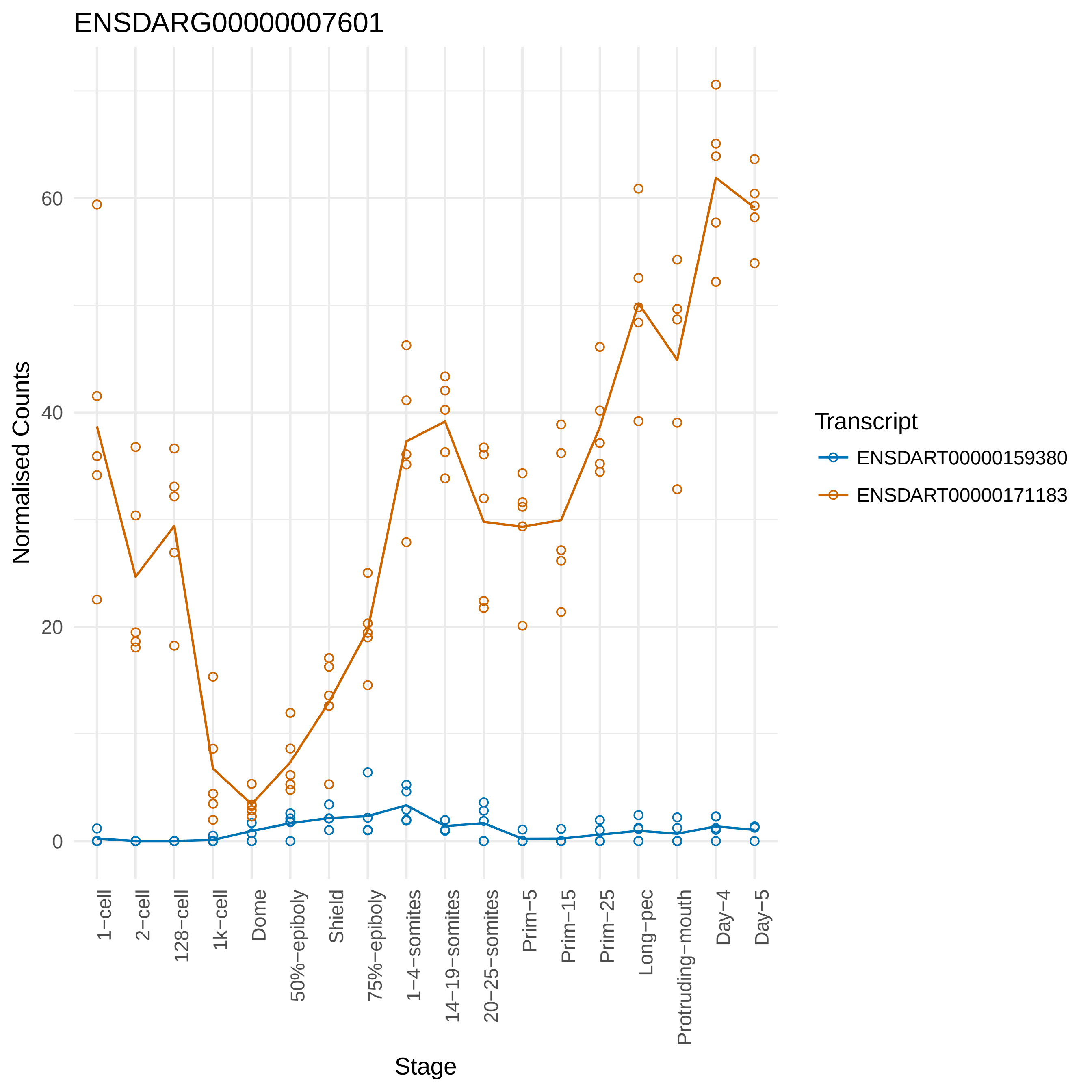

ENSDARG00000007601
Normalised Counts
0
20
40
60
Stage
1−cell
2−cell
128−cell
1k−cell
Dome
50%−epiboly
Shield
75%−epiboly
1−4−somites
14−19−somites
20−25−somites
Prim−5
Prim−15
Prim−25
Long−pec
Protruding−mouth
Day−4
Day−5
Transcript
ENSDART00000159380
ENSDART00000171183

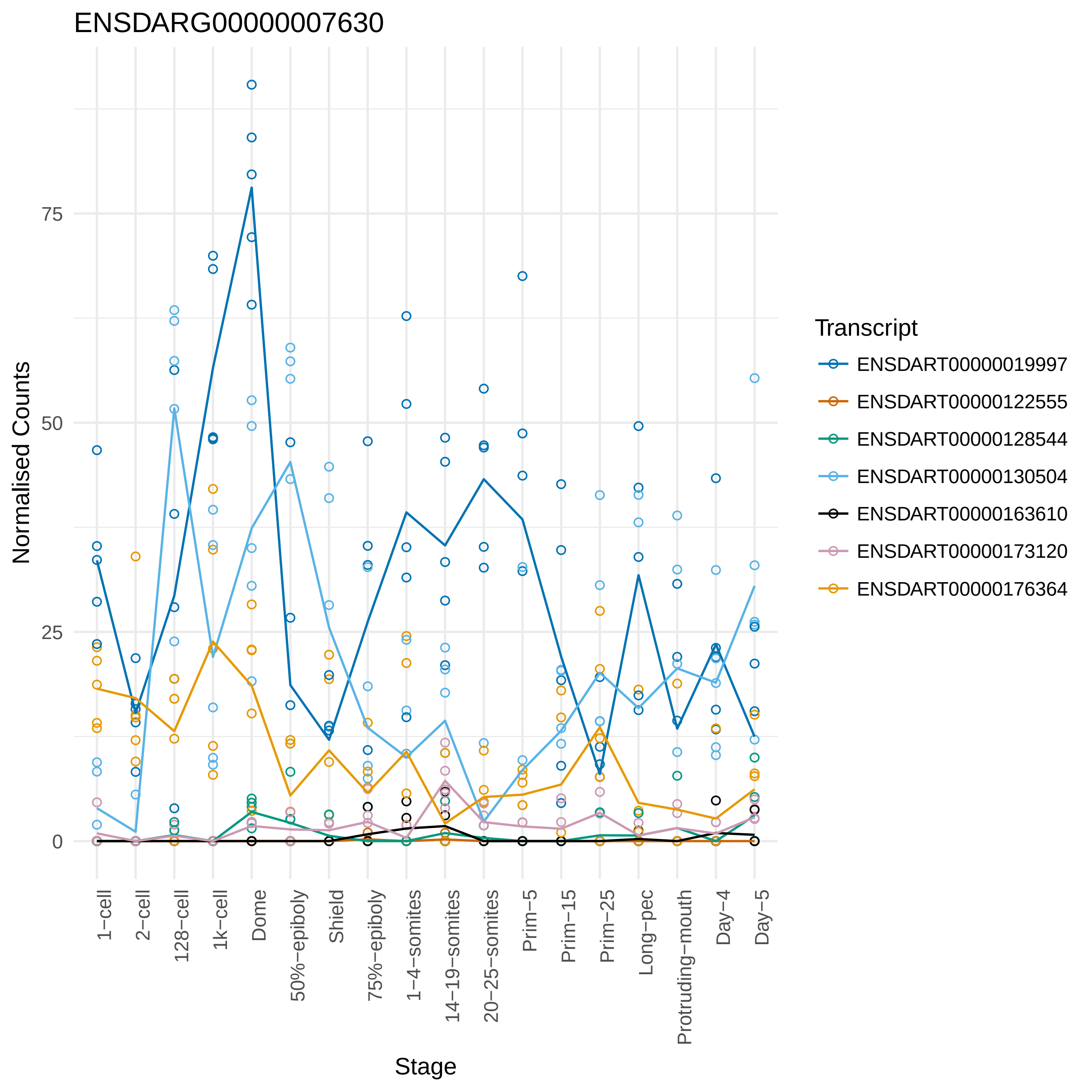

ENSDARG00000007630
Normalised Counts
0
25
50
75
Stage
1−cell
2−cell
128−cell
1k−cell
Dome
50%−epiboly
Shield
75%−epiboly
1−4−somites
14−19−somites
20−25−somites
Prim−5
Prim−15
Prim−25
Long−pec
Protruding−mouth
Day−4
Day−5
Transcript
ENSDART00000019997
ENSDART00000122555
ENSDART00000128544
ENSDART00000130504
ENSDART00000163610
ENSDART00000173120
ENSDART00000176364

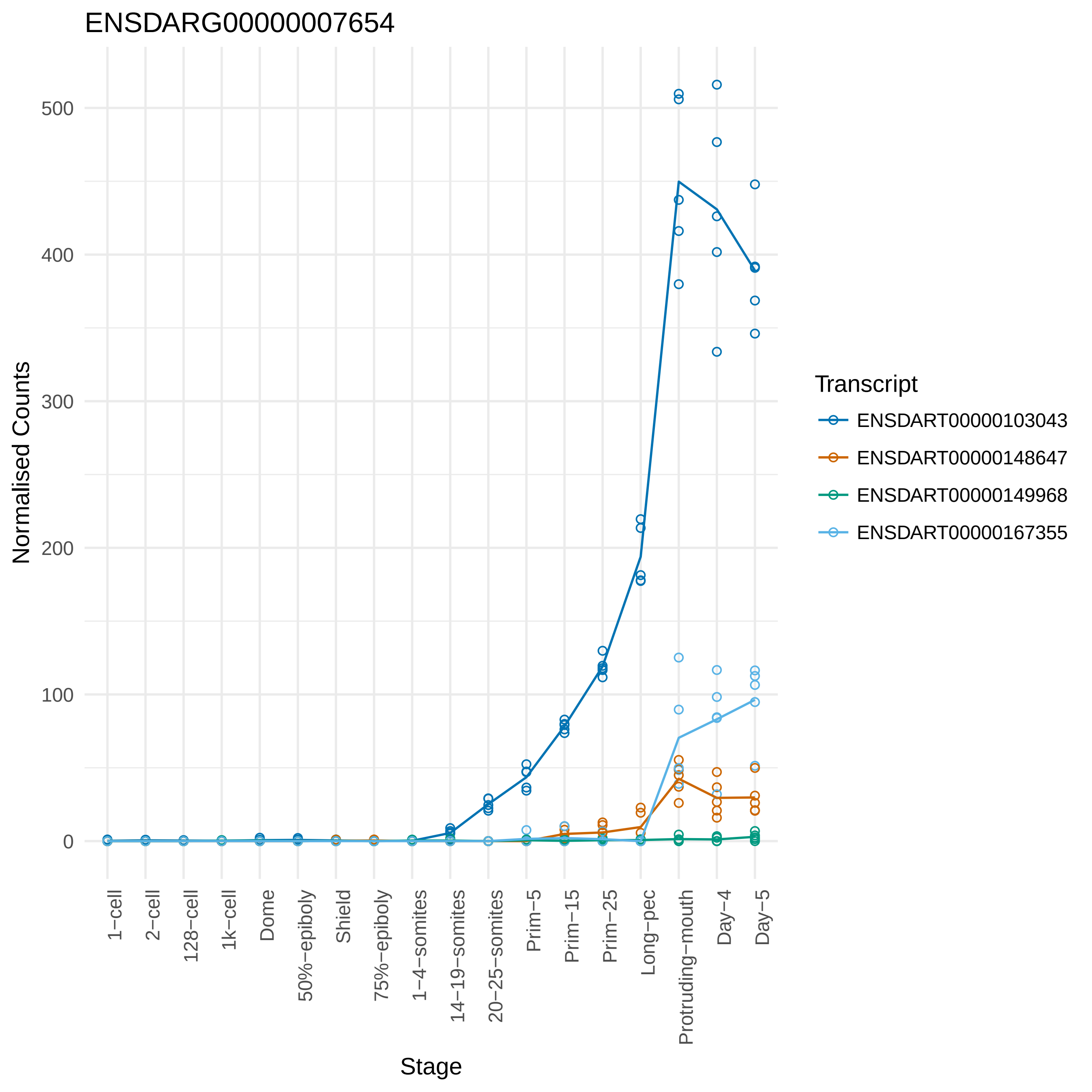

ENSDARG00000007654
Normalised Counts
0
100
200
300
400
500
1−cell
2−cell
128−cell
1k−cell
Dome
50%−epiboly
Shield
75%−epiboly
1−4−somites
14−19−somites
20−25−somites
Prim−5
Prim−15
Prim−25
Long−pec
Protruding−mouth
Day−4
Day−5
Stage
Transcript
ENSDART00000103043
ENSDART00000148647
ENSDART00000149968
ENSDART00000167355

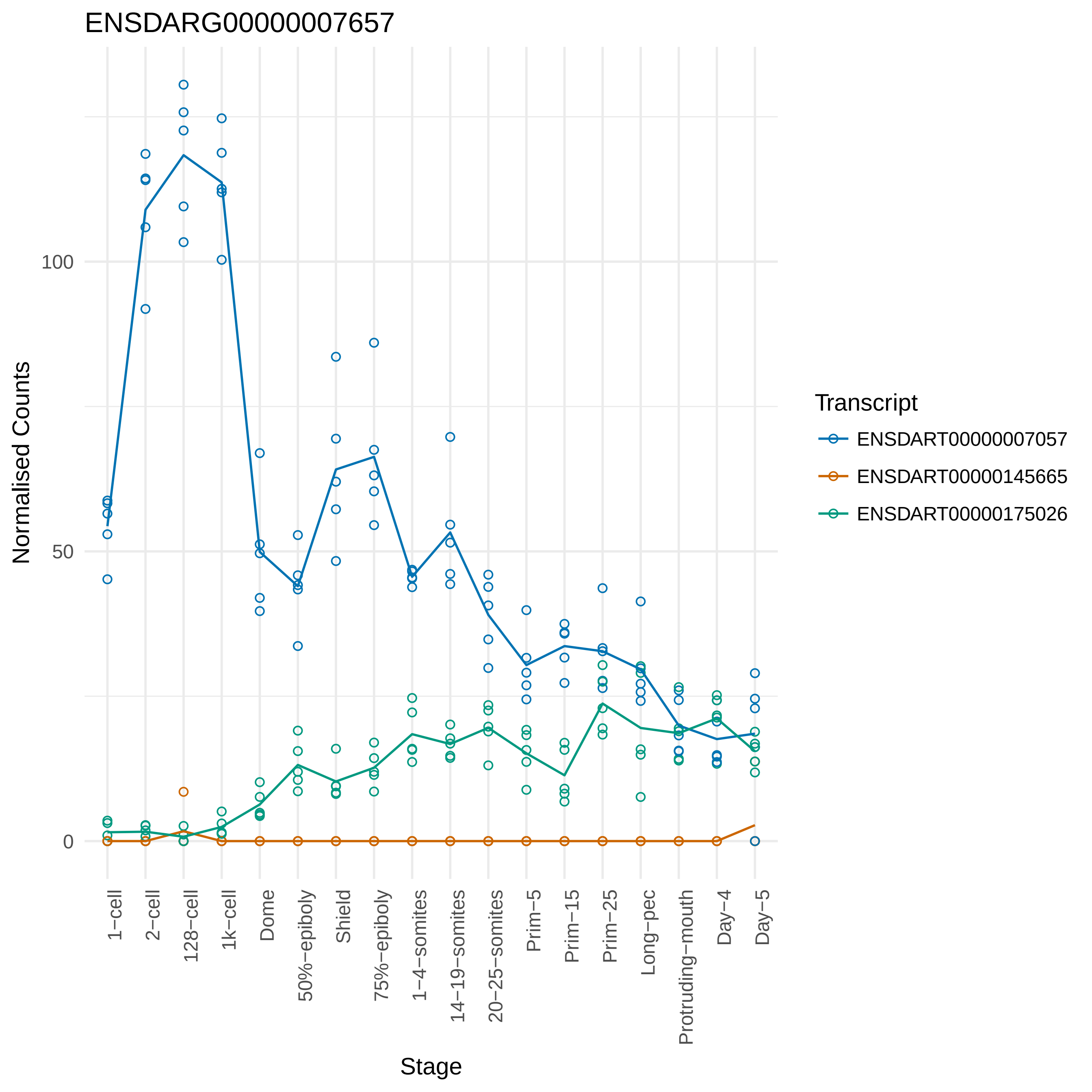
ENSDARG00000007657
Normalised Counts
0
50
100
1−cell
2−cell
128−cell
1k−cell
Dome
50%−epiboly
Shield
75%−epiboly
1−4−somites
14−19−somites
20−25−somites
Prim−5
Prim−15
Prim−25
Long−pec
Protruding−mouth
Day−4
Day−5
Stage
Transcript
ENSDART00000007057
ENSDART00000145665
ENSDART00000175026

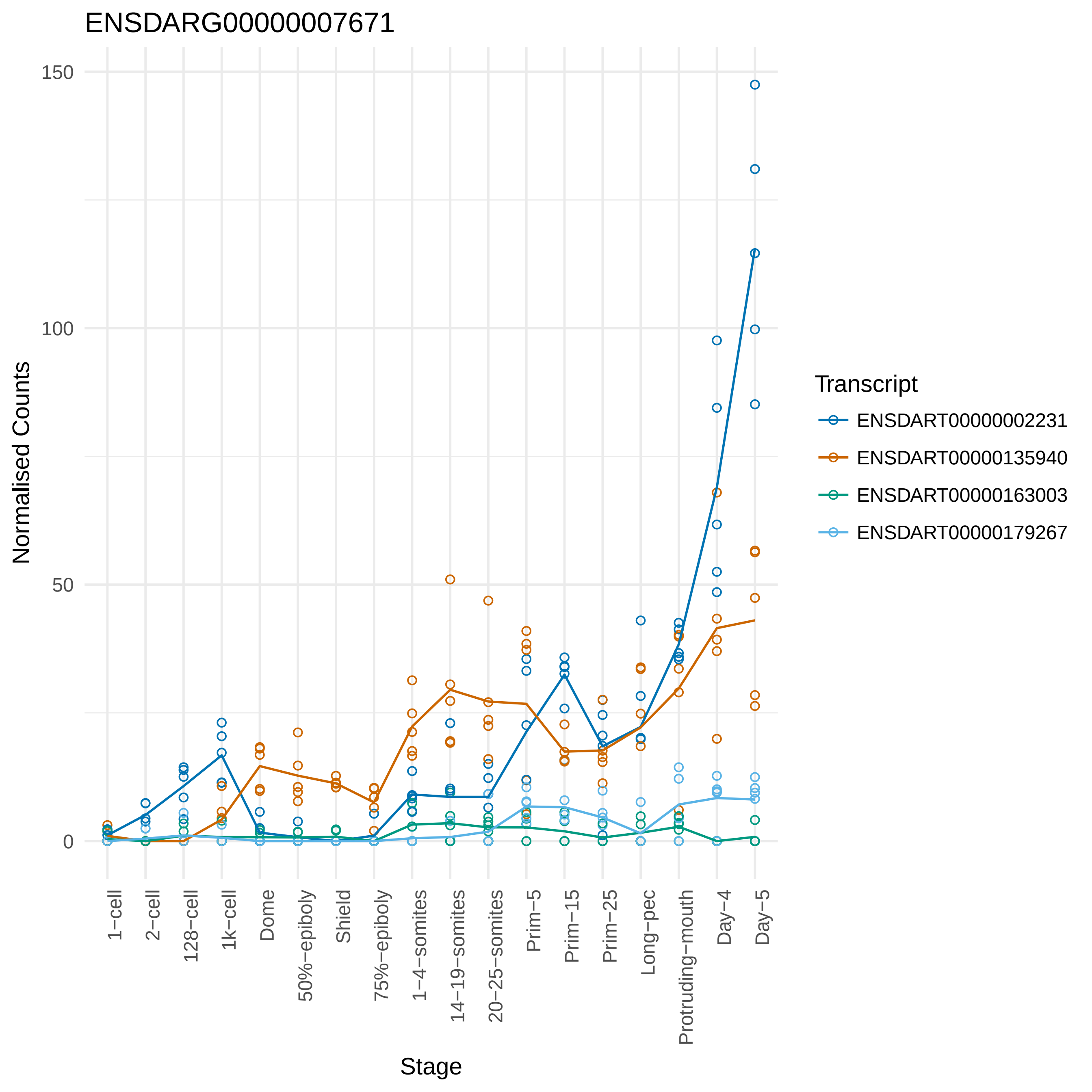

ENSDARG00000007671
Normalised Counts
150
100
50
0
1−cell
2−cell
128−cell
1k−cell
Dome
50%−epiboly
Shield
75%−epiboly
1−4−somites
14−19−somites
20−25−somites
Prim−5
Prim−15
Prim−25
Long−pec
Protruding−mouth
Day−4
Day−5
Stage
Transcript
ENSDART00000002231
ENSDART00000135940
ENSDART00000163003
ENSDART00000179267

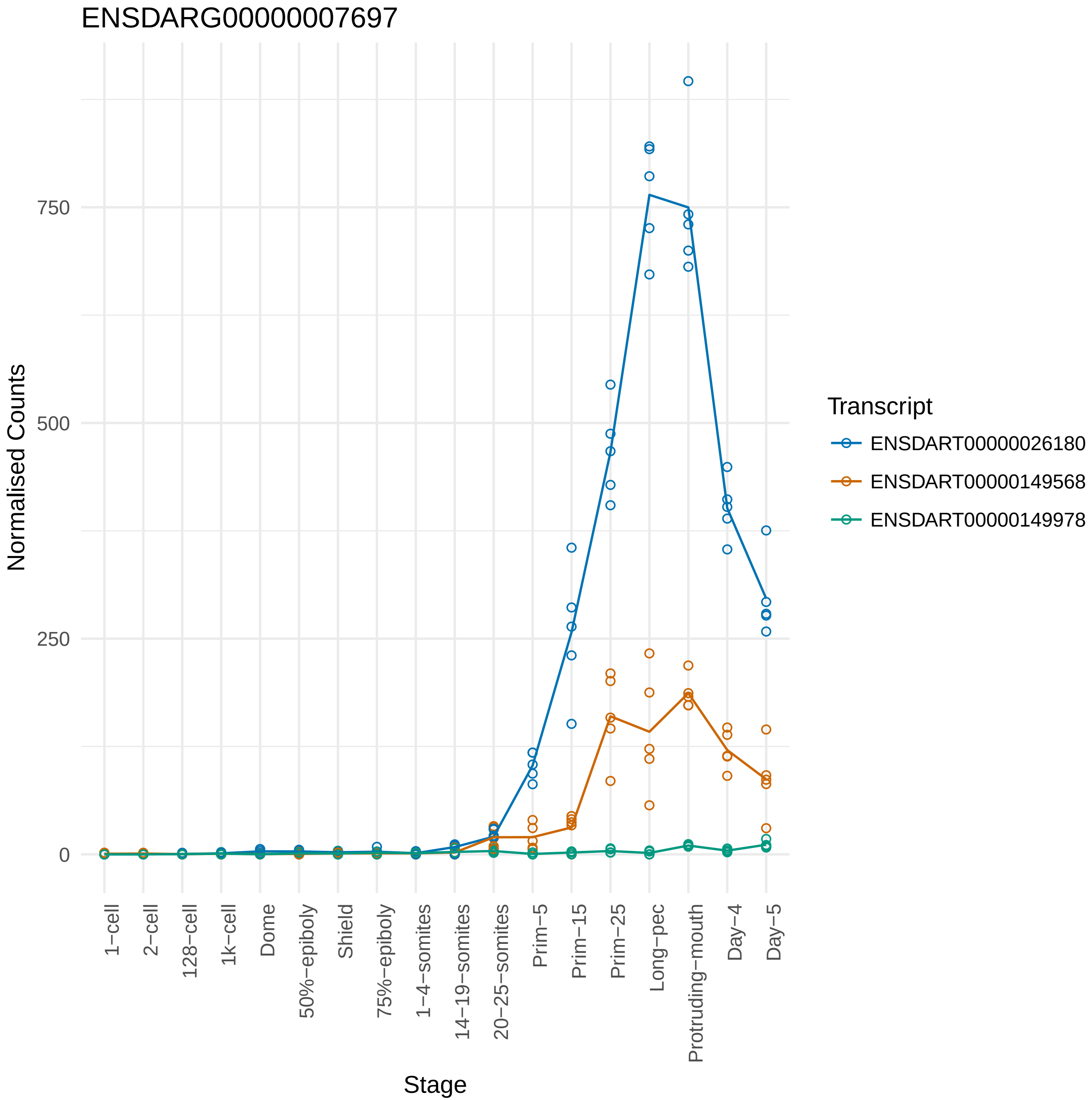

ENSDARG00000007697
Normalised Counts
750
500
250
0
1−cell
2−cell
128−cell
1k−cell
Dome
50%−epiboly
Shield
75%−epiboly
1−4−somites
14−19−somites
20−25−somites
Prim−5
Prim−15
Prim−25
Long−pec
Protruding−mouth
Day−4
Day−5
Stage
Transcript
ENSDART00000026180
ENSDART00000149568
ENSDART00000149978

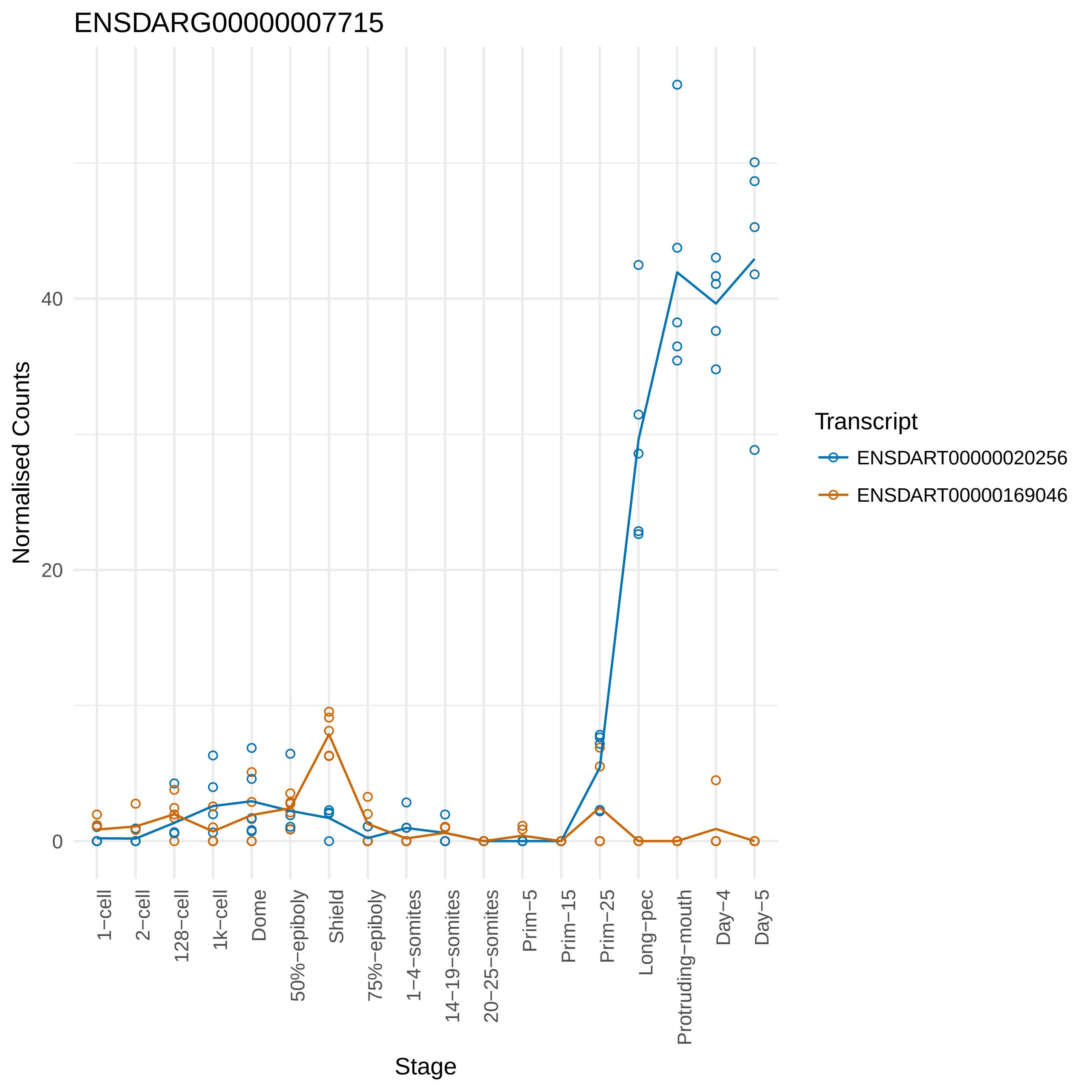
ENSDARG00000007715
Normalised Counts
40
20
0
1−cell
2−cell
128−cell
1k−cell
Dome
50%−epiboly
Shield
75%−epiboly
1−4−somites
14−19−somites
20−25−somites
Prim−5
Prim−15
Prim−25
Long−pec
Protruding−mouth
Day−4
Day−5
Stage
Transcript
ENSDART00000020256
ENSDART00000169046

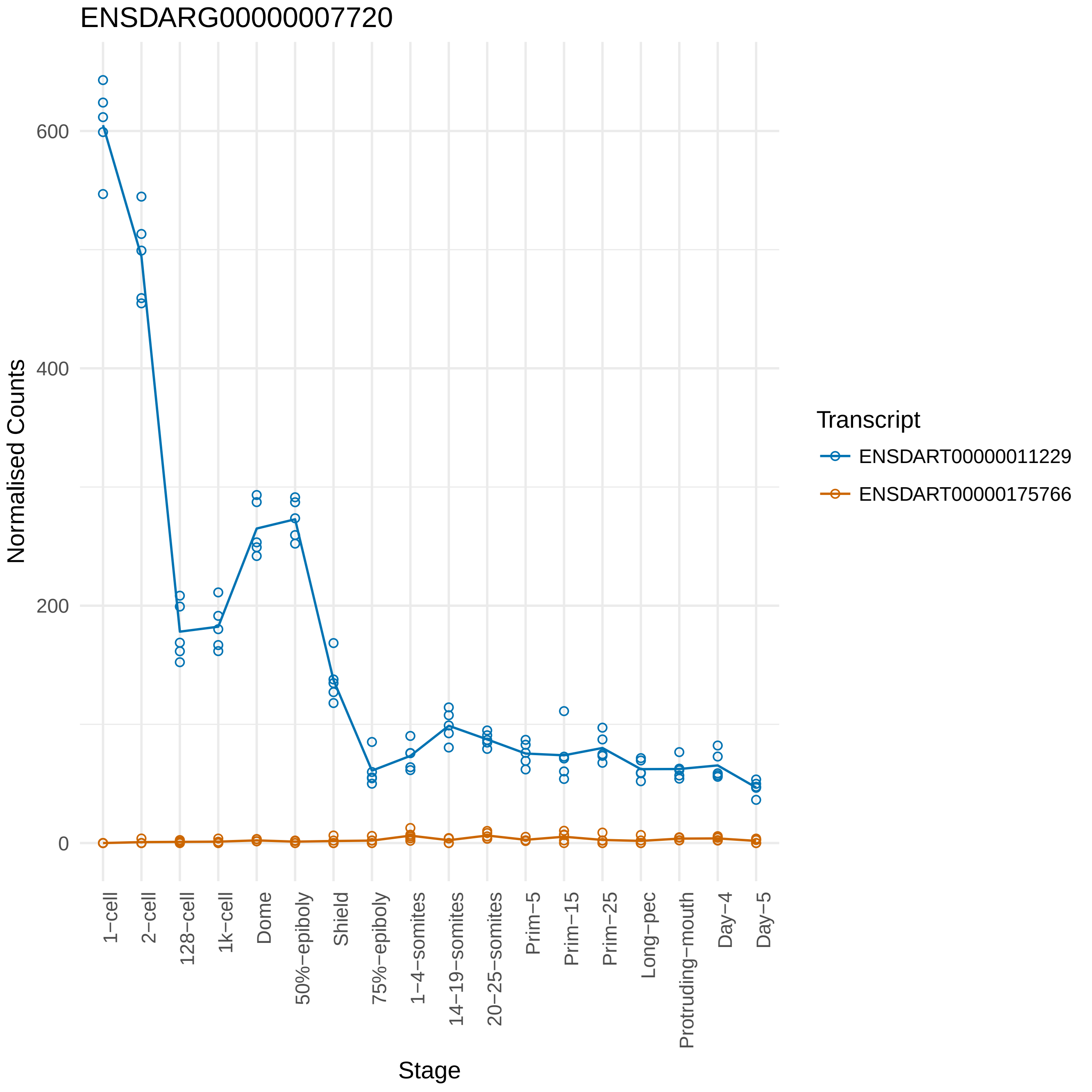

ENSDARG00000007720
Normalised Counts
600
400
200
0
1−cell
2−cell
128−cell
1k−cell
Dome
50%−epiboly
Shield
75%−epiboly
1−4−somites
14−19−somites
20−25−somites
Prim−5
Prim−15
Prim−25
Long−pec
Protruding−mouth
Day−4
Day−5
Stage
Transcript
ENSDART00000011229
ENSDART00000175766

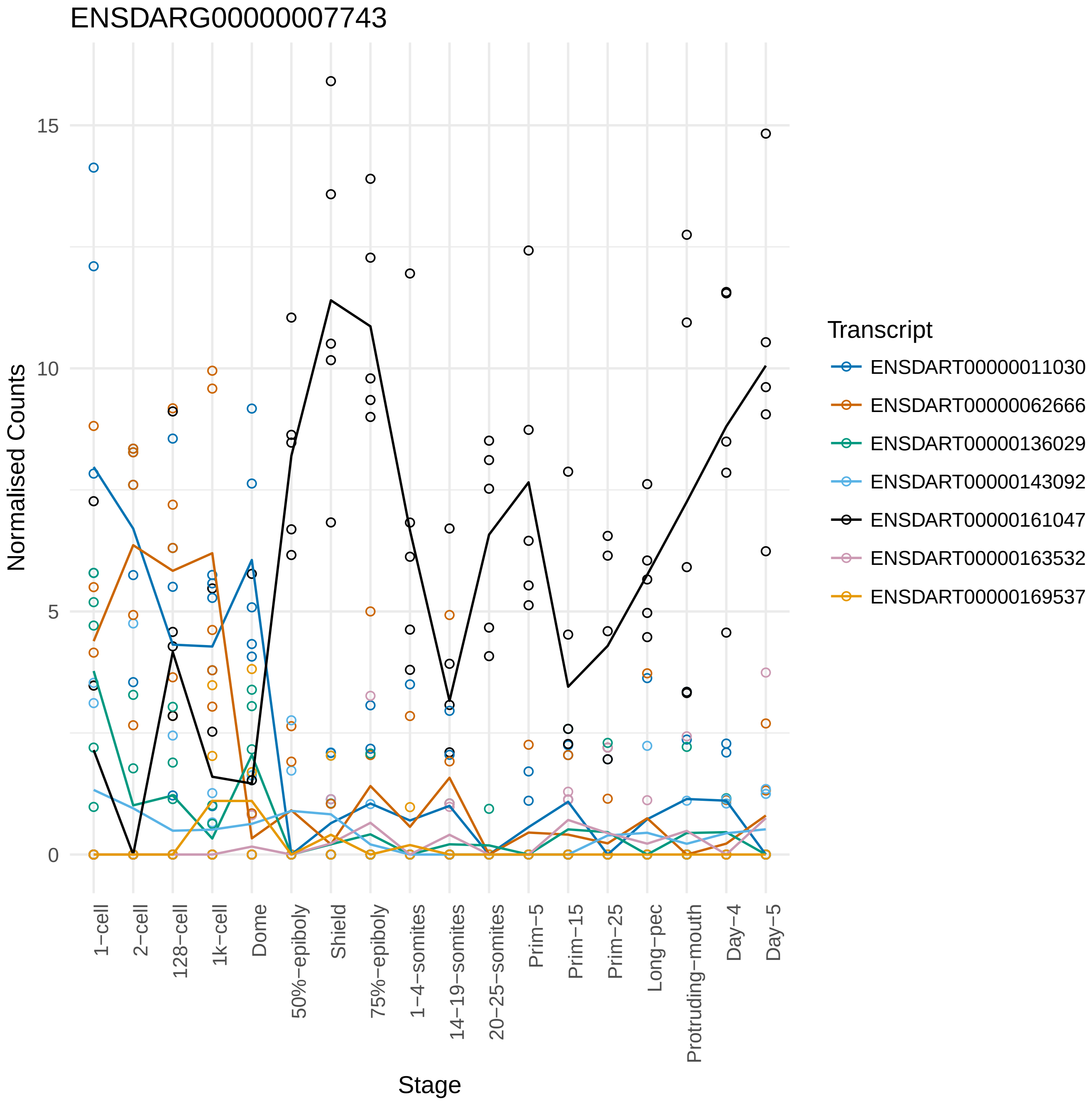

ENSDARG00000007743
Normalised Counts
15
10
5
0
1−cell
2−cell
128−cell
1k−cell
Dome
50%−epiboly
Shield
75%−epiboly
1−4−somites
14−19−somites
20−25−somites
Prim−5
Prim−15
Prim−25
Long−pec
Protruding−mouth
Day−4
Day−5
Stage
Transcript
ENSDART00000011030
ENSDART00000062666
ENSDART00000136029
ENSDART00000143092
ENSDART00000161047
ENSDART00000163532
ENSDART00000169537

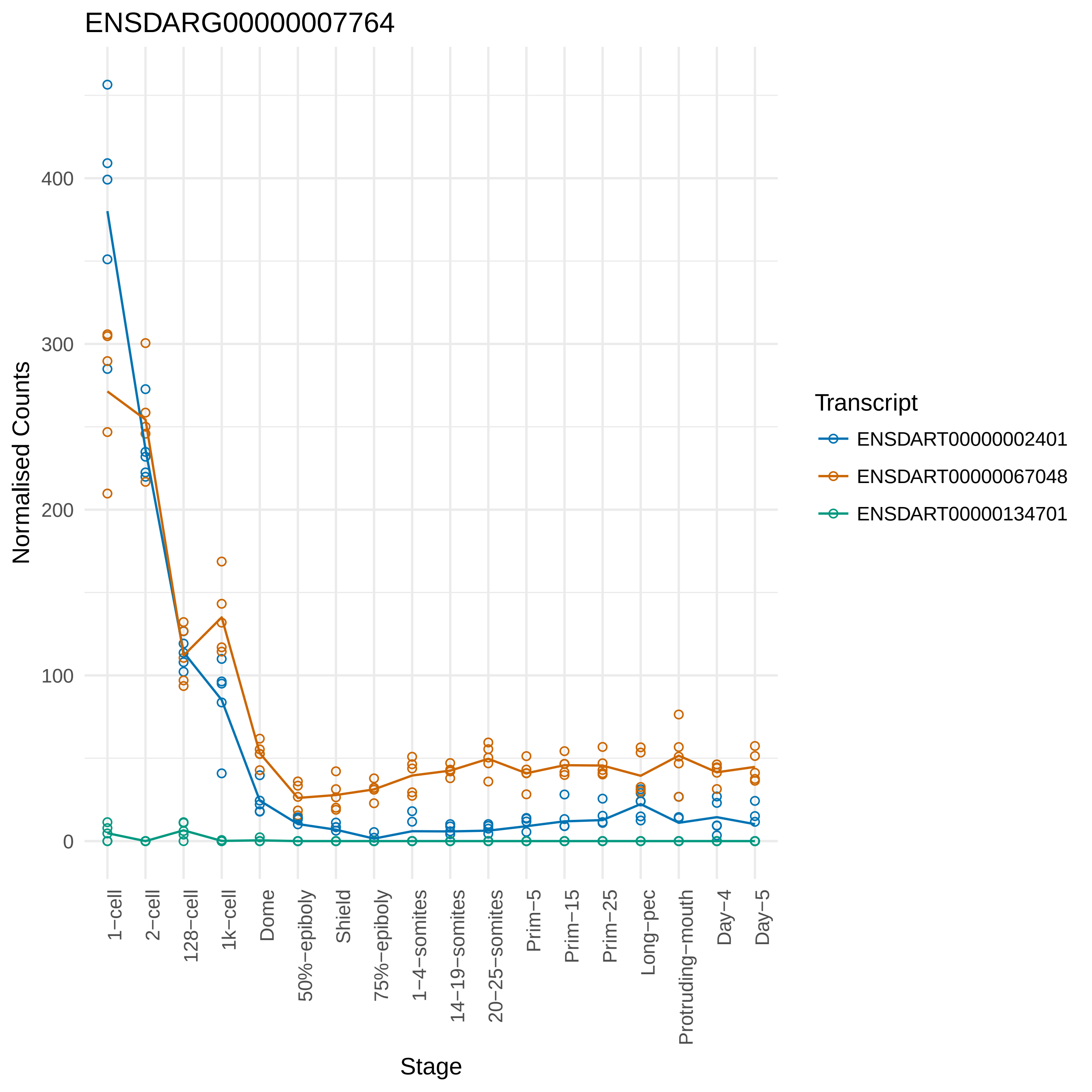
ENSDARG00000007764
Normalised Counts
0
100
200
300
400
Stage
1−cell
2−cell
128−cell
1k−cell
Dome
50%−epiboly
Shield
75%−epiboly
1−4−somites
14−19−somites
20−25−somites
Prim−5
Prim−15
Prim−25
Long−pec
Protruding−mouth
Day−4
Day−5
Transcript
ENSDART00000002401
ENSDART00000067048
ENSDART00000134701

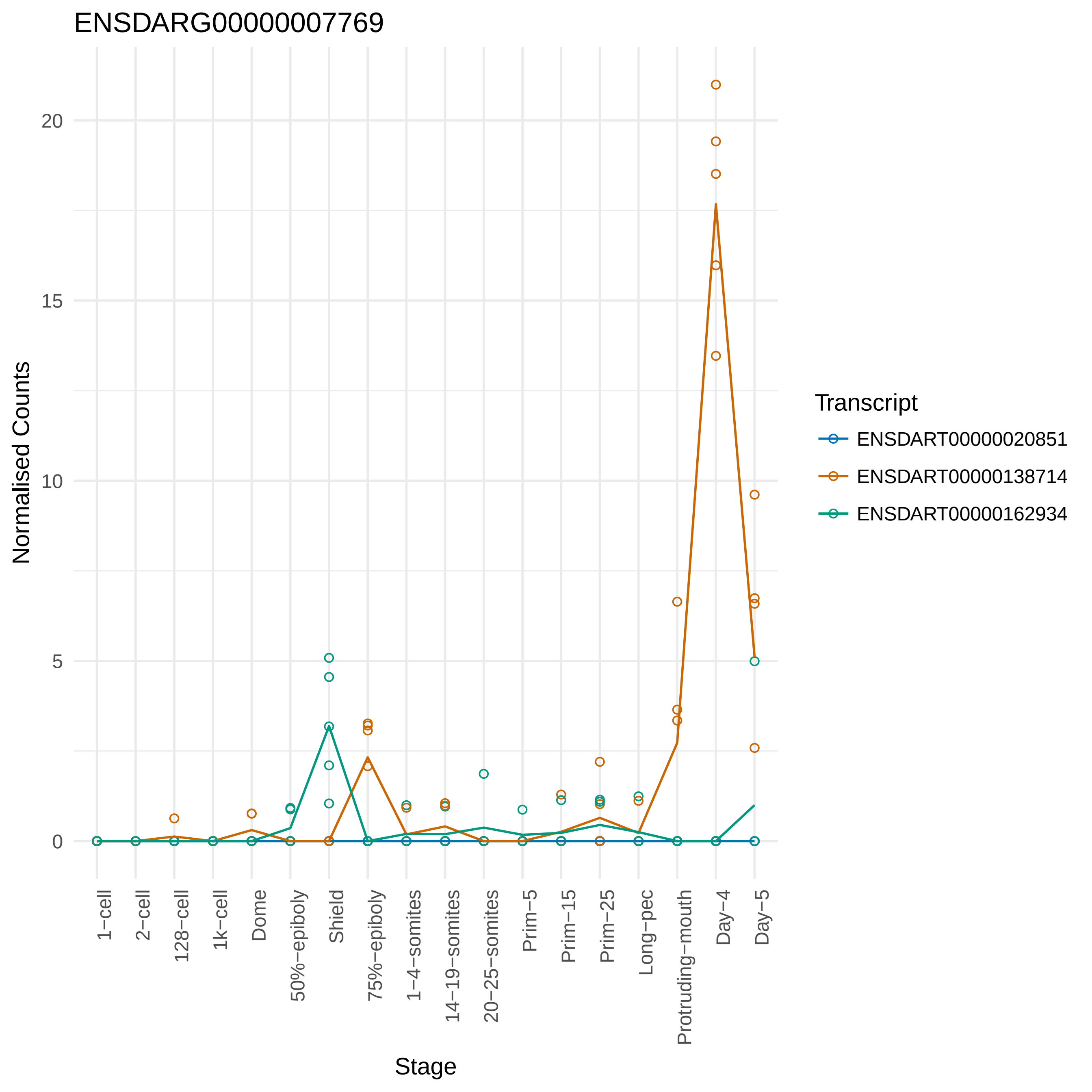

ENSDARG00000007769
Normalised Counts
0
5
10
15
20
Stage
1−cell
2−cell
128−cell
1k−cell
Dome
50%−epiboly
Shield
75%−epiboly
1−4−somites
14−19−somites
20−25−somites
Prim−5
Prim−15
Prim−25
Long−pec
Protruding−mouth
Day−4
Day−5
Transcript
ENSDART00000020851
ENSDART00000138714
ENSDART00000162934

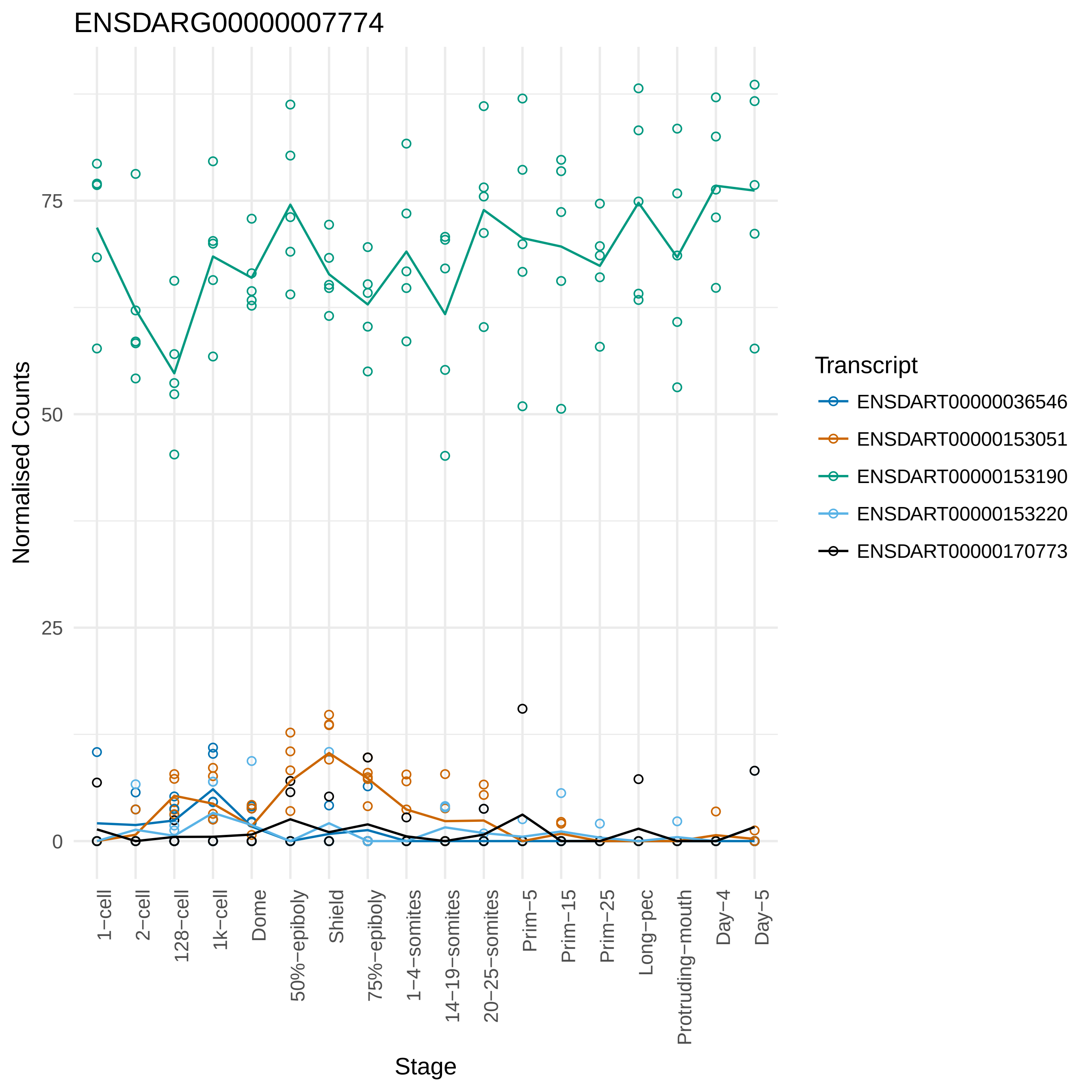

ENSDARG00000007774
Normalised Counts
75
50
25
0
1−cell
2−cell
128−cell
1k−cell
Dome
50%−epiboly
Shield
75%−epiboly
1−4−somites
14−19−somites
20−25−somites
Prim−5
Prim−15
Prim−25
Long−pec
Protruding−mouth
Day−4
Day−5
Stage
Transcript
ENSDART00000036546
ENSDART00000153051
ENSDART00000153190
ENSDART00000153220
ENSDART00000170773

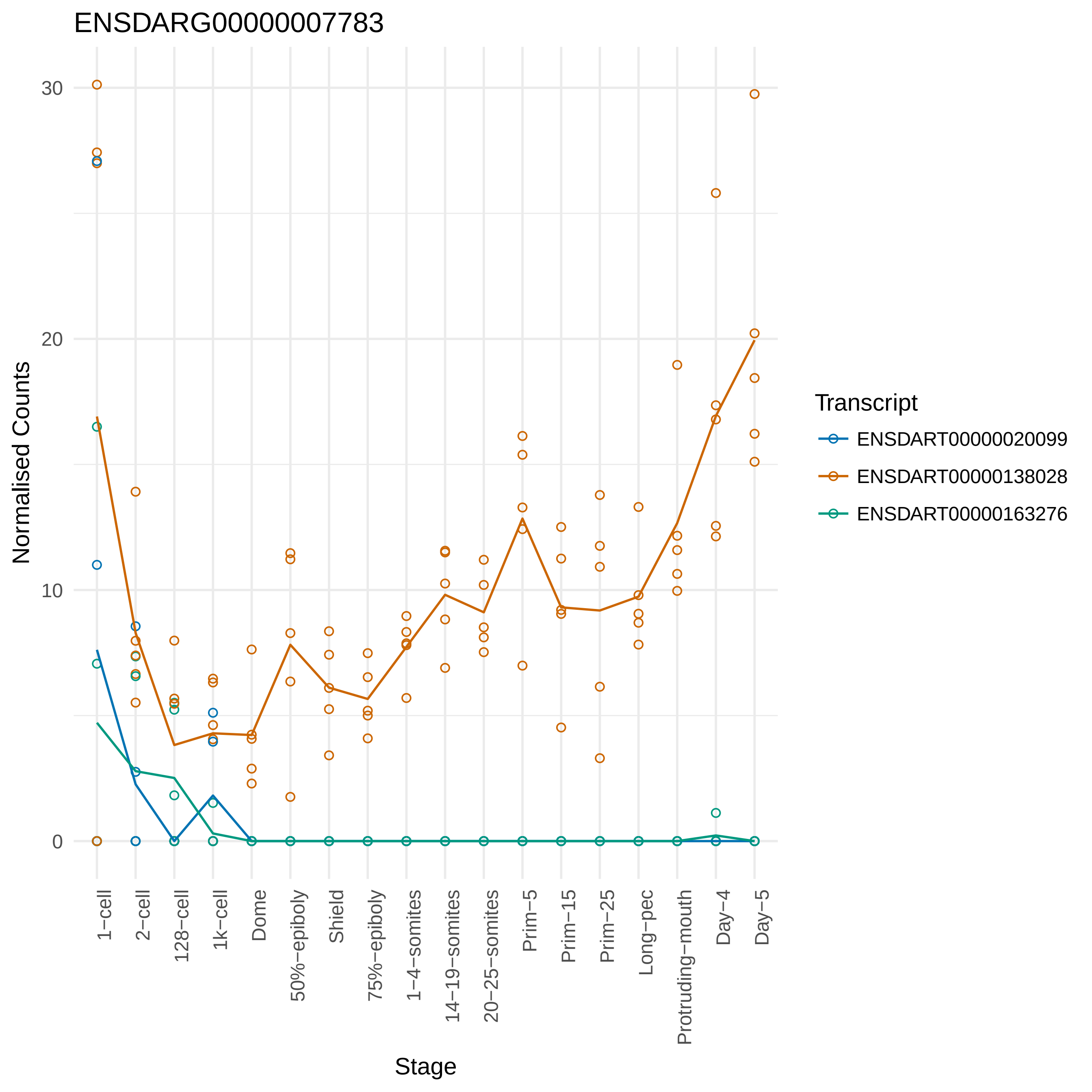
ENSDARG00000007783
Normalised Counts
30
20
10
0
Stage
1−cell
2−cell
128−cell
1k−cell
Dome
50%−epiboly
Shield
75%−epiboly
1−4−somites
14−19−somites
20−25−somites
Prim−5
Prim−15
Prim−25
Long−pec
Protruding−mouth
Day−4
Day−5
Transcript
ENSDART00000020099
ENSDART00000138028
ENSDART00000163276

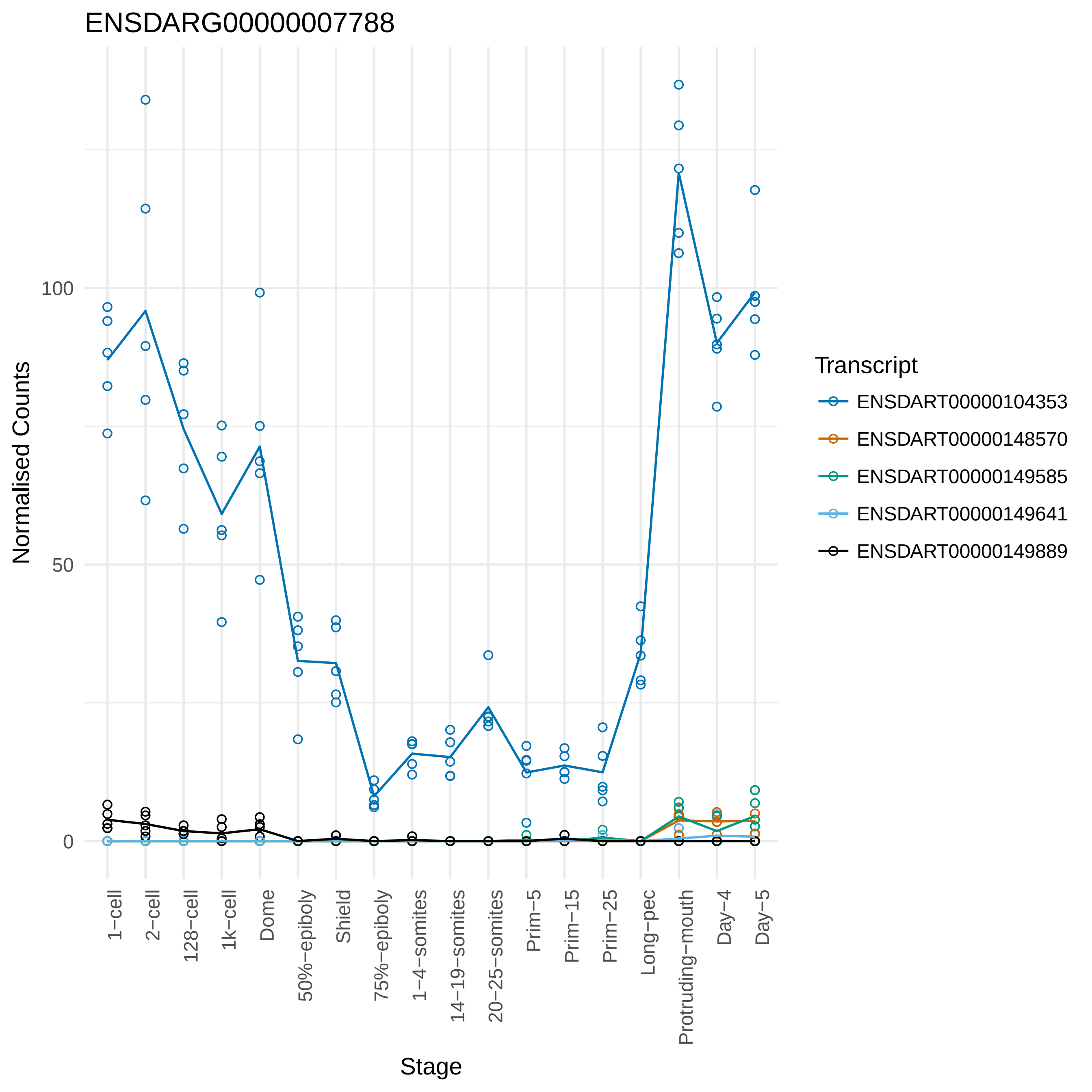
ENSDARG00000007788
Normalised Counts
100
50
0
1−cell
2−cell
128−cell
1k−cell
Dome
50%−epiboly
Shield
75%−epiboly
1−4−somites
14−19−somites
20−25−somites
Prim−5
Prim−15
Prim−25
Long−pec
Protruding−mouth
Day−4
Day−5
Stage
Transcript
ENSDART00000104353
ENSDART00000148570
ENSDART00000149585
ENSDART00000149641
ENSDART00000149889

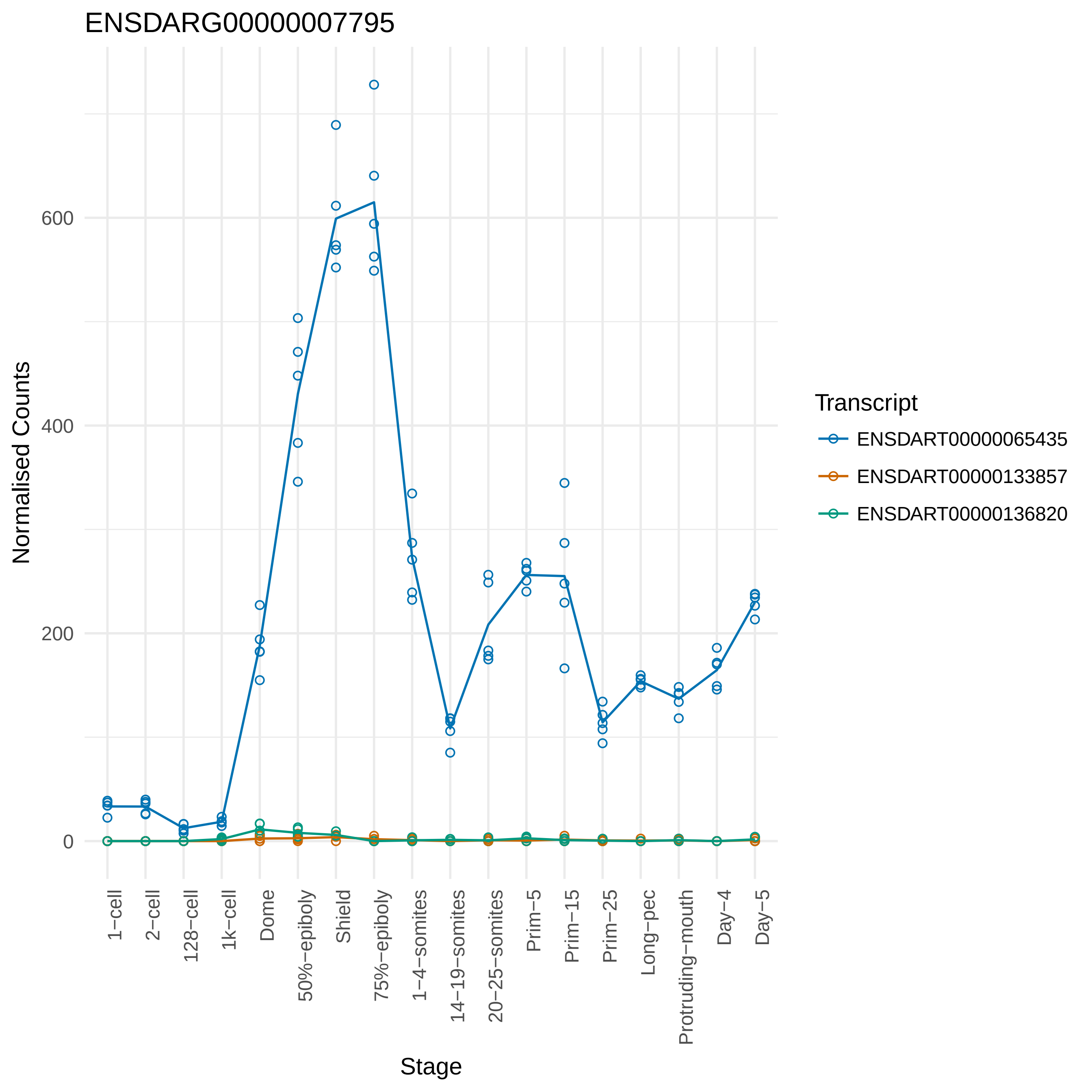

ENSDARG00000007795
Normalised Counts
0
200
400
600
1−cell
2−cell
128−cell
1k−cell
Dome
50%−epiboly
Shield
75%−epiboly
1−4−somites
14−19−somites
20−25−somites
Prim−5
Prim−15
Prim−25
Long−pec
Protruding−mouth
Day−4
Day−5
Stage
Transcript
ENSDART00000065435
ENSDART00000133857
ENSDART00000136820

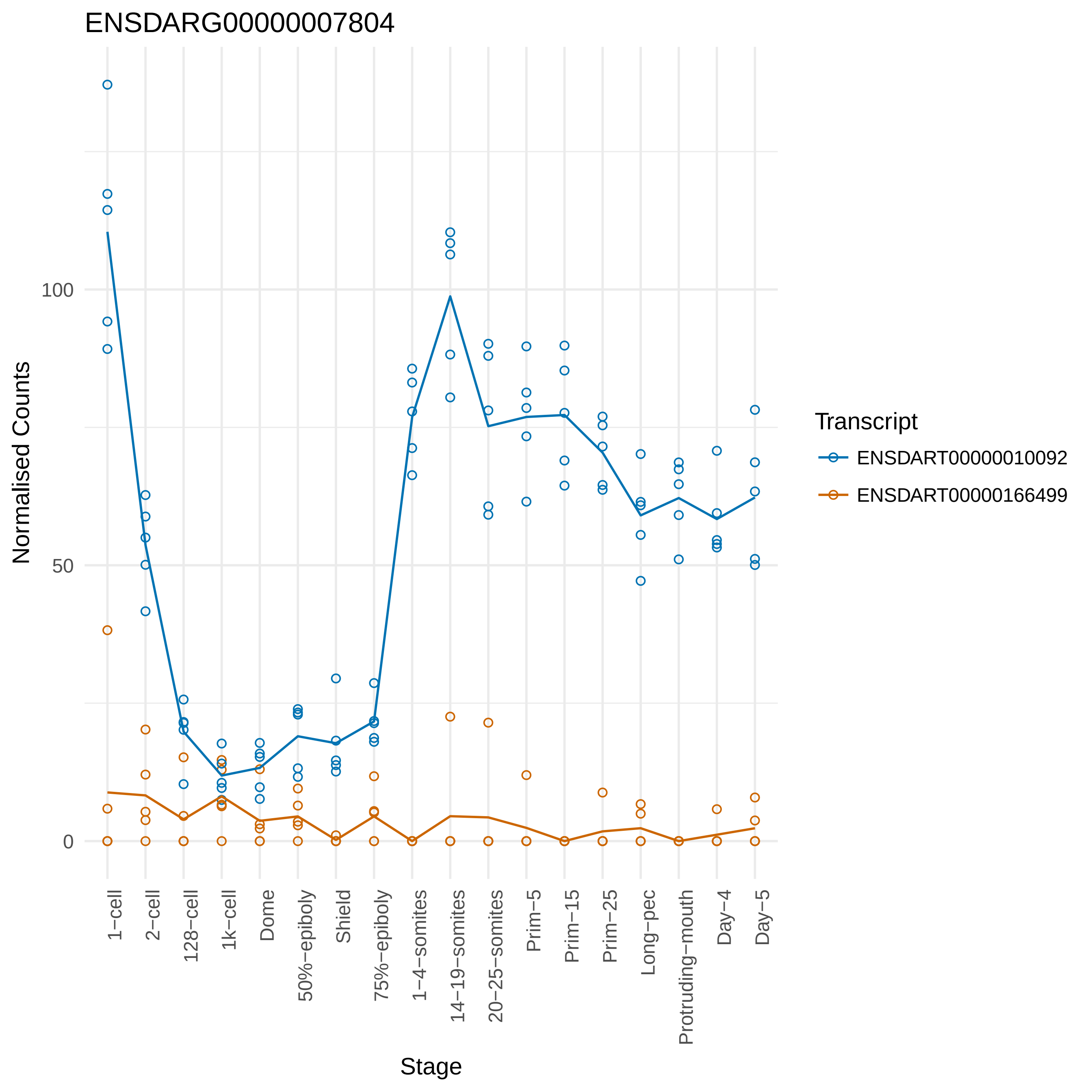

ENSDARG00000007804
Normalised Counts
100
50
0
Stage
1−cell
2−cell
128−cell
1k−cell
Dome
50%−epiboly
Shield
75%−epiboly
1−4−somites
14−19−somites
20−25−somites
Prim−5
Prim−15
Prim−25
Long−pec
Protruding−mouth
Day−4
Day−5
Transcript
ENSDART00000010092
ENSDART00000166499

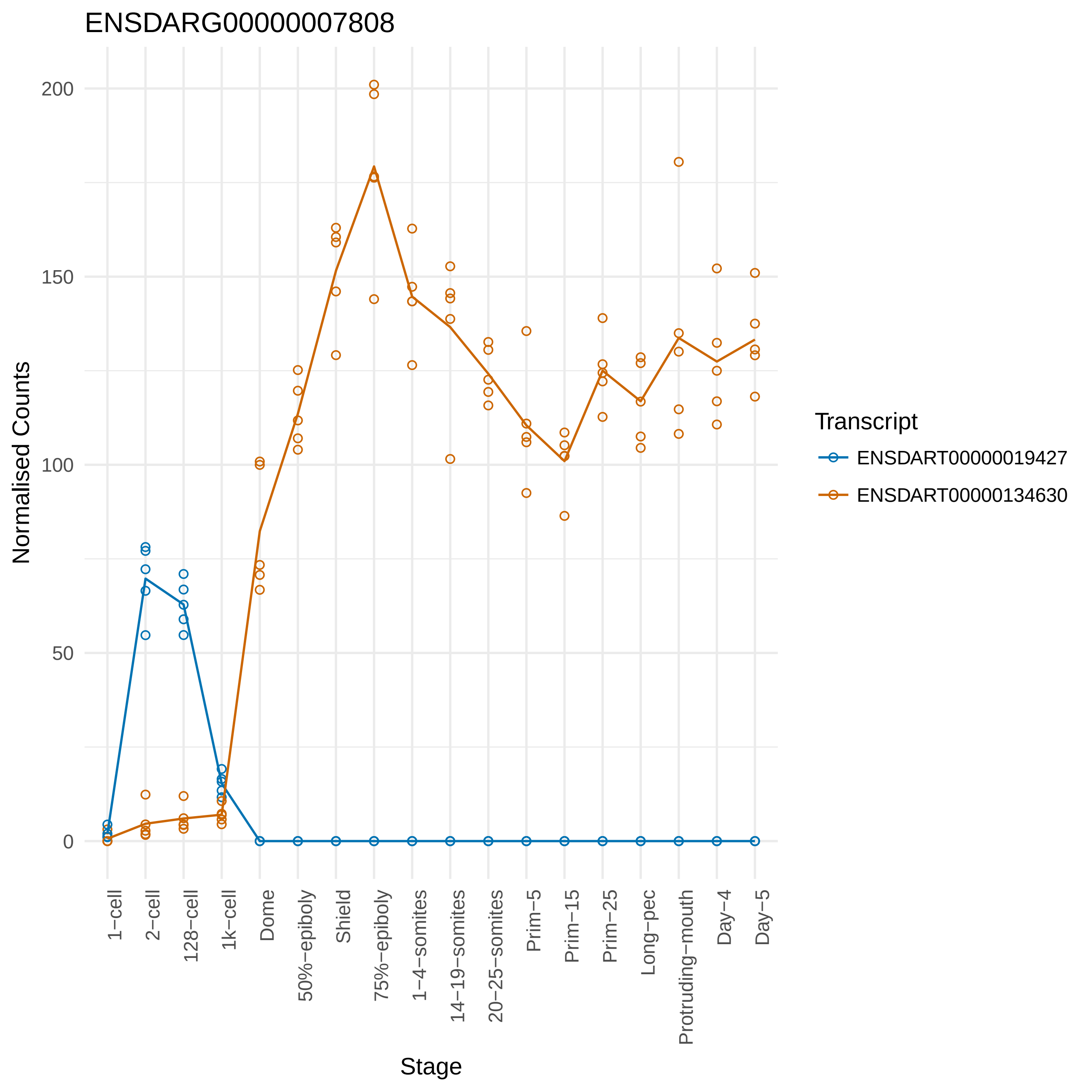
ENSDARG00000007808
Normalised Counts
200
150
100
50
0
1−cell
2−cell
128−cell
1k−cell
Dome
50%−epiboly
Shield
75%−epiboly
1−4−somites
14−19−somites
20−25−somites
Prim−5
Prim−15
Prim−25
Long−pec
Protruding−mouth
Day−4
Day−5
Stage
Transcript
ENSDART00000019427
ENSDART00000134630

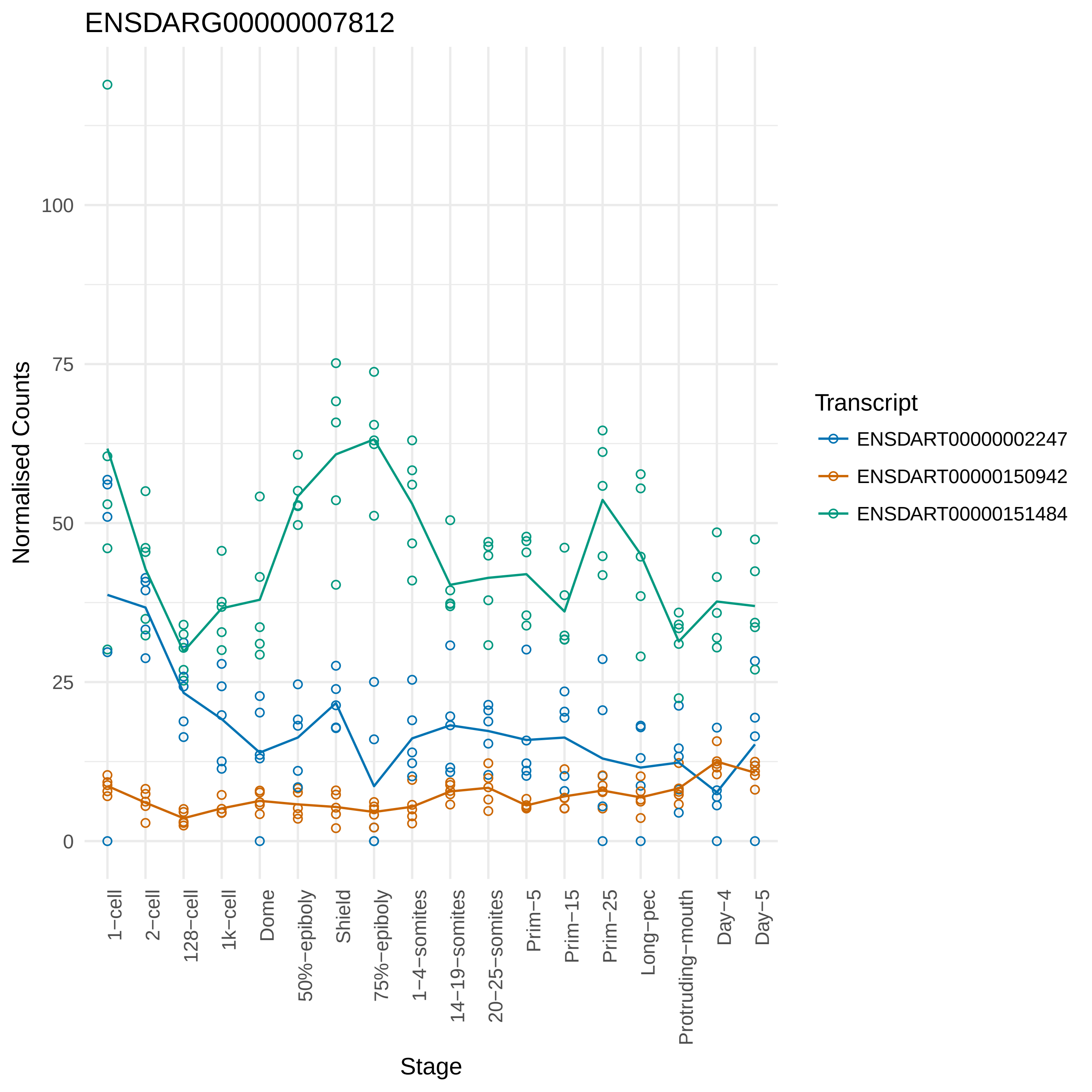

ENSDARG00000007812
Normalised Counts
0
25
50
75
100
Stage
1−cell
2−cell
128−cell
1k−cell
Dome
50%−epiboly
Shield
75%−epiboly
1−4−somites
14−19−somites
20−25−somites
Prim−5
Prim−15
Prim−25
Long−pec
Protruding−mouth
Day−4
Day−5
Transcript
ENSDART00000002247
ENSDART00000150942
ENSDART00000151484

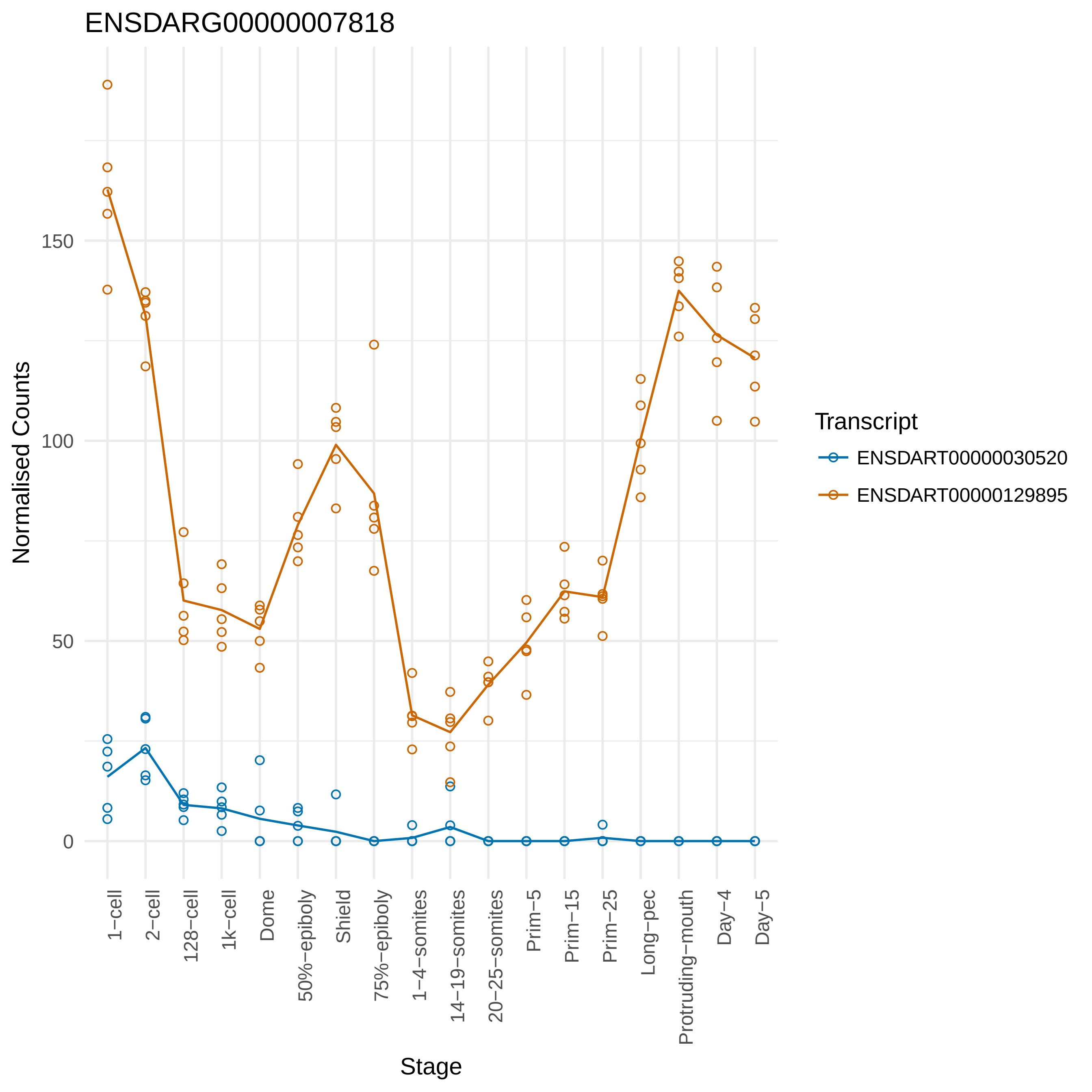

ENSDARG00000007818
Normalised Counts
150
100
50
0
1−cell
2−cell
128−cell
1k−cell
Dome
50%−epiboly
Shield
75%−epiboly
1−4−somites
14−19−somites
20−25−somites
Prim−5
Prim−15
Prim−25
Long−pec
Protruding−mouth
Day−4
Day−5
Stage
Transcript
ENSDART00000030520
ENSDART00000129895

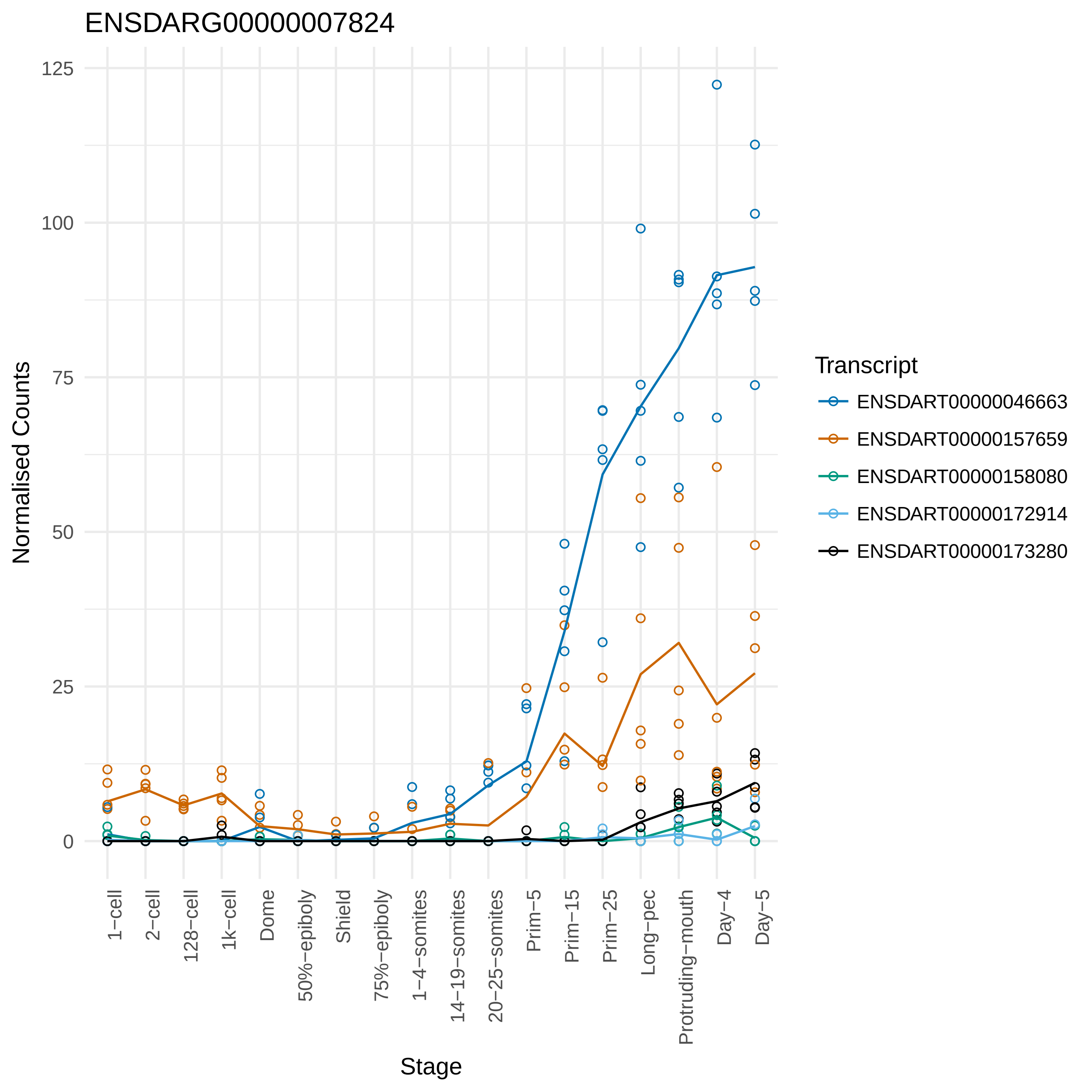

ENSDARG00000007824
Normalised Counts
125
100
75
50
25
0
1−cell
2−cell
128−cell
1k−cell
Dome
50%−epiboly
Shield
75%−epiboly
1−4−somites
14−19−somites
20−25−somites
Prim−5
Prim−15
Prim−25
Long−pec
Protruding−mouth
Day−4
Day−5
Stage
Transcript
ENSDART00000046663
ENSDART00000157659
ENSDART00000158080
ENSDART00000172914
ENSDART00000173280

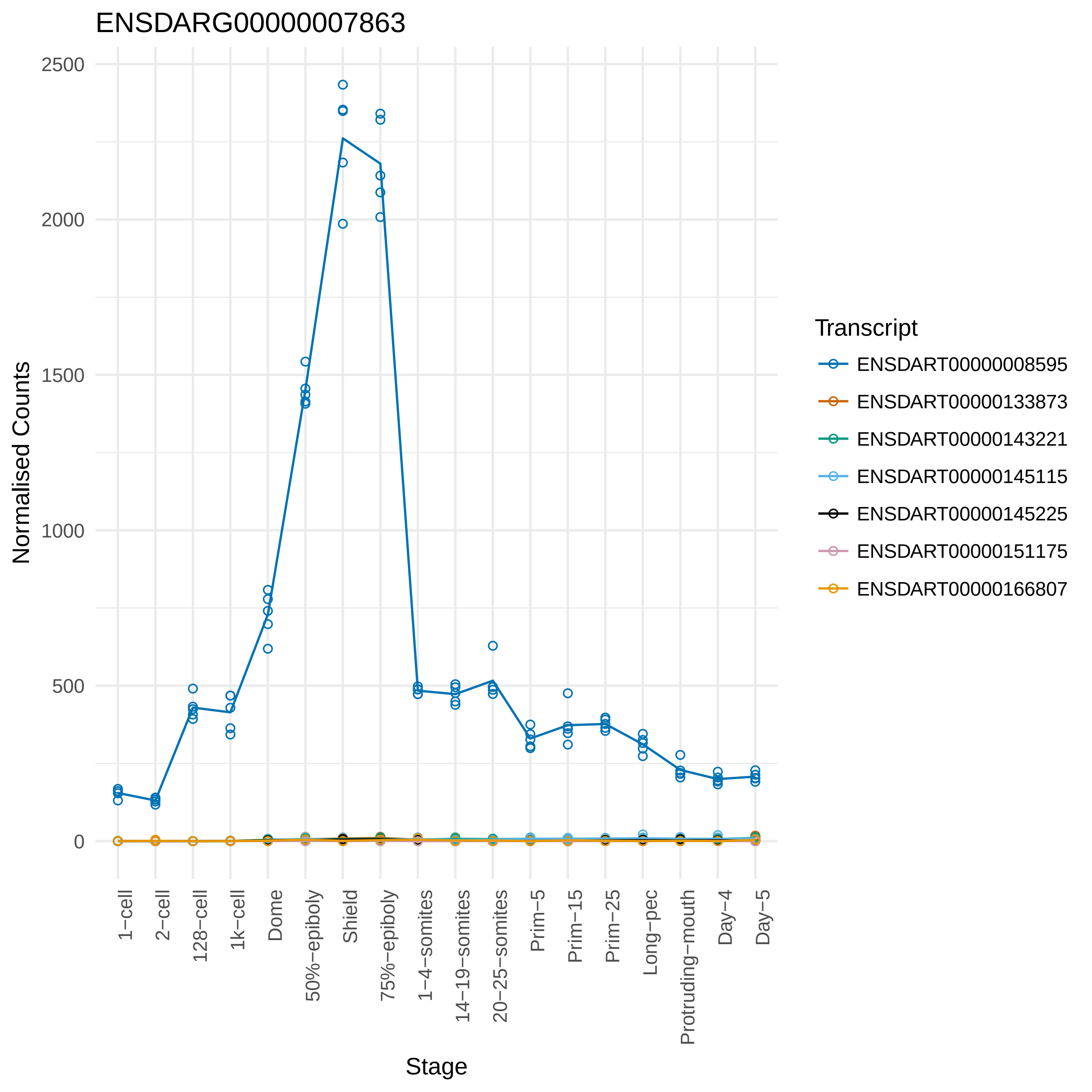
ENSDARG00000007863
Normalised Counts
0
500
1000
1500
2000
2500
Stage
1−cell
2−cell
128−cell
1k−cell
Dome
50%−epiboly
Shield
75%−epiboly
1−4−somites
14−19−somites
20−25−somites
Prim−5
Prim−15
Prim−25
Long−pec
Protruding−mouth
Day−4
Day−5
Transcript
ENSDART00000008595
ENSDART00000133873
ENSDART00000143221
ENSDART00000145115
ENSDART00000145225
ENSDART00000151175
ENSDART00000166807

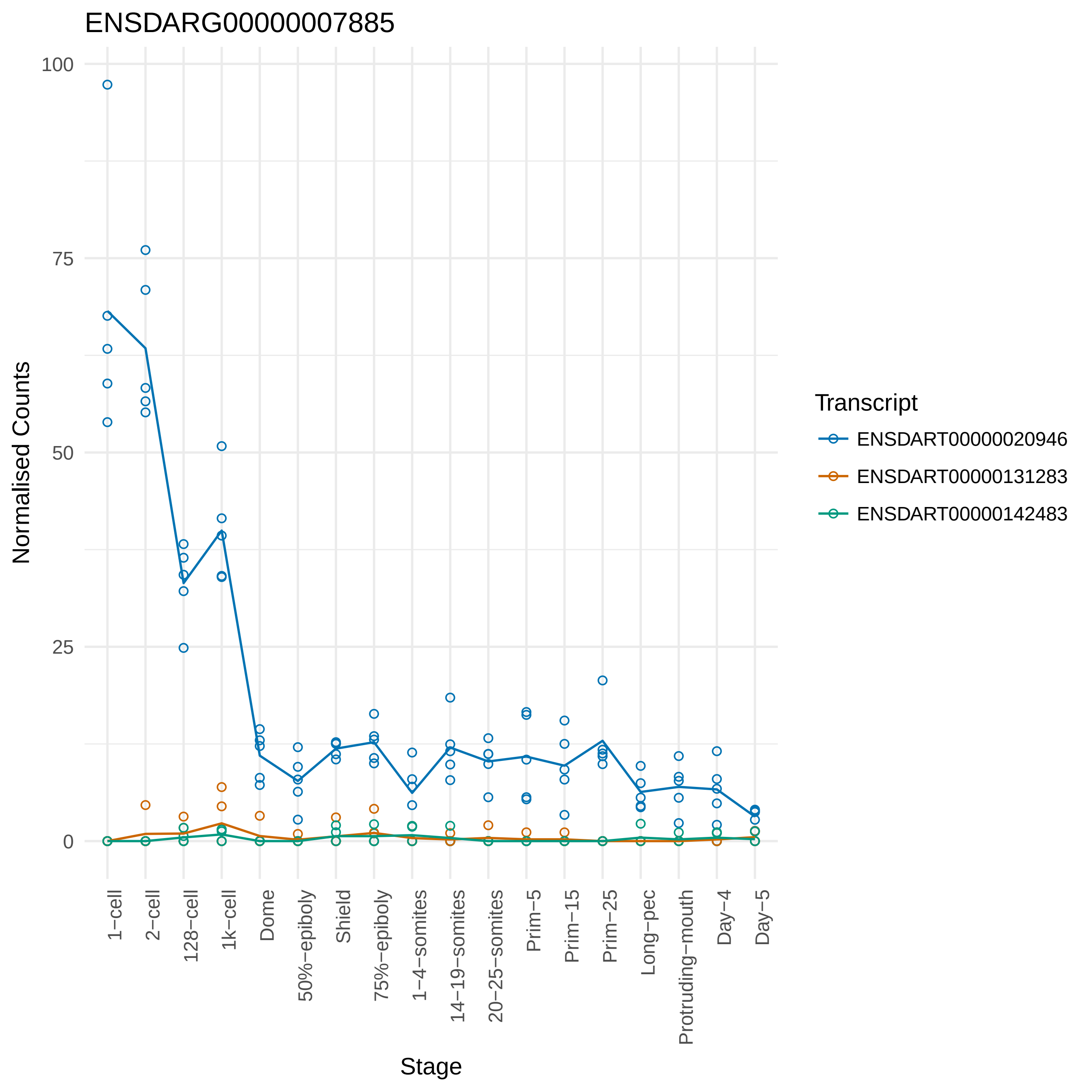
ENSDARG00000007885
Normalised Counts
100
75
50
25
0
Stage
1−cell
2−cell
128−cell
1k−cell
Dome
50%−epiboly
Shield
75%−epiboly
1−4−somites
14−19−somites
20−25−somites
Prim−5
Prim−15
Prim−25
Long−pec
Protruding−mouth
Day−4
Day−5
Transcript
ENSDART00000020946
ENSDART00000131283
ENSDART00000142483

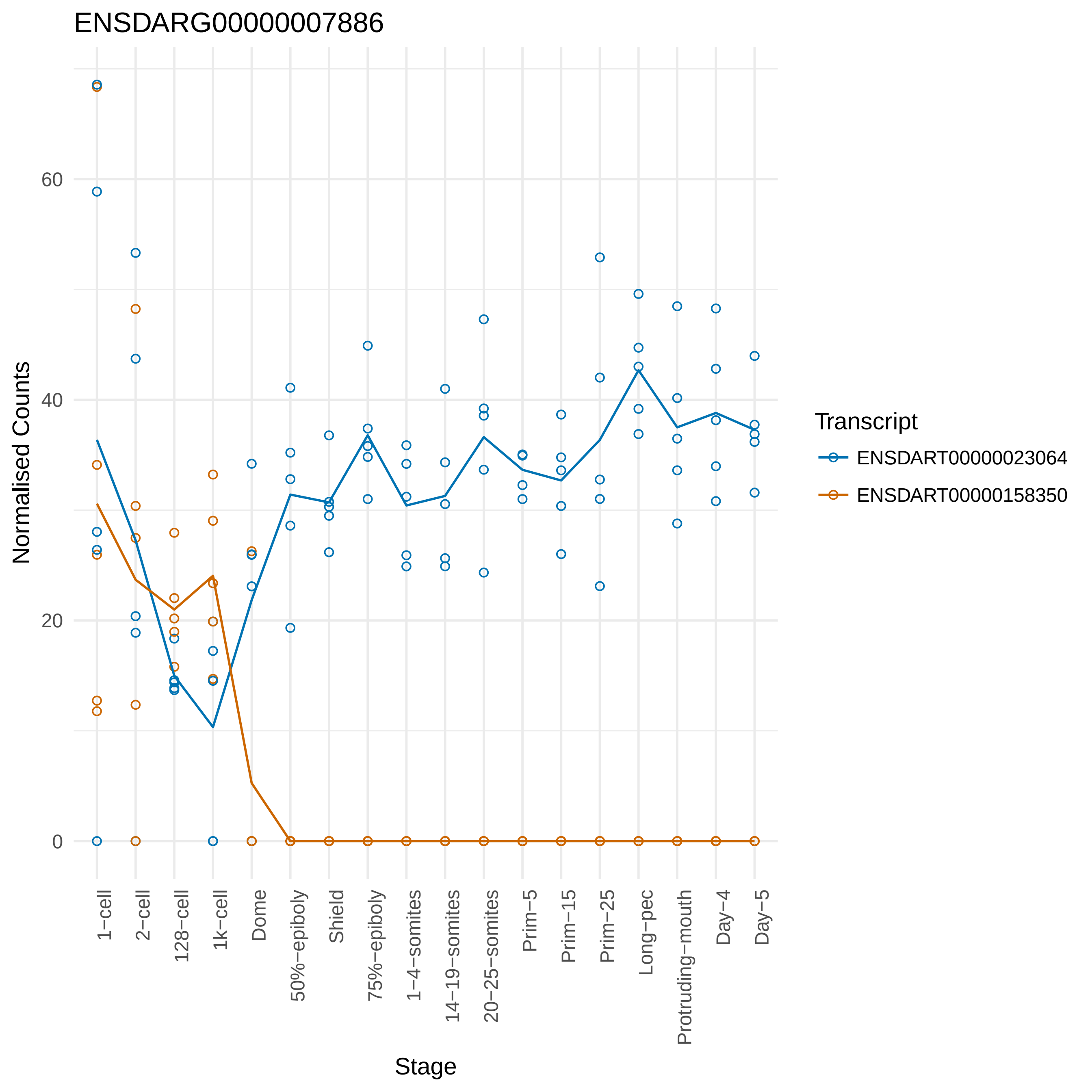
ENSDARG00000007886
Normalised Counts
0
20
40
60
Stage
1−cell
2−cell
128−cell
1k−cell
Dome
50%−epiboly
Shield
75%−epiboly
1−4−somites
14−19−somites
20−25−somites
Prim−5
Prim−15
Prim−25
Long−pec
Protruding−mouth
Day−4
Day−5
Transcript
ENSDART00000023064
ENSDART00000158350

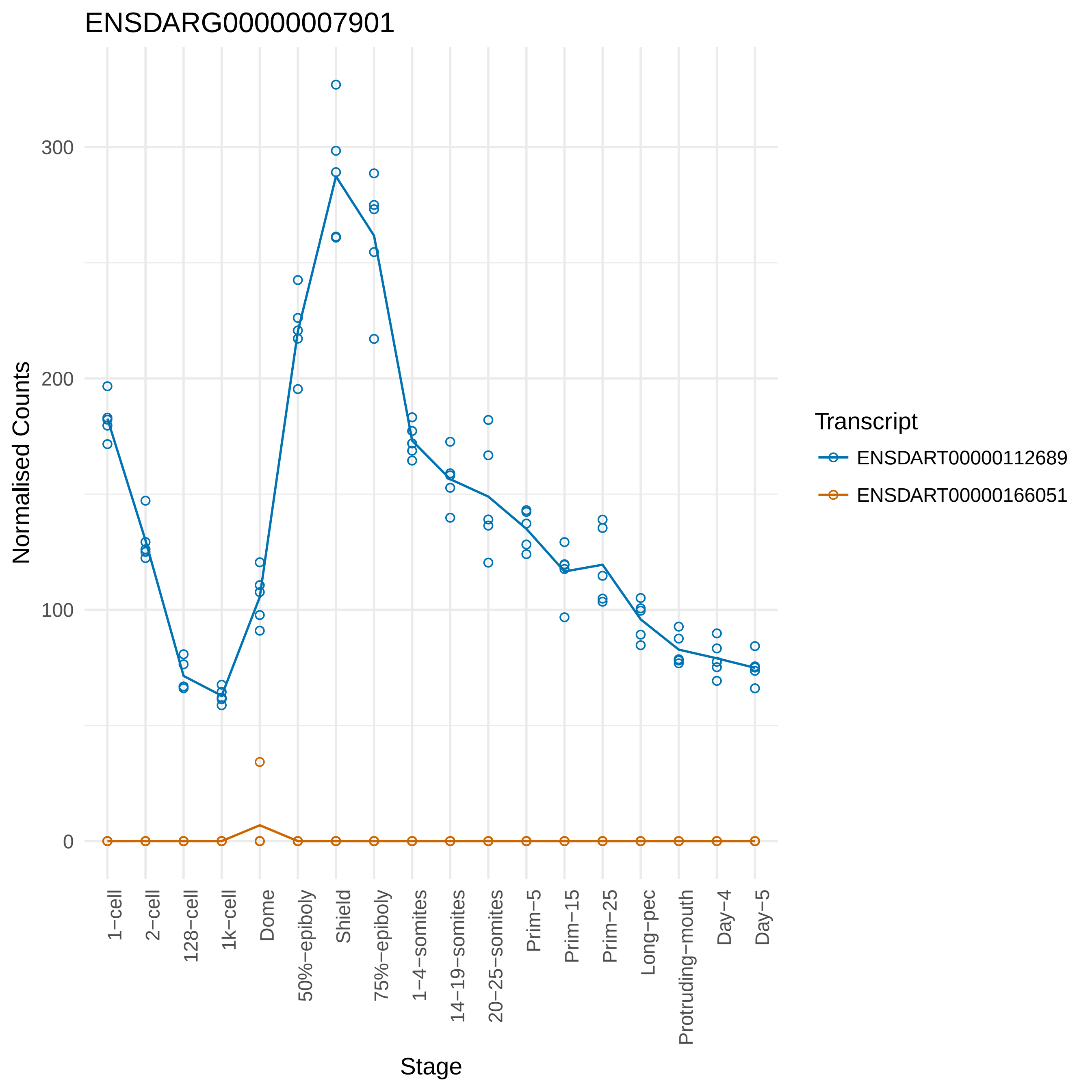
ENSDARG00000007901
Normalised Counts
300
200
100
0
1−cell
2−cell
128−cell
1k−cell
Dome
50%−epiboly
Shield
75%−epiboly
1−4−somites
14−19−somites
20−25−somites
Prim−5
Prim−15
Prim−25
Long−pec
Protruding−mouth
Day−4
Day−5
Stage
Transcript
ENSDART00000112689
ENSDART00000166051

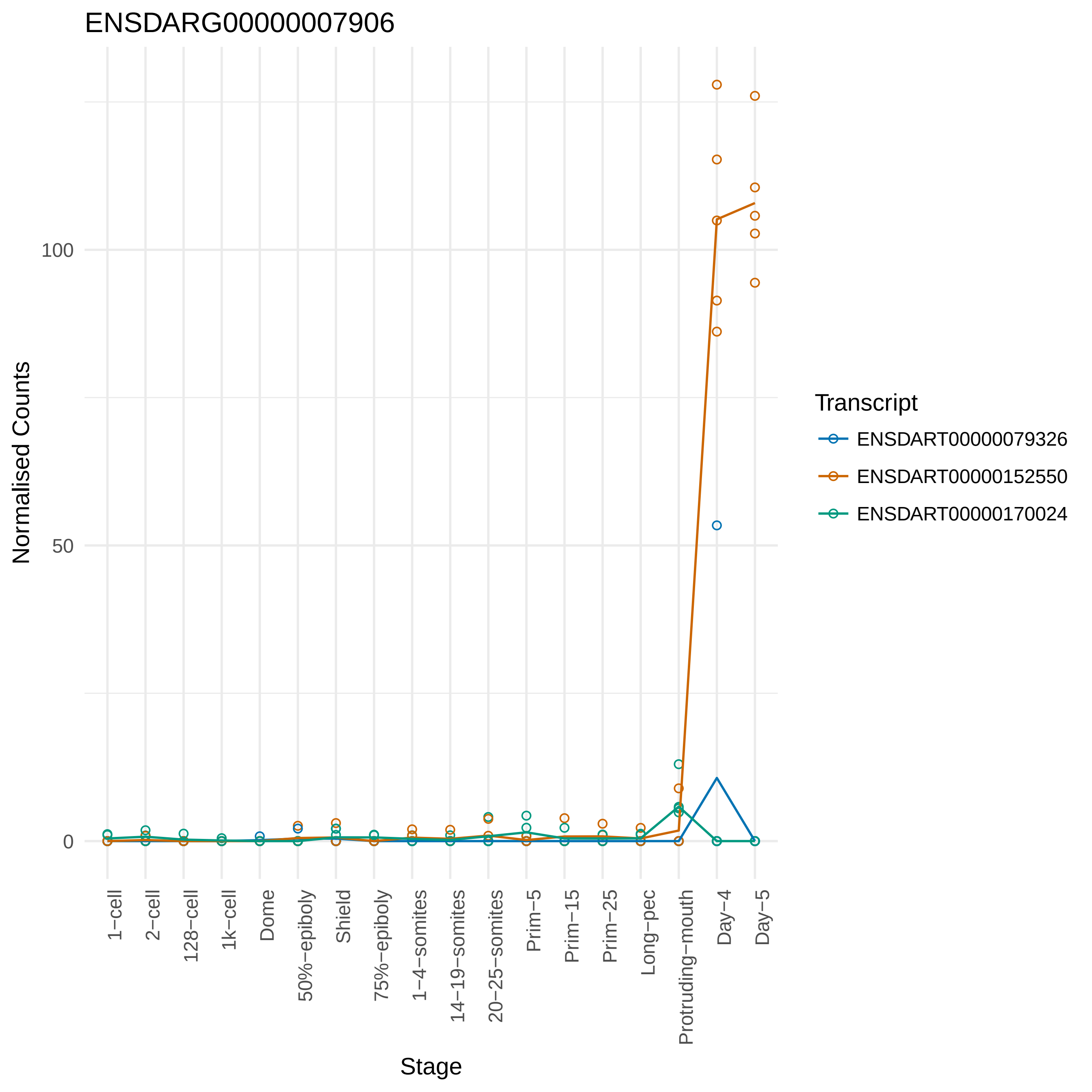

ENSDARG00000007906
Normalised Counts
100
50
0
1−cell
2−cell
128−cell
1k−cell
Dome
50%−epiboly
Shield
75%−epiboly
1−4−somites
14−19−somites
20−25−somites
Prim−5
Prim−15
Prim−25
Long−pec
Protruding−mouth
Day−4
Day−5
Stage
Transcript
ENSDART00000079326
ENSDART00000152550
ENSDART00000170024

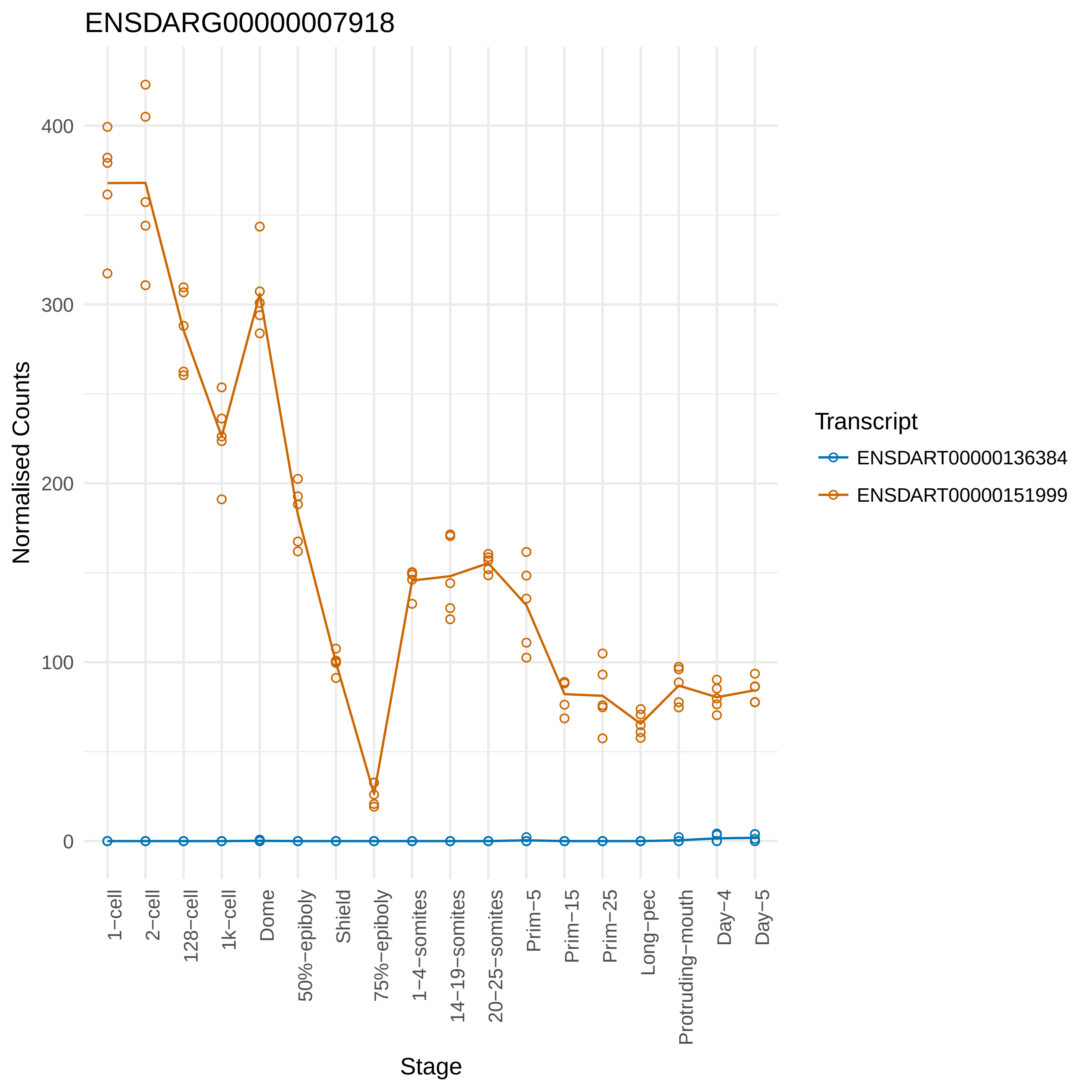

ENSDARG00000007918
Normalised Counts
400
300
200
100
0
1−cell
2−cell
128−cell
1k−cell
Dome
50%−epiboly
Shield
75%−epiboly
1−4−somites
14−19−somites
20−25−somites
Prim−5
Prim−15
Prim−25
Long−pec
Protruding−mouth
Day−4
Day−5
Stage
Transcript
ENSDART00000136384
ENSDART00000151999

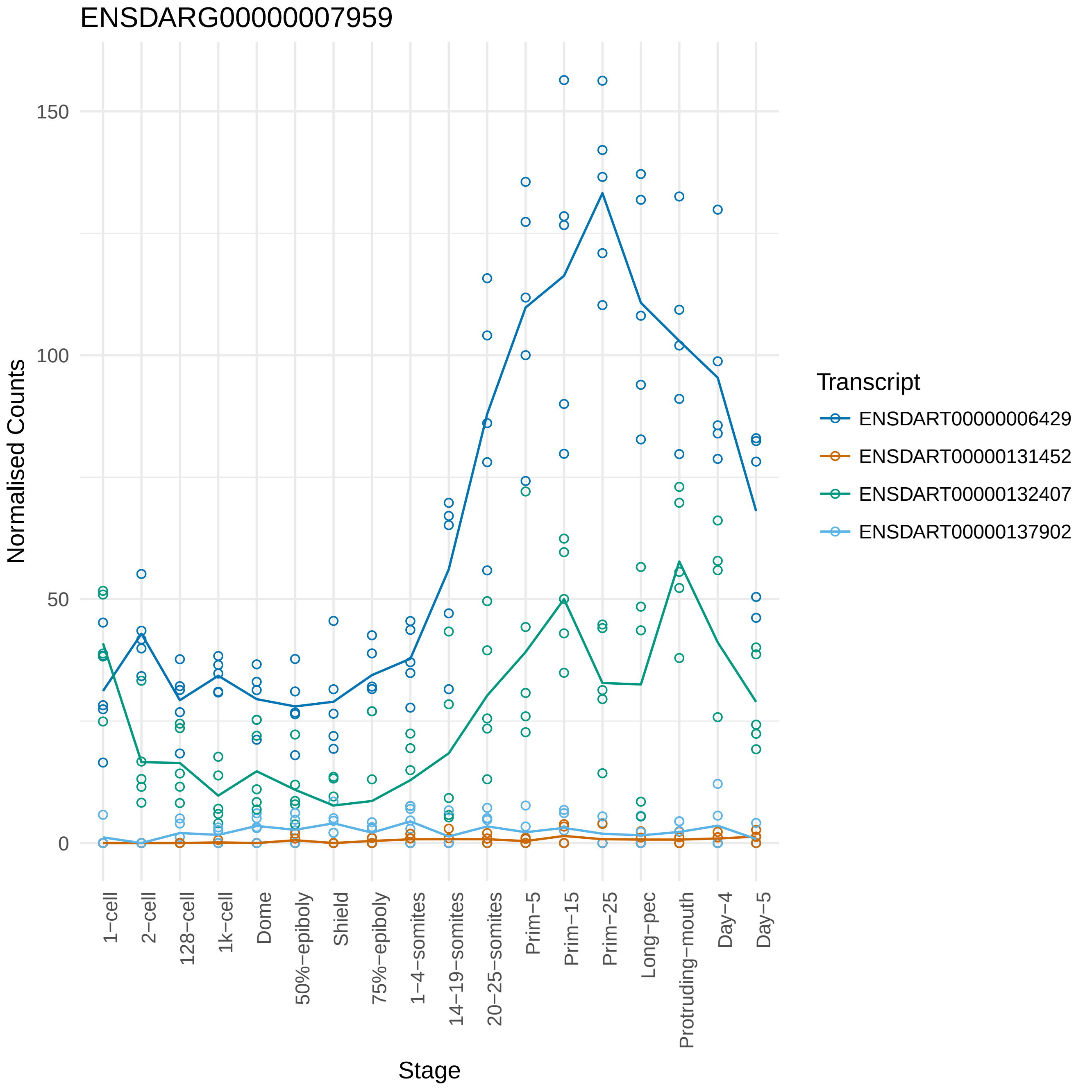

ENSDARG00000007959
Normalised Counts
150
100
50
0
Stage
1−cell
2−cell
128−cell
1k−cell
Dome
50%−epiboly
Shield
75%−epiboly
1−4−somites
14−19−somites
20−25−somites
Prim−5
Prim−15
Prim−25
Long−pec
Protruding−mouth
Day−4
Day−5
Transcript
ENSDART00000006429
ENSDART00000131452
ENSDART00000132407
ENSDART00000137902

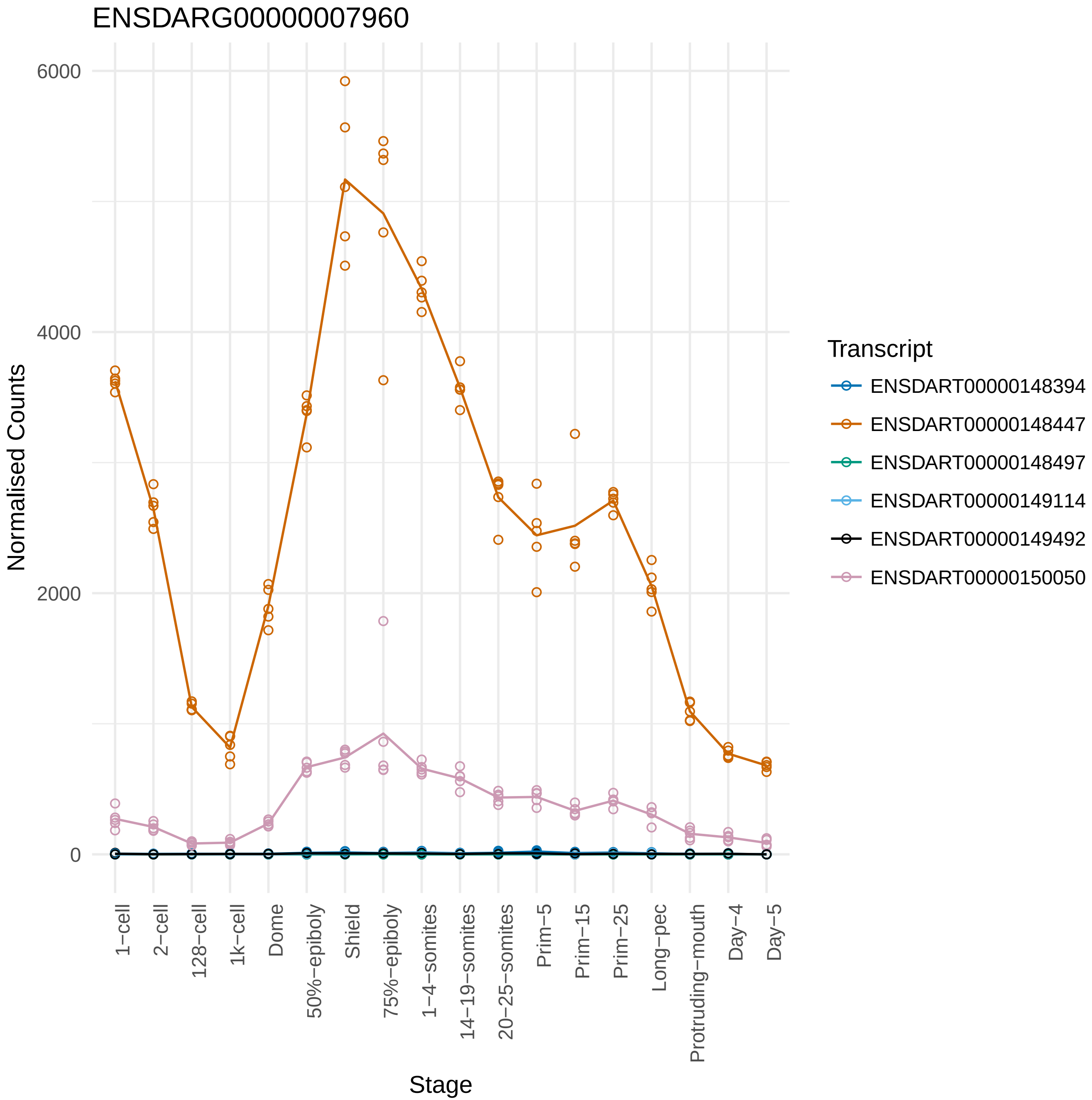
ENSDARG00000007960
Normalised Counts
6000
4000
2000
0
1−cell
2−cell
128−cell
1k−cell
Dome
50%−epiboly
Shield
75%−epiboly
1−4−somites
14−19−somites
20−25−somites
Prim−5
Prim−15
Prim−25
Long−pec
Protruding−mouth
Day−4
Day−5
Stage
Transcript
ENSDART00000148394
ENSDART00000148447
ENSDART00000148497
ENSDART00000149114
ENSDART00000149492
ENSDART00000150050

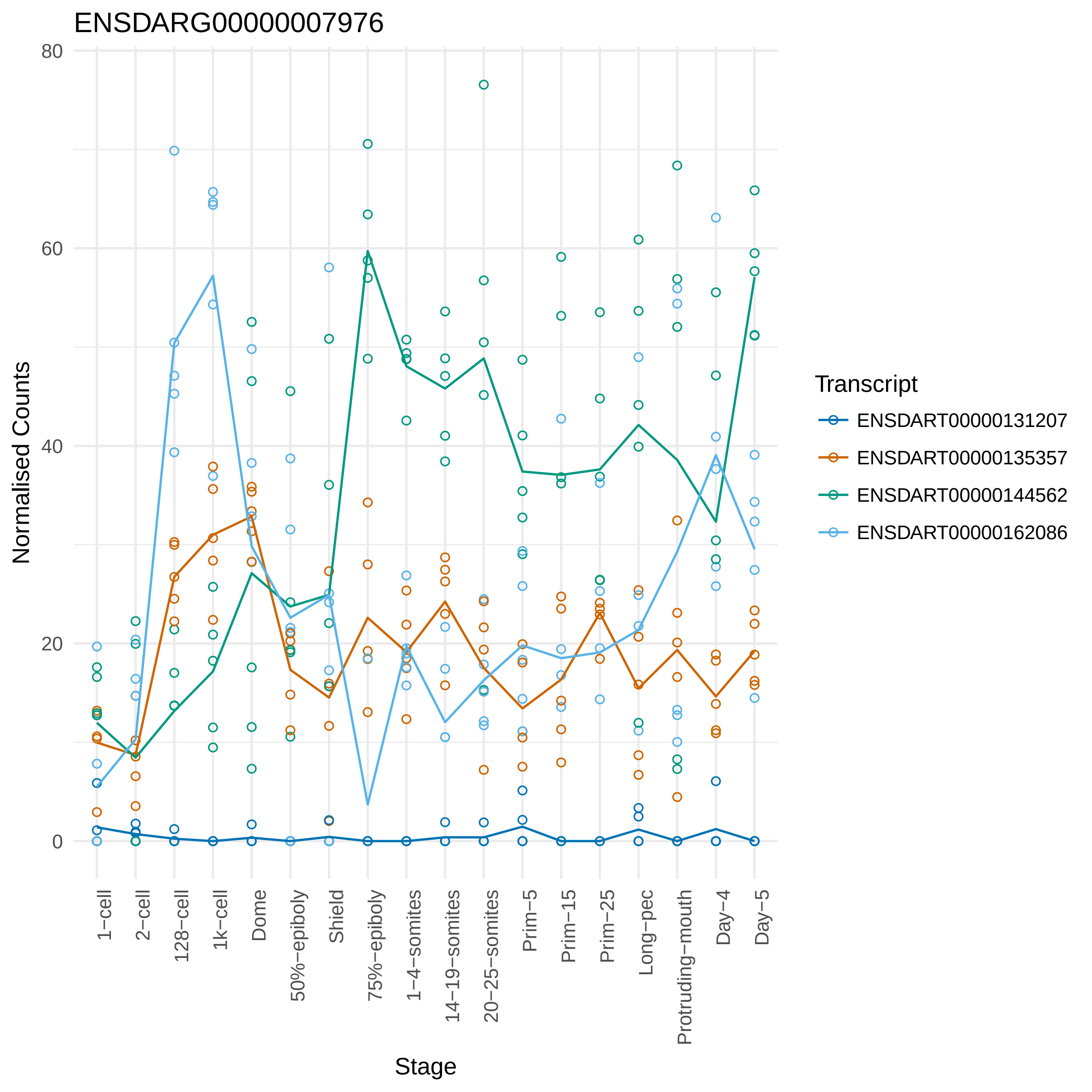

ENSDARG00000007976
Normalised Counts
80
60
40
20
0
1−cell
2−cell
128−cell
1k−cell
Dome
50%−epiboly
Shield
75%−epiboly
1−4−somites
14−19−somites
20−25−somites
Prim−5
Prim−15
Prim−25
Long−pec
Protruding−mouth
Day−4
Day−5
Stage
Transcript
ENSDART00000131207
ENSDART00000135357
ENSDART00000144562
ENSDART00000162086

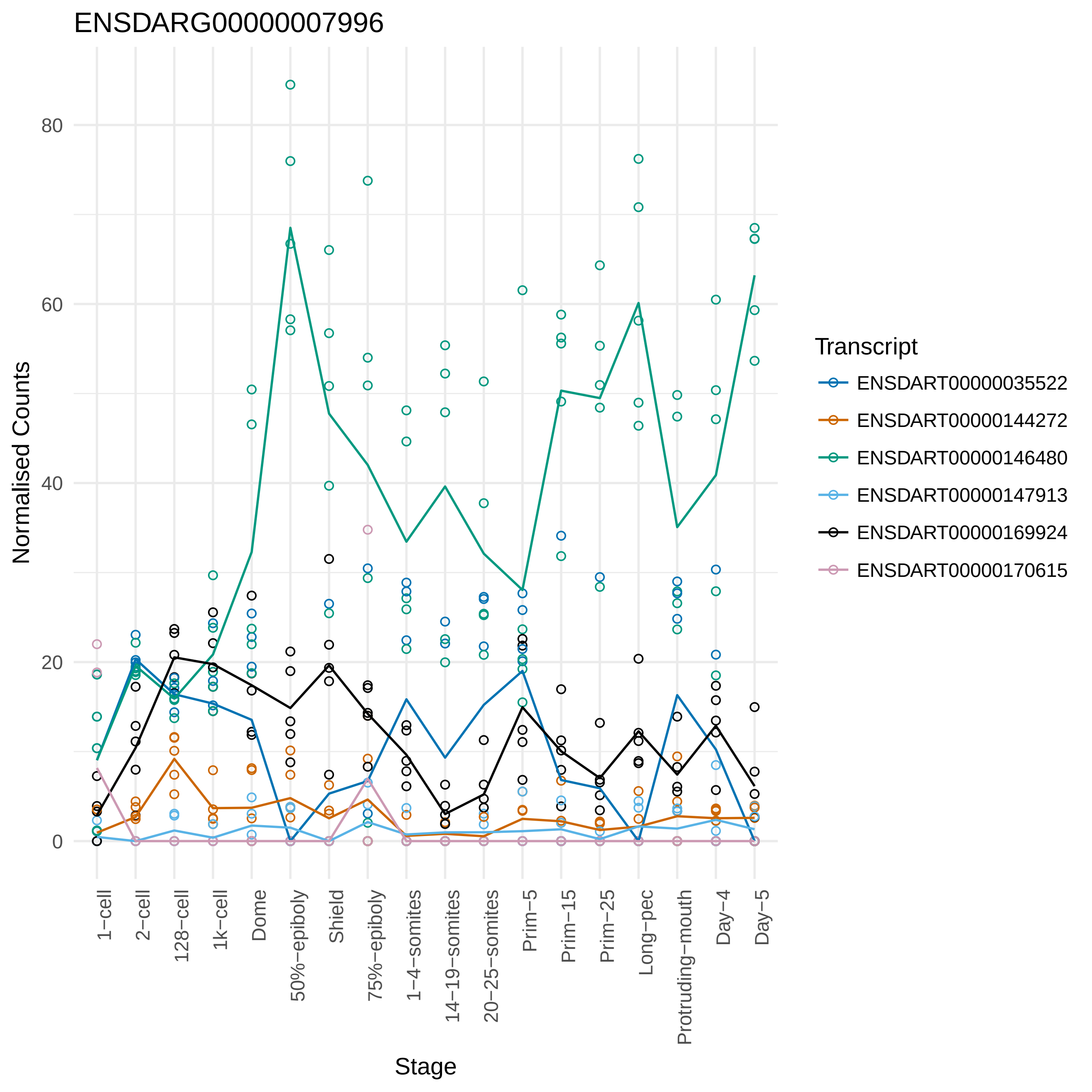
ENSDARG00000007996
Normalised Counts
80
60
40
20
0
1−cell
2−cell
128−cell
1k−cell
Dome
50%−epiboly
Shield
75%−epiboly
1−4−somites
14−19−somites
20−25−somites
Prim−5
Prim−15
Prim−25
Long−pec
Protruding−mouth
Day−4
Day−5
Stage
Transcript
ENSDART00000035522
ENSDART00000144272
ENSDART00000146480
ENSDART00000147913
ENSDART00000169924
ENSDART00000170615

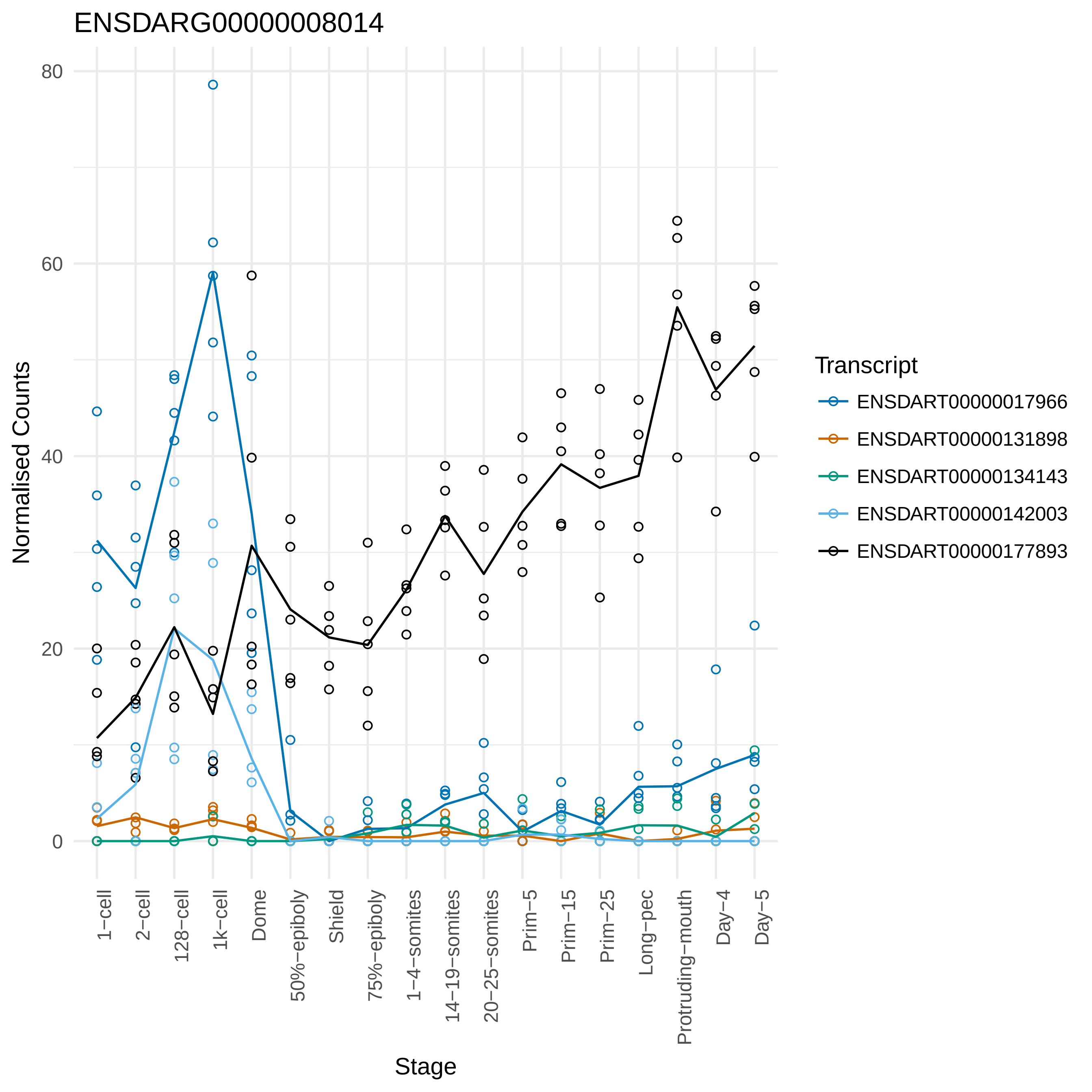
ENSDARG00000008014
Normalised Counts
80
60
40
20
0
1−cell
2−cell
128−cell
1k−cell
Dome
50%−epiboly
Shield
75%−epiboly
1−4−somites
14−19−somites
20−25−somites
Prim−5
Prim−15
Prim−25
Long−pec
Protruding−mouth
Day−4
Day−5
Stage
Transcript
ENSDART00000017966
ENSDART00000131898
ENSDART00000134143
ENSDART00000142003
ENSDART00000177893

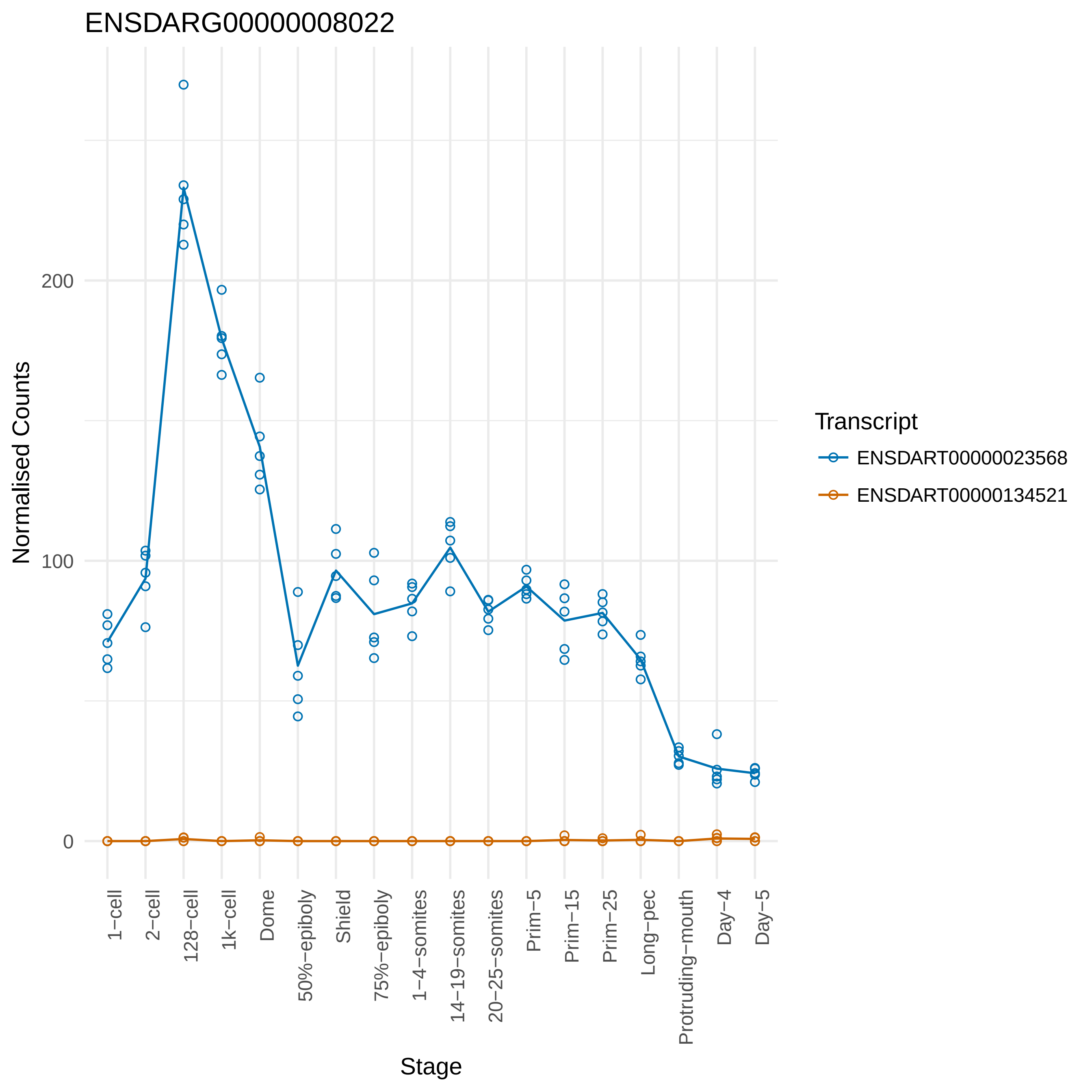
ENSDARG00000008022
Normalised Counts
200
100
0
Stage
1−cell
2−cell
128−cell
1k−cell
Dome
50%−epiboly
Shield
75%−epiboly
1−4−somites
14−19−somites
20−25−somites
Prim−5
Prim−15
Prim−25
Long−pec
Protruding−mouth
Day−4
Day−5
Transcript
ENSDART00000023568
ENSDART00000134521

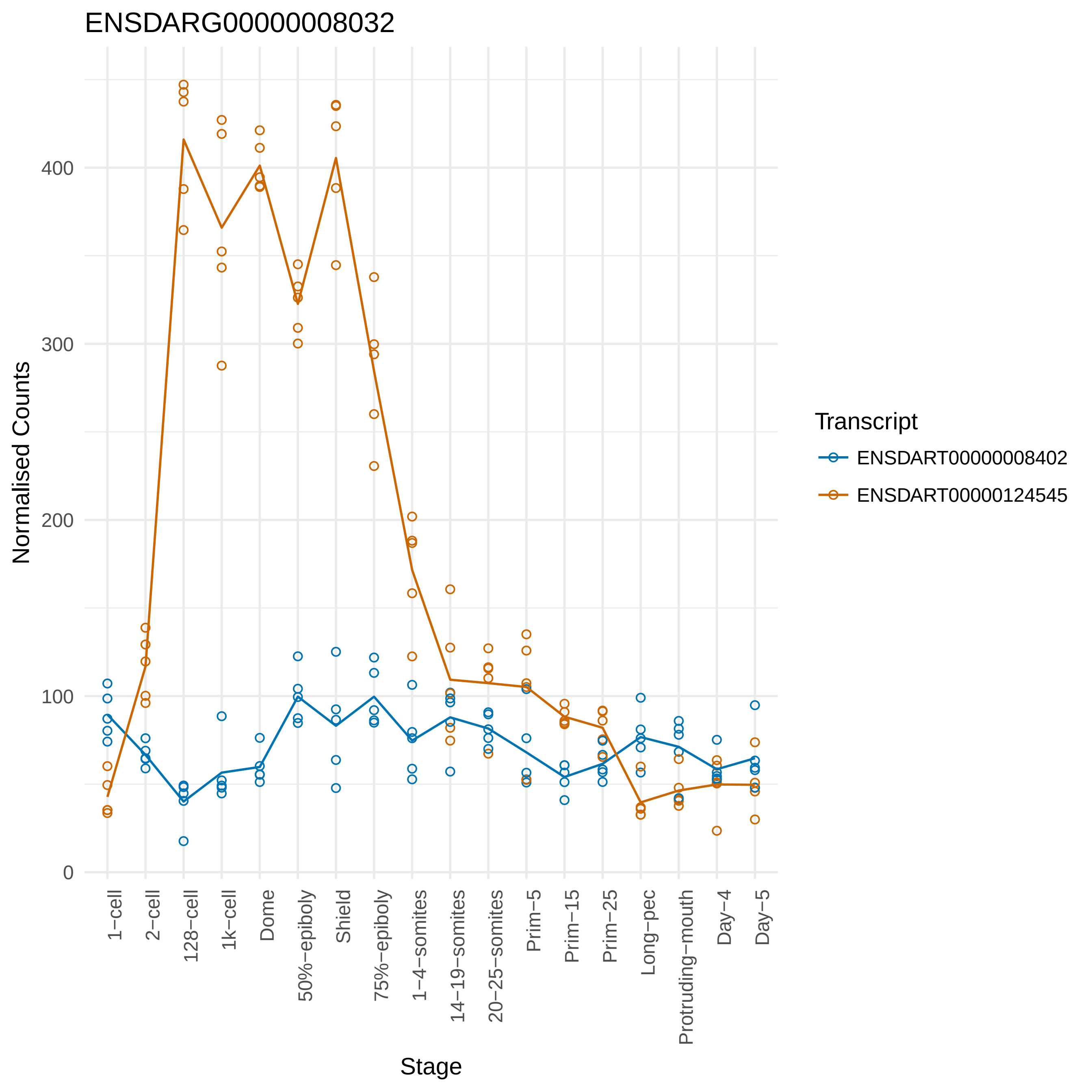

ENSDARG00000008032
Normalised Counts
400
300
200
100
0
1−cell
2−cell
128−cell
1k−cell
Dome
50%−epiboly
Shield
75%−epiboly
1−4−somites
14−19−somites
20−25−somites
Prim−5
Prim−15
Prim−25
Long−pec
Protruding−mouth
Day−4
Day−5
Stage
Transcript
ENSDART00000008402
ENSDART00000124545

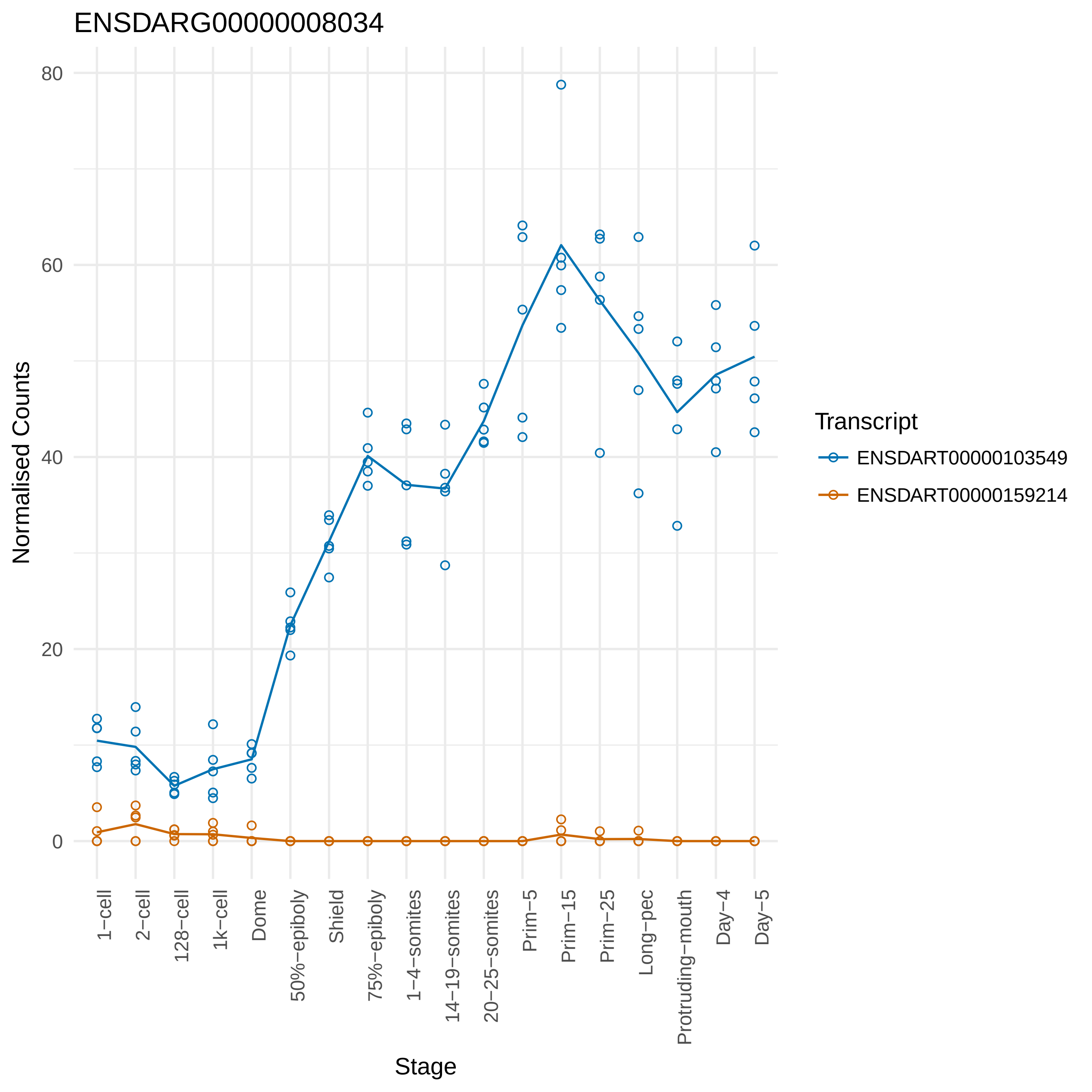

ENSDARG00000008034
Normalised Counts
80
60
40
20
0
1−cell
2−cell
128−cell
1k−cell
Dome
50%−epiboly
Shield
75%−epiboly
1−4−somites
14−19−somites
20−25−somites
Prim−5
Prim−15
Prim−25
Long−pec
Protruding−mouth
Day−4
Day−5
Stage
Transcript
ENSDART00000103549
ENSDART00000159214

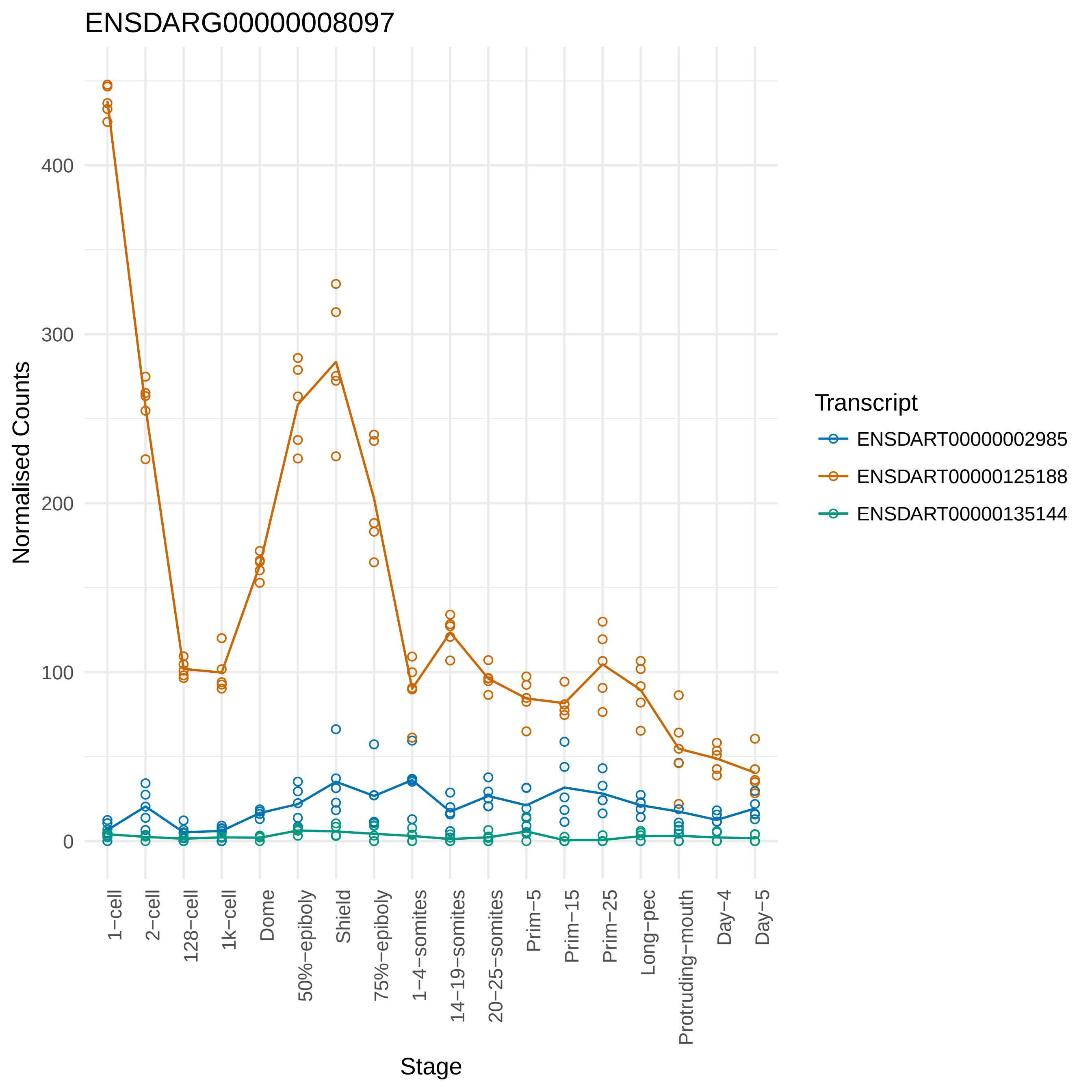

ENSDARG00000008097
Normalised Counts
0
100
200
300
400
Stage
1−cell
2−cell
128−cell
1k−cell
Dome
50%−epiboly
Shield
75%−epiboly
1−4−somites
14−19−somites
20−25−somites
Prim−5
Prim−15
Prim−25
Long−pec
Protruding−mouth
Day−4
Day−5
Transcript
ENSDART00000002985
ENSDART00000125188
ENSDART00000135144

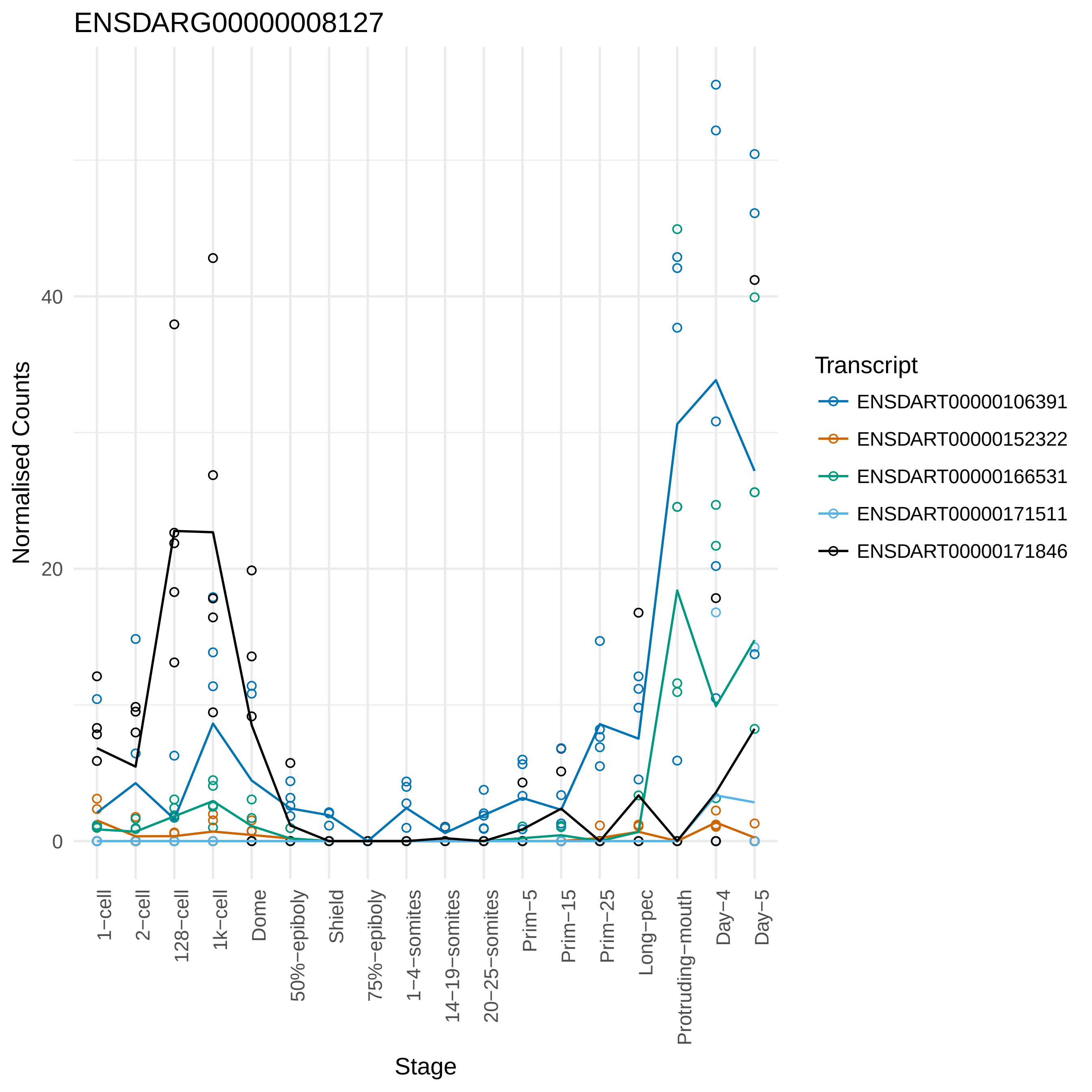
ENSDARG00000008127
Normalised Counts
0
20
40
1−cell
2−cell
128−cell
1k−cell
Dome
50%−epiboly
Shield
75%−epiboly
1−4−somites
14−19−somites
20−25−somites
Prim−5
Prim−15
Prim−25
Long−pec
Protruding−mouth
Day−4
Day−5
Stage
Transcript
ENSDART00000106391
ENSDART00000152322
ENSDART00000166531
ENSDART00000171511
ENSDART00000171846

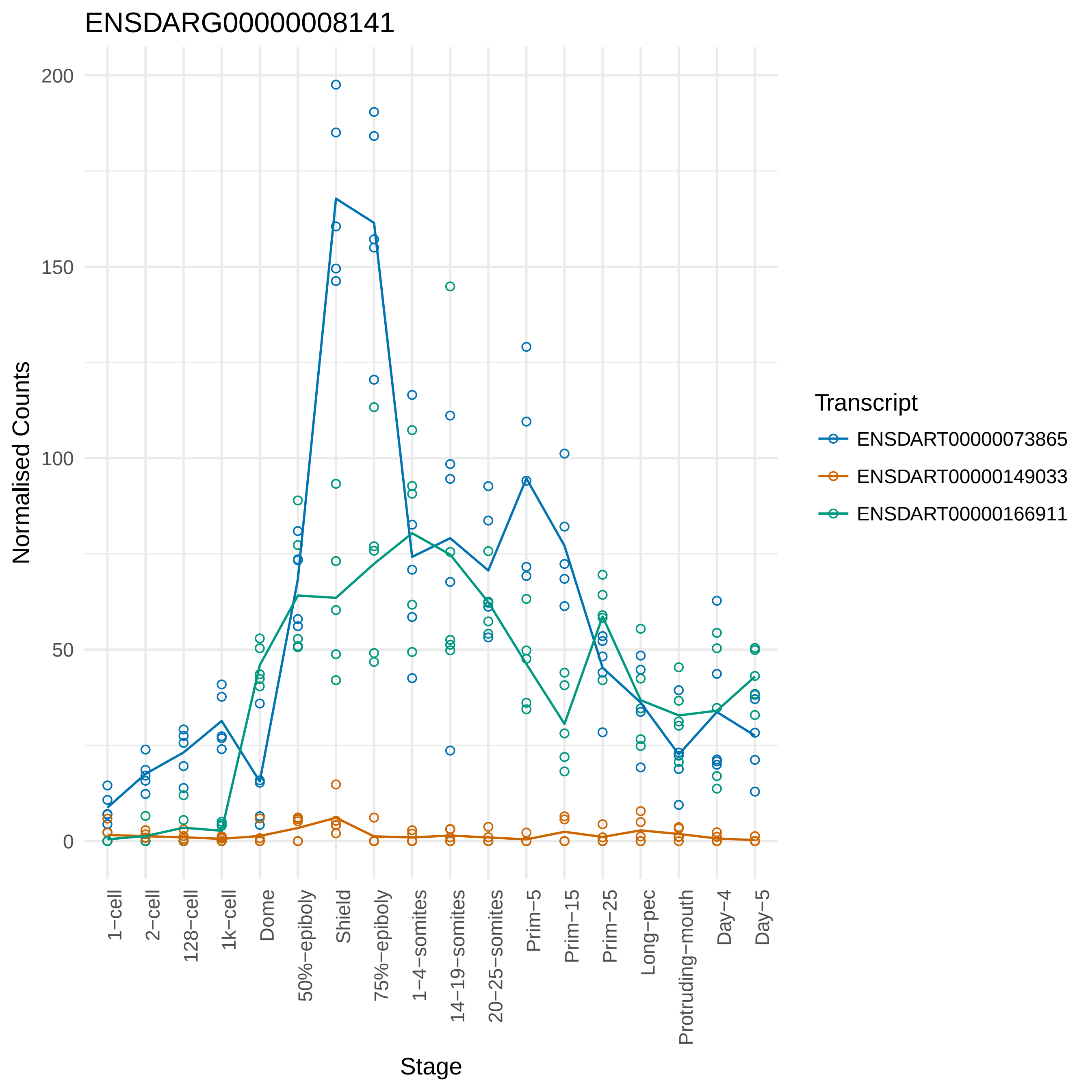

ENSDARG00000008141
Normalised Counts
200
150
100
50
0
1−cell
2−cell
128−cell
1k−cell
Dome
50%−epiboly
Shield
75%−epiboly
1−4−somites
14−19−somites
20−25−somites
Prim−5
Prim−15
Prim−25
Long−pec
Protruding−mouth
Day−4
Day−5
Stage
Transcript
ENSDART00000073865
ENSDART00000149033
ENSDART00000166911

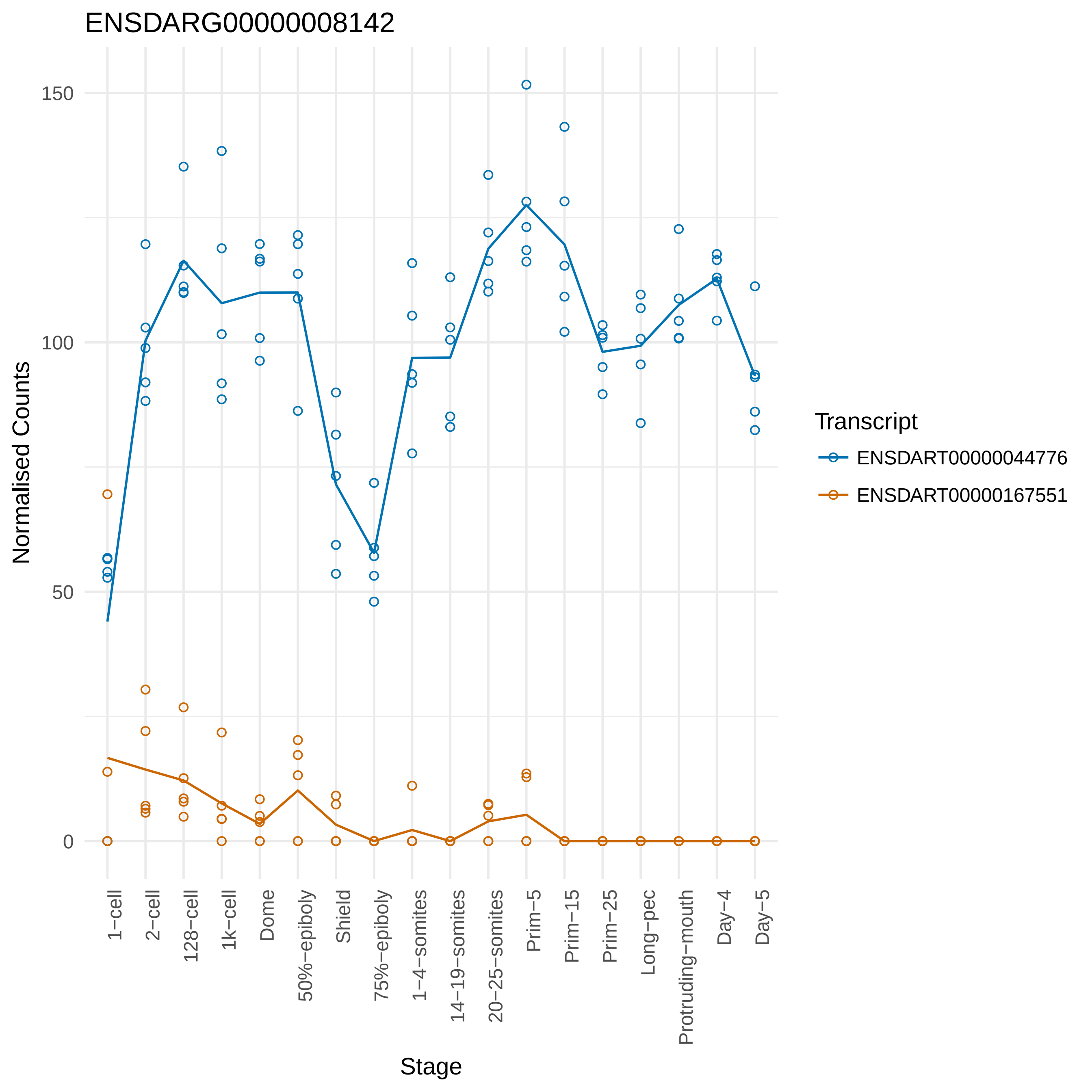

ENSDARG00000008142
Normalised Counts
150
100
50
0
1−cell
2−cell
128−cell
1k−cell
Dome
50%−epiboly
Shield
75%−epiboly
1−4−somites
14−19−somites
20−25−somites
Prim−5
Prim−15
Prim−25
Long−pec
Protruding−mouth
Day−4
Day−5
Stage
Transcript
ENSDART00000044776
ENSDART00000167551

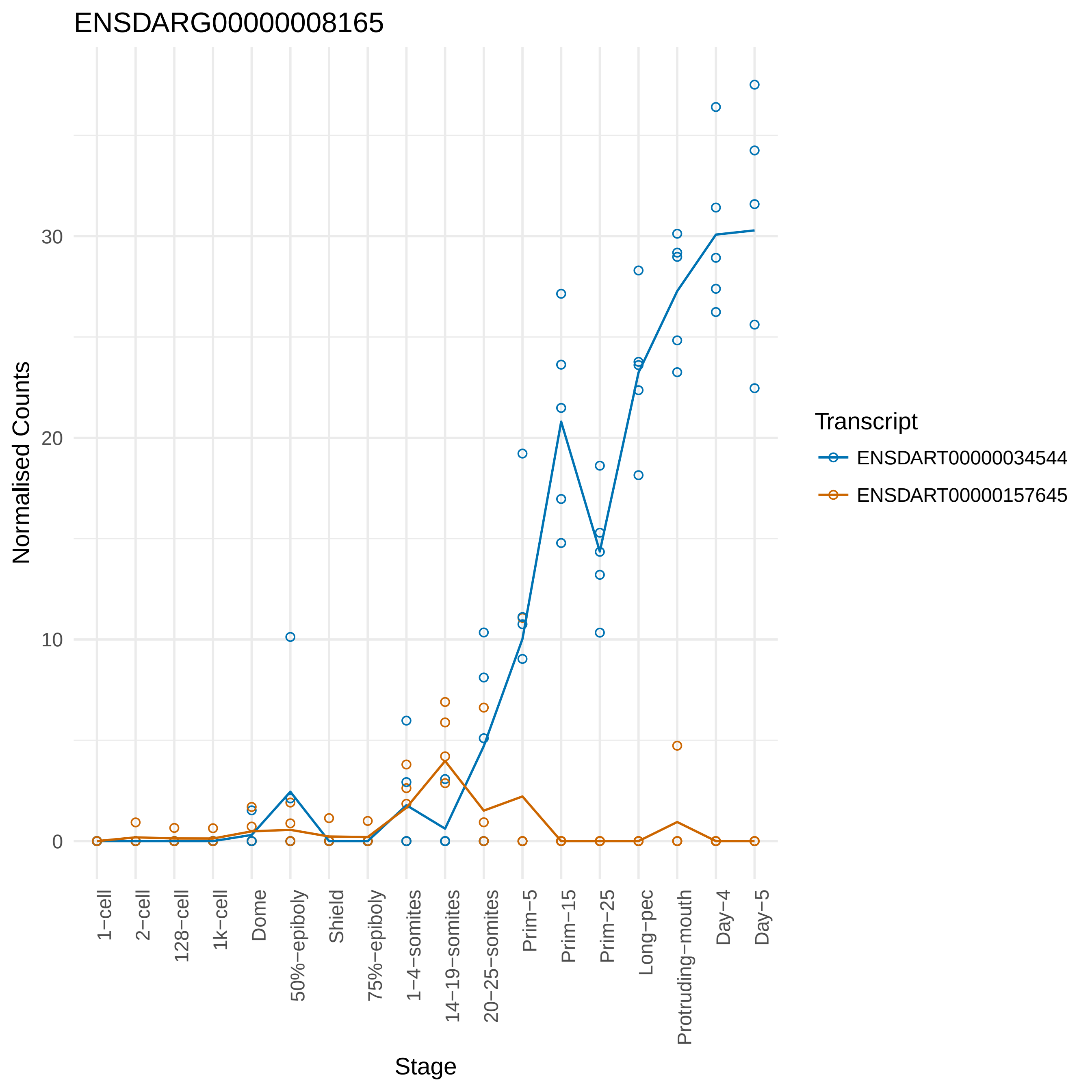

ENSDARG00000008165
Normalised Counts
0
10
20
30
Stage
1−cell
2−cell
128−cell
1k−cell
Dome
50%−epiboly
Shield
75%−epiboly
1−4−somites
14−19−somites
20−25−somites
Prim−5
Prim−15
Prim−25
Long−pec
Protruding−mouth
Day−4
Day−5
Transcript
ENSDART00000034544
ENSDART00000157645

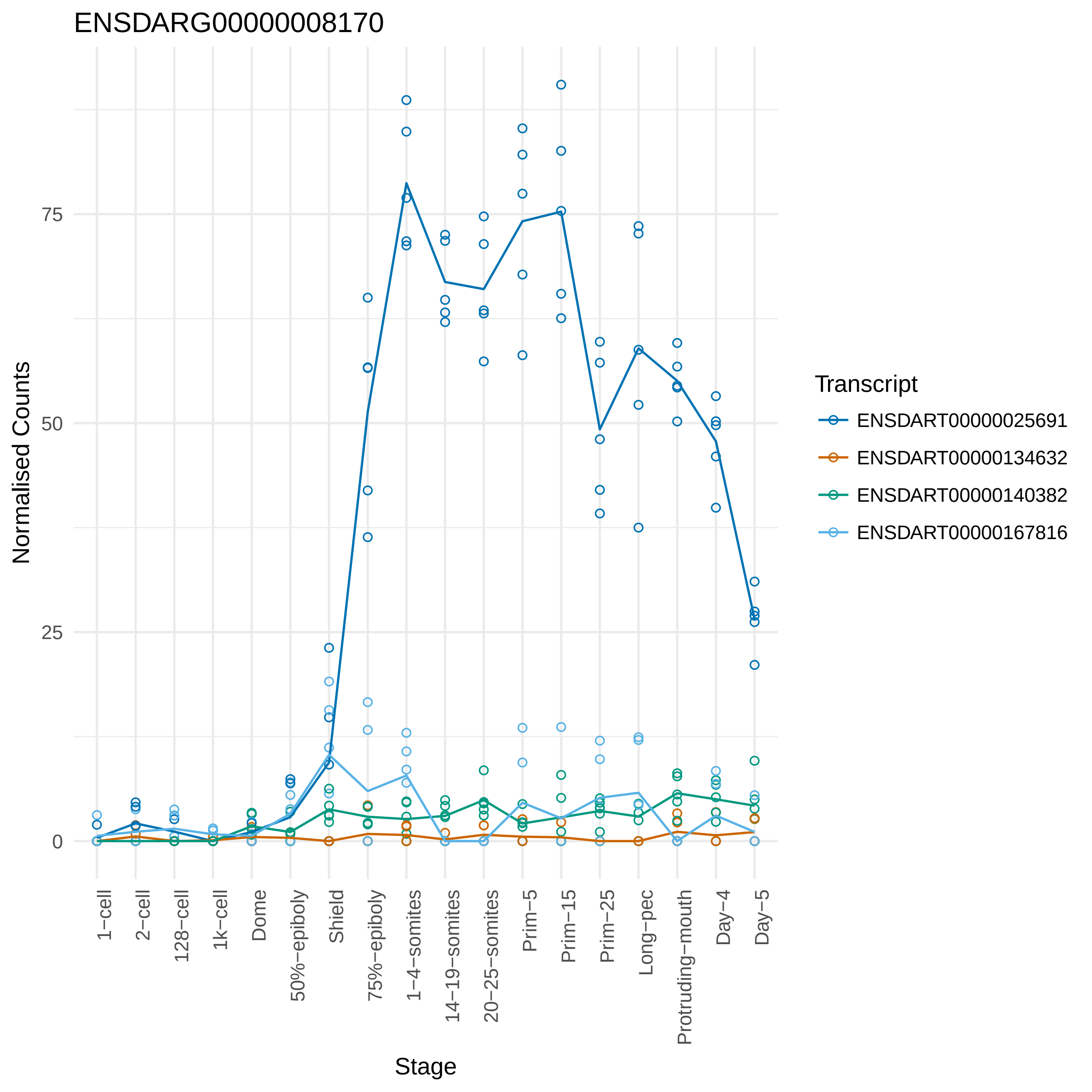

ENSDARG00000008170
Normalised Counts
0
25
50
75
Stage
1−cell
2−cell
128−cell
1k−cell
Dome
50%−epiboly
Shield
75%−epiboly
1−4−somites
14−19−somites
20−25−somites
Prim−5
Prim−15
Prim−25
Long−pec
Protruding−mouth
Day−4
Day−5
Transcript
ENSDART00000025691
ENSDART00000134632
ENSDART00000140382
ENSDART00000167816

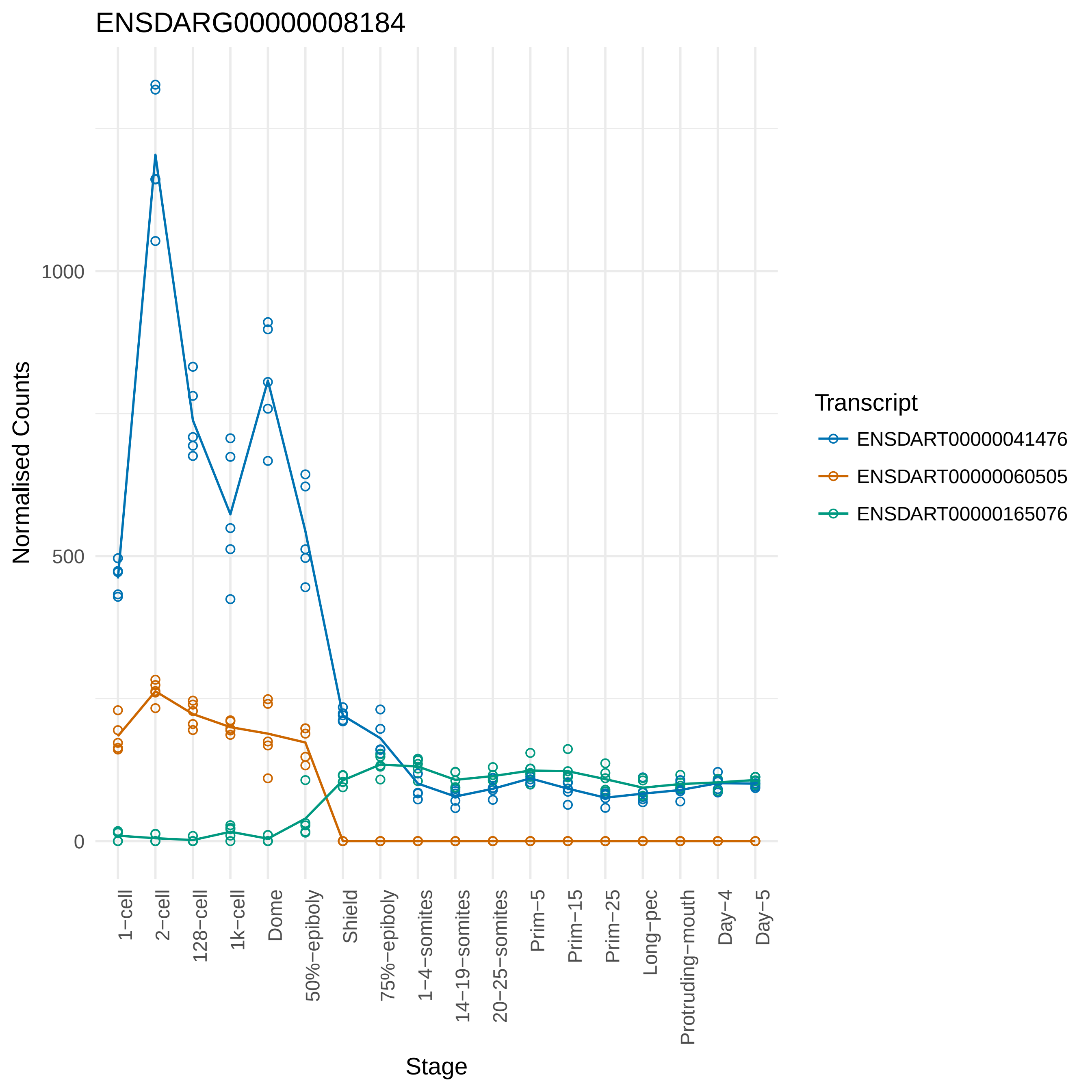

ENSDARG00000008184
Normalised Counts
1000
500
0
1−cell
2−cell
128−cell
1k−cell
Dome
50%−epiboly
Shield
75%−epiboly
1−4−somites
14−19−somites
20−25−somites
Prim−5
Prim−15
Prim−25
Long−pec
Protruding−mouth
Day−4
Day−5
Stage
Transcript
ENSDART00000041476
ENSDART00000060505
ENSDART00000165076

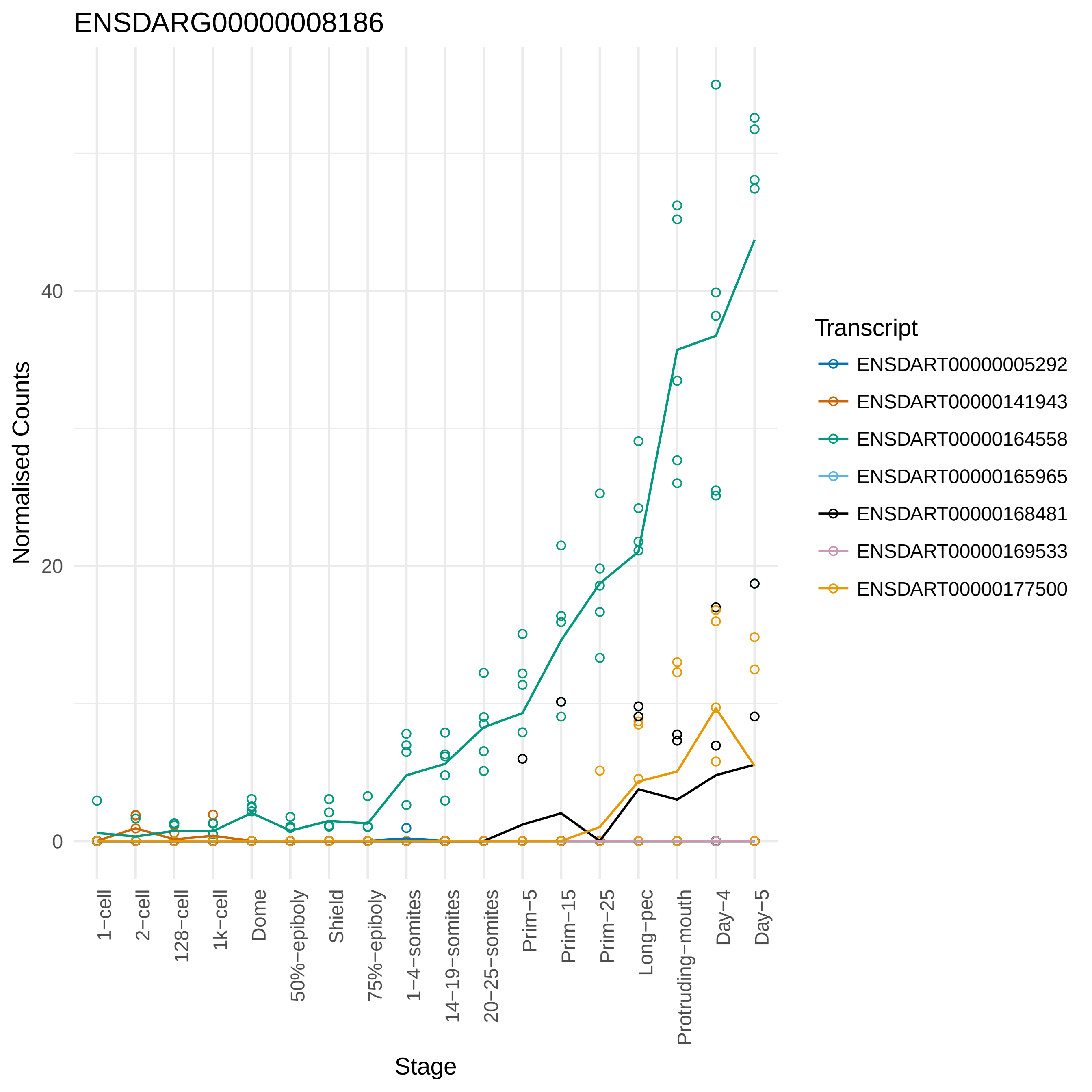
ENSDARG00000008186
Normalised Counts
0
20
40
Stage
1−cell
2−cell
128−cell
1k−cell
Dome
50%−epiboly
Shield
75%−epiboly
1−4−somites
14−19−somites
20−25−somites
Prim−5
Prim−15
Prim−25
Long−pec
Protruding−mouth
Day−4
Day−5
Transcript
ENSDART00000005292
ENSDART00000141943
ENSDART00000164558
ENSDART00000165965
ENSDART00000168481
ENSDART00000169533
ENSDART00000177500

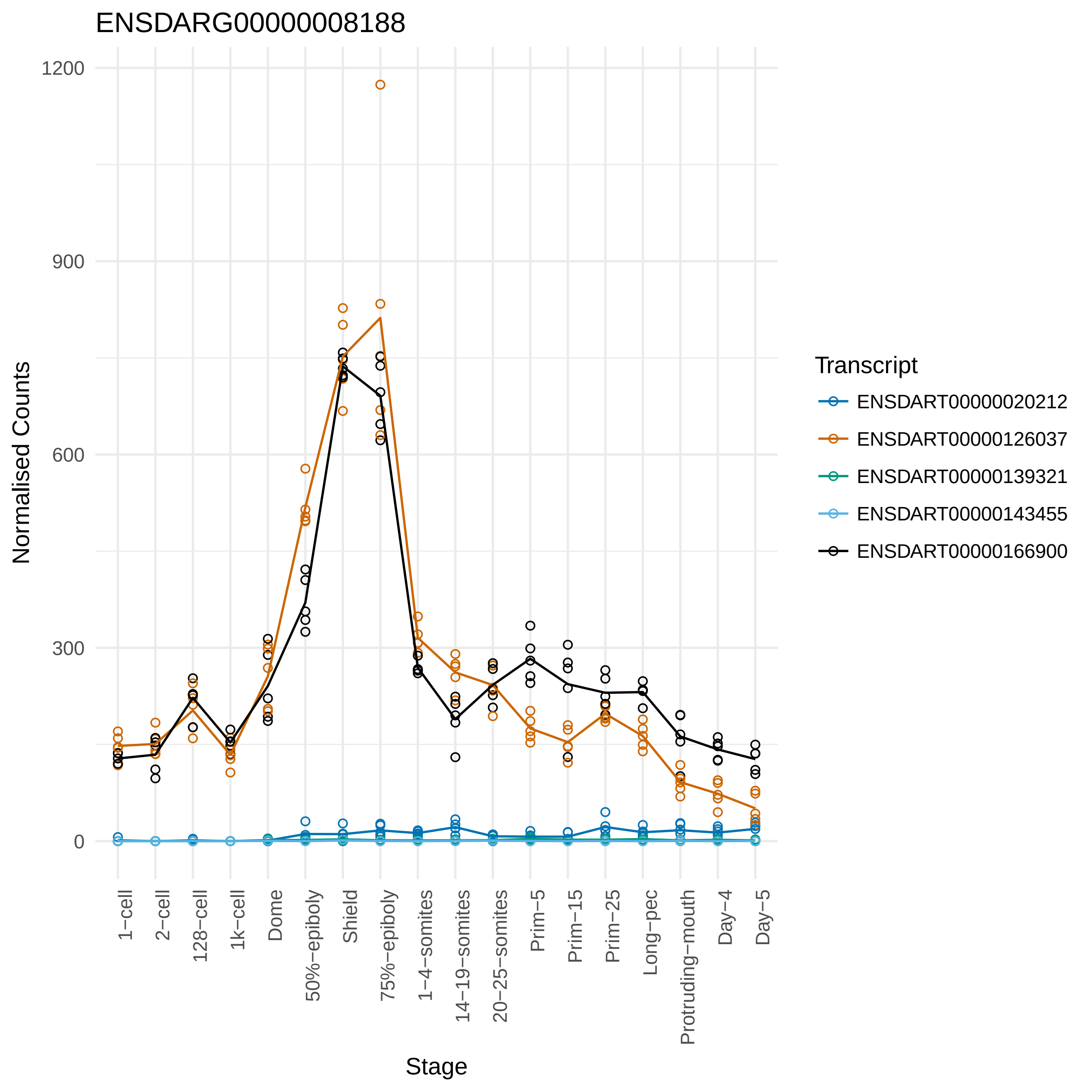

ENSDARG00000008188
Normalised Counts
1200
900
600
300
0
1−cell
2−cell
128−cell
1k−cell
Dome
50%−epiboly
Shield
75%−epiboly
1−4−somites
14−19−somites
20−25−somites
Prim−5
Prim−15
Prim−25
Long−pec
Protruding−mouth
Day−4
Day−5
Stage
Transcript
ENSDART00000020212
ENSDART00000126037
ENSDART00000139321
ENSDART00000143455
ENSDART00000166900

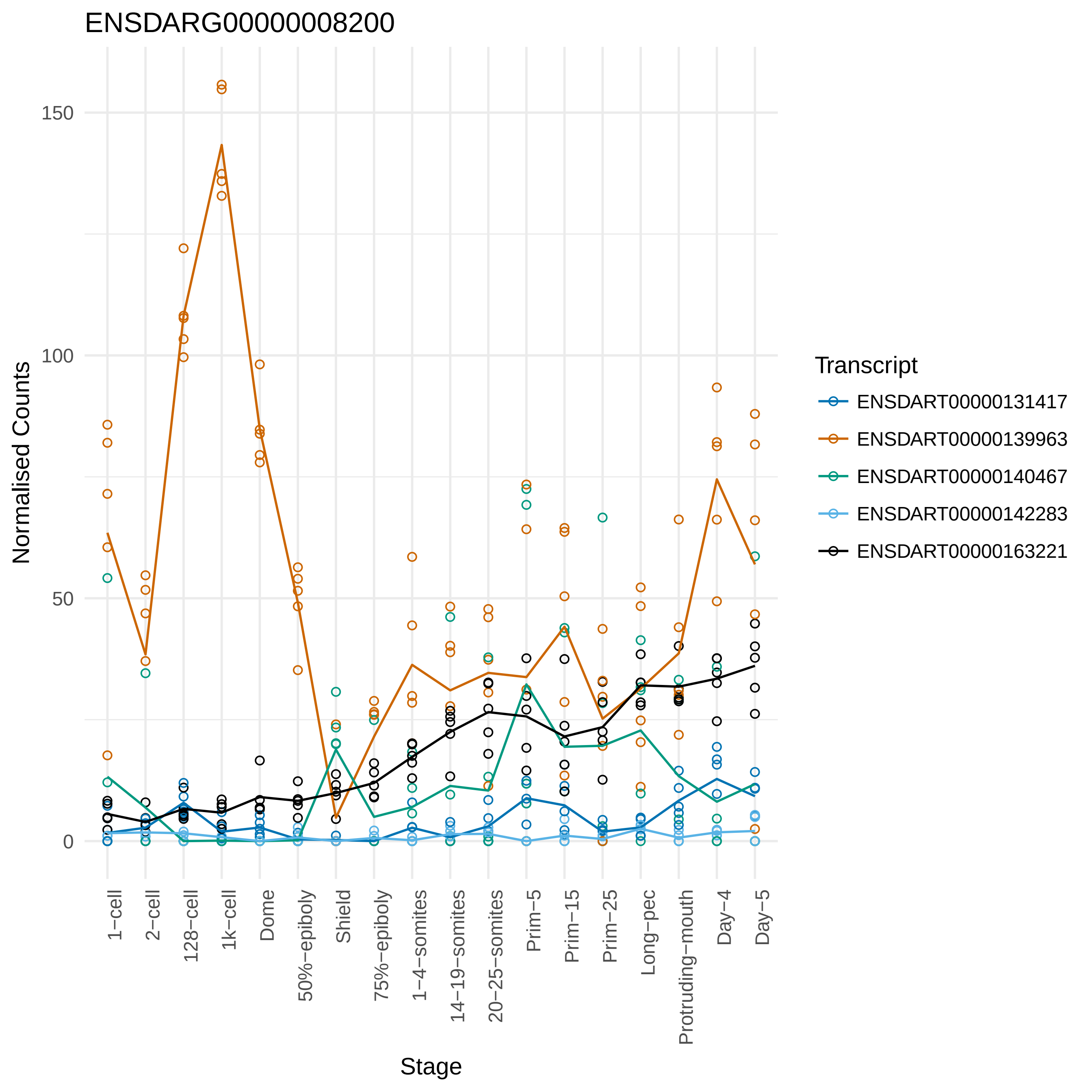

ENSDARG00000008200
Normalised Counts
150
100
50
0
1−cell
2−cell
128−cell
1k−cell
Dome
50%−epiboly
Shield
75%−epiboly
1−4−somites
14−19−somites
20−25−somites
Prim−5
Prim−15
Prim−25
Long−pec
Protruding−mouth
Day−4
Day−5
Stage
Transcript
ENSDART00000131417
ENSDART00000139963
ENSDART00000140467
ENSDART00000142283
ENSDART00000163221

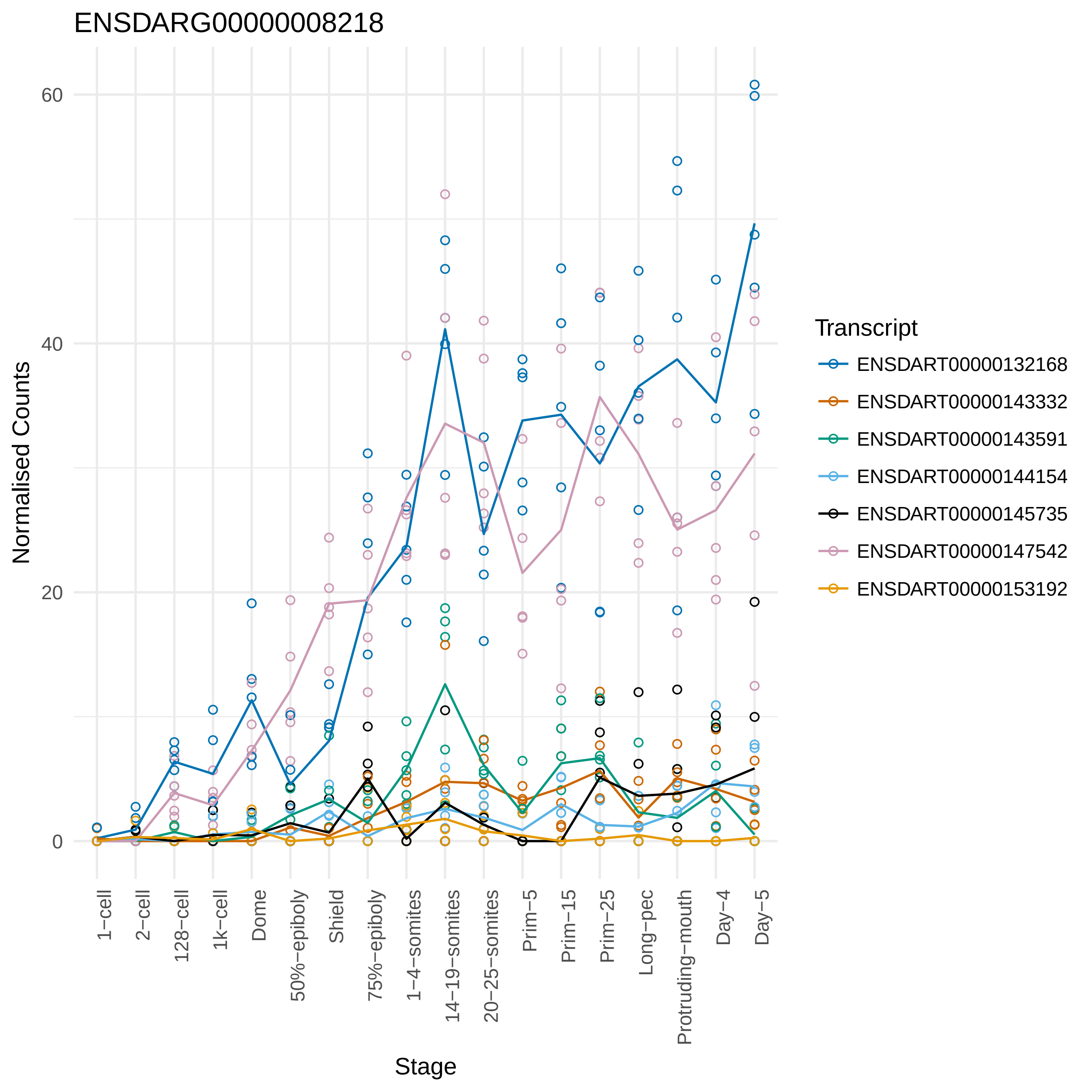
ENSDARG00000008218
Normalised Counts
0
20
40
60
Stage
1−cell
2−cell
128−cell
1k−cell
Dome
50%−epiboly
Shield
75%−epiboly
1−4−somites
14−19−somites
20−25−somites
Prim−5
Prim−15
Prim−25
Long−pec
Protruding−mouth
Day−4
Day−5
Transcript
ENSDART00000132168
ENSDART00000143332
ENSDART00000143591
ENSDART00000144154
ENSDART00000145735
ENSDART00000147542
ENSDART00000153192

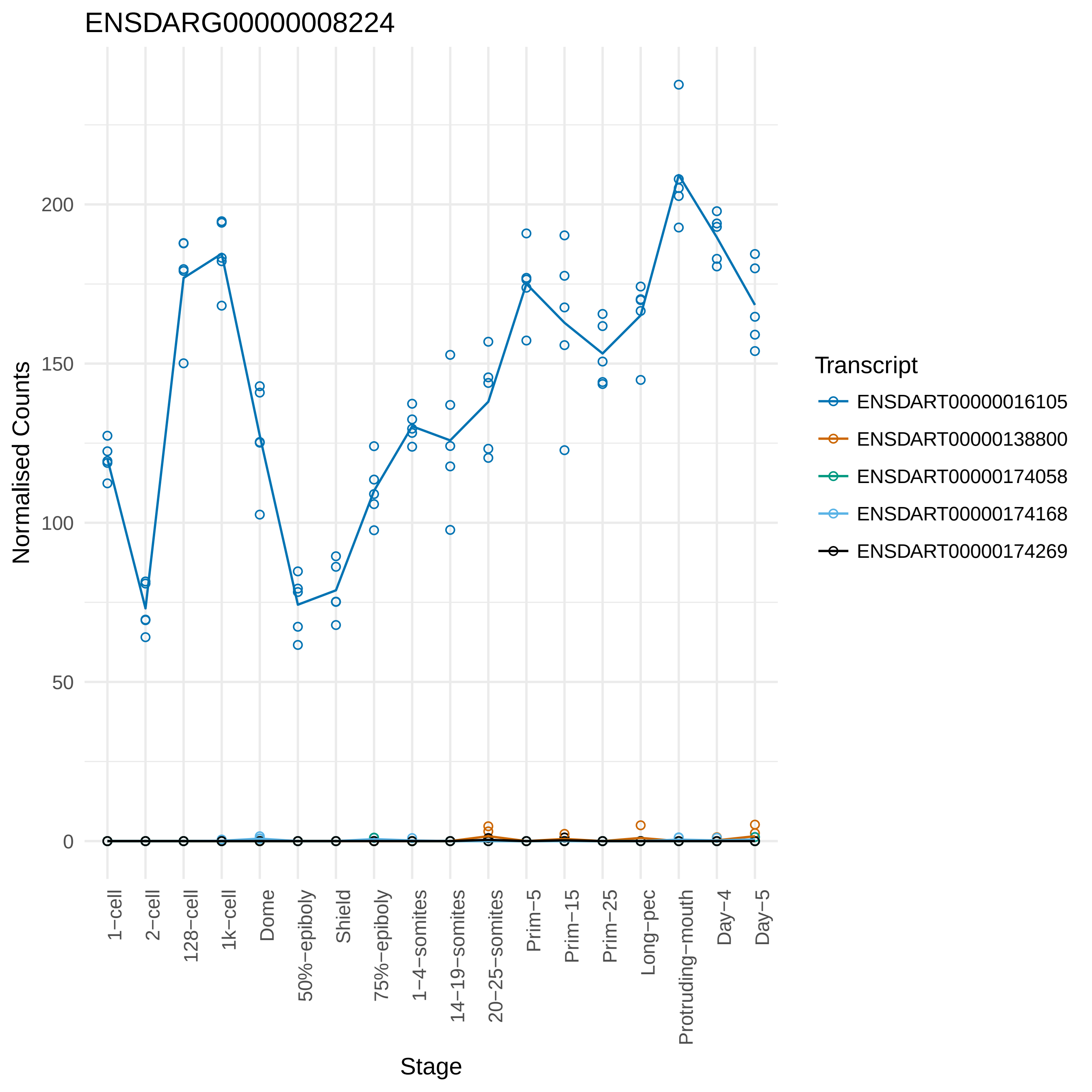

ENSDARG00000008224
Normalised Counts
0
50
100
150
200
Stage
1−cell
2−cell
128−cell
1k−cell
Dome
50%−epiboly
Shield
75%−epiboly
1−4−somites
14−19−somites
20−25−somites
Prim−5
Prim−15
Prim−25
Long−pec
Protruding−mouth
Day−4
Day−5
Transcript
ENSDART00000016105
ENSDART00000138800
ENSDART00000174058
ENSDART00000174168
ENSDART00000174269

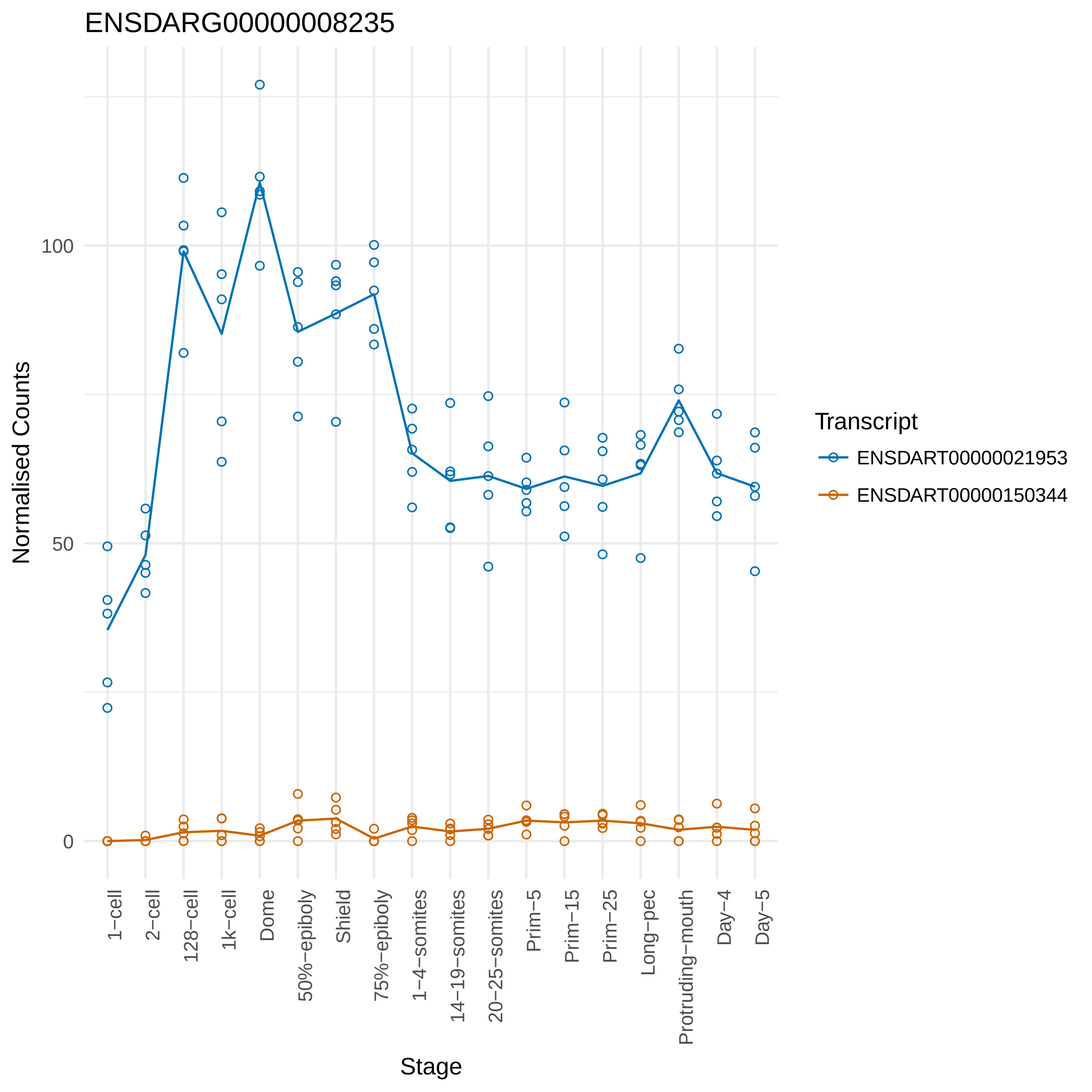
ENSDARG00000008235
Normalised Counts
100
50
0
Stage
1−cell
2−cell
128−cell
1k−cell
Dome
50%−epiboly
Shield
75%−epiboly
1−4−somites
14−19−somites
20−25−somites
Prim−5
Prim−15
Prim−25
Long−pec
Protruding−mouth
Day−4
Day−5
Transcript
ENSDART00000021953
ENSDART00000150344

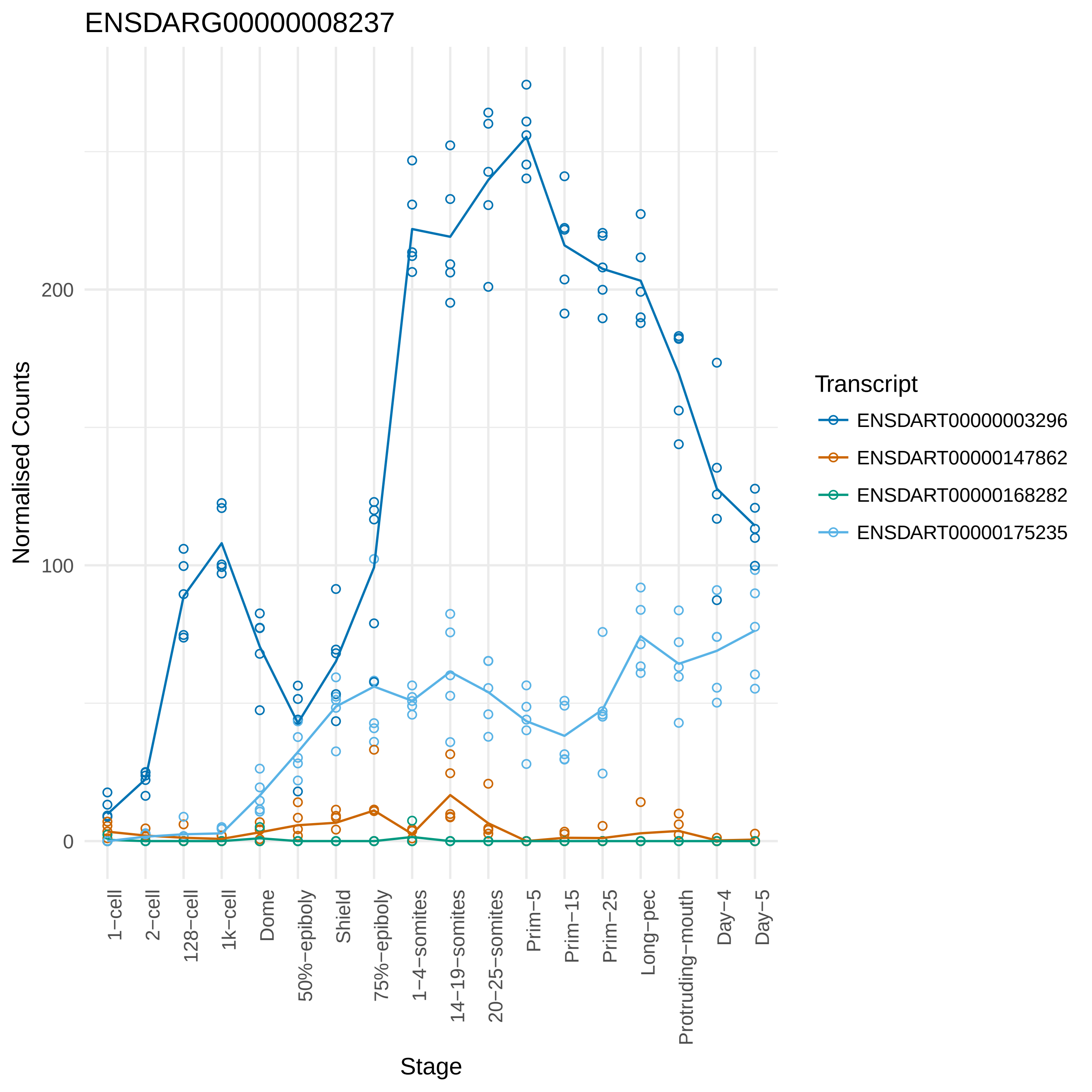

ENSDARG00000008237
Normalised Counts
200
100
0
1−cell
2−cell
128−cell
1k−cell
Dome
50%−epiboly
Shield
75%−epiboly
1−4−somites
14−19−somites
20−25−somites
Prim−5
Prim−15
Prim−25
Long−pec
Protruding−mouth
Day−4
Day−5
Stage
Transcript
ENSDART00000003296
ENSDART00000147862
ENSDART00000168282
ENSDART00000175235

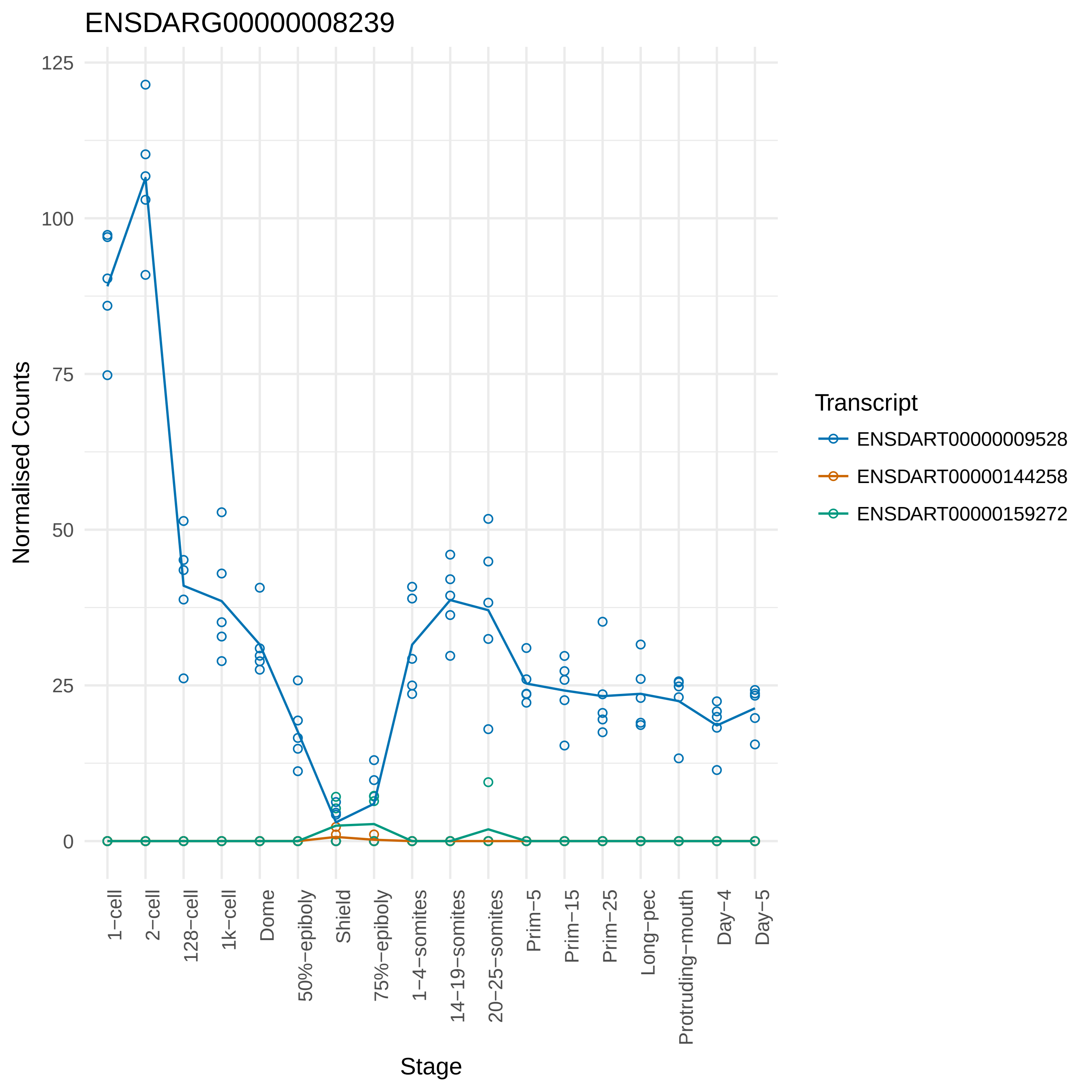

ENSDARG00000008239
Normalised Counts
125
100
75
50
25
0
1−cell
2−cell
128−cell
1k−cell
Dome
50%−epiboly
Shield
75%−epiboly
1−4−somites
14−19−somites
20−25−somites
Prim−5
Prim−15
Prim−25
Long−pec
Protruding−mouth
Day−4
Day−5
Stage
Transcript
ENSDART00000009528
ENSDART00000144258
ENSDART00000159272

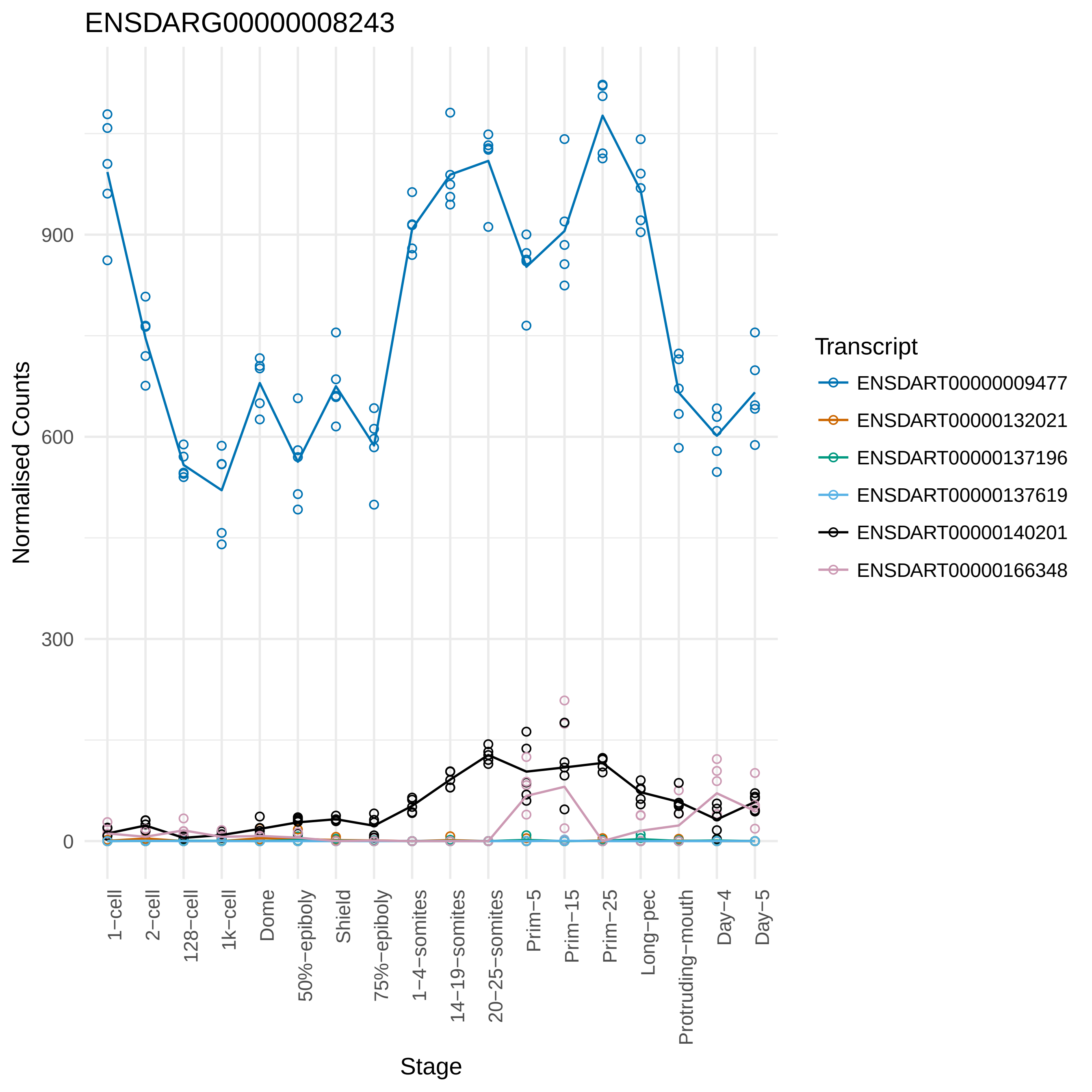

ENSDARG00000008243
Normalised Counts
900
600
300
0
1−cell
2−cell
128−cell
1k−cell
Dome
50%−epiboly
Shield
75%−epiboly
1−4−somites
14−19−somites
20−25−somites
Prim−5
Prim−15
Prim−25
Long−pec
Protruding−mouth
Day−4
Day−5
Stage
Transcript
ENSDART00000009477
ENSDART00000132021
ENSDART00000137196
ENSDART00000137619
ENSDART00000140201
ENSDART00000166348

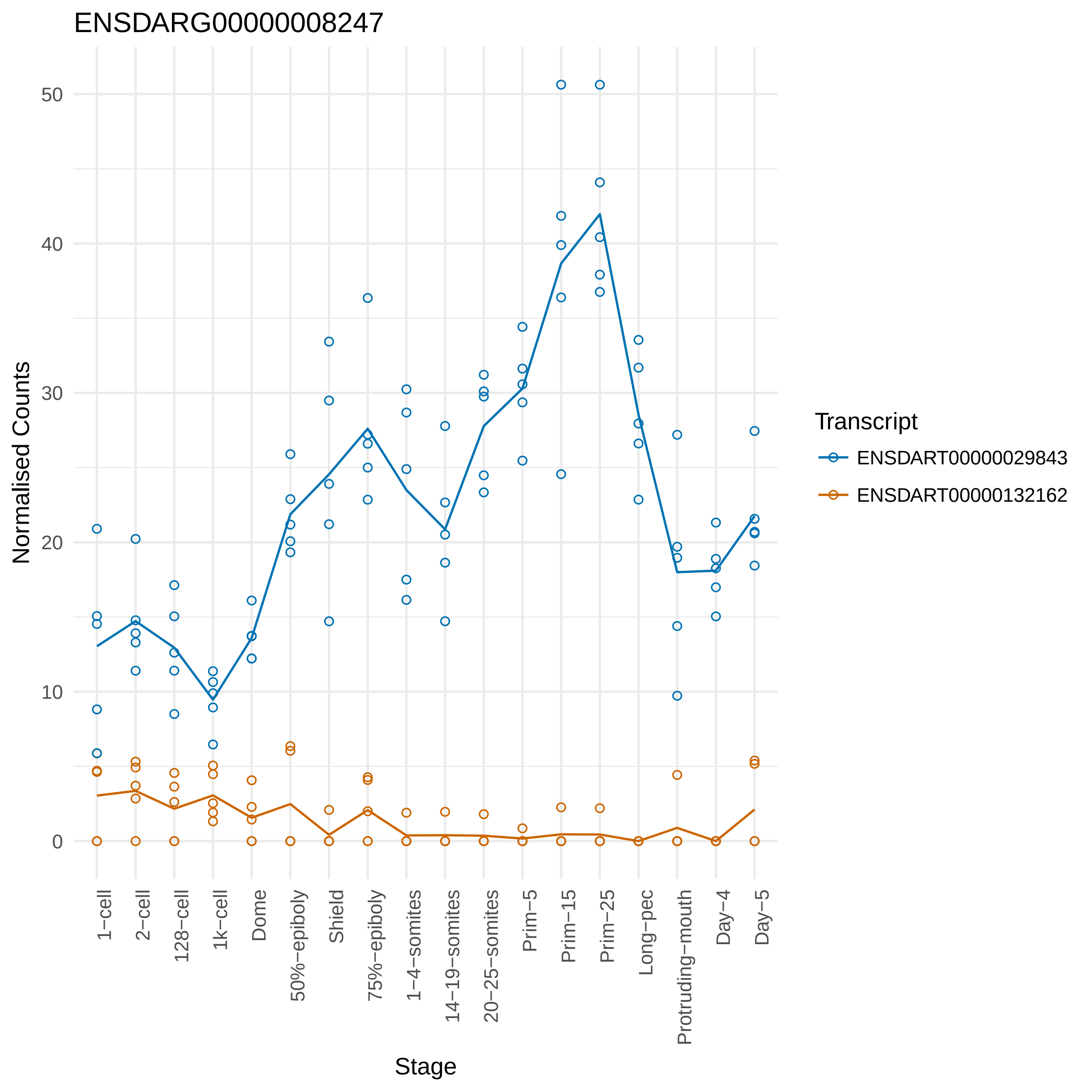

ENSDARG00000008247
Normalised Counts
0
10
20
30
40
50
Stage
1−cell
2−cell
128−cell
1k−cell
Dome
50%−epiboly
Shield
75%−epiboly
1−4−somites
14−19−somites
20−25−somites
Prim−5
Prim−15
Prim−25
Long−pec
Protruding−mouth
Day−4
Day−5
Transcript
ENSDART00000029843
ENSDART00000132162

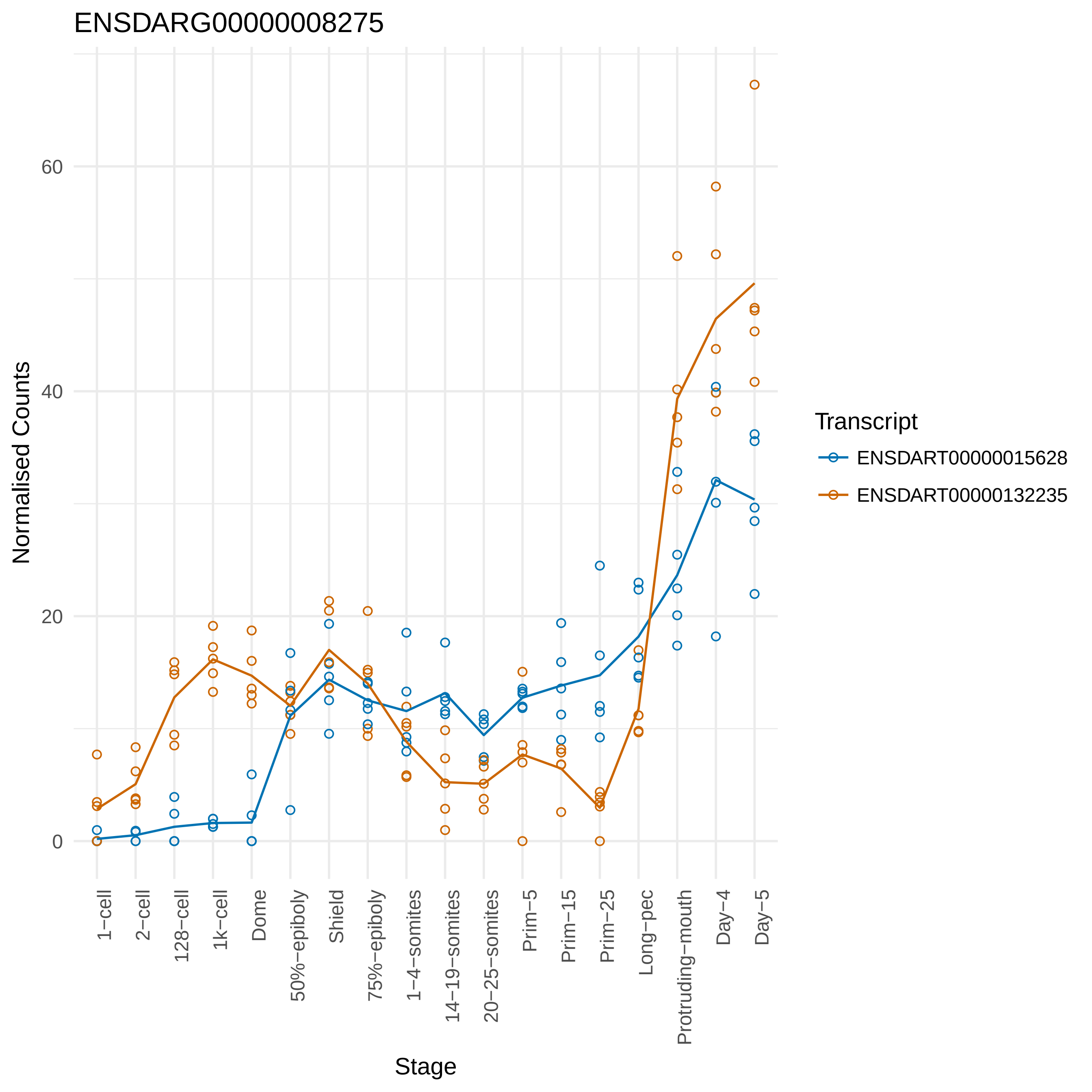

ENSDARG00000008275
Normalised Counts
0
20
40
60
1−cell
2−cell
128−cell
1k−cell
Dome
50%−epiboly
Shield
75%−epiboly
1−4−somites
14−19−somites
20−25−somites
Prim−5
Prim−15
Prim−25
Long−pec
Protruding−mouth
Day−4
Day−5
Stage
Transcript
ENSDART00000015628
ENSDART00000132235

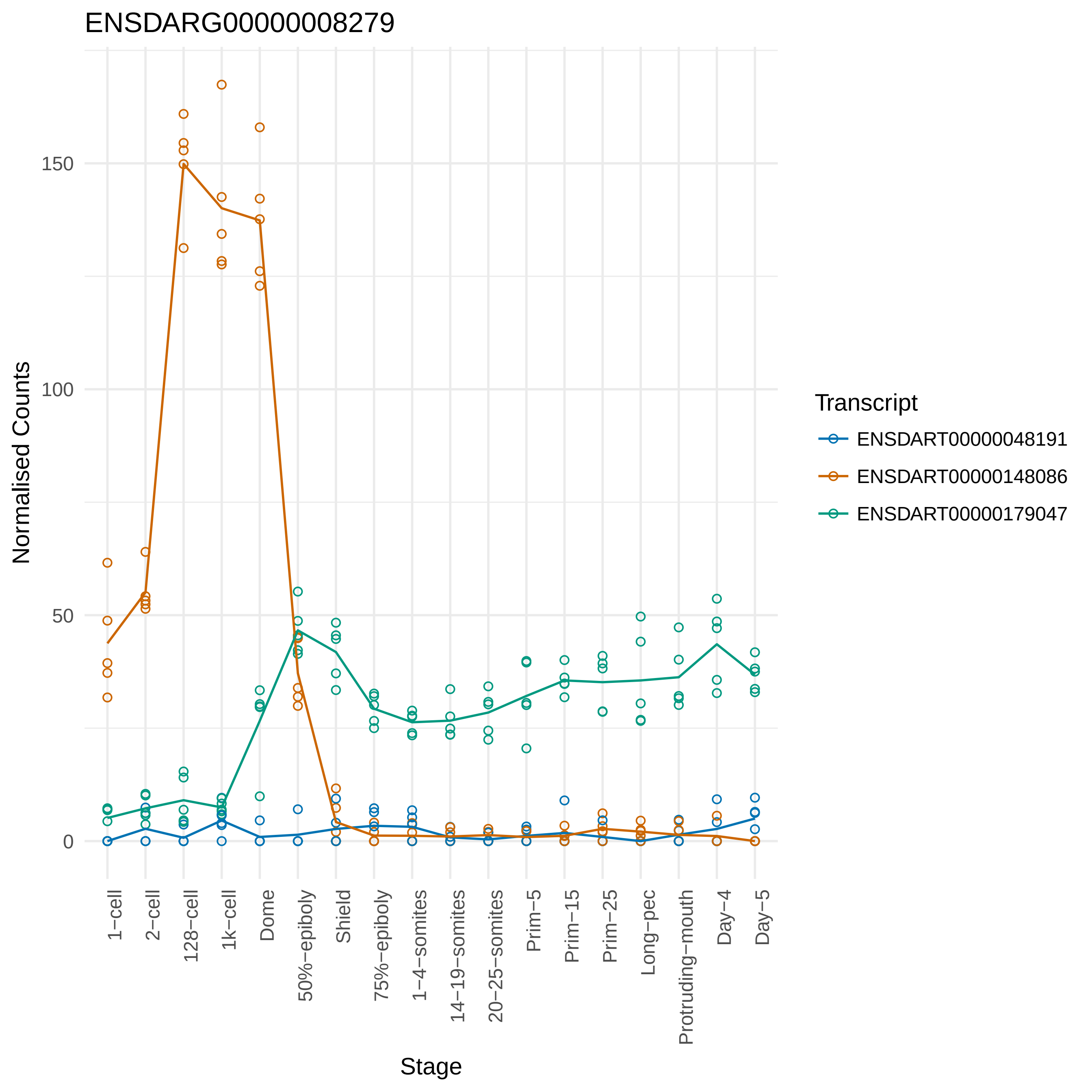

ENSDARG00000008279
Normalised Counts
0
50
100
150
1−cell
2−cell
128−cell
1k−cell
Dome
50%−epiboly
Shield
75%−epiboly
1−4−somites
14−19−somites
20−25−somites
Prim−5
Prim−15
Prim−25
Long−pec
Protruding−mouth
Day−4
Day−5
Stage
Transcript
ENSDART00000048191
ENSDART00000148086
ENSDART00000179047

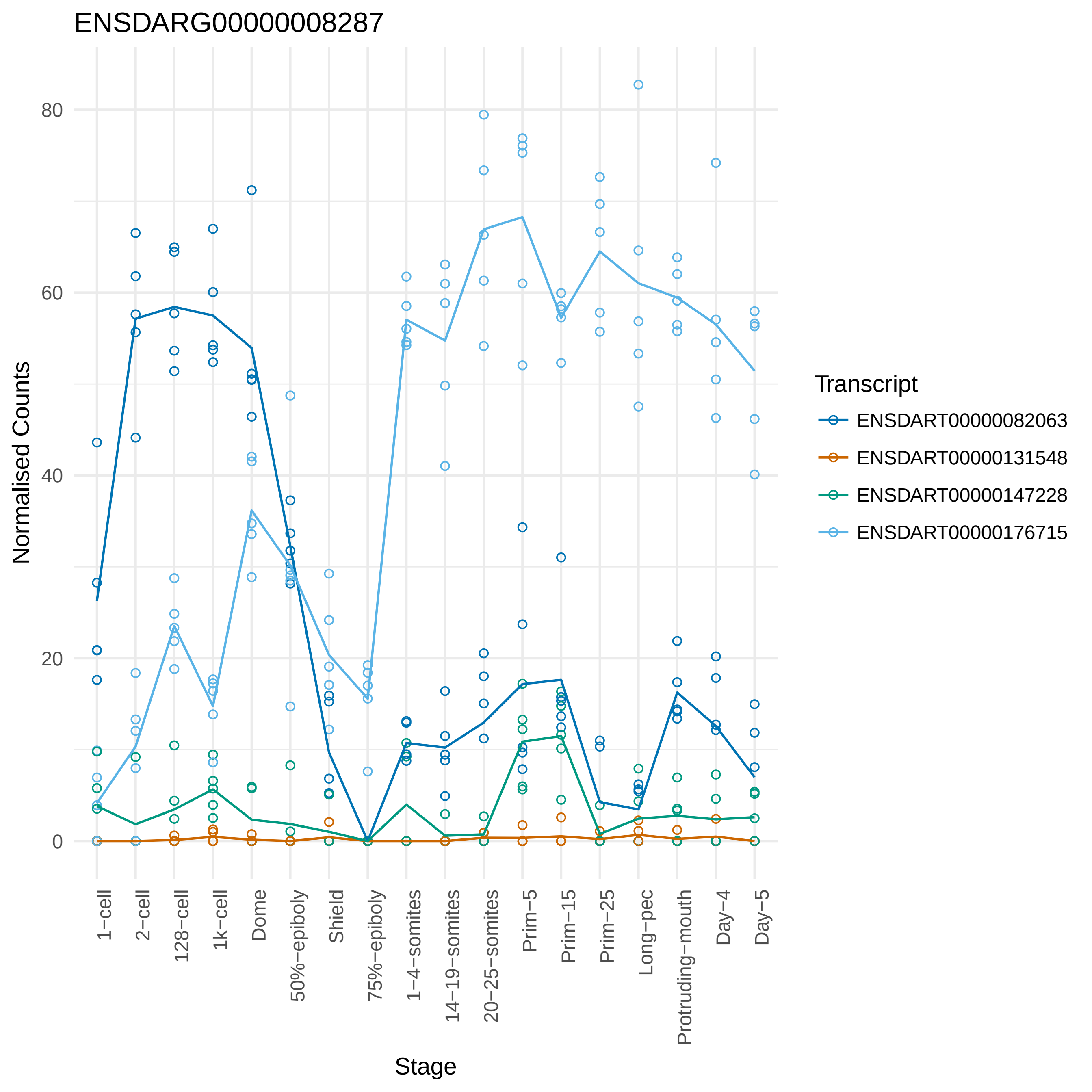

ENSDARG00000008287
Normalised Counts
0
20
40
60
80
Stage
1−cell
2−cell
128−cell
1k−cell
Dome
50%−epiboly
Shield
75%−epiboly
1−4−somites
14−19−somites
20−25−somites
Prim−5
Prim−15
Prim−25
Long−pec
Protruding−mouth
Day−4
Day−5
Transcript
ENSDART00000082063
ENSDART00000131548
ENSDART00000147228
ENSDART00000176715

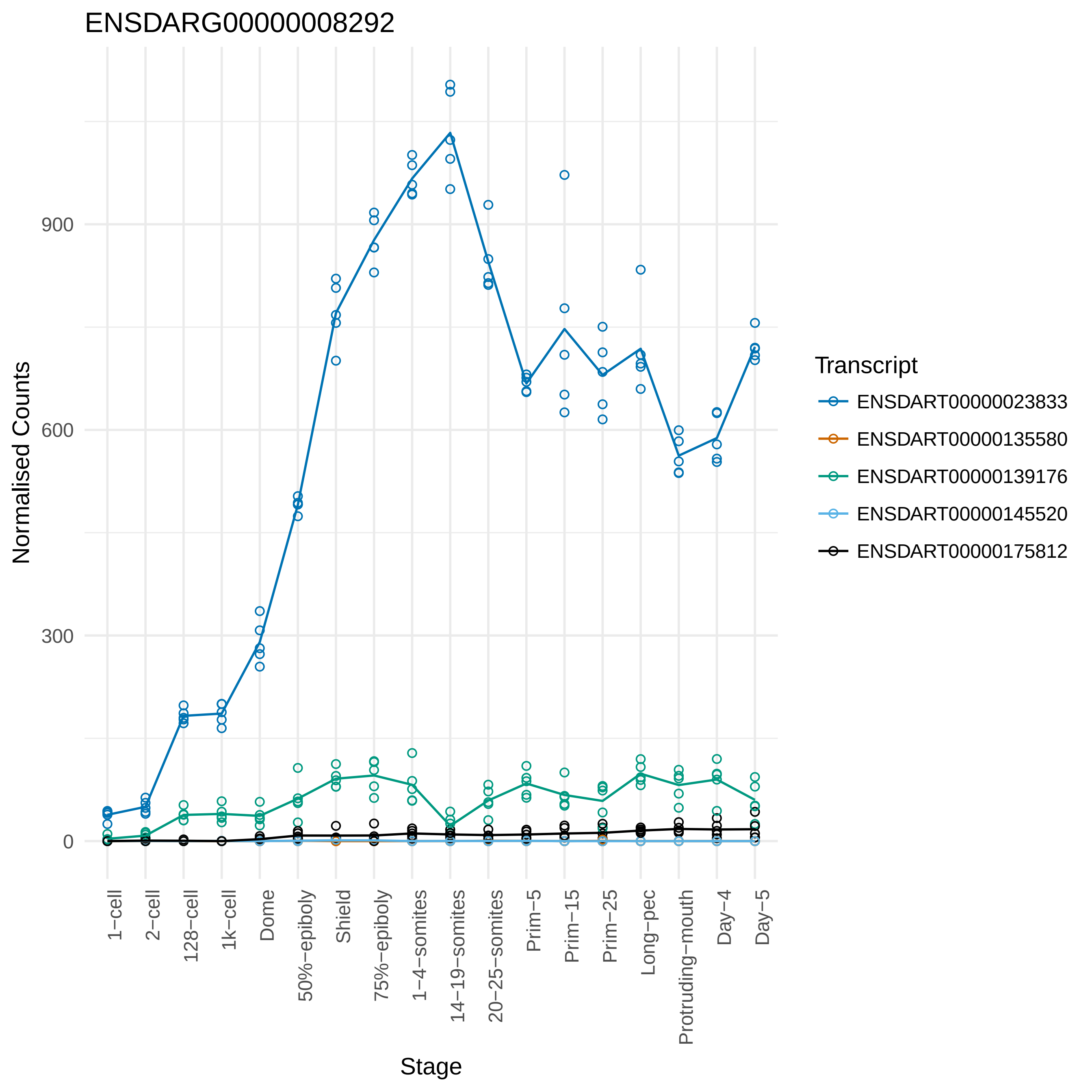
ENSDARG00000008292
Normalised Counts
0
300
600
900
Stage
1−cell
2−cell
128−cell
1k−cell
Dome
50%−epiboly
Shield
75%−epiboly
1−4−somites
14−19−somites
20−25−somites
Prim−5
Prim−15
Prim−25
Long−pec
Protruding−mouth
Day−4
Day−5
Transcript
ENSDART00000023833
ENSDART00000135580
ENSDART00000139176
ENSDART00000145520
ENSDART00000175812

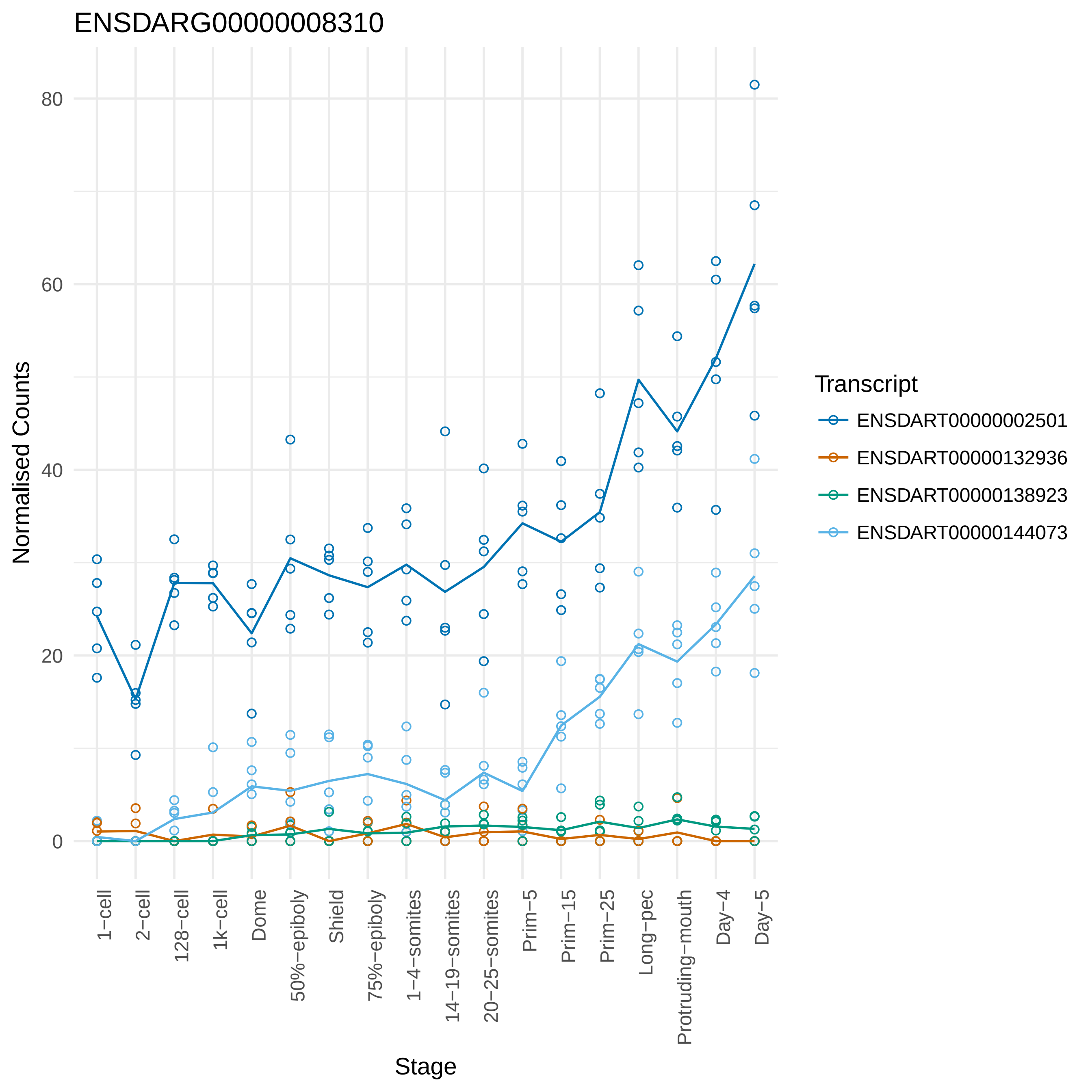
ENSDARG00000008310
Normalised Counts
0
20
40
60
80
1−cell
2−cell
128−cell
1k−cell
Dome
50%−epiboly
Shield
75%−epiboly
1−4−somites
14−19−somites
20−25−somites
Prim−5
Prim−15
Prim−25
Long−pec
Protruding−mouth
Day−4
Day−5
Stage
Transcript
ENSDART00000002501
ENSDART00000132936
ENSDART00000138923
ENSDART00000144073

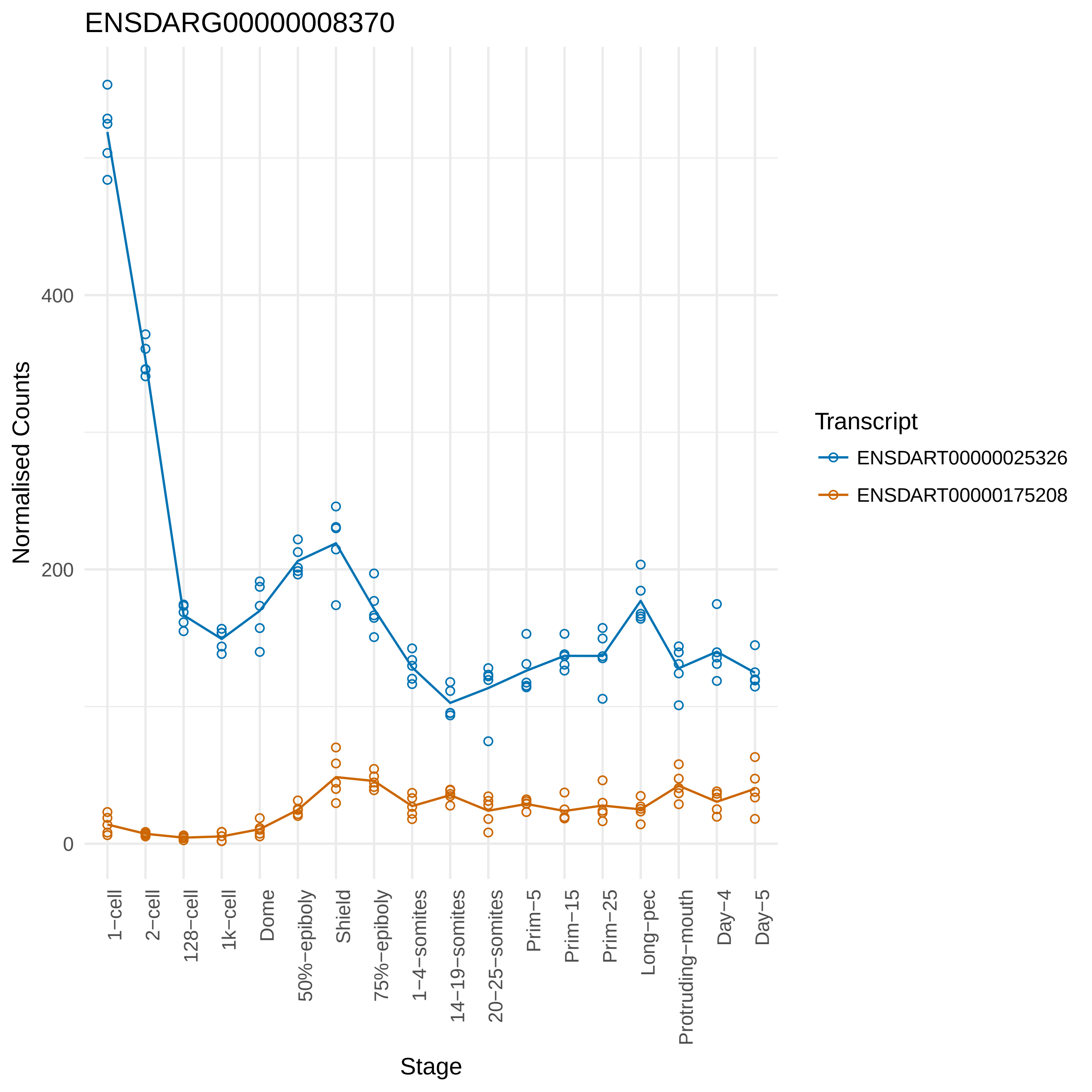
ENSDARG00000008370
Normalised Counts
400
200
0
Stage
1−cell
2−cell
128−cell
1k−cell
Dome
50%−epiboly
Shield
75%−epiboly
1−4−somites
14−19−somites
20−25−somites
Prim−5
Prim−15
Prim−25
Long−pec
Protruding−mouth
Day−4
Day−5
Transcript
ENSDART00000025326
ENSDART00000175208

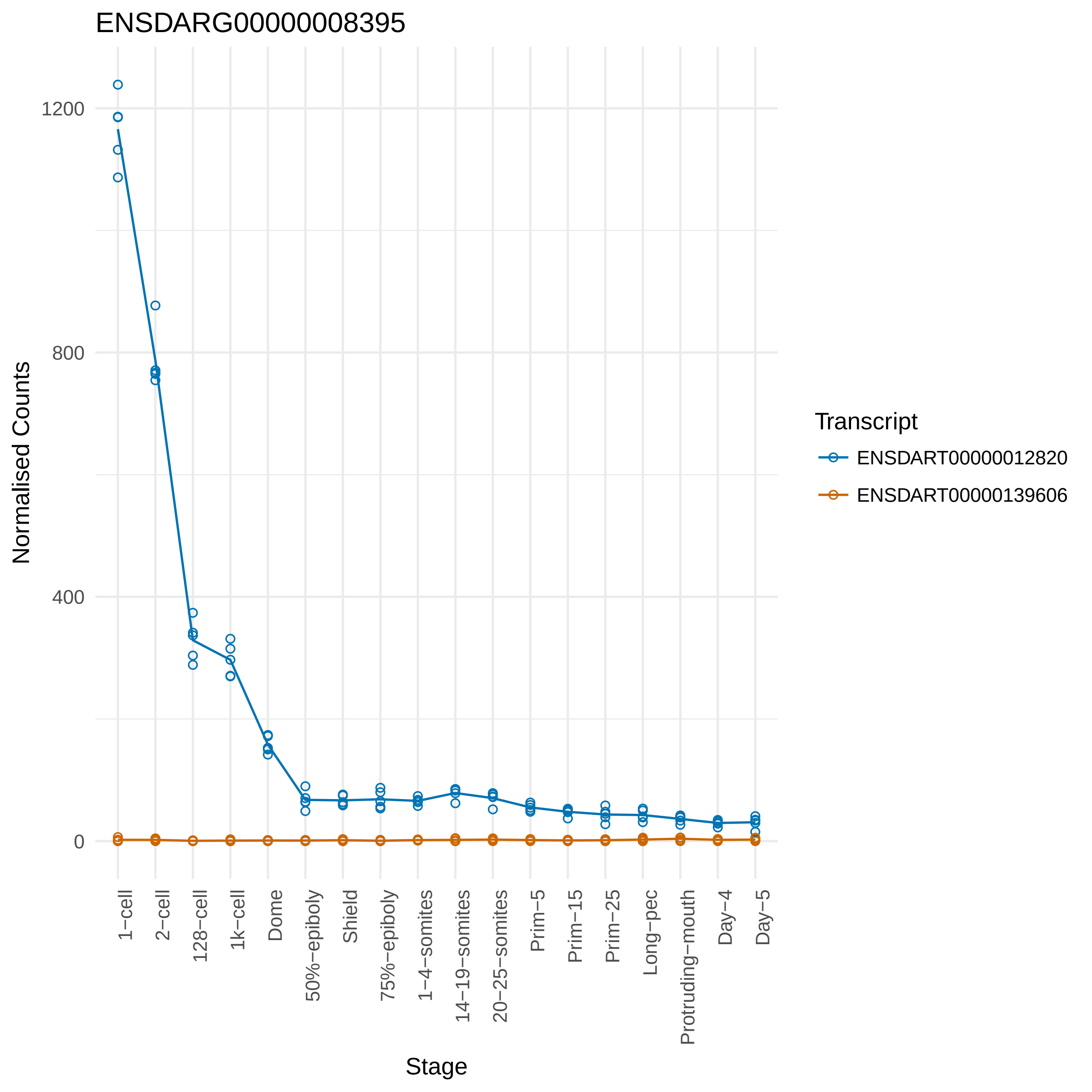

ENSDARG00000008395
Normalised Counts
0
400
800
1200
Stage
1−cell
2−cell
128−cell
1k−cell
Dome
50%−epiboly
Shield
75%−epiboly
1−4−somites
14−19−somites
20−25−somites
Prim−5
Prim−15
Prim−25
Long−pec
Protruding−mouth
Day−4
Day−5
Transcript
ENSDART00000012820
ENSDART00000139606

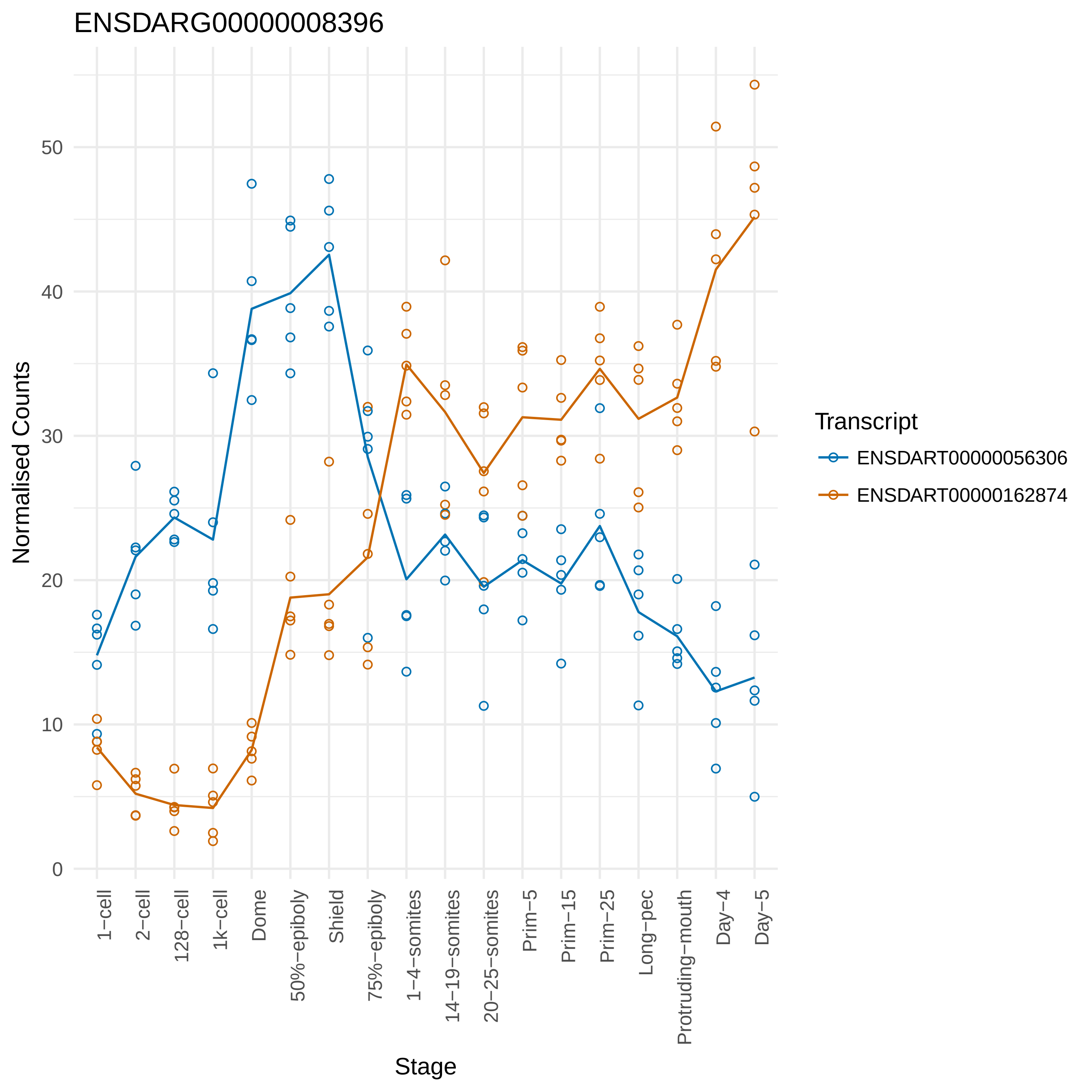

ENSDARG00000008396
Normalised Counts
0
10
20
30
40
50
1−cell
2−cell
128−cell
1k−cell
Dome
50%−epiboly
Shield
75%−epiboly
1−4−somites
14−19−somites
20−25−somites
Prim−5
Prim−15
Prim−25
Long−pec
Protruding−mouth
Day−4
Day−5
Stage
Transcript
ENSDART00000056306
ENSDART00000162874

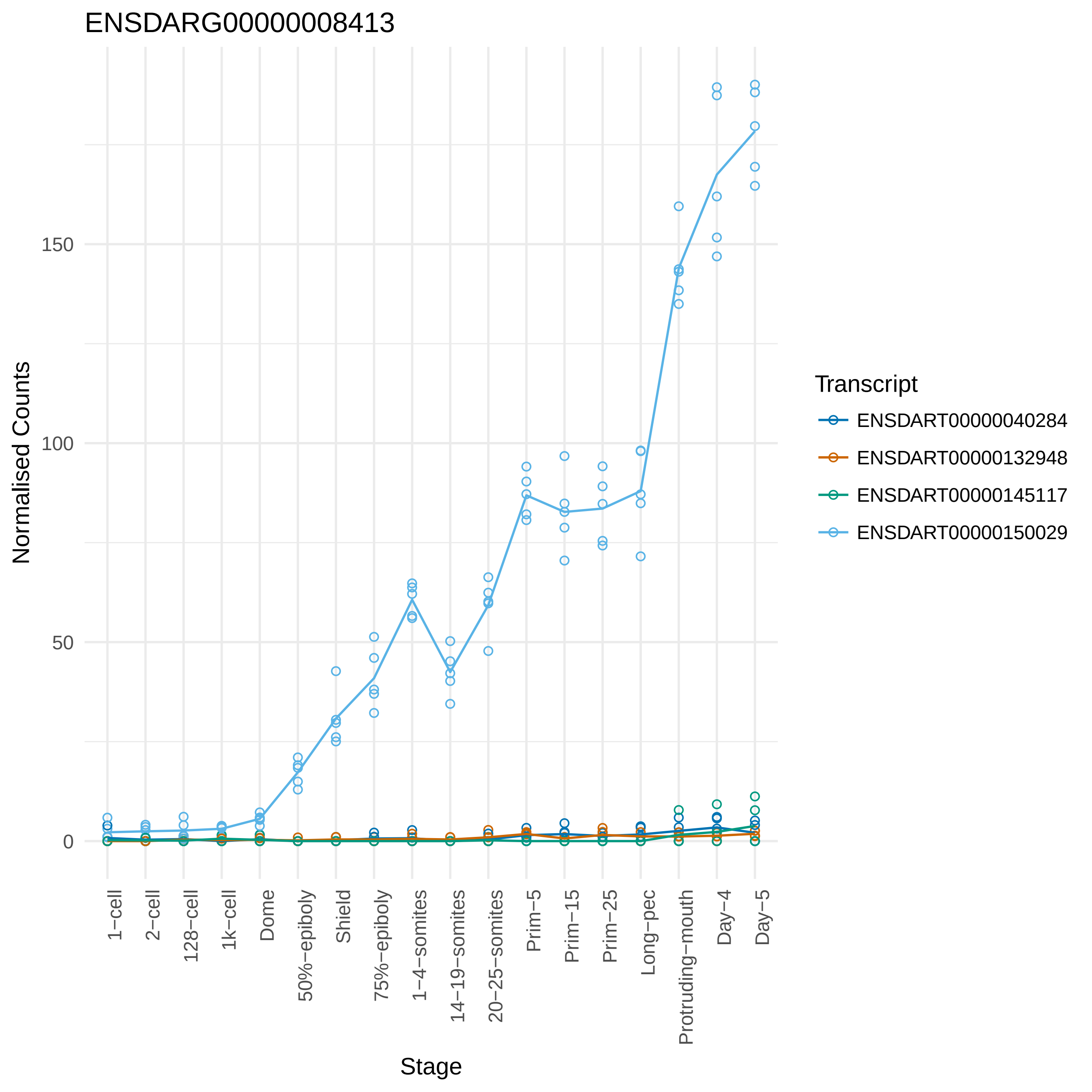

ENSDARG00000008413
Normalised Counts
150
100
50
0
1−cell
2−cell
128−cell
1k−cell
Dome
50%−epiboly
Shield
75%−epiboly
1−4−somites
14−19−somites
20−25−somites
Prim−5
Prim−15
Prim−25
Long−pec
Protruding−mouth
Day−4
Day−5
Stage
Transcript
ENSDART00000040284
ENSDART00000132948
ENSDART00000145117
ENSDART00000150029

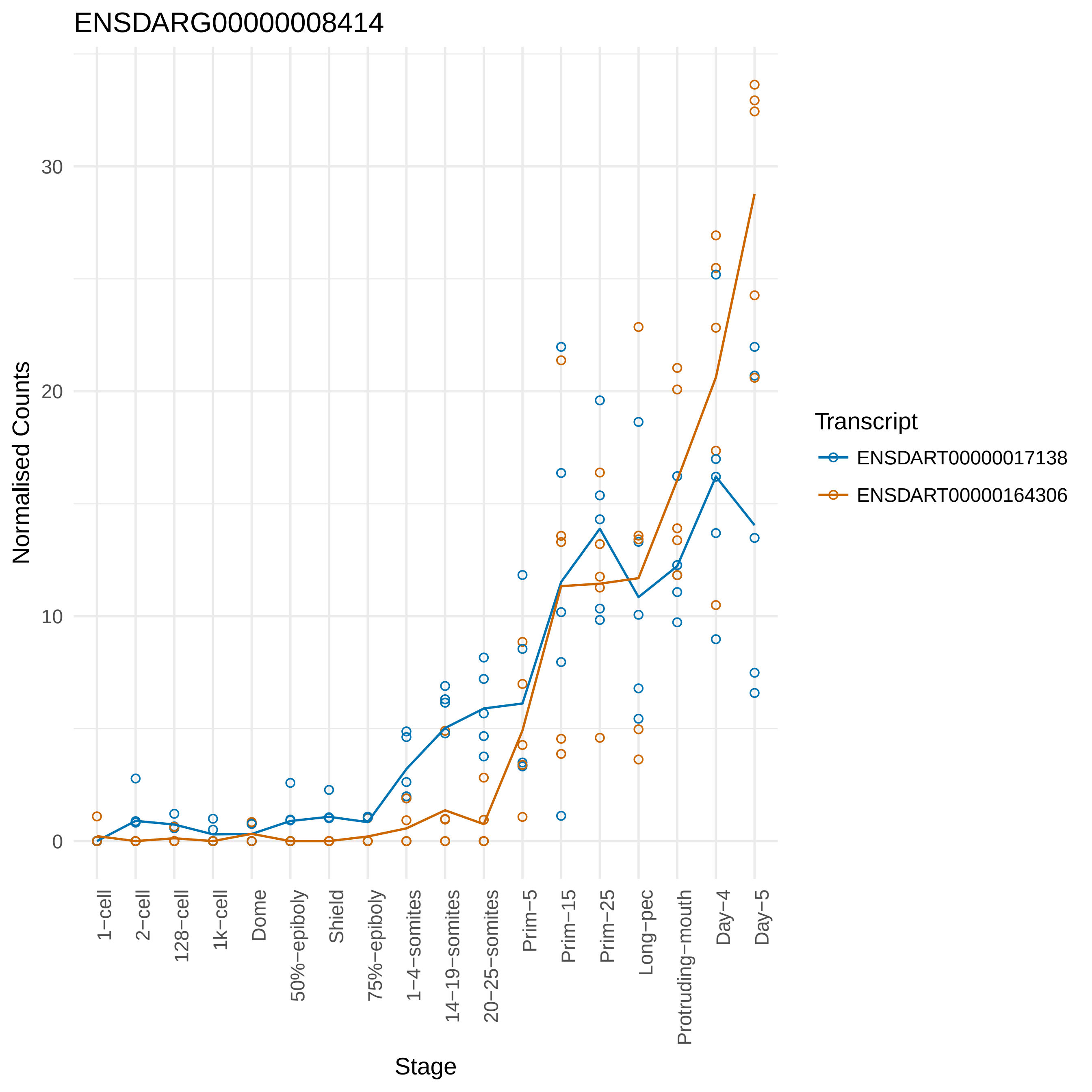

ENSDARG00000008414
Normalised Counts
0
10
20
30
Stage
1−cell
2−cell
128−cell
1k−cell
Dome
50%−epiboly
Shield
75%−epiboly
1−4−somites
14−19−somites
20−25−somites
Prim−5
Prim−15
Prim−25
Long−pec
Protruding−mouth
Day−4
Day−5
Transcript
ENSDART00000017138
ENSDART00000164306

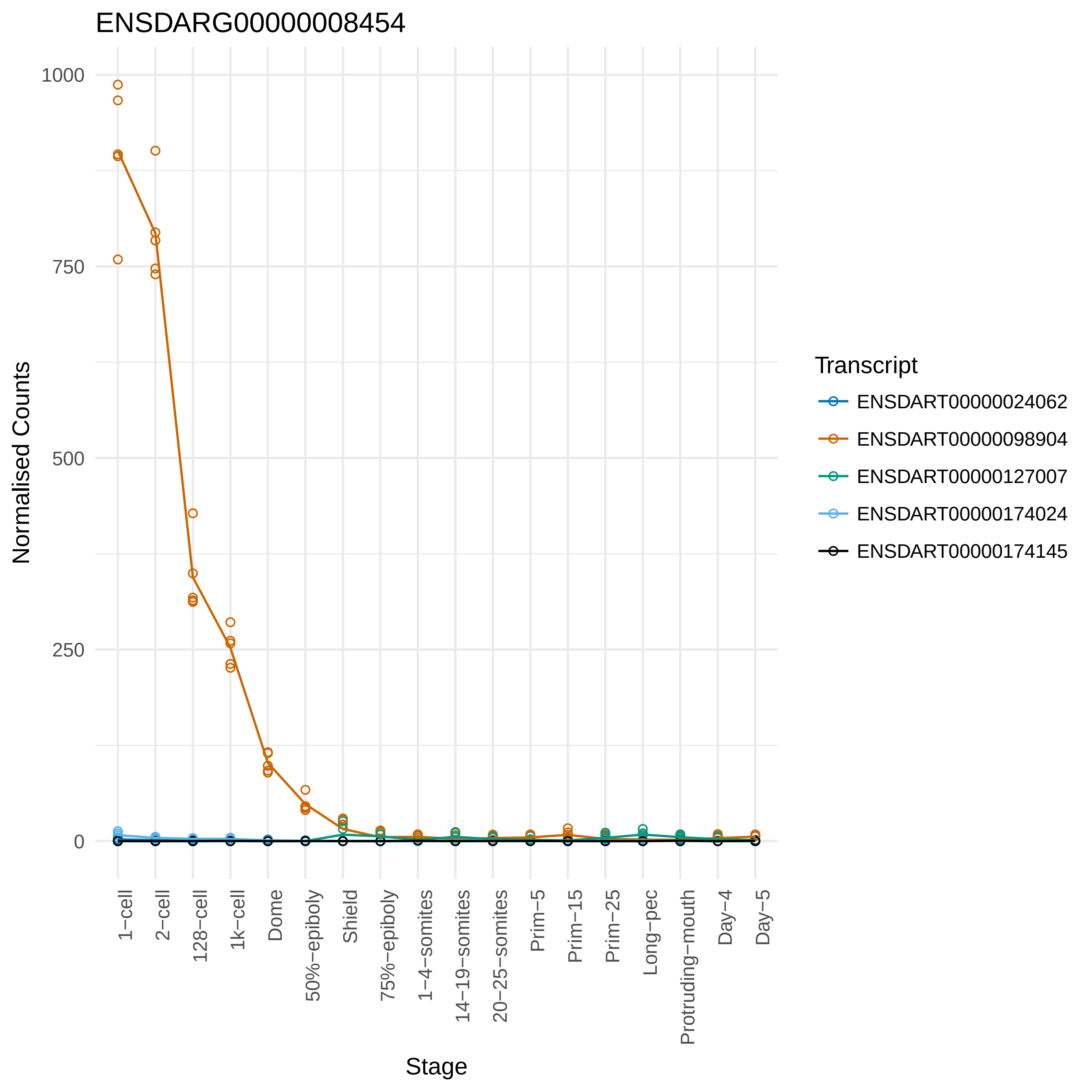

ENSDARG00000008454
Normalised Counts
1000
750
500
250
0
1−cell
2−cell
128−cell
1k−cell
Dome
50%−epiboly
Shield
75%−epiboly
1−4−somites
14−19−somites
20−25−somites
Prim−5
Prim−15
Prim−25
Long−pec
Protruding−mouth
Day−4
Day−5
Stage
Transcript
ENSDART00000024062
ENSDART00000098904
ENSDART00000127007
ENSDART00000174024
ENSDART00000174145

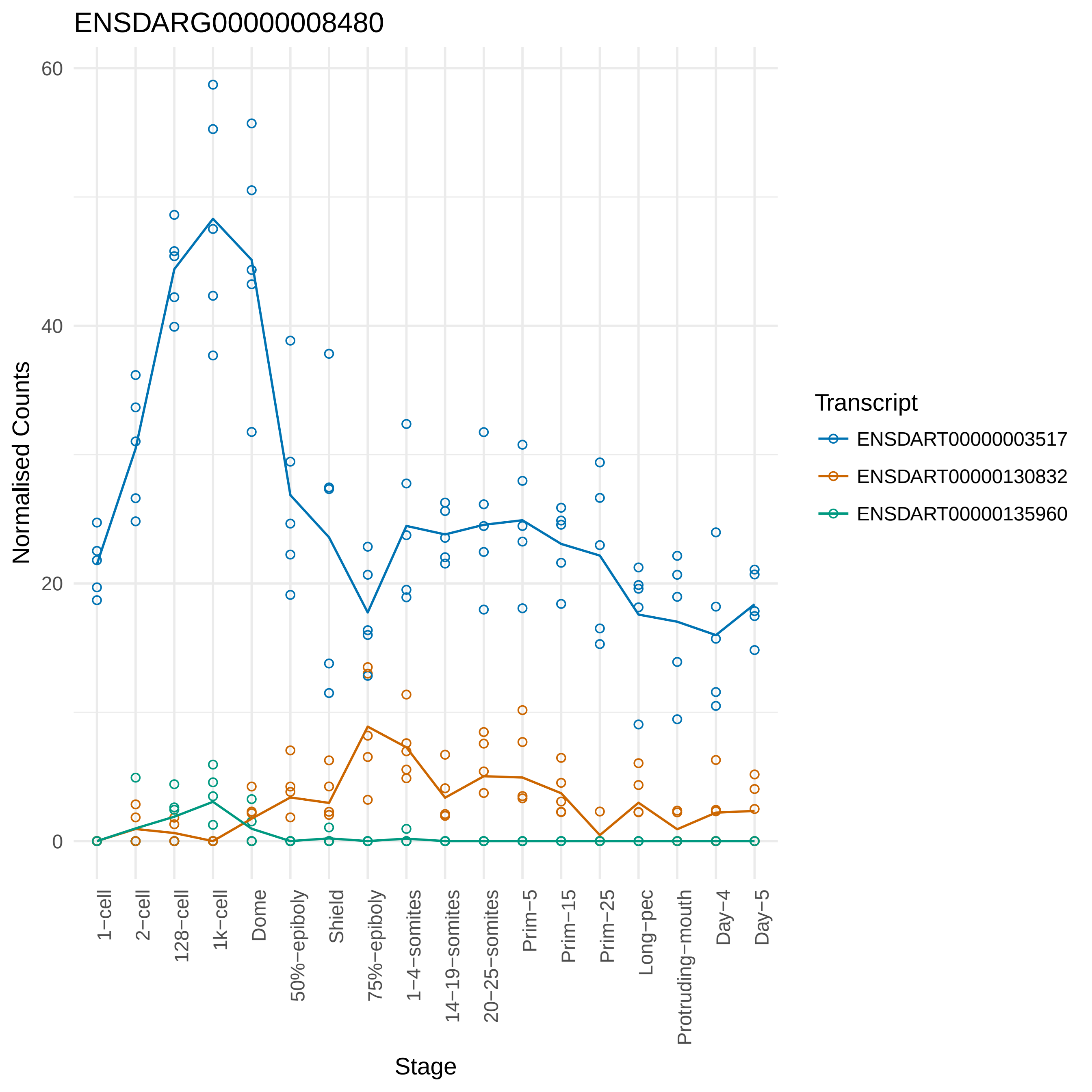
ENSDARG00000008480
Normalised Counts
60
40
20
0
1−cell
2−cell
128−cell
1k−cell
Dome
50%−epiboly
Shield
75%−epiboly
1−4−somites
14−19−somites
20−25−somites
Prim−5
Prim−15
Prim−25
Long−pec
Protruding−mouth
Day−4
Day−5
Stage
Transcript
ENSDART00000003517
ENSDART00000130832
ENSDART00000135960

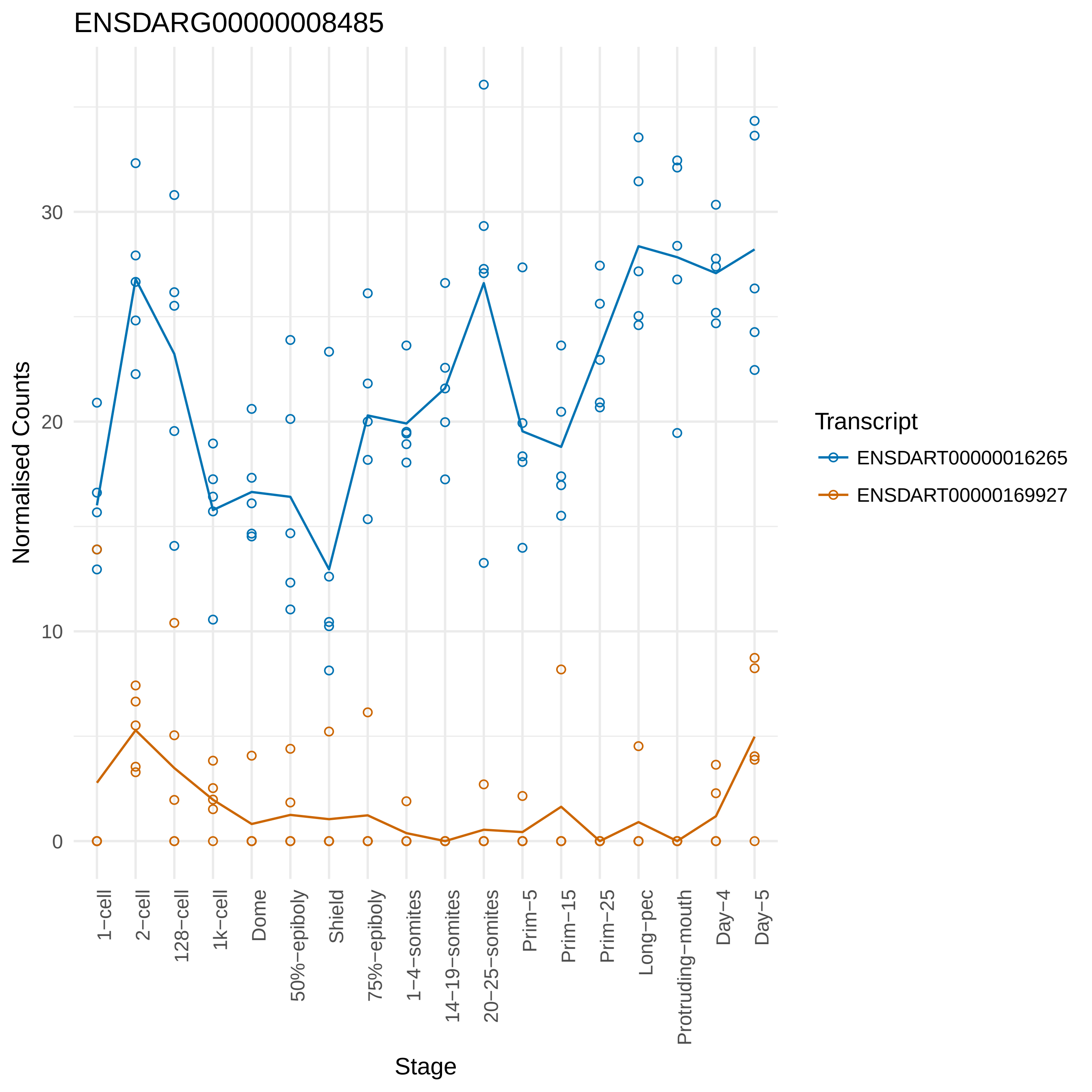
ENSDARG00000008485
Normalised Counts
0
10
20
30
Stage
1−cell
2−cell
128−cell
1k−cell
Dome
50%−epiboly
Shield
75%−epiboly
1−4−somites
14−19−somites
20−25−somites
Prim−5
Prim−15
Prim−25
Long−pec
Protruding−mouth
Day−4
Day−5
Transcript
ENSDART00000016265
ENSDART00000169927

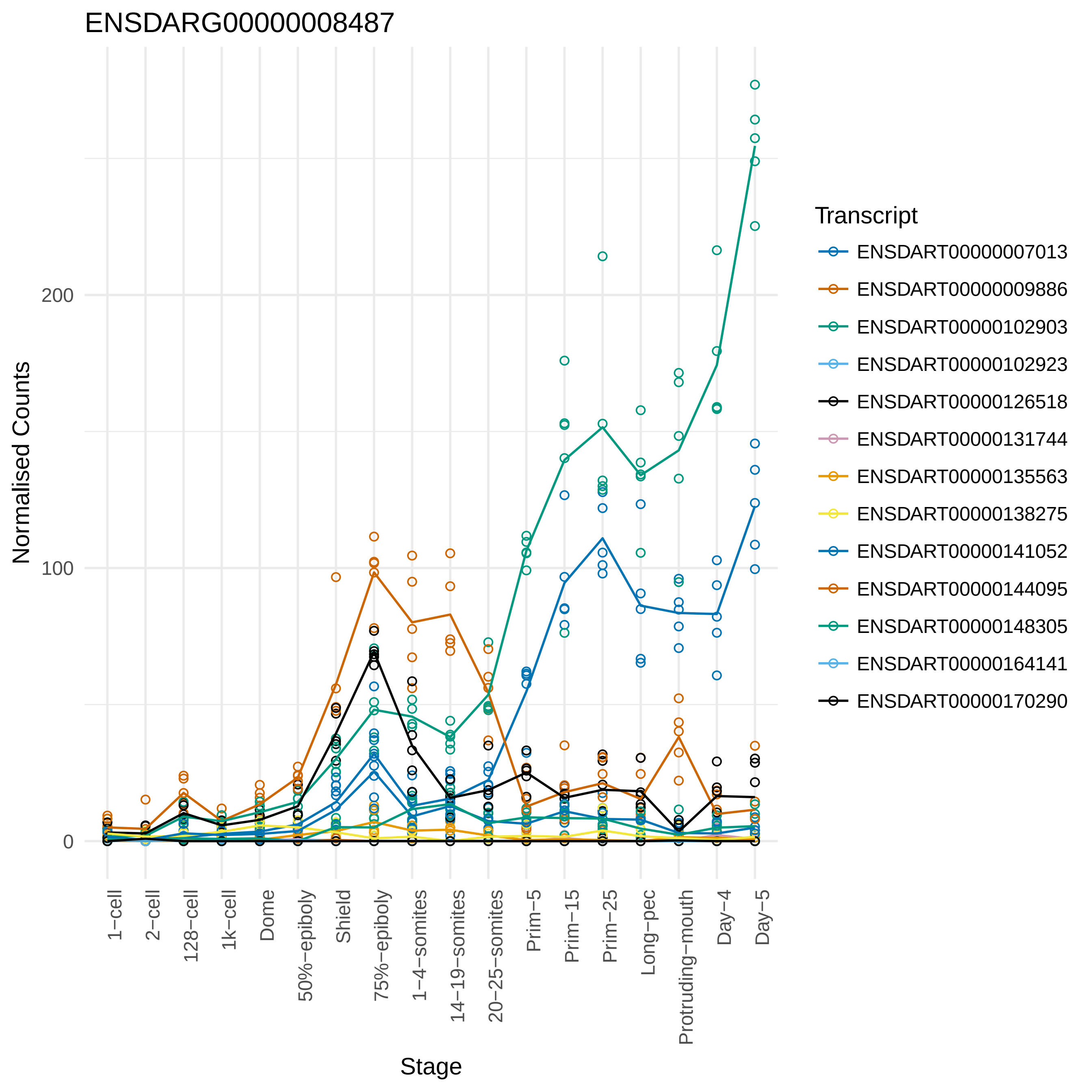
ENSDARG00000008487
Normalised Counts
0
100
200
Stage
1−cell
2−cell
128−cell
1k−cell
Dome
50%−epiboly
Shield
75%−epiboly
1−4−somites
14−19−somites
20−25−somites
Prim−5
Prim−15
Prim−25
Long−pec
Protruding−mouth
Day−4
Day−5
Transcript
ENSDART00000007013
ENSDART00000009886
ENSDART00000102903
ENSDART00000102923
ENSDART00000126518
ENSDART00000131744
ENSDART00000135563
ENSDART00000138275
ENSDART00000141052
ENSDART00000144095
ENSDART00000148305
ENSDART00000164141
ENSDART00000170290

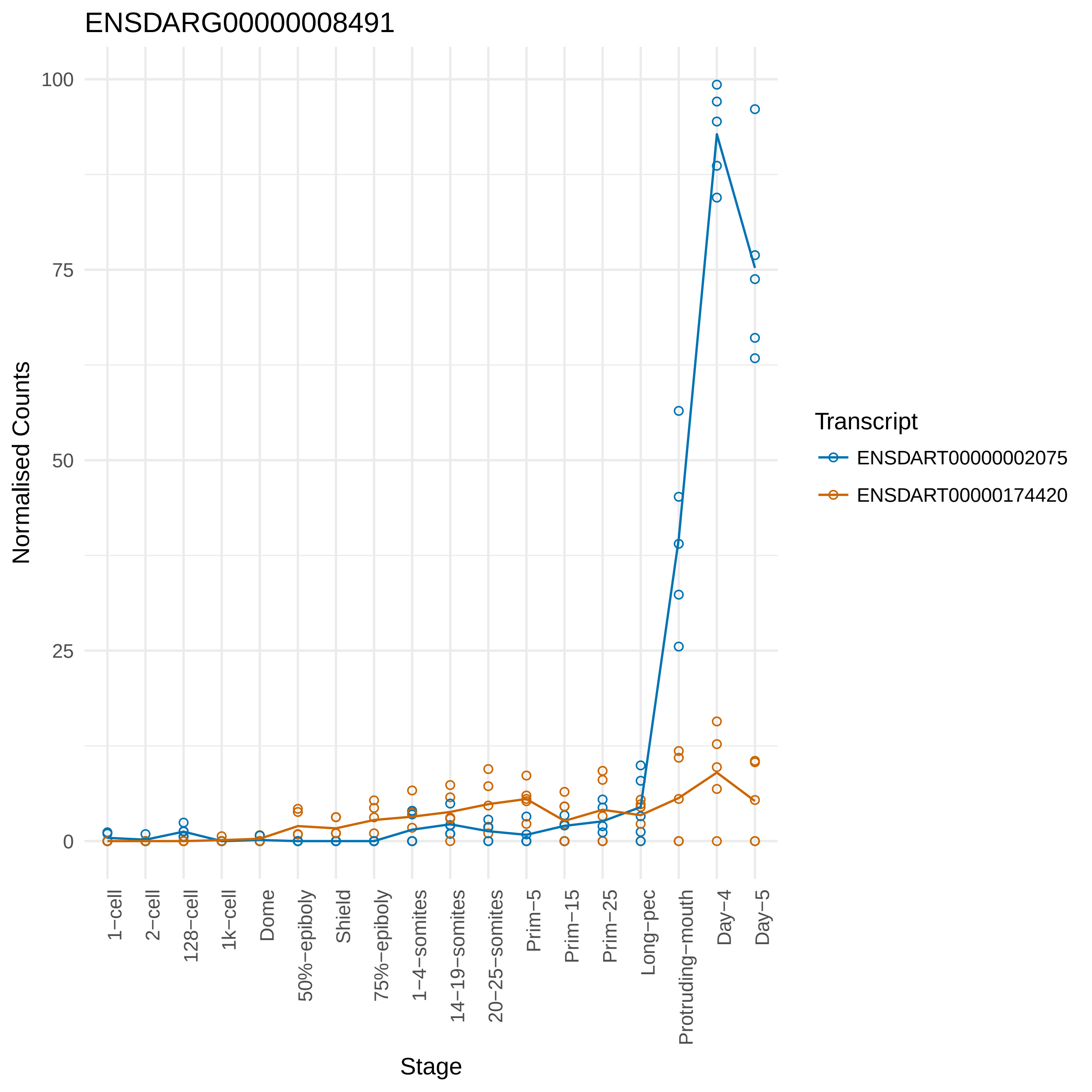

ENSDARG00000008491
Normalised Counts
100
75
50
25
0
1−cell
2−cell
128−cell
1k−cell
Dome
50%−epiboly
Shield
75%−epiboly
1−4−somites
14−19−somites
20−25−somites
Prim−5
Prim−15
Prim−25
Long−pec
Protruding−mouth
Day−4
Day−5
Stage
Transcript
ENSDART00000002075
ENSDART00000174420

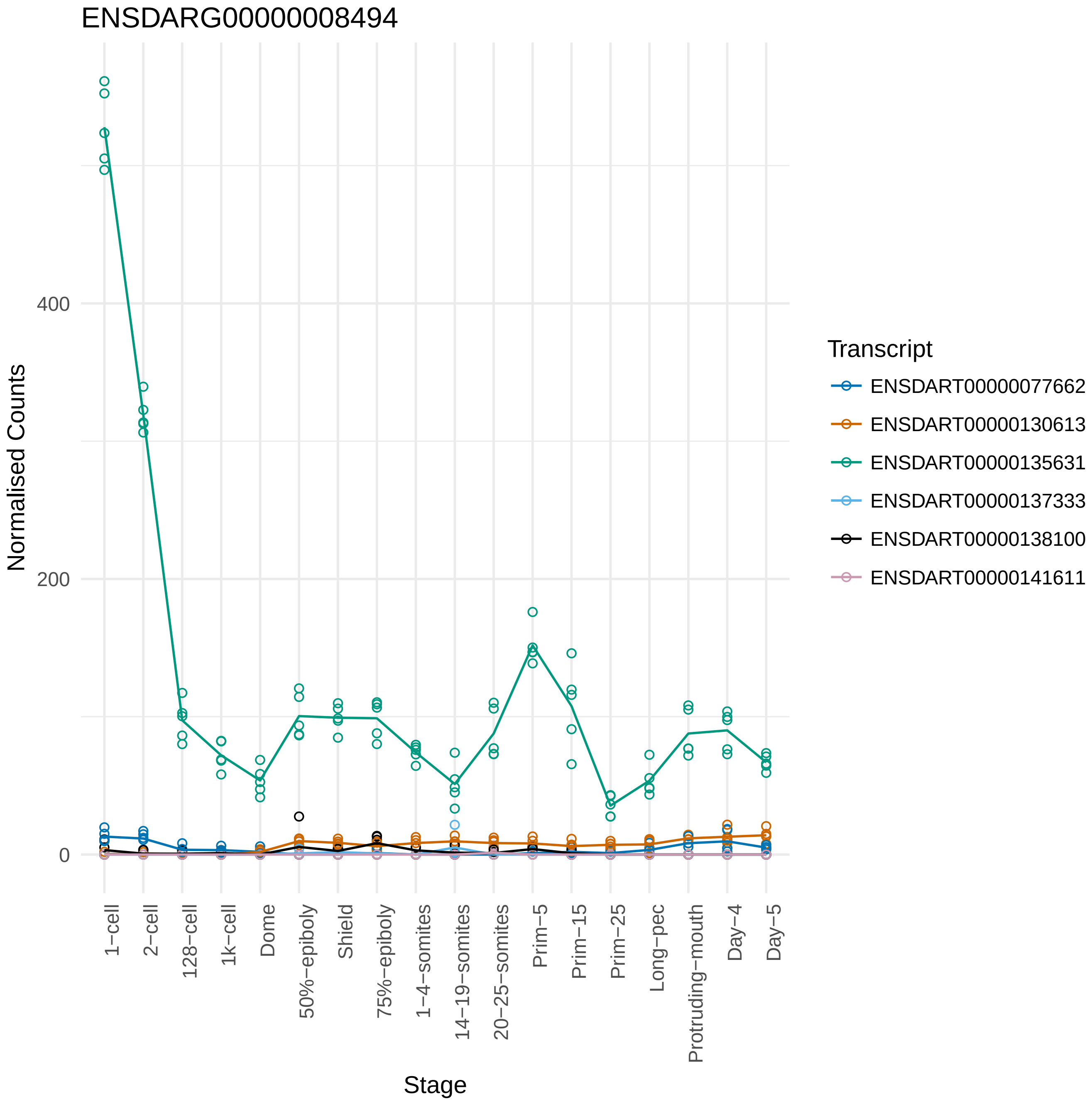
ENSDARG00000008494
Normalised Counts
400
200
0
1−cell
2−cell
128−cell
1k−cell
Dome
50%−epiboly
Shield
75%−epiboly
1−4−somites
14−19−somites
20−25−somites
Prim−5
Prim−15
Prim−25
Long−pec
Protruding−mouth
Day−4
Day−5
Stage
Transcript
ENSDART00000077662
ENSDART00000130613
ENSDART00000135631
ENSDART00000137333
ENSDART00000138100
ENSDART00000141611

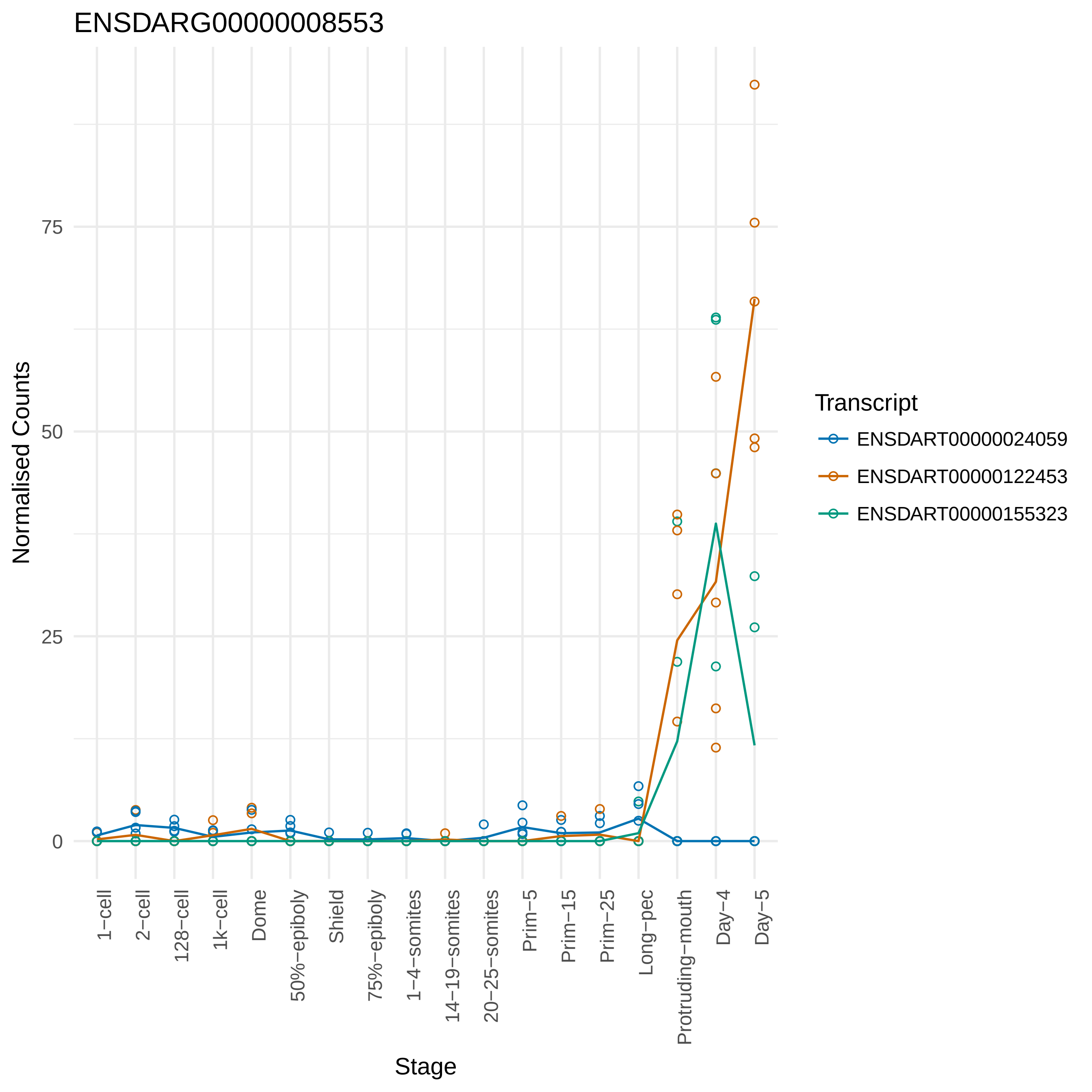

ENSDARG00000008553
Normalised Counts
0
25
50
75
1−cell
2−cell
128−cell
1k−cell
Dome
50%−epiboly
Shield
75%−epiboly
1−4−somites
14−19−somites
20−25−somites
Prim−5
Prim−15
Prim−25
Long−pec
Protruding−mouth
Day−4
Day−5
Stage
Transcript
ENSDART00000024059
ENSDART00000122453
ENSDART00000155323

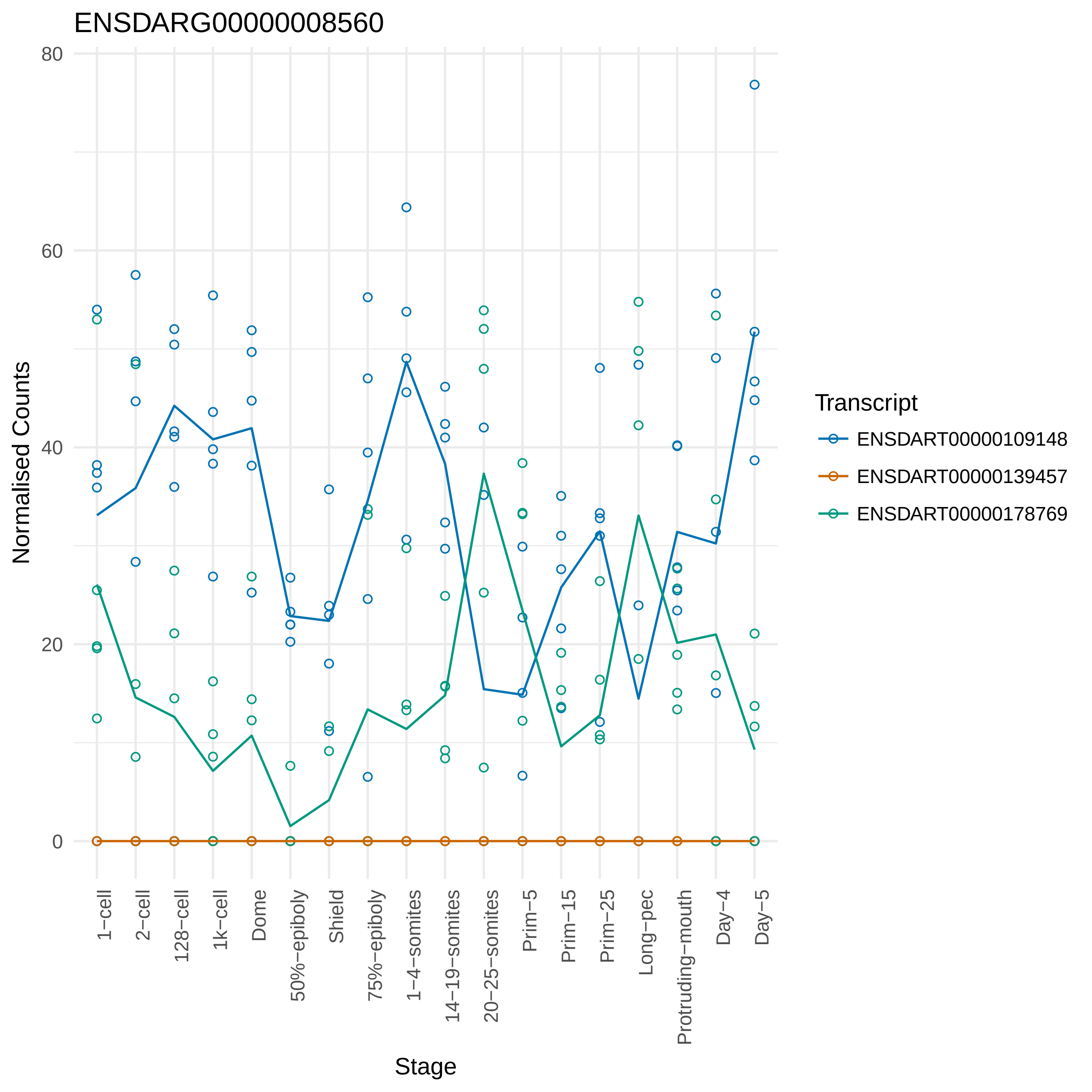
ENSDARG00000008560
Normalised Counts
0
20
40
60
80
Stage
1−cell
2−cell
128−cell
1k−cell
Dome
50%−epiboly
Shield
75%−epiboly
1−4−somites
14−19−somites
20−25−somites
Prim−5
Prim−15
Prim−25
Long−pec
Protruding−mouth
Day−4
Day−5
Transcript
ENSDART00000109148
ENSDART00000139457
ENSDART00000178769

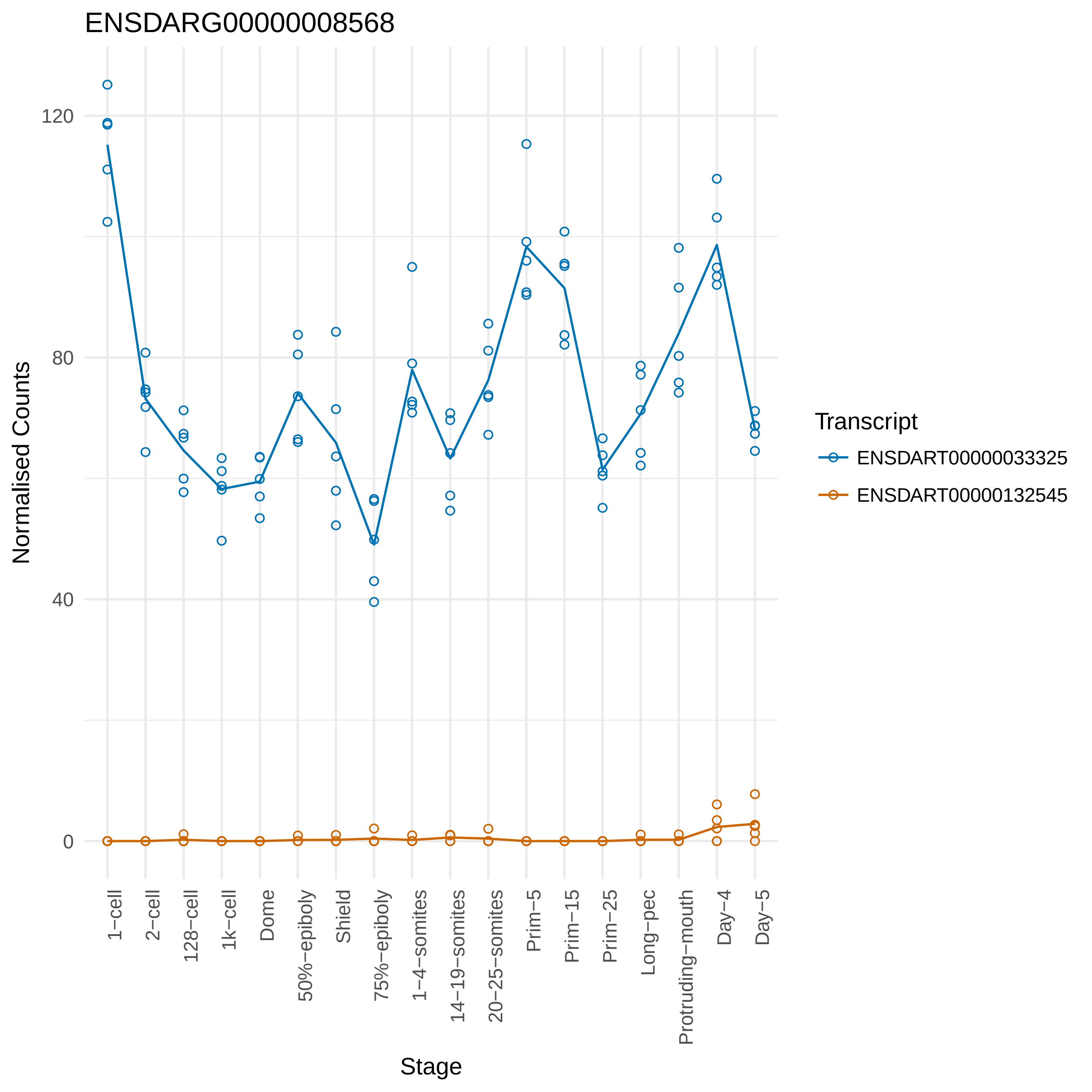

ENSDARG00000008568
Normalised Counts
120
80
40
0
1−cell
2−cell
128−cell
1k−cell
Dome
50%−epiboly
Shield
75%−epiboly
1−4−somites
14−19−somites
20−25−somites
Prim−5
Prim−15
Prim−25
Long−pec
Protruding−mouth
Day−4
Day−5
Stage
Transcript
ENSDART00000033325
ENSDART00000132545

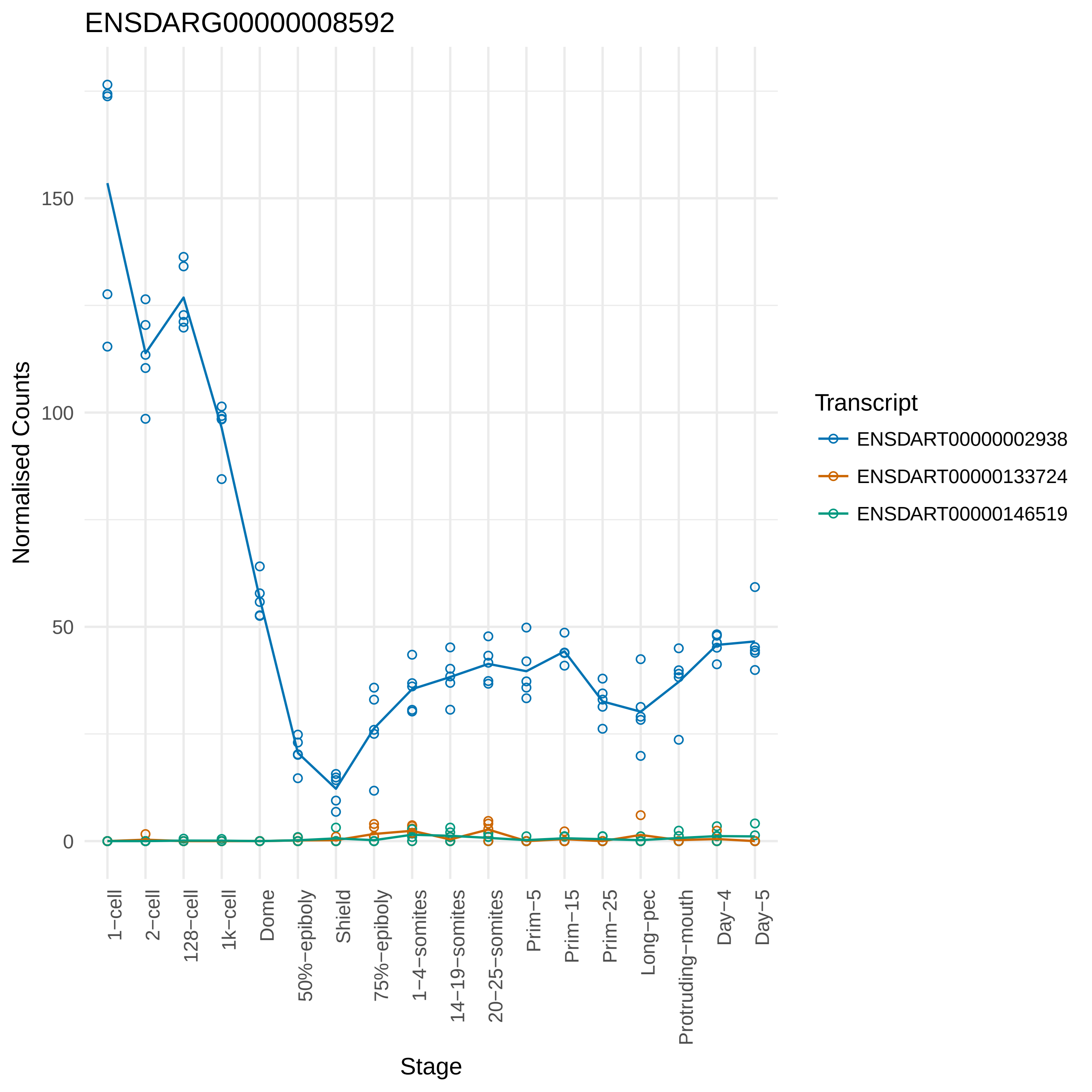
ENSDARG00000008592
Normalised Counts
150
100
50
0
1−cell
2−cell
128−cell
1k−cell
Dome
50%−epiboly
Shield
75%−epiboly
1−4−somites
14−19−somites
20−25−somites
Prim−5
Prim−15
Prim−25
Long−pec
Protruding−mouth
Day−4
Day−5
Stage
Transcript
ENSDART00000002938
ENSDART00000133724
ENSDART00000146519

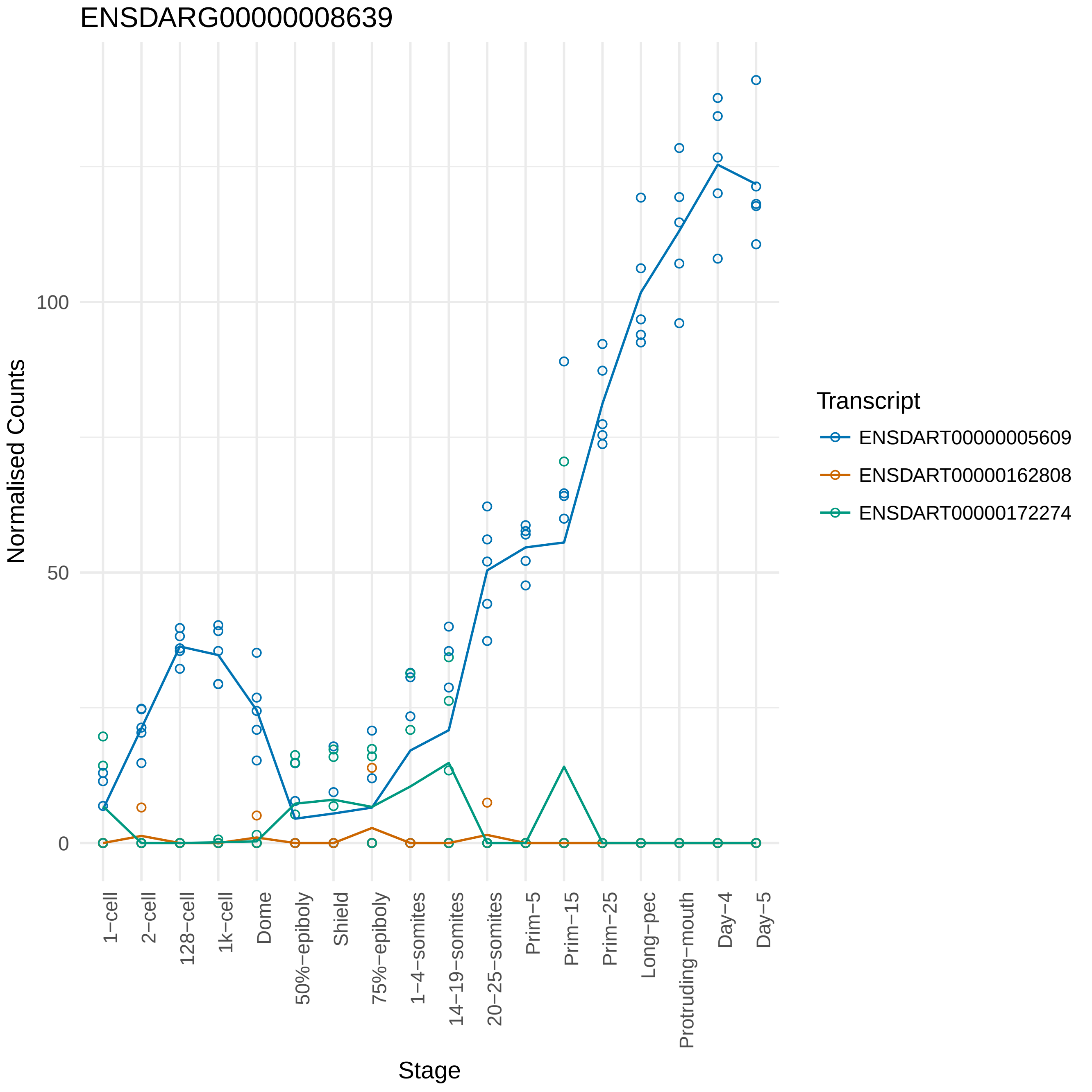
ENSDARG00000008639
Normalised Counts
0
50
100
Stage
1−cell
2−cell
128−cell
1k−cell
Dome
50%−epiboly
Shield
75%−epiboly
1−4−somites
14−19−somites
20−25−somites
Prim−5
Prim−15
Prim−25
Long−pec
Protruding−mouth
Day−4
Day−5
Transcript
ENSDART00000005609
ENSDART00000162808
ENSDART00000172274

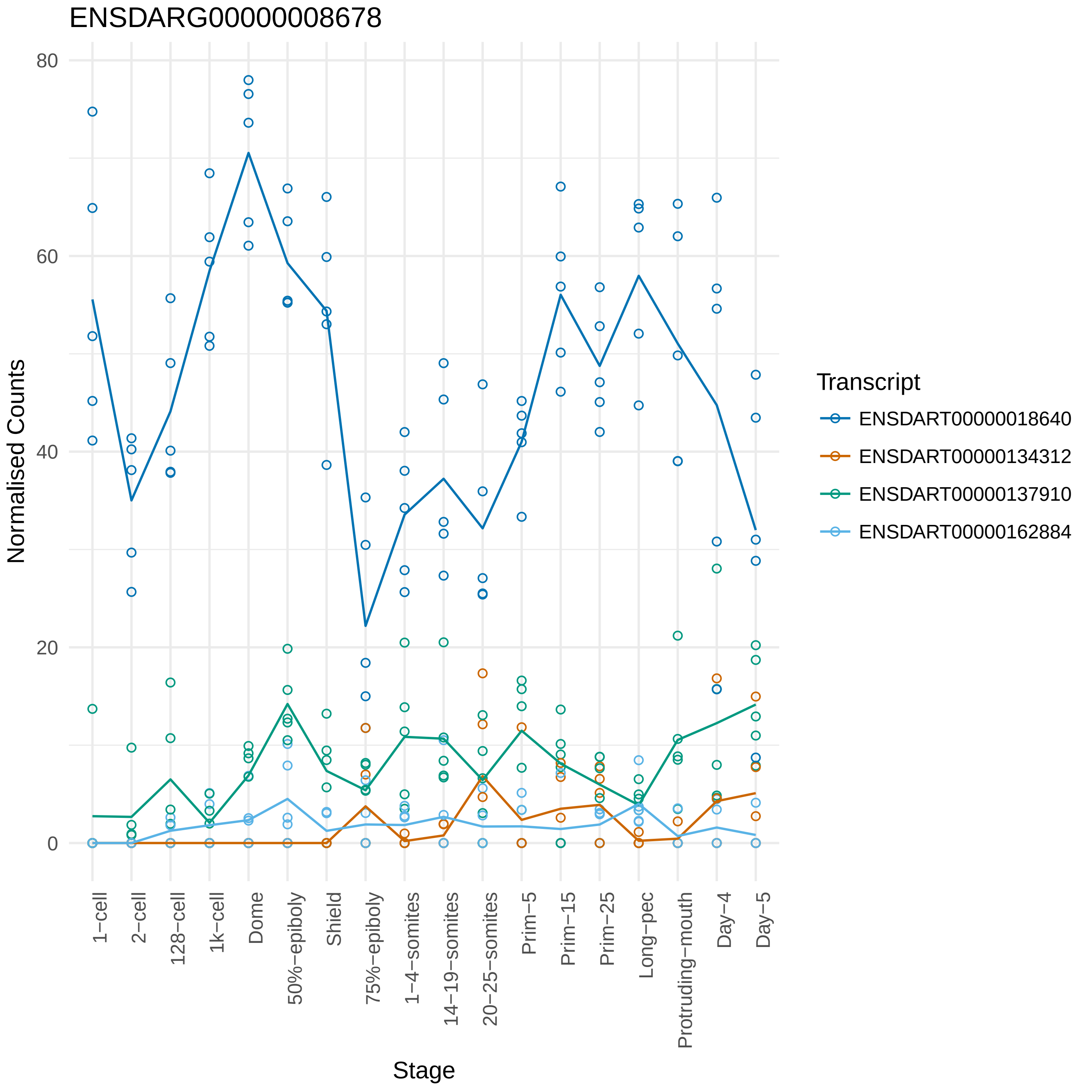

ENSDARG00000008678
Normalised Counts
0
20
40
60
80
1−cell
2−cell
128−cell
1k−cell
Dome
50%−epiboly
Shield
75%−epiboly
1−4−somites
14−19−somites
20−25−somites
Prim−5
Prim−15
Prim−25
Long−pec
Protruding−mouth
Day−4
Day−5
Stage
Transcript
ENSDART00000018640
ENSDART00000134312
ENSDART00000137910
ENSDART00000162884

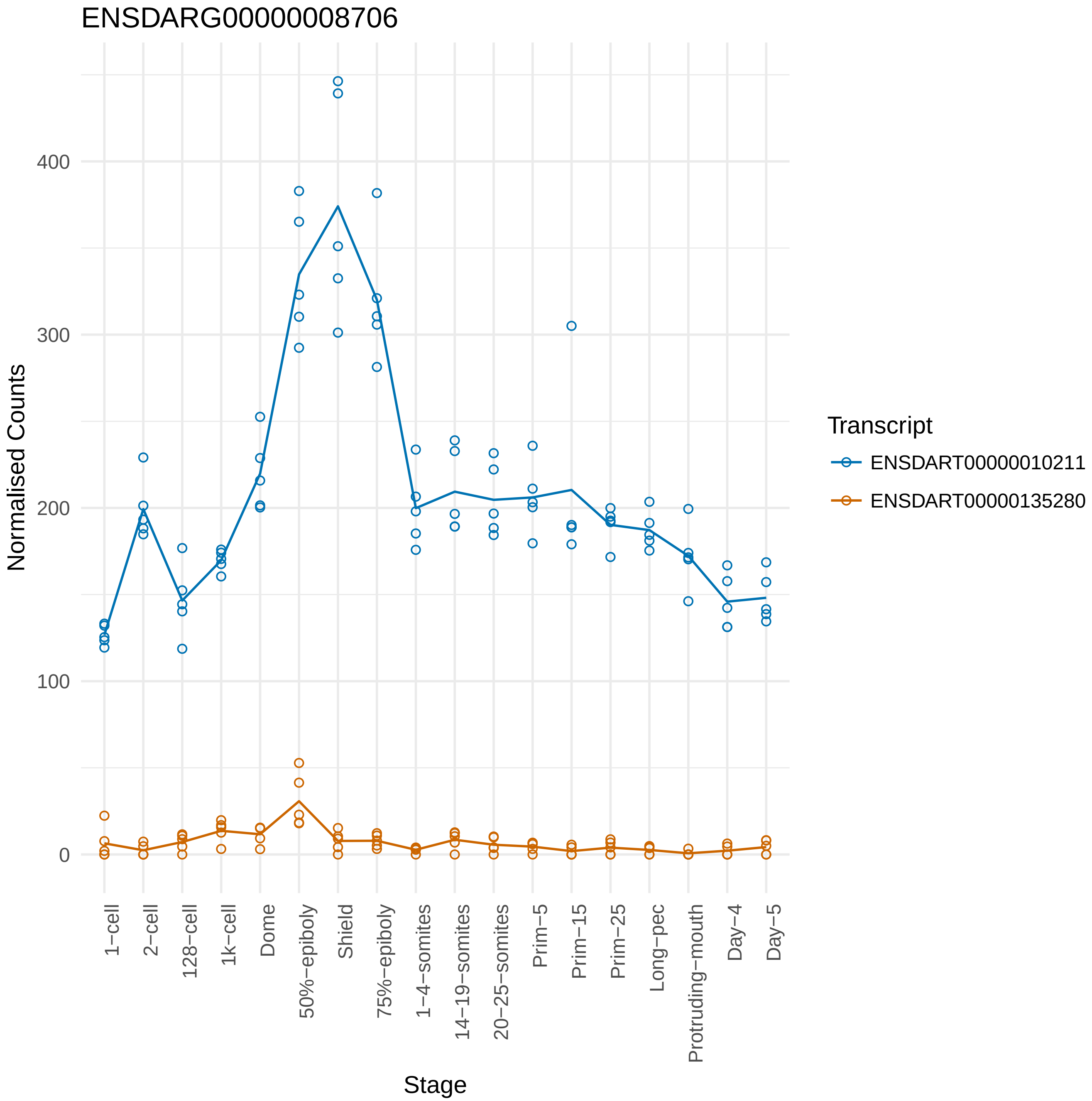

ENSDARG00000008706
Normalised Counts
0
100
200
300
400
Stage
1−cell
2−cell
128−cell
1k−cell
Dome
50%−epiboly
Shield
75%−epiboly
1−4−somites
14−19−somites
20−25−somites
Prim−5
Prim−15
Prim−25
Long−pec
Protruding−mouth
Day−4
Day−5
Transcript
ENSDART00000010211
ENSDART00000135280

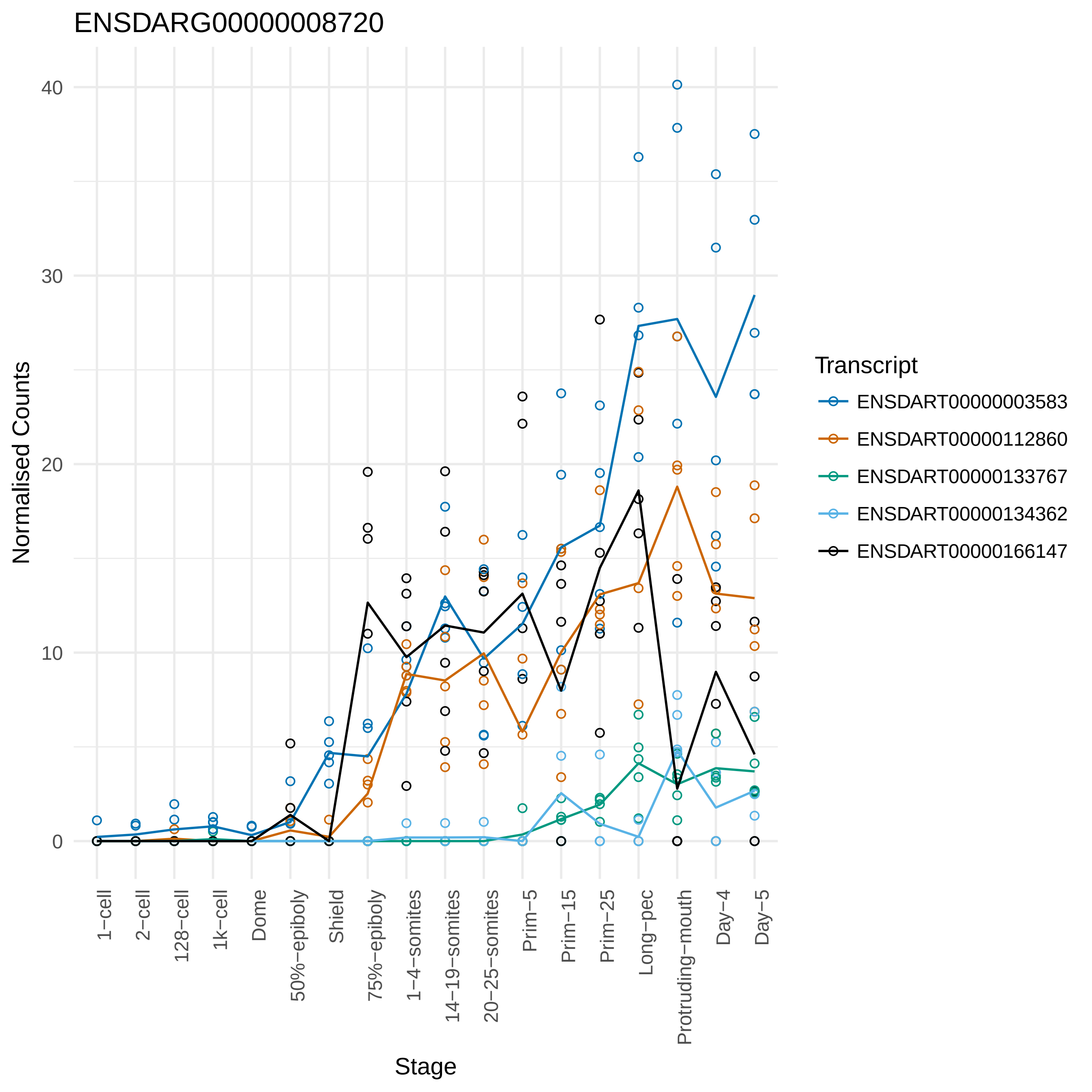

ENSDARG00000008720
Normalised Counts
0
10
20
30
40
Stage
1−cell
2−cell
128−cell
1k−cell
Dome
50%−epiboly
Shield
75%−epiboly
1−4−somites
14−19−somites
20−25−somites
Prim−5
Prim−15
Prim−25
Long−pec
Protruding−mouth
Day−4
Day−5
Transcript
ENSDART00000003583
ENSDART00000112860
ENSDART00000133767
ENSDART00000134362
ENSDART00000166147

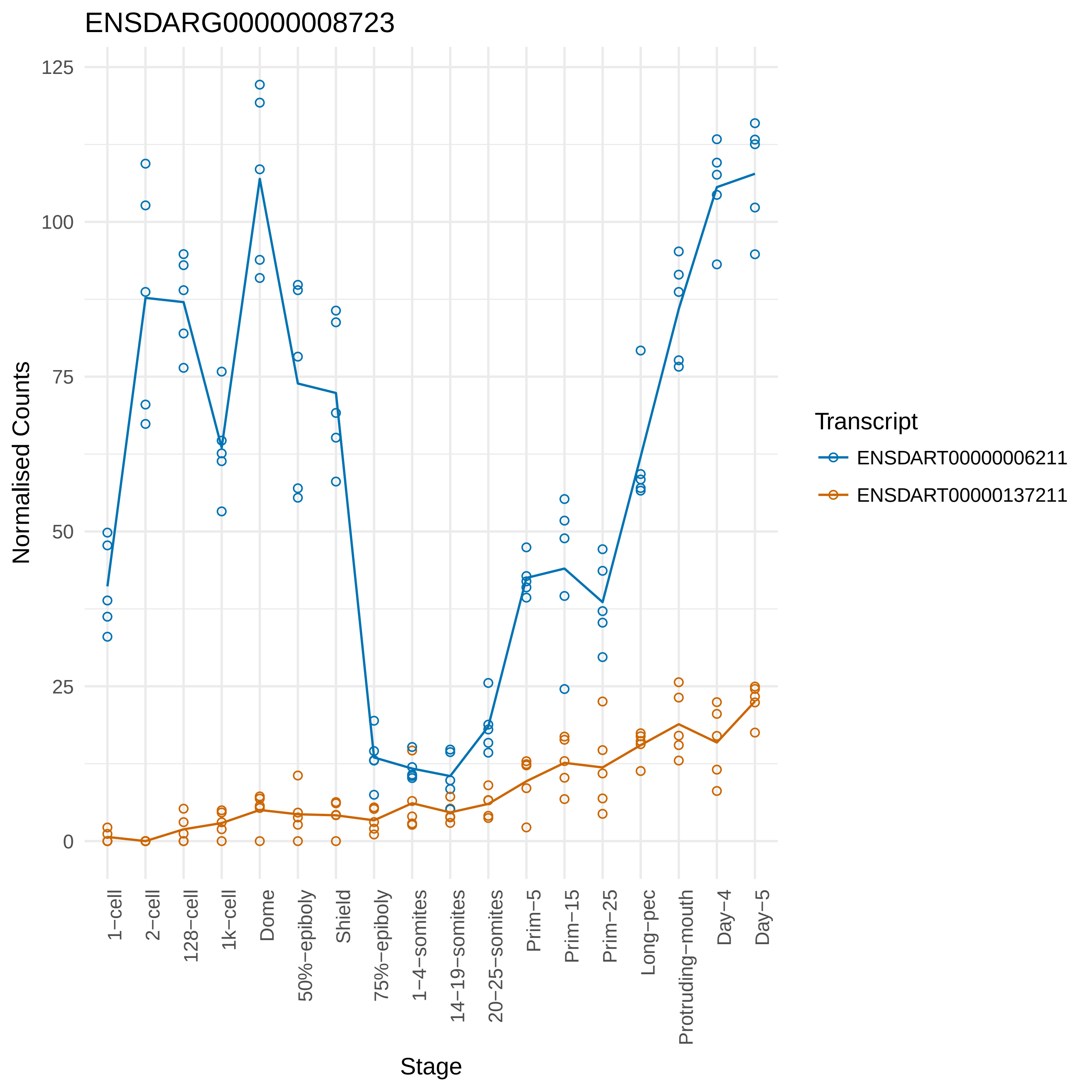
ENSDARG00000008723
Normalised Counts
125
100
75
50
25
0
1−cell
2−cell
128−cell
1k−cell
Dome
50%−epiboly
Shield
75%−epiboly
1−4−somites
14−19−somites
20−25−somites
Prim−5
Prim−15
Prim−25
Long−pec
Protruding−mouth
Day−4
Day−5
Stage
Transcript
ENSDART00000006211
ENSDART00000137211

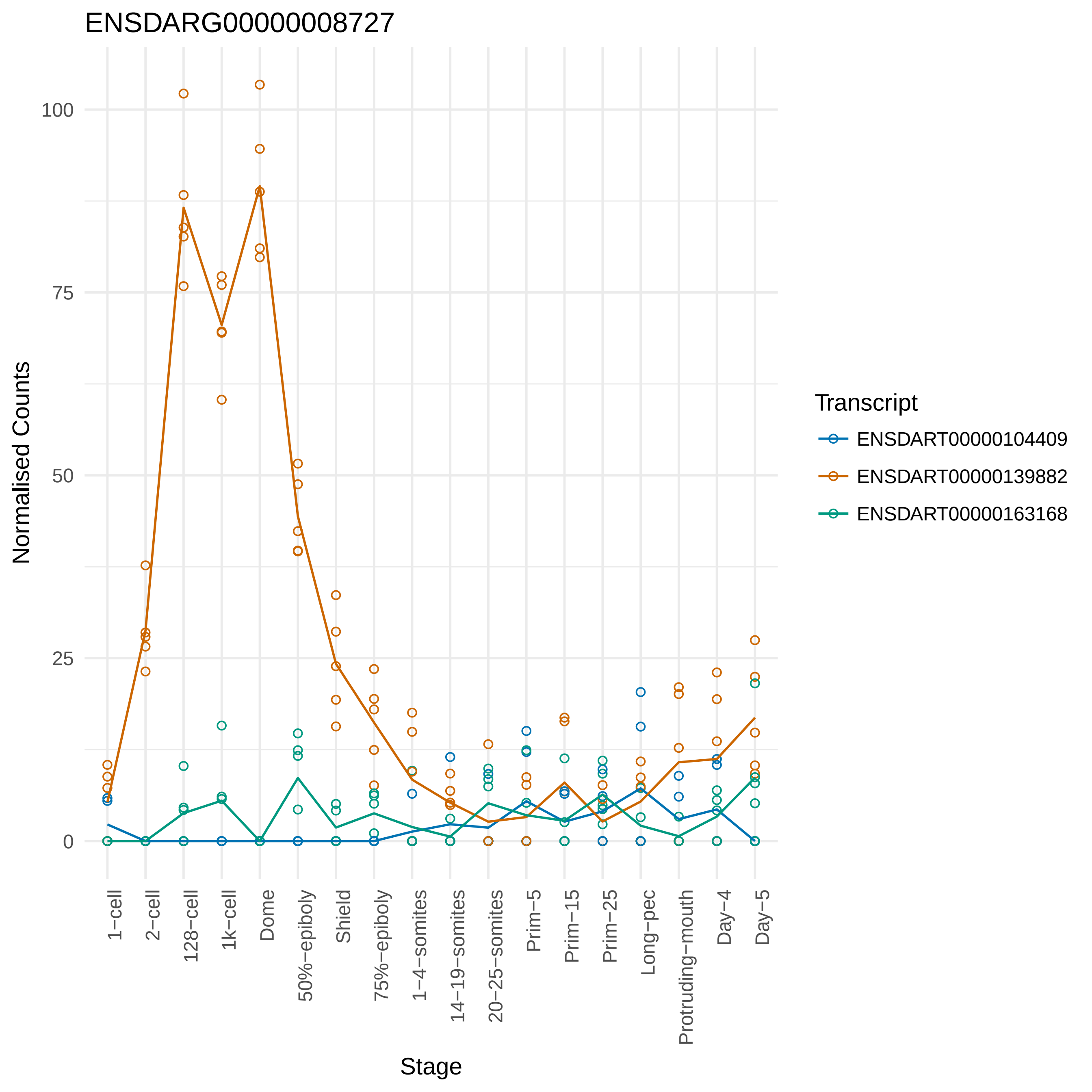

ENSDARG00000008727
Normalised Counts
100
75
50
25
0
1−cell
2−cell
128−cell
1k−cell
Dome
50%−epiboly
Shield
75%−epiboly
1−4−somites
14−19−somites
20−25−somites
Prim−5
Prim−15
Prim−25
Long−pec
Protruding−mouth
Day−4
Day−5
Stage
Transcript
ENSDART00000104409
ENSDART00000139882
ENSDART00000163168

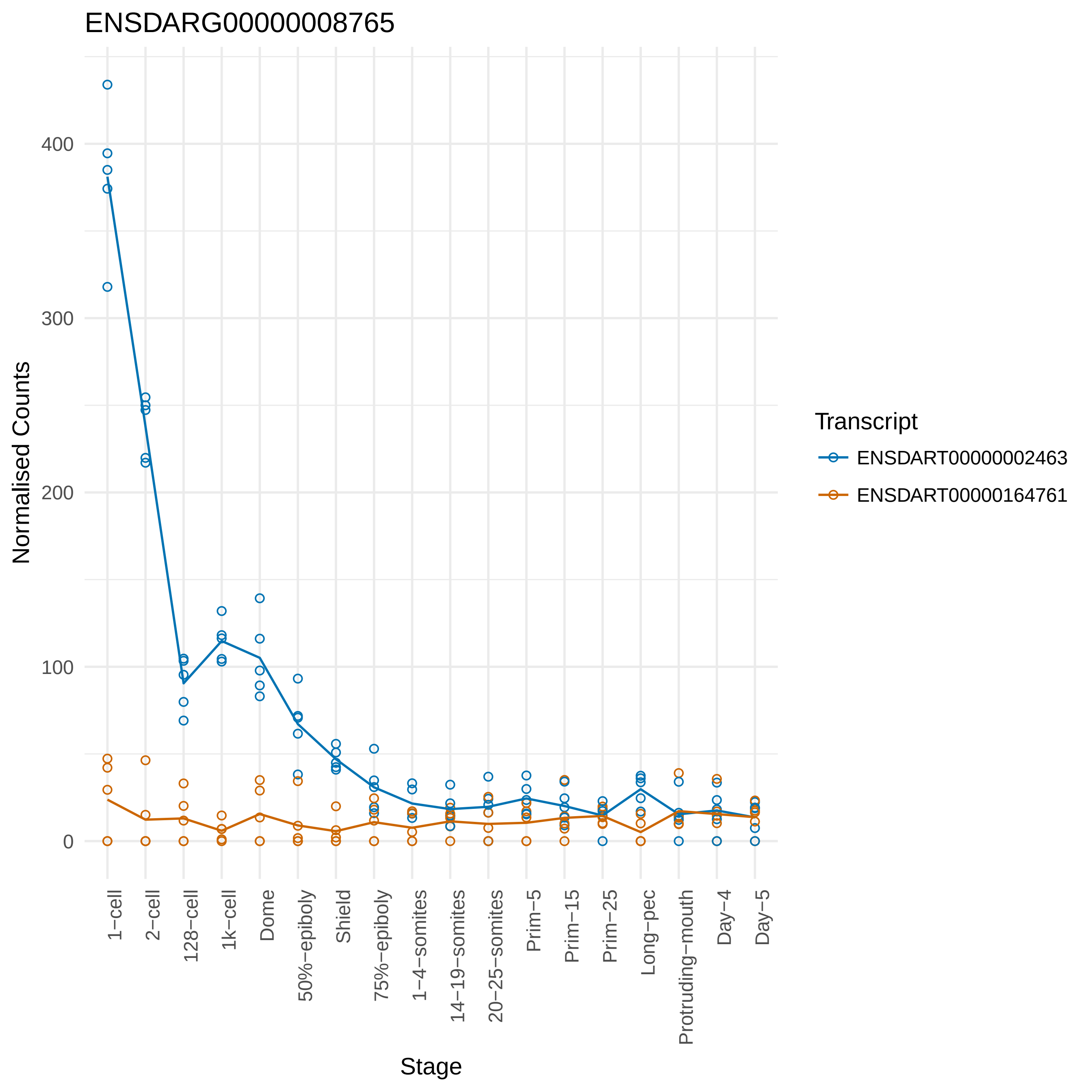

ENSDARG00000008765
Normalised Counts
400
300
200
100
0
1−cell
2−cell
128−cell
1k−cell
Dome
50%−epiboly
Shield
75%−epiboly
1−4−somites
14−19−somites
20−25−somites
Prim−5
Prim−15
Prim−25
Long−pec
Protruding−mouth
Day−4
Day−5
Stage
Transcript
ENSDART00000002463
ENSDART00000164761

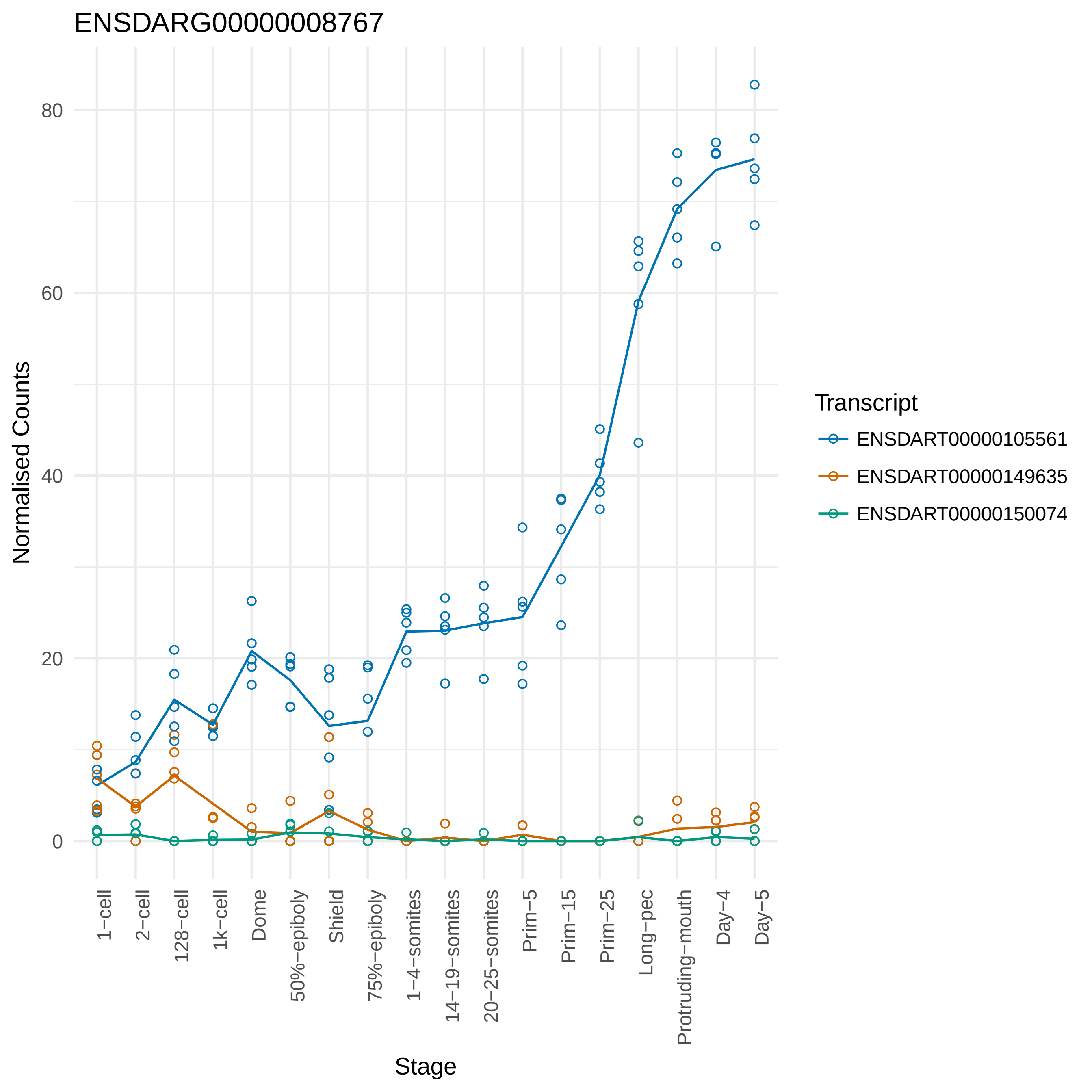
ENSDARG00000008767
Normalised Counts
0
20
40
60
80
1−cell
2−cell
128−cell
1k−cell
Dome
50%−epiboly
Shield
75%−epiboly
1−4−somites
14−19−somites
20−25−somites
Prim−5
Prim−15
Prim−25
Long−pec
Protruding−mouth
Day−4
Day−5
Stage
Transcript
ENSDART00000105561
ENSDART00000149635
ENSDART00000150074

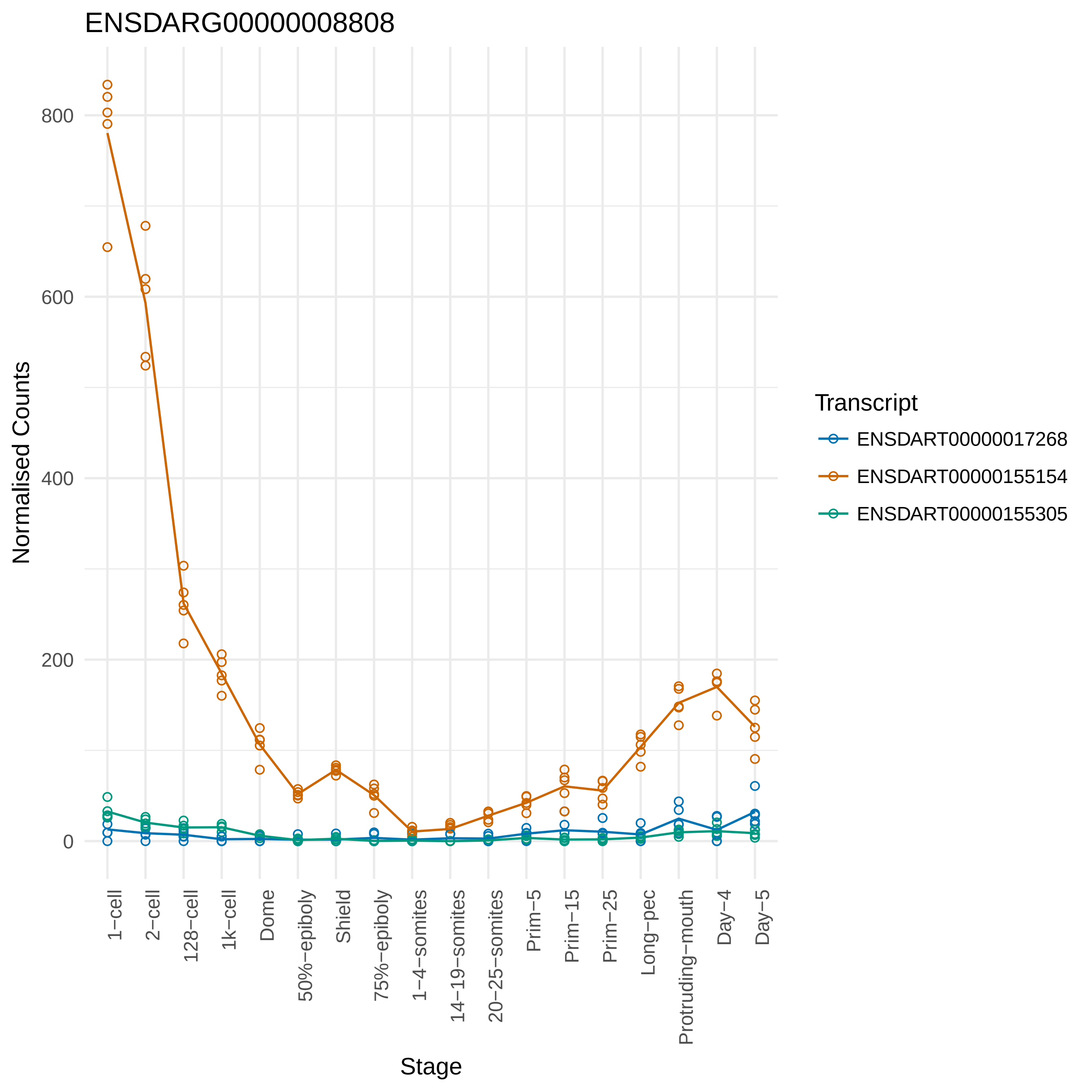

ENSDARG00000008808
Normalised Counts
800
600
400
200
0
1−cell
2−cell
128−cell
1k−cell
Dome
50%−epiboly
Shield
75%−epiboly
1−4−somites
14−19−somites
20−25−somites
Prim−5
Prim−15
Prim−25
Long−pec
Protruding−mouth
Day−4
Day−5
Stage
Transcript
ENSDART00000017268
ENSDART00000155154
ENSDART00000155305

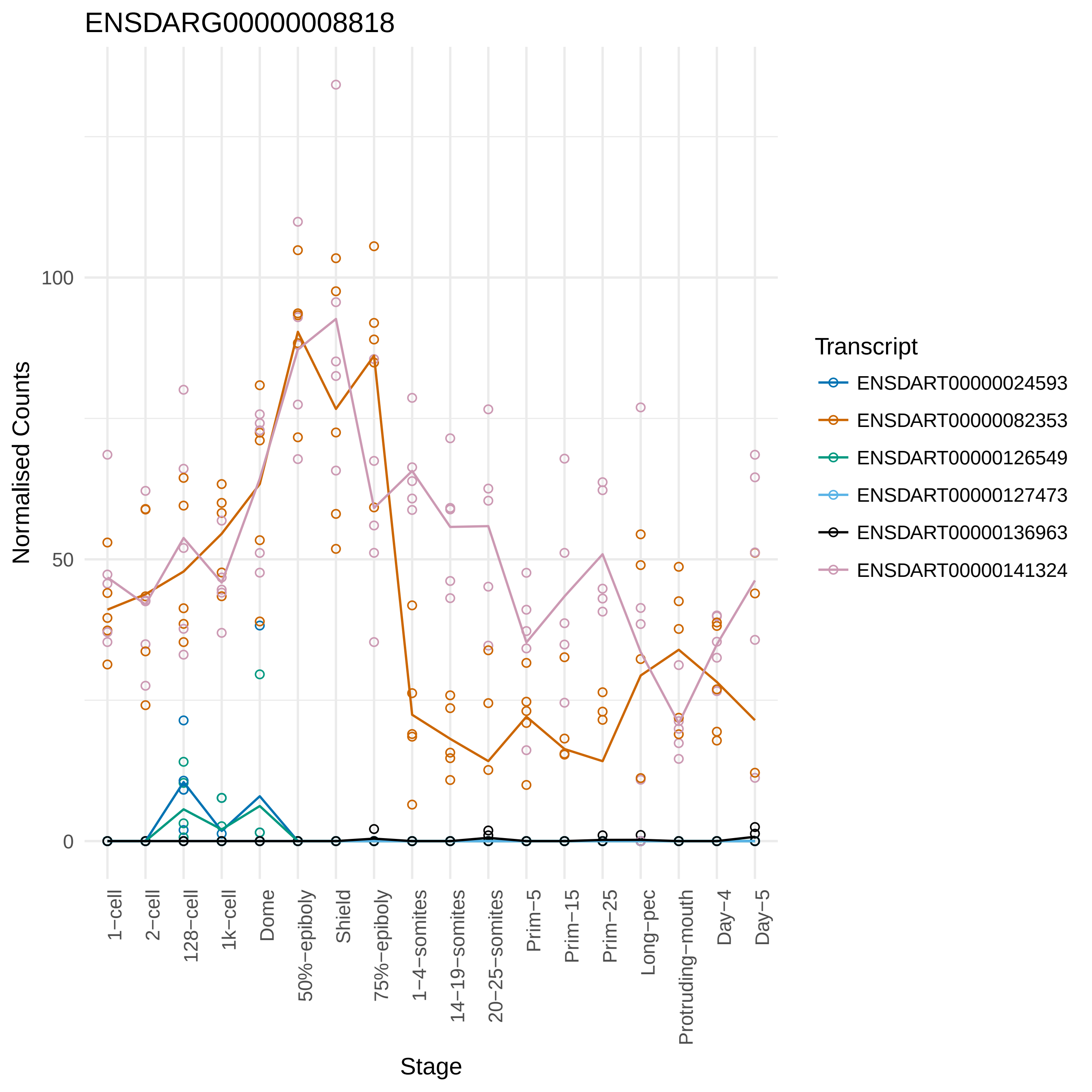

ENSDARG00000008818
Normalised Counts
100
50
0
1−cell
2−cell
128−cell
1k−cell
Dome
50%−epiboly
Shield
75%−epiboly
1−4−somites
14−19−somites
20−25−somites
Prim−5
Prim−15
Prim−25
Long−pec
Protruding−mouth
Day−4
Day−5
Stage
Transcript
ENSDART00000024593
ENSDART00000082353
ENSDART00000126549
ENSDART00000127473
ENSDART00000136963
ENSDART00000141324

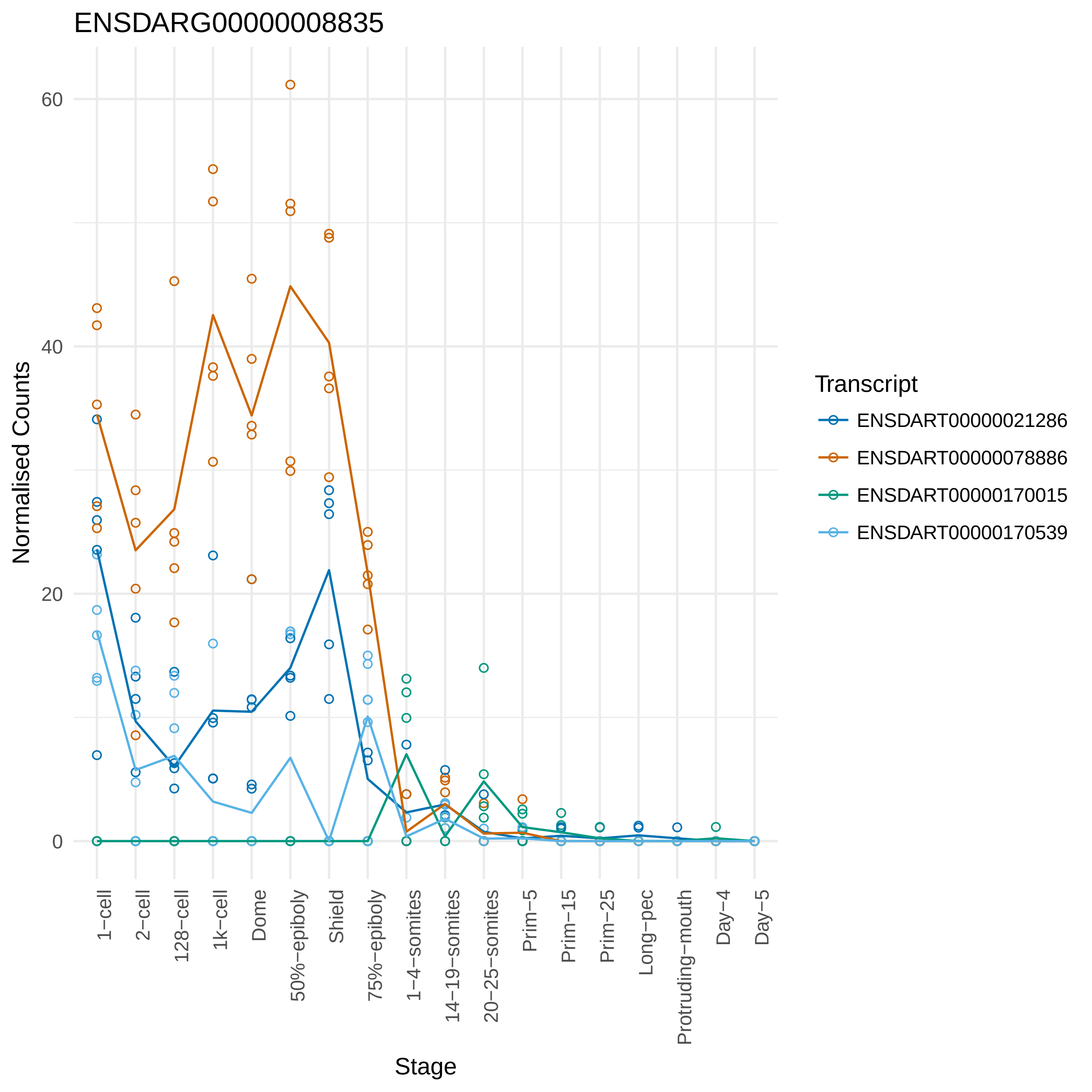

ENSDARG00000008835
Normalised Counts
60
40
20
0
1−cell
2−cell
128−cell
1k−cell
Dome
50%−epiboly
Shield
75%−epiboly
1−4−somites
14−19−somites
20−25−somites
Prim−5
Prim−15
Prim−25
Long−pec
Protruding−mouth
Day−4
Day−5
Stage
Transcript
ENSDART00000021286
ENSDART00000078886
ENSDART00000170015
ENSDART00000170539

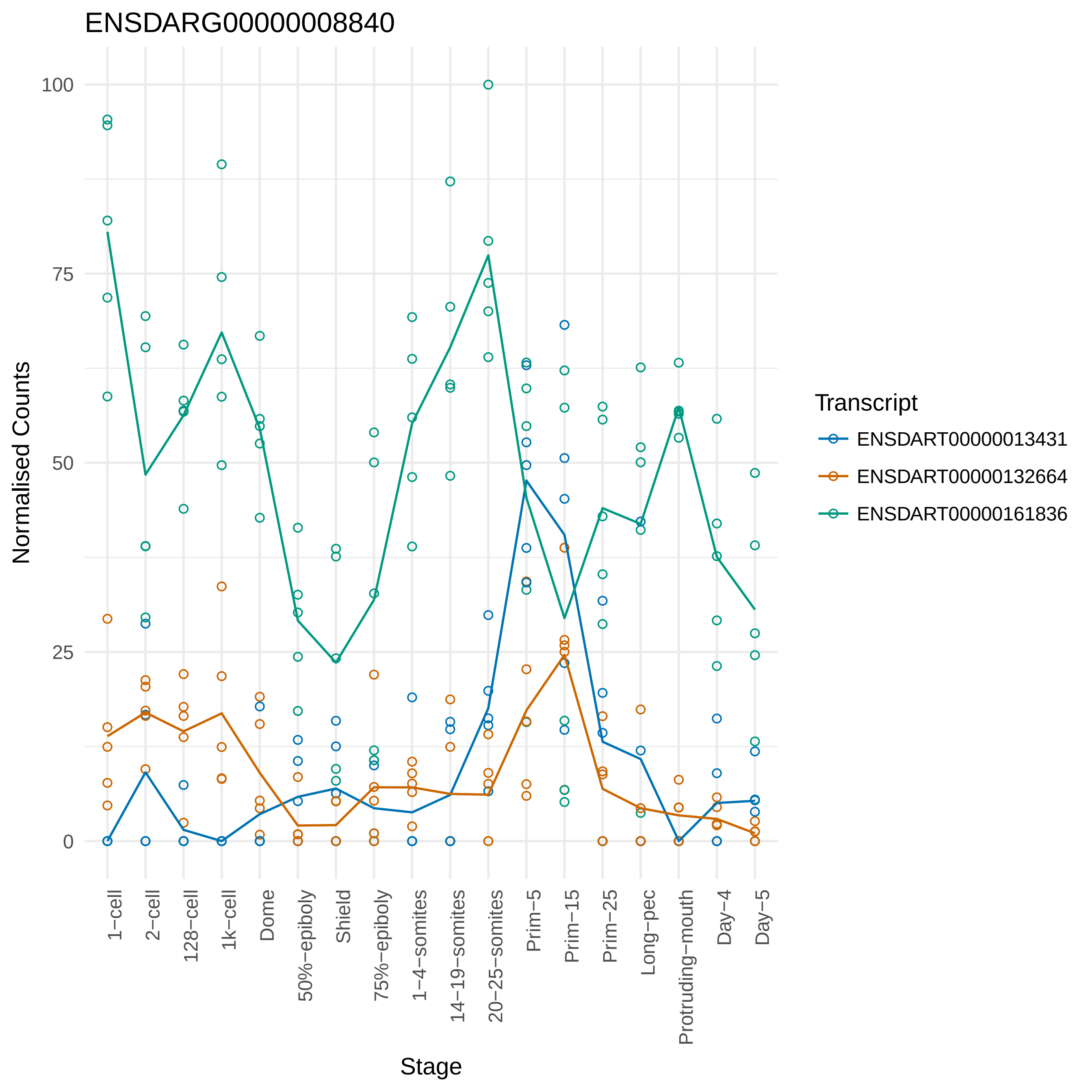
ENSDARG00000008840
Normalised Counts
100
75
50
25
0
1−cell
2−cell
128−cell
1k−cell
Dome
50%−epiboly
Shield
75%−epiboly
1−4−somites
14−19−somites
20−25−somites
Prim−5
Prim−15
Prim−25
Long−pec
Protruding−mouth
Day−4
Day−5
Stage
Transcript
ENSDART00000013431
ENSDART00000132664
ENSDART00000161836

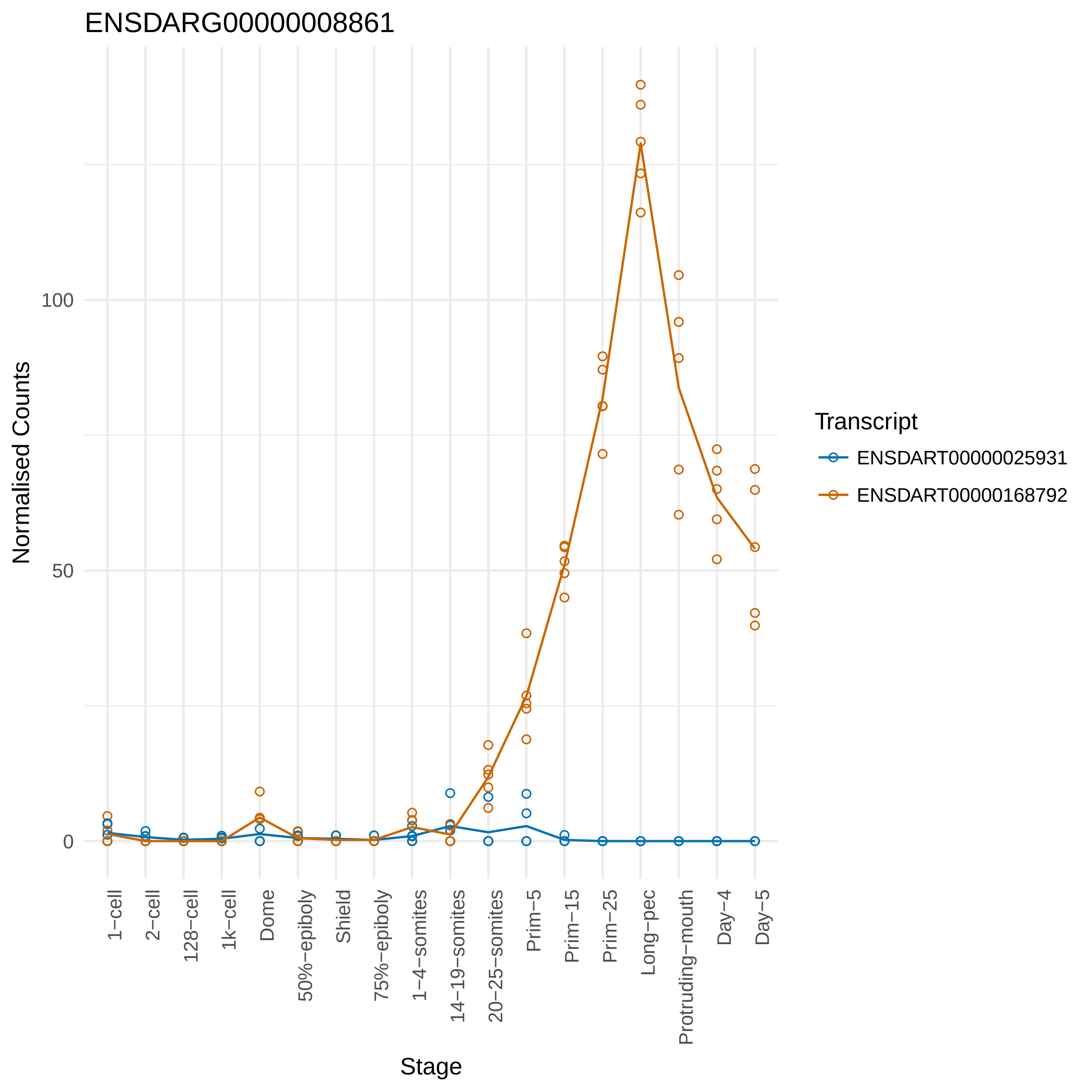

ENSDARG00000008861
Normalised Counts
100
50
0
1−cell
2−cell
128−cell
1k−cell
Dome
50%−epiboly
Shield
75%−epiboly
1−4−somites
14−19−somites
20−25−somites
Prim−5
Prim−15
Prim−25
Long−pec
Protruding−mouth
Day−4
Day−5
Stage
Transcript
ENSDART00000025931
ENSDART00000168792

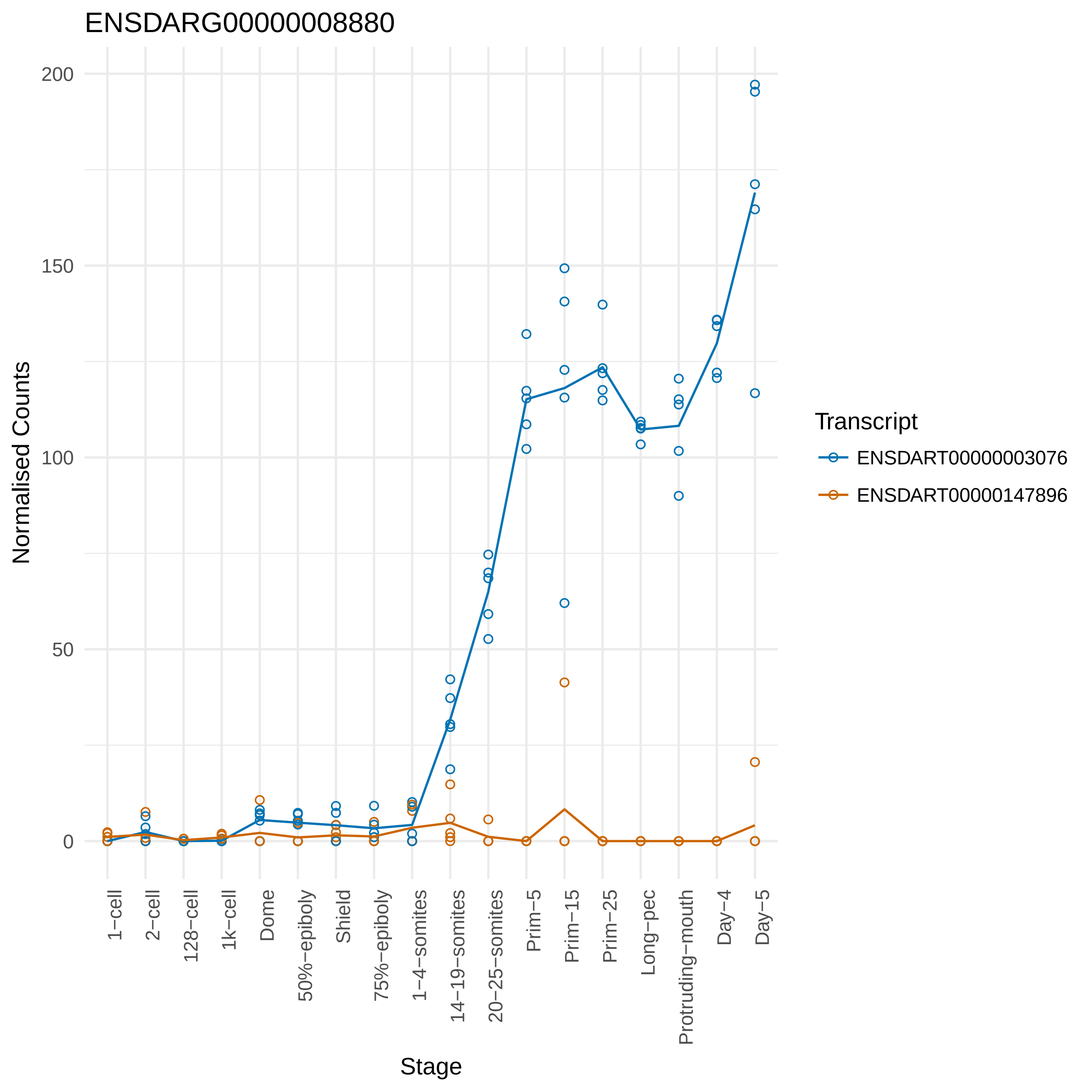
ENSDARG00000008880
Normalised Counts
200
150
100
50
0
1−cell
2−cell
128−cell
1k−cell
Dome
50%−epiboly
Shield
75%−epiboly
1−4−somites
14−19−somites
20−25−somites
Prim−5
Prim−15
Prim−25
Long−pec
Protruding−mouth
Day−4
Day−5
Stage
Transcript
ENSDART00000003076
ENSDART00000147896

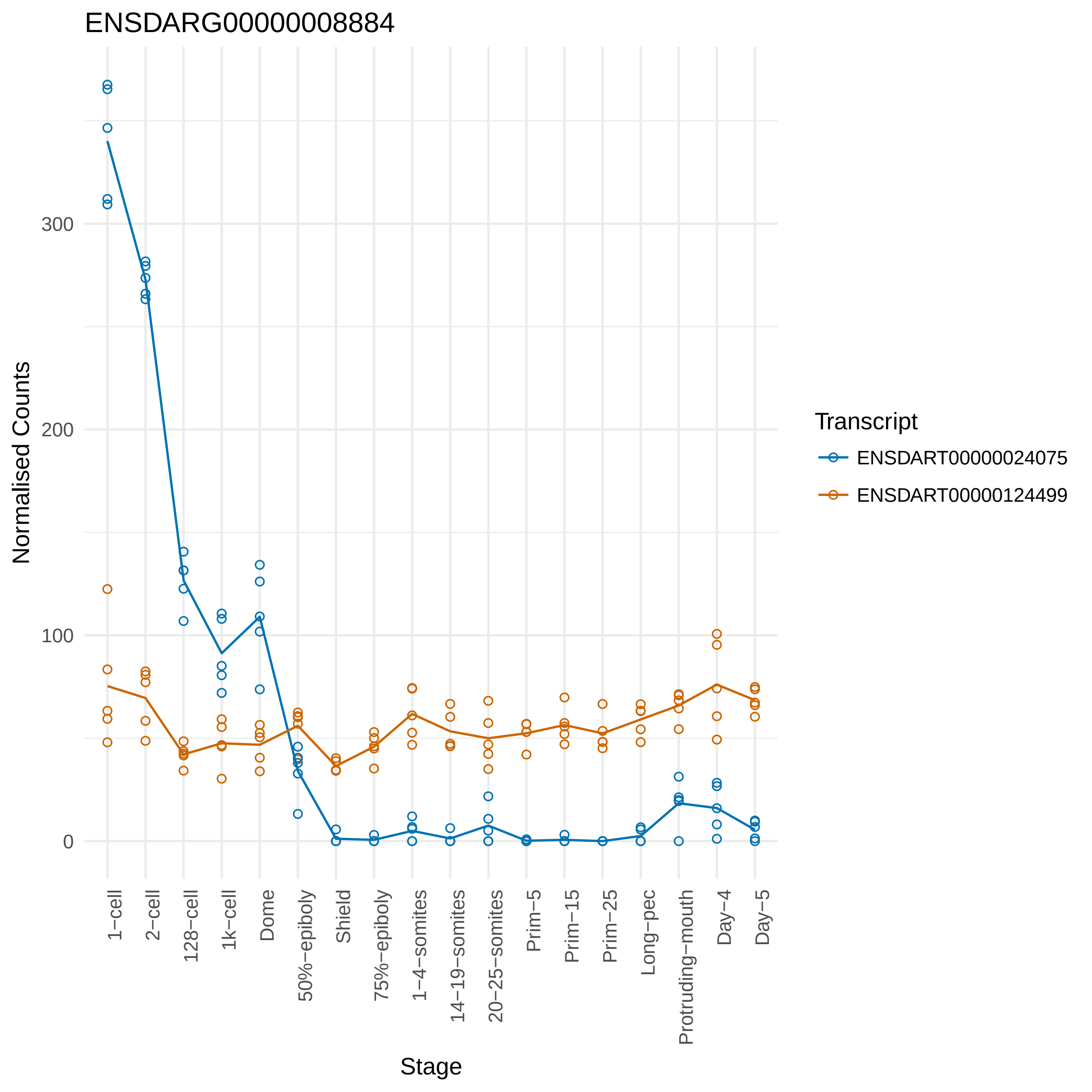
ENSDARG00000008884
Normalised Counts
300
200
100
0
1−cell
2−cell
128−cell
1k−cell
Dome
50%−epiboly
Shield
75%−epiboly
1−4−somites
14−19−somites
20−25−somites
Prim−5
Prim−15
Prim−25
Long−pec
Protruding−mouth
Day−4
Day−5
Stage
Transcript
ENSDART00000024075
ENSDART00000124499

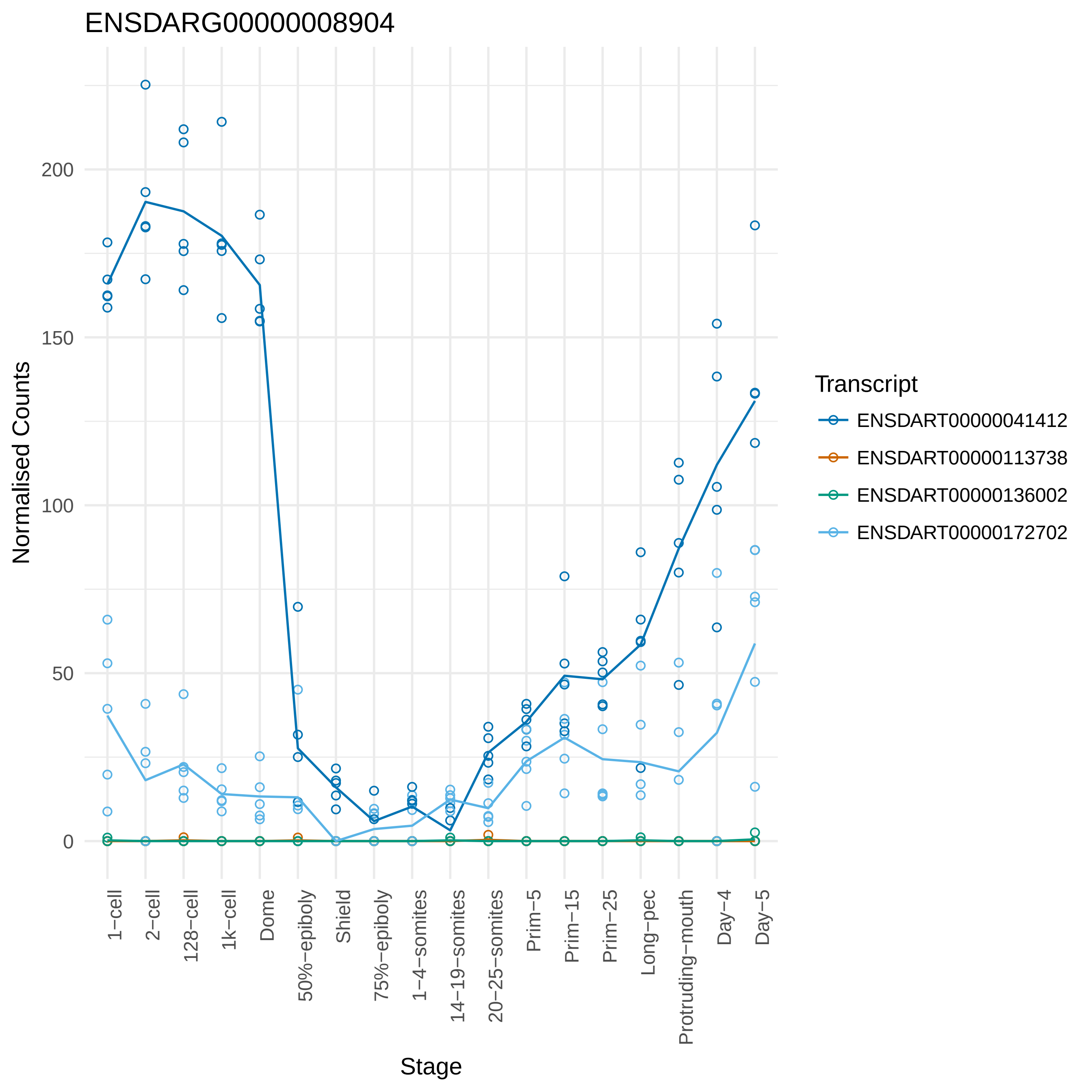
ENSDARG00000008904
Normalised Counts
200
150
100
50
0
1−cell
2−cell
128−cell
1k−cell
Dome
50%−epiboly
Shield
75%−epiboly
1−4−somites
14−19−somites
20−25−somites
Prim−5
Prim−15
Prim−25
Long−pec
Protruding−mouth
Day−4
Day−5
Stage
Transcript
ENSDART00000041412
ENSDART00000113738
ENSDART00000136002
ENSDART00000172702

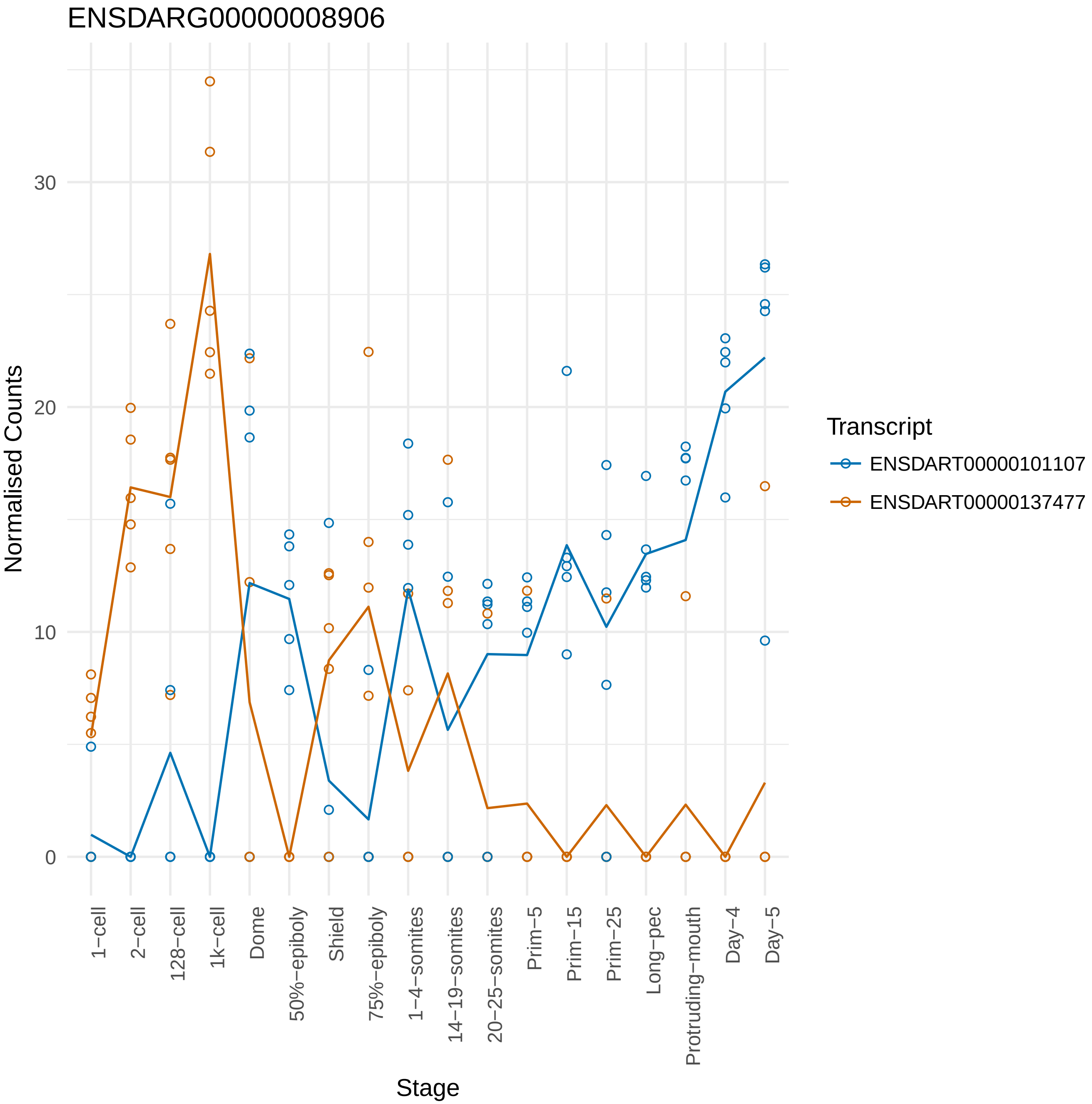
ENSDARG00000008906
Normalised Counts
0
10
20
30
1−cell
2−cell
128−cell
1k−cell
Dome
50%−epiboly
Shield
75%−epiboly
1−4−somites
14−19−somites
20−25−somites
Prim−5
Prim−15
Prim−25
Long−pec
Protruding−mouth
Day−4
Day−5
Stage
Transcript
ENSDART00000101107
ENSDART00000137477

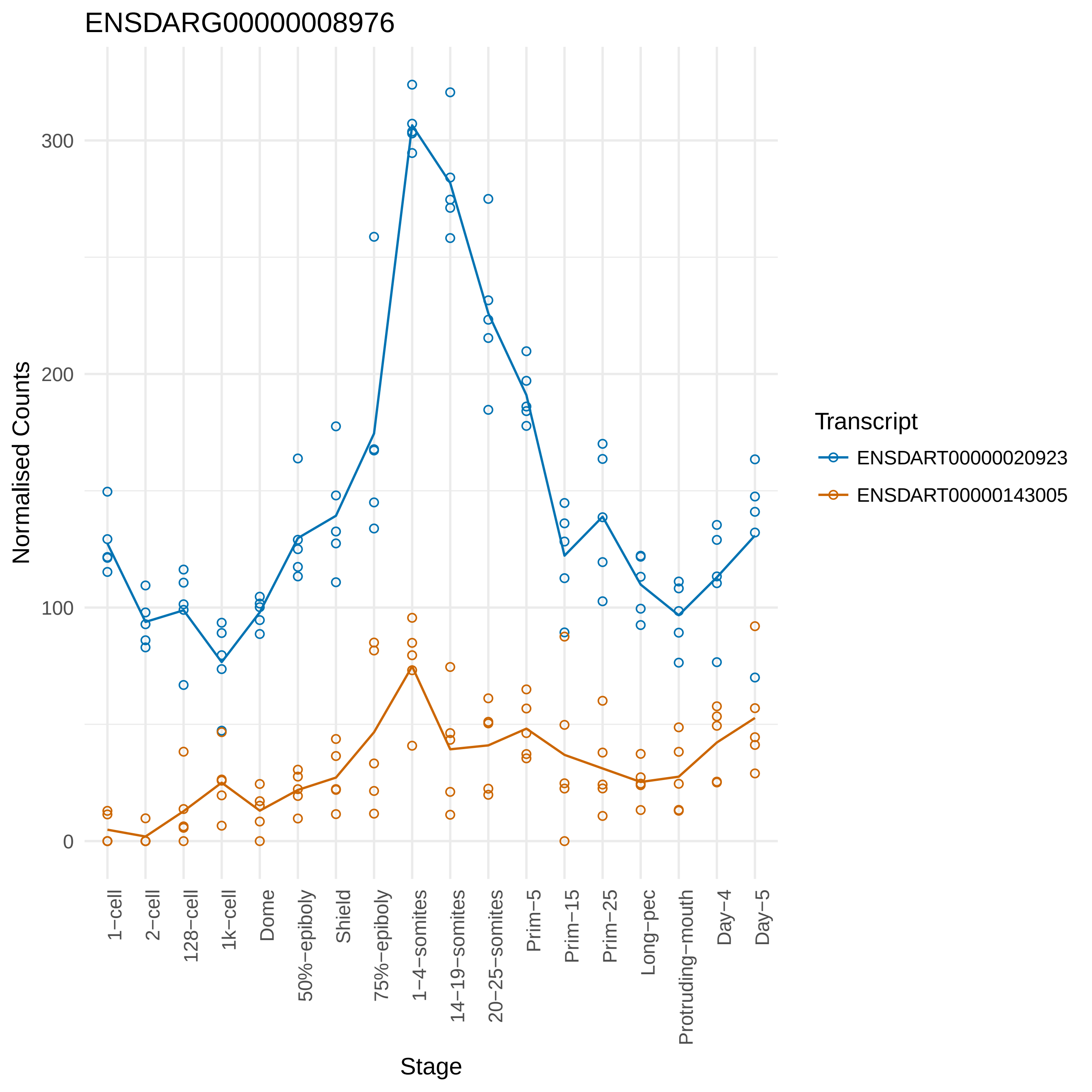

ENSDARG00000008976
Normalised Counts
300
200
100
0
1−cell
2−cell
128−cell
1k−cell
Dome
50%−epiboly
Shield
75%−epiboly
1−4−somites
14−19−somites
20−25−somites
Prim−5
Prim−15
Prim−25
Long−pec
Protruding−mouth
Day−4
Day−5
Stage
Transcript
ENSDART00000020923
ENSDART00000143005

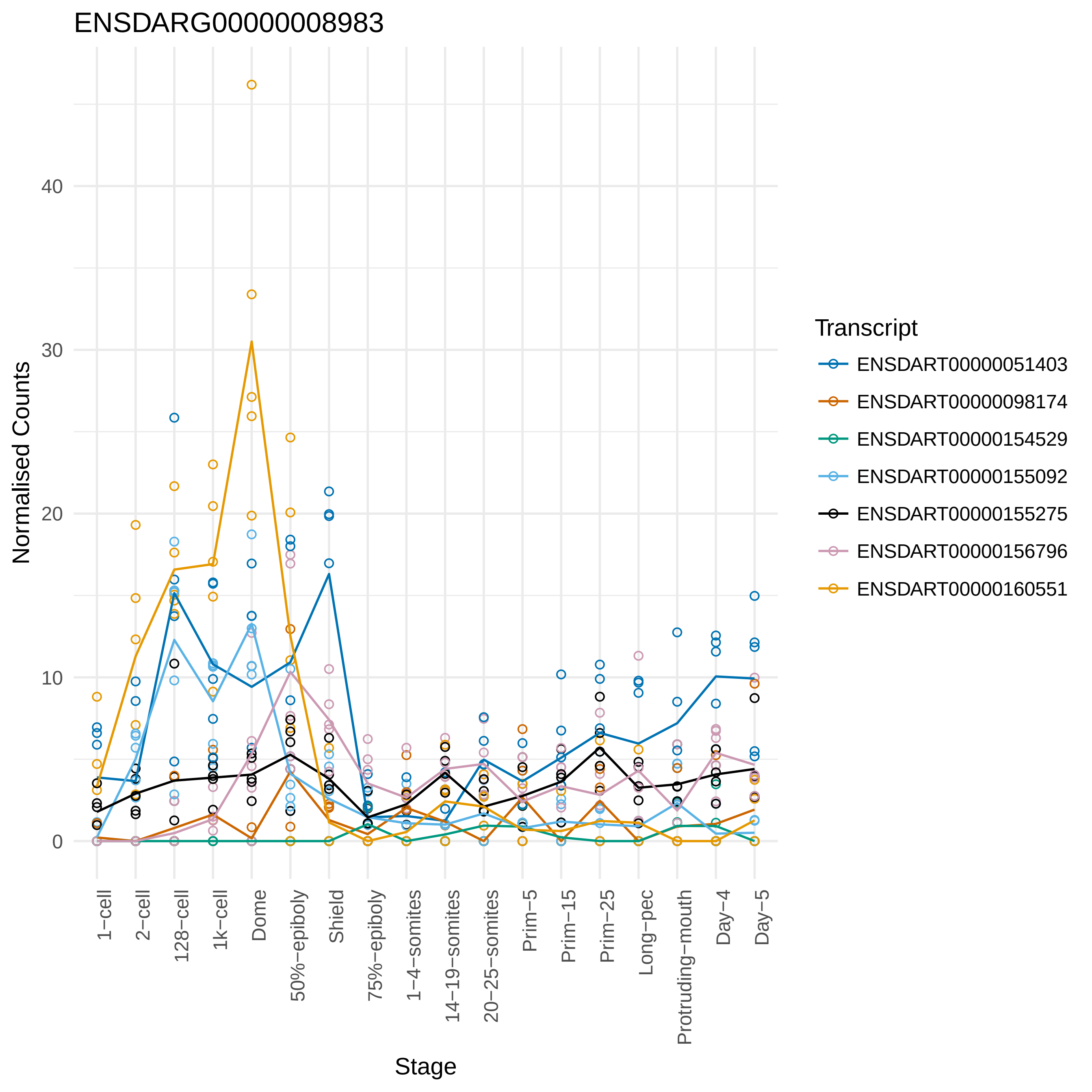
ENSDARG00000008983
Normalised Counts
0
10
20
30
40
Stage
1−cell
2−cell
128−cell
1k−cell
Dome
50%−epiboly
Shield
75%−epiboly
1−4−somites
14−19−somites
20−25−somites
Prim−5
Prim−15
Prim−25
Long−pec
Protruding−mouth
Day−4
Day−5
Transcript
ENSDART00000051403
ENSDART00000098174
ENSDART00000154529
ENSDART00000155092
ENSDART00000155275
ENSDART00000156796
ENSDART00000160551

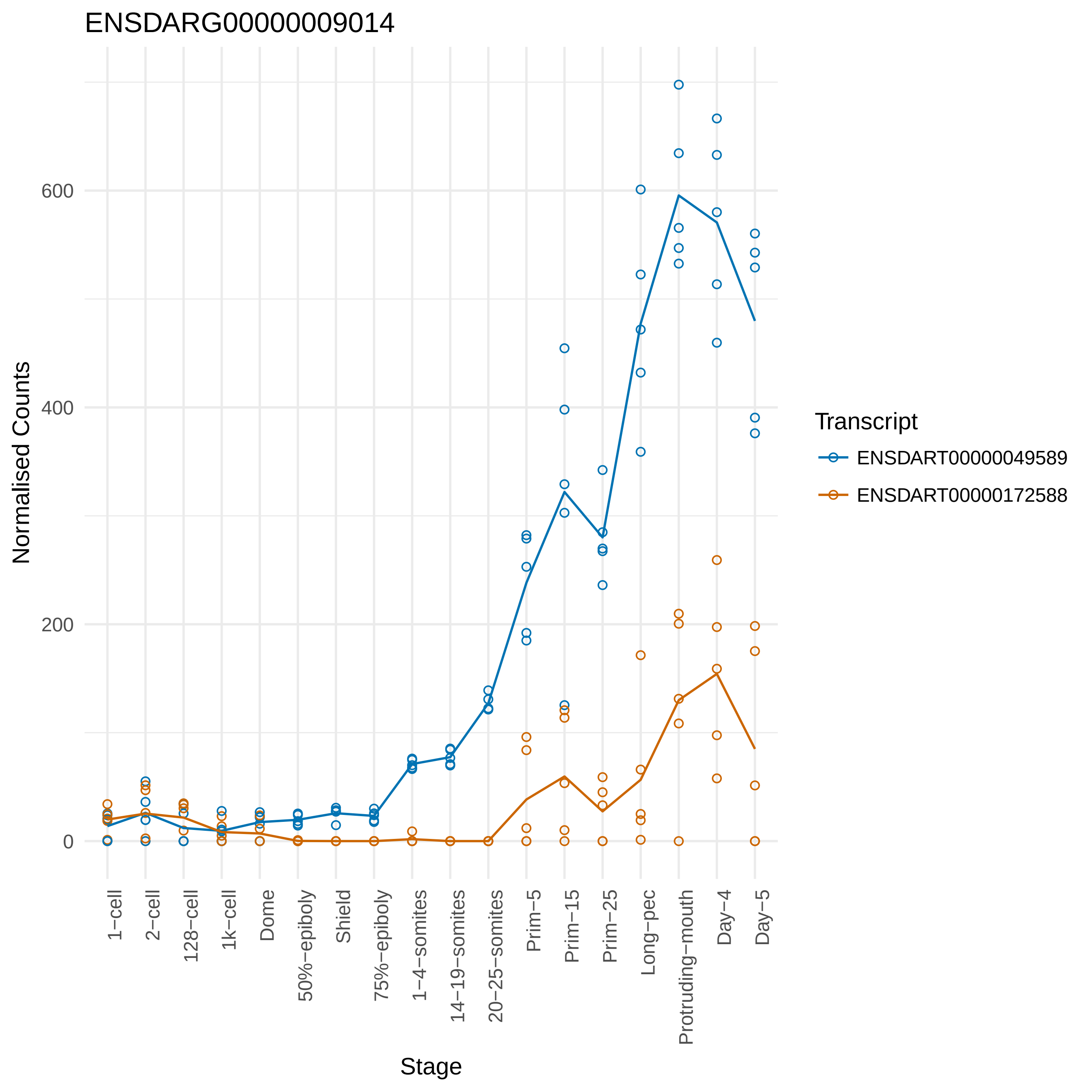

ENSDARG00000009014
Normalised Counts
600
400
200
0
1−cell
2−cell
128−cell
1k−cell
Dome
50%−epiboly
Shield
75%−epiboly
1−4−somites
14−19−somites
20−25−somites
Prim−5
Prim−15
Prim−25
Long−pec
Protruding−mouth
Day−4
Day−5
Stage
Transcript
ENSDART00000049589
ENSDART00000172588

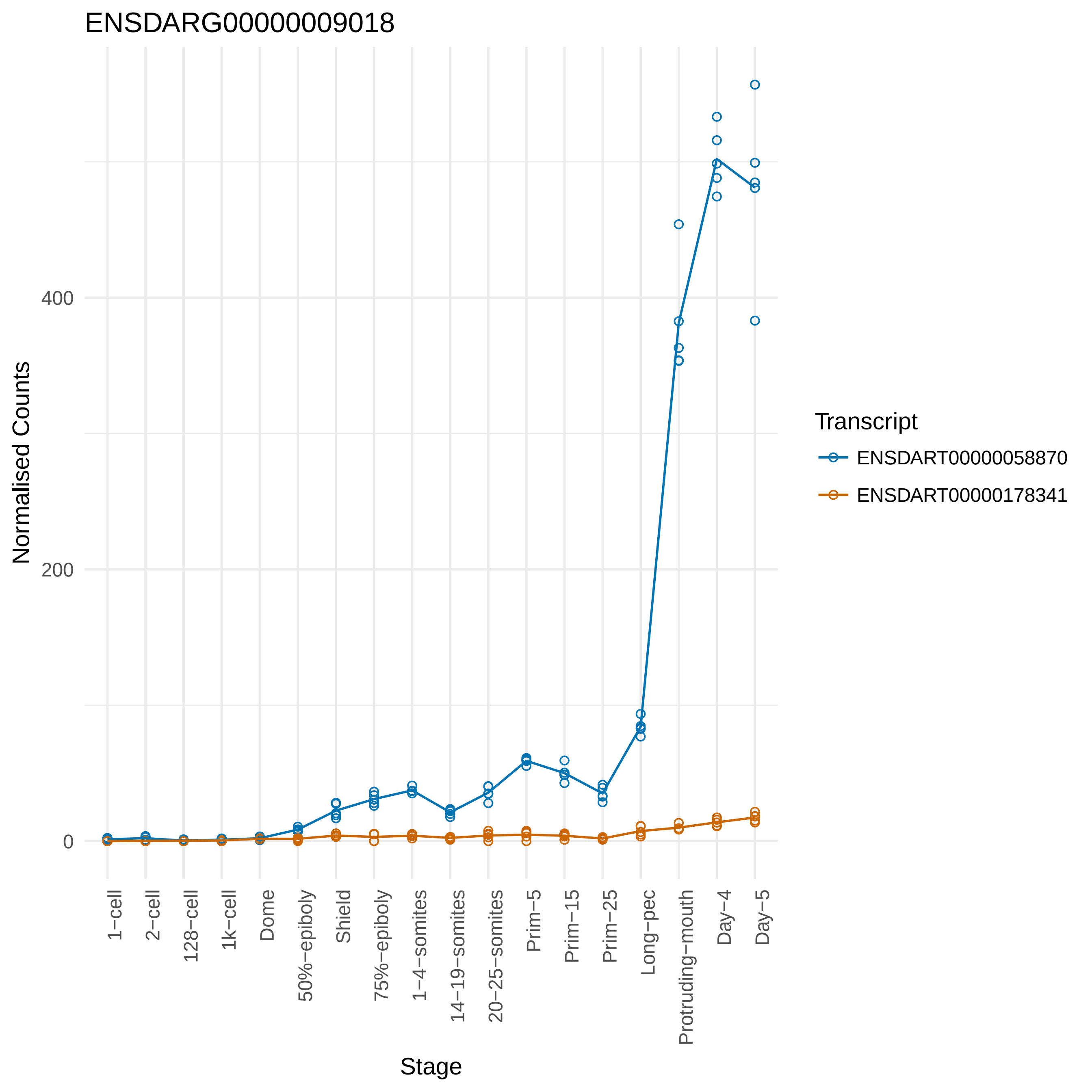

ENSDARG00000009018
Normalised Counts
0
200
400
1−cell
2−cell
128−cell
1k−cell
Dome
50%−epiboly
Shield
75%−epiboly
1−4−somites
14−19−somites
20−25−somites
Prim−5
Prim−15
Prim−25
Long−pec
Protruding−mouth
Day−4
Day−5
Stage
Transcript
ENSDART00000058870
ENSDART00000178341

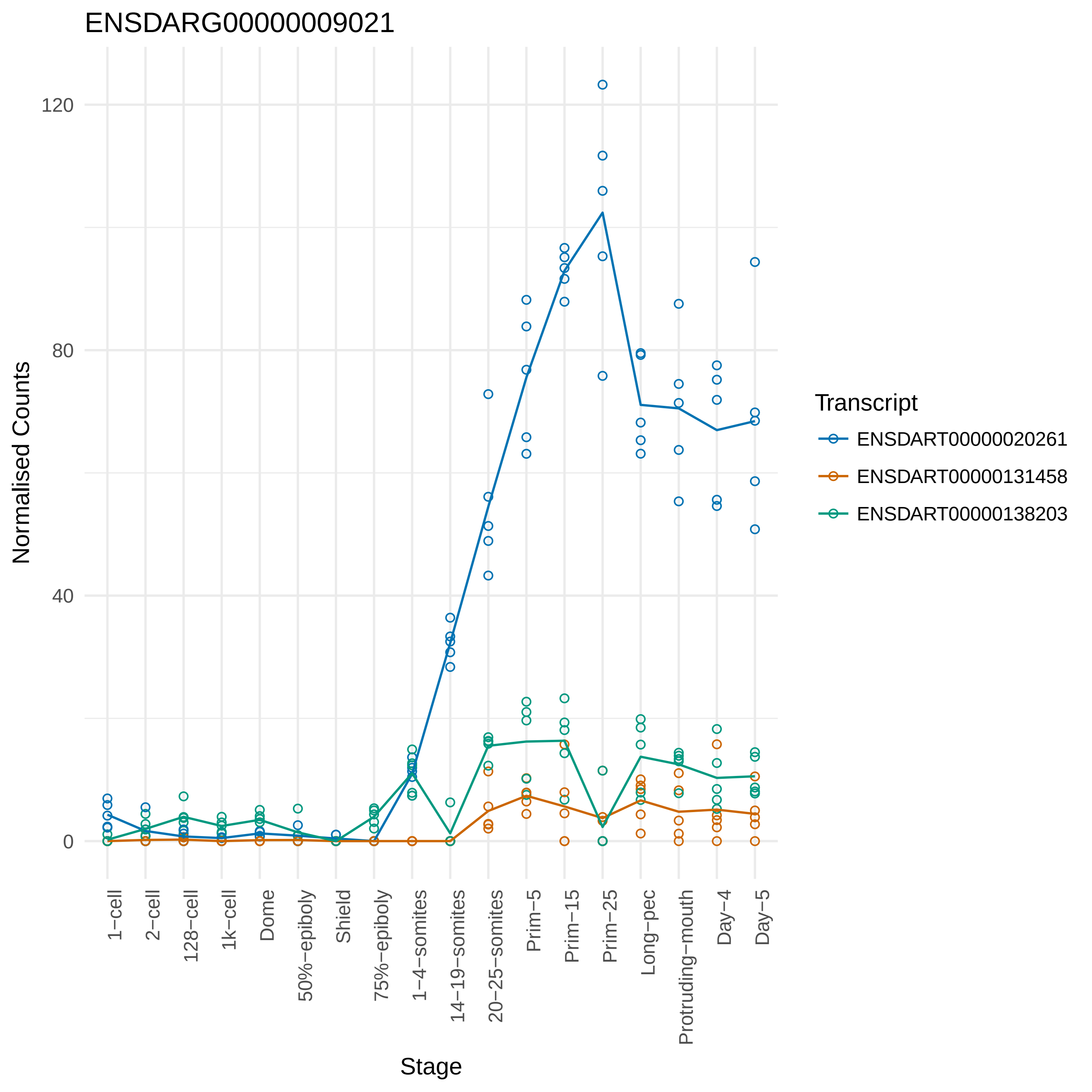

ENSDARG00000009021
Normalised Counts
120
80
40
0
1−cell
2−cell
128−cell
1k−cell
Dome
50%−epiboly
Shield
75%−epiboly
1−4−somites
14−19−somites
20−25−somites
Prim−5
Prim−15
Prim−25
Long−pec
Protruding−mouth
Day−4
Day−5
Stage
Transcript
ENSDART00000020261
ENSDART00000131458
ENSDART00000138203

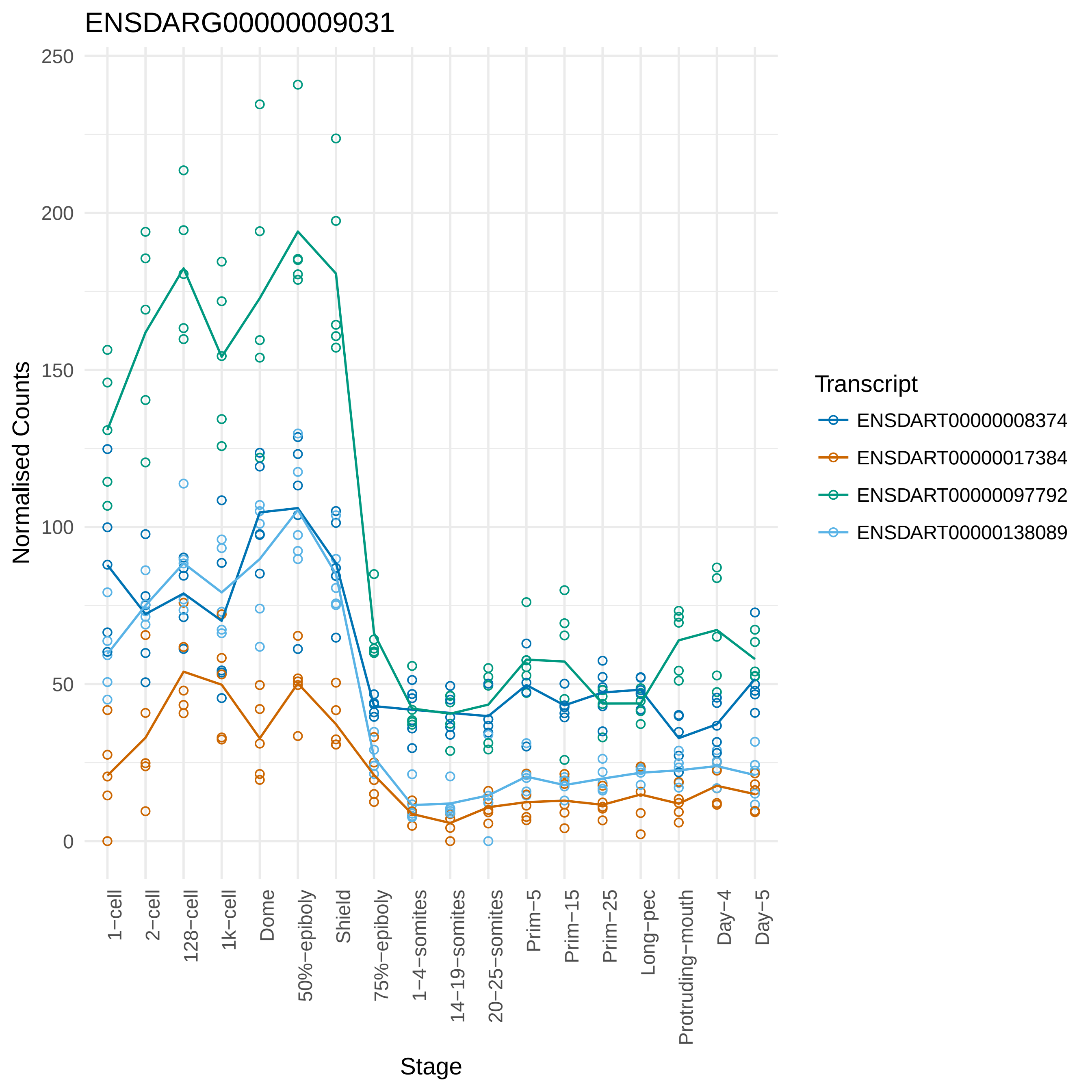

ENSDARG00000009031
Normalised Counts
250
200
150
100
50
0
1−cell
2−cell
128−cell
1k−cell
Dome
50%−epiboly
Shield
75%−epiboly
1−4−somites
14−19−somites
20−25−somites
Prim−5
Prim−15
Prim−25
Long−pec
Protruding−mouth
Day−4
Day−5
Stage
Transcript
ENSDART00000008374
ENSDART00000017384
ENSDART00000097792
ENSDART00000138089

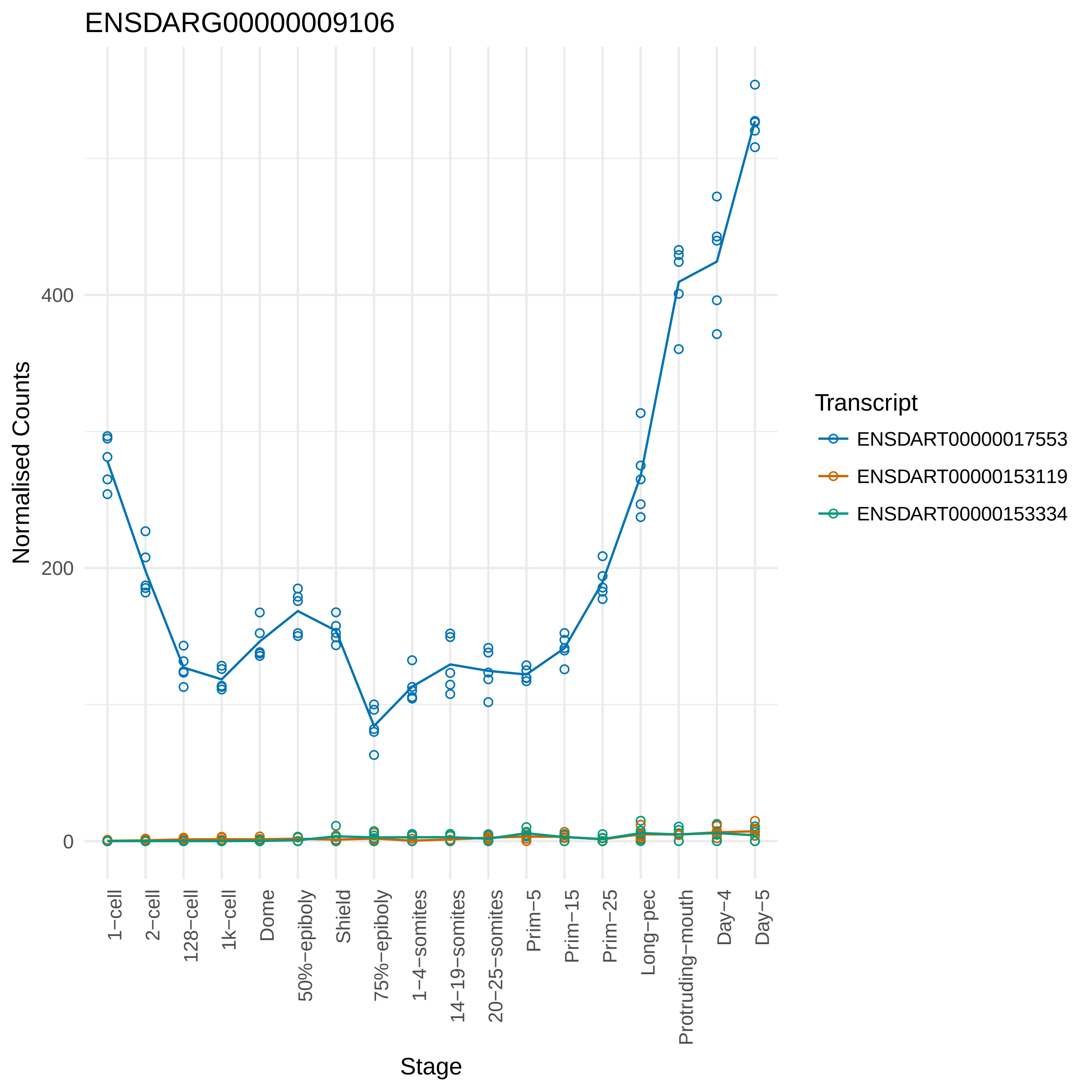

ENSDARG00000009106
Normalised Counts
0
200
400
Stage
1−cell
2−cell
128−cell
1k−cell
Dome
50%−epiboly
Shield
75%−epiboly
1−4−somites
14−19−somites
20−25−somites
Prim−5
Prim−15
Prim−25
Long−pec
Protruding−mouth
Day−4
Day−5
Transcript
ENSDART00000017553
ENSDART00000153119
ENSDART00000153334

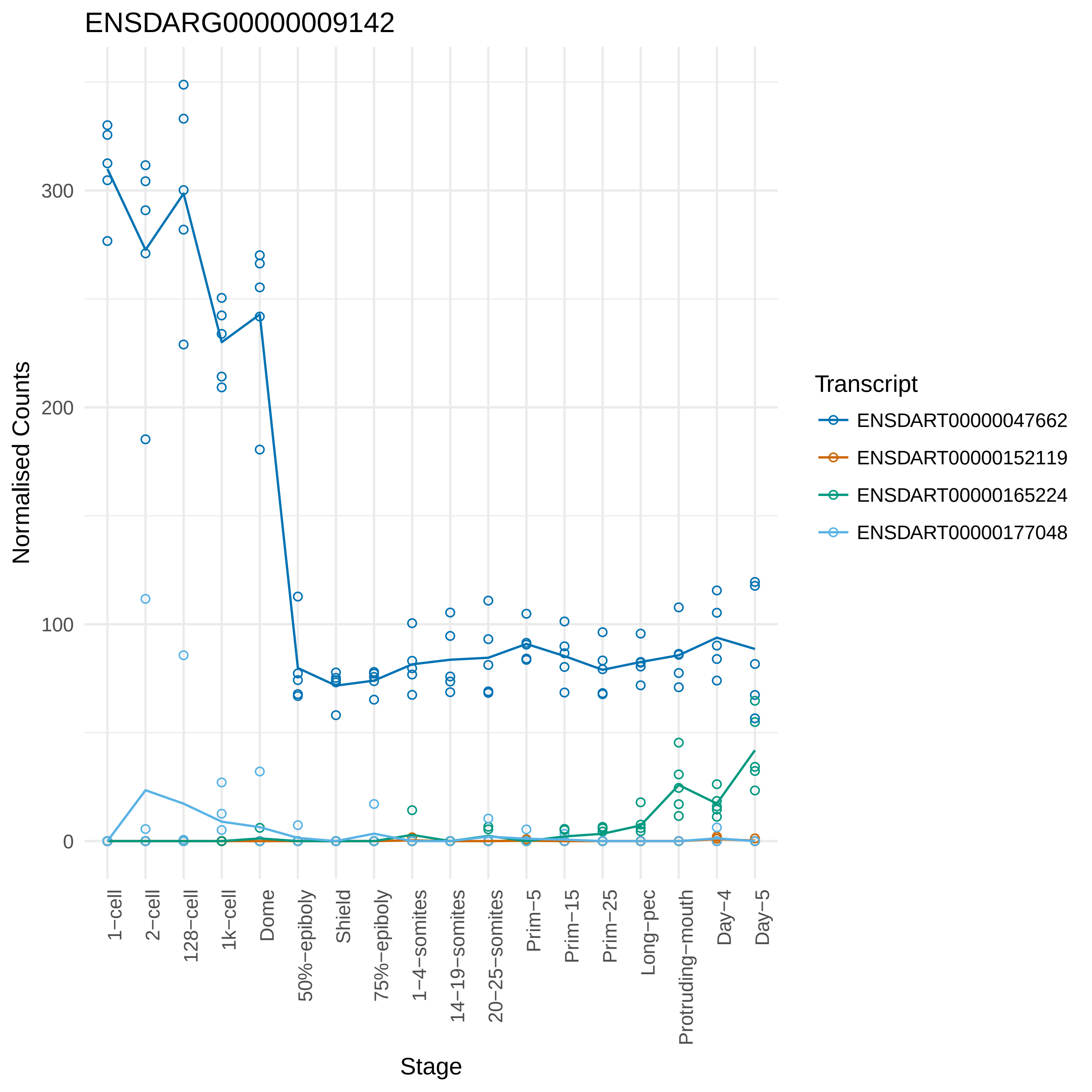
ENSDARG00000009142
Normalised Counts
300
200
100
0
1−cell
2−cell
128−cell
1k−cell
Dome
50%−epiboly
Shield
75%−epiboly
1−4−somites
14−19−somites
20−25−somites
Prim−5
Prim−15
Prim−25
Long−pec
Protruding−mouth
Day−4
Day−5
Stage
Transcript
ENSDART00000047662
ENSDART00000152119
ENSDART00000165224
ENSDART00000177048

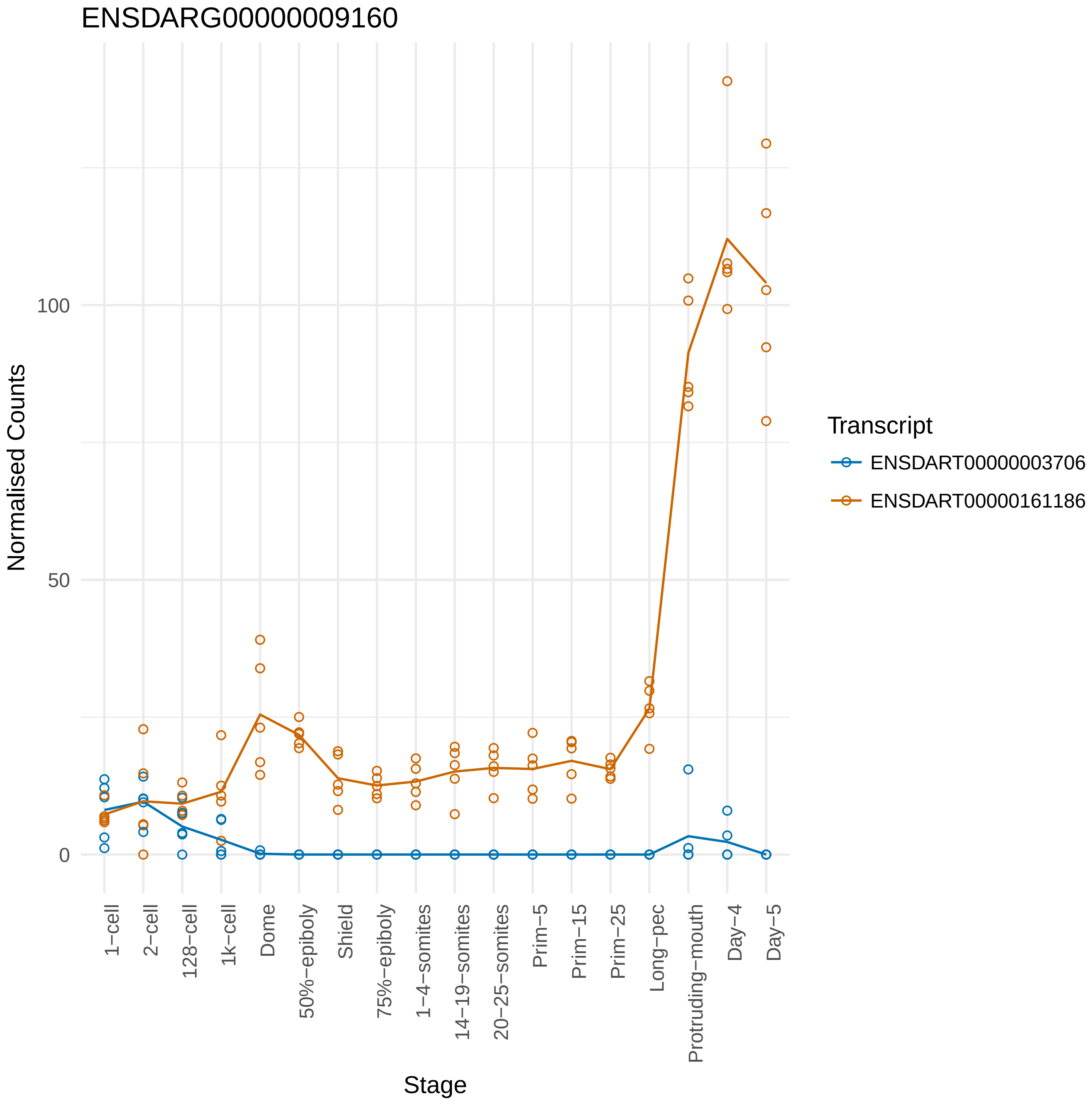

ENSDARG00000009160
Normalised Counts
0
50
100
Stage
1−cell
2−cell
128−cell
1k−cell
Dome
50%−epiboly
Shield
75%−epiboly
1−4−somites
14−19−somites
20−25−somites
Prim−5
Prim−15
Prim−25
Long−pec
Protruding−mouth
Day−4
Day−5
Transcript
ENSDART00000003706
ENSDART00000161186

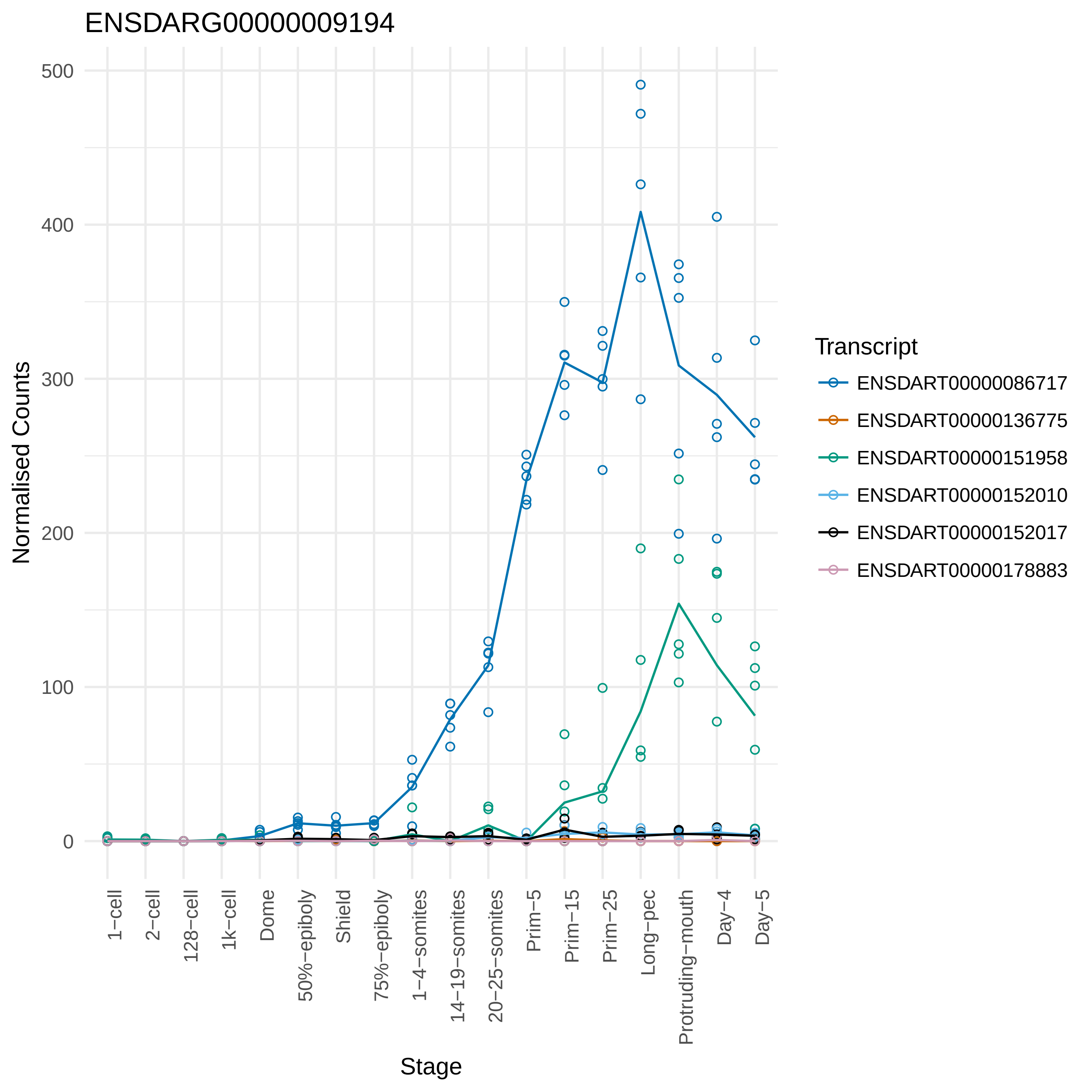

ENSDARG00000009194
Normalised Counts
0
100
200
300
400
500
1−cell
2−cell
128−cell
1k−cell
Dome
50%−epiboly
Shield
75%−epiboly
1−4−somites
14−19−somites
20−25−somites
Prim−5
Prim−15
Prim−25
Long−pec
Protruding−mouth
Day−4
Day−5
Stage
Transcript
ENSDART00000086717
ENSDART00000136775
ENSDART00000151958
ENSDART00000152010
ENSDART00000152017
ENSDART00000178883

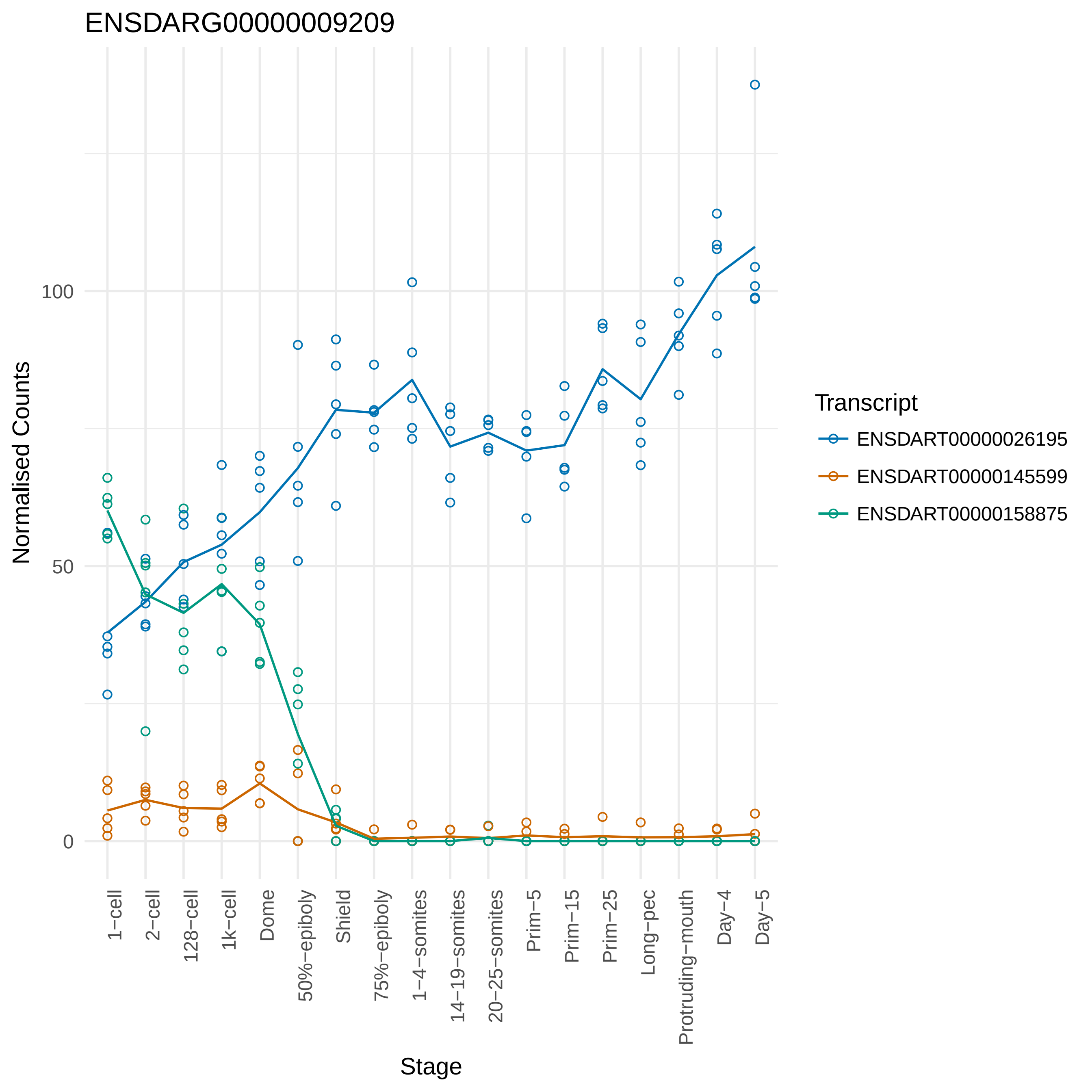
ENSDARG00000009209
Normalised Counts
100
50
0
Stage
1−cell
2−cell
128−cell
1k−cell
Dome
50%−epiboly
Shield
75%−epiboly
1−4−somites
14−19−somites
20−25−somites
Prim−5
Prim−15
Prim−25
Long−pec
Protruding−mouth
Day−4
Day−5
Transcript
ENSDART00000026195
ENSDART00000145599
ENSDART00000158875

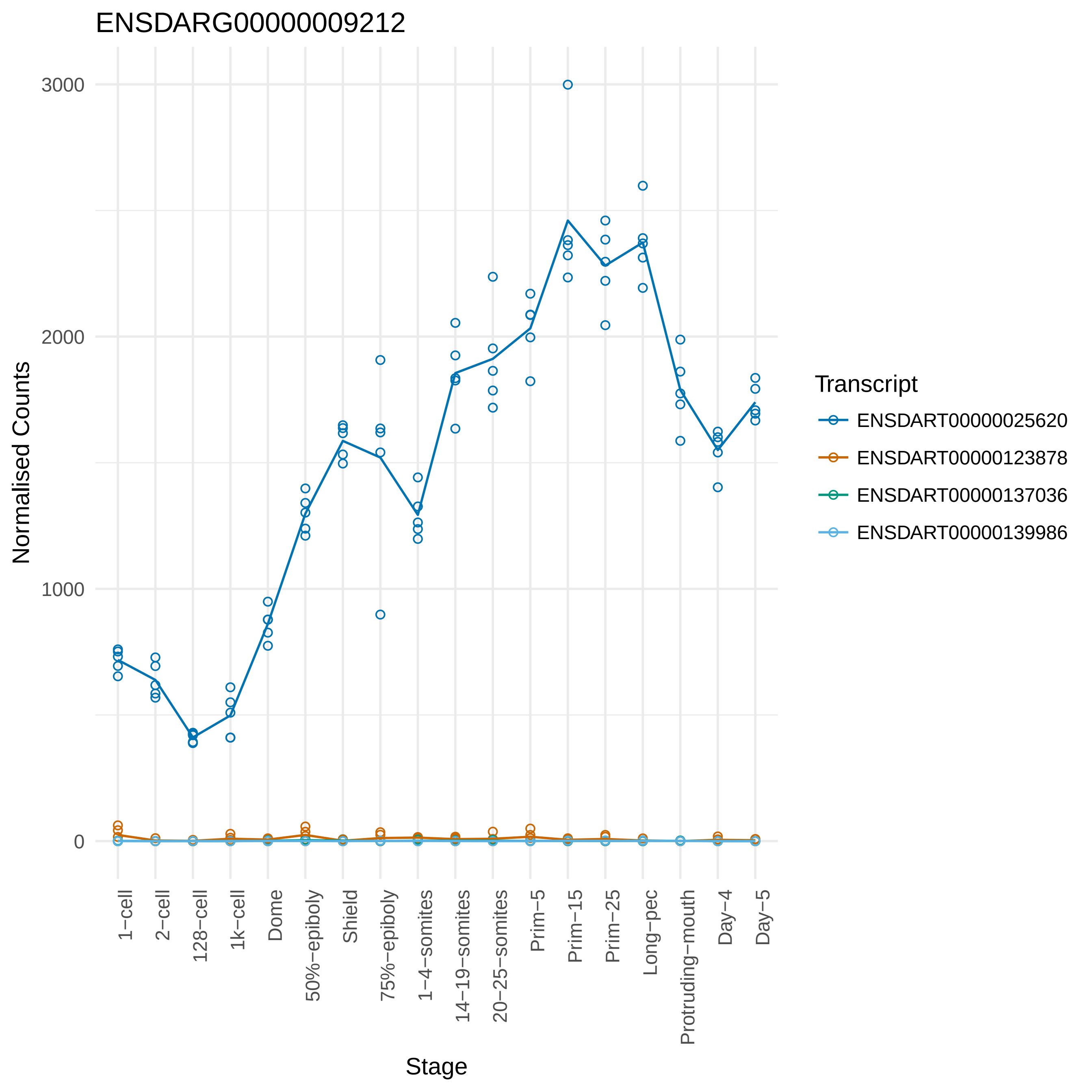
ENSDARG00000009212
Normalised Counts
3000
2000
1000
0
Stage
1−cell
2−cell
128−cell
1k−cell
Dome
50%−epiboly
Shield
75%−epiboly
1−4−somites
14−19−somites
20−25−somites
Prim−5
Prim−15
Prim−25
Long−pec
Protruding−mouth
Day−4
Day−5
Transcript
ENSDART00000025620
ENSDART00000123878
ENSDART00000137036
ENSDART00000139986

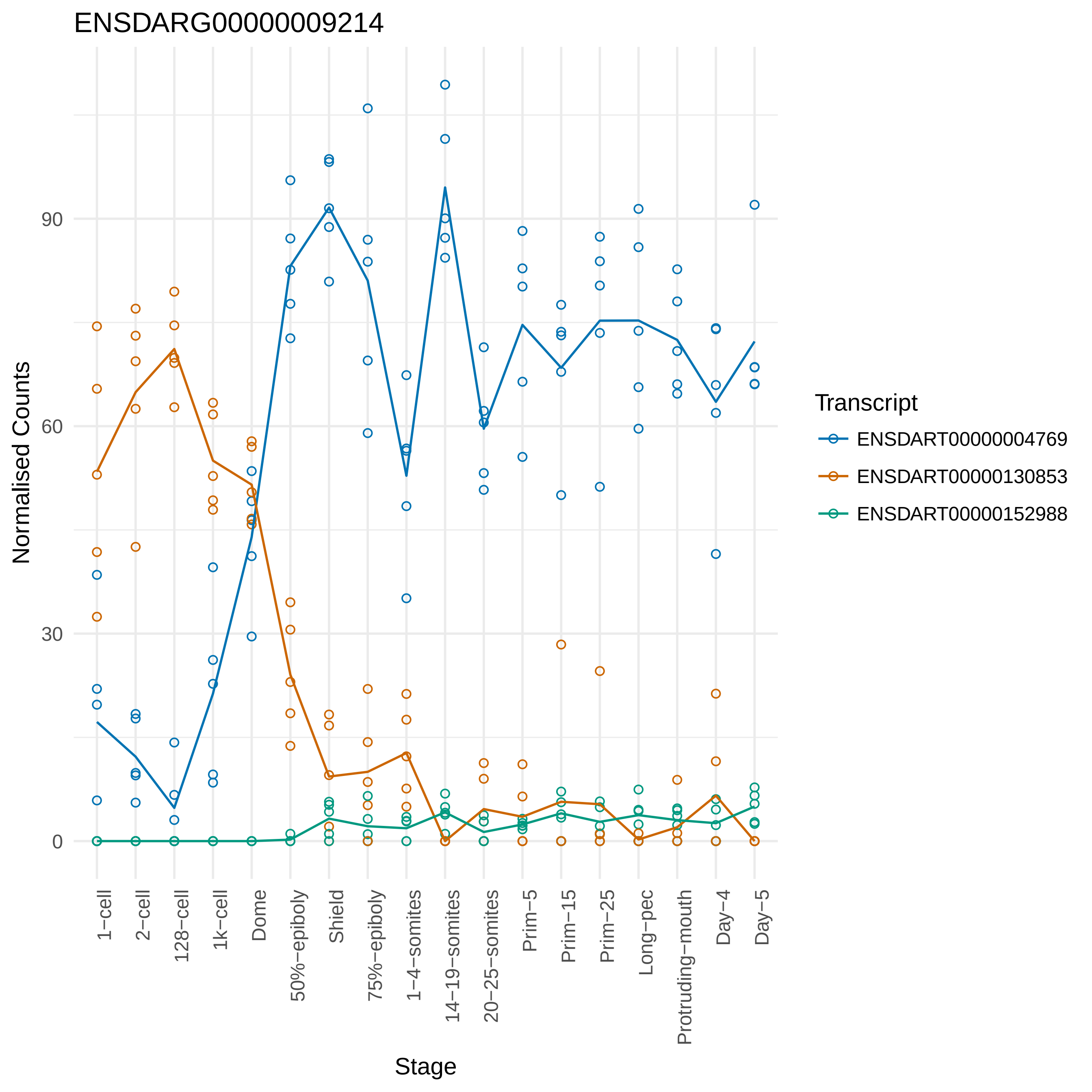
ENSDARG00000009214
Normalised Counts
0
30
60
90
Stage
1−cell
2−cell
128−cell
1k−cell
Dome
50%−epiboly
Shield
75%−epiboly
1−4−somites
14−19−somites
20−25−somites
Prim−5
Prim−15
Prim−25
Long−pec
Protruding−mouth
Day−4
Day−5
Transcript
ENSDART00000004769
ENSDART00000130853
ENSDART00000152988

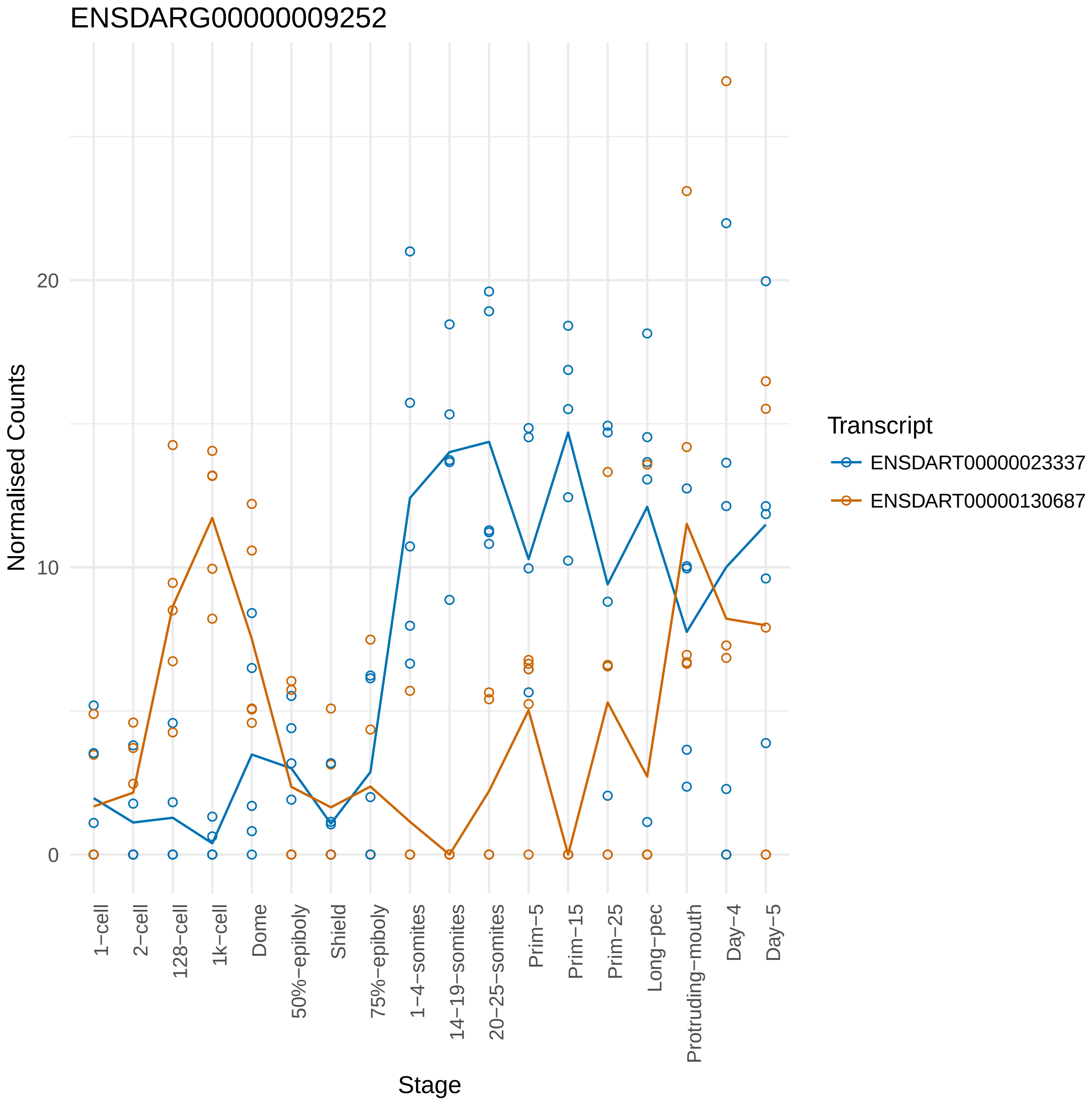
ENSDARG00000009252
Normalised Counts
0
10
20
Stage
1−cell
2−cell
128−cell
1k−cell
Dome
50%−epiboly
Shield
75%−epiboly
1−4−somites
14−19−somites
20−25−somites
Prim−5
Prim−15
Prim−25
Long−pec
Protruding−mouth
Day−4
Day−5
Transcript
ENSDART00000023337
ENSDART00000130687

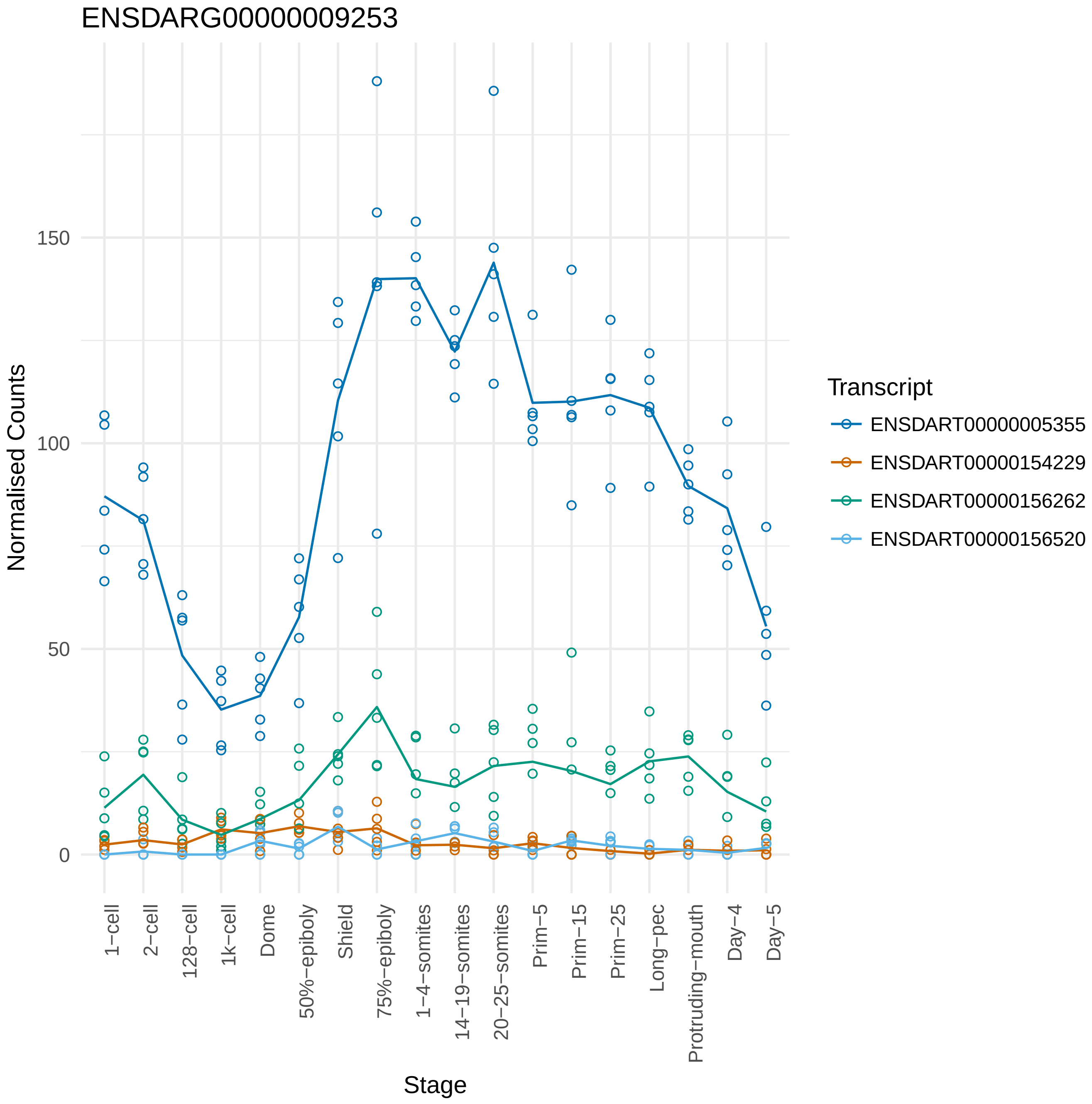
ENSDARG00000009253
Normalised Counts
0
50
100
150
Stage
1−cell
2−cell
128−cell
1k−cell
Dome
50%−epiboly
Shield
75%−epiboly
1−4−somites
14−19−somites
20−25−somites
Prim−5
Prim−15
Prim−25
Long−pec
Protruding−mouth
Day−4
Day−5
Transcript
ENSDART00000005355
ENSDART00000154229
ENSDART00000156262
ENSDART00000156520

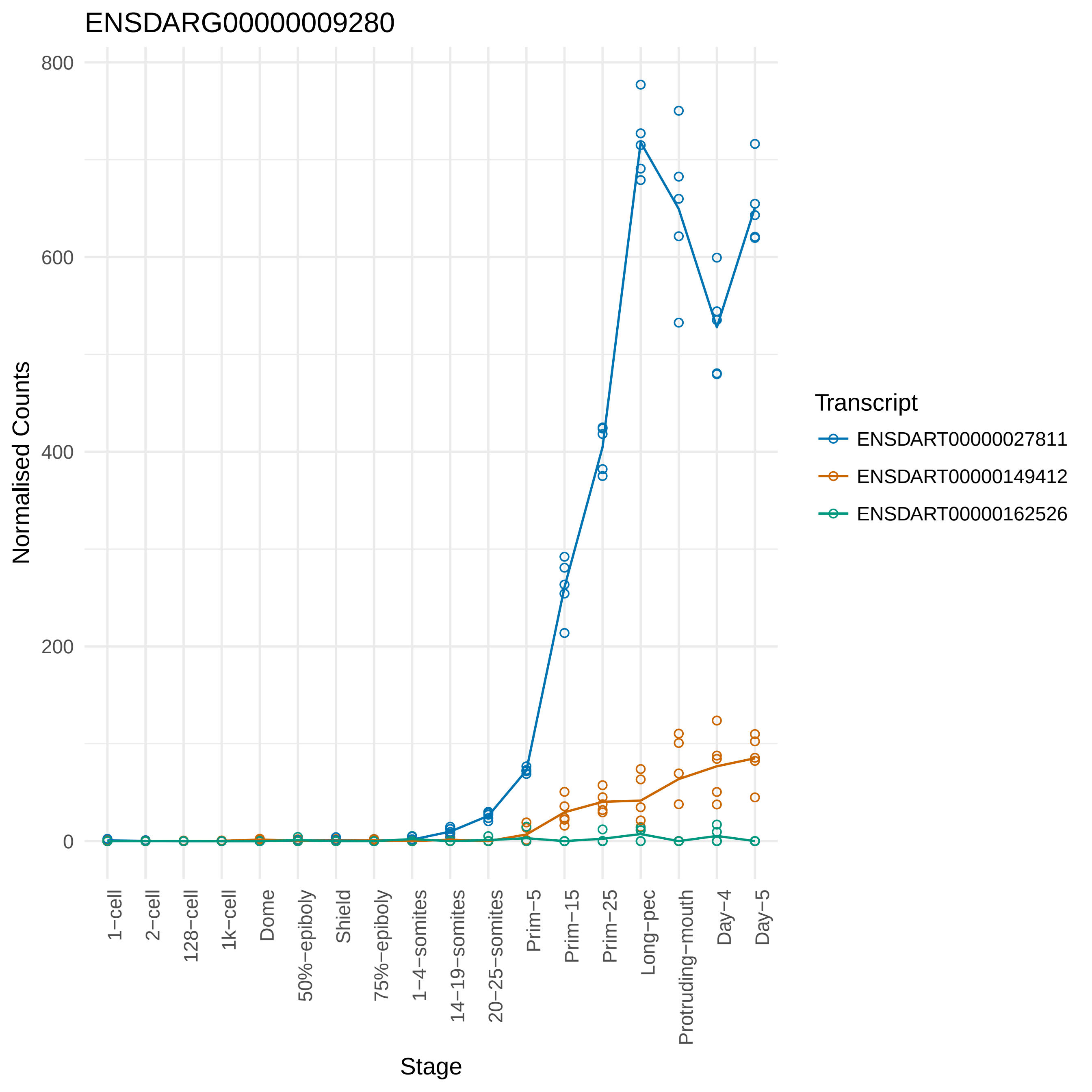

ENSDARG00000009280
Normalised Counts
800
600
400
200
0
1−cell
2−cell
128−cell
1k−cell
Dome
50%−epiboly
Shield
75%−epiboly
1−4−somites
14−19−somites
20−25−somites
Prim−5
Prim−15
Prim−25
Long−pec
Protruding−mouth
Day−4
Day−5
Stage
Transcript
ENSDART00000027811
ENSDART00000149412
ENSDART00000162526

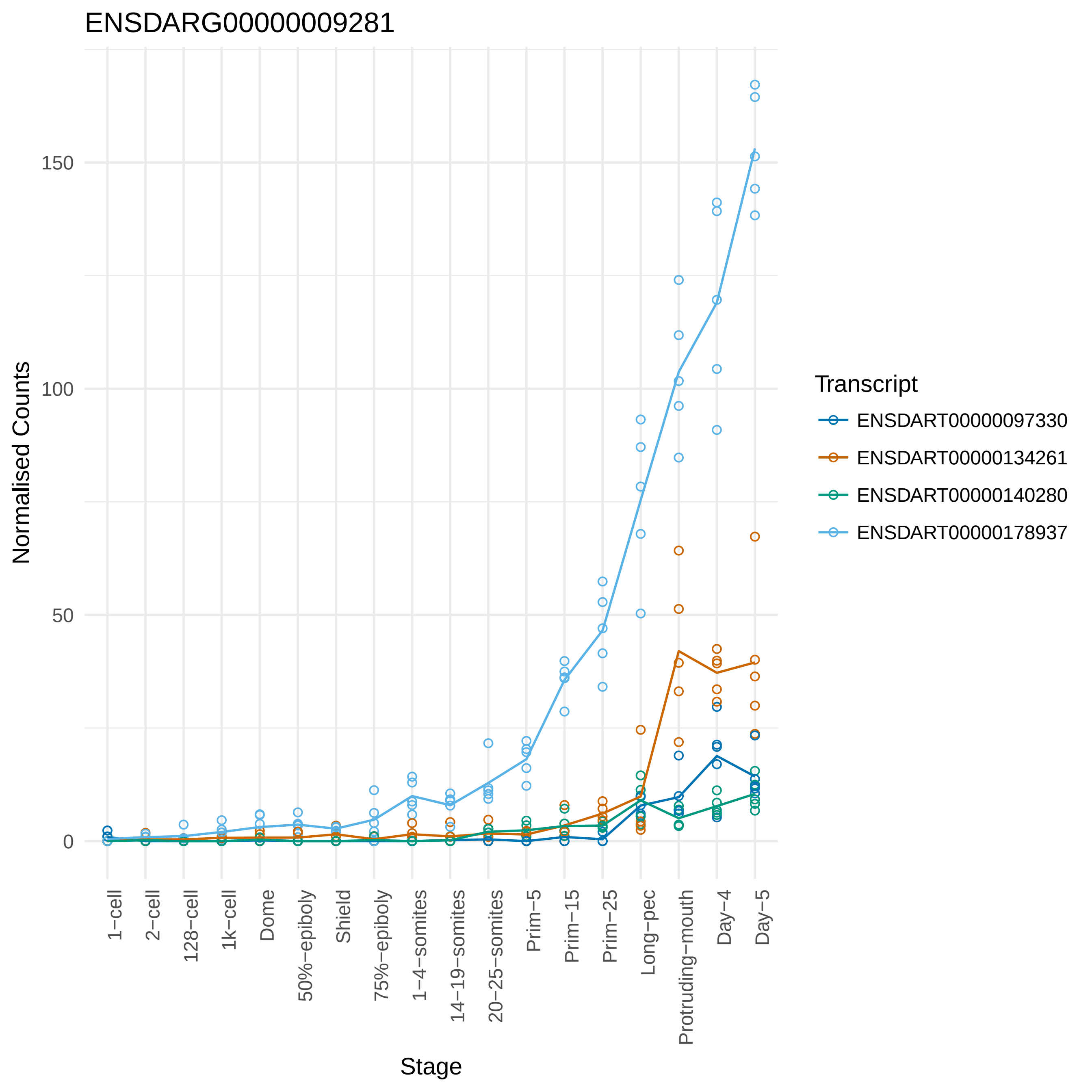
ENSDARG00000009281
Normalised Counts
150
100
50
0
1−cell
2−cell
128−cell
1k−cell
Dome
50%−epiboly
Shield
75%−epiboly
1−4−somites
14−19−somites
20−25−somites
Prim−5
Prim−15
Prim−25
Long−pec
Protruding−mouth
Day−4
Day−5
Stage
Transcript
ENSDART00000097330
ENSDART00000134261
ENSDART00000140280
ENSDART00000178937

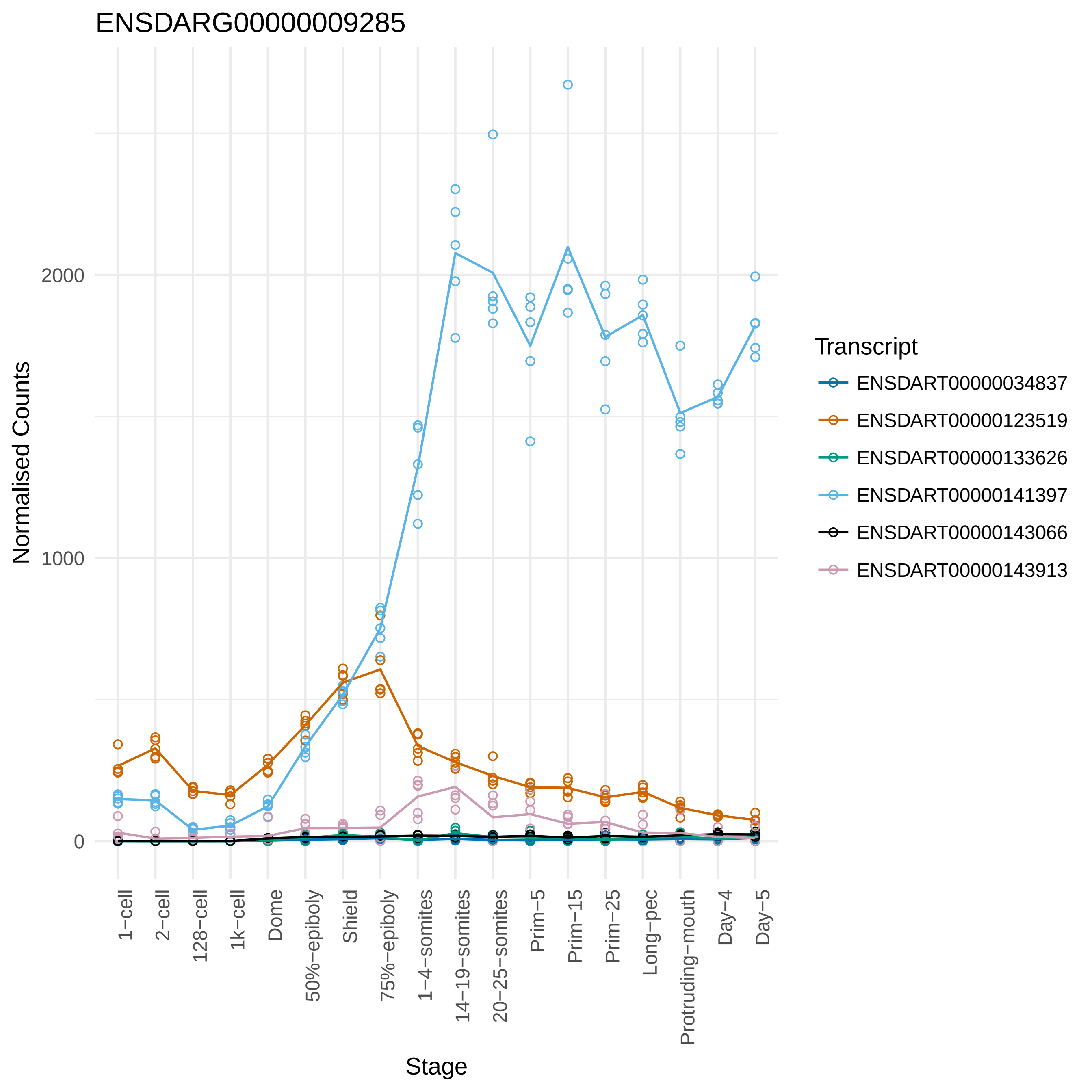

ENSDARG00000009285
Normalised Counts
2000
1000
0
Stage
1−cell
2−cell
128−cell
1k−cell
Dome
50%−epiboly
Shield
75%−epiboly
1−4−somites
14−19−somites
20−25−somites
Prim−5
Prim−15
Prim−25
Long−pec
Protruding−mouth
Day−4
Day−5
Transcript
ENSDART00000034837
ENSDART00000123519
ENSDART00000133626
ENSDART00000141397
ENSDART00000143066
ENSDART00000143913

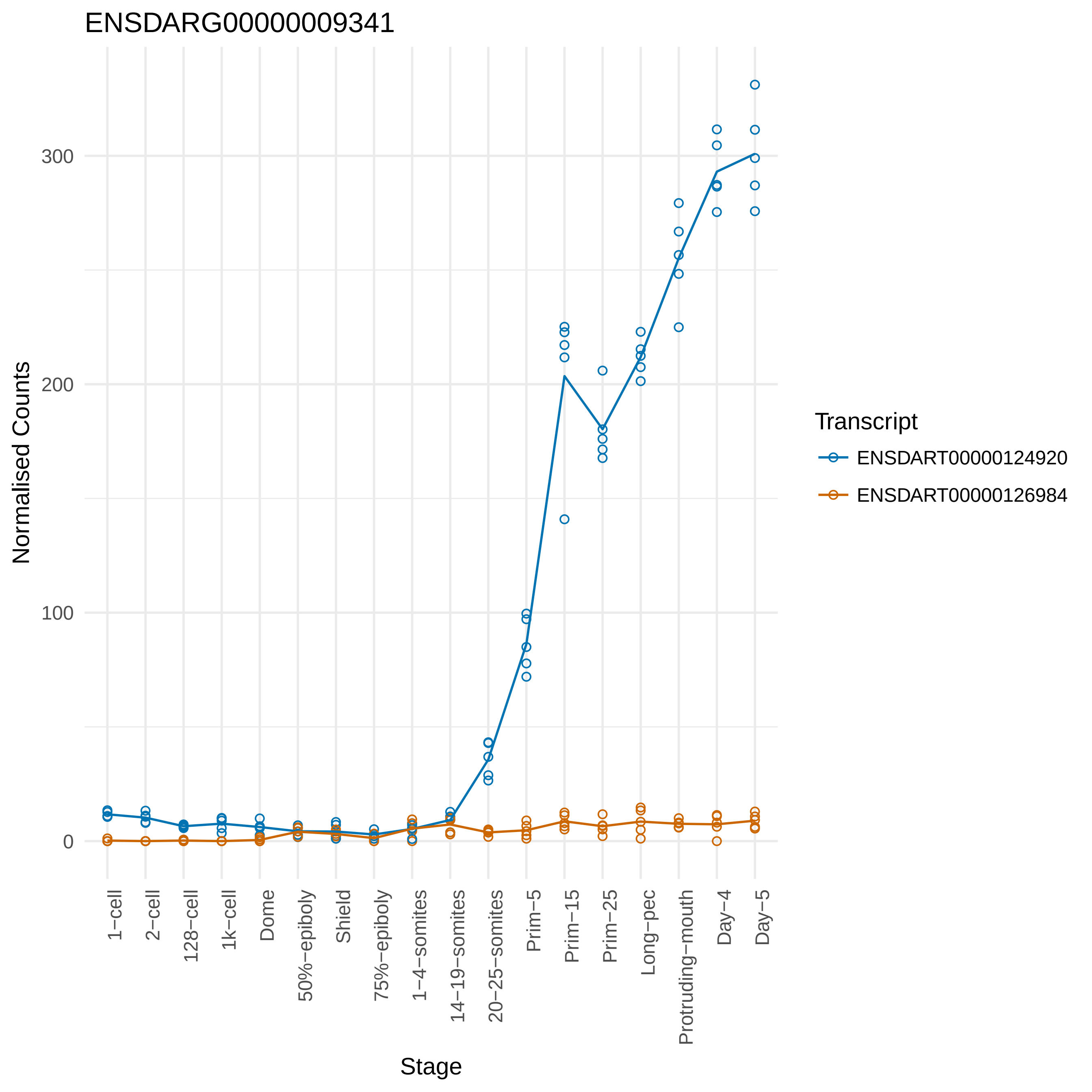

ENSDARG00000009341
Normalised Counts
300
200
100
0
1−cell
2−cell
128−cell
1k−cell
Dome
50%−epiboly
Shield
75%−epiboly
1−4−somites
14−19−somites
20−25−somites
Prim−5
Prim−15
Prim−25
Long−pec
Protruding−mouth
Day−4
Day−5
Stage
Transcript
ENSDART00000124920
ENSDART00000126984

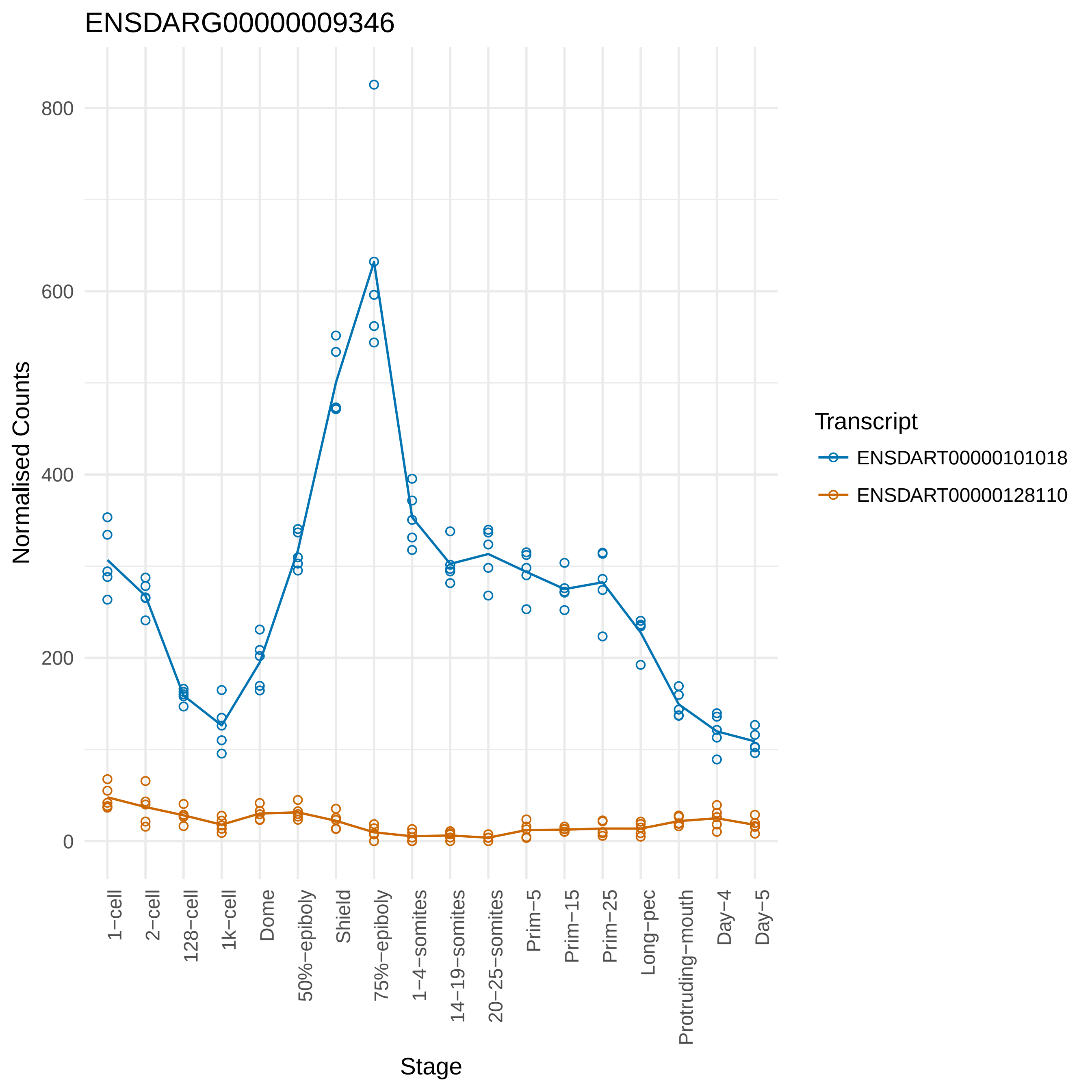

ENSDARG00000009346
Normalised Counts
0
200
400
600
800
Stage
1−cell
2−cell
128−cell
1k−cell
Dome
50%−epiboly
Shield
75%−epiboly
1−4−somites
14−19−somites
20−25−somites
Prim−5
Prim−15
Prim−25
Long−pec
Protruding−mouth
Day−4
Day−5
Transcript
ENSDART00000101018
ENSDART00000128110

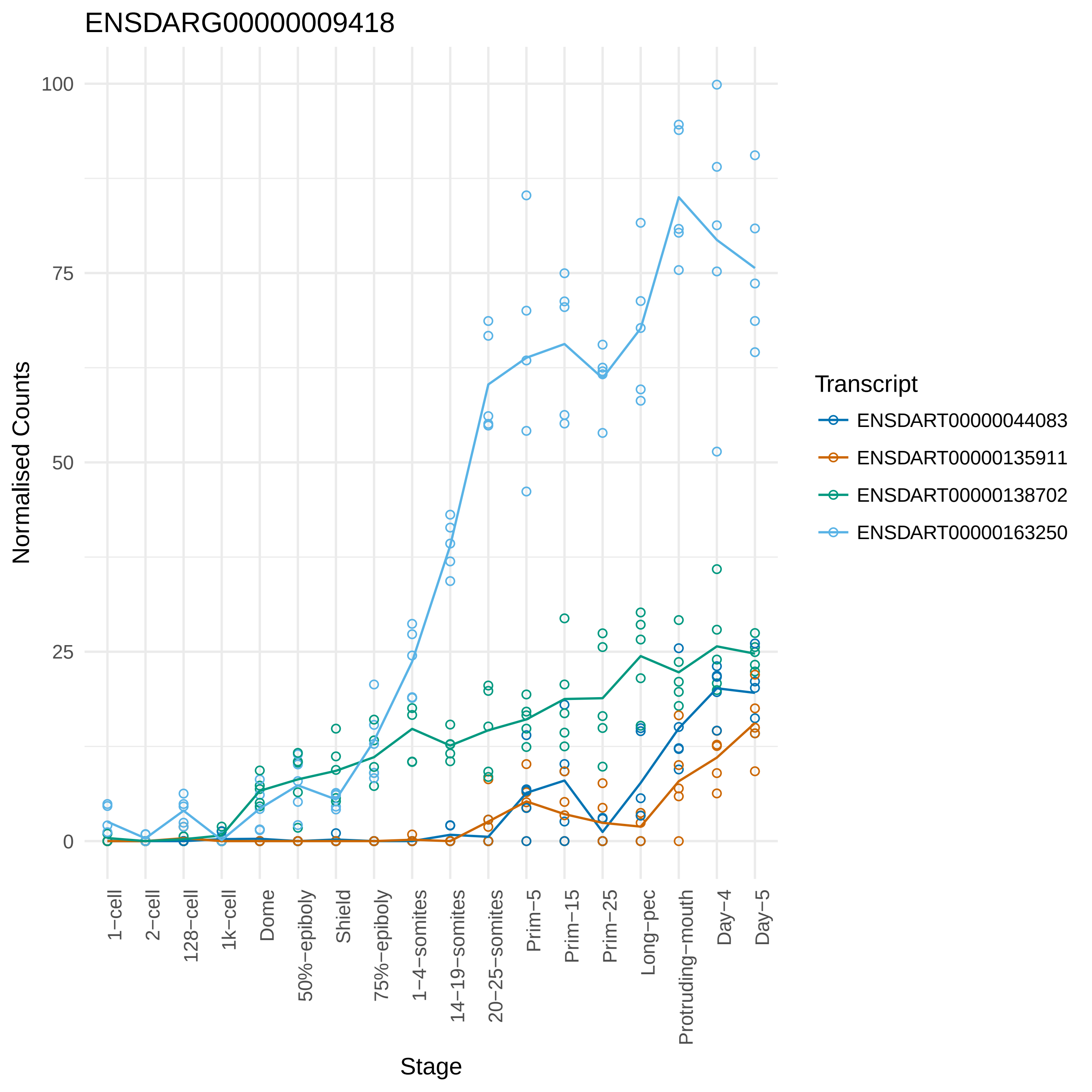
ENSDARG00000009418
Normalised Counts
100
75
50
25
0
1−cell
2−cell
128−cell
1k−cell
Dome
50%−epiboly
Shield
75%−epiboly
1−4−somites
14−19−somites
20−25−somites
Prim−5
Prim−15
Prim−25
Long−pec
Protruding−mouth
Day−4
Day−5
Stage
Transcript
ENSDART00000044083
ENSDART00000135911
ENSDART00000138702
ENSDART00000163250

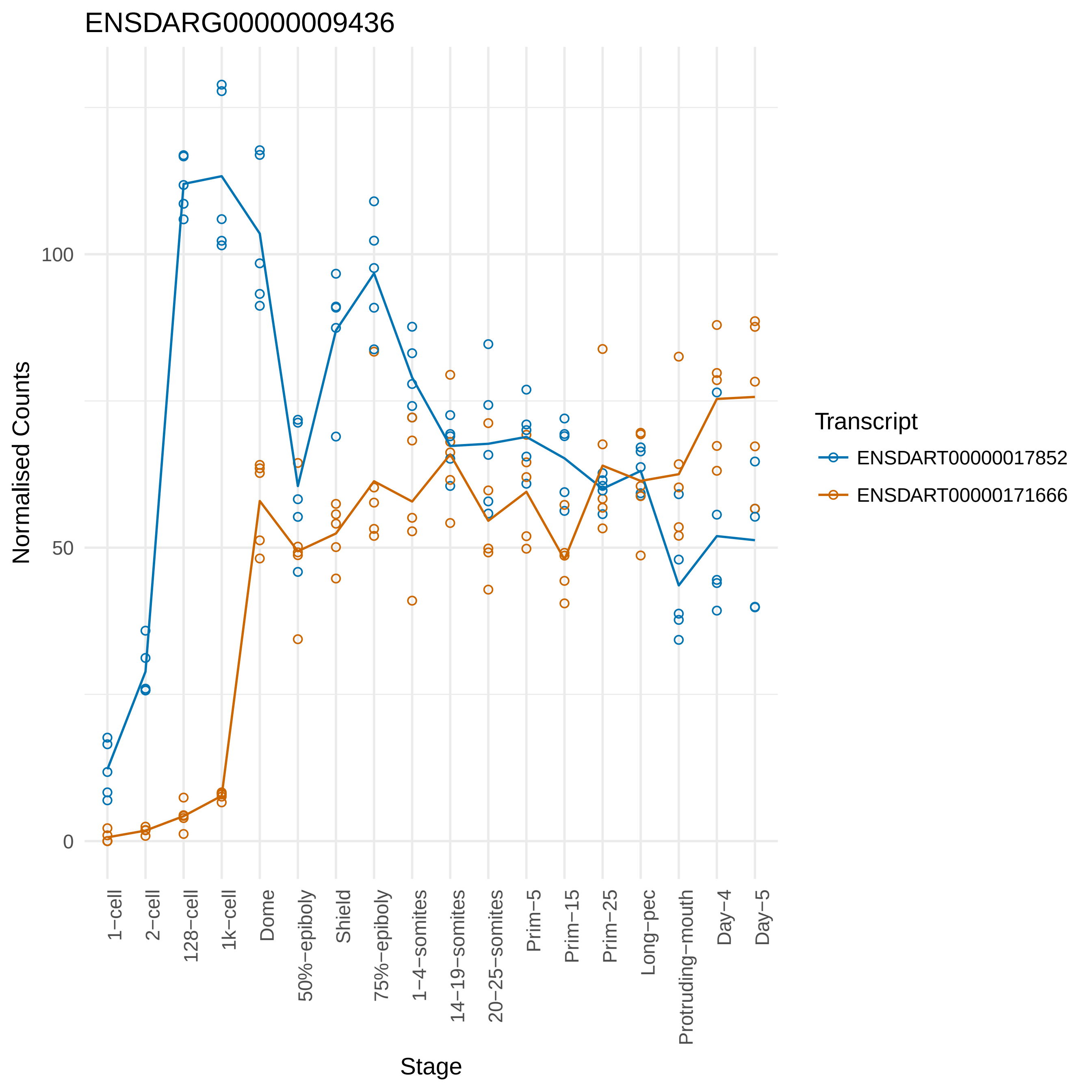

ENSDARG00000009436
Normalised Counts
100
50
0
1−cell
2−cell
128−cell
1k−cell
Dome
50%−epiboly
Shield
75%−epiboly
1−4−somites
14−19−somites
20−25−somites
Prim−5
Prim−15
Prim−25
Long−pec
Protruding−mouth
Day−4
Day−5
Stage
Transcript
ENSDART00000017852
ENSDART00000171666

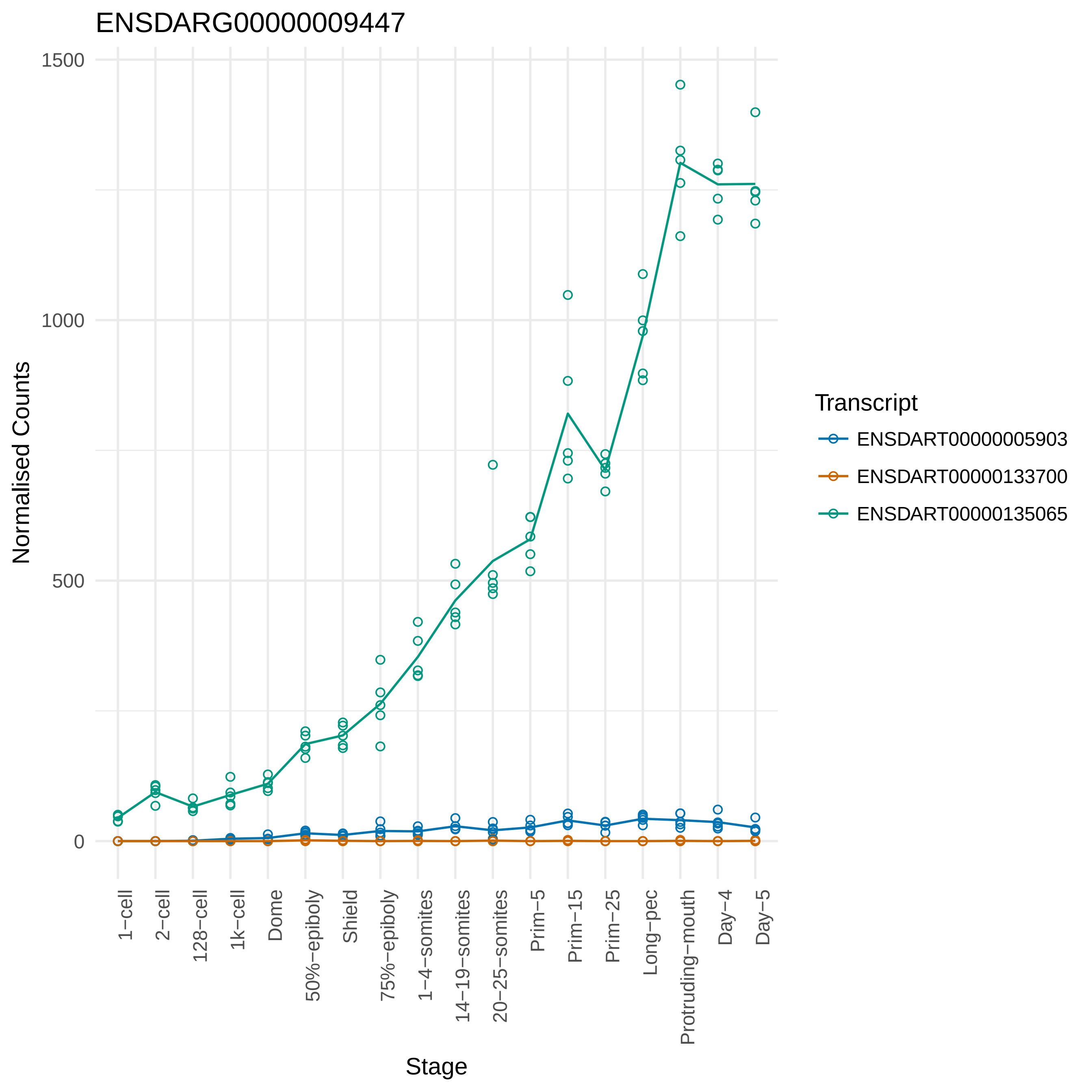
ENSDARG00000009447
Normalised Counts
1500
1000
500
0
1−cell
2−cell
128−cell
1k−cell
Dome
50%−epiboly
Shield
75%−epiboly
1−4−somites
14−19−somites
20−25−somites
Prim−5
Prim−15
Prim−25
Long−pec
Protruding−mouth
Day−4
Day−5
Stage
Transcript
ENSDART00000005903
ENSDART00000133700
ENSDART00000135065

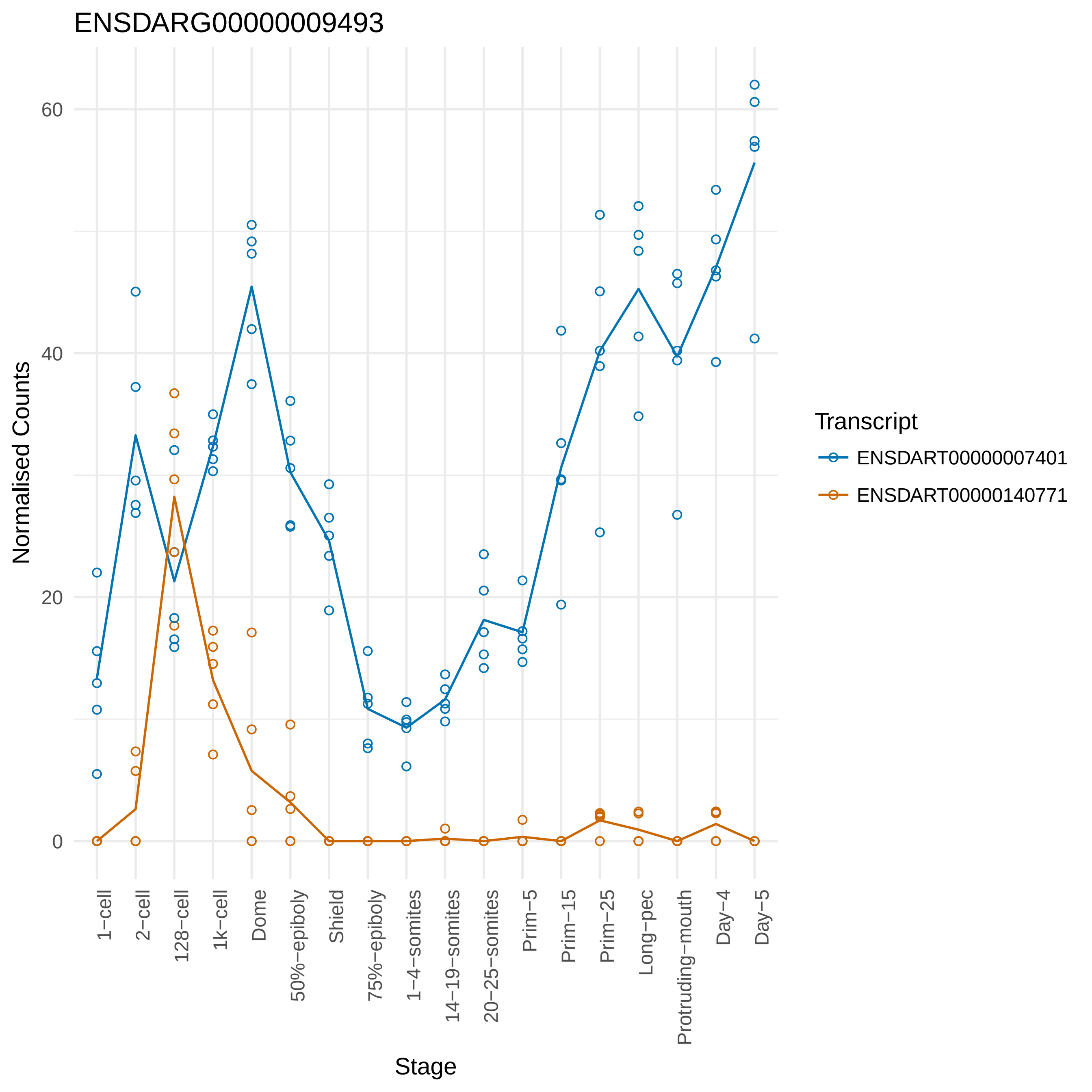

ENSDARG00000009493
Normalised Counts
60
40
20
0
1−cell
2−cell
128−cell
1k−cell
Dome
50%−epiboly
Shield
75%−epiboly
1−4−somites
14−19−somites
20−25−somites
Prim−5
Prim−15
Prim−25
Long−pec
Protruding−mouth
Day−4
Day−5
Stage
Transcript
ENSDART00000007401
ENSDART00000140771

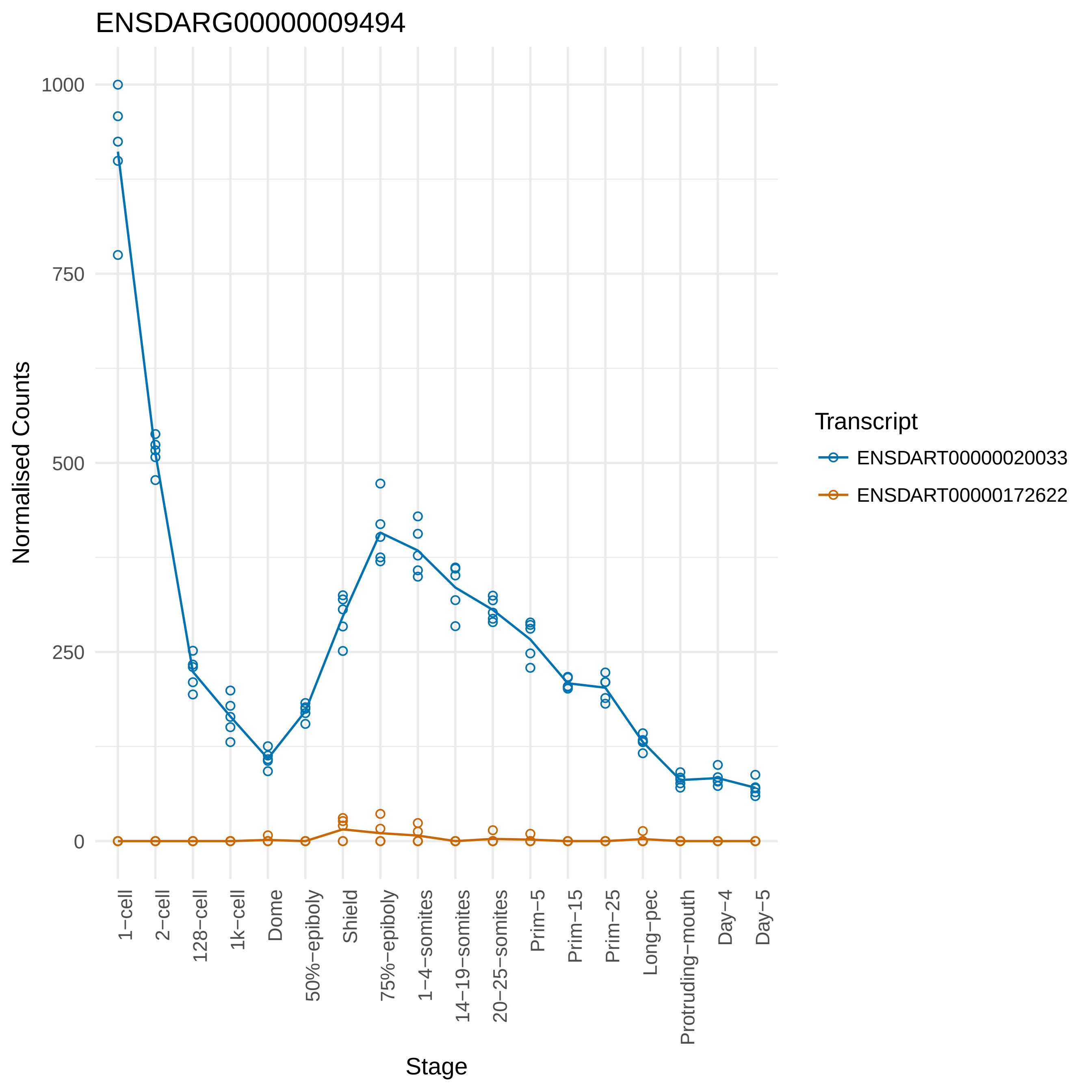
ENSDARG00000009494
Normalised Counts
1000
750
500
250
0
1−cell
2−cell
128−cell
1k−cell
Dome
50%−epiboly
Shield
75%−epiboly
1−4−somites
14−19−somites
20−25−somites
Prim−5
Prim−15
Prim−25
Long−pec
Protruding−mouth
Day−4
Day−5
Stage
Transcript
ENSDART00000020033
ENSDART00000172622

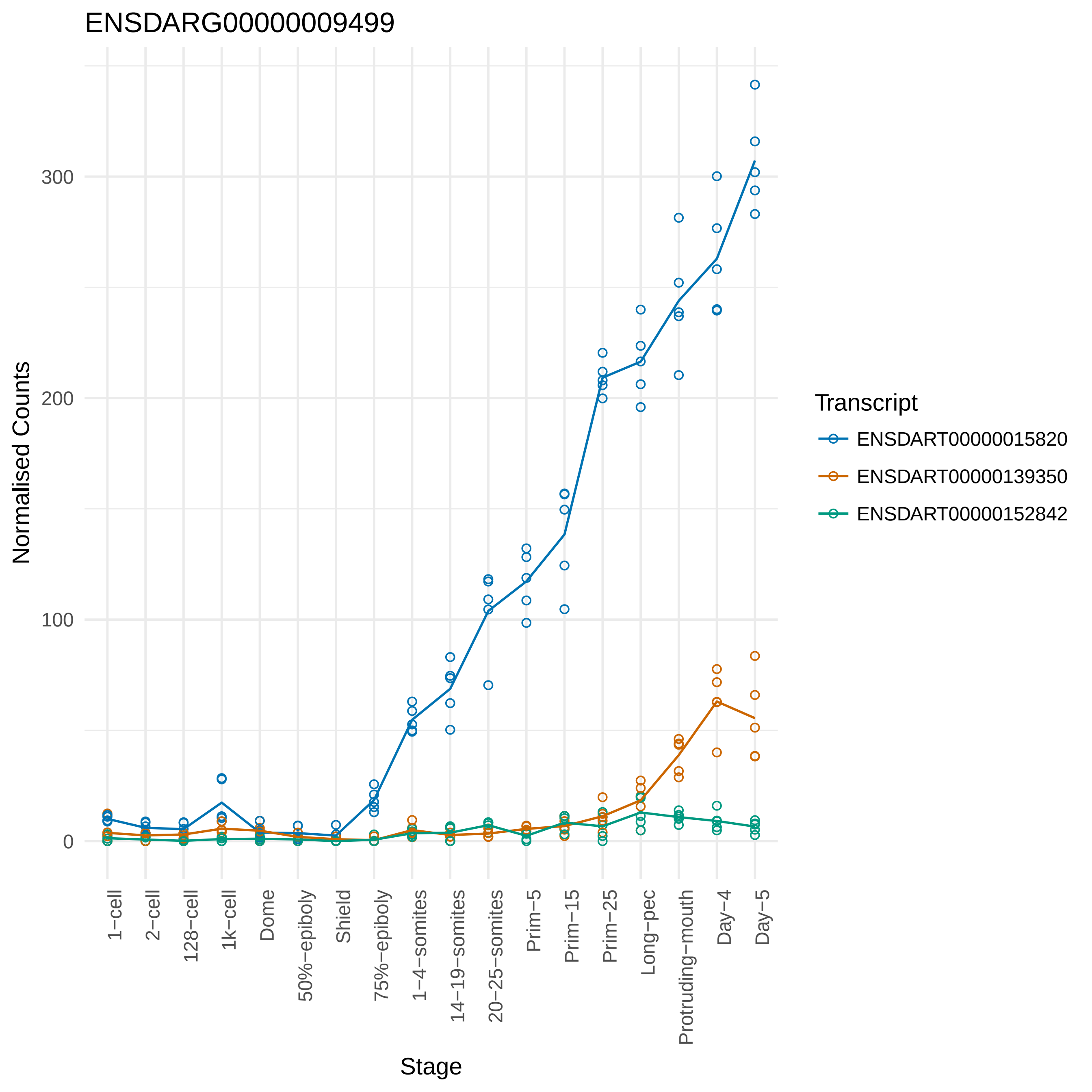
ENSDARG00000009499
Normalised Counts
300
200
100
0
1−cell
2−cell
128−cell
1k−cell
Dome
50%−epiboly
Shield
75%−epiboly
1−4−somites
14−19−somites
20−25−somites
Prim−5
Prim−15
Prim−25
Long−pec
Protruding−mouth
Day−4
Day−5
Stage
Transcript
ENSDART00000015820
ENSDART00000139350
ENSDART00000152842

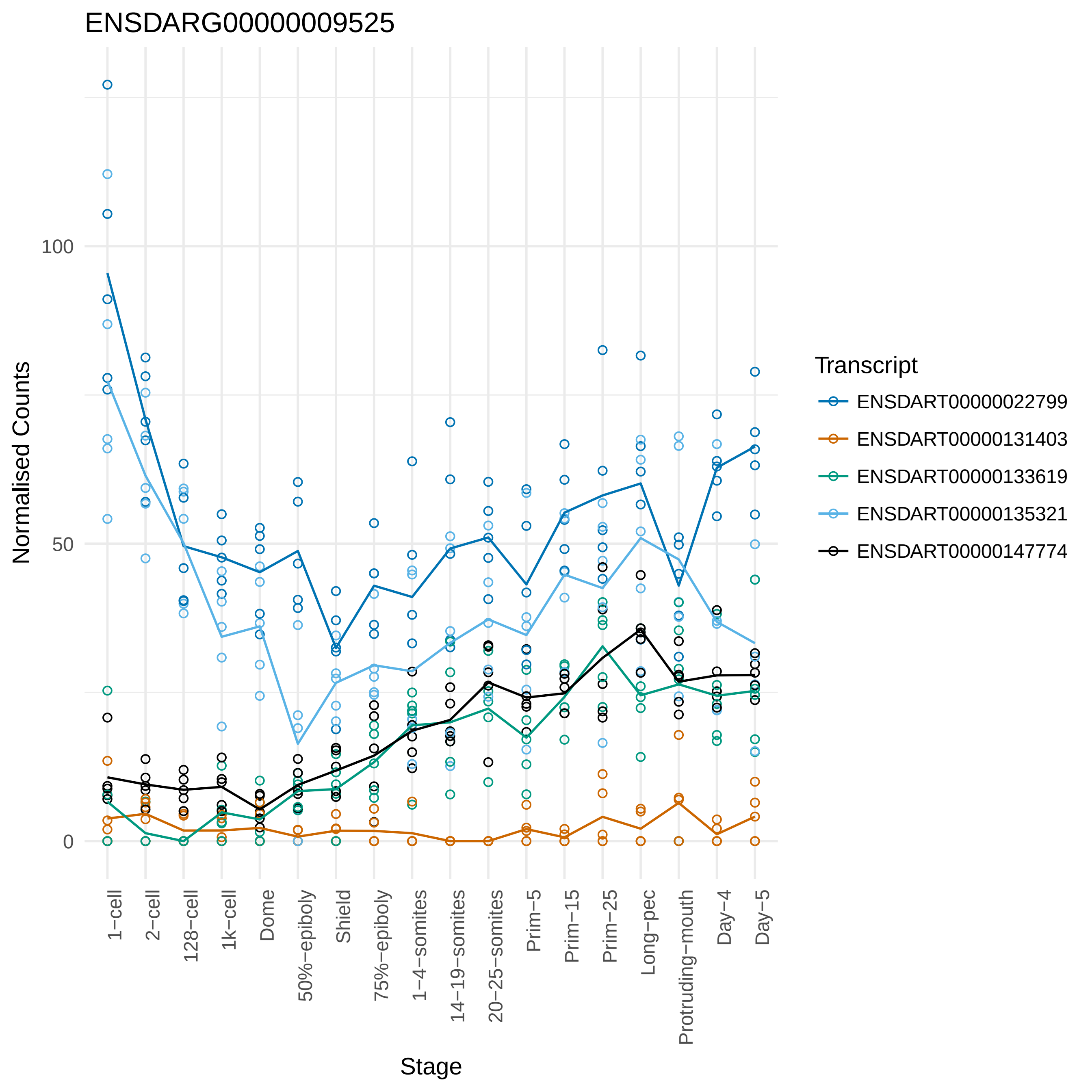

ENSDARG00000009525
Normalised Counts
100
50
0
Stage
1−cell
2−cell
128−cell
1k−cell
Dome
50%−epiboly
Shield
75%−epiboly
1−4−somites
14−19−somites
20−25−somites
Prim−5
Prim−15
Prim−25
Long−pec
Protruding−mouth
Day−4
Day−5
Transcript
ENSDART00000022799
ENSDART00000131403
ENSDART00000133619
ENSDART00000135321
ENSDART00000147774

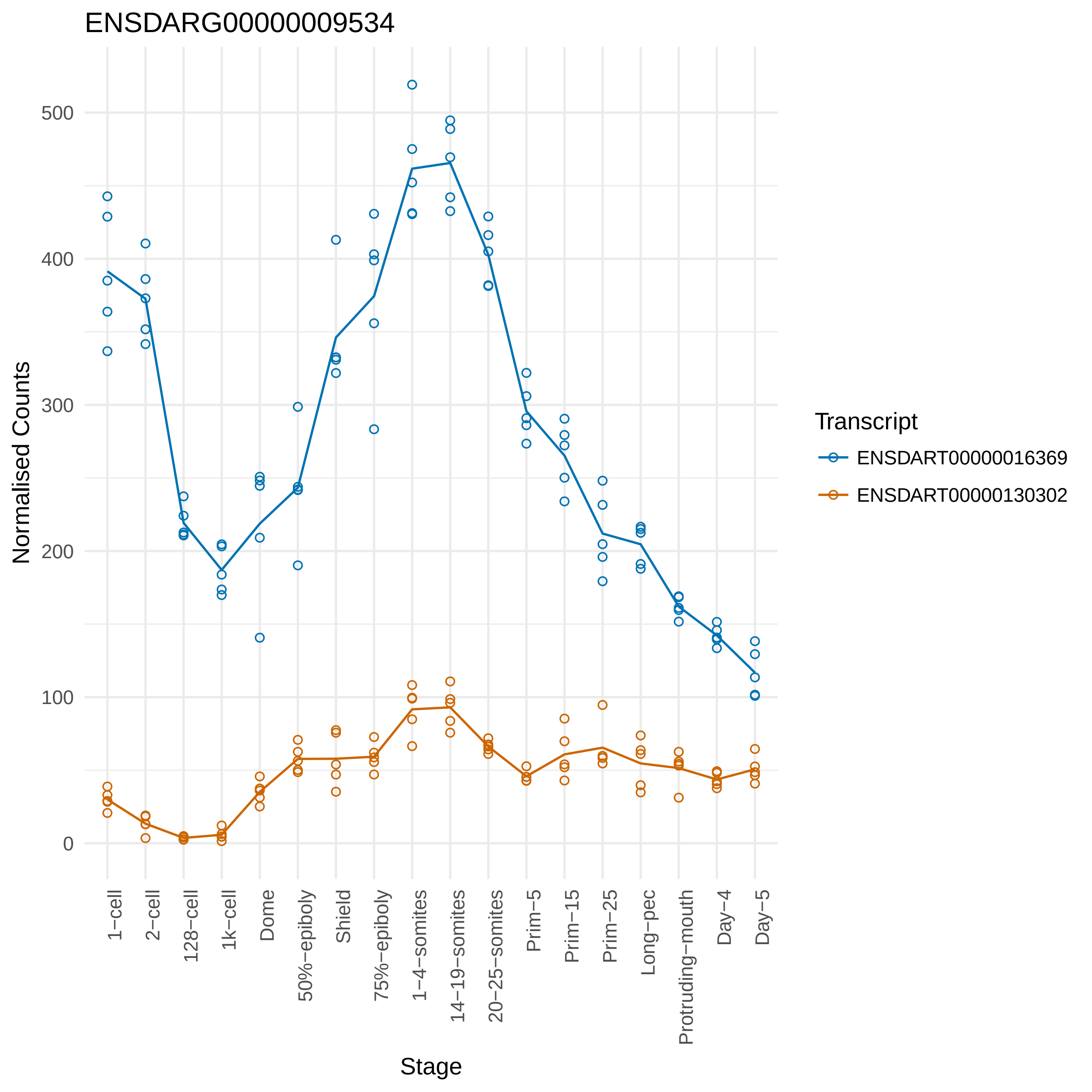

ENSDARG00000009534
Normalised Counts
500
400
300
200
100
0
1−cell
2−cell
128−cell
1k−cell
Dome
50%−epiboly
Shield
75%−epiboly
1−4−somites
14−19−somites
20−25−somites
Prim−5
Prim−15
Prim−25
Long−pec
Protruding−mouth
Day−4
Day−5
Stage
Transcript
ENSDART00000016369
ENSDART00000130302

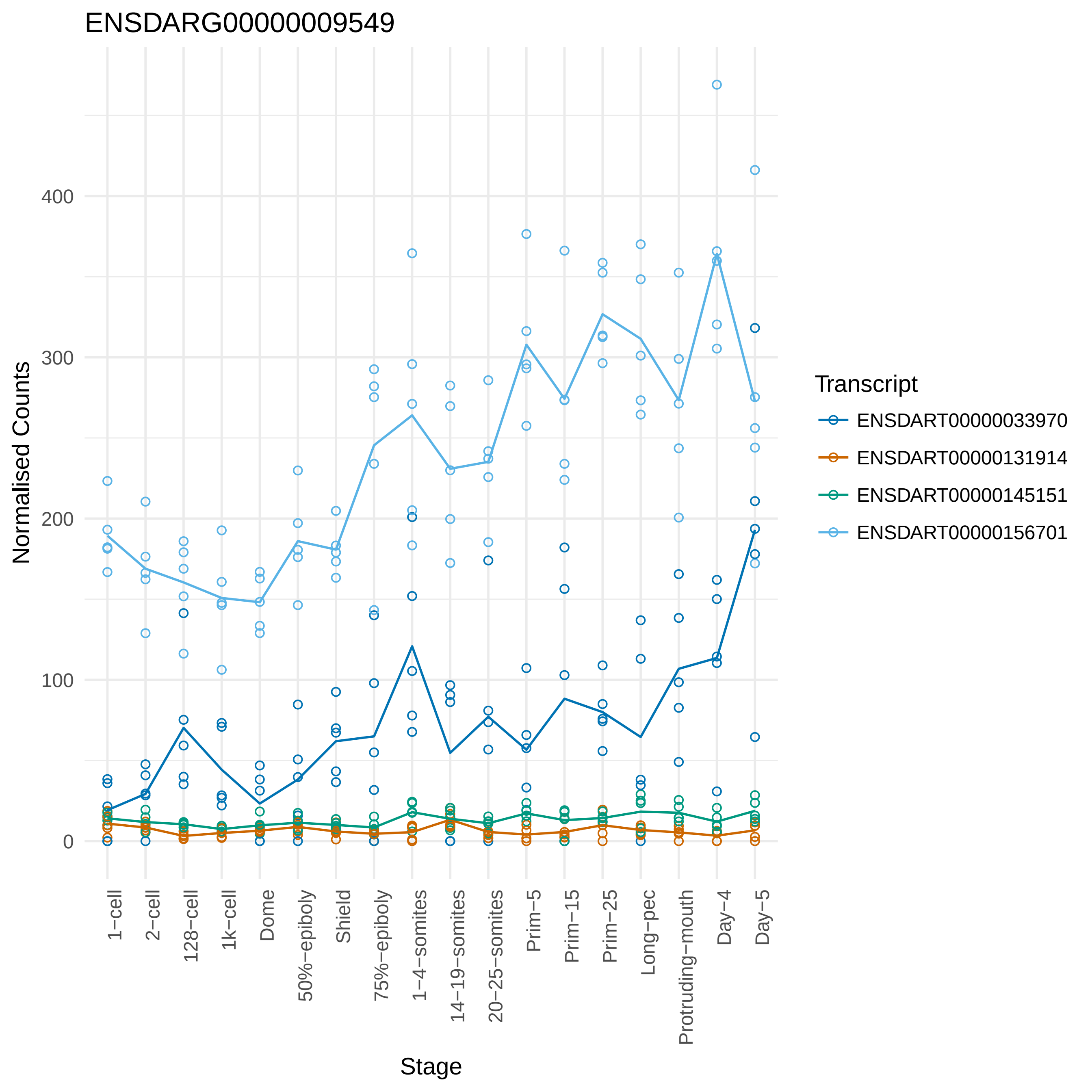

ENSDARG00000009549
Normalised Counts
0
100
200
300
400
1−cell
2−cell
128−cell
1k−cell
Dome
50%−epiboly
Shield
75%−epiboly
1−4−somites
14−19−somites
20−25−somites
Prim−5
Prim−15
Prim−25
Long−pec
Protruding−mouth
Day−4
Day−5
Stage
Transcript
ENSDART00000033970
ENSDART00000131914
ENSDART00000145151
ENSDART00000156701

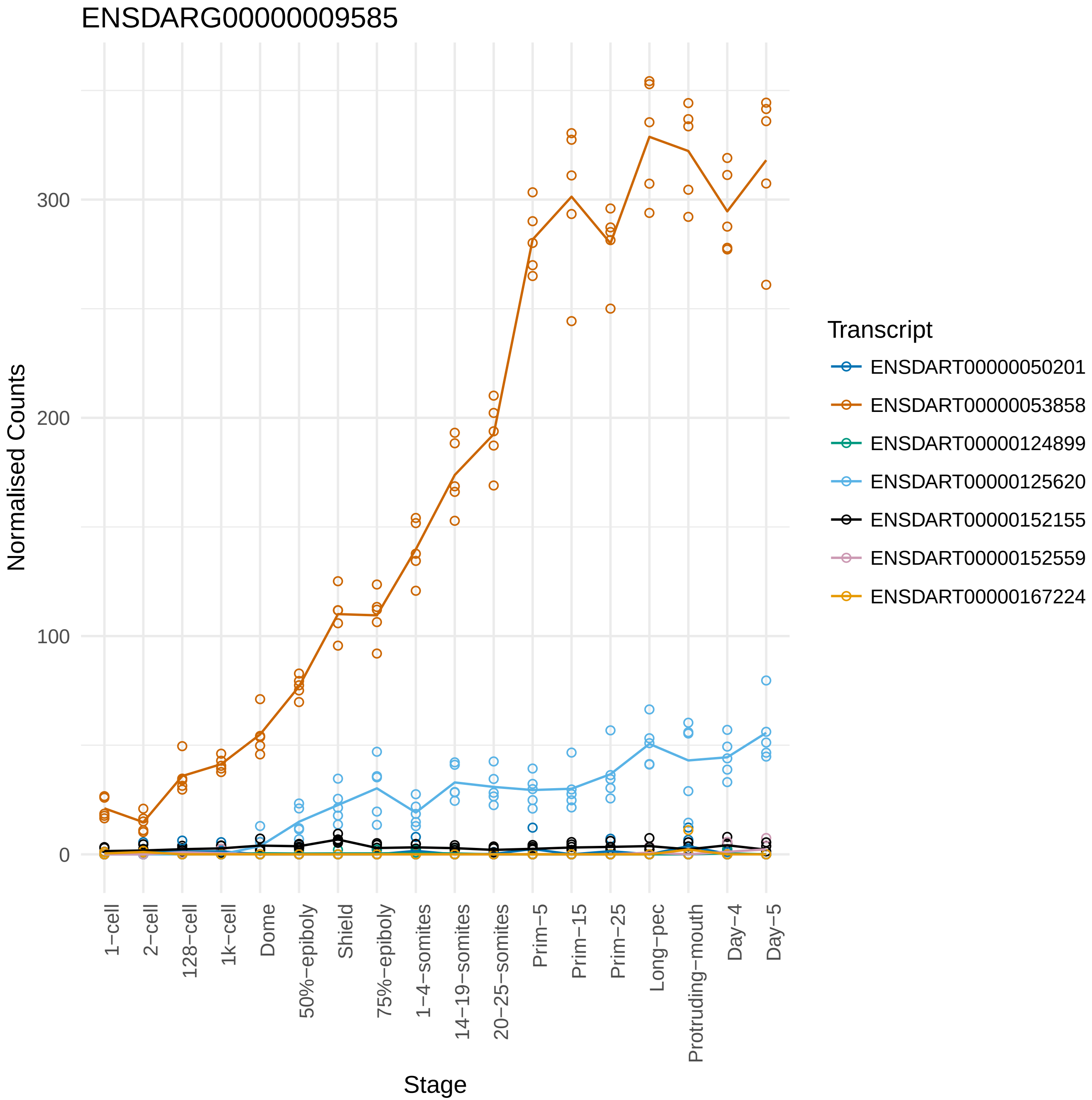

ENSDARG00000009585
Normalised Counts
300
200
100
0
1−cell
2−cell
128−cell
1k−cell
Dome
50%−epiboly
Shield
75%−epiboly
1−4−somites
14−19−somites
20−25−somites
Prim−5
Prim−15
Prim−25
Long−pec
Protruding−mouth
Day−4
Day−5
Stage
Transcript
ENSDART00000050201
ENSDART00000053858
ENSDART00000124899
ENSDART00000125620
ENSDART00000152155
ENSDART00000152559
ENSDART00000167224

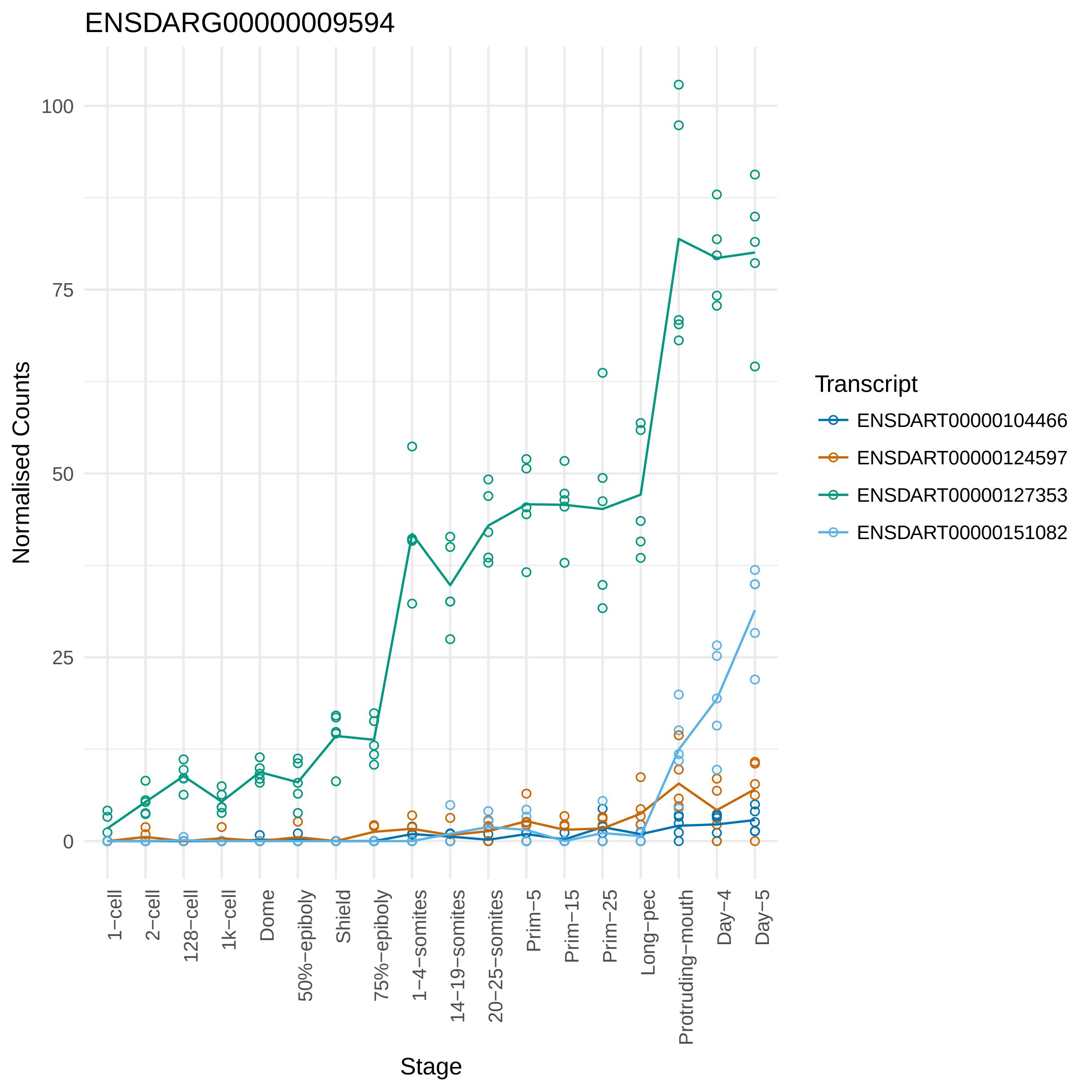
ENSDARG00000009594
Normalised Counts
100
75
50
25
0
1−cell
2−cell
128−cell
1k−cell
Dome
50%−epiboly
Shield
75%−epiboly
1−4−somites
14−19−somites
20−25−somites
Prim−5
Prim−15
Prim−25
Long−pec
Protruding−mouth
Day−4
Day−5
Stage
Transcript
ENSDART00000104466
ENSDART00000124597
ENSDART00000127353
ENSDART00000151082

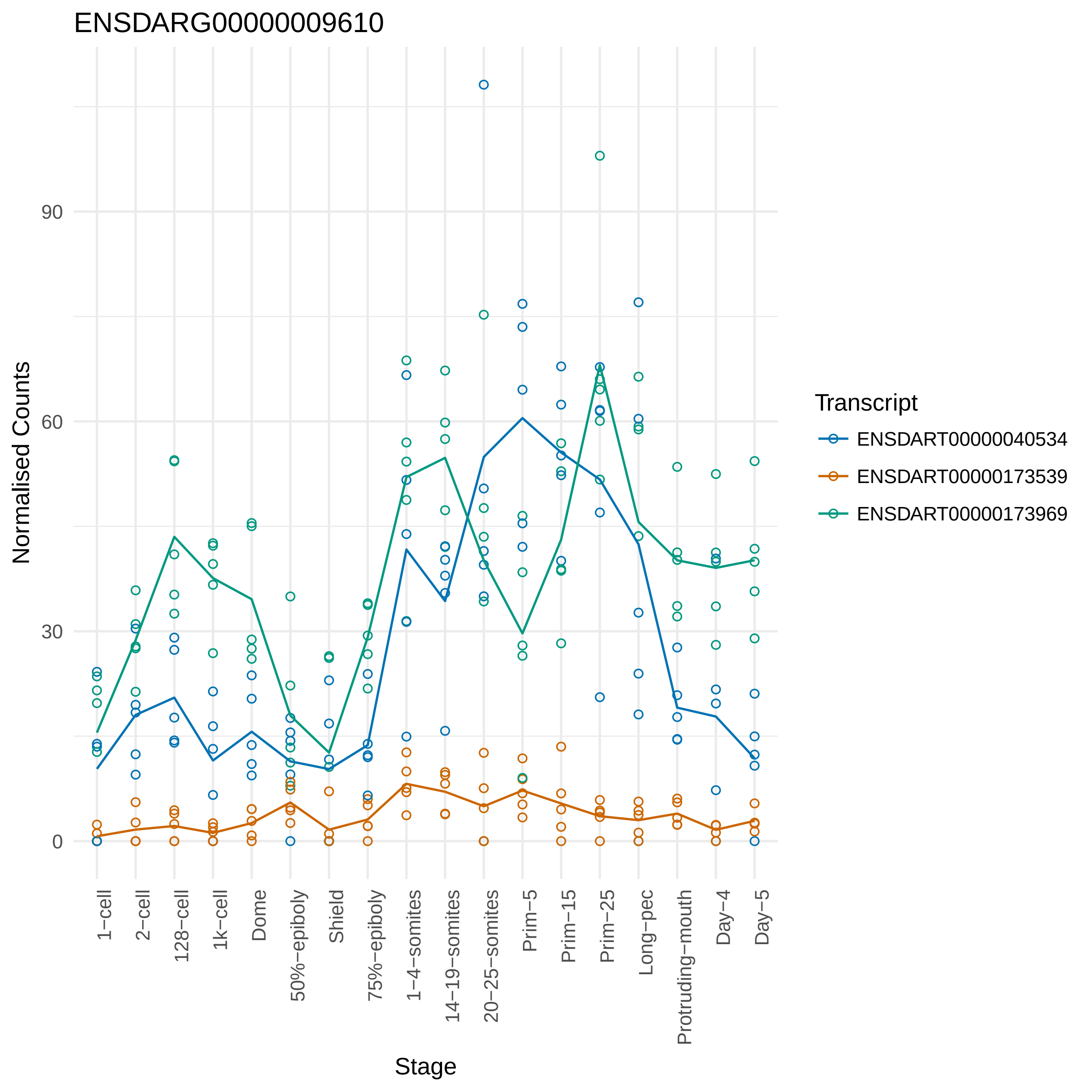

ENSDARG00000009610
Normalised Counts
0
30
60
90
Stage
1−cell
2−cell
128−cell
1k−cell
Dome
50%−epiboly
Shield
75%−epiboly
1−4−somites
14−19−somites
20−25−somites
Prim−5
Prim−15
Prim−25
Long−pec
Protruding−mouth
Day−4
Day−5
Transcript
ENSDART00000040534
ENSDART00000173539
ENSDART00000173969

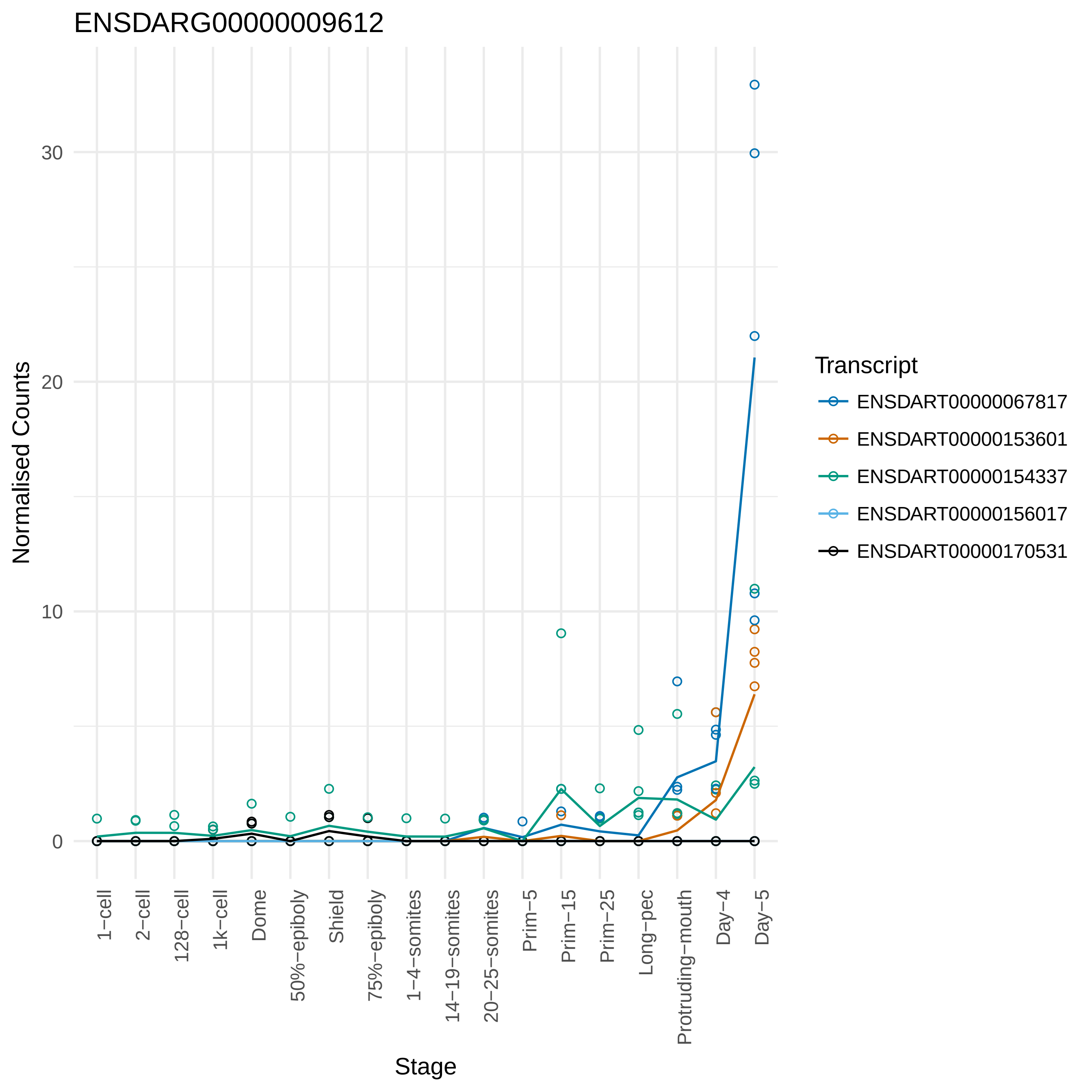
ENSDARG00000009612
Normalised Counts
0
10
20
30
1−cell
2−cell
128−cell
1k−cell
Dome
50%−epiboly
Shield
75%−epiboly
1−4−somites
14−19−somites
20−25−somites
Prim−5
Prim−15
Prim−25
Long−pec
Protruding−mouth
Day−4
Day−5
Stage
Transcript
ENSDART00000067817
ENSDART00000153601
ENSDART00000154337
ENSDART00000156017
ENSDART00000170531

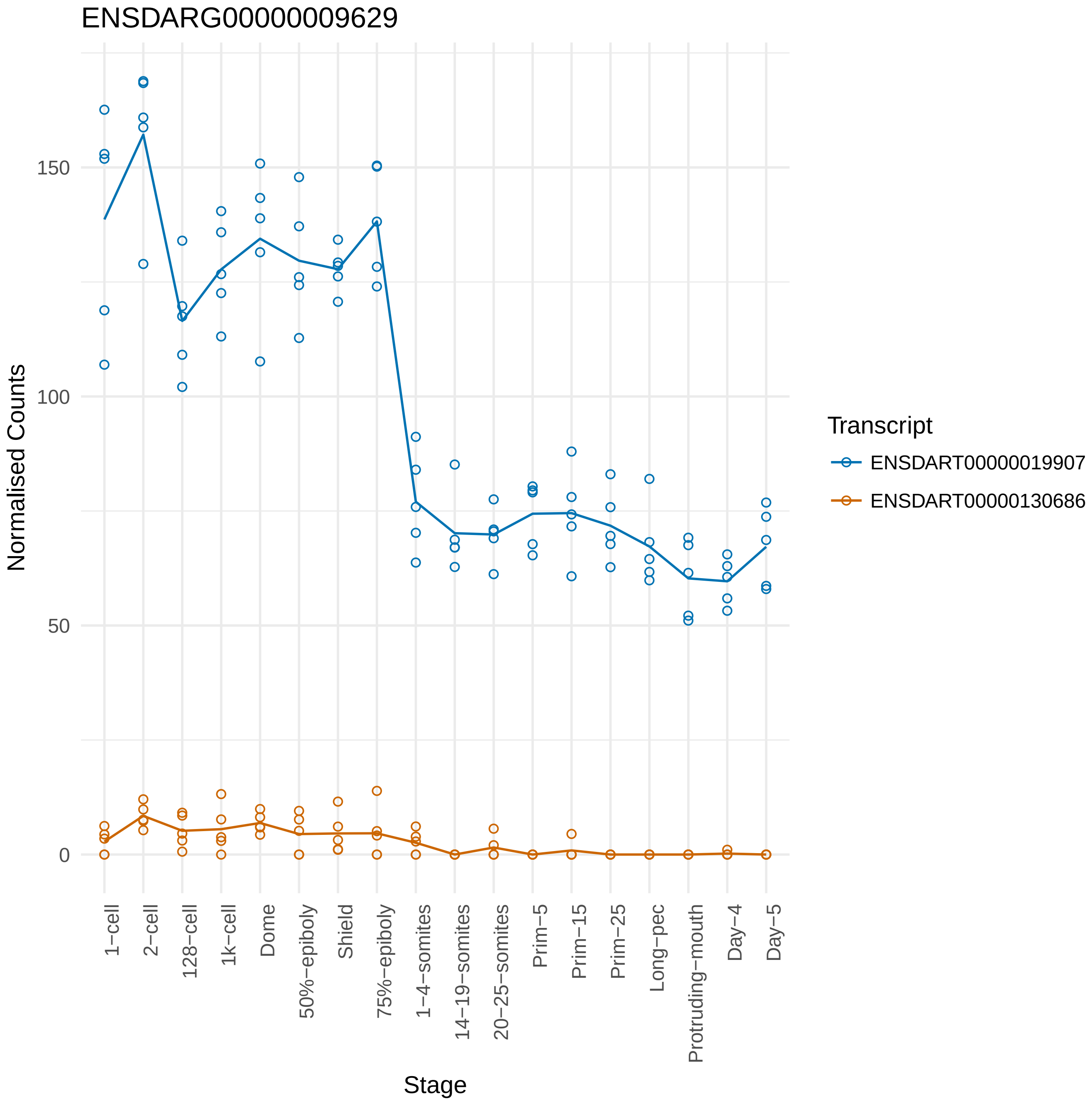
ENSDARG00000009629
Normalised Counts
150
100
50
0
1−cell
2−cell
128−cell
1k−cell
Dome
50%−epiboly
Shield
75%−epiboly
1−4−somites
14−19−somites
20−25−somites
Prim−5
Prim−15
Prim−25
Long−pec
Protruding−mouth
Day−4
Day−5
Stage
Transcript
ENSDART00000019907
ENSDART00000130686

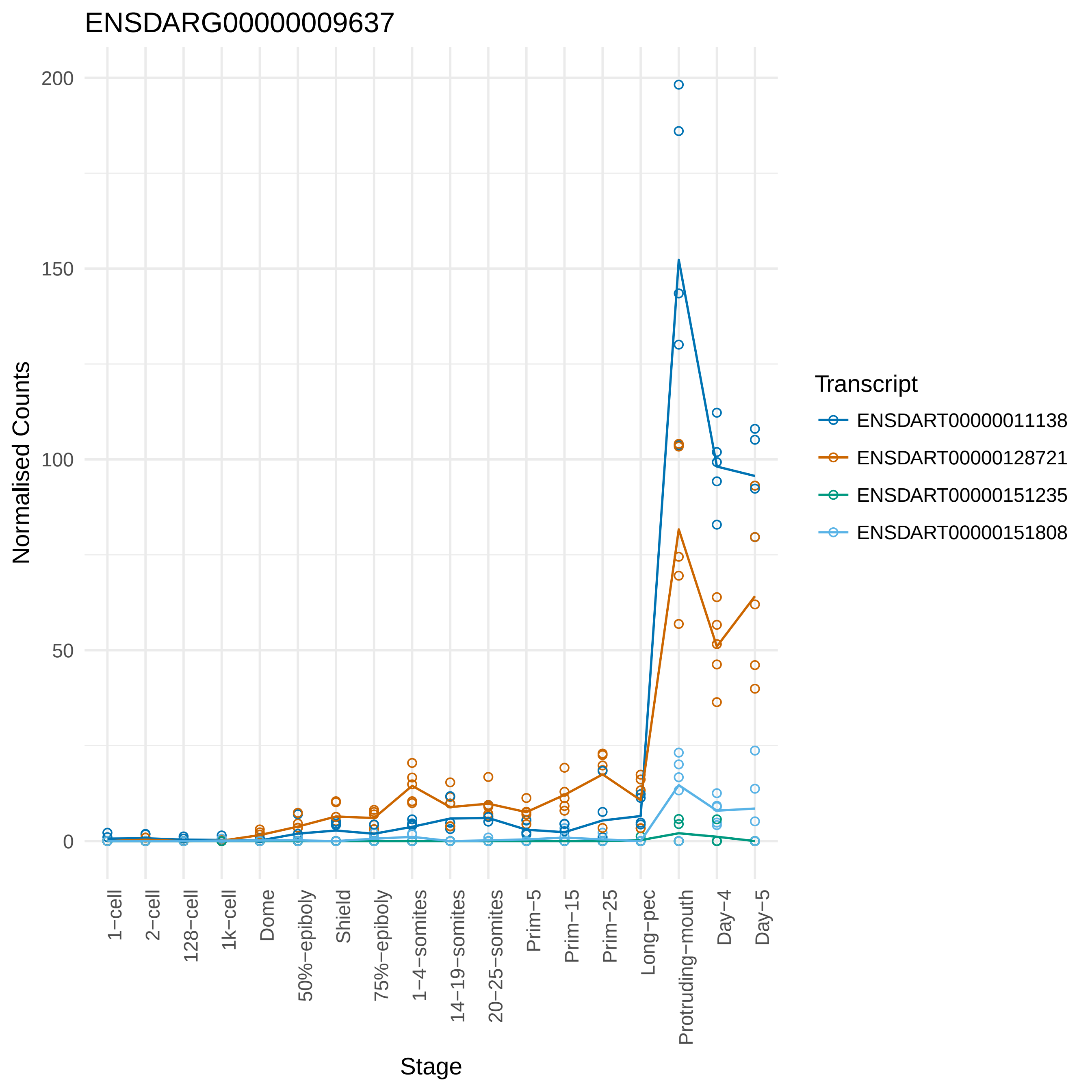
ENSDARG00000009637
Normalised Counts
200
150
100
50
0
1−cell
2−cell
128−cell
1k−cell
Dome
50%−epiboly
Shield
75%−epiboly
1−4−somites
14−19−somites
20−25−somites
Prim−5
Prim−15
Prim−25
Long−pec
Protruding−mouth
Day−4
Day−5
Stage
Transcript
ENSDART00000011138
ENSDART00000128721
ENSDART00000151235
ENSDART00000151808

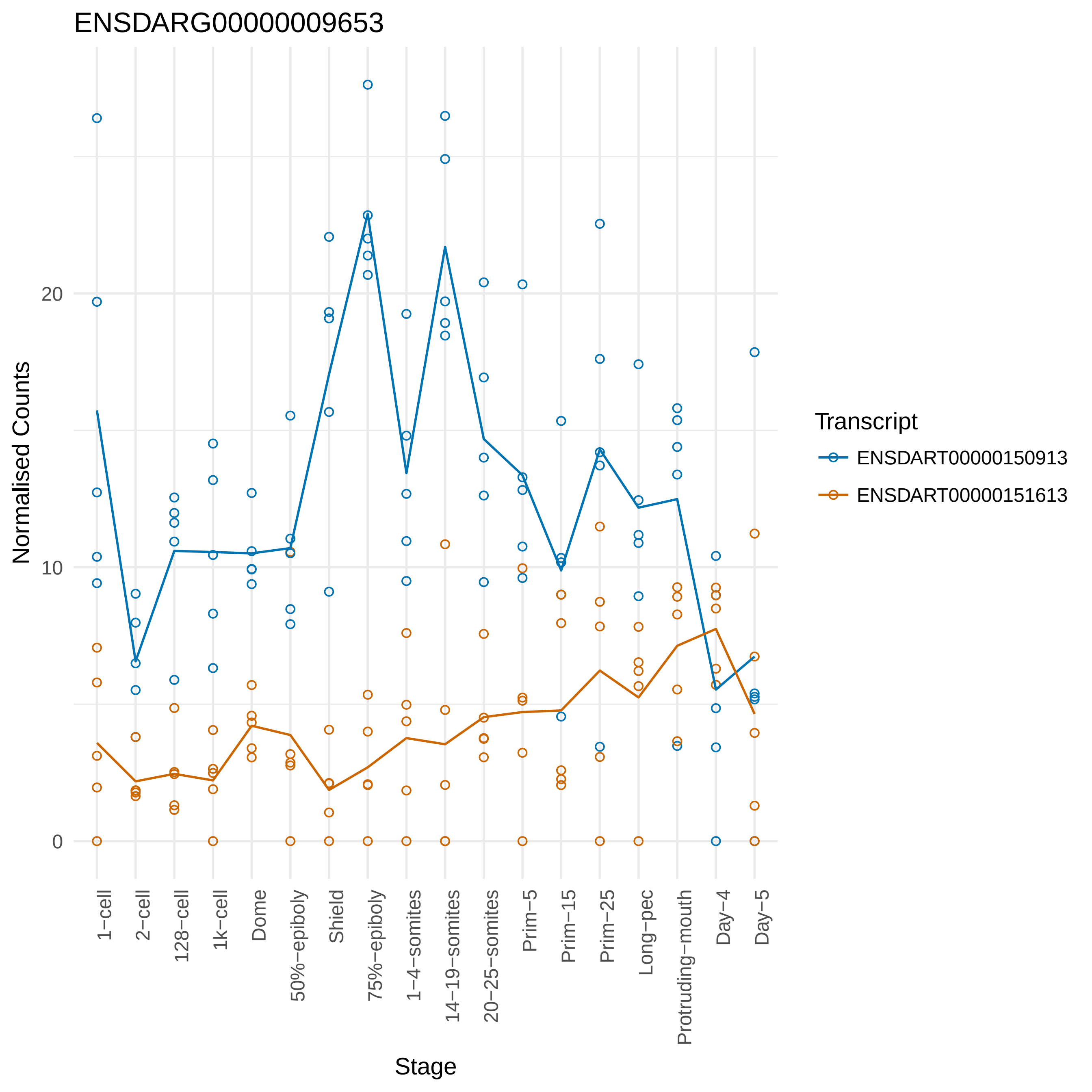

ENSDARG00000009653
Normalised Counts
0
10
20
Stage
1−cell
2−cell
128−cell
1k−cell
Dome
50%−epiboly
Shield
75%−epiboly
1−4−somites
14−19−somites
20−25−somites
Prim−5
Prim−15
Prim−25
Long−pec
Protruding−mouth
Day−4
Day−5
Transcript
ENSDART00000150913
ENSDART00000151613

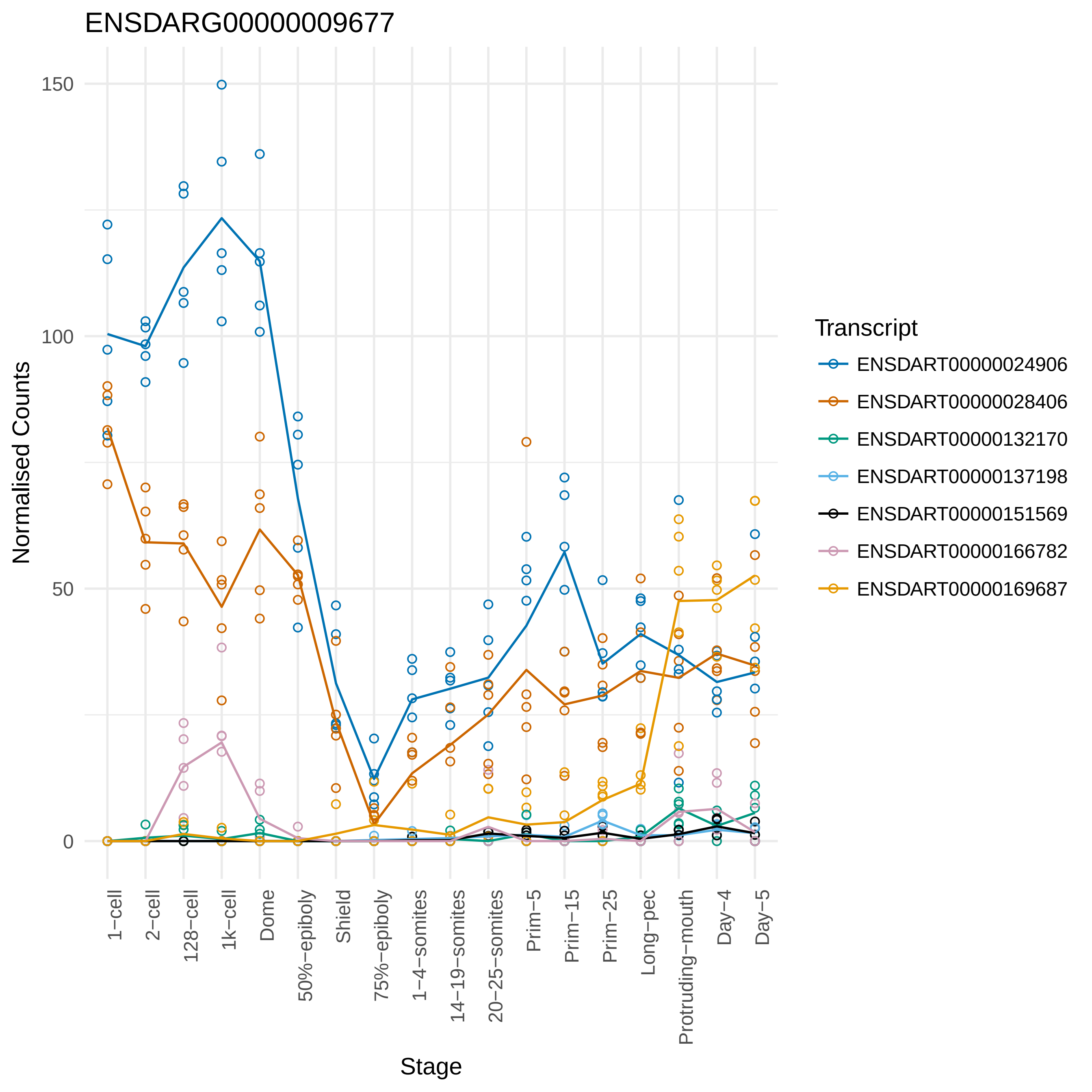

ENSDARG00000009677
Normalised Counts
150
100
50
0
1−cell
2−cell
128−cell
1k−cell
Dome
50%−epiboly
Shield
75%−epiboly
1−4−somites
14−19−somites
20−25−somites
Prim−5
Prim−15
Prim−25
Long−pec
Protruding−mouth
Day−4
Day−5
Stage
Transcript
ENSDART00000024906
ENSDART00000028406
ENSDART00000132170
ENSDART00000137198
ENSDART00000151569
ENSDART00000166782
ENSDART00000169687

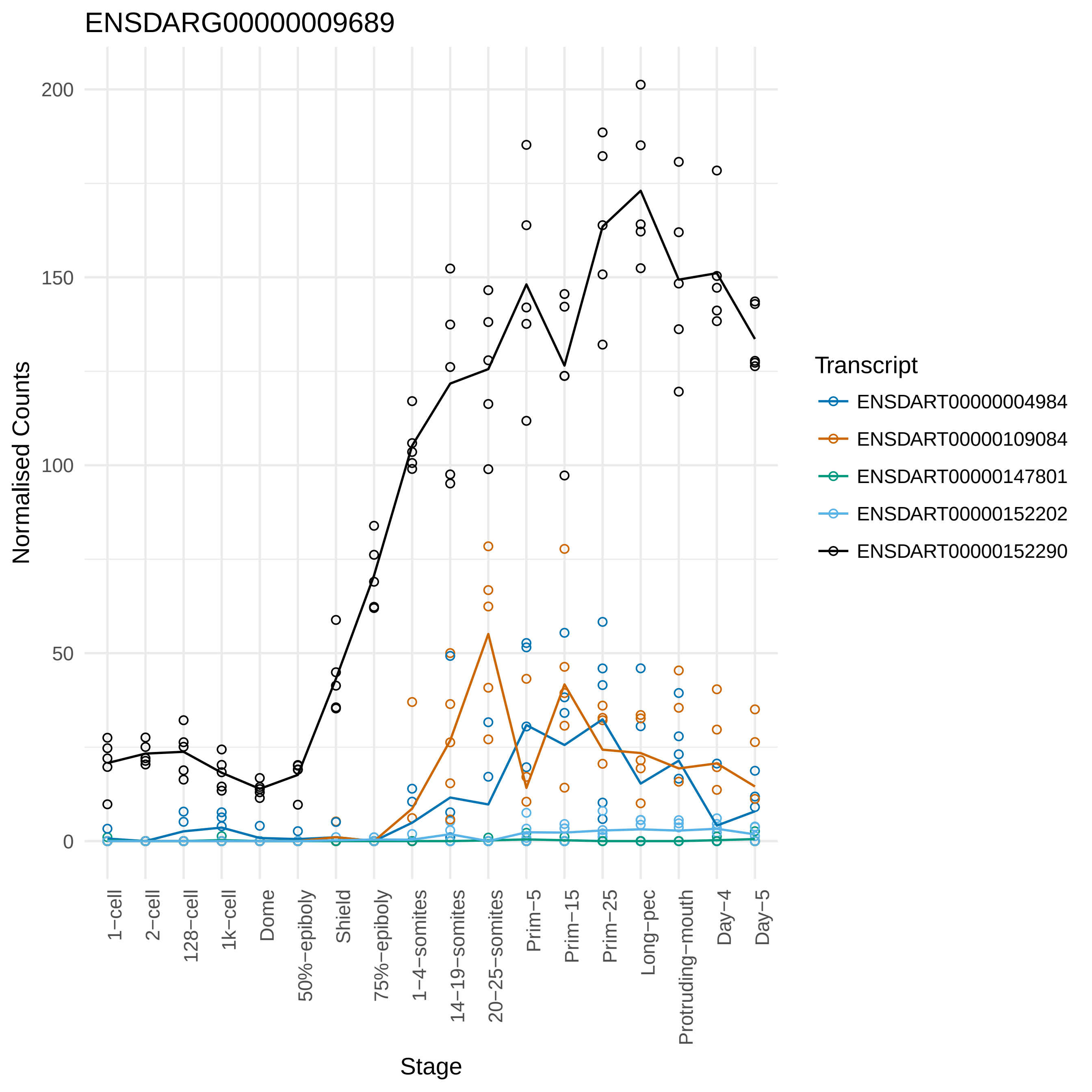

ENSDARG00000009689
Normalised Counts
0
50
100
150
200
Stage
1−cell
2−cell
128−cell
1k−cell
Dome
50%−epiboly
Shield
75%−epiboly
1−4−somites
14−19−somites
20−25−somites
Prim−5
Prim−15
Prim−25
Long−pec
Protruding−mouth
Day−4
Day−5
Transcript
ENSDART00000004984
ENSDART00000109084
ENSDART00000147801
ENSDART00000152202
ENSDART00000152290

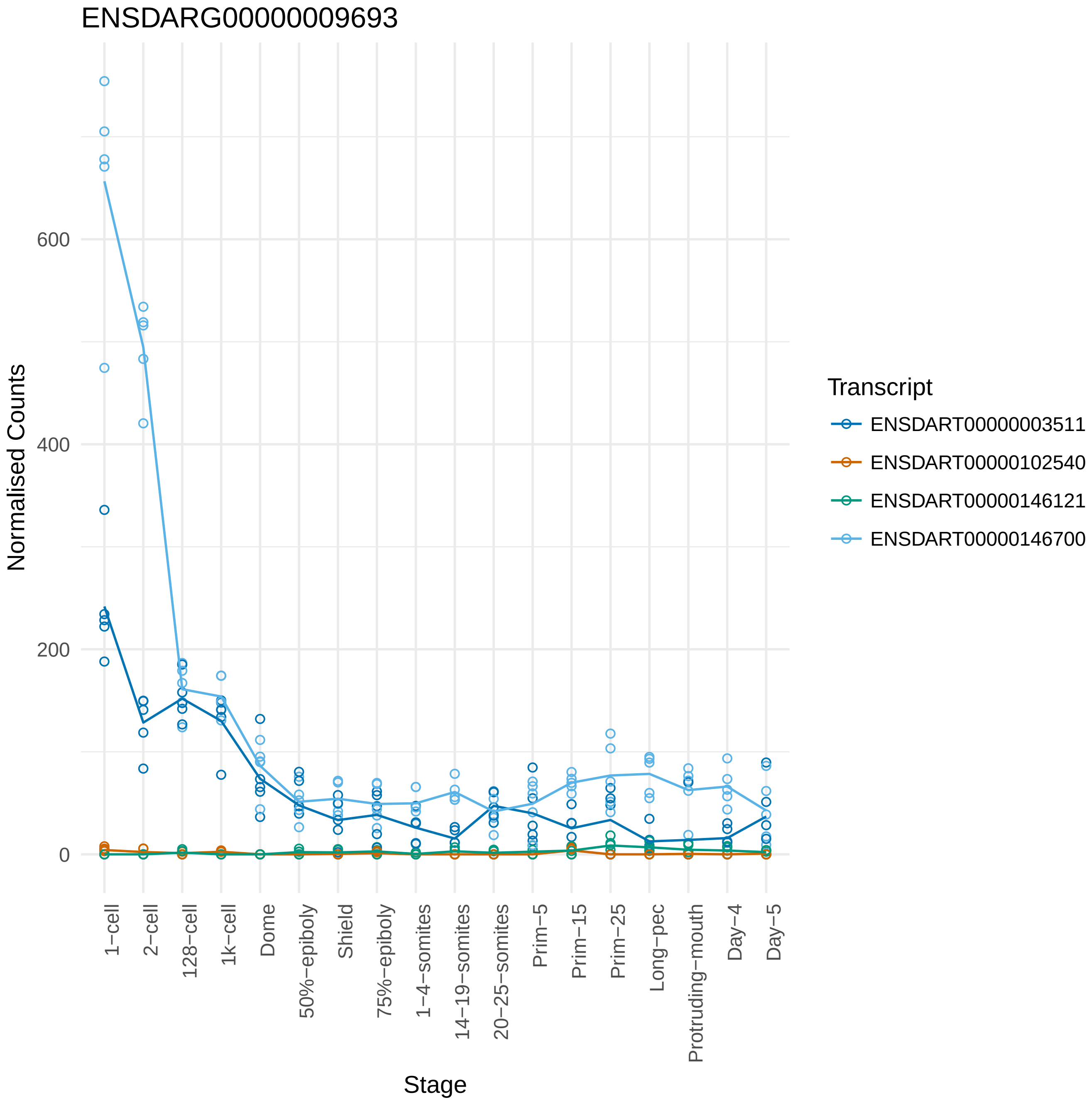

ENSDARG00000009693
Normalised Counts
600
400
200
0
1−cell
2−cell
128−cell
1k−cell
Dome
50%−epiboly
Shield
75%−epiboly
1−4−somites
14−19−somites
20−25−somites
Prim−5
Prim−15
Prim−25
Long−pec
Protruding−mouth
Day−4
Day−5
Stage
Transcript
ENSDART00000003511
ENSDART00000102540
ENSDART00000146121
ENSDART00000146700

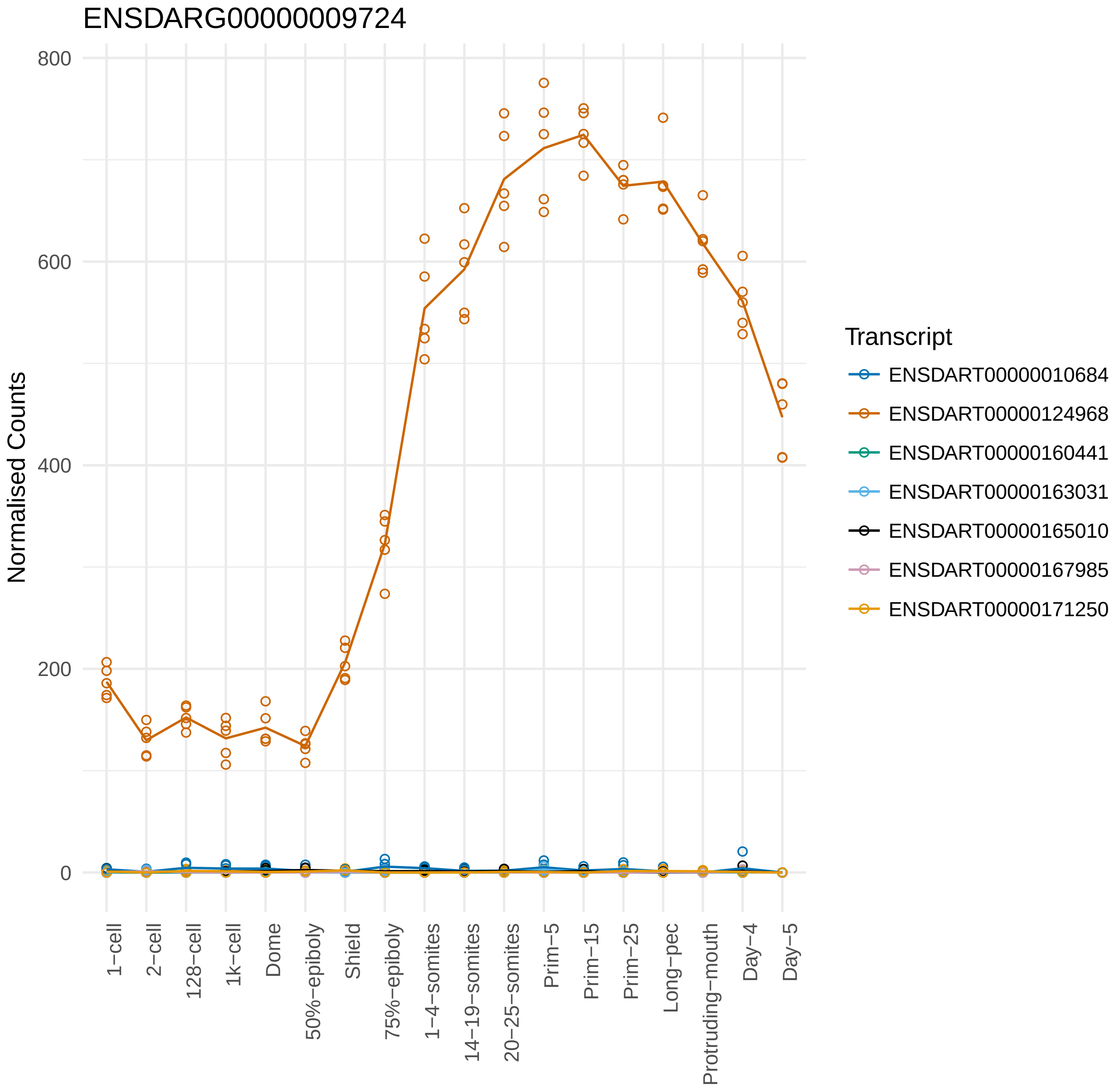

ENSDARG00000009724
Normalised Counts
800
600
400
200
0
1−cell
2−cell
128−cell
1k−cell
Dome
50%−epiboly
Shield
75%−epiboly
1−4−somites
14−19−somites
20−25−somites
Prim−5
Prim−15
Prim−25
Long−pec
Protruding−mouth
Day−4
Day−5
Stage
Transcript
ENSDART00000010684
ENSDART00000124968
ENSDART00000160441
ENSDART00000163031
ENSDART00000165010
ENSDART00000167985
ENSDART00000171250

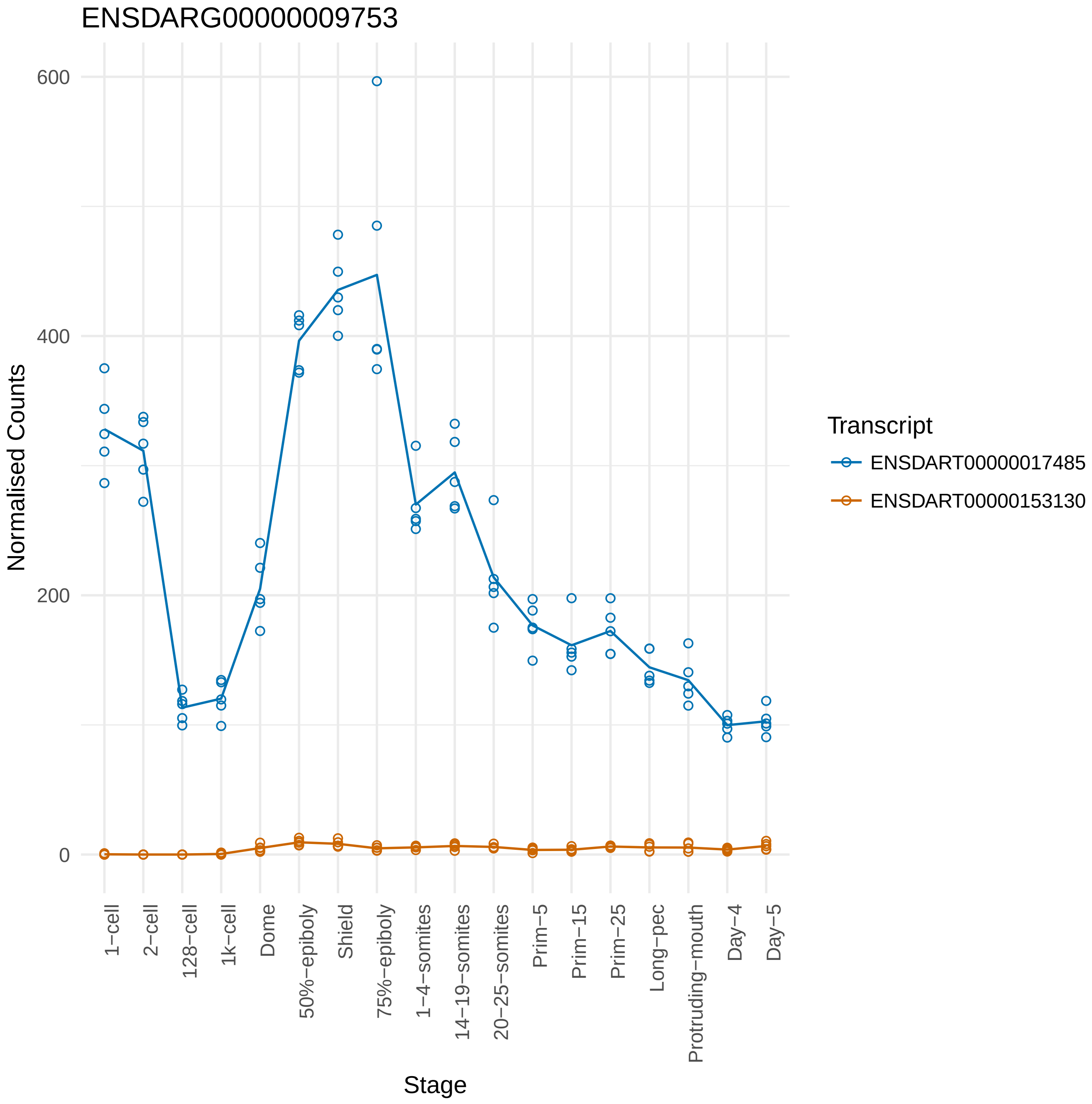

ENSDARG00000009753
Normalised Counts
600
400
200
0
1−cell
2−cell
128−cell
1k−cell
Dome
50%−epiboly
Shield
75%−epiboly
1−4−somites
14−19−somites
20−25−somites
Prim−5
Prim−15
Prim−25
Long−pec
Protruding−mouth
Day−4
Day−5
Stage
Transcript
ENSDART00000017485
ENSDART00000153130

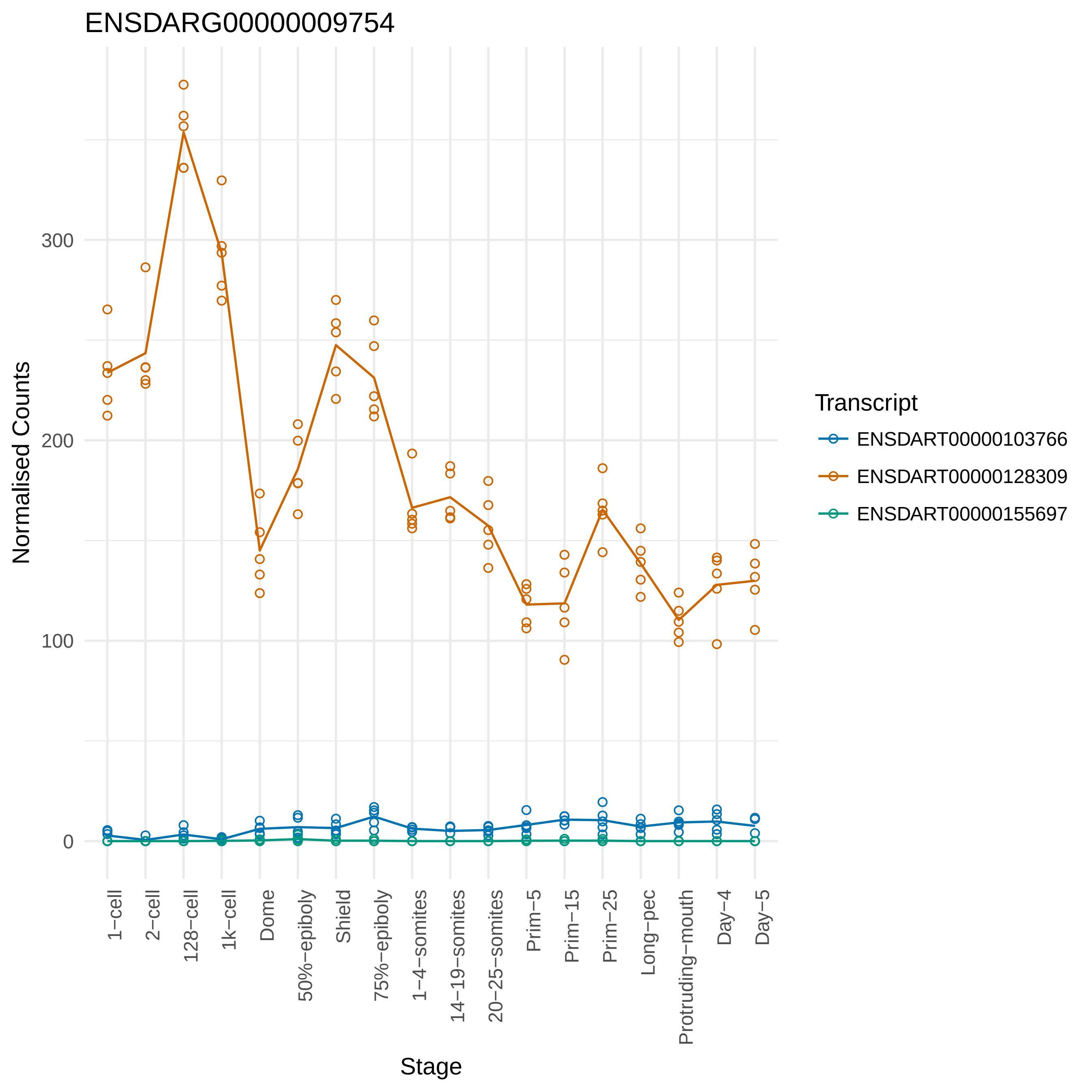

ENSDARG00000009754
Normalised Counts
300
200
100
0
1−cell
2−cell
128−cell
1k−cell
Dome
50%−epiboly
Shield
75%−epiboly
1−4−somites
14−19−somites
20−25−somites
Prim−5
Prim−15
Prim−25
Long−pec
Protruding−mouth
Day−4
Day−5
Stage
Transcript
ENSDART00000103766
ENSDART00000128309
ENSDART00000155697

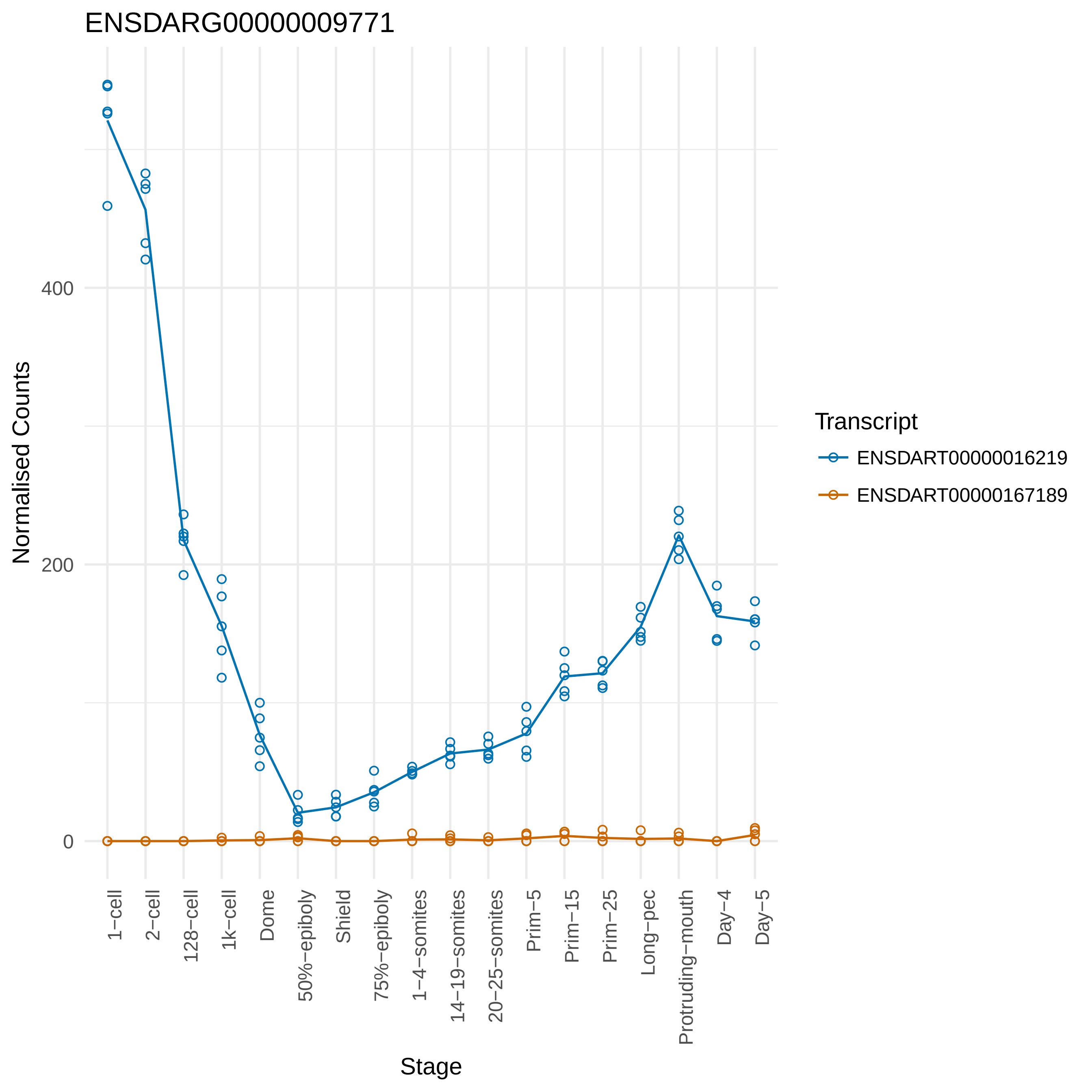

ENSDARG00000009771
Normalised Counts
400
200
0
1−cell
2−cell
128−cell
1k−cell
Dome
50%−epiboly
Shield
75%−epiboly
1−4−somites
14−19−somites
20−25−somites
Prim−5
Prim−15
Prim−25
Long−pec
Protruding−mouth
Day−4
Day−5
Stage
Transcript
ENSDART00000016219
ENSDART00000167189

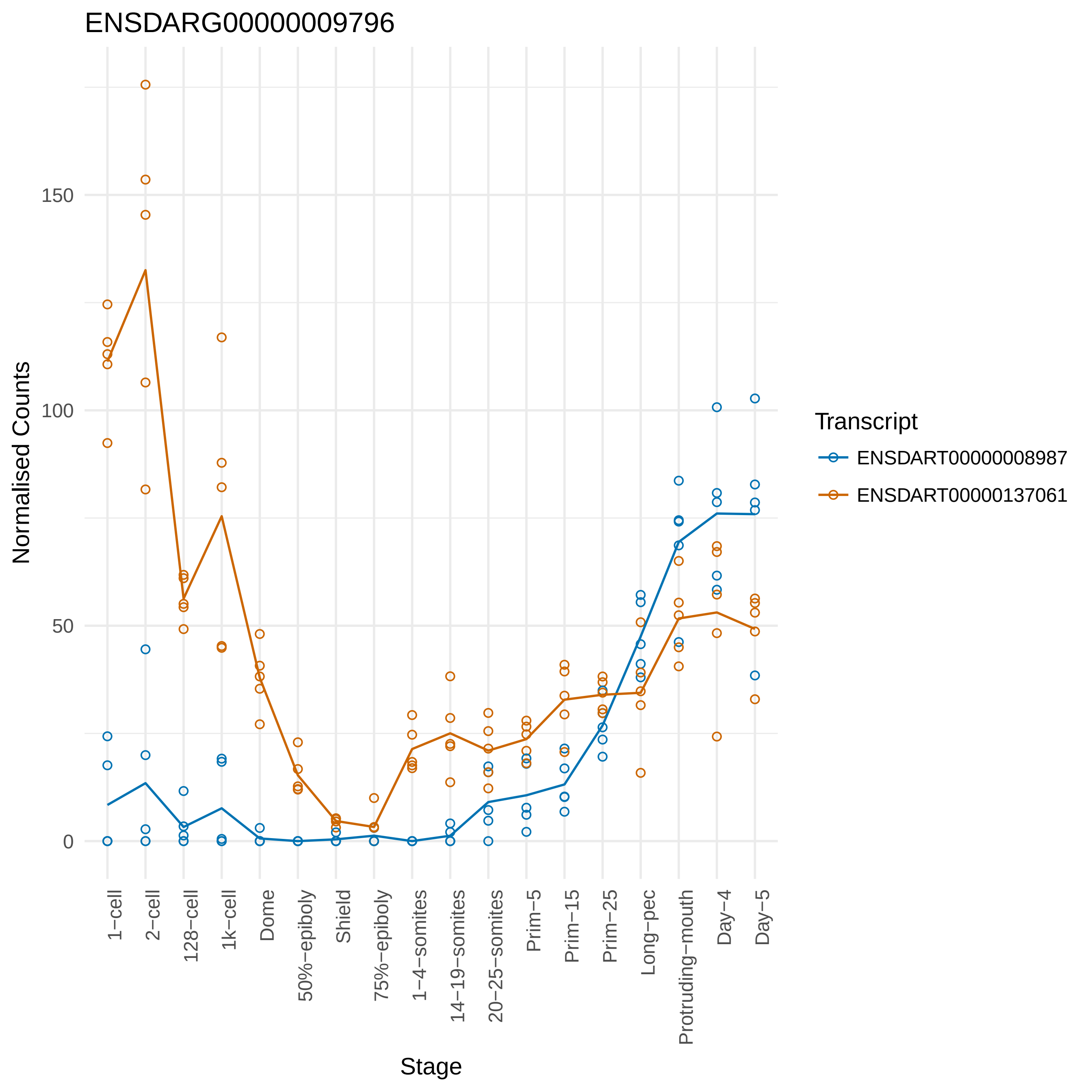

ENSDARG00000009796
Normalised Counts
150
100
50
0
1−cell
2−cell
128−cell
1k−cell
Dome
50%−epiboly
Shield
75%−epiboly
1−4−somites
14−19−somites
20−25−somites
Prim−5
Prim−15
Prim−25
Long−pec
Protruding−mouth
Day−4
Day−5
Stage
Transcript
ENSDART00000008987
ENSDART00000137061

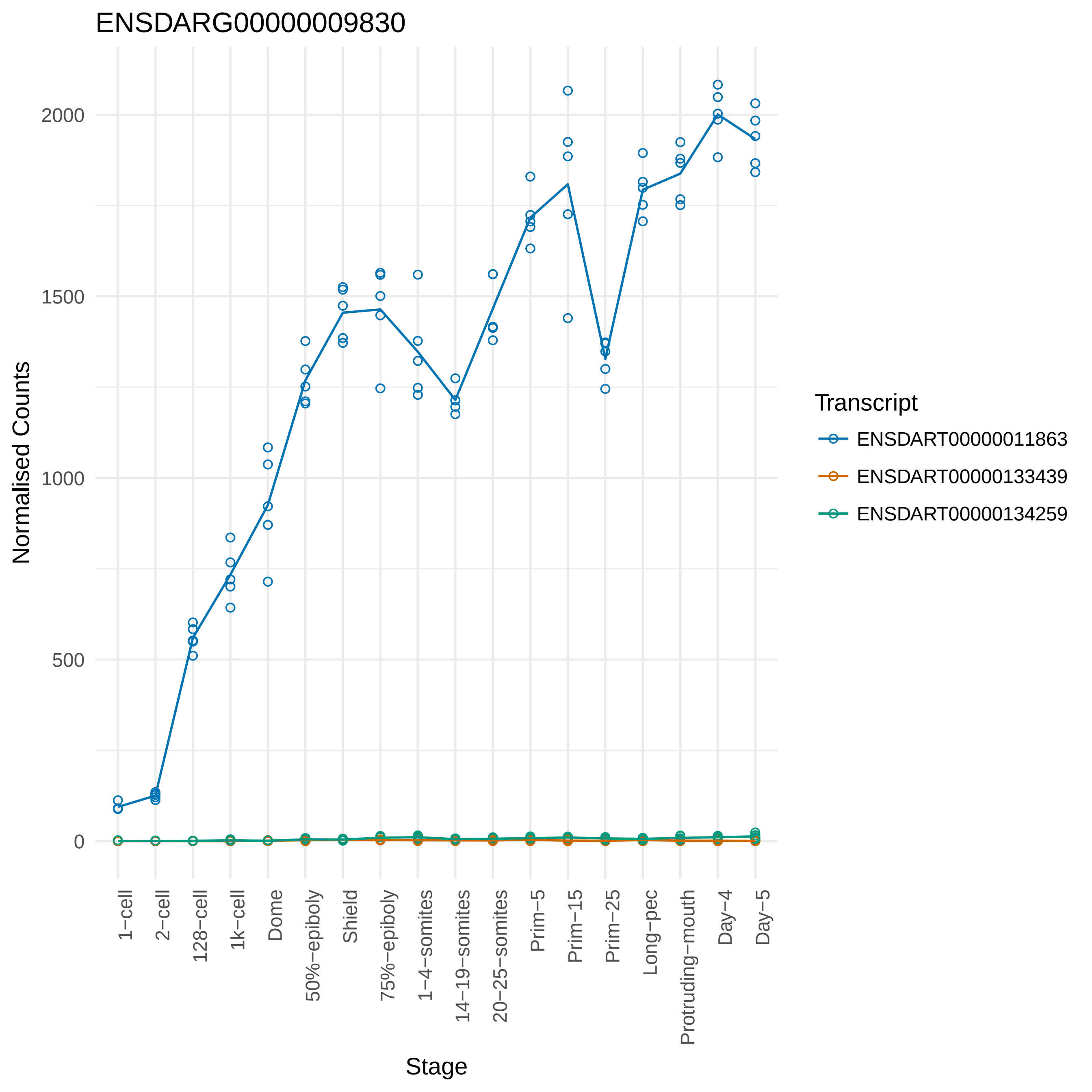

ENSDARG00000009830
Normalised Counts
2000
1500
1000
500
0
1−cell
2−cell
128−cell
1k−cell
Dome
50%−epiboly
Shield
75%−epiboly
1−4−somites
14−19−somites
20−25−somites
Prim−5
Prim−15
Prim−25
Long−pec
Protruding−mouth
Day−4
Day−5
Stage
Transcript
ENSDART00000011863
ENSDART00000133439
ENSDART00000134259

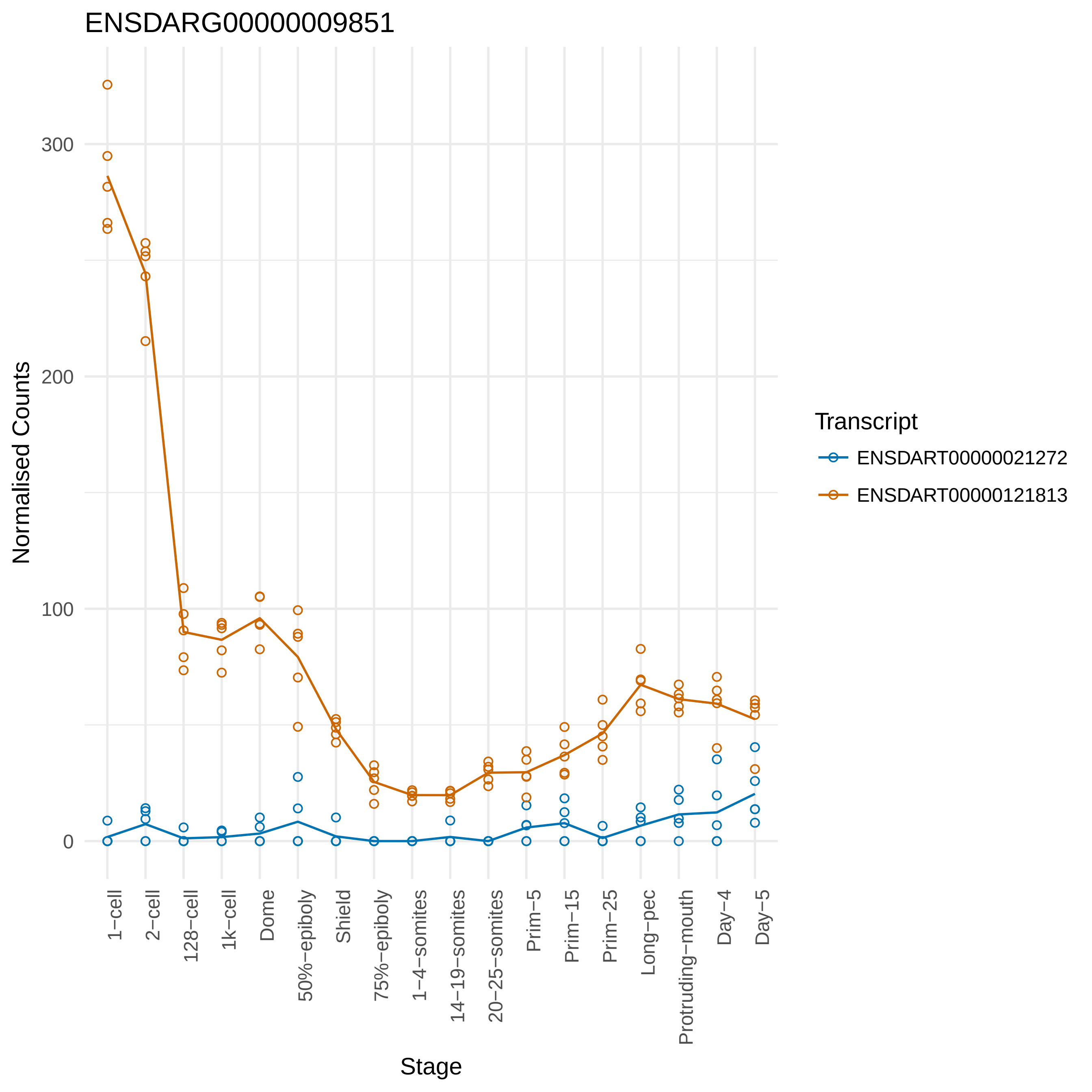

ENSDARG00000009851
Normalised Counts
300
200
100
0
1−cell
2−cell
128−cell
1k−cell
Dome
50%−epiboly
Shield
75%−epiboly
1−4−somites
14−19−somites
20−25−somites
Prim−5
Prim−15
Prim−25
Long−pec
Protruding−mouth
Day−4
Day−5
Stage
Transcript
ENSDART00000021272
ENSDART00000121813

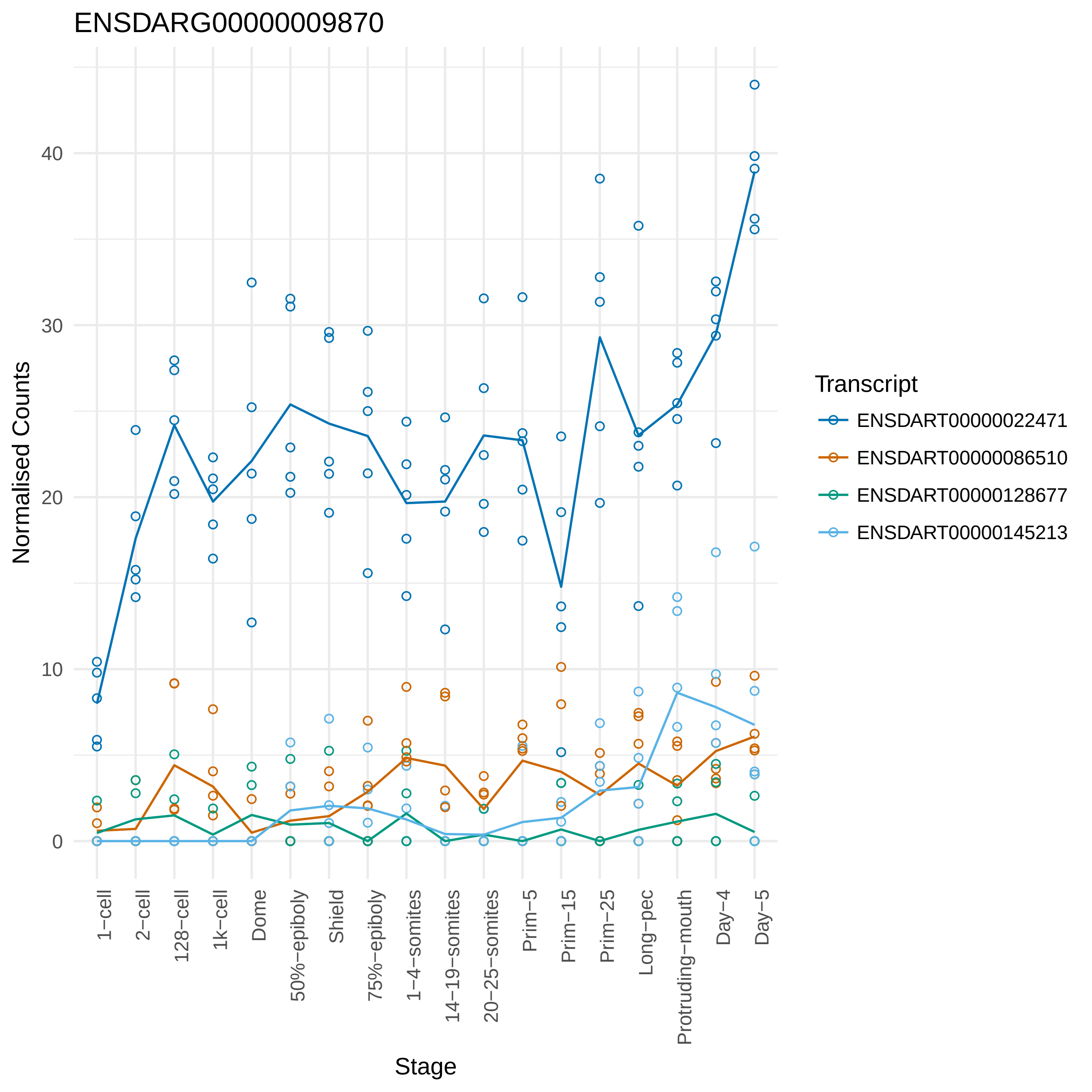

ENSDARG00000009870
Normalised Counts
0
10
20
30
40
Stage
1−cell
2−cell
128−cell
1k−cell
Dome
50%−epiboly
Shield
75%−epiboly
1−4−somites
14−19−somites
20−25−somites
Prim−5
Prim−15
Prim−25
Long−pec
Protruding−mouth
Day−4
Day−5
Transcript
ENSDART00000022471
ENSDART00000086510
ENSDART00000128677
ENSDART00000145213

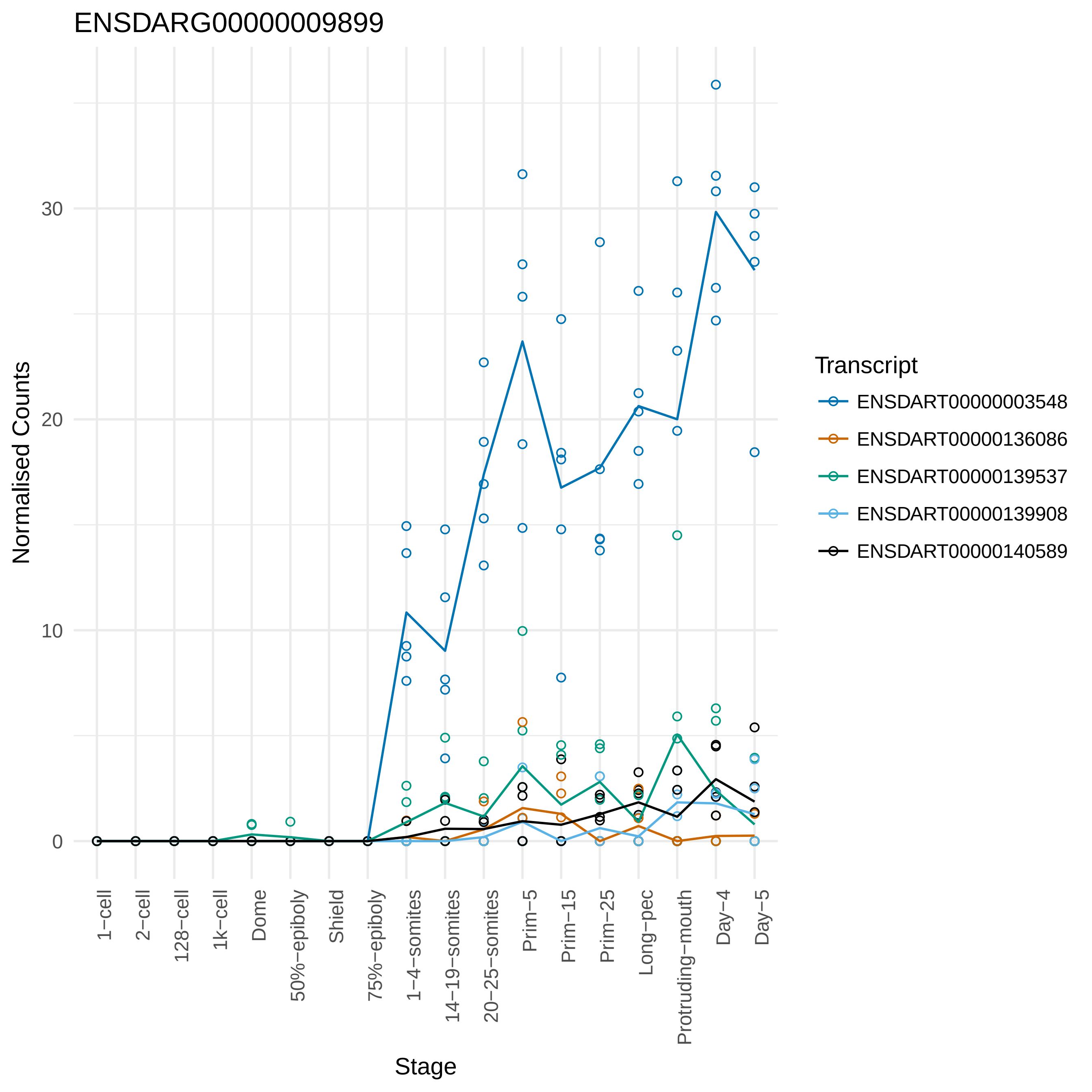

ENSDARG00000009899
Normalised Counts
30
20
10
0
1−cell
2−cell
128−cell
1k−cell
Dome
50%−epiboly
Shield
75%−epiboly
1−4−somites
14−19−somites
20−25−somites
Prim−5
Prim−15
Prim−25
Long−pec
Protruding−mouth
Day−4
Day−5
Stage
Transcript
ENSDART00000003548
ENSDART00000136086
ENSDART00000139537
ENSDART00000139908
ENSDART00000140589

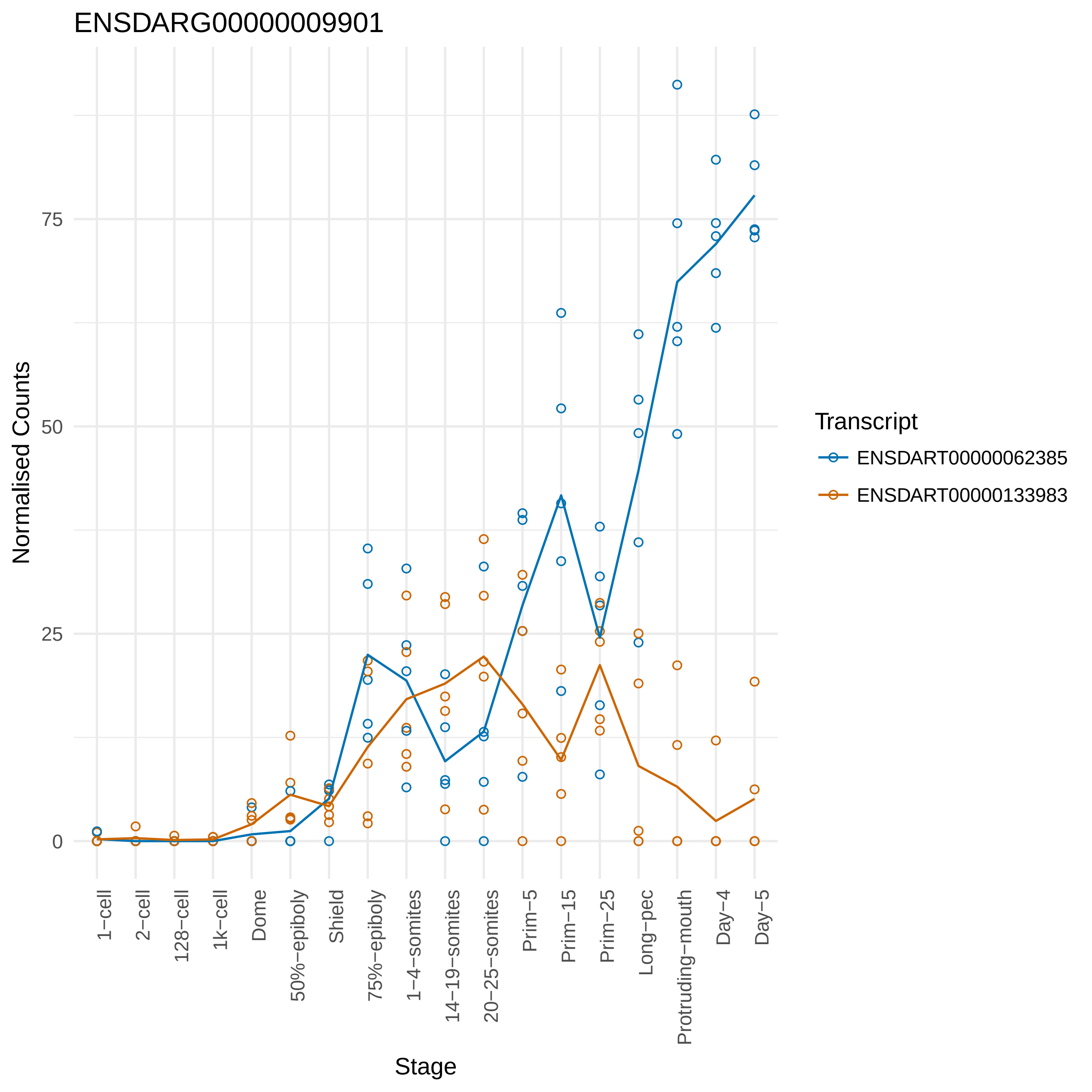
ENSDARG00000009901
Normalised Counts
75
50
25
0
1−cell
2−cell
128−cell
1k−cell
Dome
50%−epiboly
Shield
75%−epiboly
1−4−somites
14−19−somites
20−25−somites
Prim−5
Prim−15
Prim−25
Long−pec
Protruding−mouth
Day−4
Day−5
Stage
Transcript
ENSDART00000062385
ENSDART00000133983

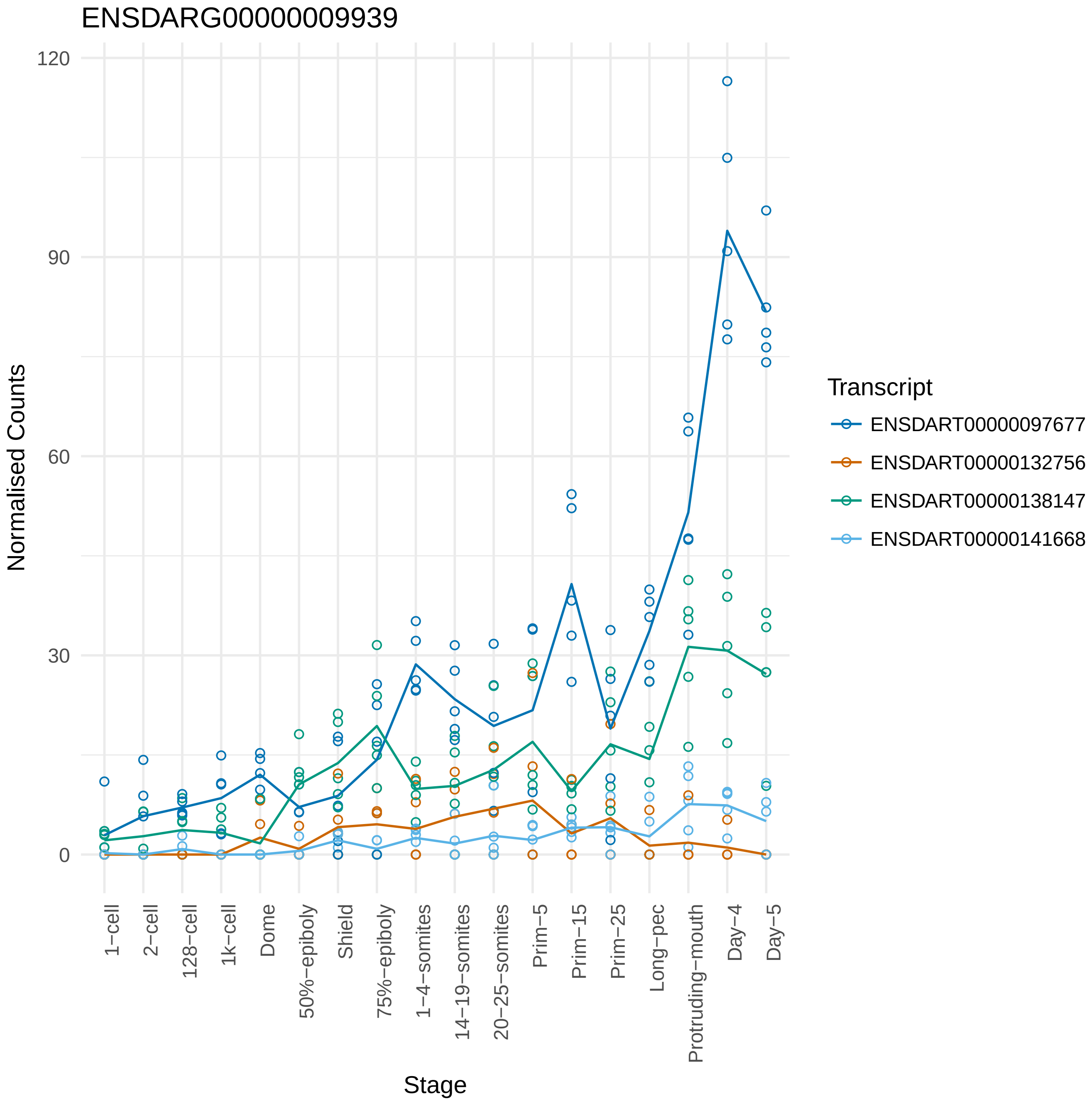

ENSDARG00000009939
Normalised Counts
120
90
60
30
0
1−cell
2−cell
128−cell
1k−cell
Dome
50%−epiboly
Shield
75%−epiboly
1−4−somites
14−19−somites
20−25−somites
Prim−5
Prim−15
Prim−25
Long−pec
Protruding−mouth
Day−4
Day−5
Stage
Transcript
ENSDART00000097677
ENSDART00000132756
ENSDART00000138147
ENSDART00000141668

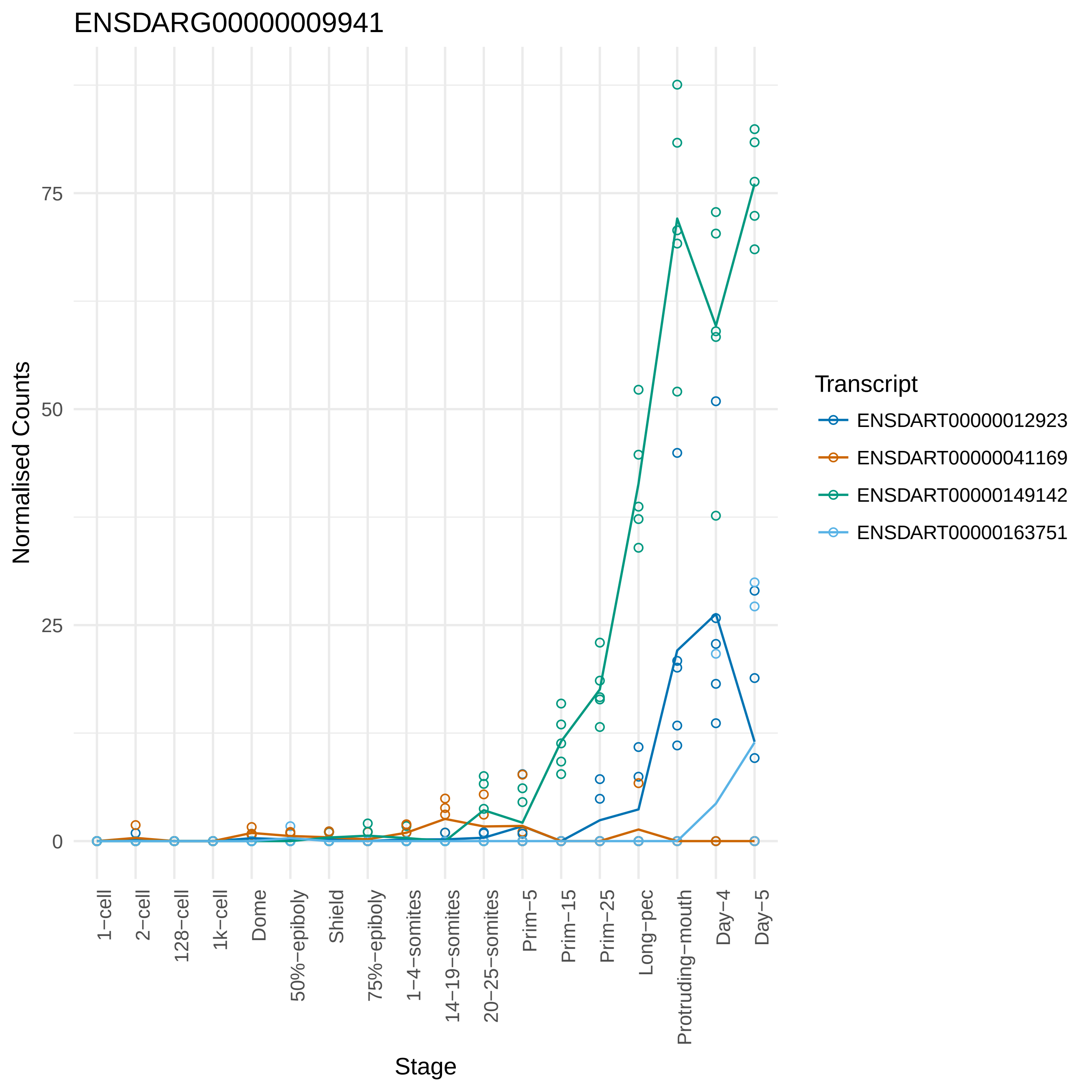
ENSDARG00000009941
Normalised Counts
0
25
50
75
Stage
1−cell
2−cell
128−cell
1k−cell
Dome
50%−epiboly
Shield
75%−epiboly
1−4−somites
14−19−somites
20−25−somites
Prim−5
Prim−15
Prim−25
Long−pec
Protruding−mouth
Day−4
Day−5
Transcript
ENSDART00000012923
ENSDART00000041169
ENSDART00000149142
ENSDART00000163751

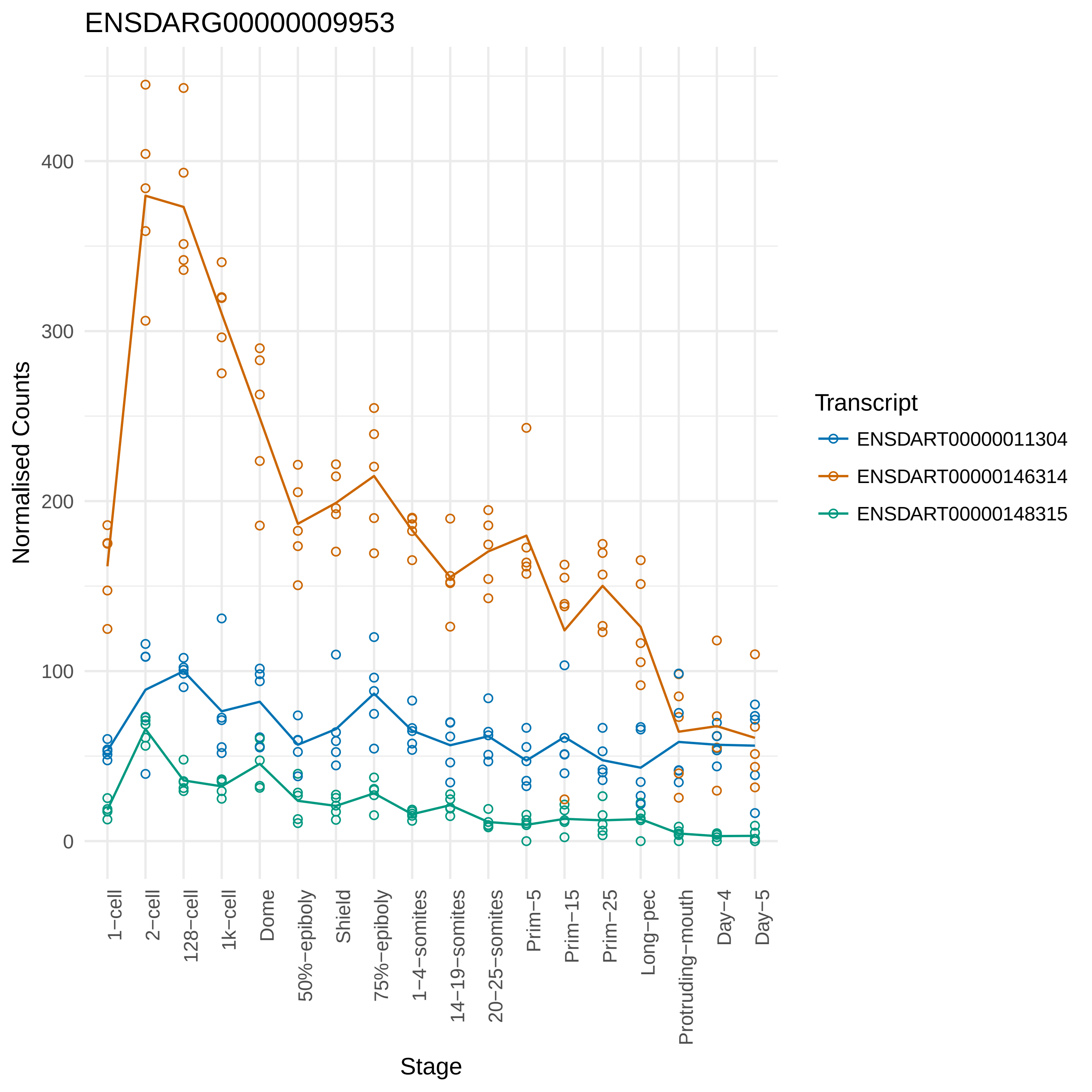

ENSDARG00000009953
Normalised Counts
0
100
200
300
400
Stage
1−cell
2−cell
128−cell
1k−cell
Dome
50%−epiboly
Shield
75%−epiboly
1−4−somites
14−19−somites
20−25−somites
Prim−5
Prim−15
Prim−25
Long−pec
Protruding−mouth
Day−4
Day−5
Transcript
ENSDART00000011304
ENSDART00000146314
ENSDART00000148315

ENSDARG00000010002

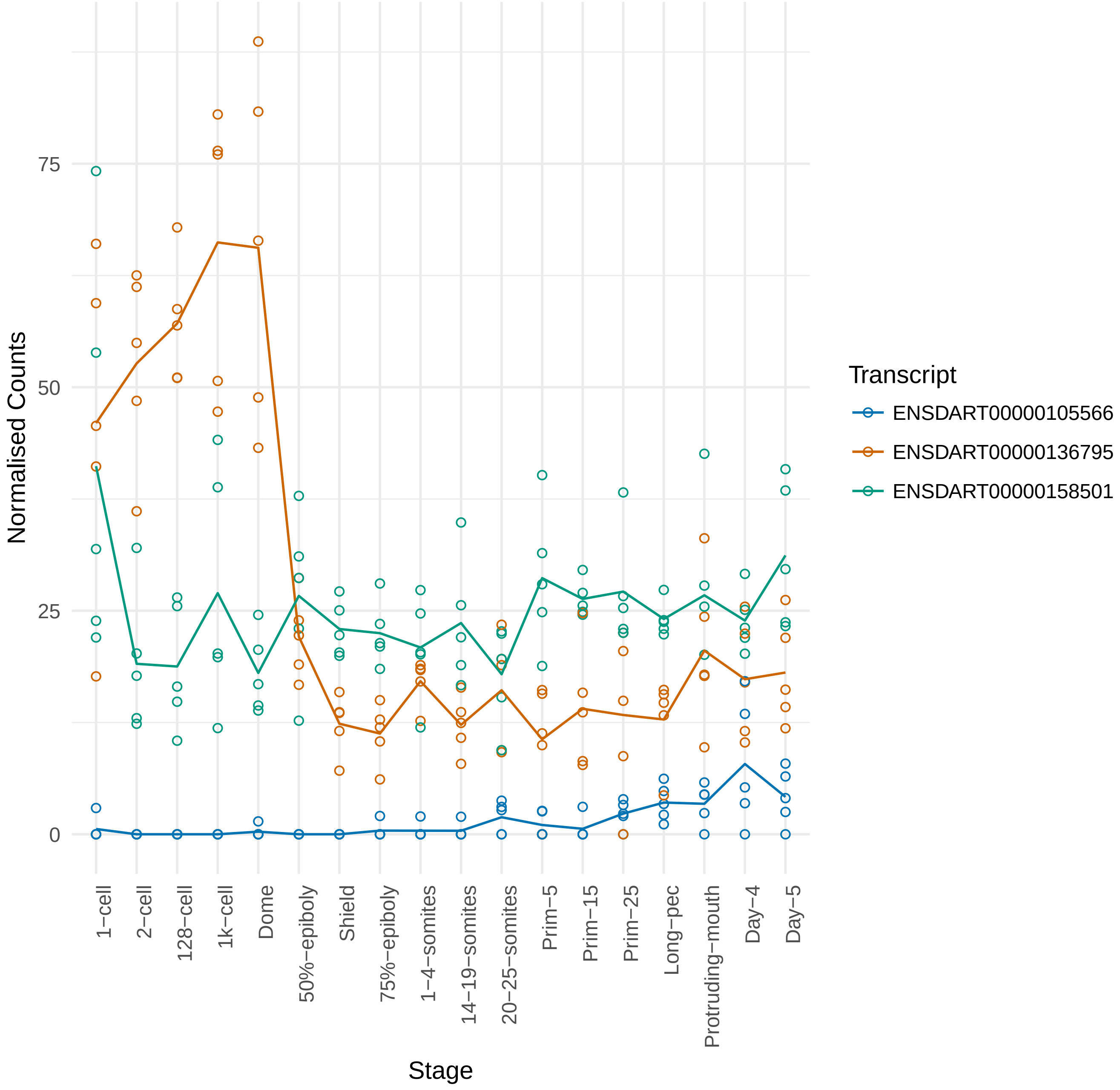

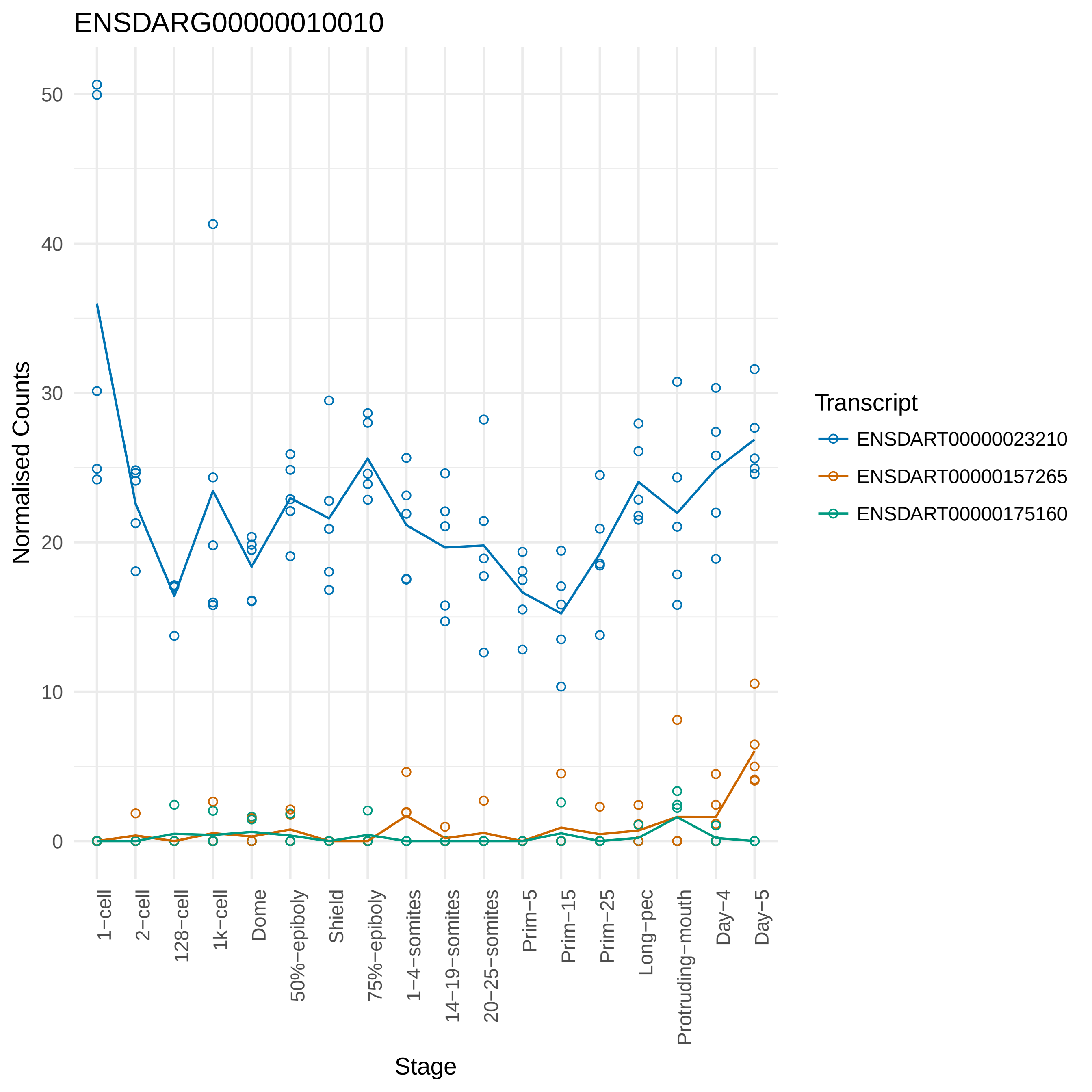

ENSDARG00000010010
Normalised Counts
50
40
30
20
10
0
Stage
1−cell
2−cell
128−cell
1k−cell
Dome
50%−epiboly
Shield
75%−epiboly
1−4−somites
14−19−somites
20−25−somites
Prim−5
Prim−15
Prim−25
Long−pec
Protruding−mouth
Day−4
Day−5
Transcript
ENSDART00000023210
ENSDART00000157265
ENSDART00000175160

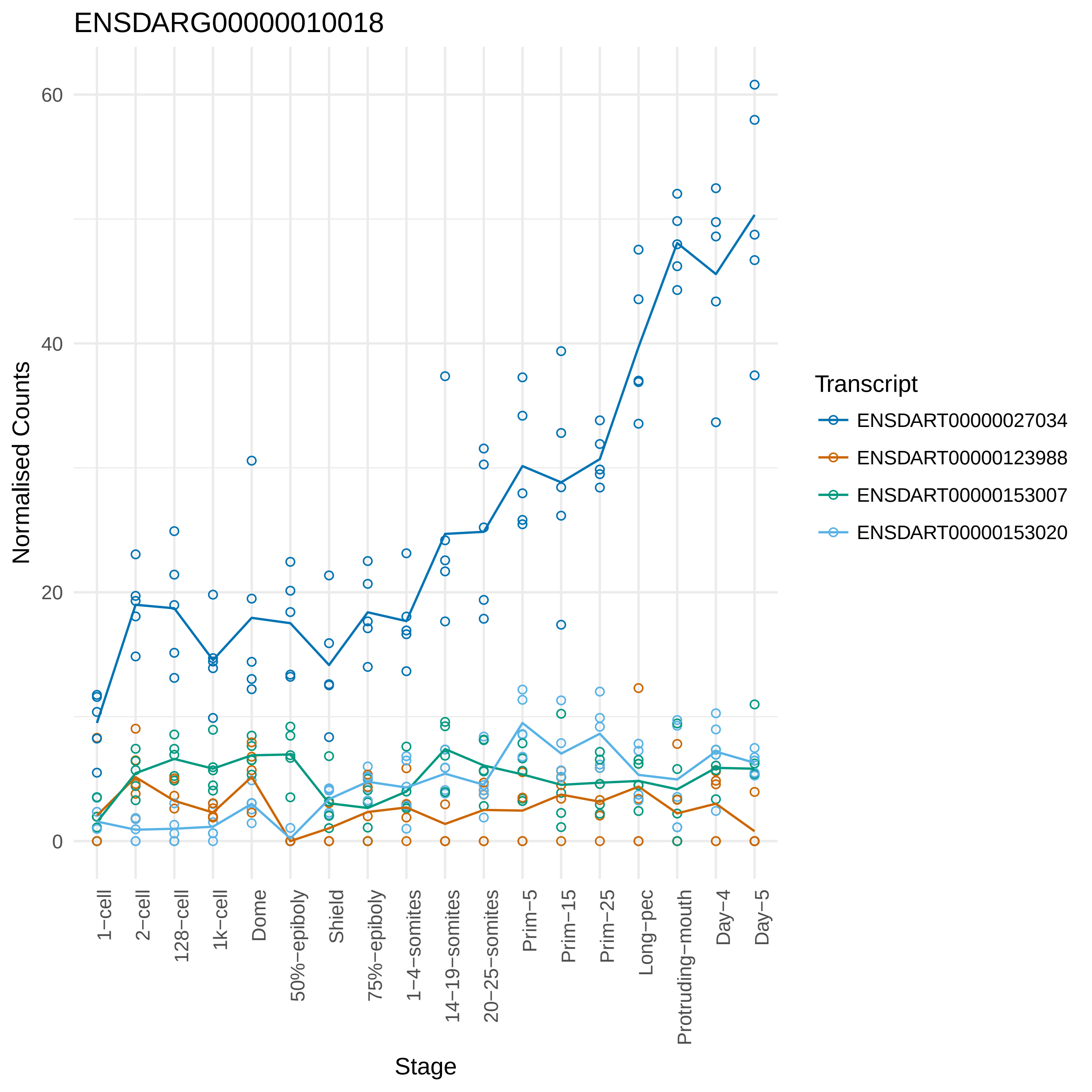

ENSDARG00000010018
Normalised Counts
0
20
40
60
1−cell
2−cell
128−cell
1k−cell
Dome
50%−epiboly
Shield
75%−epiboly
1−4−somites
14−19−somites
20−25−somites
Prim−5
Prim−15
Prim−25
Long−pec
Protruding−mouth
Day−4
Day−5
Stage
Transcript
ENSDART00000027034
ENSDART00000123988
ENSDART00000153007
ENSDART00000153020

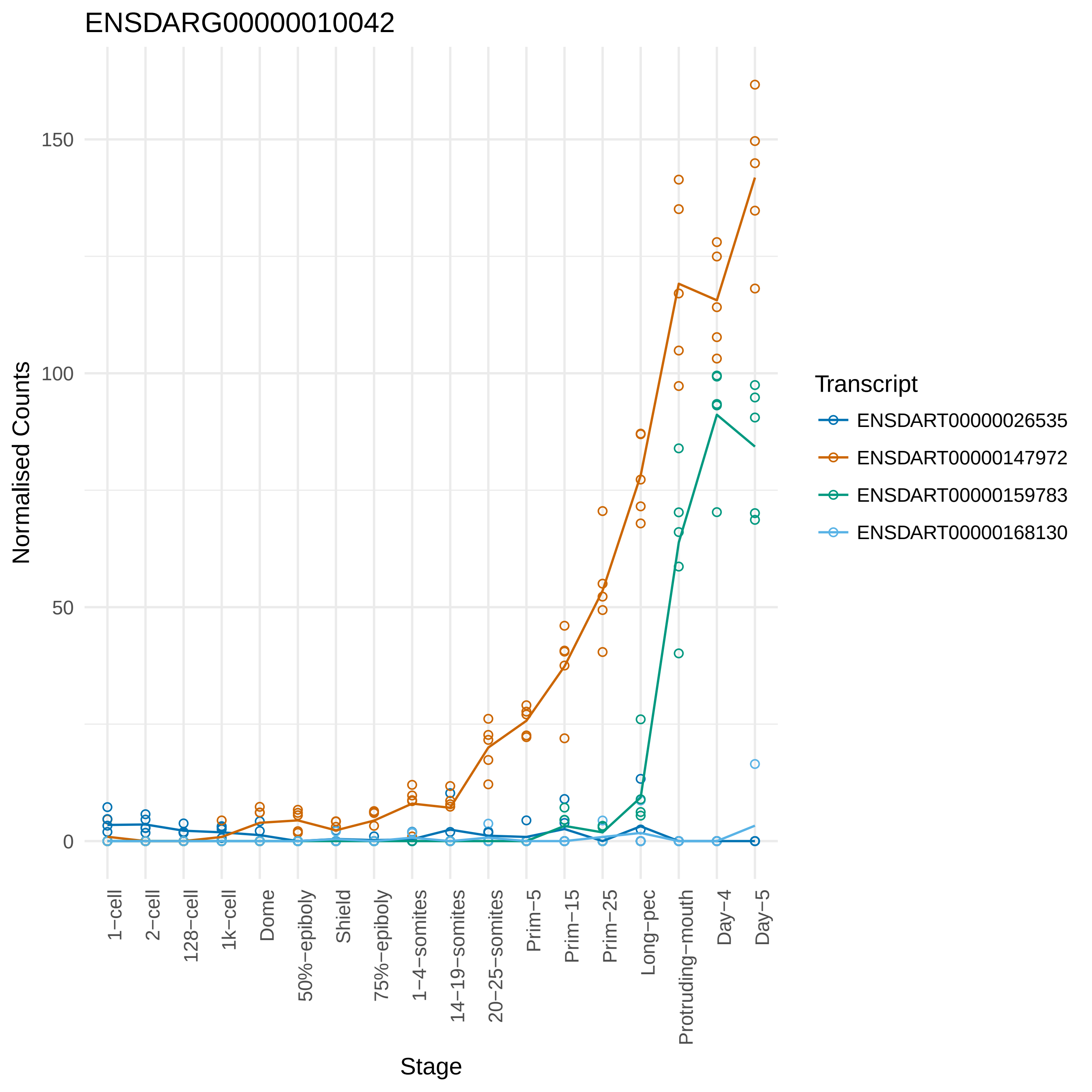
ENSDARG00000010042
Normalised Counts
150
100
50
0
1−cell
2−cell
128−cell
1k−cell
Dome
50%−epiboly
Shield
75%−epiboly
1−4−somites
14−19−somites
20−25−somites
Prim−5
Prim−15
Prim−25
Long−pec
Protruding−mouth
Day−4
Day−5
Stage
Transcript
ENSDART00000026535
ENSDART00000147972
ENSDART00000159783
ENSDART00000168130

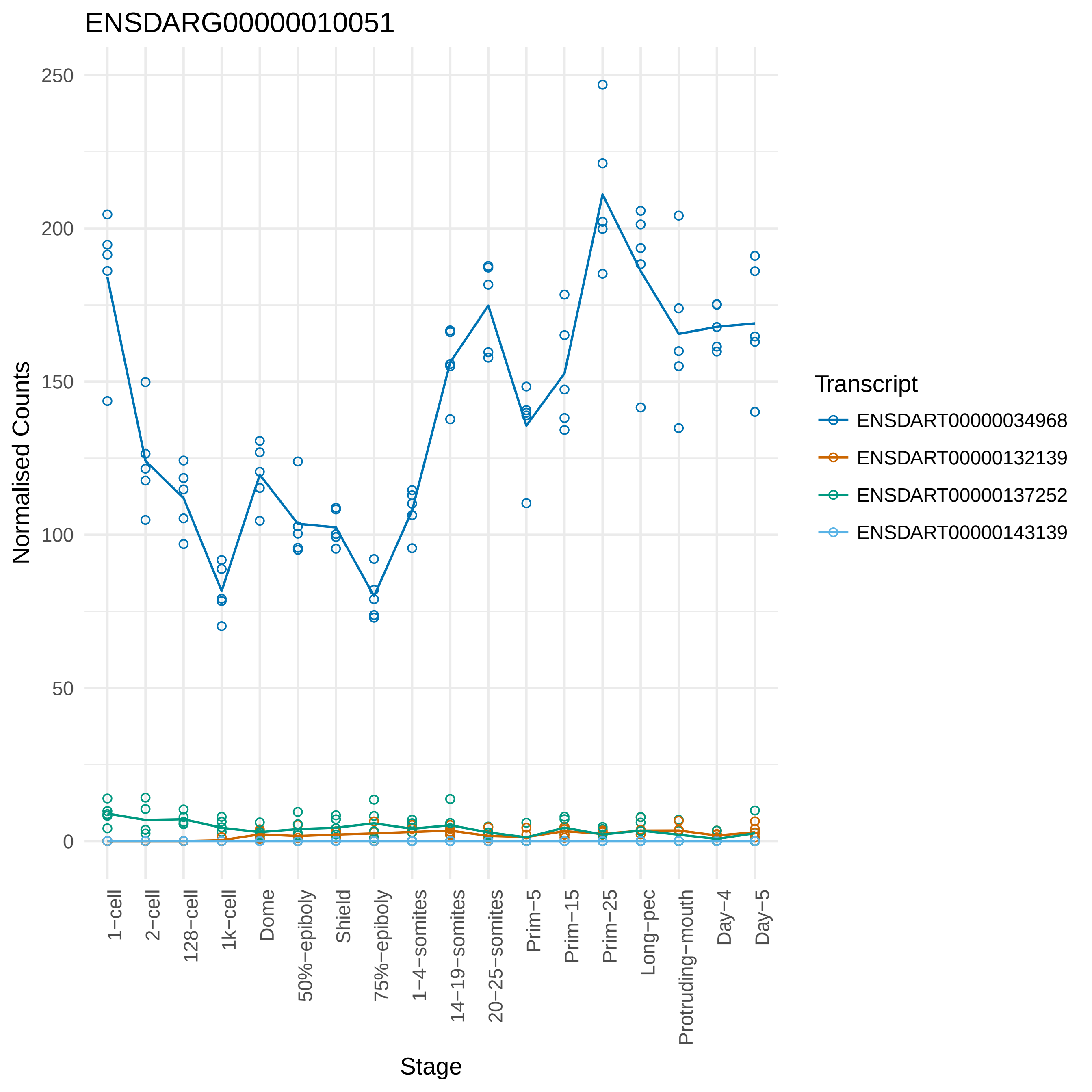

ENSDARG00000010051
Normalised Counts
250
200
150
100
50
0
Stage
1−cell
2−cell
128−cell
1k−cell
Dome
50%−epiboly
Shield
75%−epiboly
1−4−somites
14−19−somites
20−25−somites
Prim−5
Prim−15
Prim−25
Long−pec
Protruding−mouth
Day−4
Day−5
Transcript
ENSDART00000034968
ENSDART00000132139
ENSDART00000137252
ENSDART00000143139

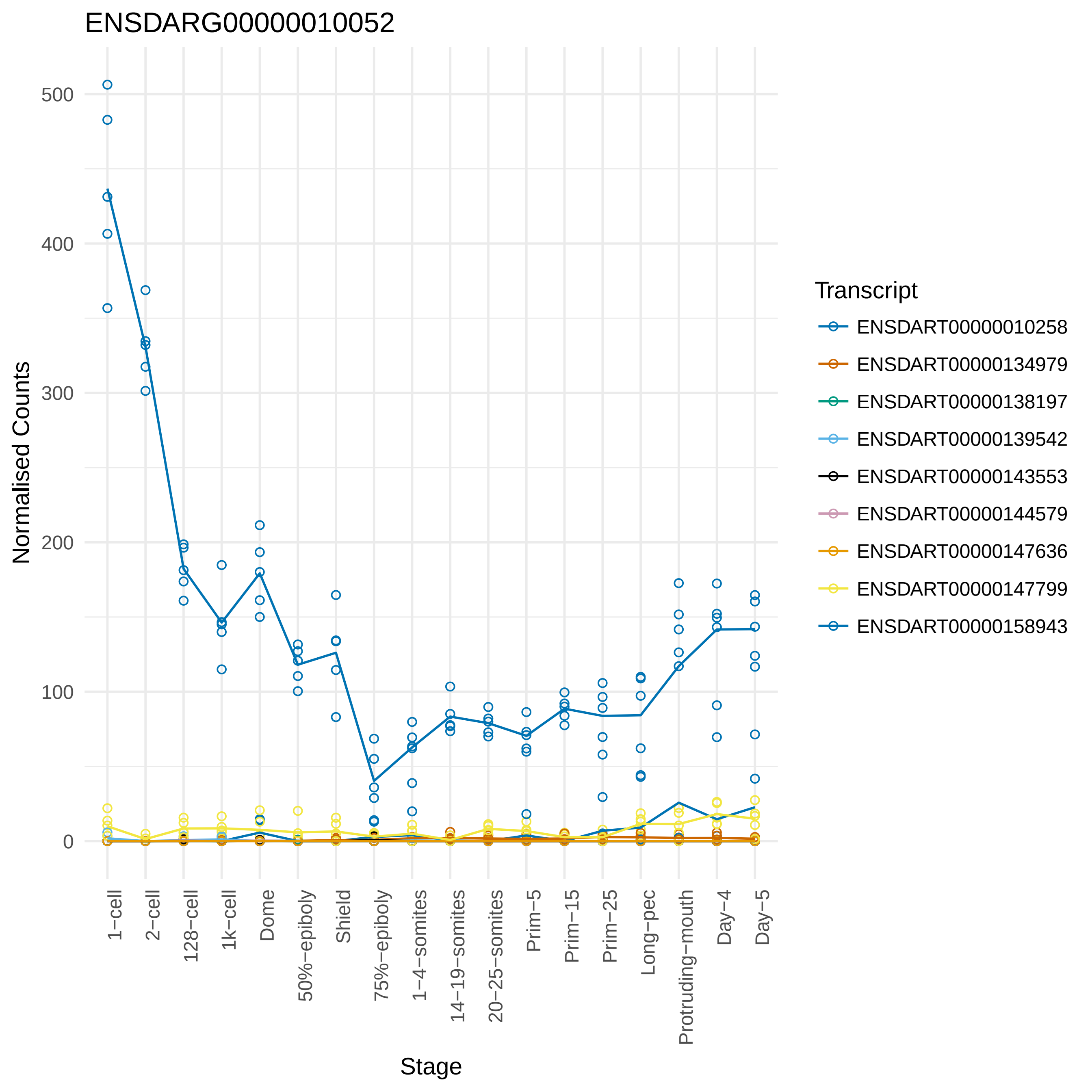
ENSDARG00000010052
Normalised Counts
500
400
300
200
100
0
Stage
1−cell
2−cell
128−cell
1k−cell
Dome
50%−epiboly
Shield
75%−epiboly
1−4−somites
14−19−somites
20−25−somites
Prim−5
Prim−15
Prim−25
Long−pec
Protruding−mouth
Day−4
Day−5
Transcript
ENSDART00000010258
ENSDART00000134979
ENSDART00000138197
ENSDART00000139542
ENSDART00000143553
ENSDART00000144579
ENSDART00000147636
ENSDART00000147799
ENSDART00000158943

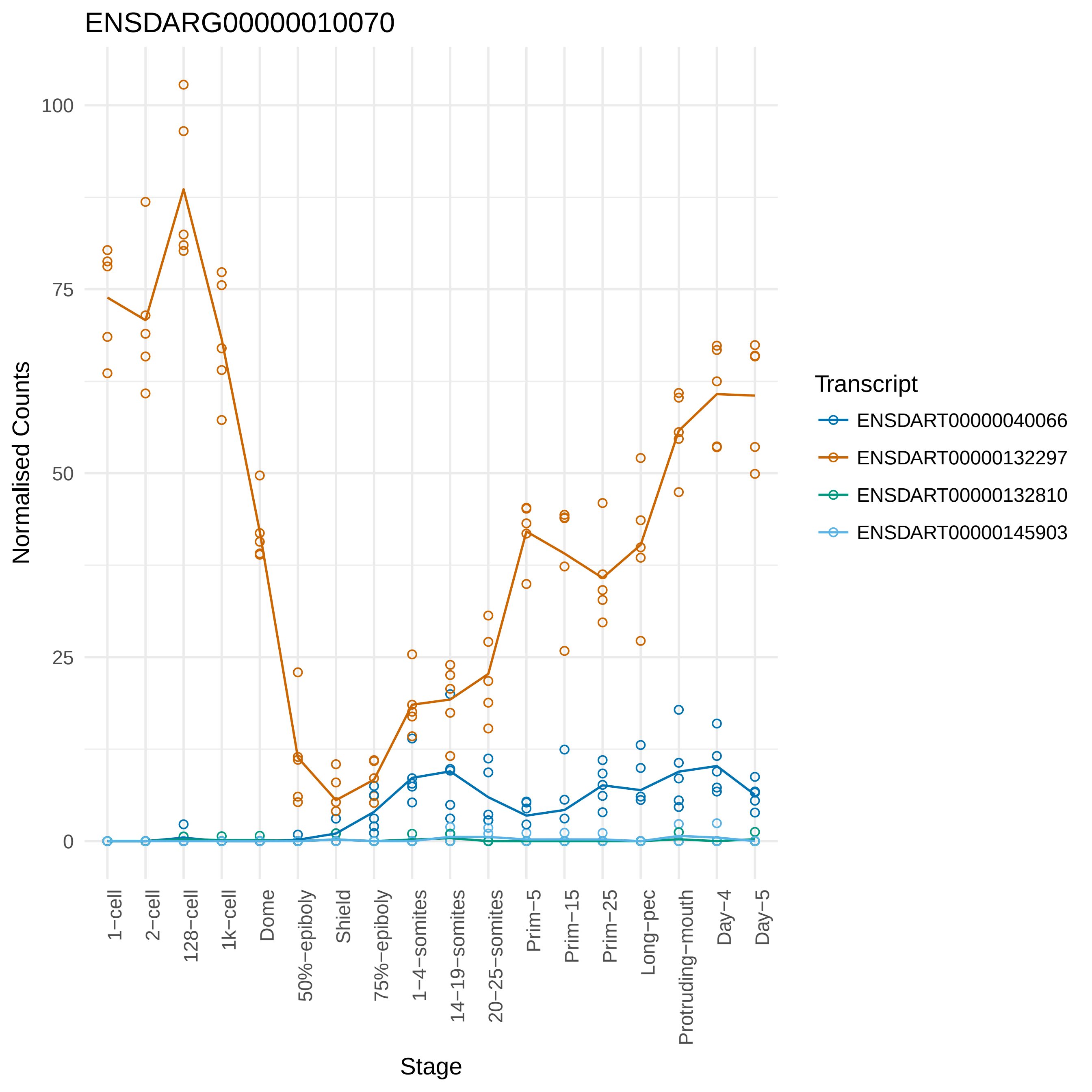

ENSDARG00000010070
Normalised Counts
100
75
50
25
0
1−cell
2−cell
128−cell
1k−cell
Dome
50%−epiboly
Shield
75%−epiboly
1−4−somites
14−19−somites
20−25−somites
Prim−5
Prim−15
Prim−25
Long−pec
Protruding−mouth
Day−4
Day−5
Stage
Transcript
ENSDART00000040066
ENSDART00000132297
ENSDART00000132810
ENSDART00000145903

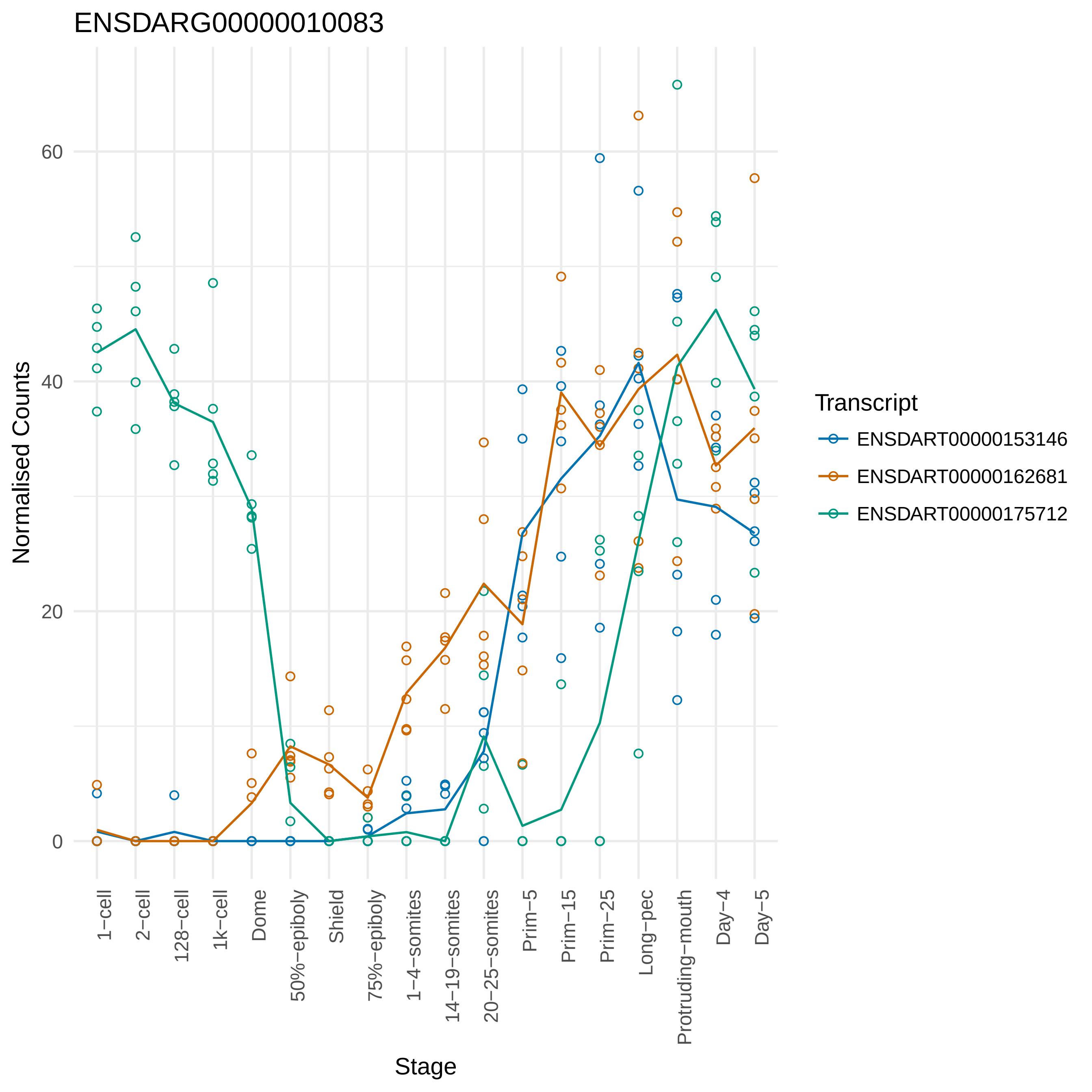

ENSDARG00000010083
Normalised Counts
60
40
20
0
1−cell
2−cell
128−cell
1k−cell
Dome
50%−epiboly
Shield
75%−epiboly
1−4−somites
14−19−somites
20−25−somites
Prim−5
Prim−15
Prim−25
Long−pec
Protruding−mouth
Day−4
Day−5
Stage
Transcript
ENSDART00000153146
ENSDART00000162681
ENSDART00000175712

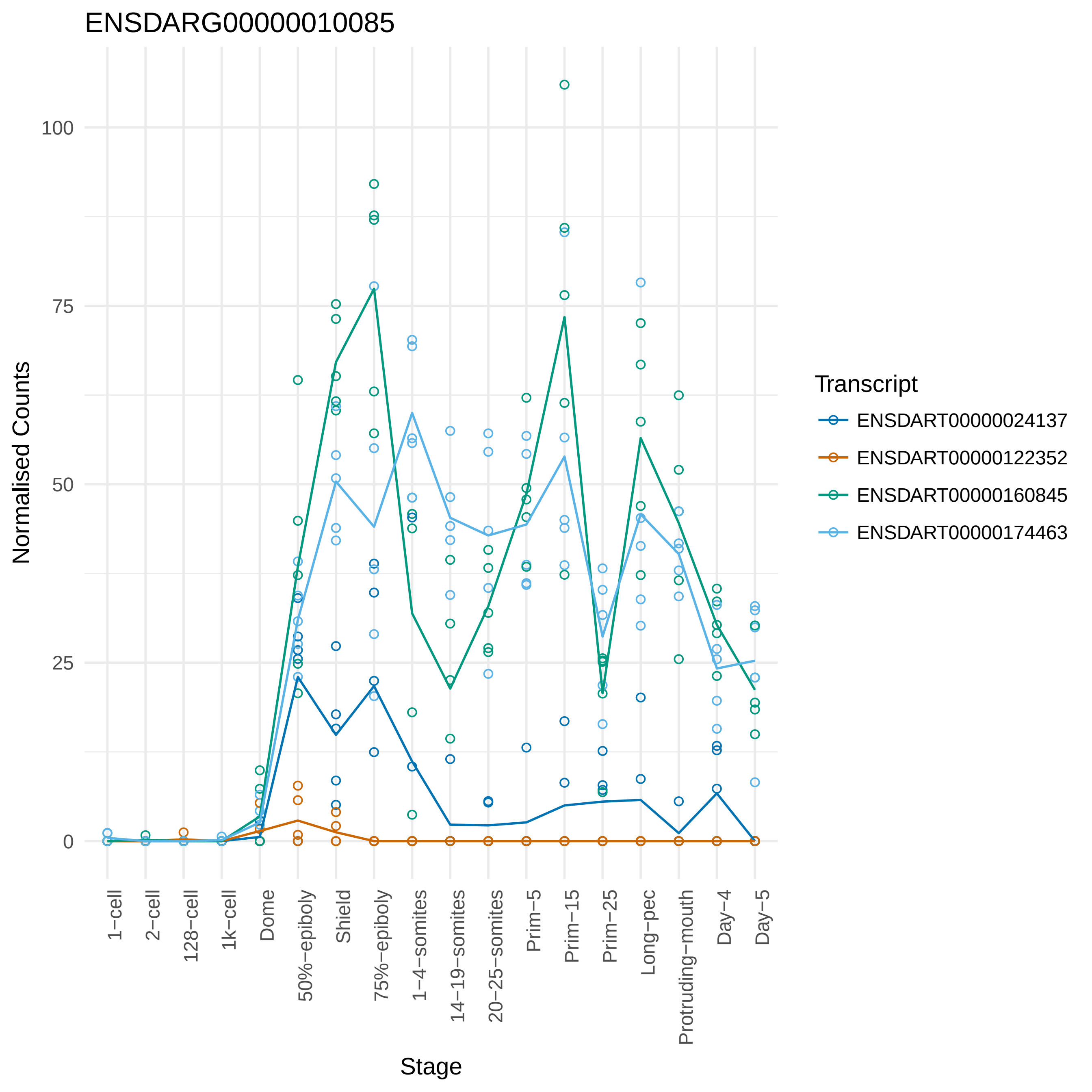

ENSDARG00000010085
Normalised Counts
0
25
50
75
100
Stage
1−cell
2−cell
128−cell
1k−cell
Dome
50%−epiboly
Shield
75%−epiboly
1−4−somites
14−19−somites
20−25−somites
Prim−5
Prim−15
Prim−25
Long−pec
Protruding−mouth
Day−4
Day−5
Transcript
ENSDART00000024137
ENSDART00000122352
ENSDART00000160845
ENSDART00000174463

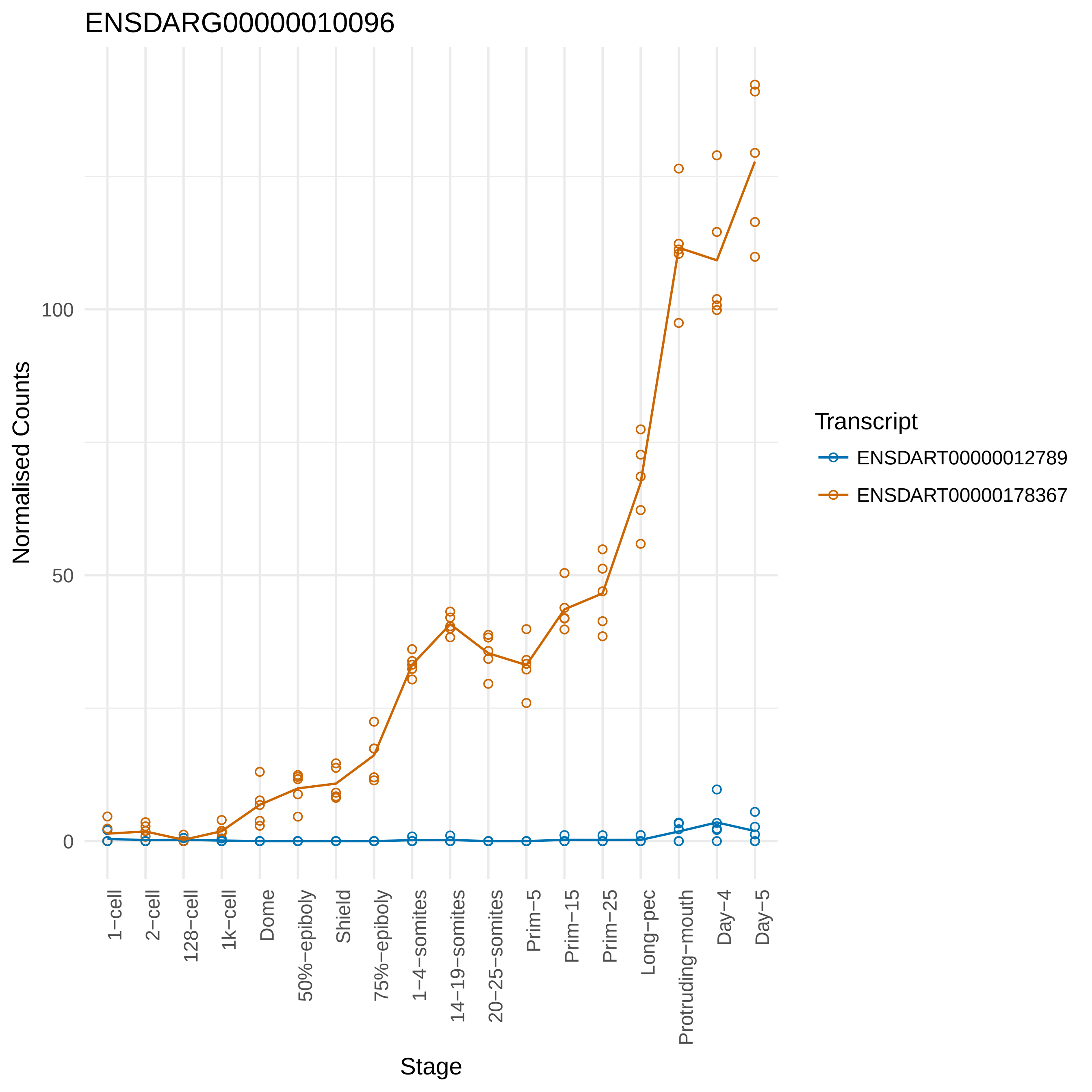

ENSDARG00000010096
Normalised Counts
0
50
100
Stage
1−cell
2−cell
128−cell
1k−cell
Dome
50%−epiboly
Shield
75%−epiboly
1−4−somites
14−19−somites
20−25−somites
Prim−5
Prim−15
Prim−25
Long−pec
Protruding−mouth
Day−4
Day−5
Transcript
ENSDART00000012789
ENSDART00000178367

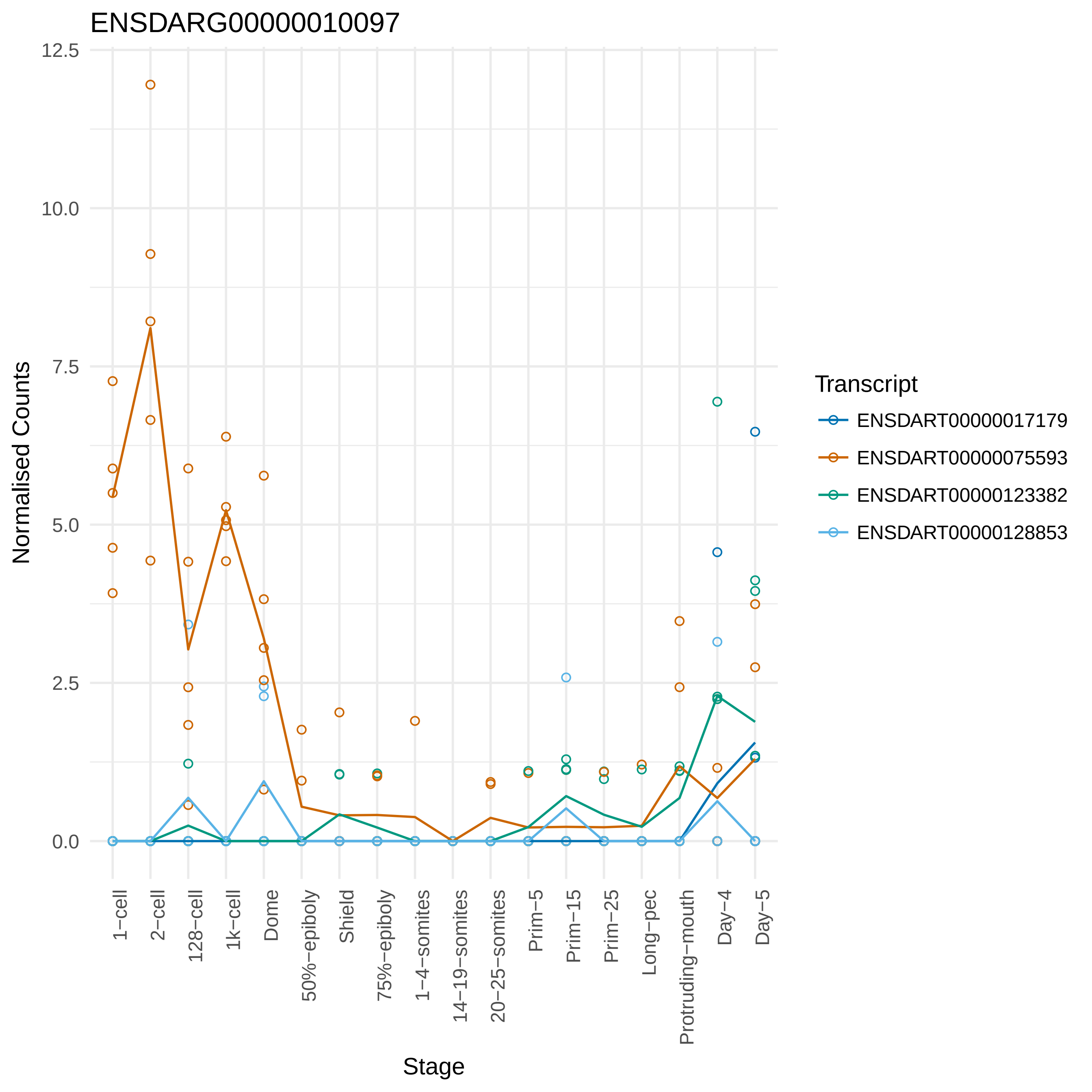

ENSDARG00000010097
Normalised Counts
12.5
10.0
7.5
5.0
2.5
0.0
1−cell
2−cell
128−cell
1k−cell
Dome
50%−epiboly
Shield
75%−epiboly
1−4−somites
14−19−somites
20−25−somites
Prim−5
Prim−15
Prim−25
Long−pec
Protruding−mouth
Day−4
Day−5
Stage
Transcript
ENSDART00000017179
ENSDART00000075593
ENSDART00000123382
ENSDART00000128853

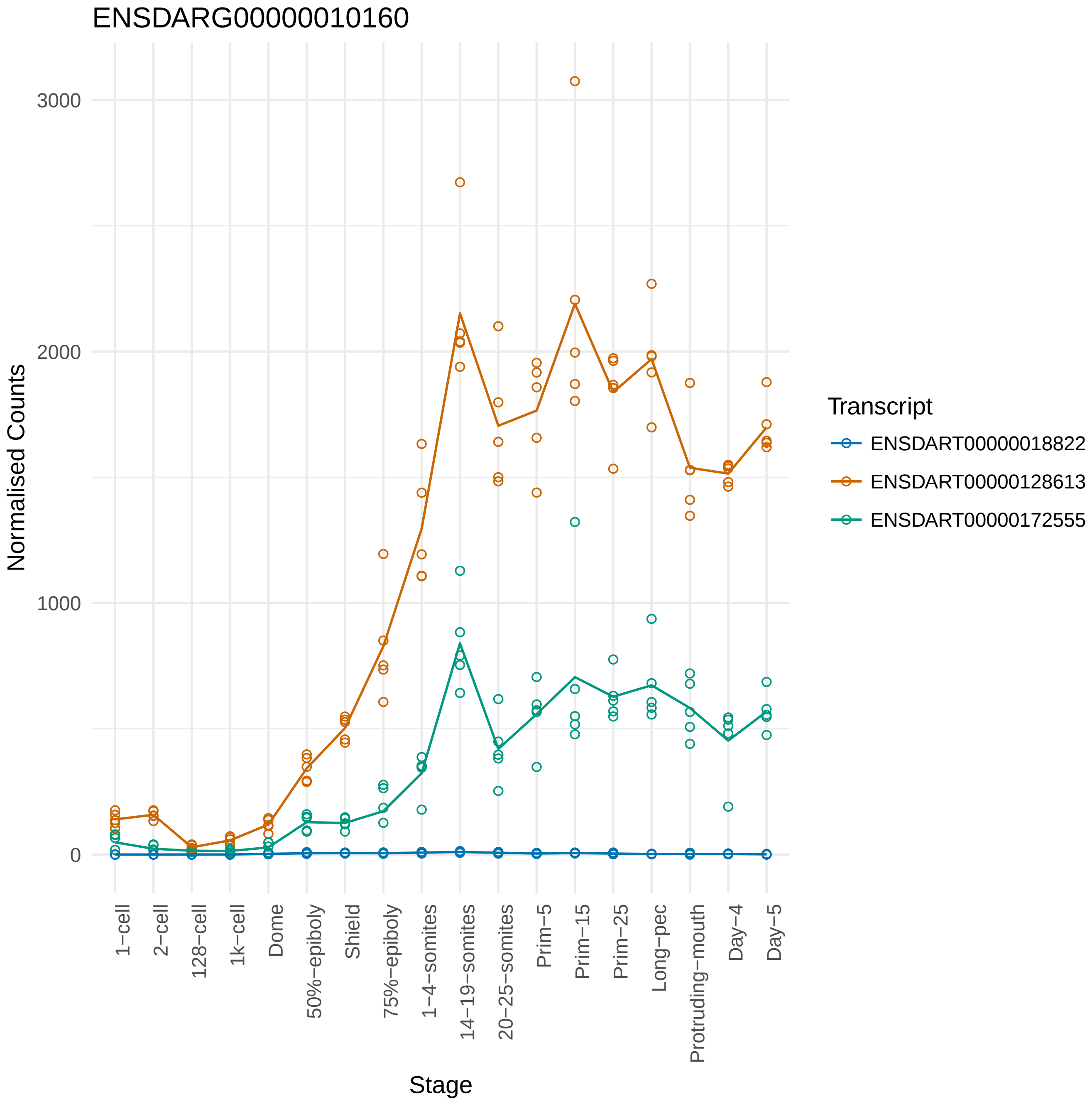

ENSDARG00000010160
Normalised Counts
3000
2000
1000
0
Stage
1−cell
2−cell
128−cell
1k−cell
Dome
50%−epiboly
Shield
75%−epiboly
1−4−somites
14−19−somites
20−25−somites
Prim−5
Prim−15
Prim−25
Long−pec
Protruding−mouth
Day−4
Day−5
Transcript
ENSDART00000018822
ENSDART00000128613
ENSDART00000172555

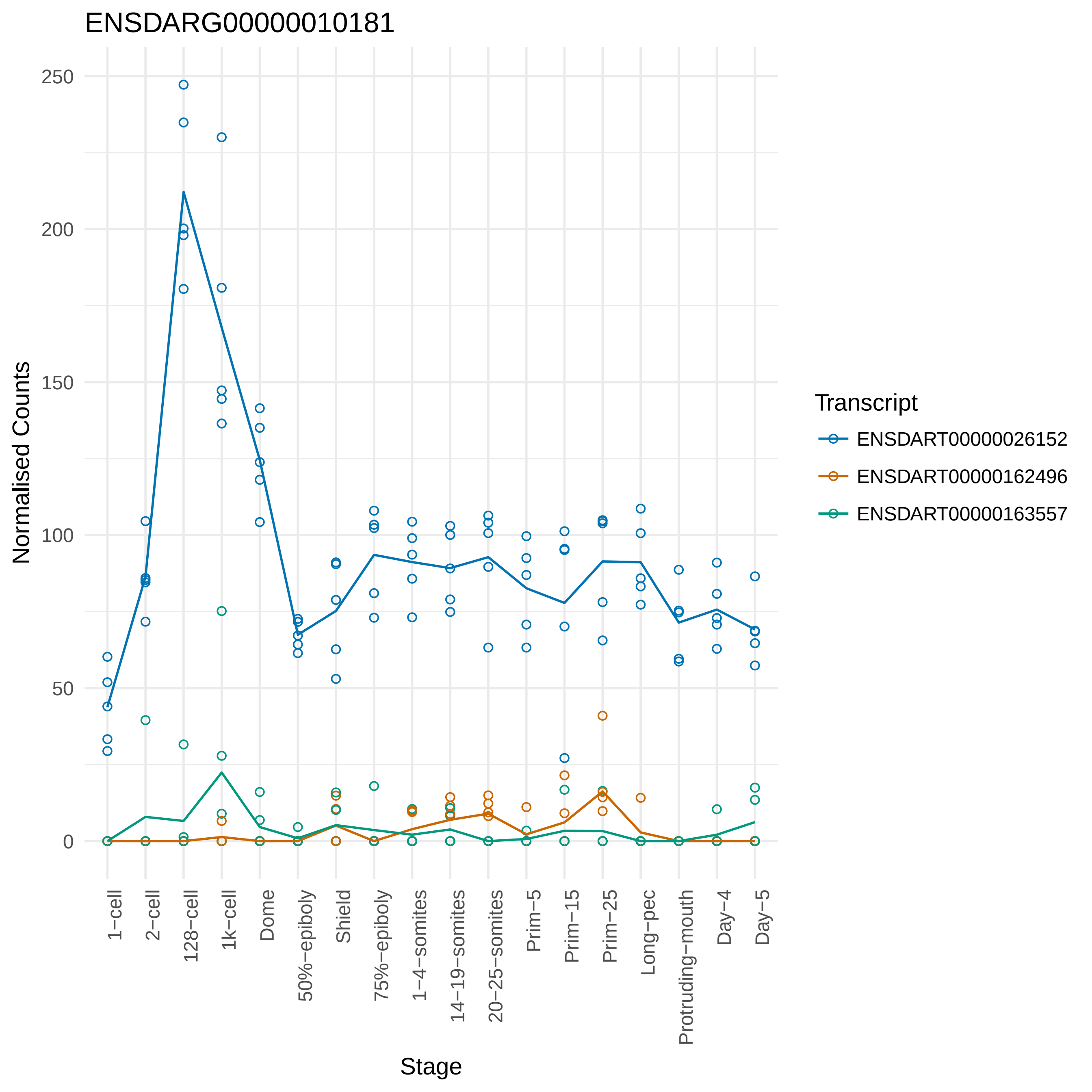
ENSDARG00000010181
Normalised Counts
250
200
150
100
50
0
1−cell
2−cell
128−cell
1k−cell
Dome
50%−epiboly
Shield
75%−epiboly
1−4−somites
14−19−somites
20−25−somites
Prim−5
Prim−15
Prim−25
Long−pec
Protruding−mouth
Day−4
Day−5
Stage
Transcript
ENSDART00000026152
ENSDART00000162496
ENSDART00000163557

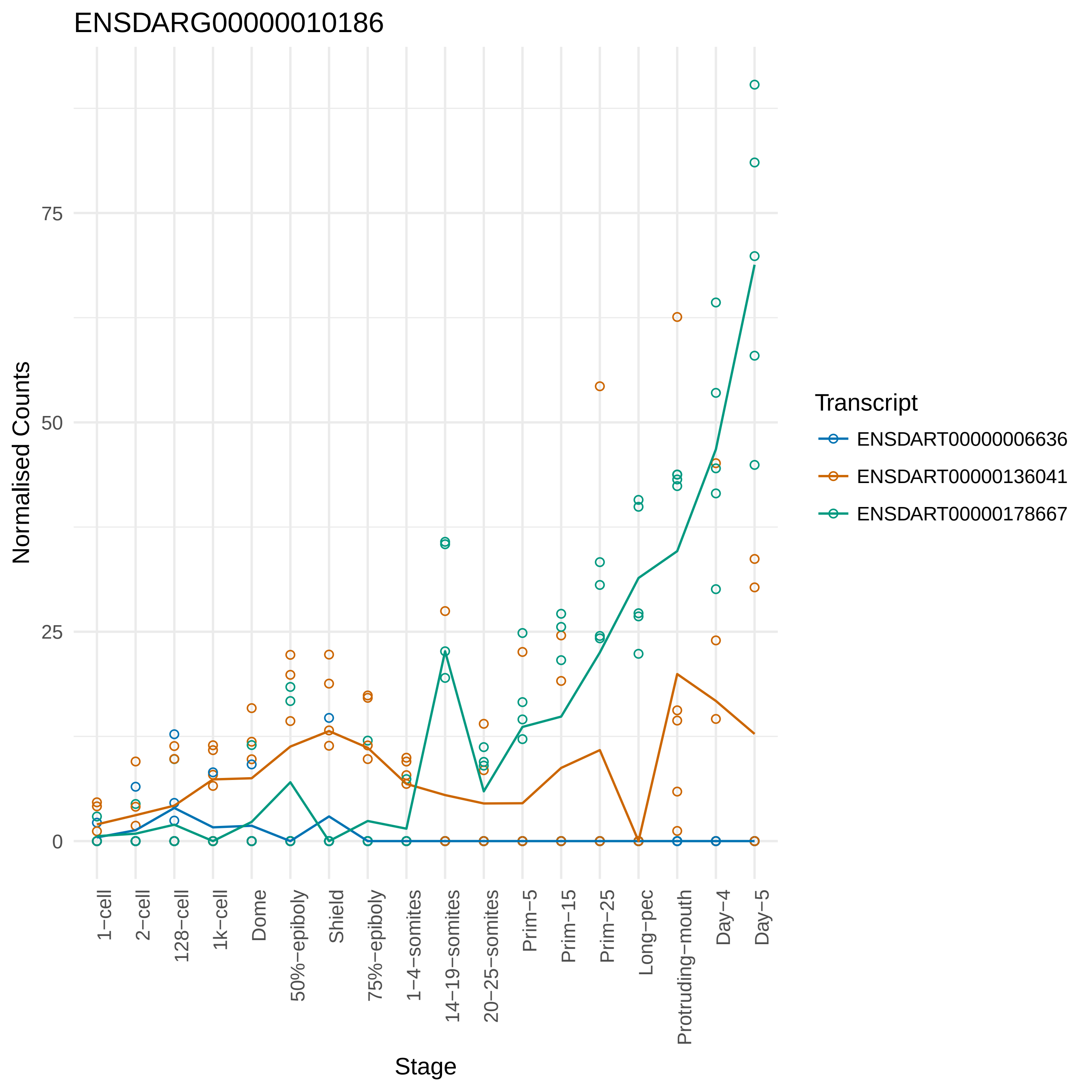

ENSDARG00000010186
Normalised Counts
0
25
50
75
Stage
1−cell
2−cell
128−cell
1k−cell
Dome
50%−epiboly
Shield
75%−epiboly
1−4−somites
14−19−somites
20−25−somites
Prim−5
Prim−15
Prim−25
Long−pec
Protruding−mouth
Day−4
Day−5
Transcript
ENSDART00000006636
ENSDART00000136041
ENSDART00000178667

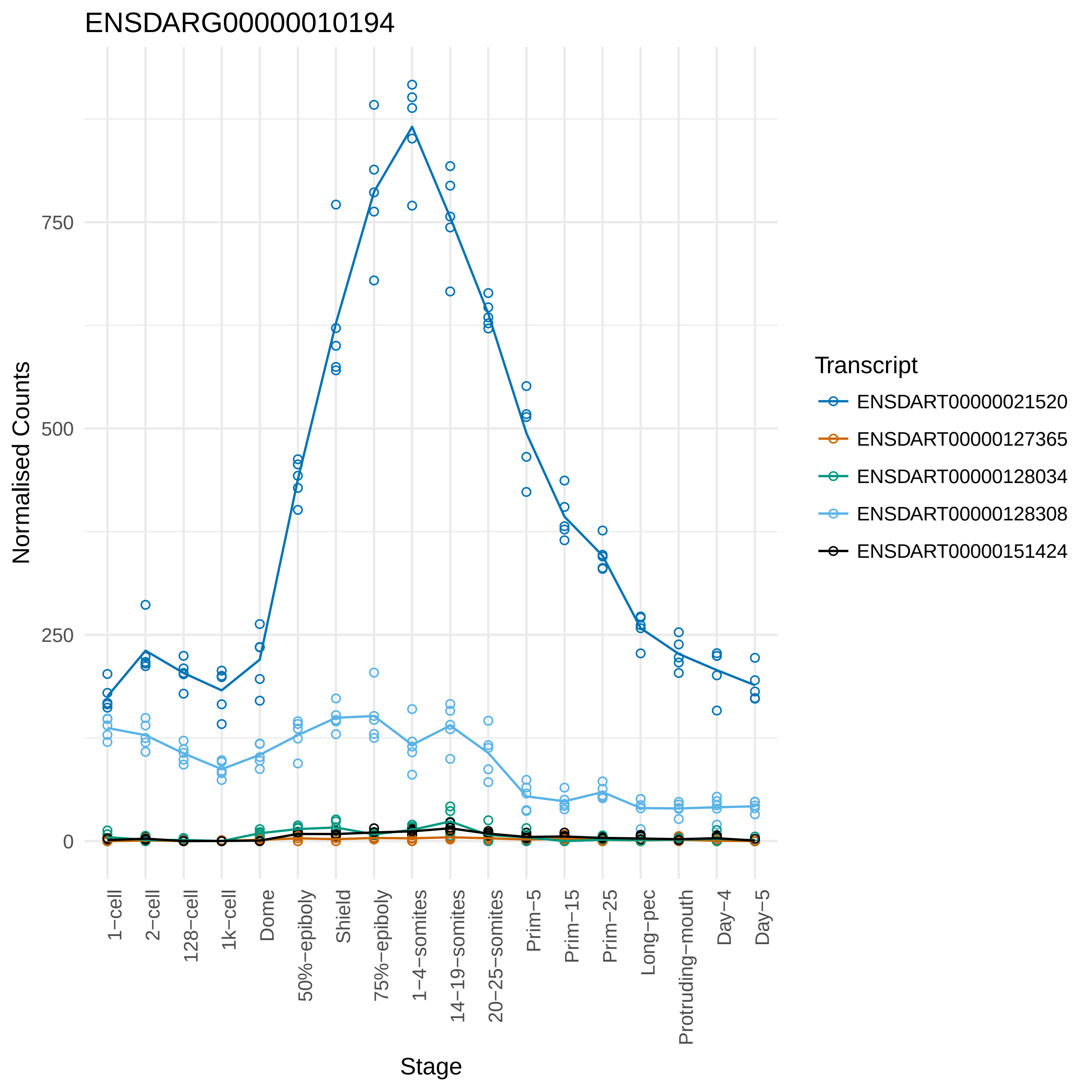
ENSDARG00000010194
Normalised Counts
0
250
500
750
Stage
1−cell
2−cell
128−cell
1k−cell
Dome
50%−epiboly
Shield
75%−epiboly
1−4−somites
14−19−somites
20−25−somites
Prim−5
Prim−15
Prim−25
Long−pec
Protruding−mouth
Day−4
Day−5
Transcript
ENSDART00000021520
ENSDART00000127365
ENSDART00000128034
ENSDART00000128308
ENSDART00000151424

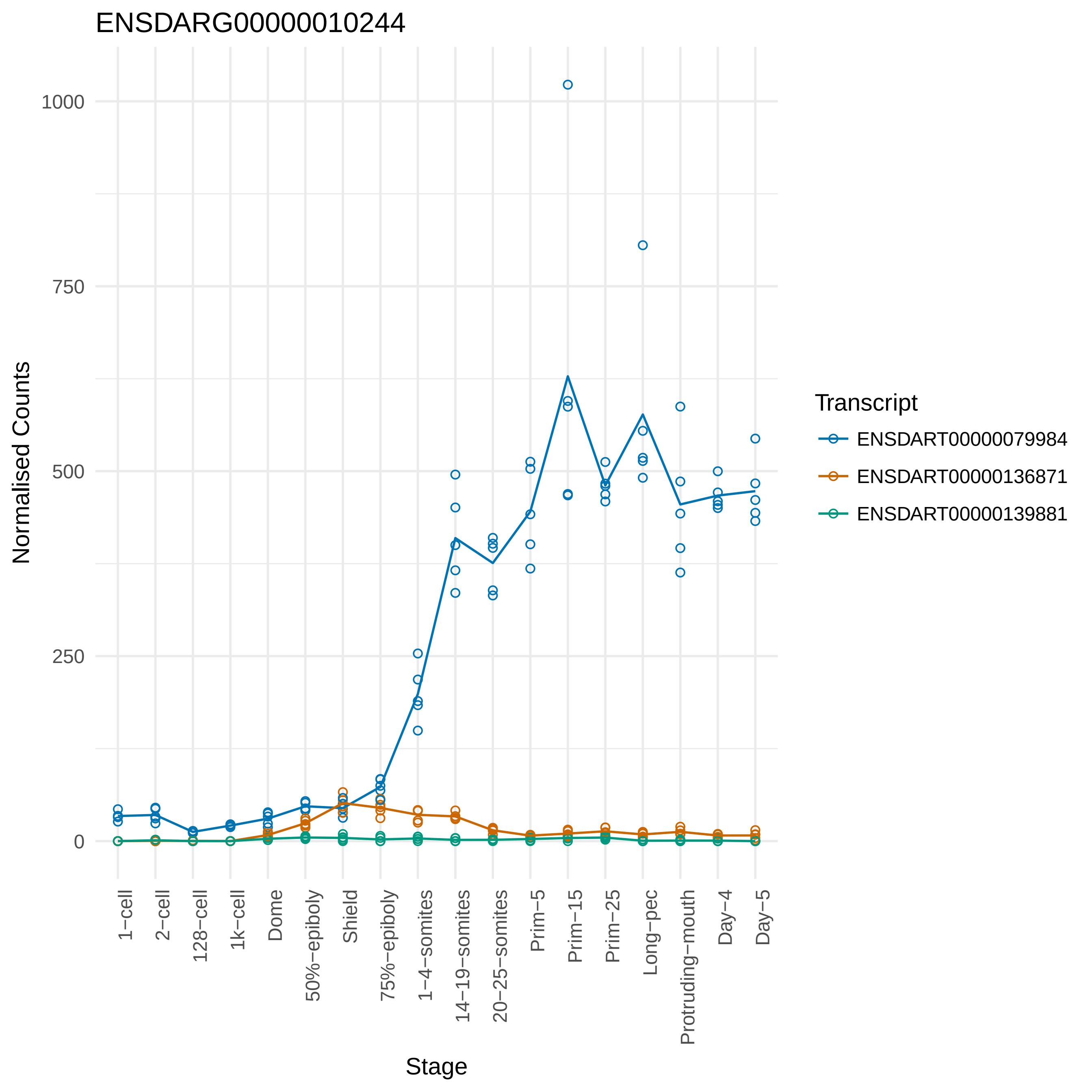
ENSDARG00000010244
Normalised Counts
1000
750
500
250
0
1−cell
2−cell
128−cell
1k−cell
Dome
50%−epiboly
Shield
75%−epiboly
1−4−somites
14−19−somites
20−25−somites
Prim−5
Prim−15
Prim−25
Long−pec
Protruding−mouth
Day−4
Day−5
Stage
Transcript
ENSDART00000079984
ENSDART00000136871
ENSDART00000139881

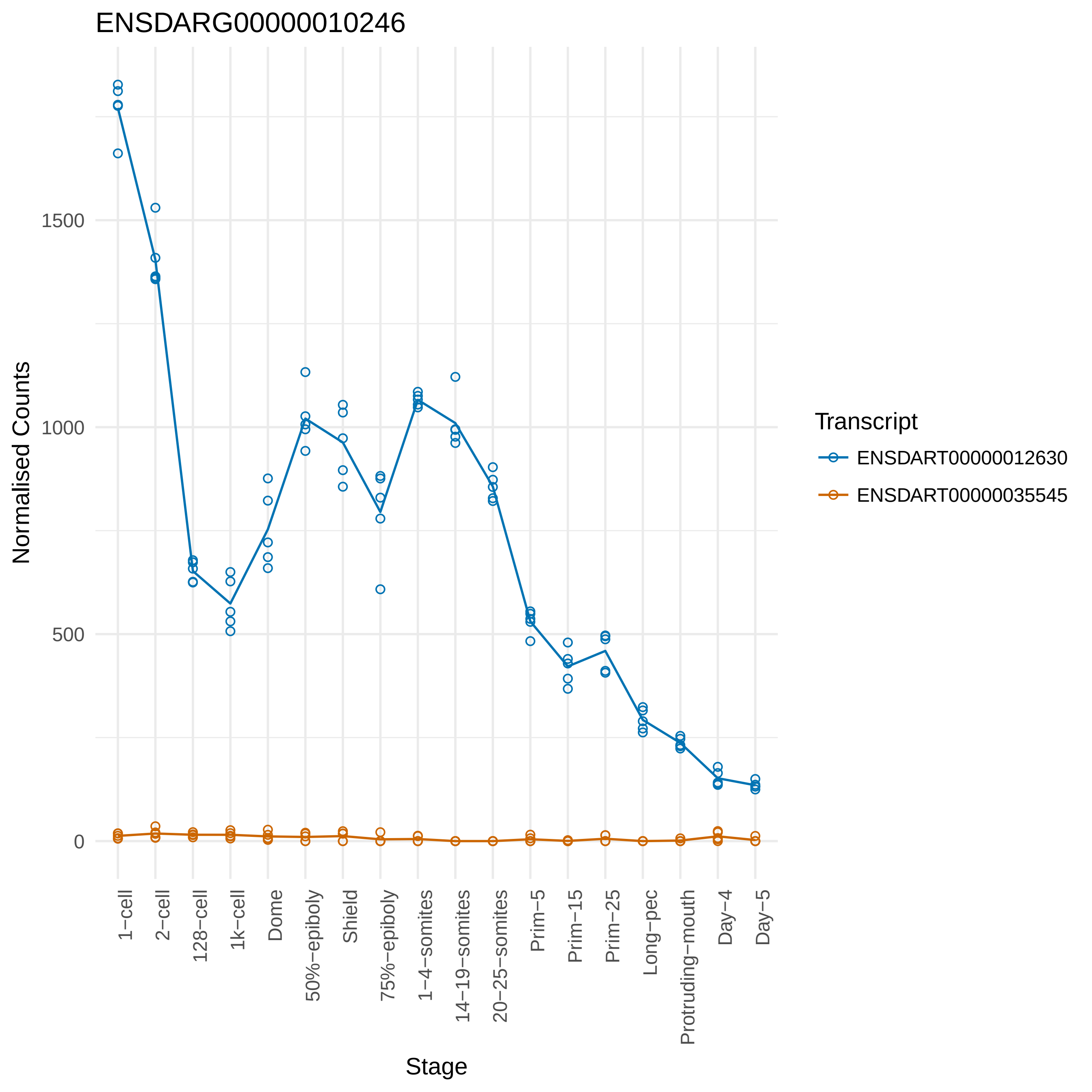

ENSDARG00000010246
Normalised Counts
1500
1000
500
0
Stage
1−cell
2−cell
128−cell
1k−cell
Dome
50%−epiboly
Shield
75%−epiboly
1−4−somites
14−19−somites
20−25−somites
Prim−5
Prim−15
Prim−25
Long−pec
Protruding−mouth
Day−4
Day−5
Transcript
ENSDART00000012630
ENSDART00000035545

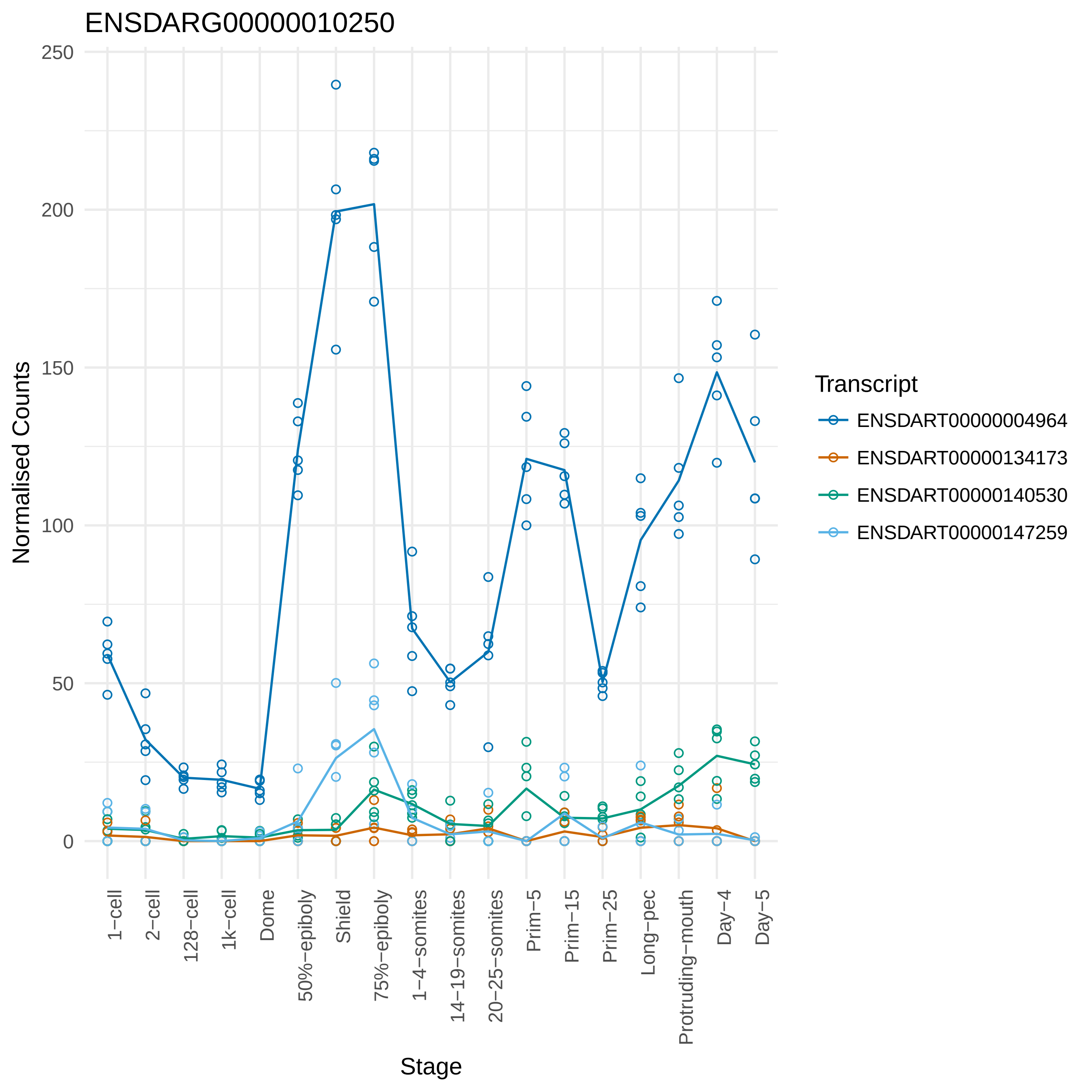

ENSDARG00000010250
Normalised Counts
250
200
150
100
50
0
1−cell
2−cell
128−cell
1k−cell
Dome
50%−epiboly
Shield
75%−epiboly
1−4−somites
14−19−somites
20−25−somites
Prim−5
Prim−15
Prim−25
Long−pec
Protruding−mouth
Day−4
Day−5
Stage
Transcript
ENSDART00000004964
ENSDART00000134173
ENSDART00000140530
ENSDART00000147259

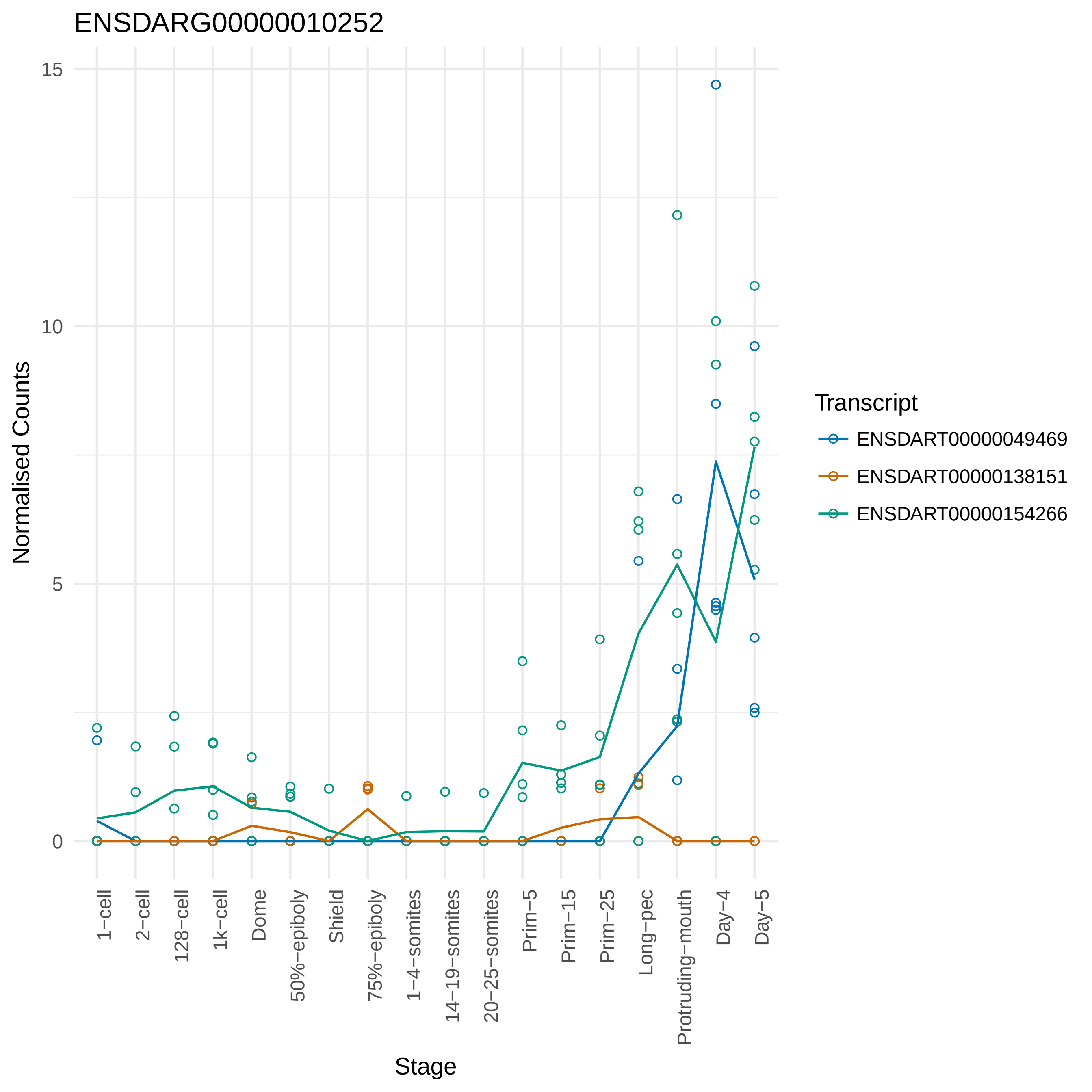
ENSDARG00000010252
Normalised Counts
0
5
10
15
1−cell
2−cell
128−cell
1k−cell
Dome
50%−epiboly
Shield
75%−epiboly
1−4−somites
14−19−somites
20−25−somites
Prim−5
Prim−15
Prim−25
Long−pec
Protruding−mouth
Day−4
Day−5
Stage
Transcript
ENSDART00000049469
ENSDART00000138151
ENSDART00000154266

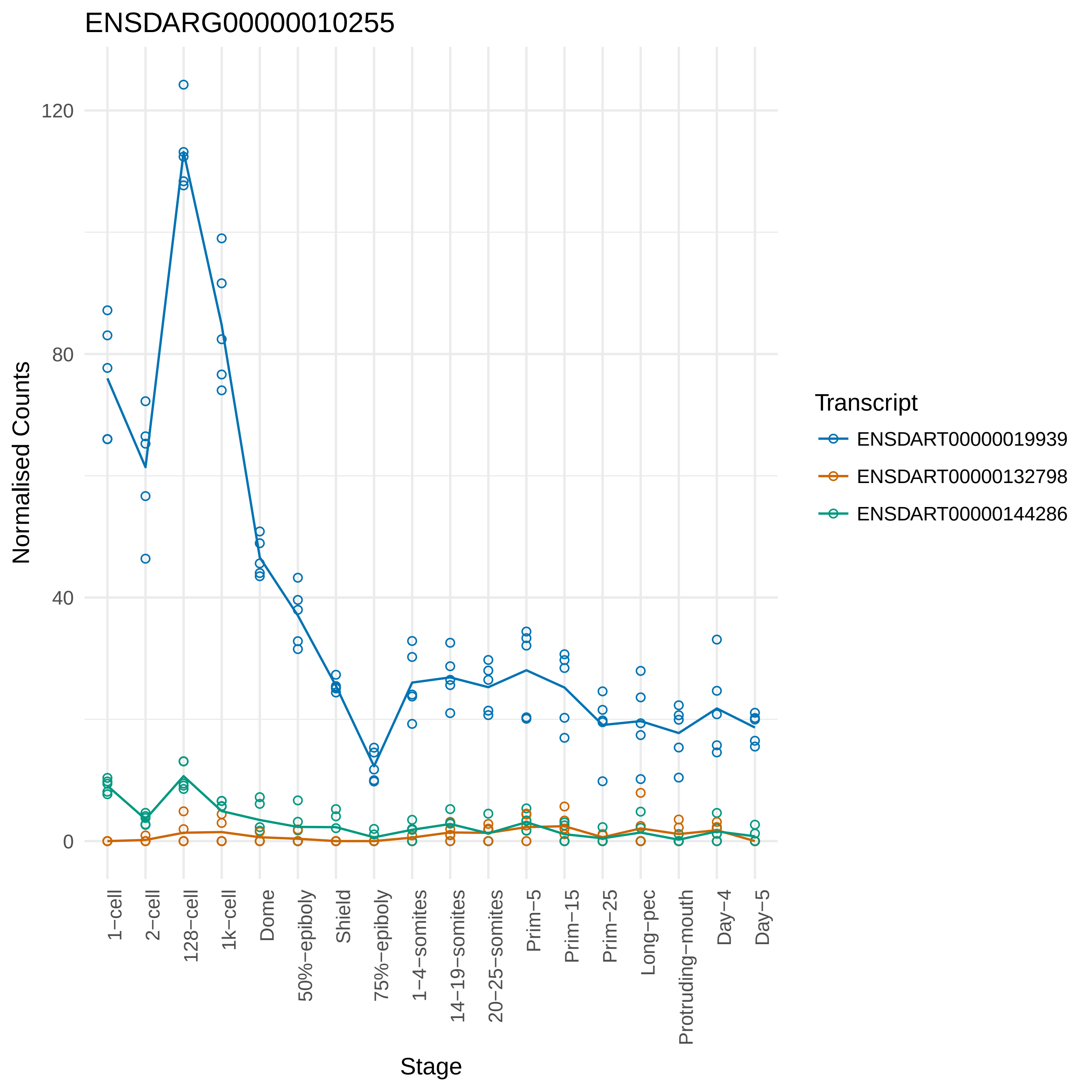

ENSDARG00000010255
Normalised Counts
120
80
40
0
1−cell
2−cell
128−cell
1k−cell
Dome
50%−epiboly
Shield
75%−epiboly
1−4−somites
14−19−somites
20−25−somites
Prim−5
Prim−15
Prim−25
Long−pec
Protruding−mouth
Day−4
Day−5
Stage
Transcript
ENSDART00000019939
ENSDART00000132798
ENSDART00000144286

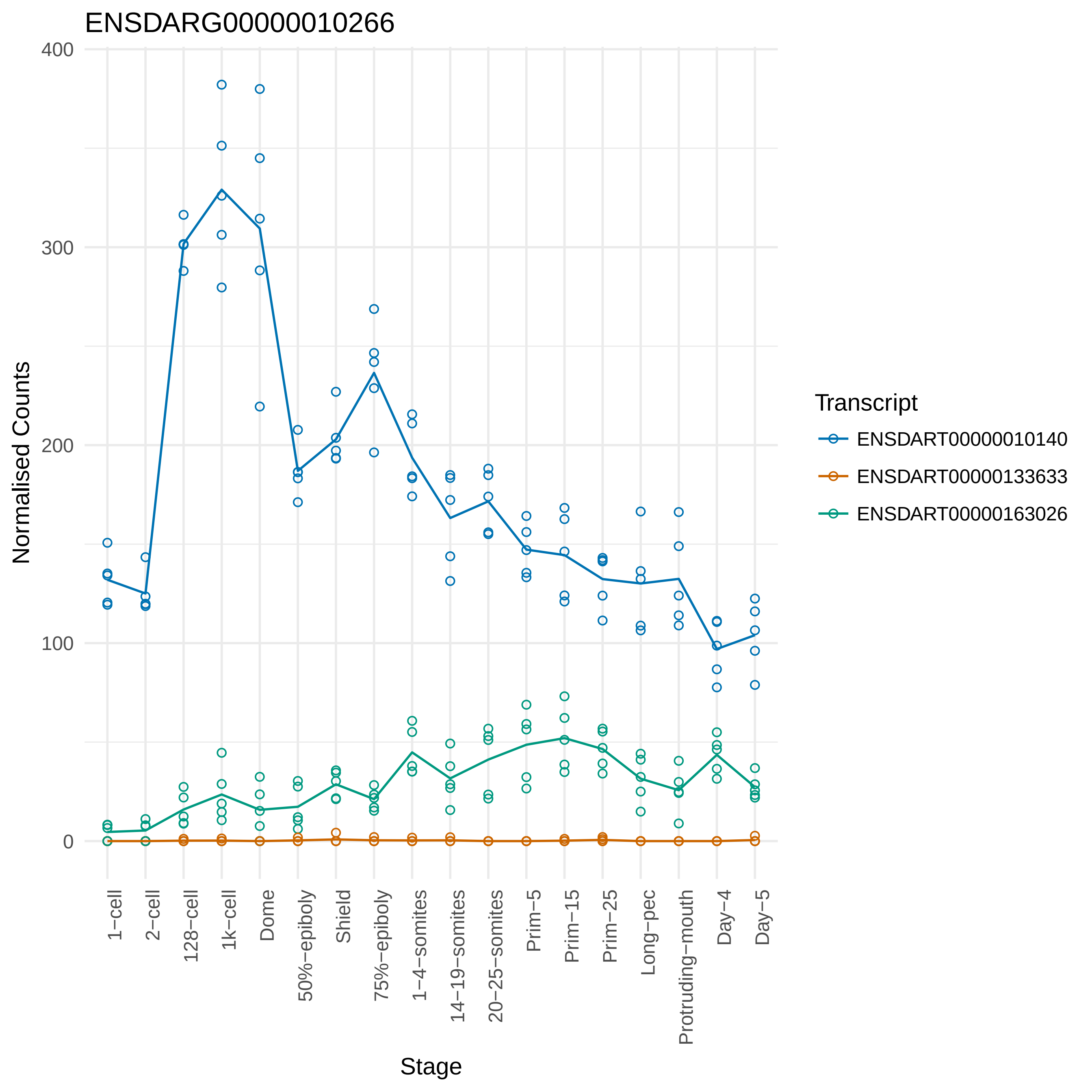
ENSDARG00000010266
Normalised Counts
400
300
200
100
0
Stage
1−cell
2−cell
128−cell
1k−cell
Dome
50%−epiboly
Shield
75%−epiboly
1−4−somites
14−19−somites
20−25−somites
Prim−5
Prim−15
Prim−25
Long−pec
Protruding−mouth
Day−4
Day−5
Transcript
ENSDART00000010140
ENSDART00000133633
ENSDART00000163026

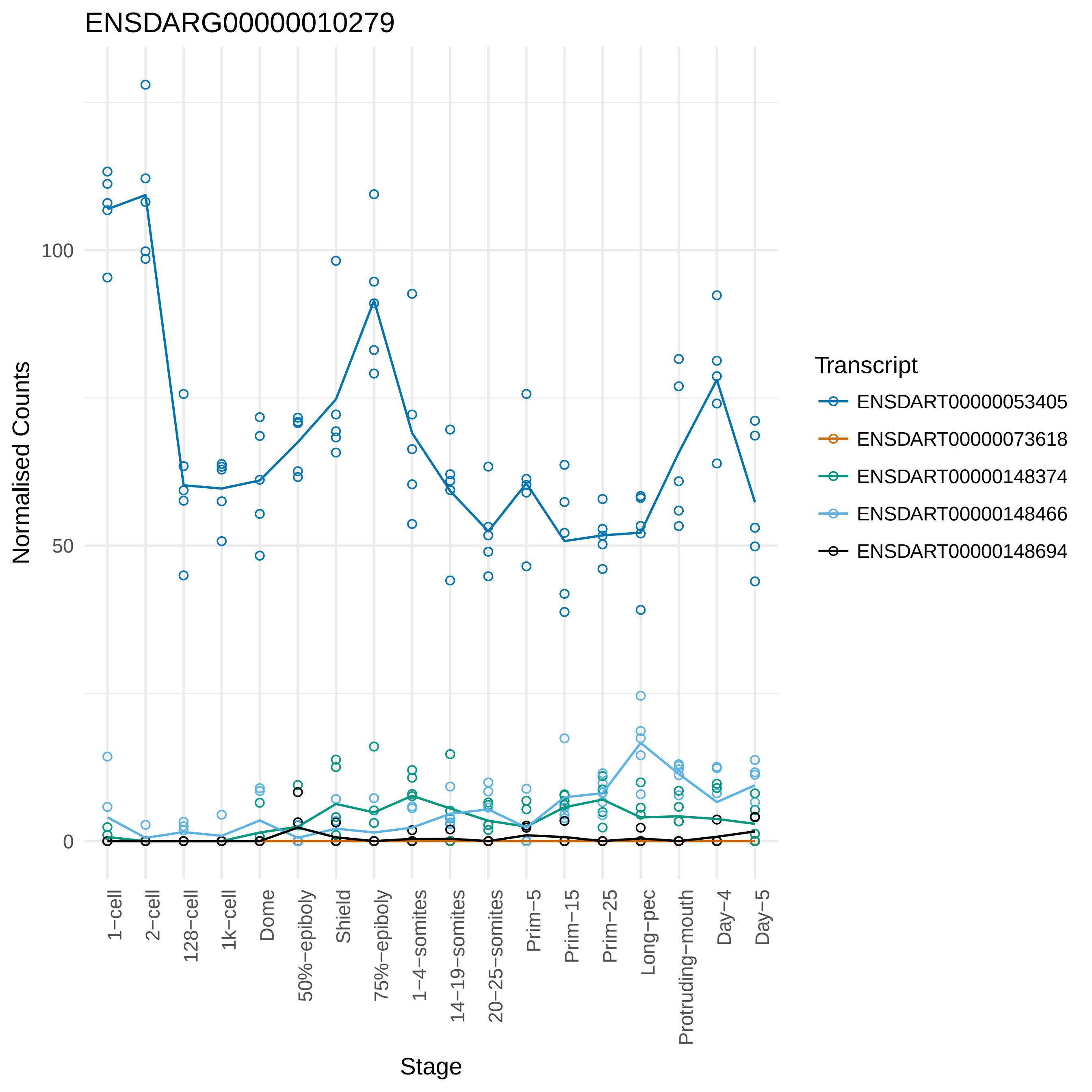

ENSDARG00000010279
Normalised Counts
100
50
0
1−cell
2−cell
128−cell
1k−cell
Dome
50%−epiboly
Shield
75%−epiboly
1−4−somites
14−19−somites
20−25−somites
Prim−5
Prim−15
Prim−25
Long−pec
Protruding−mouth
Day−4
Day−5
Stage
Transcript
ENSDART00000053405
ENSDART00000073618
ENSDART00000148374
ENSDART00000148466
ENSDART00000148694

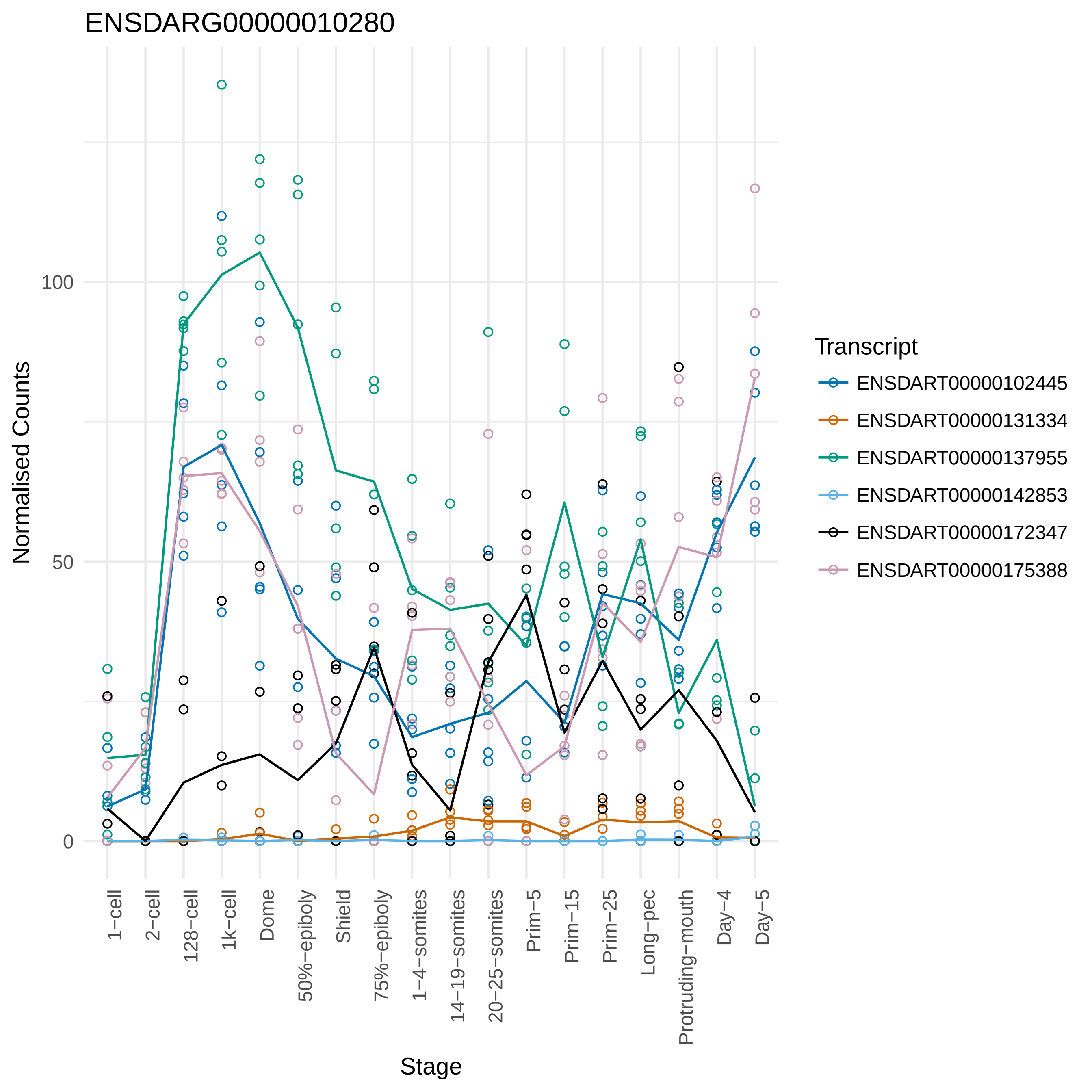
ENSDARG00000010280
Normalised Counts
100
50
0
Stage
1−cell
2−cell
128−cell
1k−cell
Dome
50%−epiboly
Shield
75%−epiboly
1−4−somites
14−19−somites
20−25−somites
Prim−5
Prim−15
Prim−25
Long−pec
Protruding−mouth
Day−4
Day−5
Transcript
ENSDART00000102445
ENSDART00000131334
ENSDART00000137955
ENSDART00000142853
ENSDART00000172347
ENSDART00000175388

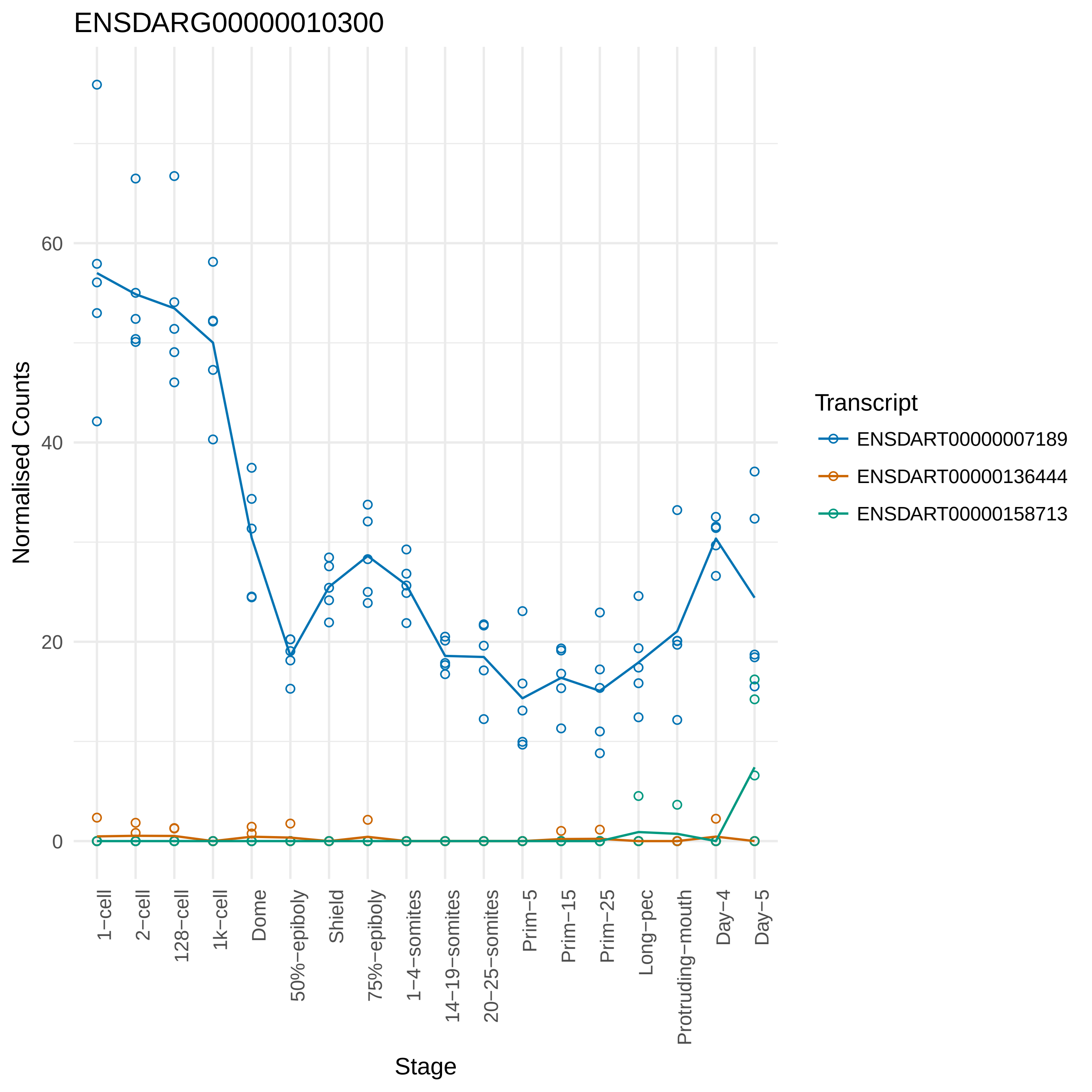

ENSDARG00000010300
Normalised Counts
0
20
40
60
Stage
1−cell
2−cell
128−cell
1k−cell
Dome
50%−epiboly
Shield
75%−epiboly
1−4−somites
14−19−somites
20−25−somites
Prim−5
Prim−15
Prim−25
Long−pec
Protruding−mouth
Day−4
Day−5
Transcript
ENSDART00000007189
ENSDART00000136444
ENSDART00000158713

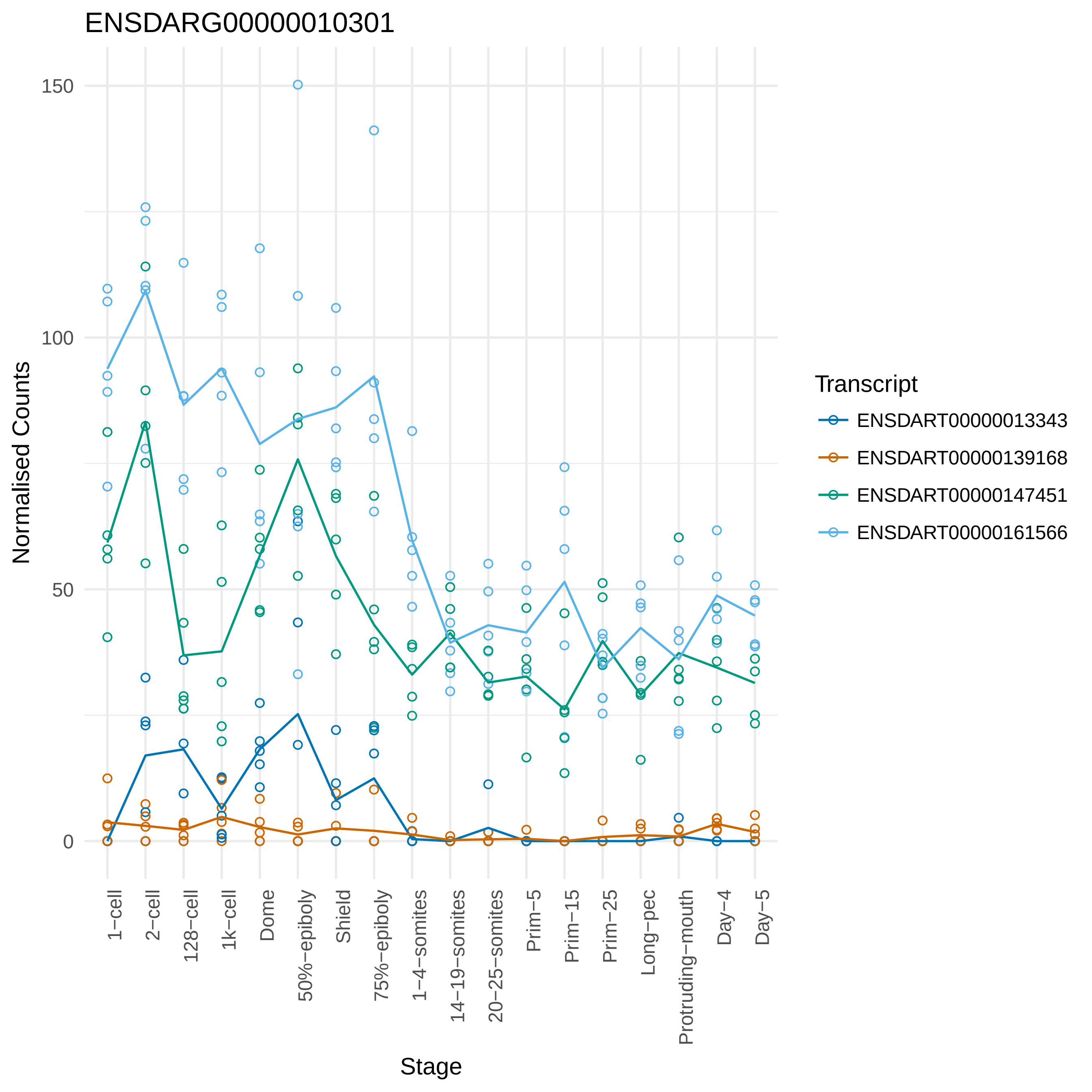

ENSDARG00000010301
Normalised Counts
150
100
50
0
1−cell
2−cell
128−cell
1k−cell
Dome
50%−epiboly
Shield
75%−epiboly
1−4−somites
14−19−somites
20−25−somites
Prim−5
Prim−15
Prim−25
Long−pec
Protruding−mouth
Day−4
Day−5
Stage
Transcript
ENSDART00000013343
ENSDART00000139168
ENSDART00000147451
ENSDART00000161566

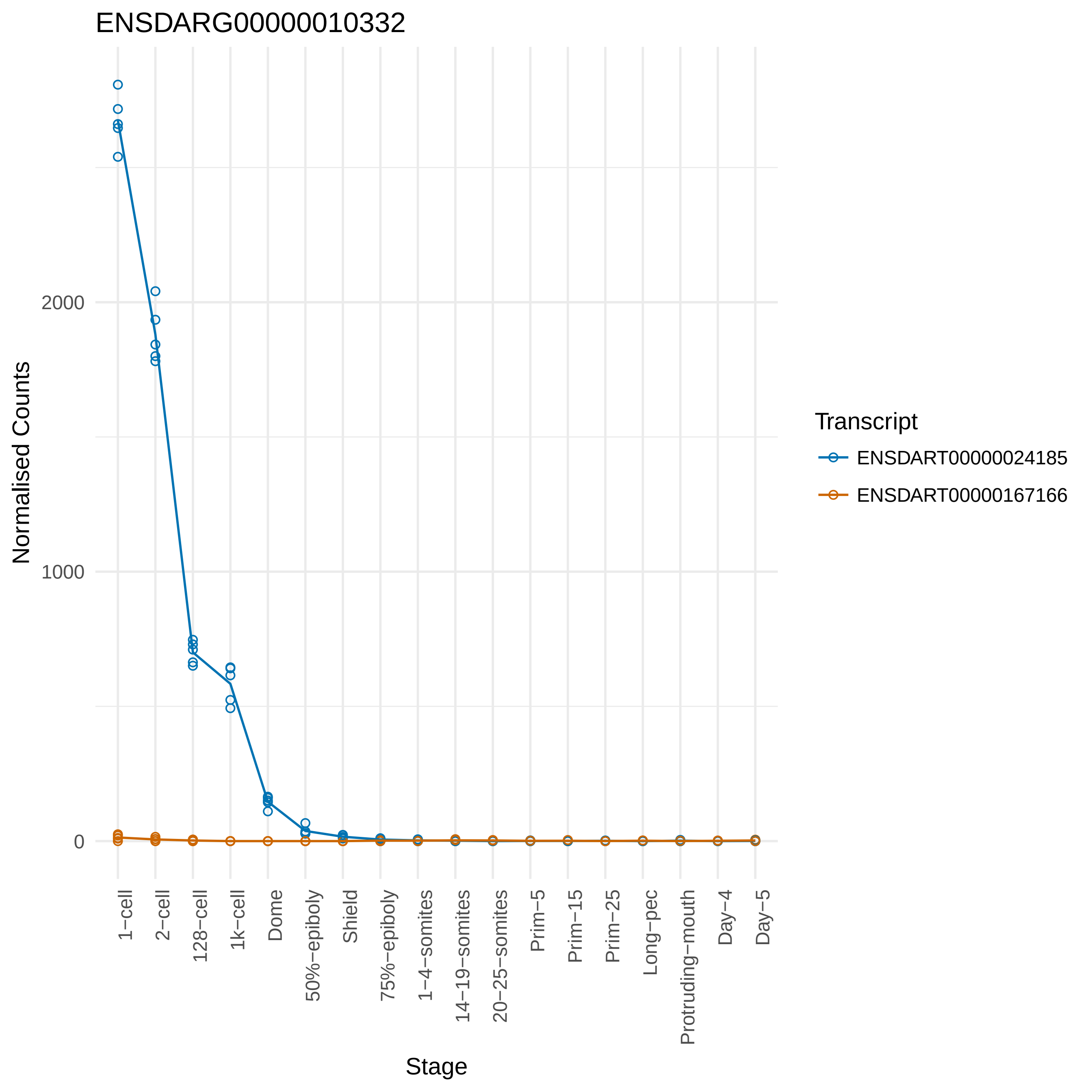

ENSDARG00000010332
Normalised Counts
2000
1000
0
1−cell
2−cell
128−cell
1k−cell
Dome
50%−epiboly
Shield
75%−epiboly
1−4−somites
14−19−somites
20−25−somites
Prim−5
Prim−15
Prim−25
Long−pec
Protruding−mouth
Day−4
Day−5
Stage
Transcript
ENSDART00000024185
ENSDART00000167166

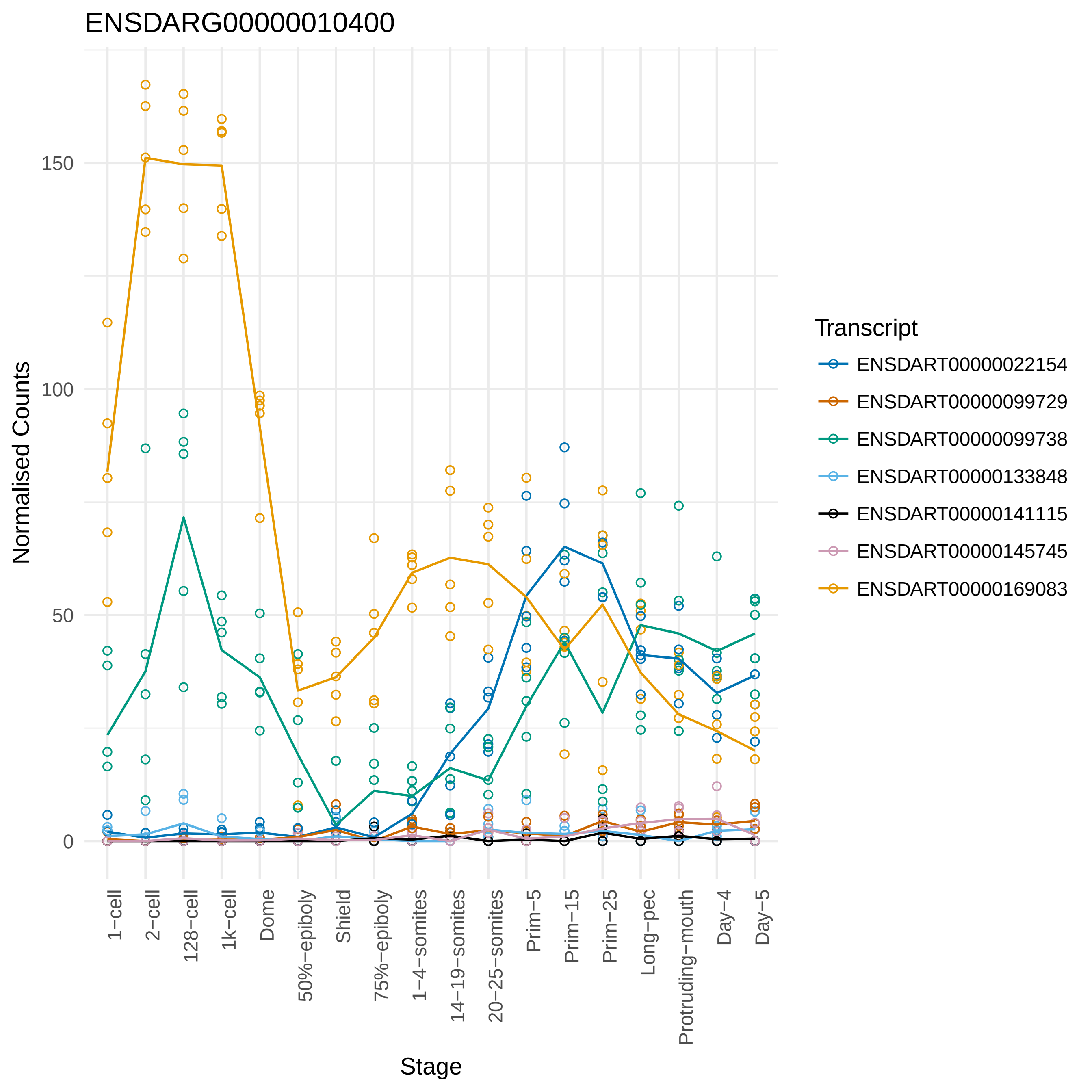

ENSDARG00000010400
Normalised Counts
150
100
50
0
1−cell
2−cell
128−cell
1k−cell
Dome
50%−epiboly
Shield
75%−epiboly
1−4−somites
14−19−somites
20−25−somites
Prim−5
Prim−15
Prim−25
Long−pec
Protruding−mouth
Day−4
Day−5
Stage
Transcript
ENSDART00000022154
ENSDART00000099729
ENSDART00000099738
ENSDART00000133848
ENSDART00000141115
ENSDART00000145745
ENSDART00000169083

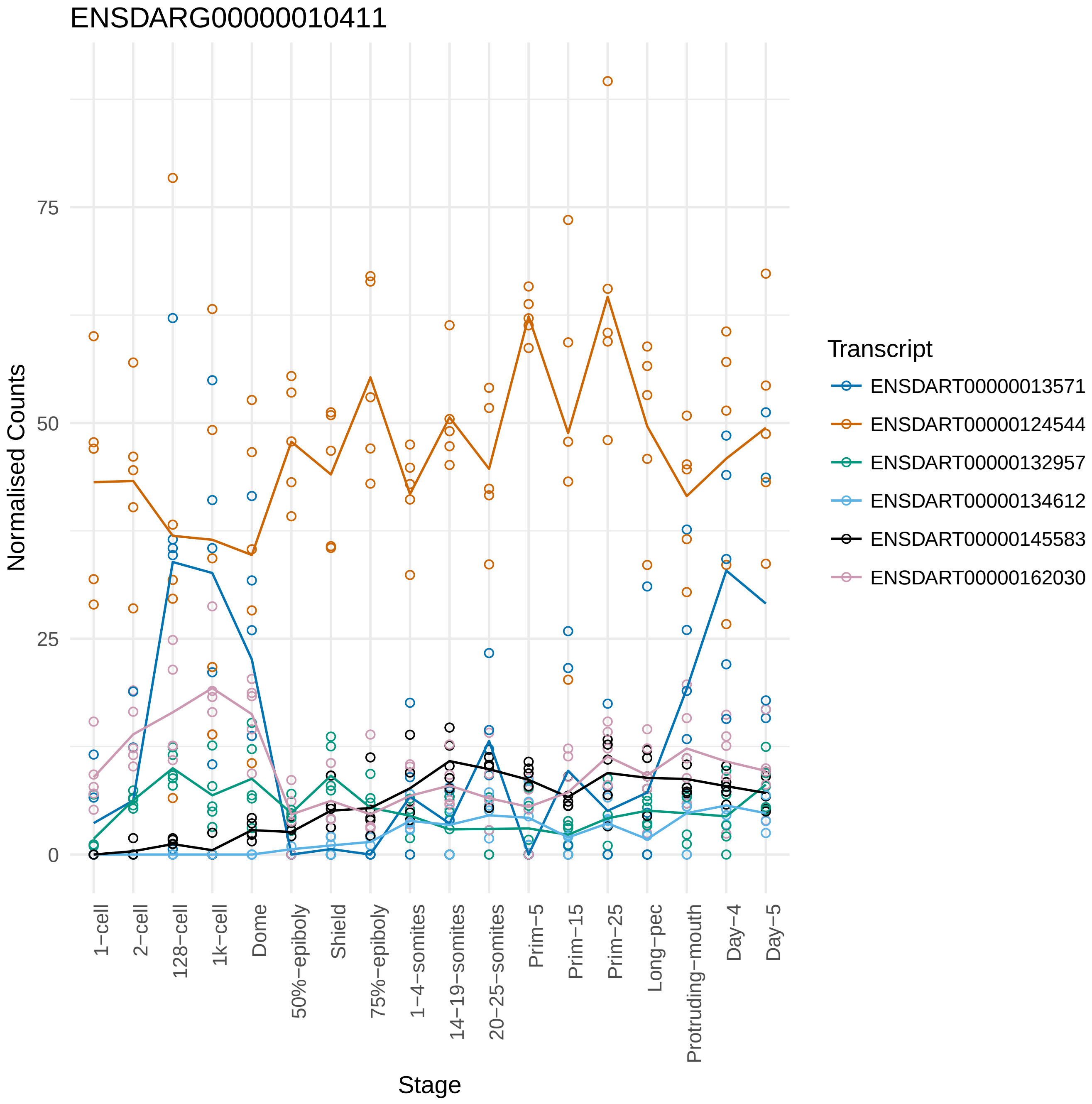

ENSDARG00000010411
Normalised Counts
0
25
50
75
Stage
1−cell
2−cell
128−cell
1k−cell
Dome
50%−epiboly
Shield
75%−epiboly
1−4−somites
14−19−somites
20−25−somites
Prim−5
Prim−15
Prim−25
Long−pec
Protruding−mouth
Day−4
Day−5
Transcript
ENSDART00000013571
ENSDART00000124544
ENSDART00000132957
ENSDART00000134612
ENSDART00000145583
ENSDART00000162030

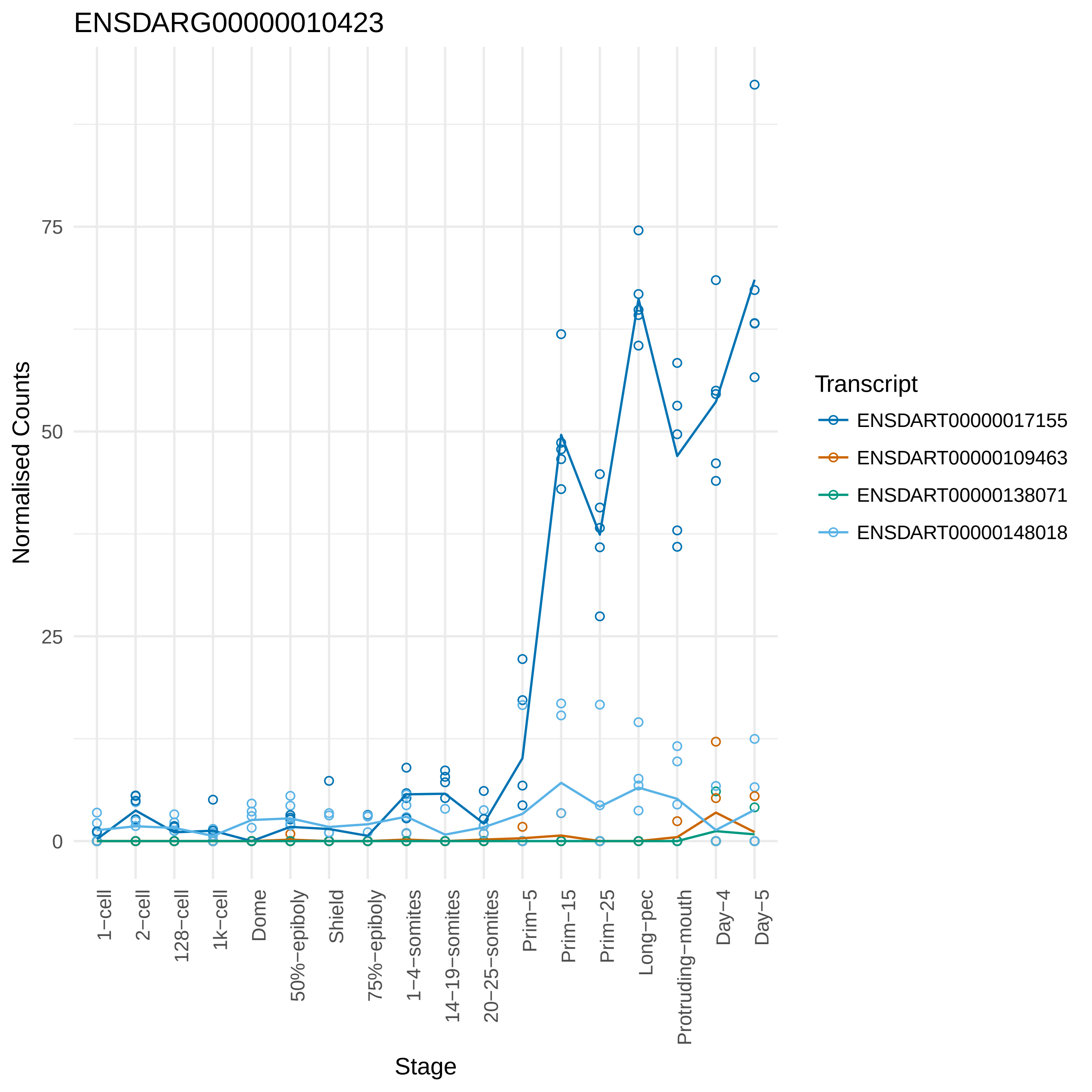
ENSDARG00000010423
Normalised Counts
0
25
50
75
Stage
1−cell
2−cell
128−cell
1k−cell
Dome
50%−epiboly
Shield
75%−epiboly
1−4−somites
14−19−somites
20−25−somites
Prim−5
Prim−15
Prim−25
Long−pec
Protruding−mouth
Day−4
Day−5
Transcript
ENSDART00000017155
ENSDART00000109463
ENSDART00000138071
ENSDART00000148018

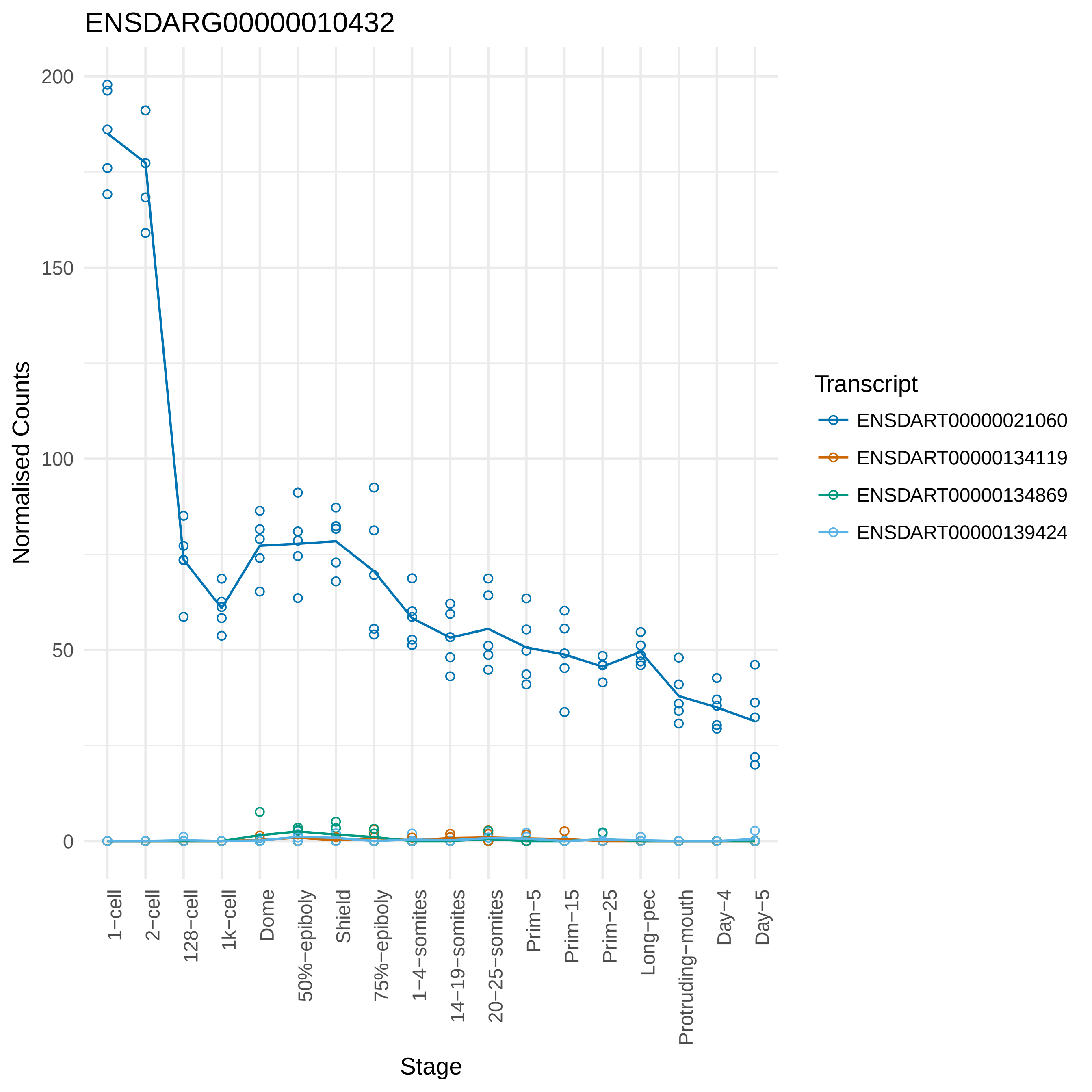
ENSDARG00000010432
Normalised Counts
200
150
100
50
0
1−cell
2−cell
128−cell
1k−cell
Dome
50%−epiboly
Shield
75%−epiboly
1−4−somites
14−19−somites
20−25−somites
Prim−5
Prim−15
Prim−25
Long−pec
Protruding−mouth
Day−4
Day−5
Stage
Transcript
ENSDART00000021060
ENSDART00000134119
ENSDART00000134869
ENSDART00000139424

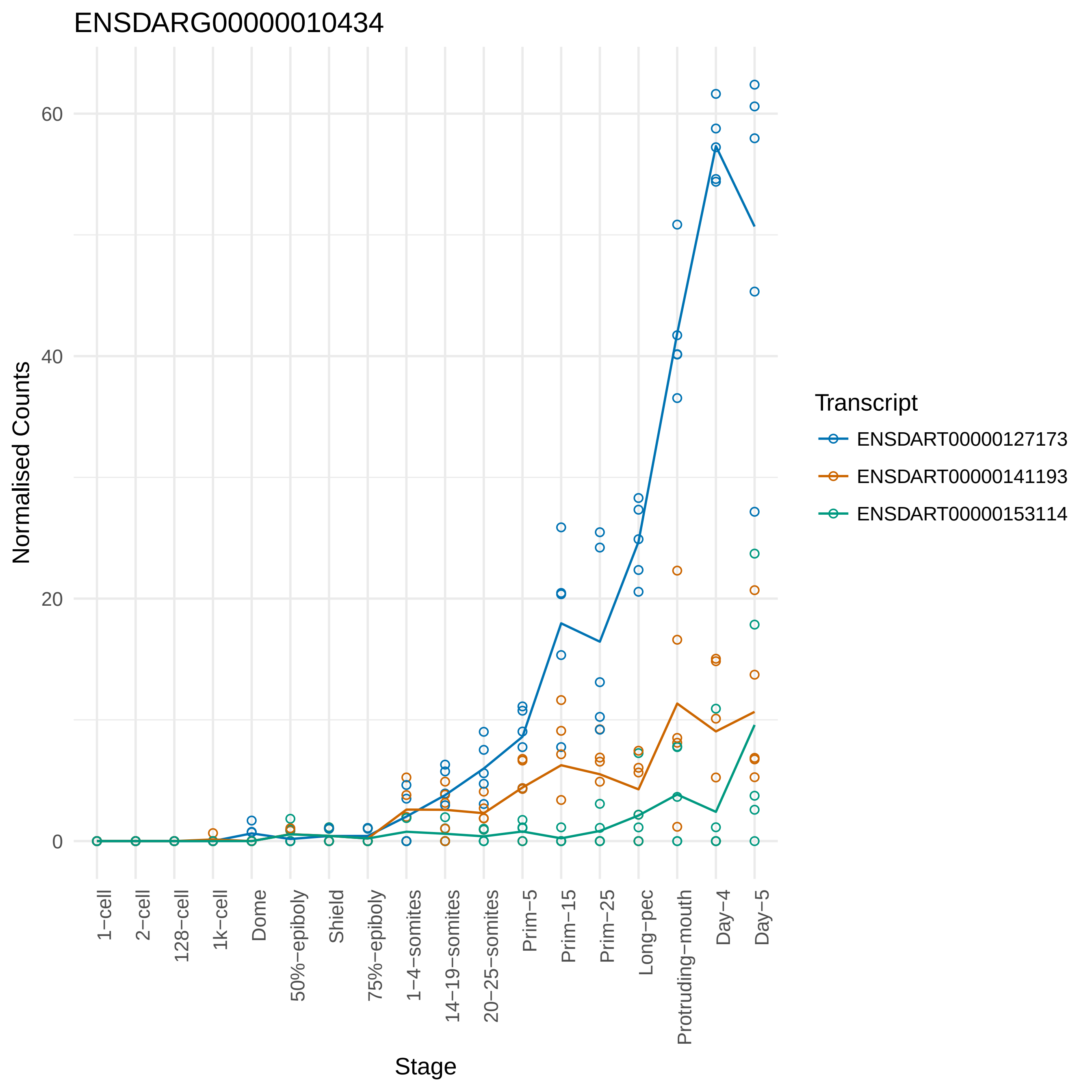
ENSDARG00000010434
Normalised Counts
0
20
40
60
Stage
1−cell
2−cell
128−cell
1k−cell
Dome
50%−epiboly
Shield
75%−epiboly
1−4−somites
14−19−somites
20−25−somites
Prim−5
Prim−15
Prim−25
Long−pec
Protruding−mouth
Day−4
Day−5
Transcript
ENSDART00000127173
ENSDART00000141193
ENSDART00000153114

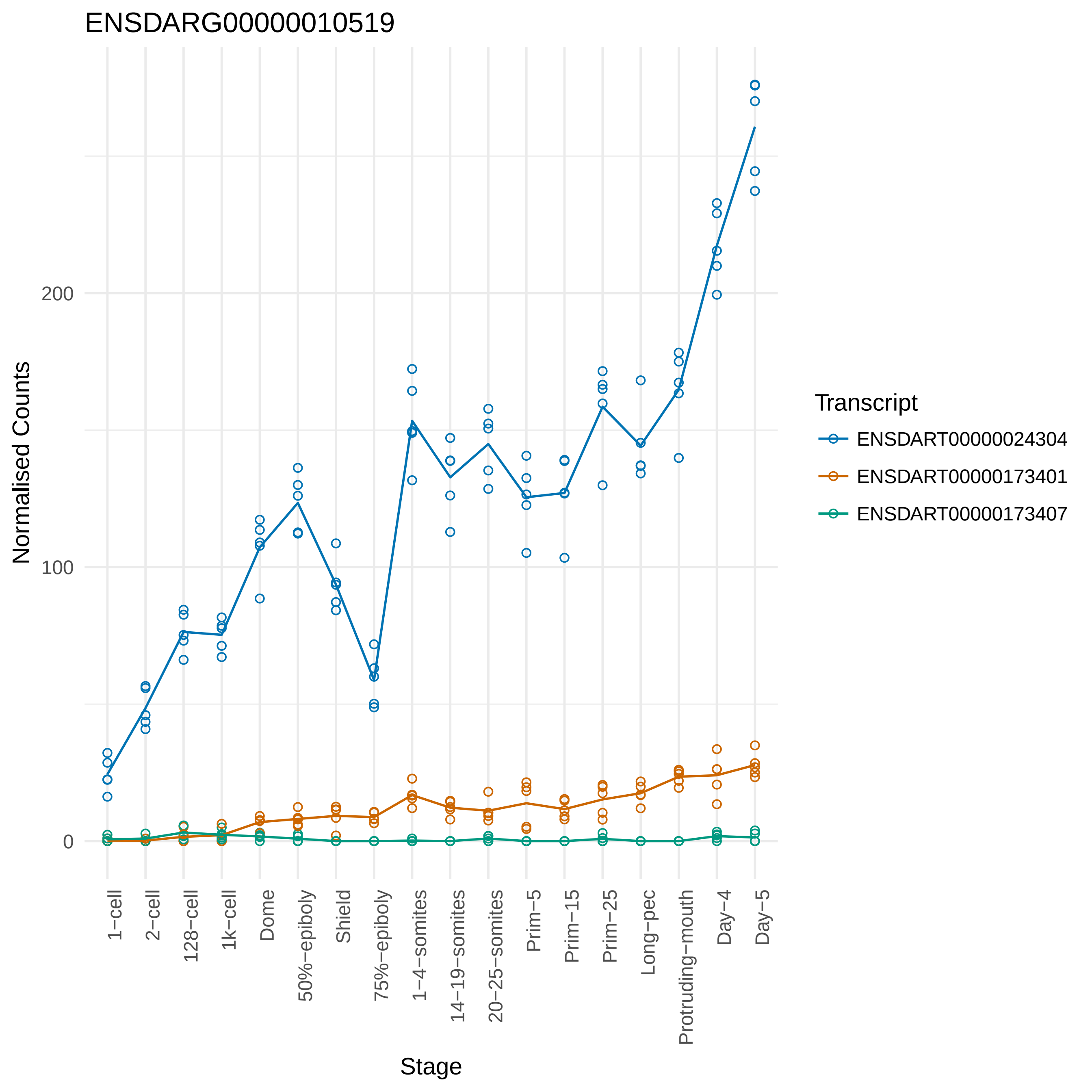

ENSDARG00000010519
Normalised Counts
200
100
0
1−cell
2−cell
128−cell
1k−cell
Dome
50%−epiboly
Shield
75%−epiboly
1−4−somites
14−19−somites
20−25−somites
Prim−5
Prim−15
Prim−25
Long−pec
Protruding−mouth
Day−4
Day−5
Stage
Transcript
ENSDART00000024304
ENSDART00000173401
ENSDART00000173407

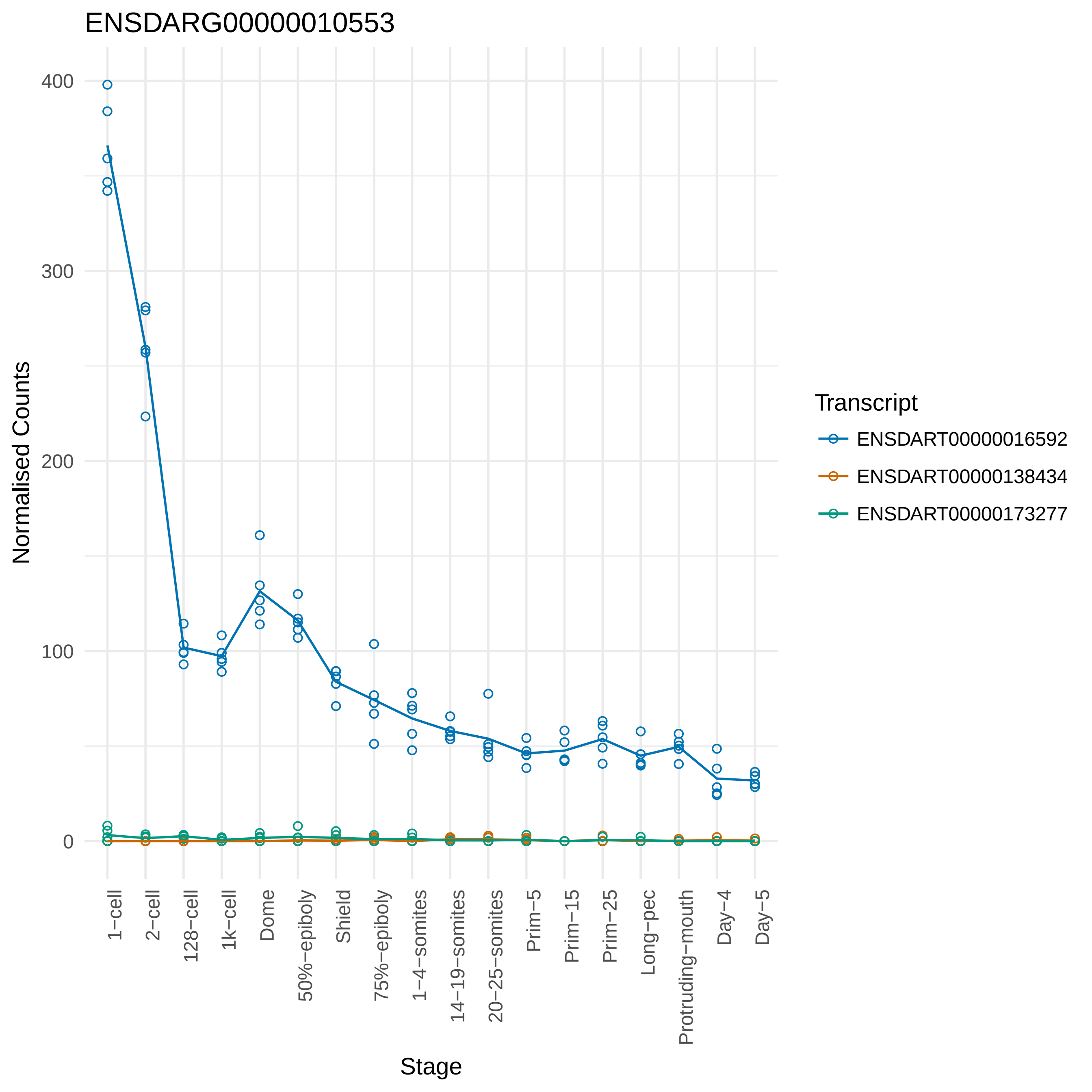

ENSDARG00000010553
Normalised Counts
400
300
200
100
0
Stage
1−cell
2−cell
128−cell
1k−cell
Dome
50%−epiboly
Shield
75%−epiboly
1−4−somites
14−19−somites
20−25−somites
Prim−5
Prim−15
Prim−25
Long−pec
Protruding−mouth
Day−4
Day−5
Transcript
ENSDART00000016592
ENSDART00000138434
ENSDART00000173277

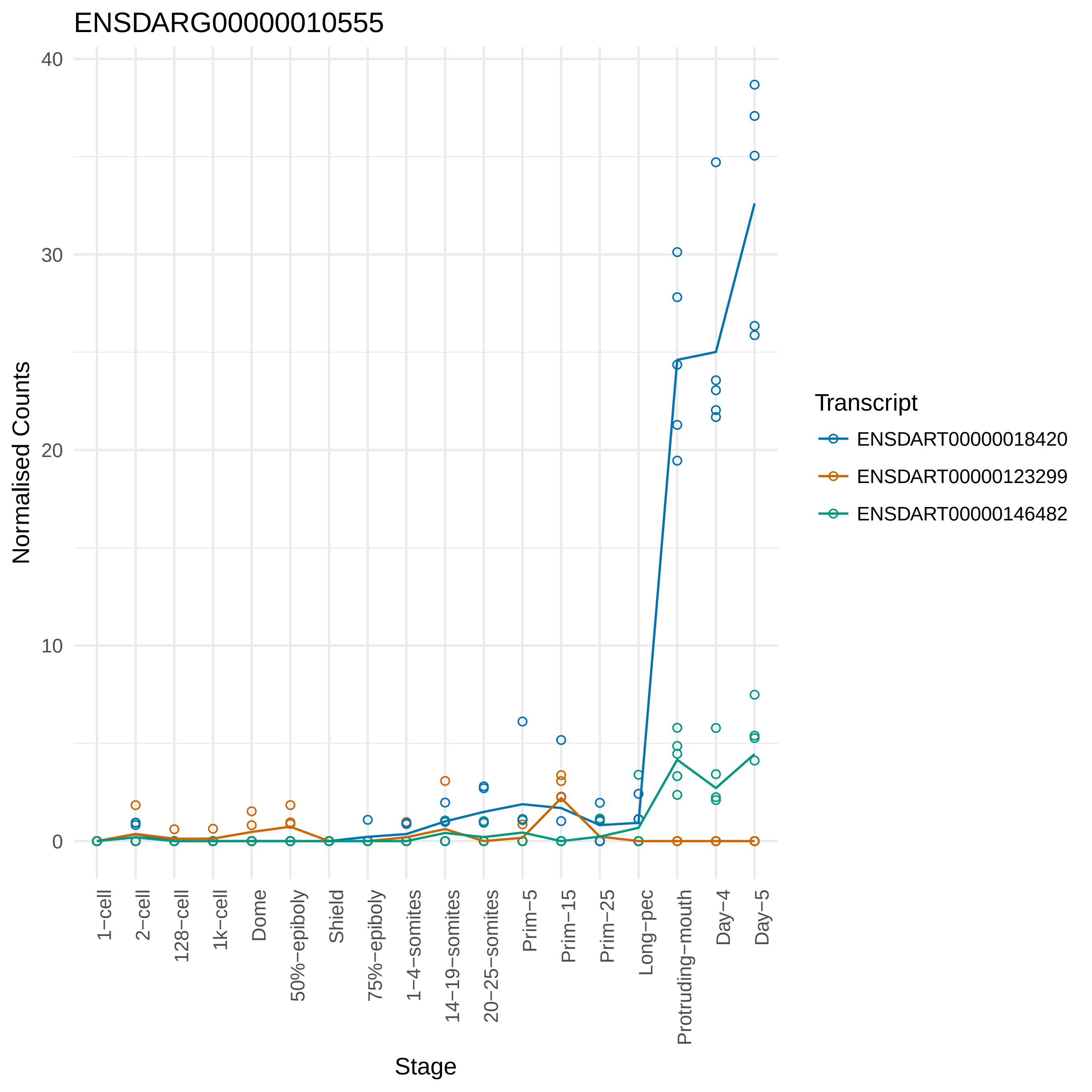
ENSDARG00000010555
Normalised Counts
40
30
20
10
0
1−cell
2−cell
128−cell
1k−cell
Dome
50%−epiboly
Shield
75%−epiboly
1−4−somites
14−19−somites
20−25−somites
Prim−5
Prim−15
Prim−25
Long−pec
Protruding−mouth
Day−4
Day−5
Stage
Transcript
ENSDART00000018420
ENSDART00000123299
ENSDART00000146482

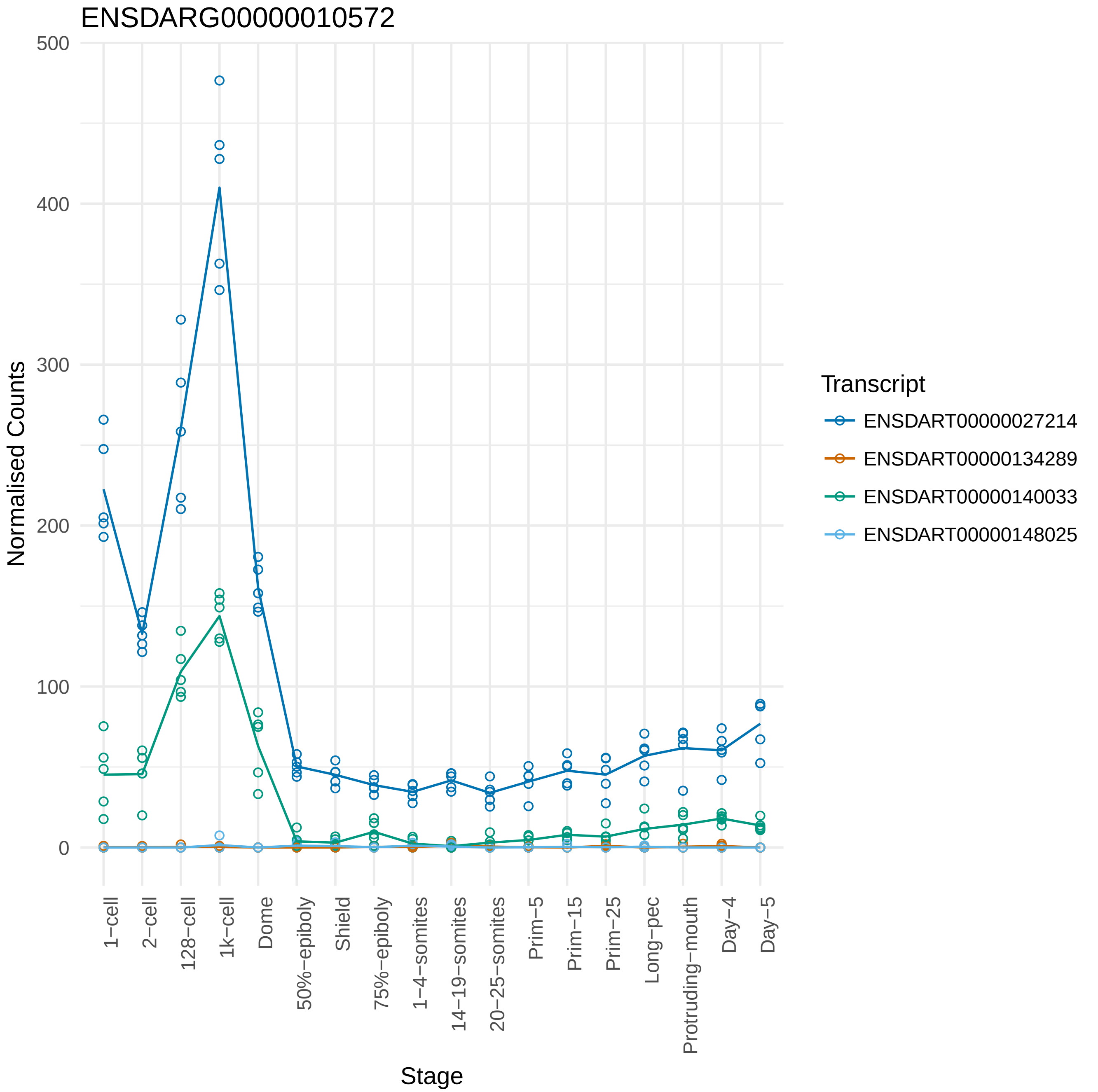

ENSDARG00000010572
Normalised Counts
0
100
200
300
400
500
Stage
1−cell
2−cell
128−cell
1k−cell
Dome
50%−epiboly
Shield
75%−epiboly
1−4−somites
14−19−somites
20−25−somites
Prim−5
Prim−15
Prim−25
Long−pec
Protruding−mouth
Day−4
Day−5
Transcript
ENSDART00000027214
ENSDART00000134289
ENSDART00000140033
ENSDART00000148025

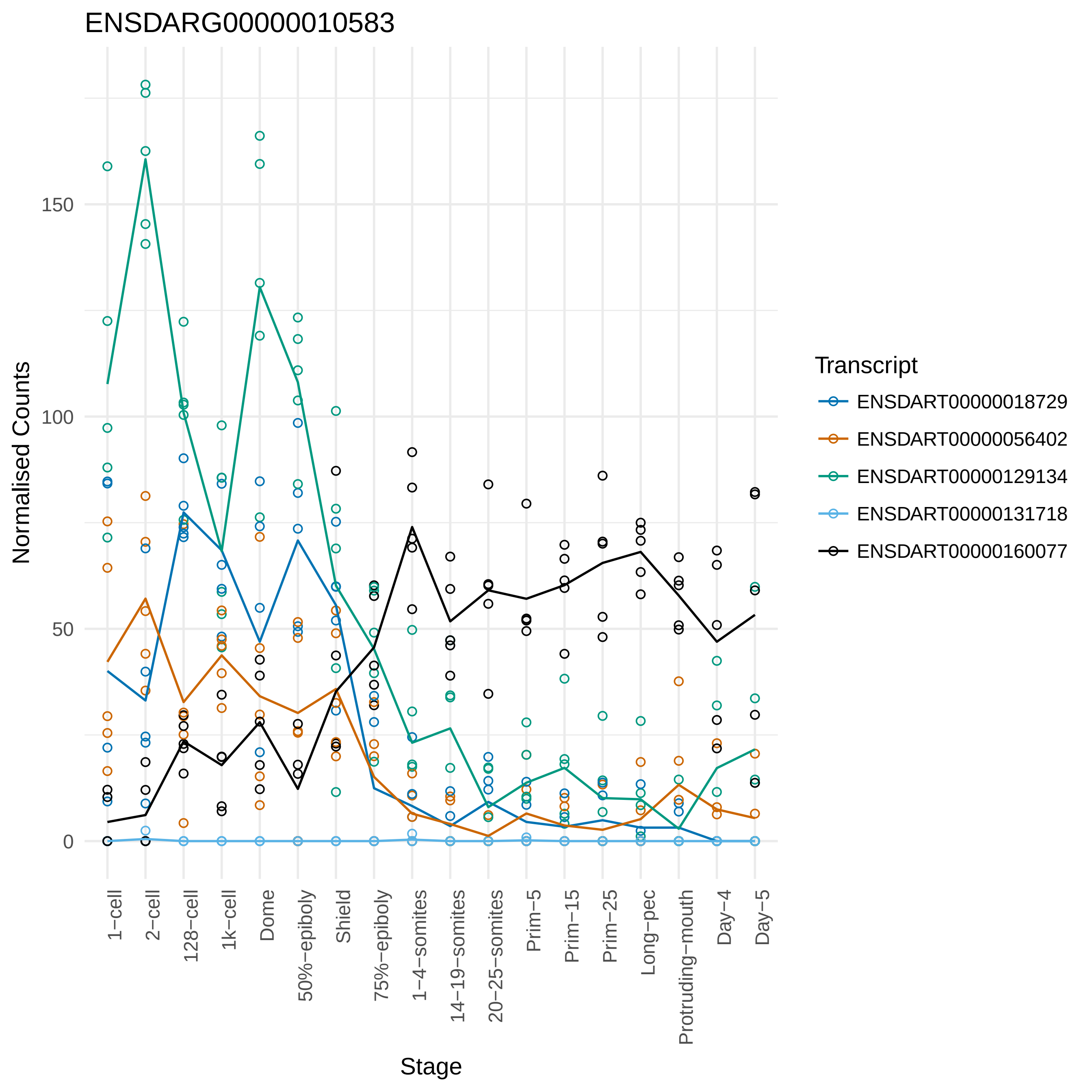

ENSDARG00000010583
Normalised Counts
150
100
50
0
1−cell
2−cell
128−cell
1k−cell
Dome
50%−epiboly
Shield
75%−epiboly
1−4−somites
14−19−somites
20−25−somites
Prim−5
Prim−15
Prim−25
Long−pec
Protruding−mouth
Day−4
Day−5
Stage
Transcript
ENSDART00000018729
ENSDART00000056402
ENSDART00000129134
ENSDART00000131718
ENSDART00000160077

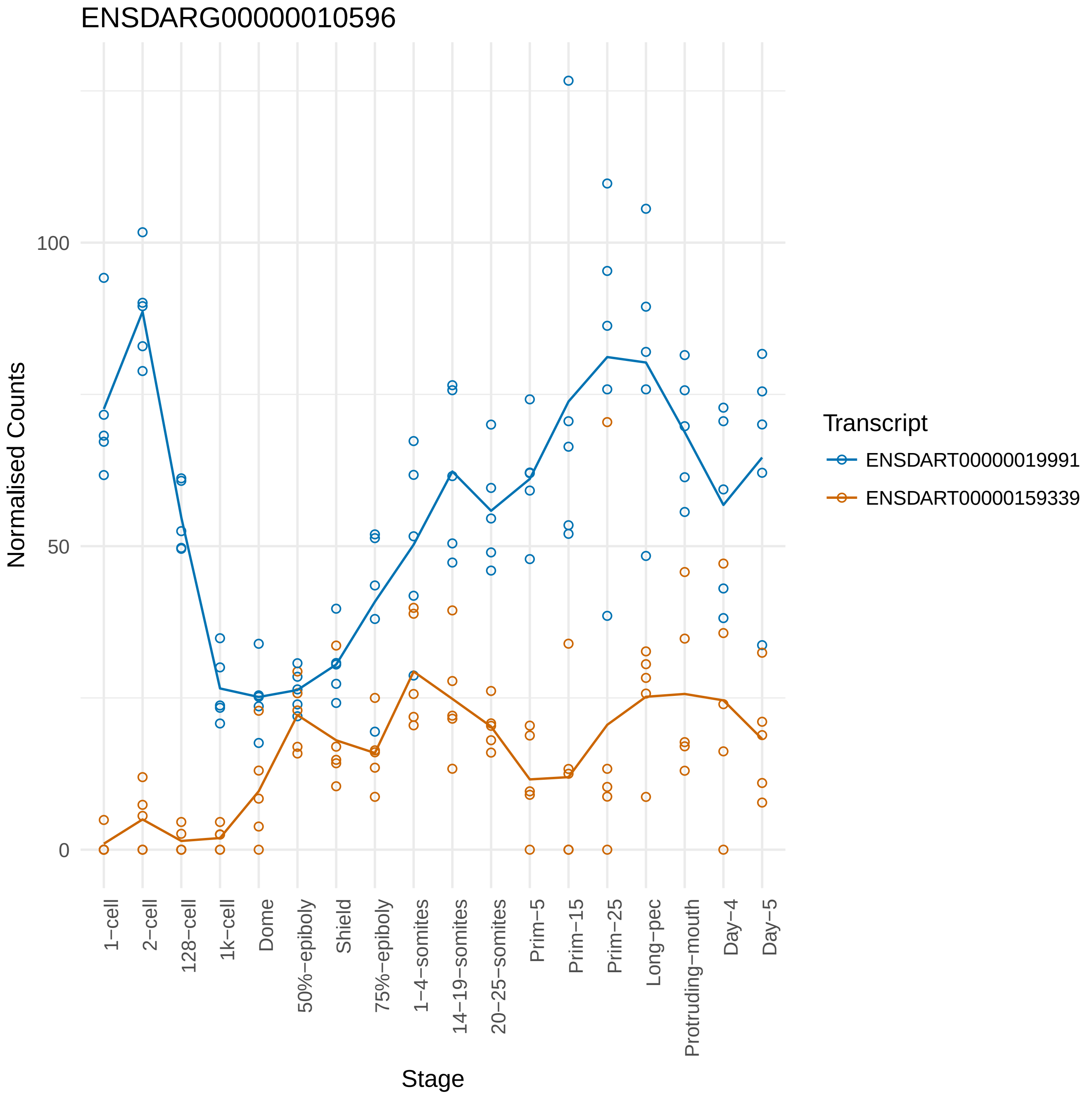

ENSDARG00000010596
Normalised Counts
100
50
0
1−cell
2−cell
128−cell
1k−cell
Dome
50%−epiboly
Shield
75%−epiboly
1−4−somites
14−19−somites
20−25−somites
Prim−5
Prim−15
Prim−25
Long−pec
Protruding−mouth
Day−4
Day−5
Stage
Transcript
ENSDART00000019991
ENSDART00000159339

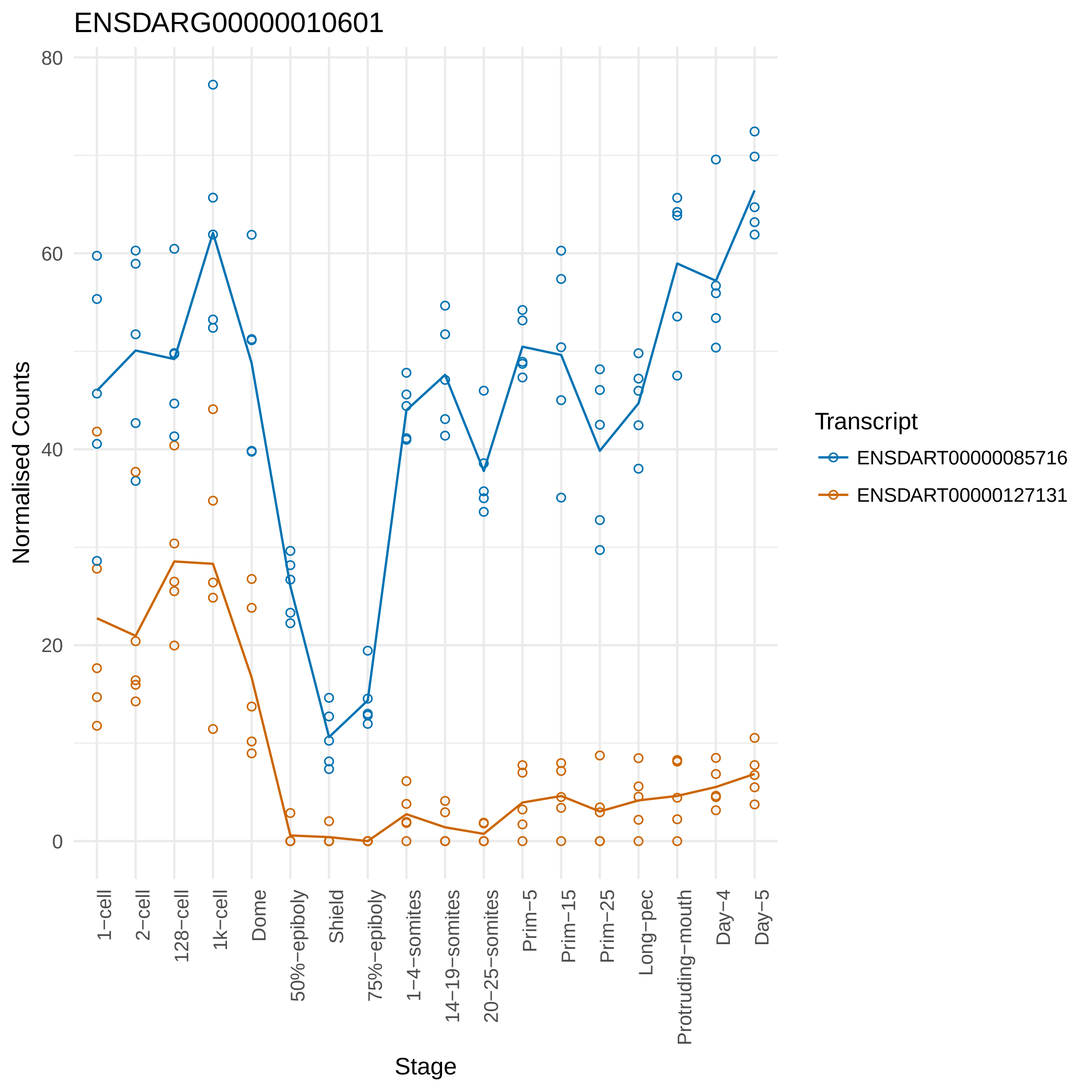
ENSDARG00000010601
Normalised Counts
0
20
40
60
80
1−cell
2−cell
128−cell
1k−cell
Dome
50%−epiboly
Shield
75%−epiboly
1−4−somites
14−19−somites
20−25−somites
Prim−5
Prim−15
Prim−25
Long−pec
Protruding−mouth
Day−4
Day−5
Stage
Transcript
ENSDART00000085716
ENSDART00000127131

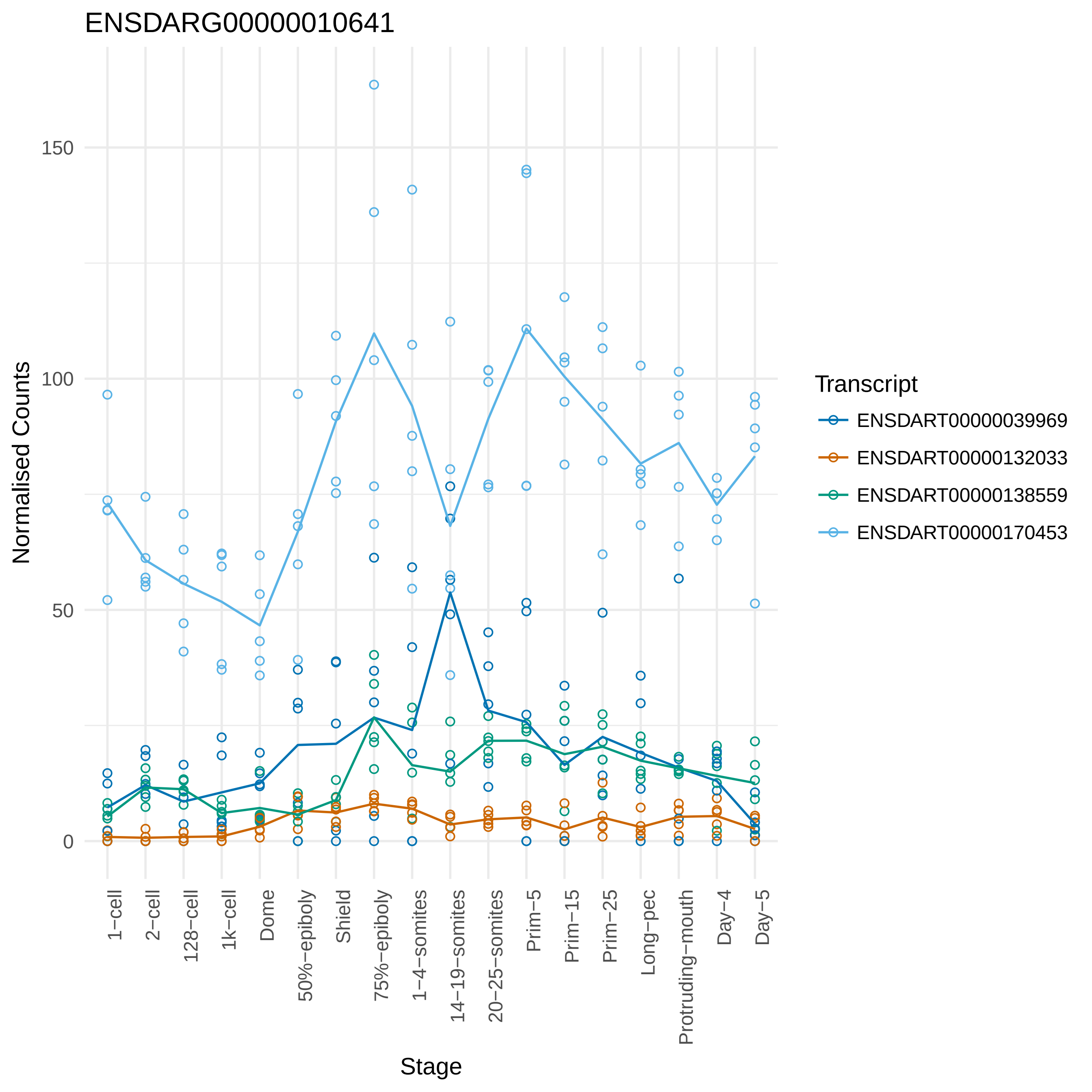

ENSDARG00000010641
Normalised Counts
150
100
50
0
1−cell
2−cell
128−cell
1k−cell
Dome
50%−epiboly
Shield
75%−epiboly
1−4−somites
14−19−somites
20−25−somites
Prim−5
Prim−15
Prim−25
Long−pec
Protruding−mouth
Day−4
Day−5
Stage
Transcript
ENSDART00000039969
ENSDART00000132033
ENSDART00000138559
ENSDART00000170453

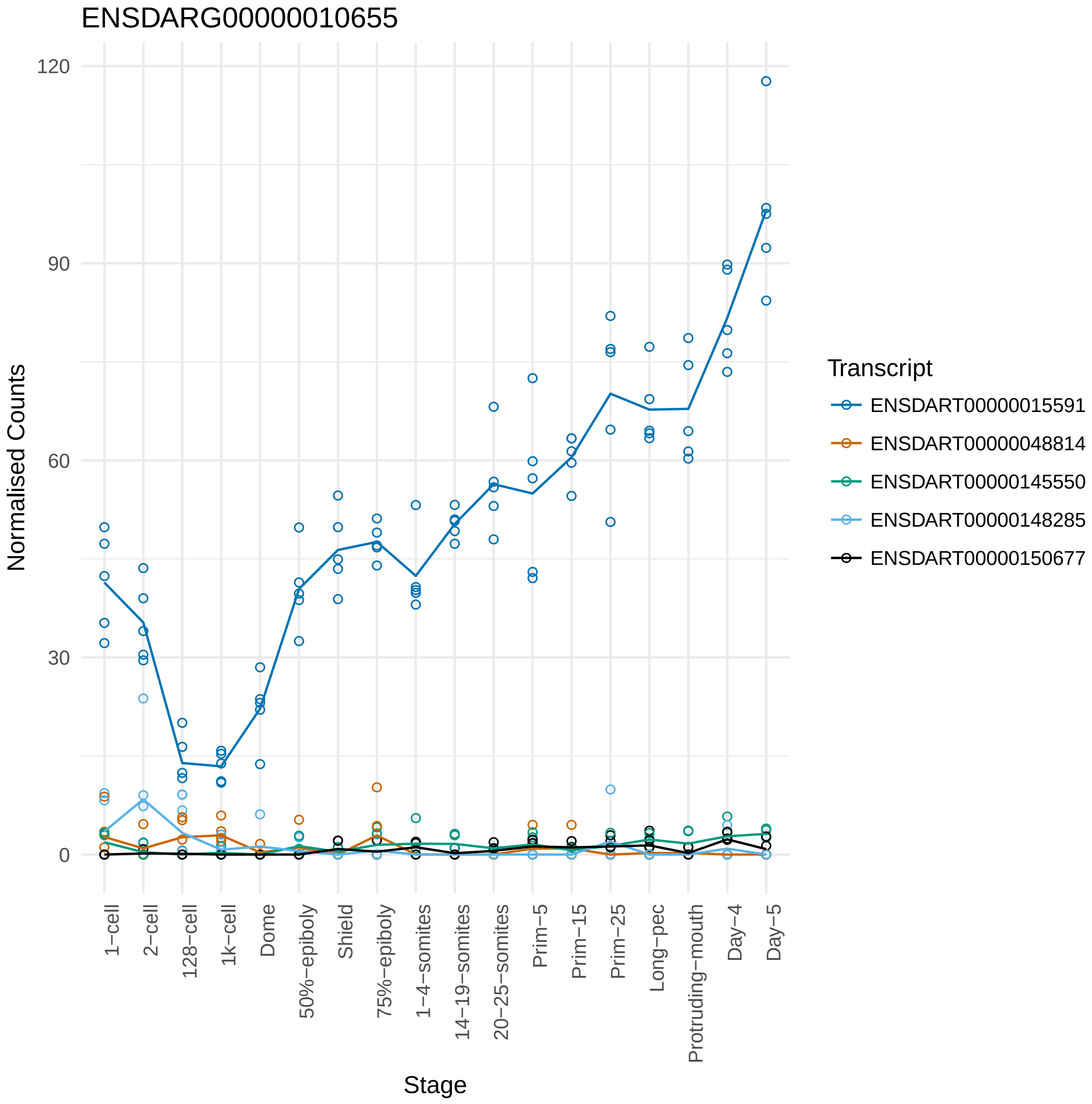

ENSDARG00000010655
Normalised Counts
0
30
60
90
120
Stage
1−cell
2−cell
128−cell
1k−cell
Dome
50%−epiboly
Shield
75%−epiboly
1−4−somites
14−19−somites
20−25−somites
Prim−5
Prim−15
Prim−25
Long−pec
Protruding−mouth
Day−4
Day−5
Transcript
ENSDART00000015591
ENSDART00000048814
ENSDART00000145550
ENSDART00000148285
ENSDART00000150677

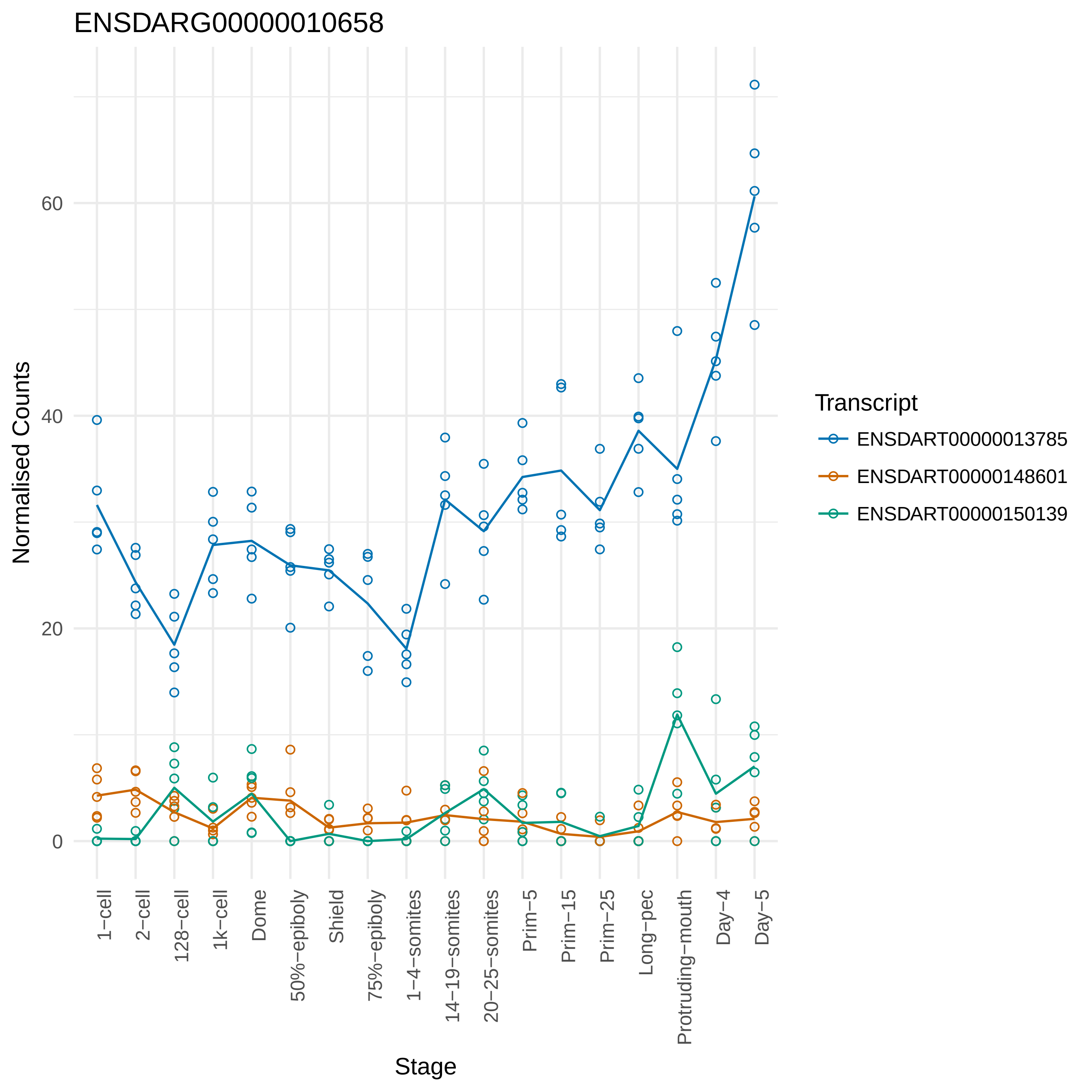
ENSDARG00000010658
Normalised Counts
0
20
40
60
Stage
1−cell
2−cell
128−cell
1k−cell
Dome
50%−epiboly
Shield
75%−epiboly
1−4−somites
14−19−somites
20−25−somites
Prim−5
Prim−15
Prim−25
Long−pec
Protruding−mouth
Day−4
Day−5
Transcript
ENSDART00000013785
ENSDART00000148601
ENSDART00000150139

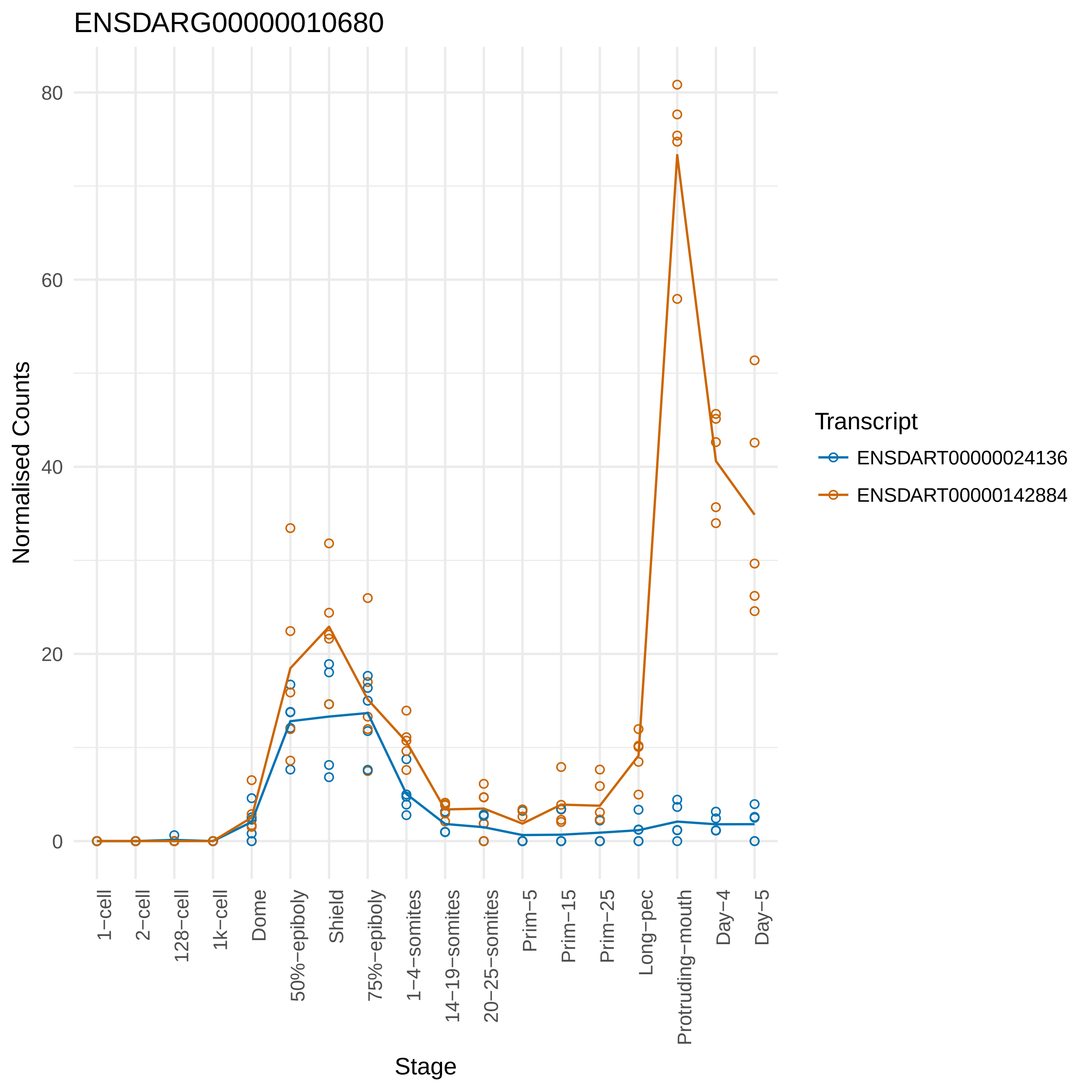
ENSDARG00000010680
Normalised Counts
80
60
40
20
0
Stage
1−cell
2−cell
128−cell
1k−cell
Dome
50%−epiboly
Shield
75%−epiboly
1−4−somites
14−19−somites
20−25−somites
Prim−5
Prim−15
Prim−25
Long−pec
Protruding−mouth
Day−4
Day−5
Transcript
ENSDART00000024136
ENSDART00000142884

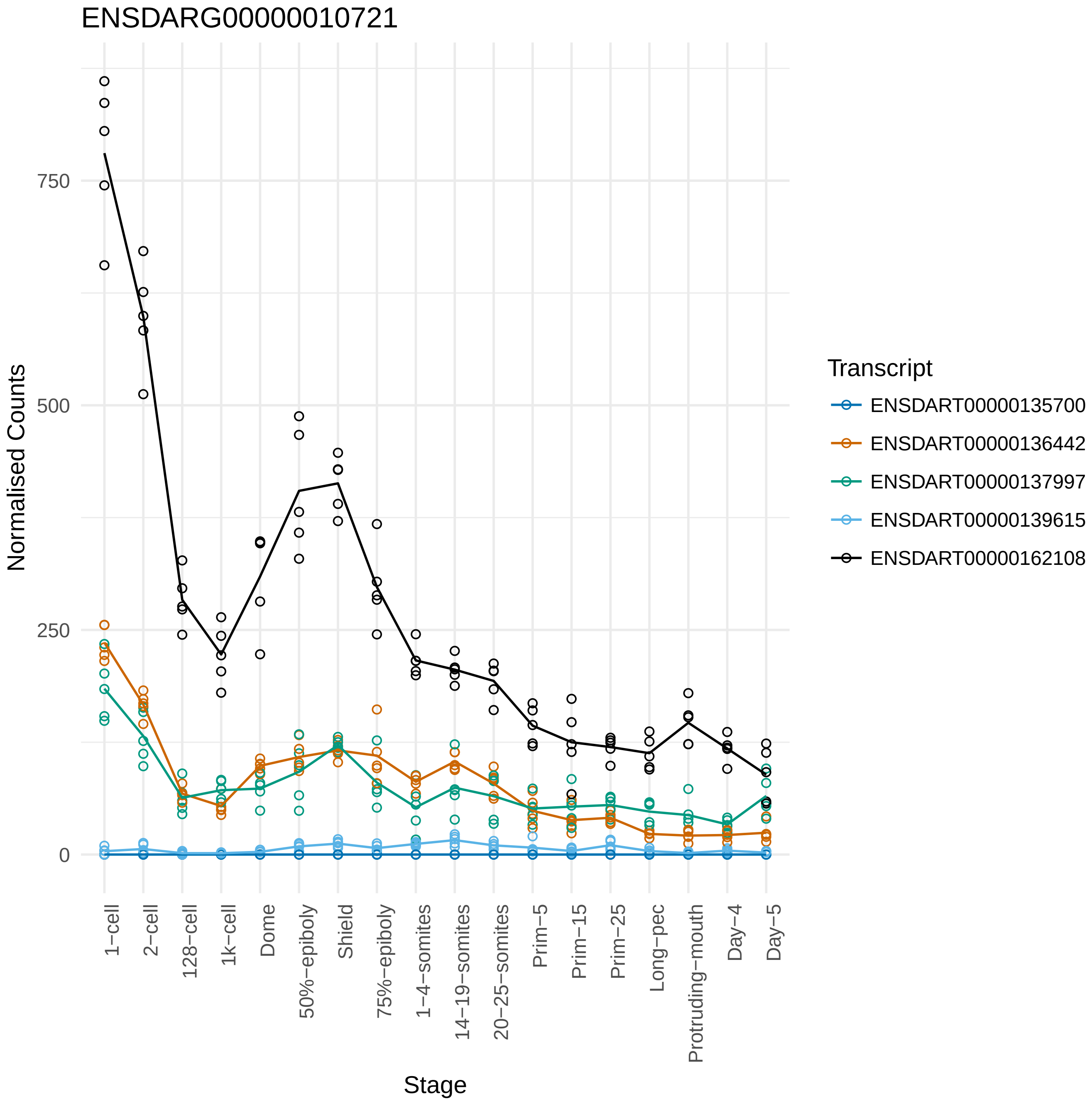

ENSDARG00000010721
Normalised Counts
750
500
250
0
1−cell
2−cell
128−cell
1k−cell
Dome
50%−epiboly
Shield
75%−epiboly
1−4−somites
14−19−somites
20−25−somites
Prim−5
Prim−15
Prim−25
Long−pec
Protruding−mouth
Day−4
Day−5
Stage
Transcript
ENSDART00000135700
ENSDART00000136442
ENSDART00000137997
ENSDART00000139615
ENSDART00000162108

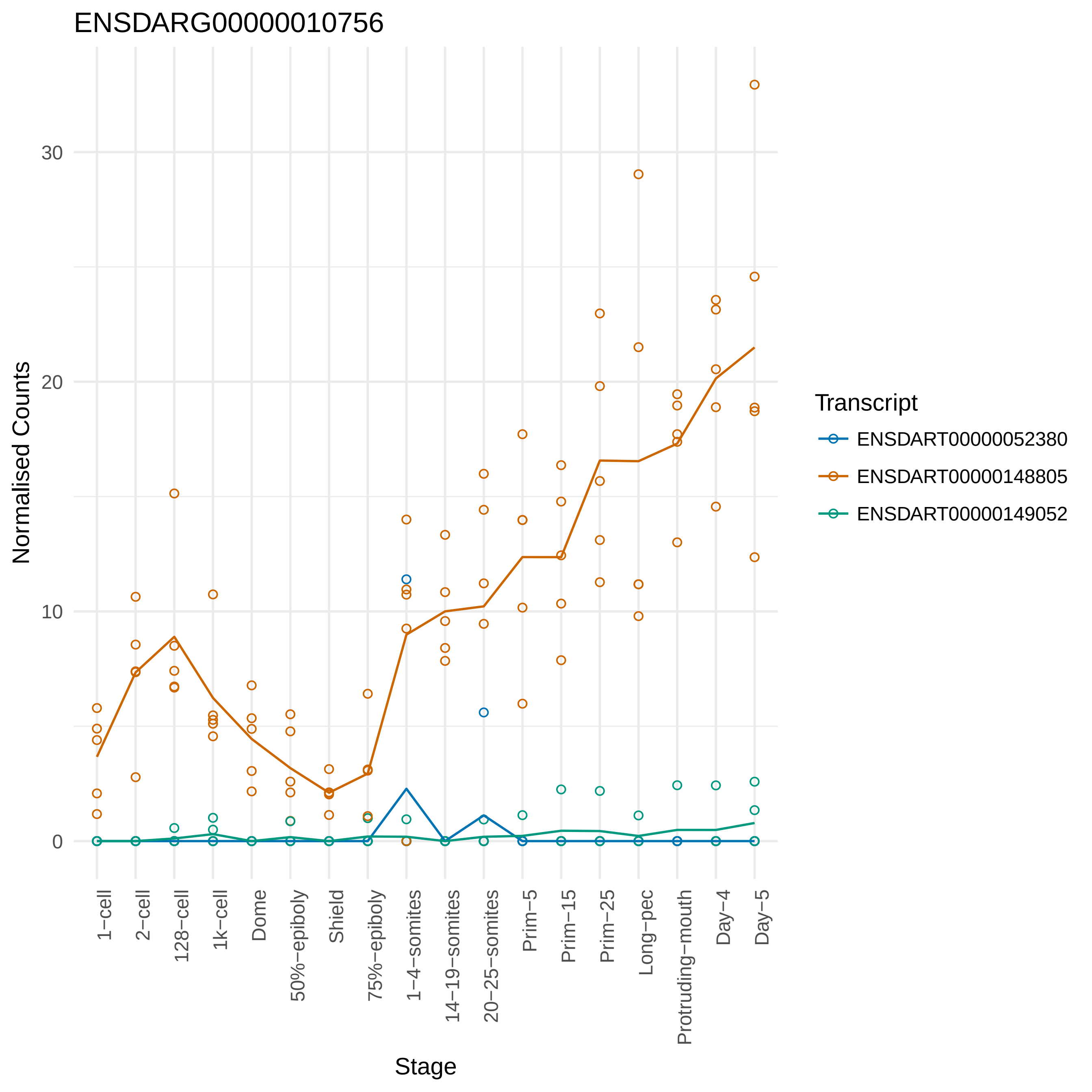

ENSDARG00000010756
Normalised Counts
30
20
10
0
1−cell
2−cell
128−cell
1k−cell
Dome
50%−epiboly
Shield
75%−epiboly
1−4−somites
14−19−somites
20−25−somites
Prim−5
Prim−15
Prim−25
Long−pec
Protruding−mouth
Day−4
Day−5
Stage
Transcript
ENSDART00000052380
ENSDART00000148805
ENSDART00000149052

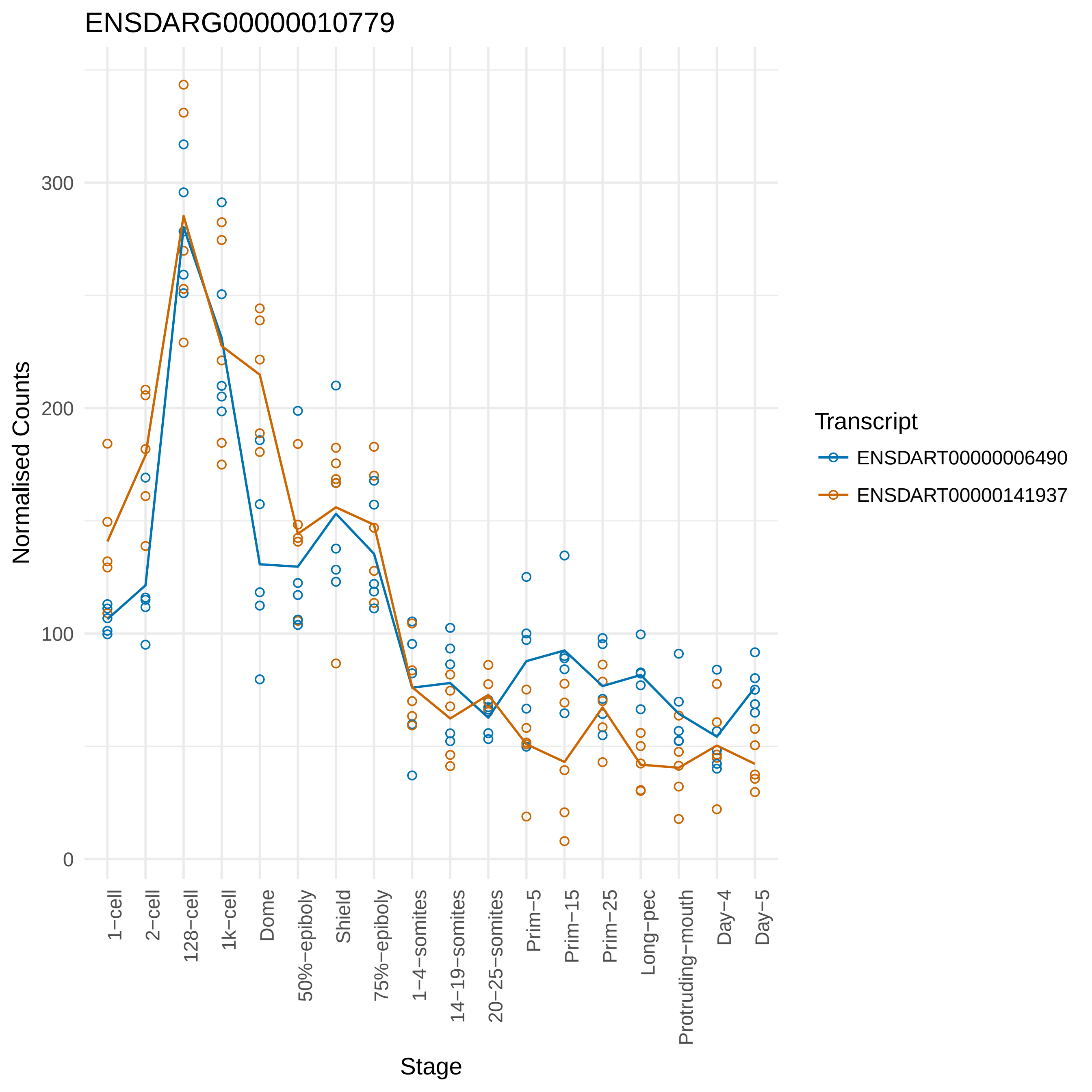
ENSDARG00000010779
Normalised Counts
300
200
100
0
1−cell
2−cell
128−cell
1k−cell
Dome
50%−epiboly
Shield
75%−epiboly
1−4−somites
14−19−somites
20−25−somites
Prim−5
Prim−15
Prim−25
Long−pec
Protruding−mouth
Day−4
Day−5
Stage
Transcript
ENSDART00000006490
ENSDART00000141937

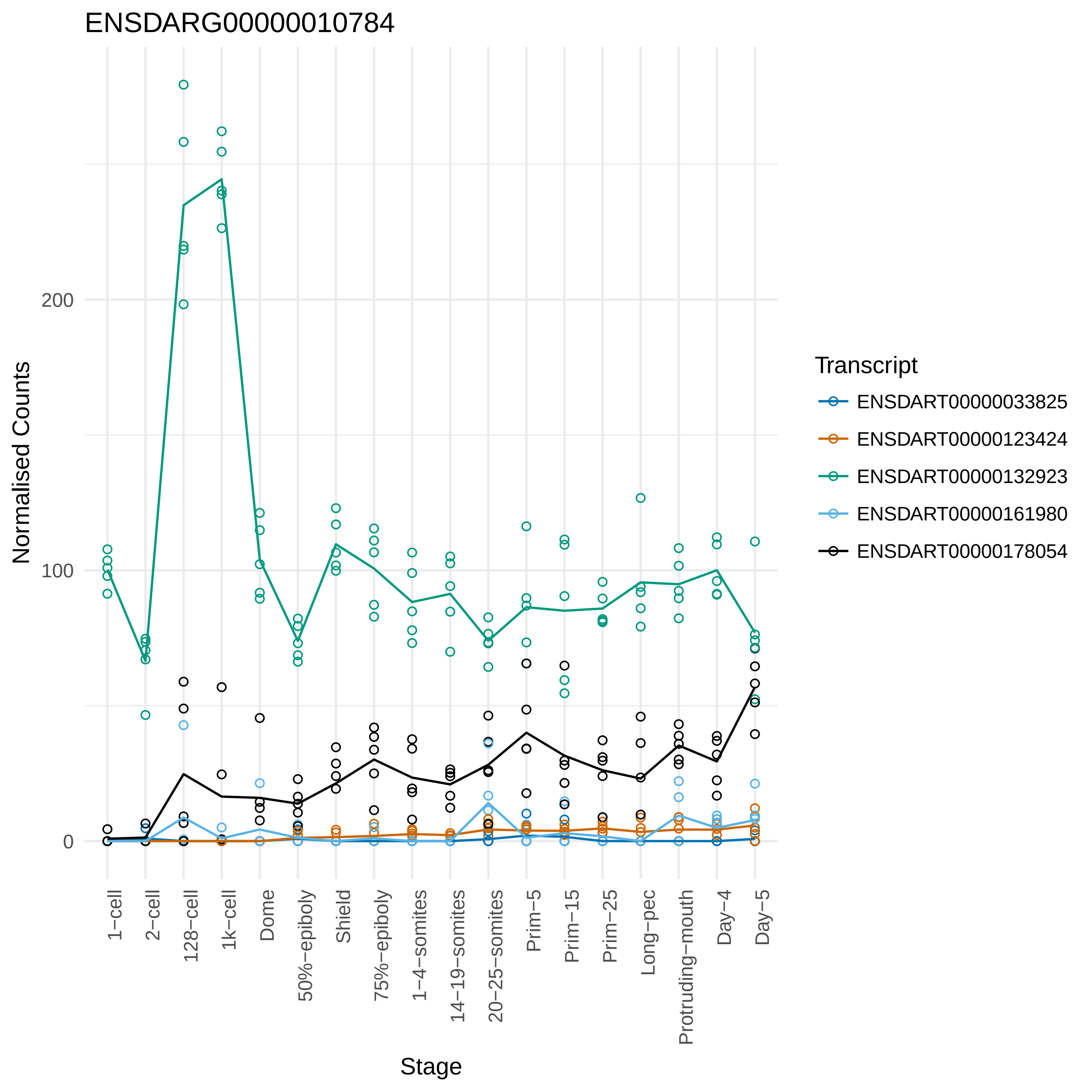

ENSDARG00000010784
Normalised Counts
0
100
200
Stage
1−cell
2−cell
128−cell
1k−cell
Dome
50%−epiboly
Shield
75%−epiboly
1−4−somites
14−19−somites
20−25−somites
Prim−5
Prim−15
Prim−25
Long−pec
Protruding−mouth
Day−4
Day−5
Transcript
ENSDART00000033825
ENSDART00000123424
ENSDART00000132923
ENSDART00000161980
ENSDART00000178054

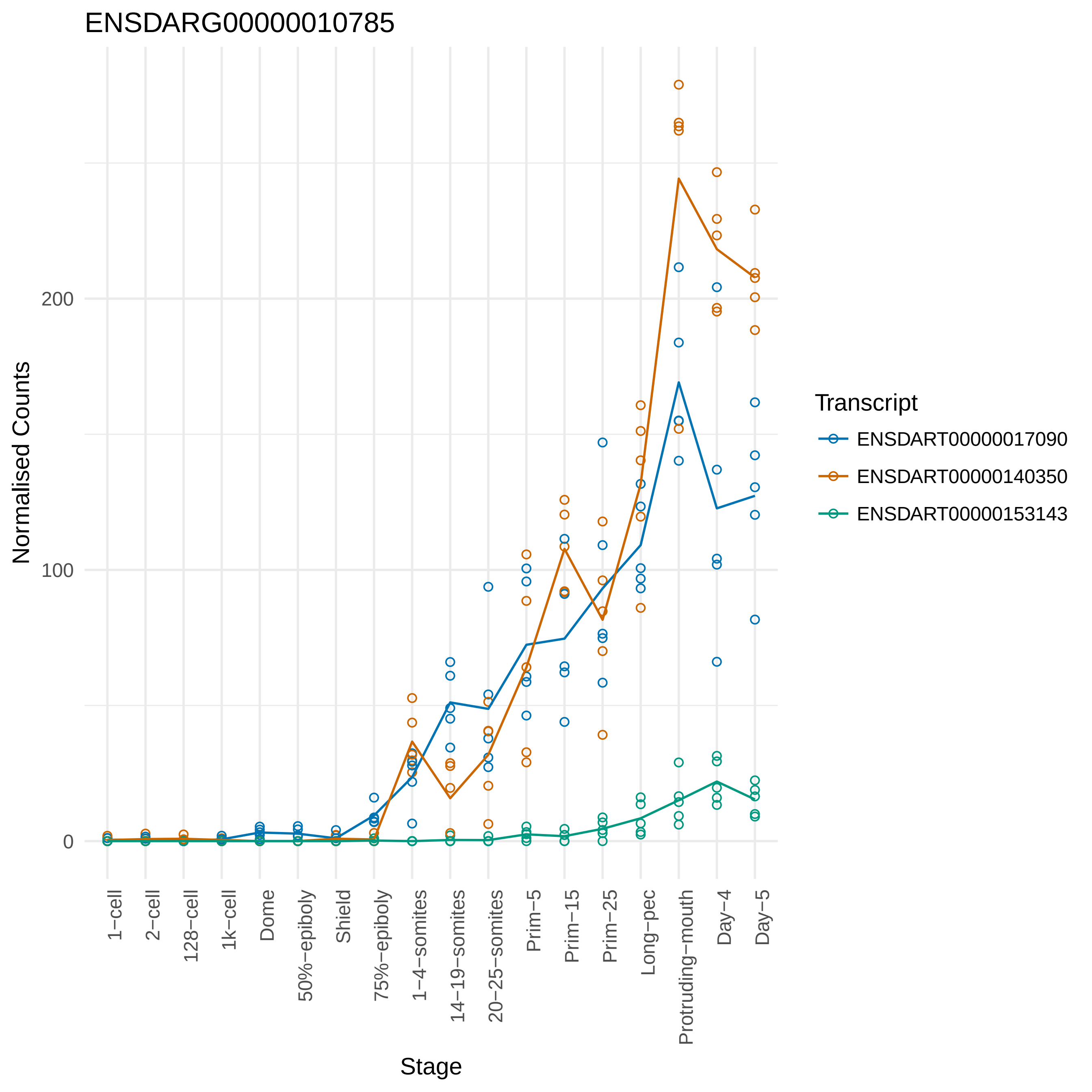
ENSDARG00000010785
Normalised Counts
0
100
200
Stage
1−cell
2−cell
128−cell
1k−cell
Dome
50%−epiboly
Shield
75%−epiboly
1−4−somites
14−19−somites
20−25−somites
Prim−5
Prim−15
Prim−25
Long−pec
Protruding−mouth
Day−4
Day−5
Transcript
ENSDART00000017090
ENSDART00000140350
ENSDART00000153143

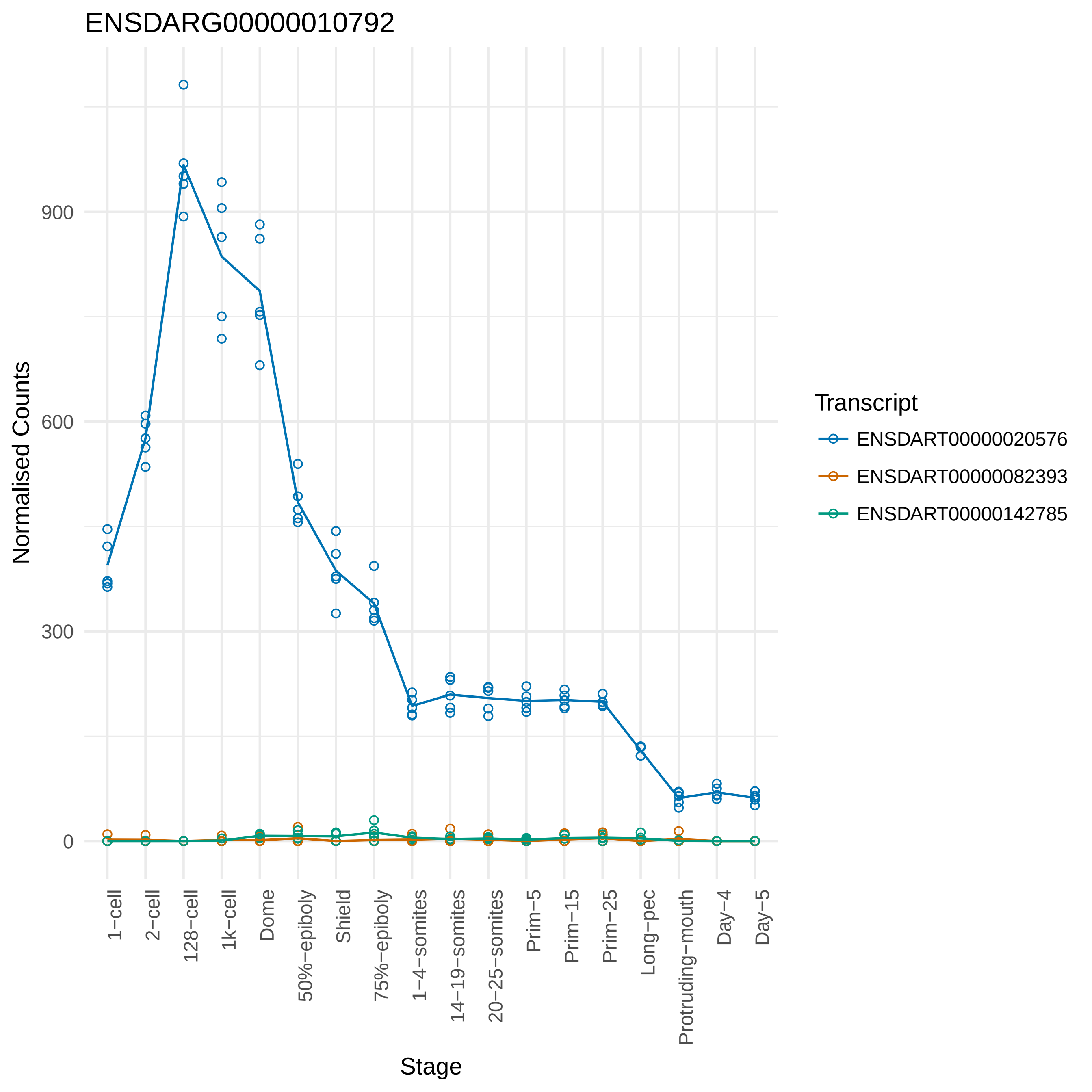

ENSDARG00000010792
Normalised Counts
900
600
300
0
1−cell
2−cell
128−cell
1k−cell
Dome
50%−epiboly
Shield
75%−epiboly
1−4−somites
14−19−somites
20−25−somites
Prim−5
Prim−15
Prim−25
Long−pec
Protruding−mouth
Day−4
Day−5
Stage
Transcript
ENSDART00000020576
ENSDART00000082393
ENSDART00000142785

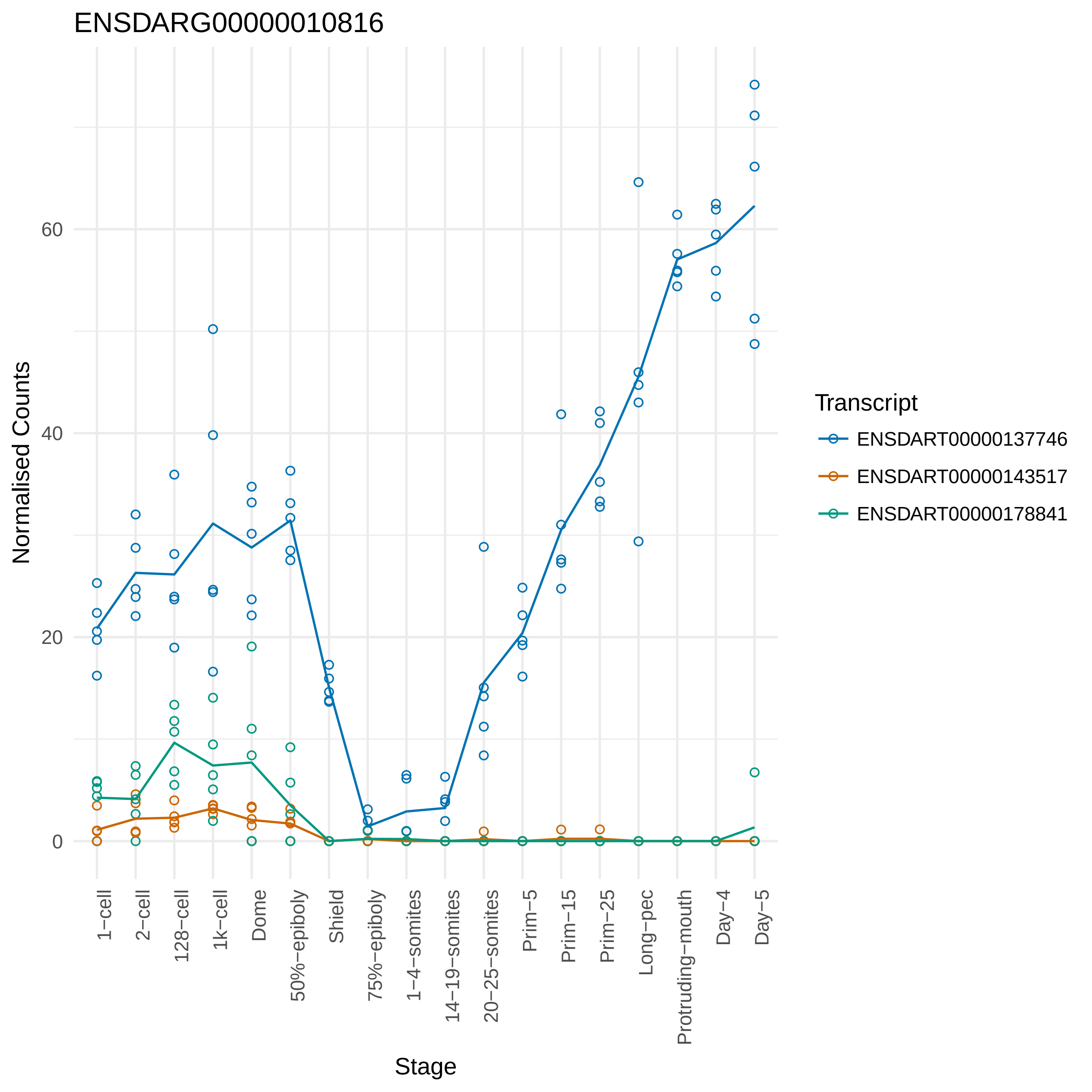
ENSDARG00000010816
Normalised Counts
0
20
40
60
1−cell
2−cell
128−cell
1k−cell
Dome
50%−epiboly
Shield
75%−epiboly
1−4−somites
14−19−somites
20−25−somites
Prim−5
Prim−15
Prim−25
Long−pec
Protruding−mouth
Day−4
Day−5
Stage
Transcript
ENSDART00000137746
ENSDART00000143517
ENSDART00000178841

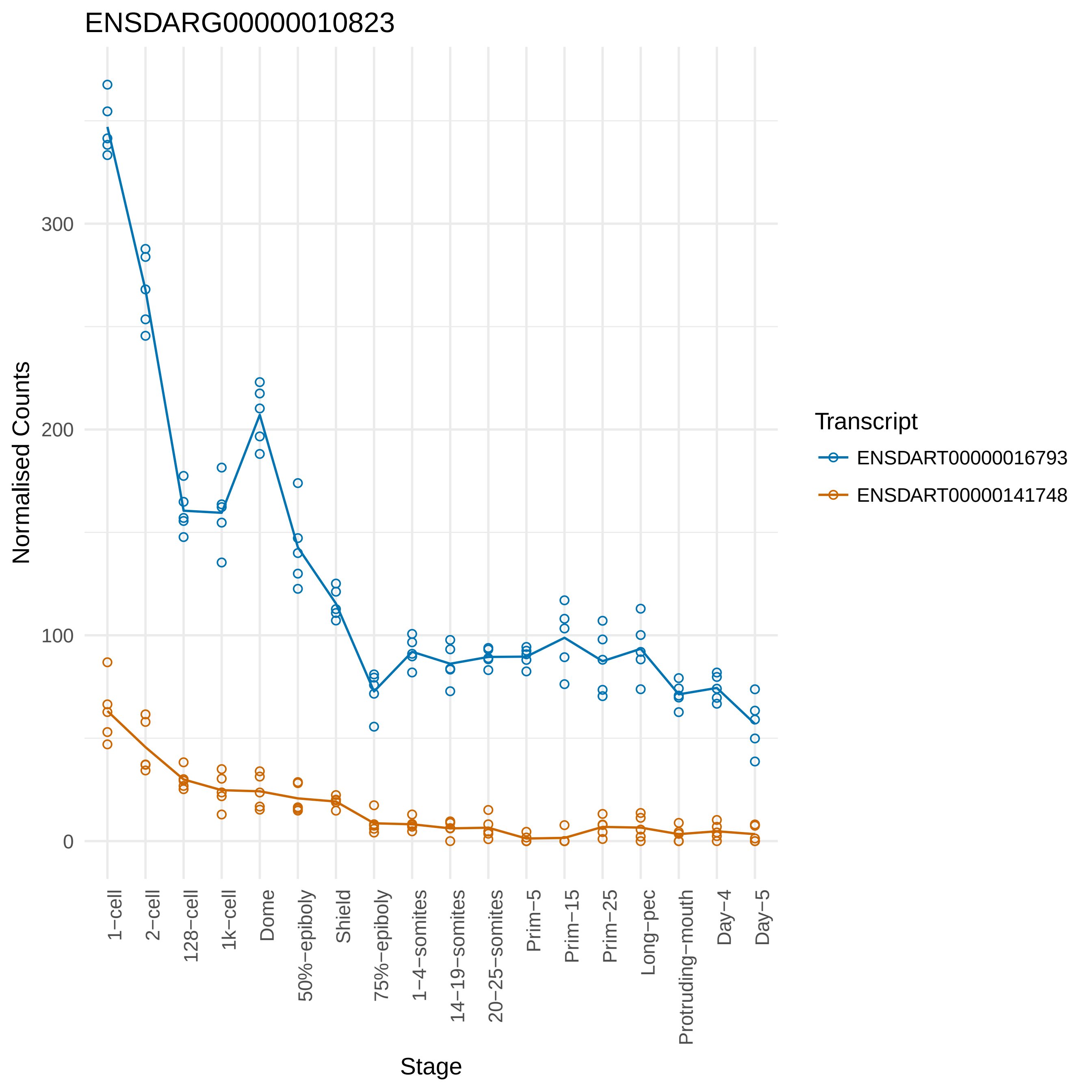

ENSDARG00000010823
Normalised Counts
300
200
100
0
1−cell
2−cell
128−cell
1k−cell
Dome
50%−epiboly
Shield
75%−epiboly
1−4−somites
14−19−somites
20−25−somites
Prim−5
Prim−15
Prim−25
Long−pec
Protruding−mouth
Day−4
Day−5
Stage
Transcript
ENSDART00000016793
ENSDART00000141748

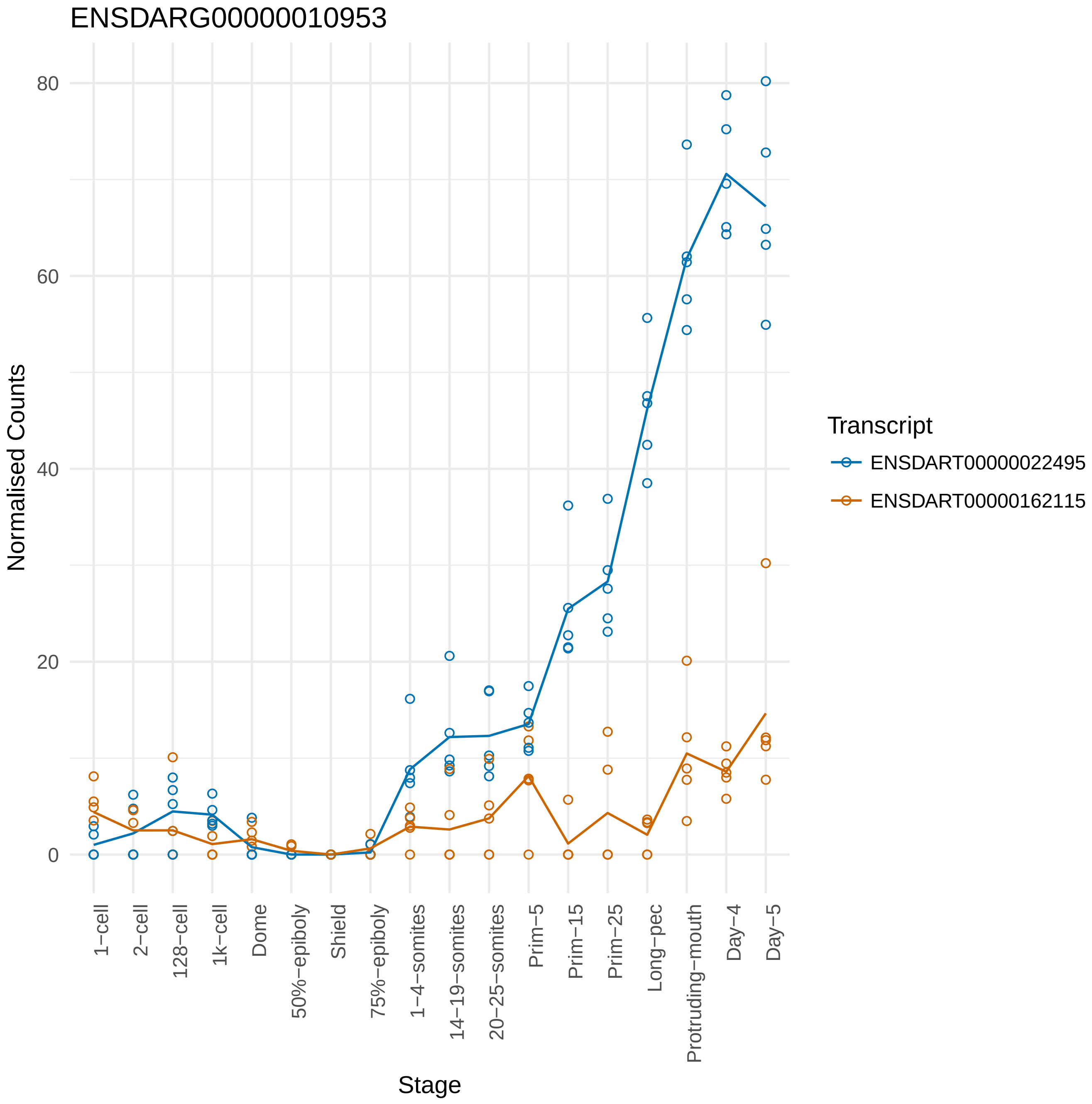

ENSDARG00000010953
Normalised Counts
80
60
40
20
0
Stage
1−cell
2−cell
128−cell
1k−cell
Dome
50%−epiboly
Shield
75%−epiboly
1−4−somites
14−19−somites
20−25−somites
Prim−5
Prim−15
Prim−25
Long−pec
Protruding−mouth
Day−4
Day−5
Transcript
ENSDART00000022495
ENSDART00000162115

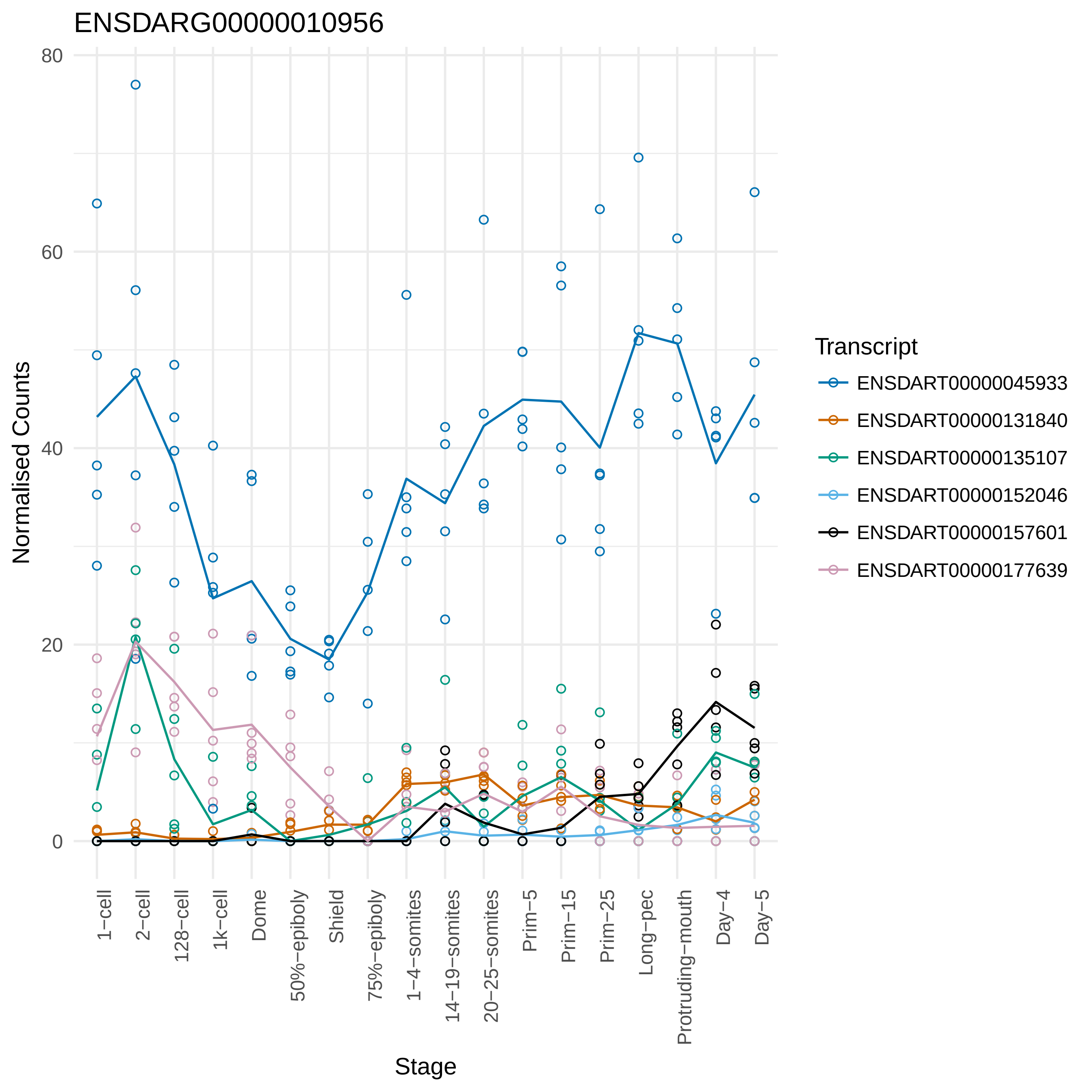
ENSDARG00000010956
Normalised Counts
80
60
40
20
0
1−cell
2−cell
128−cell
1k−cell
Dome
50%−epiboly
Shield
75%−epiboly
1−4−somites
14−19−somites
20−25−somites
Prim−5
Prim−15
Prim−25
Long−pec
Protruding−mouth
Day−4
Day−5
Stage
Transcript
ENSDART00000045933
ENSDART00000131840
ENSDART00000135107
ENSDART00000152046
ENSDART00000157601
ENSDART00000177639

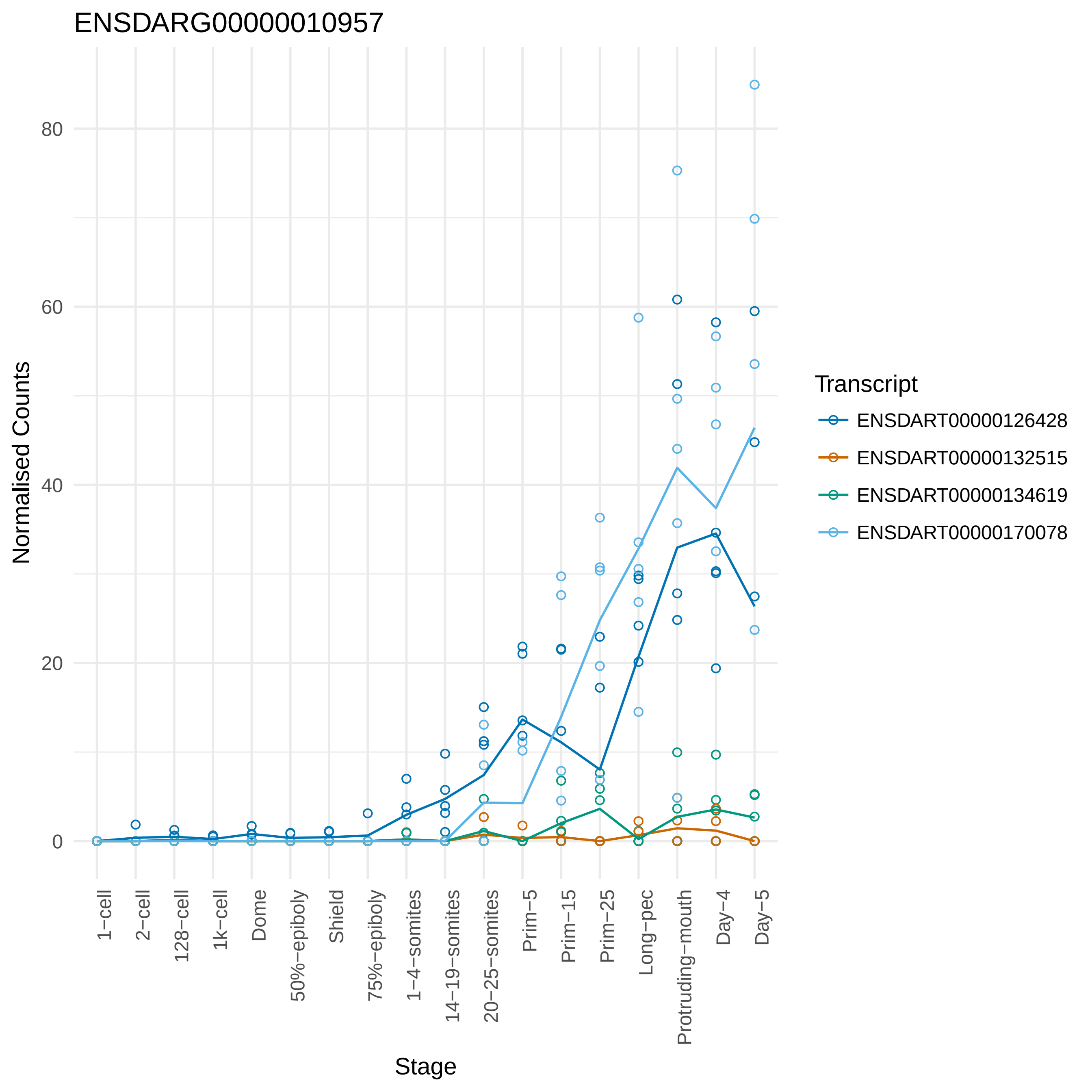
ENSDARG00000010957
Normalised Counts
0
20
40
60
80
Stage
1−cell
2−cell
128−cell
1k−cell
Dome
50%−epiboly
Shield
75%−epiboly
1−4−somites
14−19−somites
20−25−somites
Prim−5
Prim−15
Prim−25
Long−pec
Protruding−mouth
Day−4
Day−5
Transcript
ENSDART00000126428
ENSDART00000132515
ENSDART00000134619
ENSDART00000170078

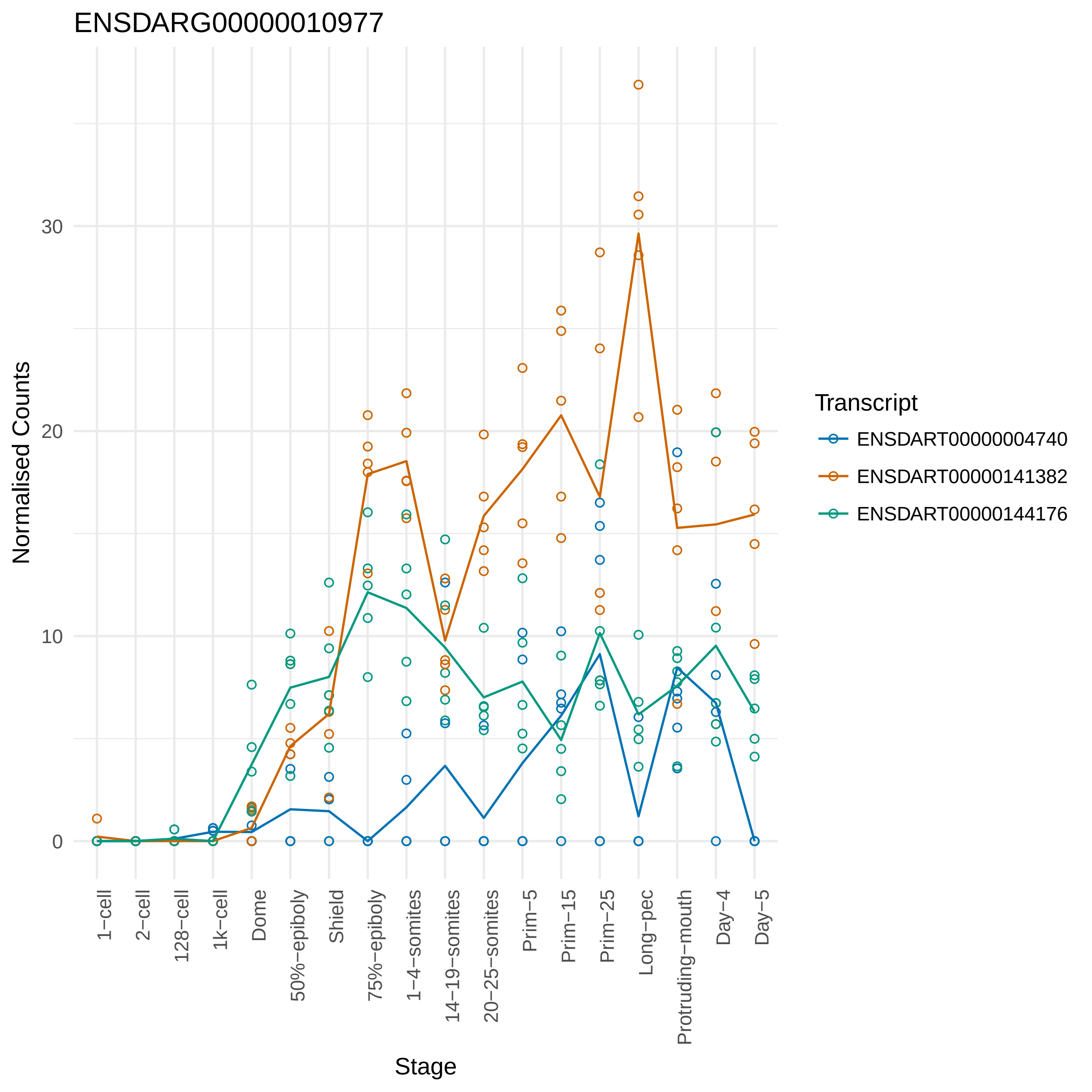

ENSDARG00000010977
Normalised Counts
0
10
20
30
1−cell
2−cell
128−cell
1k−cell
Dome
50%−epiboly
Shield
75%−epiboly
1−4−somites
14−19−somites
20−25−somites
Prim−5
Prim−15
Prim−25
Long−pec
Protruding−mouth
Day−4
Day−5
Stage
Transcript
ENSDART00000004740
ENSDART00000141382
ENSDART00000144176

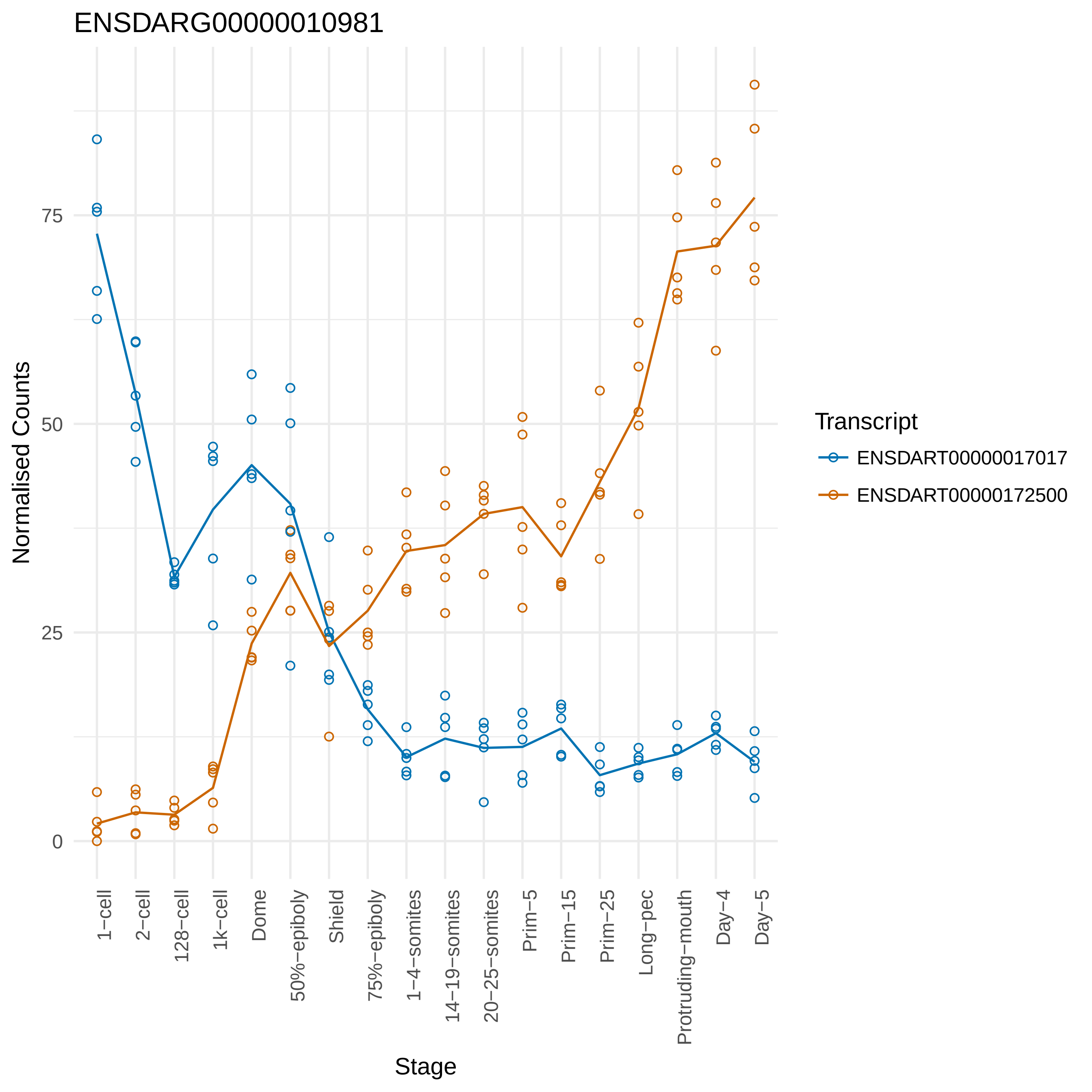
ENSDARG00000010981
Normalised Counts
0
25
50
75
1−cell
2−cell
128−cell
1k−cell
Dome
50%−epiboly
Shield
75%−epiboly
1−4−somites
14−19−somites
20−25−somites
Prim−5
Prim−15
Prim−25
Long−pec
Protruding−mouth
Day−4
Day−5
Stage
Transcript
ENSDART00000017017
ENSDART00000172500

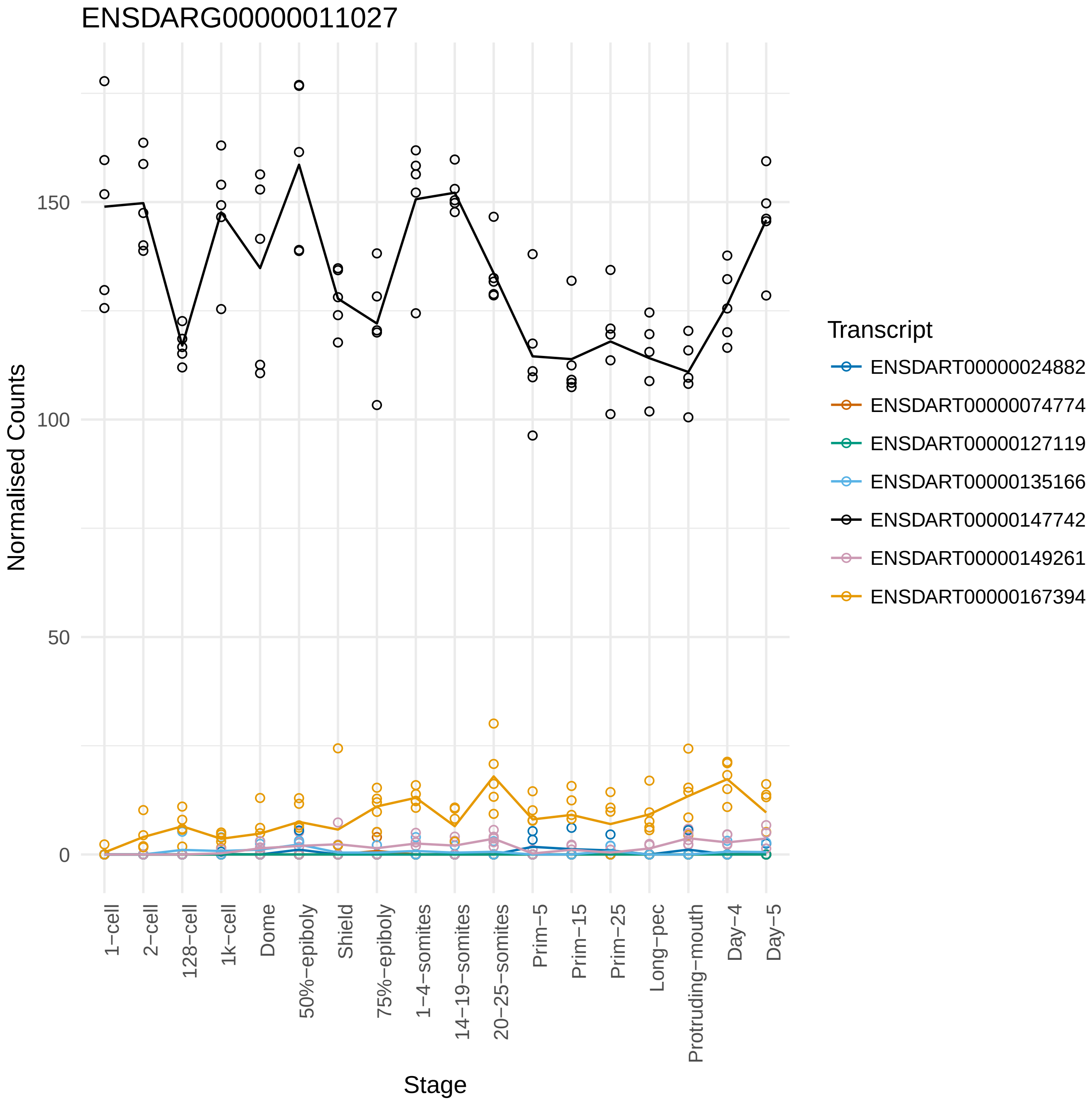

ENSDARG00000011027
Normalised Counts
150
100
50
0
1−cell
2−cell
128−cell
1k−cell
Dome
50%−epiboly
Shield
75%−epiboly
1−4−somites
14−19−somites
20−25−somites
Prim−5
Prim−15
Prim−25
Long−pec
Protruding−mouth
Day−4
Day−5
Stage
Transcript
ENSDART00000024882
ENSDART00000074774
ENSDART00000127119
ENSDART00000135166
ENSDART00000147742
ENSDART00000149261
ENSDART00000167394

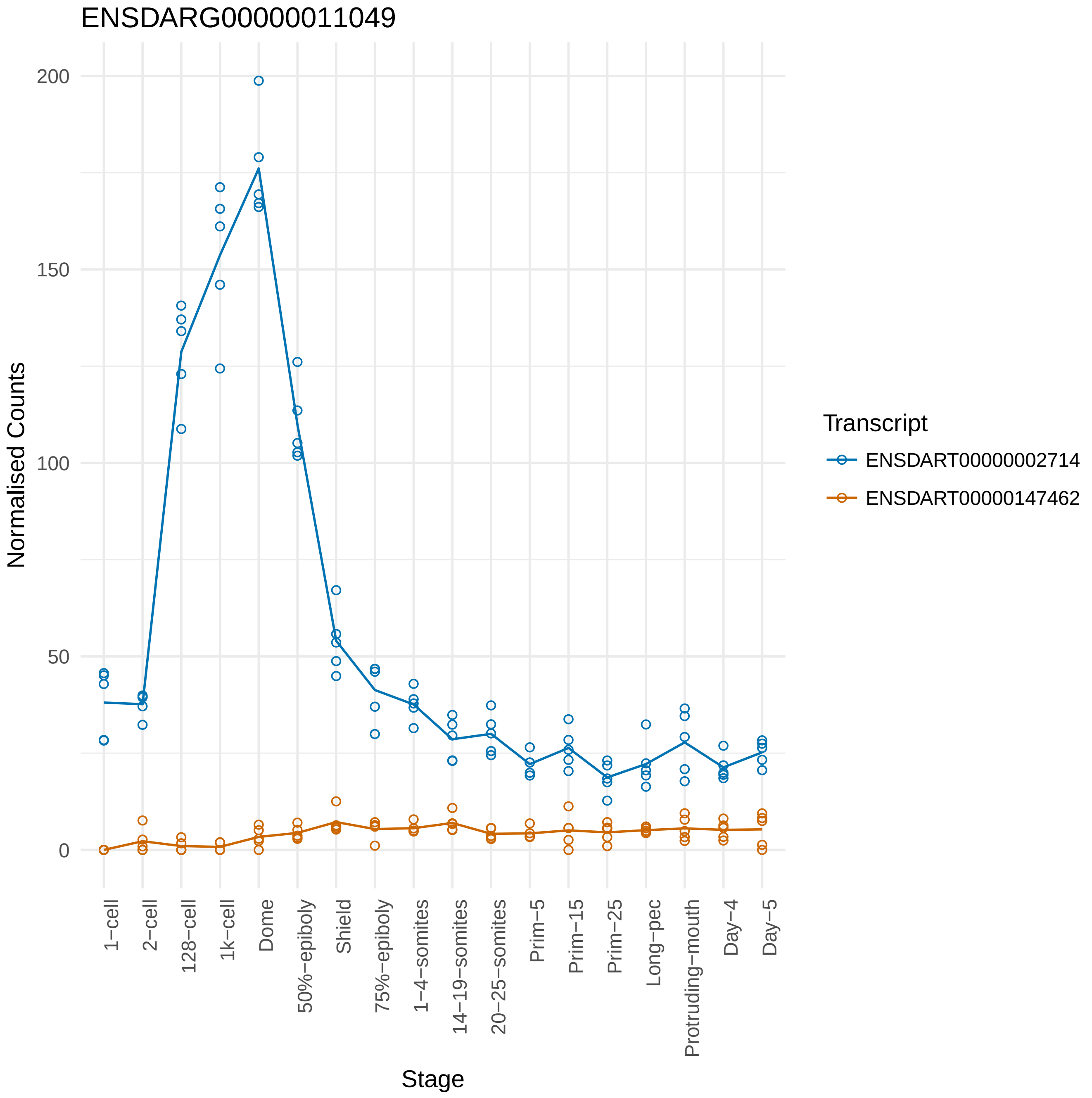

ENSDARG00000011049
Normalised Counts
200
150
100
50
0
1−cell
2−cell
128−cell
1k−cell
Dome
50%−epiboly
Shield
75%−epiboly
1−4−somites
14−19−somites
20−25−somites
Prim−5
Prim−15
Prim−25
Long−pec
Protruding−mouth
Day−4
Day−5
Stage
Transcript
ENSDART00000002714
ENSDART00000147462

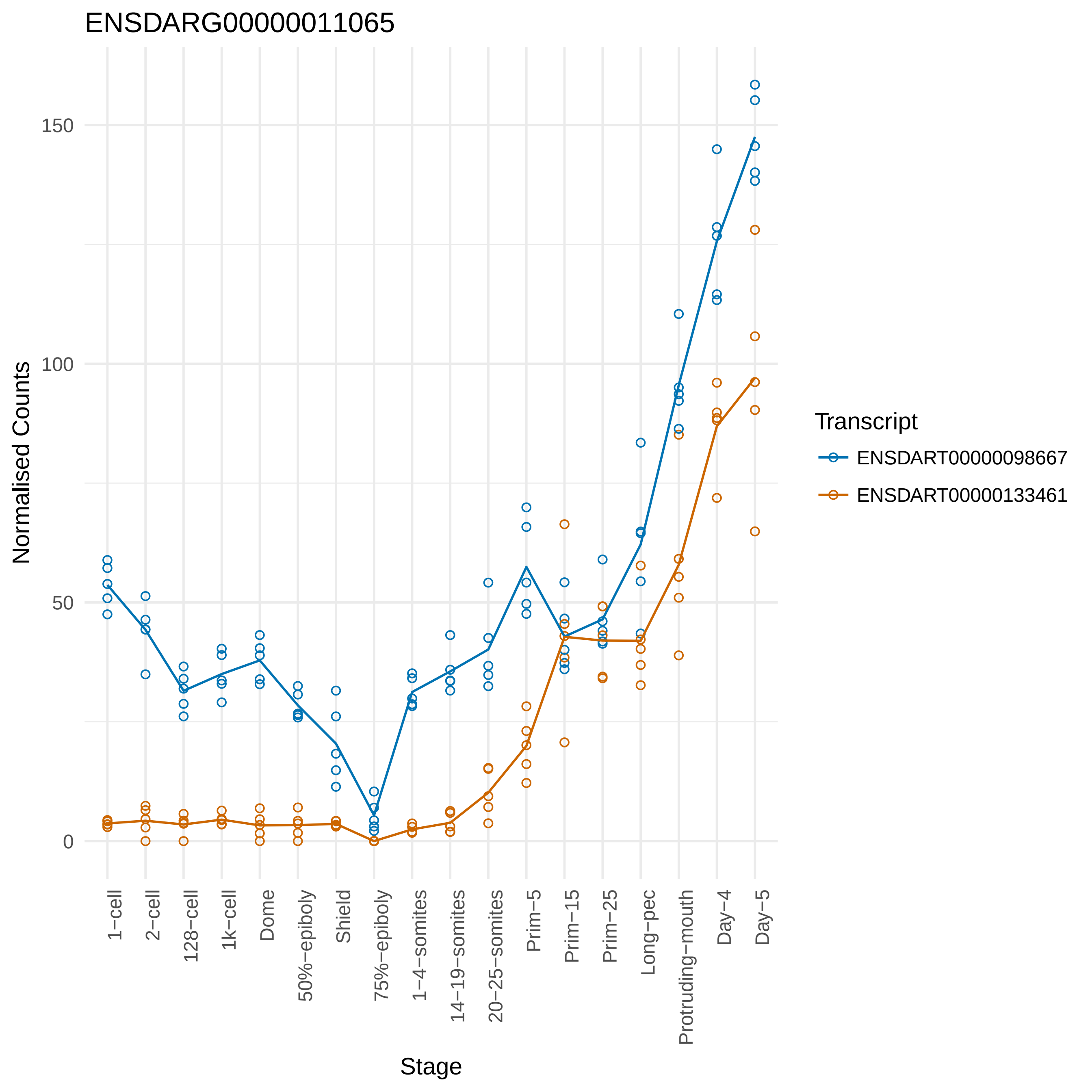

ENSDARG00000011065
Normalised Counts
150
100
50
0
1−cell
2−cell
128−cell
1k−cell
Dome
50%−epiboly
Shield
75%−epiboly
1−4−somites
14−19−somites
20−25−somites
Prim−5
Prim−15
Prim−25
Long−pec
Protruding−mouth
Day−4
Day−5
Stage
Transcript
ENSDART00000098667
ENSDART00000133461

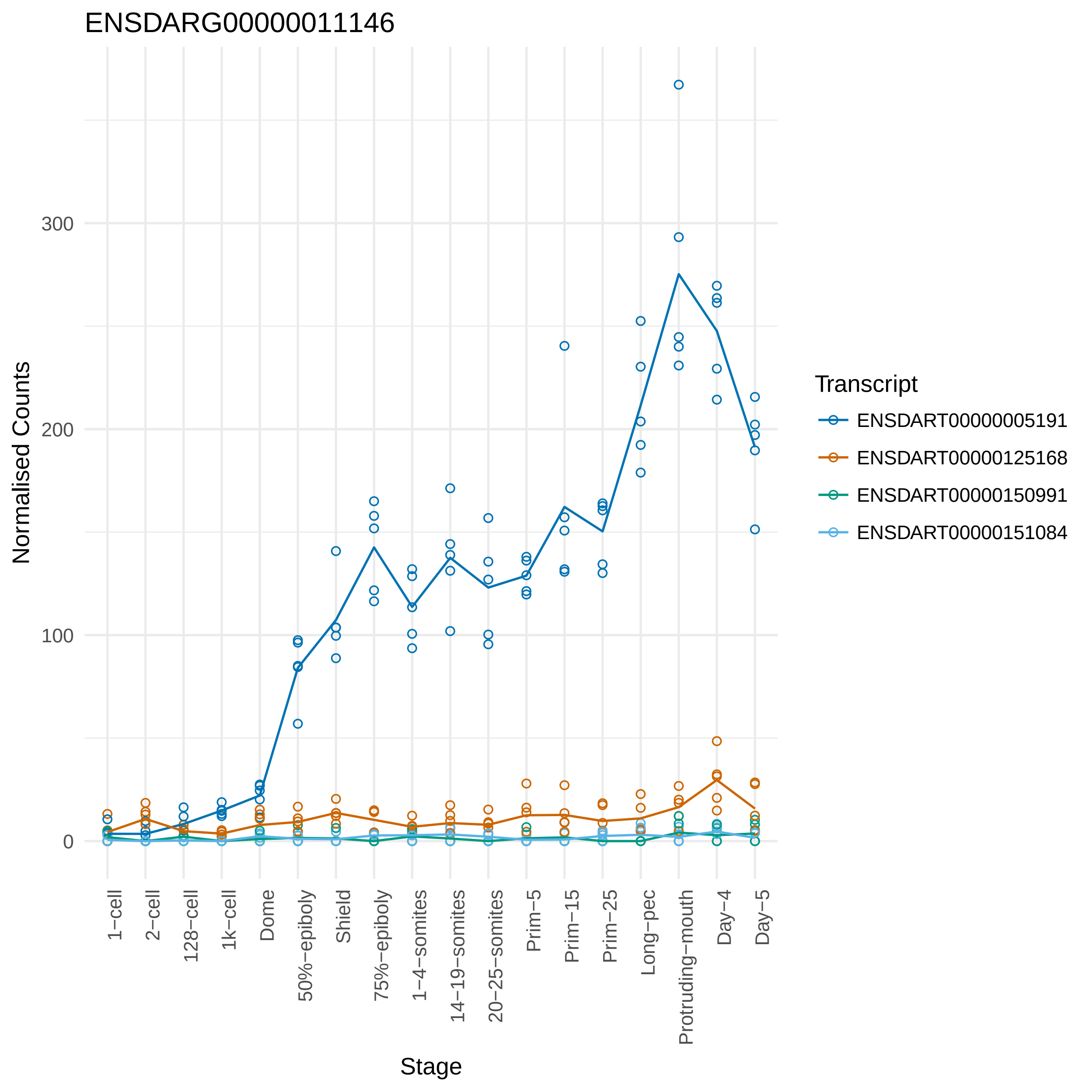

ENSDARG00000011146
Normalised Counts
300
200
100
0
1−cell
2−cell
128−cell
1k−cell
Dome
50%−epiboly
Shield
75%−epiboly
1−4−somites
14−19−somites
20−25−somites
Prim−5
Prim−15
Prim−25
Long−pec
Protruding−mouth
Day−4
Day−5
Stage
Transcript
ENSDART00000005191
ENSDART00000125168
ENSDART00000150991
ENSDART00000151084

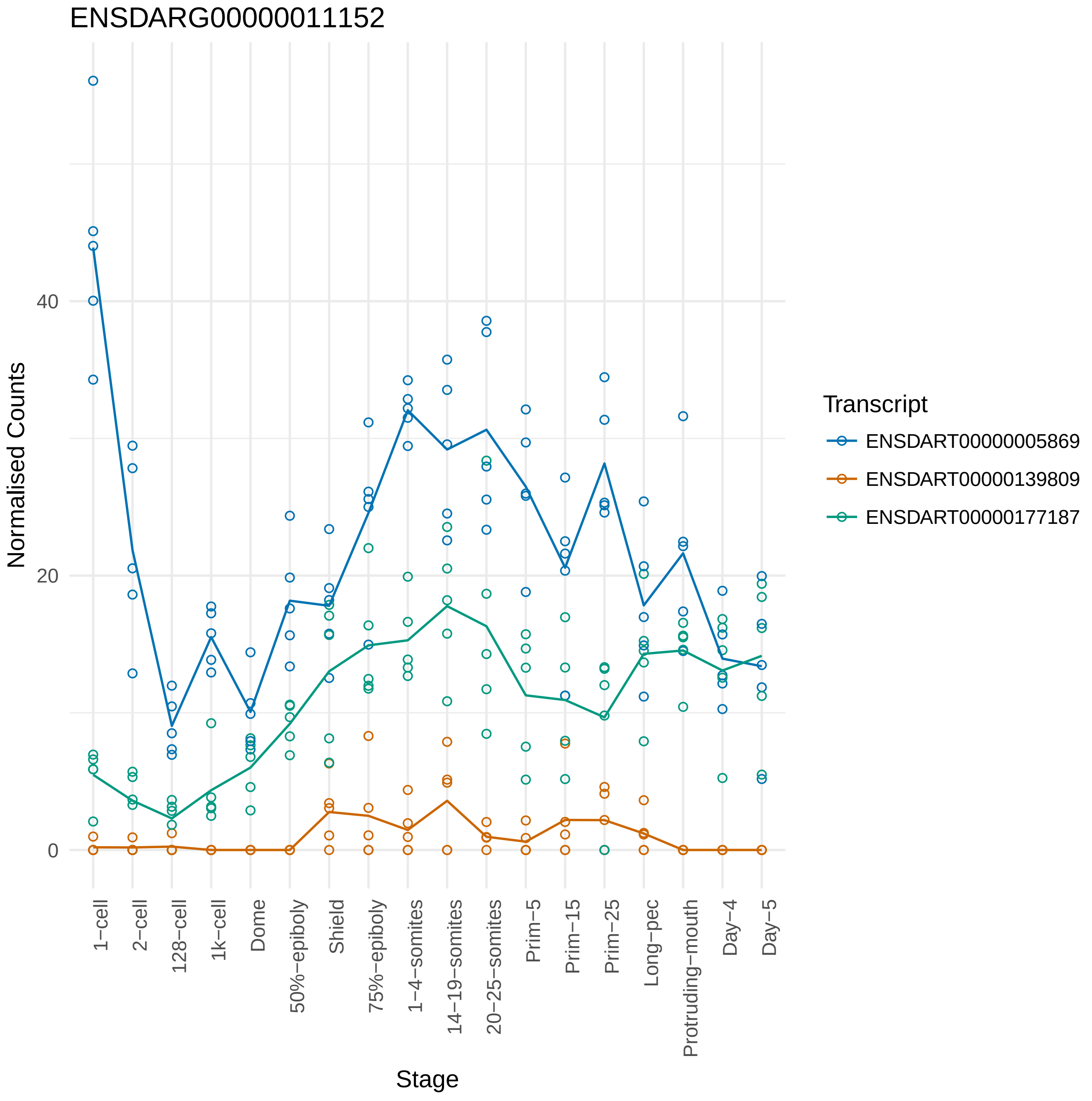

ENSDARG00000011152
Normalised Counts
0
20
40
Stage
1−cell
2−cell
128−cell
1k−cell
Dome
50%−epiboly
Shield
75%−epiboly
1−4−somites
14−19−somites
20−25−somites
Prim−5
Prim−15
Prim−25
Long−pec
Protruding−mouth
Day−4
Day−5
Transcript
ENSDART00000005869
ENSDART00000139809
ENSDART00000177187

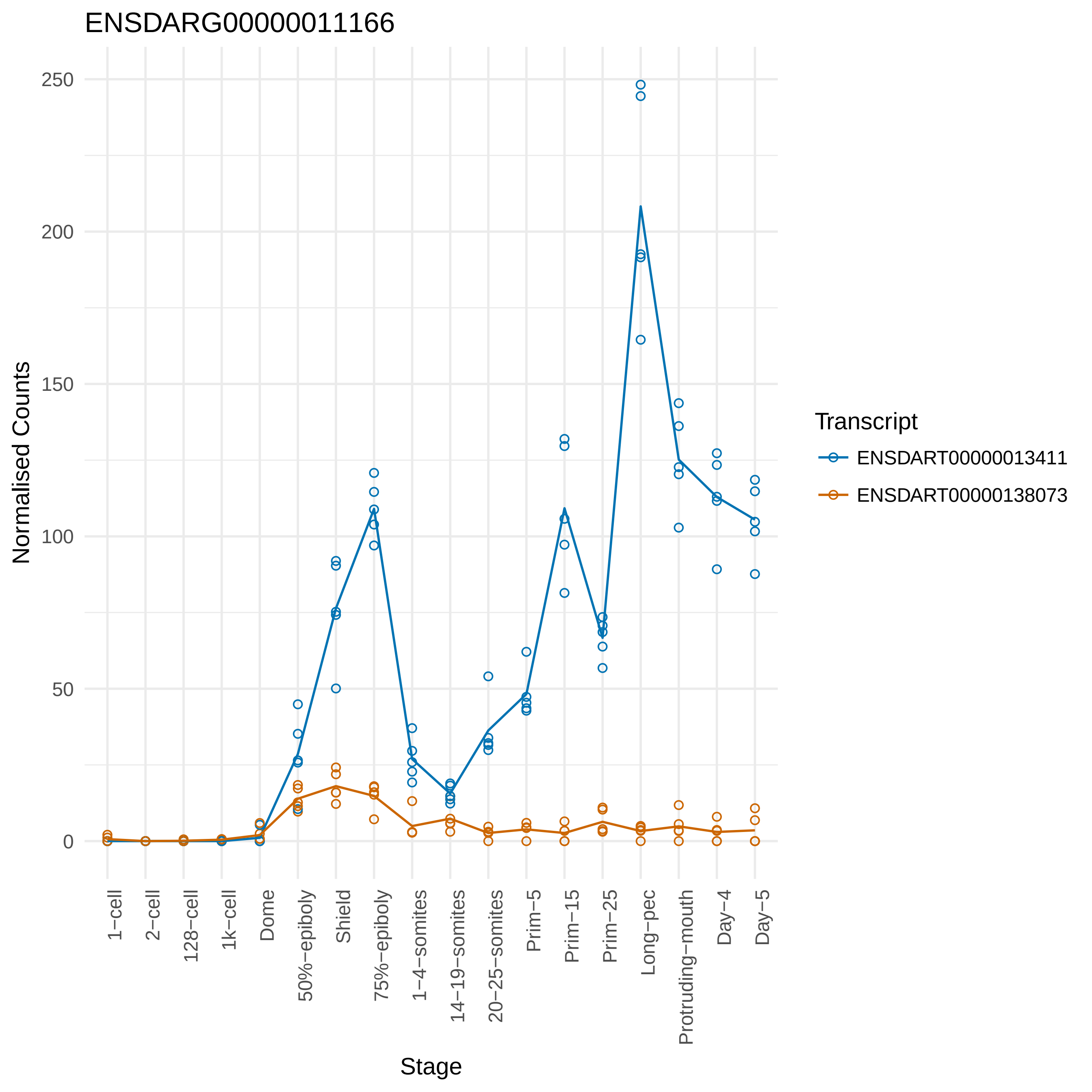

ENSDARG00000011166
Normalised Counts
0
50
100
150
200
250
Stage
1−cell
2−cell
128−cell
1k−cell
Dome
50%−epiboly
Shield
75%−epiboly
1−4−somites
14−19−somites
20−25−somites
Prim−5
Prim−15
Prim−25
Long−pec
Protruding−mouth
Day−4
Day−5
Transcript
ENSDART00000013411
ENSDART00000138073

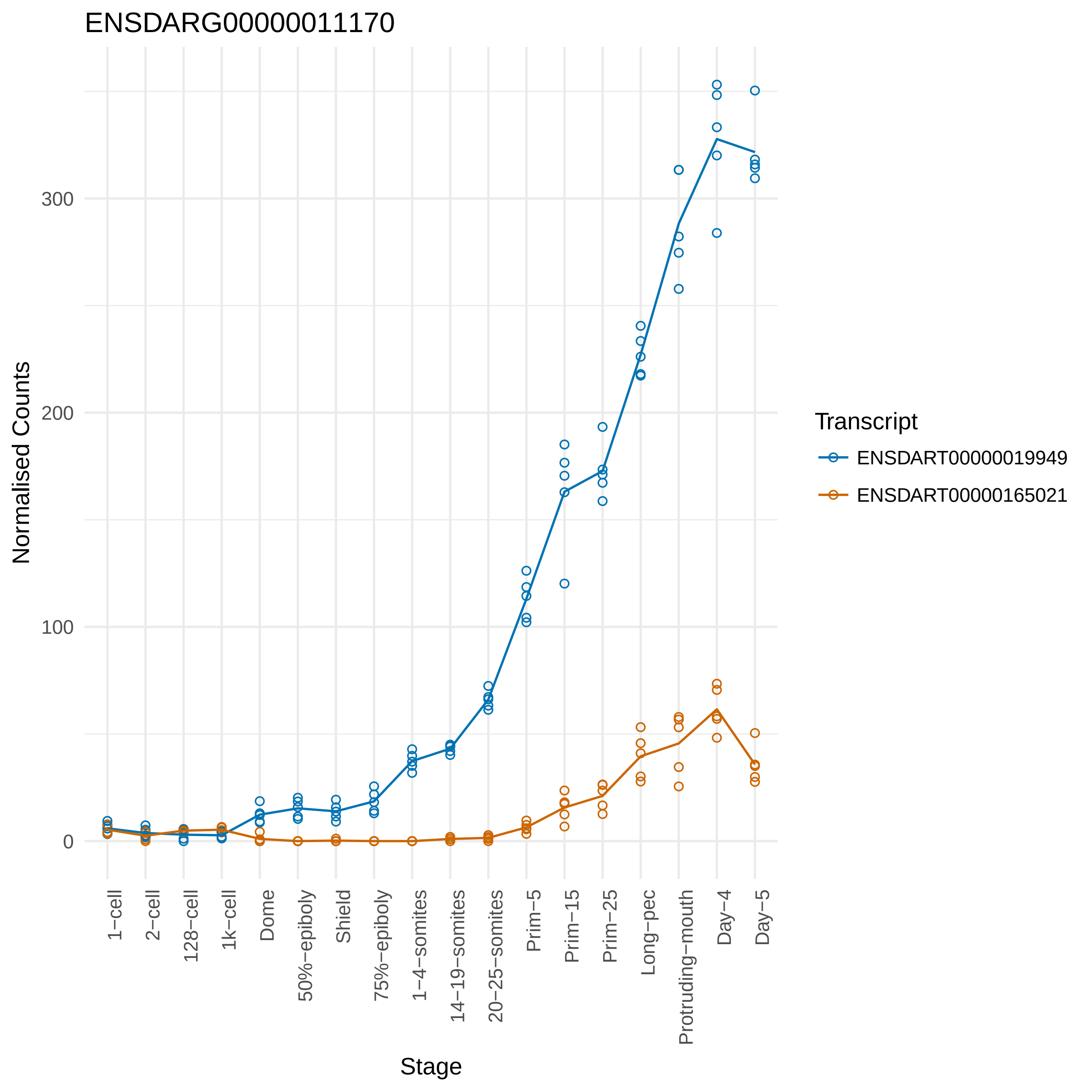

ENSDARG00000011170
Normalised Counts
0
100
200
300
Stage
1−cell
2−cell
128−cell
1k−cell
Dome
50%−epiboly
Shield
75%−epiboly
1−4−somites
14−19−somites
20−25−somites
Prim−5
Prim−15
Prim−25
Long−pec
Protruding−mouth
Day−4
Day−5
Transcript
ENSDART00000019949
ENSDART00000165021

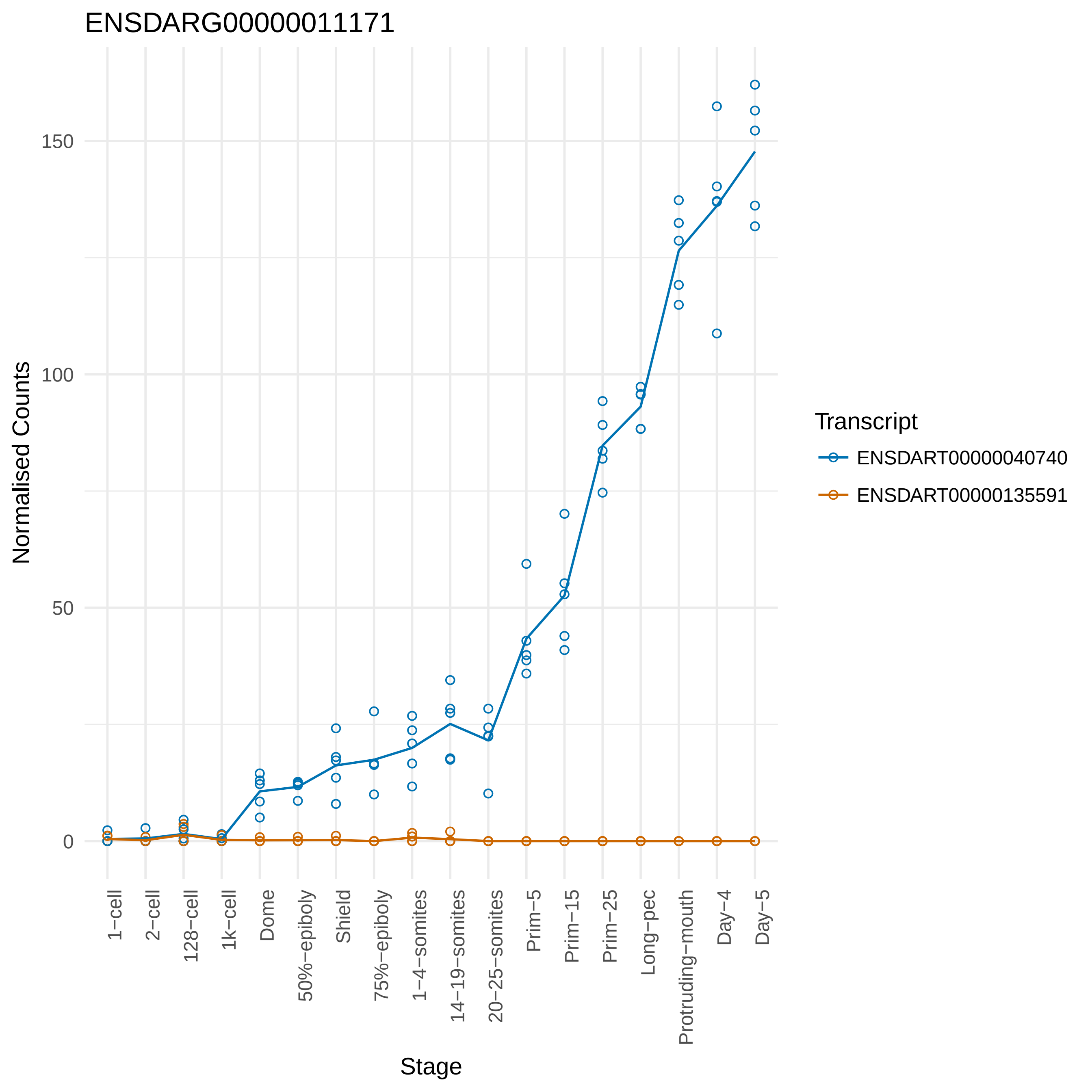

ENSDARG00000011171
Normalised Counts
150
100
50
0
1−cell
2−cell
128−cell
1k−cell
Dome
50%−epiboly
Shield
75%−epiboly
1−4−somites
14−19−somites
20−25−somites
Prim−5
Prim−15
Prim−25
Long−pec
Protruding−mouth
Day−4
Day−5
Stage
Transcript
ENSDART00000040740
ENSDART00000135591

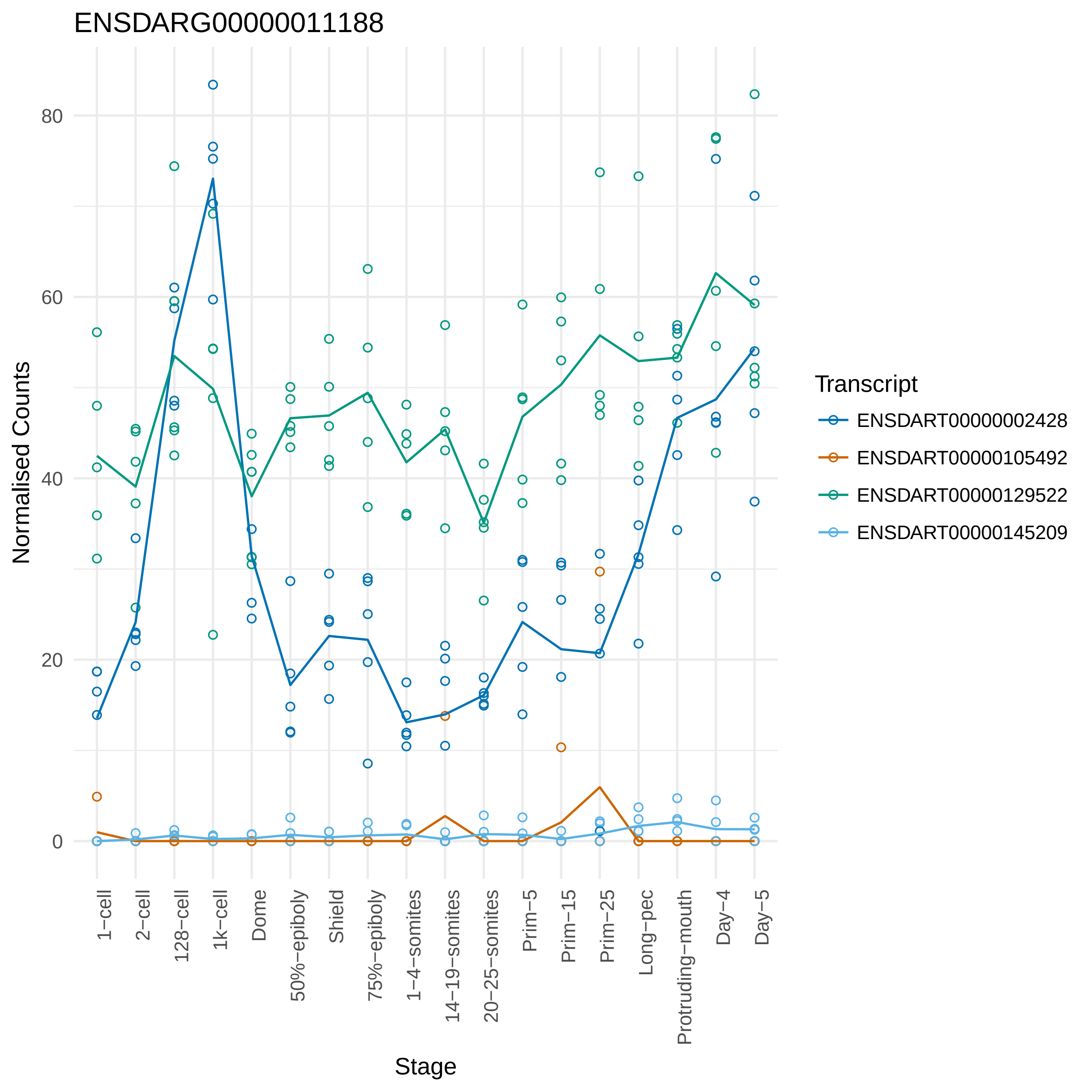

ENSDARG00000011188
Normalised Counts
0
20
40
60
80
1−cell
2−cell
128−cell
1k−cell
Dome
50%−epiboly
Shield
75%−epiboly
1−4−somites
14−19−somites
20−25−somites
Prim−5
Prim−15
Prim−25
Long−pec
Protruding−mouth
Day−4
Day−5
Stage
Transcript
ENSDART00000002428
ENSDART00000105492
ENSDART00000129522
ENSDART00000145209

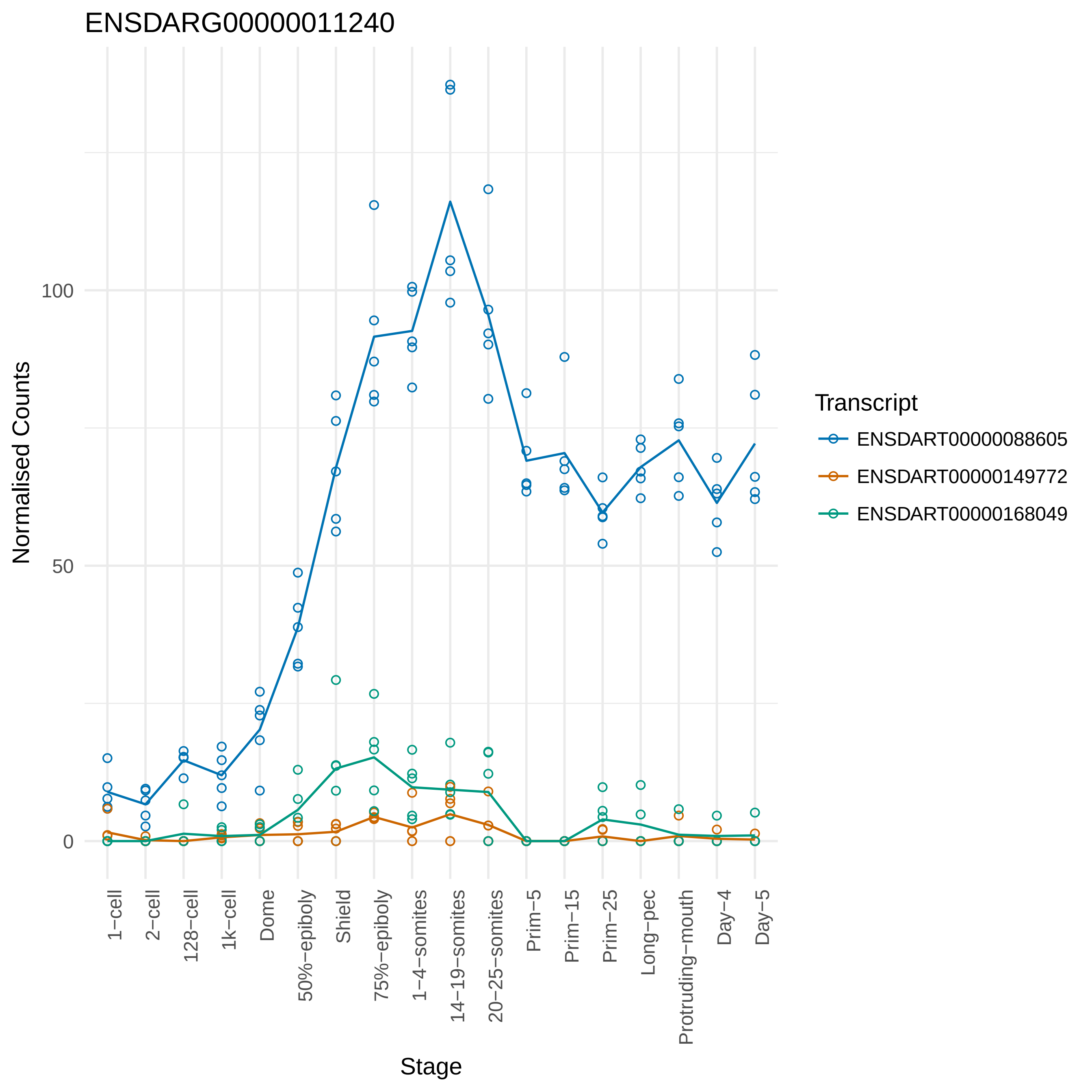
ENSDARG00000011240
Normalised Counts
100
50
0
1−cell
2−cell
128−cell
1k−cell
Dome
50%−epiboly
Shield
75%−epiboly
1−4−somites
14−19−somites
20−25−somites
Prim−5
Prim−15
Prim−25
Long−pec
Protruding−mouth
Day−4
Day−5
Stage
Transcript
ENSDART00000088605
ENSDART00000149772
ENSDART00000168049

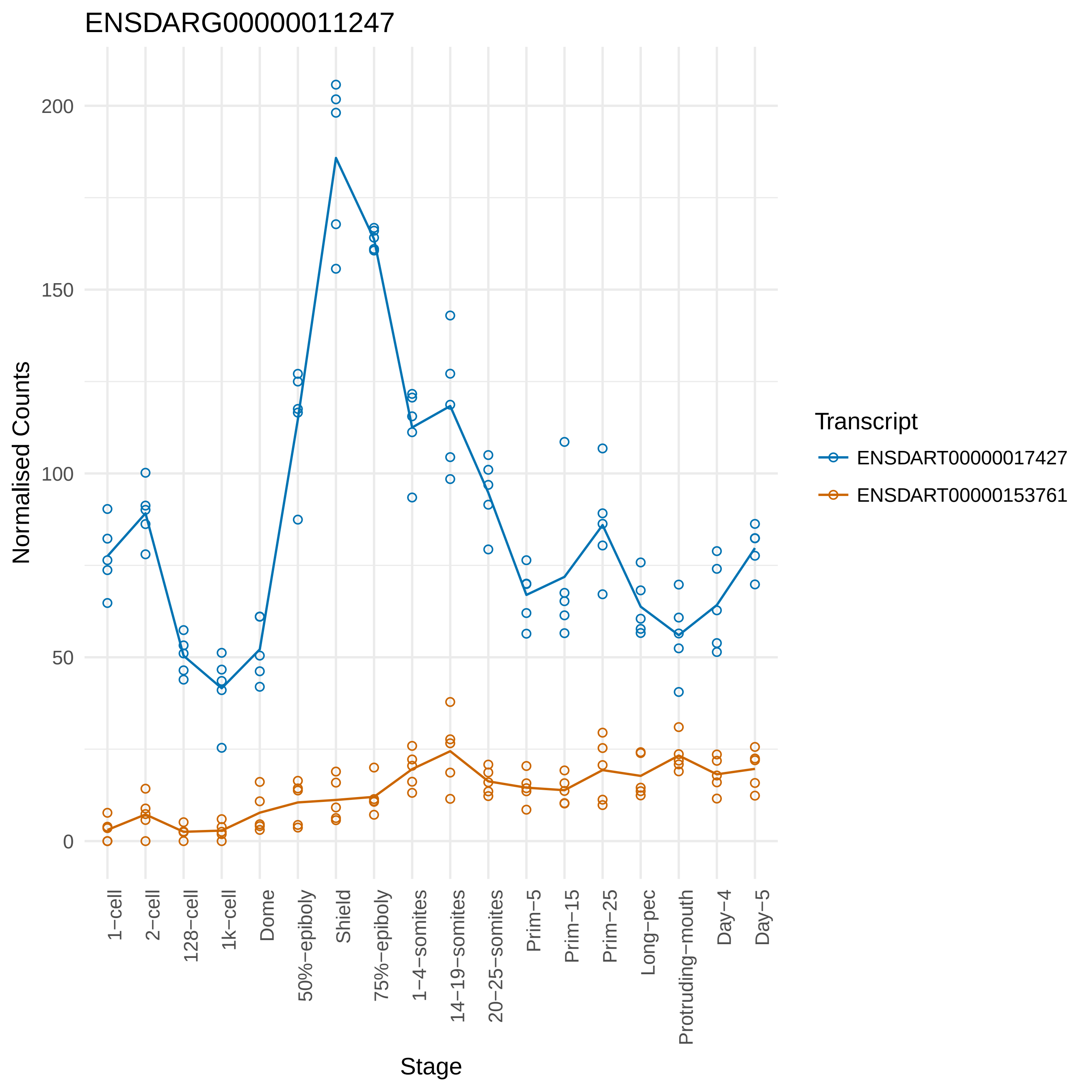
ENSDARG00000011247
Normalised Counts
0
50
100
150
200
Stage
1−cell
2−cell
128−cell
1k−cell
Dome
50%−epiboly
Shield
75%−epiboly
1−4−somites
14−19−somites
20−25−somites
Prim−5
Prim−15
Prim−25
Long−pec
Protruding−mouth
Day−4
Day−5
Transcript
ENSDART00000017427
ENSDART00000153761

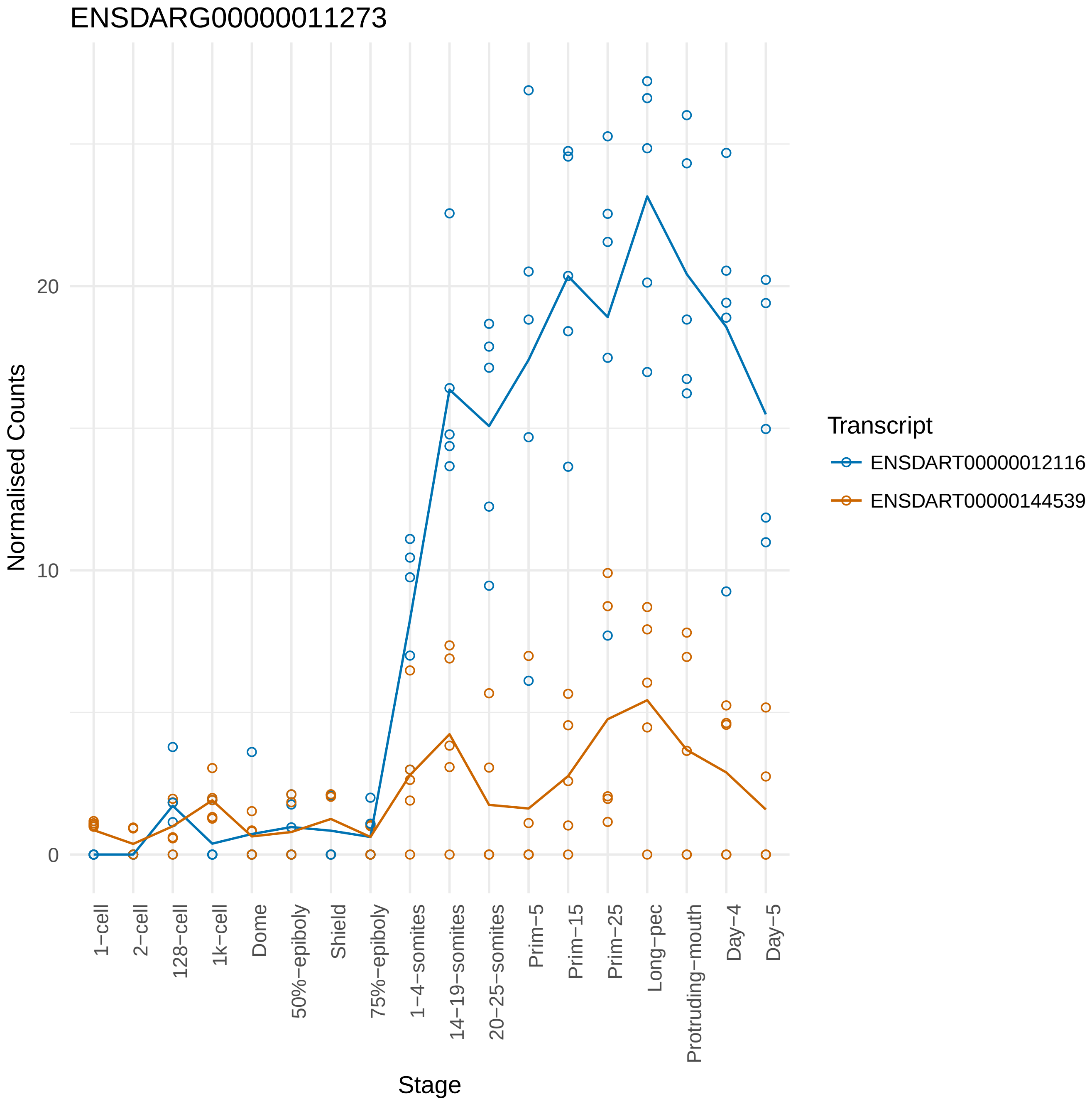
ENSDARG00000011273
Normalised Counts
0
10
20
Stage
1−cell
2−cell
128−cell
1k−cell
Dome
50%−epiboly
Shield
75%−epiboly
1−4−somites
14−19−somites
20−25−somites
Prim−5
Prim−15
Prim−25
Long−pec
Protruding−mouth
Day−4
Day−5
Transcript
ENSDART00000012116
ENSDART00000144539

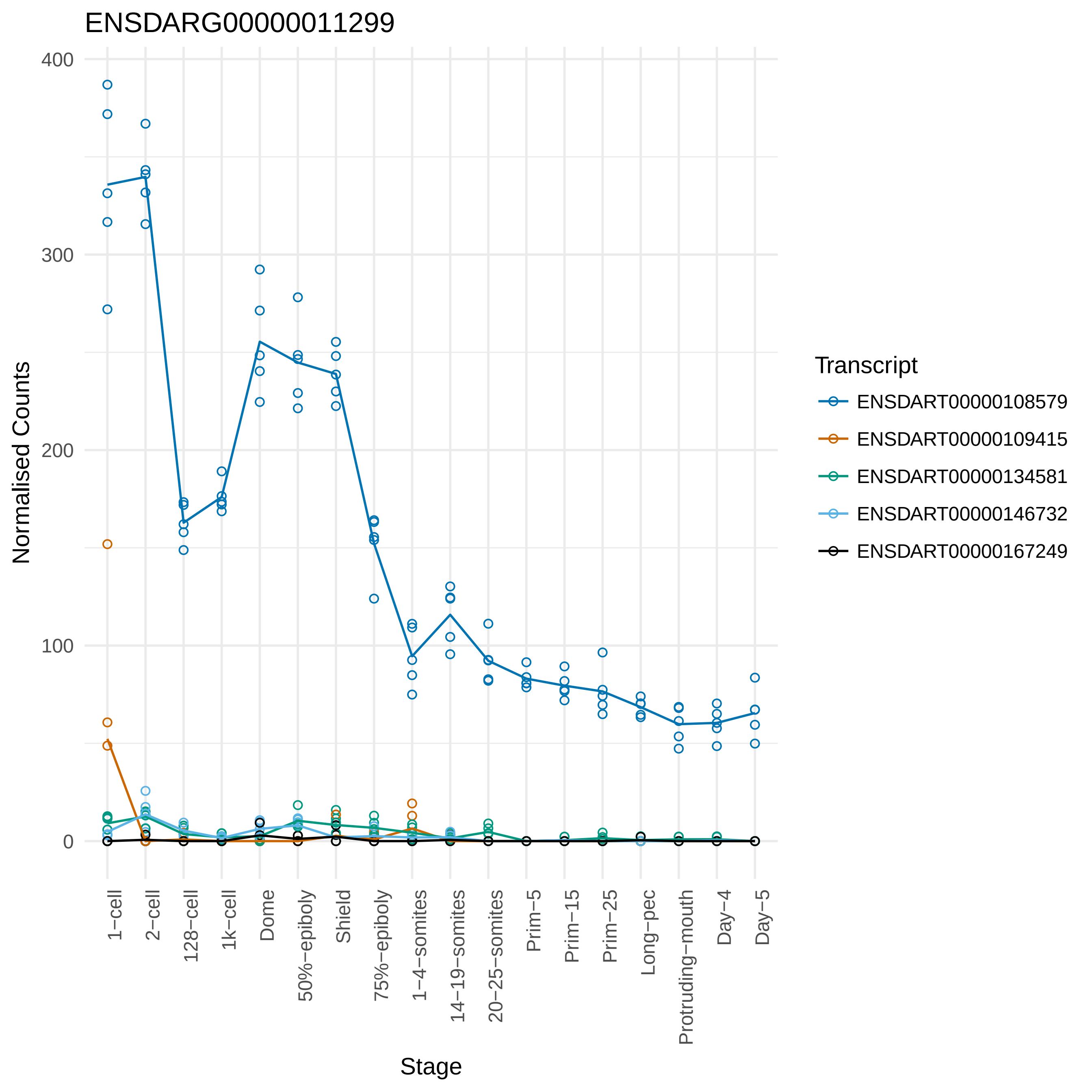
ENSDARG00000011299
Normalised Counts
400
300
200
100
0
Stage
1−cell
2−cell
128−cell
1k−cell
Dome
50%−epiboly
Shield
75%−epiboly
1−4−somites
14−19−somites
20−25−somites
Prim−5
Prim−15
Prim−25
Long−pec
Protruding−mouth
Day−4
Day−5
Transcript
ENSDART00000108579
ENSDART00000109415
ENSDART00000134581
ENSDART00000146732
ENSDART00000167249

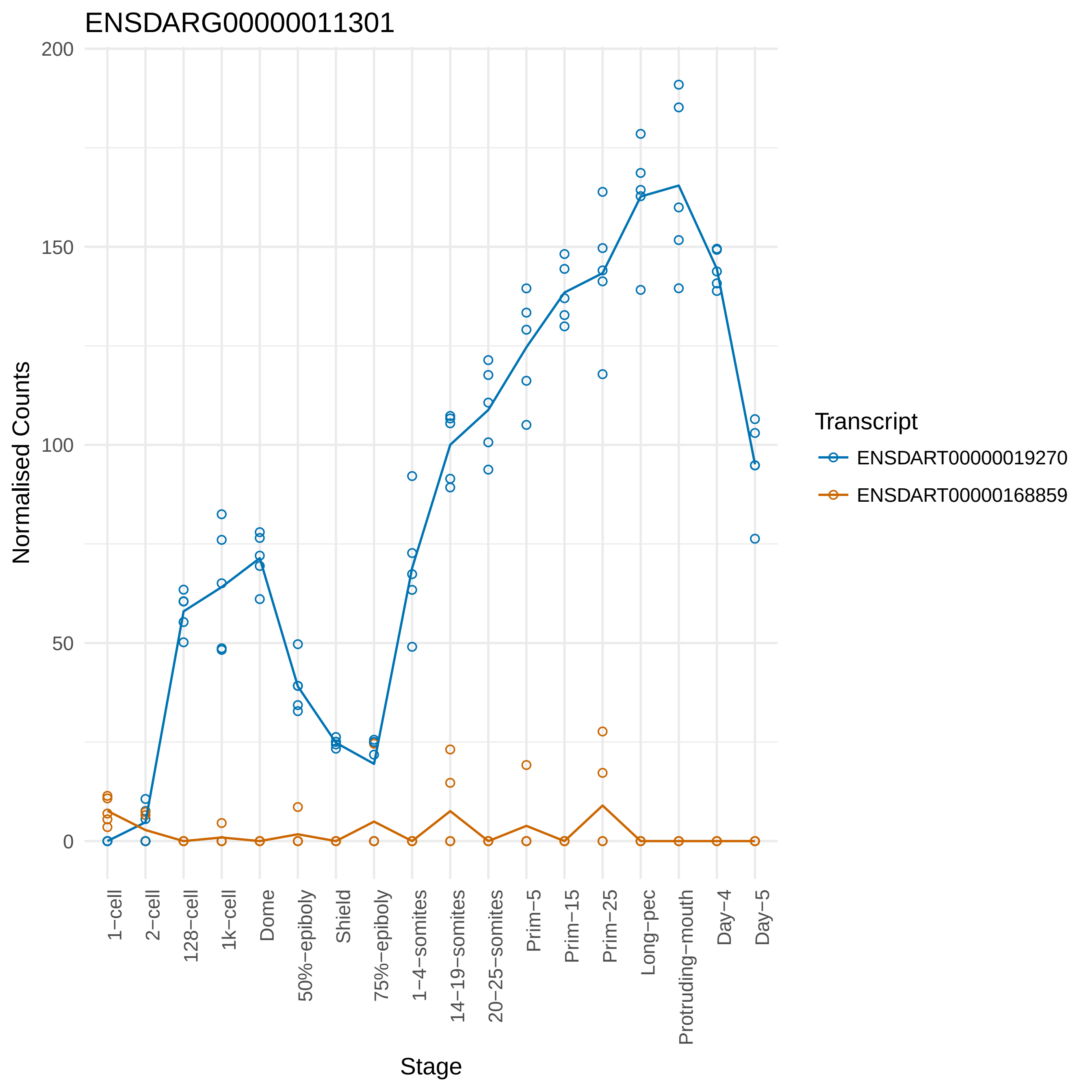

ENSDARG00000011301
Normalised Counts
0
50
100
150
200
Stage
1−cell
2−cell
128−cell
1k−cell
Dome
50%−epiboly
Shield
75%−epiboly
1−4−somites
14−19−somites
20−25−somites
Prim−5
Prim−15
Prim−25
Long−pec
Protruding−mouth
Day−4
Day−5
Transcript
ENSDART00000019270
ENSDART00000168859

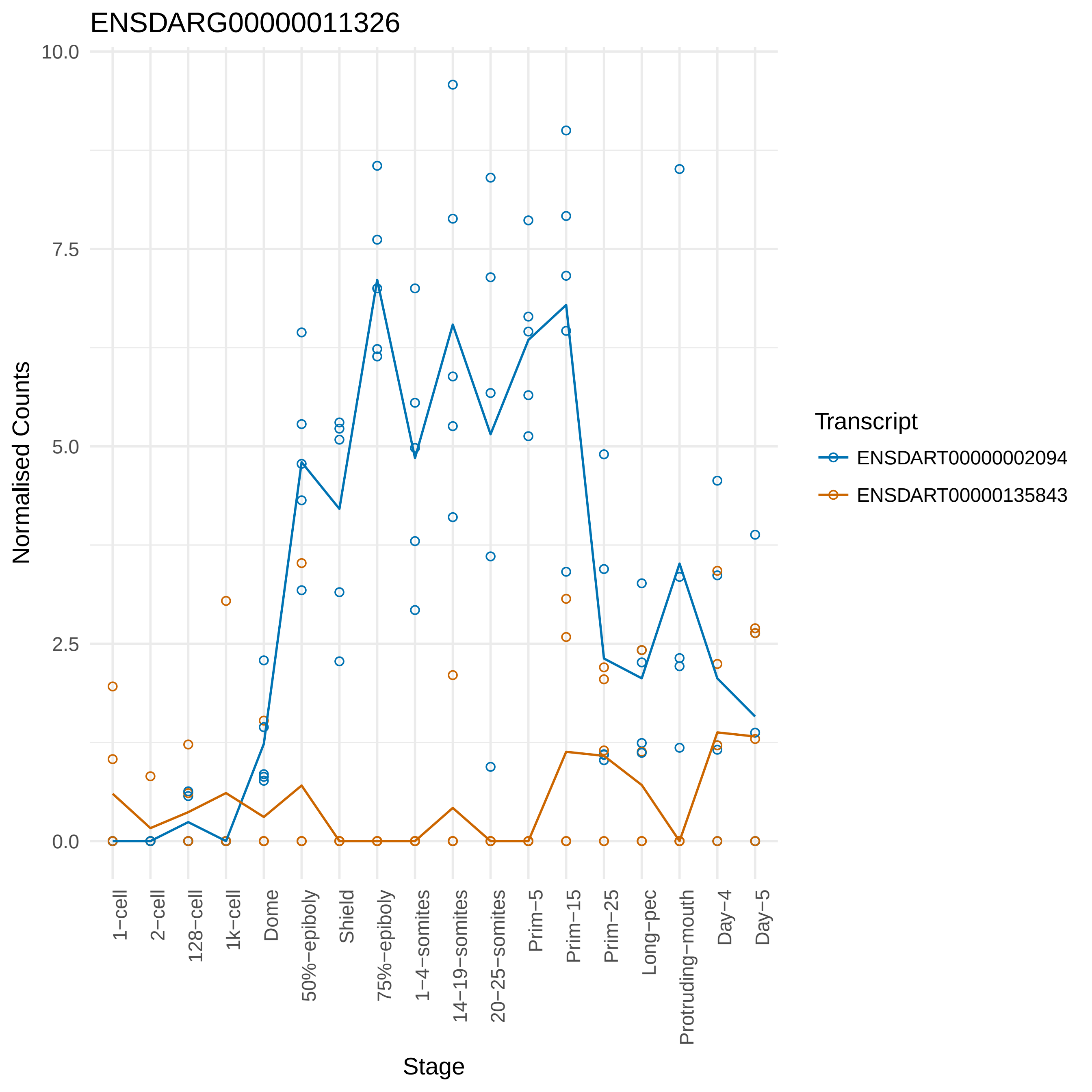

ENSDARG00000011326
10.0
7.5
5.0
2.5
0.0
Normalised Counts
1−cell
2−cell
128−cell
1k−cell
Dome
50%−epiboly
Shield
75%−epiboly
1−4−somites
14−19−somites
20−25−somites
Prim−5
Prim−15
Prim−25
Long−pec
Protruding−mouth
Day−4
Day−5
Stage
Transcript
ENSDART00000002094
ENSDART00000135843

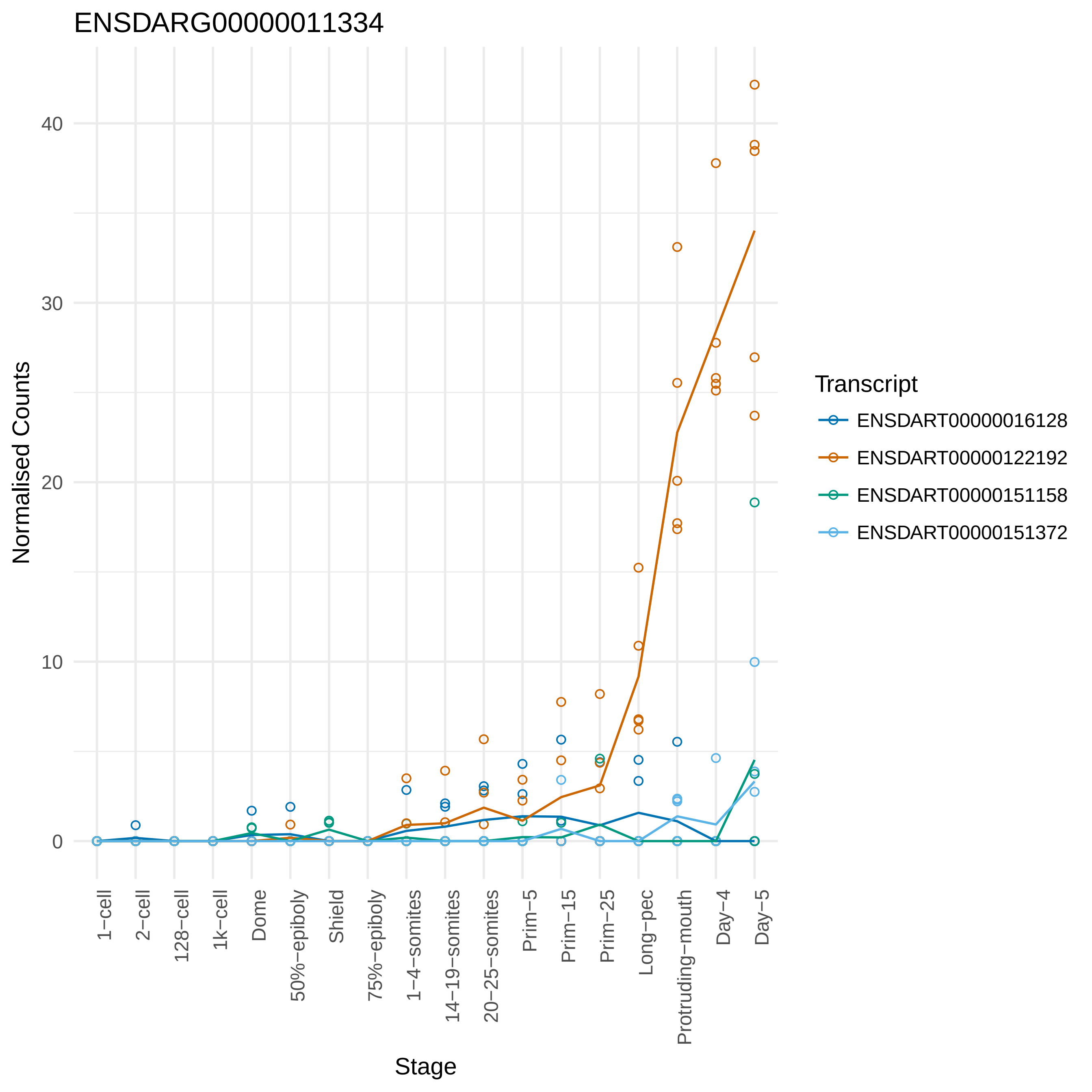
ENSDARG00000011334
Normalised Counts
0
10
20
30
40
Stage
1−cell
2−cell
128−cell
1k−cell
Dome
50%−epiboly
Shield
75%−epiboly
1−4−somites
14−19−somites
20−25−somites
Prim−5
Prim−15
Prim−25
Long−pec
Protruding−mouth
Day−4
Day−5
Transcript
ENSDART00000016128
ENSDART00000122192
ENSDART00000151158
ENSDART00000151372

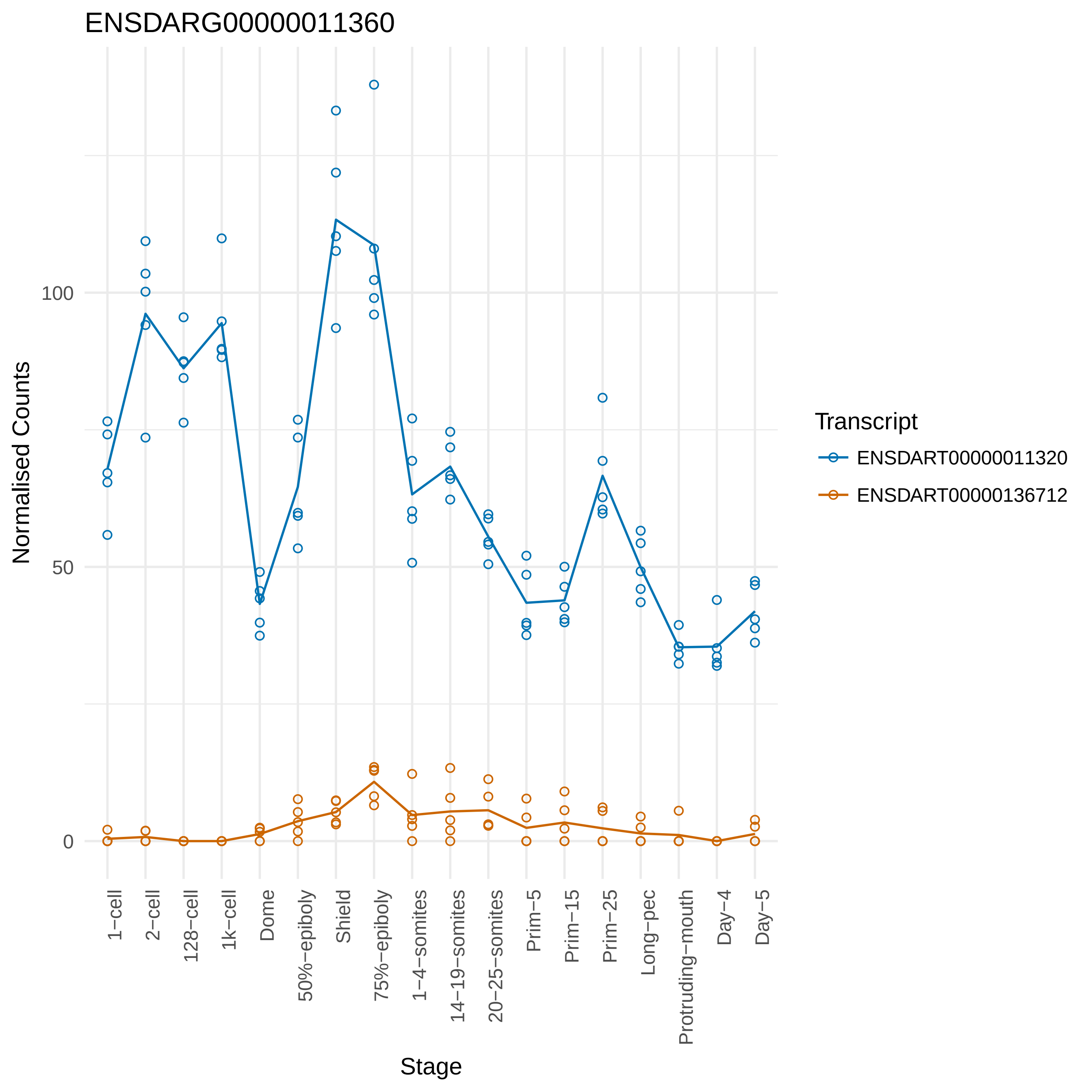
ENSDARG00000011360
Normalised Counts
0
50
100
Stage
1−cell
2−cell
128−cell
1k−cell
Dome
50%−epiboly
Shield
75%−epiboly
1−4−somites
14−19−somites
20−25−somites
Prim−5
Prim−15
Prim−25
Long−pec
Protruding−mouth
Day−4
Day−5
Transcript
ENSDART00000011320
ENSDART00000136712

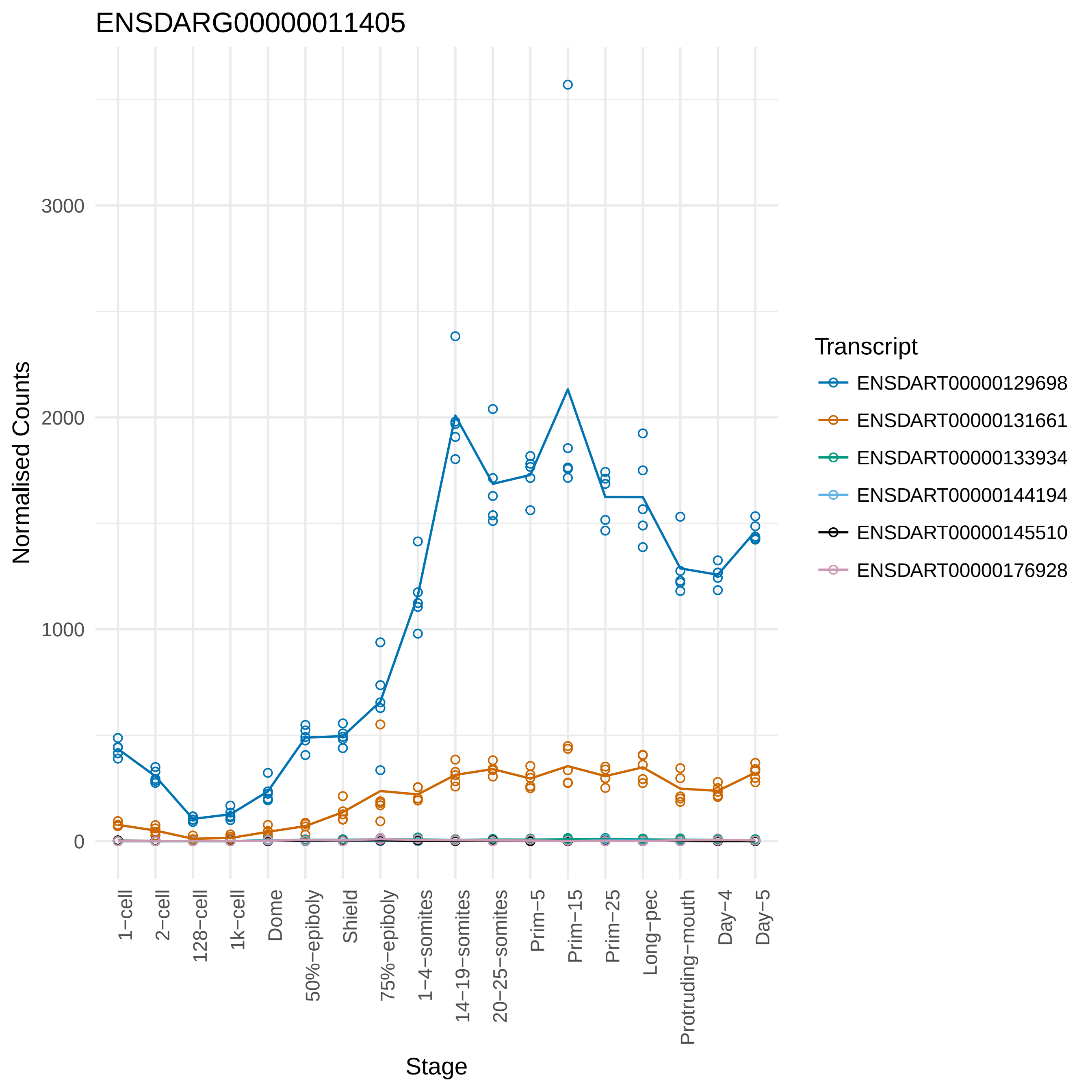

ENSDARG00000011405
Normalised Counts
3000
2000
1000
0
Stage
1−cell
2−cell
128−cell
1k−cell
Dome
50%−epiboly
Shield
75%−epiboly
1−4−somites
14−19−somites
20−25−somites
Prim−5
Prim−15
Prim−25
Long−pec
Protruding−mouth
Day−4
Day−5
Transcript
ENSDART00000129698
ENSDART00000131661
ENSDART00000133934
ENSDART00000144194
ENSDART00000145510
ENSDART00000176928

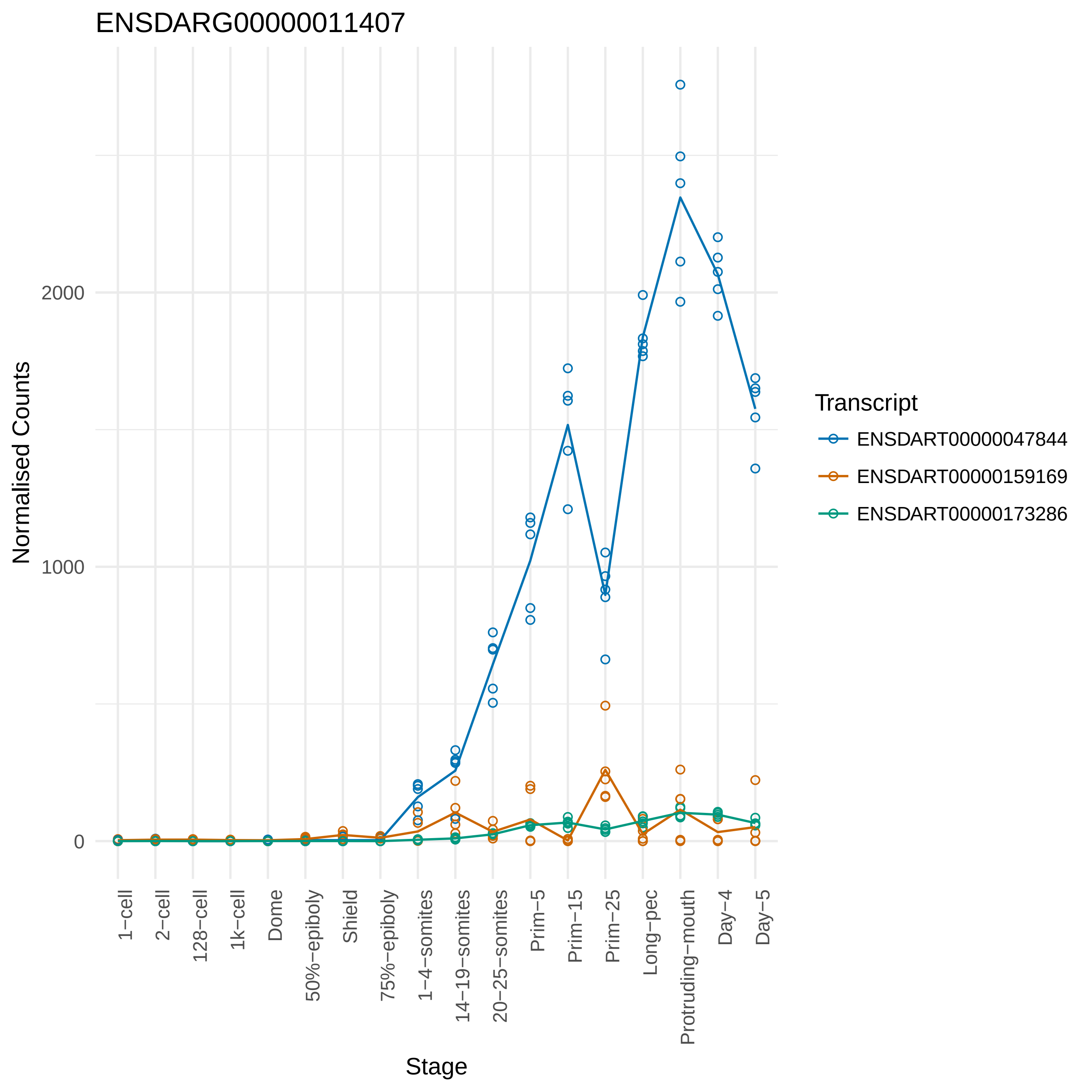

ENSDARG00000011407
Normalised Counts
0
1000
2000
Stage
1−cell
2−cell
128−cell
1k−cell
Dome
50%−epiboly
Shield
75%−epiboly
1−4−somites
14−19−somites
20−25−somites
Prim−5
Prim−15
Prim−25
Long−pec
Protruding−mouth
Day−4
Day−5
Transcript
ENSDART00000047844
ENSDART00000159169
ENSDART00000173286

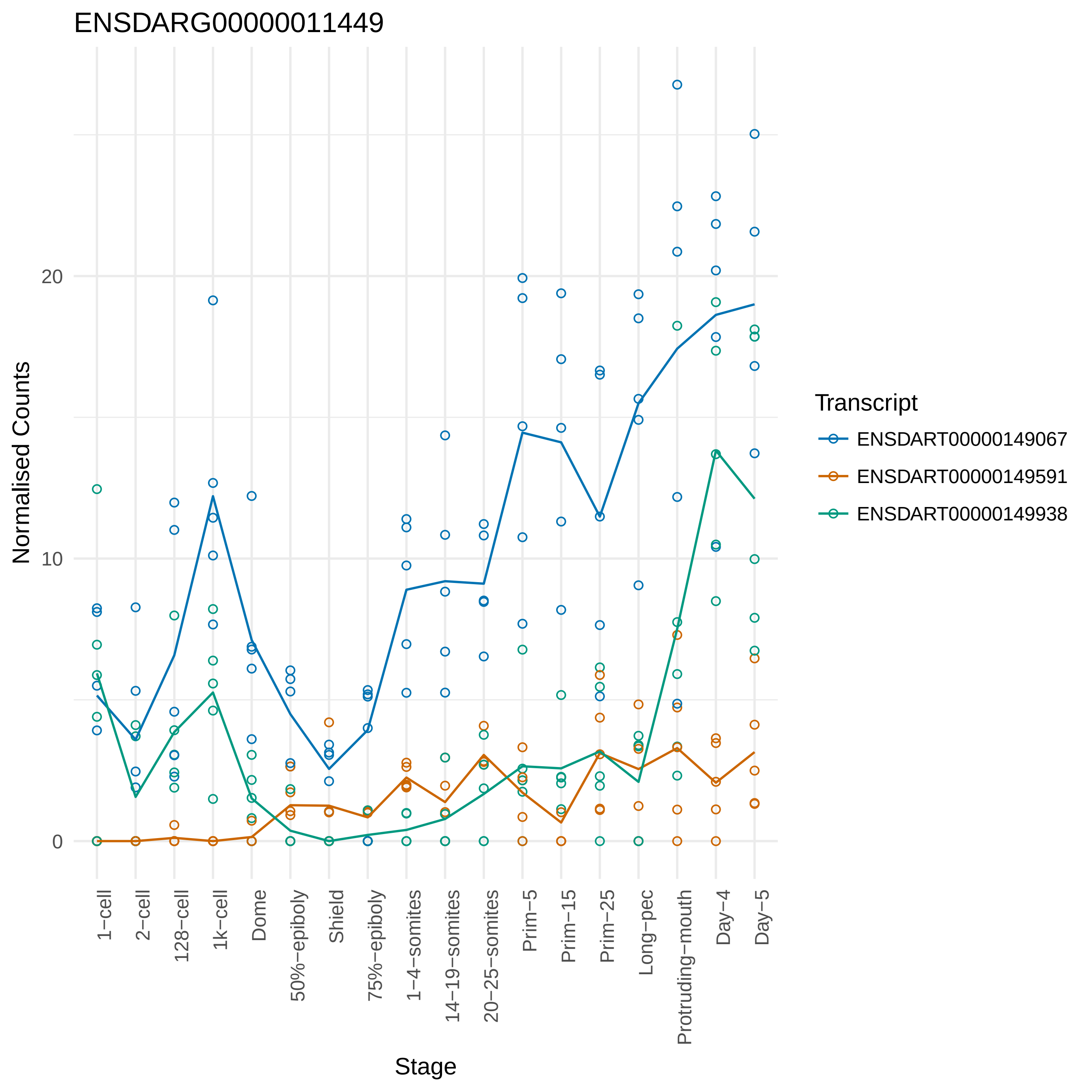

ENSDARG00000011449
Normalised Counts
0
10
20
Stage
1−cell
2−cell
128−cell
1k−cell
Dome
50%−epiboly
Shield
75%−epiboly
1−4−somites
14−19−somites
20−25−somites
Prim−5
Prim−15
Prim−25
Long−pec
Protruding−mouth
Day−4
Day−5
Transcript
ENSDART00000149067
ENSDART00000149591
ENSDART00000149938

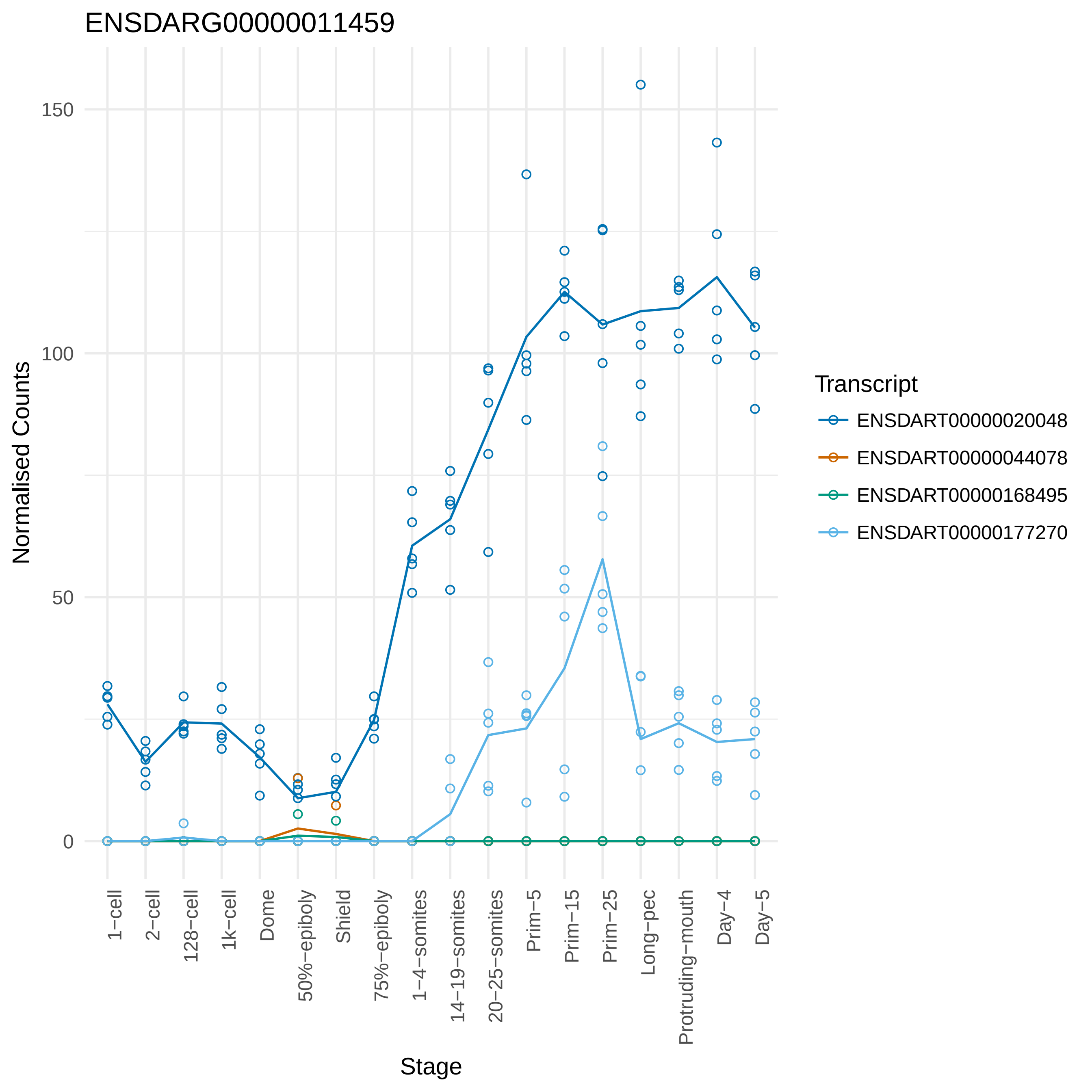

ENSDARG00000011459
Normalised Counts
150
100
50
0
1−cell
2−cell
128−cell
1k−cell
Dome
50%−epiboly
Shield
75%−epiboly
1−4−somites
14−19−somites
20−25−somites
Prim−5
Prim−15
Prim−25
Long−pec
Protruding−mouth
Day−4
Day−5
Stage
Transcript
ENSDART00000020048
ENSDART00000044078
ENSDART00000168495
ENSDART00000177270

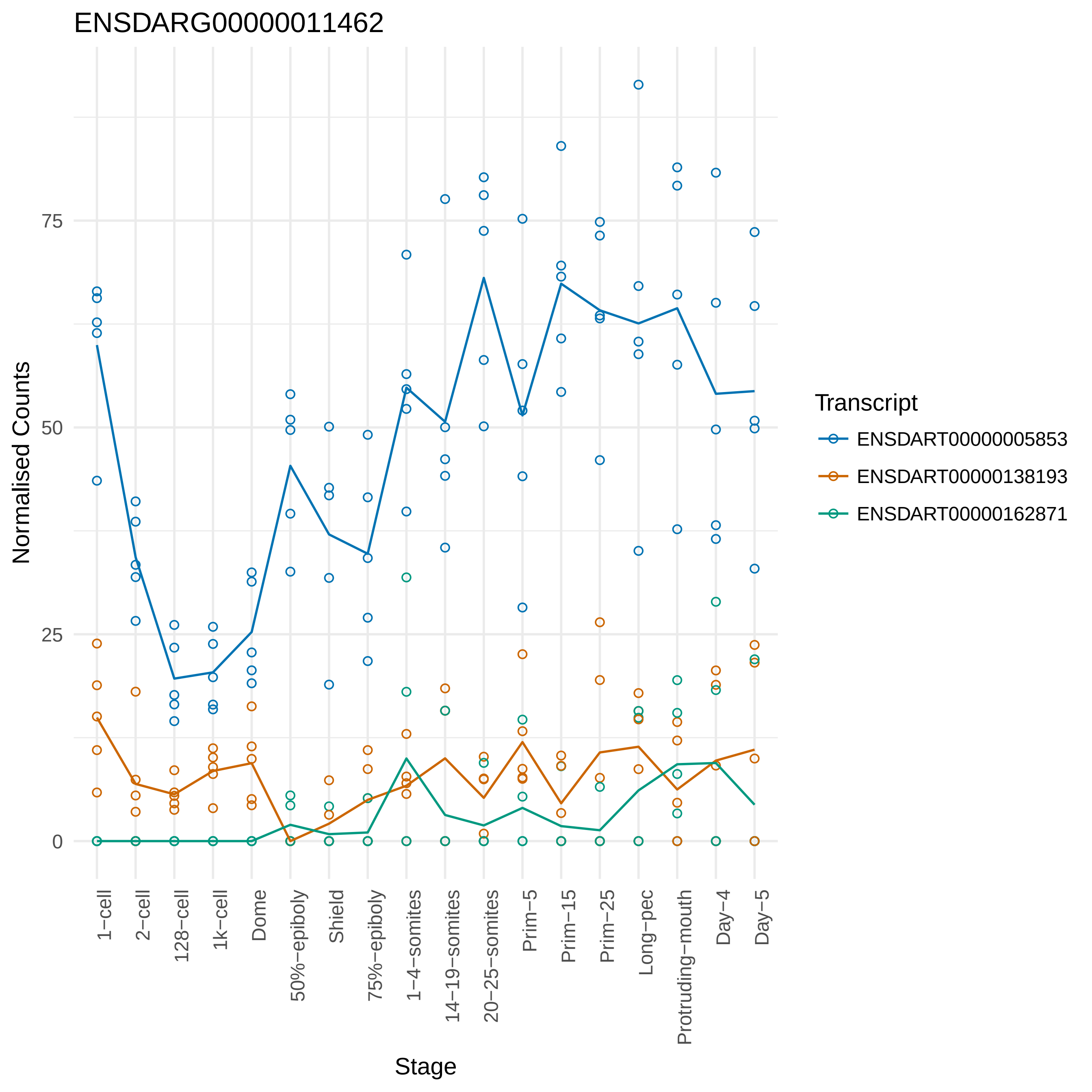

ENSDARG00000011462
Normalised Counts
75
50
25
0
1−cell
2−cell
128−cell
1k−cell
Dome
50%−epiboly
Shield
75%−epiboly
1−4−somites
14−19−somites
20−25−somites
Prim−5
Prim−15
Prim−25
Long−pec
Protruding−mouth
Day−4
Day−5
Stage
Transcript
ENSDART00000005853
ENSDART00000138193
ENSDART00000162871

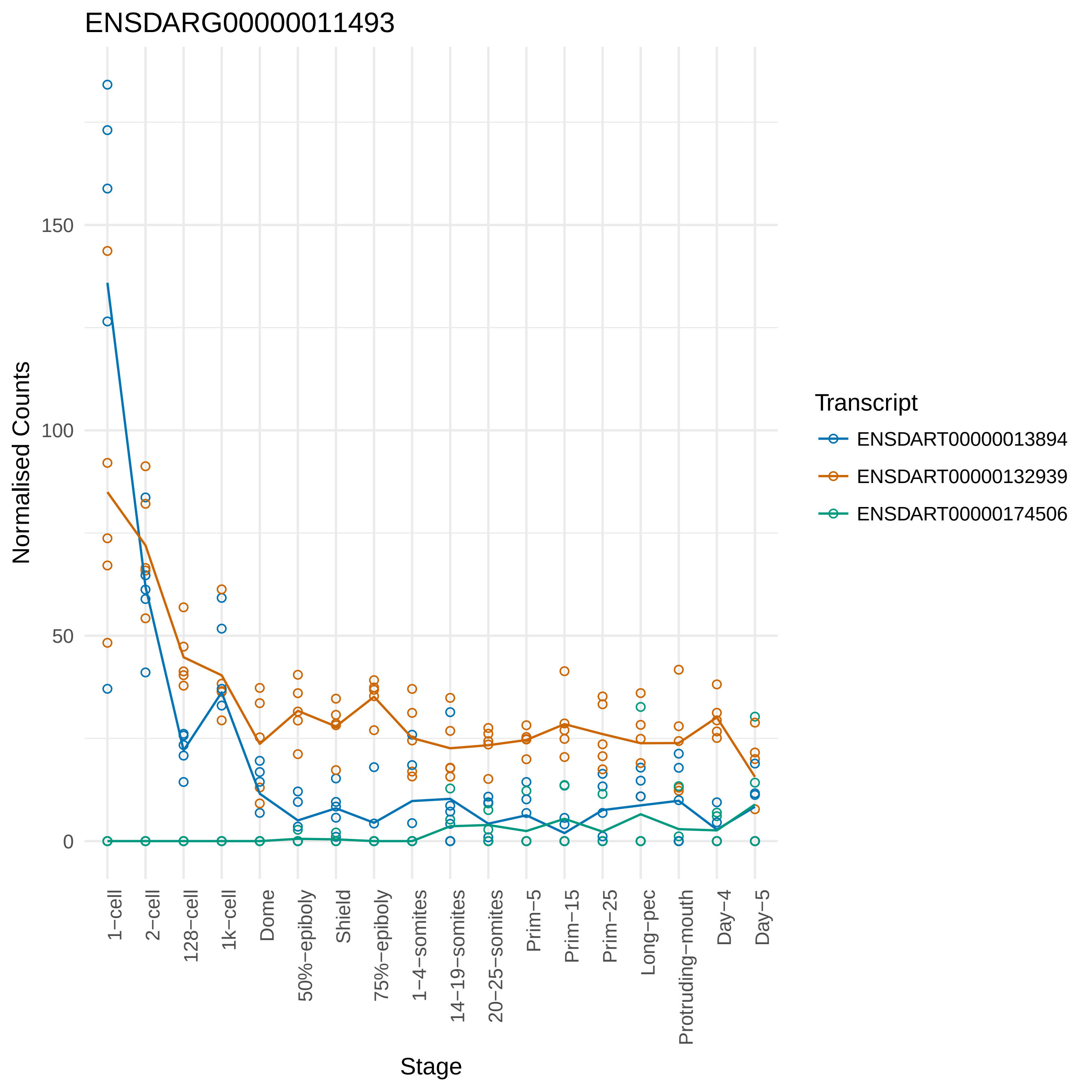

ENSDARG00000011493
Normalised Counts
150
100
50
0
1−cell
2−cell
128−cell
1k−cell
Dome
50%−epiboly
Shield
75%−epiboly
1−4−somites
14−19−somites
20−25−somites
Prim−5
Prim−15
Prim−25
Long−pec
Protruding−mouth
Day−4
Day−5
Stage
Transcript
ENSDART00000013894
ENSDART00000132939
ENSDART00000174506

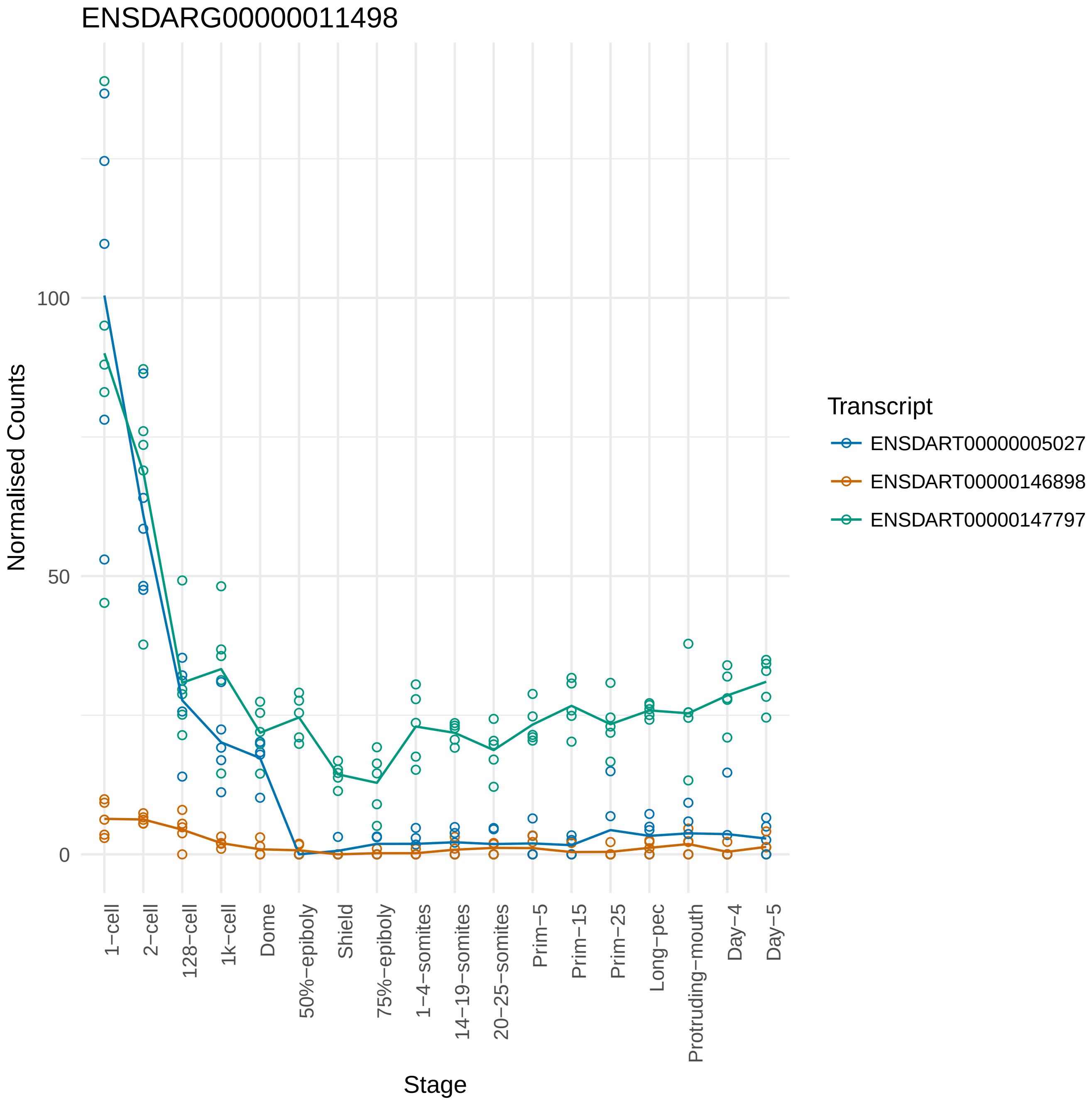

ENSDARG00000011498
Normalised Counts
100
50
0
1−cell
2−cell
128−cell
1k−cell
Dome
50%−epiboly
Shield
75%−epiboly
1−4−somites
14−19−somites
20−25−somites
Prim−5
Prim−15
Prim−25
Long−pec
Protruding−mouth
Day−4
Day−5
Stage
Transcript
ENSDART00000005027
ENSDART00000146898
ENSDART00000147797

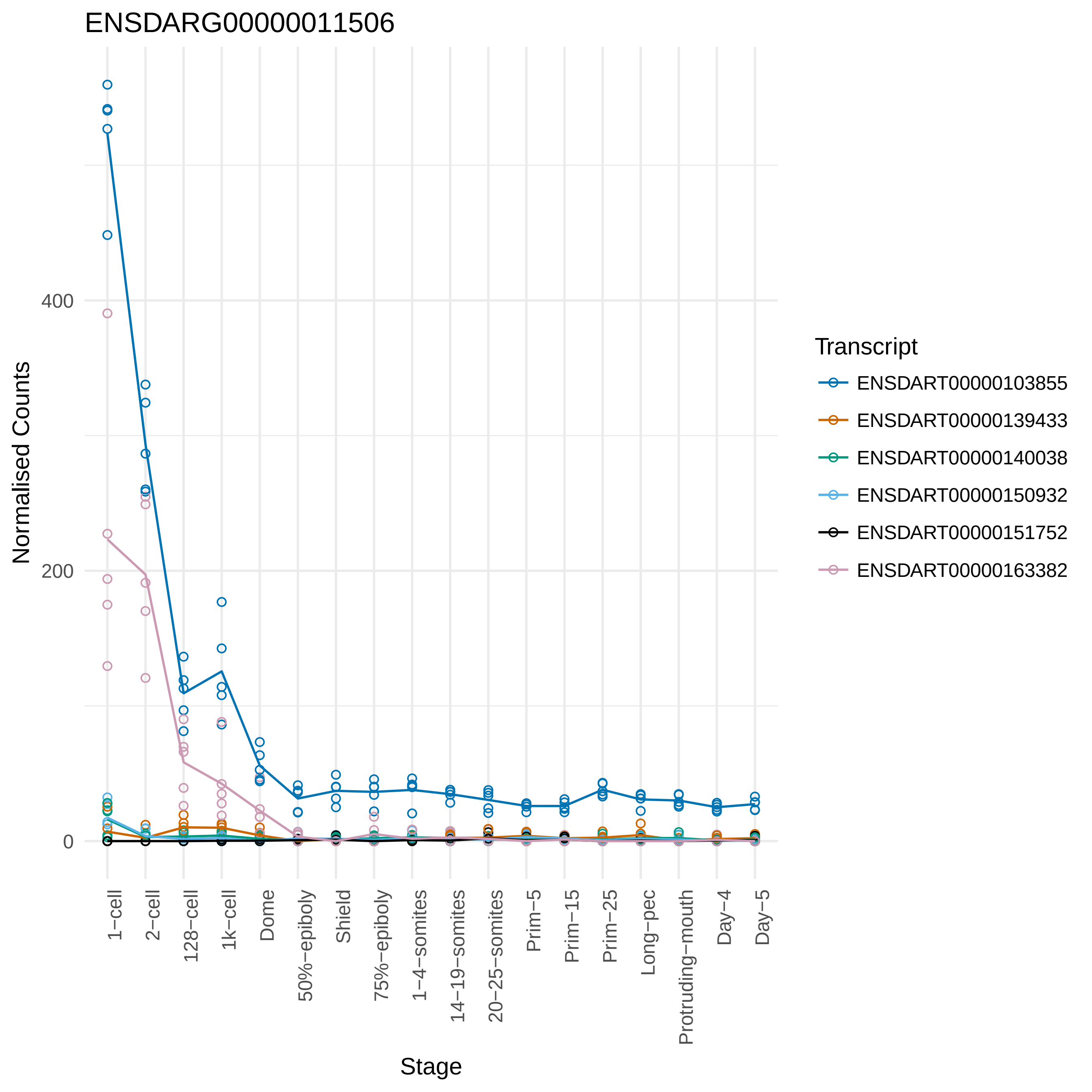
ENSDARG00000011506
Normalised Counts
0
200
400
Stage
1−cell
2−cell
128−cell
1k−cell
Dome
50%−epiboly
Shield
75%−epiboly
1−4−somites
14−19−somites
20−25−somites
Prim−5
Prim−15
Prim−25
Long−pec
Protruding−mouth
Day−4
Day−5
Transcript
ENSDART00000103855
ENSDART00000139433
ENSDART00000140038
ENSDART00000150932
ENSDART00000151752
ENSDART00000163382

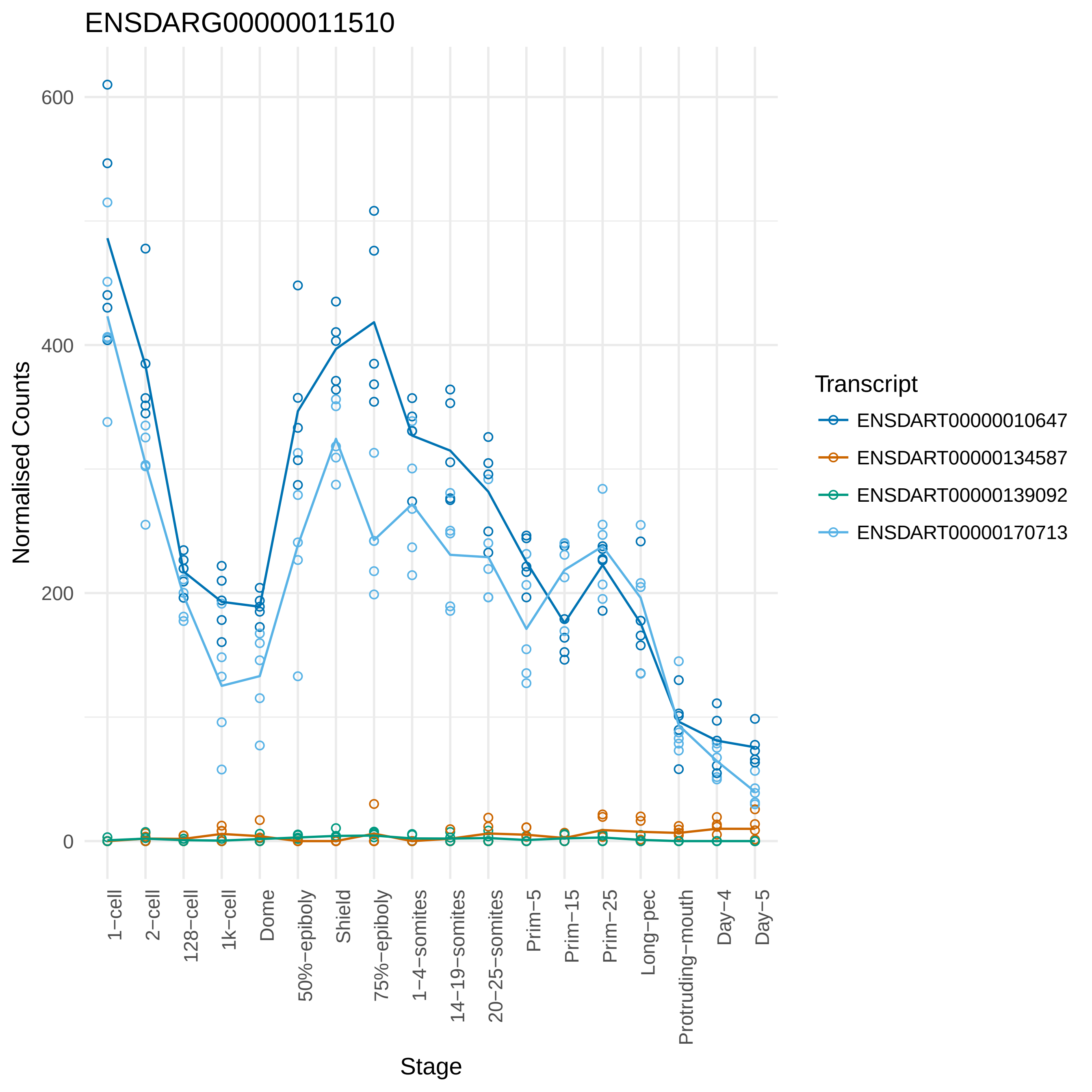

ENSDARG00000011510
Normalised Counts
600
400
200
0
Stage
1−cell
2−cell
128−cell
1k−cell
Dome
50%−epiboly
Shield
75%−epiboly
1−4−somites
14−19−somites
20−25−somites
Prim−5
Prim−15
Prim−25
Long−pec
Protruding−mouth
Day−4
Day−5
Transcript
ENSDART00000010647
ENSDART00000134587
ENSDART00000139092
ENSDART00000170713

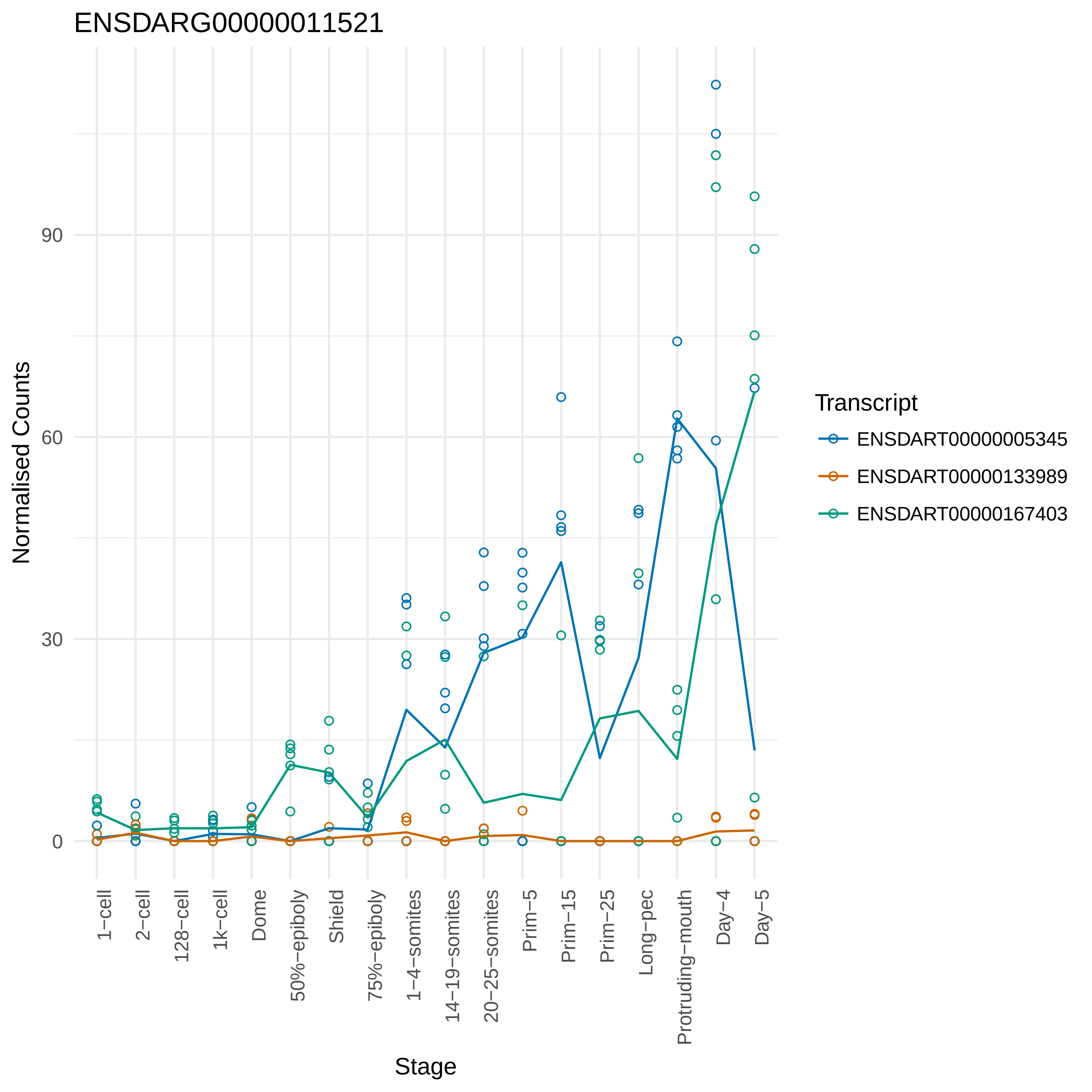

ENSDARG00000011521
Normalised Counts
0
30
60
90
1−cell
2−cell
128−cell
1k−cell
Dome
50%−epiboly
Shield
75%−epiboly
1−4−somites
14−19−somites
20−25−somites
Prim−5
Prim−15
Prim−25
Long−pec
Protruding−mouth
Day−4
Day−5
Stage
Transcript
ENSDART00000005345
ENSDART00000133989
ENSDART00000167403

ENSDARG00000011537

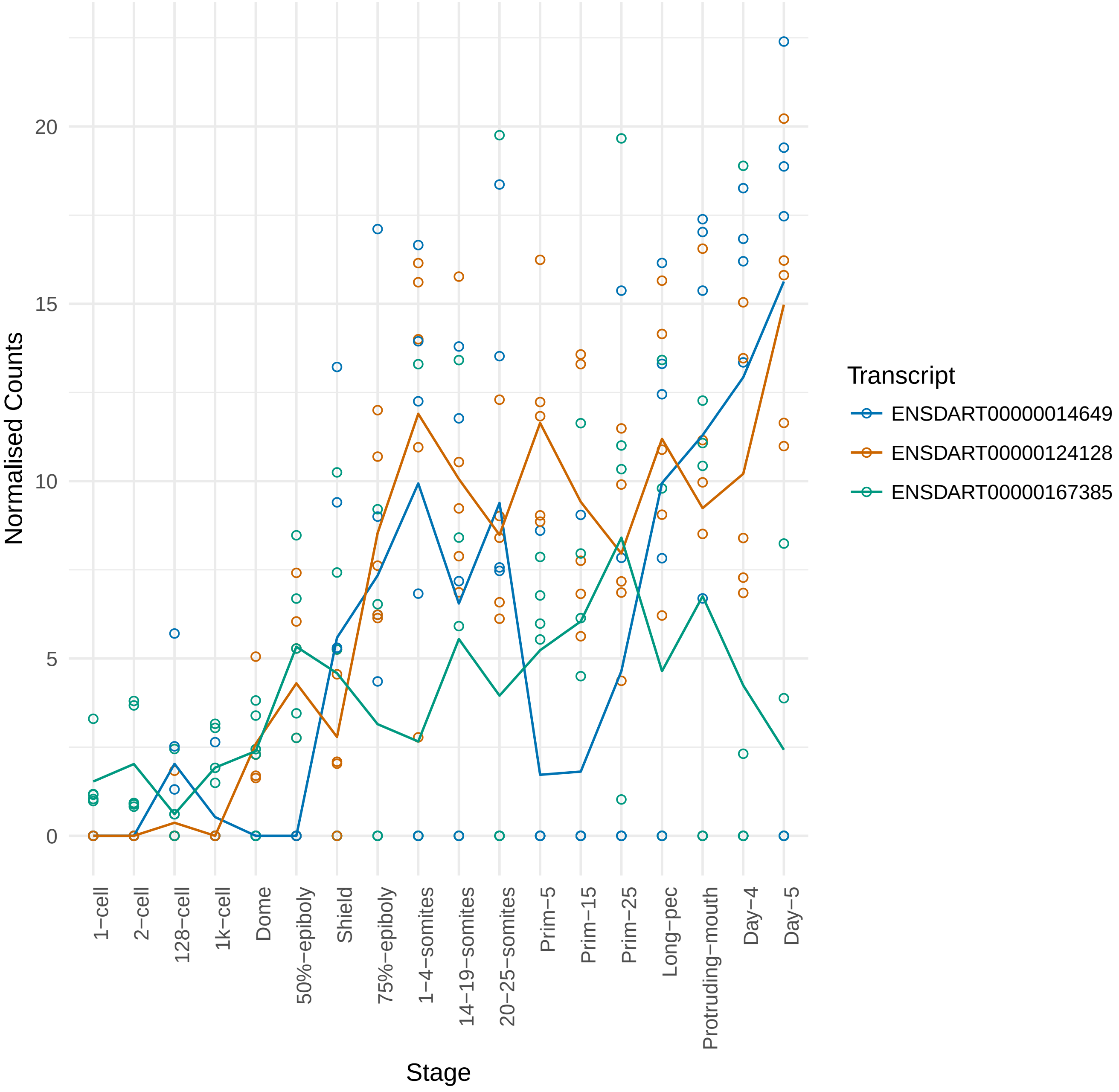

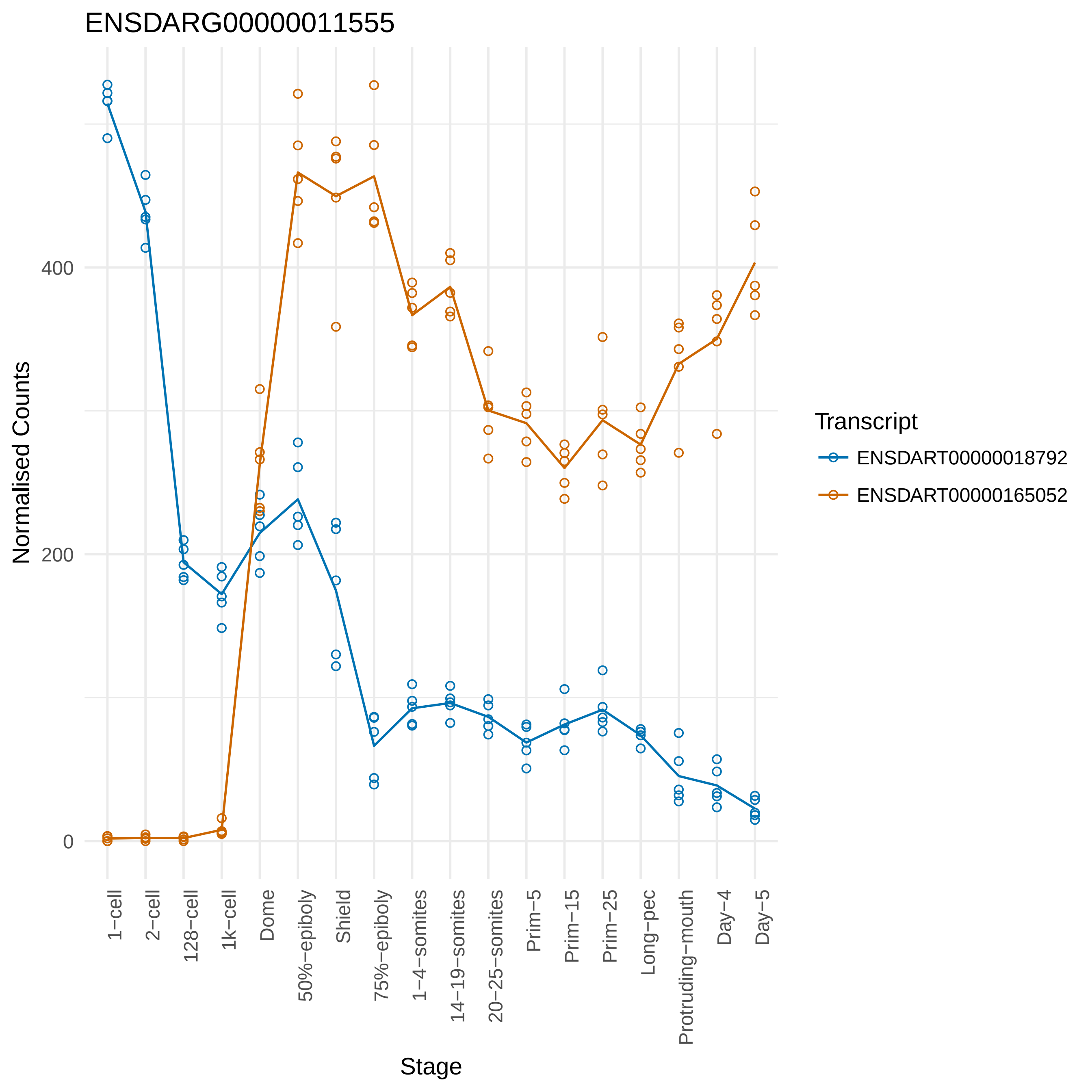

ENSDARG00000011555
Normalised Counts
400
200
0
1−cell
2−cell
128−cell
1k−cell
Dome
50%−epiboly
Shield
75%−epiboly
1−4−somites
14−19−somites
20−25−somites
Prim−5
Prim−15
Prim−25
Long−pec
Protruding−mouth
Day−4
Day−5
Stage
Transcript
ENSDART00000018792
ENSDART00000165052

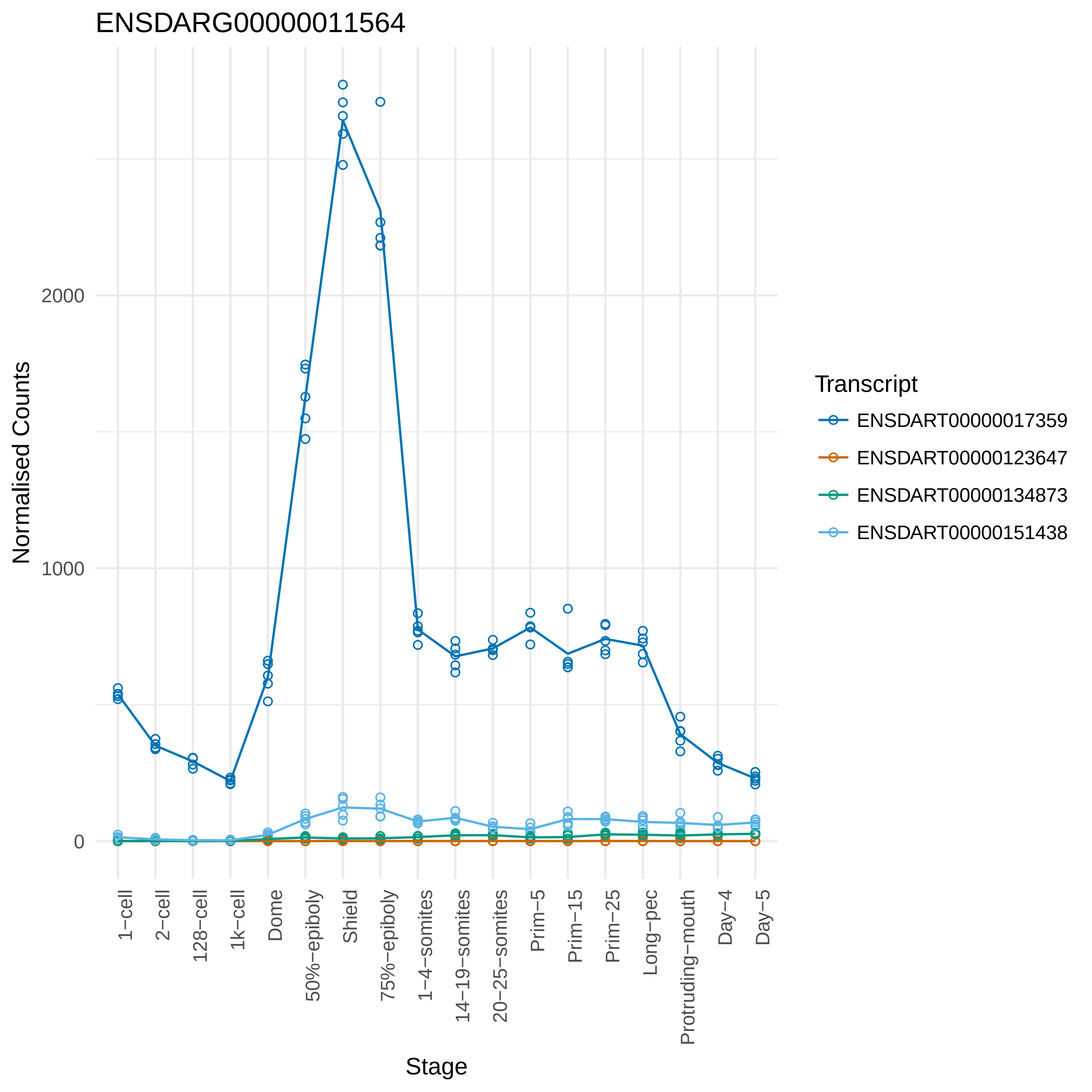

ENSDARG00000011564
Normalised Counts
2000
1000
0
1−cell
2−cell
128−cell
1k−cell
Dome
50%−epiboly
Shield
75%−epiboly
1−4−somites
14−19−somites
20−25−somites
Prim−5
Prim−15
Prim−25
Long−pec
Protruding−mouth
Day−4
Day−5
Stage
Transcript
ENSDART00000017359
ENSDART00000123647
ENSDART00000134873
ENSDART00000151438

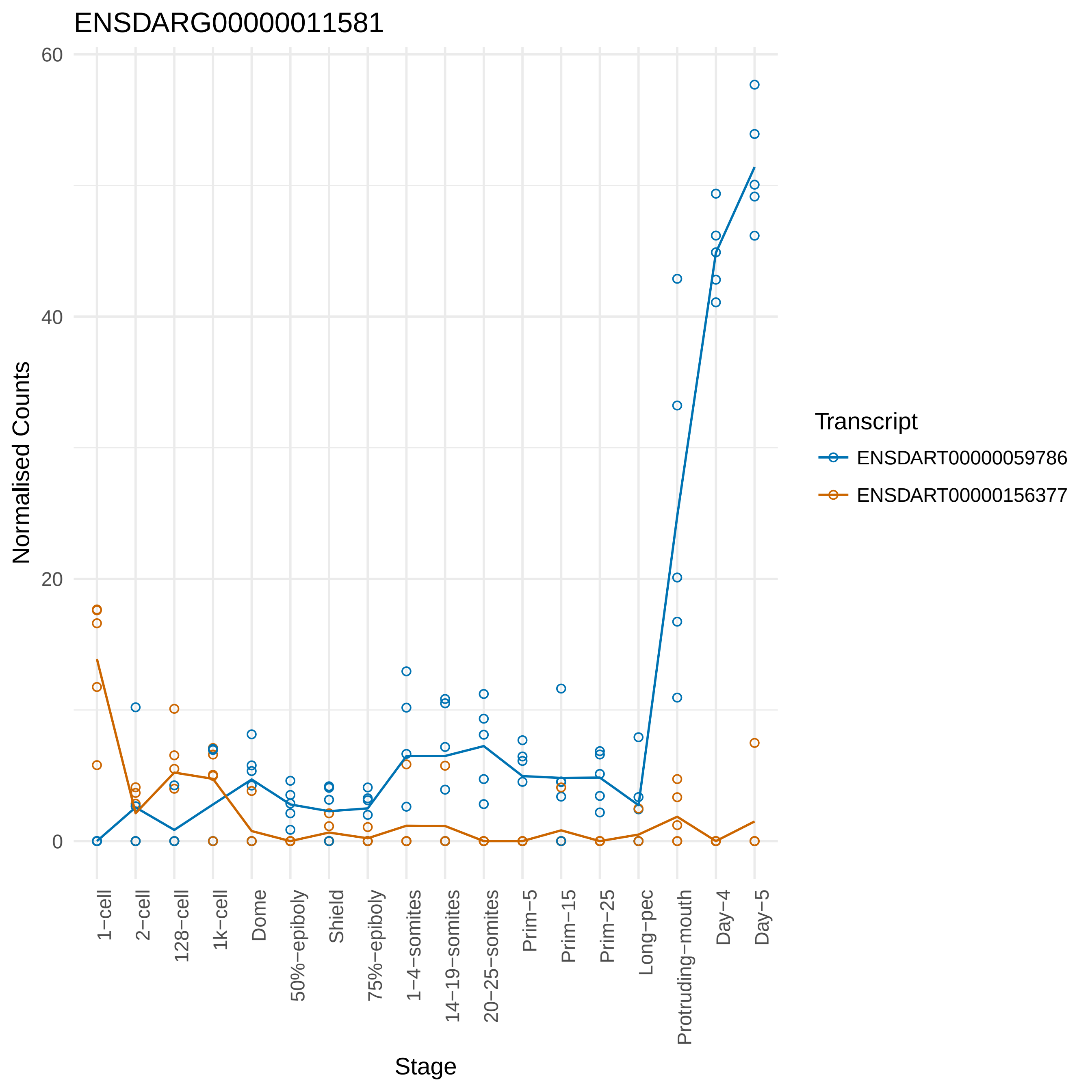
ENSDARG00000011581
Normalised Counts
60
40
20
0
1−cell
2−cell
128−cell
1k−cell
Dome
50%−epiboly
Shield
75%−epiboly
1−4−somites
14−19−somites
20−25−somites
Prim−5
Prim−15
Prim−25
Long−pec
Protruding−mouth
Day−4
Day−5
Stage
Transcript
ENSDART00000059786
ENSDART00000156377

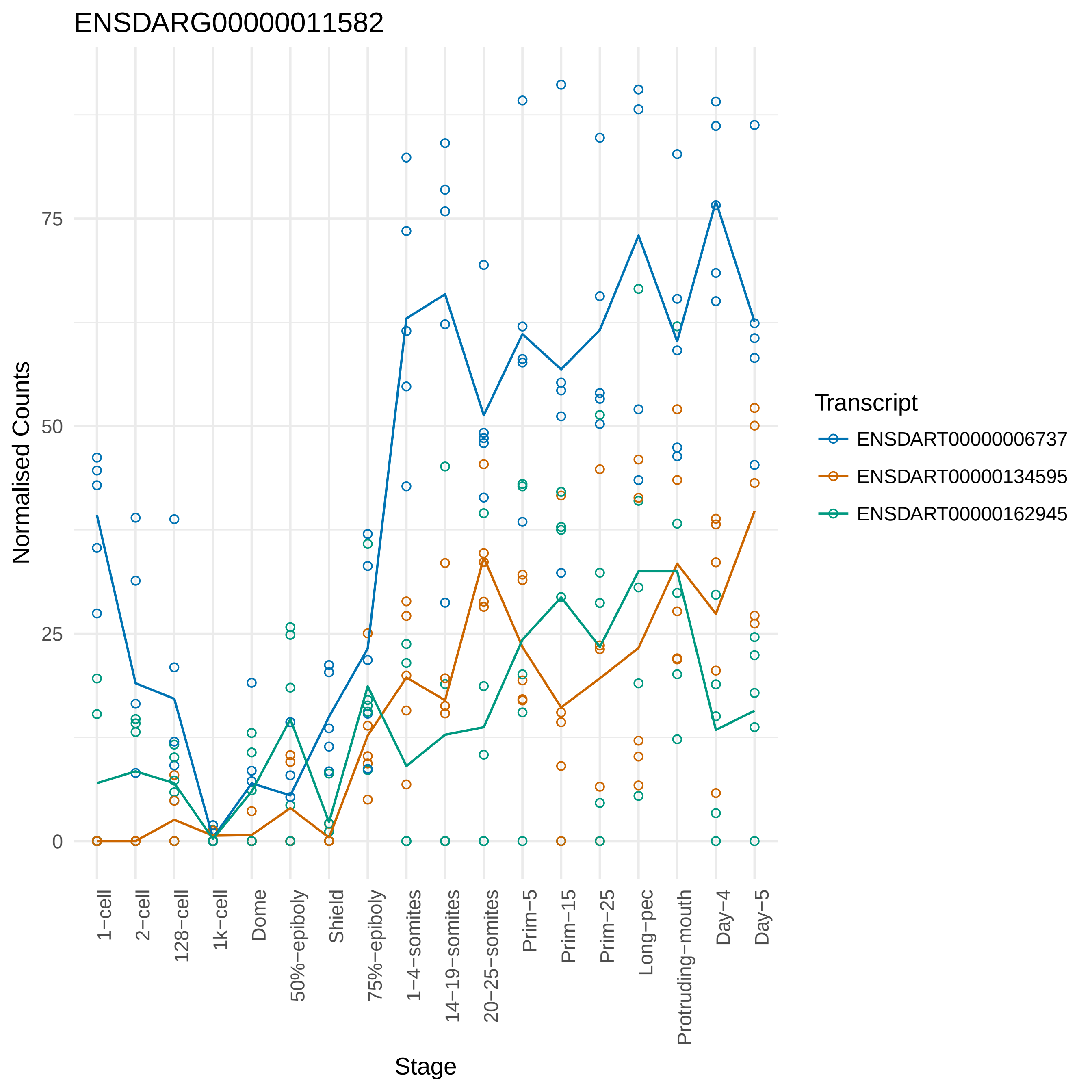

ENSDARG00000011582
Normalised Counts
0
25
50
75
Stage
1−cell
2−cell
128−cell
1k−cell
Dome
50%−epiboly
Shield
75%−epiboly
1−4−somites
14−19−somites
20−25−somites
Prim−5
Prim−15
Prim−25
Long−pec
Protruding−mouth
Day−4
Day−5
Transcript
ENSDART00000006737
ENSDART00000134595
ENSDART00000162945

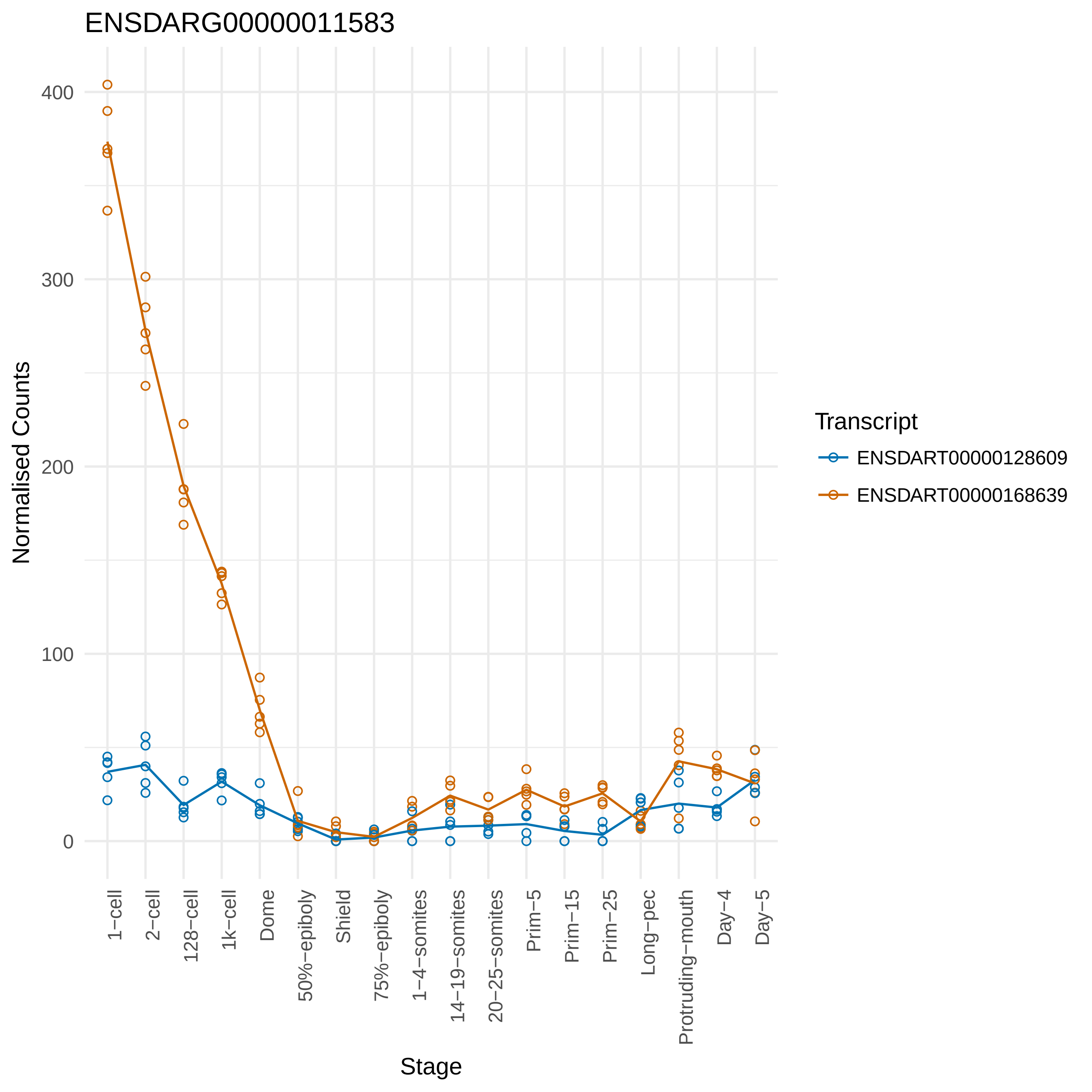

ENSDARG00000011583
Normalised Counts
400
300
200
100
0
1−cell
2−cell
128−cell
1k−cell
Dome
50%−epiboly
Shield
75%−epiboly
1−4−somites
14−19−somites
20−25−somites
Prim−5
Prim−15
Prim−25
Long−pec
Protruding−mouth
Day−4
Day−5
Stage
Transcript
ENSDART00000128609
ENSDART00000168639

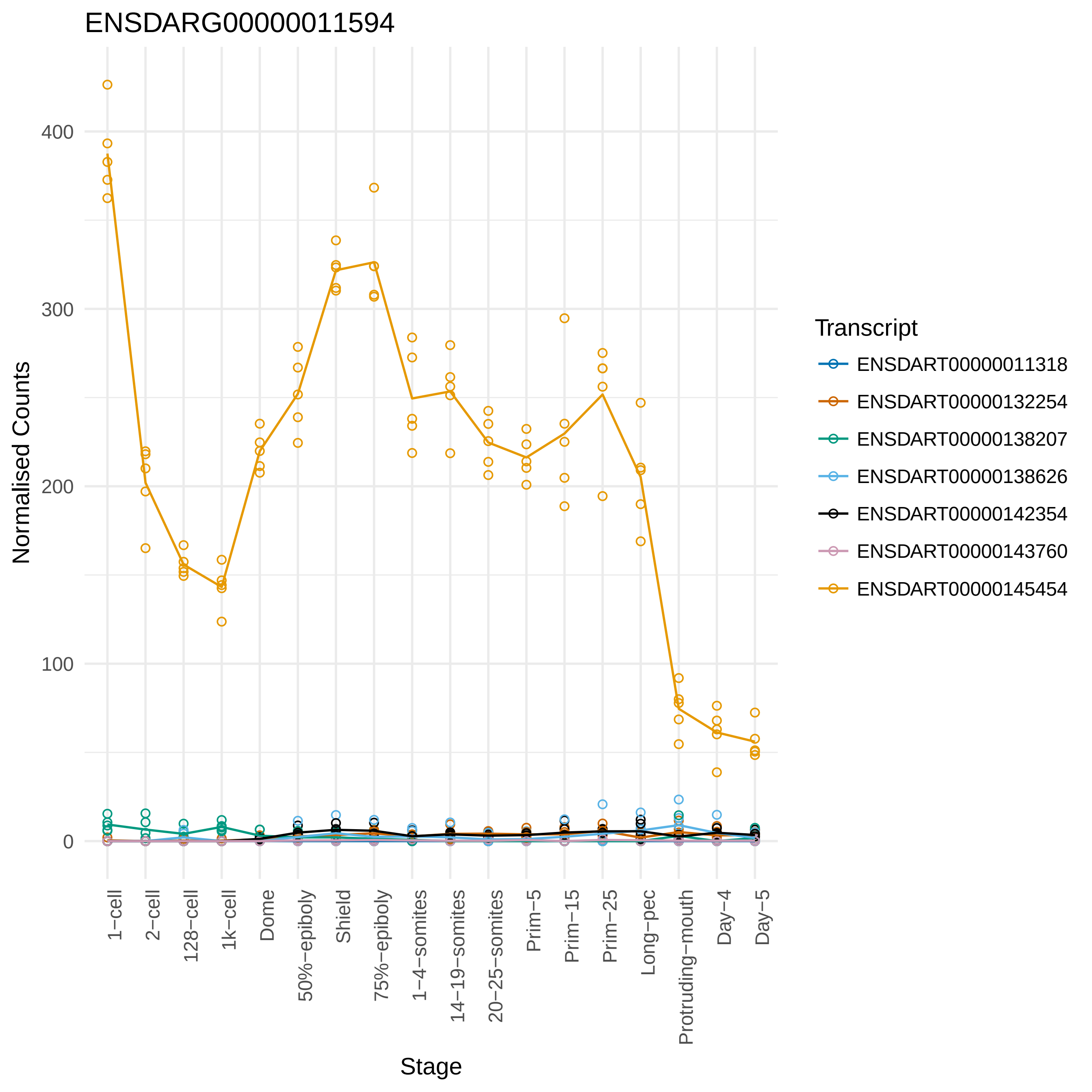

ENSDARG00000011594
Normalised Counts
0
100
200
300
400
Stage
1−cell
2−cell
128−cell
1k−cell
Dome
50%−epiboly
Shield
75%−epiboly
1−4−somites
14−19−somites
20−25−somites
Prim−5
Prim−15
Prim−25
Long−pec
Protruding−mouth
Day−4
Day−5
Transcript
ENSDART00000011318
ENSDART00000132254
ENSDART00000138207
ENSDART00000138626
ENSDART00000142354
ENSDART00000143760
ENSDART00000145454

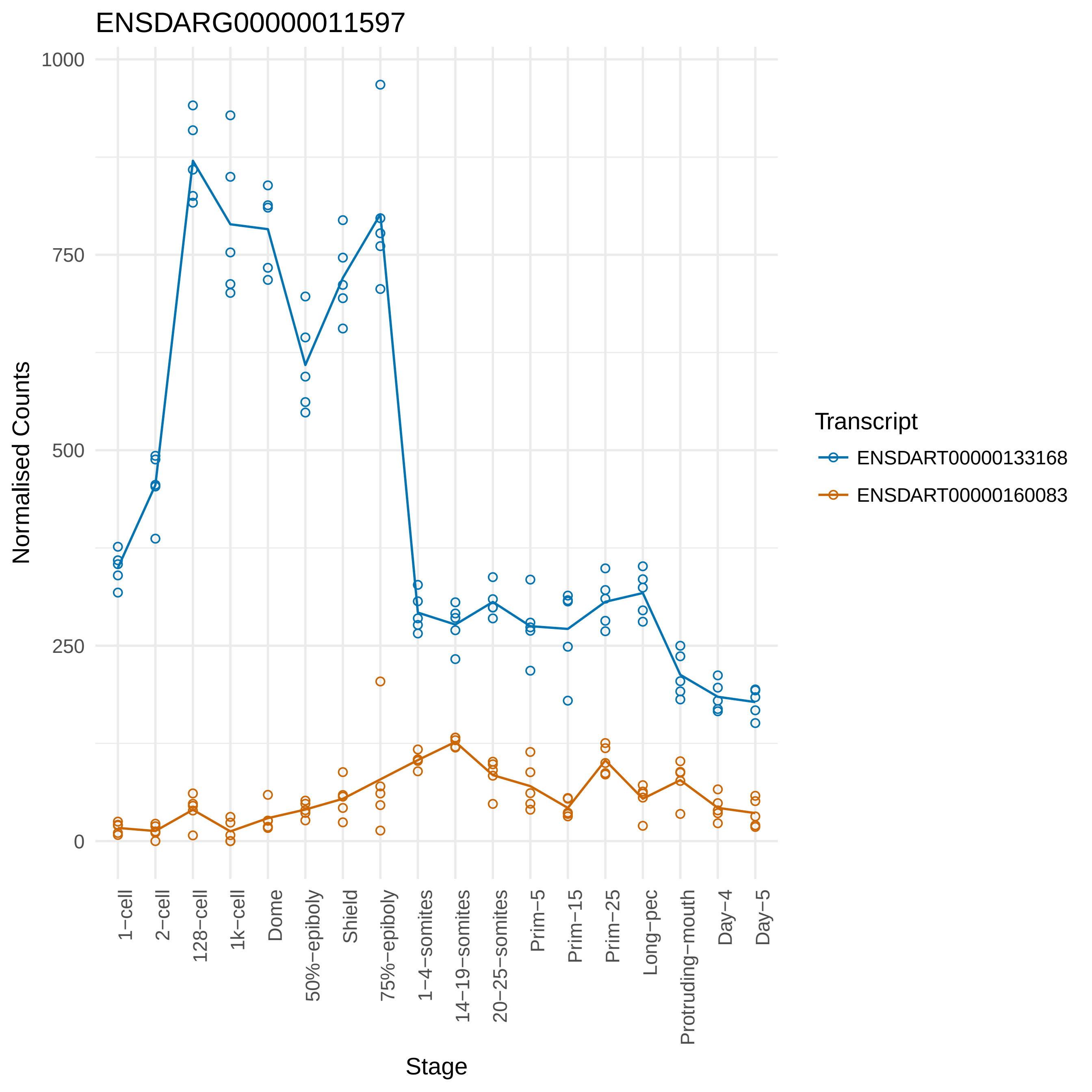
ENSDARG00000011597
1000
750
500
250
0
Normalised Counts
1−cell
2−cell
128−cell
1k−cell
Dome
50%−epiboly
Shield
75%−epiboly
1−4−somites
14−19−somites
20−25−somites
Prim−5
Prim−15
Prim−25
Long−pec
Protruding−mouth
Day−4
Day−5
Stage
Transcript
ENSDART00000133168
ENSDART00000160083

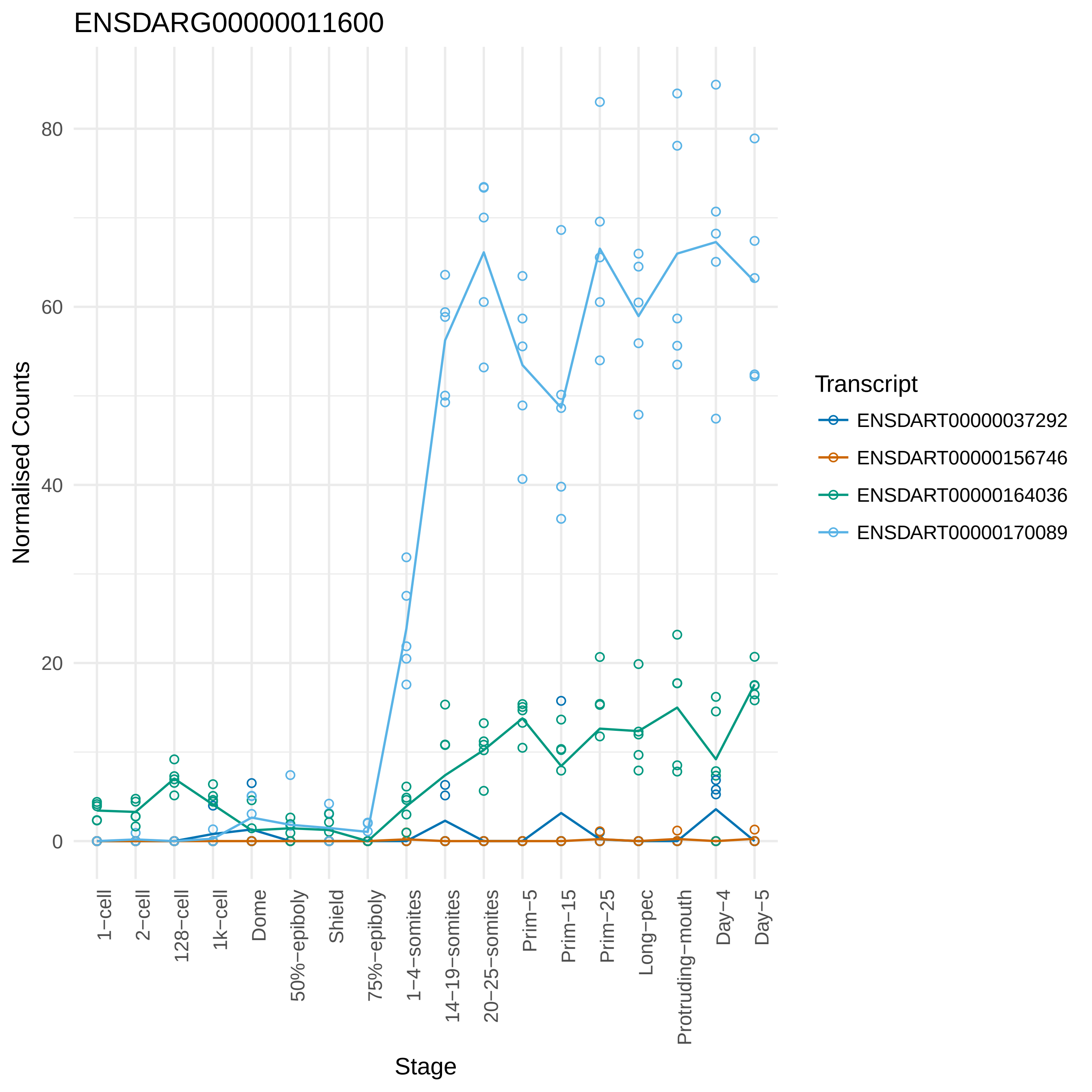
ENSDARG00000011600
Normalised Counts
0
20
40
60
80
1−cell
2−cell
128−cell
1k−cell
Dome
50%−epiboly
Shield
75%−epiboly
1−4−somites
14−19−somites
20−25−somites
Prim−5
Prim−15
Prim−25
Long−pec
Protruding−mouth
Day−4
Day−5
Stage
Transcript
ENSDART00000037292
ENSDART00000156746
ENSDART00000164036
ENSDART00000170089

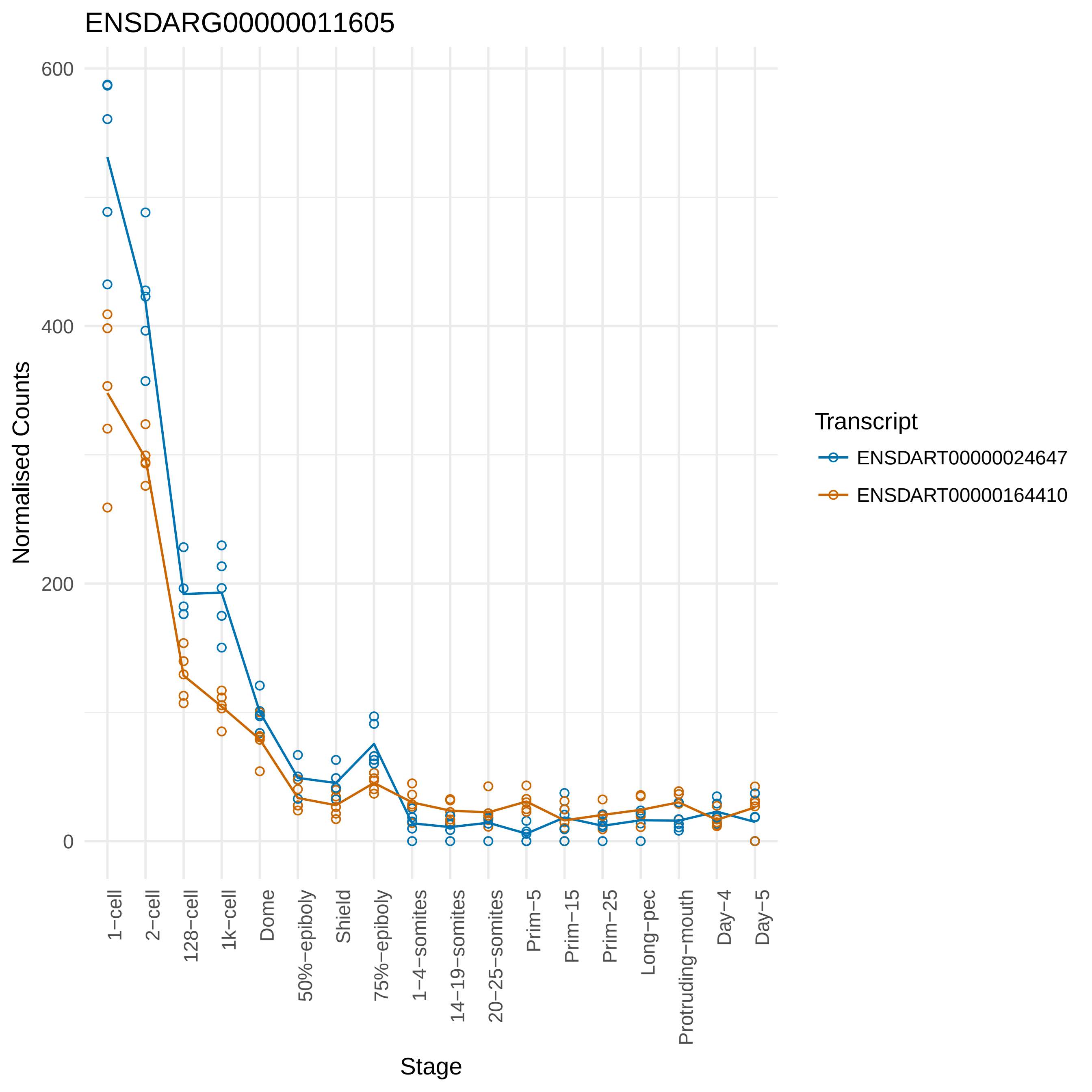

ENSDARG00000011605
Normalised Counts
600
400
200
0
1−cell
2−cell
128−cell
1k−cell
Dome
50%−epiboly
Shield
75%−epiboly
1−4−somites
14−19−somites
20−25−somites
Prim−5
Prim−15
Prim−25
Long−pec
Protruding−mouth
Day−4
Day−5
Stage
Transcript
ENSDART00000024647
ENSDART00000164410

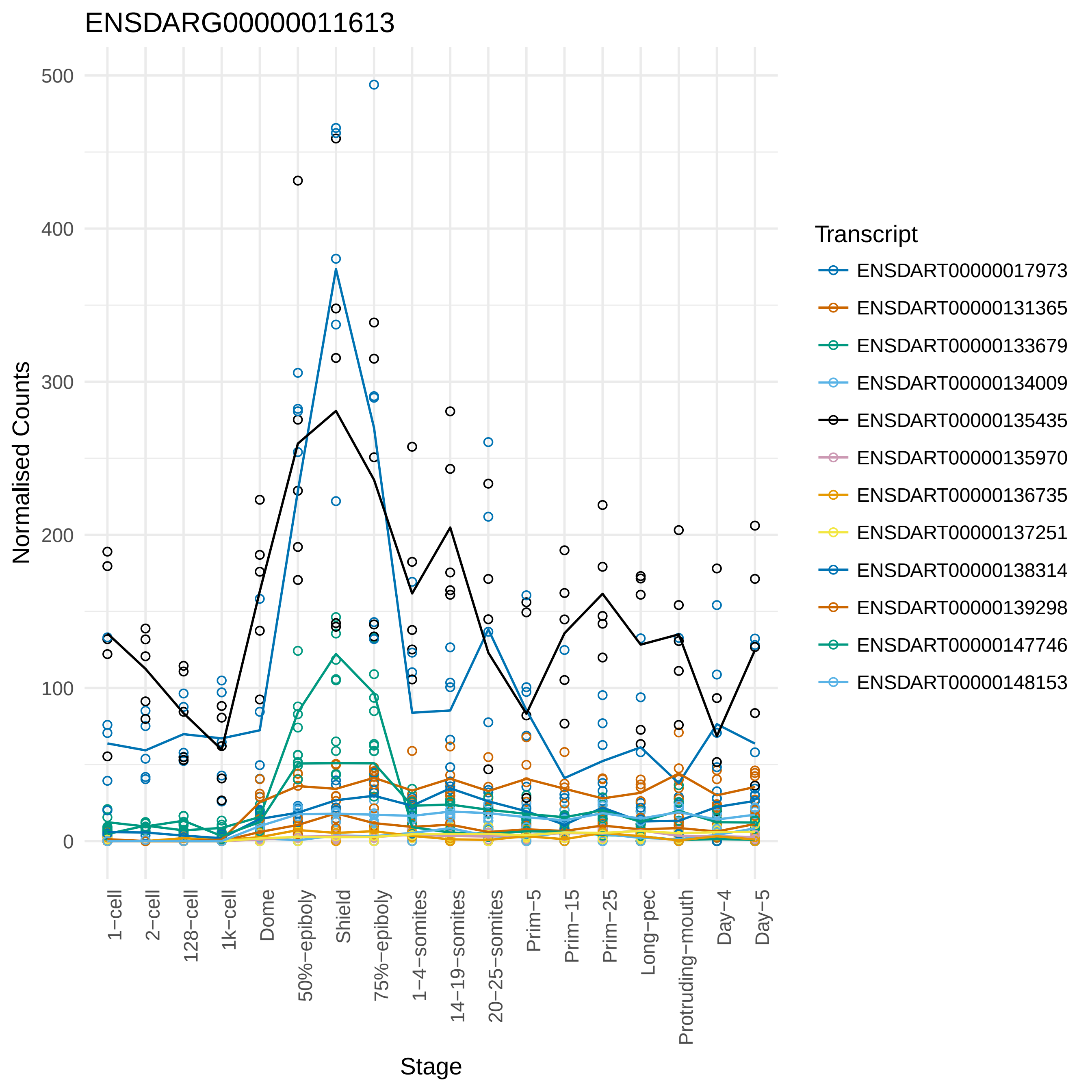

ENSDARG00000011613
Normalised Counts
0
100
200
300
400
500
1−cell
2−cell
128−cell
1k−cell
Dome
50%−epiboly
Shield
75%−epiboly
1−4−somites
14−19−somites
20−25−somites
Prim−5
Prim−15
Prim−25
Long−pec
Protruding−mouth
Day−4
Day−5
Stage
Transcript
ENSDART00000017973
ENSDART00000131365
ENSDART00000133679
ENSDART00000134009
ENSDART00000135435
ENSDART00000135970
ENSDART00000136735
ENSDART00000137251
ENSDART00000138314
ENSDART00000139298
ENSDART00000147746
ENSDART00000148153

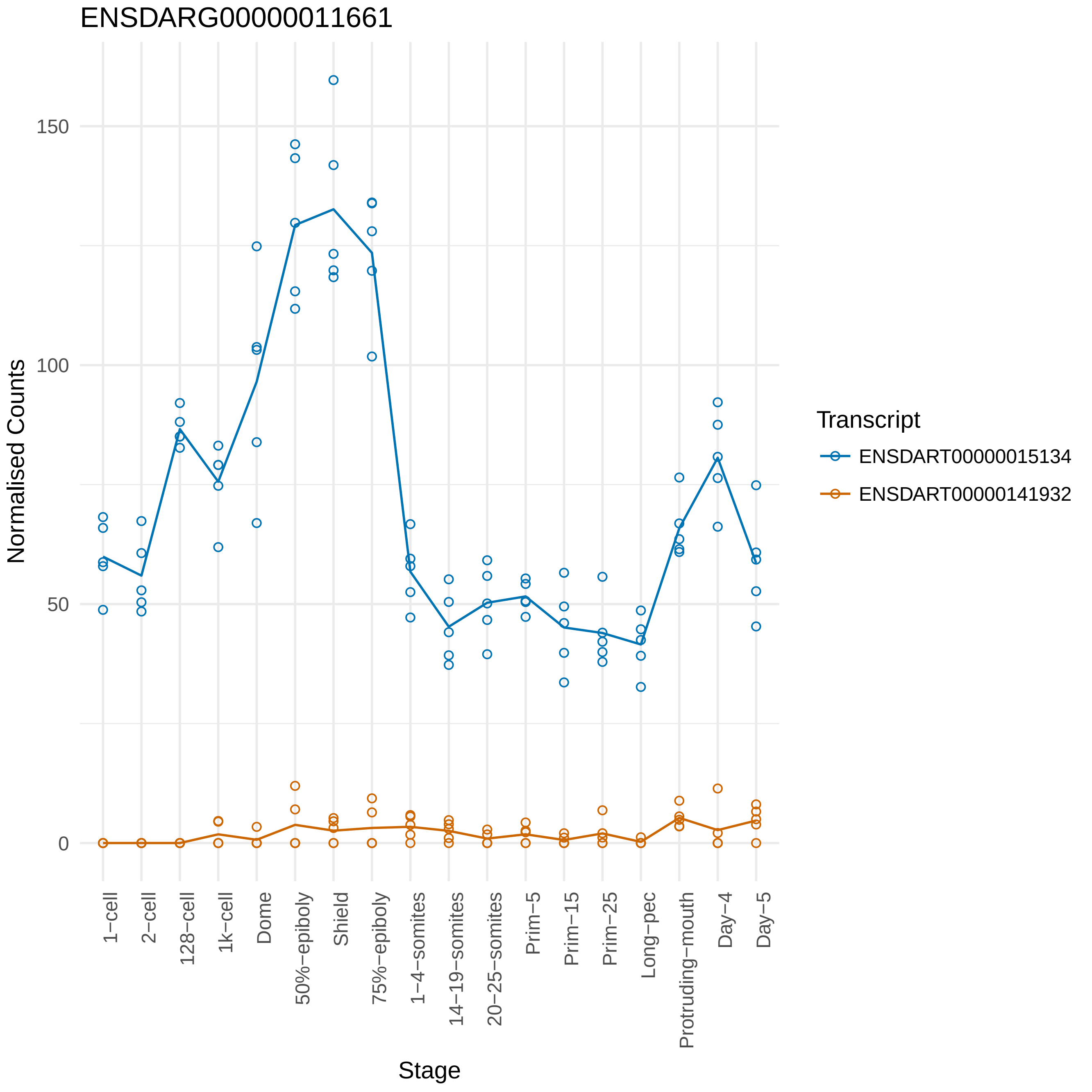
ENSDARG00000011661
Normalised Counts
150
100
50
0
1−cell
2−cell
128−cell
1k−cell
Dome
50%−epiboly
Shield
75%−epiboly
1−4−somites
14−19−somites
20−25−somites
Prim−5
Prim−15
Prim−25
Long−pec
Protruding−mouth
Day−4
Day−5
Stage
Transcript
ENSDART00000015134
ENSDART00000141932

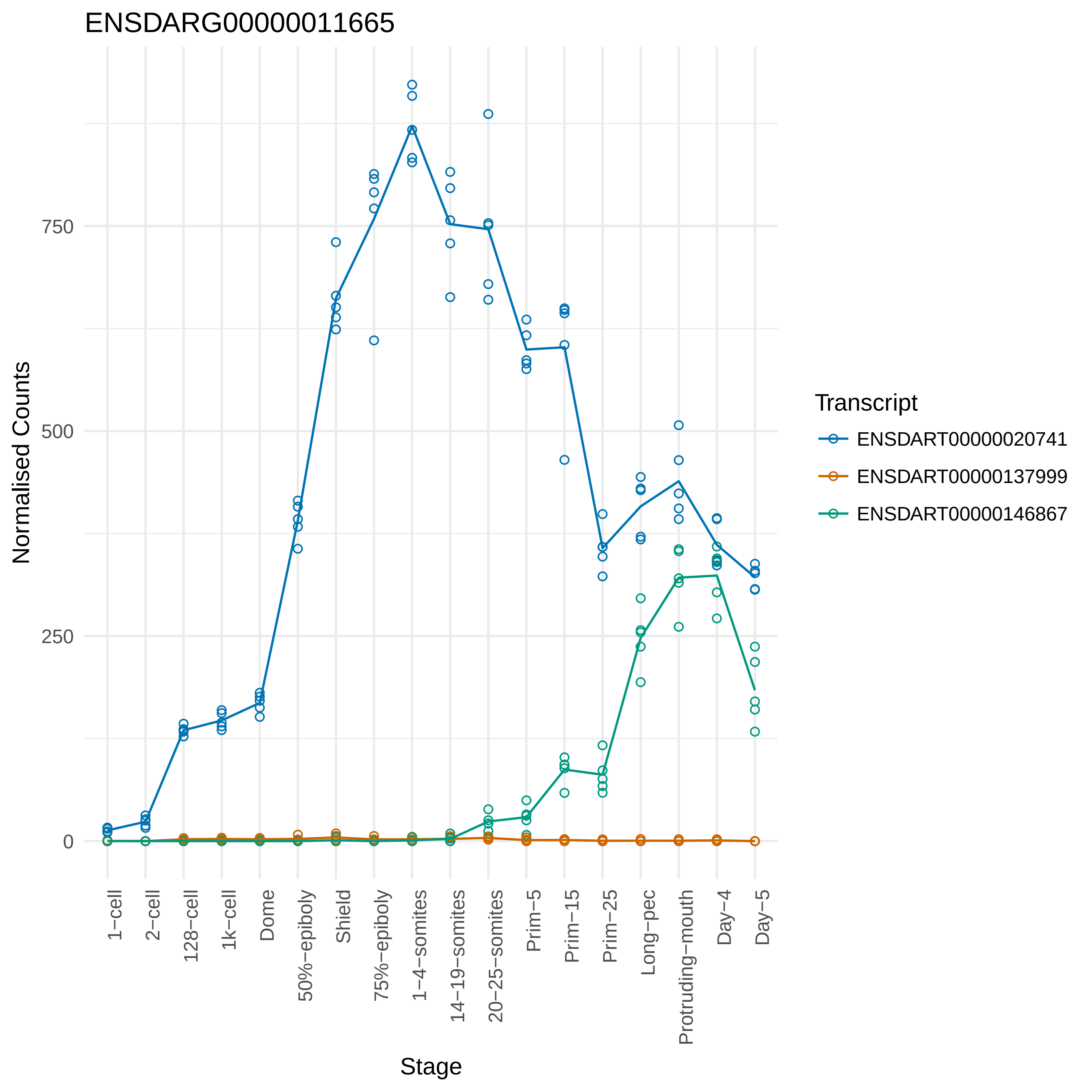

ENSDARG00000011665
Normalised Counts
750
500
250
0
1−cell
2−cell
128−cell
1k−cell
Dome
50%−epiboly
Shield
75%−epiboly
1−4−somites
14−19−somites
20−25−somites
Prim−5
Prim−15
Prim−25
Long−pec
Protruding−mouth
Day−4
Day−5
Stage
Transcript
ENSDART00000020741
ENSDART00000137999
ENSDART00000146867

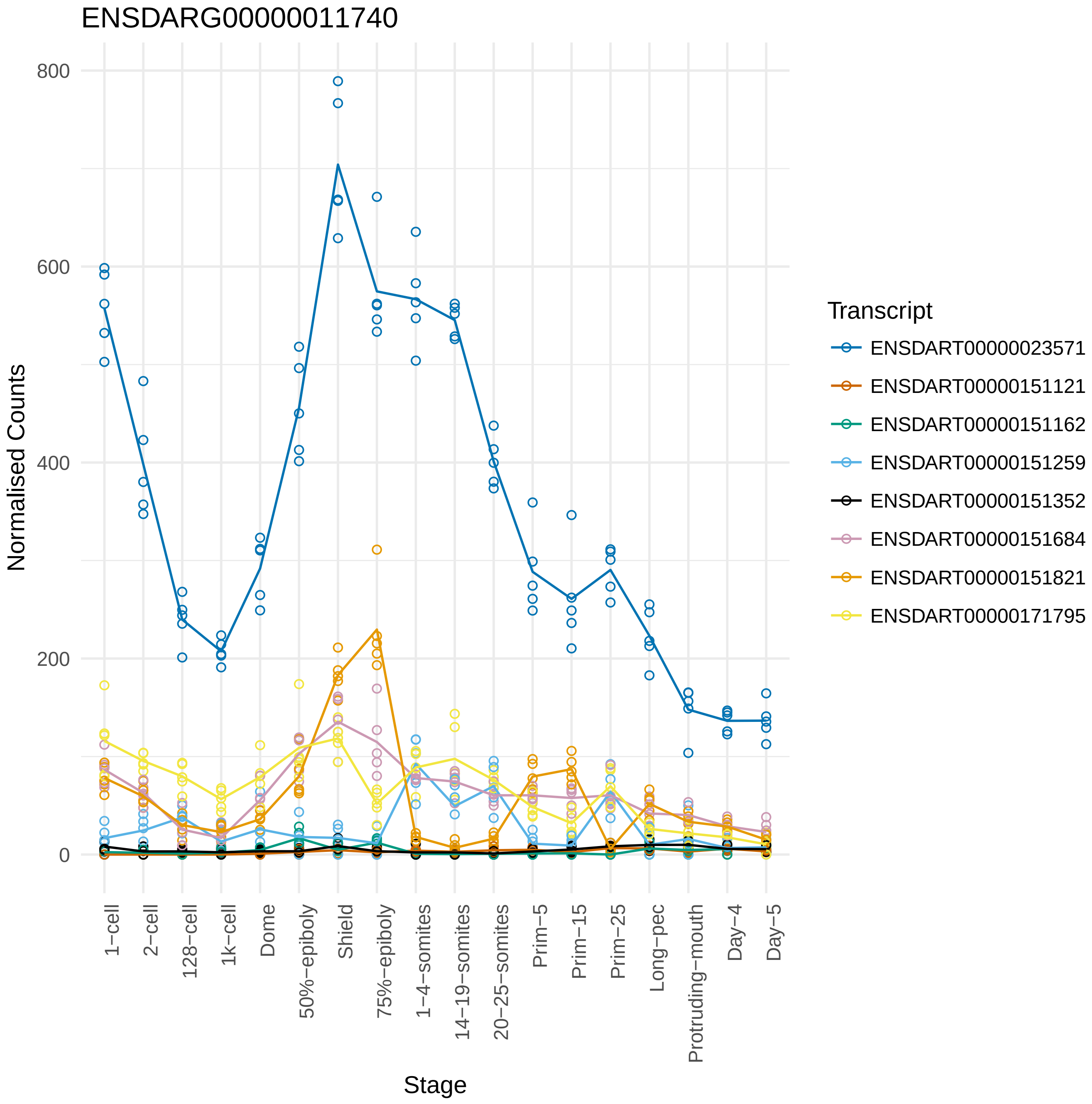
ENSDARG00000011740
Normalised Counts
800
600
400
200
0
1−cell
2−cell
128−cell
1k−cell
Dome
50%−epiboly
Shield
75%−epiboly
1−4−somites
14−19−somites
20−25−somites
Prim−5
Prim−15
Prim−25
Long−pec
Protruding−mouth
Day−4
Day−5
Stage
Transcript
ENSDART00000023571
ENSDART00000151121
ENSDART00000151162
ENSDART00000151259
ENSDART00000151352
ENSDART00000151684
ENSDART00000151821
ENSDART00000171795

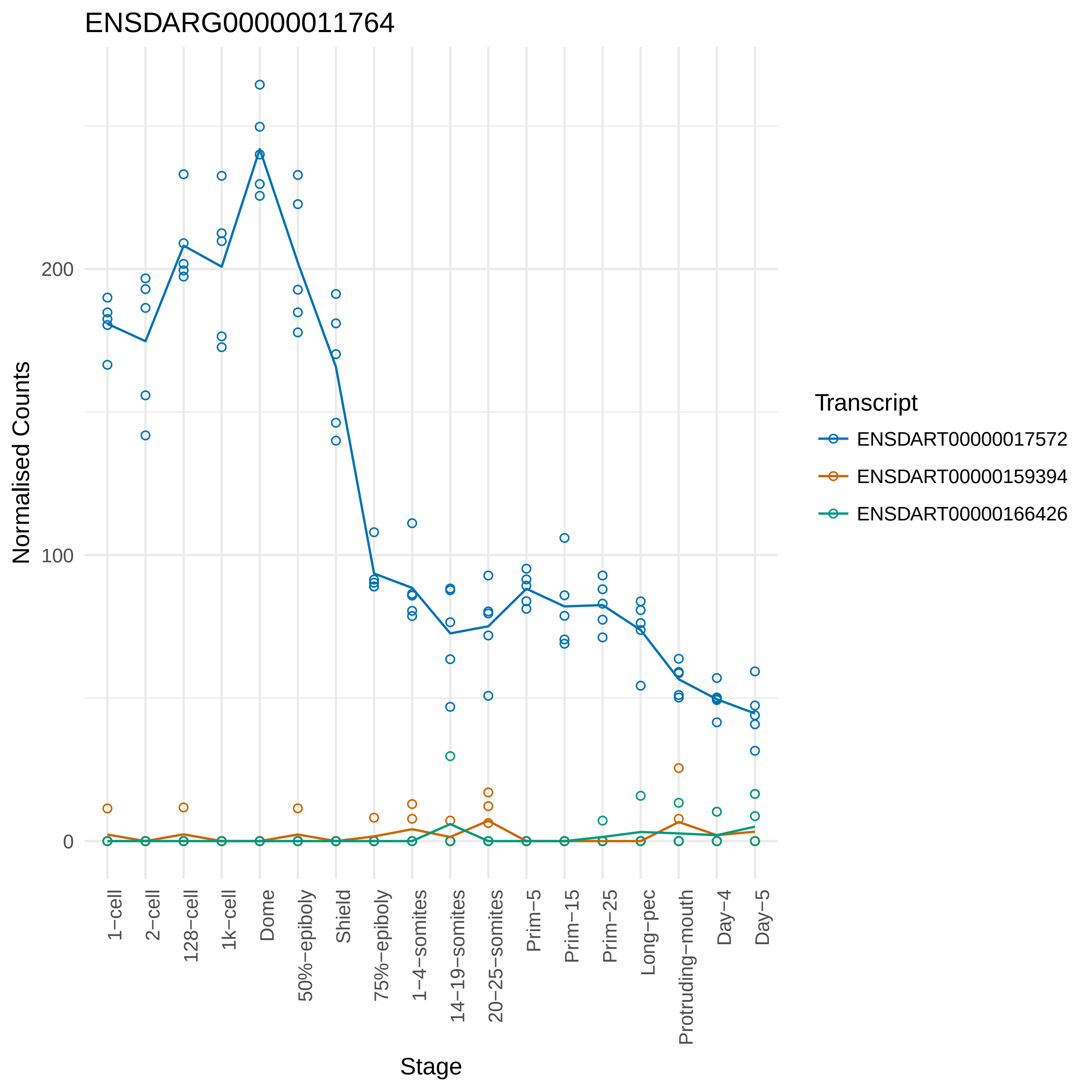

ENSDARG00000011764
Normalised Counts
200
100
0
1−cell
2−cell
128−cell
1k−cell
Dome
50%−epiboly
Shield
75%−epiboly
1−4−somites
14−19−somites
20−25−somites
Prim−5
Prim−15
Prim−25
Long−pec
Protruding−mouth
Day−4
Day−5
Stage
Transcript
ENSDART00000017572
ENSDART00000159394
ENSDART00000166426

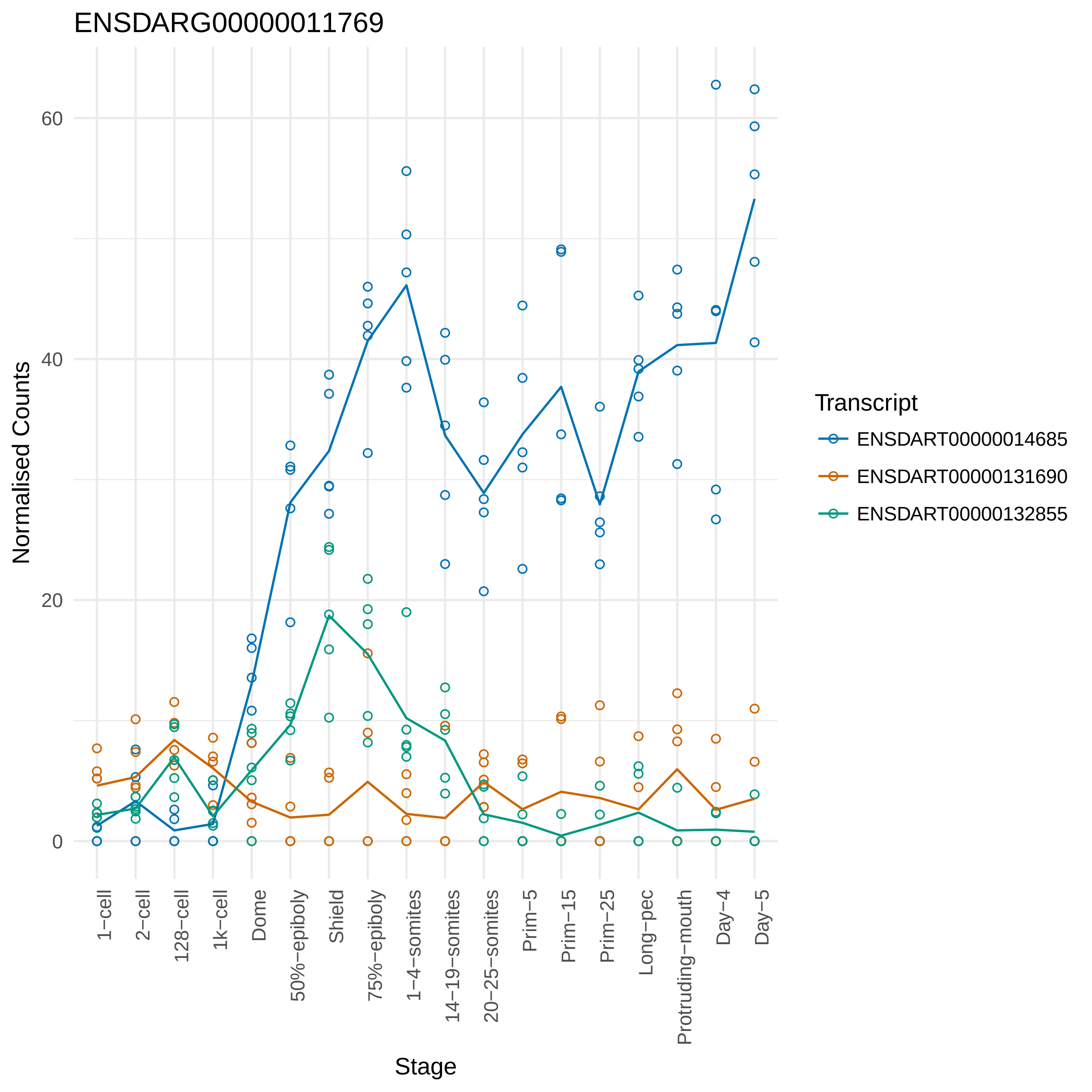

ENSDARG00000011769
Normalised Counts
60
40
20
0
1−cell
2−cell
128−cell
1k−cell
Dome
50%−epiboly
Shield
75%−epiboly
1−4−somites
14−19−somites
20−25−somites
Prim−5
Prim−15
Prim−25
Long−pec
Protruding−mouth
Day−4
Day−5
Stage
Transcript
ENSDART00000014685
ENSDART00000131690
ENSDART00000132855

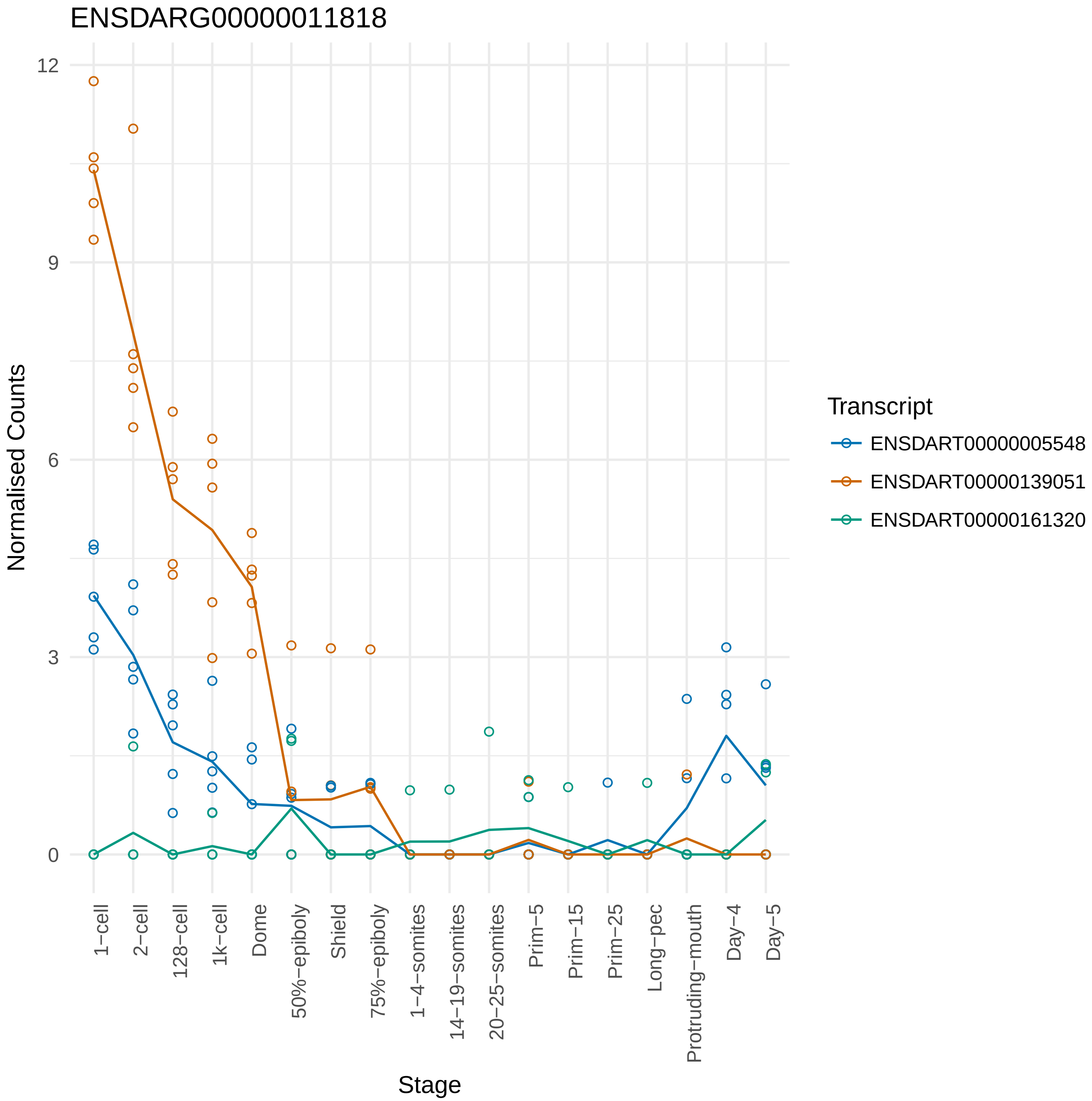
ENSDARG00000011818
Normalised Counts
12
9
6
3
0
1−cell
2−cell
128−cell
1k−cell
Dome
50%−epiboly
Shield
75%−epiboly
1−4−somites
14−19−somites
20−25−somites
Prim−5
Prim−15
Prim−25
Long−pec
Protruding−mouth
Day−4
Day−5
Stage
Transcript
ENSDART00000005548
ENSDART00000139051
ENSDART00000161320

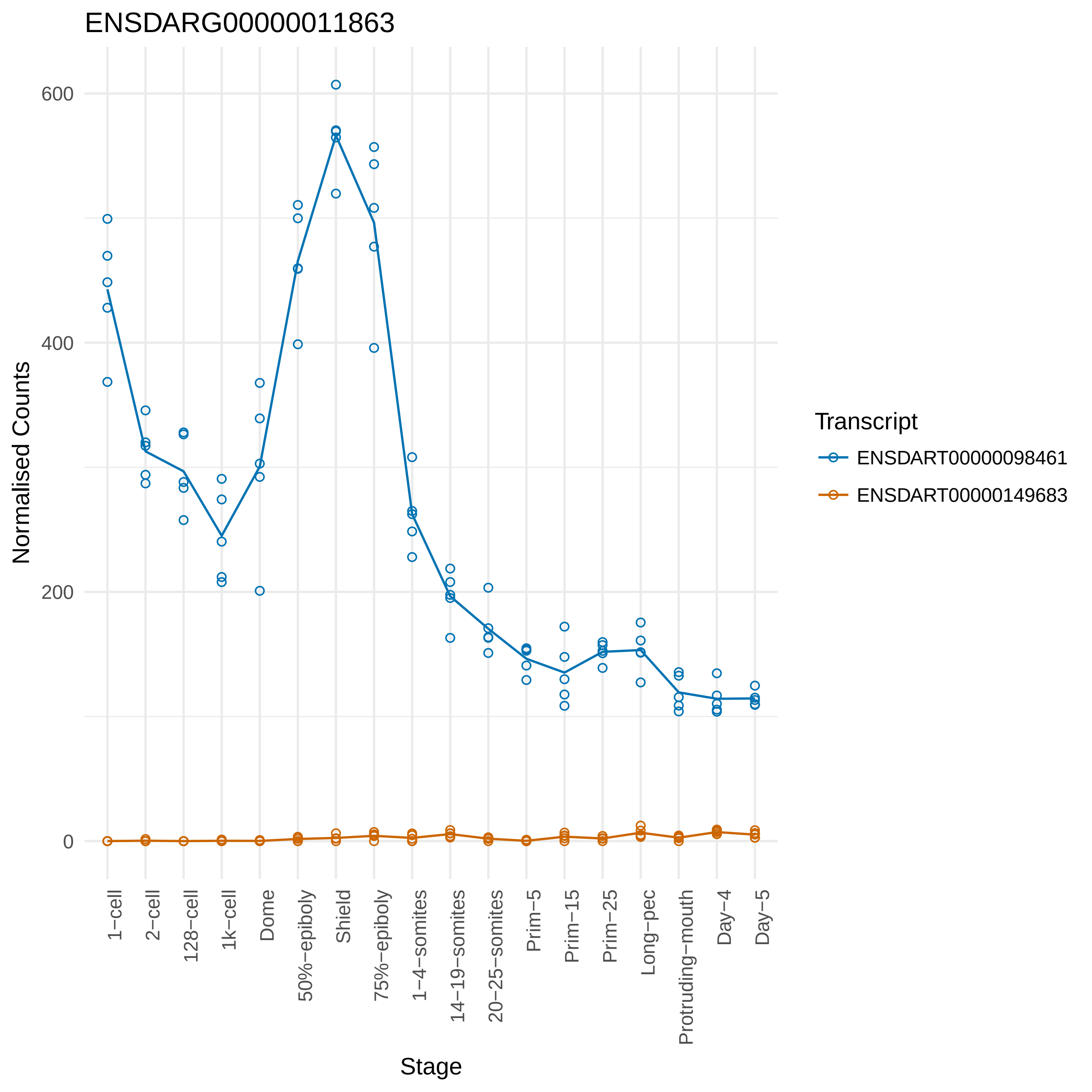

ENSDARG00000011863
Normalised Counts
600
400
200
0
1−cell
2−cell
128−cell
1k−cell
Dome
50%−epiboly
Shield
75%−epiboly
1−4−somites
14−19−somites
20−25−somites
Prim−5
Prim−15
Prim−25
Long−pec
Protruding−mouth
Day−4
Day−5
Stage
Transcript
ENSDART00000098461
ENSDART00000149683

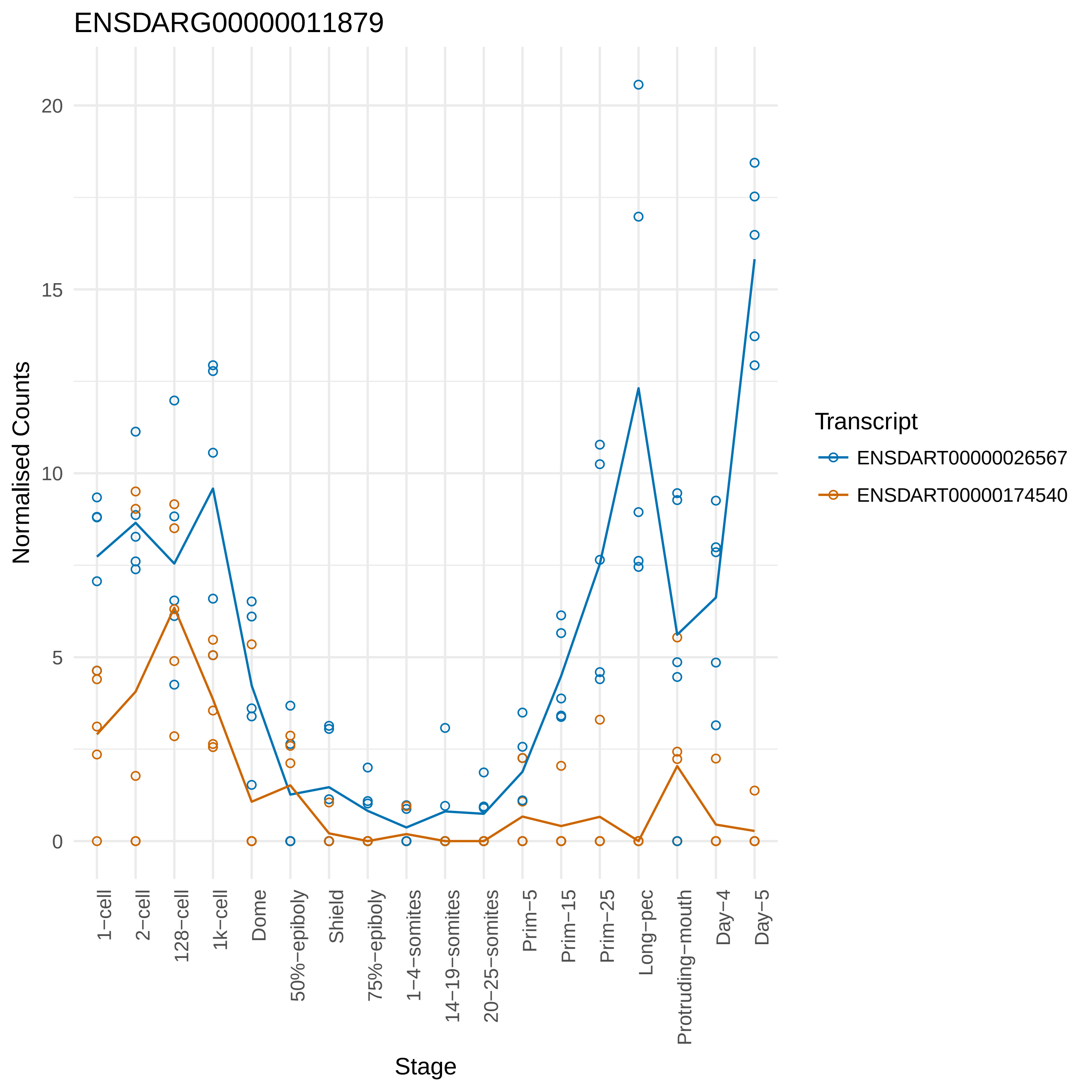
ENSDARG00000011879
Normalised Counts
Stage
Transcript
ENSDART00000026567
ENSDART00000174540
0
5
10
15
20
1−cell
2−cell
128−cell
1k−cell
Dome
50%−epiboly
Shield
75%−epiboly
1−4−somites
14−19−somites
20−25−somites
Prim−5
Prim−15
Prim−25
Long−pec
Protruding−mouth
Day−4
Day−5

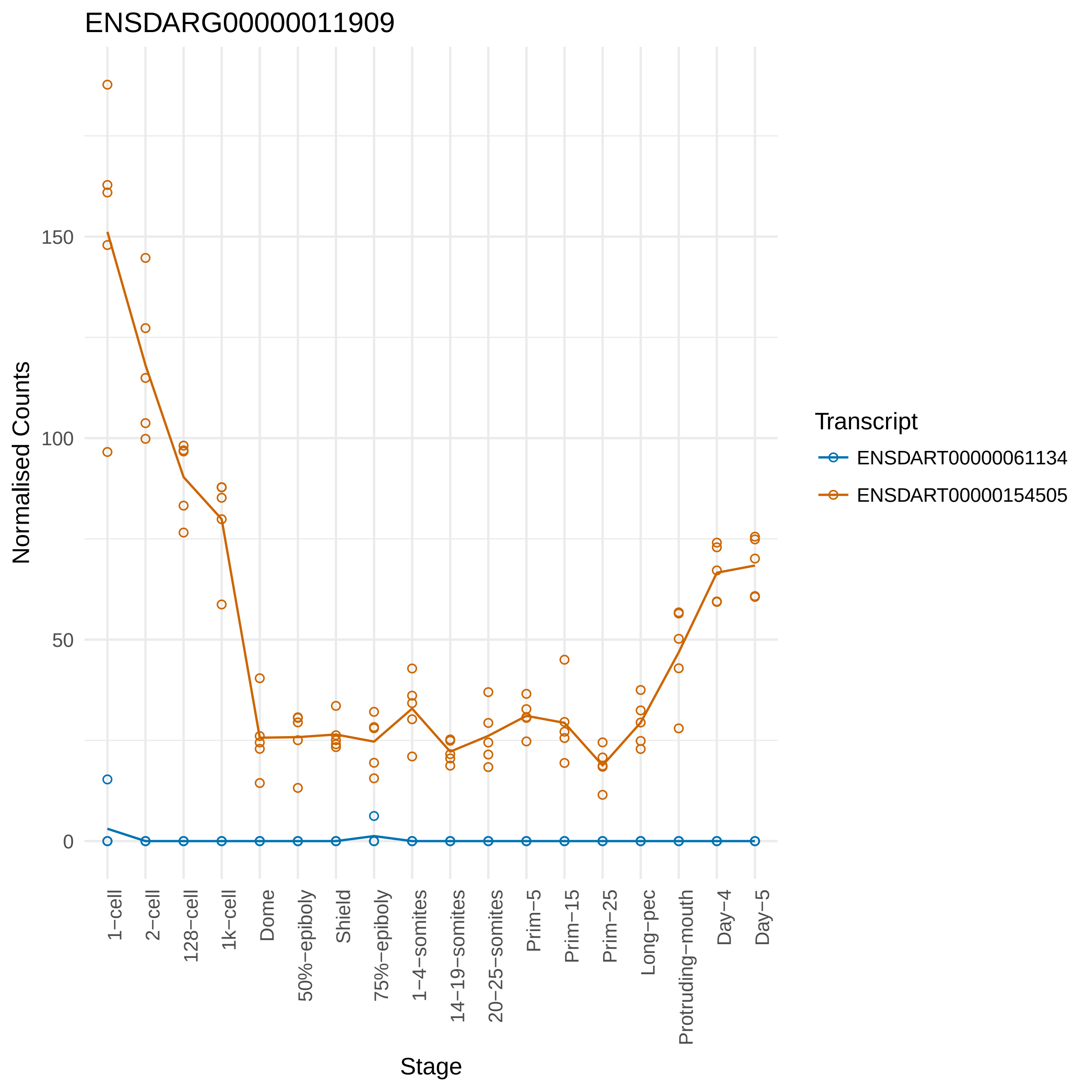

ENSDARG00000011909
Normalised Counts
150
100
50
0
Stage
1−cell
2−cell
128−cell
1k−cell
Dome
50%−epiboly
Shield
75%−epiboly
1−4−somites
14−19−somites
20−25−somites
Prim−5
Prim−15
Prim−25
Long−pec
Protruding−mouth
Day−4
Day−5
Transcript
ENSDART00000061134
ENSDART00000154505

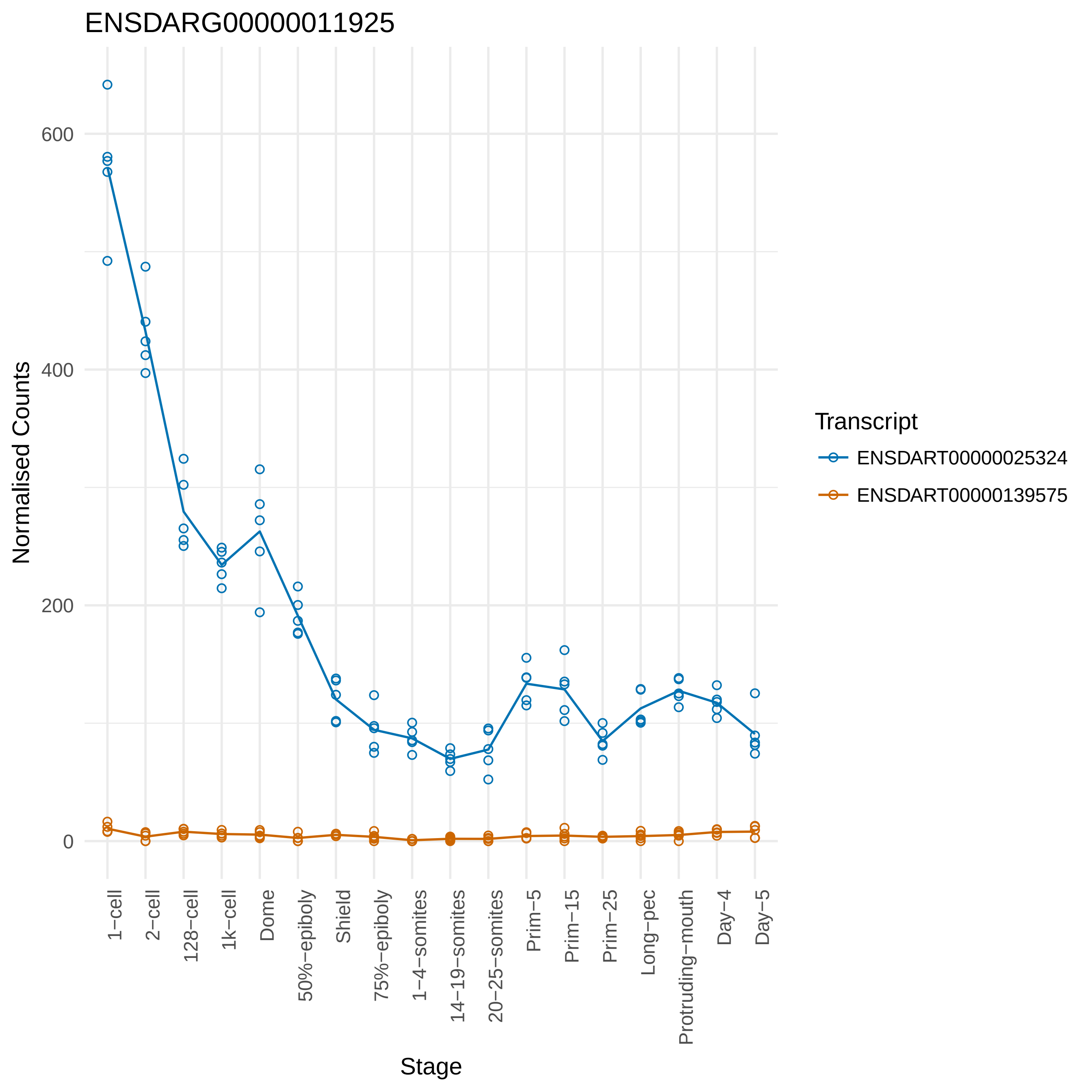
ENSDARG00000011925
Normalised Counts
600
400
200
0
1−cell
2−cell
128−cell
1k−cell
Dome
50%−epiboly
Shield
75%−epiboly
1−4−somites
14−19−somites
20−25−somites
Prim−5
Prim−15
Prim−25
Long−pec
Protruding−mouth
Day−4
Day−5
Stage
Transcript
ENSDART00000025324
ENSDART00000139575

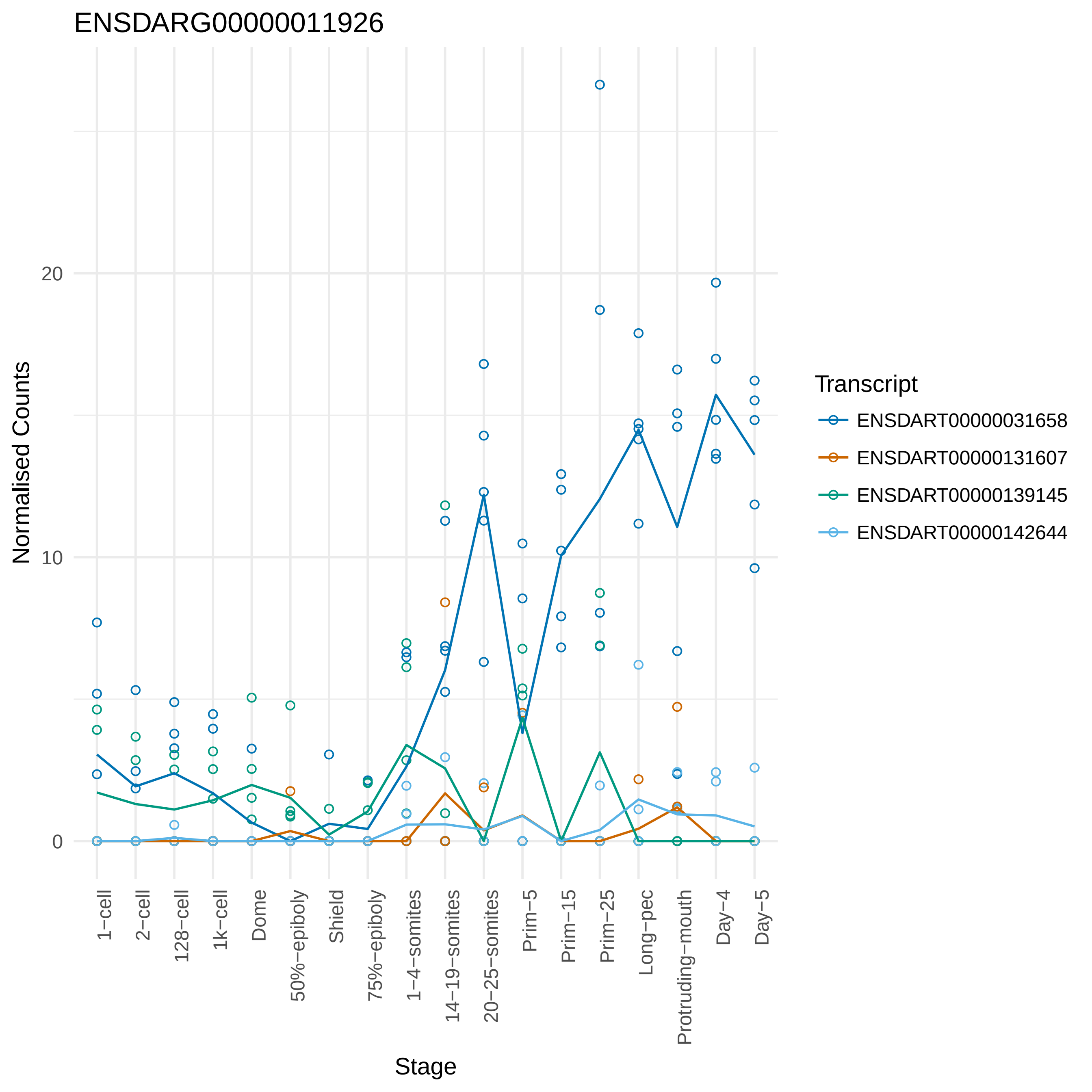

ENSDARG00000011926
Normalised Counts
0
10
20
1−cell
2−cell
128−cell
1k−cell
Dome
50%−epiboly
Shield
75%−epiboly
1−4−somites
14−19−somites
20−25−somites
Prim−5
Prim−15
Prim−25
Long−pec
Protruding−mouth
Day−4
Day−5
Stage
Transcript
ENSDART00000031658
ENSDART00000131607
ENSDART00000139145
ENSDART00000142644

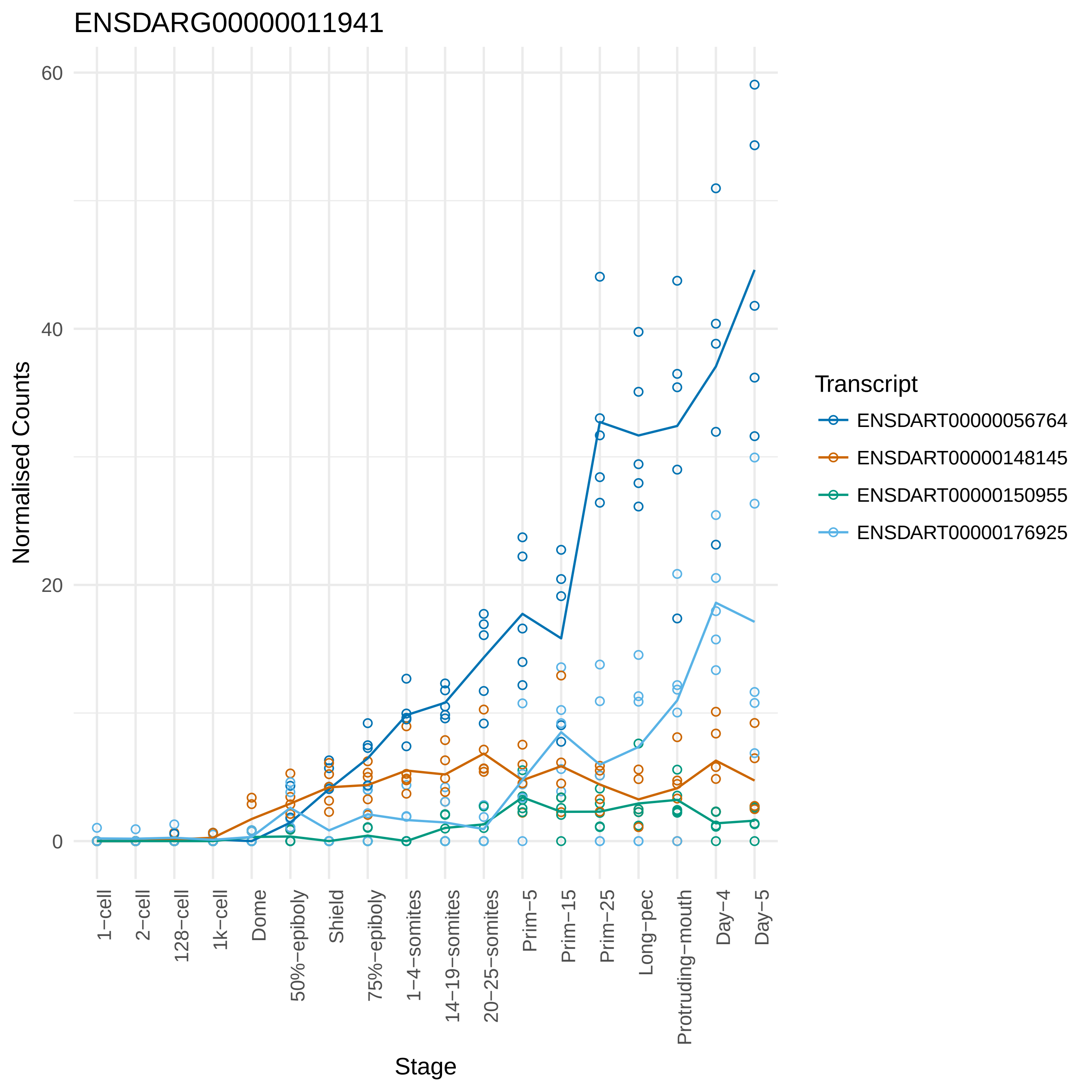
ENSDARG00000011941
Normalised Counts
0
20
40
60
1−cell
2−cell
128−cell
1k−cell
Dome
50%−epiboly
Shield
75%−epiboly
1−4−somites
14−19−somites
20−25−somites
Prim−5
Prim−15
Prim−25
Long−pec
Protruding−mouth
Day−4
Day−5
Stage
Transcript
ENSDART00000056764
ENSDART00000148145
ENSDART00000150955
ENSDART00000176925

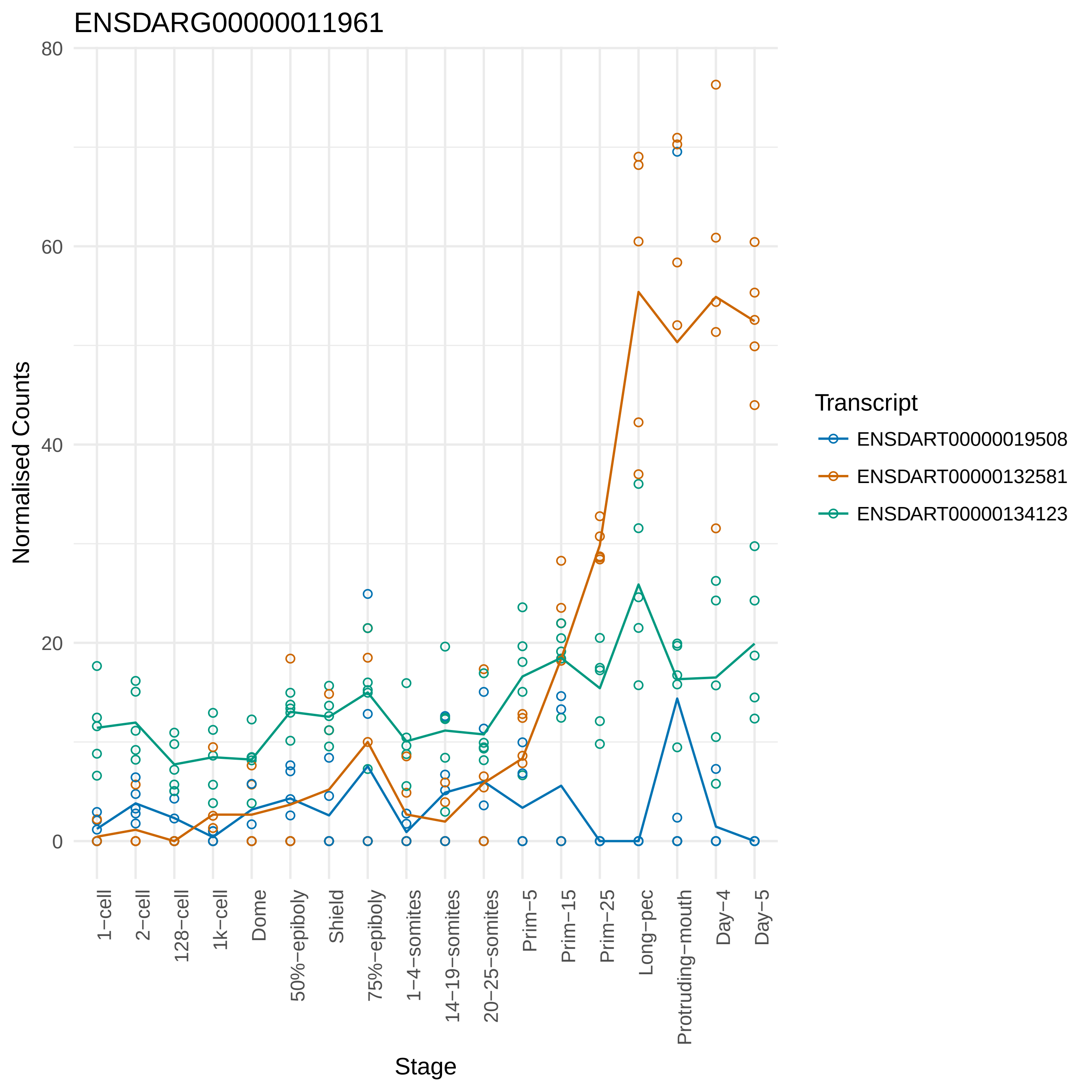
ENSDARG00000011961
Normalised Counts
80
60
40
20
0
1−cell
2−cell
128−cell
1k−cell
Dome
50%−epiboly
Shield
75%−epiboly
1−4−somites
14−19−somites
20−25−somites
Prim−5
Prim−15
Prim−25
Long−pec
Protruding−mouth
Day−4
Day−5
Stage
Transcript
ENSDART00000019508
ENSDART00000132581
ENSDART00000134123

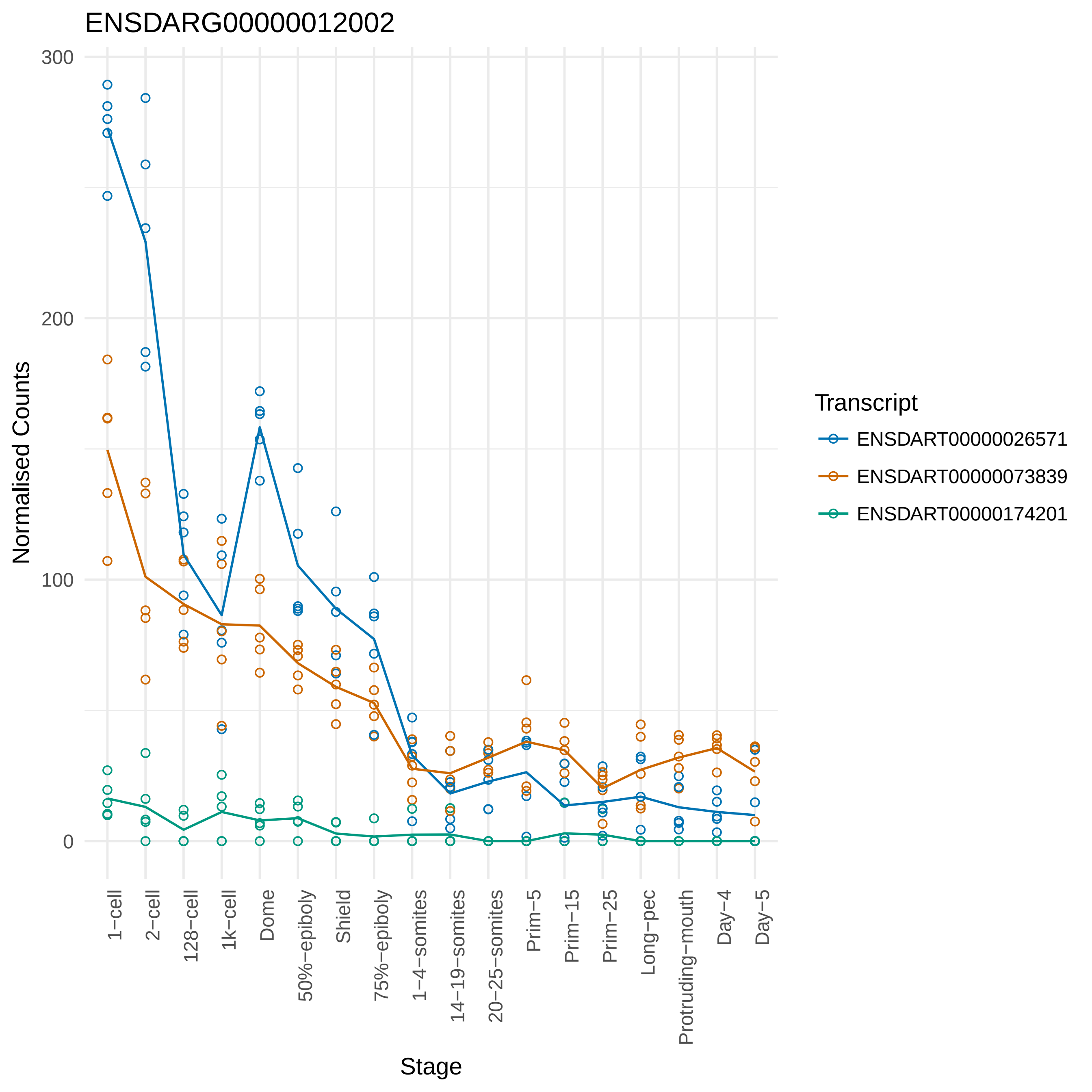

ENSDARG00000012002
Normalised Counts
300
200
100
0
1−cell
2−cell
128−cell
1k−cell
Dome
50%−epiboly
Shield
75%−epiboly
1−4−somites
14−19−somites
20−25−somites
Prim−5
Prim−15
Prim−25
Long−pec
Protruding−mouth
Day−4
Day−5
Stage
Transcript
ENSDART00000026571
ENSDART00000073839
ENSDART00000174201

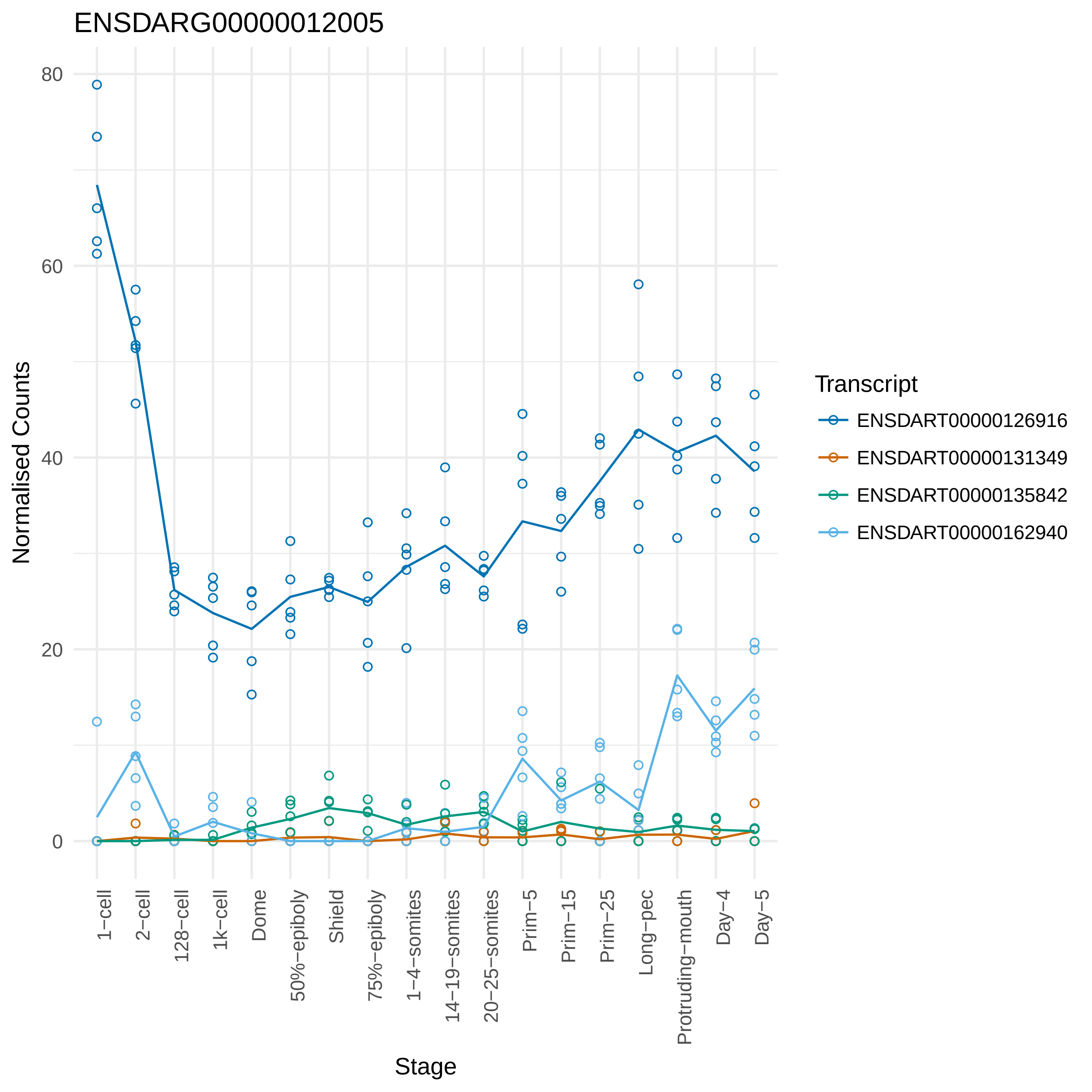
ENSDARG00000012005
Normalised Counts
80
60
40
20
0
Stage
1−cell
2−cell
128−cell
1k−cell
Dome
50%−epiboly
Shield
75%−epiboly
1−4−somites
14−19−somites
20−25−somites
Prim−5
Prim−15
Prim−25
Long−pec
Protruding−mouth
Day−4
Day−5
Transcript
ENSDART00000126916
ENSDART00000131349
ENSDART00000135842
ENSDART00000162940

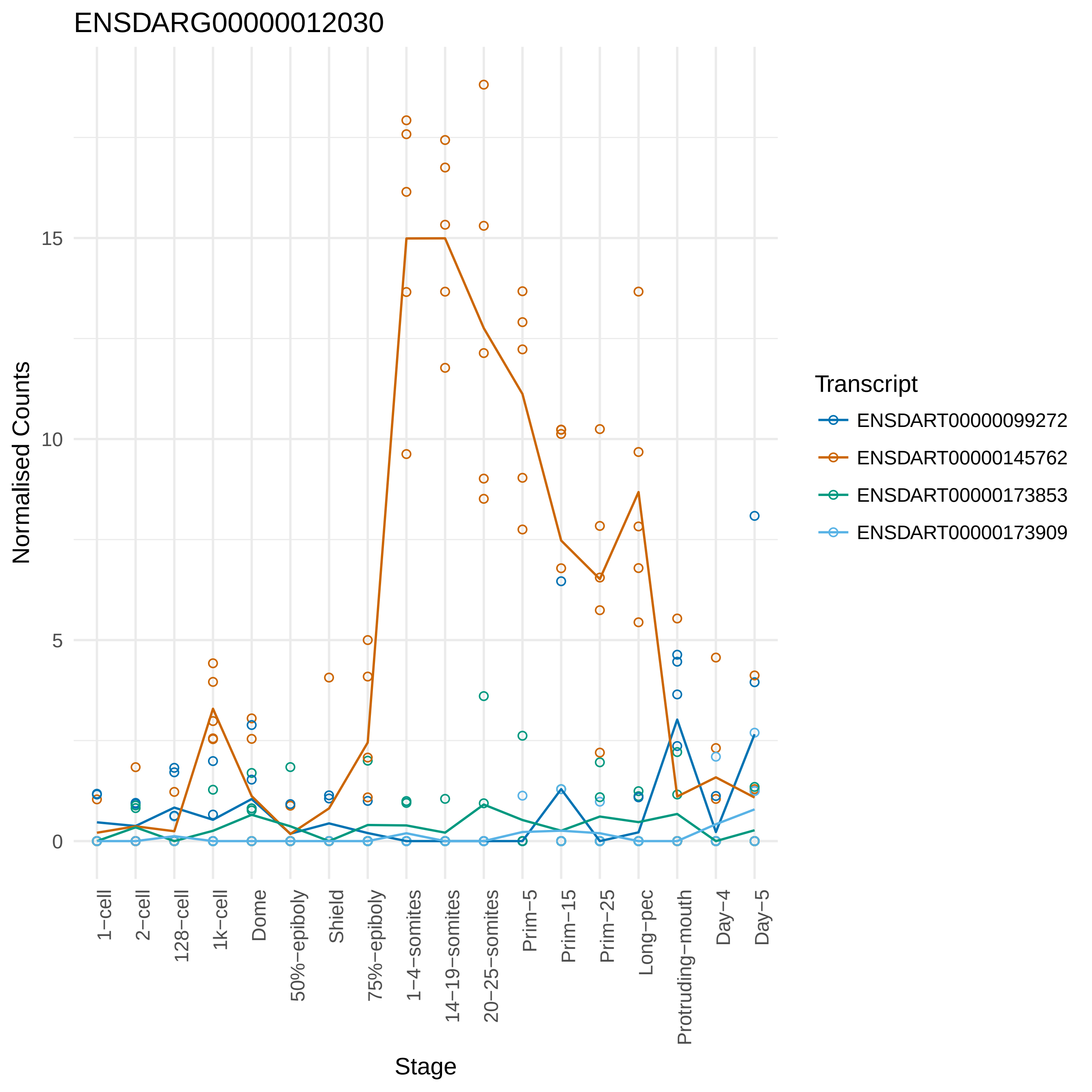

ENSDARG00000012030
Normalised Counts
0
5
10
15
1−cell
2−cell
128−cell
1k−cell
Dome
50%−epiboly
Shield
75%−epiboly
1−4−somites
14−19−somites
20−25−somites
Prim−5
Prim−15
Prim−25
Long−pec
Protruding−mouth
Day−4
Day−5
Stage
Transcript
ENSDART00000099272
ENSDART00000145762
ENSDART00000173853
ENSDART00000173909

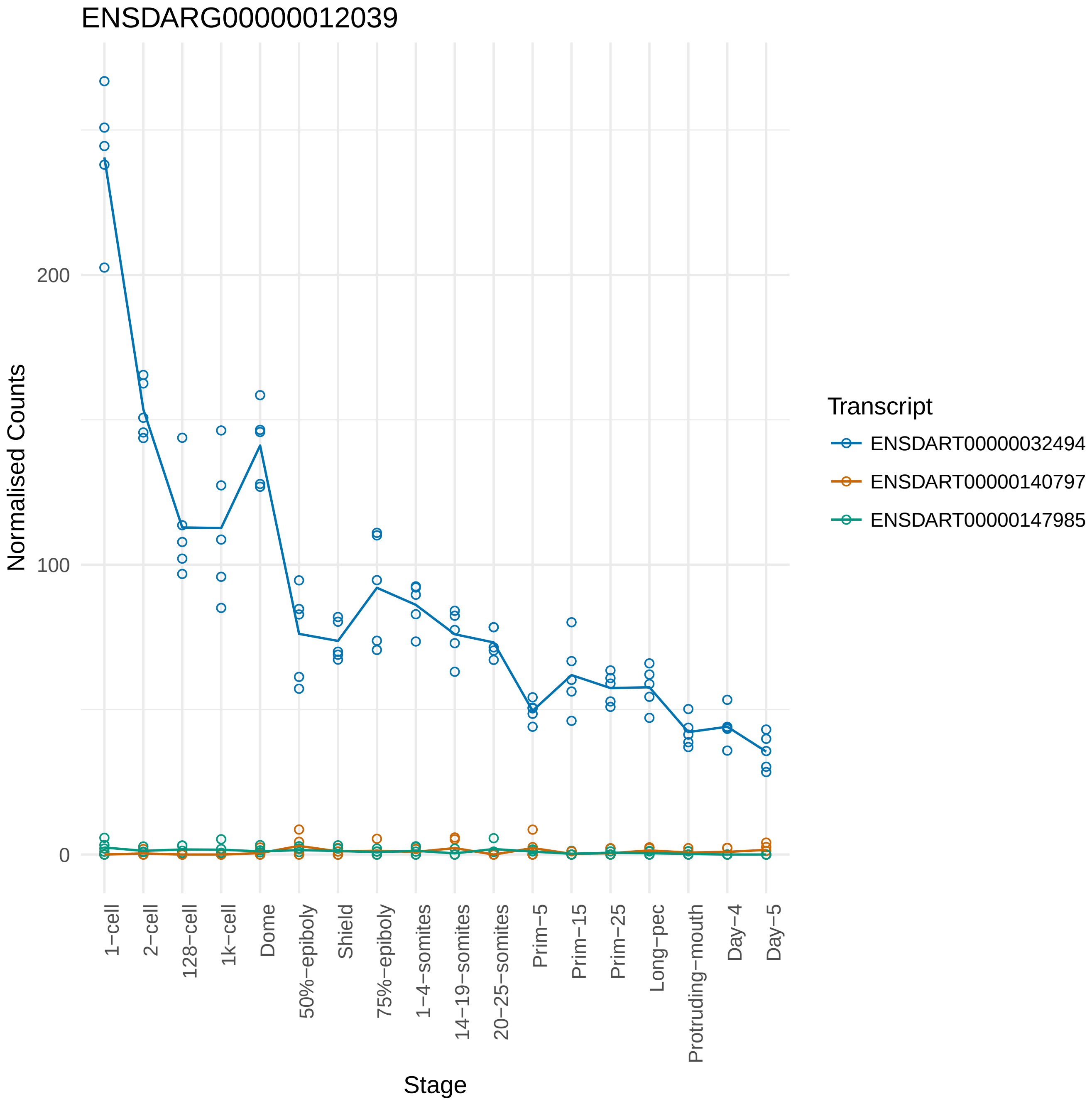

ENSDARG00000012039
Normalised Counts
200
100
0
1−cell
2−cell
128−cell
1k−cell
Dome
50%−epiboly
Shield
75%−epiboly
1−4−somites
14−19−somites
20−25−somites
Prim−5
Prim−15
Prim−25
Long−pec
Protruding−mouth
Day−4
Day−5
Stage
Transcript
ENSDART00000032494
ENSDART00000140797
ENSDART00000147985

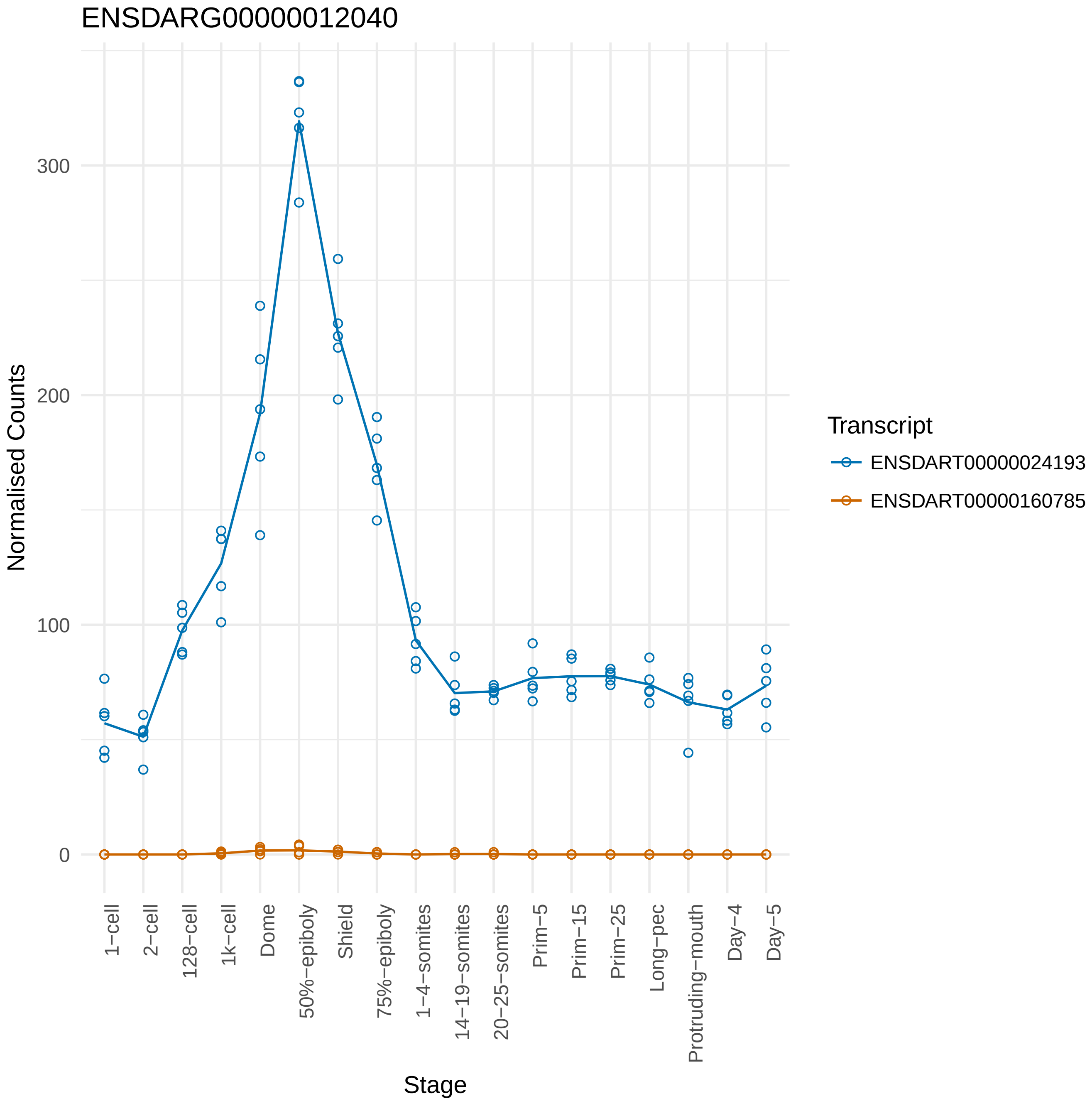

ENSDARG00000012040
Normalised Counts
300
200
100
0
1−cell
2−cell
128−cell
1k−cell
Dome
50%−epiboly
Shield
75%−epiboly
1−4−somites
14−19−somites
20−25−somites
Prim−5
Prim−15
Prim−25
Long−pec
Protruding−mouth
Day−4
Day−5
Stage
Transcript
ENSDART00000024193
ENSDART00000160785

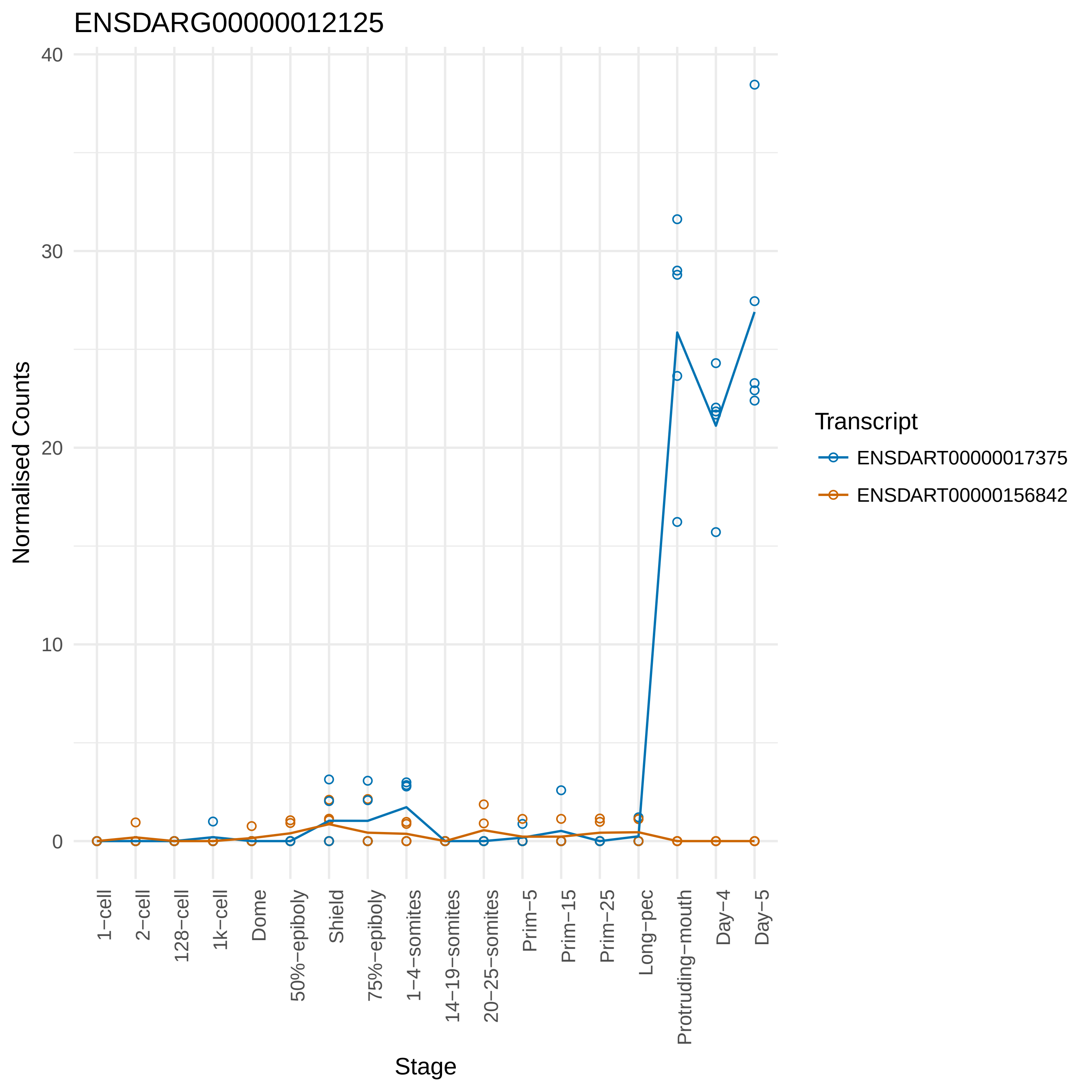

ENSDARG00000012125
Normalised Counts
0
10
20
30
40
Stage
1−cell
2−cell
128−cell
1k−cell
Dome
50%−epiboly
Shield
75%−epiboly
1−4−somites
14−19−somites
20−25−somites
Prim−5
Prim−15
Prim−25
Long−pec
Protruding−mouth
Day−4
Day−5
Transcript
ENSDART00000017375
ENSDART00000156842

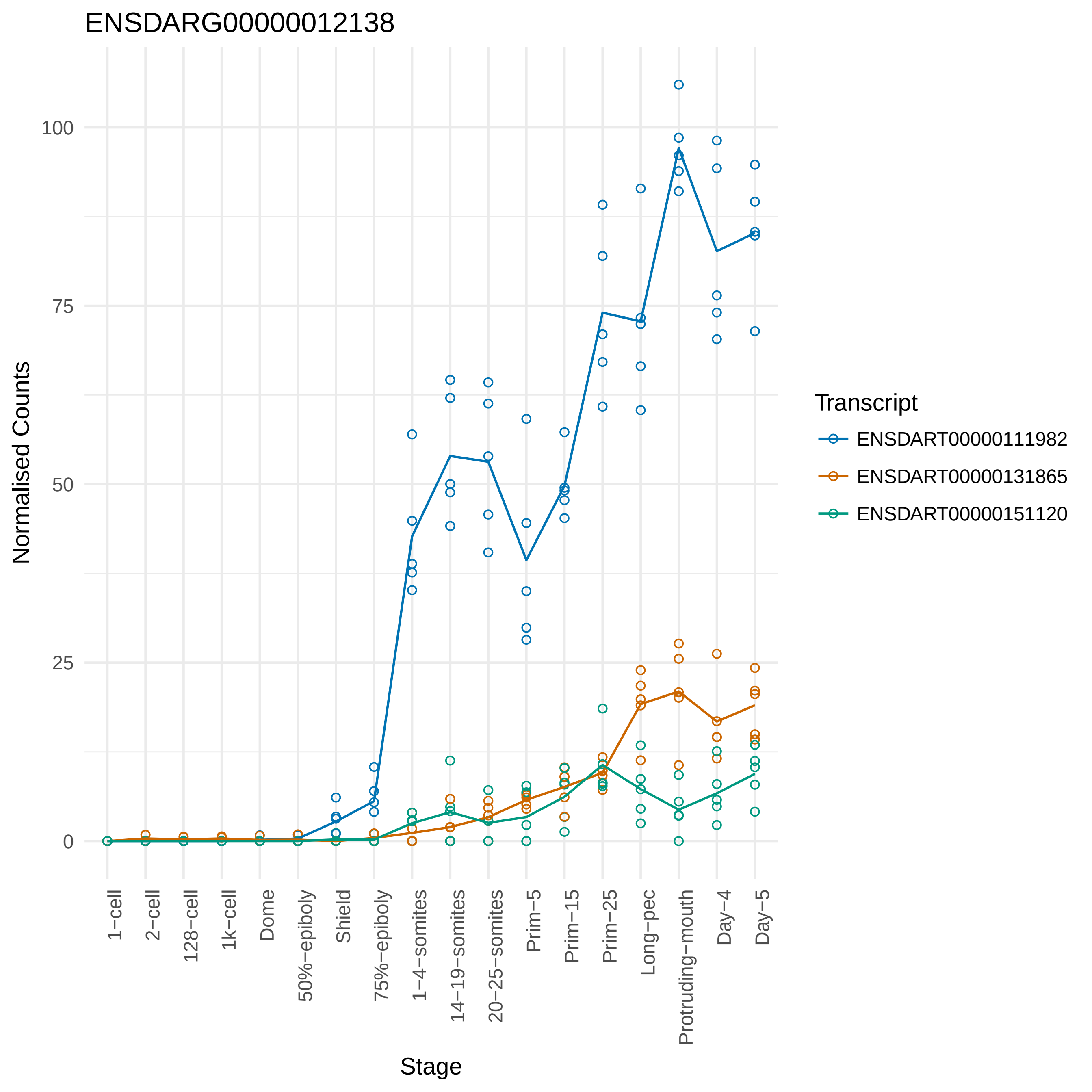

ENSDARG00000012138
Normalised Counts
100
75
50
25
0
1−cell
2−cell
128−cell
1k−cell
Dome
50%−epiboly
Shield
75%−epiboly
1−4−somites
14−19−somites
20−25−somites
Prim−5
Prim−15
Prim−25
Long−pec
Protruding−mouth
Day−4
Day−5
Stage
Transcript
ENSDART00000111982
ENSDART00000131865
ENSDART00000151120

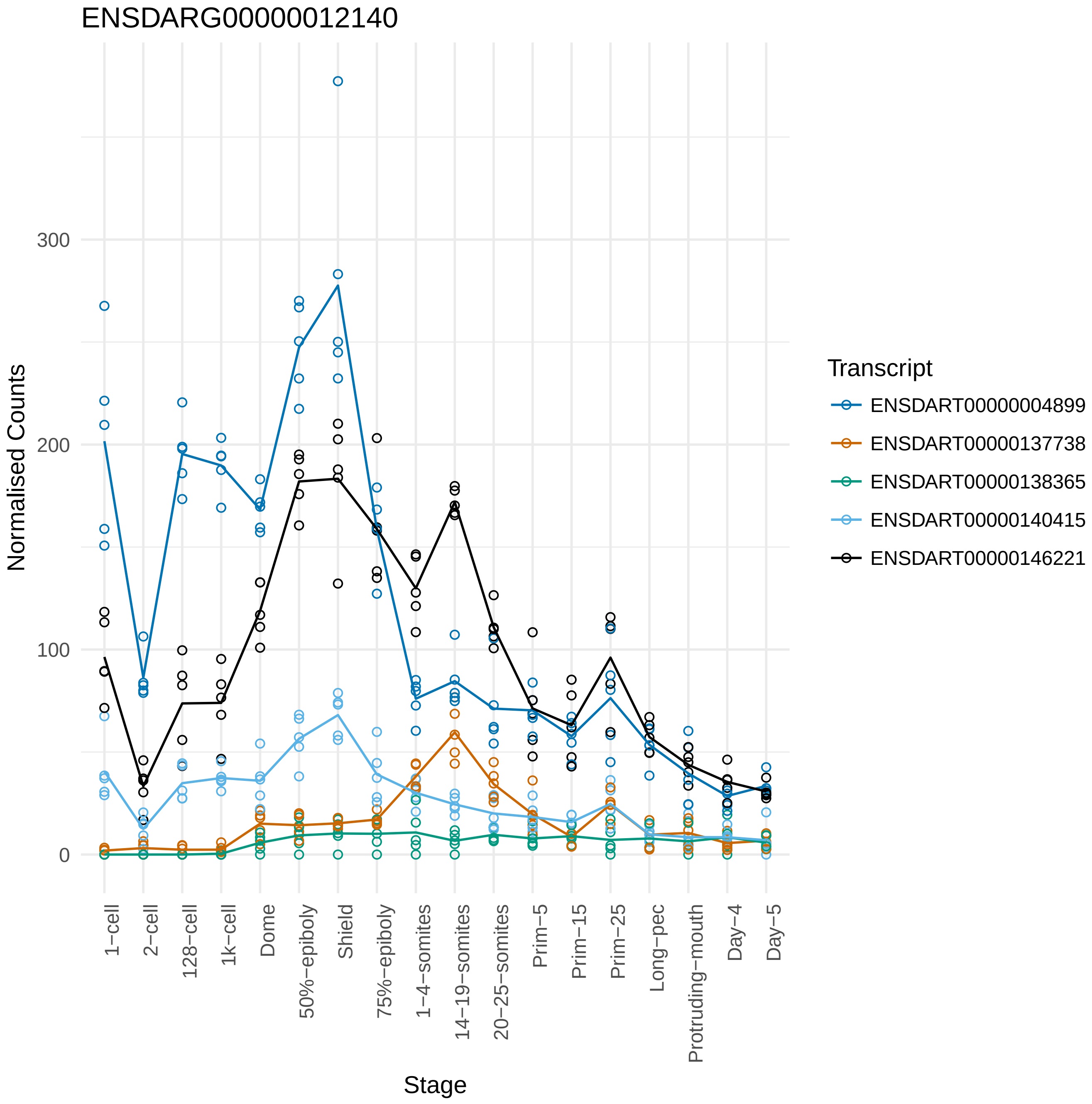

ENSDARG00000012140
Normalised Counts
0
100
200
300
1−cell
2−cell
128−cell
1k−cell
Dome
50%−epiboly
Shield
75%−epiboly
1−4−somites
14−19−somites
20−25−somites
Prim−5
Prim−15
Prim−25
Long−pec
Protruding−mouth
Day−4
Day−5
Stage
Transcript
ENSDART00000004899
ENSDART00000137738
ENSDART00000138365
ENSDART00000140415
ENSDART00000146221

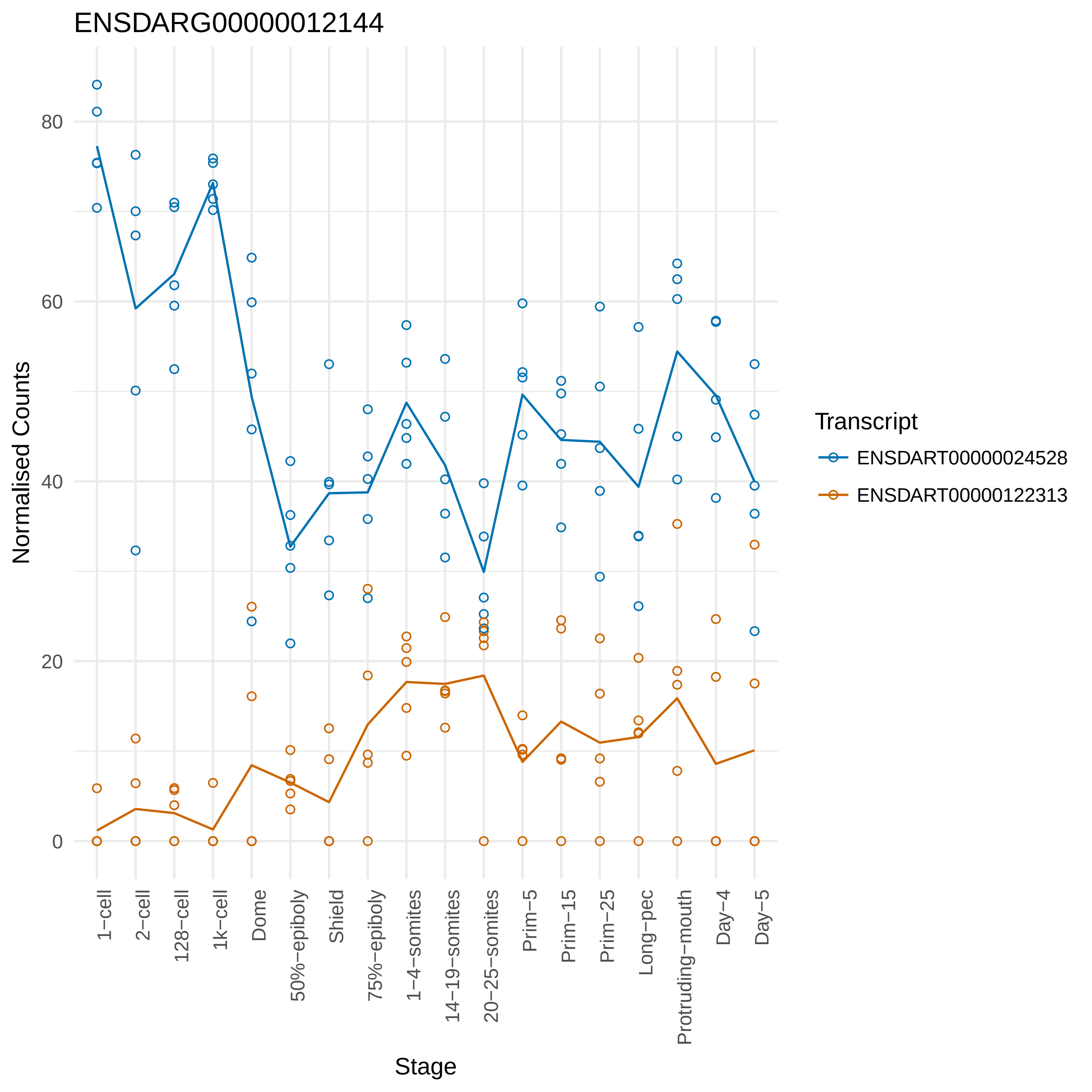

ENSDARG00000012144
Normalised Counts
80
60
40
20
0
1−cell
2−cell
128−cell
1k−cell
Dome
50%−epiboly
Shield
75%−epiboly
1−4−somites
14−19−somites
20−25−somites
Prim−5
Prim−15
Prim−25
Long−pec
Protruding−mouth
Day−4
Day−5
Stage
Transcript
ENSDART00000024528
ENSDART00000122313

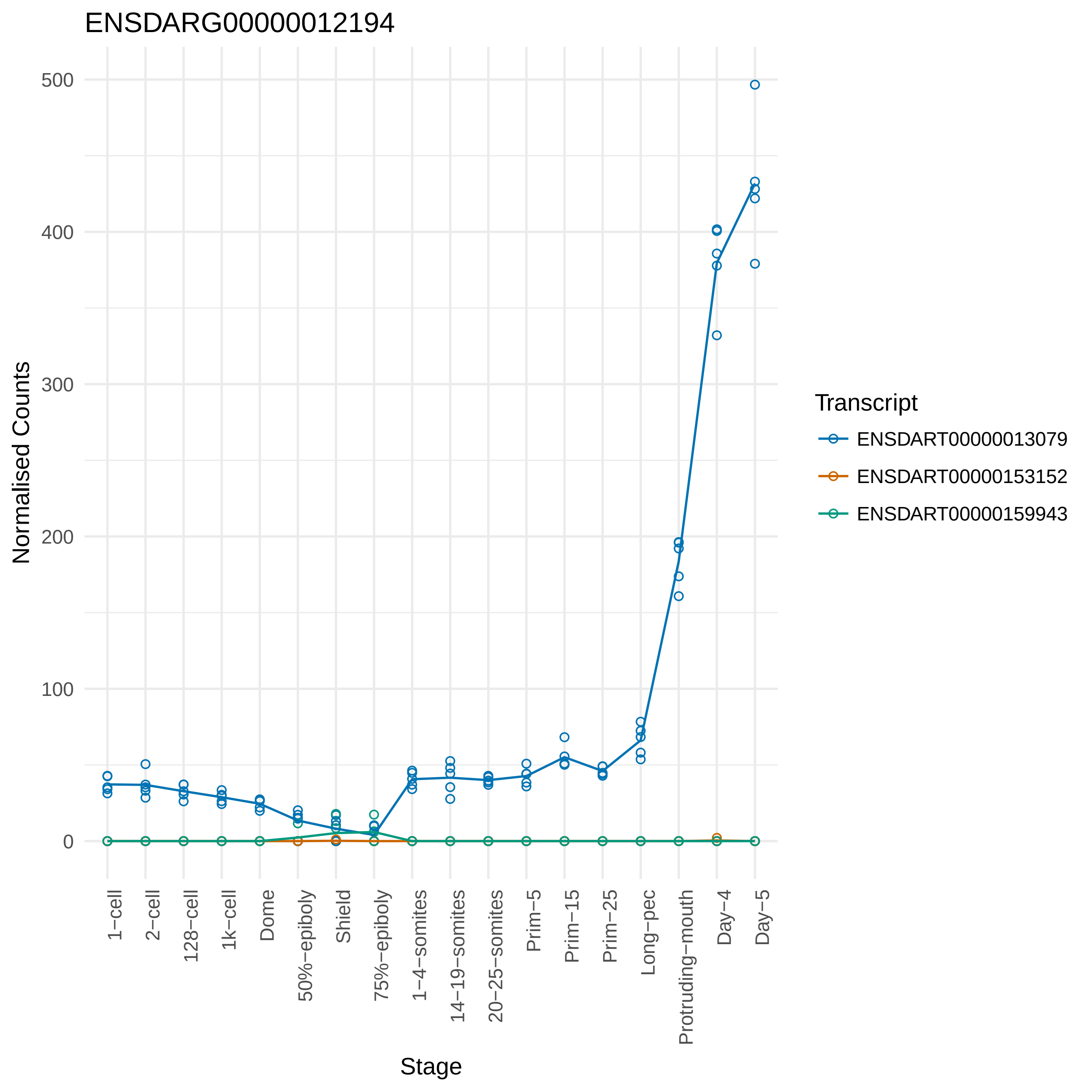
ENSDARG00000012194
Normalised Counts
500
400
300
200
100
0
1−cell
2−cell
128−cell
1k−cell
Dome
50%−epiboly
Shield
75%−epiboly
1−4−somites
14−19−somites
20−25−somites
Prim−5
Prim−15
Prim−25
Long−pec
Protruding−mouth
Day−4
Day−5
Stage
Transcript
ENSDART00000013079
ENSDART00000153152
ENSDART00000159943

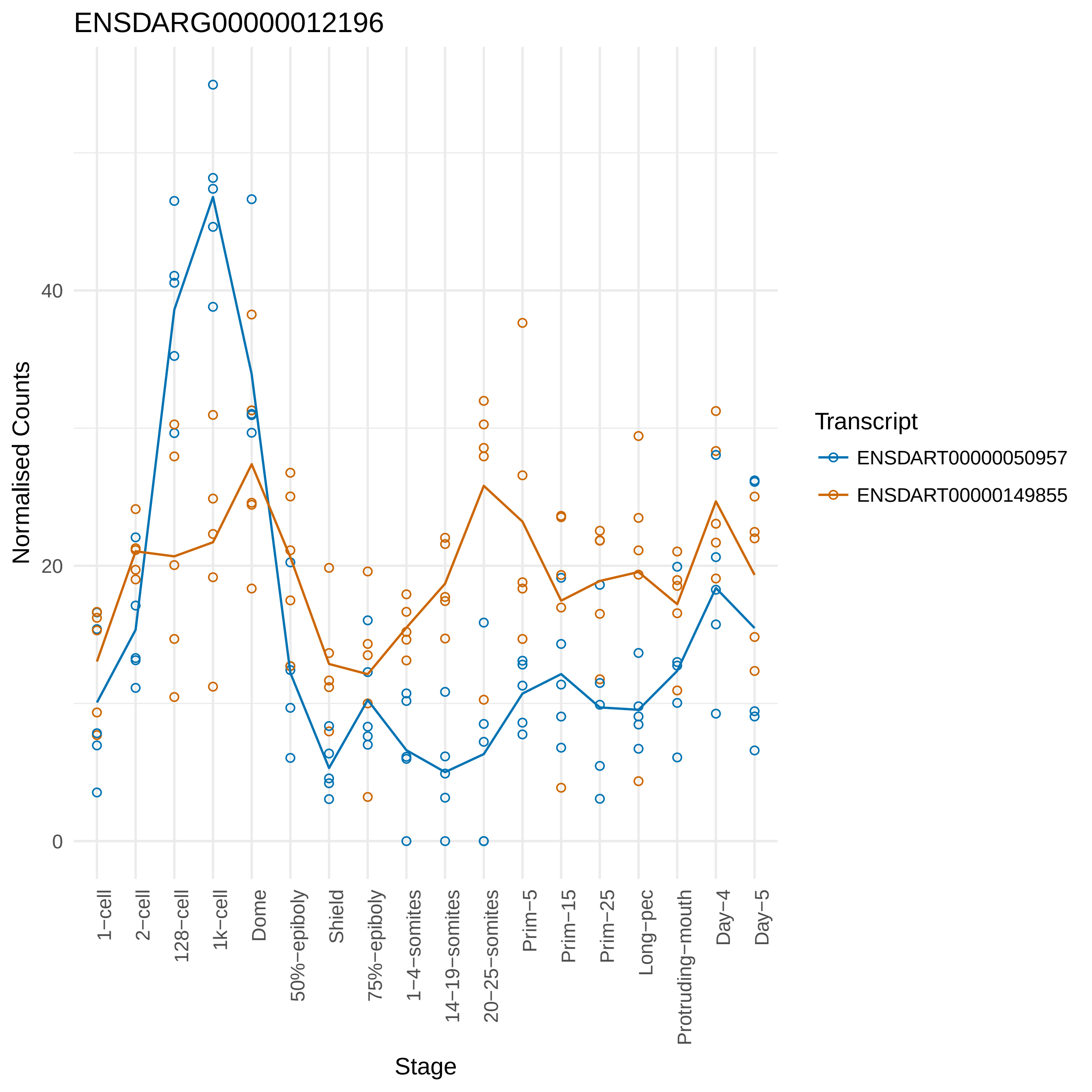
ENSDARG00000012196
Normalised Counts
0
20
40
Stage
1−cell
2−cell
128−cell
1k−cell
Dome
50%−epiboly
Shield
75%−epiboly
1−4−somites
14−19−somites
20−25−somites
Prim−5
Prim−15
Prim−25
Long−pec
Protruding−mouth
Day−4
Day−5
Transcript
ENSDART00000050957
ENSDART00000149855

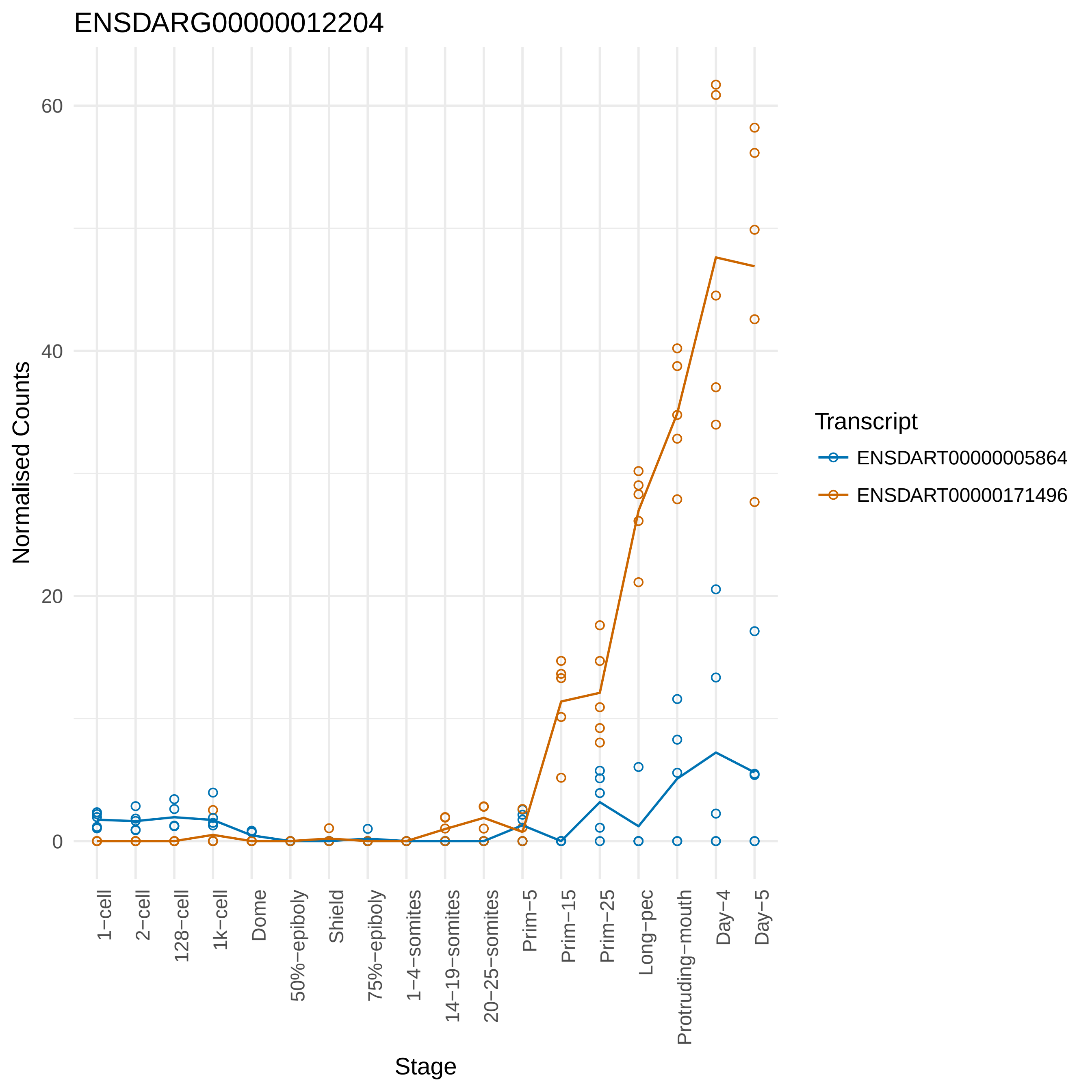

ENSDARG00000012204
Normalised Counts
60
40
20
0
1−cell
2−cell
128−cell
1k−cell
Dome
50%−epiboly
Shield
75%−epiboly
1−4−somites
14−19−somites
20−25−somites
Prim−5
Prim−15
Prim−25
Long−pec
Protruding−mouth
Day−4
Day−5
Stage
Transcript
ENSDART00000005864
ENSDART00000171496

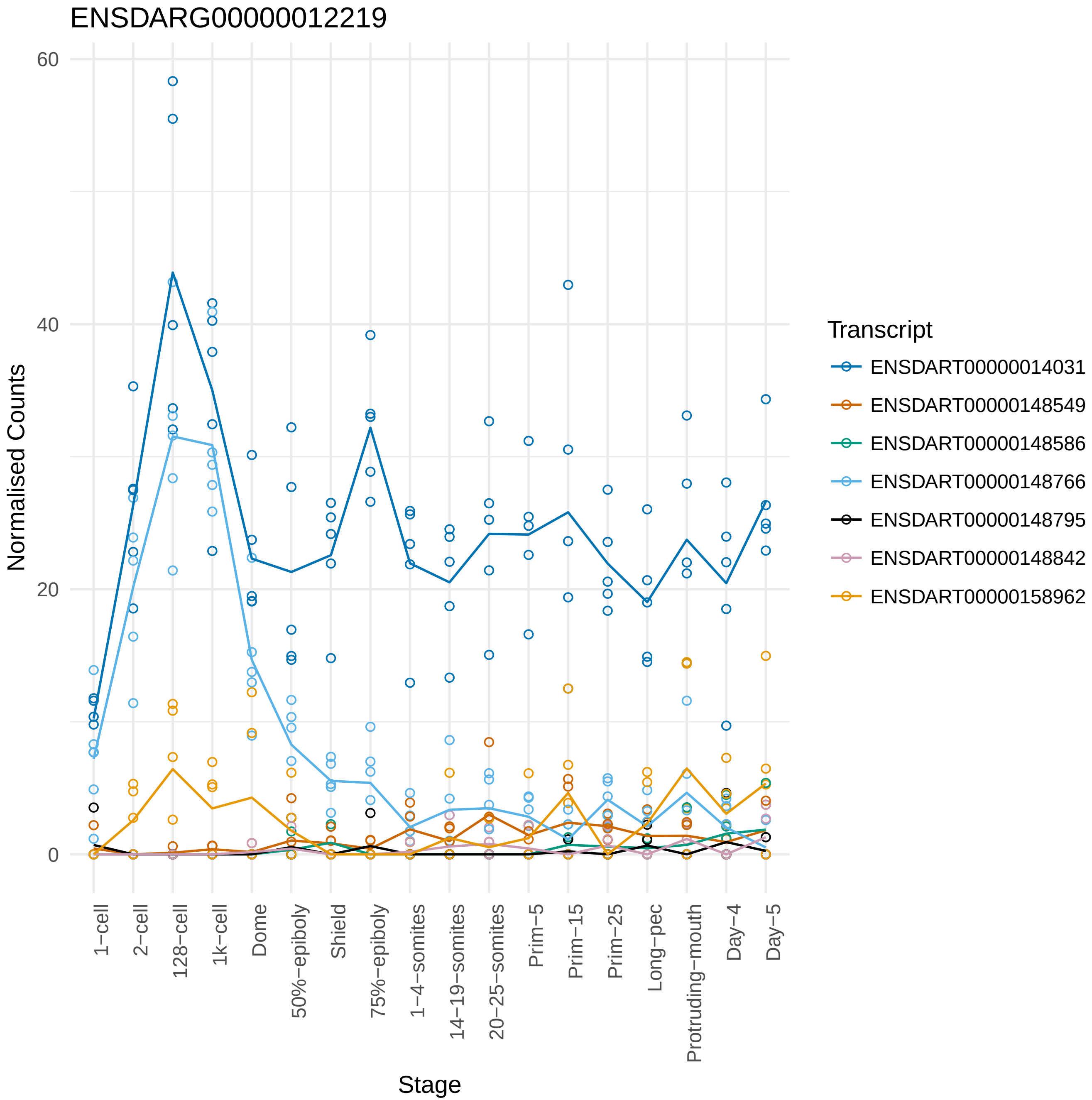

ENSDARG00000012219
Normalised Counts
0
20
40
60
1−cell
2−cell
128−cell
1k−cell
Dome
50%−epiboly
Shield
75%−epiboly
1−4−somites
14−19−somites
20−25−somites
Prim−5
Prim−15
Prim−25
Long−pec
Protruding−mouth
Day−4
Day−5
Stage
Transcript
ENSDART00000014031
ENSDART00000148549
ENSDART00000148586
ENSDART00000148766
ENSDART00000148795
ENSDART00000148842
ENSDART00000158962

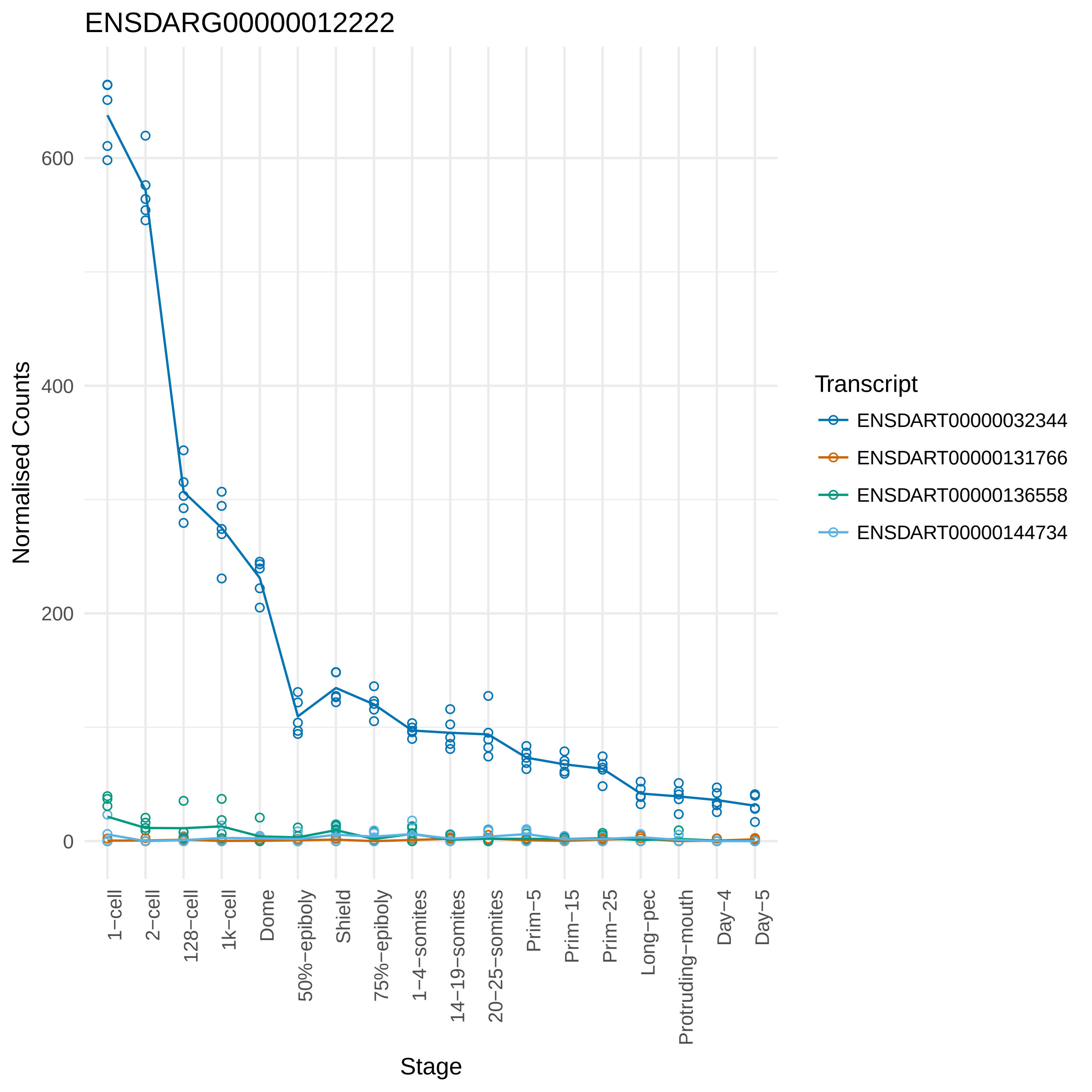

ENSDARG00000012222
Normalised Counts
600
400
200
0
1−cell
2−cell
128−cell
1k−cell
Dome
50%−epiboly
Shield
75%−epiboly
1−4−somites
14−19−somites
20−25−somites
Prim−5
Prim−15
Prim−25
Long−pec
Protruding−mouth
Day−4
Day−5
Stage
Transcript
ENSDART00000032344
ENSDART00000131766
ENSDART00000136558
ENSDART00000144734

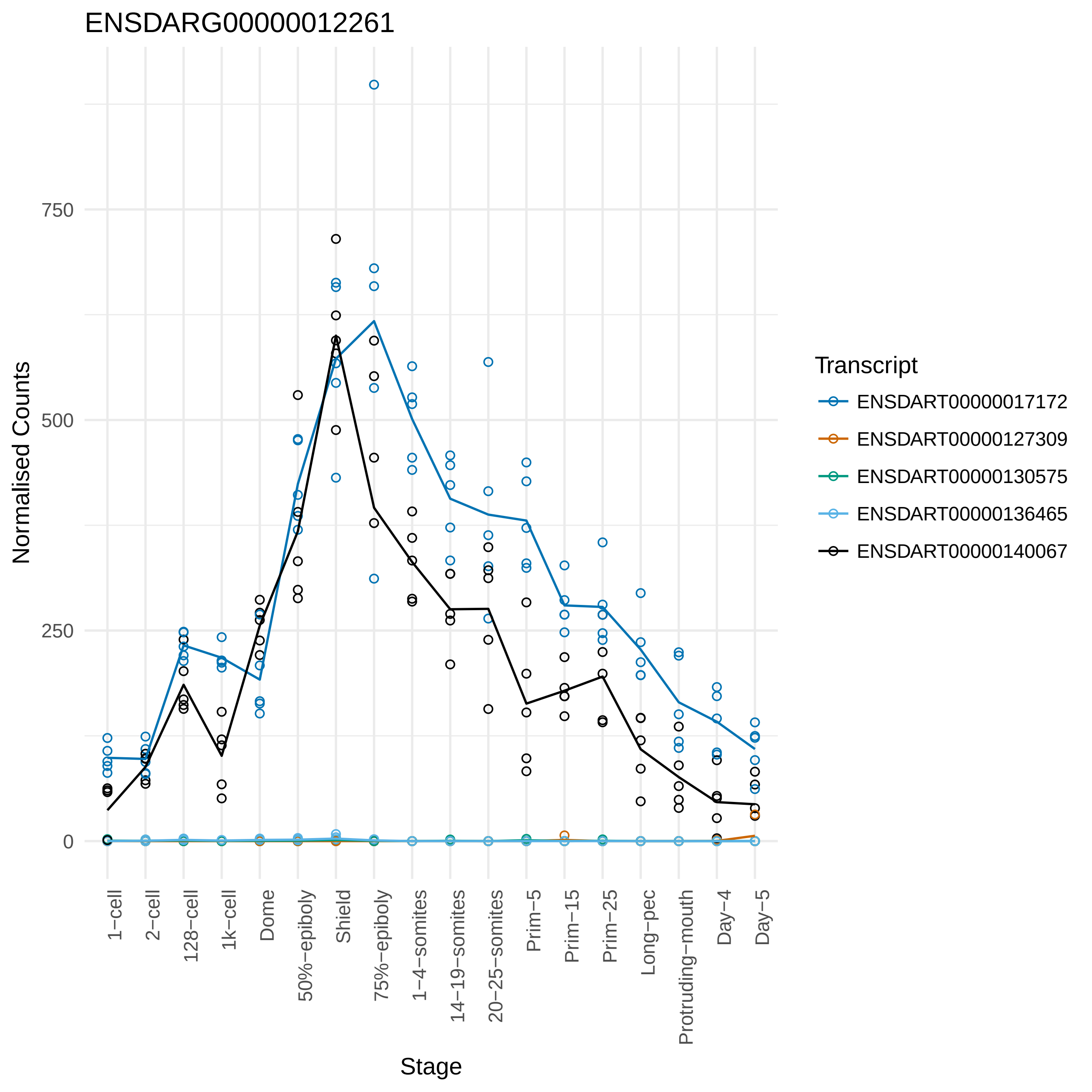

ENSDARG00000012261
Normalised Counts
750
500
250
0
1−cell
2−cell
128−cell
1k−cell
Dome
50%−epiboly
Shield
75%−epiboly
1−4−somites
14−19−somites
20−25−somites
Prim−5
Prim−15
Prim−25
Long−pec
Protruding−mouth
Day−4
Day−5
Stage
Transcript
ENSDART00000017172
ENSDART00000127309
ENSDART00000130575
ENSDART00000136465
ENSDART00000140067

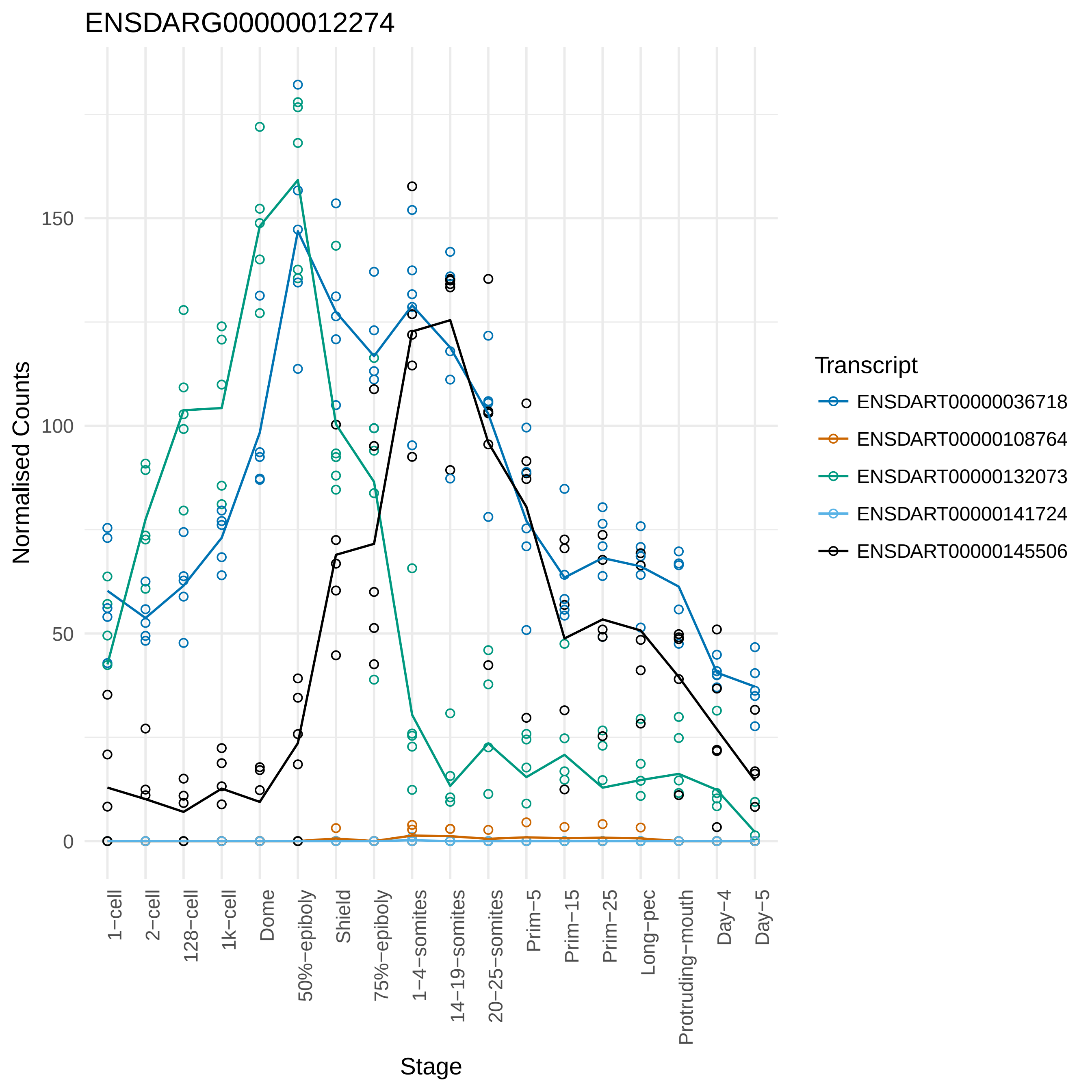
ENSDARG00000012274
Normalised Counts
150
100
50
0
1−cell
2−cell
128−cell
1k−cell
Dome
50%−epiboly
Shield
75%−epiboly
1−4−somites
14−19−somites
20−25−somites
Prim−5
Prim−15
Prim−25
Long−pec
Protruding−mouth
Day−4
Day−5
Stage
Transcript
ENSDART00000036718
ENSDART00000108764
ENSDART00000132073
ENSDART00000141724
ENSDART00000145506

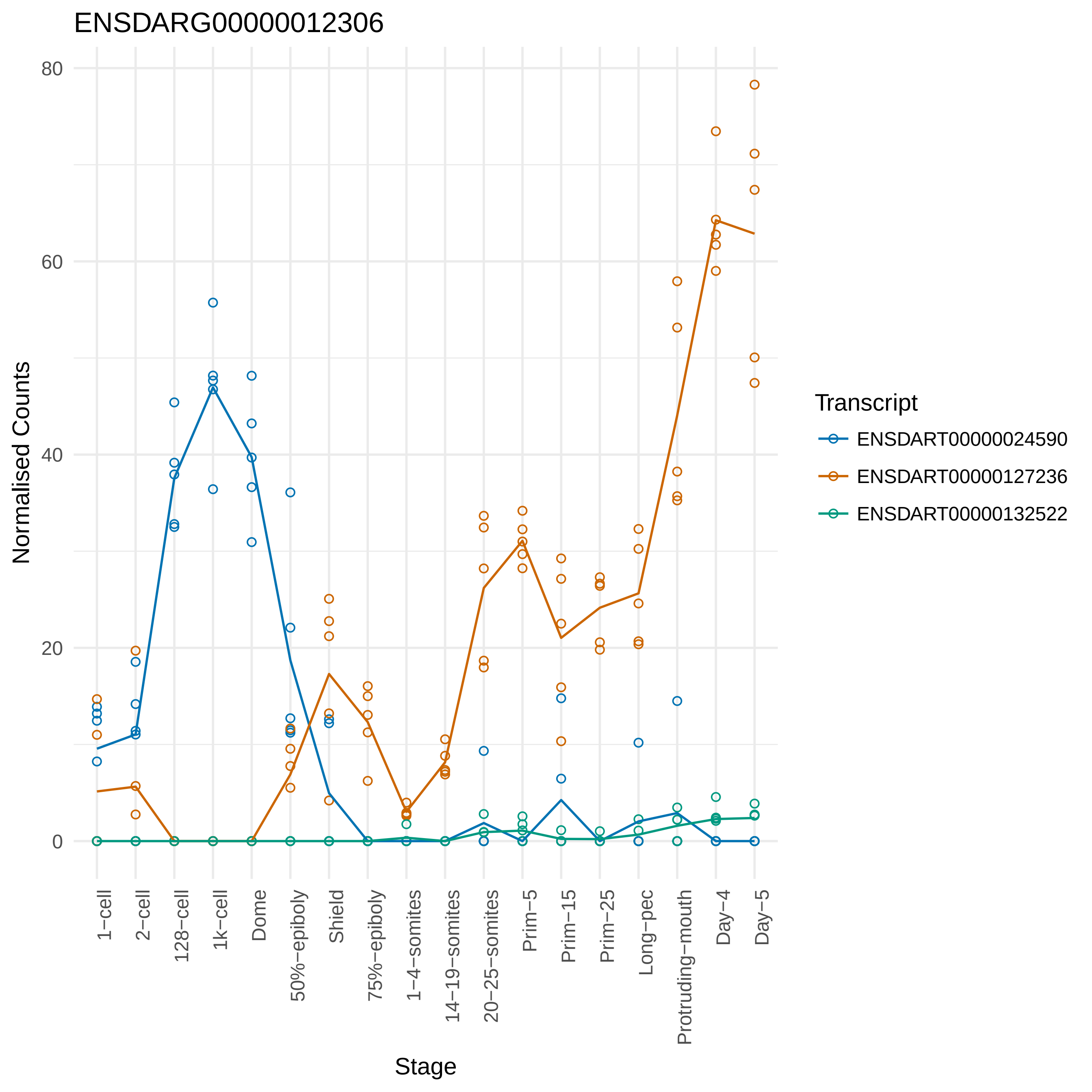
ENSDARG00000012306
Normalised Counts
0
20
40
60
80
1−cell
2−cell
128−cell
1k−cell
Dome
50%−epiboly
Shield
75%−epiboly
1−4−somites
14−19−somites
20−25−somites
Prim−5
Prim−15
Prim−25
Long−pec
Protruding−mouth
Day−4
Day−5
Stage
Transcript
ENSDART00000024590
ENSDART00000127236
ENSDART00000132522

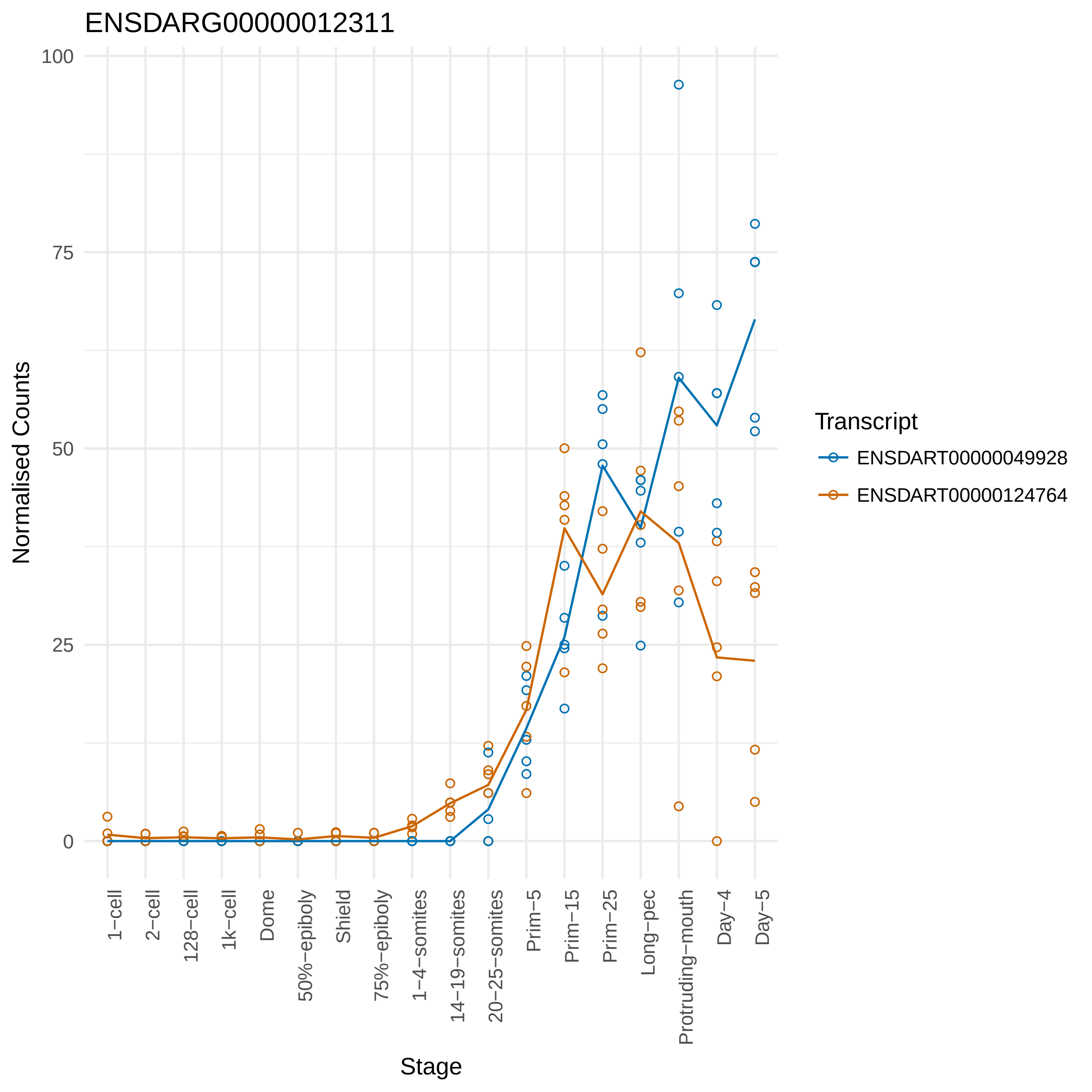

ENSDARG00000012311
Normalised Counts
100
75
50
25
0
1−cell
2−cell
128−cell
1k−cell
Dome
50%−epiboly
Shield
75%−epiboly
1−4−somites
14−19−somites
20−25−somites
Prim−5
Prim−15
Prim−25
Long−pec
Protruding−mouth
Day−4
Day−5
Stage
Transcript
ENSDART00000049928
ENSDART00000124764

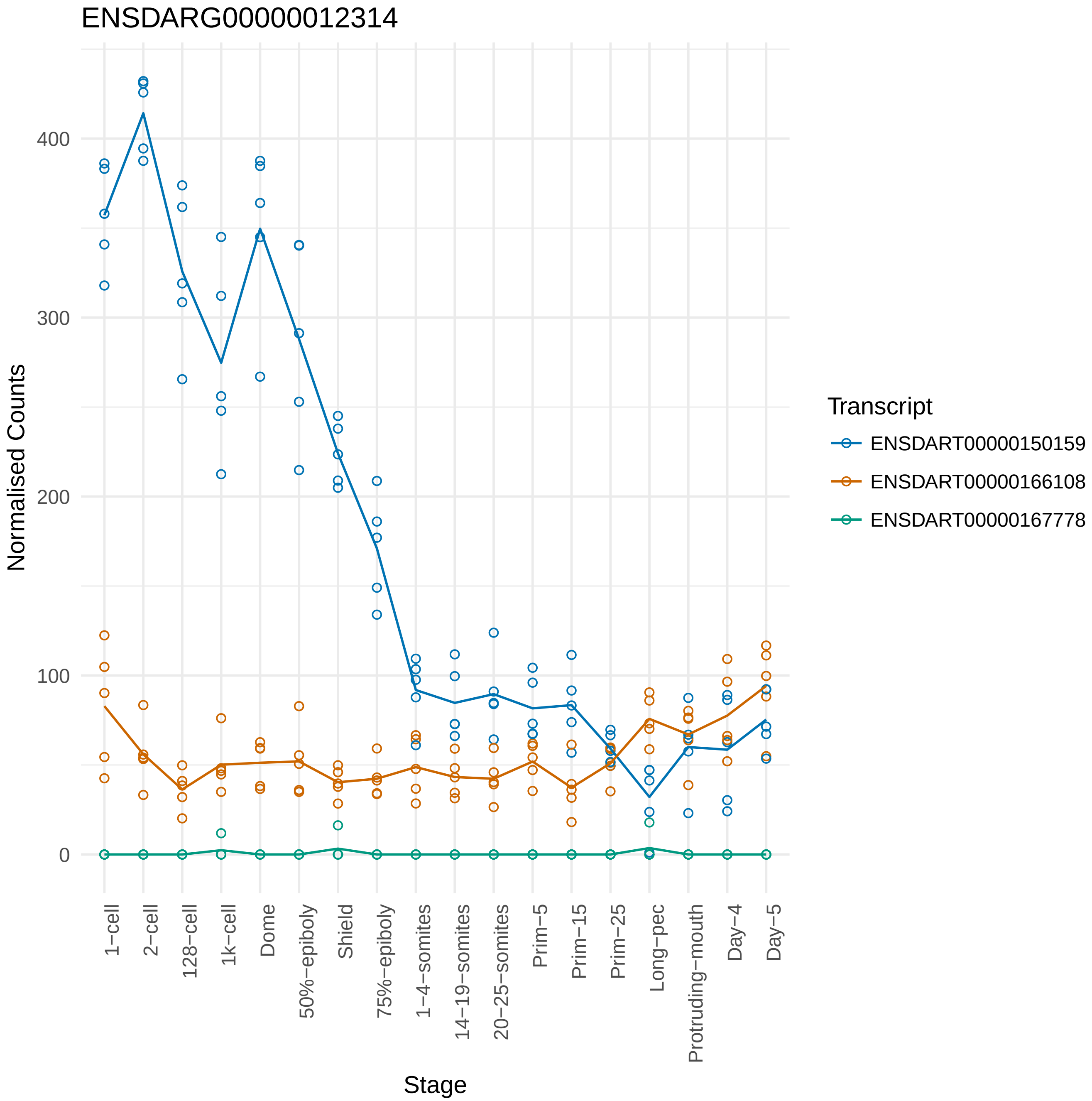

ENSDARG00000012314
Normalised Counts
0
100
200
300
400
Stage
1−cell
2−cell
128−cell
1k−cell
Dome
50%−epiboly
Shield
75%−epiboly
1−4−somites
14−19−somites
20−25−somites
Prim−5
Prim−15
Prim−25
Long−pec
Protruding−mouth
Day−4
Day−5
Transcript
ENSDART00000150159
ENSDART00000166108
ENSDART00000167778

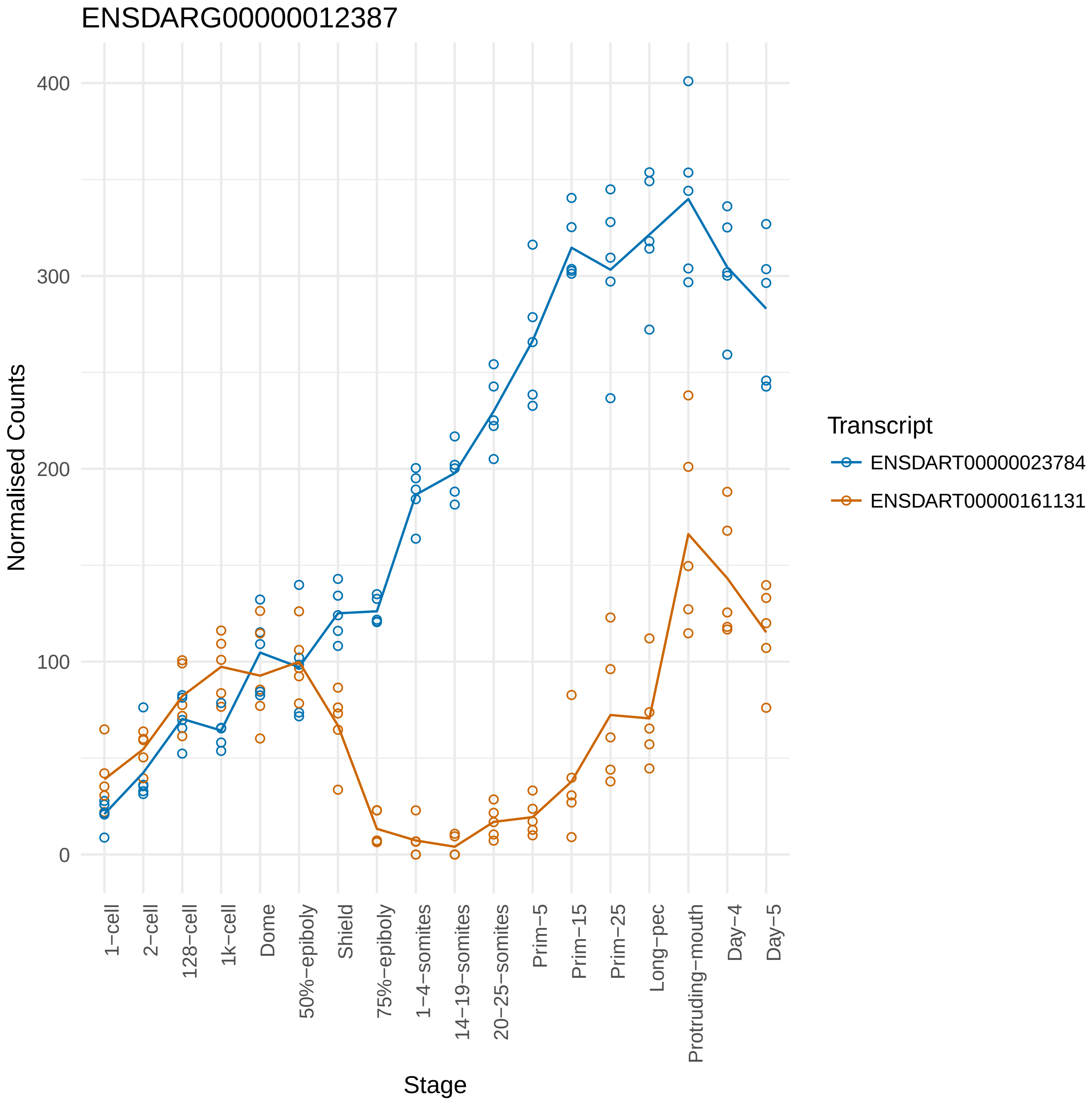

ENSDARG00000012387
Normalised Counts
400
300
200
100
0
1−cell
2−cell
128−cell
1k−cell
Dome
50%−epiboly
Shield
75%−epiboly
1−4−somites
14−19−somites
20−25−somites
Prim−5
Prim−15
Prim−25
Long−pec
Protruding−mouth
Day−4
Day−5
Stage
Transcript
ENSDART00000023784
ENSDART00000161131

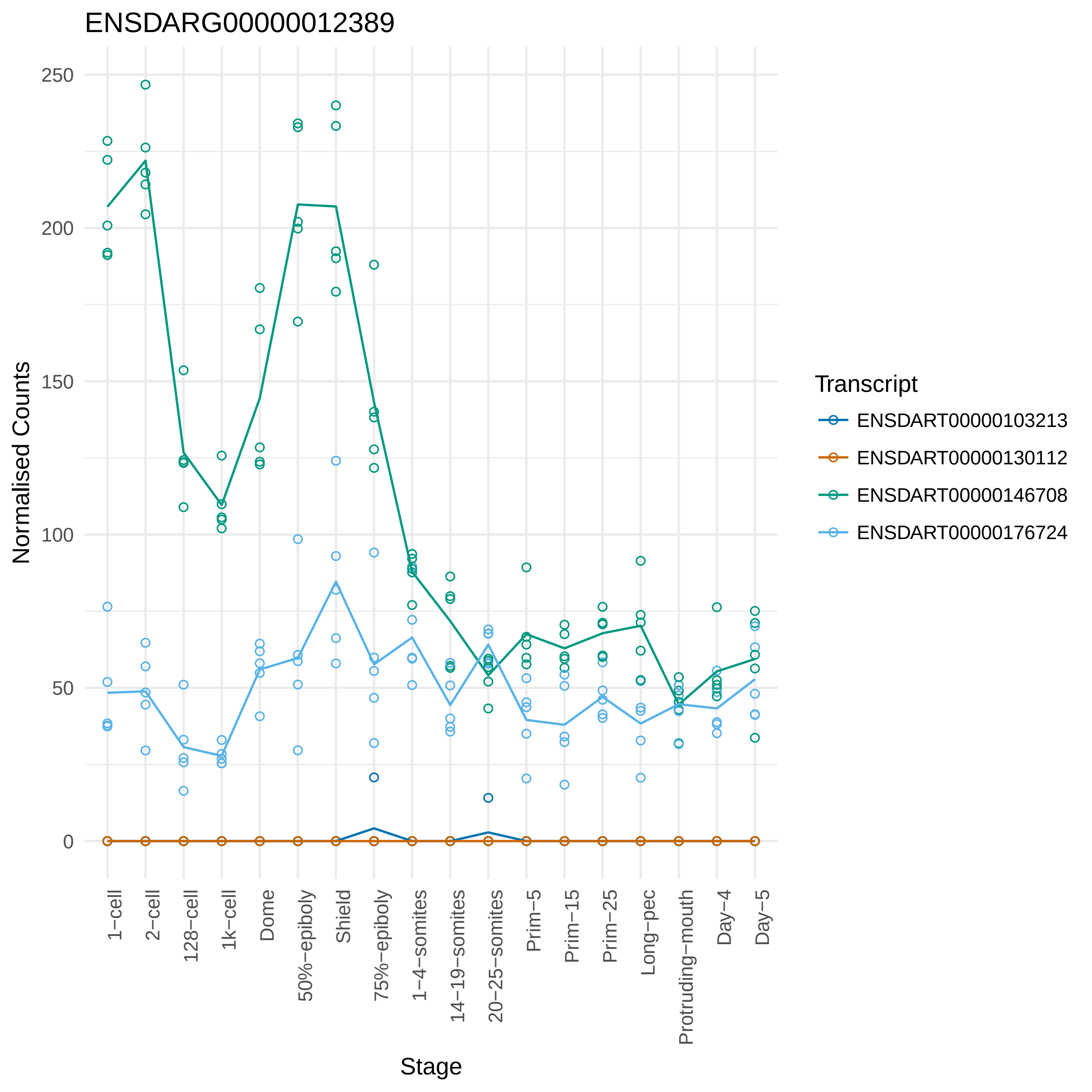
ENSDARG00000012389
Normalised Counts
250
200
150
100
50
0
1−cell
2−cell
128−cell
1k−cell
Dome
50%−epiboly
Shield
75%−epiboly
1−4−somites
14−19−somites
20−25−somites
Prim−5
Prim−15
Prim−25
Long−pec
Protruding−mouth
Day−4
Day−5
Stage
Transcript
ENSDART00000103213
ENSDART00000130112
ENSDART00000146708
ENSDART00000176724

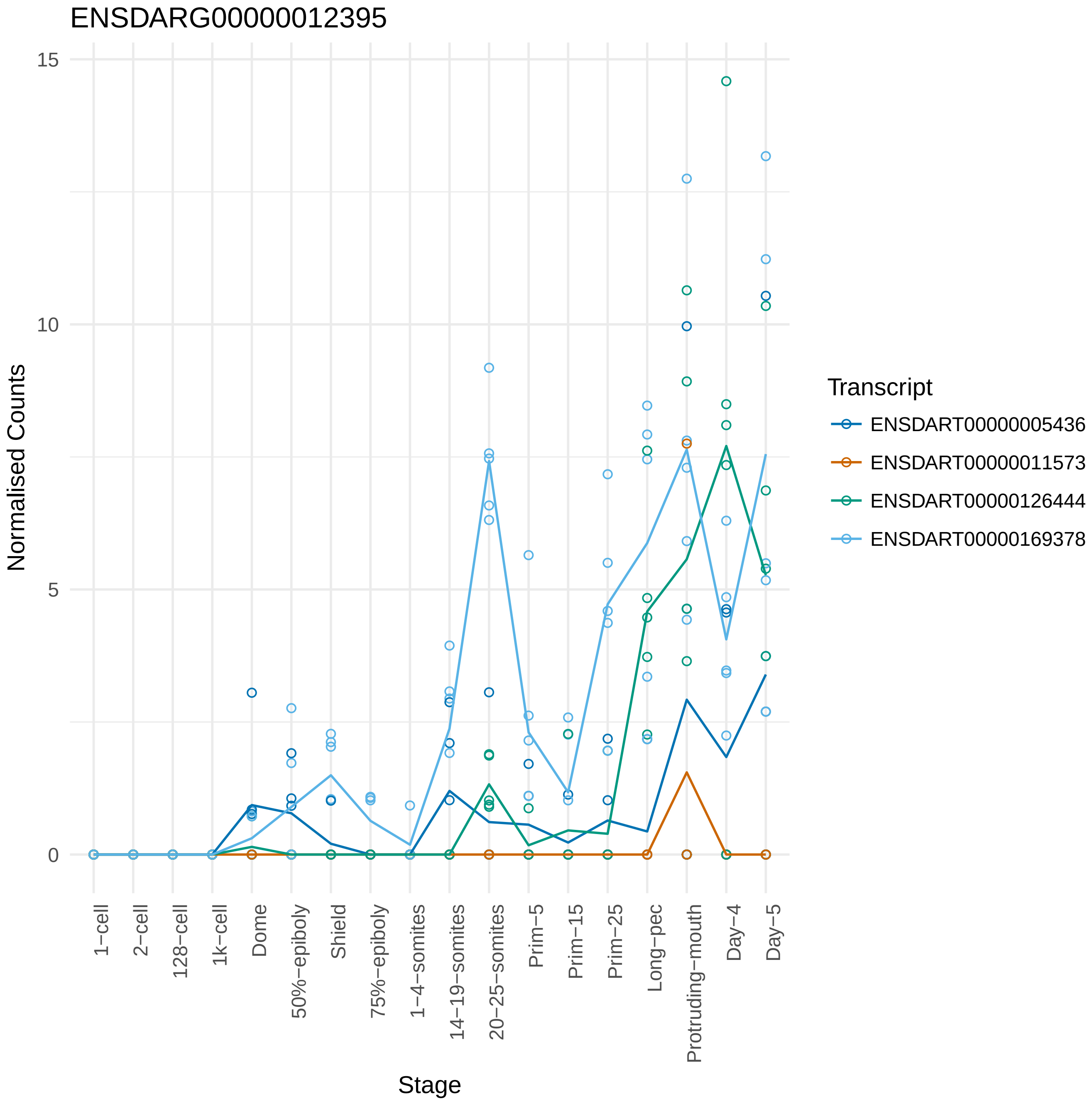

ENSDARG00000012395
Normalised Counts
15
10
5
0
Stage
1−cell
2−cell
128−cell
1k−cell
Dome
50%−epiboly
Shield
75%−epiboly
1−4−somites
14−19−somites
20−25−somites
Prim−5
Prim−15
Prim−25
Long−pec
Protruding−mouth
Day−4
Day−5
Transcript
ENSDART00000005436
ENSDART00000011573
ENSDART00000126444
ENSDART00000169378

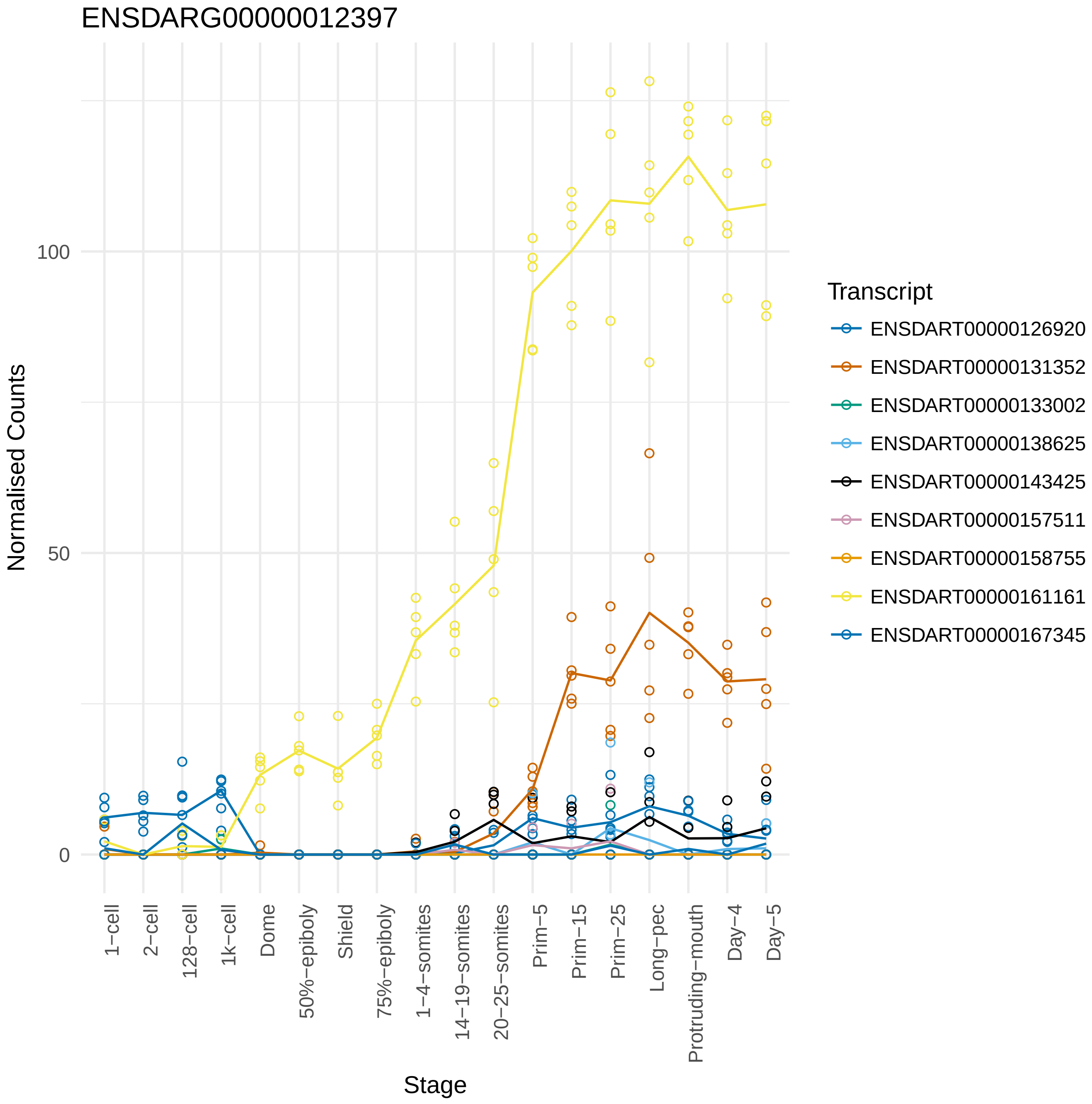

ENSDARG00000012397
Normalised Counts
100
50
0
1−cell
2−cell
128−cell
1k−cell
Dome
50%−epiboly
Shield
75%−epiboly
1−4−somites
14−19−somites
20−25−somites
Prim−5
Prim−15
Prim−25
Long−pec
Protruding−mouth
Day−4
Day−5
Stage
Transcript
ENSDART00000126920
ENSDART00000131352
ENSDART00000133002
ENSDART00000138625
ENSDART00000143425
ENSDART00000157511
ENSDART00000158755
ENSDART00000161161
ENSDART00000167345

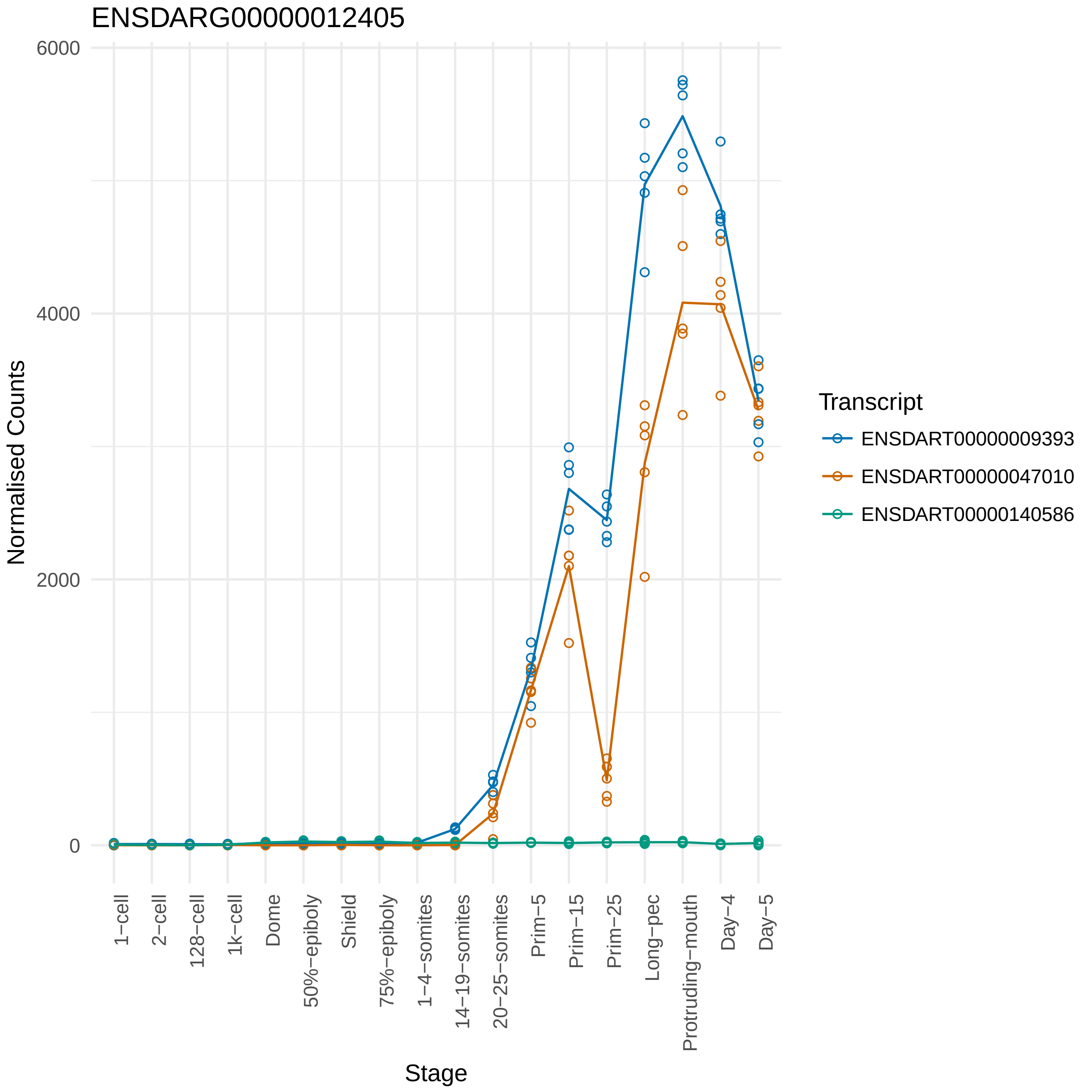

ENSDARG00000012405
Normalised Counts
6000
4000
2000
0
1−cell
2−cell
128−cell
1k−cell
Dome
50%−epiboly
Shield
75%−epiboly
1−4−somites
14−19−somites
20−25−somites
Prim−5
Prim−15
Prim−25
Long−pec
Protruding−mouth
Day−4
Day−5
Stage
Transcript
ENSDART00000009393
ENSDART00000047010
ENSDART00000140586

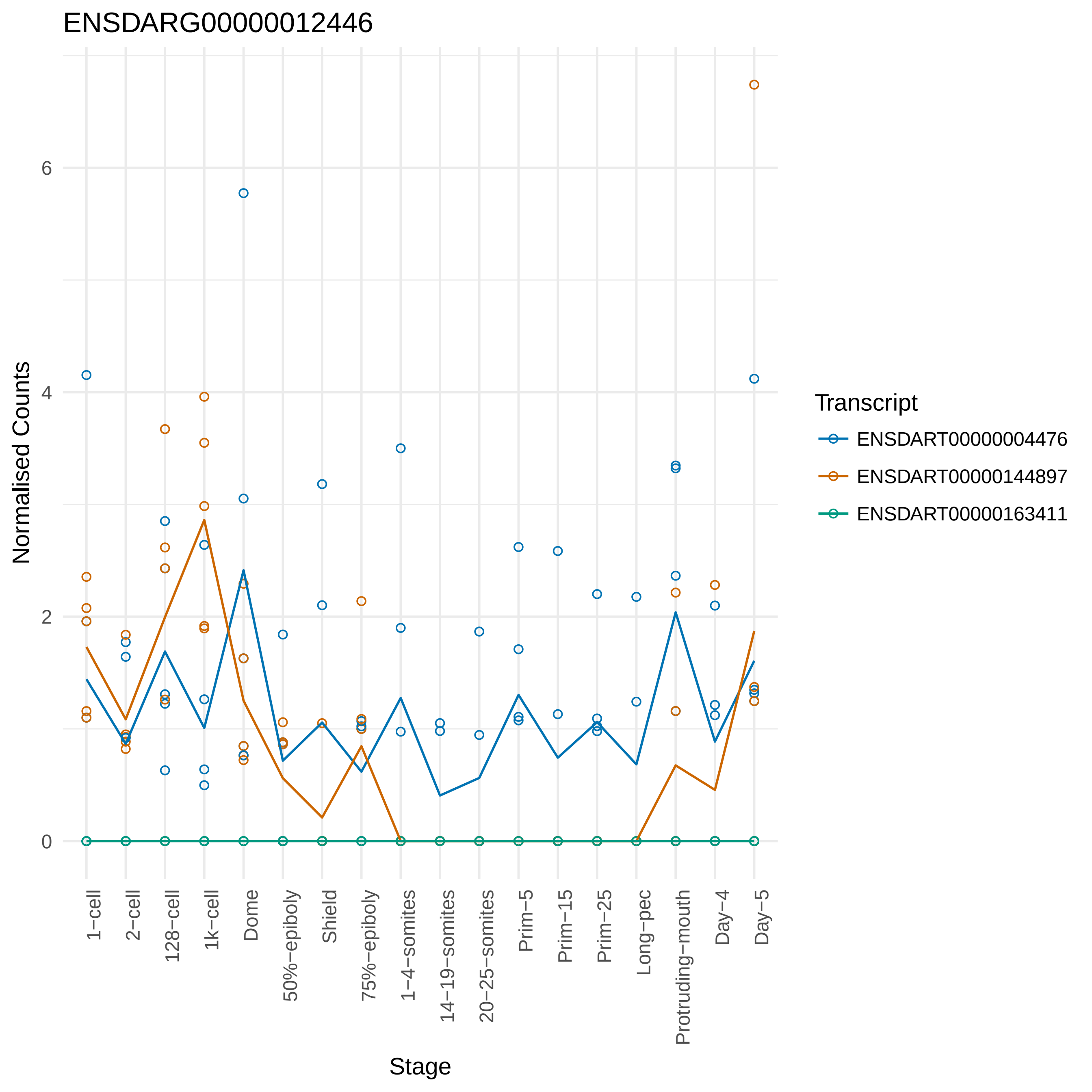

ENSDARG00000012446
Normalised Counts
0
2
4
6
Stage
1−cell
2−cell
128−cell
1k−cell
Dome
50%−epiboly
Shield
75%−epiboly
1−4−somites
14−19−somites
20−25−somites
Prim−5
Prim−15
Prim−25
Long−pec
Protruding−mouth
Day−4
Day−5
Transcript
ENSDART00000004476
ENSDART00000144897
ENSDART00000163411

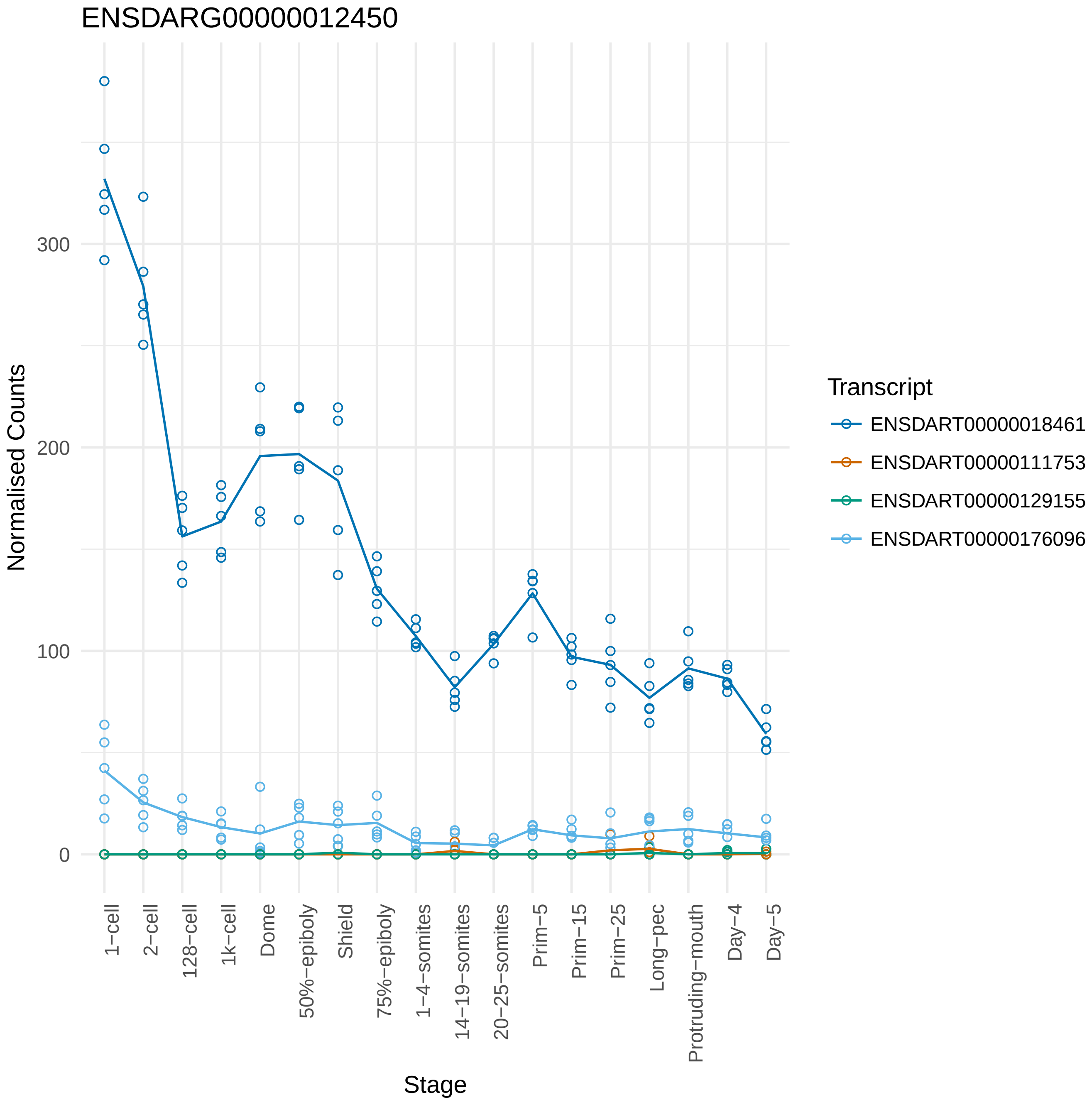
ENSDARG00000012450
Normalised Counts
300
200
100
0
Stage
1−cell
2−cell
128−cell
1k−cell
Dome
50%−epiboly
Shield
75%−epiboly
1−4−somites
14−19−somites
20−25−somites
Prim−5
Prim−15
Prim−25
Long−pec
Protruding−mouth
Day−4
Day−5
Transcript
ENSDART00000018461
ENSDART00000111753
ENSDART00000129155
ENSDART00000176096

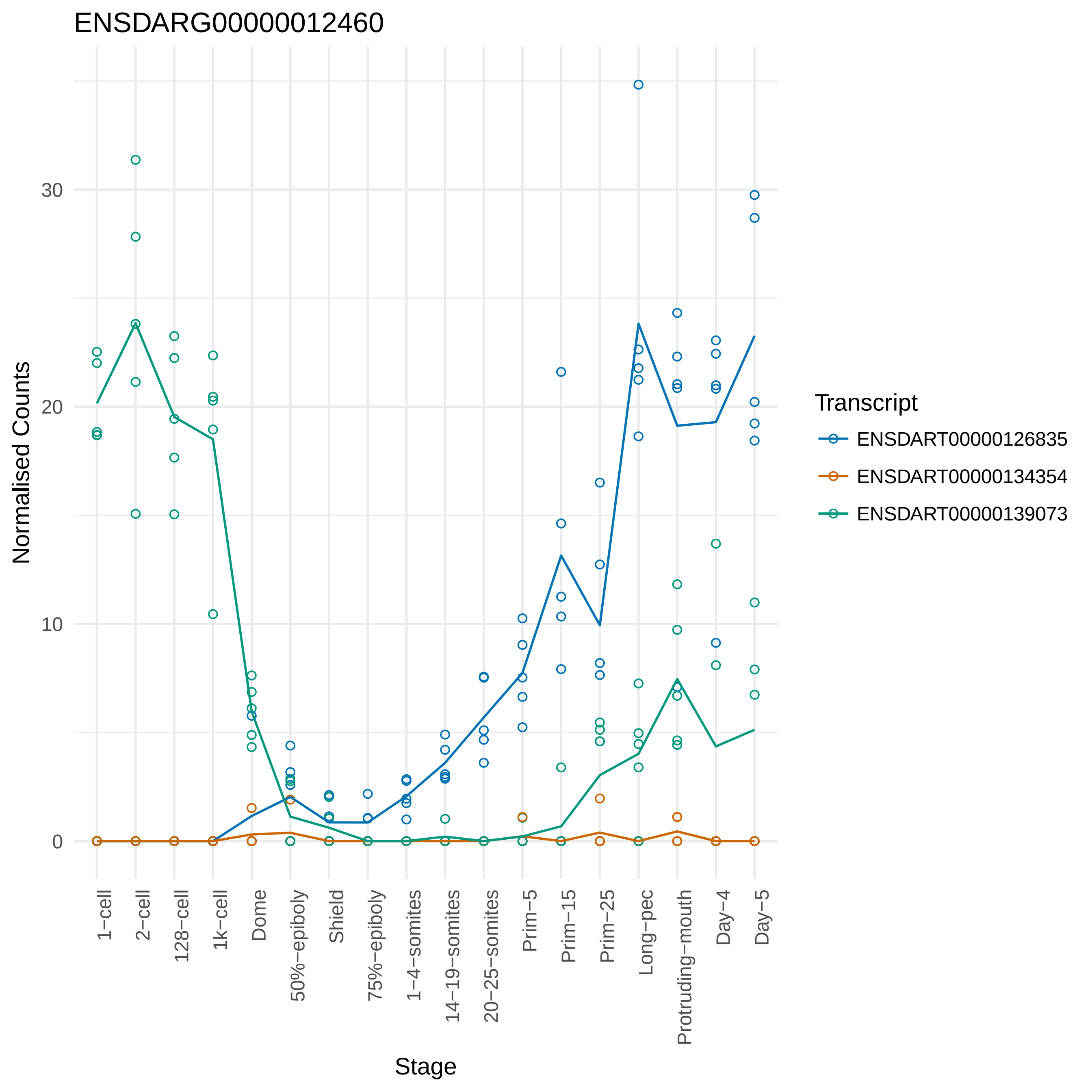

ENSDARG00000012460
Normalised Counts
30
20
10
0
1−cell
2−cell
128−cell
1k−cell
Dome
50%−epiboly
Shield
75%−epiboly
1−4−somites
14−19−somites
20−25−somites
Prim−5
Prim−15
Prim−25
Long−pec
Protruding−mouth
Day−4
Day−5
Stage
Transcript
ENSDART00000126835
ENSDART00000134354
ENSDART00000139073

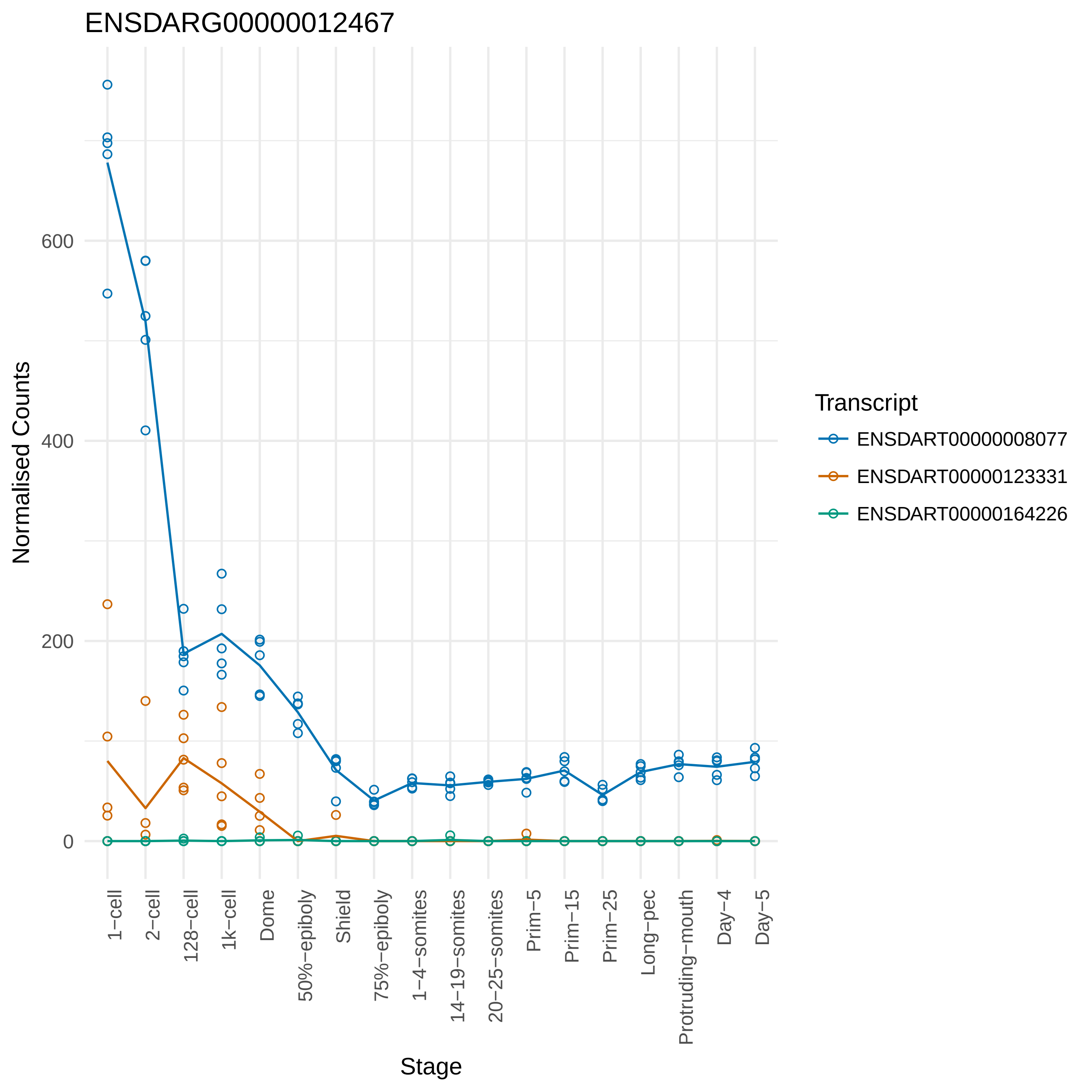

ENSDARG00000012467
Normalised Counts
0
200
400
600
Stage
1−cell
2−cell
128−cell
1k−cell
Dome
50%−epiboly
Shield
75%−epiboly
1−4−somites
14−19−somites
20−25−somites
Prim−5
Prim−15
Prim−25
Long−pec
Protruding−mouth
Day−4
Day−5
Transcript
ENSDART00000008077
ENSDART00000123331
ENSDART00000164226

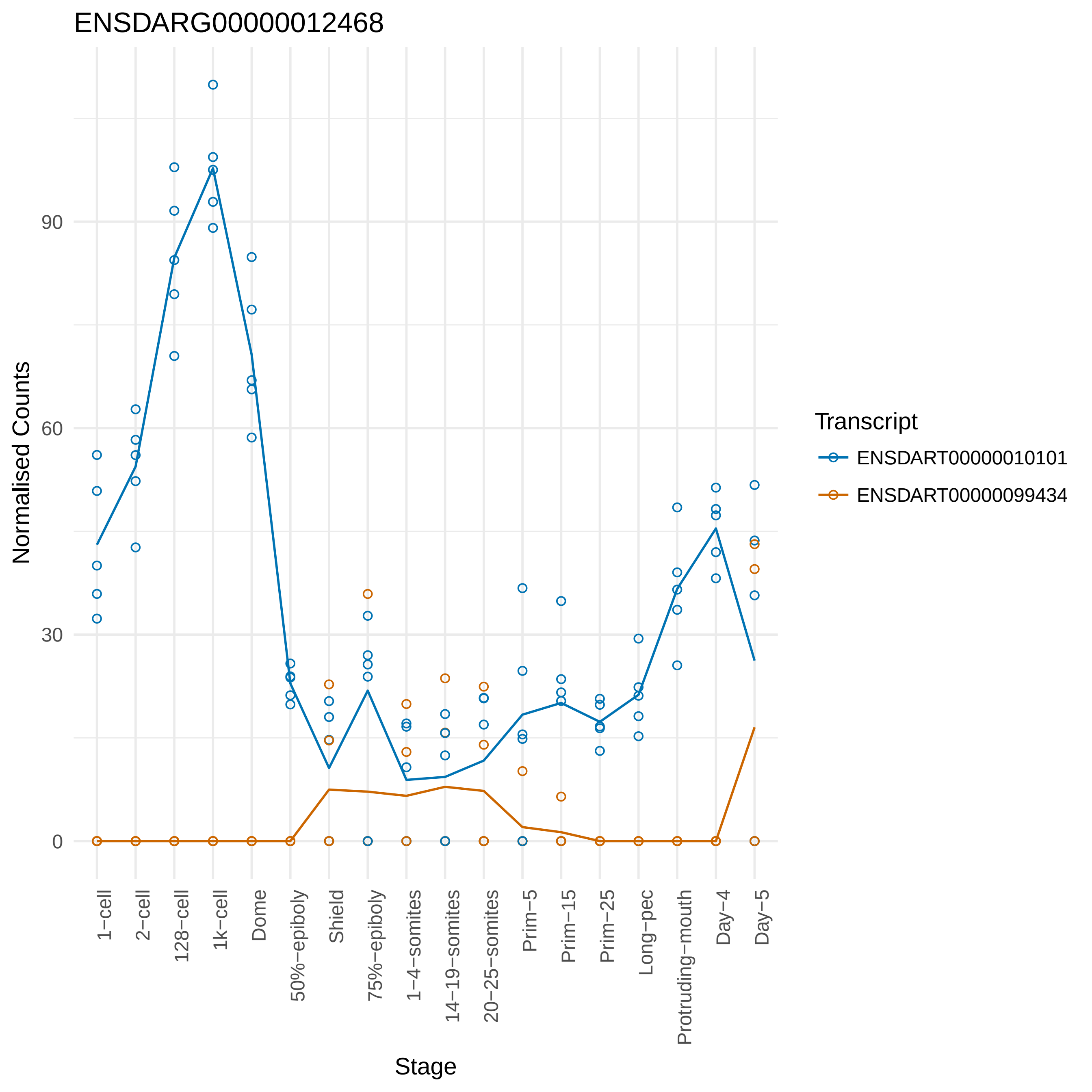
ENSDARG00000012468
Normalised Counts
0
30
60
90
Stage
1−cell
2−cell
128−cell
1k−cell
Dome
50%−epiboly
Shield
75%−epiboly
1−4−somites
14−19−somites
20−25−somites
Prim−5
Prim−15
Prim−25
Long−pec
Protruding−mouth
Day−4
Day−5
Transcript
ENSDART00000010101
ENSDART00000099434

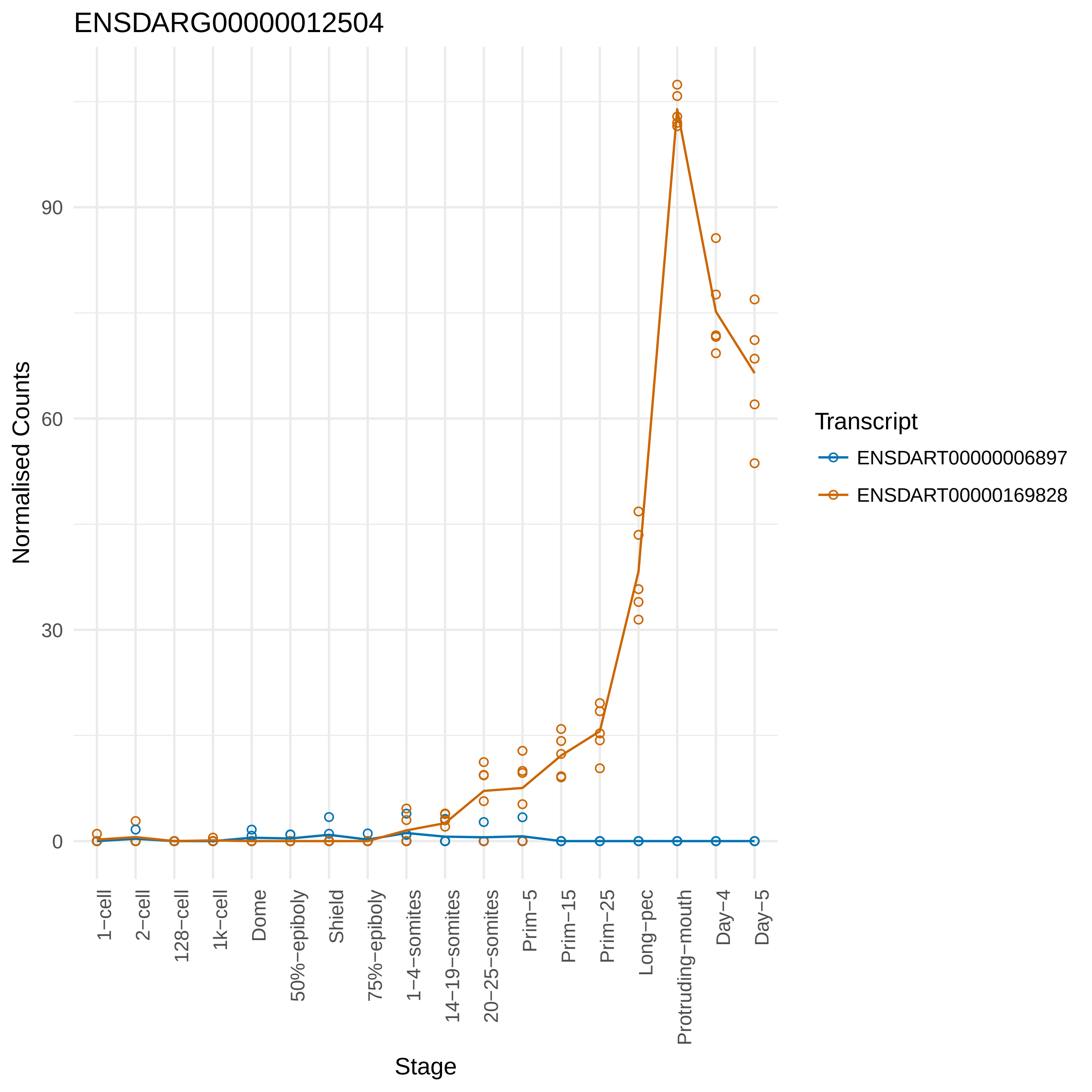

ENSDARG00000012504
Normalised Counts
90
60
30
0
1−cell
2−cell
128−cell
1k−cell
Dome
50%−epiboly
Shield
75%−epiboly
1−4−somites
14−19−somites
20−25−somites
Prim−5
Prim−15
Prim−25
Long−pec
Protruding−mouth
Day−4
Day−5
Stage
Transcript
ENSDART00000006897
ENSDART00000169828

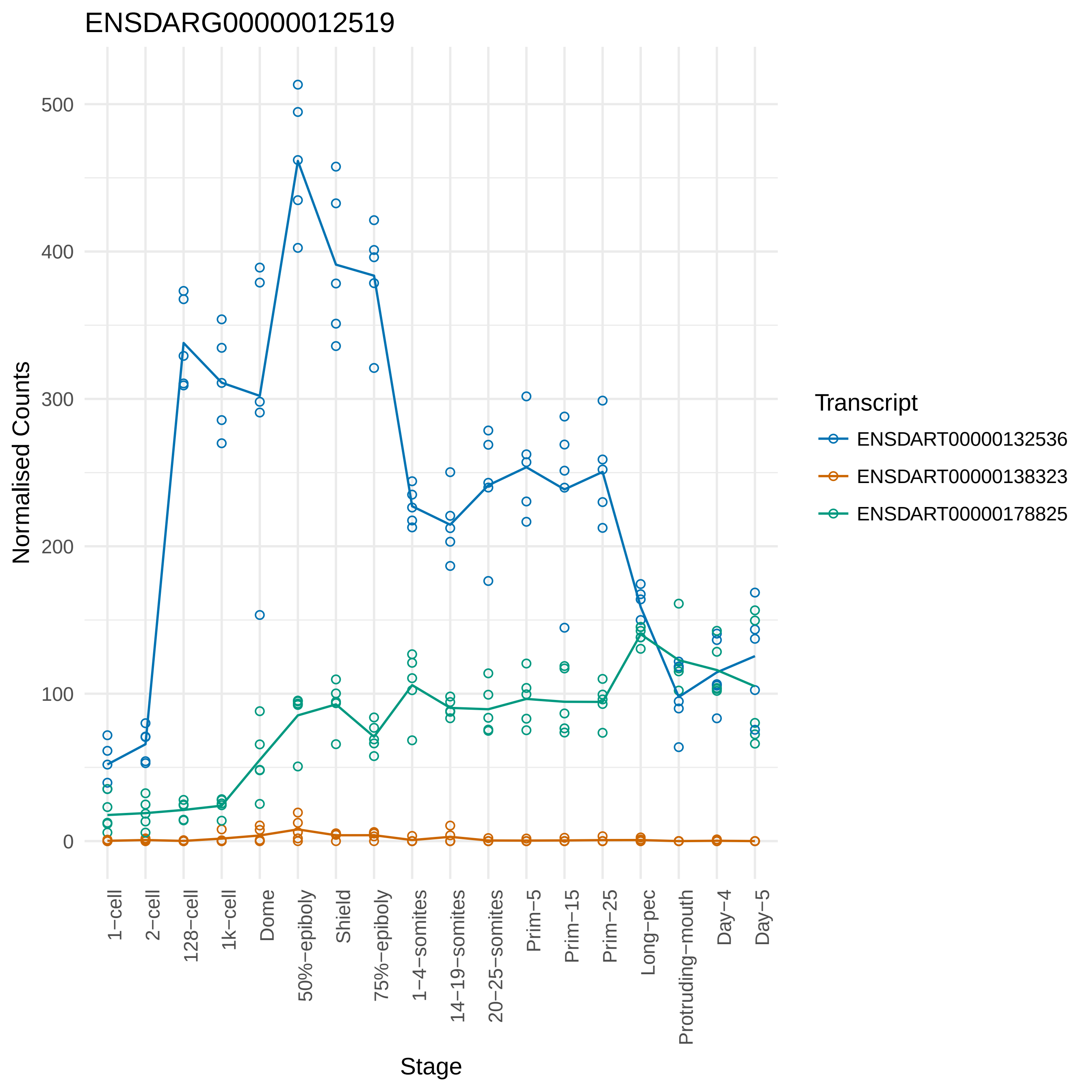

ENSDARG00000012519
Normalised Counts
500
400
300
200
100
0
1−cell
2−cell
128−cell
1k−cell
Dome
50%−epiboly
Shield
75%−epiboly
1−4−somites
14−19−somites
20−25−somites
Prim−5
Prim−15
Prim−25
Long−pec
Protruding−mouth
Day−4
Day−5
Stage
Transcript
ENSDART00000132536
ENSDART00000138323
ENSDART00000178825

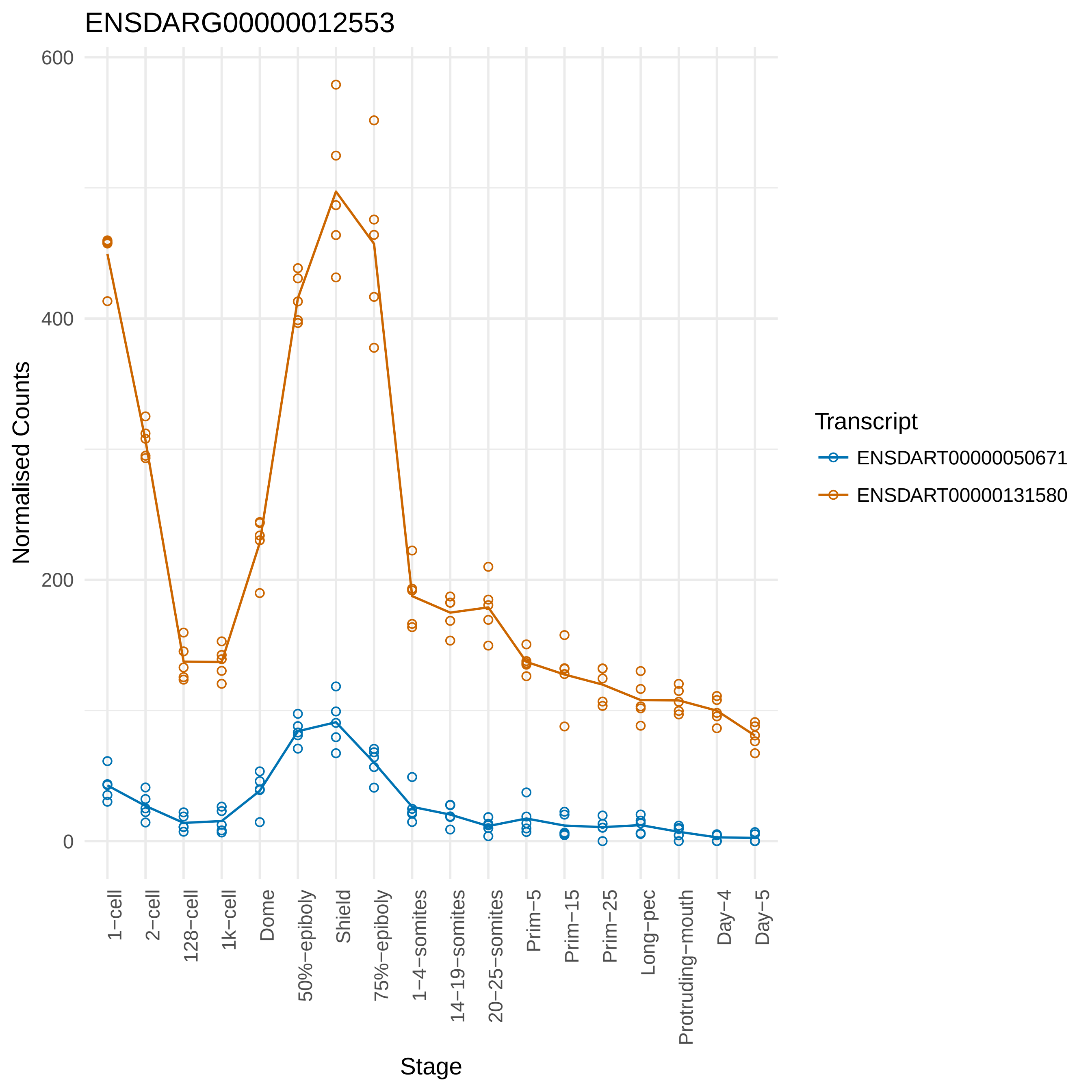
ENSDARG00000012553
Normalised Counts
600
400
200
0
1−cell
2−cell
128−cell
1k−cell
Dome
50%−epiboly
Shield
75%−epiboly
1−4−somites
14−19−somites
20−25−somites
Prim−5
Prim−15
Prim−25
Long−pec
Protruding−mouth
Day−4
Day−5
Stage
Transcript
ENSDART00000050671
ENSDART00000131580

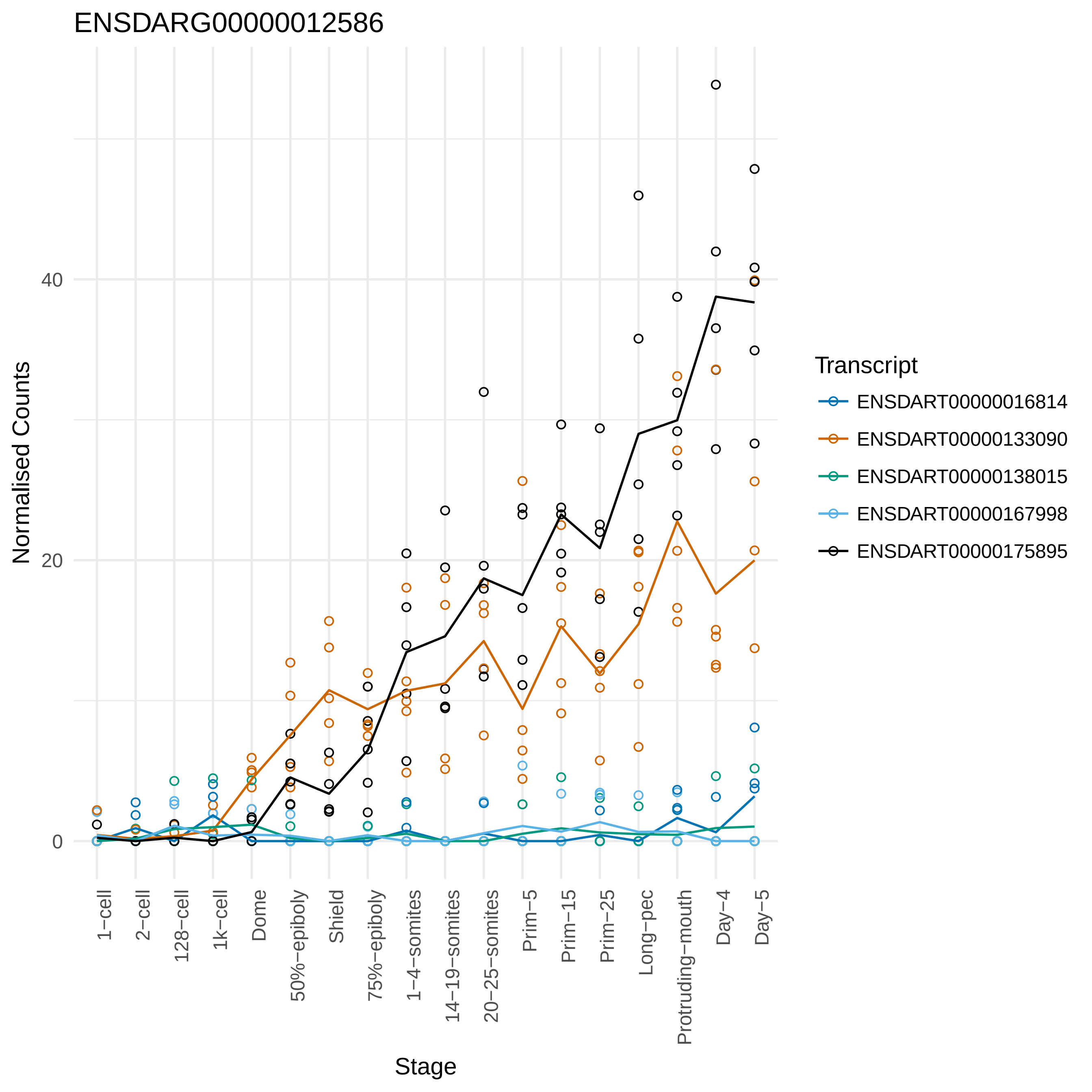
ENSDARG00000012586
Normalised Counts
0
20
40
Stage
1−cell
2−cell
128−cell
1k−cell
Dome
50%−epiboly
Shield
75%−epiboly
1−4−somites
14−19−somites
20−25−somites
Prim−5
Prim−15
Prim−25
Long−pec
Protruding−mouth
Day−4
Day−5
Transcript
ENSDART00000016814
ENSDART00000133090
ENSDART00000138015
ENSDART00000167998
ENSDART00000175895

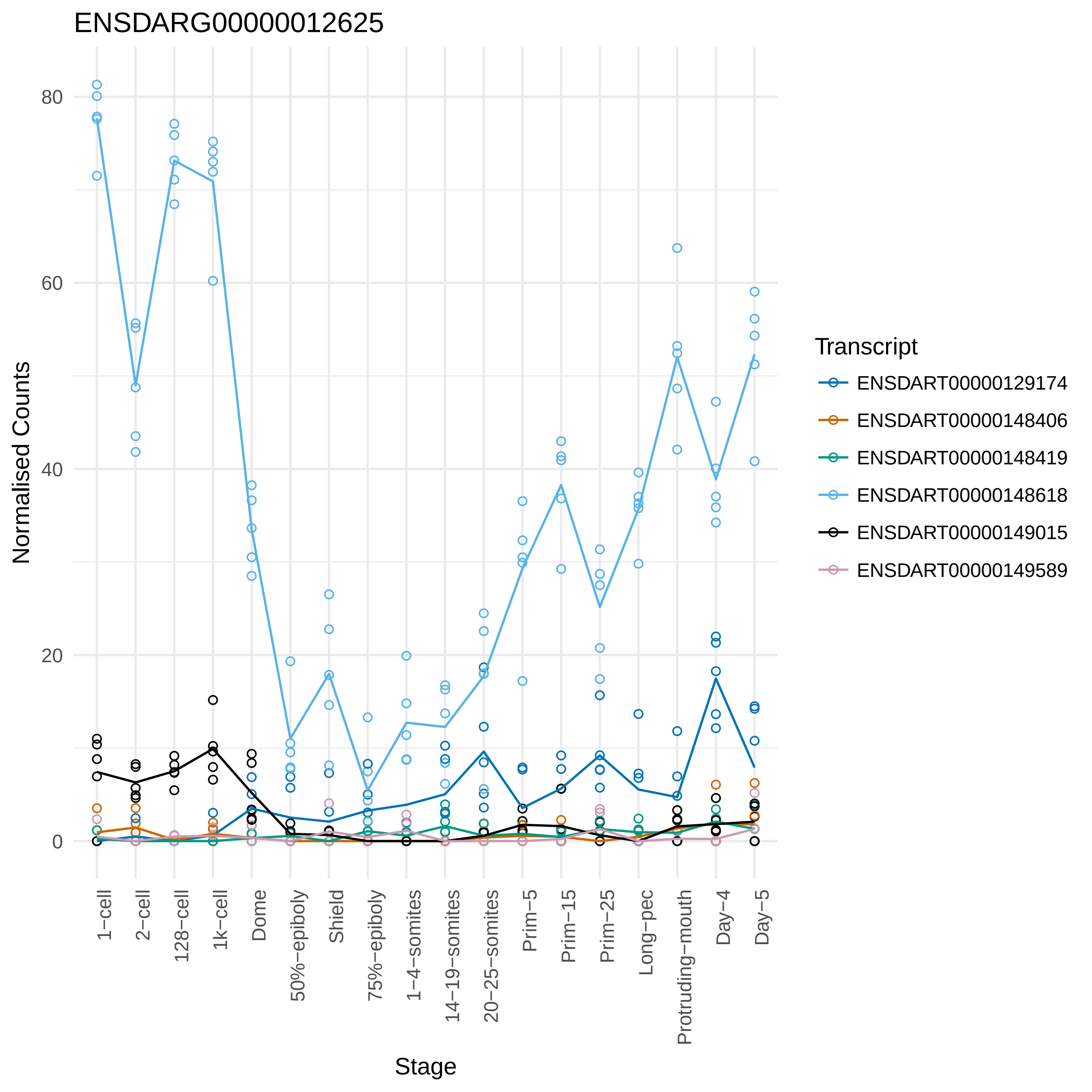

ENSDARG00000012625
Normalised Counts
Stage
0
20
40
60
80
1−cell
2−cell
128−cell
1k−cell
Dome
50%−epiboly
Shield
75%−epiboly
1−4−somites
14−19−somites
20−25−somites
Prim−5
Prim−15
Prim−25
Long−pec
Protruding−mouth
Day−4
Day−5
Transcript
ENSDART00000129174
ENSDART00000148406
ENSDART00000148419
ENSDART00000148618
ENSDART00000149015
ENSDART00000149589

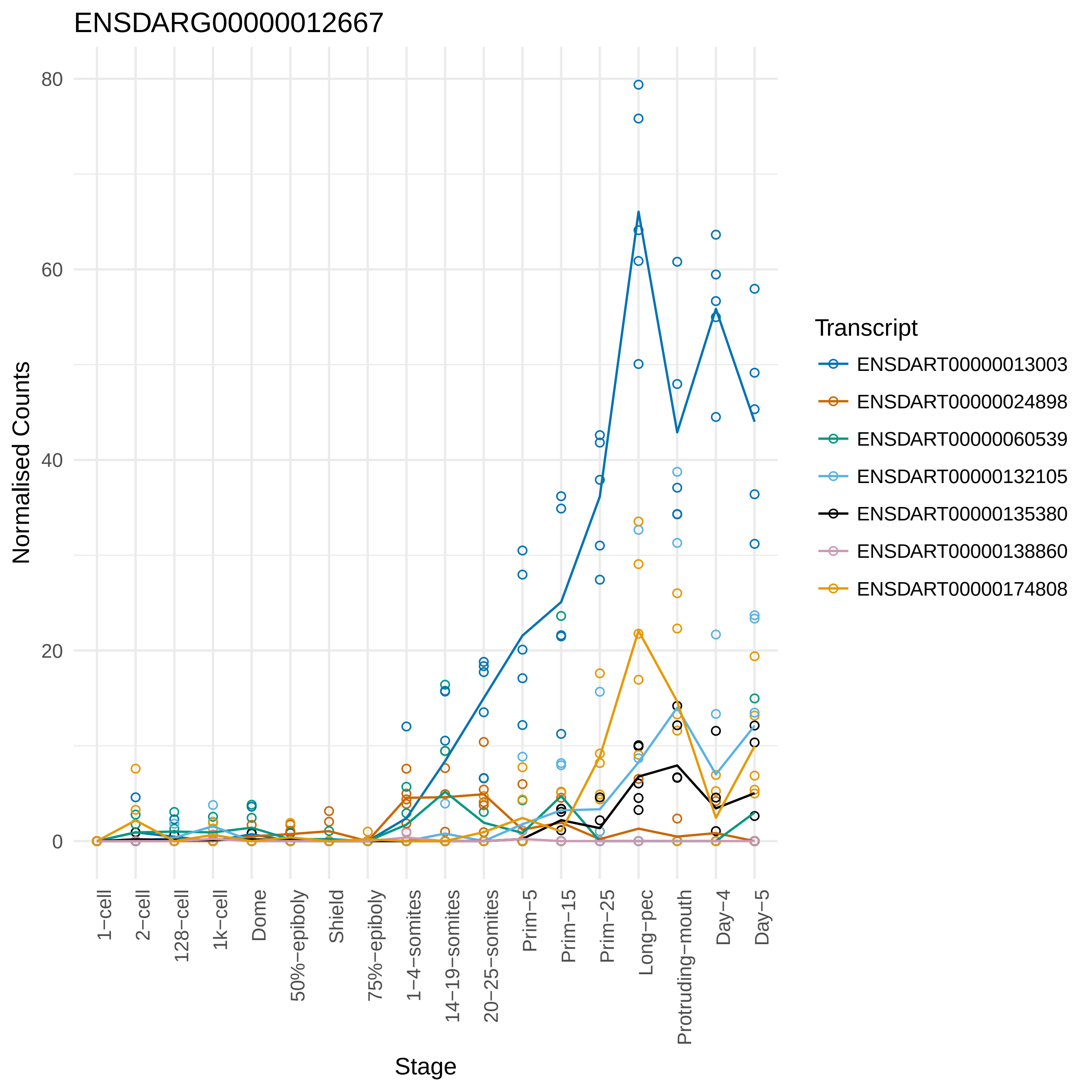
ENSDARG00000012667
Normalised Counts
80
60
40
20
0
1−cell
2−cell
128−cell
1k−cell
Dome
50%−epiboly
Shield
75%−epiboly
1−4−somites
14−19−somites
20−25−somites
Prim−5
Prim−15
Prim−25
Long−pec
Protruding−mouth
Day−4
Day−5
Stage
Transcript
ENSDART00000013003
ENSDART00000024898
ENSDART00000060539
ENSDART00000132105
ENSDART00000135380
ENSDART00000138860
ENSDART00000174808

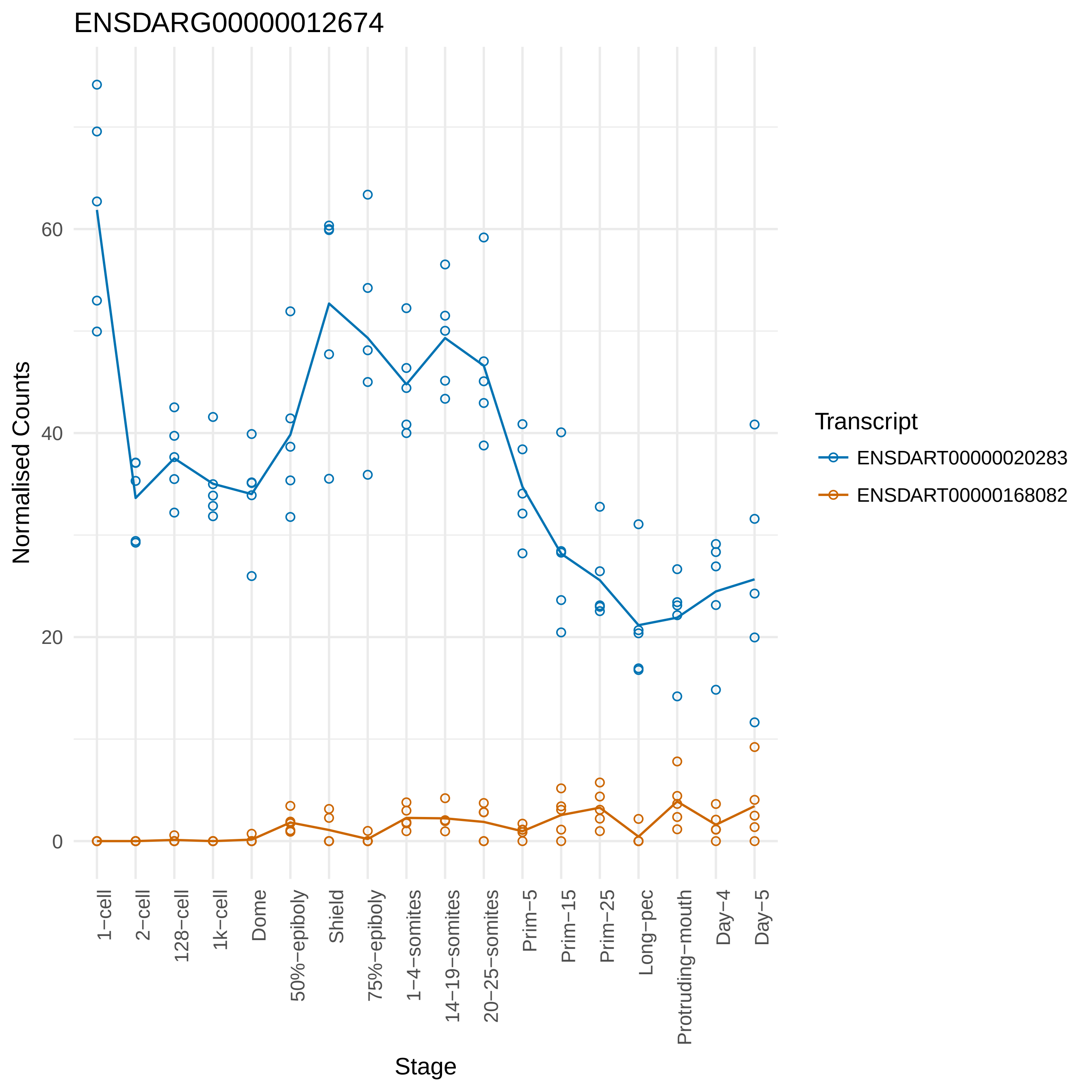

ENSDARG00000012674
Normalised Counts
0
20
40
60
Stage
1−cell
2−cell
128−cell
1k−cell
Dome
50%−epiboly
Shield
75%−epiboly
1−4−somites
14−19−somites
20−25−somites
Prim−5
Prim−15
Prim−25
Long−pec
Protruding−mouth
Day−4
Day−5
Transcript
ENSDART00000020283
ENSDART00000168082

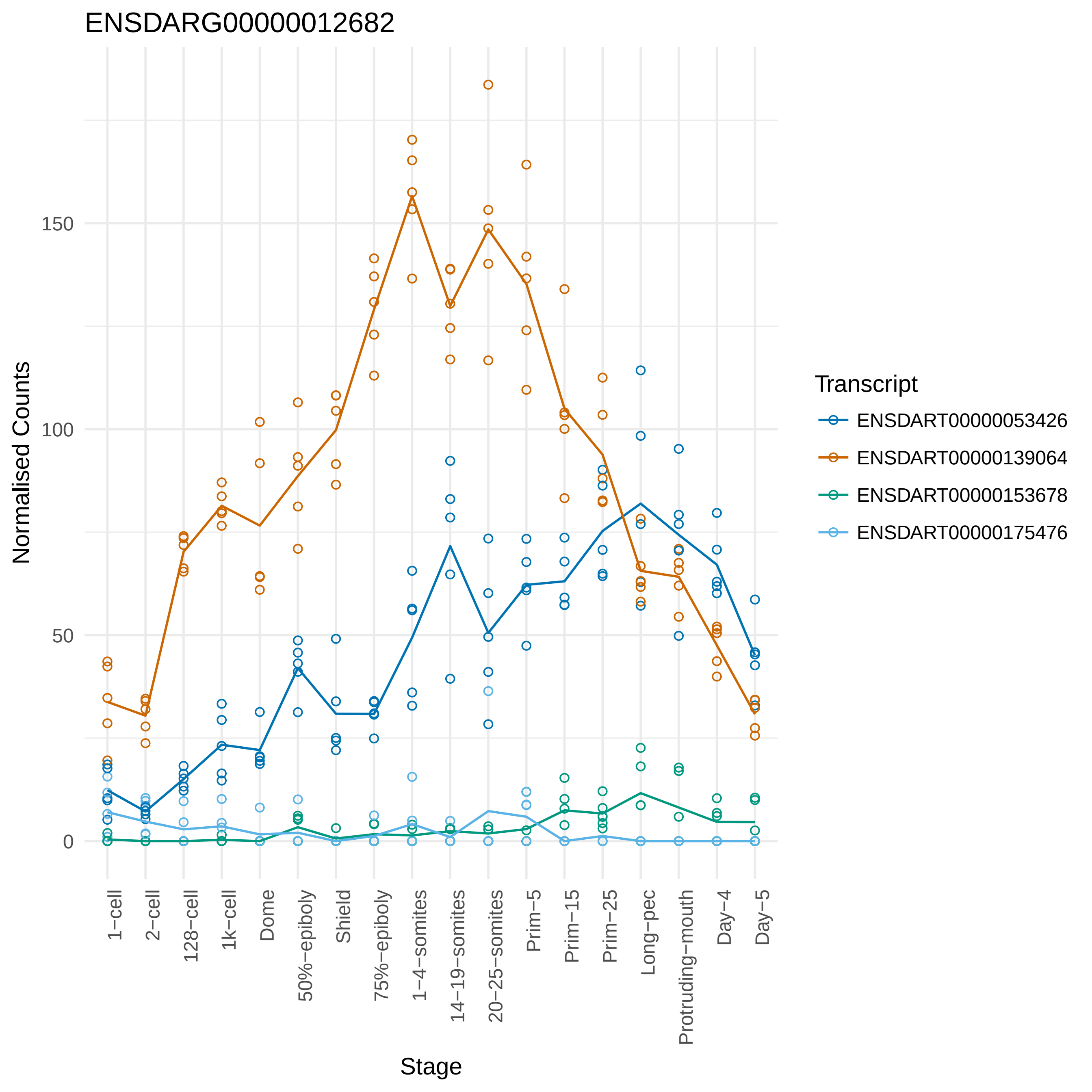

ENSDARG00000012682
Normalised Counts
0
50
100
150
Stage
1−cell
2−cell
128−cell
1k−cell
Dome
50%−epiboly
Shield
75%−epiboly
1−4−somites
14−19−somites
20−25−somites
Prim−5
Prim−15
Prim−25
Long−pec
Protruding−mouth
Day−4
Day−5
Transcript
ENSDART00000053426
ENSDART00000139064
ENSDART00000153678
ENSDART00000175476

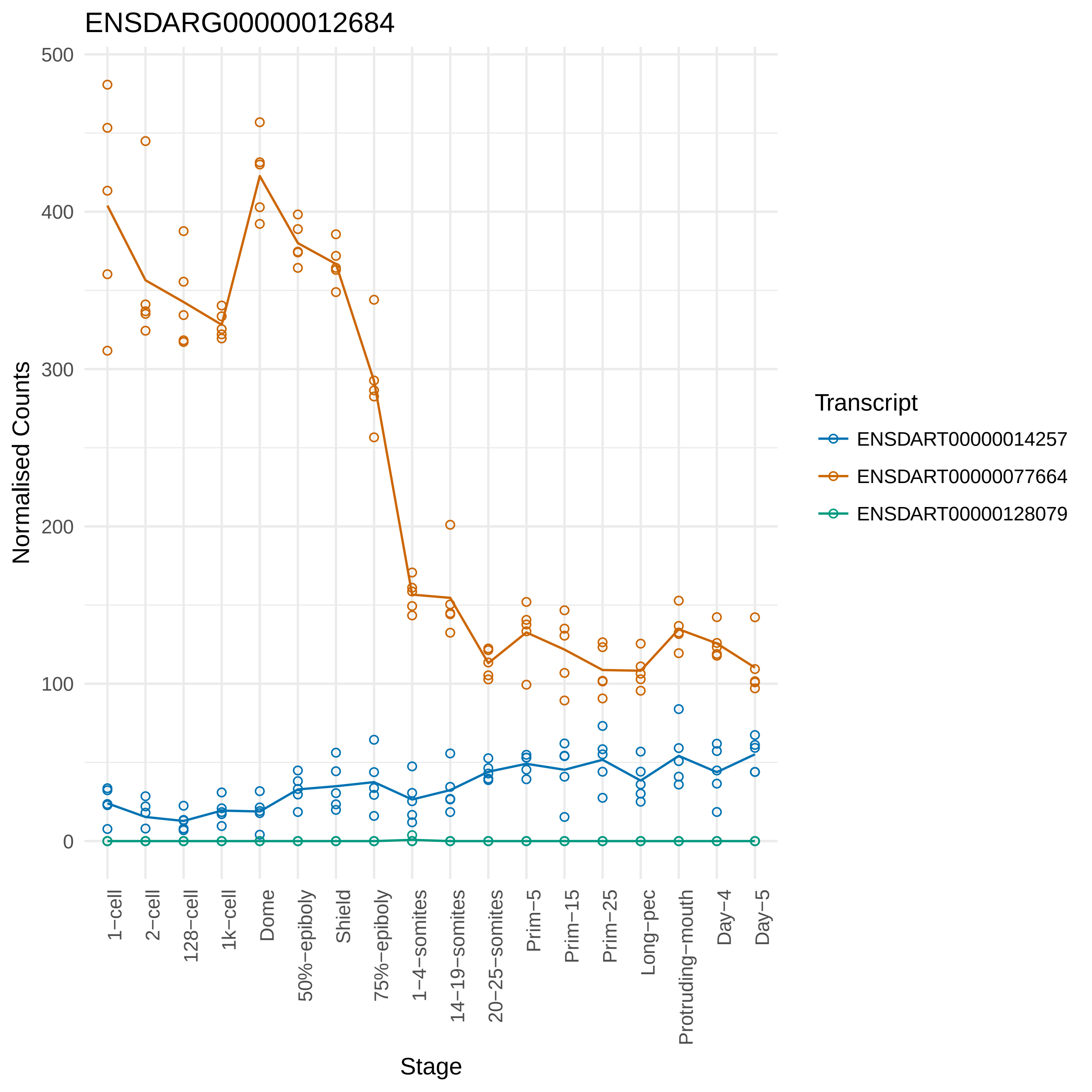

ENSDARG00000012684
Normalised Counts
500
400
300
200
100
0
1−cell
2−cell
128−cell
1k−cell
Dome
50%−epiboly
Shield
75%−epiboly
1−4−somites
14−19−somites
20−25−somites
Prim−5
Prim−15
Prim−25
Long−pec
Protruding−mouth
Day−4
Day−5
Stage
Transcript
ENSDART00000014257
ENSDART00000077664
ENSDART00000128079

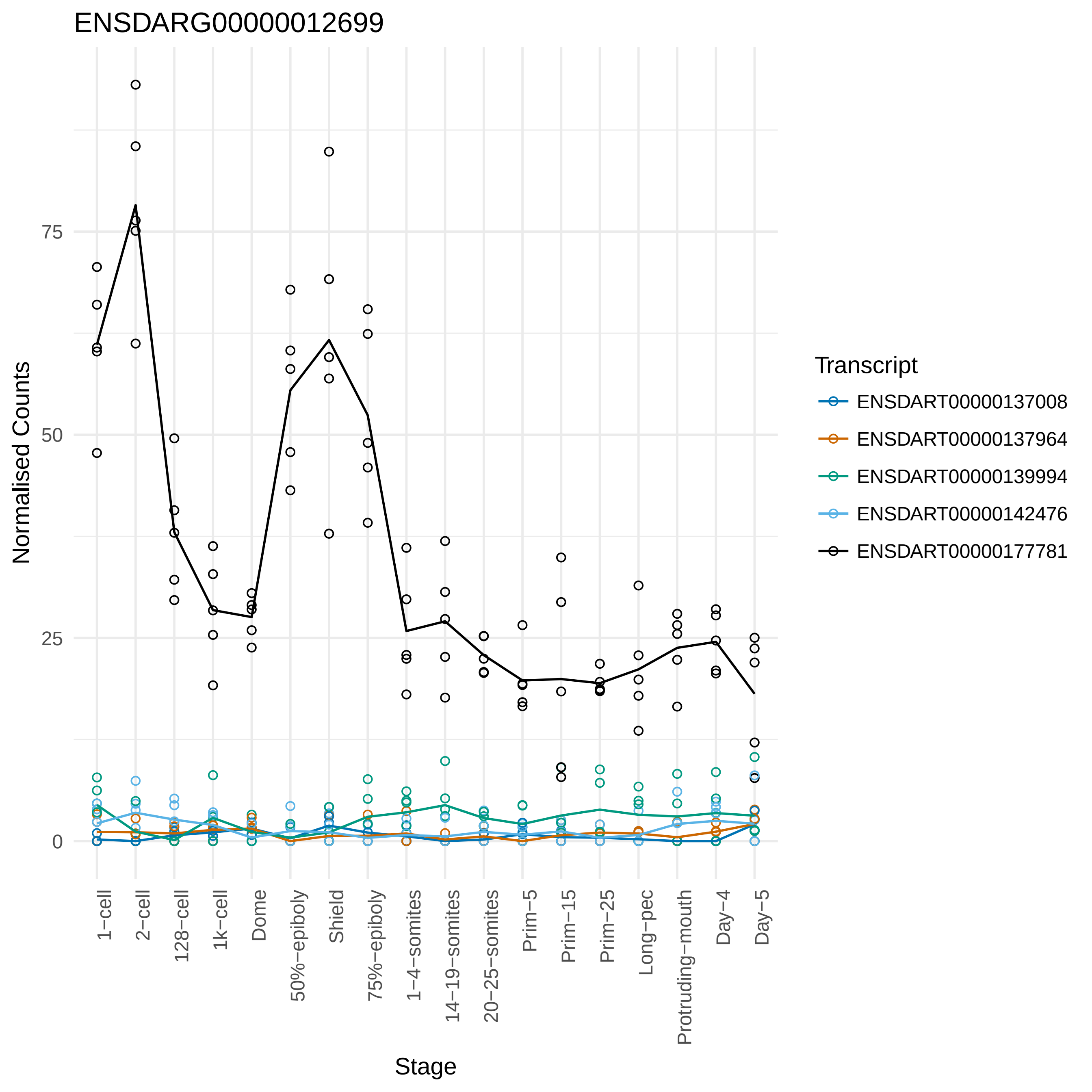

ENSDARG00000012699
Normalised Counts
75
50
25
0
1−cell
2−cell
128−cell
1k−cell
Dome
50%−epiboly
Shield
75%−epiboly
1−4−somites
14−19−somites
20−25−somites
Prim−5
Prim−15
Prim−25
Long−pec
Protruding−mouth
Day−4
Day−5
Stage
Transcript
ENSDART00000137008
ENSDART00000137964
ENSDART00000139994
ENSDART00000142476
ENSDART00000177781

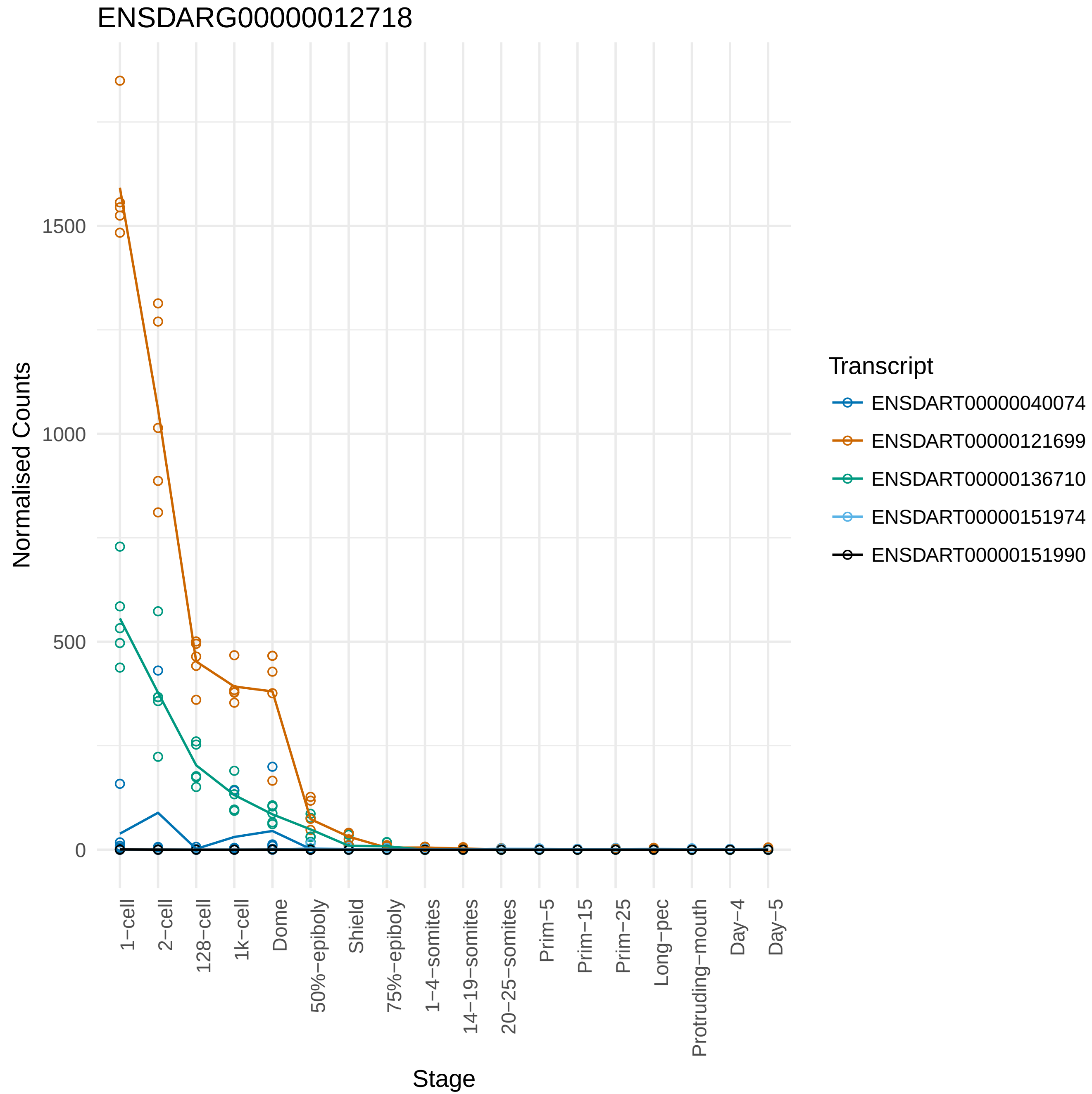

ENSDARG00000012718
Normalised Counts
1500
1000
500
0
1−cell
2−cell
128−cell
1k−cell
Dome
50%−epiboly
Shield
75%−epiboly
1−4−somites
14−19−somites
20−25−somites
Prim−5
Prim−15
Prim−25
Long−pec
Protruding−mouth
Day−4
Day−5
Stage
Transcript
ENSDART00000040074
ENSDART00000121699
ENSDART00000136710
ENSDART00000151974
ENSDART00000151990

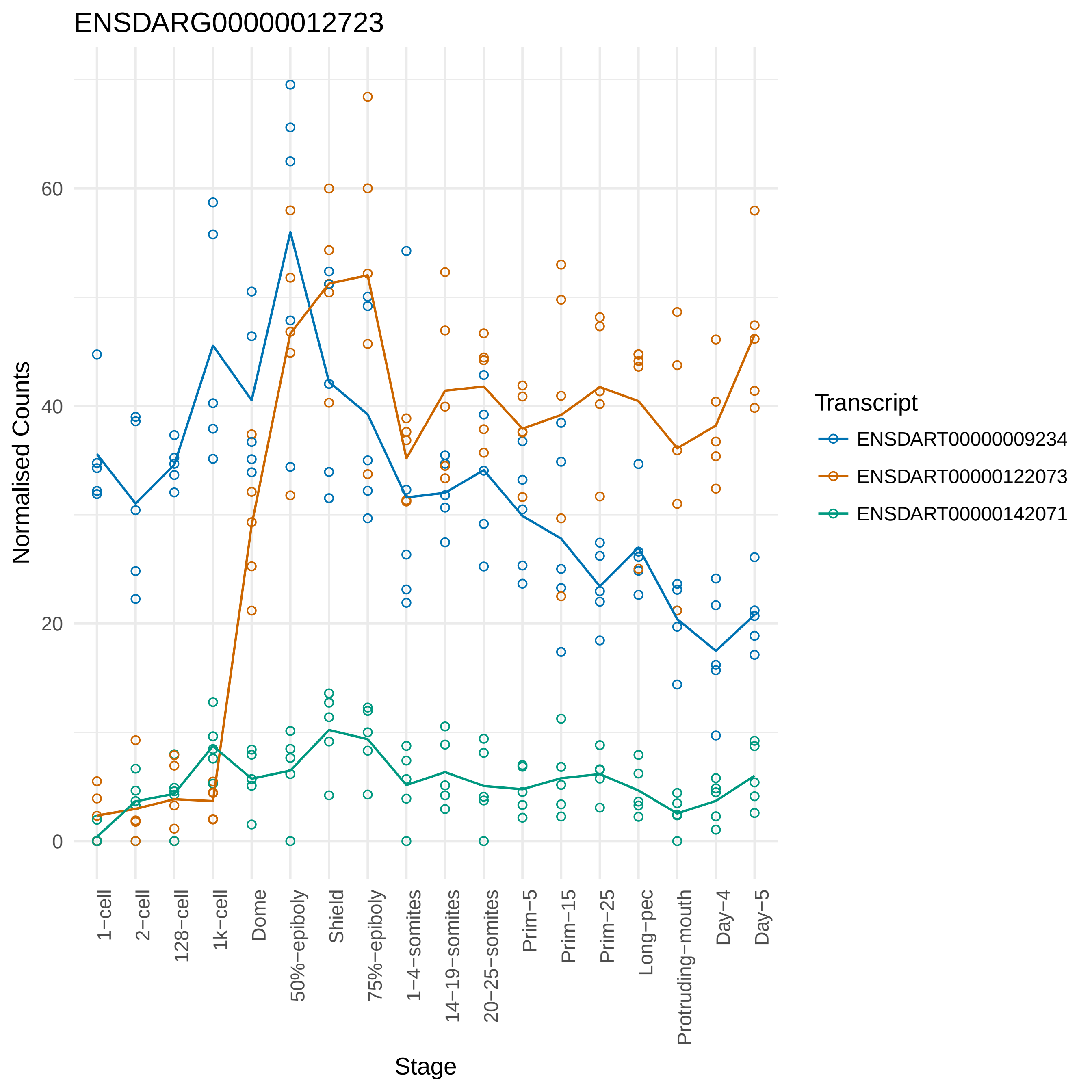

ENSDARG00000012723
Normalised Counts
0
20
40
60
1−cell
2−cell
128−cell
1k−cell
Dome
50%−epiboly
Shield
75%−epiboly
1−4−somites
14−19−somites
20−25−somites
Prim−5
Prim−15
Prim−25
Long−pec
Protruding−mouth
Day−4
Day−5
Stage
Transcript
ENSDART00000009234
ENSDART00000122073
ENSDART00000142071

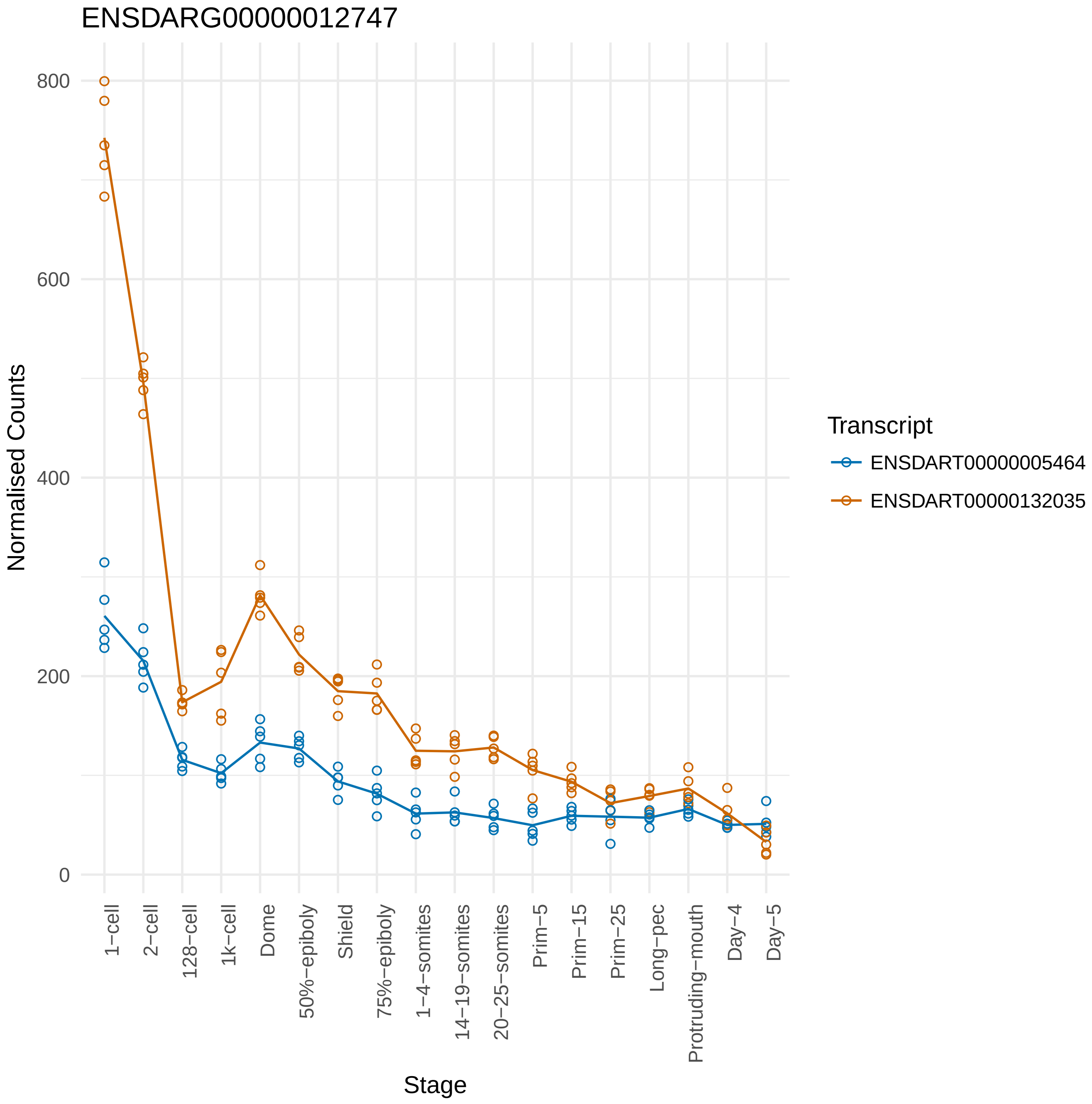
ENSDARG00000012747
Normalised Counts
800
600
400
200
0
1−cell
2−cell
128−cell
1k−cell
Dome
50%−epiboly
Shield
75%−epiboly
1−4−somites
14−19−somites
20−25−somites
Prim−5
Prim−15
Prim−25
Long−pec
Protruding−mouth
Day−4
Day−5
Stage
Transcript
ENSDART00000005464
ENSDART00000132035

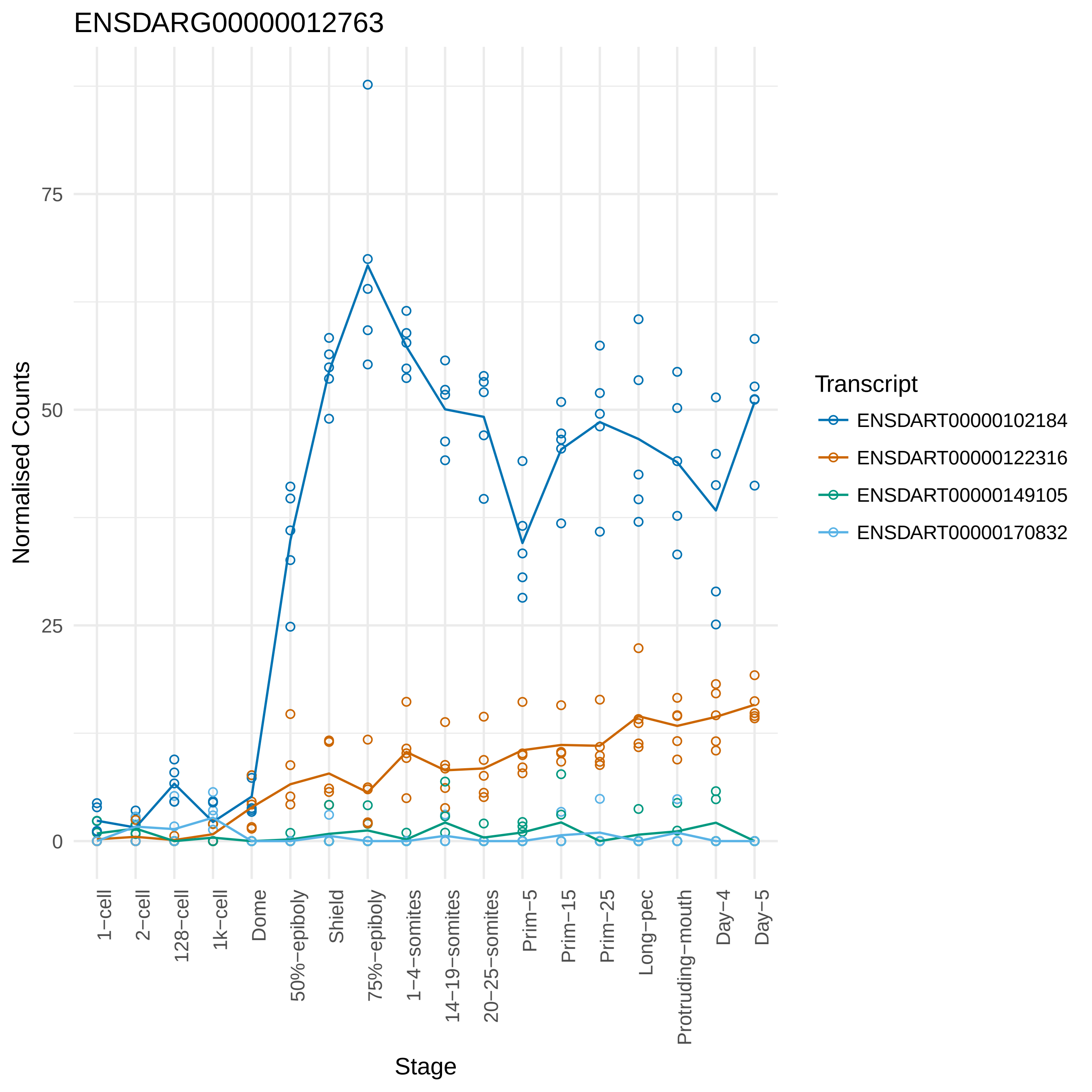
ENSDARG00000012763
Normalised Counts
75
50
25
0
1−cell
2−cell
128−cell
1k−cell
Dome
50%−epiboly
Shield
75%−epiboly
1−4−somites
14−19−somites
20−25−somites
Prim−5
Prim−15
Prim−25
Long−pec
Protruding−mouth
Day−4
Day−5
Stage
Transcript
ENSDART00000102184
ENSDART00000122316
ENSDART00000149105
ENSDART00000170832

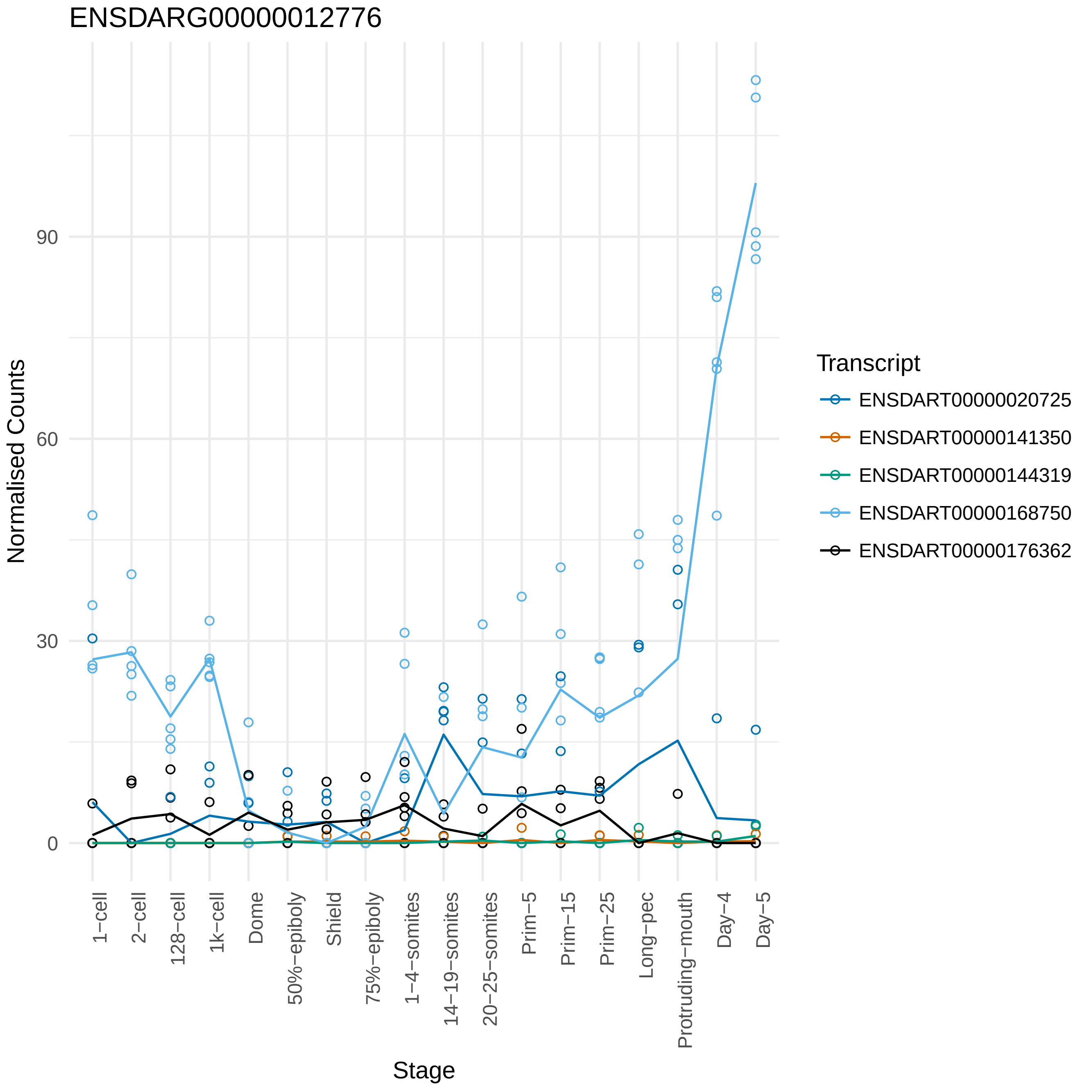

ENSDARG00000012776
Normalised Counts
90
60
30
0
1−cell
2−cell
128−cell
1k−cell
Dome
50%−epiboly
Shield
75%−epiboly
1−4−somites
14−19−somites
20−25−somites
Prim−5
Prim−15
Prim−25
Long−pec
Protruding−mouth
Day−4
Day−5
Stage
Transcript
ENSDART00000020725
ENSDART00000141350
ENSDART00000144319
ENSDART00000168750
ENSDART00000176362

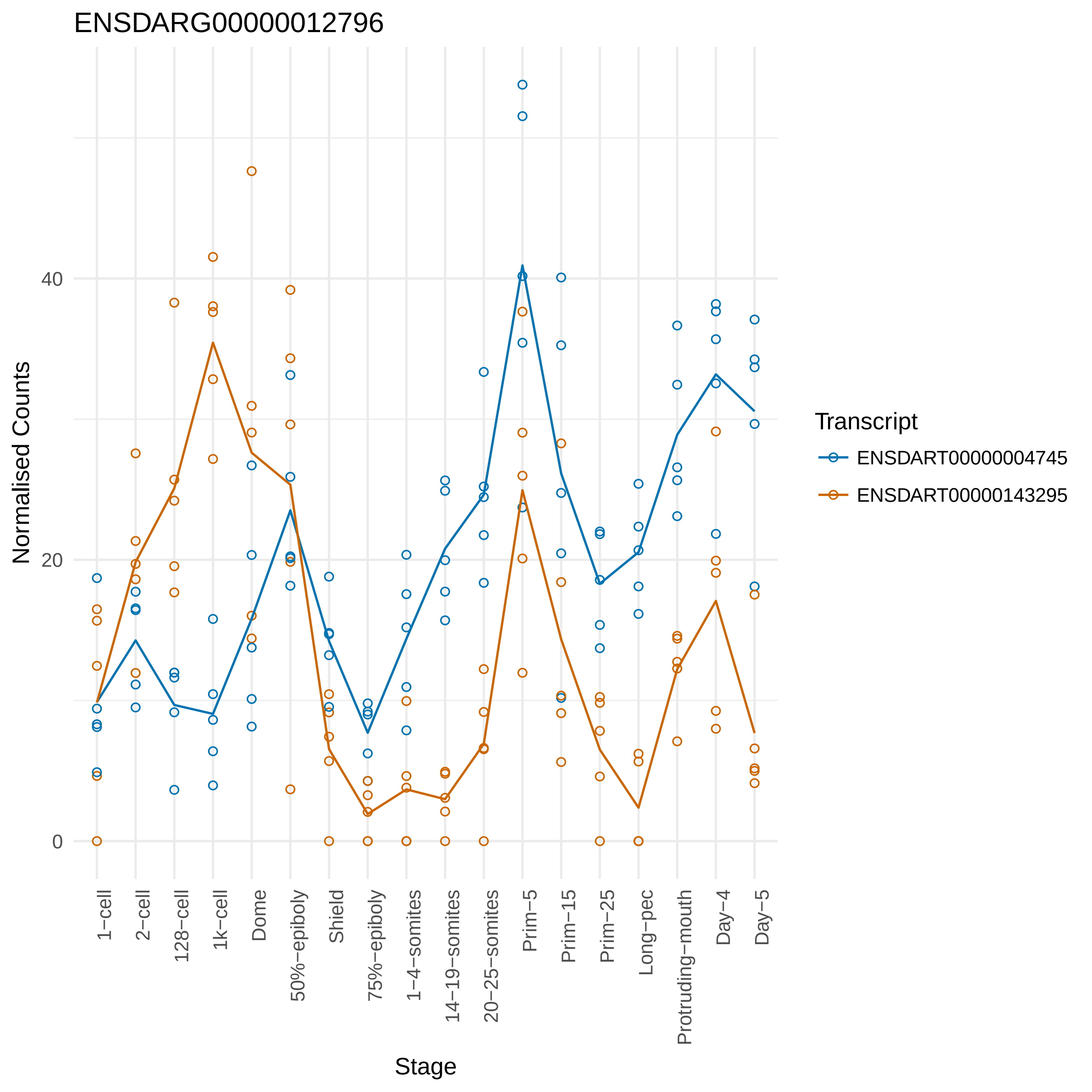

ENSDARG00000012796
Normalised Counts
0
20
40
Stage
1−cell
2−cell
128−cell
1k−cell
Dome
50%−epiboly
Shield
75%−epiboly
1−4−somites
14−19−somites
20−25−somites
Prim−5
Prim−15
Prim−25
Long−pec
Protruding−mouth
Day−4
Day−5
Transcript
ENSDART00000004745
ENSDART00000143295

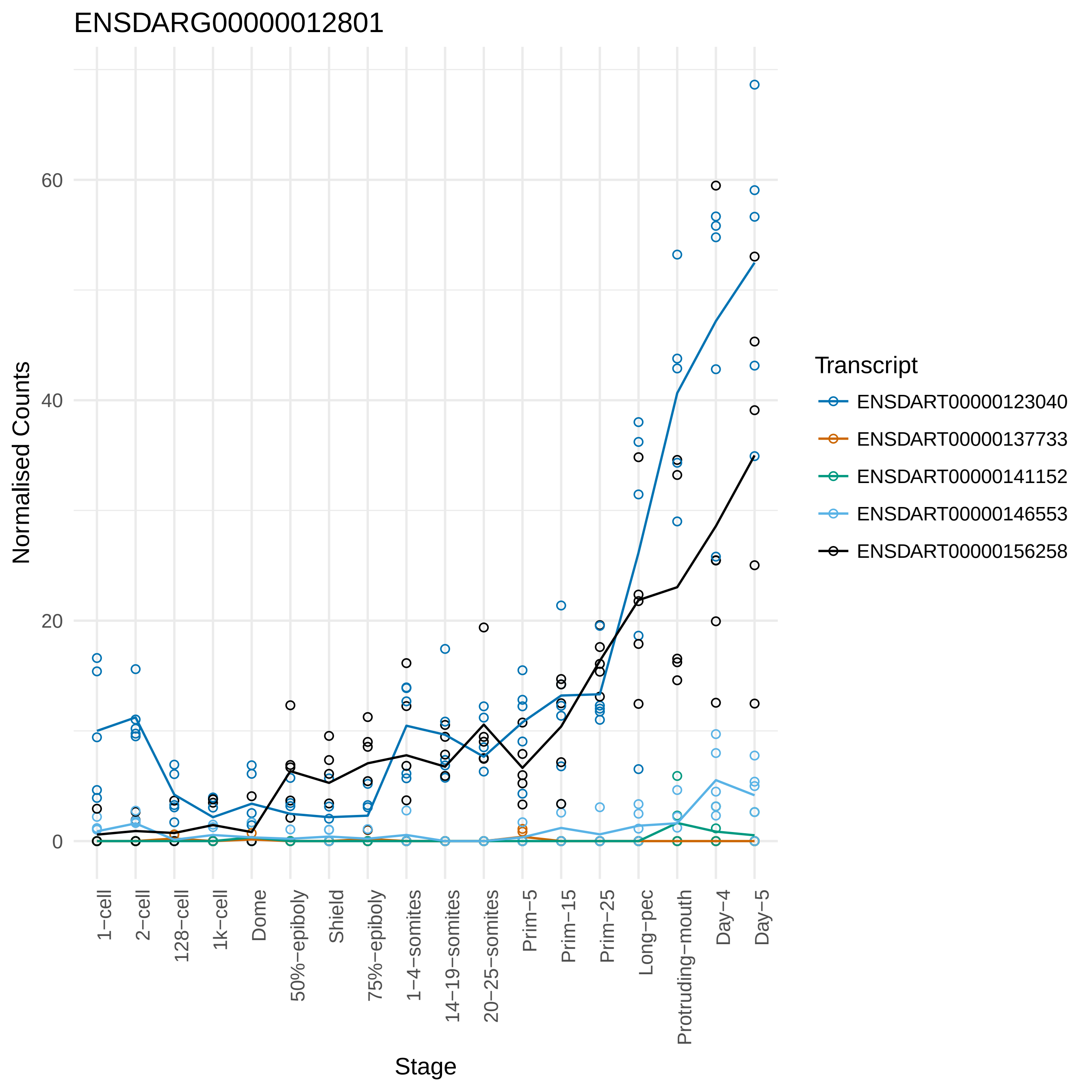

ENSDARG00000012801
Normalised Counts
0
20
40
60
Stage
1−cell
2−cell
128−cell
1k−cell
Dome
50%−epiboly
Shield
75%−epiboly
1−4−somites
14−19−somites
20−25−somites
Prim−5
Prim−15
Prim−25
Long−pec
Protruding−mouth
Day−4
Day−5
Transcript
ENSDART00000123040
ENSDART00000137733
ENSDART00000141152
ENSDART00000146553
ENSDART00000156258

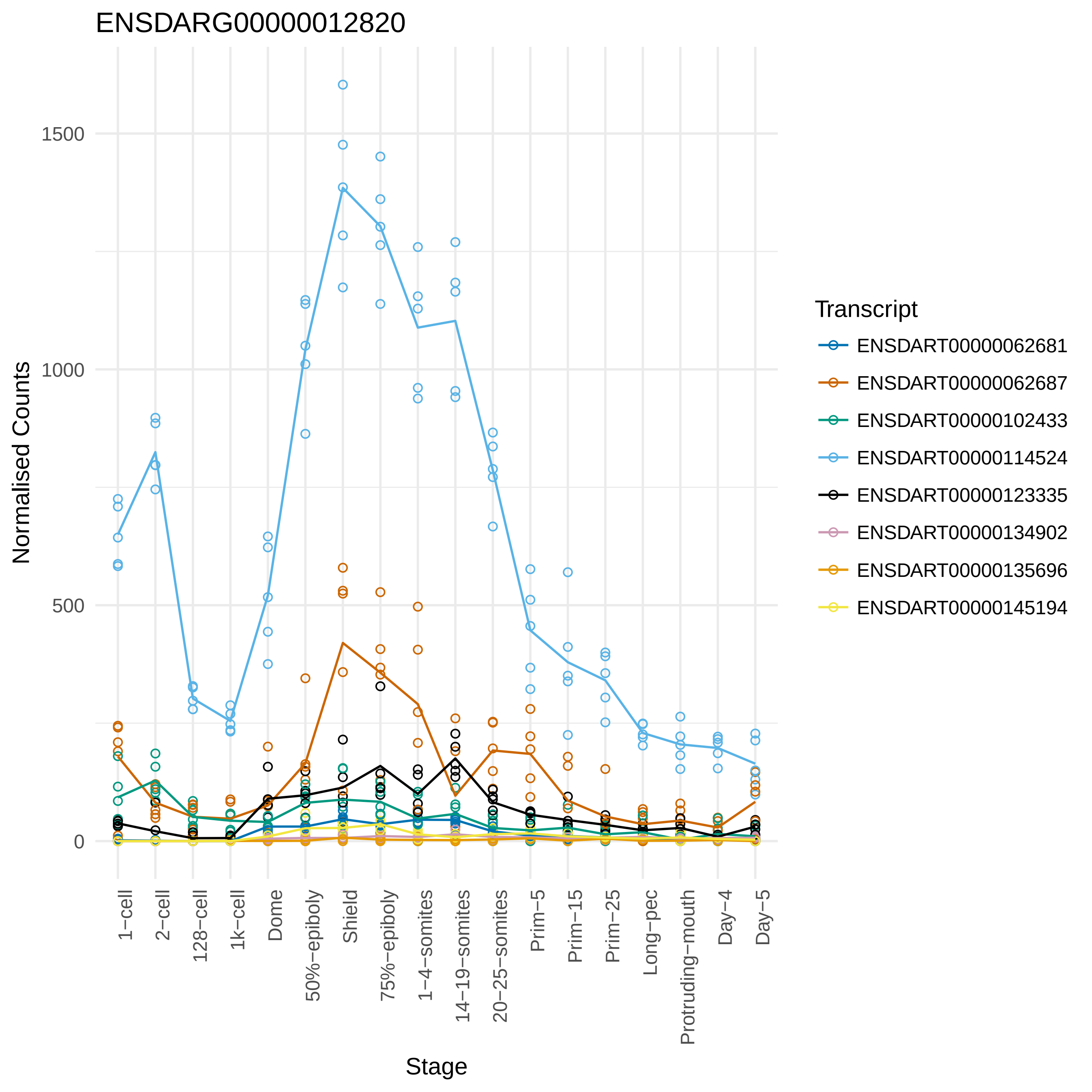

ENSDARG00000012820
Normalised Counts
1500
1000
500
0
1−cell
2−cell
128−cell
1k−cell
Dome
50%−epiboly
Shield
75%−epiboly
1−4−somites
14−19−somites
20−25−somites
Prim−5
Prim−15
Prim−25
Long−pec
Protruding−mouth
Day−4
Day−5
Stage
Transcript
ENSDART00000062681
ENSDART00000062687
ENSDART00000102433
ENSDART00000114524
ENSDART00000123335
ENSDART00000134902
ENSDART00000135696
ENSDART00000145194

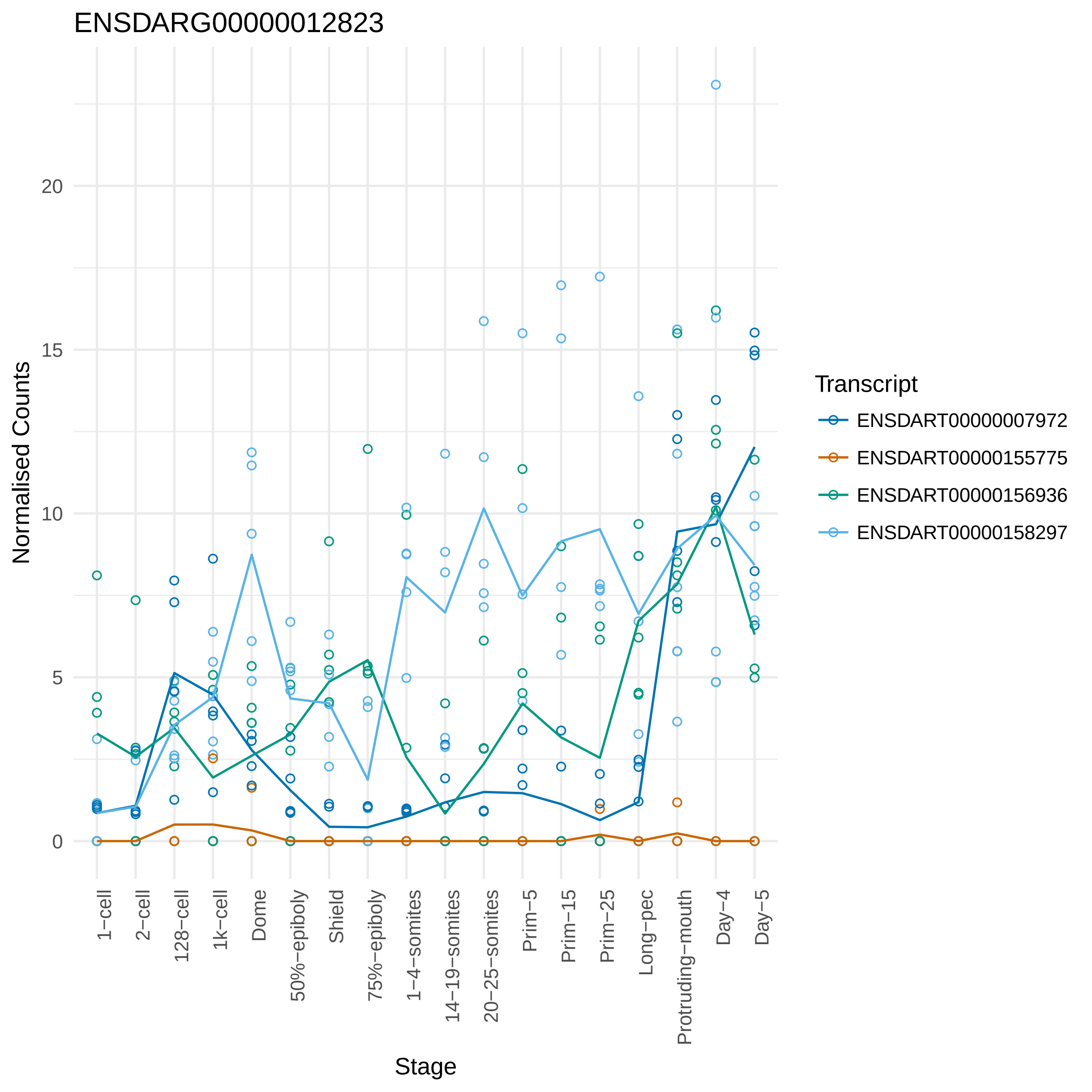

ENSDARG00000012823
Normalised Counts
0
5
10
15
20
Stage
1−cell
2−cell
128−cell
1k−cell
Dome
50%−epiboly
Shield
75%−epiboly
1−4−somites
14−19−somites
20−25−somites
Prim−5
Prim−15
Prim−25
Long−pec
Protruding−mouth
Day−4
Day−5
Transcript
ENSDART00000007972
ENSDART00000155775
ENSDART00000156936
ENSDART00000158297

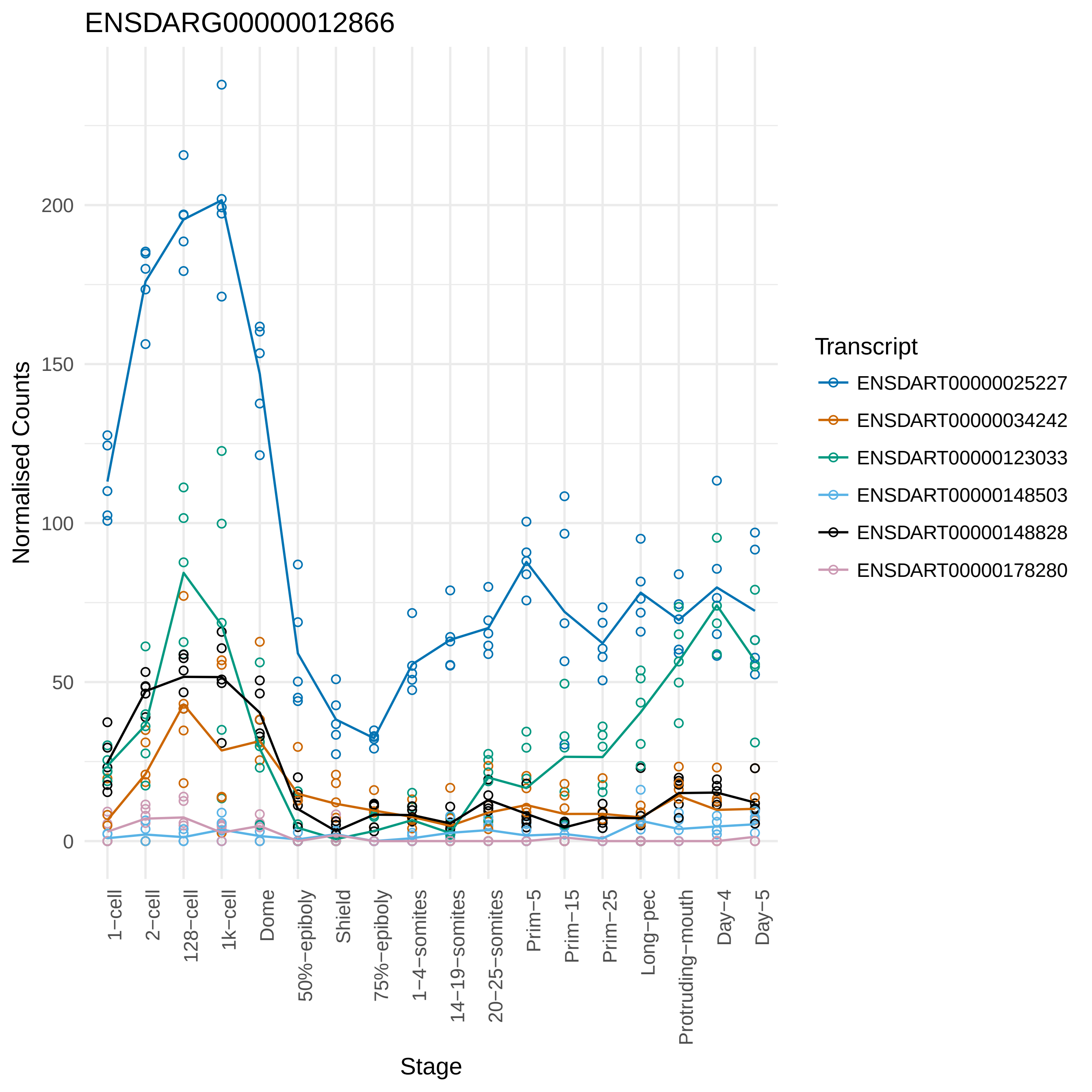
ENSDARG00000012866
Normalised Counts
0
50
100
150
200
Stage
1−cell
2−cell
128−cell
1k−cell
Dome
50%−epiboly
Shield
75%−epiboly
1−4−somites
14−19−somites
20−25−somites
Prim−5
Prim−15
Prim−25
Long−pec
Protruding−mouth
Day−4
Day−5
Transcript
ENSDART00000025227
ENSDART00000034242
ENSDART00000123033
ENSDART00000148503
ENSDART00000148828
ENSDART00000178280

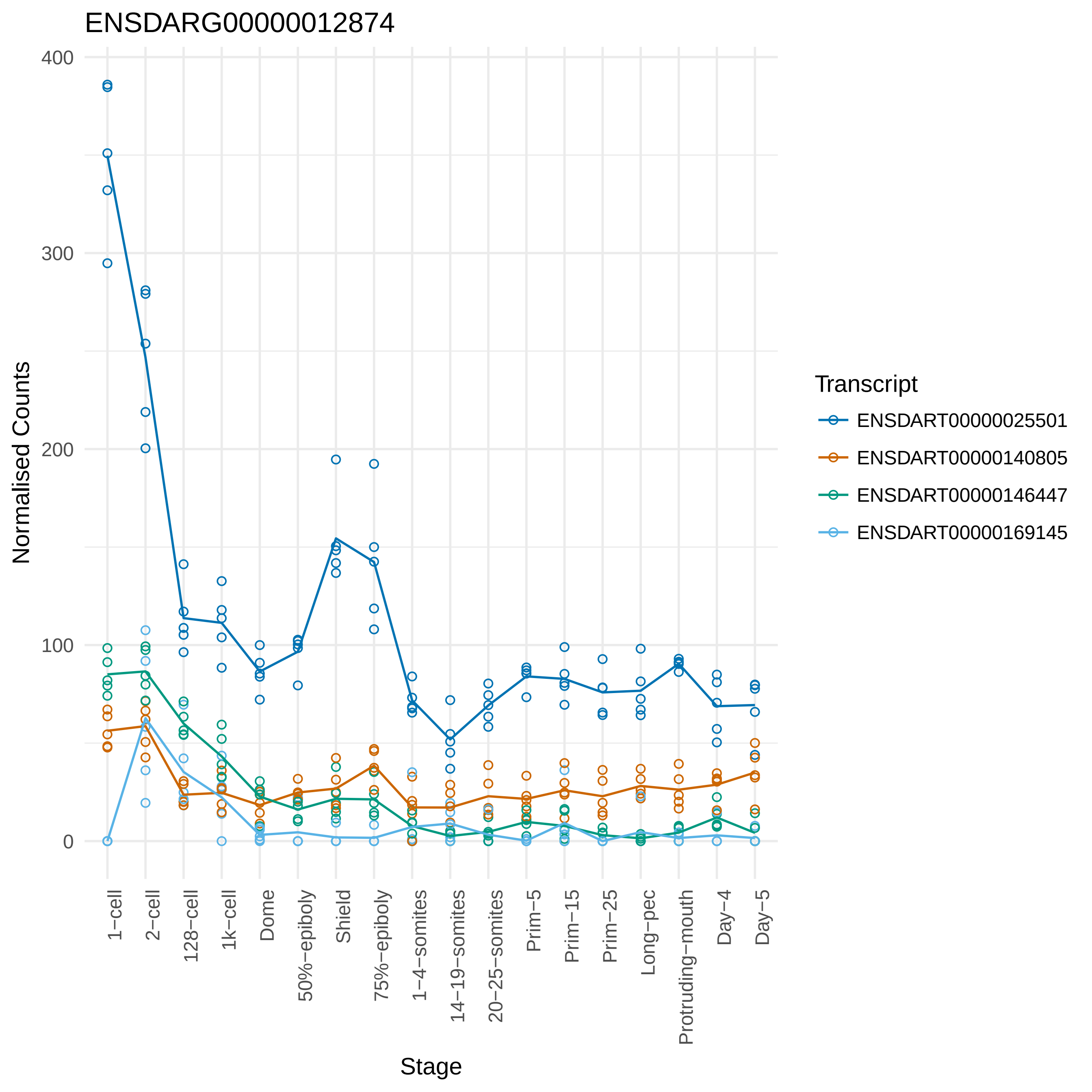

ENSDARG00000012874
Normalised Counts
400
300
200
100
0
1−cell
2−cell
128−cell
1k−cell
Dome
50%−epiboly
Shield
75%−epiboly
1−4−somites
14−19−somites
20−25−somites
Prim−5
Prim−15
Prim−25
Long−pec
Protruding−mouth
Day−4
Day−5
Stage
Transcript
ENSDART00000025501
ENSDART00000140805
ENSDART00000146447
ENSDART00000169145

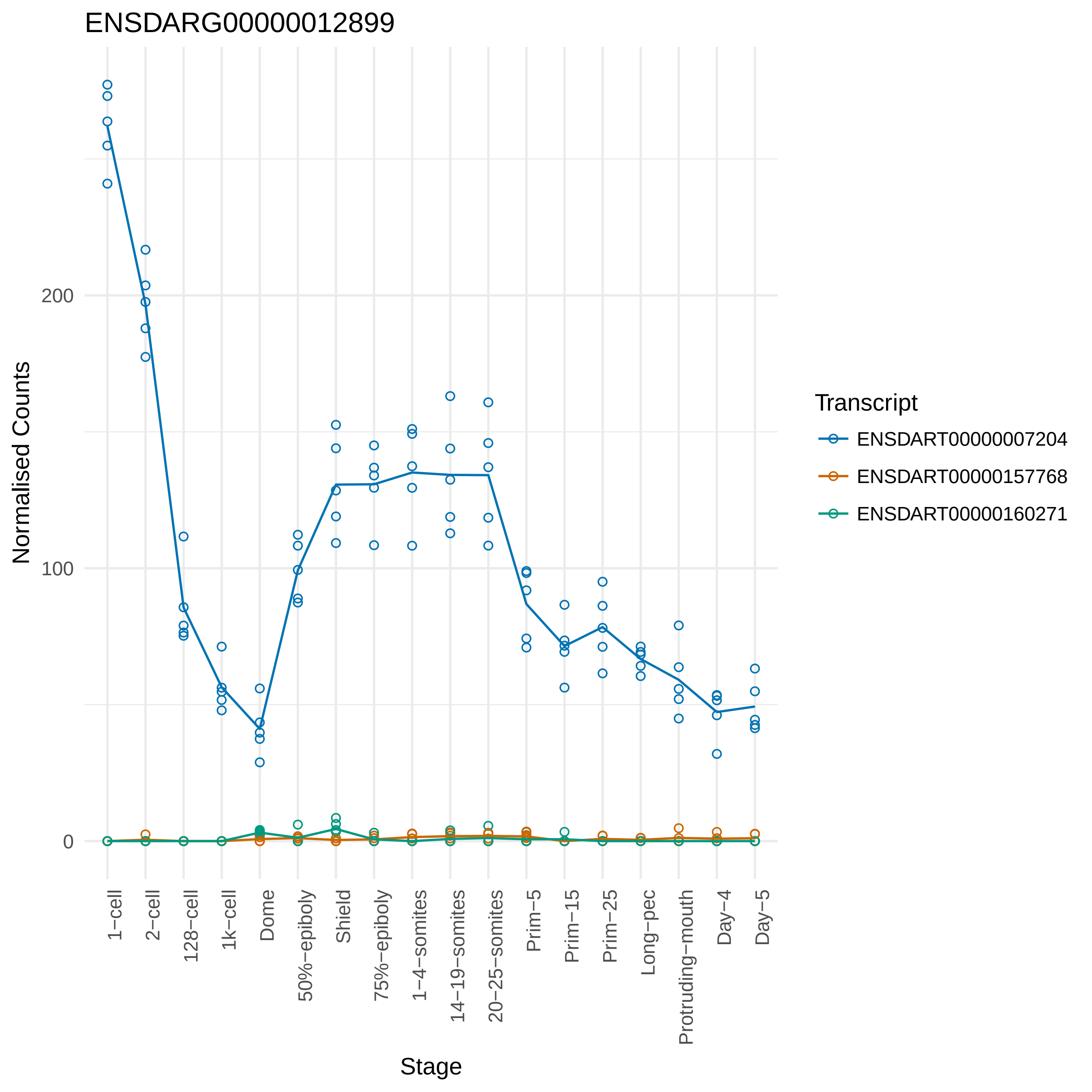
ENSDARG00000012899
Normalised Counts
0
100
200
1−cell
2−cell
128−cell
1k−cell
Dome
50%−epiboly
Shield
75%−epiboly
1−4−somites
14−19−somites
20−25−somites
Prim−5
Prim−15
Prim−25
Long−pec
Protruding−mouth
Day−4
Day−5
Stage
Transcript
ENSDART00000007204
ENSDART00000157768
ENSDART00000160271

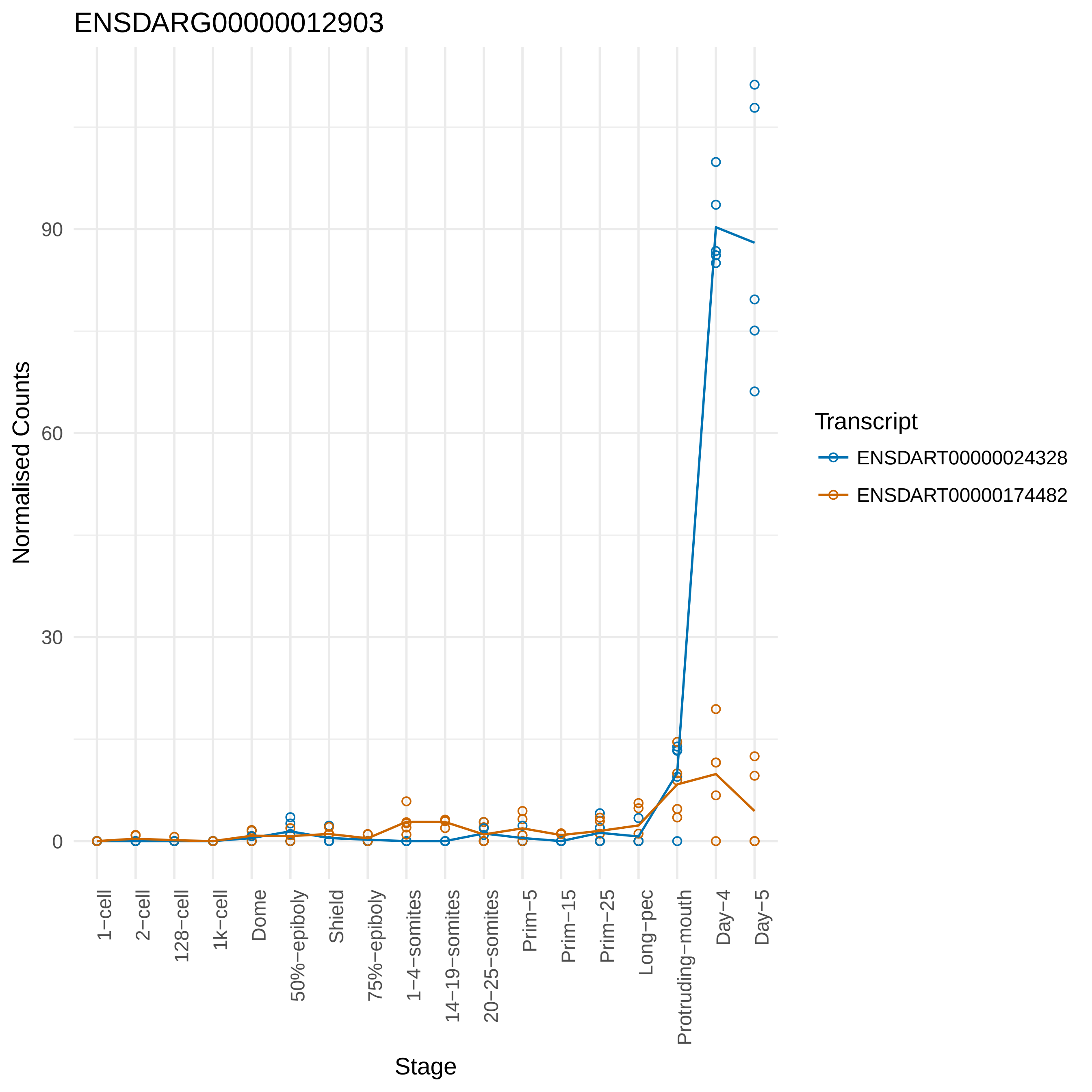

ENSDARG00000012903
Normalised Counts
90
60
30
0
1−cell
2−cell
128−cell
1k−cell
Dome
50%−epiboly
Shield
75%−epiboly
1−4−somites
14−19−somites
20−25−somites
Prim−5
Prim−15
Prim−25
Long−pec
Protruding−mouth
Day−4
Day−5
Stage
Transcript
ENSDART00000024328
ENSDART00000174482

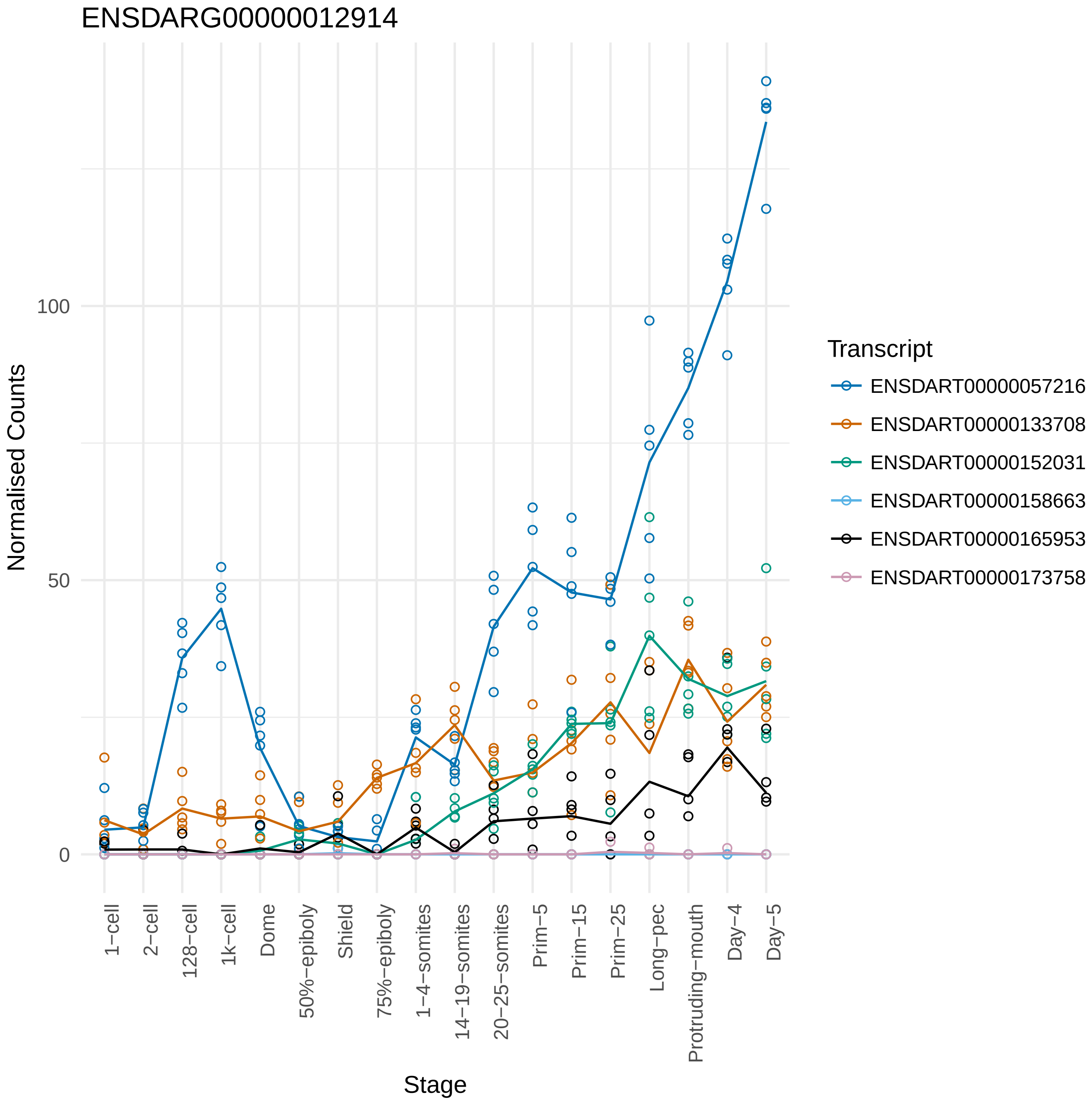

ENSDARG00000012914
Normalised Counts
0
50
100
Stage
1−cell
2−cell
128−cell
1k−cell
Dome
50%−epiboly
Shield
75%−epiboly
1−4−somites
14−19−somites
20−25−somites
Prim−5
Prim−15
Prim−25
Long−pec
Protruding−mouth
Day−4
Day−5
Transcript
ENSDART00000057216
ENSDART00000133708
ENSDART00000152031
ENSDART00000158663
ENSDART00000165953
ENSDART00000173758

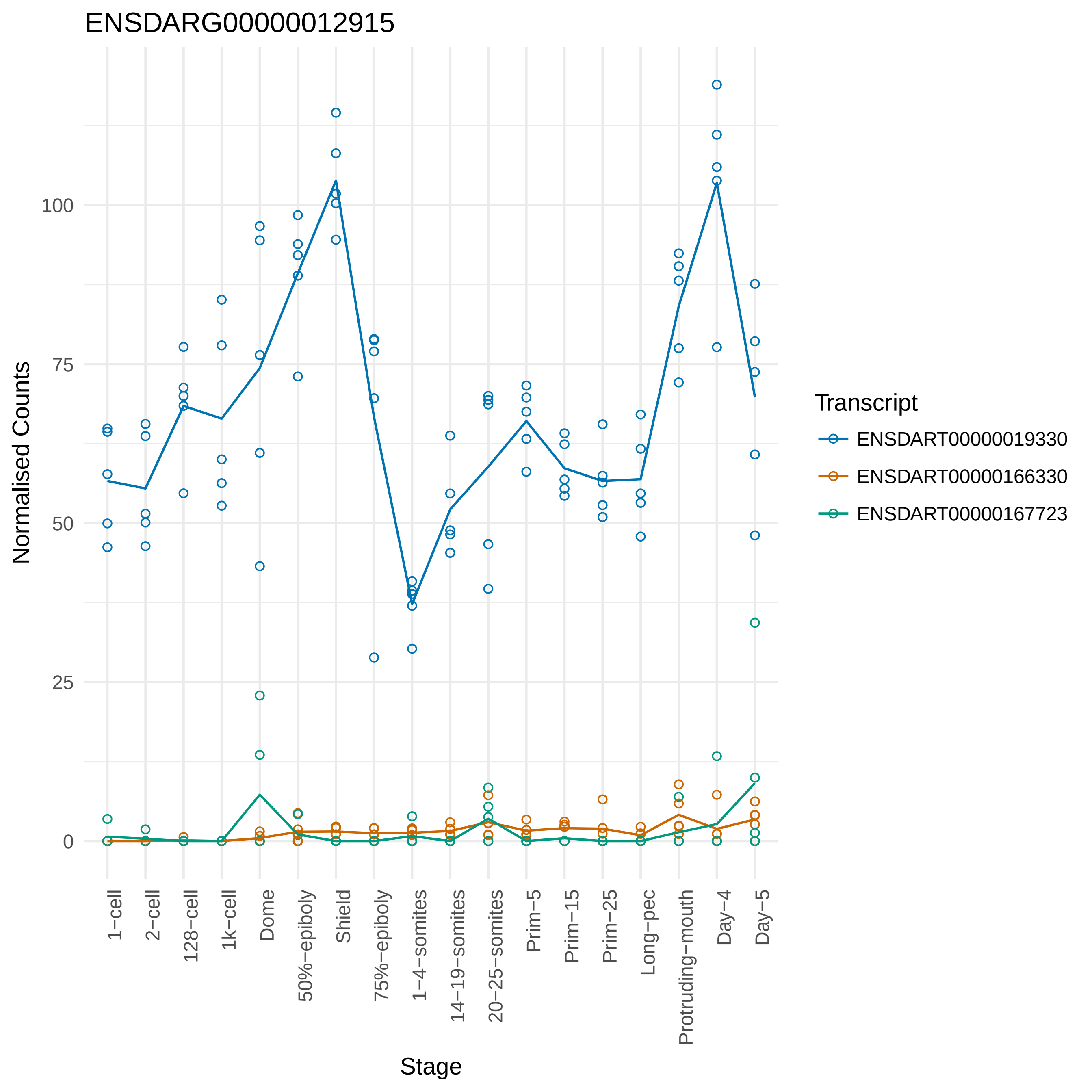

ENSDARG00000012915
Normalised Counts
0
25
50
75
100
1−cell
2−cell
128−cell
1k−cell
Dome
50%−epiboly
Shield
75%−epiboly
1−4−somites
14−19−somites
20−25−somites
Prim−5
Prim−15
Prim−25
Long−pec
Protruding−mouth
Day−4
Day−5
Stage
Transcript
ENSDART00000019330
ENSDART00000166330
ENSDART00000167723

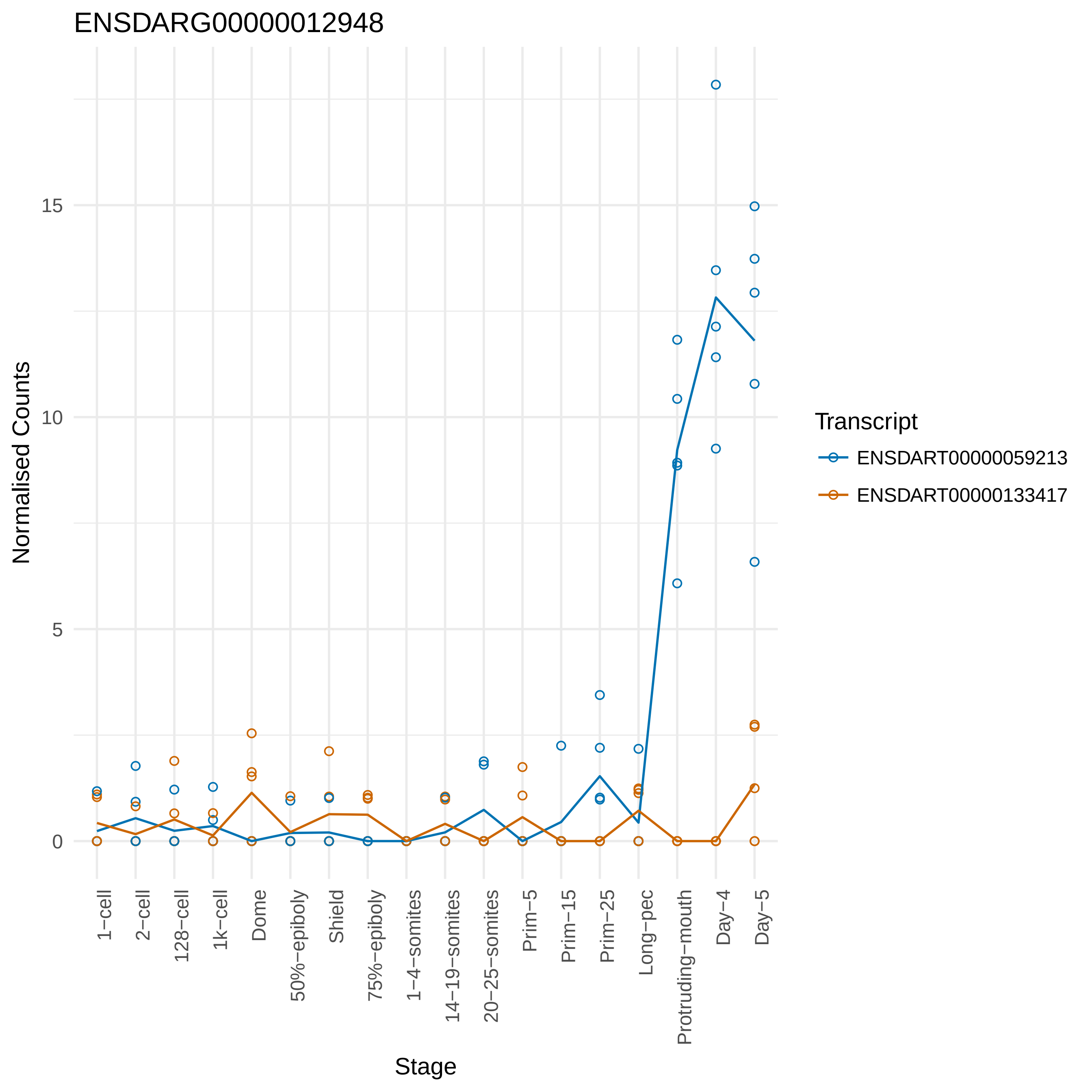

ENSDARG00000012948
Normalised Counts
0
5
10
15
Stage
1−cell
2−cell
128−cell
1k−cell
Dome
50%−epiboly
Shield
75%−epiboly
1−4−somites
14−19−somites
20−25−somites
Prim−5
Prim−15
Prim−25
Long−pec
Protruding−mouth
Day−4
Day−5
Transcript
ENSDART00000059213
ENSDART00000133417

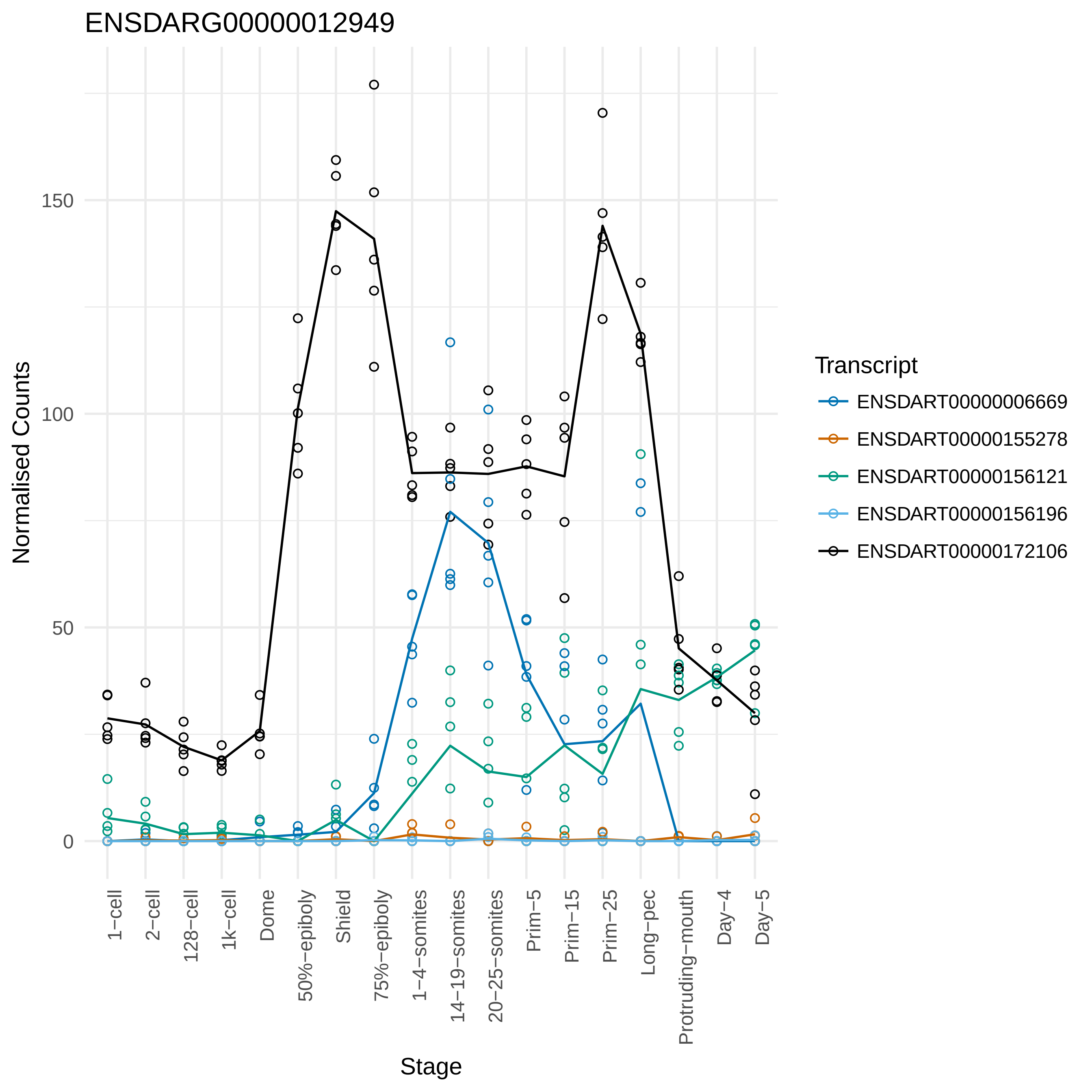

ENSDARG00000012949
Normalised Counts
150
100
50
0
1−cell
2−cell
128−cell
1k−cell
Dome
50%−epiboly
Shield
75%−epiboly
1−4−somites
14−19−somites
20−25−somites
Prim−5
Prim−15
Prim−25
Long−pec
Protruding−mouth
Day−4
Day−5
Stage
Transcript
ENSDART00000006669
ENSDART00000155278
ENSDART00000156121
ENSDART00000156196
ENSDART00000172106

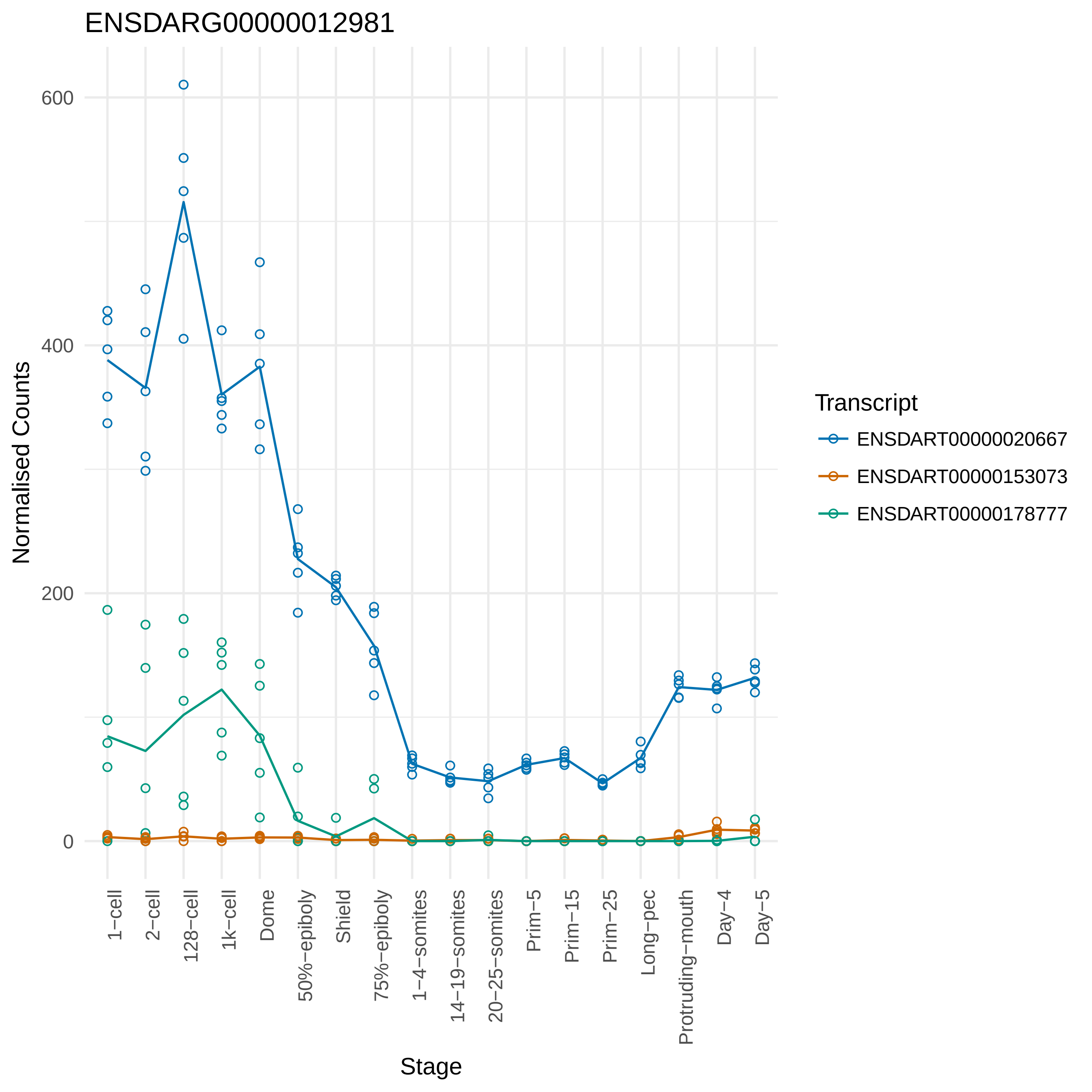

ENSDARG00000012981
Normalised Counts
600
400
200
0
Stage
1−cell
2−cell
128−cell
1k−cell
Dome
50%−epiboly
Shield
75%−epiboly
1−4−somites
14−19−somites
20−25−somites
Prim−5
Prim−15
Prim−25
Long−pec
Protruding−mouth
Day−4
Day−5
Transcript
ENSDART00000020667
ENSDART00000153073
ENSDART00000178777

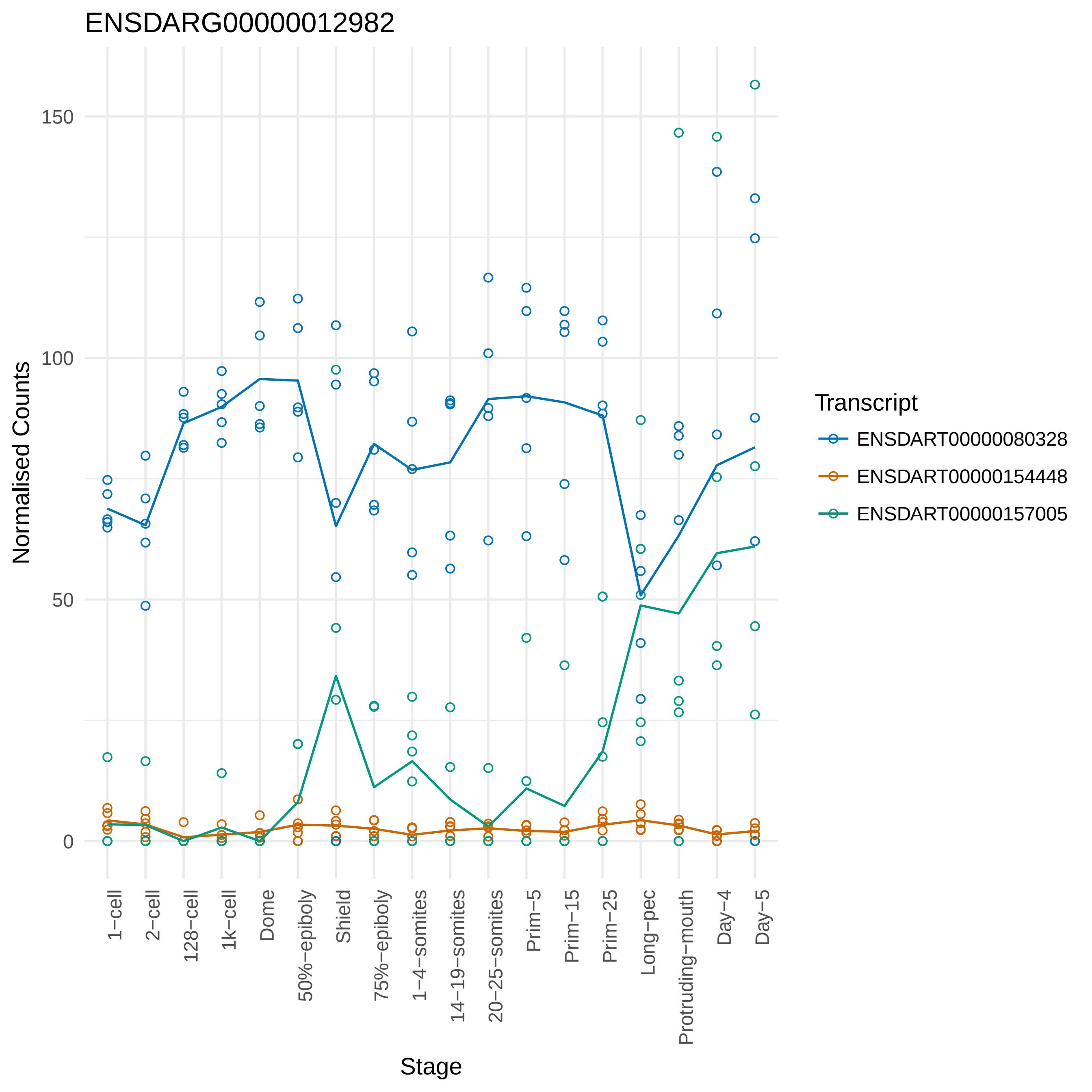

ENSDARG00000012982
Normalised Counts
150
100
50
0
1−cell
2−cell
128−cell
1k−cell
Dome
50%−epiboly
Shield
75%−epiboly
1−4−somites
14−19−somites
20−25−somites
Prim−5
Prim−15
Prim−25
Long−pec
Protruding−mouth
Day−4
Day−5
Stage
Transcript
ENSDART00000080328
ENSDART00000154448
ENSDART00000157005

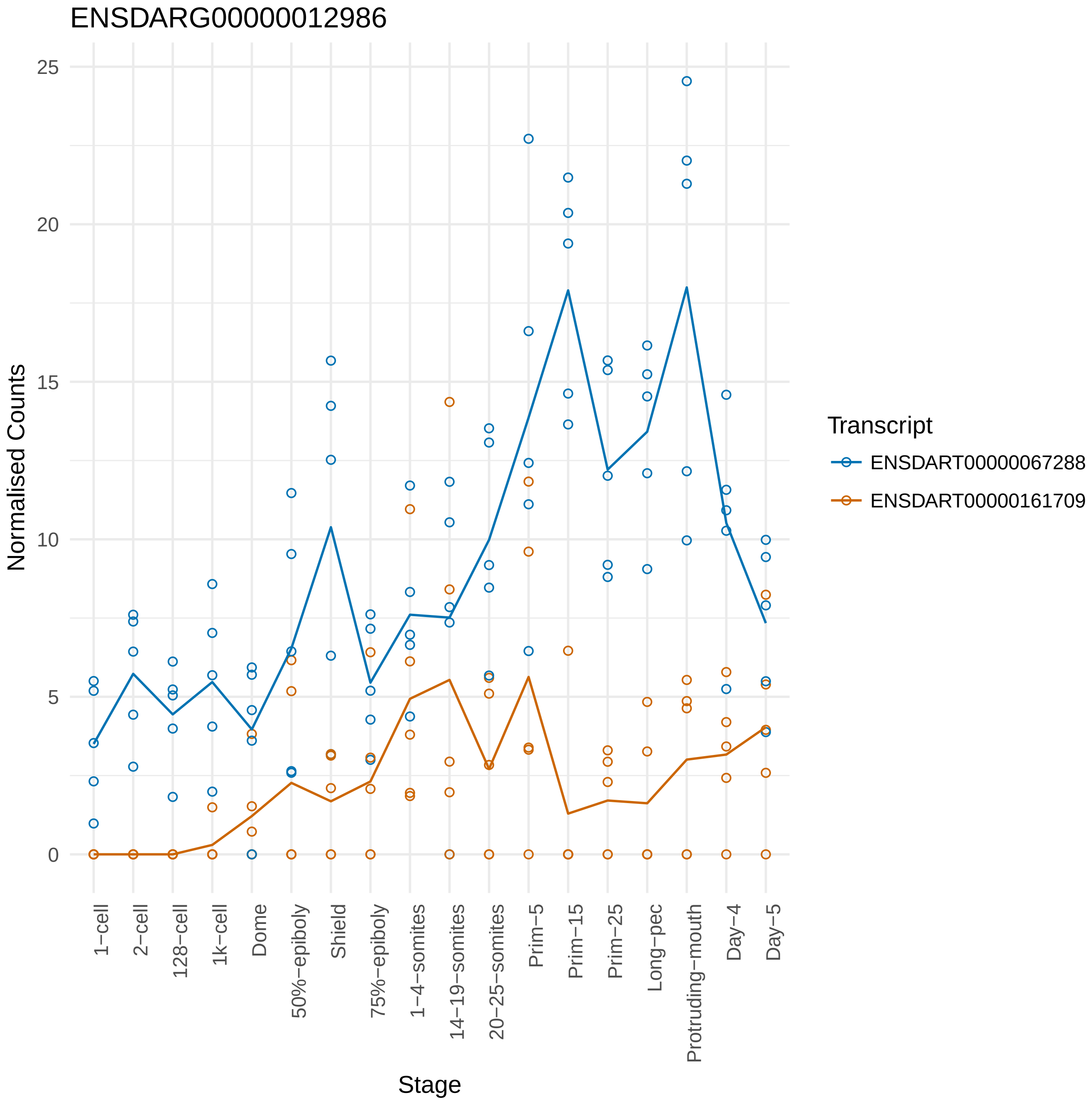
ENSDARG00000012986
Normalised Counts
0
5
10
15
20
25
Stage
1−cell
2−cell
128−cell
1k−cell
Dome
50%−epiboly
Shield
75%−epiboly
1−4−somites
14−19−somites
20−25−somites
Prim−5
Prim−15
Prim−25
Long−pec
Protruding−mouth
Day−4
Day−5
Transcript
ENSDART00000067288
ENSDART00000161709

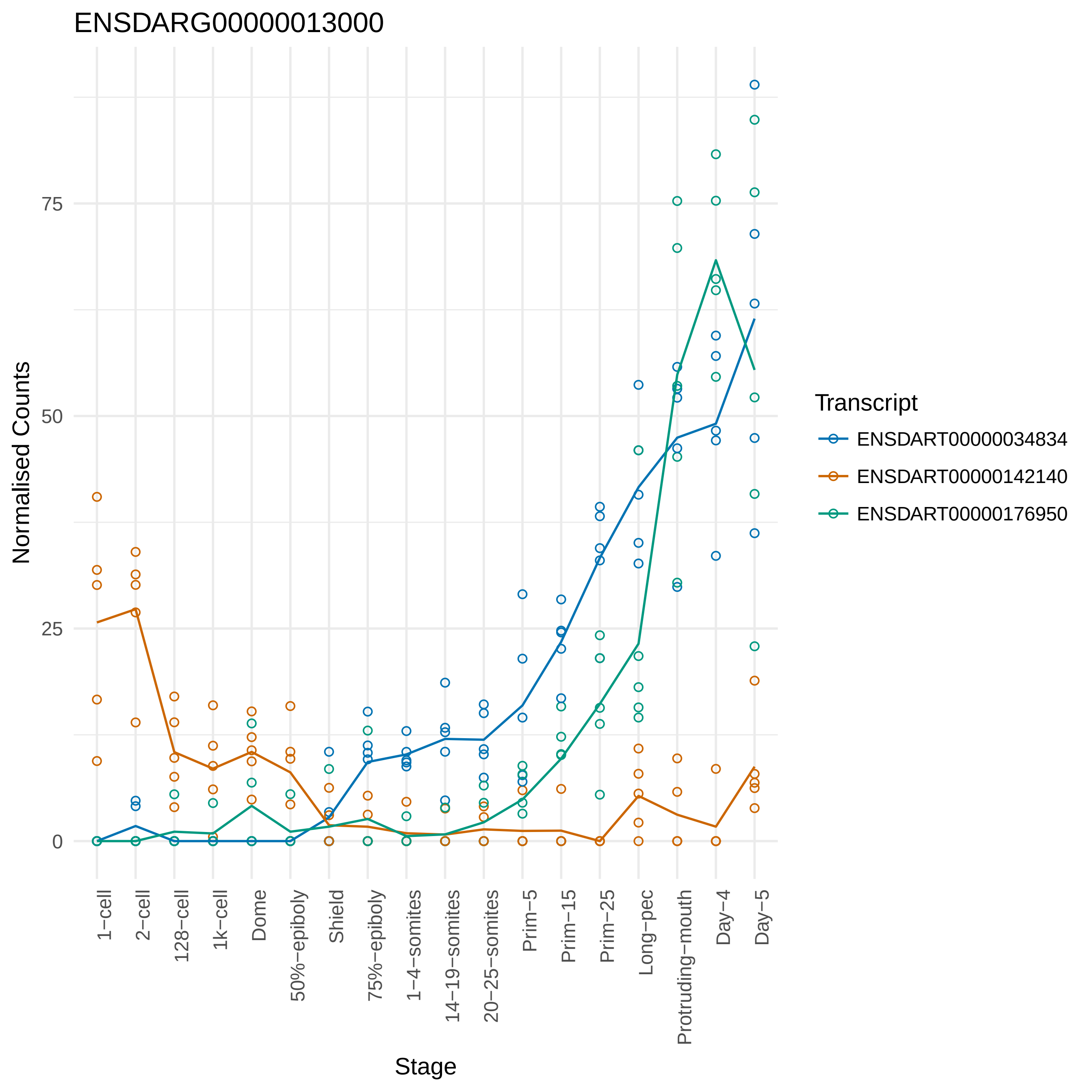

ENSDARG00000013000
Normalised Counts
0
25
50
75
1−cell
2−cell
128−cell
1k−cell
Dome
50%−epiboly
Shield
75%−epiboly
1−4−somites
14−19−somites
20−25−somites
Prim−5
Prim−15
Prim−25
Long−pec
Protruding−mouth
Day−4
Day−5
Stage
Transcript
ENSDART00000034834
ENSDART00000142140
ENSDART00000176950

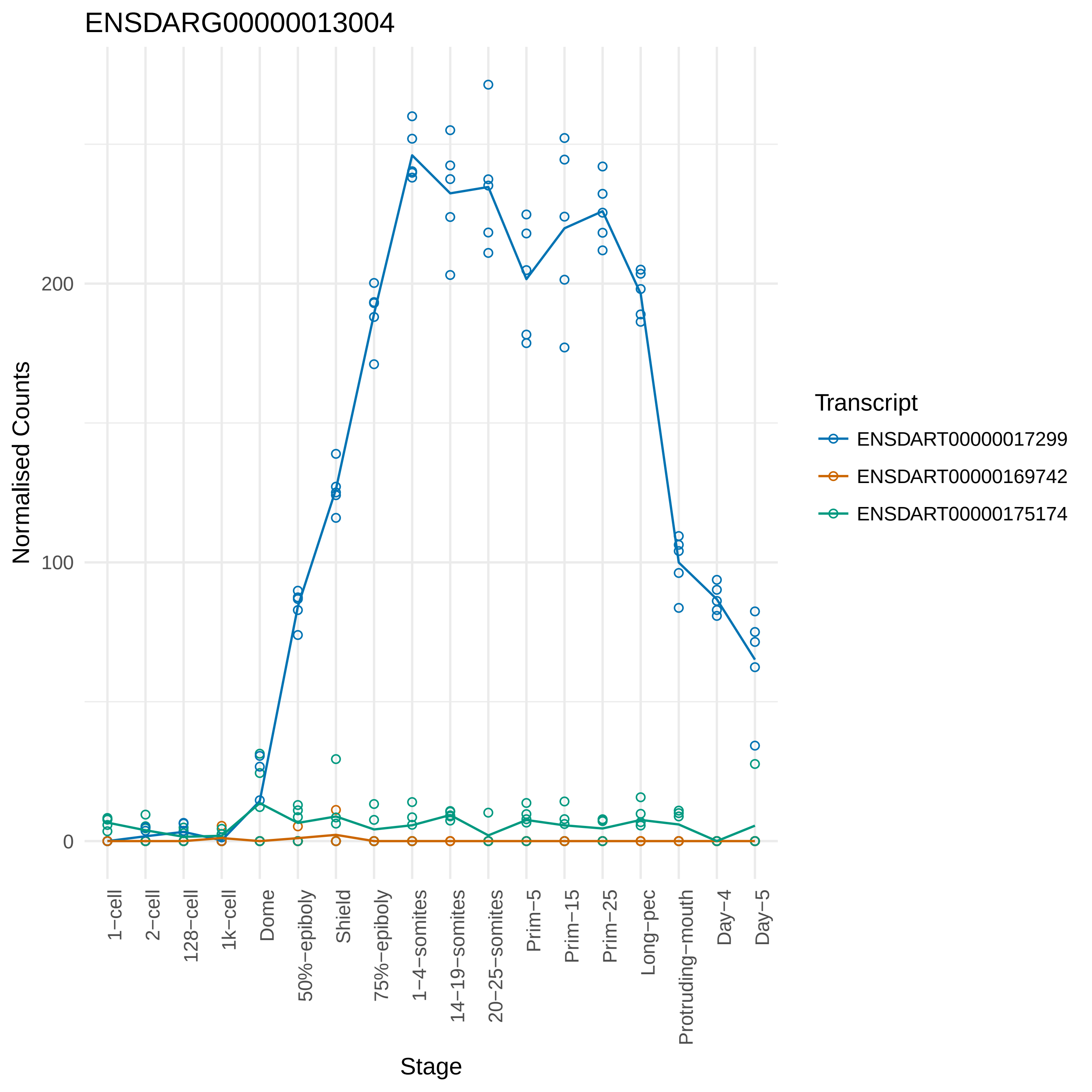

ENSDARG00000013004
Normalised Counts
200
100
0
1−cell
2−cell
128−cell
1k−cell
Dome
50%−epiboly
Shield
75%−epiboly
1−4−somites
14−19−somites
20−25−somites
Prim−5
Prim−15
Prim−25
Long−pec
Protruding−mouth
Day−4
Day−5
Stage
Transcript
ENSDART00000017299
ENSDART00000169742
ENSDART00000175174

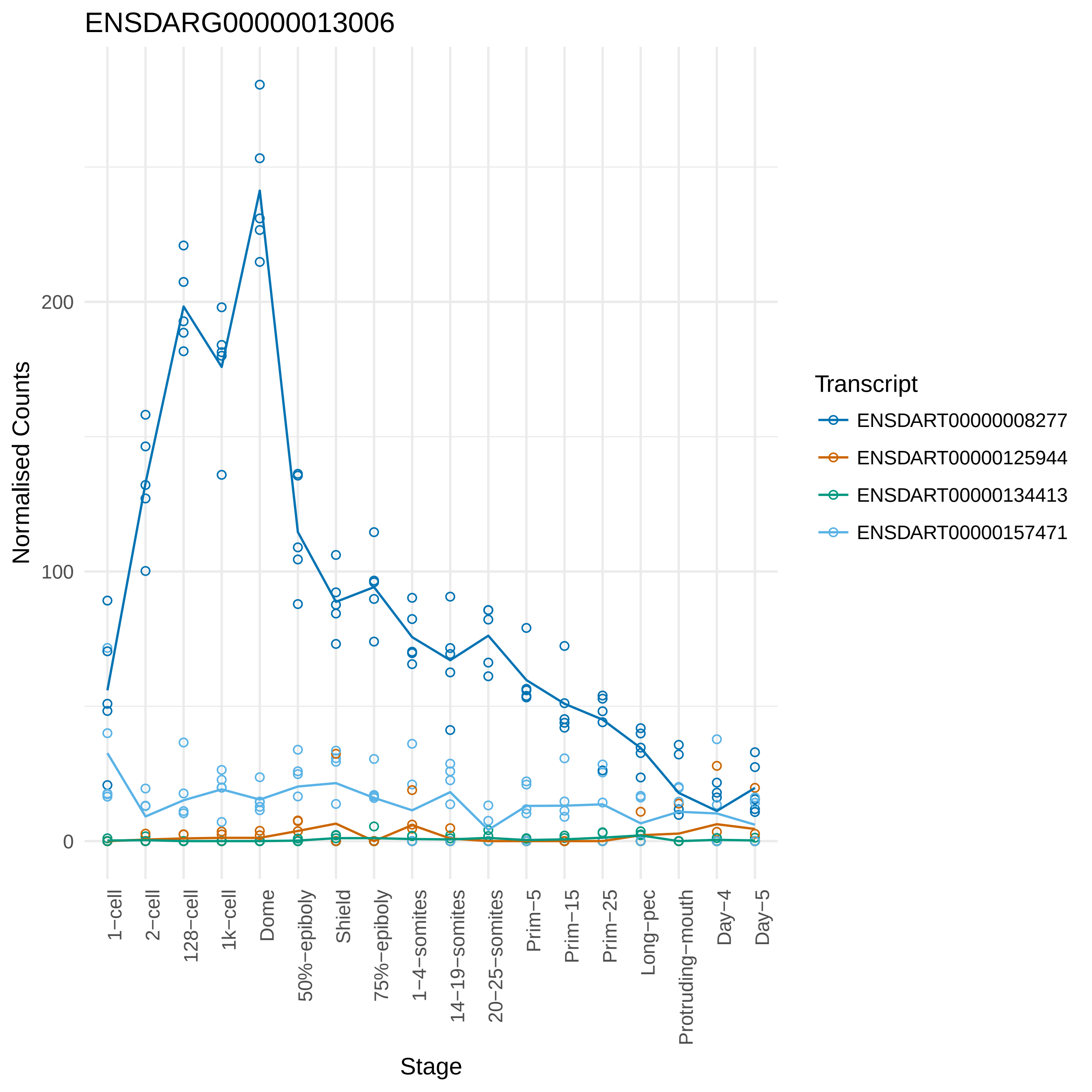
ENSDARG00000013006
Normalised Counts
200
100
0
1−cell
2−cell
128−cell
1k−cell
Dome
50%−epiboly
Shield
75%−epiboly
1−4−somites
14−19−somites
20−25−somites
Prim−5
Prim−15
Prim−25
Long−pec
Protruding−mouth
Day−4
Day−5
Stage
Transcript
ENSDART00000008277
ENSDART00000125944
ENSDART00000134413
ENSDART00000157471

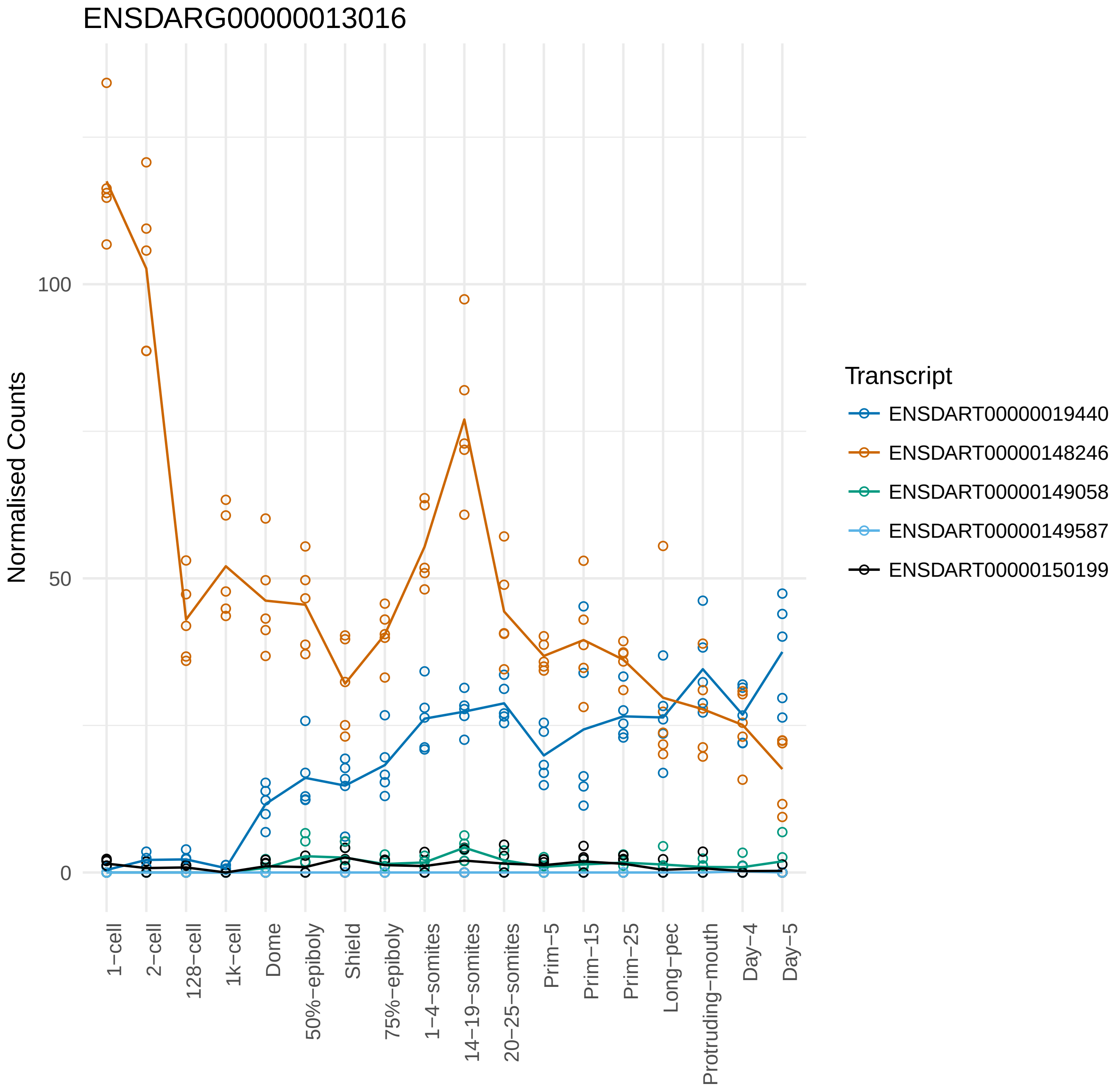

ENSDARG00000013016
Normalised Counts
100
50
0
1−cell
2−cell
128−cell
1k−cell
Dome
50%−epiboly
Shield
75%−epiboly
1−4−somites
14−19−somites
20−25−somites
Prim−5
Prim−15
Prim−25
Long−pec
Protruding−mouth
Day−4
Day−5
Stage
Transcript
ENSDART00000019440
ENSDART00000148246
ENSDART00000149058
ENSDART00000149587
ENSDART00000150199

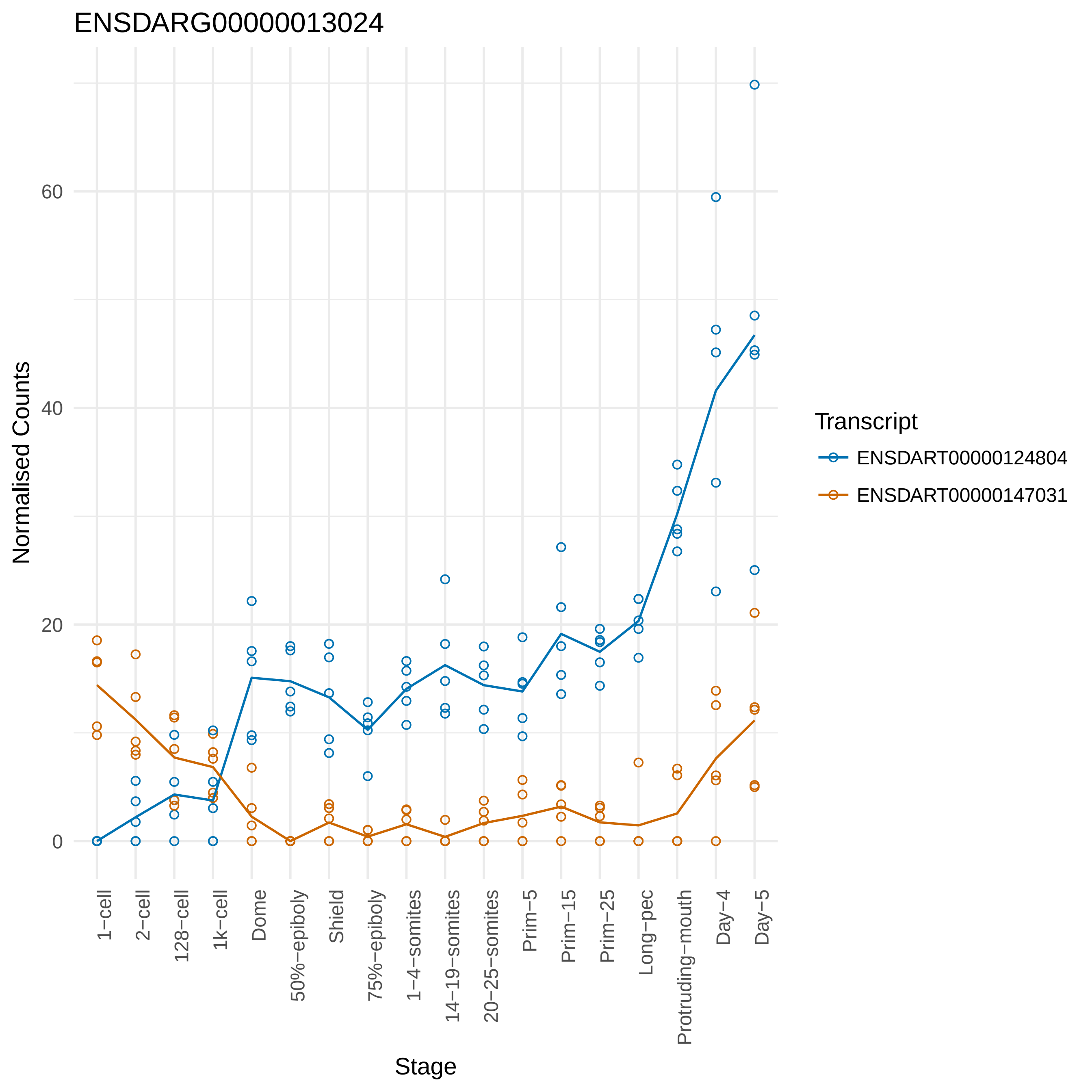
ENSDARG00000013024
Normalised Counts
0
20
40
60
1−cell
2−cell
128−cell
1k−cell
Dome
50%−epiboly
Shield
75%−epiboly
1−4−somites
14−19−somites
20−25−somites
Prim−5
Prim−15
Prim−25
Long−pec
Protruding−mouth
Day−4
Day−5
Stage
Transcript
ENSDART00000124804
ENSDART00000147031

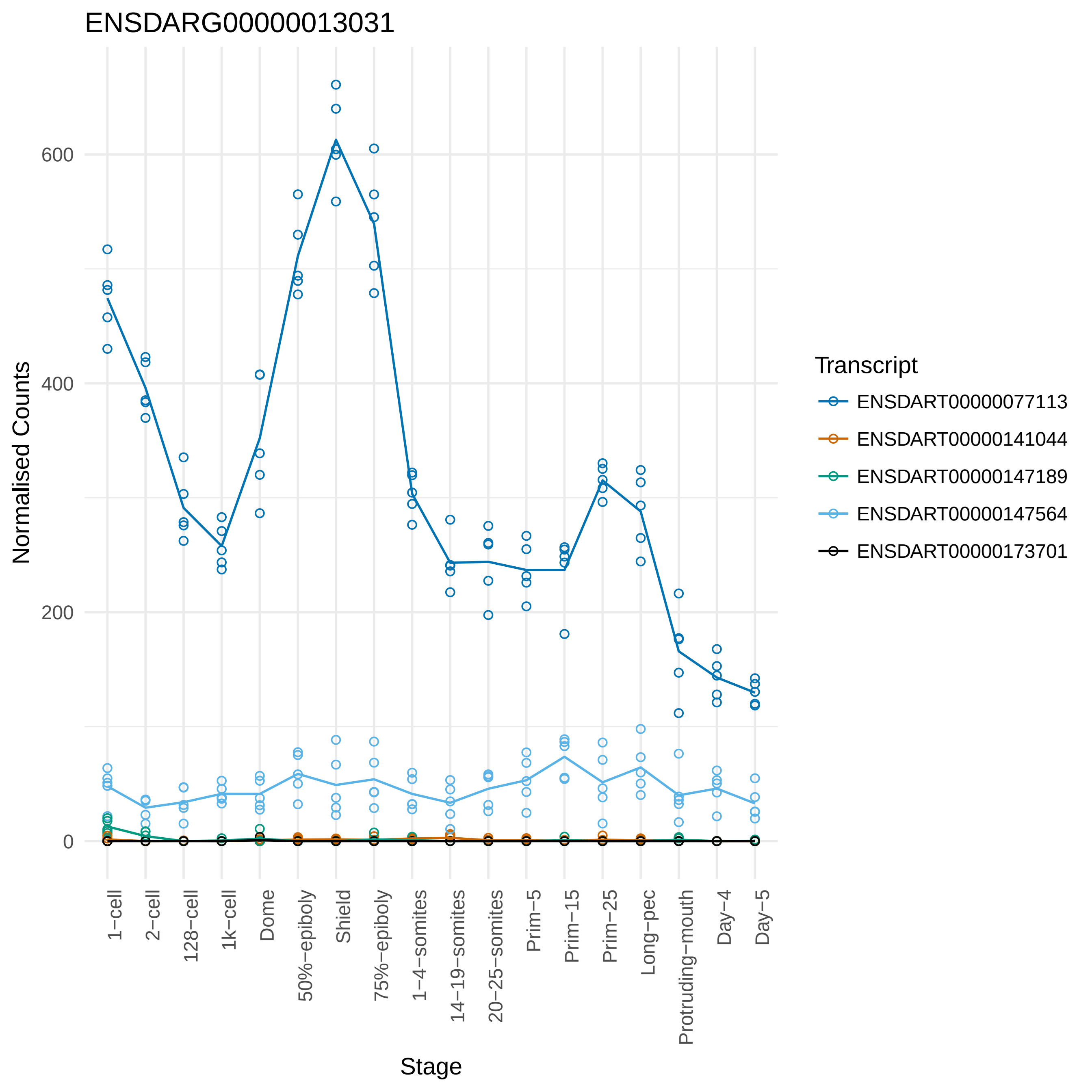

ENSDARG00000013031
Normalised Counts
0
200
400
600
1−cell
2−cell
128−cell
1k−cell
Dome
50%−epiboly
Shield
75%−epiboly
1−4−somites
14−19−somites
20−25−somites
Prim−5
Prim−15
Prim−25
Long−pec
Protruding−mouth
Day−4
Day−5
Stage
Transcript
ENSDART00000077113
ENSDART00000141044
ENSDART00000147189
ENSDART00000147564
ENSDART00000173701

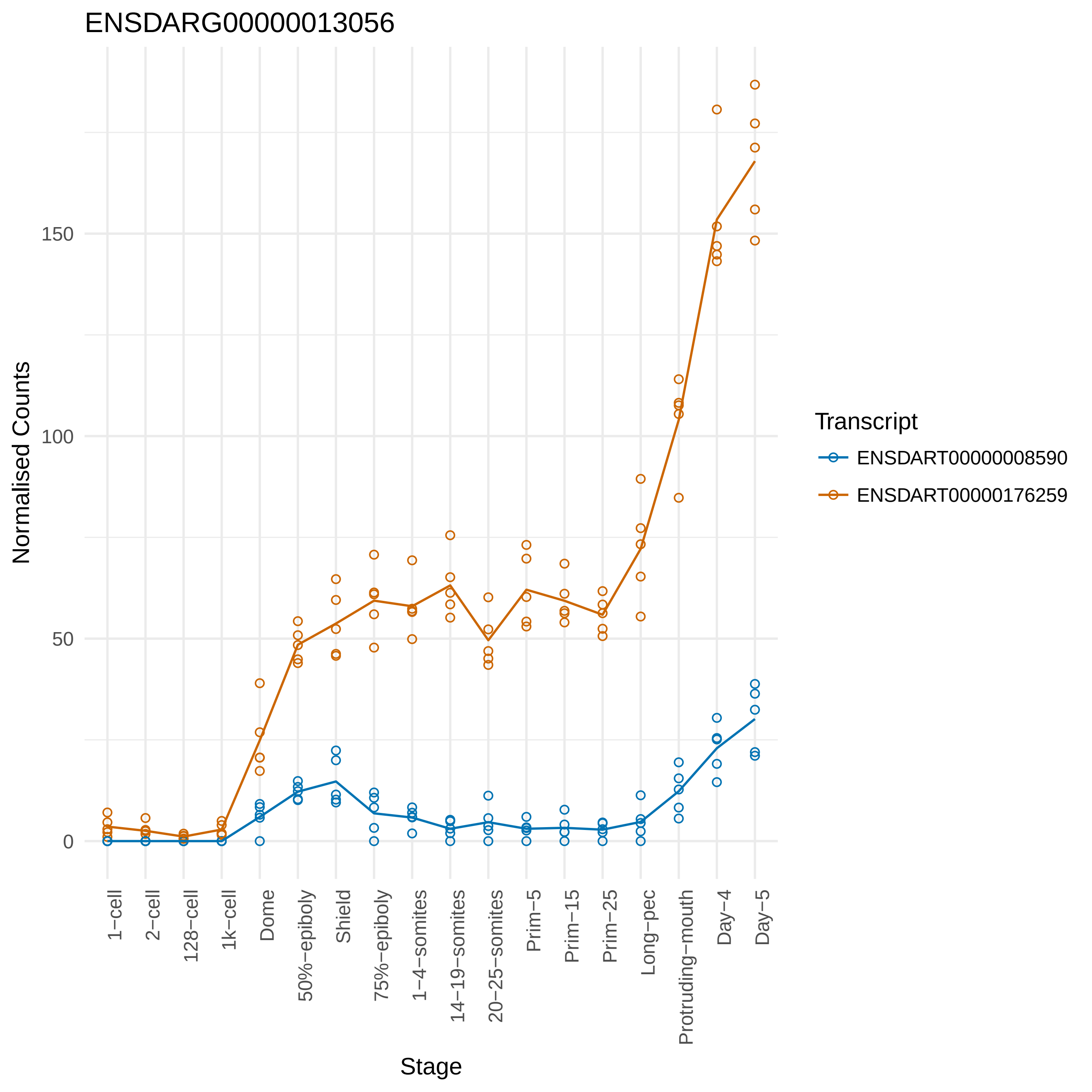

ENSDARG00000013056
Normalised Counts
0
50
100
150
Stage
1−cell
2−cell
128−cell
1k−cell
Dome
50%−epiboly
Shield
75%−epiboly
1−4−somites
14−19−somites
20−25−somites
Prim−5
Prim−15
Prim−25
Long−pec
Protruding−mouth
Day−4
Day−5
Transcript
ENSDART00000008590
ENSDART00000176259

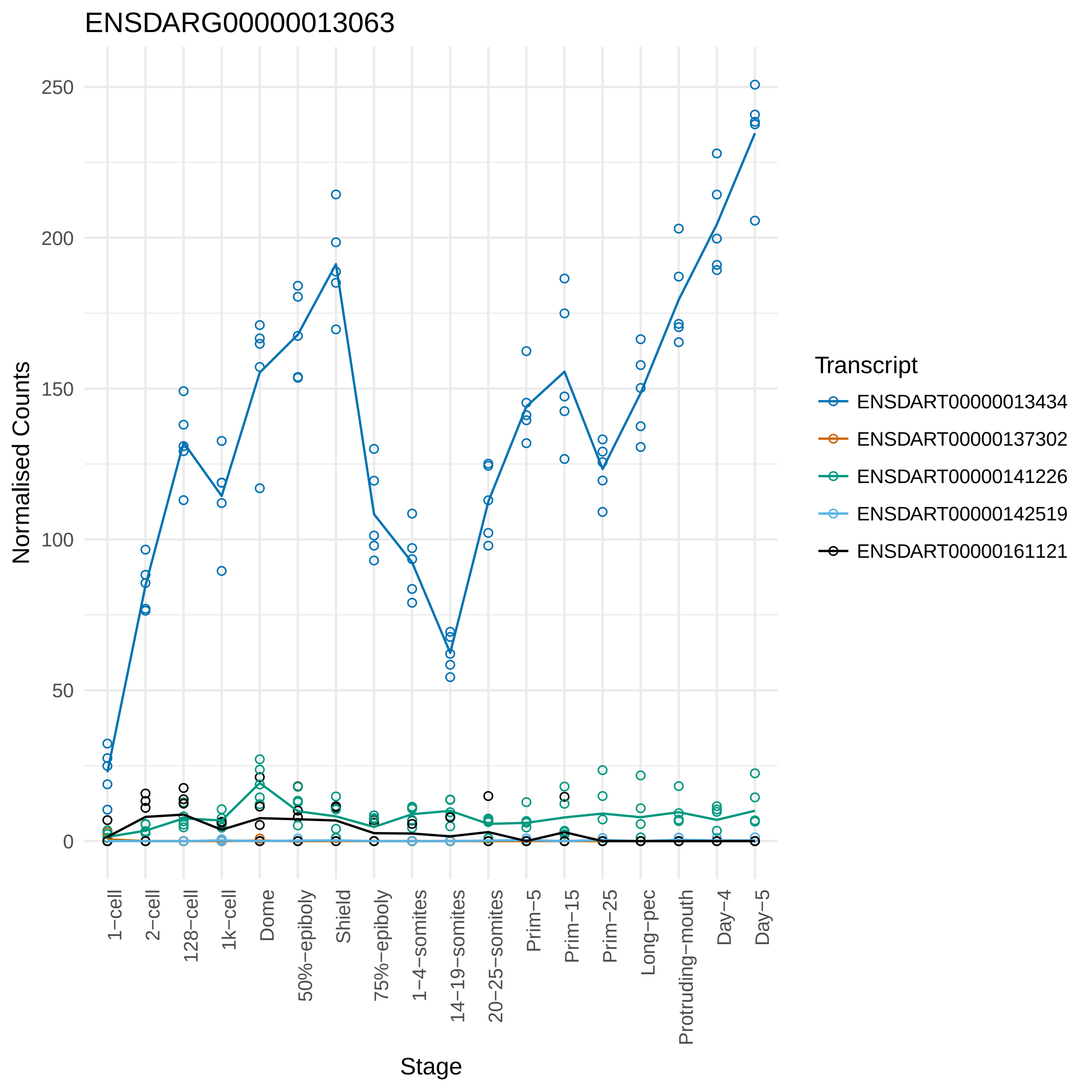

ENSDARG00000013063
Normalised Counts
250
200
150
100
50
0
1−cell
2−cell
128−cell
1k−cell
Dome
50%−epiboly
Shield
75%−epiboly
1−4−somites
14−19−somites
20−25−somites
Prim−5
Prim−15
Prim−25
Long−pec
Protruding−mouth
Day−4
Day−5
Stage
Transcript
ENSDART00000013434
ENSDART00000137302
ENSDART00000141226
ENSDART00000142519
ENSDART00000161121

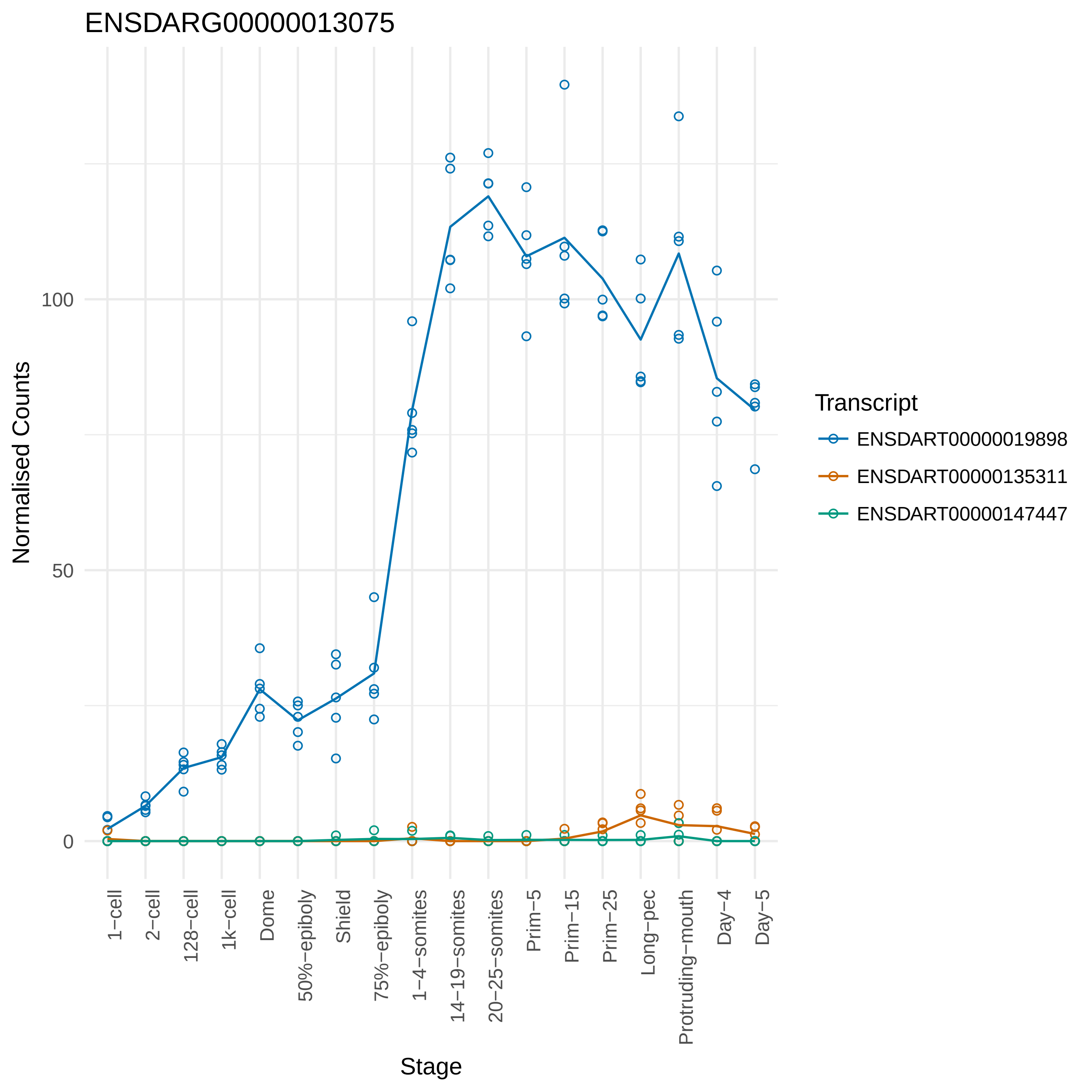

ENSDARG00000013075
Normalised Counts
100
50
0
Stage
1−cell
2−cell
128−cell
1k−cell
Dome
50%−epiboly
Shield
75%−epiboly
1−4−somites
14−19−somites
20−25−somites
Prim−5
Prim−15
Prim−25
Long−pec
Protruding−mouth
Day−4
Day−5
Transcript
ENSDART00000019898
ENSDART00000135311
ENSDART00000147447

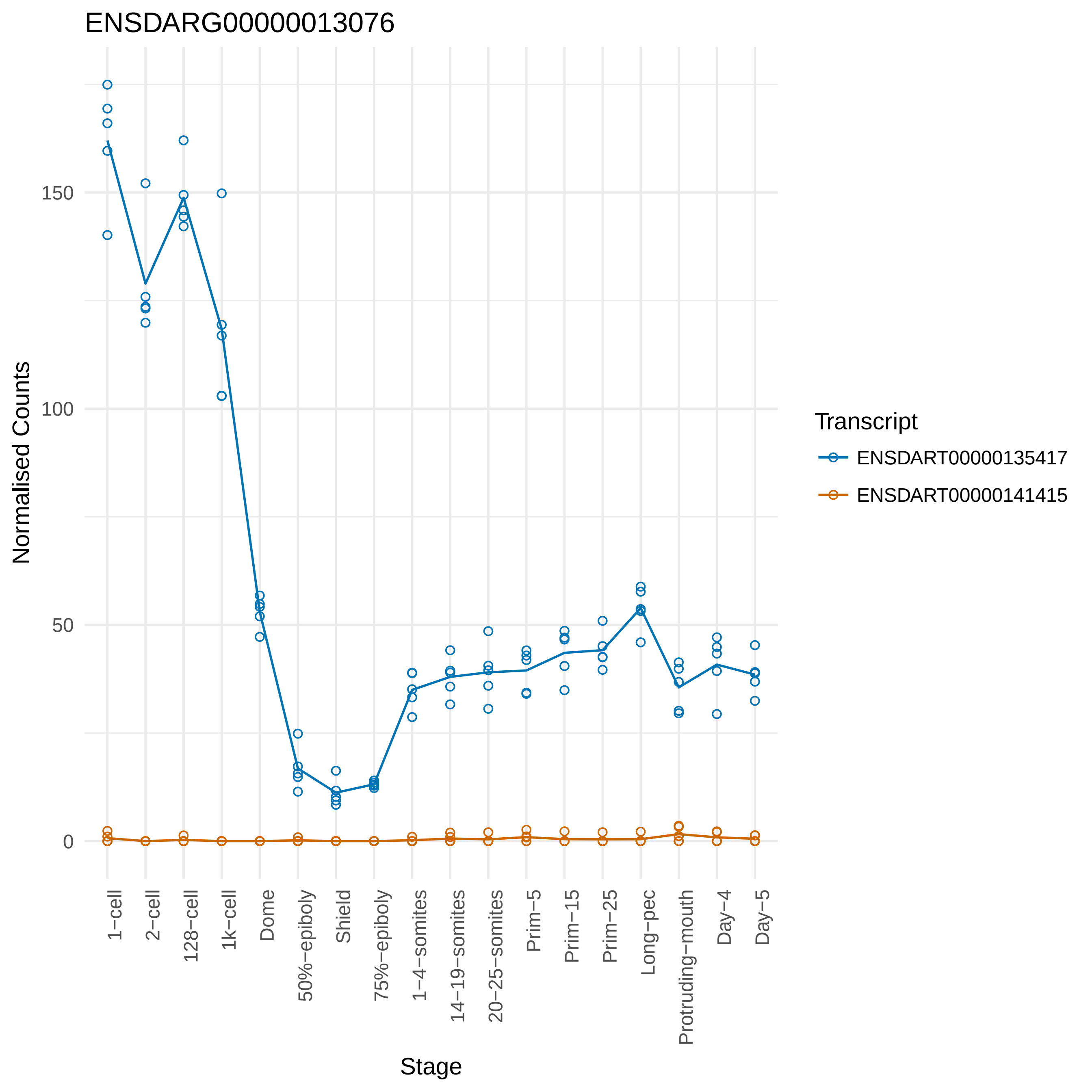
ENSDARG00000013076
Normalised Counts
150
100
50
0
1−cell
2−cell
128−cell
1k−cell
Dome
50%−epiboly
Shield
75%−epiboly
1−4−somites
14−19−somites
20−25−somites
Prim−5
Prim−15
Prim−25
Long−pec
Protruding−mouth
Day−4
Day−5
Stage
Transcript
ENSDART00000135417
ENSDART00000141415

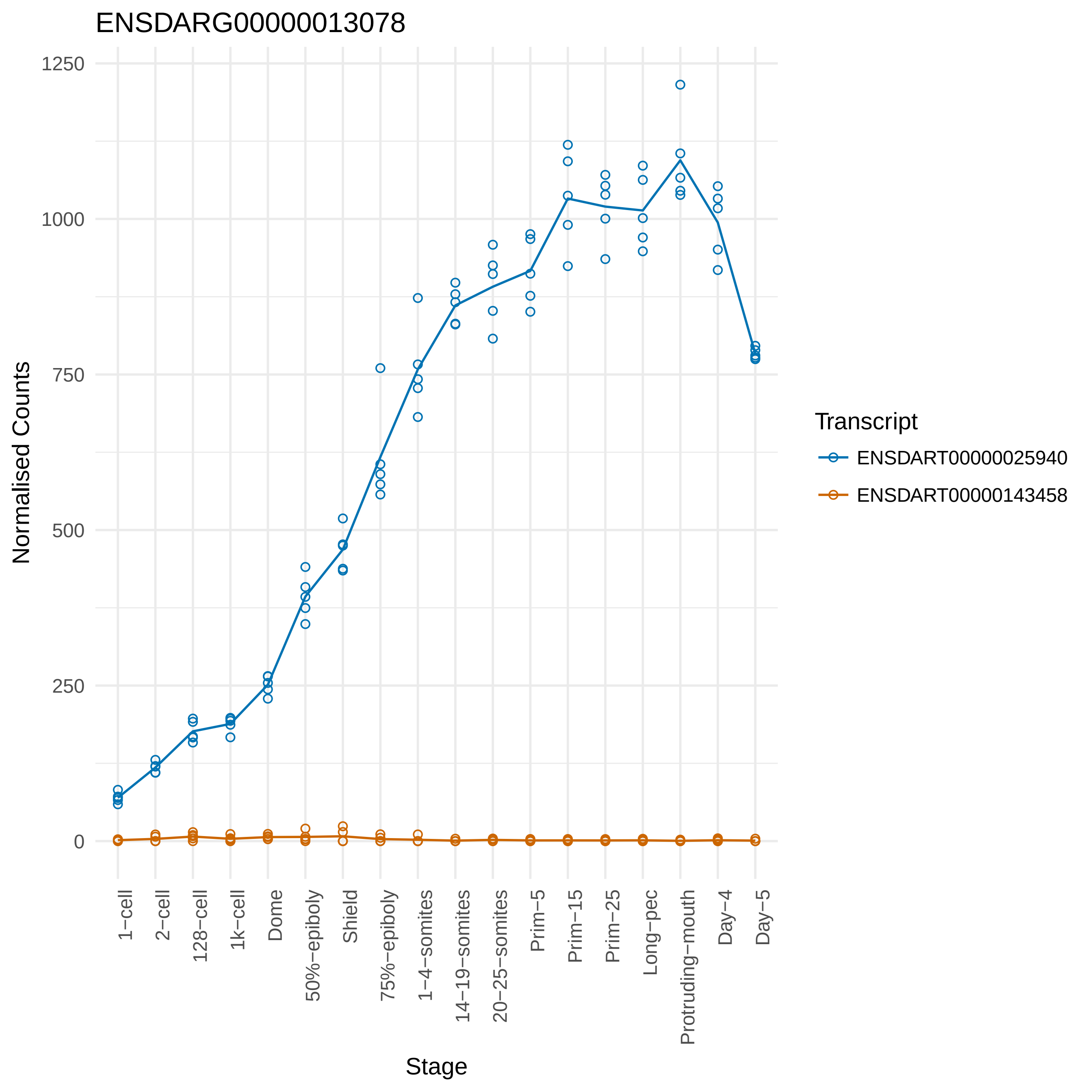
ENSDARG00000013078
Normalised Counts
0
250
500
750
1000
1250
Stage
1−cell
2−cell
128−cell
1k−cell
Dome
50%−epiboly
Shield
75%−epiboly
1−4−somites
14−19−somites
20−25−somites
Prim−5
Prim−15
Prim−25
Long−pec
Protruding−mouth
Day−4
Day−5
Transcript
ENSDART00000025940
ENSDART00000143458

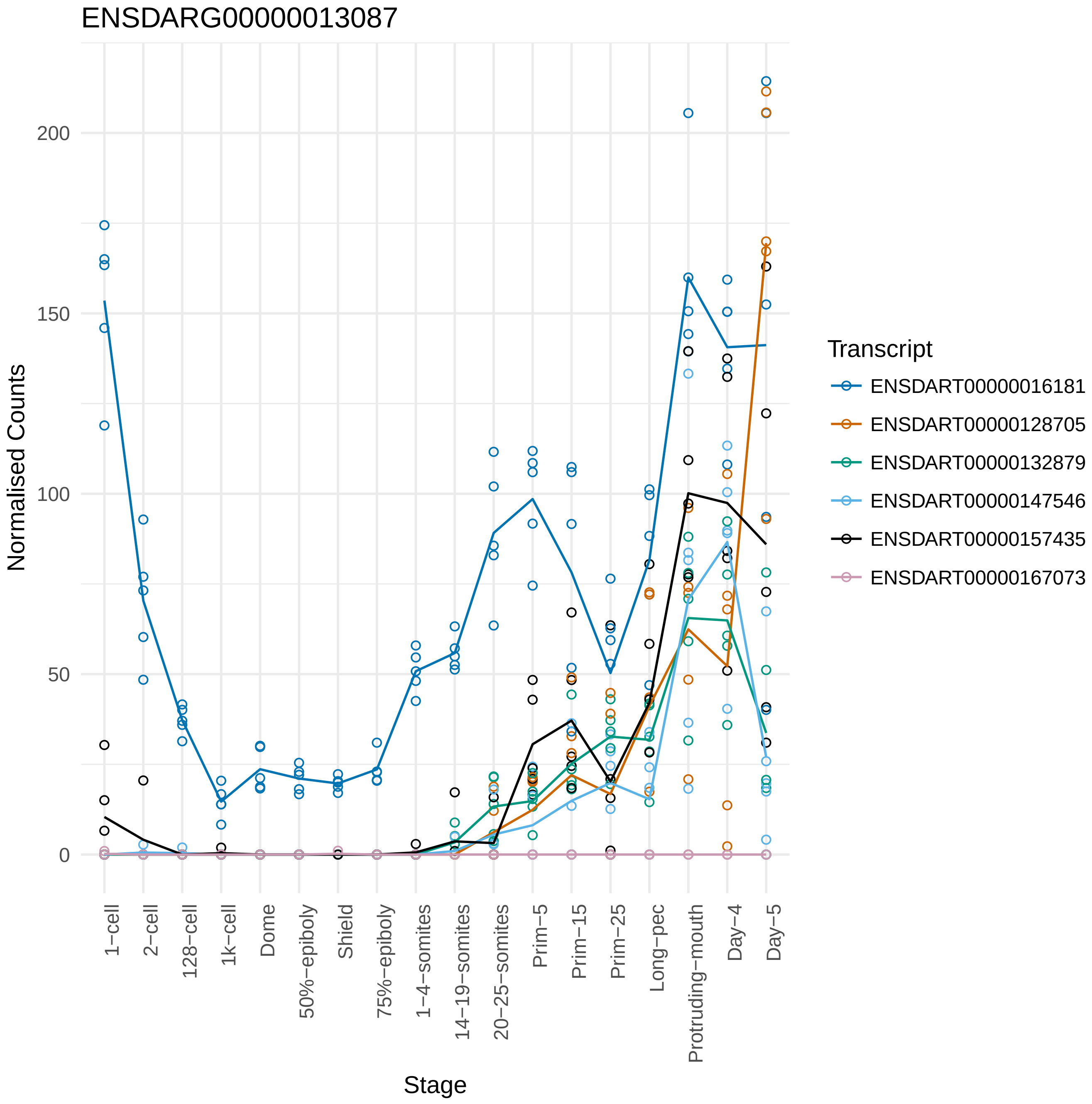
ENSDARG00000013087
Normalised Counts
0
50
100
150
200
Stage
1−cell
2−cell
128−cell
1k−cell
Dome
50%−epiboly
Shield
75%−epiboly
1−4−somites
14−19−somites
20−25−somites
Prim−5
Prim−15
Prim−25
Long−pec
Protruding−mouth
Day−4
Day−5
Transcript
ENSDART00000016181
ENSDART00000128705
ENSDART00000132879
ENSDART00000147546
ENSDART00000157435
ENSDART00000167073

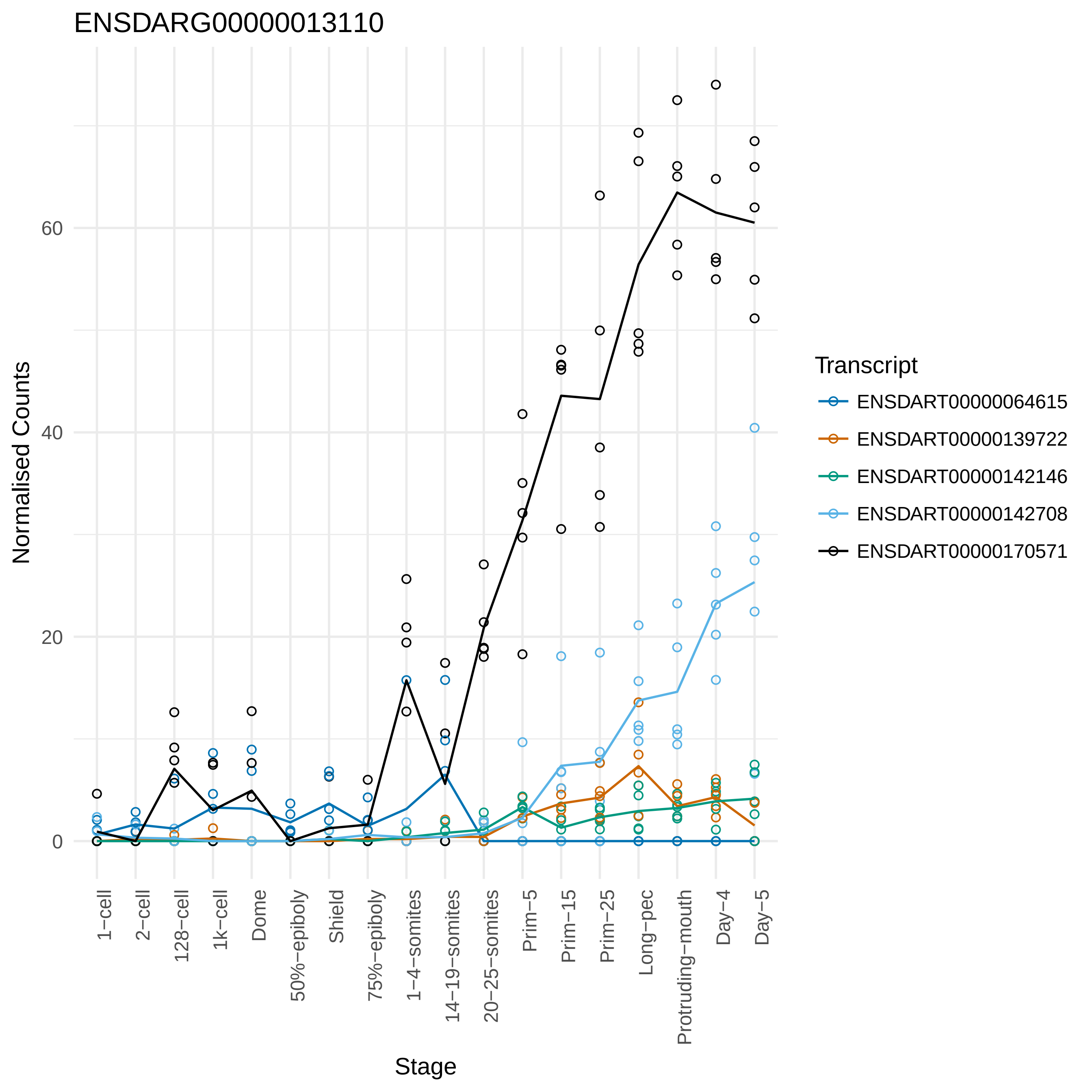

ENSDARG00000013110
Normalised Counts
60
40
20
0
1−cell
2−cell
128−cell
1k−cell
Dome
50%−epiboly
Shield
75%−epiboly
1−4−somites
14−19−somites
20−25−somites
Prim−5
Prim−15
Prim−25
Long−pec
Protruding−mouth
Day−4
Day−5
Stage
Transcript
ENSDART00000064615
ENSDART00000139722
ENSDART00000142146
ENSDART00000142708
ENSDART00000170571

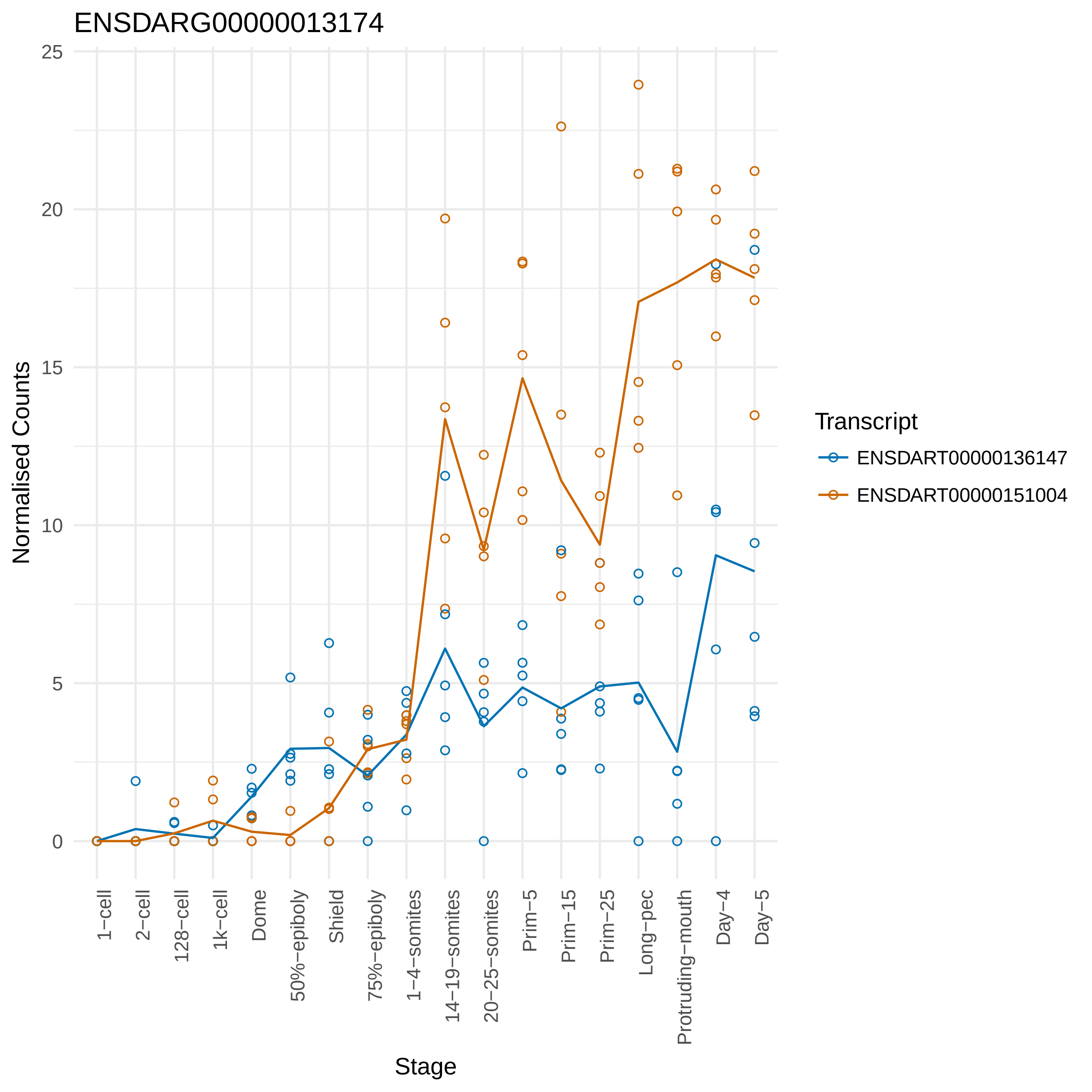

ENSDARG00000013174
Normalised Counts
0
5
10
15
20
25
1−cell
2−cell
128−cell
1k−cell
Dome
50%−epiboly
Shield
75%−epiboly
1−4−somites
14−19−somites
20−25−somites
Prim−5
Prim−15
Prim−25
Long−pec
Protruding−mouth
Day−4
Day−5
Stage
Transcript
ENSDART00000136147
ENSDART00000151004

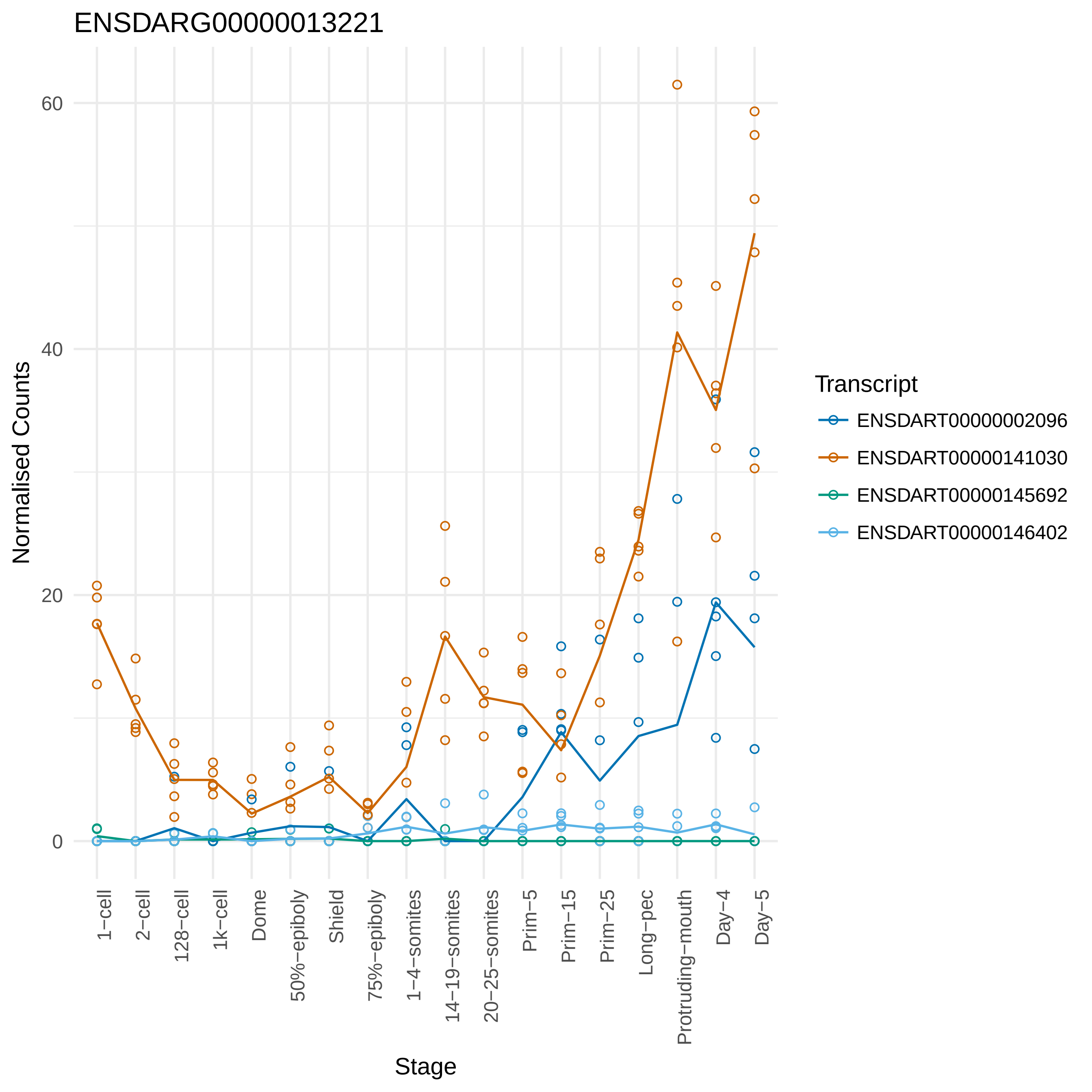

ENSDARG00000013221
Normalised Counts
60
40
20
0
1−cell
2−cell
128−cell
1k−cell
Dome
50%−epiboly
Shield
75%−epiboly
1−4−somites
14−19−somites
20−25−somites
Prim−5
Prim−15
Prim−25
Long−pec
Protruding−mouth
Day−4
Day−5
Stage
Transcript
ENSDART00000002096
ENSDART00000141030
ENSDART00000145692
ENSDART00000146402

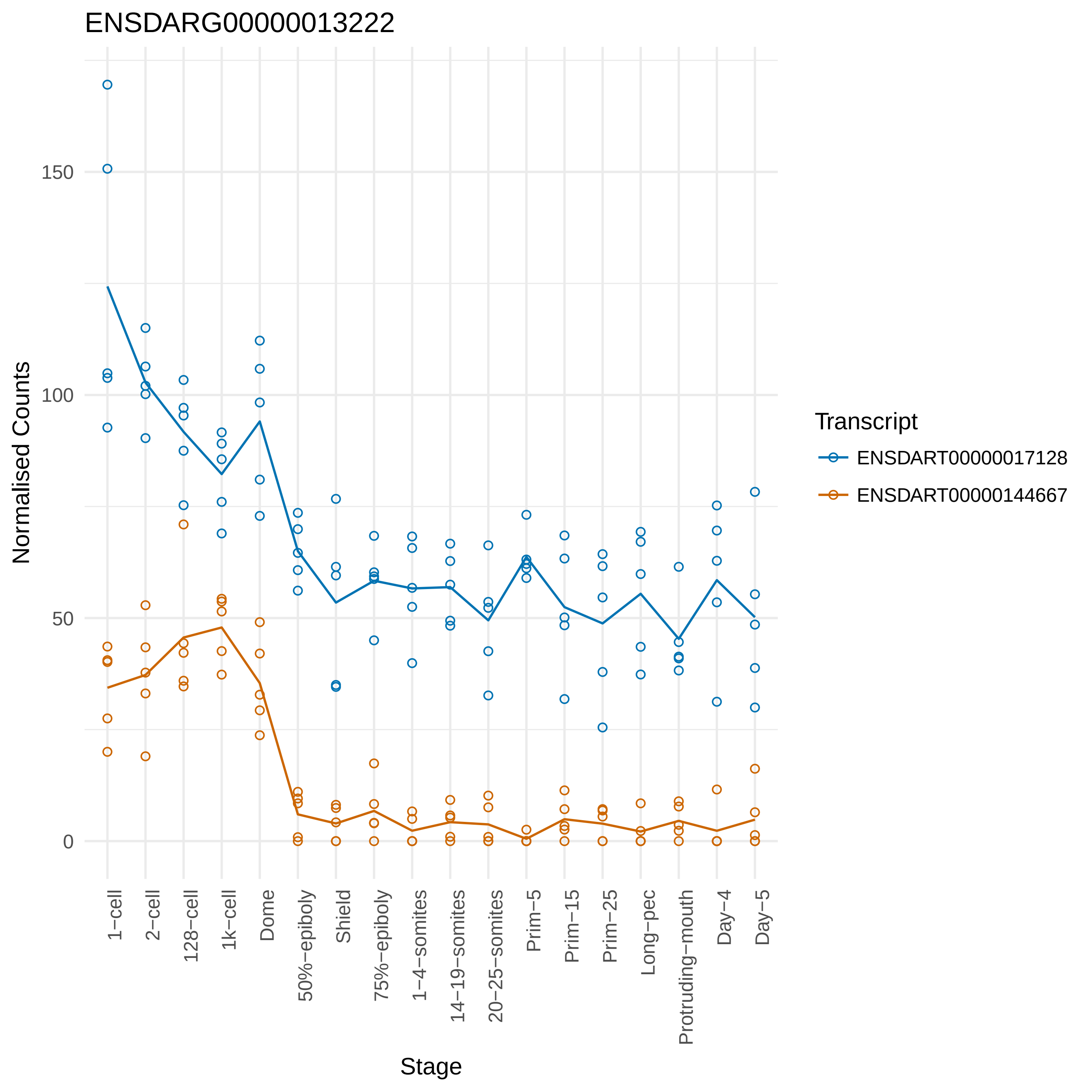
ENSDARG00000013222
Normalised Counts
150
100
50
0
1−cell
2−cell
128−cell
1k−cell
Dome
50%−epiboly
Shield
75%−epiboly
1−4−somites
14−19−somites
20−25−somites
Prim−5
Prim−15
Prim−25
Long−pec
Protruding−mouth
Day−4
Day−5
Stage
Transcript
ENSDART00000017128
ENSDART00000144667

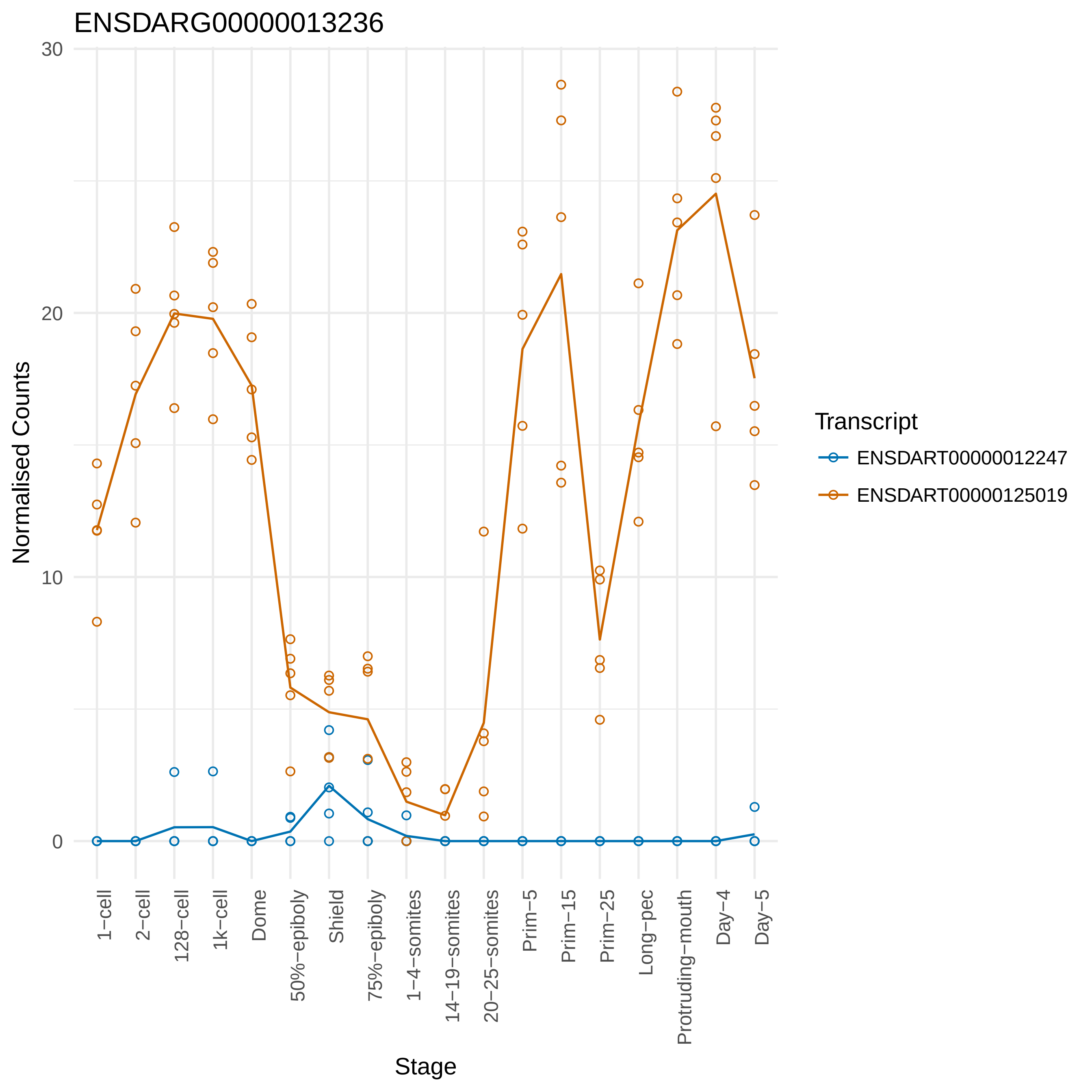

ENSDARG00000013236
Normalised Counts
30
20
10
0
1−cell
2−cell
128−cell
1k−cell
Dome
50%−epiboly
Shield
75%−epiboly
1−4−somites
14−19−somites
20−25−somites
Prim−5
Prim−15
Prim−25
Long−pec
Protruding−mouth
Day−4
Day−5
Stage
Transcript
ENSDART00000012247
ENSDART00000125019

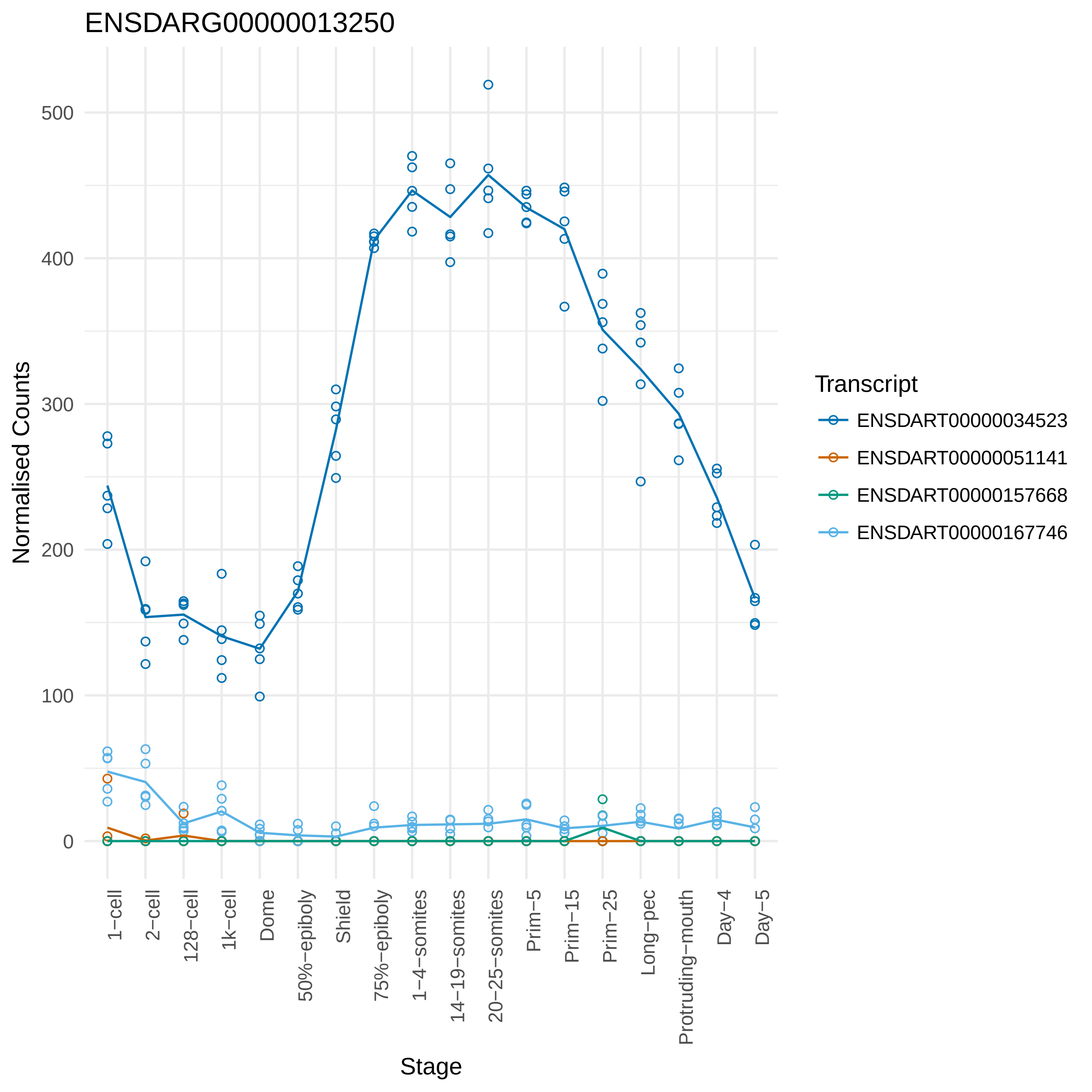

ENSDARG00000013250
Normalised Counts
0
100
200
300
400
500
Stage
1−cell
2−cell
128−cell
1k−cell
Dome
50%−epiboly
Shield
75%−epiboly
1−4−somites
14−19−somites
20−25−somites
Prim−5
Prim−15
Prim−25
Long−pec
Protruding−mouth
Day−4
Day−5
Transcript
ENSDART00000034523
ENSDART00000051141
ENSDART00000157668
ENSDART00000167746

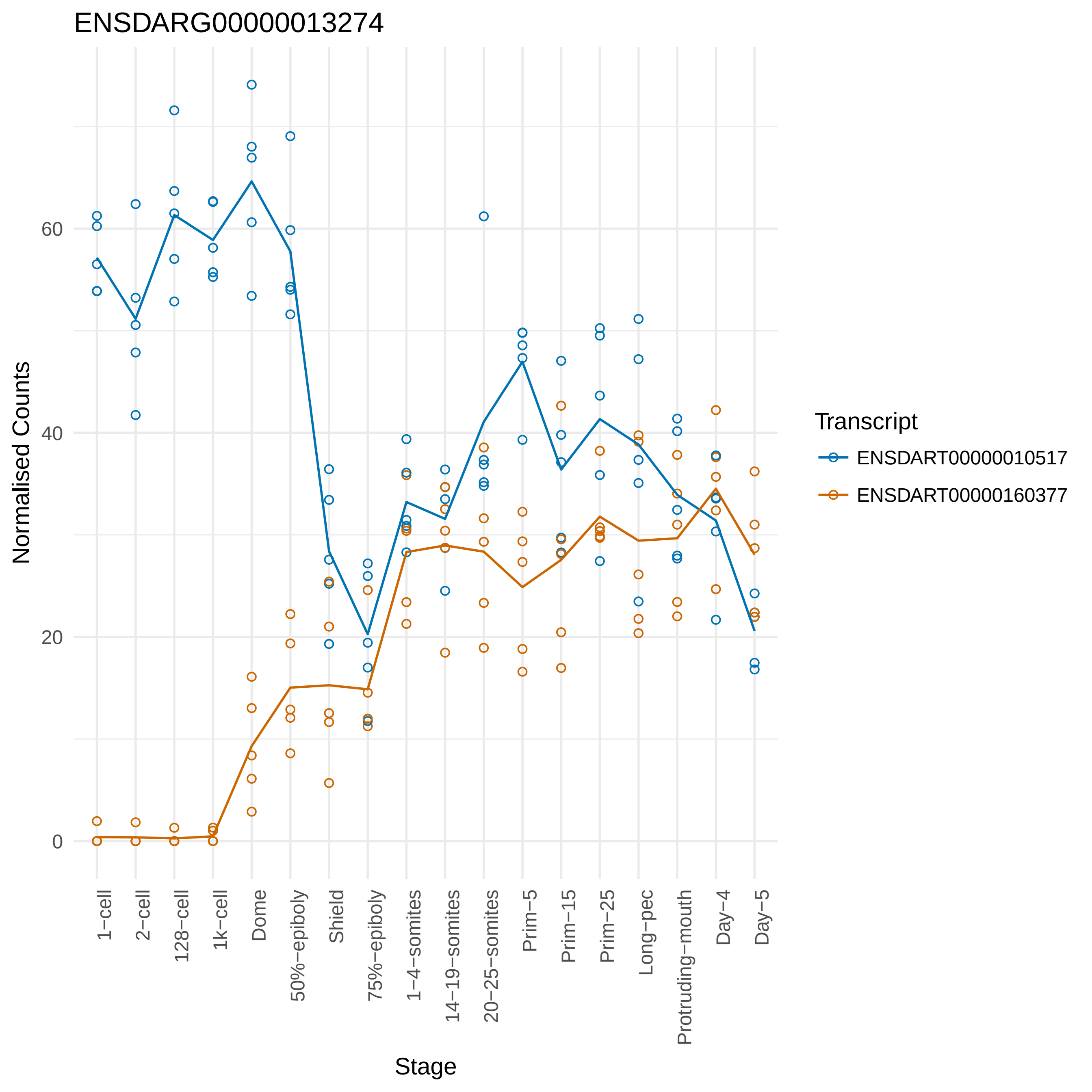

ENSDARG00000013274
Normalised Counts
0
20
40
60
1−cell
2−cell
128−cell
1k−cell
Dome
50%−epiboly
Shield
75%−epiboly
1−4−somites
14−19−somites
20−25−somites
Prim−5
Prim−15
Prim−25
Long−pec
Protruding−mouth
Day−4
Day−5
Stage
Transcript
ENSDART00000010517
ENSDART00000160377

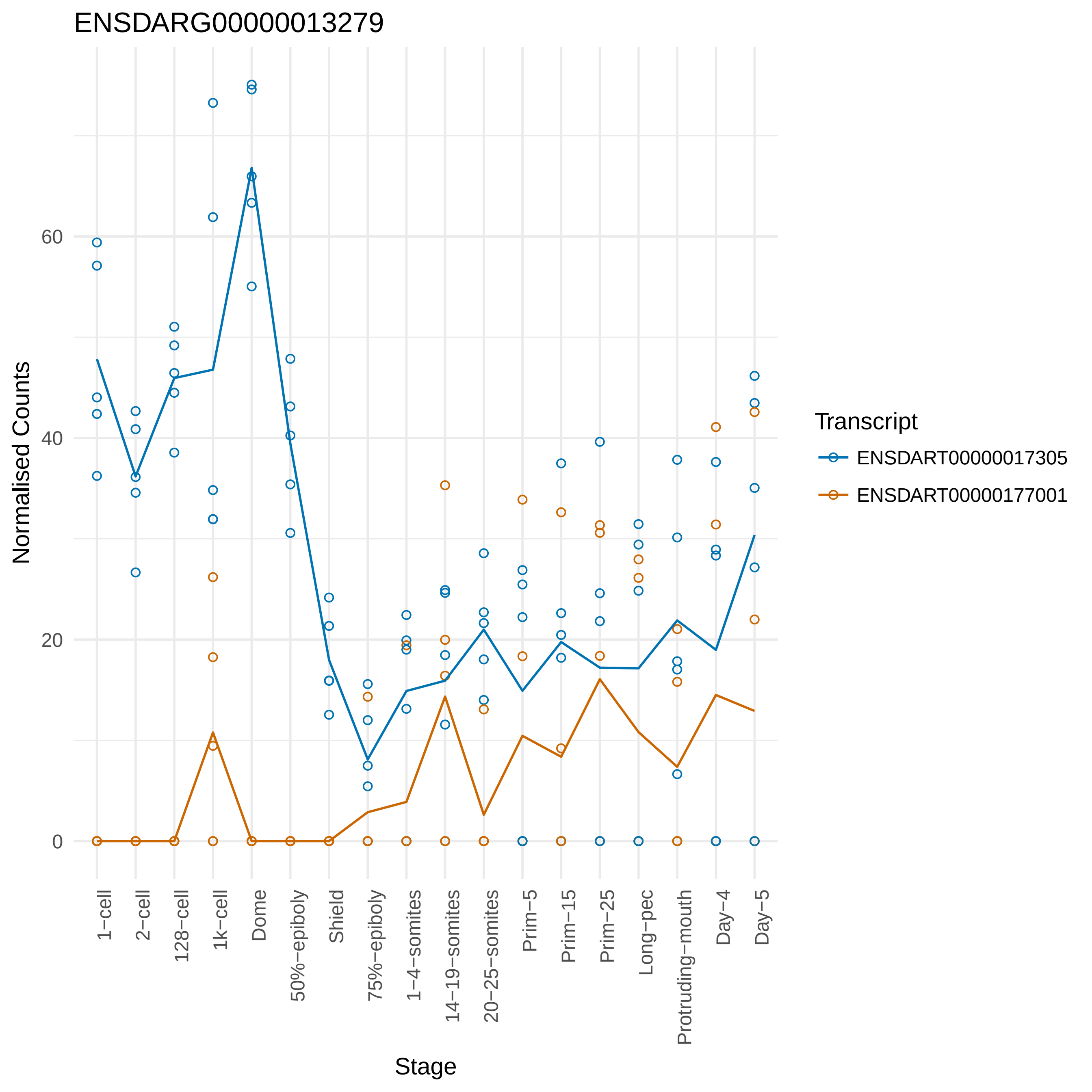
ENSDARG00000013279
Normalised Counts
0
20
40
60
1−cell
2−cell
128−cell
1k−cell
Dome
50%−epiboly
Shield
75%−epiboly
1−4−somites
14−19−somites
20−25−somites
Prim−5
Prim−15
Prim−25
Long−pec
Protruding−mouth
Day−4
Day−5
Stage
Transcript
ENSDART00000017305
ENSDART00000177001

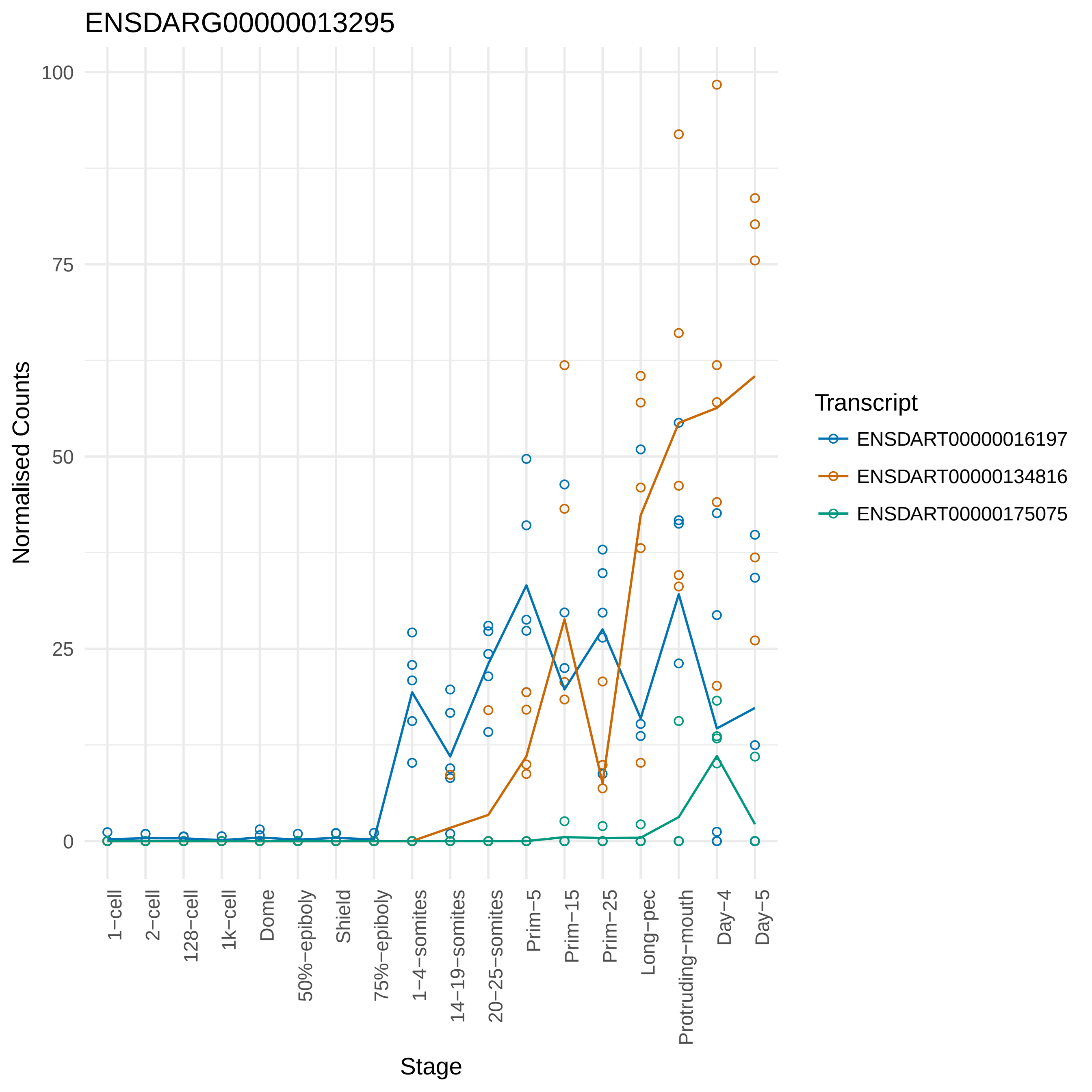

ENSDARG00000013295
Normalised Counts
100
75
50
25
0
1−cell
2−cell
128−cell
1k−cell
Dome
50%−epiboly
Shield
75%−epiboly
1−4−somites
14−19−somites
20−25−somites
Prim−5
Prim−15
Prim−25
Long−pec
Protruding−mouth
Day−4
Day−5
Stage
Transcript
ENSDART00000016197
ENSDART00000134816
ENSDART00000175075

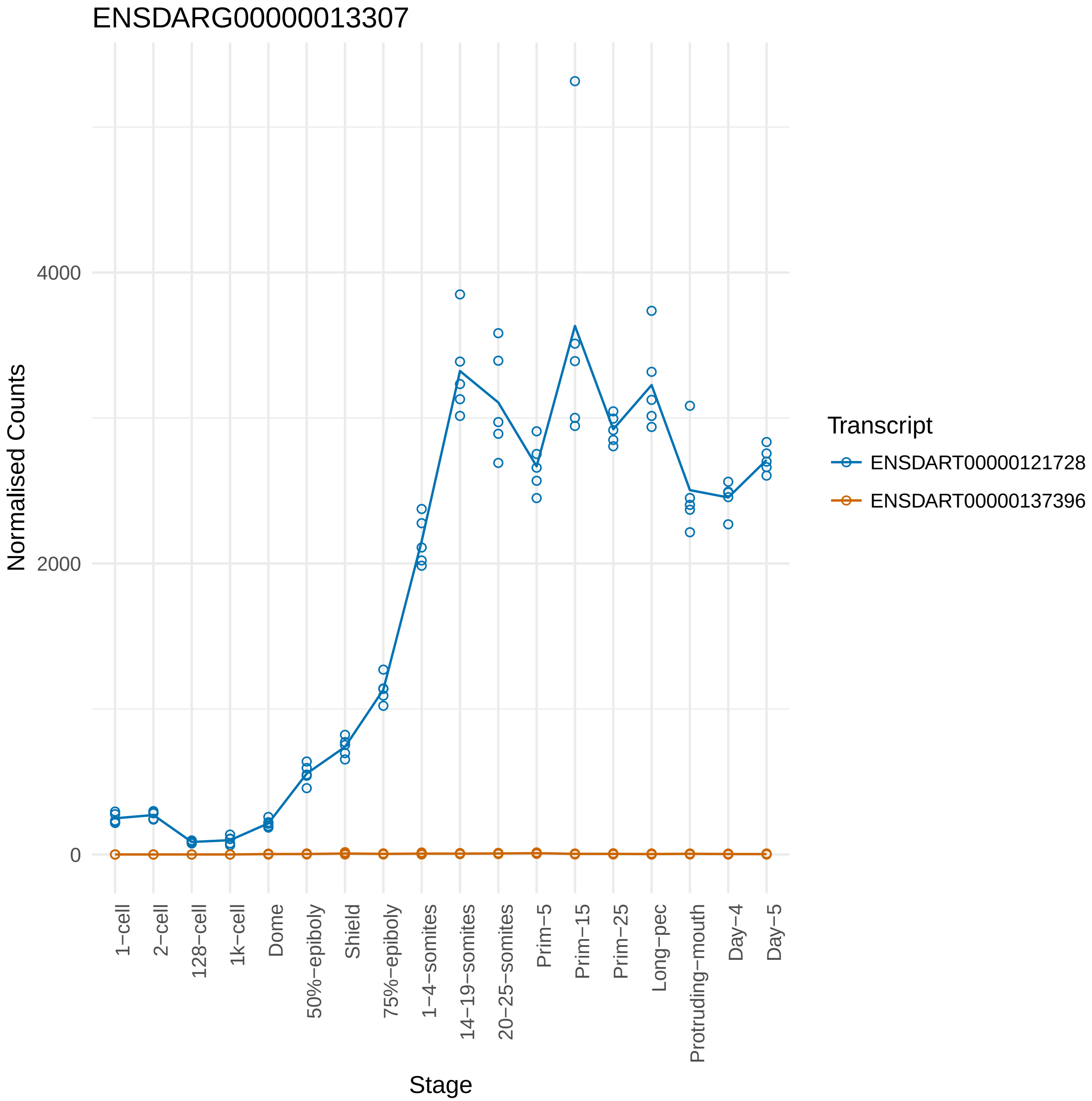

ENSDARG00000013307
Normalised Counts
0
2000
4000
Stage
1−cell
2−cell
128−cell
1k−cell
Dome
50%−epiboly
Shield
75%−epiboly
1−4−somites
14−19−somites
20−25−somites
Prim−5
Prim−15
Prim−25
Long−pec
Protruding−mouth
Day−4
Day−5
Transcript
ENSDART00000121728
ENSDART00000137396

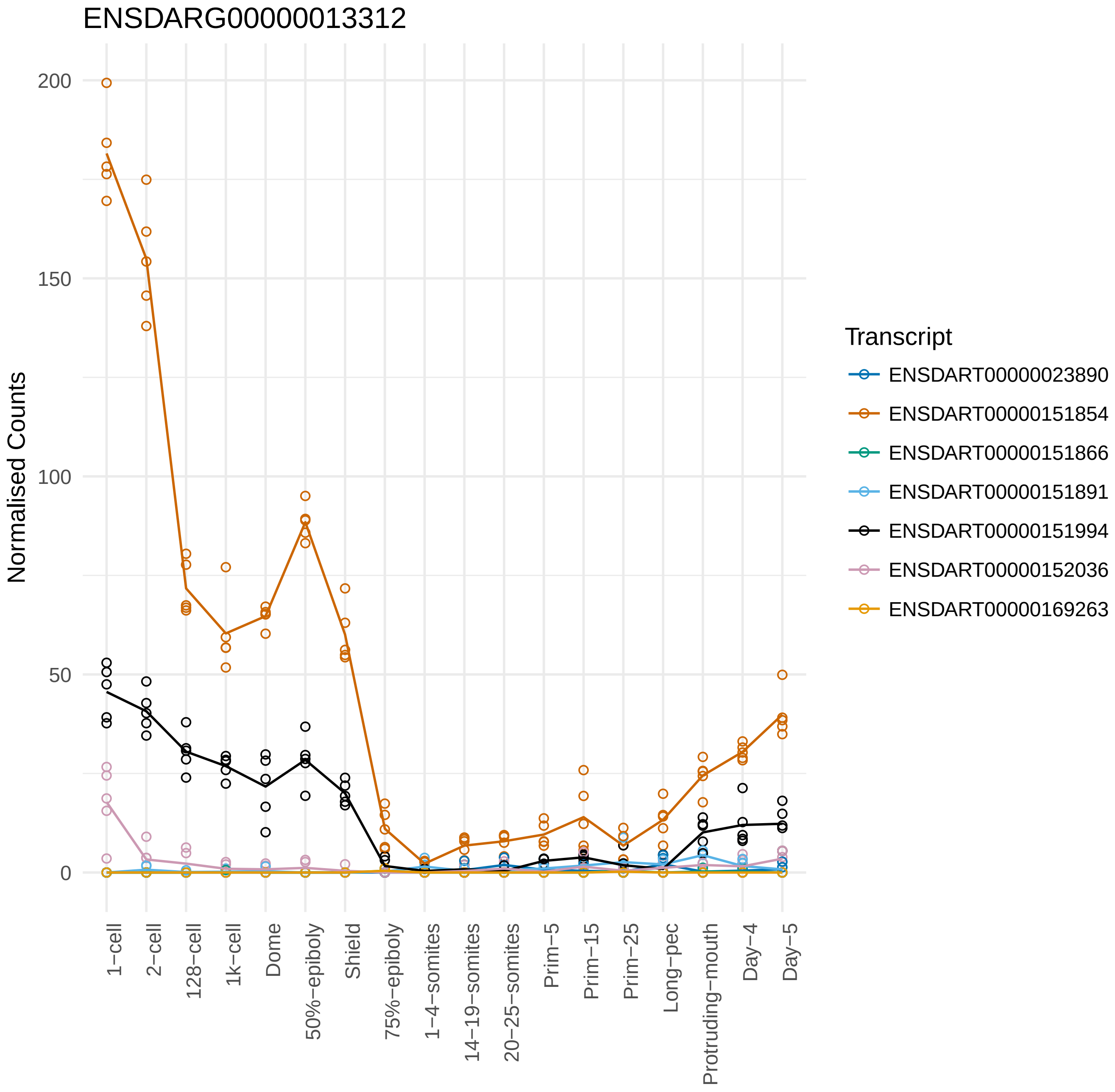
ENSDARG00000013312
Normalised Counts
200
150
100
50
0
1−cell
2−cell
128−cell
1k−cell
Dome
50%−epiboly
Shield
75%−epiboly
1−4−somites
14−19−somites
20−25−somites
Prim−5
Prim−15
Prim−25
Long−pec
Protruding−mouth
Day−4
Day−5
Stage
Transcript
ENSDART00000023890
ENSDART00000151854
ENSDART00000151866
ENSDART00000151891
ENSDART00000151994
ENSDART00000152036
ENSDART00000169263

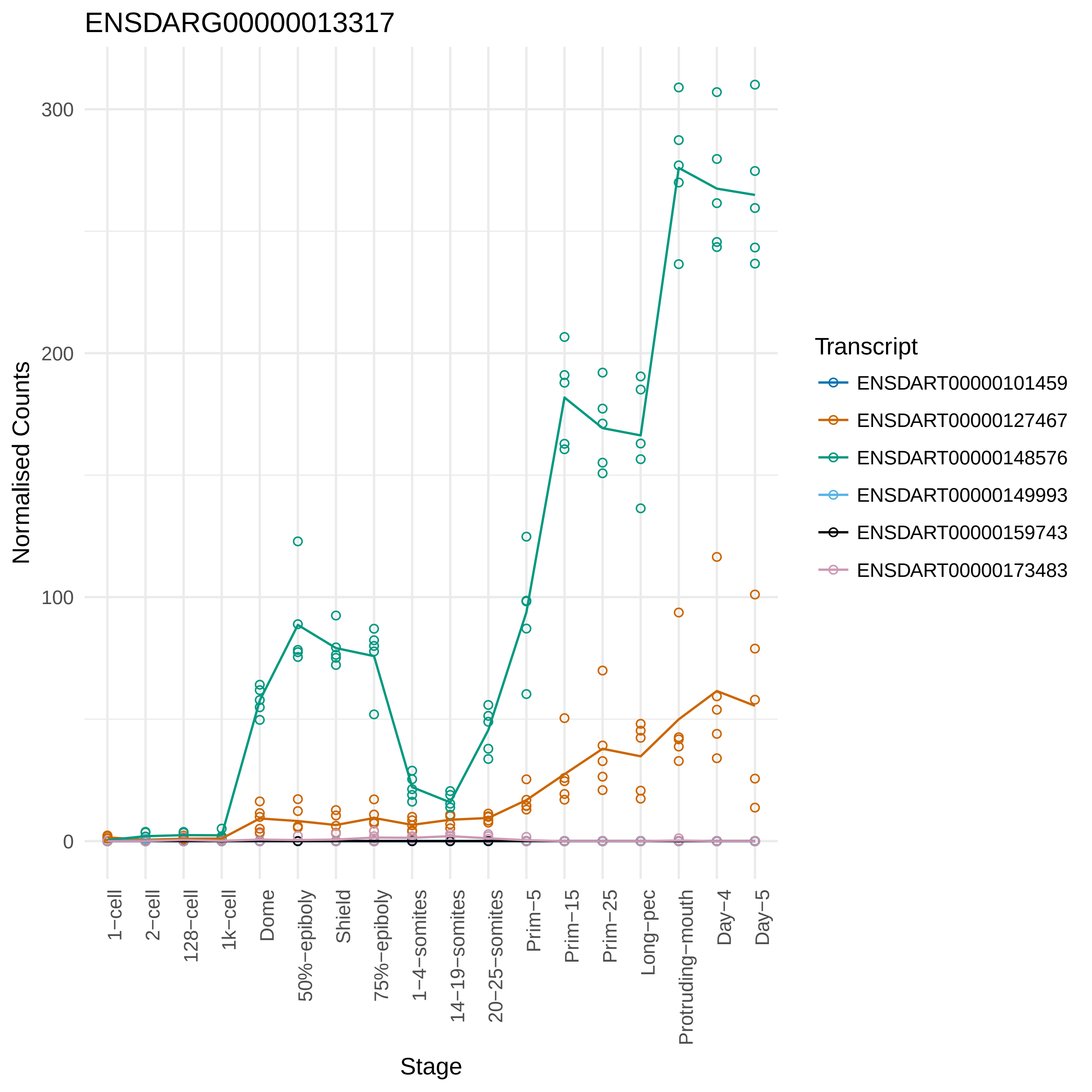

ENSDARG00000013317
Normalised Counts
300
200
100
0
Stage
1−cell
2−cell
128−cell
1k−cell
Dome
50%−epiboly
Shield
75%−epiboly
1−4−somites
14−19−somites
20−25−somites
Prim−5
Prim−15
Prim−25
Long−pec
Protruding−mouth
Day−4
Day−5
Transcript
ENSDART00000101459
ENSDART00000127467
ENSDART00000148576
ENSDART00000149993
ENSDART00000159743
ENSDART00000173483

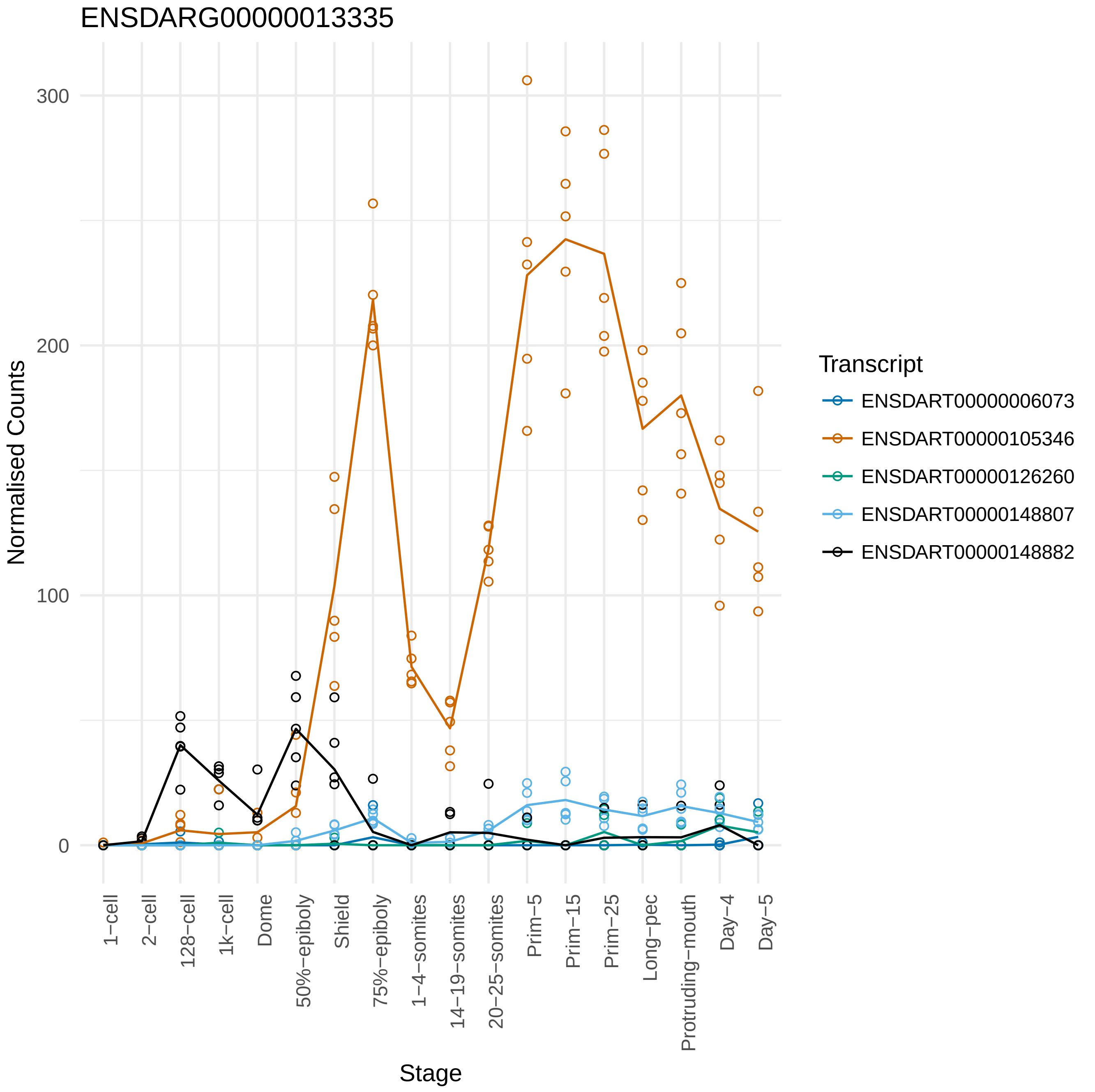
ENSDARG00000013335
Normalised Counts
300
200
100
0
Stage
1−cell
2−cell
128−cell
1k−cell
Dome
50%−epiboly
Shield
75%−epiboly
1−4−somites
14−19−somites
20−25−somites
Prim−5
Prim−15
Prim−25
Long−pec
Protruding−mouth
Day−4
Day−5
Transcript
ENSDART00000006073
ENSDART00000105346
ENSDART00000126260
ENSDART00000148807
ENSDART00000148882

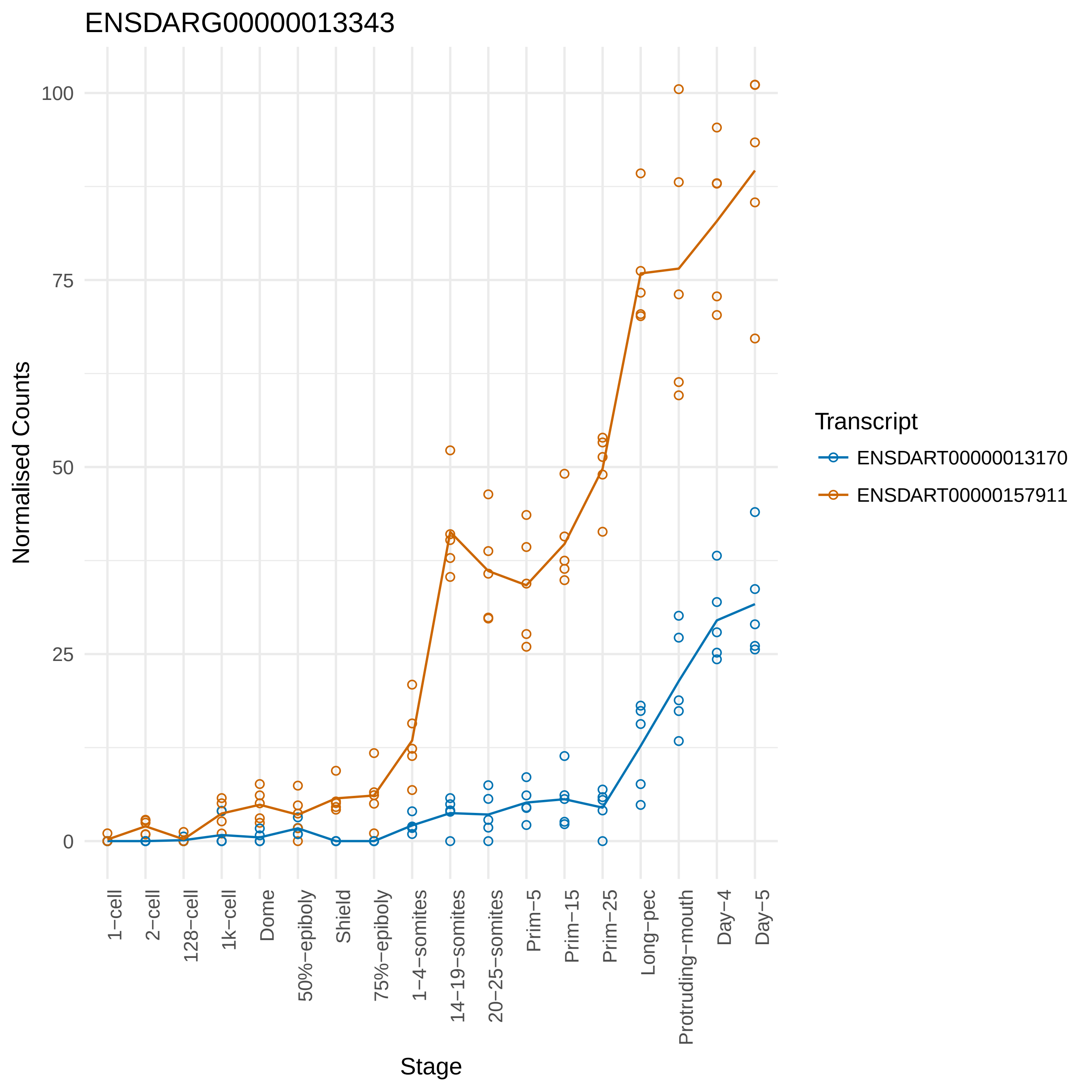
ENSDARG00000013343
Normalised Counts
100
75
50
25
0
1−cell
2−cell
128−cell
1k−cell
Dome
50%−epiboly
Shield
75%−epiboly
1−4−somites
14−19−somites
20−25−somites
Prim−5
Prim−15
Prim−25
Long−pec
Protruding−mouth
Day−4
Day−5
Stage
Transcript
ENSDART00000013170
ENSDART00000157911

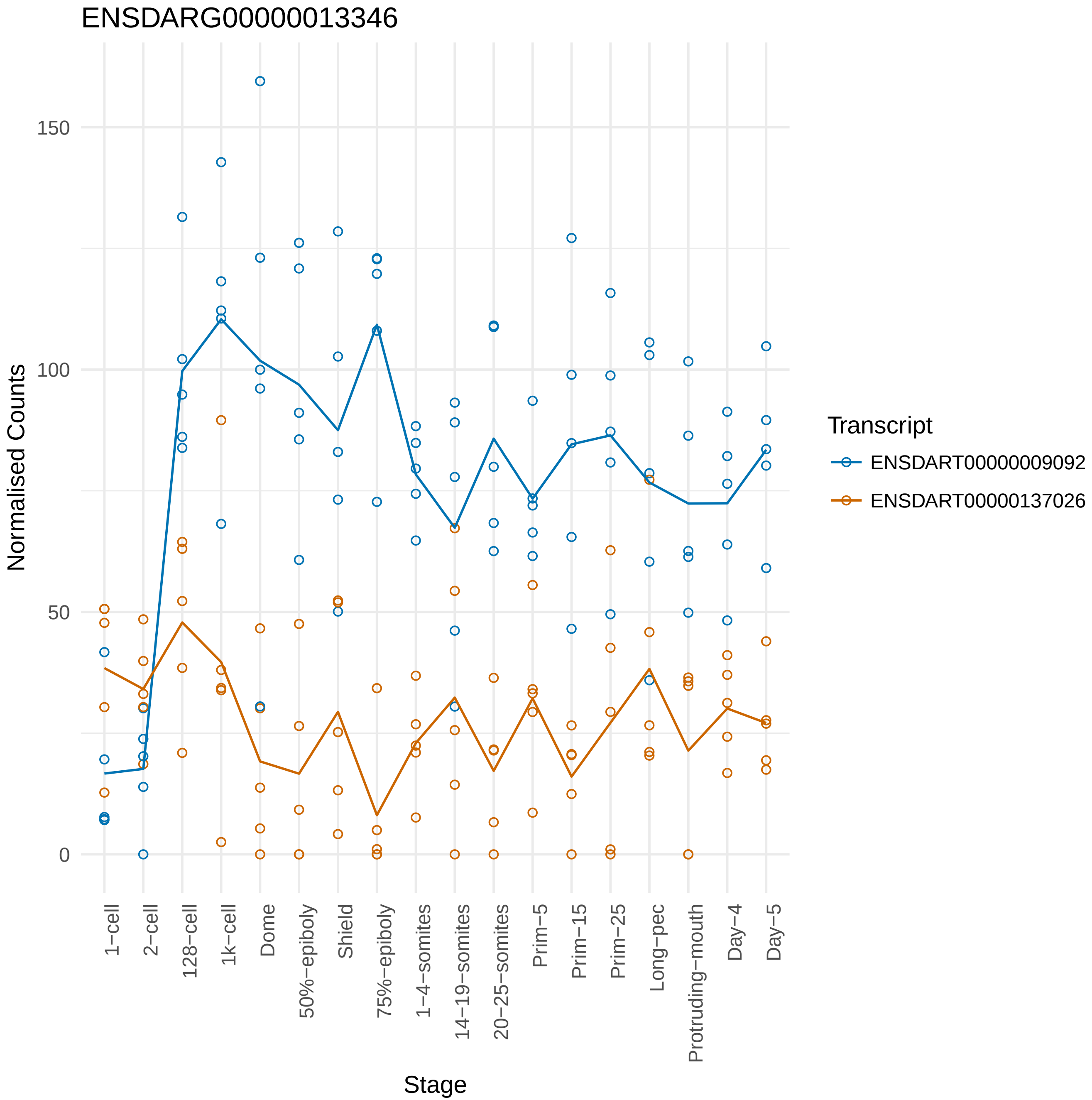

ENSDARG00000013346
Normalised Counts
150
100
50
0
1−cell
2−cell
128−cell
1k−cell
Dome
50%−epiboly
Shield
75%−epiboly
1−4−somites
14−19−somites
20−25−somites
Prim−5
Prim−15
Prim−25
Long−pec
Protruding−mouth
Day−4
Day−5
Stage
Transcript
ENSDART00000009092
ENSDART00000137026

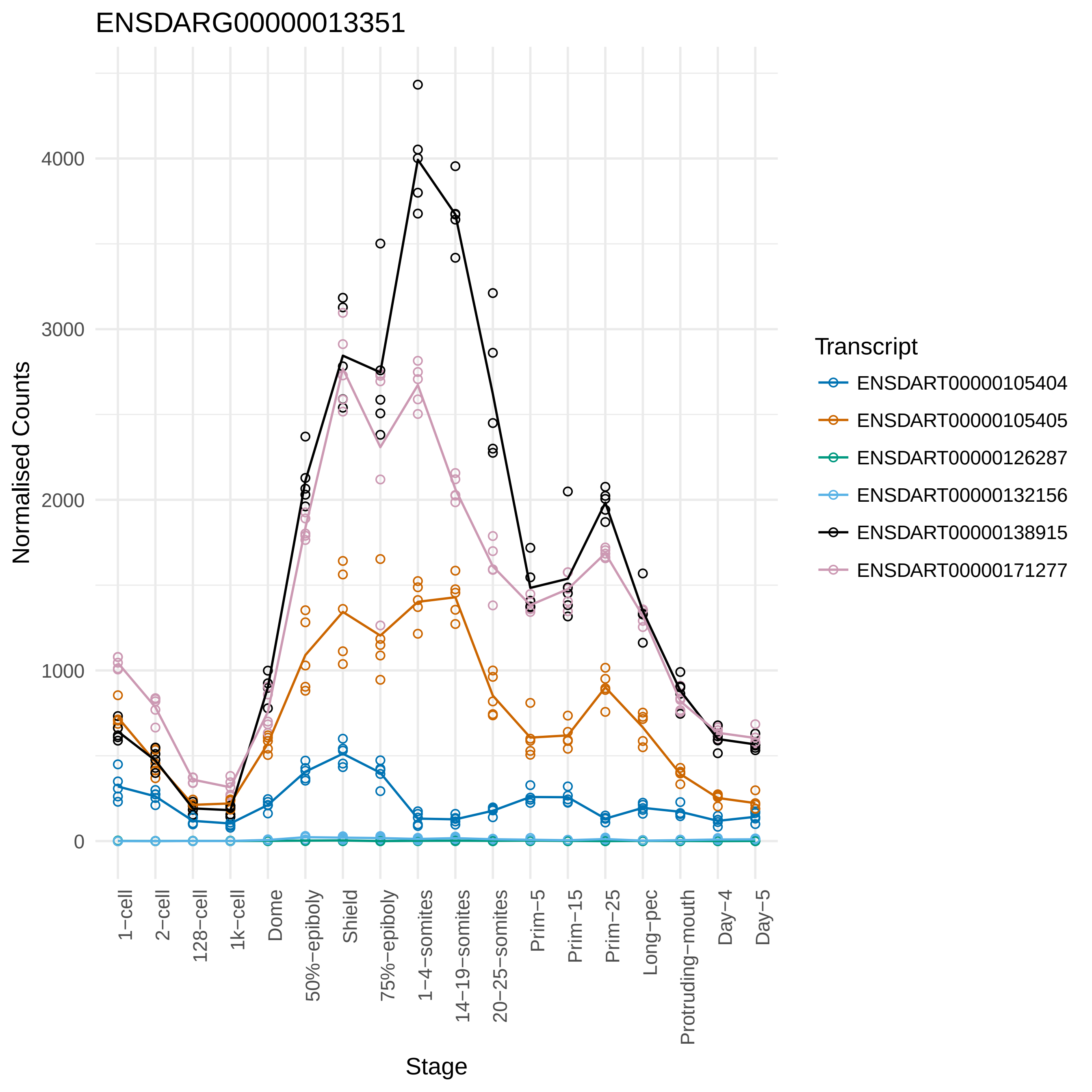
ENSDARG00000013351
Normalised Counts
4000
3000
2000
1000
0
1−cell
2−cell
128−cell
1k−cell
Dome
50%−epiboly
Shield
75%−epiboly
1−4−somites
14−19−somites
20−25−somites
Prim−5
Prim−15
Prim−25
Long−pec
Protruding−mouth
Day−4
Day−5
Stage
Transcript
ENSDART00000105404
ENSDART00000105405
ENSDART00000126287
ENSDART00000132156
ENSDART00000138915
ENSDART00000171277

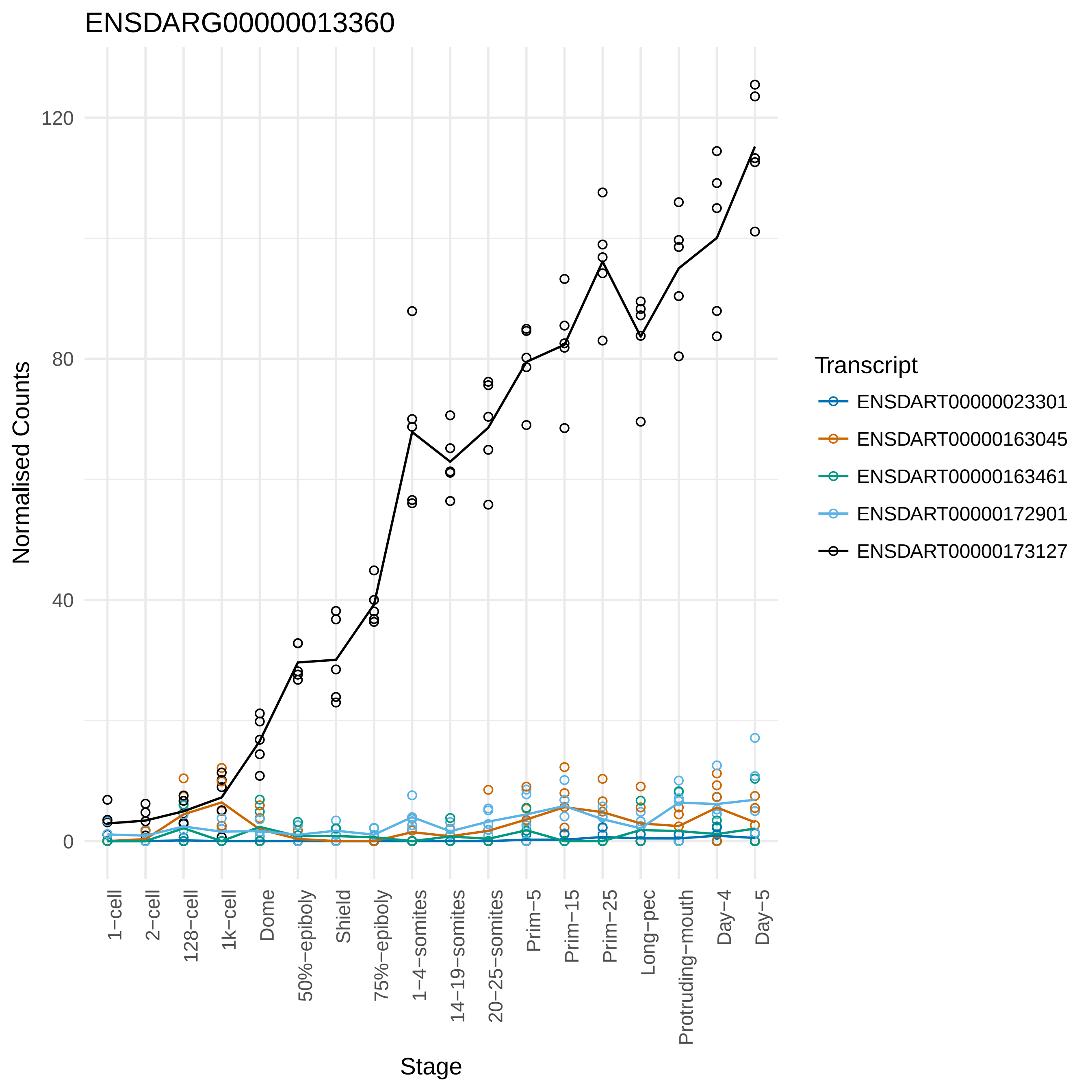

ENSDARG00000013360
Normalised Counts
0
40
80
120
1−cell
2−cell
128−cell
1k−cell
Dome
50%−epiboly
Shield
75%−epiboly
1−4−somites
14−19−somites
20−25−somites
Prim−5
Prim−15
Prim−25
Long−pec
Protruding−mouth
Day−4
Day−5
Stage
Transcript
ENSDART00000023301
ENSDART00000163045
ENSDART00000163461
ENSDART00000172901
ENSDART00000173127

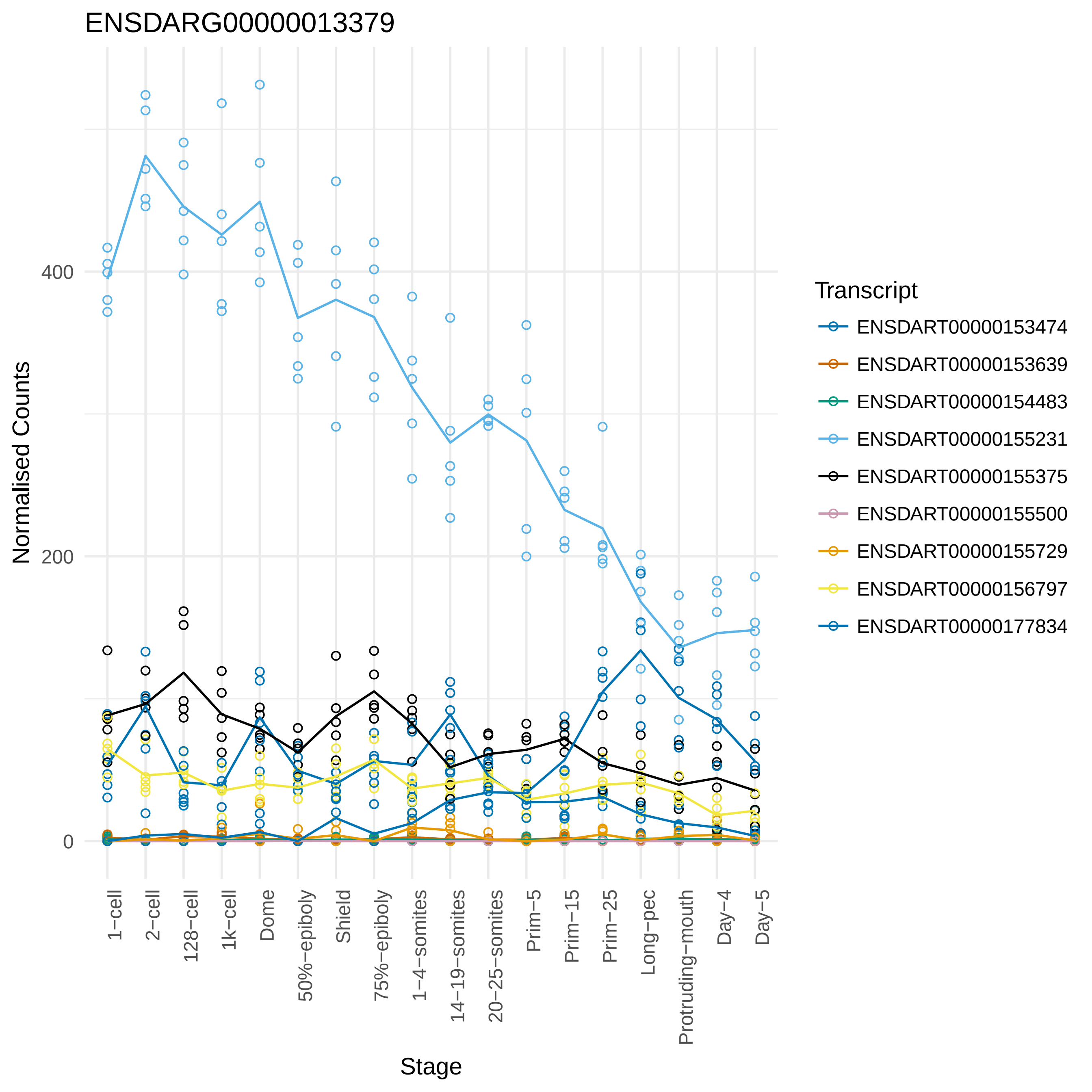

ENSDARG00000013379
Normalised Counts
400
200
0
1−cell
2−cell
128−cell
1k−cell
Dome
50%−epiboly
Shield
75%−epiboly
1−4−somites
14−19−somites
20−25−somites
Prim−5
Prim−15
Prim−25
Long−pec
Protruding−mouth
Day−4
Day−5
Stage
Transcript
ENSDART00000153474
ENSDART00000153639
ENSDART00000154483
ENSDART00000155231
ENSDART00000155375
ENSDART00000155500
ENSDART00000155729
ENSDART00000156797
ENSDART00000177834

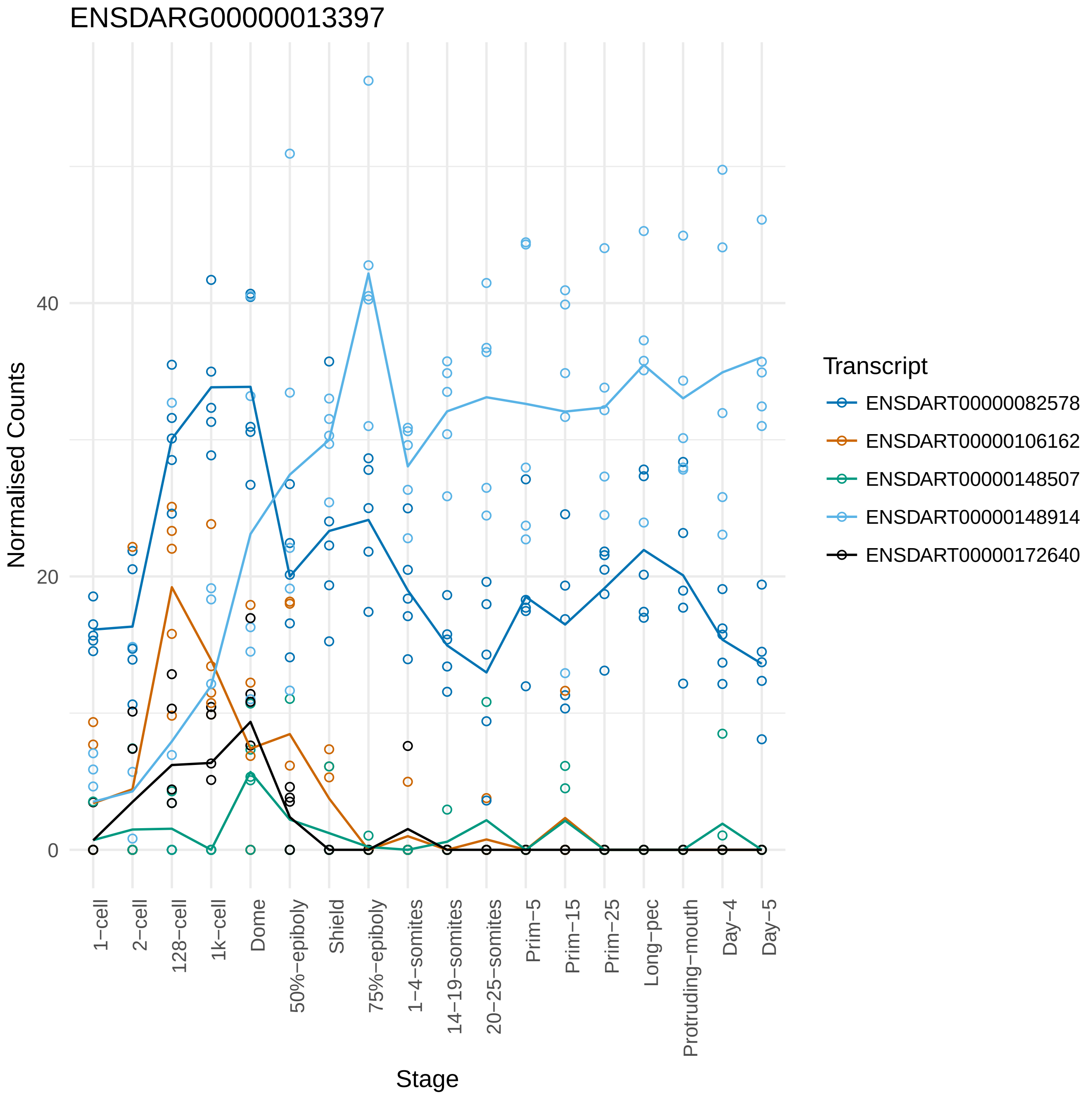
ENSDARG00000013397
Normalised Counts
0
20
40
1−cell
2−cell
128−cell
1k−cell
Dome
50%−epiboly
Shield
75%−epiboly
1−4−somites
14−19−somites
20−25−somites
Prim−5
Prim−15
Prim−25
Long−pec
Protruding−mouth
Day−4
Day−5
Stage
Transcript
ENSDART00000082578
ENSDART00000106162
ENSDART00000148507
ENSDART00000148914
ENSDART00000172640

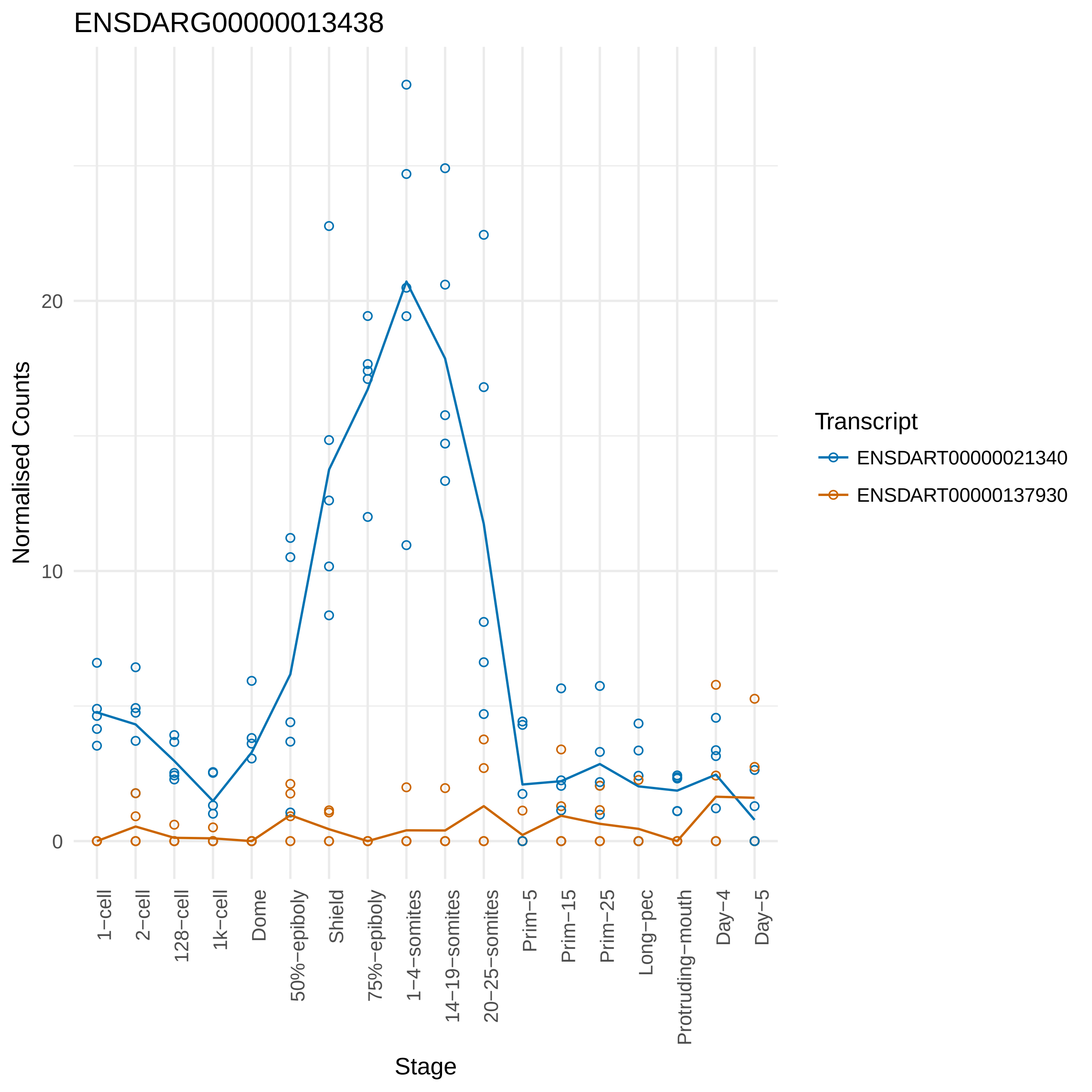
ENSDARG00000013438
Normalised Counts
0
10
20
Stage
1−cell
2−cell
128−cell
1k−cell
Dome
50%−epiboly
Shield
75%−epiboly
1−4−somites
14−19−somites
20−25−somites
Prim−5
Prim−15
Prim−25
Long−pec
Protruding−mouth
Day−4
Day−5
Transcript
ENSDART00000021340
ENSDART00000137930

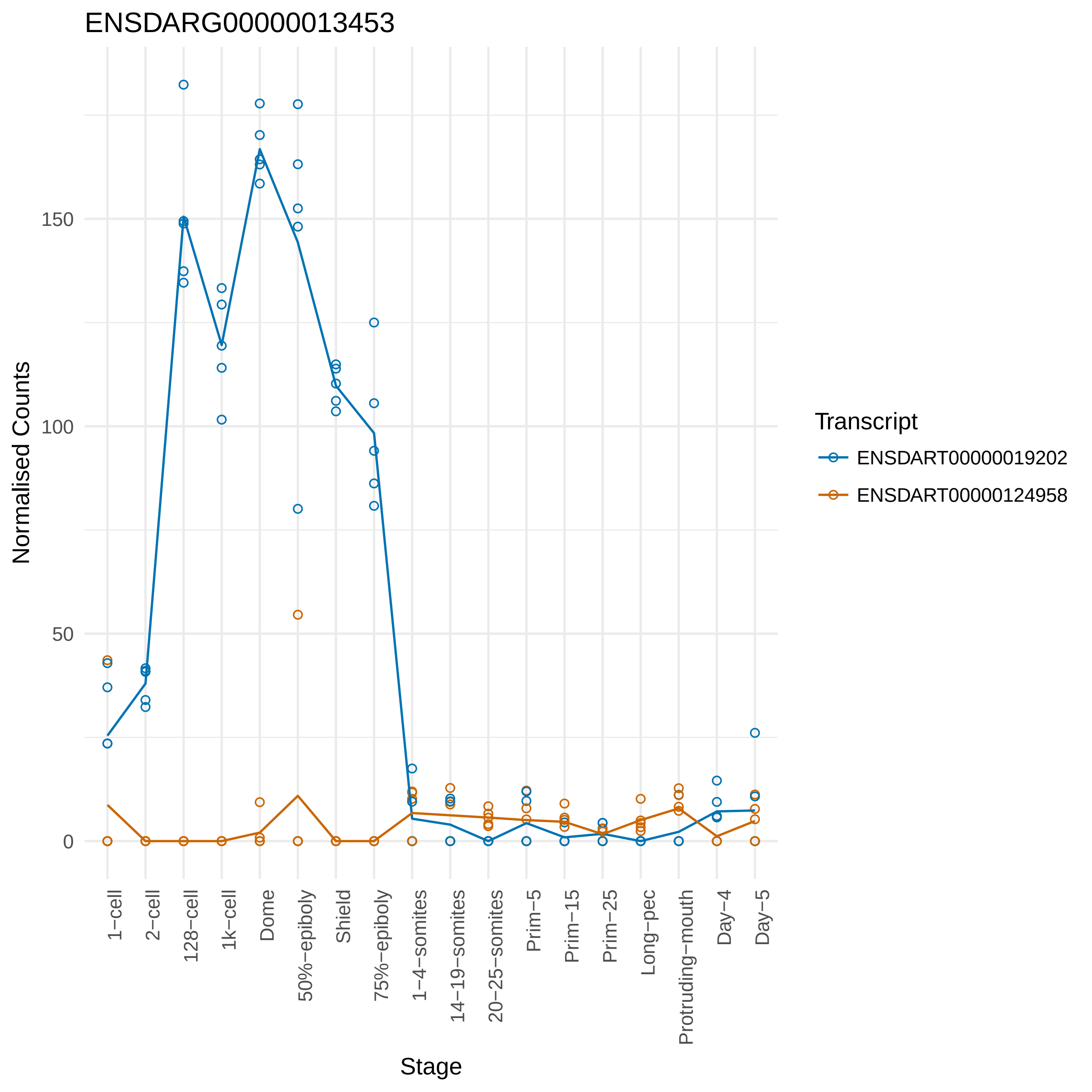

ENSDARG00000013453
Normalised Counts
150
100
50
0
Stage
1−cell
2−cell
128−cell
1k−cell
Dome
50%−epiboly
Shield
75%−epiboly
1−4−somites
14−19−somites
20−25−somites
Prim−5
Prim−15
Prim−25
Long−pec
Protruding−mouth
Day−4
Day−5
Transcript
ENSDART00000019202
ENSDART00000124958

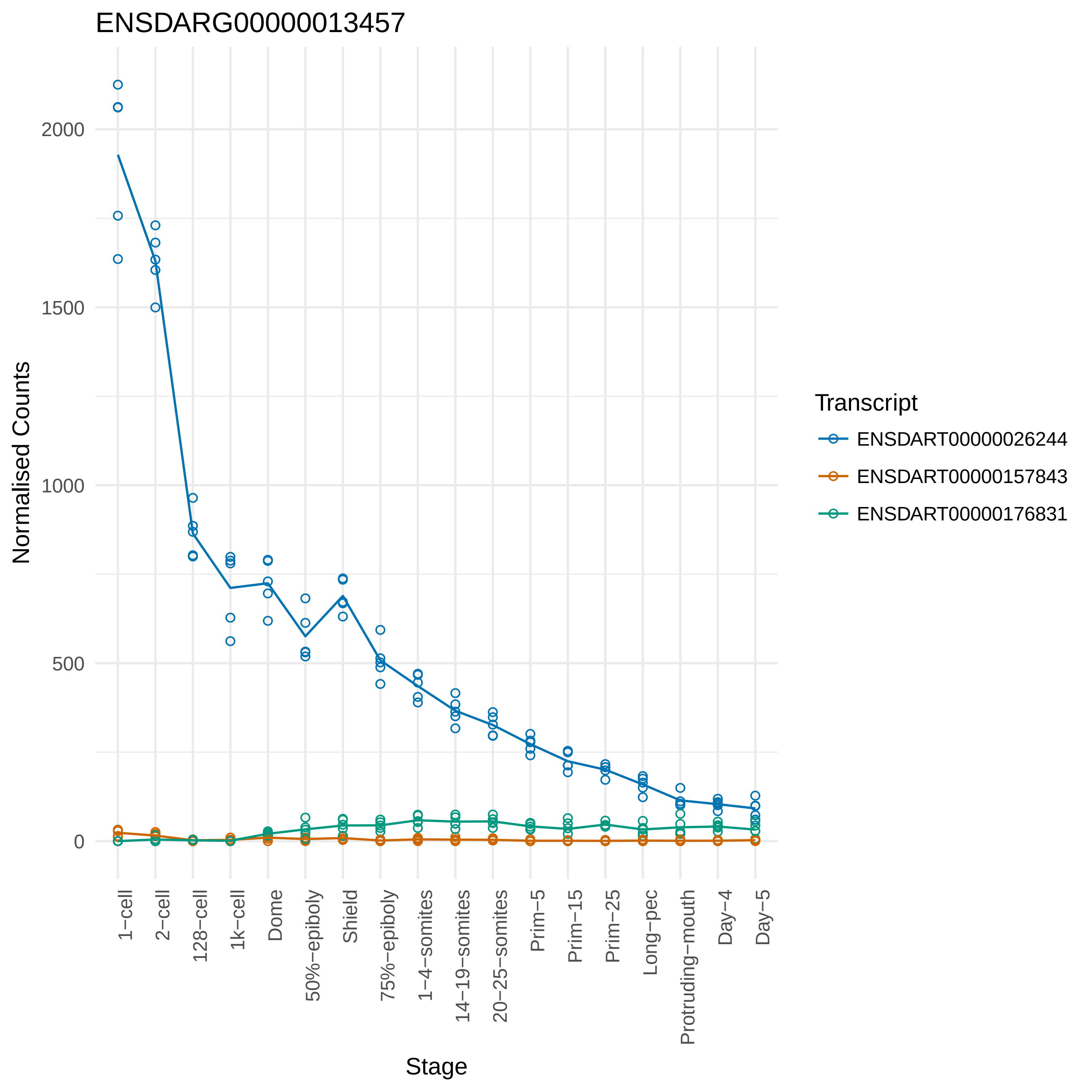
ENSDARG00000013457
Normalised Counts
2000
1500
1000
500
0
1−cell
2−cell
128−cell
1k−cell
Dome
50%−epiboly
Shield
75%−epiboly
1−4−somites
14−19−somites
20−25−somites
Prim−5
Prim−15
Prim−25
Long−pec
Protruding−mouth
Day−4
Day−5
Stage
Transcript
ENSDART00000026244
ENSDART00000157843
ENSDART00000176831

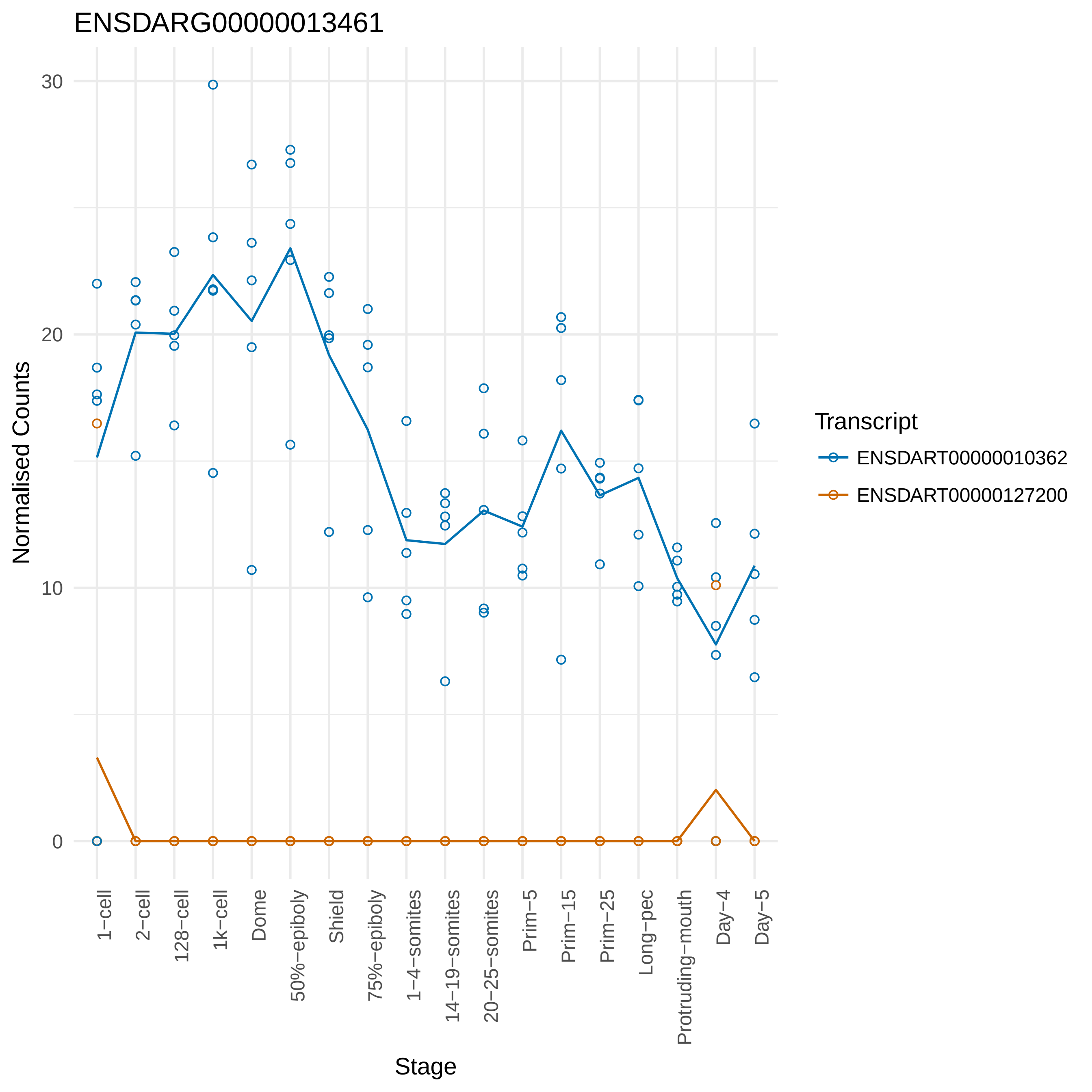

ENSDARG00000013461
Normalised Counts
30
20
10
0
1−cell
2−cell
128−cell
1k−cell
Dome
50%−epiboly
Shield
75%−epiboly
1−4−somites
14−19−somites
20−25−somites
Prim−5
Prim−15
Prim−25
Long−pec
Protruding−mouth
Day−4
Day−5
Stage
Transcript
ENSDART00000010362
ENSDART00000127200

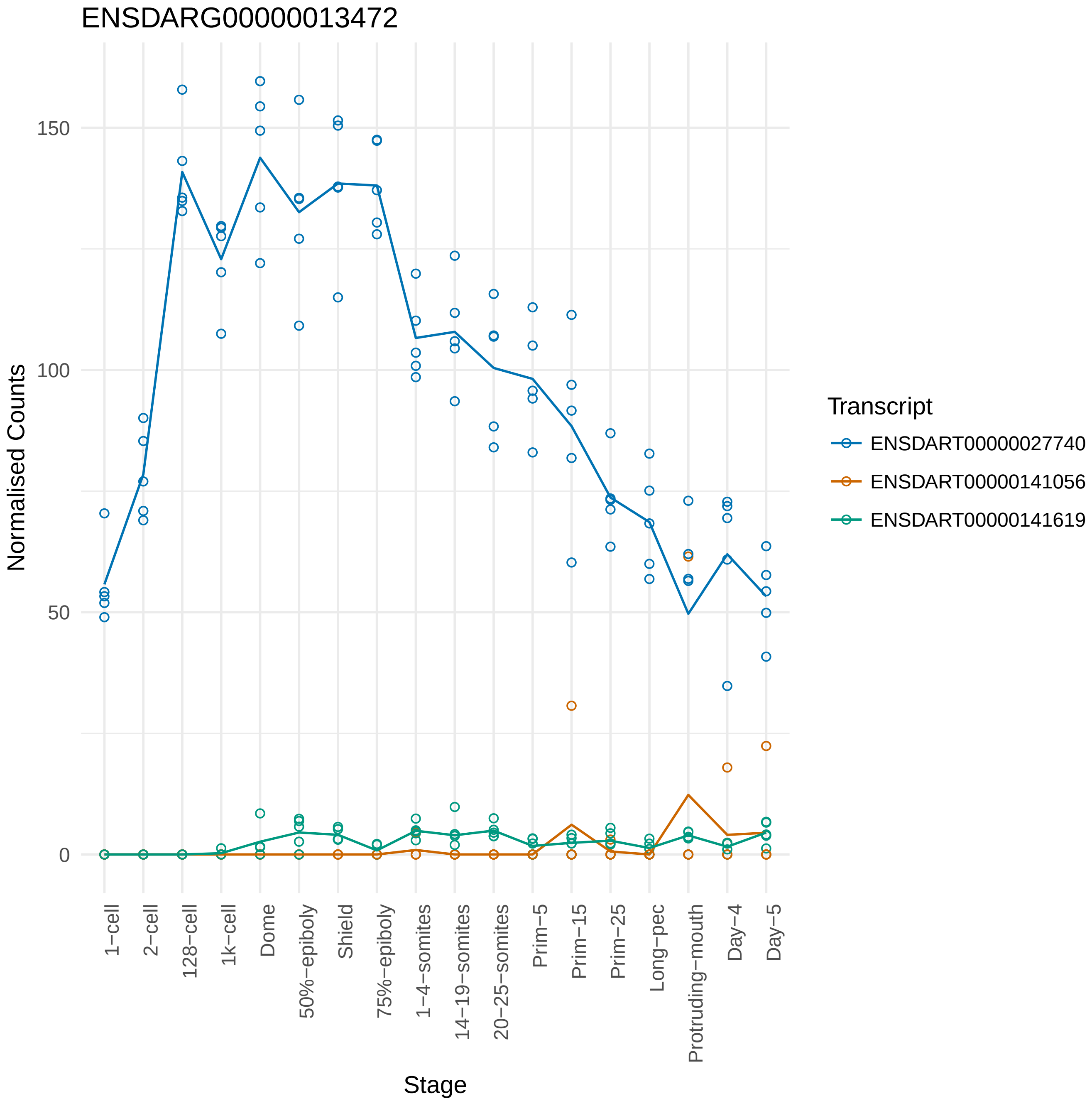

ENSDARG00000013472
Normalised Counts
150
100
50
0
1−cell
2−cell
128−cell
1k−cell
Dome
50%−epiboly
Shield
75%−epiboly
1−4−somites
14−19−somites
20−25−somites
Prim−5
Prim−15
Prim−25
Long−pec
Protruding−mouth
Day−4
Day−5
Stage
Transcript
ENSDART00000027740
ENSDART00000141056
ENSDART00000141619

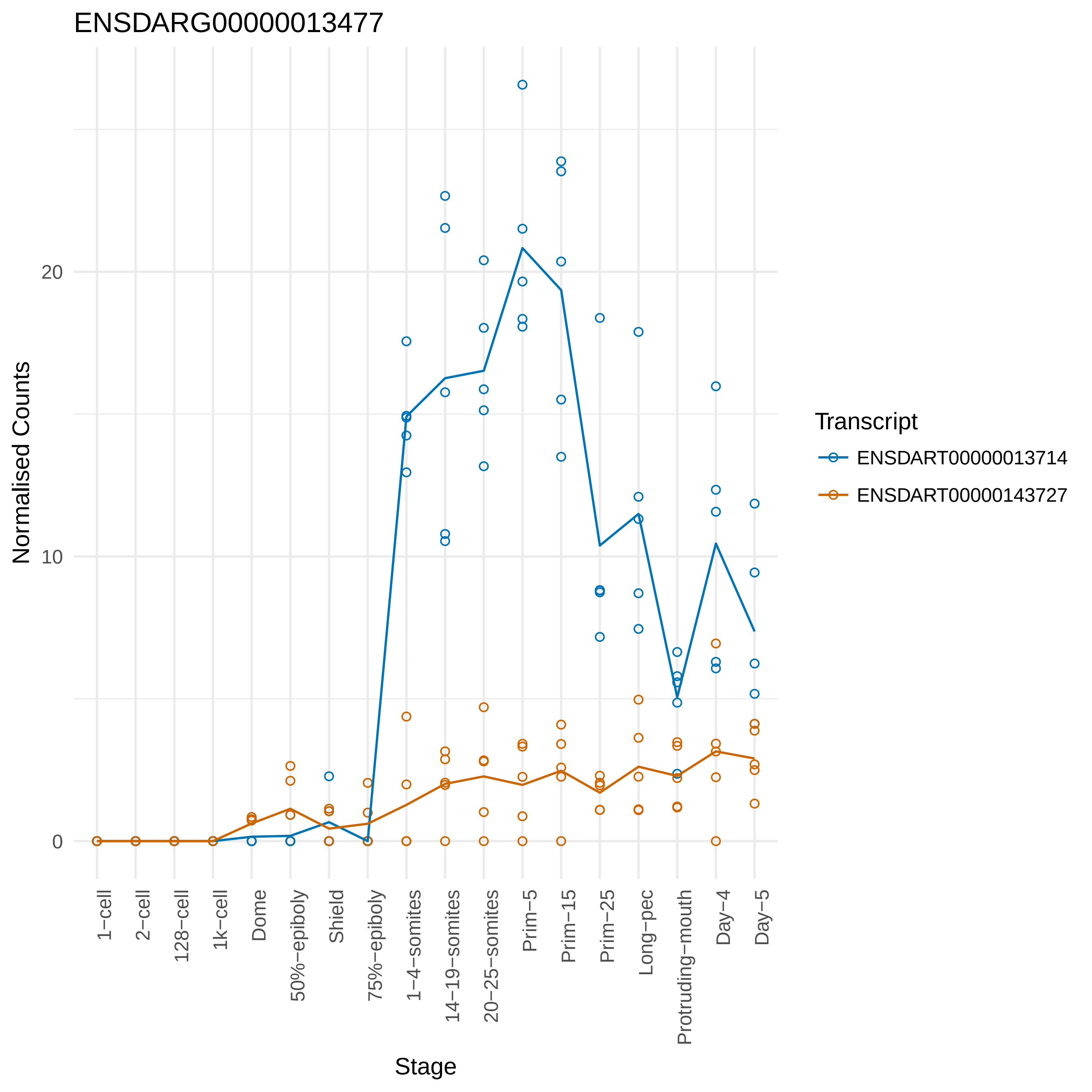

ENSDARG00000013477
Normalised Counts
20
10
0
1−cell
2−cell
128−cell
1k−cell
Dome
50%−epiboly
Shield
75%−epiboly
1−4−somites
14−19−somites
20−25−somites
Prim−5
Prim−15
Prim−25
Long−pec
Protruding−mouth
Day−4
Day−5
Stage
Transcript
ENSDART00000013714
ENSDART00000143727

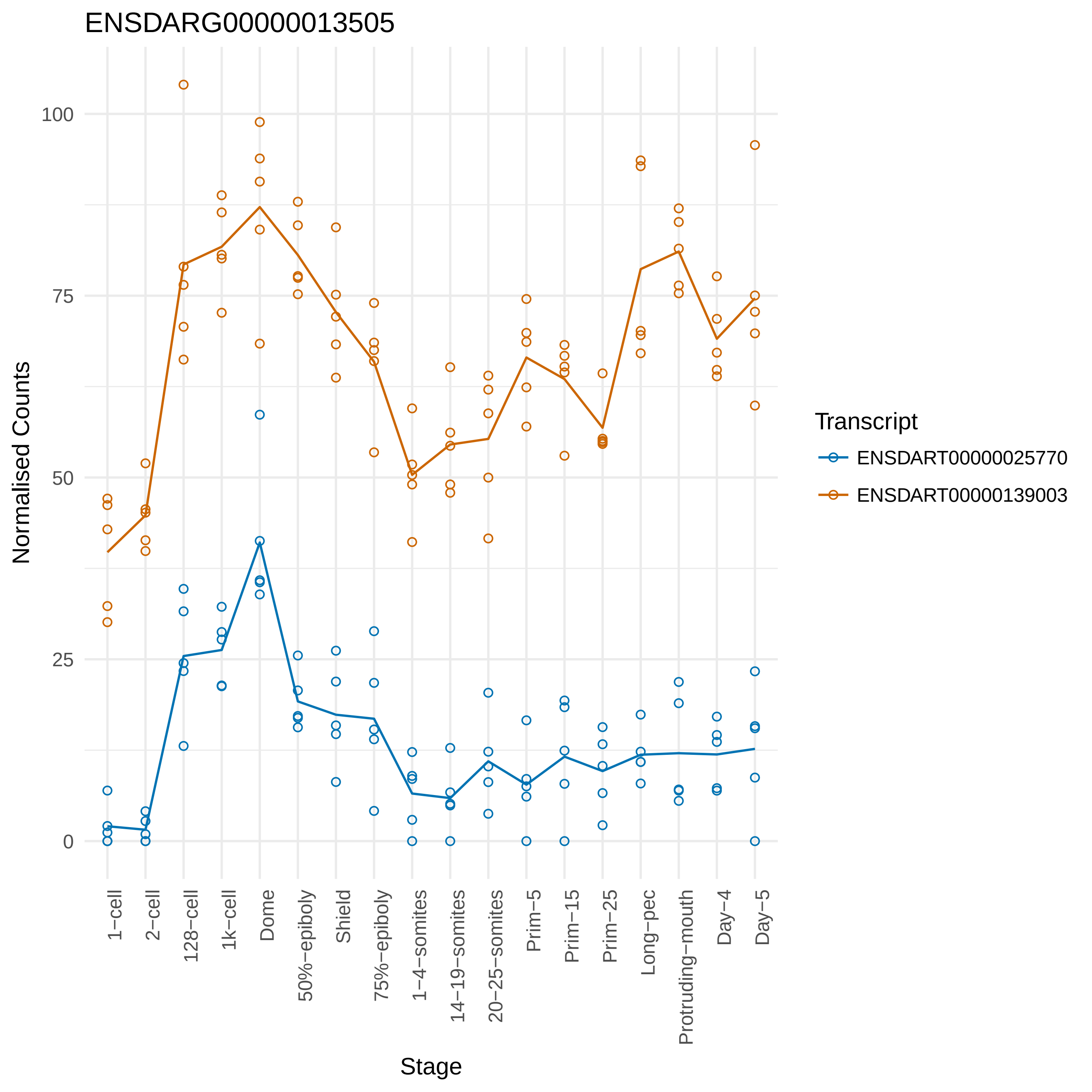
ENSDARG00000013505
Normalised Counts
0
25
50
75
100
Stage
1−cell
2−cell
128−cell
1k−cell
Dome
50%−epiboly
Shield
75%−epiboly
1−4−somites
14−19−somites
20−25−somites
Prim−5
Prim−15
Prim−25
Long−pec
Protruding−mouth
Day−4
Day−5
Transcript
ENSDART00000025770
ENSDART00000139003

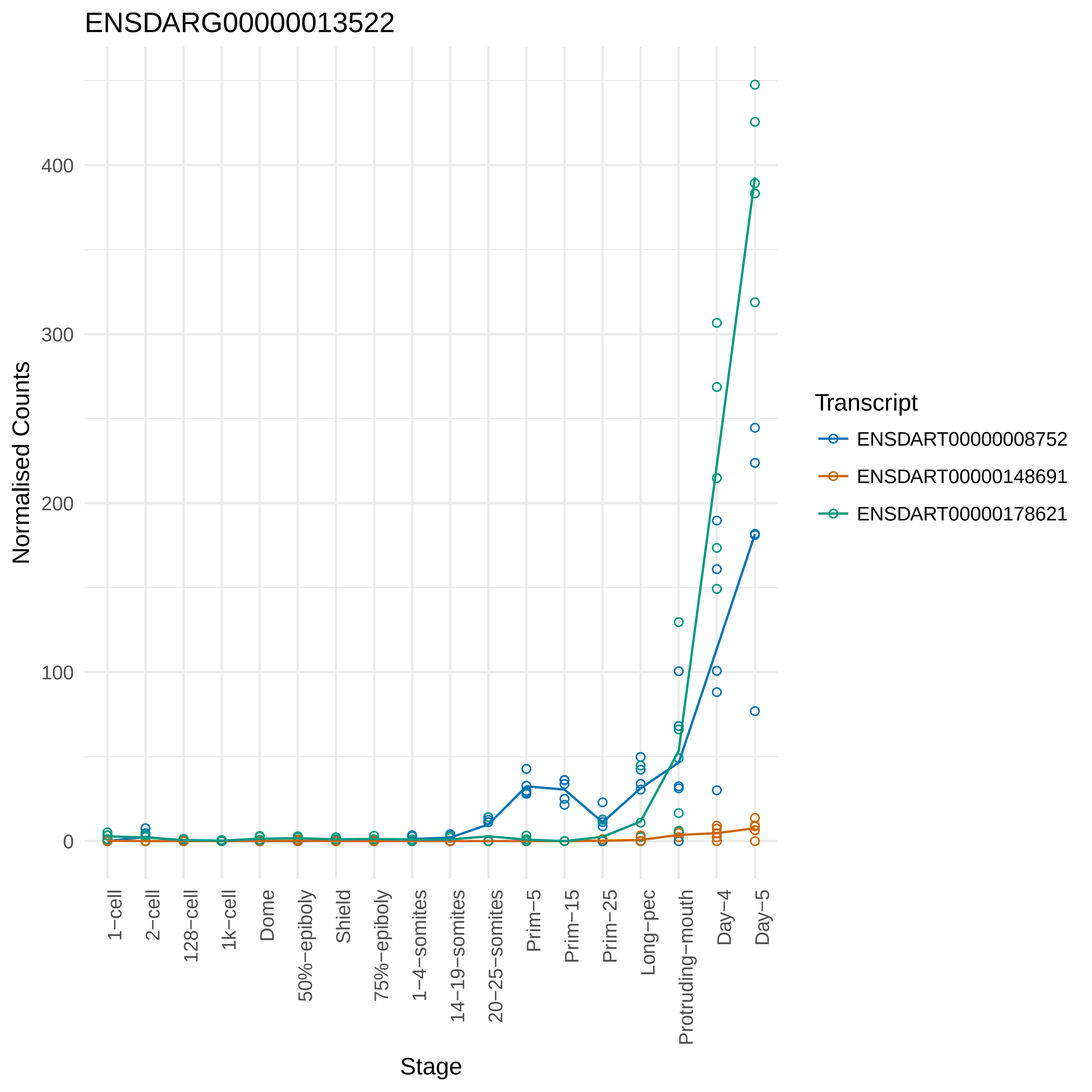

ENSDARG00000013522
Normalised Counts
400
300
200
100
0
1−cell
2−cell
128−cell
1k−cell
Dome
50%−epiboly
Shield
75%−epiboly
1−4−somites
14−19−somites
20−25−somites
Prim−5
Prim−15
Prim−25
Long−pec
Protruding−mouth
Day−4
Day−5
Stage
Transcript
ENSDART00000008752
ENSDART00000148691
ENSDART00000178621

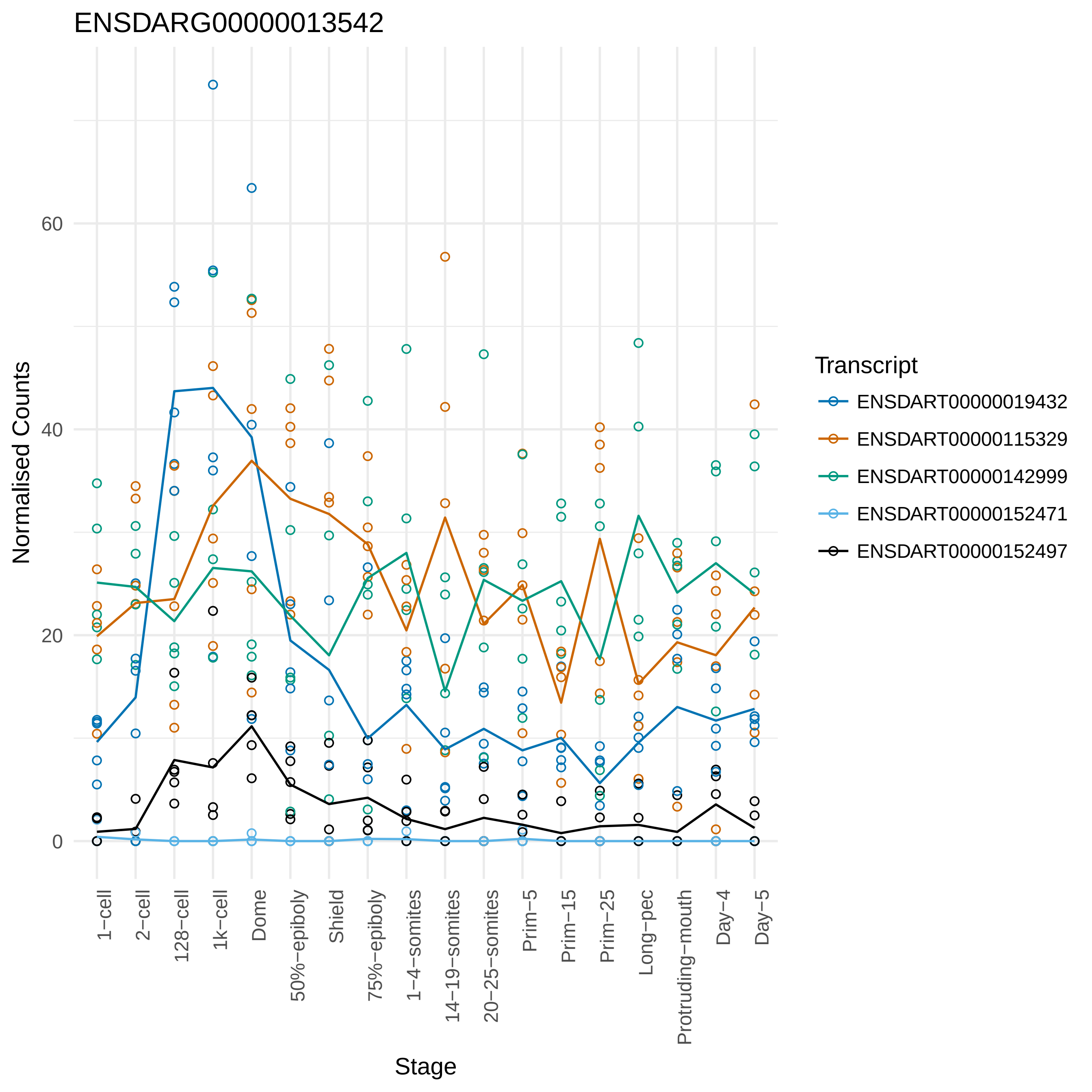

ENSDARG00000013542
Normalised Counts
0
20
40
60
1−cell
2−cell
128−cell
1k−cell
Dome
50%−epiboly
Shield
75%−epiboly
1−4−somites
14−19−somites
20−25−somites
Prim−5
Prim−15
Prim−25
Long−pec
Protruding−mouth
Day−4
Day−5
Stage
Transcript
ENSDART00000019432
ENSDART00000115329
ENSDART00000142999
ENSDART00000152471
ENSDART00000152497

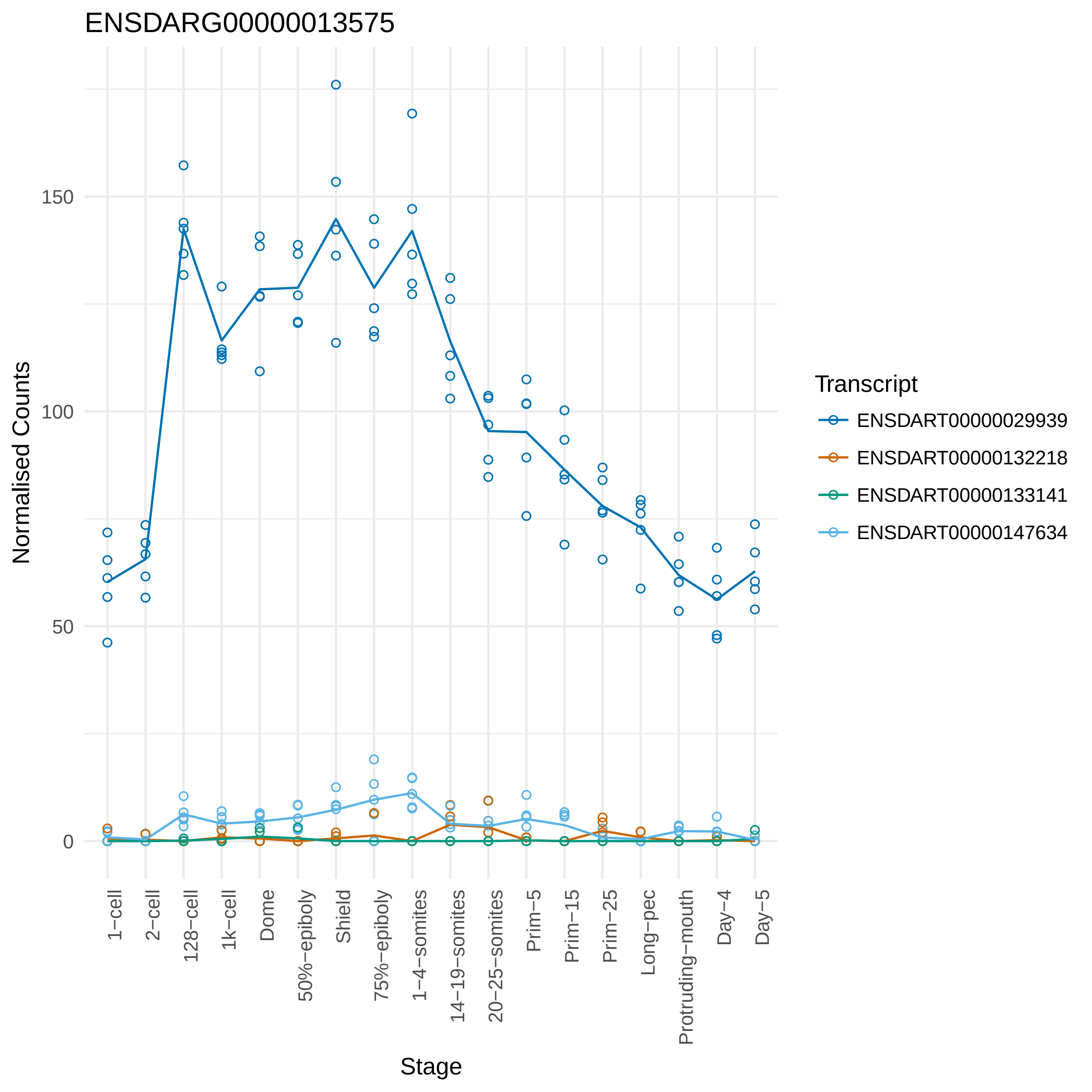
ENSDARG00000013575
Normalised Counts
150
100
50
0
Stage
1−cell
2−cell
128−cell
1k−cell
Dome
50%−epiboly
Shield
75%−epiboly
1−4−somites
14−19−somites
20−25−somites
Prim−5
Prim−15
Prim−25
Long−pec
Protruding−mouth
Day−4
Day−5
Transcript
ENSDART00000029939
ENSDART00000132218
ENSDART00000133141
ENSDART00000147634

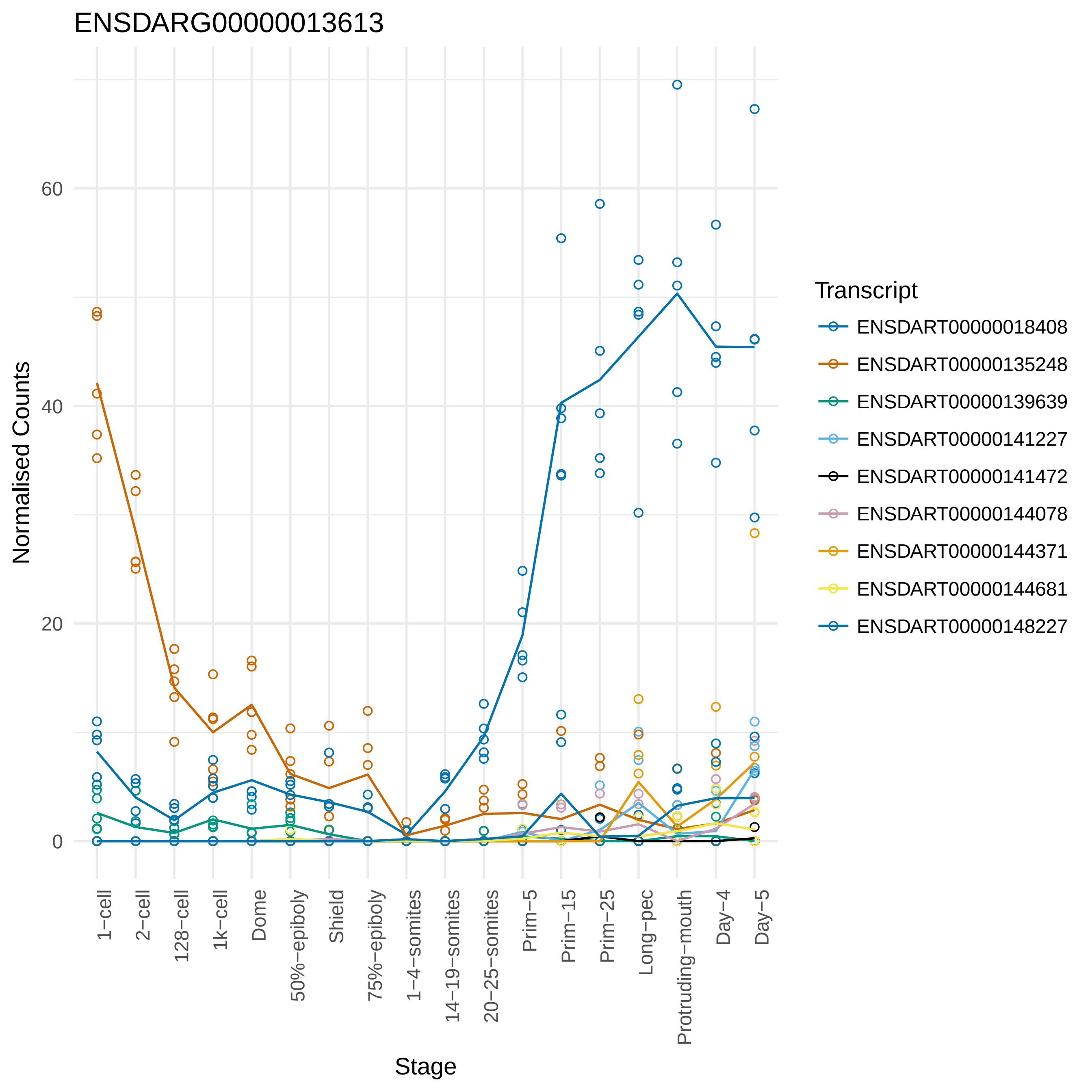

ENSDARG00000013613
Normalised Counts
Stage
0
20
40
60
1−cell
2−cell
128−cell
1k−cell
Dome
50%−epiboly
Shield
75%−epiboly
1−4−somites
14−19−somites
20−25−somites
Prim−5
Prim−15
Prim−25
Long−pec
Protruding−mouth
Day−4
Day−5
Transcript
ENSDART00000018408
ENSDART00000135248
ENSDART00000139639
ENSDART00000141227
ENSDART00000141472
ENSDART00000144078
ENSDART00000144371
ENSDART00000144681
ENSDART00000148227

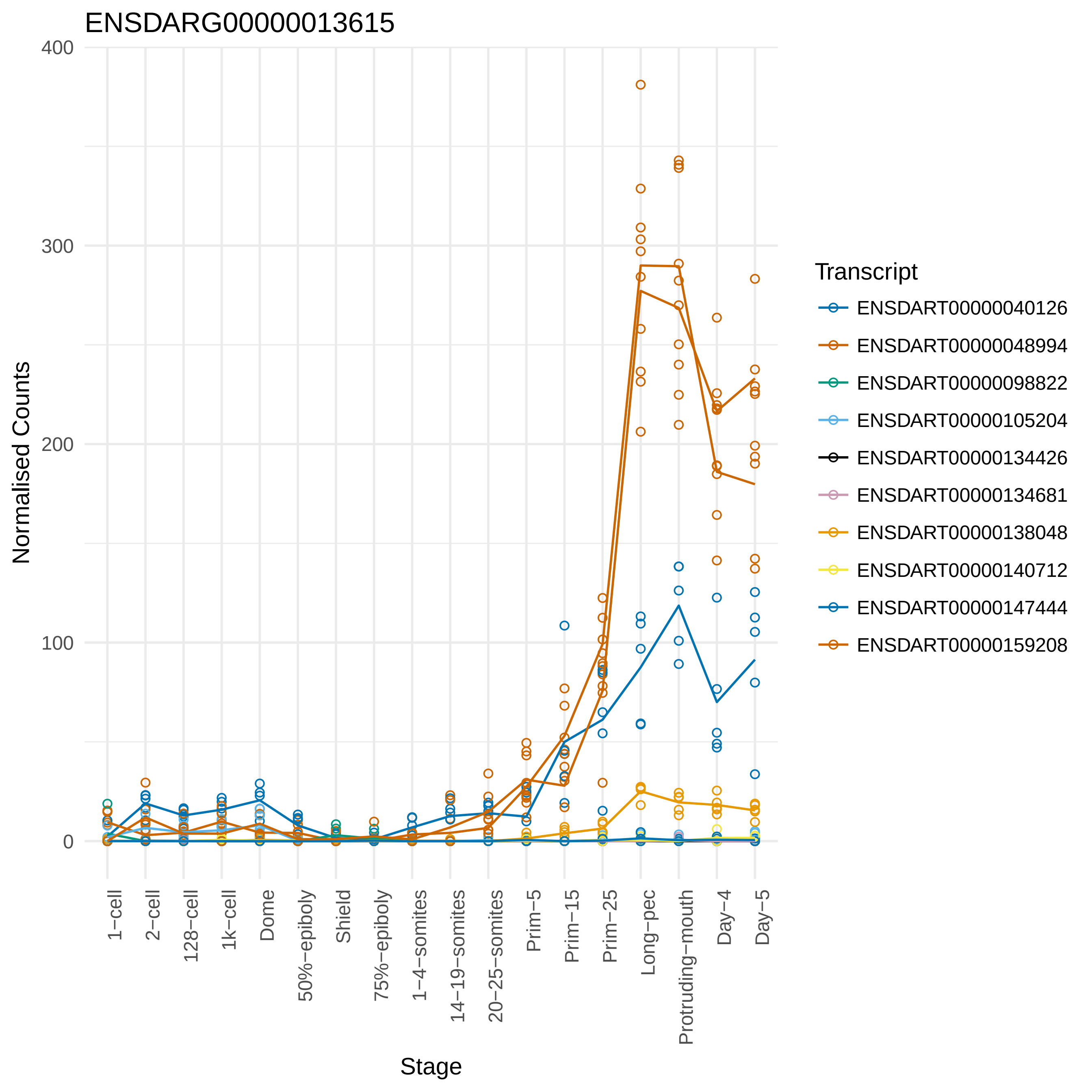

ENSDARG00000013615
Normalised Counts
400
300
200
100
0
1−cell
2−cell
128−cell
1k−cell
Dome
50%−epiboly
Shield
75%−epiboly
1−4−somites
14−19−somites
20−25−somites
Prim−5
Prim−15
Prim−25
Long−pec
Protruding−mouth
Day−4
Day−5
Stage
Transcript
ENSDART00000040126
ENSDART00000048994
ENSDART00000098822
ENSDART00000105204
ENSDART00000134426
ENSDART00000134681
ENSDART00000138048
ENSDART00000140712
ENSDART00000147444
ENSDART00000159208

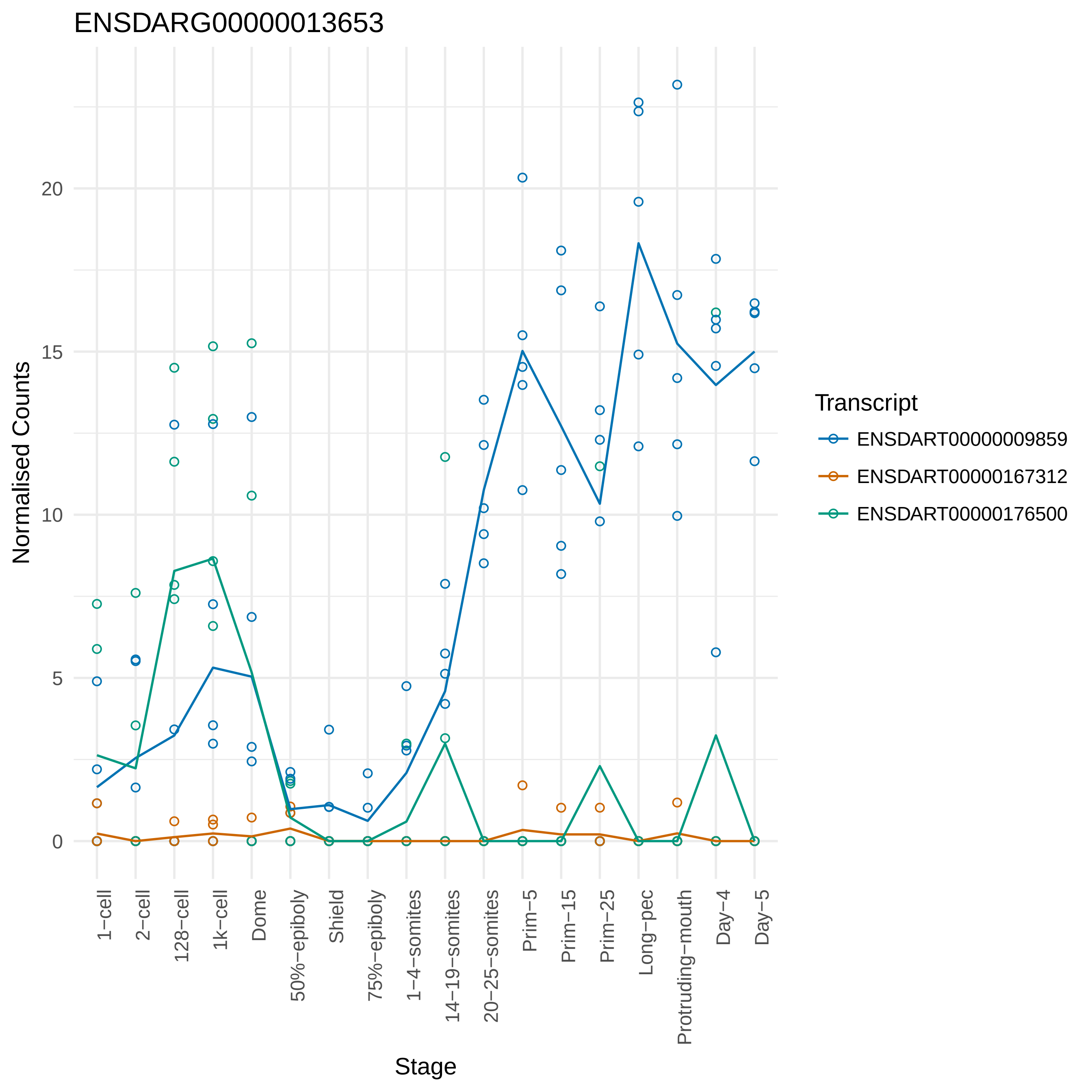

ENSDARG00000013653
Normalised Counts
0
5
10
15
20
Stage
1−cell
2−cell
128−cell
1k−cell
Dome
50%−epiboly
Shield
75%−epiboly
1−4−somites
14−19−somites
20−25−somites
Prim−5
Prim−15
Prim−25
Long−pec
Protruding−mouth
Day−4
Day−5
Transcript
ENSDART00000009859
ENSDART00000167312
ENSDART00000176500

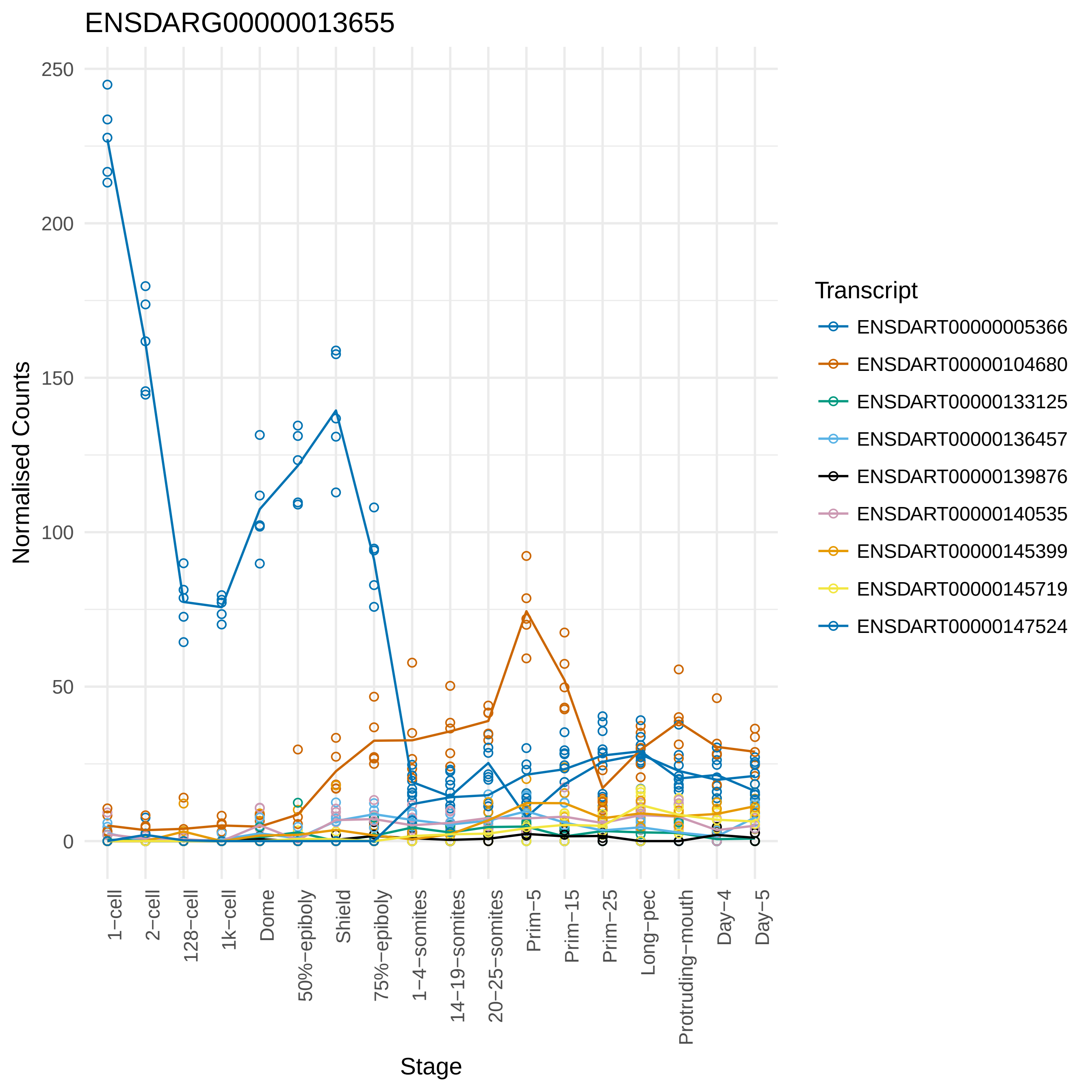
ENSDARG00000013655
Normalised Counts
250
200
150
100
50
0
1−cell
2−cell
128−cell
1k−cell
Dome
50%−epiboly
Shield
75%−epiboly
1−4−somites
14−19−somites
20−25−somites
Prim−5
Prim−15
Prim−25
Long−pec
Protruding−mouth
Day−4
Day−5
Stage
Transcript
ENSDART00000005366
ENSDART00000104680
ENSDART00000133125
ENSDART00000136457
ENSDART00000139876
ENSDART00000140535
ENSDART00000145399
ENSDART00000145719
ENSDART00000147524

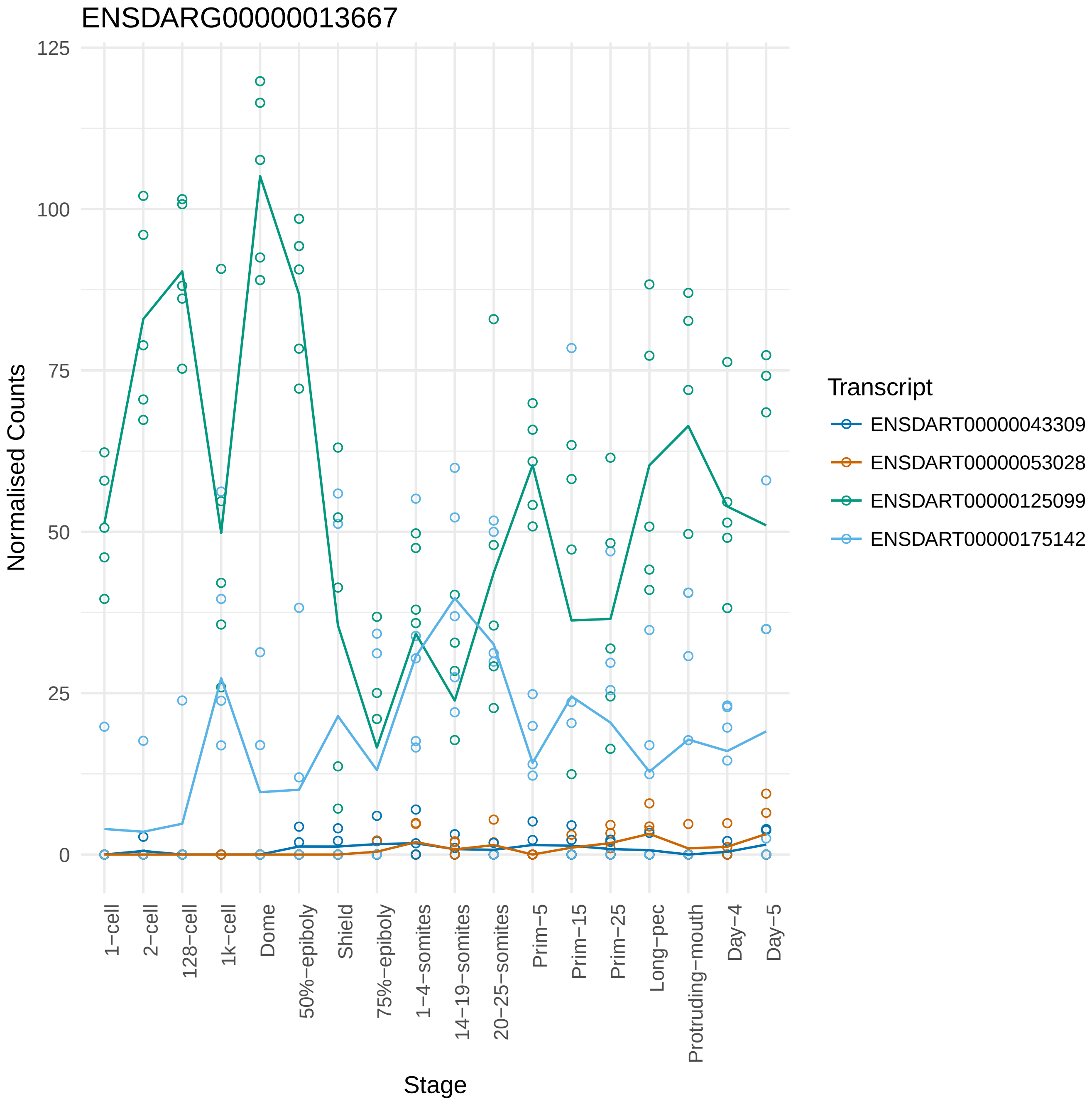

ENSDARG00000013667
Normalised Counts
125
100
75
50
25
0
1−cell
2−cell
128−cell
1k−cell
Dome
50%−epiboly
Shield
75%−epiboly
1−4−somites
14−19−somites
20−25−somites
Prim−5
Prim−15
Prim−25
Long−pec
Protruding−mouth
Day−4
Day−5
Stage
Transcript
ENSDART00000043309
ENSDART00000053028
ENSDART00000125099
ENSDART00000175142

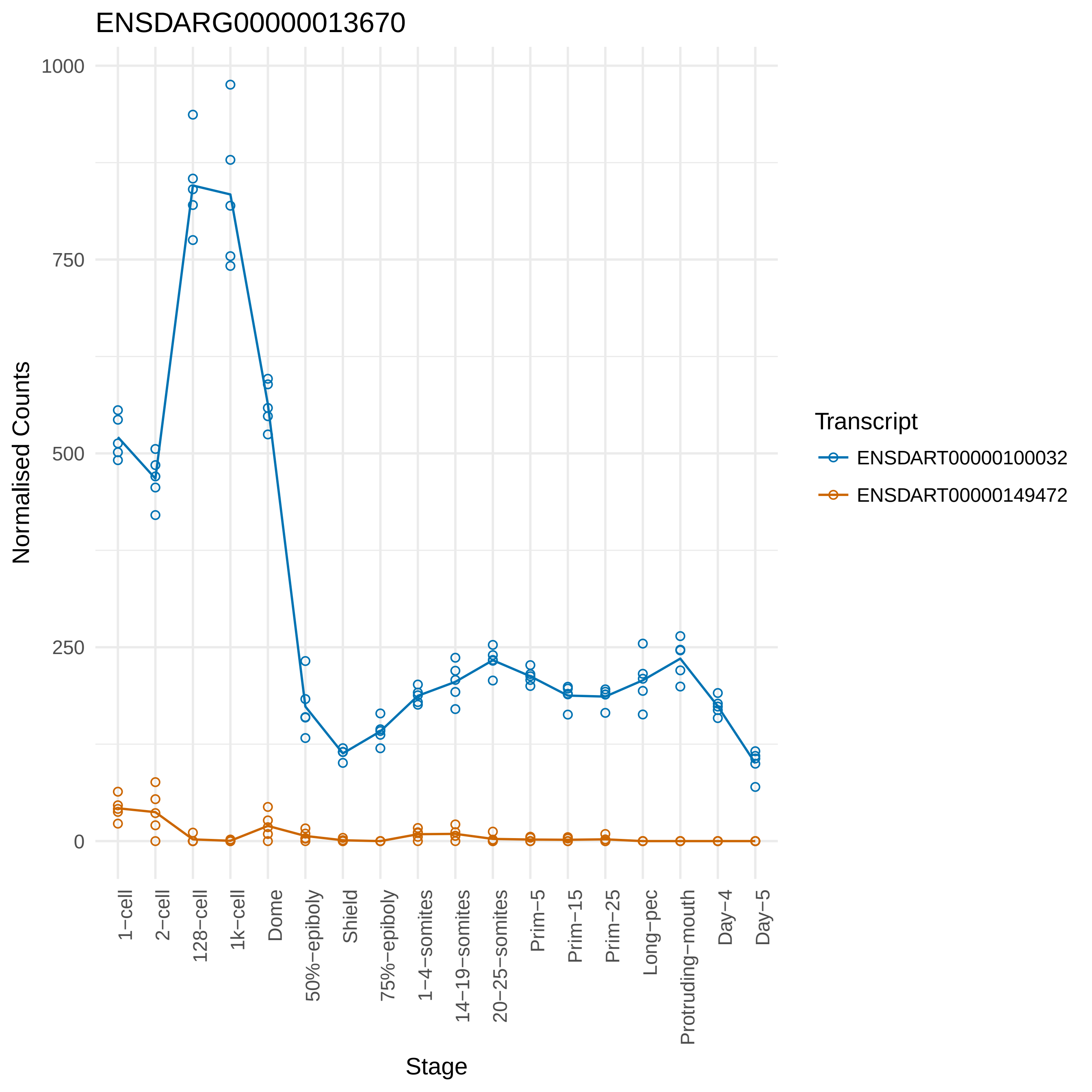
ENSDARG00000013670
Normalised Counts
1000
750
500
250
0
1−cell
2−cell
128−cell
1k−cell
Dome
50%−epiboly
Shield
75%−epiboly
1−4−somites
14−19−somites
20−25−somites
Prim−5
Prim−15
Prim−25
Long−pec
Protruding−mouth
Day−4
Day−5
Stage
Transcript
ENSDART00000100032
ENSDART00000149472

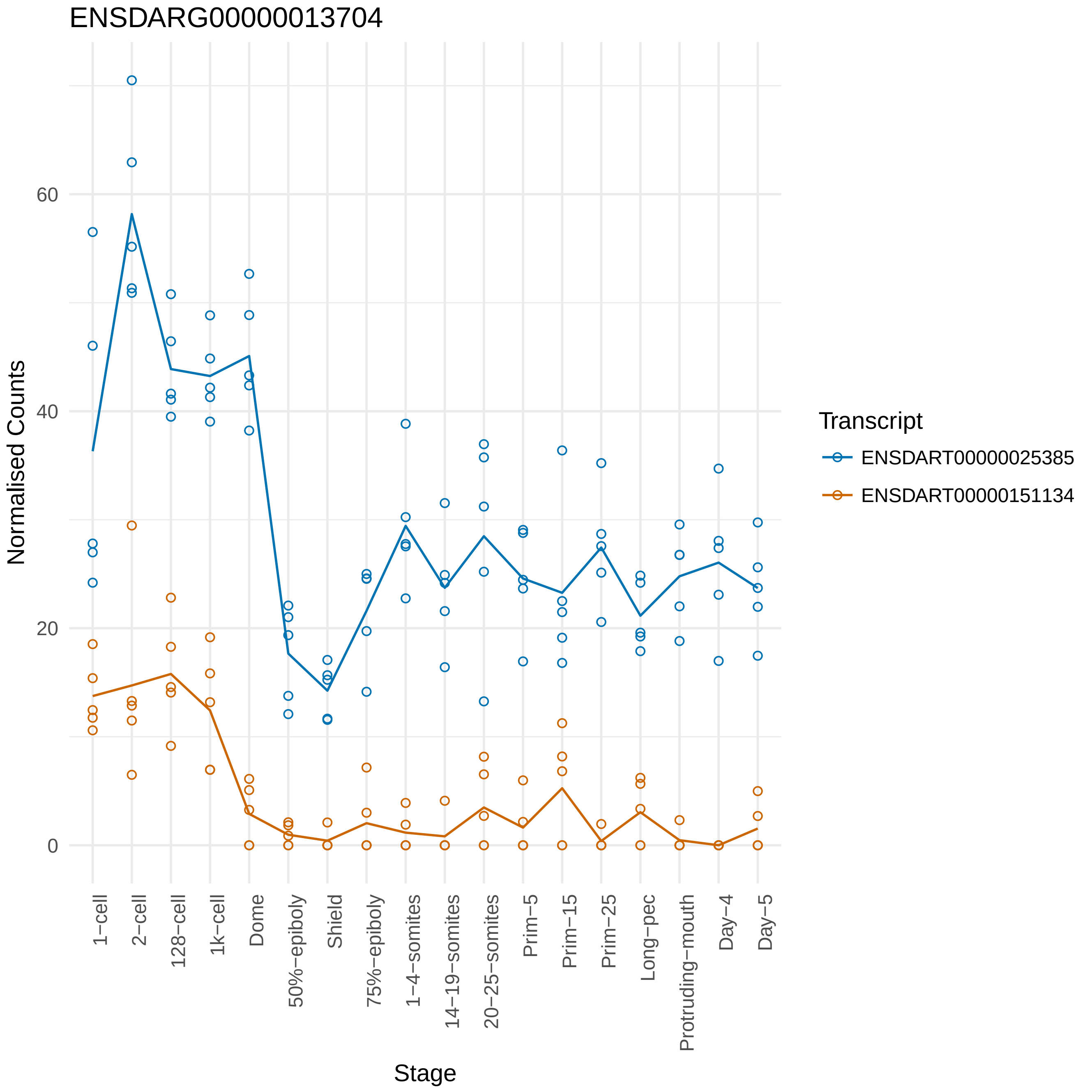

ENSDARG00000013704
Normalised Counts
0
20
40
60
Stage
1−cell
2−cell
128−cell
1k−cell
Dome
50%−epiboly
Shield
75%−epiboly
1−4−somites
14−19−somites
20−25−somites
Prim−5
Prim−15
Prim−25
Long−pec
Protruding−mouth
Day−4
Day−5
Transcript
ENSDART00000025385
ENSDART00000151134

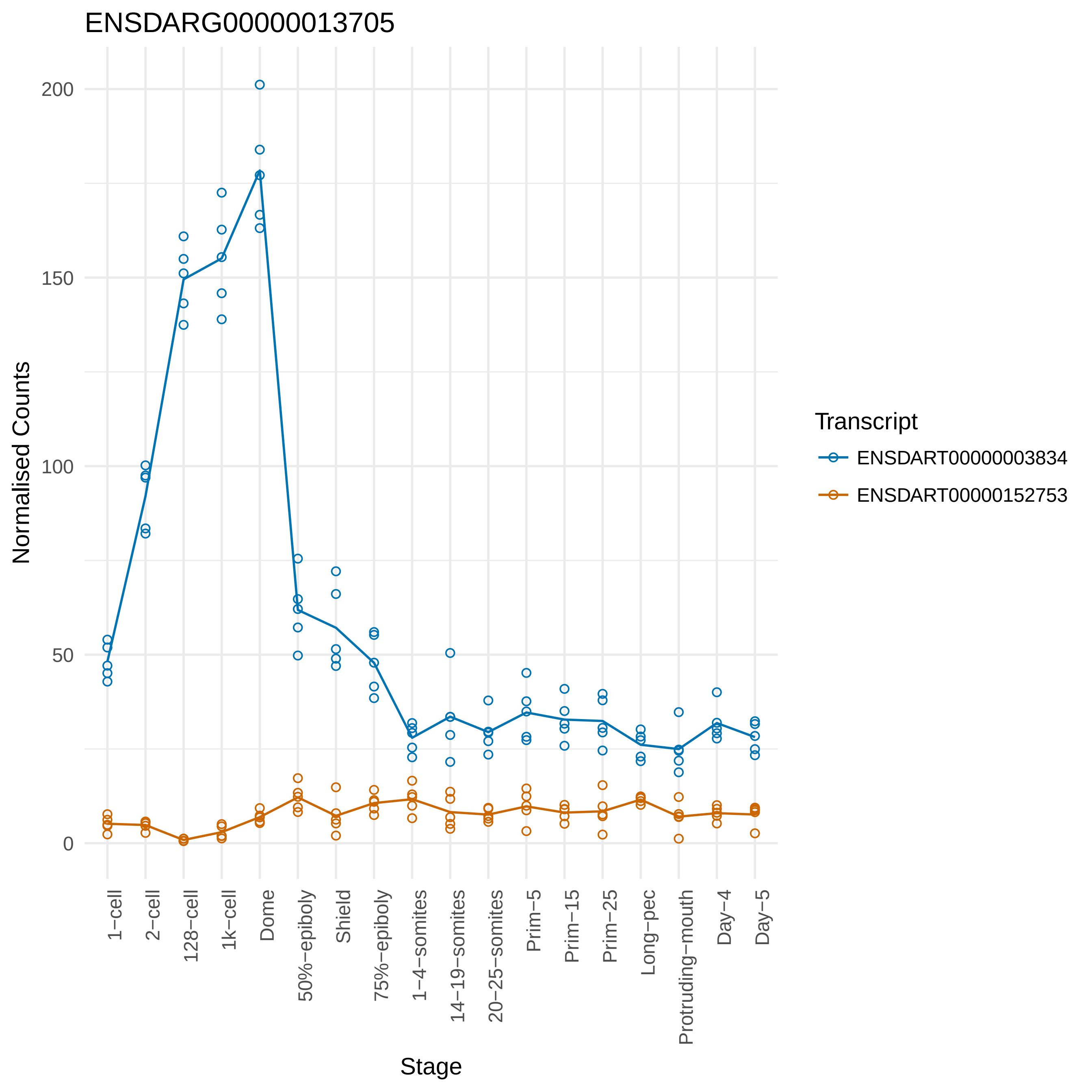

ENSDARG00000013705
Normalised Counts
200
150
100
50
0
1−cell
2−cell
128−cell
1k−cell
Dome
50%−epiboly
Shield
75%−epiboly
1−4−somites
14−19−somites
20−25−somites
Prim−5
Prim−15
Prim−25
Long−pec
Protruding−mouth
Day−4
Day−5
Stage
Transcript
ENSDART00000003834
ENSDART00000152753

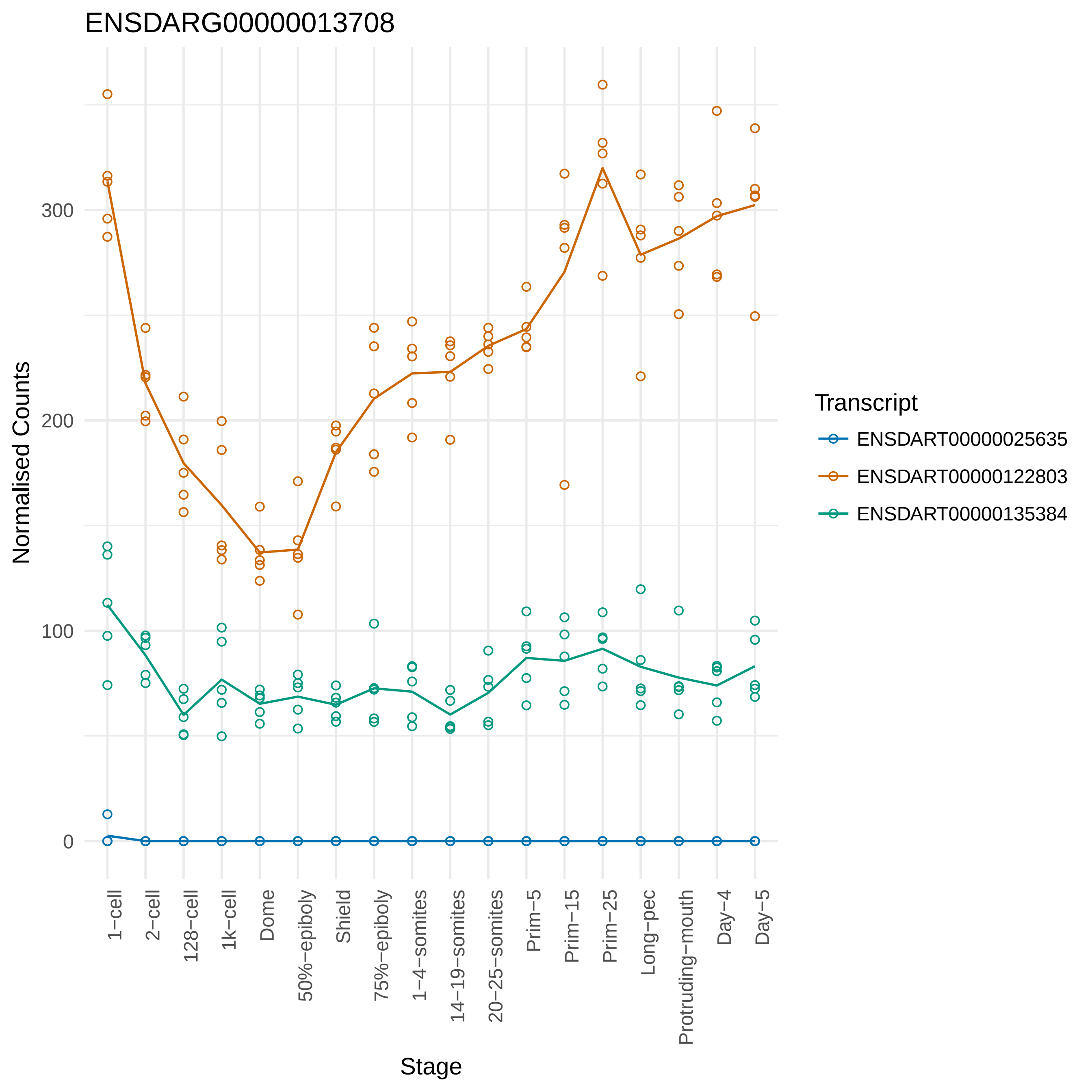

ENSDARG00000013708
Normalised Counts
300
200
100
0
1−cell
2−cell
128−cell
1k−cell
Dome
50%−epiboly
Shield
75%−epiboly
1−4−somites
14−19−somites
20−25−somites
Prim−5
Prim−15
Prim−25
Long−pec
Protruding−mouth
Day−4
Day−5
Stage
Transcript
ENSDART00000025635
ENSDART00000122803
ENSDART00000135384

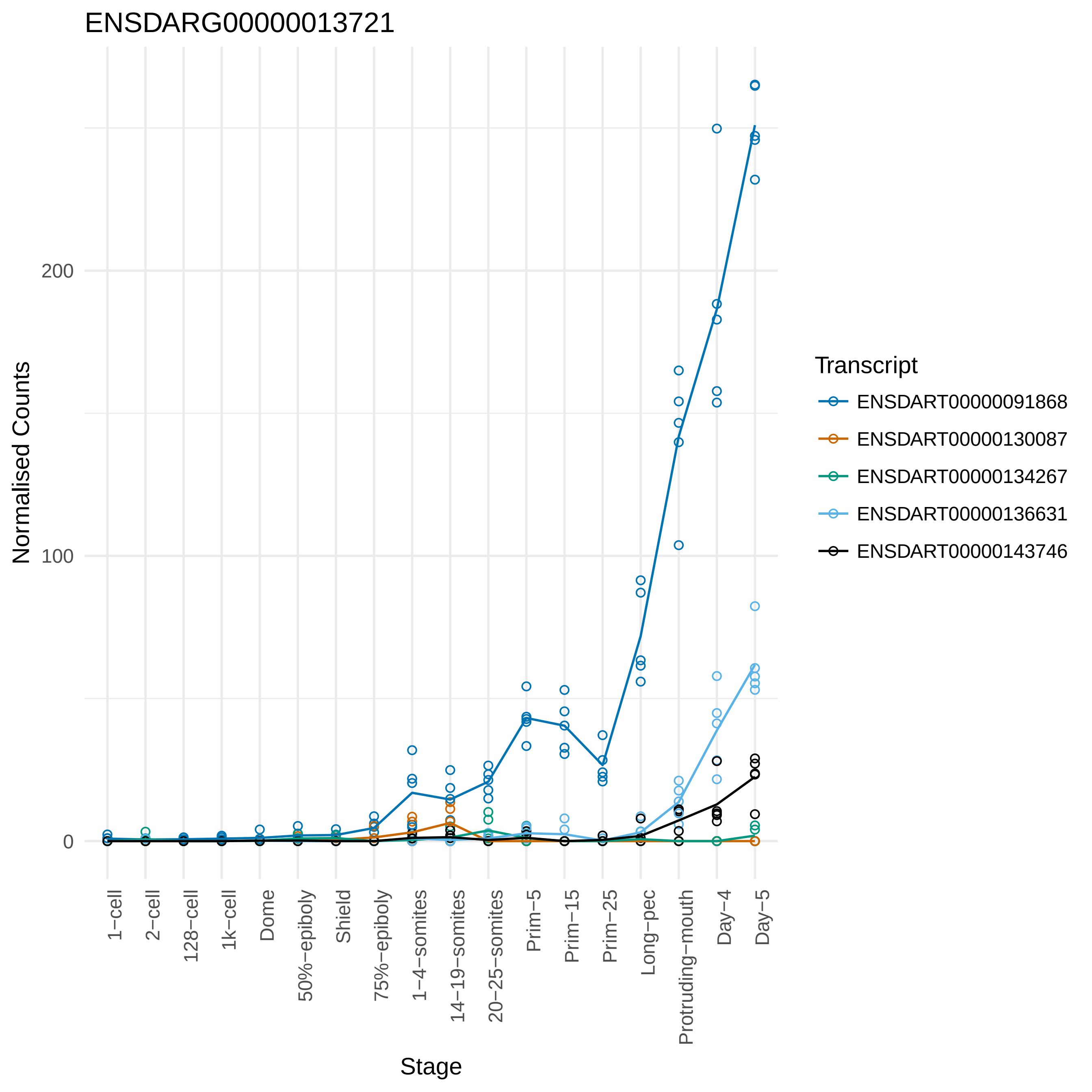
ENSDARG00000013721
Normalised Counts
0
100
200
1−cell
2−cell
128−cell
1k−cell
Dome
50%−epiboly
Shield
75%−epiboly
1−4−somites
14−19−somites
20−25−somites
Prim−5
Prim−15
Prim−25
Long−pec
Protruding−mouth
Day−4
Day−5
Stage
Transcript
ENSDART00000091868
ENSDART00000130087
ENSDART00000134267
ENSDART00000136631
ENSDART00000143746

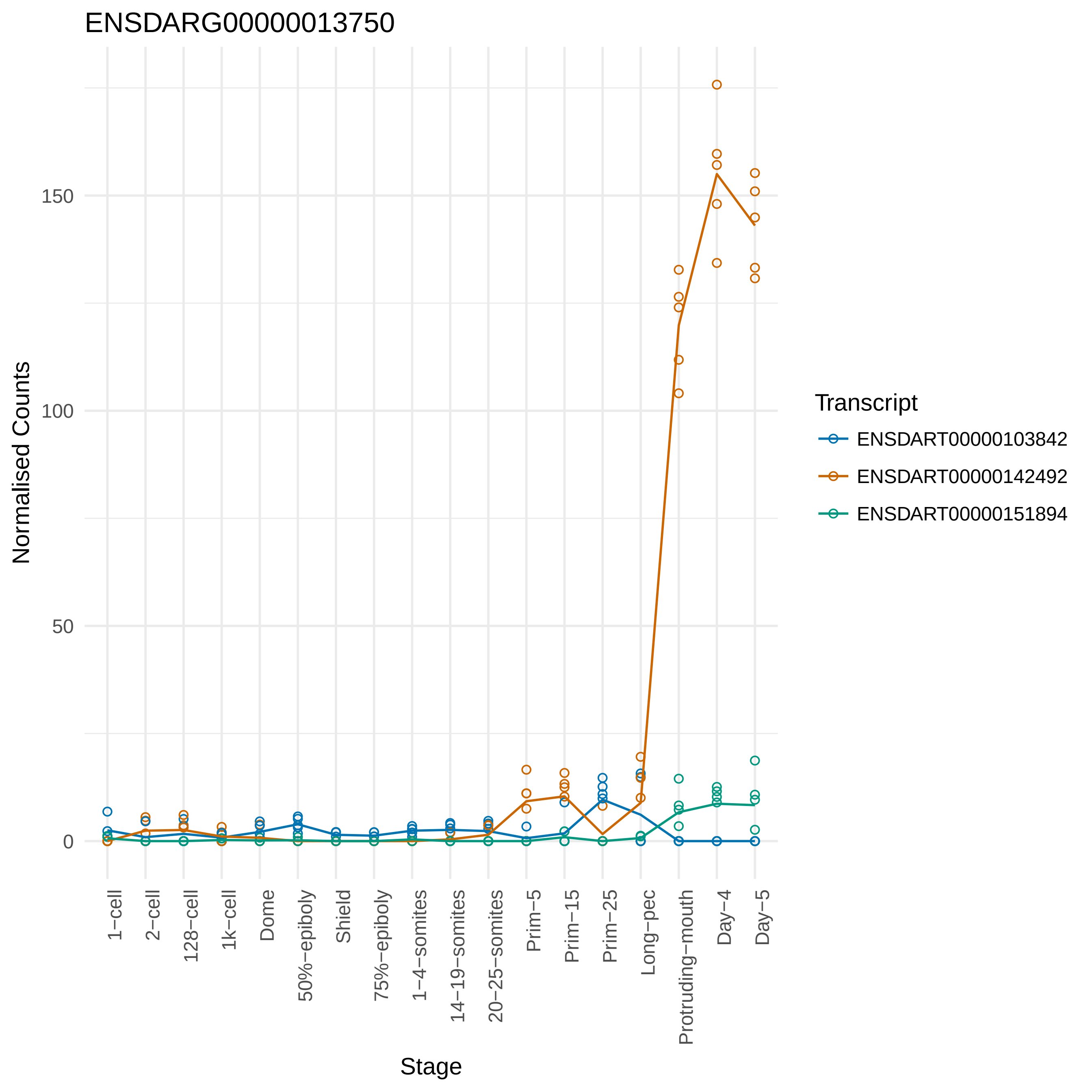

ENSDARG00000013750
Normalised Counts
150
100
50
0
1−cell
2−cell
128−cell
1k−cell
Dome
50%−epiboly
Shield
75%−epiboly
1−4−somites
14−19−somites
20−25−somites
Prim−5
Prim−15
Prim−25
Long−pec
Protruding−mouth
Day−4
Day−5
Stage
Transcript
ENSDART00000103842
ENSDART00000142492
ENSDART00000151894

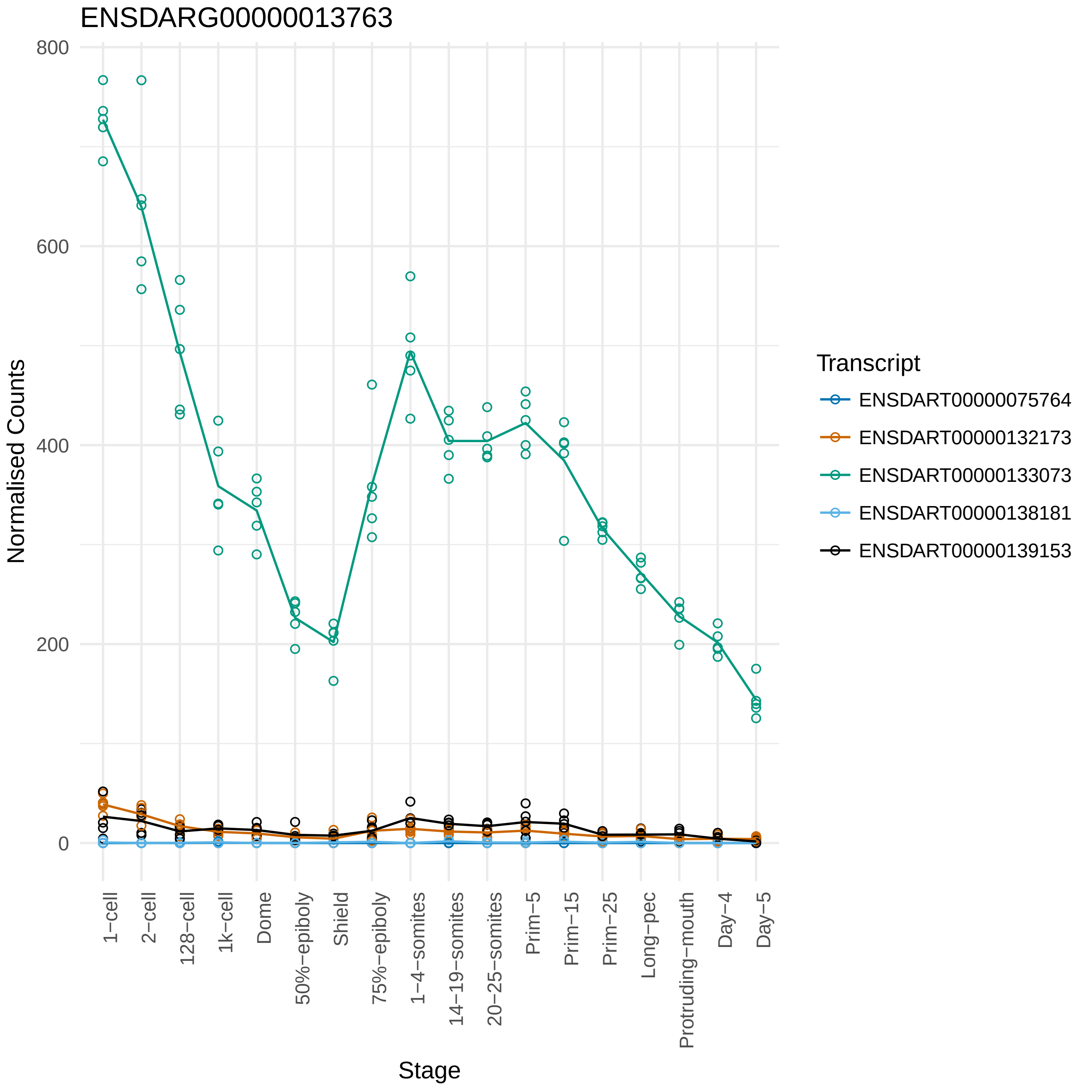

ENSDARG00000013763
Normalised Counts
800
600
400
200
0
1−cell
2−cell
128−cell
1k−cell
Dome
50%−epiboly
Shield
75%−epiboly
1−4−somites
14−19−somites
20−25−somites
Prim−5
Prim−15
Prim−25
Long−pec
Protruding−mouth
Day−4
Day−5
Stage
Transcript
ENSDART00000075764
ENSDART00000132173
ENSDART00000133073
ENSDART00000138181
ENSDART00000139153

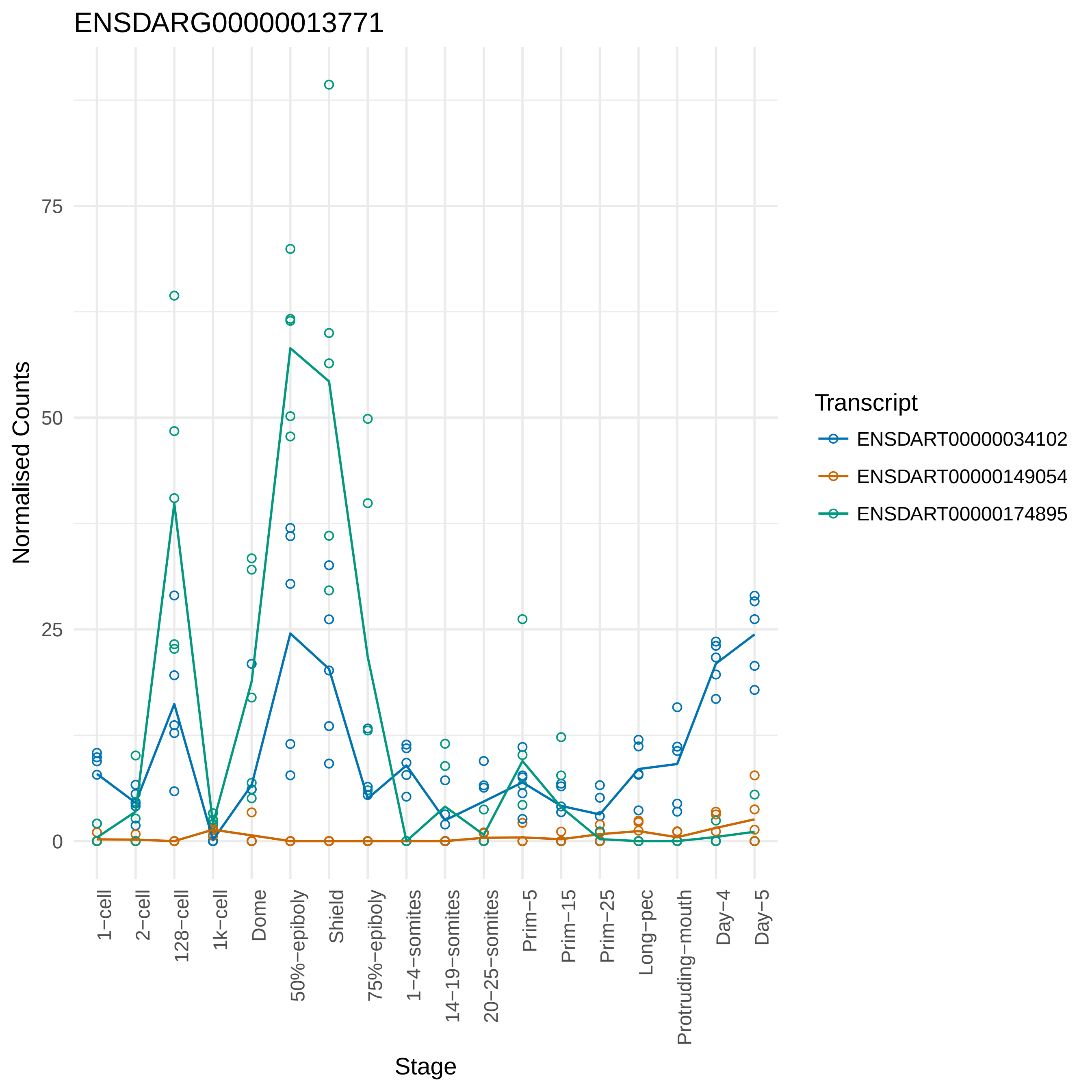

ENSDARG00000013771
Normalised Counts
75
50
25
0
1−cell
2−cell
128−cell
1k−cell
Dome
50%−epiboly
Shield
75%−epiboly
1−4−somites
14−19−somites
20−25−somites
Prim−5
Prim−15
Prim−25
Long−pec
Protruding−mouth
Day−4
Day−5
Stage
Transcript
ENSDART00000034102
ENSDART00000149054
ENSDART00000174895

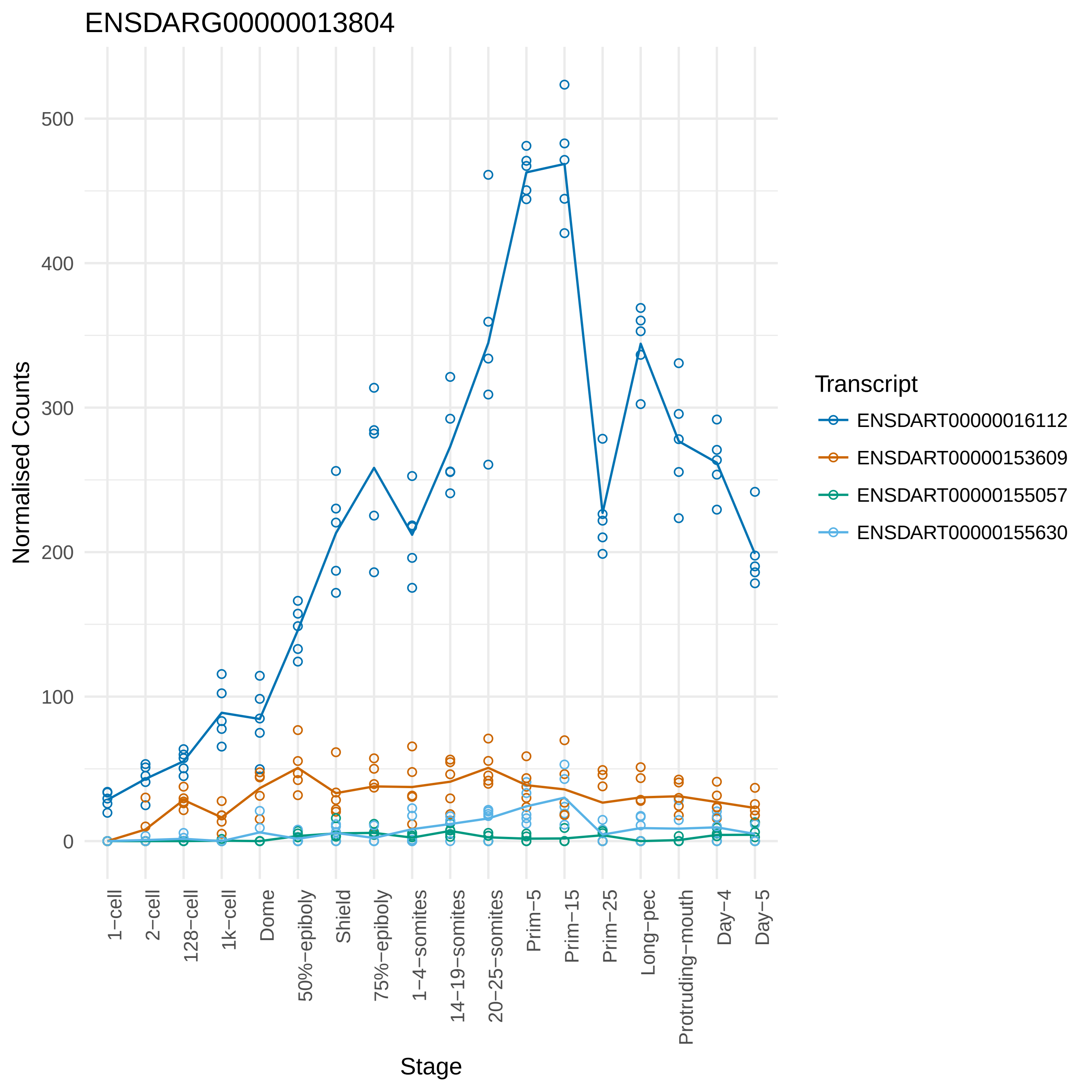

ENSDARG00000013804
Normalised Counts
0
100
200
300
400
500
Stage
1−cell
2−cell
128−cell
1k−cell
Dome
50%−epiboly
Shield
75%−epiboly
1−4−somites
14−19−somites
20−25−somites
Prim−5
Prim−15
Prim−25
Long−pec
Protruding−mouth
Day−4
Day−5
Transcript
ENSDART00000016112
ENSDART00000153609
ENSDART00000155057
ENSDART00000155630

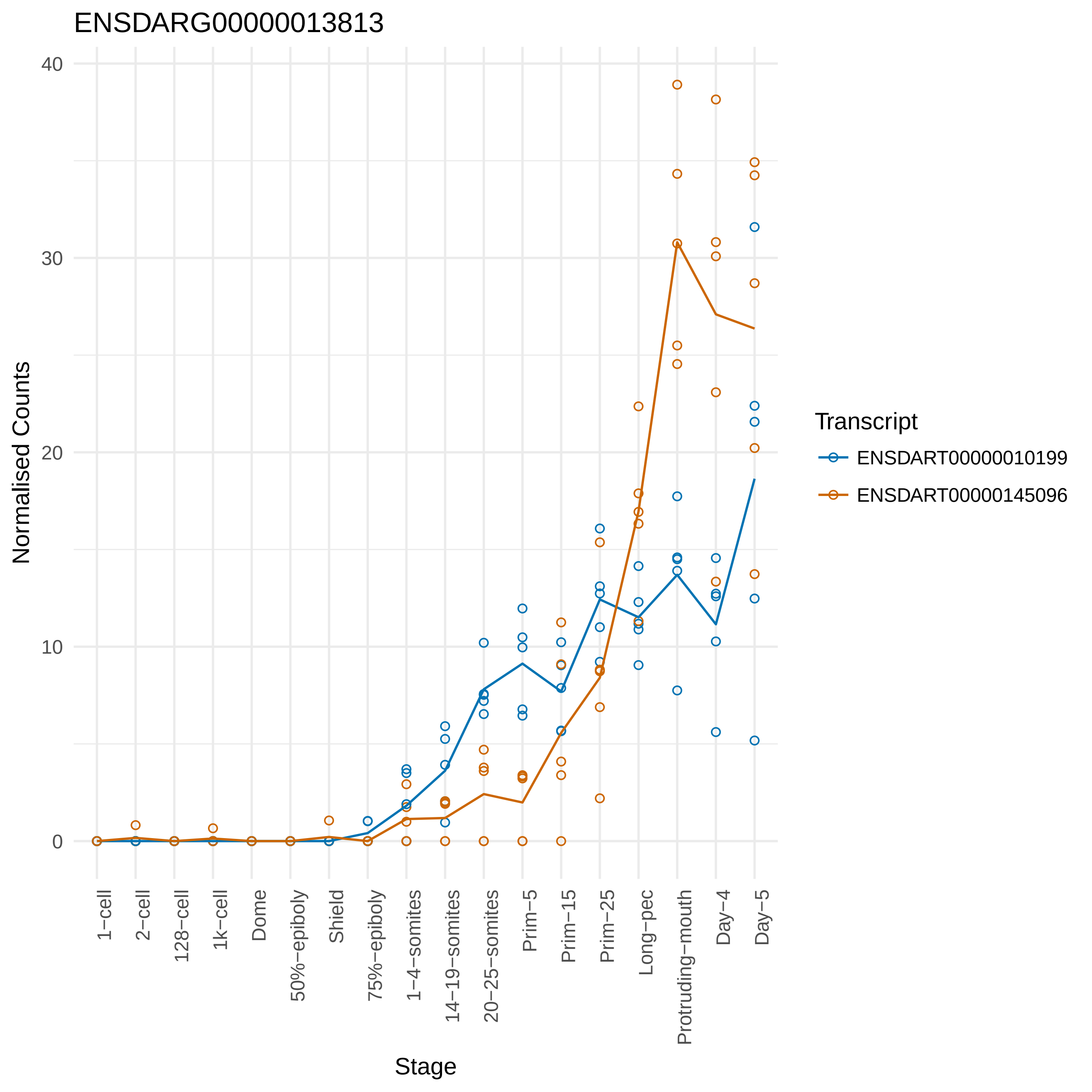

ENSDARG00000013813
Normalised Counts
0
10
20
30
40
1−cell
2−cell
128−cell
1k−cell
Dome
50%−epiboly
Shield
75%−epiboly
1−4−somites
14−19−somites
20−25−somites
Prim−5
Prim−15
Prim−25
Long−pec
Protruding−mouth
Day−4
Day−5
Stage
Transcript
ENSDART00000010199
ENSDART00000145096

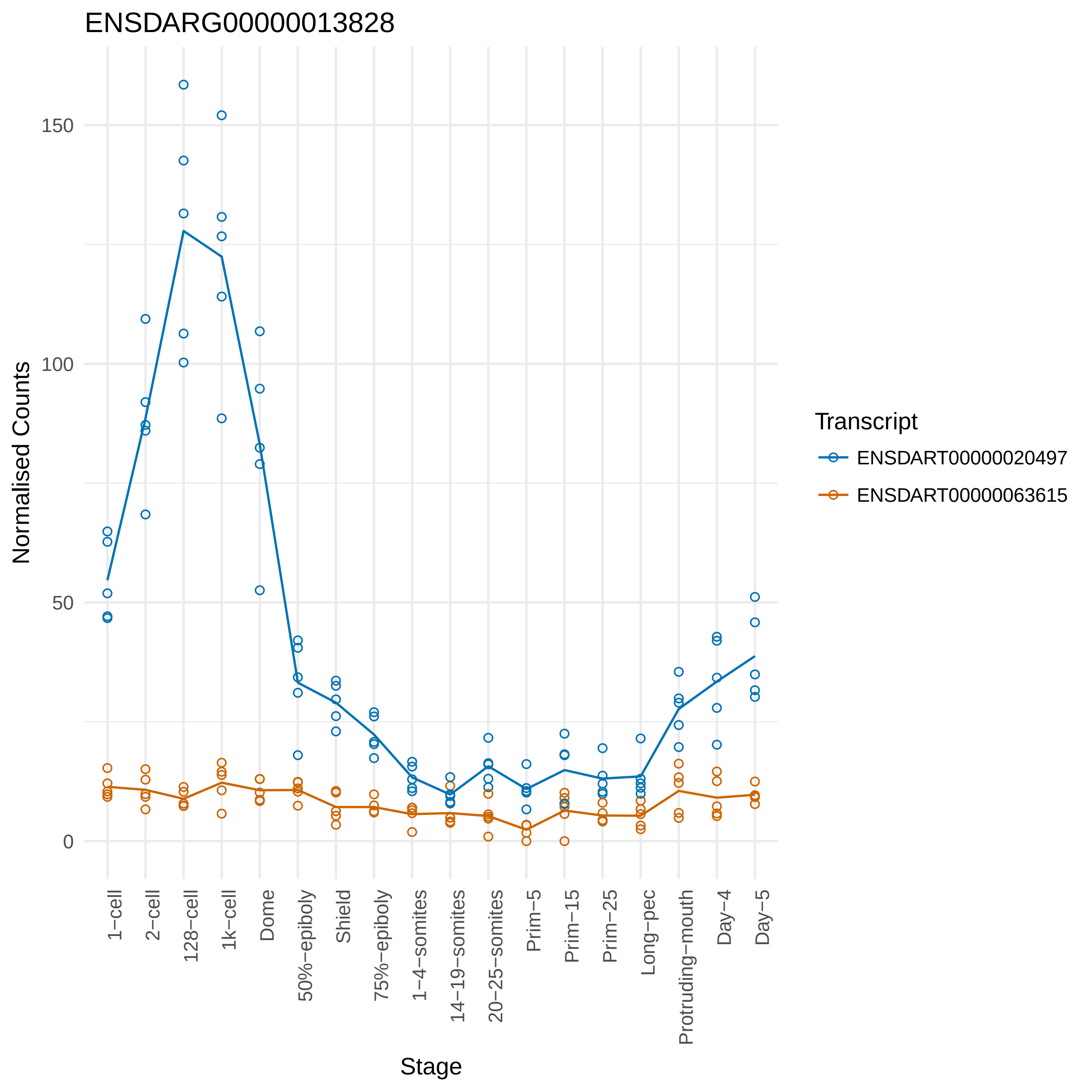

ENSDARG00000013828
Normalised Counts
150
100
50
0
1−cell
2−cell
128−cell
1k−cell
Dome
50%−epiboly
Shield
75%−epiboly
1−4−somites
14−19−somites
20−25−somites
Prim−5
Prim−15
Prim−25
Long−pec
Protruding−mouth
Day−4
Day−5
Stage
Transcript
ENSDART00000020497
ENSDART00000063615

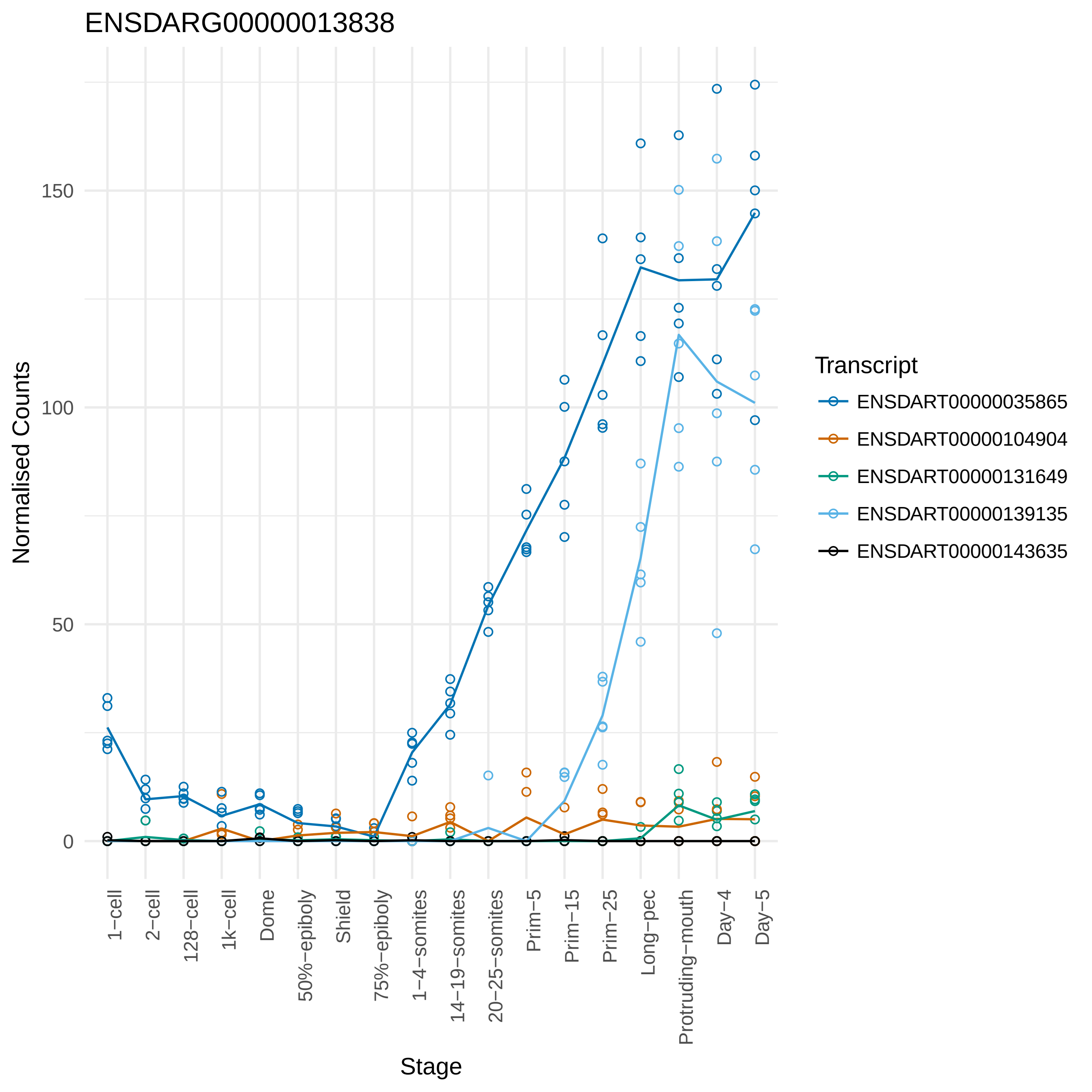

ENSDARG00000013838
Normalised Counts
0
50
100
150
Stage
1−cell
2−cell
128−cell
1k−cell
Dome
50%−epiboly
Shield
75%−epiboly
1−4−somites
14−19−somites
20−25−somites
Prim−5
Prim−15
Prim−25
Long−pec
Protruding−mouth
Day−4
Day−5
Transcript
ENSDART00000035865
ENSDART00000104904
ENSDART00000131649
ENSDART00000139135
ENSDART00000143635

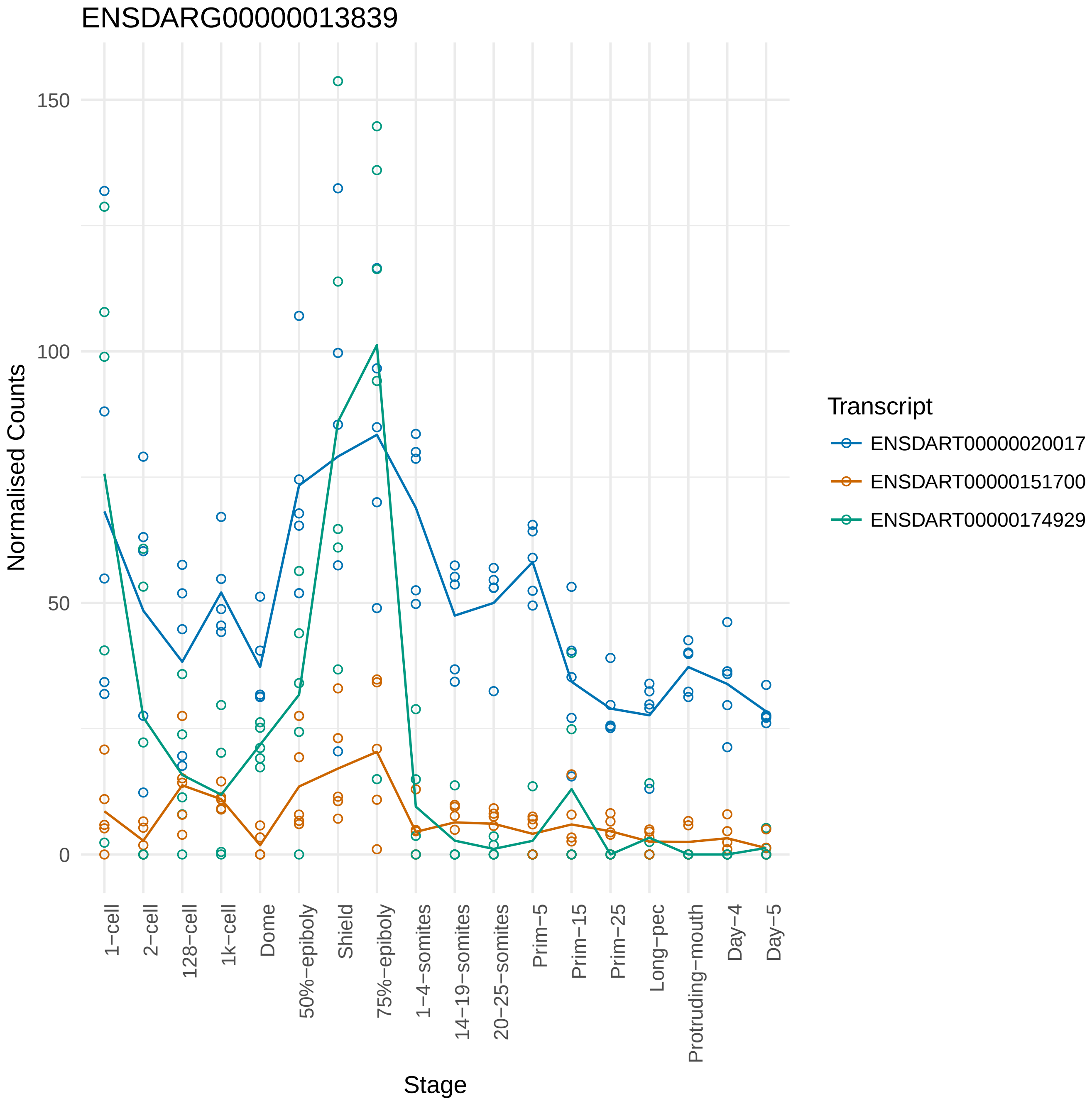
ENSDARG00000013839
Normalised Counts
150
100
50
0
1−cell
2−cell
128−cell
1k−cell
Dome
50%−epiboly
Shield
75%−epiboly
1−4−somites
14−19−somites
20−25−somites
Prim−5
Prim−15
Prim−25
Long−pec
Protruding−mouth
Day−4
Day−5
Stage
Transcript
ENSDART00000020017
ENSDART00000151700
ENSDART00000174929

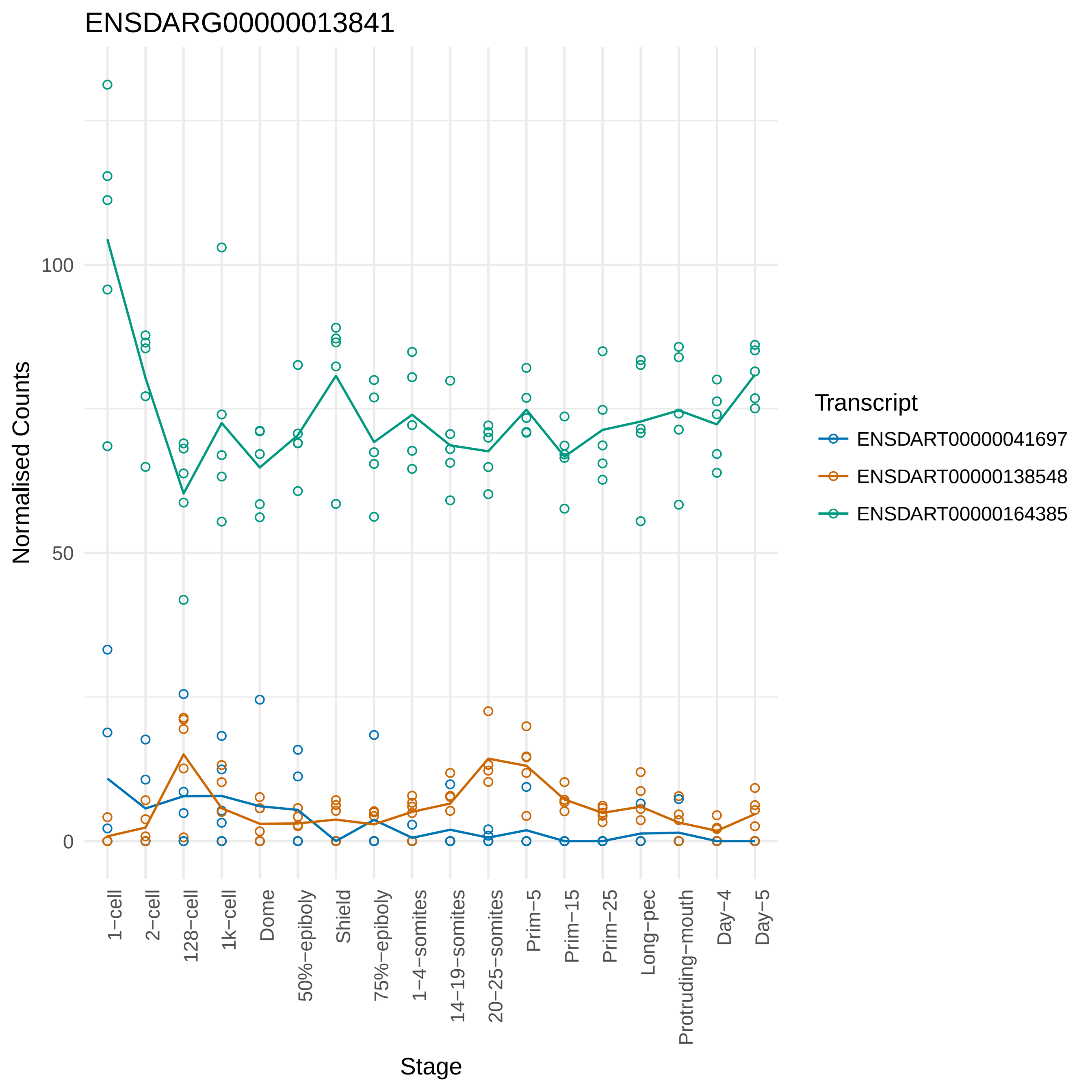
ENSDARG00000013841
Normalised Counts
100
50
0
Stage
1−cell
2−cell
128−cell
1k−cell
Dome
50%−epiboly
Shield
75%−epiboly
1−4−somites
14−19−somites
20−25−somites
Prim−5
Prim−15
Prim−25
Long−pec
Protruding−mouth
Day−4
Day−5
Transcript
ENSDART00000041697
ENSDART00000138548
ENSDART00000164385

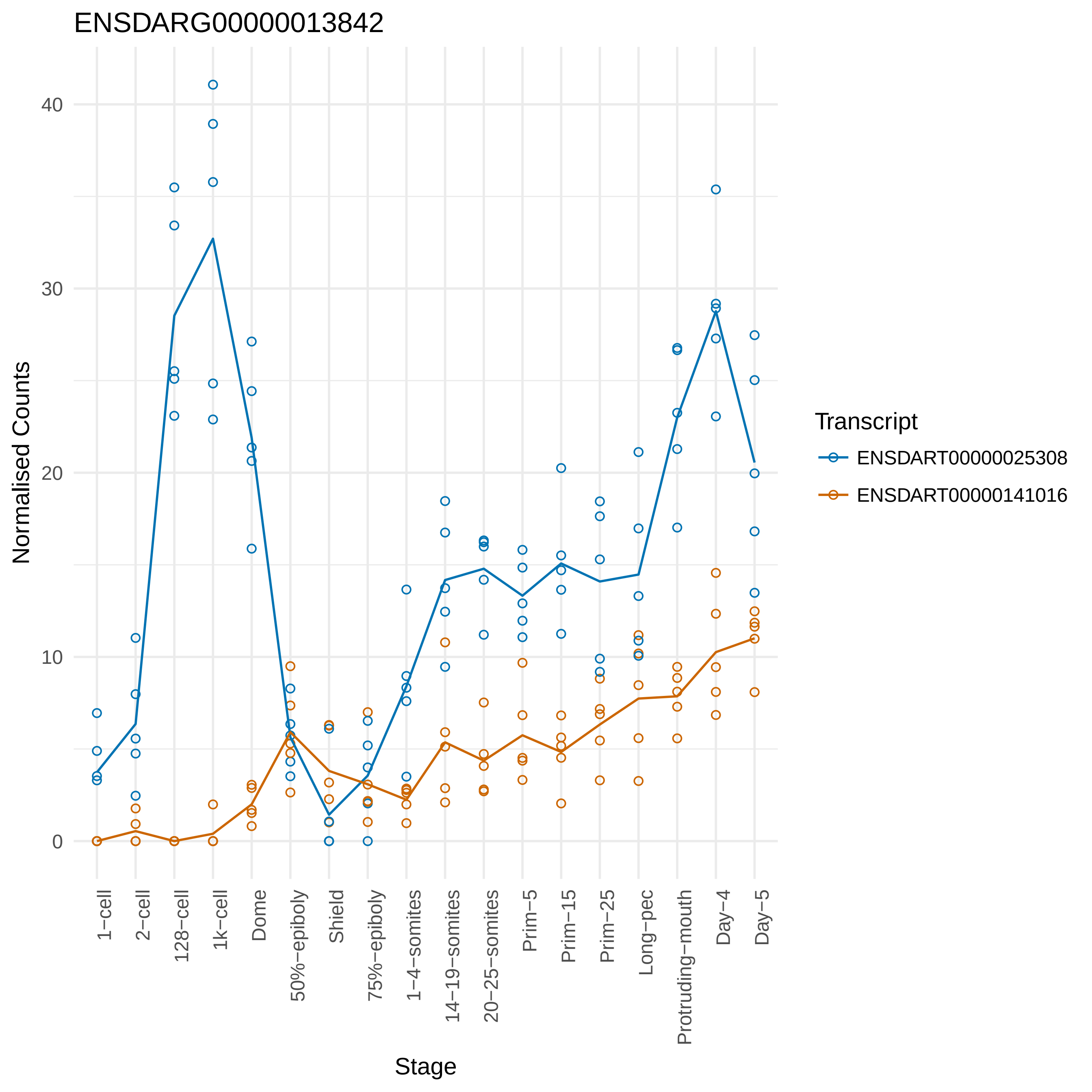

ENSDARG00000013842
Normalised Counts
40
30
20
10
0
Stage
1−cell
2−cell
128−cell
1k−cell
Dome
50%−epiboly
Shield
75%−epiboly
1−4−somites
14−19−somites
20−25−somites
Prim−5
Prim−15
Prim−25
Long−pec
Protruding−mouth
Day−4
Day−5
Transcript
ENSDART00000025308
ENSDART00000141016

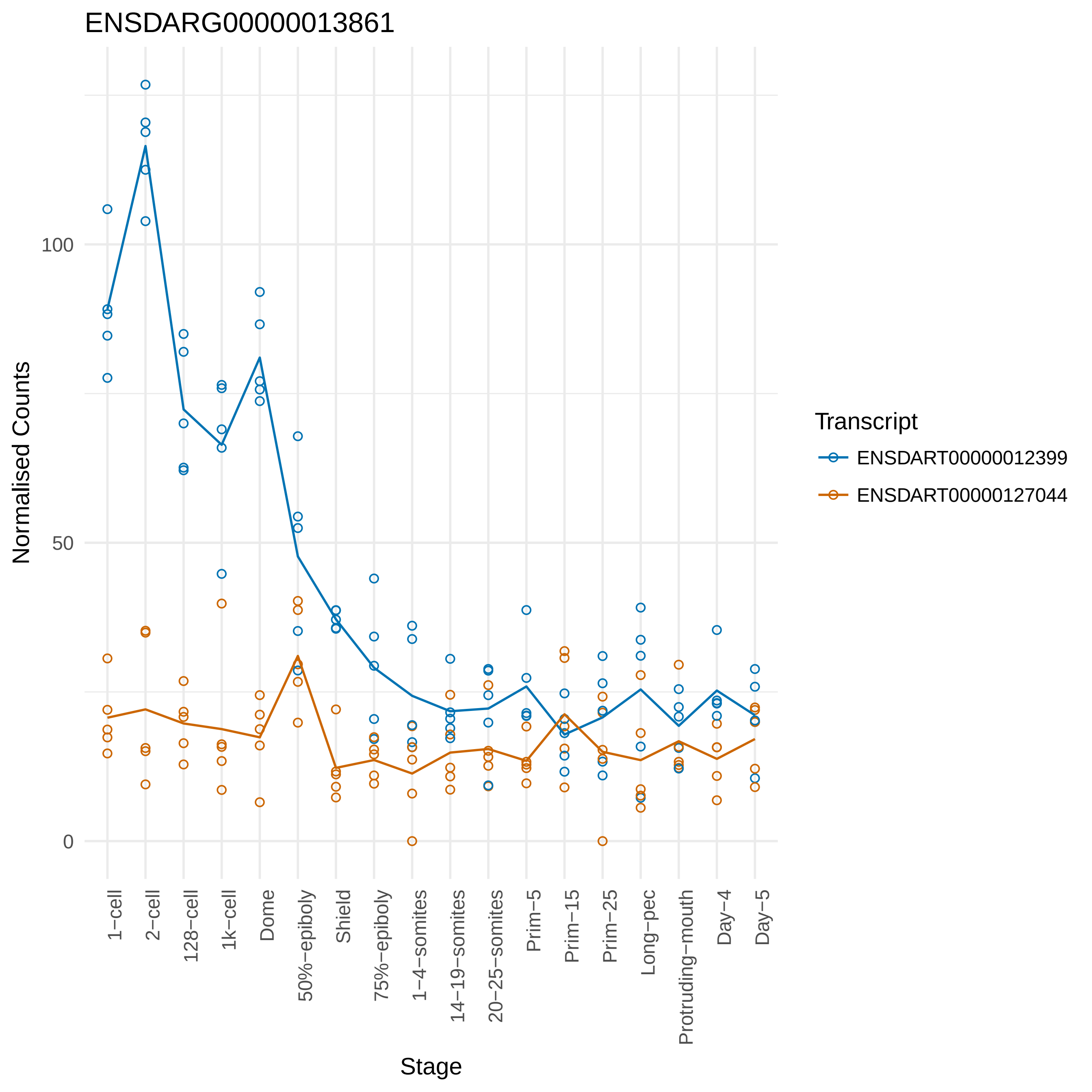

ENSDARG00000013861
Normalised Counts
100
50
0
1−cell
2−cell
128−cell
1k−cell
Dome
50%−epiboly
Shield
75%−epiboly
1−4−somites
14−19−somites
20−25−somites
Prim−5
Prim−15
Prim−25
Long−pec
Protruding−mouth
Day−4
Day−5
Stage
Transcript
ENSDART00000012399
ENSDART00000127044

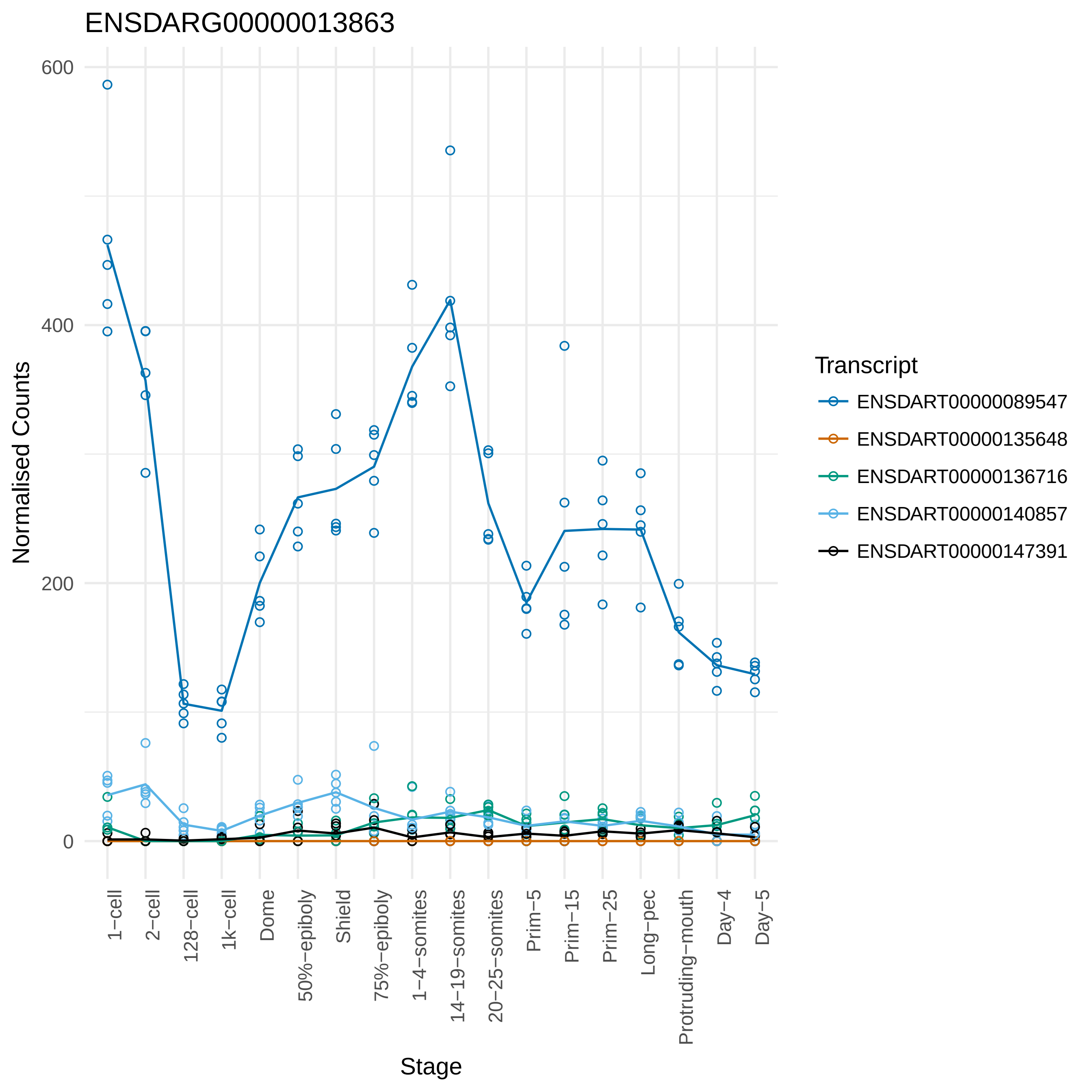

ENSDARG00000013863
Normalised Counts
600
400
200
0
1−cell
2−cell
128−cell
1k−cell
Dome
50%−epiboly
Shield
75%−epiboly
1−4−somites
14−19−somites
20−25−somites
Prim−5
Prim−15
Prim−25
Long−pec
Protruding−mouth
Day−4
Day−5
Stage
Transcript
ENSDART00000089547
ENSDART00000135648
ENSDART00000136716
ENSDART00000140857
ENSDART00000147391

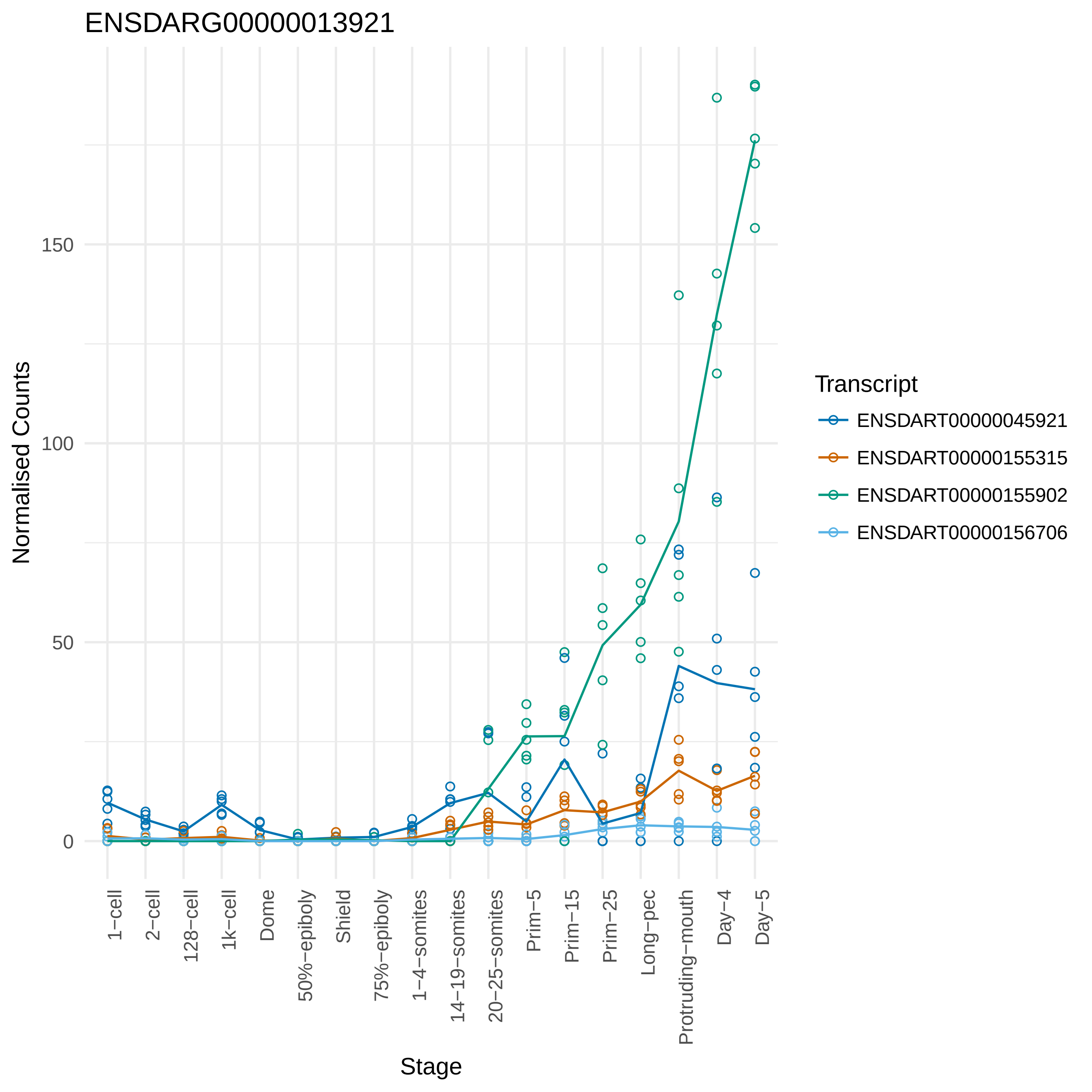

ENSDARG00000013921
Normalised Counts
150
100
50
0
1−cell
2−cell
128−cell
1k−cell
Dome
50%−epiboly
Shield
75%−epiboly
1−4−somites
14−19−somites
20−25−somites
Prim−5
Prim−15
Prim−25
Long−pec
Protruding−mouth
Day−4
Day−5
Stage
Transcript
ENSDART00000045921
ENSDART00000155315
ENSDART00000155902
ENSDART00000156706

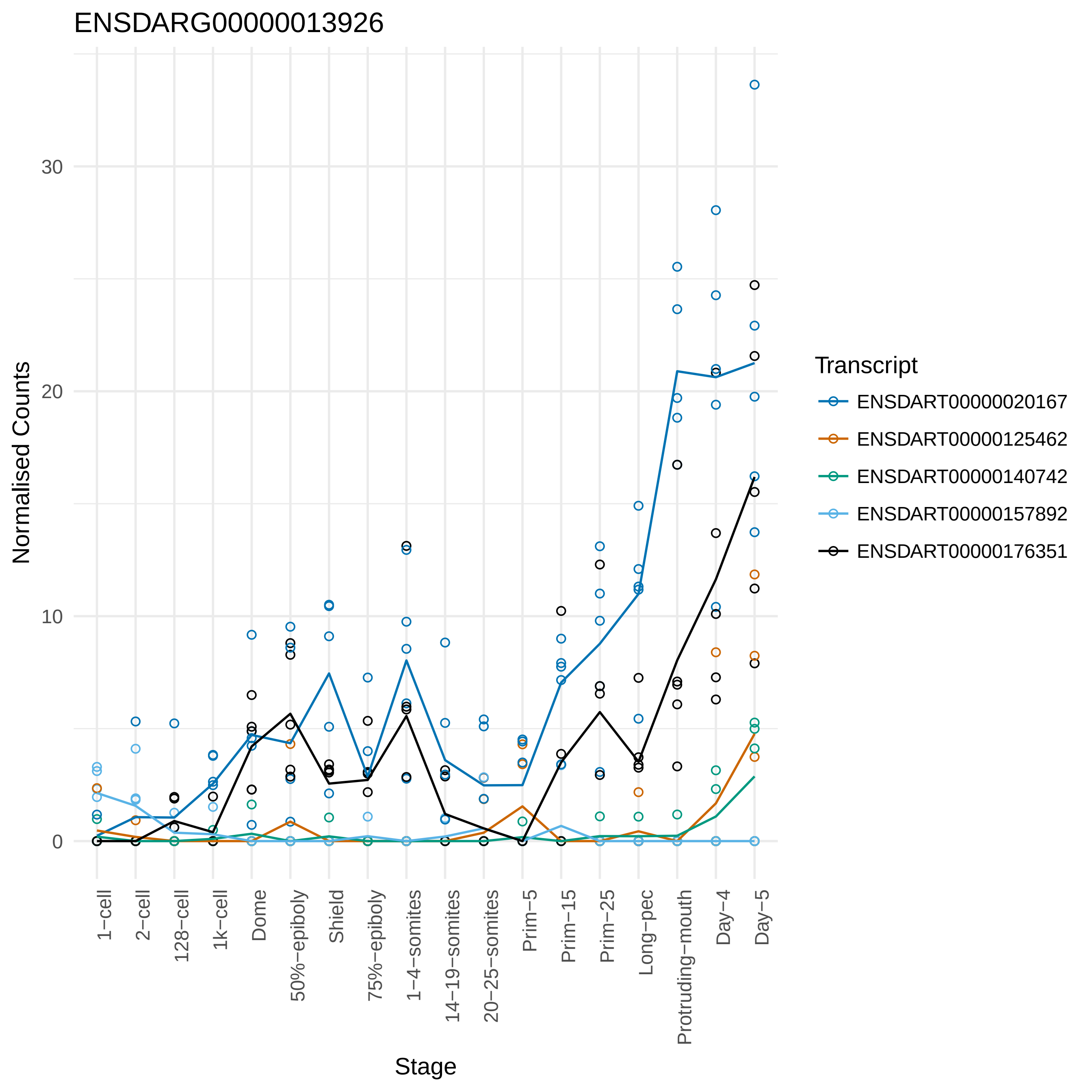
ENSDARG00000013926
Normalised Counts
0
10
20
30
Stage
1−cell
2−cell
128−cell
1k−cell
Dome
50%−epiboly
Shield
75%−epiboly
1−4−somites
14−19−somites
20−25−somites
Prim−5
Prim−15
Prim−25
Long−pec
Protruding−mouth
Day−4
Day−5
Transcript
ENSDART00000020167
ENSDART00000125462
ENSDART00000140742
ENSDART00000157892
ENSDART00000176351

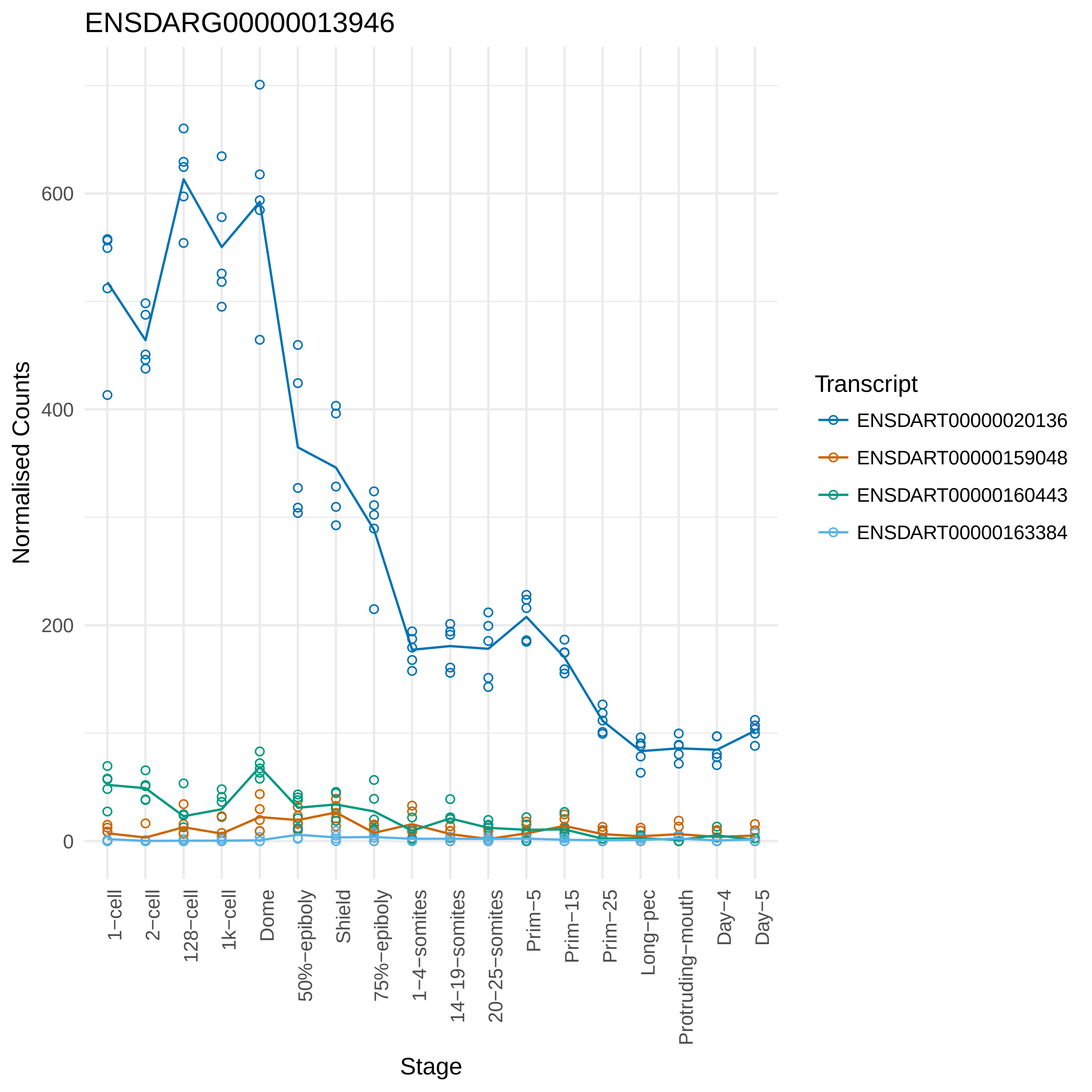

ENSDARG00000013946
Normalised Counts
0
200
400
600
Stage
1−cell
2−cell
128−cell
1k−cell
Dome
50%−epiboly
Shield
75%−epiboly
1−4−somites
14−19−somites
20−25−somites
Prim−5
Prim−15
Prim−25
Long−pec
Protruding−mouth
Day−4
Day−5
Transcript
ENSDART00000020136
ENSDART00000159048
ENSDART00000160443
ENSDART00000163384

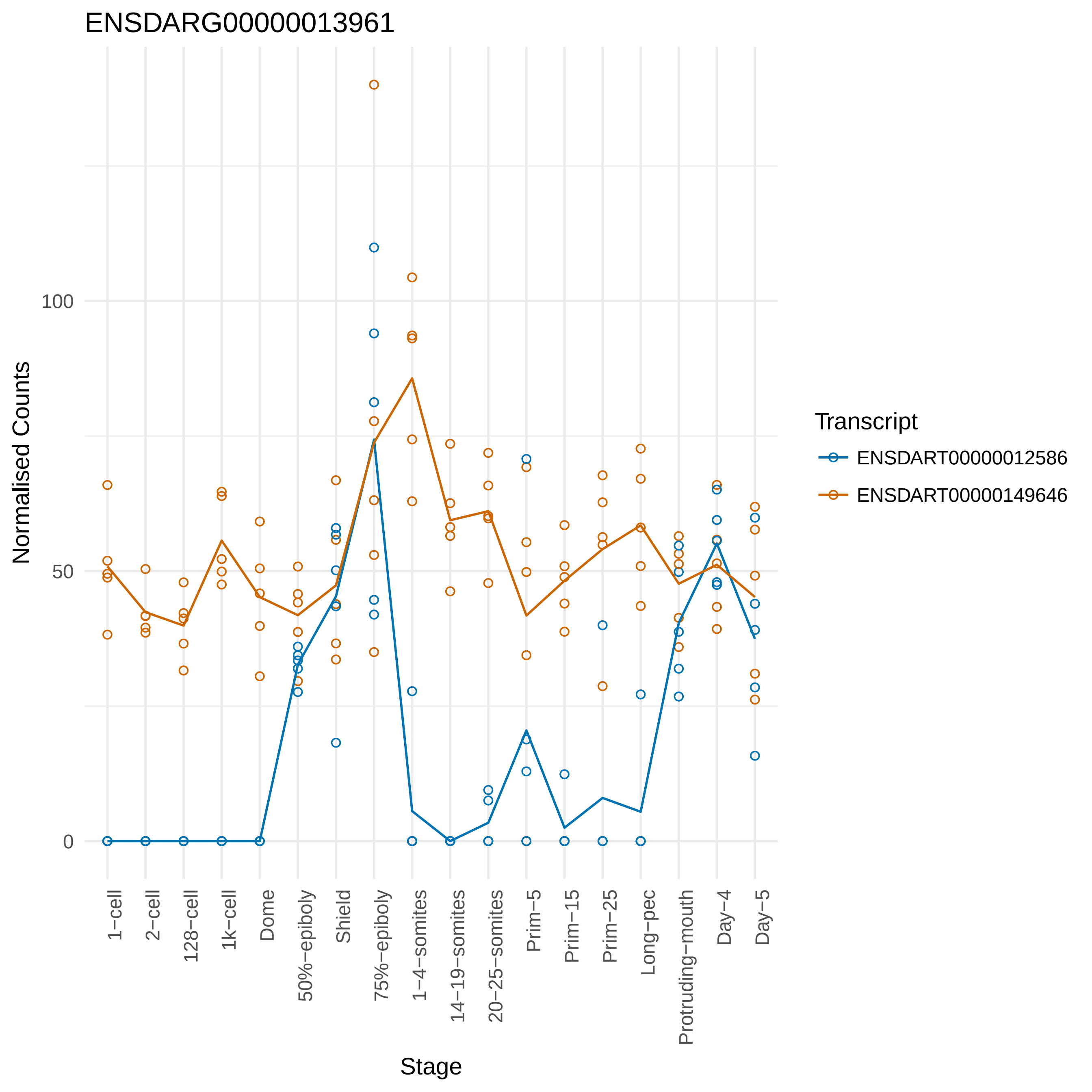
ENSDARG00000013961
Normalised Counts
100
50
0
Stage
1−cell
2−cell
128−cell
1k−cell
Dome
50%−epiboly
Shield
75%−epiboly
1−4−somites
14−19−somites
20−25−somites
Prim−5
Prim−15
Prim−25
Long−pec
Protruding−mouth
Day−4
Day−5
Transcript
ENSDART00000012586
ENSDART00000149646

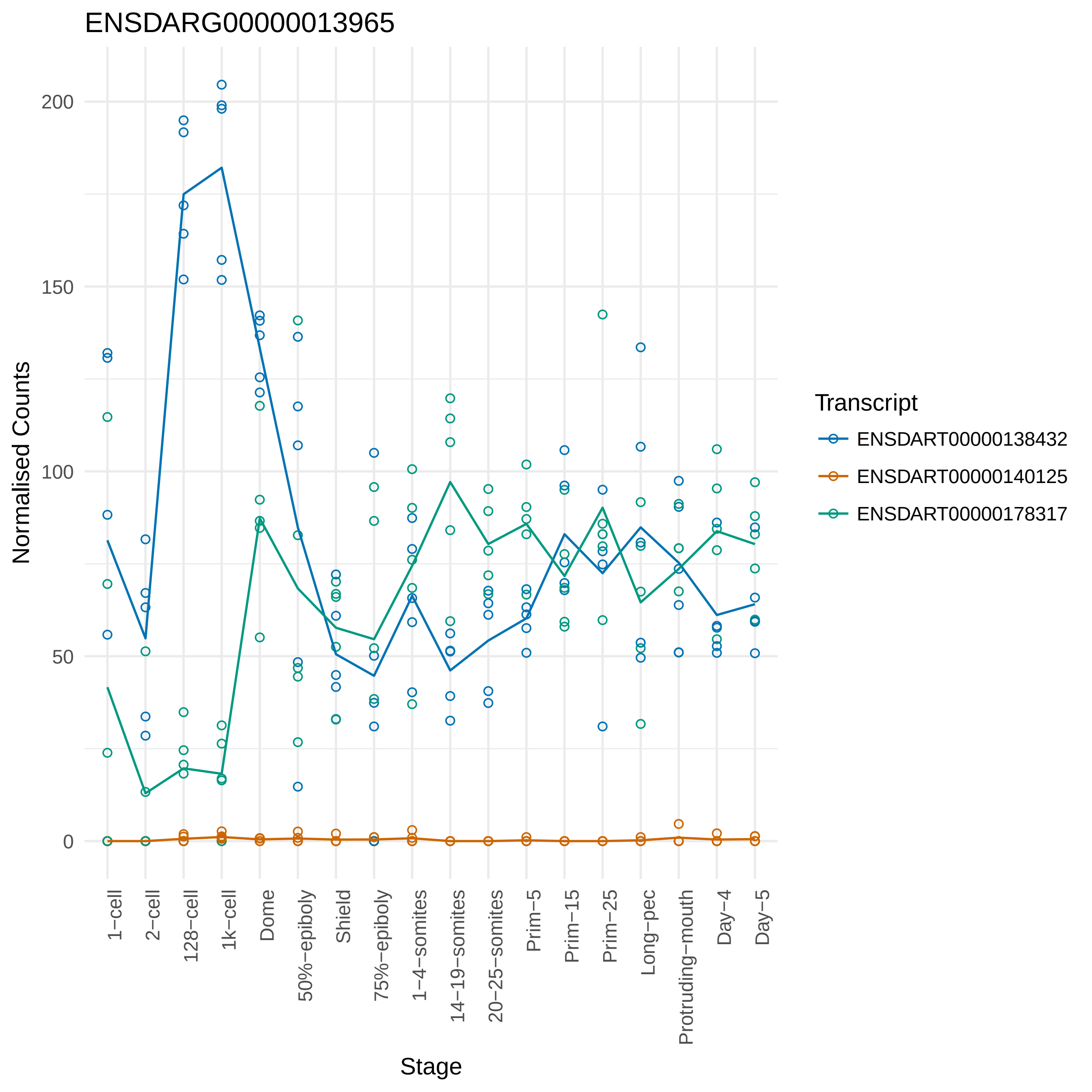
ENSDARG00000013965
Normalised Counts
200
150
100
50
0
1−cell
2−cell
128−cell
1k−cell
Dome
50%−epiboly
Shield
75%−epiboly
1−4−somites
14−19−somites
20−25−somites
Prim−5
Prim−15
Prim−25
Long−pec
Protruding−mouth
Day−4
Day−5
Stage
Transcript
ENSDART00000138432
ENSDART00000140125
ENSDART00000178317

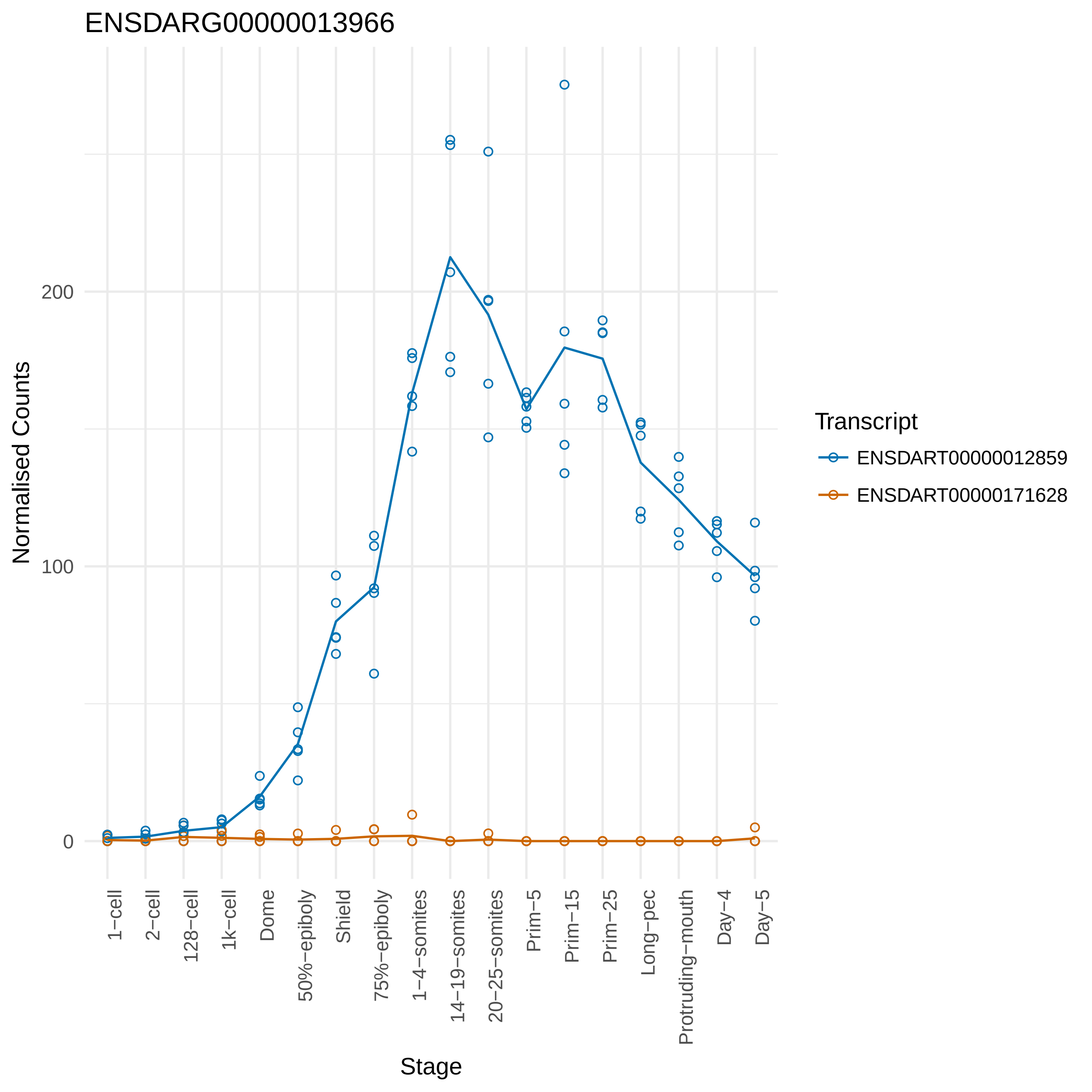

ENSDARG00000013966
Normalised Counts
200
100
0
Stage
1−cell
2−cell
128−cell
1k−cell
Dome
50%−epiboly
Shield
75%−epiboly
1−4−somites
14−19−somites
20−25−somites
Prim−5
Prim−15
Prim−25
Long−pec
Protruding−mouth
Day−4
Day−5
Transcript
ENSDART00000012859
ENSDART00000171628

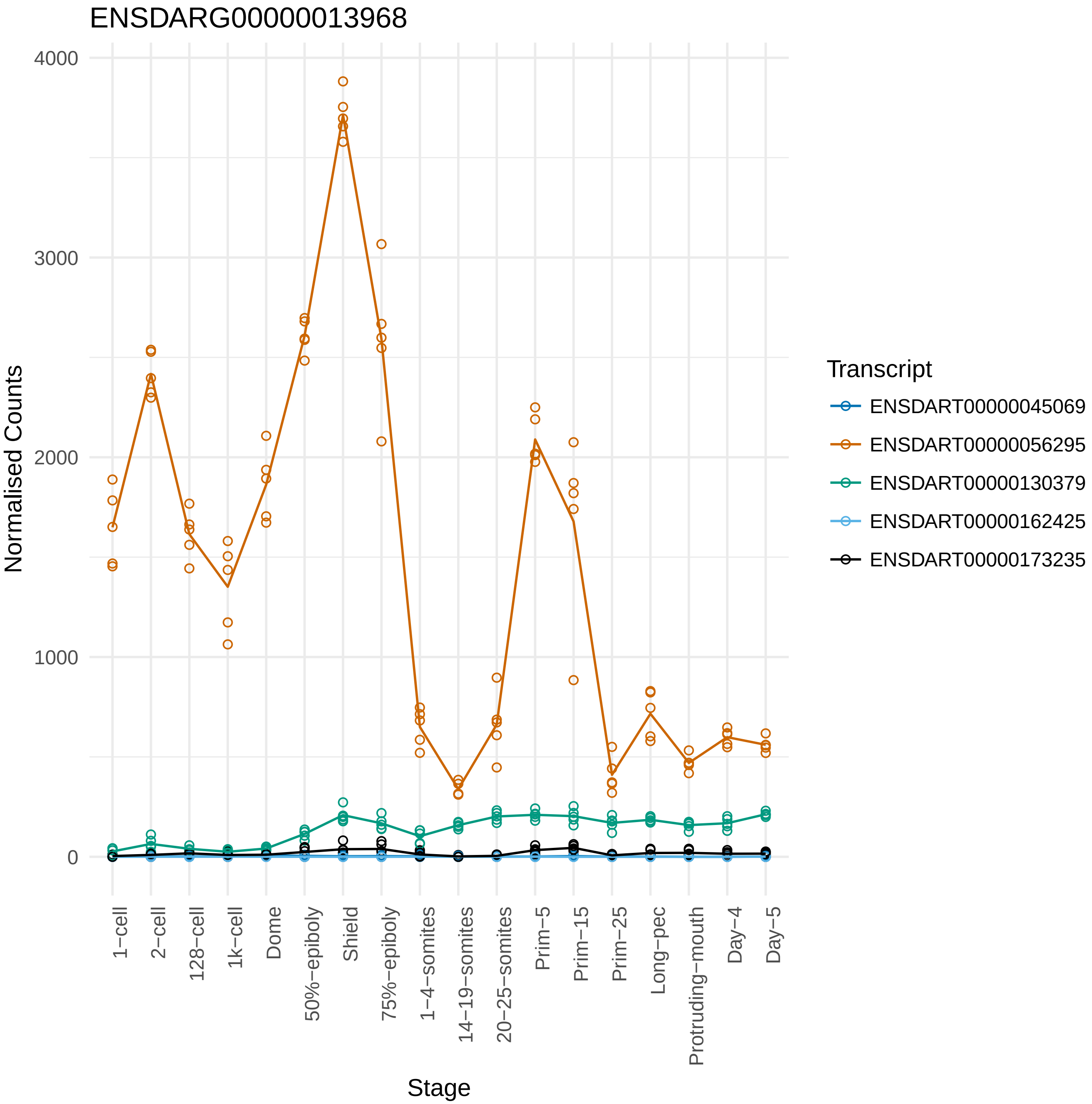
ENSDARG00000013968
Normalised Counts
4000
3000
2000
1000
0
1−cell
2−cell
128−cell
1k−cell
Dome
50%−epiboly
Shield
75%−epiboly
1−4−somites
14−19−somites
20−25−somites
Prim−5
Prim−15
Prim−25
Long−pec
Protruding−mouth
Day−4
Day−5
Stage
Transcript
ENSDART00000045069
ENSDART00000056295
ENSDART00000130379
ENSDART00000162425
ENSDART00000173235

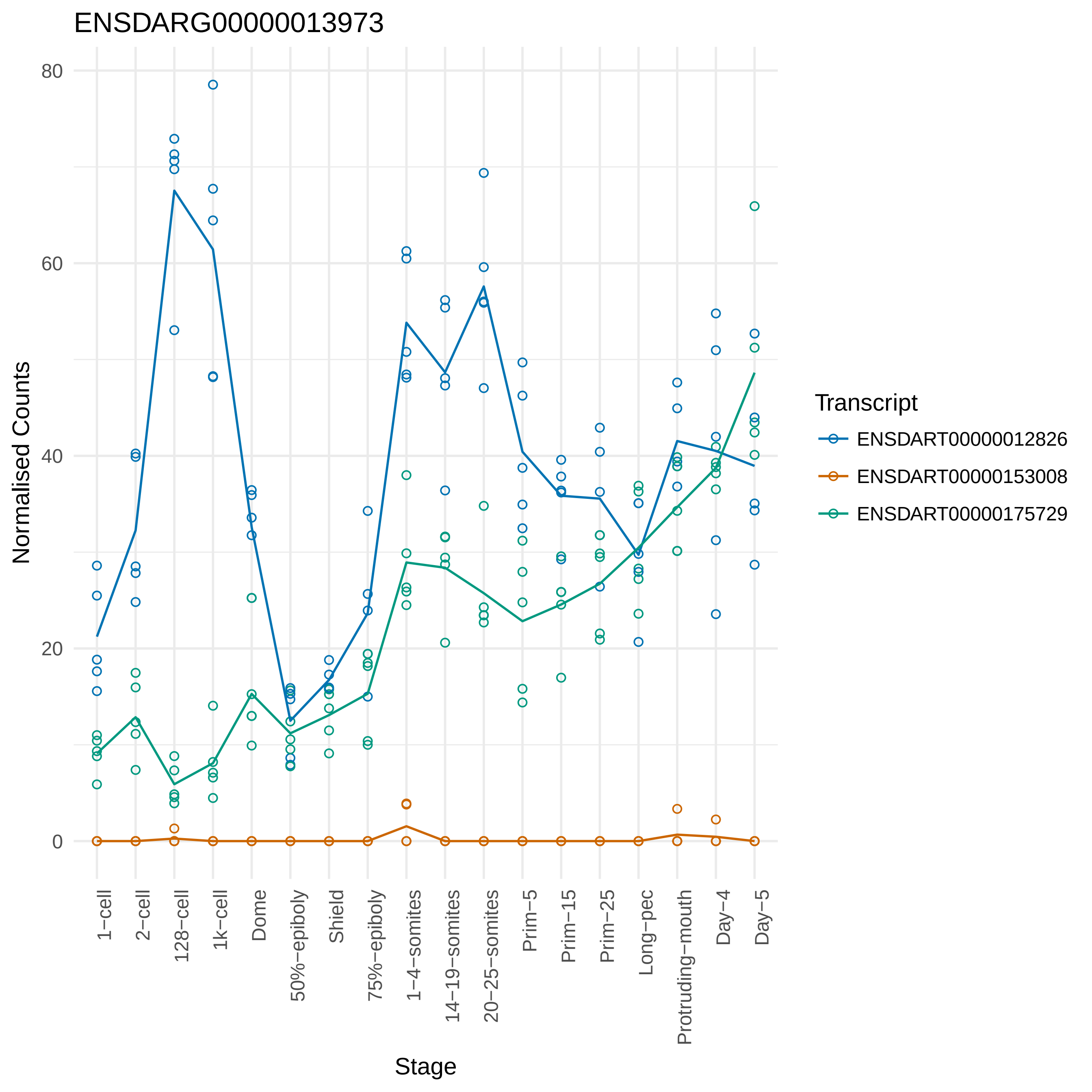

ENSDARG00000013973
Normalised Counts
0
20
40
60
80
Stage
1−cell
2−cell
128−cell
1k−cell
Dome
50%−epiboly
Shield
75%−epiboly
1−4−somites
14−19−somites
20−25−somites
Prim−5
Prim−15
Prim−25
Long−pec
Protruding−mouth
Day−4
Day−5
Transcript
ENSDART00000012826
ENSDART00000153008
ENSDART00000175729

ENSDARG00000013979

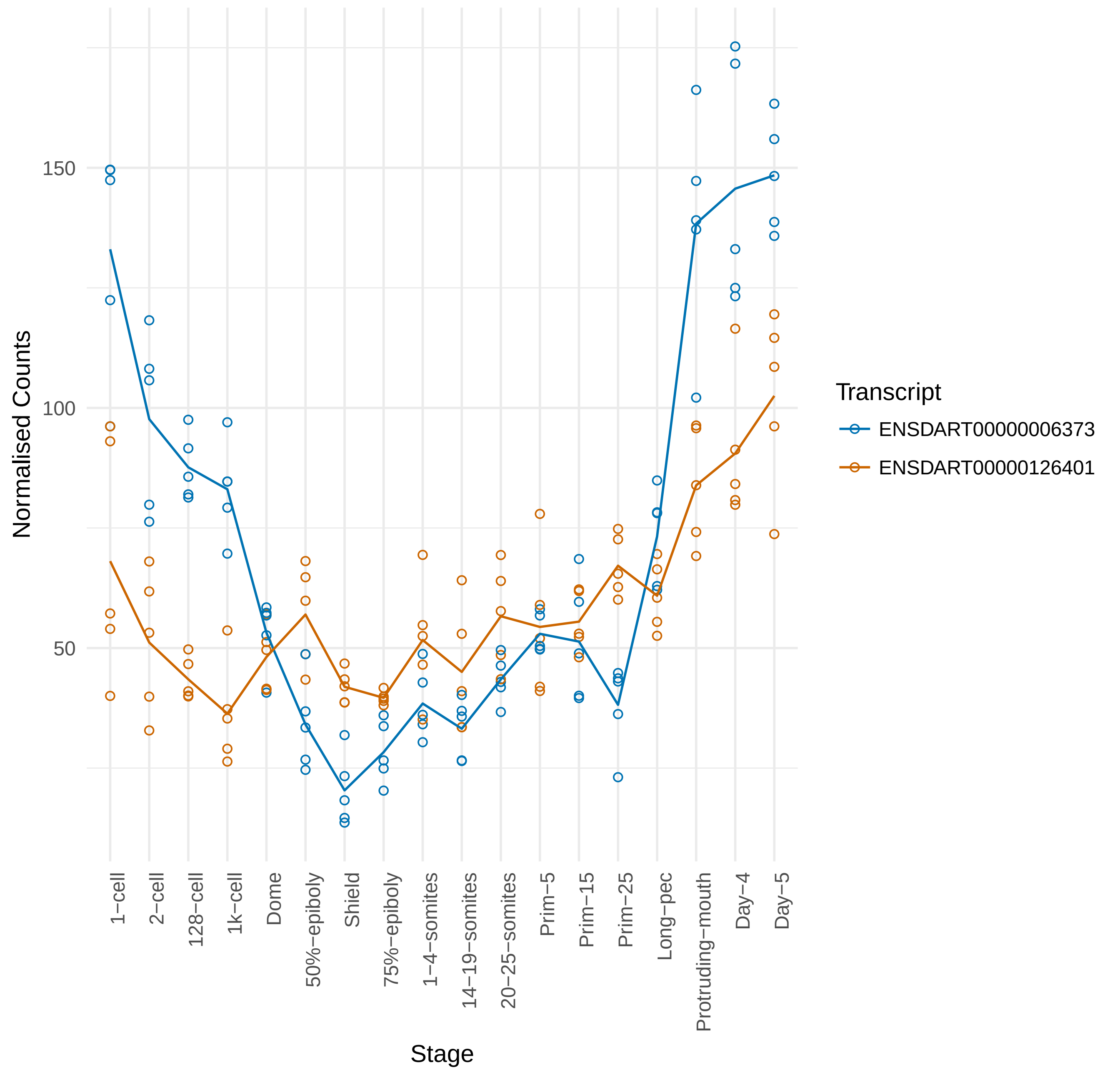

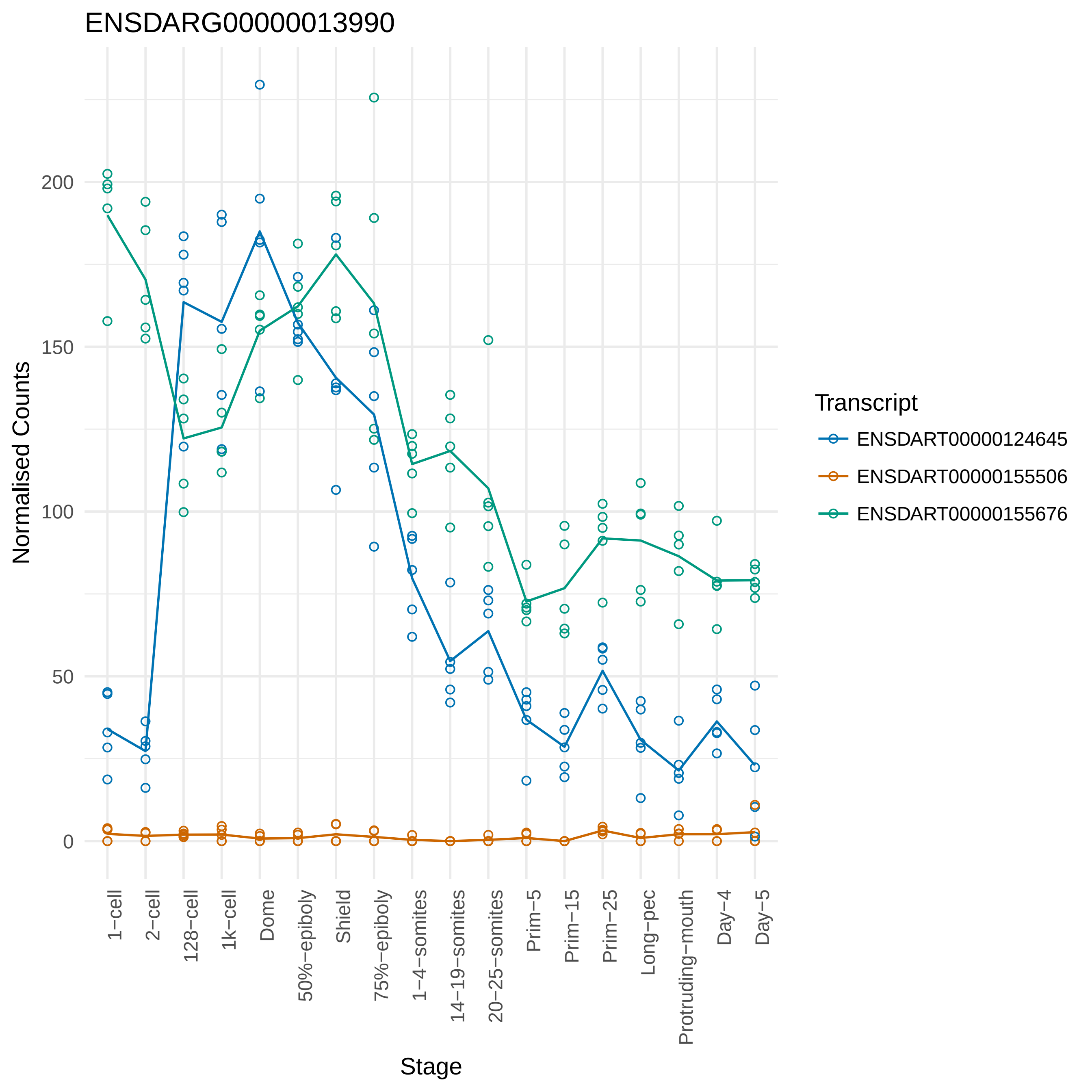

ENSDARG00000013990
Normalised Counts
200
150
100
50
0
1−cell
2−cell
128−cell
1k−cell
Dome
50%−epiboly
Shield
75%−epiboly
1−4−somites
14−19−somites
20−25−somites
Prim−5
Prim−15
Prim−25
Long−pec
Protruding−mouth
Day−4
Day−5
Stage
Transcript
ENSDART00000124645
ENSDART00000155506
ENSDART00000155676

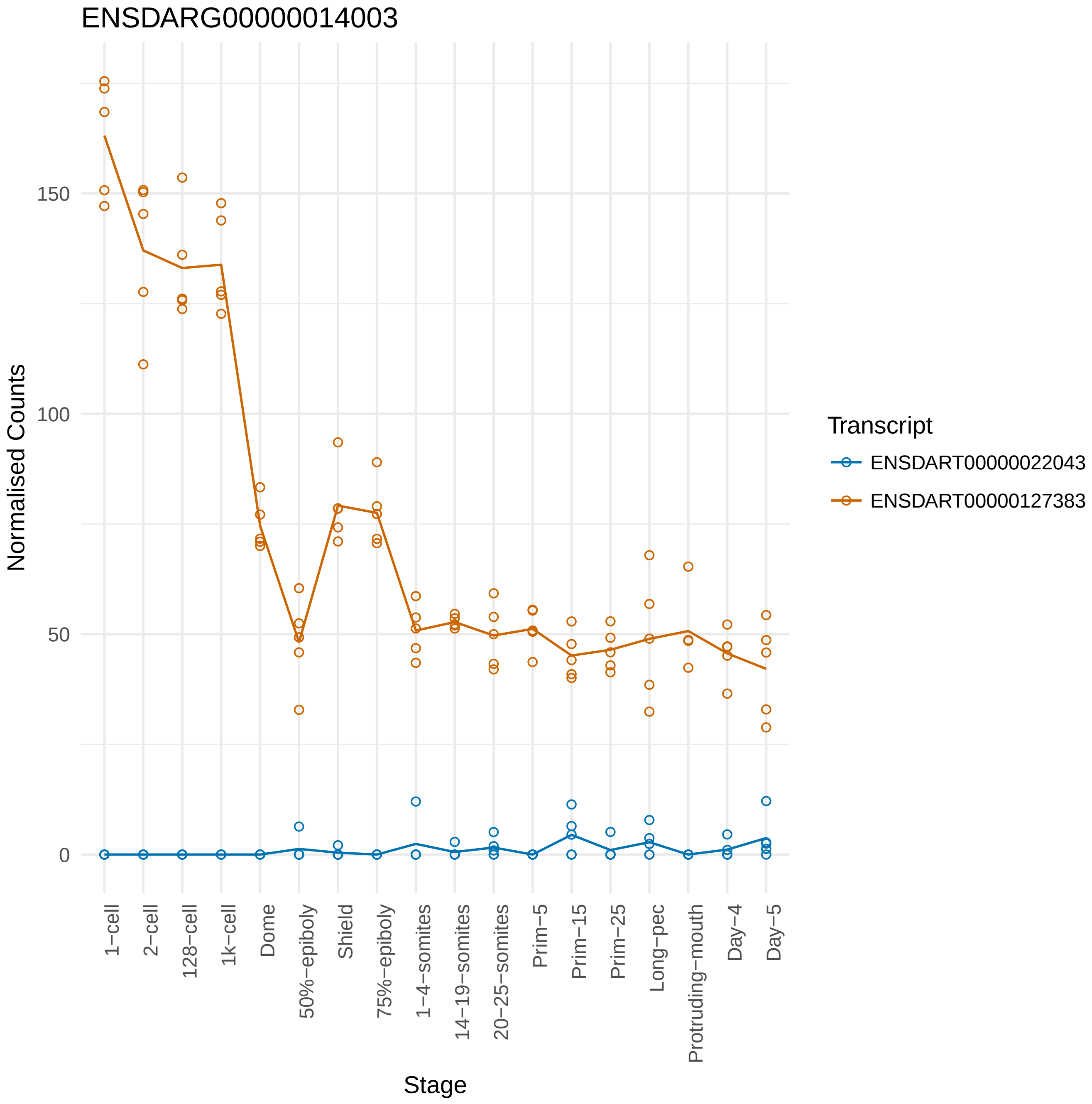

ENSDARG00000014003
Normalised Counts
150
100
50
0
1−cell
2−cell
128−cell
1k−cell
Dome
50%−epiboly
Shield
75%−epiboly
1−4−somites
14−19−somites
20−25−somites
Prim−5
Prim−15
Prim−25
Long−pec
Protruding−mouth
Day−4
Day−5
Stage
Transcript
ENSDART00000022043
ENSDART00000127383

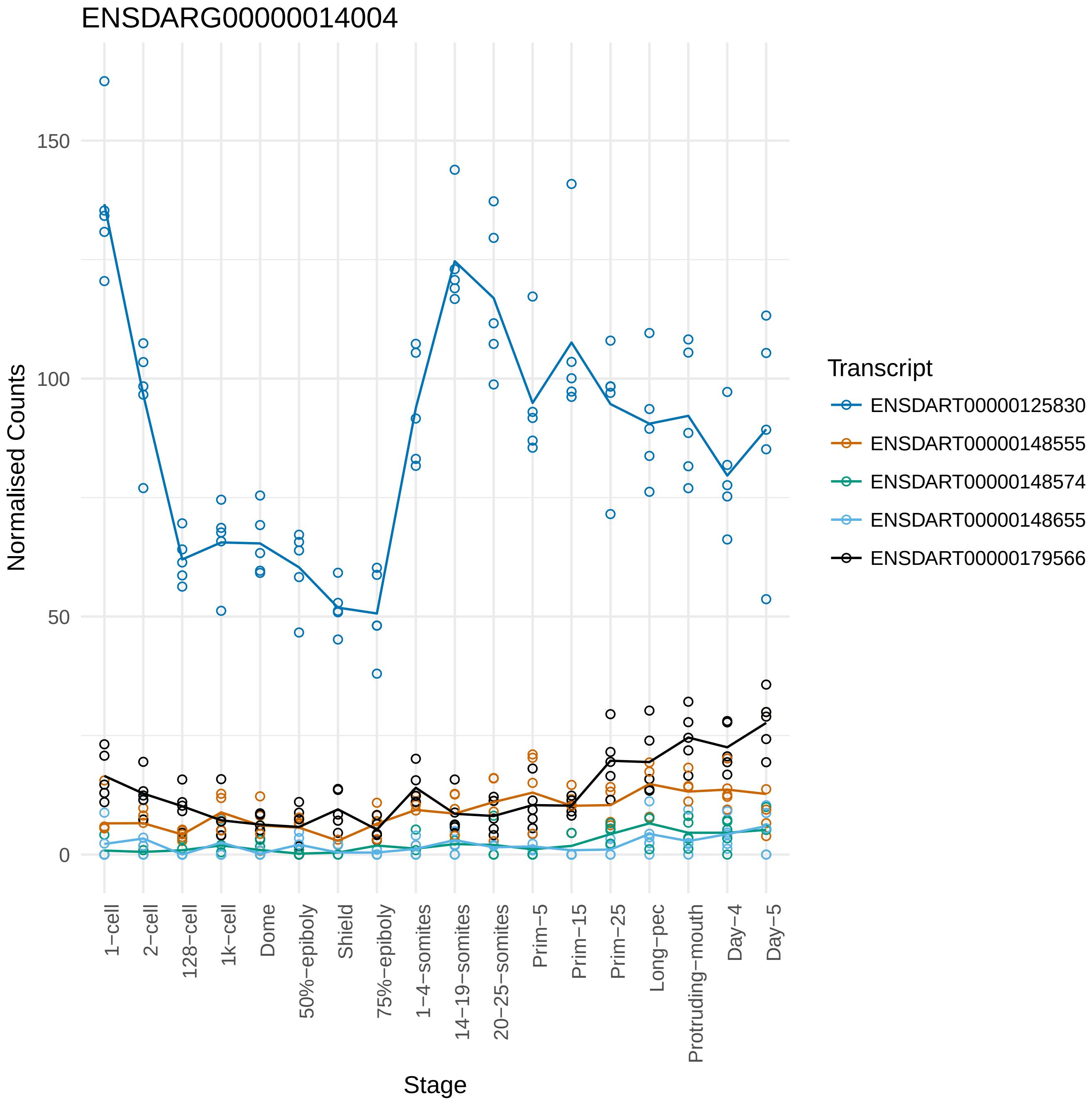

ENSDARG00000014004
Normalised Counts
150
100
50
0
1−cell
2−cell
128−cell
1k−cell
Dome
50%−epiboly
Shield
75%−epiboly
1−4−somites
14−19−somites
20−25−somites
Prim−5
Prim−15
Prim−25
Long−pec
Protruding−mouth
Day−4
Day−5
Stage
Transcript
ENSDART00000125830
ENSDART00000148555
ENSDART00000148574
ENSDART00000148655
ENSDART00000179566

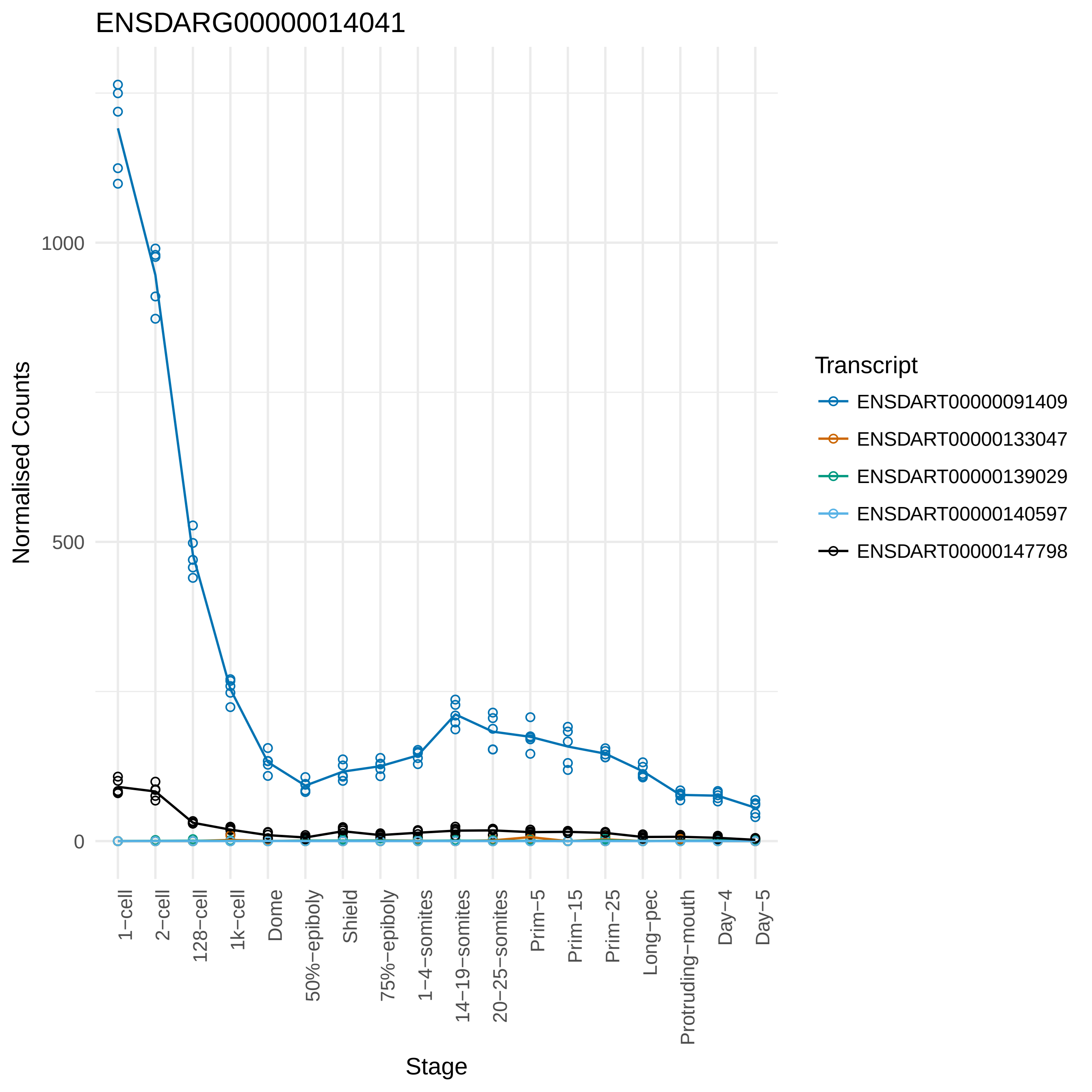

ENSDARG00000014041
Normalised Counts
1000
500
0
1−cell
2−cell
128−cell
1k−cell
Dome
50%−epiboly
Shield
75%−epiboly
1−4−somites
14−19−somites
20−25−somites
Prim−5
Prim−15
Prim−25
Long−pec
Protruding−mouth
Day−4
Day−5
Stage
Transcript
ENSDART00000091409
ENSDART00000133047
ENSDART00000139029
ENSDART00000140597
ENSDART00000147798

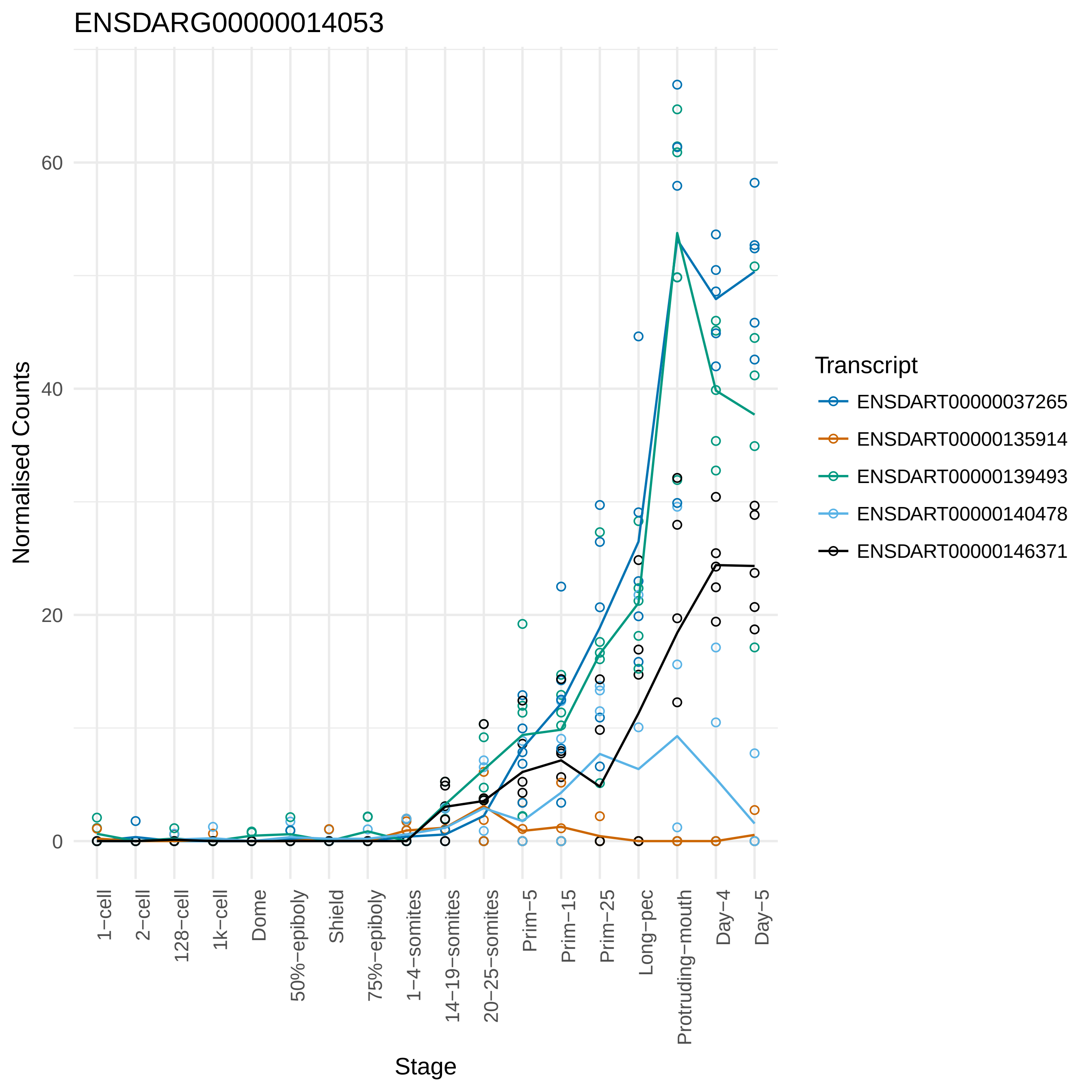

ENSDARG00000014053
Normalised Counts
60
40
20
0
1−cell
2−cell
128−cell
1k−cell
Dome
50%−epiboly
Shield
75%−epiboly
1−4−somites
14−19−somites
20−25−somites
Prim−5
Prim−15
Prim−25
Long−pec
Protruding−mouth
Day−4
Day−5
Stage
Transcript
ENSDART00000037265
ENSDART00000135914
ENSDART00000139493
ENSDART00000140478
ENSDART00000146371

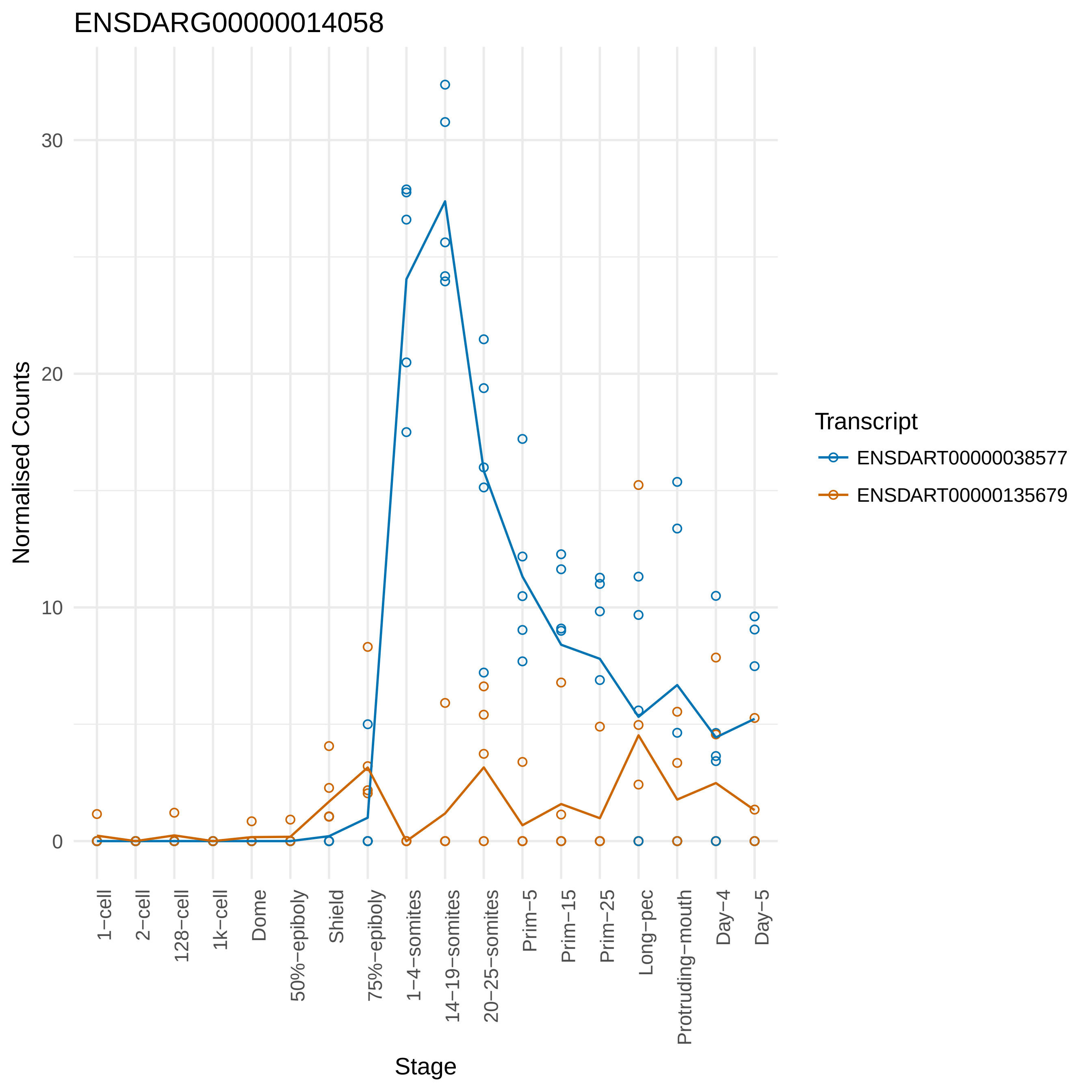
ENSDARG00000014058
Normalised Counts
30
20
10
0
1−cell
2−cell
128−cell
1k−cell
Dome
50%−epiboly
Shield
75%−epiboly
1−4−somites
14−19−somites
20−25−somites
Prim−5
Prim−15
Prim−25
Long−pec
Protruding−mouth
Day−4
Day−5
Stage
Transcript
ENSDART00000038577
ENSDART00000135679

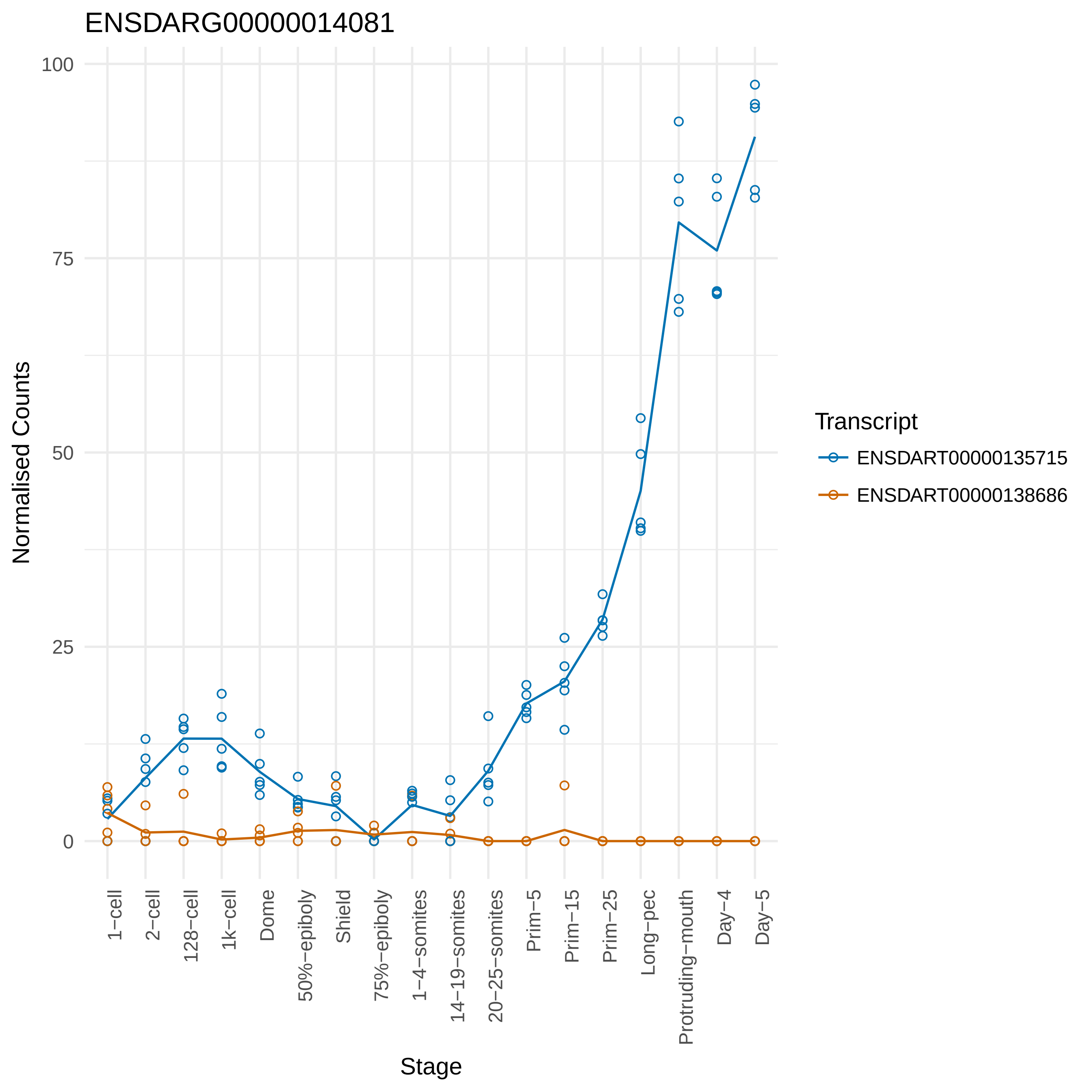

ENSDARG00000014081
Normalised Counts
100
75
50
25
0
1−cell
2−cell
128−cell
1k−cell
Dome
50%−epiboly
Shield
75%−epiboly
1−4−somites
14−19−somites
20−25−somites
Prim−5
Prim−15
Prim−25
Long−pec
Protruding−mouth
Day−4
Day−5
Stage
Transcript
ENSDART00000135715
ENSDART00000138686

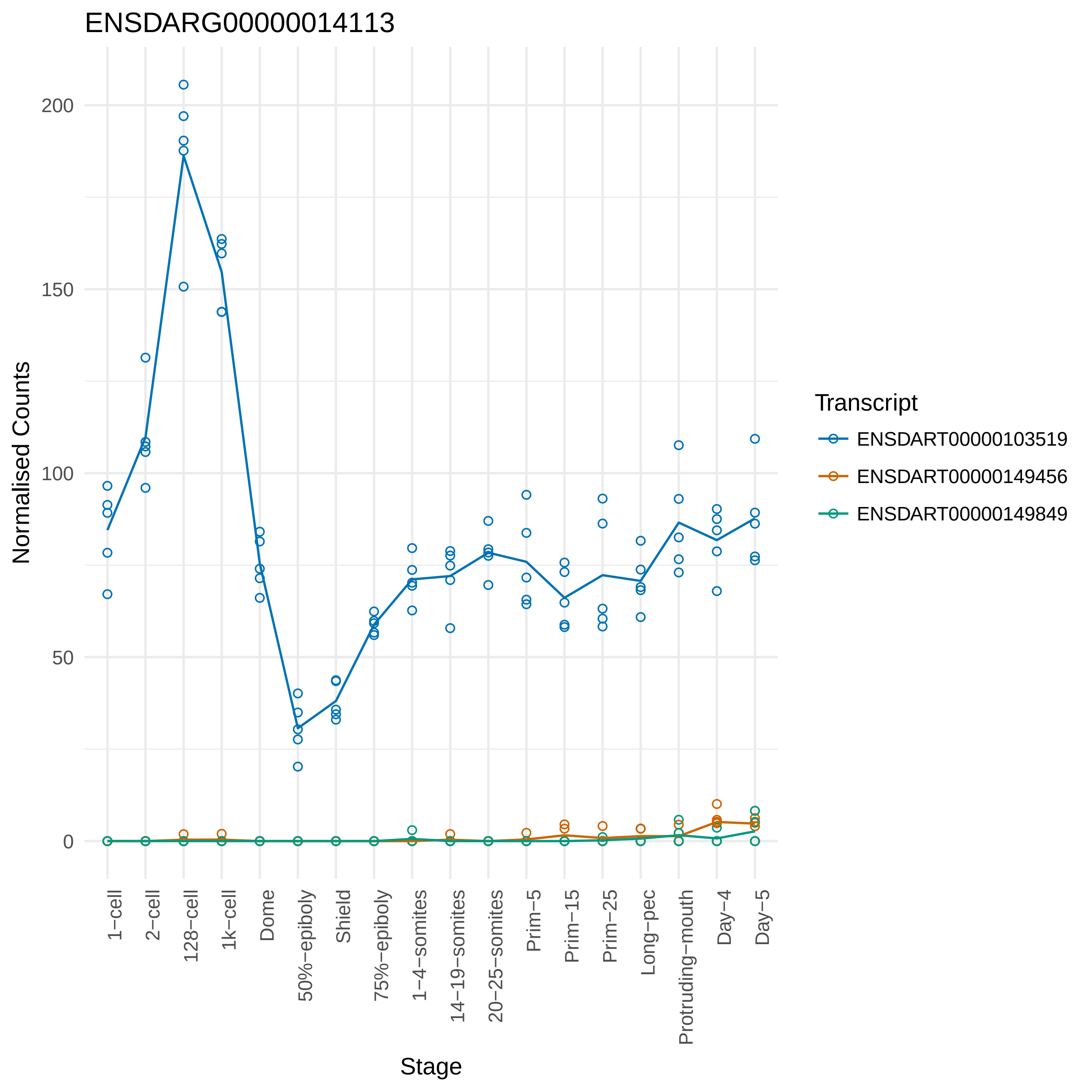

ENSDARG00000014113
Normalised Counts
200
150
100
50
0
1−cell
2−cell
128−cell
1k−cell
Dome
50%−epiboly
Shield
75%−epiboly
1−4−somites
14−19−somites
20−25−somites
Prim−5
Prim−15
Prim−25
Long−pec
Protruding−mouth
Day−4
Day−5
Stage
Transcript
ENSDART00000103519
ENSDART00000149456
ENSDART00000149849

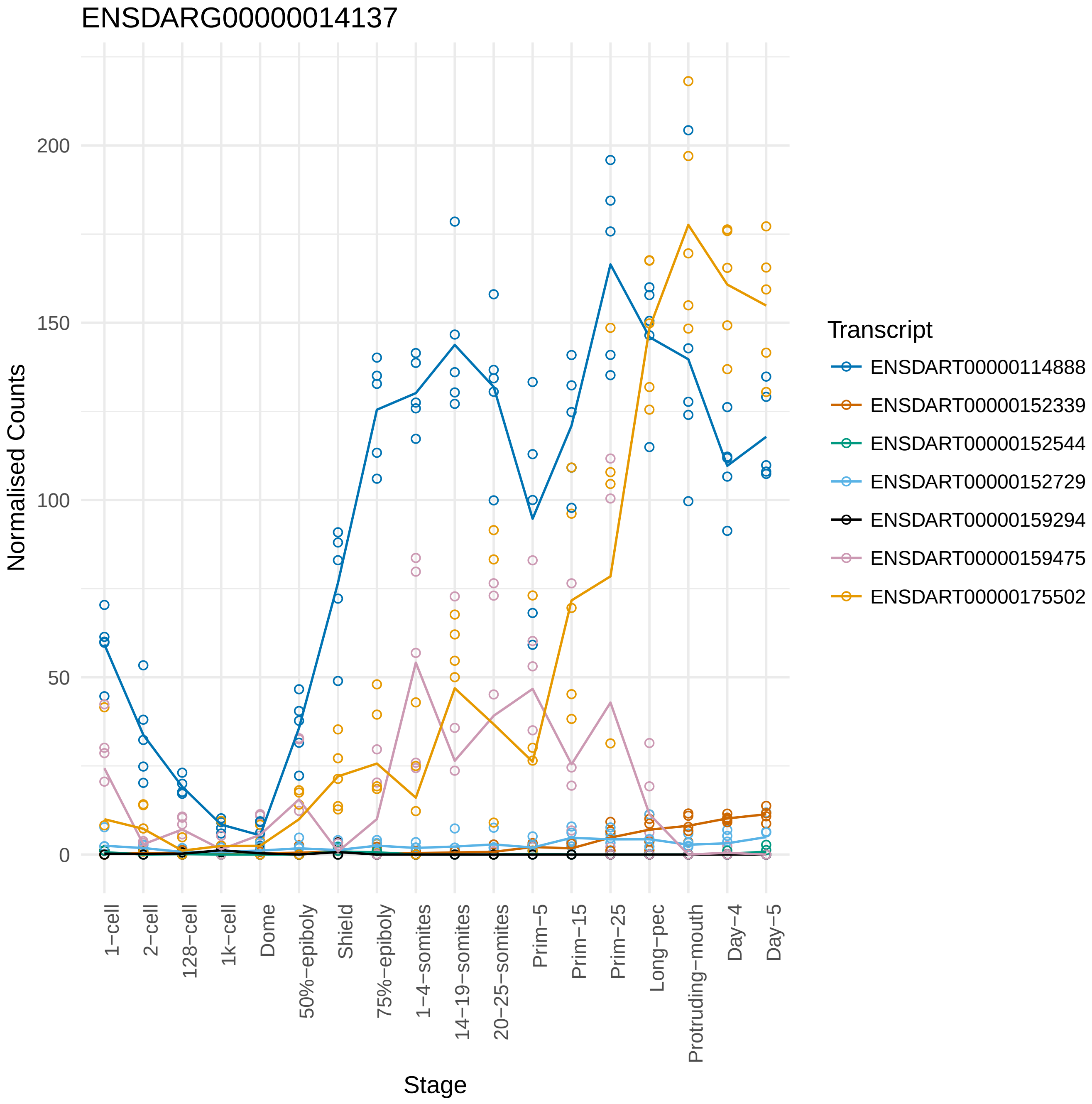

ENSDARG00000014137
Normalised Counts
0
50
100
150
200
Stage
1−cell
2−cell
128−cell
1k−cell
Dome
50%−epiboly
Shield
75%−epiboly
1−4−somites
14−19−somites
20−25−somites
Prim−5
Prim−15
Prim−25
Long−pec
Protruding−mouth
Day−4
Day−5
Transcript
ENSDART00000114888
ENSDART00000152339
ENSDART00000152544
ENSDART00000152729
ENSDART00000159294
ENSDART00000159475
ENSDART00000175502

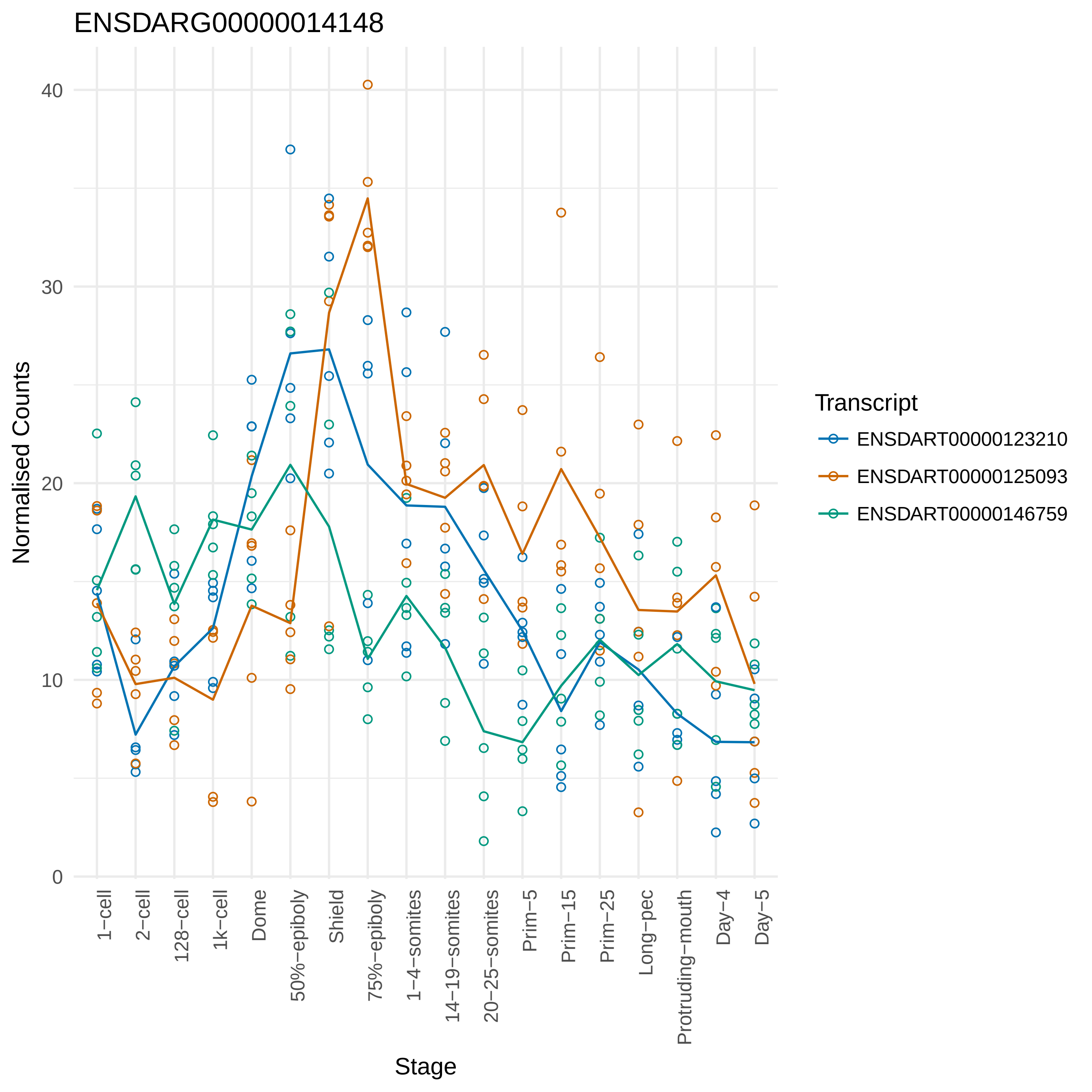

ENSDARG00000014148
Normalised Counts
40
30
20
10
0
1−cell
2−cell
128−cell
1k−cell
Dome
50%−epiboly
Shield
75%−epiboly
1−4−somites
14−19−somites
20−25−somites
Prim−5
Prim−15
Prim−25
Long−pec
Protruding−mouth
Day−4
Day−5
Stage
Transcript
ENSDART00000123210
ENSDART00000125093
ENSDART00000146759

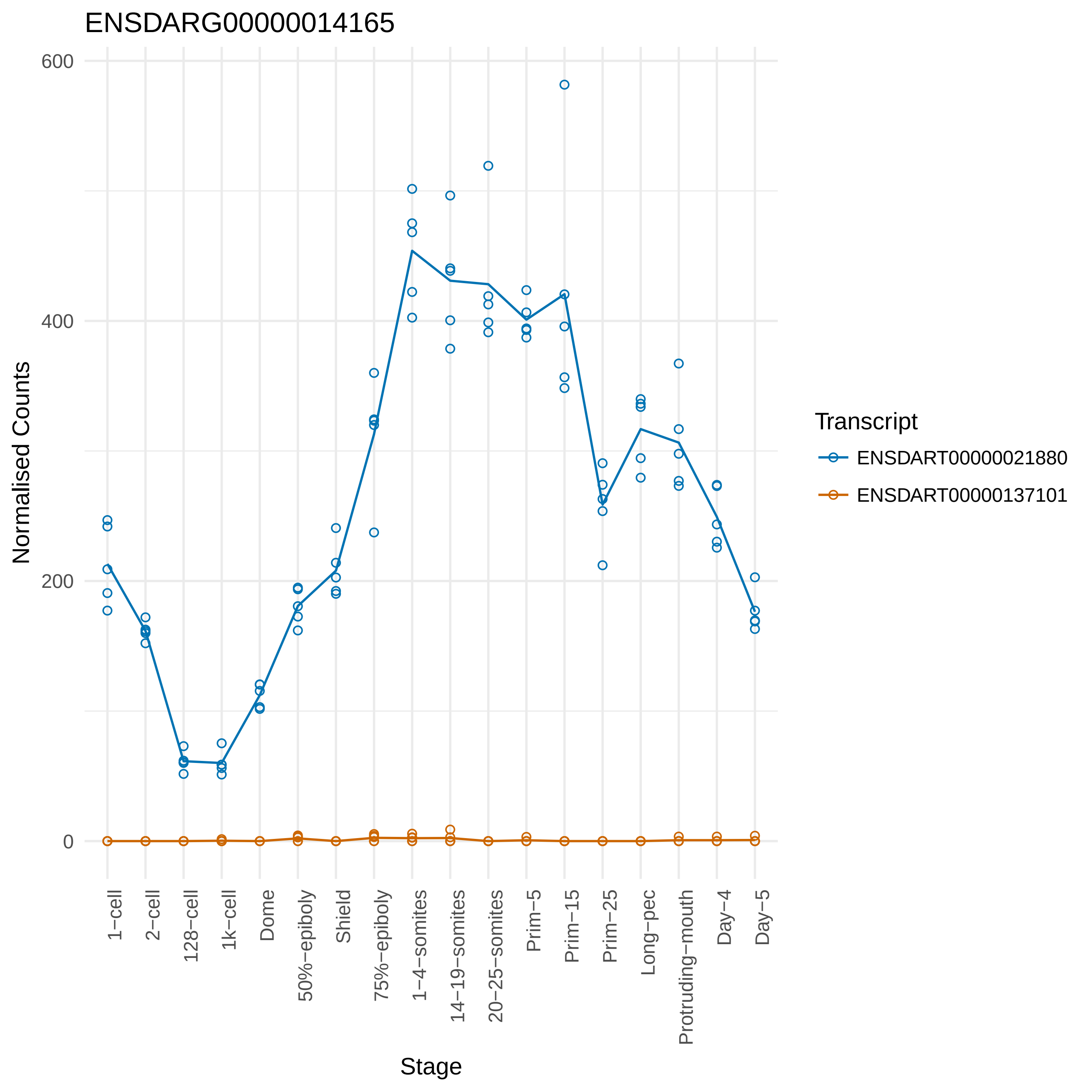
ENSDARG00000014165
Normalised Counts
600
400
200
0
Stage
1−cell
2−cell
128−cell
1k−cell
Dome
50%−epiboly
Shield
75%−epiboly
1−4−somites
14−19−somites
20−25−somites
Prim−5
Prim−15
Prim−25
Long−pec
Protruding−mouth
Day−4
Day−5
Transcript
ENSDART00000021880
ENSDART00000137101

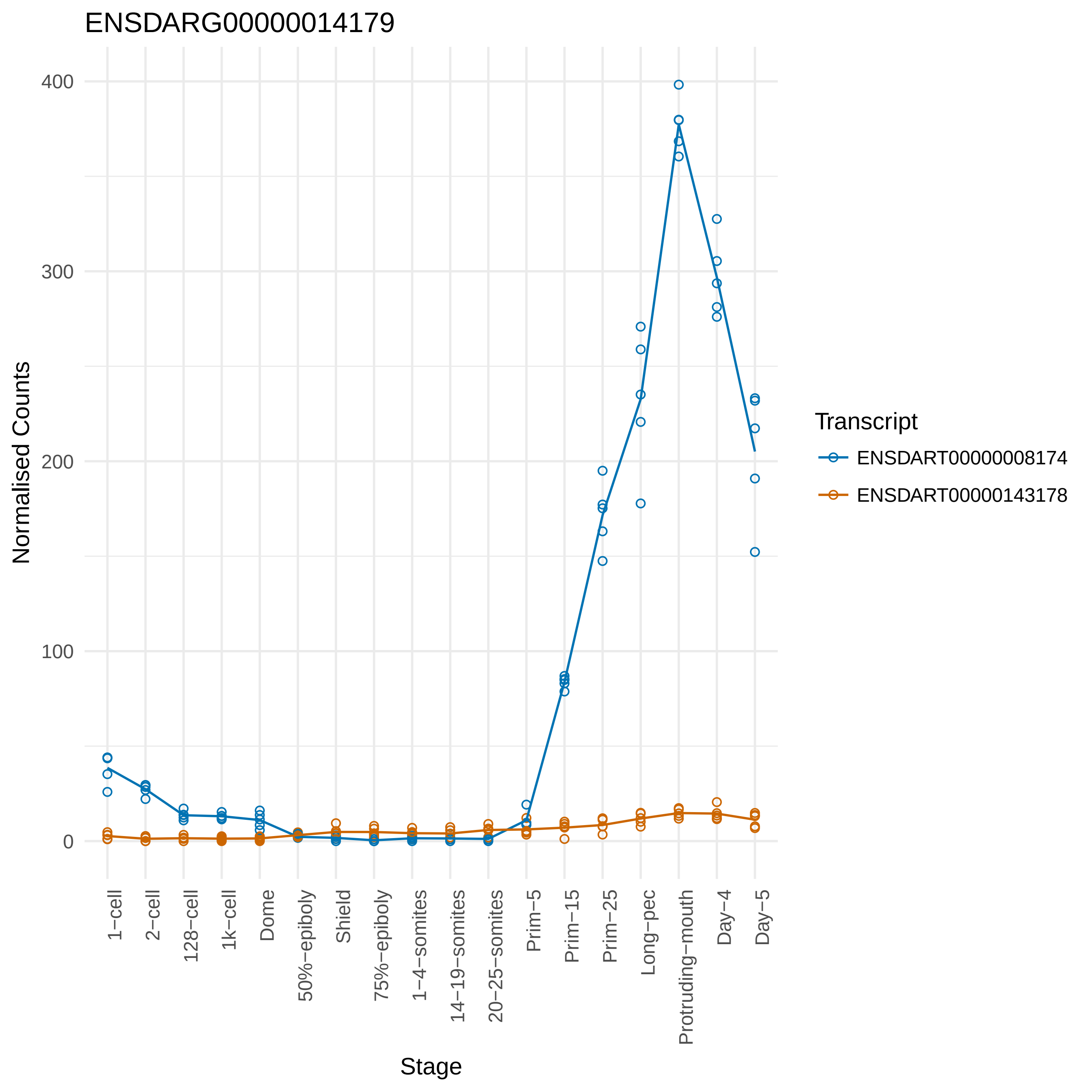

ENSDARG00000014179
Normalised Counts
400
300
200
100
0
1−cell
2−cell
128−cell
1k−cell
Dome
50%−epiboly
Shield
75%−epiboly
1−4−somites
14−19−somites
20−25−somites
Prim−5
Prim−15
Prim−25
Long−pec
Protruding−mouth
Day−4
Day−5
Stage
Transcript
ENSDART00000008174
ENSDART00000143178

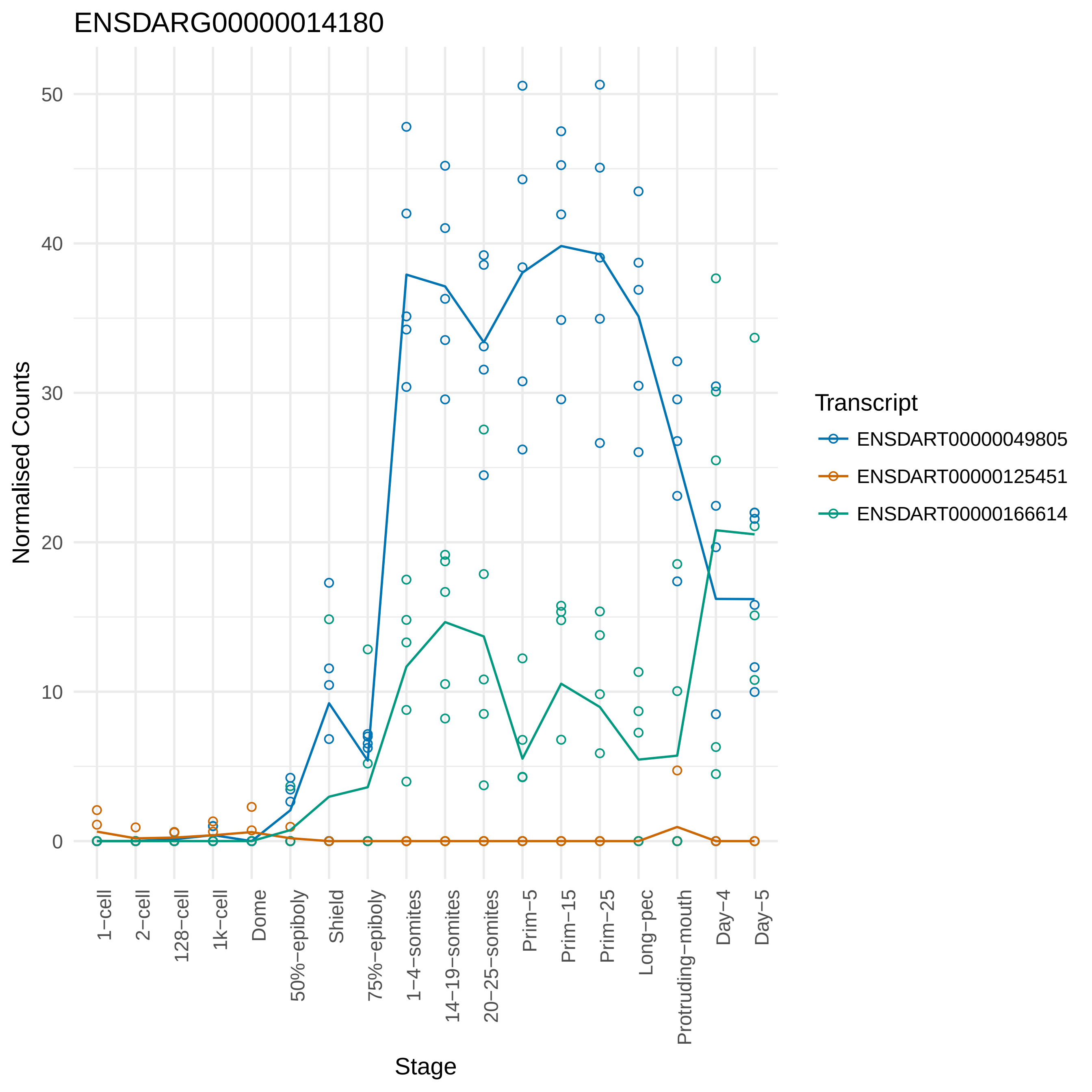

ENSDARG00000014180
Normalised Counts
0
10
20
30
40
50
1−cell
2−cell
128−cell
1k−cell
Dome
50%−epiboly
Shield
75%−epiboly
1−4−somites
14−19−somites
20−25−somites
Prim−5
Prim−15
Prim−25
Long−pec
Protruding−mouth
Day−4
Day−5
Stage
Transcript
ENSDART00000049805
ENSDART00000125451
ENSDART00000166614

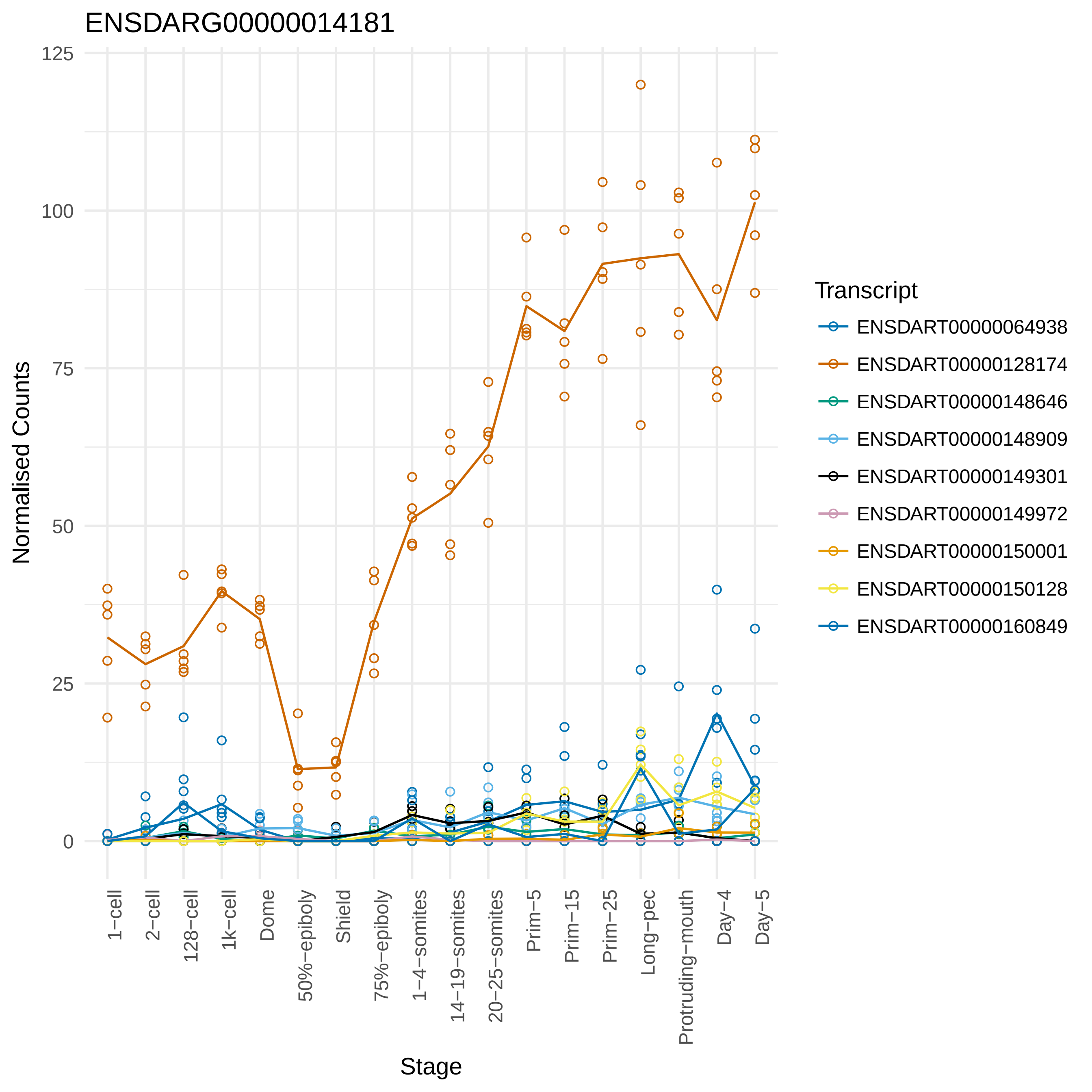
ENSDARG00000014181
Normalised Counts
125
100
75
50
25
0
1−cell
2−cell
128−cell
1k−cell
Dome
50%−epiboly
Shield
75%−epiboly
1−4−somites
14−19−somites
20−25−somites
Prim−5
Prim−15
Prim−25
Long−pec
Protruding−mouth
Day−4
Day−5
Stage
Transcript
ENSDART00000064938
ENSDART00000128174
ENSDART00000148646
ENSDART00000148909
ENSDART00000149301
ENSDART00000149972
ENSDART00000150001
ENSDART00000150128
ENSDART00000160849

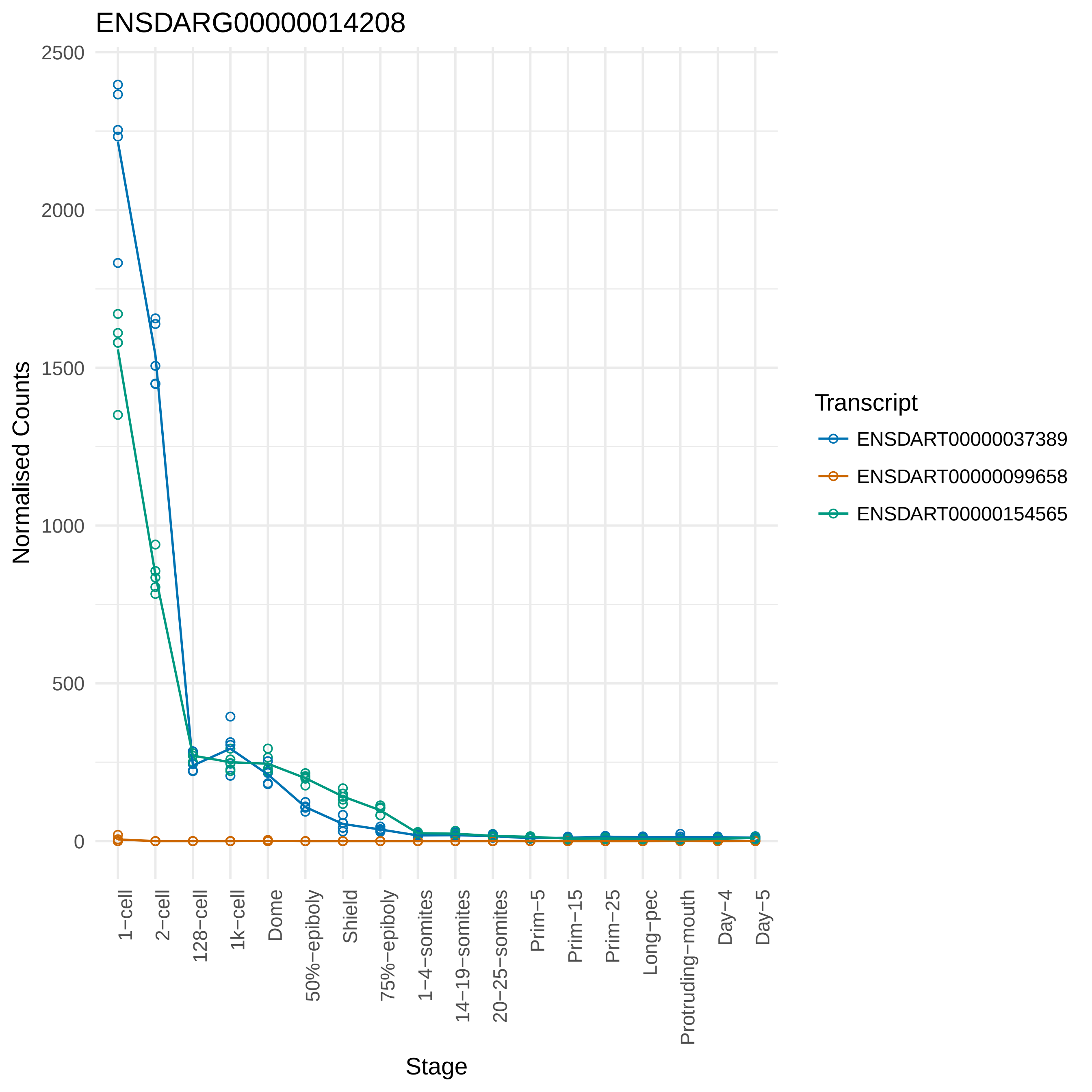
ENSDARG00000014208
Normalised Counts
2500
2000
1500
1000
500
0
Stage
1−cell
2−cell
128−cell
1k−cell
Dome
50%−epiboly
Shield
75%−epiboly
1−4−somites
14−19−somites
20−25−somites
Prim−5
Prim−15
Prim−25
Long−pec
Protruding−mouth
Day−4
Day−5
Transcript
ENSDART00000037389
ENSDART00000099658
ENSDART00000154565

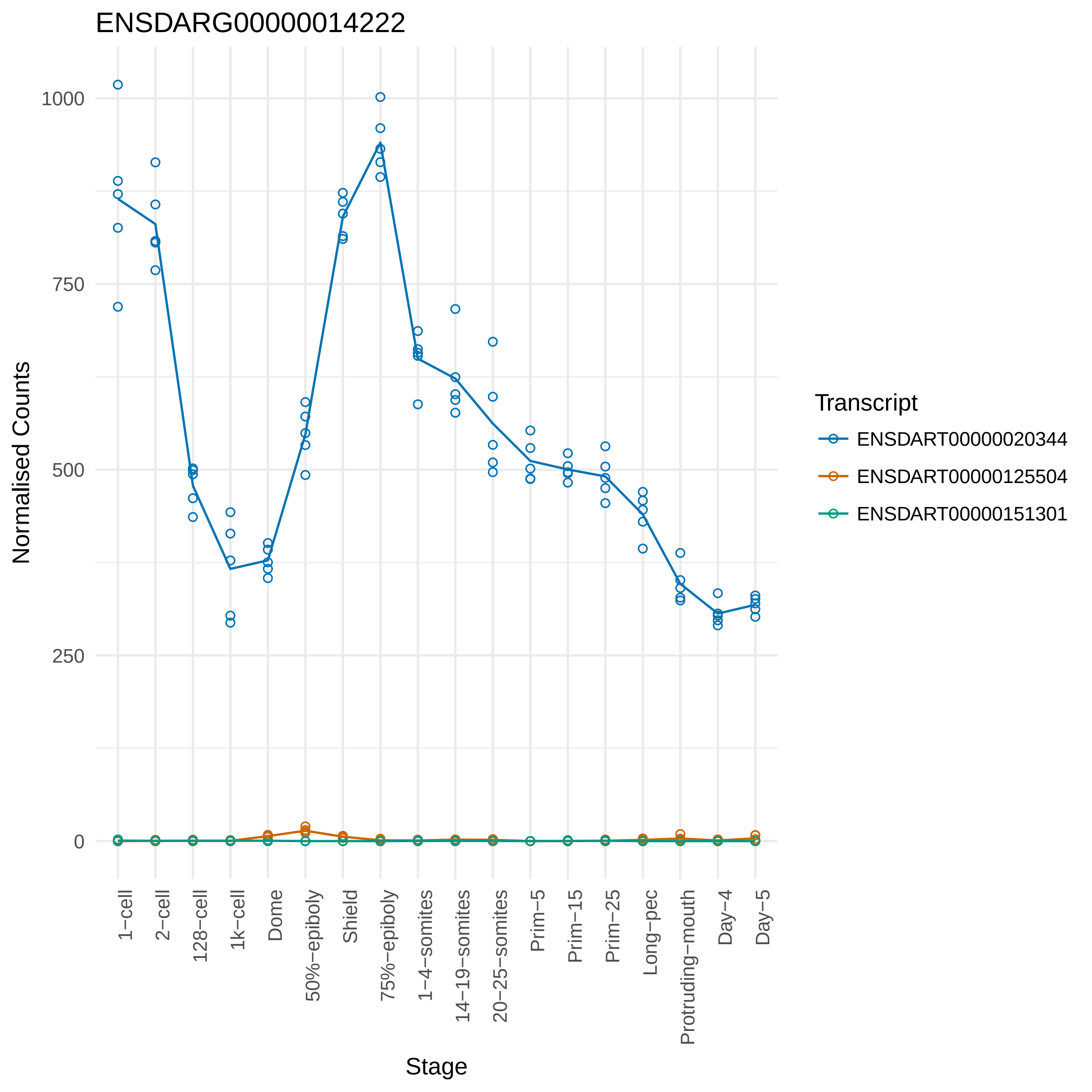
ENSDARG00000014222
Normalised Counts
1000
750
500
250
0
1−cell
2−cell
128−cell
1k−cell
Dome
50%−epiboly
Shield
75%−epiboly
1−4−somites
14−19−somites
20−25−somites
Prim−5
Prim−15
Prim−25
Long−pec
Protruding−mouth
Day−4
Day−5
Stage
Transcript
ENSDART00000020344
ENSDART00000125504
ENSDART00000151301

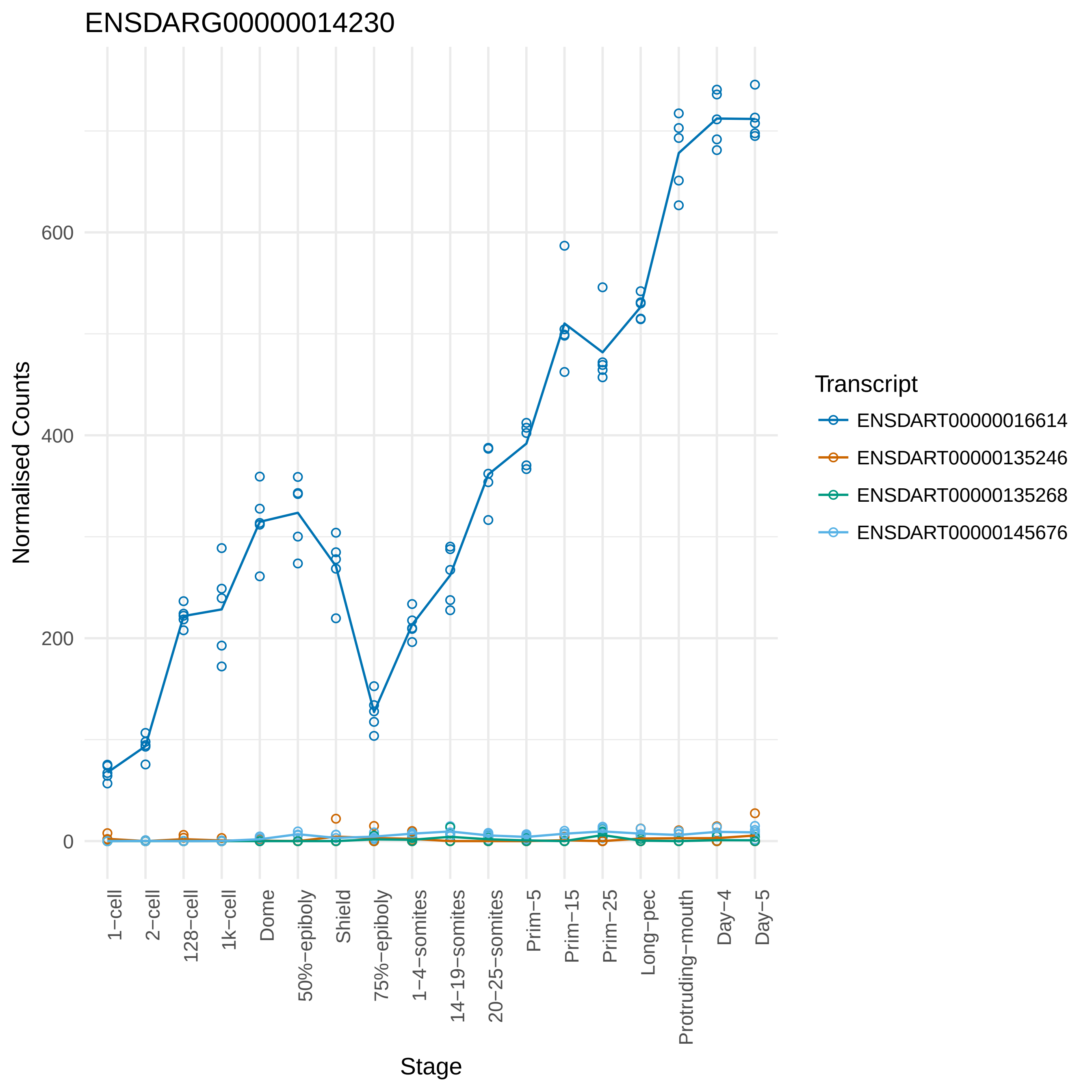
ENSDARG00000014230
Normalised Counts
600
400
200
0
1−cell
2−cell
128−cell
1k−cell
Dome
50%−epiboly
Shield
75%−epiboly
1−4−somites
14−19−somites
20−25−somites
Prim−5
Prim−15
Prim−25
Long−pec
Protruding−mouth
Day−4
Day−5
Stage
Transcript
ENSDART00000016614
ENSDART00000135246
ENSDART00000135268
ENSDART00000145676

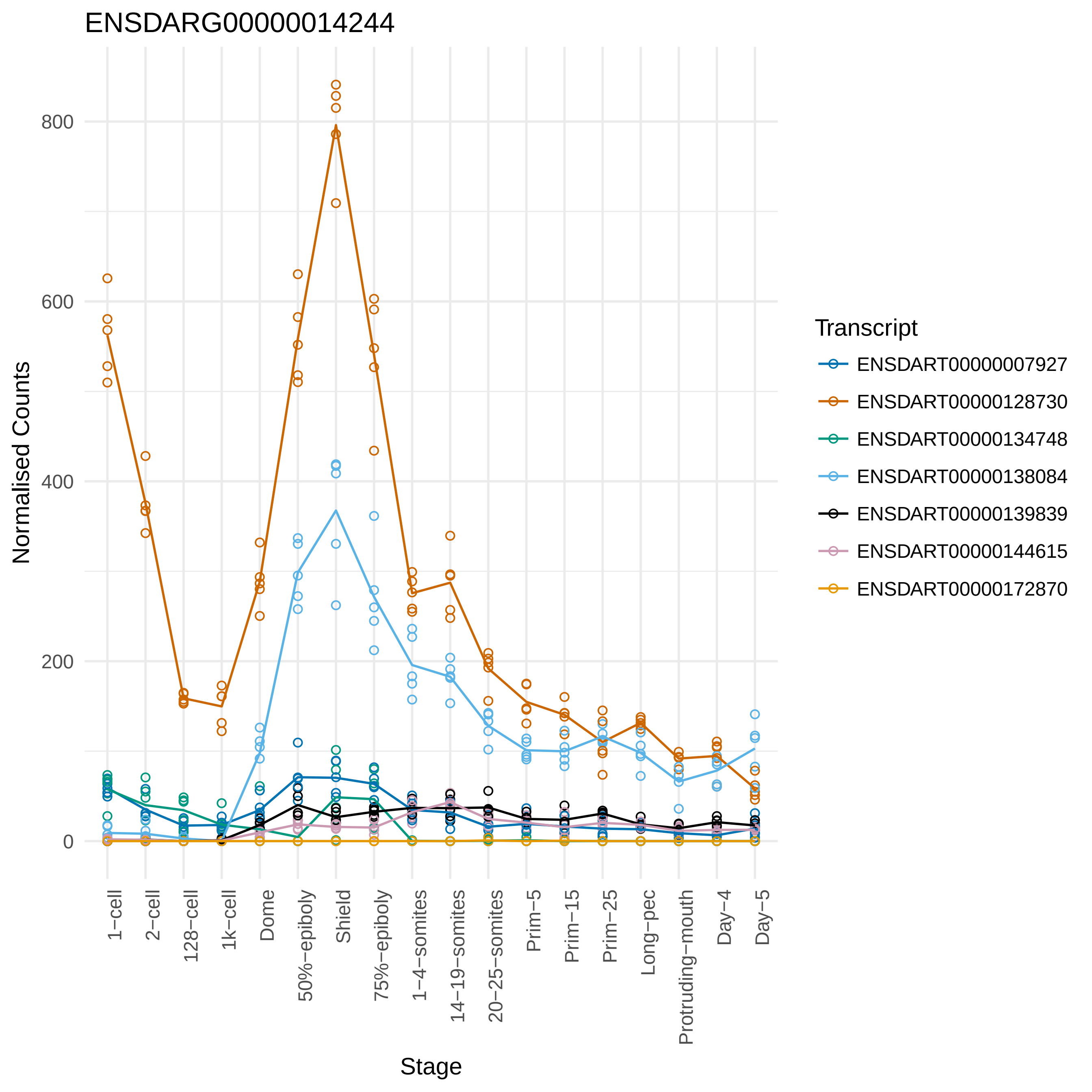

ENSDARG00000014244
Normalised Counts
800
600
400
200
0
1−cell
2−cell
128−cell
1k−cell
Dome
50%−epiboly
Shield
75%−epiboly
1−4−somites
14−19−somites
20−25−somites
Prim−5
Prim−15
Prim−25
Long−pec
Protruding−mouth
Day−4
Day−5
Stage
Transcript
ENSDART00000007927
ENSDART00000128730
ENSDART00000134748
ENSDART00000138084
ENSDART00000139839
ENSDART00000144615
ENSDART00000172870

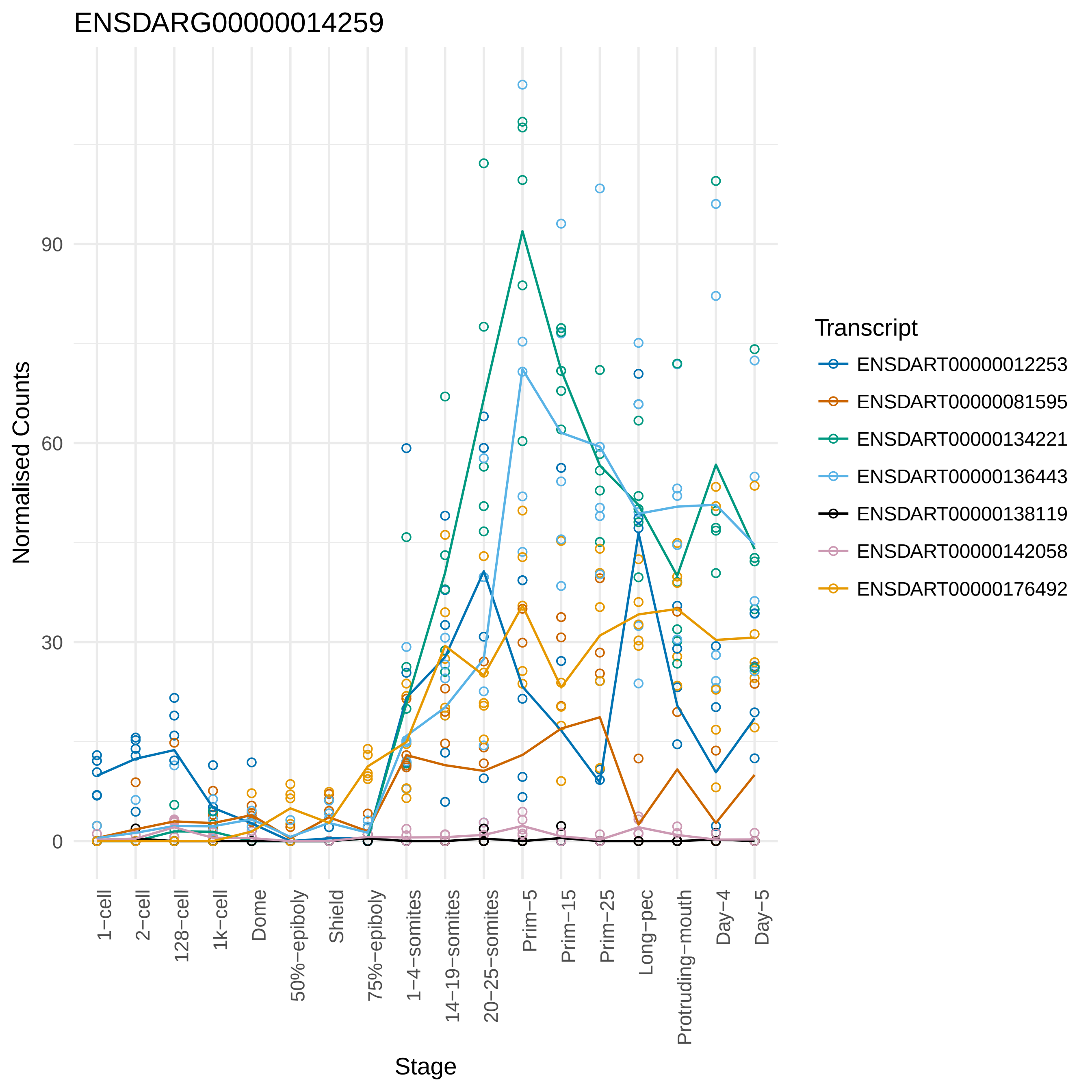

ENSDARG00000014259
Normalised Counts
90
60
30
0
1−cell
2−cell
128−cell
1k−cell
Dome
50%−epiboly
Shield
75%−epiboly
1−4−somites
14−19−somites
20−25−somites
Prim−5
Prim−15
Prim−25
Long−pec
Protruding−mouth
Day−4
Day−5
Stage
Transcript
ENSDART00000012253
ENSDART00000081595
ENSDART00000134221
ENSDART00000136443
ENSDART00000138119
ENSDART00000142058
ENSDART00000176492

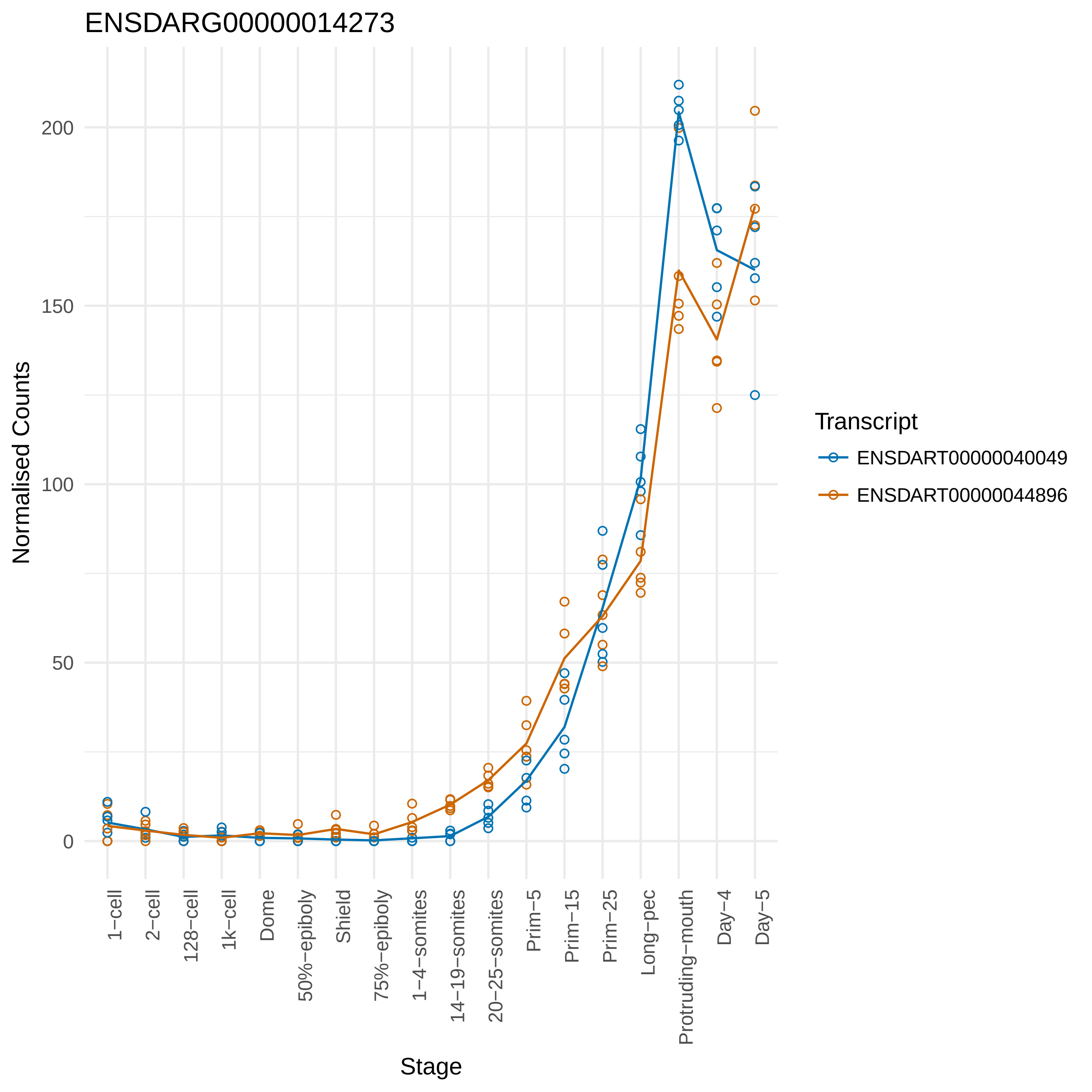

ENSDARG00000014273
Normalised Counts
0
50
100
150
200
Stage
1−cell
2−cell
128−cell
1k−cell
Dome
50%−epiboly
Shield
75%−epiboly
1−4−somites
14−19−somites
20−25−somites
Prim−5
Prim−15
Prim−25
Long−pec
Protruding−mouth
Day−4
Day−5
Transcript
ENSDART00000040049
ENSDART00000044896

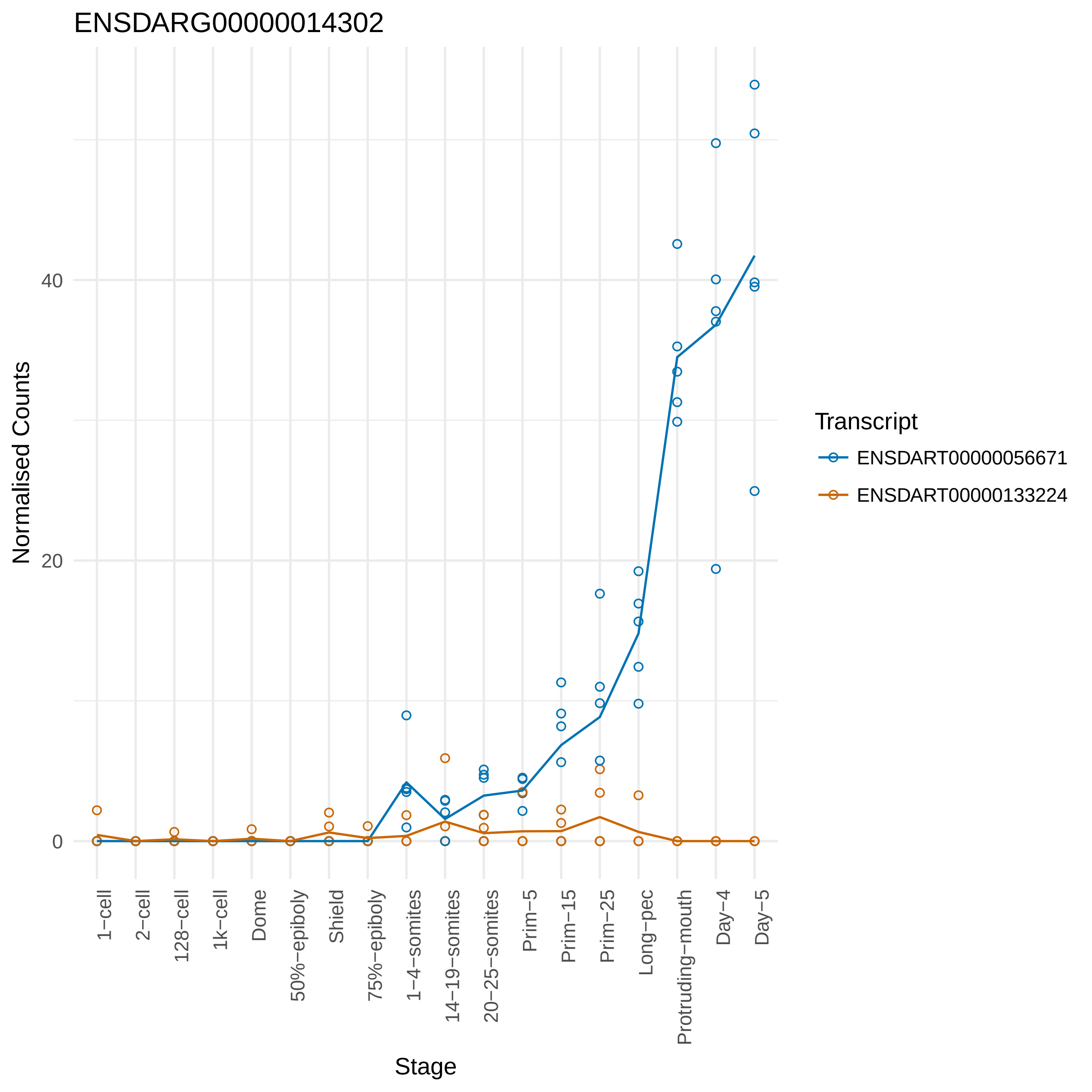
ENSDARG00000014302
Normalised Counts
0
20
40
1−cell
2−cell
128−cell
1k−cell
Dome
50%−epiboly
Shield
75%−epiboly
1−4−somites
14−19−somites
20−25−somites
Prim−5
Prim−15
Prim−25
Long−pec
Protruding−mouth
Day−4
Day−5
Stage
Transcript
ENSDART00000056671
ENSDART00000133224

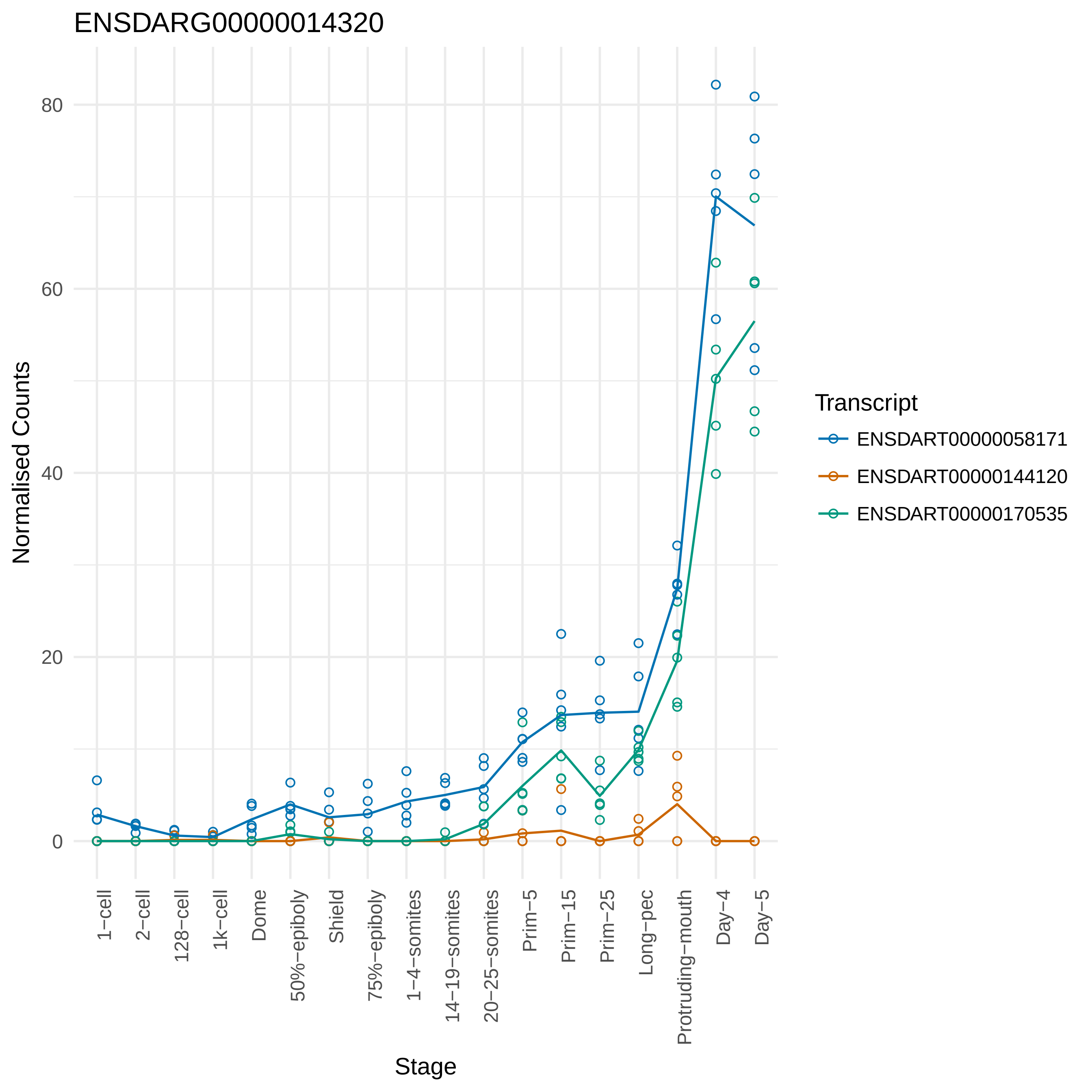

ENSDARG00000014320
Normalised Counts
0
20
40
60
80
1−cell
2−cell
128−cell
1k−cell
Dome
50%−epiboly
Shield
75%−epiboly
1−4−somites
14−19−somites
20−25−somites
Prim−5
Prim−15
Prim−25
Long−pec
Protruding−mouth
Day−4
Day−5
Stage
Transcript
ENSDART00000058171
ENSDART00000144120
ENSDART00000170535

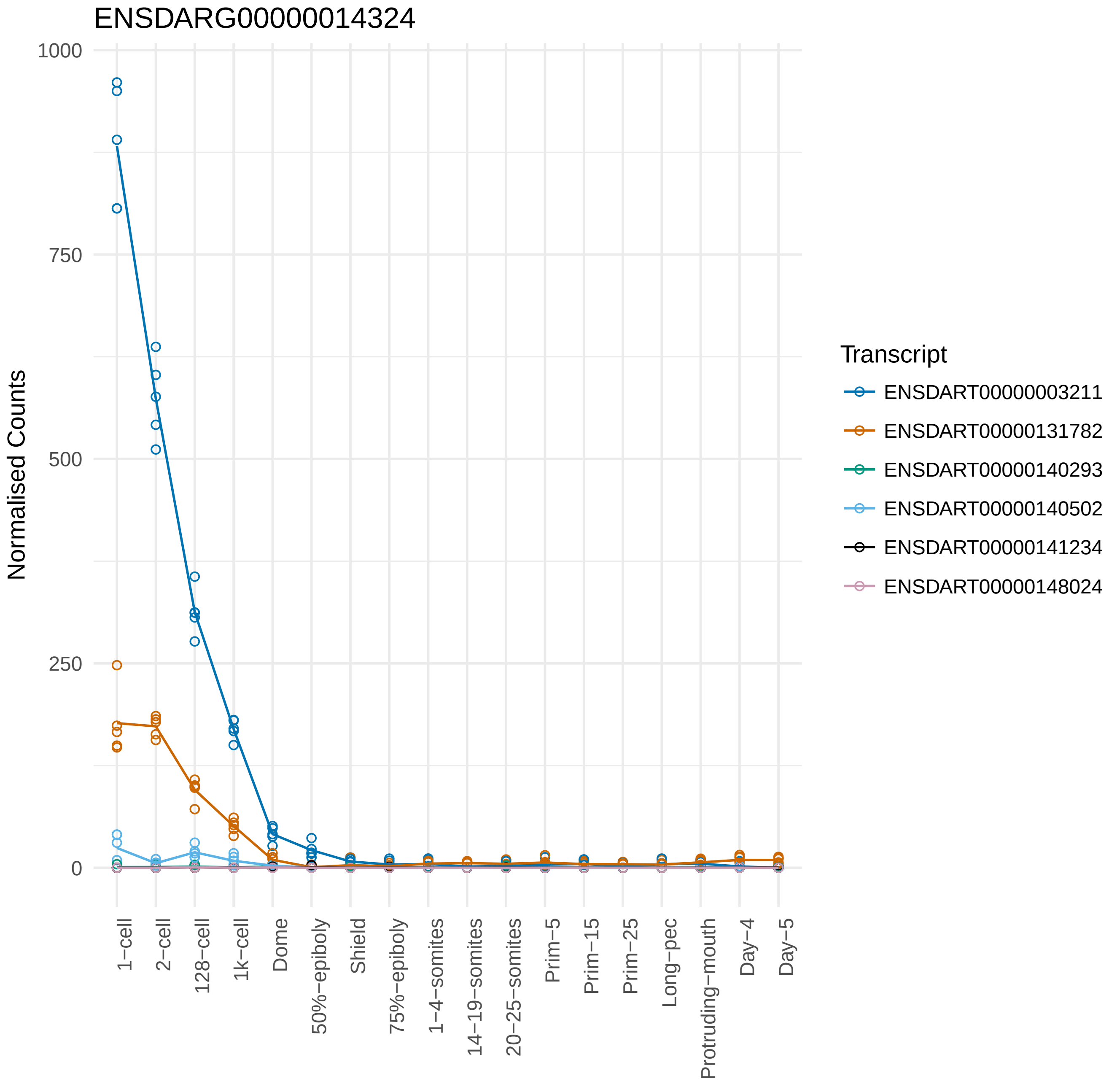

ENSDARG00000014324
Normalised Counts
1000
750
500
250
0
1−cell
2−cell
128−cell
1k−cell
Dome
50%−epiboly
Shield
75%−epiboly
1−4−somites
14−19−somites
20−25−somites
Prim−5
Prim−15
Prim−25
Long−pec
Protruding−mouth
Day−4
Day−5
Stage
Transcript
ENSDART00000003211
ENSDART00000131782
ENSDART00000140293
ENSDART00000140502
ENSDART00000141234
ENSDART00000148024

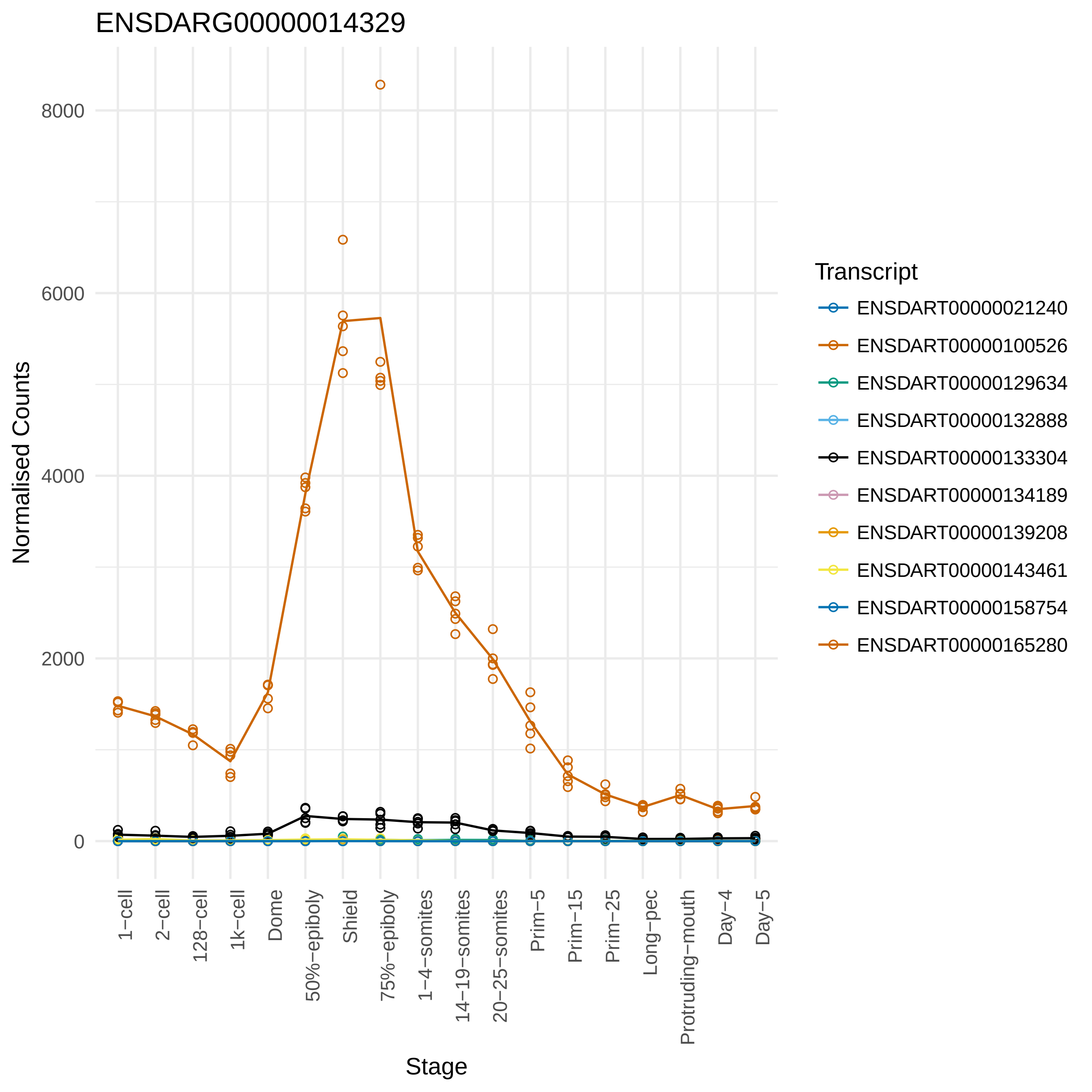
ENSDARG00000014329
Normalised Counts
0
2000
4000
6000
8000
Stage
1−cell
2−cell
128−cell
1k−cell
Dome
50%−epiboly
Shield
75%−epiboly
1−4−somites
14−19−somites
20−25−somites
Prim−5
Prim−15
Prim−25
Long−pec
Protruding−mouth
Day−4
Day−5
Transcript
ENSDART00000021240
ENSDART00000100526
ENSDART00000129634
ENSDART00000132888
ENSDART00000133304
ENSDART00000134189
ENSDART00000139208
ENSDART00000143461
ENSDART00000158754
ENSDART00000165280

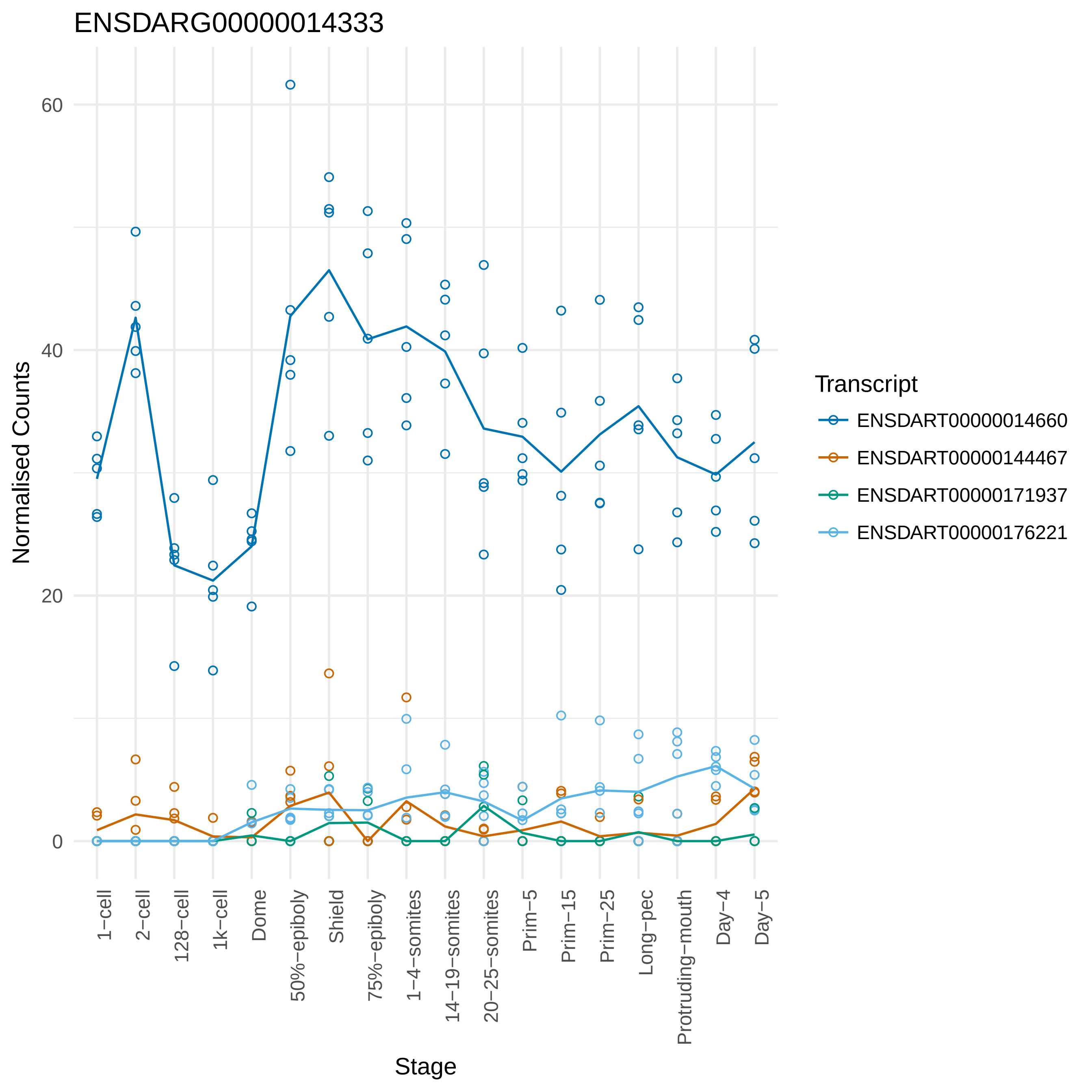

ENSDARG00000014333
Normalised Counts
0
20
40
60
1−cell
2−cell
128−cell
1k−cell
Dome
50%−epiboly
Shield
75%−epiboly
1−4−somites
14−19−somites
20−25−somites
Prim−5
Prim−15
Prim−25
Long−pec
Protruding−mouth
Day−4
Day−5
Stage
Transcript
ENSDART00000014660
ENSDART00000144467
ENSDART00000171937
ENSDART00000176221

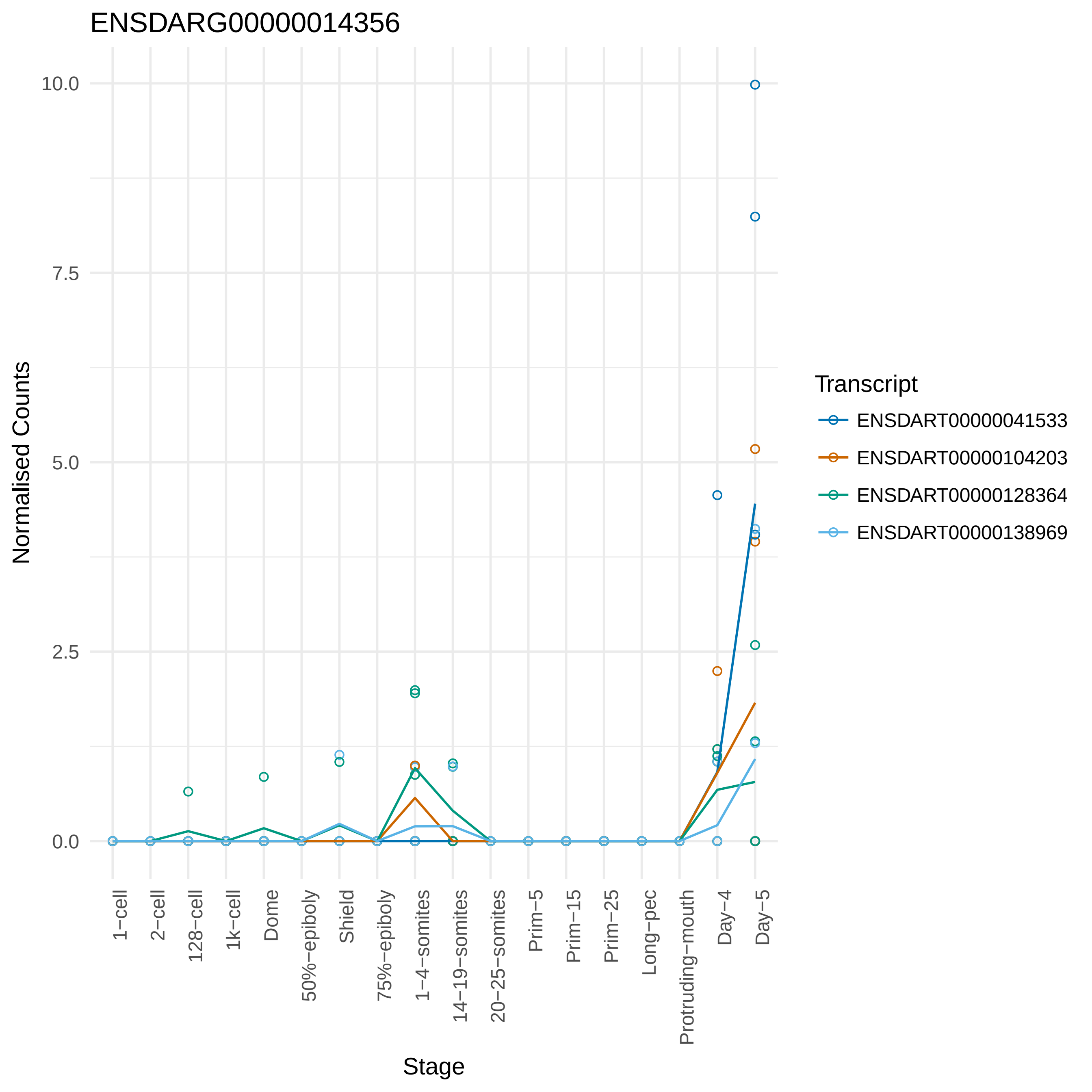

ENSDARG00000014356
Normalised Counts
10.0
7.5
5.0
2.5
0.0
Stage
1−cell
2−cell
128−cell
1k−cell
Dome
50%−epiboly
Shield
75%−epiboly
1−4−somites
14−19−somites
20−25−somites
Prim−5
Prim−15
Prim−25
Long−pec
Protruding−mouth
Day−4
Day−5
Transcript
ENSDART00000041533
ENSDART00000104203
ENSDART00000128364
ENSDART00000138969

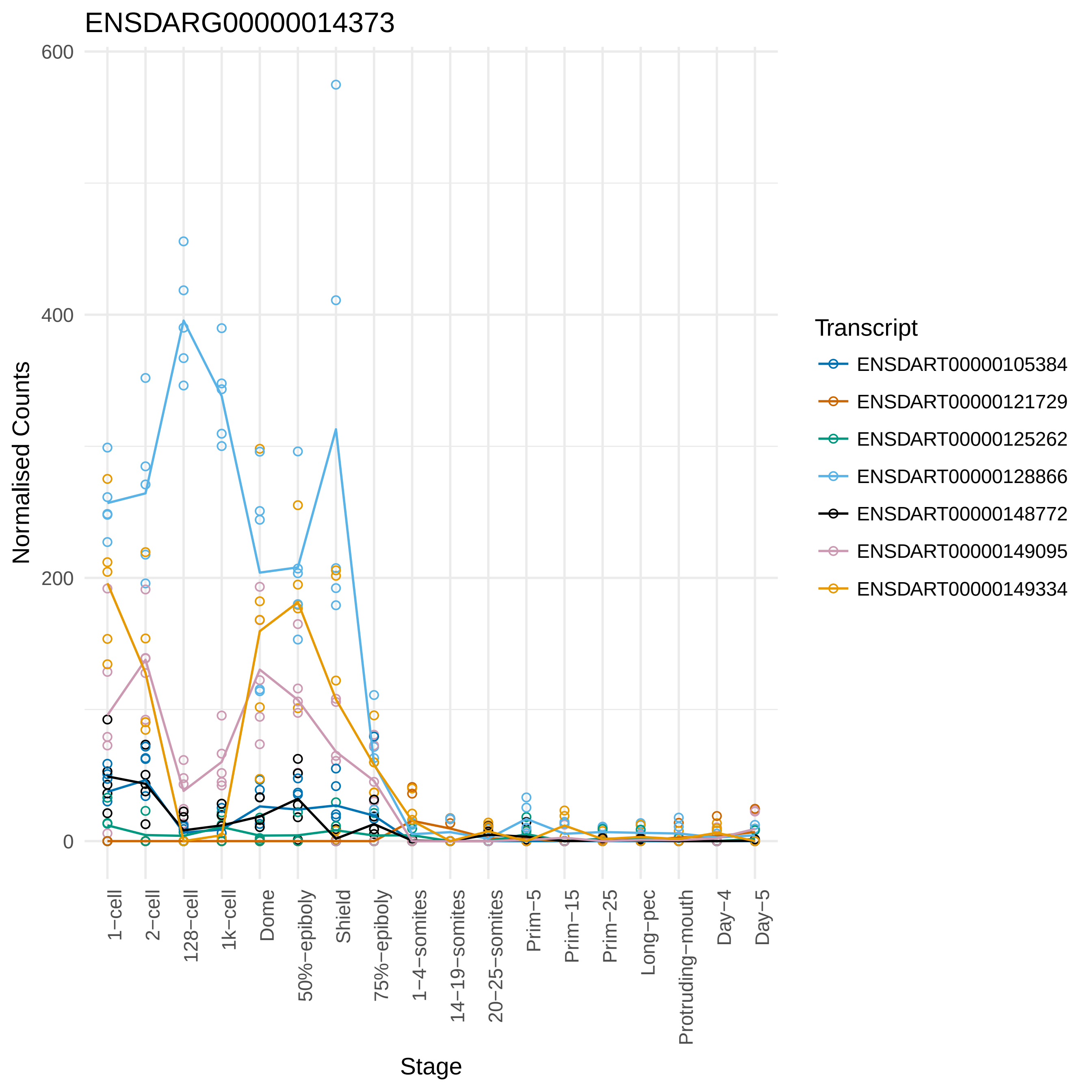
ENSDARG00000014373
Normalised Counts
600
400
200
0
1−cell
2−cell
128−cell
1k−cell
Dome
50%−epiboly
Shield
75%−epiboly
1−4−somites
14−19−somites
20−25−somites
Prim−5
Prim−15
Prim−25
Long−pec
Protruding−mouth
Day−4
Day−5
Stage
Transcript
ENSDART00000105384
ENSDART00000121729
ENSDART00000125262
ENSDART00000128866
ENSDART00000148772
ENSDART00000149095
ENSDART00000149334

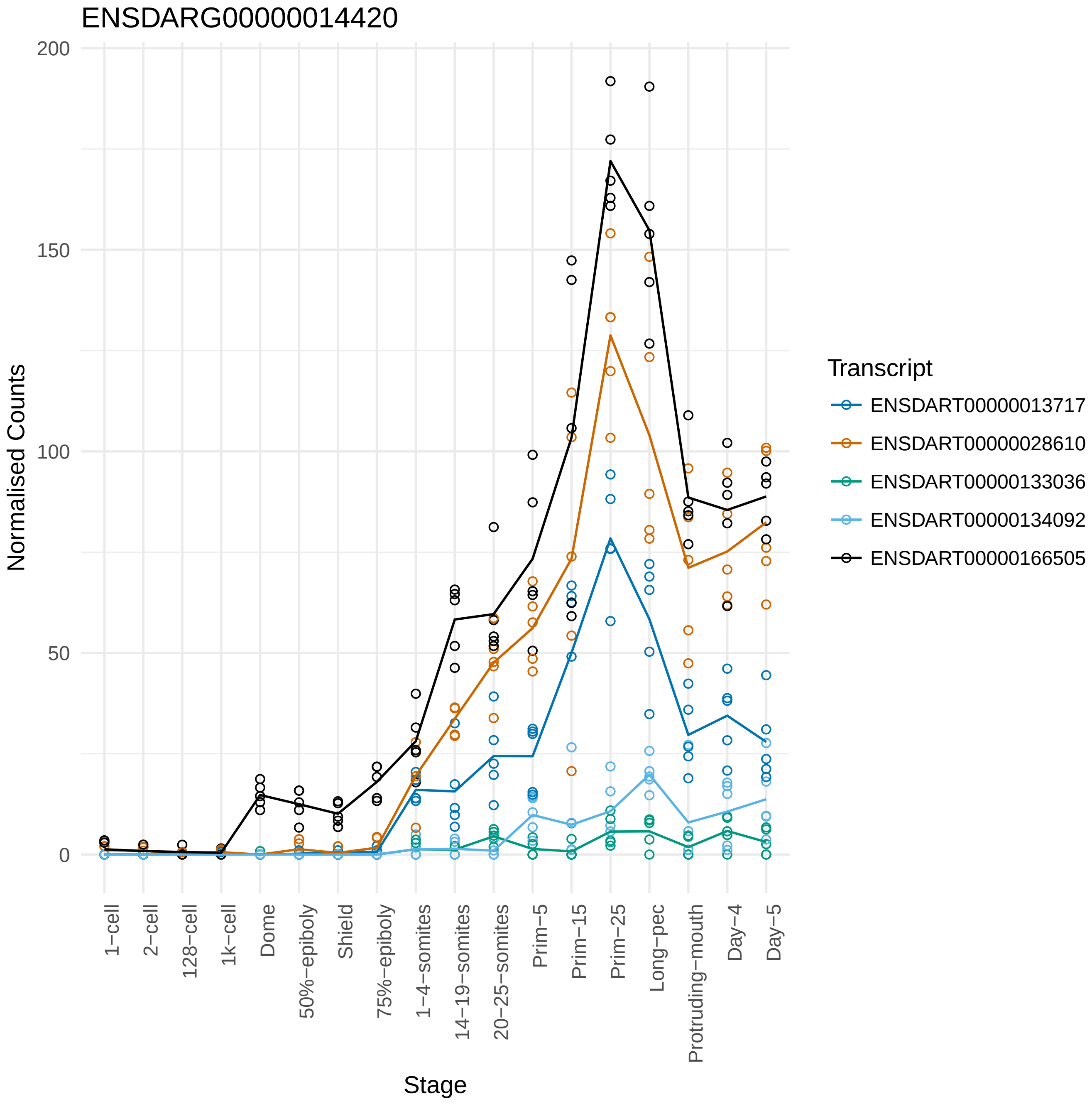

ENSDARG00000014420
Normalised Counts
200
150
100
50
0
1−cell
2−cell
128−cell
1k−cell
Dome
50%−epiboly
Shield
75%−epiboly
1−4−somites
14−19−somites
20−25−somites
Prim−5
Prim−15
Prim−25
Long−pec
Protruding−mouth
Day−4
Day−5
Stage
Transcript
ENSDART00000013717
ENSDART00000028610
ENSDART00000133036
ENSDART00000134092
ENSDART00000166505

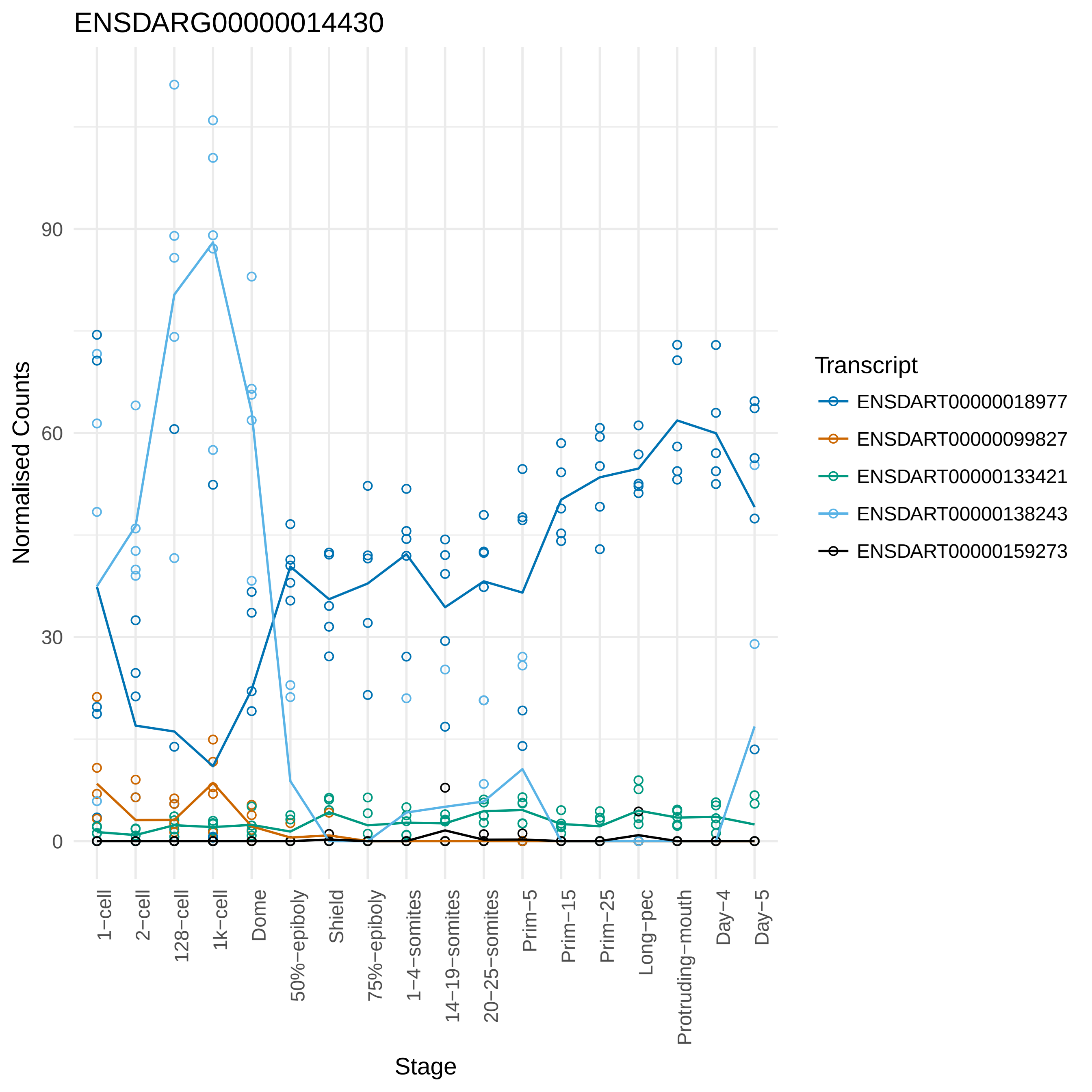

ENSDARG00000014430
Normalised Counts
90
60
30
0
1−cell
2−cell
128−cell
1k−cell
Dome
50%−epiboly
Shield
75%−epiboly
1−4−somites
14−19−somites
20−25−somites
Prim−5
Prim−15
Prim−25
Long−pec
Protruding−mouth
Day−4
Day−5
Stage
Transcript
ENSDART00000018977
ENSDART00000099827
ENSDART00000133421
ENSDART00000138243
ENSDART00000159273

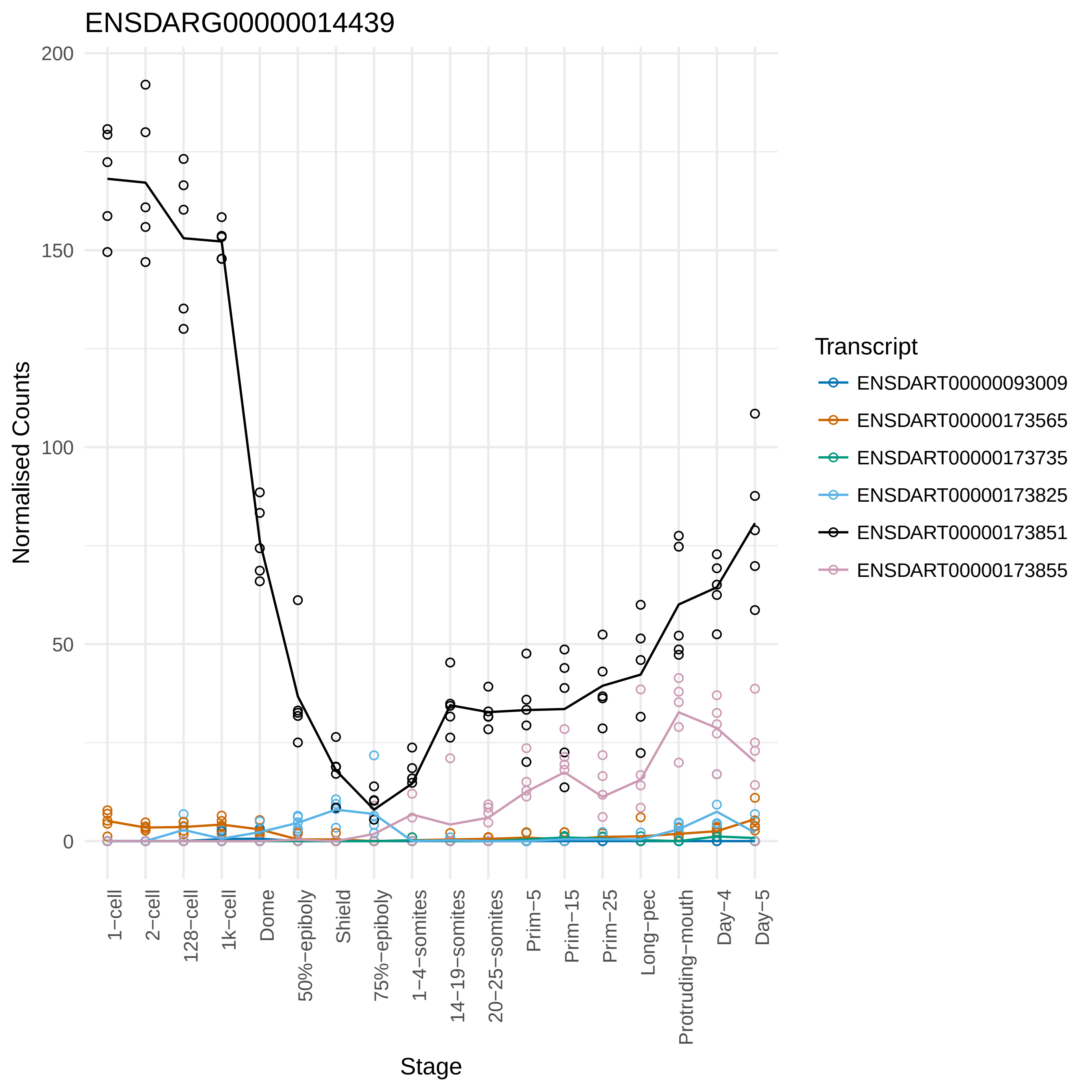

ENSDARG00000014439
Normalised Counts
200
150
100
50
0
1−cell
2−cell
128−cell
1k−cell
Dome
50%−epiboly
Shield
75%−epiboly
1−4−somites
14−19−somites
20−25−somites
Prim−5
Prim−15
Prim−25
Long−pec
Protruding−mouth
Day−4
Day−5
Stage
Transcript
ENSDART00000093009
ENSDART00000173565
ENSDART00000173735
ENSDART00000173825
ENSDART00000173851
ENSDART00000173855

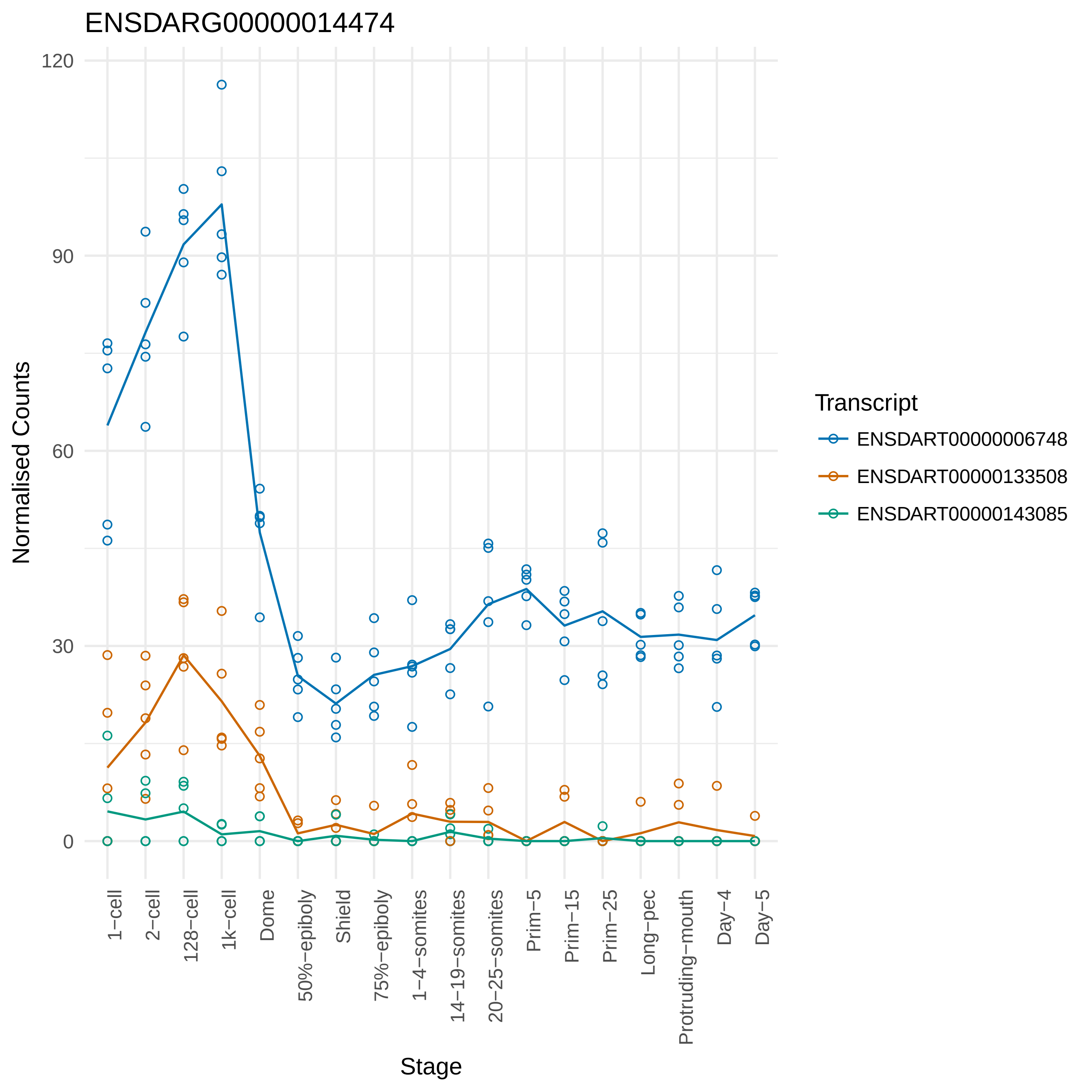
ENSDARG00000014474
Normalised Counts
0
30
60
90
120
1−cell
2−cell
128−cell
1k−cell
Dome
50%−epiboly
Shield
75%−epiboly
1−4−somites
14−19−somites
20−25−somites
Prim−5
Prim−15
Prim−25
Long−pec
Protruding−mouth
Day−4
Day−5
Stage
Transcript
ENSDART00000006748
ENSDART00000133508
ENSDART00000143085

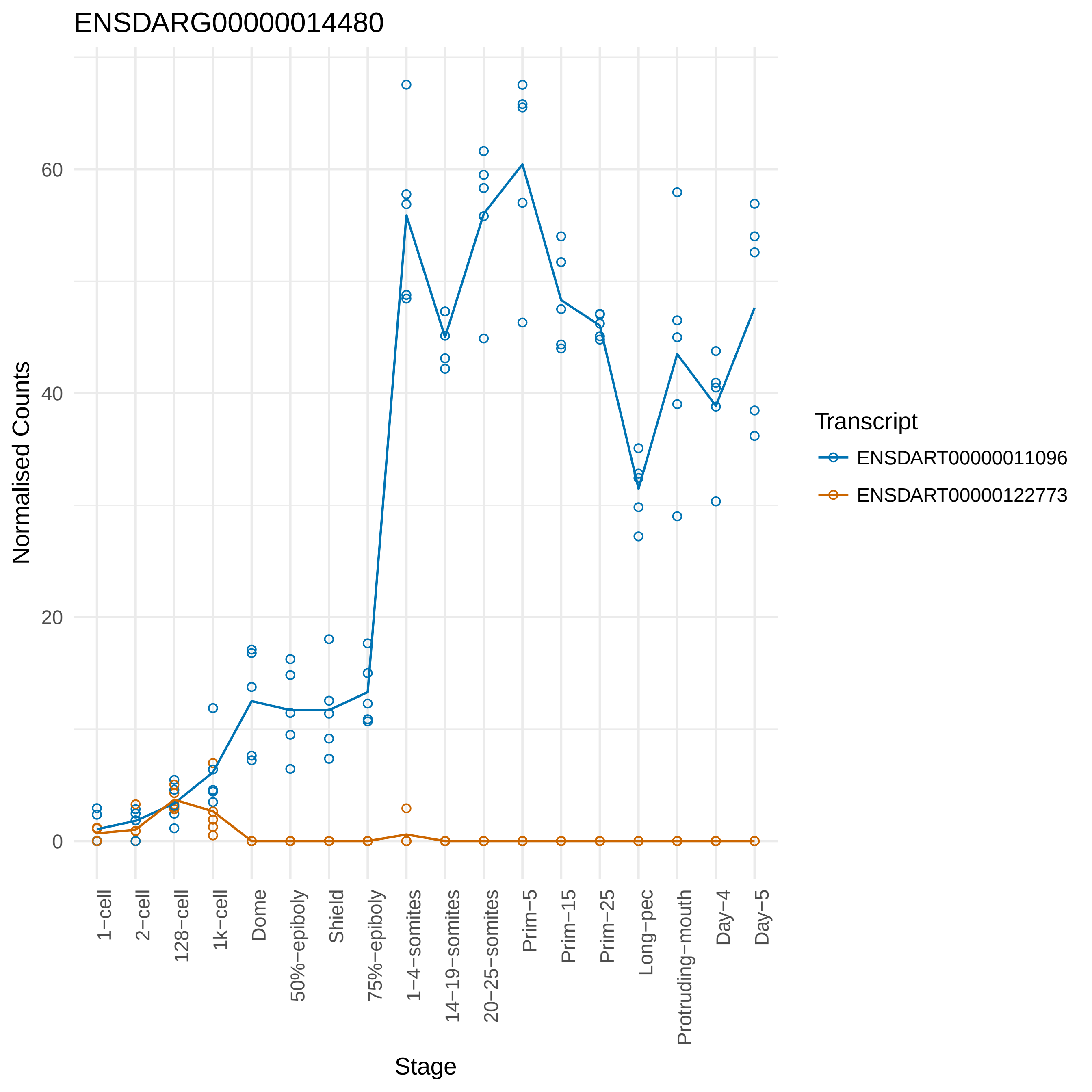
ENSDARG00000014480
Normalised Counts
60
40
20
0
1−cell
2−cell
128−cell
1k−cell
Dome
50%−epiboly
Shield
75%−epiboly
1−4−somites
14−19−somites
20−25−somites
Prim−5
Prim−15
Prim−25
Long−pec
Protruding−mouth
Day−4
Day−5
Stage
Transcript
ENSDART00000011096
ENSDART00000122773

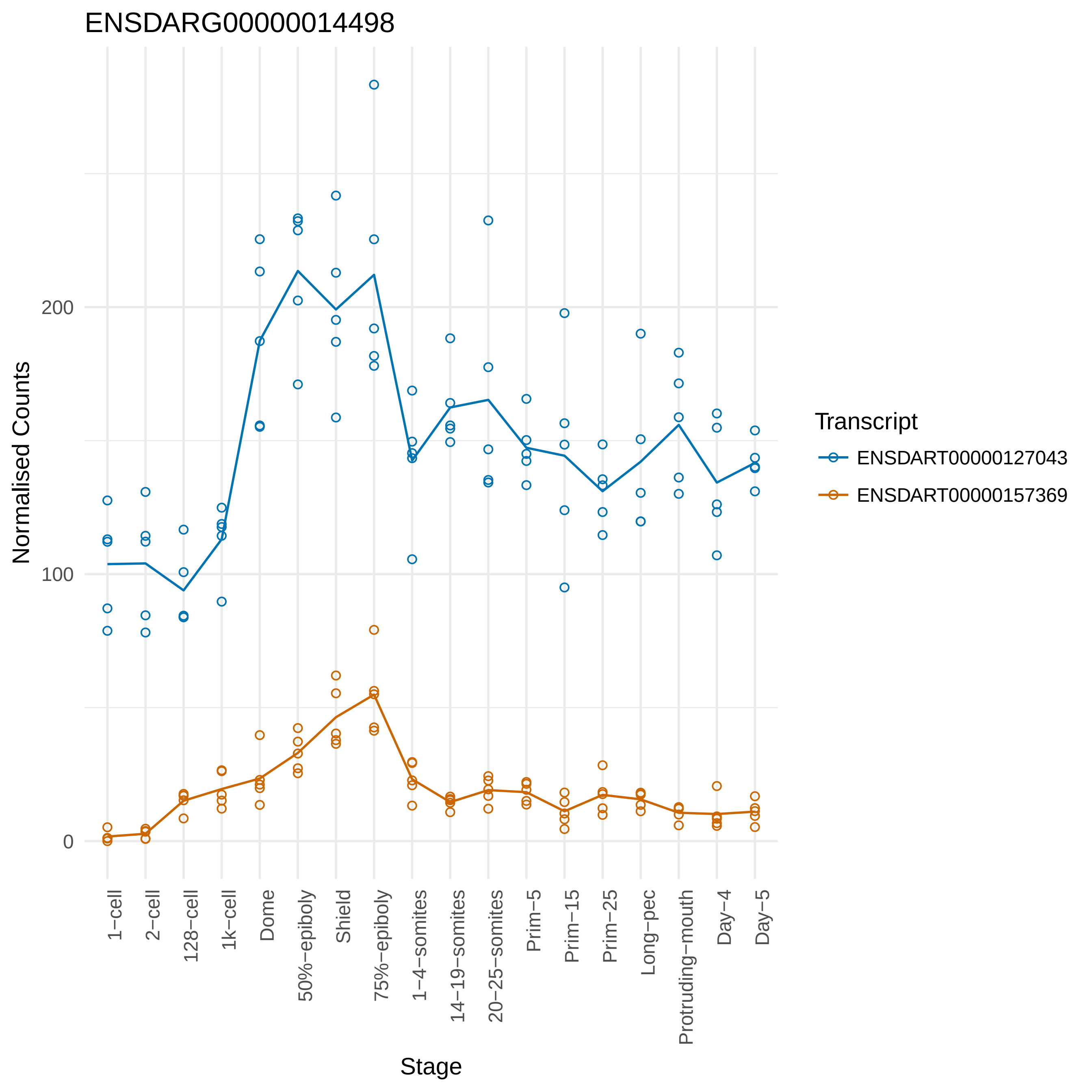

ENSDARG00000014498
Normalised Counts
0
100
200
Stage
1−cell
2−cell
128−cell
1k−cell
Dome
50%−epiboly
Shield
75%−epiboly
1−4−somites
14−19−somites
20−25−somites
Prim−5
Prim−15
Prim−25
Long−pec
Protruding−mouth
Day−4
Day−5
Transcript
ENSDART00000127043
ENSDART00000157369

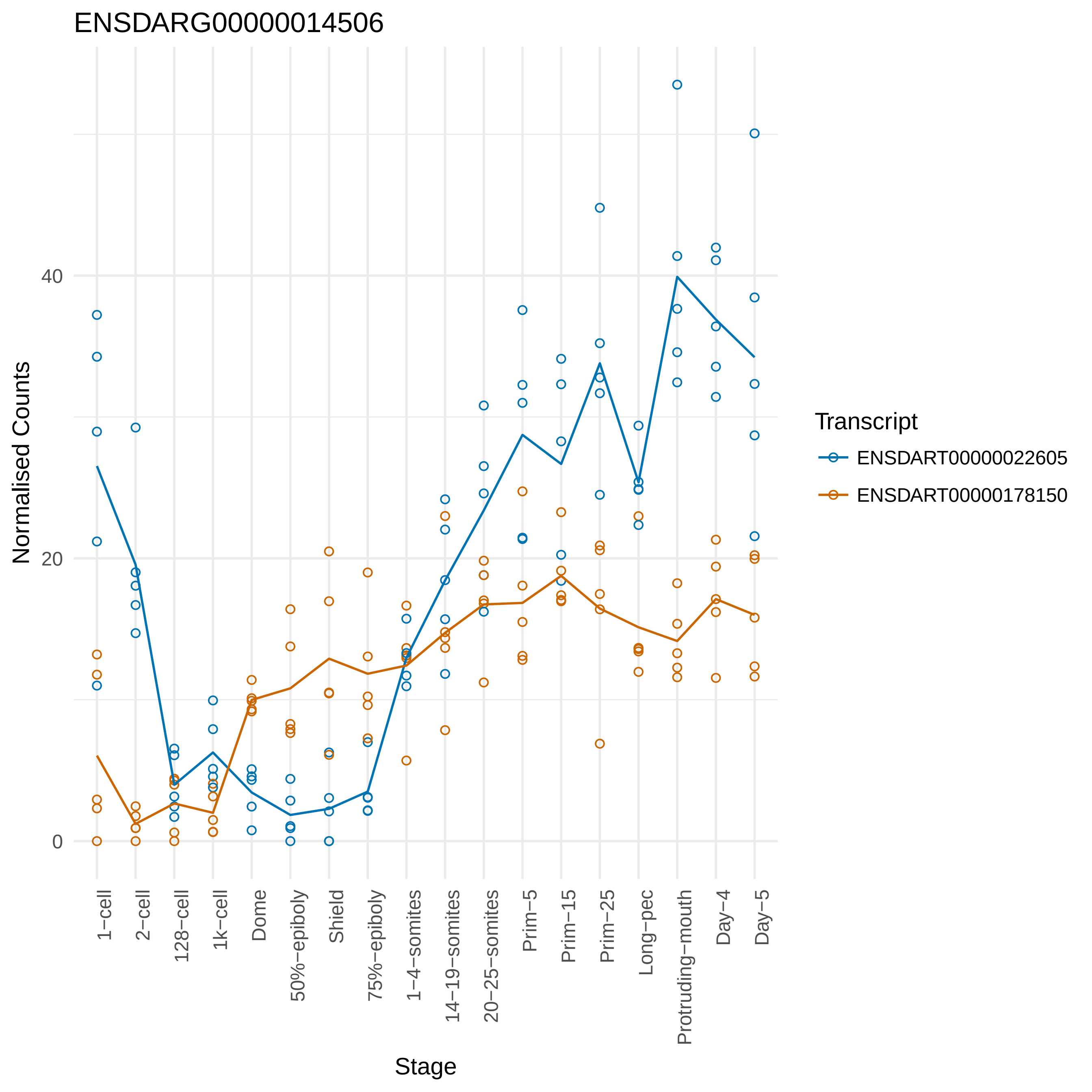
ENSDARG00000014506
Normalised Counts
0
20
40
Stage
1−cell
2−cell
128−cell
1k−cell
Dome
50%−epiboly
Shield
75%−epiboly
1−4−somites
14−19−somites
20−25−somites
Prim−5
Prim−15
Prim−25
Long−pec
Protruding−mouth
Day−4
Day−5
Transcript
ENSDART00000022605
ENSDART00000178150

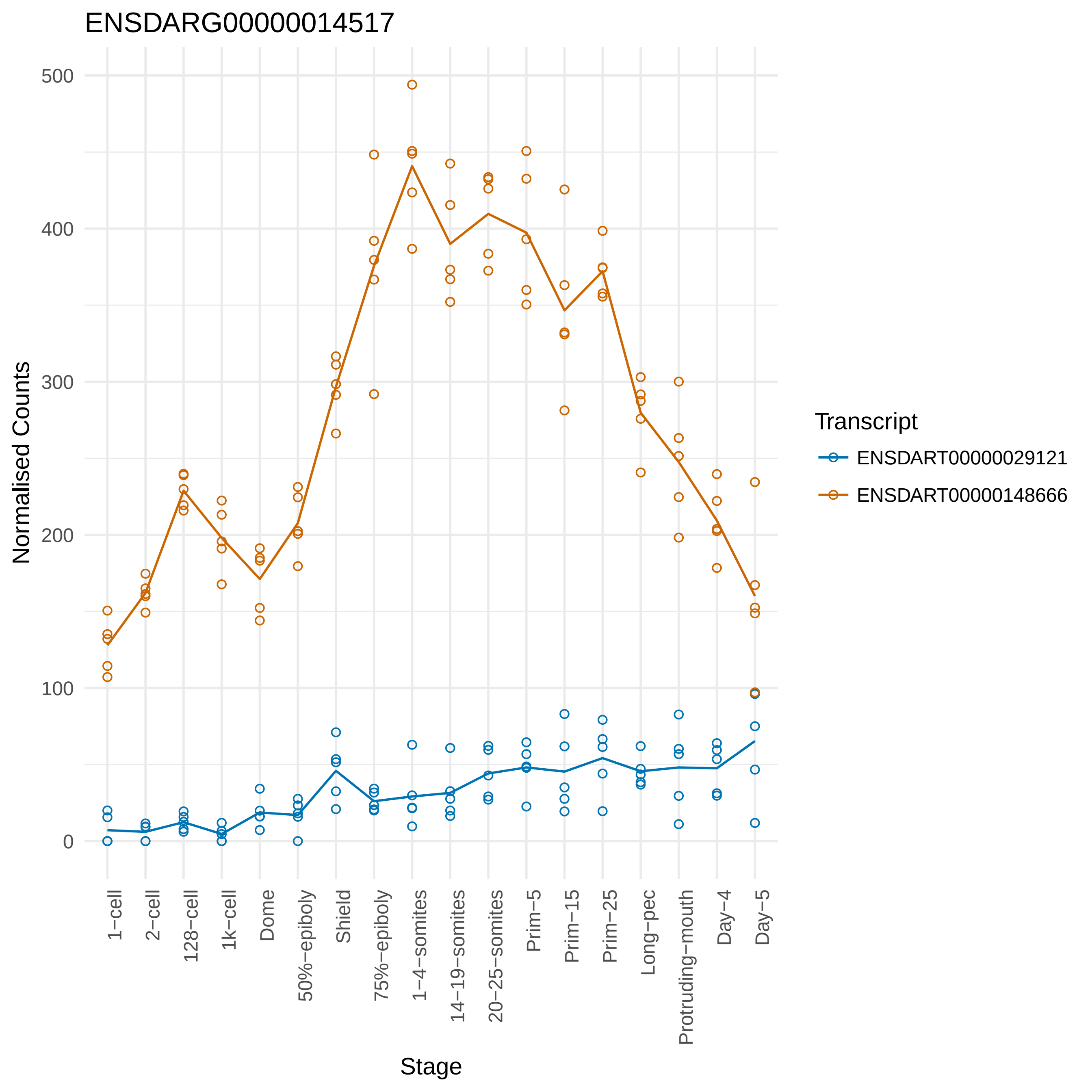

ENSDARG00000014517
Normalised Counts
500
400
300
200
100
0
1−cell
2−cell
128−cell
1k−cell
Dome
50%−epiboly
Shield
75%−epiboly
1−4−somites
14−19−somites
20−25−somites
Prim−5
Prim−15
Prim−25
Long−pec
Protruding−mouth
Day−4
Day−5
Stage
Transcript
ENSDART00000029121
ENSDART00000148666

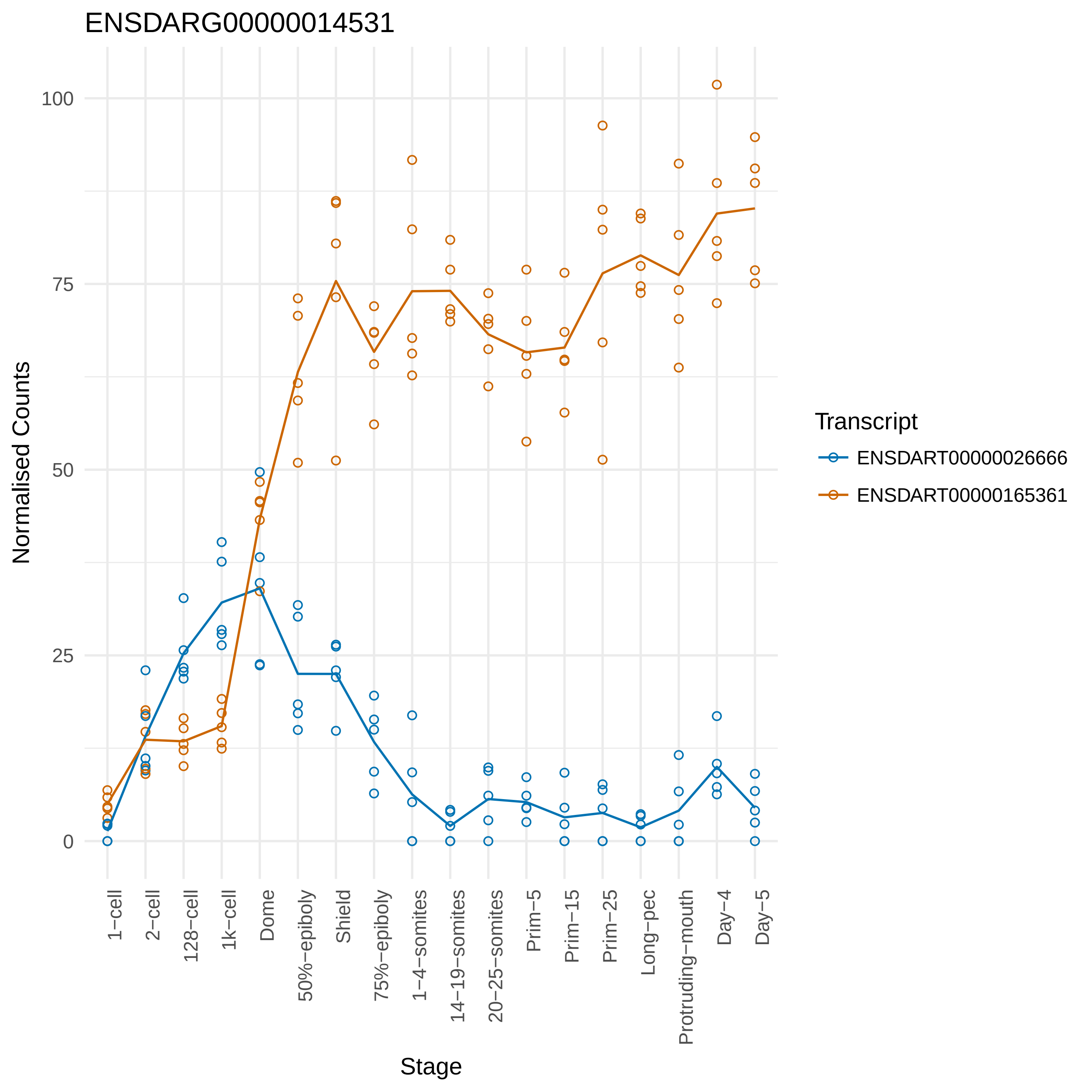
ENSDARG00000014531
Normalised Counts
100
75
50
25
0
1−cell
2−cell
128−cell
1k−cell
Dome
50%−epiboly
Shield
75%−epiboly
1−4−somites
14−19−somites
20−25−somites
Prim−5
Prim−15
Prim−25
Long−pec
Protruding−mouth
Day−4
Day−5
Stage
Transcript
ENSDART00000026666
ENSDART00000165361

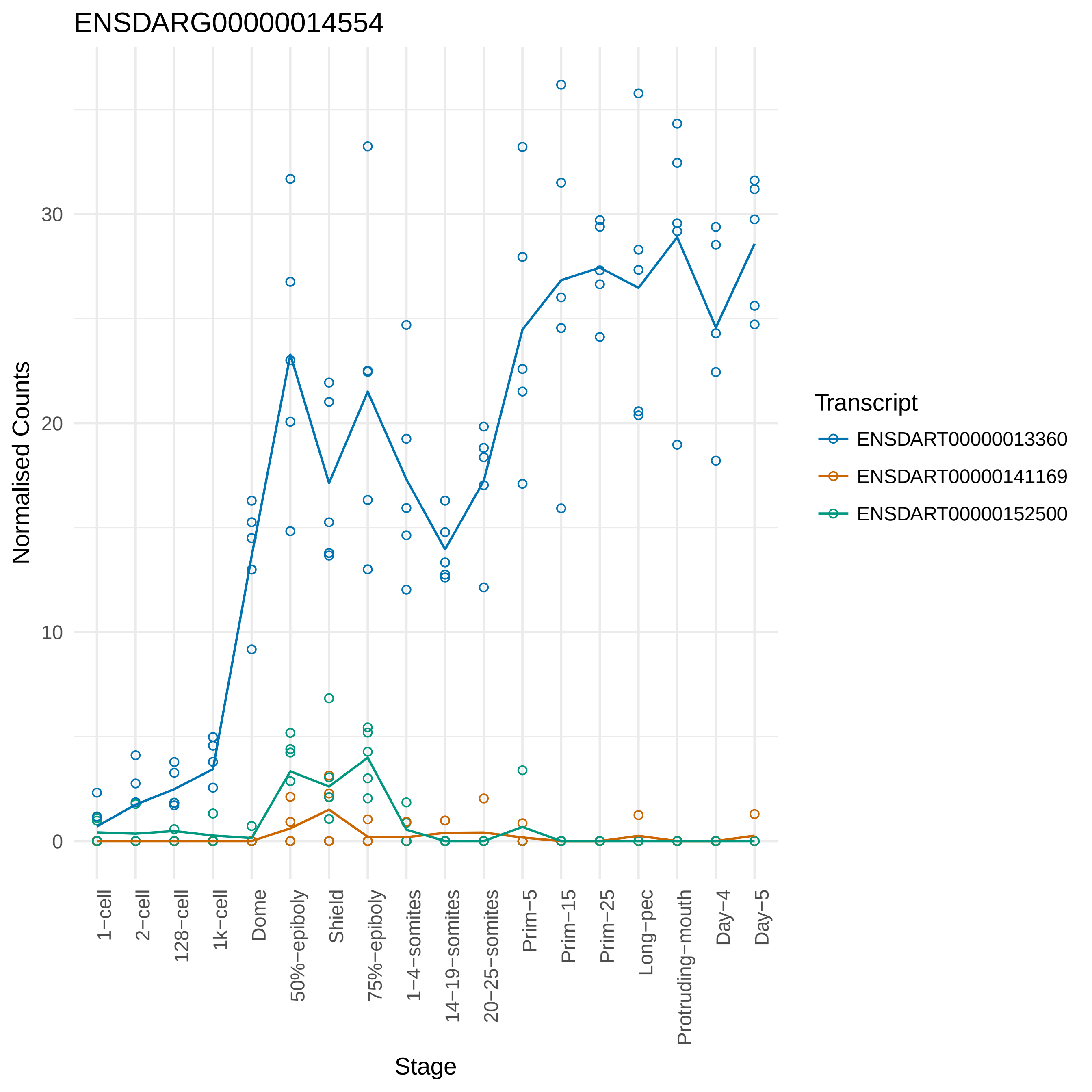

ENSDARG00000014554
Normalised Counts
0
10
20
30
1−cell
2−cell
128−cell
1k−cell
Dome
50%−epiboly
Shield
75%−epiboly
1−4−somites
14−19−somites
20−25−somites
Prim−5
Prim−15
Prim−25
Long−pec
Protruding−mouth
Day−4
Day−5
Stage
Transcript
ENSDART00000013360
ENSDART00000141169
ENSDART00000152500

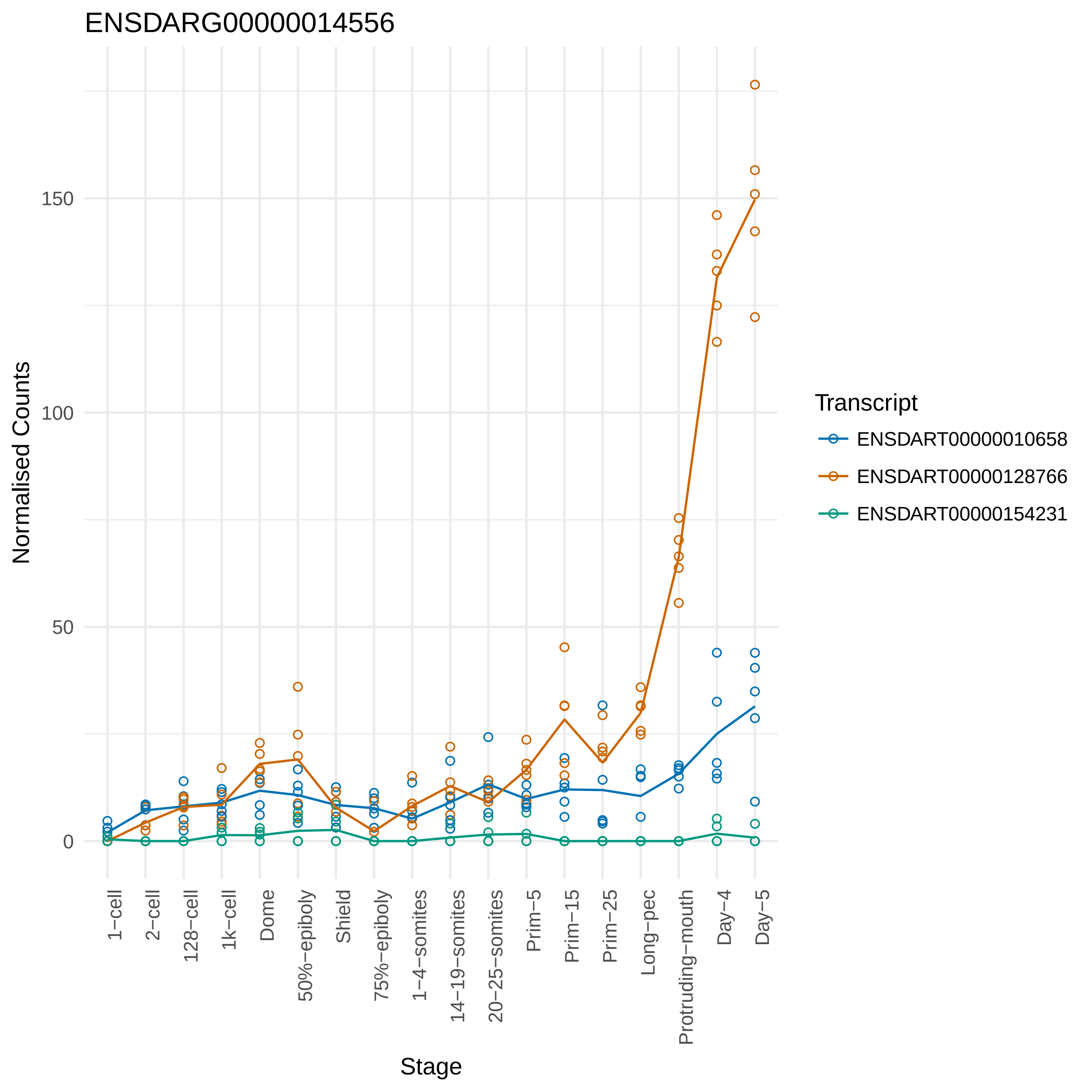

ENSDARG00000014556
Normalised Counts
150
100
50
0
1−cell
2−cell
128−cell
1k−cell
Dome
50%−epiboly
Shield
75%−epiboly
1−4−somites
14−19−somites
20−25−somites
Prim−5
Prim−15
Prim−25
Long−pec
Protruding−mouth
Day−4
Day−5
Stage
Transcript
ENSDART00000010658
ENSDART00000128766
ENSDART00000154231

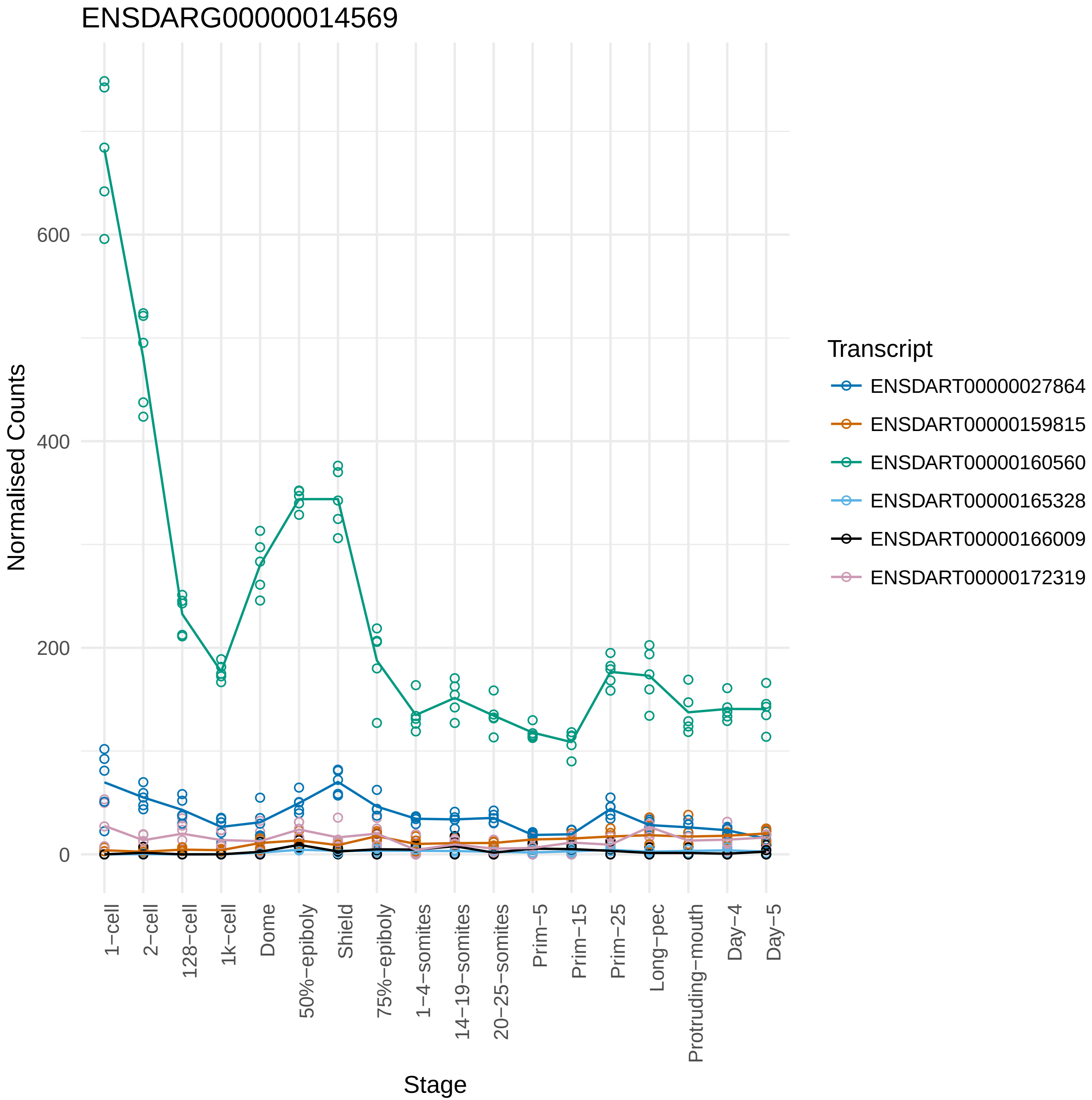
ENSDARG00000014569
Normalised Counts
0
200
400
600
Stage
1−cell
2−cell
128−cell
1k−cell
Dome
50%−epiboly
Shield
75%−epiboly
1−4−somites
14−19−somites
20−25−somites
Prim−5
Prim−15
Prim−25
Long−pec
Protruding−mouth
Day−4
Day−5
Transcript
ENSDART00000027864
ENSDART00000159815
ENSDART00000160560
ENSDART00000165328
ENSDART00000166009
ENSDART00000172319

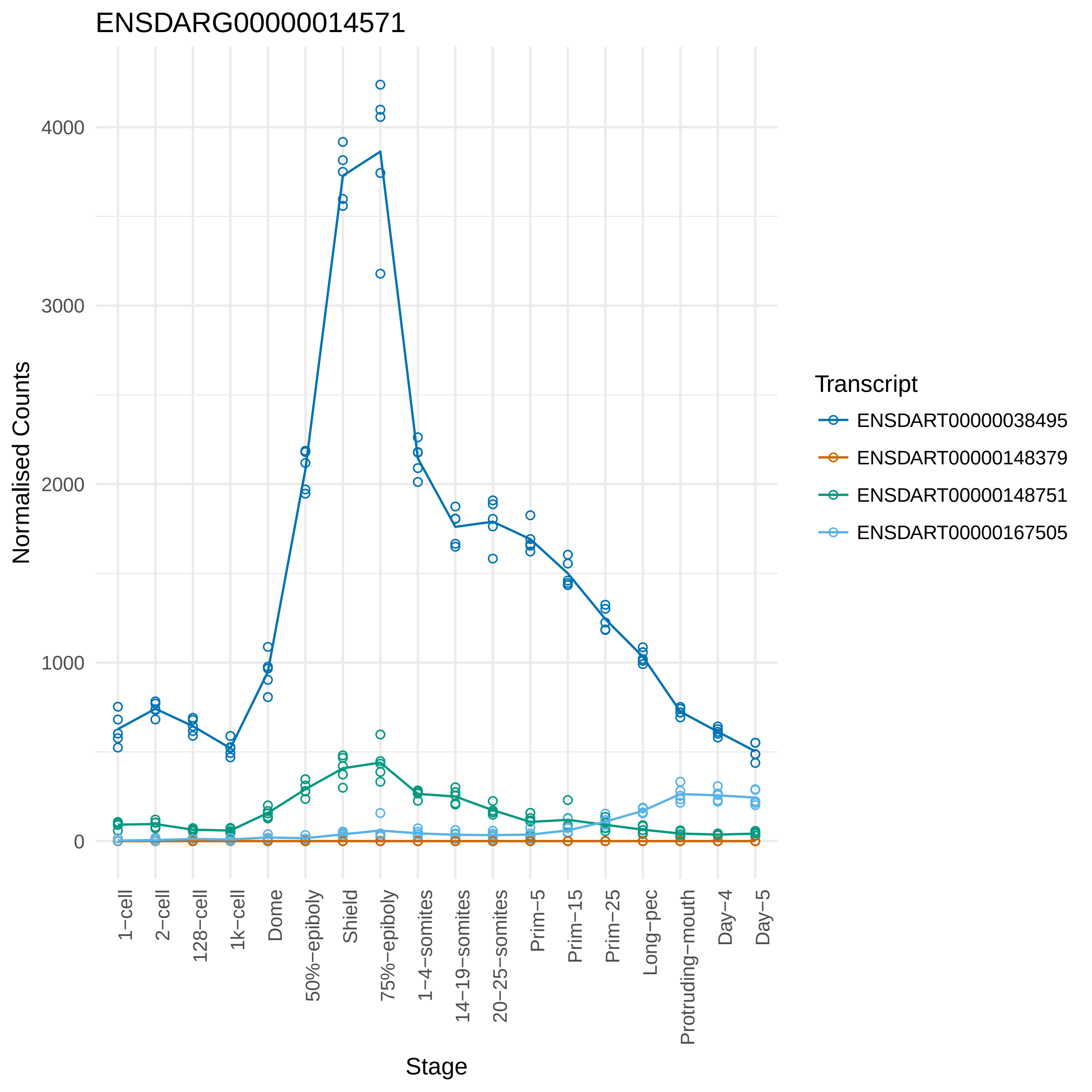

ENSDARG00000014571
Normalised Counts
4000
3000
2000
1000
0
1−cell
2−cell
128−cell
1k−cell
Dome
50%−epiboly
Shield
75%−epiboly
1−4−somites
14−19−somites
20−25−somites
Prim−5
Prim−15
Prim−25
Long−pec
Protruding−mouth
Day−4
Day−5
Stage
Transcript
ENSDART00000038495
ENSDART00000148379
ENSDART00000148751
ENSDART00000167505

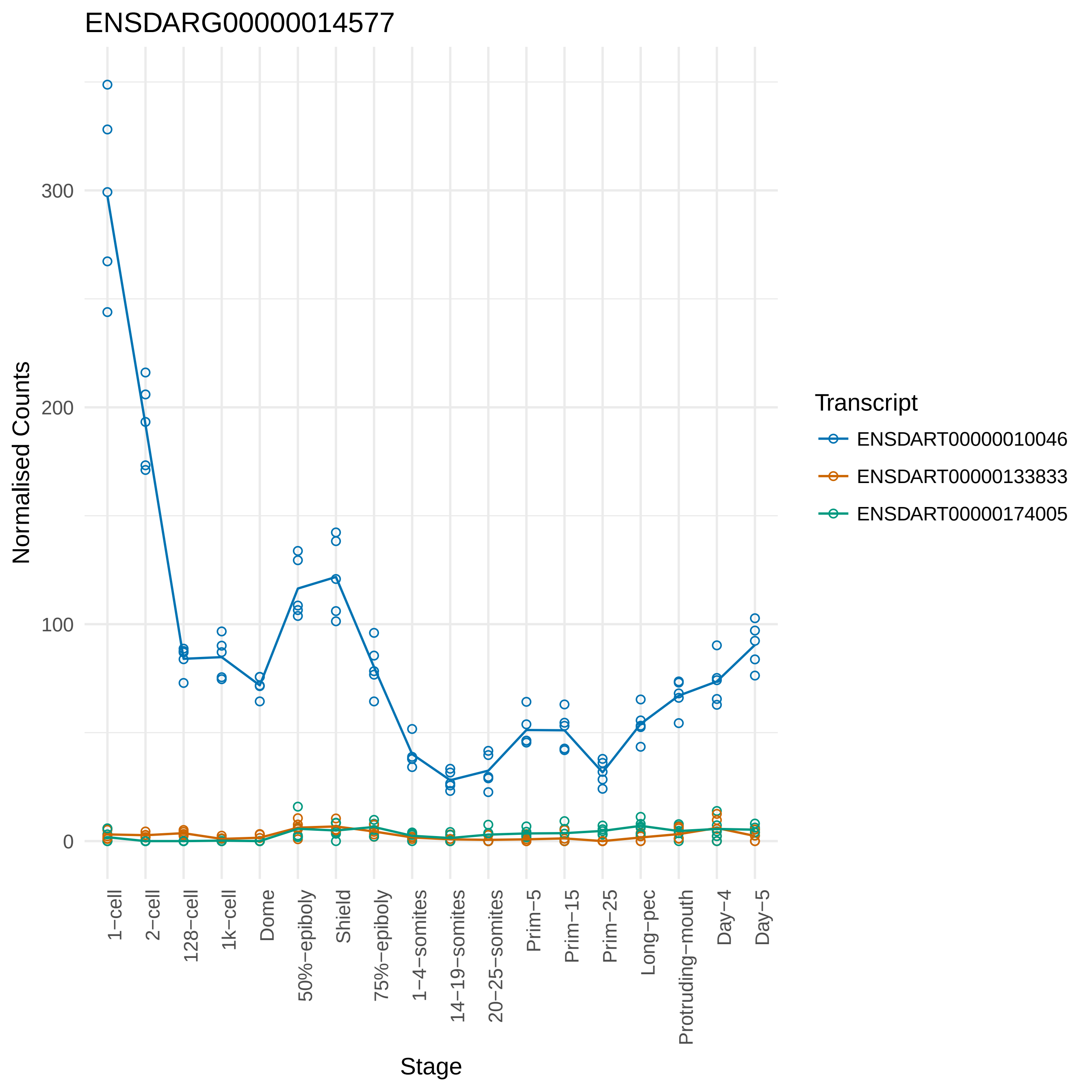
ENSDARG00000014577
Normalised Counts
300
200
100
0
Stage
1−cell
2−cell
128−cell
1k−cell
Dome
50%−epiboly
Shield
75%−epiboly
1−4−somites
14−19−somites
20−25−somites
Prim−5
Prim−15
Prim−25
Long−pec
Protruding−mouth
Day−4
Day−5
Transcript
ENSDART00000010046
ENSDART00000133833
ENSDART00000174005

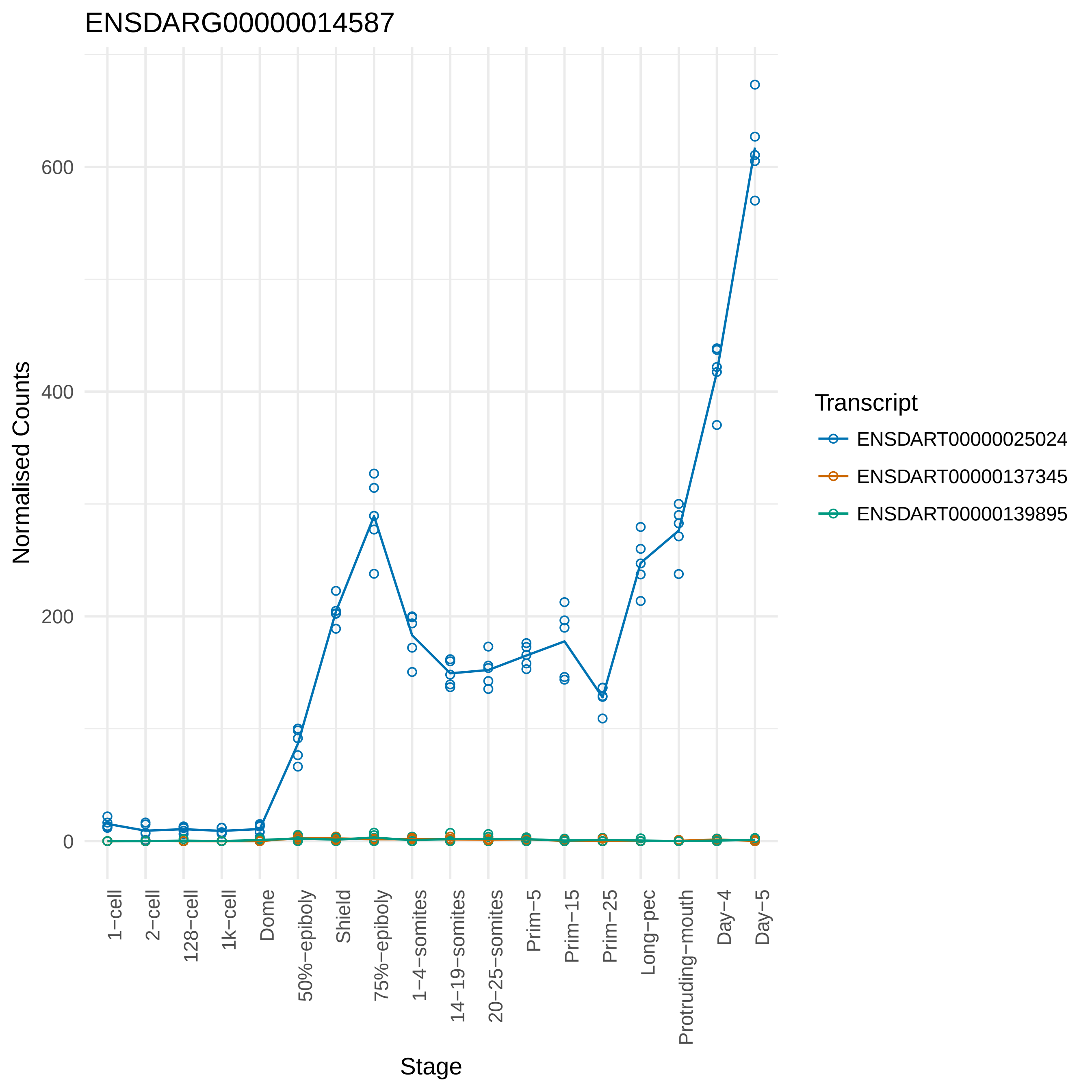

ENSDARG00000014587
Normalised Counts
0
200
400
600
Stage
1−cell
2−cell
128−cell
1k−cell
Dome
50%−epiboly
Shield
75%−epiboly
1−4−somites
14−19−somites
20−25−somites
Prim−5
Prim−15
Prim−25
Long−pec
Protruding−mouth
Day−4
Day−5
Transcript
ENSDART00000025024
ENSDART00000137345
ENSDART00000139895

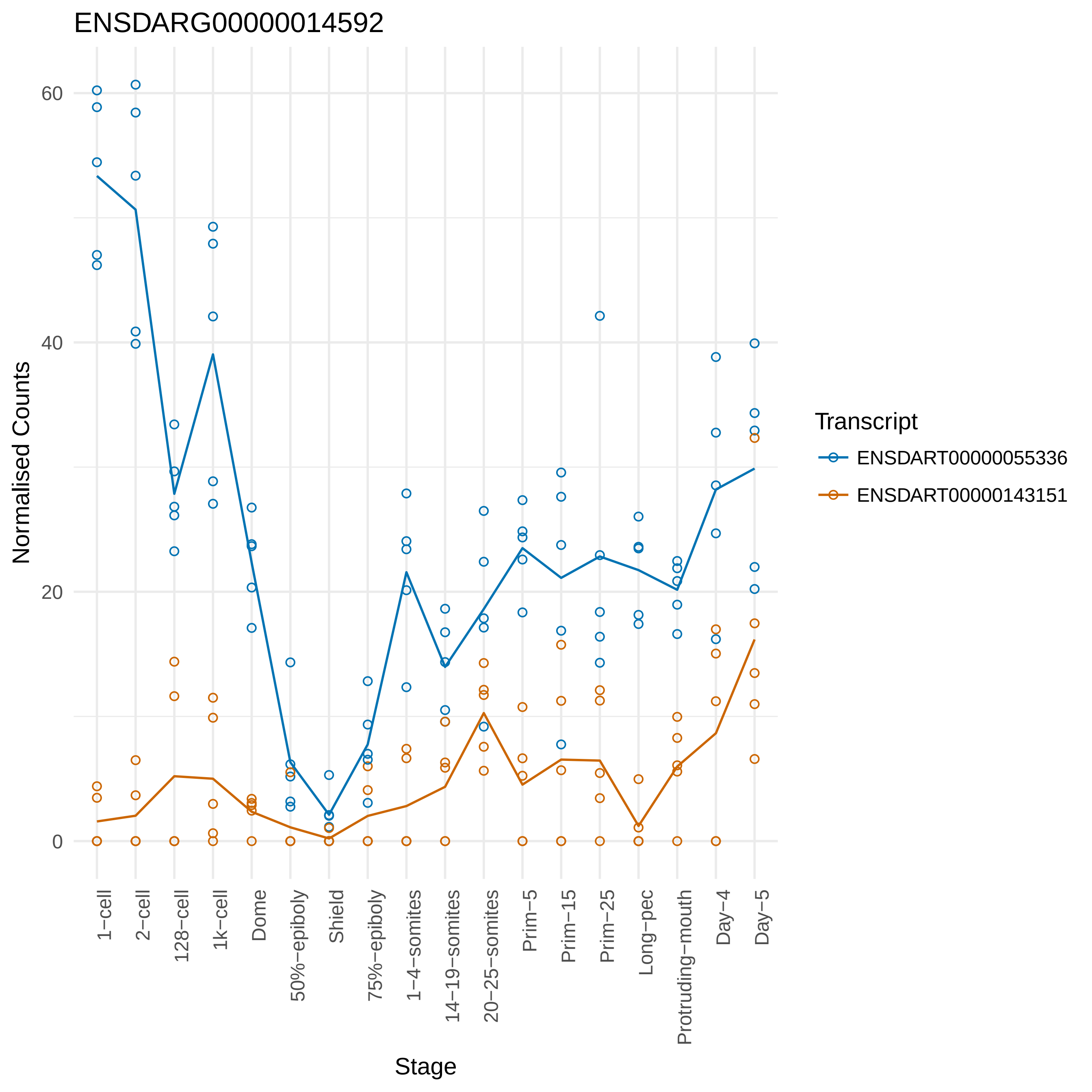
ENSDARG00000014592
Normalised Counts
60
40
20
0
1−cell
2−cell
128−cell
1k−cell
Dome
50%−epiboly
Shield
75%−epiboly
1−4−somites
14−19−somites
20−25−somites
Prim−5
Prim−15
Prim−25
Long−pec
Protruding−mouth
Day−4
Day−5
Stage
Transcript
ENSDART00000055336
ENSDART00000143151

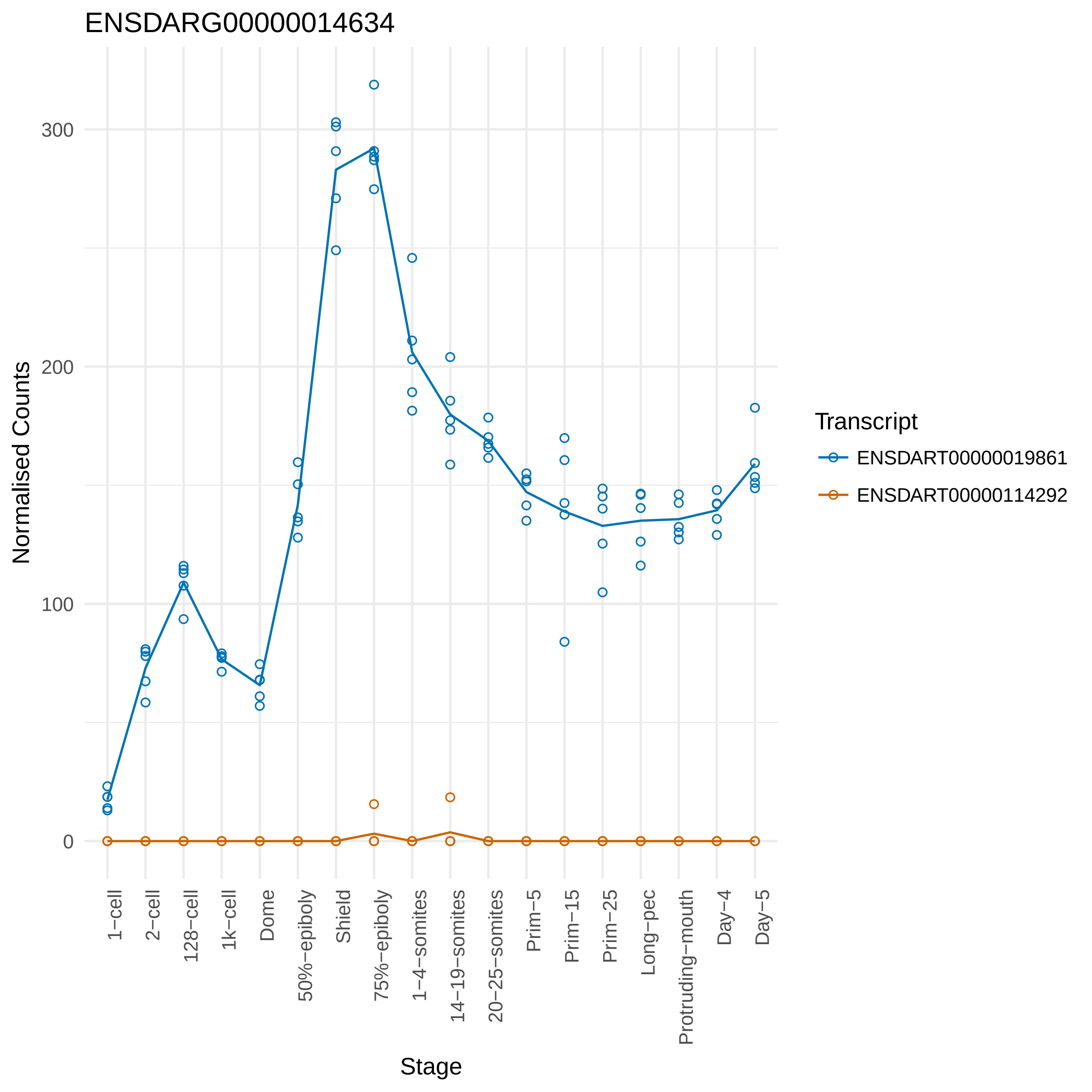

ENSDARG00000014634
Normalised Counts
300
200
100
0
1−cell
2−cell
128−cell
1k−cell
Dome
50%−epiboly
Shield
75%−epiboly
1−4−somites
14−19−somites
20−25−somites
Prim−5
Prim−15
Prim−25
Long−pec
Protruding−mouth
Day−4
Day−5
Stage
Transcript
ENSDART00000019861
ENSDART00000114292

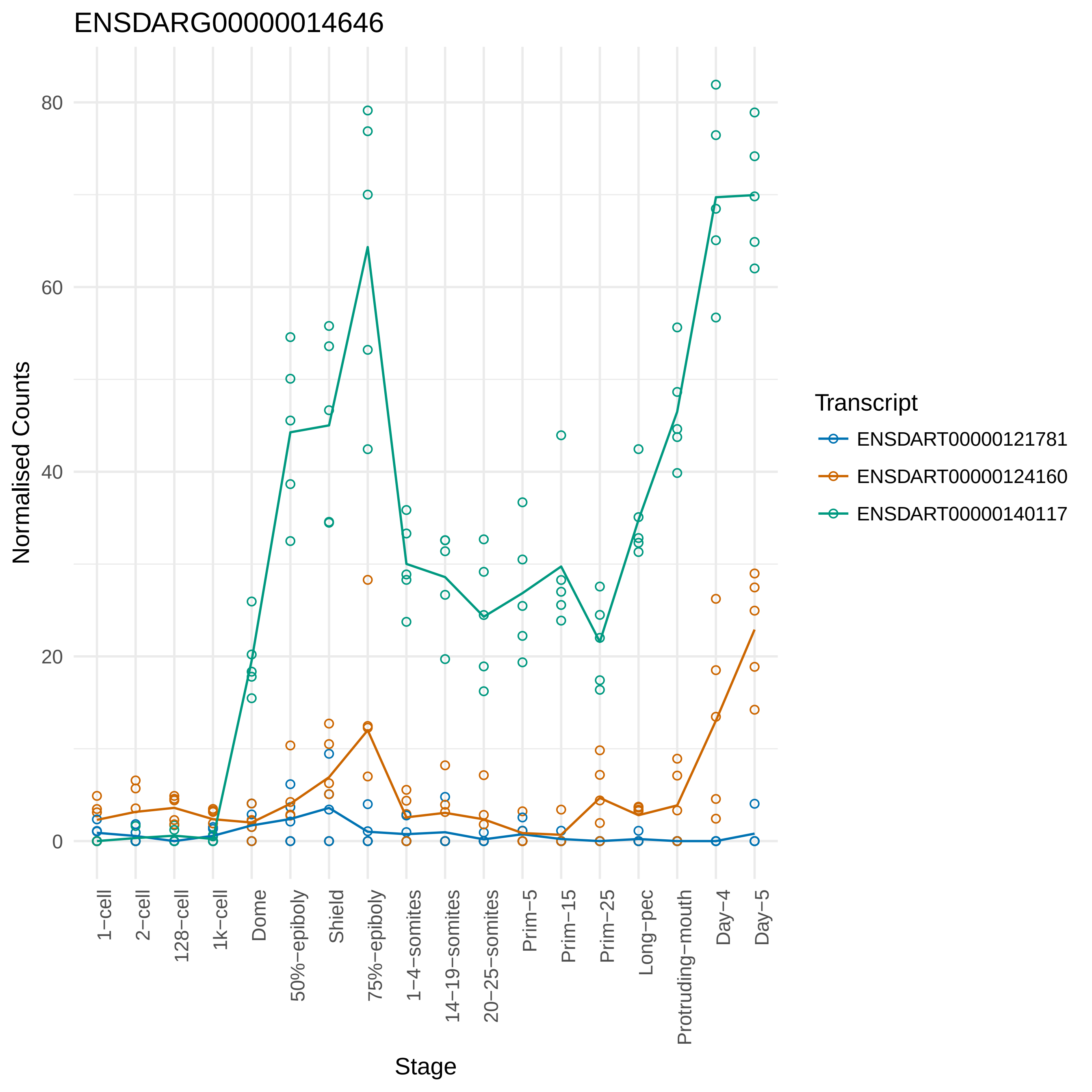
ENSDARG00000014646
Normalised Counts
0
20
40
60
80
Stage
1−cell
2−cell
128−cell
1k−cell
Dome
50%−epiboly
Shield
75%−epiboly
1−4−somites
14−19−somites
20−25−somites
Prim−5
Prim−15
Prim−25
Long−pec
Protruding−mouth
Day−4
Day−5
Transcript
ENSDART00000121781
ENSDART00000124160
ENSDART00000140117

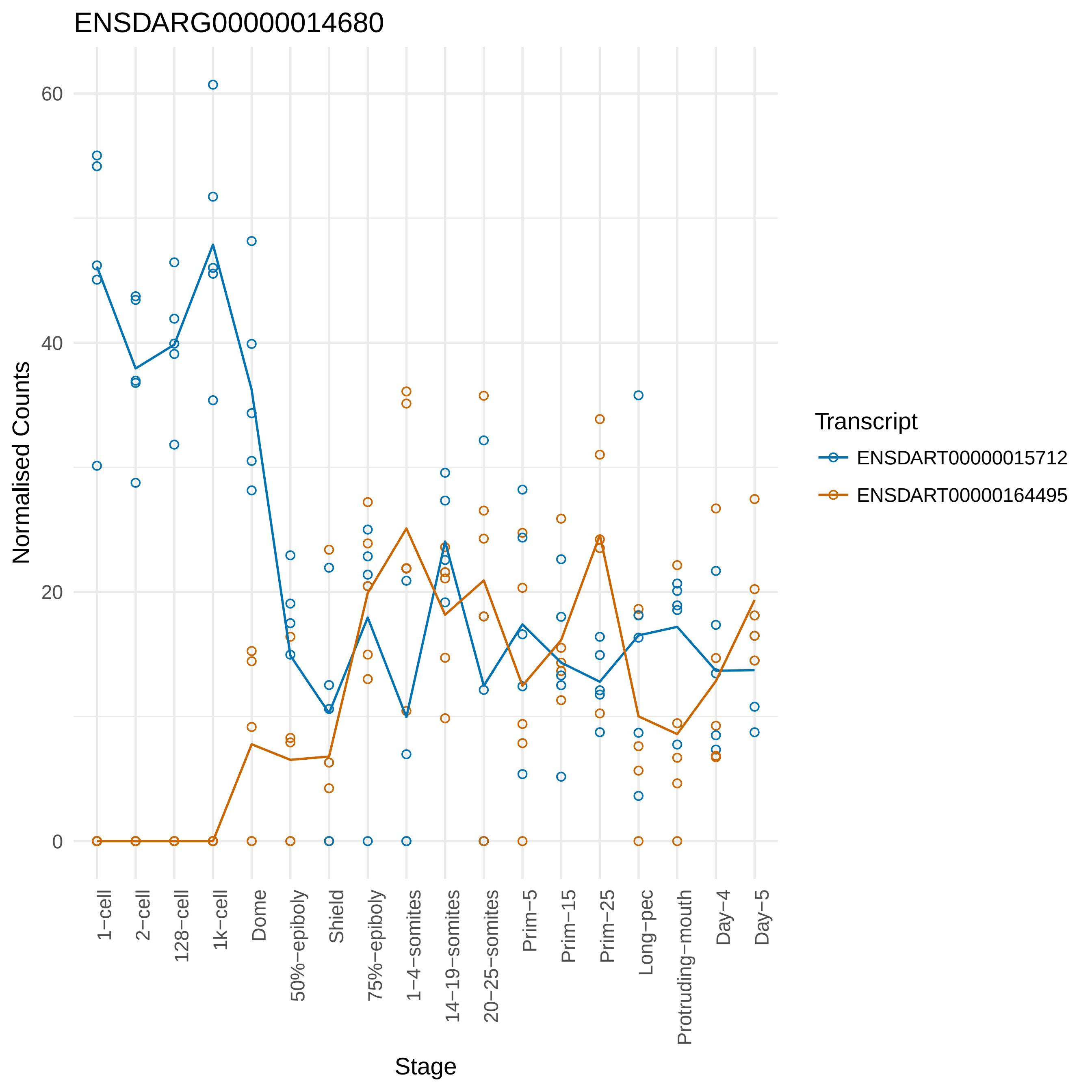

ENSDARG00000014680
Normalised Counts
0
20
40
60
1−cell
2−cell
128−cell
1k−cell
Dome
50%−epiboly
Shield
75%−epiboly
1−4−somites
14−19−somites
20−25−somites
Prim−5
Prim−15
Prim−25
Long−pec
Protruding−mouth
Day−4
Day−5
Stage
Transcript
ENSDART00000015712
ENSDART00000164495

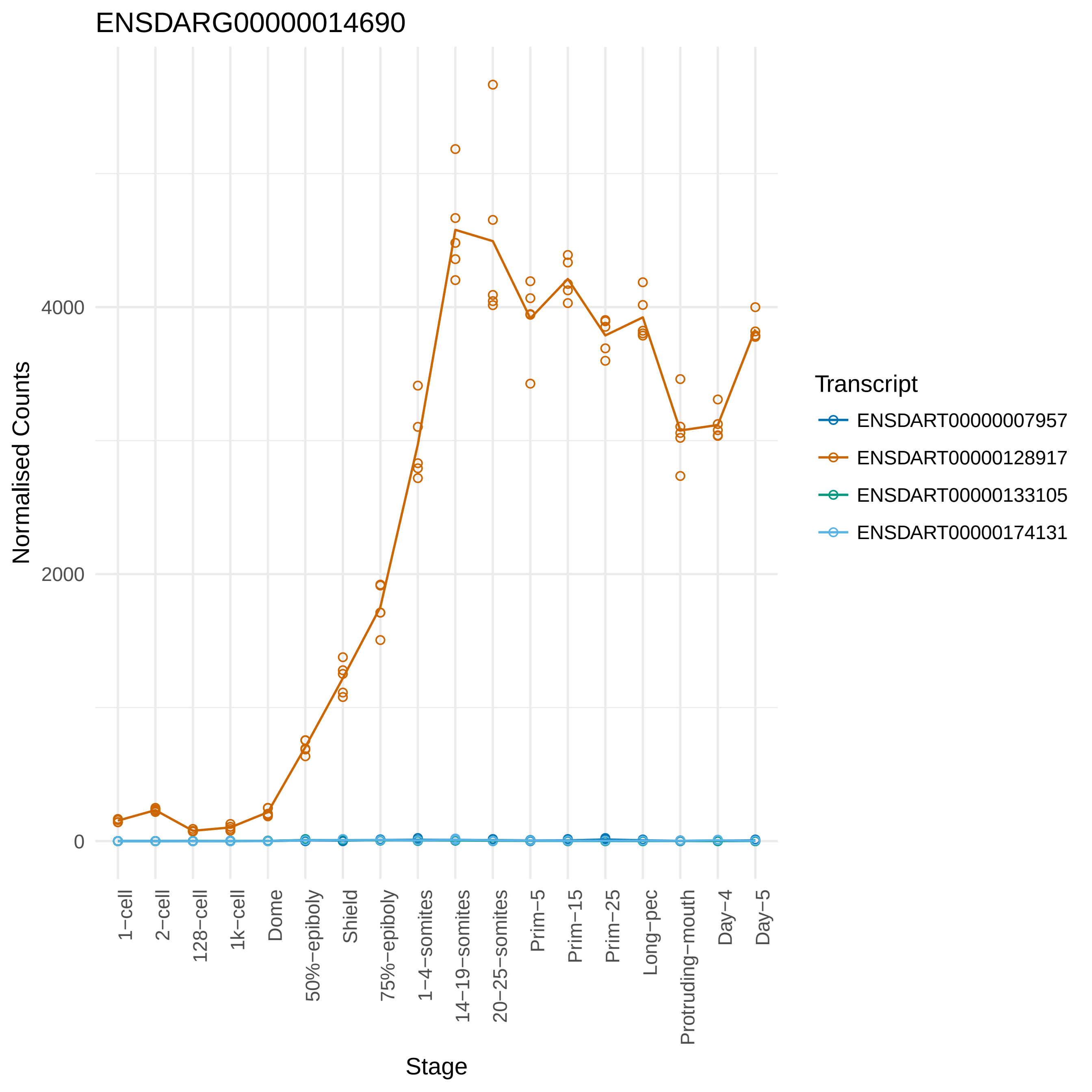

ENSDARG00000014690
Normalised Counts
4000
2000
0
1−cell
2−cell
128−cell
1k−cell
Dome
50%−epiboly
Shield
75%−epiboly
1−4−somites
14−19−somites
20−25−somites
Prim−5
Prim−15
Prim−25
Long−pec
Protruding−mouth
Day−4
Day−5
Stage
Transcript
ENSDART00000007957
ENSDART00000128917
ENSDART00000133105
ENSDART00000174131

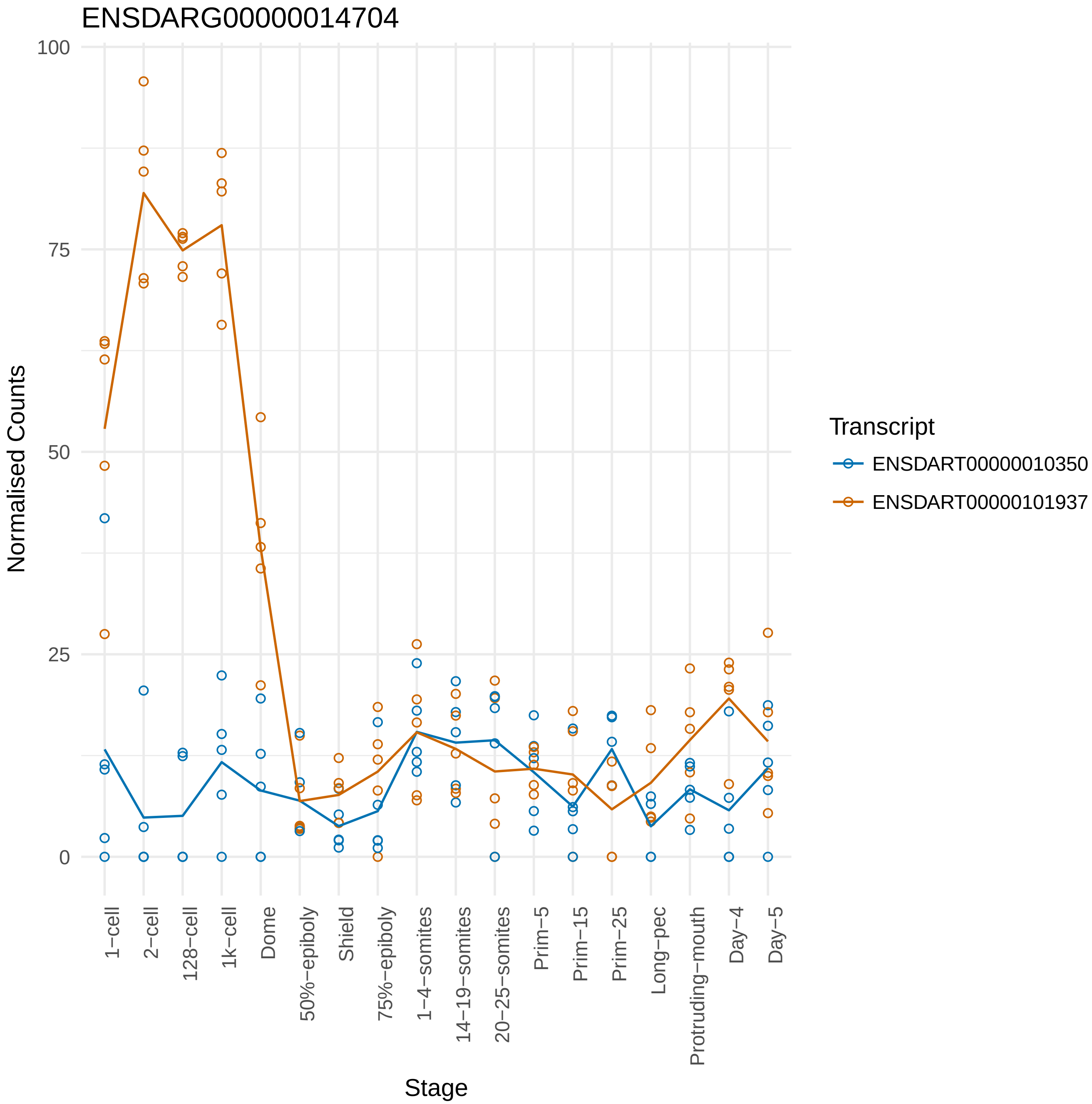

ENSDARG00000014704
Normalised Counts
100
75
50
25
0
1−cell
2−cell
128−cell
1k−cell
Dome
50%−epiboly
Shield
75%−epiboly
1−4−somites
14−19−somites
20−25−somites
Prim−5
Prim−15
Prim−25
Long−pec
Protruding−mouth
Day−4
Day−5
Stage
Transcript
ENSDART00000010350
ENSDART00000101937

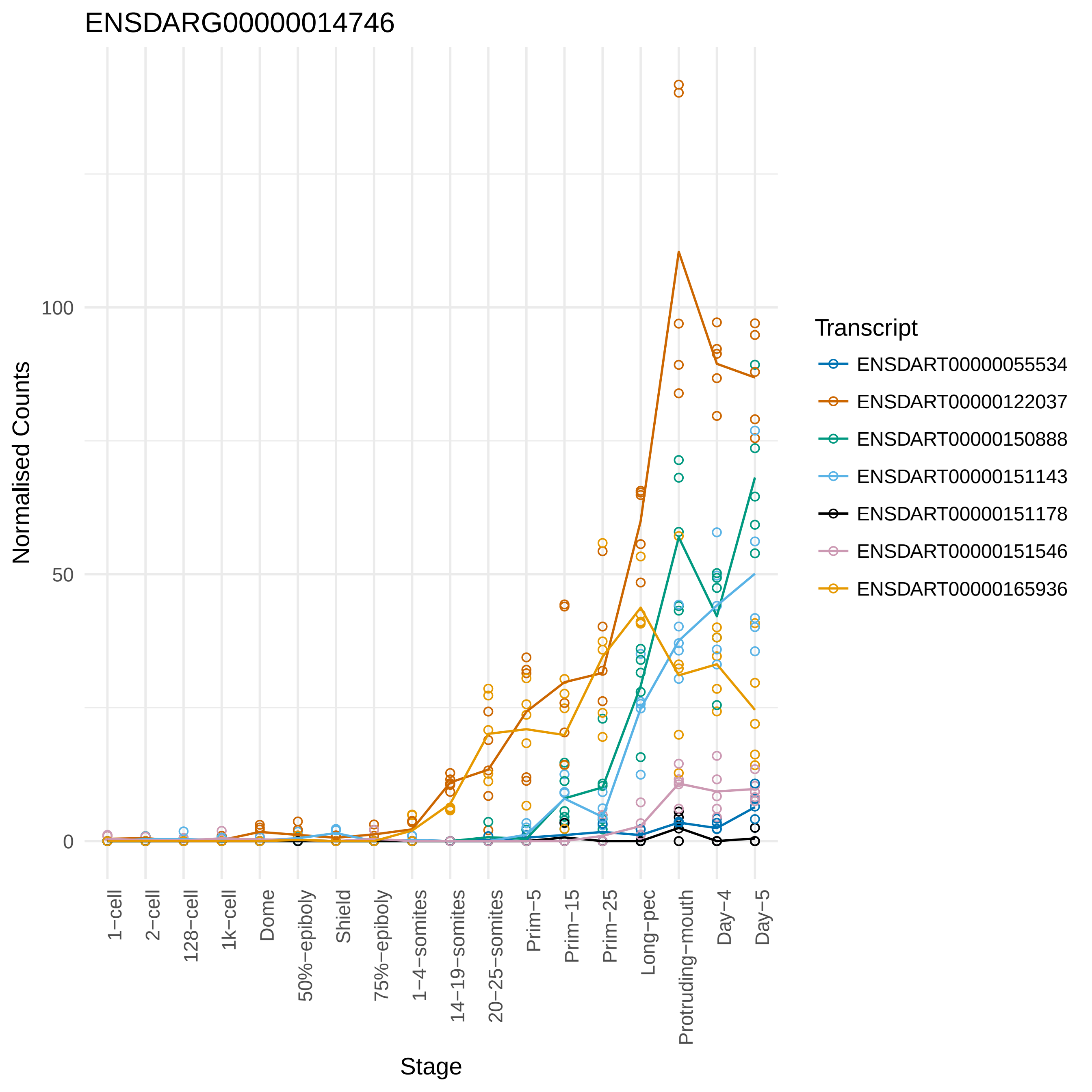
ENSDARG00000014746
Normalised Counts
100
50
0
1−cell
2−cell
128−cell
1k−cell
Dome
50%−epiboly
Shield
75%−epiboly
1−4−somites
14−19−somites
20−25−somites
Prim−5
Prim−15
Prim−25
Long−pec
Protruding−mouth
Day−4
Day−5
Stage
Transcript
ENSDART00000055534
ENSDART00000122037
ENSDART00000150888
ENSDART00000151143
ENSDART00000151178
ENSDART00000151546
ENSDART00000165936

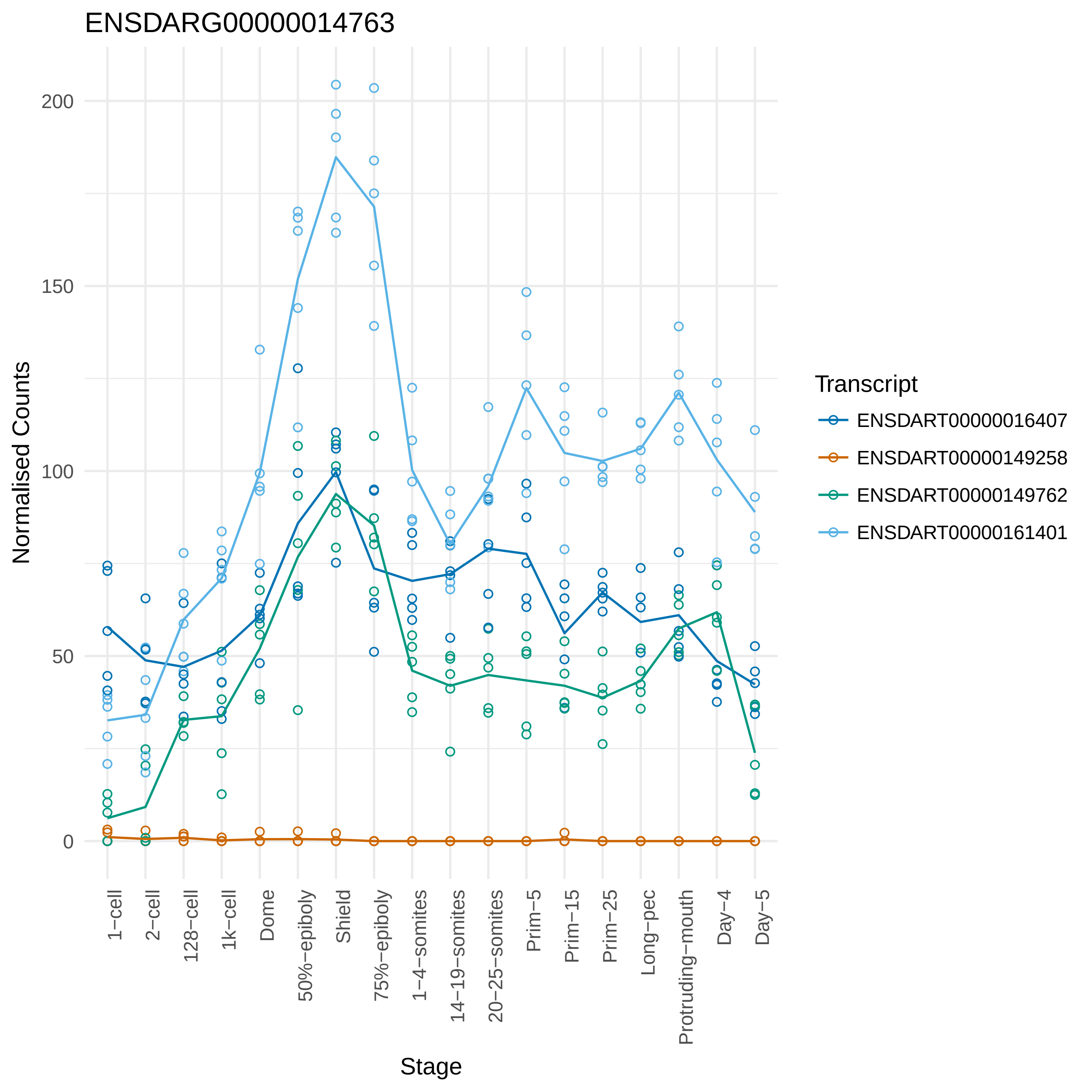

ENSDARG00000014763
Normalised Counts
200
150
100
50
0
1−cell
2−cell
128−cell
1k−cell
Dome
50%−epiboly
Shield
75%−epiboly
1−4−somites
14−19−somites
20−25−somites
Prim−5
Prim−15
Prim−25
Long−pec
Protruding−mouth
Day−4
Day−5
Stage
Transcript
ENSDART00000016407
ENSDART00000149258
ENSDART00000149762
ENSDART00000161401

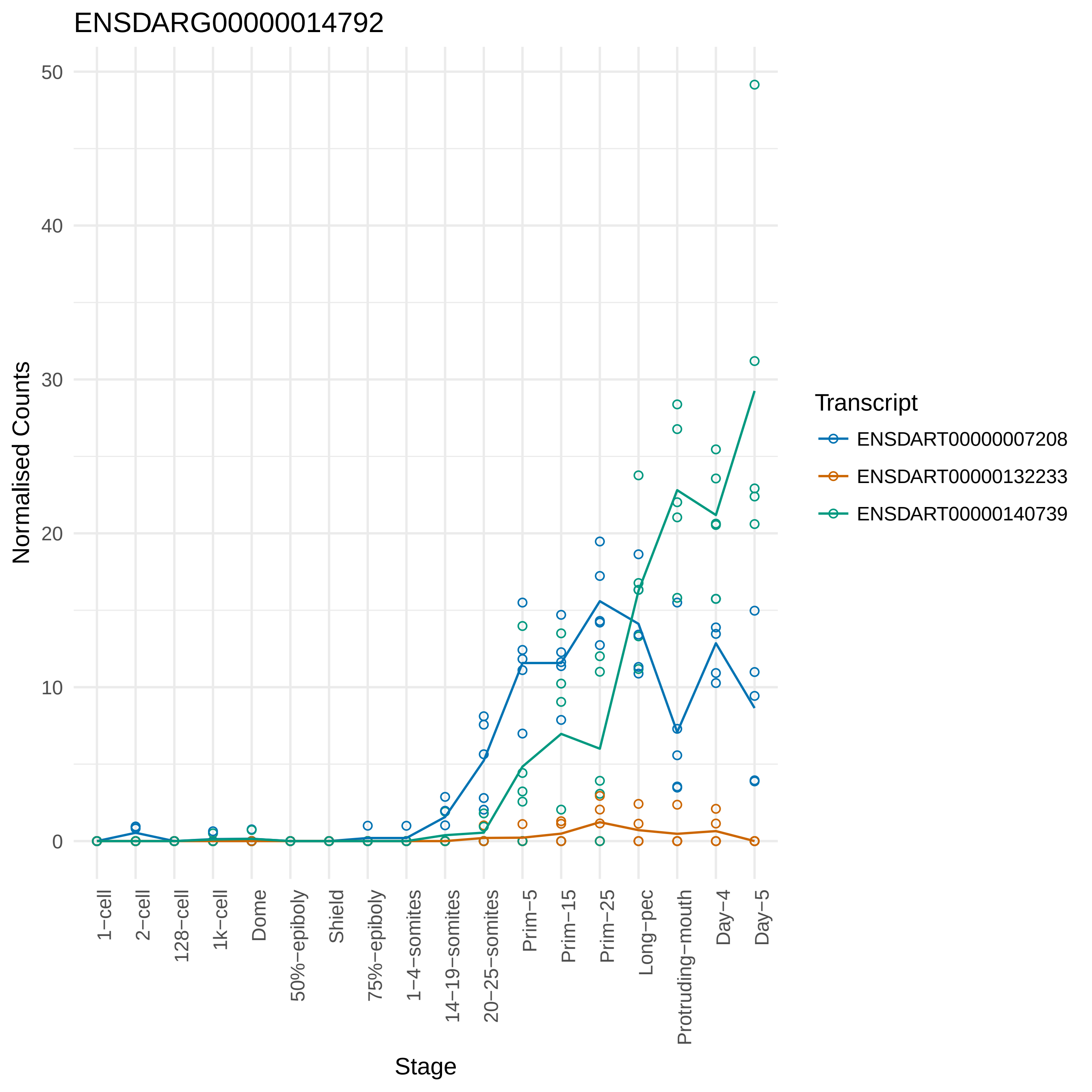
ENSDARG00000014792
Normalised Counts
0
10
20
30
40
50
1−cell
2−cell
128−cell
1k−cell
Dome
50%−epiboly
Shield
75%−epiboly
1−4−somites
14−19−somites
20−25−somites
Prim−5
Prim−15
Prim−25
Long−pec
Protruding−mouth
Day−4
Day−5
Stage
Transcript
ENSDART00000007208
ENSDART00000132233
ENSDART00000140739

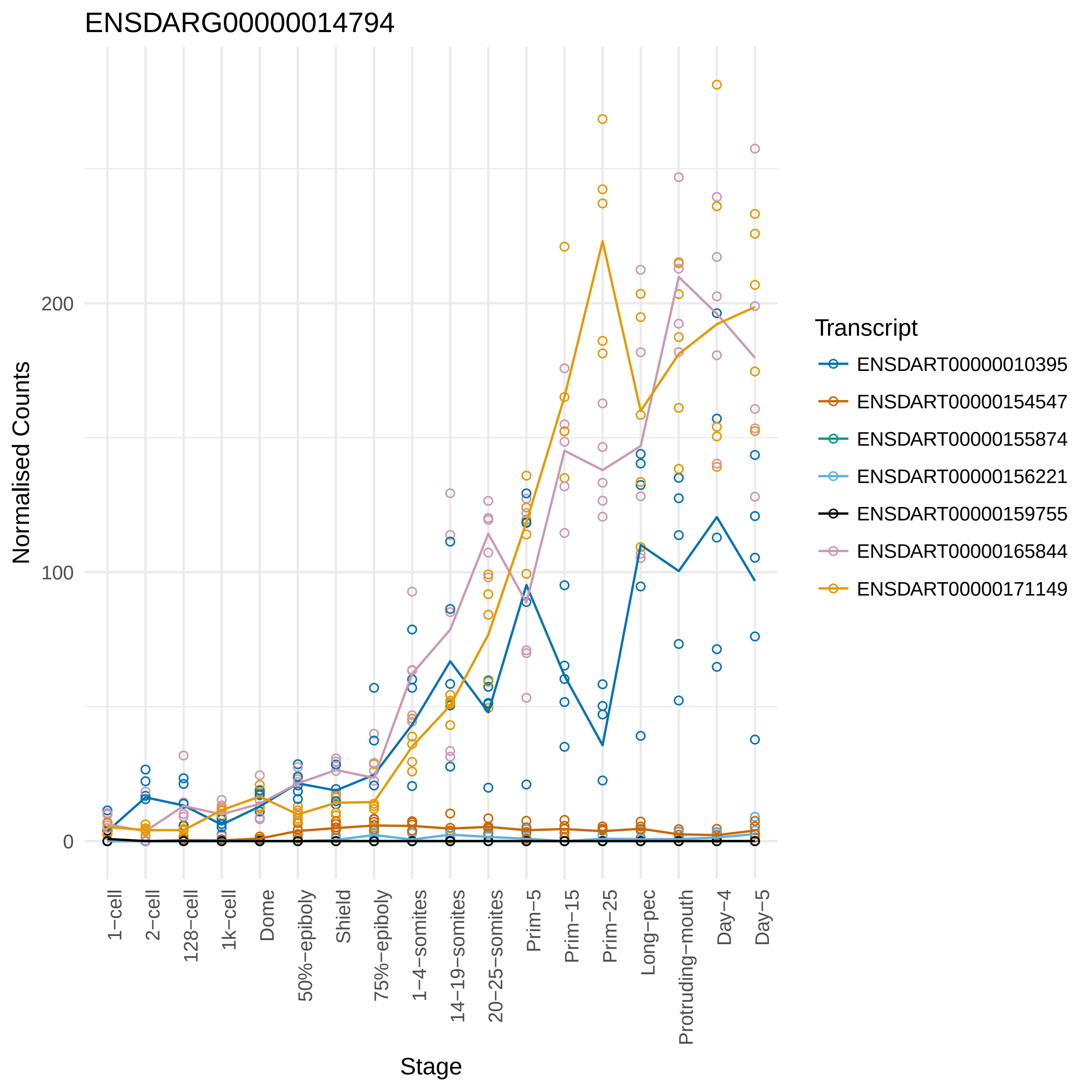

ENSDARG00000014794
Normalised Counts
0
100
200
Stage
1−cell
2−cell
128−cell
1k−cell
Dome
50%−epiboly
Shield
75%−epiboly
1−4−somites
14−19−somites
20−25−somites
Prim−5
Prim−15
Prim−25
Long−pec
Protruding−mouth
Day−4
Day−5
Transcript
ENSDART00000010395
ENSDART00000154547
ENSDART00000155874
ENSDART00000156221
ENSDART00000159755
ENSDART00000165844
ENSDART00000171149

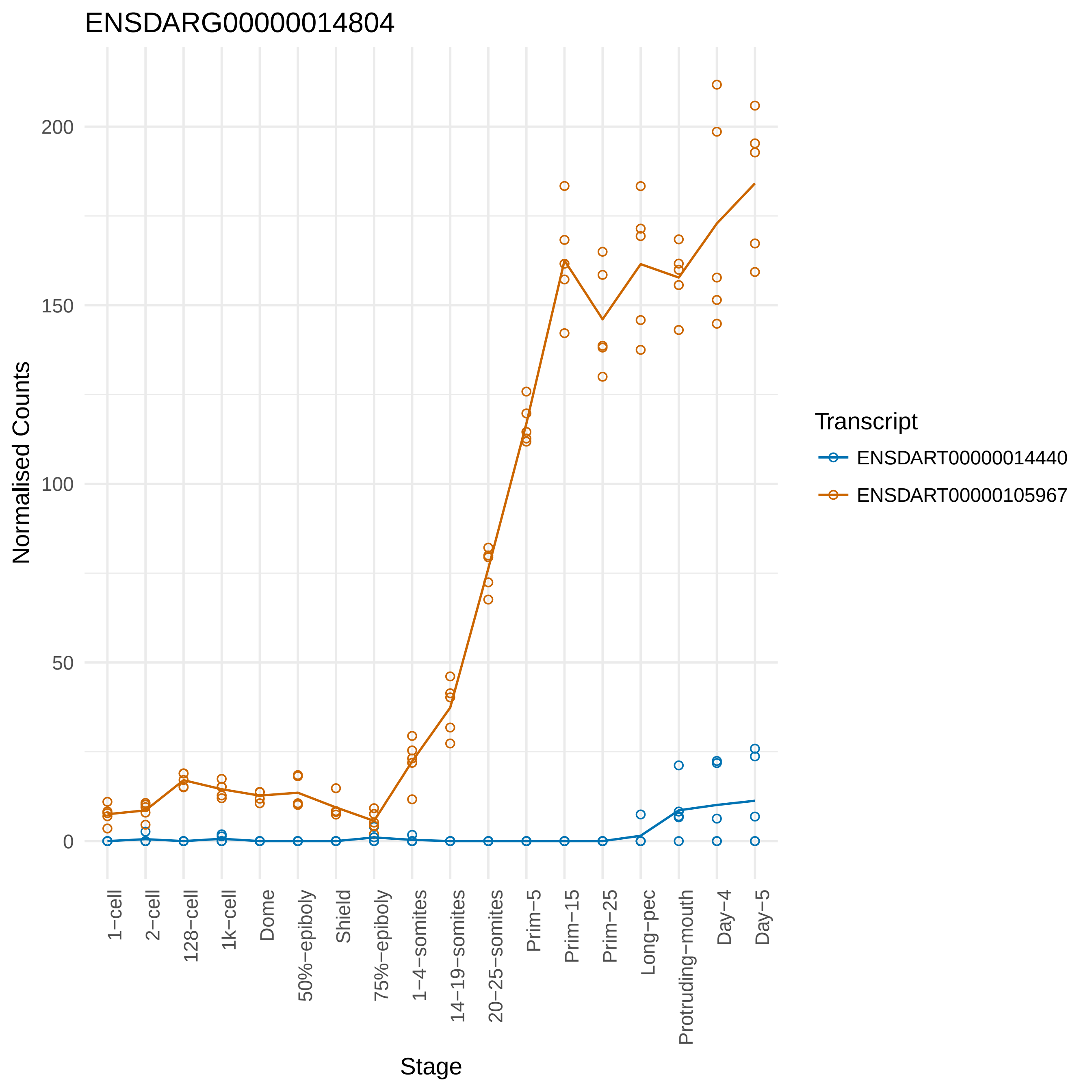

ENSDARG00000014804
Normalised Counts
0
50
100
150
200
1−cell
2−cell
128−cell
1k−cell
Dome
50%−epiboly
Shield
75%−epiboly
1−4−somites
14−19−somites
20−25−somites
Prim−5
Prim−15
Prim−25
Long−pec
Protruding−mouth
Day−4
Day−5
Stage
Transcript
ENSDART00000014440
ENSDART00000105967

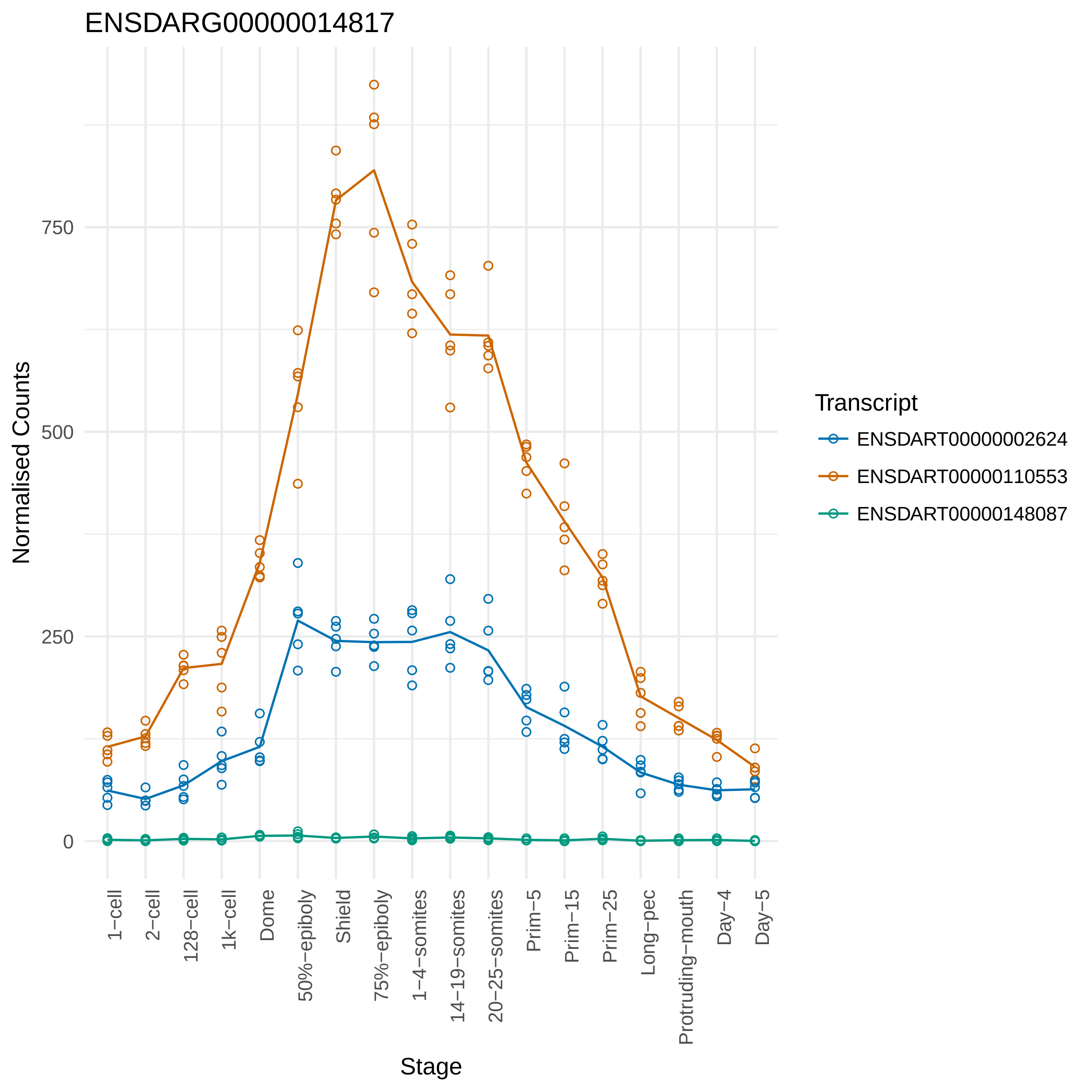

ENSDARG00000014817
Normalised Counts
750
500
250
0
Stage
1−cell
2−cell
128−cell
1k−cell
Dome
50%−epiboly
Shield
75%−epiboly
1−4−somites
14−19−somites
20−25−somites
Prim−5
Prim−15
Prim−25
Long−pec
Protruding−mouth
Day−4
Day−5
Transcript
ENSDART00000002624
ENSDART00000110553
ENSDART00000148087

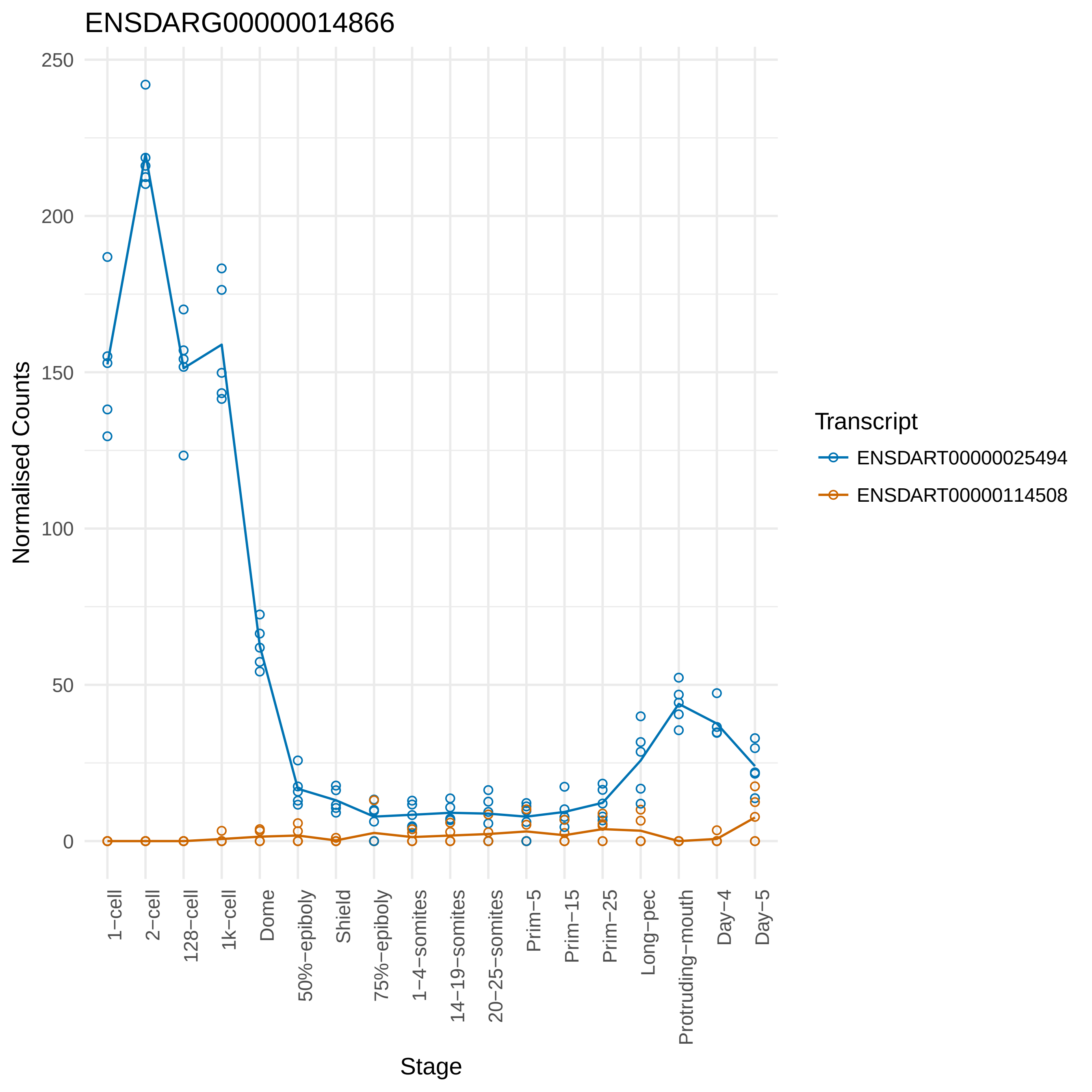

ENSDARG00000014866
Normalised Counts
250
200
150
100
50
0
1−cell
2−cell
128−cell
1k−cell
Dome
50%−epiboly
Shield
75%−epiboly
1−4−somites
14−19−somites
20−25−somites
Prim−5
Prim−15
Prim−25
Long−pec
Protruding−mouth
Day−4
Day−5
Stage
Transcript
ENSDART00000025494
ENSDART00000114508

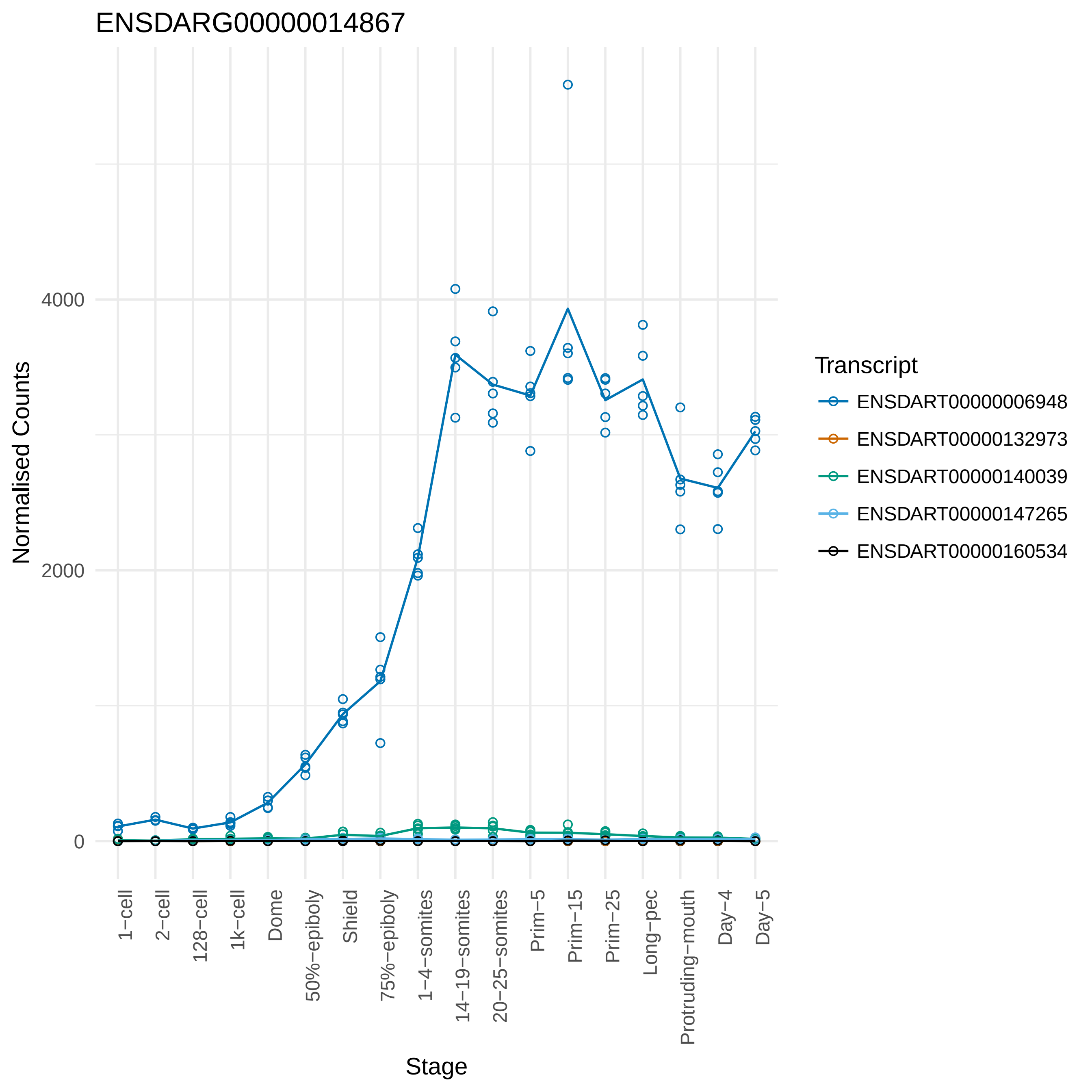
ENSDARG00000014867
Normalised Counts
4000
2000
0
1−cell
2−cell
128−cell
1k−cell
Dome
50%−epiboly
Shield
75%−epiboly
1−4−somites
14−19−somites
20−25−somites
Prim−5
Prim−15
Prim−25
Long−pec
Protruding−mouth
Day−4
Day−5
Stage
Transcript
ENSDART00000006948
ENSDART00000132973
ENSDART00000140039
ENSDART00000147265
ENSDART00000160534

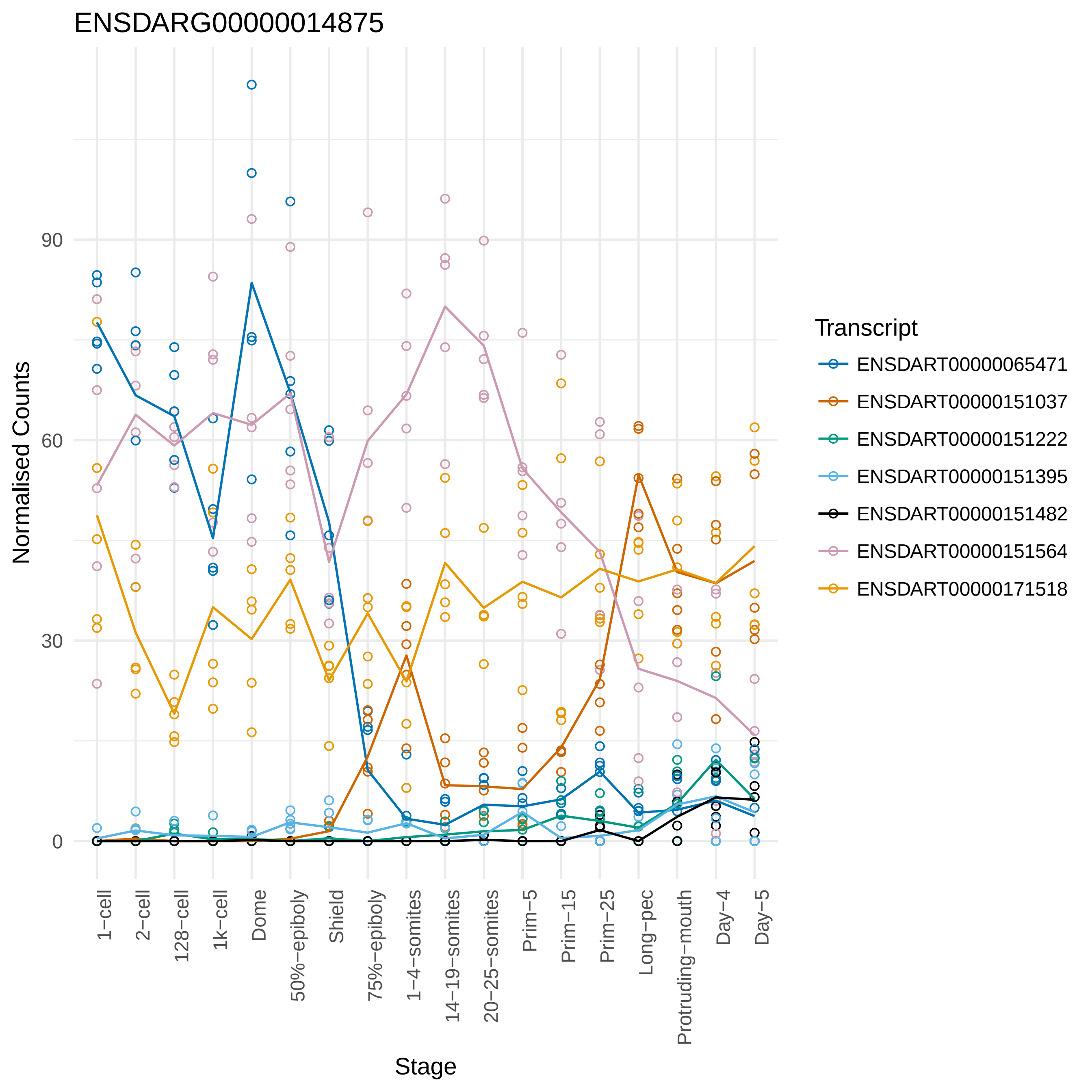

ENSDARG00000014875
Normalised Counts
0
30
60
90
Stage
1−cell
2−cell
128−cell
1k−cell
Dome
50%−epiboly
Shield
75%−epiboly
1−4−somites
14−19−somites
20−25−somites
Prim−5
Prim−15
Prim−25
Long−pec
Protruding−mouth
Day−4
Day−5
Transcript
ENSDART00000065471
ENSDART00000151037
ENSDART00000151222
ENSDART00000151395
ENSDART00000151482
ENSDART00000151564
ENSDART00000171518

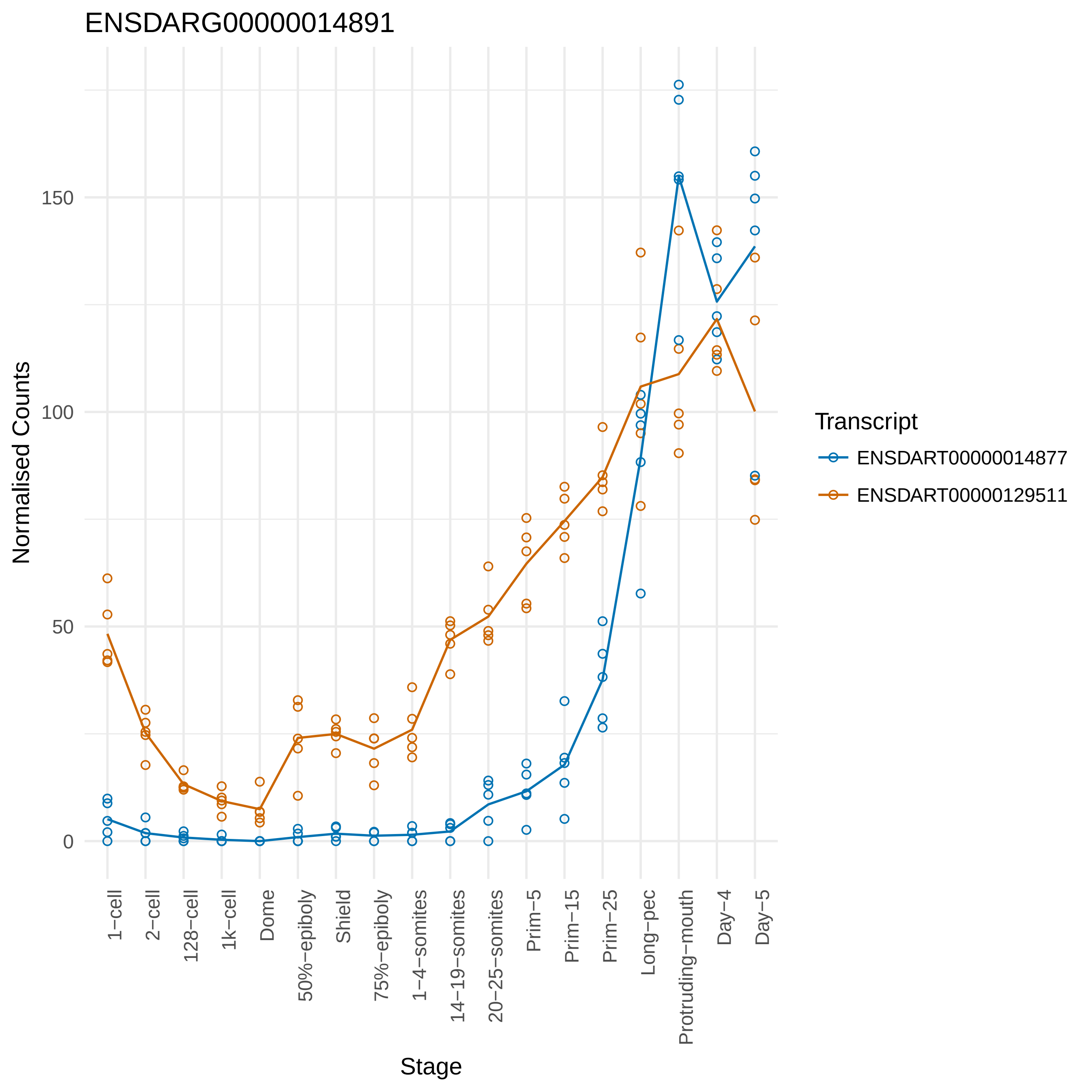

ENSDARG00000014891
Normalised Counts
150
100
50
0
1−cell
2−cell
128−cell
1k−cell
Dome
50%−epiboly
Shield
75%−epiboly
1−4−somites
14−19−somites
20−25−somites
Prim−5
Prim−15
Prim−25
Long−pec
Protruding−mouth
Day−4
Day−5
Stage
Transcript
ENSDART00000014877
ENSDART00000129511

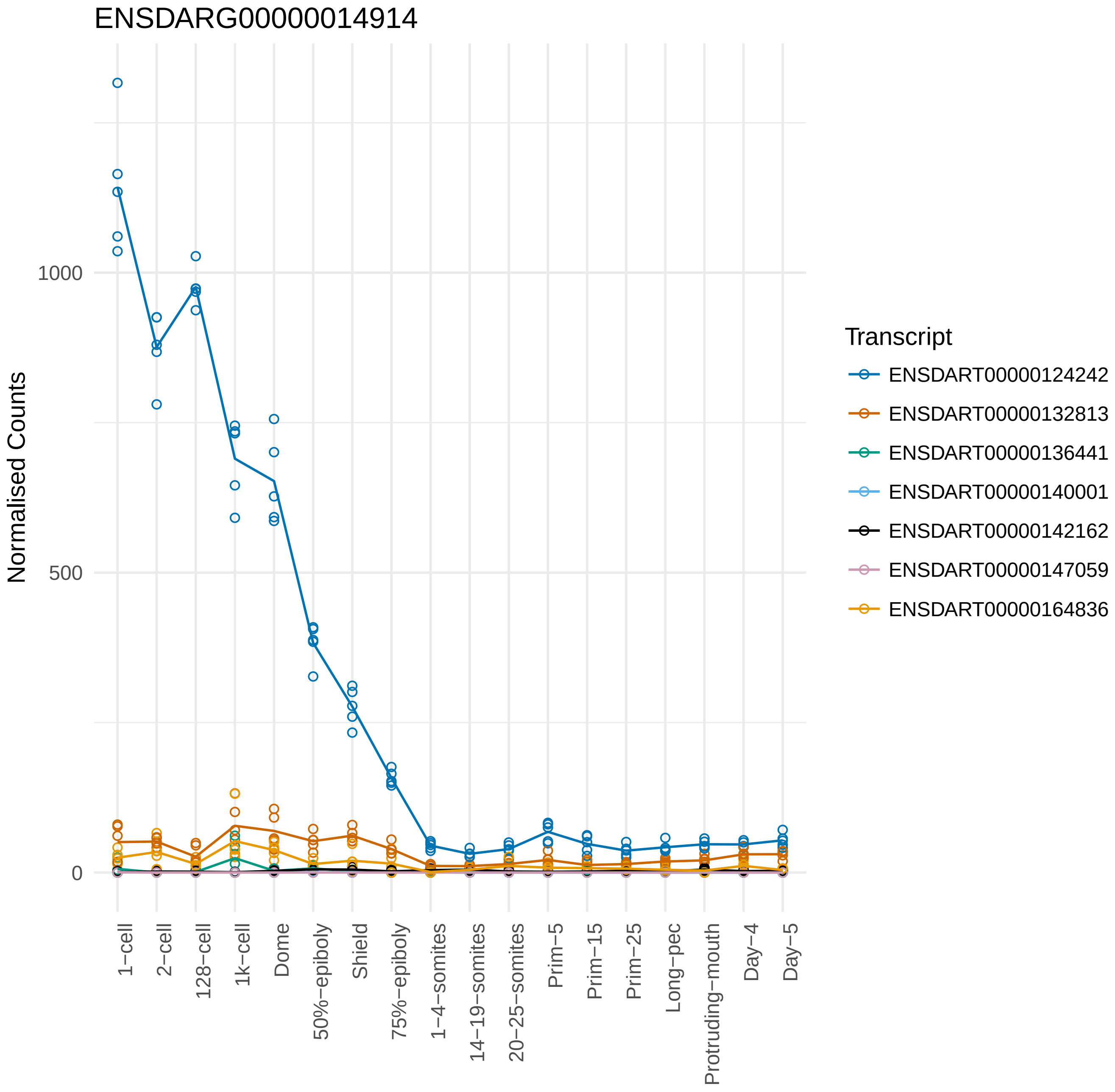

ENSDARG00000014914
Normalised Counts
1000
500
0
Stage
1−cell
2−cell
128−cell
1k−cell
Dome
50%−epiboly
Shield
75%−epiboly
1−4−somites
14−19−somites
20−25−somites
Prim−5
Prim−15
Prim−25
Long−pec
Protruding−mouth
Day−4
Day−5
Transcript
ENSDART00000124242
ENSDART00000132813
ENSDART00000136441
ENSDART00000140001
ENSDART00000142162
ENSDART00000147059
ENSDART00000164836

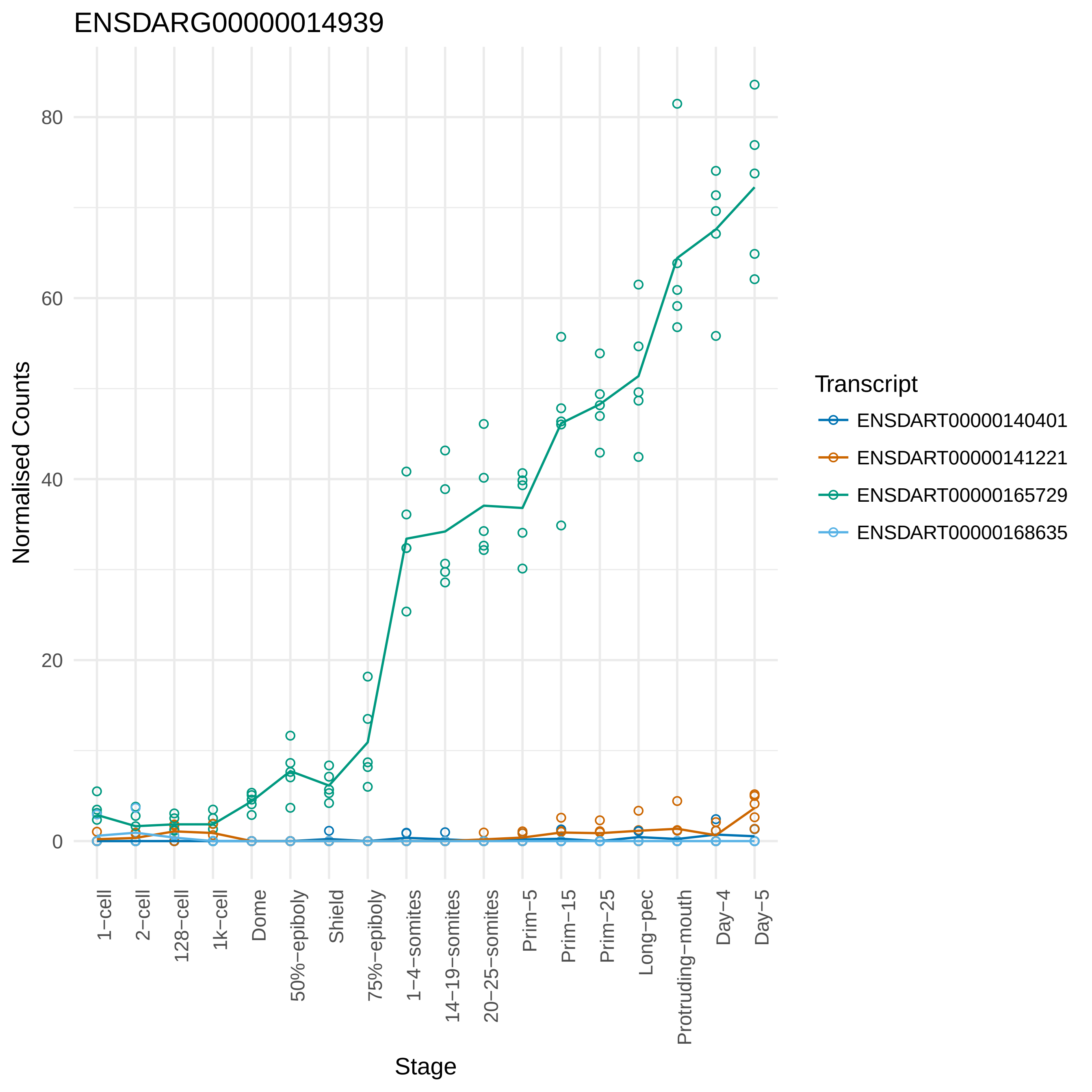
ENSDARG00000014939
Normalised Counts
0
20
40
60
80
1−cell
2−cell
128−cell
1k−cell
Dome
50%−epiboly
Shield
75%−epiboly
1−4−somites
14−19−somites
20−25−somites
Prim−5
Prim−15
Prim−25
Long−pec
Protruding−mouth
Day−4
Day−5
Stage
Transcript
ENSDART00000140401
ENSDART00000141221
ENSDART00000165729
ENSDART00000168635

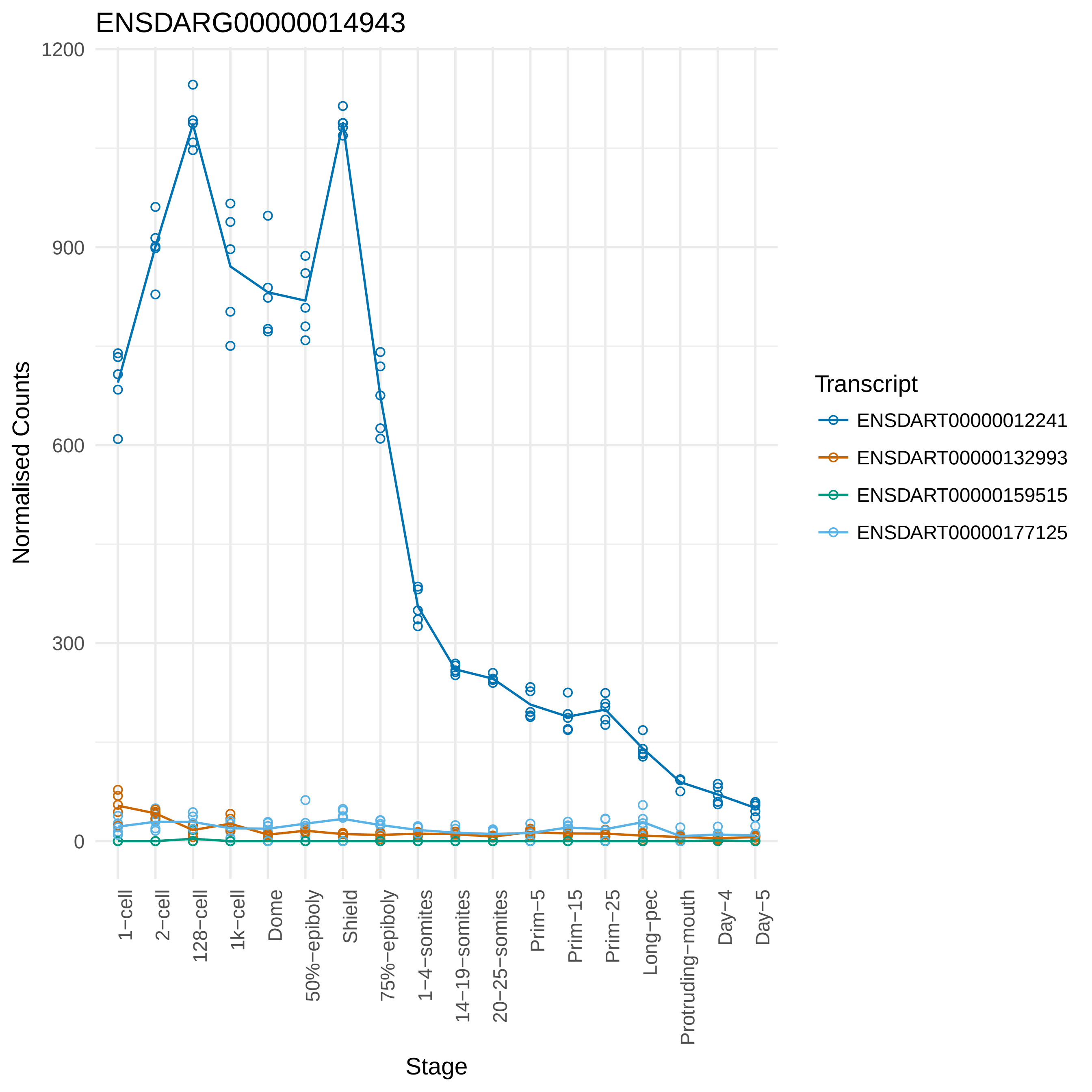
ENSDARG00000014943
1200
900
600
300
0
Normalised Counts
1−cell
2−cell
128−cell
1k−cell
Dome
50%−epiboly
Shield
75%−epiboly
1−4−somites
14−19−somites
20−25−somites
Prim−5
Prim−15
Prim−25
Long−pec
Protruding−mouth
Day−4
Day−5
Stage
Transcript
ENSDART00000012241
ENSDART00000132993
ENSDART00000159515
ENSDART00000177125

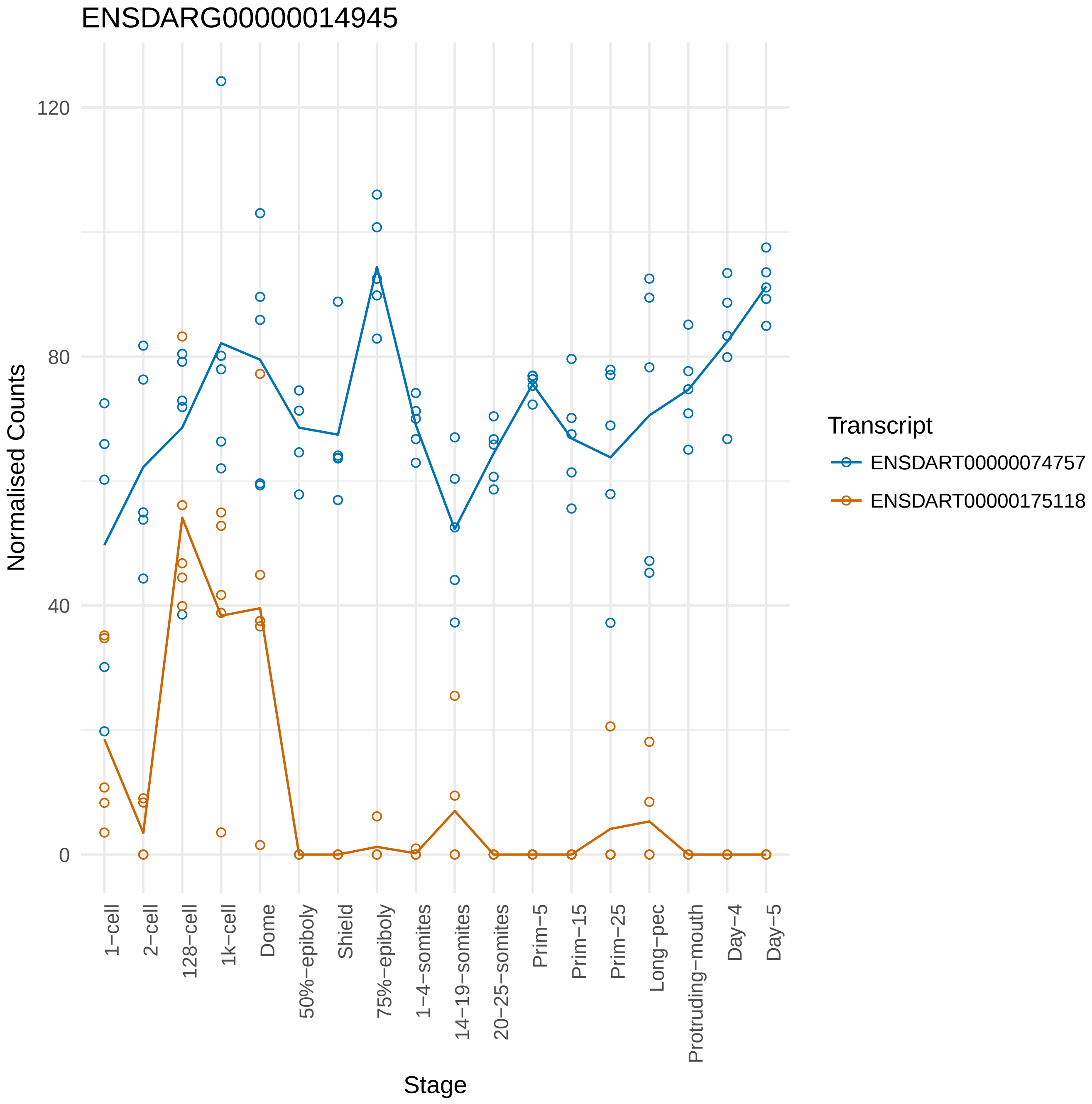

ENSDARG00000014945
Normalised Counts
120
80
40
0
1−cell
2−cell
128−cell
1k−cell
Dome
50%−epiboly
Shield
75%−epiboly
1−4−somites
14−19−somites
20−25−somites
Prim−5
Prim−15
Prim−25
Long−pec
Protruding−mouth
Day−4
Day−5
Stage
Transcript
ENSDART00000074757
ENSDART00000175118

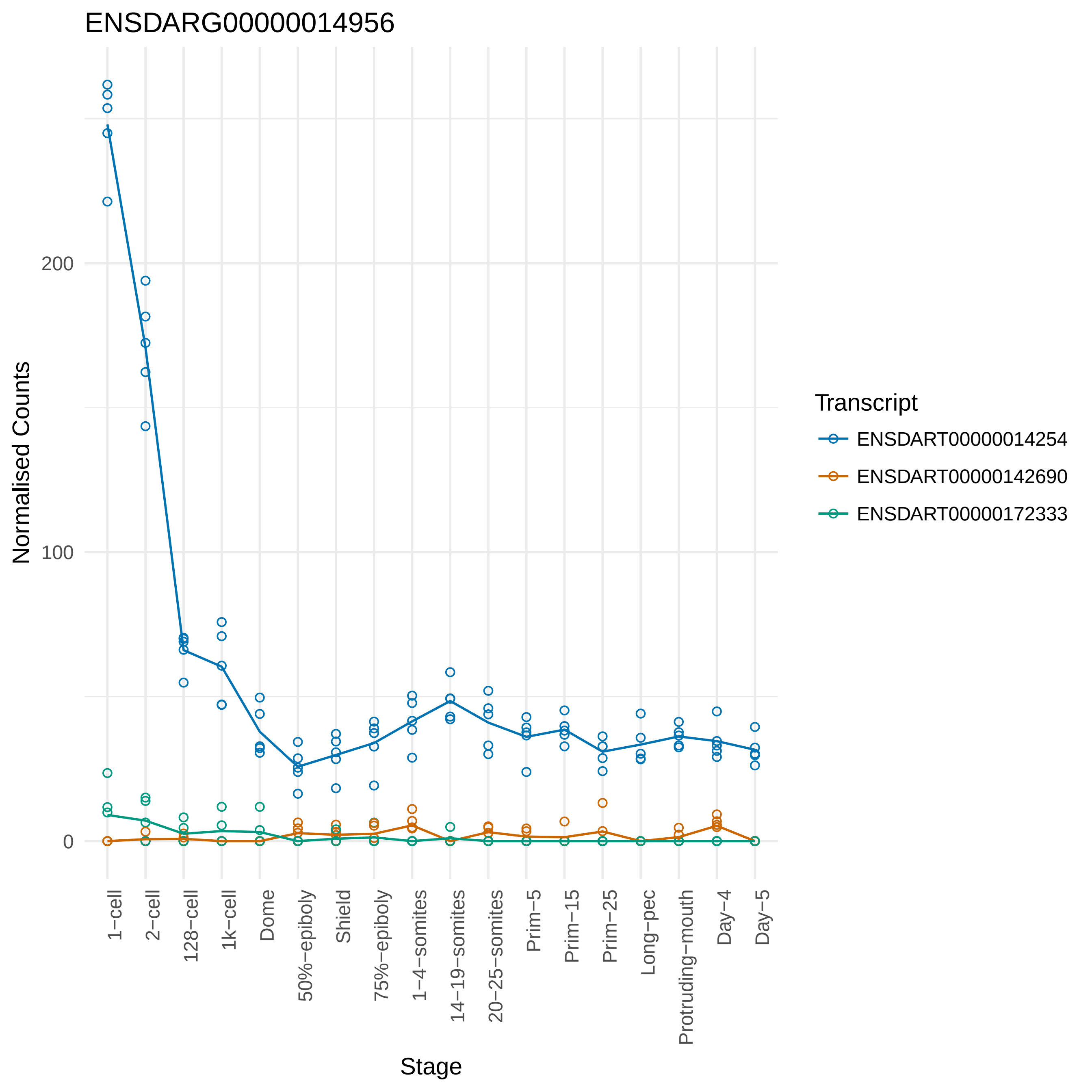
ENSDARG00000014956
Normalised Counts
200
100
0
1−cell
2−cell
128−cell
1k−cell
Dome
50%−epiboly
Shield
75%−epiboly
1−4−somites
14−19−somites
20−25−somites
Prim−5
Prim−15
Prim−25
Long−pec
Protruding−mouth
Day−4
Day−5
Stage
Transcript
ENSDART00000014254
ENSDART00000142690
ENSDART00000172333

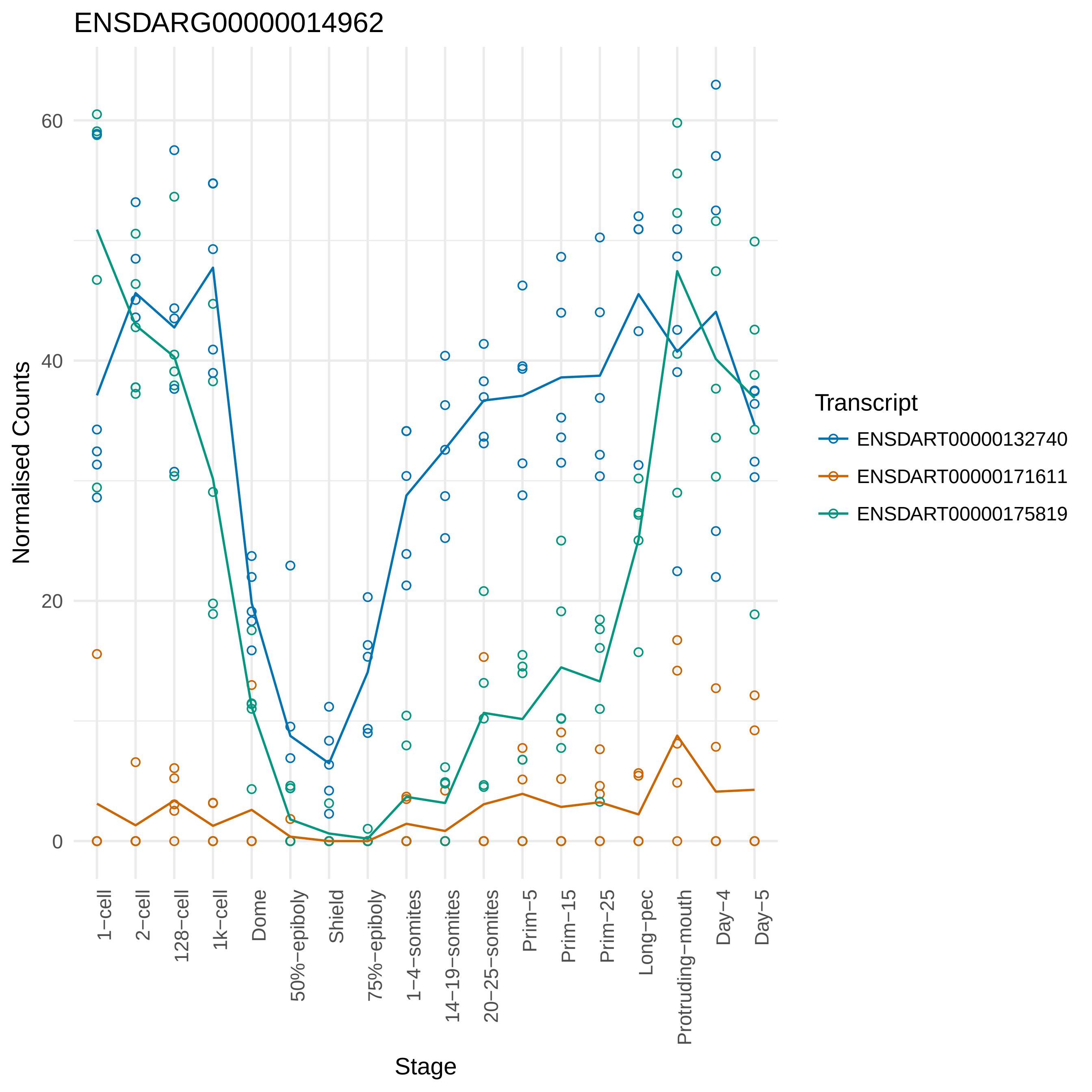
ENSDARG00000014962
Normalised Counts
0
20
40
60
Stage
1−cell
2−cell
128−cell
1k−cell
Dome
50%−epiboly
Shield
75%−epiboly
1−4−somites
14−19−somites
20−25−somites
Prim−5
Prim−15
Prim−25
Long−pec
Protruding−mouth
Day−4
Day−5
Transcript
ENSDART00000132740
ENSDART00000171611
ENSDART00000175819

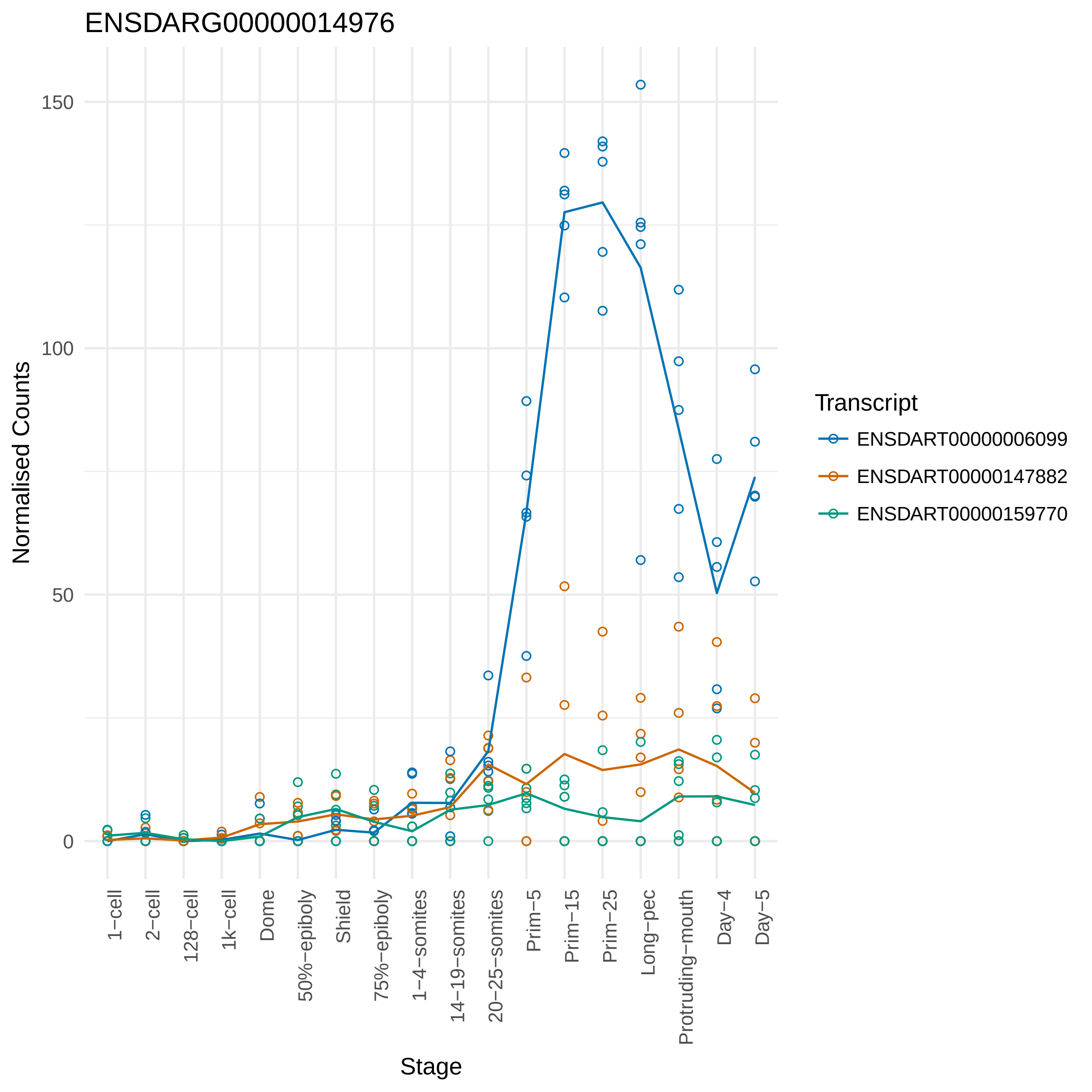
ENSDARG00000014976
Normalised Counts
150
100
50
0
1−cell
2−cell
128−cell
1k−cell
Dome
50%−epiboly
Shield
75%−epiboly
1−4−somites
14−19−somites
20−25−somites
Prim−5
Prim−15
Prim−25
Long−pec
Protruding−mouth
Day−4
Day−5
Stage
Transcript
ENSDART00000006099
ENSDART00000147882
ENSDART00000159770

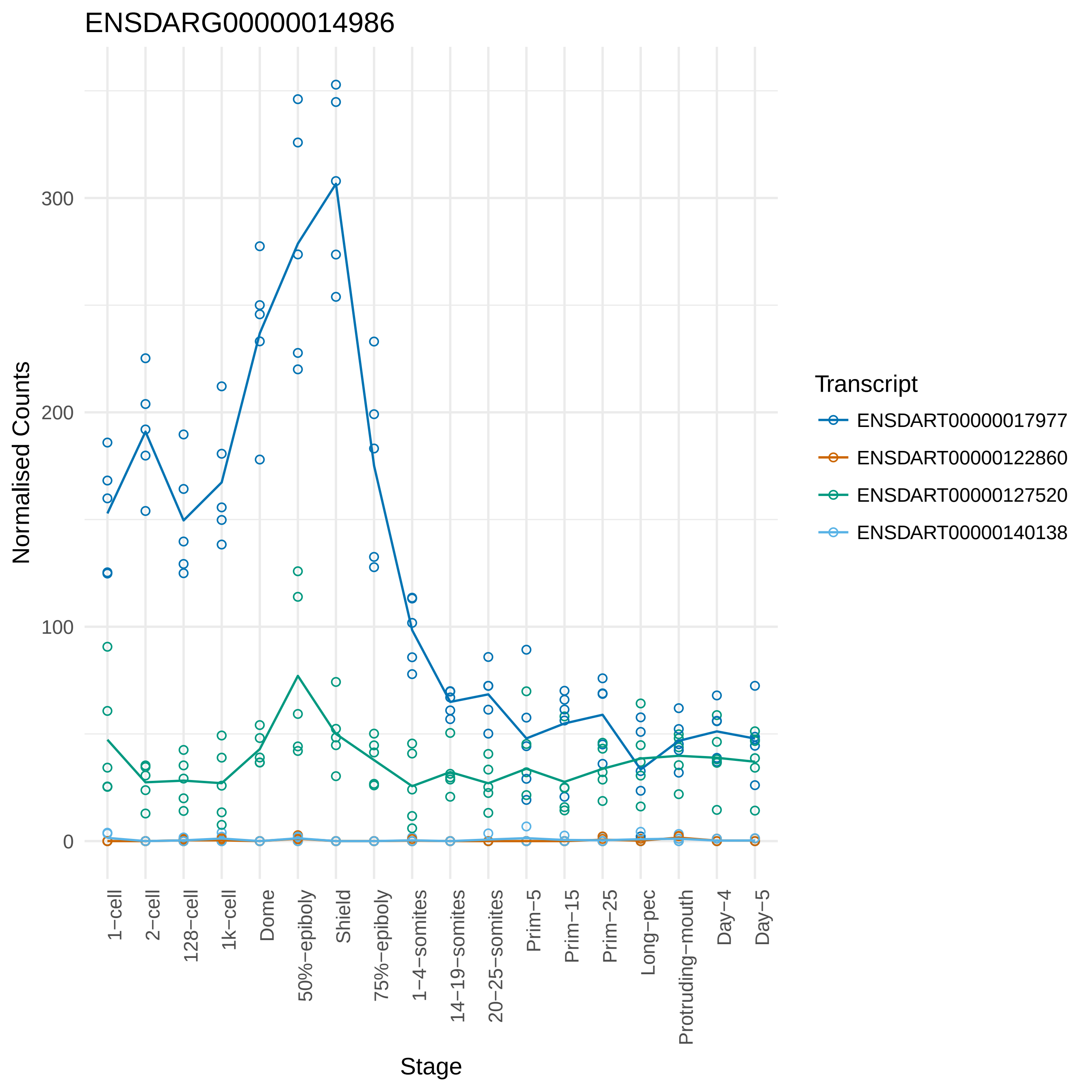

ENSDARG00000014986
Normalised Counts
300
200
100
0
1−cell
2−cell
128−cell
1k−cell
Dome
50%−epiboly
Shield
75%−epiboly
1−4−somites
14−19−somites
20−25−somites
Prim−5
Prim−15
Prim−25
Long−pec
Protruding−mouth
Day−4
Day−5
Stage
Transcript
ENSDART00000017977
ENSDART00000122860
ENSDART00000127520
ENSDART00000140138

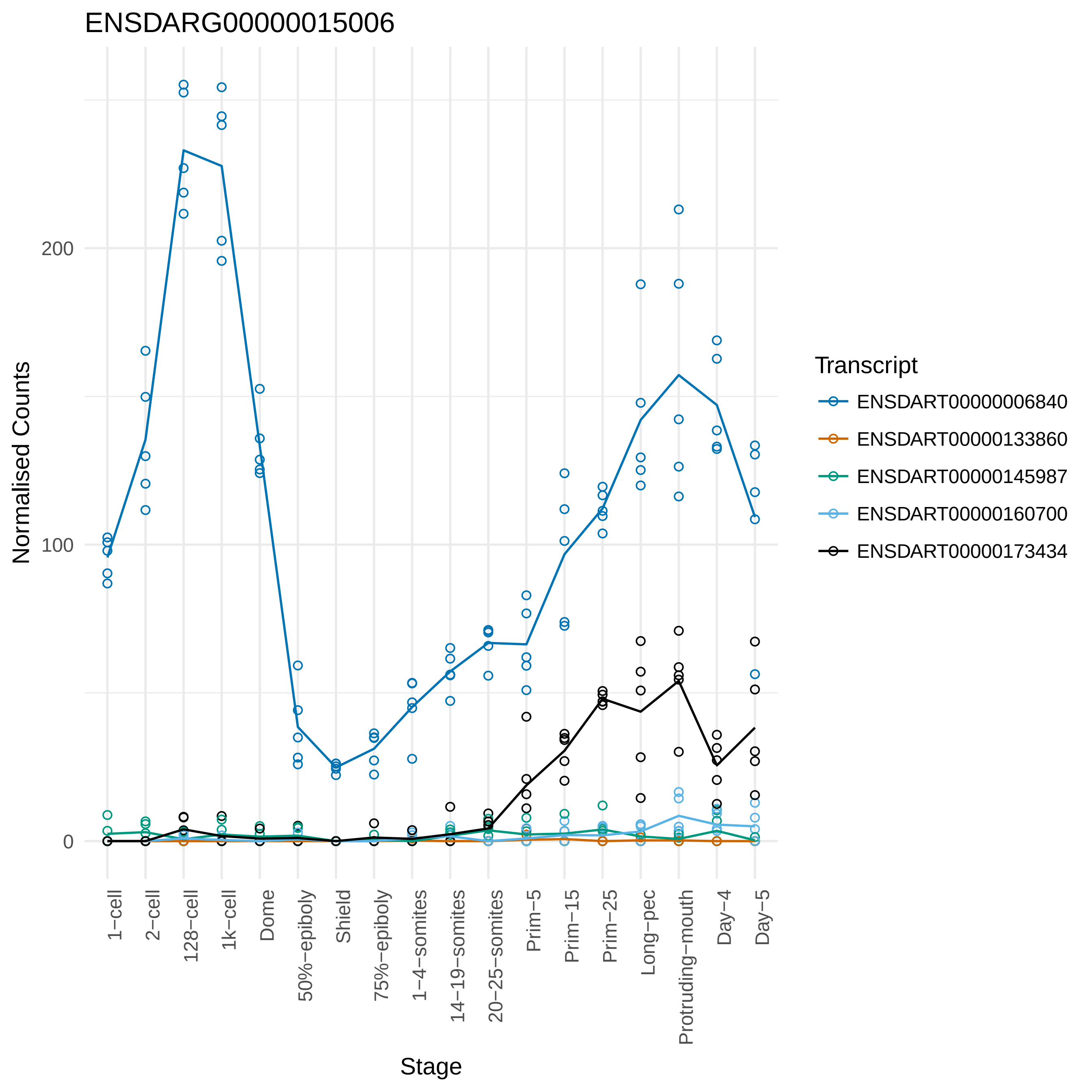

ENSDARG00000015006
Normalised Counts
0
100
200
1−cell
2−cell
128−cell
1k−cell
Dome
50%−epiboly
Shield
75%−epiboly
1−4−somites
14−19−somites
20−25−somites
Prim−5
Prim−15
Prim−25
Long−pec
Protruding−mouth
Day−4
Day−5
Stage
Transcript
ENSDART00000006840
ENSDART00000133860
ENSDART00000145987
ENSDART00000160700
ENSDART00000173434

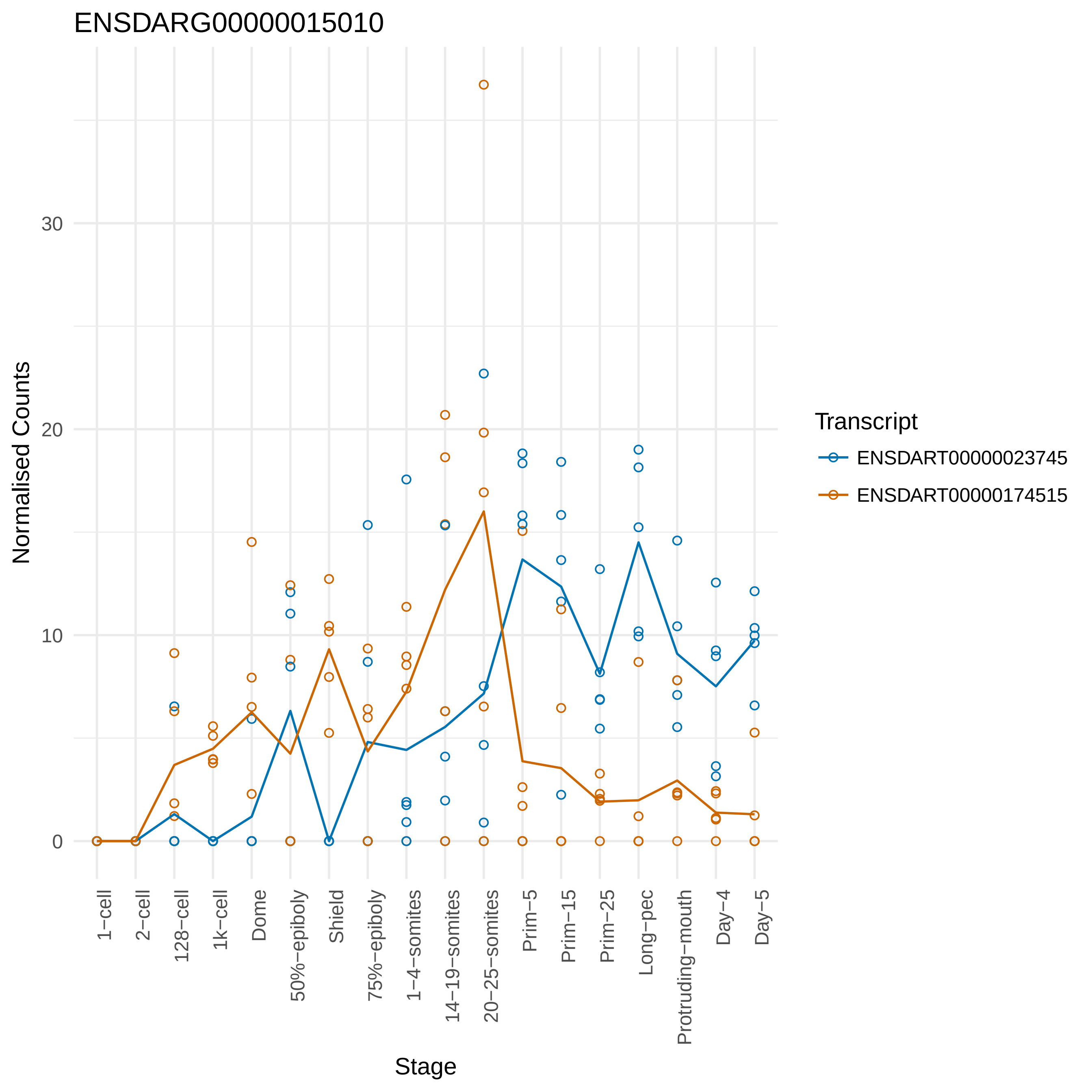

ENSDARG00000015010
Normalised Counts
0
10
20
30
Stage
1−cell
2−cell
128−cell
1k−cell
Dome
50%−epiboly
Shield
75%−epiboly
1−4−somites
14−19−somites
20−25−somites
Prim−5
Prim−15
Prim−25
Long−pec
Protruding−mouth
Day−4
Day−5
Transcript
ENSDART00000023745
ENSDART00000174515

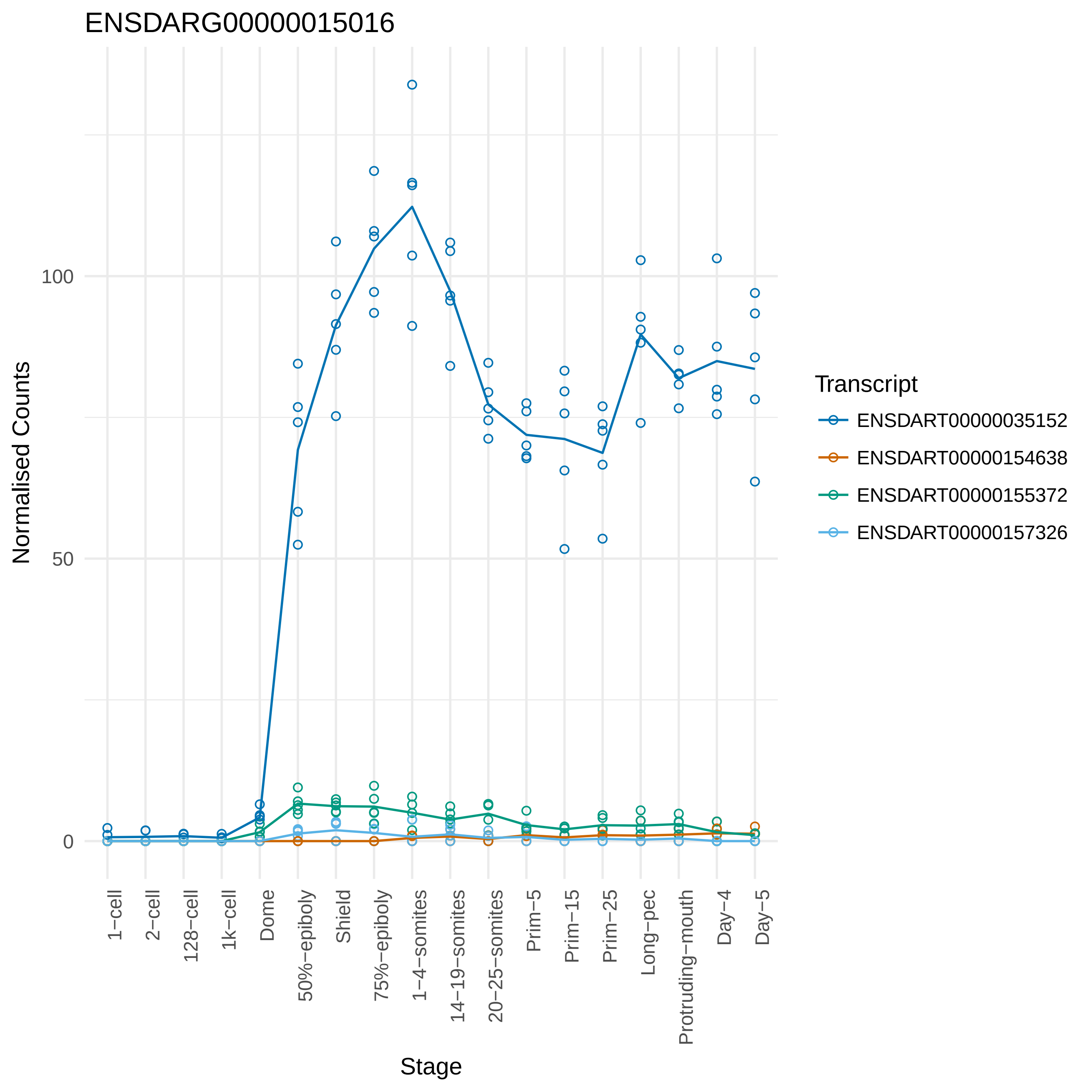
ENSDARG00000015016
Normalised Counts
100
50
0
1−cell
2−cell
128−cell
1k−cell
Dome
50%−epiboly
Shield
75%−epiboly
1−4−somites
14−19−somites
20−25−somites
Prim−5
Prim−15
Prim−25
Long−pec
Protruding−mouth
Day−4
Day−5
Stage
Transcript
ENSDART00000035152
ENSDART00000154638
ENSDART00000155372
ENSDART00000157326

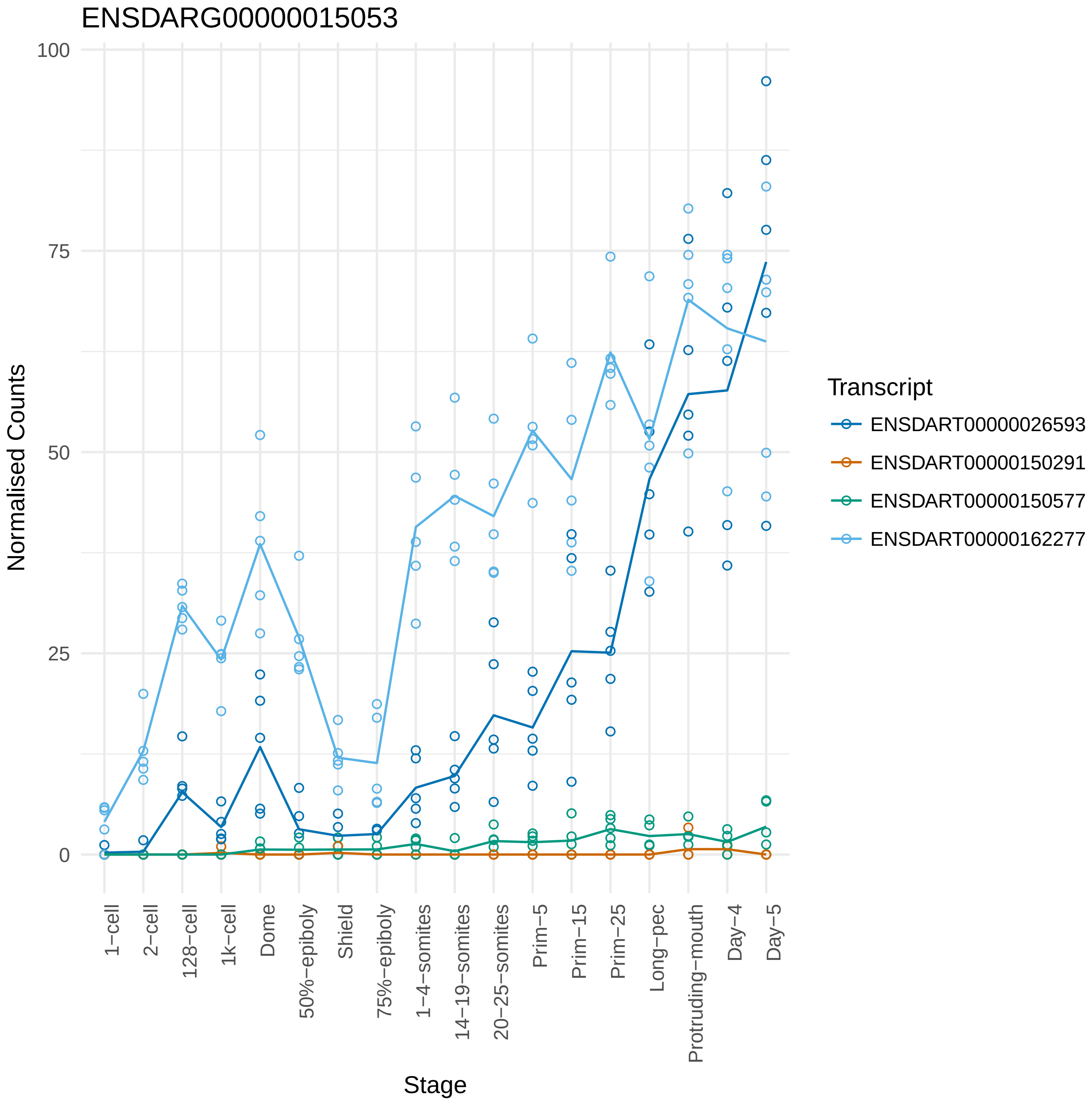

ENSDARG00000015053
Normalised Counts
100
75
50
25
0
1−cell
2−cell
128−cell
1k−cell
Dome
50%−epiboly
Shield
75%−epiboly
1−4−somites
14−19−somites
20−25−somites
Prim−5
Prim−15
Prim−25
Long−pec
Protruding−mouth
Day−4
Day−5
Stage
Transcript
ENSDART00000026593
ENSDART00000150291
ENSDART00000150577
ENSDART00000162277

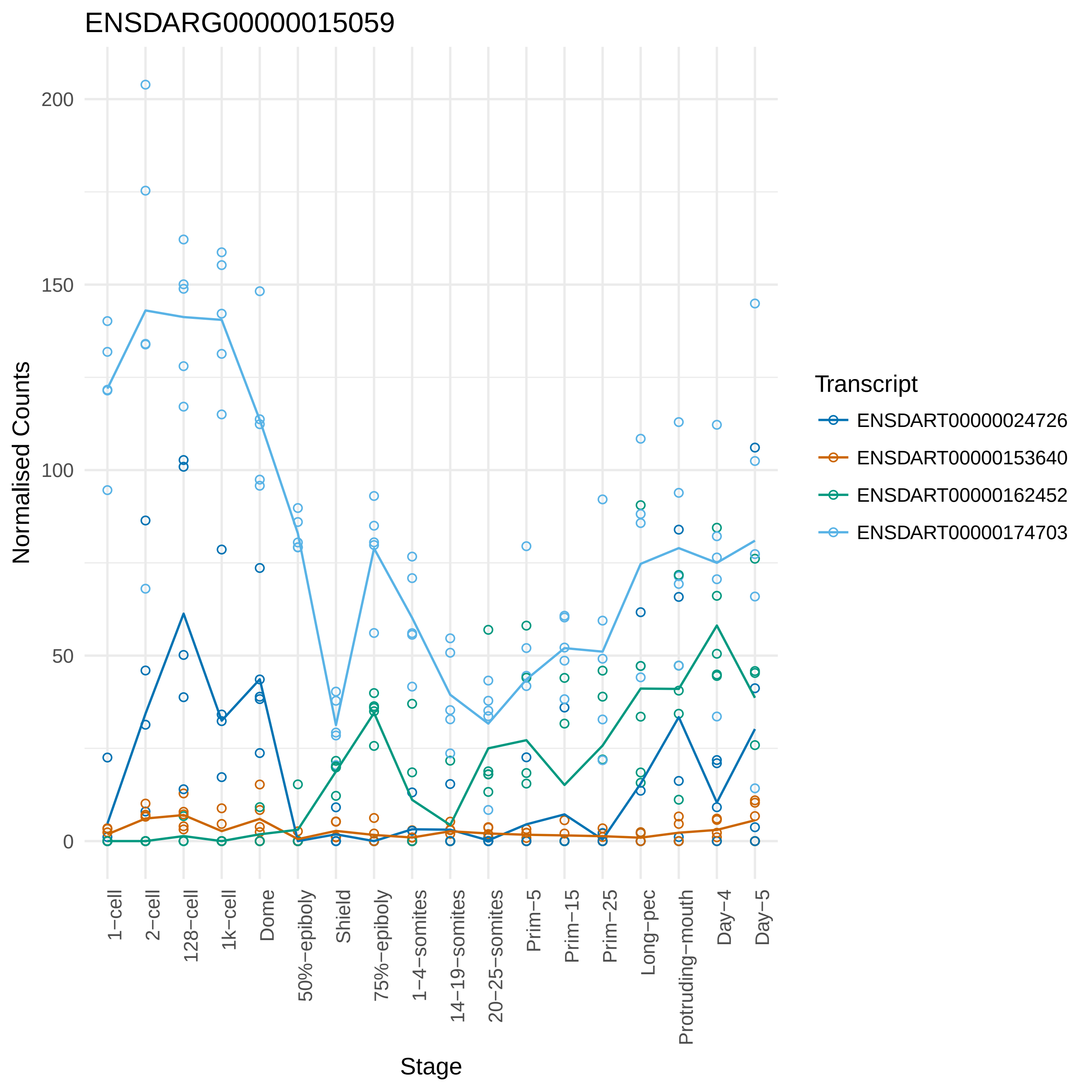
ENSDARG00000015059
Normalised Counts
200
150
100
50
0
1−cell
2−cell
128−cell
1k−cell
Dome
50%−epiboly
Shield
75%−epiboly
1−4−somites
14−19−somites
20−25−somites
Prim−5
Prim−15
Prim−25
Long−pec
Protruding−mouth
Day−4
Day−5
Stage
Transcript
ENSDART00000024726
ENSDART00000153640
ENSDART00000162452
ENSDART00000174703

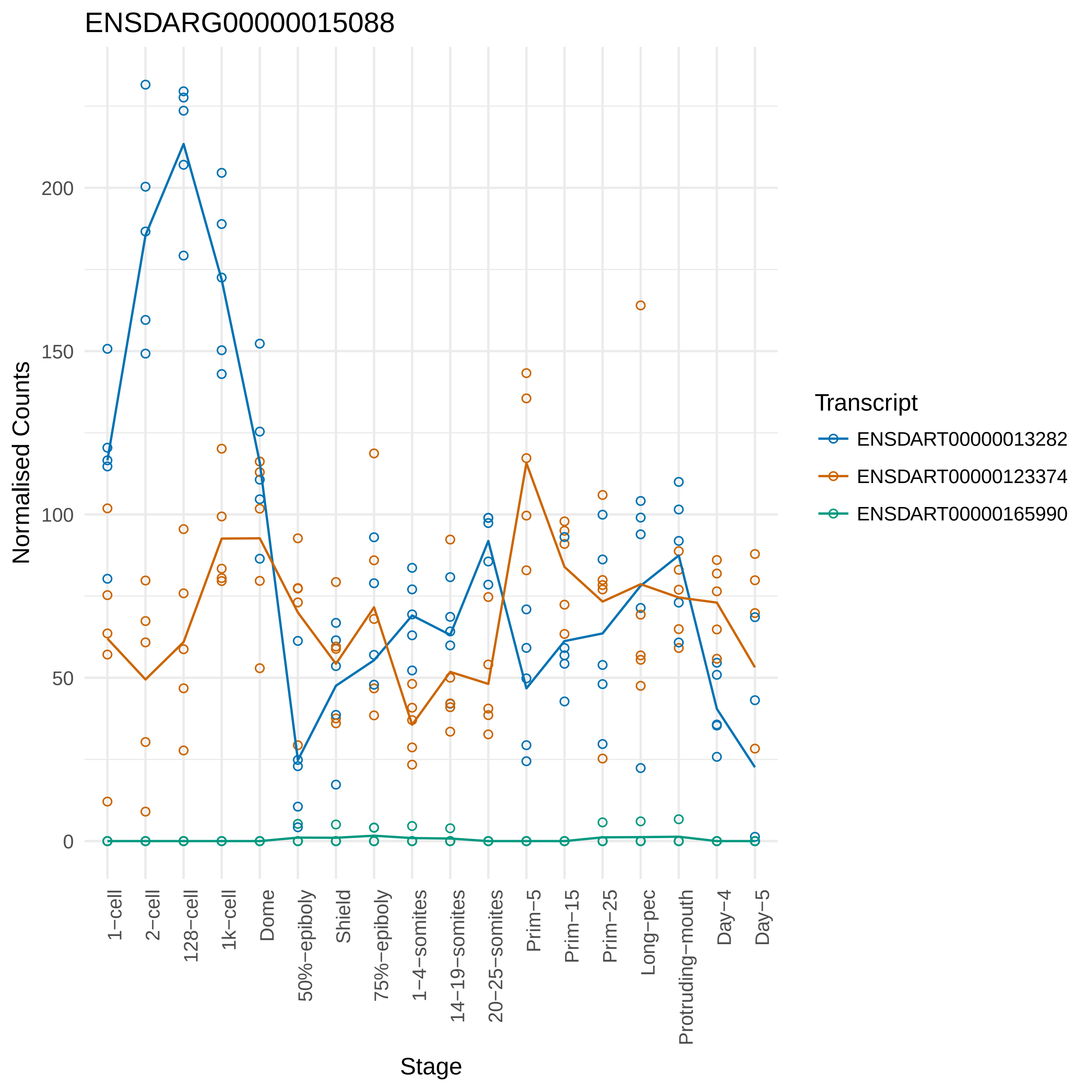
ENSDARG00000015088
Normalised Counts
0
50
100
150
200
1−cell
2−cell
128−cell
1k−cell
Dome
50%−epiboly
Shield
75%−epiboly
1−4−somites
14−19−somites
20−25−somites
Prim−5
Prim−15
Prim−25
Long−pec
Protruding−mouth
Day−4
Day−5
Stage
Transcript
ENSDART00000013282
ENSDART00000123374
ENSDART00000165990

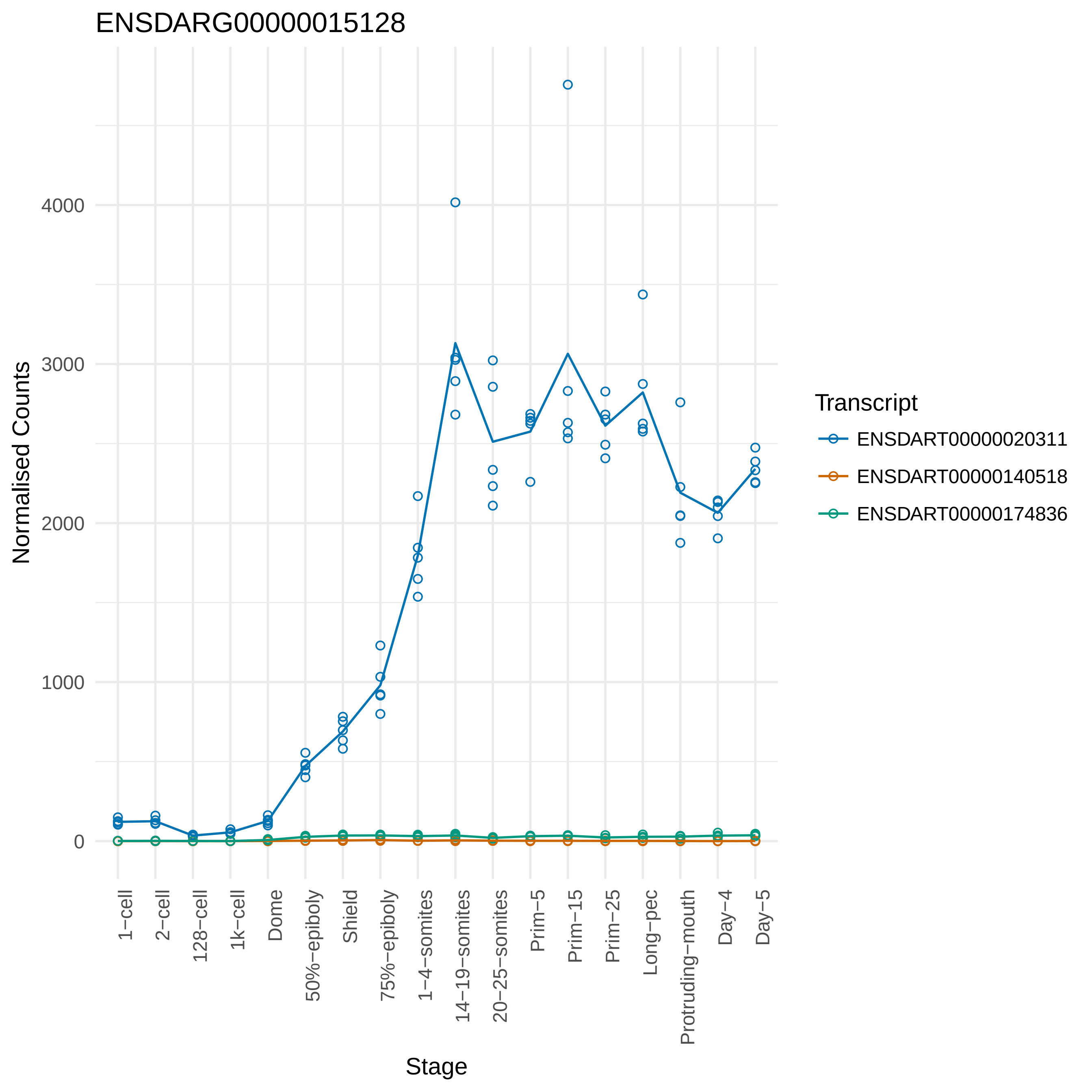

ENSDARG00000015128
Normalised Counts
4000
3000
2000
1000
0
1−cell
2−cell
128−cell
1k−cell
Dome
50%−epiboly
Shield
75%−epiboly
1−4−somites
14−19−somites
20−25−somites
Prim−5
Prim−15
Prim−25
Long−pec
Protruding−mouth
Day−4
Day−5
Stage
Transcript
ENSDART00000020311
ENSDART00000140518
ENSDART00000174836

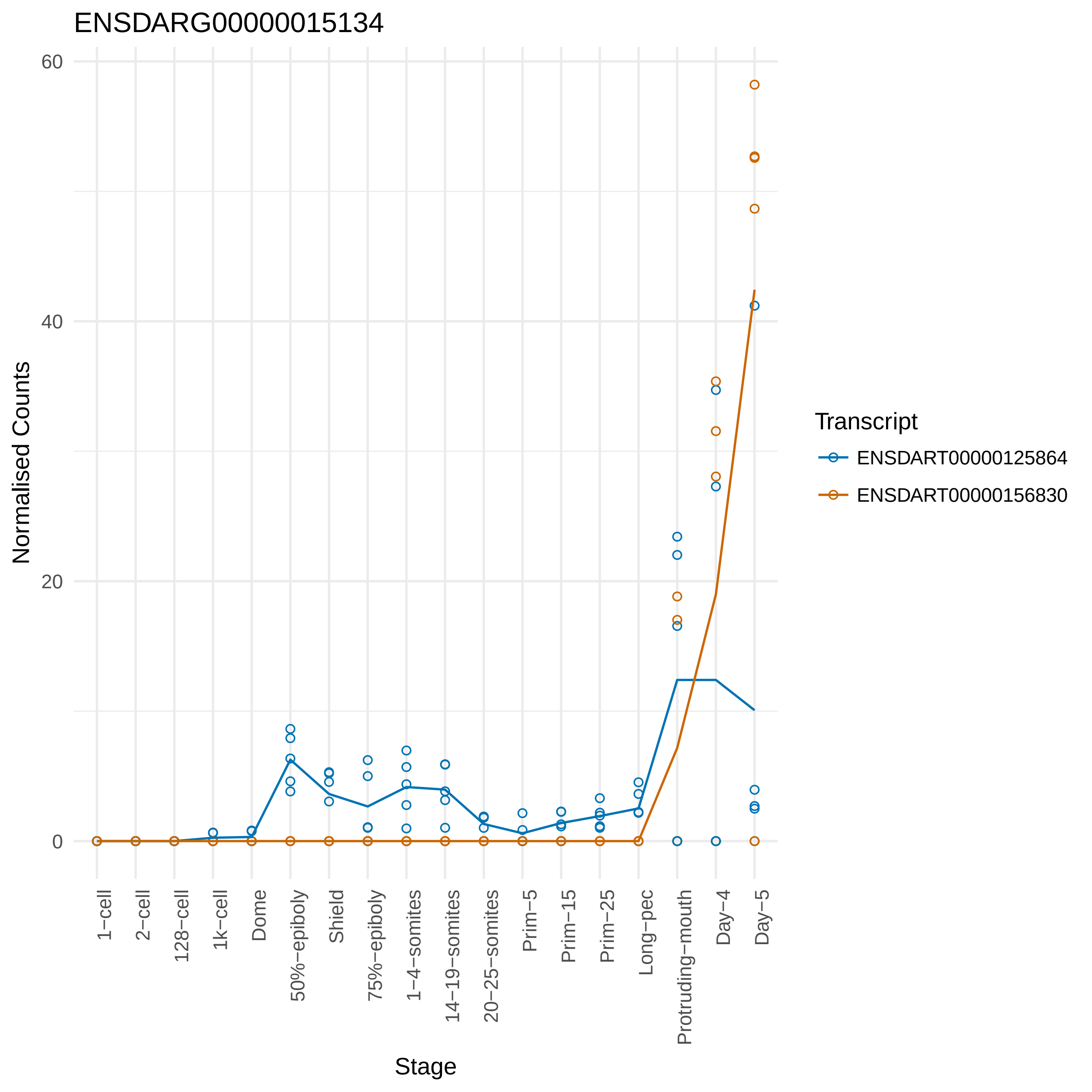

ENSDARG00000015134
Normalised Counts
60
40
20
0
1−cell
2−cell
128−cell
1k−cell
Dome
50%−epiboly
Shield
75%−epiboly
1−4−somites
14−19−somites
20−25−somites
Prim−5
Prim−15
Prim−25
Long−pec
Protruding−mouth
Day−4
Day−5
Stage
Transcript
ENSDART00000125864
ENSDART00000156830

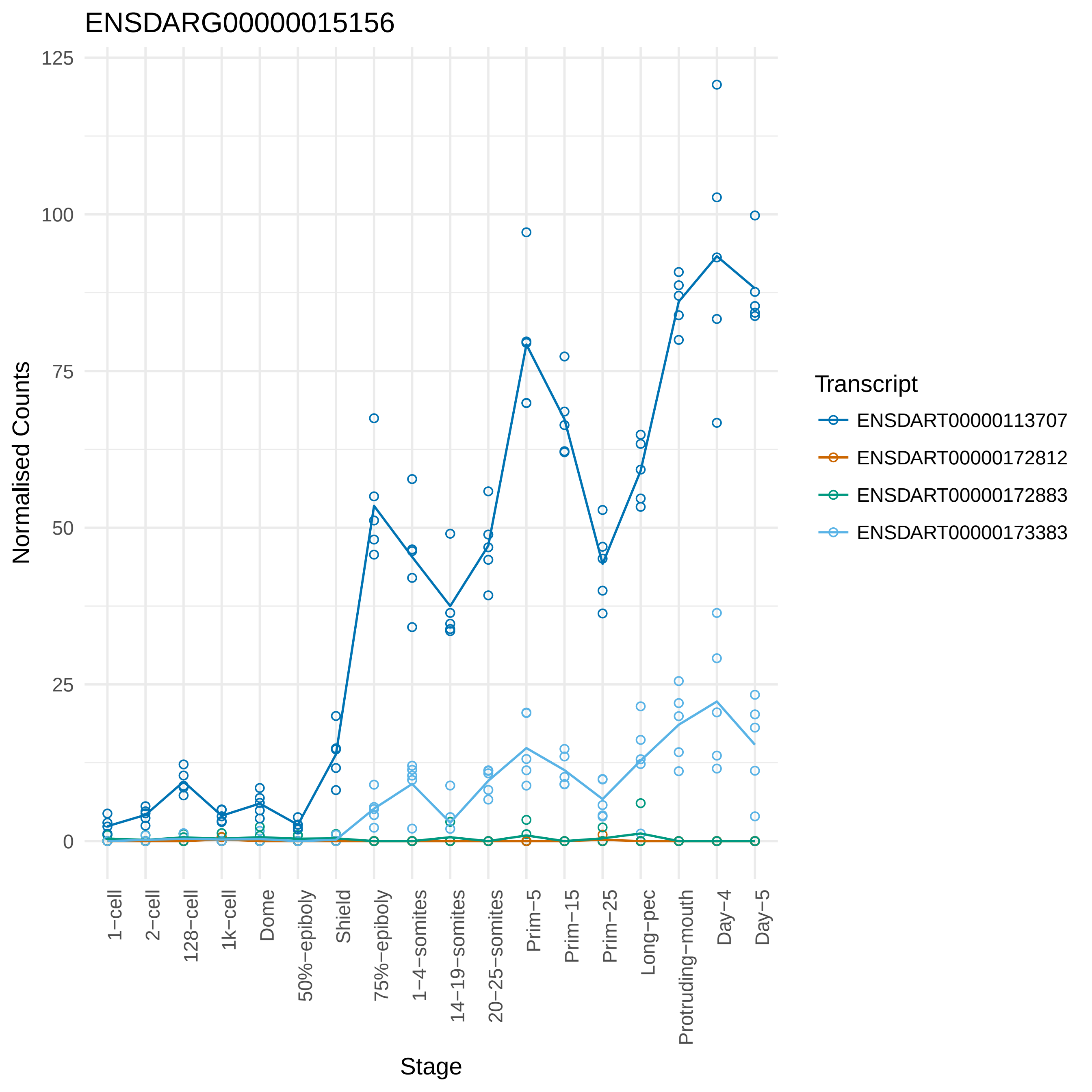

ENSDARG00000015156
Normalised Counts
125
100
75
50
25
0
1−cell
2−cell
128−cell
1k−cell
Dome
50%−epiboly
Shield
75%−epiboly
1−4−somites
14−19−somites
20−25−somites
Prim−5
Prim−15
Prim−25
Long−pec
Protruding−mouth
Day−4
Day−5
Stage
Transcript
ENSDART00000113707
ENSDART00000172812
ENSDART00000172883
ENSDART00000173383

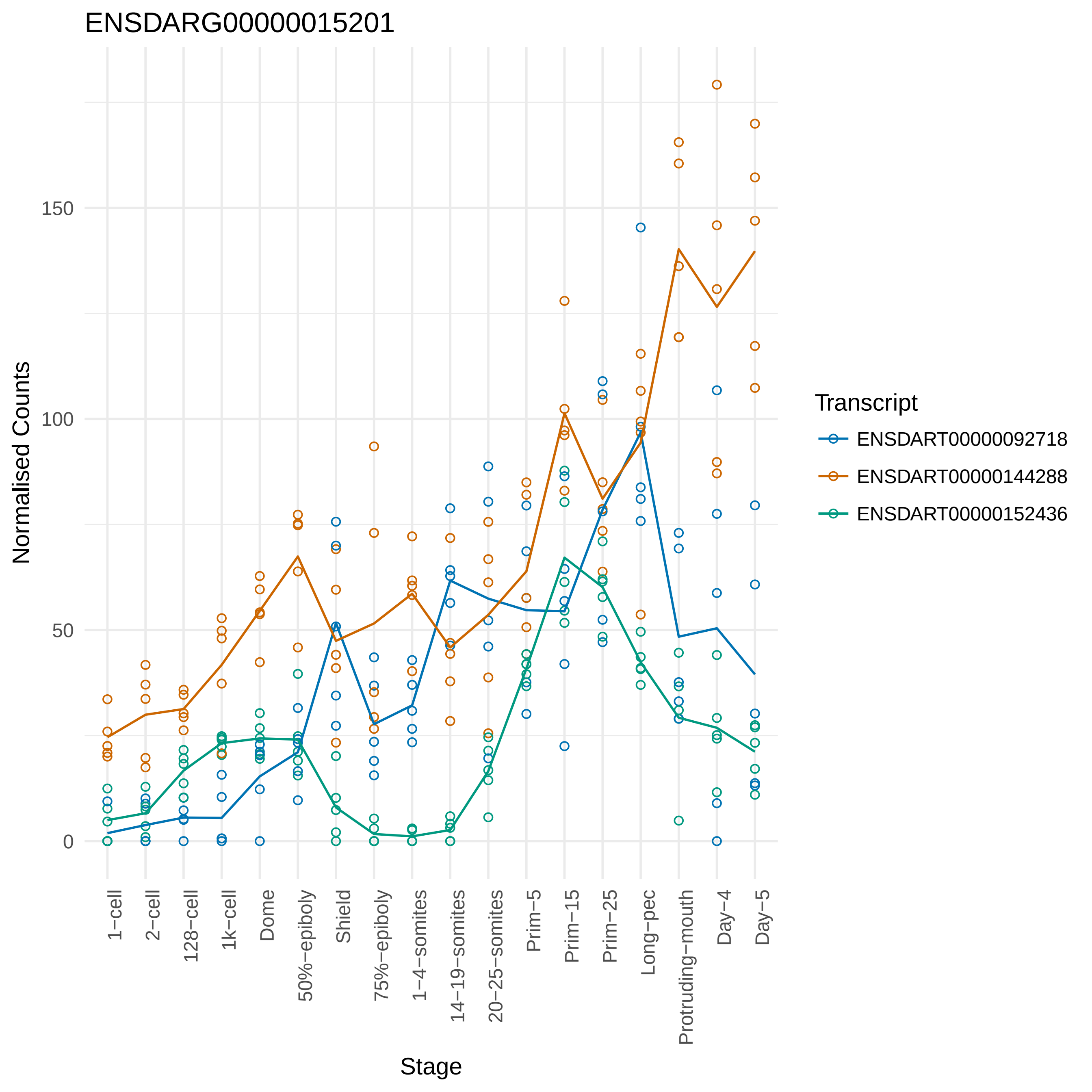

ENSDARG00000015201
Normalised Counts
0
50
100
150
1−cell
2−cell
128−cell
1k−cell
Dome
50%−epiboly
Shield
75%−epiboly
1−4−somites
14−19−somites
20−25−somites
Prim−5
Prim−15
Prim−25
Long−pec
Protruding−mouth
Day−4
Day−5
Stage
Transcript
ENSDART00000092718
ENSDART00000144288
ENSDART00000152436

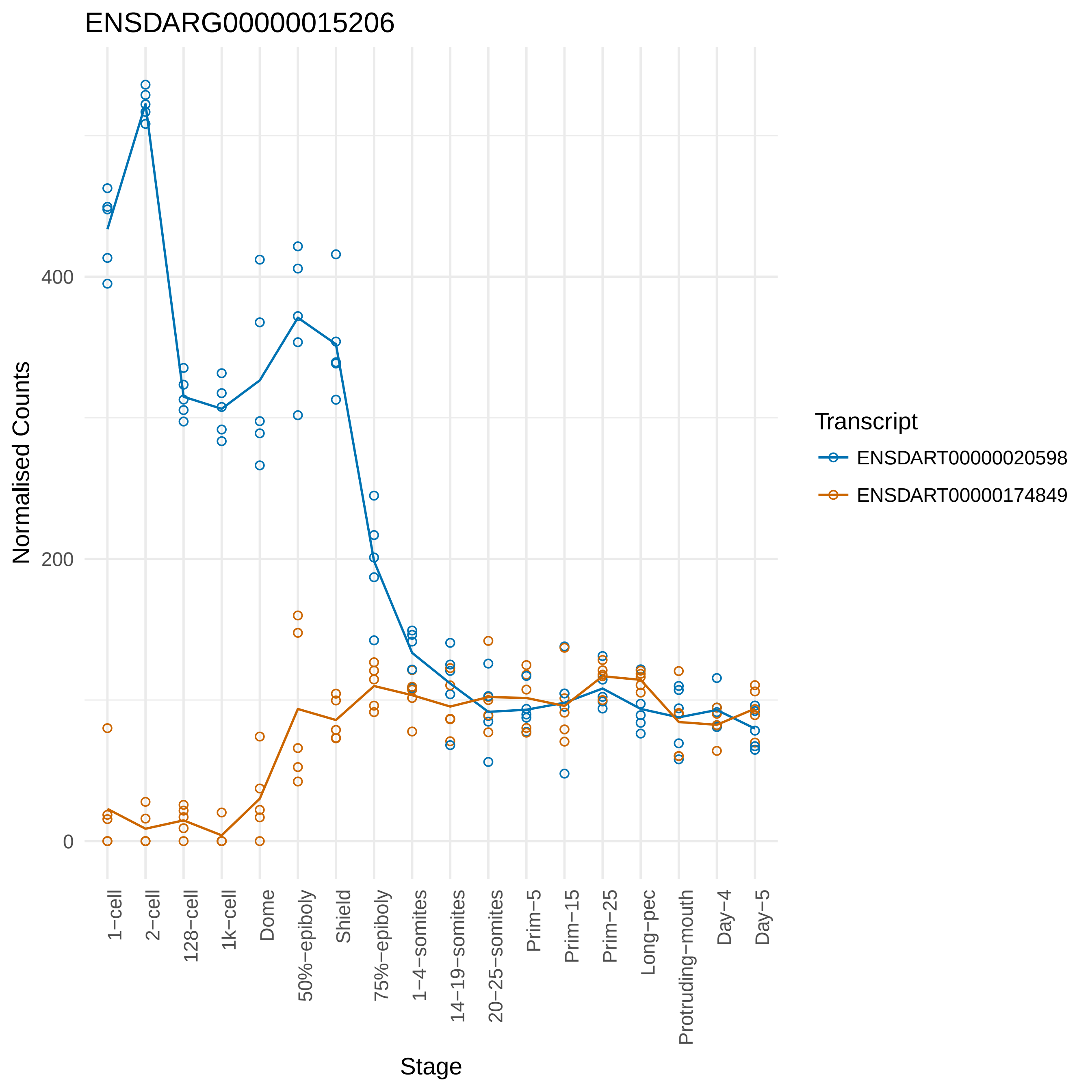

ENSDARG00000015206
Normalised Counts
400
200
0
1−cell
2−cell
128−cell
1k−cell
Dome
50%−epiboly
Shield
75%−epiboly
1−4−somites
14−19−somites
20−25−somites
Prim−5
Prim−15
Prim−25
Long−pec
Protruding−mouth
Day−4
Day−5
Stage
Transcript
ENSDART00000020598
ENSDART00000174849

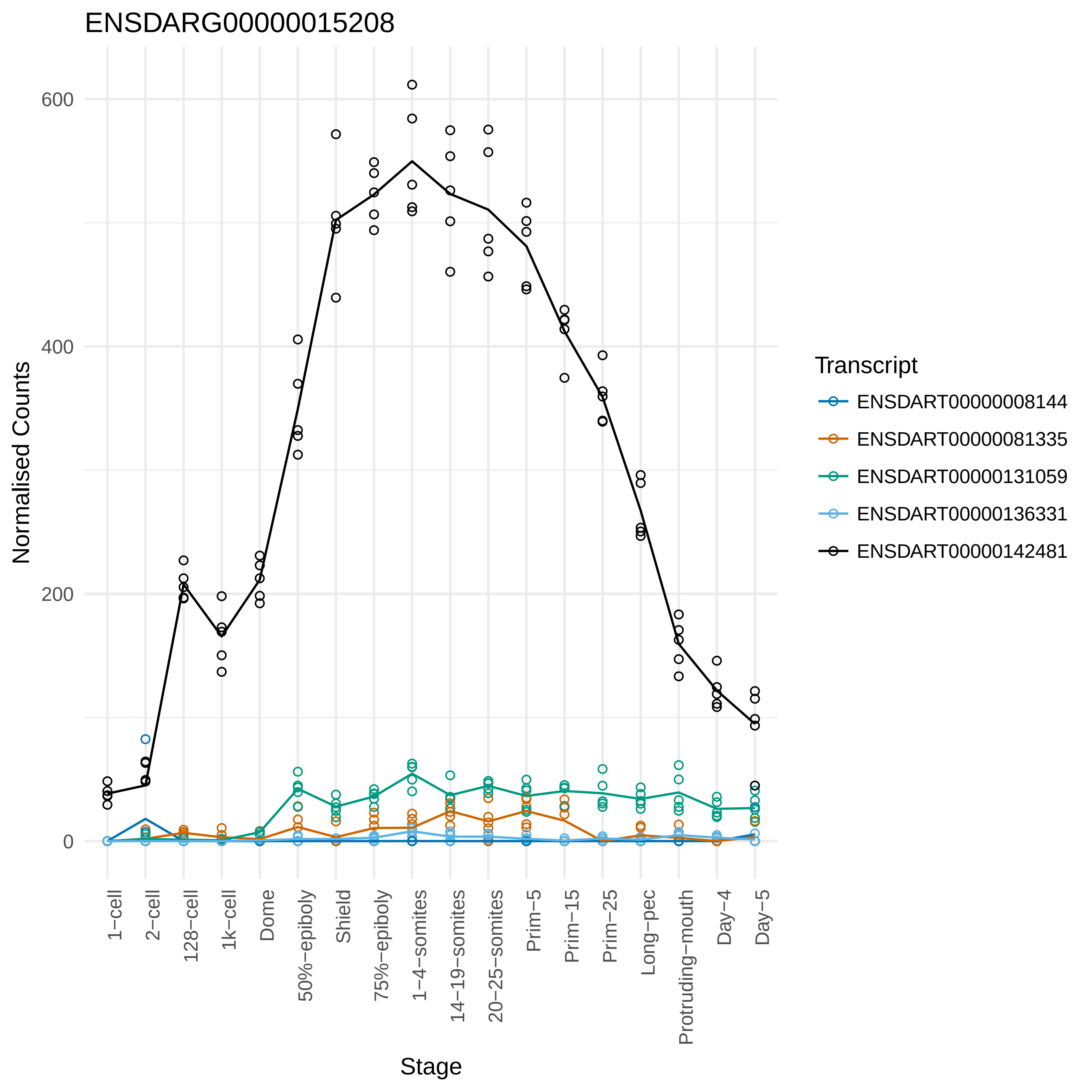

ENSDARG00000015208
Normalised Counts
0
200
400
600
Stage
1−cell
2−cell
128−cell
1k−cell
Dome
50%−epiboly
Shield
75%−epiboly
1−4−somites
14−19−somites
20−25−somites
Prim−5
Prim−15
Prim−25
Long−pec
Protruding−mouth
Day−4
Day−5
Transcript
ENSDART00000008144
ENSDART00000081335
ENSDART00000131059
ENSDART00000136331
ENSDART00000142481

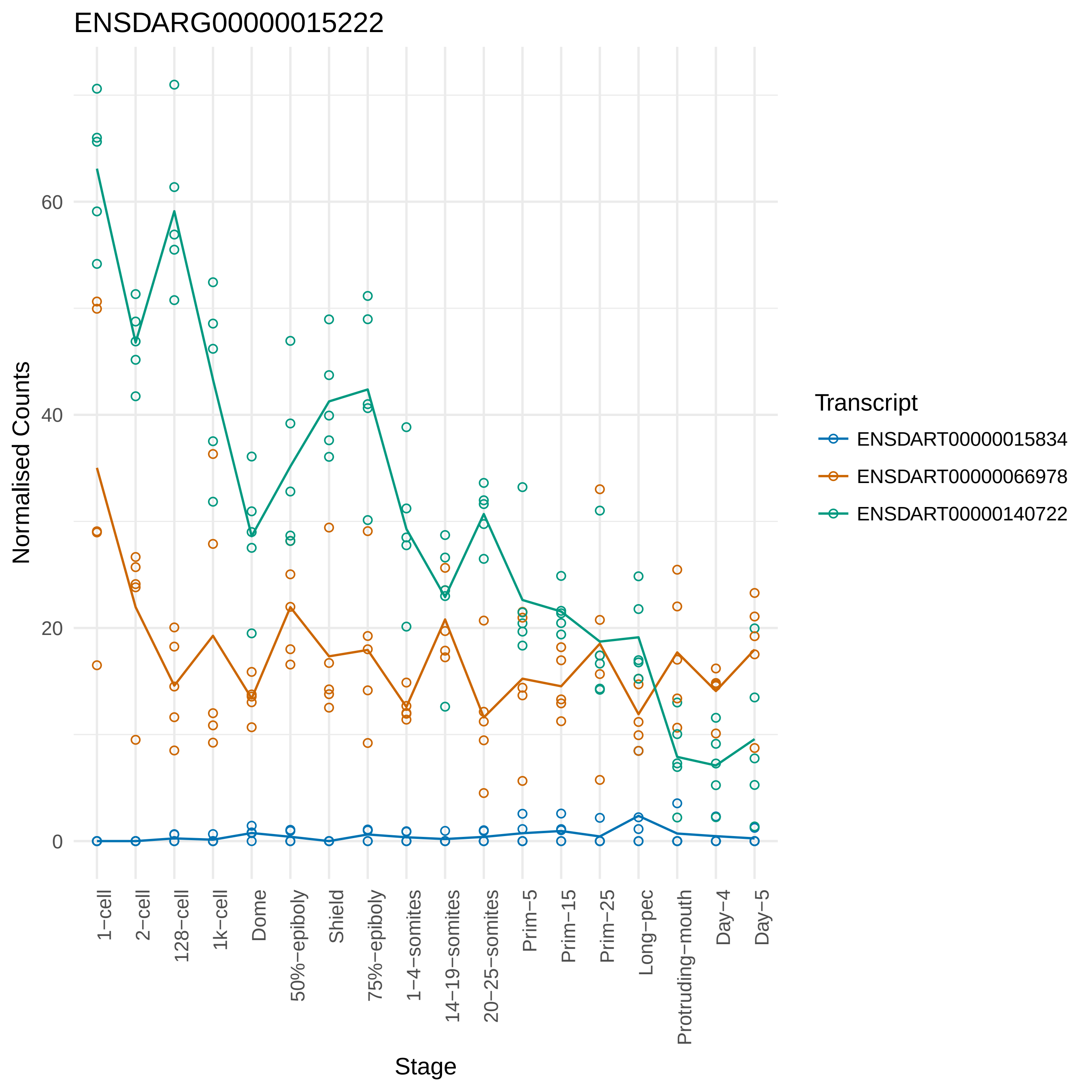
ENSDARG00000015222
Normalised Counts
60
40
20
0
Stage
1−cell
2−cell
128−cell
1k−cell
Dome
50%−epiboly
Shield
75%−epiboly
1−4−somites
14−19−somites
20−25−somites
Prim−5
Prim−15
Prim−25
Long−pec
Protruding−mouth
Day−4
Day−5
Transcript
ENSDART00000015834
ENSDART00000066978
ENSDART00000140722

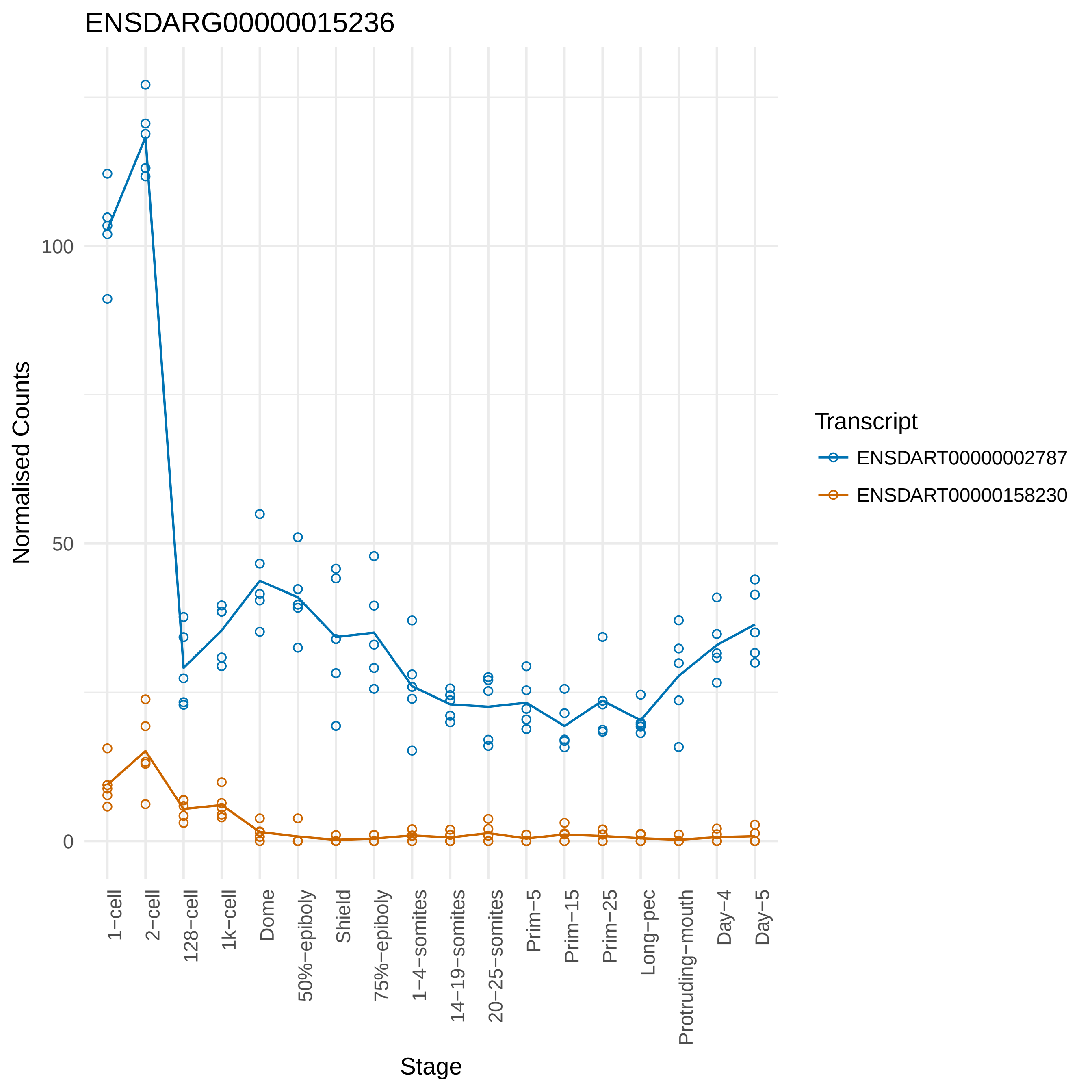
ENSDARG00000015236
Normalised Counts
100
50
0
1−cell
2−cell
128−cell
1k−cell
Dome
50%−epiboly
Shield
75%−epiboly
1−4−somites
14−19−somites
20−25−somites
Prim−5
Prim−15
Prim−25
Long−pec
Protruding−mouth
Day−4
Day−5
Stage
Transcript
ENSDART00000002787
ENSDART00000158230

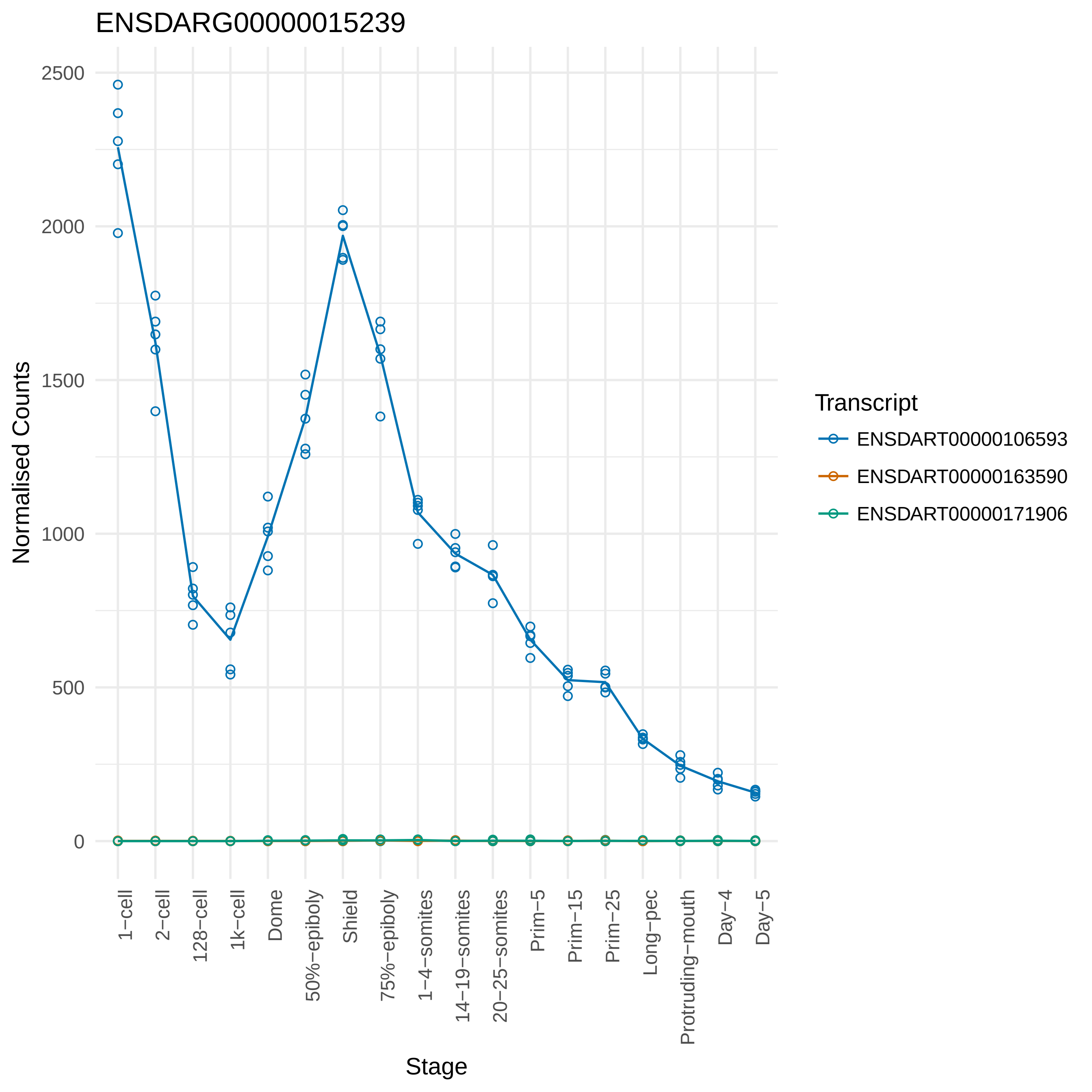
ENSDARG00000015239
Normalised Counts
2500
2000
1500
1000
500
0
1−cell
2−cell
128−cell
1k−cell
Dome
50%−epiboly
Shield
75%−epiboly
1−4−somites
14−19−somites
20−25−somites
Prim−5
Prim−15
Prim−25
Long−pec
Protruding−mouth
Day−4
Day−5
Stage
Transcript
ENSDART00000106593
ENSDART00000163590
ENSDART00000171906

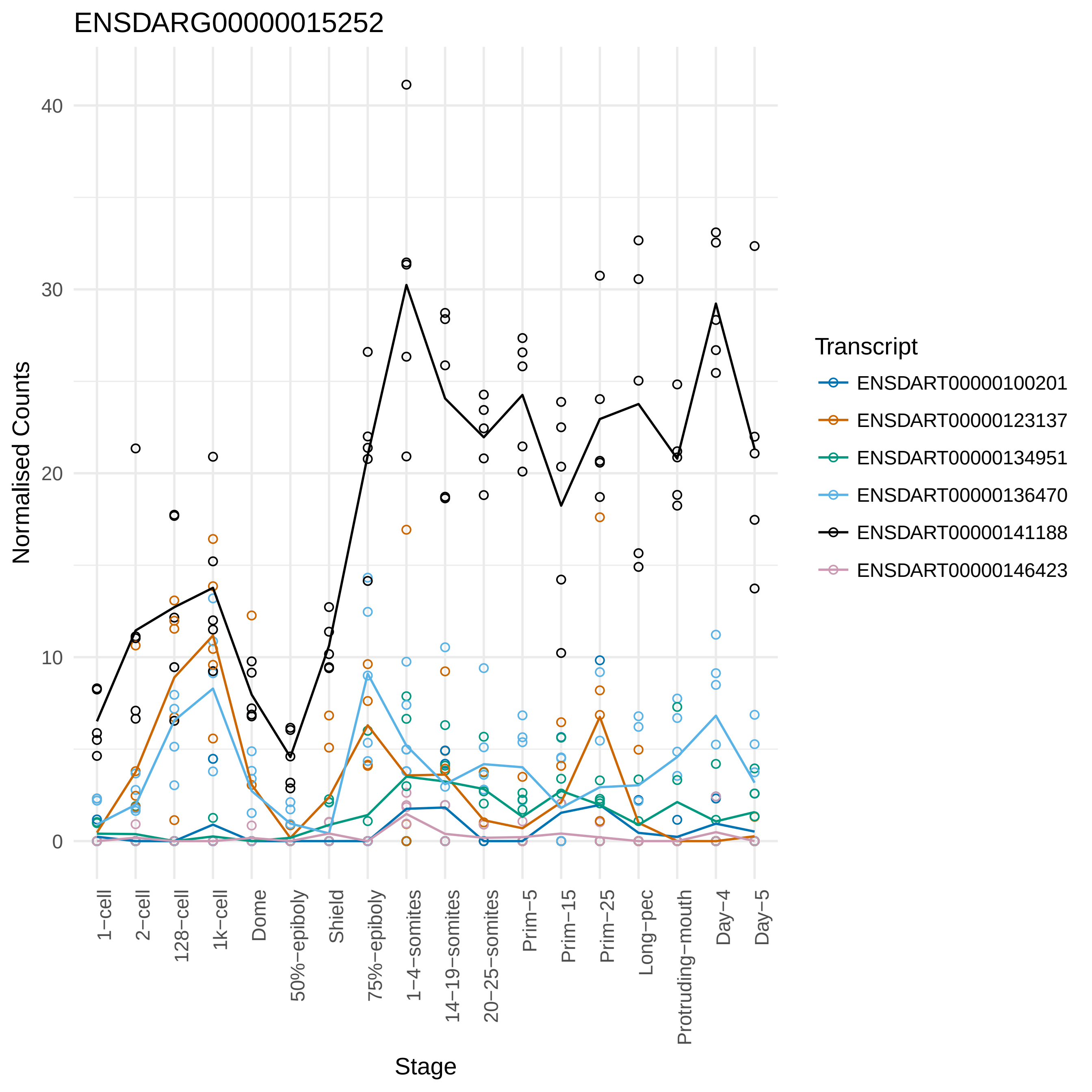

ENSDARG00000015252
Normalised Counts
0
10
20
30
40
Stage
1−cell
2−cell
128−cell
1k−cell
Dome
50%−epiboly
Shield
75%−epiboly
1−4−somites
14−19−somites
20−25−somites
Prim−5
Prim−15
Prim−25
Long−pec
Protruding−mouth
Day−4
Day−5
Transcript
ENSDART00000100201
ENSDART00000123137
ENSDART00000134951
ENSDART00000136470
ENSDART00000141188
ENSDART00000146423

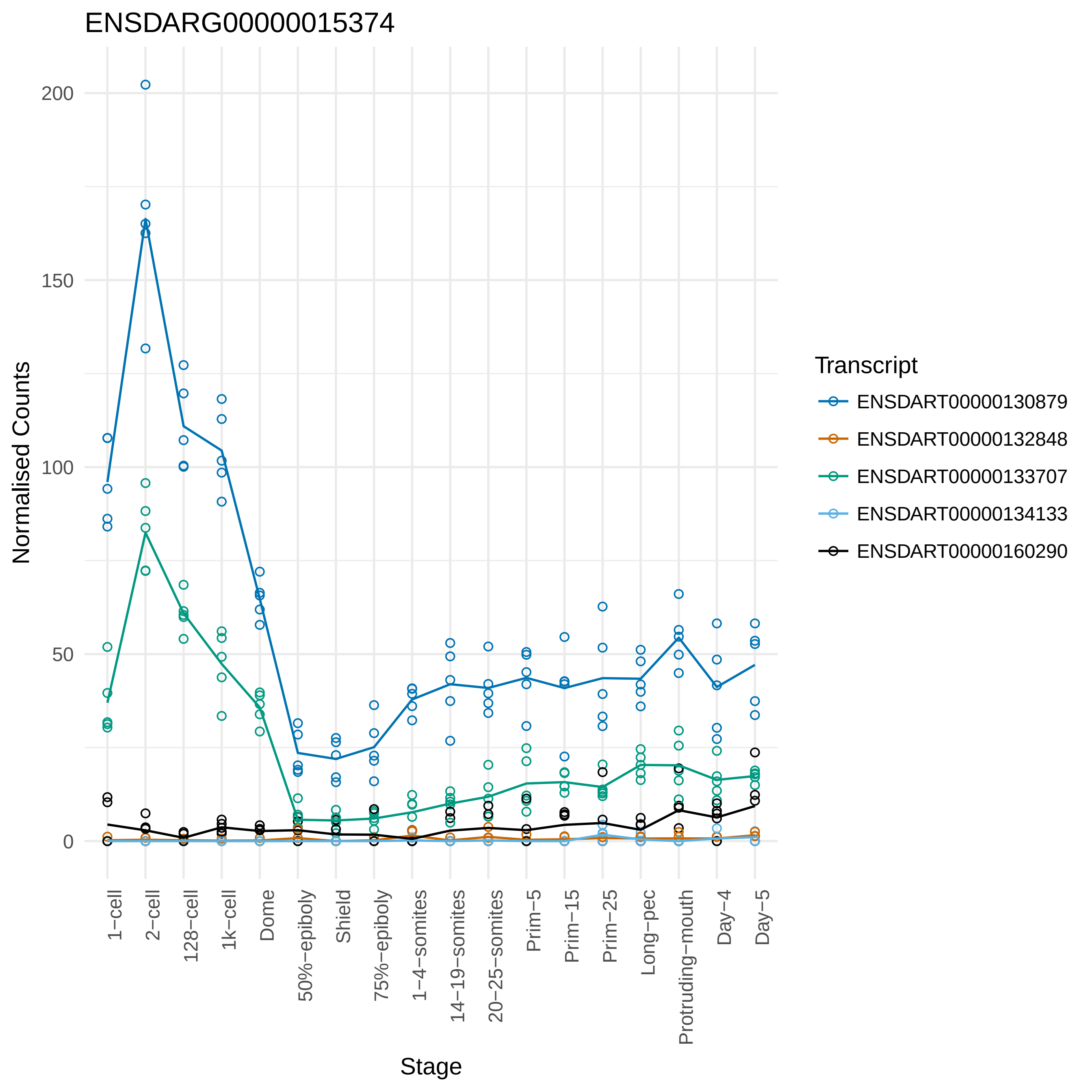

ENSDARG00000015374
Normalised Counts
0
50
100
150
200
1−cell
2−cell
128−cell
1k−cell
Dome
50%−epiboly
Shield
75%−epiboly
1−4−somites
14−19−somites
20−25−somites
Prim−5
Prim−15
Prim−25
Long−pec
Protruding−mouth
Day−4
Day−5
Stage
Transcript
ENSDART00000130879
ENSDART00000132848
ENSDART00000133707
ENSDART00000134133
ENSDART00000160290

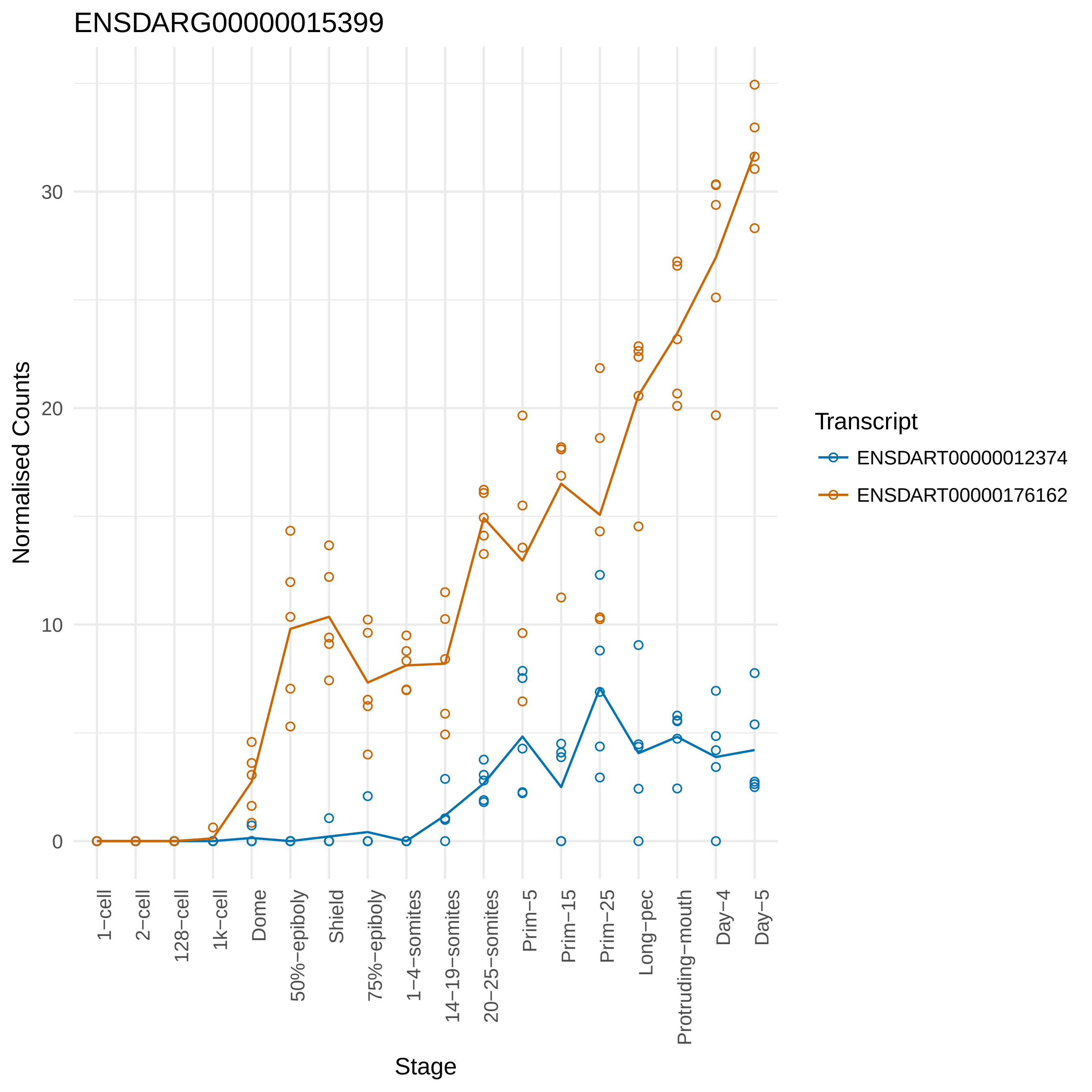

ENSDARG00000015399
Normalised Counts
0
10
20
30
Stage
1−cell
2−cell
128−cell
1k−cell
Dome
50%−epiboly
Shield
75%−epiboly
1−4−somites
14−19−somites
20−25−somites
Prim−5
Prim−15
Prim−25
Long−pec
Protruding−mouth
Day−4
Day−5
Transcript
ENSDART00000012374
ENSDART00000176162

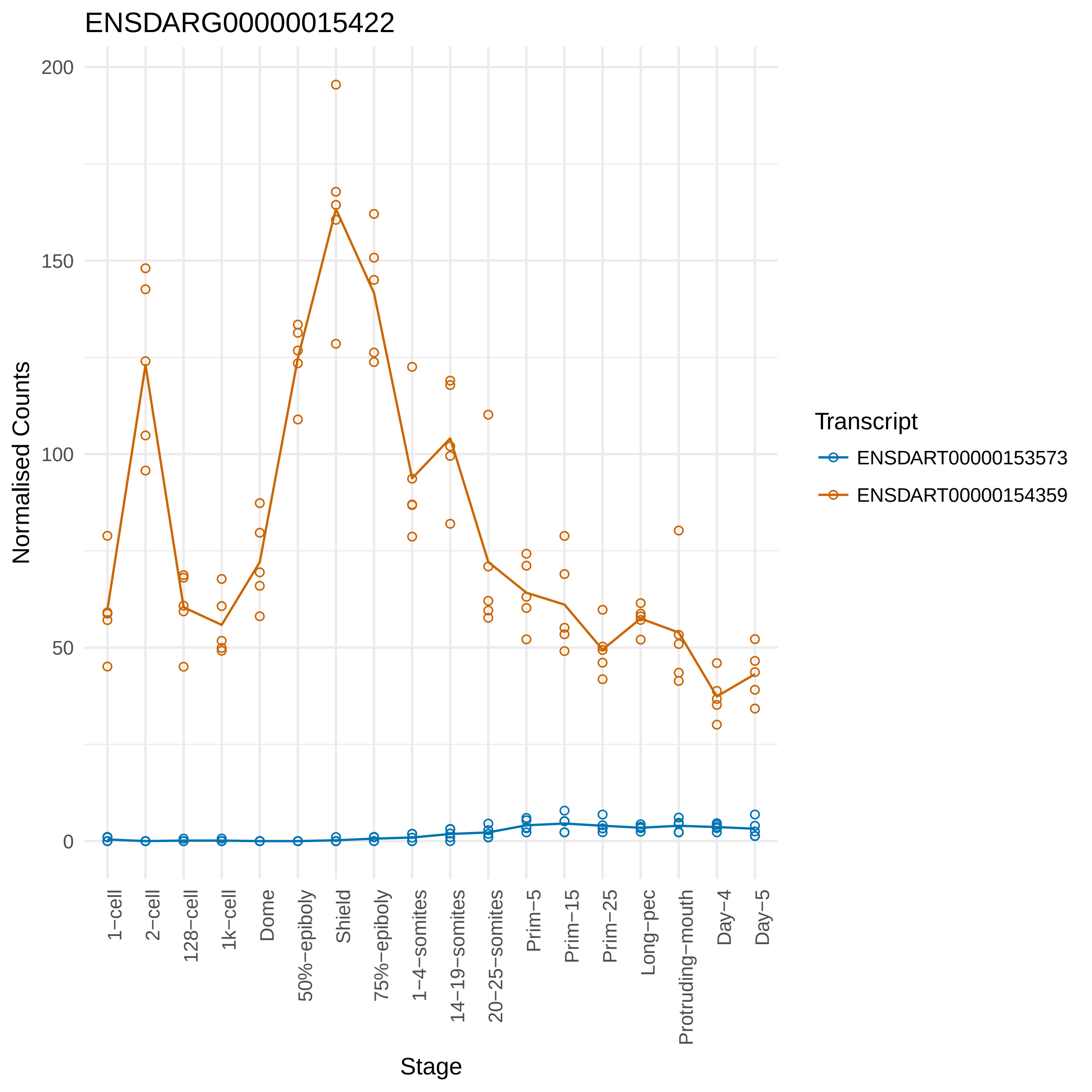

ENSDARG00000015422
Normalised Counts
200
150
100
50
0
1−cell
2−cell
128−cell
1k−cell
Dome
50%−epiboly
Shield
75%−epiboly
1−4−somites
14−19−somites
20−25−somites
Prim−5
Prim−15
Prim−25
Long−pec
Protruding−mouth
Day−4
Day−5
Stage
Transcript
ENSDART00000153573
ENSDART00000154359

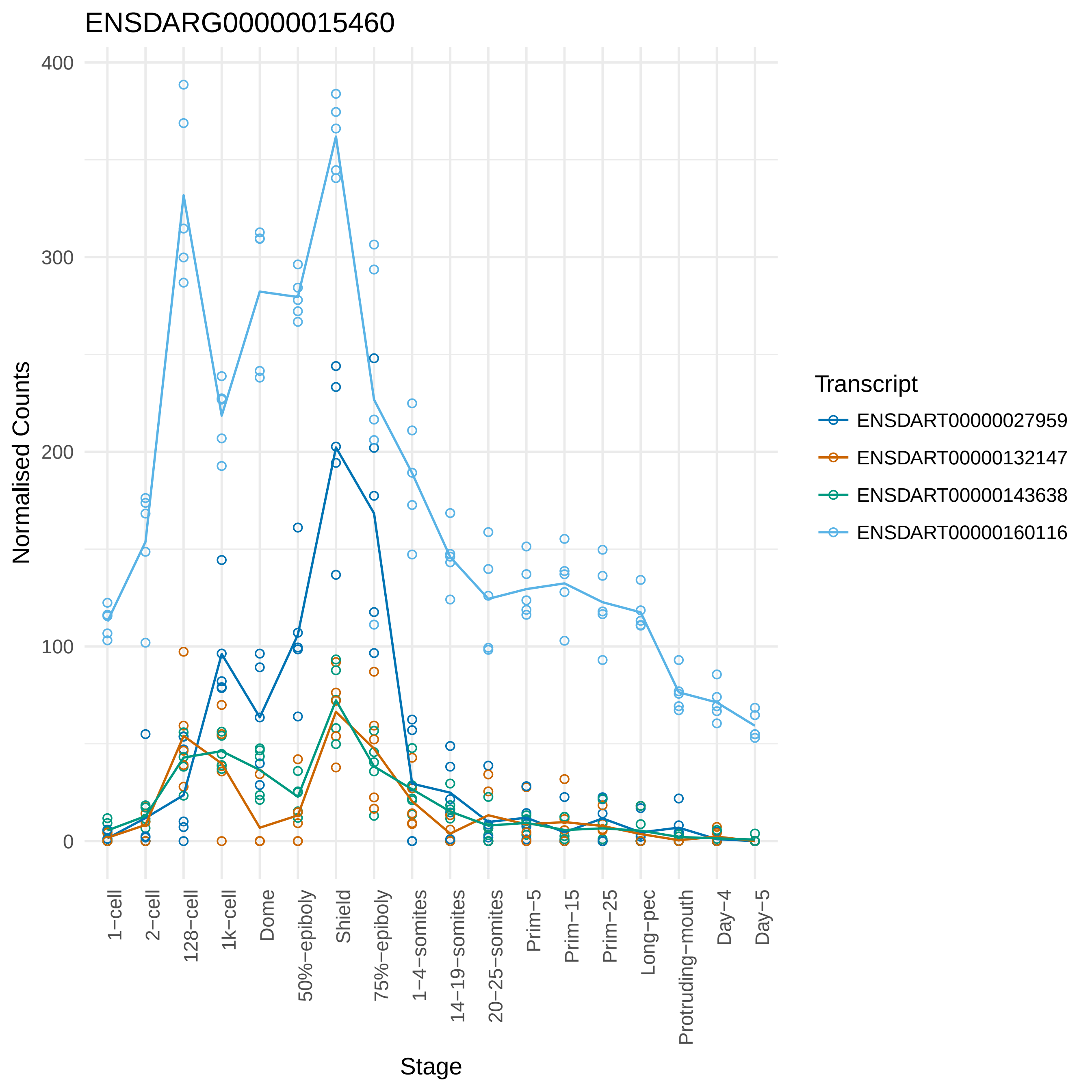
ENSDARG00000015460
Normalised Counts
400
300
200
100
0
1−cell
2−cell
128−cell
1k−cell
Dome
50%−epiboly
Shield
75%−epiboly
1−4−somites
14−19−somites
20−25−somites
Prim−5
Prim−15
Prim−25
Long−pec
Protruding−mouth
Day−4
Day−5
Stage
Transcript
ENSDART00000027959
ENSDART00000132147
ENSDART00000143638
ENSDART00000160116

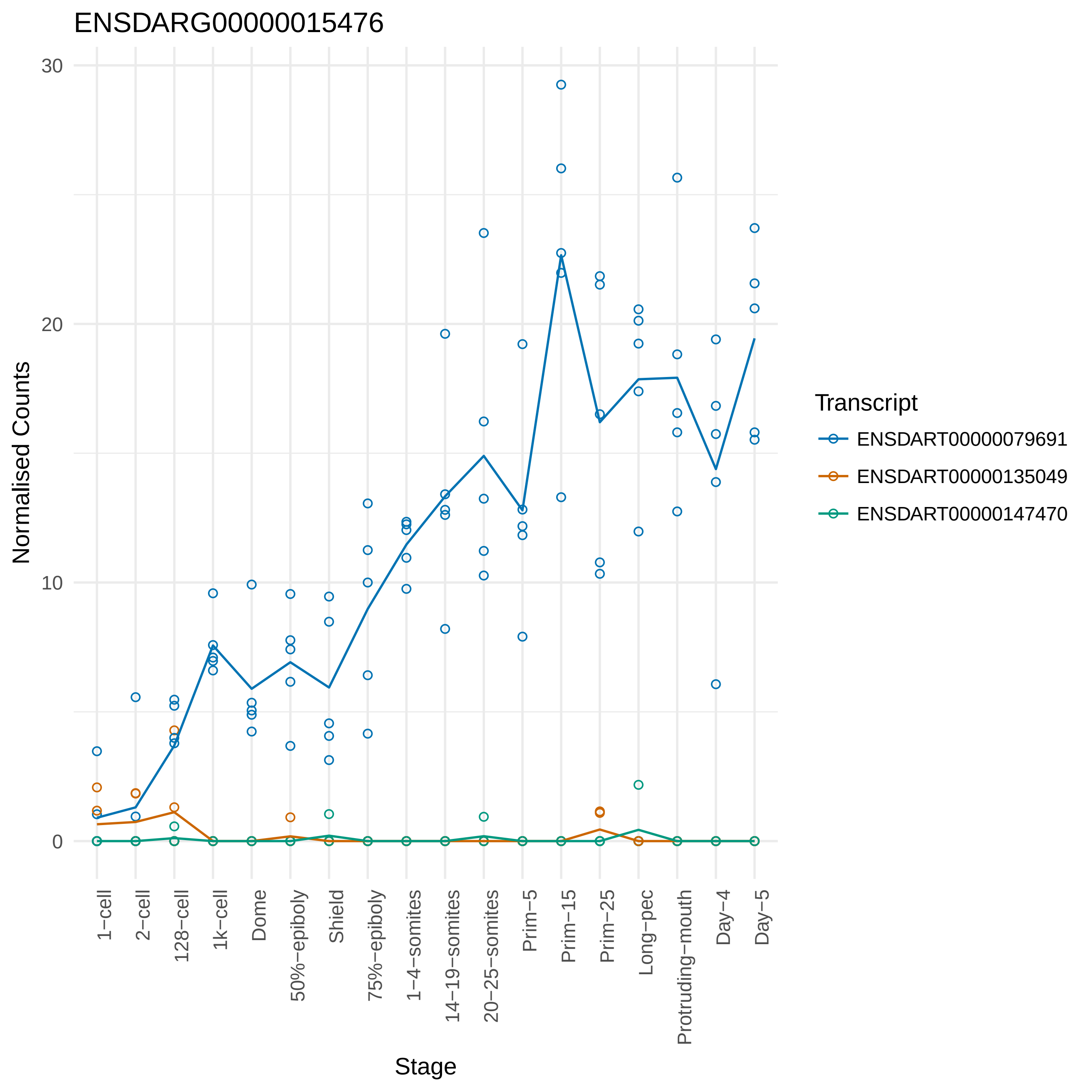

ENSDARG00000015476
Normalised Counts
30
20
10
0
1−cell
2−cell
128−cell
1k−cell
Dome
50%−epiboly
Shield
75%−epiboly
1−4−somites
14−19−somites
20−25−somites
Prim−5
Prim−15
Prim−25
Long−pec
Protruding−mouth
Day−4
Day−5
Stage
Transcript
ENSDART00000079691
ENSDART00000135049
ENSDART00000147470

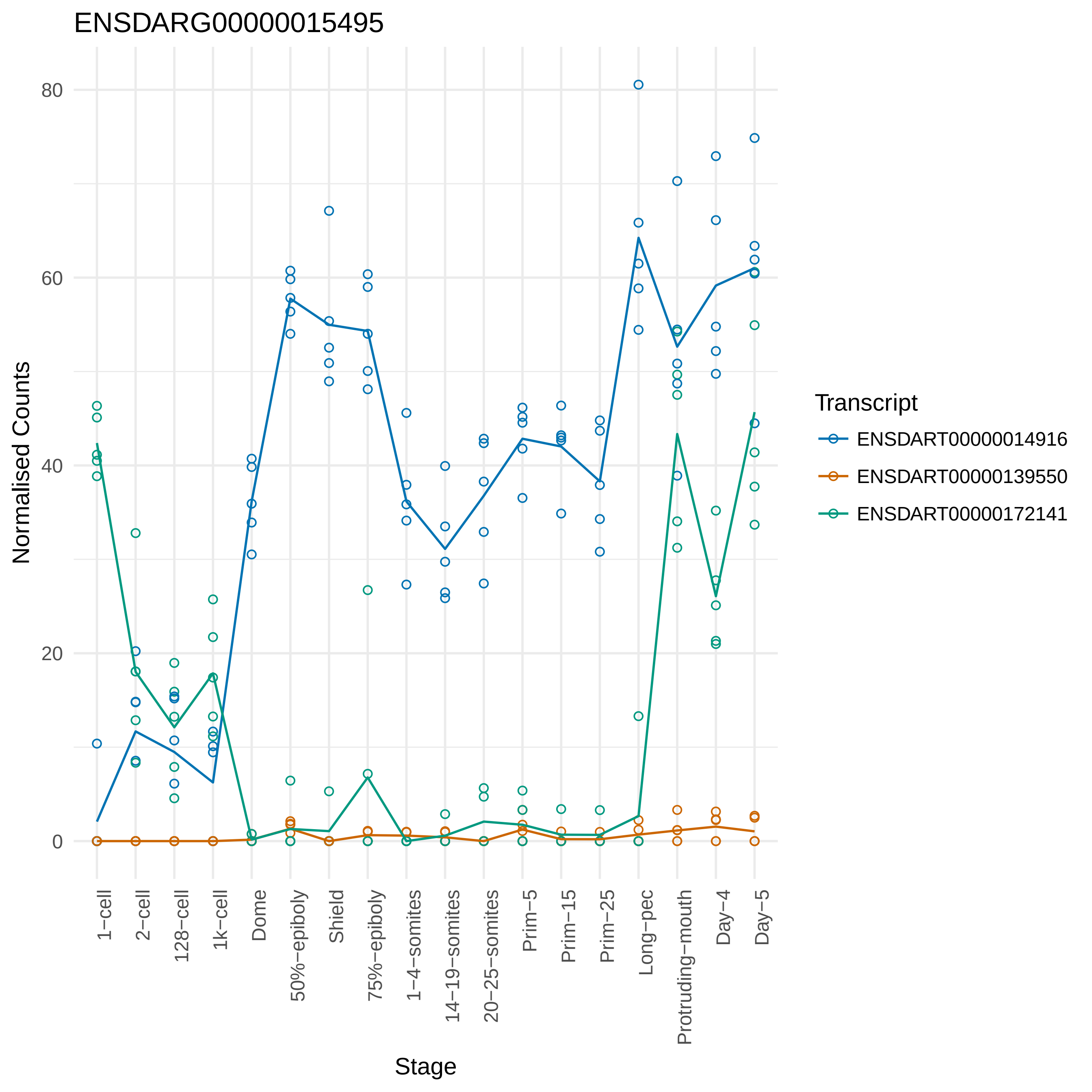
ENSDARG00000015495
Normalised Counts
80
60
40
20
0
Stage
1−cell
2−cell
128−cell
1k−cell
Dome
50%−epiboly
Shield
75%−epiboly
1−4−somites
14−19−somites
20−25−somites
Prim−5
Prim−15
Prim−25
Long−pec
Protruding−mouth
Day−4
Day−5
Transcript
ENSDART00000014916
ENSDART00000139550
ENSDART00000172141

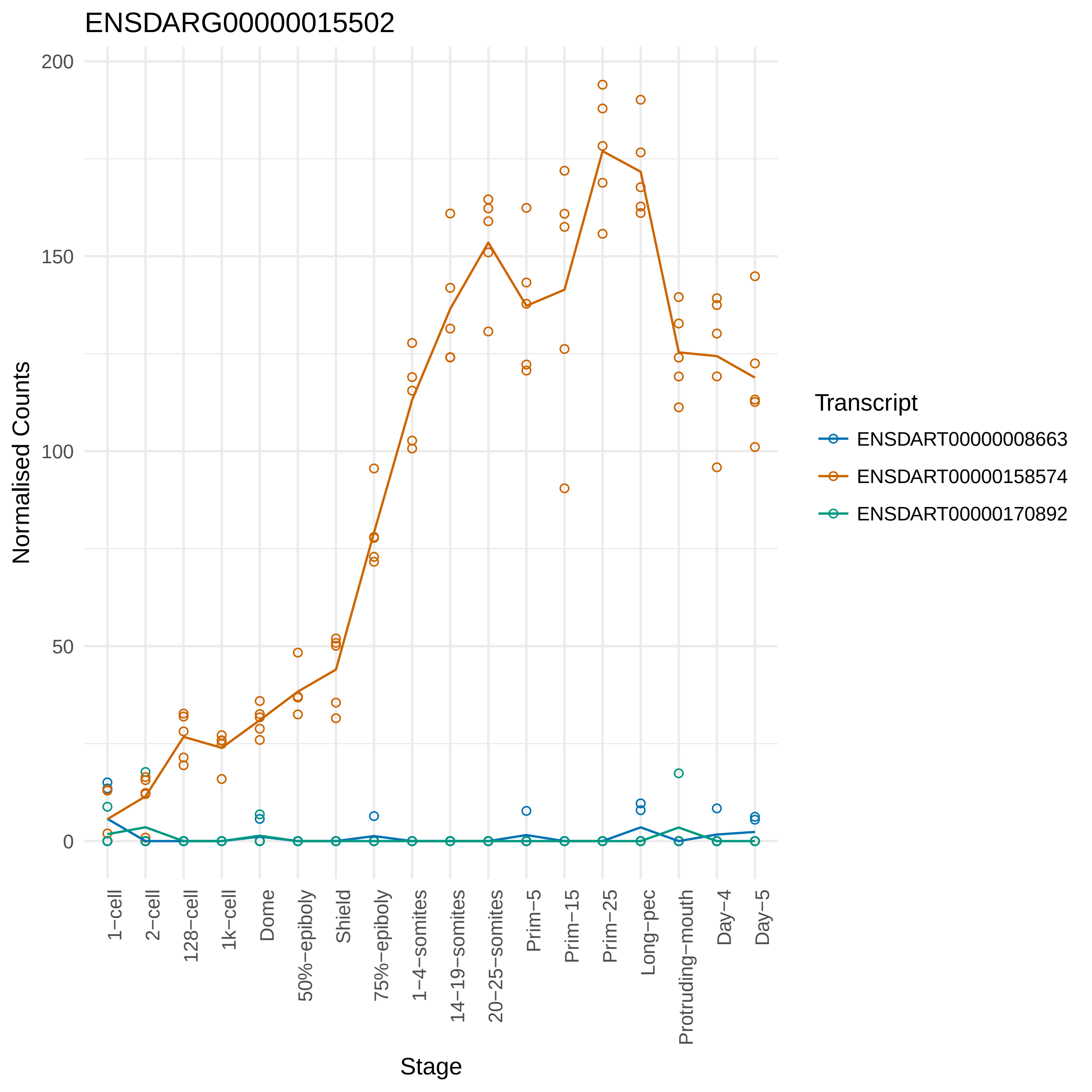

ENSDARG00000015502
Normalised Counts
200
150
100
50
0
1−cell
2−cell
128−cell
1k−cell
Dome
50%−epiboly
Shield
75%−epiboly
1−4−somites
14−19−somites
20−25−somites
Prim−5
Prim−15
Prim−25
Long−pec
Protruding−mouth
Day−4
Day−5
Stage
Transcript
ENSDART00000008663
ENSDART00000158574
ENSDART00000170892

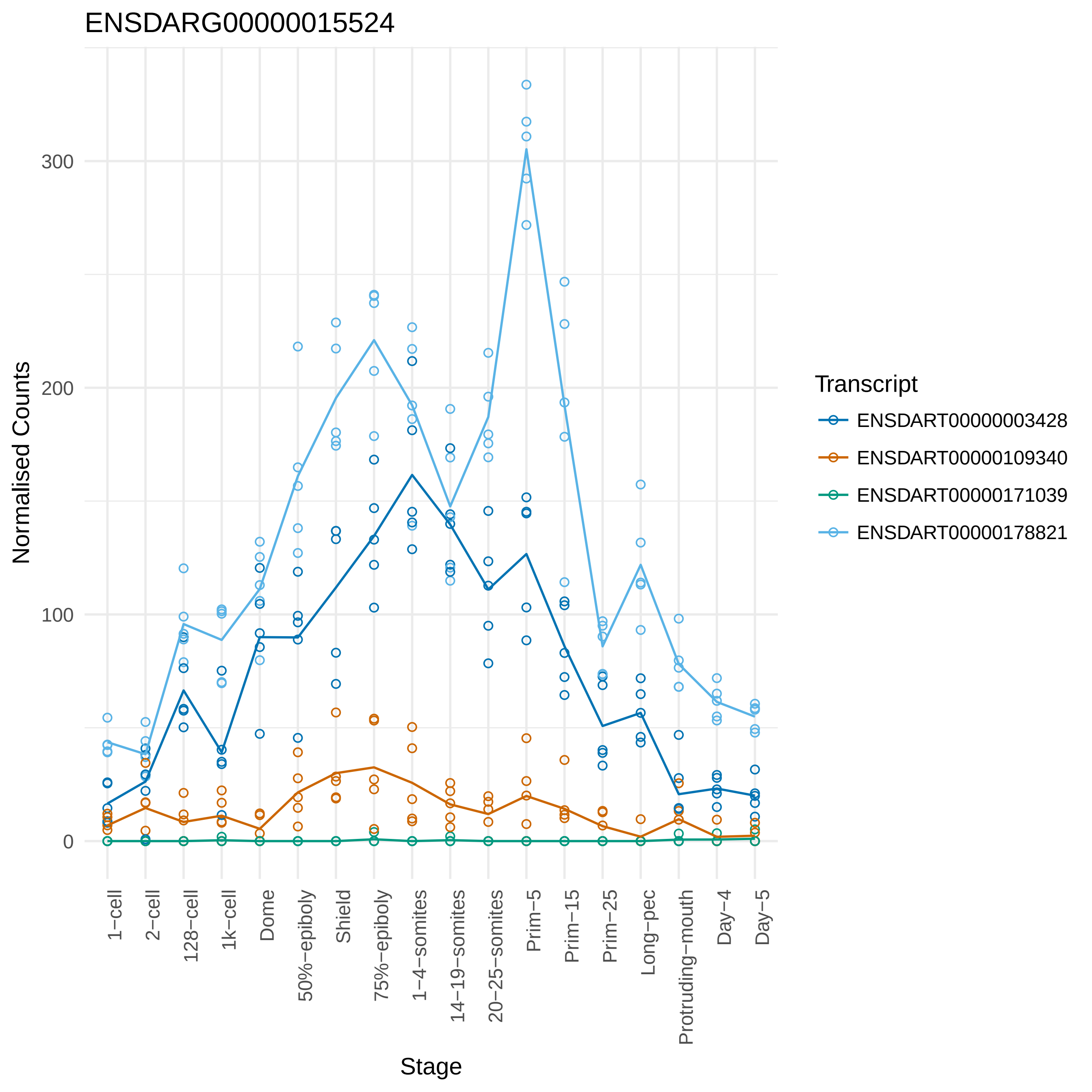

ENSDARG00000015524
Normalised Counts
300
200
100
0
1−cell
2−cell
128−cell
1k−cell
Dome
50%−epiboly
Shield
75%−epiboly
1−4−somites
14−19−somites
20−25−somites
Prim−5
Prim−15
Prim−25
Long−pec
Protruding−mouth
Day−4
Day−5
Stage
Transcript
ENSDART00000003428
ENSDART00000109340
ENSDART00000171039
ENSDART00000178821

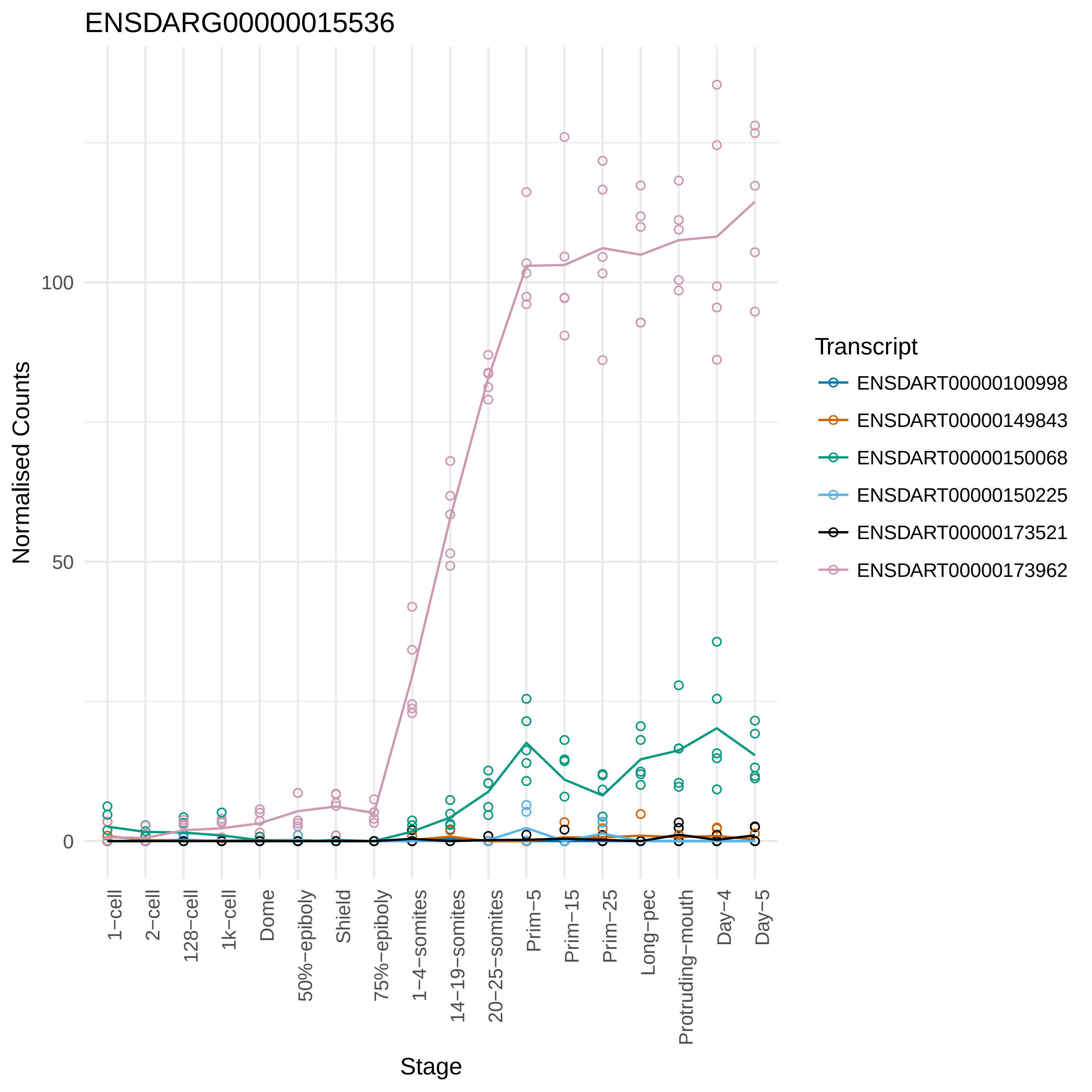

ENSDARG00000015536
Normalised Counts
100
50
0
1−cell
2−cell
128−cell
1k−cell
Dome
50%−epiboly
Shield
75%−epiboly
1−4−somites
14−19−somites
20−25−somites
Prim−5
Prim−15
Prim−25
Long−pec
Protruding−mouth
Day−4
Day−5
Stage
Transcript
ENSDART00000100998
ENSDART00000149843
ENSDART00000150068
ENSDART00000150225
ENSDART00000173521
ENSDART00000173962

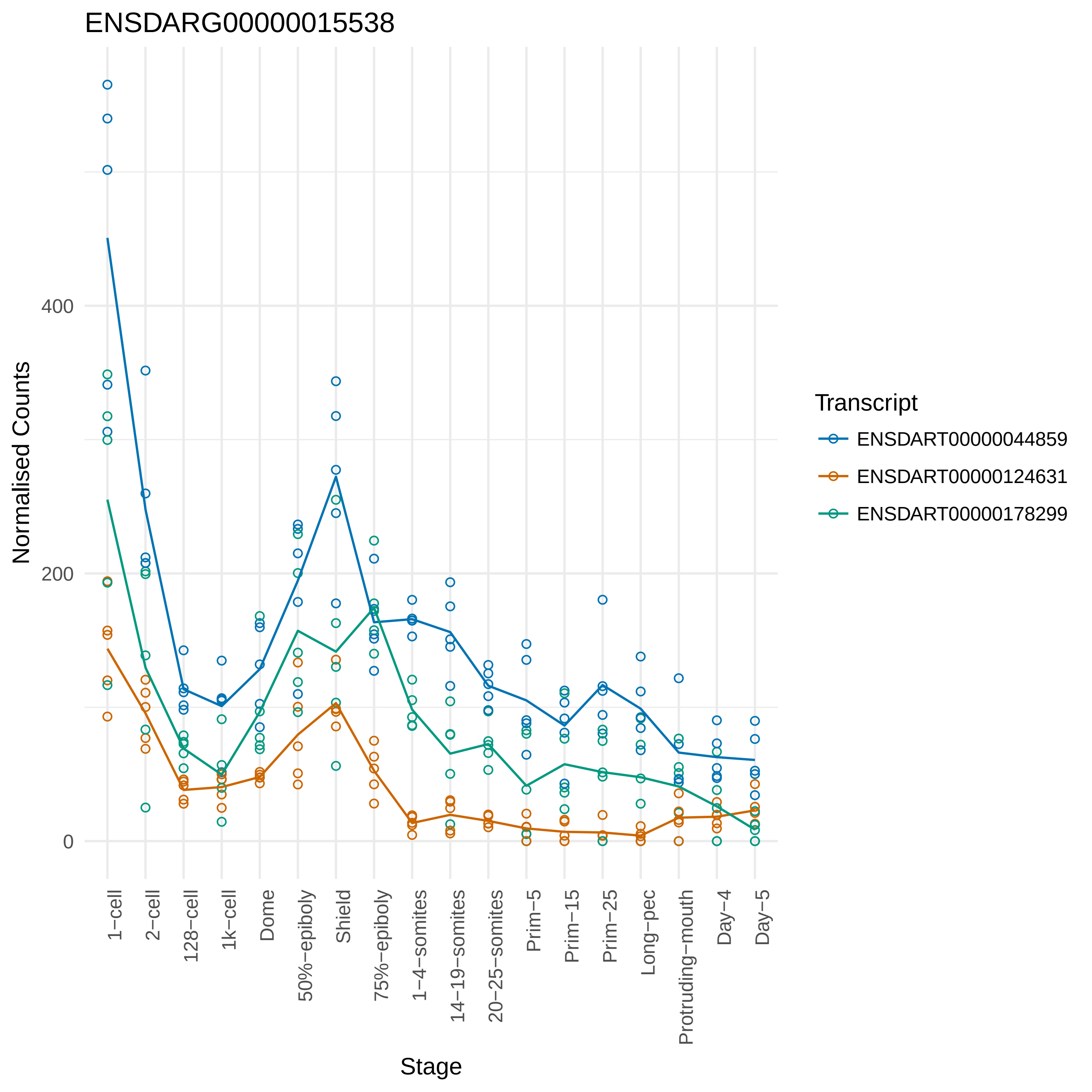

ENSDARG00000015538
Normalised Counts
0
200
400
1−cell
2−cell
128−cell
1k−cell
Dome
50%−epiboly
Shield
75%−epiboly
1−4−somites
14−19−somites
20−25−somites
Prim−5
Prim−15
Prim−25
Long−pec
Protruding−mouth
Day−4
Day−5
Stage
Transcript
ENSDART00000044859
ENSDART00000124631
ENSDART00000178299

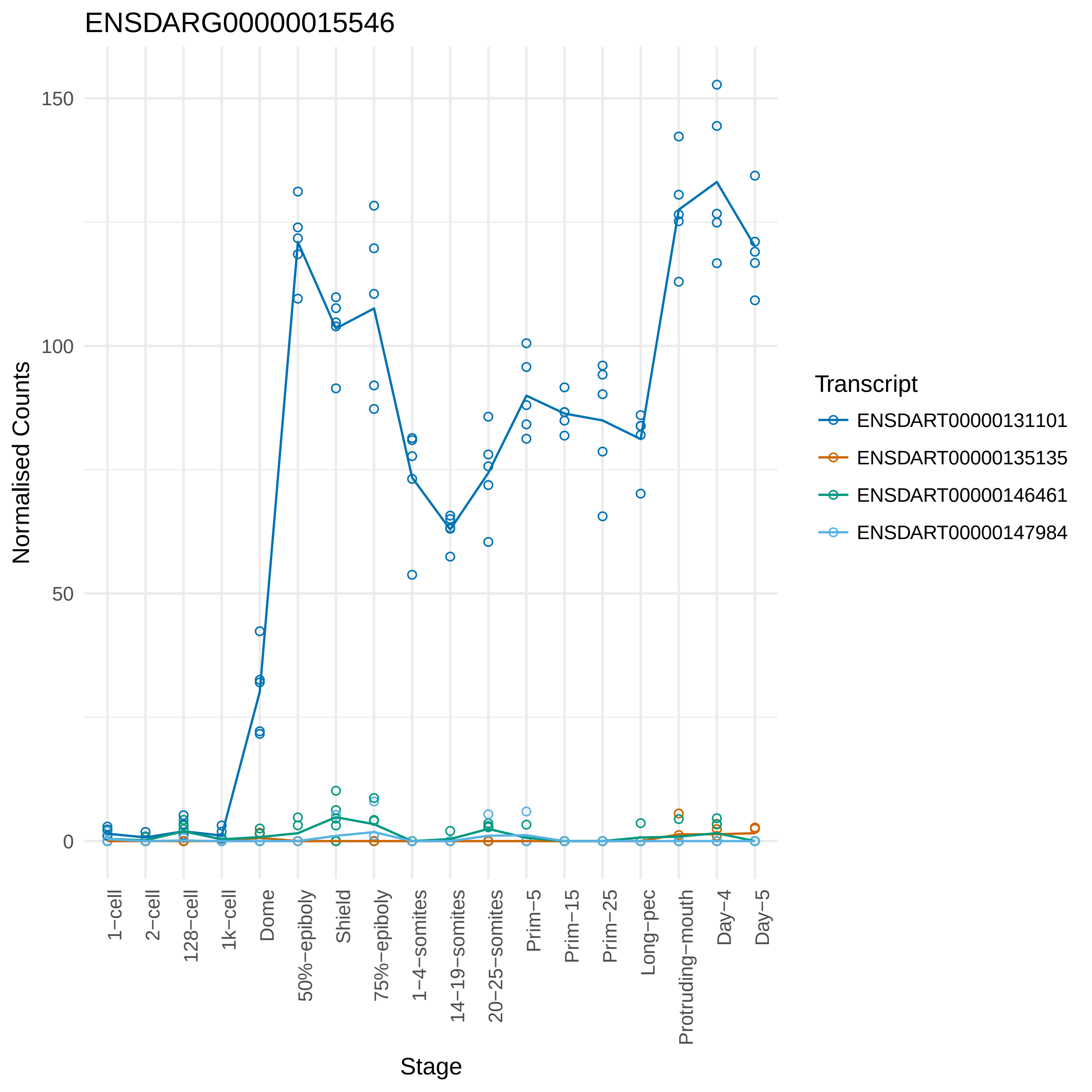
ENSDARG00000015546
Normalised Counts
150
100
50
0
1−cell
2−cell
128−cell
1k−cell
Dome
50%−epiboly
Shield
75%−epiboly
1−4−somites
14−19−somites
20−25−somites
Prim−5
Prim−15
Prim−25
Long−pec
Protruding−mouth
Day−4
Day−5
Stage
Transcript
ENSDART00000131101
ENSDART00000135135
ENSDART00000146461
ENSDART00000147984

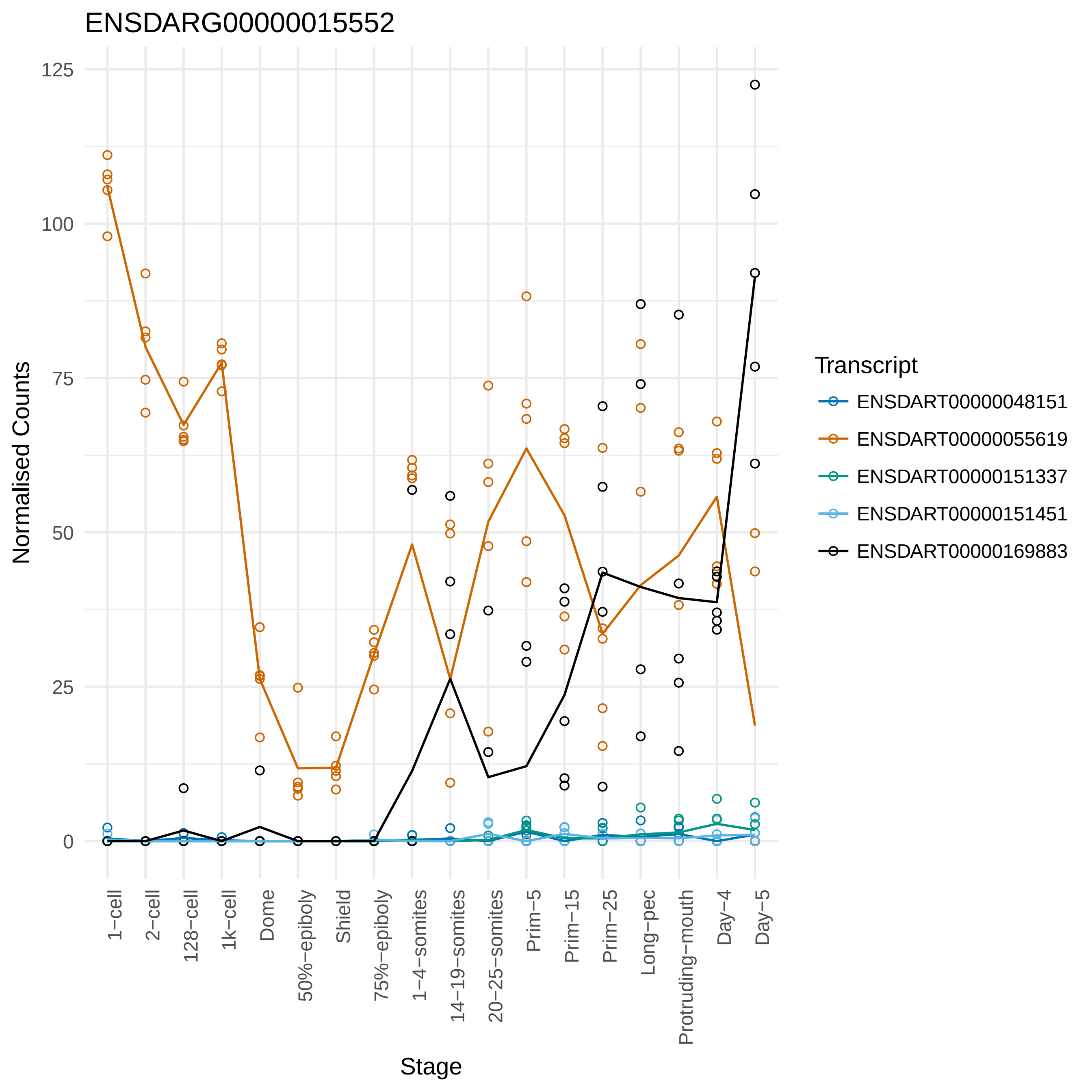

ENSDARG00000015552
Normalised Counts
125
100
75
50
25
0
1−cell
2−cell
128−cell
1k−cell
Dome
50%−epiboly
Shield
75%−epiboly
1−4−somites
14−19−somites
20−25−somites
Prim−5
Prim−15
Prim−25
Long−pec
Protruding−mouth
Day−4
Day−5
Stage
Transcript
ENSDART00000048151
ENSDART00000055619
ENSDART00000151337
ENSDART00000151451
ENSDART00000169883

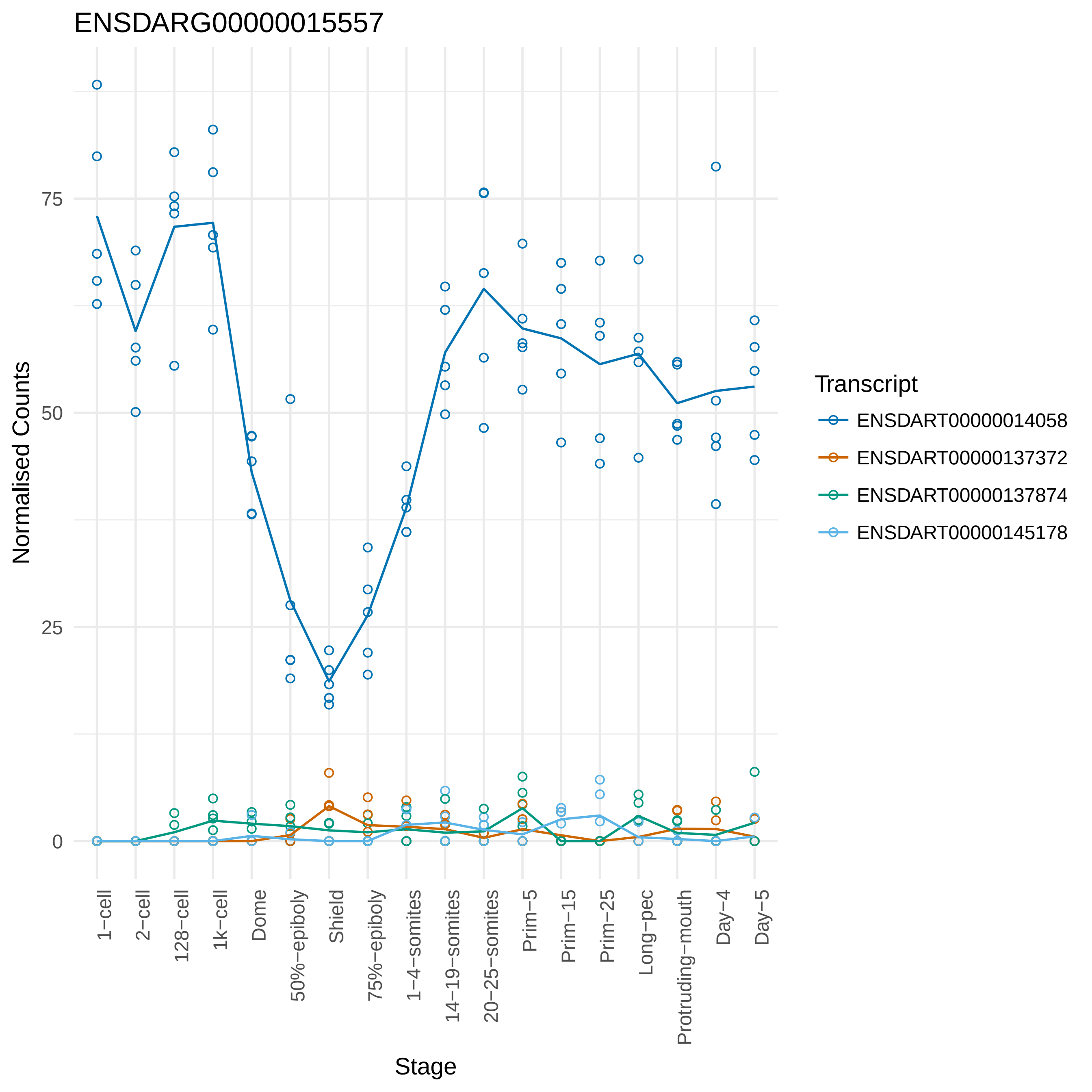

ENSDARG00000015557
Normalised Counts
0
25
50
75
Stage
1−cell
2−cell
128−cell
1k−cell
Dome
50%−epiboly
Shield
75%−epiboly
1−4−somites
14−19−somites
20−25−somites
Prim−5
Prim−15
Prim−25
Long−pec
Protruding−mouth
Day−4
Day−5
Transcript
ENSDART00000014058
ENSDART00000137372
ENSDART00000137874
ENSDART00000145178

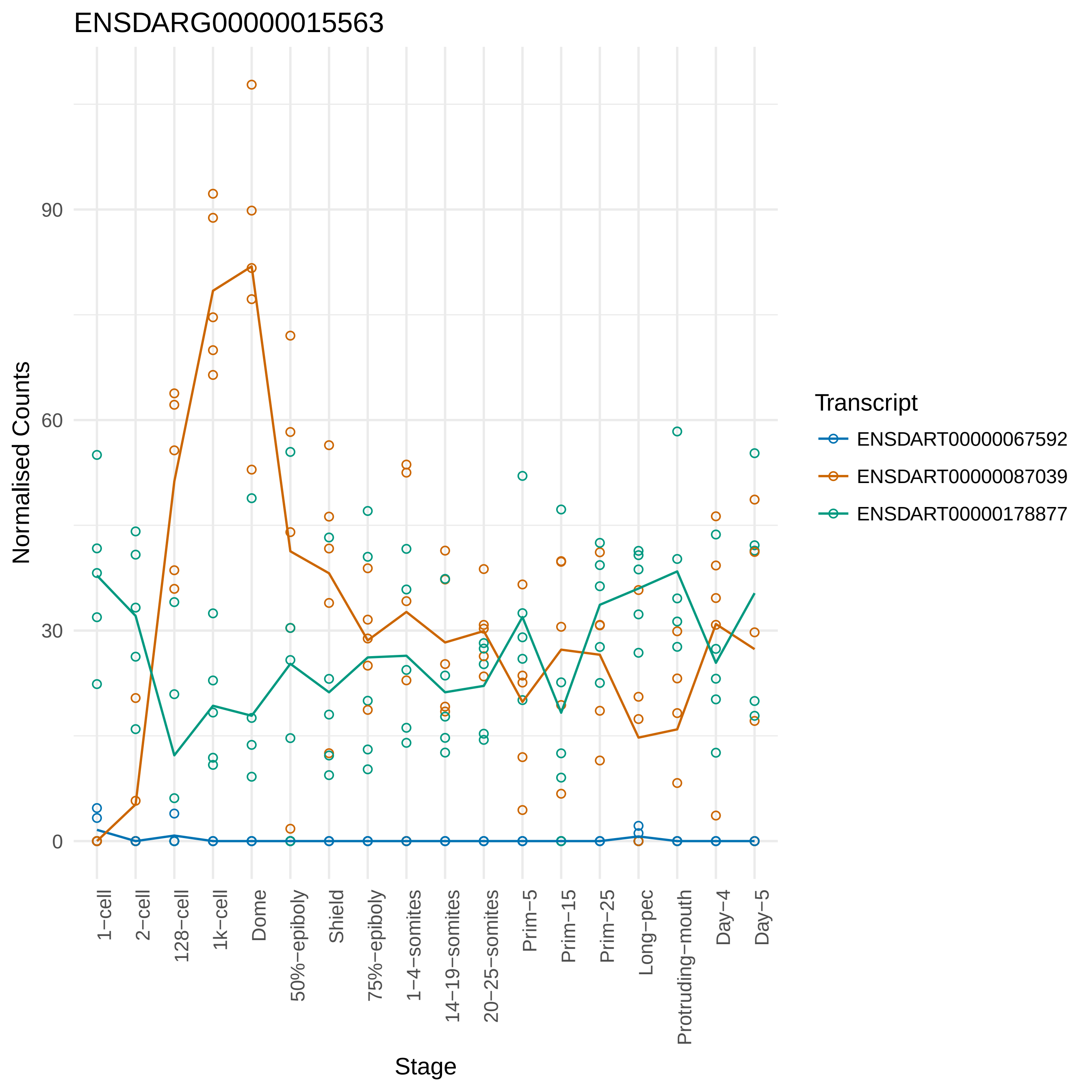

ENSDARG00000015563
Normalised Counts
0
30
60
90
Stage
1−cell
2−cell
128−cell
1k−cell
Dome
50%−epiboly
Shield
75%−epiboly
1−4−somites
14−19−somites
20−25−somites
Prim−5
Prim−15
Prim−25
Long−pec
Protruding−mouth
Day−4
Day−5
Transcript
ENSDART00000067592
ENSDART00000087039
ENSDART00000178877

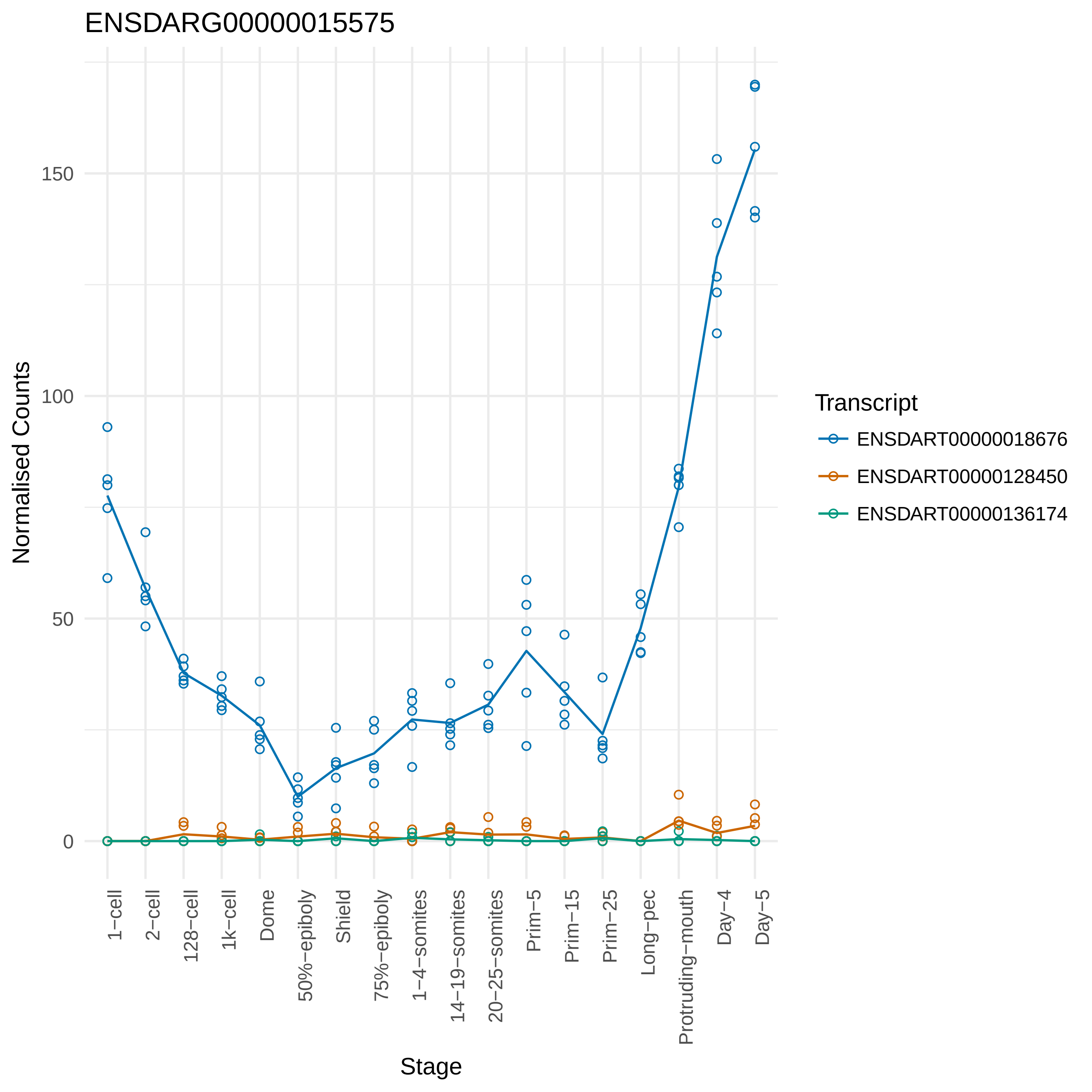

ENSDARG00000015575
Normalised Counts
150
100
50
0
1−cell
2−cell
128−cell
1k−cell
Dome
50%−epiboly
Shield
75%−epiboly
1−4−somites
14−19−somites
20−25−somites
Prim−5
Prim−15
Prim−25
Long−pec
Protruding−mouth
Day−4
Day−5
Stage
Transcript
ENSDART00000018676
ENSDART00000128450
ENSDART00000136174

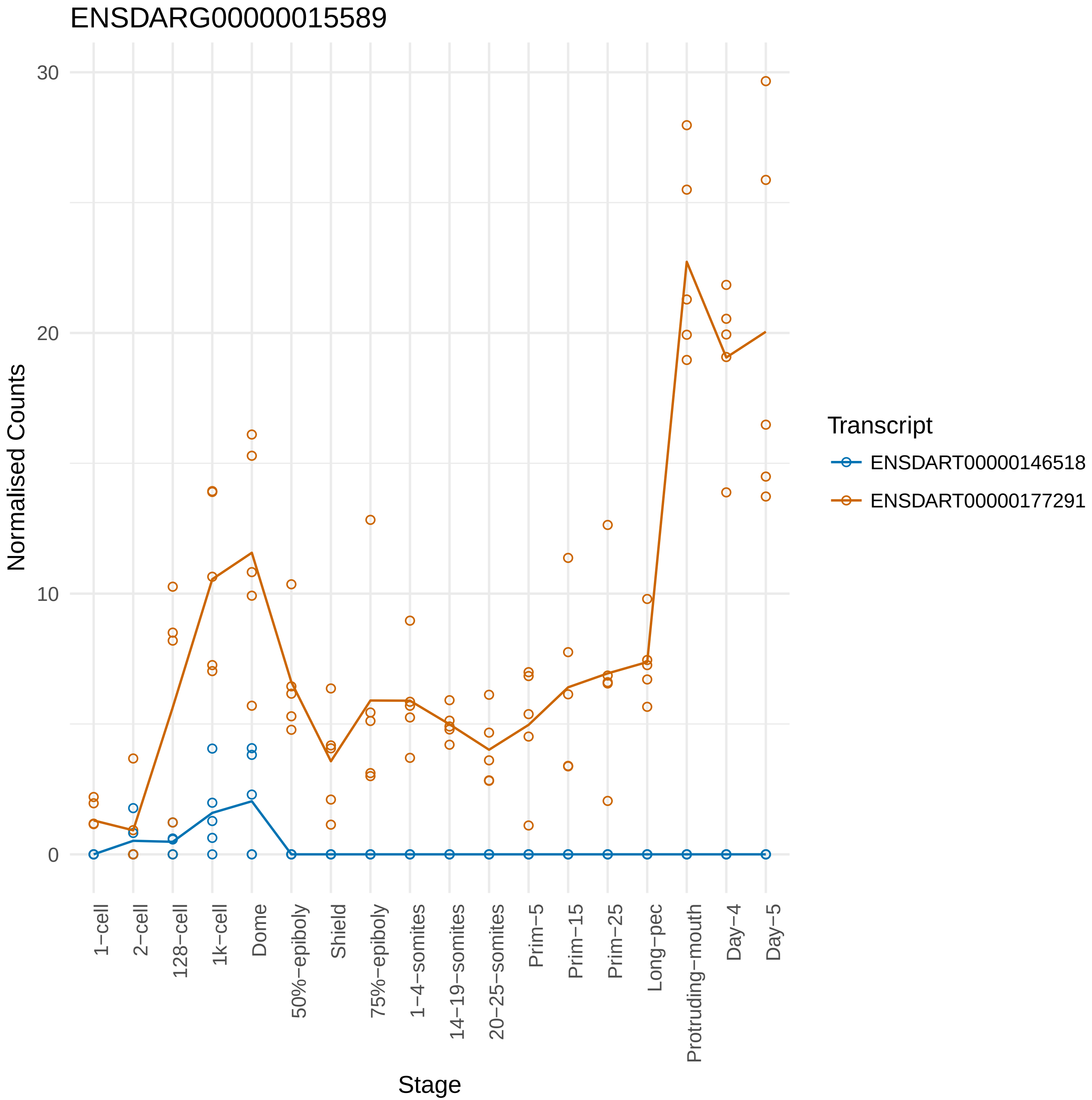
ENSDARG00000015589
Normalised Counts
0
10
20
30
Stage
1−cell
2−cell
128−cell
1k−cell
Dome
50%−epiboly
Shield
75%−epiboly
1−4−somites
14−19−somites
20−25−somites
Prim−5
Prim−15
Prim−25
Long−pec
Protruding−mouth
Day−4
Day−5
Transcript
ENSDART00000146518
ENSDART00000177291

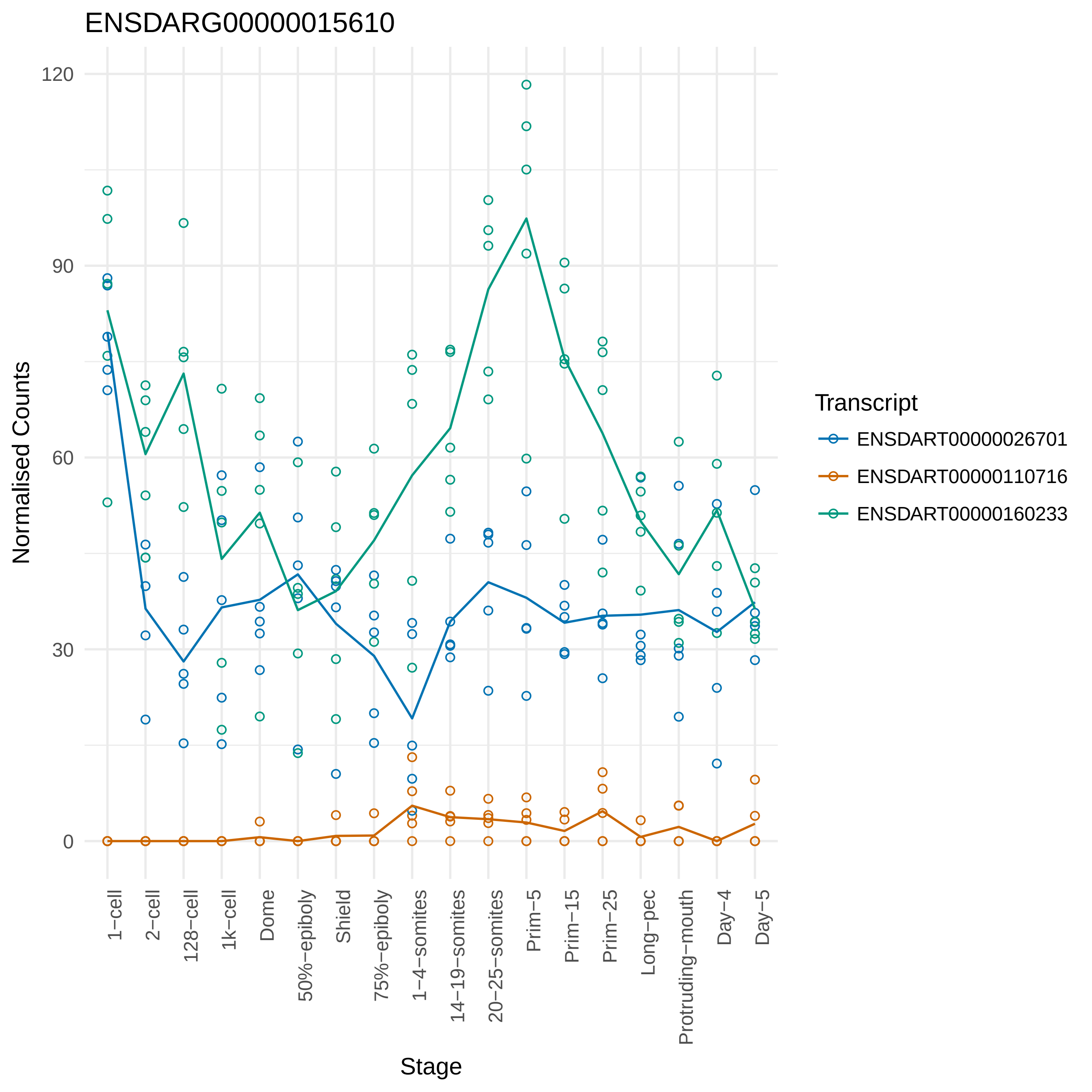

ENSDARG00000015610
Normalised Counts
0
30
60
90
120
1−cell
2−cell
128−cell
1k−cell
Dome
50%−epiboly
Shield
75%−epiboly
1−4−somites
14−19−somites
20−25−somites
Prim−5
Prim−15
Prim−25
Long−pec
Protruding−mouth
Day−4
Day−5
Stage
Transcript
ENSDART00000026701
ENSDART00000110716
ENSDART00000160233

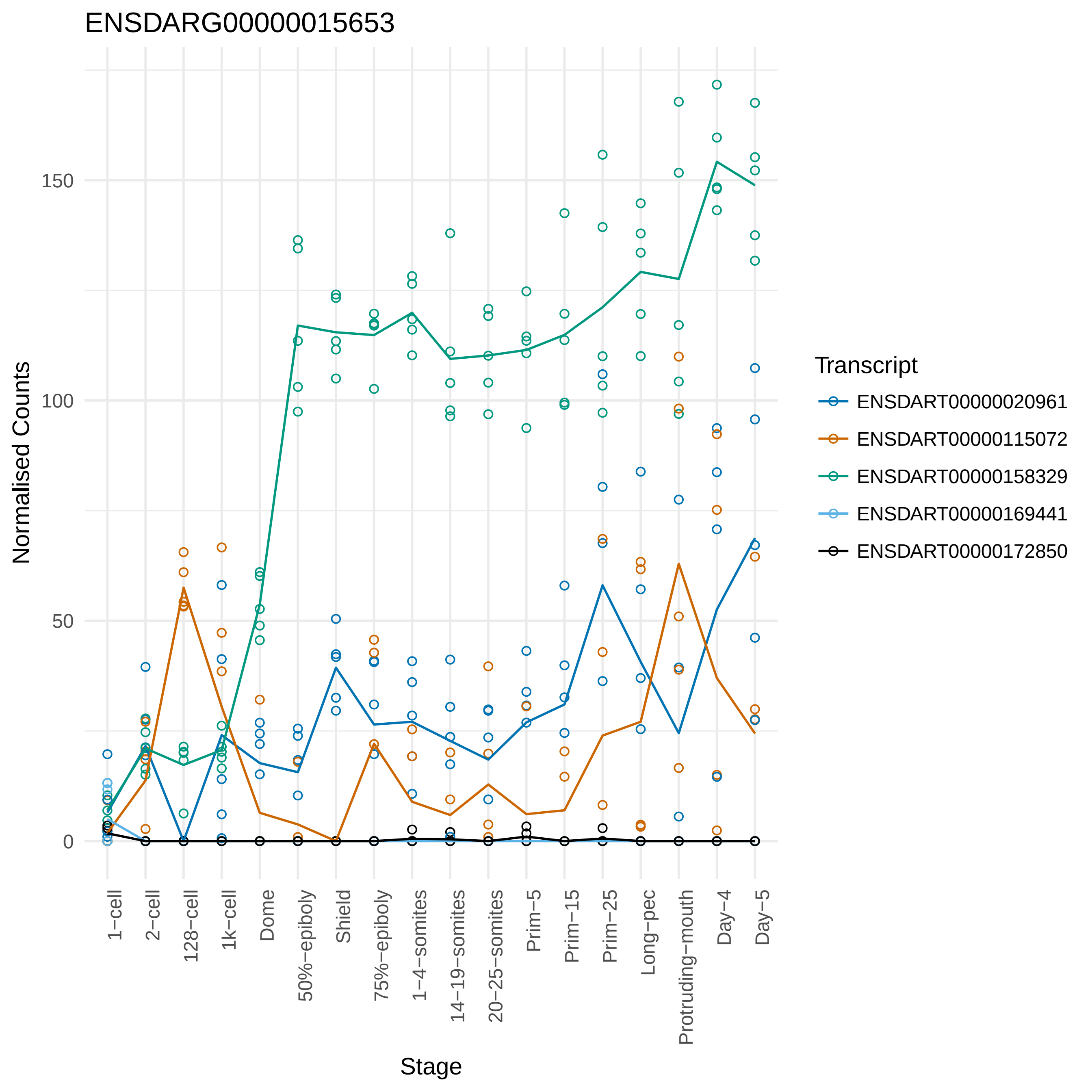

ENSDARG00000015653
Normalised Counts
150
100
50
0
1−cell
2−cell
128−cell
1k−cell
Dome
50%−epiboly
Shield
75%−epiboly
1−4−somites
14−19−somites
20−25−somites
Prim−5
Prim−15
Prim−25
Long−pec
Protruding−mouth
Day−4
Day−5
Stage
Transcript
ENSDART00000020961
ENSDART00000115072
ENSDART00000158329
ENSDART00000169441
ENSDART00000172850

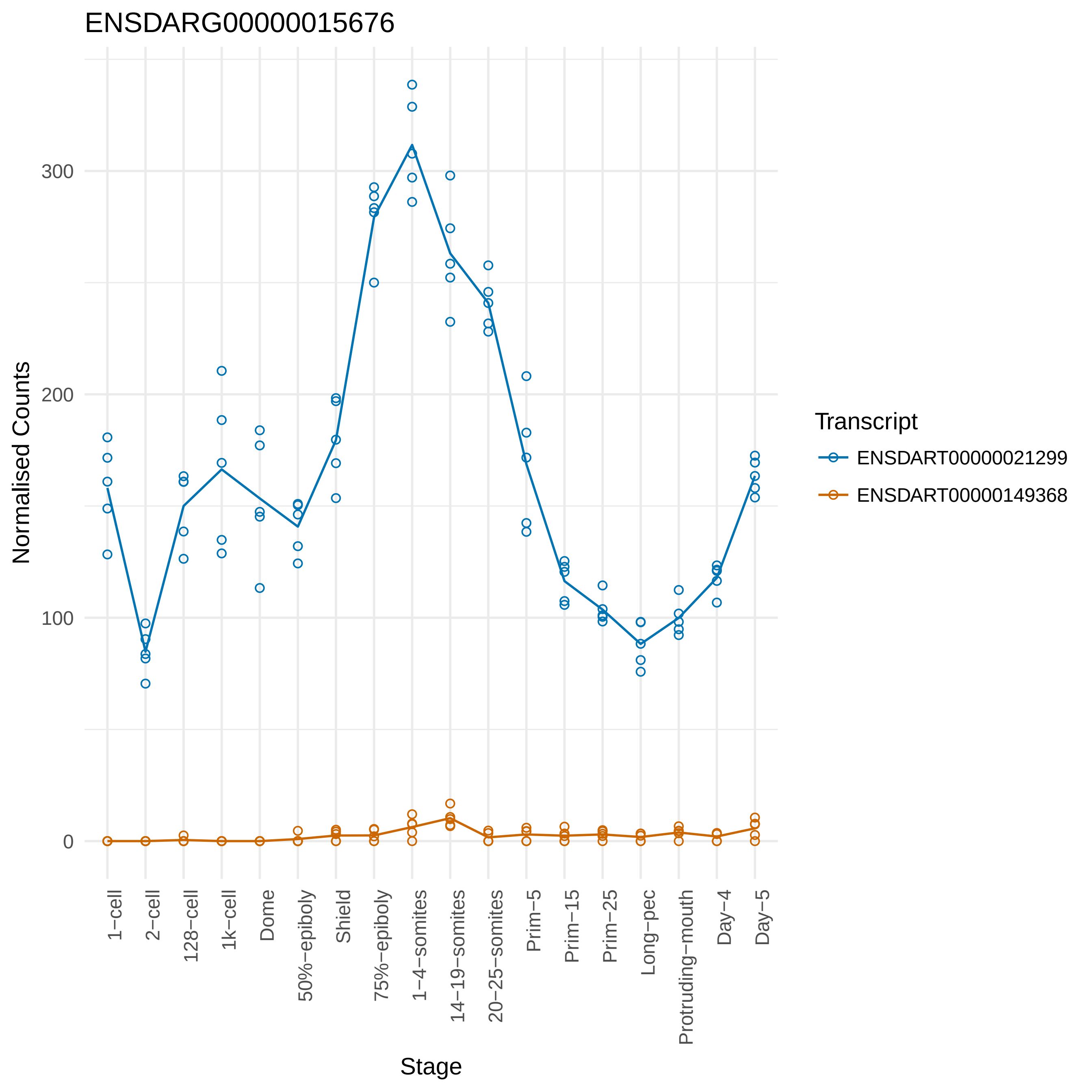

ENSDARG00000015676
Normalised Counts
300
200
100
0
Stage
1−cell
2−cell
128−cell
1k−cell
Dome
50%−epiboly
Shield
75%−epiboly
1−4−somites
14−19−somites
20−25−somites
Prim−5
Prim−15
Prim−25
Long−pec
Protruding−mouth
Day−4
Day−5
Transcript
ENSDART00000021299
ENSDART00000149368

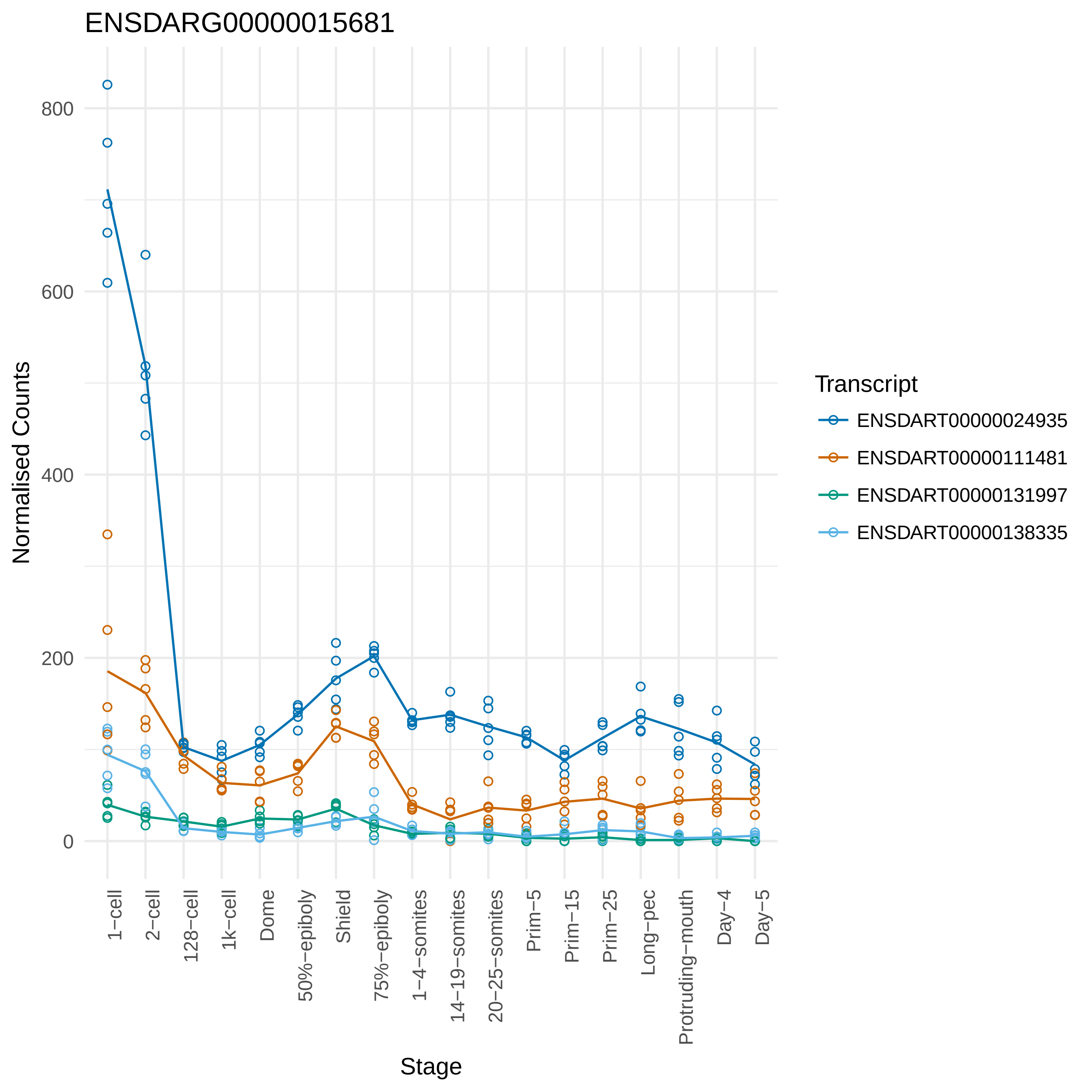

ENSDARG00000015681
Normalised Counts
0
200
400
600
800
1−cell
2−cell
128−cell
1k−cell
Dome
50%−epiboly
Shield
75%−epiboly
1−4−somites
14−19−somites
20−25−somites
Prim−5
Prim−15
Prim−25
Long−pec
Protruding−mouth
Day−4
Day−5
Stage
Transcript
ENSDART00000024935
ENSDART00000111481
ENSDART00000131997
ENSDART00000138335

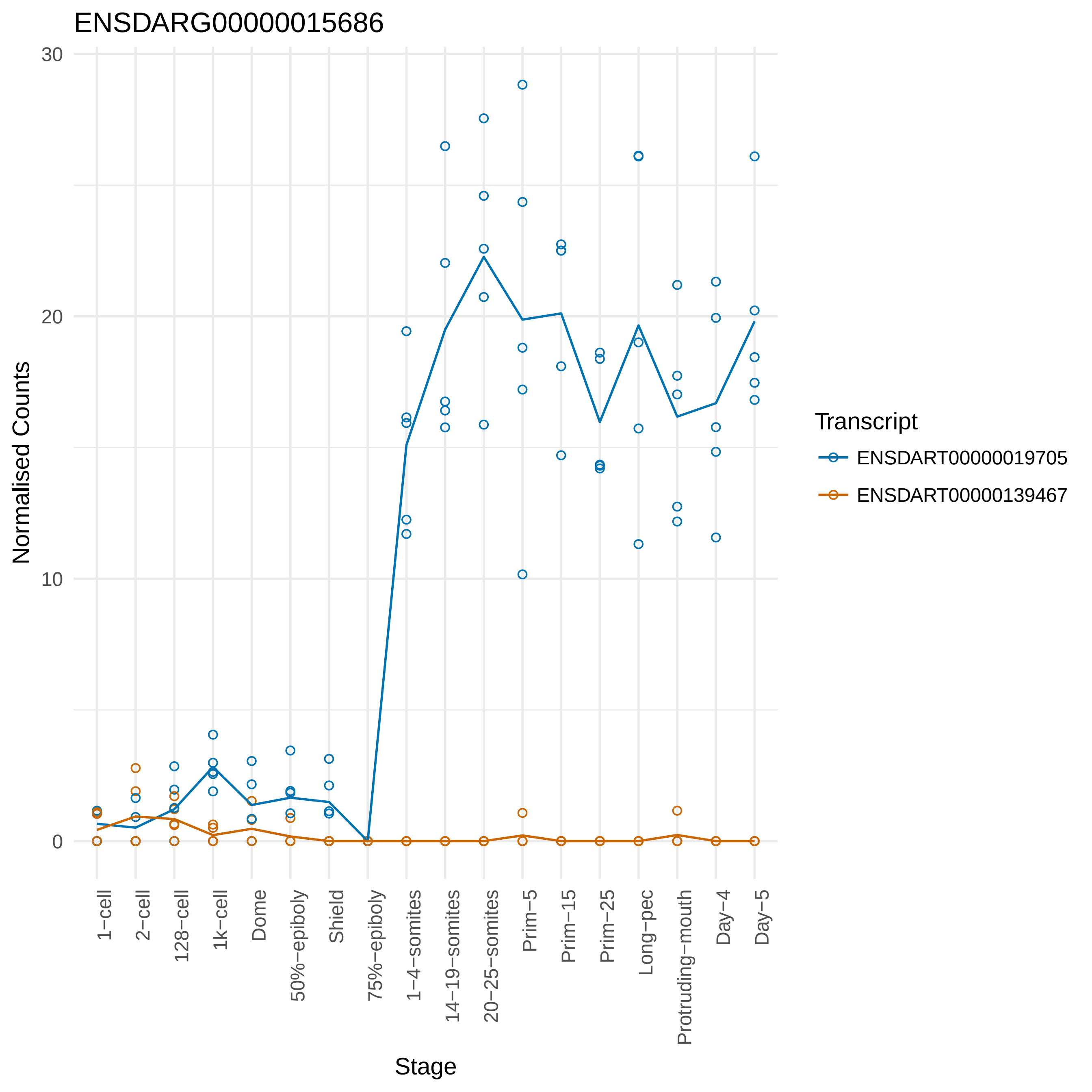

ENSDARG00000015686
Normalised Counts
30
20
10
0
1−cell
2−cell
128−cell
1k−cell
Dome
50%−epiboly
Shield
75%−epiboly
1−4−somites
14−19−somites
20−25−somites
Prim−5
Prim−15
Prim−25
Long−pec
Protruding−mouth
Day−4
Day−5
Stage
Transcript
ENSDART00000019705
ENSDART00000139467

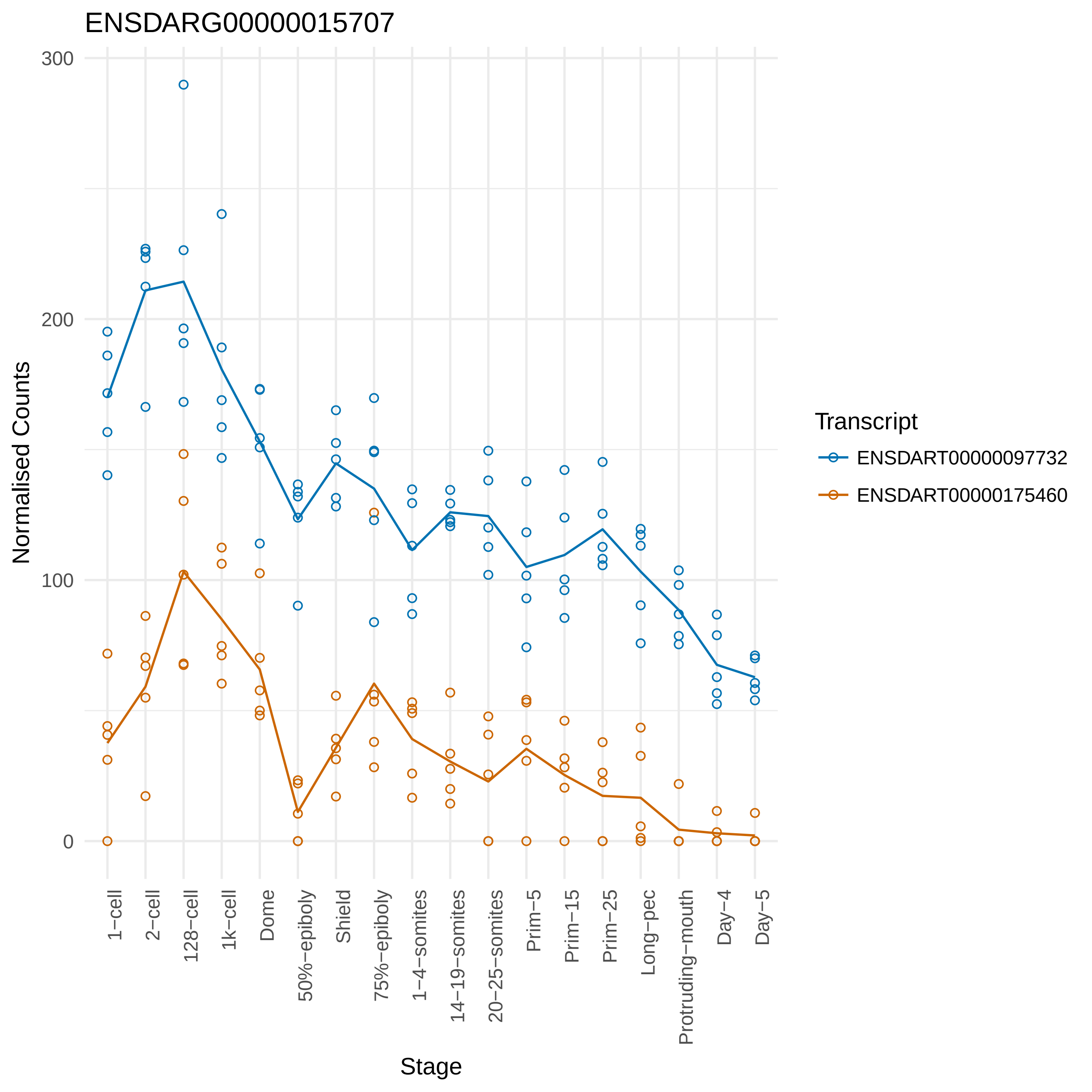

ENSDARG00000015707
Normalised Counts
300
200
100
0
1−cell
2−cell
128−cell
1k−cell
Dome
50%−epiboly
Shield
75%−epiboly
1−4−somites
14−19−somites
20−25−somites
Prim−5
Prim−15
Prim−25
Long−pec
Protruding−mouth
Day−4
Day−5
Stage
Transcript
ENSDART00000097732
ENSDART00000175460

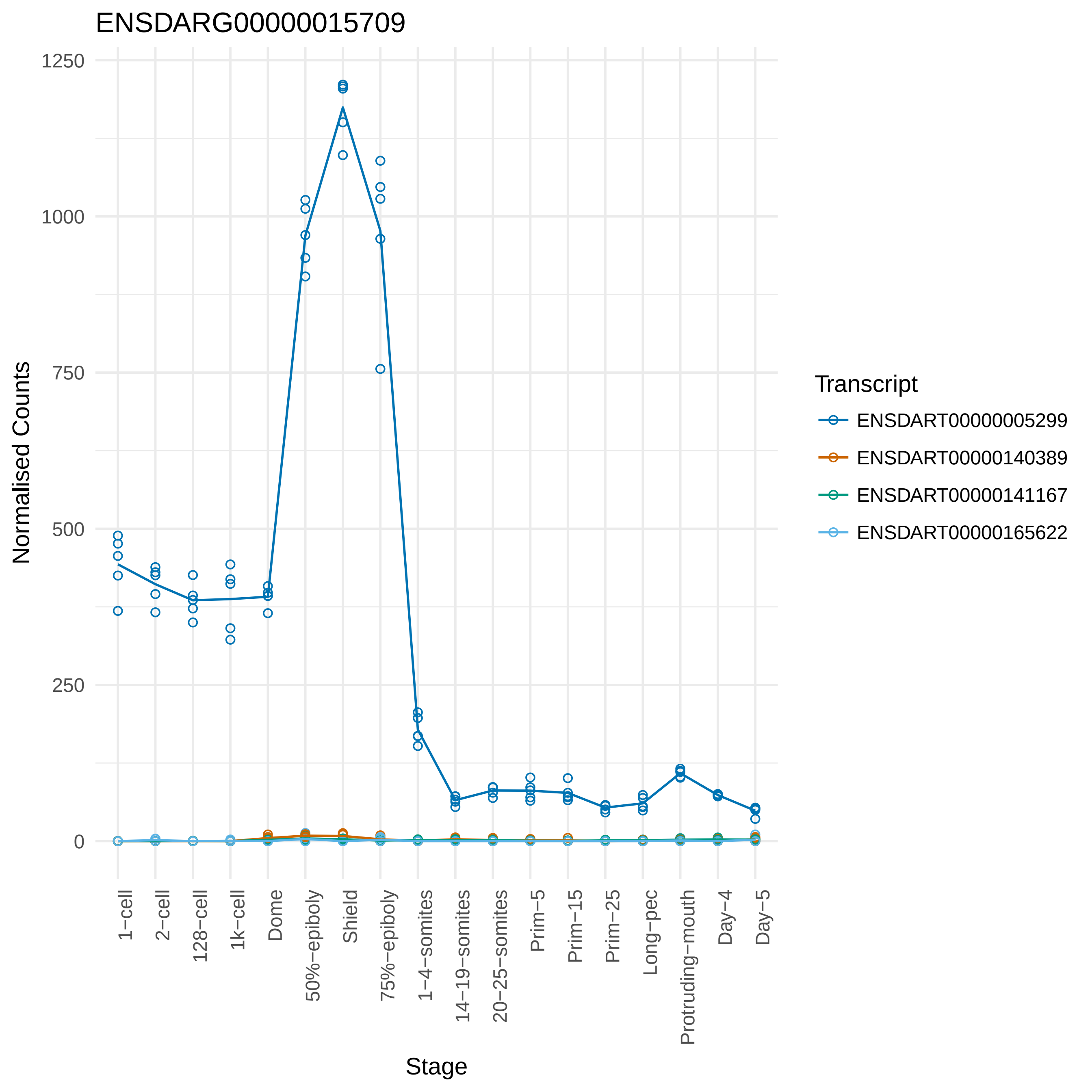

ENSDARG00000015709
Normalised Counts
1250
1000
750
500
250
0
1−cell
2−cell
128−cell
1k−cell
Dome
50%−epiboly
Shield
75%−epiboly
1−4−somites
14−19−somites
20−25−somites
Prim−5
Prim−15
Prim−25
Long−pec
Protruding−mouth
Day−4
Day−5
Stage
Transcript
ENSDART00000005299
ENSDART00000140389
ENSDART00000141167
ENSDART00000165622

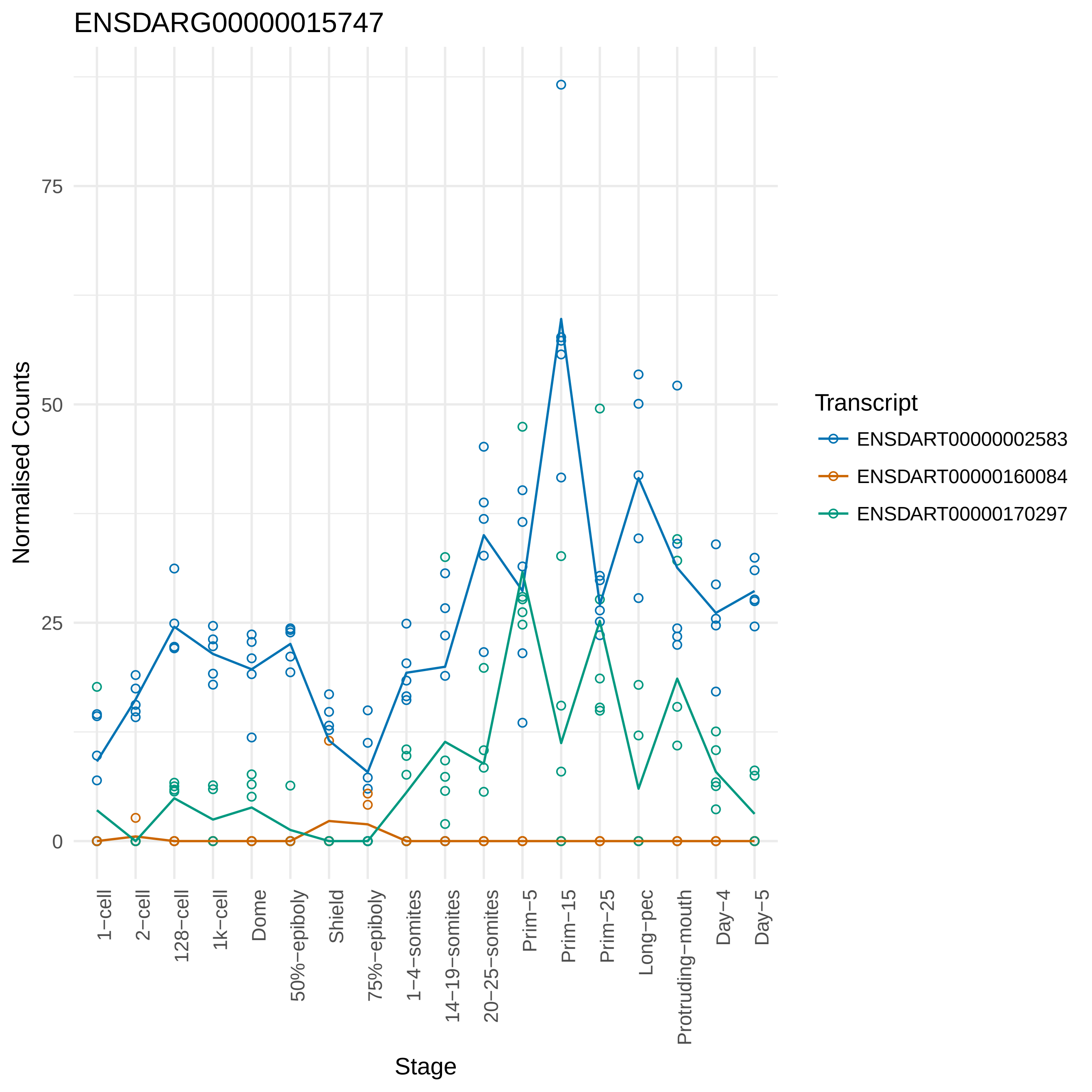

ENSDARG00000015747
Normalised Counts
75
50
25
0
1−cell
2−cell
128−cell
1k−cell
Dome
50%−epiboly
Shield
75%−epiboly
1−4−somites
14−19−somites
20−25−somites
Prim−5
Prim−15
Prim−25
Long−pec
Protruding−mouth
Day−4
Day−5
Stage
Transcript
ENSDART00000002583
ENSDART00000160084
ENSDART00000170297

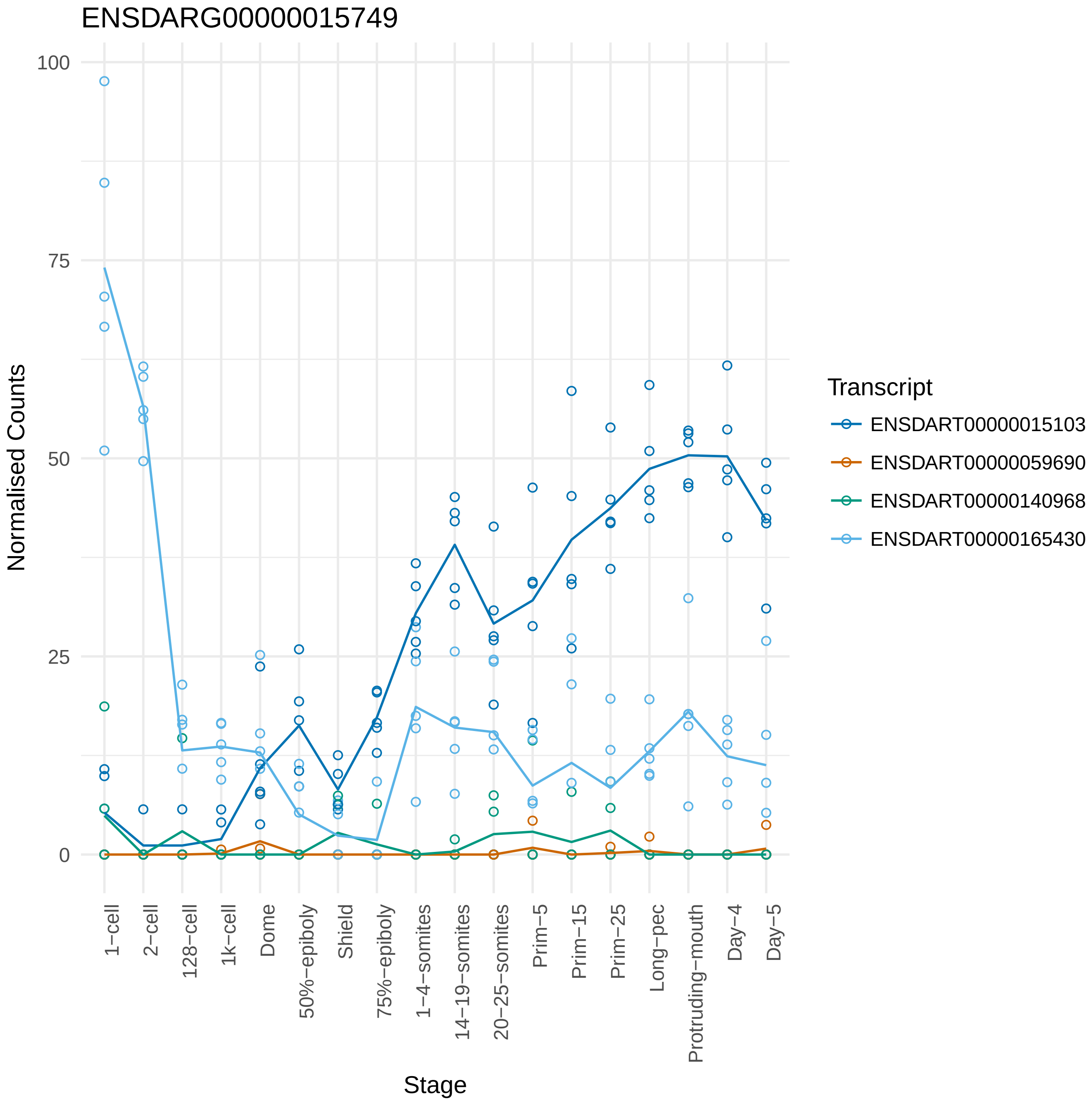

ENSDARG00000015749
Normalised Counts
100
75
50
25
0
1−cell
2−cell
128−cell
1k−cell
Dome
50%−epiboly
Shield
75%−epiboly
1−4−somites
14−19−somites
20−25−somites
Prim−5
Prim−15
Prim−25
Long−pec
Protruding−mouth
Day−4
Day−5
Stage
Transcript
ENSDART00000015103
ENSDART00000059690
ENSDART00000140968
ENSDART00000165430

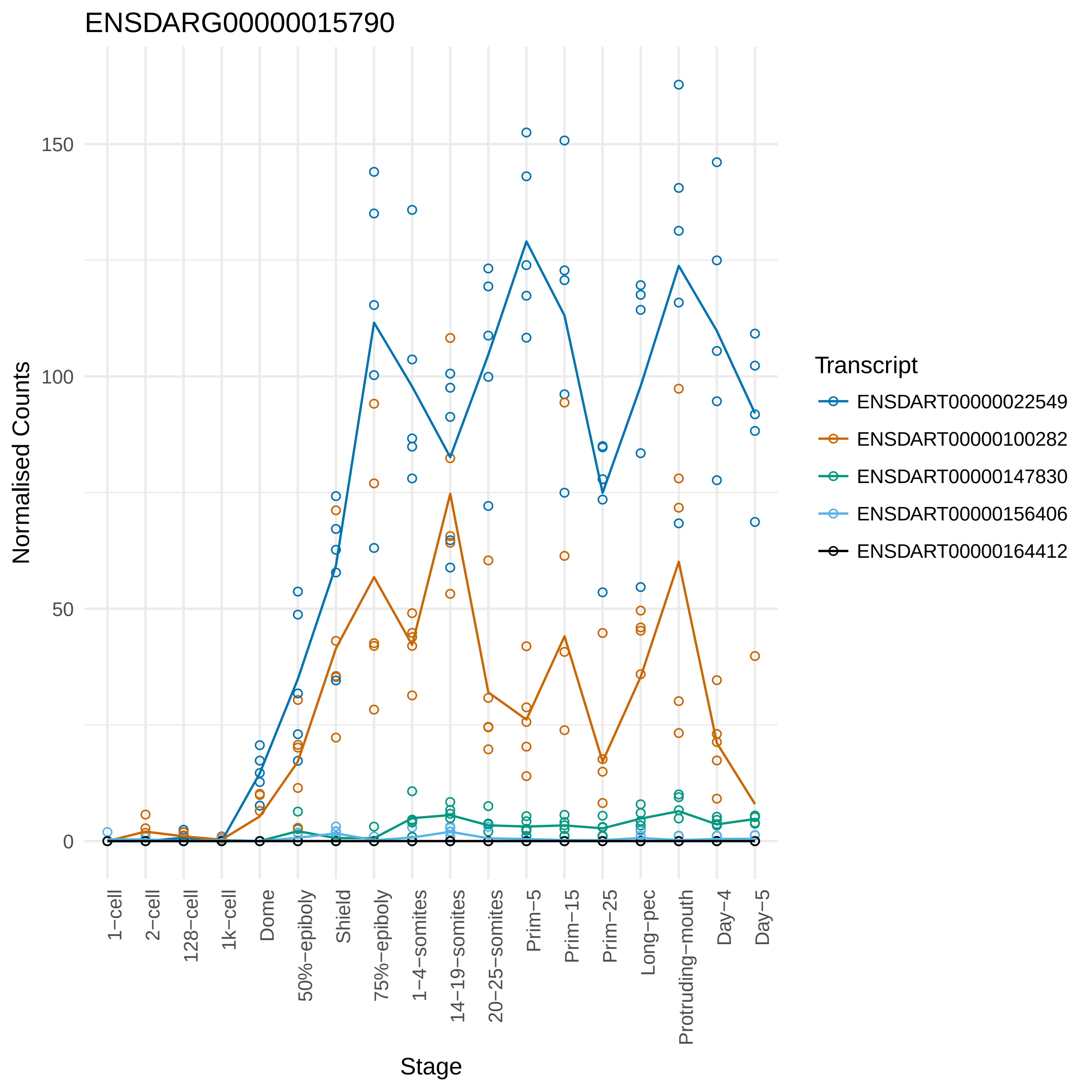

ENSDARG00000015790
Normalised Counts
0
50
100
150
Stage
1−cell
2−cell
128−cell
1k−cell
Dome
50%−epiboly
Shield
75%−epiboly
1−4−somites
14−19−somites
20−25−somites
Prim−5
Prim−15
Prim−25
Long−pec
Protruding−mouth
Day−4
Day−5
Transcript
ENSDART00000022549
ENSDART00000100282
ENSDART00000147830
ENSDART00000156406
ENSDART00000164412

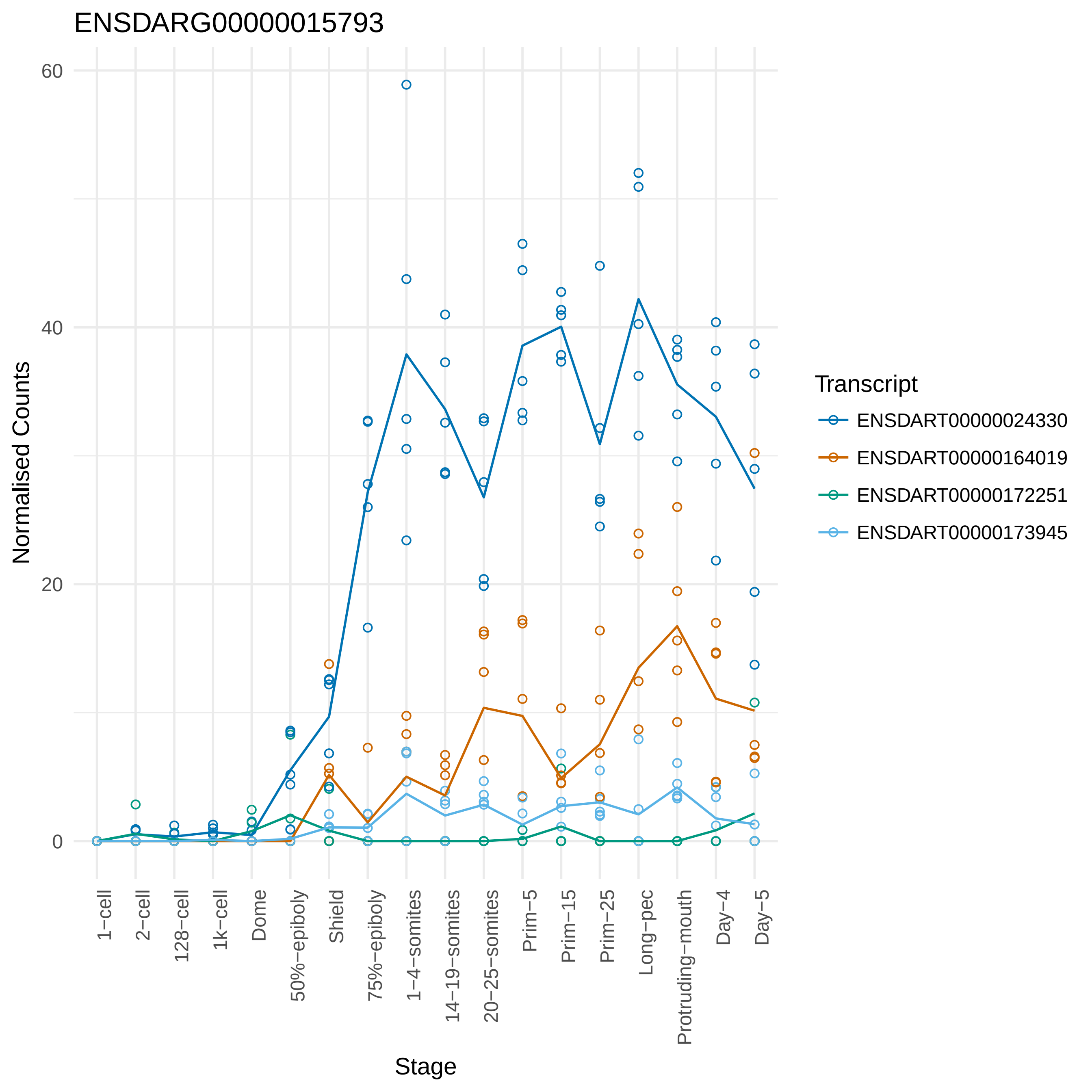

ENSDARG00000015793
Normalised Counts
60
40
20
0
1−cell
2−cell
128−cell
1k−cell
Dome
50%−epiboly
Shield
75%−epiboly
1−4−somites
14−19−somites
20−25−somites
Prim−5
Prim−15
Prim−25
Long−pec
Protruding−mouth
Day−4
Day−5
Stage
Transcript
ENSDART00000024330
ENSDART00000164019
ENSDART00000172251
ENSDART00000173945

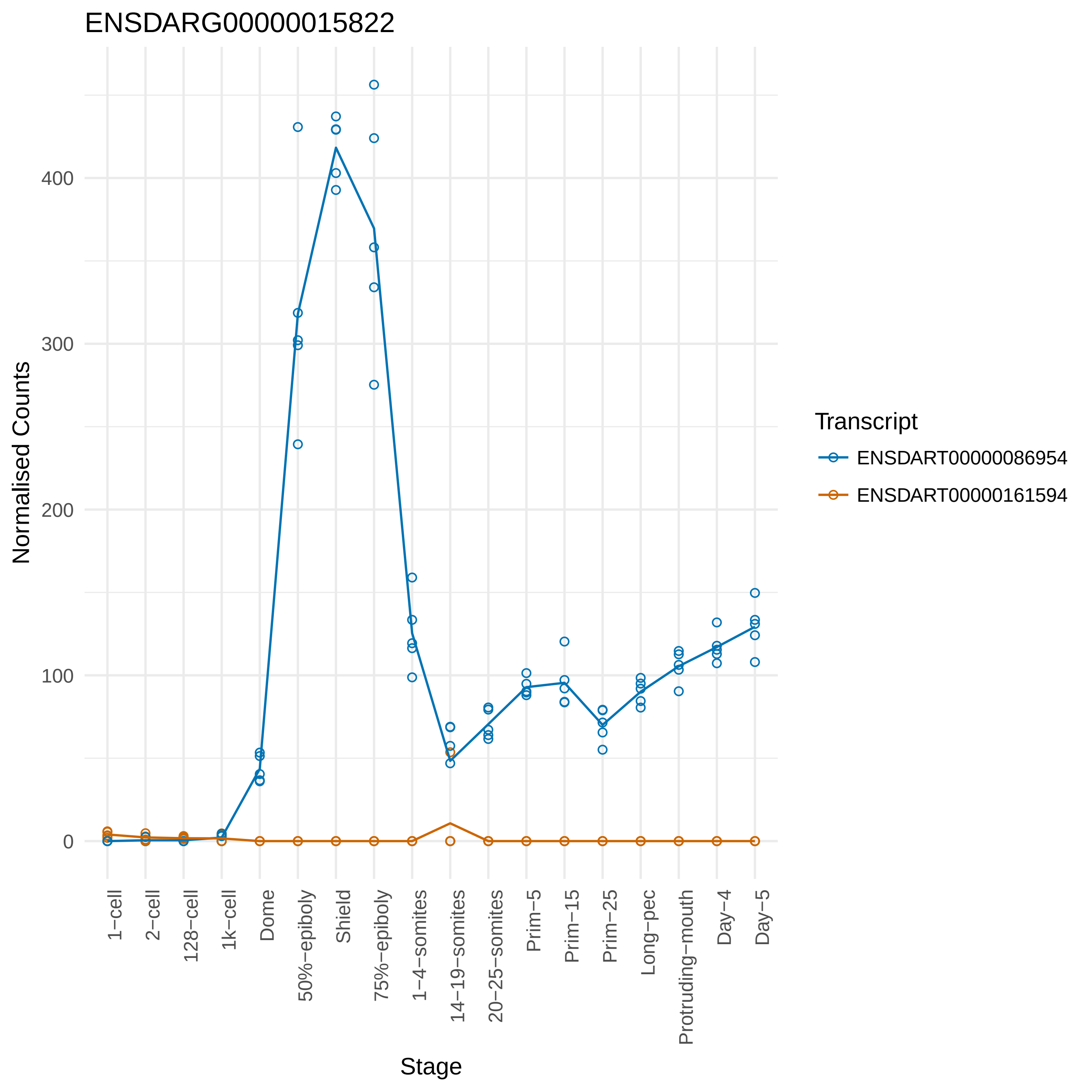
ENSDARG00000015822
Normalised Counts
0
100
200
300
400
Stage
1−cell
2−cell
128−cell
1k−cell
Dome
50%−epiboly
Shield
75%−epiboly
1−4−somites
14−19−somites
20−25−somites
Prim−5
Prim−15
Prim−25
Long−pec
Protruding−mouth
Day−4
Day−5
Transcript
ENSDART00000086954
ENSDART00000161594

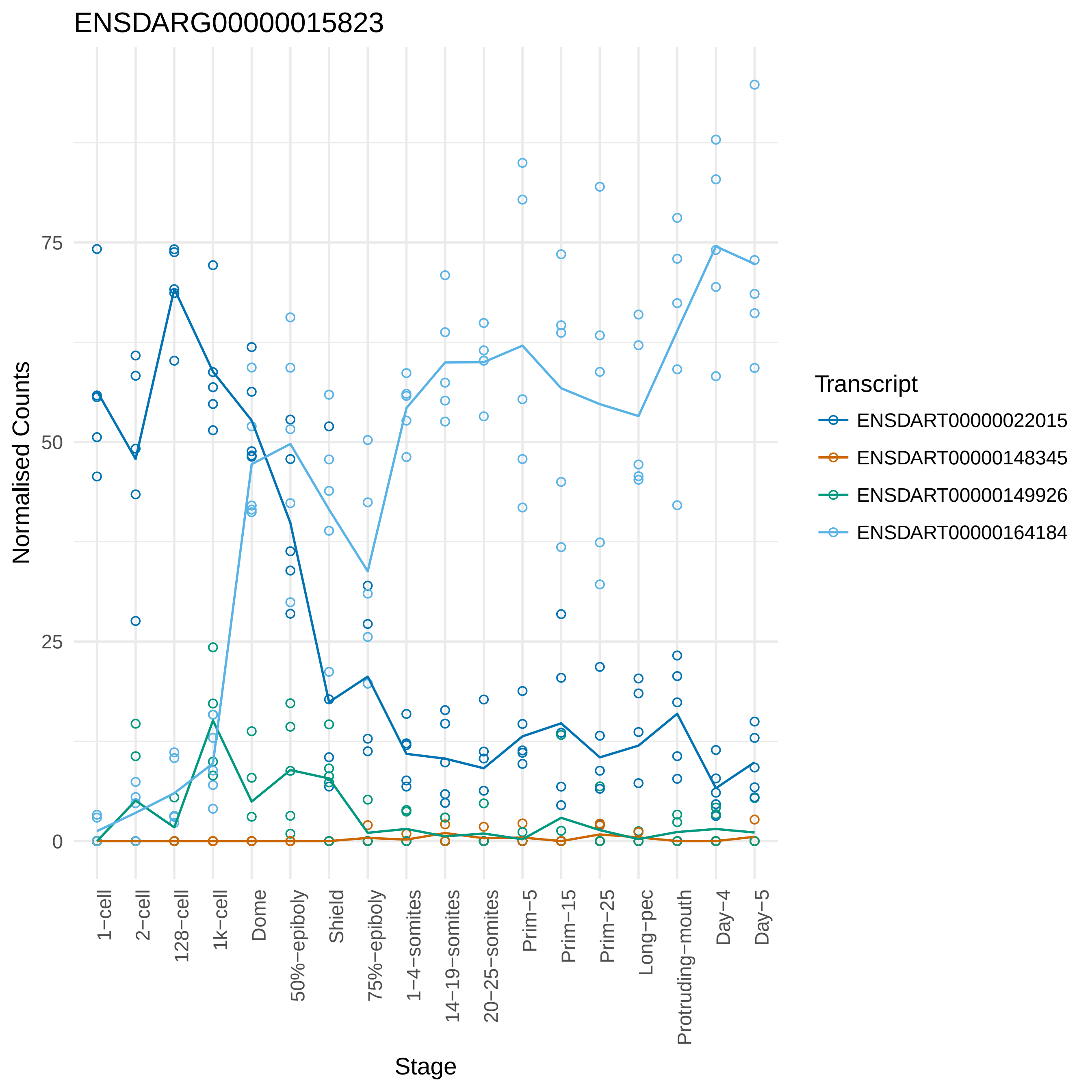

ENSDARG00000015823
Normalised Counts
75
50
25
0
1−cell
2−cell
128−cell
1k−cell
Dome
50%−epiboly
Shield
75%−epiboly
1−4−somites
14−19−somites
20−25−somites
Prim−5
Prim−15
Prim−25
Long−pec
Protruding−mouth
Day−4
Day−5
Stage
Transcript
ENSDART00000022015
ENSDART00000148345
ENSDART00000149926
ENSDART00000164184

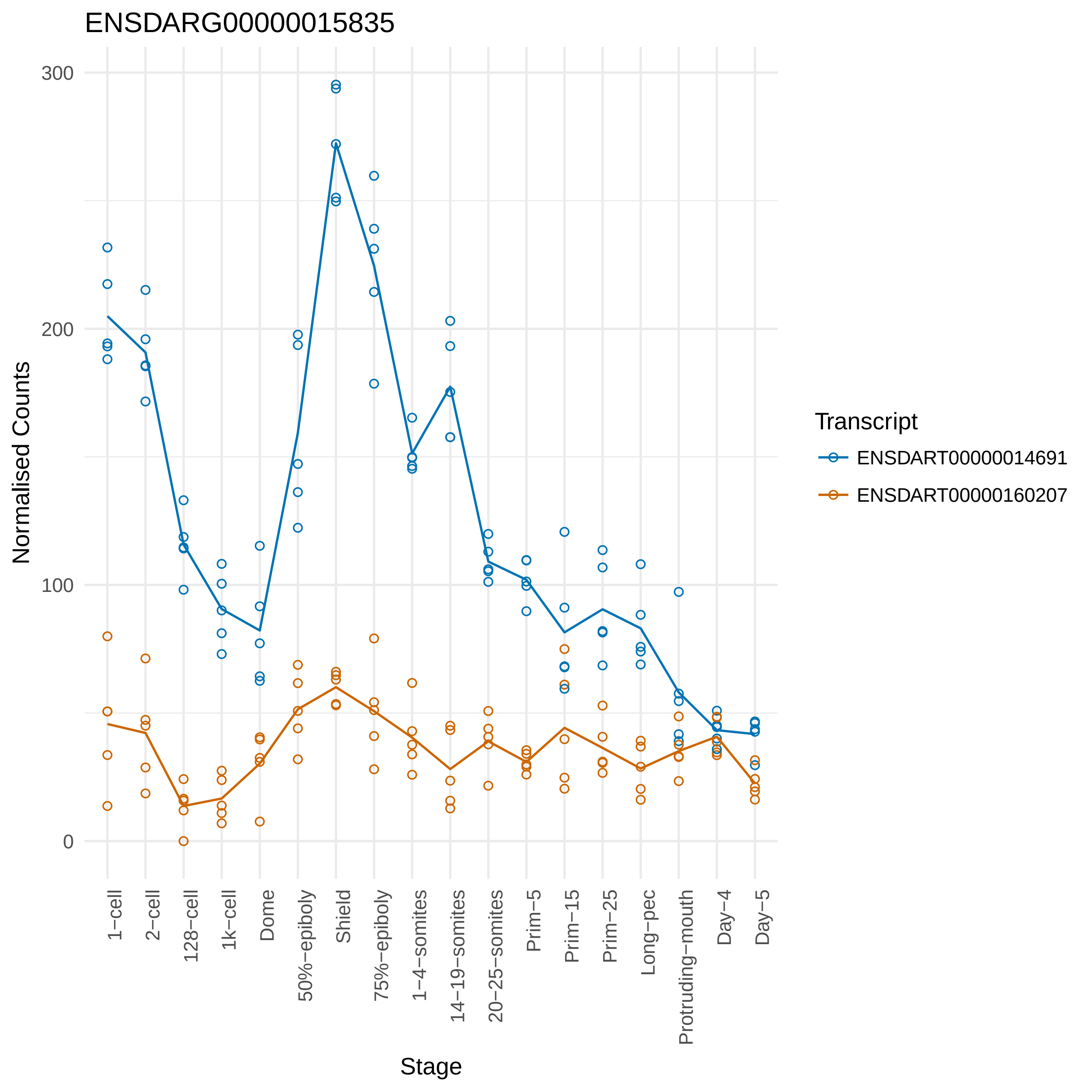

ENSDARG00000015835
Normalised Counts
300
200
100
0
Stage
1−cell
2−cell
128−cell
1k−cell
Dome
50%−epiboly
Shield
75%−epiboly
1−4−somites
14−19−somites
20−25−somites
Prim−5
Prim−15
Prim−25
Long−pec
Protruding−mouth
Day−4
Day−5
Transcript
ENSDART00000014691
ENSDART00000160207

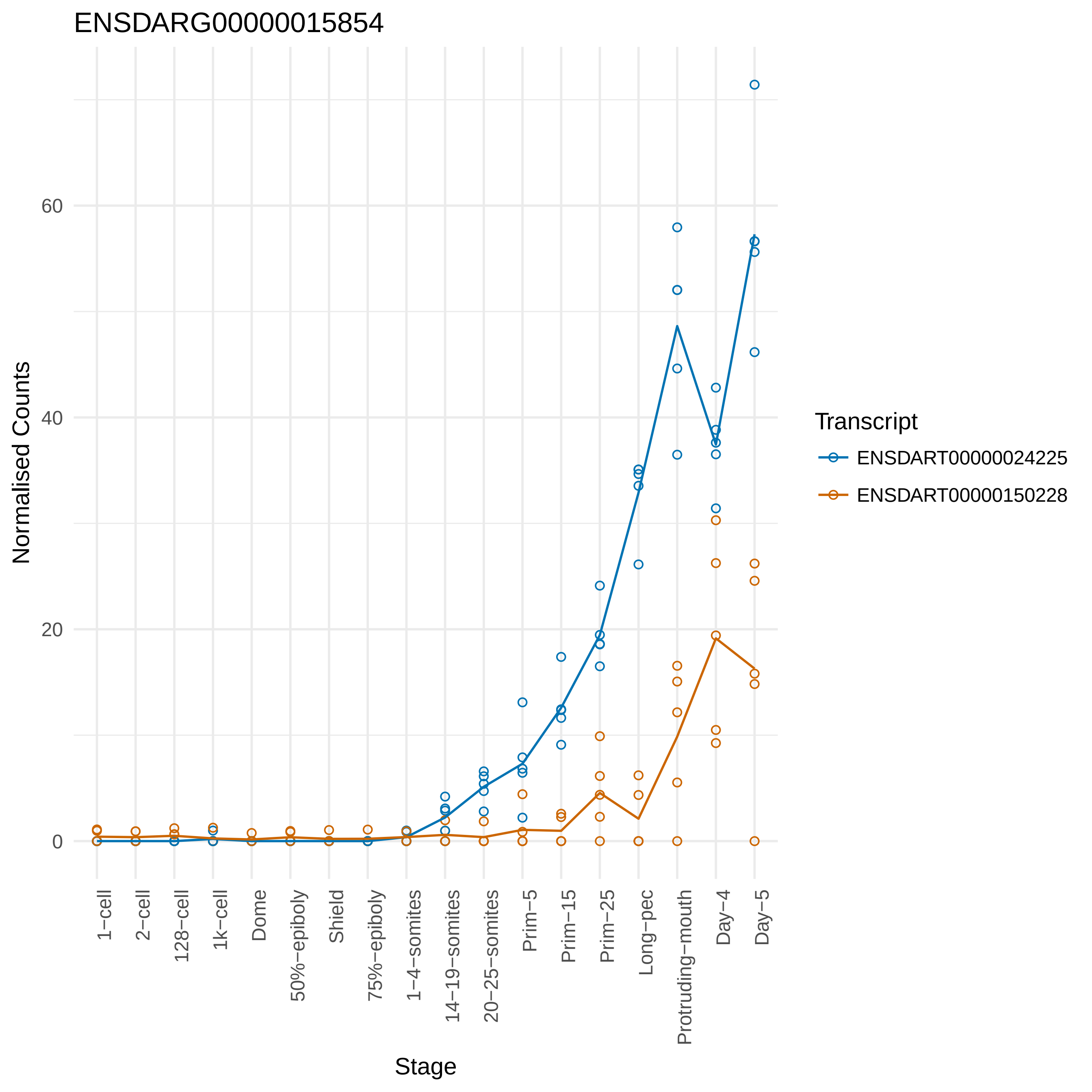
ENSDARG00000015854
Normalised Counts
0
20
40
60
Stage
1−cell
2−cell
128−cell
1k−cell
Dome
50%−epiboly
Shield
75%−epiboly
1−4−somites
14−19−somites
20−25−somites
Prim−5
Prim−15
Prim−25
Long−pec
Protruding−mouth
Day−4
Day−5
Transcript
ENSDART00000024225
ENSDART00000150228

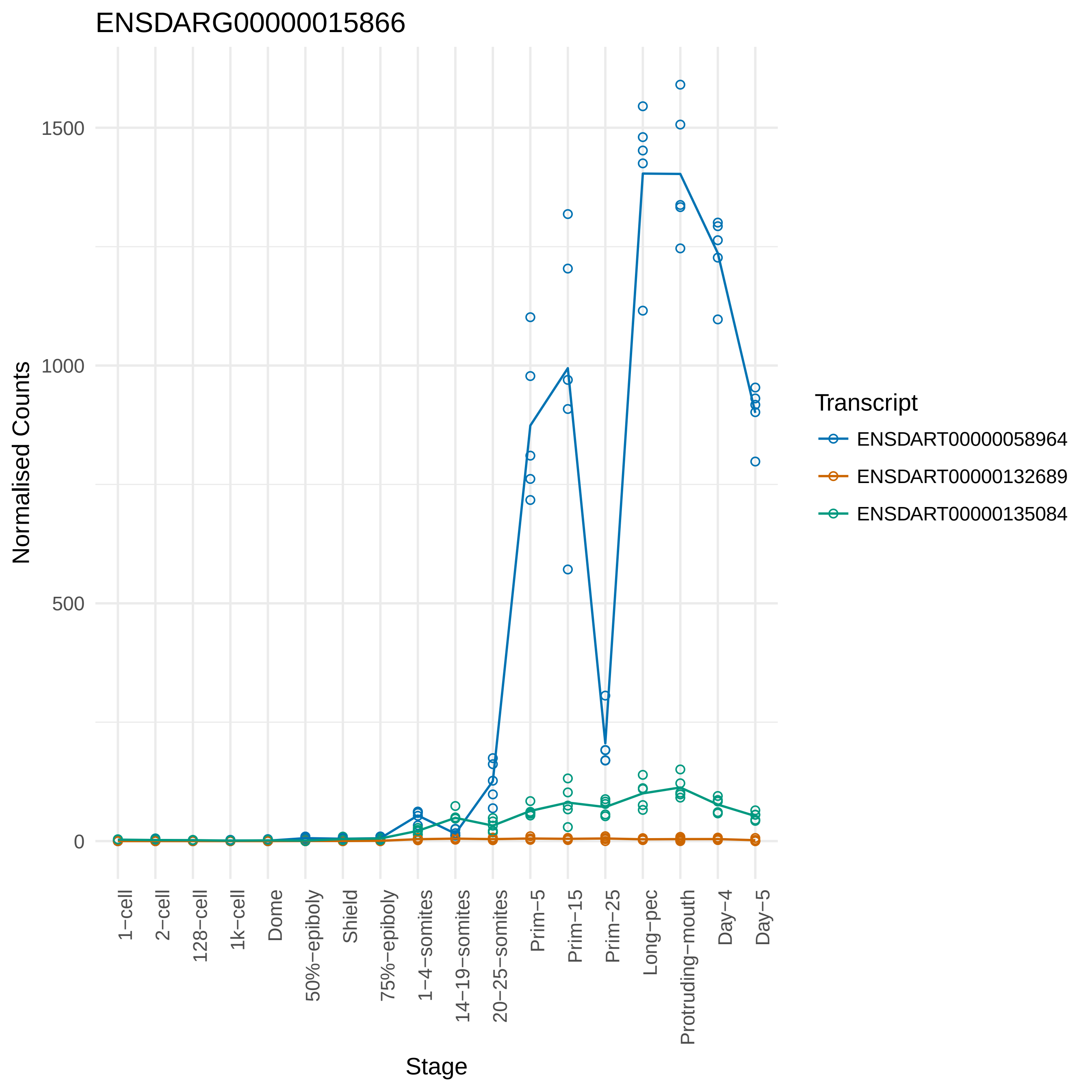
ENSDARG00000015866
Normalised Counts
1500
1000
500
0
1−cell
2−cell
128−cell
1k−cell
Dome
50%−epiboly
Shield
75%−epiboly
1−4−somites
14−19−somites
20−25−somites
Prim−5
Prim−15
Prim−25
Long−pec
Protruding−mouth
Day−4
Day−5
Stage
Transcript
ENSDART00000058964
ENSDART00000132689
ENSDART00000135084

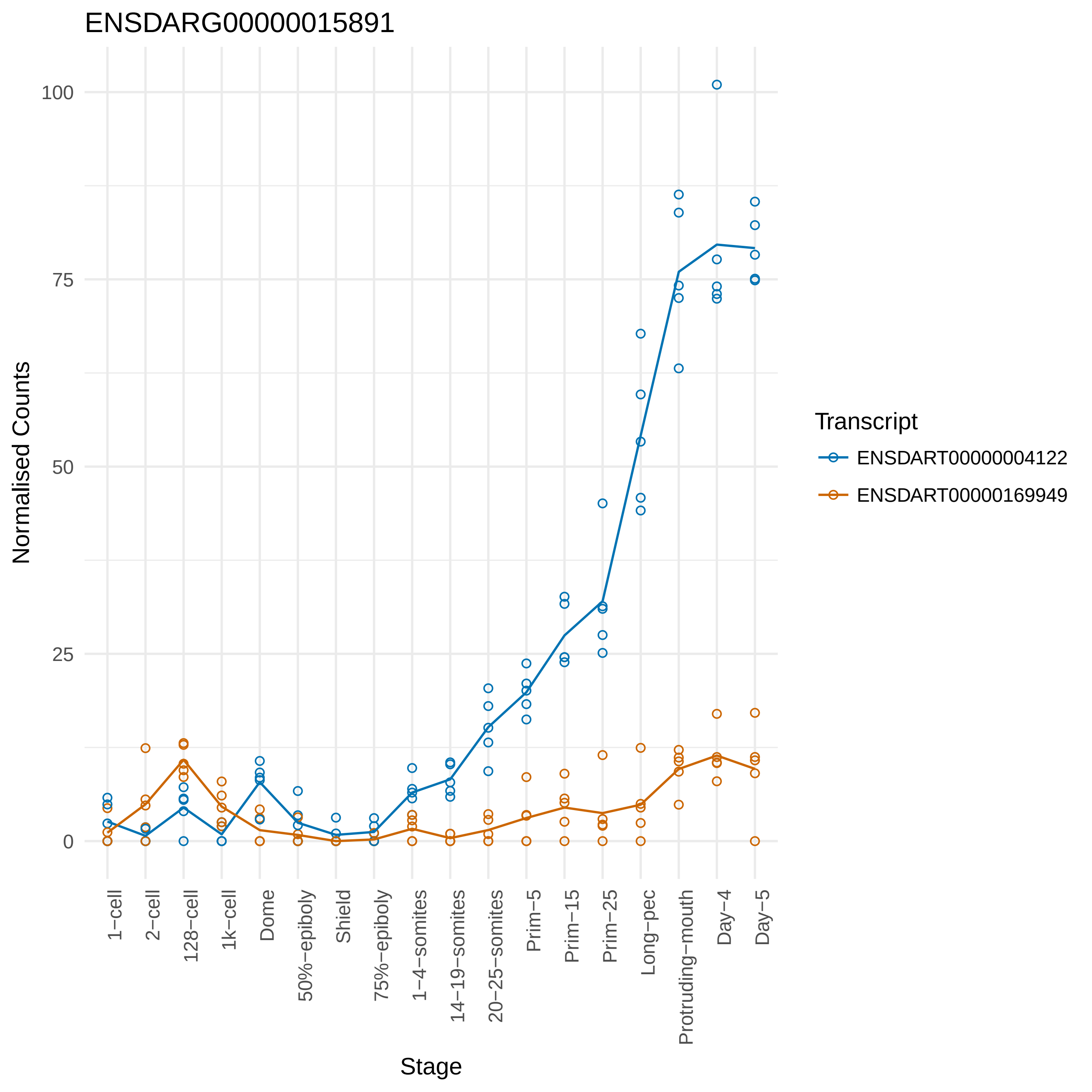

ENSDARG00000015891
Normalised Counts
100
75
50
25
0
1−cell
2−cell
128−cell
1k−cell
Dome
50%−epiboly
Shield
75%−epiboly
1−4−somites
14−19−somites
20−25−somites
Prim−5
Prim−15
Prim−25
Long−pec
Protruding−mouth
Day−4
Day−5
Stage
Transcript
ENSDART00000004122
ENSDART00000169949

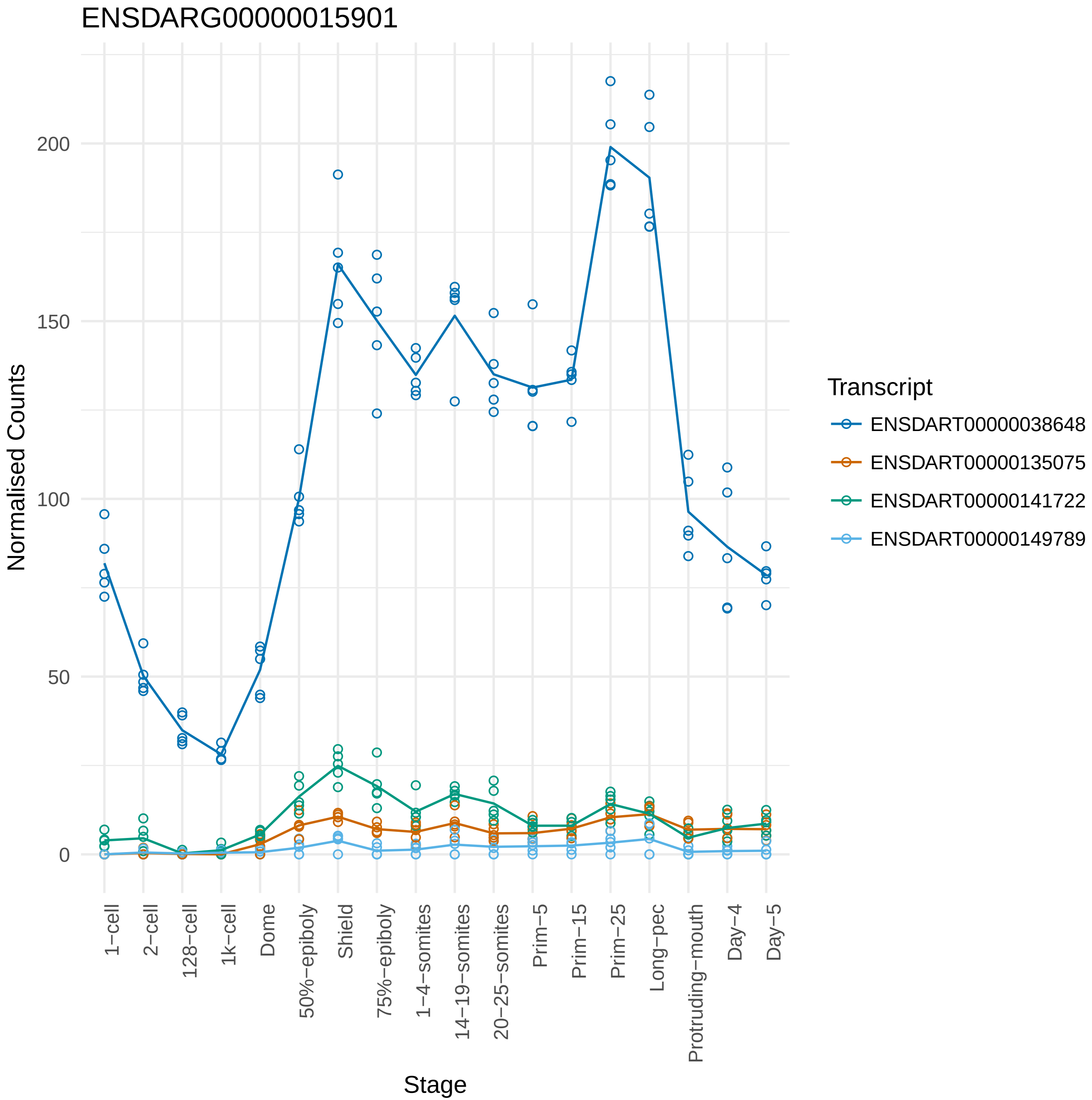

ENSDARG00000015901
Normalised Counts
0
50
100
150
200
Stage
1−cell
2−cell
128−cell
1k−cell
Dome
50%−epiboly
Shield
75%−epiboly
1−4−somites
14−19−somites
20−25−somites
Prim−5
Prim−15
Prim−25
Long−pec
Protruding−mouth
Day−4
Day−5
Transcript
ENSDART00000038648
ENSDART00000135075
ENSDART00000141722
ENSDART00000149789

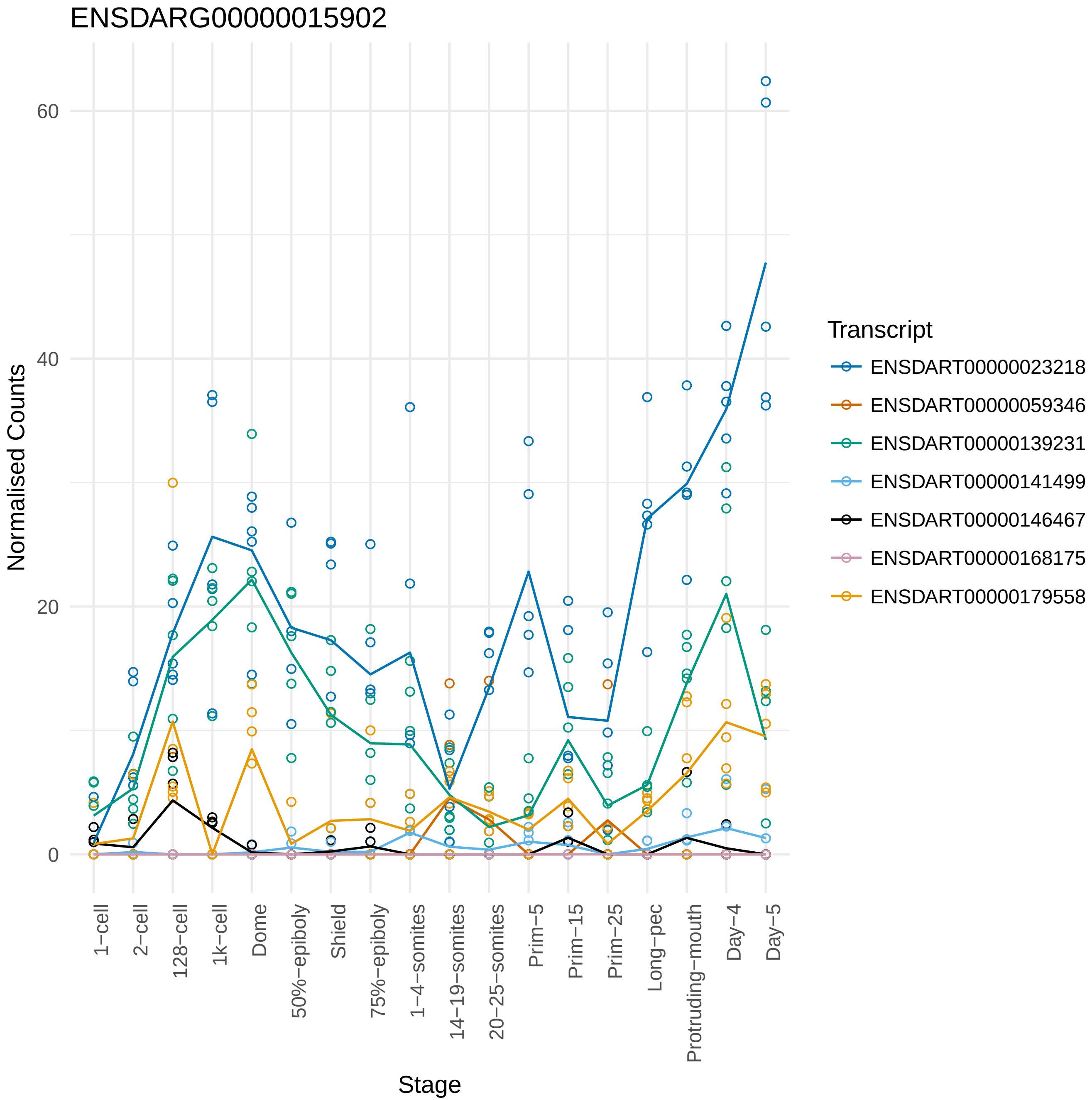

ENSDARG00000015902
Normalised Counts
0
20
40
60
Stage
1−cell
2−cell
128−cell
1k−cell
Dome
50%−epiboly
Shield
75%−epiboly
1−4−somites
14−19−somites
20−25−somites
Prim−5
Prim−15
Prim−25
Long−pec
Protruding−mouth
Day−4
Day−5
Transcript
ENSDART00000023218
ENSDART00000059346
ENSDART00000139231
ENSDART00000141499
ENSDART00000146467
ENSDART00000168175
ENSDART00000179558

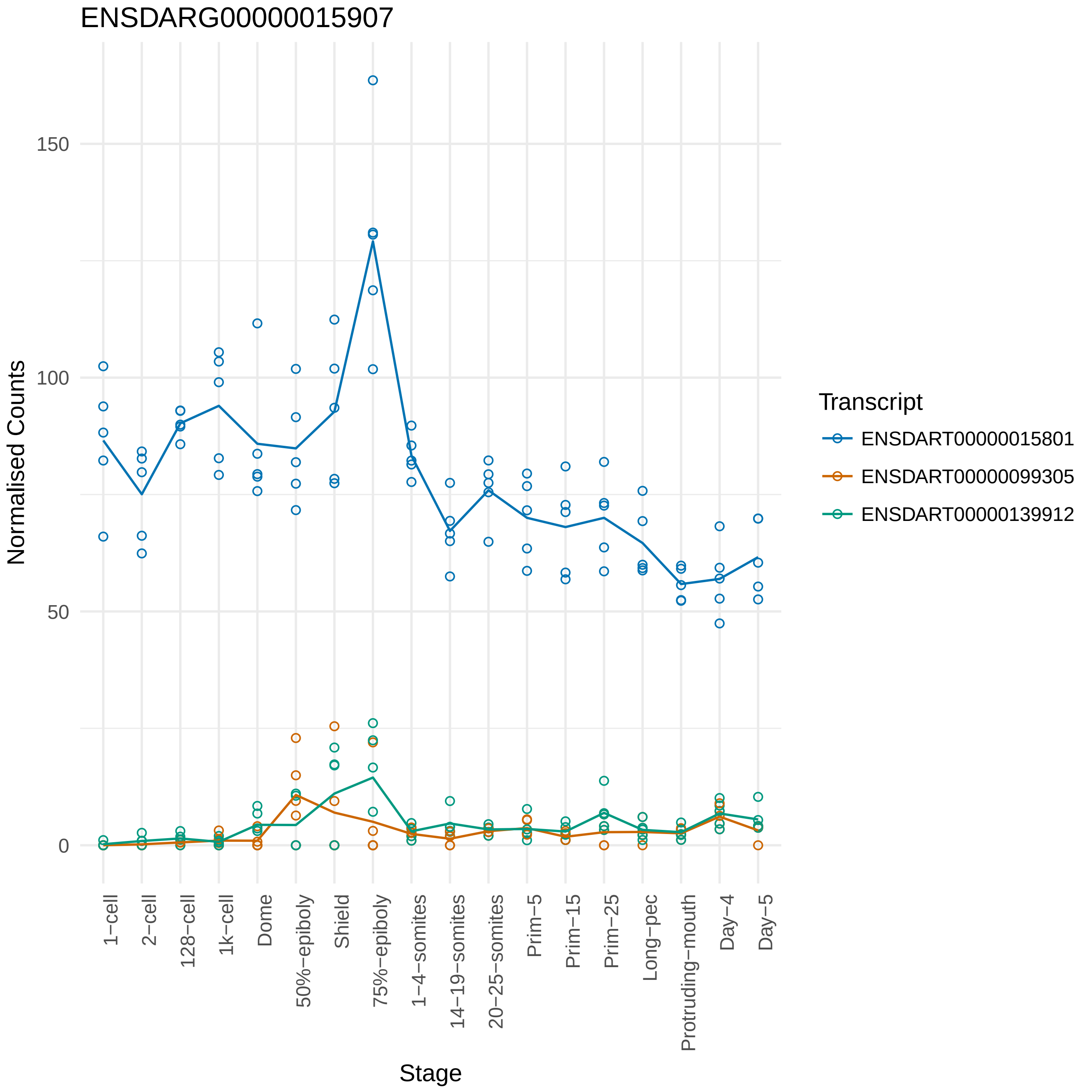
ENSDARG00000015907
Normalised Counts
150
100
50
0
Stage
1−cell
2−cell
128−cell
1k−cell
Dome
50%−epiboly
Shield
75%−epiboly
1−4−somites
14−19−somites
20−25−somites
Prim−5
Prim−15
Prim−25
Long−pec
Protruding−mouth
Day−4
Day−5
Transcript
ENSDART00000015801
ENSDART00000099305
ENSDART00000139912

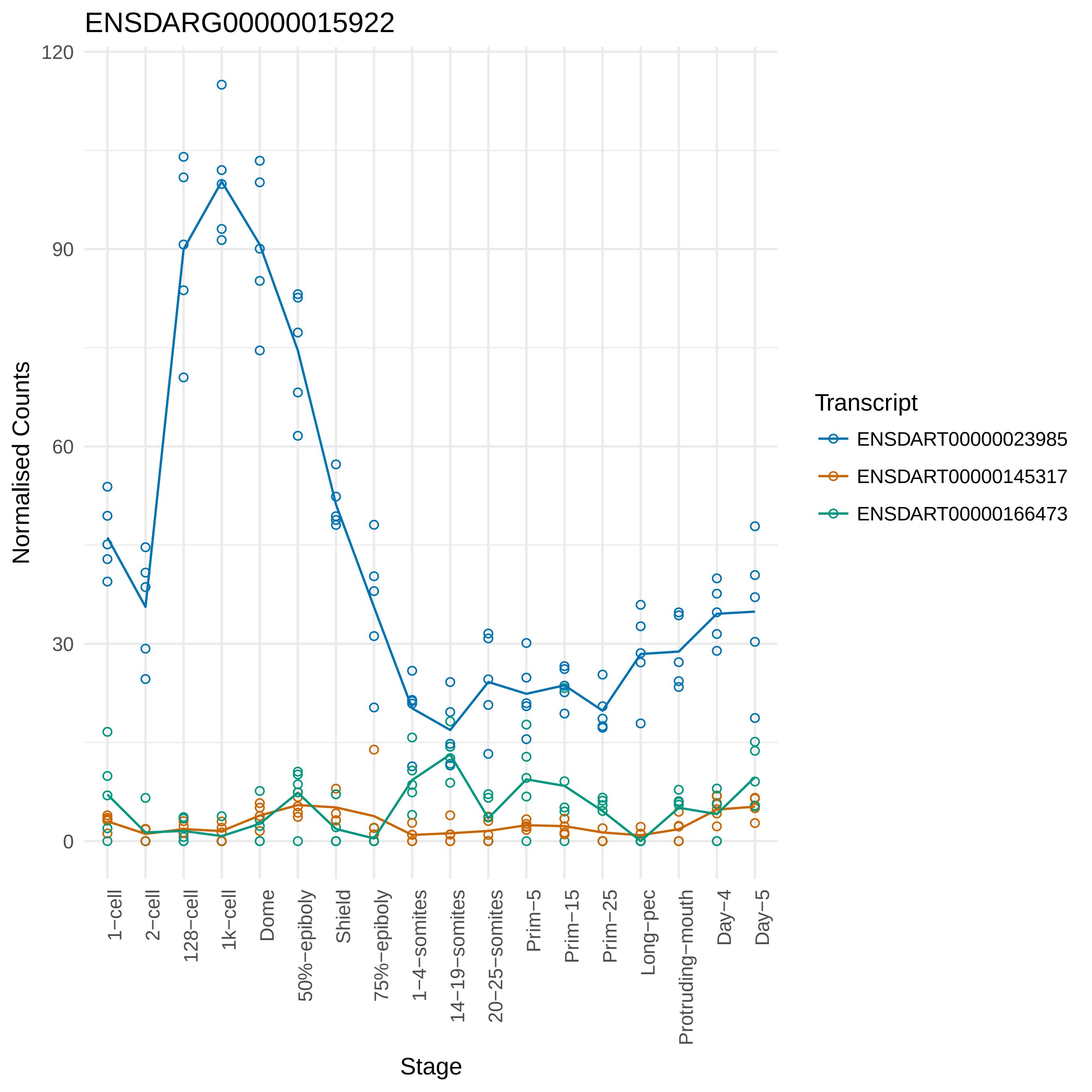
ENSDARG00000015922
Normalised Counts
120
90
60
30
0
Stage
1−cell
2−cell
128−cell
1k−cell
Dome
50%−epiboly
Shield
75%−epiboly
1−4−somites
14−19−somites
20−25−somites
Prim−5
Prim−15
Prim−25
Long−pec
Protruding−mouth
Day−4
Day−5
Transcript
ENSDART00000023985
ENSDART00000145317
ENSDART00000166473

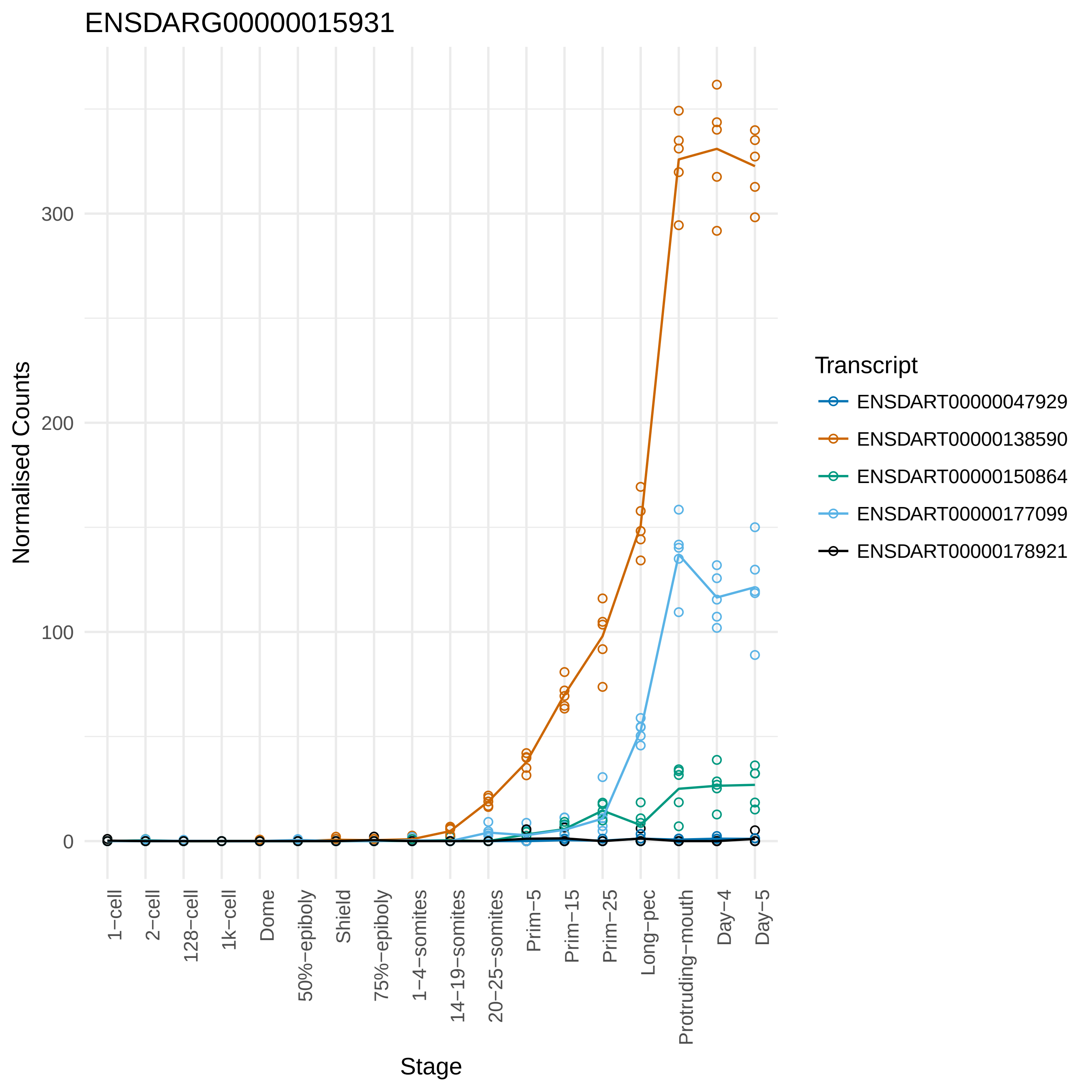
ENSDARG00000015931
Normalised Counts
0
100
200
300
1−cell
2−cell
128−cell
1k−cell
Dome
50%−epiboly
Shield
75%−epiboly
1−4−somites
14−19−somites
20−25−somites
Prim−5
Prim−15
Prim−25
Long−pec
Protruding−mouth
Day−4
Day−5
Stage
Transcript
ENSDART00000047929
ENSDART00000138590
ENSDART00000150864
ENSDART00000177099
ENSDART00000178921

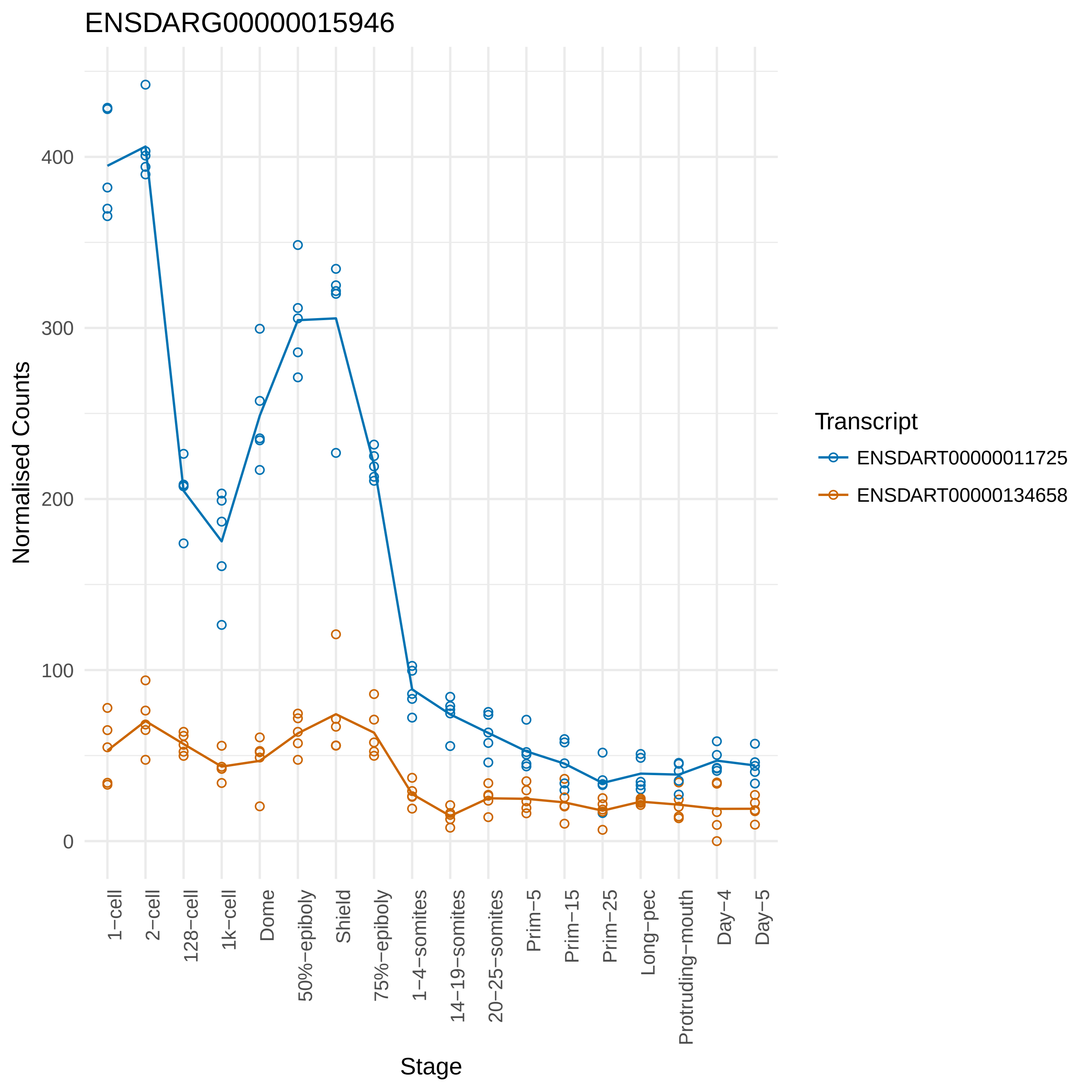

ENSDARG00000015946
Normalised Counts
400
300
200
100
0
Stage
1−cell
2−cell
128−cell
1k−cell
Dome
50%−epiboly
Shield
75%−epiboly
1−4−somites
14−19−somites
20−25−somites
Prim−5
Prim−15
Prim−25
Long−pec
Protruding−mouth
Day−4
Day−5
Transcript
ENSDART00000011725
ENSDART00000134658

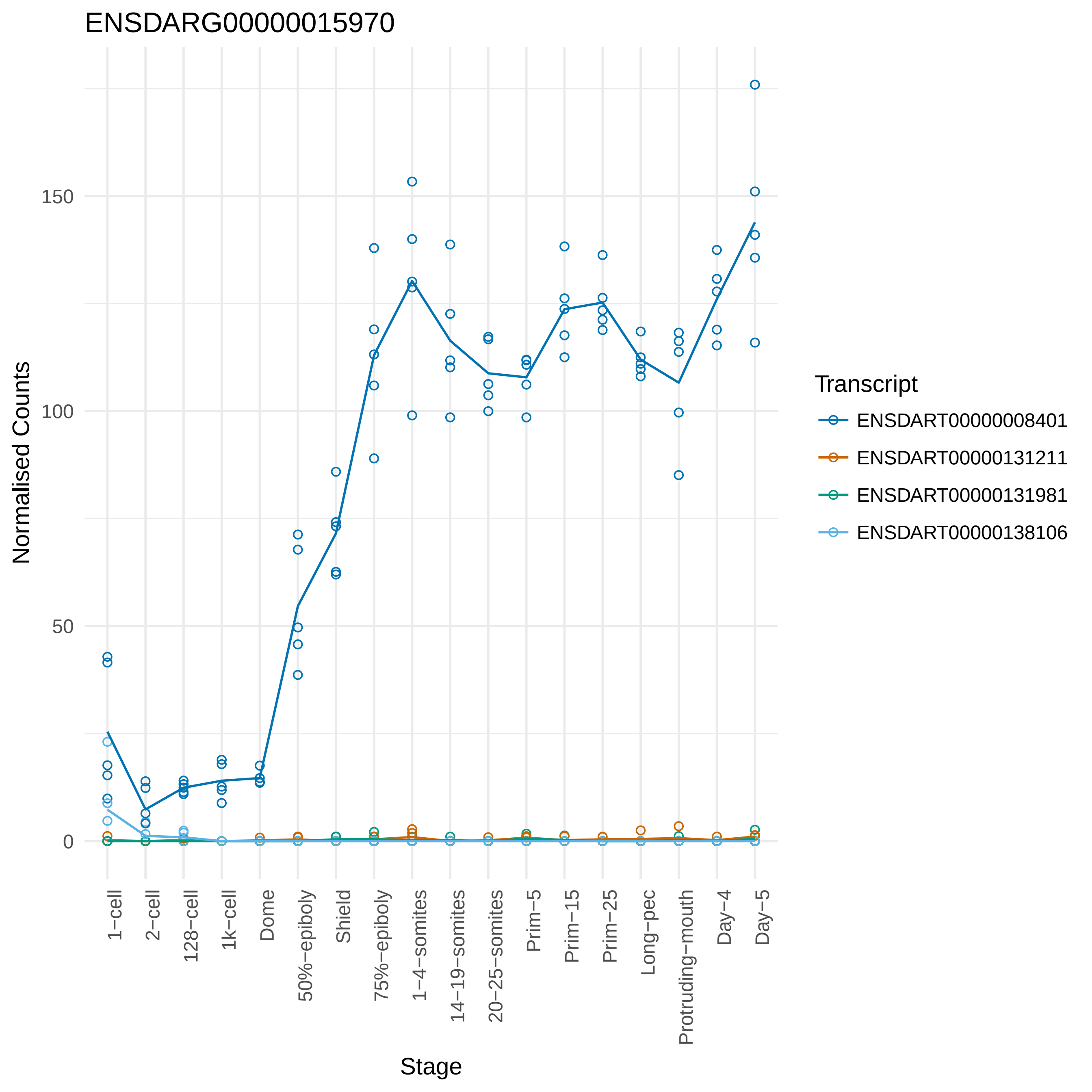

ENSDARG00000015970
Normalised Counts
150
100
50
0
1−cell
2−cell
128−cell
1k−cell
Dome
50%−epiboly
Shield
75%−epiboly
1−4−somites
14−19−somites
20−25−somites
Prim−5
Prim−15
Prim−25
Long−pec
Protruding−mouth
Day−4
Day−5
Stage
Transcript
ENSDART00000008401
ENSDART00000131211
ENSDART00000131981
ENSDART00000138106

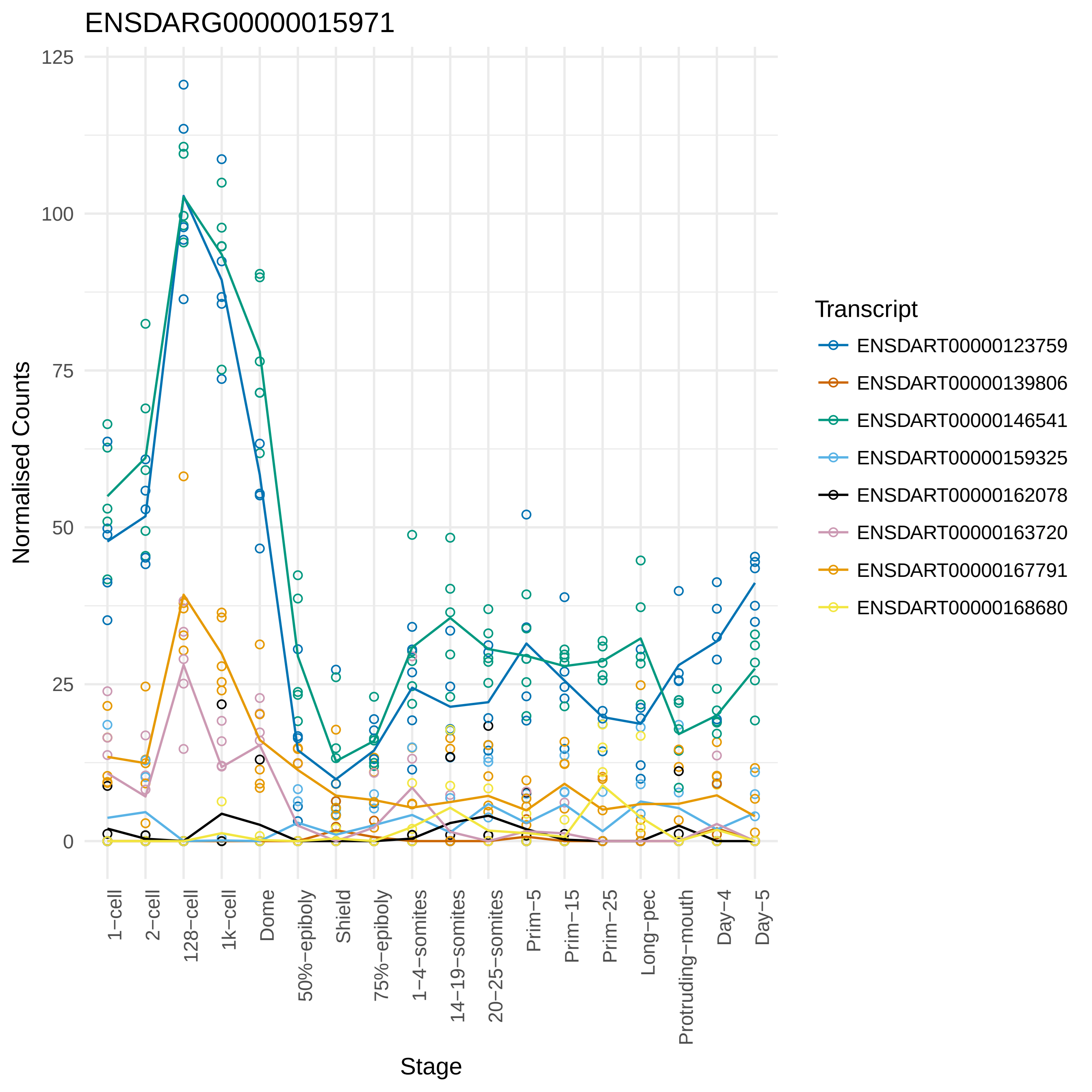
ENSDARG00000015971
Normalised Counts
125
100
75
50
25
0
1−cell
2−cell
128−cell
1k−cell
Dome
50%−epiboly
Shield
75%−epiboly
1−4−somites
14−19−somites
20−25−somites
Prim−5
Prim−15
Prim−25
Long−pec
Protruding−mouth
Day−4
Day−5
Stage
Transcript
ENSDART00000123759
ENSDART00000139806
ENSDART00000146541
ENSDART00000159325
ENSDART00000162078
ENSDART00000163720
ENSDART00000167791
ENSDART00000168680

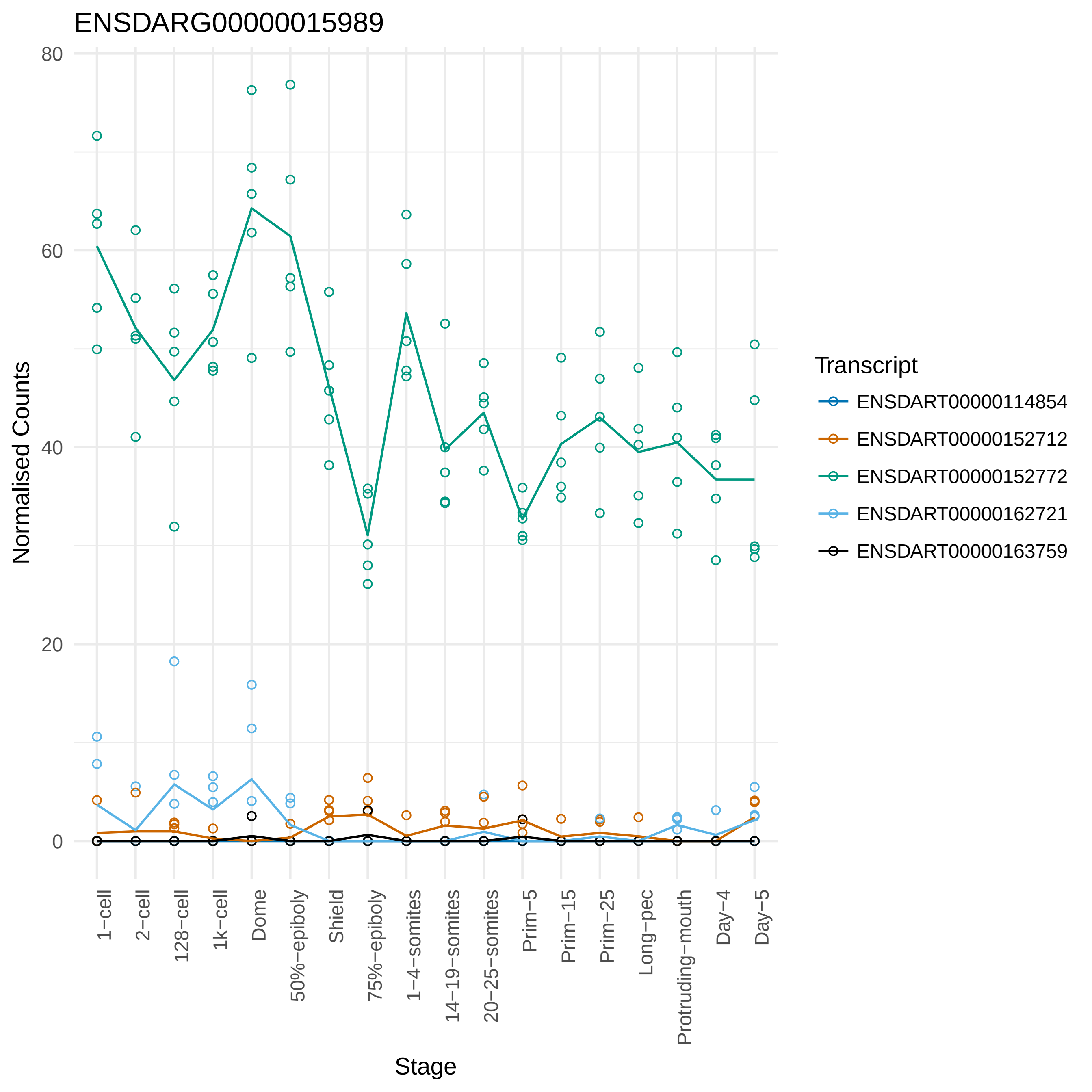

ENSDARG00000015989
Normalised Counts
0
20
40
60
80
Stage
1−cell
2−cell
128−cell
1k−cell
Dome
50%−epiboly
Shield
75%−epiboly
1−4−somites
14−19−somites
20−25−somites
Prim−5
Prim−15
Prim−25
Long−pec
Protruding−mouth
Day−4
Day−5
Transcript
ENSDART00000114854
ENSDART00000152712
ENSDART00000152772
ENSDART00000162721
ENSDART00000163759

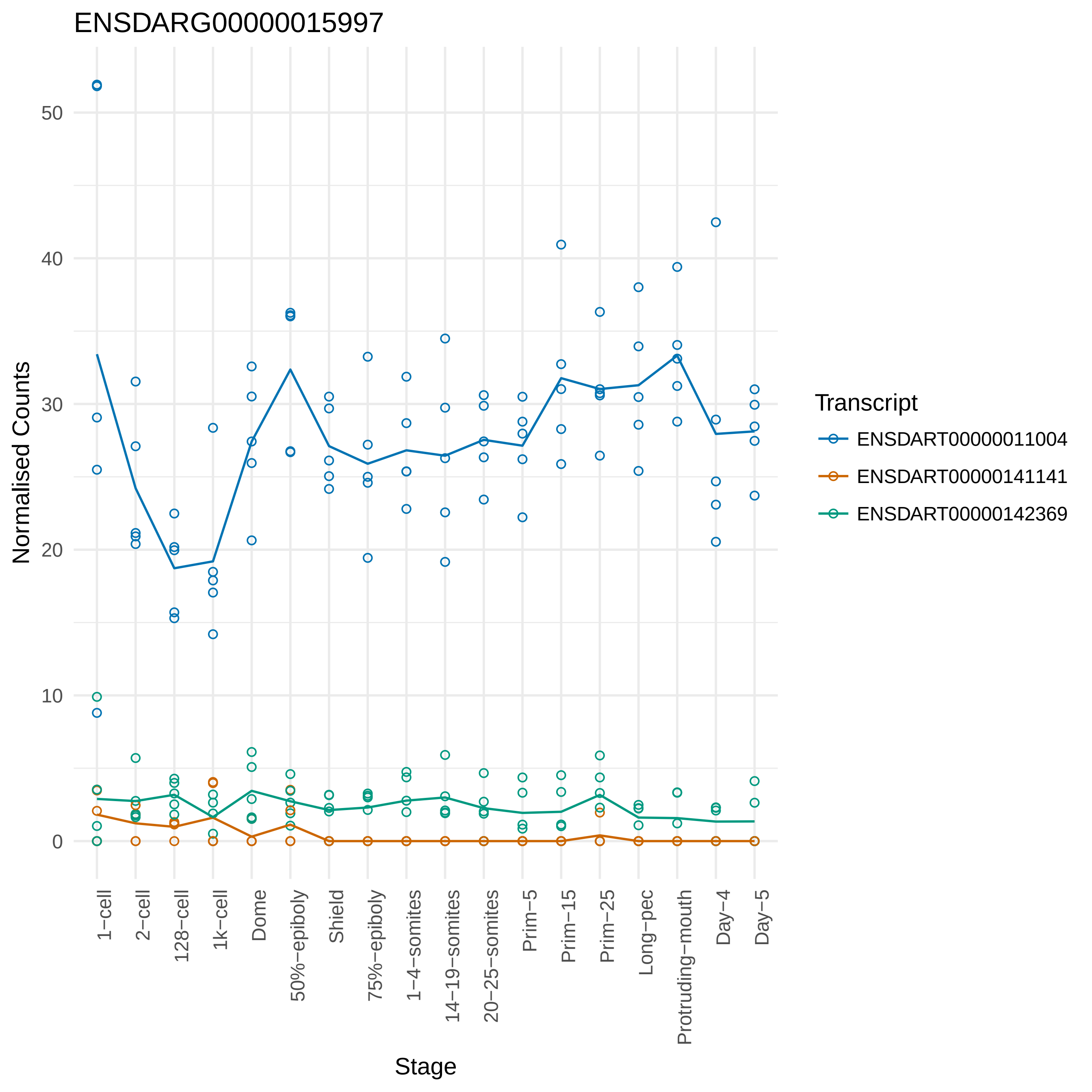
ENSDARG00000015997
Normalised Counts
0
10
20
30
40
50
1−cell
2−cell
128−cell
1k−cell
Dome
50%−epiboly
Shield
75%−epiboly
1−4−somites
14−19−somites
20−25−somites
Prim−5
Prim−15
Prim−25
Long−pec
Protruding−mouth
Day−4
Day−5
Stage
Transcript
ENSDART00000011004
ENSDART00000141141
ENSDART00000142369

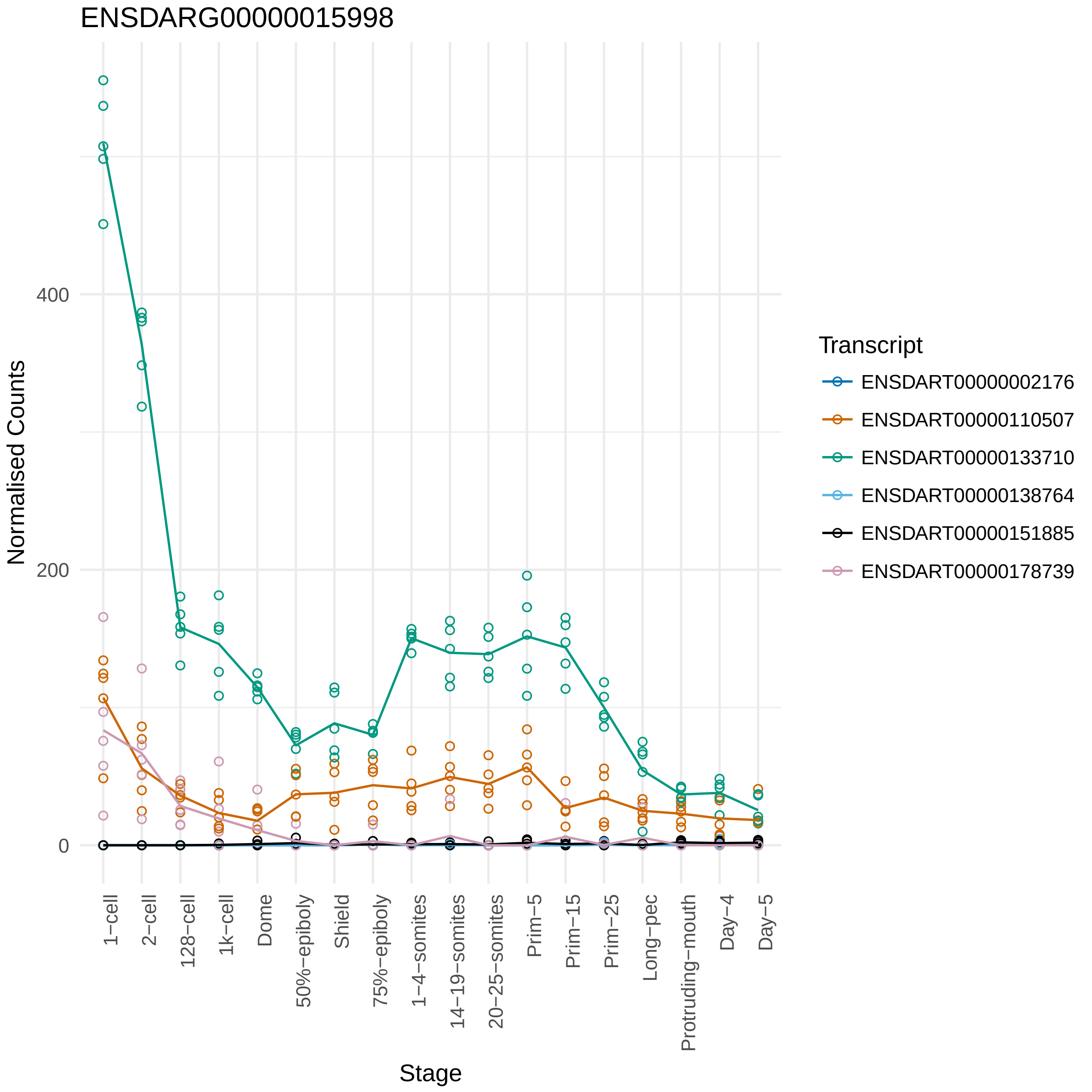

ENSDARG00000015998
Normalised Counts
400
200
0
1−cell
2−cell
128−cell
1k−cell
Dome
50%−epiboly
Shield
75%−epiboly
1−4−somites
14−19−somites
20−25−somites
Prim−5
Prim−15
Prim−25
Long−pec
Protruding−mouth
Day−4
Day−5
Stage
Transcript
ENSDART00000002176
ENSDART00000110507
ENSDART00000133710
ENSDART00000138764
ENSDART00000151885
ENSDART00000178739

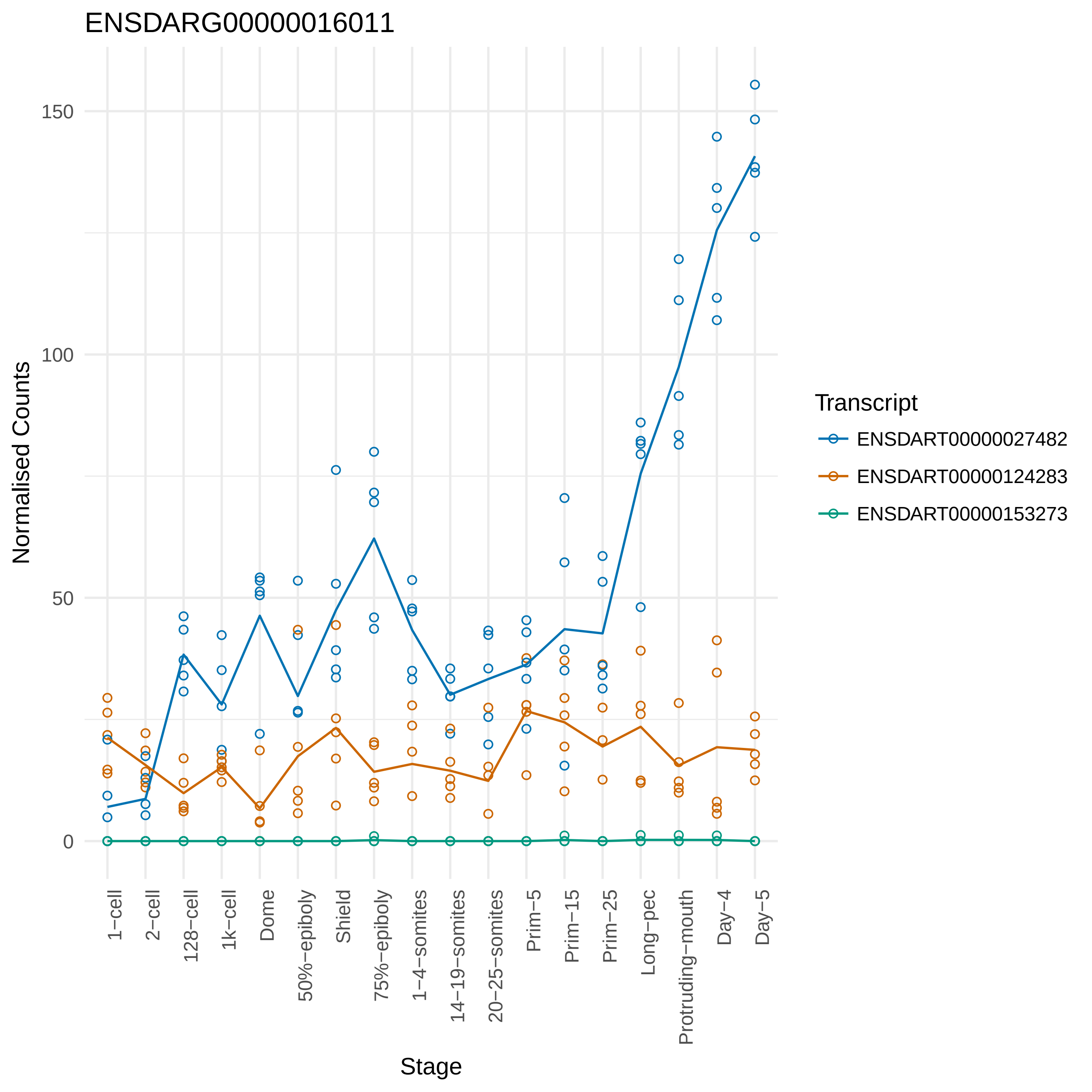
ENSDARG00000016011
Normalised Counts
150
100
50
0
1−cell
2−cell
128−cell
1k−cell
Dome
50%−epiboly
Shield
75%−epiboly
1−4−somites
14−19−somites
20−25−somites
Prim−5
Prim−15
Prim−25
Long−pec
Protruding−mouth
Day−4
Day−5
Stage
Transcript
ENSDART00000027482
ENSDART00000124283
ENSDART00000153273

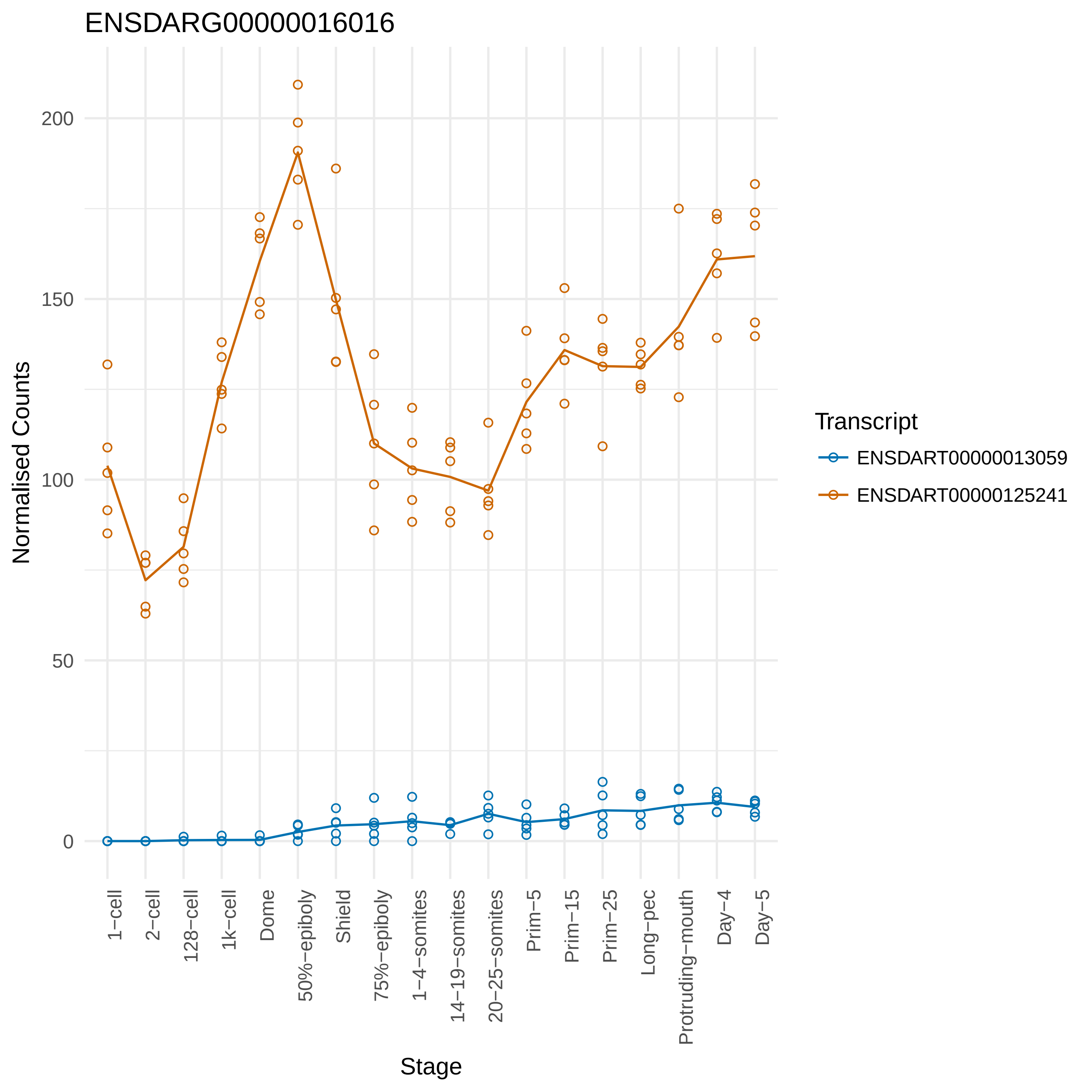

ENSDARG00000016016
Normalised Counts
200
150
100
50
0
1−cell
2−cell
128−cell
1k−cell
Dome
50%−epiboly
Shield
75%−epiboly
1−4−somites
14−19−somites
20−25−somites
Prim−5
Prim−15
Prim−25
Long−pec
Protruding−mouth
Day−4
Day−5
Stage
Transcript
ENSDART00000013059
ENSDART00000125241

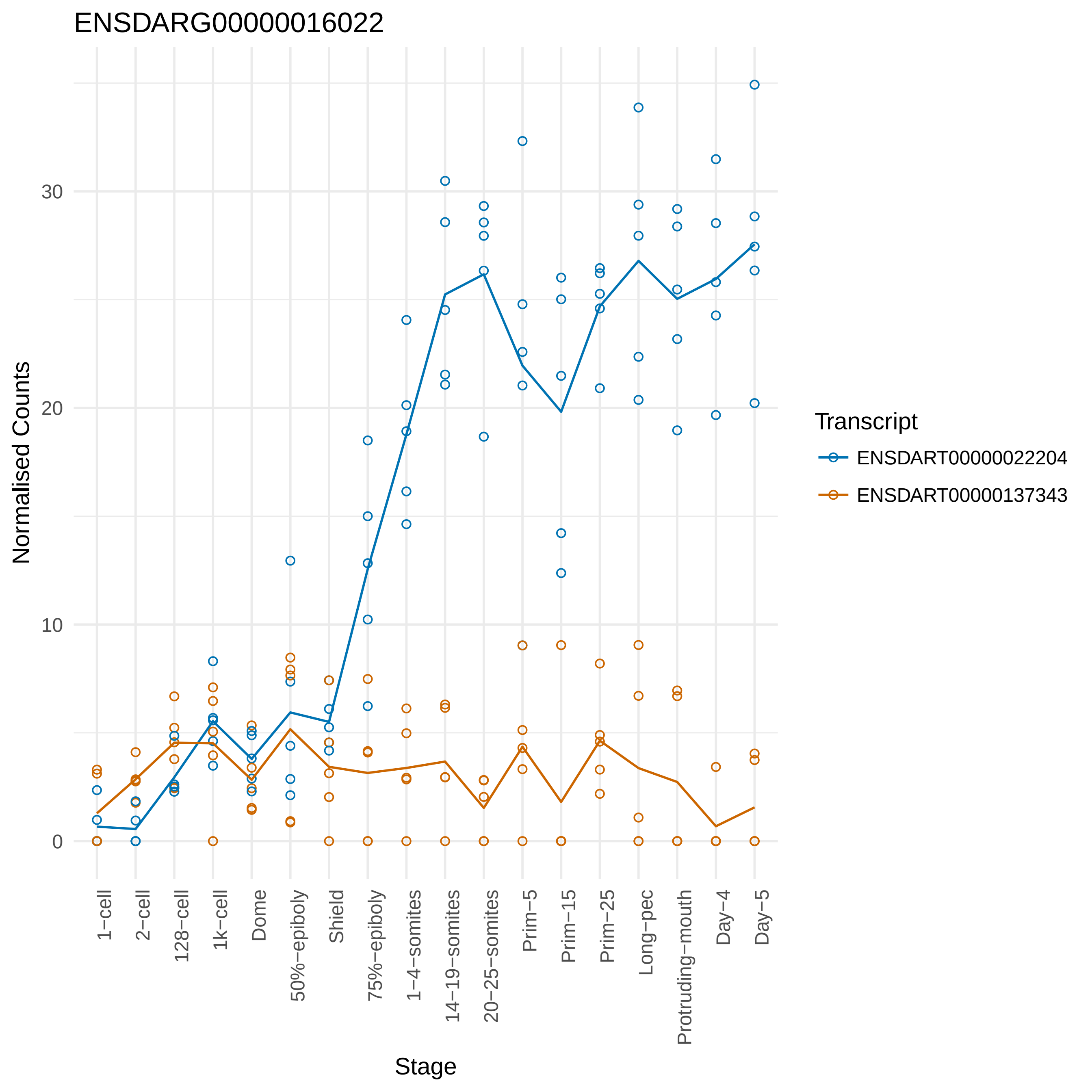

ENSDARG00000016022
Normalised Counts
0
10
20
30
Stage
1−cell
2−cell
128−cell
1k−cell
Dome
50%−epiboly
Shield
75%−epiboly
1−4−somites
14−19−somites
20−25−somites
Prim−5
Prim−15
Prim−25
Long−pec
Protruding−mouth
Day−4
Day−5
Transcript
ENSDART00000022204
ENSDART00000137343

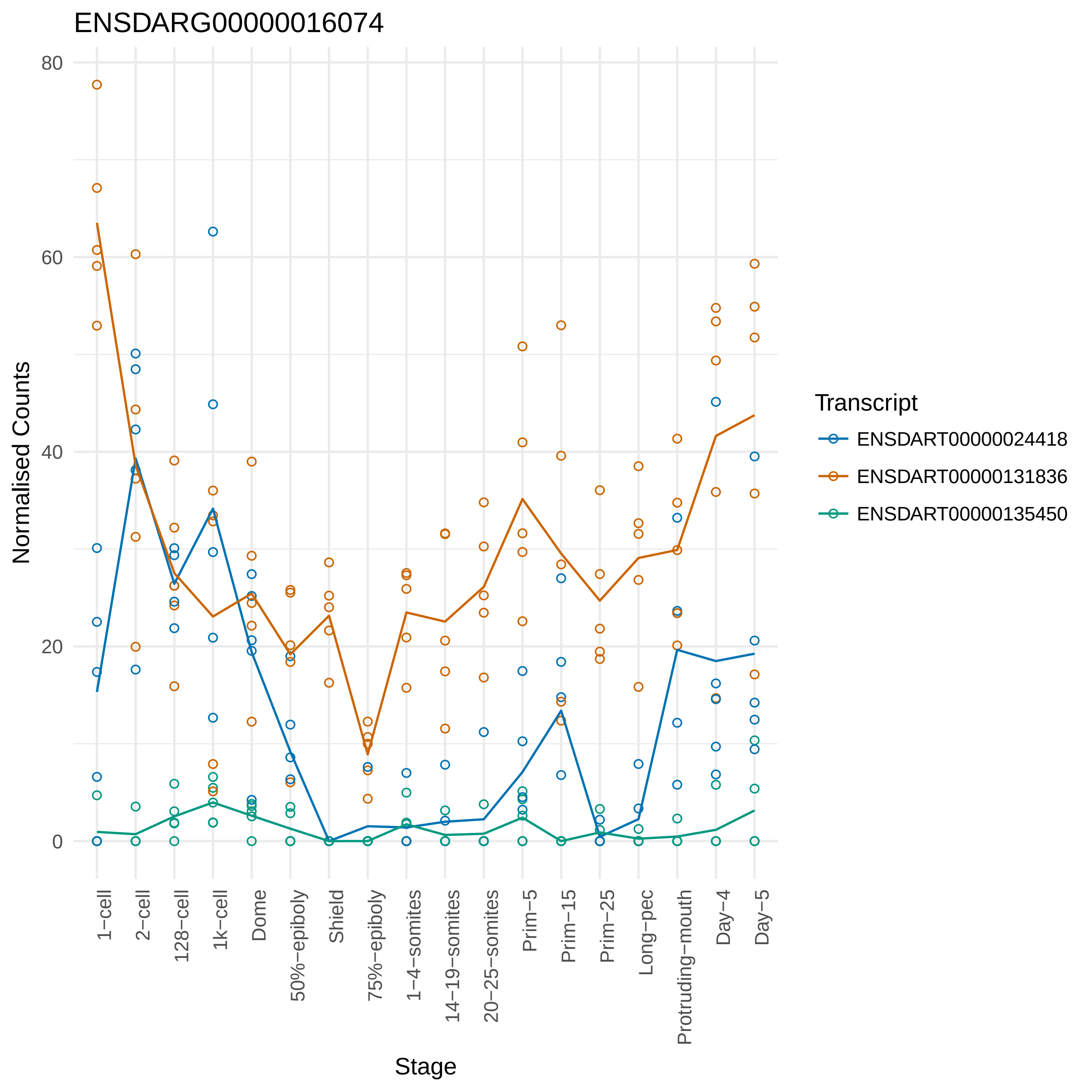

ENSDARG00000016074
Normalised Counts
80
60
40
20
0
1−cell
2−cell
128−cell
1k−cell
Dome
50%−epiboly
Shield
75%−epiboly
1−4−somites
14−19−somites
20−25−somites
Prim−5
Prim−15
Prim−25
Long−pec
Protruding−mouth
Day−4
Day−5
Stage
Transcript
ENSDART00000024418
ENSDART00000131836
ENSDART00000135450

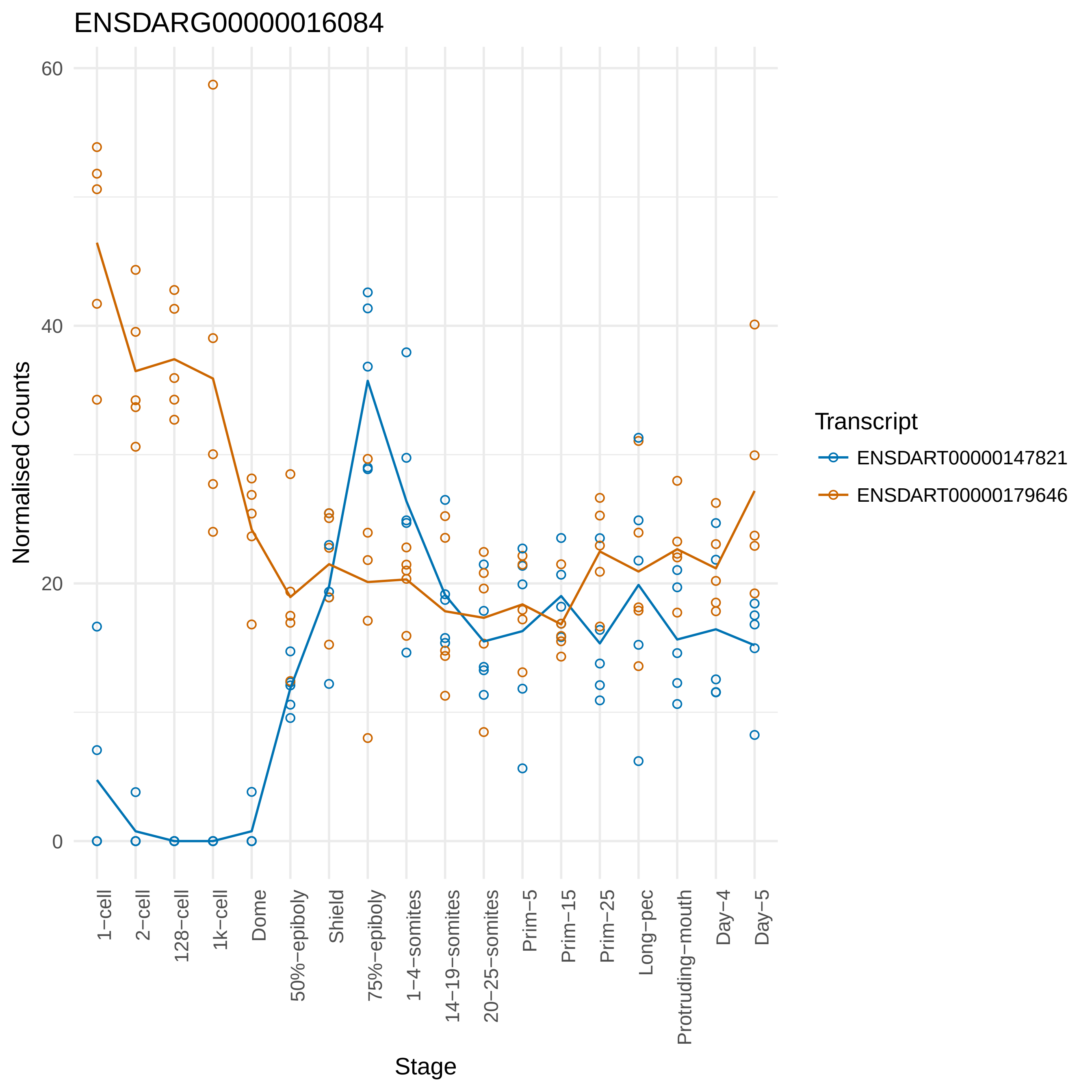

ENSDARG00000016084
Normalised Counts
60
40
20
0
1−cell
2−cell
128−cell
1k−cell
Dome
50%−epiboly
Shield
75%−epiboly
1−4−somites
14−19−somites
20−25−somites
Prim−5
Prim−15
Prim−25
Long−pec
Protruding−mouth
Day−4
Day−5
Stage
Transcript
ENSDART00000147821
ENSDART00000179646

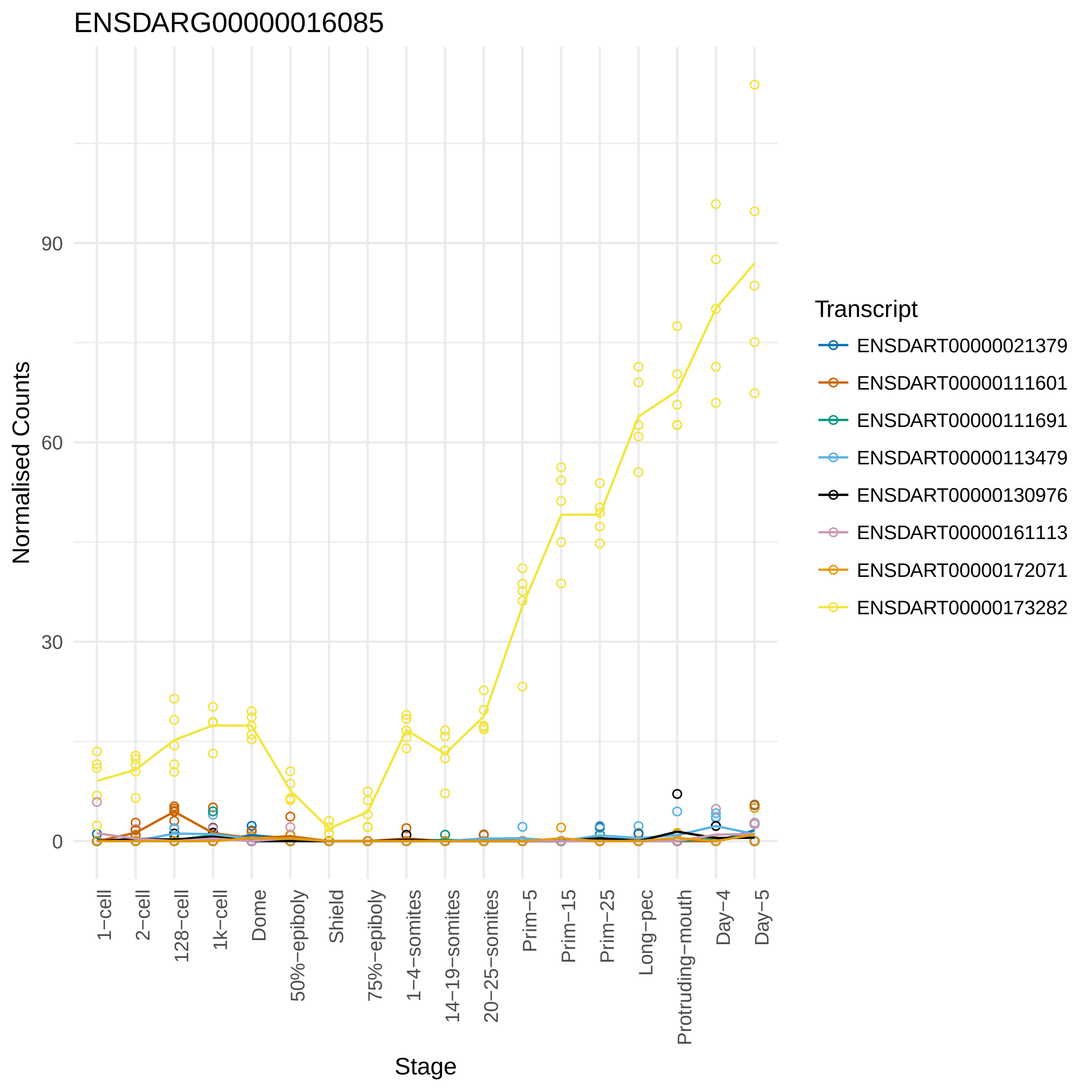

ENSDARG00000016085
Normalised Counts
0
30
60
90
1−cell
2−cell
128−cell
1k−cell
Dome
50%−epiboly
Shield
75%−epiboly
1−4−somites
14−19−somites
20−25−somites
Prim−5
Prim−15
Prim−25
Long−pec
Protruding−mouth
Day−4
Day−5
Stage
Transcript
ENSDART00000021379
ENSDART00000111601
ENSDART00000111691
ENSDART00000113479
ENSDART00000130976
ENSDART00000161113
ENSDART00000172071
ENSDART00000173282

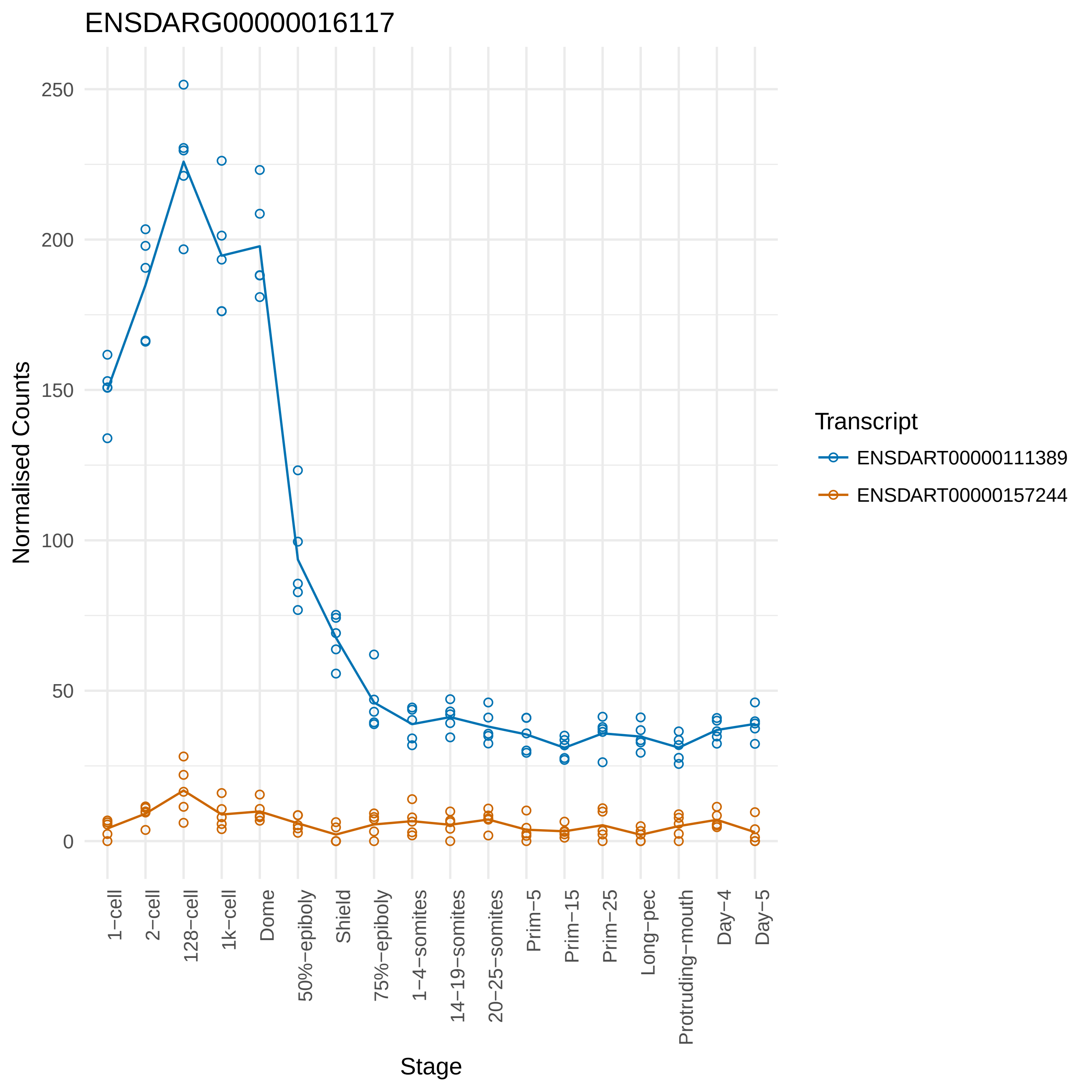

ENSDARG00000016117
Normalised Counts
250
200
150
100
50
0
1−cell
2−cell
128−cell
1k−cell
Dome
50%−epiboly
Shield
75%−epiboly
1−4−somites
14−19−somites
20−25−somites
Prim−5
Prim−15
Prim−25
Long−pec
Protruding−mouth
Day−4
Day−5
Stage
Transcript
ENSDART00000111389
ENSDART00000157244

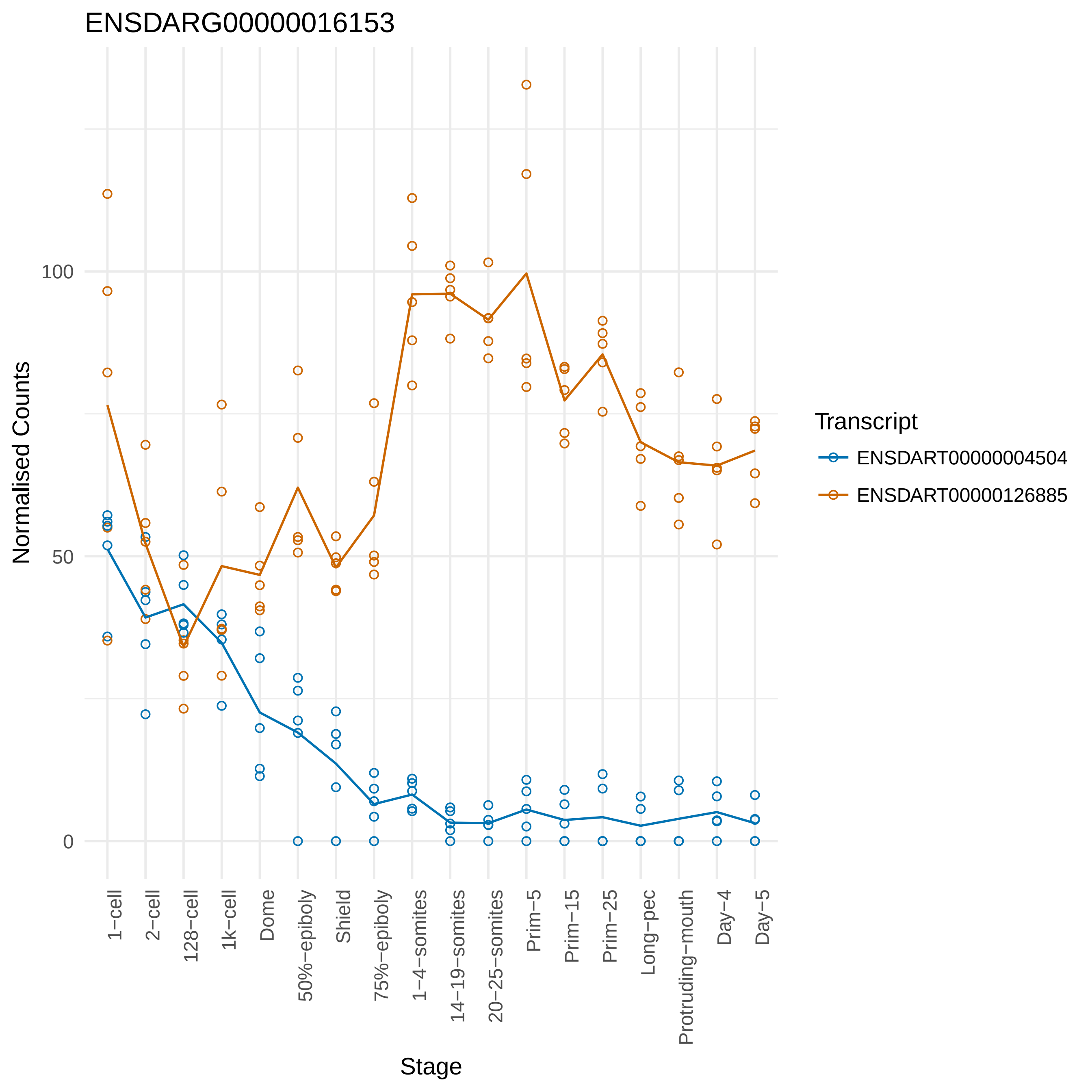

ENSDARG00000016153
Normalised Counts
100
50
0
1−cell
2−cell
128−cell
1k−cell
Dome
50%−epiboly
Shield
75%−epiboly
1−4−somites
14−19−somites
20−25−somites
Prim−5
Prim−15
Prim−25
Long−pec
Protruding−mouth
Day−4
Day−5
Stage
Transcript
ENSDART00000004504
ENSDART00000126885

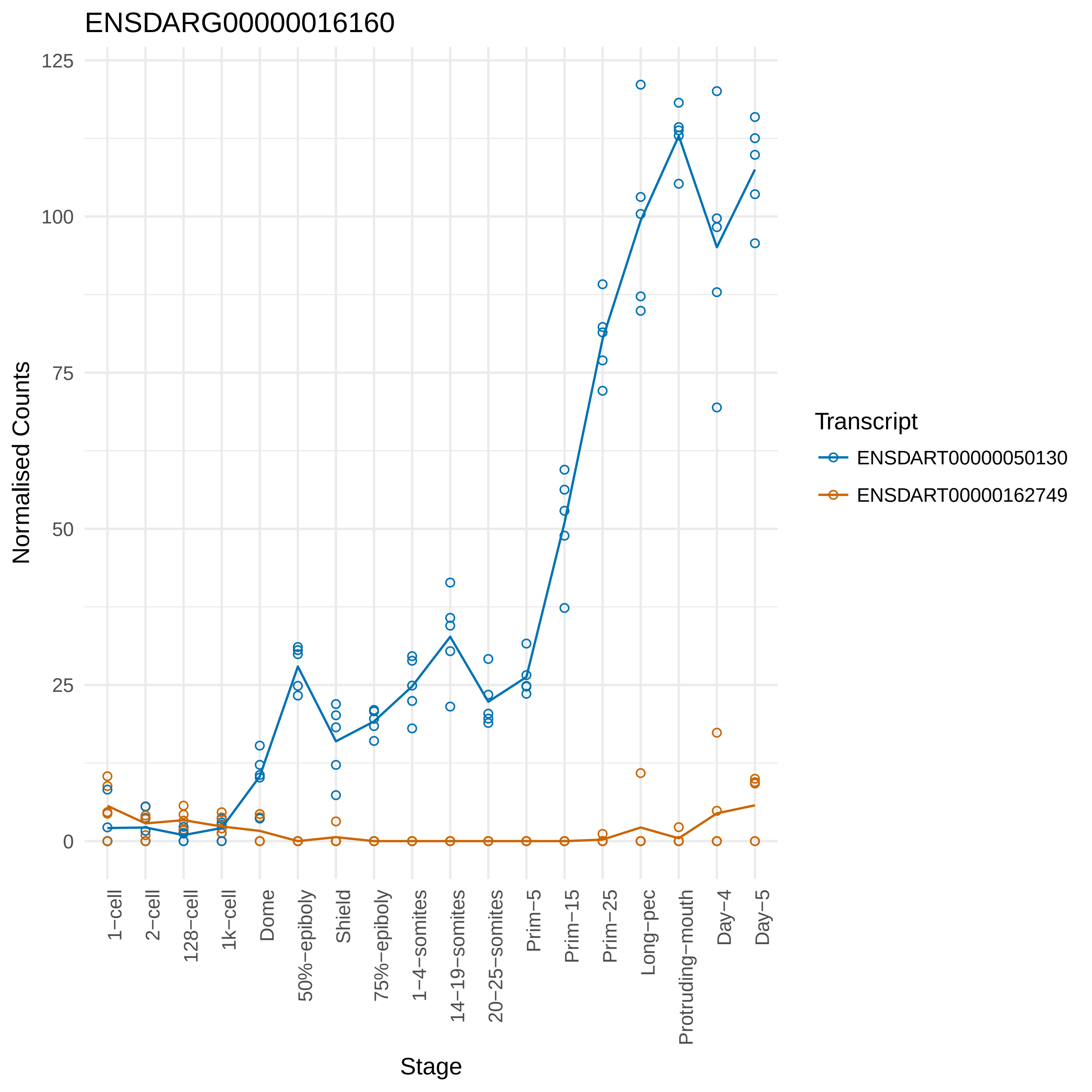

ENSDARG00000016160
Normalised Counts
125
100
75
50
25
0
1−cell
2−cell
128−cell
1k−cell
Dome
50%−epiboly
Shield
75%−epiboly
1−4−somites
14−19−somites
20−25−somites
Prim−5
Prim−15
Prim−25
Long−pec
Protruding−mouth
Day−4
Day−5
Stage
Transcript
ENSDART00000050130
ENSDART00000162749

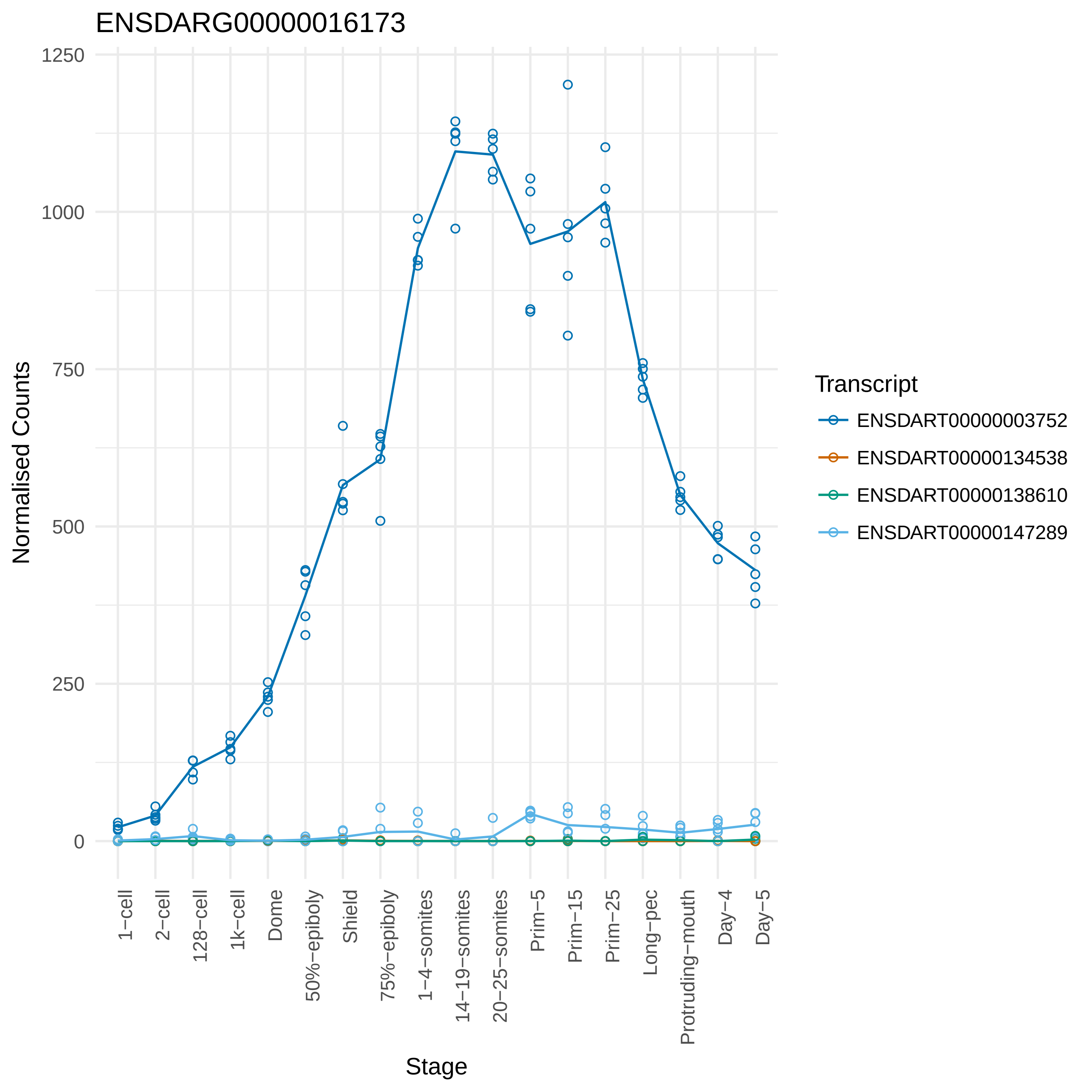
ENSDARG00000016173
Normalised Counts
1250
1000
750
500
250
0
1−cell
2−cell
128−cell
1k−cell
Dome
50%−epiboly
Shield
75%−epiboly
1−4−somites
14−19−somites
20−25−somites
Prim−5
Prim−15
Prim−25
Long−pec
Protruding−mouth
Day−4
Day−5
Stage
Transcript
ENSDART00000003752
ENSDART00000134538
ENSDART00000138610
ENSDART00000147289

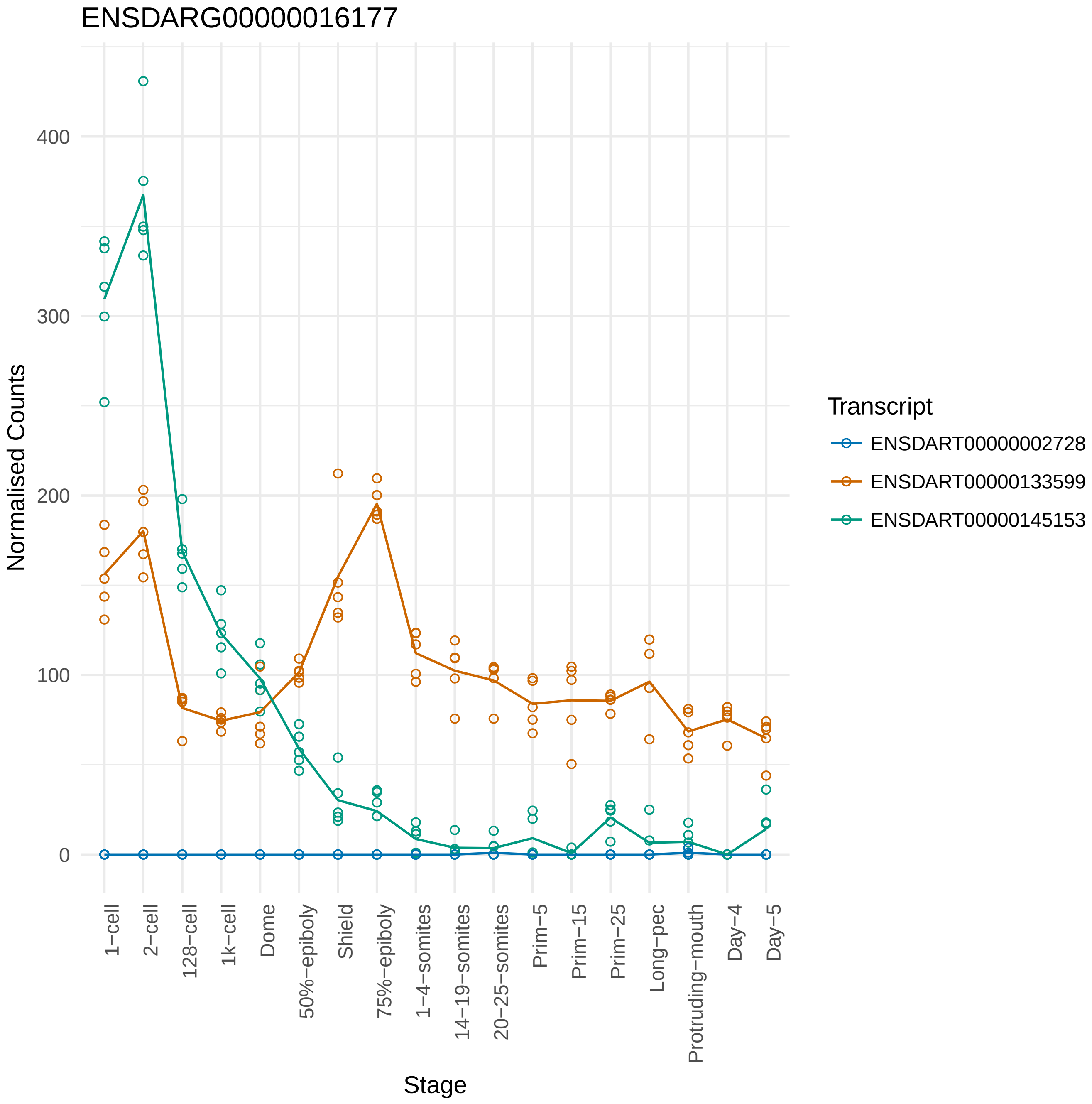

ENSDARG00000016177
Normalised Counts
400
300
200
100
0
1−cell
2−cell
128−cell
1k−cell
Dome
50%−epiboly
Shield
75%−epiboly
1−4−somites
14−19−somites
20−25−somites
Prim−5
Prim−15
Prim−25
Long−pec
Protruding−mouth
Day−4
Day−5
Stage
Transcript
ENSDART00000002728
ENSDART00000133599
ENSDART00000145153

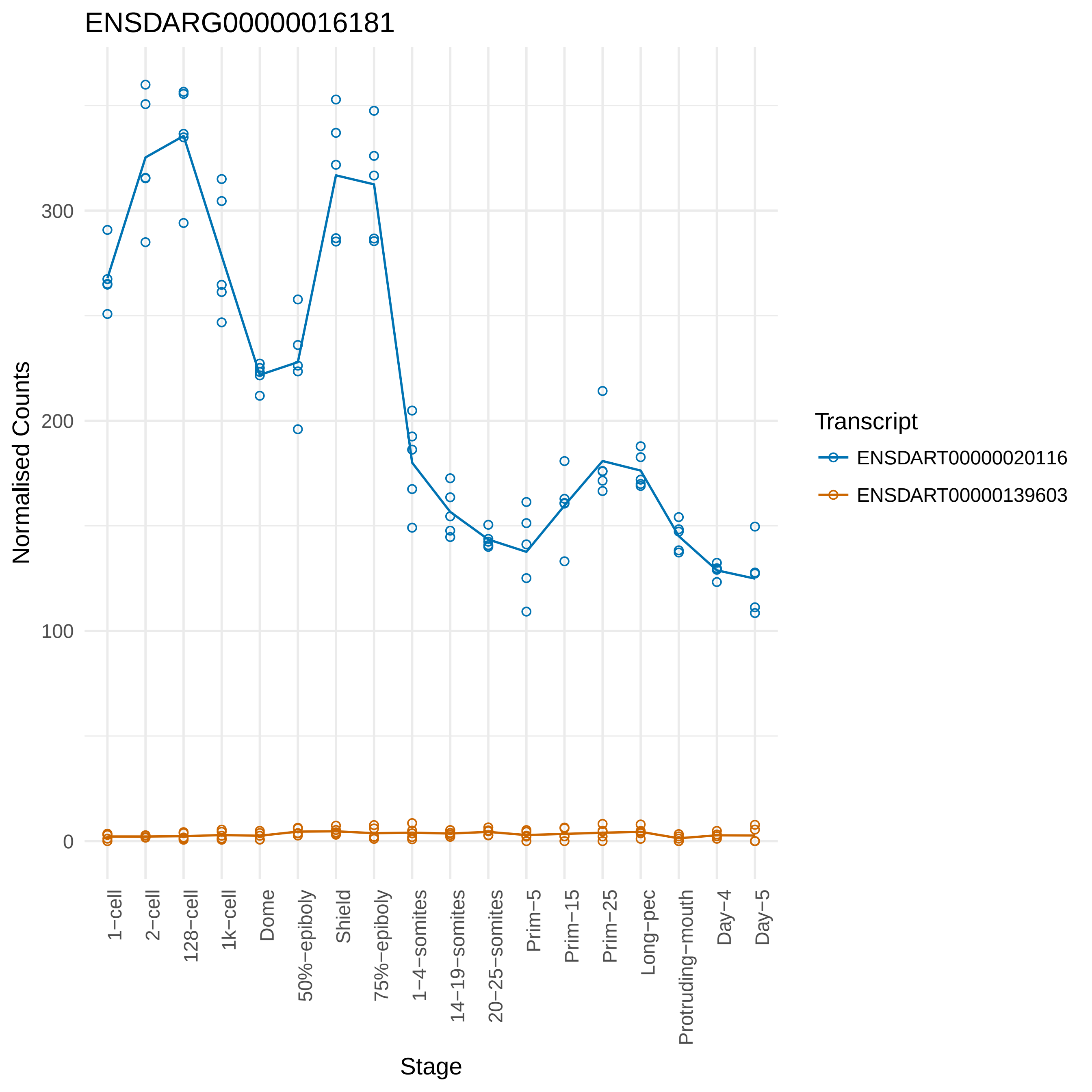

ENSDARG00000016181
Normalised Counts
300
200
100
0
1−cell
2−cell
128−cell
1k−cell
Dome
50%−epiboly
Shield
75%−epiboly
1−4−somites
14−19−somites
20−25−somites
Prim−5
Prim−15
Prim−25
Long−pec
Protruding−mouth
Day−4
Day−5
Stage
Transcript
ENSDART00000020116
ENSDART00000139603

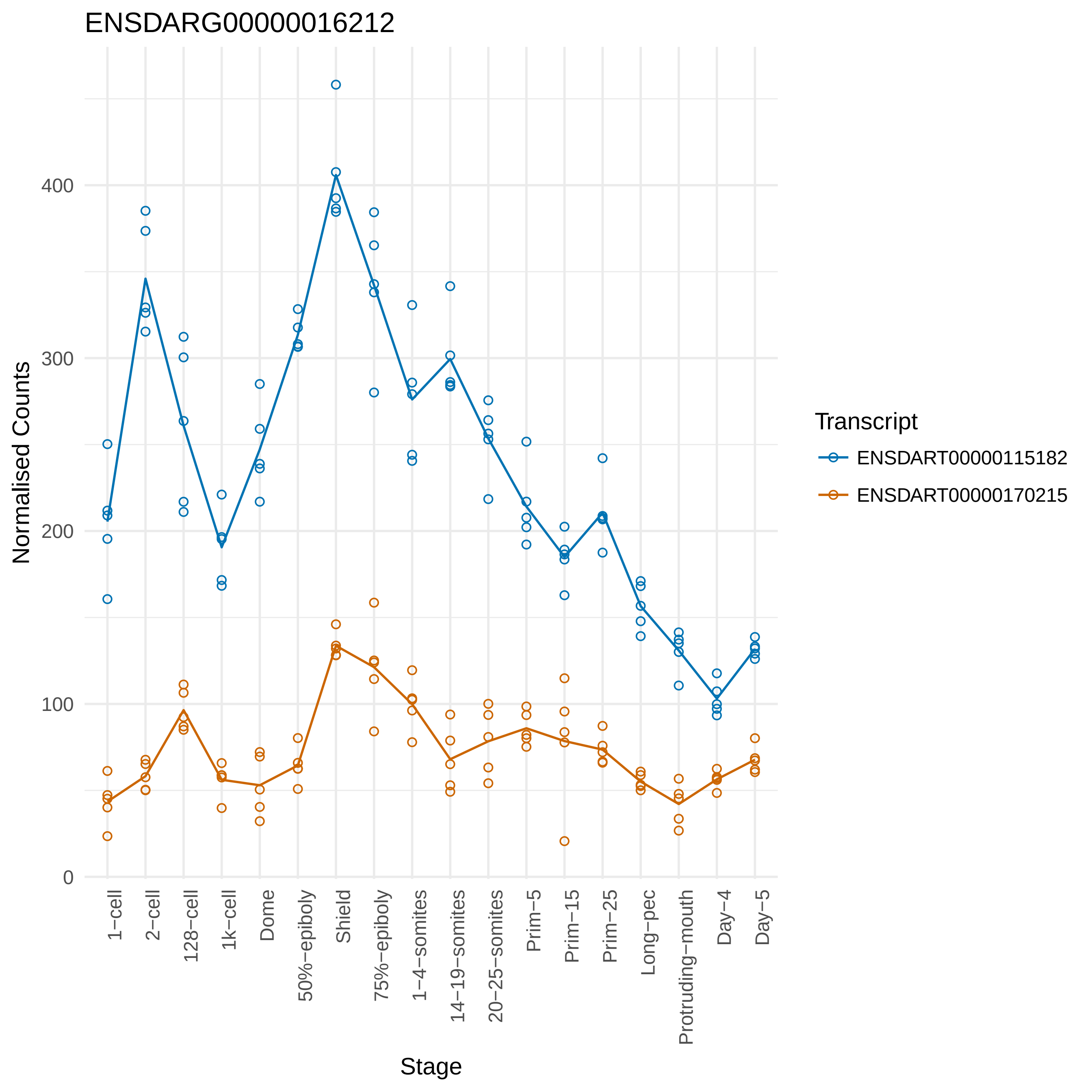

ENSDARG00000016212
Normalised Counts
400
300
200
100
0
1−cell
2−cell
128−cell
1k−cell
Dome
50%−epiboly
Shield
75%−epiboly
1−4−somites
14−19−somites
20−25−somites
Prim−5
Prim−15
Prim−25
Long−pec
Protruding−mouth
Day−4
Day−5
Stage
Transcript
ENSDART00000115182
ENSDART00000170215

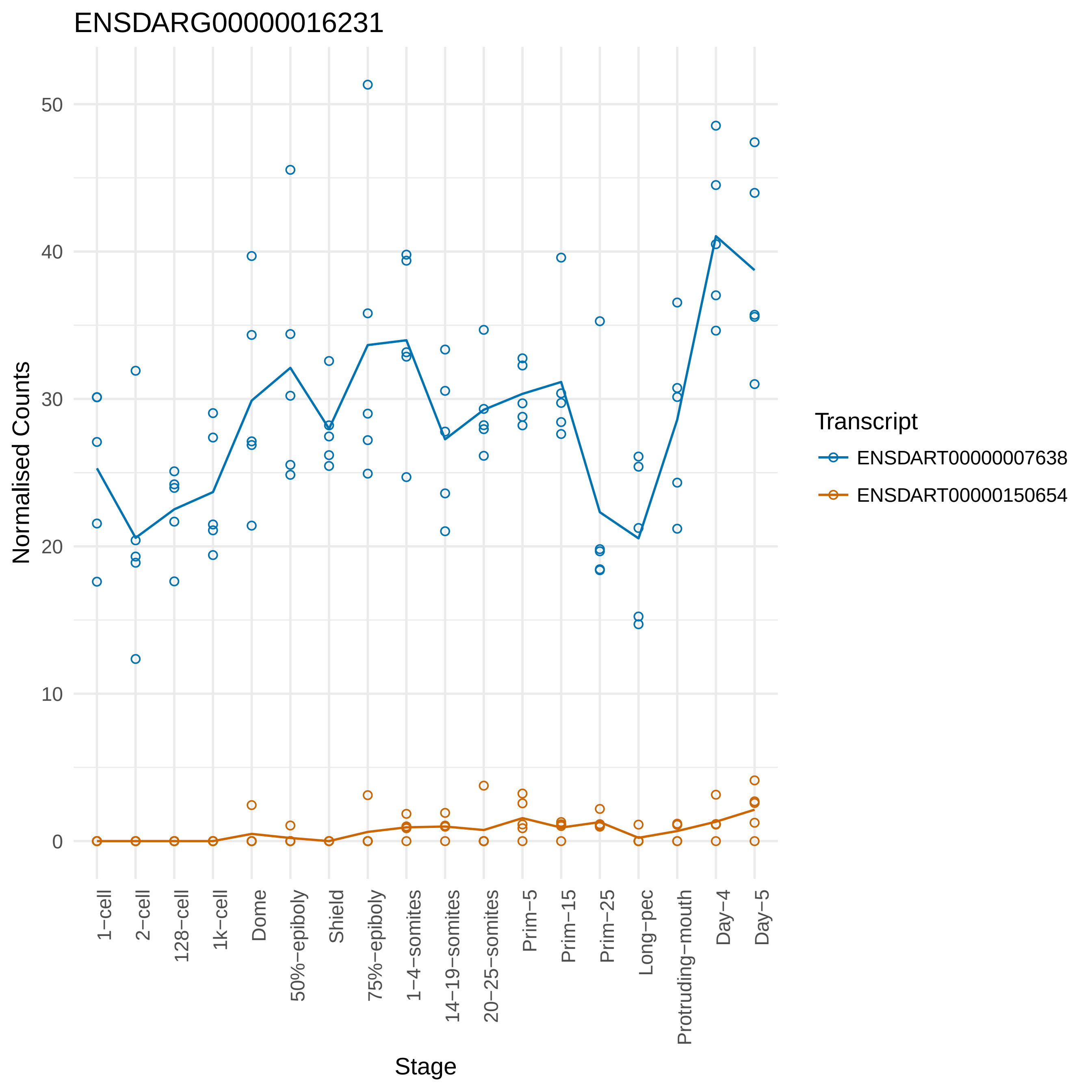

ENSDARG00000016231
Normalised Counts
0
10
20
30
40
50
1−cell
2−cell
128−cell
1k−cell
Dome
50%−epiboly
Shield
75%−epiboly
1−4−somites
14−19−somites
20−25−somites
Prim−5
Prim−15
Prim−25
Long−pec
Protruding−mouth
Day−4
Day−5
Stage
Transcript
ENSDART00000007638
ENSDART00000150654

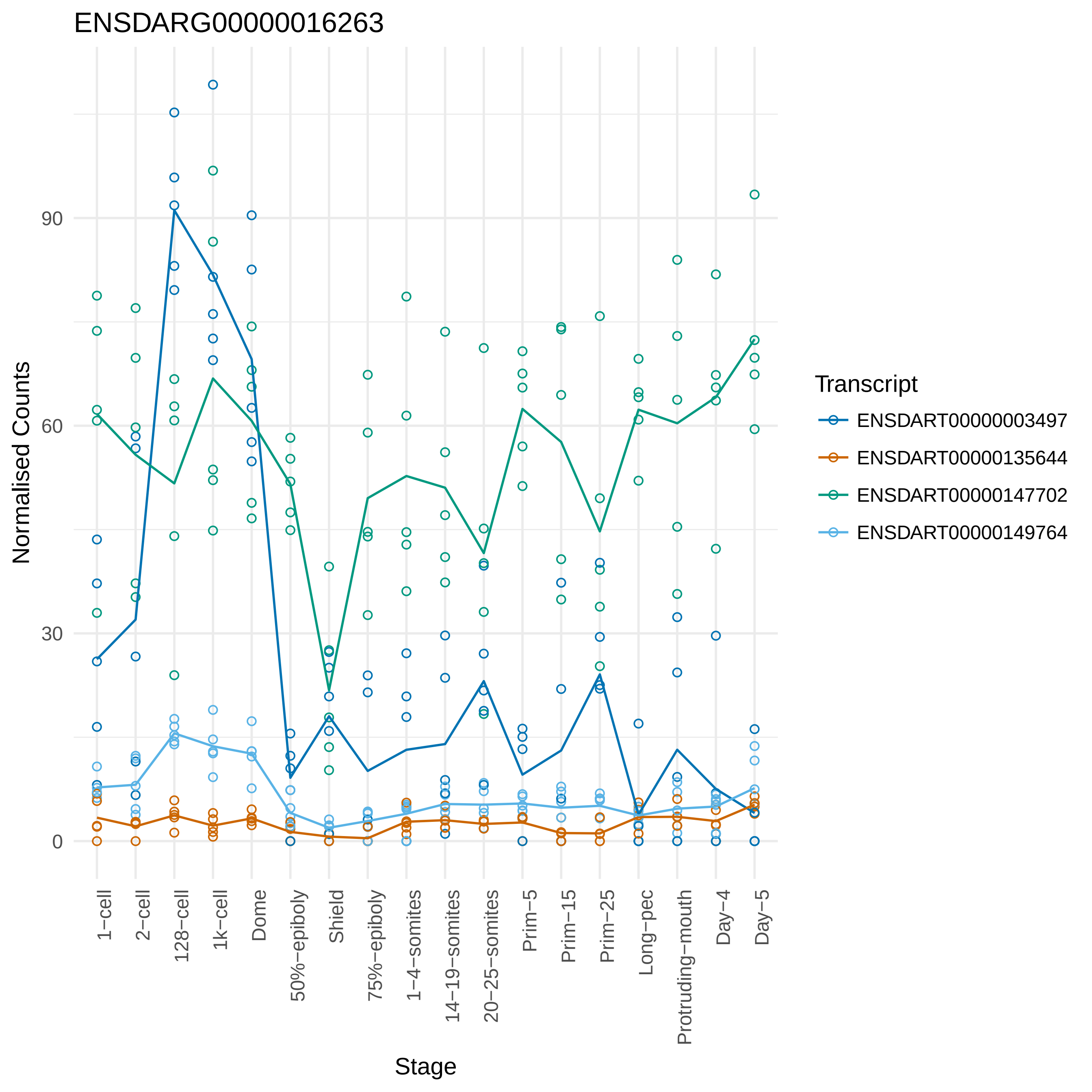

ENSDARG00000016263
Normalised Counts
0
30
60
90
1−cell
2−cell
128−cell
1k−cell
Dome
50%−epiboly
Shield
75%−epiboly
1−4−somites
14−19−somites
20−25−somites
Prim−5
Prim−15
Prim−25
Long−pec
Protruding−mouth
Day−4
Day−5
Stage
Transcript
ENSDART00000003497
ENSDART00000135644
ENSDART00000147702
ENSDART00000149764

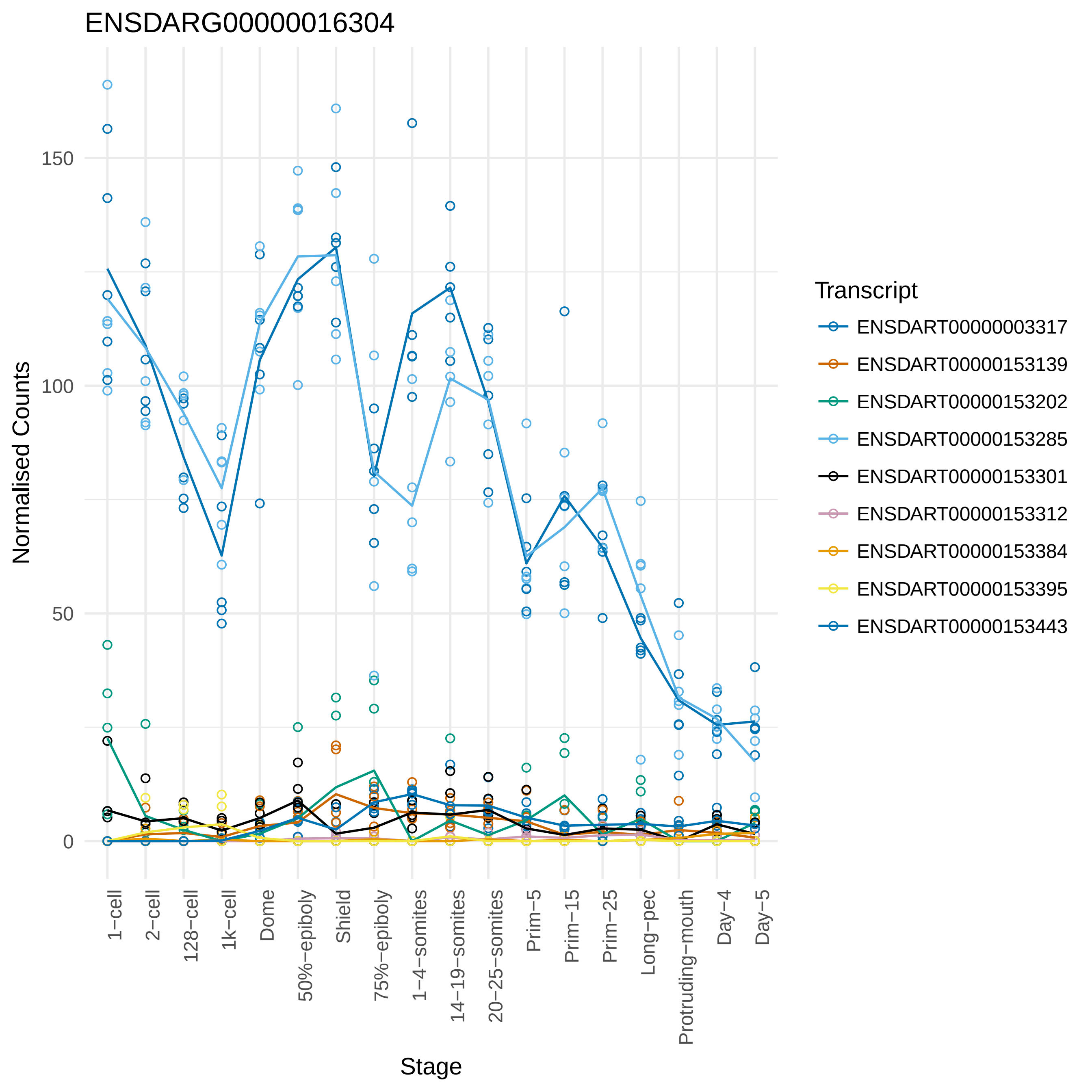

ENSDARG00000016304
Normalised Counts
0
50
100
150
Stage
1−cell
2−cell
128−cell
1k−cell
Dome
50%−epiboly
Shield
75%−epiboly
1−4−somites
14−19−somites
20−25−somites
Prim−5
Prim−15
Prim−25
Long−pec
Protruding−mouth
Day−4
Day−5
Transcript
ENSDART00000003317
ENSDART00000153139
ENSDART00000153202
ENSDART00000153285
ENSDART00000153301
ENSDART00000153312
ENSDART00000153384
ENSDART00000153395
ENSDART00000153443

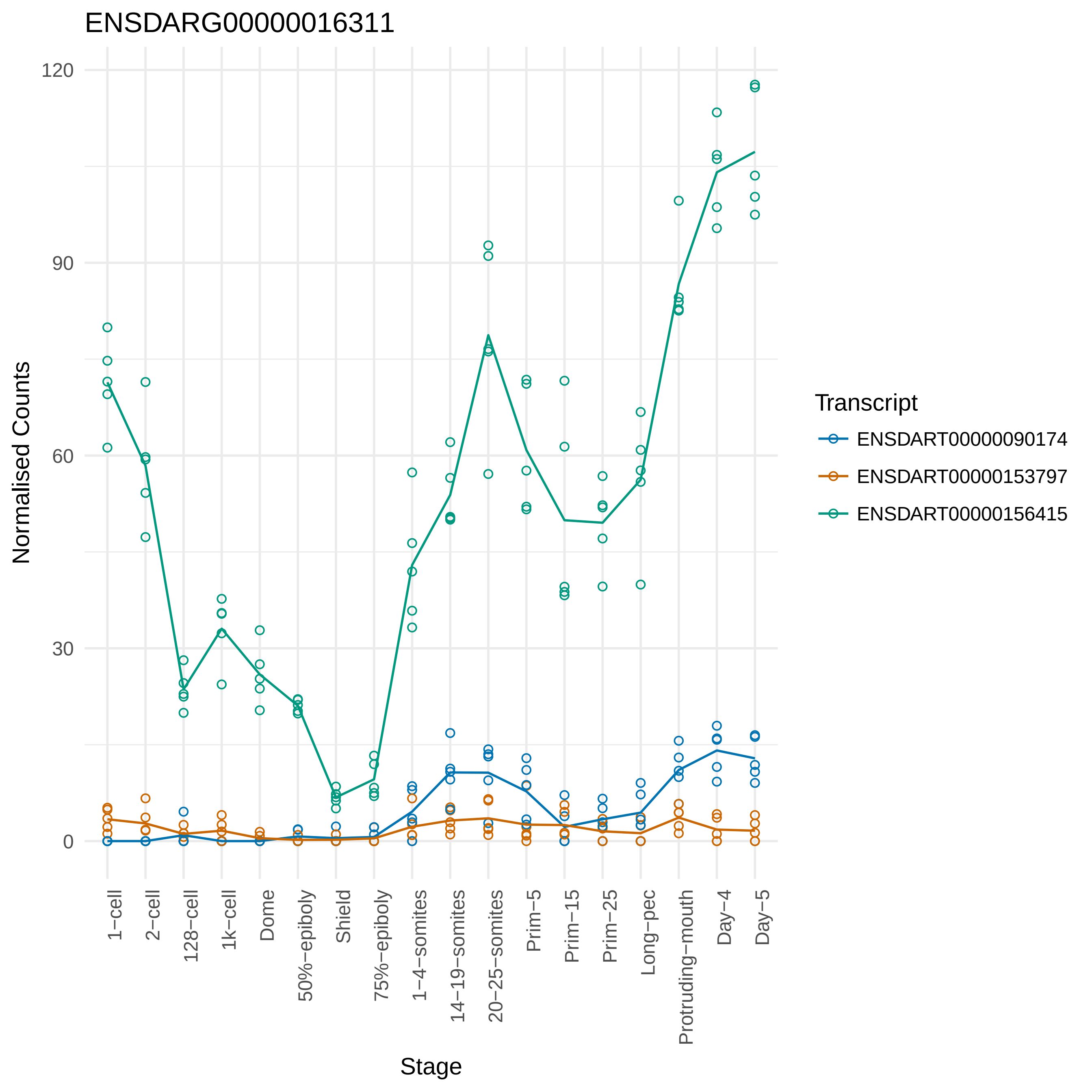

ENSDARG00000016311
Normalised Counts
120
90
60
30
0
1−cell
2−cell
128−cell
1k−cell
Dome
50%−epiboly
Shield
75%−epiboly
1−4−somites
14−19−somites
20−25−somites
Prim−5
Prim−15
Prim−25
Long−pec
Protruding−mouth
Day−4
Day−5
Stage
Transcript
ENSDART00000090174
ENSDART00000153797
ENSDART00000156415

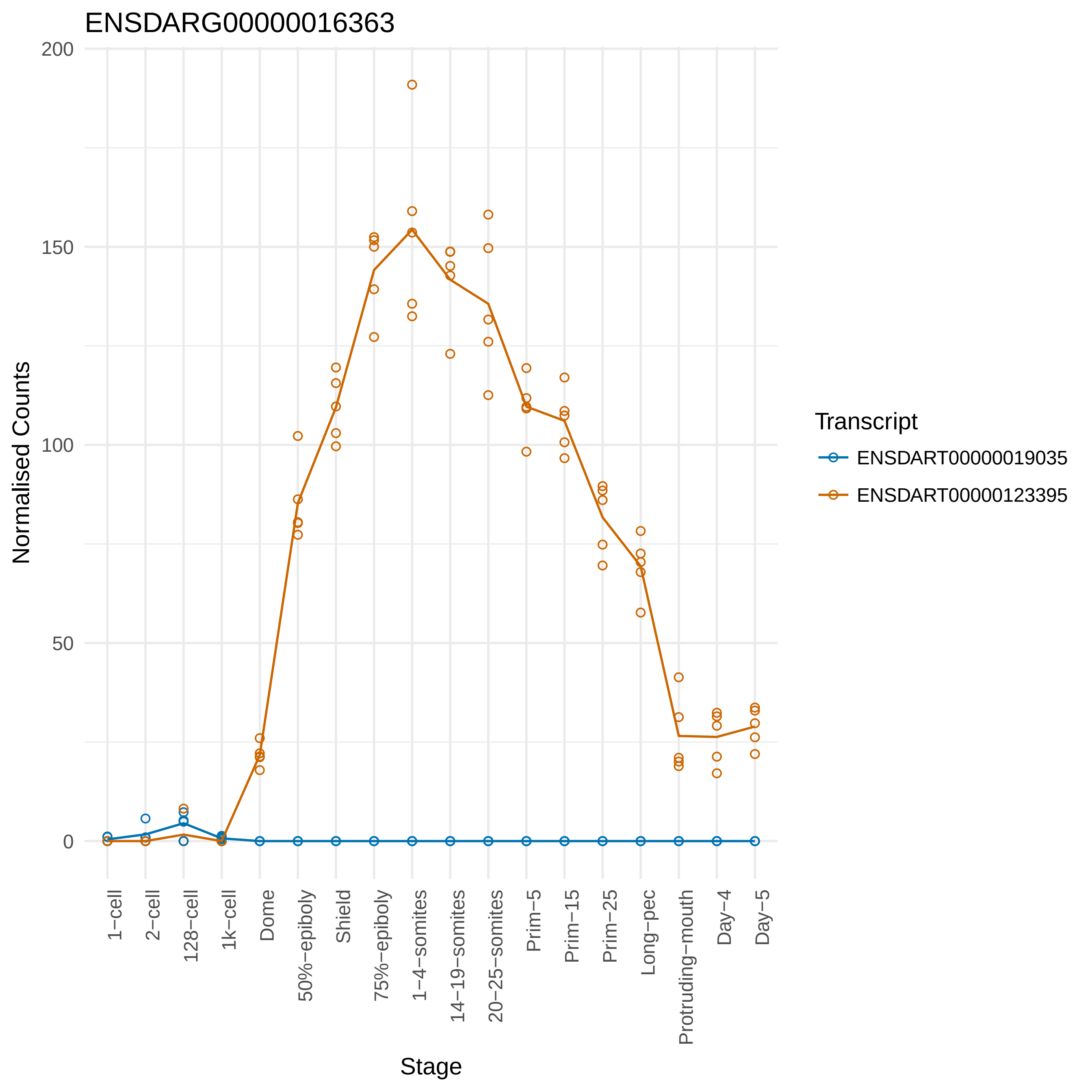
ENSDARG00000016363
Normalised Counts
200
150
100
50
0
1−cell
2−cell
128−cell
1k−cell
Dome
50%−epiboly
Shield
75%−epiboly
1−4−somites
14−19−somites
20−25−somites
Prim−5
Prim−15
Prim−25
Long−pec
Protruding−mouth
Day−4
Day−5
Stage
Transcript
ENSDART00000019035
ENSDART00000123395

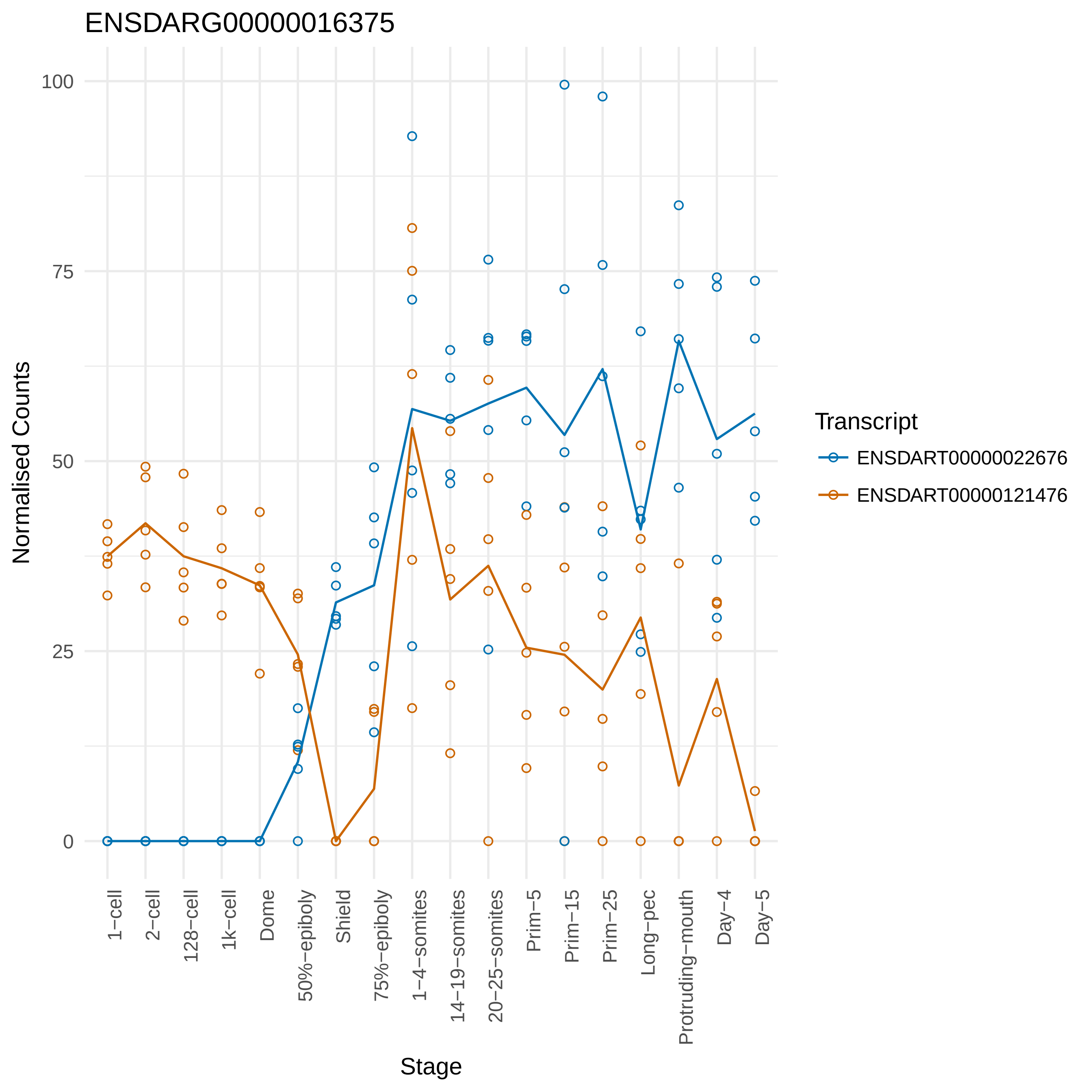

ENSDARG00000016375
Normalised Counts
100
75
50
25
0
1−cell
2−cell
128−cell
1k−cell
Dome
50%−epiboly
Shield
75%−epiboly
1−4−somites
14−19−somites
20−25−somites
Prim−5
Prim−15
Prim−25
Long−pec
Protruding−mouth
Day−4
Day−5
Stage
Transcript
ENSDART00000022676
ENSDART00000121476

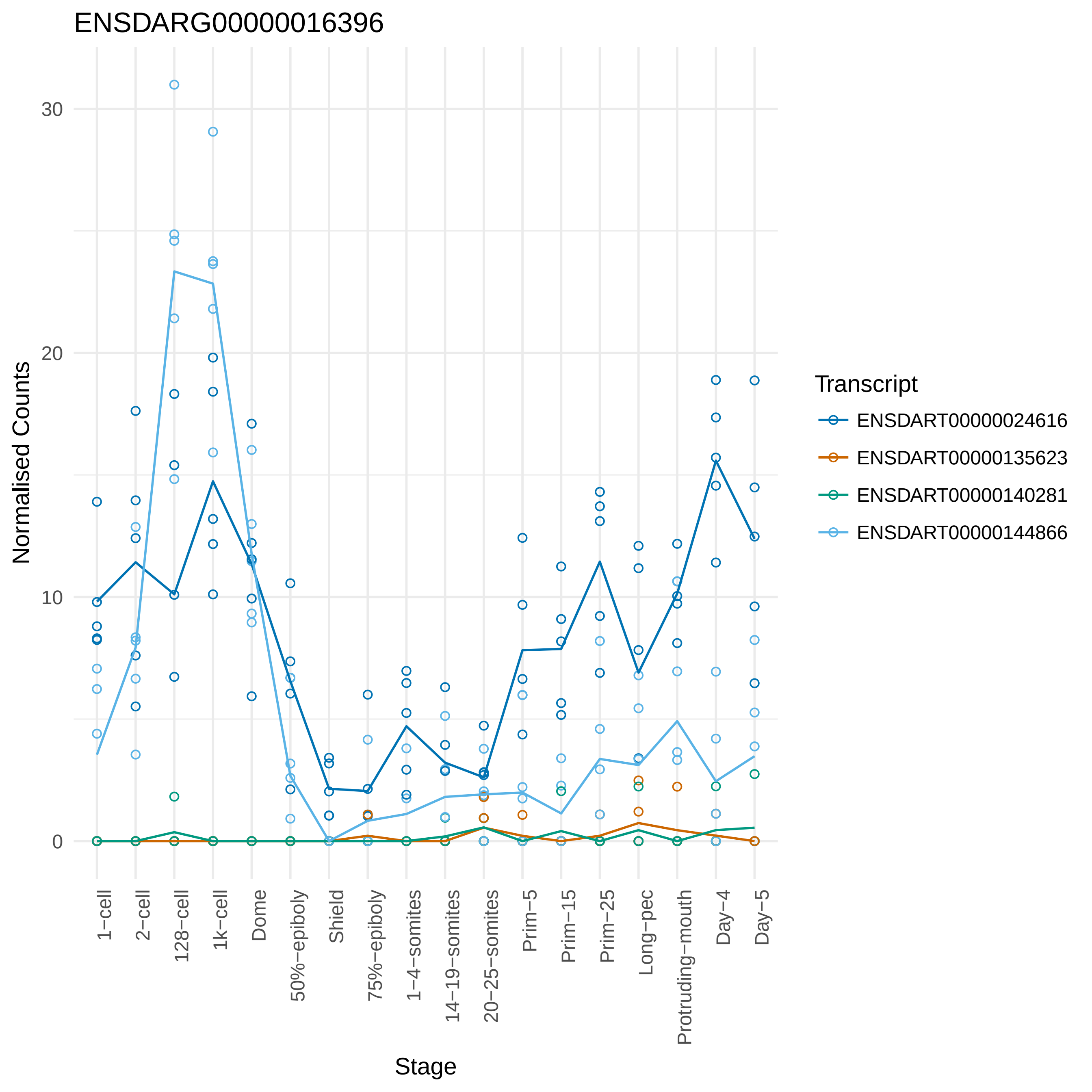

ENSDARG00000016396
Normalised Counts
0
10
20
30
Stage
1−cell
2−cell
128−cell
1k−cell
Dome
50%−epiboly
Shield
75%−epiboly
1−4−somites
14−19−somites
20−25−somites
Prim−5
Prim−15
Prim−25
Long−pec
Protruding−mouth
Day−4
Day−5
Transcript
ENSDART00000024616
ENSDART00000135623
ENSDART00000140281
ENSDART00000144866

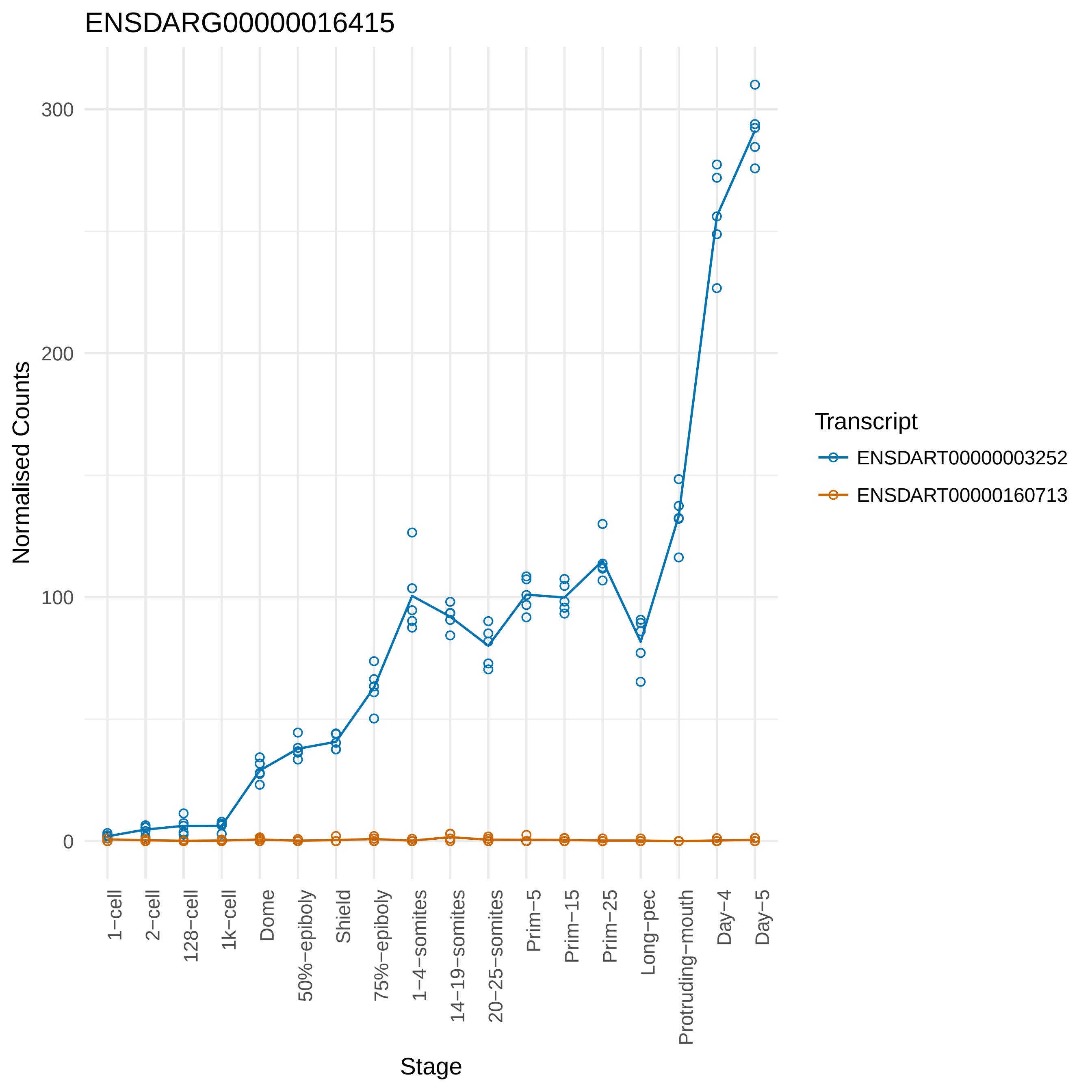

ENSDARG00000016415
Normalised Counts
300
200
100
0
1−cell
2−cell
128−cell
1k−cell
Dome
50%−epiboly
Shield
75%−epiboly
1−4−somites
14−19−somites
20−25−somites
Prim−5
Prim−15
Prim−25
Long−pec
Protruding−mouth
Day−4
Day−5
Stage
Transcript
ENSDART00000003252
ENSDART00000160713

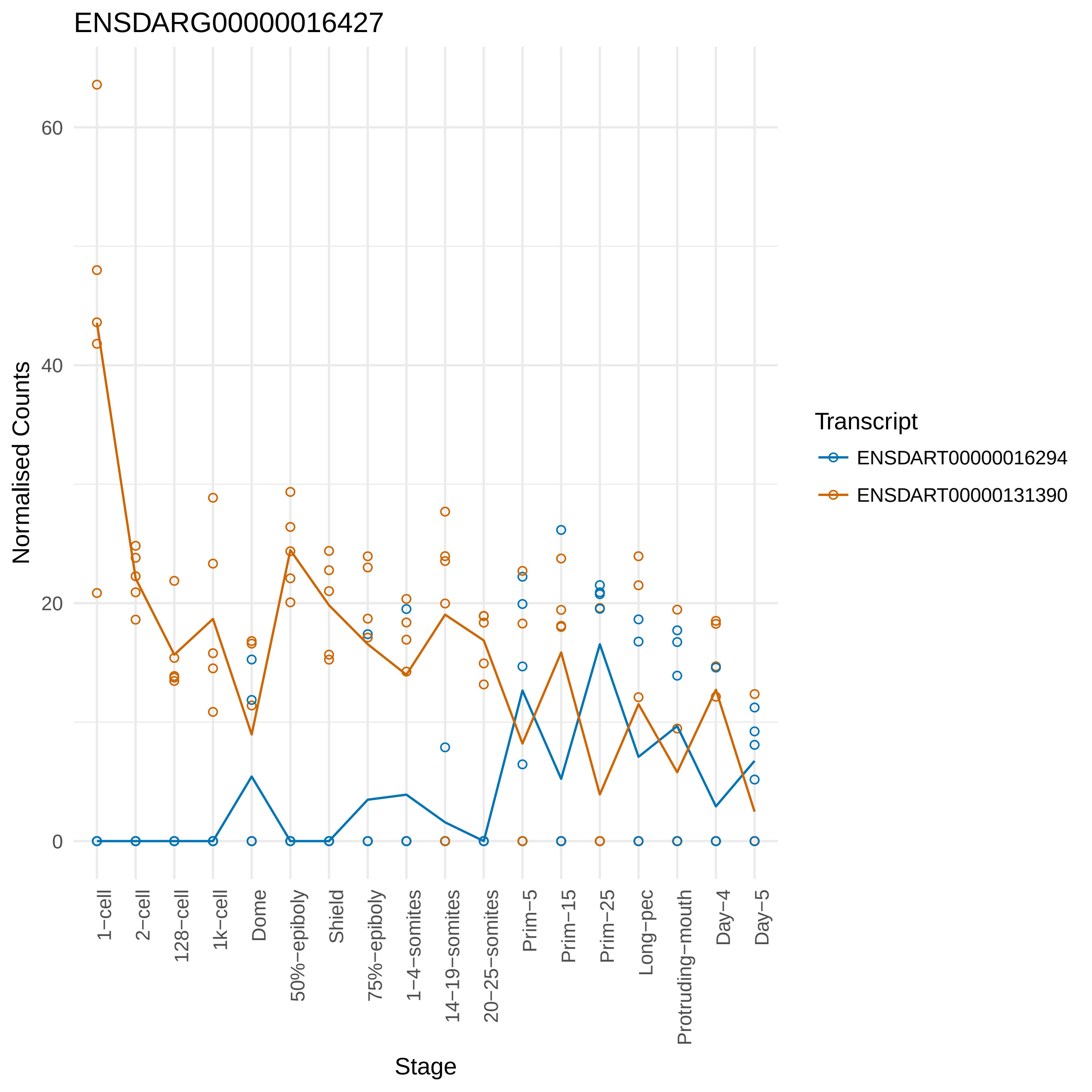

ENSDARG00000016427
Normalised Counts
60
40
20
0
1−cell
2−cell
128−cell
1k−cell
Dome
50%−epiboly
Shield
75%−epiboly
1−4−somites
14−19−somites
20−25−somites
Prim−5
Prim−15
Prim−25
Long−pec
Protruding−mouth
Day−4
Day−5
Stage
Transcript
ENSDART00000016294
ENSDART00000131390

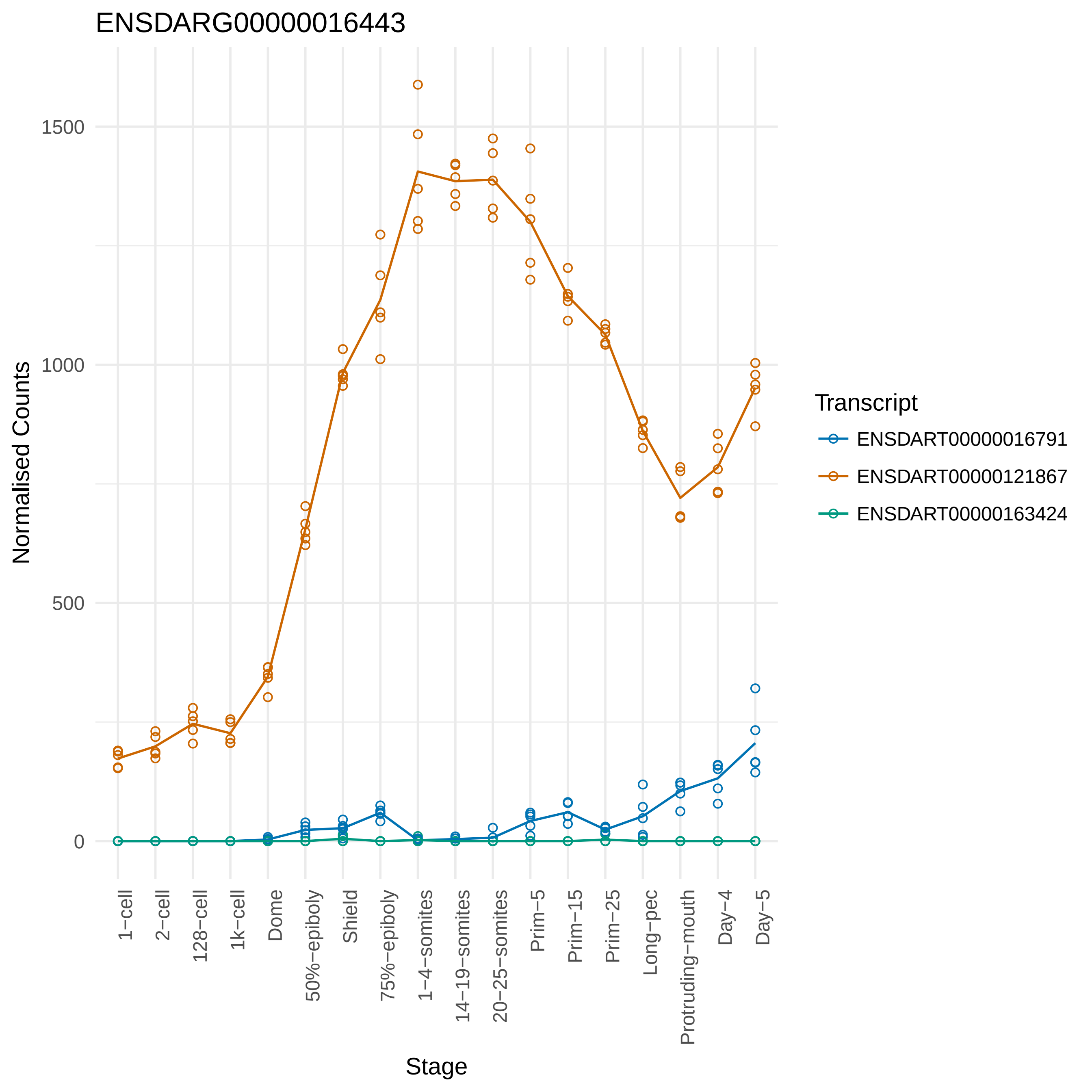
ENSDARG00000016443
Normalised Counts
1500
1000
500
0
1−cell
2−cell
128−cell
1k−cell
Dome
50%−epiboly
Shield
75%−epiboly
1−4−somites
14−19−somites
20−25−somites
Prim−5
Prim−15
Prim−25
Long−pec
Protruding−mouth
Day−4
Day−5
Stage
Transcript
ENSDART00000016791
ENSDART00000121867
ENSDART00000163424

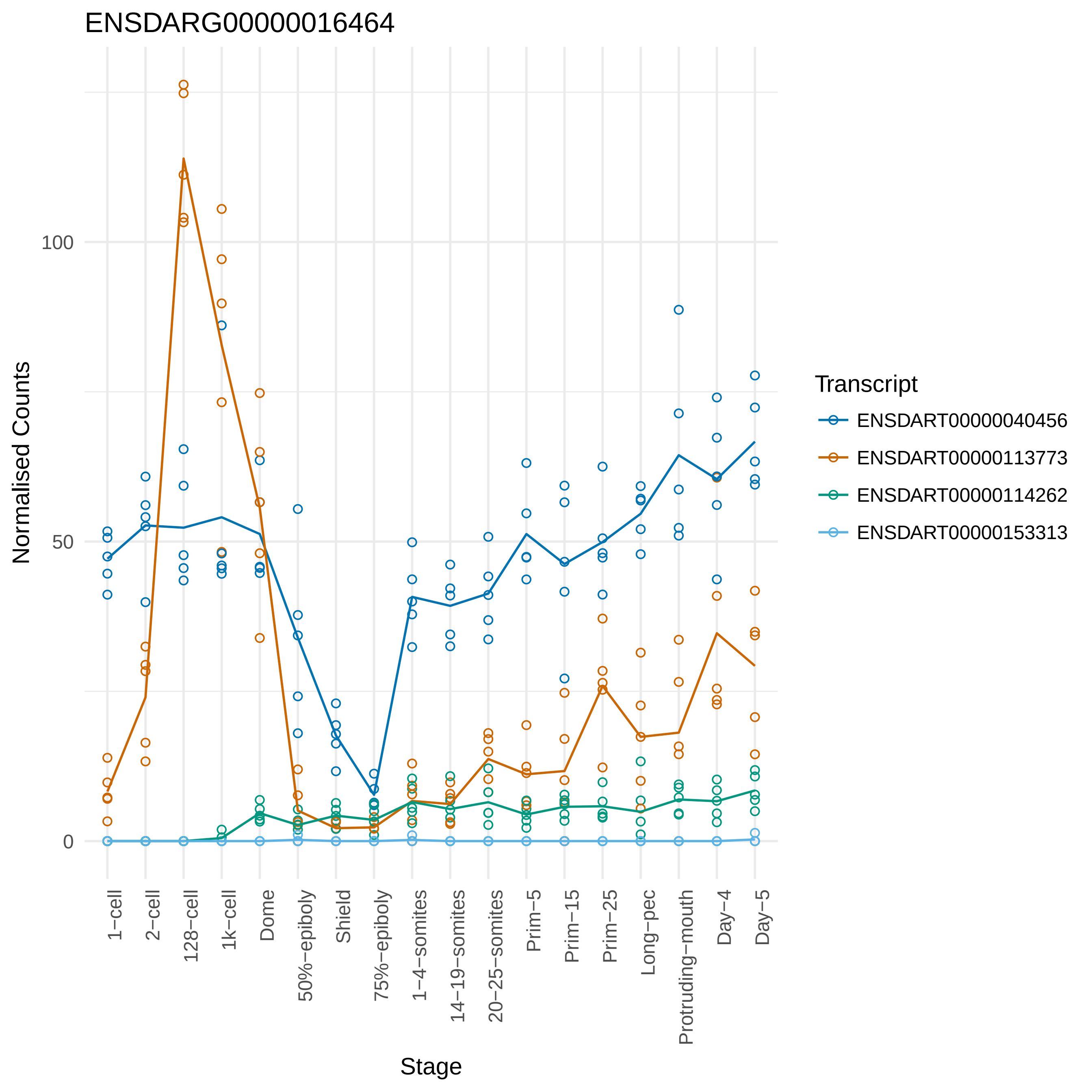
ENSDARG00000016464
Normalised Counts
100
50
0
Stage
1−cell
2−cell
128−cell
1k−cell
Dome
50%−epiboly
Shield
75%−epiboly
1−4−somites
14−19−somites
20−25−somites
Prim−5
Prim−15
Prim−25
Long−pec
Protruding−mouth
Day−4
Day−5
Transcript
ENSDART00000040456
ENSDART00000113773
ENSDART00000114262
ENSDART00000153313

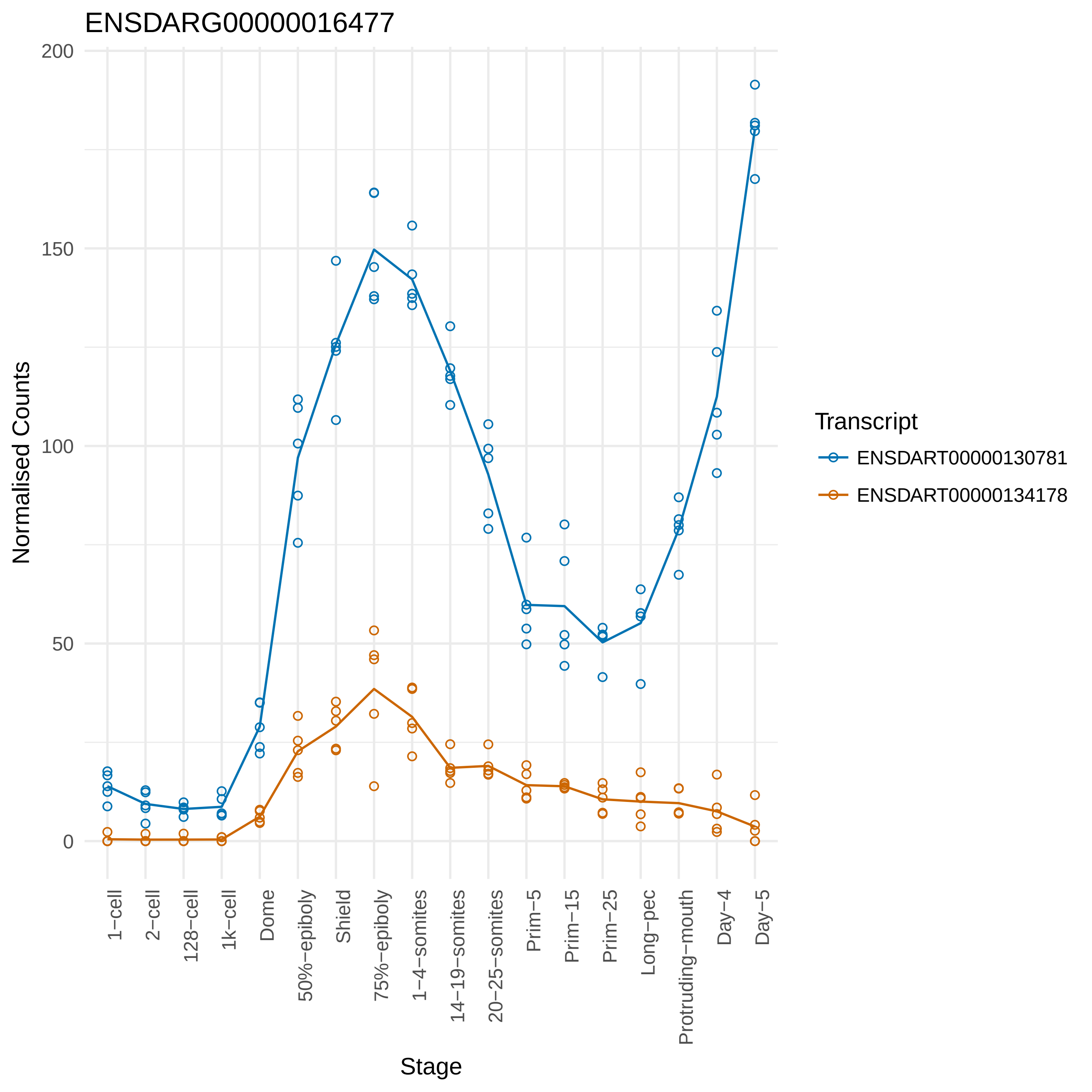

ENSDARG00000016477
Normalised Counts
200
150
100
50
0
1−cell
2−cell
128−cell
1k−cell
Dome
50%−epiboly
Shield
75%−epiboly
1−4−somites
14−19−somites
20−25−somites
Prim−5
Prim−15
Prim−25
Long−pec
Protruding−mouth
Day−4
Day−5
Stage
Transcript
ENSDART00000130781
ENSDART00000134178

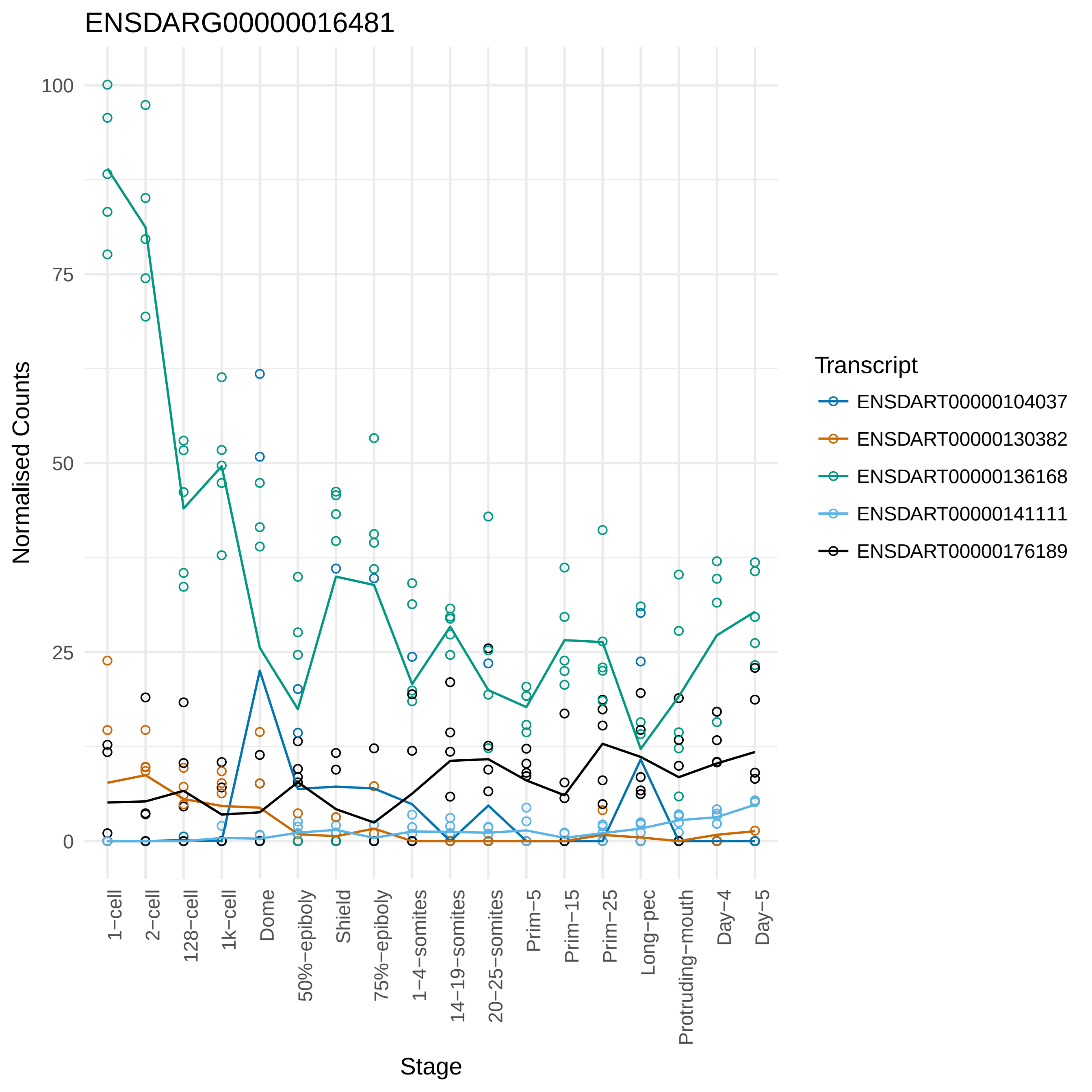

ENSDARG00000016481
Normalised Counts
Stage
0
25
50
75
100
1−cell
2−cell
128−cell
1k−cell
Dome
50%−epiboly
Shield
75%−epiboly
1−4−somites
14−19−somites
20−25−somites
Prim−5
Prim−15
Prim−25
Long−pec
Protruding−mouth
Day−4
Day−5
Transcript
ENSDART00000104037
ENSDART00000130382
ENSDART00000136168
ENSDART00000141111
ENSDART00000176189

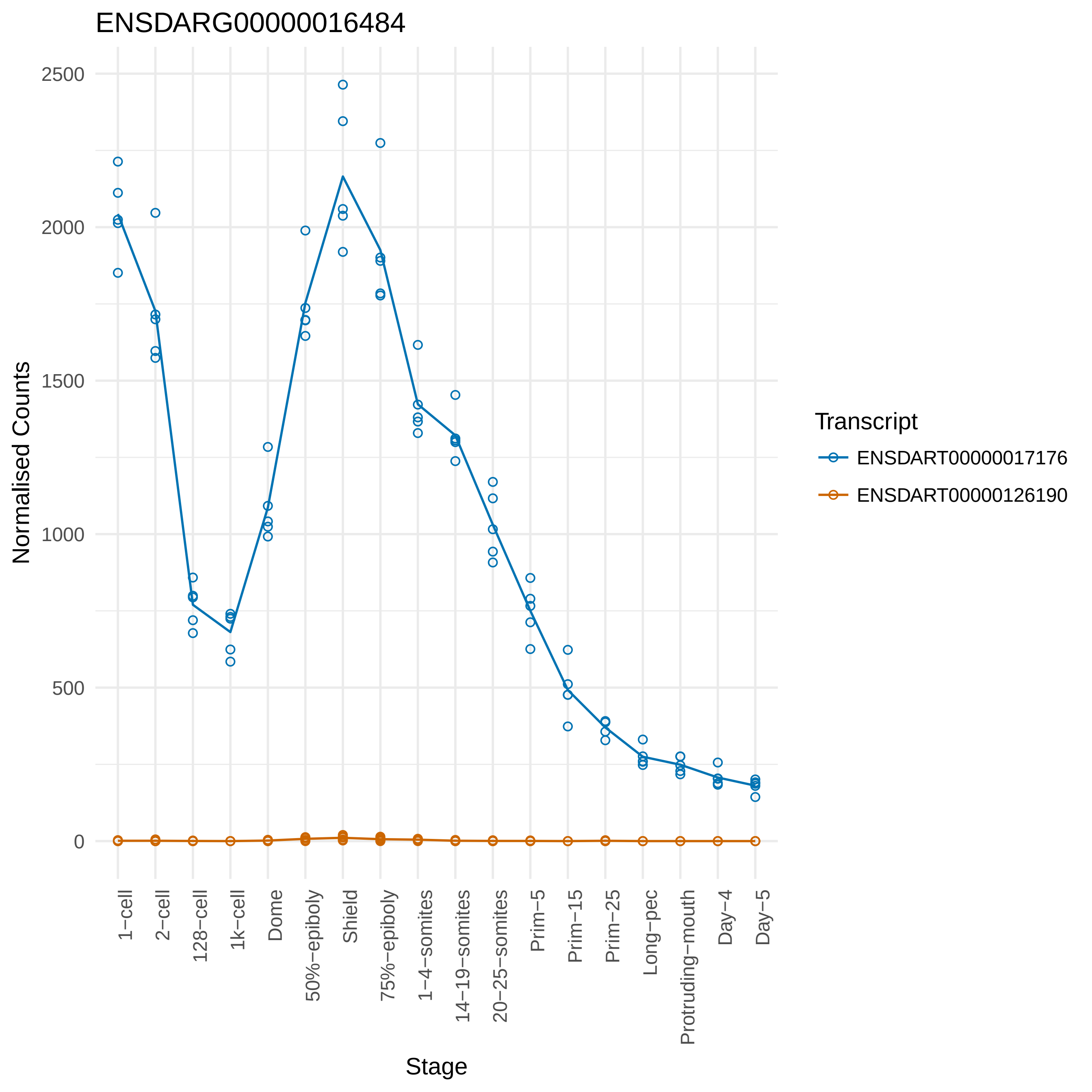

ENSDARG00000016484
Normalised Counts
2500
2000
1500
1000
500
0
Stage
1−cell
2−cell
128−cell
1k−cell
Dome
50%−epiboly
Shield
75%−epiboly
1−4−somites
14−19−somites
20−25−somites
Prim−5
Prim−15
Prim−25
Long−pec
Protruding−mouth
Day−4
Day−5
Transcript
ENSDART00000017176
ENSDART00000126190

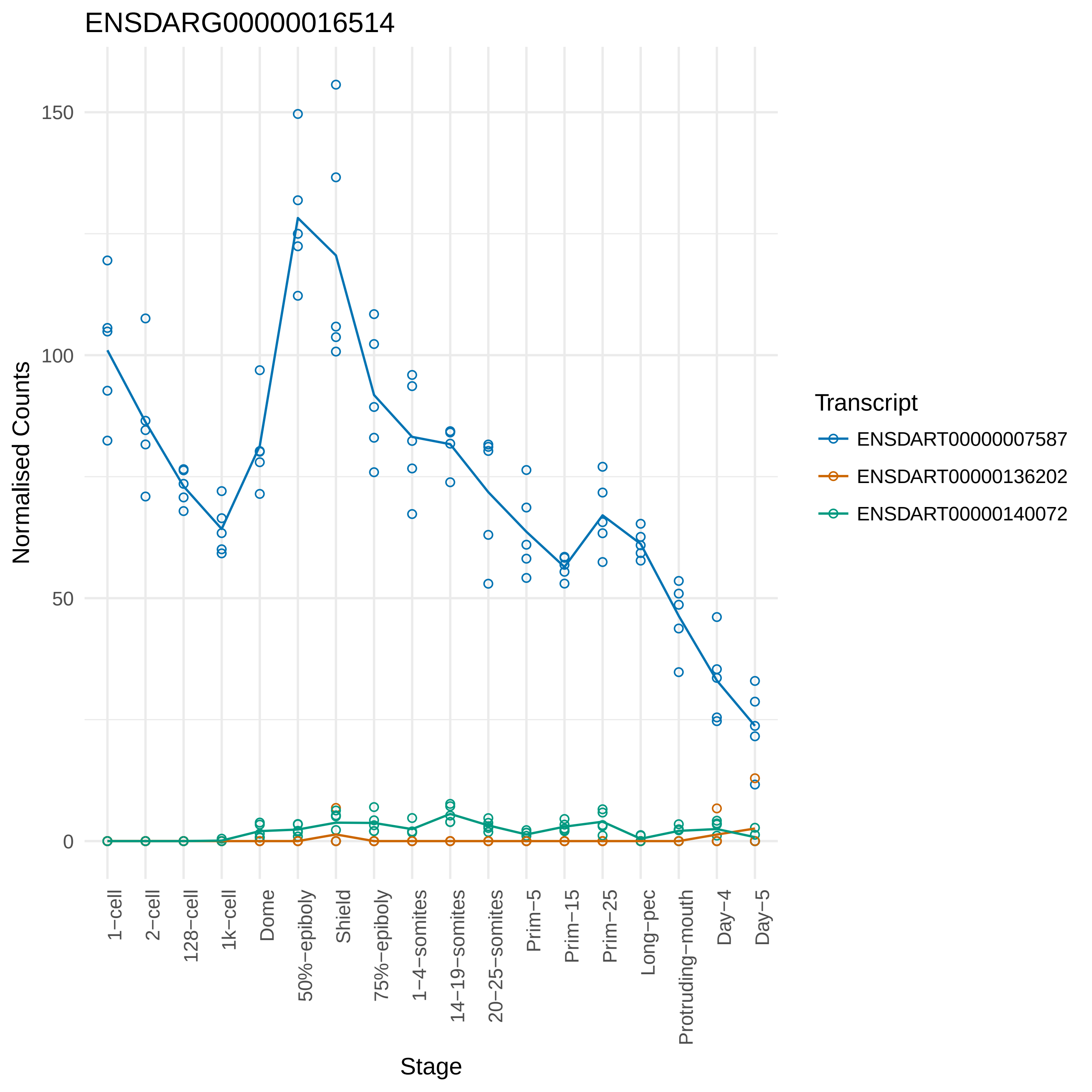

ENSDARG00000016514
Normalised Counts
150
100
50
0
1−cell
2−cell
128−cell
1k−cell
Dome
50%−epiboly
Shield
75%−epiboly
1−4−somites
14−19−somites
20−25−somites
Prim−5
Prim−15
Prim−25
Long−pec
Protruding−mouth
Day−4
Day−5
Stage
Transcript
ENSDART00000007587
ENSDART00000136202
ENSDART00000140072

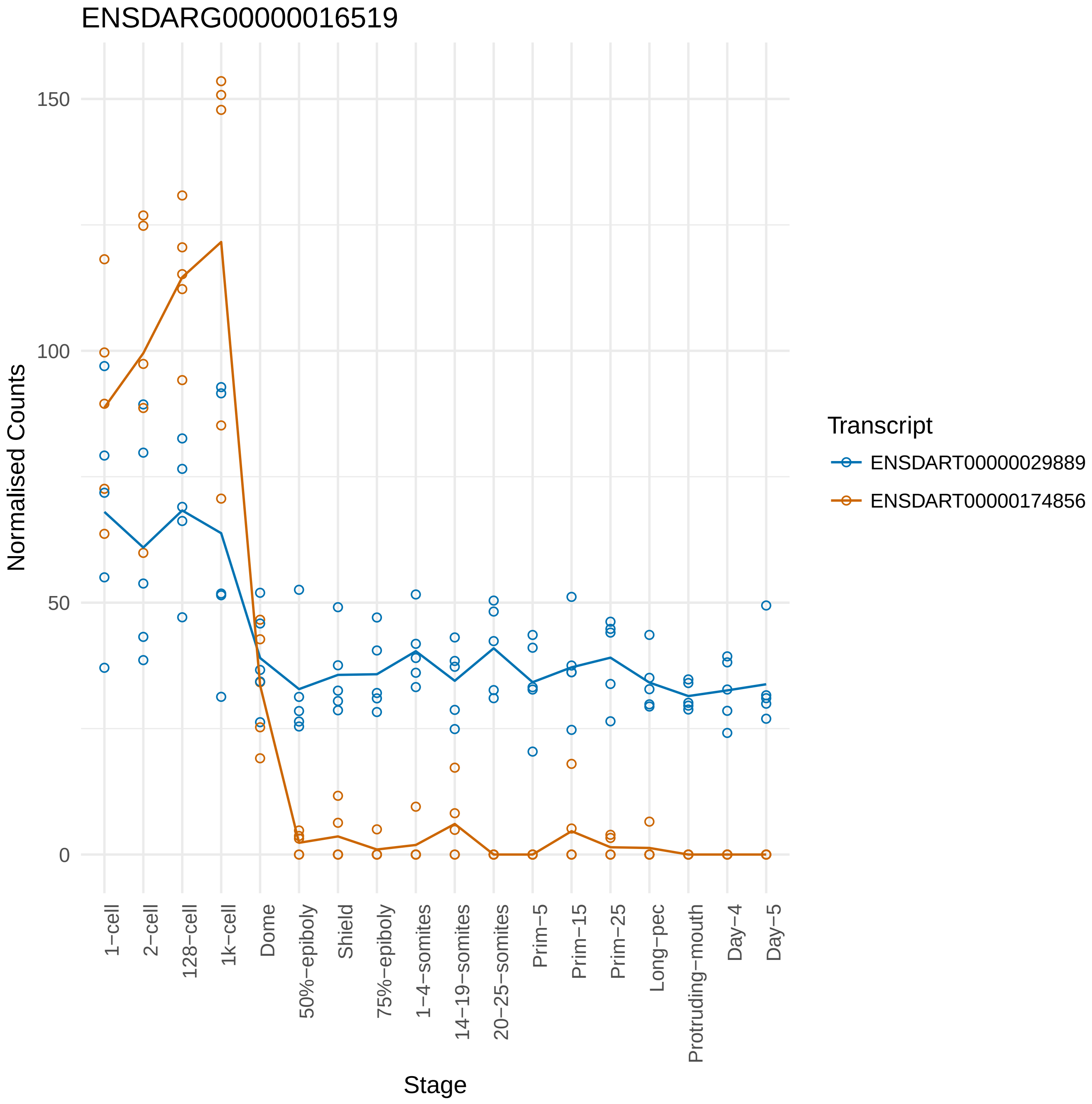

ENSDARG00000016519
Normalised Counts
150
100
50
0
1−cell
2−cell
128−cell
1k−cell
Dome
50%−epiboly
Shield
75%−epiboly
1−4−somites
14−19−somites
20−25−somites
Prim−5
Prim−15
Prim−25
Long−pec
Protruding−mouth
Day−4
Day−5
Stage
Transcript
ENSDART00000029889
ENSDART00000174856

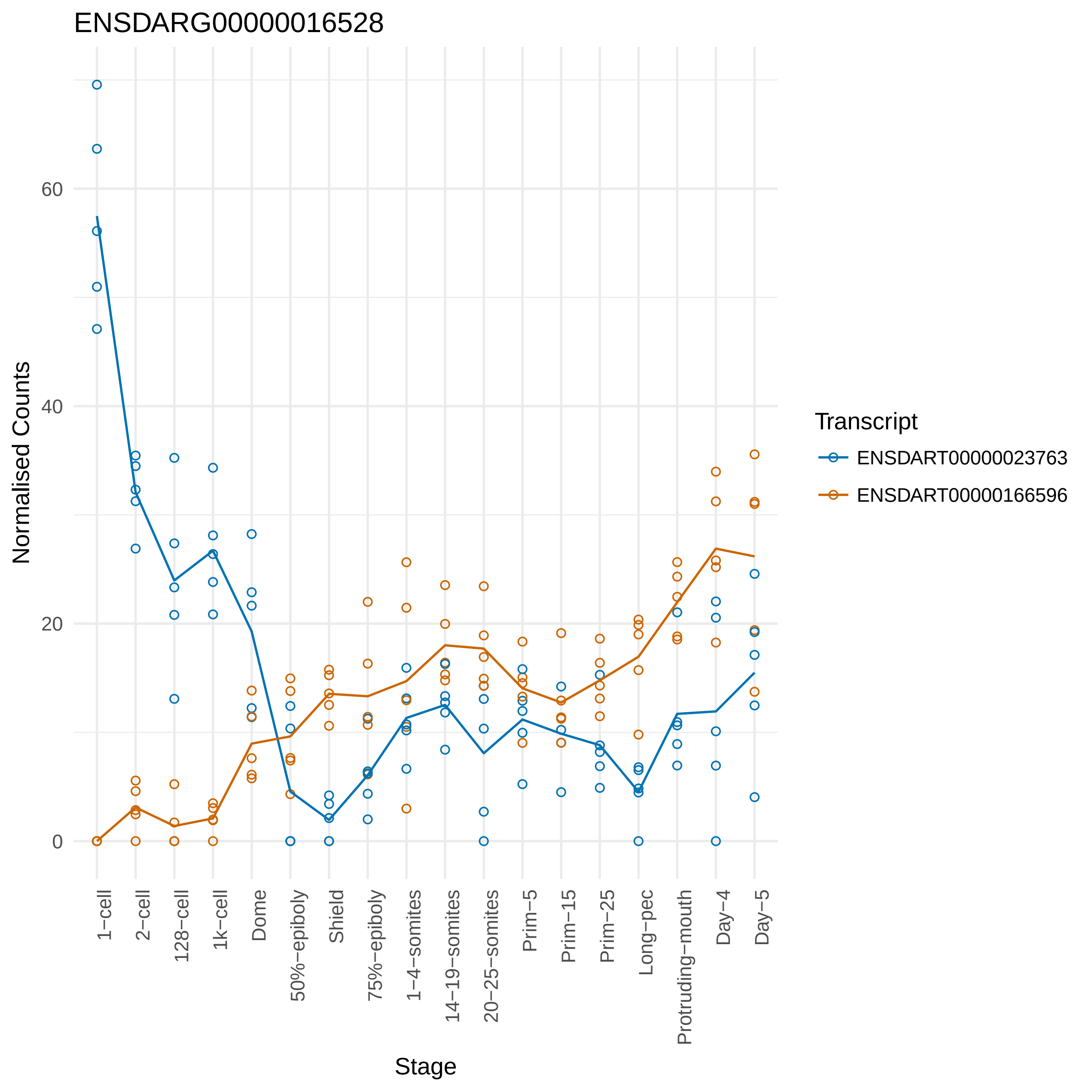

ENSDARG00000016528
Normalised Counts
60
40
20
0
1−cell
2−cell
128−cell
1k−cell
Dome
50%−epiboly
Shield
75%−epiboly
1−4−somites
14−19−somites
20−25−somites
Prim−5
Prim−15
Prim−25
Long−pec
Protruding−mouth
Day−4
Day−5
Stage
Transcript
ENSDART00000023763
ENSDART00000166596

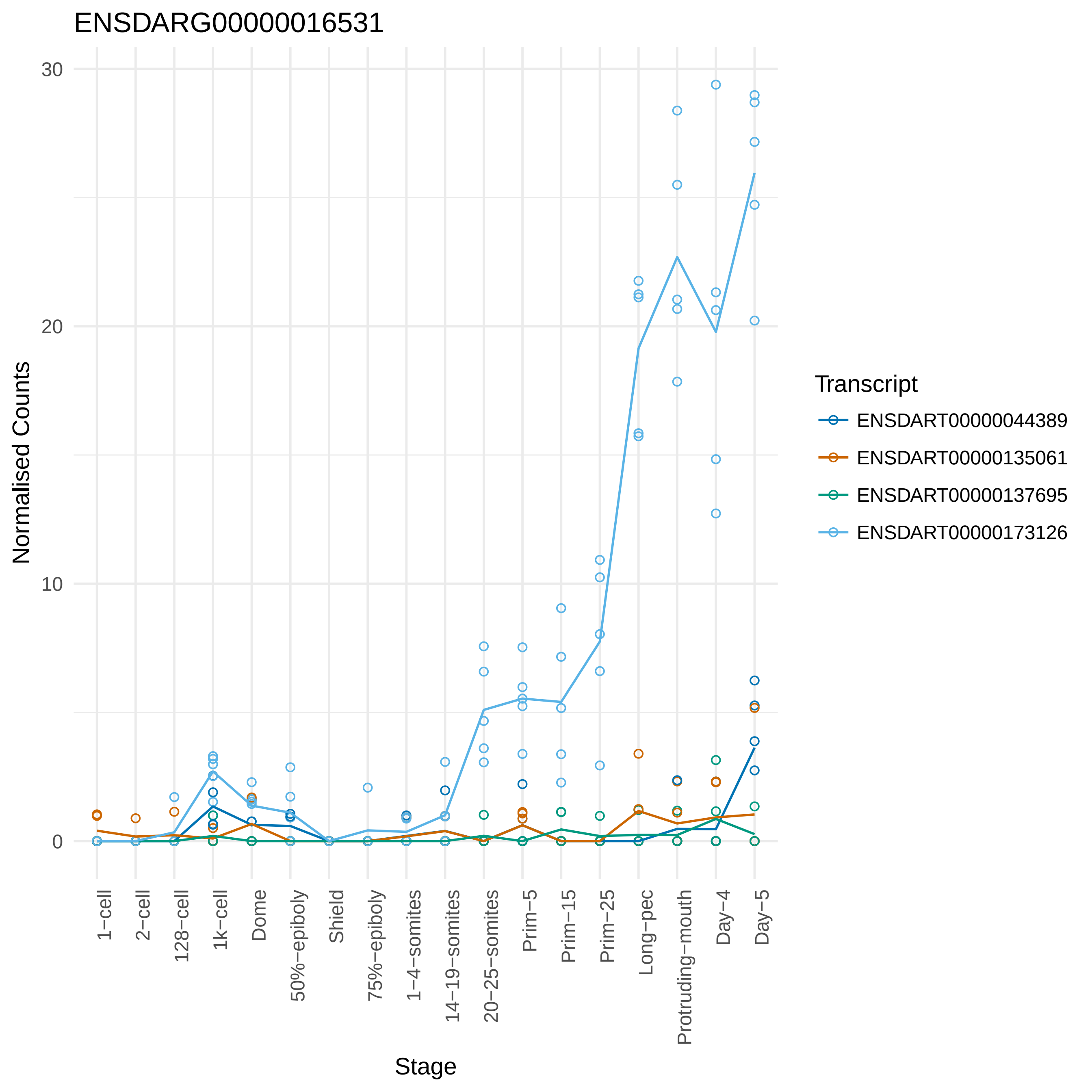

ENSDARG00000016531
Normalised Counts
30
20
10
0
1−cell
2−cell
128−cell
1k−cell
Dome
50%−epiboly
Shield
75%−epiboly
1−4−somites
14−19−somites
20−25−somites
Prim−5
Prim−15
Prim−25
Long−pec
Protruding−mouth
Day−4
Day−5
Stage
Transcript
ENSDART00000044389
ENSDART00000135061
ENSDART00000137695
ENSDART00000173126

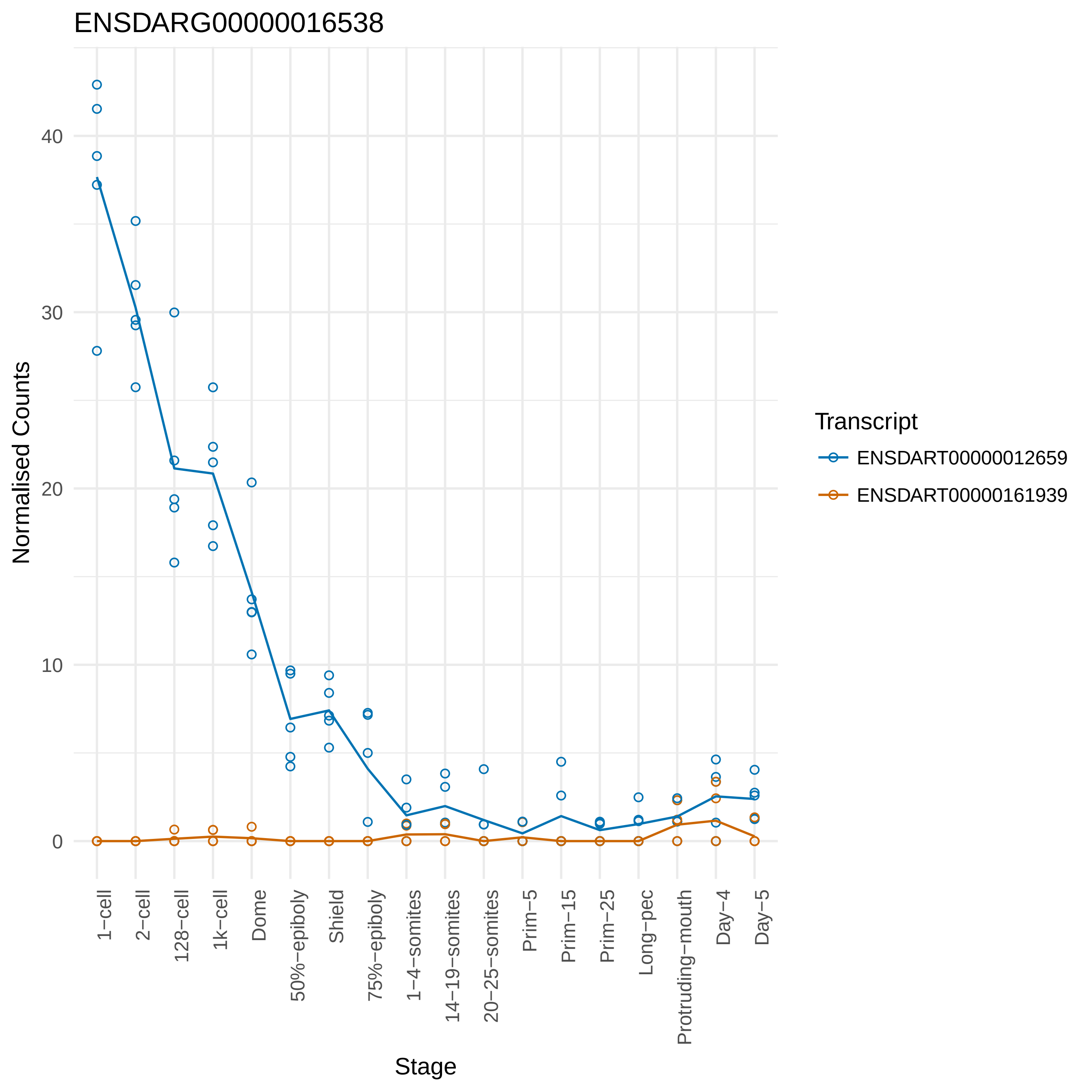

ENSDARG00000016538
Normalised Counts
40
30
20
10
0
1−cell
2−cell
128−cell
1k−cell
Dome
50%−epiboly
Shield
75%−epiboly
1−4−somites
14−19−somites
20−25−somites
Prim−5
Prim−15
Prim−25
Long−pec
Protruding−mouth
Day−4
Day−5
Stage
Transcript
ENSDART00000012659
ENSDART00000161939

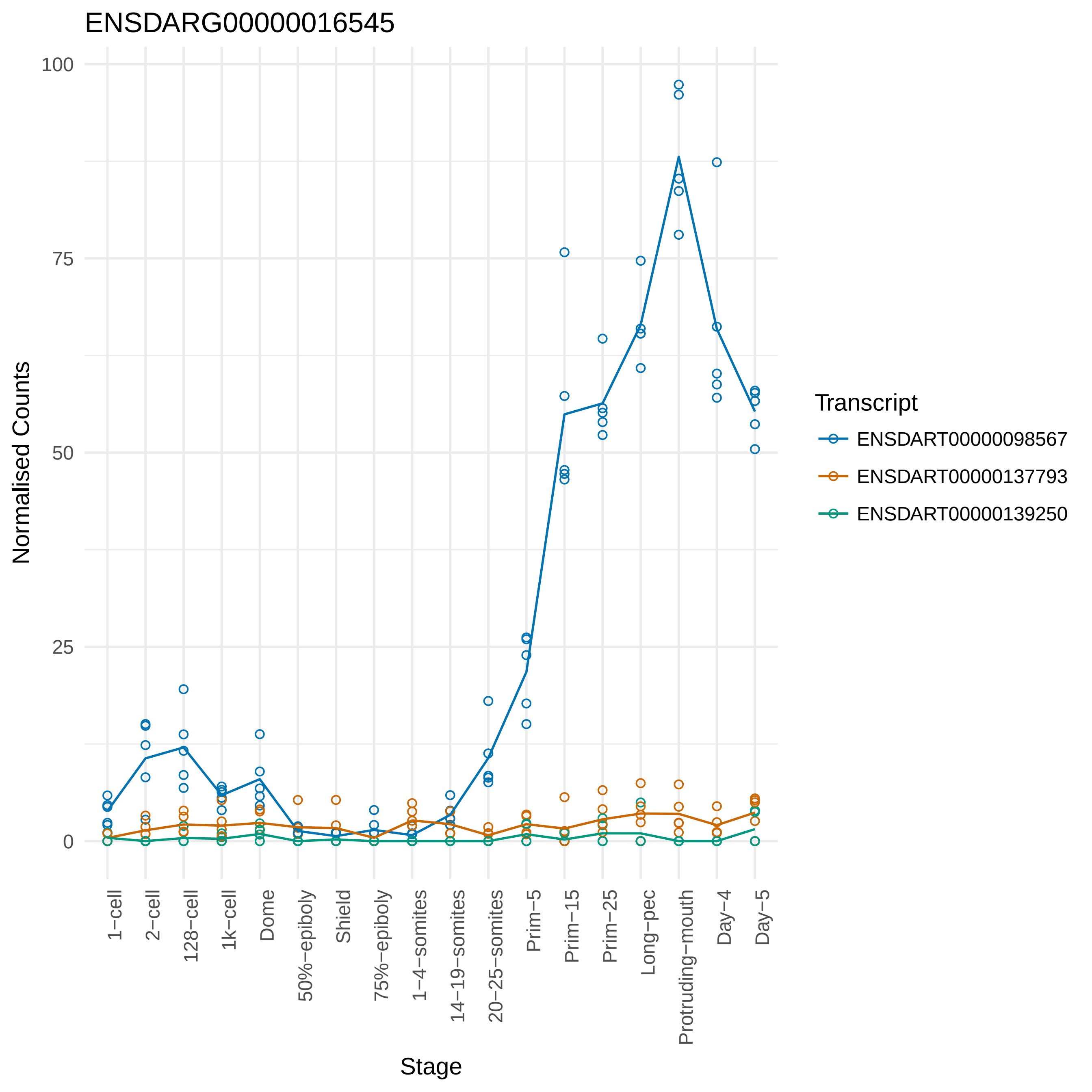

ENSDARG00000016545
Normalised Counts
100
75
50
25
0
1−cell
2−cell
128−cell
1k−cell
Dome
50%−epiboly
Shield
75%−epiboly
1−4−somites
14−19−somites
20−25−somites
Prim−5
Prim−15
Prim−25
Long−pec
Protruding−mouth
Day−4
Day−5
Stage
Transcript
ENSDART00000098567
ENSDART00000137793
ENSDART00000139250

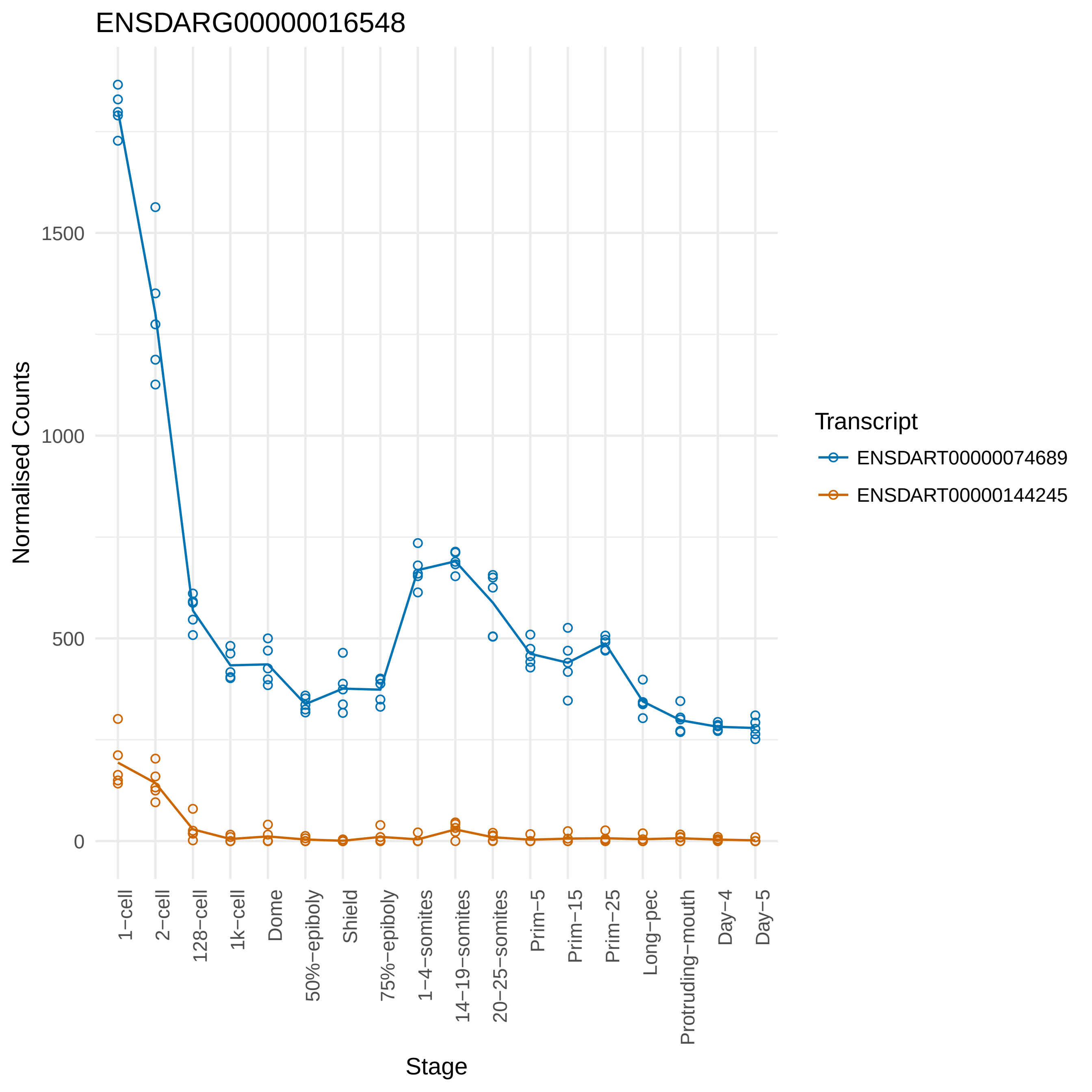

ENSDARG00000016548
Normalised Counts
1500
1000
500
0
Stage
1−cell
2−cell
128−cell
1k−cell
Dome
50%−epiboly
Shield
75%−epiboly
1−4−somites
14−19−somites
20−25−somites
Prim−5
Prim−15
Prim−25
Long−pec
Protruding−mouth
Day−4
Day−5
Transcript
ENSDART00000074689
ENSDART00000144245

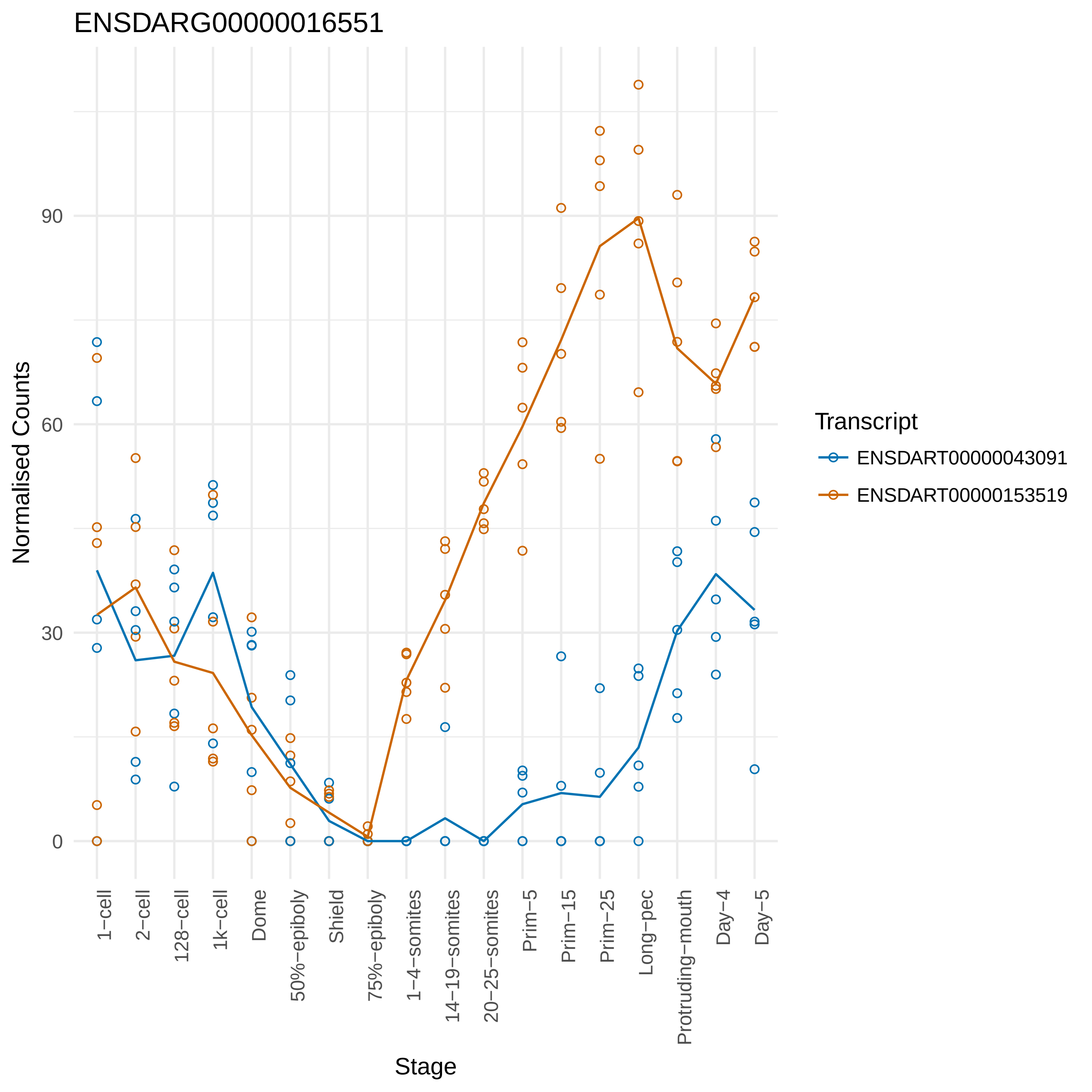

ENSDARG00000016551
Normalised Counts
0
30
60
90
Stage
1−cell
2−cell
128−cell
1k−cell
Dome
50%−epiboly
Shield
75%−epiboly
1−4−somites
14−19−somites
20−25−somites
Prim−5
Prim−15
Prim−25
Long−pec
Protruding−mouth
Day−4
Day−5
Transcript
ENSDART00000043091
ENSDART00000153519

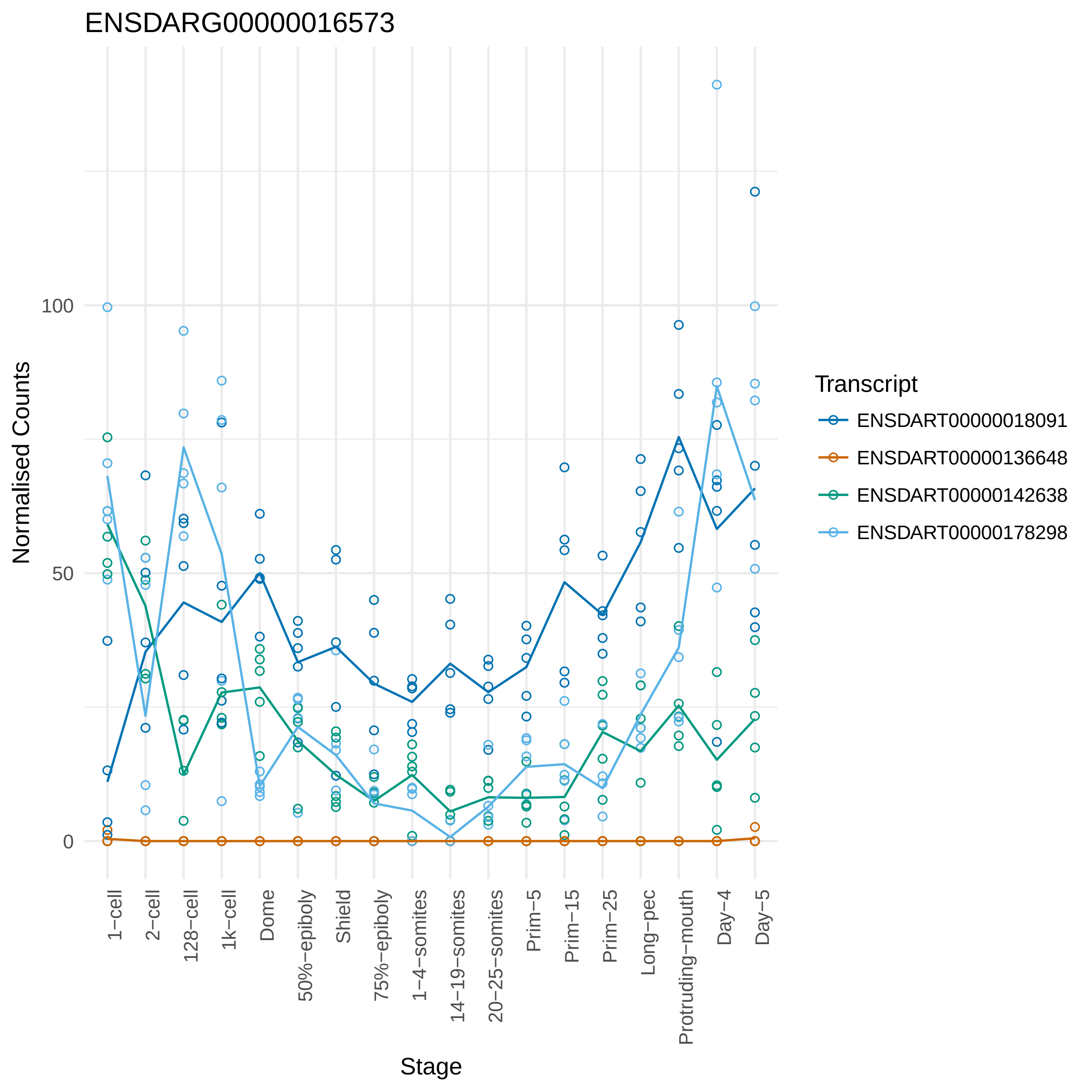

ENSDARG00000016573
Normalised Counts
0
50
100
Stage
1−cell
2−cell
128−cell
1k−cell
Dome
50%−epiboly
Shield
75%−epiboly
1−4−somites
14−19−somites
20−25−somites
Prim−5
Prim−15
Prim−25
Long−pec
Protruding−mouth
Day−4
Day−5
Transcript
ENSDART00000018091
ENSDART00000136648
ENSDART00000142638
ENSDART00000178298

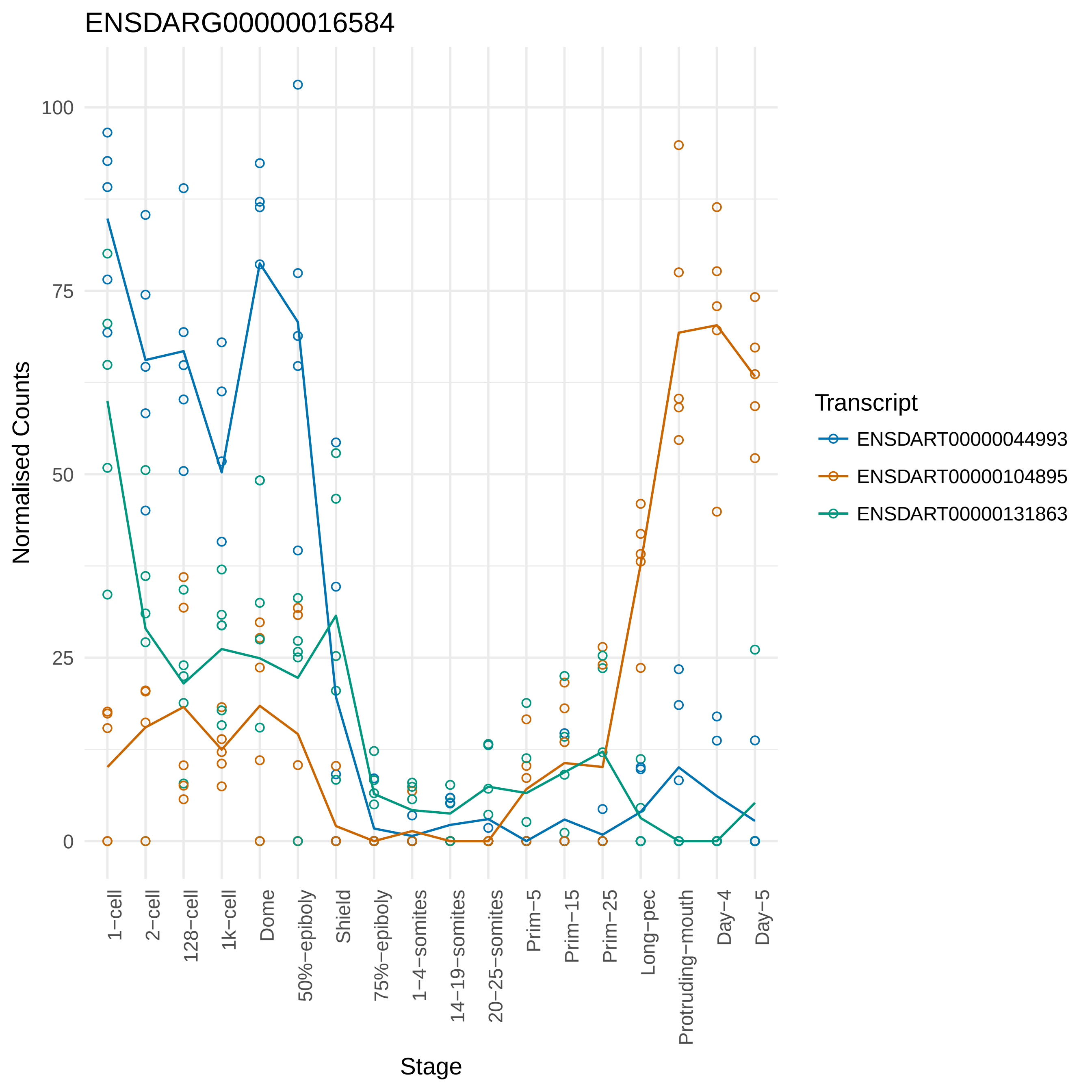

ENSDARG00000016584
Normalised Counts
100
75
50
25
0
1−cell
2−cell
128−cell
1k−cell
Dome
50%−epiboly
Shield
75%−epiboly
1−4−somites
14−19−somites
20−25−somites
Prim−5
Prim−15
Prim−25
Long−pec
Protruding−mouth
Day−4
Day−5
Stage
Transcript
ENSDART00000044993
ENSDART00000104895
ENSDART00000131863

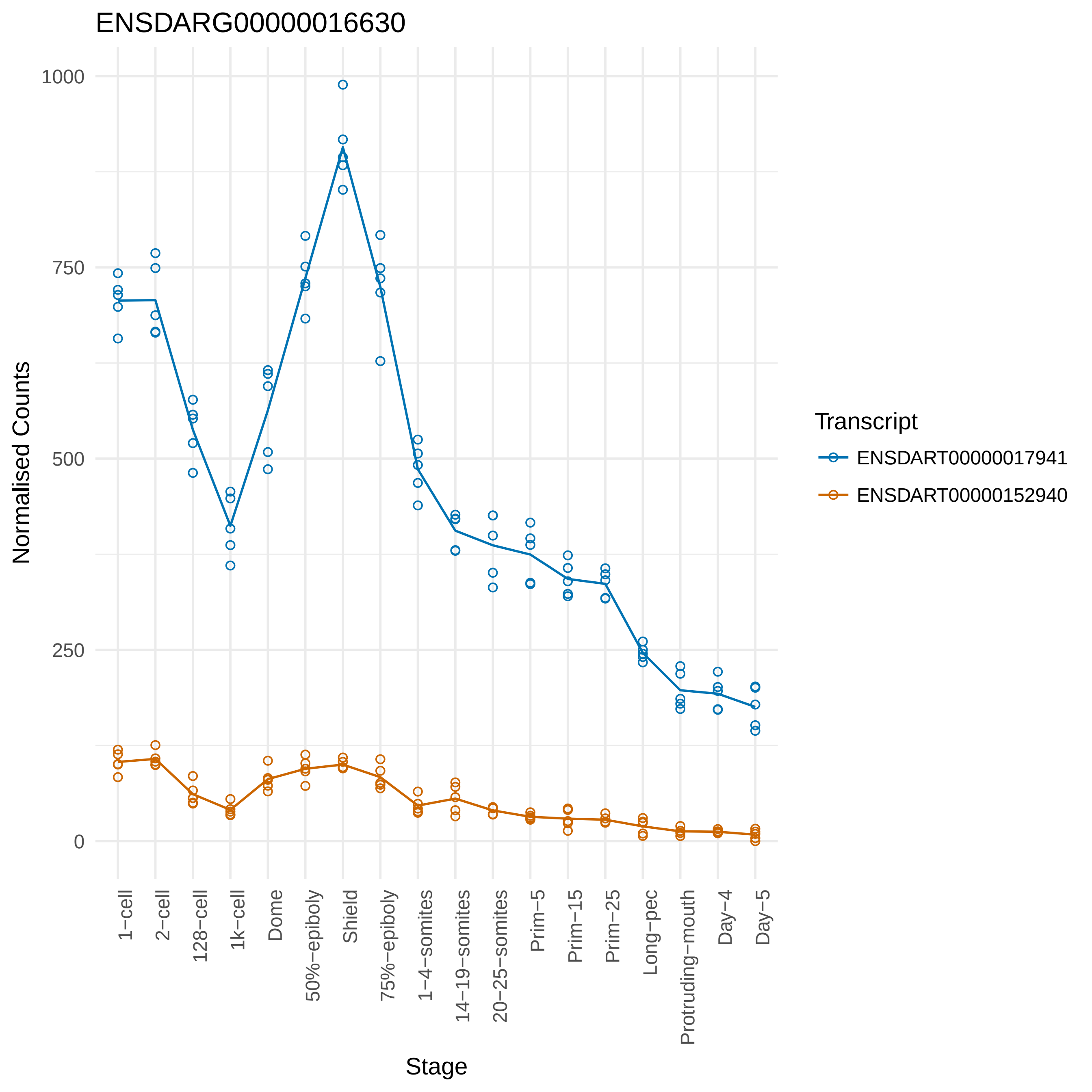

ENSDARG00000016630
Normalised Counts
1000
750
500
250
0
1−cell
2−cell
128−cell
1k−cell
Dome
50%−epiboly
Shield
75%−epiboly
1−4−somites
14−19−somites
20−25−somites
Prim−5
Prim−15
Prim−25
Long−pec
Protruding−mouth
Day−4
Day−5
Stage
Transcript
ENSDART00000017941
ENSDART00000152940

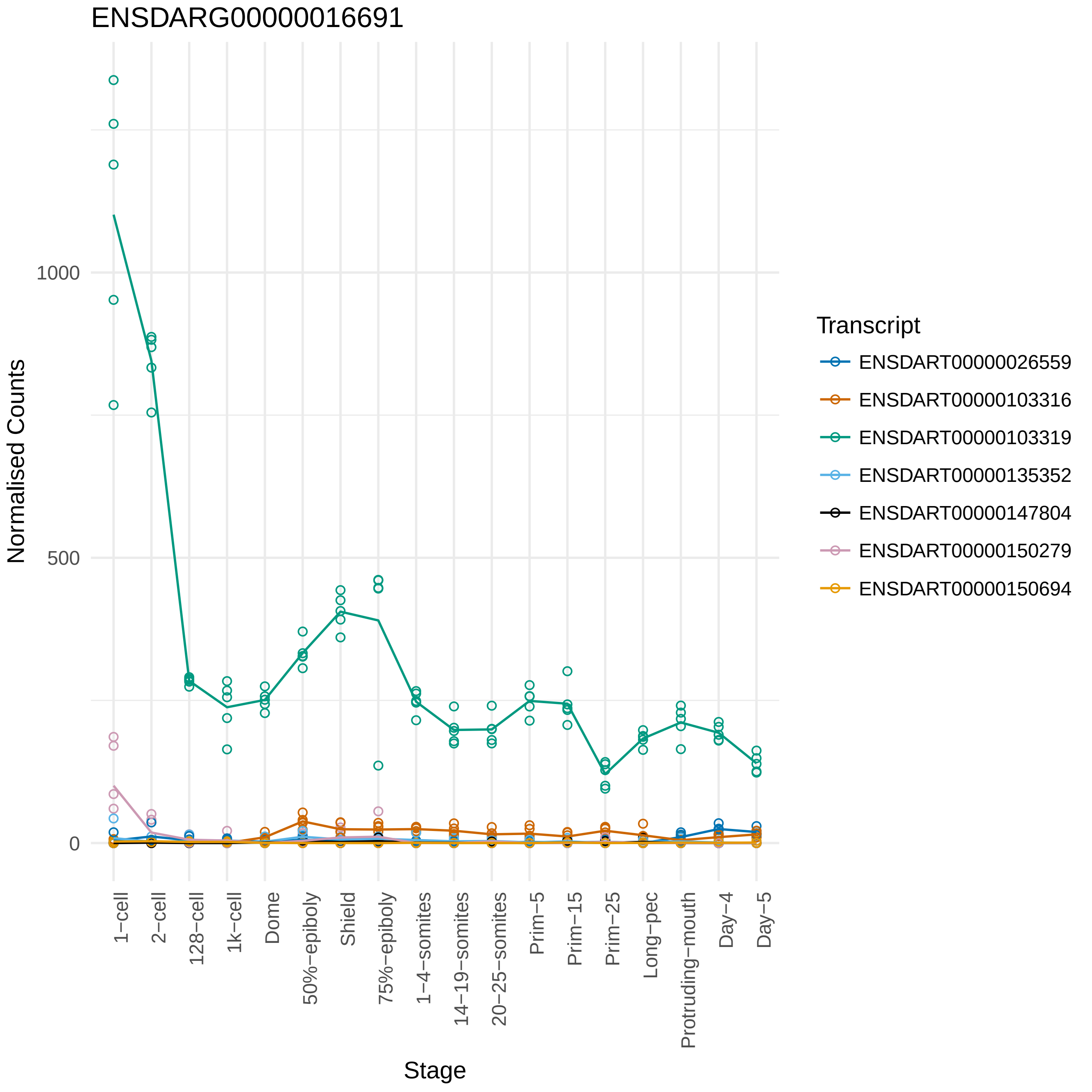

ENSDARG00000016691
Normalised Counts
0
500
1000
Stage
1−cell
2−cell
128−cell
1k−cell
Dome
50%−epiboly
Shield
75%−epiboly
1−4−somites
14−19−somites
20−25−somites
Prim−5
Prim−15
Prim−25
Long−pec
Protruding−mouth
Day−4
Day−5
Transcript
ENSDART00000026559
ENSDART00000103316
ENSDART00000103319
ENSDART00000135352
ENSDART00000147804
ENSDART00000150279
ENSDART00000150694

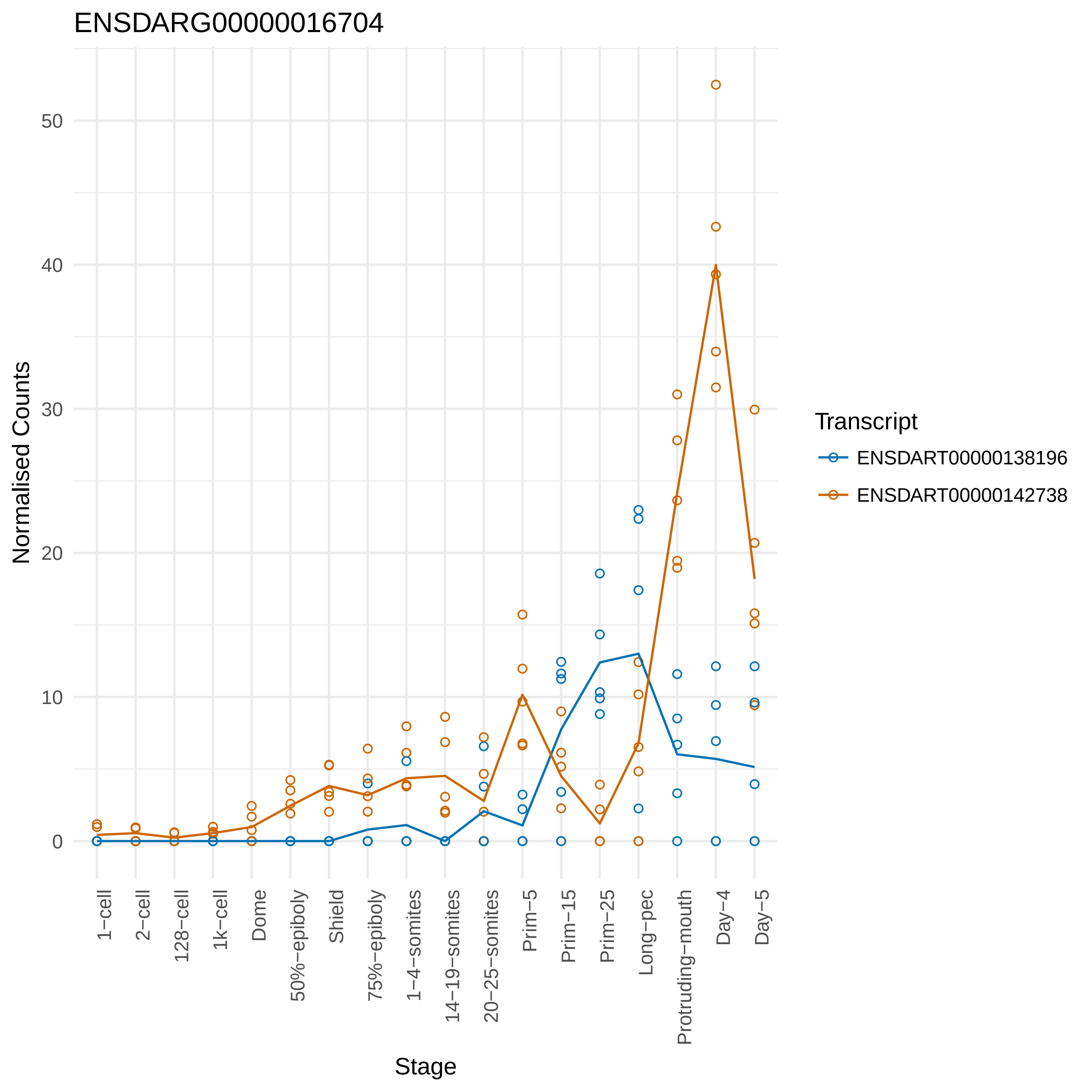

ENSDARG00000016704
Normalised Counts
0
10
20
30
40
50
Stage
1−cell
2−cell
128−cell
1k−cell
Dome
50%−epiboly
Shield
75%−epiboly
1−4−somites
14−19−somites
20−25−somites
Prim−5
Prim−15
Prim−25
Long−pec
Protruding−mouth
Day−4
Day−5
Transcript
ENSDART00000138196
ENSDART00000142738

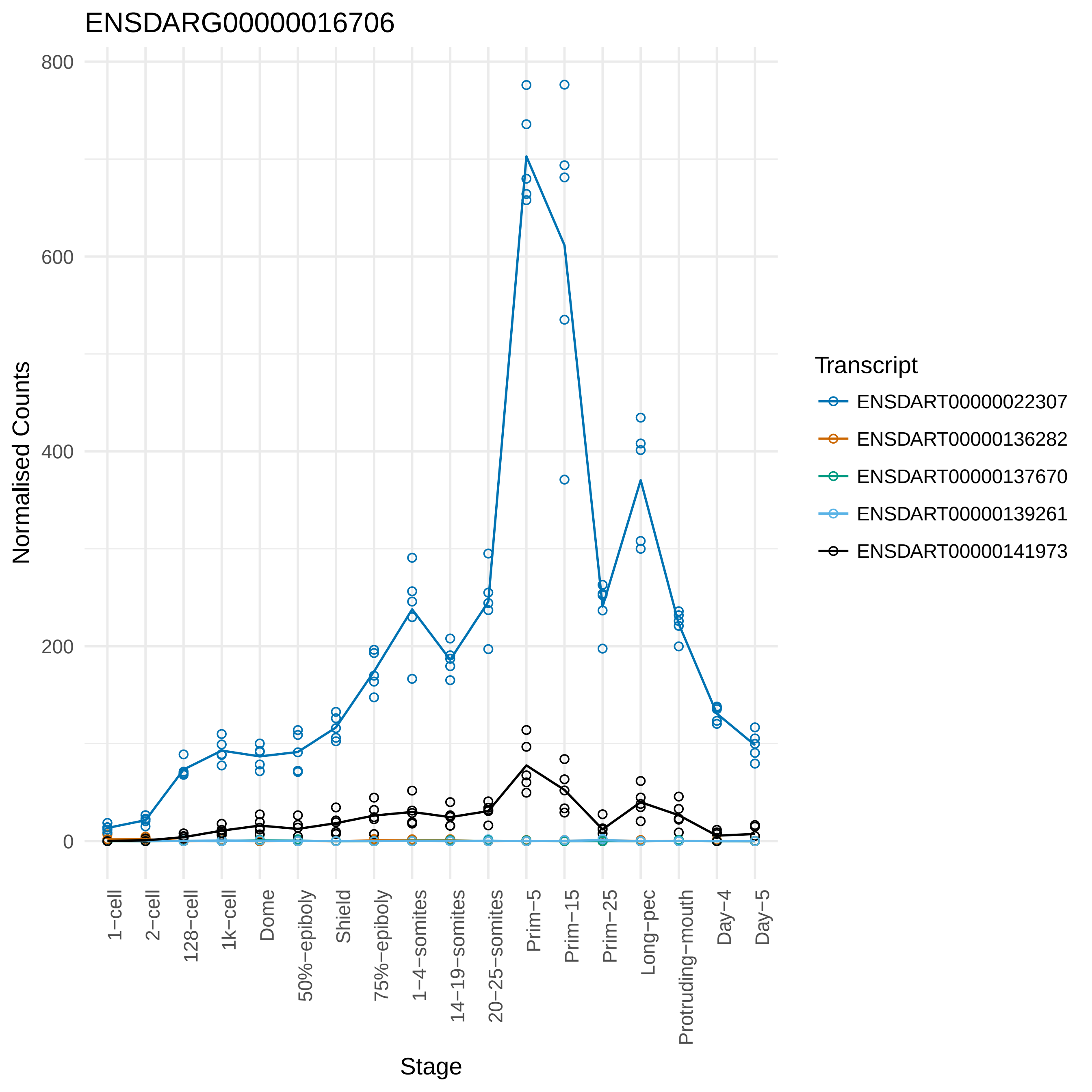
ENSDARG00000016706
Normalised Counts
800
600
400
200
0
1−cell
2−cell
128−cell
1k−cell
Dome
50%−epiboly
Shield
75%−epiboly
1−4−somites
14−19−somites
20−25−somites
Prim−5
Prim−15
Prim−25
Long−pec
Protruding−mouth
Day−4
Day−5
Stage
Transcript
ENSDART00000022307
ENSDART00000136282
ENSDART00000137670
ENSDART00000139261
ENSDART00000141973

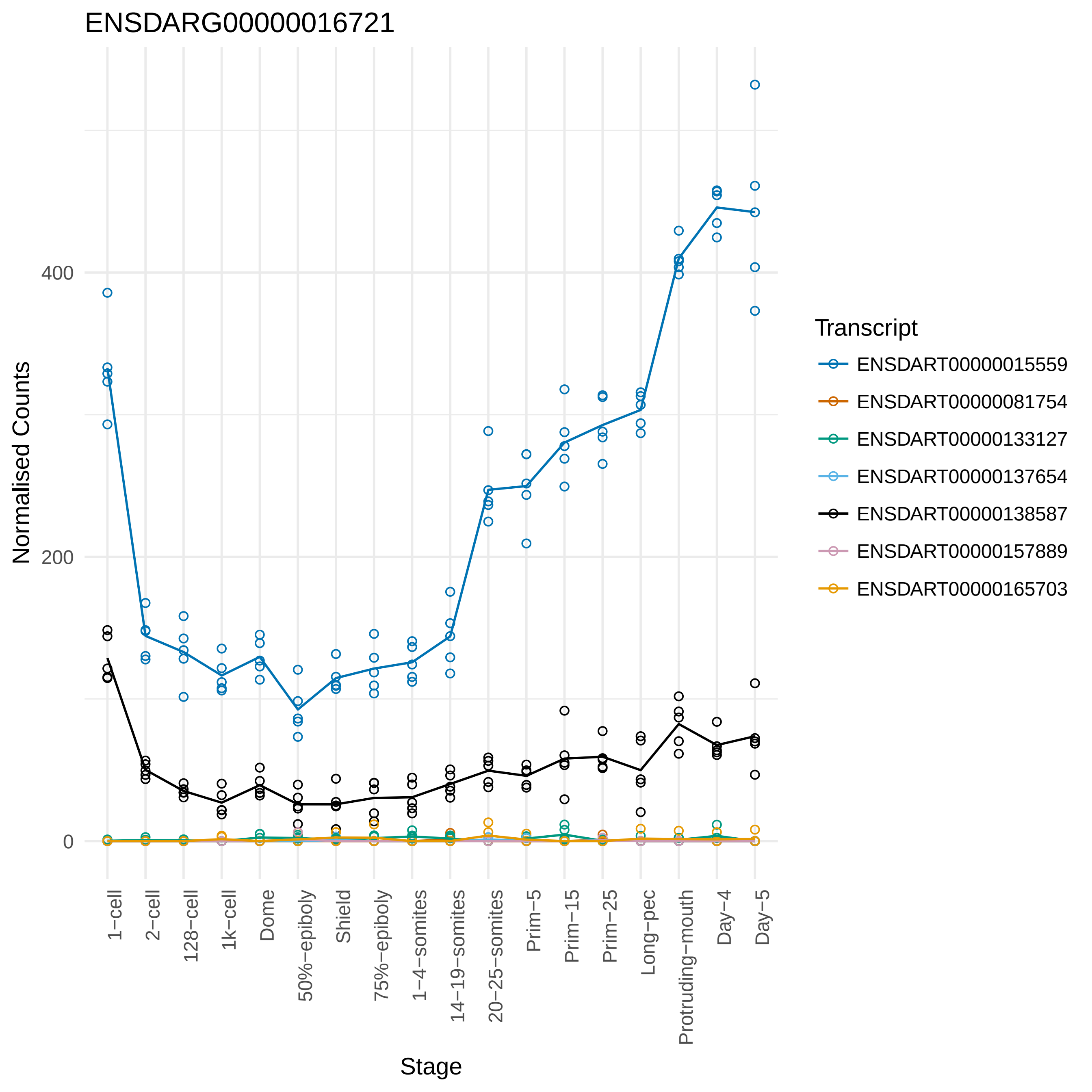

ENSDARG00000016721
Normalised Counts
400
200
0
Stage
1−cell
2−cell
128−cell
1k−cell
Dome
50%−epiboly
Shield
75%−epiboly
1−4−somites
14−19−somites
20−25−somites
Prim−5
Prim−15
Prim−25
Long−pec
Protruding−mouth
Day−4
Day−5
Transcript
ENSDART00000015559
ENSDART00000081754
ENSDART00000133127
ENSDART00000137654
ENSDART00000138587
ENSDART00000157889
ENSDART00000165703

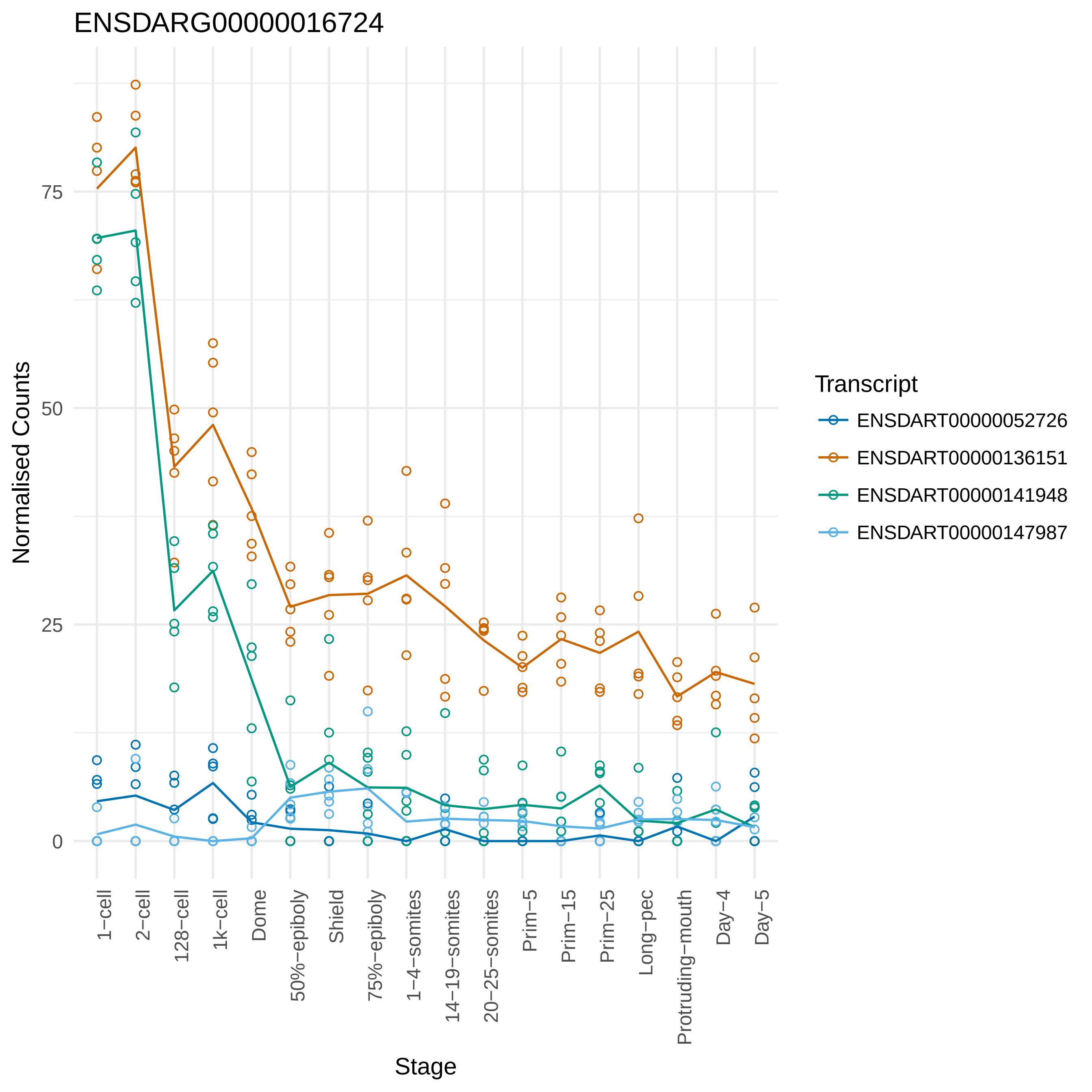

ENSDARG00000016724
Normalised Counts
0
25
50
75
Stage
1−cell
2−cell
128−cell
1k−cell
Dome
50%−epiboly
Shield
75%−epiboly
1−4−somites
14−19−somites
20−25−somites
Prim−5
Prim−15
Prim−25
Long−pec
Protruding−mouth
Day−4
Day−5
Transcript
ENSDART00000052726
ENSDART00000136151
ENSDART00000141948
ENSDART00000147987

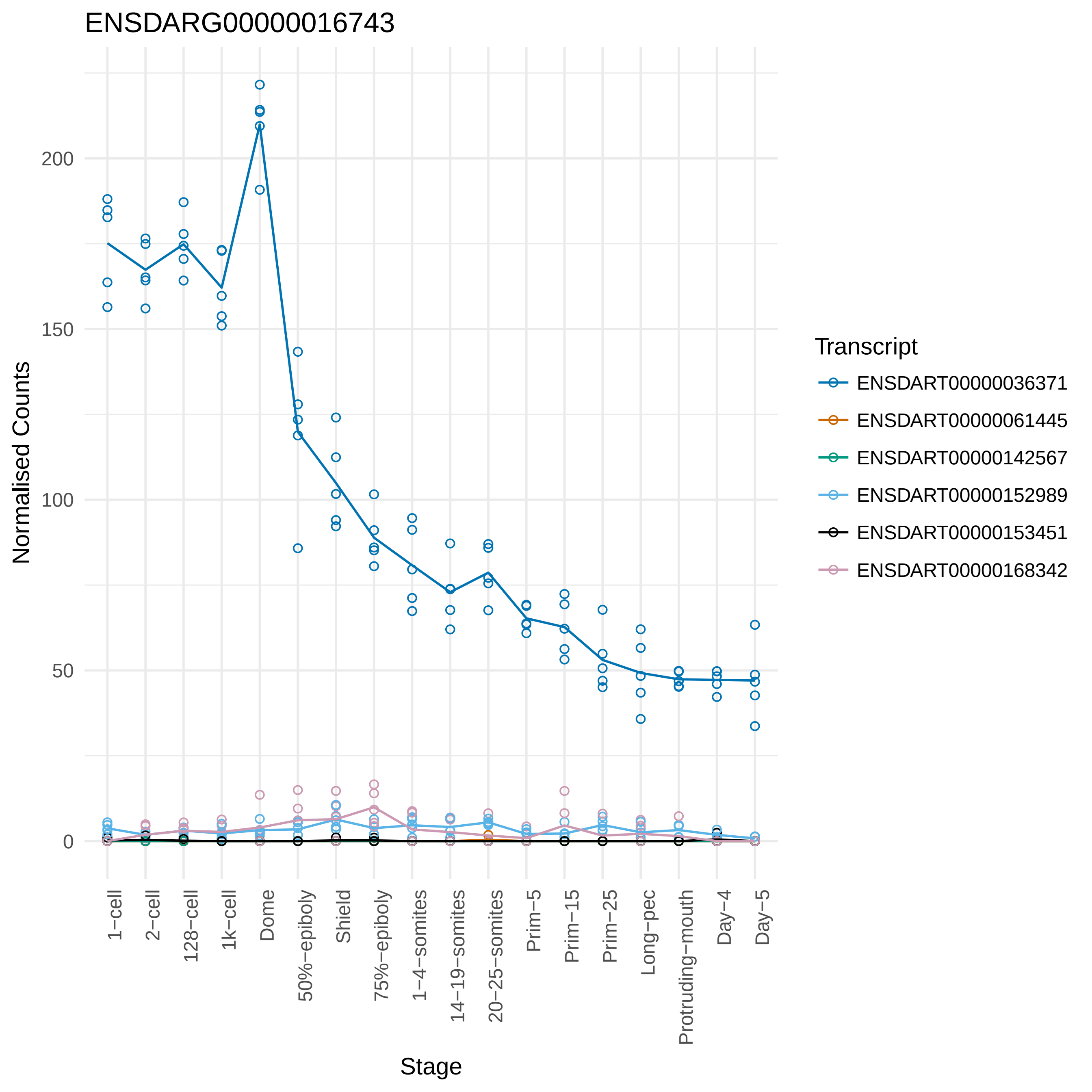

ENSDARG00000016743
Normalised Counts
0
50
100
150
200
1−cell
2−cell
128−cell
1k−cell
Dome
50%−epiboly
Shield
75%−epiboly
1−4−somites
14−19−somites
20−25−somites
Prim−5
Prim−15
Prim−25
Long−pec
Protruding−mouth
Day−4
Day−5
Stage
Transcript
ENSDART00000036371
ENSDART00000061445
ENSDART00000142567
ENSDART00000152989
ENSDART00000153451
ENSDART00000168342

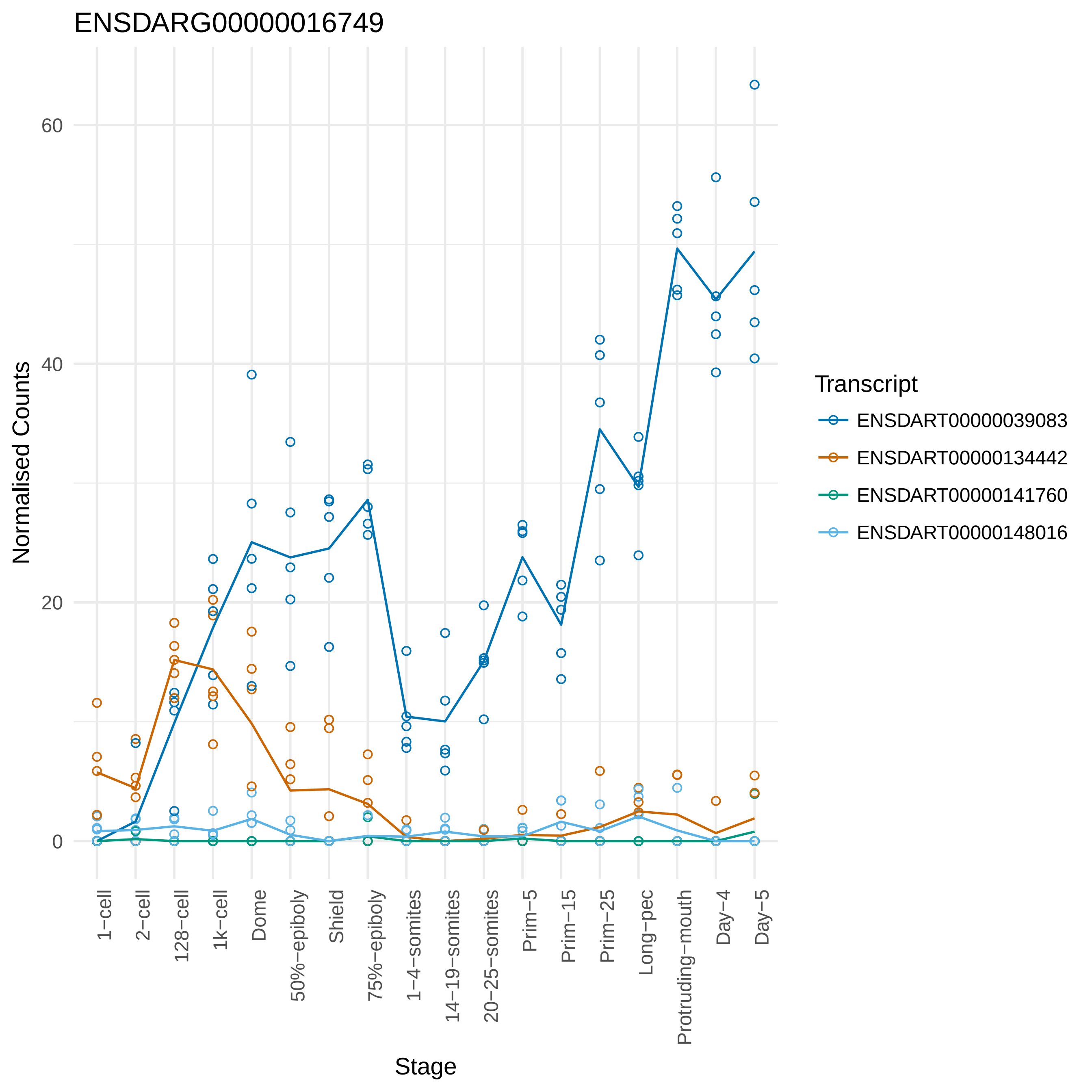

ENSDARG00000016749
Normalised Counts
60
40
20
0
1−cell
2−cell
128−cell
1k−cell
Dome
50%−epiboly
Shield
75%−epiboly
1−4−somites
14−19−somites
20−25−somites
Prim−5
Prim−15
Prim−25
Long−pec
Protruding−mouth
Day−4
Day−5
Stage
Transcript
ENSDART00000039083
ENSDART00000134442
ENSDART00000141760
ENSDART00000148016

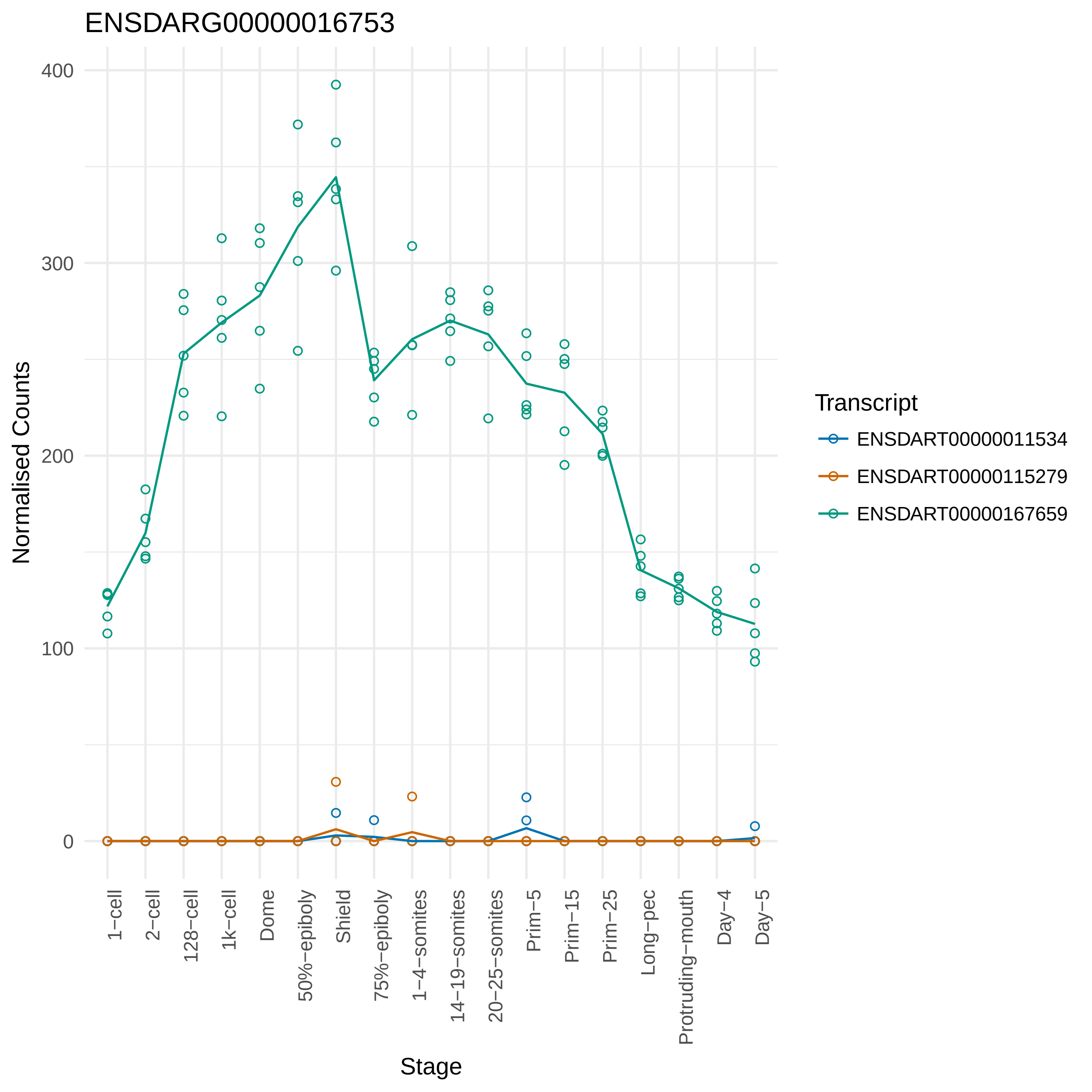

ENSDARG00000016753
Normalised Counts
400
300
200
100
0
1−cell
2−cell
128−cell
1k−cell
Dome
50%−epiboly
Shield
75%−epiboly
1−4−somites
14−19−somites
20−25−somites
Prim−5
Prim−15
Prim−25
Long−pec
Protruding−mouth
Day−4
Day−5
Stage
Transcript
ENSDART00000011534
ENSDART00000115279
ENSDART00000167659

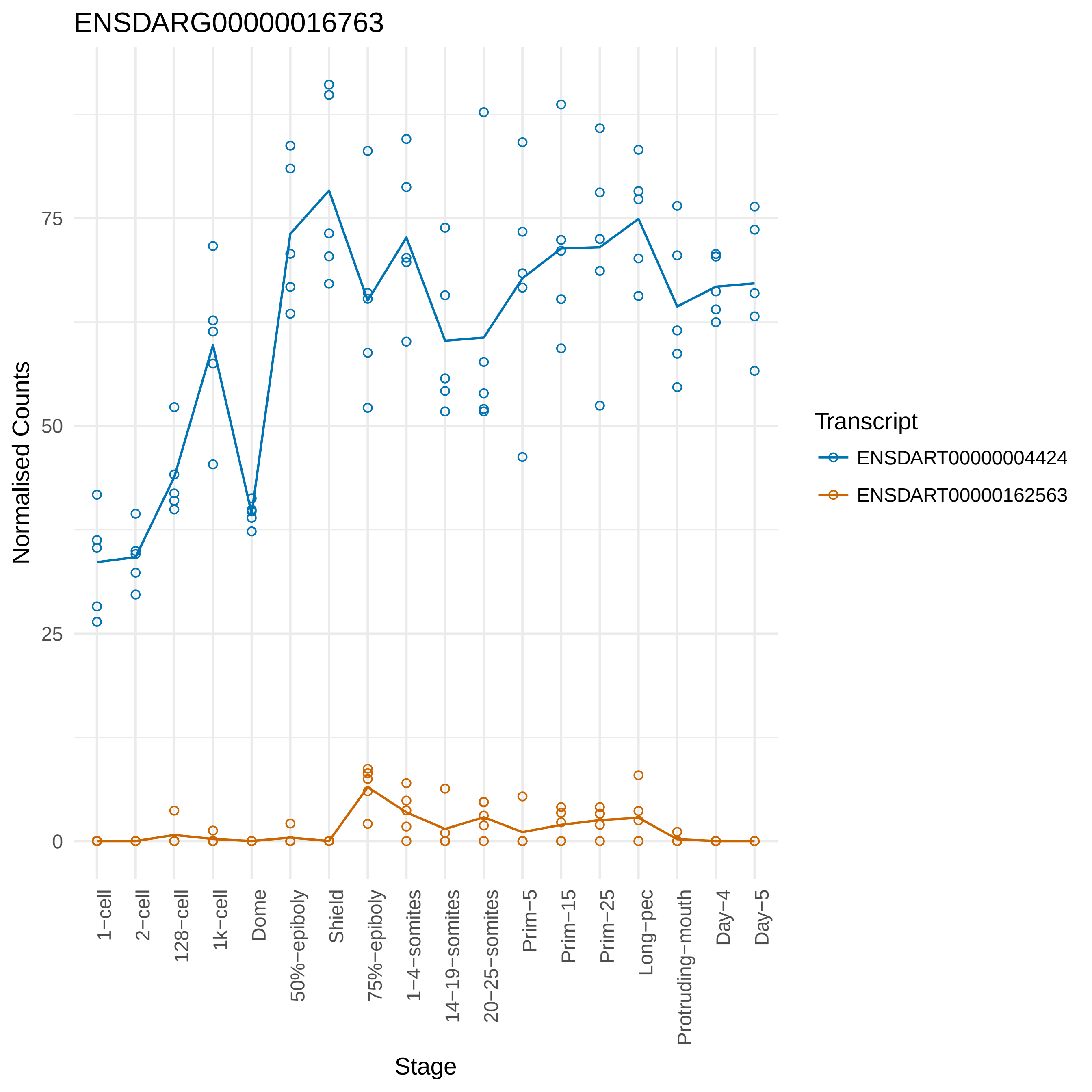
ENSDARG00000016763
Normalised Counts
0
25
50
75
Stage
1−cell
2−cell
128−cell
1k−cell
Dome
50%−epiboly
Shield
75%−epiboly
1−4−somites
14−19−somites
20−25−somites
Prim−5
Prim−15
Prim−25
Long−pec
Protruding−mouth
Day−4
Day−5
Transcript
ENSDART00000004424
ENSDART00000162563

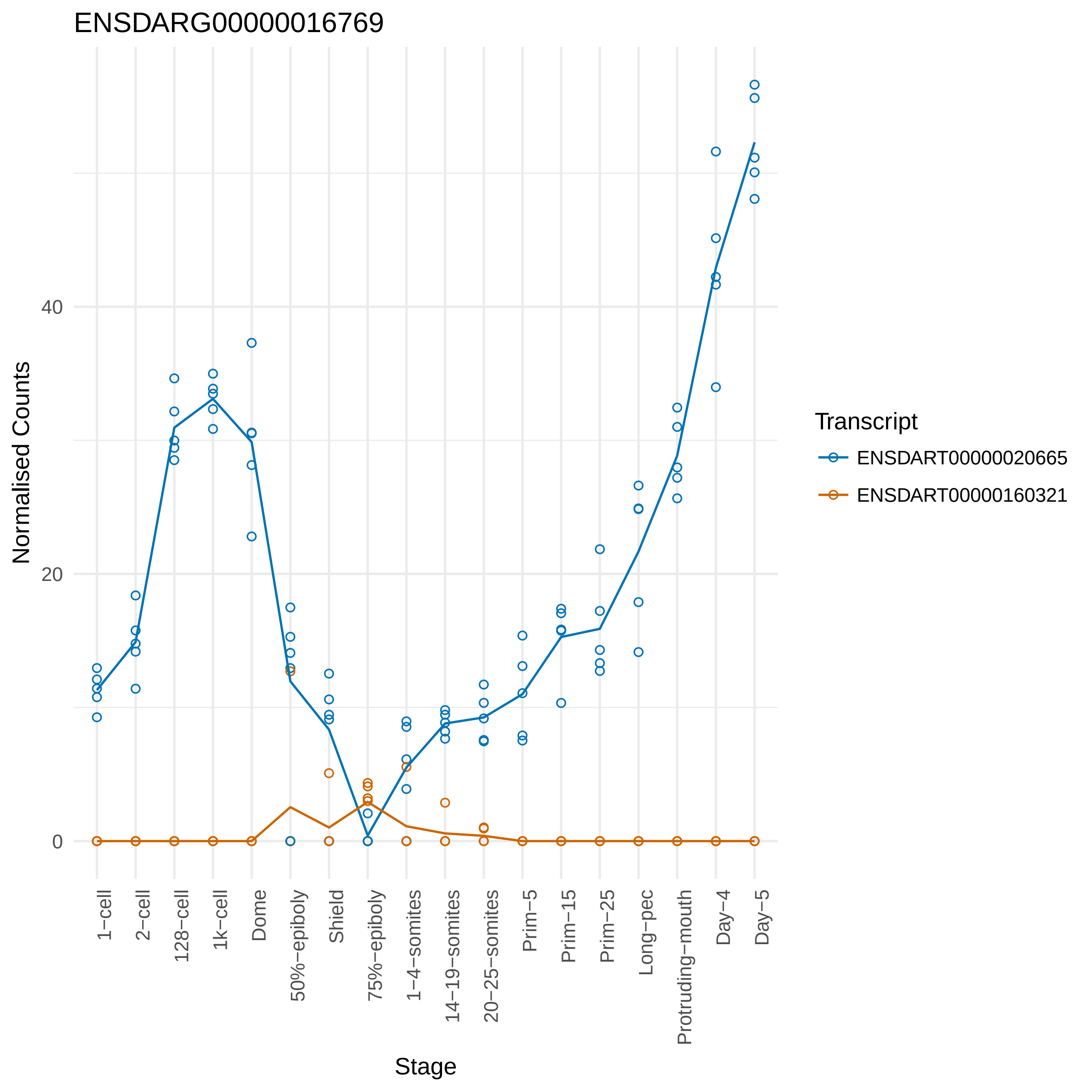
ENSDARG00000016769
Normalised Counts
0
20
40
Stage
1−cell
2−cell
128−cell
1k−cell
Dome
50%−epiboly
Shield
75%−epiboly
1−4−somites
14−19−somites
20−25−somites
Prim−5
Prim−15
Prim−25
Long−pec
Protruding−mouth
Day−4
Day−5
Transcript
ENSDART00000020665
ENSDART00000160321

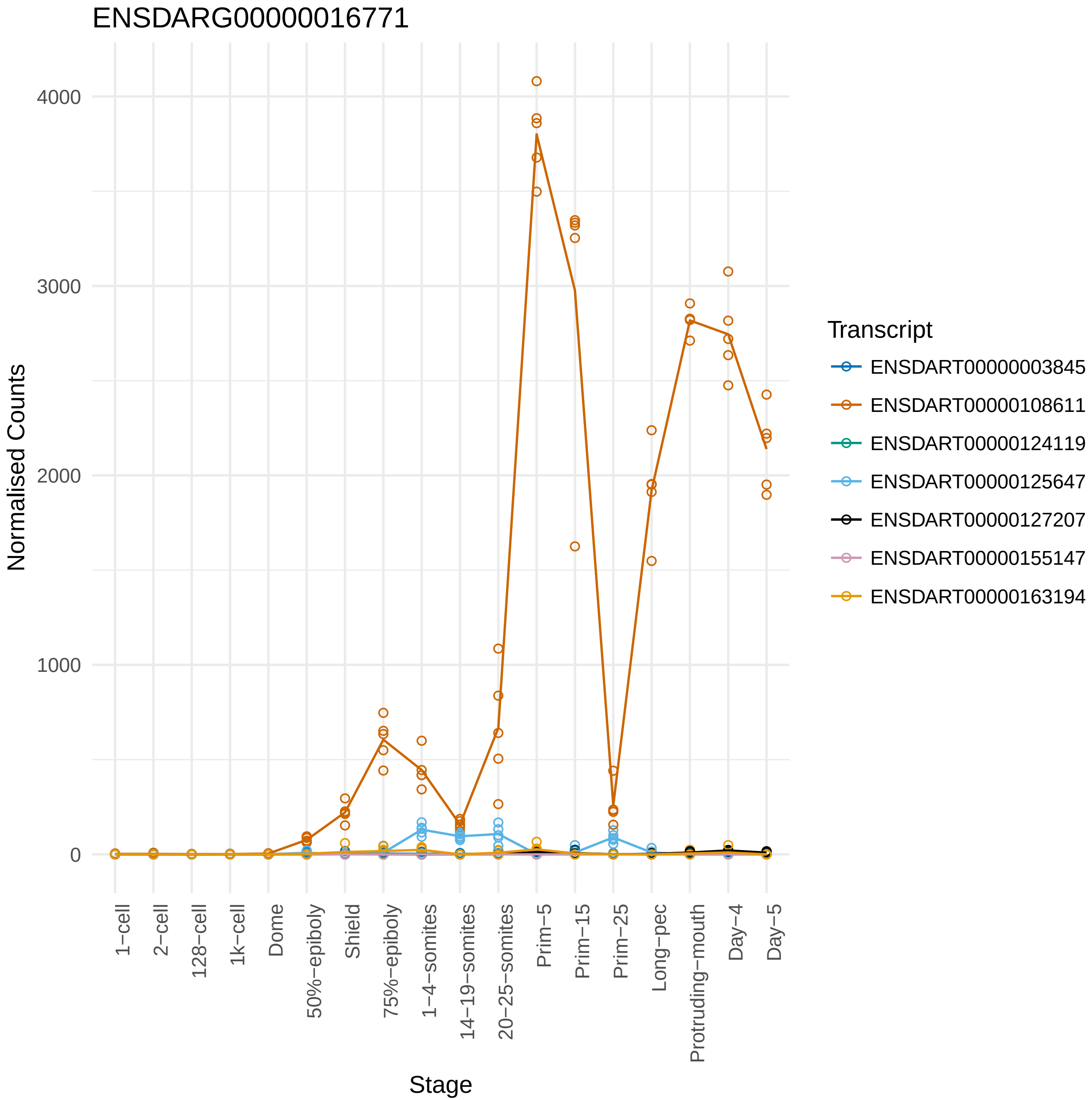

ENSDARG00000016771
Normalised Counts
4000
3000
2000
1000
0
1−cell
2−cell
128−cell
1k−cell
Dome
50%−epiboly
Shield
75%−epiboly
1−4−somites
14−19−somites
20−25−somites
Prim−5
Prim−15
Prim−25
Long−pec
Protruding−mouth
Day−4
Day−5
Stage
Transcript
ENSDART00000003845
ENSDART00000108611
ENSDART00000124119
ENSDART00000125647
ENSDART00000127207
ENSDART00000155147
ENSDART00000163194

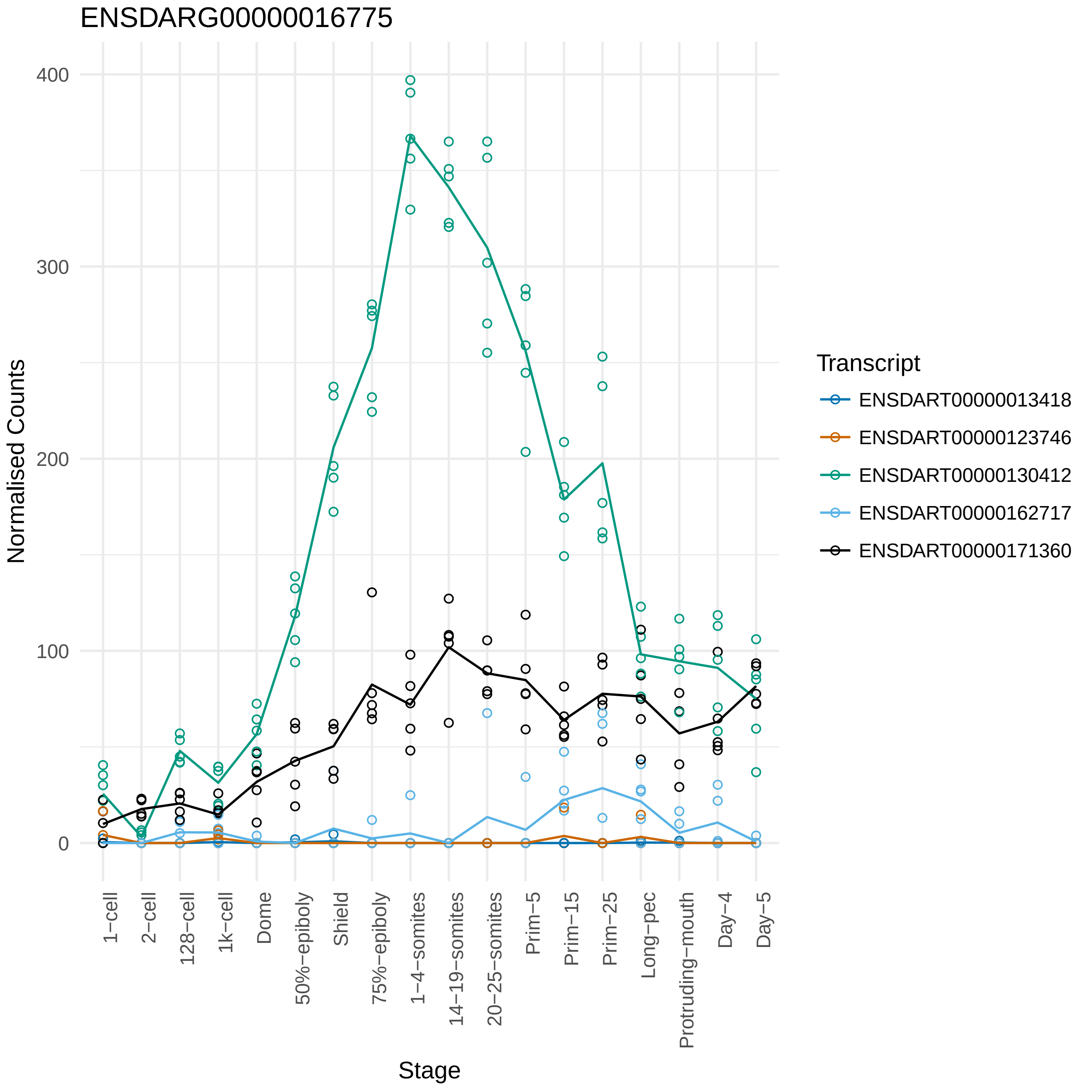
ENSDARG00000016775
Normalised Counts
400
300
200
100
0
1−cell
2−cell
128−cell
1k−cell
Dome
50%−epiboly
Shield
75%−epiboly
1−4−somites
14−19−somites
20−25−somites
Prim−5
Prim−15
Prim−25
Long−pec
Protruding−mouth
Day−4
Day−5
Stage
Transcript
ENSDART00000013418
ENSDART00000123746
ENSDART00000130412
ENSDART00000162717
ENSDART00000171360

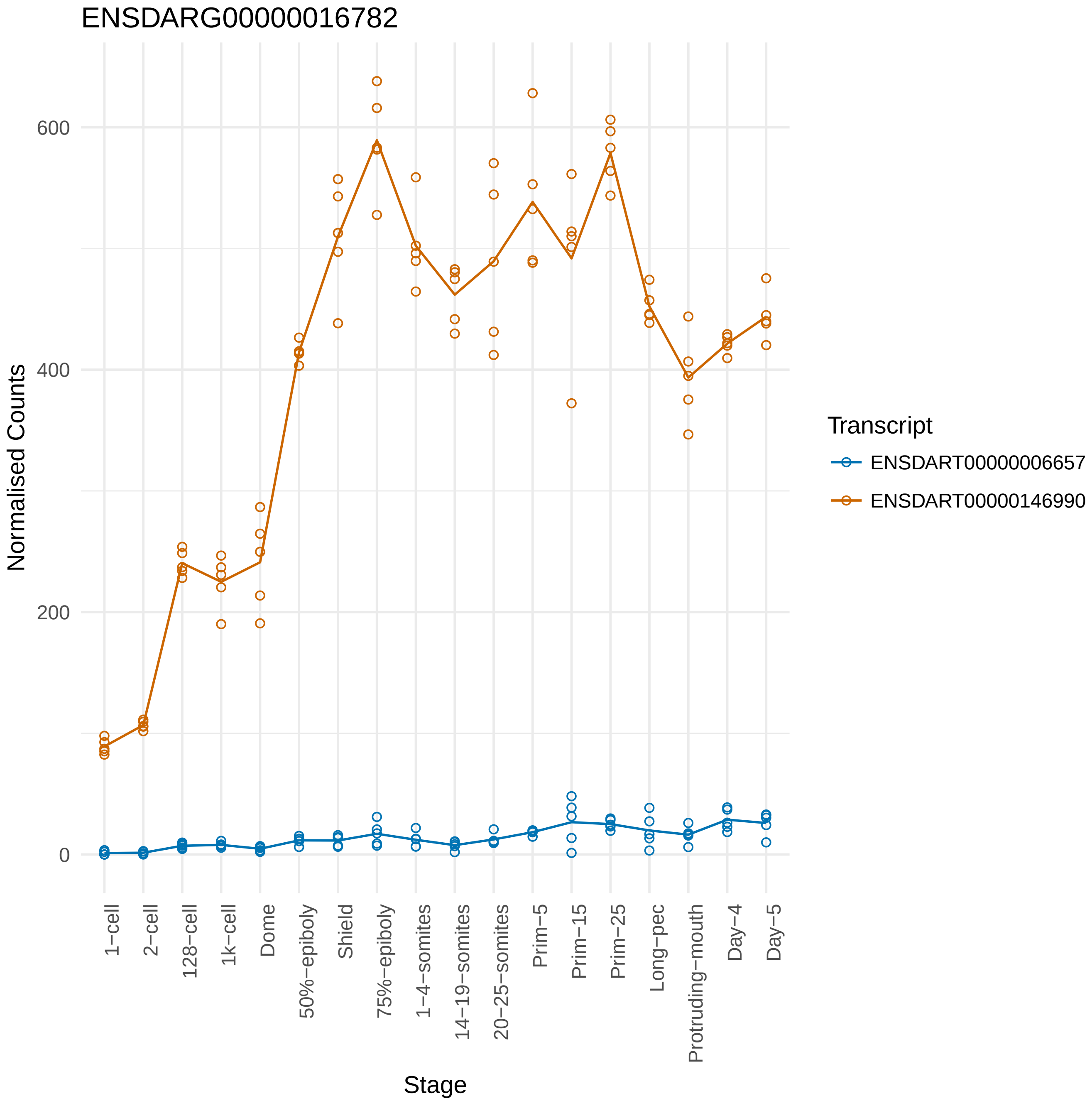

ENSDARG00000016782
Normalised Counts
600
400
200
0
1−cell
2−cell
128−cell
1k−cell
Dome
50%−epiboly
Shield
75%−epiboly
1−4−somites
14−19−somites
20−25−somites
Prim−5
Prim−15
Prim−25
Long−pec
Protruding−mouth
Day−4
Day−5
Stage
Transcript
ENSDART00000006657
ENSDART00000146990

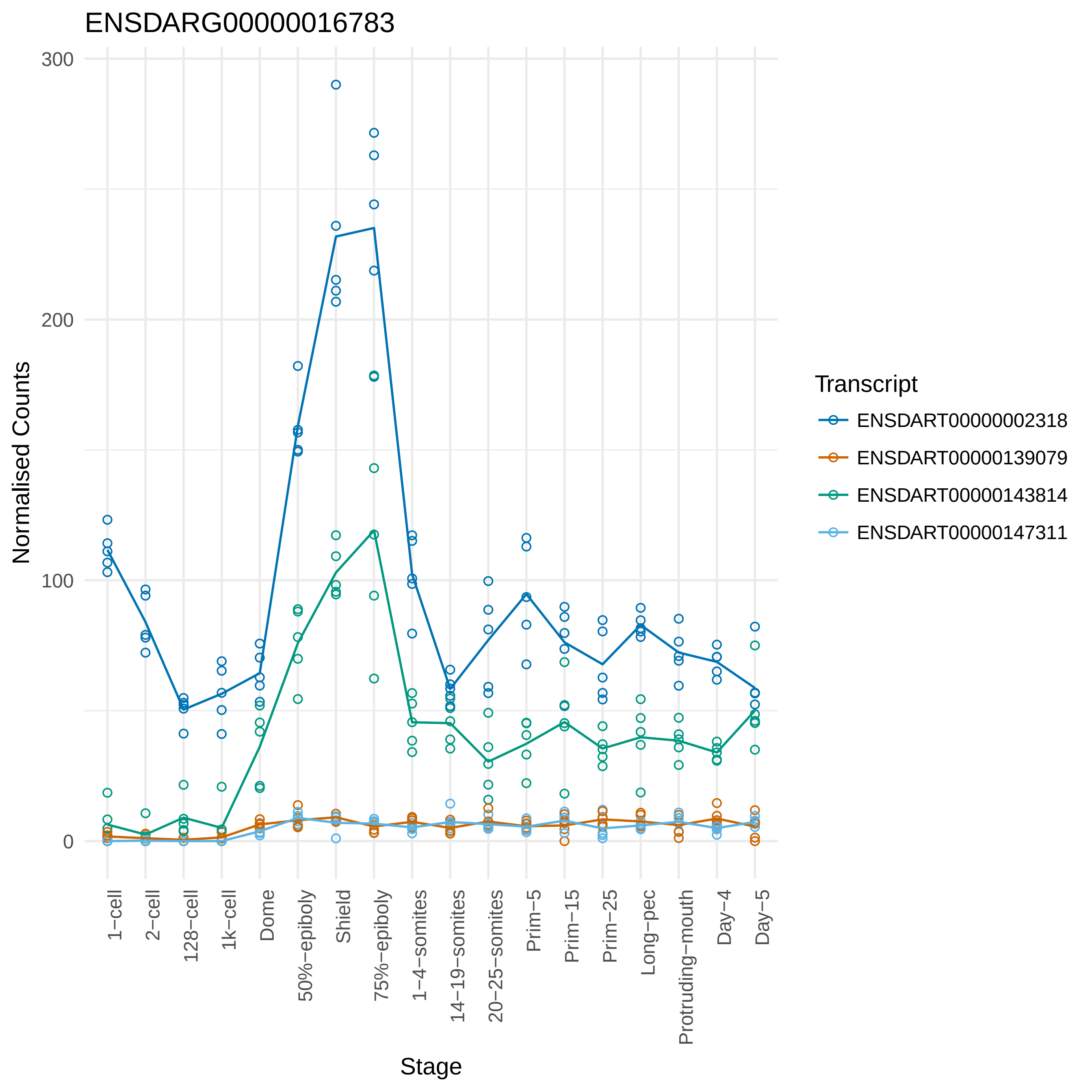

ENSDARG00000016783
300
200
100
0
Normalised Counts
1−cell
2−cell
128−cell
1k−cell
Dome
50%−epiboly
Shield
75%−epiboly
1−4−somites
14−19−somites
20−25−somites
Prim−5
Prim−15
Prim−25
Long−pec
Protruding−mouth
Day−4
Day−5
Stage
Transcript
ENSDART00000002318
ENSDART00000139079
ENSDART00000143814
ENSDART00000147311

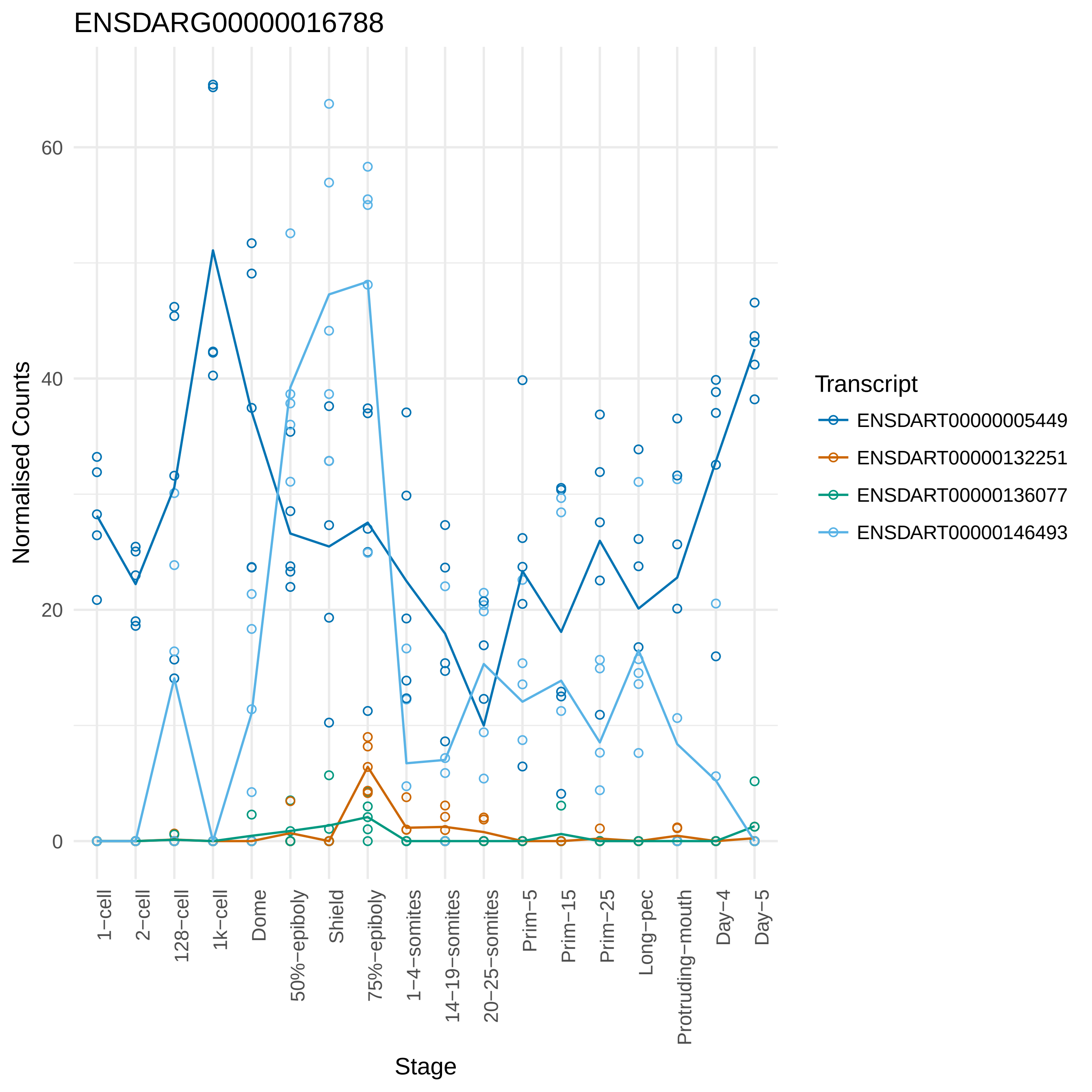

ENSDARG00000016788
Normalised Counts
60
40
20
0
1−cell
2−cell
128−cell
1k−cell
Dome
50%−epiboly
Shield
75%−epiboly
1−4−somites
14−19−somites
20−25−somites
Prim−5
Prim−15
Prim−25
Long−pec
Protruding−mouth
Day−4
Day−5
Stage
Transcript
ENSDART00000005449
ENSDART00000132251
ENSDART00000136077
ENSDART00000146493

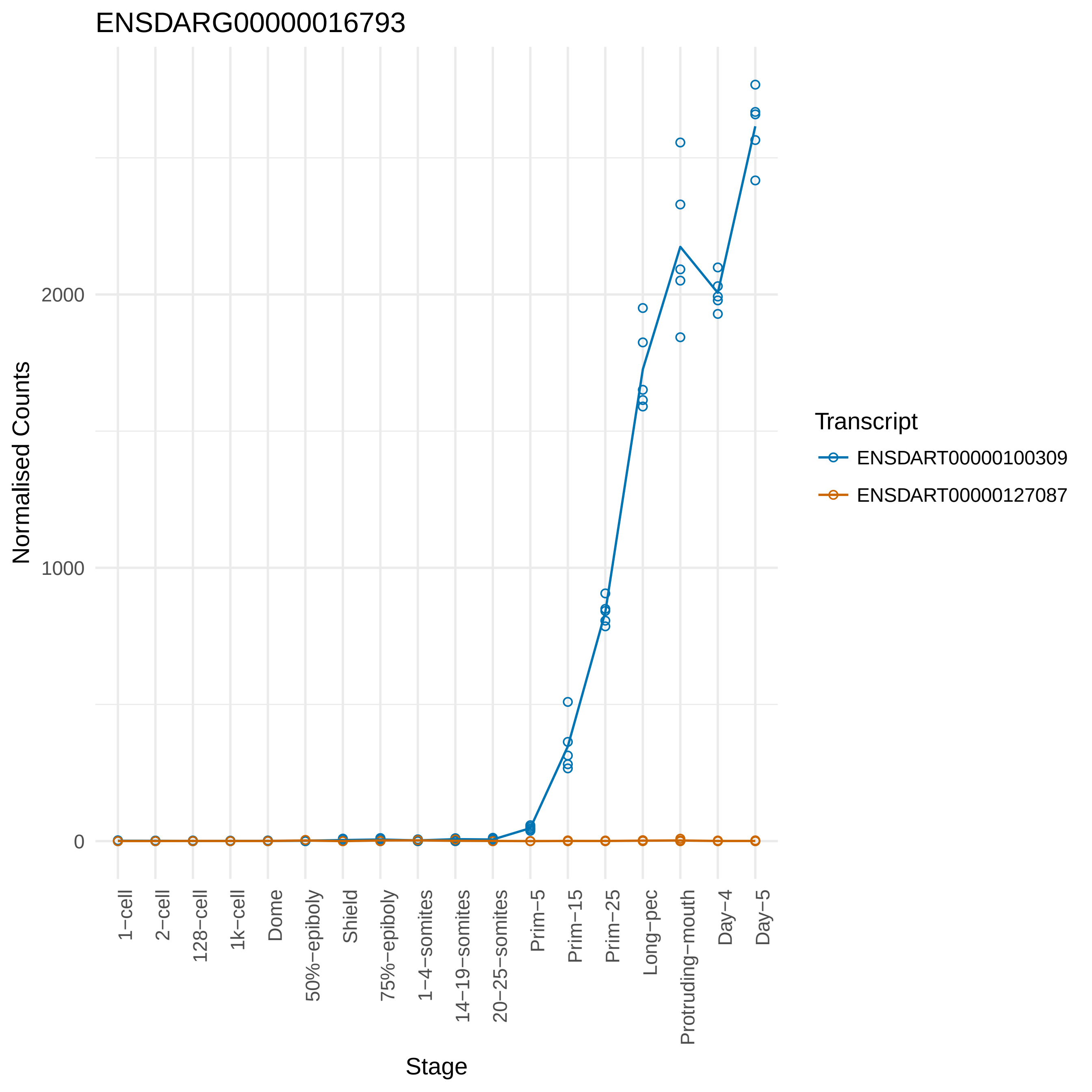

ENSDARG00000016793
Normalised Counts
2000
1000
0
1−cell
2−cell
128−cell
1k−cell
Dome
50%−epiboly
Shield
75%−epiboly
1−4−somites
14−19−somites
20−25−somites
Prim−5
Prim−15
Prim−25
Long−pec
Protruding−mouth
Day−4
Day−5
Stage
Transcript
ENSDART00000100309
ENSDART00000127087

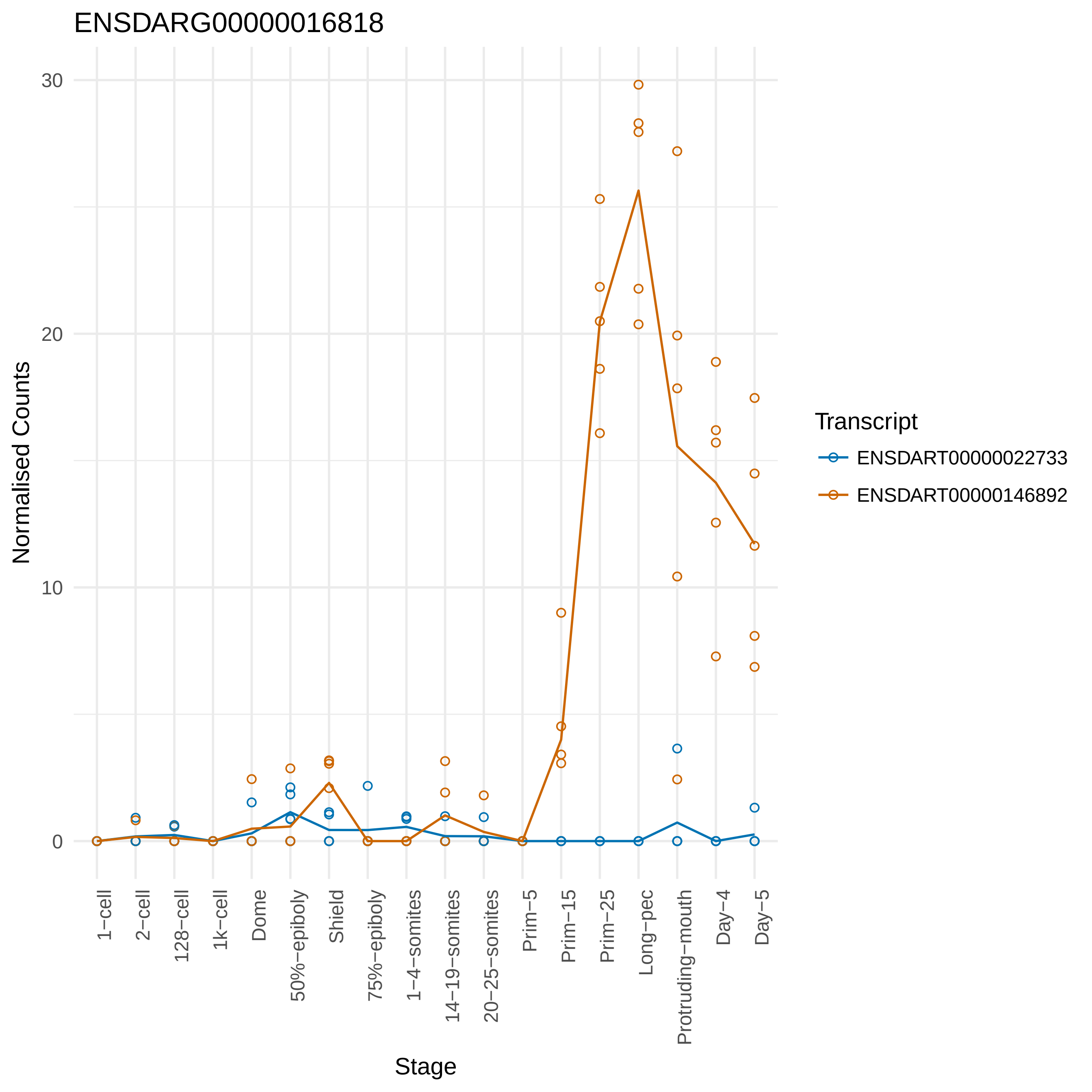

ENSDARG00000016818
Normalised Counts
30
20
10
0
1−cell
2−cell
128−cell
1k−cell
Dome
50%−epiboly
Shield
75%−epiboly
1−4−somites
14−19−somites
20−25−somites
Prim−5
Prim−15
Prim−25
Long−pec
Protruding−mouth
Day−4
Day−5
Stage
Transcript
ENSDART00000022733
ENSDART00000146892

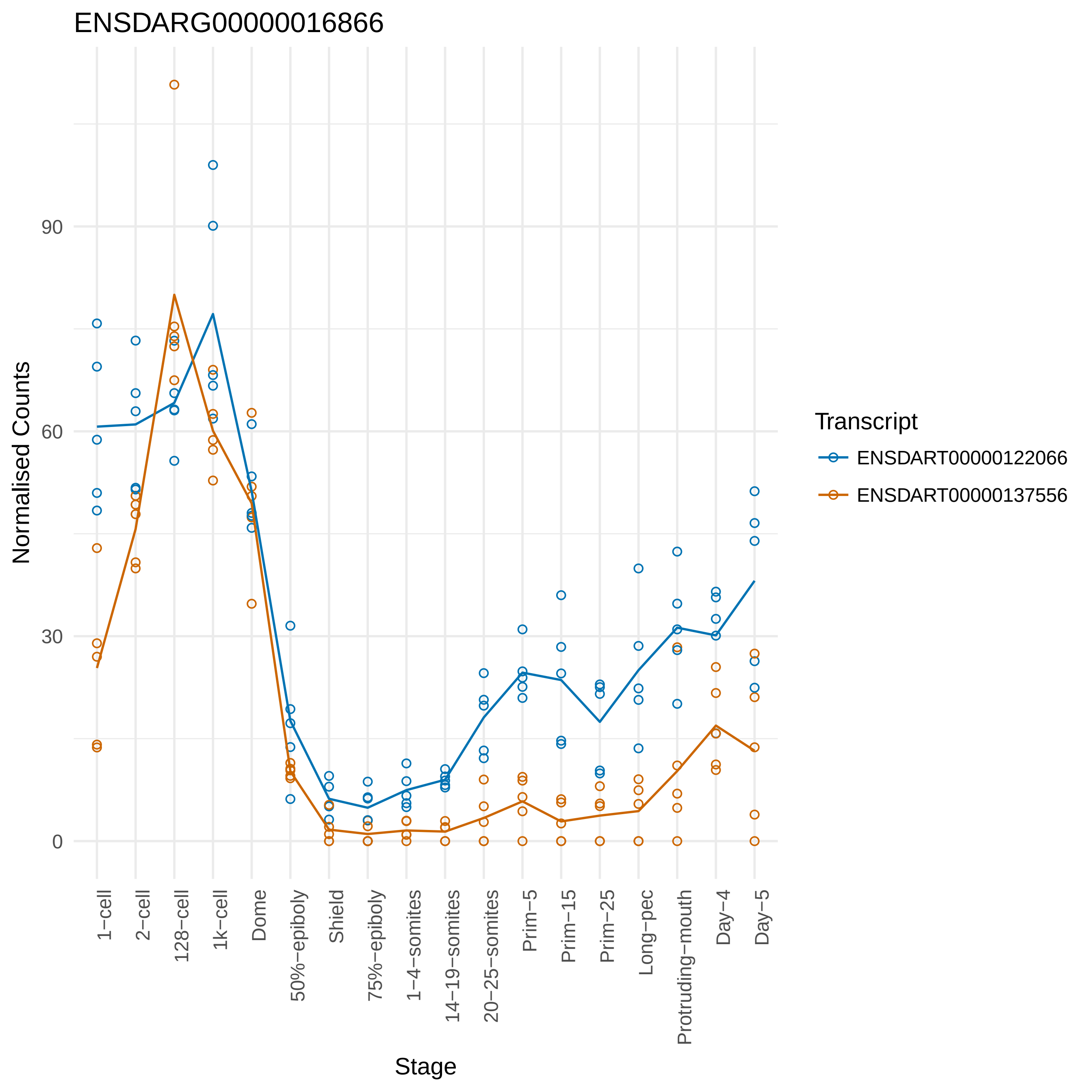

ENSDARG00000016866
Normalised Counts
90
60
30
0
1−cell
2−cell
128−cell
1k−cell
Dome
50%−epiboly
Shield
75%−epiboly
1−4−somites
14−19−somites
20−25−somites
Prim−5
Prim−15
Prim−25
Long−pec
Protruding−mouth
Day−4
Day−5
Stage
Transcript
ENSDART00000122066
ENSDART00000137556

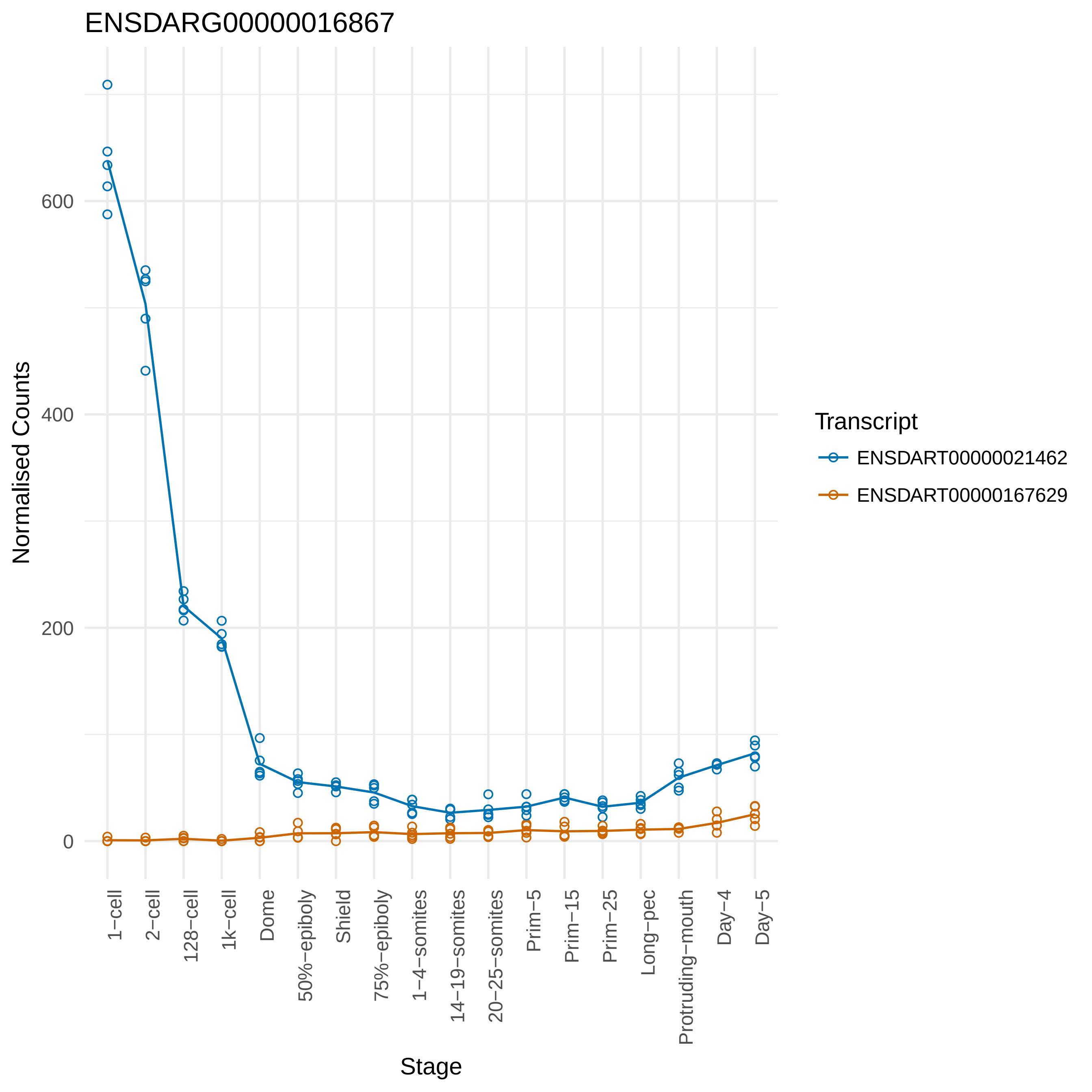

ENSDARG00000016867
Normalised Counts
600
400
200
0
Stage
1−cell
2−cell
128−cell
1k−cell
Dome
50%−epiboly
Shield
75%−epiboly
1−4−somites
14−19−somites
20−25−somites
Prim−5
Prim−15
Prim−25
Long−pec
Protruding−mouth
Day−4
Day−5
Transcript
ENSDART00000021462
ENSDART00000167629

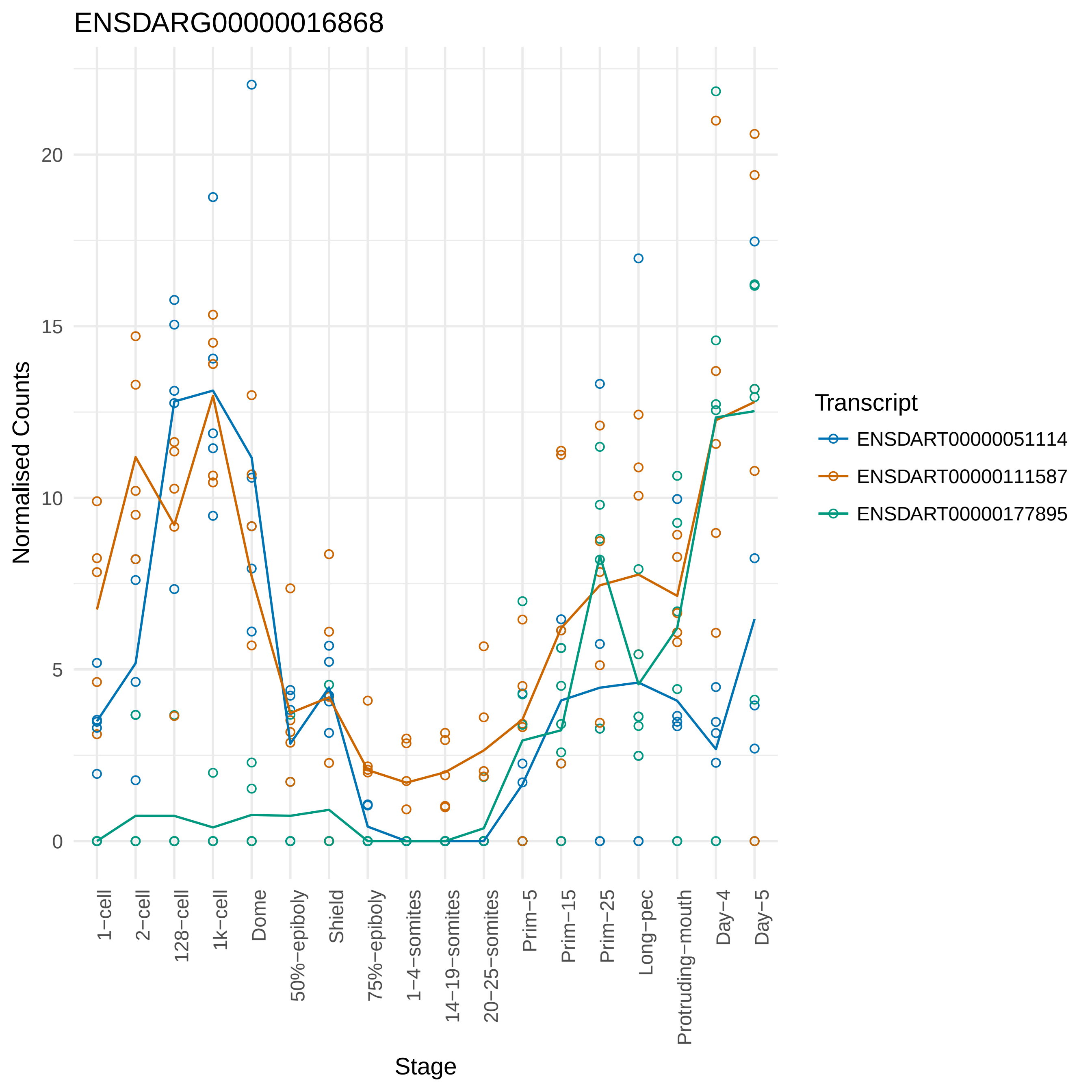

ENSDARG00000016868
Normalised Counts
0
5
10
15
20
1−cell
2−cell
128−cell
1k−cell
Dome
50%−epiboly
Shield
75%−epiboly
1−4−somites
14−19−somites
20−25−somites
Prim−5
Prim−15
Prim−25
Long−pec
Protruding−mouth
Day−4
Day−5
Stage
Transcript
ENSDART00000051114
ENSDART00000111587
ENSDART00000177895

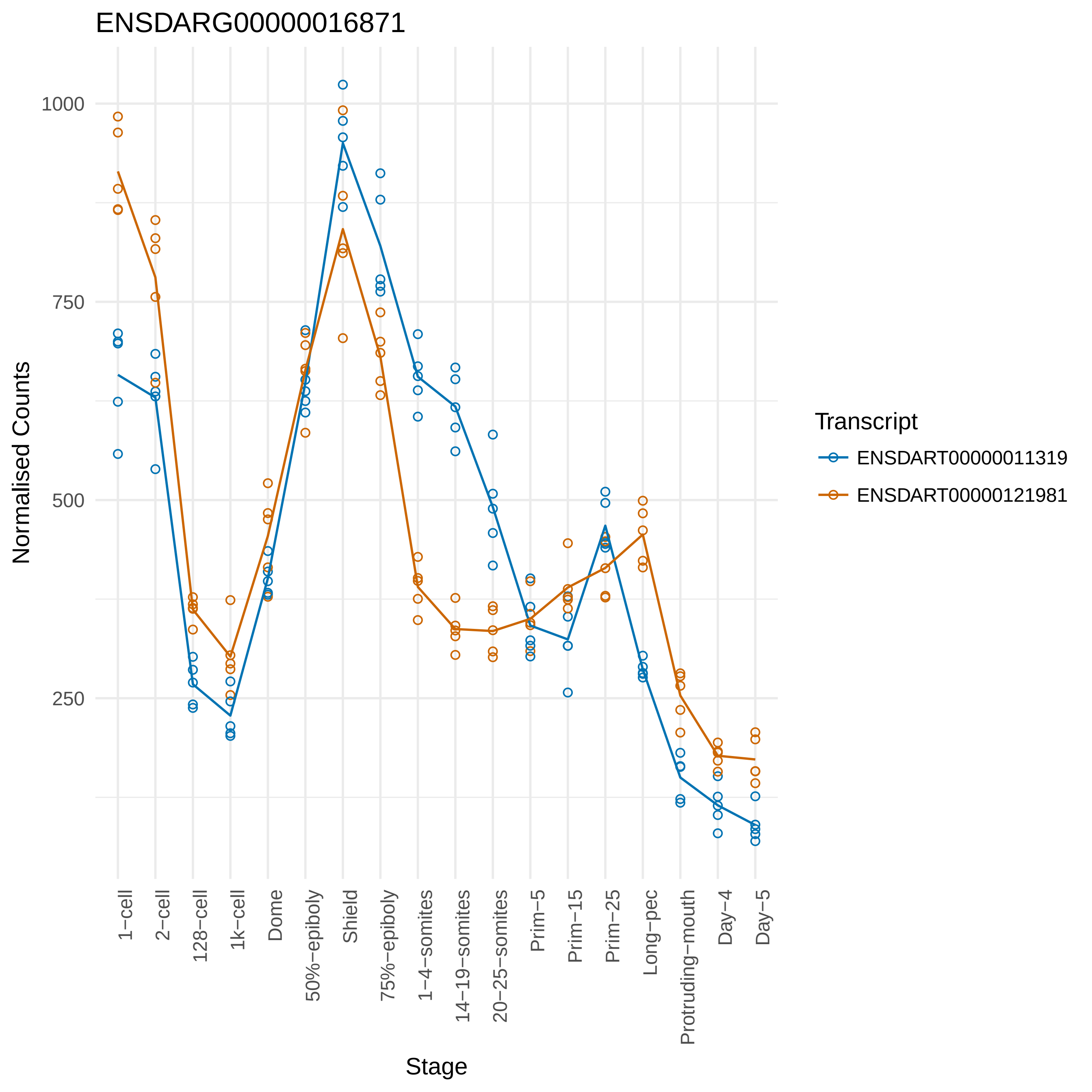
ENSDARG00000016871
Normalised Counts
1000
750
500
250
Stage
1−cell
2−cell
128−cell
1k−cell
Dome
50%−epiboly
Shield
75%−epiboly
1−4−somites
14−19−somites
20−25−somites
Prim−5
Prim−15
Prim−25
Long−pec
Protruding−mouth
Day−4
Day−5
Transcript
ENSDART00000011319
ENSDART00000121981

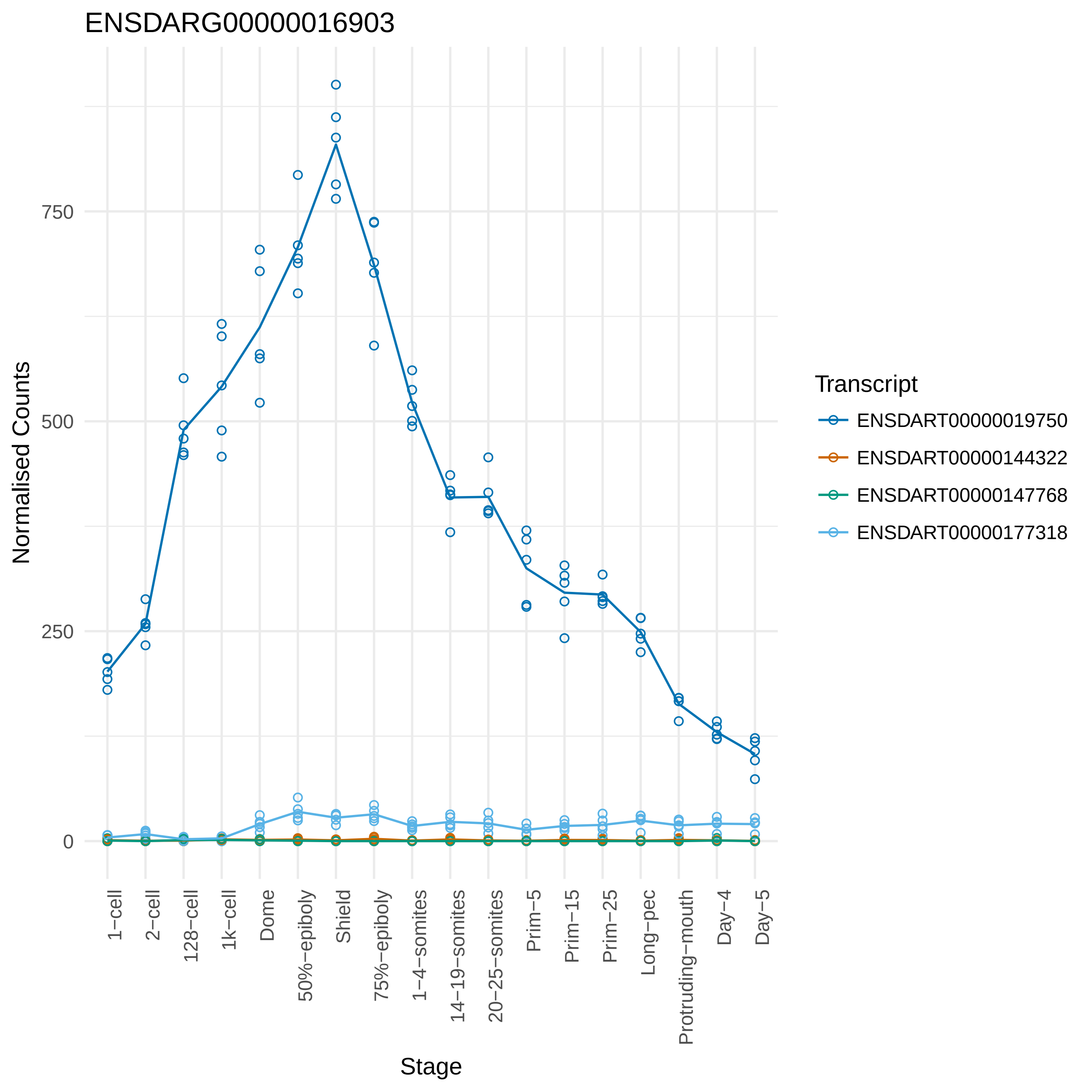
ENSDARG00000016903
Normalised Counts
750
500
250
0
1−cell
2−cell
128−cell
1k−cell
Dome
50%−epiboly
Shield
75%−epiboly
1−4−somites
14−19−somites
20−25−somites
Prim−5
Prim−15
Prim−25
Long−pec
Protruding−mouth
Day−4
Day−5
Stage
Transcript
ENSDART00000019750
ENSDART00000144322
ENSDART00000147768
ENSDART00000177318

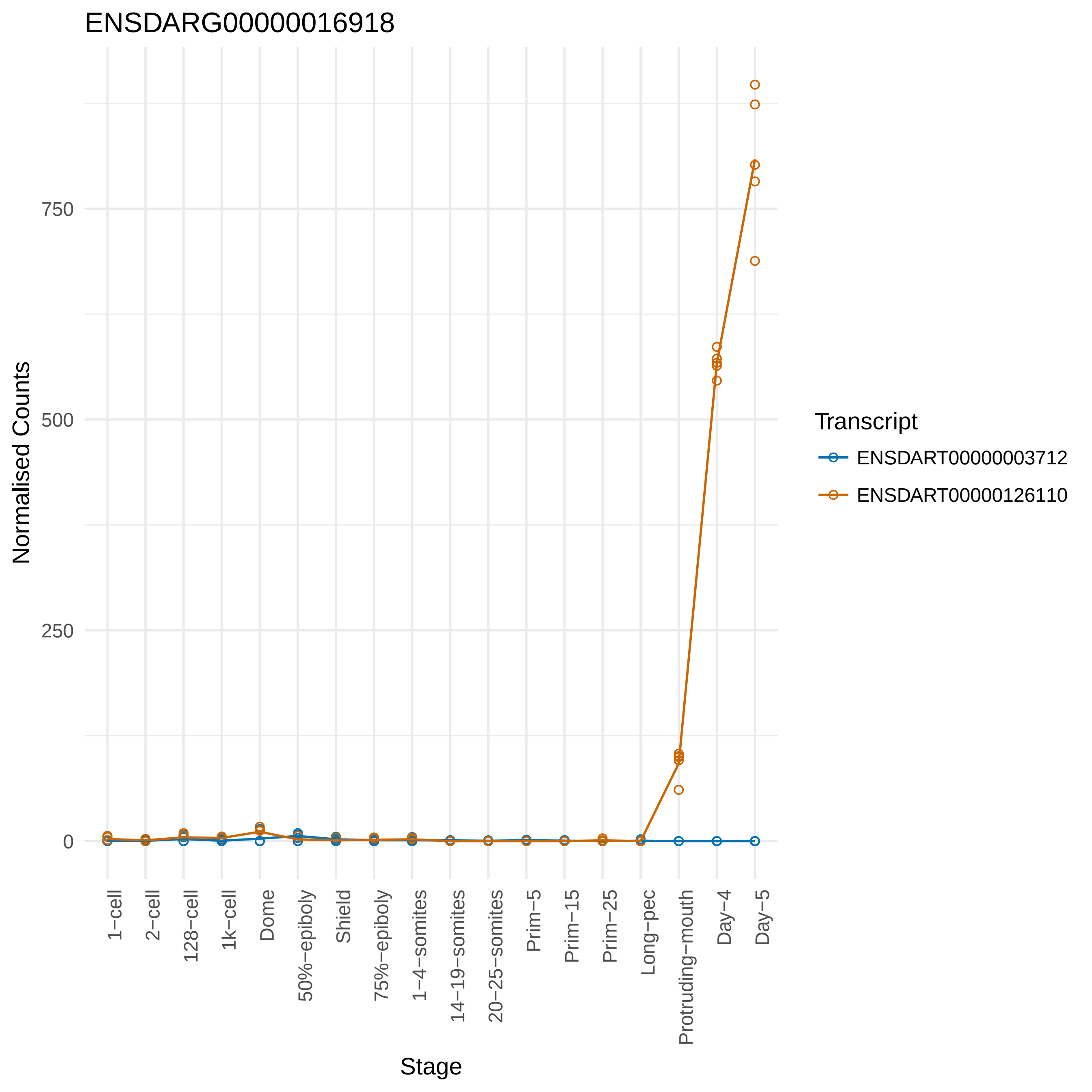

ENSDARG00000016918
Normalised Counts
750
500
250
0
1−cell
2−cell
128−cell
1k−cell
Dome
50%−epiboly
Shield
75%−epiboly
1−4−somites
14−19−somites
20−25−somites
Prim−5
Prim−15
Prim−25
Long−pec
Protruding−mouth
Day−4
Day−5
Stage
Transcript
ENSDART00000003712
ENSDART00000126110

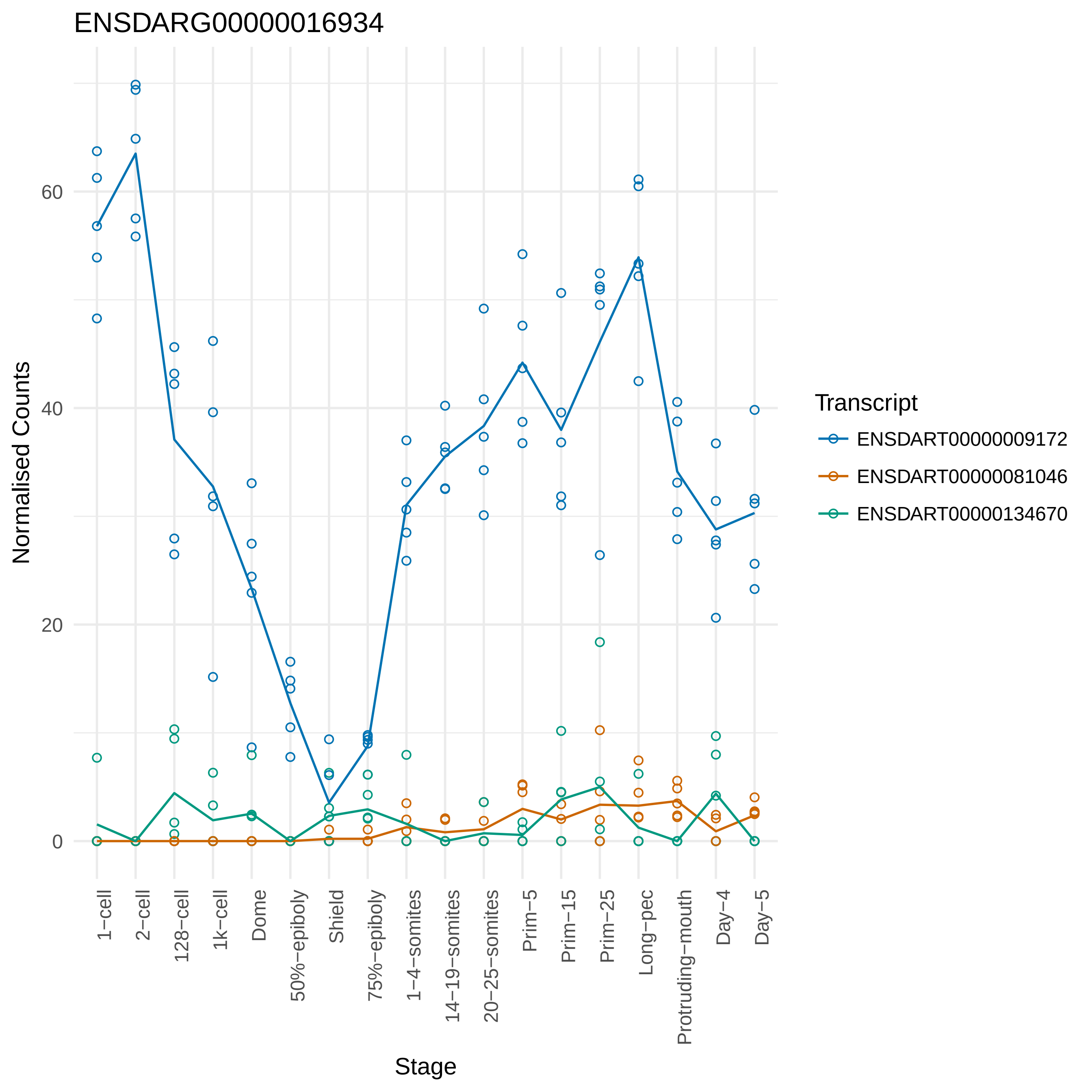

ENSDARG00000016934
Normalised Counts
60
40
20
0
1−cell
2−cell
128−cell
1k−cell
Dome
50%−epiboly
Shield
75%−epiboly
1−4−somites
14−19−somites
20−25−somites
Prim−5
Prim−15
Prim−25
Long−pec
Protruding−mouth
Day−4
Day−5
Stage
Transcript
ENSDART00000009172
ENSDART00000081046
ENSDART00000134670

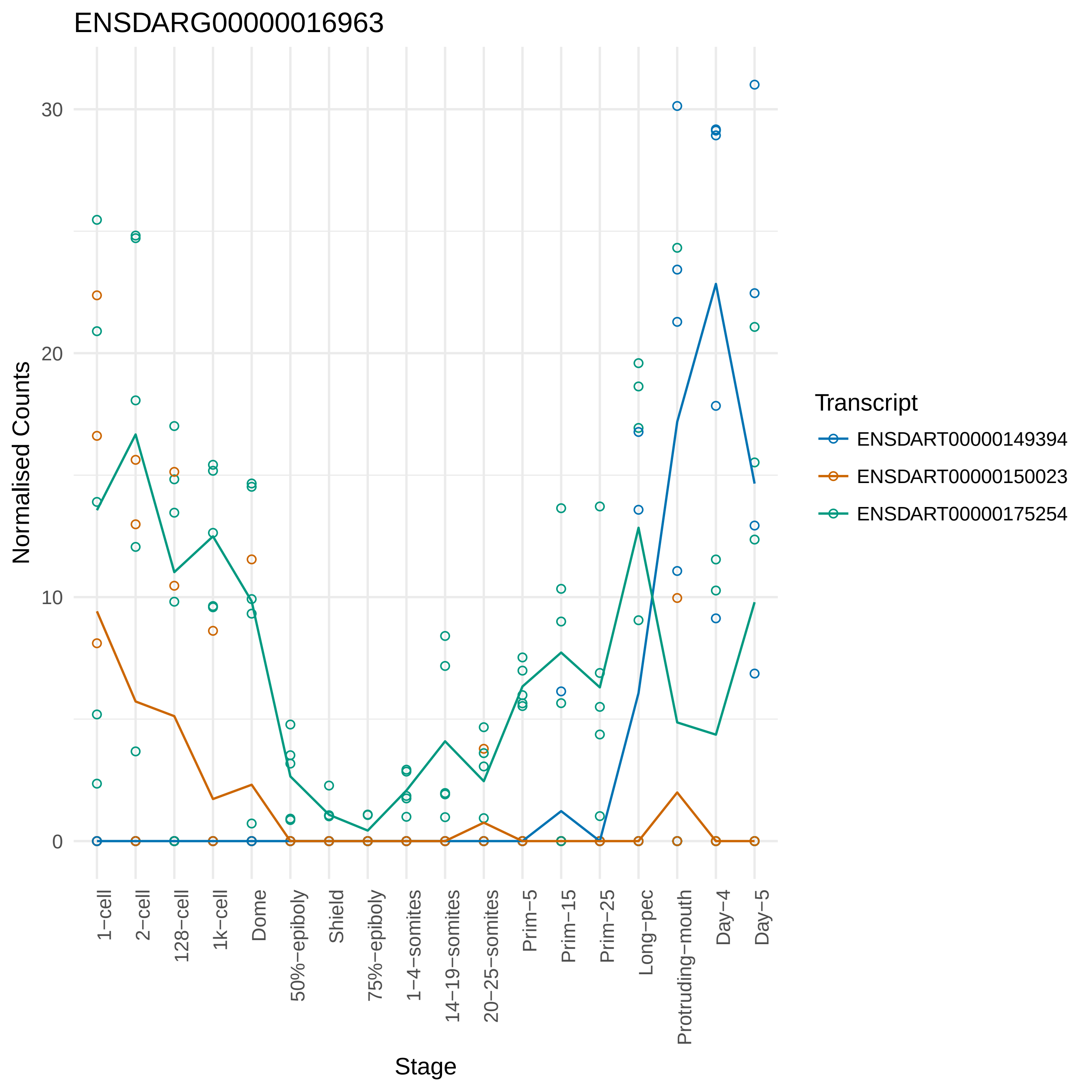

ENSDARG00000016963
Normalised Counts
0
10
20
30
1−cell
2−cell
128−cell
1k−cell
Dome
50%−epiboly
Shield
75%−epiboly
1−4−somites
14−19−somites
20−25−somites
Prim−5
Prim−15
Prim−25
Long−pec
Protruding−mouth
Day−4
Day−5
Stage
Transcript
ENSDART00000149394
ENSDART00000150023
ENSDART00000175254

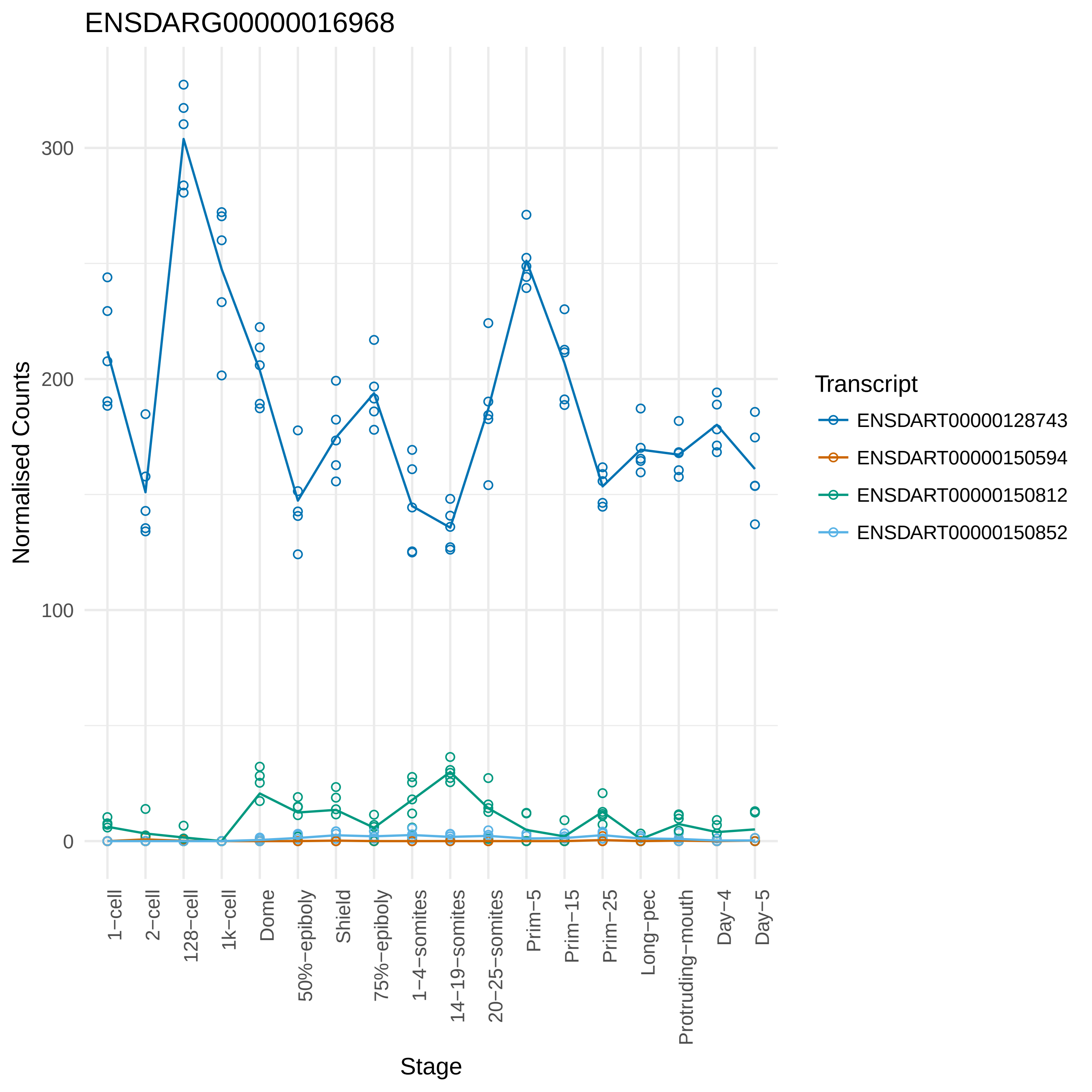

ENSDARG00000016968
Normalised Counts
300
200
100
0
1−cell
2−cell
128−cell
1k−cell
Dome
50%−epiboly
Shield
75%−epiboly
1−4−somites
14−19−somites
20−25−somites
Prim−5
Prim−15
Prim−25
Long−pec
Protruding−mouth
Day−4
Day−5
Stage
Transcript
ENSDART00000128743
ENSDART00000150594
ENSDART00000150812
ENSDART00000150852

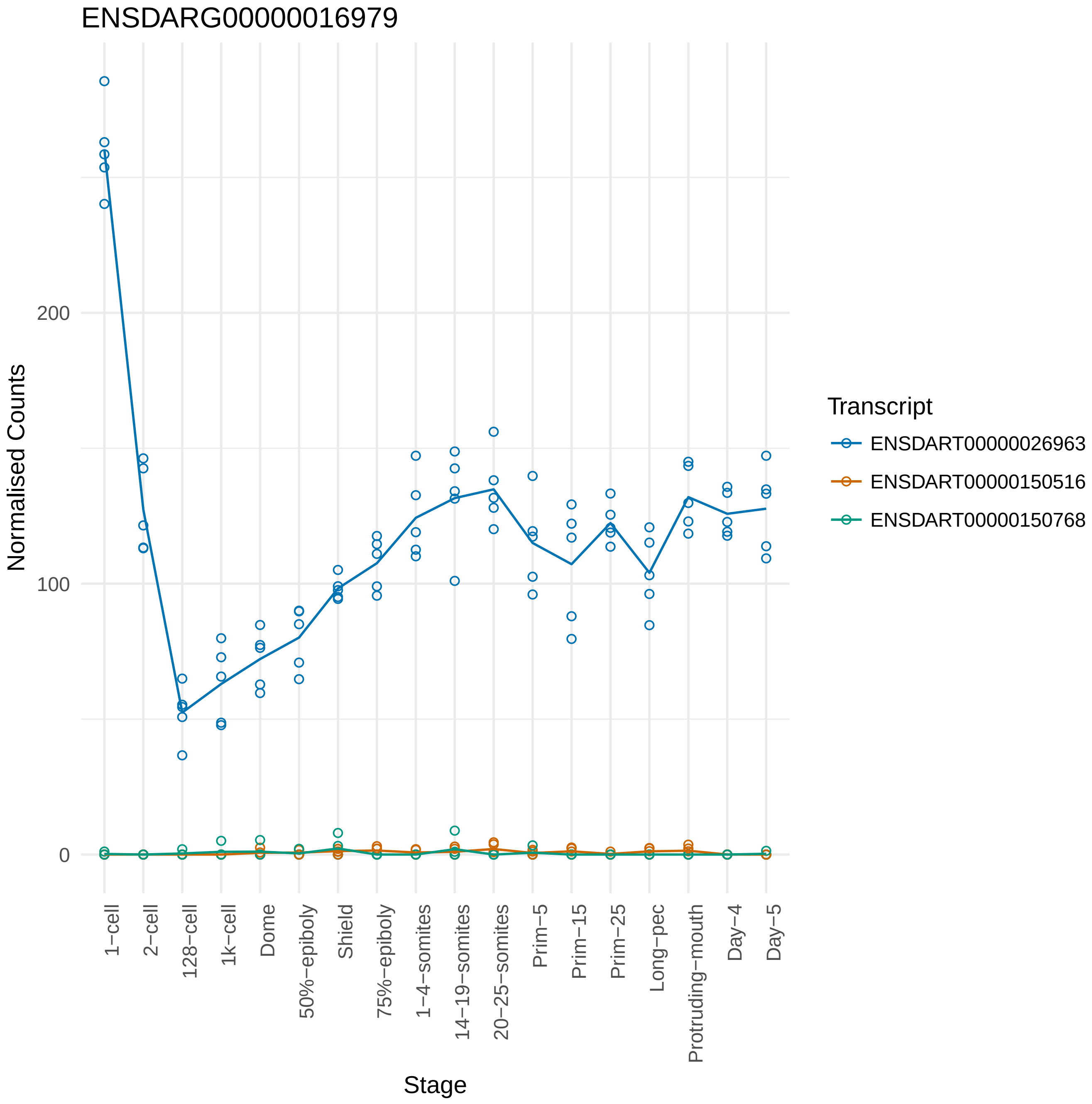
ENSDARG00000016979
Normalised Counts
0
100
200
Stage
1−cell
2−cell
128−cell
1k−cell
Dome
50%−epiboly
Shield
75%−epiboly
1−4−somites
14−19−somites
20−25−somites
Prim−5
Prim−15
Prim−25
Long−pec
Protruding−mouth
Day−4
Day−5
Transcript
ENSDART00000026963
ENSDART00000150516
ENSDART00000150768

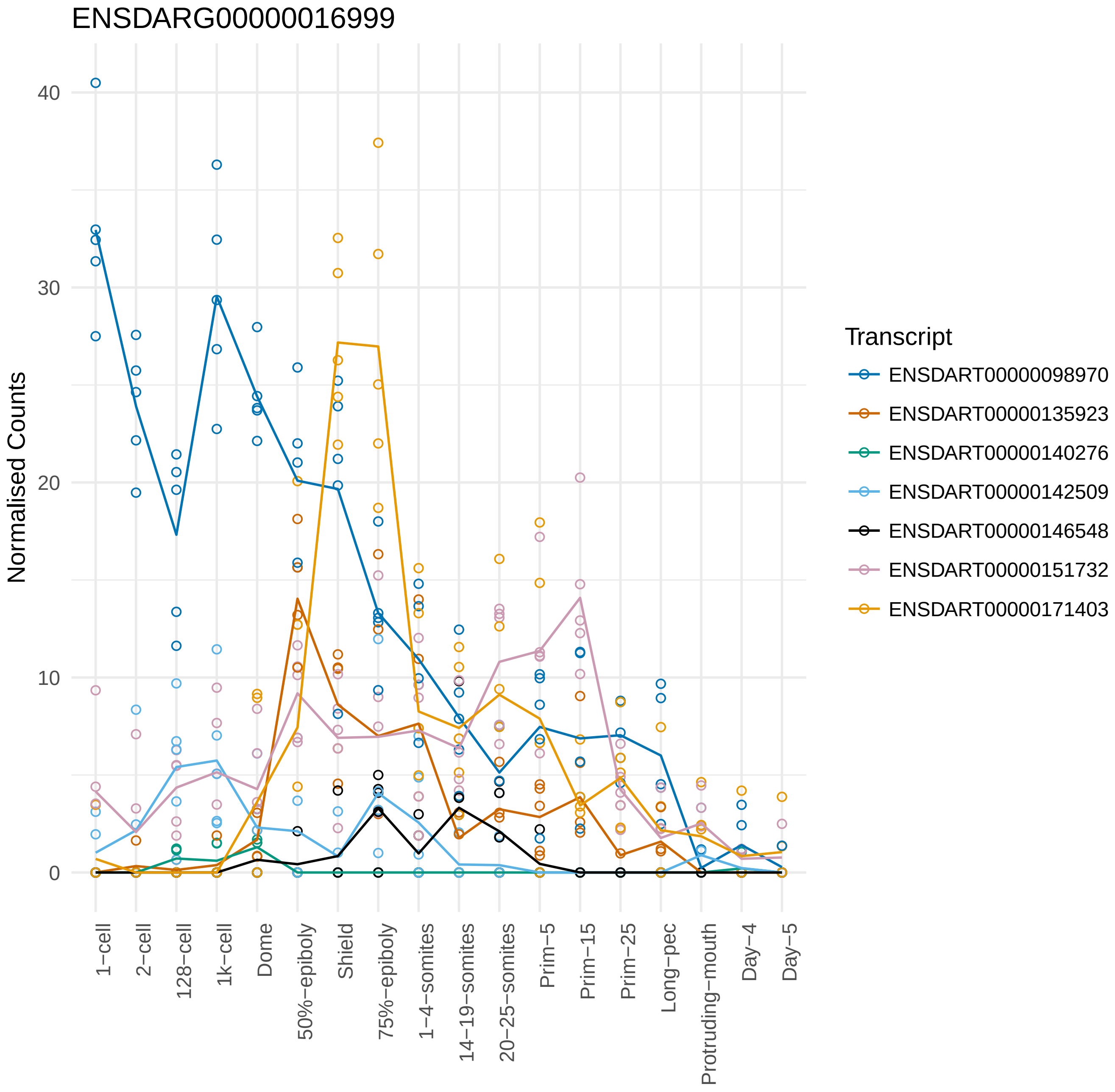

ENSDARG00000016999
Normalised Counts
0
10
20
30
40
Stage
1−cell
2−cell
128−cell
1k−cell
Dome
50%−epiboly
Shield
75%−epiboly
1−4−somites
14−19−somites
20−25−somites
Prim−5
Prim−15
Prim−25
Long−pec
Protruding−mouth
Day−4
Day−5
Transcript
ENSDART00000098970
ENSDART00000135923
ENSDART00000140276
ENSDART00000142509
ENSDART00000146548
ENSDART00000151732
ENSDART00000171403

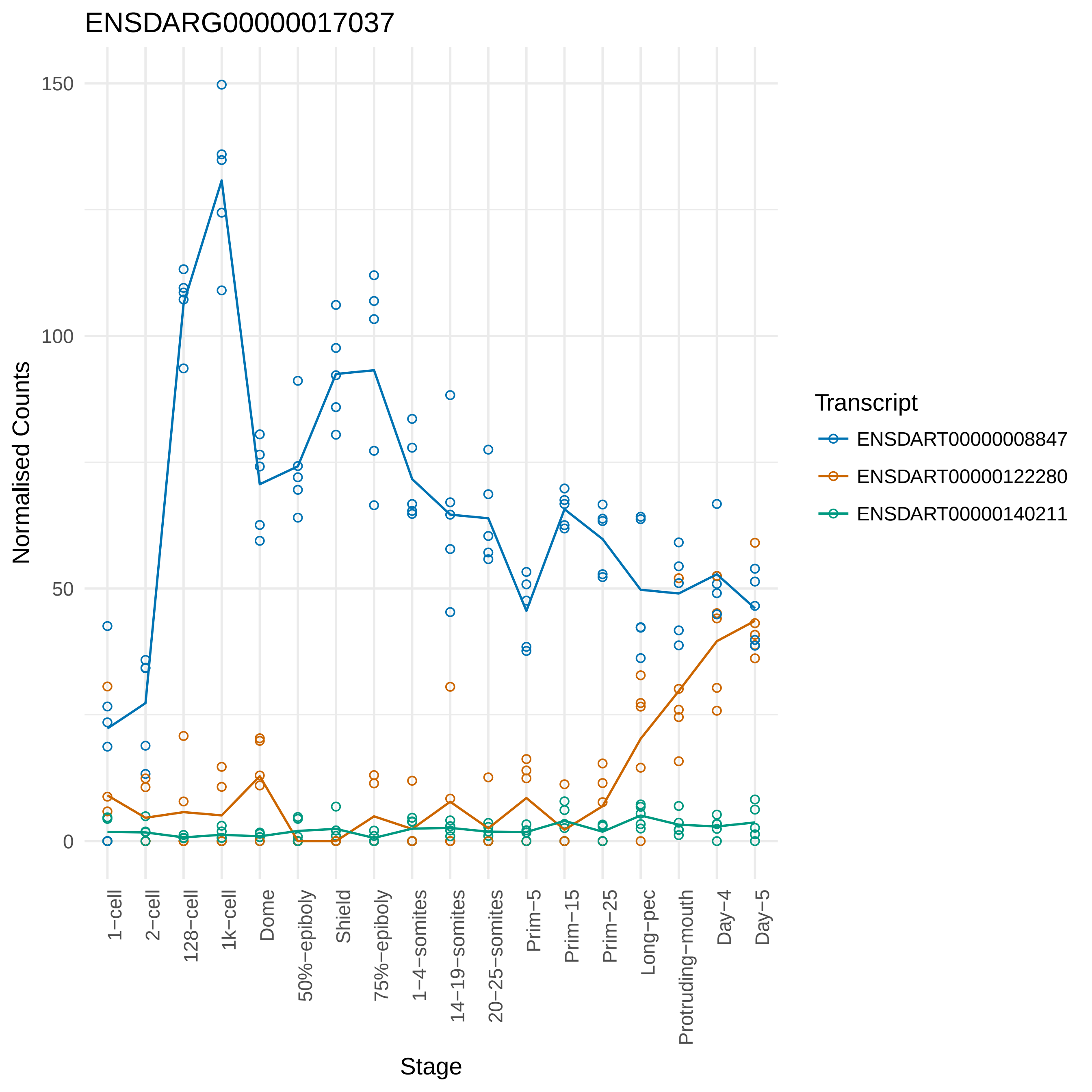

ENSDARG00000017037
Normalised Counts
150
100
50
0
1−cell
2−cell
128−cell
1k−cell
Dome
50%−epiboly
Shield
75%−epiboly
1−4−somites
14−19−somites
20−25−somites
Prim−5
Prim−15
Prim−25
Long−pec
Protruding−mouth
Day−4
Day−5
Stage
Transcript
ENSDART00000008847
ENSDART00000122280
ENSDART00000140211

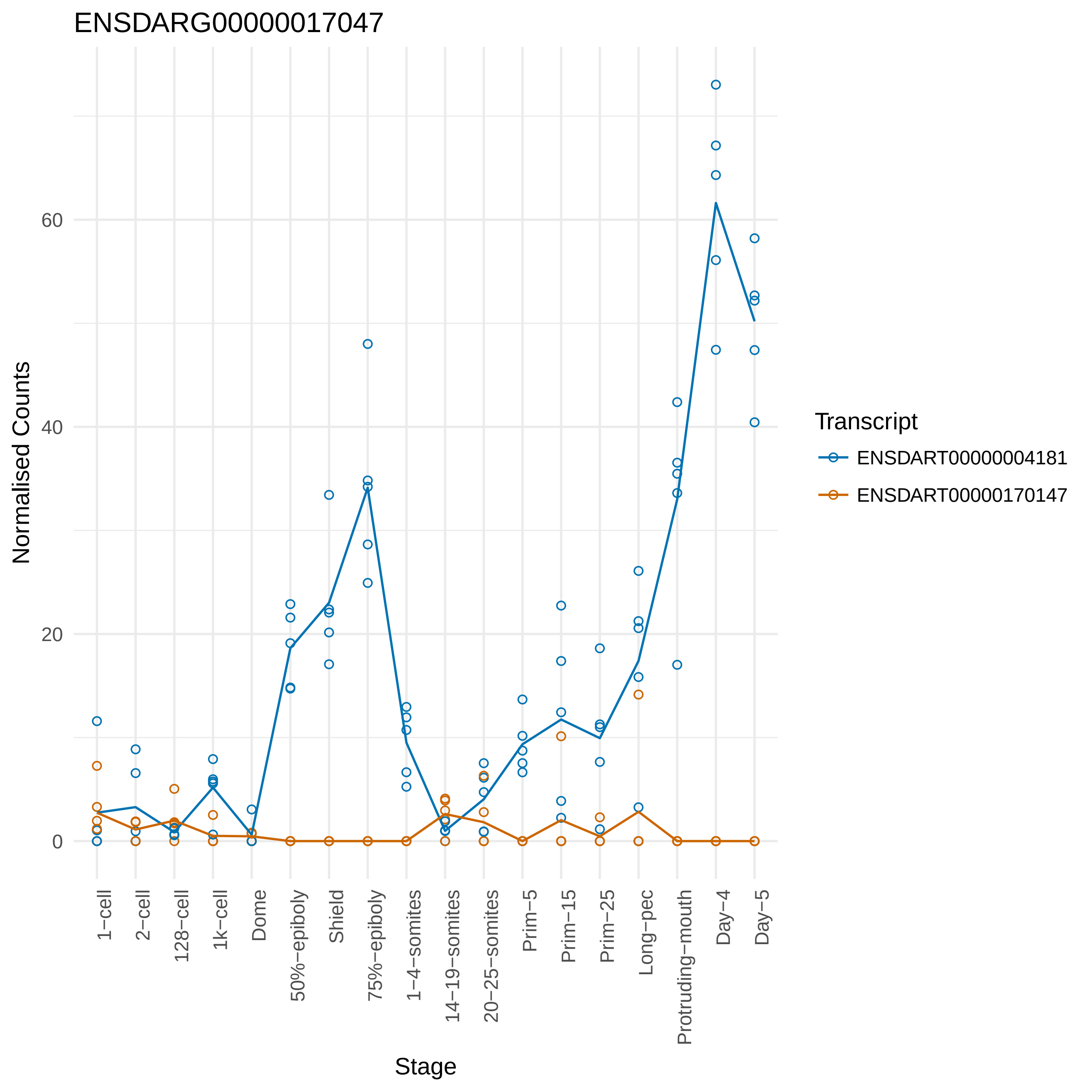

ENSDARG00000017047
Normalised Counts
0
20
40
60
Stage
1−cell
2−cell
128−cell
1k−cell
Dome
50%−epiboly
Shield
75%−epiboly
1−4−somites
14−19−somites
20−25−somites
Prim−5
Prim−15
Prim−25
Long−pec
Protruding−mouth
Day−4
Day−5
Transcript
ENSDART00000004181
ENSDART00000170147

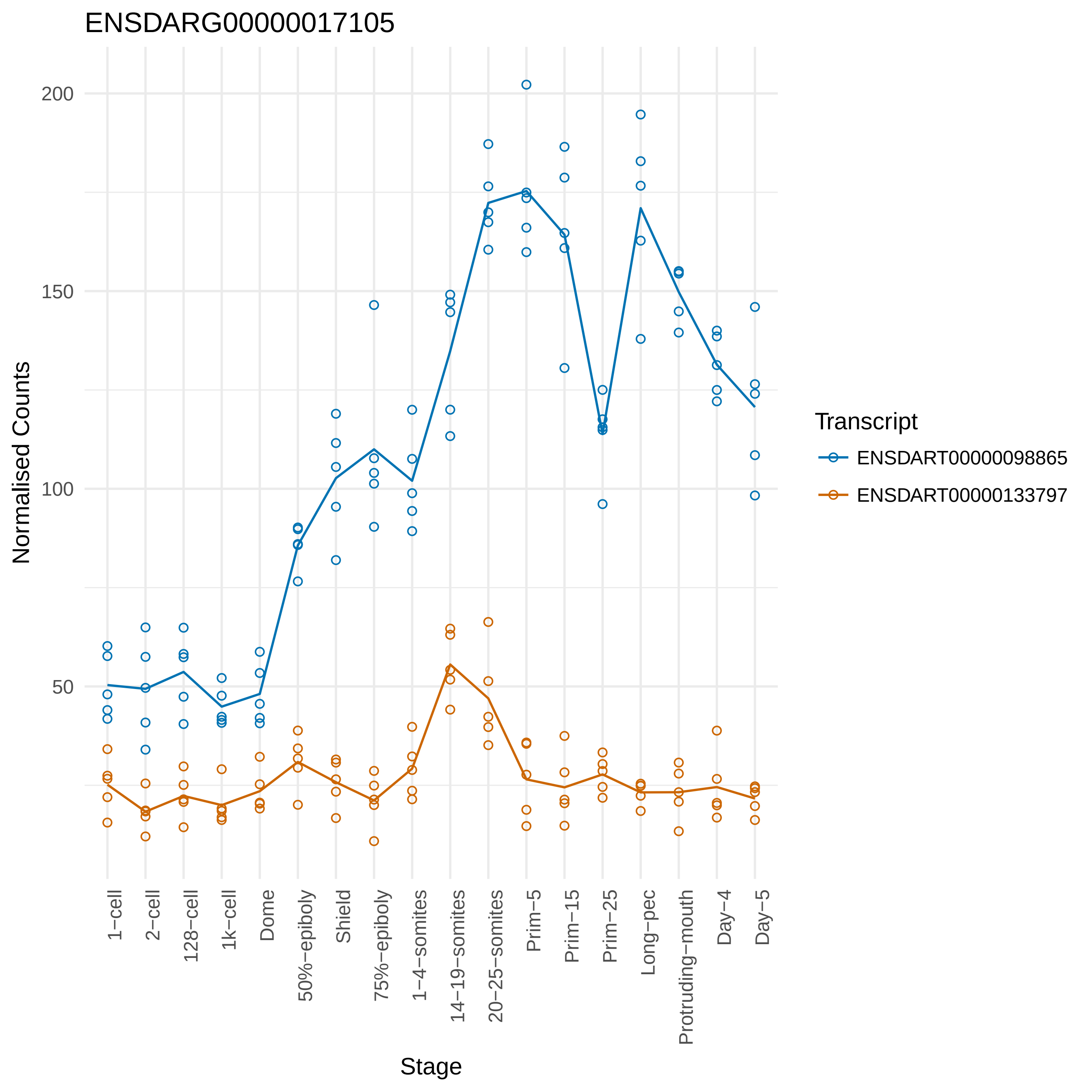

ENSDARG00000017105
Normalised Counts
200
150
100
50
Stage
1−cell
2−cell
128−cell
1k−cell
Dome
50%−epiboly
Shield
75%−epiboly
1−4−somites
14−19−somites
20−25−somites
Prim−5
Prim−15
Prim−25
Long−pec
Protruding−mouth
Day−4
Day−5
Transcript
ENSDART00000098865
ENSDART00000133797

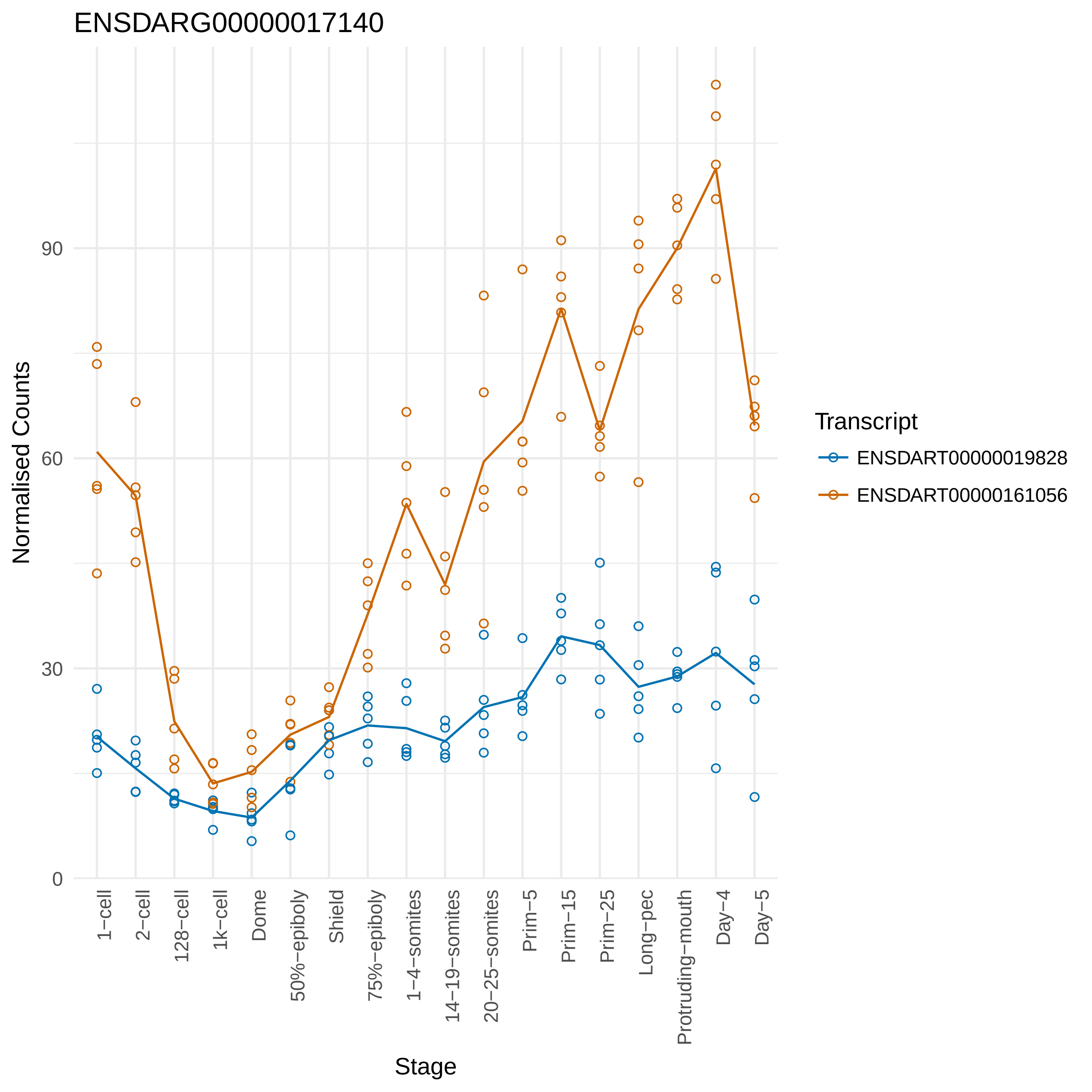
ENSDARG00000017140
Normalised Counts
90
60
30
0
1−cell
2−cell
128−cell
1k−cell
Dome
50%−epiboly
Shield
75%−epiboly
1−4−somites
14−19−somites
20−25−somites
Prim−5
Prim−15
Prim−25
Long−pec
Protruding−mouth
Day−4
Day−5
Stage
Transcript
ENSDART00000019828
ENSDART00000161056

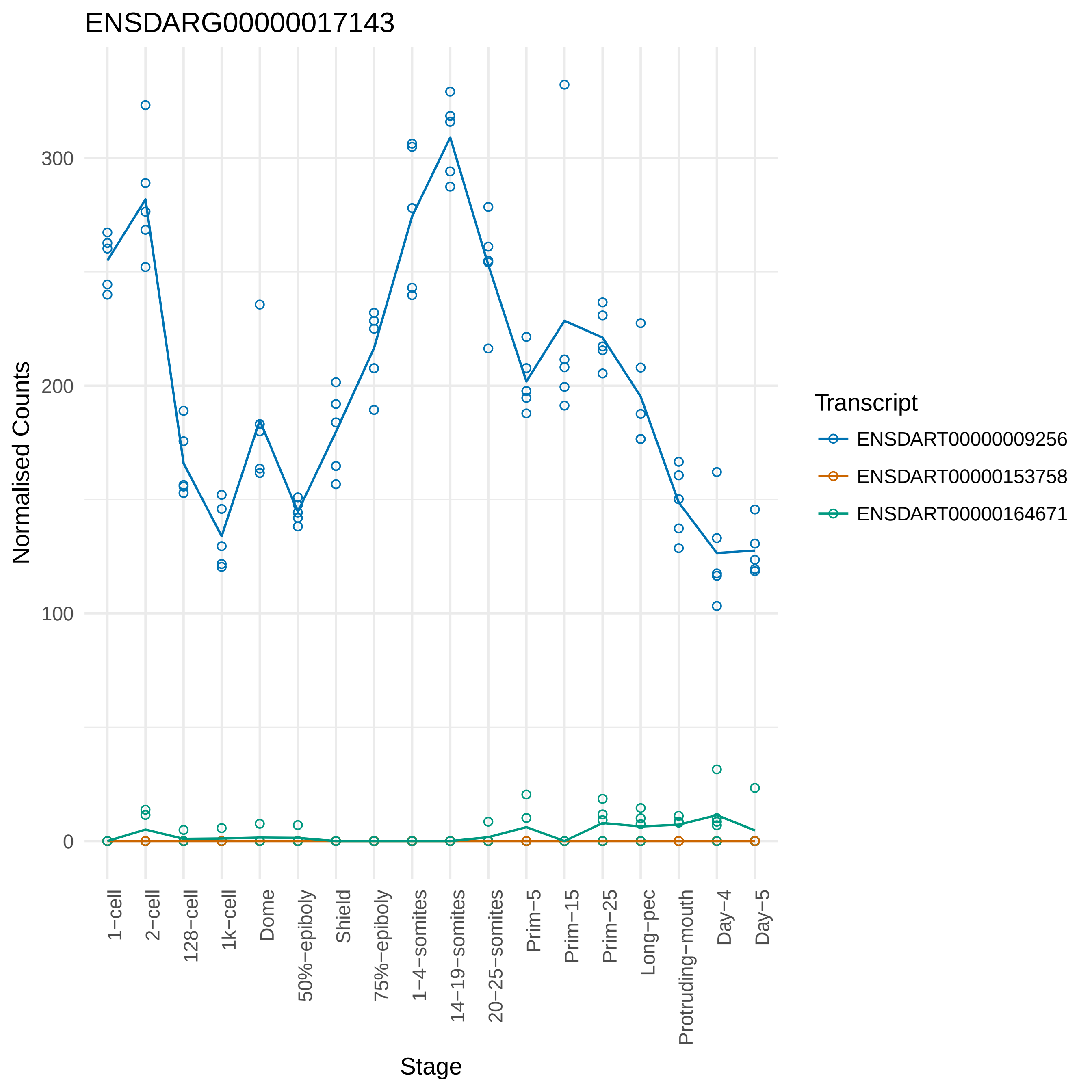
ENSDARG00000017143
Normalised Counts
300
200
100
0
1−cell
2−cell
128−cell
1k−cell
Dome
50%−epiboly
Shield
75%−epiboly
1−4−somites
14−19−somites
20−25−somites
Prim−5
Prim−15
Prim−25
Long−pec
Protruding−mouth
Day−4
Day−5
Stage
Transcript
ENSDART00000009256
ENSDART00000153758
ENSDART00000164671

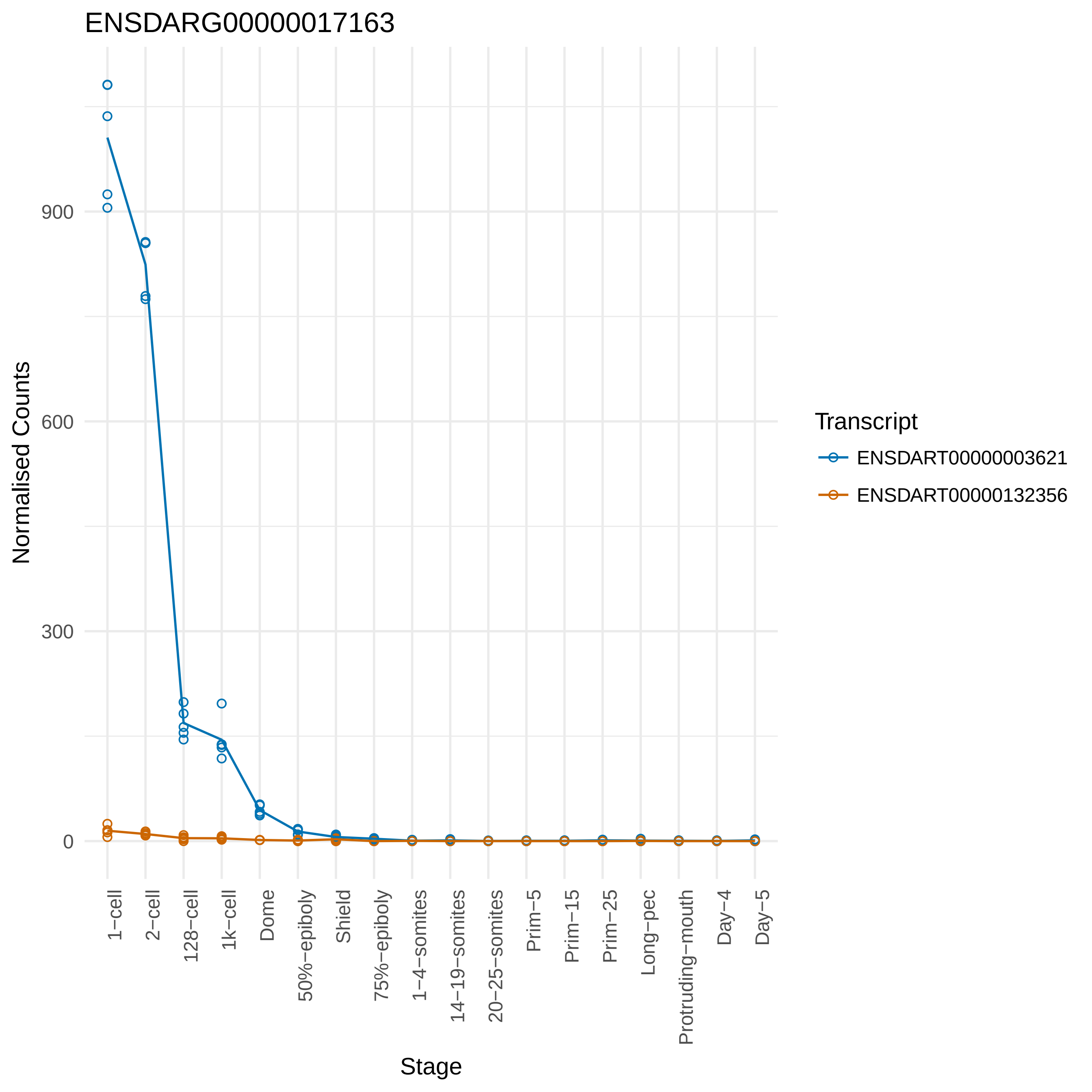
ENSDARG00000017163
Normalised Counts
900
600
300
0
1−cell
2−cell
128−cell
1k−cell
Dome
50%−epiboly
Shield
75%−epiboly
1−4−somites
14−19−somites
20−25−somites
Prim−5
Prim−15
Prim−25
Long−pec
Protruding−mouth
Day−4
Day−5
Stage
Transcript
ENSDART00000003621
ENSDART00000132356

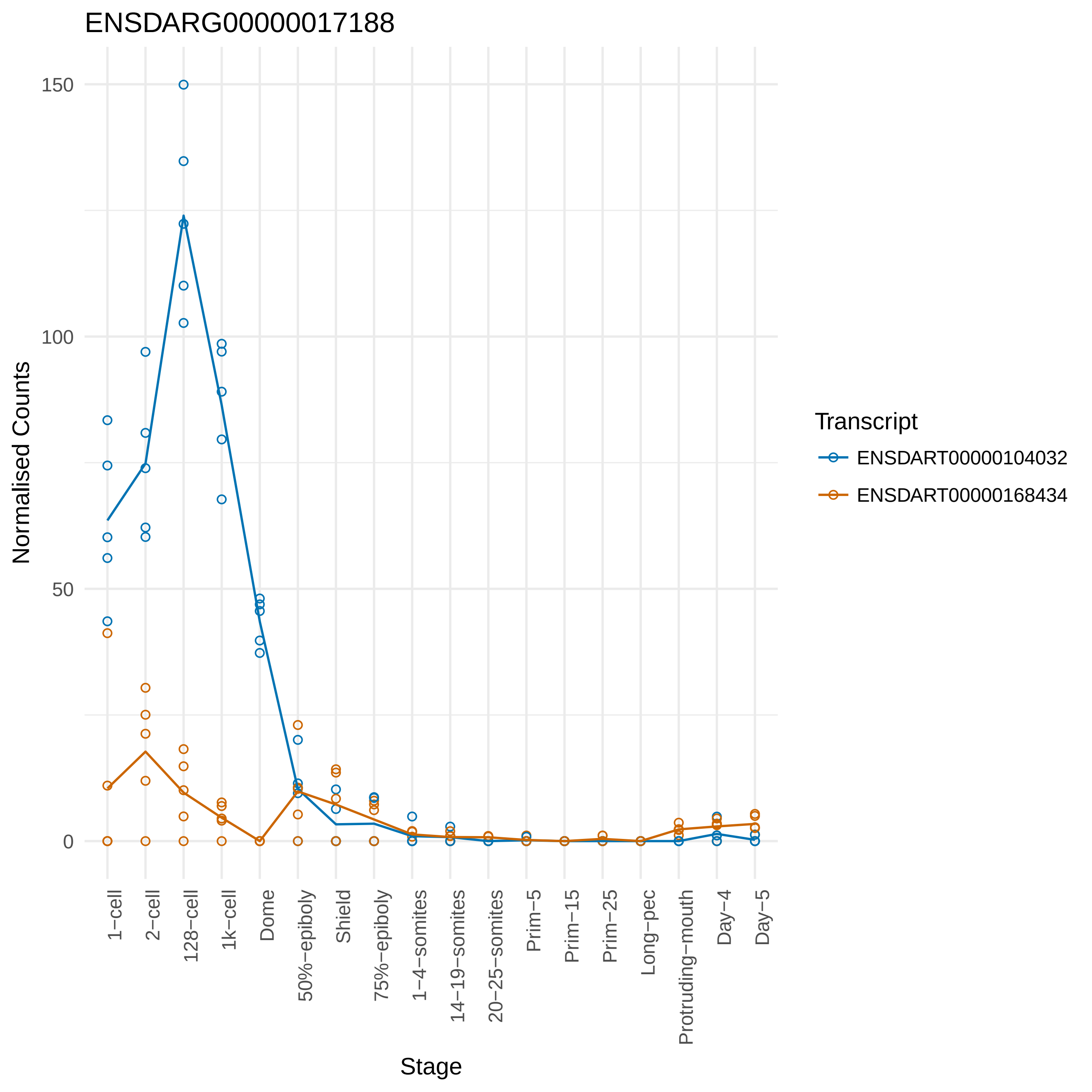
ENSDARG00000017188
Normalised Counts
150
100
50
0
1−cell
2−cell
128−cell
1k−cell
Dome
50%−epiboly
Shield
75%−epiboly
1−4−somites
14−19−somites
20−25−somites
Prim−5
Prim−15
Prim−25
Long−pec
Protruding−mouth
Day−4
Day−5
Stage
Transcript
ENSDART00000104032
ENSDART00000168434

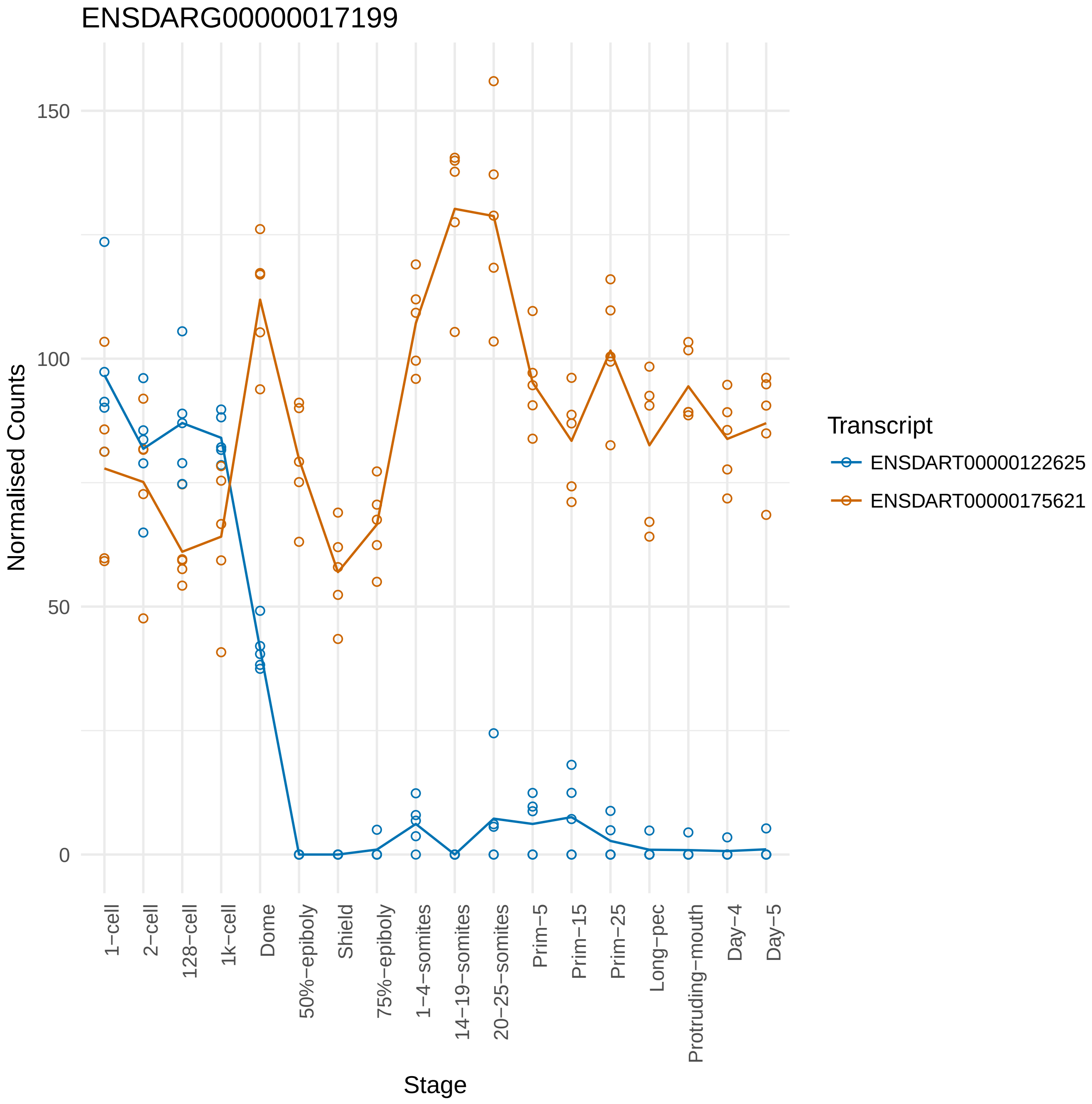

ENSDARG00000017199
Normalised Counts
150
100
50
0
1−cell
2−cell
128−cell
1k−cell
Dome
50%−epiboly
Shield
75%−epiboly
1−4−somites
14−19−somites
20−25−somites
Prim−5
Prim−15
Prim−25
Long−pec
Protruding−mouth
Day−4
Day−5
Stage
Transcript
ENSDART00000122625
ENSDART00000175621

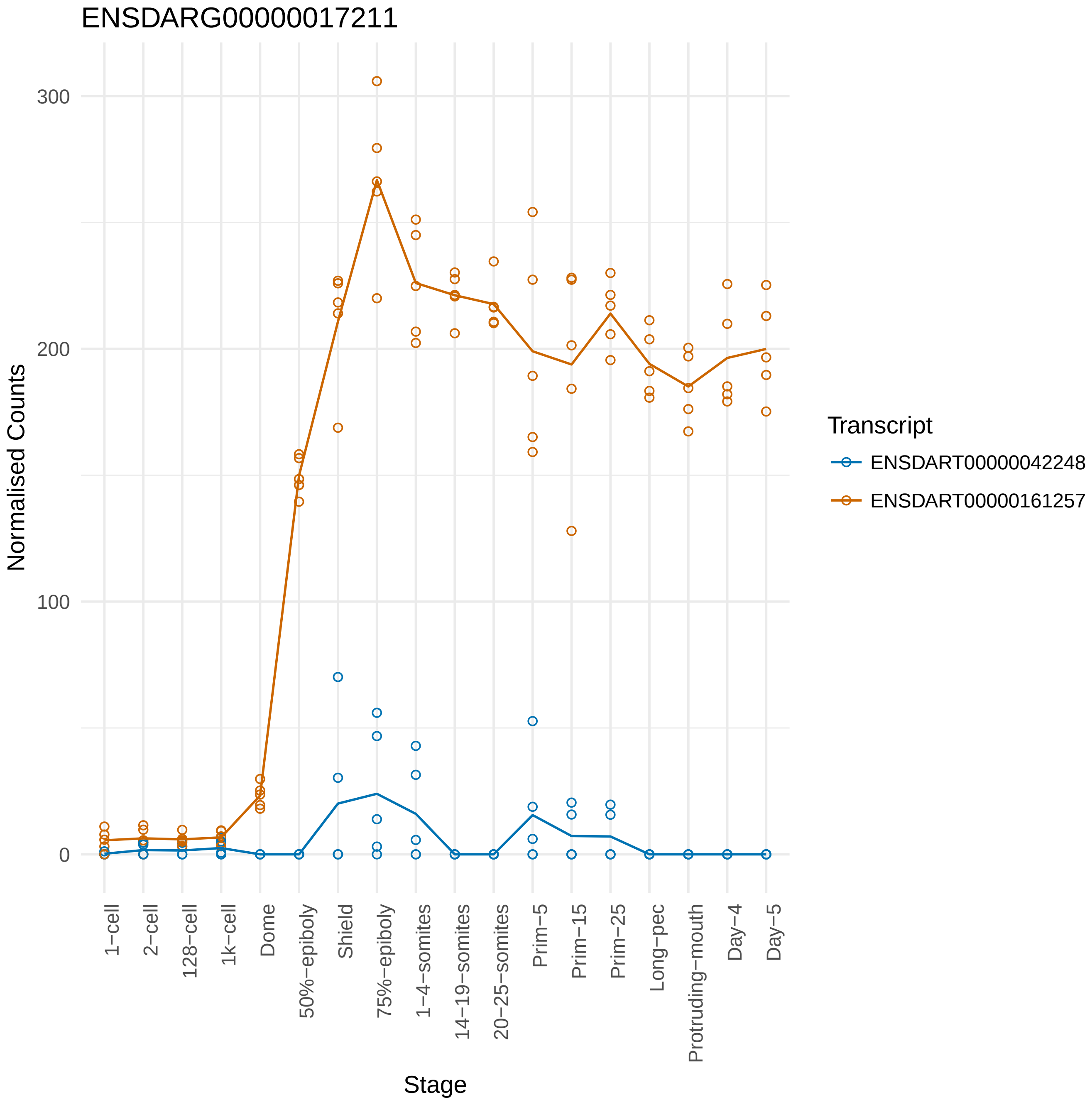

ENSDARG00000017211
Normalised Counts
300
200
100
0
1−cell
2−cell
128−cell
1k−cell
Dome
50%−epiboly
Shield
75%−epiboly
1−4−somites
14−19−somites
20−25−somites
Prim−5
Prim−15
Prim−25
Long−pec
Protruding−mouth
Day−4
Day−5
Stage
Transcript
ENSDART00000042248
ENSDART00000161257

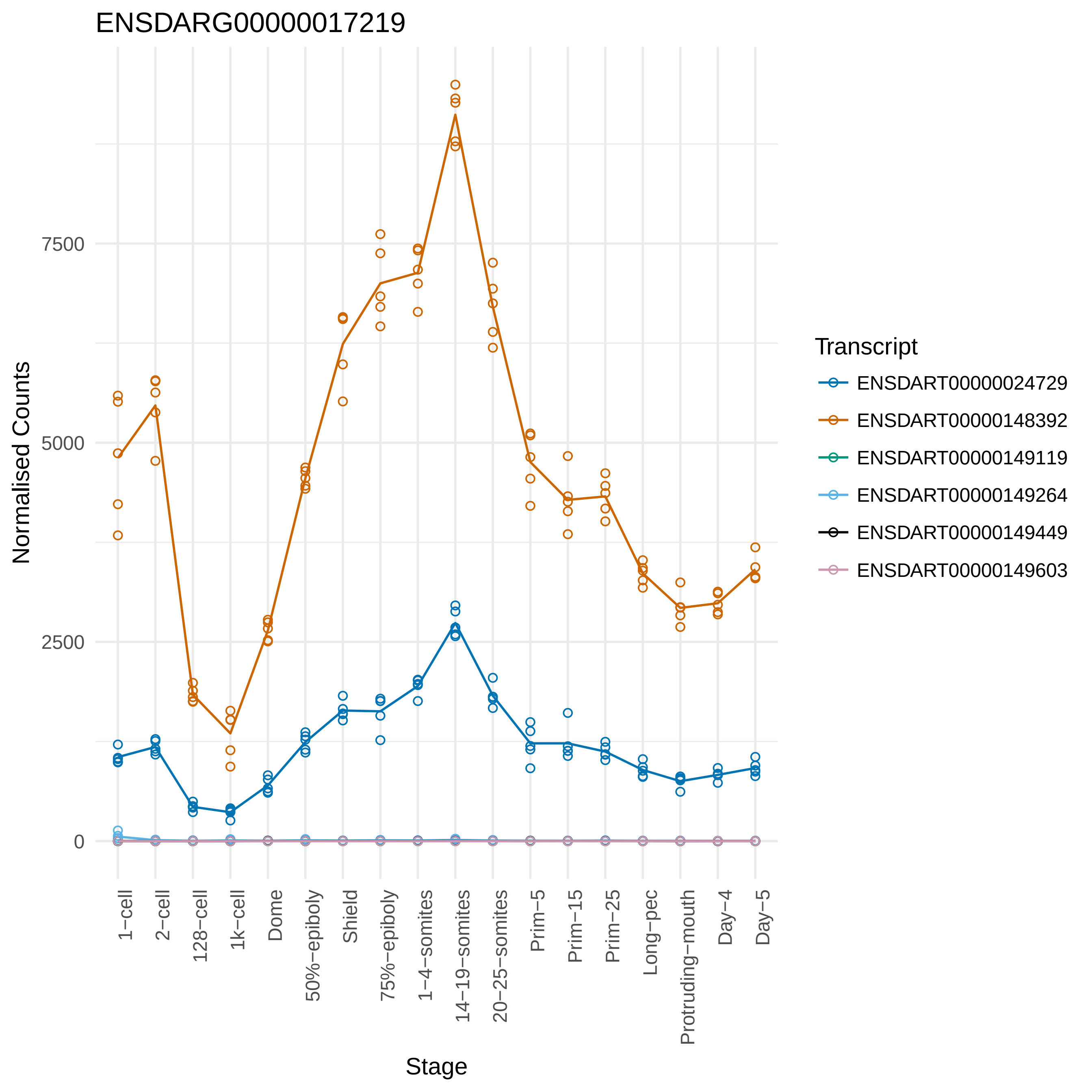

ENSDARG00000017219
Normalised Counts
7500
5000
2500
0
1−cell
2−cell
128−cell
1k−cell
Dome
50%−epiboly
Shield
75%−epiboly
1−4−somites
14−19−somites
20−25−somites
Prim−5
Prim−15
Prim−25
Long−pec
Protruding−mouth
Day−4
Day−5
Stage
Transcript
ENSDART00000024729
ENSDART00000148392
ENSDART00000149119
ENSDART00000149264
ENSDART00000149449
ENSDART00000149603

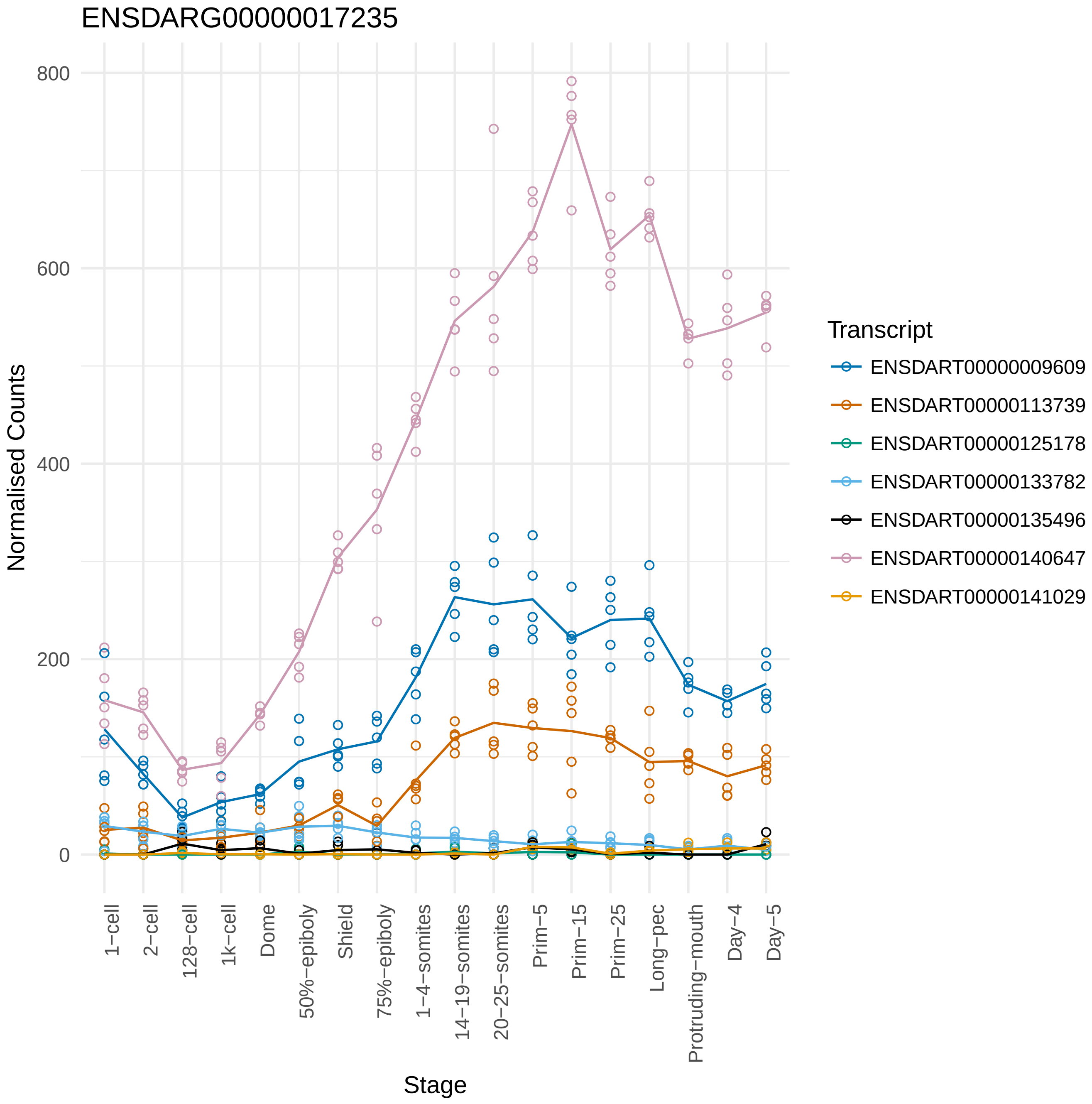

ENSDARG00000017235
Normalised Counts
800
600
400
200
0
1−cell
2−cell
128−cell
1k−cell
Dome
50%−epiboly
Shield
75%−epiboly
1−4−somites
14−19−somites
20−25−somites
Prim−5
Prim−15
Prim−25
Long−pec
Protruding−mouth
Day−4
Day−5
Stage
Transcript
ENSDART00000009609
ENSDART00000113739
ENSDART00000125178
ENSDART00000133782
ENSDART00000135496
ENSDART00000140647
ENSDART00000141029

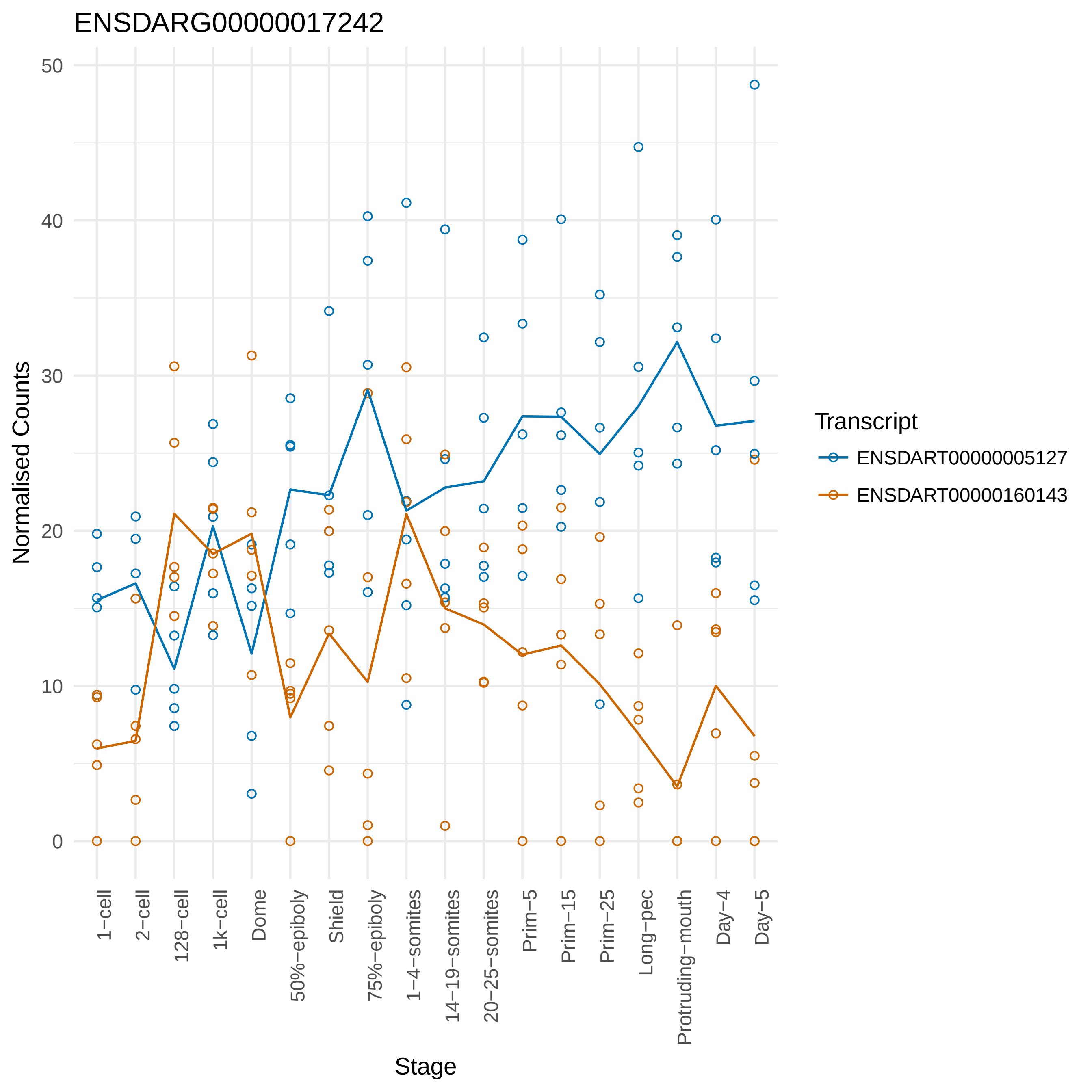

ENSDARG00000017242
Normalised Counts
0
10
20
30
40
50
1−cell
2−cell
128−cell
1k−cell
Dome
50%−epiboly
Shield
75%−epiboly
1−4−somites
14−19−somites
20−25−somites
Prim−5
Prim−15
Prim−25
Long−pec
Protruding−mouth
Day−4
Day−5
Stage
Transcript
ENSDART00000005127
ENSDART00000160143

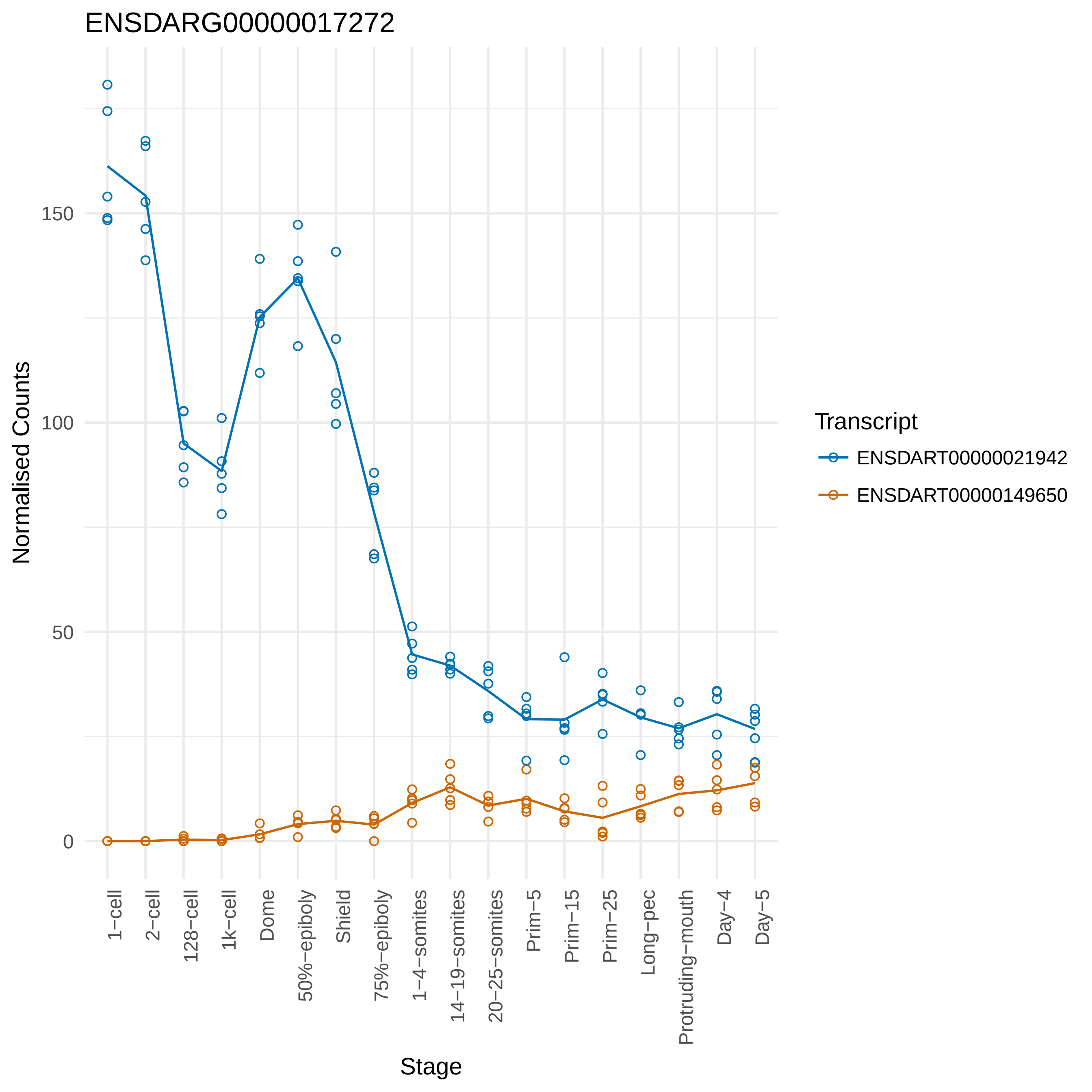
ENSDARG00000017272
Normalised Counts
0
50
100
150
1−cell
2−cell
128−cell
1k−cell
Dome
50%−epiboly
Shield
75%−epiboly
1−4−somites
14−19−somites
20−25−somites
Prim−5
Prim−15
Prim−25
Long−pec
Protruding−mouth
Day−4
Day−5
Stage
Transcript
ENSDART00000021942
ENSDART00000149650

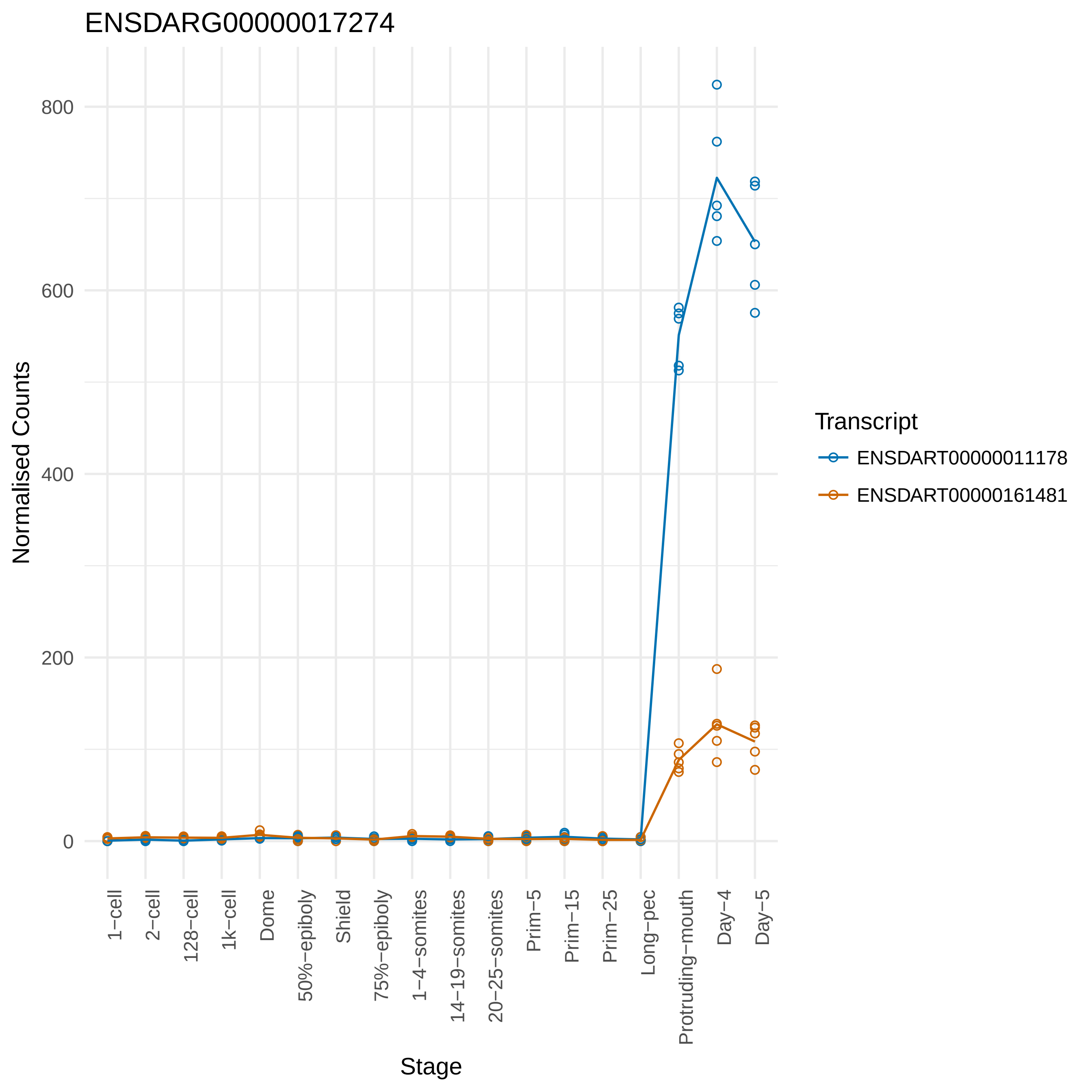
ENSDARG00000017274
Normalised Counts
800
600
400
200
0
1−cell
2−cell
128−cell
1k−cell
Dome
50%−epiboly
Shield
75%−epiboly
1−4−somites
14−19−somites
20−25−somites
Prim−5
Prim−15
Prim−25
Long−pec
Protruding−mouth
Day−4
Day−5
Stage
Transcript
ENSDART00000011178
ENSDART00000161481

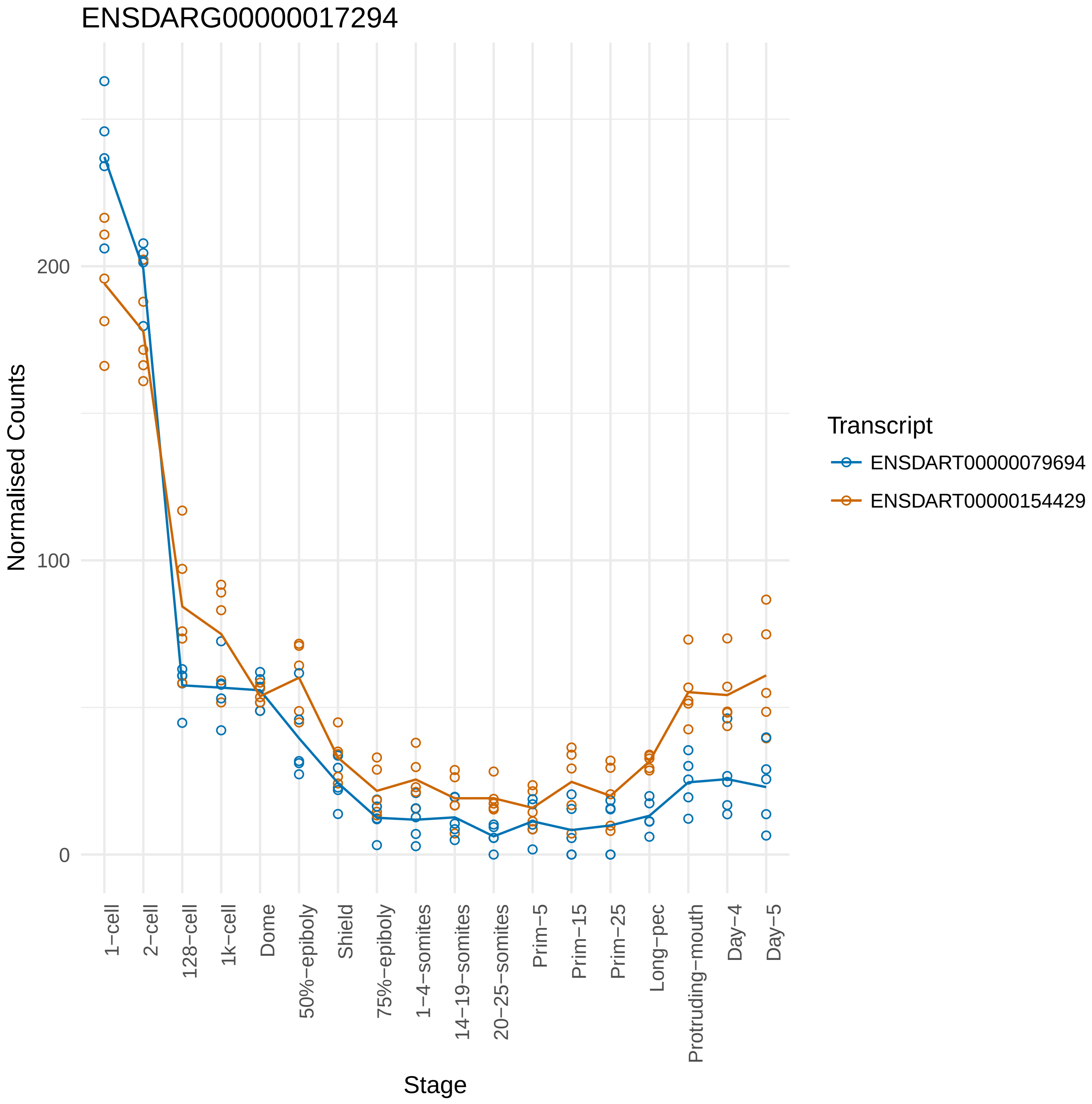

ENSDARG00000017294
Normalised Counts
200
100
0
1−cell
2−cell
128−cell
1k−cell
Dome
50%−epiboly
Shield
75%−epiboly
1−4−somites
14−19−somites
20−25−somites
Prim−5
Prim−15
Prim−25
Long−pec
Protruding−mouth
Day−4
Day−5
Stage
Transcript
ENSDART00000079694
ENSDART00000154429

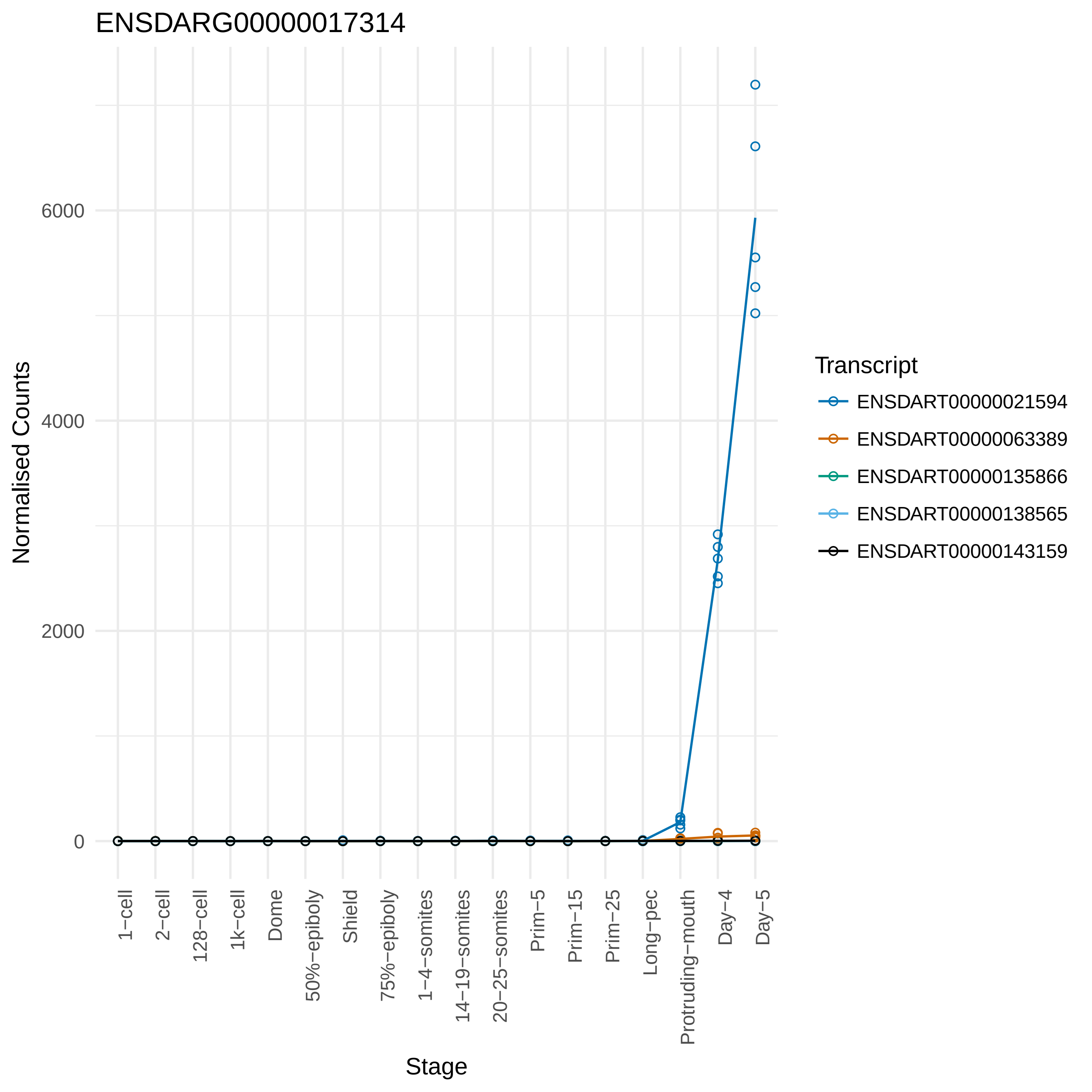

ENSDARG00000017314
Normalised Counts
6000
4000
2000
0
1−cell
2−cell
128−cell
1k−cell
Dome
50%−epiboly
Shield
75%−epiboly
1−4−somites
14−19−somites
20−25−somites
Prim−5
Prim−15
Prim−25
Long−pec
Protruding−mouth
Day−4
Day−5
Stage
Transcript
ENSDART00000021594
ENSDART00000063389
ENSDART00000135866
ENSDART00000138565
ENSDART00000143159

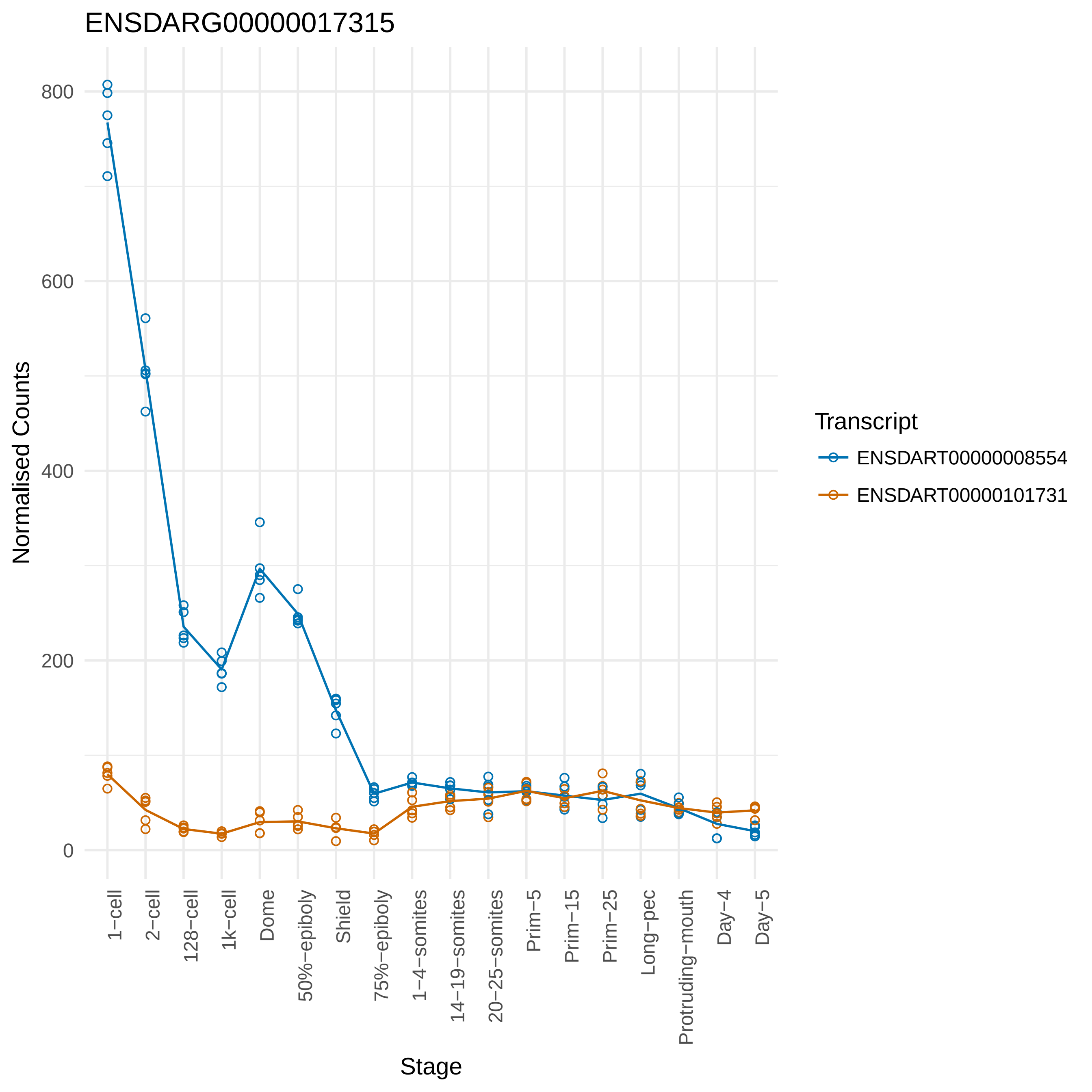

ENSDARG00000017315
Normalised Counts
800
600
400
200
0
1−cell
2−cell
128−cell
1k−cell
Dome
50%−epiboly
Shield
75%−epiboly
1−4−somites
14−19−somites
20−25−somites
Prim−5
Prim−15
Prim−25
Long−pec
Protruding−mouth
Day−4
Day−5
Stage
Transcript
ENSDART00000008554
ENSDART00000101731

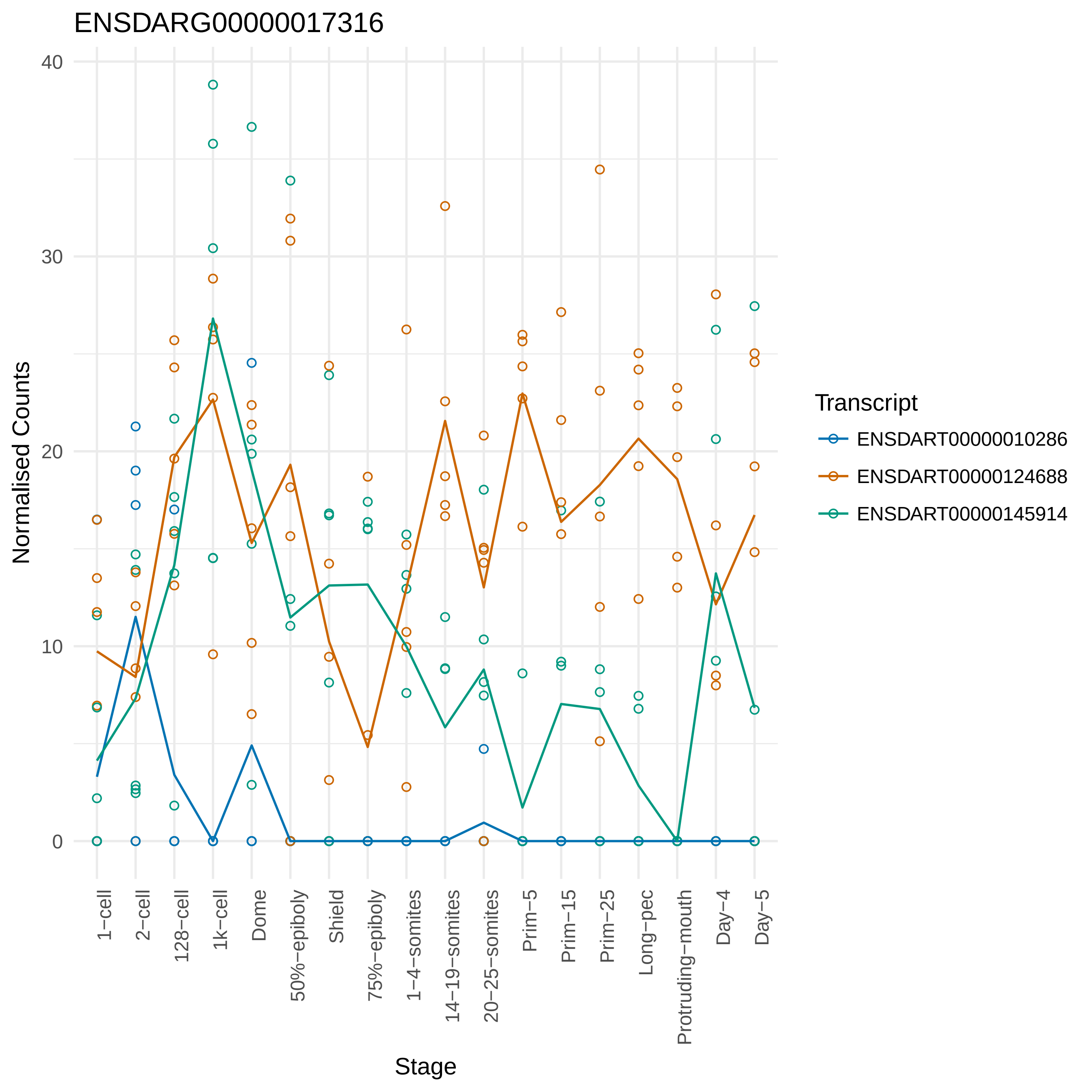
ENSDARG00000017316
Normalised Counts
40
30
20
10
0
Stage
1−cell
2−cell
128−cell
1k−cell
Dome
50%−epiboly
Shield
75%−epiboly
1−4−somites
14−19−somites
20−25−somites
Prim−5
Prim−15
Prim−25
Long−pec
Protruding−mouth
Day−4
Day−5
Transcript
ENSDART00000010286
ENSDART00000124688
ENSDART00000145914

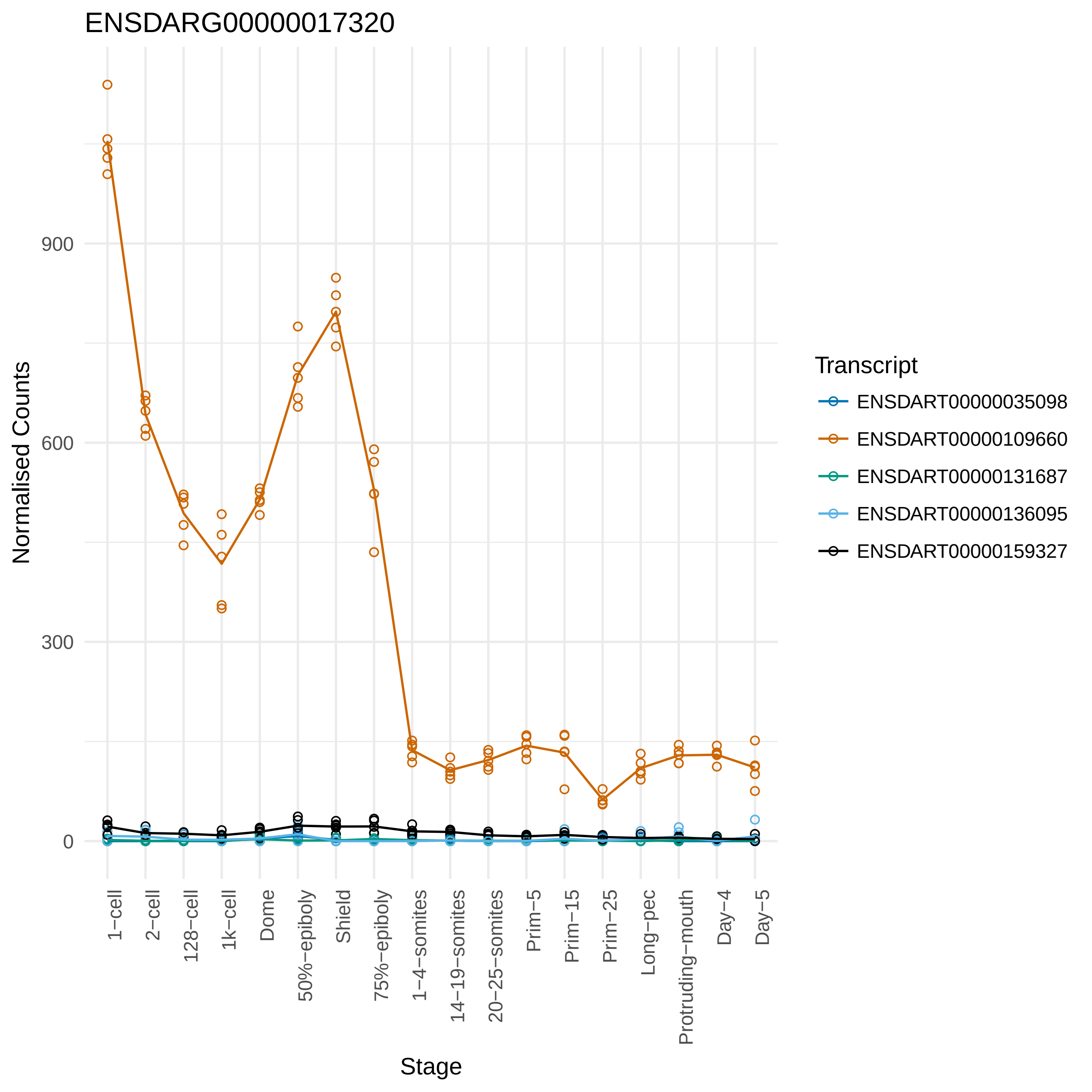
ENSDARG00000017320
Normalised Counts
900
600
300
0
1−cell
2−cell
128−cell
1k−cell
Dome
50%−epiboly
Shield
75%−epiboly
1−4−somites
14−19−somites
20−25−somites
Prim−5
Prim−15
Prim−25
Long−pec
Protruding−mouth
Day−4
Day−5
Stage
Transcript
ENSDART00000035098
ENSDART00000109660
ENSDART00000131687
ENSDART00000136095
ENSDART00000159327

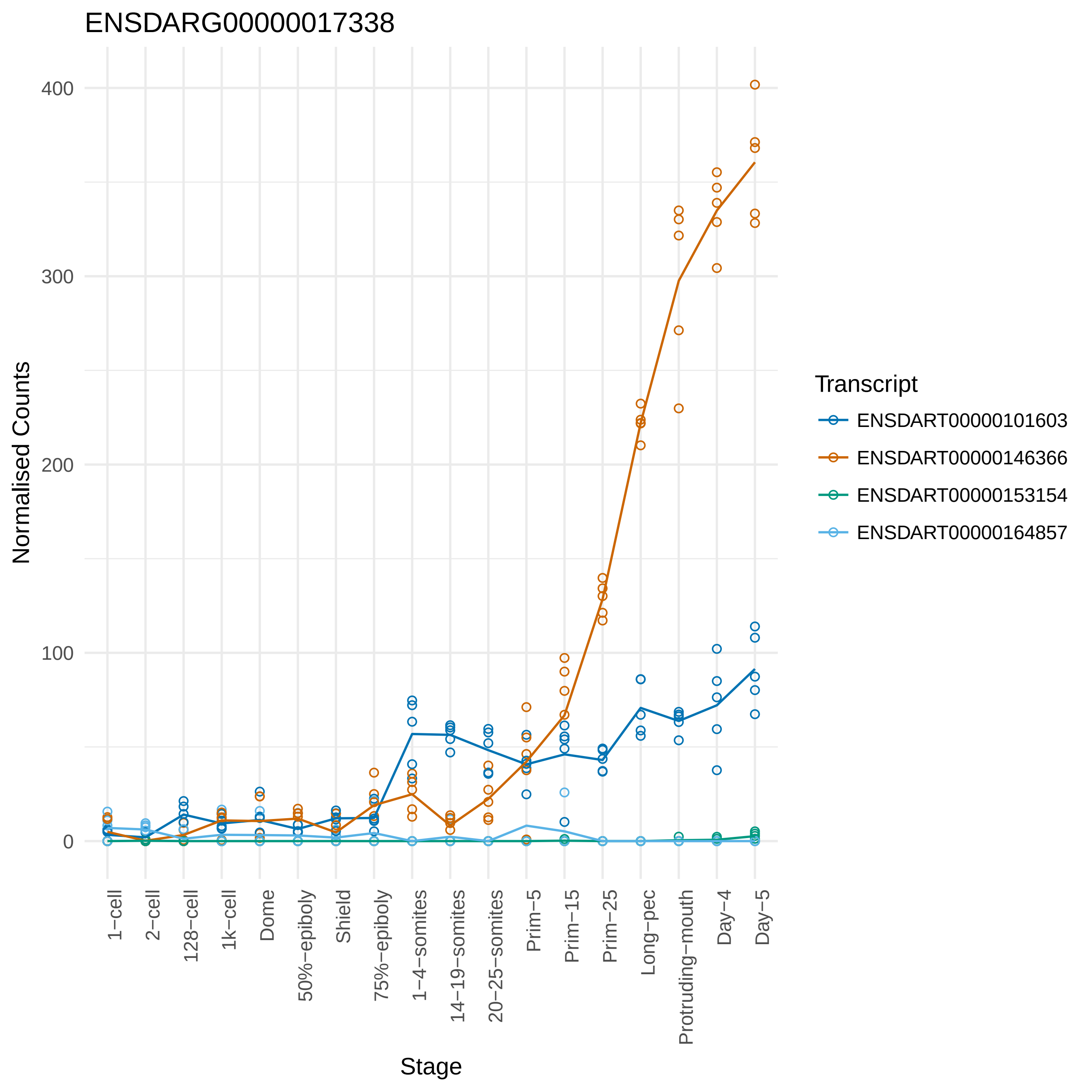
ENSDARG00000017338
Normalised Counts
400
300
200
100
0
1−cell
2−cell
128−cell
1k−cell
Dome
50%−epiboly
Shield
75%−epiboly
1−4−somites
14−19−somites
20−25−somites
Prim−5
Prim−15
Prim−25
Long−pec
Protruding−mouth
Day−4
Day−5
Stage
Transcript
ENSDART00000101603
ENSDART00000146366
ENSDART00000153154
ENSDART00000164857

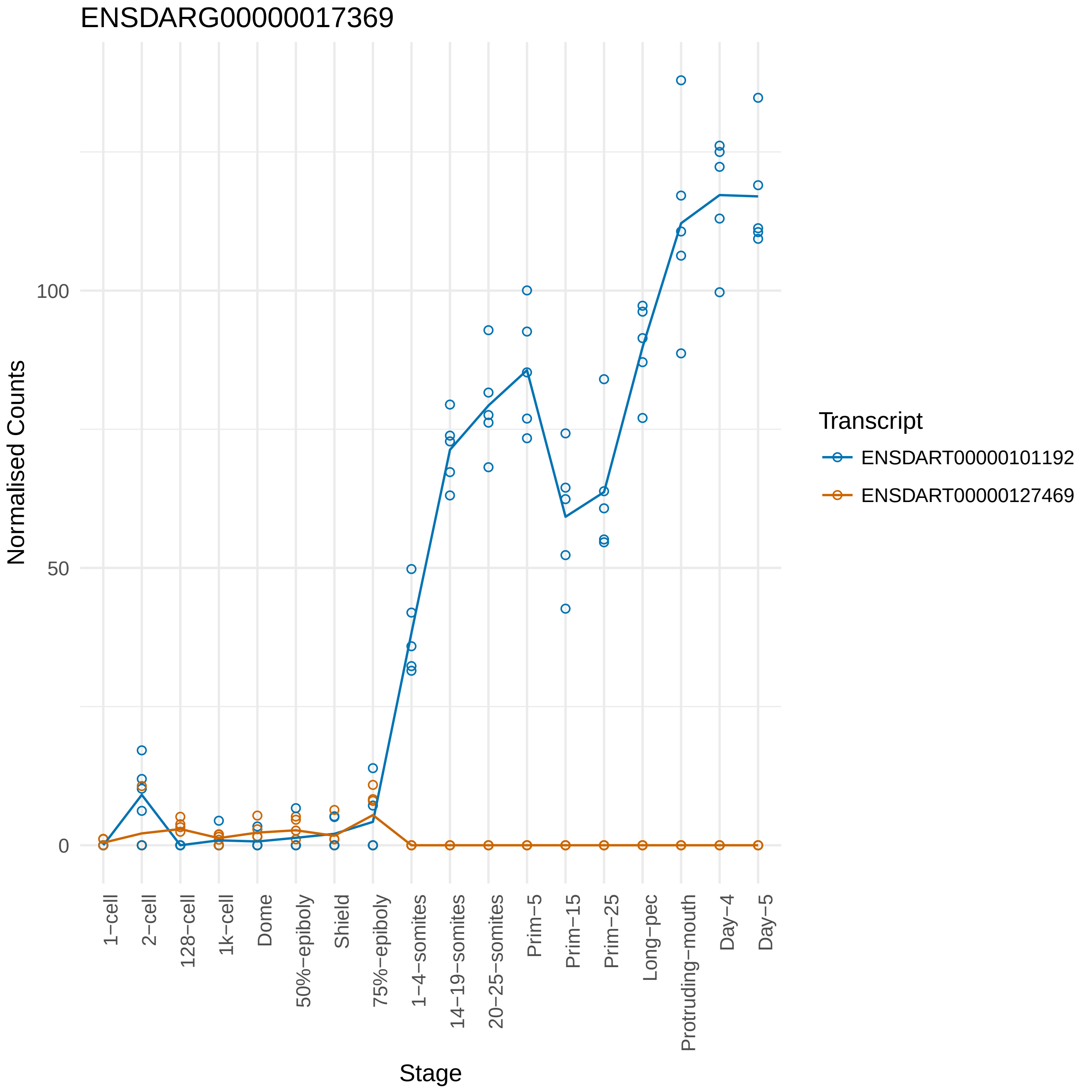
ENSDARG00000017369
Normalised Counts
0
50
100
Stage
1−cell
2−cell
128−cell
1k−cell
Dome
50%−epiboly
Shield
75%−epiboly
1−4−somites
14−19−somites
20−25−somites
Prim−5
Prim−15
Prim−25
Long−pec
Protruding−mouth
Day−4
Day−5
Transcript
ENSDART00000101192
ENSDART00000127469

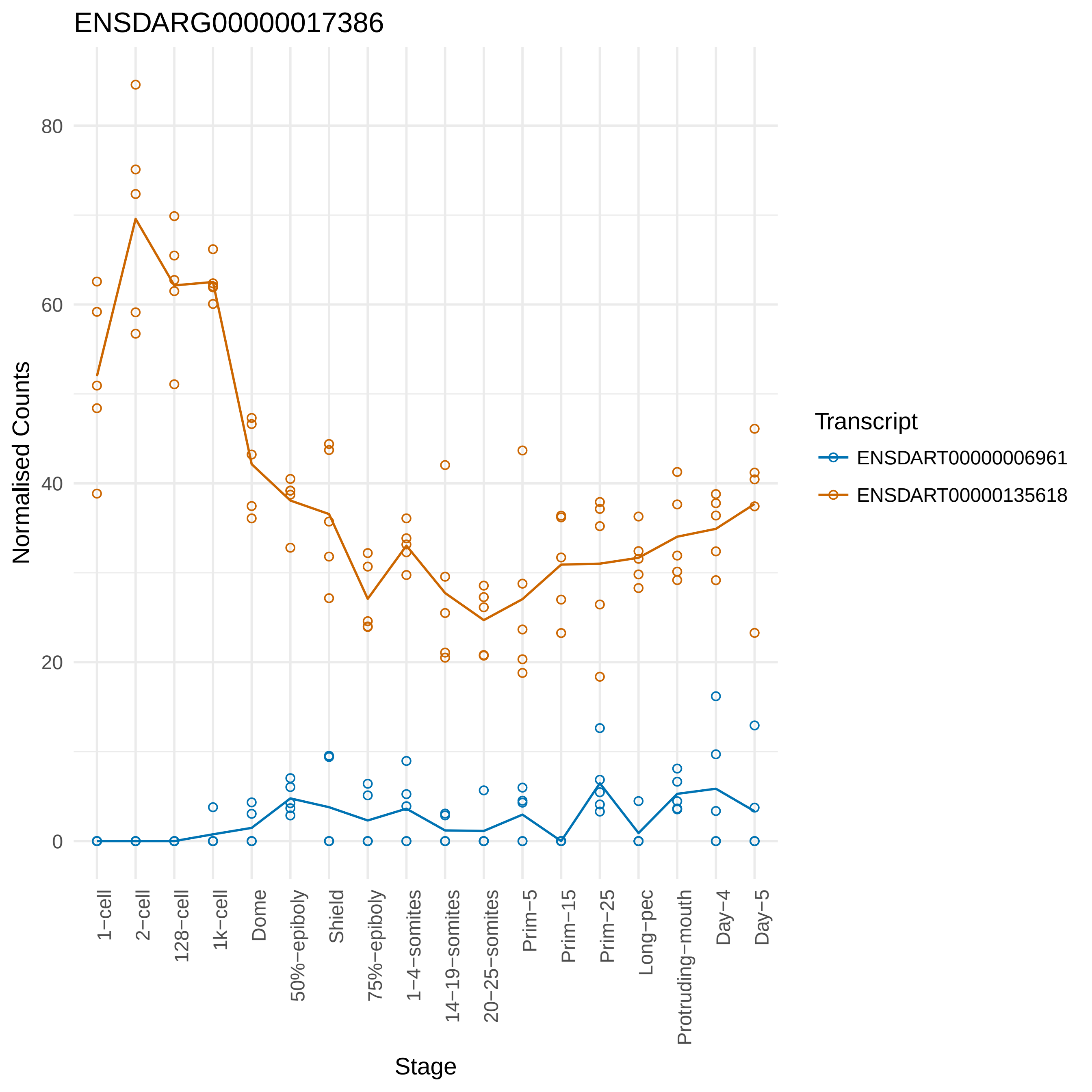

ENSDARG00000017386
Normalised Counts
0
20
40
60
80
Stage
1−cell
2−cell
128−cell
1k−cell
Dome
50%−epiboly
Shield
75%−epiboly
1−4−somites
14−19−somites
20−25−somites
Prim−5
Prim−15
Prim−25
Long−pec
Protruding−mouth
Day−4
Day−5
Transcript
ENSDART00000006961
ENSDART00000135618

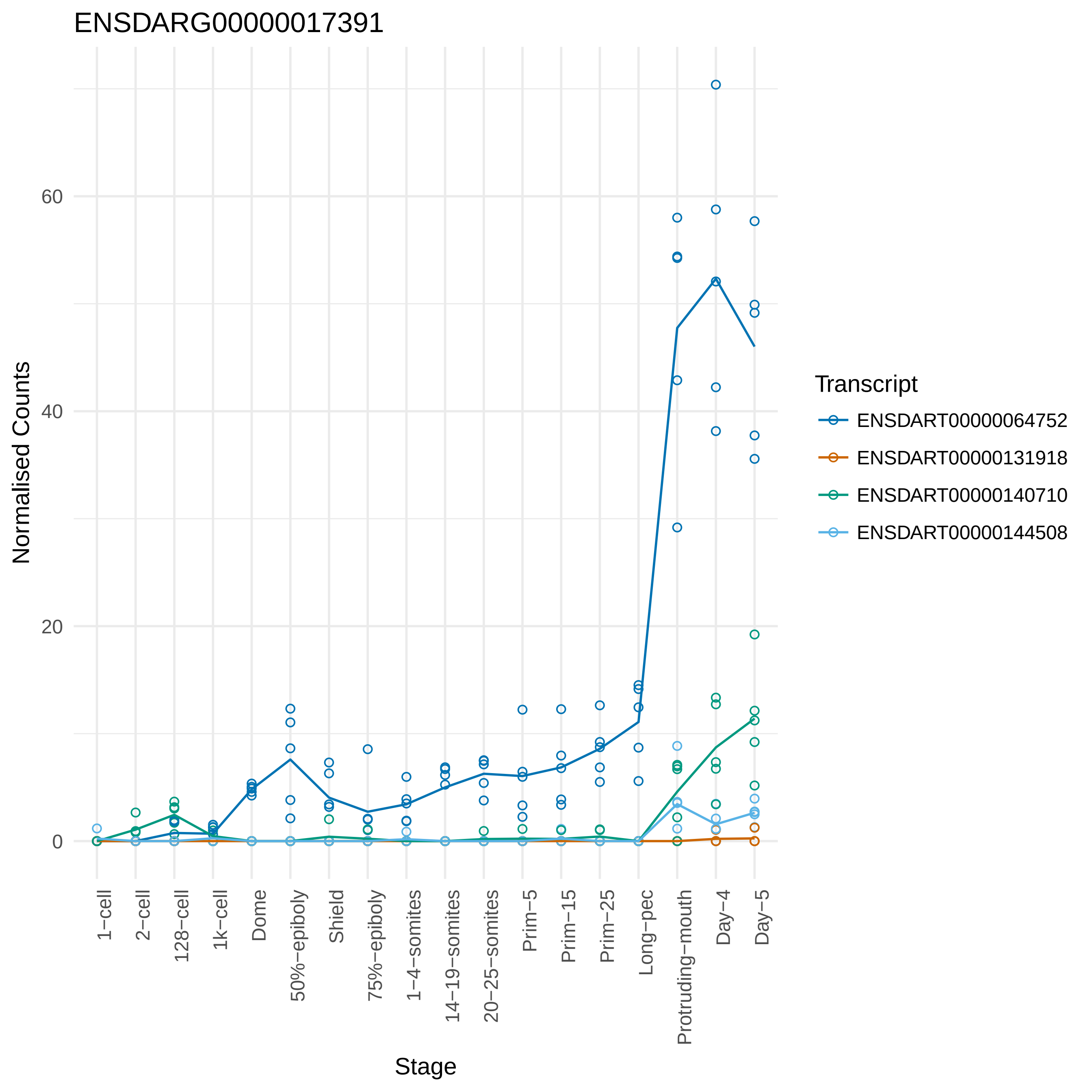
ENSDARG00000017391
Normalised Counts
60
40
20
0
1−cell
2−cell
128−cell
1k−cell
Dome
50%−epiboly
Shield
75%−epiboly
1−4−somites
14−19−somites
20−25−somites
Prim−5
Prim−15
Prim−25
Long−pec
Protruding−mouth
Day−4
Day−5
Stage
Transcript
ENSDART00000064752
ENSDART00000131918
ENSDART00000140710
ENSDART00000144508

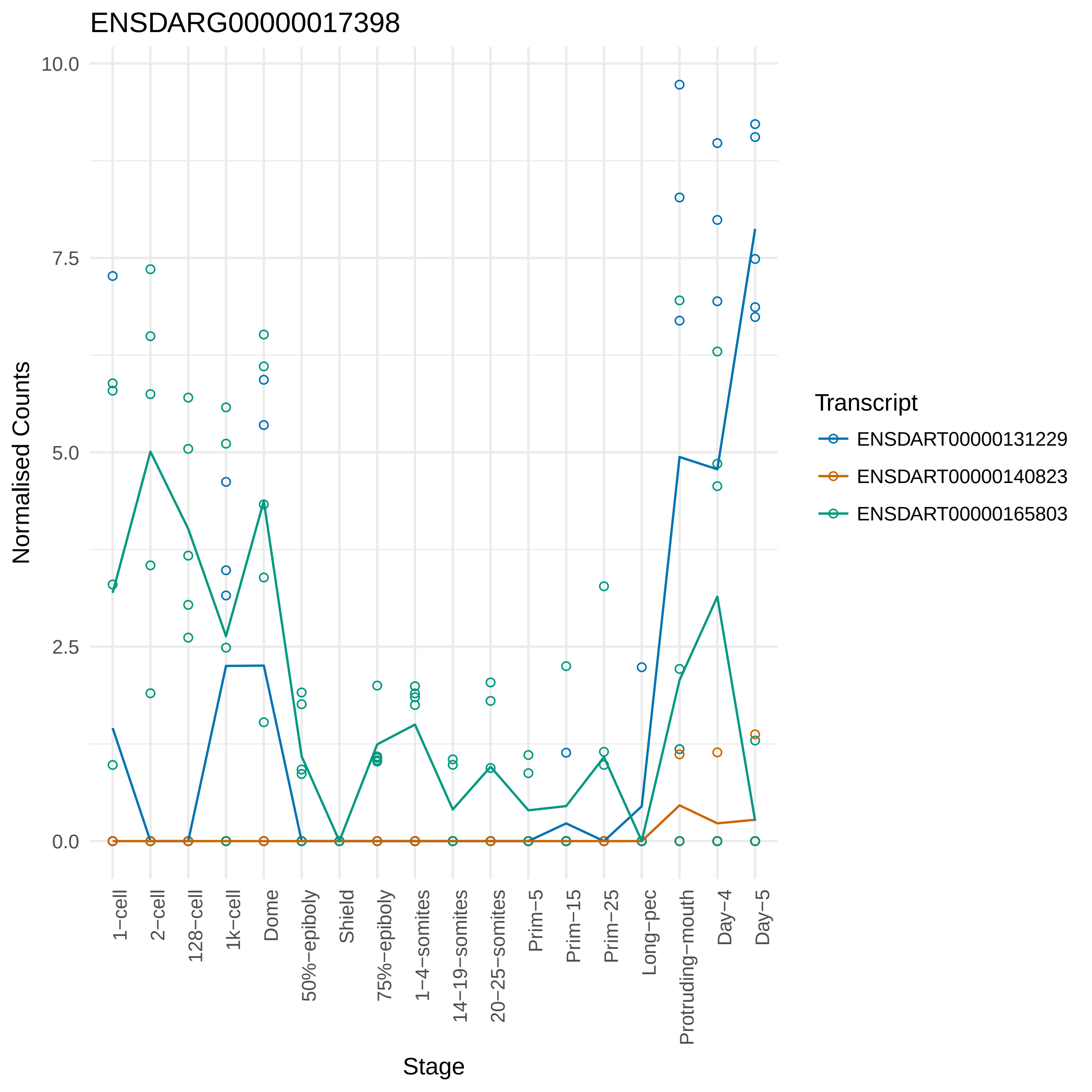

ENSDARG00000017398
Normalised Counts
10.0
7.5
5.0
2.5
0.0
1−cell
2−cell
128−cell
1k−cell
Dome
50%−epiboly
Shield
75%−epiboly
1−4−somites
14−19−somites
20−25−somites
Prim−5
Prim−15
Prim−25
Long−pec
Protruding−mouth
Day−4
Day−5
Stage
Transcript
ENSDART00000131229
ENSDART00000140823
ENSDART00000165803

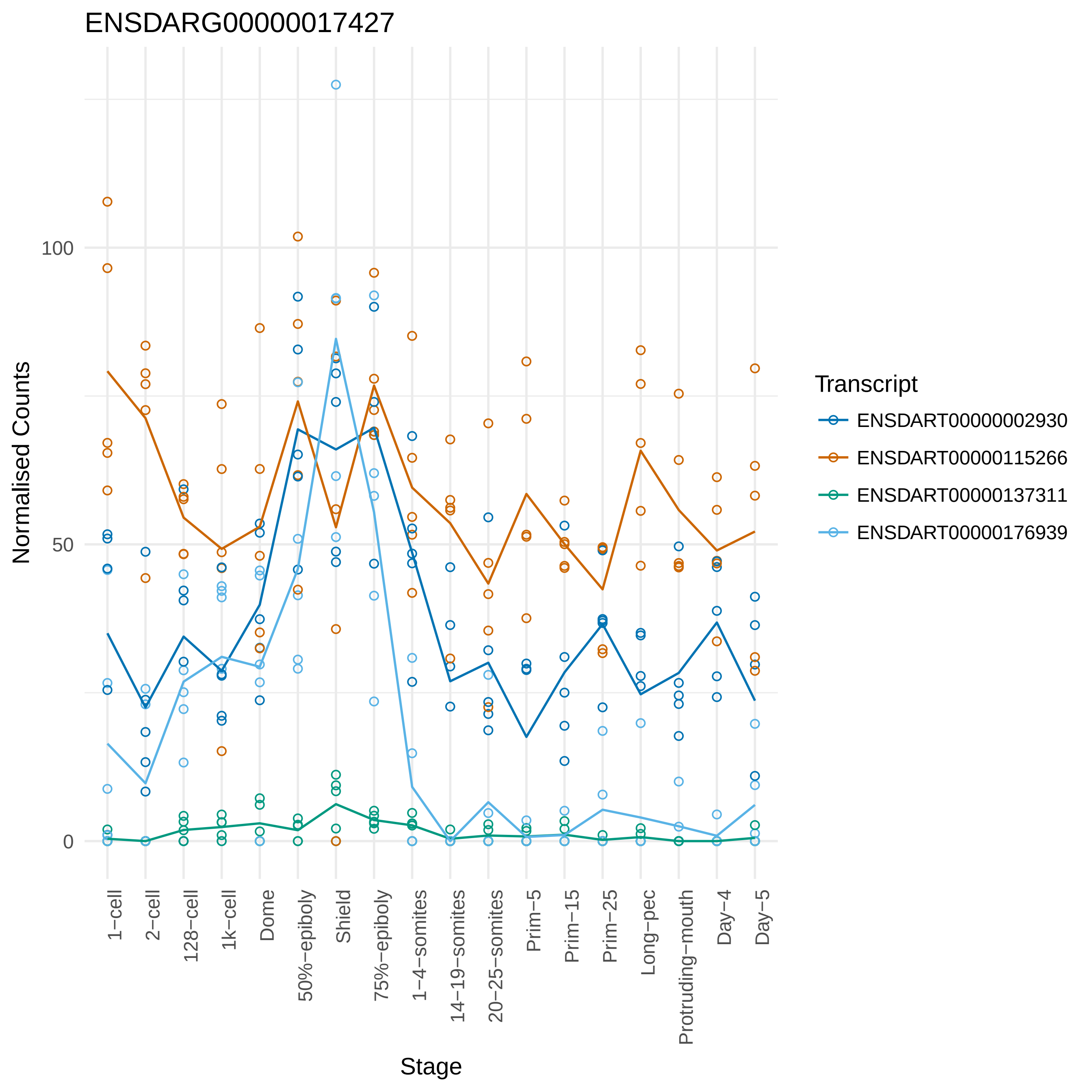

ENSDARG00000017427
Normalised Counts
100
50
0
1−cell
2−cell
128−cell
1k−cell
Dome
50%−epiboly
Shield
75%−epiboly
1−4−somites
14−19−somites
20−25−somites
Prim−5
Prim−15
Prim−25
Long−pec
Protruding−mouth
Day−4
Day−5
Stage
Transcript
ENSDART00000002930
ENSDART00000115266
ENSDART00000137311
ENSDART00000176939

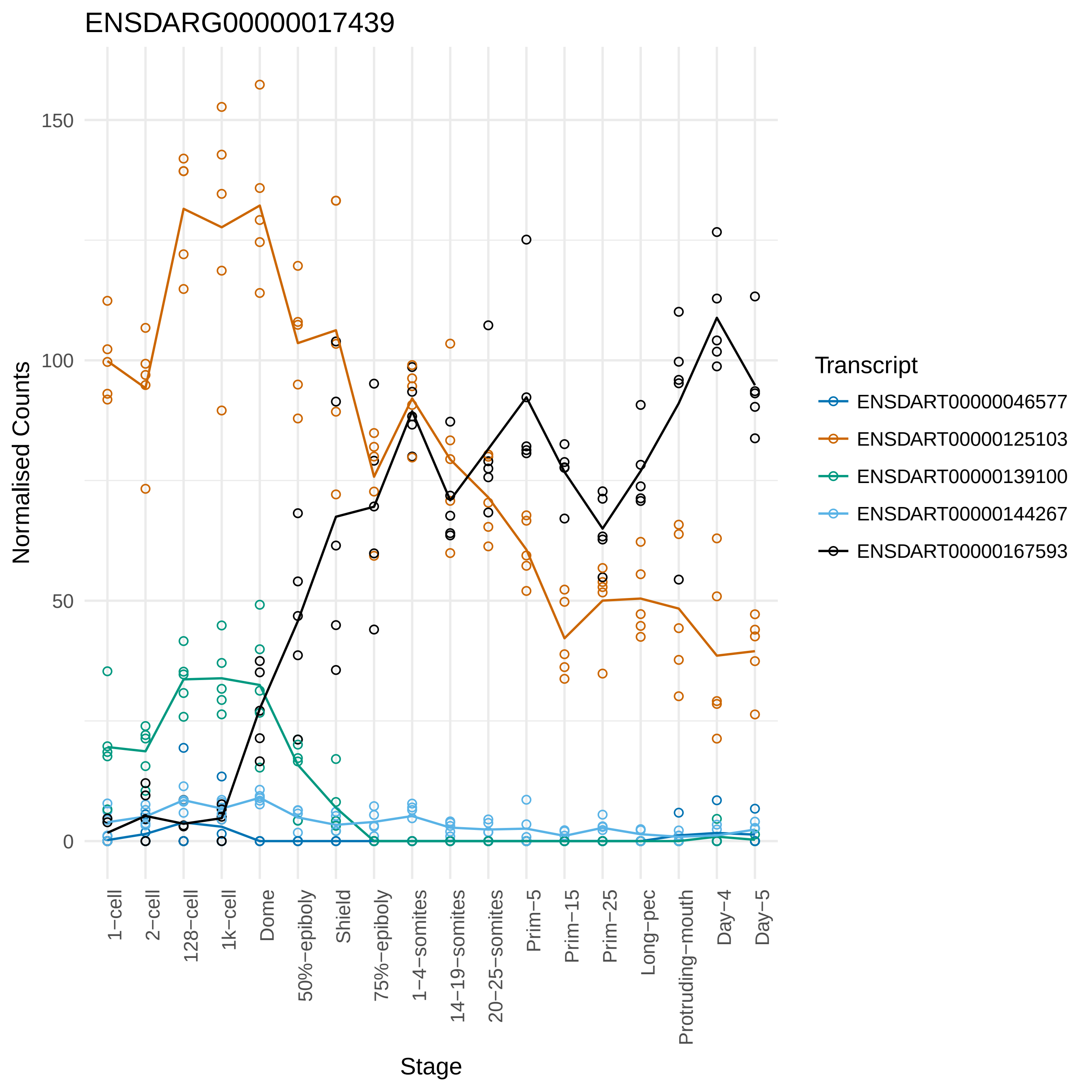
ENSDARG00000017439
Normalised Counts
150
100
50
0
1−cell
2−cell
128−cell
1k−cell
Dome
50%−epiboly
Shield
75%−epiboly
1−4−somites
14−19−somites
20−25−somites
Prim−5
Prim−15
Prim−25
Long−pec
Protruding−mouth
Day−4
Day−5
Stage
Transcript
ENSDART00000046577
ENSDART00000125103
ENSDART00000139100
ENSDART00000144267
ENSDART00000167593

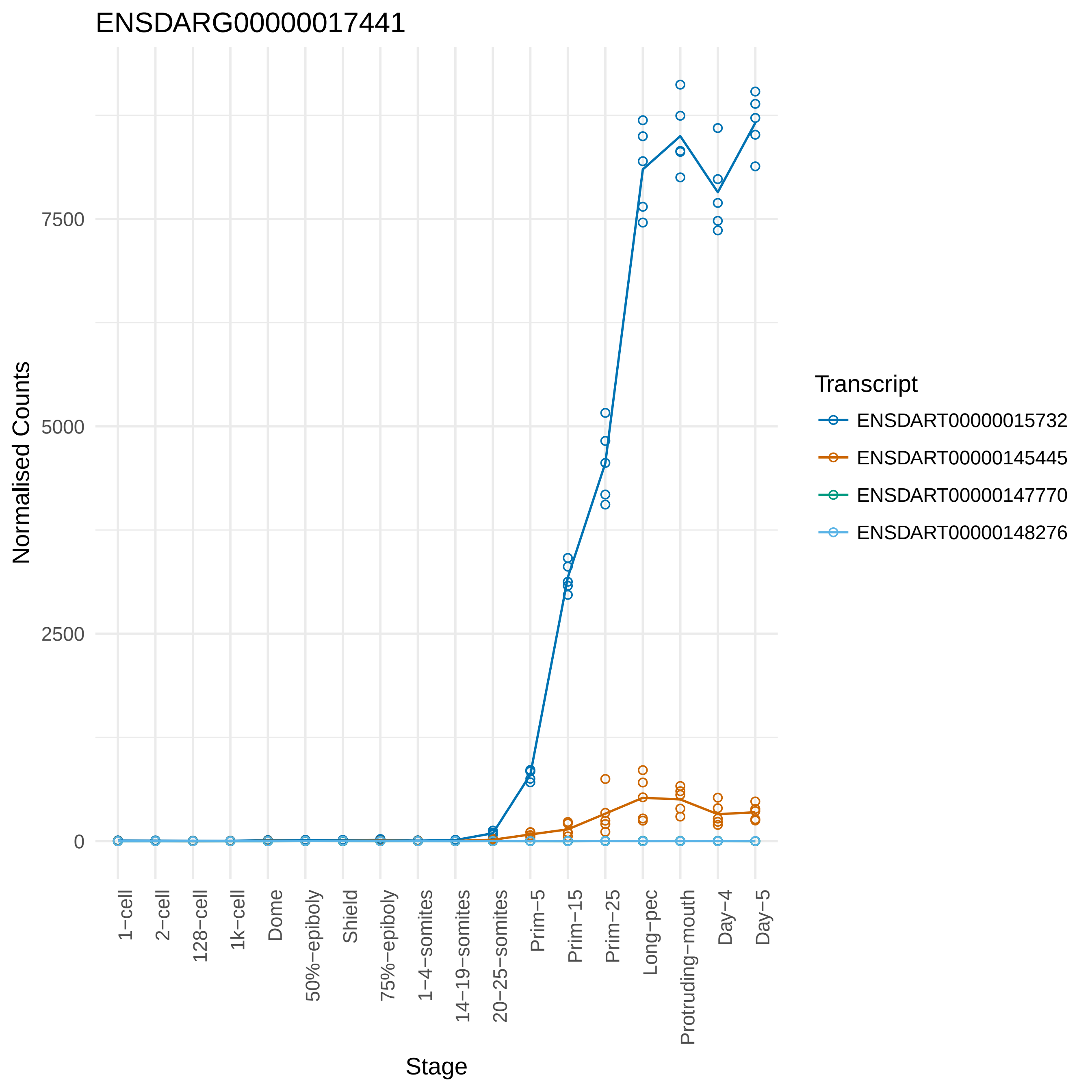

ENSDARG00000017441
Normalised Counts
0
2500
5000
7500
Stage
1−cell
2−cell
128−cell
1k−cell
Dome
50%−epiboly
Shield
75%−epiboly
1−4−somites
14−19−somites
20−25−somites
Prim−5
Prim−15
Prim−25
Long−pec
Protruding−mouth
Day−4
Day−5
Transcript
ENSDART00000015732
ENSDART00000145445
ENSDART00000147770
ENSDART00000148276

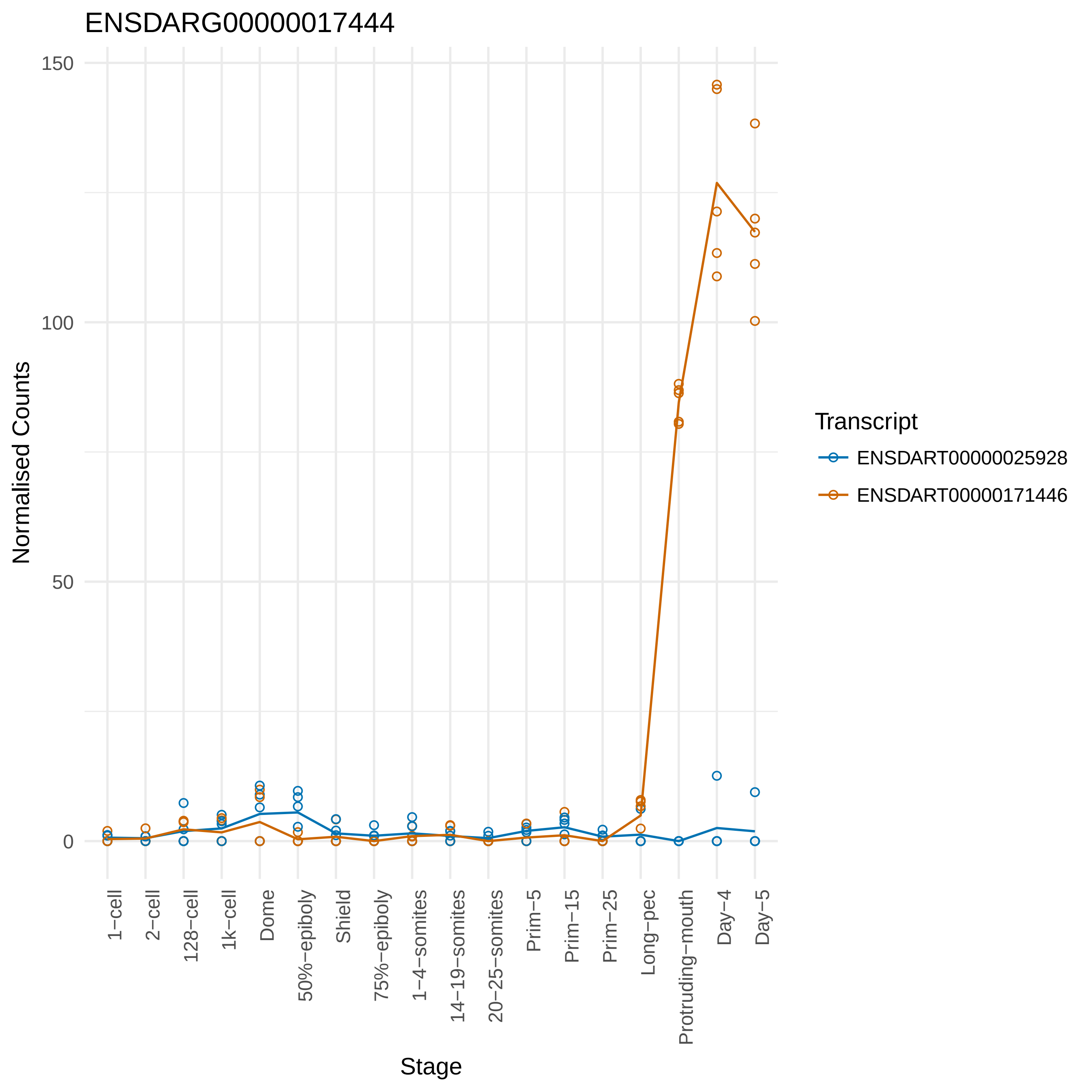

ENSDARG00000017444
Normalised Counts
0
50
100
150
Stage
1−cell
2−cell
128−cell
1k−cell
Dome
50%−epiboly
Shield
75%−epiboly
1−4−somites
14−19−somites
20−25−somites
Prim−5
Prim−15
Prim−25
Long−pec
Protruding−mouth
Day−4
Day−5
Transcript
ENSDART00000025928
ENSDART00000171446

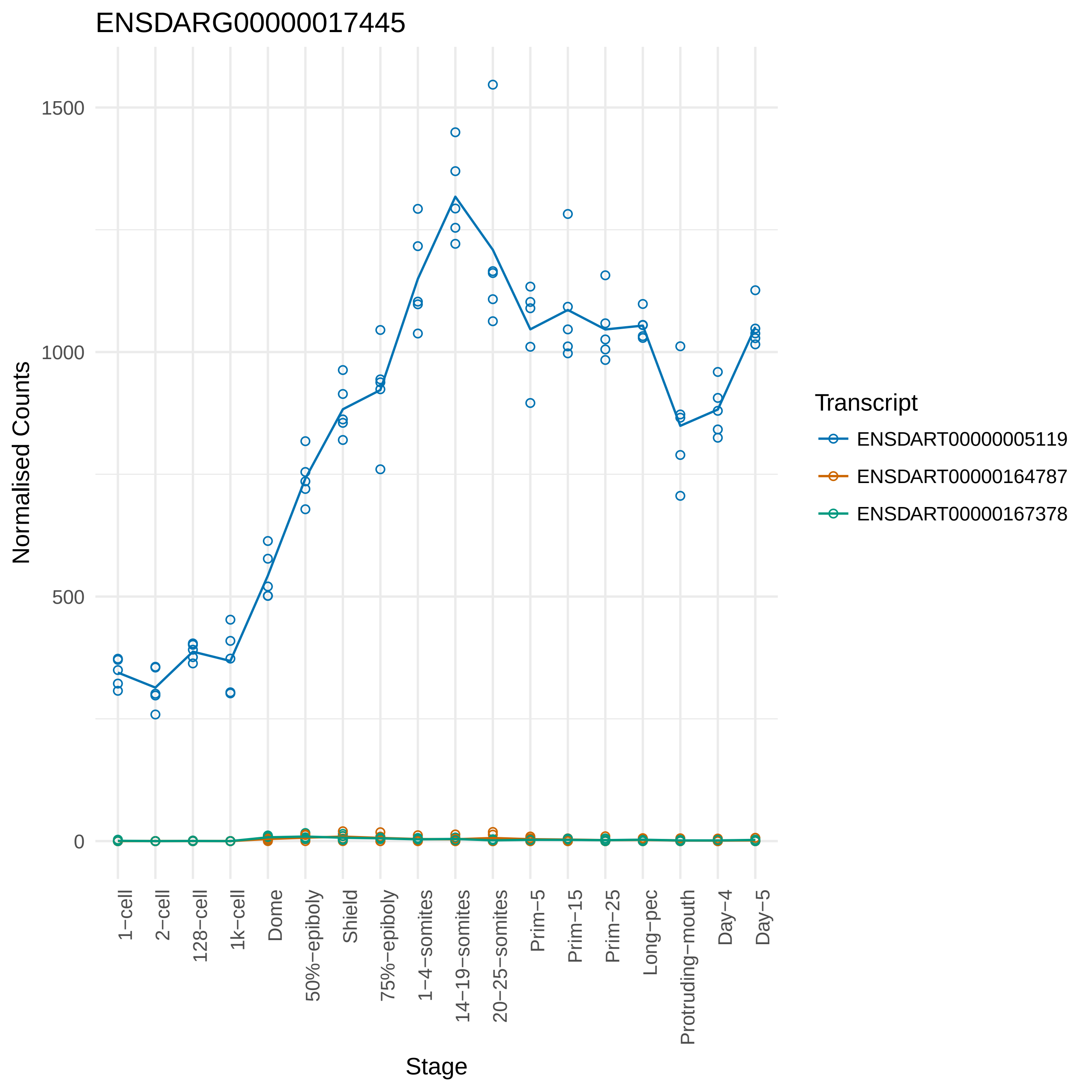

ENSDARG00000017445
Normalised Counts
1500
1000
500
0
1−cell
2−cell
128−cell
1k−cell
Dome
50%−epiboly
Shield
75%−epiboly
1−4−somites
14−19−somites
20−25−somites
Prim−5
Prim−15
Prim−25
Long−pec
Protruding−mouth
Day−4
Day−5
Stage
Transcript
ENSDART00000005119
ENSDART00000164787
ENSDART00000167378

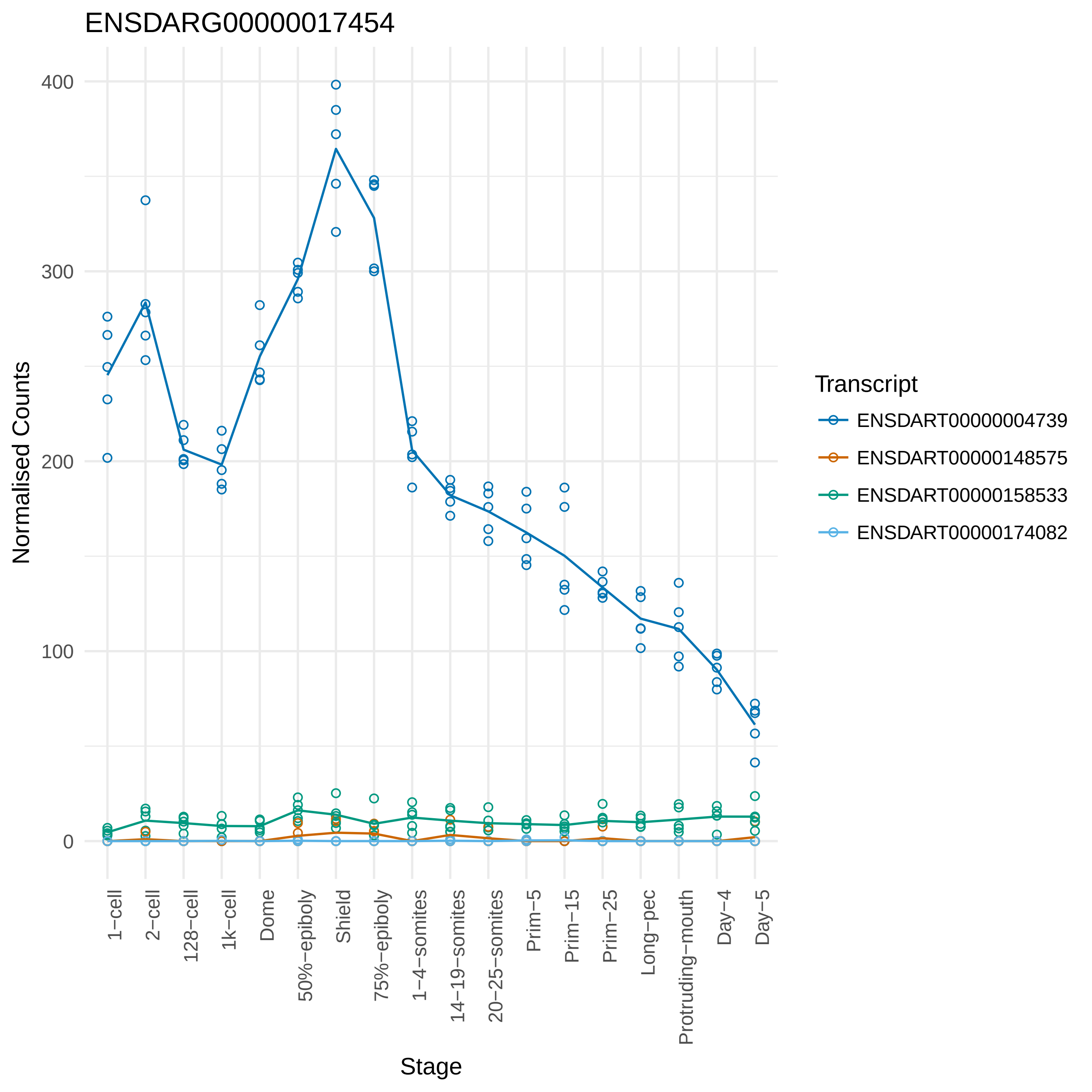
ENSDARG00000017454
Normalised Counts
400
300
200
100
0
Stage
1−cell
2−cell
128−cell
1k−cell
Dome
50%−epiboly
Shield
75%−epiboly
1−4−somites
14−19−somites
20−25−somites
Prim−5
Prim−15
Prim−25
Long−pec
Protruding−mouth
Day−4
Day−5
Transcript
ENSDART00000004739
ENSDART00000148575
ENSDART00000158533
ENSDART00000174082

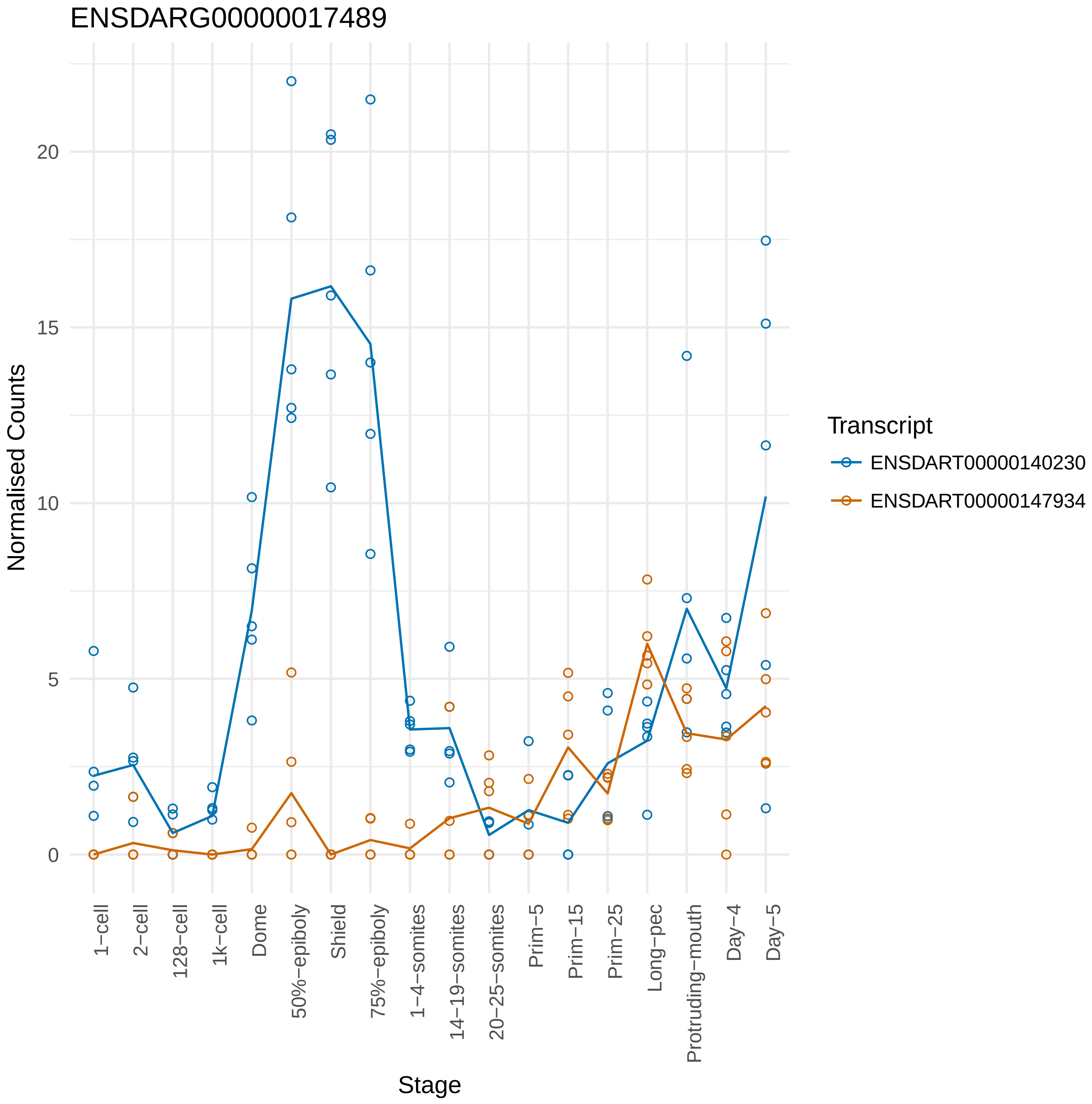

ENSDARG00000017489
Normalised Counts
0
5
10
15
20
1−cell
2−cell
128−cell
1k−cell
Dome
50%−epiboly
Shield
75%−epiboly
1−4−somites
14−19−somites
20−25−somites
Prim−5
Prim−15
Prim−25
Long−pec
Protruding−mouth
Day−4
Day−5
Stage
Transcript
ENSDART00000140230
ENSDART00000147934

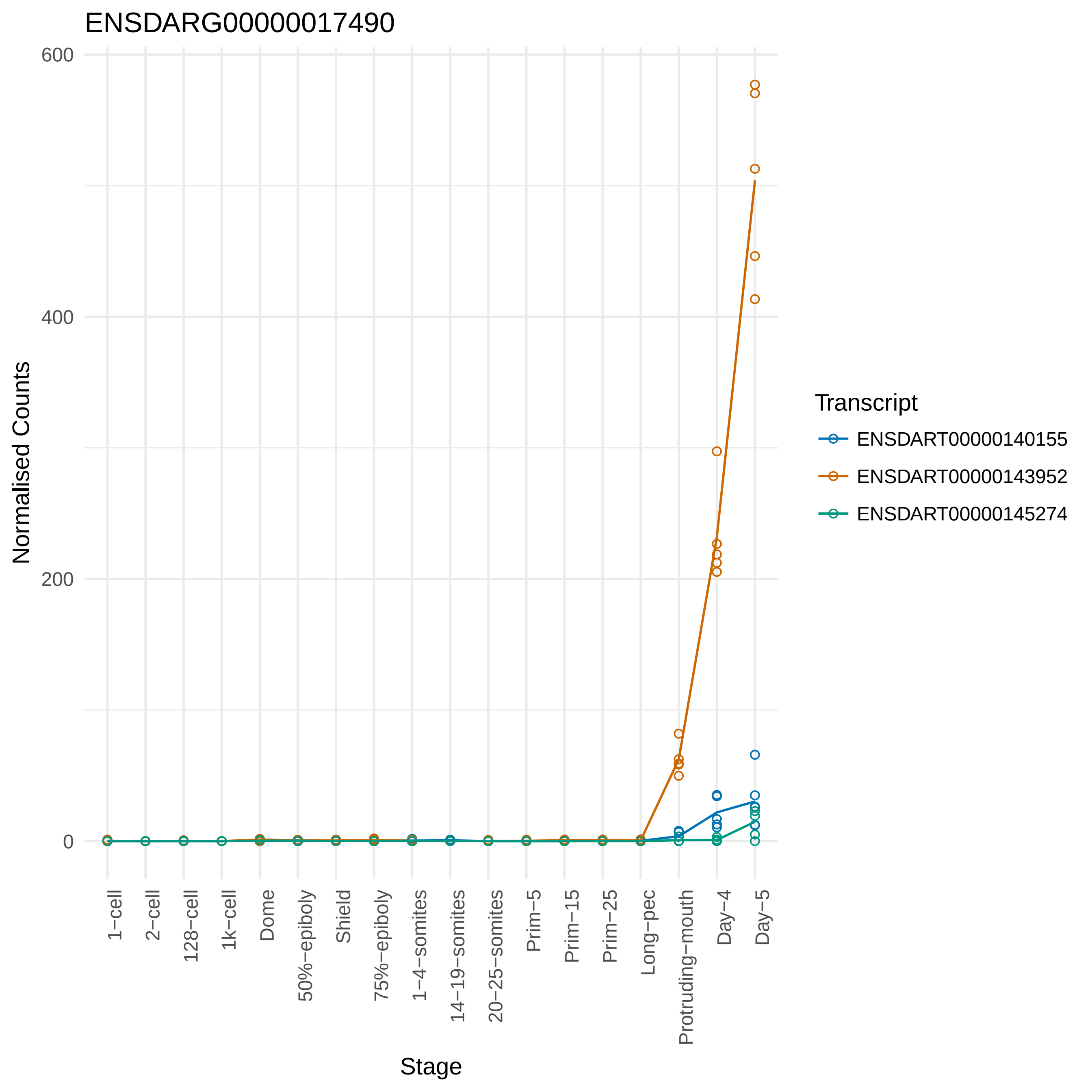

ENSDARG00000017490
600
400
200
0
Normalised Counts
1−cell
2−cell
128−cell
1k−cell
Dome
50%−epiboly
Shield
75%−epiboly
1−4−somites
14−19−somites
20−25−somites
Prim−5
Prim−15
Prim−25
Long−pec
Protruding−mouth
Day−4
Day−5
Stage
Transcript
ENSDART00000140155
ENSDART00000143952
ENSDART00000145274

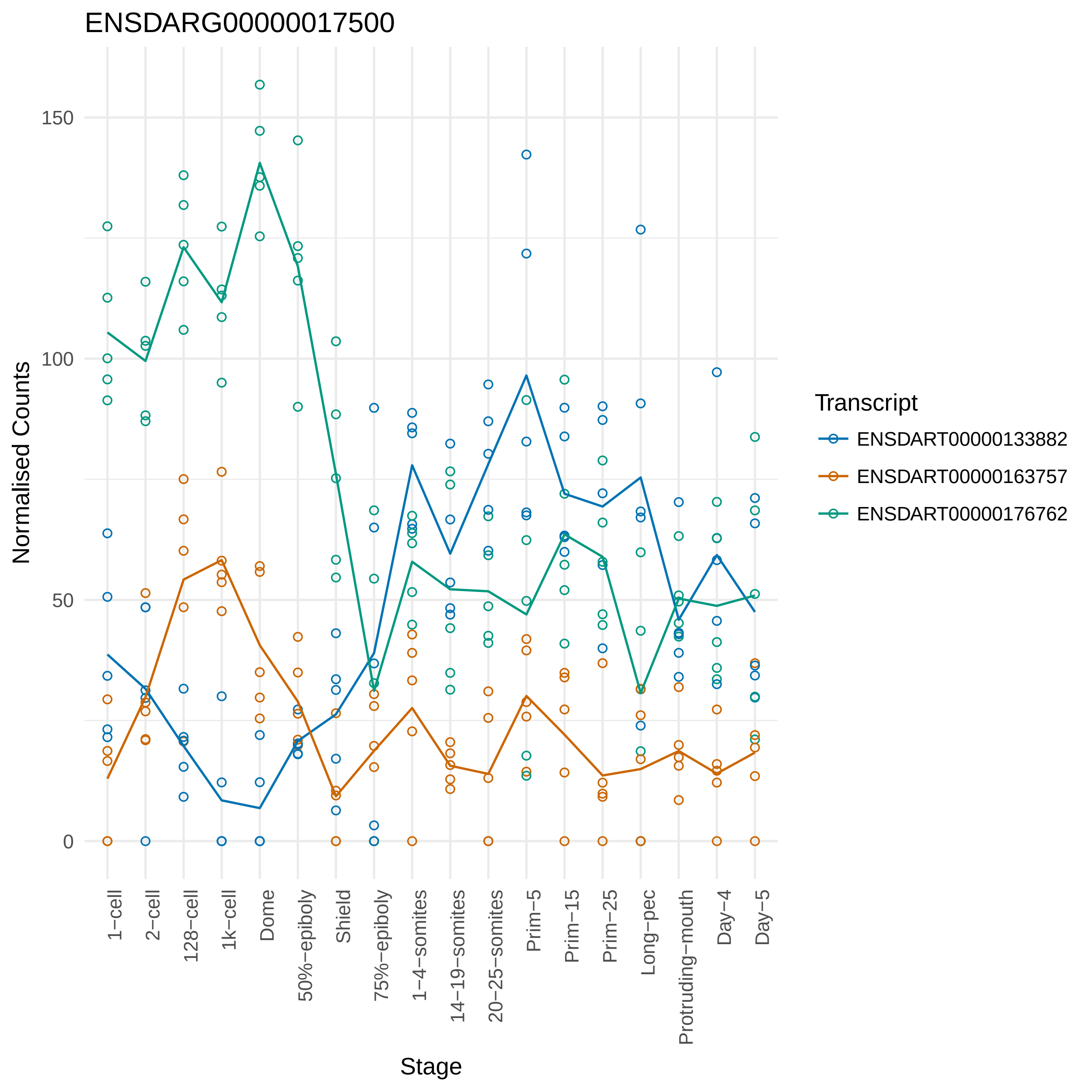

ENSDARG00000017500
Normalised Counts
150
100
50
0
1−cell
2−cell
128−cell
1k−cell
Dome
50%−epiboly
Shield
75%−epiboly
1−4−somites
14−19−somites
20−25−somites
Prim−5
Prim−15
Prim−25
Long−pec
Protruding−mouth
Day−4
Day−5
Stage
Transcript
ENSDART00000133882
ENSDART00000163757
ENSDART00000176762

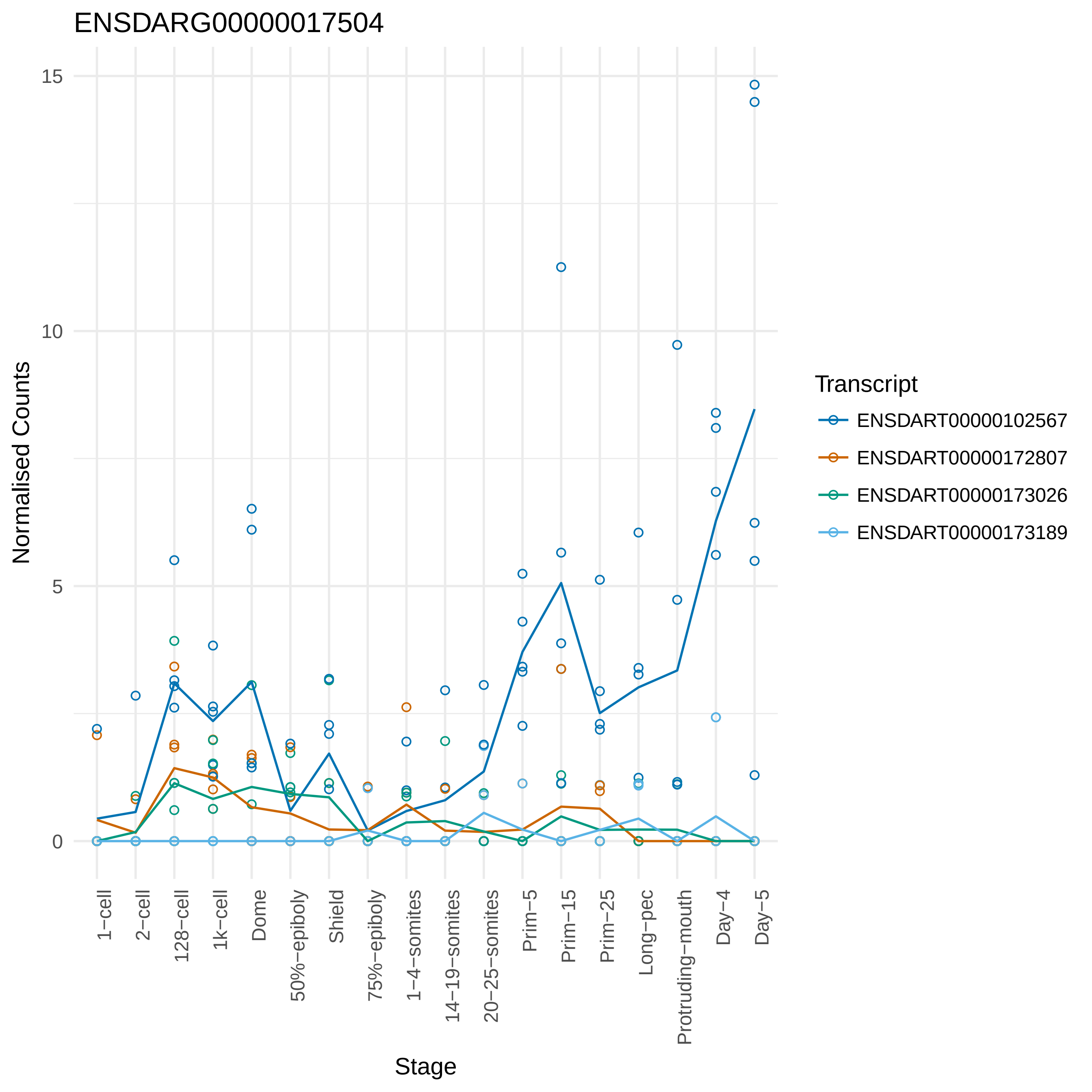
ENSDARG00000017504
Normalised Counts
15
10
5
0
Stage
1−cell
2−cell
128−cell
1k−cell
Dome
50%−epiboly
Shield
75%−epiboly
1−4−somites
14−19−somites
20−25−somites
Prim−5
Prim−15
Prim−25
Long−pec
Protruding−mouth
Day−4
Day−5
Transcript
ENSDART00000102567
ENSDART00000172807
ENSDART00000173026
ENSDART00000173189

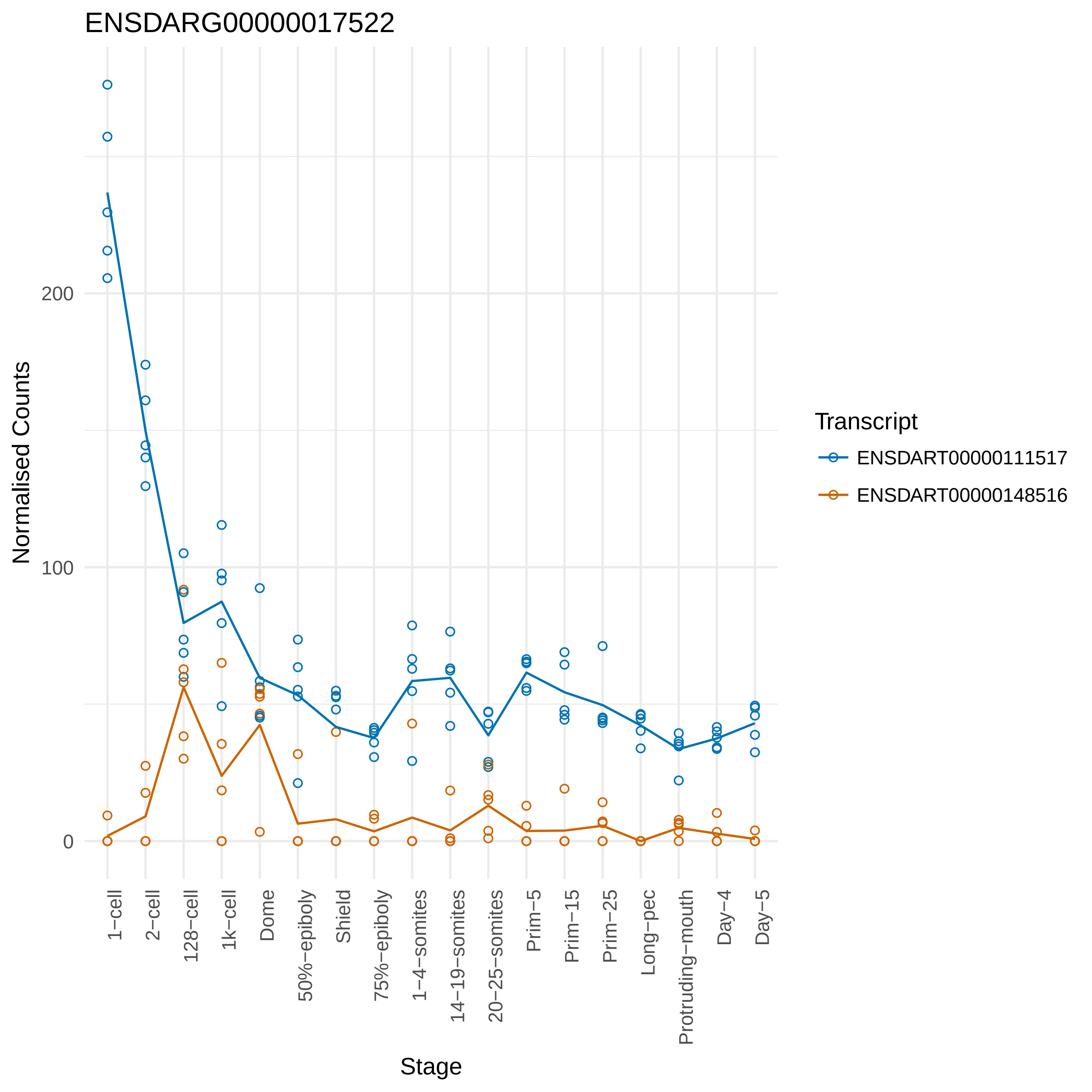

ENSDARG00000017522
Normalised Counts
0
100
200
Stage
1−cell
2−cell
128−cell
1k−cell
Dome
50%−epiboly
Shield
75%−epiboly
1−4−somites
14−19−somites
20−25−somites
Prim−5
Prim−15
Prim−25
Long−pec
Protruding−mouth
Day−4
Day−5
Transcript
ENSDART00000111517
ENSDART00000148516

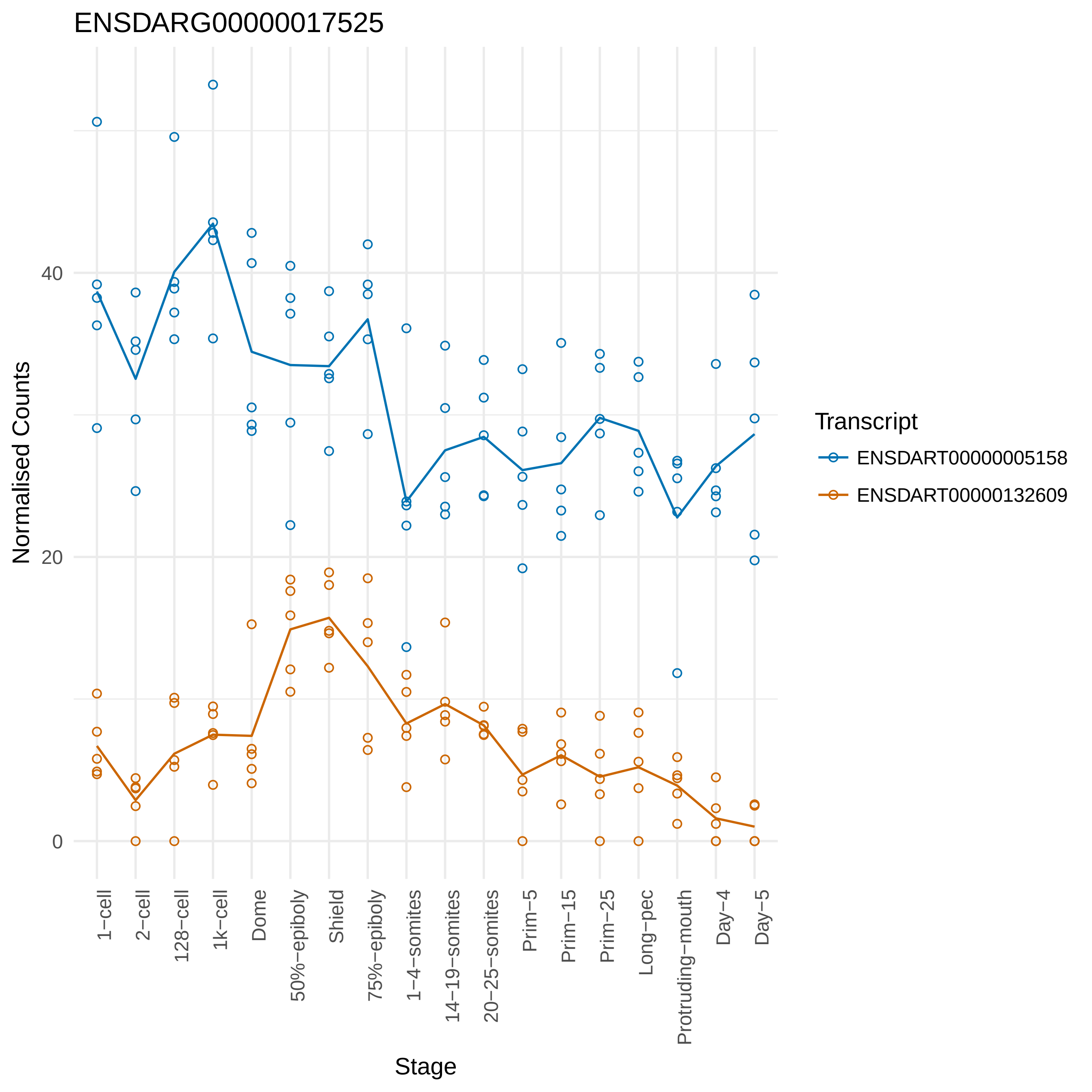
ENSDARG00000017525
Normalised Counts
0
20
40
Stage
1−cell
2−cell
128−cell
1k−cell
Dome
50%−epiboly
Shield
75%−epiboly
1−4−somites
14−19−somites
20−25−somites
Prim−5
Prim−15
Prim−25
Long−pec
Protruding−mouth
Day−4
Day−5
Transcript
ENSDART00000005158
ENSDART00000132609

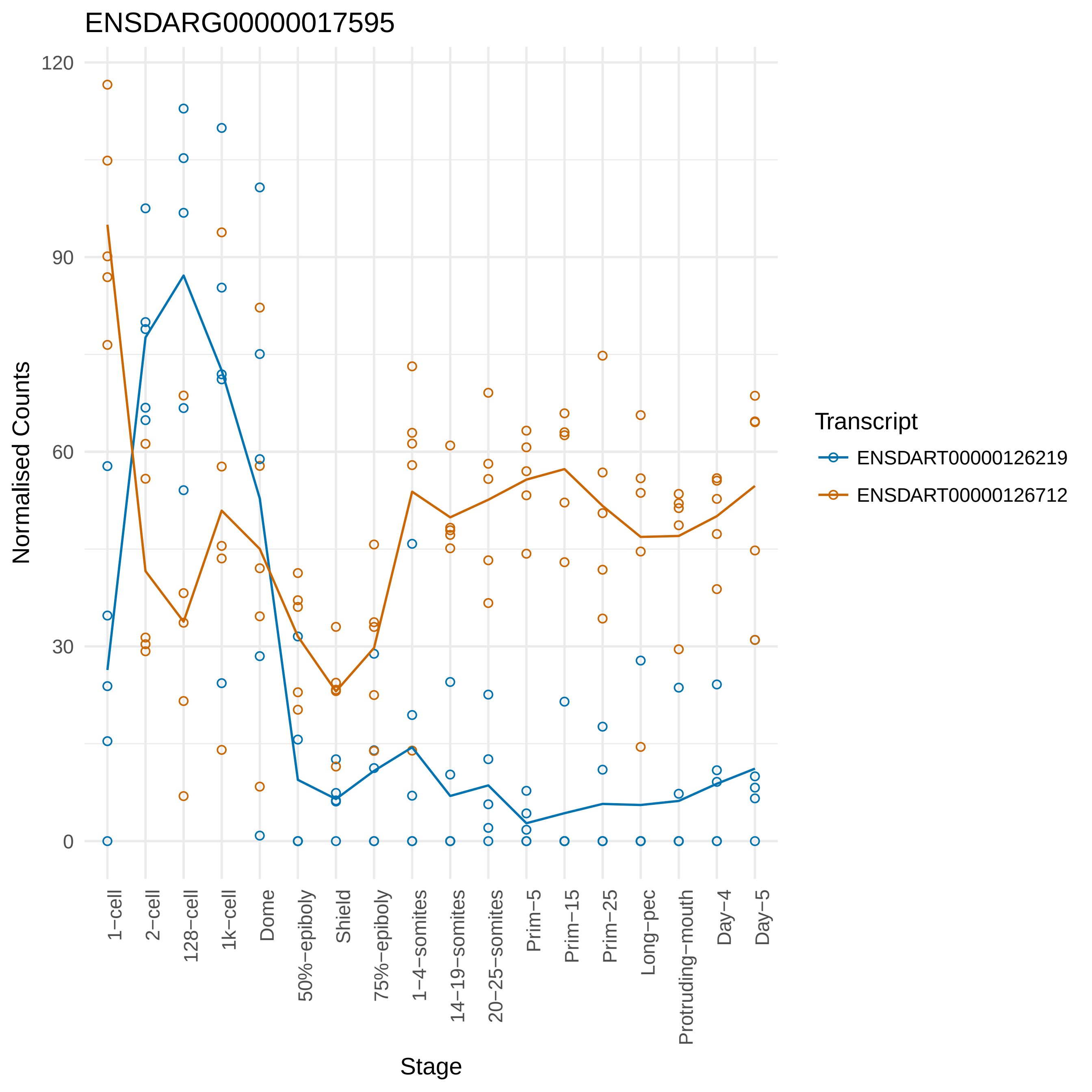

ENSDARG00000017595
Normalised Counts
120
90
60
30
0
1−cell
2−cell
128−cell
1k−cell
Dome
50%−epiboly
Shield
75%−epiboly
1−4−somites
14−19−somites
20−25−somites
Prim−5
Prim−15
Prim−25
Long−pec
Protruding−mouth
Day−4
Day−5
Stage
Transcript
ENSDART00000126219
ENSDART00000126712

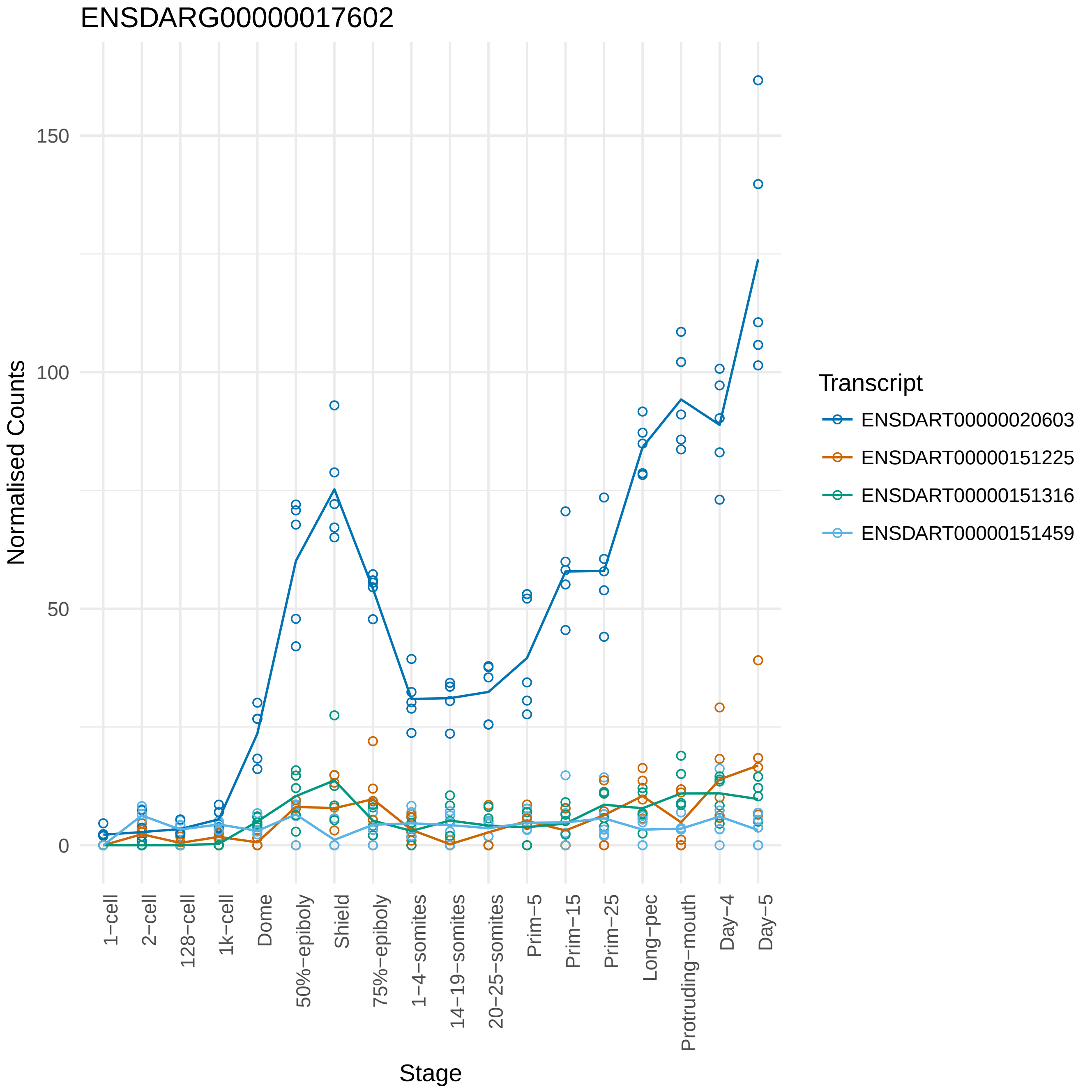
ENSDARG00000017602
Normalised Counts
0
50
100
150
Stage
1−cell
2−cell
128−cell
1k−cell
Dome
50%−epiboly
Shield
75%−epiboly
1−4−somites
14−19−somites
20−25−somites
Prim−5
Prim−15
Prim−25
Long−pec
Protruding−mouth
Day−4
Day−5
Transcript
ENSDART00000020603
ENSDART00000151225
ENSDART00000151316
ENSDART00000151459

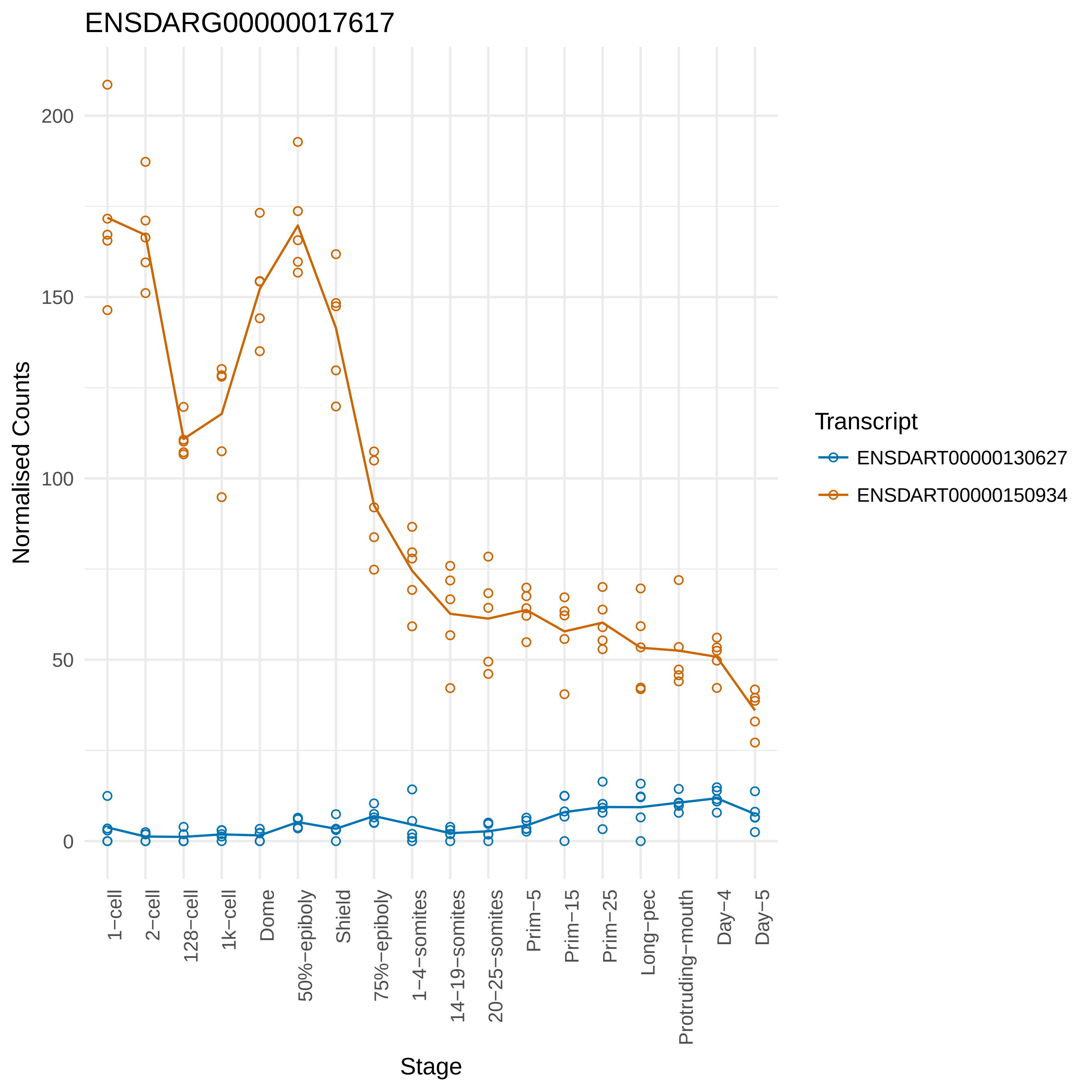

ENSDARG00000017617
Normalised Counts
200
150
100
50
0
1−cell
2−cell
128−cell
1k−cell
Dome
50%−epiboly
Shield
75%−epiboly
1−4−somites
14−19−somites
20−25−somites
Prim−5
Prim−15
Prim−25
Long−pec
Protruding−mouth
Day−4
Day−5
Stage
Transcript
ENSDART00000130627
ENSDART00000150934

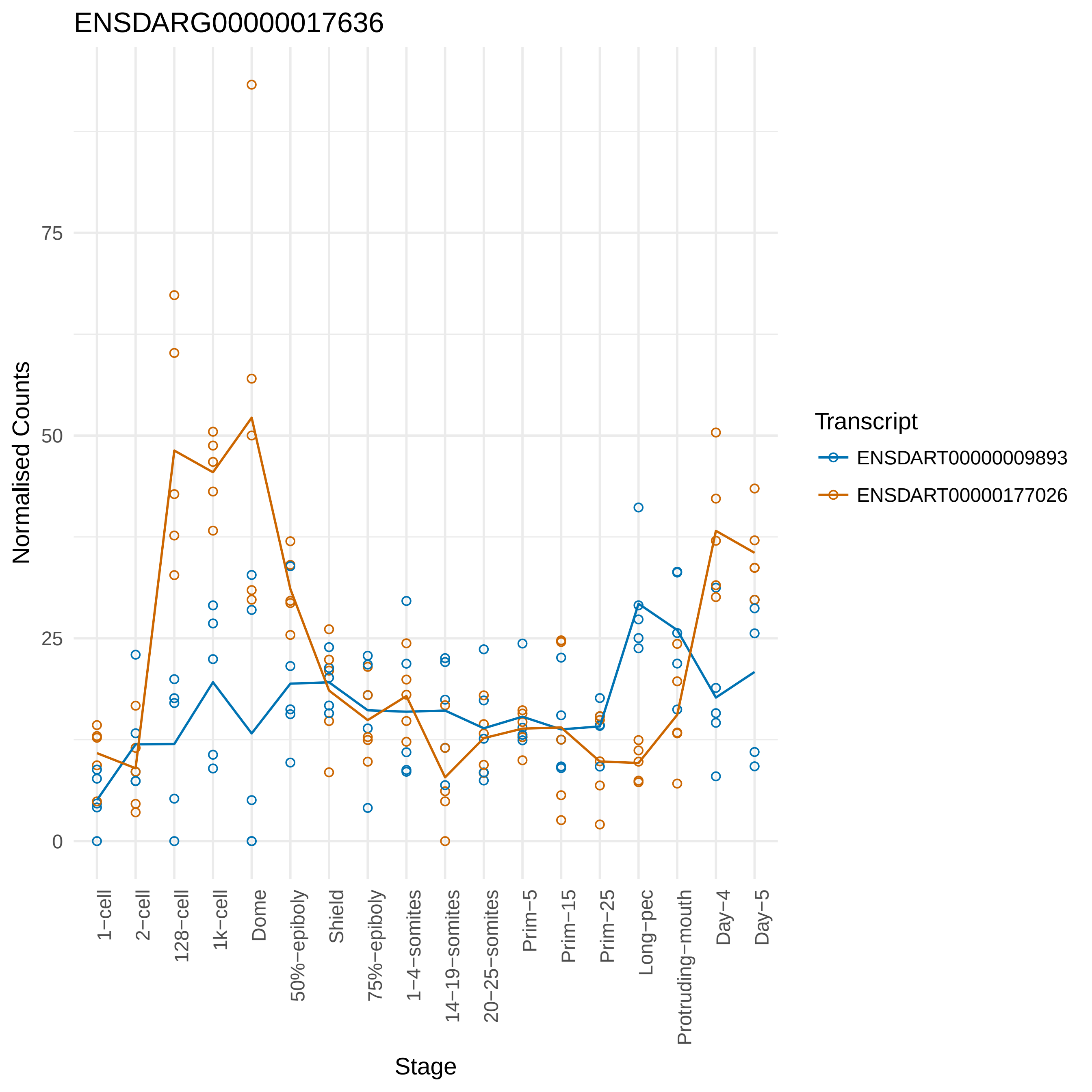

ENSDARG00000017636
Normalised Counts
0
25
50
75
1−cell
2−cell
128−cell
1k−cell
Dome
50%−epiboly
Shield
75%−epiboly
1−4−somites
14−19−somites
20−25−somites
Prim−5
Prim−15
Prim−25
Long−pec
Protruding−mouth
Day−4
Day−5
Stage
Transcript
ENSDART00000009893
ENSDART00000177026

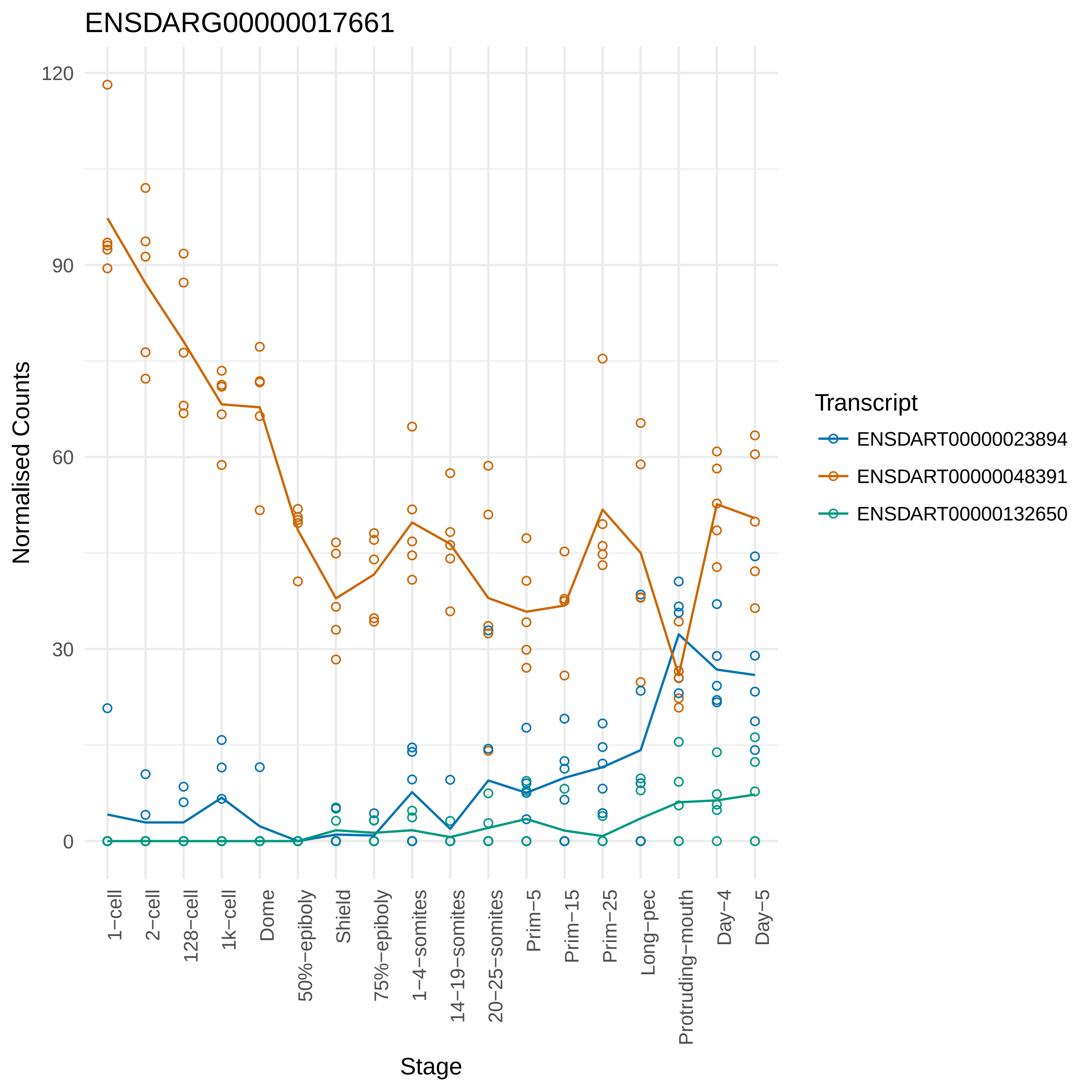

ENSDARG00000017661
Normalised Counts
120
90
60
30
0
1−cell
2−cell
128−cell
1k−cell
Dome
50%−epiboly
Shield
75%−epiboly
1−4−somites
14−19−somites
20−25−somites
Prim−5
Prim−15
Prim−25
Long−pec
Protruding−mouth
Day−4
Day−5
Stage
Transcript
ENSDART00000023894
ENSDART00000048391
ENSDART00000132650

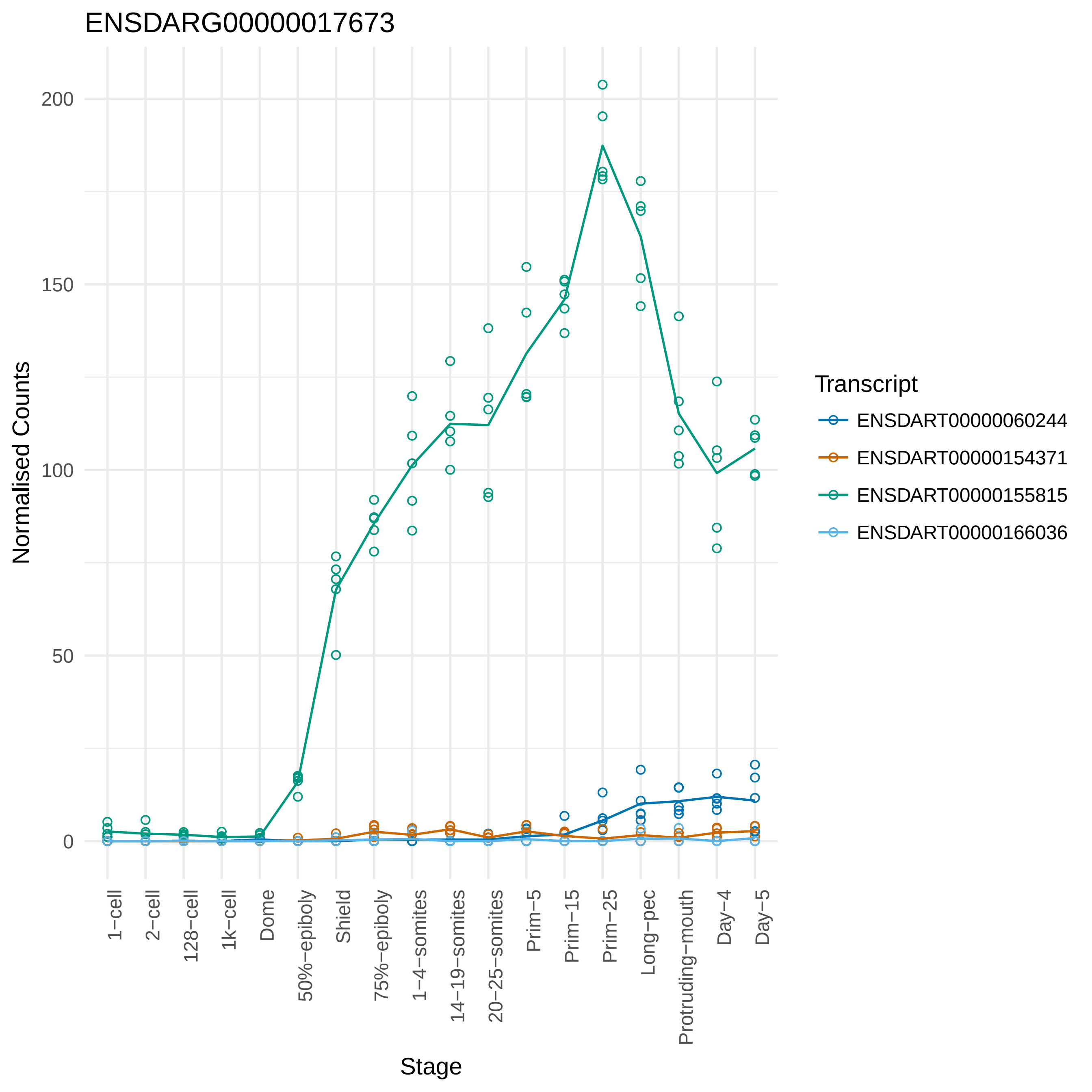

ENSDARG00000017673
Normalised Counts
0
50
100
150
200
Stage
1−cell
2−cell
128−cell
1k−cell
Dome
50%−epiboly
Shield
75%−epiboly
1−4−somites
14−19−somites
20−25−somites
Prim−5
Prim−15
Prim−25
Long−pec
Protruding−mouth
Day−4
Day−5
Transcript
ENSDART00000060244
ENSDART00000154371
ENSDART00000155815
ENSDART00000166036

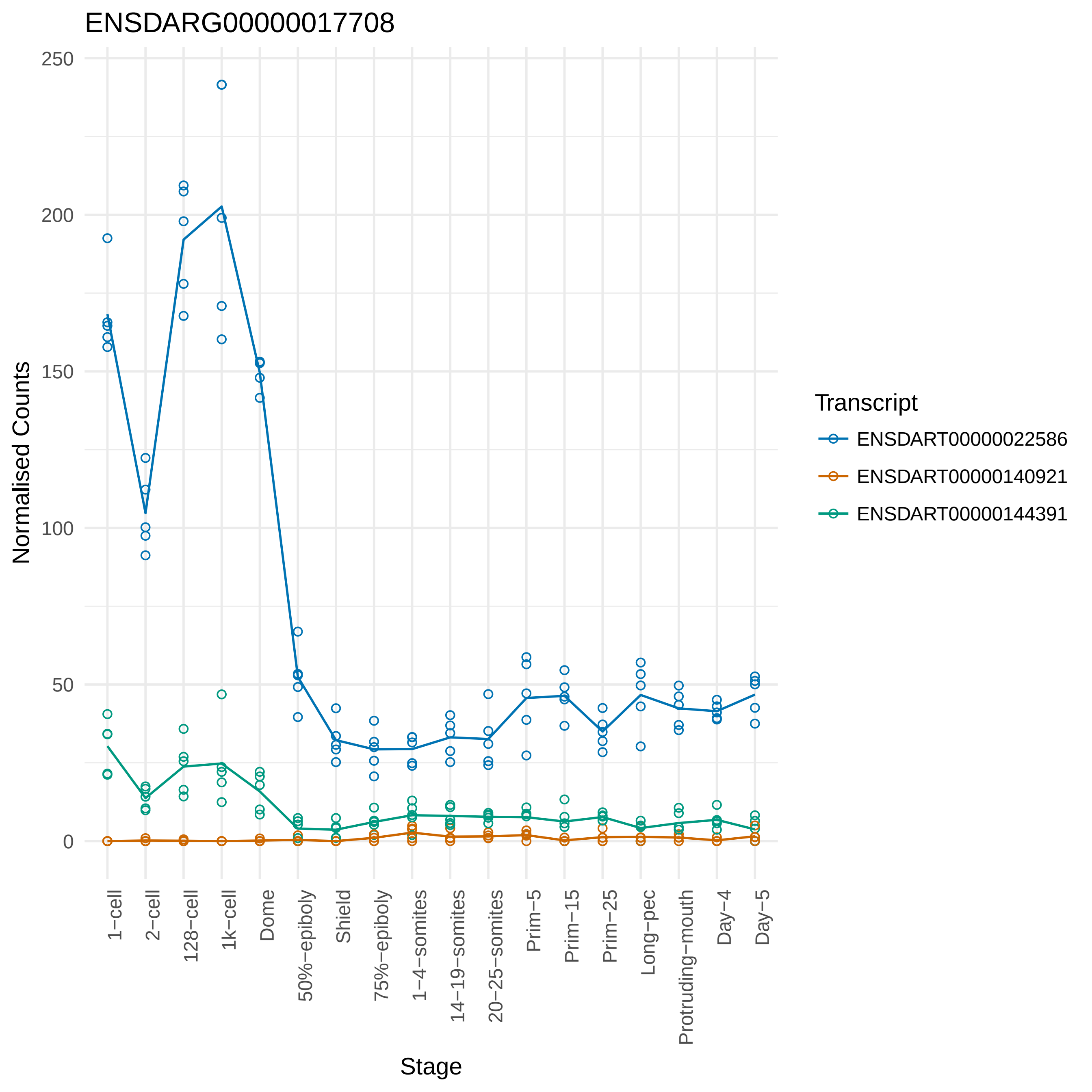

ENSDARG00000017708
Normalised Counts
250
200
150
100
50
0
1−cell
2−cell
128−cell
1k−cell
Dome
50%−epiboly
Shield
75%−epiboly
1−4−somites
14−19−somites
20−25−somites
Prim−5
Prim−15
Prim−25
Long−pec
Protruding−mouth
Day−4
Day−5
Stage
Transcript
ENSDART00000022586
ENSDART00000140921
ENSDART00000144391

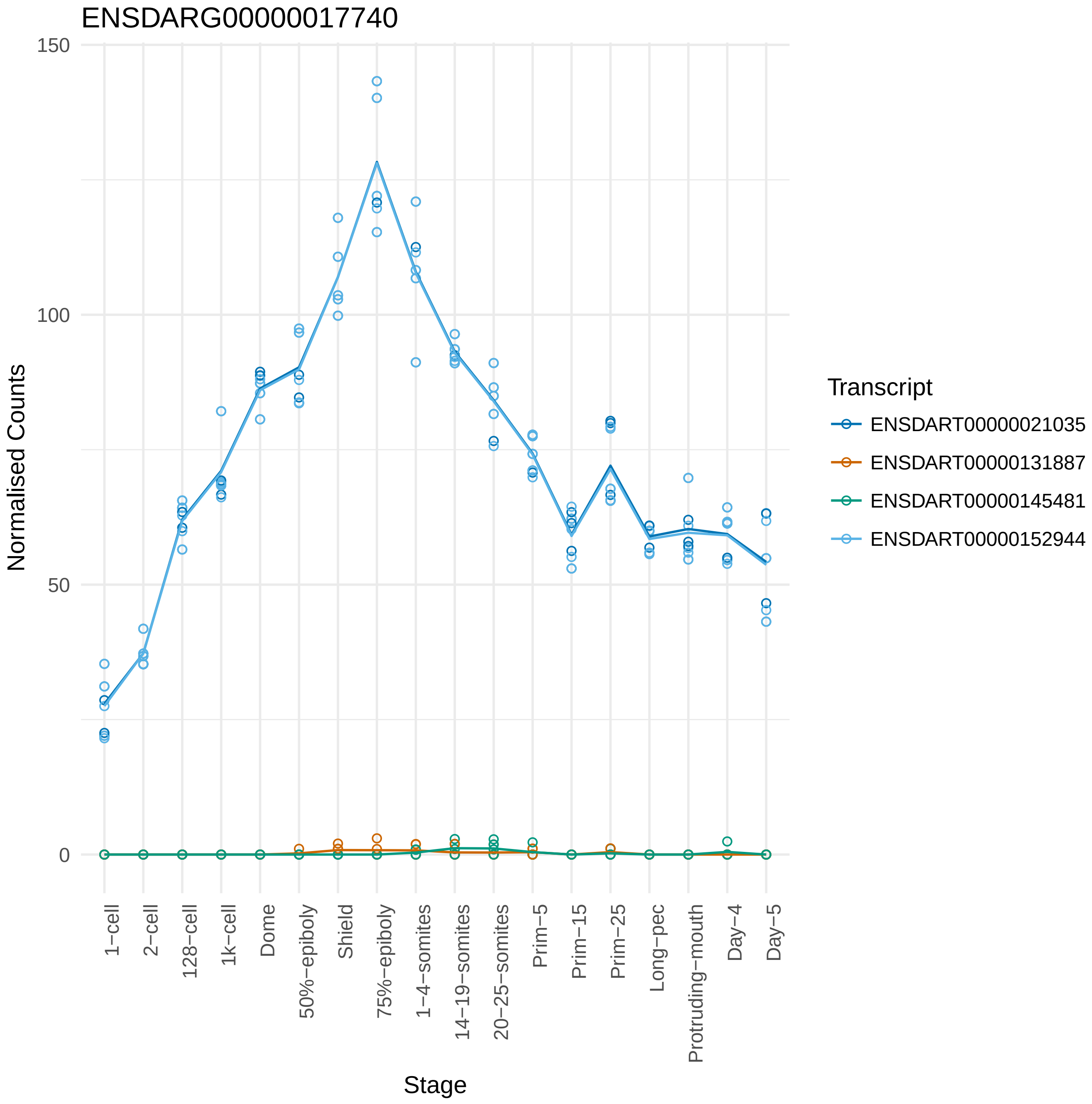
ENSDARG00000017740
Normalised Counts
150
100
50
0
1−cell
2−cell
128−cell
1k−cell
Dome
50%−epiboly
Shield
75%−epiboly
1−4−somites
14−19−somites
20−25−somites
Prim−5
Prim−15
Prim−25
Long−pec
Protruding−mouth
Day−4
Day−5
Stage
Transcript
ENSDART00000021035
ENSDART00000131887
ENSDART00000145481
ENSDART00000152944

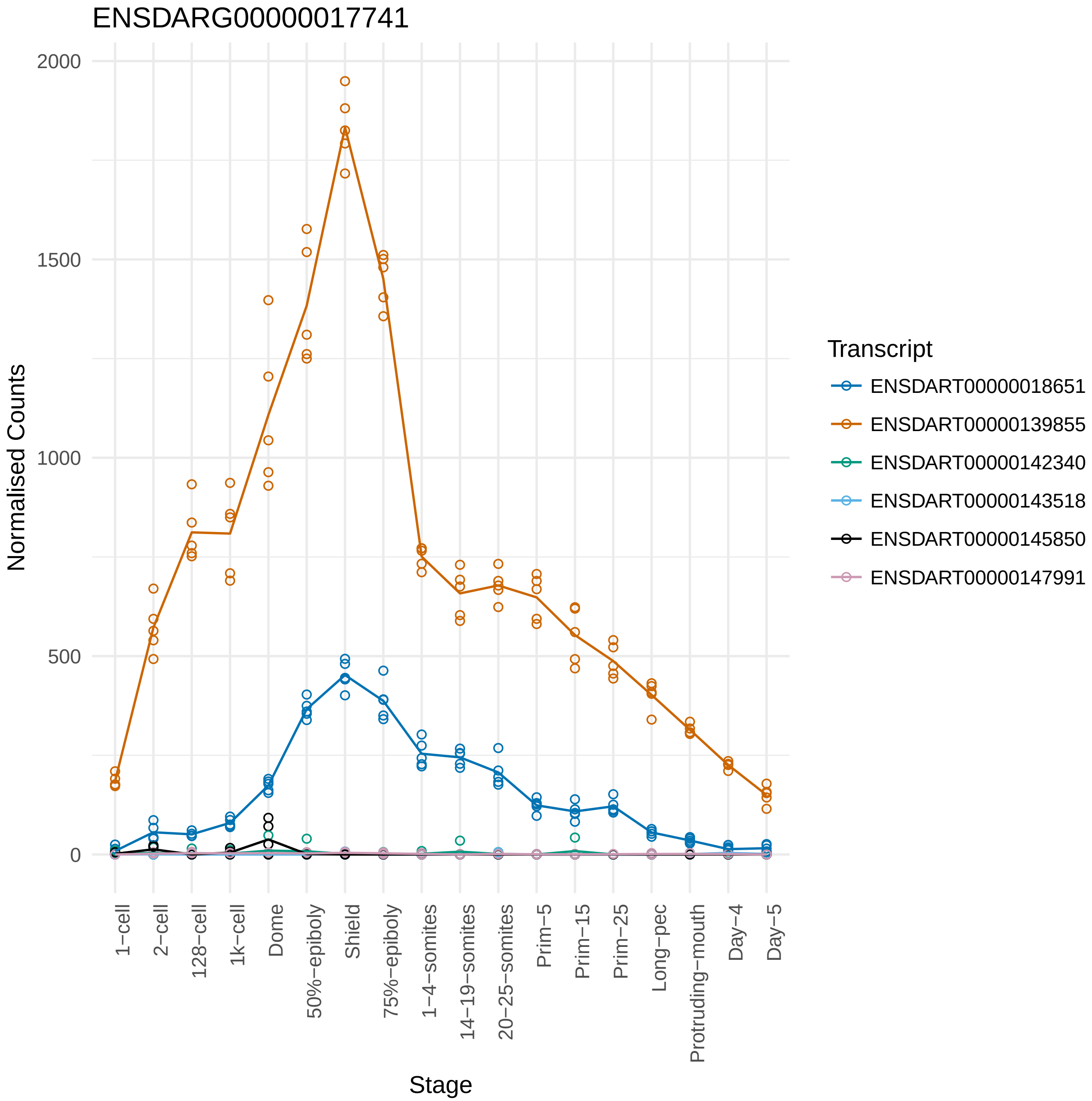

ENSDARG00000017741
Normalised Counts
2000
1500
1000
500
0
1−cell
2−cell
128−cell
1k−cell
Dome
50%−epiboly
Shield
75%−epiboly
1−4−somites
14−19−somites
20−25−somites
Prim−5
Prim−15
Prim−25
Long−pec
Protruding−mouth
Day−4
Day−5
Stage
Transcript
ENSDART00000018651
ENSDART00000139855
ENSDART00000142340
ENSDART00000143518
ENSDART00000145850
ENSDART00000147991

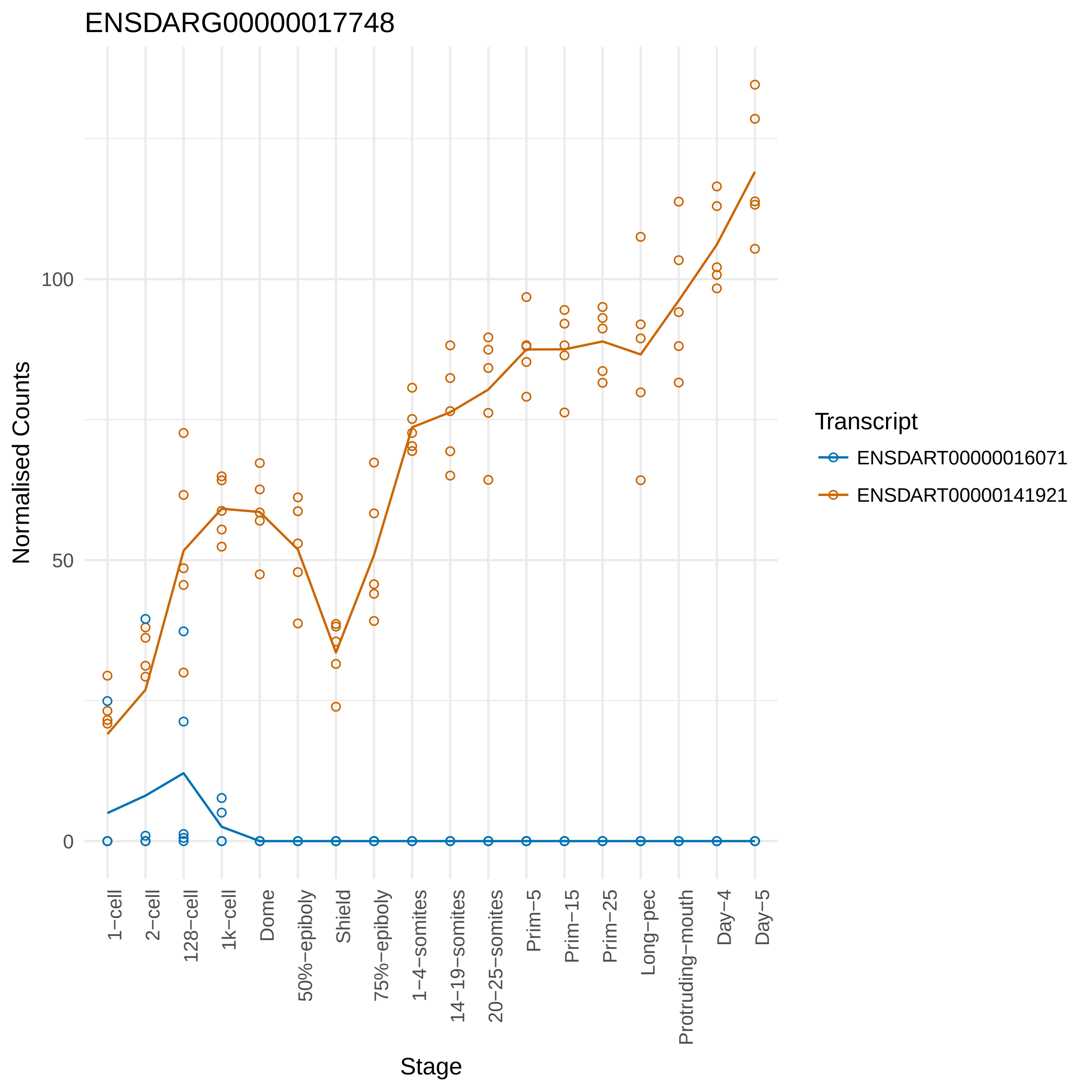

ENSDARG00000017748
Normalised Counts
100
50
0
Stage
1−cell
2−cell
128−cell
1k−cell
Dome
50%−epiboly
Shield
75%−epiboly
1−4−somites
14−19−somites
20−25−somites
Prim−5
Prim−15
Prim−25
Long−pec
Protruding−mouth
Day−4
Day−5
Transcript
ENSDART00000016071
ENSDART00000141921

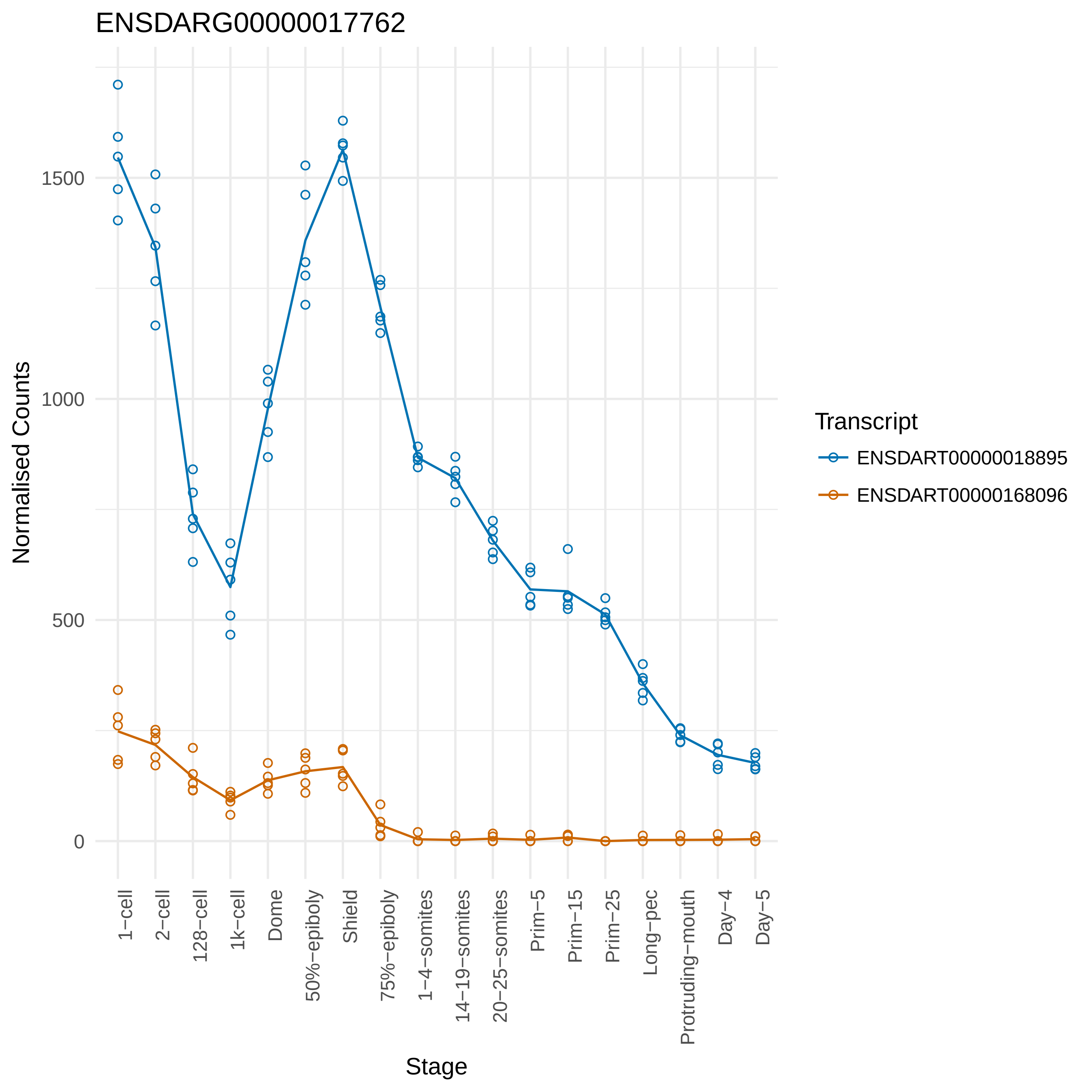

ENSDARG00000017762
Normalised Counts
1500
1000
500
0
Stage
1−cell
2−cell
128−cell
1k−cell
Dome
50%−epiboly
Shield
75%−epiboly
1−4−somites
14−19−somites
20−25−somites
Prim−5
Prim−15
Prim−25
Long−pec
Protruding−mouth
Day−4
Day−5
Transcript
ENSDART00000018895
ENSDART00000168096

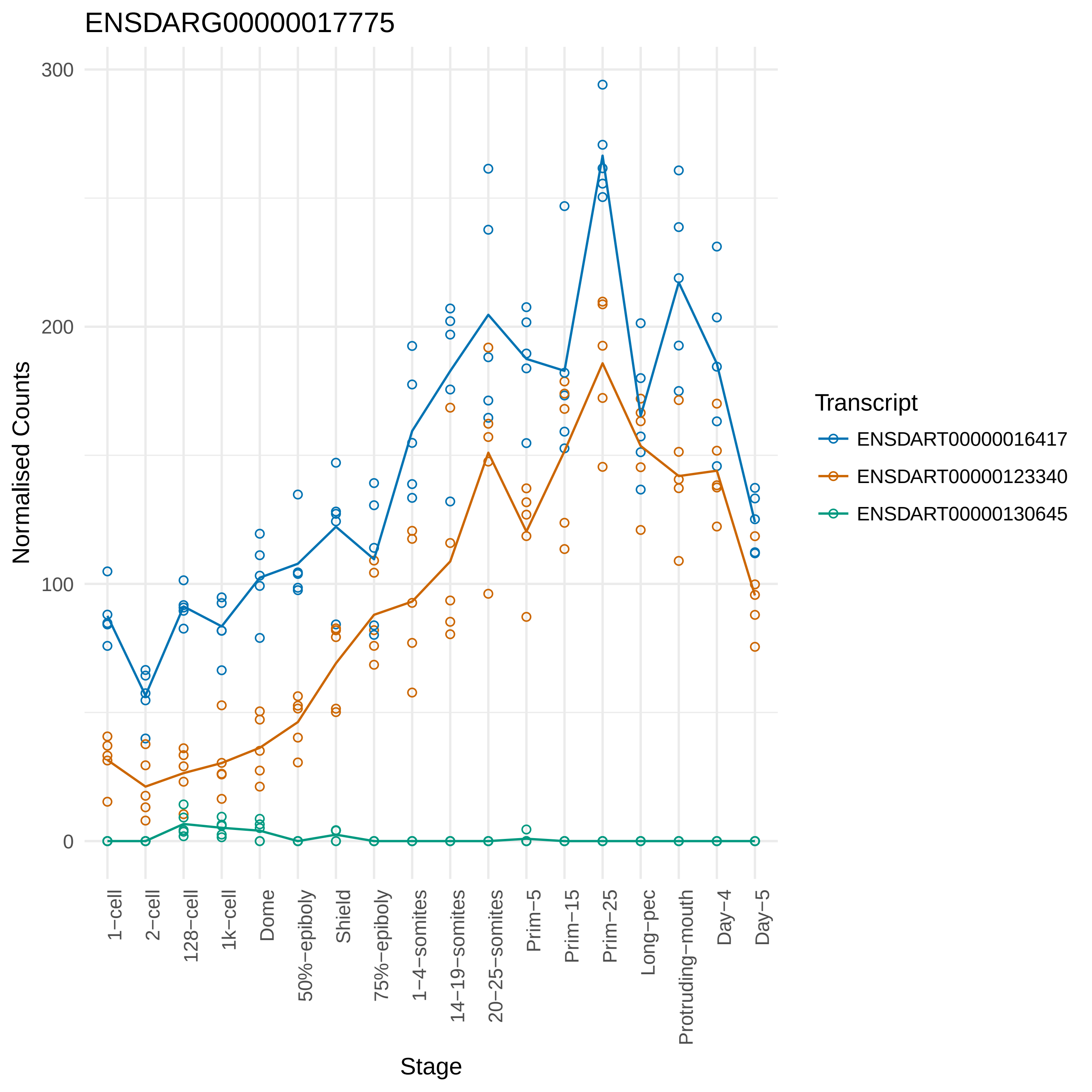
ENSDARG00000017775
Normalised Counts
300
200
100
0
1−cell
2−cell
128−cell
1k−cell
Dome
50%−epiboly
Shield
75%−epiboly
1−4−somites
14−19−somites
20−25−somites
Prim−5
Prim−15
Prim−25
Long−pec
Protruding−mouth
Day−4
Day−5
Stage
Transcript
ENSDART00000016417
ENSDART00000123340
ENSDART00000130645

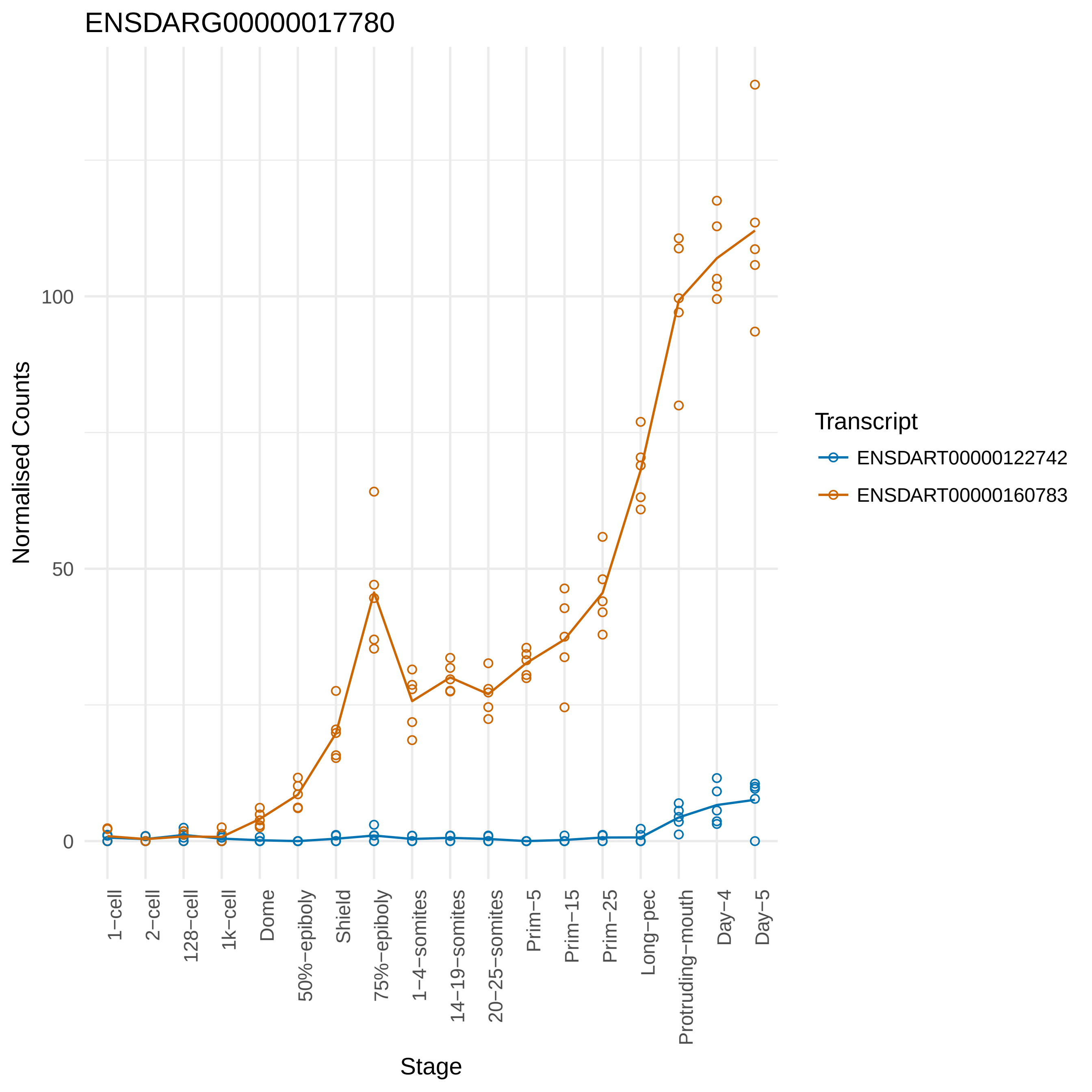

ENSDARG00000017780
Normalised Counts
100
50
0
1−cell
2−cell
128−cell
1k−cell
Dome
50%−epiboly
Shield
75%−epiboly
1−4−somites
14−19−somites
20−25−somites
Prim−5
Prim−15
Prim−25
Long−pec
Protruding−mouth
Day−4
Day−5
Stage
Transcript
ENSDART00000122742
ENSDART00000160783

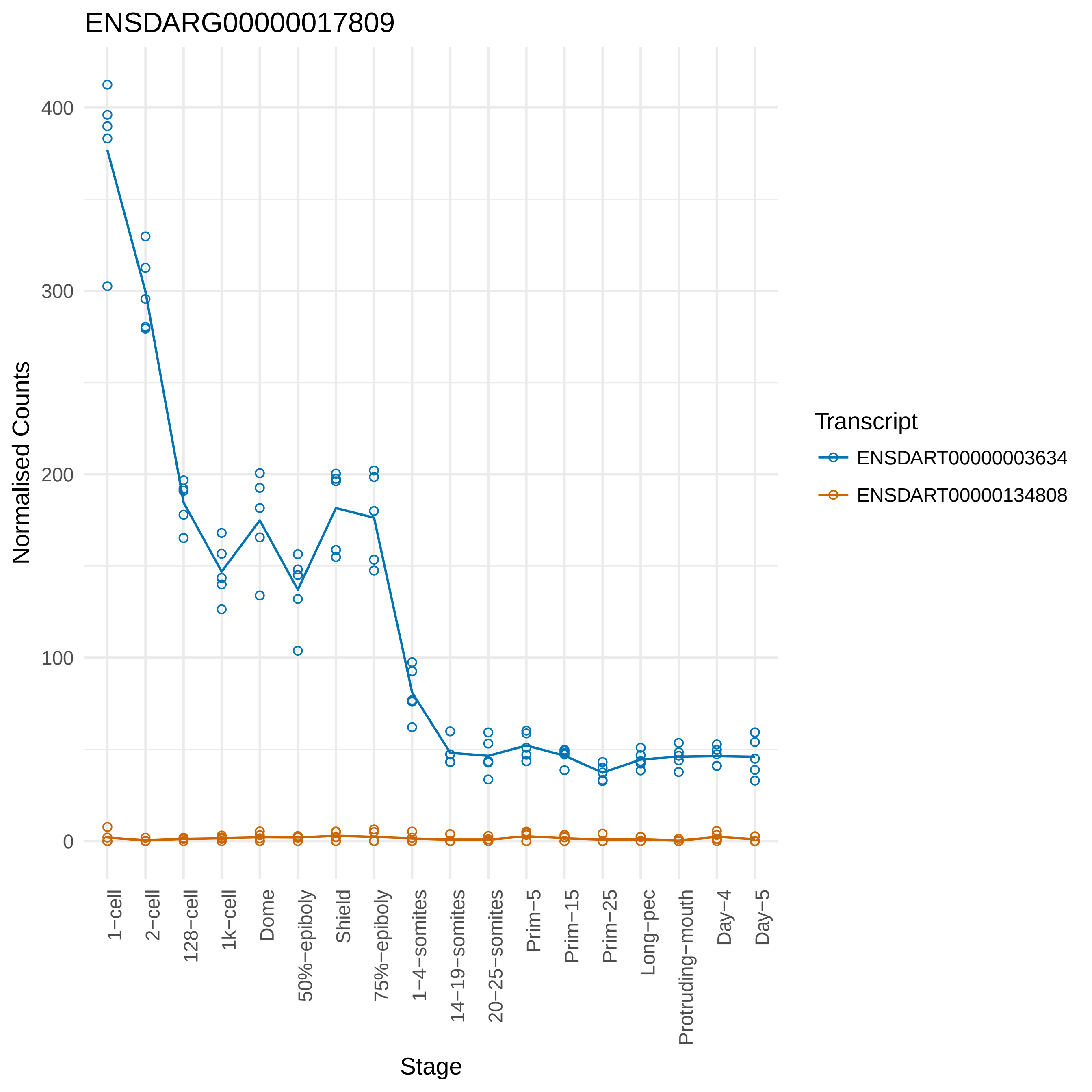
ENSDARG00000017809
Normalised Counts
0
100
200
300
400
Stage
1−cell
2−cell
128−cell
1k−cell
Dome
50%−epiboly
Shield
75%−epiboly
1−4−somites
14−19−somites
20−25−somites
Prim−5
Prim−15
Prim−25
Long−pec
Protruding−mouth
Day−4
Day−5
Transcript
ENSDART00000003634
ENSDART00000134808

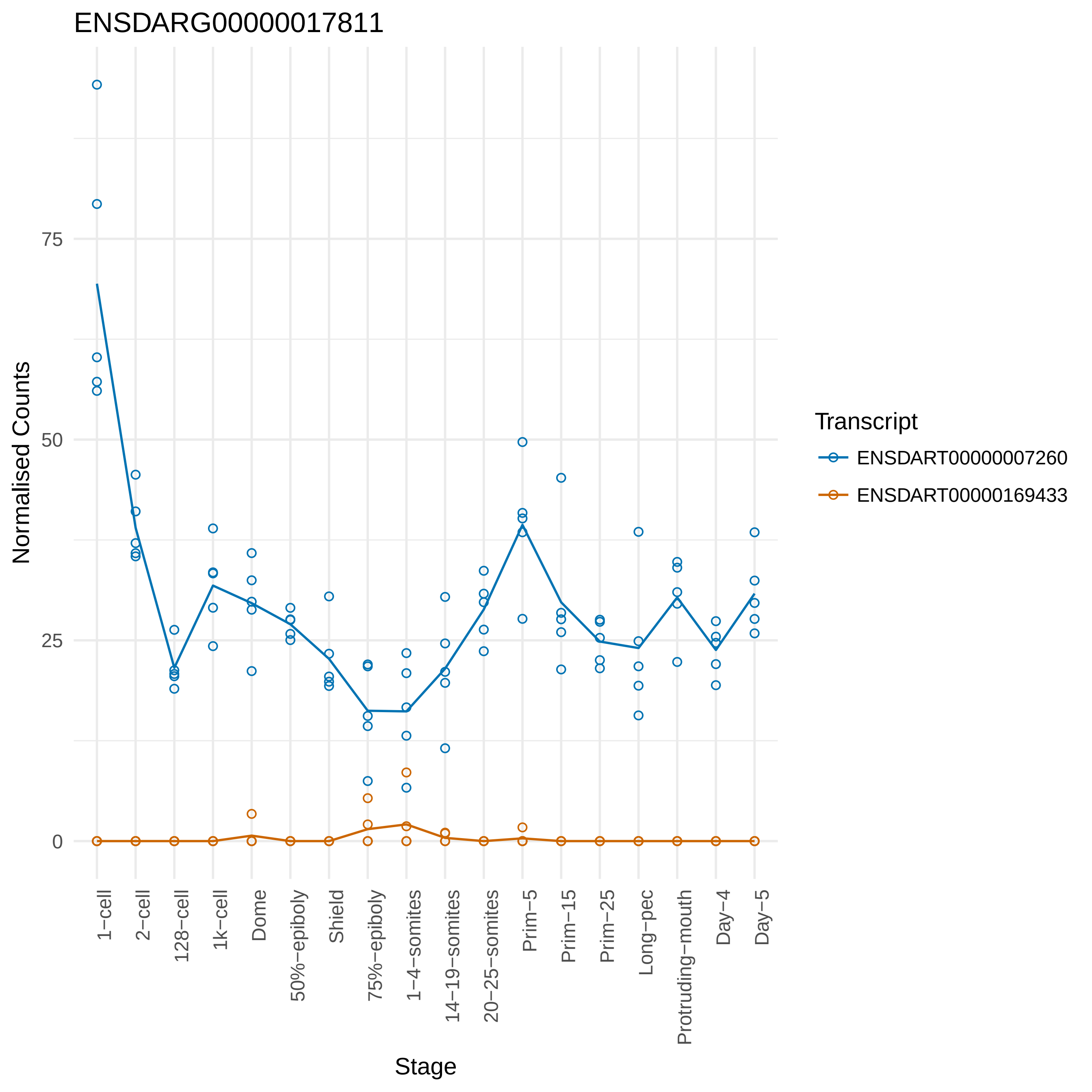
ENSDARG00000017811
Normalised Counts
75
50
25
0
1−cell
2−cell
128−cell
1k−cell
Dome
50%−epiboly
Shield
75%−epiboly
1−4−somites
14−19−somites
20−25−somites
Prim−5
Prim−15
Prim−25
Long−pec
Protruding−mouth
Day−4
Day−5
Stage
Transcript
ENSDART00000007260
ENSDART00000169433

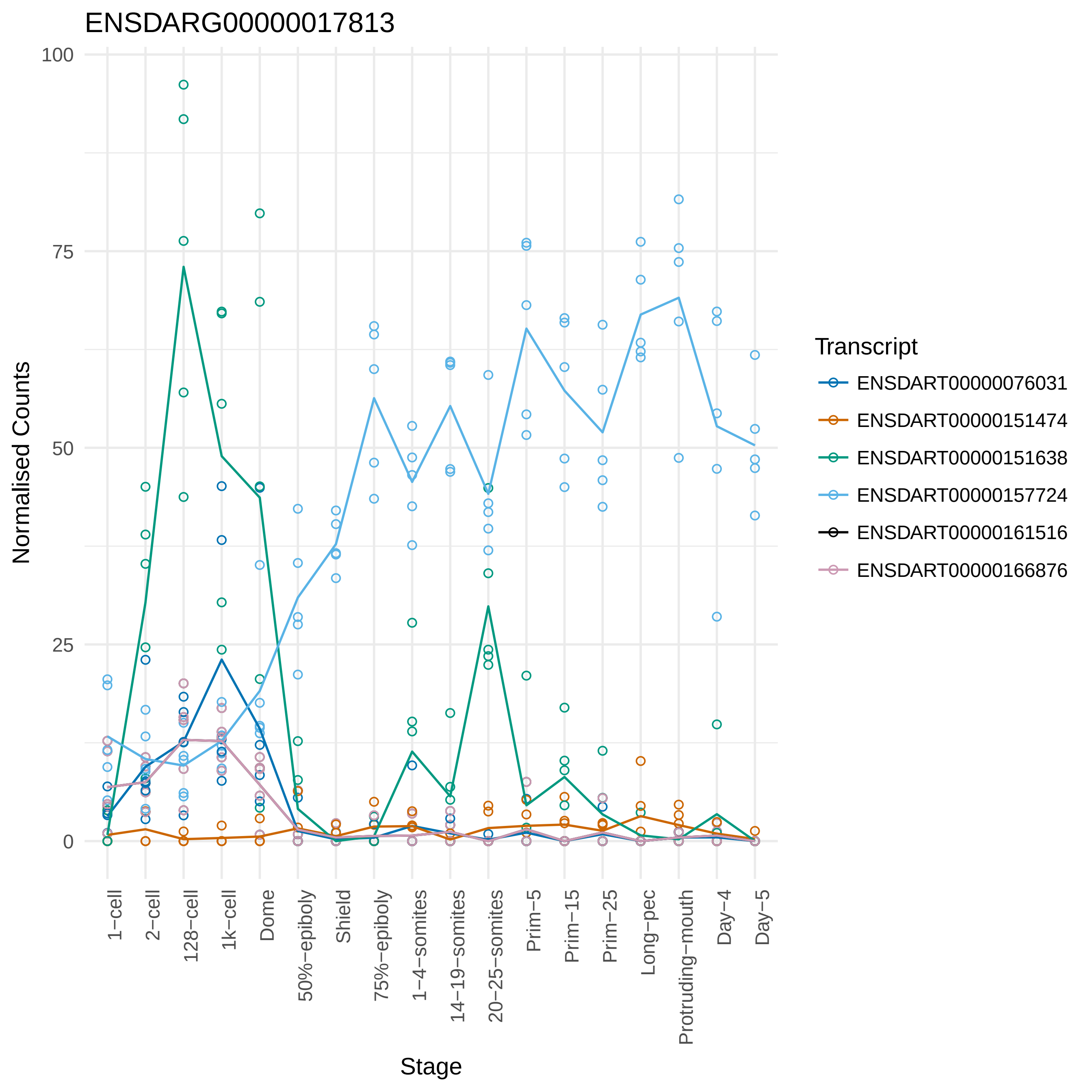
ENSDARG00000017813
Normalised Counts
100
75
50
25
0
1−cell
2−cell
128−cell
1k−cell
Dome
50%−epiboly
Shield
75%−epiboly
1−4−somites
14−19−somites
20−25−somites
Prim−5
Prim−15
Prim−25
Long−pec
Protruding−mouth
Day−4
Day−5
Stage
Transcript
ENSDART00000076031
ENSDART00000151474
ENSDART00000151638
ENSDART00000157724
ENSDART00000161516
ENSDART00000166876

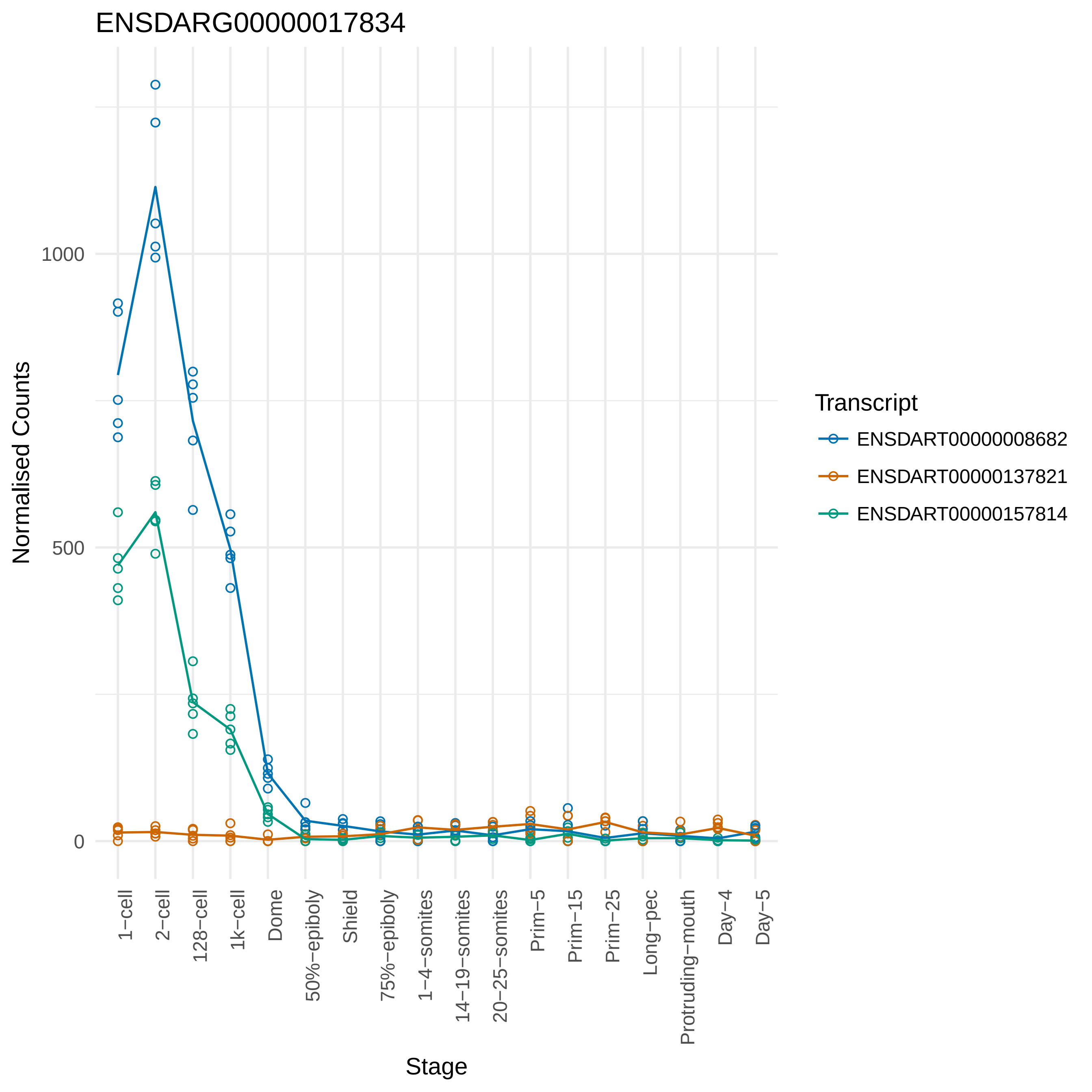

ENSDARG00000017834
Normalised Counts
1000
500
0
1−cell
2−cell
128−cell
1k−cell
Dome
50%−epiboly
Shield
75%−epiboly
1−4−somites
14−19−somites
20−25−somites
Prim−5
Prim−15
Prim−25
Long−pec
Protruding−mouth
Day−4
Day−5
Stage
Transcript
ENSDART00000008682
ENSDART00000137821
ENSDART00000157814

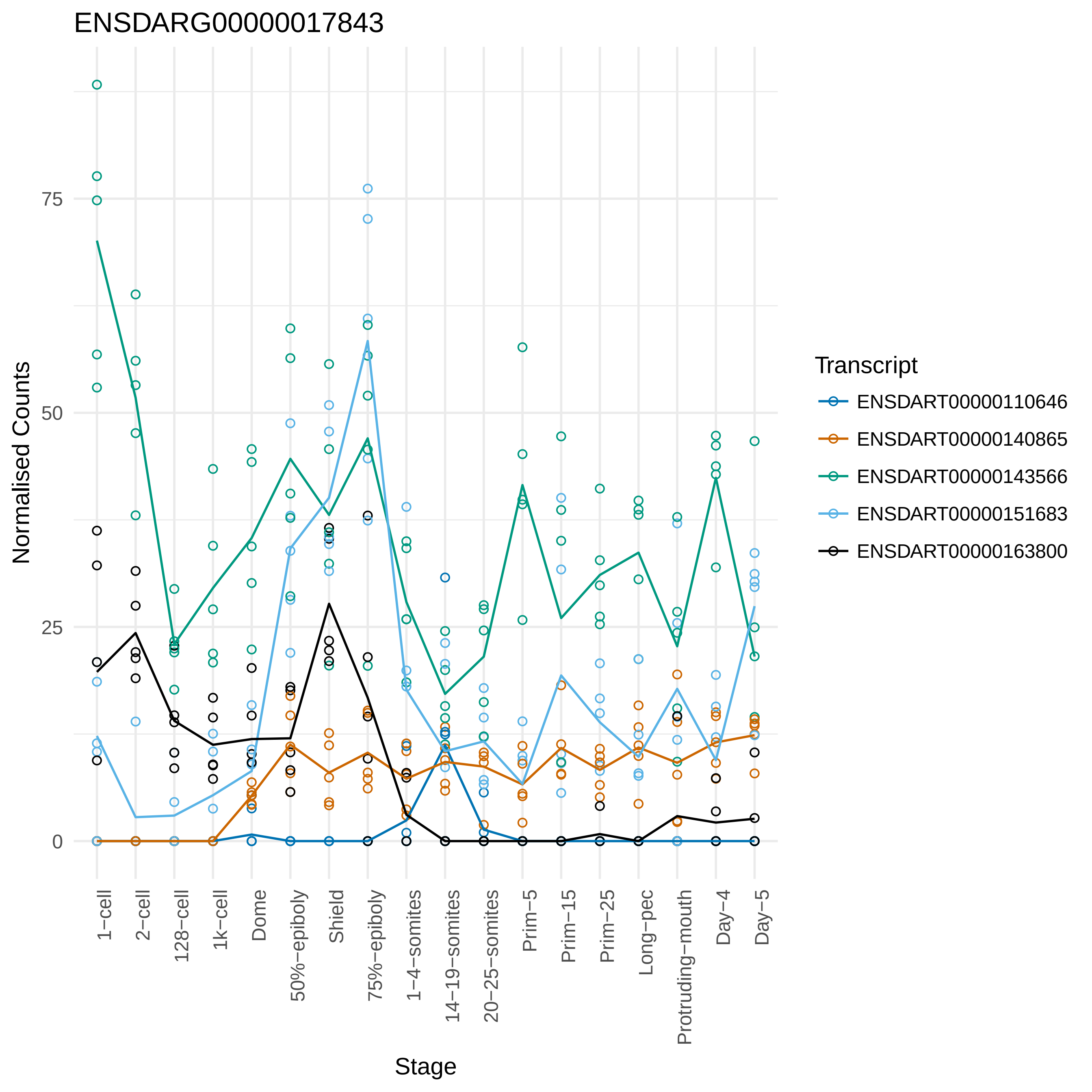
ENSDARG00000017843
Normalised Counts
0
25
50
75
1−cell
2−cell
128−cell
1k−cell
Dome
50%−epiboly
Shield
75%−epiboly
1−4−somites
14−19−somites
20−25−somites
Prim−5
Prim−15
Prim−25
Long−pec
Protruding−mouth
Day−4
Day−5
Stage
Transcript
ENSDART00000110646
ENSDART00000140865
ENSDART00000143566
ENSDART00000151683
ENSDART00000163800

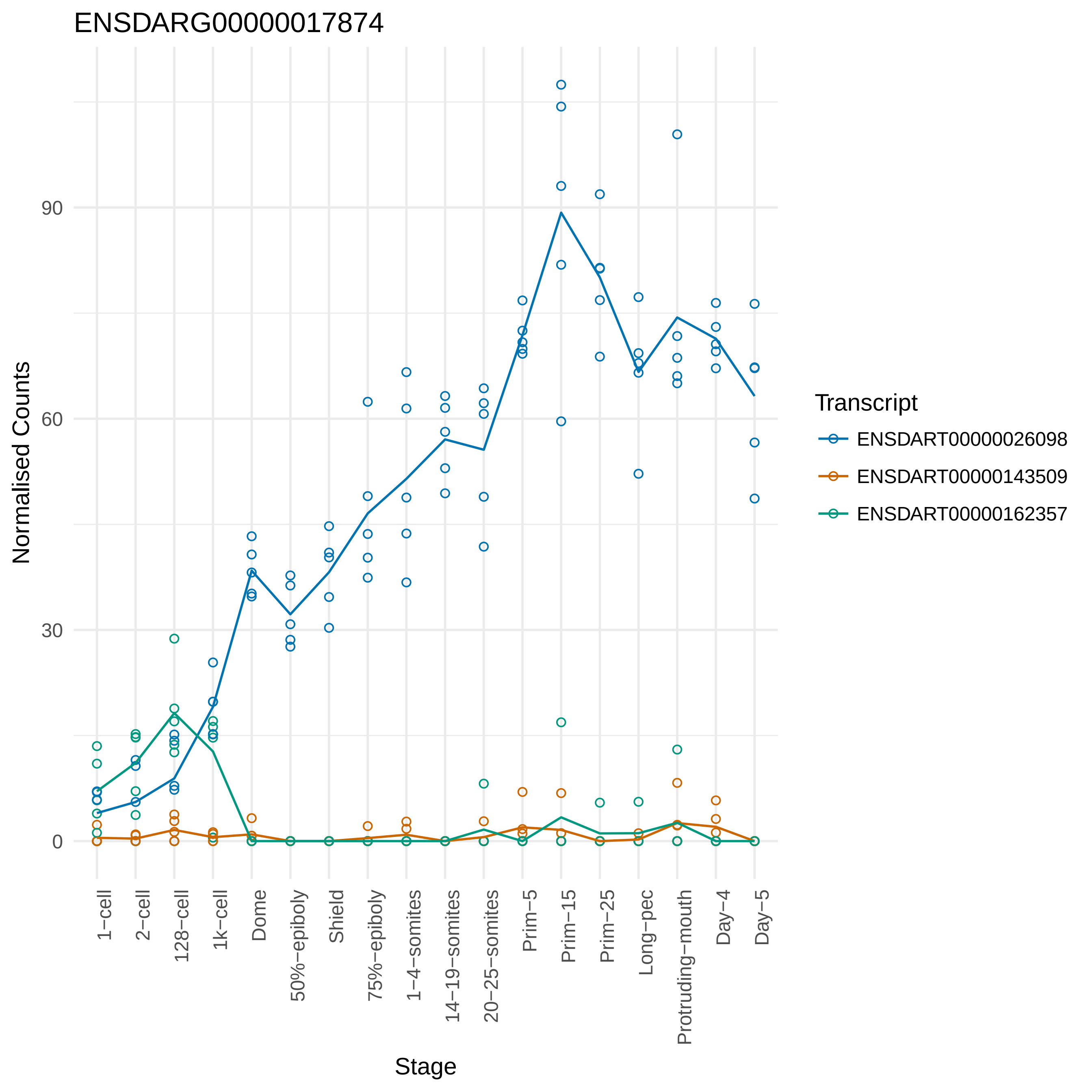
ENSDARG00000017874
Normalised Counts
0
30
60
90
1−cell
2−cell
128−cell
1k−cell
Dome
50%−epiboly
Shield
75%−epiboly
1−4−somites
14−19−somites
20−25−somites
Prim−5
Prim−15
Prim−25
Long−pec
Protruding−mouth
Day−4
Day−5
Stage
Transcript
ENSDART00000026098
ENSDART00000143509
ENSDART00000162357

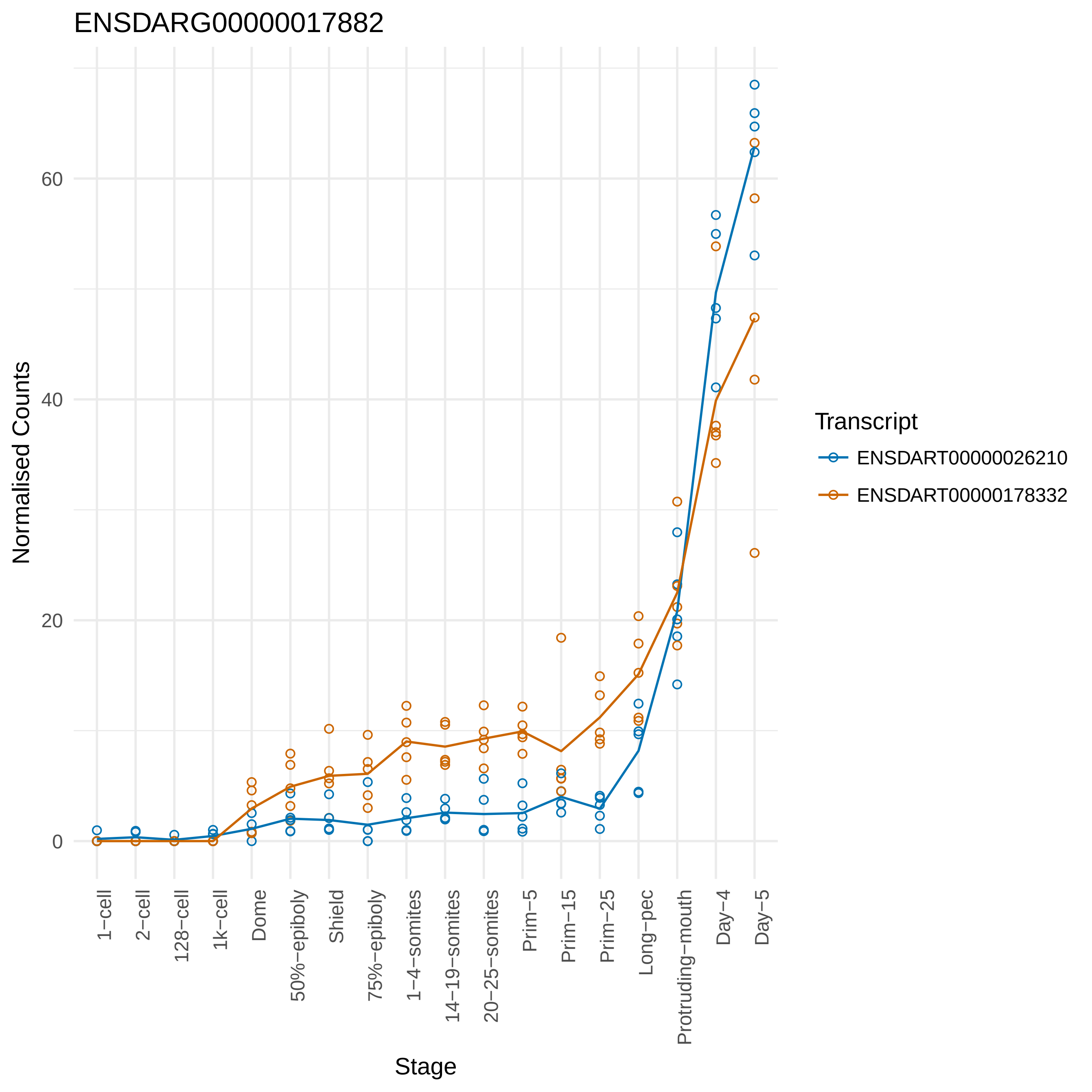

ENSDARG00000017882
Normalised Counts
0
20
40
60
Stage
1−cell
2−cell
128−cell
1k−cell
Dome
50%−epiboly
Shield
75%−epiboly
1−4−somites
14−19−somites
20−25−somites
Prim−5
Prim−15
Prim−25
Long−pec
Protruding−mouth
Day−4
Day−5
Transcript
ENSDART00000026210
ENSDART00000178332

ENSDARG00000017886

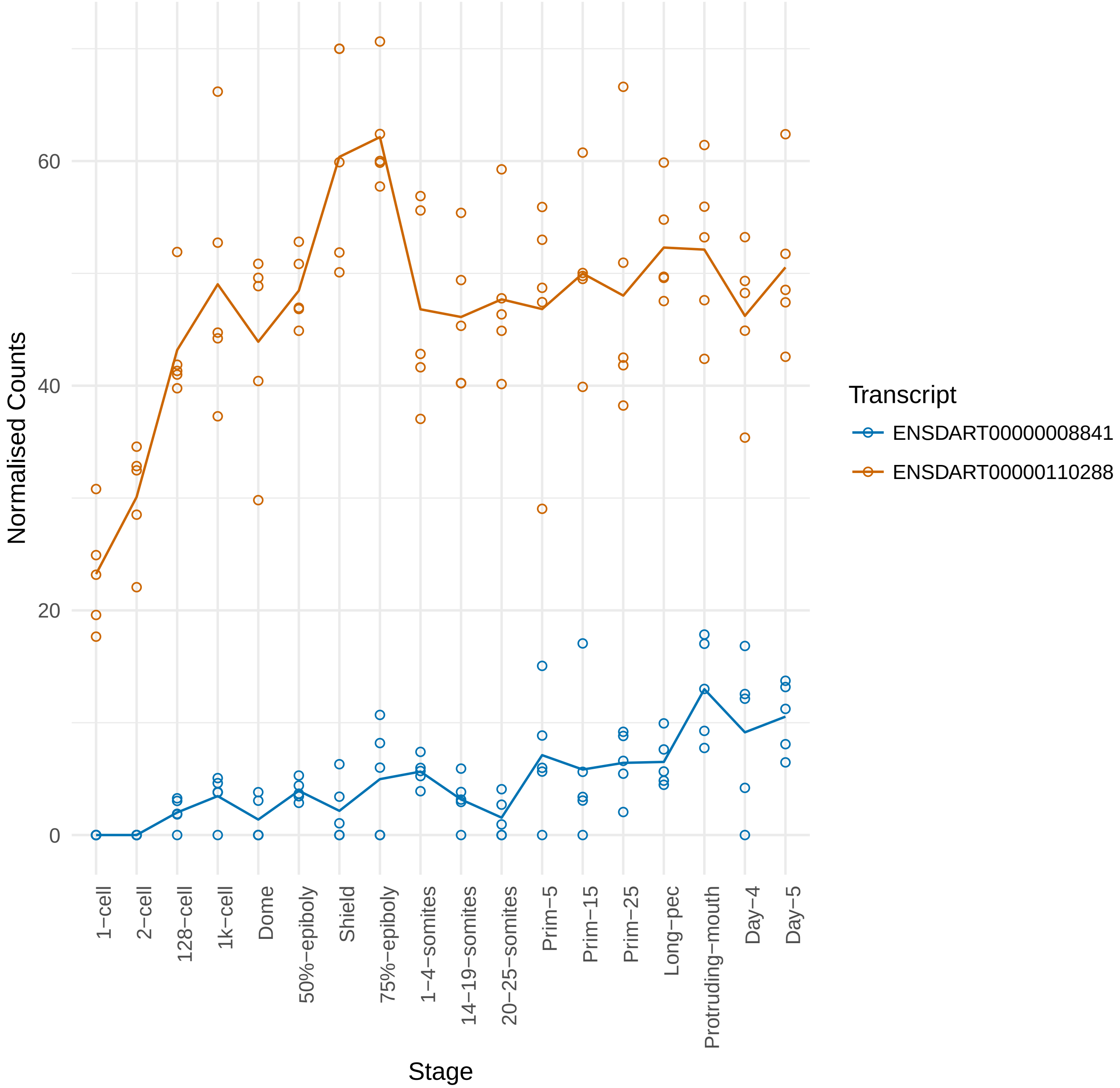

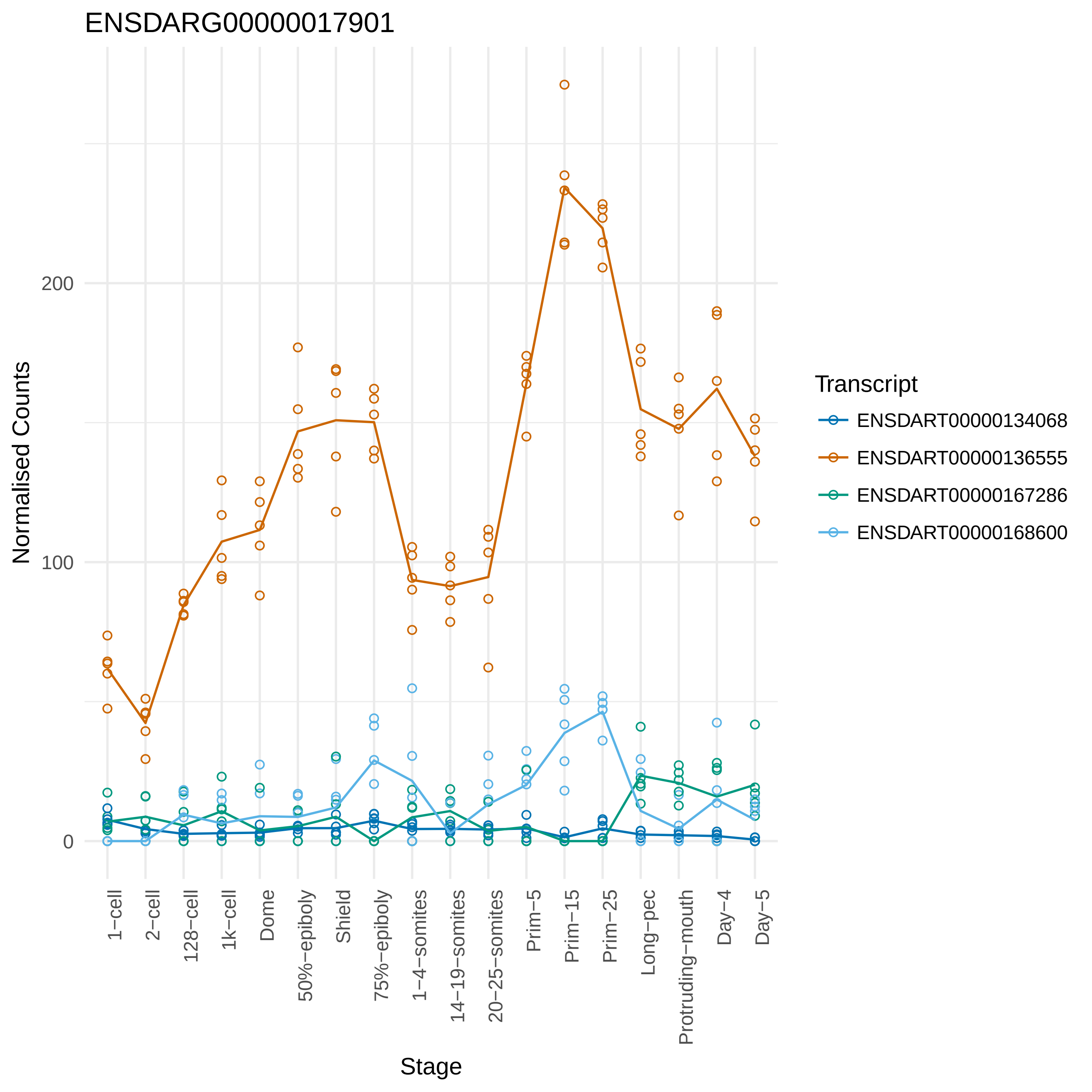

ENSDARG00000017901
Normalised Counts
0
100
200
Stage
1−cell
2−cell
128−cell
1k−cell
Dome
50%−epiboly
Shield
75%−epiboly
1−4−somites
14−19−somites
20−25−somites
Prim−5
Prim−15
Prim−25
Long−pec
Protruding−mouth
Day−4
Day−5
Transcript
ENSDART00000134068
ENSDART00000136555
ENSDART00000167286
ENSDART00000168600

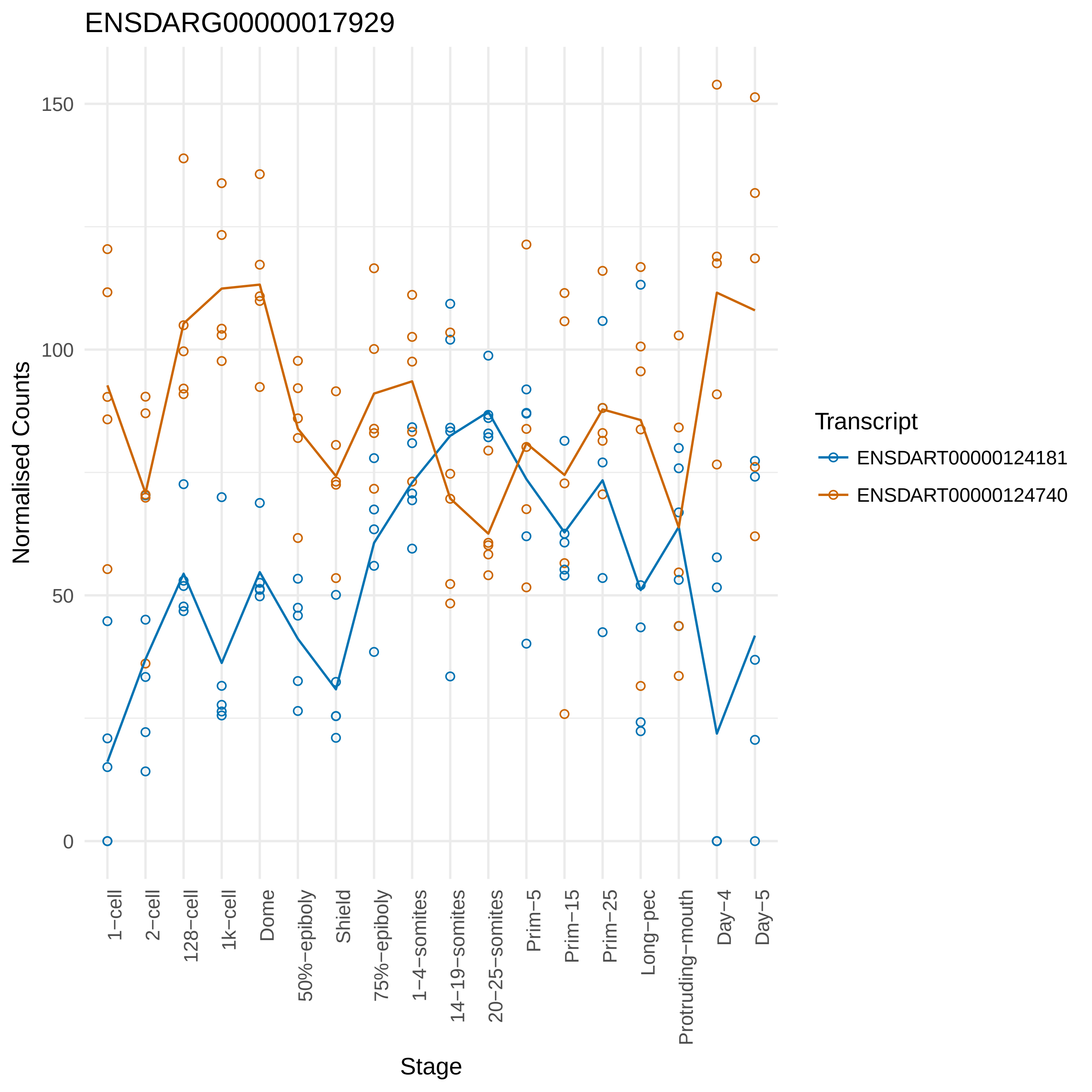

ENSDARG00000017929
Normalised Counts
150
100
50
0
1−cell
2−cell
128−cell
1k−cell
Dome
50%−epiboly
Shield
75%−epiboly
1−4−somites
14−19−somites
20−25−somites
Prim−5
Prim−15
Prim−25
Long−pec
Protruding−mouth
Day−4
Day−5
Stage
Transcript
ENSDART00000124181
ENSDART00000124740

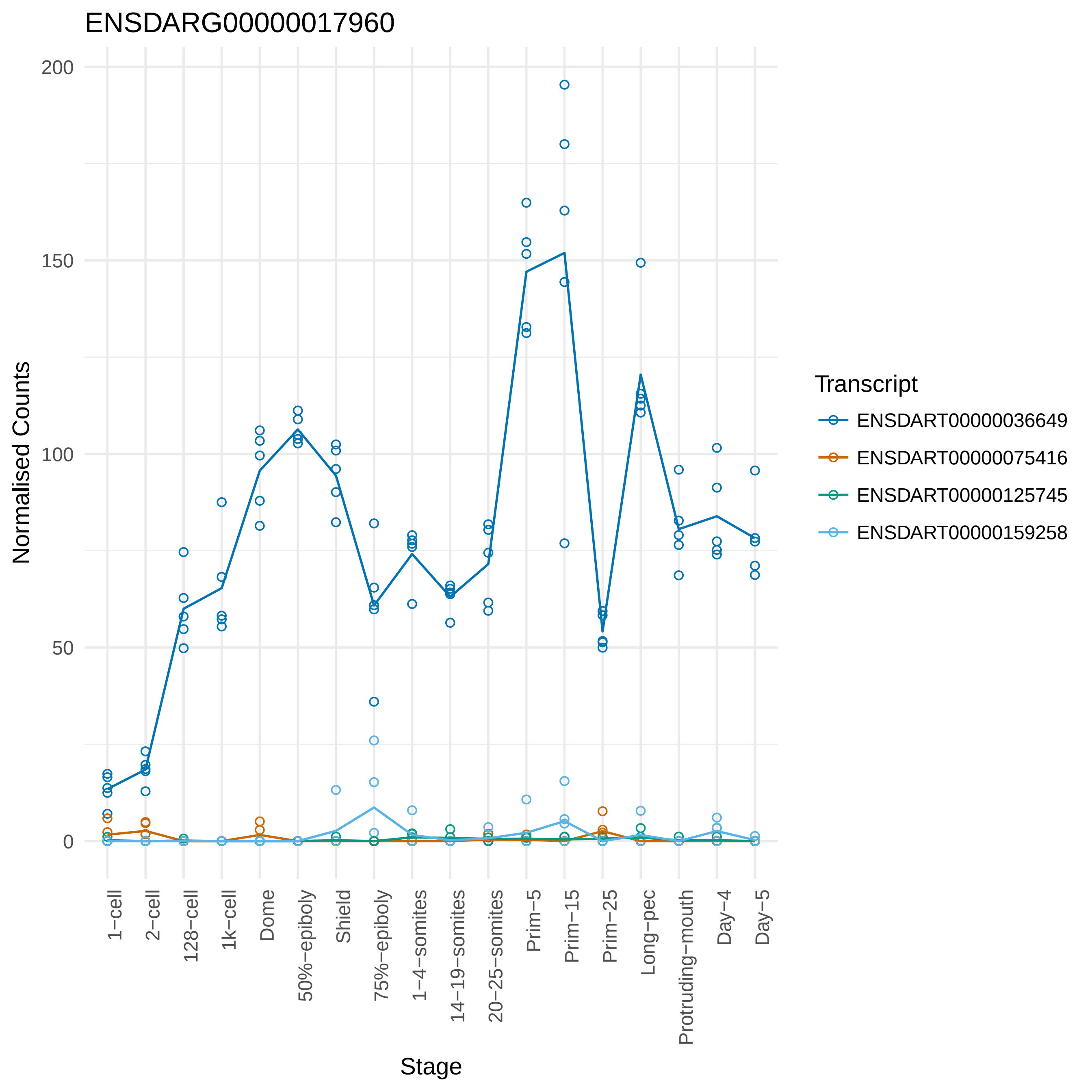

ENSDARG00000017960
Normalised Counts
200
150
100
50
0
Stage
1−cell
2−cell
128−cell
1k−cell
Dome
50%−epiboly
Shield
75%−epiboly
1−4−somites
14−19−somites
20−25−somites
Prim−5
Prim−15
Prim−25
Long−pec
Protruding−mouth
Day−4
Day−5
Transcript
ENSDART00000036649
ENSDART00000075416
ENSDART00000125745
ENSDART00000159258

ENSDARG00000017985

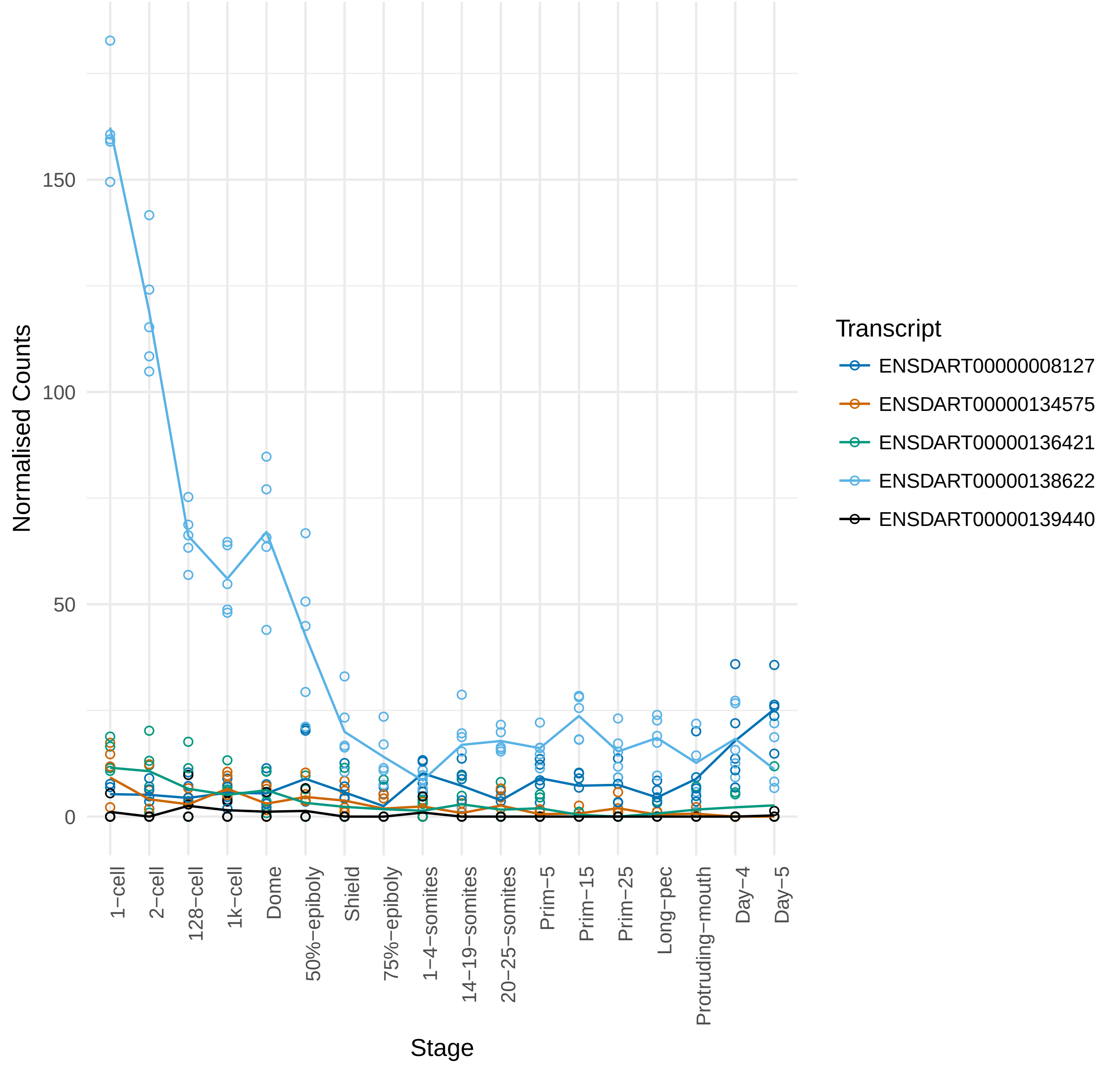

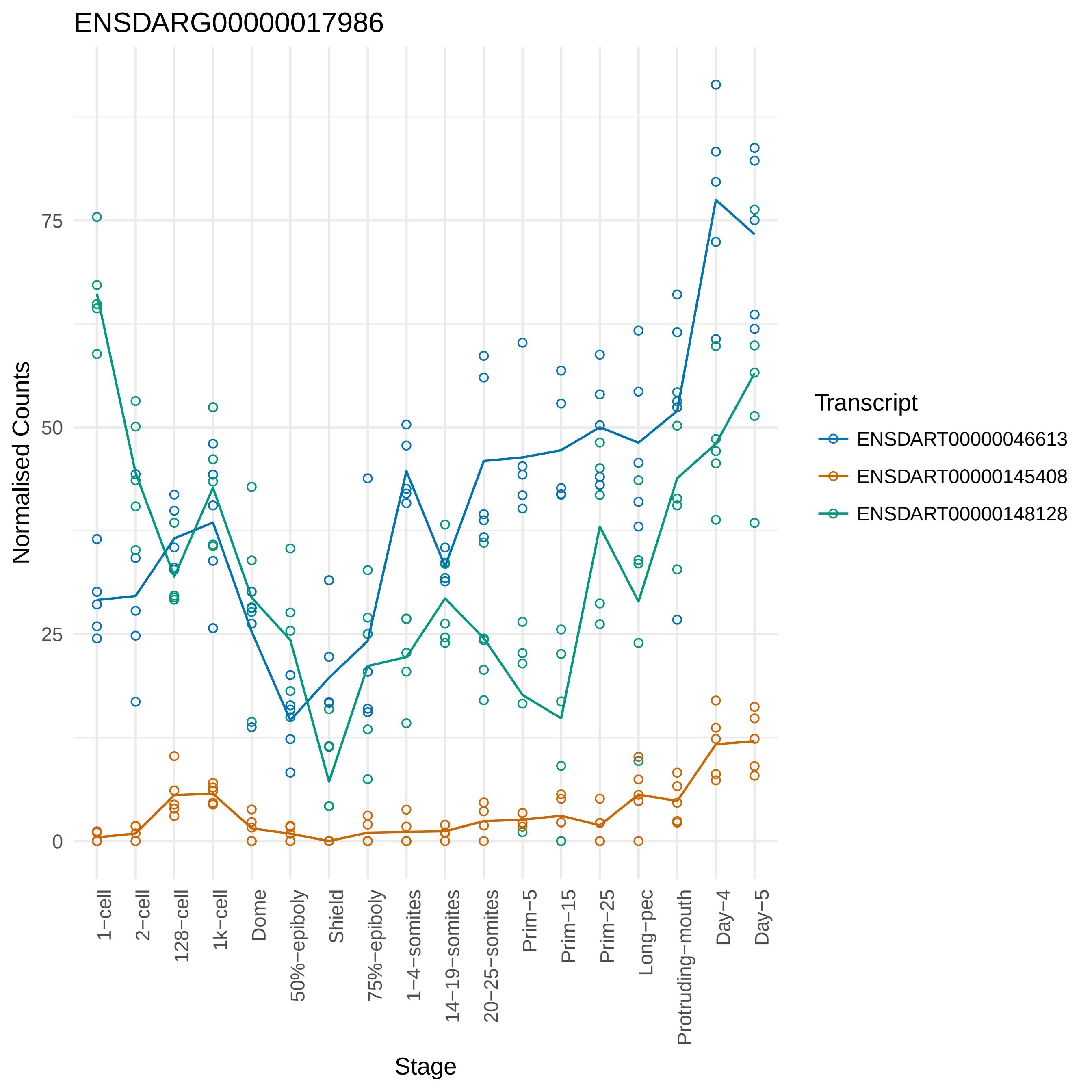

ENSDARG00000017986
Normalised Counts
0
25
50
75
Stage
1−cell
2−cell
128−cell
1k−cell
Dome
50%−epiboly
Shield
75%−epiboly
1−4−somites
14−19−somites
20−25−somites
Prim−5
Prim−15
Prim−25
Long−pec
Protruding−mouth
Day−4
Day−5
Transcript
ENSDART00000046613
ENSDART00000145408
ENSDART00000148128

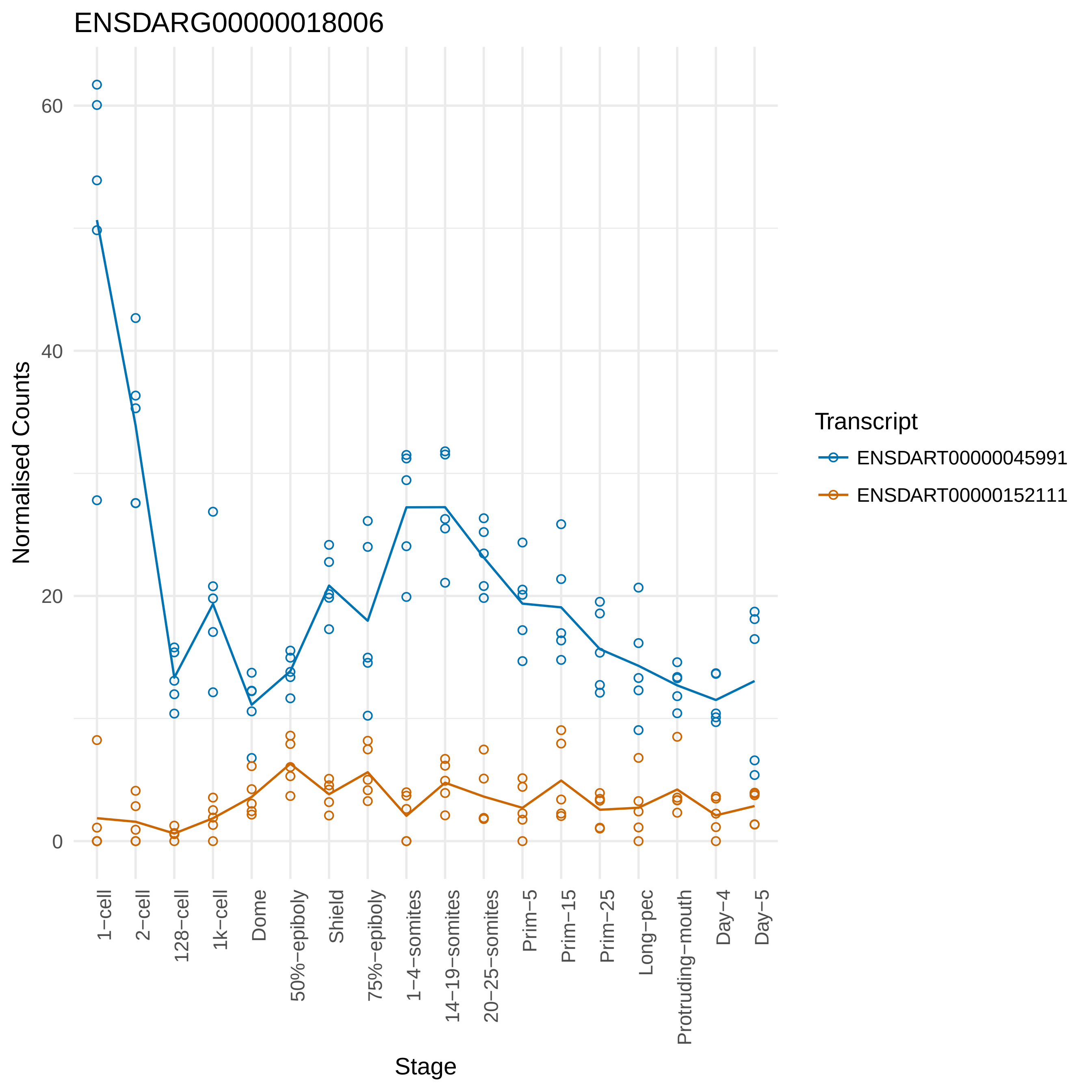
ENSDARG00000018006
Normalised Counts
0
20
40
60
1−cell
2−cell
128−cell
1k−cell
Dome
50%−epiboly
Shield
75%−epiboly
1−4−somites
14−19−somites
20−25−somites
Prim−5
Prim−15
Prim−25
Long−pec
Protruding−mouth
Day−4
Day−5
Stage
Transcript
ENSDART00000045991
ENSDART00000152111

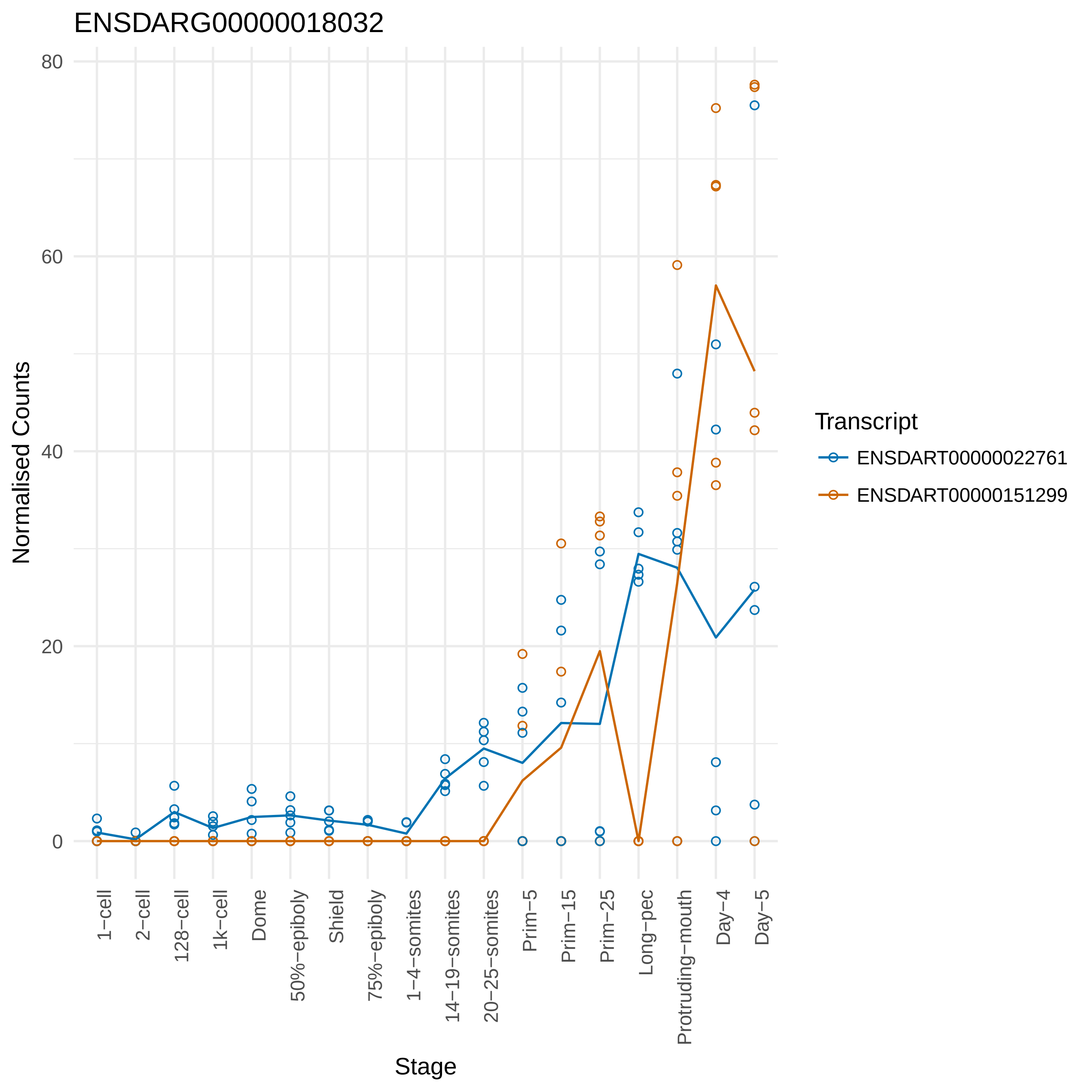

ENSDARG00000018032
Normalised Counts
0
20
40
60
80
Stage
1−cell
2−cell
128−cell
1k−cell
Dome
50%−epiboly
Shield
75%−epiboly
1−4−somites
14−19−somites
20−25−somites
Prim−5
Prim−15
Prim−25
Long−pec
Protruding−mouth
Day−4
Day−5
Transcript
ENSDART00000022761
ENSDART00000151299

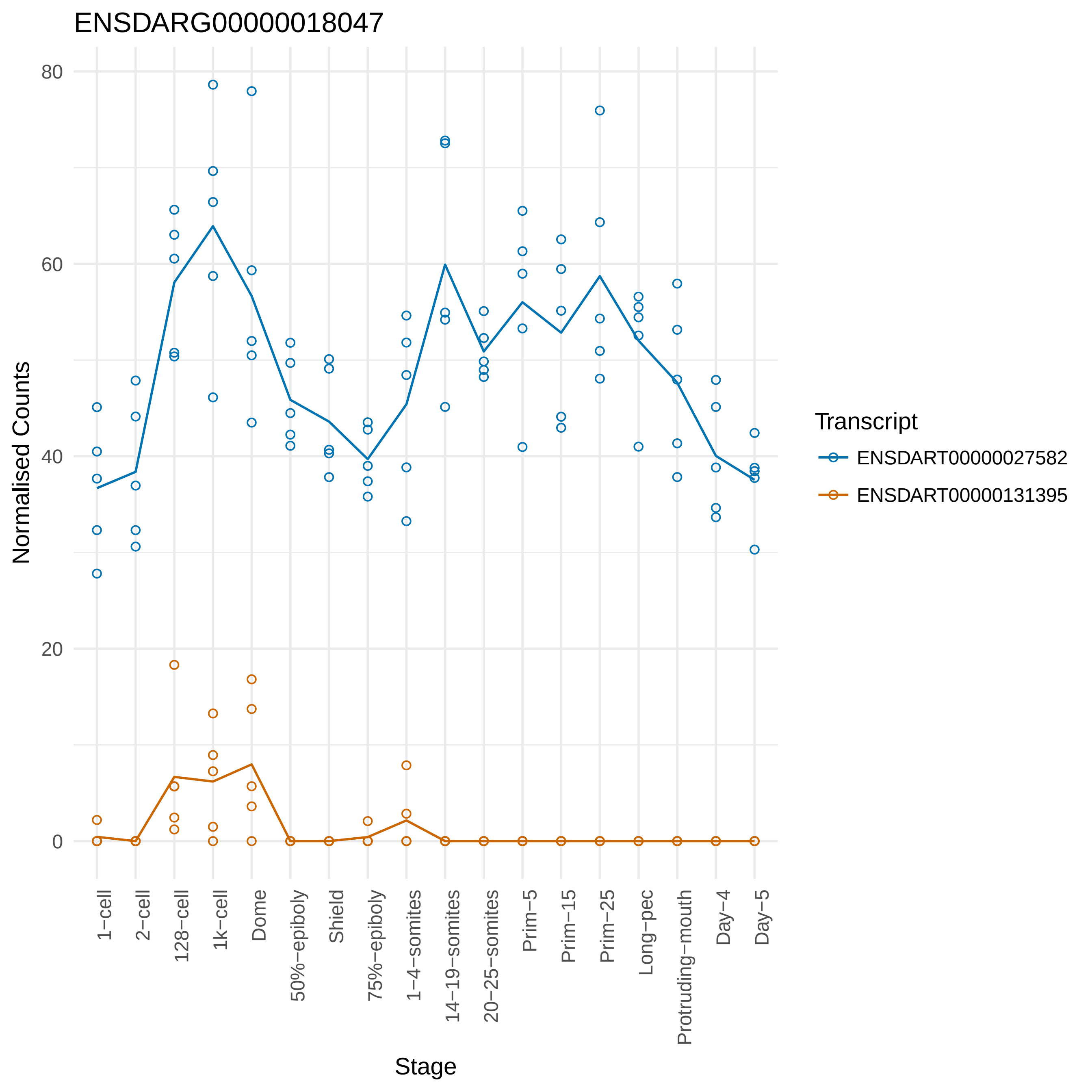

ENSDARG00000018047
Normalised Counts
80
60
40
20
0
1−cell
2−cell
128−cell
1k−cell
Dome
50%−epiboly
Shield
75%−epiboly
1−4−somites
14−19−somites
20−25−somites
Prim−5
Prim−15
Prim−25
Long−pec
Protruding−mouth
Day−4
Day−5
Stage
Transcript
ENSDART00000027582
ENSDART00000131395

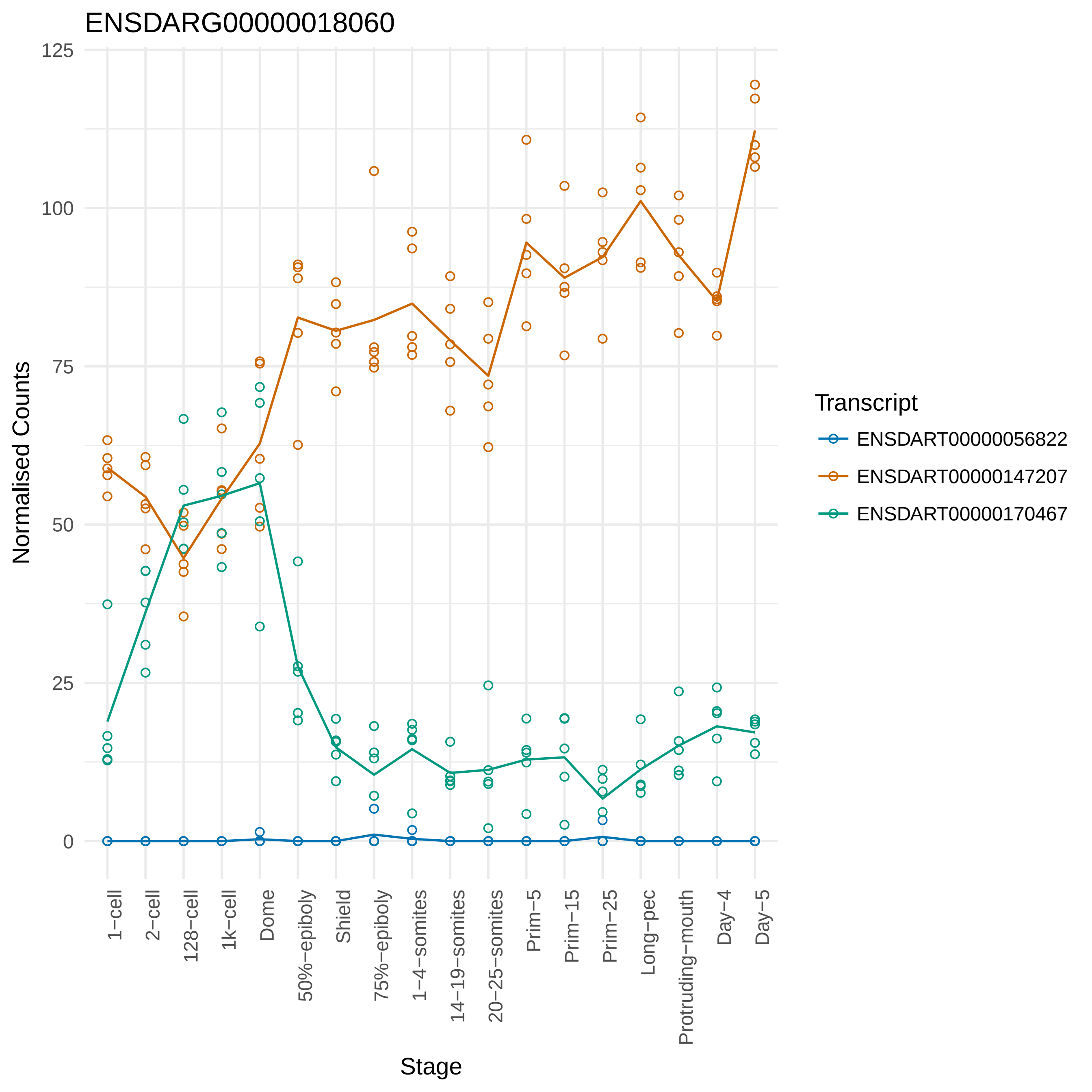

ENSDARG00000018060
Normalised Counts
125
100
75
50
25
0
1−cell
2−cell
128−cell
1k−cell
Dome
50%−epiboly
Shield
75%−epiboly
1−4−somites
14−19−somites
20−25−somites
Prim−5
Prim−15
Prim−25
Long−pec
Protruding−mouth
Day−4
Day−5
Stage
Transcript
ENSDART00000056822
ENSDART00000147207
ENSDART00000170467

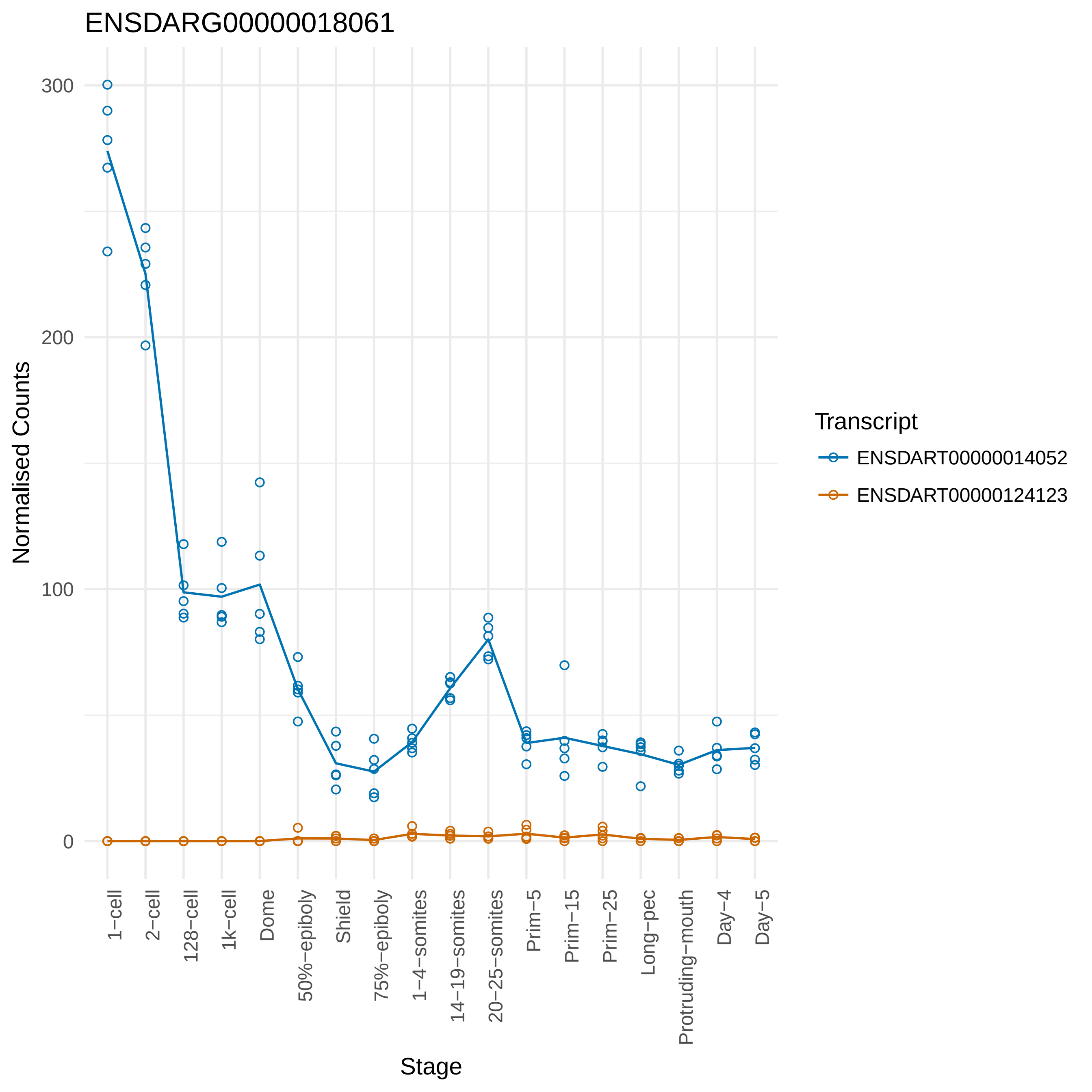
ENSDARG00000018061
Normalised Counts
300
200
100
0
1−cell
2−cell
128−cell
1k−cell
Dome
50%−epiboly
Shield
75%−epiboly
1−4−somites
14−19−somites
20−25−somites
Prim−5
Prim−15
Prim−25
Long−pec
Protruding−mouth
Day−4
Day−5
Stage
Transcript
ENSDART00000014052
ENSDART00000124123

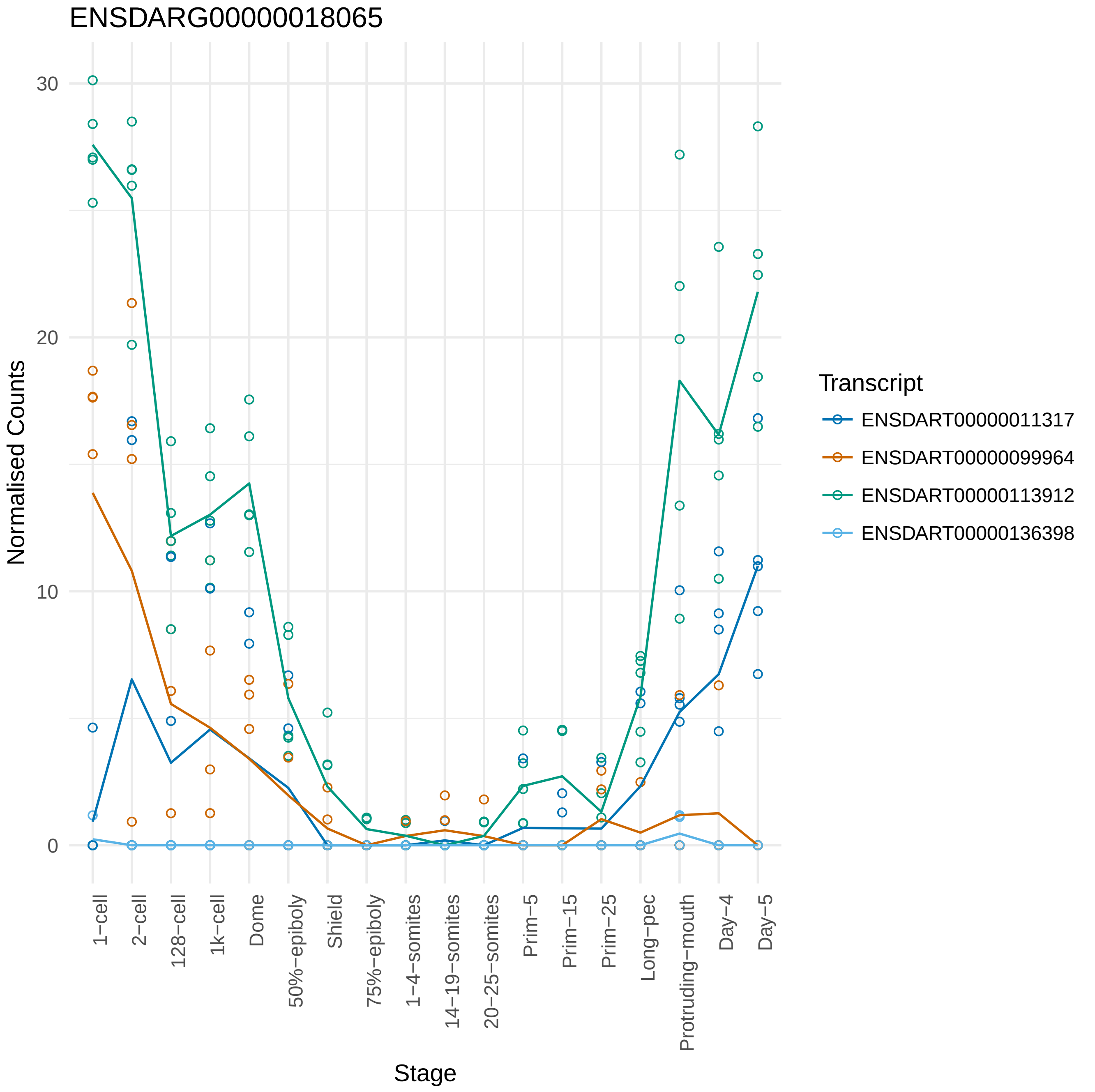
ENSDARG00000018065
Normalised Counts
30
20
10
0
1−cell
2−cell
128−cell
1k−cell
Dome
50%−epiboly
Shield
75%−epiboly
1−4−somites
14−19−somites
20−25−somites
Prim−5
Prim−15
Prim−25
Long−pec
Protruding−mouth
Day−4
Day−5
Stage
Transcript
ENSDART00000011317
ENSDART00000099964
ENSDART00000113912
ENSDART00000136398

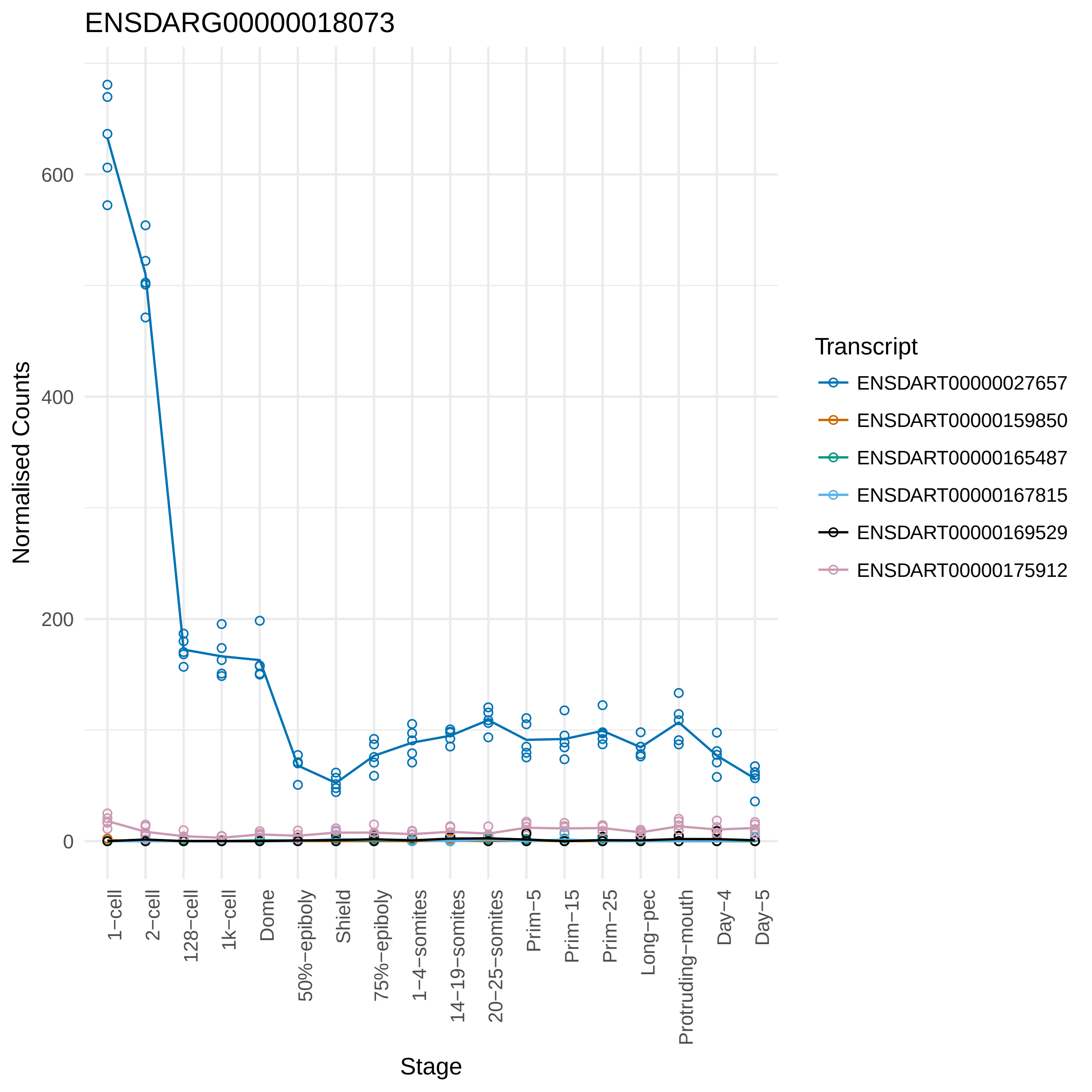

ENSDARG00000018073
Normalised Counts
600
400
200
0
1−cell
2−cell
128−cell
1k−cell
Dome
50%−epiboly
Shield
75%−epiboly
1−4−somites
14−19−somites
20−25−somites
Prim−5
Prim−15
Prim−25
Long−pec
Protruding−mouth
Day−4
Day−5
Stage
Transcript
ENSDART00000027657
ENSDART00000159850
ENSDART00000165487
ENSDART00000167815
ENSDART00000169529
ENSDART00000175912

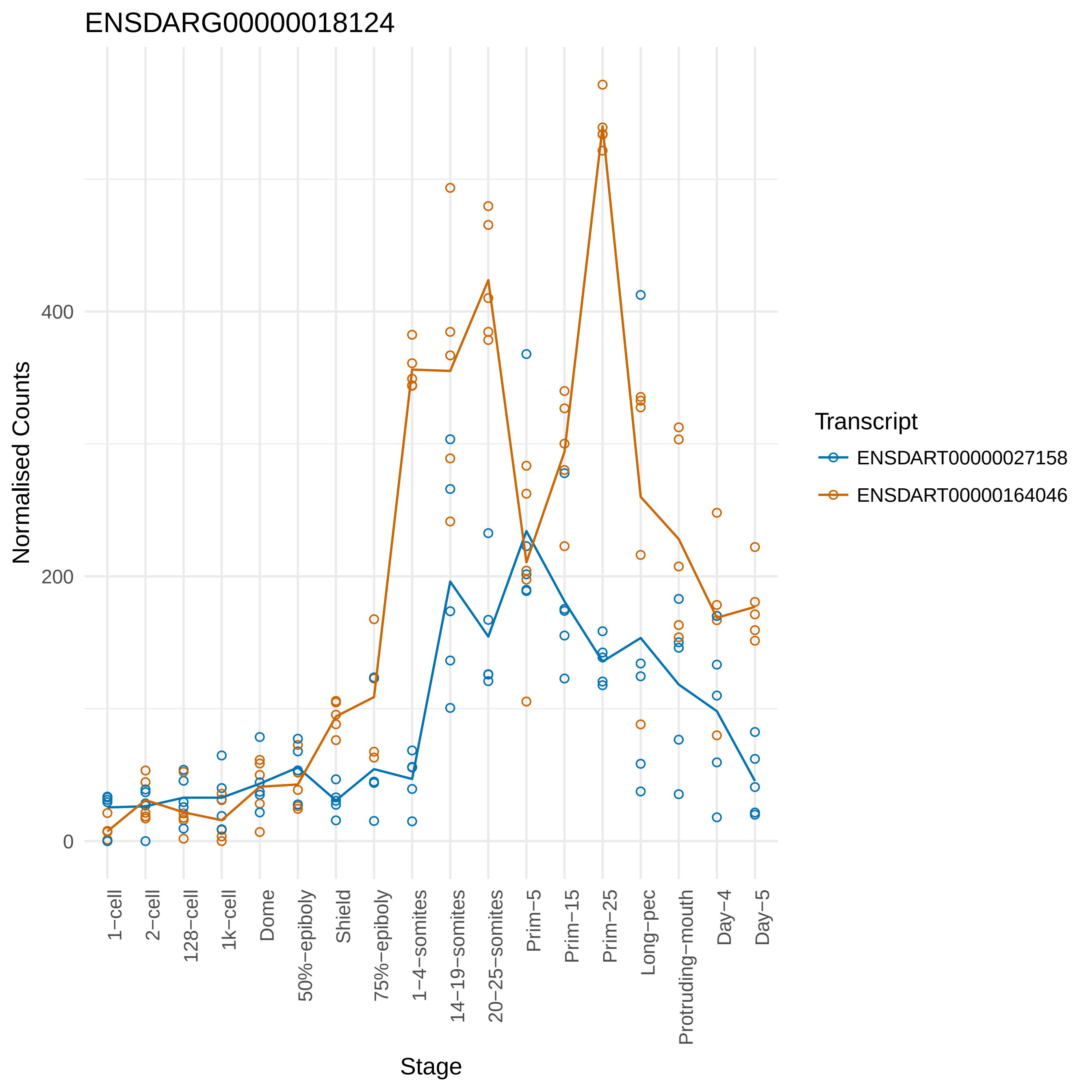
ENSDARG00000018124
Normalised Counts
400
200
0
1−cell
2−cell
128−cell
1k−cell
Dome
50%−epiboly
Shield
75%−epiboly
1−4−somites
14−19−somites
20−25−somites
Prim−5
Prim−15
Prim−25
Long−pec
Protruding−mouth
Day−4
Day−5
Stage
Transcript
ENSDART00000027158
ENSDART00000164046

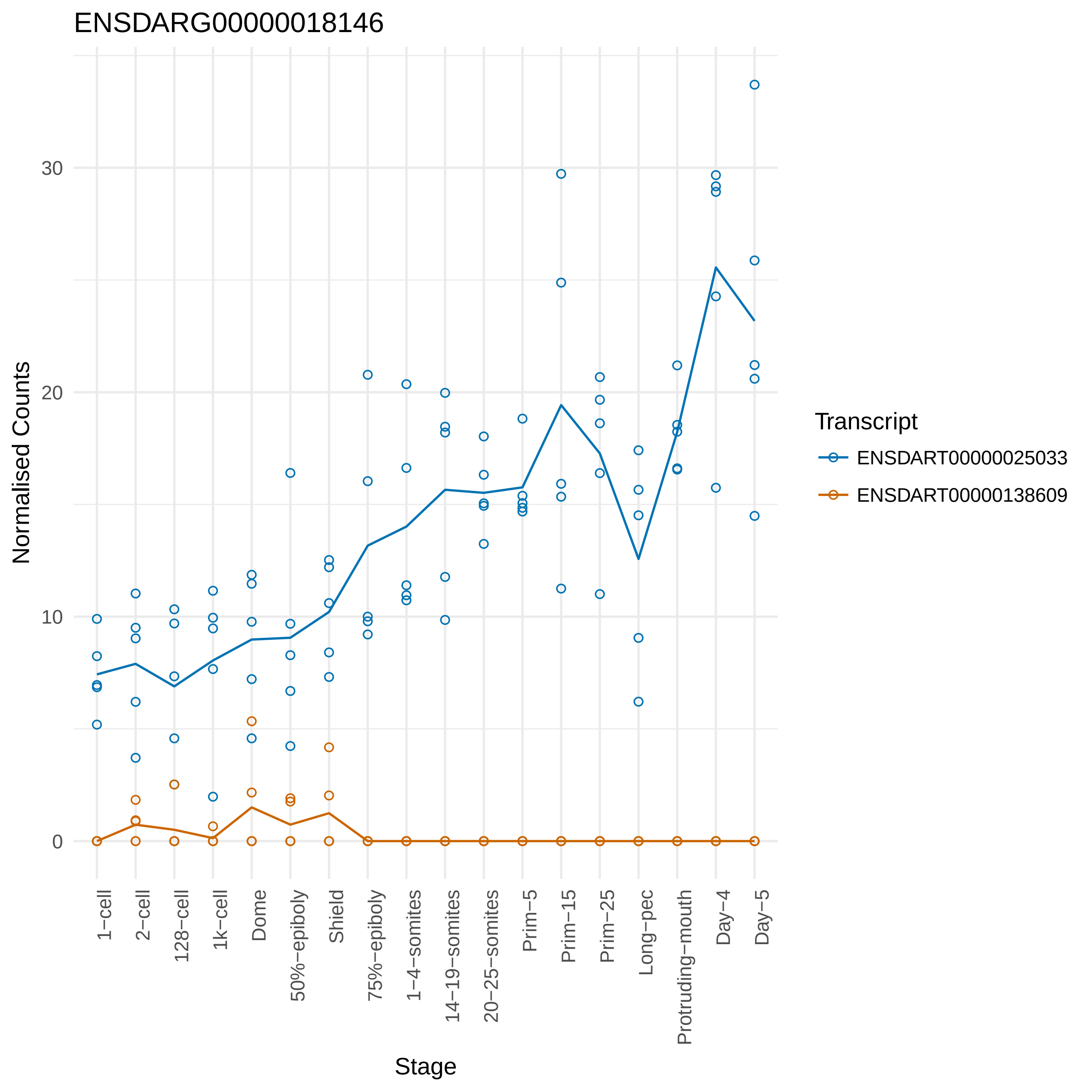
ENSDARG00000018146
Normalised Counts
30
20
10
0
1−cell
2−cell
128−cell
1k−cell
Dome
50%−epiboly
Shield
75%−epiboly
1−4−somites
14−19−somites
20−25−somites
Prim−5
Prim−15
Prim−25
Long−pec
Protruding−mouth
Day−4
Day−5
Stage
Transcript
ENSDART00000025033
ENSDART00000138609

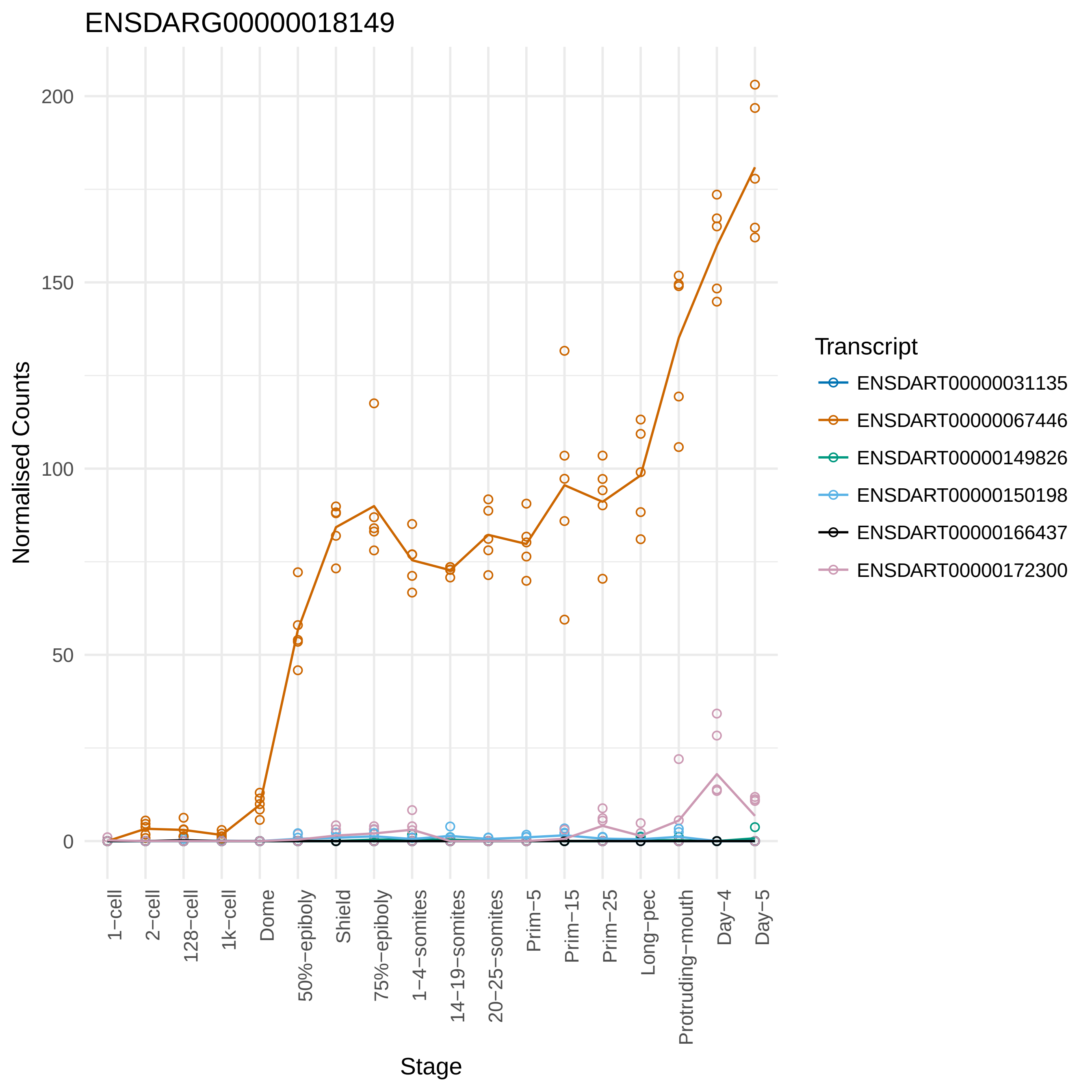
ENSDARG00000018149
Normalised Counts
0
50
100
150
200
Stage
1−cell
2−cell
128−cell
1k−cell
Dome
50%−epiboly
Shield
75%−epiboly
1−4−somites
14−19−somites
20−25−somites
Prim−5
Prim−15
Prim−25
Long−pec
Protruding−mouth
Day−4
Day−5
Transcript
ENSDART00000031135
ENSDART00000067446
ENSDART00000149826
ENSDART00000150198
ENSDART00000166437
ENSDART00000172300

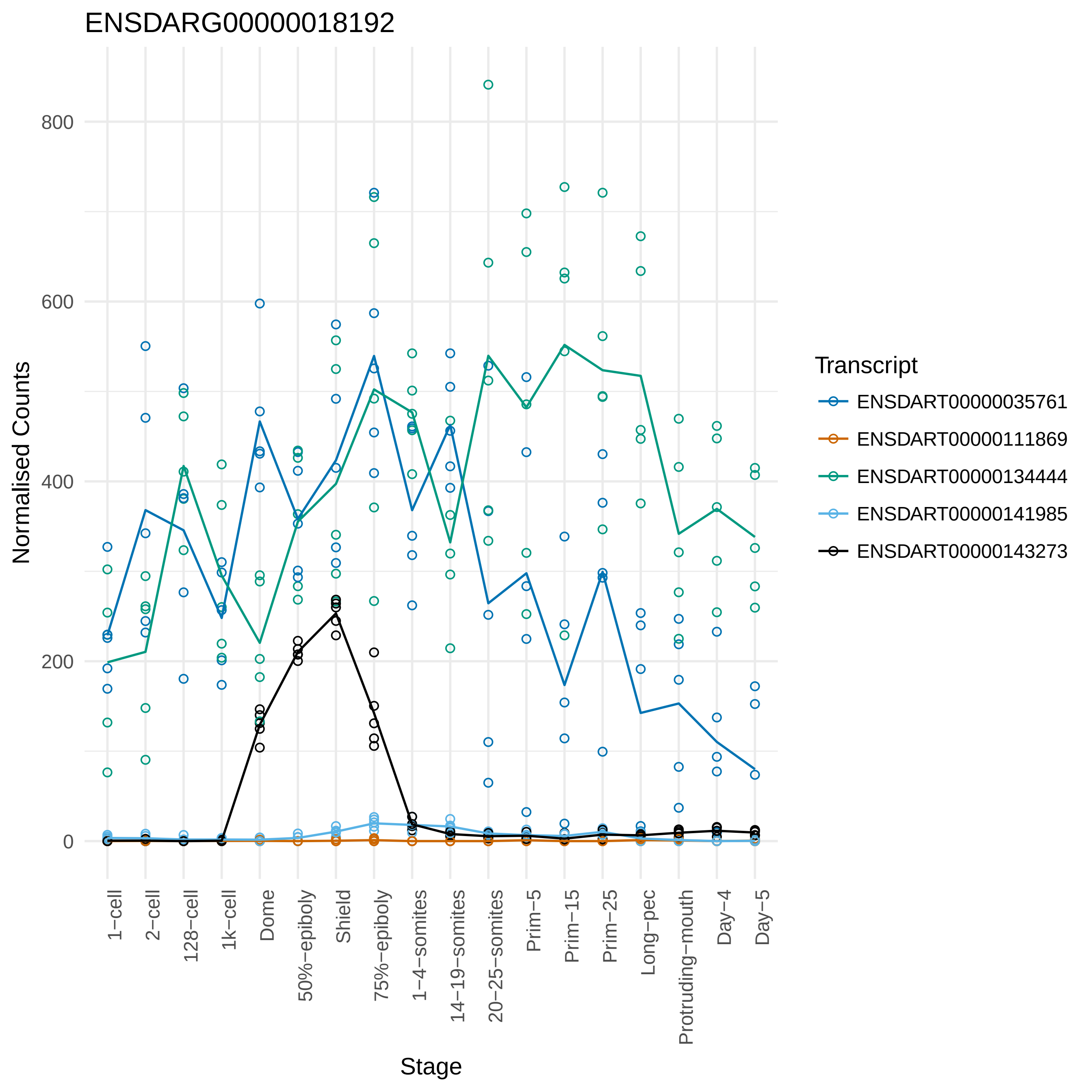

ENSDARG00000018192
Normalised Counts
0
200
400
600
800
Stage
1−cell
2−cell
128−cell
1k−cell
Dome
50%−epiboly
Shield
75%−epiboly
1−4−somites
14−19−somites
20−25−somites
Prim−5
Prim−15
Prim−25
Long−pec
Protruding−mouth
Day−4
Day−5
Transcript
ENSDART00000035761
ENSDART00000111869
ENSDART00000134444
ENSDART00000141985
ENSDART00000143273

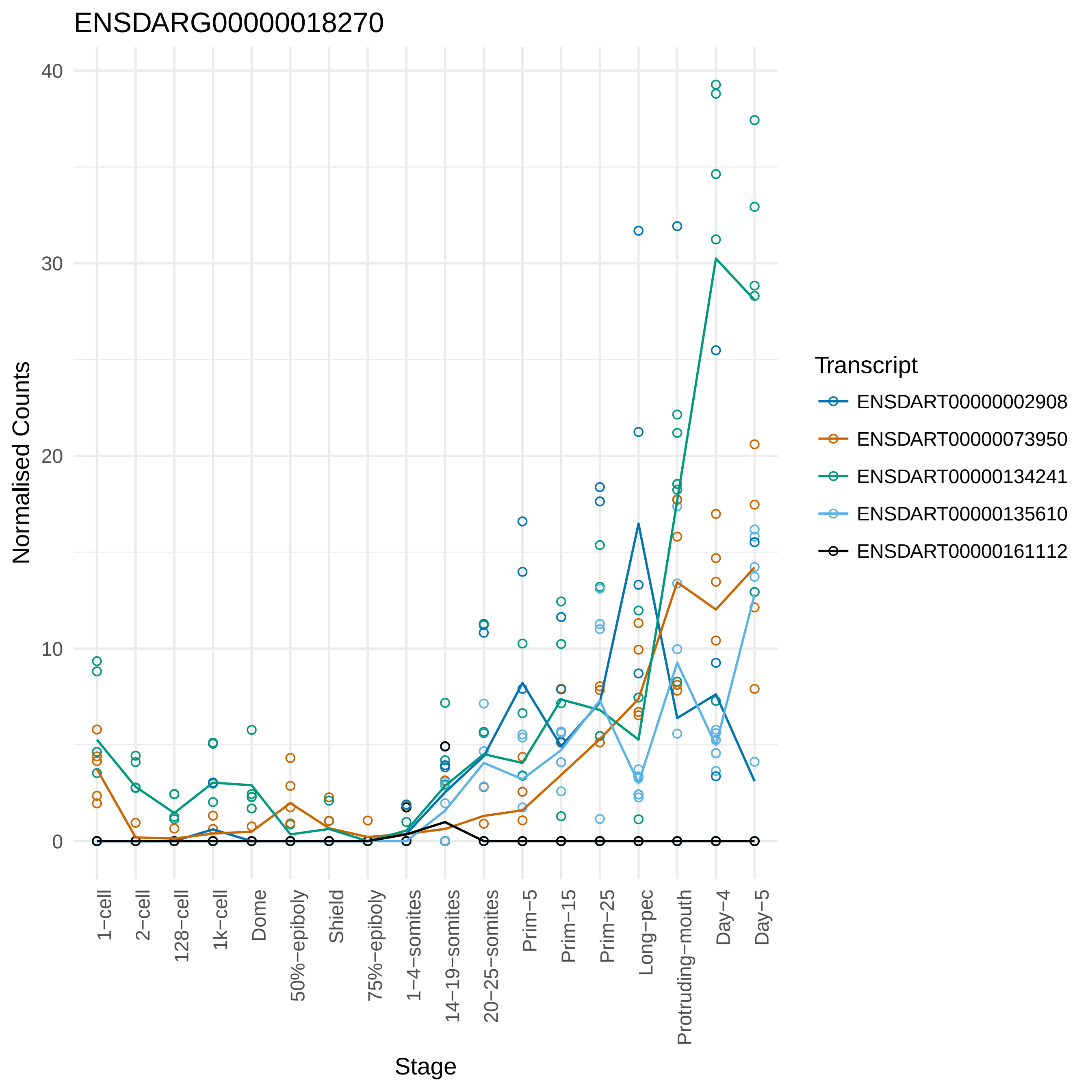
ENSDARG00000018270
Normalised Counts
0
10
20
30
40
1−cell
2−cell
128−cell
1k−cell
Dome
50%−epiboly
Shield
75%−epiboly
1−4−somites
14−19−somites
20−25−somites
Prim−5
Prim−15
Prim−25
Long−pec
Protruding−mouth
Day−4
Day−5
Stage
Transcript
ENSDART00000002908
ENSDART00000073950
ENSDART00000134241
ENSDART00000135610
ENSDART00000161112

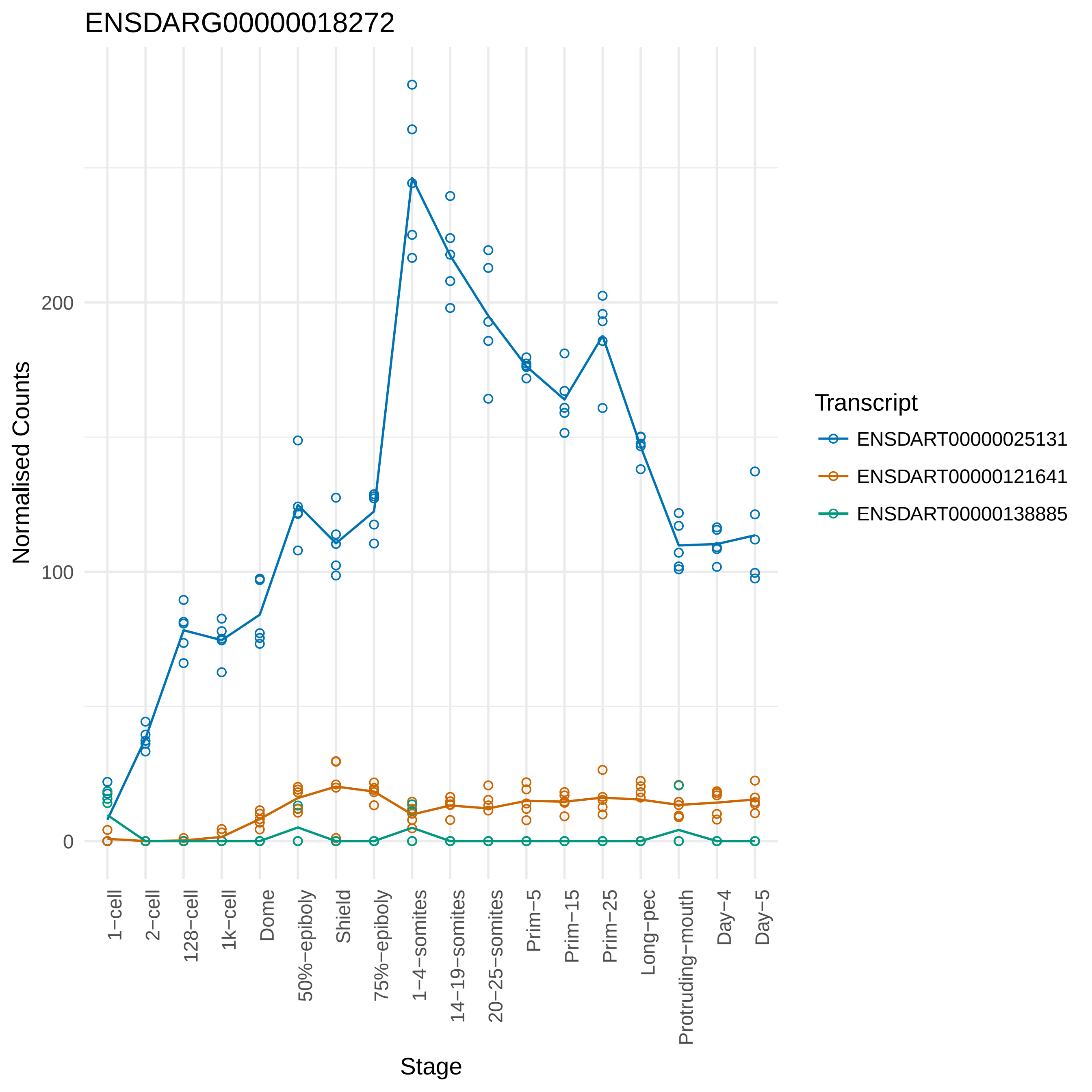

ENSDARG00000018272
Normalised Counts
200
100
0
1−cell
2−cell
128−cell
1k−cell
Dome
50%−epiboly
Shield
75%−epiboly
1−4−somites
14−19−somites
20−25−somites
Prim−5
Prim−15
Prim−25
Long−pec
Protruding−mouth
Day−4
Day−5
Stage
Transcript
ENSDART00000025131
ENSDART00000121641
ENSDART00000138885

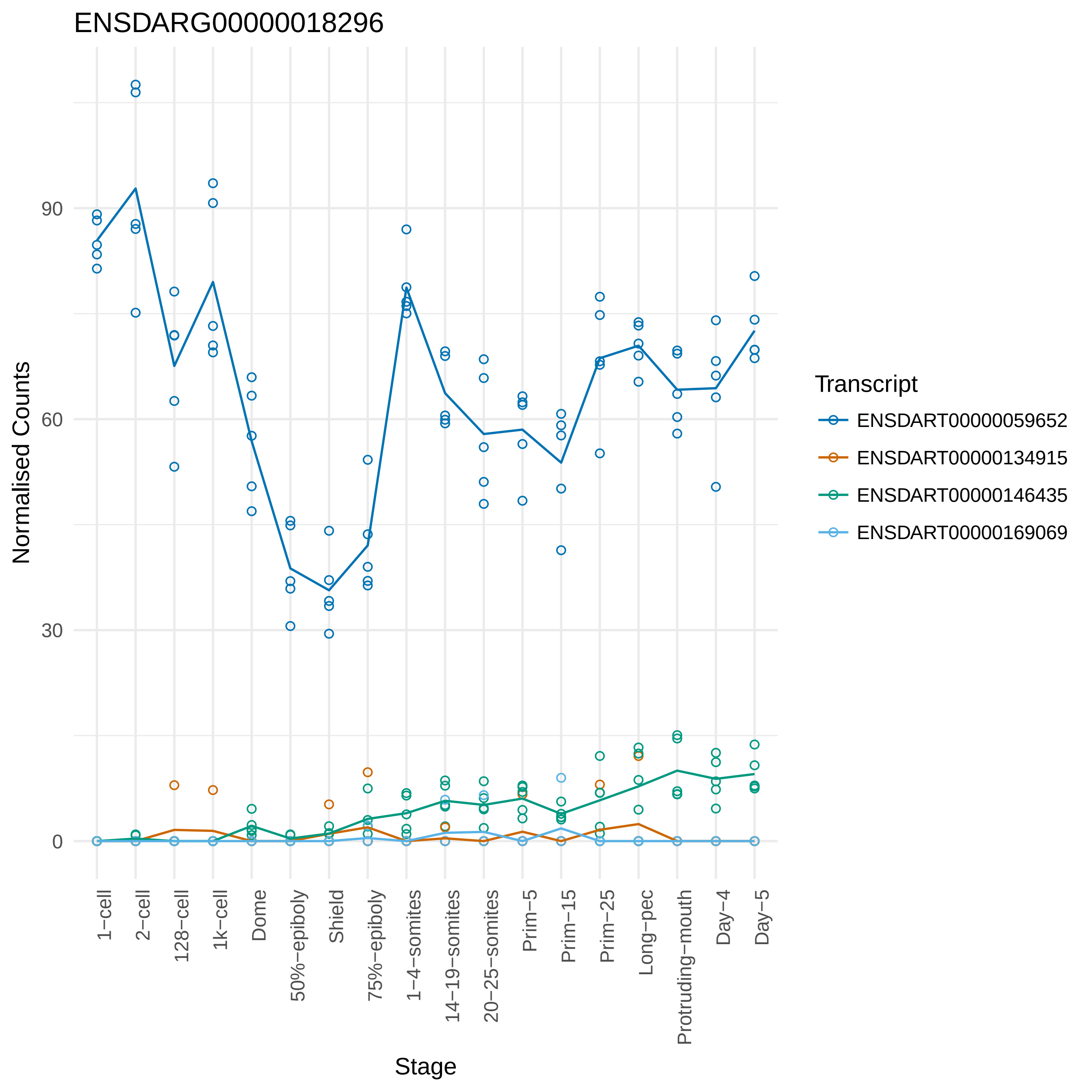
ENSDARG00000018296
Normalised Counts
0
30
60
90
Stage
1−cell
2−cell
128−cell
1k−cell
Dome
50%−epiboly
Shield
75%−epiboly
1−4−somites
14−19−somites
20−25−somites
Prim−5
Prim−15
Prim−25
Long−pec
Protruding−mouth
Day−4
Day−5
Transcript
ENSDART00000059652
ENSDART00000134915
ENSDART00000146435
ENSDART00000169069

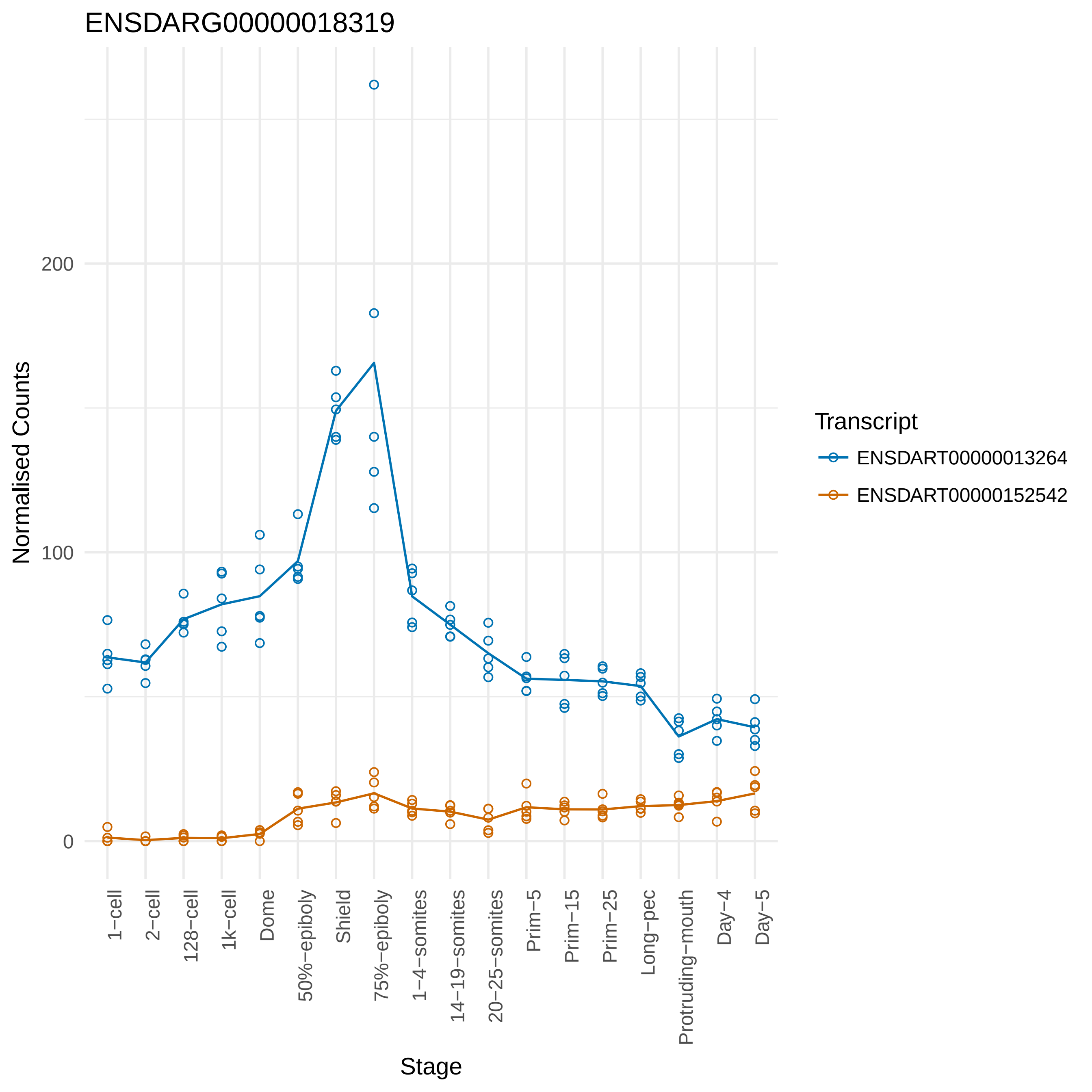
ENSDARG00000018319
Normalised Counts
200
100
0
Stage
1−cell
2−cell
128−cell
1k−cell
Dome
50%−epiboly
Shield
75%−epiboly
1−4−somites
14−19−somites
20−25−somites
Prim−5
Prim−15
Prim−25
Long−pec
Protruding−mouth
Day−4
Day−5
Transcript
ENSDART00000013264
ENSDART00000152542

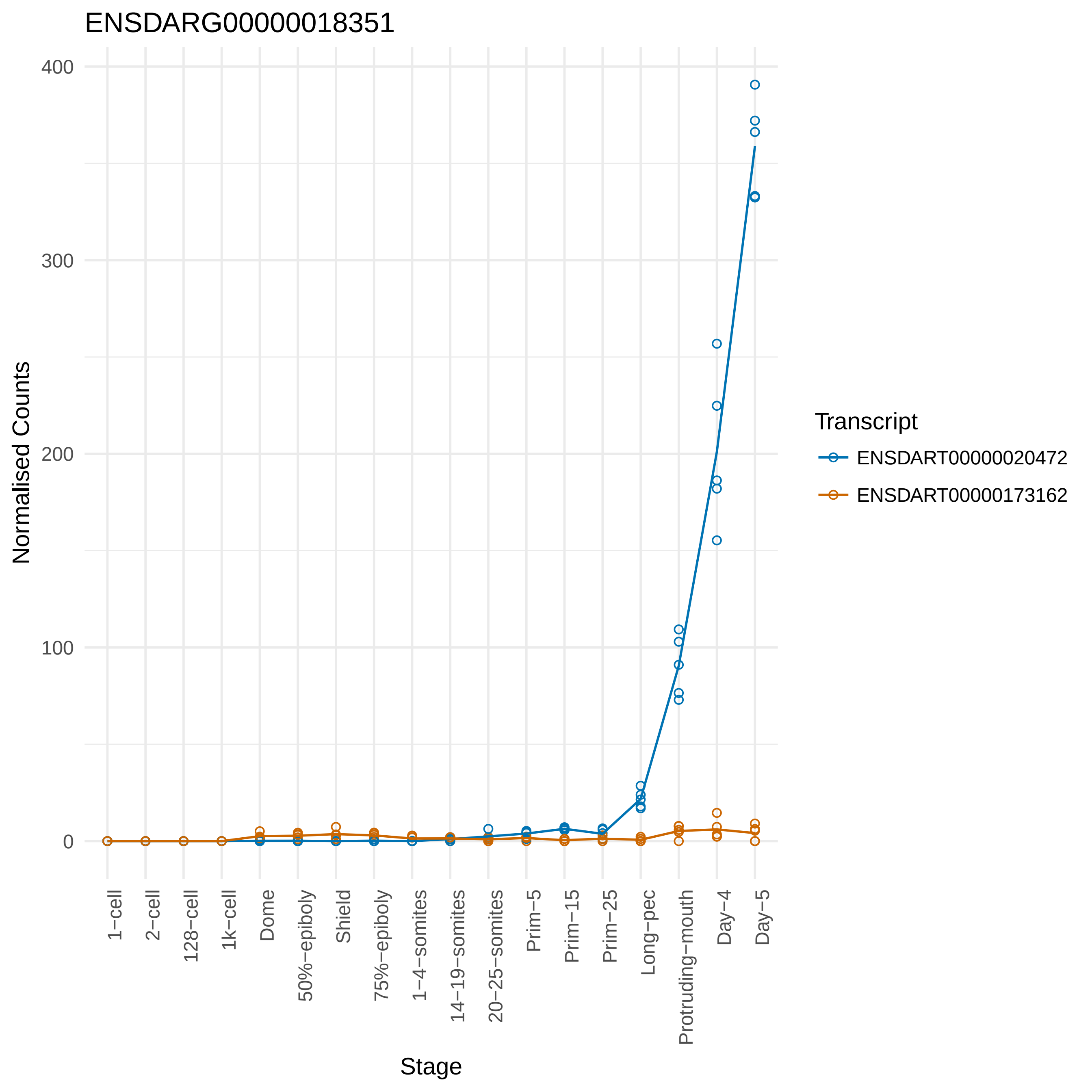
ENSDARG00000018351
Normalised Counts
400
300
200
100
0
Stage
1−cell
2−cell
128−cell
1k−cell
Dome
50%−epiboly
Shield
75%−epiboly
1−4−somites
14−19−somites
20−25−somites
Prim−5
Prim−15
Prim−25
Long−pec
Protruding−mouth
Day−4
Day−5
Transcript
ENSDART00000020472
ENSDART00000173162

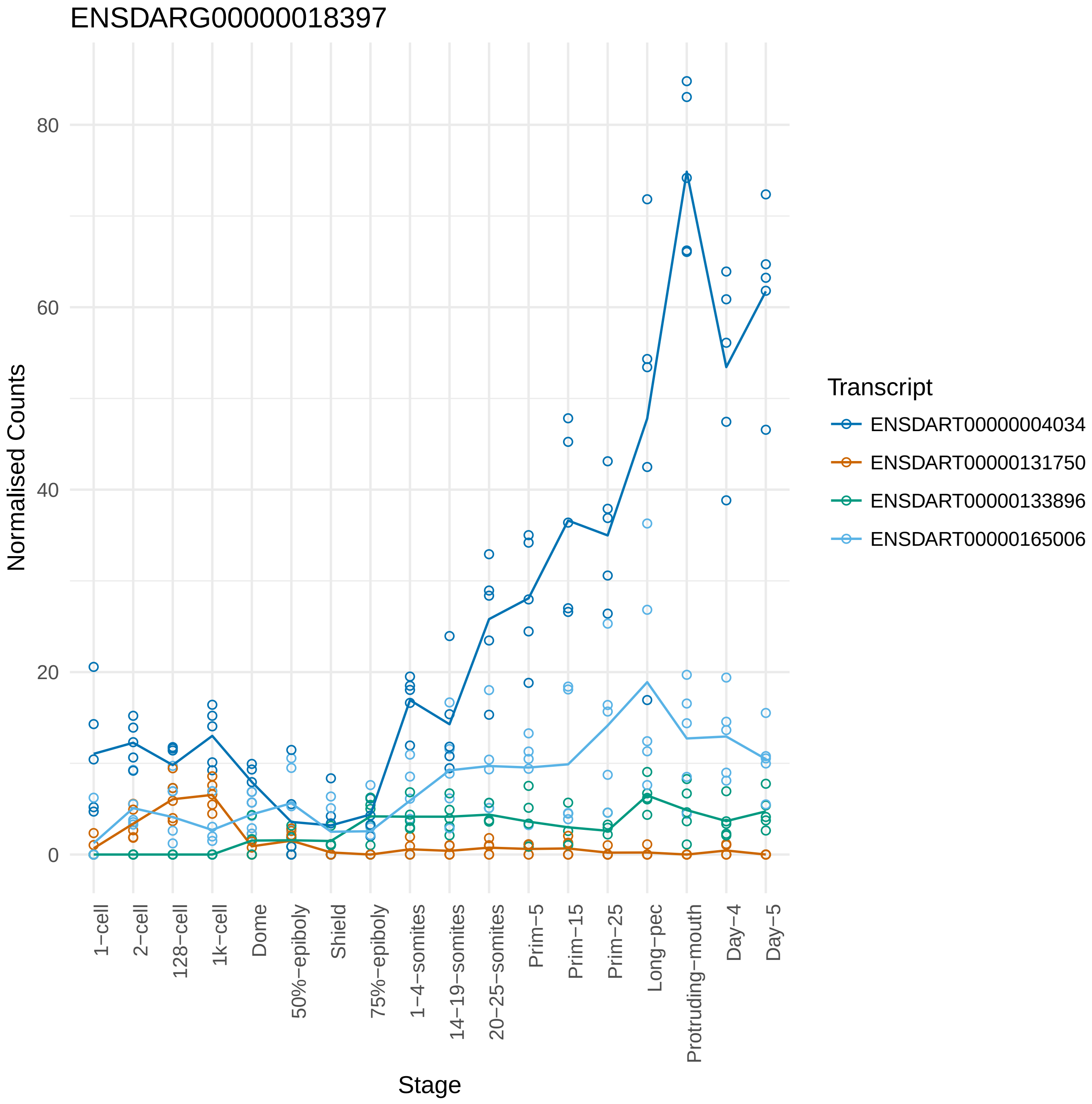
ENSDARG00000018397
Normalised Counts
0
20
40
60
80
1−cell
2−cell
128−cell
1k−cell
Dome
50%−epiboly
Shield
75%−epiboly
1−4−somites
14−19−somites
20−25−somites
Prim−5
Prim−15
Prim−25
Long−pec
Protruding−mouth
Day−4
Day−5
Stage
Transcript
ENSDART00000004034
ENSDART00000131750
ENSDART00000133896
ENSDART00000165006

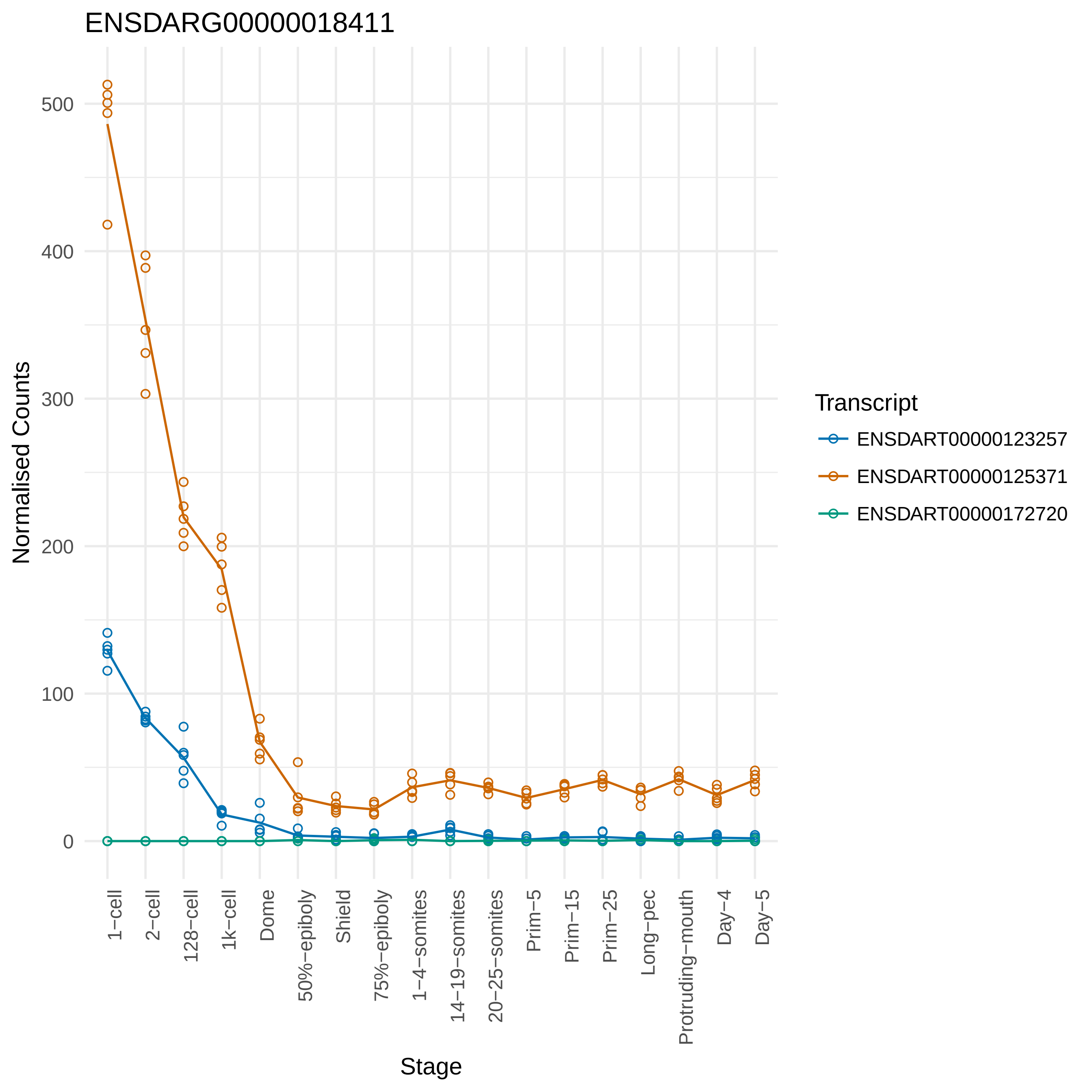

ENSDARG00000018411
Normalised Counts
500
400
300
200
100
0
1−cell
2−cell
128−cell
1k−cell
Dome
50%−epiboly
Shield
75%−epiboly
1−4−somites
14−19−somites
20−25−somites
Prim−5
Prim−15
Prim−25
Long−pec
Protruding−mouth
Day−4
Day−5
Stage
Transcript
ENSDART00000123257
ENSDART00000125371
ENSDART00000172720

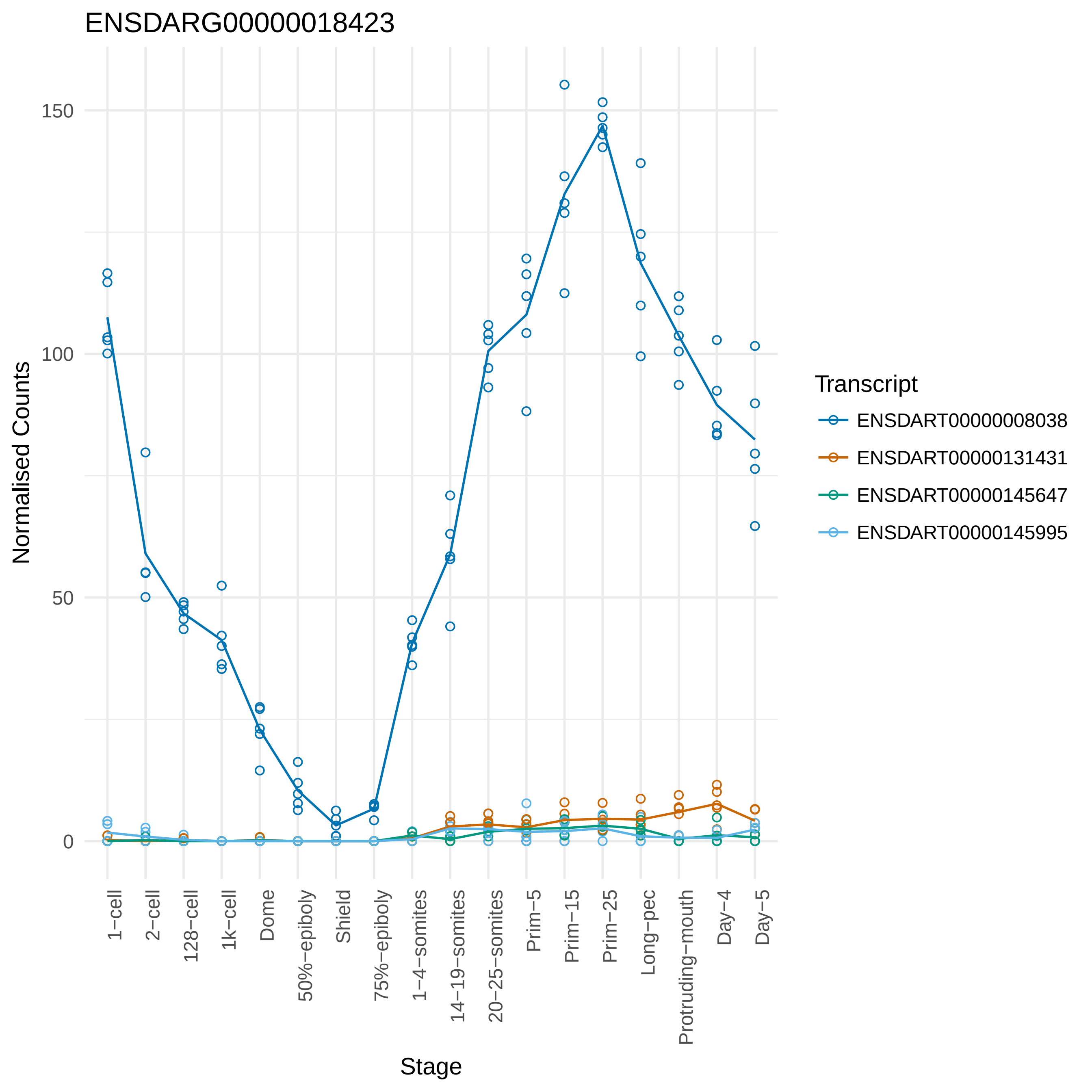
ENSDARG00000018423
Normalised Counts
150
100
50
0
1−cell
2−cell
128−cell
1k−cell
Dome
50%−epiboly
Shield
75%−epiboly
1−4−somites
14−19−somites
20−25−somites
Prim−5
Prim−15
Prim−25
Long−pec
Protruding−mouth
Day−4
Day−5
Stage
Transcript
ENSDART00000008038
ENSDART00000131431
ENSDART00000145647
ENSDART00000145995

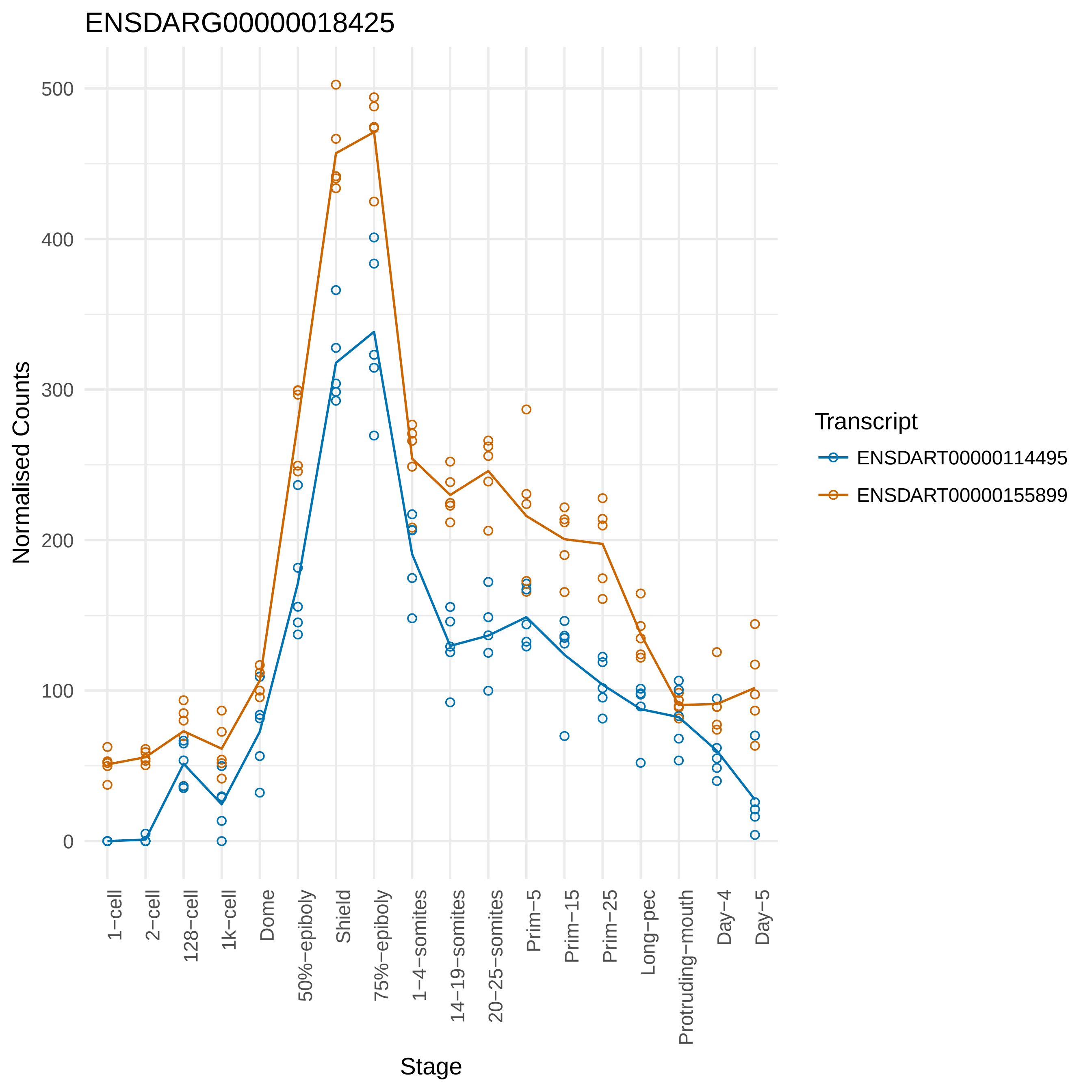

ENSDARG00000018425
Normalised Counts
0
100
200
300
400
500
1−cell
2−cell
128−cell
1k−cell
Dome
50%−epiboly
Shield
75%−epiboly
1−4−somites
14−19−somites
20−25−somites
Prim−5
Prim−15
Prim−25
Long−pec
Protruding−mouth
Day−4
Day−5
Stage
Transcript
ENSDART00000114495
ENSDART00000155899

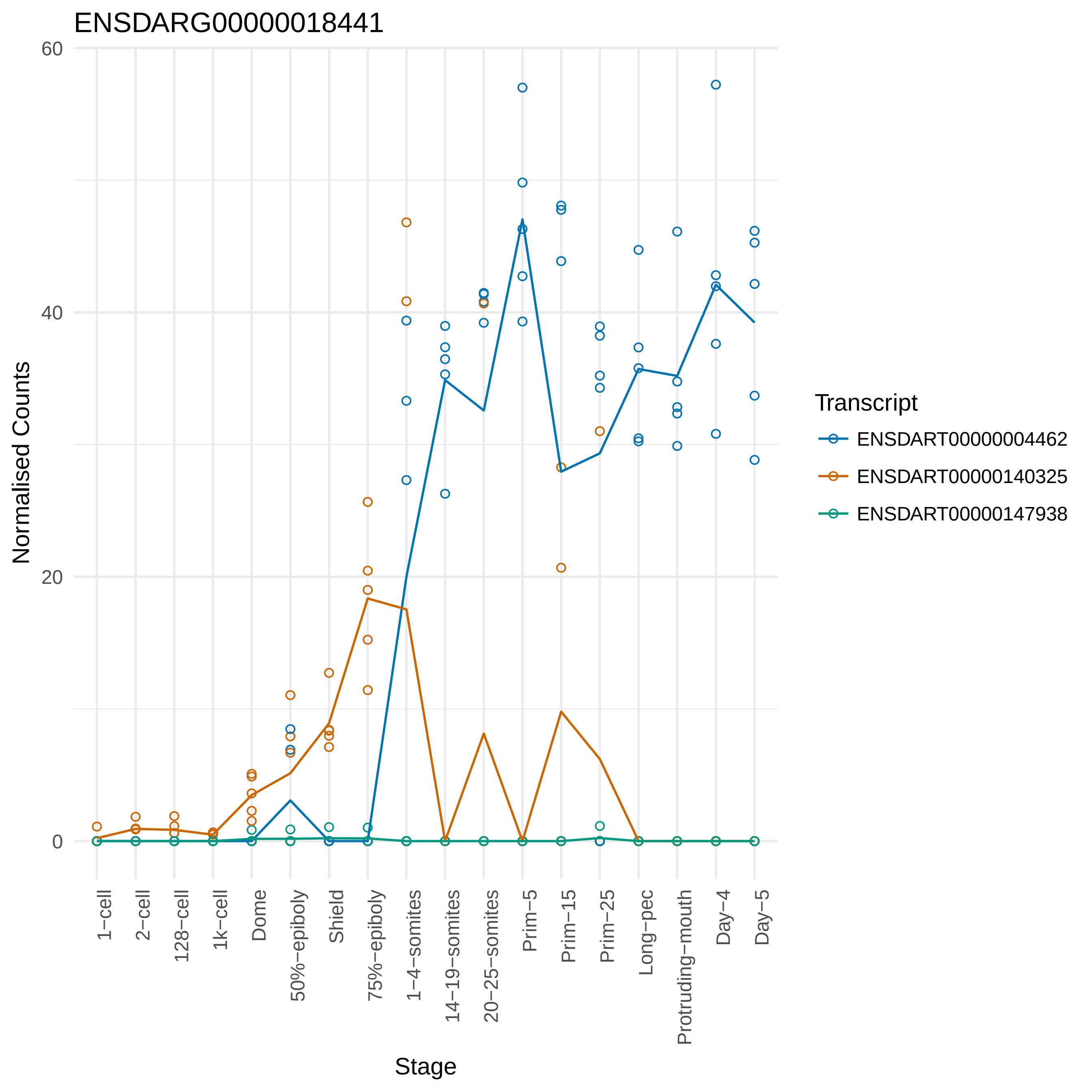
ENSDARG00000018441
60
40
20
0
Normalised Counts
1−cell
2−cell
128−cell
1k−cell
Dome
50%−epiboly
Shield
75%−epiboly
1−4−somites
14−19−somites
20−25−somites
Prim−5
Prim−15
Prim−25
Long−pec
Protruding−mouth
Day−4
Day−5
Stage
Transcript
ENSDART00000004462
ENSDART00000140325
ENSDART00000147938

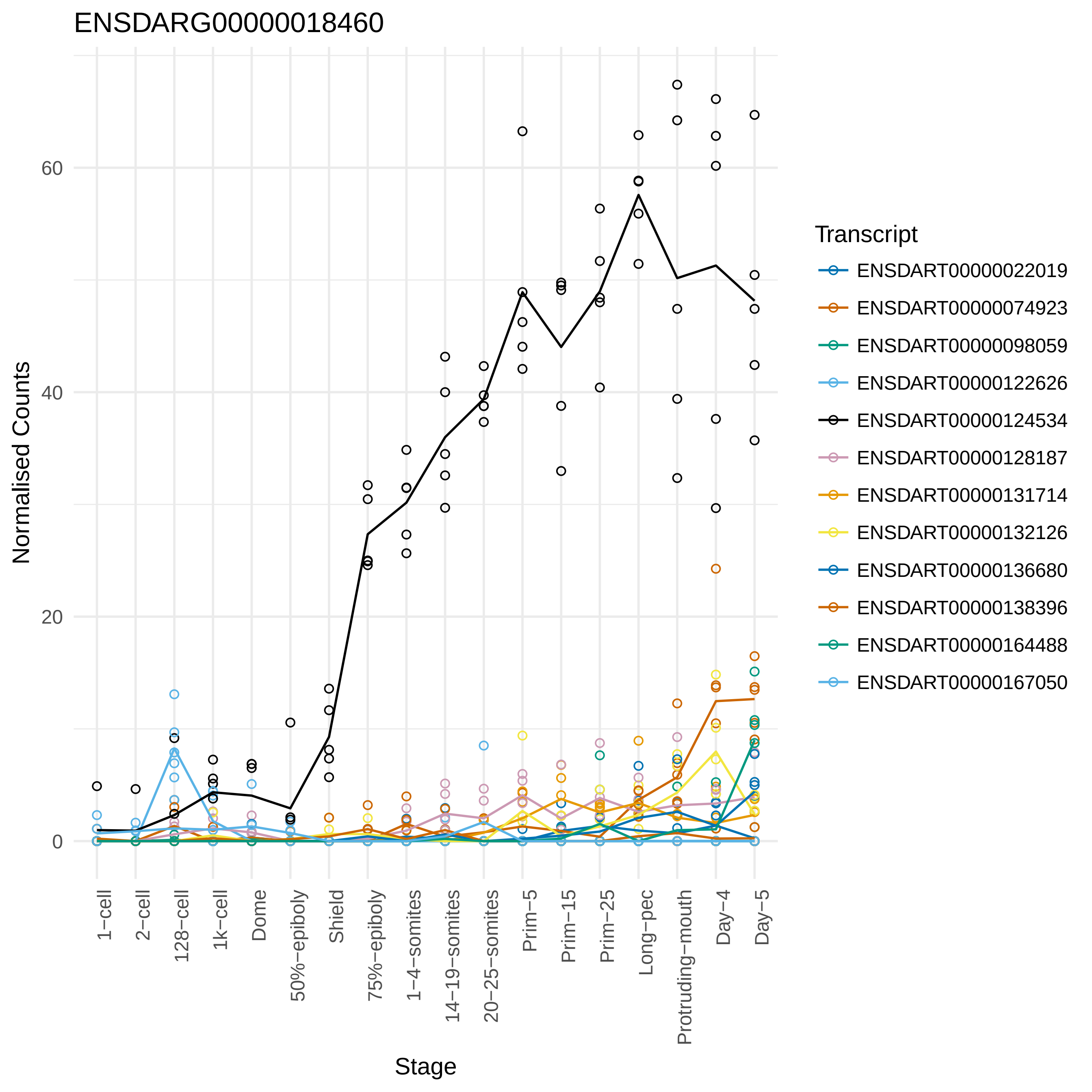

ENSDARG00000018460
Normalised Counts
0
20
40
60
Stage
1−cell
2−cell
128−cell
1k−cell
Dome
50%−epiboly
Shield
75%−epiboly
1−4−somites
14−19−somites
20−25−somites
Prim−5
Prim−15
Prim−25
Long−pec
Protruding−mouth
Day−4
Day−5
Transcript
ENSDART00000022019
ENSDART00000074923
ENSDART00000098059
ENSDART00000122626
ENSDART00000124534
ENSDART00000128187
ENSDART00000131714
ENSDART00000132126
ENSDART00000136680
ENSDART00000138396
ENSDART00000164488
ENSDART00000167050

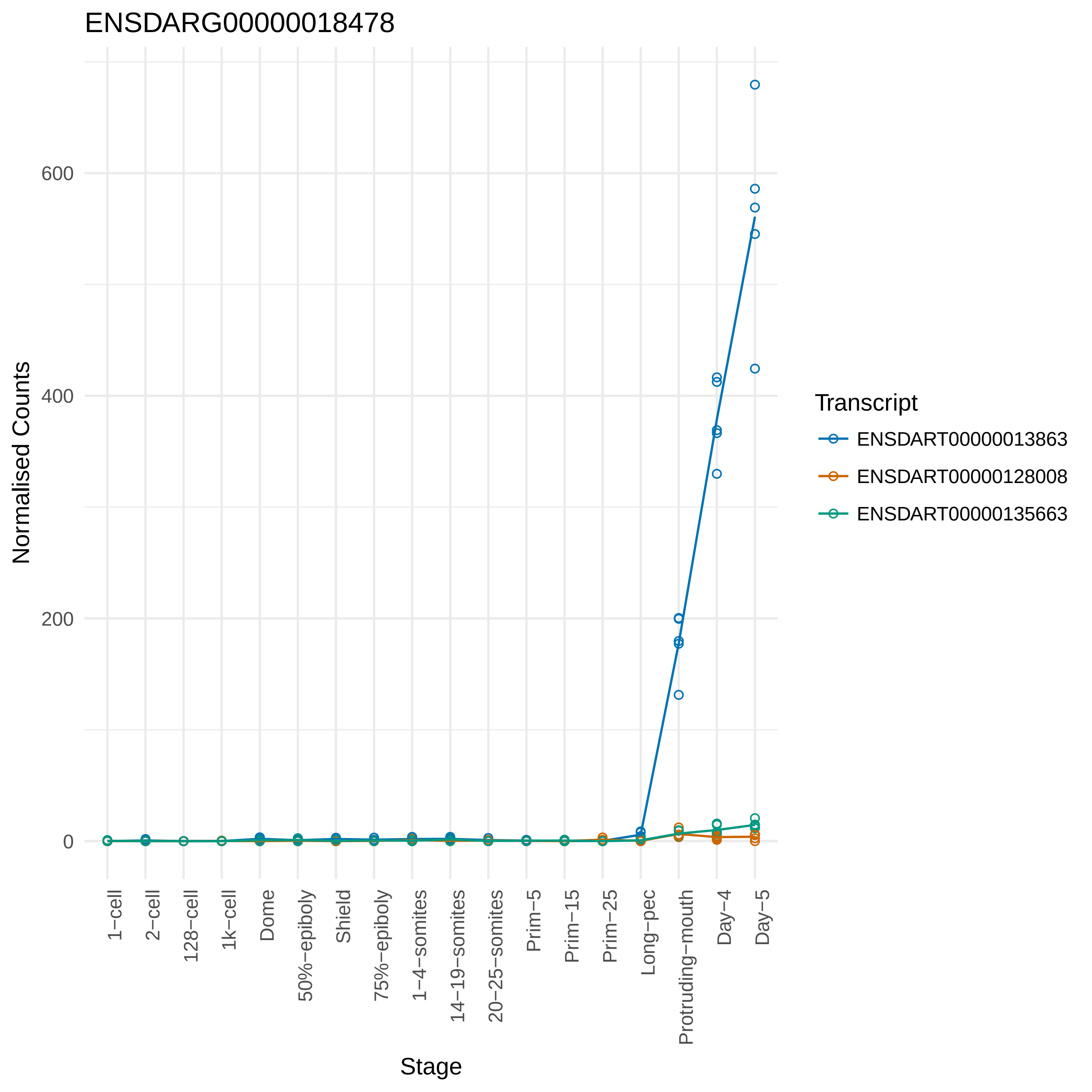
ENSDARG00000018478
Normalised Counts
0
200
400
600
1−cell
2−cell
128−cell
1k−cell
Dome
50%−epiboly
Shield
75%−epiboly
1−4−somites
14−19−somites
20−25−somites
Prim−5
Prim−15
Prim−25
Long−pec
Protruding−mouth
Day−4
Day−5
Stage
Transcript
ENSDART00000013863
ENSDART00000128008
ENSDART00000135663

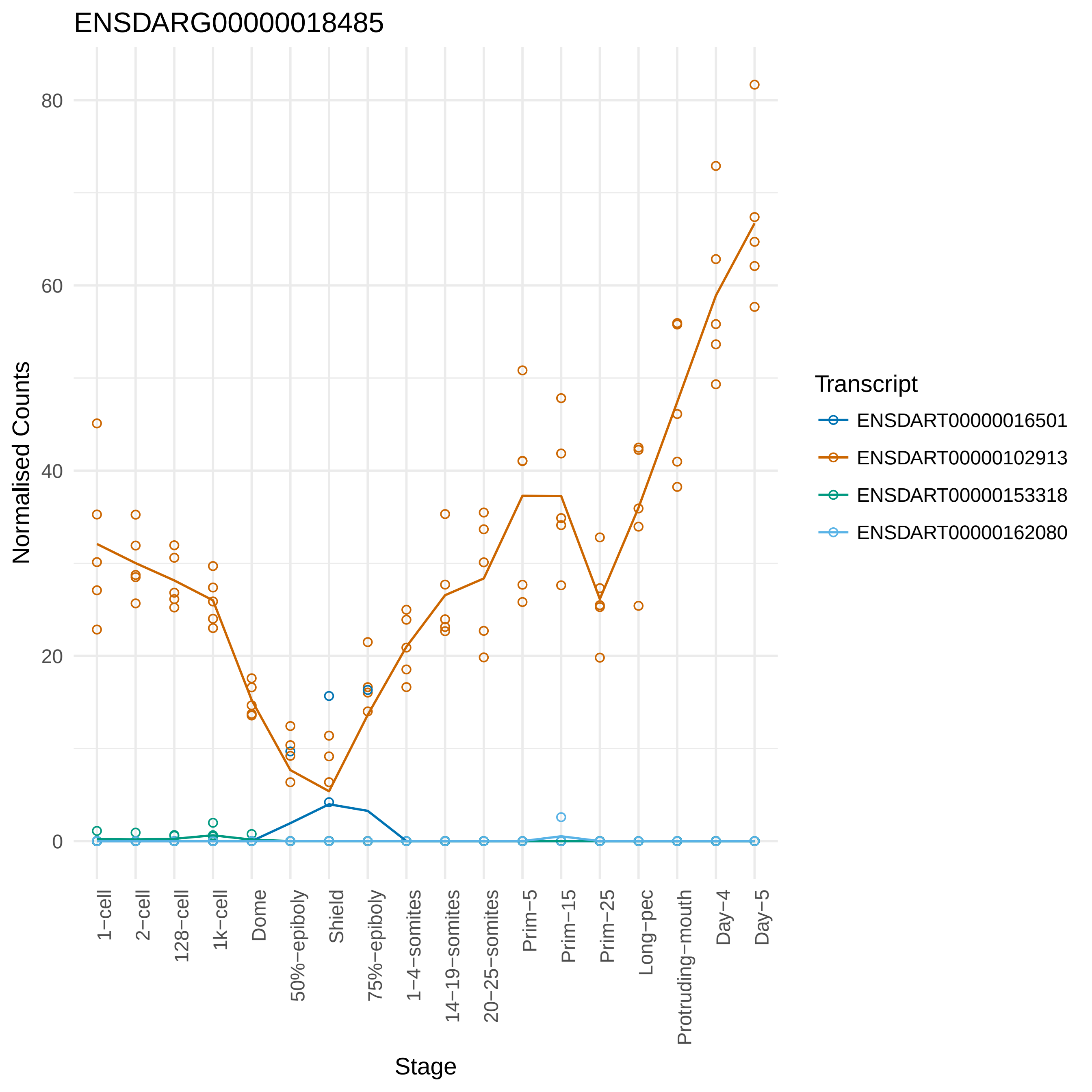

ENSDARG00000018485
Normalised Counts
0
20
40
60
80
Stage
1−cell
2−cell
128−cell
1k−cell
Dome
50%−epiboly
Shield
75%−epiboly
1−4−somites
14−19−somites
20−25−somites
Prim−5
Prim−15
Prim−25
Long−pec
Protruding−mouth
Day−4
Day−5
Transcript
ENSDART00000016501
ENSDART00000102913
ENSDART00000153318
ENSDART00000162080

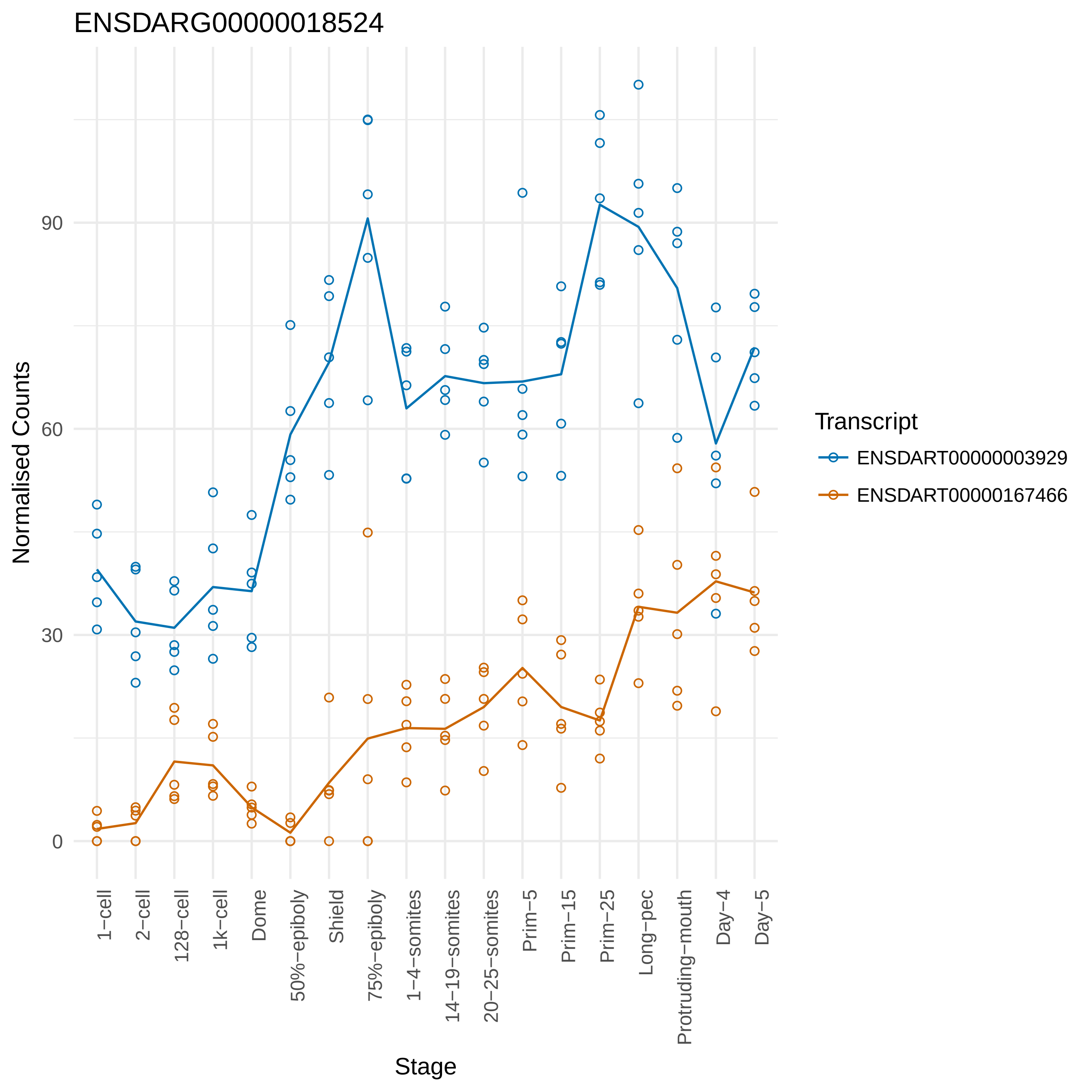

ENSDARG00000018524
Normalised Counts
90
60
30
0
1−cell
2−cell
128−cell
1k−cell
Dome
50%−epiboly
Shield
75%−epiboly
1−4−somites
14−19−somites
20−25−somites
Prim−5
Prim−15
Prim−25
Long−pec
Protruding−mouth
Day−4
Day−5
Stage
Transcript
ENSDART00000003929
ENSDART00000167466

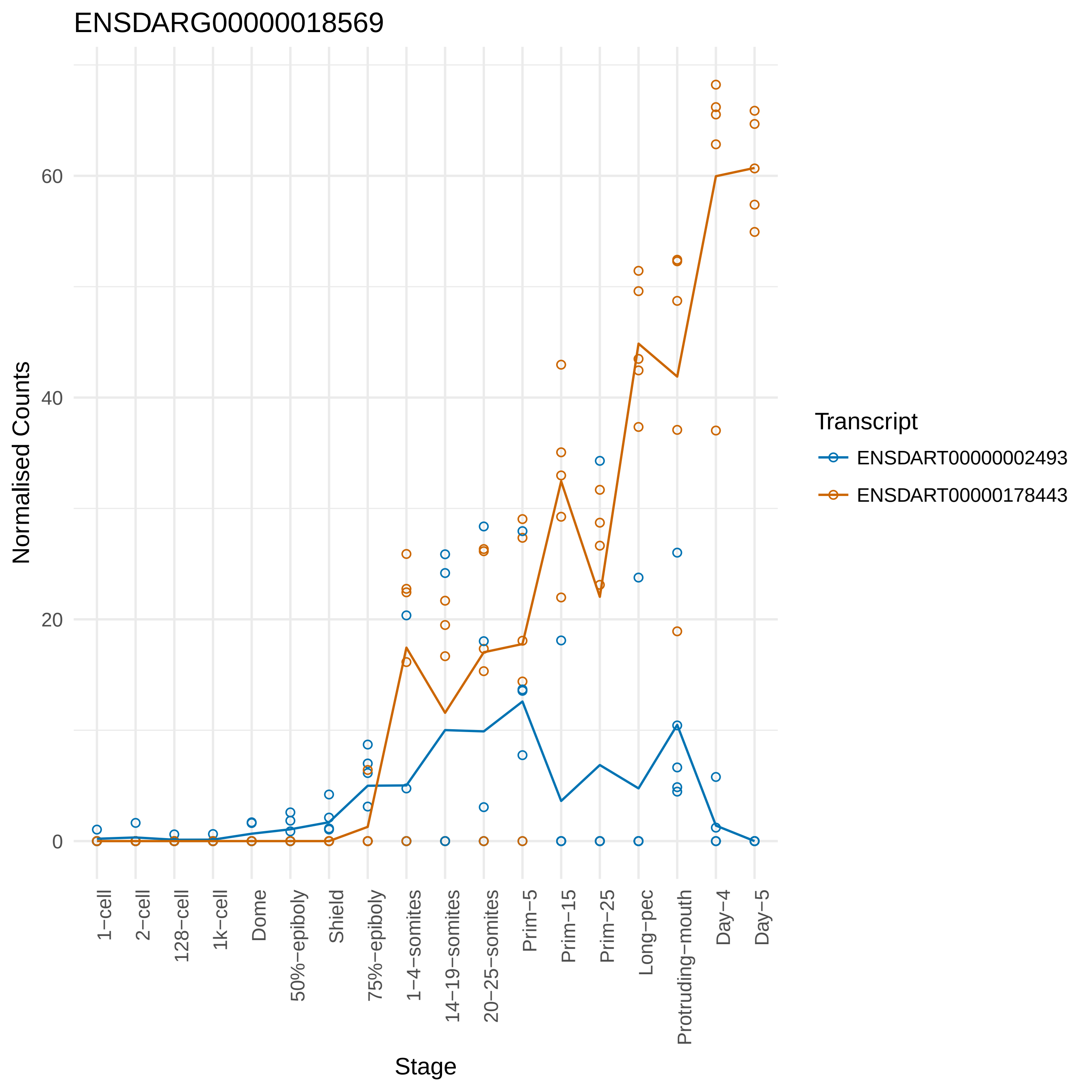
ENSDARG00000018569
Normalised Counts
0
20
40
60
Stage
1−cell
2−cell
128−cell
1k−cell
Dome
50%−epiboly
Shield
75%−epiboly
1−4−somites
14−19−somites
20−25−somites
Prim−5
Prim−15
Prim−25
Long−pec
Protruding−mouth
Day−4
Day−5
Transcript
ENSDART00000002493
ENSDART00000178443

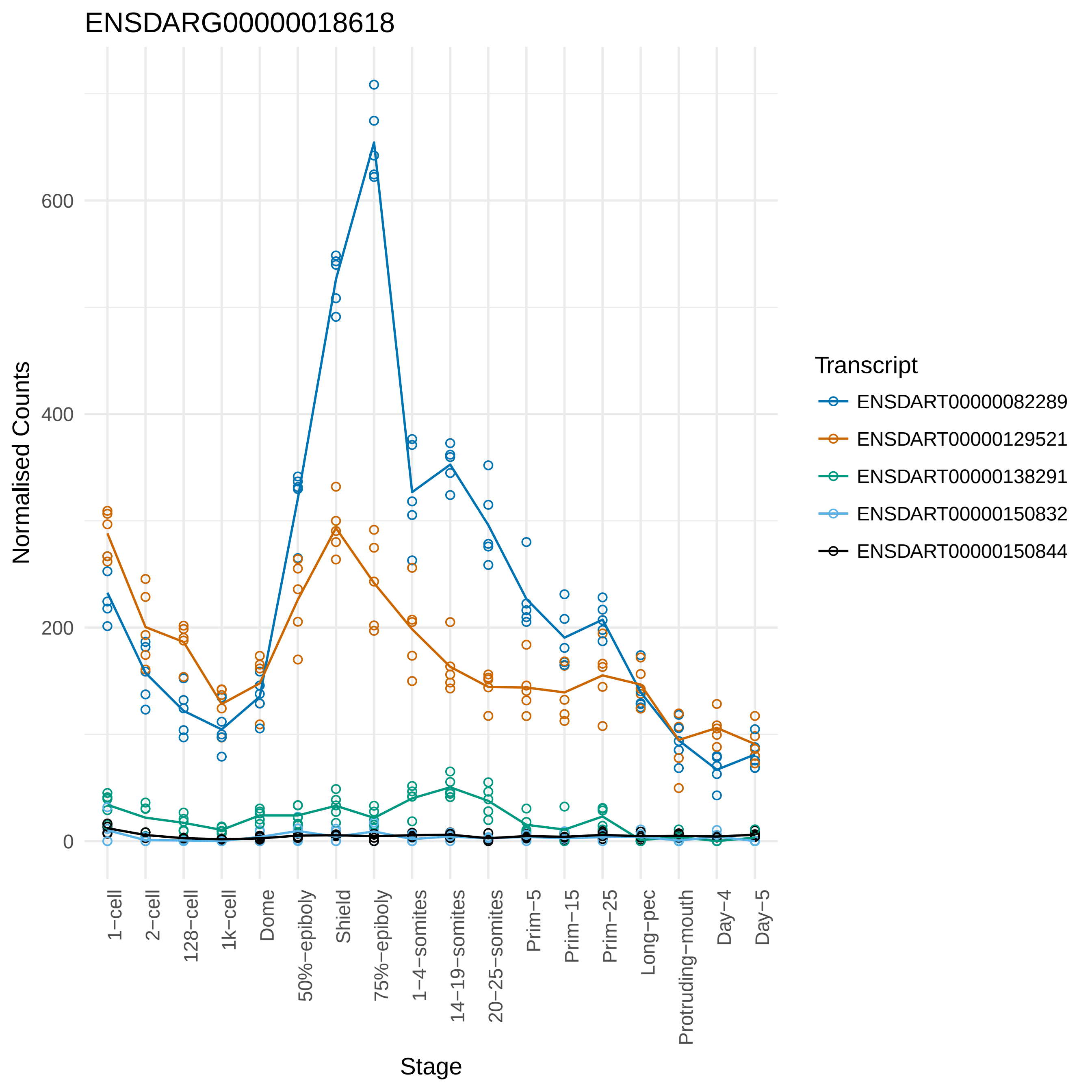
ENSDARG00000018618
Normalised Counts
600
400
200
0
Stage
1−cell
2−cell
128−cell
1k−cell
Dome
50%−epiboly
Shield
75%−epiboly
1−4−somites
14−19−somites
20−25−somites
Prim−5
Prim−15
Prim−25
Long−pec
Protruding−mouth
Day−4
Day−5
Transcript
ENSDART00000082289
ENSDART00000129521
ENSDART00000138291
ENSDART00000150832
ENSDART00000150844

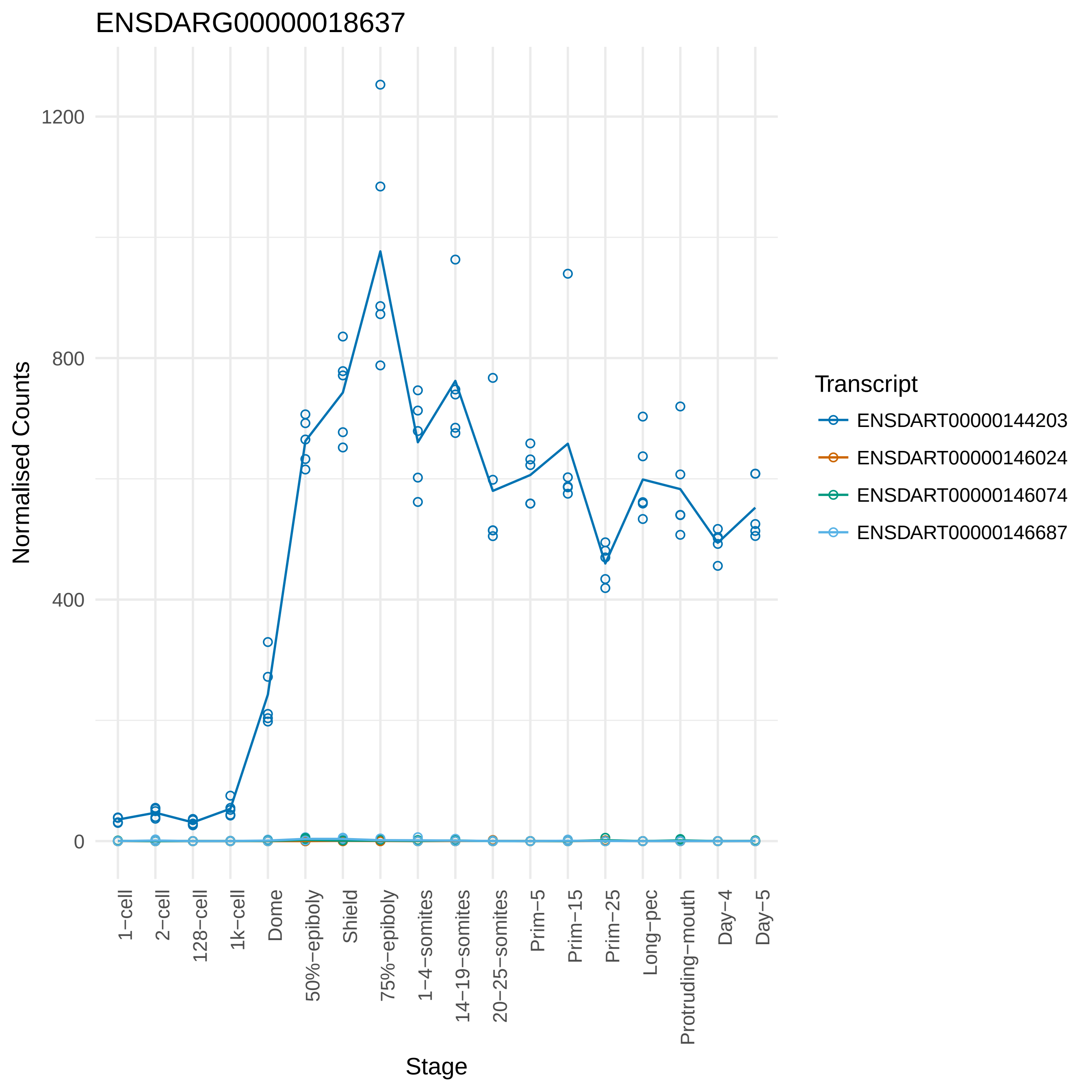

ENSDARG00000018637
Normalised Counts
1200
800
400
0
1−cell
2−cell
128−cell
1k−cell
Dome
50%−epiboly
Shield
75%−epiboly
1−4−somites
14−19−somites
20−25−somites
Prim−5
Prim−15
Prim−25
Long−pec
Protruding−mouth
Day−4
Day−5
Stage
Transcript
ENSDART00000144203
ENSDART00000146024
ENSDART00000146074
ENSDART00000146687

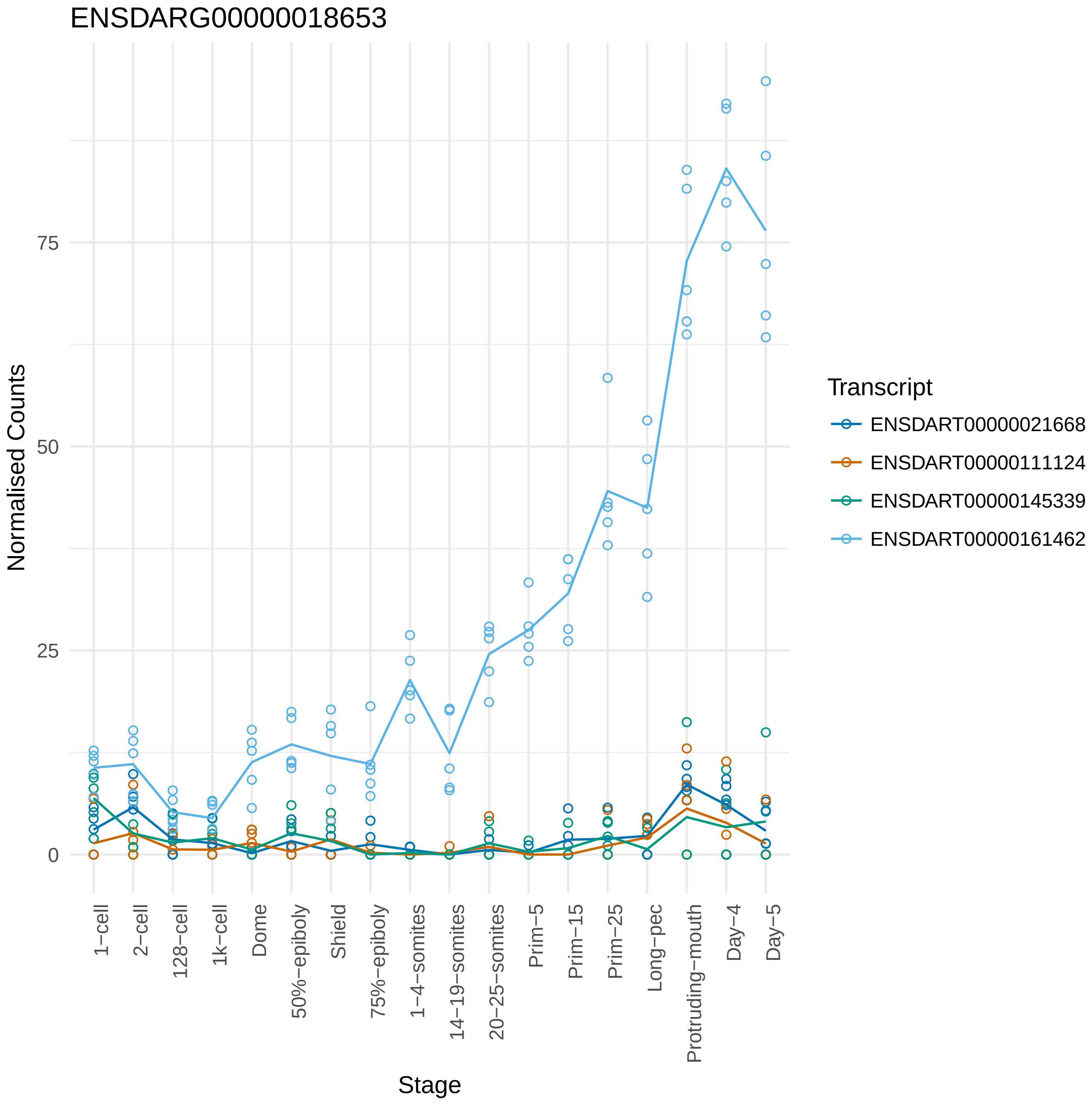
ENSDARG00000018653
Normalised Counts
0
25
50
75
1−cell
2−cell
128−cell
1k−cell
Dome
50%−epiboly
Shield
75%−epiboly
1−4−somites
14−19−somites
20−25−somites
Prim−5
Prim−15
Prim−25
Long−pec
Protruding−mouth
Day−4
Day−5
Stage
Transcript
ENSDART00000021668
ENSDART00000111124
ENSDART00000145339
ENSDART00000161462

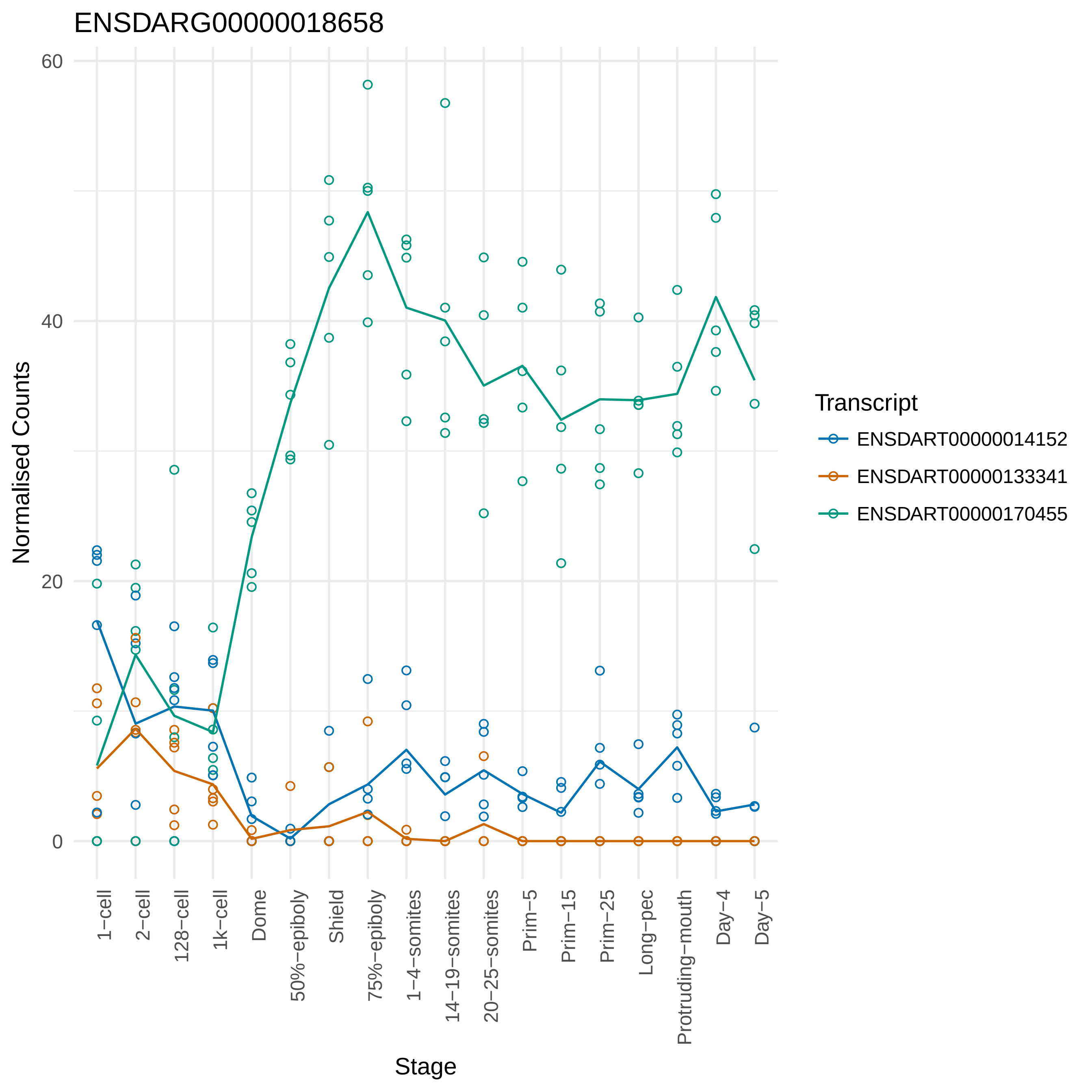

ENSDARG00000018658
Normalised Counts
60
40
20
0
Stage
1−cell
2−cell
128−cell
1k−cell
Dome
50%−epiboly
Shield
75%−epiboly
1−4−somites
14−19−somites
20−25−somites
Prim−5
Prim−15
Prim−25
Long−pec
Protruding−mouth
Day−4
Day−5
Transcript
ENSDART00000014152
ENSDART00000133341
ENSDART00000170455

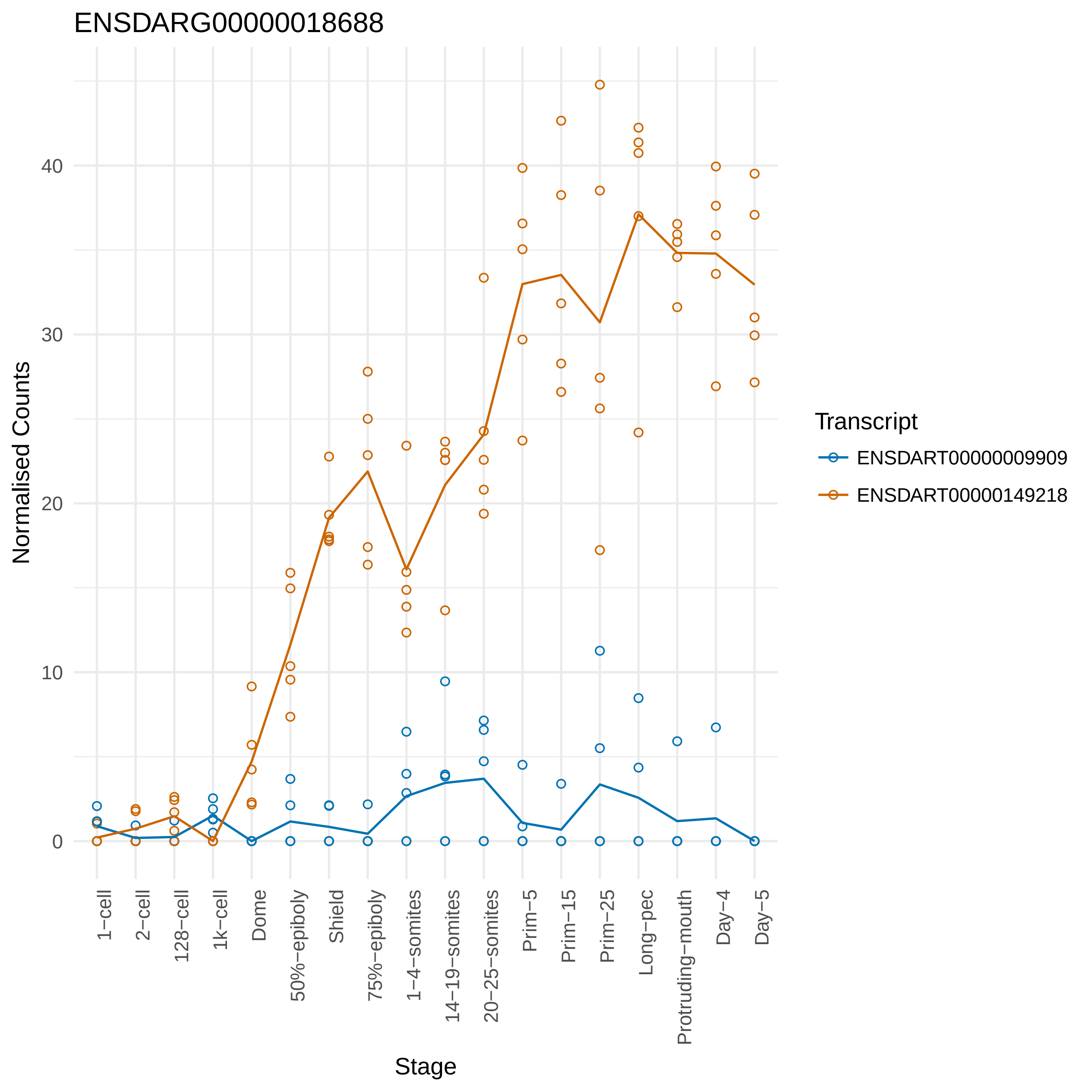
ENSDARG00000018688
Normalised Counts
0
10
20
30
40
Stage
1−cell
2−cell
128−cell
1k−cell
Dome
50%−epiboly
Shield
75%−epiboly
1−4−somites
14−19−somites
20−25−somites
Prim−5
Prim−15
Prim−25
Long−pec
Protruding−mouth
Day−4
Day−5
Transcript
ENSDART00000009909
ENSDART00000149218

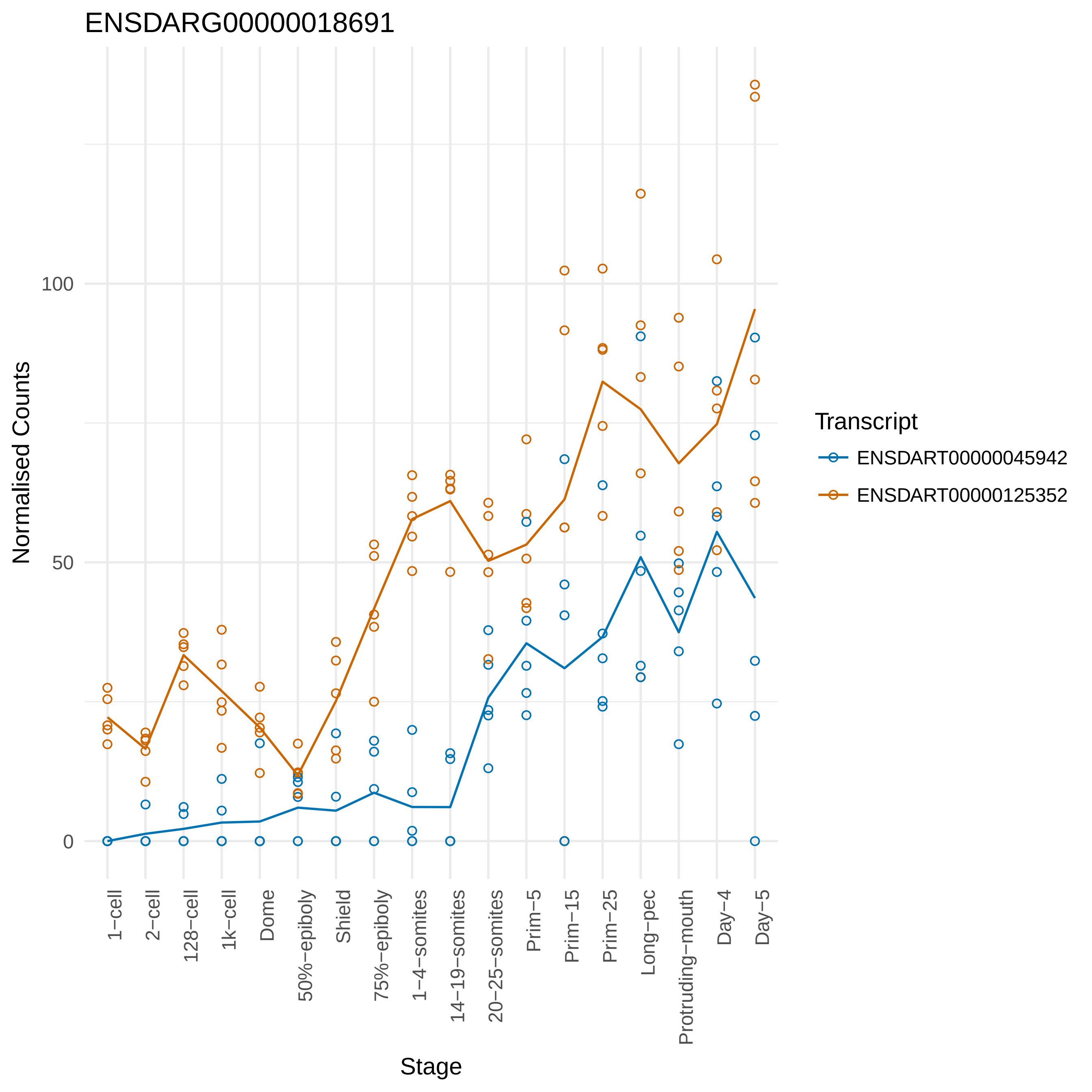
ENSDARG00000018691
Normalised Counts
100
50
0
1−cell
2−cell
128−cell
1k−cell
Dome
50%−epiboly
Shield
75%−epiboly
1−4−somites
14−19−somites
20−25−somites
Prim−5
Prim−15
Prim−25
Long−pec
Protruding−mouth
Day−4
Day−5
Stage
Transcript
ENSDART00000045942
ENSDART00000125352

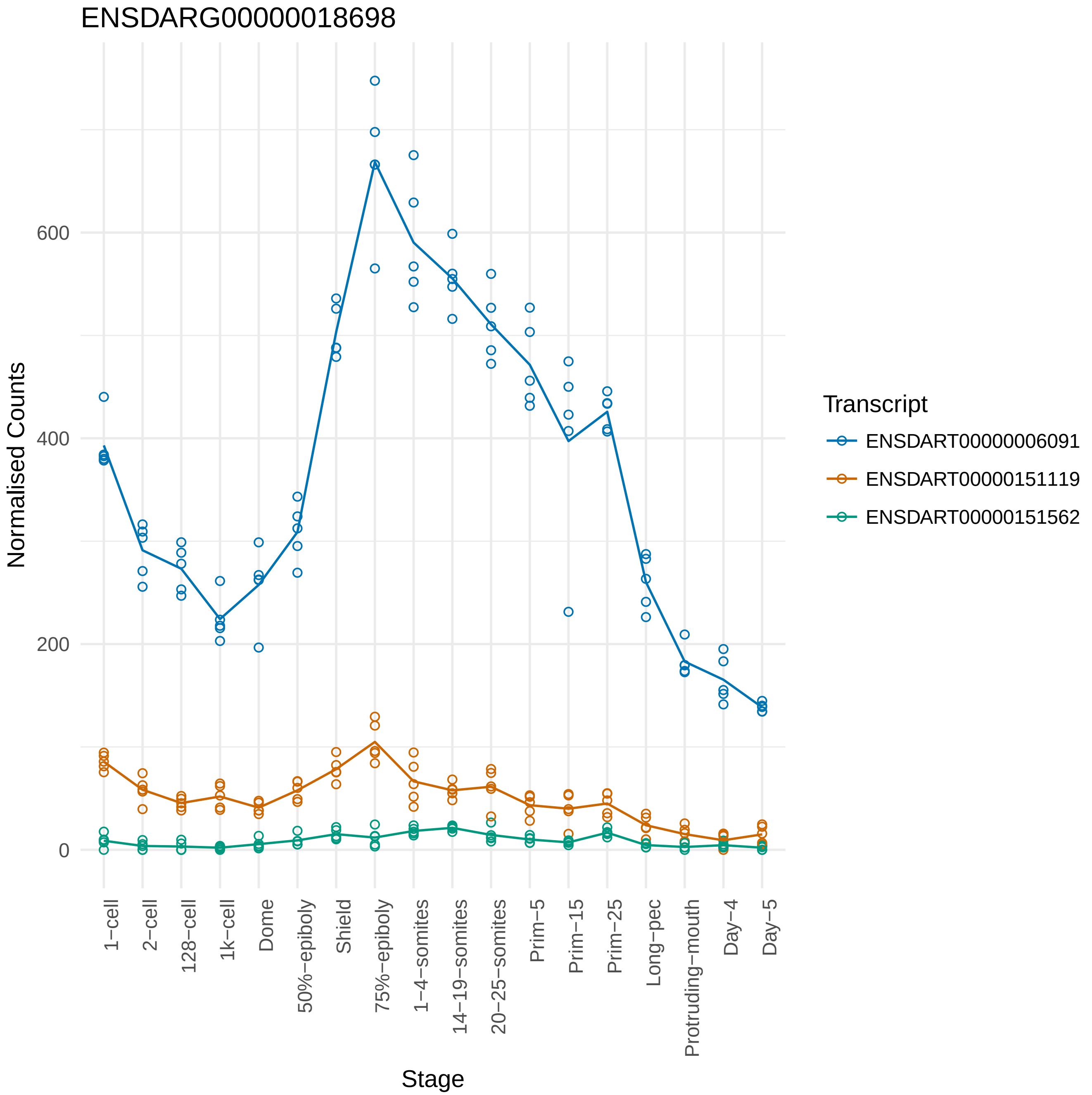
ENSDARG00000018698
Normalised Counts
600
400
200
0
1−cell
2−cell
128−cell
1k−cell
Dome
50%−epiboly
Shield
75%−epiboly
1−4−somites
14−19−somites
20−25−somites
Prim−5
Prim−15
Prim−25
Long−pec
Protruding−mouth
Day−4
Day−5
Stage
Transcript
ENSDART00000006091
ENSDART00000151119
ENSDART00000151562

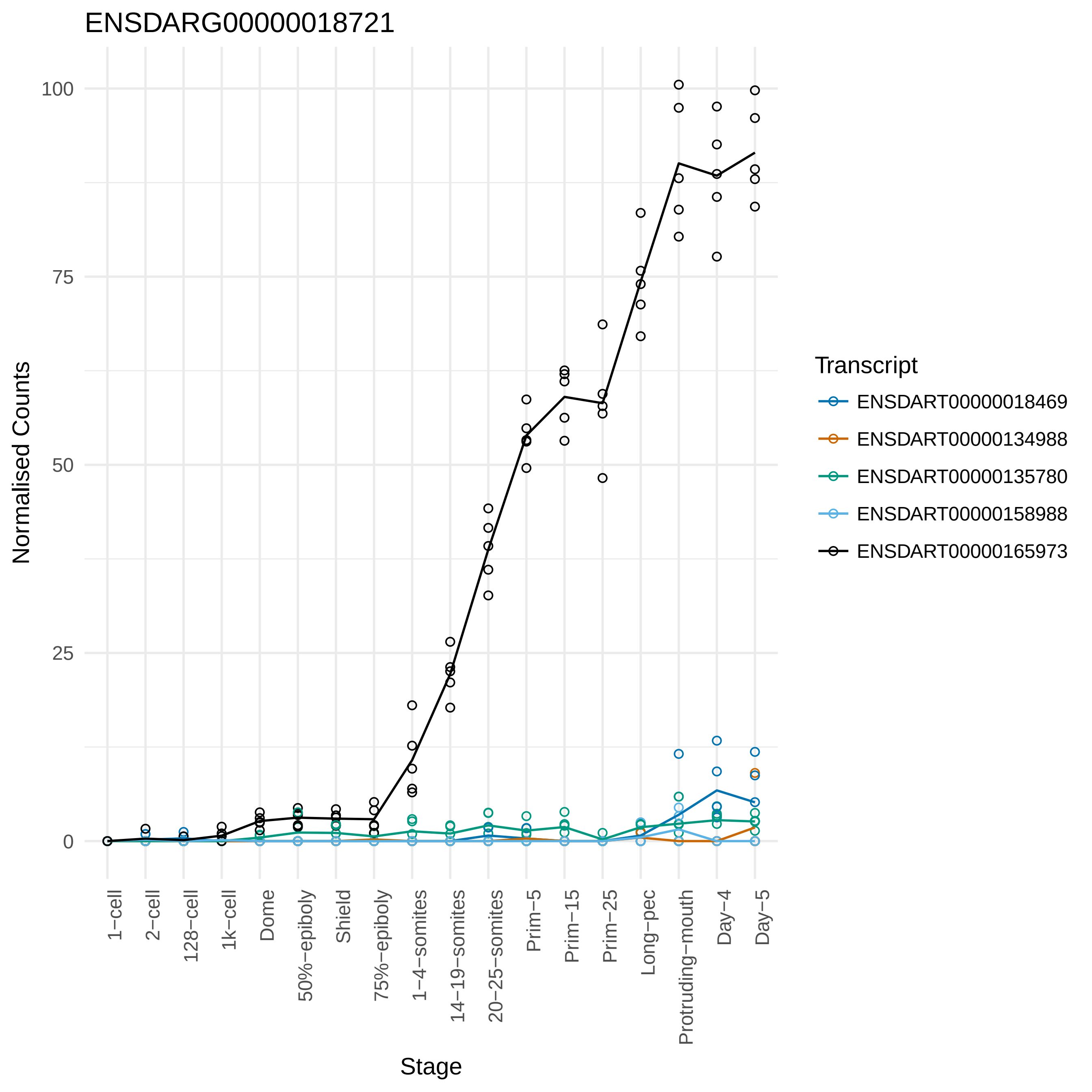
ENSDARG00000018721
Normalised Counts
100
75
50
25
0
Stage
1−cell
2−cell
128−cell
1k−cell
Dome
50%−epiboly
Shield
75%−epiboly
1−4−somites
14−19−somites
20−25−somites
Prim−5
Prim−15
Prim−25
Long−pec
Protruding−mouth
Day−4
Day−5
Transcript
ENSDART00000018469
ENSDART00000134988
ENSDART00000135780
ENSDART00000158988
ENSDART00000165973

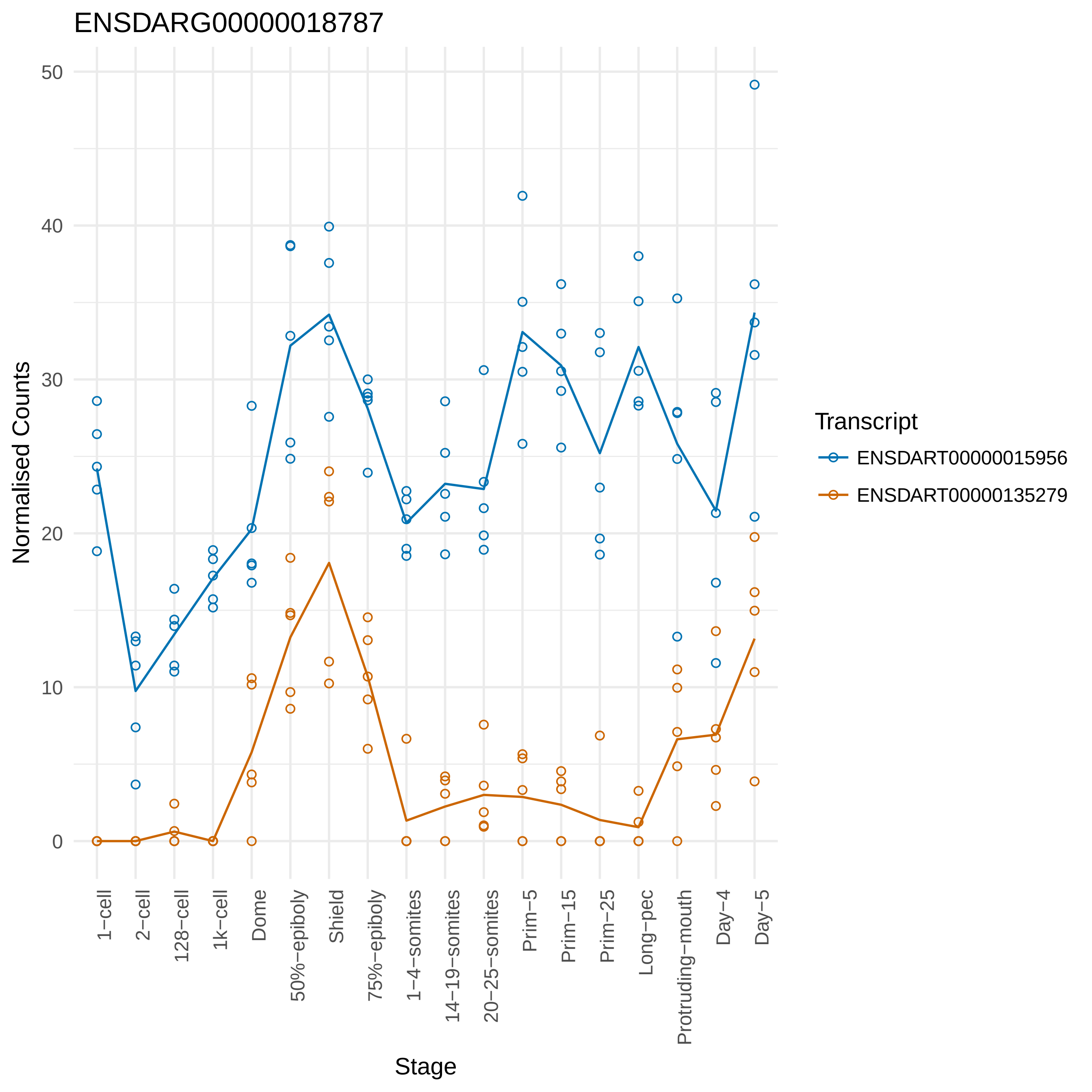

ENSDARG00000018787
Normalised Counts
0
10
20
30
40
50
1−cell
2−cell
128−cell
1k−cell
Dome
50%−epiboly
Shield
75%−epiboly
1−4−somites
14−19−somites
20−25−somites
Prim−5
Prim−15
Prim−25
Long−pec
Protruding−mouth
Day−4
Day−5
Stage
Transcript
ENSDART00000015956
ENSDART00000135279

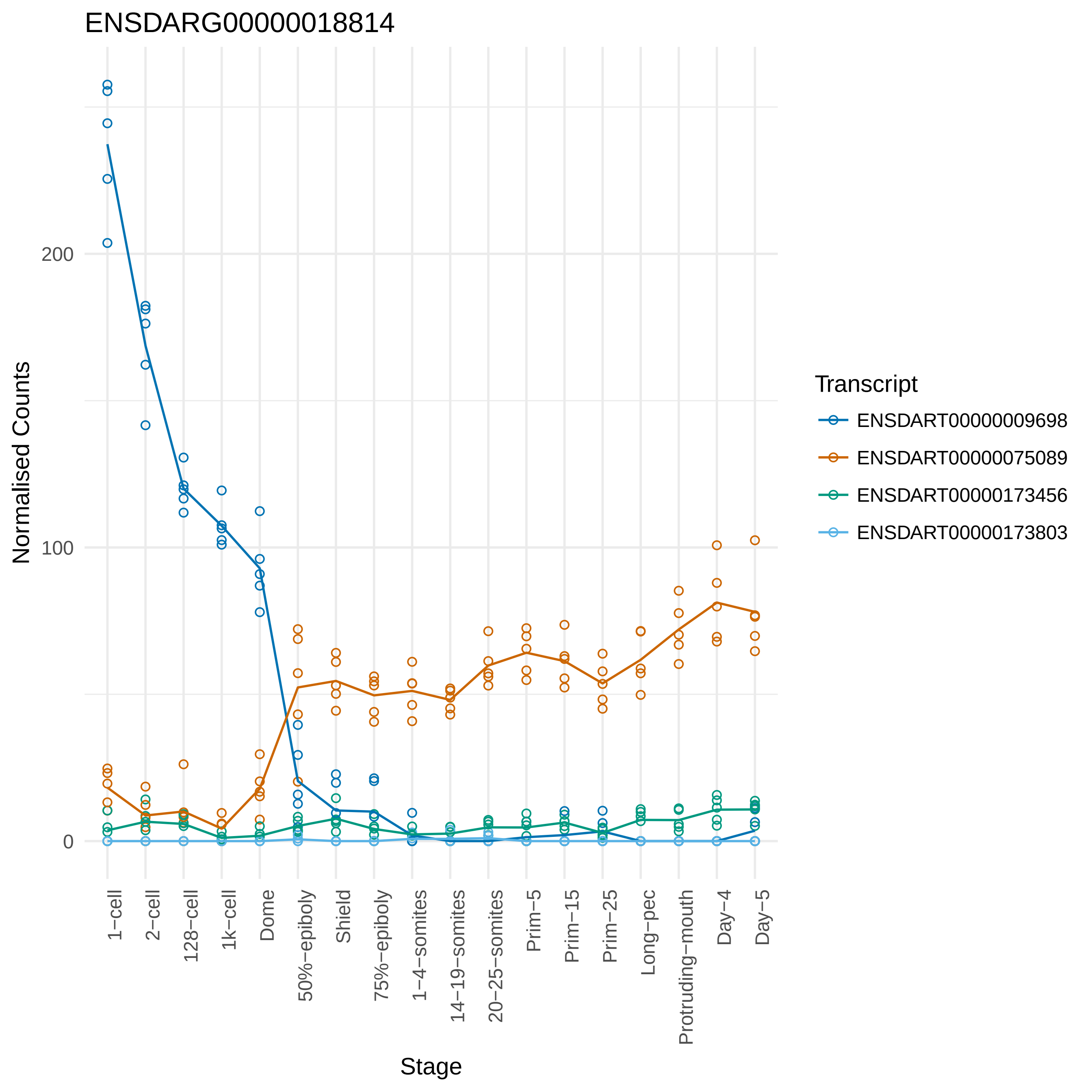

ENSDARG00000018814
Normalised Counts
200
100
0
1−cell
2−cell
128−cell
1k−cell
Dome
50%−epiboly
Shield
75%−epiboly
1−4−somites
14−19−somites
20−25−somites
Prim−5
Prim−15
Prim−25
Long−pec
Protruding−mouth
Day−4
Day−5
Stage
Transcript
ENSDART00000009698
ENSDART00000075089
ENSDART00000173456
ENSDART00000173803

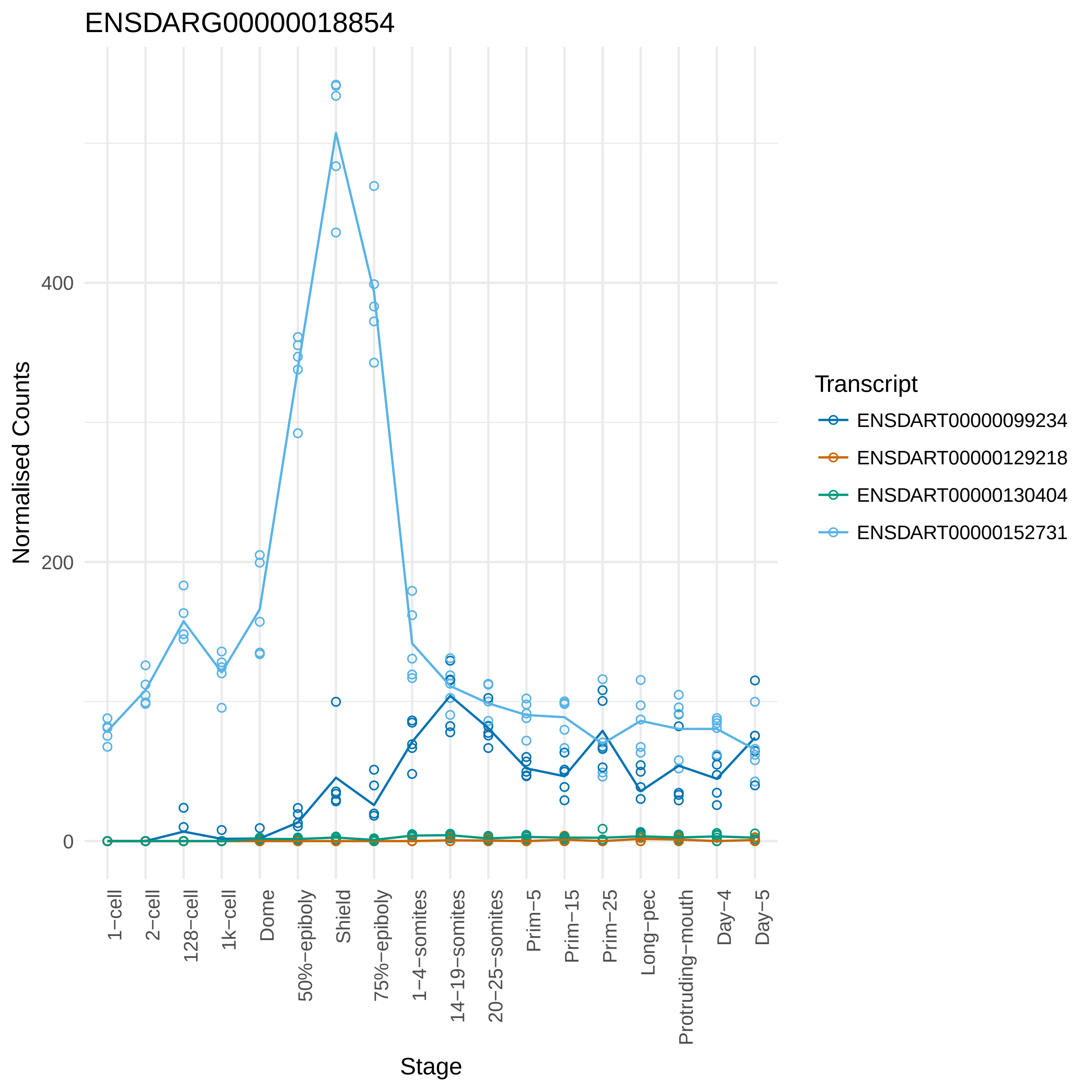

ENSDARG00000018854
Normalised Counts
0
200
400
1−cell
2−cell
128−cell
1k−cell
Dome
50%−epiboly
Shield
75%−epiboly
1−4−somites
14−19−somites
20−25−somites
Prim−5
Prim−15
Prim−25
Long−pec
Protruding−mouth
Day−4
Day−5
Stage
Transcript
ENSDART00000099234
ENSDART00000129218
ENSDART00000130404
ENSDART00000152731

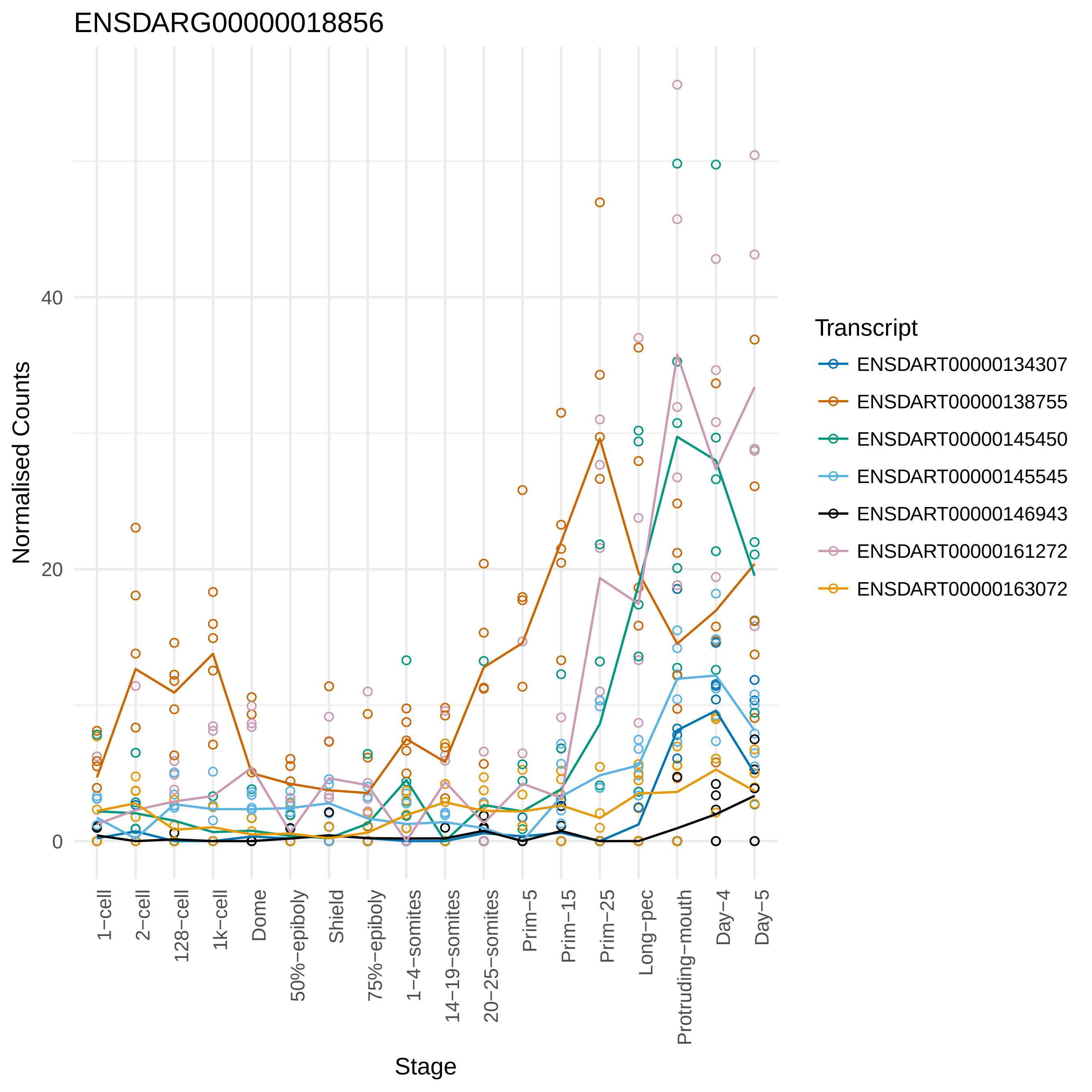

ENSDARG00000018856
Normalised Counts
0
20
40
1−cell
2−cell
128−cell
1k−cell
Dome
50%−epiboly
Shield
75%−epiboly
1−4−somites
14−19−somites
20−25−somites
Prim−5
Prim−15
Prim−25
Long−pec
Protruding−mouth
Day−4
Day−5
Stage
Transcript
ENSDART00000134307
ENSDART00000138755
ENSDART00000145450
ENSDART00000145545
ENSDART00000146943
ENSDART00000161272
ENSDART00000163072

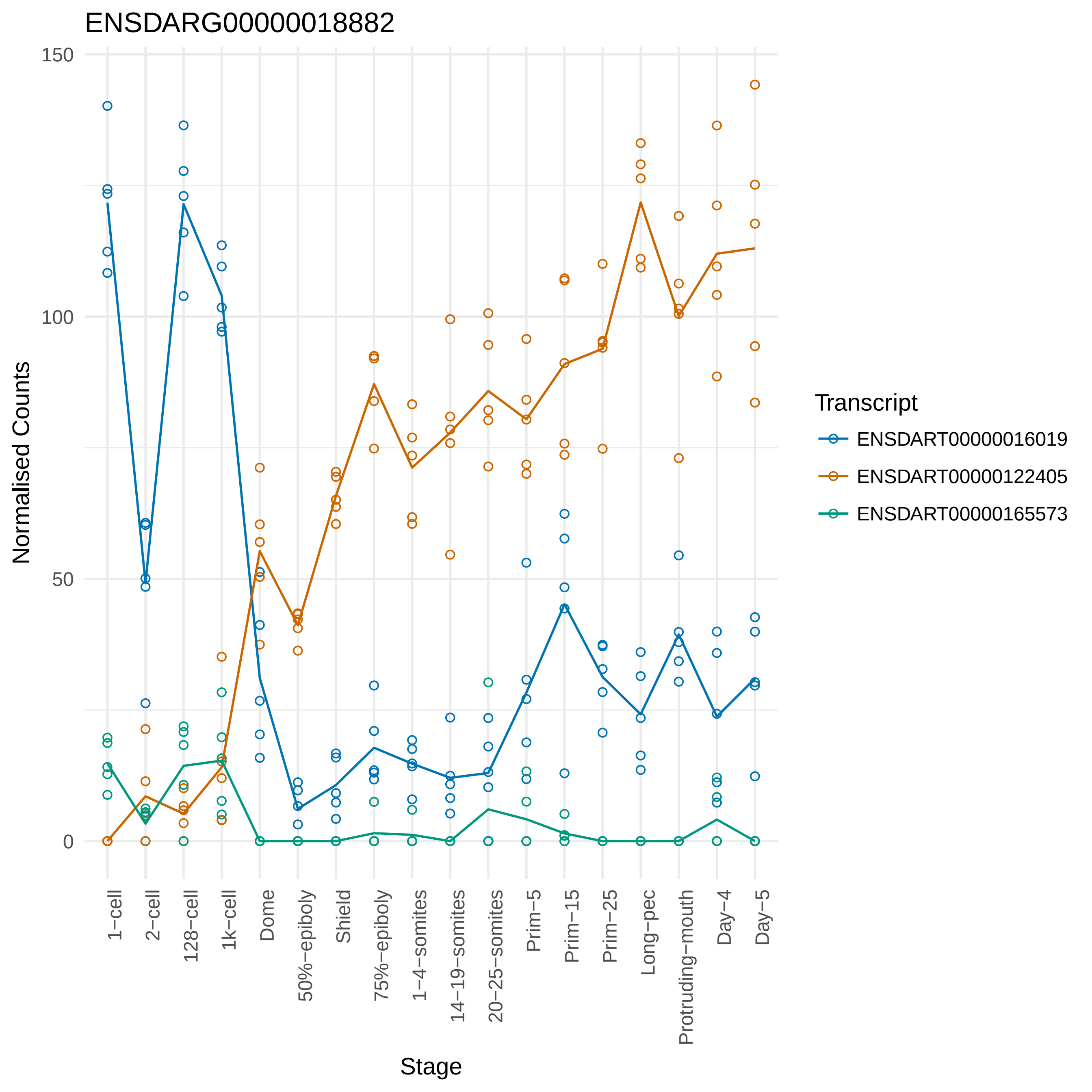

ENSDARG00000018882
Normalised Counts
150
100
50
0
1−cell
2−cell
128−cell
1k−cell
Dome
50%−epiboly
Shield
75%−epiboly
1−4−somites
14−19−somites
20−25−somites
Prim−5
Prim−15
Prim−25
Long−pec
Protruding−mouth
Day−4
Day−5
Stage
Transcript
ENSDART00000016019
ENSDART00000122405
ENSDART00000165573

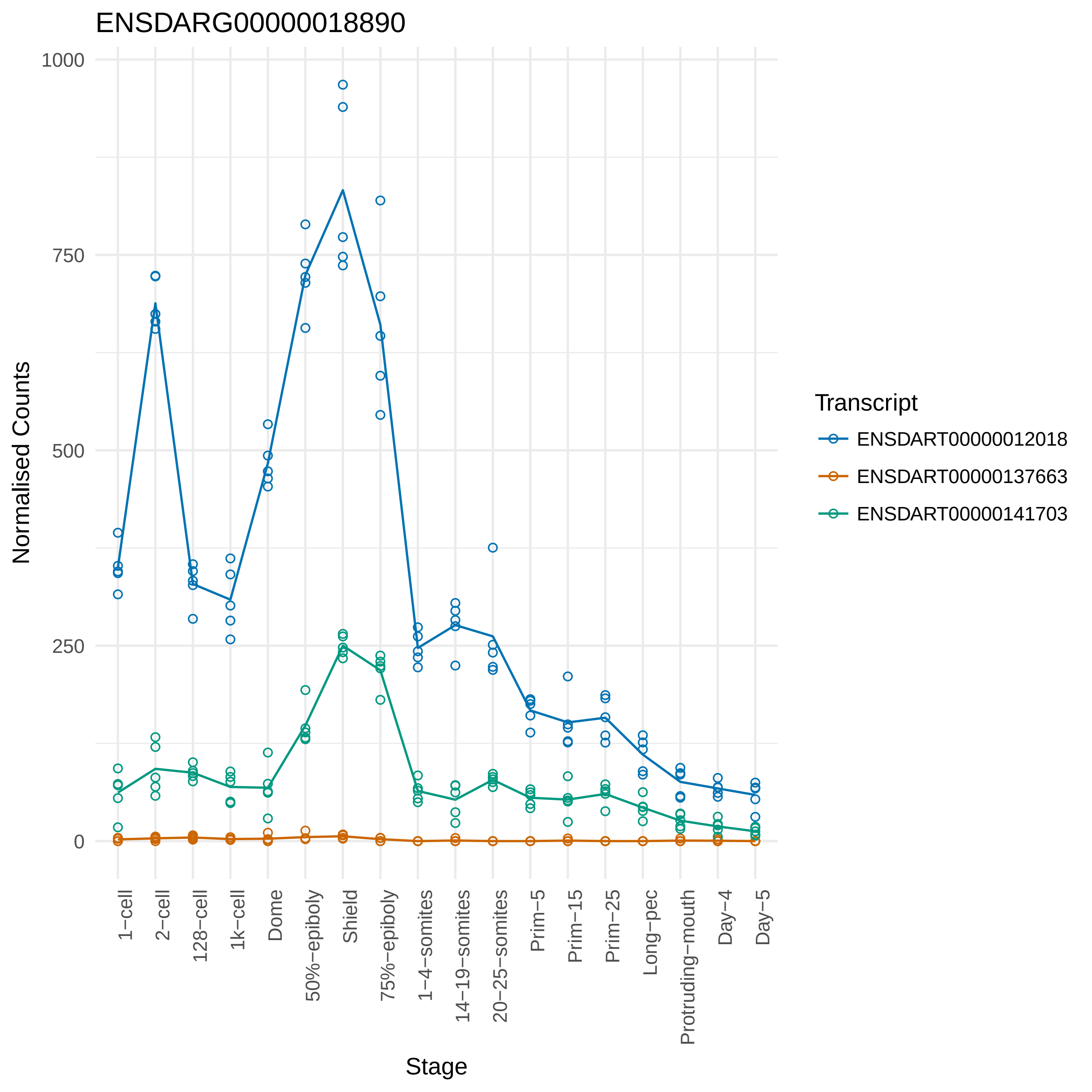

ENSDARG00000018890
Normalised Counts
1000
750
500
250
0
1−cell
2−cell
128−cell
1k−cell
Dome
50%−epiboly
Shield
75%−epiboly
1−4−somites
14−19−somites
20−25−somites
Prim−5
Prim−15
Prim−25
Long−pec
Protruding−mouth
Day−4
Day−5
Stage
Transcript
ENSDART00000012018
ENSDART00000137663
ENSDART00000141703

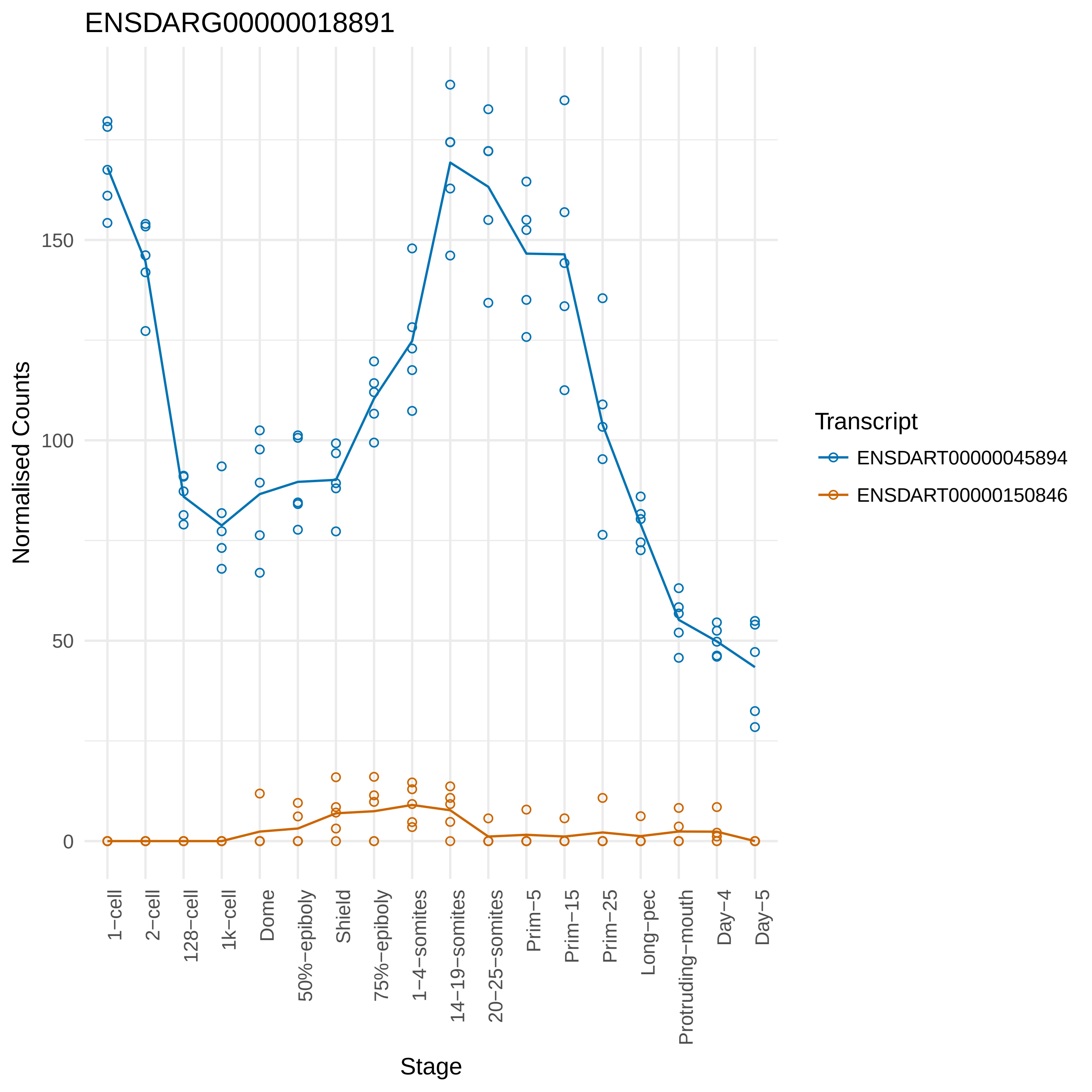

ENSDARG00000018891
Normalised Counts
150
100
50
0
1−cell
2−cell
128−cell
1k−cell
Dome
50%−epiboly
Shield
75%−epiboly
1−4−somites
14−19−somites
20−25−somites
Prim−5
Prim−15
Prim−25
Long−pec
Protruding−mouth
Day−4
Day−5
Stage
Transcript
ENSDART00000045894
ENSDART00000150846

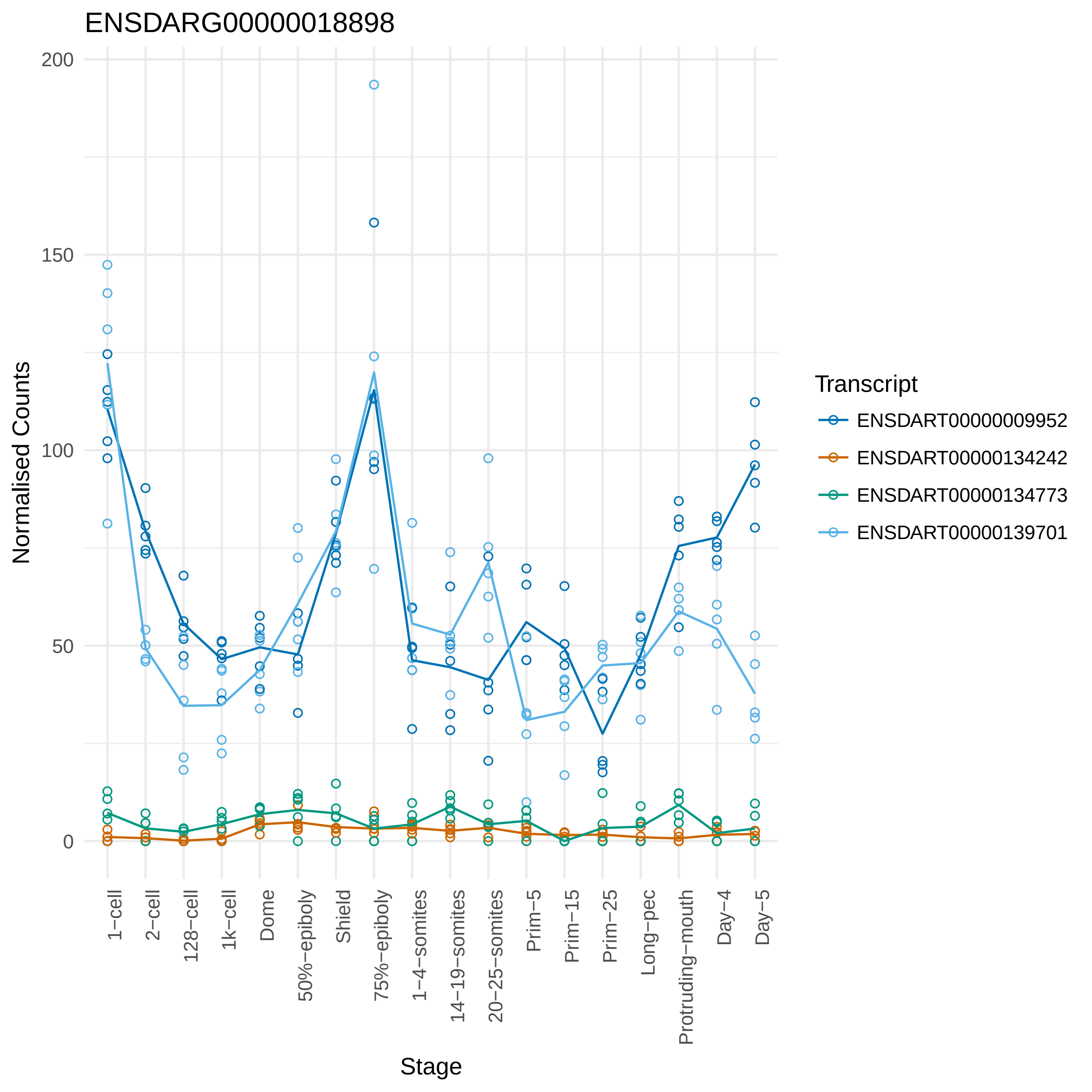
ENSDARG00000018898
Normalised Counts
200
150
100
50
0
1−cell
2−cell
128−cell
1k−cell
Dome
50%−epiboly
Shield
75%−epiboly
1−4−somites
14−19−somites
20−25−somites
Prim−5
Prim−15
Prim−25
Long−pec
Protruding−mouth
Day−4
Day−5
Stage
Transcript
ENSDART00000009952
ENSDART00000134242
ENSDART00000134773
ENSDART00000139701

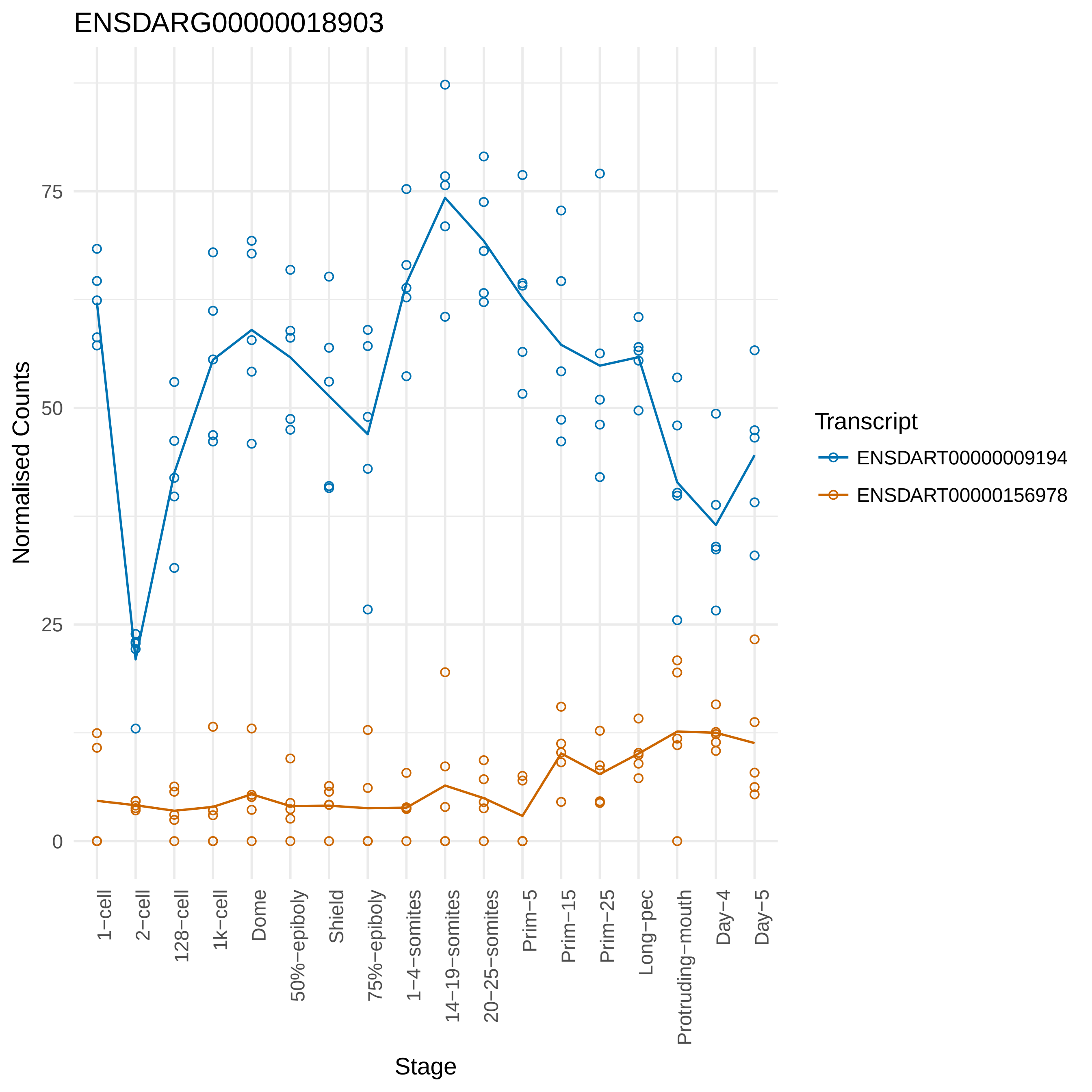
ENSDARG00000018903
Normalised Counts
0
25
50
75
1−cell
2−cell
128−cell
1k−cell
Dome
50%−epiboly
Shield
75%−epiboly
1−4−somites
14−19−somites
20−25−somites
Prim−5
Prim−15
Prim−25
Long−pec
Protruding−mouth
Day−4
Day−5
Stage
Transcript
ENSDART00000009194
ENSDART00000156978

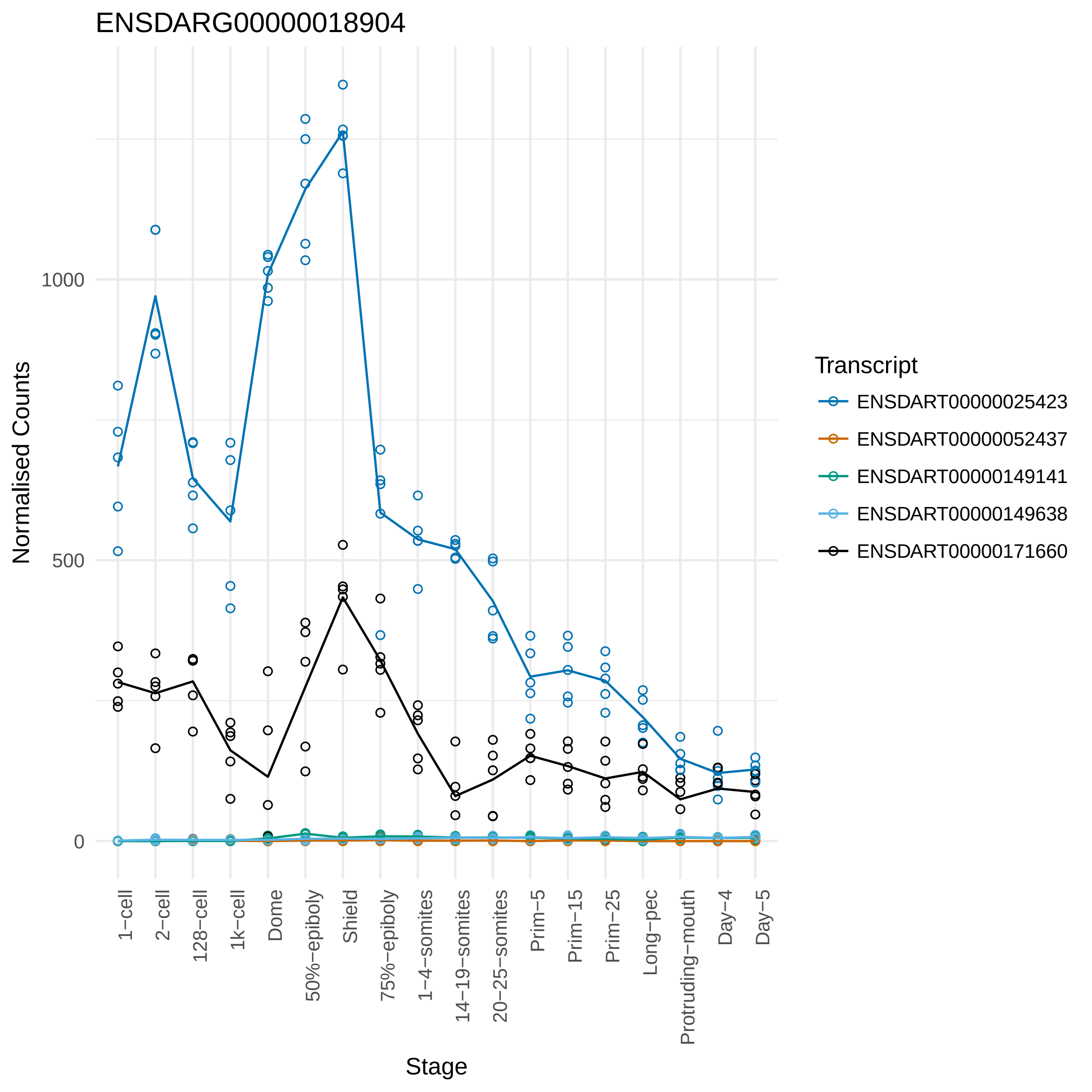
ENSDARG00000018904
Normalised Counts
0
500
1000
1−cell
2−cell
128−cell
1k−cell
Dome
50%−epiboly
Shield
75%−epiboly
1−4−somites
14−19−somites
20−25−somites
Prim−5
Prim−15
Prim−25
Long−pec
Protruding−mouth
Day−4
Day−5
Stage
Transcript
ENSDART00000025423
ENSDART00000052437
ENSDART00000149141
ENSDART00000149638
ENSDART00000171660

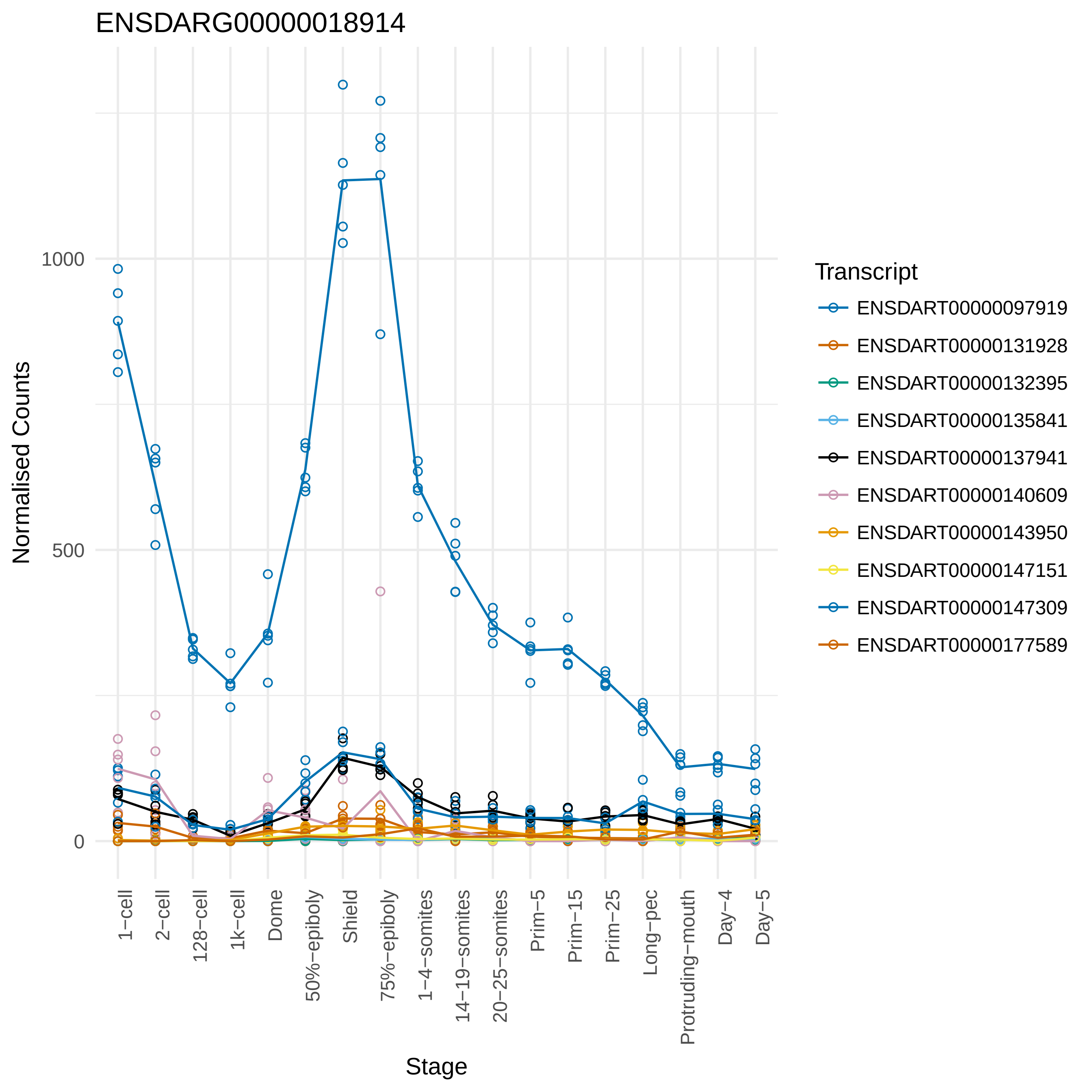
ENSDARG00000018914
Normalised Counts
1000
500
0
1−cell
2−cell
128−cell
1k−cell
Dome
50%−epiboly
Shield
75%−epiboly
1−4−somites
14−19−somites
20−25−somites
Prim−5
Prim−15
Prim−25
Long−pec
Protruding−mouth
Day−4
Day−5
Stage
Transcript
ENSDART00000097919
ENSDART00000131928
ENSDART00000132395
ENSDART00000135841
ENSDART00000137941
ENSDART00000140609
ENSDART00000143950
ENSDART00000147151
ENSDART00000147309
ENSDART00000177589

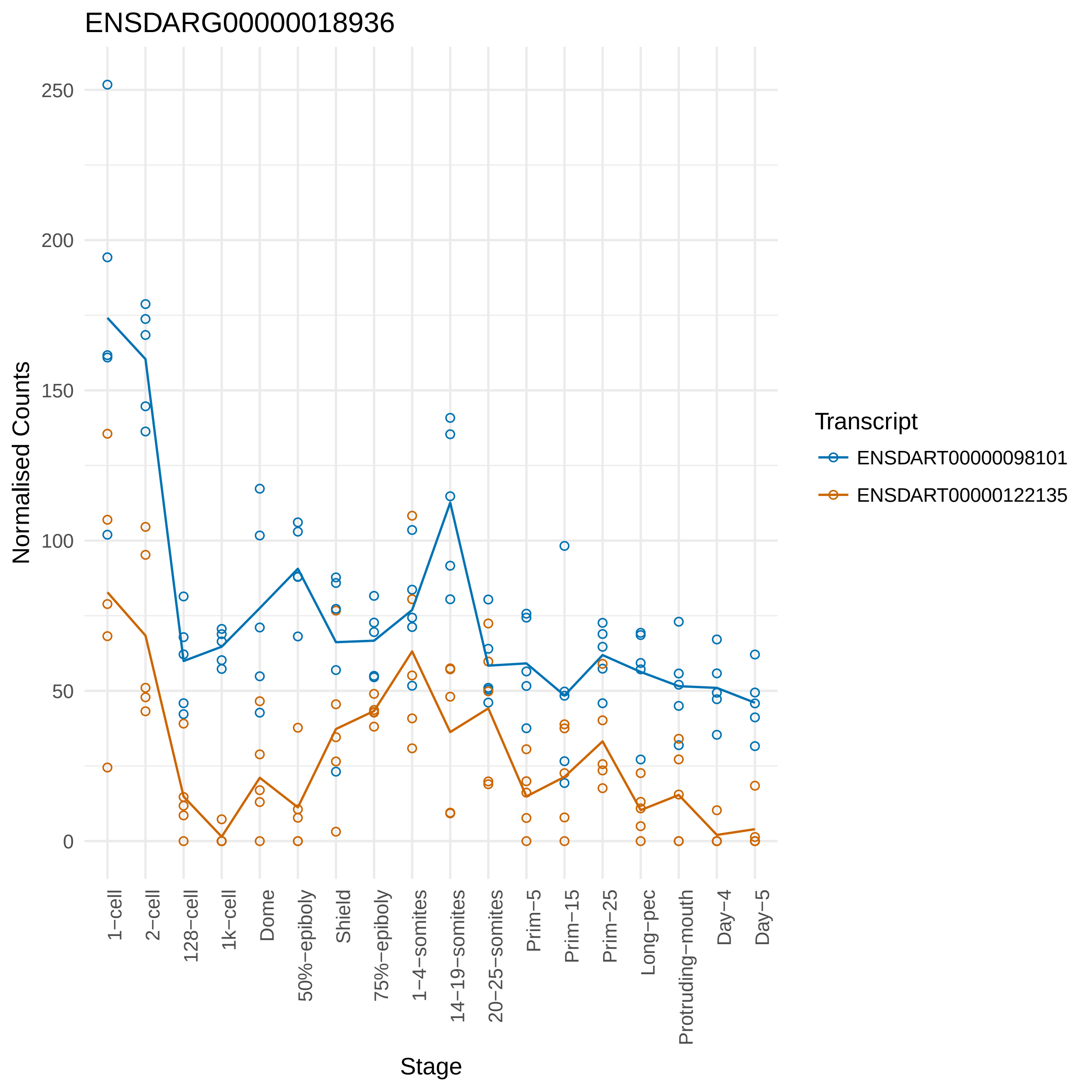

ENSDARG00000018936
Normalised Counts
250
200
150
100
50
0
1−cell
2−cell
128−cell
1k−cell
Dome
50%−epiboly
Shield
75%−epiboly
1−4−somites
14−19−somites
20−25−somites
Prim−5
Prim−15
Prim−25
Long−pec
Protruding−mouth
Day−4
Day−5
Stage
Transcript
ENSDART00000098101
ENSDART00000122135

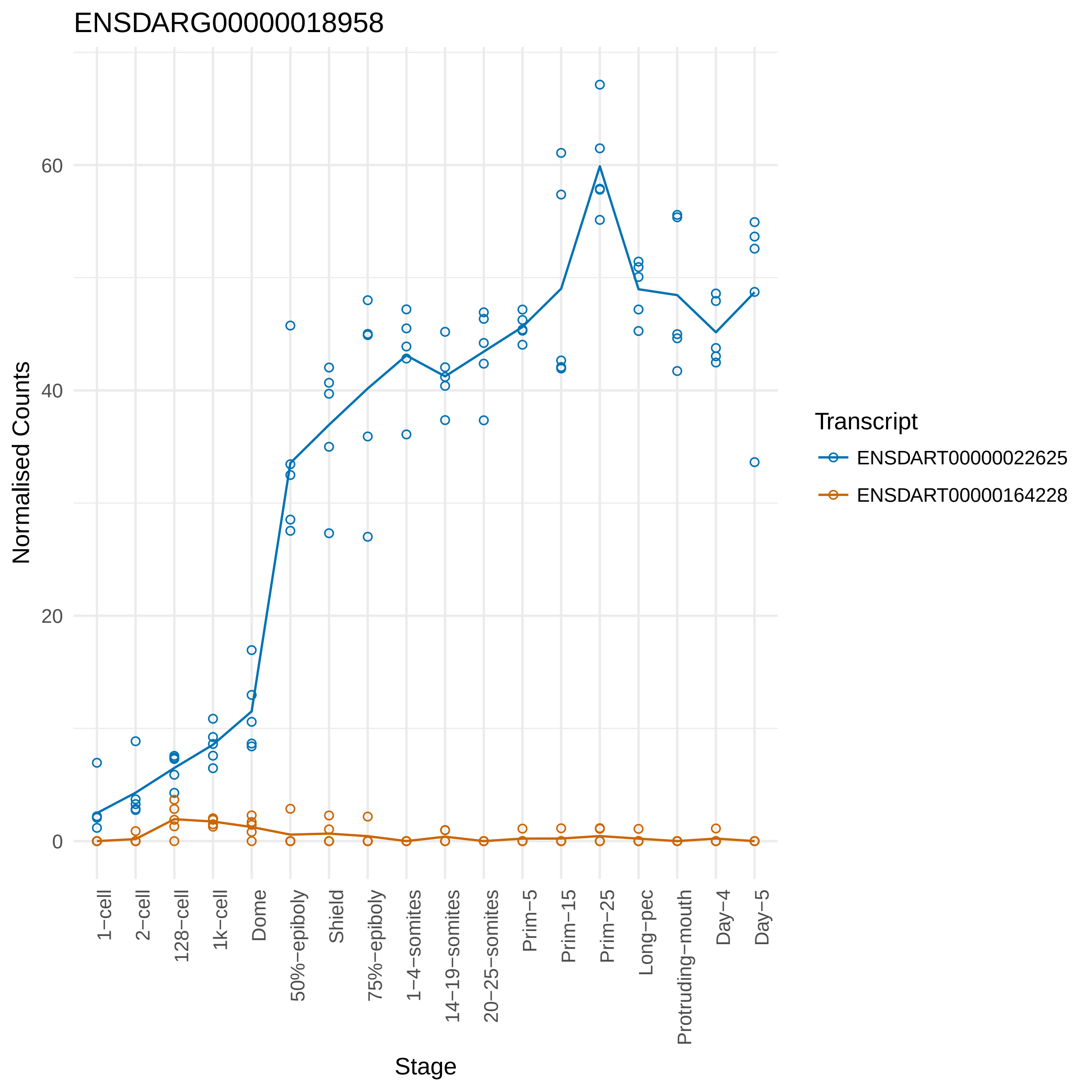
ENSDARG00000018958
Normalised Counts
0
20
40
60
Stage
1−cell
2−cell
128−cell
1k−cell
Dome
50%−epiboly
Shield
75%−epiboly
1−4−somites
14−19−somites
20−25−somites
Prim−5
Prim−15
Prim−25
Long−pec
Protruding−mouth
Day−4
Day−5
Transcript
ENSDART00000022625
ENSDART00000164228

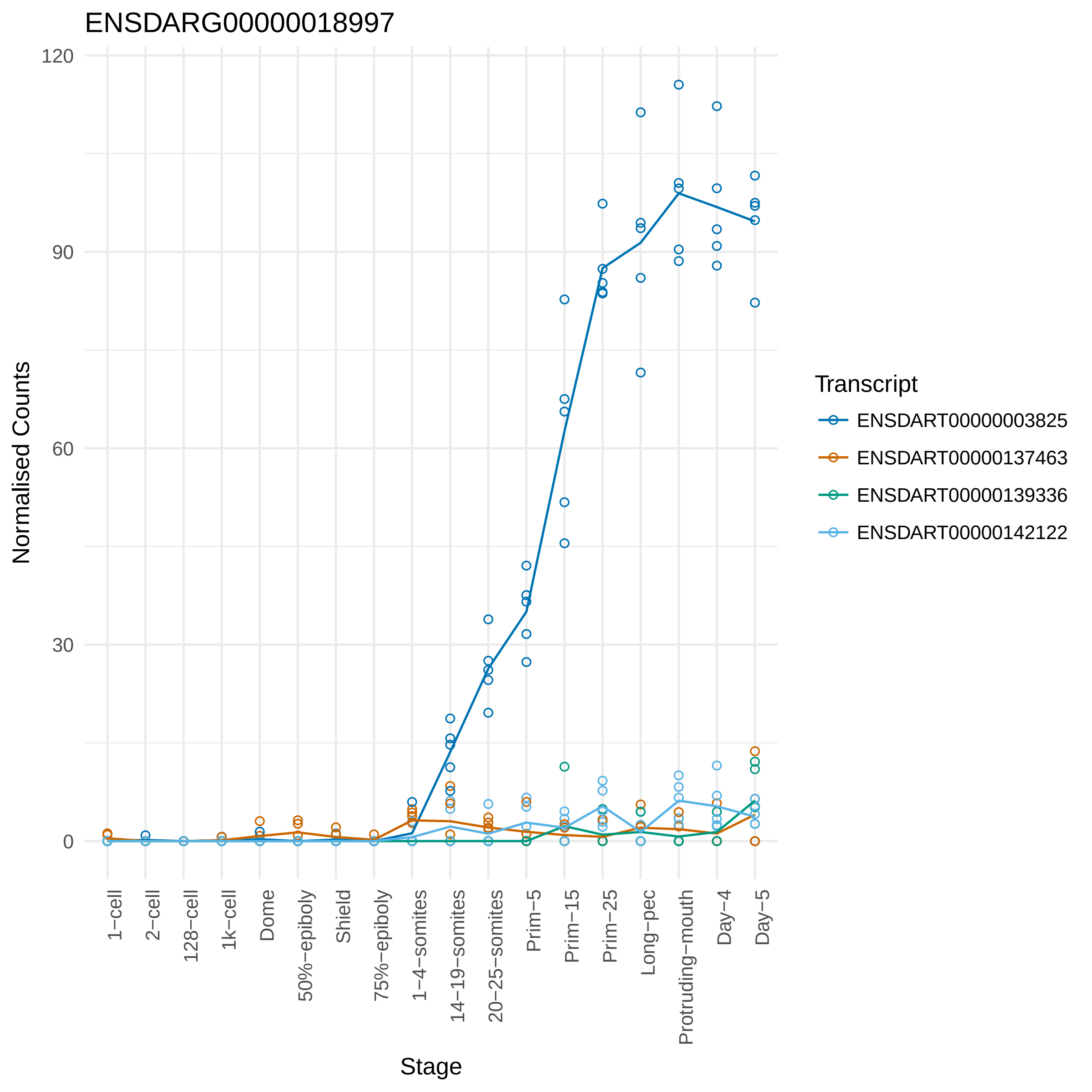

ENSDARG00000018997
Normalised Counts
120
90
60
30
0
1−cell
2−cell
128−cell
1k−cell
Dome
50%−epiboly
Shield
75%−epiboly
1−4−somites
14−19−somites
20−25−somites
Prim−5
Prim−15
Prim−25
Long−pec
Protruding−mouth
Day−4
Day−5
Stage
Transcript
ENSDART00000003825
ENSDART00000137463
ENSDART00000139336
ENSDART00000142122

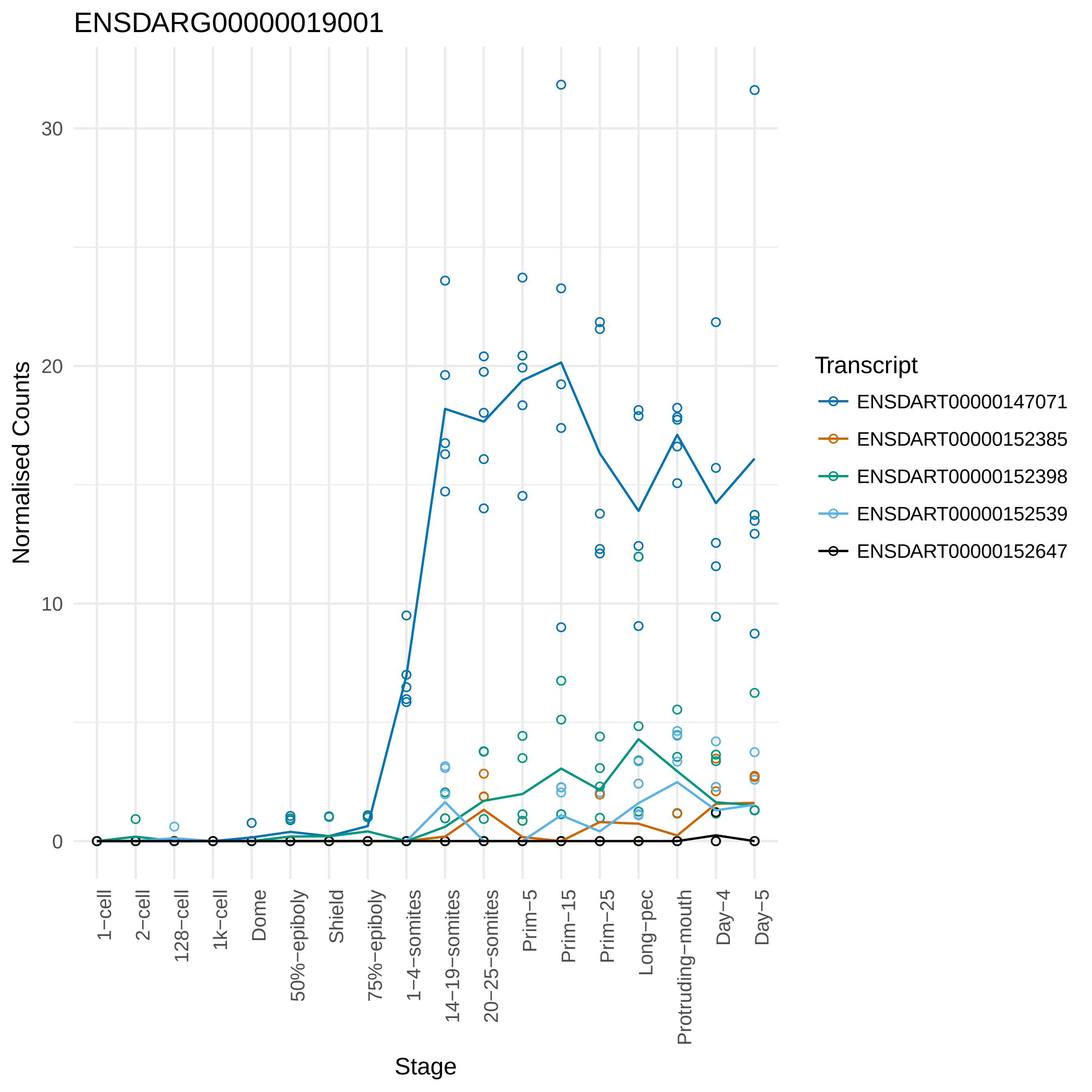

ENSDARG00000019001
Normalised Counts
30
20
10
0
1−cell
2−cell
128−cell
1k−cell
Dome
50%−epiboly
Shield
75%−epiboly
1−4−somites
14−19−somites
20−25−somites
Prim−5
Prim−15
Prim−25
Long−pec
Protruding−mouth
Day−4
Day−5
Stage
Transcript
ENSDART00000147071
ENSDART00000152385
ENSDART00000152398
ENSDART00000152539
ENSDART00000152647

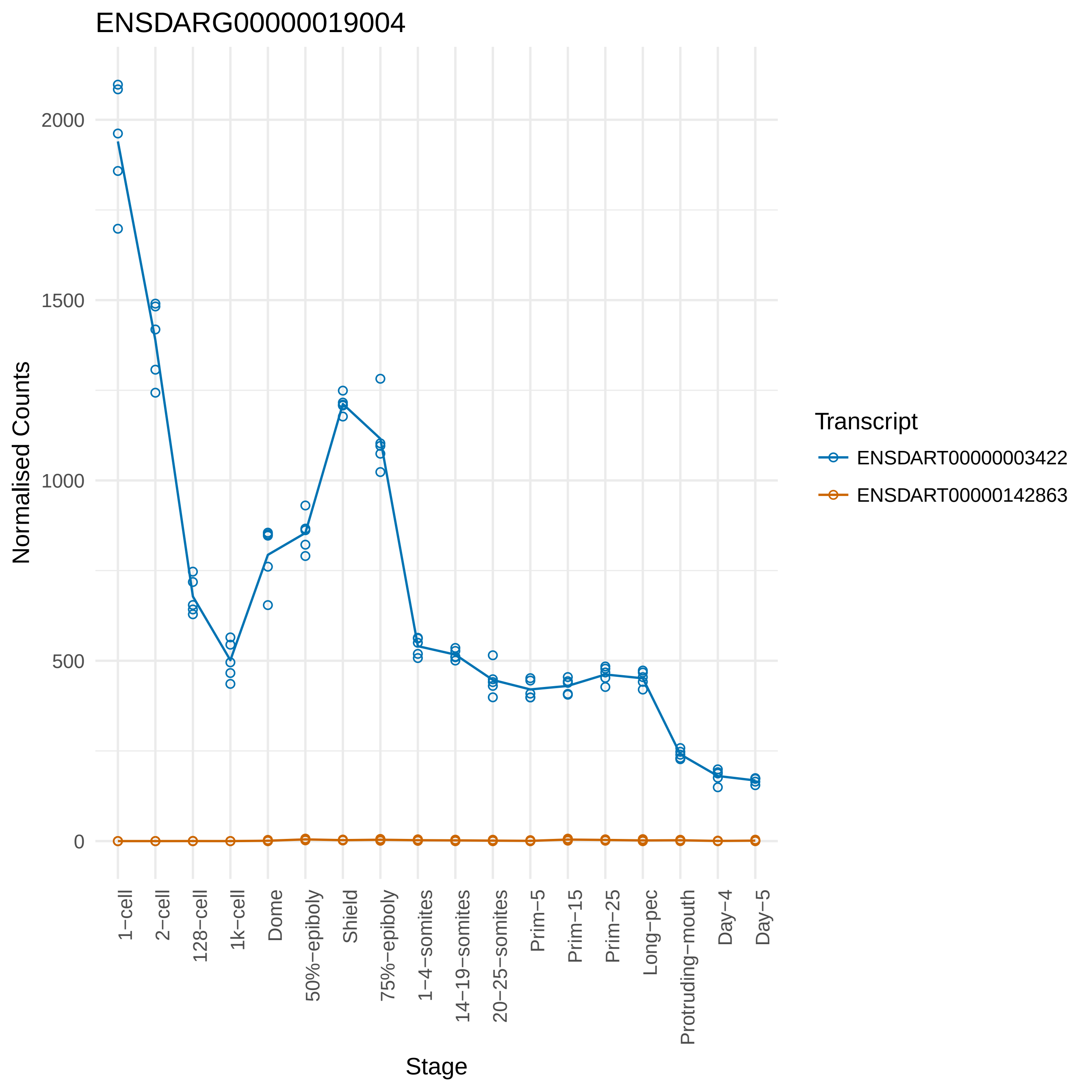

ENSDARG00000019004
Normalised Counts
2000
1500
1000
500
0
1−cell
2−cell
128−cell
1k−cell
Dome
50%−epiboly
Shield
75%−epiboly
1−4−somites
14−19−somites
20−25−somites
Prim−5
Prim−15
Prim−25
Long−pec
Protruding−mouth
Day−4
Day−5
Stage
Transcript
ENSDART00000003422
ENSDART00000142863

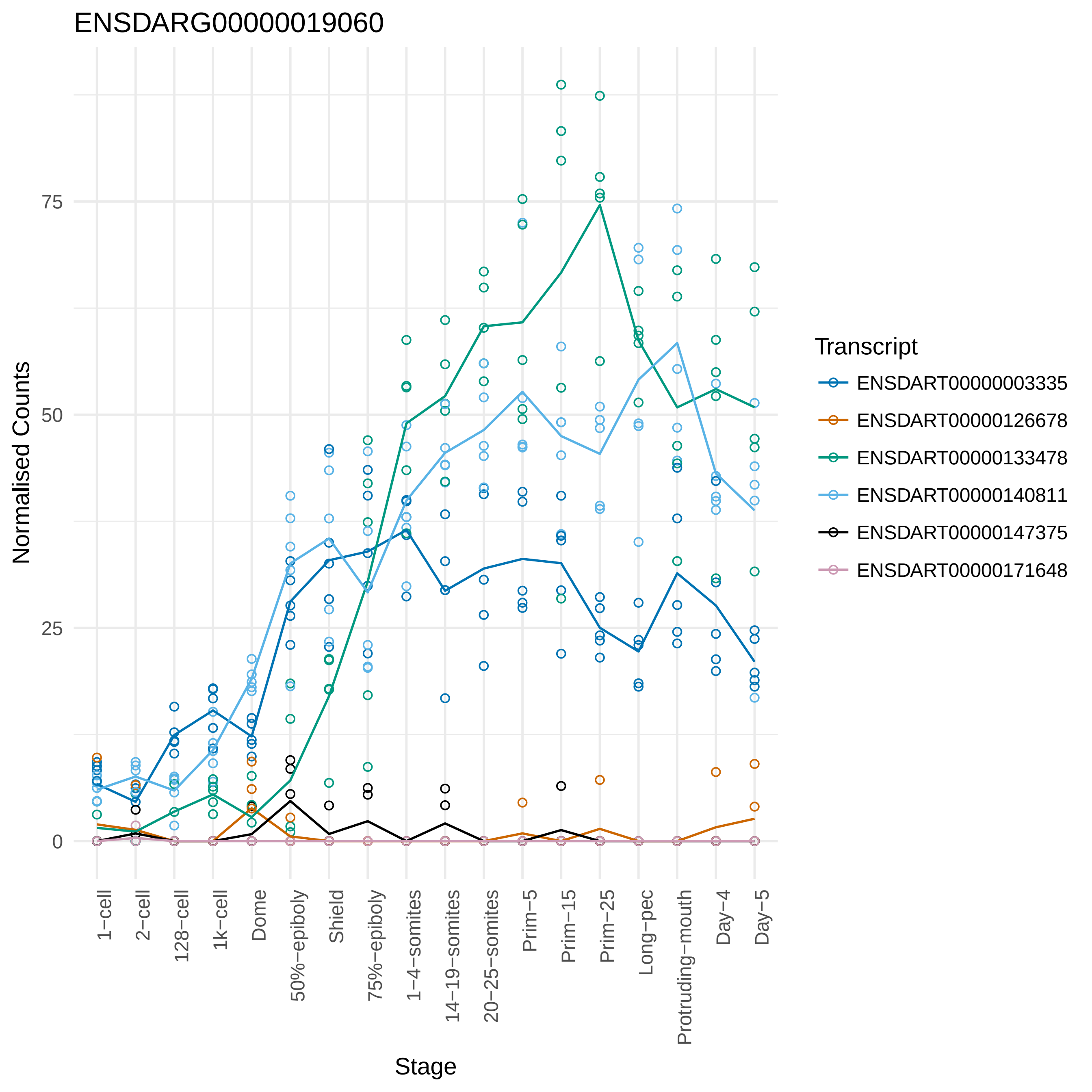
ENSDARG00000019060
Normalised Counts
0
25
50
75
1−cell
2−cell
128−cell
1k−cell
Dome
50%−epiboly
Shield
75%−epiboly
1−4−somites
14−19−somites
20−25−somites
Prim−5
Prim−15
Prim−25
Long−pec
Protruding−mouth
Day−4
Day−5
Stage
Transcript
ENSDART00000003335
ENSDART00000126678
ENSDART00000133478
ENSDART00000140811
ENSDART00000147375
ENSDART00000171648

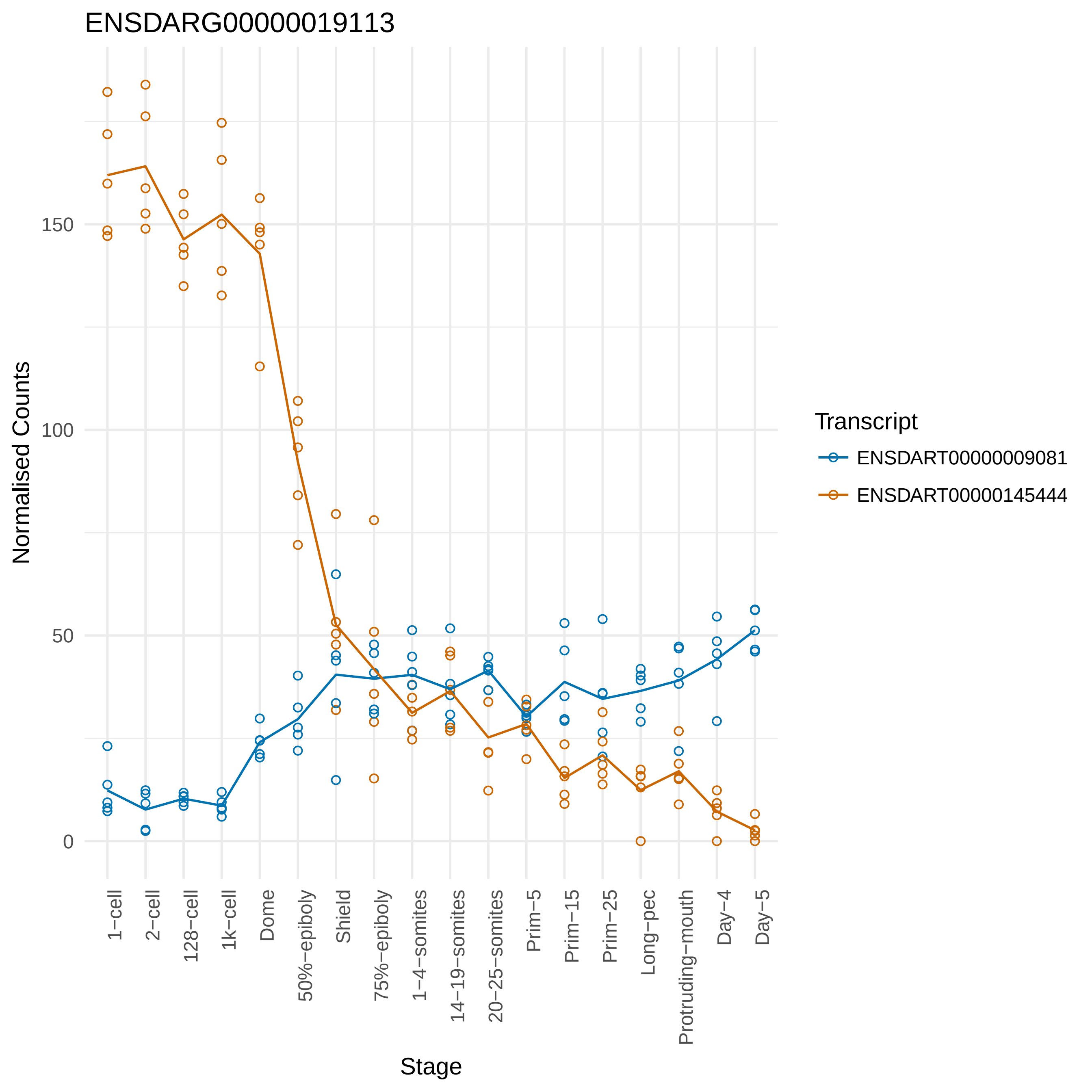

ENSDARG00000019113
Normalised Counts
150
100
50
0
1−cell
2−cell
128−cell
1k−cell
Dome
50%−epiboly
Shield
75%−epiboly
1−4−somites
14−19−somites
20−25−somites
Prim−5
Prim−15
Prim−25
Long−pec
Protruding−mouth
Day−4
Day−5
Stage
Transcript
ENSDART00000009081
ENSDART00000145444

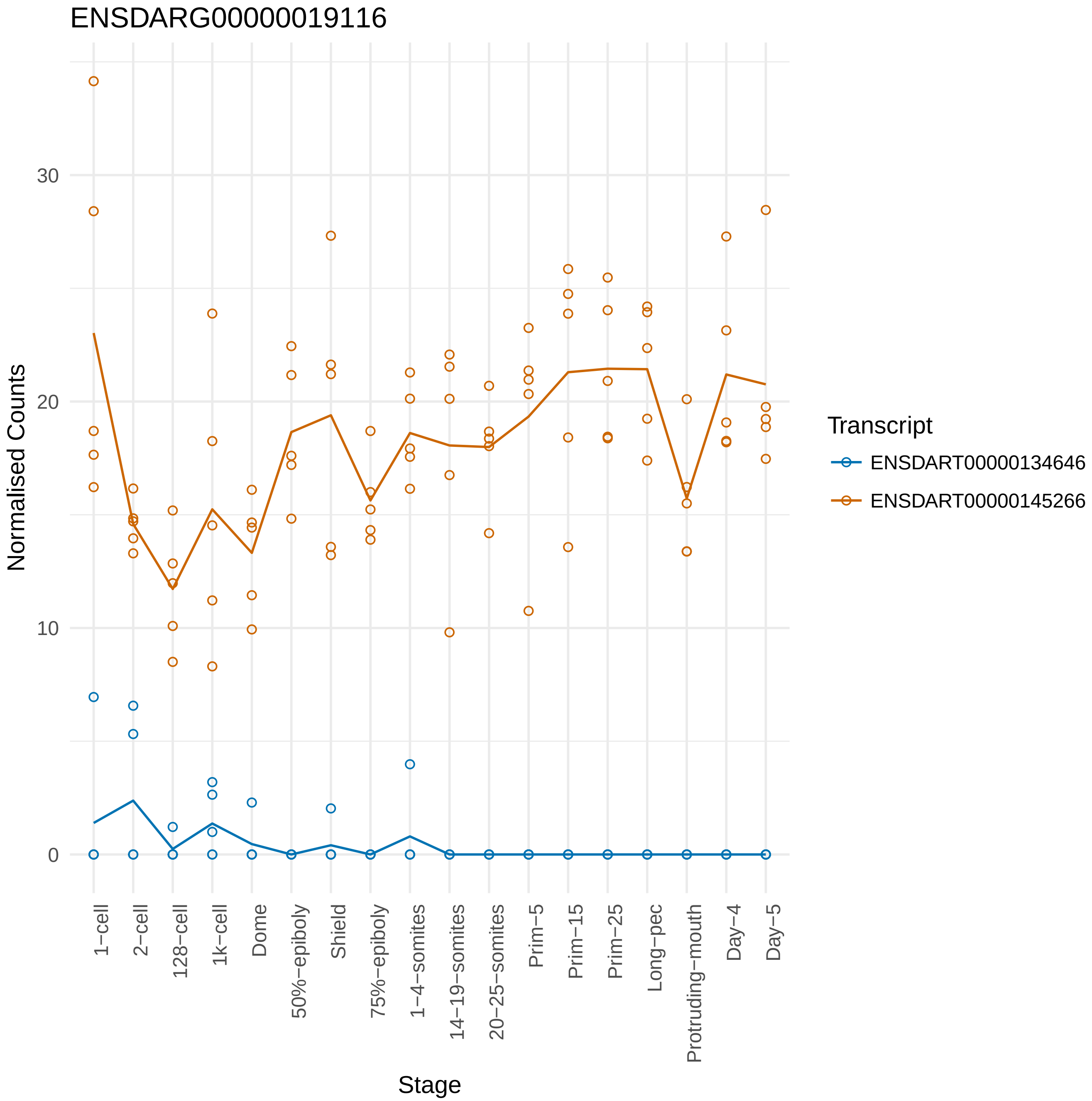
ENSDARG00000019116
Normalised Counts
0
10
20
30
1−cell
2−cell
128−cell
1k−cell
Dome
50%−epiboly
Shield
75%−epiboly
1−4−somites
14−19−somites
20−25−somites
Prim−5
Prim−15
Prim−25
Long−pec
Protruding−mouth
Day−4
Day−5
Stage
Transcript
ENSDART00000134646
ENSDART00000145266

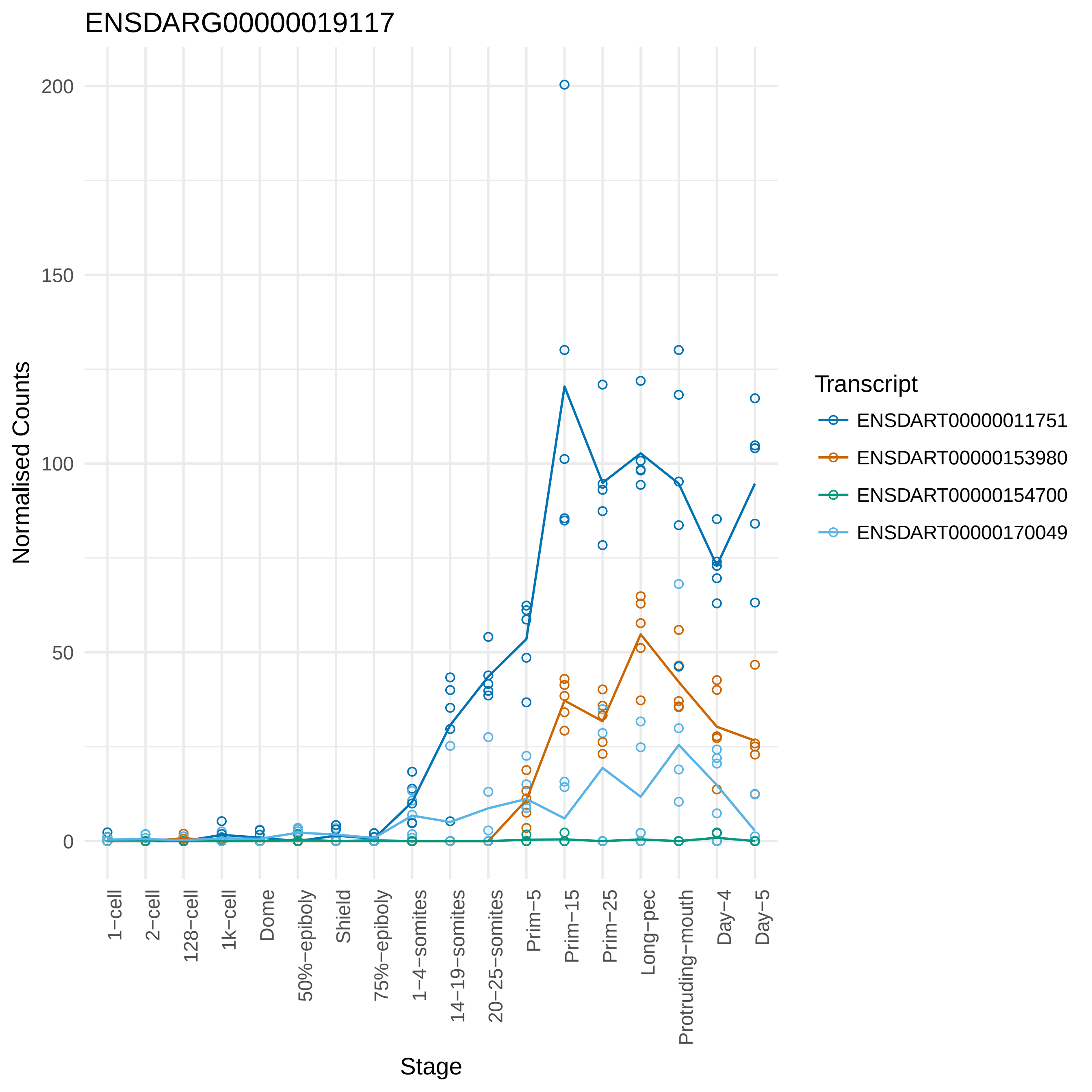

ENSDARG00000019117
Normalised Counts
200
150
100
50
0
1−cell
2−cell
128−cell
1k−cell
Dome
50%−epiboly
Shield
75%−epiboly
1−4−somites
14−19−somites
20−25−somites
Prim−5
Prim−15
Prim−25
Long−pec
Protruding−mouth
Day−4
Day−5
Stage
Transcript
ENSDART00000011751
ENSDART00000153980
ENSDART00000154700
ENSDART00000170049

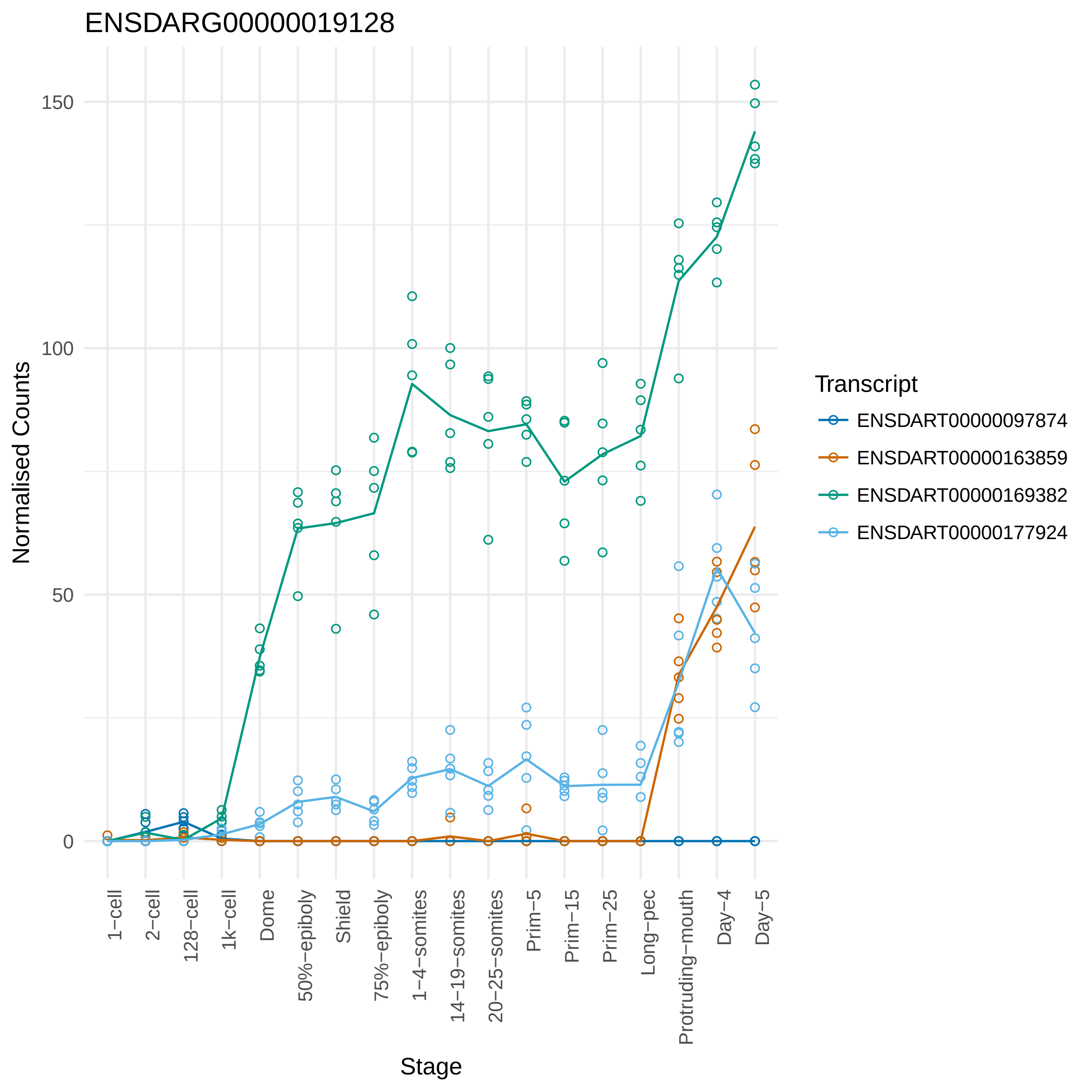

ENSDARG00000019128
Normalised Counts
150
100
50
0
1−cell
2−cell
128−cell
1k−cell
Dome
50%−epiboly
Shield
75%−epiboly
1−4−somites
14−19−somites
20−25−somites
Prim−5
Prim−15
Prim−25
Long−pec
Protruding−mouth
Day−4
Day−5
Stage
Transcript
ENSDART00000097874
ENSDART00000163859
ENSDART00000169382
ENSDART00000177924

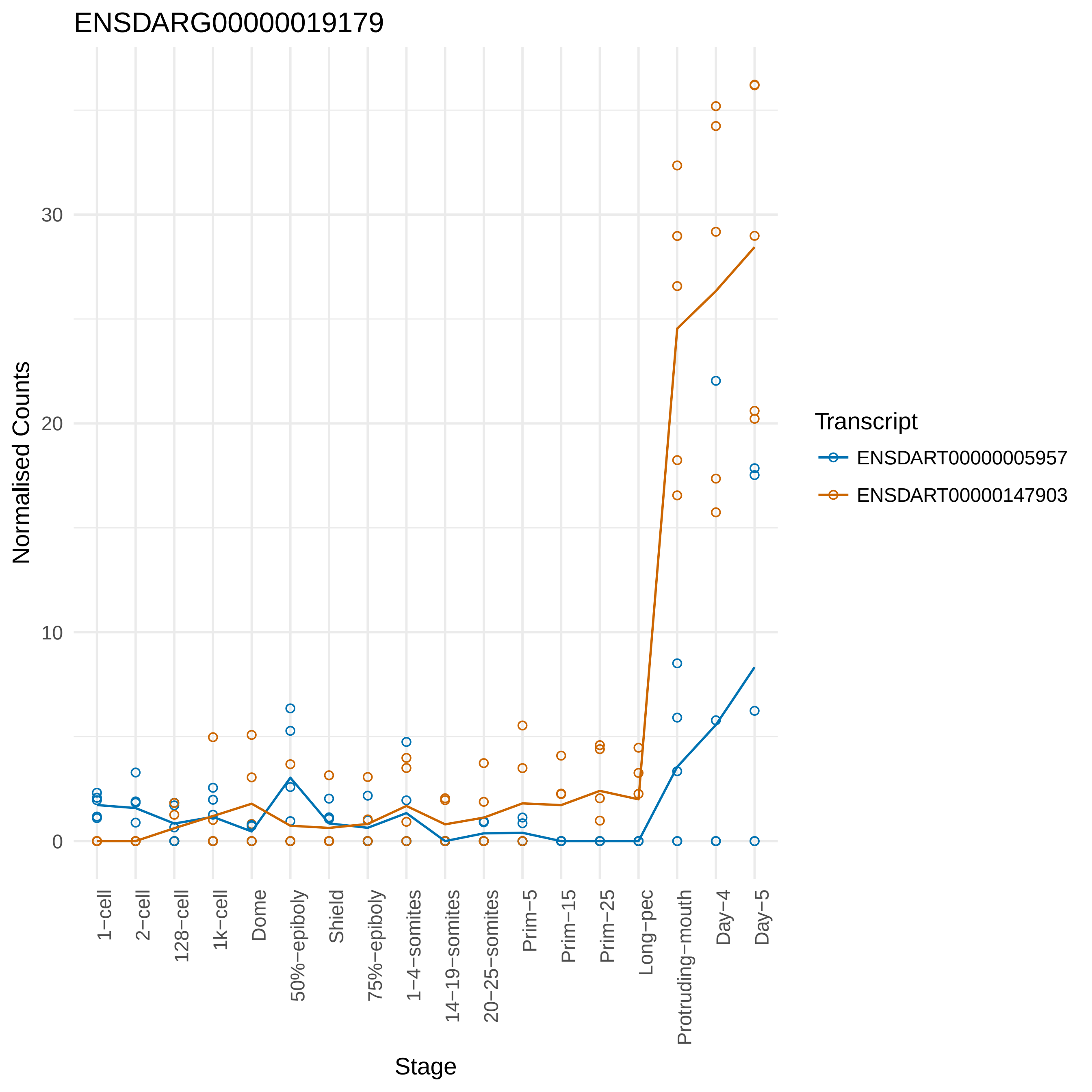
ENSDARG00000019179
Normalised Counts
0
10
20
30
Stage
1−cell
2−cell
128−cell
1k−cell
Dome
50%−epiboly
Shield
75%−epiboly
1−4−somites
14−19−somites
20−25−somites
Prim−5
Prim−15
Prim−25
Long−pec
Protruding−mouth
Day−4
Day−5
Transcript
ENSDART00000005957
ENSDART00000147903

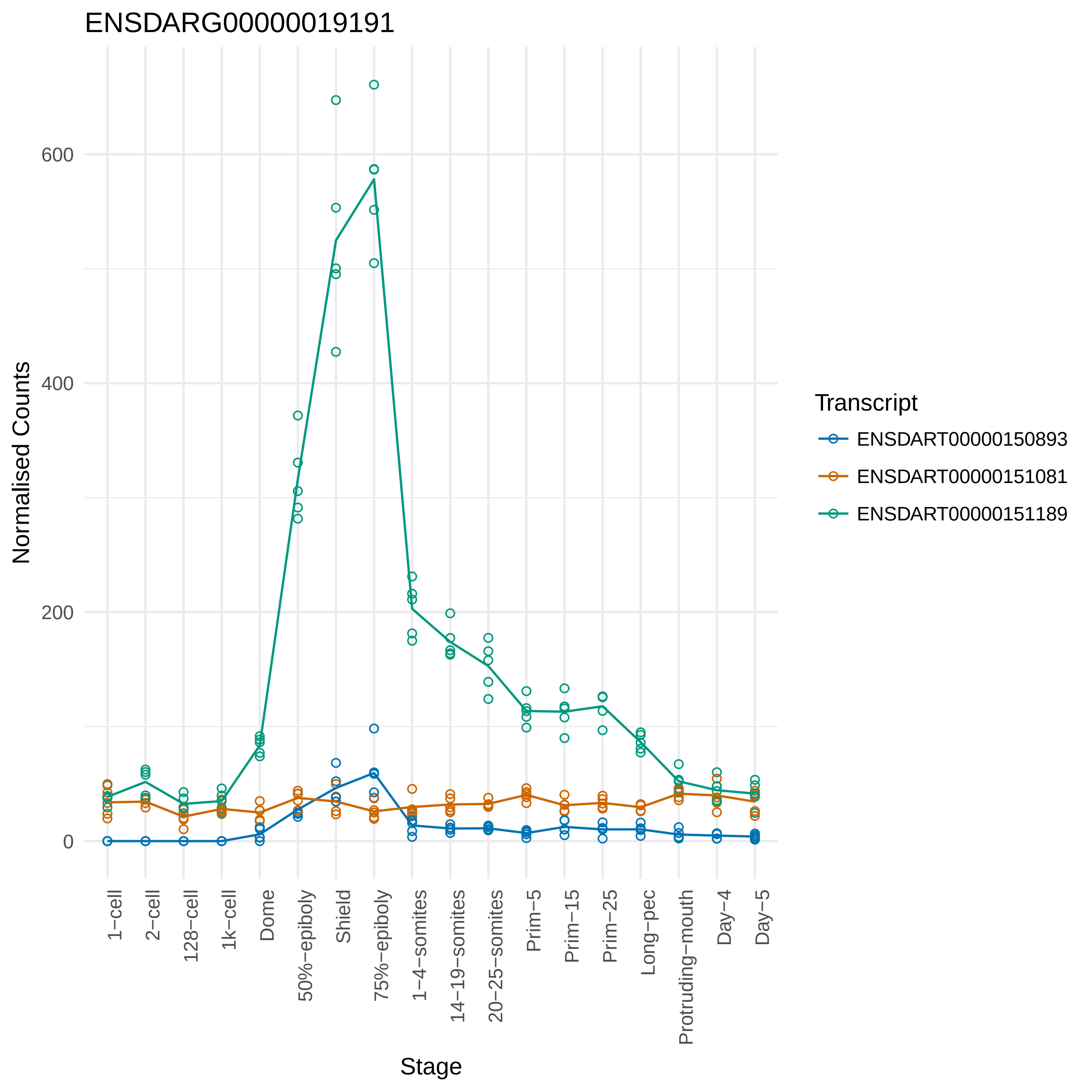

ENSDARG00000019191
Normalised Counts
600
400
200
0
1−cell
2−cell
128−cell
1k−cell
Dome
50%−epiboly
Shield
75%−epiboly
1−4−somites
14−19−somites
20−25−somites
Prim−5
Prim−15
Prim−25
Long−pec
Protruding−mouth
Day−4
Day−5
Stage
Transcript
ENSDART00000150893
ENSDART00000151081
ENSDART00000151189

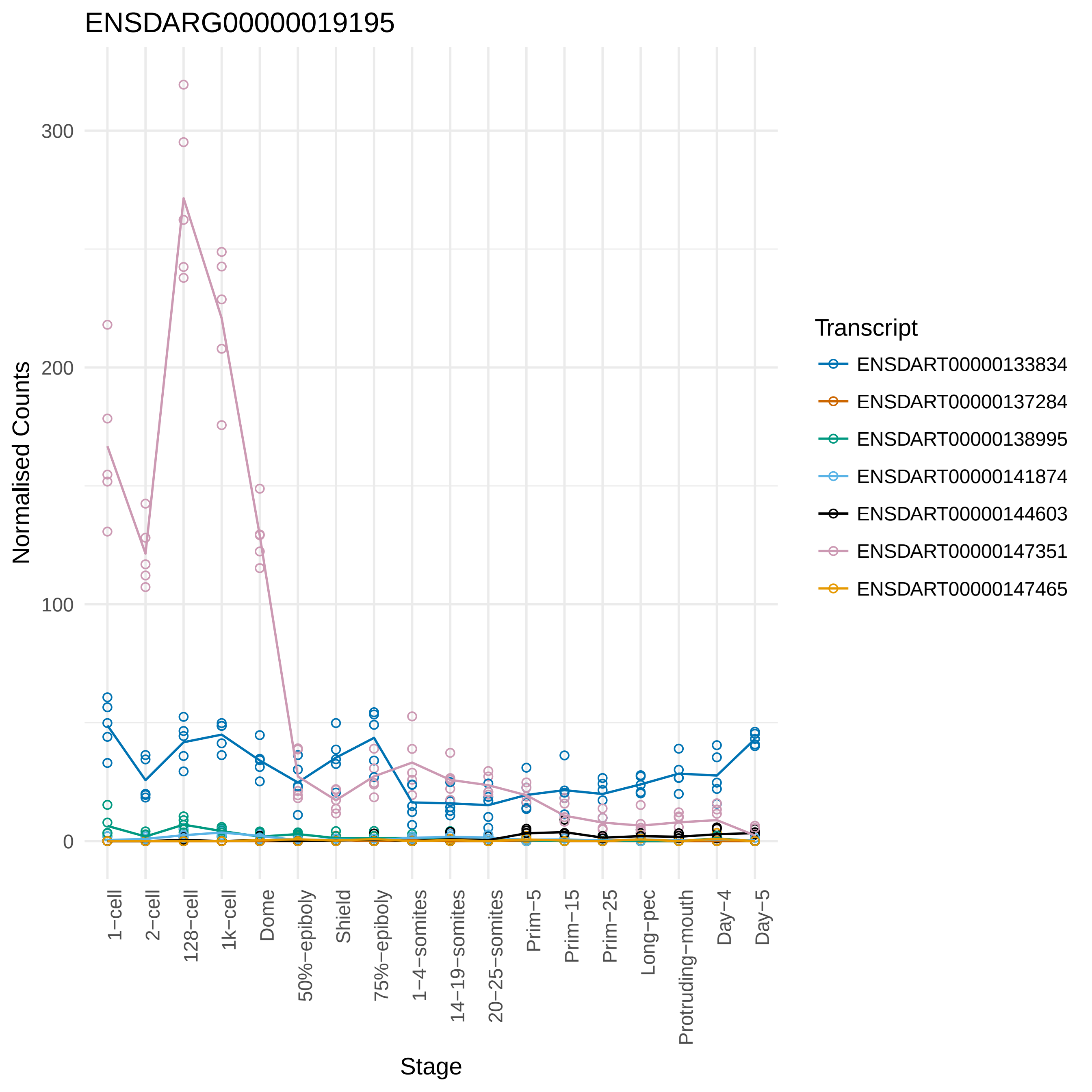

ENSDARG00000019195
Normalised Counts
300
200
100
0
1−cell
2−cell
128−cell
1k−cell
Dome
50%−epiboly
Shield
75%−epiboly
1−4−somites
14−19−somites
20−25−somites
Prim−5
Prim−15
Prim−25
Long−pec
Protruding−mouth
Day−4
Day−5
Stage
Transcript
ENSDART00000133834
ENSDART00000137284
ENSDART00000138995
ENSDART00000141874
ENSDART00000144603
ENSDART00000147351
ENSDART00000147465

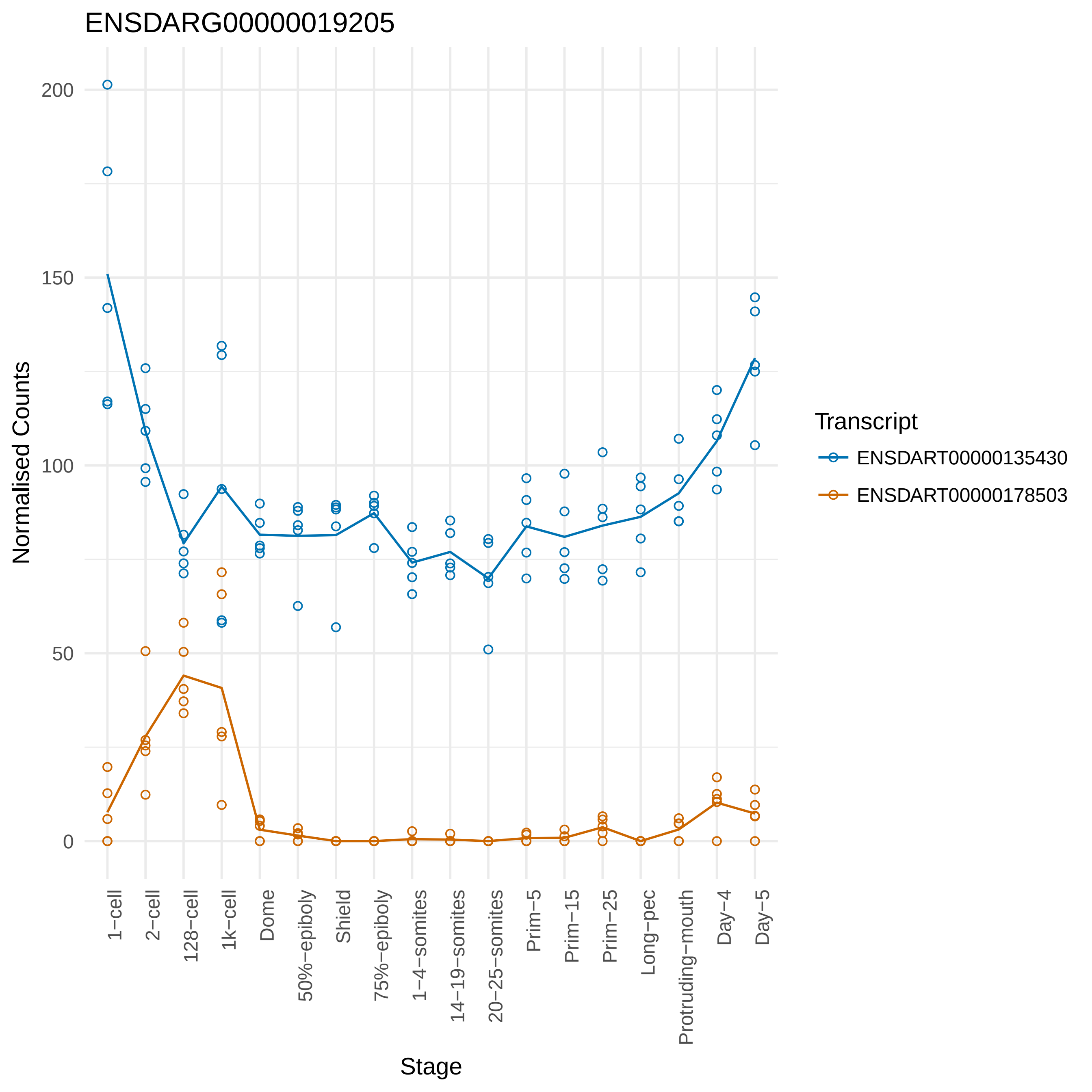

ENSDARG00000019205
Normalised Counts
200
150
100
50
0
1−cell
2−cell
128−cell
1k−cell
Dome
50%−epiboly
Shield
75%−epiboly
1−4−somites
14−19−somites
20−25−somites
Prim−5
Prim−15
Prim−25
Long−pec
Protruding−mouth
Day−4
Day−5
Stage
Transcript
ENSDART00000135430
ENSDART00000178503

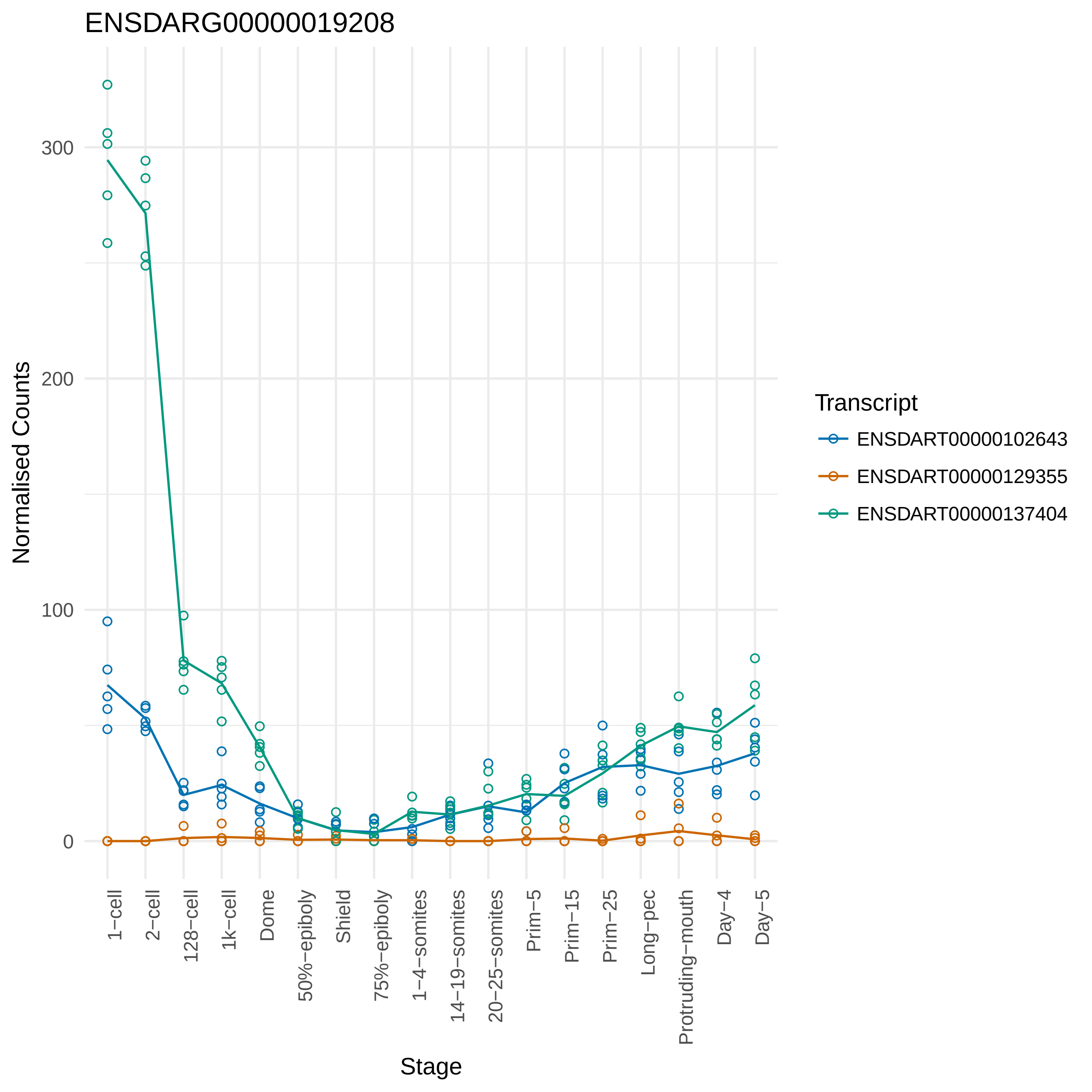

ENSDARG00000019208
Normalised Counts
300
200
100
0
1−cell
2−cell
128−cell
1k−cell
Dome
50%−epiboly
Shield
75%−epiboly
1−4−somites
14−19−somites
20−25−somites
Prim−5
Prim−15
Prim−25
Long−pec
Protruding−mouth
Day−4
Day−5
Stage
Transcript
ENSDART00000102643
ENSDART00000129355
ENSDART00000137404

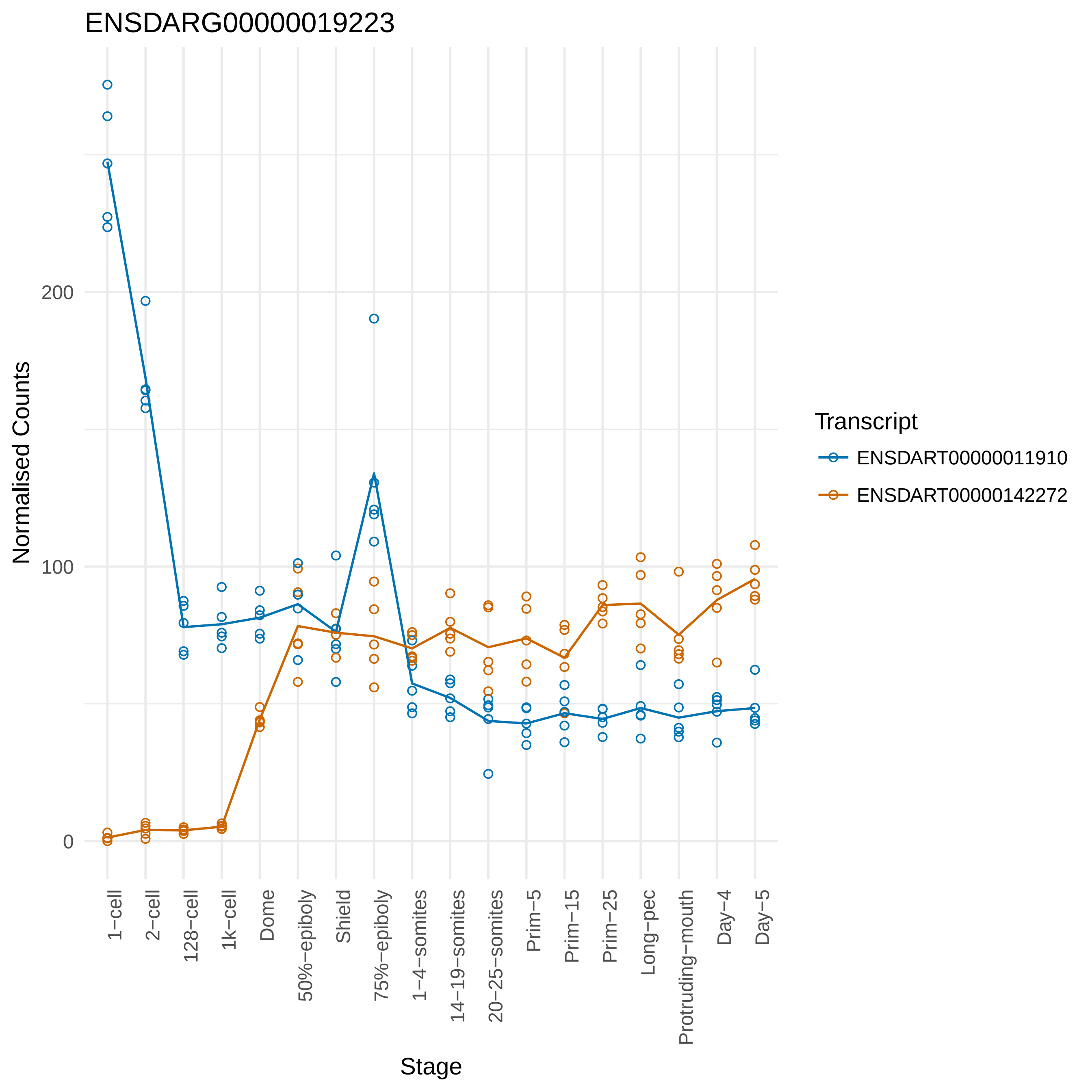

ENSDARG00000019223
Normalised Counts
200
100
0
1−cell
2−cell
128−cell
1k−cell
Dome
50%−epiboly
Shield
75%−epiboly
1−4−somites
14−19−somites
20−25−somites
Prim−5
Prim−15
Prim−25
Long−pec
Protruding−mouth
Day−4
Day−5
Stage
Transcript
ENSDART00000011910
ENSDART00000142272

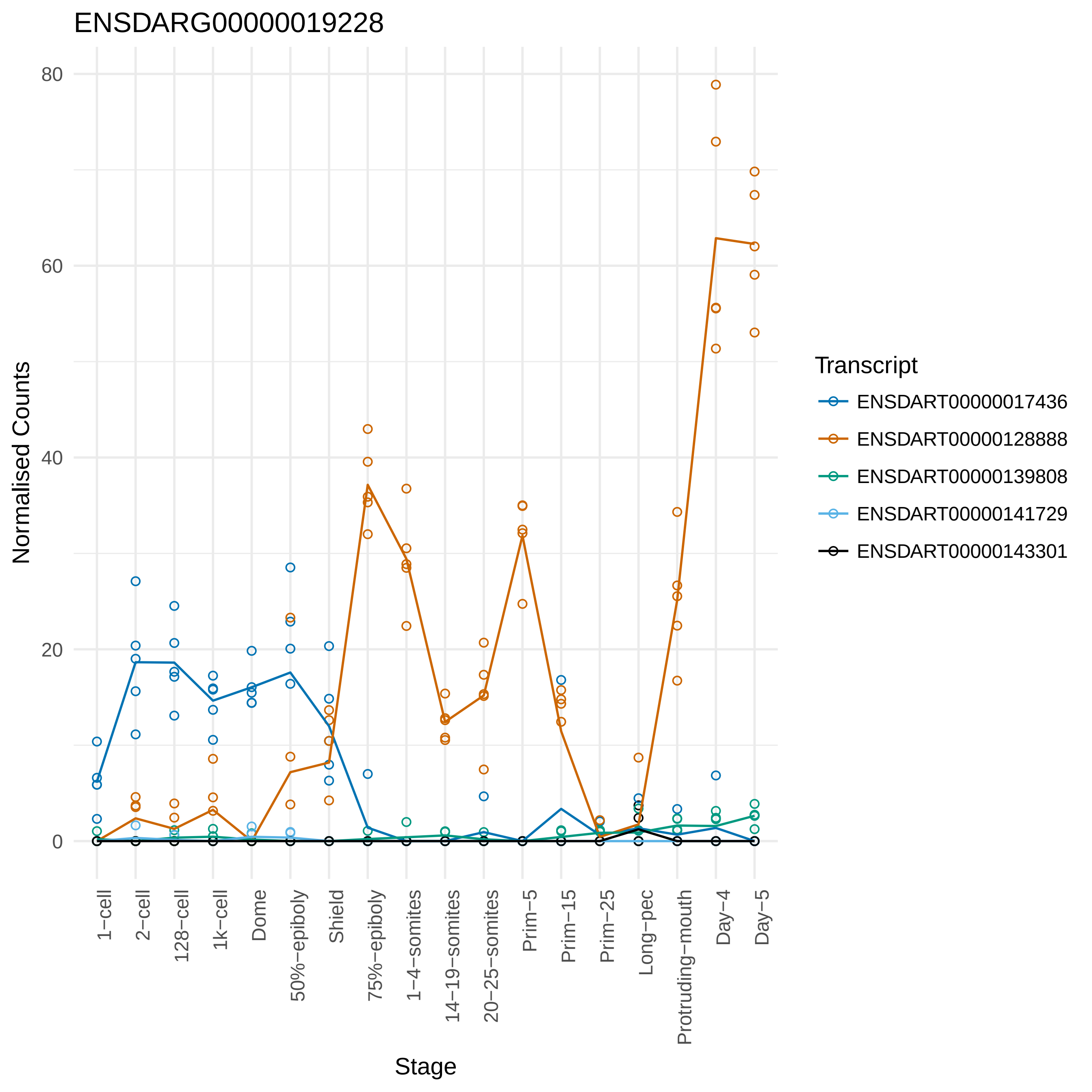

ENSDARG00000019228
Normalised Counts
80
60
40
20
0
1−cell
2−cell
128−cell
1k−cell
Dome
50%−epiboly
Shield
75%−epiboly
1−4−somites
14−19−somites
20−25−somites
Prim−5
Prim−15
Prim−25
Long−pec
Protruding−mouth
Day−4
Day−5
Stage
Transcript
ENSDART00000017436
ENSDART00000128888
ENSDART00000139808
ENSDART00000141729
ENSDART00000143301

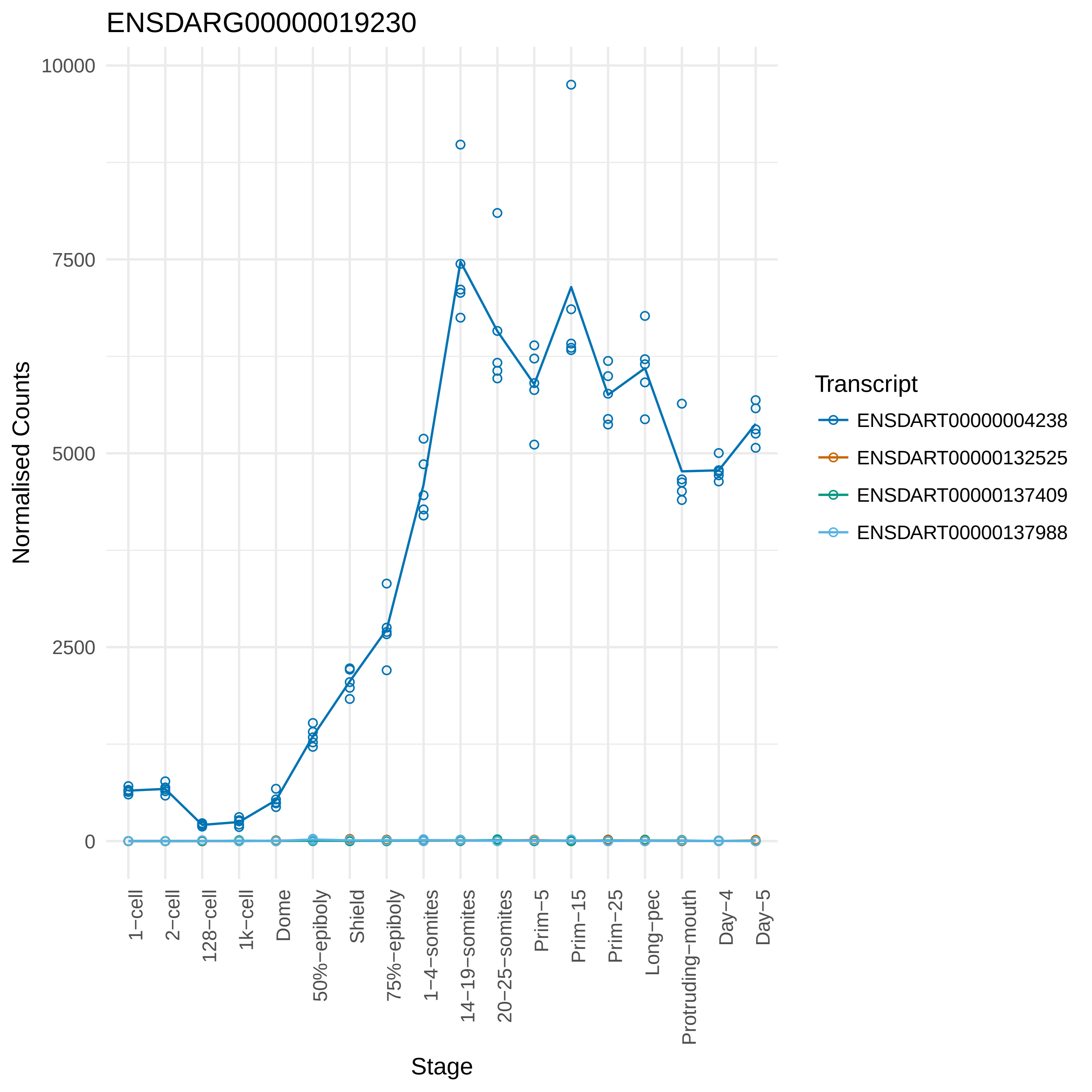
ENSDARG00000019230
Normalised Counts
10000
7500
5000
2500
0
1−cell
2−cell
128−cell
1k−cell
Dome
50%−epiboly
Shield
75%−epiboly
1−4−somites
14−19−somites
20−25−somites
Prim−5
Prim−15
Prim−25
Long−pec
Protruding−mouth
Day−4
Day−5
Stage
Transcript
ENSDART00000004238
ENSDART00000132525
ENSDART00000137409
ENSDART00000137988

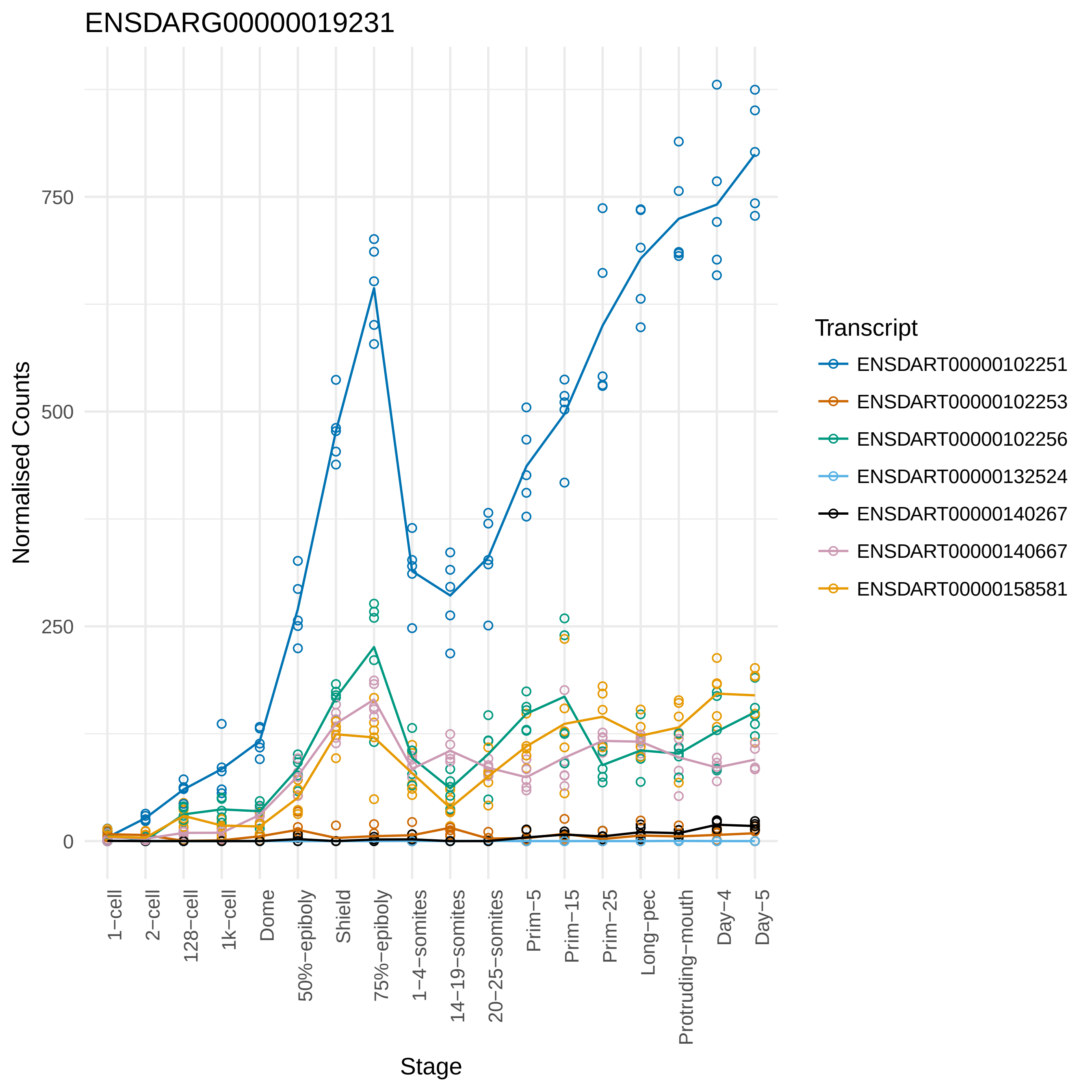

ENSDARG00000019231
Normalised Counts
750
500
250
0
1−cell
2−cell
128−cell
1k−cell
Dome
50%−epiboly
Shield
75%−epiboly
1−4−somites
14−19−somites
20−25−somites
Prim−5
Prim−15
Prim−25
Long−pec
Protruding−mouth
Day−4
Day−5
Stage
Transcript
ENSDART00000102251
ENSDART00000102253
ENSDART00000102256
ENSDART00000132524
ENSDART00000140267
ENSDART00000140667
ENSDART00000158581

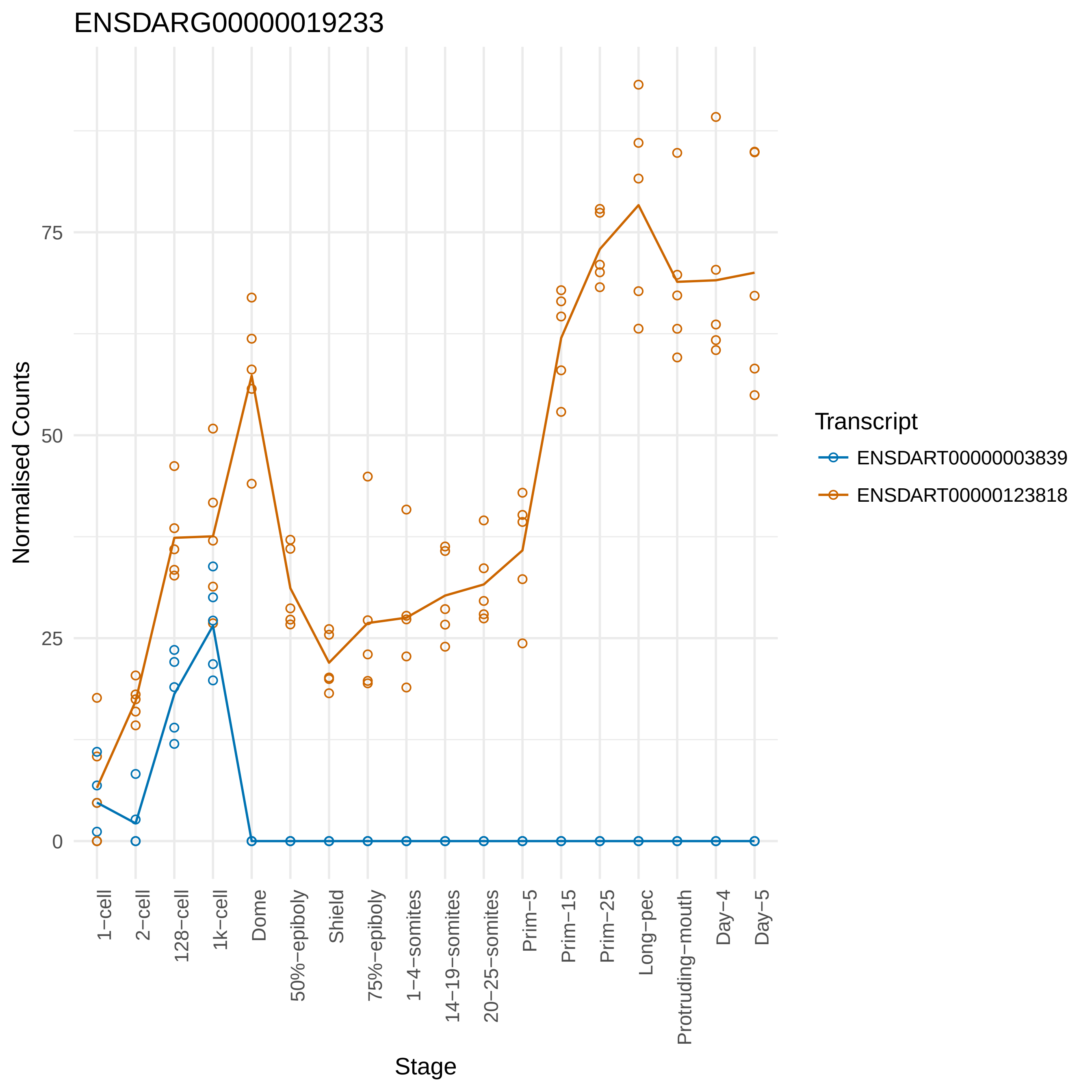
ENSDARG00000019233
Normalised Counts
75
50
25
0
1−cell
2−cell
128−cell
1k−cell
Dome
50%−epiboly
Shield
75%−epiboly
1−4−somites
14−19−somites
20−25−somites
Prim−5
Prim−15
Prim−25
Long−pec
Protruding−mouth
Day−4
Day−5
Stage
Transcript
ENSDART00000003839
ENSDART00000123818

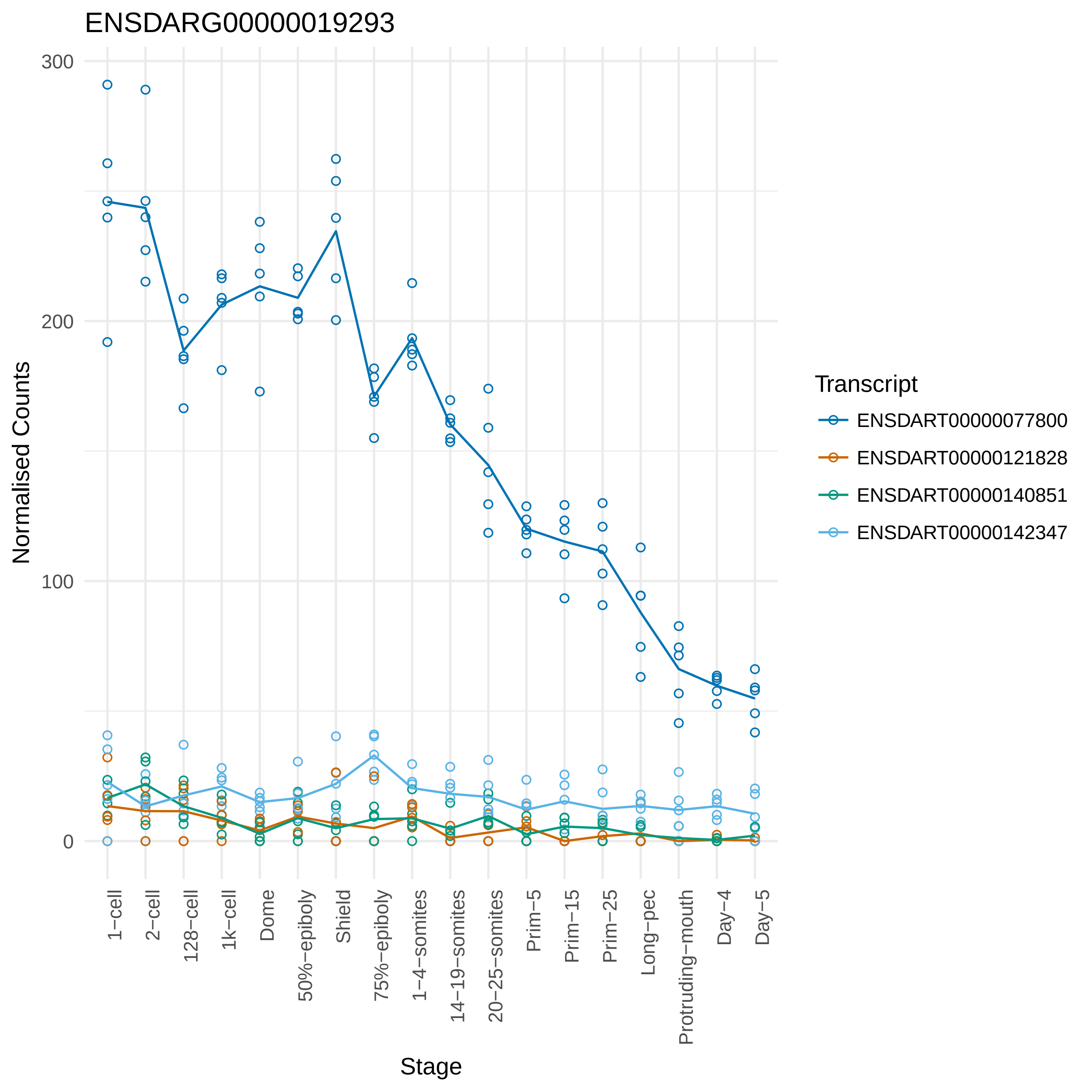
ENSDARG00000019293
Normalised Counts
300
200
100
0
1−cell
2−cell
128−cell
1k−cell
Dome
50%−epiboly
Shield
75%−epiboly
1−4−somites
14−19−somites
20−25−somites
Prim−5
Prim−15
Prim−25
Long−pec
Protruding−mouth
Day−4
Day−5
Stage
Transcript
ENSDART00000077800
ENSDART00000121828
ENSDART00000140851
ENSDART00000142347

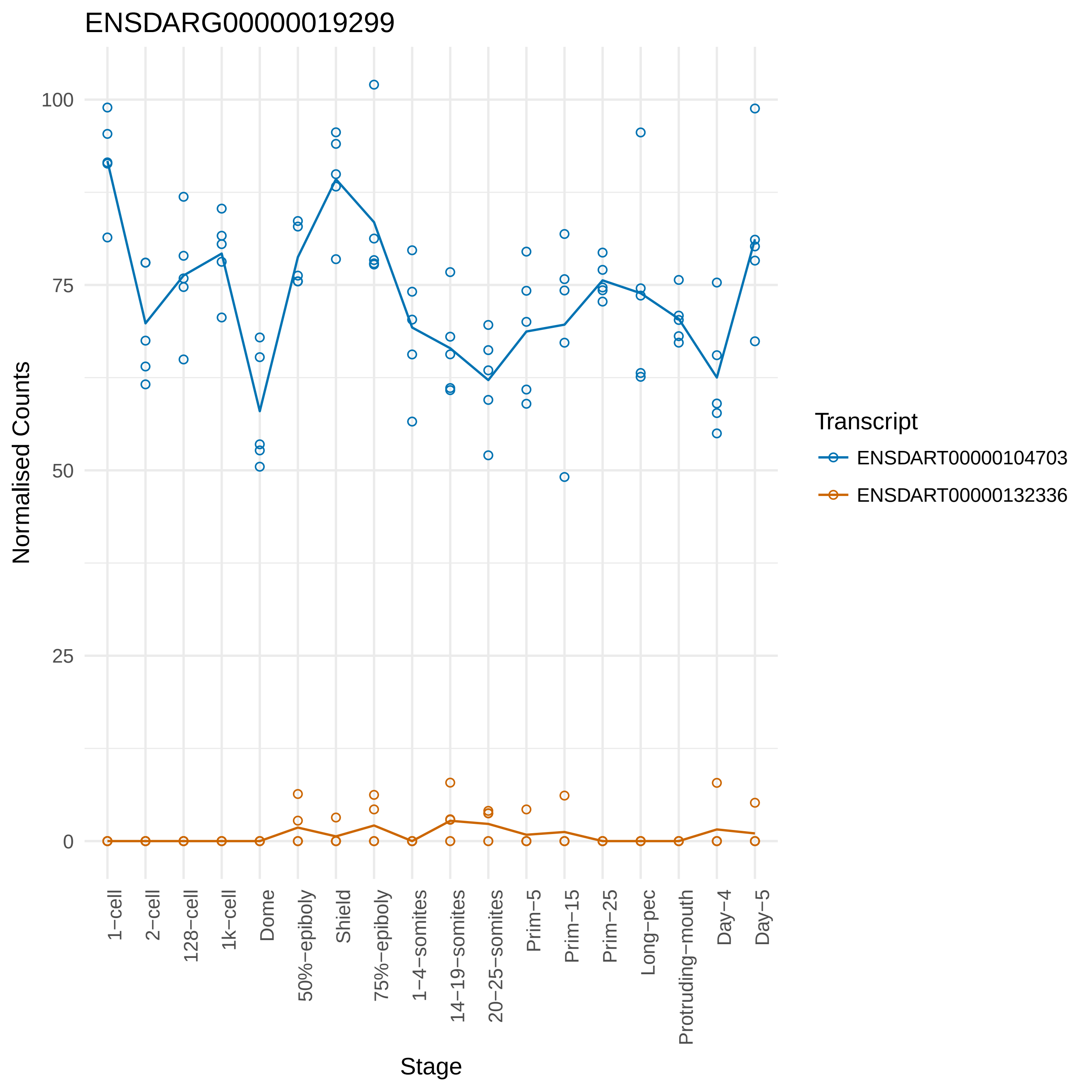
ENSDARG00000019299
Normalised Counts
100
75
50
25
0
1−cell
2−cell
128−cell
1k−cell
Dome
50%−epiboly
Shield
75%−epiboly
1−4−somites
14−19−somites
20−25−somites
Prim−5
Prim−15
Prim−25
Long−pec
Protruding−mouth
Day−4
Day−5
Stage
Transcript
ENSDART00000104703
ENSDART00000132336

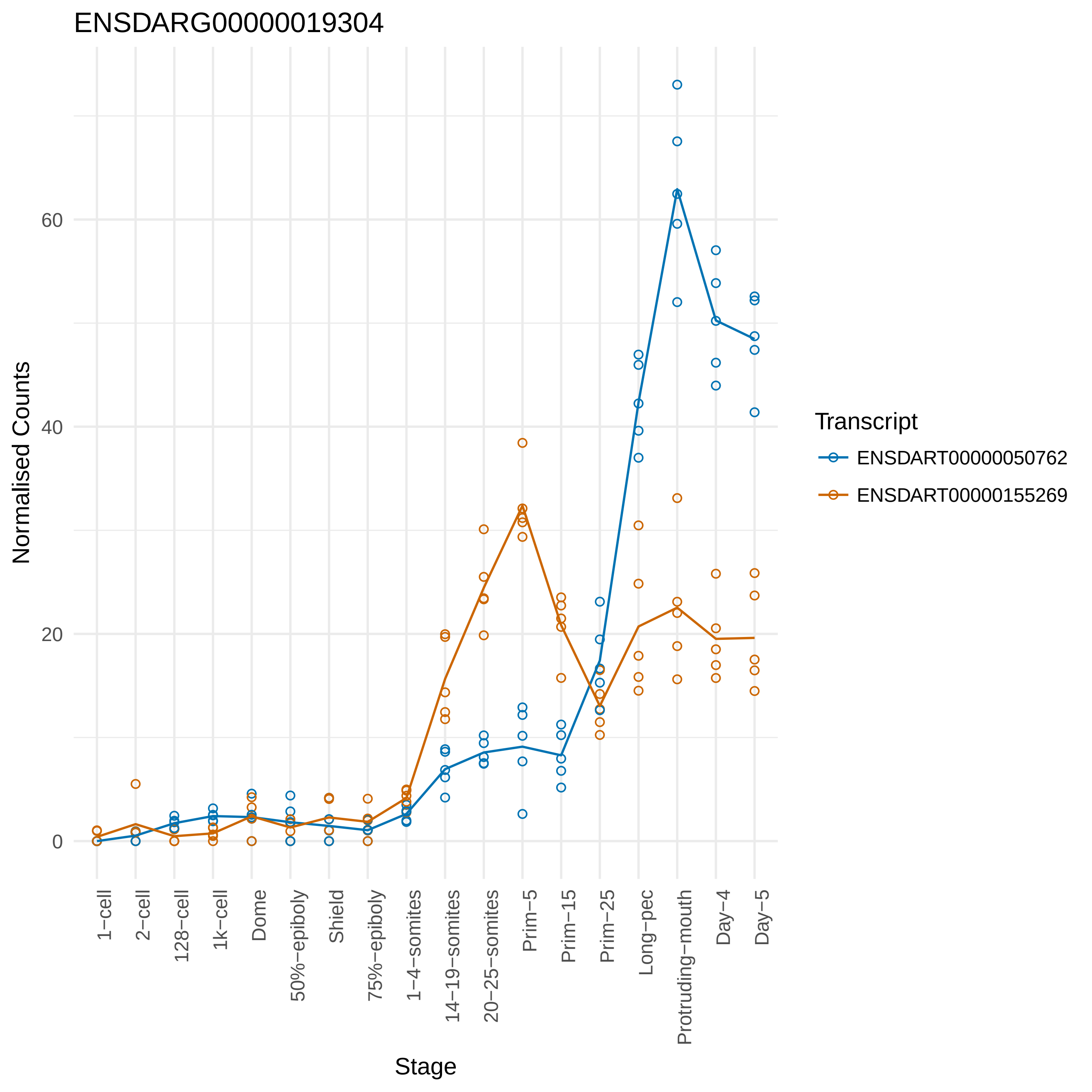

ENSDARG00000019304
Normalised Counts
0
20
40
60
Stage
1−cell
2−cell
128−cell
1k−cell
Dome
50%−epiboly
Shield
75%−epiboly
1−4−somites
14−19−somites
20−25−somites
Prim−5
Prim−15
Prim−25
Long−pec
Protruding−mouth
Day−4
Day−5
Transcript
ENSDART00000050762
ENSDART00000155269

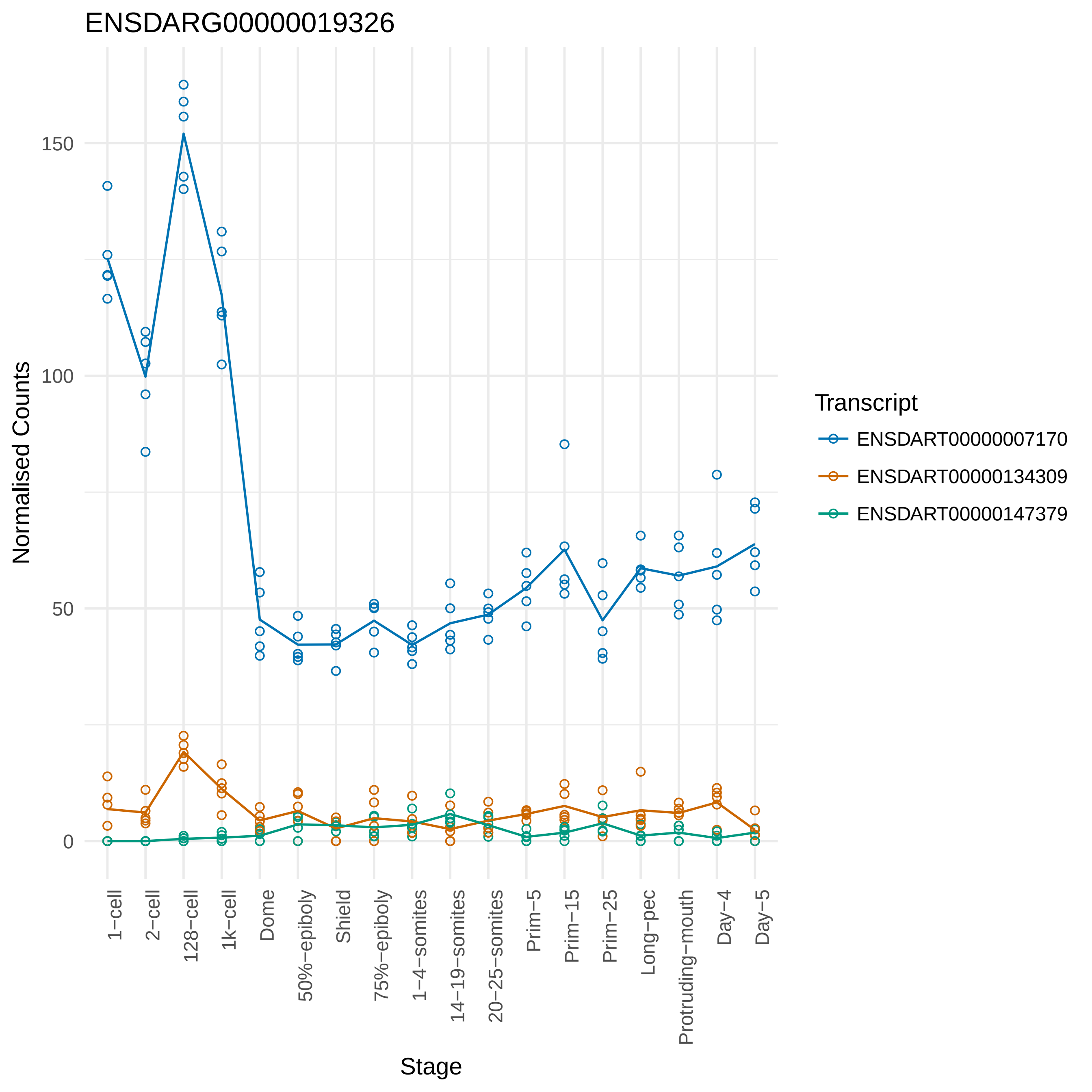

ENSDARG00000019326
Normalised Counts
150
100
50
0
Stage
1−cell
2−cell
128−cell
1k−cell
Dome
50%−epiboly
Shield
75%−epiboly
1−4−somites
14−19−somites
20−25−somites
Prim−5
Prim−15
Prim−25
Long−pec
Protruding−mouth
Day−4
Day−5
Transcript
ENSDART00000007170
ENSDART00000134309
ENSDART00000147379

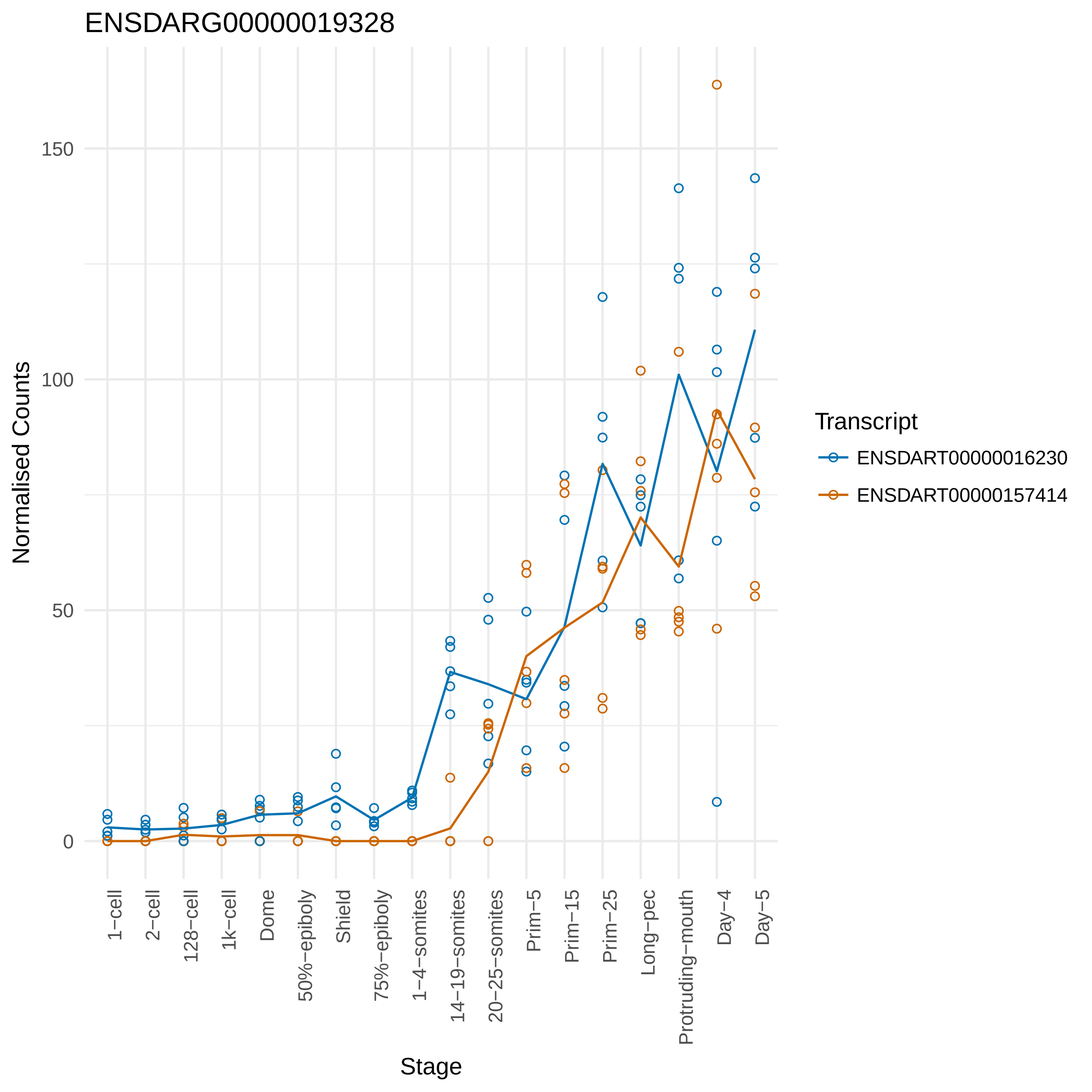
ENSDARG00000019328
Normalised Counts
150
100
50
0
1−cell
2−cell
128−cell
1k−cell
Dome
50%−epiboly
Shield
75%−epiboly
1−4−somites
14−19−somites
20−25−somites
Prim−5
Prim−15
Prim−25
Long−pec
Protruding−mouth
Day−4
Day−5
Stage
Transcript
ENSDART00000016230
ENSDART00000157414

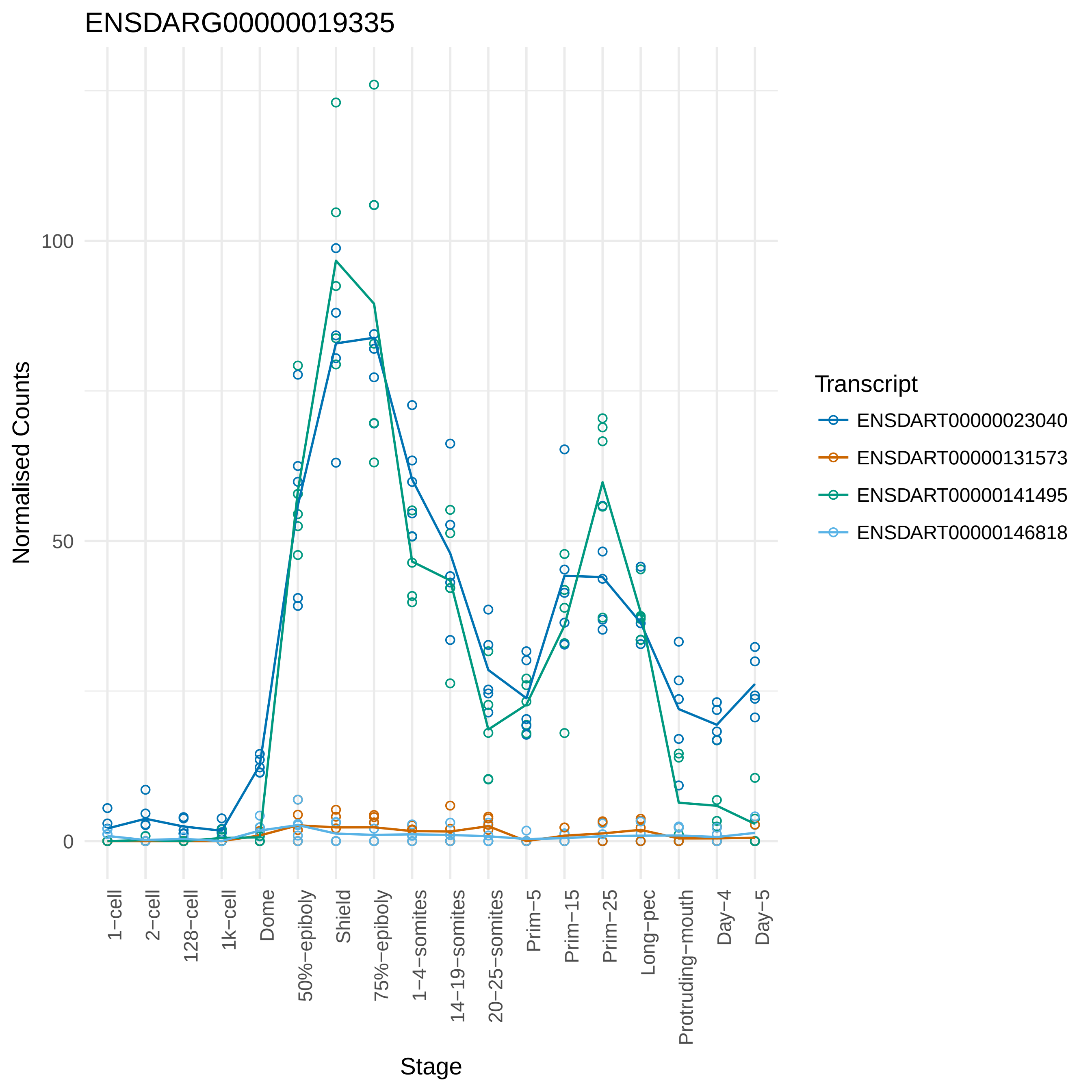
ENSDARG00000019335
Normalised Counts
100
50
0
1−cell
2−cell
128−cell
1k−cell
Dome
50%−epiboly
Shield
75%−epiboly
1−4−somites
14−19−somites
20−25−somites
Prim−5
Prim−15
Prim−25
Long−pec
Protruding−mouth
Day−4
Day−5
Stage
Transcript
ENSDART00000023040
ENSDART00000131573
ENSDART00000141495
ENSDART00000146818

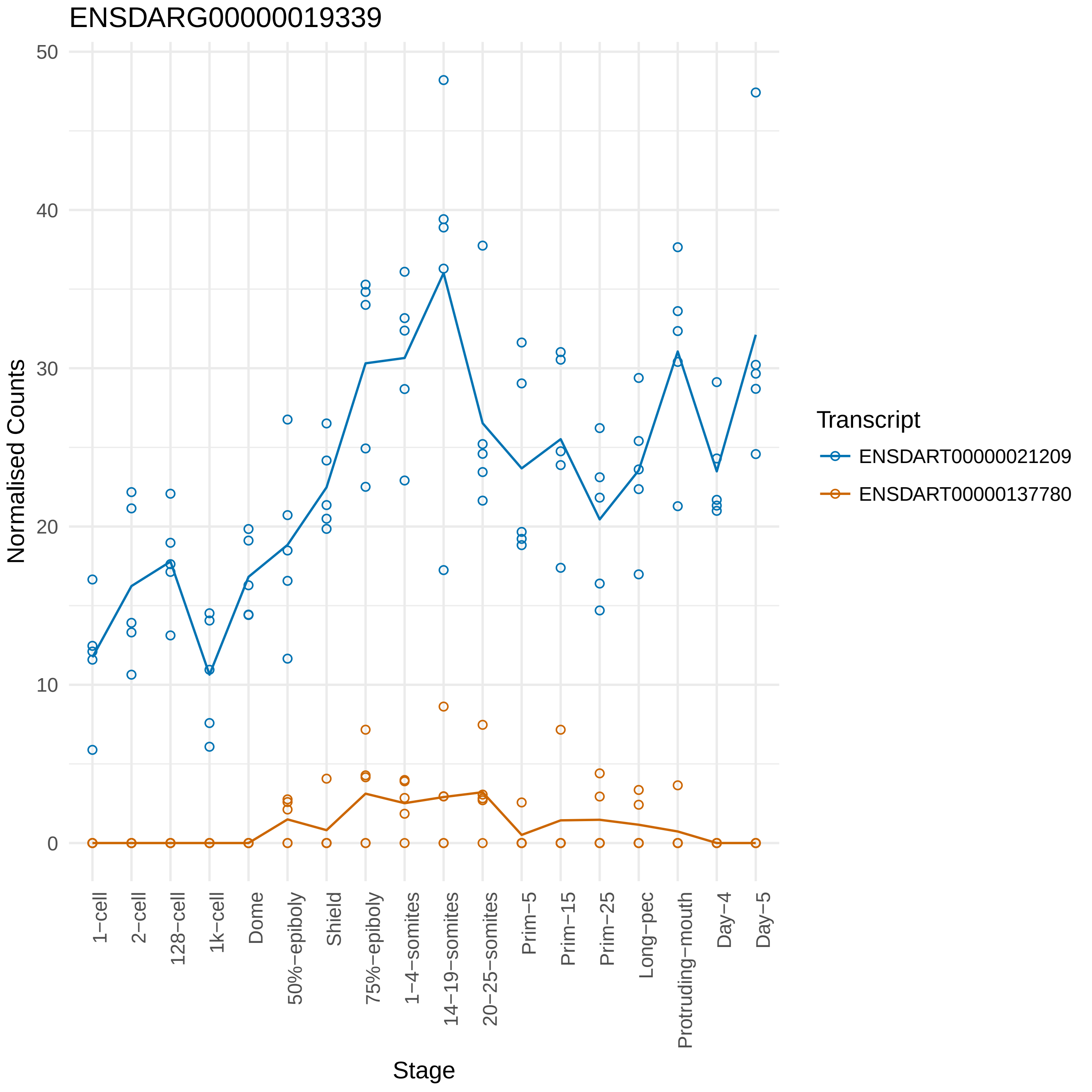
ENSDARG00000019339
Normalised Counts
50
40
30
20
10
0
1−cell
2−cell
128−cell
1k−cell
Dome
50%−epiboly
Shield
75%−epiboly
1−4−somites
14−19−somites
20−25−somites
Prim−5
Prim−15
Prim−25
Long−pec
Protruding−mouth
Day−4
Day−5
Stage
Transcript
ENSDART00000021209
ENSDART00000137780

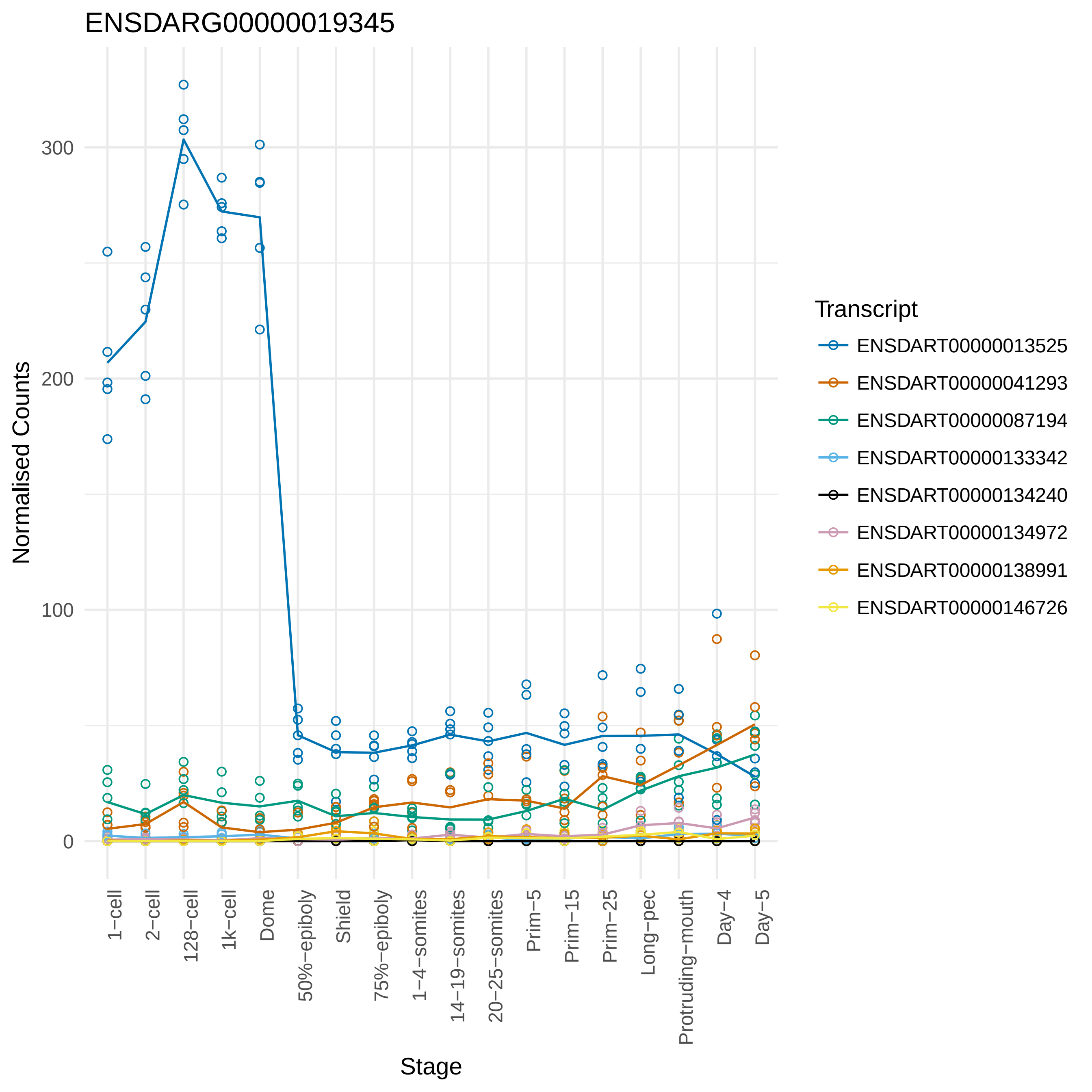

ENSDARG00000019345
Normalised Counts
0
100
200
300
1−cell
2−cell
128−cell
1k−cell
Dome
50%−epiboly
Shield
75%−epiboly
1−4−somites
14−19−somites
20−25−somites
Prim−5
Prim−15
Prim−25
Long−pec
Protruding−mouth
Day−4
Day−5
Stage
Transcript
ENSDART00000013525
ENSDART00000041293
ENSDART00000087194
ENSDART00000133342
ENSDART00000134240
ENSDART00000134972
ENSDART00000138991
ENSDART00000146726

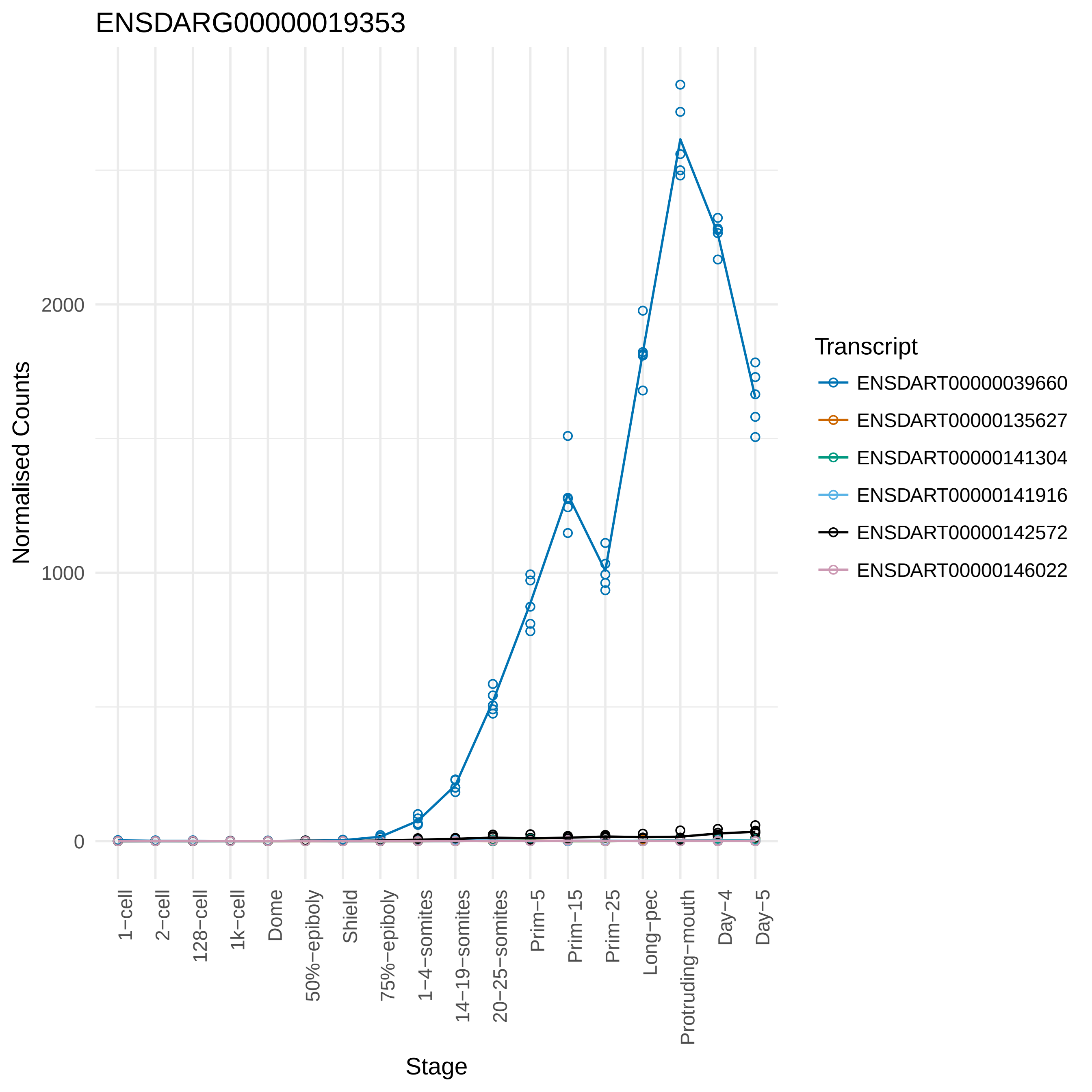

ENSDARG00000019353
Normalised Counts
0
1000
2000
Stage
1−cell
2−cell
128−cell
1k−cell
Dome
50%−epiboly
Shield
75%−epiboly
1−4−somites
14−19−somites
20−25−somites
Prim−5
Prim−15
Prim−25
Long−pec
Protruding−mouth
Day−4
Day−5
Transcript
ENSDART00000039660
ENSDART00000135627
ENSDART00000141304
ENSDART00000141916
ENSDART00000142572
ENSDART00000146022

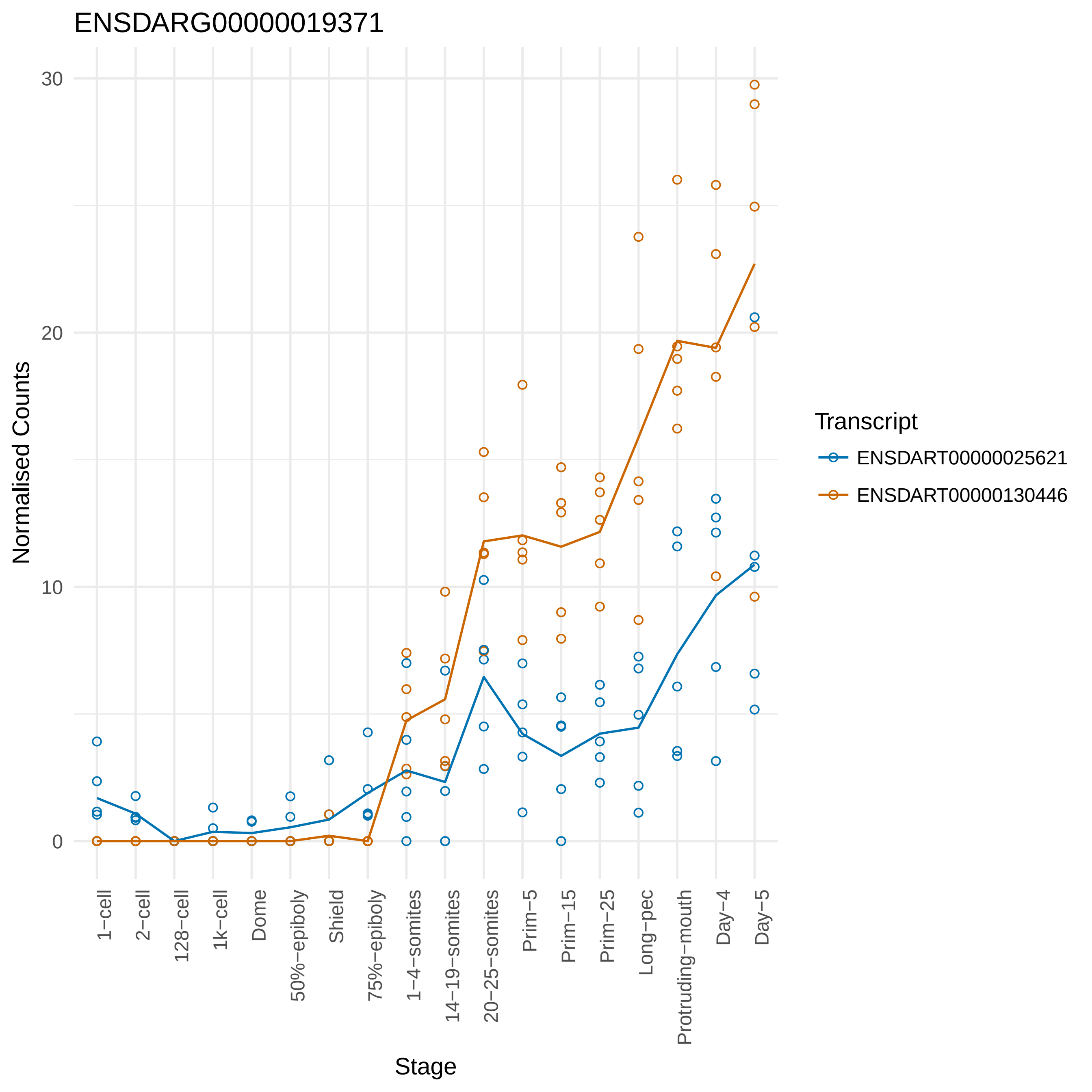
ENSDARG00000019371
Normalised Counts
30
20
10
0
1−cell
2−cell
128−cell
1k−cell
Dome
50%−epiboly
Shield
75%−epiboly
1−4−somites
14−19−somites
20−25−somites
Prim−5
Prim−15
Prim−25
Long−pec
Protruding−mouth
Day−4
Day−5
Stage
Transcript
ENSDART00000025621
ENSDART00000130446

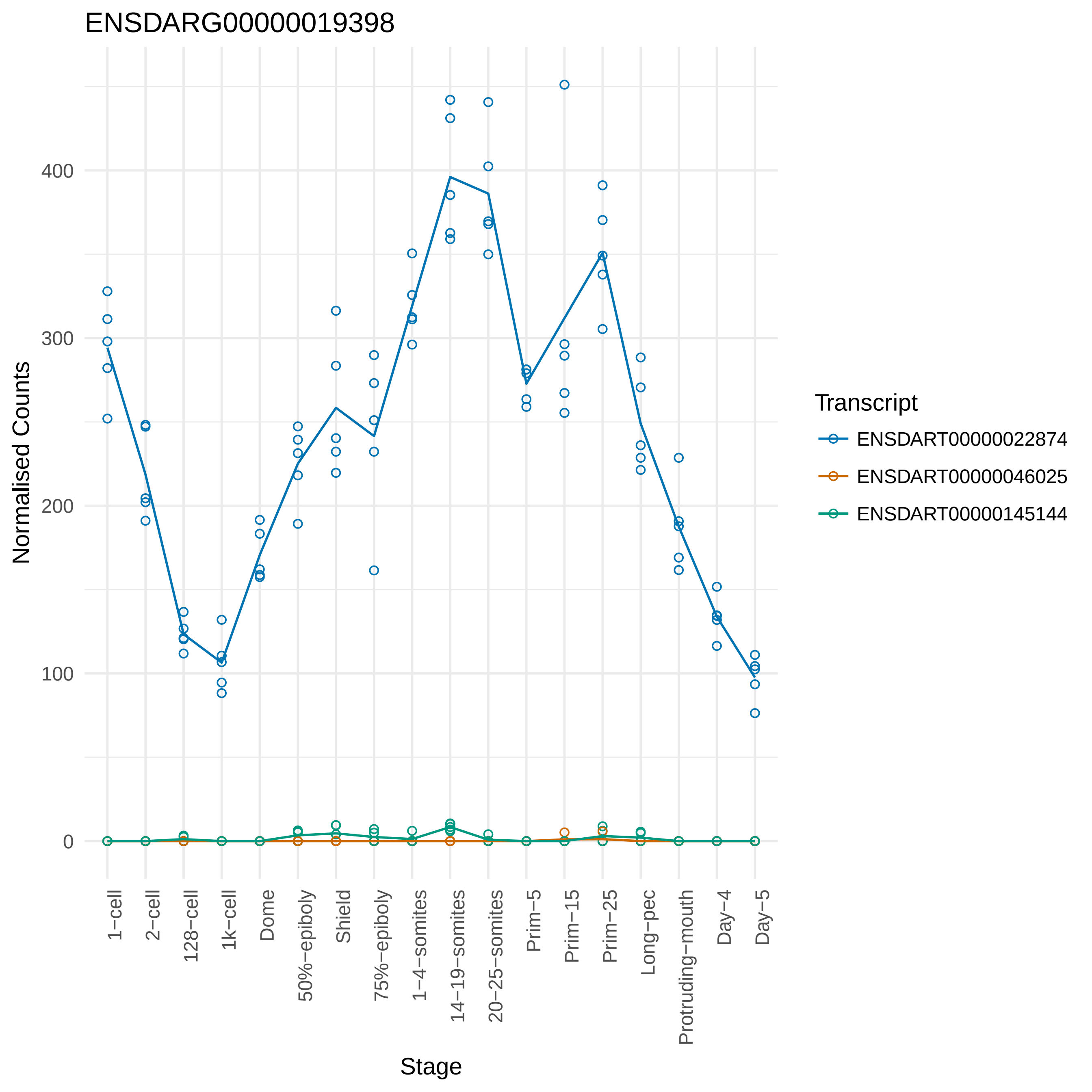
ENSDARG00000019398
Normalised Counts
400
300
200
100
0
1−cell
2−cell
128−cell
1k−cell
Dome
50%−epiboly
Shield
75%−epiboly
1−4−somites
14−19−somites
20−25−somites
Prim−5
Prim−15
Prim−25
Long−pec
Protruding−mouth
Day−4
Day−5
Stage
Transcript
ENSDART00000022874
ENSDART00000046025
ENSDART00000145144

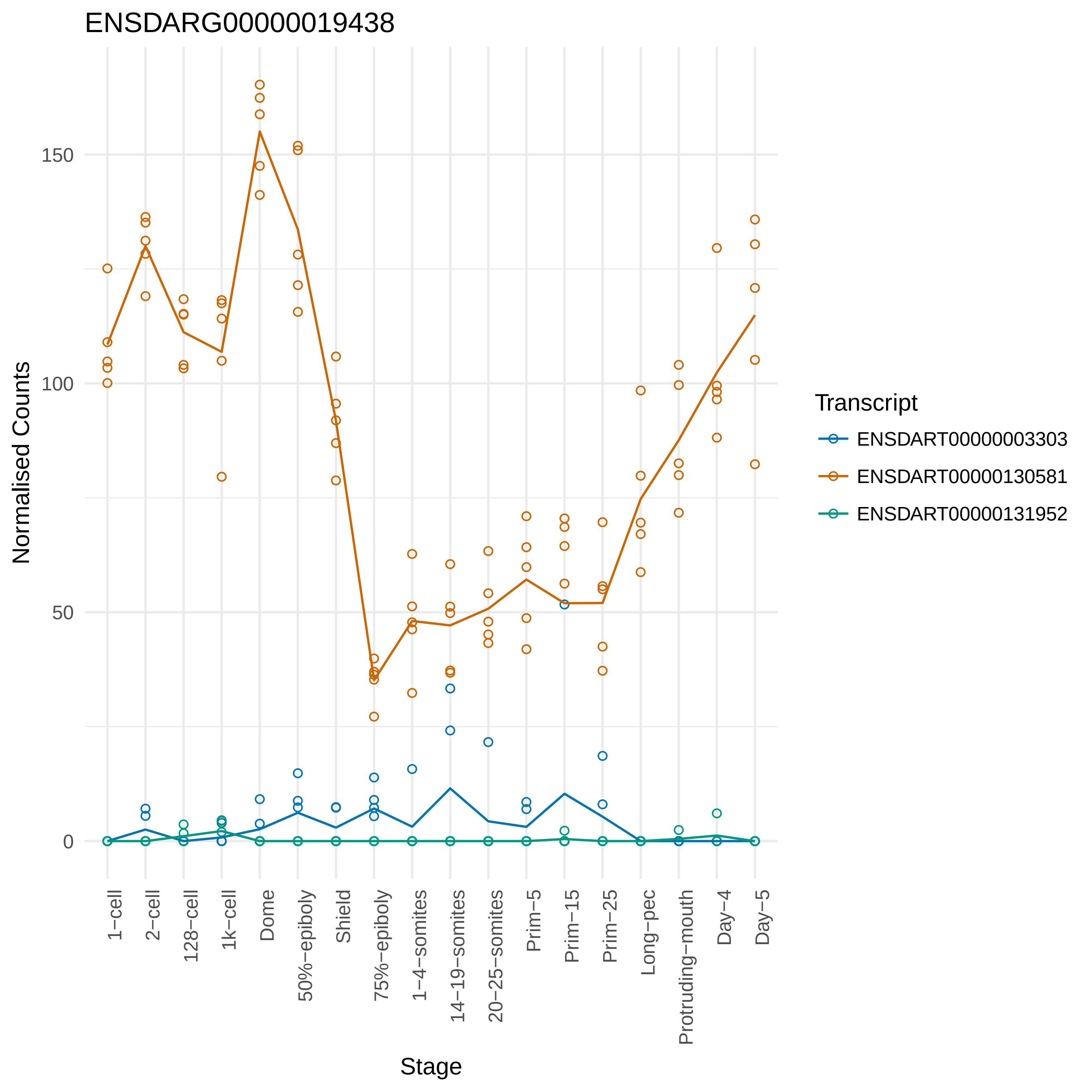
ENSDARG00000019438
Normalised Counts
150
100
50
0
1−cell
2−cell
128−cell
1k−cell
Dome
50%−epiboly
Shield
75%−epiboly
1−4−somites
14−19−somites
20−25−somites
Prim−5
Prim−15
Prim−25
Long−pec
Protruding−mouth
Day−4
Day−5
Stage
Transcript
ENSDART00000003303
ENSDART00000130581
ENSDART00000131952

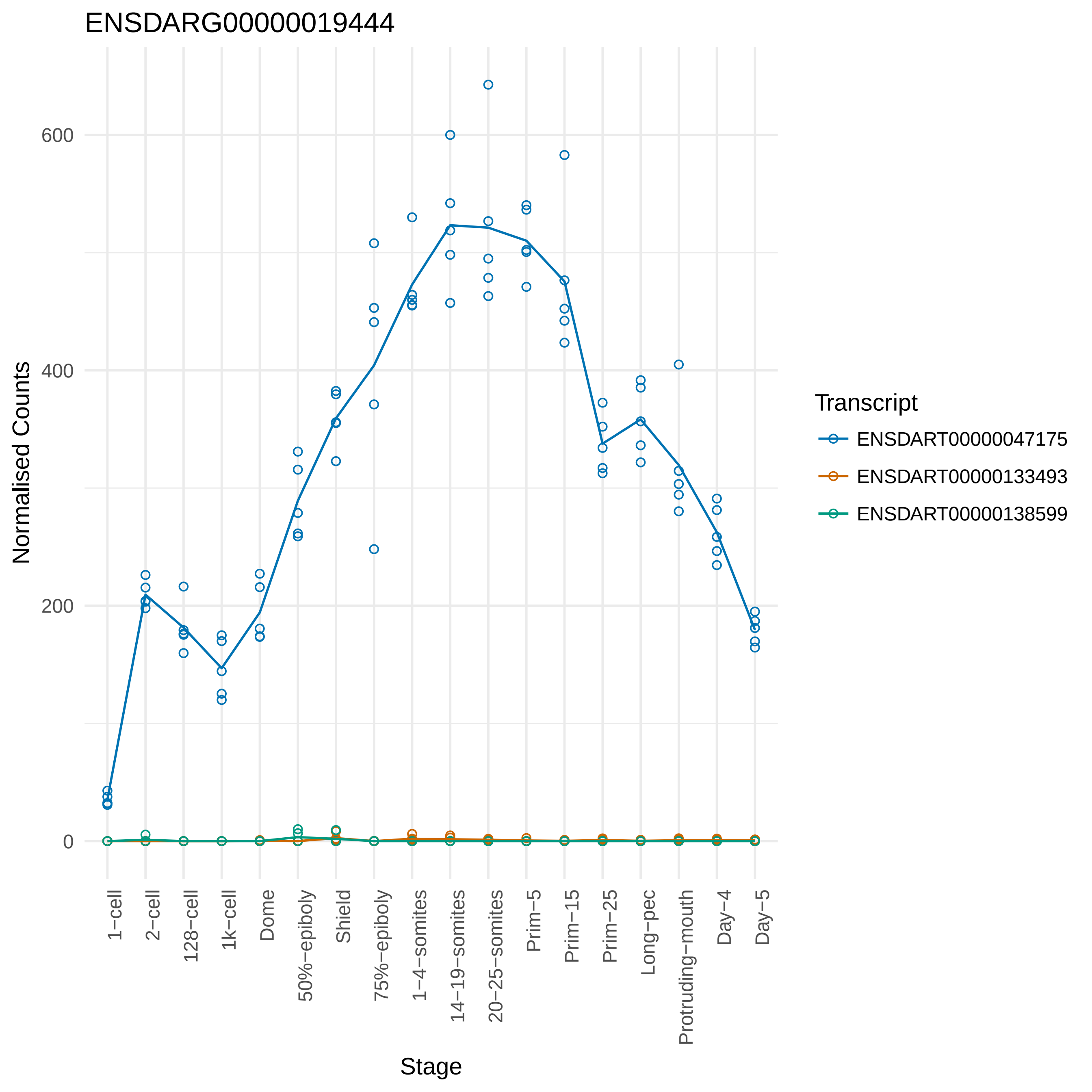
ENSDARG00000019444
Normalised Counts
600
400
200
0
1−cell
2−cell
128−cell
1k−cell
Dome
50%−epiboly
Shield
75%−epiboly
1−4−somites
14−19−somites
20−25−somites
Prim−5
Prim−15
Prim−25
Long−pec
Protruding−mouth
Day−4
Day−5
Stage
Transcript
ENSDART00000047175
ENSDART00000133493
ENSDART00000138599

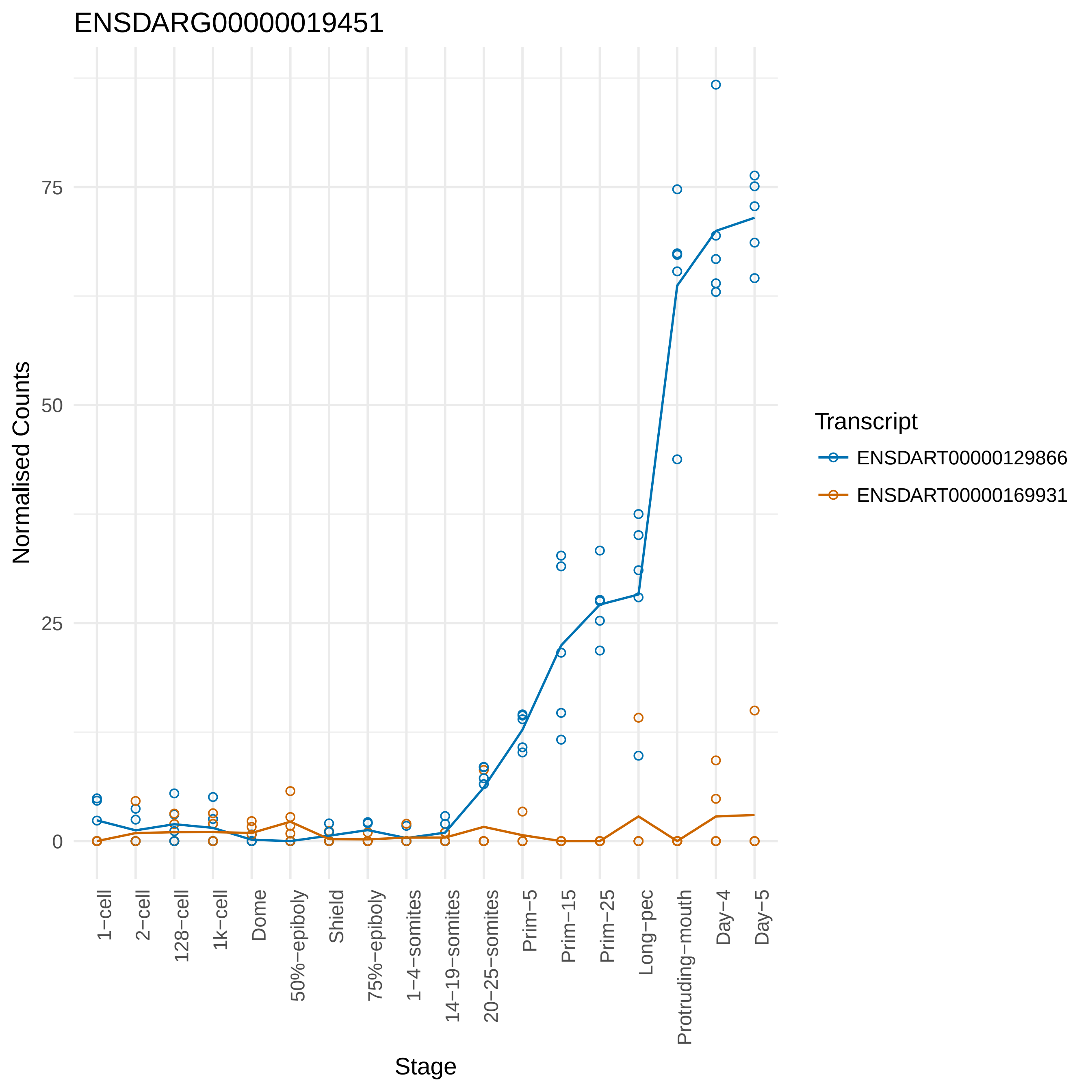
ENSDARG00000019451
Normalised Counts
0
25
50
75
Stage
1−cell
2−cell
128−cell
1k−cell
Dome
50%−epiboly
Shield
75%−epiboly
1−4−somites
14−19−somites
20−25−somites
Prim−5
Prim−15
Prim−25
Long−pec
Protruding−mouth
Day−4
Day−5
Transcript
ENSDART00000129866
ENSDART00000169931

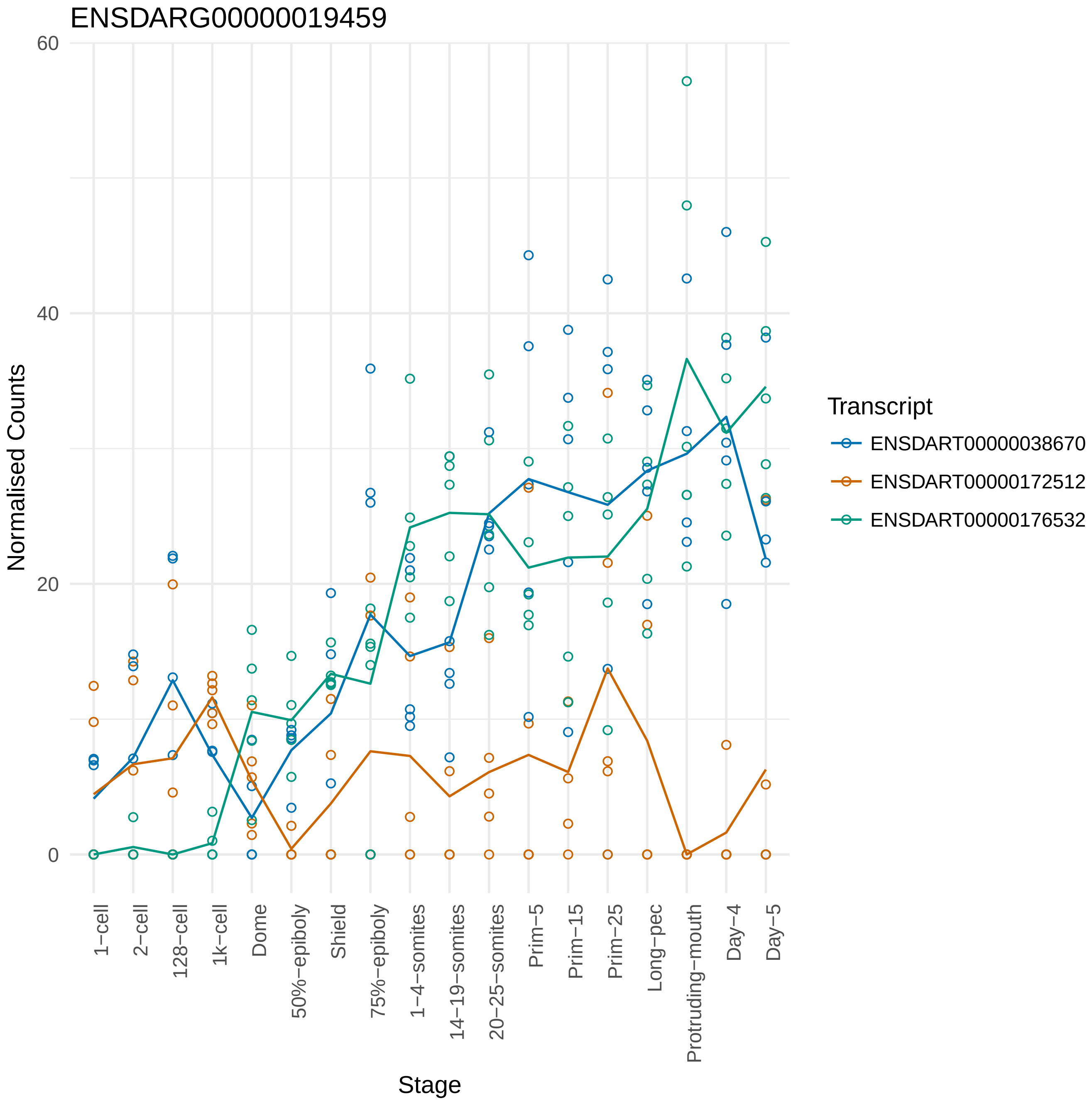

ENSDARG00000019459
Normalised Counts
0
20
40
60
1−cell
2−cell
128−cell
1k−cell
Dome
50%−epiboly
Shield
75%−epiboly
1−4−somites
14−19−somites
20−25−somites
Prim−5
Prim−15
Prim−25
Long−pec
Protruding−mouth
Day−4
Day−5
Stage
Transcript
ENSDART00000038670
ENSDART00000172512
ENSDART00000176532

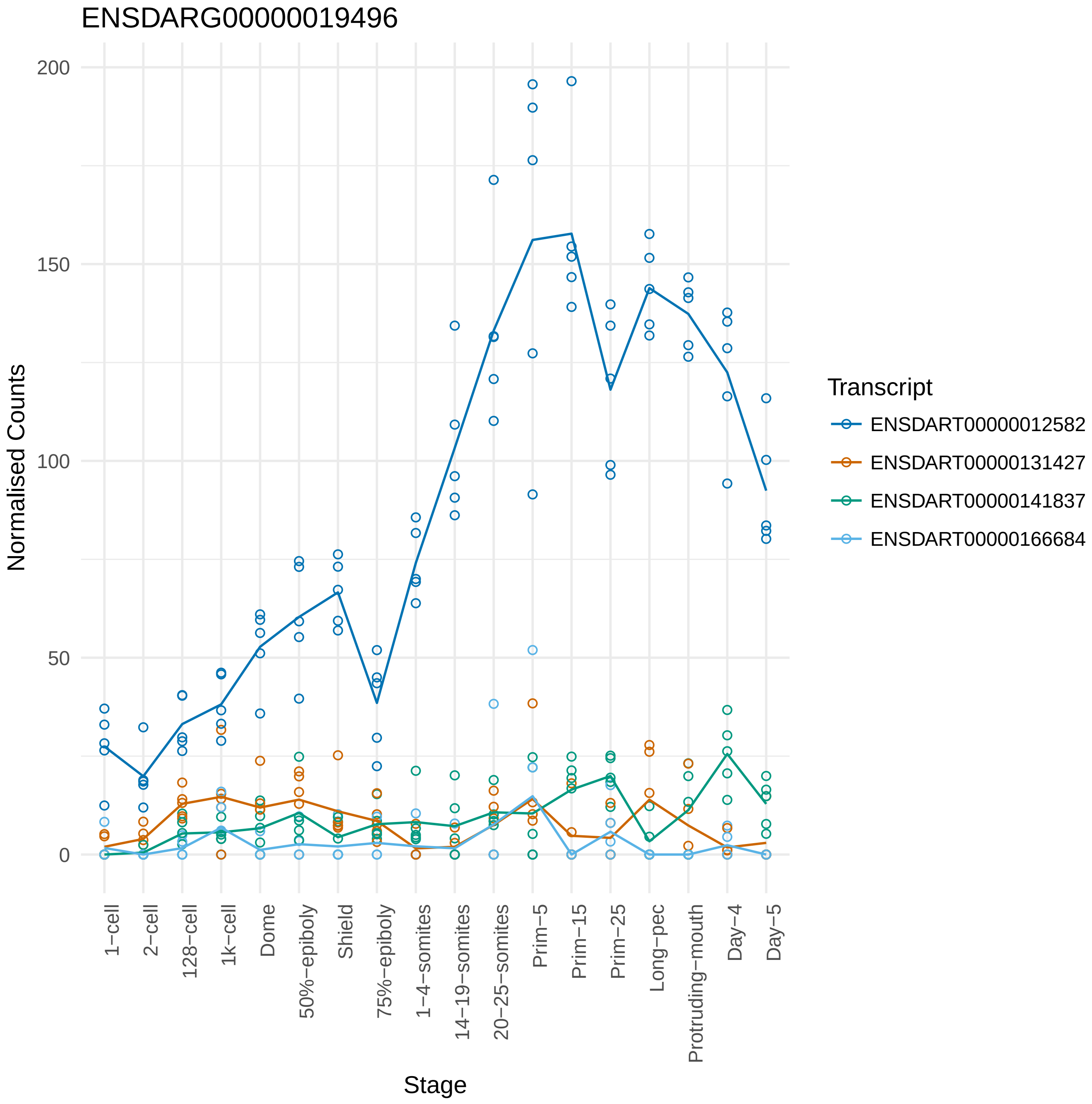

ENSDARG00000019496
Normalised Counts
200
150
100
50
0
1−cell
2−cell
128−cell
1k−cell
Dome
50%−epiboly
Shield
75%−epiboly
1−4−somites
14−19−somites
20−25−somites
Prim−5
Prim−15
Prim−25
Long−pec
Protruding−mouth
Day−4
Day−5
Stage
Transcript
ENSDART00000012582
ENSDART00000131427
ENSDART00000141837
ENSDART00000166684

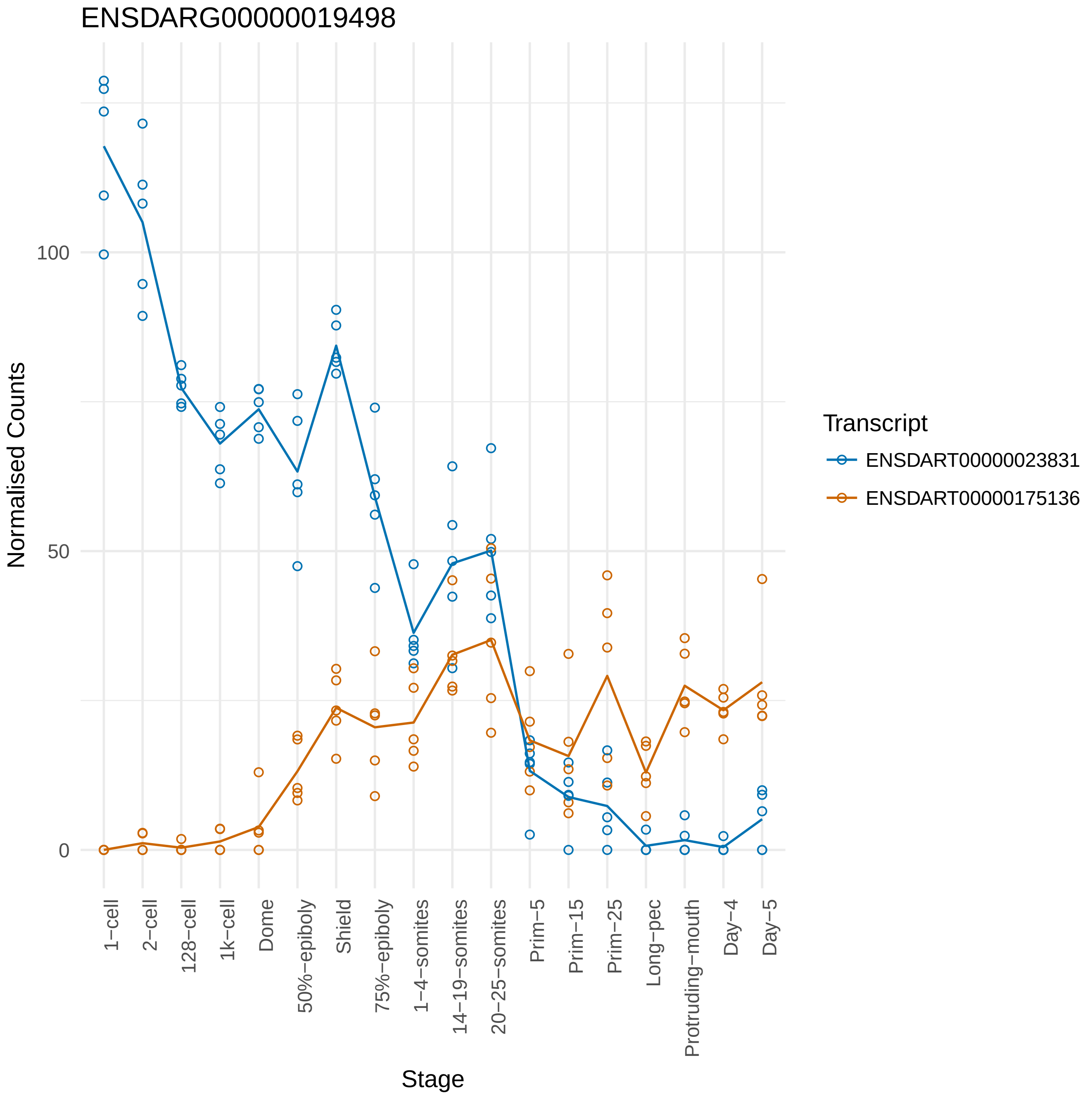
ENSDARG00000019498
Normalised Counts
100
50
0
Stage
1−cell
2−cell
128−cell
1k−cell
Dome
50%−epiboly
Shield
75%−epiboly
1−4−somites
14−19−somites
20−25−somites
Prim−5
Prim−15
Prim−25
Long−pec
Protruding−mouth
Day−4
Day−5
Transcript
ENSDART00000023831
ENSDART00000175136

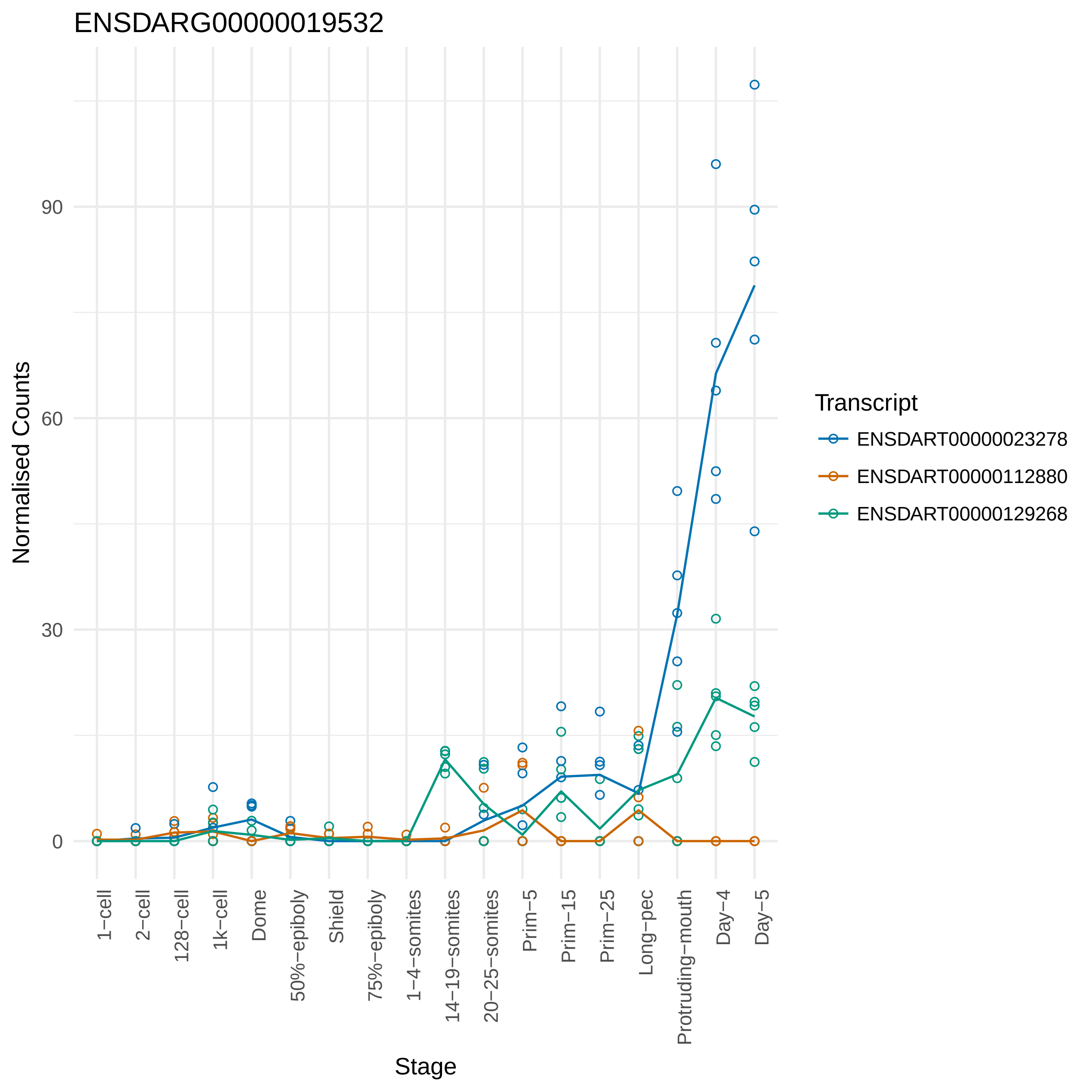

ENSDARG00000019532
Normalised Counts
0
30
60
90
Stage
1−cell
2−cell
128−cell
1k−cell
Dome
50%−epiboly
Shield
75%−epiboly
1−4−somites
14−19−somites
20−25−somites
Prim−5
Prim−15
Prim−25
Long−pec
Protruding−mouth
Day−4
Day−5
Transcript
ENSDART00000023278
ENSDART00000112880
ENSDART00000129268

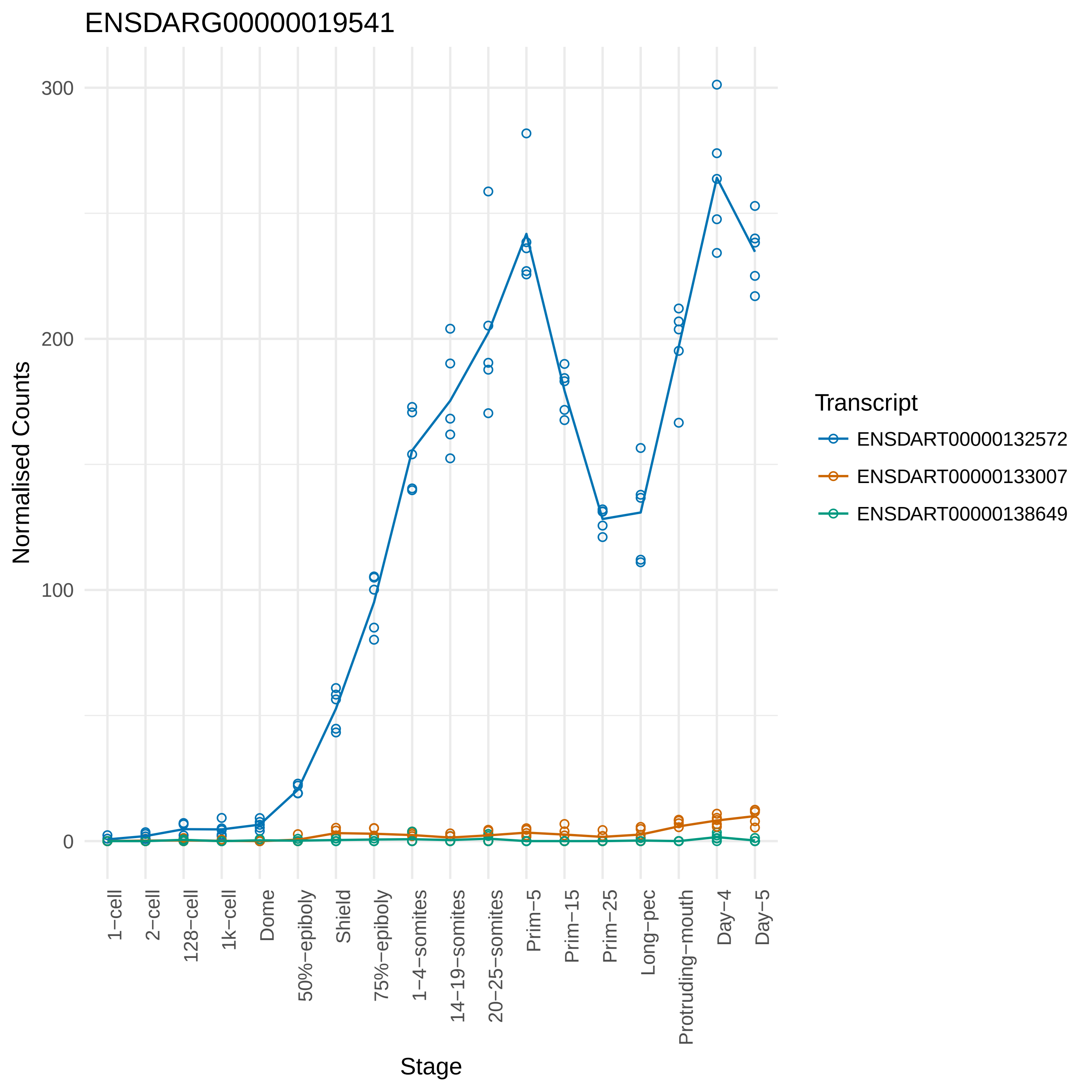

ENSDARG00000019541
Normalised Counts
300
200
100
0
1−cell
2−cell
128−cell
1k−cell
Dome
50%−epiboly
Shield
75%−epiboly
1−4−somites
14−19−somites
20−25−somites
Prim−5
Prim−15
Prim−25
Long−pec
Protruding−mouth
Day−4
Day−5
Stage
Transcript
ENSDART00000132572
ENSDART00000133007
ENSDART00000138649

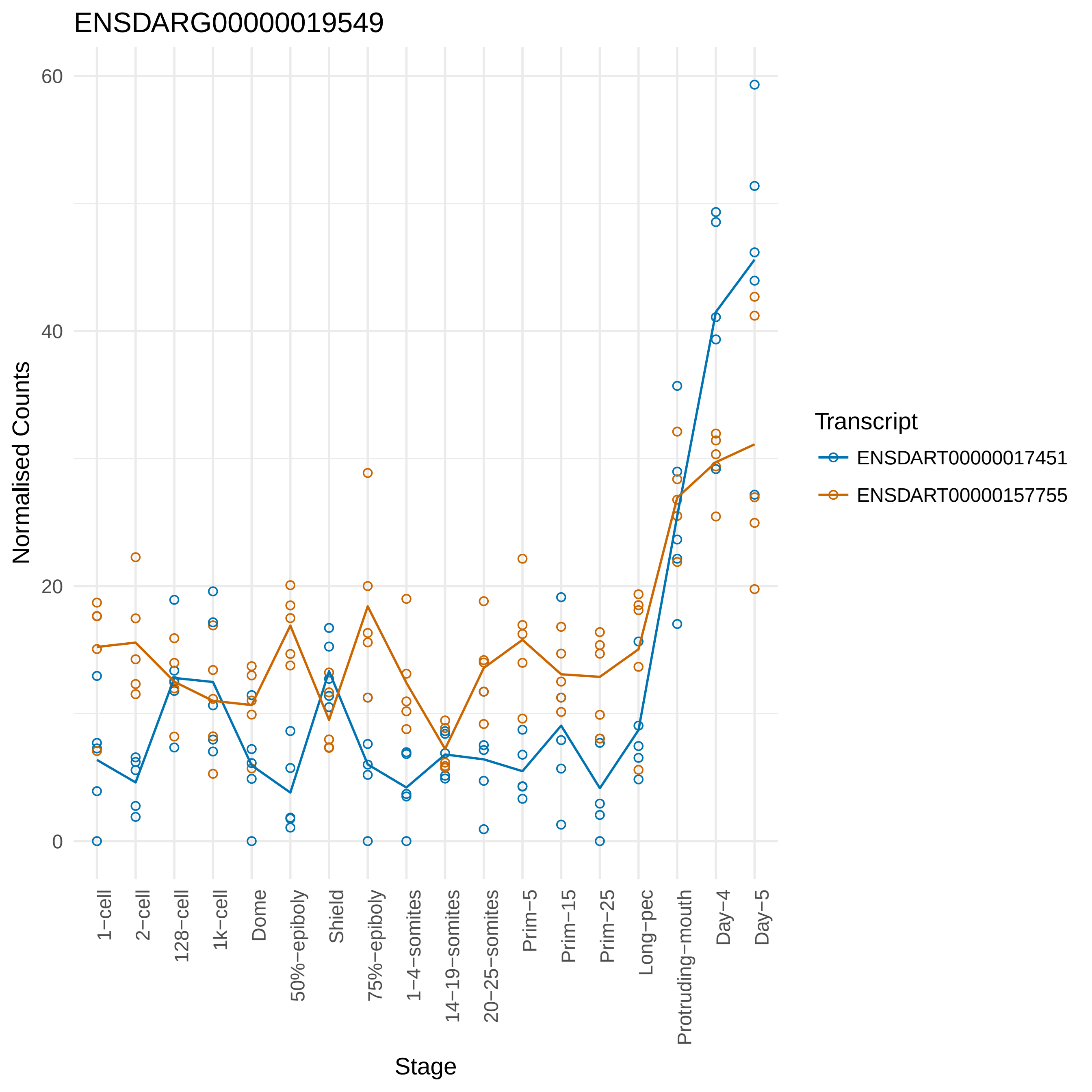

ENSDARG00000019549
Normalised Counts
60
40
20
0
1−cell
2−cell
128−cell
1k−cell
Dome
50%−epiboly
Shield
75%−epiboly
1−4−somites
14−19−somites
20−25−somites
Prim−5
Prim−15
Prim−25
Long−pec
Protruding−mouth
Day−4
Day−5
Stage
Transcript
ENSDART00000017451
ENSDART00000157755

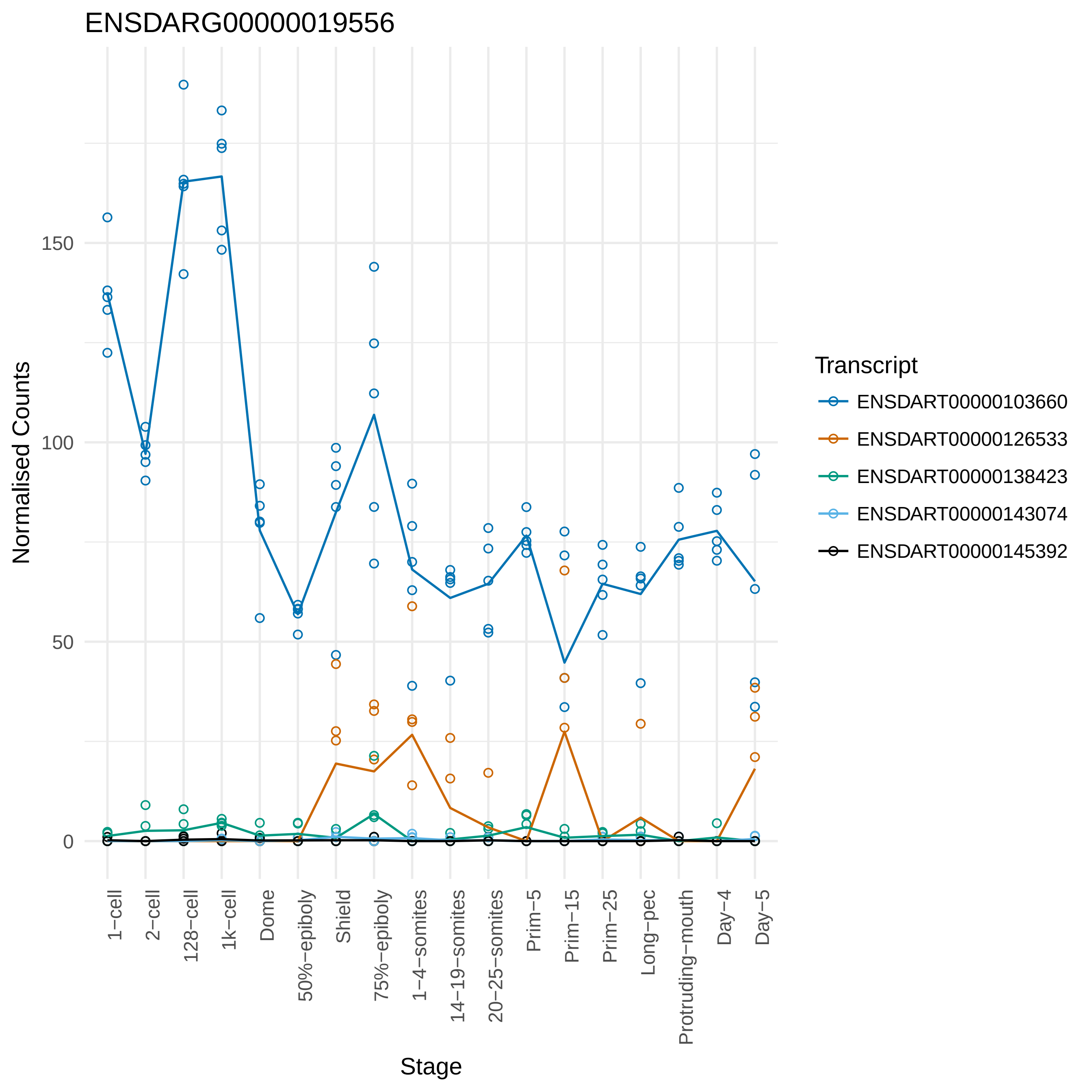

ENSDARG00000019556
Normalised Counts
150
100
50
0
1−cell
2−cell
128−cell
1k−cell
Dome
50%−epiboly
Shield
75%−epiboly
1−4−somites
14−19−somites
20−25−somites
Prim−5
Prim−15
Prim−25
Long−pec
Protruding−mouth
Day−4
Day−5
Stage
Transcript
ENSDART00000103660
ENSDART00000126533
ENSDART00000138423
ENSDART00000143074
ENSDART00000145392

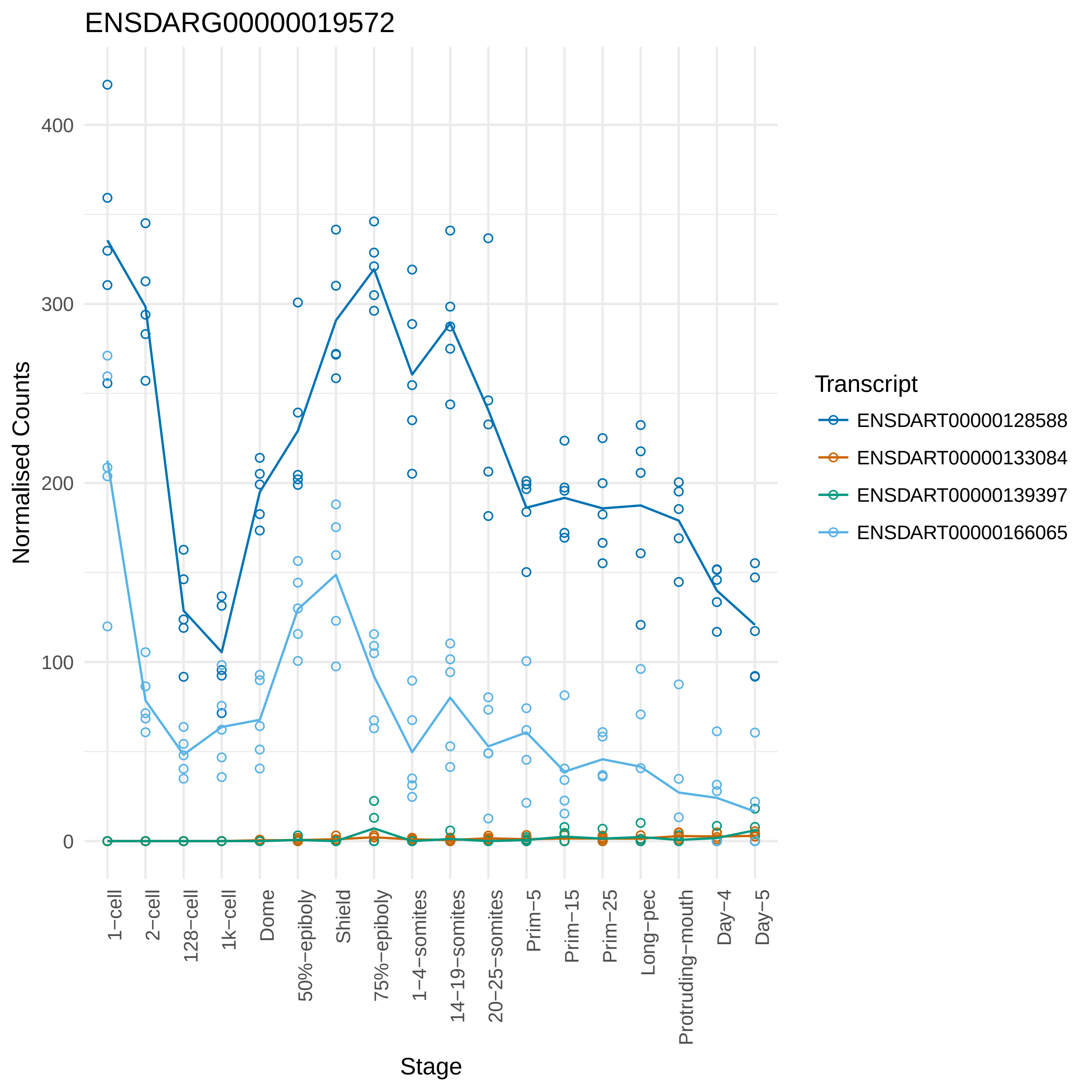

ENSDARG00000019572
Normalised Counts
400
300
200
100
0
1−cell
2−cell
128−cell
1k−cell
Dome
50%−epiboly
Shield
75%−epiboly
1−4−somites
14−19−somites
20−25−somites
Prim−5
Prim−15
Prim−25
Long−pec
Protruding−mouth
Day−4
Day−5
Stage
Transcript
ENSDART00000128588
ENSDART00000133084
ENSDART00000139397
ENSDART00000166065

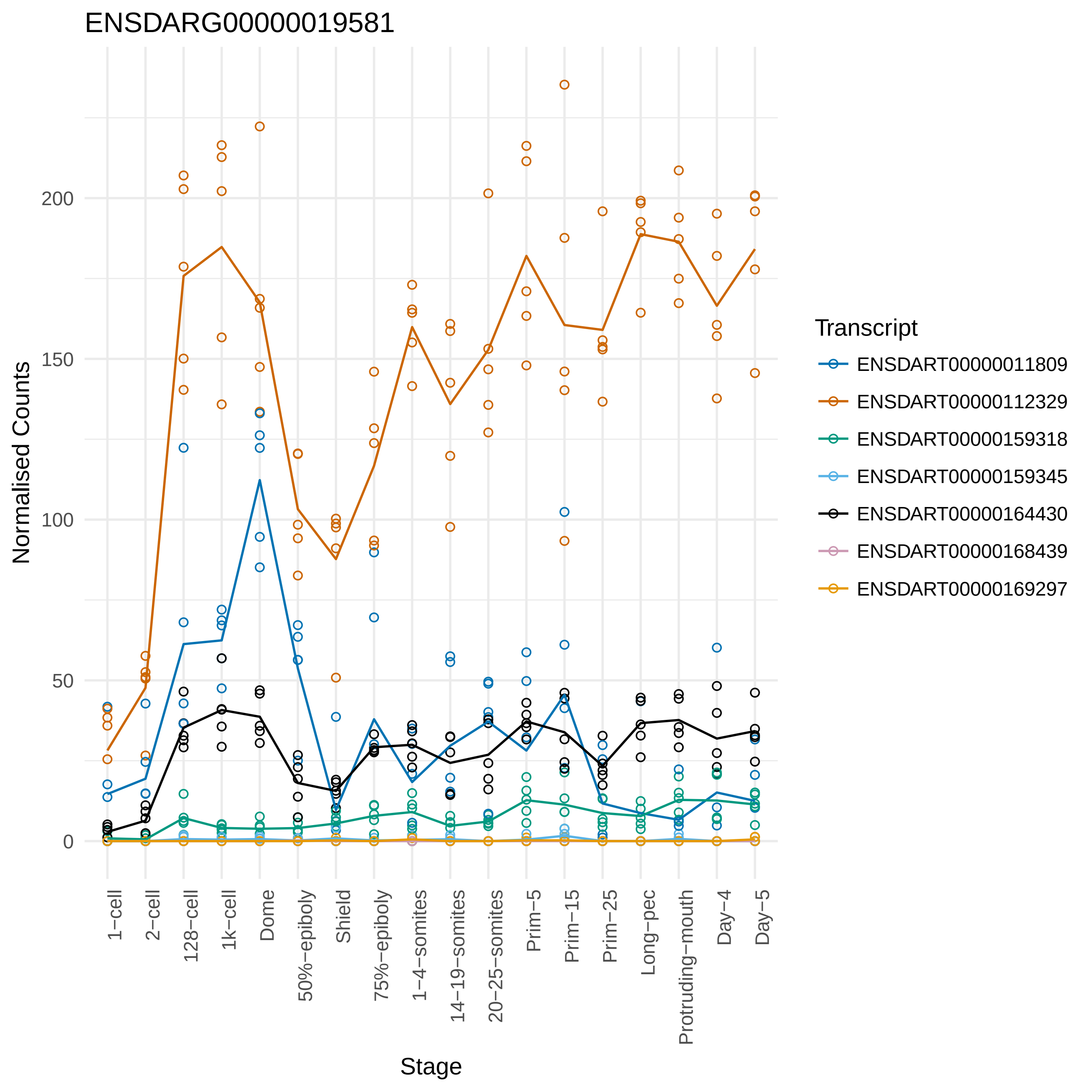
ENSDARG00000019581
Normalised Counts
200
150
100
50
0
1−cell
2−cell
128−cell
1k−cell
Dome
50%−epiboly
Shield
75%−epiboly
1−4−somites
14−19−somites
20−25−somites
Prim−5
Prim−15
Prim−25
Long−pec
Protruding−mouth
Day−4
Day−5
Stage
Transcript
ENSDART00000011809
ENSDART00000112329
ENSDART00000159318
ENSDART00000159345
ENSDART00000164430
ENSDART00000168439
ENSDART00000169297

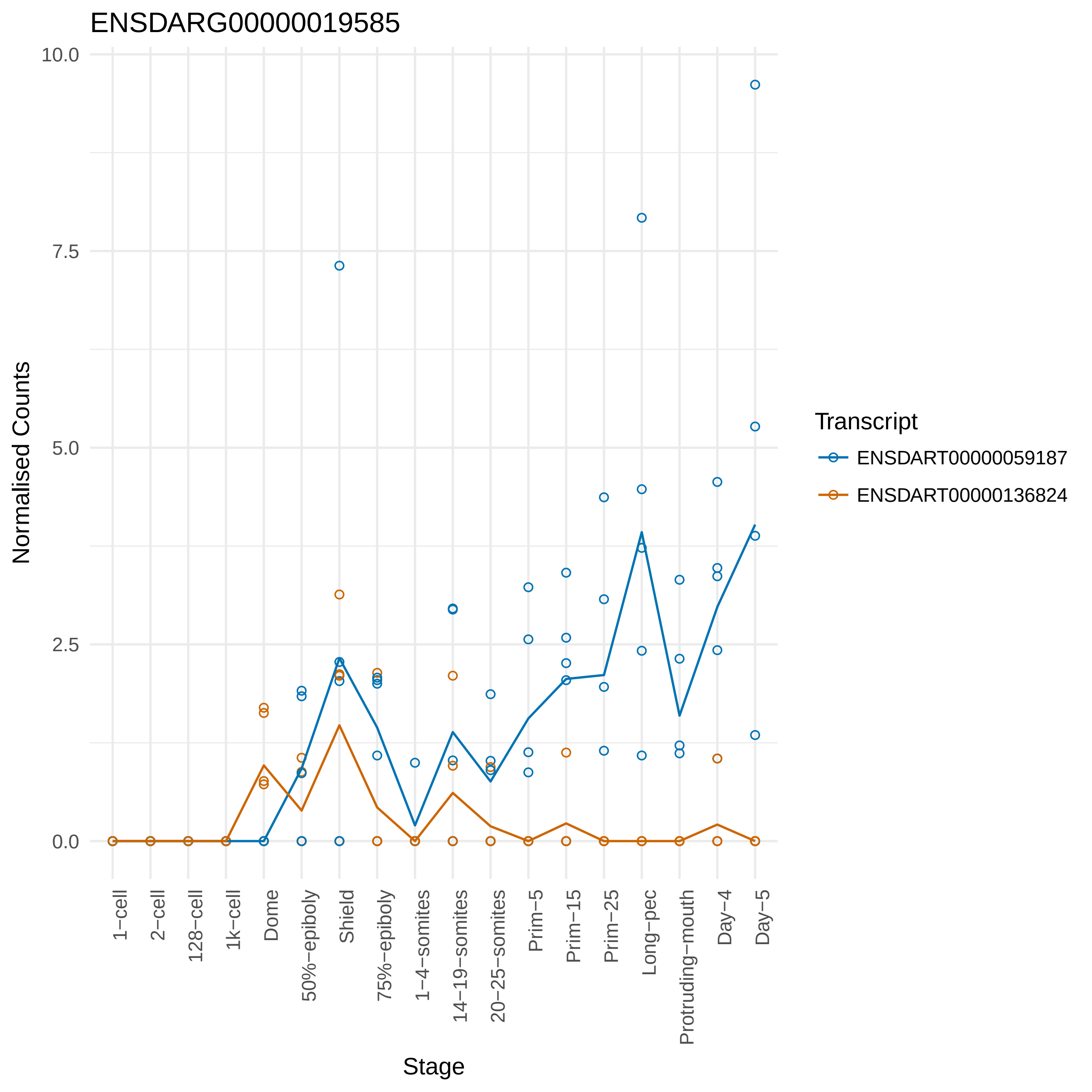

ENSDARG00000019585
Normalised Counts
10.0
7.5
5.0
2.5
0.0
1−cell
2−cell
128−cell
1k−cell
Dome
50%−epiboly
Shield
75%−epiboly
1−4−somites
14−19−somites
20−25−somites
Prim−5
Prim−15
Prim−25
Long−pec
Protruding−mouth
Day−4
Day−5
Stage
Transcript
ENSDART00000059187
ENSDART00000136824

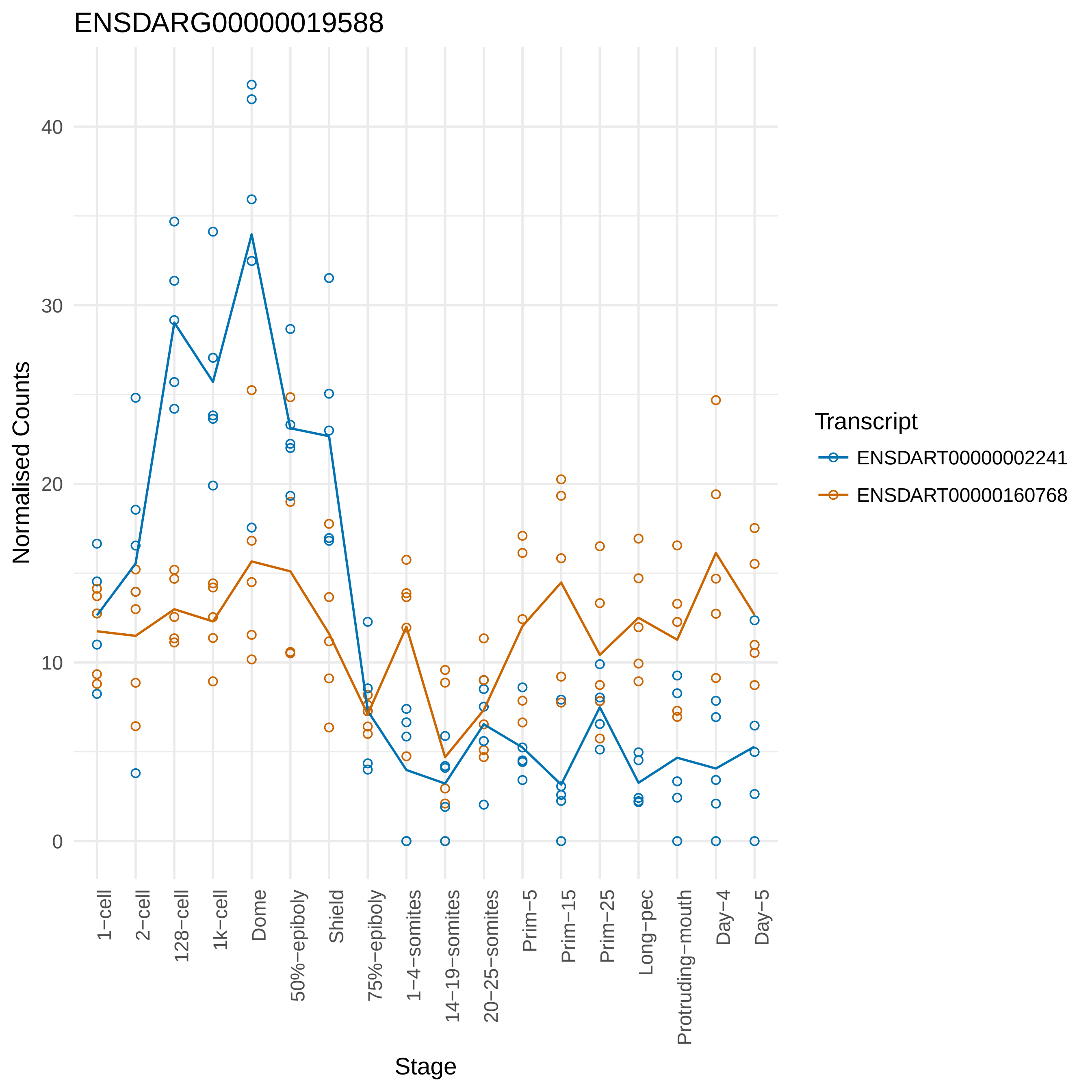
ENSDARG00000019588
Normalised Counts
0
10
20
30
40
Stage
1−cell
2−cell
128−cell
1k−cell
Dome
50%−epiboly
Shield
75%−epiboly
1−4−somites
14−19−somites
20−25−somites
Prim−5
Prim−15
Prim−25
Long−pec
Protruding−mouth
Day−4
Day−5
Transcript
ENSDART00000002241
ENSDART00000160768

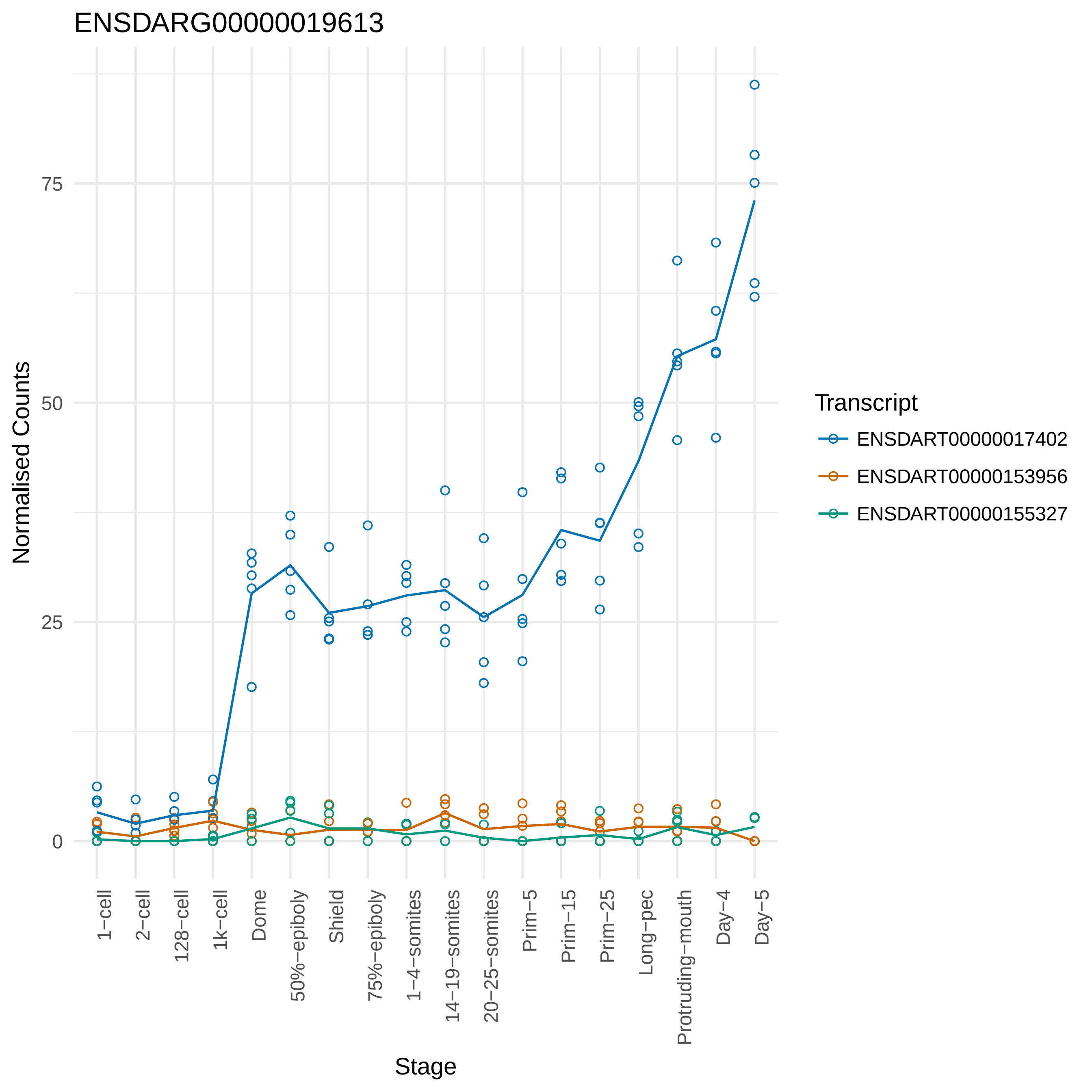

ENSDARG00000019613
Normalised Counts
75
50
25
0
1−cell
2−cell
128−cell
1k−cell
Dome
50%−epiboly
Shield
75%−epiboly
1−4−somites
14−19−somites
20−25−somites
Prim−5
Prim−15
Prim−25
Long−pec
Protruding−mouth
Day−4
Day−5
Stage
Transcript
ENSDART00000017402
ENSDART00000153956
ENSDART00000155327

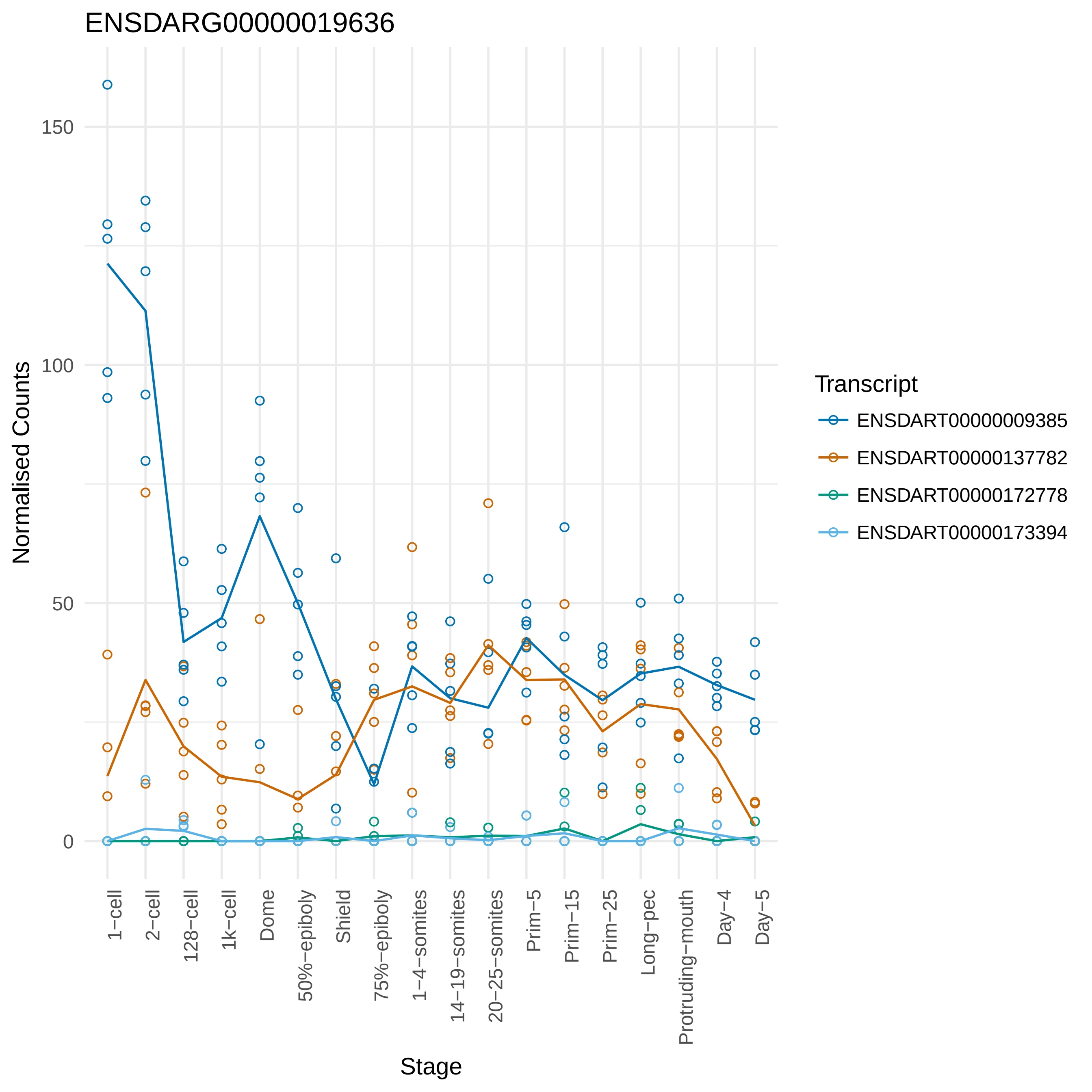

ENSDARG00000019636
Normalised Counts
150
100
50
0
1−cell
2−cell
128−cell
1k−cell
Dome
50%−epiboly
Shield
75%−epiboly
1−4−somites
14−19−somites
20−25−somites
Prim−5
Prim−15
Prim−25
Long−pec
Protruding−mouth
Day−4
Day−5
Stage
Transcript
ENSDART00000009385
ENSDART00000137782
ENSDART00000172778
ENSDART00000173394

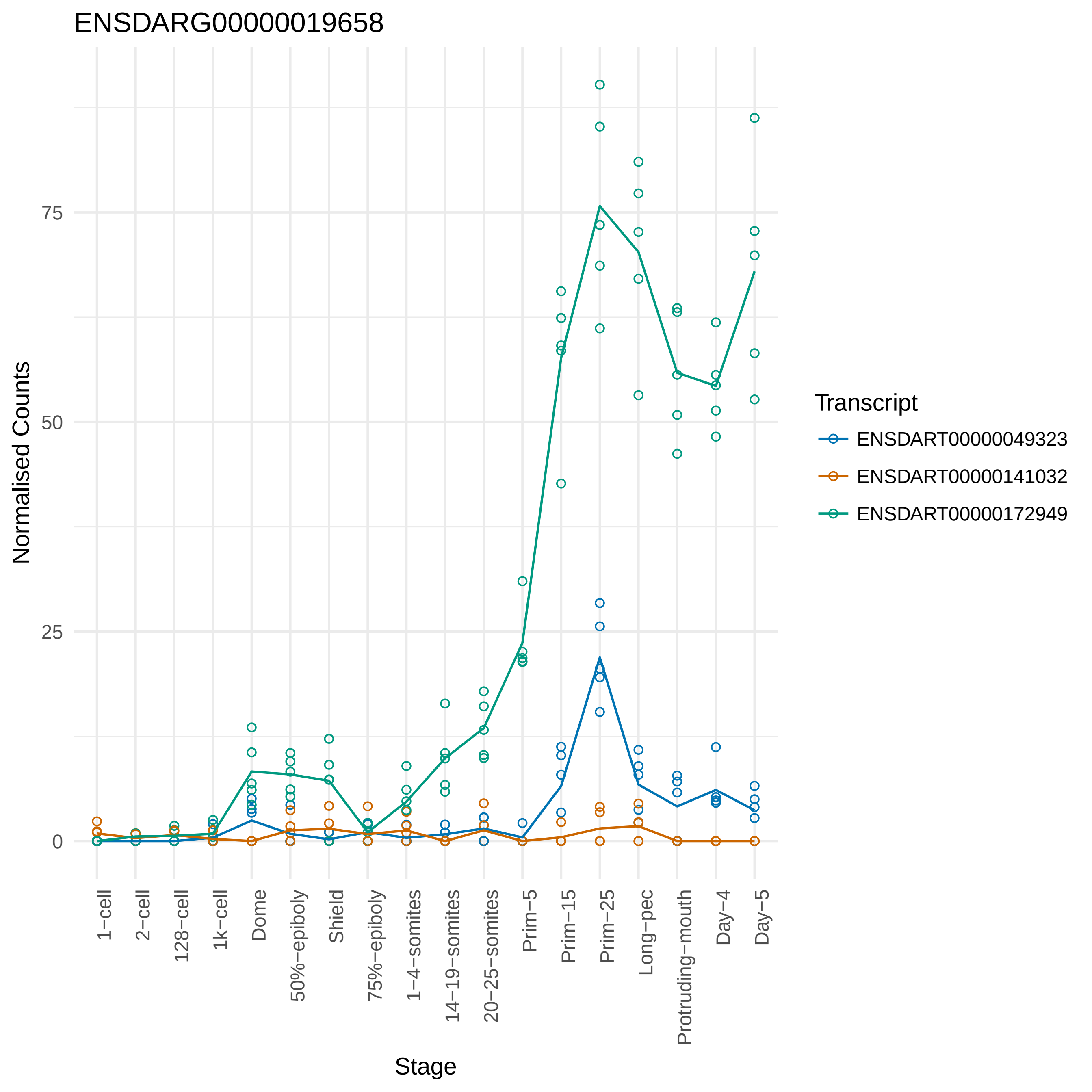
ENSDARG00000019658
Normalised Counts
75
50
25
0
1−cell
2−cell
128−cell
1k−cell
Dome
50%−epiboly
Shield
75%−epiboly
1−4−somites
14−19−somites
20−25−somites
Prim−5
Prim−15
Prim−25
Long−pec
Protruding−mouth
Day−4
Day−5
Stage
Transcript
ENSDART00000049323
ENSDART00000141032
ENSDART00000172949

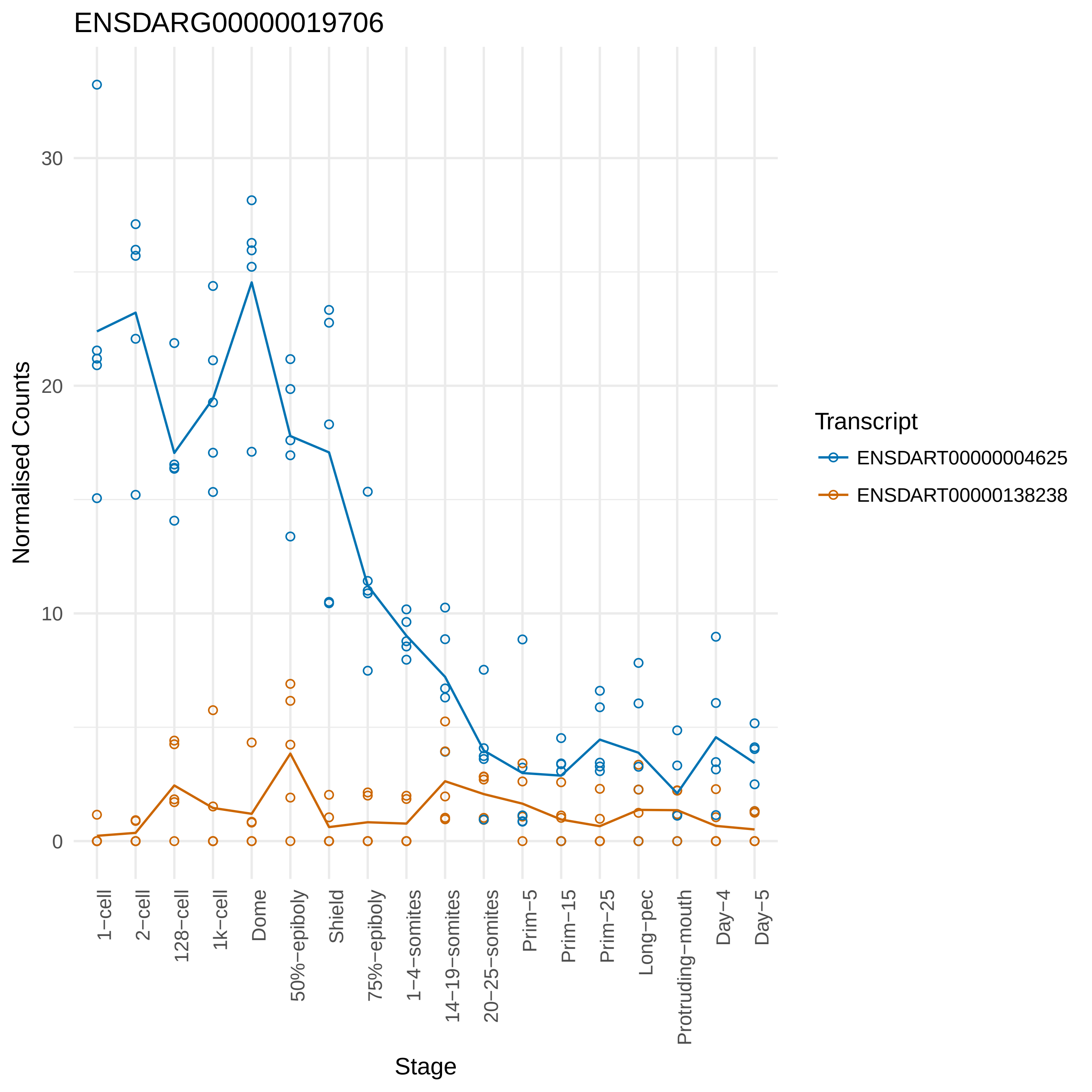
ENSDARG00000019706
Normalised Counts
30
20
10
0
1−cell
2−cell
128−cell
1k−cell
Dome
50%−epiboly
Shield
75%−epiboly
1−4−somites
14−19−somites
20−25−somites
Prim−5
Prim−15
Prim−25
Long−pec
Protruding−mouth
Day−4
Day−5
Stage
Transcript
ENSDART00000004625
ENSDART00000138238

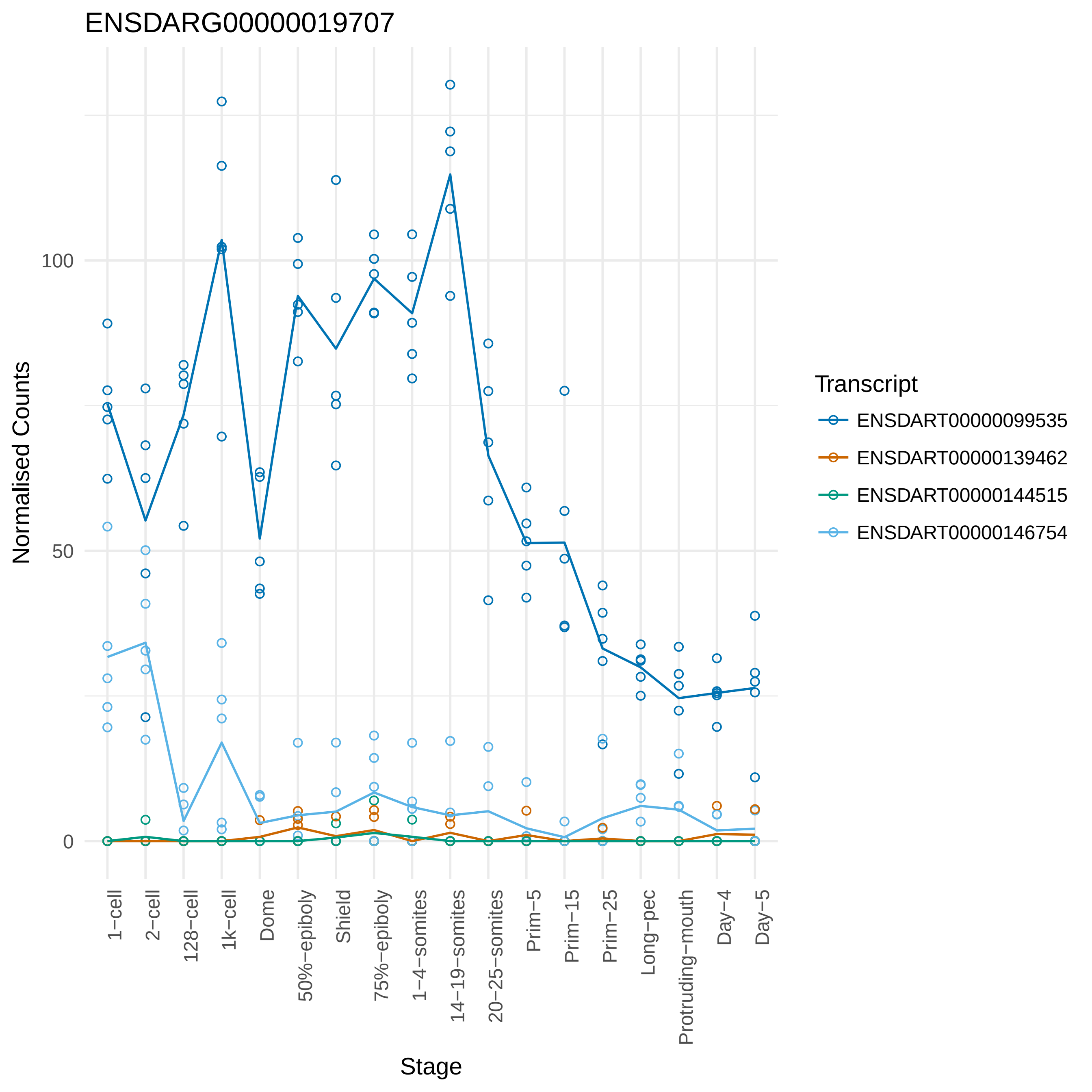

ENSDARG00000019707
Normalised Counts
100
50
0
1−cell
2−cell
128−cell
1k−cell
Dome
50%−epiboly
Shield
75%−epiboly
1−4−somites
14−19−somites
20−25−somites
Prim−5
Prim−15
Prim−25
Long−pec
Protruding−mouth
Day−4
Day−5
Stage
Transcript
ENSDART00000099535
ENSDART00000139462
ENSDART00000144515
ENSDART00000146754

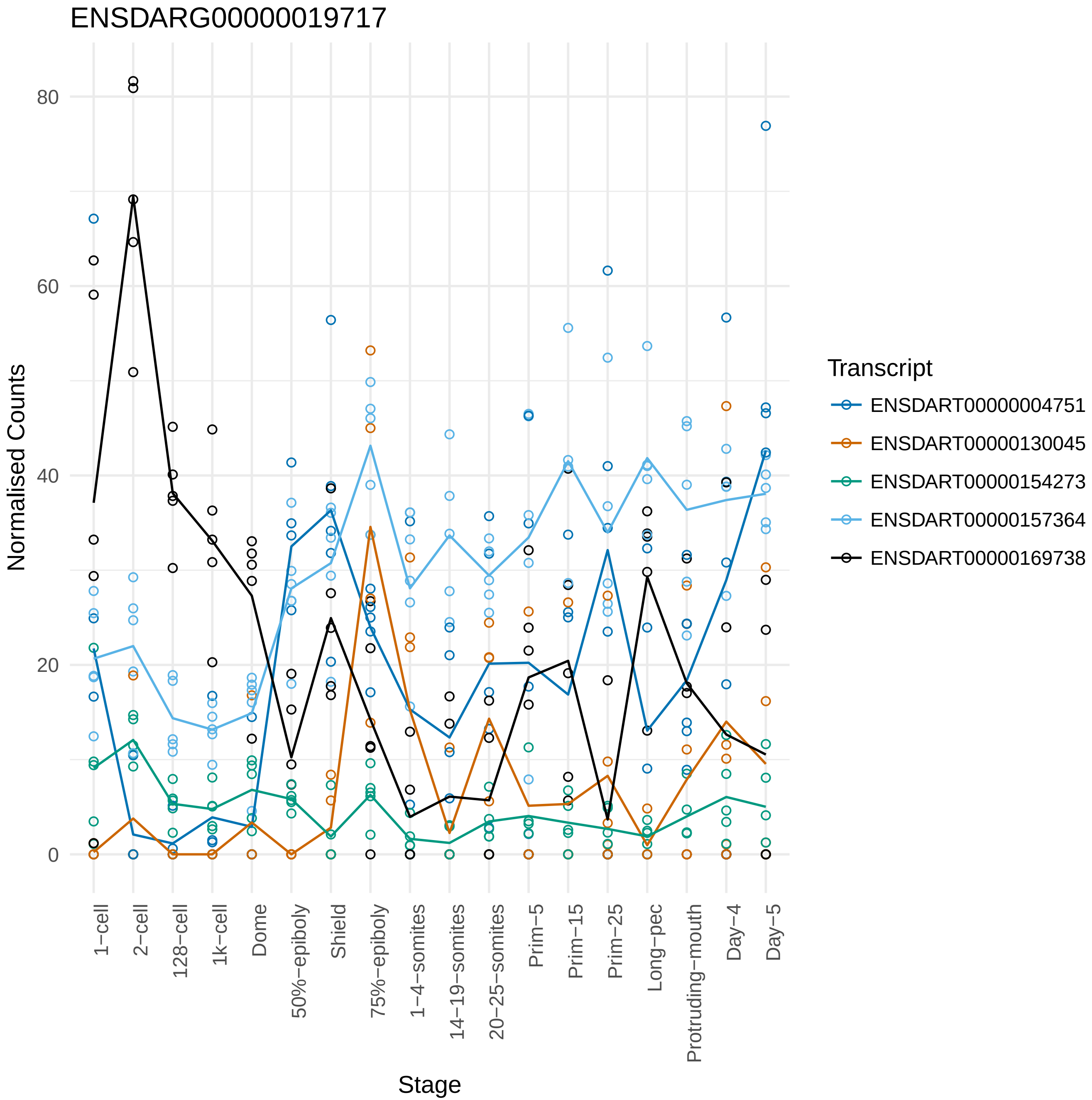

ENSDARG00000019717
Normalised Counts
0
20
40
60
80
1−cell
2−cell
128−cell
1k−cell
Dome
50%−epiboly
Shield
75%−epiboly
1−4−somites
14−19−somites
20−25−somites
Prim−5
Prim−15
Prim−25
Long−pec
Protruding−mouth
Day−4
Day−5
Stage
Transcript
ENSDART00000004751
ENSDART00000130045
ENSDART00000154273
ENSDART00000157364
ENSDART00000169738

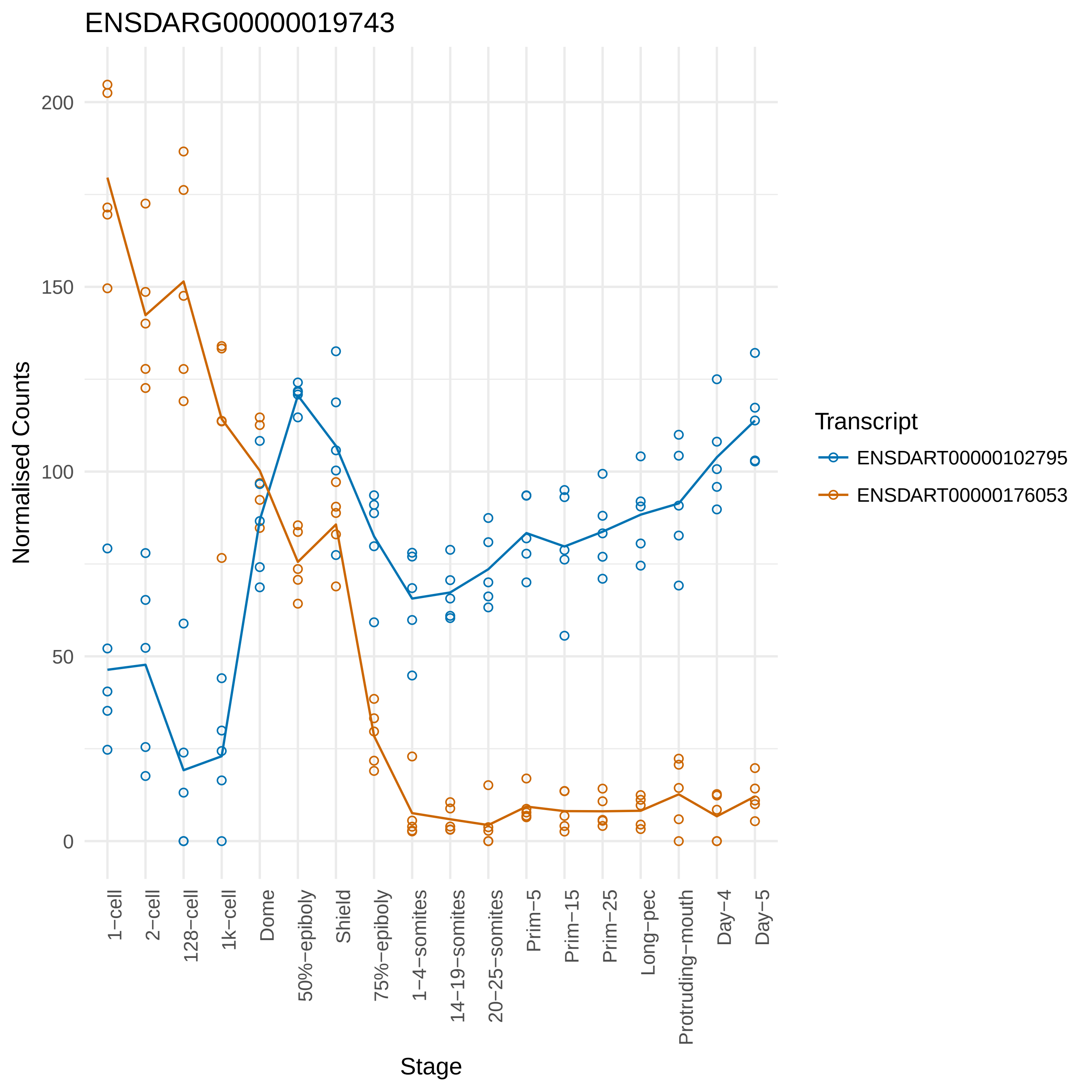

ENSDARG00000019743
Normalised Counts
200
150
100
50
0
1−cell
2−cell
128−cell
1k−cell
Dome
50%−epiboly
Shield
75%−epiboly
1−4−somites
14−19−somites
20−25−somites
Prim−5
Prim−15
Prim−25
Long−pec
Protruding−mouth
Day−4
Day−5
Stage
Transcript
ENSDART00000102795
ENSDART00000176053

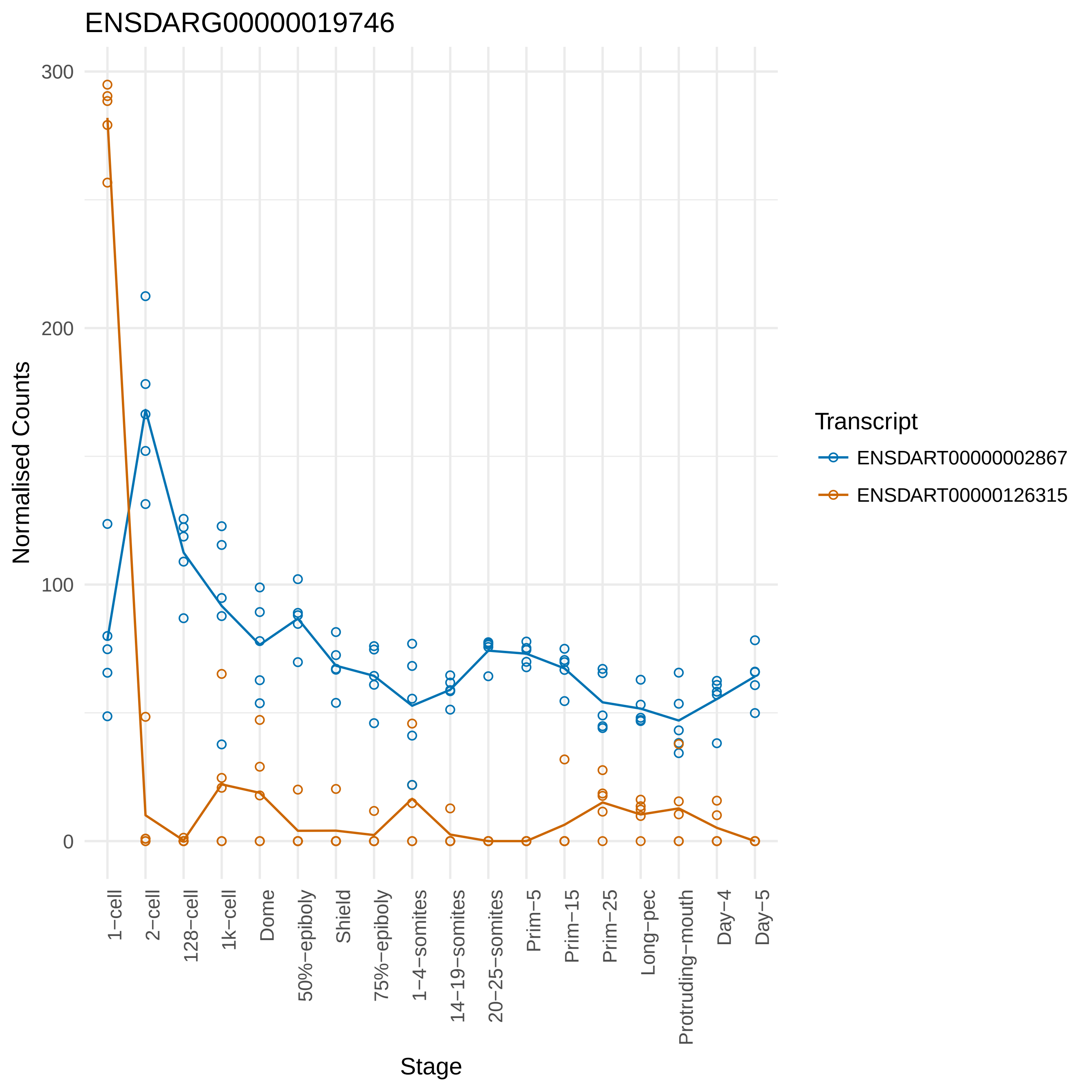
ENSDARG00000019746
Normalised Counts
300
200
100
0
1−cell
2−cell
128−cell
1k−cell
Dome
50%−epiboly
Shield
75%−epiboly
1−4−somites
14−19−somites
20−25−somites
Prim−5
Prim−15
Prim−25
Long−pec
Protruding−mouth
Day−4
Day−5
Stage
Transcript
ENSDART00000002867
ENSDART00000126315

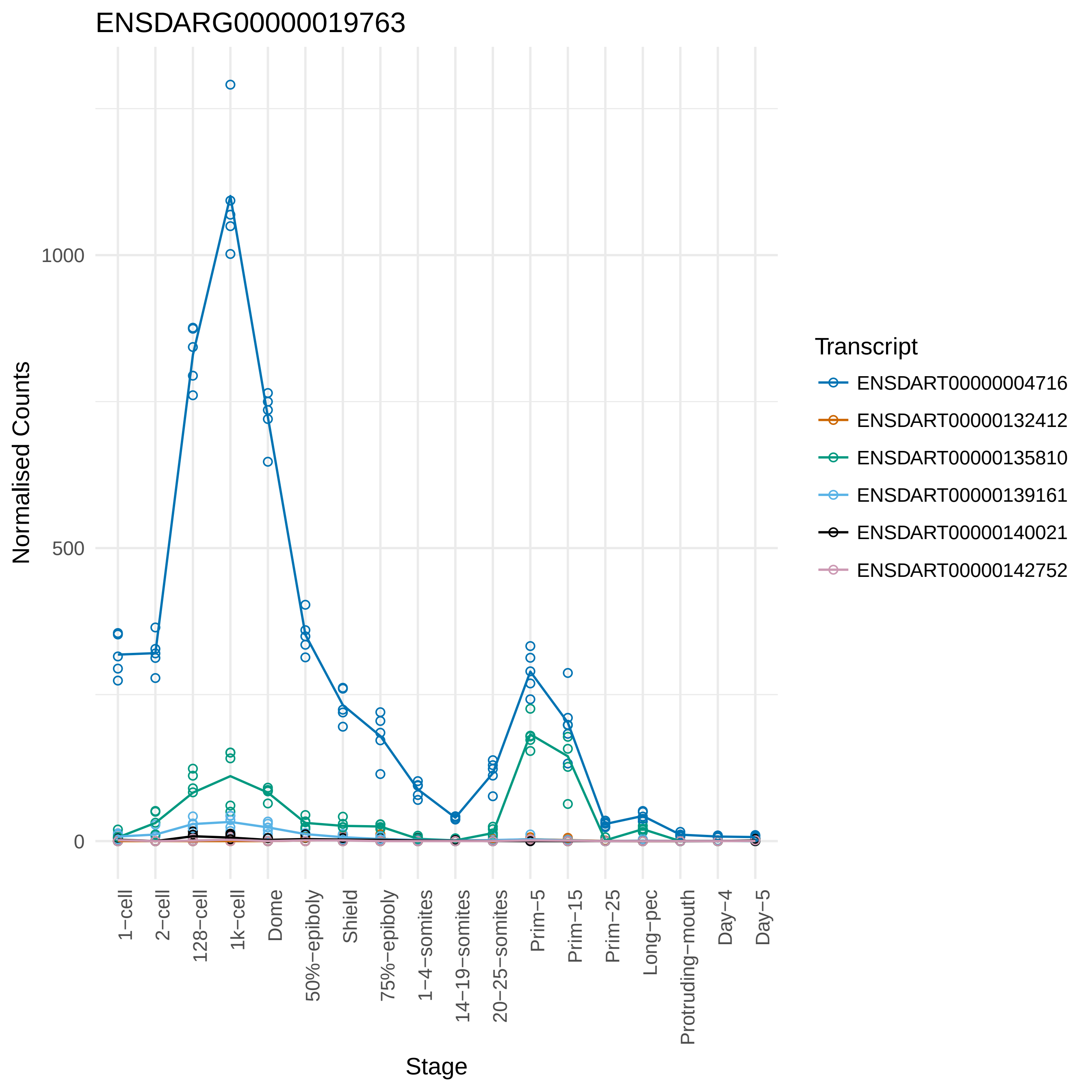

ENSDARG00000019763
Normalised Counts
1000
500
0
1−cell
2−cell
128−cell
1k−cell
Dome
50%−epiboly
Shield
75%−epiboly
1−4−somites
14−19−somites
20−25−somites
Prim−5
Prim−15
Prim−25
Long−pec
Protruding−mouth
Day−4
Day−5
Stage
Transcript
ENSDART00000004716
ENSDART00000132412
ENSDART00000135810
ENSDART00000139161
ENSDART00000140021
ENSDART00000142752

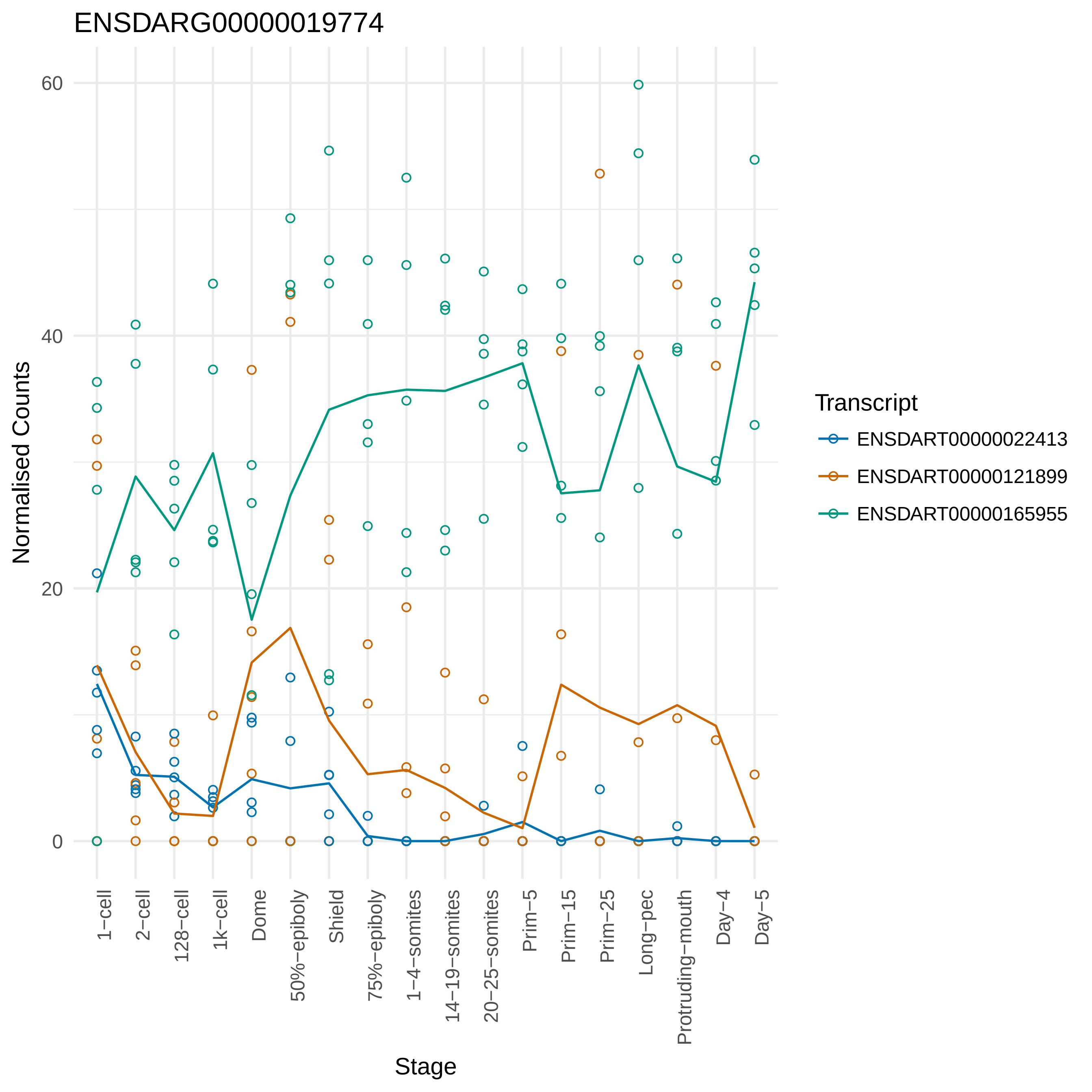
ENSDARG00000019774
Normalised Counts
0
20
40
60
Stage
1−cell
2−cell
128−cell
1k−cell
Dome
50%−epiboly
Shield
75%−epiboly
1−4−somites
14−19−somites
20−25−somites
Prim−5
Prim−15
Prim−25
Long−pec
Protruding−mouth
Day−4
Day−5
Transcript
ENSDART00000022413
ENSDART00000121899
ENSDART00000165955

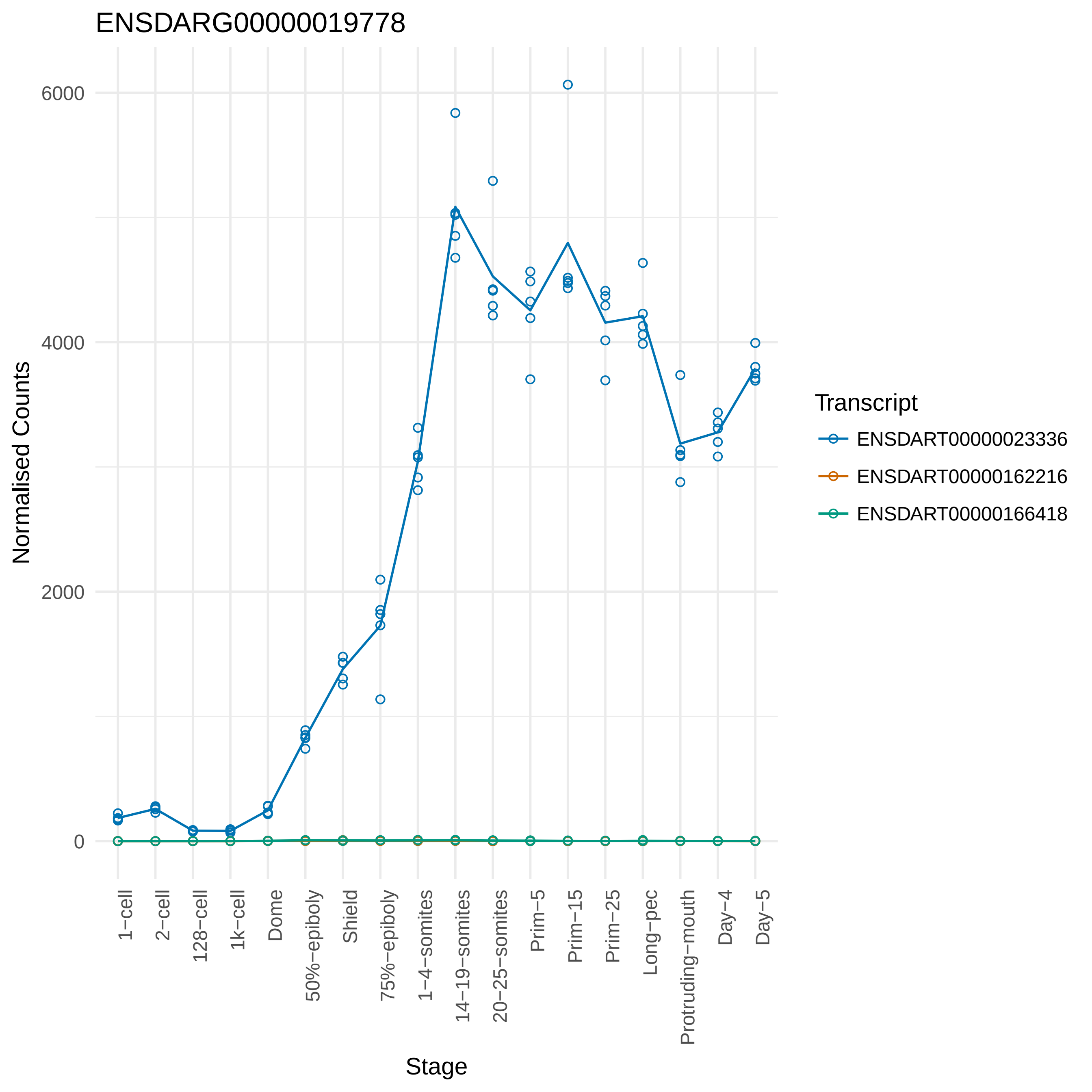

ENSDARG00000019778
Normalised Counts
6000
4000
2000
0
1−cell
2−cell
128−cell
1k−cell
Dome
50%−epiboly
Shield
75%−epiboly
1−4−somites
14−19−somites
20−25−somites
Prim−5
Prim−15
Prim−25
Long−pec
Protruding−mouth
Day−4
Day−5
Stage
Transcript
ENSDART00000023336
ENSDART00000162216
ENSDART00000166418

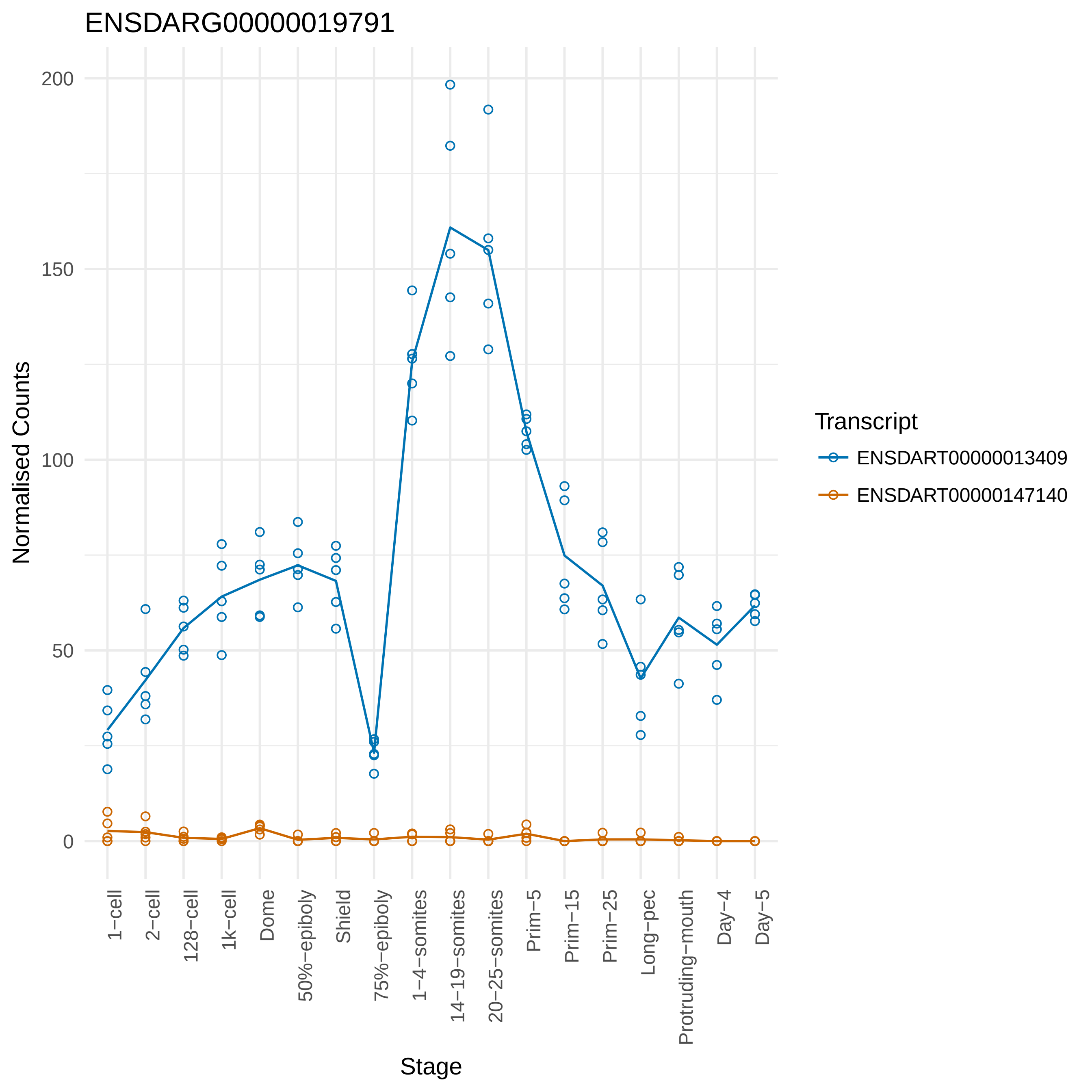

ENSDARG00000019791
Normalised Counts
200
150
100
50
0
1−cell
2−cell
128−cell
1k−cell
Dome
50%−epiboly
Shield
75%−epiboly
1−4−somites
14−19−somites
20−25−somites
Prim−5
Prim−15
Prim−25
Long−pec
Protruding−mouth
Day−4
Day−5
Stage
Transcript
ENSDART00000013409
ENSDART00000147140

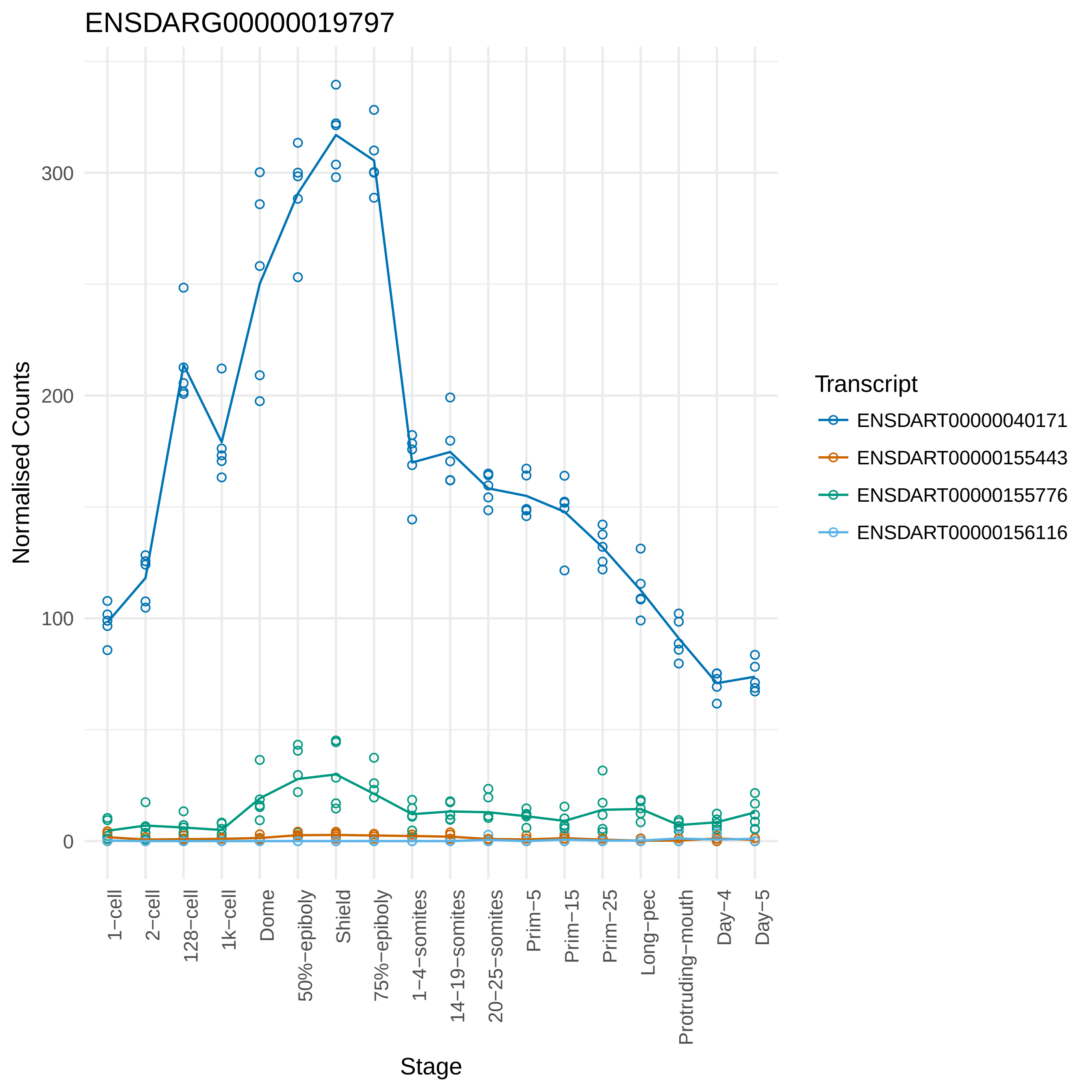
ENSDARG00000019797
Normalised Counts
300
200
100
0
1−cell
2−cell
128−cell
1k−cell
Dome
50%−epiboly
Shield
75%−epiboly
1−4−somites
14−19−somites
20−25−somites
Prim−5
Prim−15
Prim−25
Long−pec
Protruding−mouth
Day−4
Day−5
Stage
Transcript
ENSDART00000040171
ENSDART00000155443
ENSDART00000155776
ENSDART00000156116

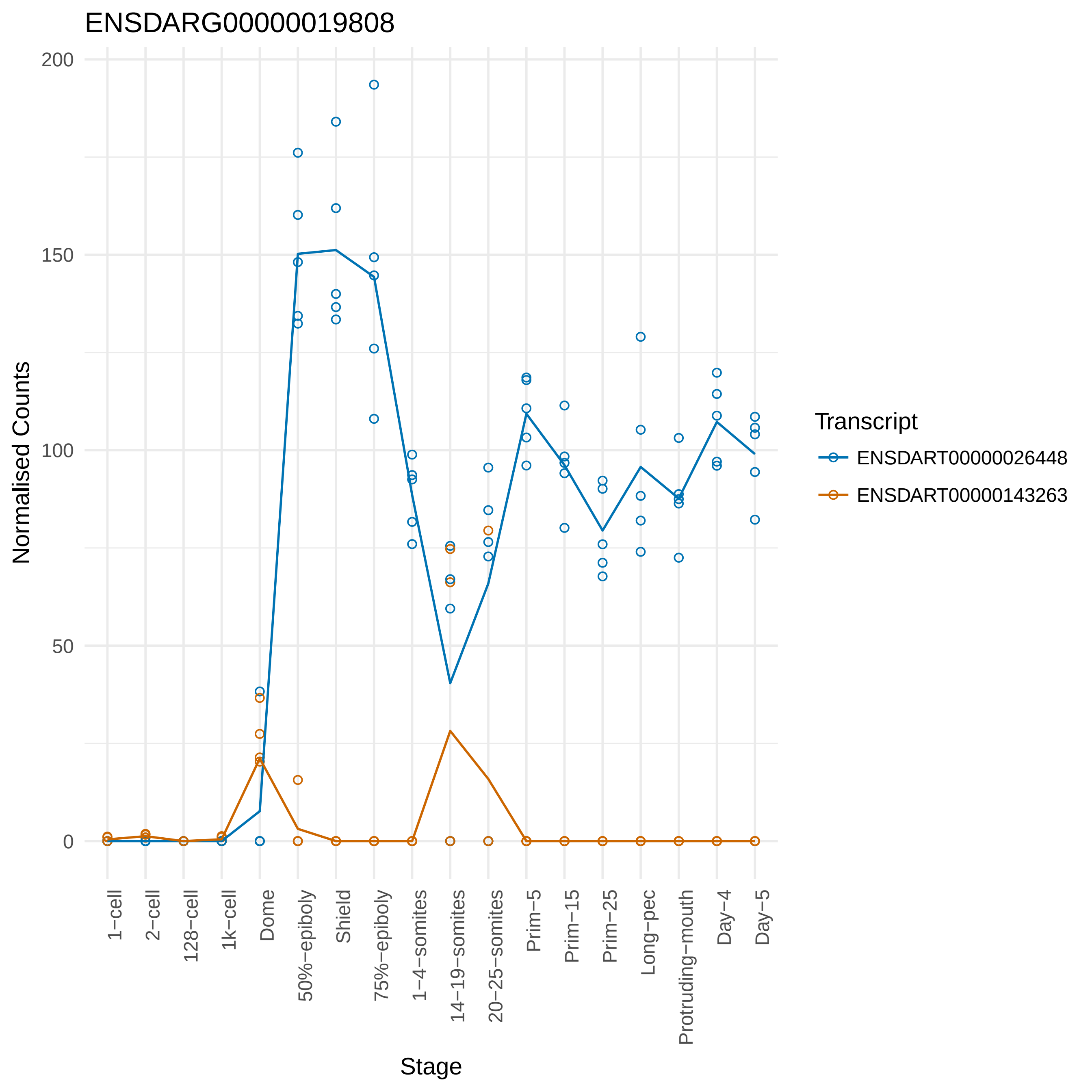

ENSDARG00000019808
Normalised Counts
200
150
100
50
0
1−cell
2−cell
128−cell
1k−cell
Dome
50%−epiboly
Shield
75%−epiboly
1−4−somites
14−19−somites
20−25−somites
Prim−5
Prim−15
Prim−25
Long−pec
Protruding−mouth
Day−4
Day−5
Stage
Transcript
ENSDART00000026448
ENSDART00000143263

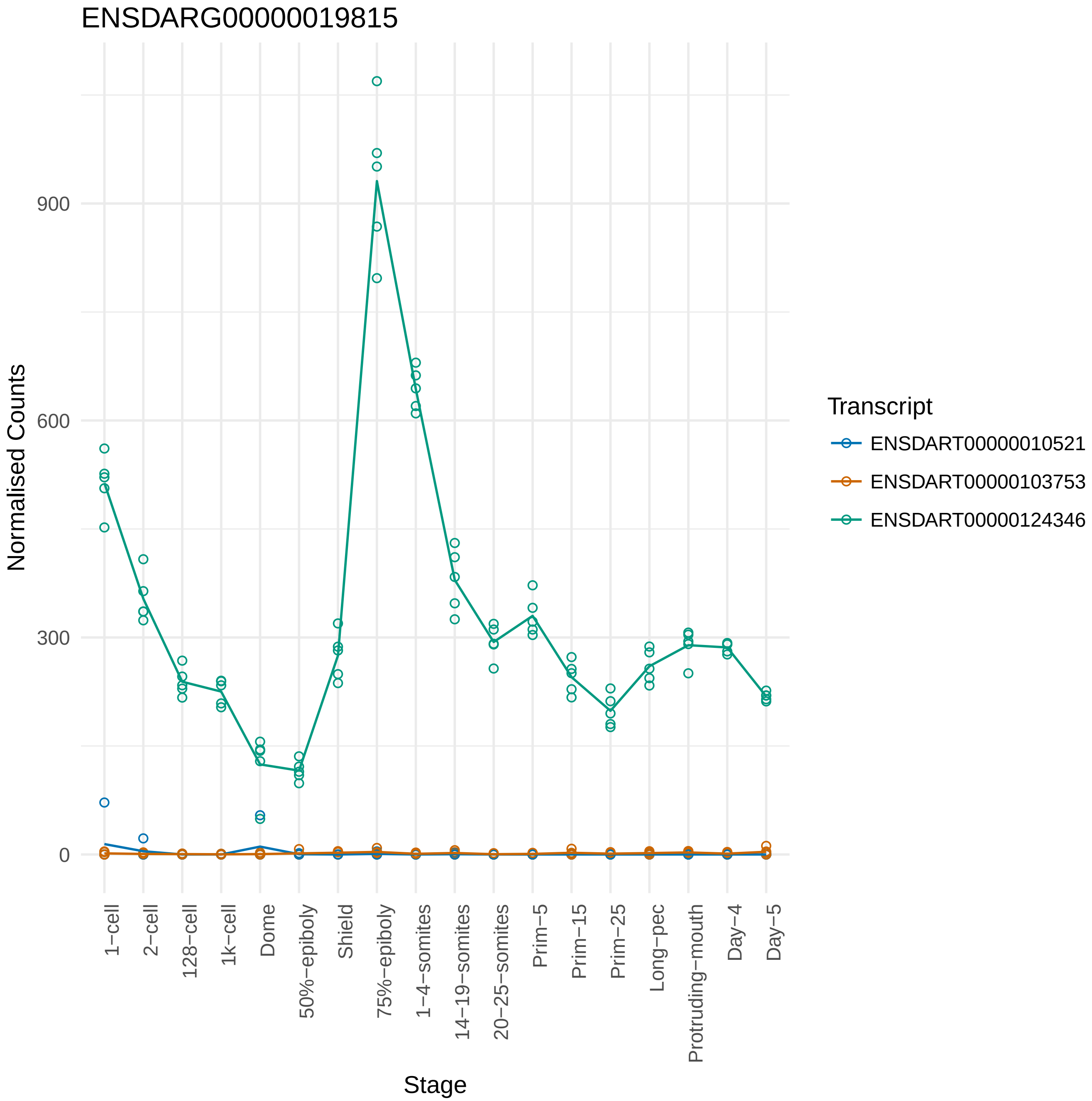

ENSDARG00000019815
Normalised Counts
900
600
300
0
1−cell
2−cell
128−cell
1k−cell
Dome
50%−epiboly
Shield
75%−epiboly
1−4−somites
14−19−somites
20−25−somites
Prim−5
Prim−15
Prim−25
Long−pec
Protruding−mouth
Day−4
Day−5
Stage
Transcript
ENSDART00000010521
ENSDART00000103753
ENSDART00000124346

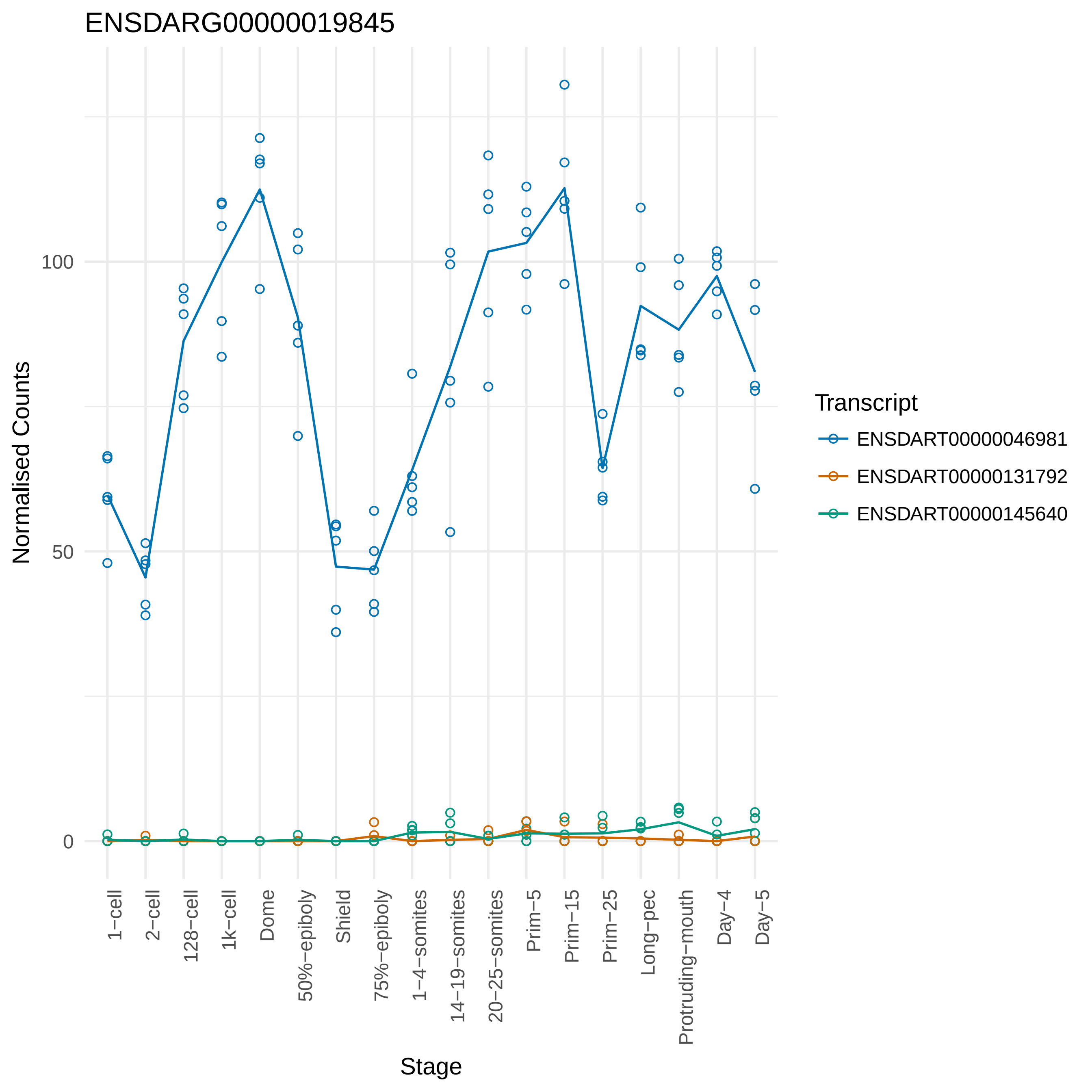
ENSDARG00000019845
Normalised Counts
100
50
0
1−cell
2−cell
128−cell
1k−cell
Dome
50%−epiboly
Shield
75%−epiboly
1−4−somites
14−19−somites
20−25−somites
Prim−5
Prim−15
Prim−25
Long−pec
Protruding−mouth
Day−4
Day−5
Stage
Transcript
ENSDART00000046981
ENSDART00000131792
ENSDART00000145640

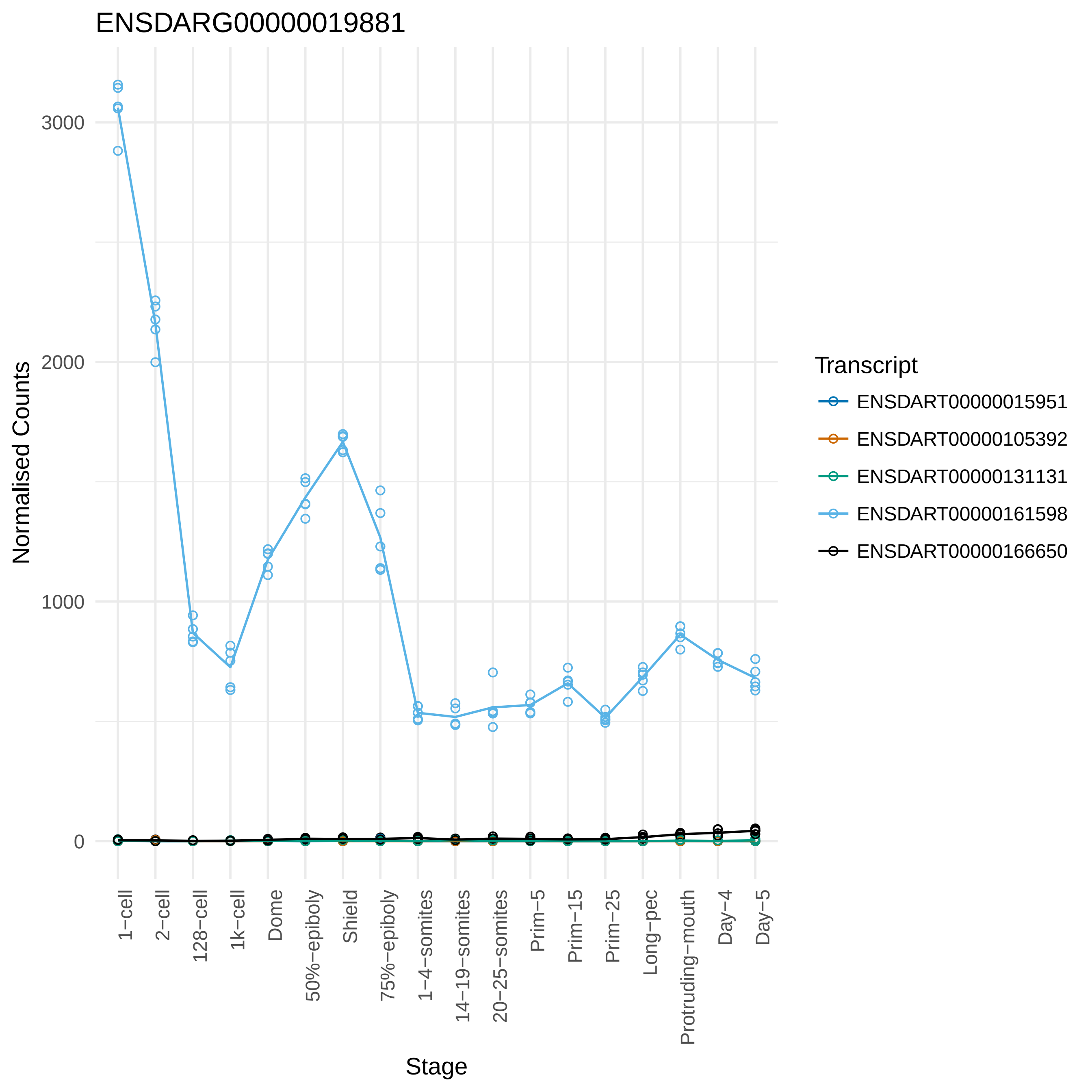

ENSDARG00000019881
Normalised Counts
3000
2000
1000
0
1−cell
2−cell
128−cell
1k−cell
Dome
50%−epiboly
Shield
75%−epiboly
1−4−somites
14−19−somites
20−25−somites
Prim−5
Prim−15
Prim−25
Long−pec
Protruding−mouth
Day−4
Day−5
Stage
Transcript
ENSDART00000015951
ENSDART00000105392
ENSDART00000131131
ENSDART00000161598
ENSDART00000166650

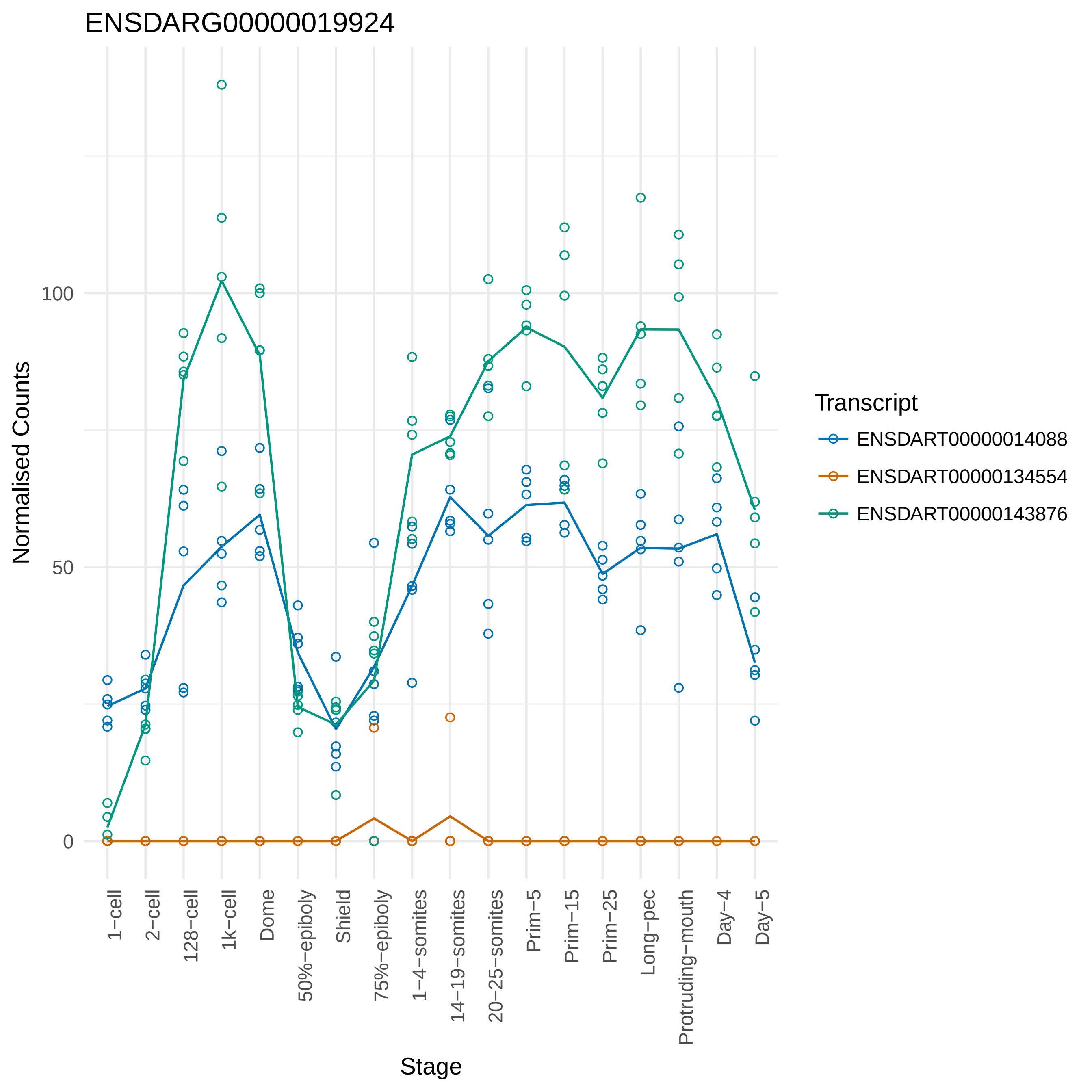

ENSDARG00000019924
Normalised Counts
100
50
0
1−cell
2−cell
128−cell
1k−cell
Dome
50%−epiboly
Shield
75%−epiboly
1−4−somites
14−19−somites
20−25−somites
Prim−5
Prim−15
Prim−25
Long−pec
Protruding−mouth
Day−4
Day−5
Stage
Transcript
ENSDART00000014088
ENSDART00000134554
ENSDART00000143876

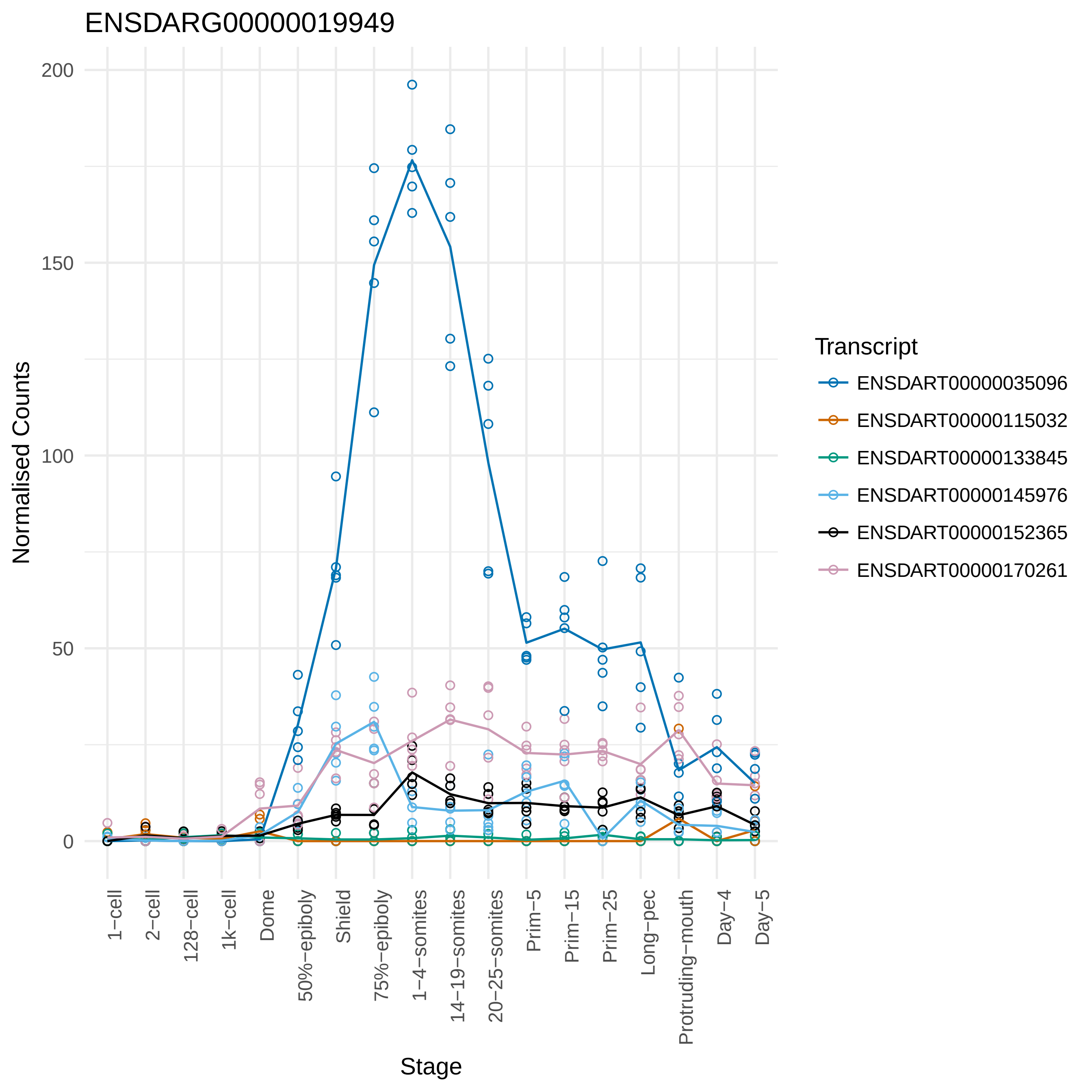

ENSDARG00000019949
Normalised Counts
200
150
100
50
0
1−cell
2−cell
128−cell
1k−cell
Dome
50%−epiboly
Shield
75%−epiboly
1−4−somites
14−19−somites
20−25−somites
Prim−5
Prim−15
Prim−25
Long−pec
Protruding−mouth
Day−4
Day−5
Stage
Transcript
ENSDART00000035096
ENSDART00000115032
ENSDART00000133845
ENSDART00000145976
ENSDART00000152365
ENSDART00000170261

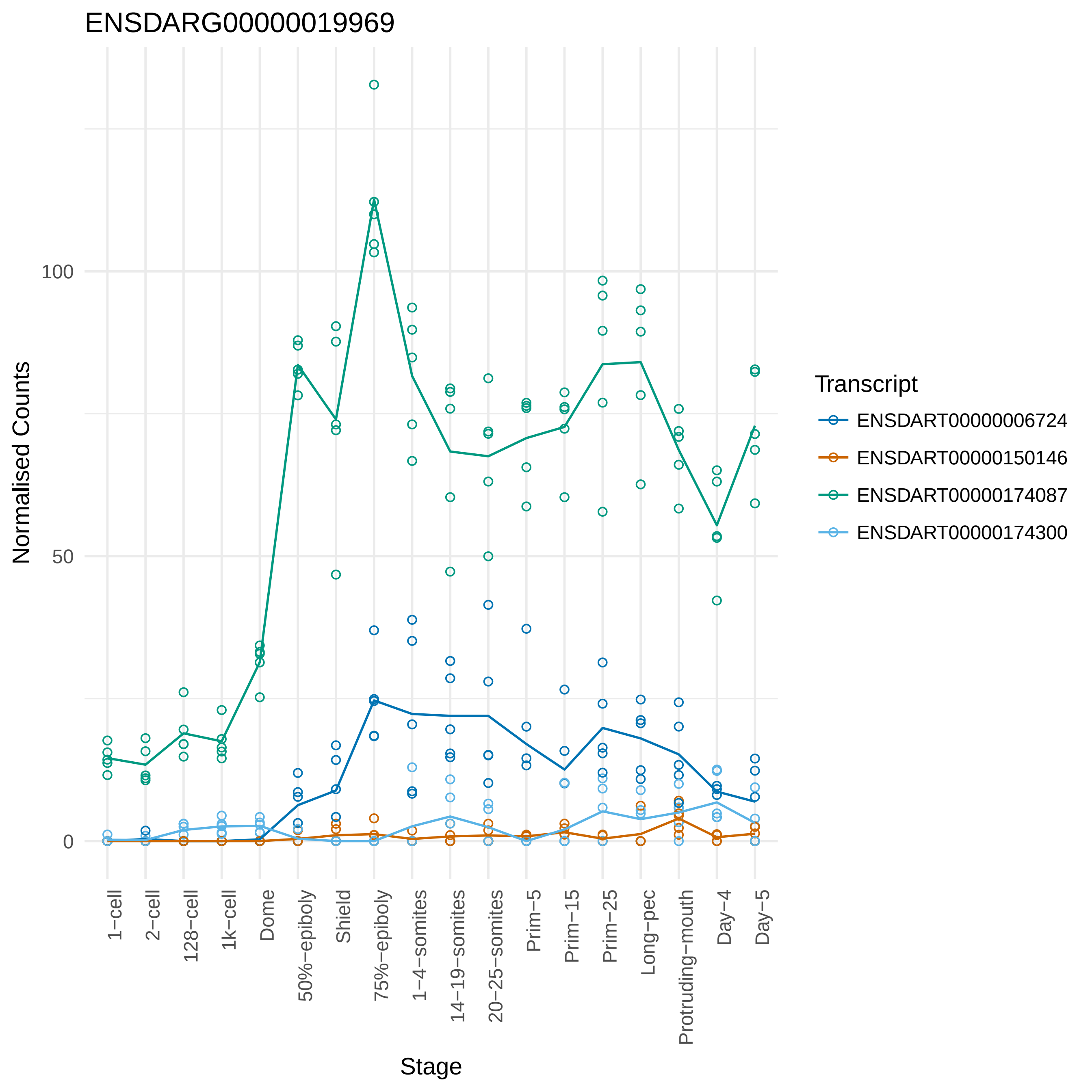

ENSDARG00000019969
Normalised Counts
100
50
0
1−cell
2−cell
128−cell
1k−cell
Dome
50%−epiboly
Shield
75%−epiboly
1−4−somites
14−19−somites
20−25−somites
Prim−5
Prim−15
Prim−25
Long−pec
Protruding−mouth
Day−4
Day−5
Stage
Transcript
ENSDART00000006724
ENSDART00000150146
ENSDART00000174087
ENSDART00000174300

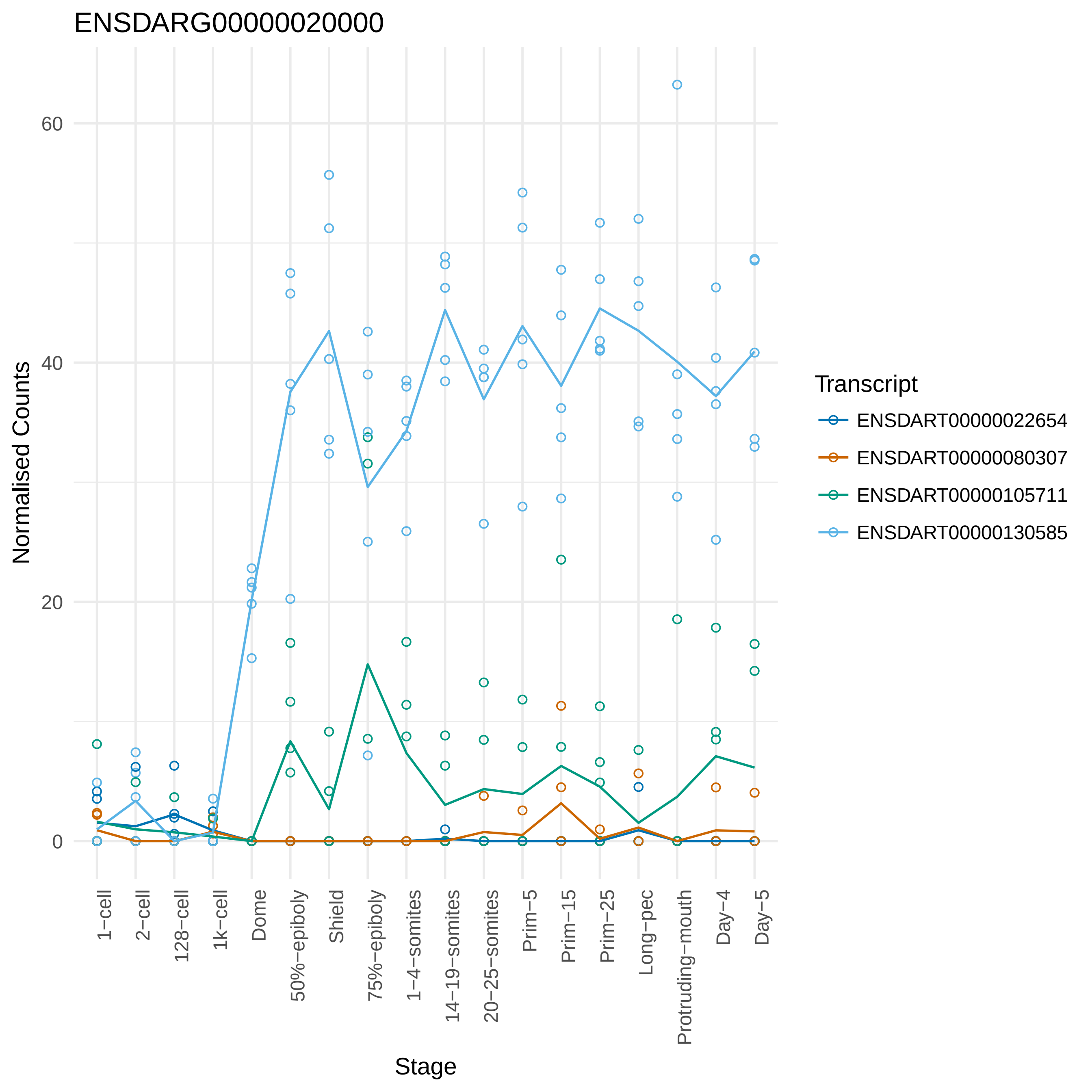

ENSDARG00000020000
Normalised Counts
0
20
40
60
1−cell
2−cell
128−cell
1k−cell
Dome
50%−epiboly
Shield
75%−epiboly
1−4−somites
14−19−somites
20−25−somites
Prim−5
Prim−15
Prim−25
Long−pec
Protruding−mouth
Day−4
Day−5
Stage
Transcript
ENSDART00000022654
ENSDART00000080307
ENSDART00000105711
ENSDART00000130585

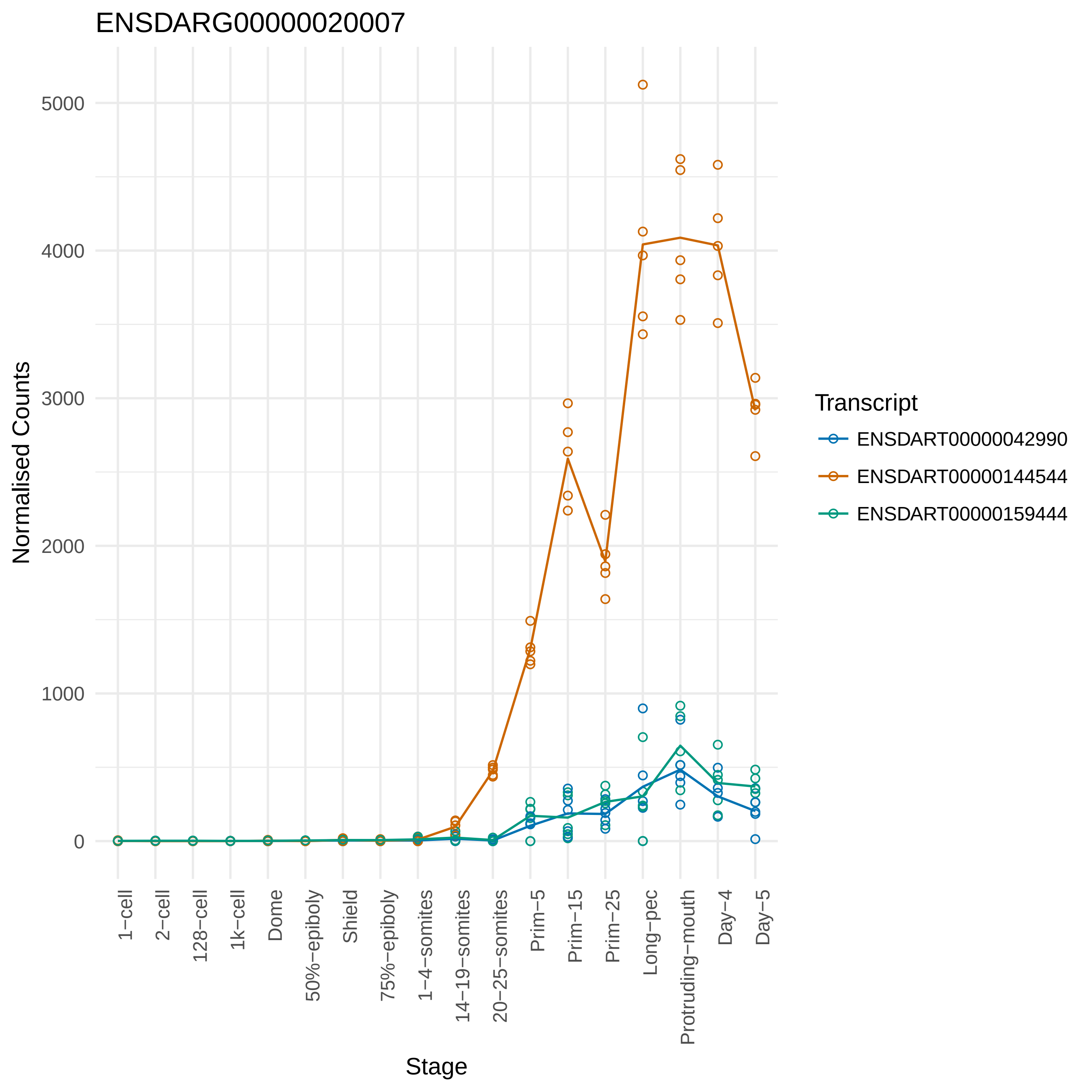
ENSDARG00000020007
Normalised Counts
0
1000
2000
3000
4000
5000
Stage
1−cell
2−cell
128−cell
1k−cell
Dome
50%−epiboly
Shield
75%−epiboly
1−4−somites
14−19−somites
20−25−somites
Prim−5
Prim−15
Prim−25
Long−pec
Protruding−mouth
Day−4
Day−5
Transcript
ENSDART00000042990
ENSDART00000144544
ENSDART00000159444

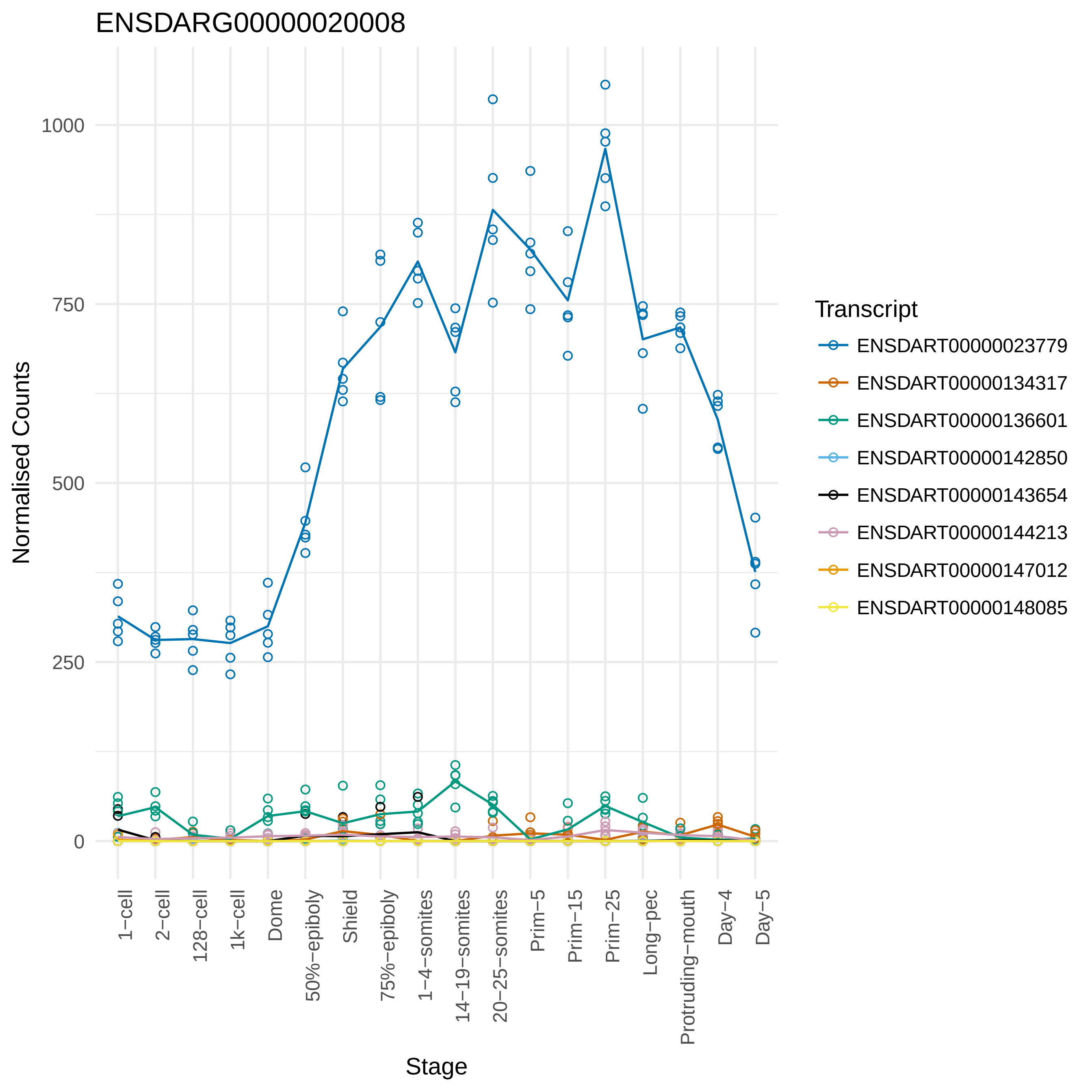

ENSDARG00000020008
Normalised Counts
1000
750
500
250
0
Stage
1−cell
2−cell
128−cell
1k−cell
Dome
50%−epiboly
Shield
75%−epiboly
1−4−somites
14−19−somites
20−25−somites
Prim−5
Prim−15
Prim−25
Long−pec
Protruding−mouth
Day−4
Day−5
Transcript
ENSDART00000023779
ENSDART00000134317
ENSDART00000136601
ENSDART00000142850
ENSDART00000143654
ENSDART00000144213
ENSDART00000147012
ENSDART00000148085

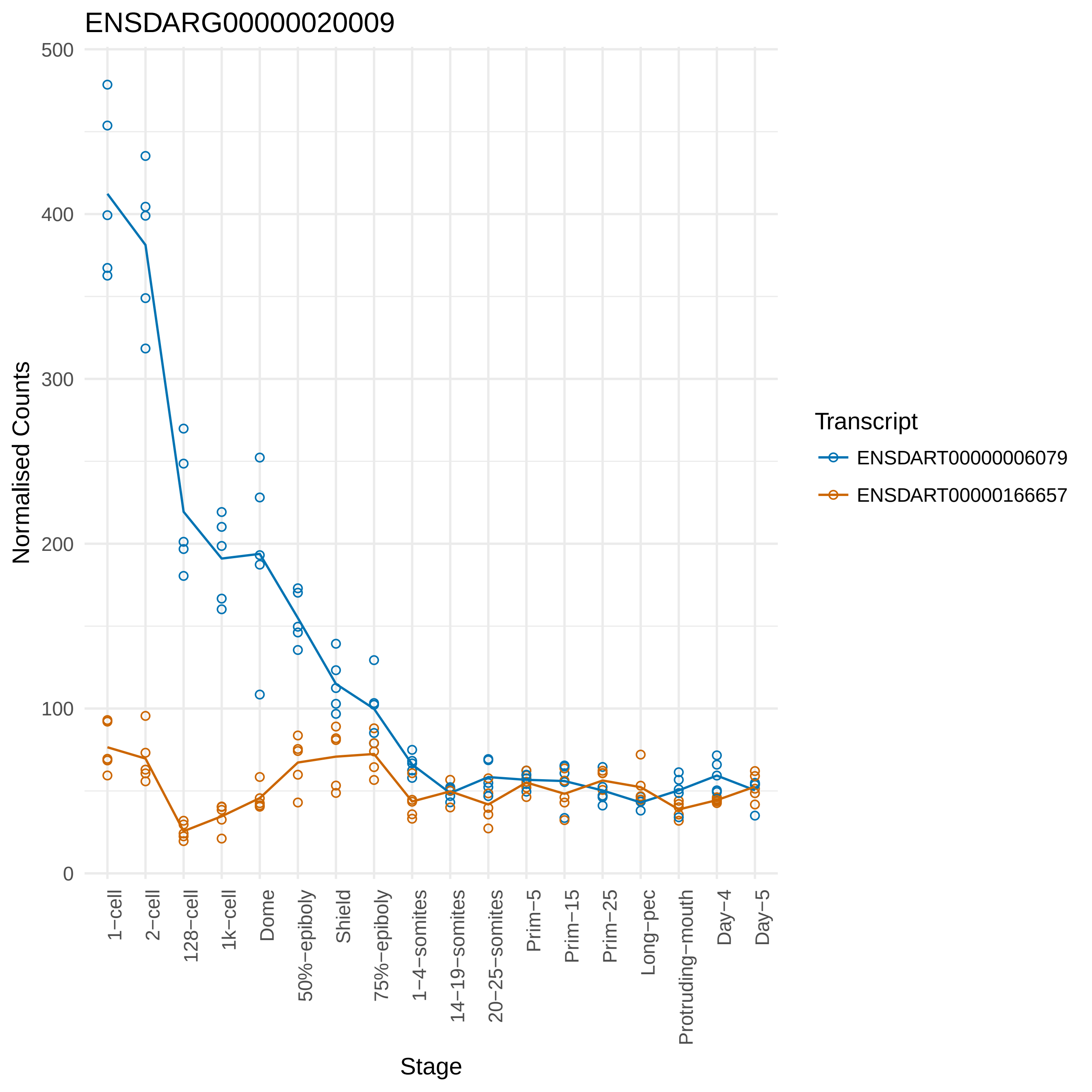

ENSDARG00000020009
Normalised Counts
500
400
300
200
100
0
1−cell
2−cell
128−cell
1k−cell
Dome
50%−epiboly
Shield
75%−epiboly
1−4−somites
14−19−somites
20−25−somites
Prim−5
Prim−15
Prim−25
Long−pec
Protruding−mouth
Day−4
Day−5
Stage
Transcript
ENSDART00000006079
ENSDART00000166657

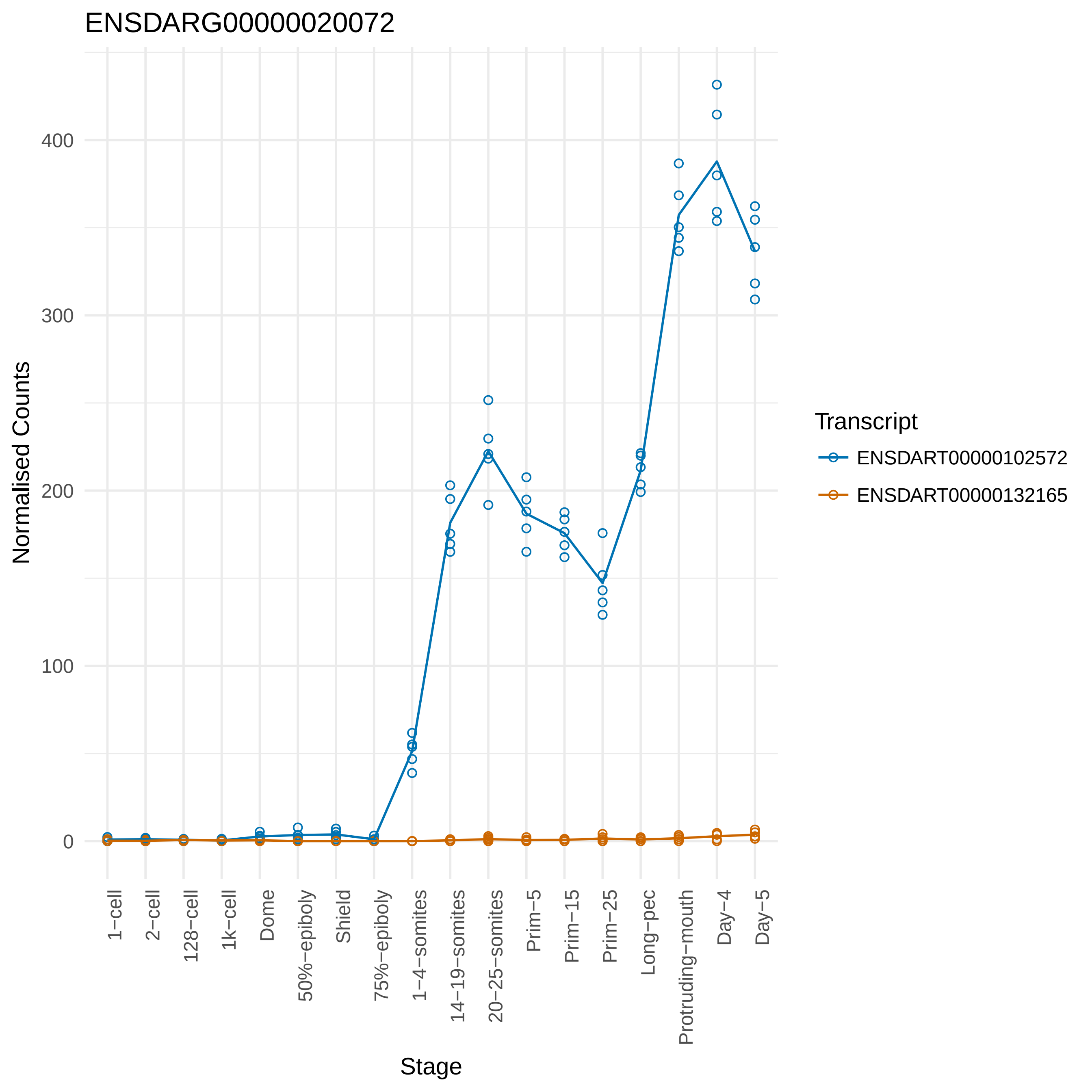
ENSDARG00000020072
Normalised Counts
400
300
200
100
0
1−cell
2−cell
128−cell
1k−cell
Dome
50%−epiboly
Shield
75%−epiboly
1−4−somites
14−19−somites
20−25−somites
Prim−5
Prim−15
Prim−25
Long−pec
Protruding−mouth
Day−4
Day−5
Stage
Transcript
ENSDART00000102572
ENSDART00000132165

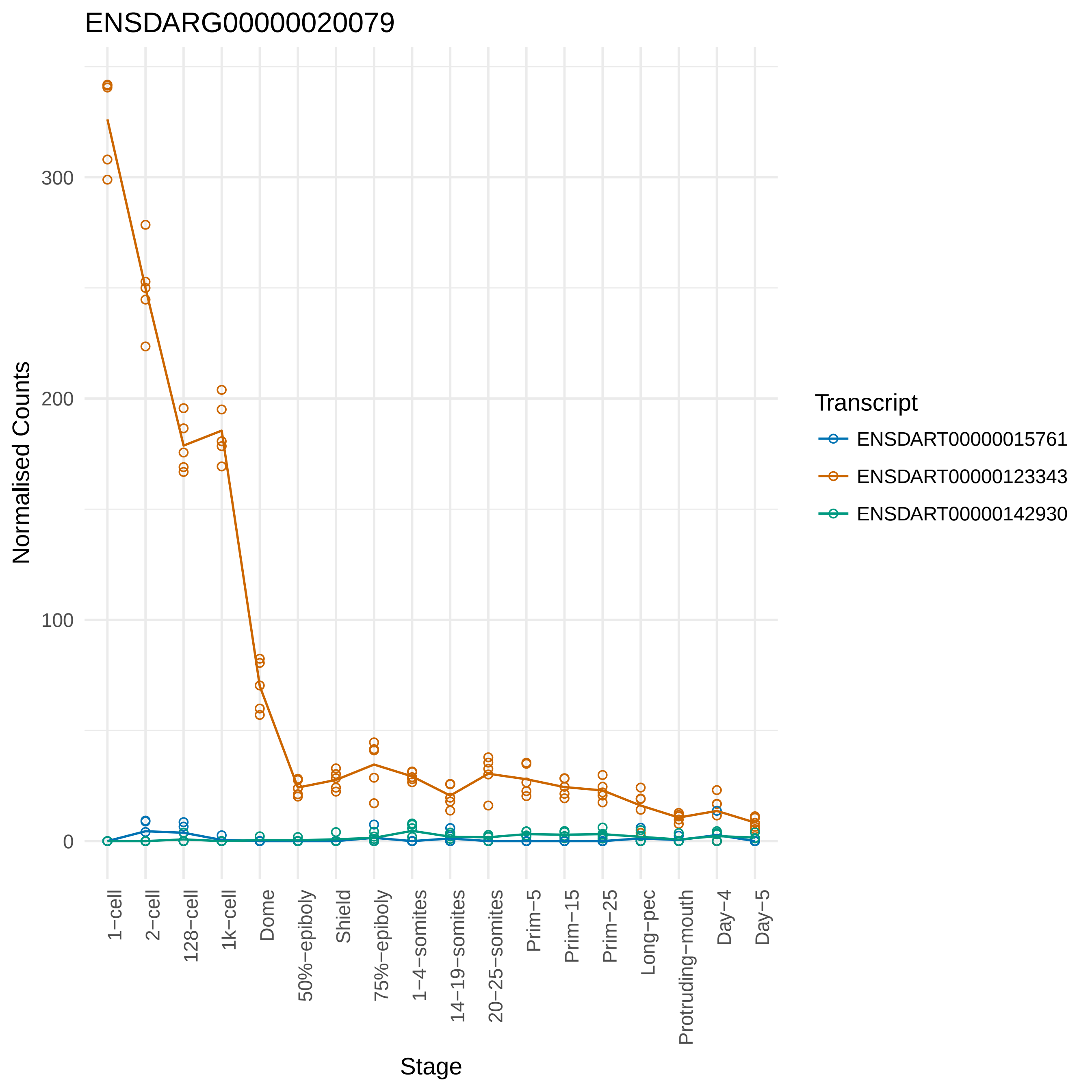

ENSDARG00000020079
Normalised Counts
300
200
100
0
1−cell
2−cell
128−cell
1k−cell
Dome
50%−epiboly
Shield
75%−epiboly
1−4−somites
14−19−somites
20−25−somites
Prim−5
Prim−15
Prim−25
Long−pec
Protruding−mouth
Day−4
Day−5
Stage
Transcript
ENSDART00000015761
ENSDART00000123343
ENSDART00000142930

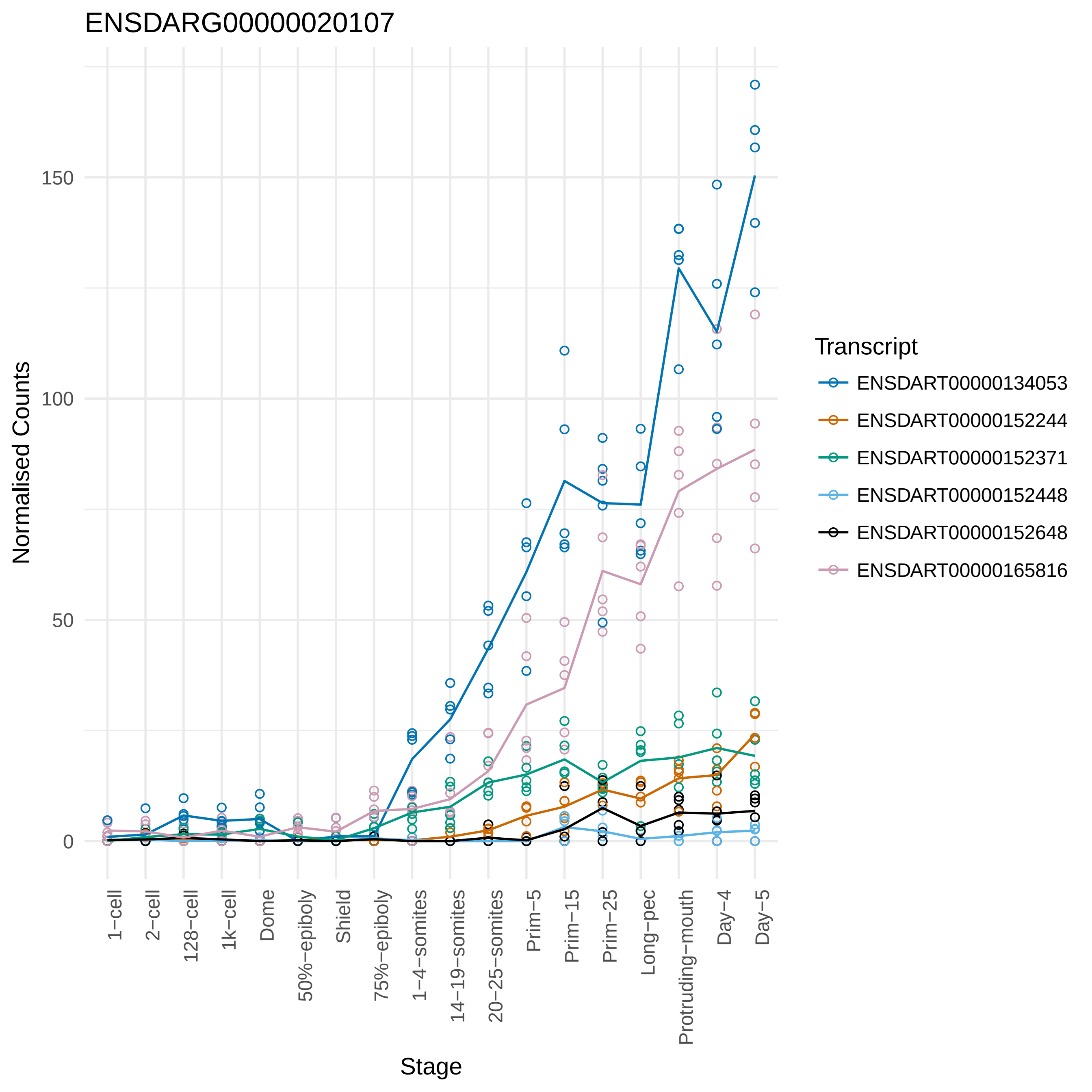

ENSDARG00000020107
Normalised Counts
Stage
Transcript
ENSDART00000134053
ENSDART00000152244
ENSDART00000152371
ENSDART00000152448
ENSDART00000152648
ENSDART00000165816
0
50
100
150
1−cell
2−cell
128−cell
1k−cell
Dome
50%−epiboly
Shield
75%−epiboly
1−4−somites
14−19−somites
20−25−somites
Prim−5
Prim−15
Prim−25
Long−pec
Protruding−mouth
Day−4
Day−5

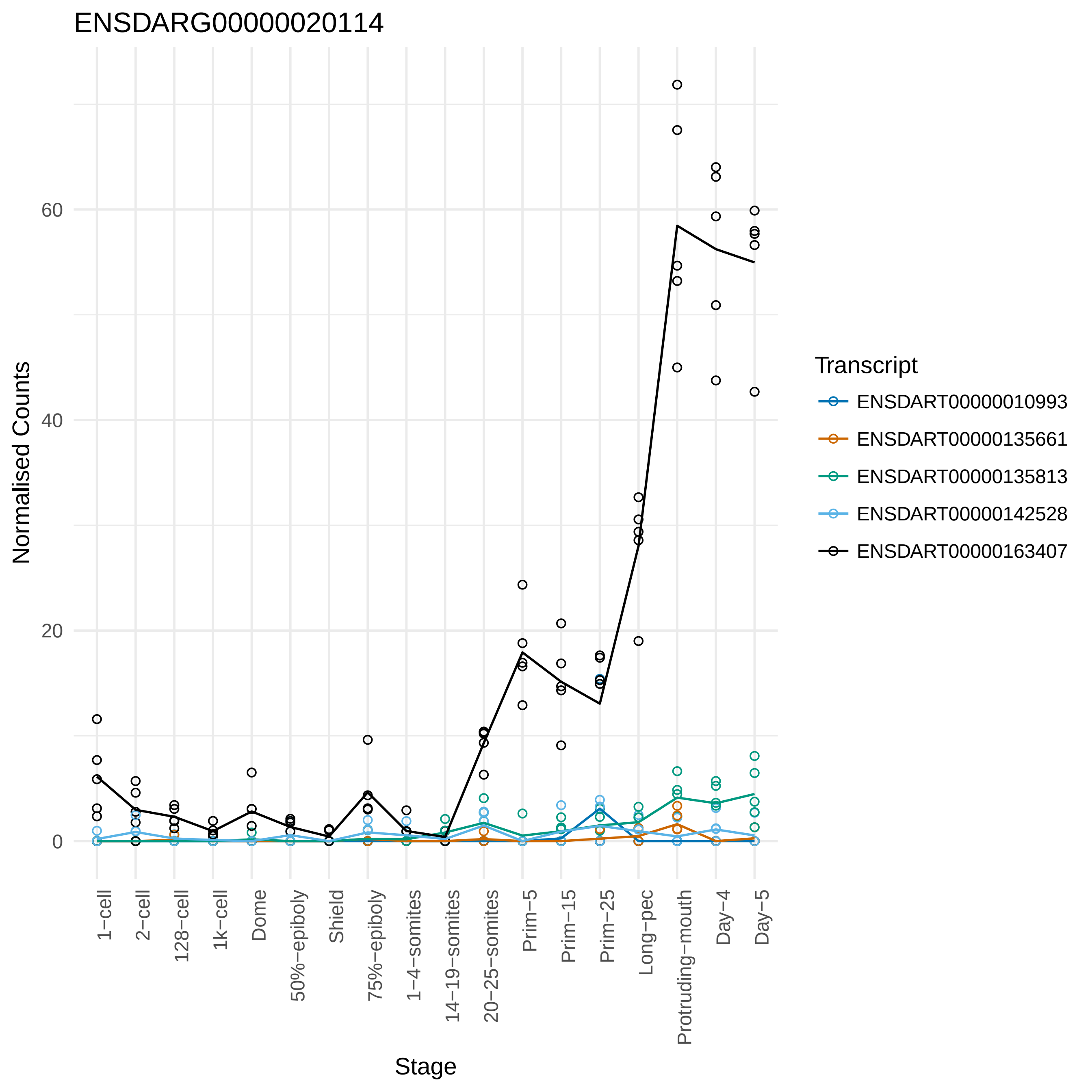
ENSDARG00000020114
Normalised Counts
0
20
40
60
1−cell
2−cell
128−cell
1k−cell
Dome
50%−epiboly
Shield
75%−epiboly
1−4−somites
14−19−somites
20−25−somites
Prim−5
Prim−15
Prim−25
Long−pec
Protruding−mouth
Day−4
Day−5
Stage
Transcript
ENSDART00000010993
ENSDART00000135661
ENSDART00000135813
ENSDART00000142528
ENSDART00000163407

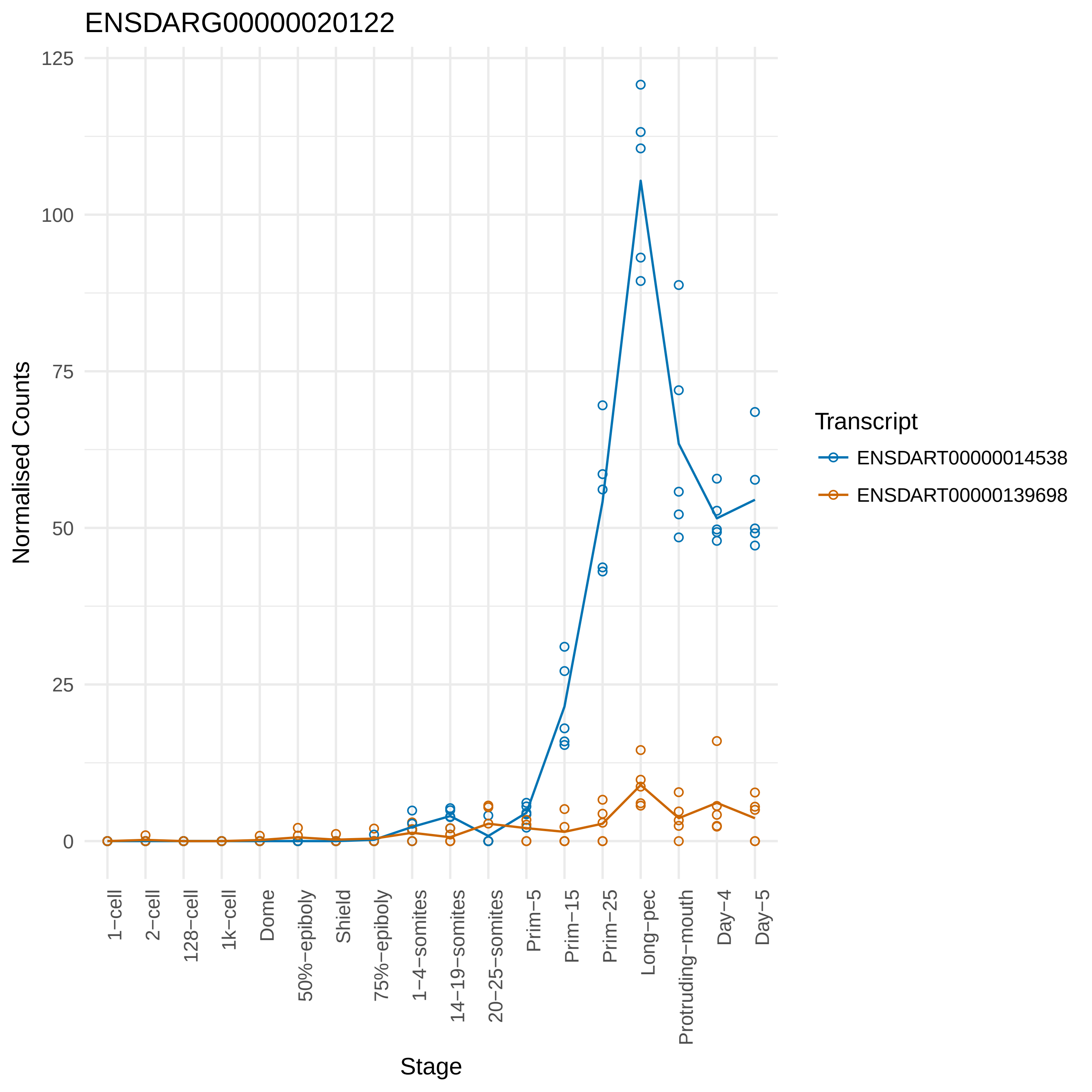
ENSDARG00000020122
Normalised Counts
125
100
75
50
25
0
1−cell
2−cell
128−cell
1k−cell
Dome
50%−epiboly
Shield
75%−epiboly
1−4−somites
14−19−somites
20−25−somites
Prim−5
Prim−15
Prim−25
Long−pec
Protruding−mouth
Day−4
Day−5
Stage
Transcript
ENSDART00000014538
ENSDART00000139698

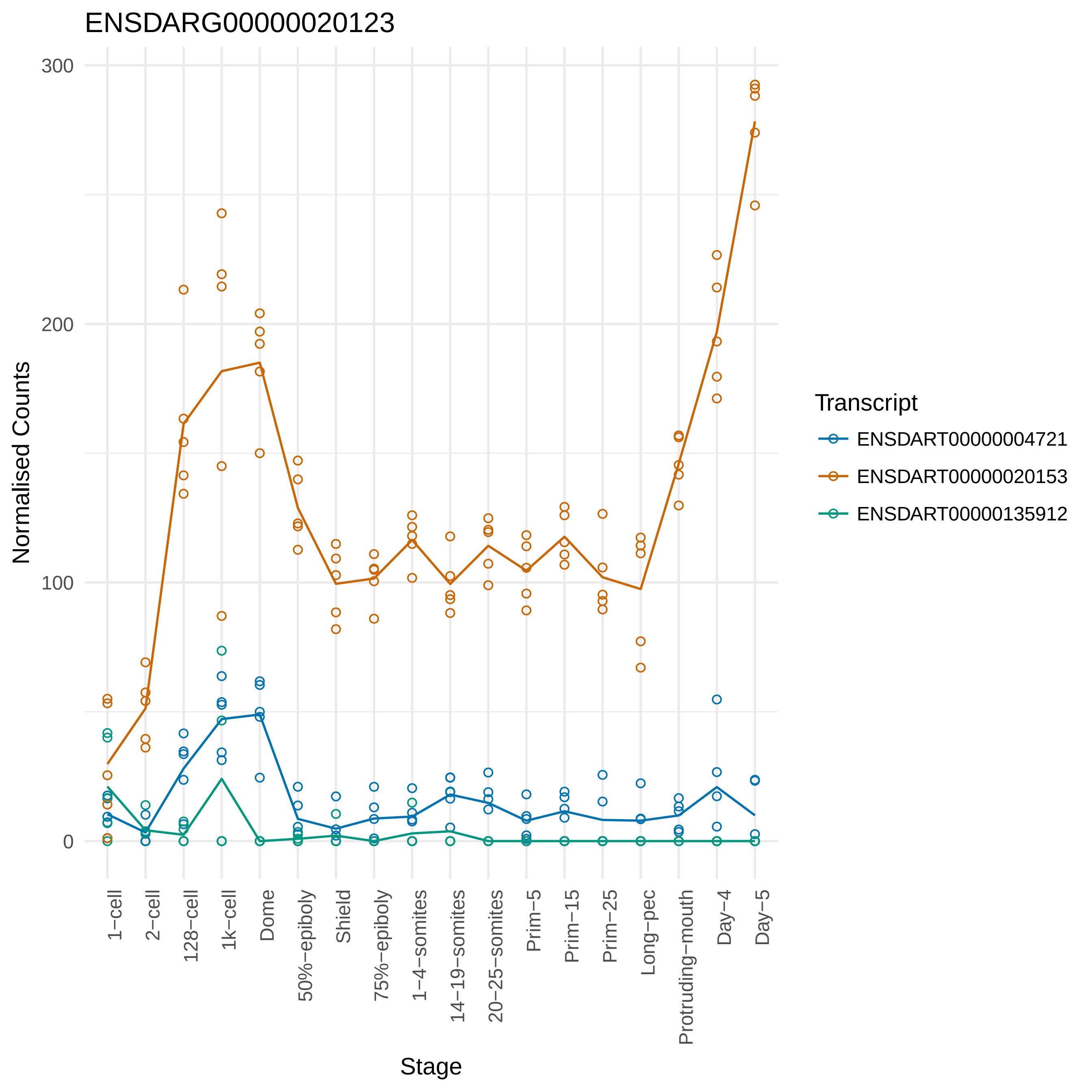
ENSDARG00000020123
Normalised Counts
300
200
100
0
1−cell
2−cell
128−cell
1k−cell
Dome
50%−epiboly
Shield
75%−epiboly
1−4−somites
14−19−somites
20−25−somites
Prim−5
Prim−15
Prim−25
Long−pec
Protruding−mouth
Day−4
Day−5
Stage
Transcript
ENSDART00000004721
ENSDART00000020153
ENSDART00000135912

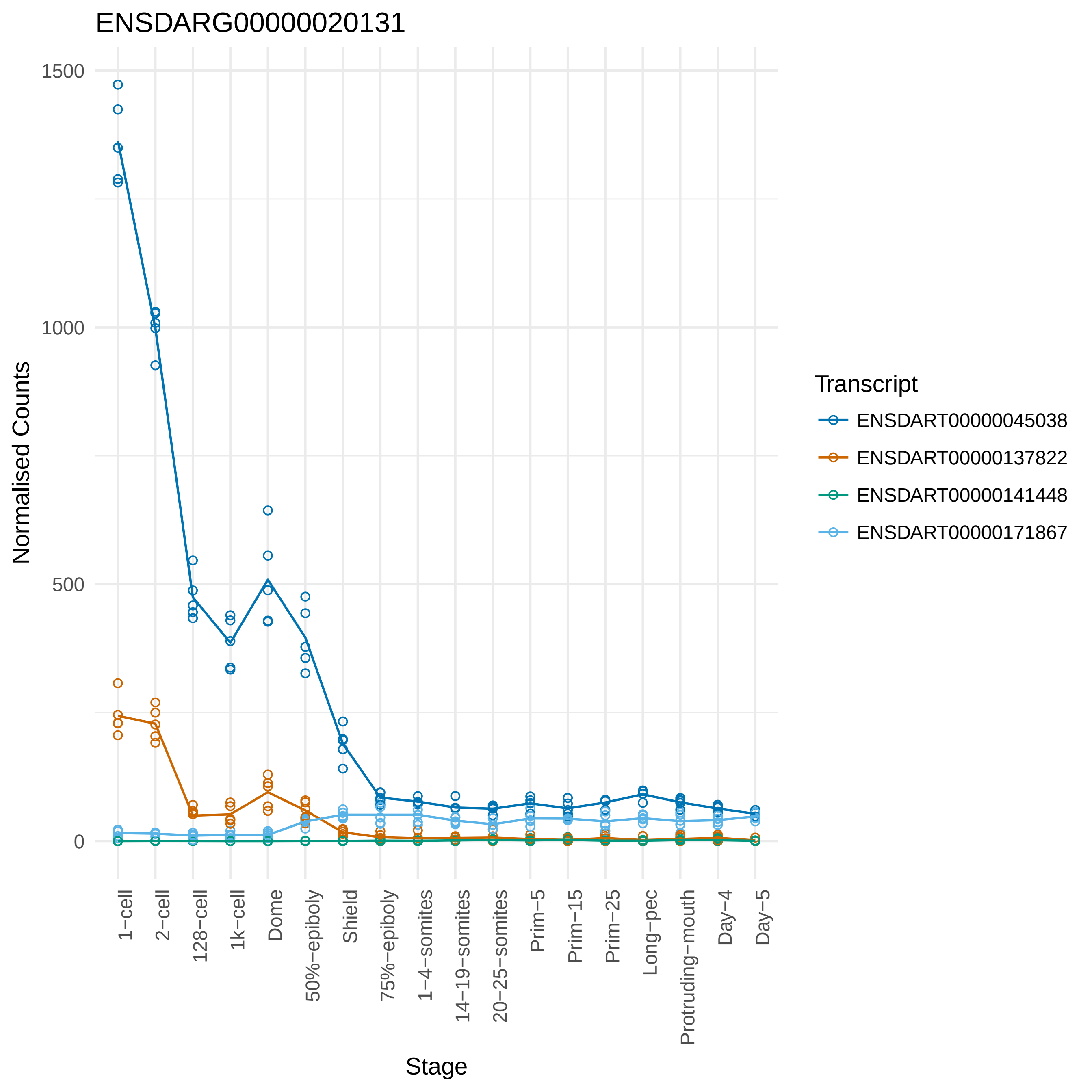

ENSDARG00000020131
Normalised Counts
1500
1000
500
0
1−cell
2−cell
128−cell
1k−cell
Dome
50%−epiboly
Shield
75%−epiboly
1−4−somites
14−19−somites
20−25−somites
Prim−5
Prim−15
Prim−25
Long−pec
Protruding−mouth
Day−4
Day−5
Stage
Transcript
ENSDART00000045038
ENSDART00000137822
ENSDART00000141448
ENSDART00000171867

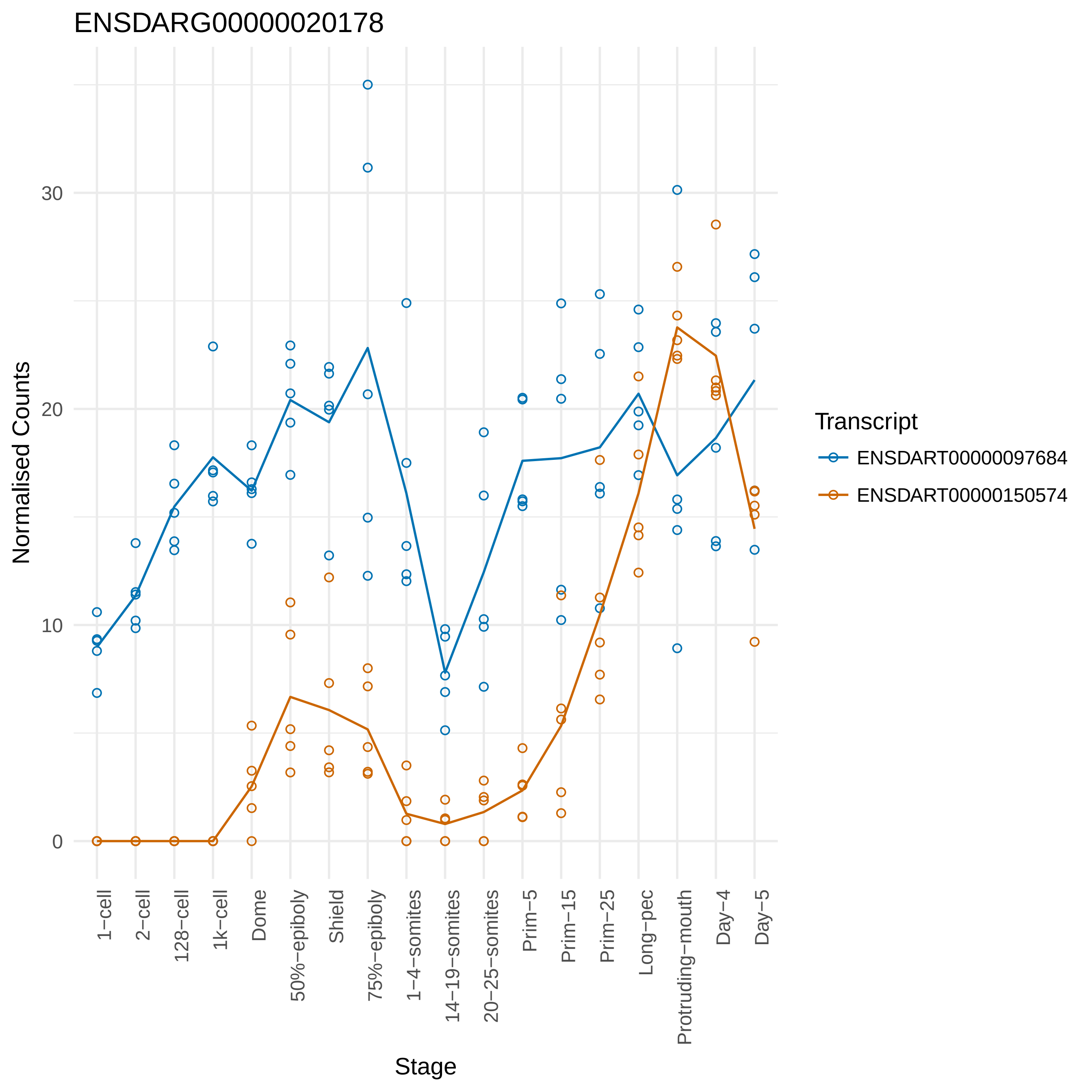

ENSDARG00000020178
Normalised Counts
0
10
20
30
1−cell
2−cell
128−cell
1k−cell
Dome
50%−epiboly
Shield
75%−epiboly
1−4−somites
14−19−somites
20−25−somites
Prim−5
Prim−15
Prim−25
Long−pec
Protruding−mouth
Day−4
Day−5
Stage
Transcript
ENSDART00000097684
ENSDART00000150574

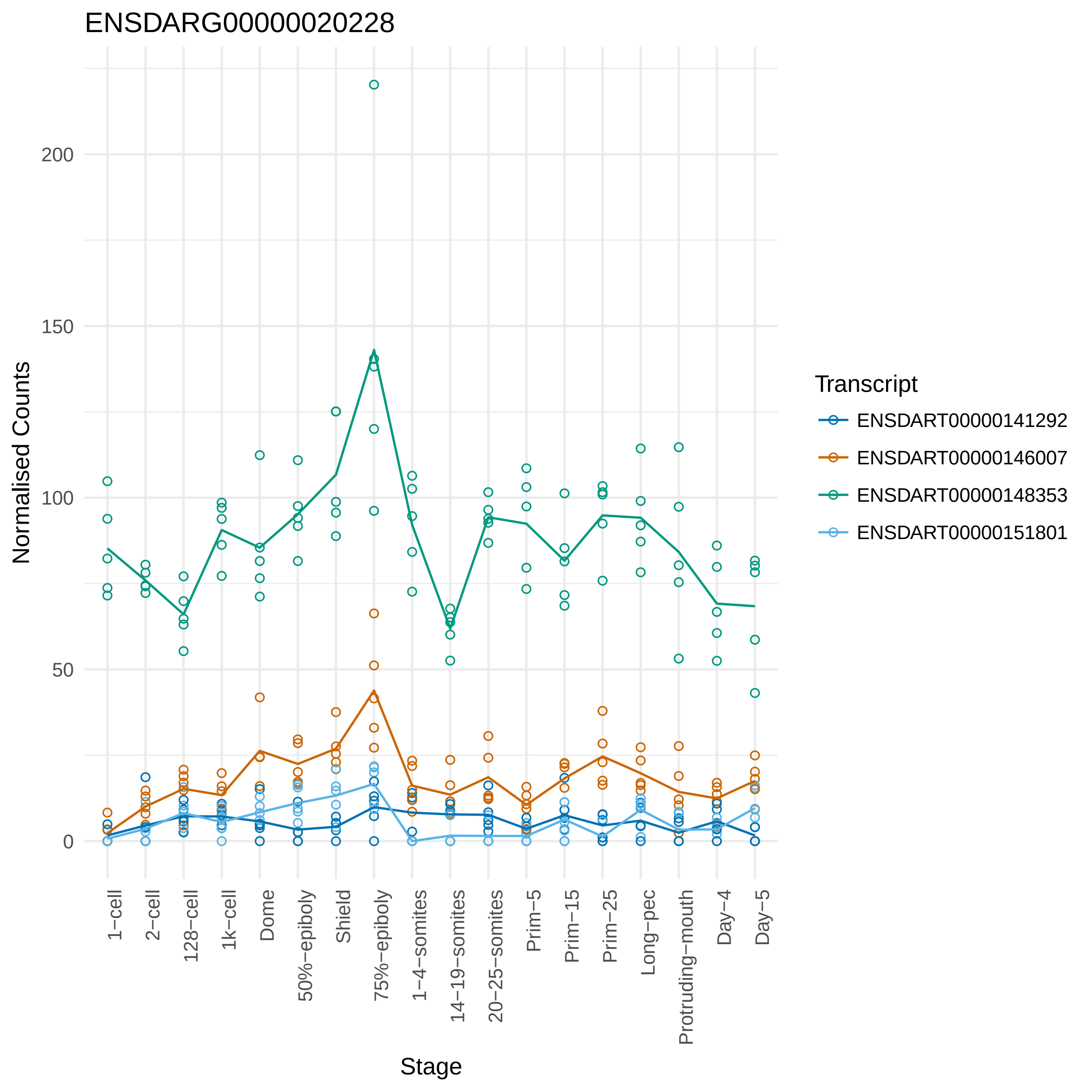

ENSDARG00000020228
Normalised Counts
0
50
100
150
200
1−cell
2−cell
128−cell
1k−cell
Dome
50%−epiboly
Shield
75%−epiboly
1−4−somites
14−19−somites
20−25−somites
Prim−5
Prim−15
Prim−25
Long−pec
Protruding−mouth
Day−4
Day−5
Stage
Transcript
ENSDART00000141292
ENSDART00000146007
ENSDART00000148353
ENSDART00000151801

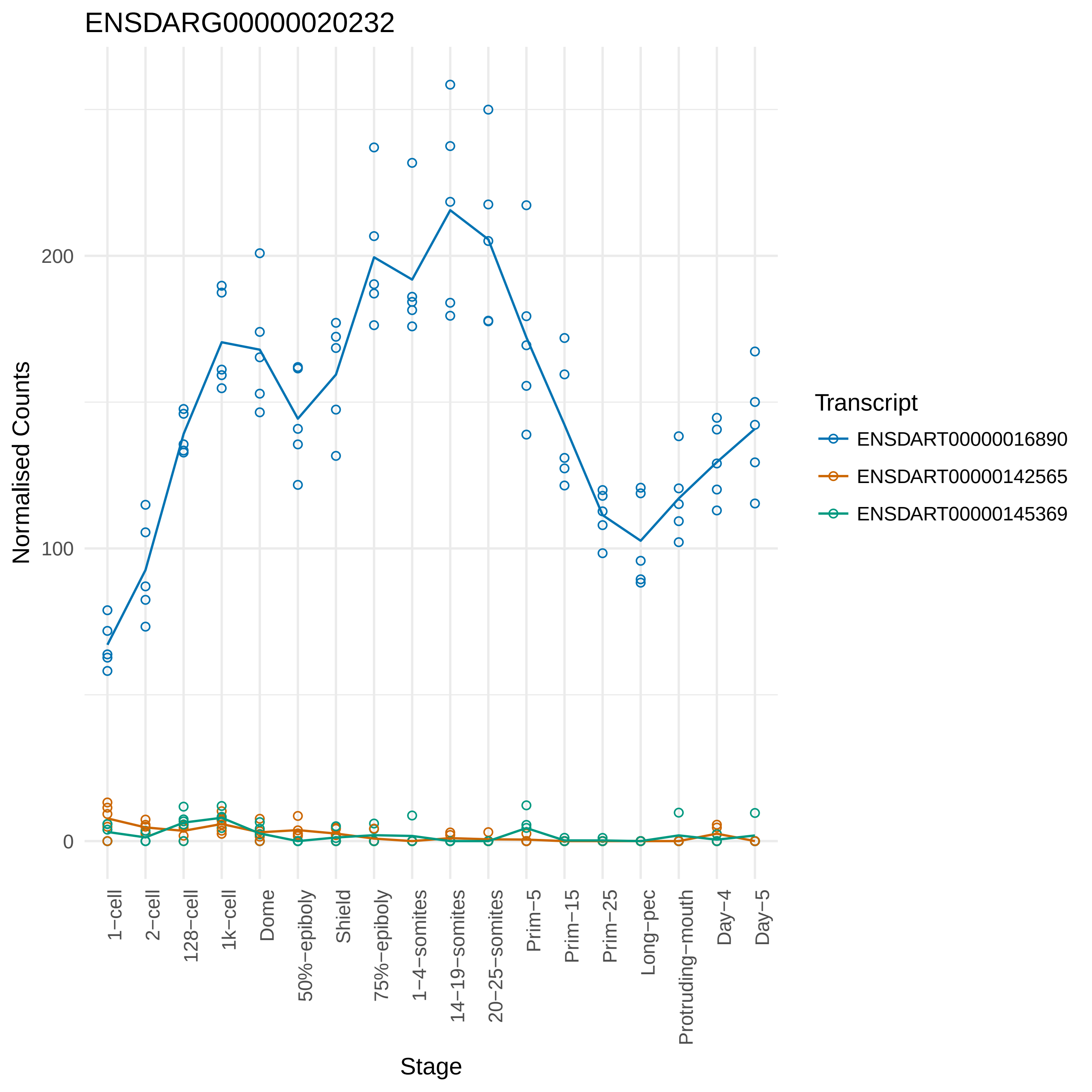

ENSDARG00000020232
Normalised Counts
200
100
0
Stage
1−cell
2−cell
128−cell
1k−cell
Dome
50%−epiboly
Shield
75%−epiboly
1−4−somites
14−19−somites
20−25−somites
Prim−5
Prim−15
Prim−25
Long−pec
Protruding−mouth
Day−4
Day−5
Transcript
ENSDART00000016890
ENSDART00000142565
ENSDART00000145369

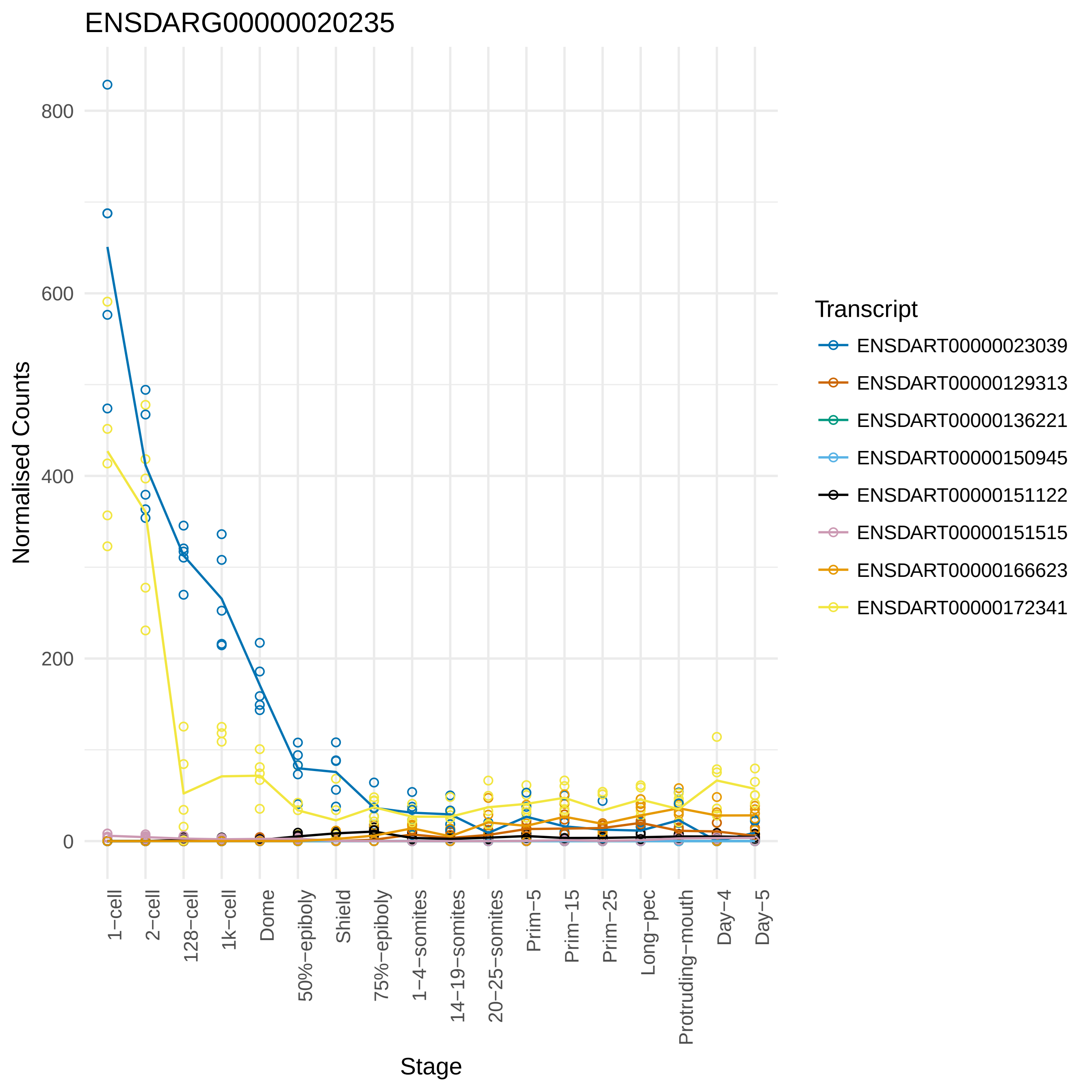
ENSDARG00000020235
Normalised Counts
800
600
400
200
0
1−cell
2−cell
128−cell
1k−cell
Dome
50%−epiboly
Shield
75%−epiboly
1−4−somites
14−19−somites
20−25−somites
Prim−5
Prim−15
Prim−25
Long−pec
Protruding−mouth
Day−4
Day−5
Stage
Transcript
ENSDART00000023039
ENSDART00000129313
ENSDART00000136221
ENSDART00000150945
ENSDART00000151122
ENSDART00000151515
ENSDART00000166623
ENSDART00000172341

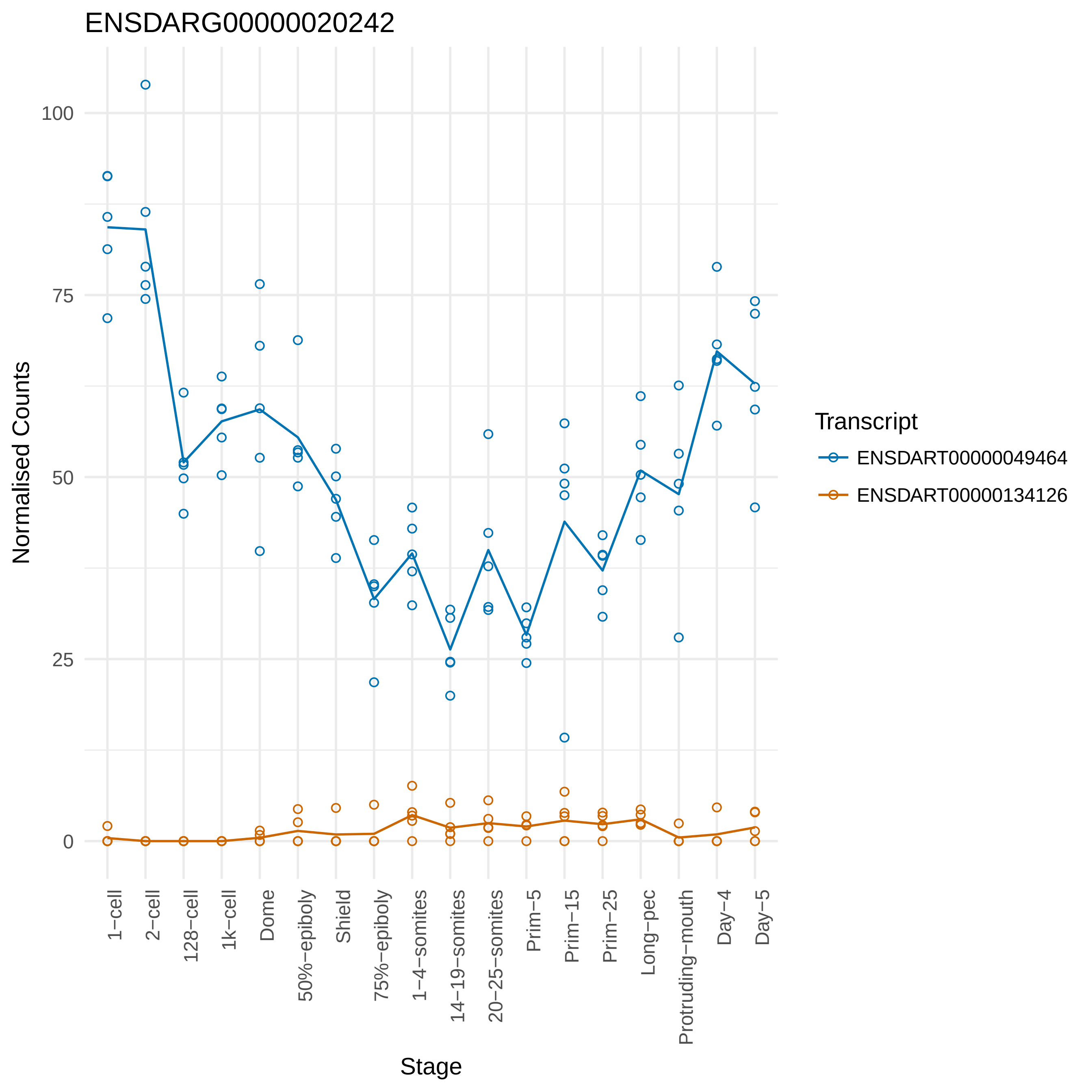

ENSDARG00000020242
Normalised Counts
100
75
50
25
0
1−cell
2−cell
128−cell
1k−cell
Dome
50%−epiboly
Shield
75%−epiboly
1−4−somites
14−19−somites
20−25−somites
Prim−5
Prim−15
Prim−25
Long−pec
Protruding−mouth
Day−4
Day−5
Stage
Transcript
ENSDART00000049464
ENSDART00000134126

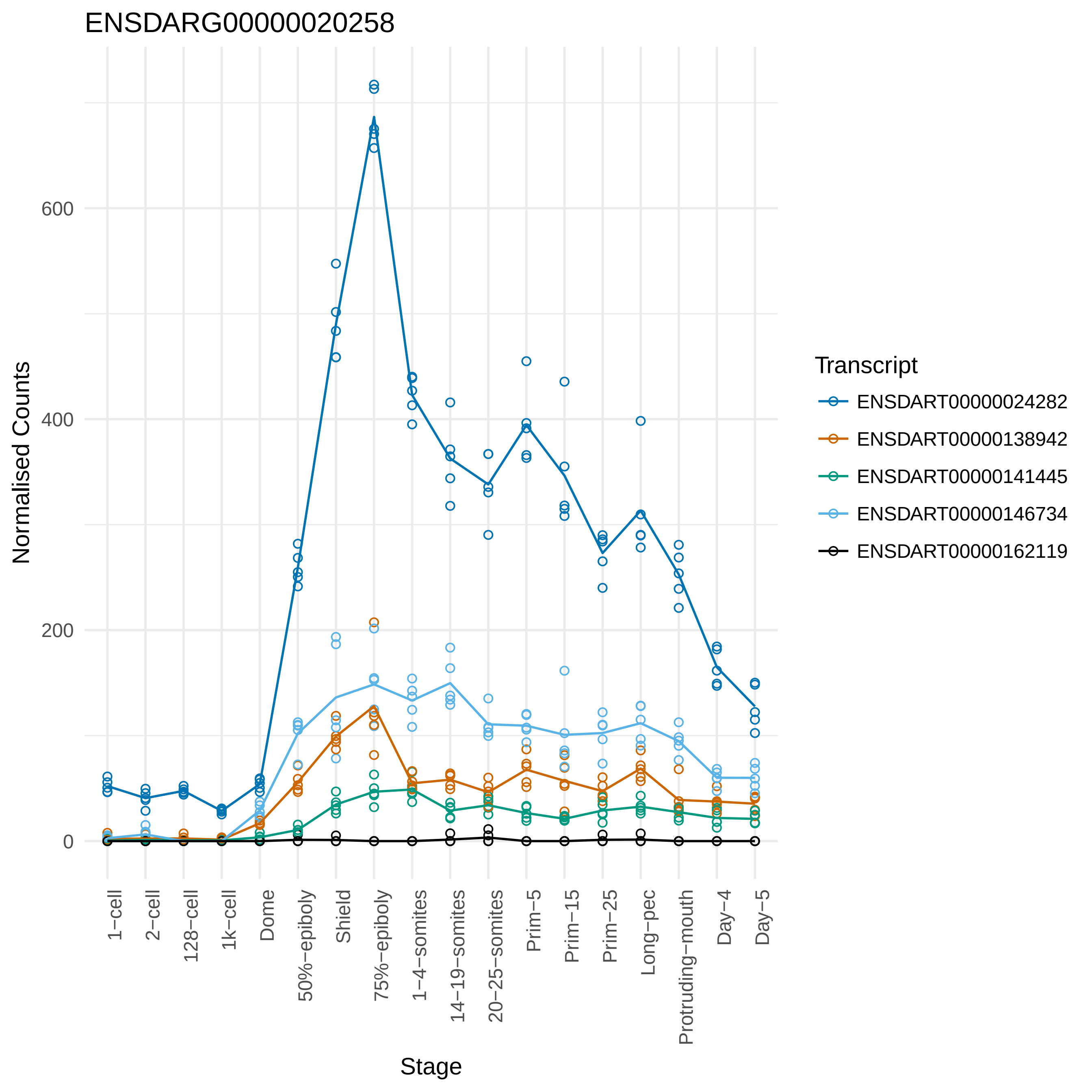

ENSDARG00000020258
Normalised Counts
600
400
200
0
1−cell
2−cell
128−cell
1k−cell
Dome
50%−epiboly
Shield
75%−epiboly
1−4−somites
14−19−somites
20−25−somites
Prim−5
Prim−15
Prim−25
Long−pec
Protruding−mouth
Day−4
Day−5
Stage
Transcript
ENSDART00000024282
ENSDART00000138942
ENSDART00000141445
ENSDART00000146734
ENSDART00000162119

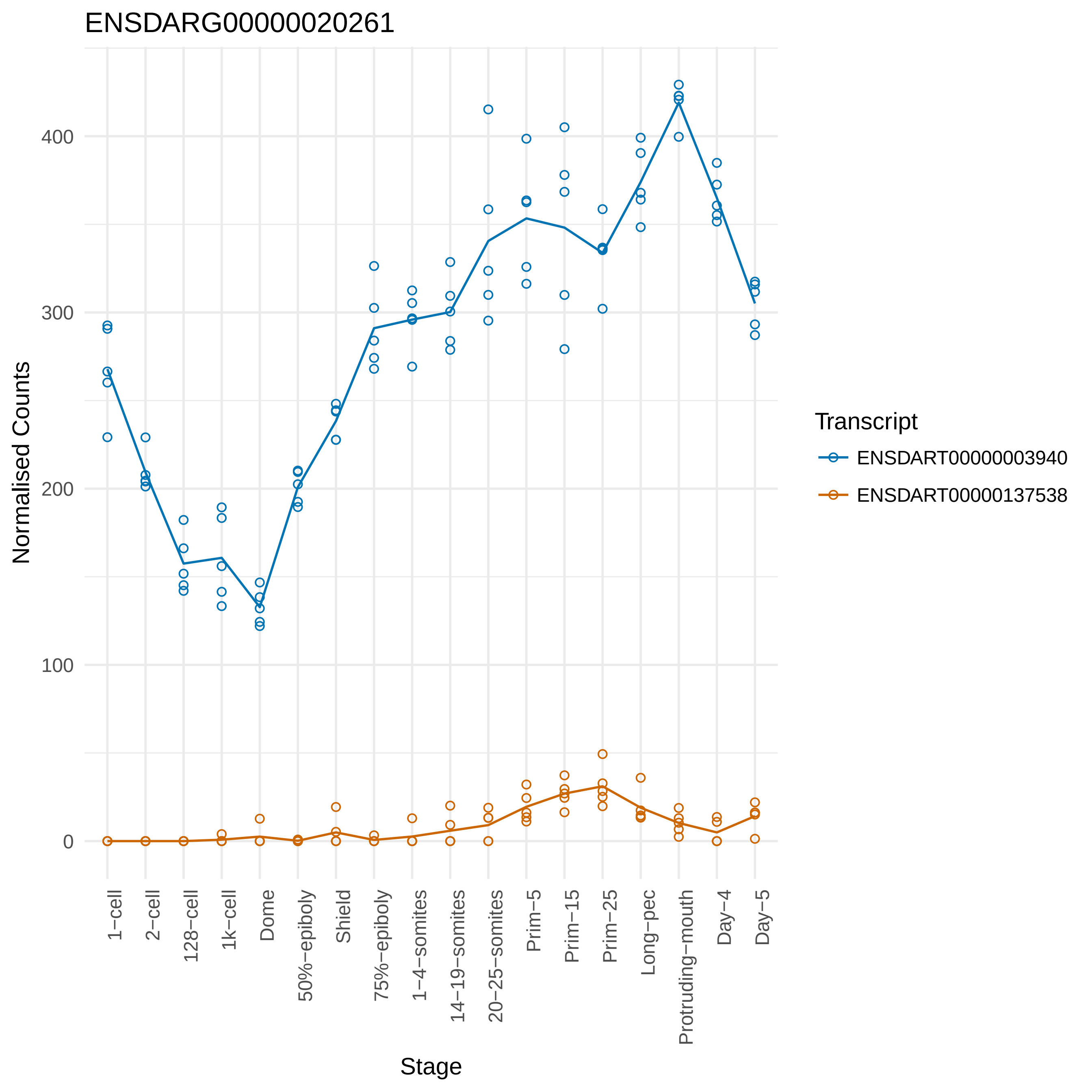
ENSDARG00000020261
Normalised Counts
400
300
200
100
0
1−cell
2−cell
128−cell
1k−cell
Dome
50%−epiboly
Shield
75%−epiboly
1−4−somites
14−19−somites
20−25−somites
Prim−5
Prim−15
Prim−25
Long−pec
Protruding−mouth
Day−4
Day−5
Stage
Transcript
ENSDART00000003940
ENSDART00000137538

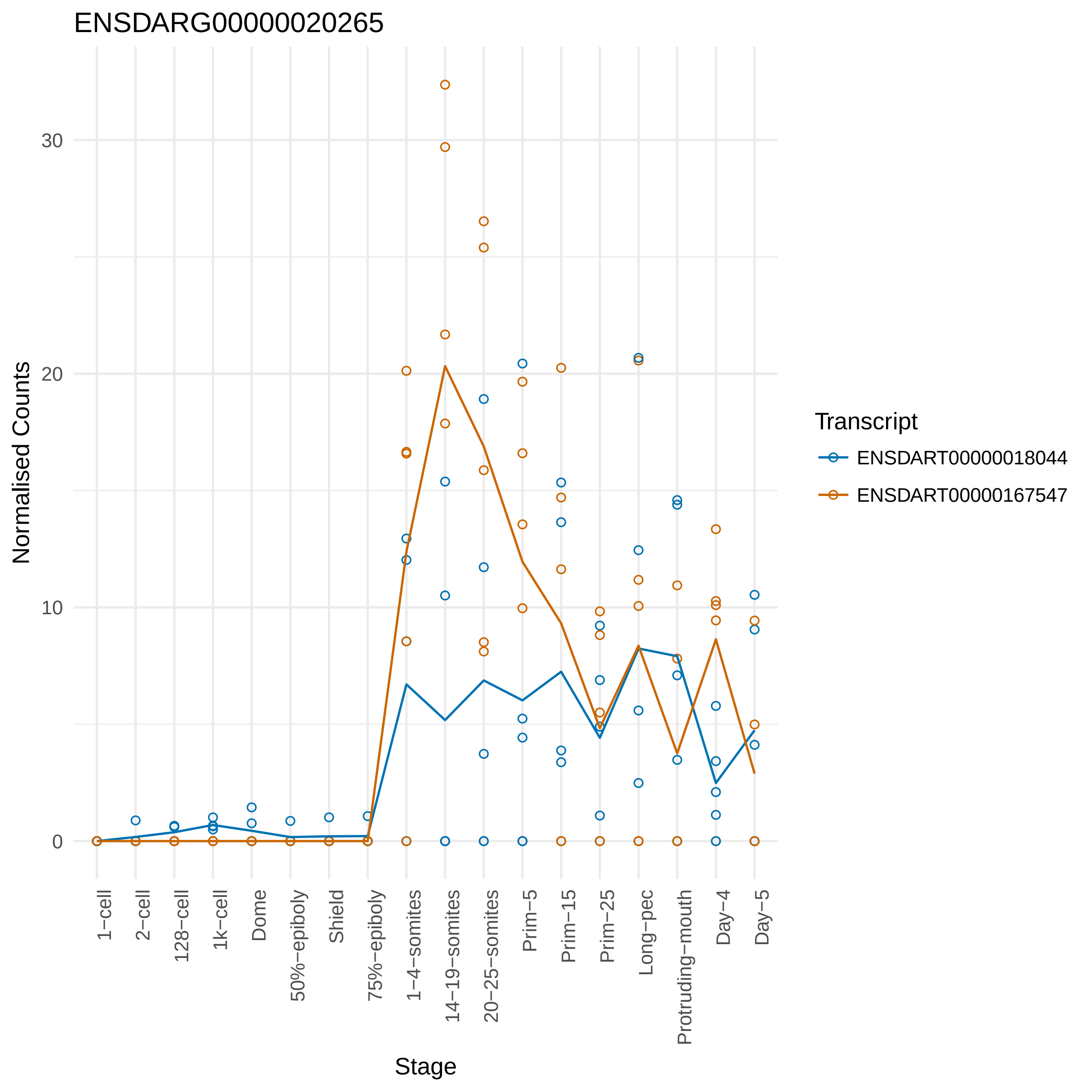

ENSDARG00000020265
Normalised Counts
0
10
20
30
Stage
1−cell
2−cell
128−cell
1k−cell
Dome
50%−epiboly
Shield
75%−epiboly
1−4−somites
14−19−somites
20−25−somites
Prim−5
Prim−15
Prim−25
Long−pec
Protruding−mouth
Day−4
Day−5
Transcript
ENSDART00000018044
ENSDART00000167547

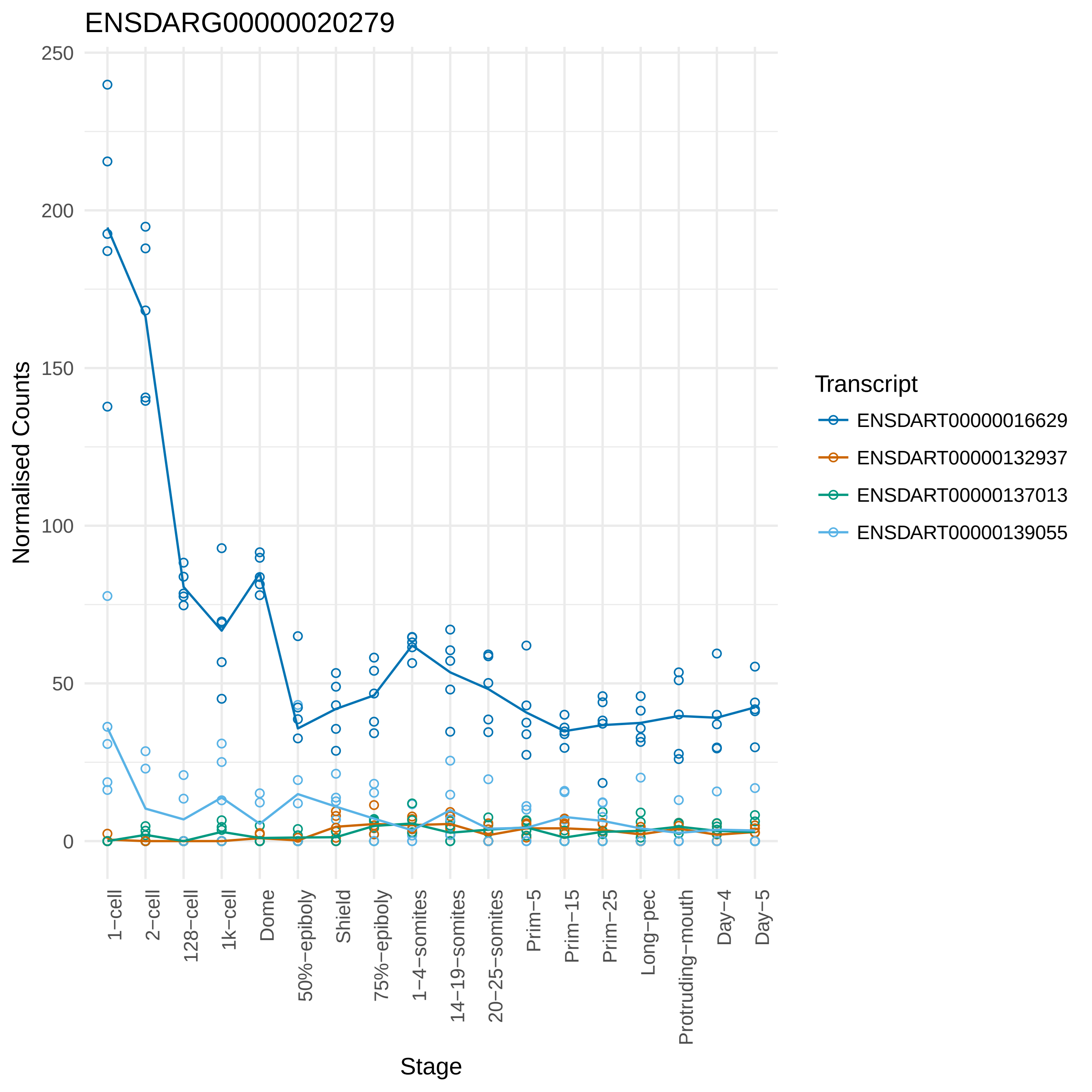

ENSDARG00000020279
Normalised Counts
250
200
150
100
50
0
1−cell
2−cell
128−cell
1k−cell
Dome
50%−epiboly
Shield
75%−epiboly
1−4−somites
14−19−somites
20−25−somites
Prim−5
Prim−15
Prim−25
Long−pec
Protruding−mouth
Day−4
Day−5
Stage
Transcript
ENSDART00000016629
ENSDART00000132937
ENSDART00000137013
ENSDART00000139055

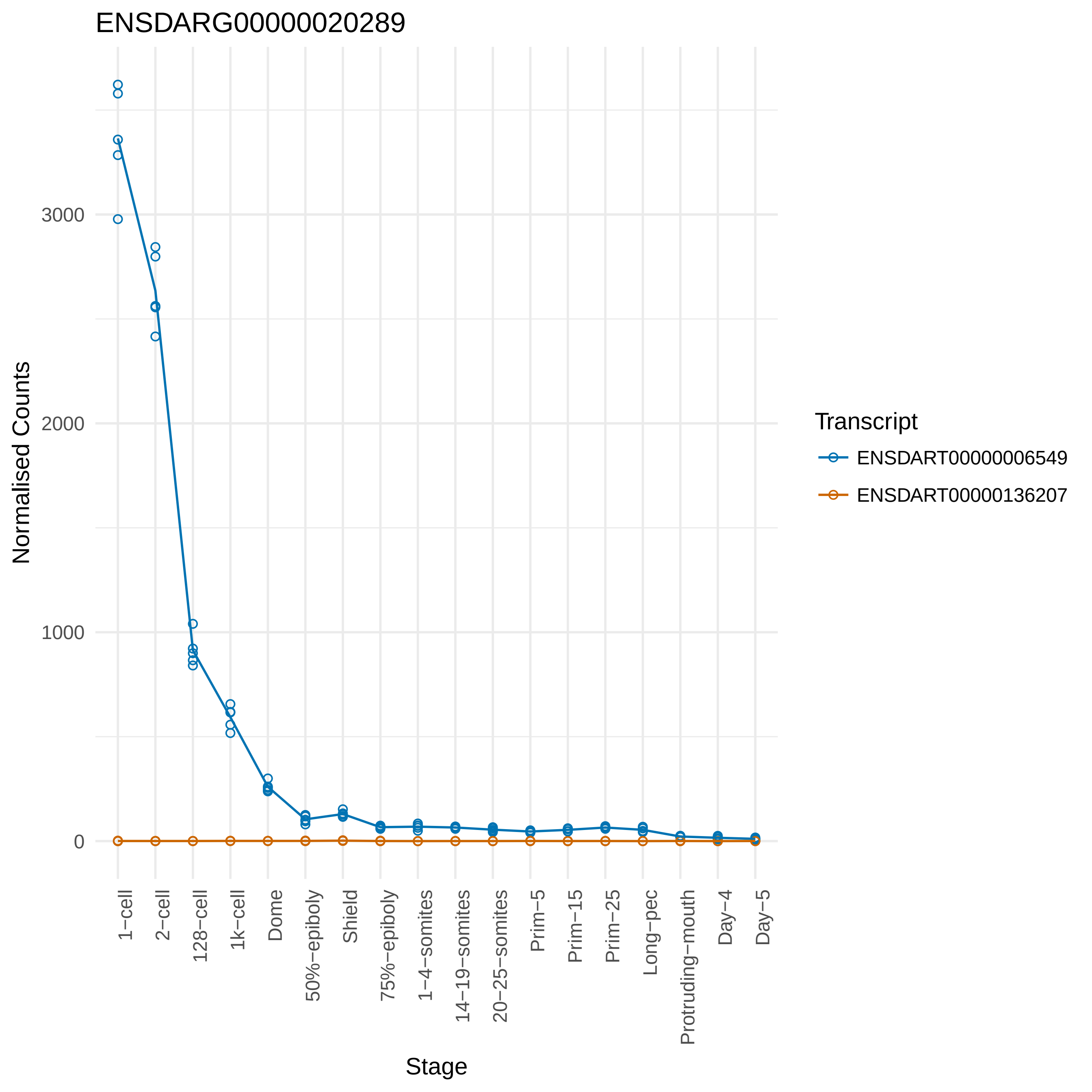
ENSDARG00000020289
Normalised Counts
3000
2000
1000
0
1−cell
2−cell
128−cell
1k−cell
Dome
50%−epiboly
Shield
75%−epiboly
1−4−somites
14−19−somites
20−25−somites
Prim−5
Prim−15
Prim−25
Long−pec
Protruding−mouth
Day−4
Day−5
Stage
Transcript
ENSDART00000006549
ENSDART00000136207

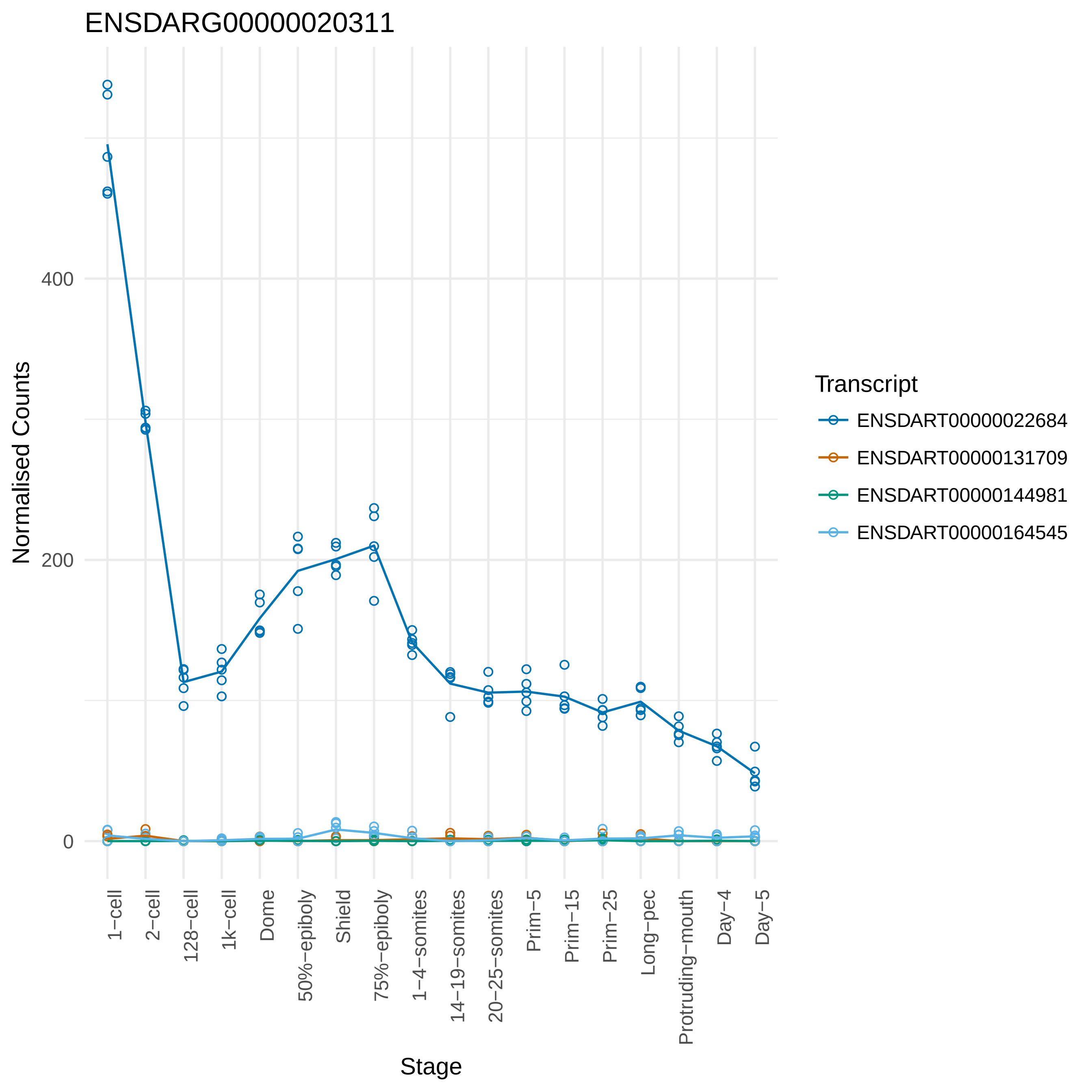
ENSDARG00000020311
Normalised Counts
400
200
0
Stage
1−cell
2−cell
128−cell
1k−cell
Dome
50%−epiboly
Shield
75%−epiboly
1−4−somites
14−19−somites
20−25−somites
Prim−5
Prim−15
Prim−25
Long−pec
Protruding−mouth
Day−4
Day−5
Transcript
ENSDART00000022684
ENSDART00000131709
ENSDART00000144981
ENSDART00000164545

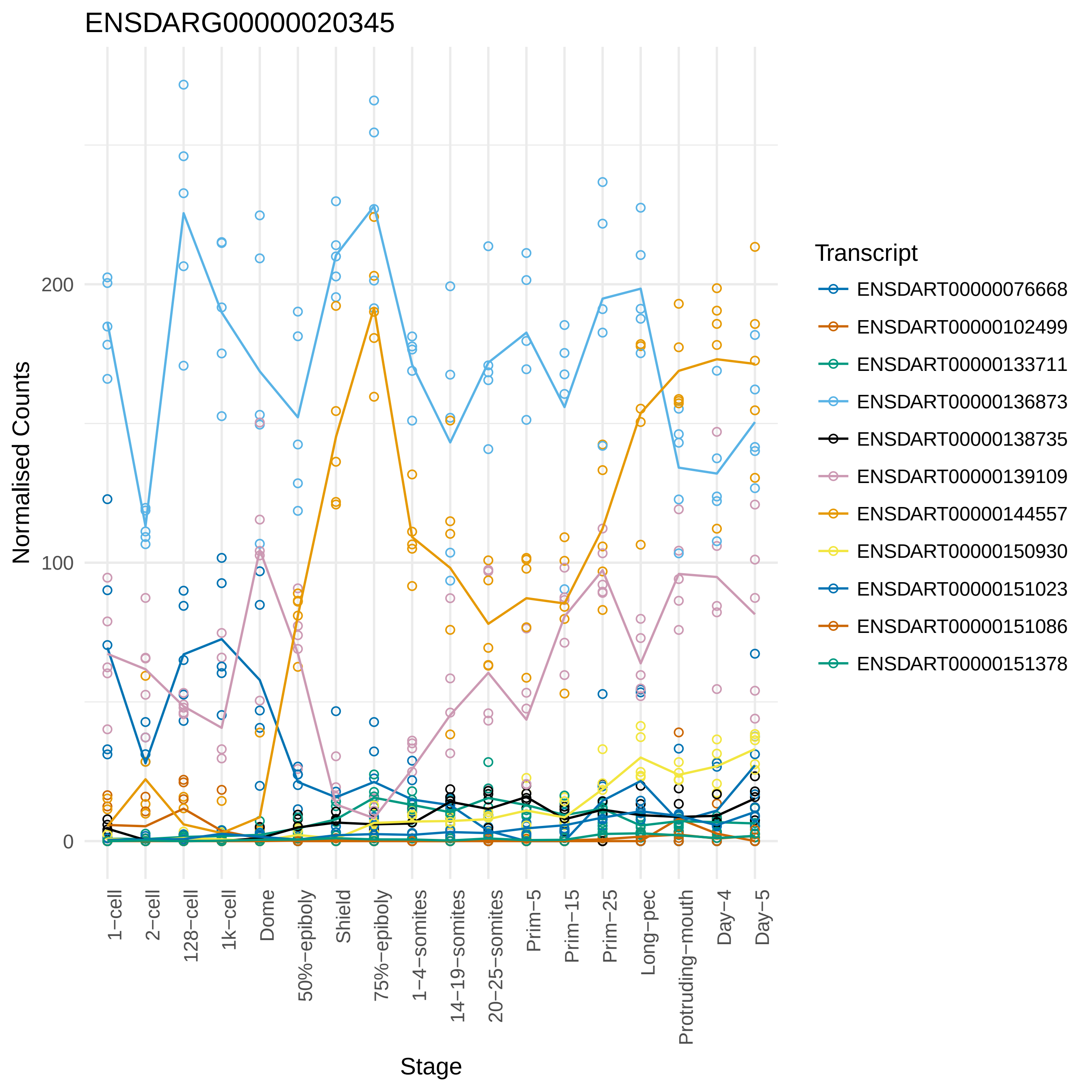

ENSDARG00000020345
Normalised Counts
0
100
200
Stage
1−cell
2−cell
128−cell
1k−cell
Dome
50%−epiboly
Shield
75%−epiboly
1−4−somites
14−19−somites
20−25−somites
Prim−5
Prim−15
Prim−25
Long−pec
Protruding−mouth
Day−4
Day−5
Transcript
ENSDART00000076668
ENSDART00000102499
ENSDART00000133711
ENSDART00000136873
ENSDART00000138735
ENSDART00000139109
ENSDART00000144557
ENSDART00000150930
ENSDART00000151023
ENSDART00000151086
ENSDART00000151378

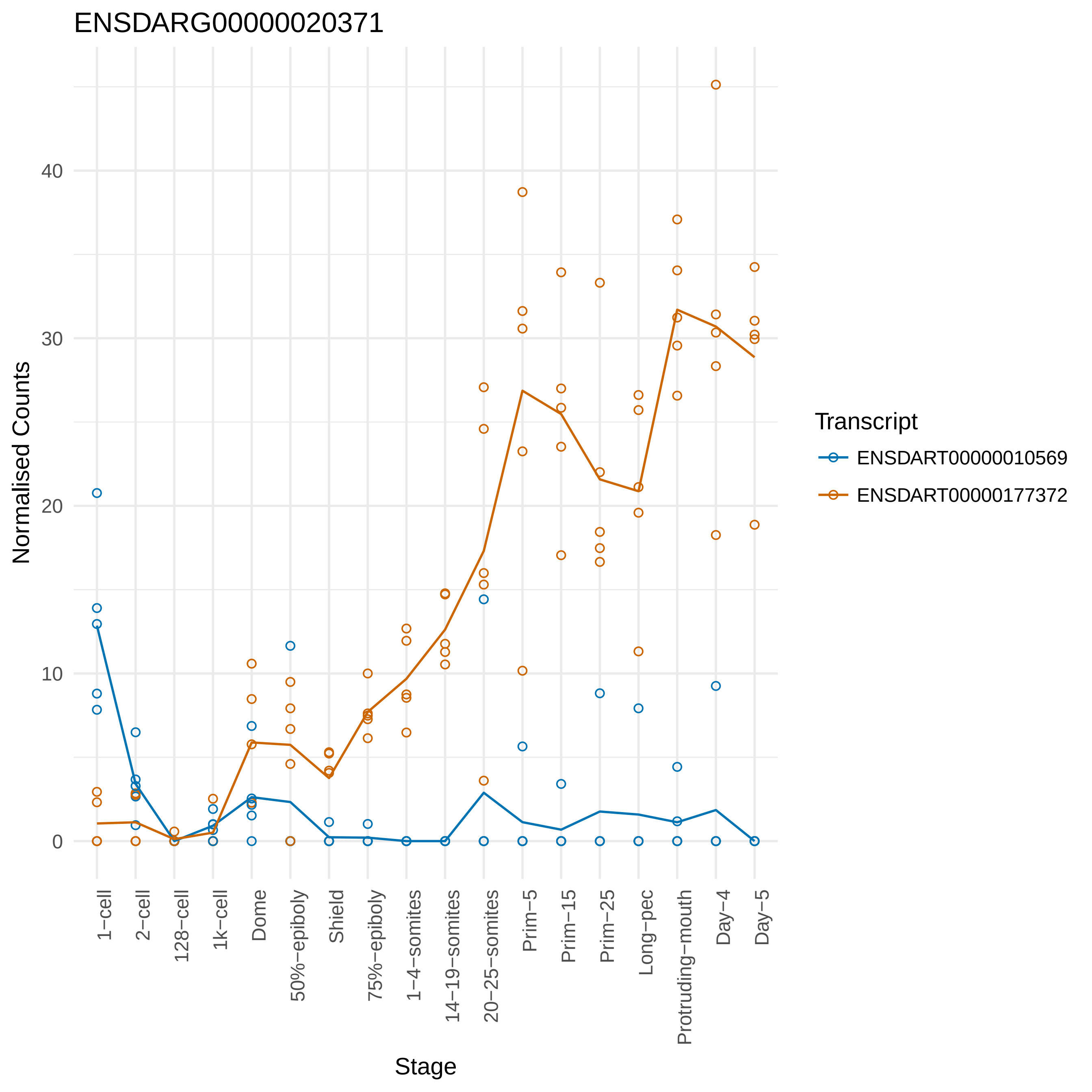

ENSDARG00000020371
Normalised Counts
0
10
20
30
40
1−cell
2−cell
128−cell
1k−cell
Dome
50%−epiboly
Shield
75%−epiboly
1−4−somites
14−19−somites
20−25−somites
Prim−5
Prim−15
Prim−25
Long−pec
Protruding−mouth
Day−4
Day−5
Stage
Transcript
ENSDART00000010569
ENSDART00000177372

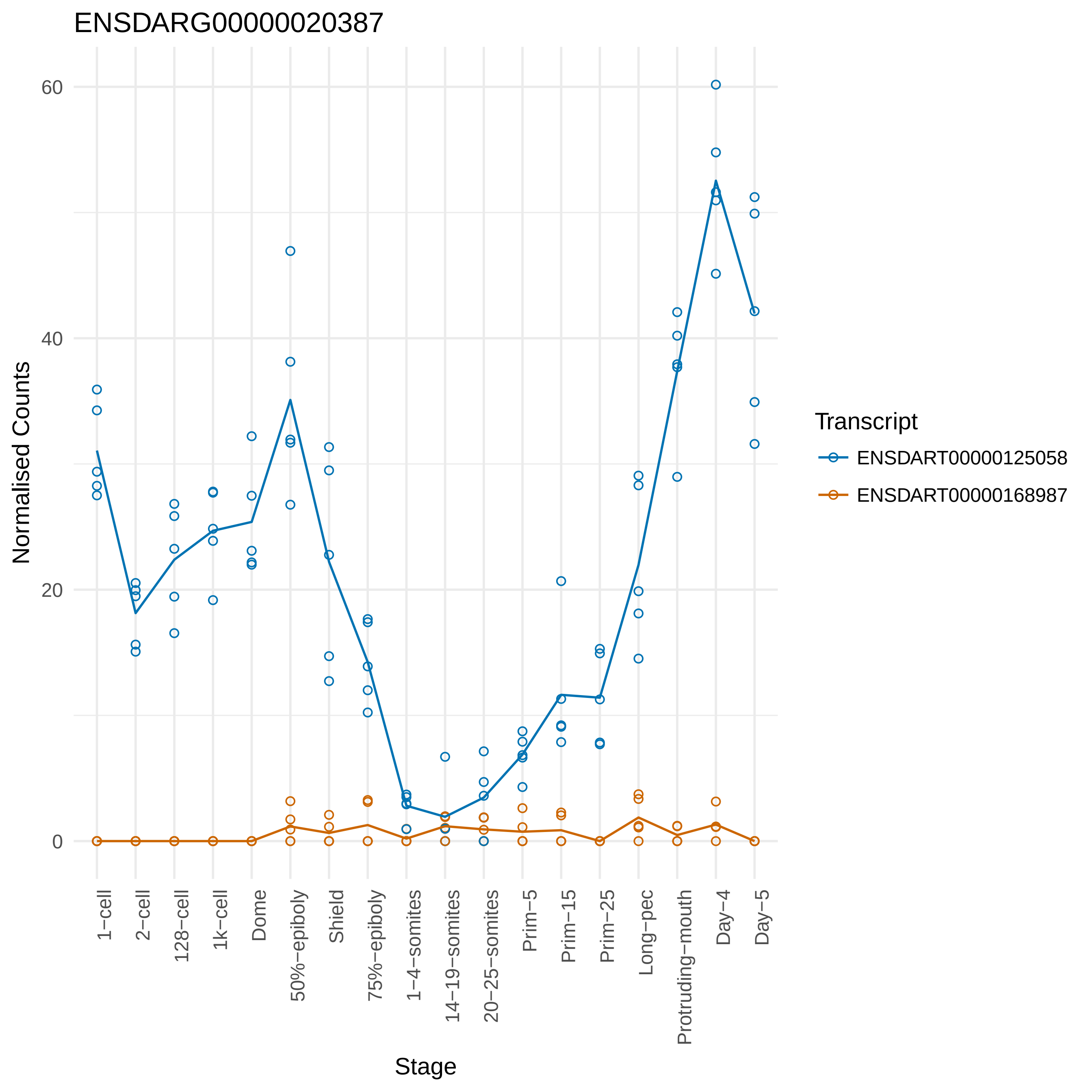
ENSDARG00000020387
Normalised Counts
0
20
40
60
Stage
1−cell
2−cell
128−cell
1k−cell
Dome
50%−epiboly
Shield
75%−epiboly
1−4−somites
14−19−somites
20−25−somites
Prim−5
Prim−15
Prim−25
Long−pec
Protruding−mouth
Day−4
Day−5
Transcript
ENSDART00000125058
ENSDART00000168987

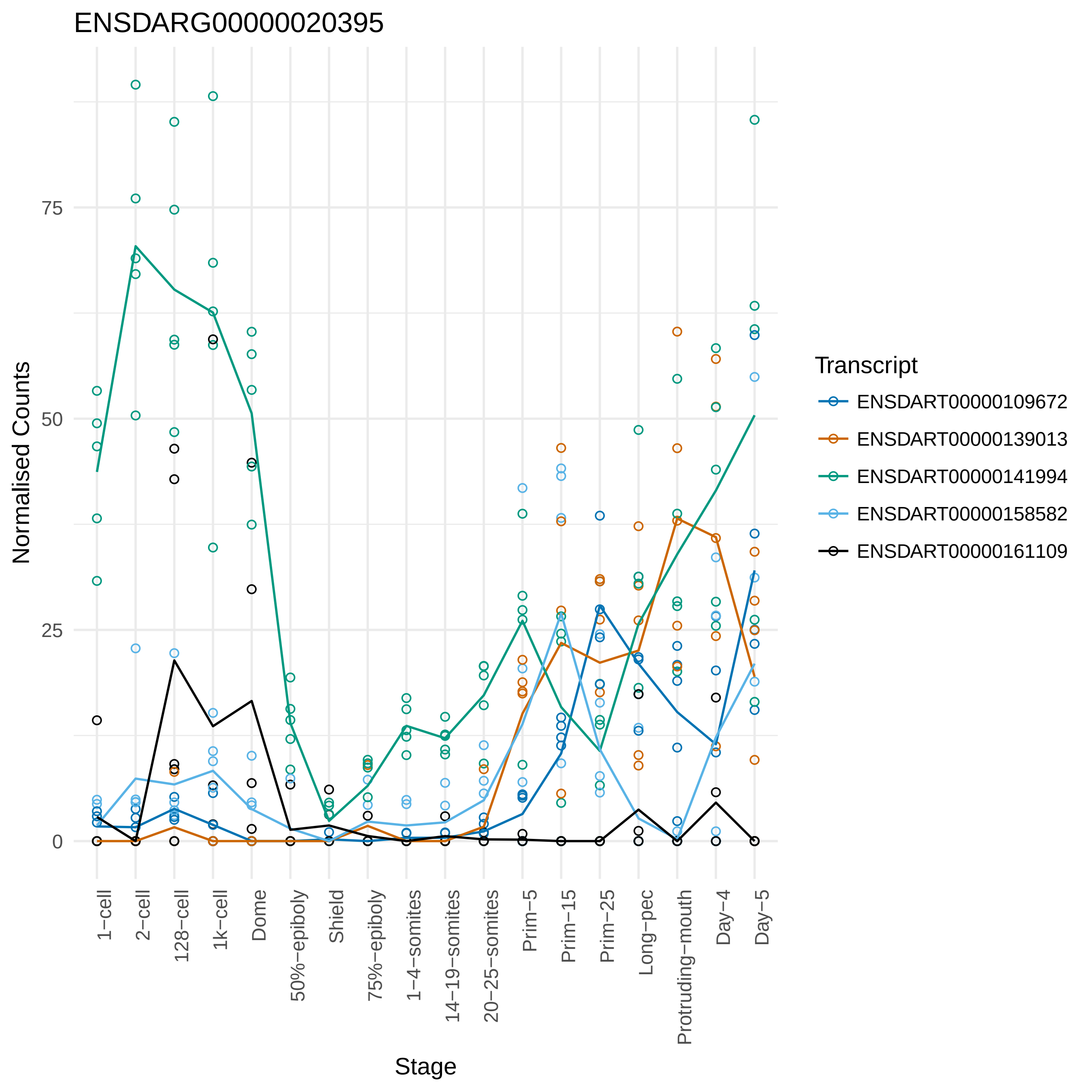

ENSDARG00000020395
Normalised Counts
0
25
50
75
1−cell
2−cell
128−cell
1k−cell
Dome
50%−epiboly
Shield
75%−epiboly
1−4−somites
14−19−somites
20−25−somites
Prim−5
Prim−15
Prim−25
Long−pec
Protruding−mouth
Day−4
Day−5
Stage
Transcript
ENSDART00000109672
ENSDART00000139013
ENSDART00000141994
ENSDART00000158582
ENSDART00000161109

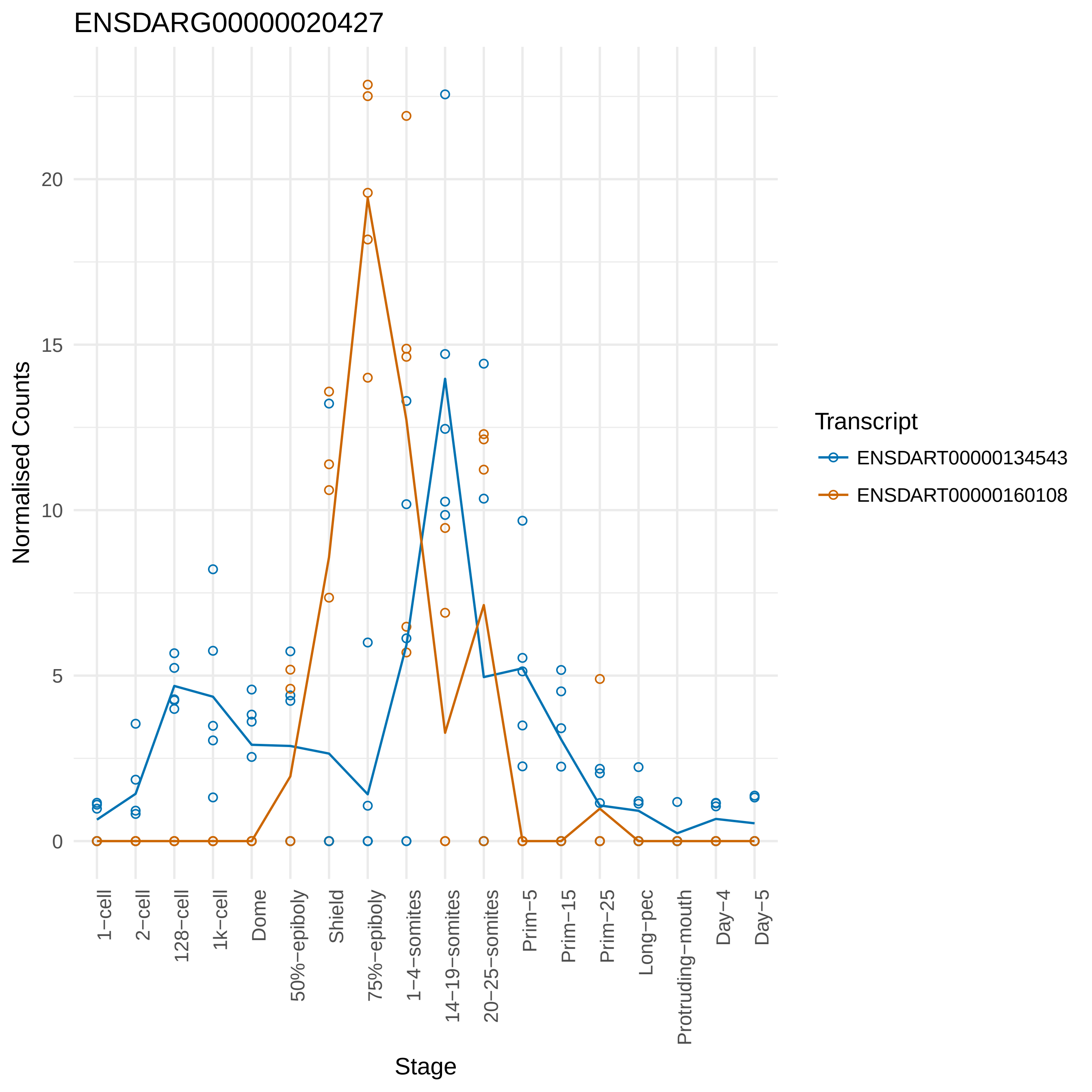

ENSDARG00000020427
Normalised Counts
0
5
10
15
20
Stage
1−cell
2−cell
128−cell
1k−cell
Dome
50%−epiboly
Shield
75%−epiboly
1−4−somites
14−19−somites
20−25−somites
Prim−5
Prim−15
Prim−25
Long−pec
Protruding−mouth
Day−4
Day−5
Transcript
ENSDART00000134543
ENSDART00000160108

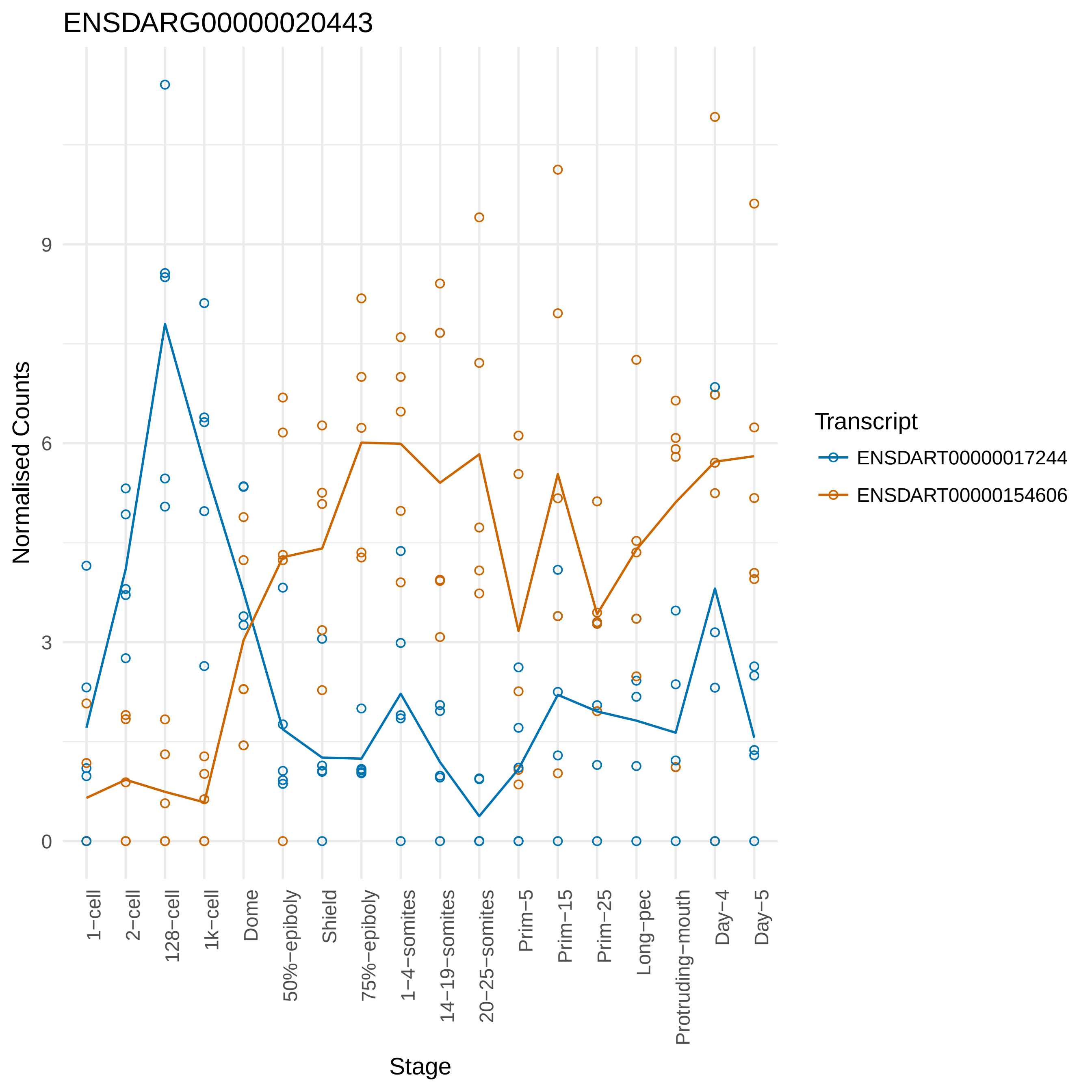
ENSDARG00000020443
Normalised Counts
0
3
6
9
Stage
1−cell
2−cell
128−cell
1k−cell
Dome
50%−epiboly
Shield
75%−epiboly
1−4−somites
14−19−somites
20−25−somites
Prim−5
Prim−15
Prim−25
Long−pec
Protruding−mouth
Day−4
Day−5
Transcript
ENSDART00000017244
ENSDART00000154606

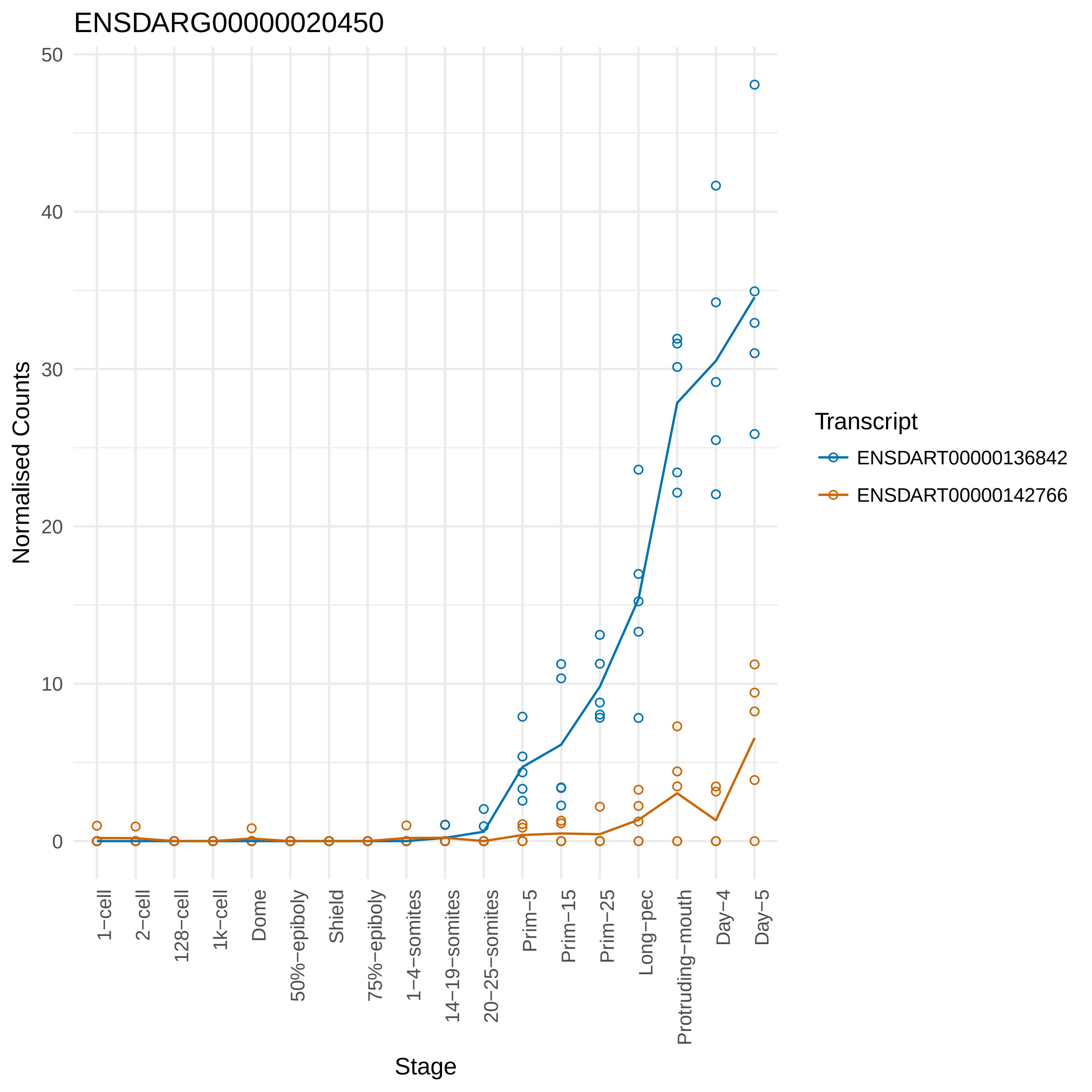

ENSDARG00000020450
Normalised Counts
0
10
20
30
40
50
1−cell
2−cell
128−cell
1k−cell
Dome
50%−epiboly
Shield
75%−epiboly
1−4−somites
14−19−somites
20−25−somites
Prim−5
Prim−15
Prim−25
Long−pec
Protruding−mouth
Day−4
Day−5
Stage
Transcript
ENSDART00000136842
ENSDART00000142766

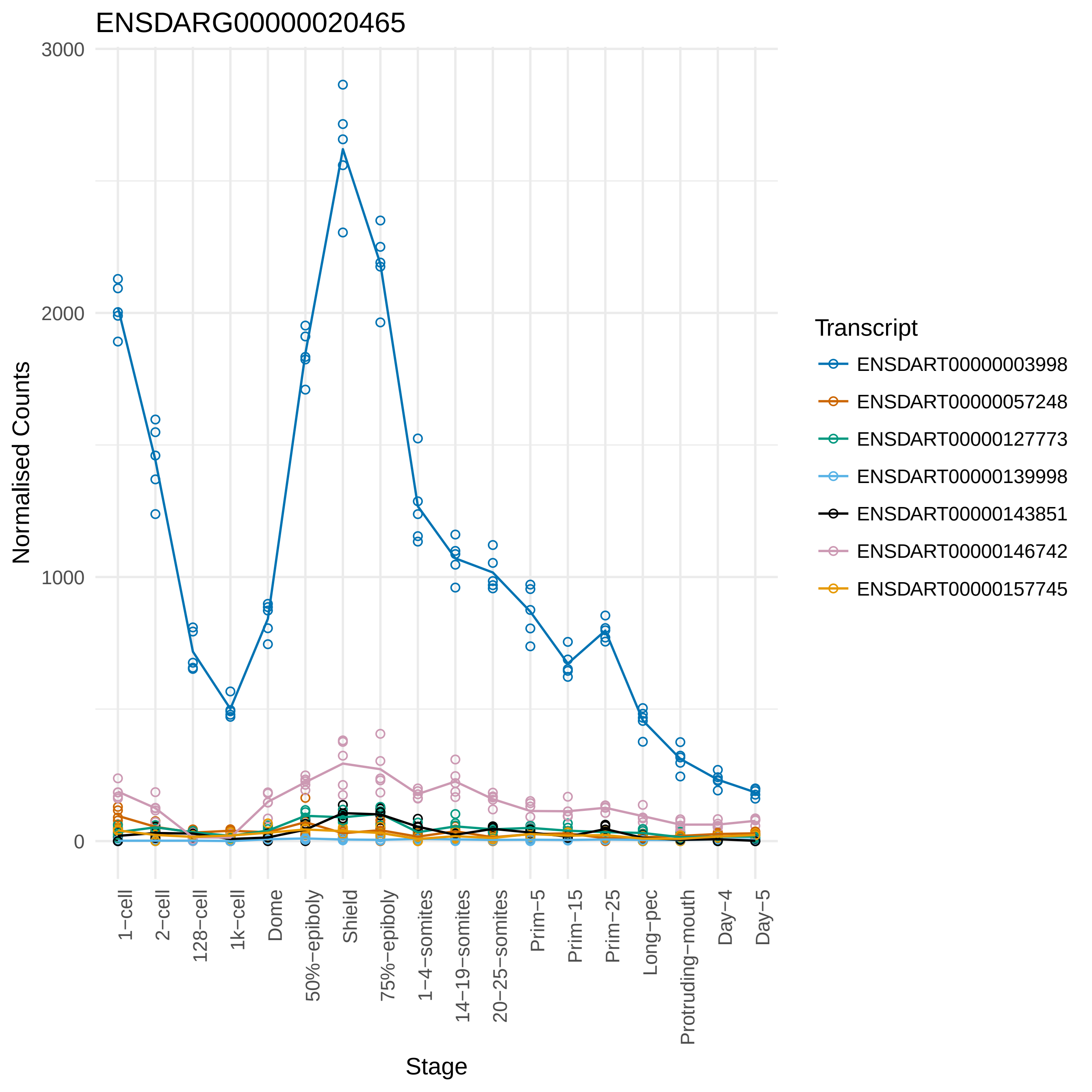
ENSDARG00000020465
Normalised Counts
3000
2000
1000
0
1−cell
2−cell
128−cell
1k−cell
Dome
50%−epiboly
Shield
75%−epiboly
1−4−somites
14−19−somites
20−25−somites
Prim−5
Prim−15
Prim−25
Long−pec
Protruding−mouth
Day−4
Day−5
Stage
Transcript
ENSDART00000003998
ENSDART00000057248
ENSDART00000127773
ENSDART00000139998
ENSDART00000143851
ENSDART00000146742
ENSDART00000157745

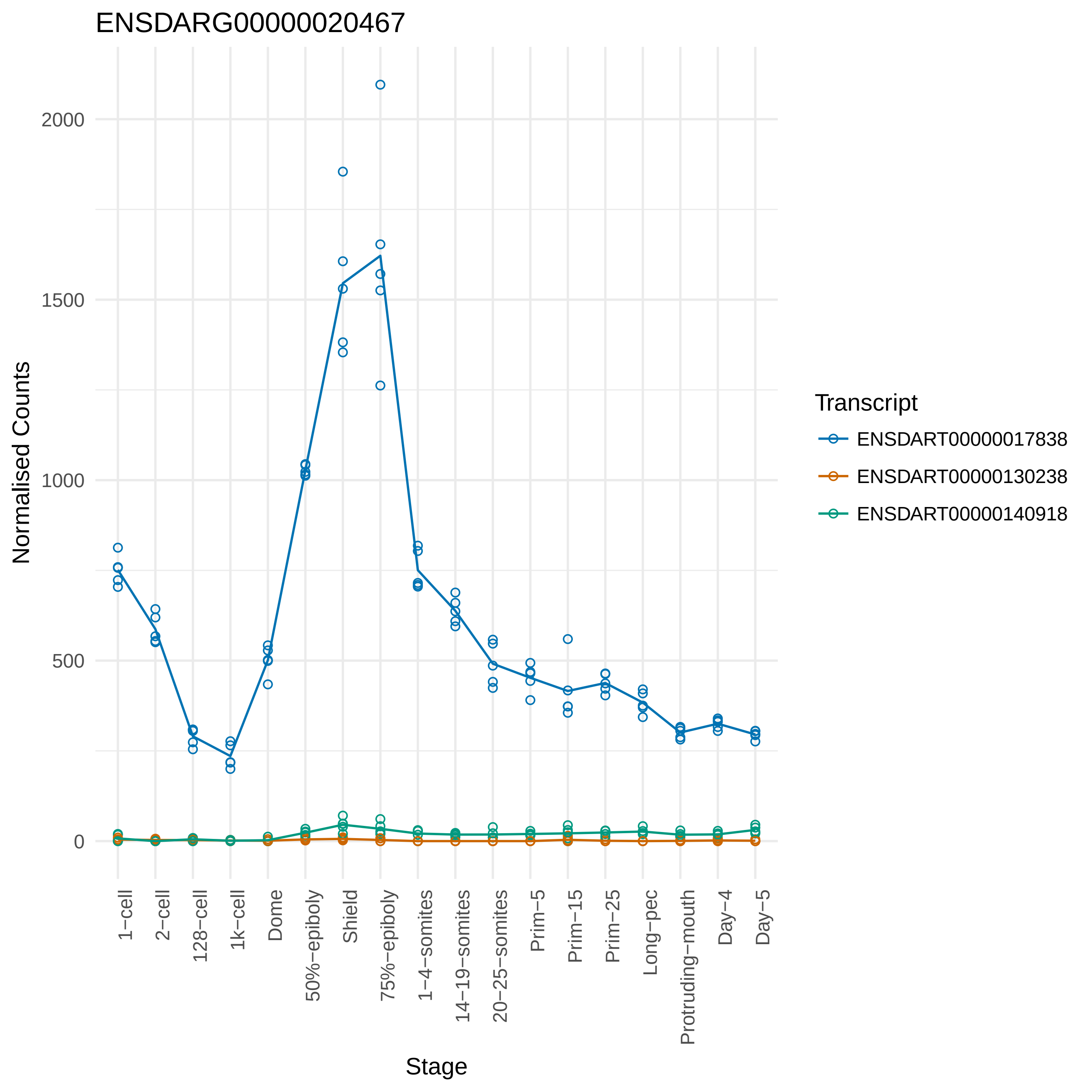

ENSDARG00000020467
Normalised Counts
2000
1500
1000
500
0
1−cell
2−cell
128−cell
1k−cell
Dome
50%−epiboly
Shield
75%−epiboly
1−4−somites
14−19−somites
20−25−somites
Prim−5
Prim−15
Prim−25
Long−pec
Protruding−mouth
Day−4
Day−5
Stage
Transcript
ENSDART00000017838
ENSDART00000130238
ENSDART00000140918

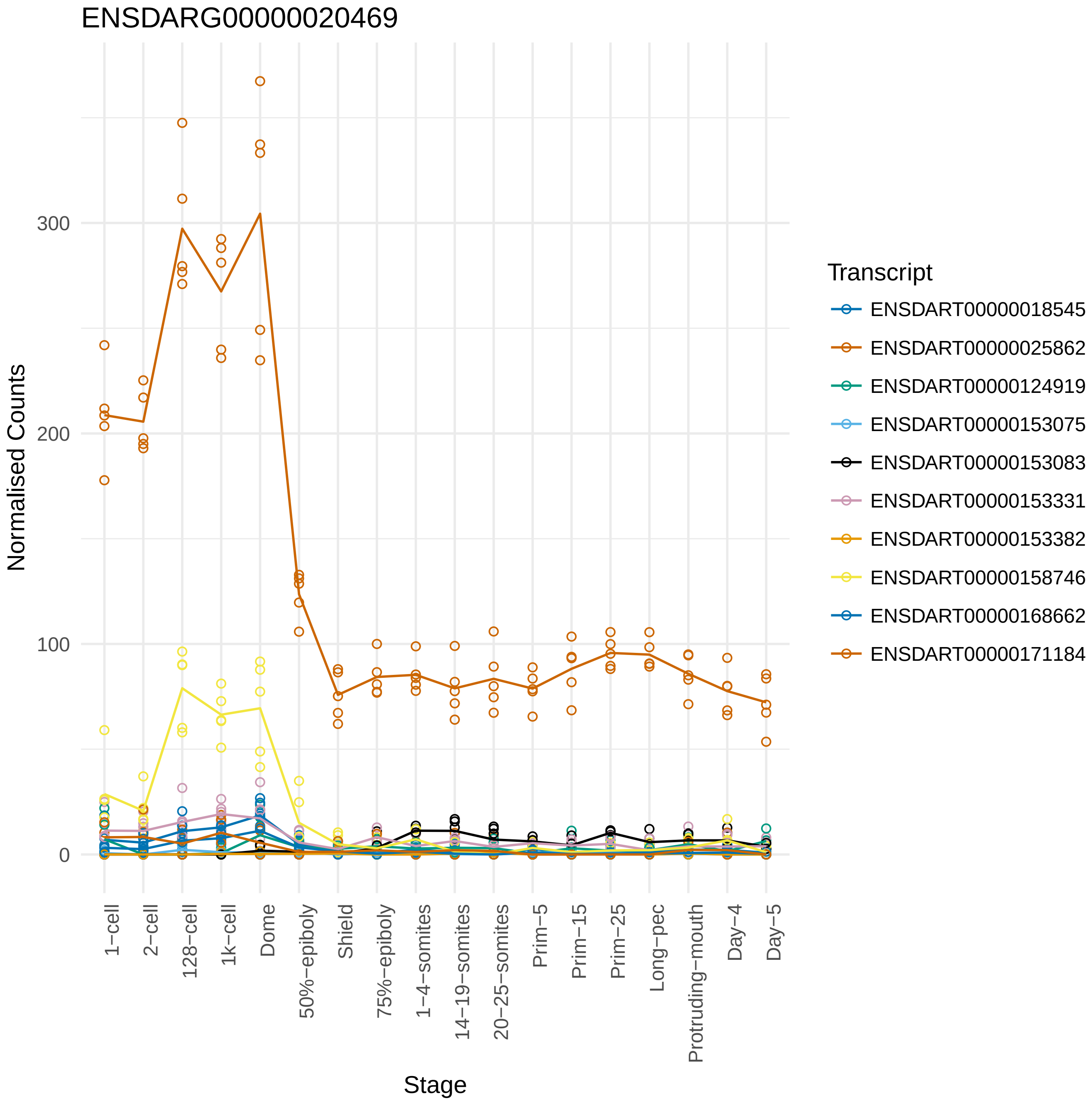

ENSDARG00000020469
Normalised Counts
300
200
100
0
1−cell
2−cell
128−cell
1k−cell
Dome
50%−epiboly
Shield
75%−epiboly
1−4−somites
14−19−somites
20−25−somites
Prim−5
Prim−15
Prim−25
Long−pec
Protruding−mouth
Day−4
Day−5
Stage
Transcript
ENSDART00000018545
ENSDART00000025862
ENSDART00000124919
ENSDART00000153075
ENSDART00000153083
ENSDART00000153331
ENSDART00000153382
ENSDART00000158746
ENSDART00000168662
ENSDART00000171184

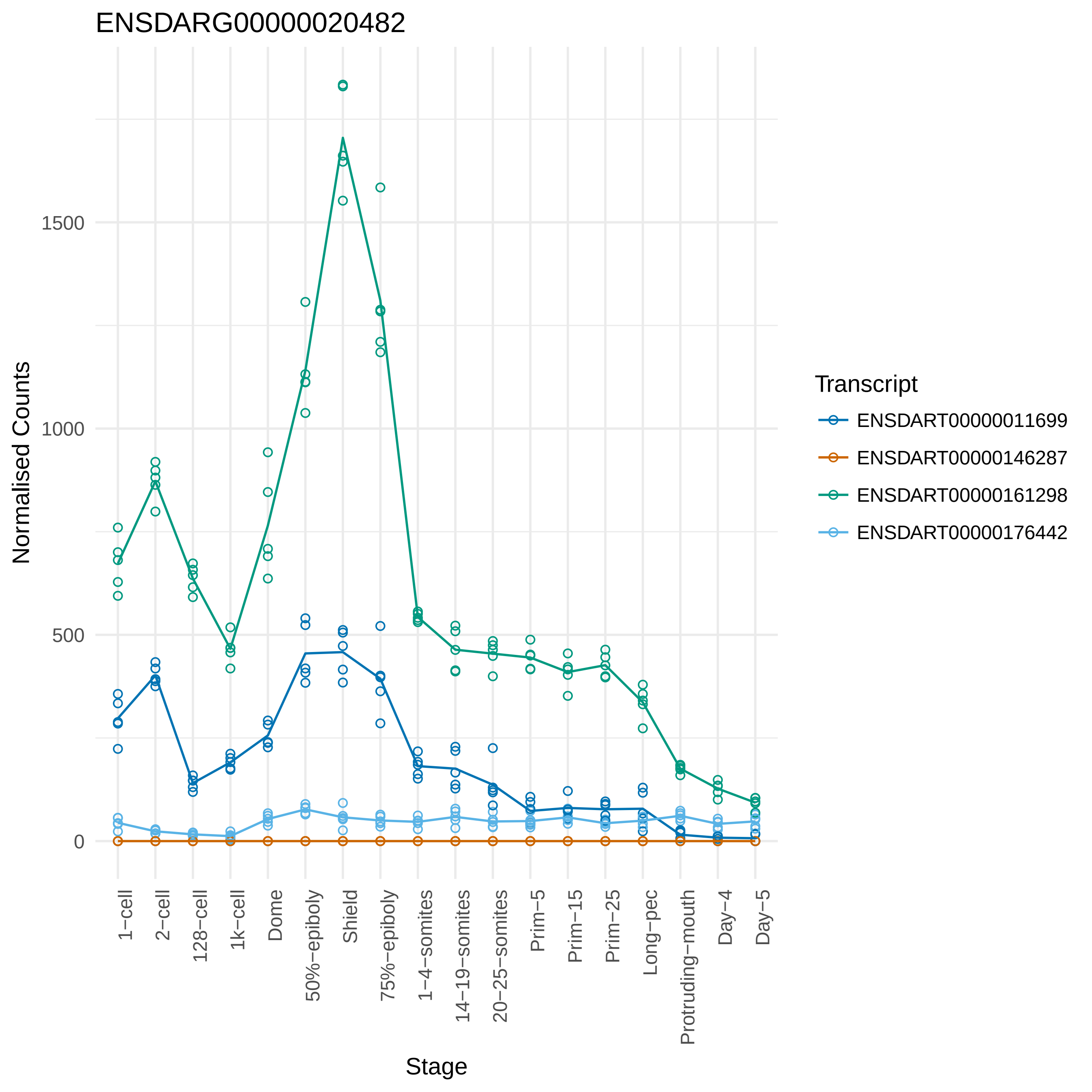
ENSDARG00000020482
Normalised Counts
0
500
1000
1500
Stage
1−cell
2−cell
128−cell
1k−cell
Dome
50%−epiboly
Shield
75%−epiboly
1−4−somites
14−19−somites
20−25−somites
Prim−5
Prim−15
Prim−25
Long−pec
Protruding−mouth
Day−4
Day−5
Transcript
ENSDART00000011699
ENSDART00000146287
ENSDART00000161298
ENSDART00000176442

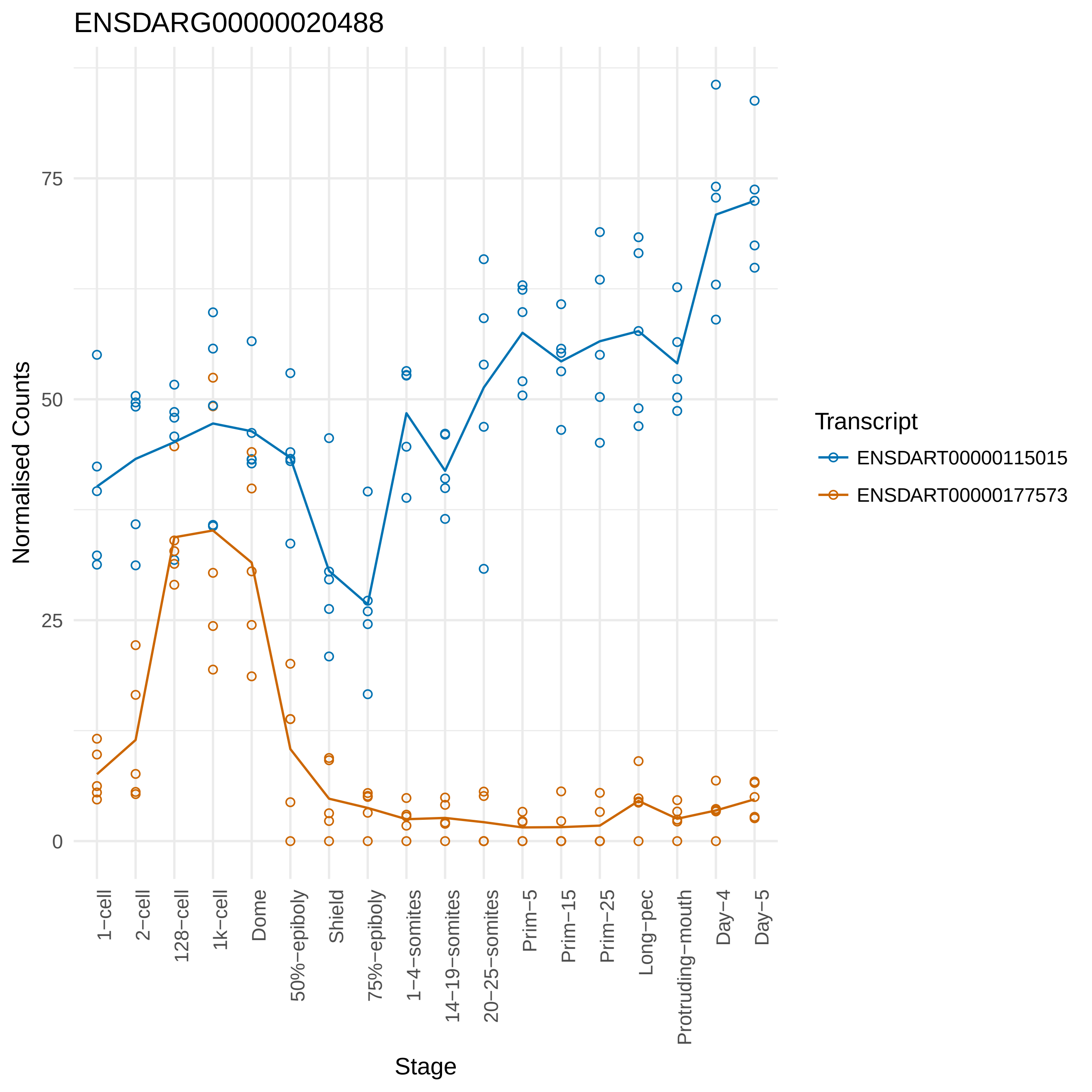

ENSDARG00000020488
Normalised Counts
0
25
50
75
Stage
1−cell
2−cell
128−cell
1k−cell
Dome
50%−epiboly
Shield
75%−epiboly
1−4−somites
14−19−somites
20−25−somites
Prim−5
Prim−15
Prim−25
Long−pec
Protruding−mouth
Day−4
Day−5
Transcript
ENSDART00000115015
ENSDART00000177573

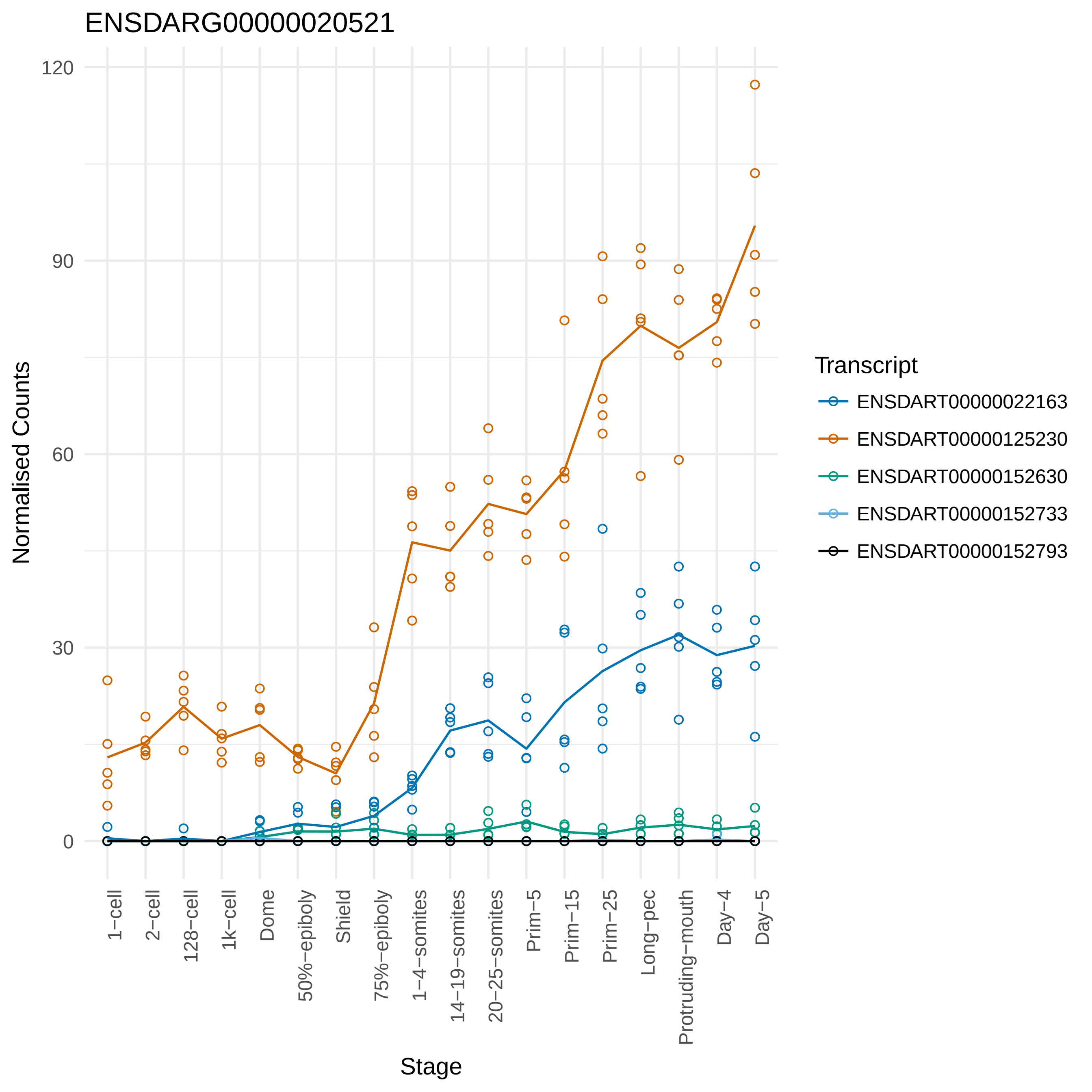
ENSDARG00000020521
Normalised Counts
120
90
60
30
0
Stage
1−cell
2−cell
128−cell
1k−cell
Dome
50%−epiboly
Shield
75%−epiboly
1−4−somites
14−19−somites
20−25−somites
Prim−5
Prim−15
Prim−25
Long−pec
Protruding−mouth
Day−4
Day−5
Transcript
ENSDART00000022163
ENSDART00000125230
ENSDART00000152630
ENSDART00000152733
ENSDART00000152793

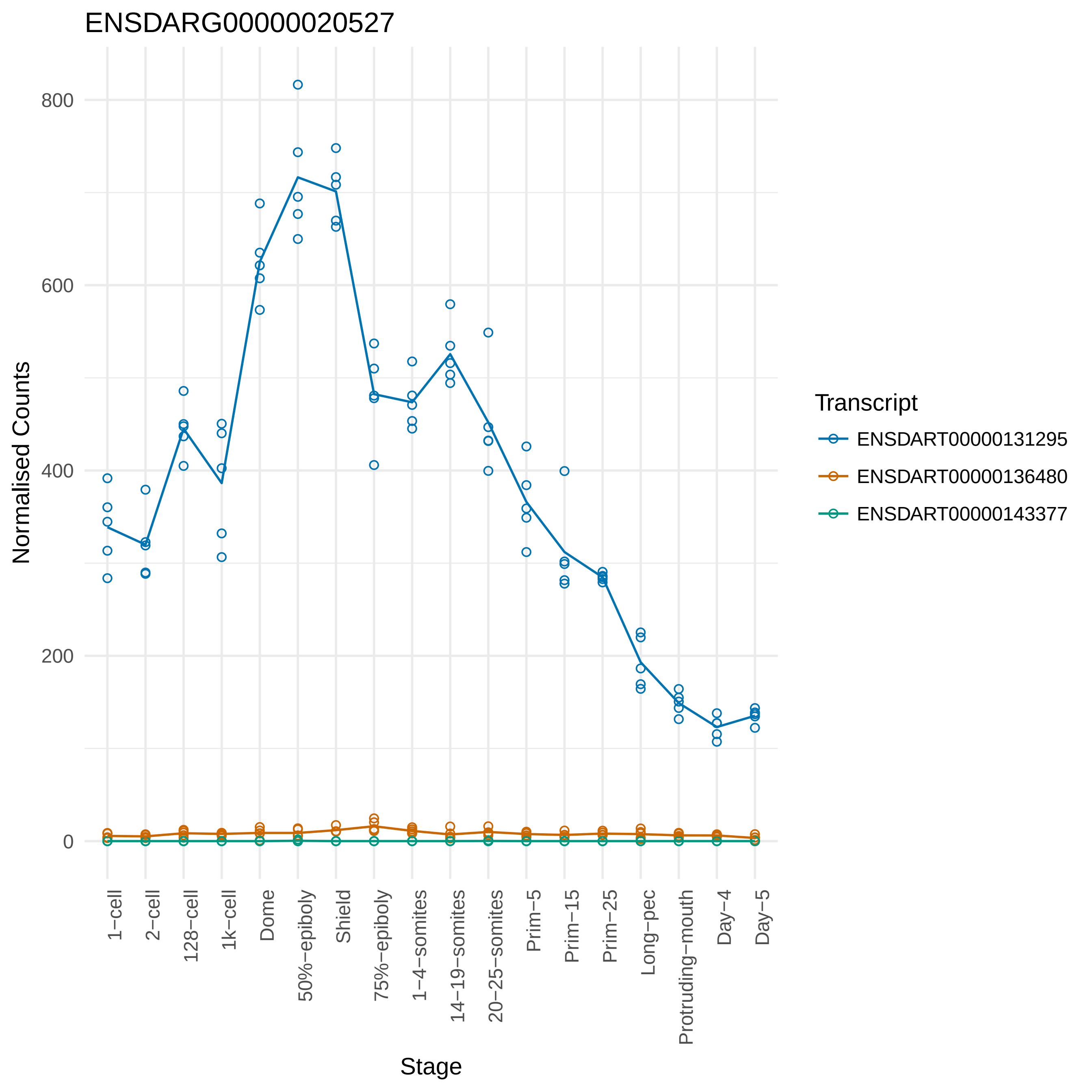

ENSDARG00000020527
Normalised Counts
800
600
400
200
0
1−cell
2−cell
128−cell
1k−cell
Dome
50%−epiboly
Shield
75%−epiboly
1−4−somites
14−19−somites
20−25−somites
Prim−5
Prim−15
Prim−25
Long−pec
Protruding−mouth
Day−4
Day−5
Stage
Transcript
ENSDART00000131295
ENSDART00000136480
ENSDART00000143377

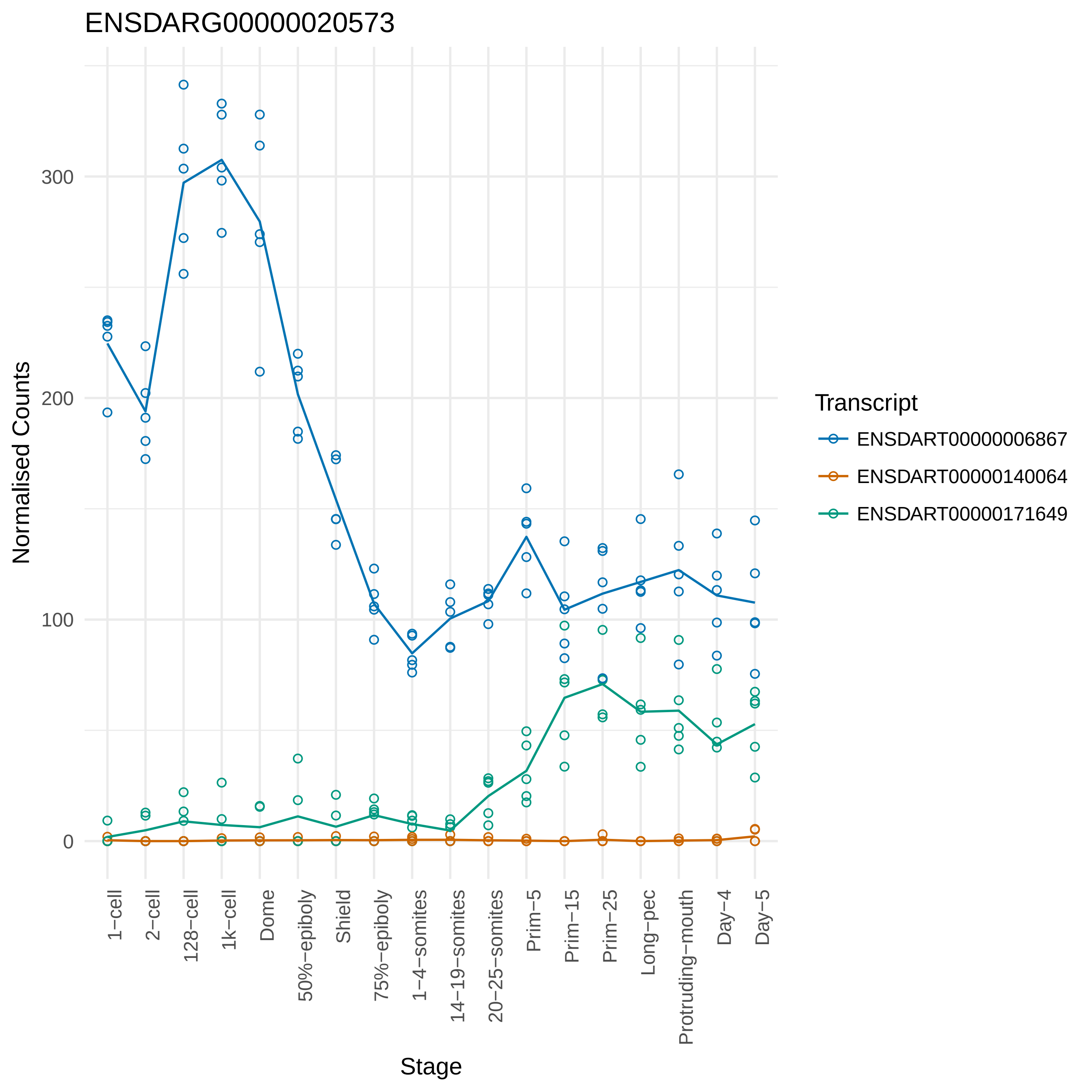

ENSDARG00000020573
Normalised Counts
300
200
100
0
1−cell
2−cell
128−cell
1k−cell
Dome
50%−epiboly
Shield
75%−epiboly
1−4−somites
14−19−somites
20−25−somites
Prim−5
Prim−15
Prim−25
Long−pec
Protruding−mouth
Day−4
Day−5
Stage
Transcript
ENSDART00000006867
ENSDART00000140064
ENSDART00000171649

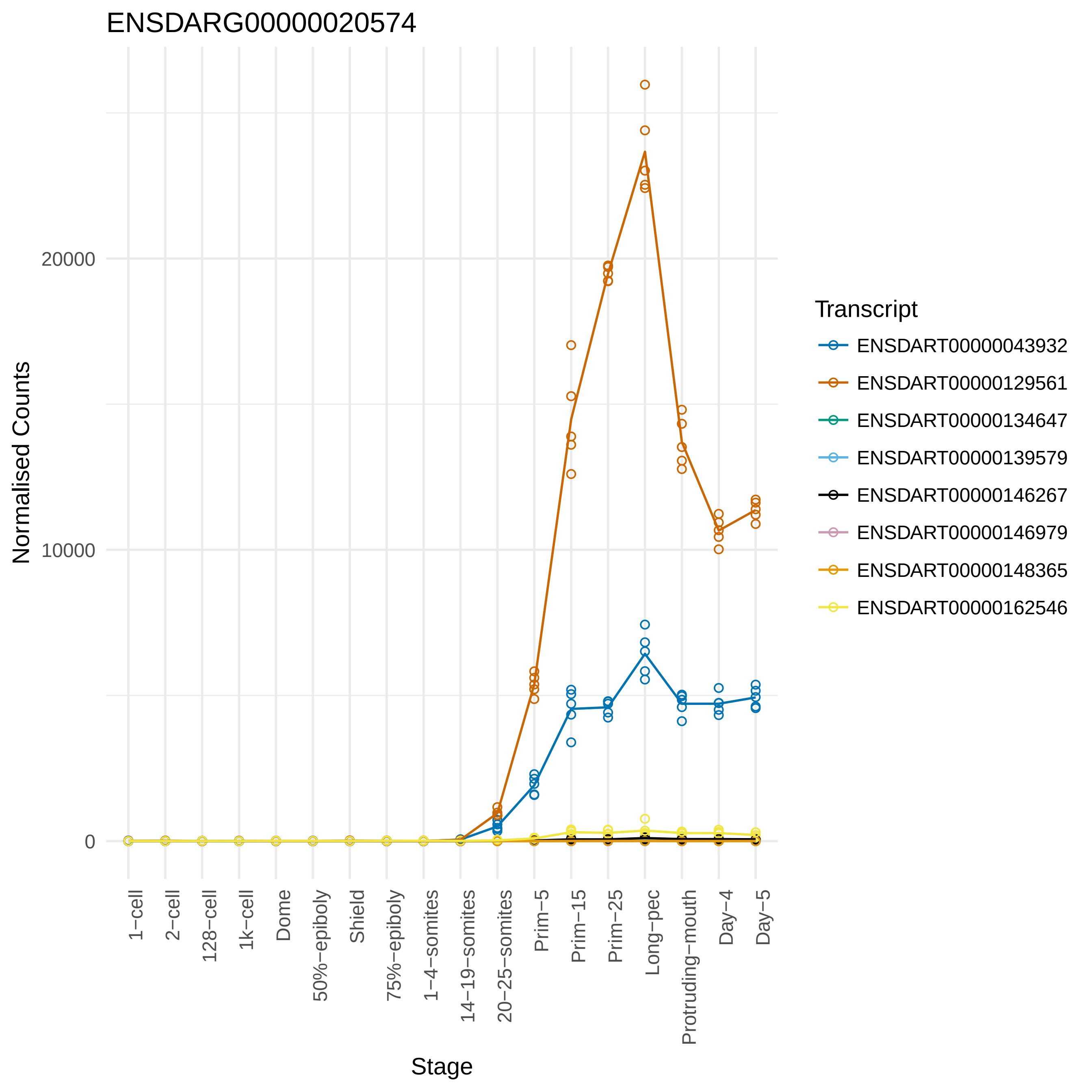

ENSDARG00000020574
Normalised Counts
20000
10000
0
1−cell
2−cell
128−cell
1k−cell
Dome
50%−epiboly
Shield
75%−epiboly
1−4−somites
14−19−somites
20−25−somites
Prim−5
Prim−15
Prim−25
Long−pec
Protruding−mouth
Day−4
Day−5
Stage
Transcript
ENSDART00000043932
ENSDART00000129561
ENSDART00000134647
ENSDART00000139579
ENSDART00000146267
ENSDART00000146979
ENSDART00000148365
ENSDART00000162546

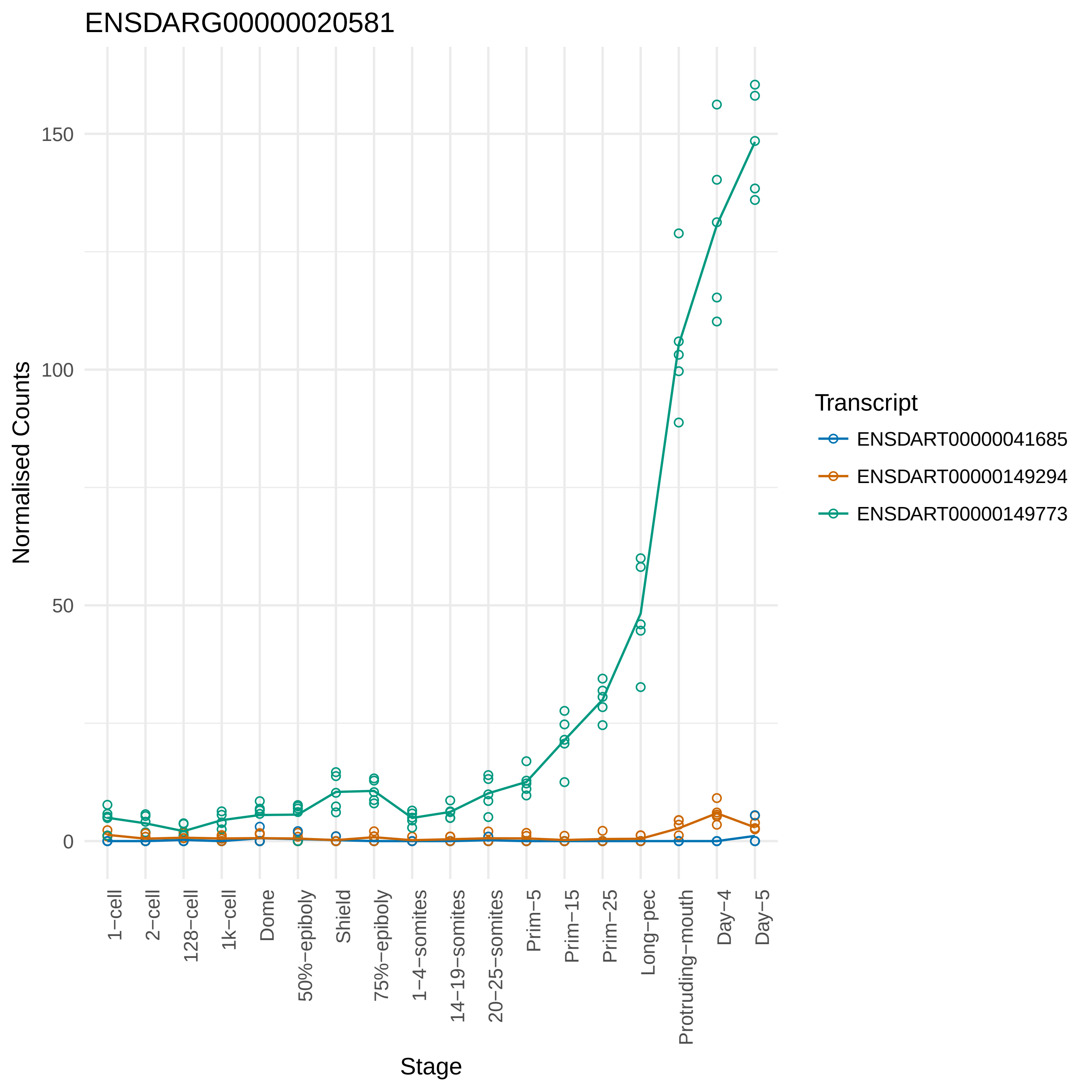

ENSDARG00000020581
Normalised Counts
150
100
50
0
Stage
1−cell
2−cell
128−cell
1k−cell
Dome
50%−epiboly
Shield
75%−epiboly
1−4−somites
14−19−somites
20−25−somites
Prim−5
Prim−15
Prim−25
Long−pec
Protruding−mouth
Day−4
Day−5
Transcript
ENSDART00000041685
ENSDART00000149294
ENSDART00000149773

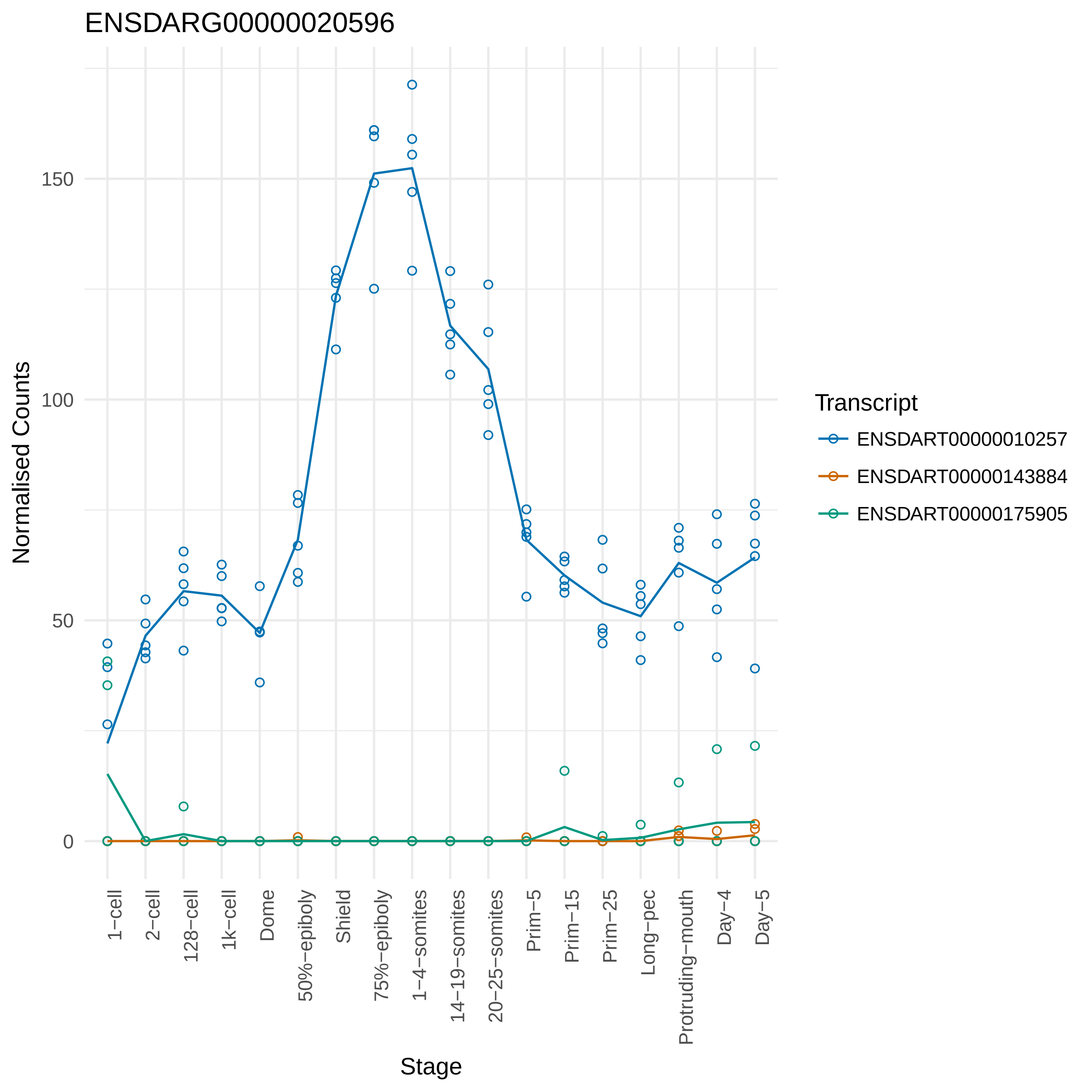
ENSDARG00000020596
Normalised Counts
150
100
50
0
1−cell
2−cell
128−cell
1k−cell
Dome
50%−epiboly
Shield
75%−epiboly
1−4−somites
14−19−somites
20−25−somites
Prim−5
Prim−15
Prim−25
Long−pec
Protruding−mouth
Day−4
Day−5
Stage
Transcript
ENSDART00000010257
ENSDART00000143884
ENSDART00000175905

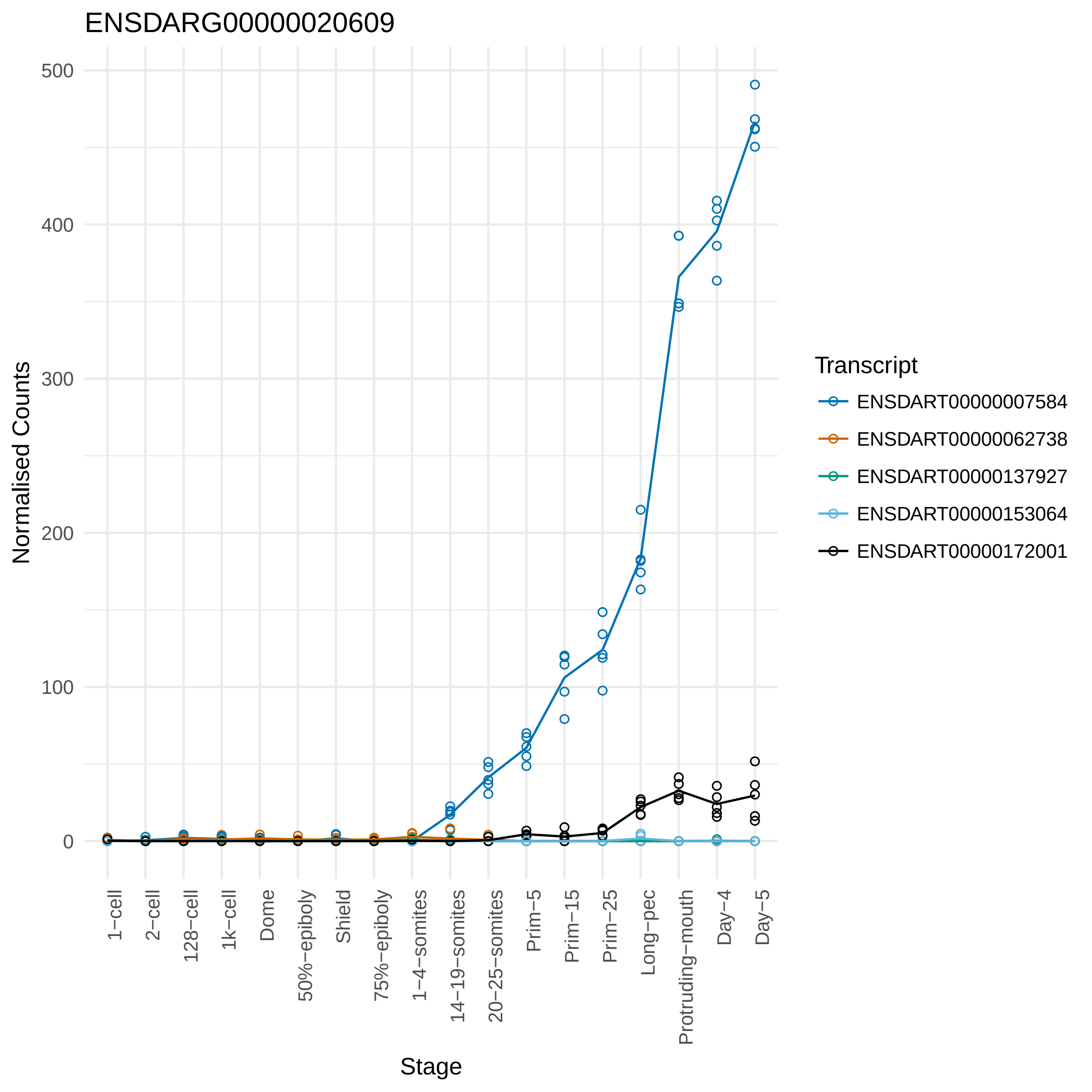
ENSDARG00000020609
Normalised Counts
500
400
300
200
100
0
1−cell
2−cell
128−cell
1k−cell
Dome
50%−epiboly
Shield
75%−epiboly
1−4−somites
14−19−somites
20−25−somites
Prim−5
Prim−15
Prim−25
Long−pec
Protruding−mouth
Day−4
Day−5
Stage
Transcript
ENSDART00000007584
ENSDART00000062738
ENSDART00000137927
ENSDART00000153064
ENSDART00000172001

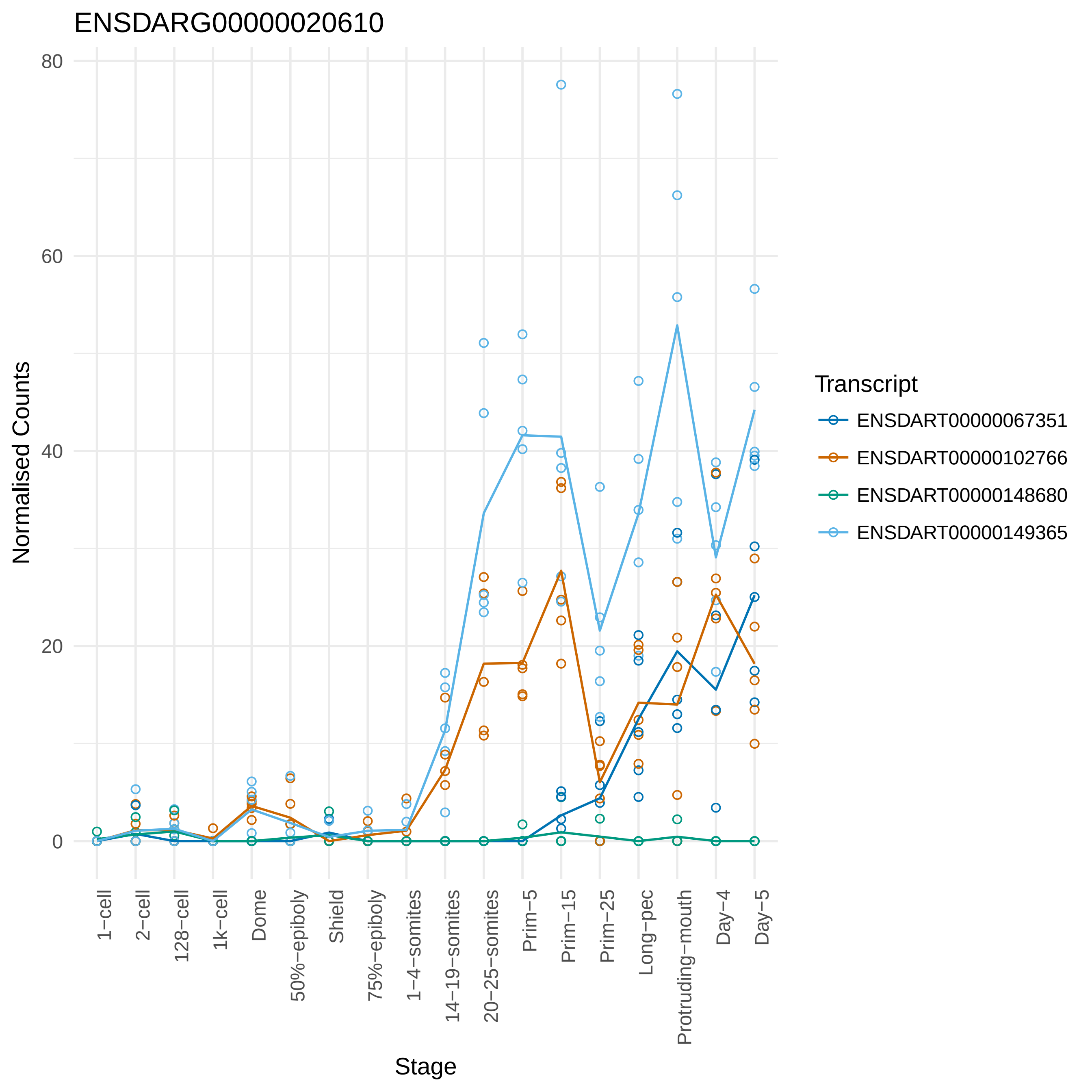

ENSDARG00000020610
Normalised Counts
Stage
0
20
40
60
80
1−cell
2−cell
128−cell
1k−cell
Dome
50%−epiboly
Shield
75%−epiboly
1−4−somites
14−19−somites
20−25−somites
Prim−5
Prim−15
Prim−25
Long−pec
Protruding−mouth
Day−4
Day−5
Transcript
ENSDART00000067351
ENSDART00000102766
ENSDART00000148680
ENSDART00000149365

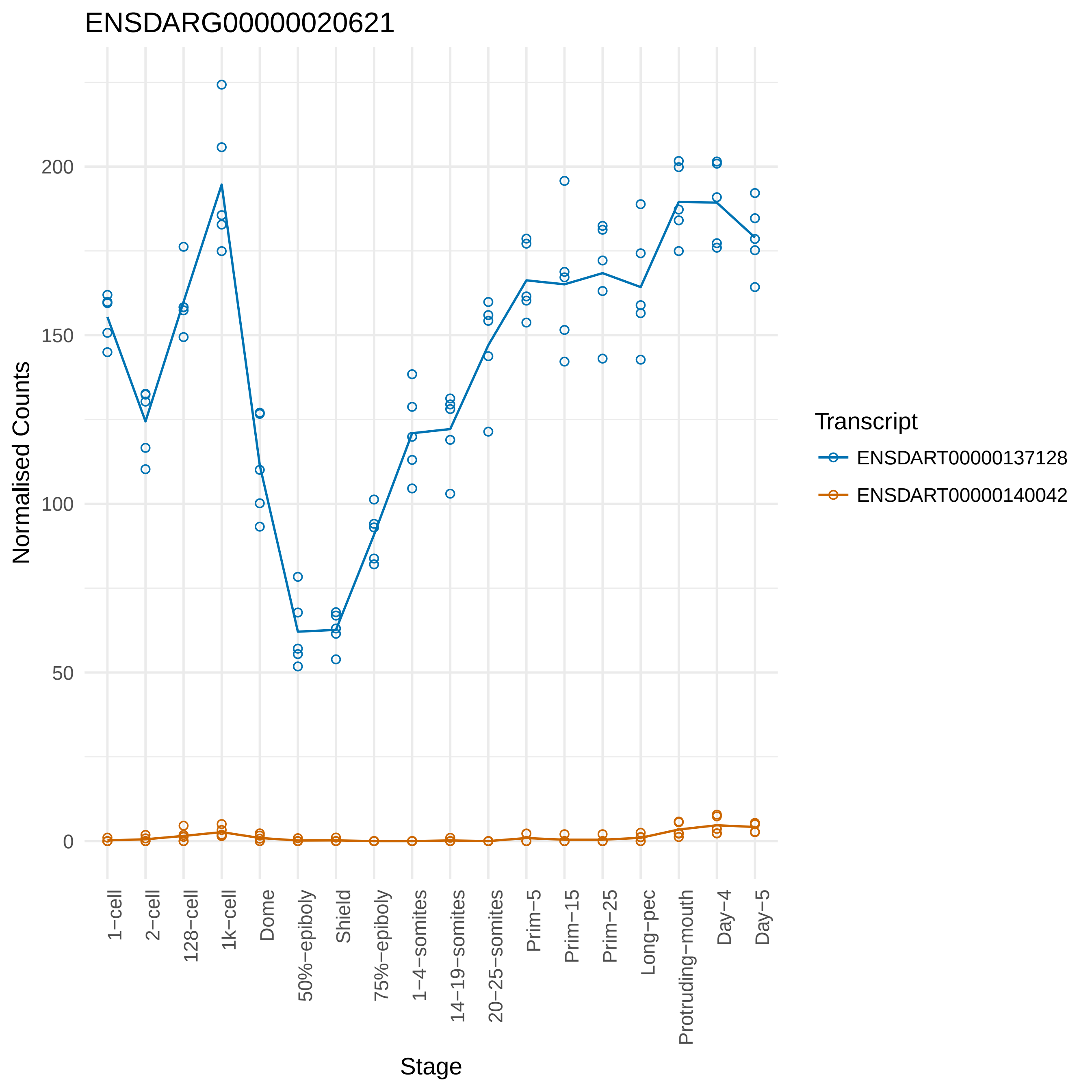

ENSDARG00000020621
Normalised Counts
200
150
100
50
0
1−cell
2−cell
128−cell
1k−cell
Dome
50%−epiboly
Shield
75%−epiboly
1−4−somites
14−19−somites
20−25−somites
Prim−5
Prim−15
Prim−25
Long−pec
Protruding−mouth
Day−4
Day−5
Stage
Transcript
ENSDART00000137128
ENSDART00000140042

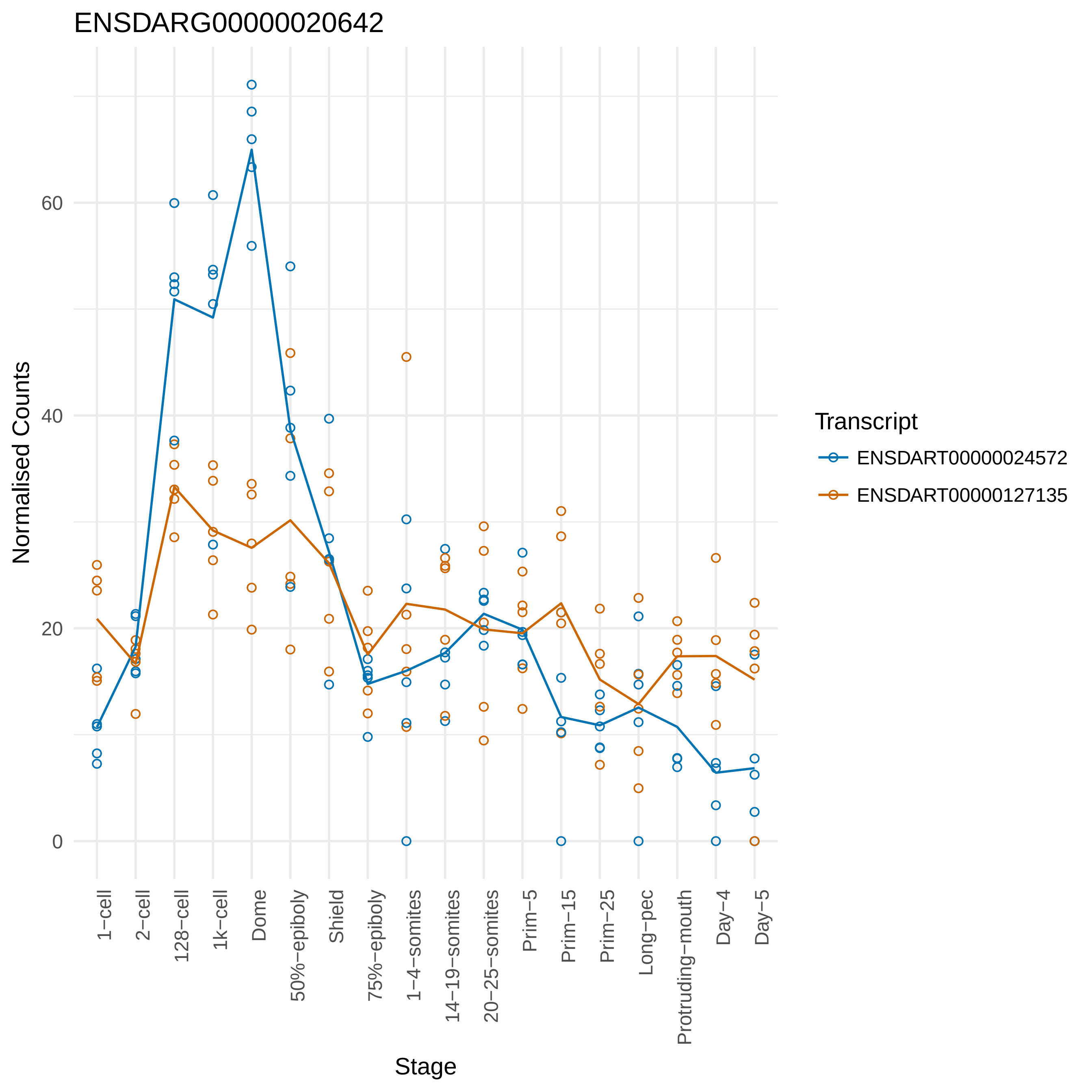

ENSDARG00000020642
Normalised Counts
0
20
40
60
Stage
1−cell
2−cell
128−cell
1k−cell
Dome
50%−epiboly
Shield
75%−epiboly
1−4−somites
14−19−somites
20−25−somites
Prim−5
Prim−15
Prim−25
Long−pec
Protruding−mouth
Day−4
Day−5
Transcript
ENSDART00000024572
ENSDART00000127135

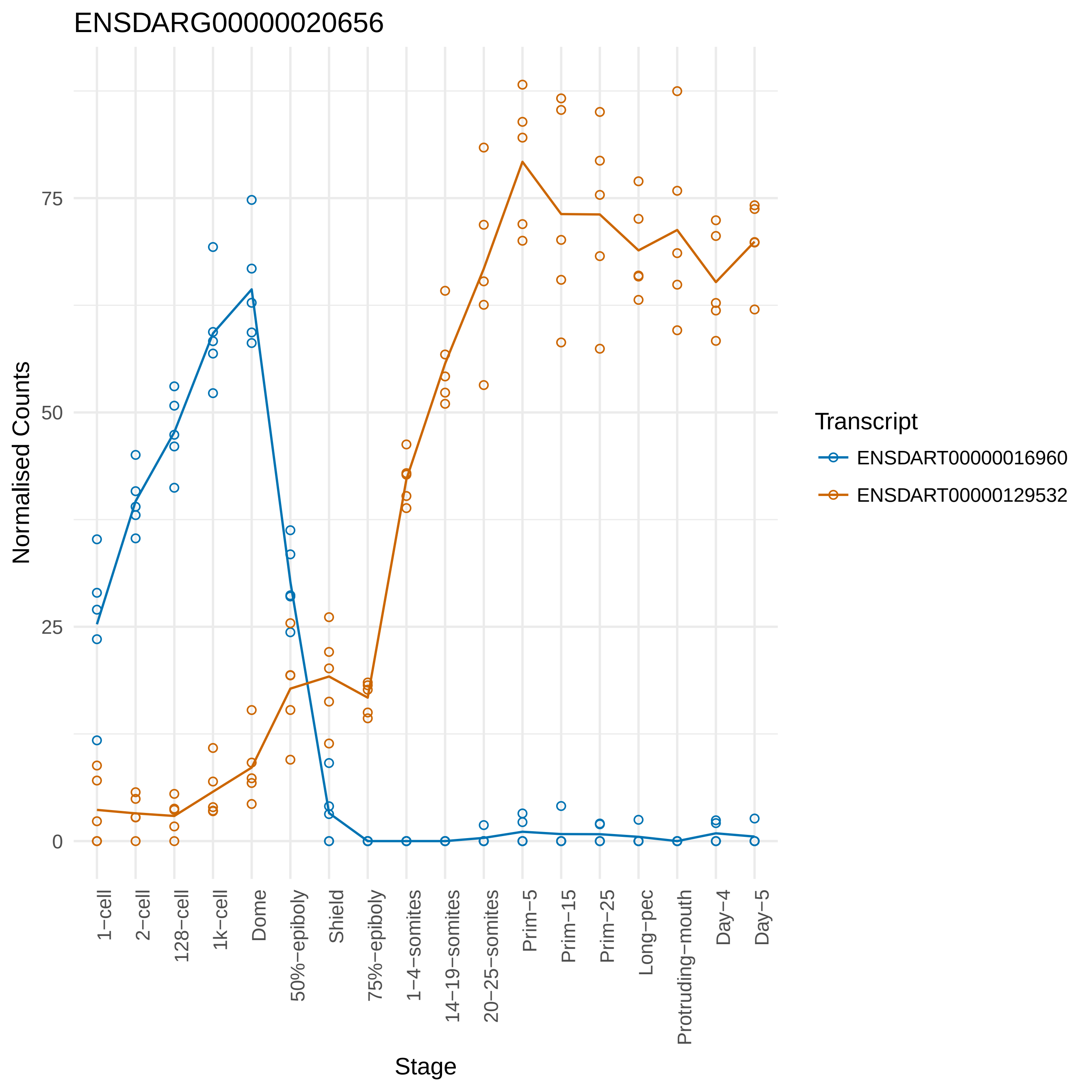

ENSDARG00000020656
Normalised Counts
0
25
50
75
Stage
1−cell
2−cell
128−cell
1k−cell
Dome
50%−epiboly
Shield
75%−epiboly
1−4−somites
14−19−somites
20−25−somites
Prim−5
Prim−15
Prim−25
Long−pec
Protruding−mouth
Day−4
Day−5
Transcript
ENSDART00000016960
ENSDART00000129532

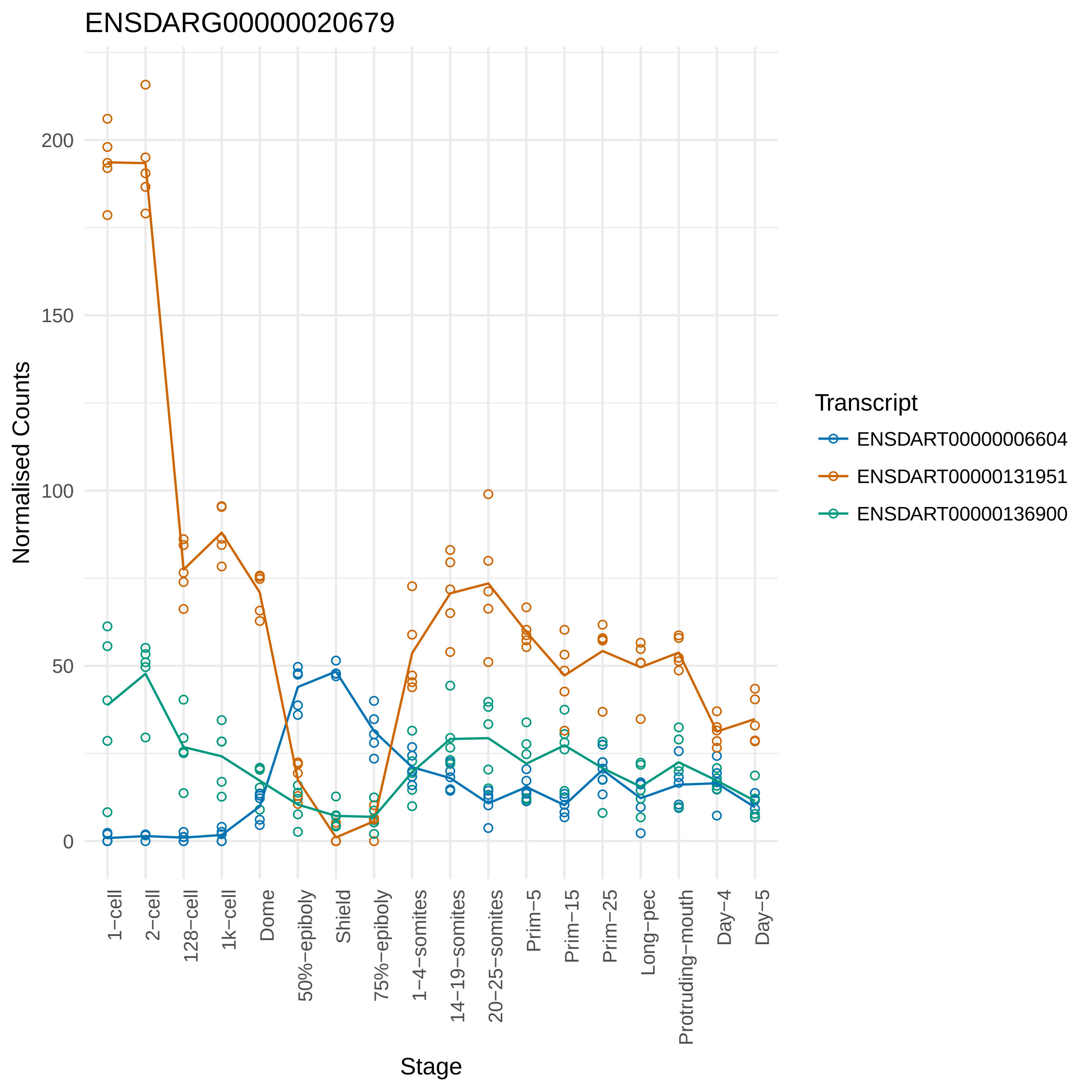

ENSDARG00000020679
Normalised Counts
200
150
100
50
0
1−cell
2−cell
128−cell
1k−cell
Dome
50%−epiboly
Shield
75%−epiboly
1−4−somites
14−19−somites
20−25−somites
Prim−5
Prim−15
Prim−25
Long−pec
Protruding−mouth
Day−4
Day−5
Stage
Transcript
ENSDART00000006604
ENSDART00000131951
ENSDART00000136900

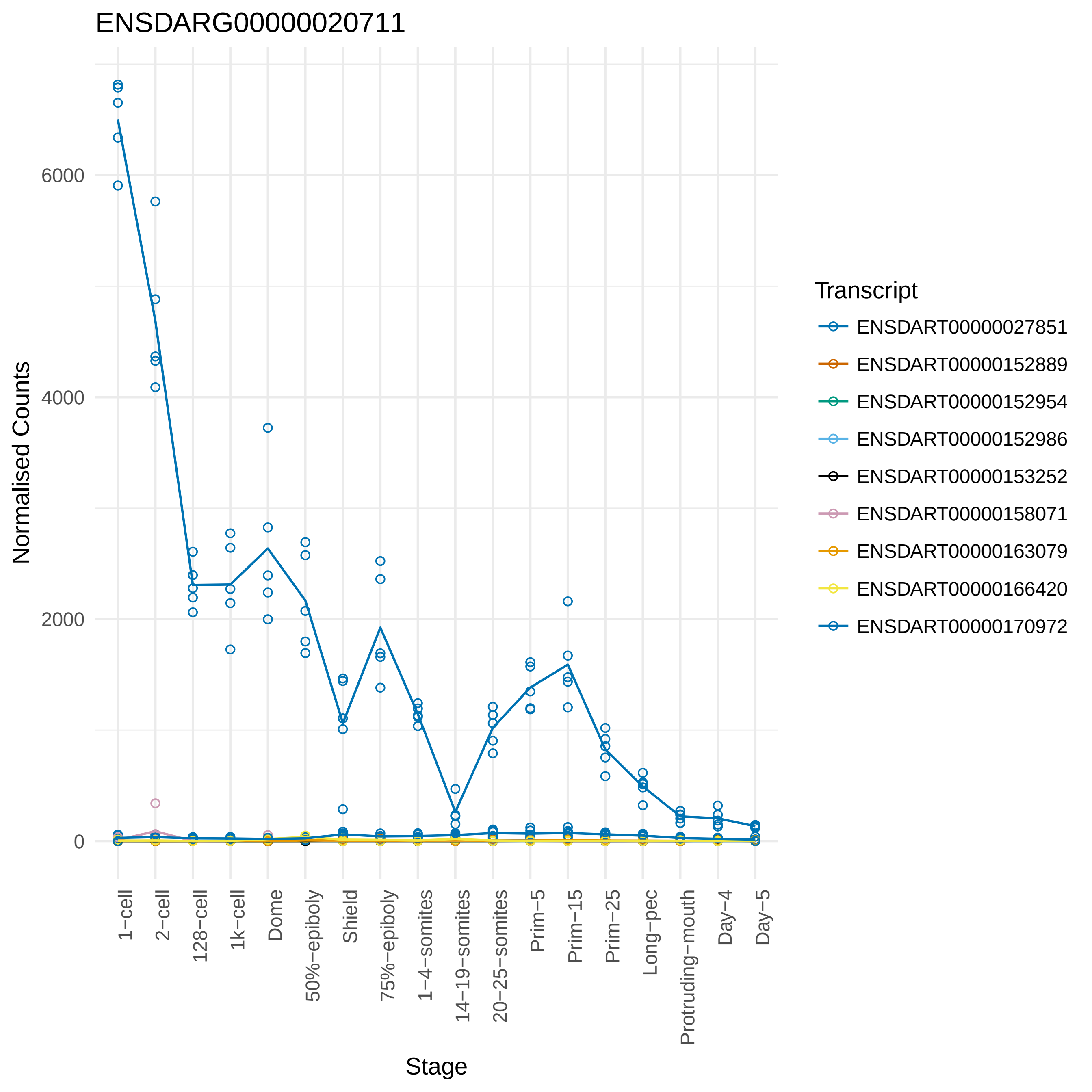

ENSDARG00000020711
Normalised Counts
6000
4000
2000
0
1−cell
2−cell
128−cell
1k−cell
Dome
50%−epiboly
Shield
75%−epiboly
1−4−somites
14−19−somites
20−25−somites
Prim−5
Prim−15
Prim−25
Long−pec
Protruding−mouth
Day−4
Day−5
Stage
Transcript
ENSDART00000027851
ENSDART00000152889
ENSDART00000152954
ENSDART00000152986
ENSDART00000153252
ENSDART00000158071
ENSDART00000163079
ENSDART00000166420
ENSDART00000170972

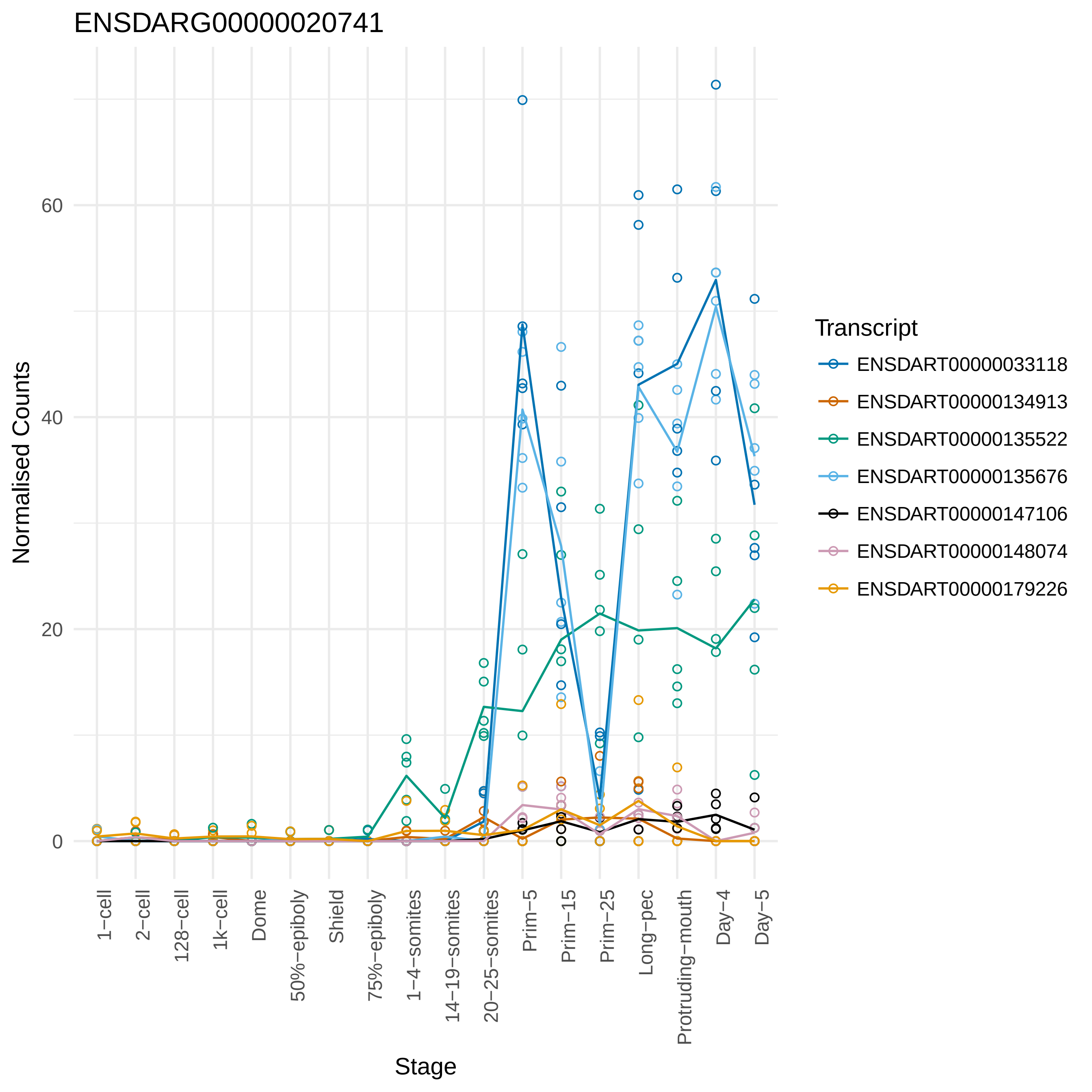
ENSDARG00000020741
Normalised Counts
60
40
20
0
Stage
1−cell
2−cell
128−cell
1k−cell
Dome
50%−epiboly
Shield
75%−epiboly
1−4−somites
14−19−somites
20−25−somites
Prim−5
Prim−15
Prim−25
Long−pec
Protruding−mouth
Day−4
Day−5
Transcript
ENSDART00000033118
ENSDART00000134913
ENSDART00000135522
ENSDART00000135676
ENSDART00000147106
ENSDART00000148074
ENSDART00000179226

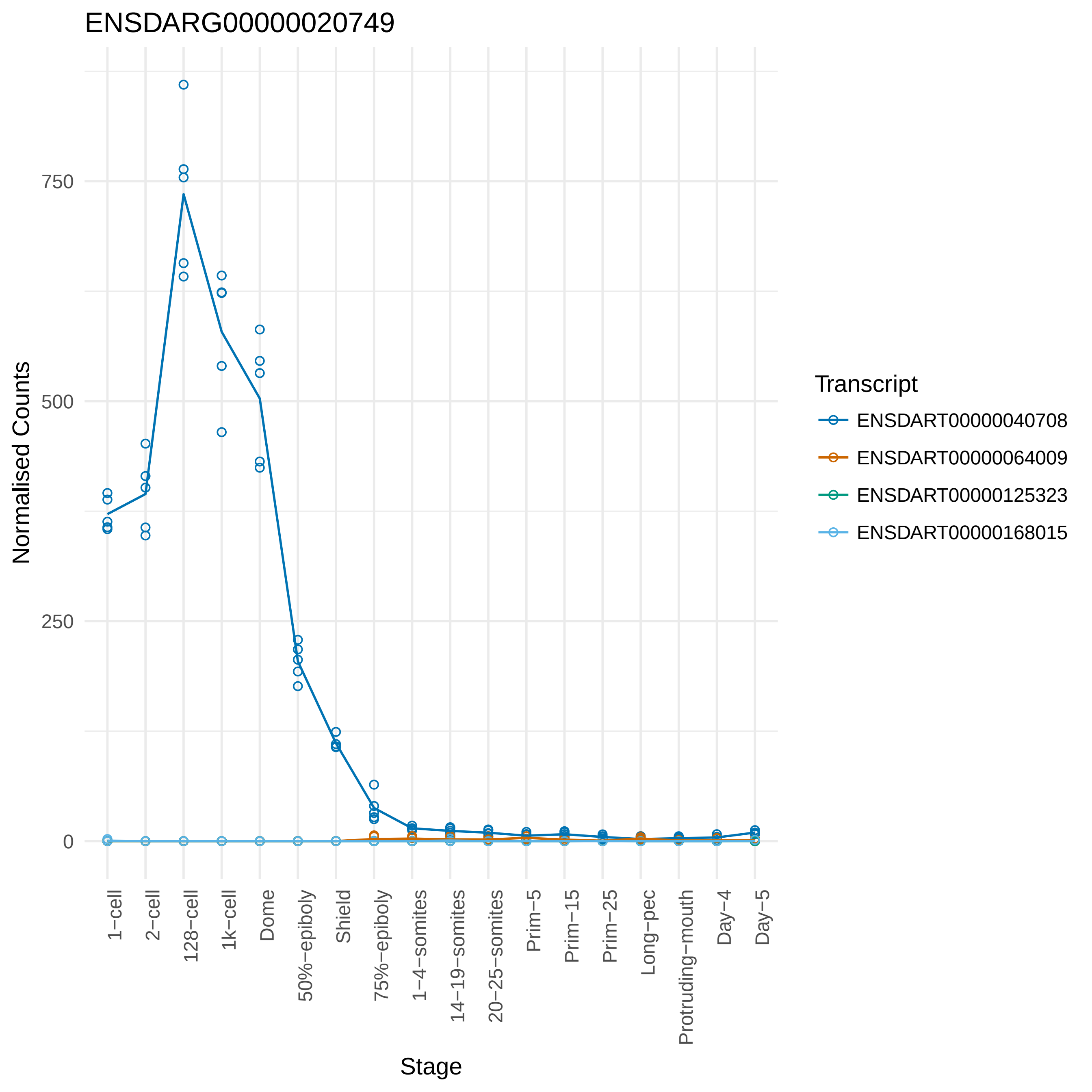

ENSDARG00000020749
Normalised Counts
750
500
250
0
1−cell
2−cell
128−cell
1k−cell
Dome
50%−epiboly
Shield
75%−epiboly
1−4−somites
14−19−somites
20−25−somites
Prim−5
Prim−15
Prim−25
Long−pec
Protruding−mouth
Day−4
Day−5
Stage
Transcript
ENSDART00000040708
ENSDART00000064009
ENSDART00000125323
ENSDART00000168015

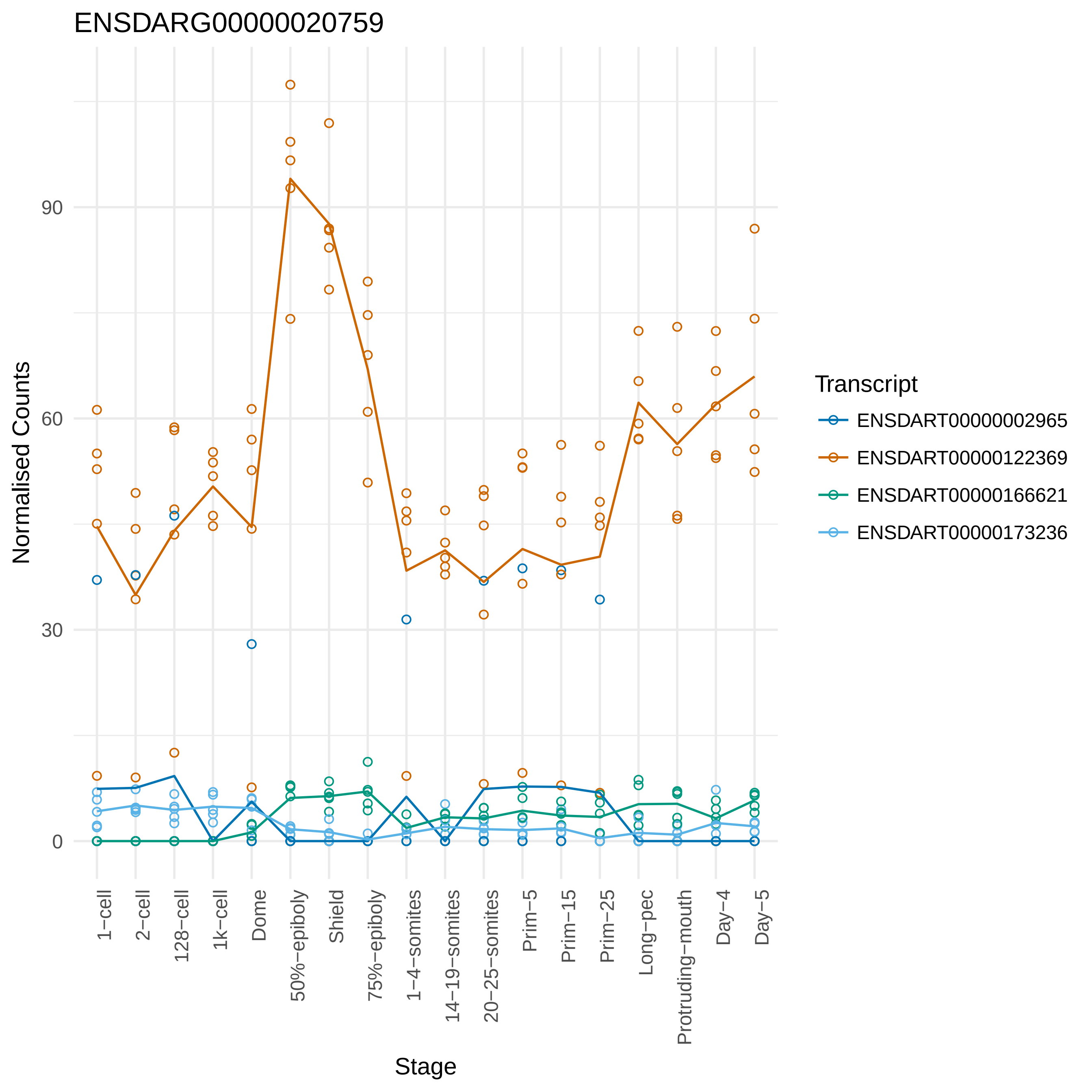

ENSDARG00000020759
Normalised Counts
0
30
60
90
Stage
1−cell
2−cell
128−cell
1k−cell
Dome
50%−epiboly
Shield
75%−epiboly
1−4−somites
14−19−somites
20−25−somites
Prim−5
Prim−15
Prim−25
Long−pec
Protruding−mouth
Day−4
Day−5
Transcript
ENSDART00000002965
ENSDART00000122369
ENSDART00000166621
ENSDART00000173236

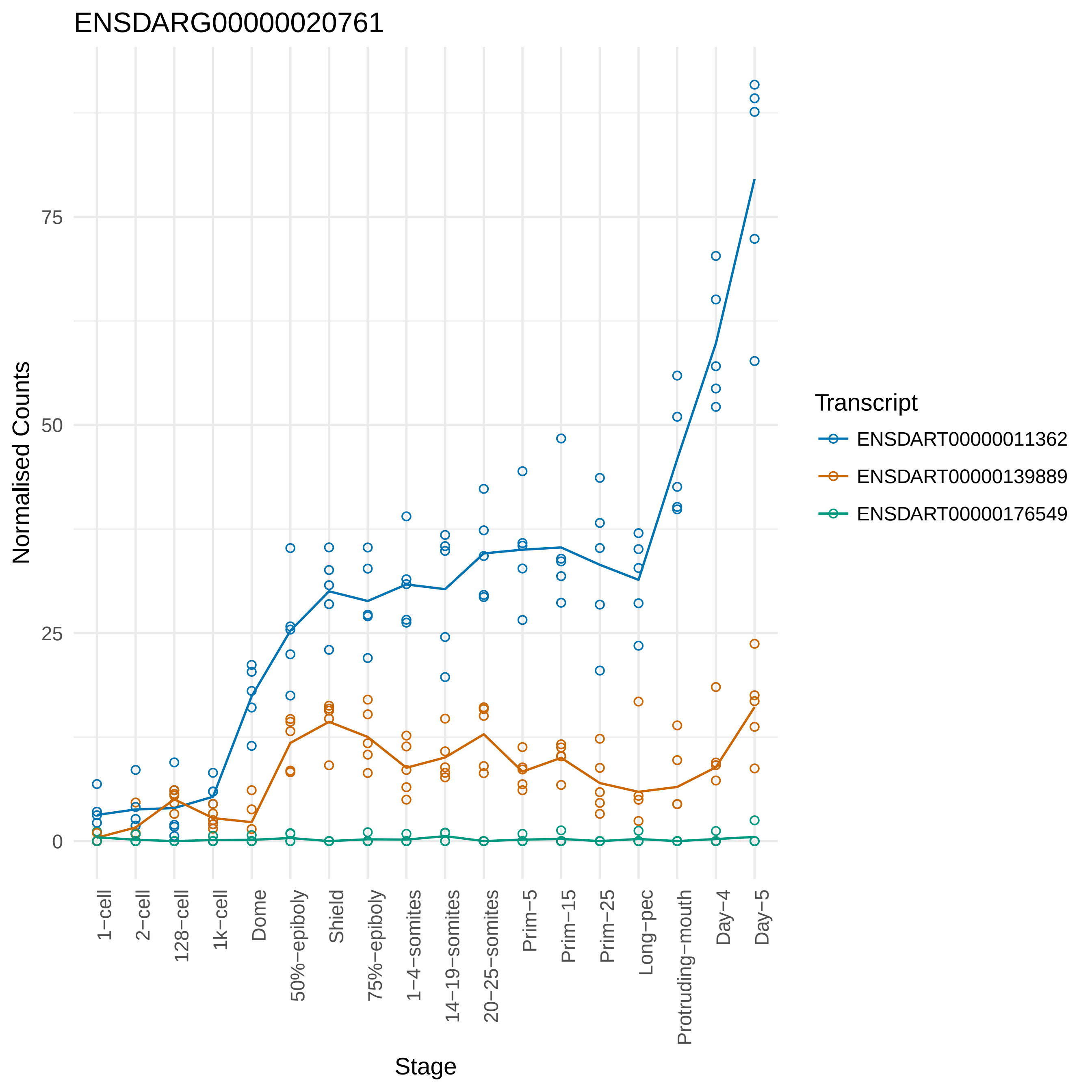

ENSDARG00000020761
Normalised Counts
0
25
50
75
Stage
1−cell
2−cell
128−cell
1k−cell
Dome
50%−epiboly
Shield
75%−epiboly
1−4−somites
14−19−somites
20−25−somites
Prim−5
Prim−15
Prim−25
Long−pec
Protruding−mouth
Day−4
Day−5
Transcript
ENSDART00000011362
ENSDART00000139889
ENSDART00000176549

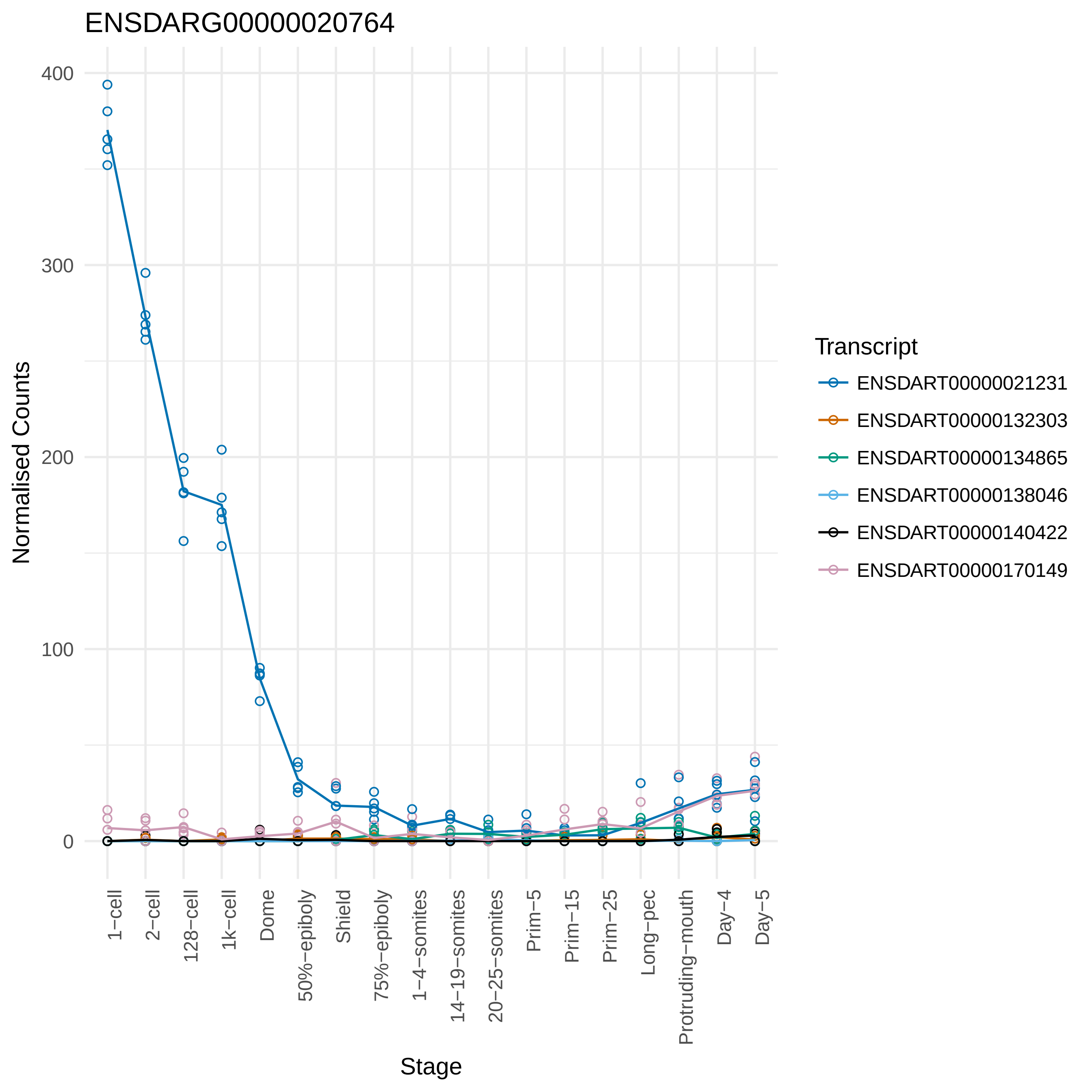
ENSDARG00000020764
Normalised Counts
400
300
200
100
0
1−cell
2−cell
128−cell
1k−cell
Dome
50%−epiboly
Shield
75%−epiboly
1−4−somites
14−19−somites
20−25−somites
Prim−5
Prim−15
Prim−25
Long−pec
Protruding−mouth
Day−4
Day−5
Stage
Transcript
ENSDART00000021231
ENSDART00000132303
ENSDART00000134865
ENSDART00000138046
ENSDART00000140422
ENSDART00000170149

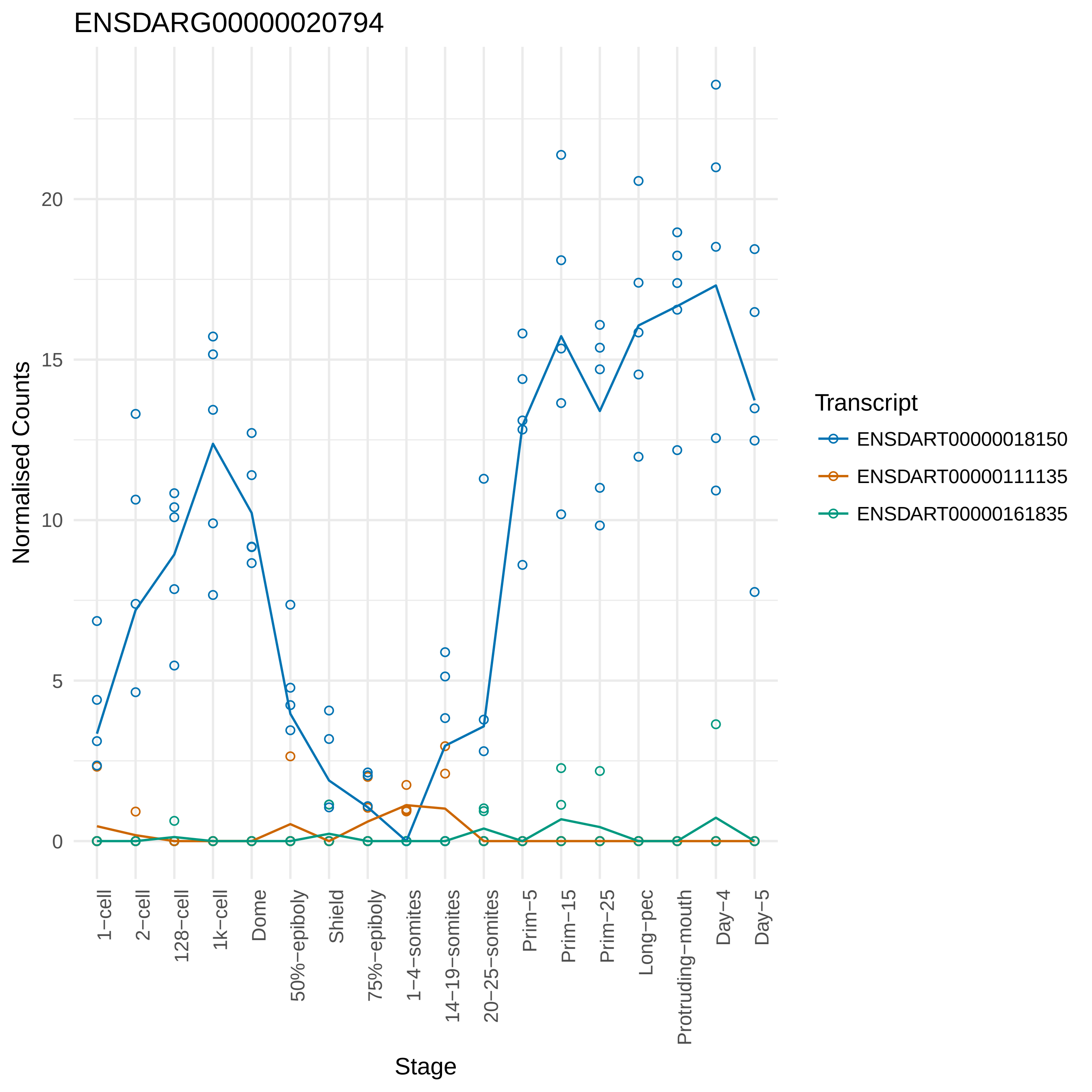

ENSDARG00000020794
Normalised Counts
0
5
10
15
20
Stage
1−cell
2−cell
128−cell
1k−cell
Dome
50%−epiboly
Shield
75%−epiboly
1−4−somites
14−19−somites
20−25−somites
Prim−5
Prim−15
Prim−25
Long−pec
Protruding−mouth
Day−4
Day−5
Transcript
ENSDART00000018150
ENSDART00000111135
ENSDART00000161835

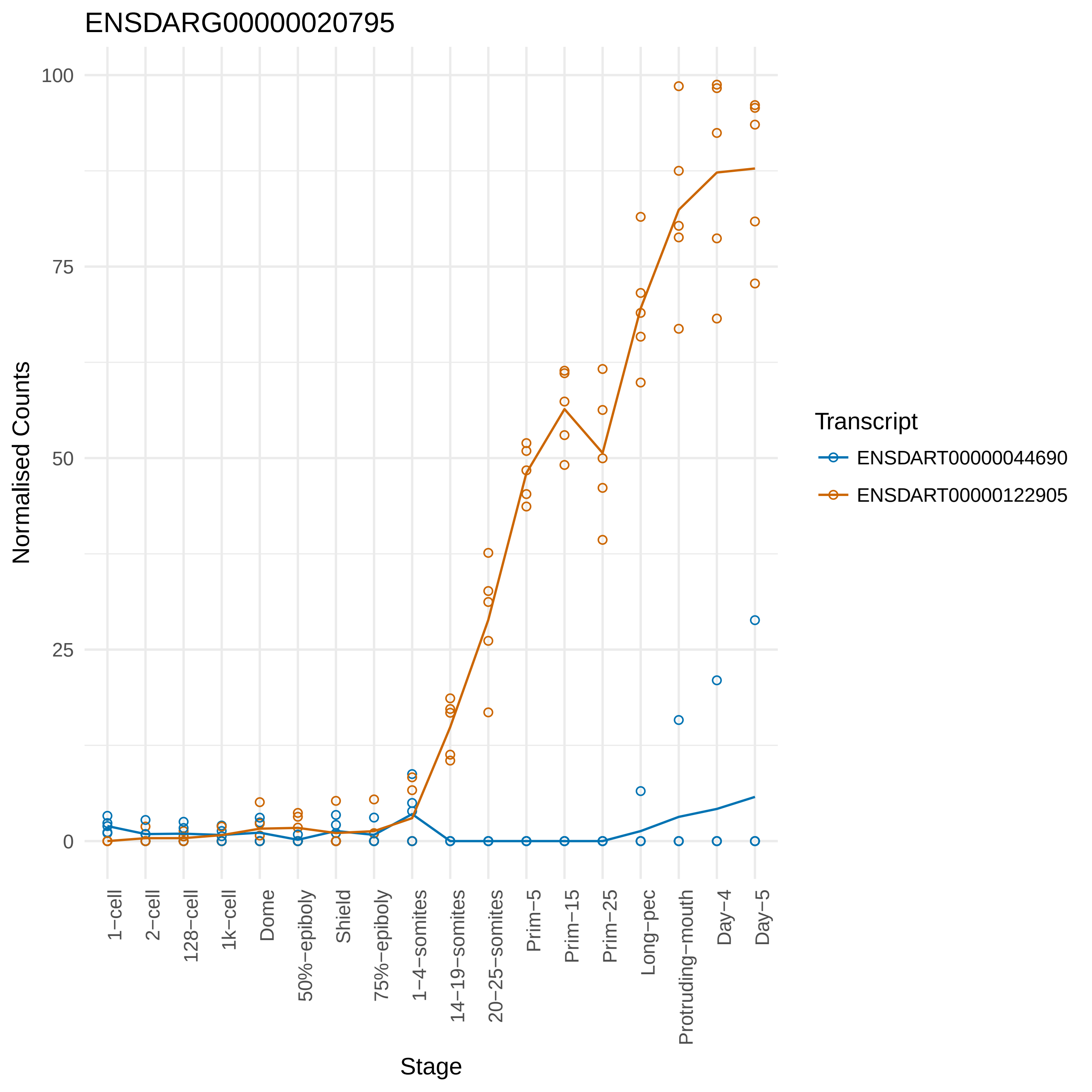
ENSDARG00000020795
Normalised Counts
100
75
50
25
0
1−cell
2−cell
128−cell
1k−cell
Dome
50%−epiboly
Shield
75%−epiboly
1−4−somites
14−19−somites
20−25−somites
Prim−5
Prim−15
Prim−25
Long−pec
Protruding−mouth
Day−4
Day−5
Stage
Transcript
ENSDART00000044690
ENSDART00000122905

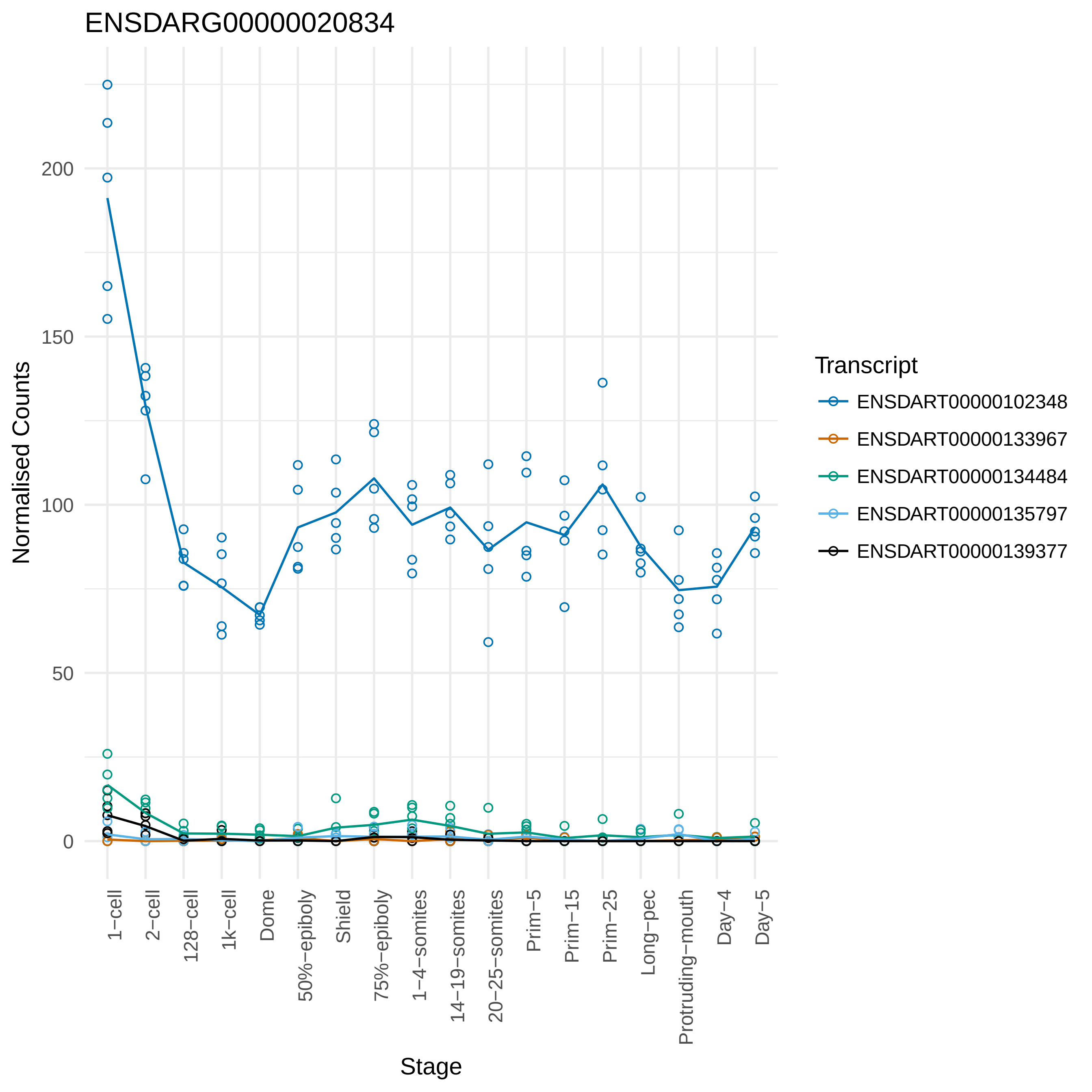
ENSDARG00000020834
Normalised Counts
0
50
100
150
200
Stage
1−cell
2−cell
128−cell
1k−cell
Dome
50%−epiboly
Shield
75%−epiboly
1−4−somites
14−19−somites
20−25−somites
Prim−5
Prim−15
Prim−25
Long−pec
Protruding−mouth
Day−4
Day−5
Transcript
ENSDART00000102348
ENSDART00000133967
ENSDART00000134484
ENSDART00000135797
ENSDART00000139377

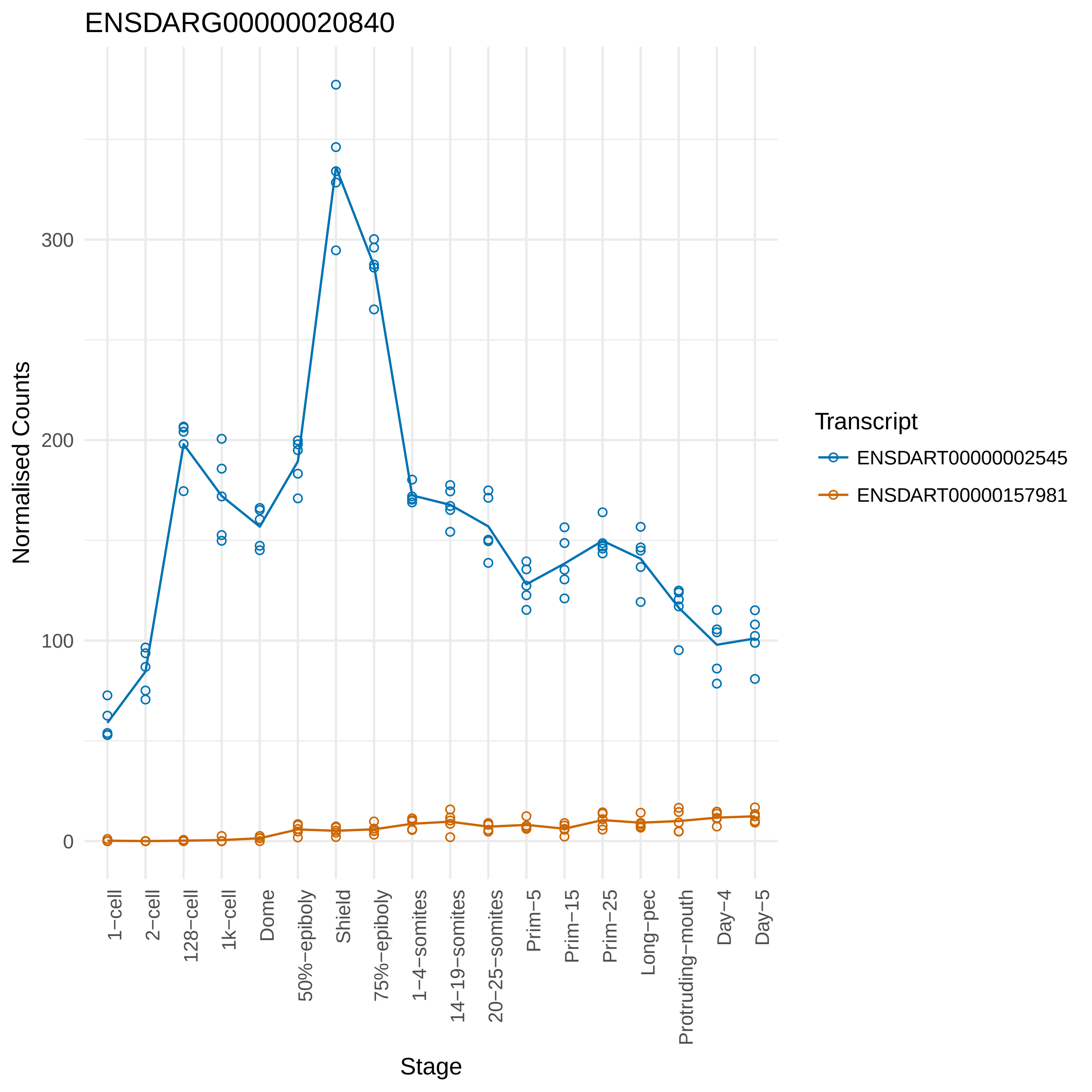

ENSDARG00000020840
Normalised Counts
300
200
100
0
1−cell
2−cell
128−cell
1k−cell
Dome
50%−epiboly
Shield
75%−epiboly
1−4−somites
14−19−somites
20−25−somites
Prim−5
Prim−15
Prim−25
Long−pec
Protruding−mouth
Day−4
Day−5
Stage
Transcript
ENSDART00000002545
ENSDART00000157981

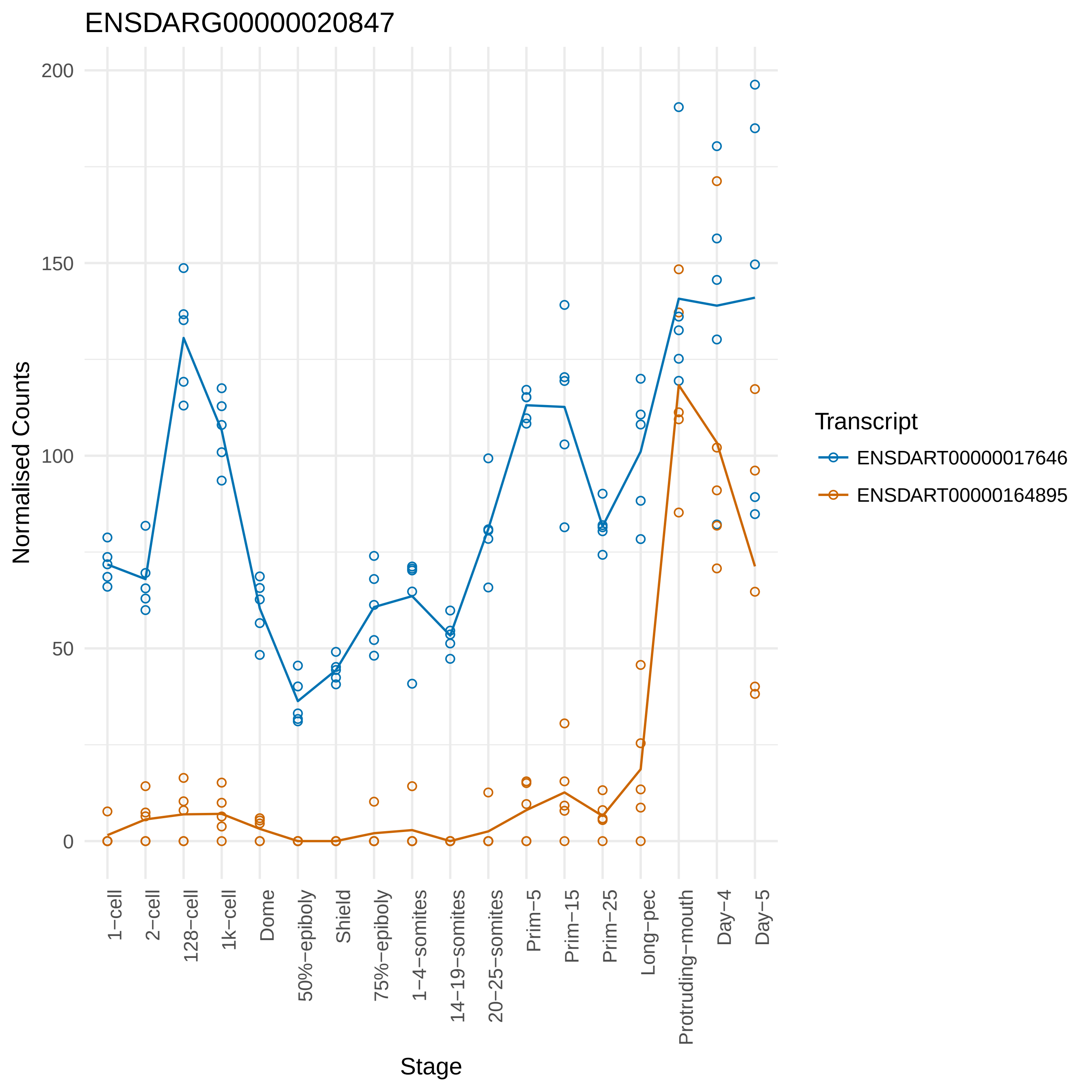
ENSDARG00000020847
Normalised Counts
200
150
100
50
0
1−cell
2−cell
128−cell
1k−cell
Dome
50%−epiboly
Shield
75%−epiboly
1−4−somites
14−19−somites
20−25−somites
Prim−5
Prim−15
Prim−25
Long−pec
Protruding−mouth
Day−4
Day−5
Stage
Transcript
ENSDART00000017646
ENSDART00000164895

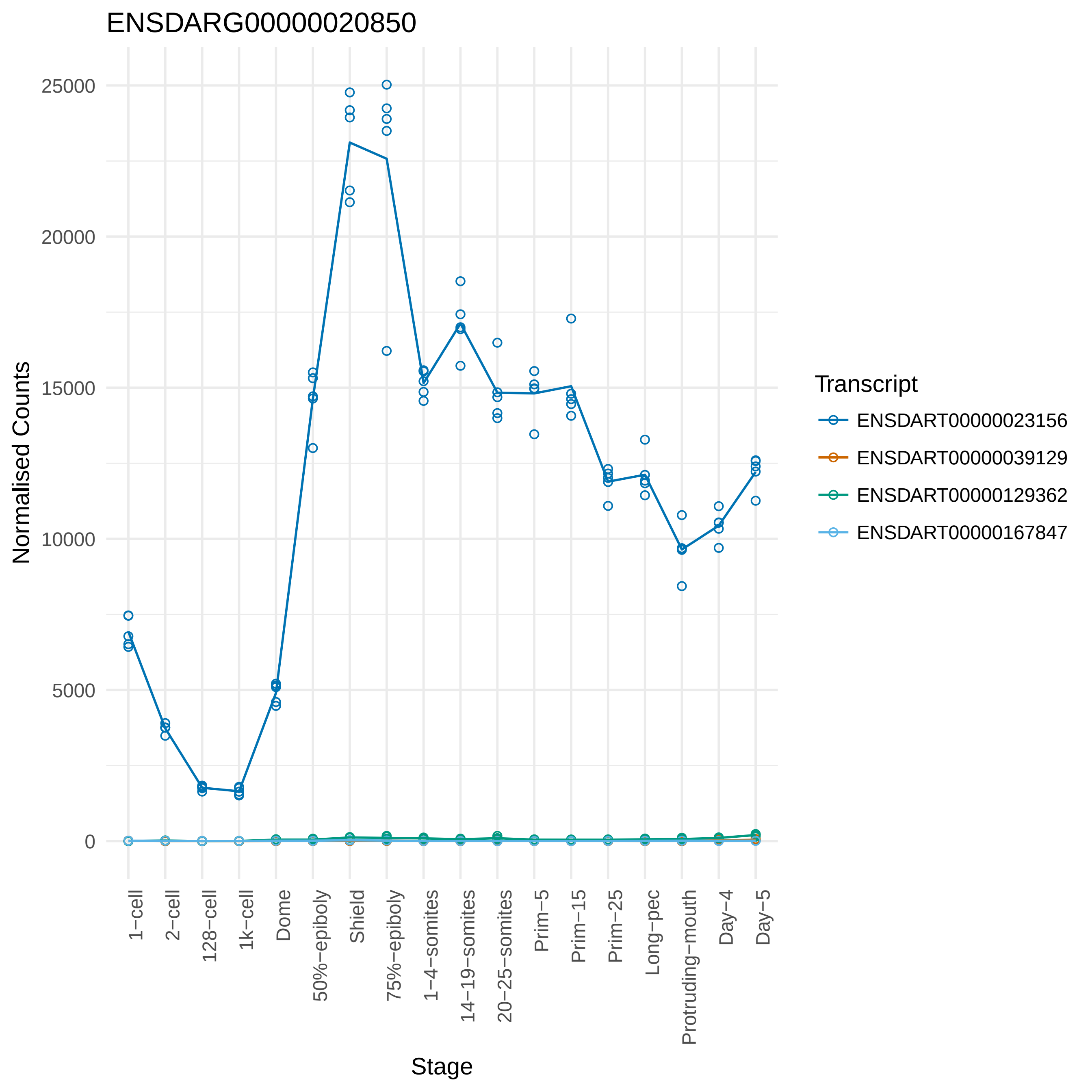

ENSDARG00000020850
Normalised Counts
25000
20000
15000
10000
5000
0
1−cell
2−cell
128−cell
1k−cell
Dome
50%−epiboly
Shield
75%−epiboly
1−4−somites
14−19−somites
20−25−somites
Prim−5
Prim−15
Prim−25
Long−pec
Protruding−mouth
Day−4
Day−5
Stage
Transcript
ENSDART00000023156
ENSDART00000039129
ENSDART00000129362
ENSDART00000167847

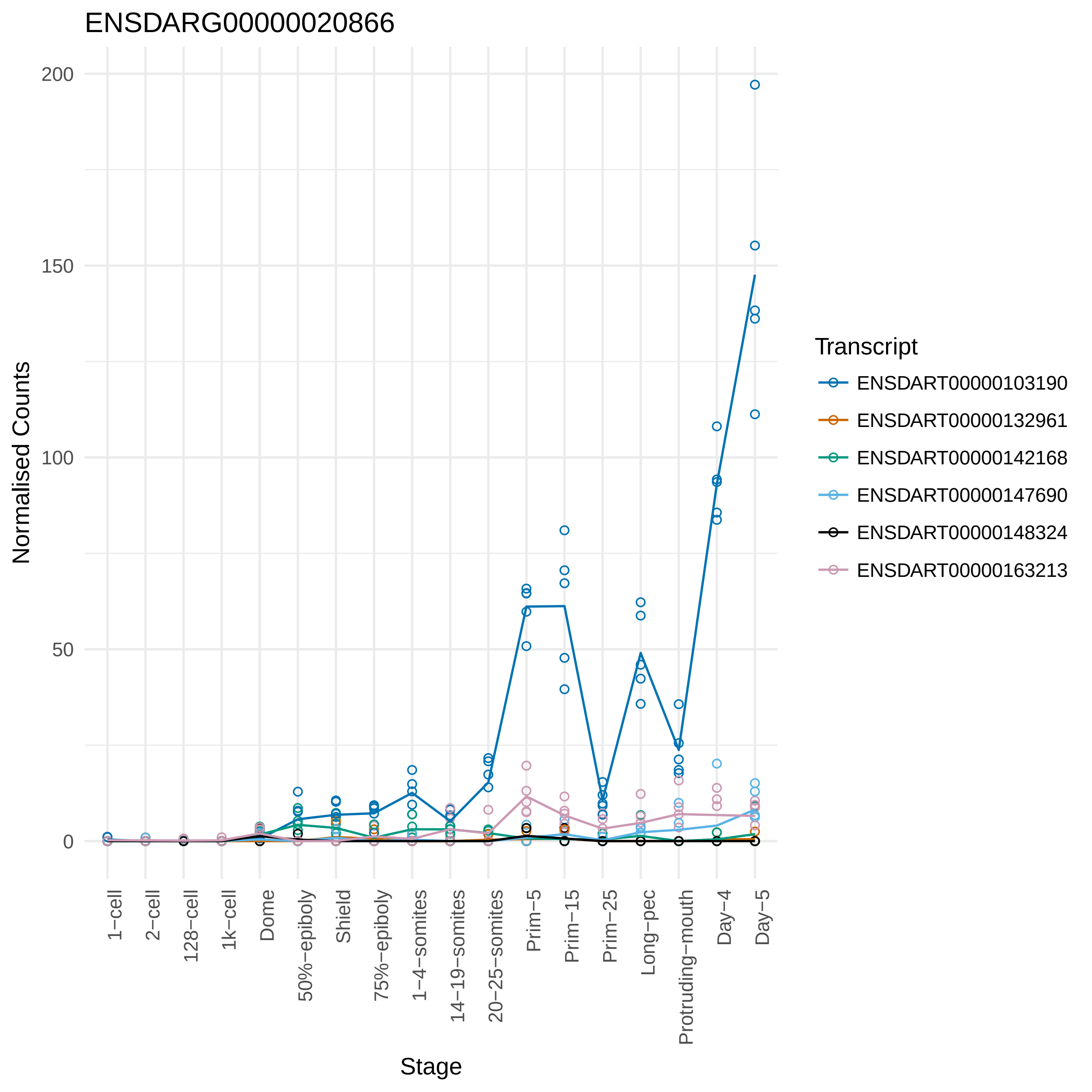

ENSDARG00000020866
Normalised Counts
200
150
100
50
0
1−cell
2−cell
128−cell
1k−cell
Dome
50%−epiboly
Shield
75%−epiboly
1−4−somites
14−19−somites
20−25−somites
Prim−5
Prim−15
Prim−25
Long−pec
Protruding−mouth
Day−4
Day−5
Stage
Transcript
ENSDART00000103190
ENSDART00000132961
ENSDART00000142168
ENSDART00000147690
ENSDART00000148324
ENSDART00000163213

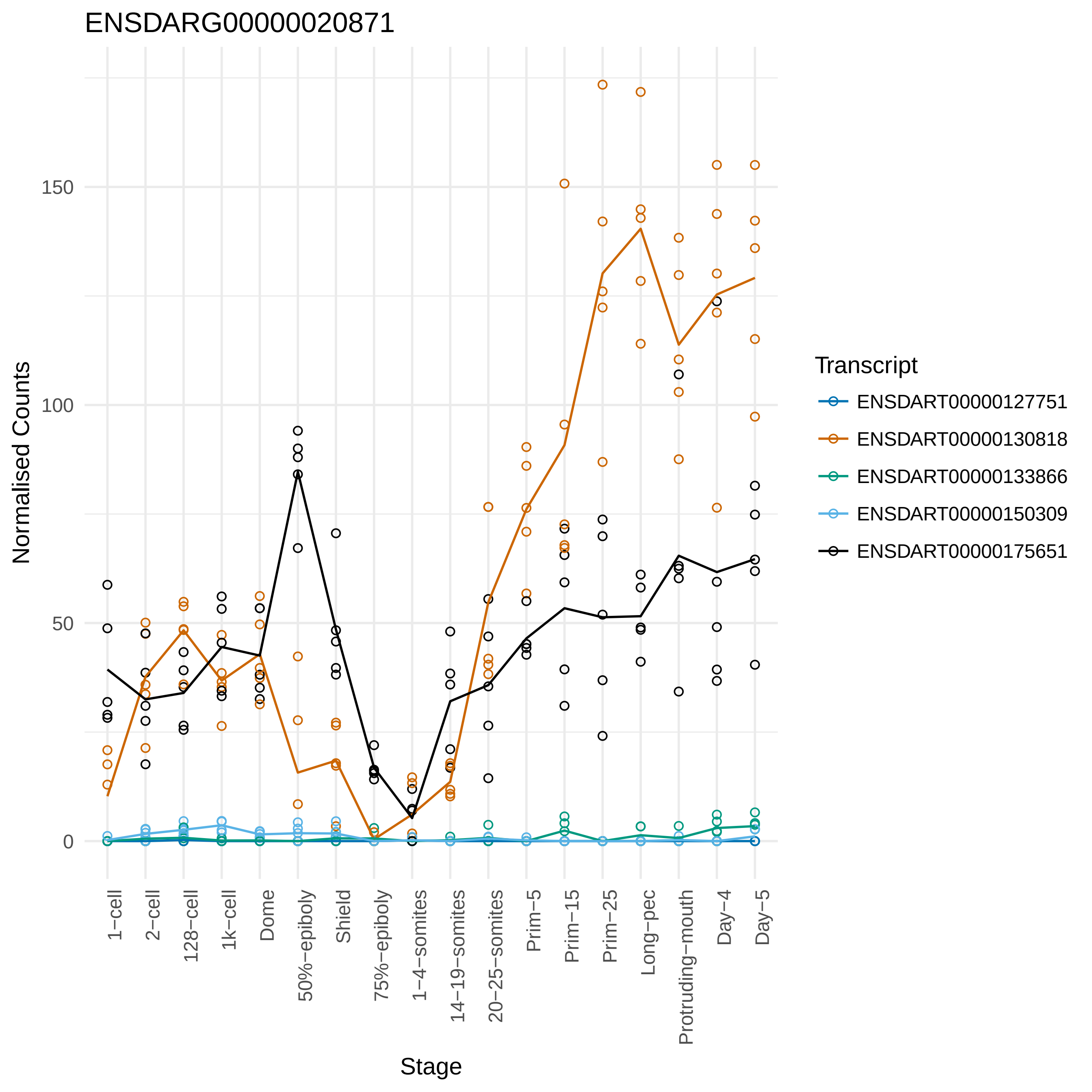
ENSDARG00000020871
Normalised Counts
0
50
100
150
Stage
1−cell
2−cell
128−cell
1k−cell
Dome
50%−epiboly
Shield
75%−epiboly
1−4−somites
14−19−somites
20−25−somites
Prim−5
Prim−15
Prim−25
Long−pec
Protruding−mouth
Day−4
Day−5
Transcript
ENSDART00000127751
ENSDART00000130818
ENSDART00000133866
ENSDART00000150309
ENSDART00000175651

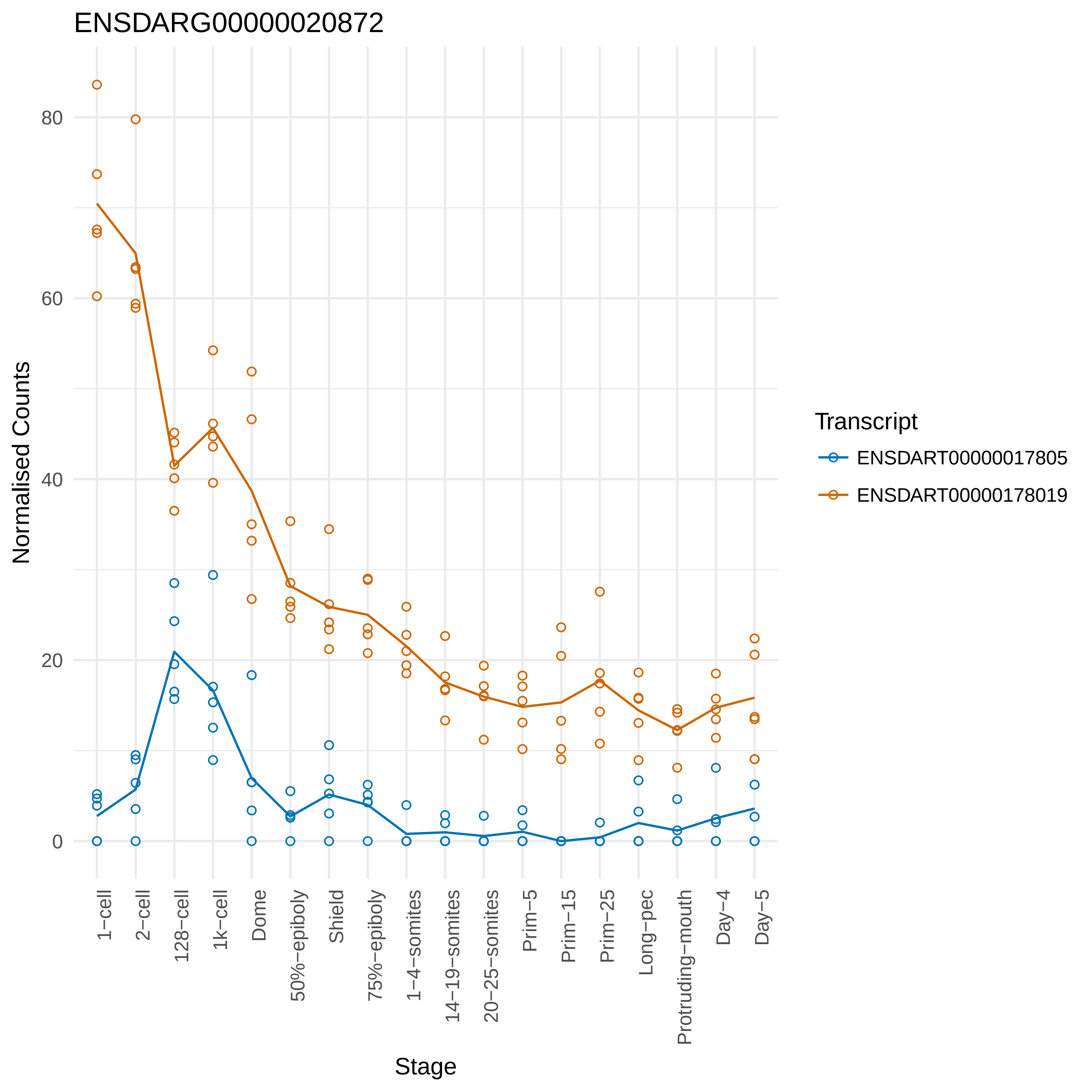

ENSDARG00000020872
Normalised Counts
80
60
40
20
0
1−cell
2−cell
128−cell
1k−cell
Dome
50%−epiboly
Shield
75%−epiboly
1−4−somites
14−19−somites
20−25−somites
Prim−5
Prim−15
Prim−25
Long−pec
Protruding−mouth
Day−4
Day−5
Stage
Transcript
ENSDART00000017805
ENSDART00000178019

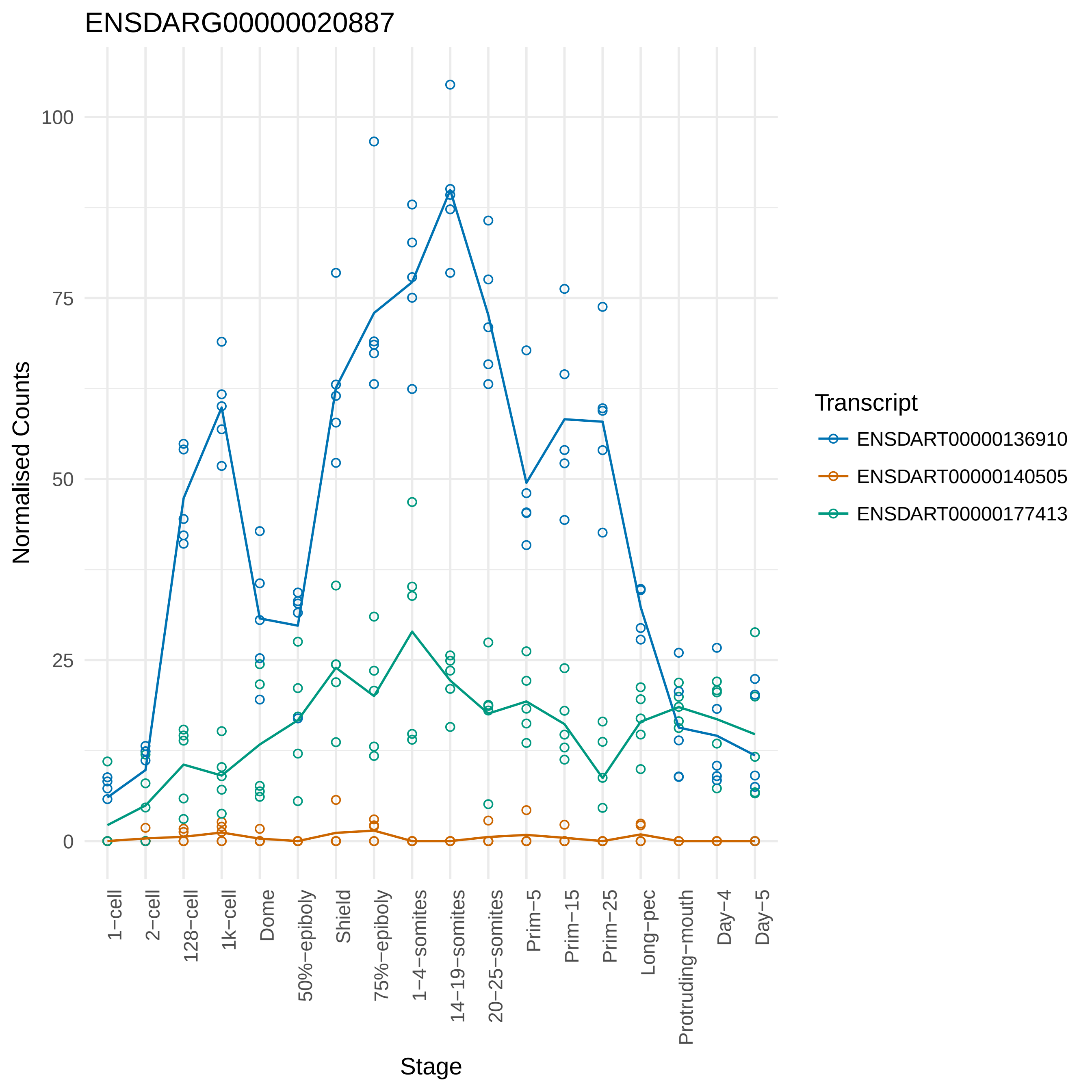

ENSDARG00000020887
Normalised Counts
100
75
50
25
0
1−cell
2−cell
128−cell
1k−cell
Dome
50%−epiboly
Shield
75%−epiboly
1−4−somites
14−19−somites
20−25−somites
Prim−5
Prim−15
Prim−25
Long−pec
Protruding−mouth
Day−4
Day−5
Stage
Transcript
ENSDART00000136910
ENSDART00000140505
ENSDART00000177413

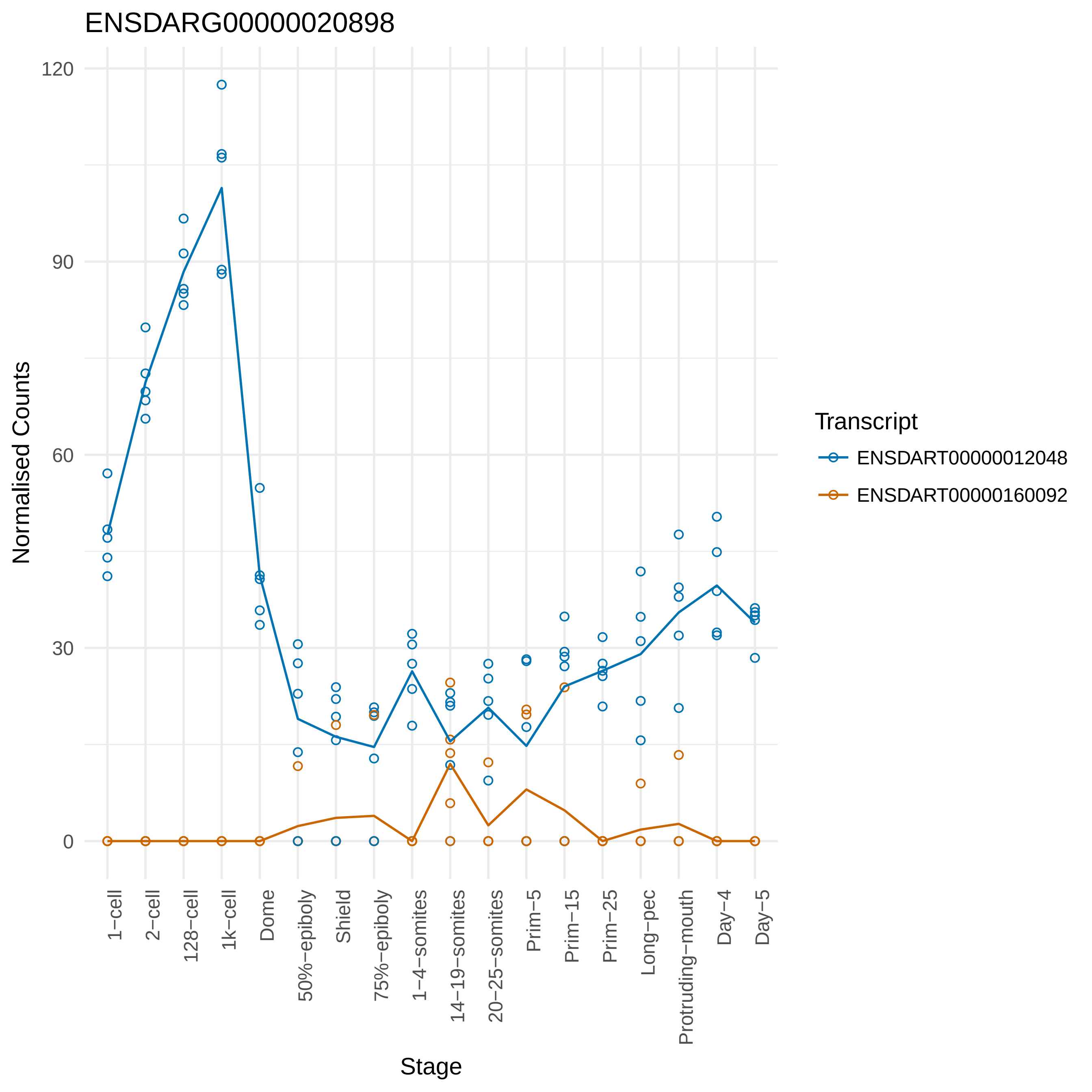
ENSDARG00000020898
Normalised Counts
120
90
60
30
0
1−cell
2−cell
128−cell
1k−cell
Dome
50%−epiboly
Shield
75%−epiboly
1−4−somites
14−19−somites
20−25−somites
Prim−5
Prim−15
Prim−25
Long−pec
Protruding−mouth
Day−4
Day−5
Stage
Transcript
ENSDART00000012048
ENSDART00000160092

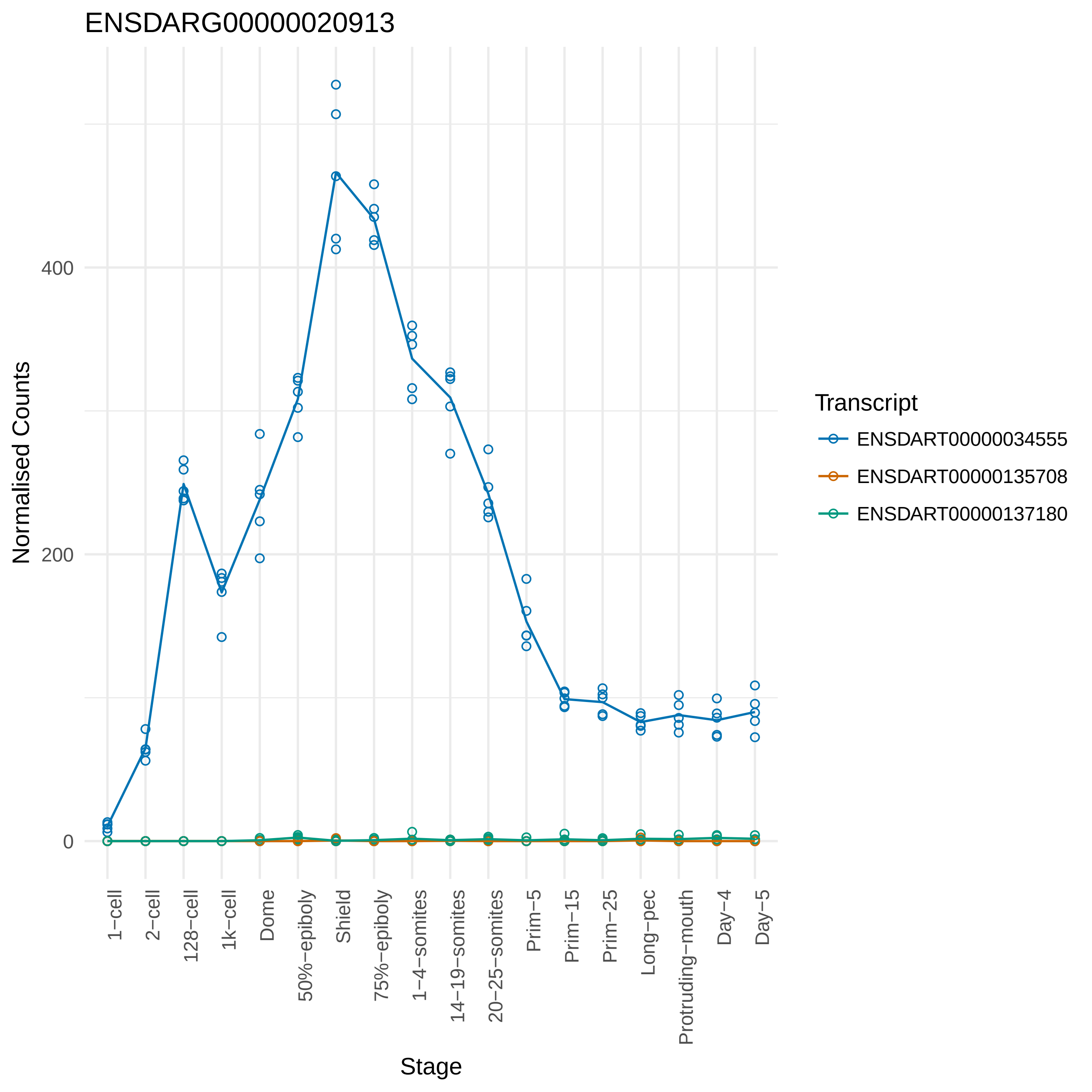

ENSDARG00000020913
Normalised Counts
400
200
0
1−cell
2−cell
128−cell
1k−cell
Dome
50%−epiboly
Shield
75%−epiboly
1−4−somites
14−19−somites
20−25−somites
Prim−5
Prim−15
Prim−25
Long−pec
Protruding−mouth
Day−4
Day−5
Stage
Transcript
ENSDART00000034555
ENSDART00000135708
ENSDART00000137180

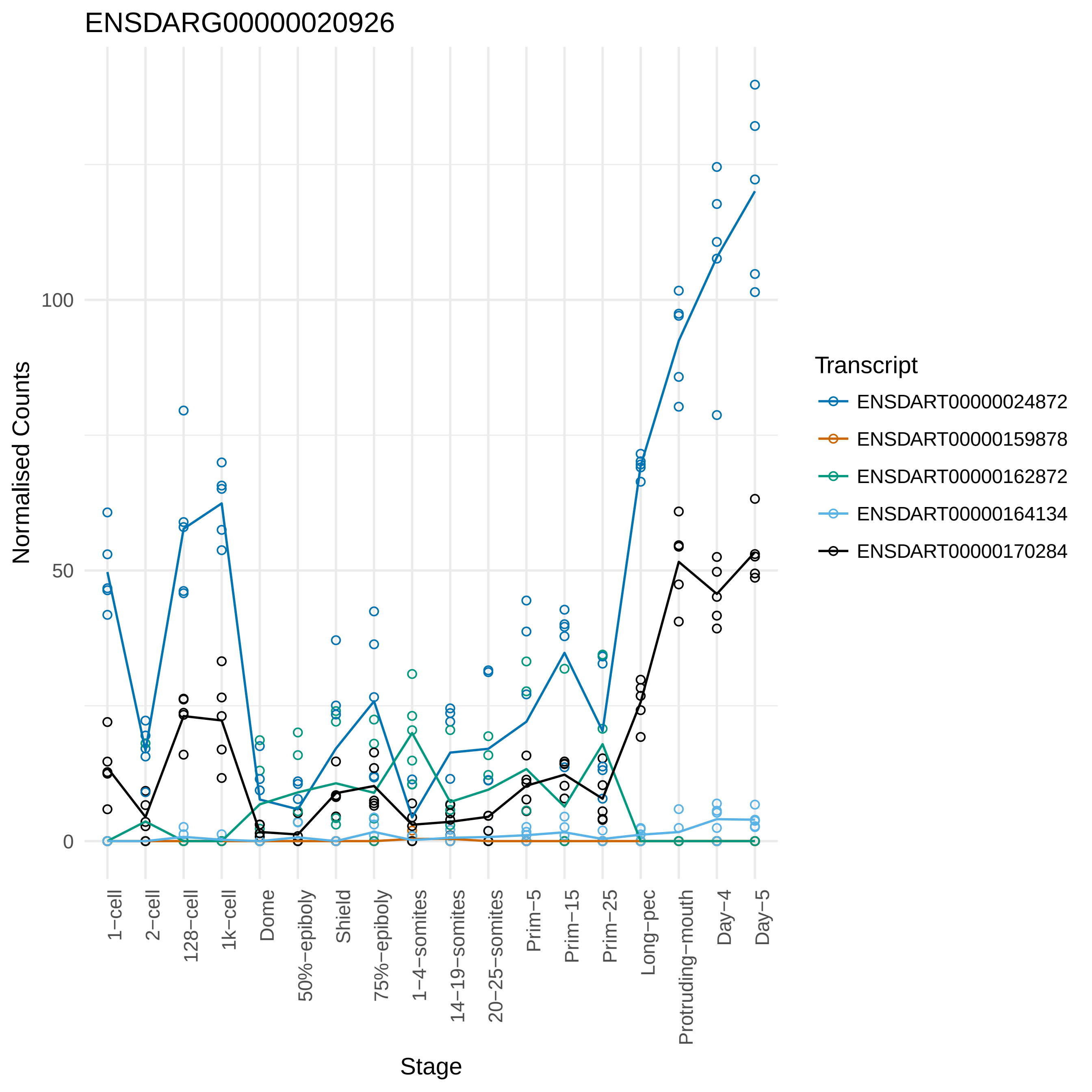

ENSDARG00000020926
Normalised Counts
0
50
100
1−cell
2−cell
128−cell
1k−cell
Dome
50%−epiboly
Shield
75%−epiboly
1−4−somites
14−19−somites
20−25−somites
Prim−5
Prim−15
Prim−25
Long−pec
Protruding−mouth
Day−4
Day−5
Stage
Transcript
ENSDART00000024872
ENSDART00000159878
ENSDART00000162872
ENSDART00000164134
ENSDART00000170284

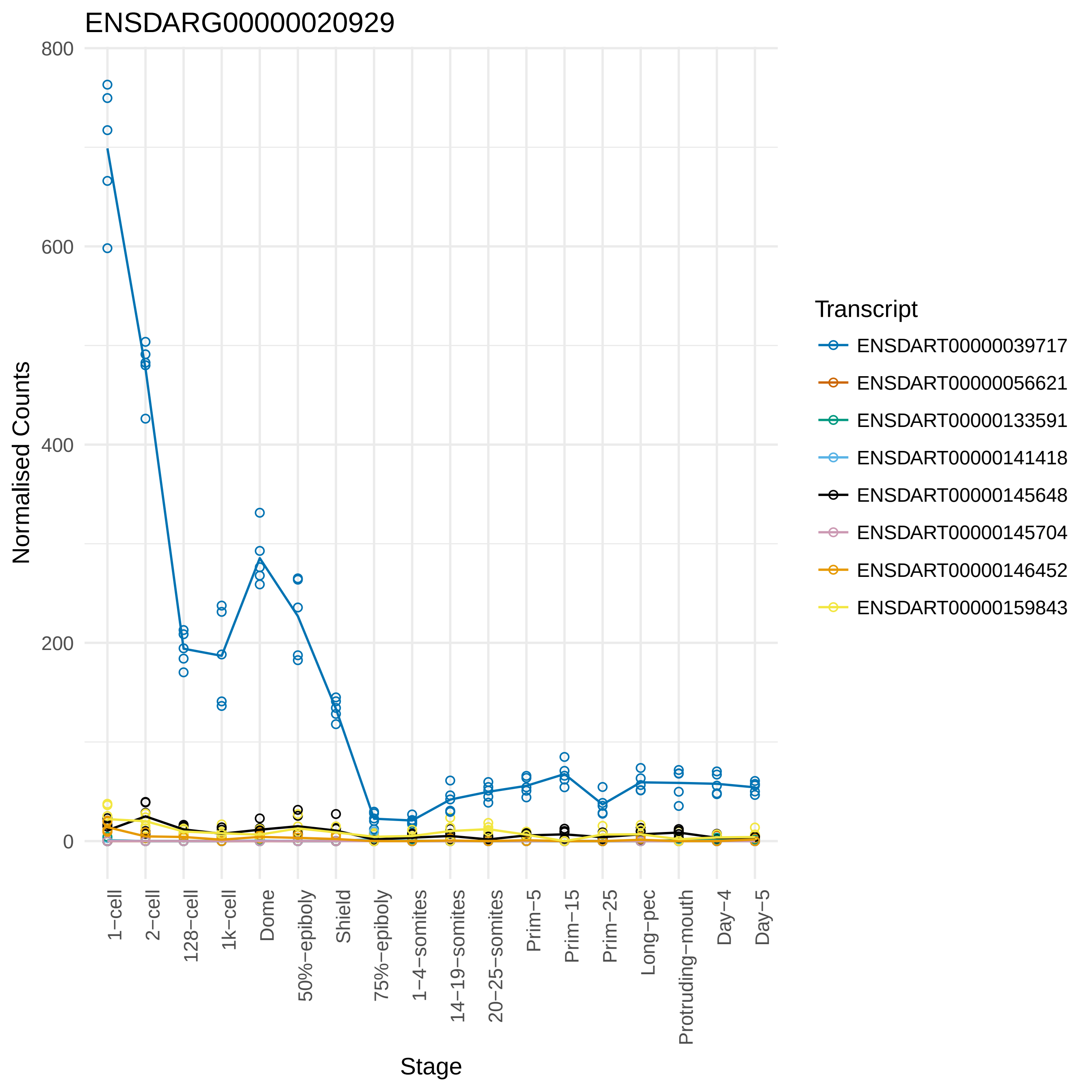

ENSDARG00000020929
Normalised Counts
800
600
400
200
0
1−cell
2−cell
128−cell
1k−cell
Dome
50%−epiboly
Shield
75%−epiboly
1−4−somites
14−19−somites
20−25−somites
Prim−5
Prim−15
Prim−25
Long−pec
Protruding−mouth
Day−4
Day−5
Stage
Transcript
ENSDART00000039717
ENSDART00000056621
ENSDART00000133591
ENSDART00000141418
ENSDART00000145648
ENSDART00000145704
ENSDART00000146452
ENSDART00000159843

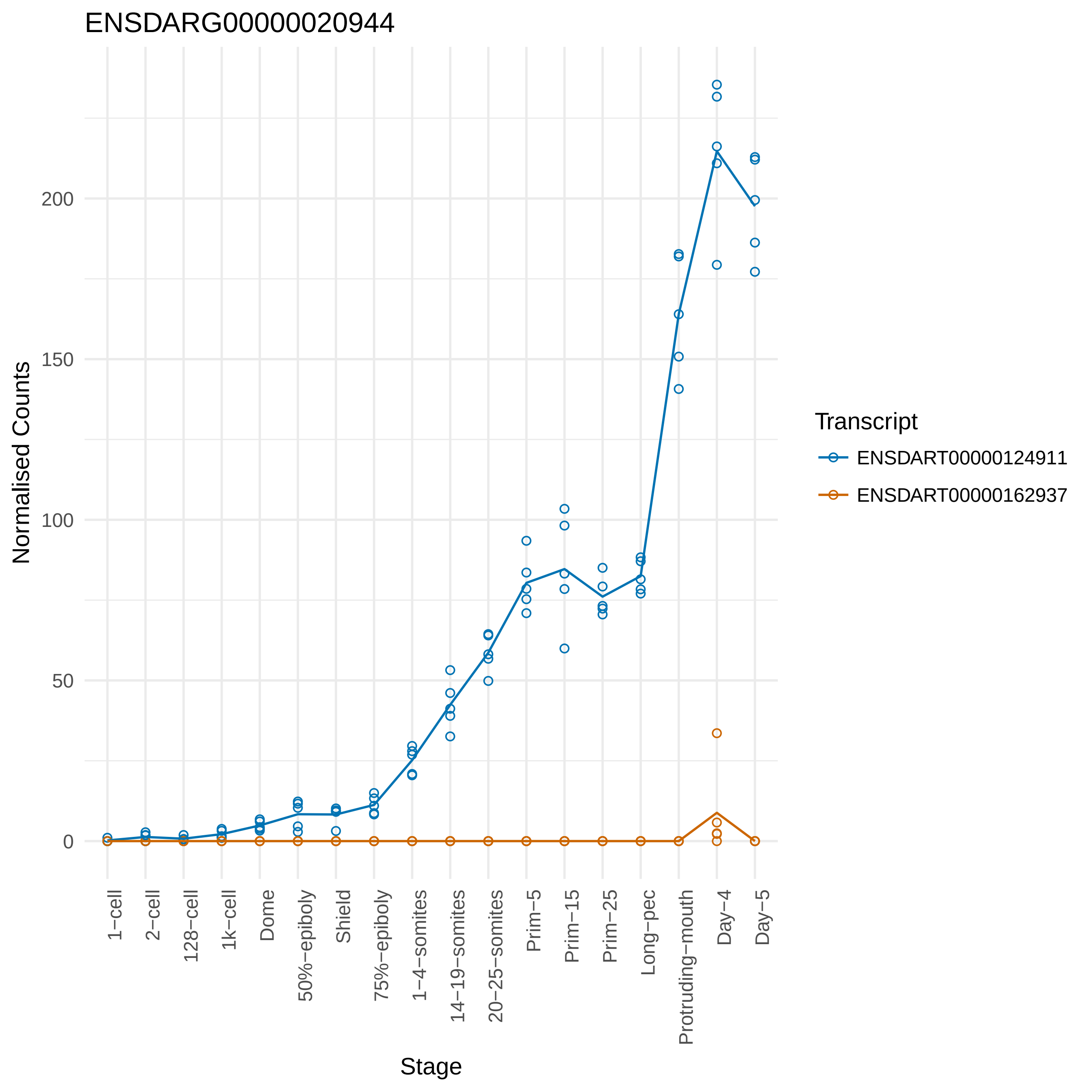

ENSDARG00000020944
Normalised Counts
0
50
100
150
200
1−cell
2−cell
128−cell
1k−cell
Dome
50%−epiboly
Shield
75%−epiboly
1−4−somites
14−19−somites
20−25−somites
Prim−5
Prim−15
Prim−25
Long−pec
Protruding−mouth
Day−4
Day−5
Stage
Transcript
ENSDART00000124911
ENSDART00000162937

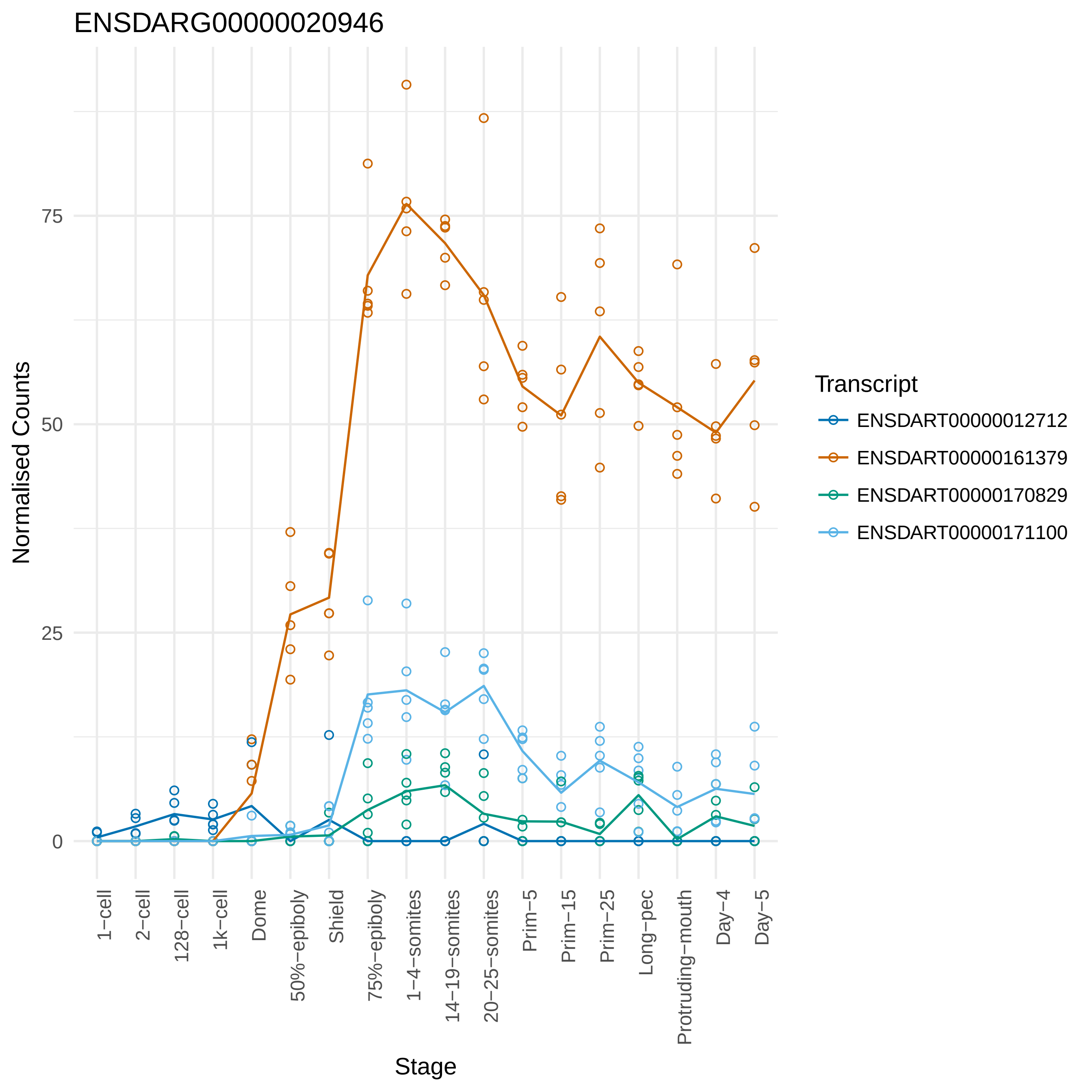
ENSDARG00000020946
Normalised Counts
0
25
50
75
Stage
1−cell
2−cell
128−cell
1k−cell
Dome
50%−epiboly
Shield
75%−epiboly
1−4−somites
14−19−somites
20−25−somites
Prim−5
Prim−15
Prim−25
Long−pec
Protruding−mouth
Day−4
Day−5
Transcript
ENSDART00000012712
ENSDART00000161379
ENSDART00000170829
ENSDART00000171100

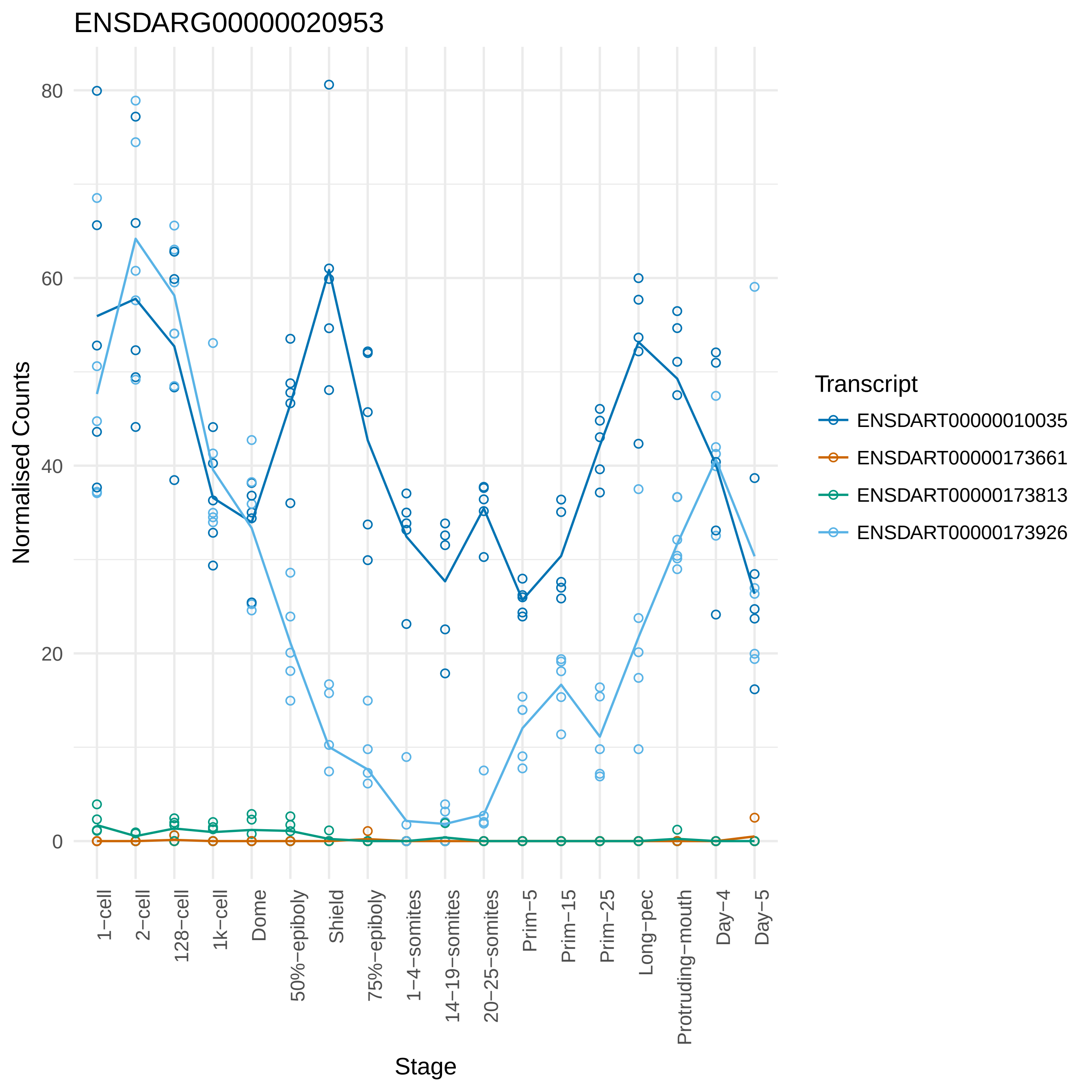

ENSDARG00000020953
Normalised Counts
80
60
40
20
0
1−cell
2−cell
128−cell
1k−cell
Dome
50%−epiboly
Shield
75%−epiboly
1−4−somites
14−19−somites
20−25−somites
Prim−5
Prim−15
Prim−25
Long−pec
Protruding−mouth
Day−4
Day−5
Stage
Transcript
ENSDART00000010035
ENSDART00000173661
ENSDART00000173813
ENSDART00000173926

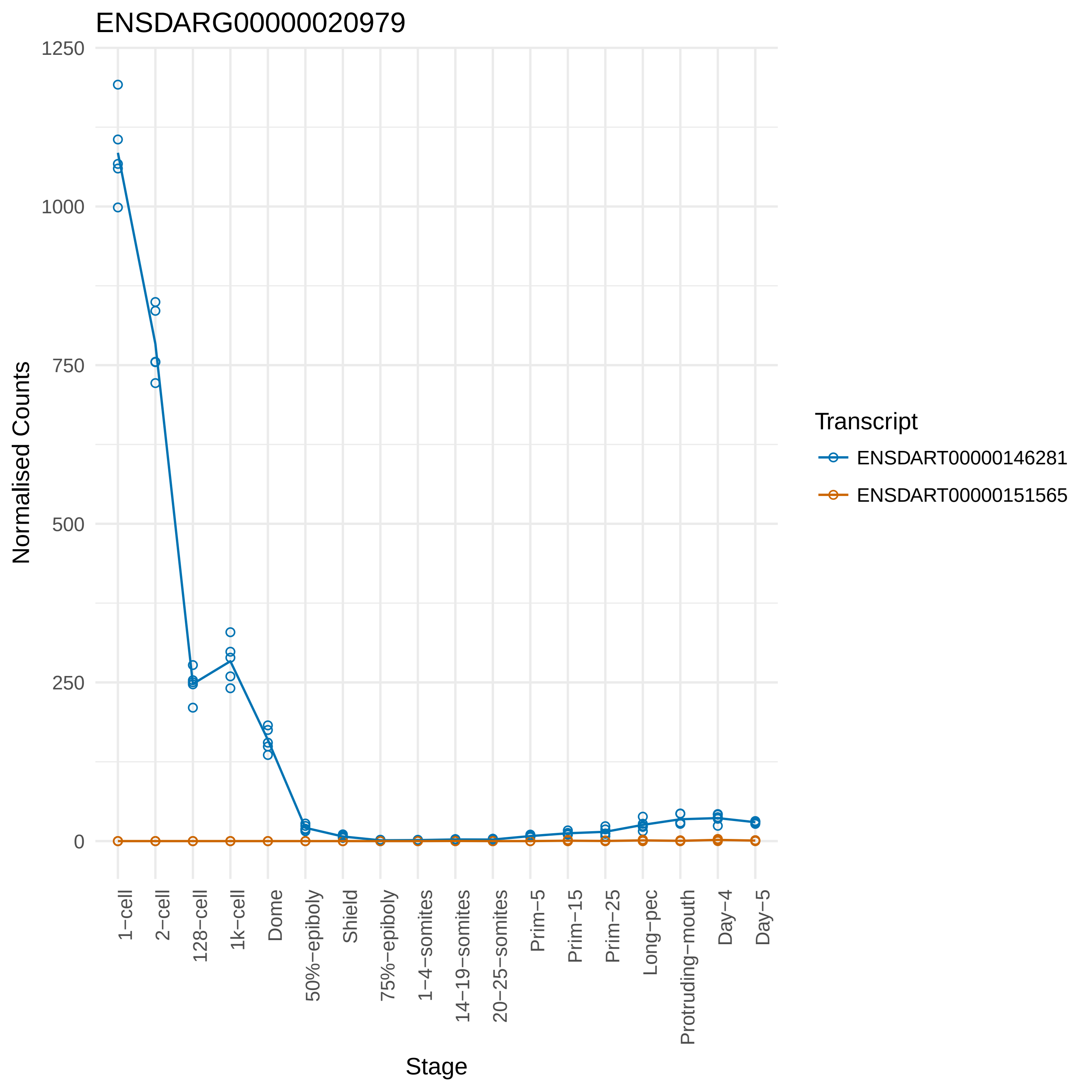

ENSDARG00000020979
Normalised Counts
1250
1000
750
500
250
0
1−cell
2−cell
128−cell
1k−cell
Dome
50%−epiboly
Shield
75%−epiboly
1−4−somites
14−19−somites
20−25−somites
Prim−5
Prim−15
Prim−25
Long−pec
Protruding−mouth
Day−4
Day−5
Stage
Transcript
ENSDART00000146281
ENSDART00000151565

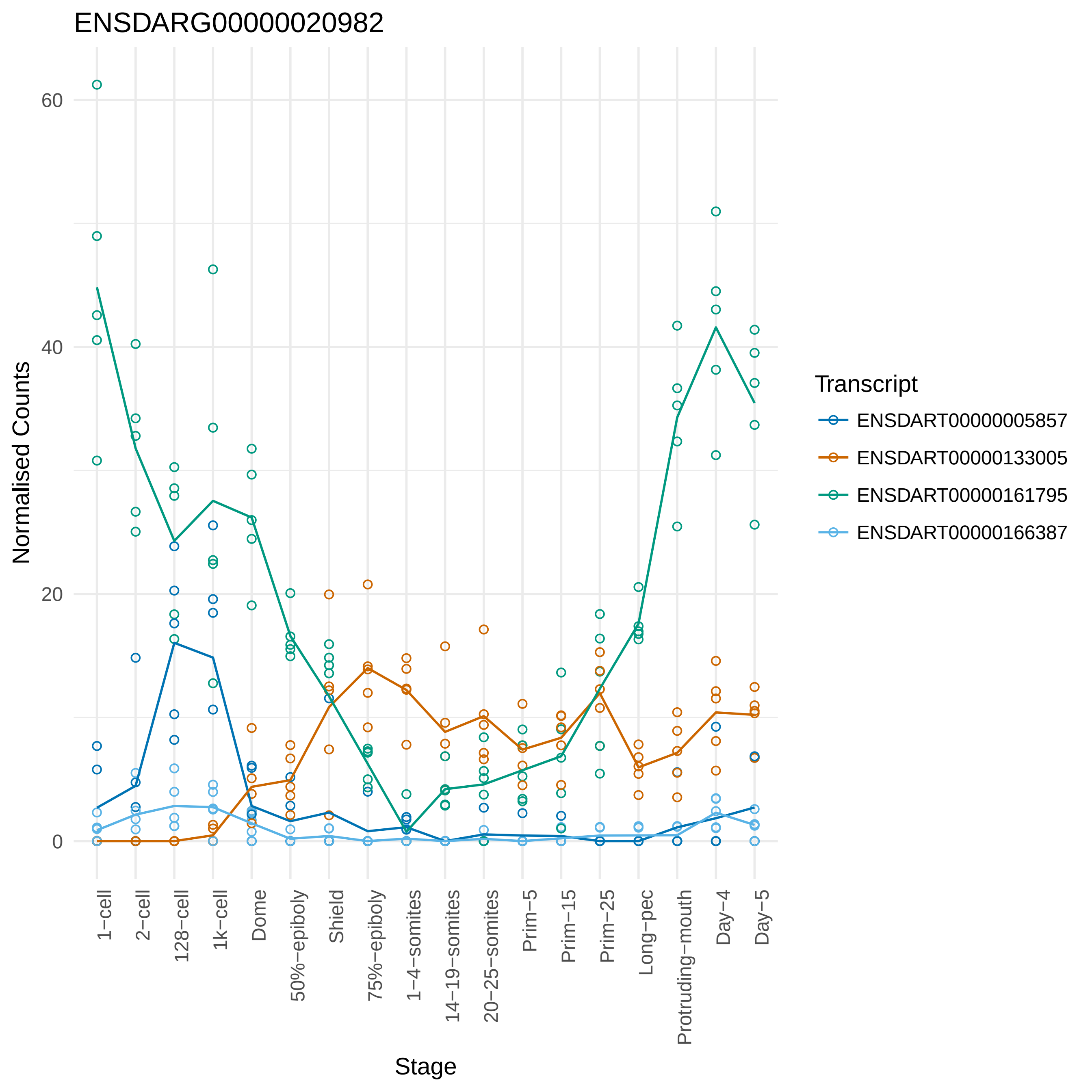
ENSDARG00000020982
Normalised Counts
0
20
40
60
Stage
1−cell
2−cell
128−cell
1k−cell
Dome
50%−epiboly
Shield
75%−epiboly
1−4−somites
14−19−somites
20−25−somites
Prim−5
Prim−15
Prim−25
Long−pec
Protruding−mouth
Day−4
Day−5
Transcript
ENSDART00000005857
ENSDART00000133005
ENSDART00000161795
ENSDART00000166387

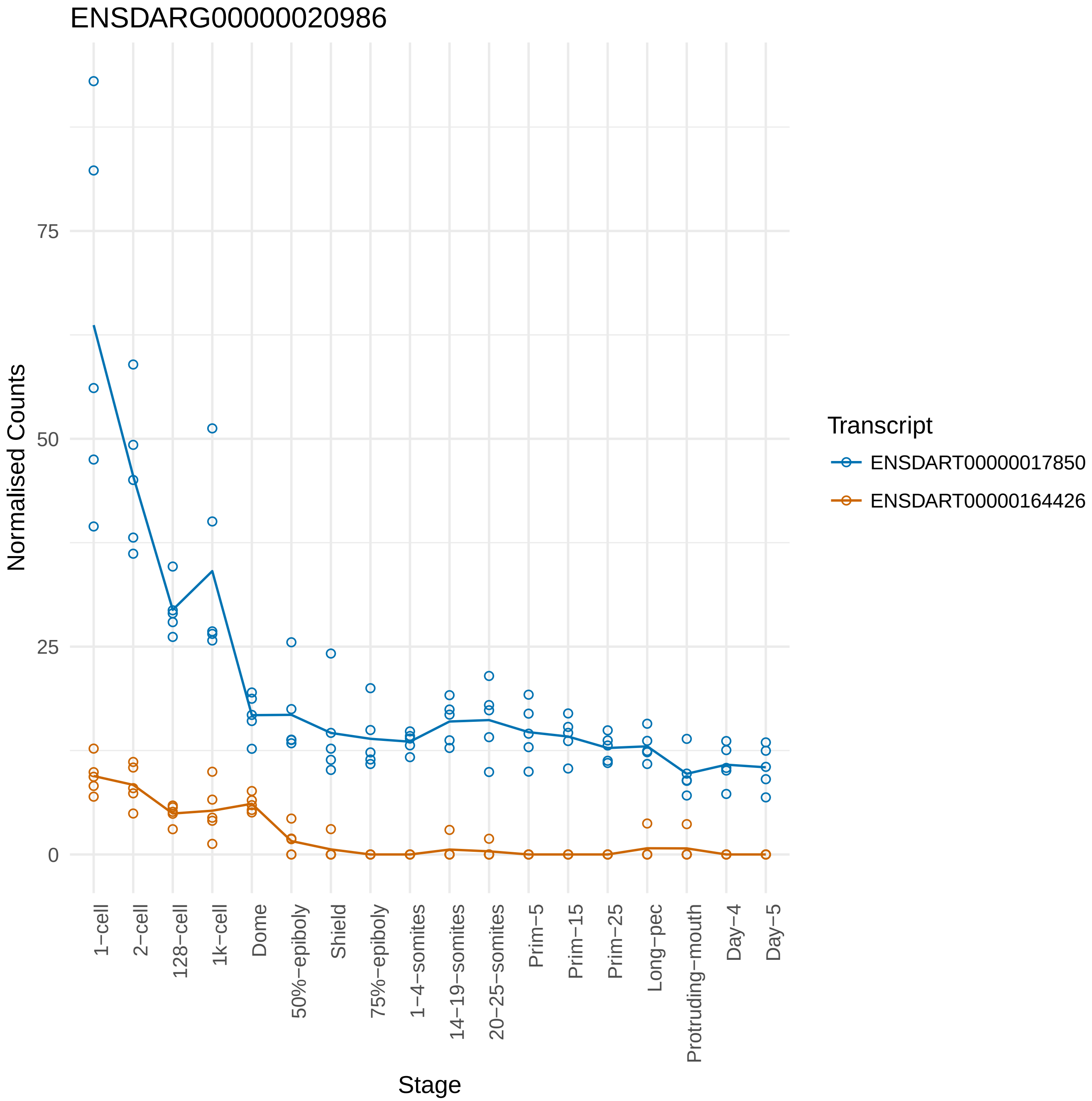

ENSDARG00000020986
Normalised Counts
75
50
25
0
1−cell
2−cell
128−cell
1k−cell
Dome
50%−epiboly
Shield
75%−epiboly
1−4−somites
14−19−somites
20−25−somites
Prim−5
Prim−15
Prim−25
Long−pec
Protruding−mouth
Day−4
Day−5
Stage
Transcript
ENSDART00000017850
ENSDART00000164426

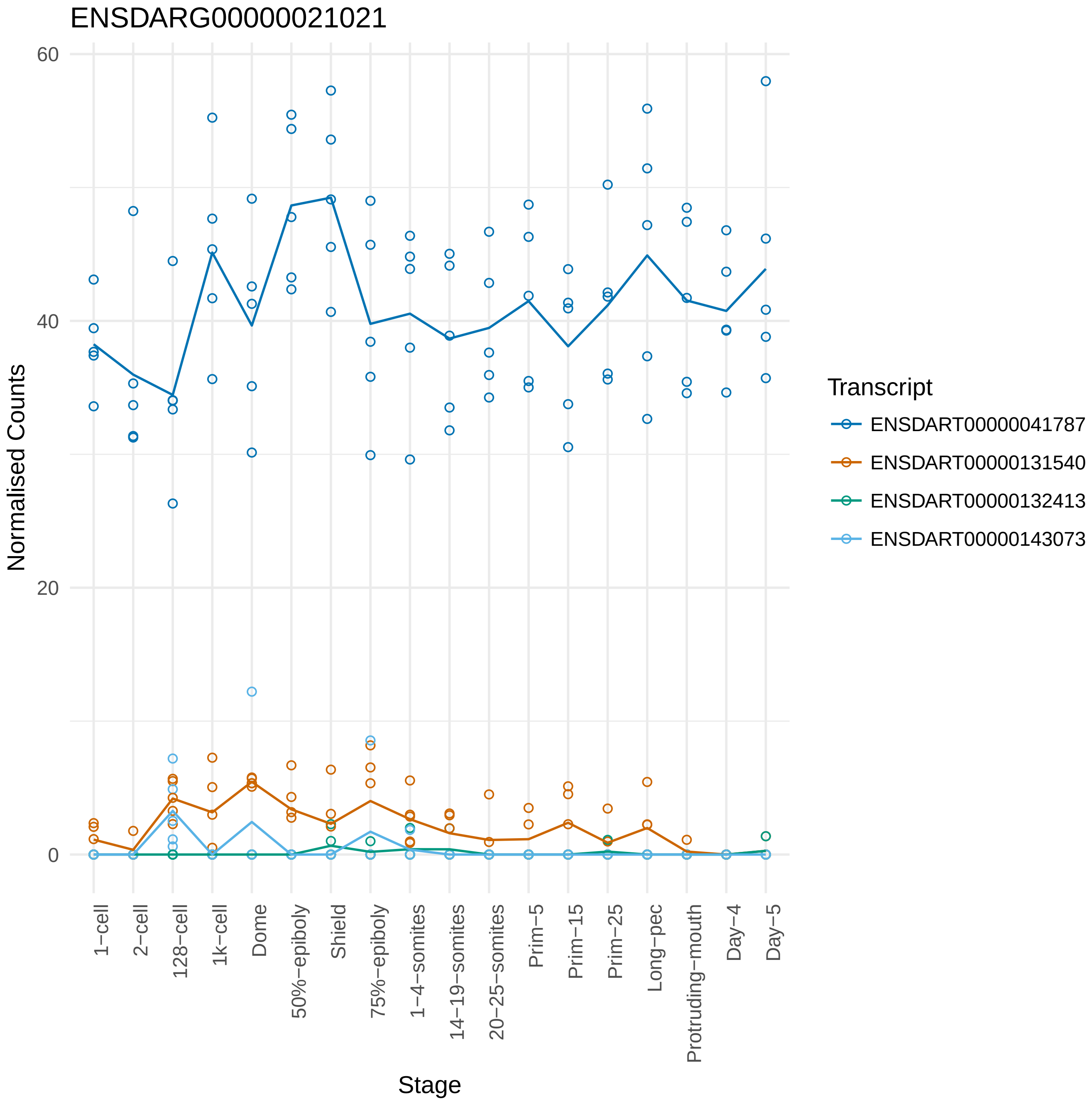

ENSDARG00000021021
Normalised Counts
60
40
20
0
1−cell
2−cell
128−cell
1k−cell
Dome
50%−epiboly
Shield
75%−epiboly
1−4−somites
14−19−somites
20−25−somites
Prim−5
Prim−15
Prim−25
Long−pec
Protruding−mouth
Day−4
Day−5
Stage
Transcript
ENSDART00000041787
ENSDART00000131540
ENSDART00000132413
ENSDART00000143073

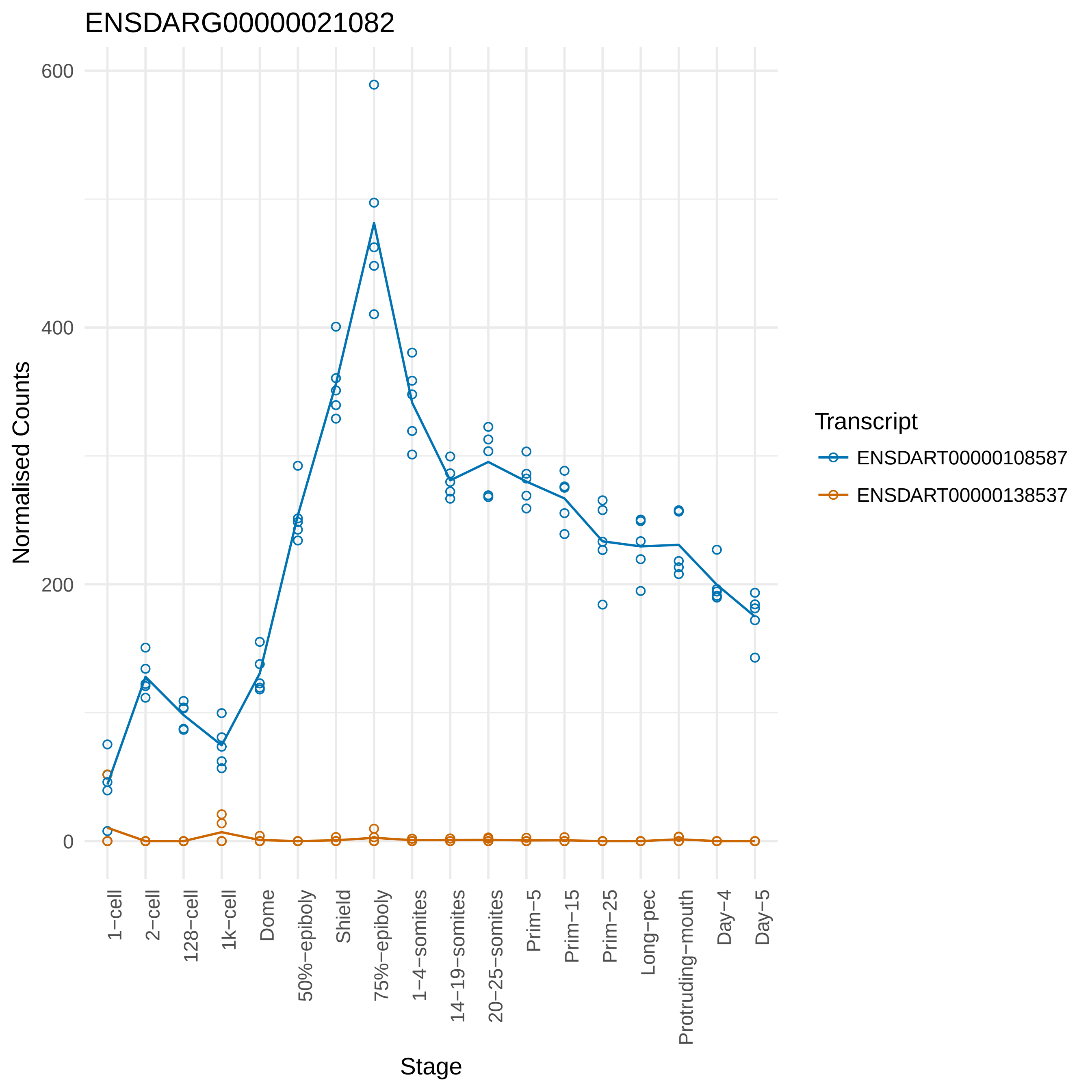

ENSDARG00000021082
Normalised Counts
600
400
200
0
1−cell
2−cell
128−cell
1k−cell
Dome
50%−epiboly
Shield
75%−epiboly
1−4−somites
14−19−somites
20−25−somites
Prim−5
Prim−15
Prim−25
Long−pec
Protruding−mouth
Day−4
Day−5
Stage
Transcript
ENSDART00000108587
ENSDART00000138537

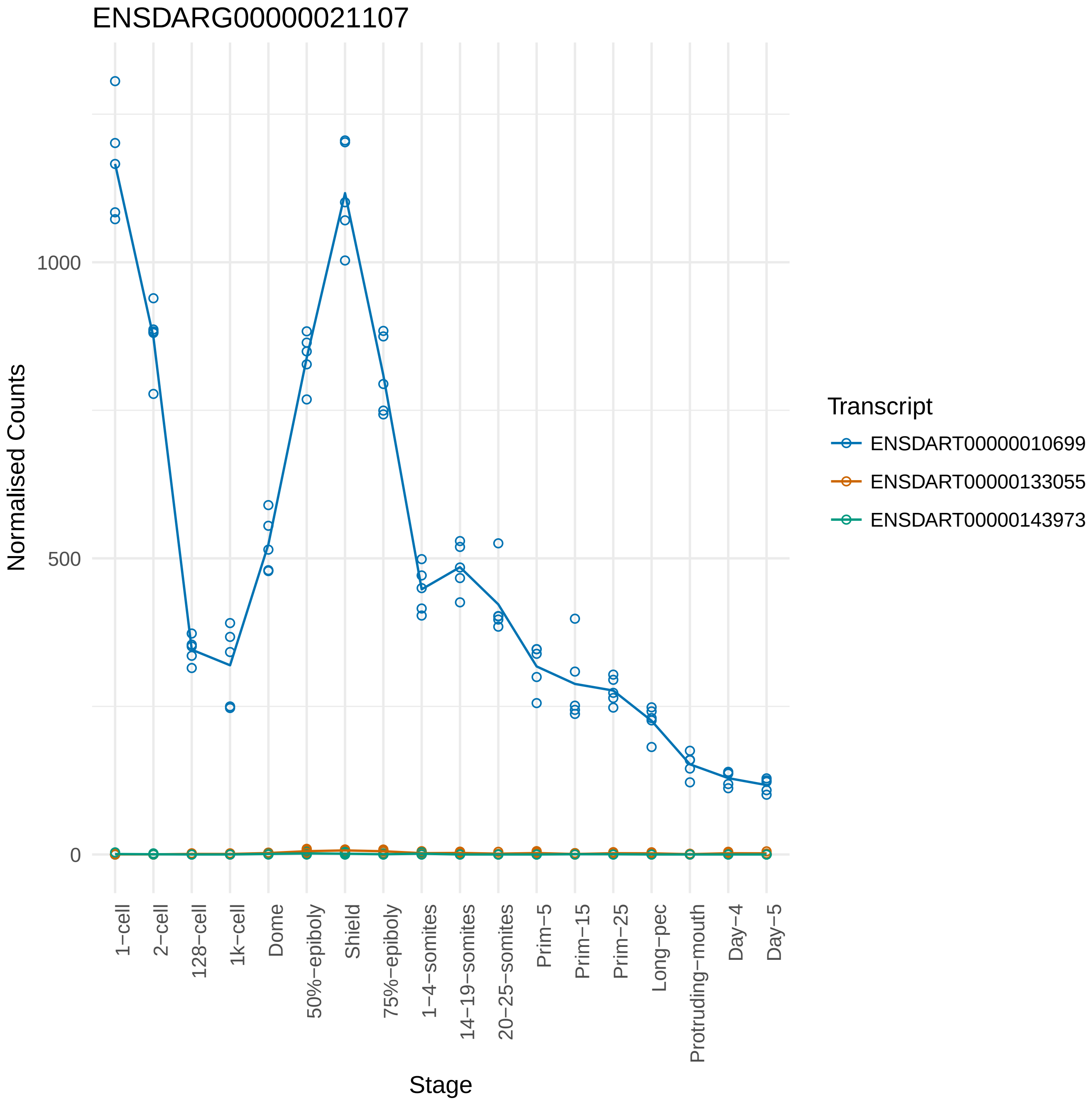

ENSDARG00000021107
Normalised Counts
1000
500
0
1−cell
2−cell
128−cell
1k−cell
Dome
50%−epiboly
Shield
75%−epiboly
1−4−somites
14−19−somites
20−25−somites
Prim−5
Prim−15
Prim−25
Long−pec
Protruding−mouth
Day−4
Day−5
Stage
Transcript
ENSDART00000010699
ENSDART00000133055
ENSDART00000143973

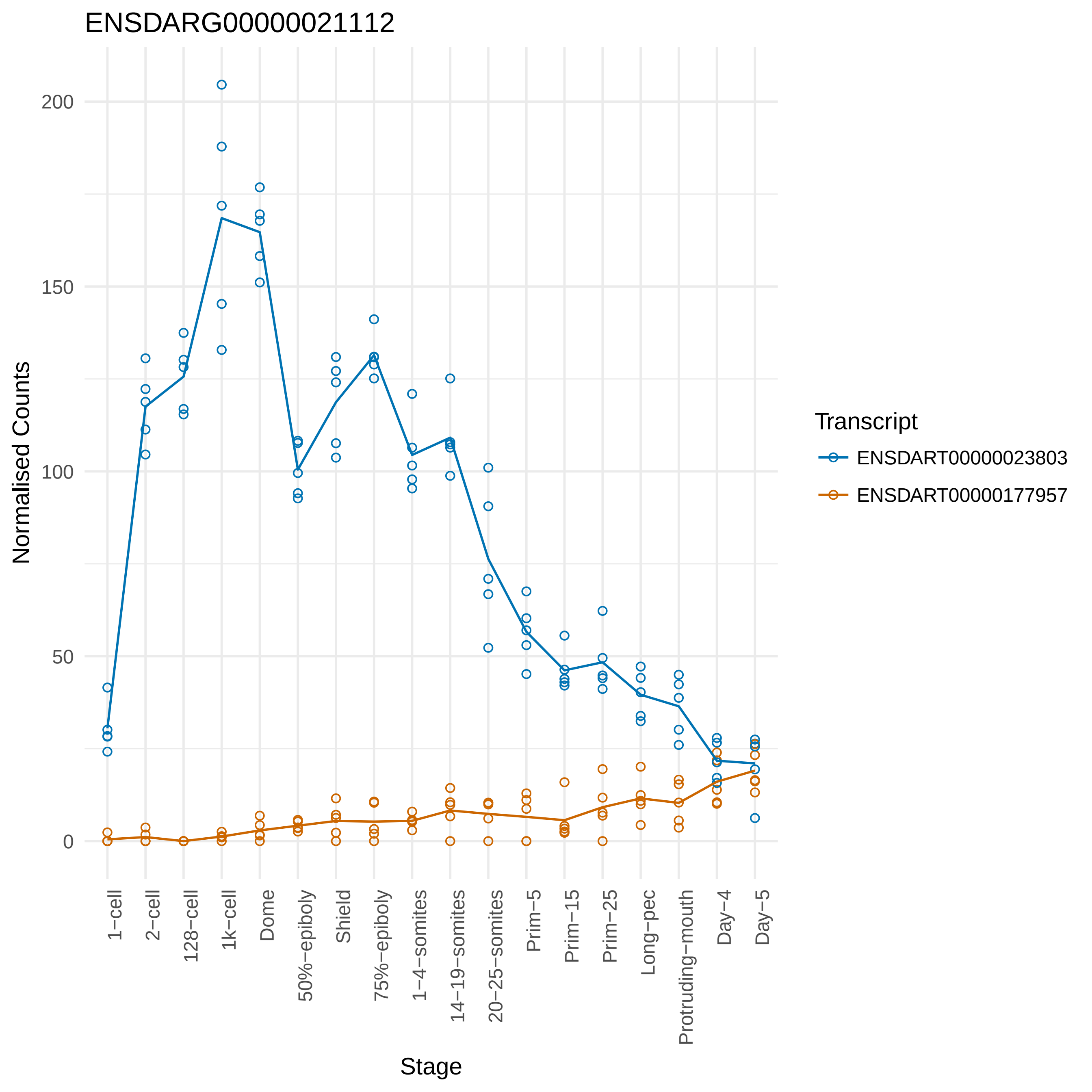

ENSDARG00000021112
Normalised Counts
200
150
100
50
0
1−cell
2−cell
128−cell
1k−cell
Dome
50%−epiboly
Shield
75%−epiboly
1−4−somites
14−19−somites
20−25−somites
Prim−5
Prim−15
Prim−25
Long−pec
Protruding−mouth
Day−4
Day−5
Stage
Transcript
ENSDART00000023803
ENSDART00000177957

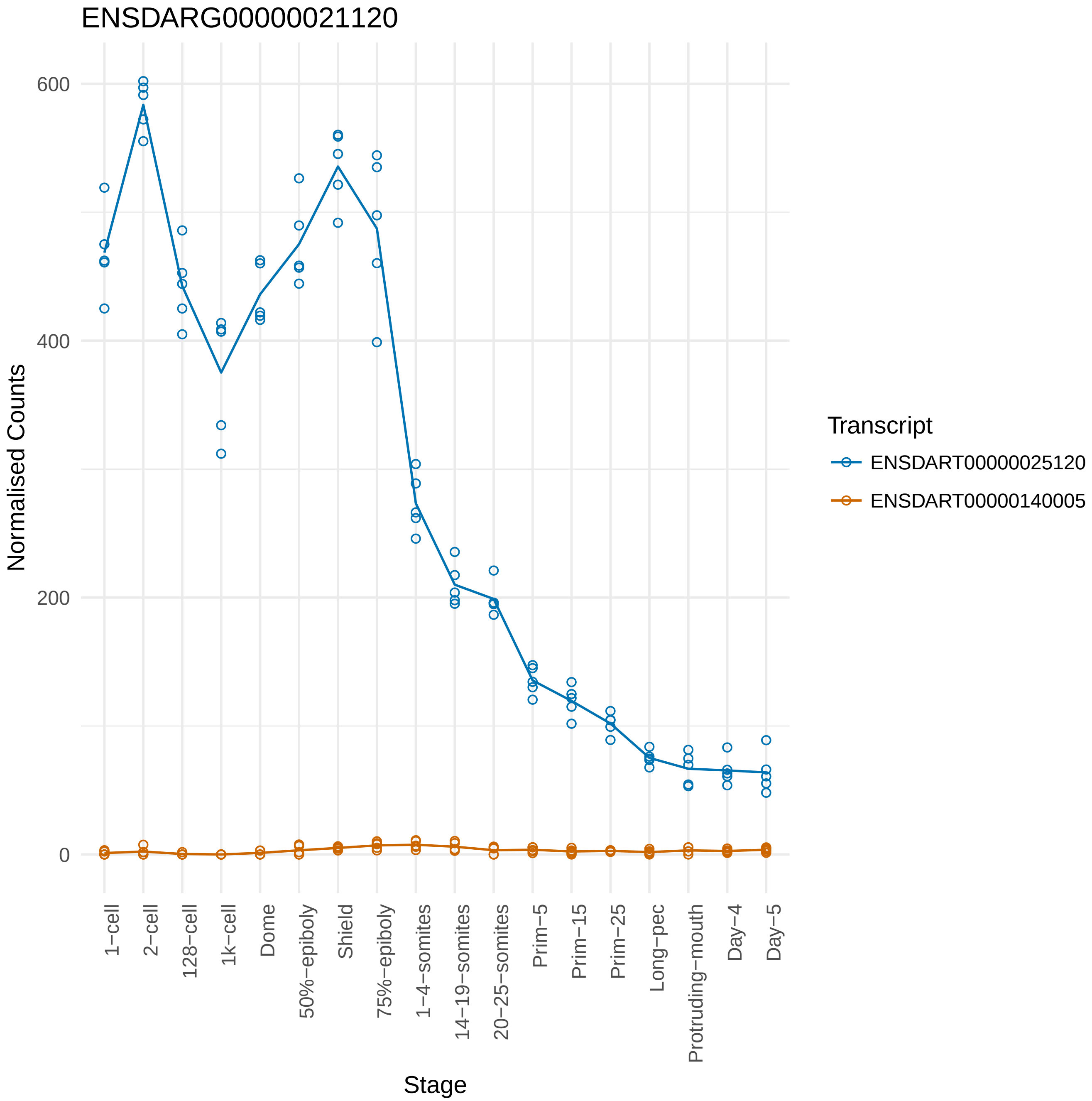
ENSDARG00000021120
600
400
200
0
Normalised Counts
1−cell
2−cell
128−cell
1k−cell
Dome
50%−epiboly
Shield
75%−epiboly
1−4−somites
14−19−somites
20−25−somites
Prim−5
Prim−15
Prim−25
Long−pec
Protruding−mouth
Day−4
Day−5
Stage
Transcript
ENSDART00000025120
ENSDART00000140005

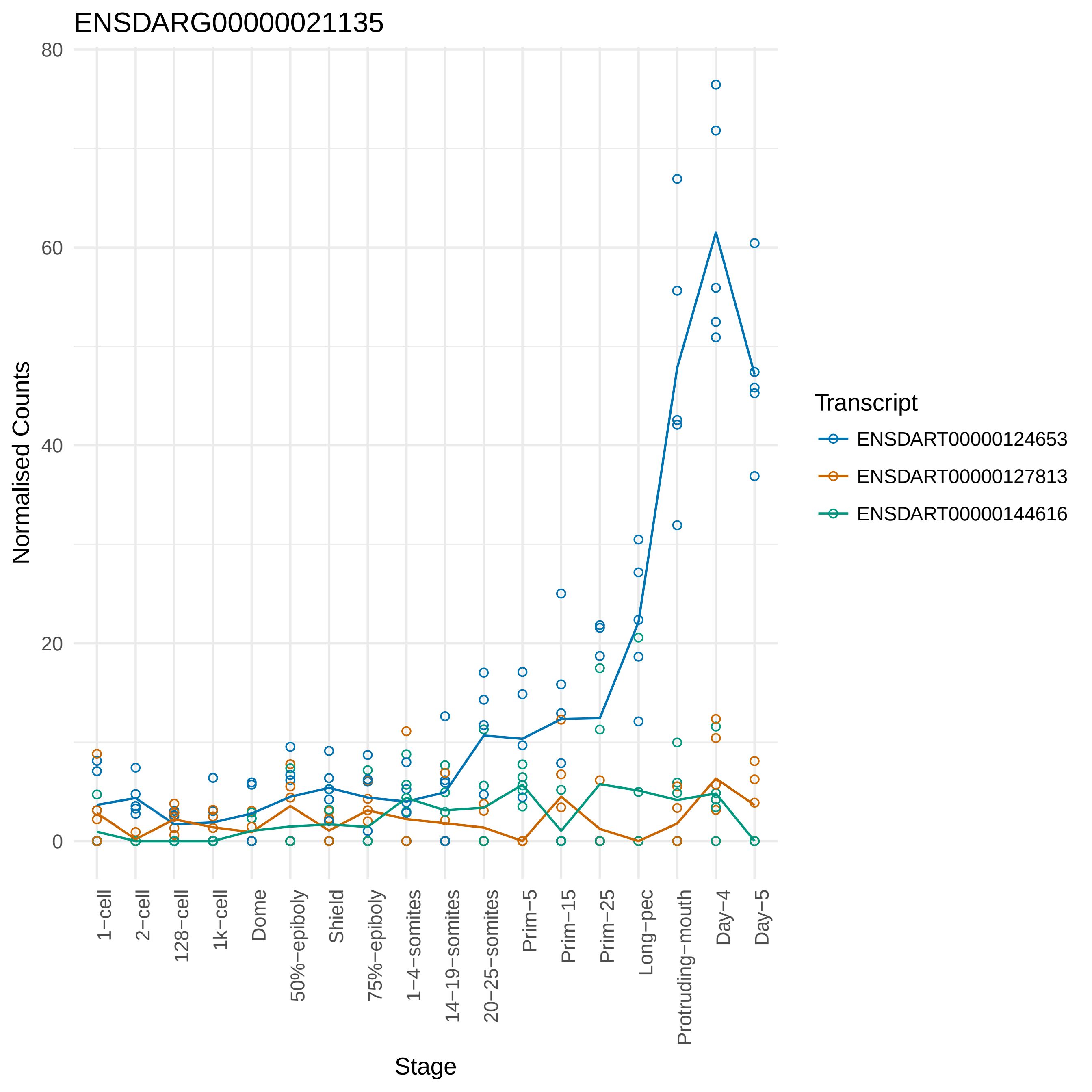
ENSDARG00000021135
Normalised Counts
80
60
40
20
0
Stage
1−cell
2−cell
128−cell
1k−cell
Dome
50%−epiboly
Shield
75%−epiboly
1−4−somites
14−19−somites
20−25−somites
Prim−5
Prim−15
Prim−25
Long−pec
Protruding−mouth
Day−4
Day−5
Transcript
ENSDART00000124653
ENSDART00000127813
ENSDART00000144616

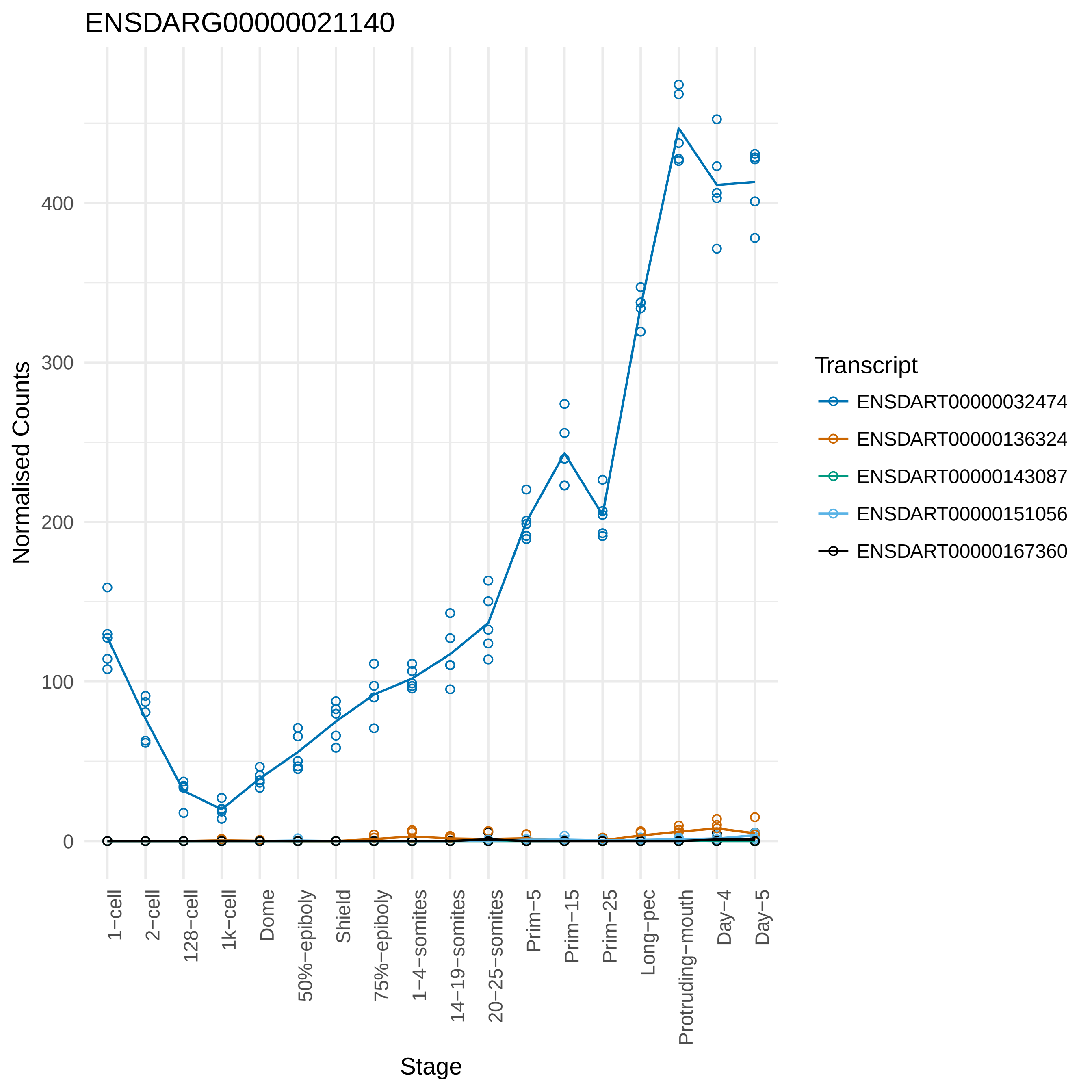
ENSDARG00000021140
Normalised Counts
0
100
200
300
400
Stage
1−cell
2−cell
128−cell
1k−cell
Dome
50%−epiboly
Shield
75%−epiboly
1−4−somites
14−19−somites
20−25−somites
Prim−5
Prim−15
Prim−25
Long−pec
Protruding−mouth
Day−4
Day−5
Transcript
ENSDART00000032474
ENSDART00000136324
ENSDART00000143087
ENSDART00000151056
ENSDART00000167360

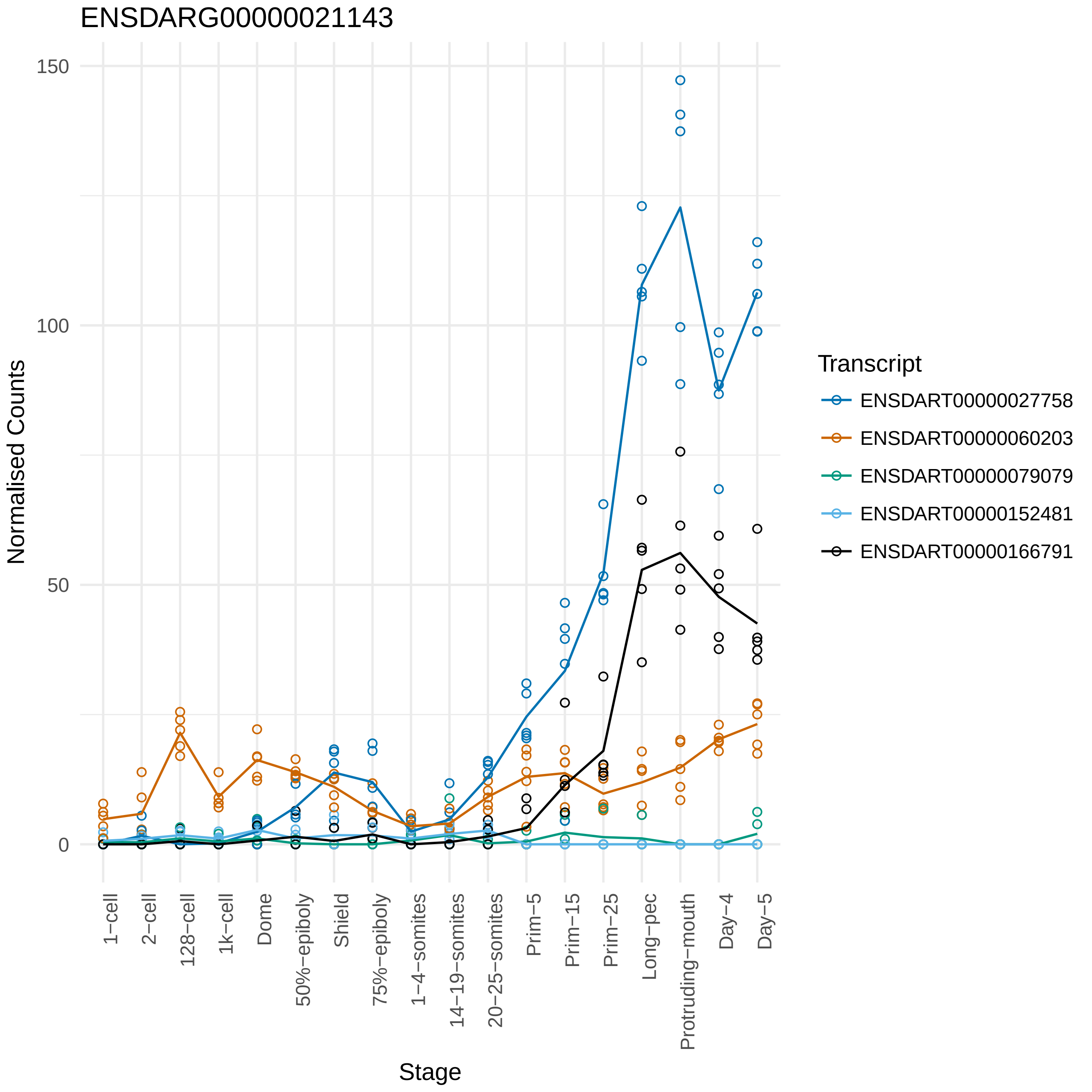
ENSDARG00000021143
Normalised Counts
150
100
50
0
Stage
1−cell
2−cell
128−cell
1k−cell
Dome
50%−epiboly
Shield
75%−epiboly
1−4−somites
14−19−somites
20−25−somites
Prim−5
Prim−15
Prim−25
Long−pec
Protruding−mouth
Day−4
Day−5
Transcript
ENSDART00000027758
ENSDART00000060203
ENSDART00000079079
ENSDART00000152481
ENSDART00000166791

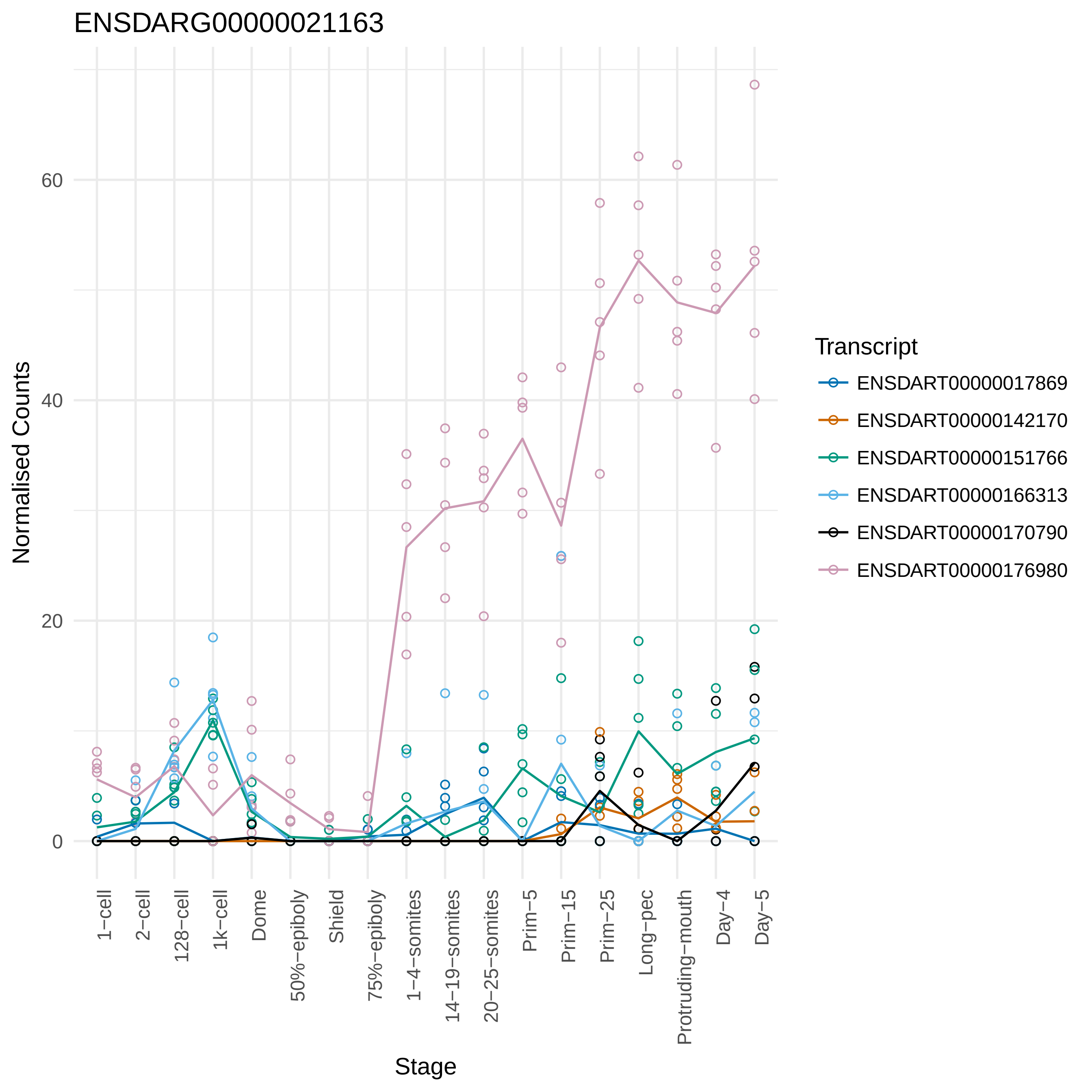

ENSDARG00000021163
Normalised Counts
0
20
40
60
1−cell
2−cell
128−cell
1k−cell
Dome
50%−epiboly
Shield
75%−epiboly
1−4−somites
14−19−somites
20−25−somites
Prim−5
Prim−15
Prim−25
Long−pec
Protruding−mouth
Day−4
Day−5
Stage
Transcript
ENSDART00000017869
ENSDART00000142170
ENSDART00000151766
ENSDART00000166313
ENSDART00000170790
ENSDART00000176980

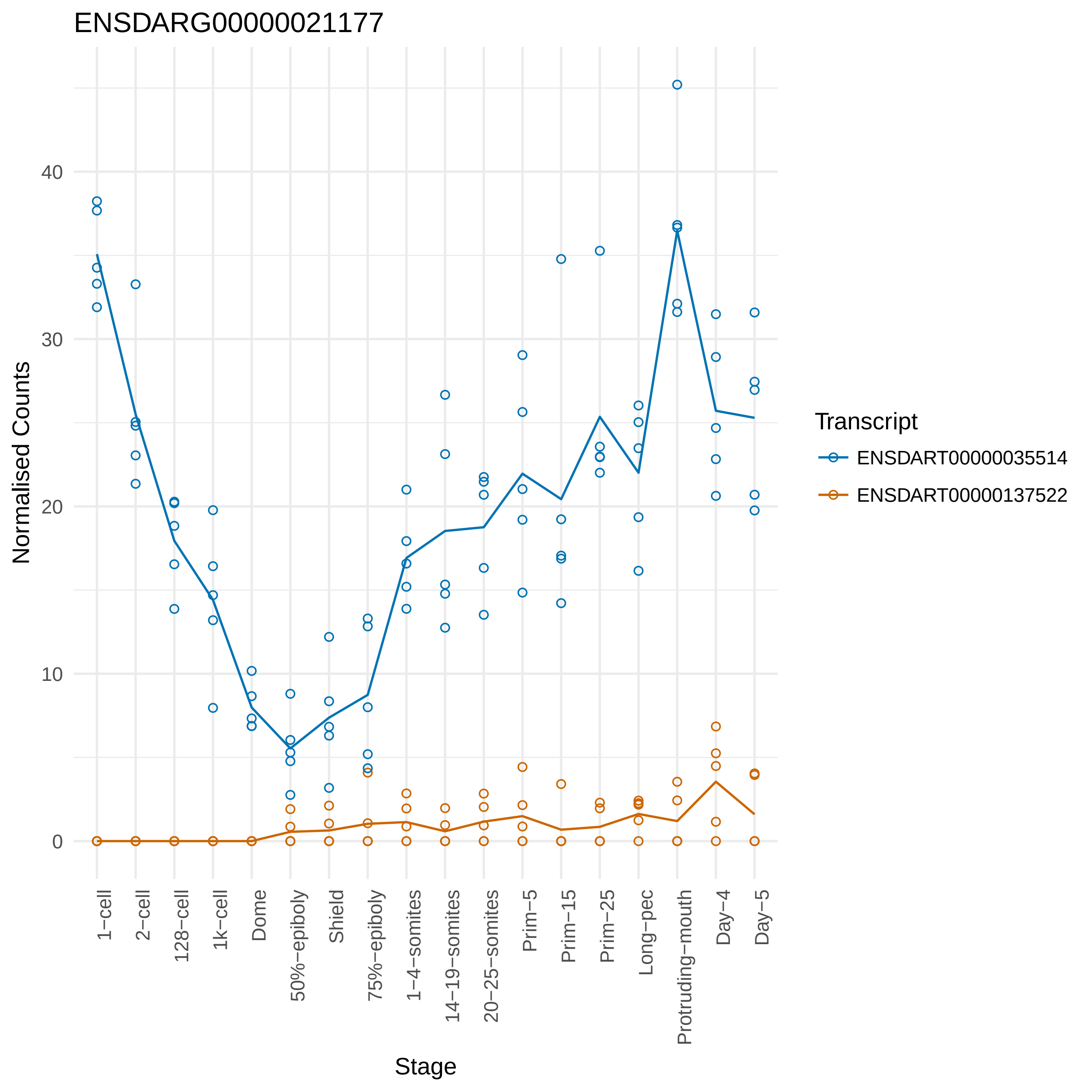

ENSDARG00000021177
Normalised Counts
0
10
20
30
40
1−cell
2−cell
128−cell
1k−cell
Dome
50%−epiboly
Shield
75%−epiboly
1−4−somites
14−19−somites
20−25−somites
Prim−5
Prim−15
Prim−25
Long−pec
Protruding−mouth
Day−4
Day−5
Stage
Transcript
ENSDART00000035514
ENSDART00000137522

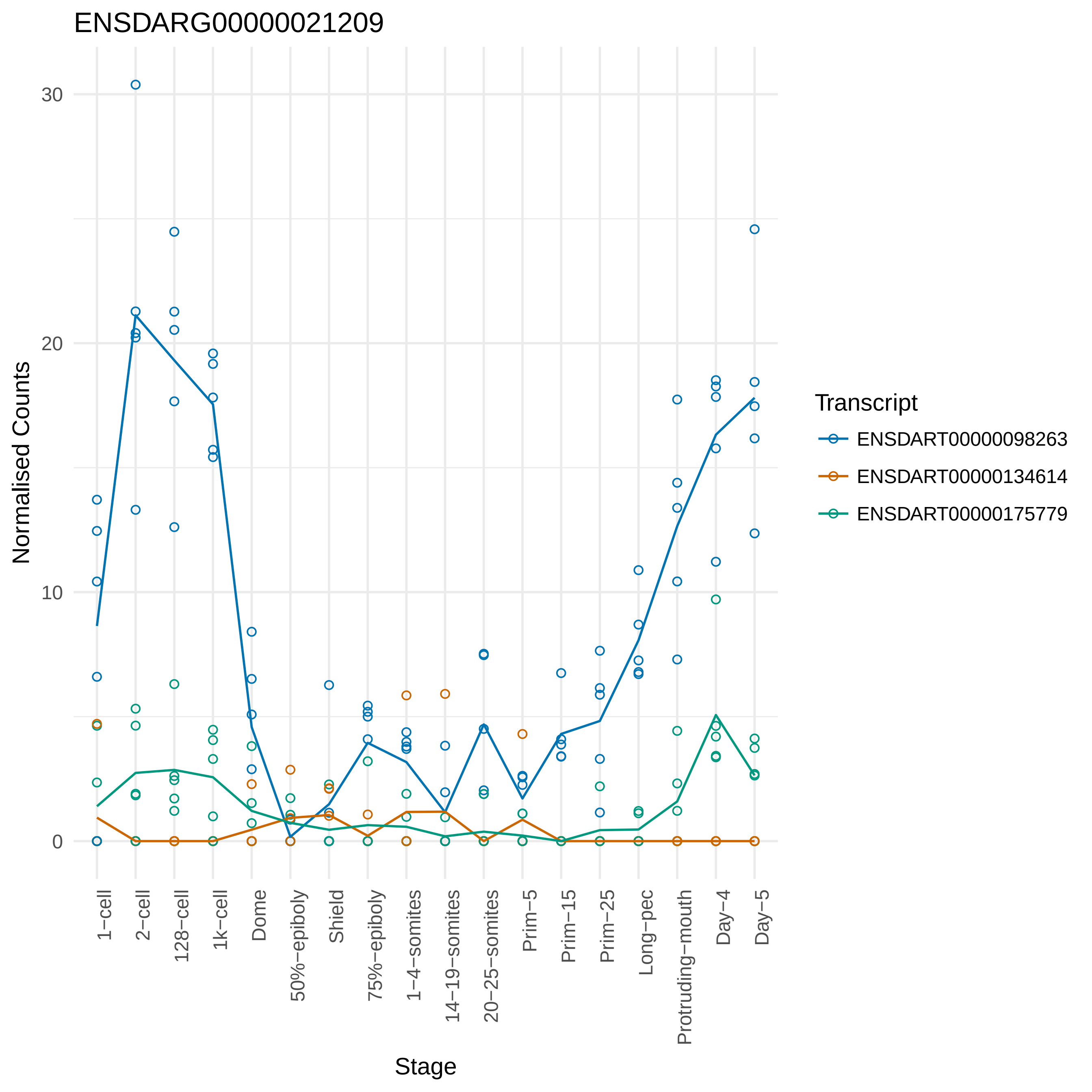
ENSDARG00000021209
Normalised Counts
30
20
10
0
1−cell
2−cell
128−cell
1k−cell
Dome
50%−epiboly
Shield
75%−epiboly
1−4−somites
14−19−somites
20−25−somites
Prim−5
Prim−15
Prim−25
Long−pec
Protruding−mouth
Day−4
Day−5
Stage
Transcript
ENSDART00000098263
ENSDART00000134614
ENSDART00000175779

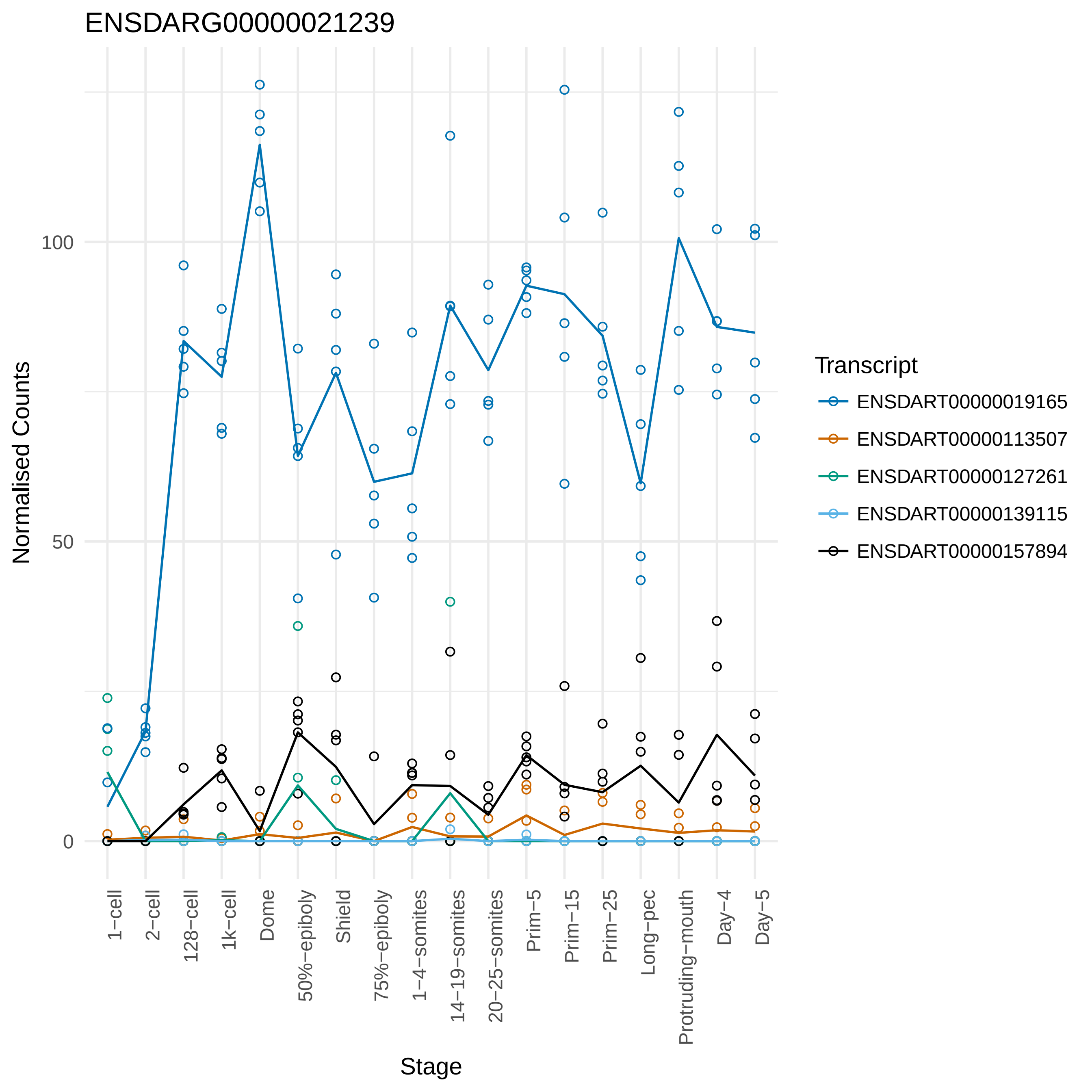

ENSDARG00000021239
Normalised Counts
100
50
0
1−cell
2−cell
128−cell
1k−cell
Dome
50%−epiboly
Shield
75%−epiboly
1−4−somites
14−19−somites
20−25−somites
Prim−5
Prim−15
Prim−25
Long−pec
Protruding−mouth
Day−4
Day−5
Stage
Transcript
ENSDART00000019165
ENSDART00000113507
ENSDART00000127261
ENSDART00000139115
ENSDART00000157894

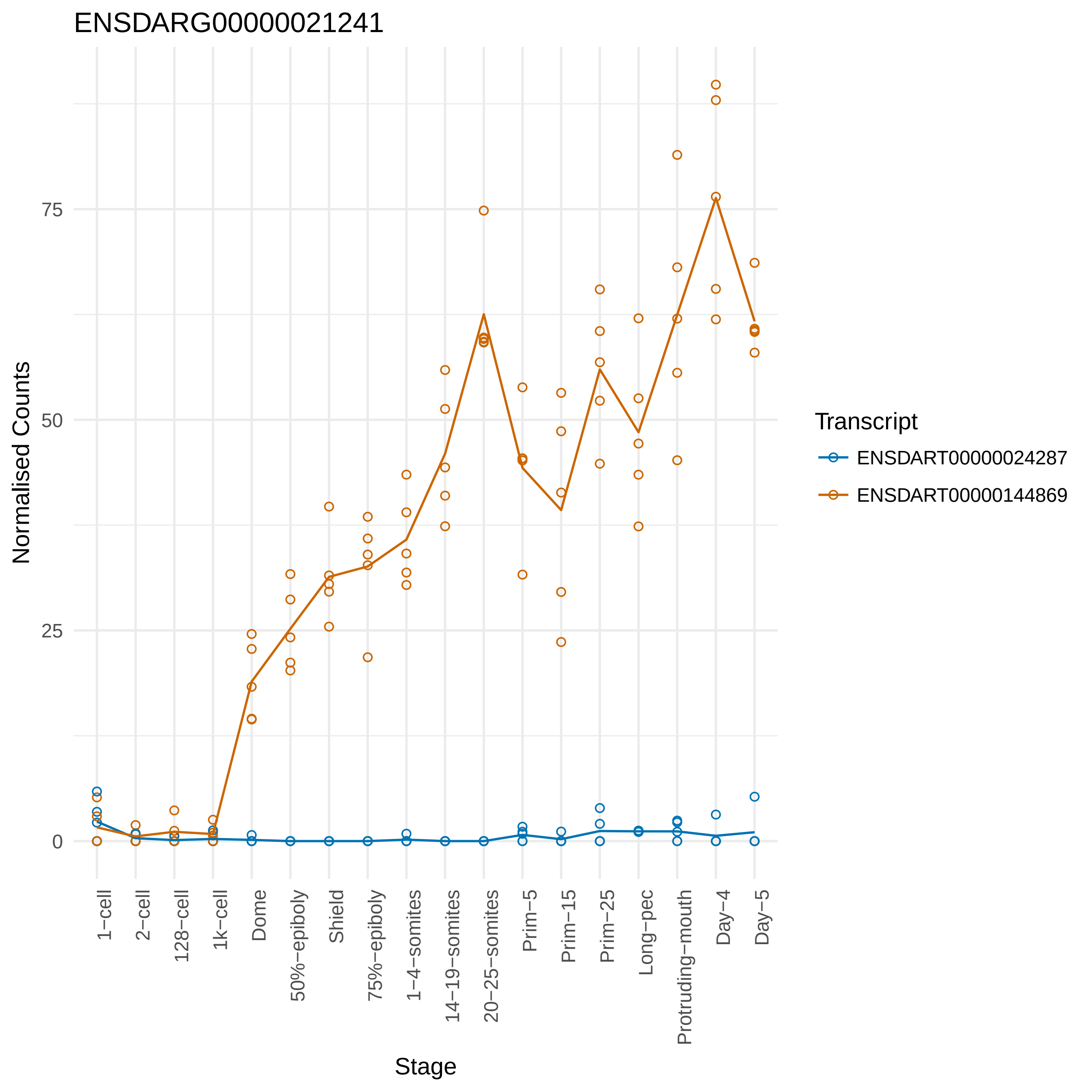
ENSDARG00000021241
Normalised Counts
0
25
50
75
1−cell
2−cell
128−cell
1k−cell
Dome
50%−epiboly
Shield
75%−epiboly
1−4−somites
14−19−somites
20−25−somites
Prim−5
Prim−15
Prim−25
Long−pec
Protruding−mouth
Day−4
Day−5
Stage
Transcript
ENSDART00000024287
ENSDART00000144869

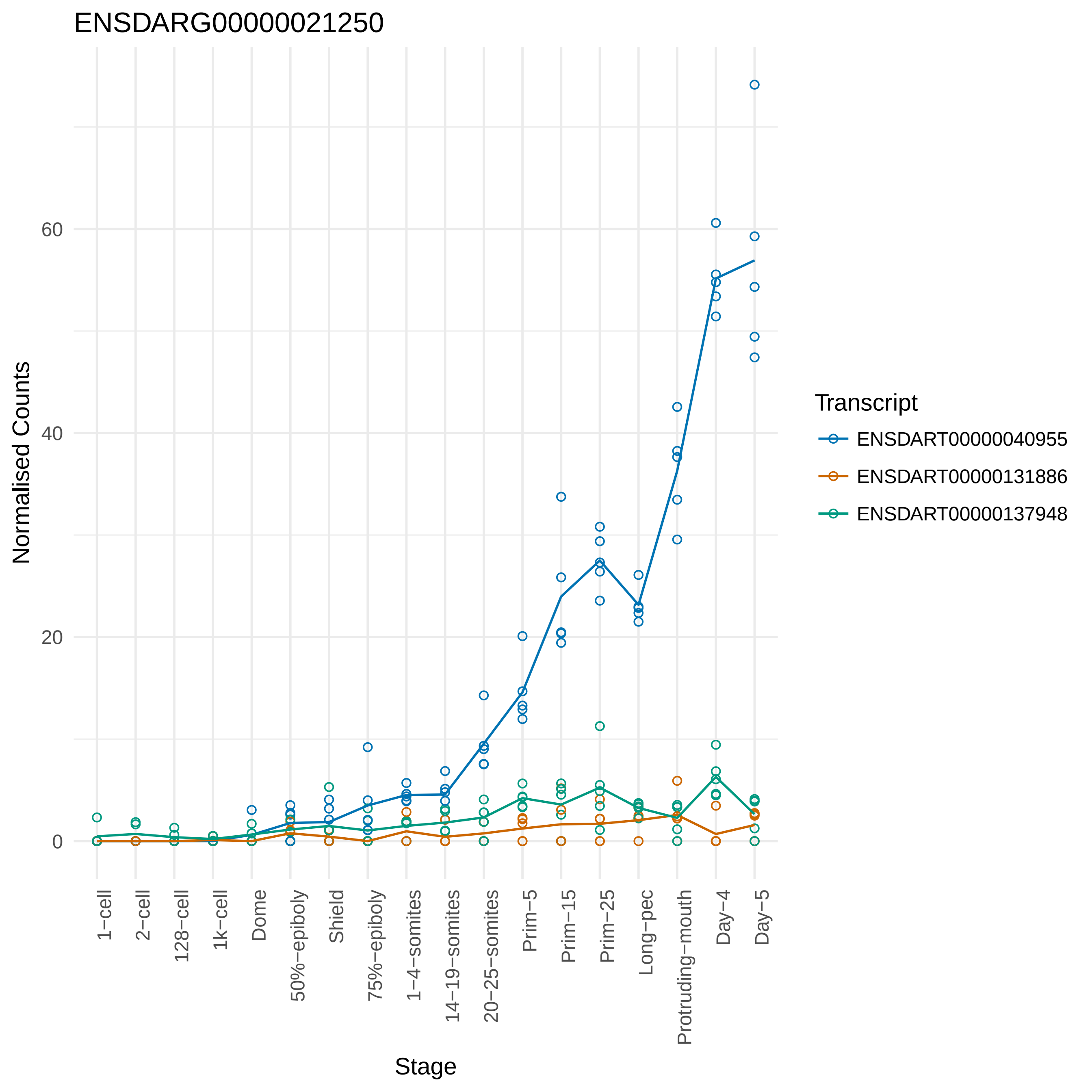
ENSDARG00000021250
Normalised Counts
60
40
20
0
1−cell
2−cell
128−cell
1k−cell
Dome
50%−epiboly
Shield
75%−epiboly
1−4−somites
14−19−somites
20−25−somites
Prim−5
Prim−15
Prim−25
Long−pec
Protruding−mouth
Day−4
Day−5
Stage
Transcript
ENSDART00000040955
ENSDART00000131886
ENSDART00000137948

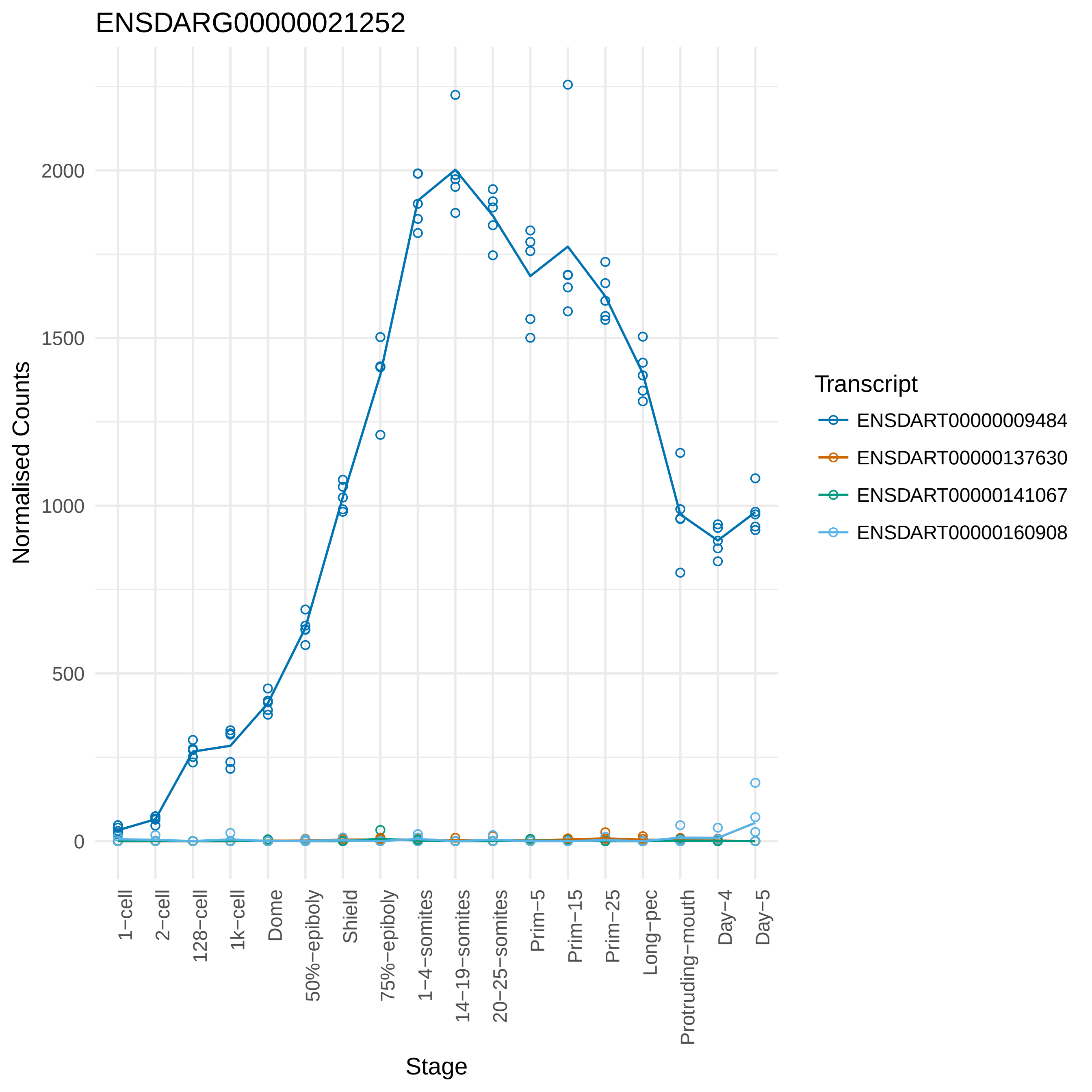
ENSDARG00000021252
Normalised Counts
0
500
1000
1500
2000
Stage
1−cell
2−cell
128−cell
1k−cell
Dome
50%−epiboly
Shield
75%−epiboly
1−4−somites
14−19−somites
20−25−somites
Prim−5
Prim−15
Prim−25
Long−pec
Protruding−mouth
Day−4
Day−5
Transcript
ENSDART00000009484
ENSDART00000137630
ENSDART00000141067
ENSDART00000160908

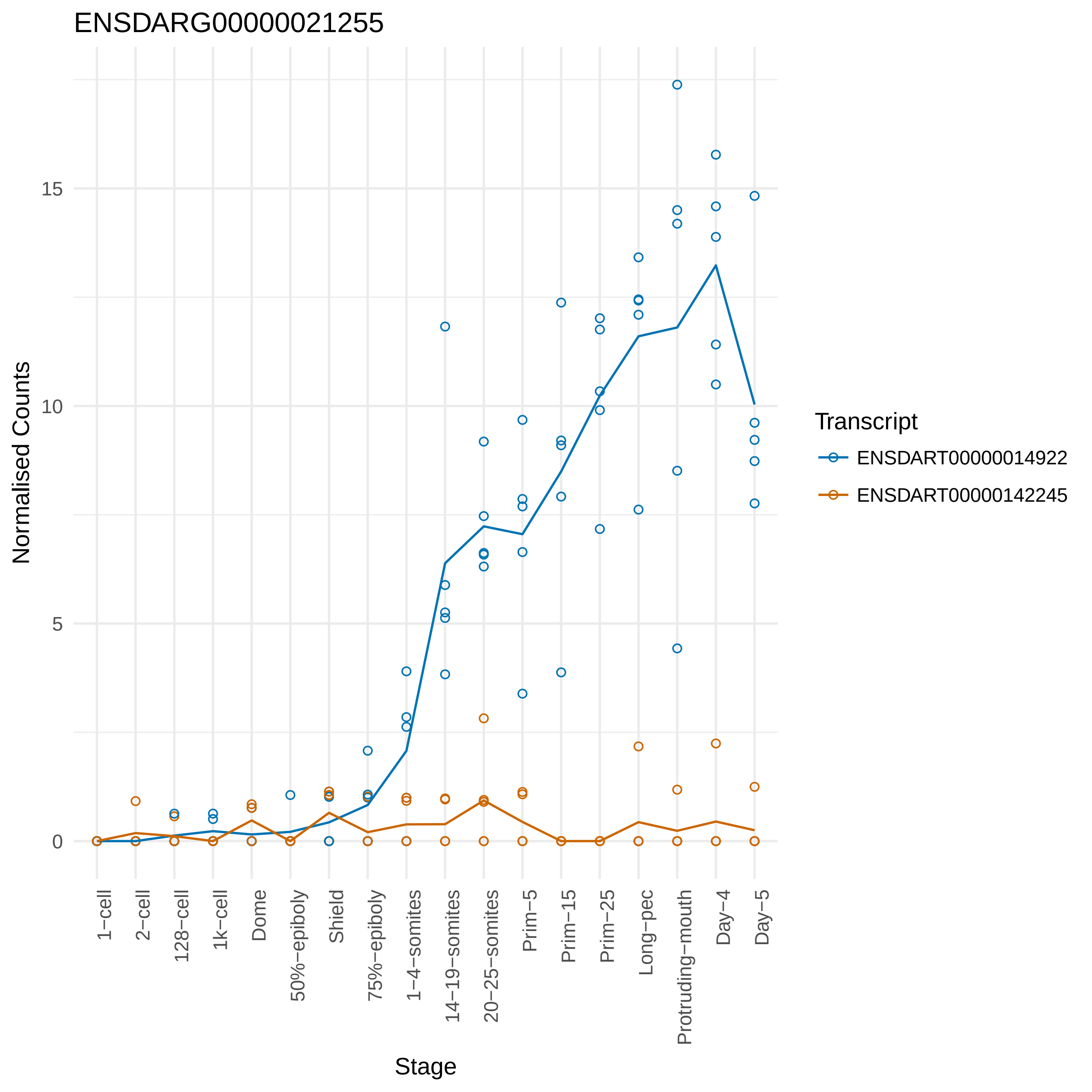

ENSDARG00000021255
Normalised Counts
15
10
5
0
1−cell
2−cell
128−cell
1k−cell
Dome
50%−epiboly
Shield
75%−epiboly
1−4−somites
14−19−somites
20−25−somites
Prim−5
Prim−15
Prim−25
Long−pec
Protruding−mouth
Day−4
Day−5
Stage
Transcript
ENSDART00000014922
ENSDART00000142245

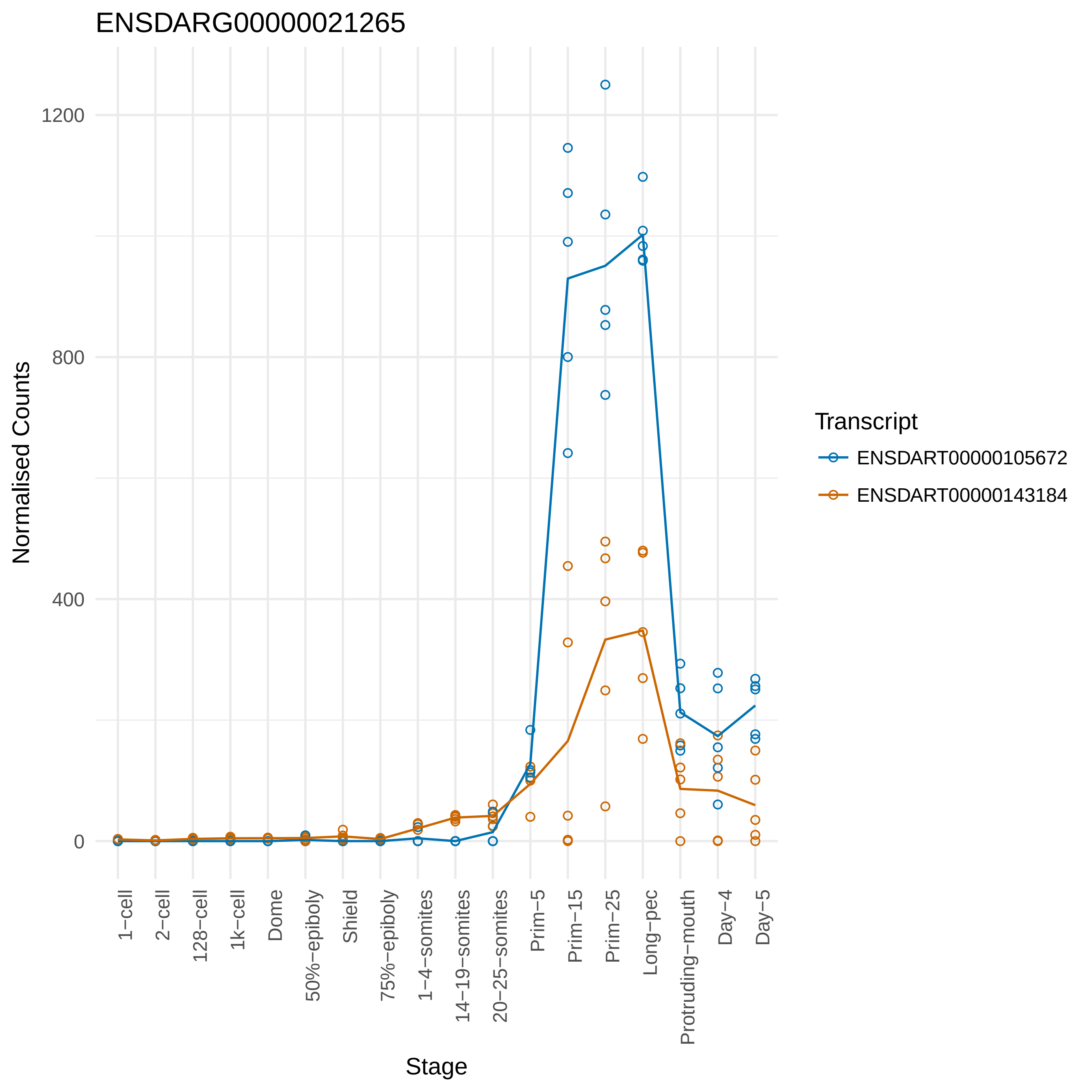

ENSDARG00000021265
Normalised Counts
1200
800
400
0
Stage
1−cell
2−cell
128−cell
1k−cell
Dome
50%−epiboly
Shield
75%−epiboly
1−4−somites
14−19−somites
20−25−somites
Prim−5
Prim−15
Prim−25
Long−pec
Protruding−mouth
Day−4
Day−5
Transcript
ENSDART00000105672
ENSDART00000143184

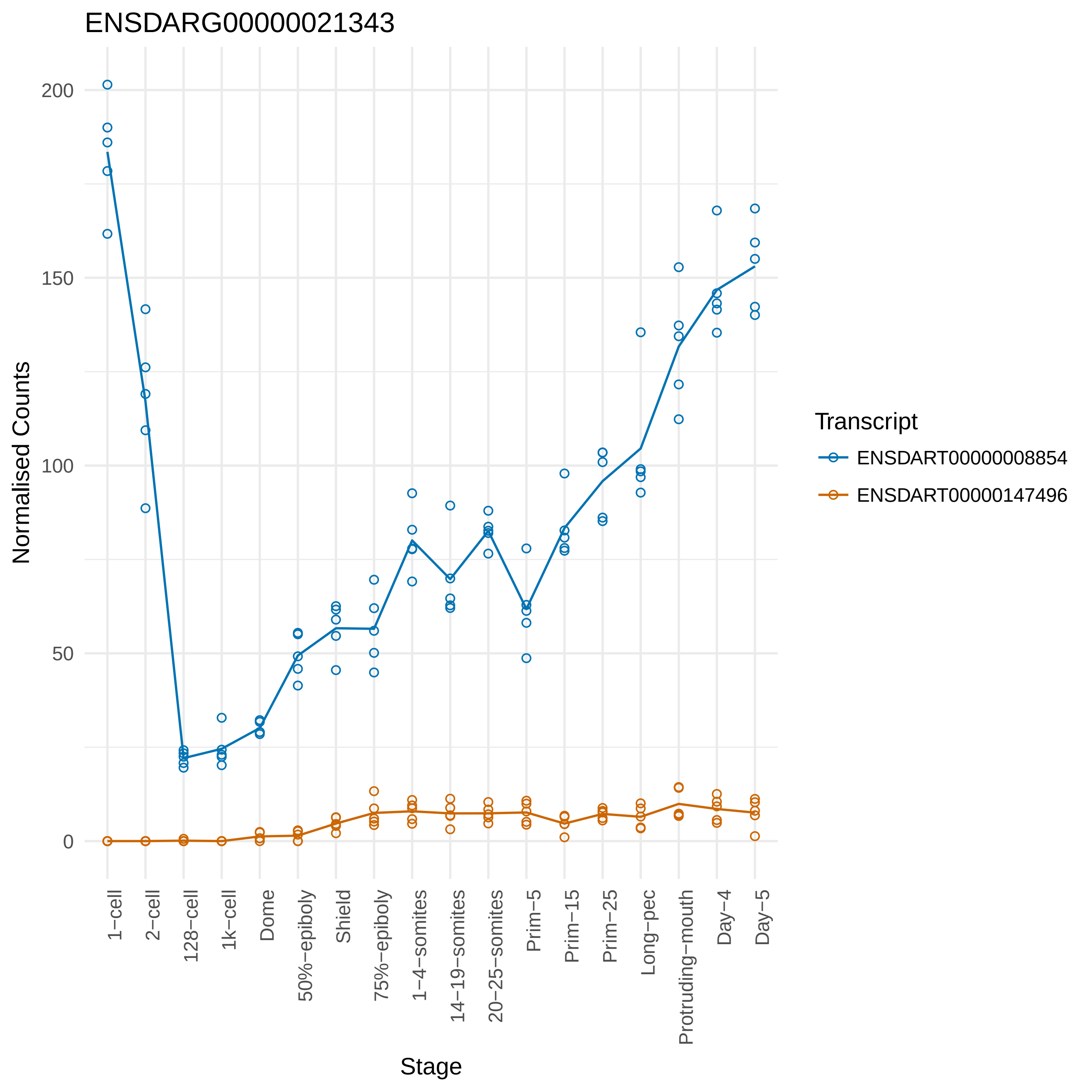

ENSDARG00000021343
Normalised Counts
200
150
100
50
0
1−cell
2−cell
128−cell
1k−cell
Dome
50%−epiboly
Shield
75%−epiboly
1−4−somites
14−19−somites
20−25−somites
Prim−5
Prim−15
Prim−25
Long−pec
Protruding−mouth
Day−4
Day−5
Stage
Transcript
ENSDART00000008854
ENSDART00000147496

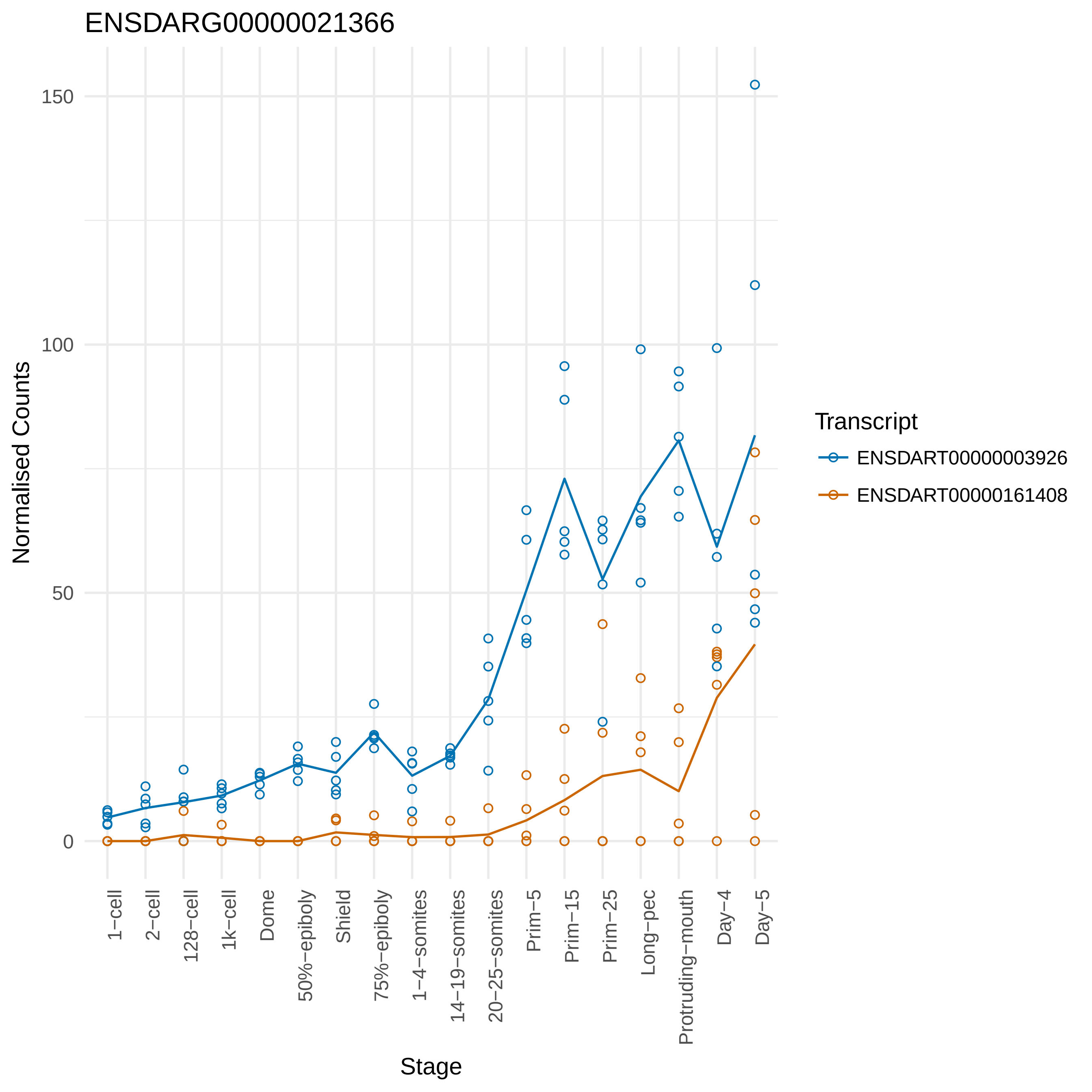

ENSDARG00000021366
Normalised Counts
150
100
50
0
1−cell
2−cell
128−cell
1k−cell
Dome
50%−epiboly
Shield
75%−epiboly
1−4−somites
14−19−somites
20−25−somites
Prim−5
Prim−15
Prim−25
Long−pec
Protruding−mouth
Day−4
Day−5
Stage
Transcript
ENSDART00000003926
ENSDART00000161408

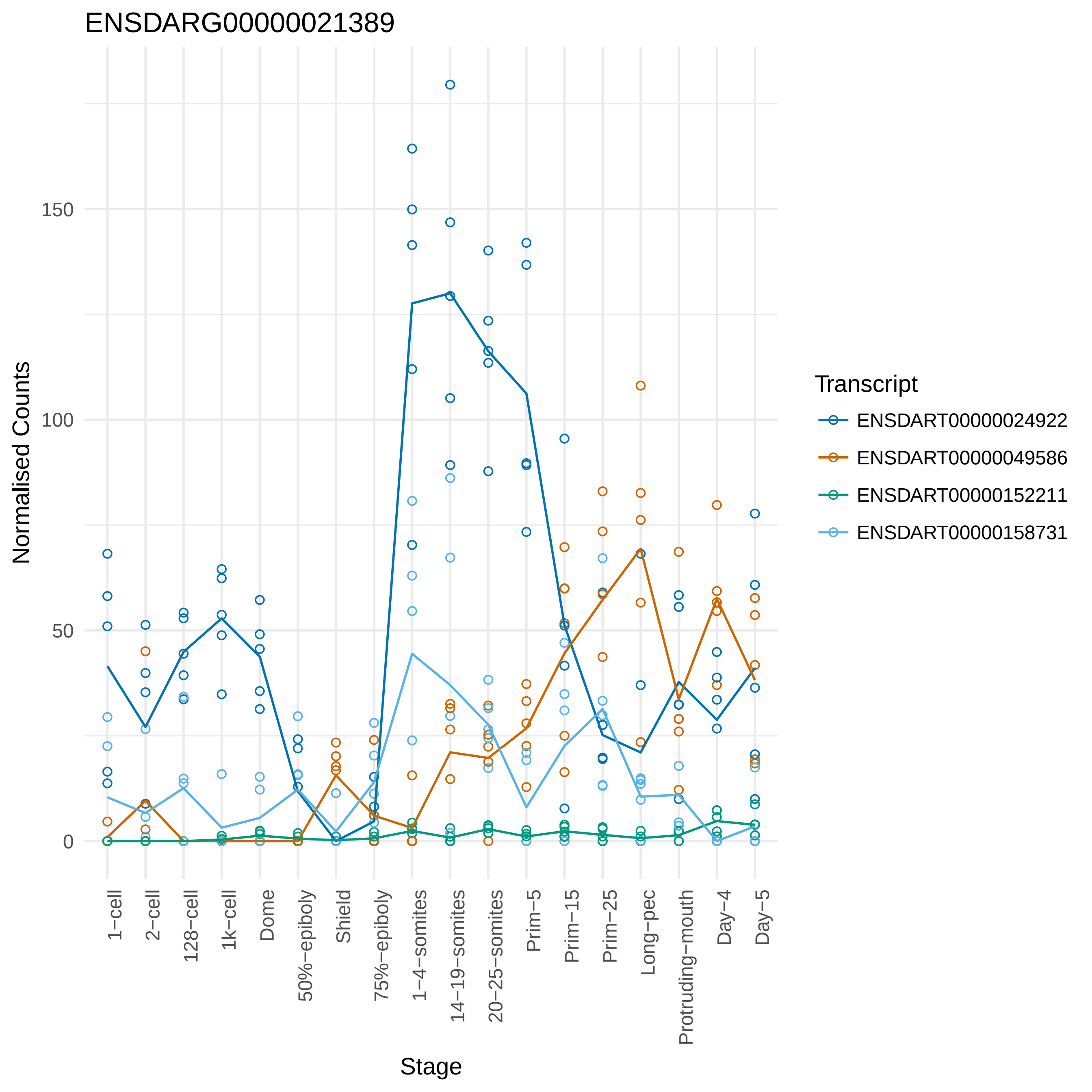
ENSDARG00000021389
Normalised Counts
0
50
100
150
Stage
1−cell
2−cell
128−cell
1k−cell
Dome
50%−epiboly
Shield
75%−epiboly
1−4−somites
14−19−somites
20−25−somites
Prim−5
Prim−15
Prim−25
Long−pec
Protruding−mouth
Day−4
Day−5
Transcript
ENSDART00000024922
ENSDART00000049586
ENSDART00000152211
ENSDART00000158731

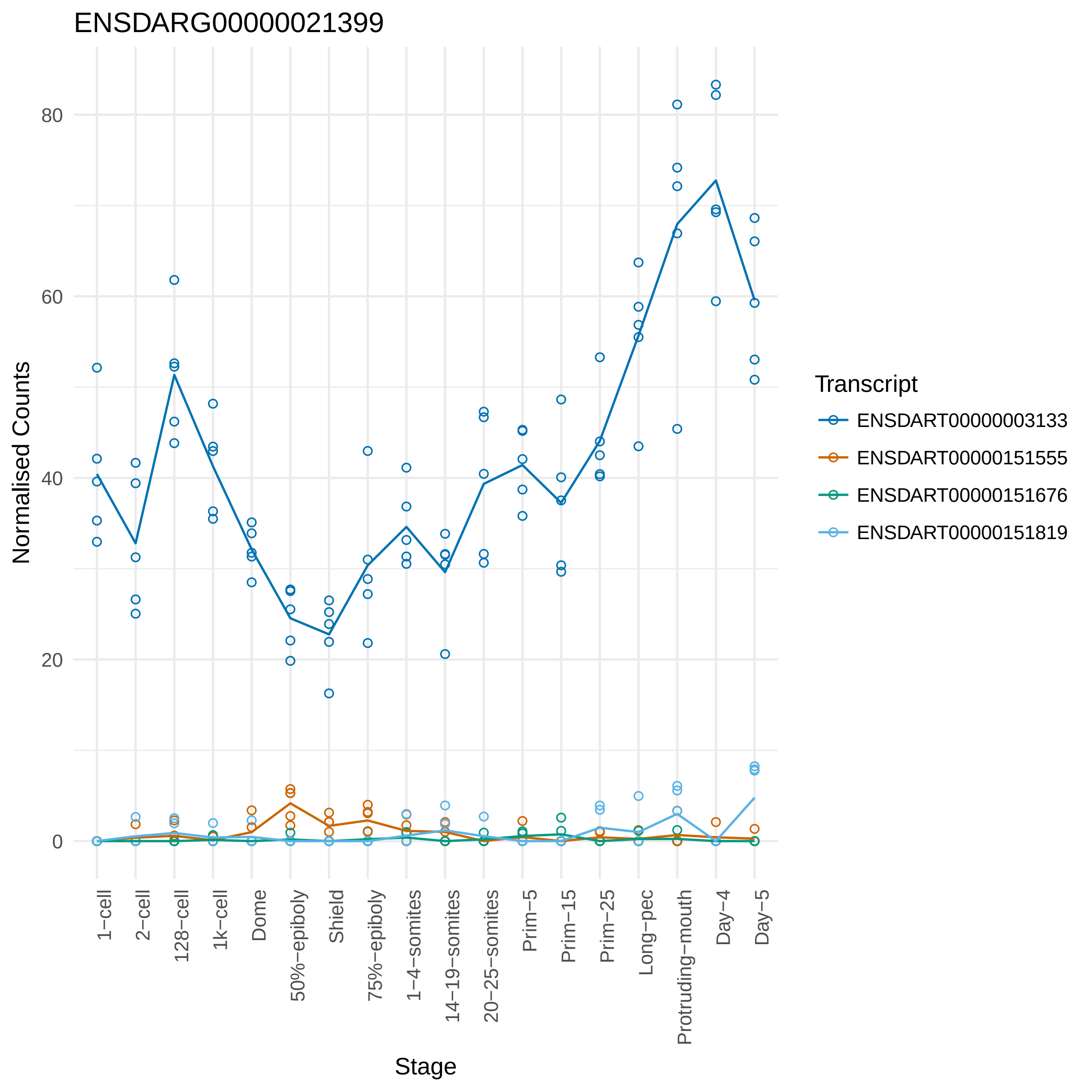

ENSDARG00000021399
Normalised Counts
0
20
40
60
80
1−cell
2−cell
128−cell
1k−cell
Dome
50%−epiboly
Shield
75%−epiboly
1−4−somites
14−19−somites
20−25−somites
Prim−5
Prim−15
Prim−25
Long−pec
Protruding−mouth
Day−4
Day−5
Stage
Transcript
ENSDART00000003133
ENSDART00000151555
ENSDART00000151676
ENSDART00000151819

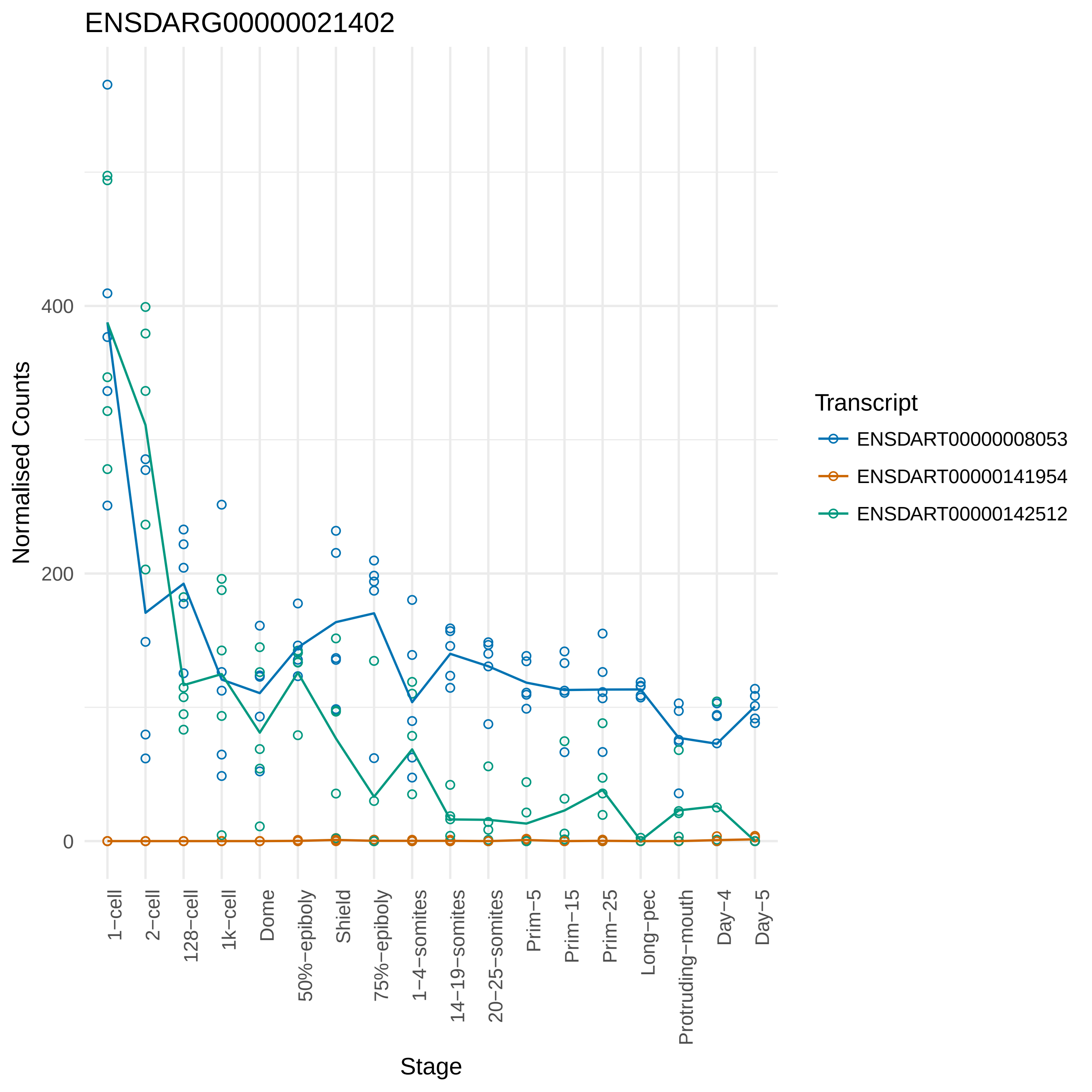

ENSDARG00000021402
Normalised Counts
400
200
0
1−cell
2−cell
128−cell
1k−cell
Dome
50%−epiboly
Shield
75%−epiboly
1−4−somites
14−19−somites
20−25−somites
Prim−5
Prim−15
Prim−25
Long−pec
Protruding−mouth
Day−4
Day−5
Stage
Transcript
ENSDART00000008053
ENSDART00000141954
ENSDART00000142512

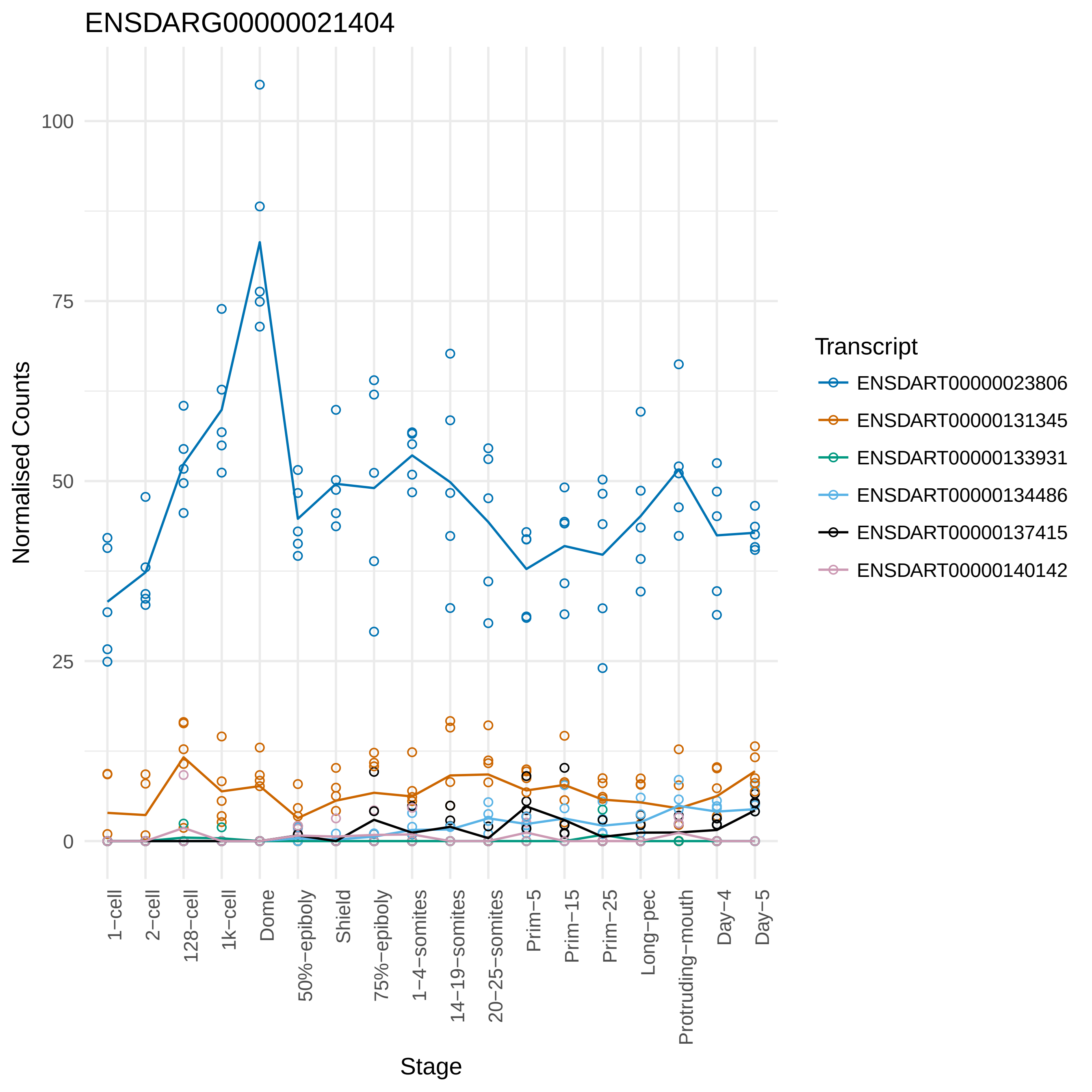
ENSDARG00000021404
Normalised Counts
100
75
50
25
0
1−cell
2−cell
128−cell
1k−cell
Dome
50%−epiboly
Shield
75%−epiboly
1−4−somites
14−19−somites
20−25−somites
Prim−5
Prim−15
Prim−25
Long−pec
Protruding−mouth
Day−4
Day−5
Stage
Transcript
ENSDART00000023806
ENSDART00000131345
ENSDART00000133931
ENSDART00000134486
ENSDART00000137415
ENSDART00000140142

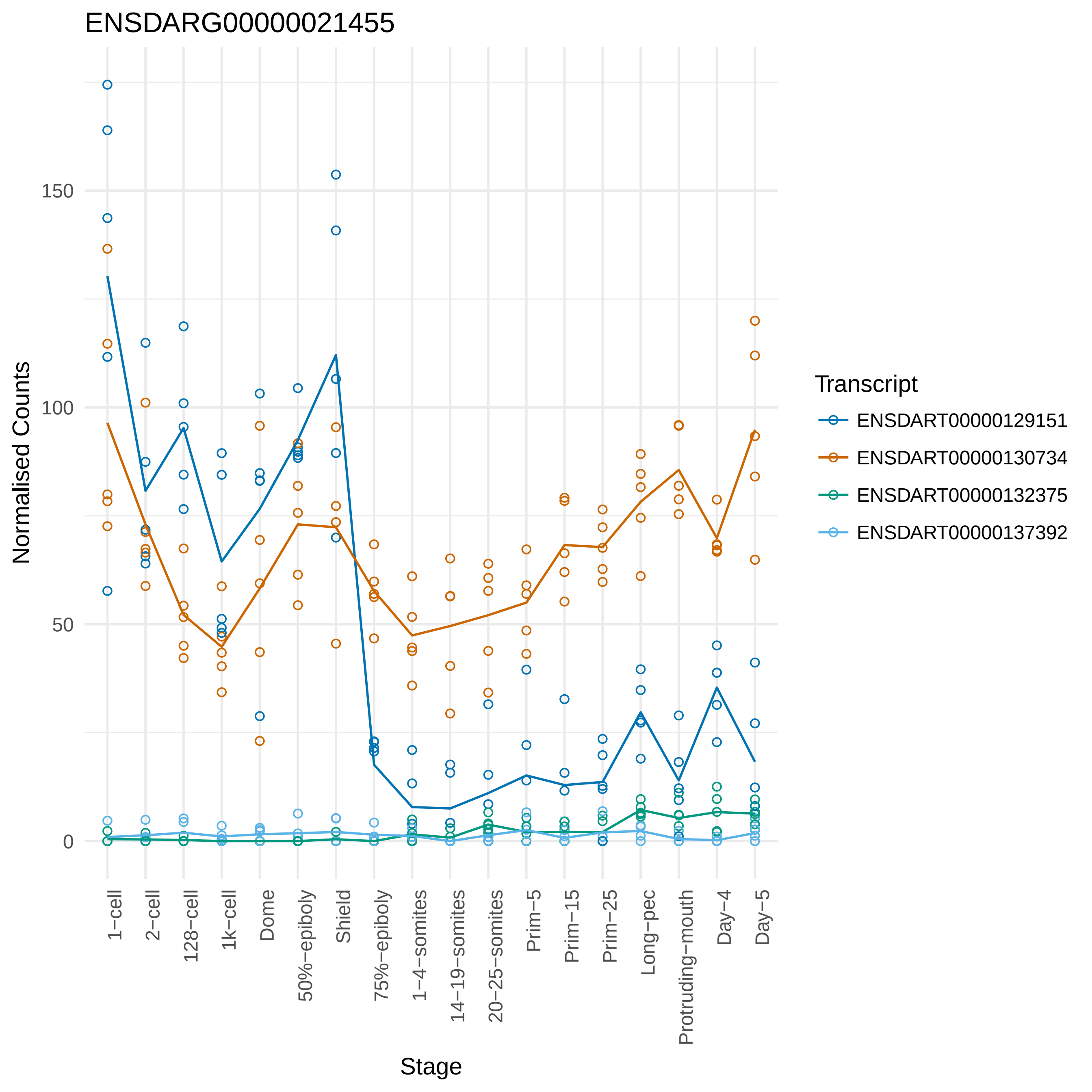
ENSDARG00000021455
Normalised Counts
150
100
50
0
1−cell
2−cell
128−cell
1k−cell
Dome
50%−epiboly
Shield
75%−epiboly
1−4−somites
14−19−somites
20−25−somites
Prim−5
Prim−15
Prim−25
Long−pec
Protruding−mouth
Day−4
Day−5
Stage
Transcript
ENSDART00000129151
ENSDART00000130734
ENSDART00000132375
ENSDART00000137392

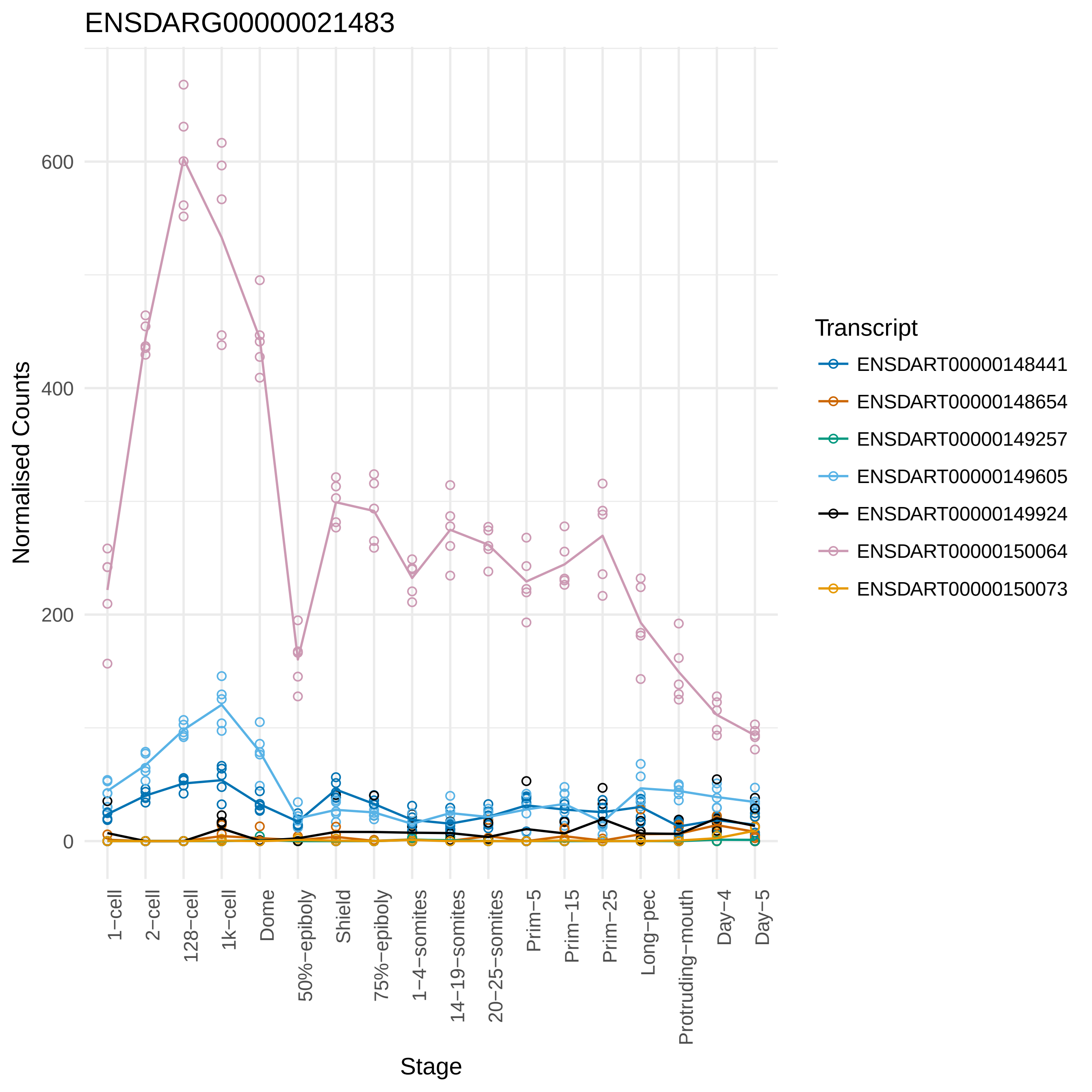
ENSDARG00000021483
Normalised Counts
600
400
200
0
1−cell
2−cell
128−cell
1k−cell
Dome
50%−epiboly
Shield
75%−epiboly
1−4−somites
14−19−somites
20−25−somites
Prim−5
Prim−15
Prim−25
Long−pec
Protruding−mouth
Day−4
Day−5
Stage
Transcript
ENSDART00000148441
ENSDART00000148654
ENSDART00000149257
ENSDART00000149605
ENSDART00000149924
ENSDART00000150064
ENSDART00000150073

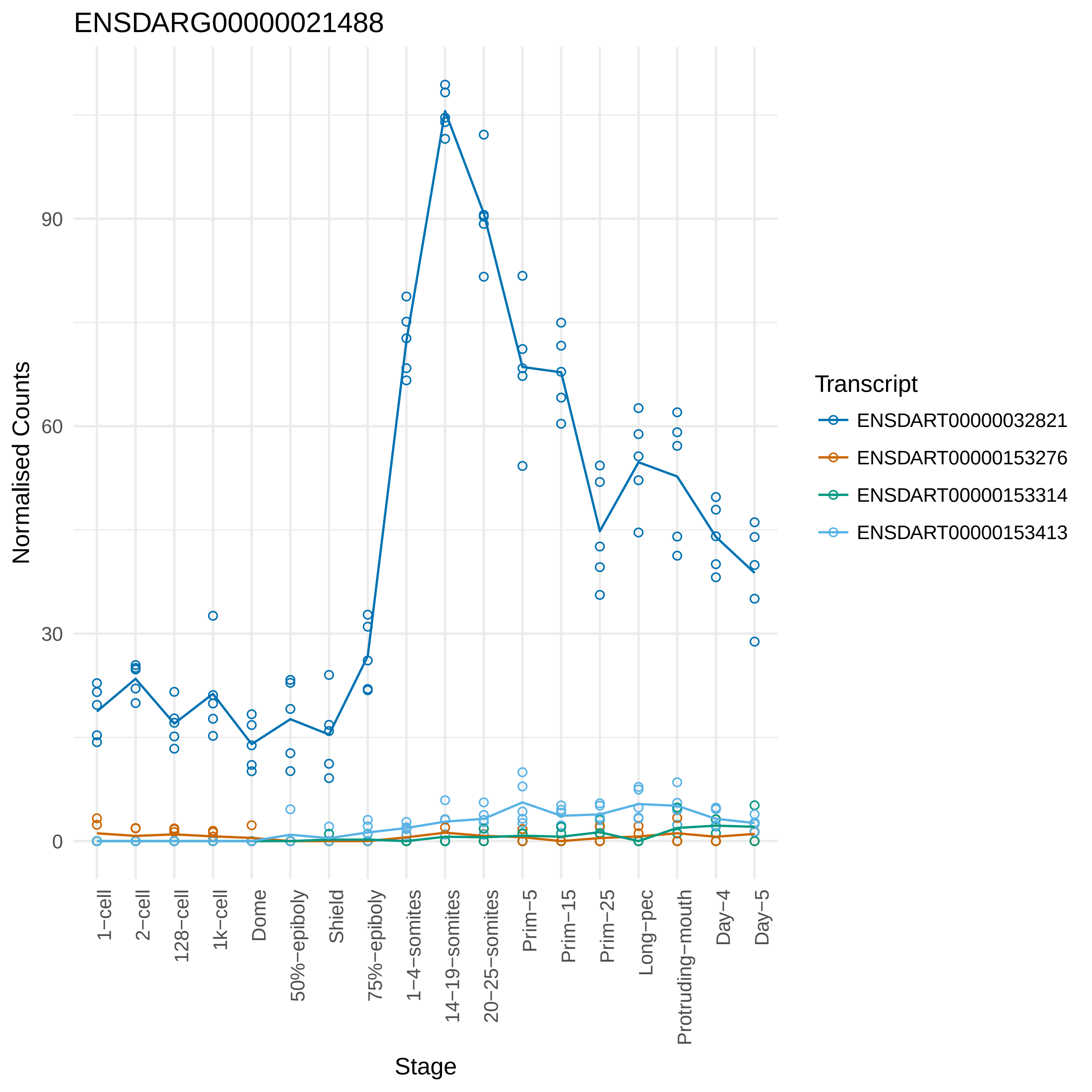

ENSDARG00000021488
Normalised Counts
0
30
60
90
Stage
1−cell
2−cell
128−cell
1k−cell
Dome
50%−epiboly
Shield
75%−epiboly
1−4−somites
14−19−somites
20−25−somites
Prim−5
Prim−15
Prim−25
Long−pec
Protruding−mouth
Day−4
Day−5
Transcript
ENSDART00000032821
ENSDART00000153276
ENSDART00000153314
ENSDART00000153413

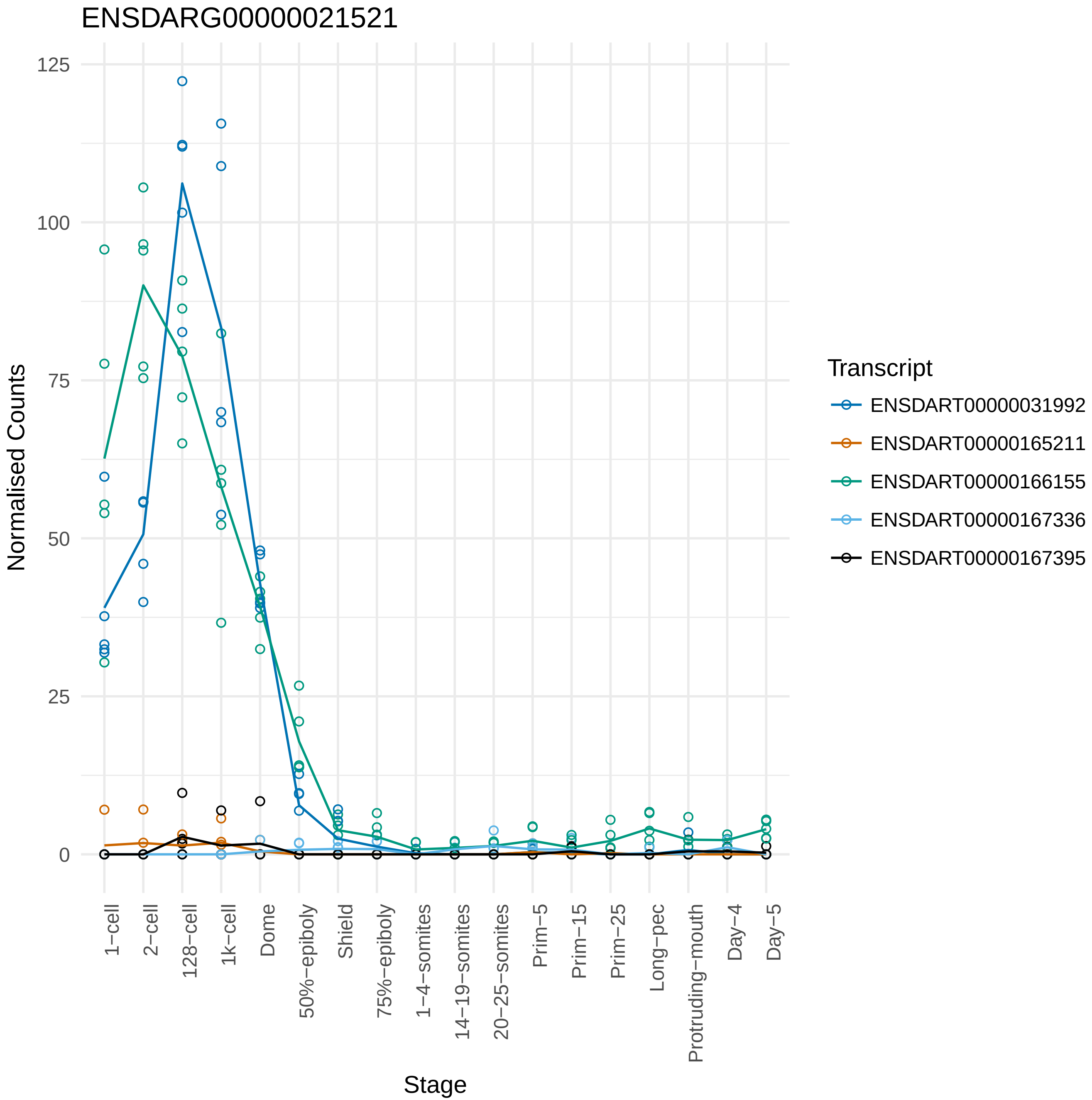
ENSDARG00000021521
Normalised Counts
125
100
75
50
25
0
1−cell
2−cell
128−cell
1k−cell
Dome
50%−epiboly
Shield
75%−epiboly
1−4−somites
14−19−somites
20−25−somites
Prim−5
Prim−15
Prim−25
Long−pec
Protruding−mouth
Day−4
Day−5
Stage
Transcript
ENSDART00000031992
ENSDART00000165211
ENSDART00000166155
ENSDART00000167336
ENSDART00000167395

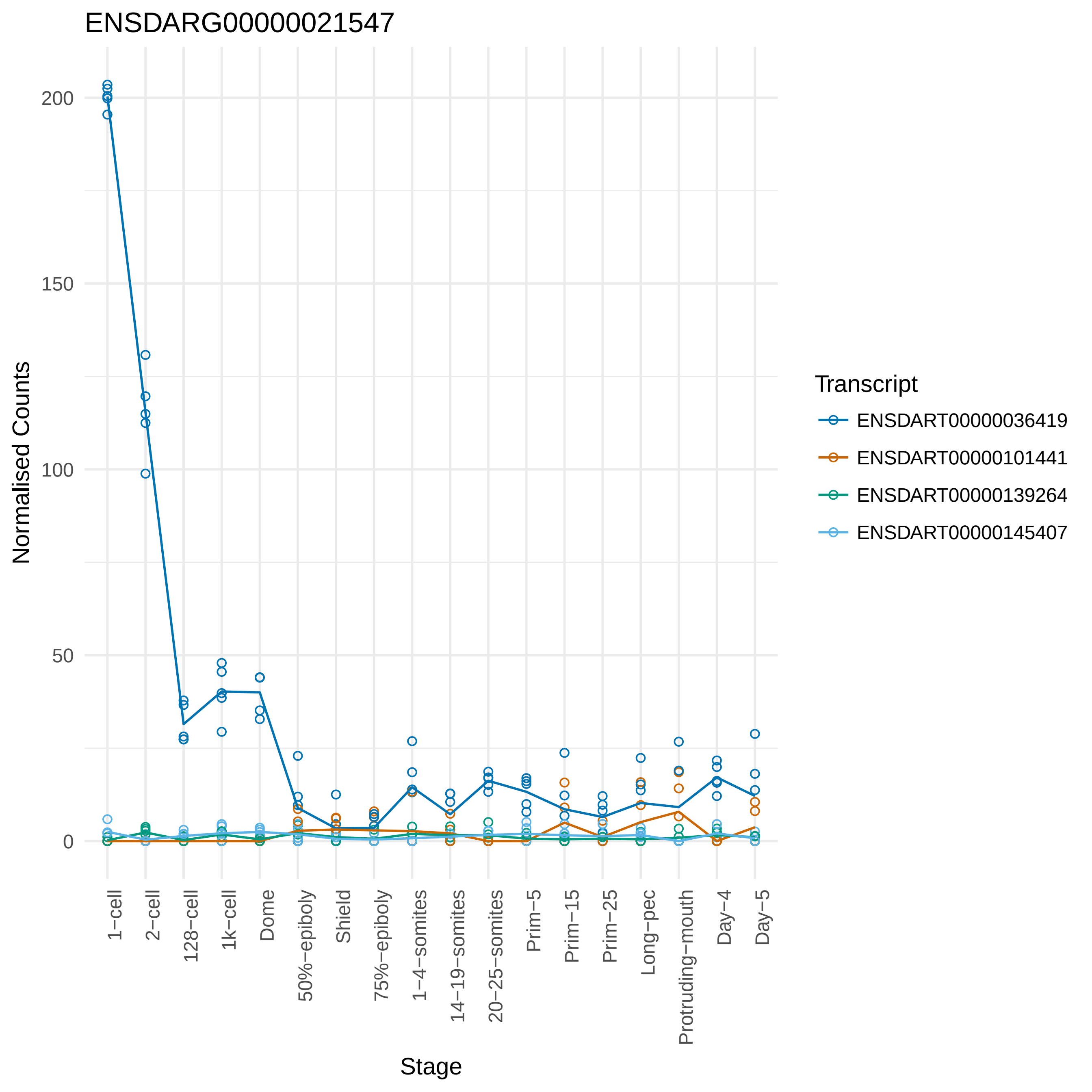
ENSDARG00000021547
Normalised Counts
200
150
100
50
0
1−cell
2−cell
128−cell
1k−cell
Dome
50%−epiboly
Shield
75%−epiboly
1−4−somites
14−19−somites
20−25−somites
Prim−5
Prim−15
Prim−25
Long−pec
Protruding−mouth
Day−4
Day−5
Stage
Transcript
ENSDART00000036419
ENSDART00000101441
ENSDART00000139264
ENSDART00000145407

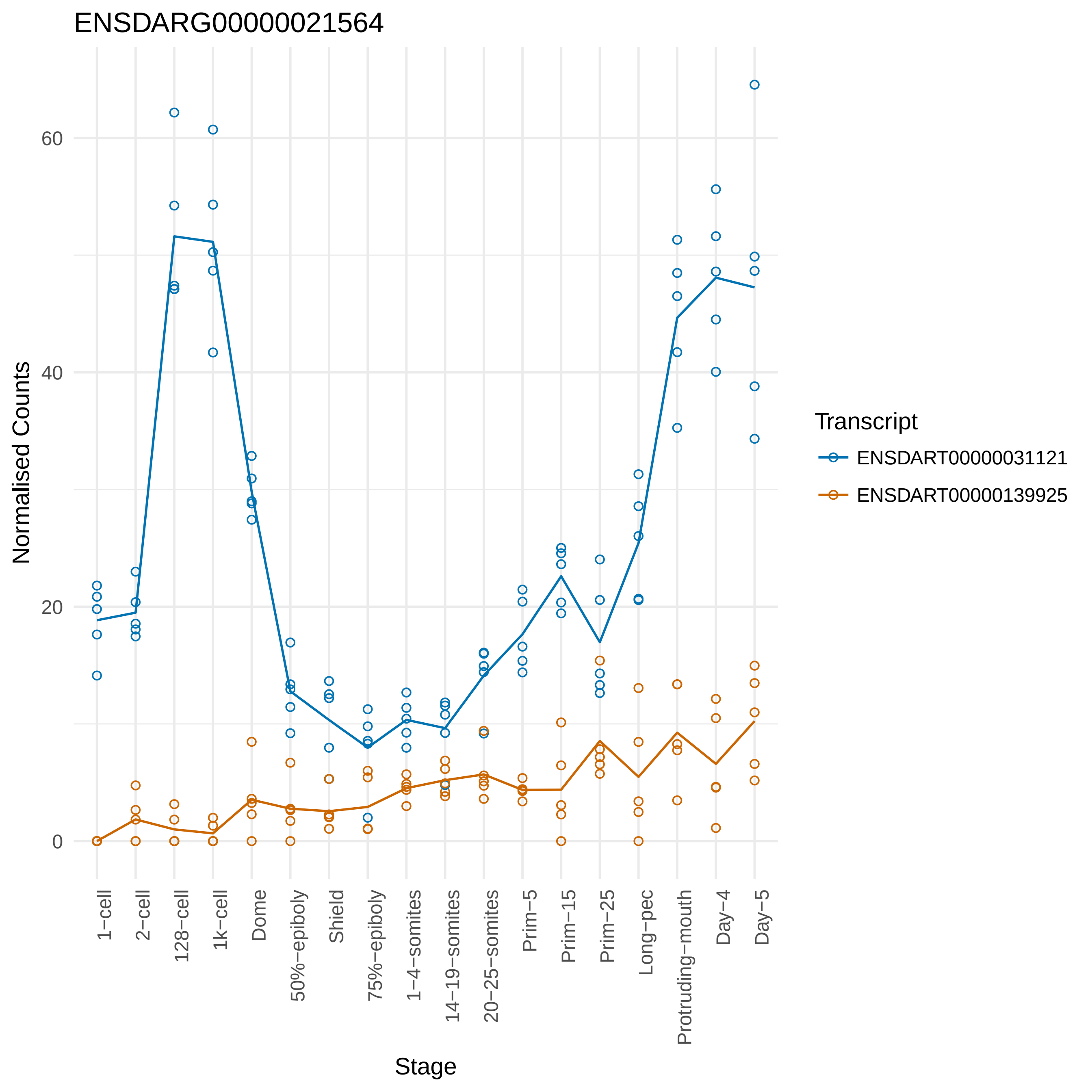

ENSDARG00000021564
Normalised Counts
60
40
20
0
1−cell
2−cell
128−cell
1k−cell
Dome
50%−epiboly
Shield
75%−epiboly
1−4−somites
14−19−somites
20−25−somites
Prim−5
Prim−15
Prim−25
Long−pec
Protruding−mouth
Day−4
Day−5
Stage
Transcript
ENSDART00000031121
ENSDART00000139925

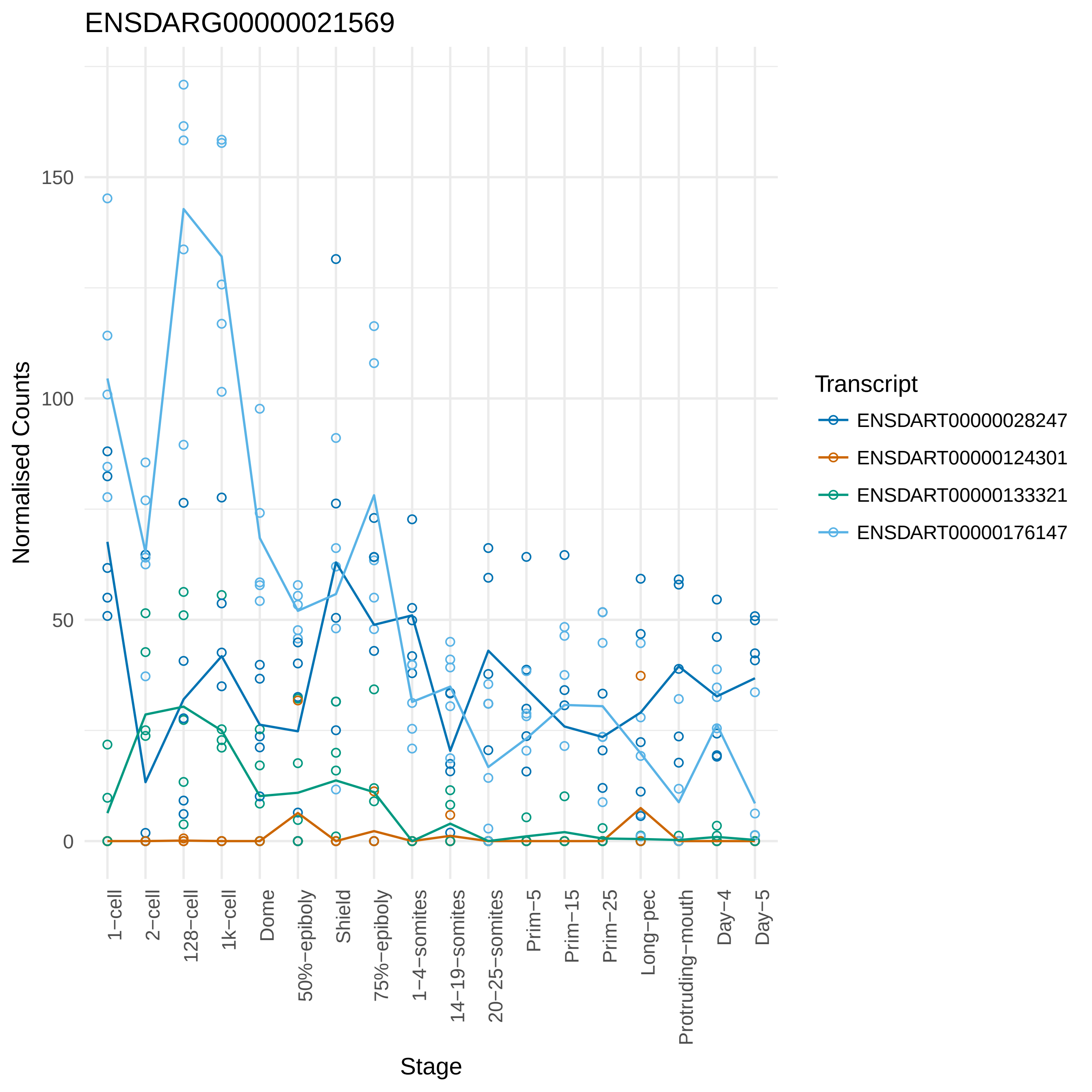

ENSDARG00000021569
Normalised Counts
150
100
50
0
1−cell
2−cell
128−cell
1k−cell
Dome
50%−epiboly
Shield
75%−epiboly
1−4−somites
14−19−somites
20−25−somites
Prim−5
Prim−15
Prim−25
Long−pec
Protruding−mouth
Day−4
Day−5
Stage
Transcript
ENSDART00000028247
ENSDART00000124301
ENSDART00000133321
ENSDART00000176147

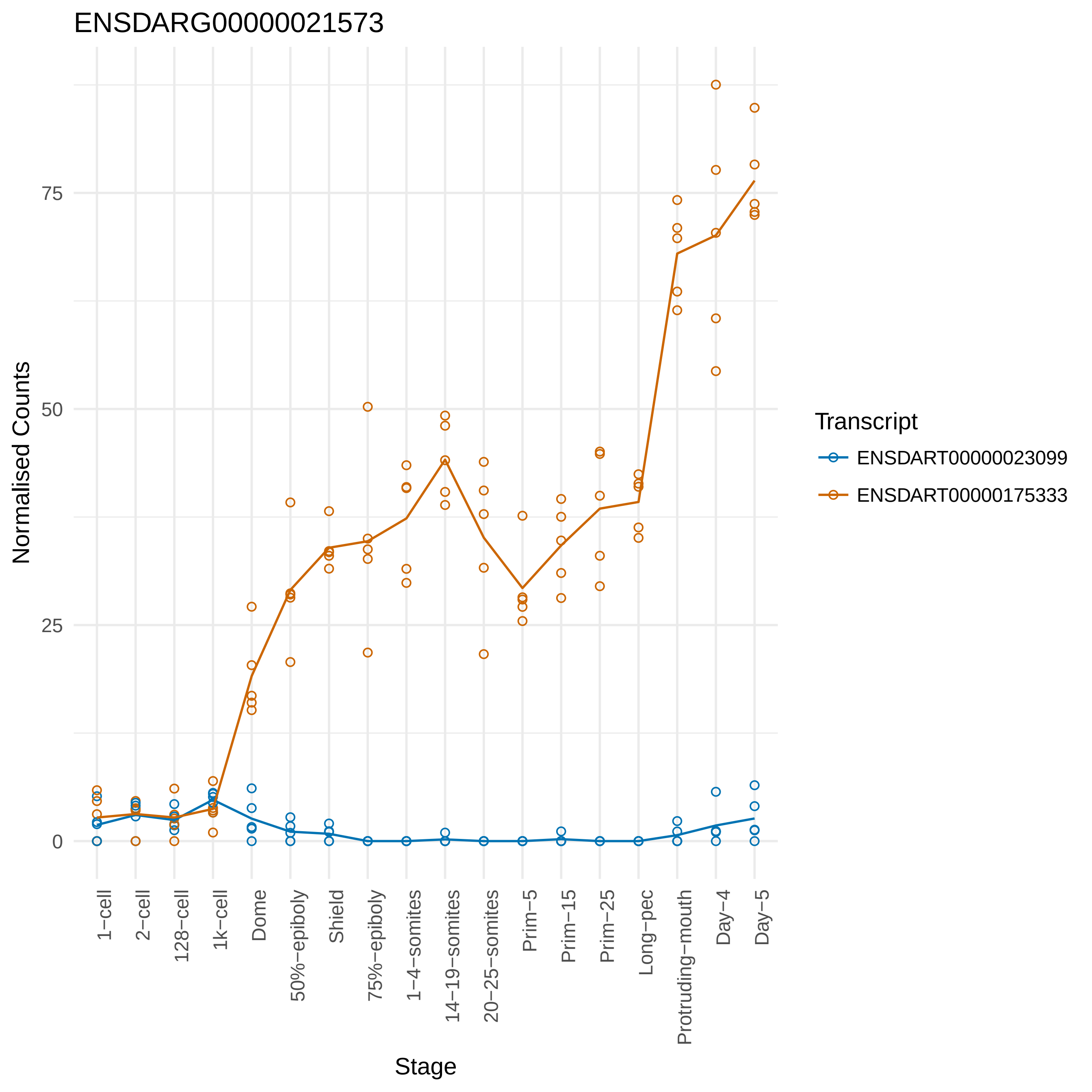

ENSDARG00000021573
Normalised Counts
0
25
50
75
1−cell
2−cell
128−cell
1k−cell
Dome
50%−epiboly
Shield
75%−epiboly
1−4−somites
14−19−somites
20−25−somites
Prim−5
Prim−15
Prim−25
Long−pec
Protruding−mouth
Day−4
Day−5
Stage
Transcript
ENSDART00000023099
ENSDART00000175333

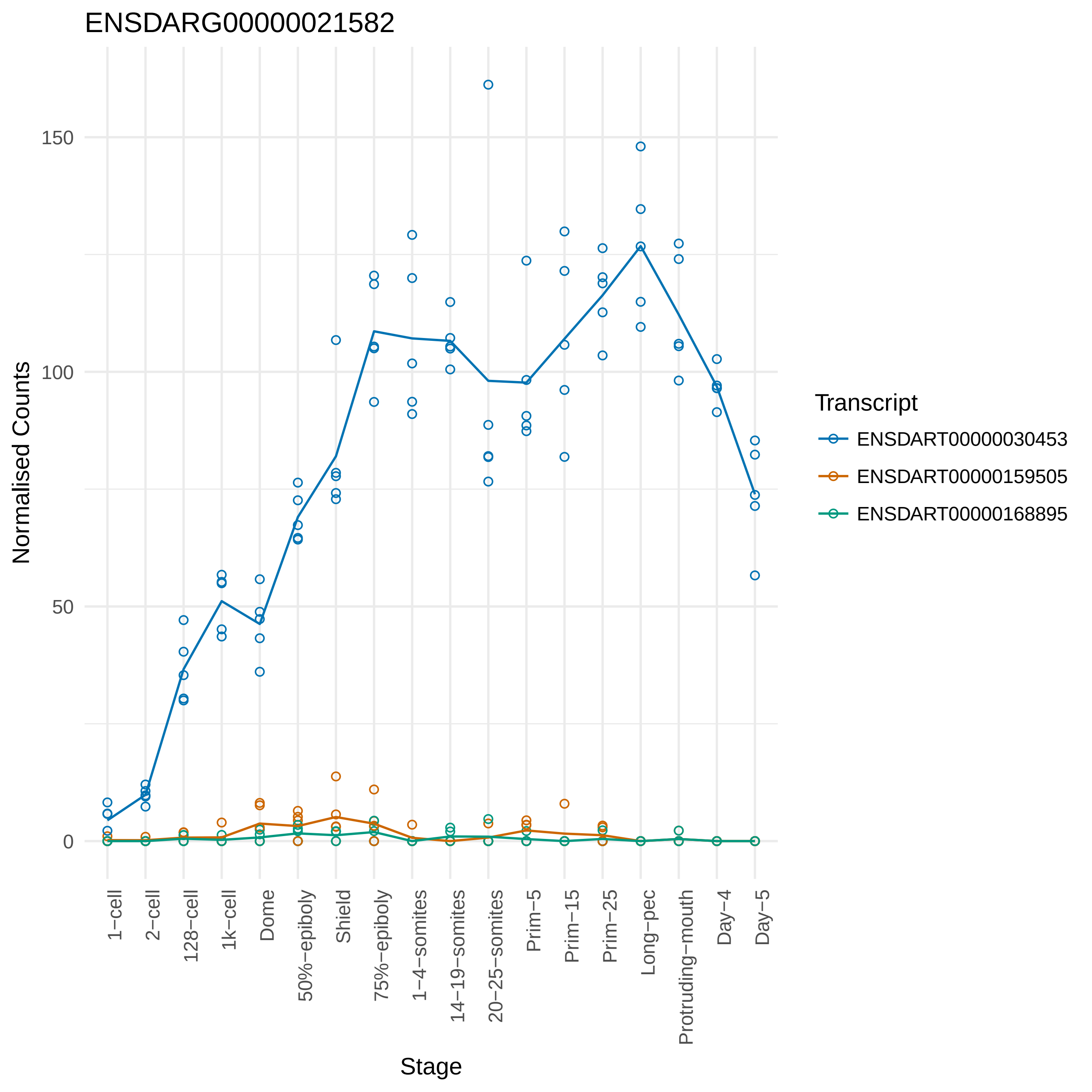

ENSDARG00000021582
Normalised Counts
150
100
50
0
1−cell
2−cell
128−cell
1k−cell
Dome
50%−epiboly
Shield
75%−epiboly
1−4−somites
14−19−somites
20−25−somites
Prim−5
Prim−15
Prim−25
Long−pec
Protruding−mouth
Day−4
Day−5
Stage
Transcript
ENSDART00000030453
ENSDART00000159505
ENSDART00000168895

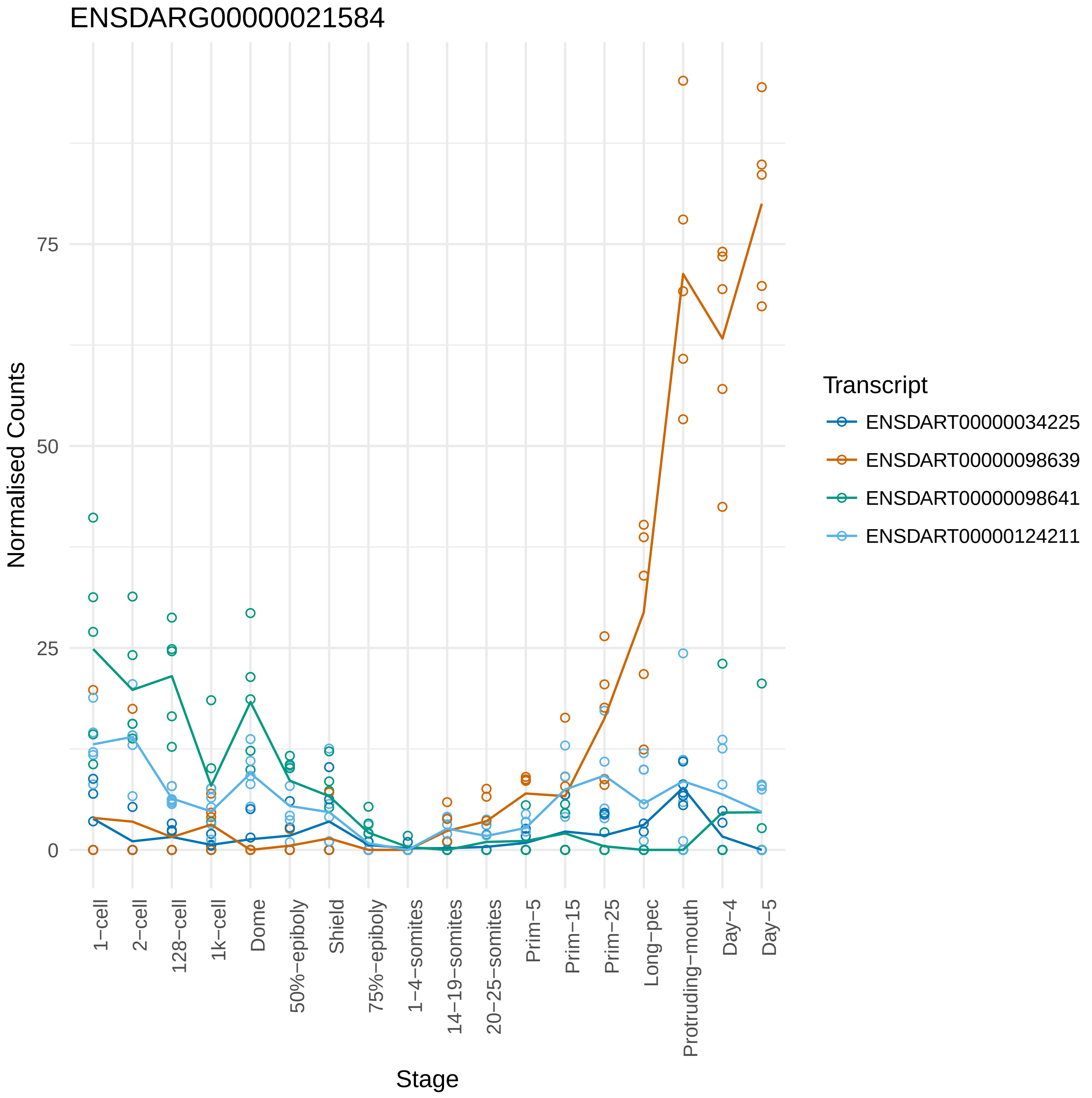

ENSDARG00000021584
Normalised Counts
0
25
50
75
Stage
1−cell
2−cell
128−cell
1k−cell
Dome
50%−epiboly
Shield
75%−epiboly
1−4−somites
14−19−somites
20−25−somites
Prim−5
Prim−15
Prim−25
Long−pec
Protruding−mouth
Day−4
Day−5
Transcript
ENSDART00000034225
ENSDART00000098639
ENSDART00000098641
ENSDART00000124211

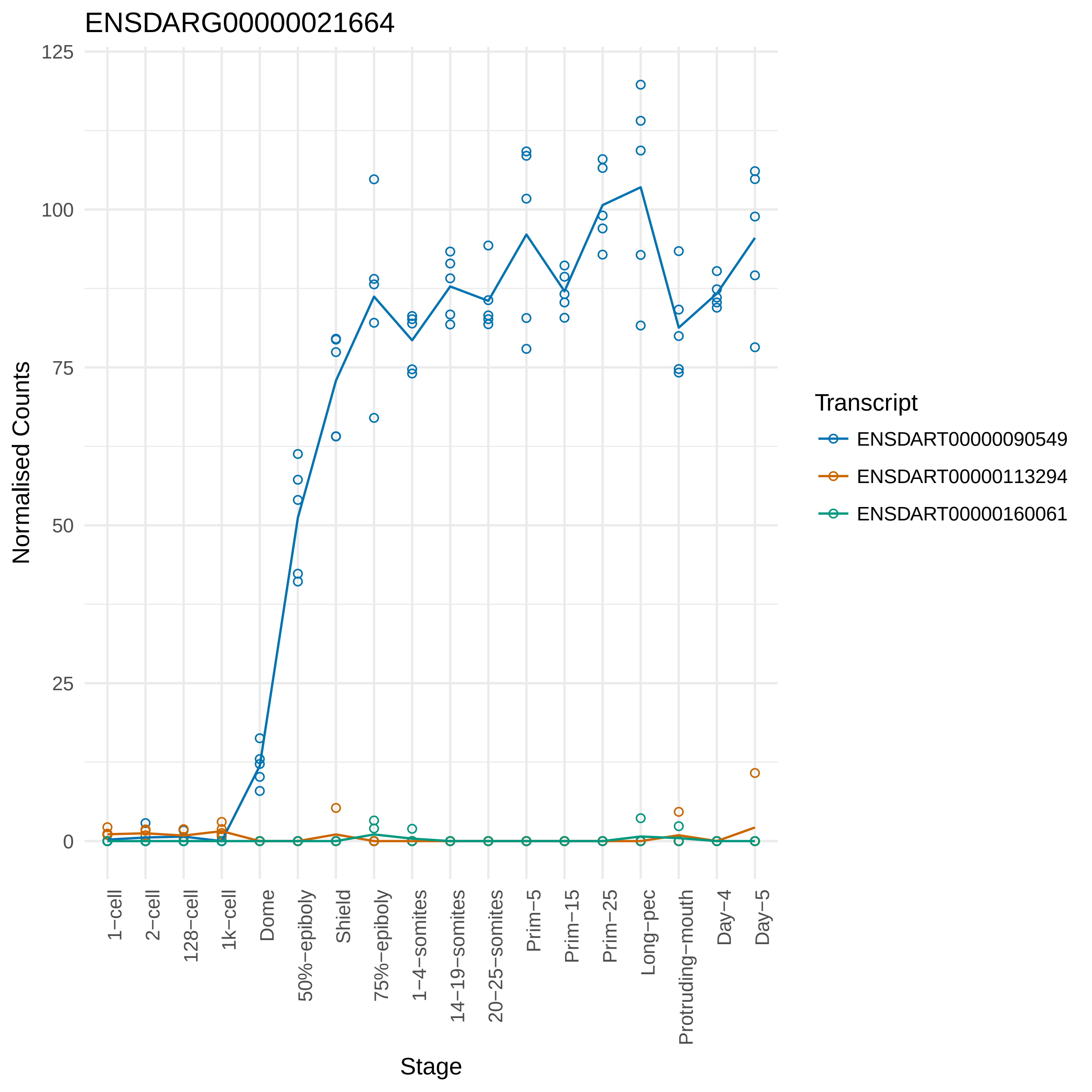

ENSDARG00000021664
Normalised Counts
125
100
75
50
25
0
1−cell
2−cell
128−cell
1k−cell
Dome
50%−epiboly
Shield
75%−epiboly
1−4−somites
14−19−somites
20−25−somites
Prim−5
Prim−15
Prim−25
Long−pec
Protruding−mouth
Day−4
Day−5
Stage
Transcript
ENSDART00000090549
ENSDART00000113294
ENSDART00000160061

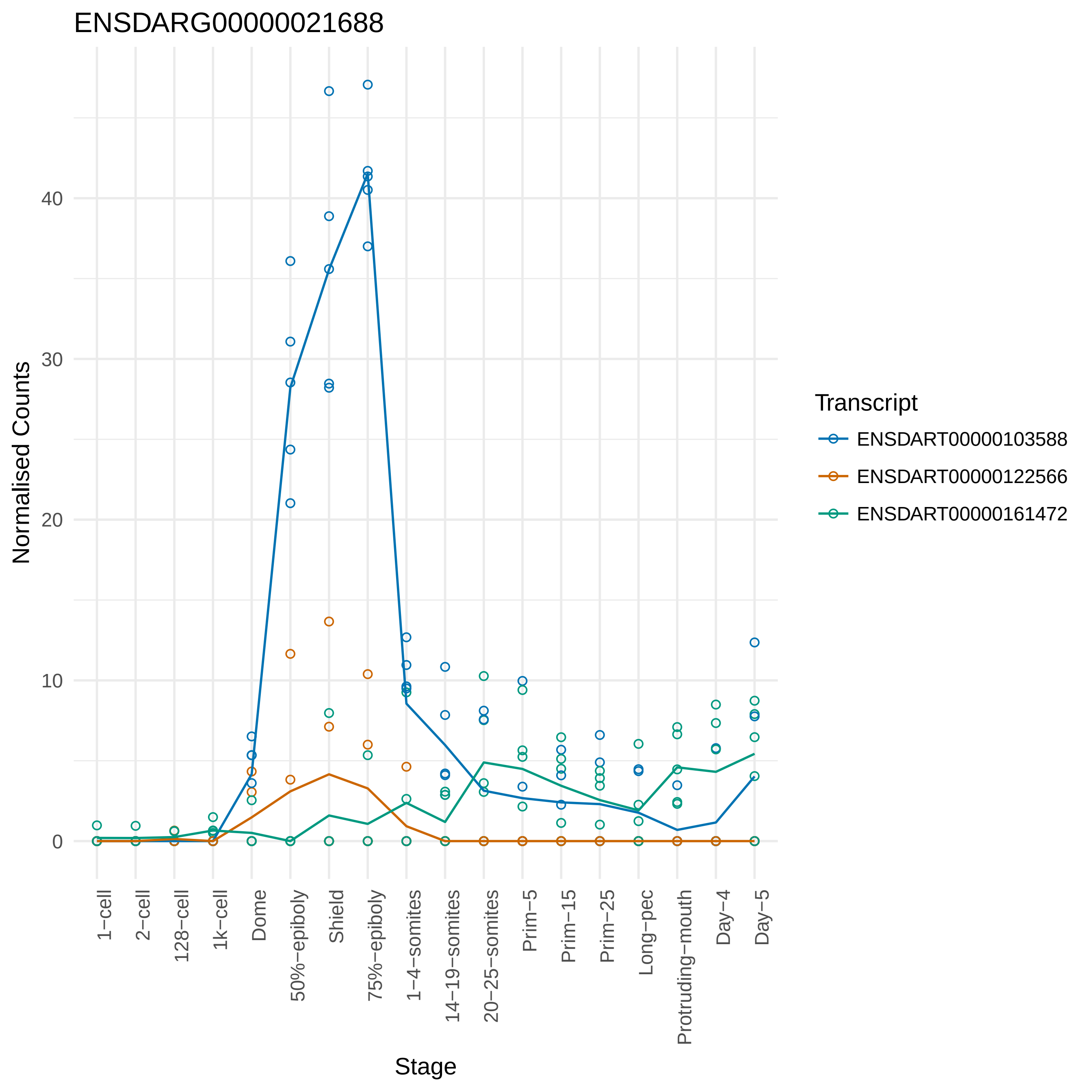
ENSDARG00000021688
Normalised Counts
0
10
20
30
40
Stage
1−cell
2−cell
128−cell
1k−cell
Dome
50%−epiboly
Shield
75%−epiboly
1−4−somites
14−19−somites
20−25−somites
Prim−5
Prim−15
Prim−25
Long−pec
Protruding−mouth
Day−4
Day−5
Transcript
ENSDART00000103588
ENSDART00000122566
ENSDART00000161472

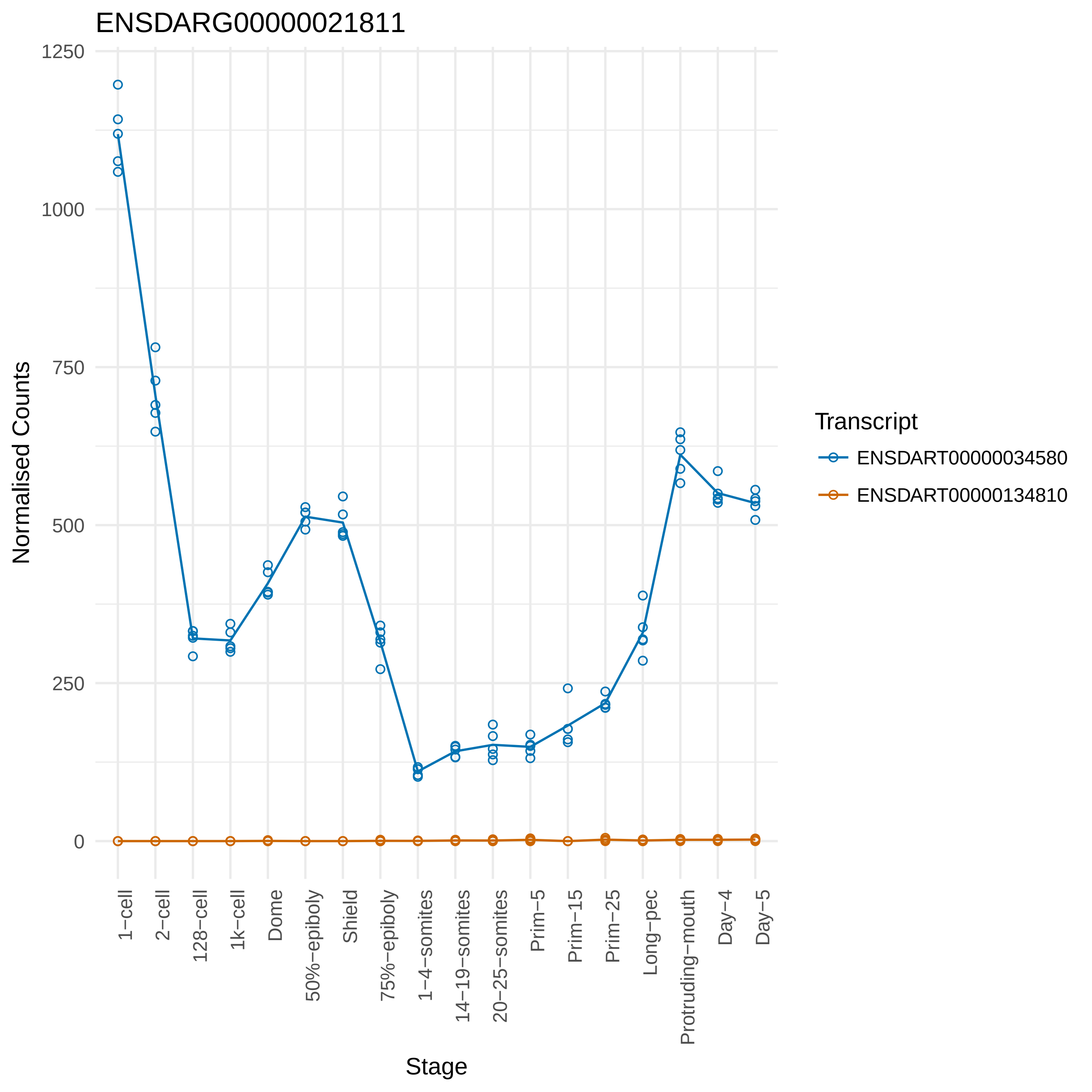
ENSDARG00000021811
Normalised Counts
1250
1000
750
500
250
0
Stage
1−cell
2−cell
128−cell
1k−cell
Dome
50%−epiboly
Shield
75%−epiboly
1−4−somites
14−19−somites
20−25−somites
Prim−5
Prim−15
Prim−25
Long−pec
Protruding−mouth
Day−4
Day−5
Transcript
ENSDART00000034580
ENSDART00000134810

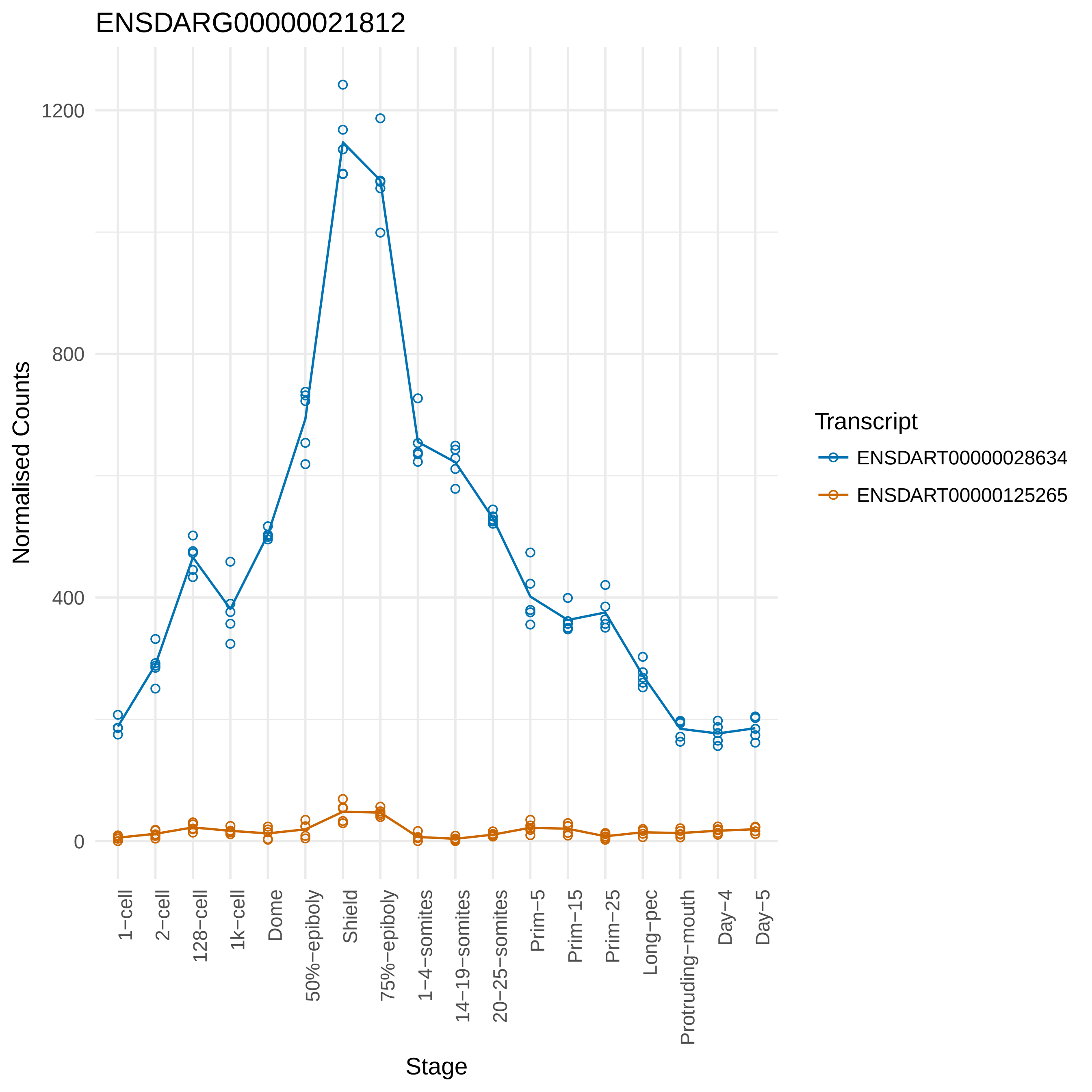

ENSDARG00000021812
Normalised Counts
1200
800
400
0
Stage
1−cell
2−cell
128−cell
1k−cell
Dome
50%−epiboly
Shield
75%−epiboly
1−4−somites
14−19−somites
20−25−somites
Prim−5
Prim−15
Prim−25
Long−pec
Protruding−mouth
Day−4
Day−5
Transcript
ENSDART00000028634
ENSDART00000125265

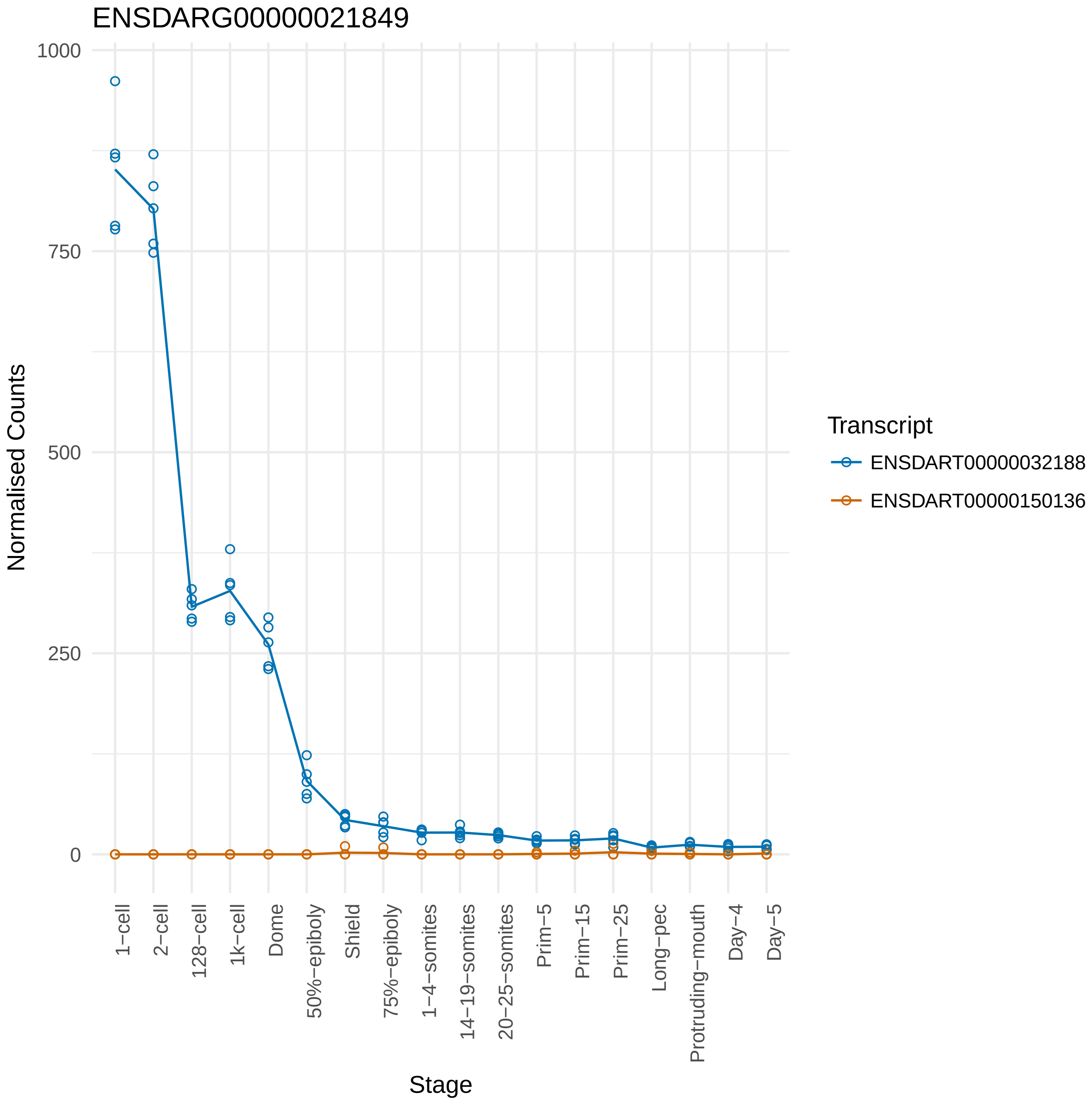

ENSDARG00000021849
1000
750
500
250
0
Normalised Counts
1−cell
2−cell
128−cell
1k−cell
Dome
50%−epiboly
Shield
75%−epiboly
1−4−somites
14−19−somites
20−25−somites
Prim−5
Prim−15
Prim−25
Long−pec
Protruding−mouth
Day−4
Day−5
Stage
Transcript
ENSDART00000032188
ENSDART00000150136

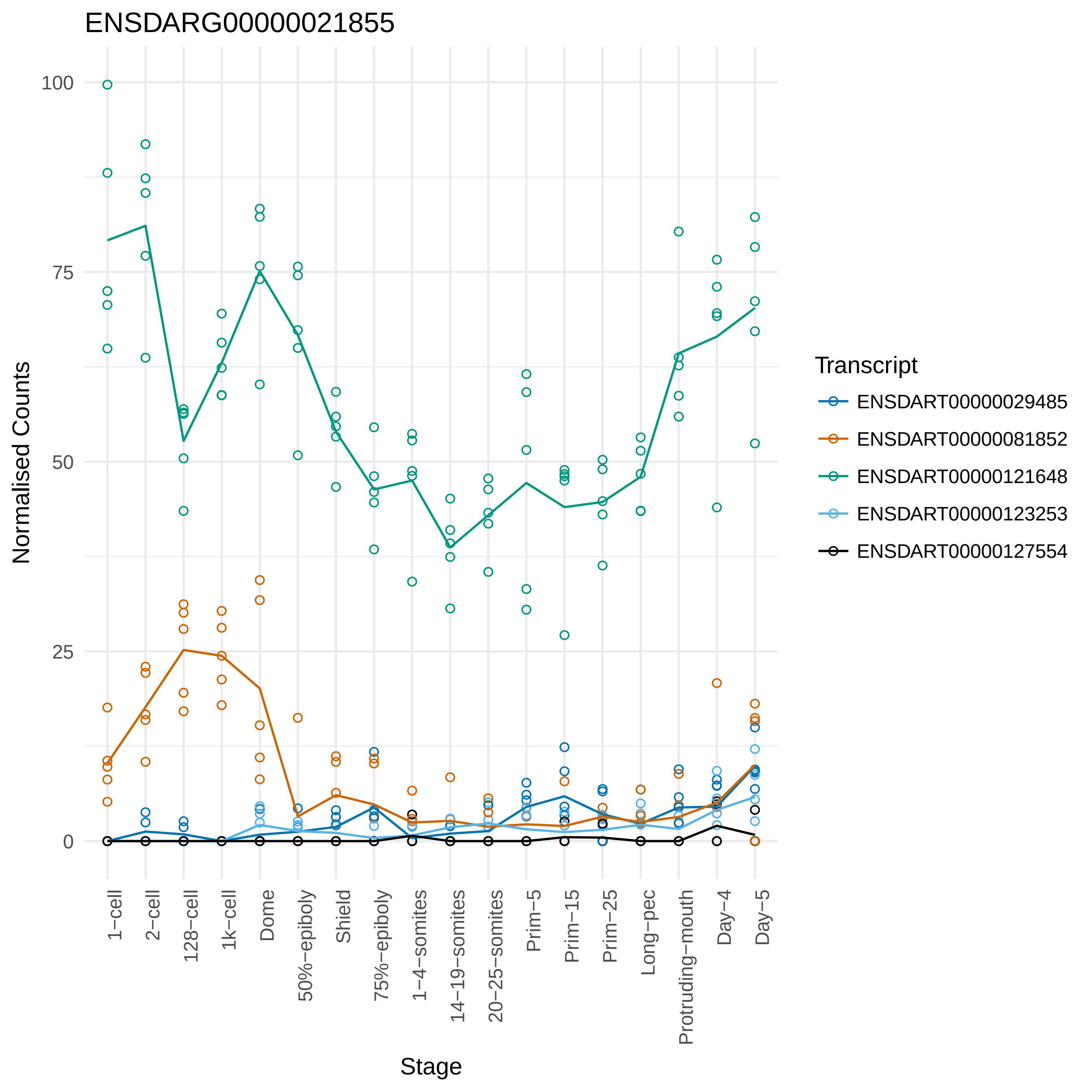
ENSDARG00000021855
Normalised Counts
100
75
50
25
0
Stage
1−cell
2−cell
128−cell
1k−cell
Dome
50%−epiboly
Shield
75%−epiboly
1−4−somites
14−19−somites
20−25−somites
Prim−5
Prim−15
Prim−25
Long−pec
Protruding−mouth
Day−4
Day−5
Transcript
ENSDART00000029485
ENSDART00000081852
ENSDART00000121648
ENSDART00000123253
ENSDART00000127554

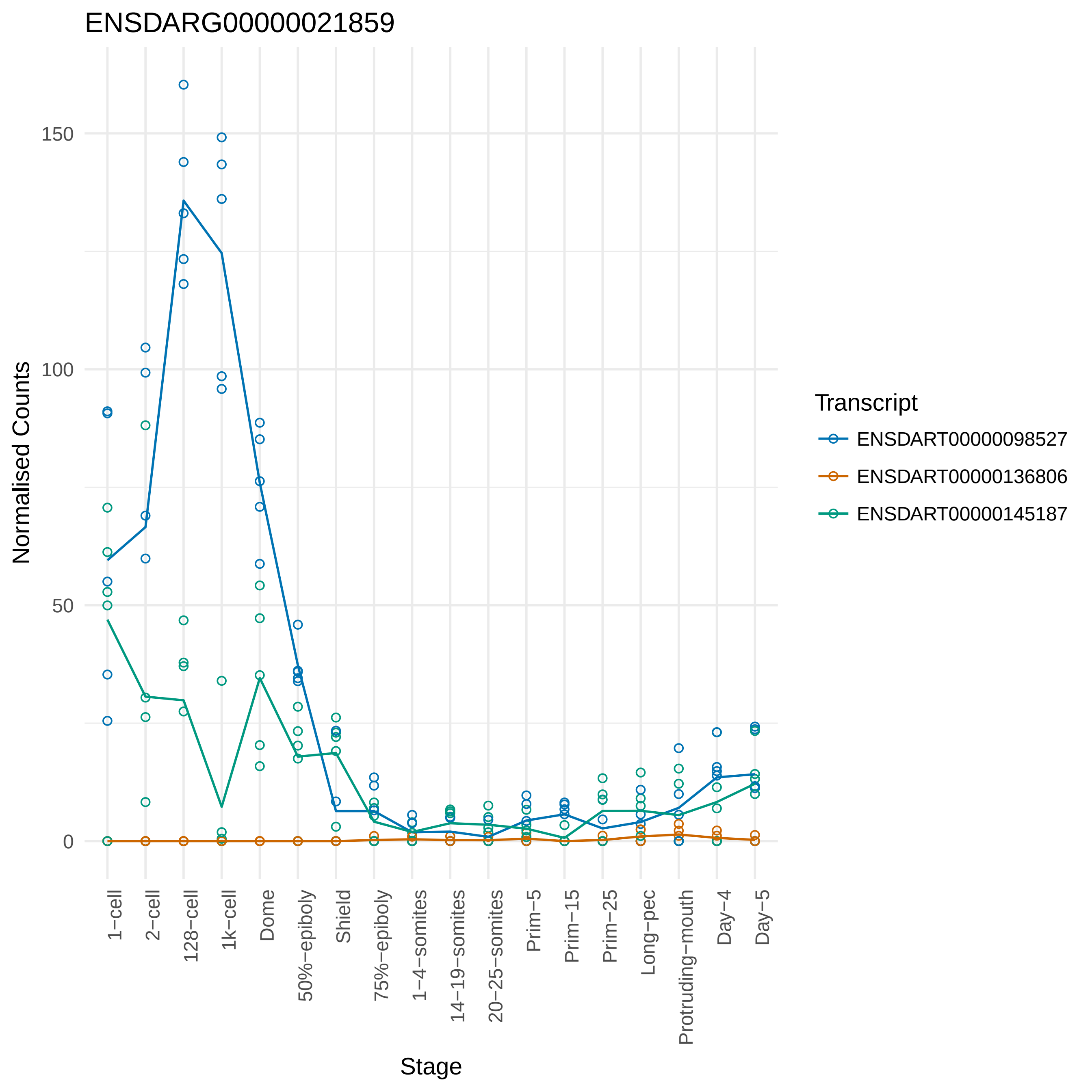
ENSDARG00000021859
Normalised Counts
150
100
50
0
1−cell
2−cell
128−cell
1k−cell
Dome
50%−epiboly
Shield
75%−epiboly
1−4−somites
14−19−somites
20−25−somites
Prim−5
Prim−15
Prim−25
Long−pec
Protruding−mouth
Day−4
Day−5
Stage
Transcript
ENSDART00000098527
ENSDART00000136806
ENSDART00000145187

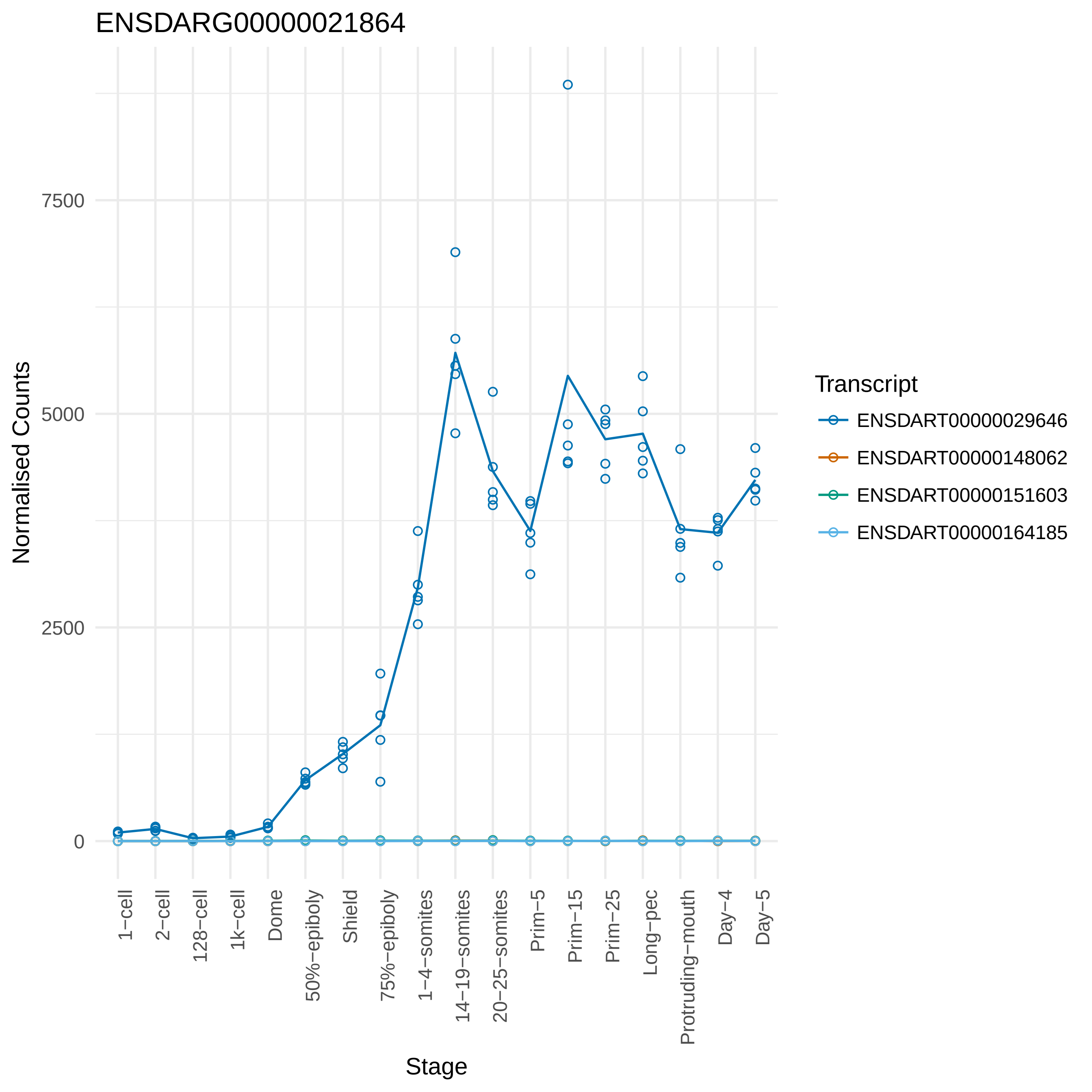

ENSDARG00000021864
Normalised Counts
7500
5000
2500
0
1−cell
2−cell
128−cell
1k−cell
Dome
50%−epiboly
Shield
75%−epiboly
1−4−somites
14−19−somites
20−25−somites
Prim−5
Prim−15
Prim−25
Long−pec
Protruding−mouth
Day−4
Day−5
Stage
Transcript
ENSDART00000029646
ENSDART00000148062
ENSDART00000151603
ENSDART00000164185

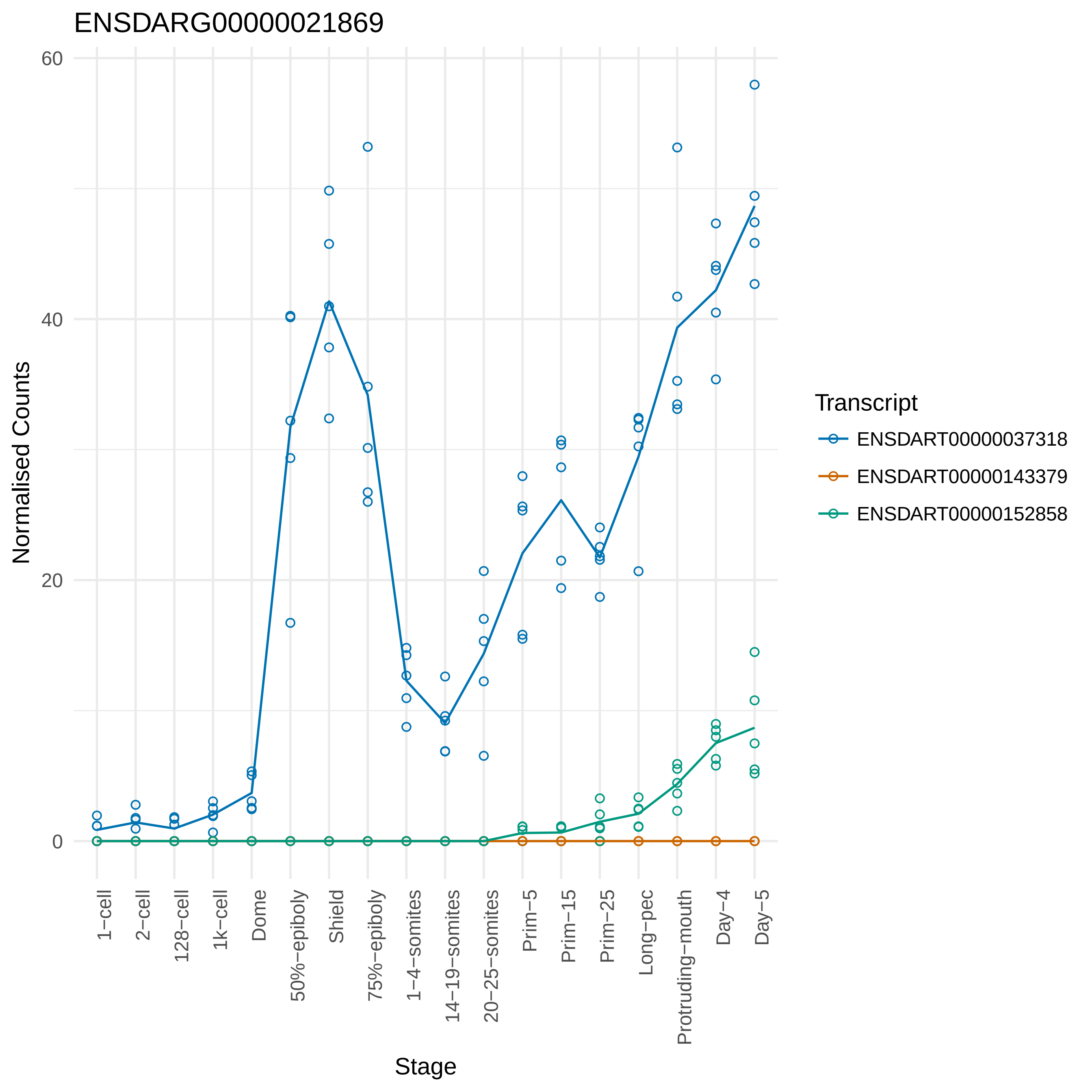

ENSDARG00000021869
Normalised Counts
60
40
20
0
1−cell
2−cell
128−cell
1k−cell
Dome
50%−epiboly
Shield
75%−epiboly
1−4−somites
14−19−somites
20−25−somites
Prim−5
Prim−15
Prim−25
Long−pec
Protruding−mouth
Day−4
Day−5
Stage
Transcript
ENSDART00000037318
ENSDART00000143379
ENSDART00000152858

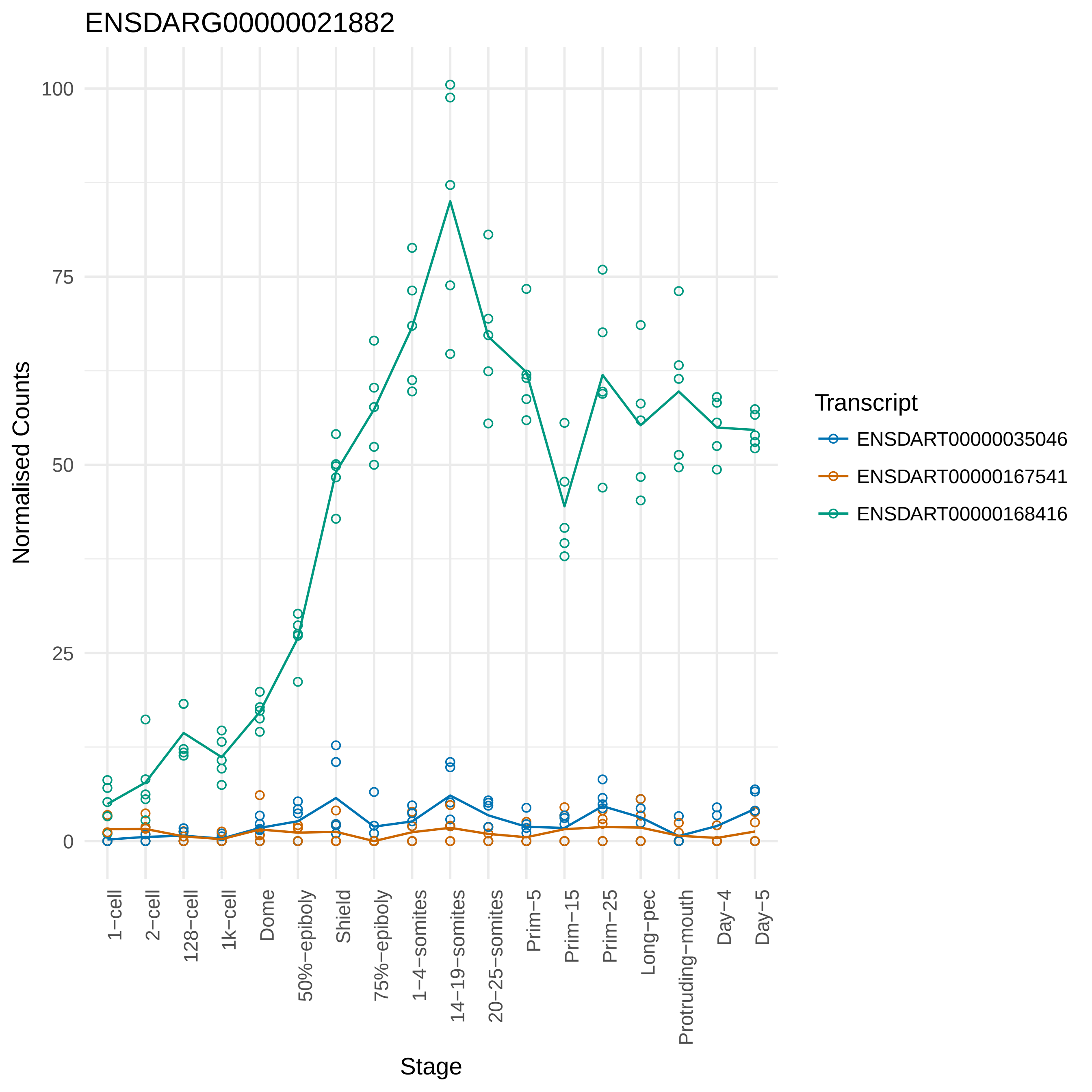

ENSDARG00000021882
Normalised Counts
100
75
50
25
0
1−cell
2−cell
128−cell
1k−cell
Dome
50%−epiboly
Shield
75%−epiboly
1−4−somites
14−19−somites
20−25−somites
Prim−5
Prim−15
Prim−25
Long−pec
Protruding−mouth
Day−4
Day−5
Stage
Transcript
ENSDART00000035046
ENSDART00000167541
ENSDART00000168416

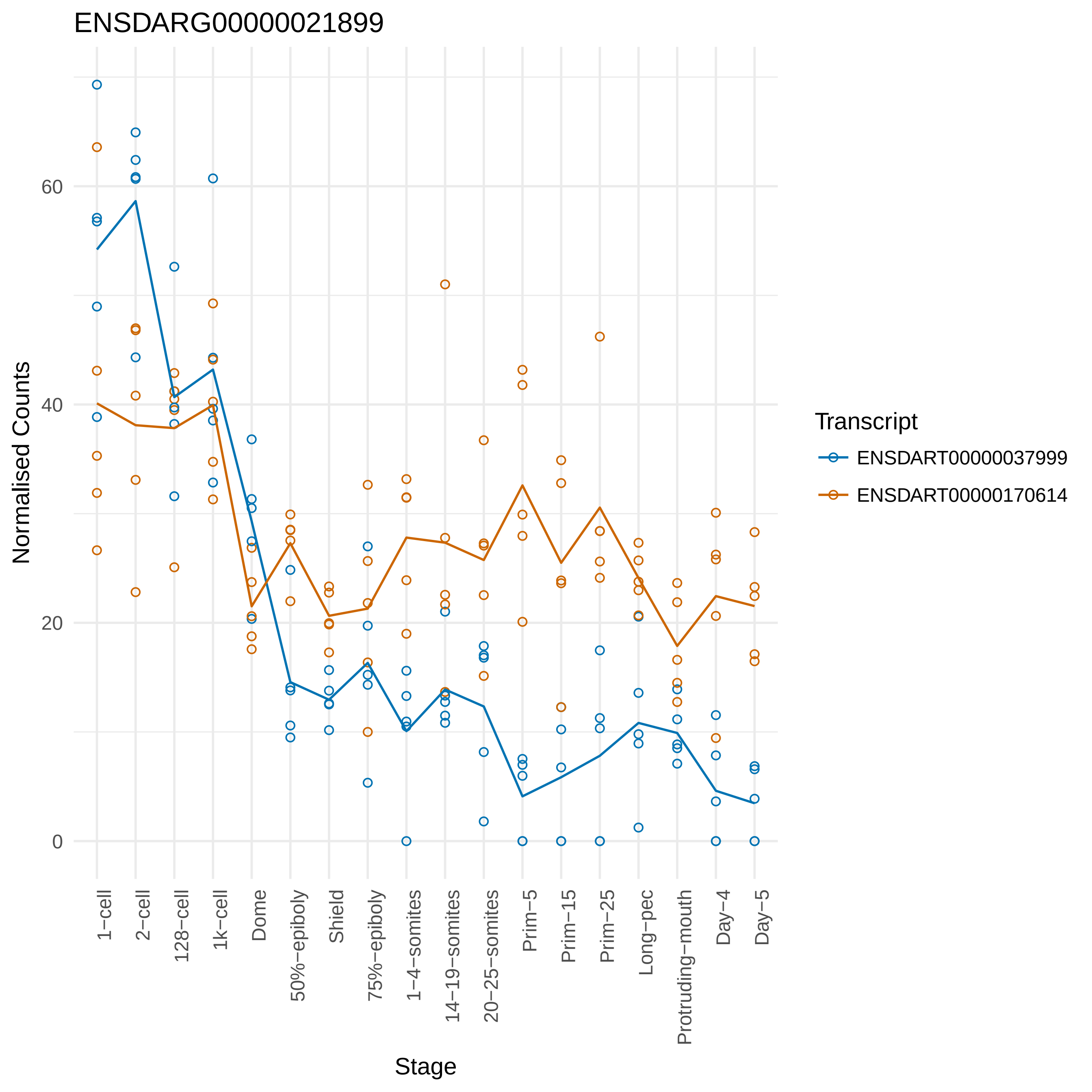

ENSDARG00000021899
Normalised Counts
0
20
40
60
Stage
1−cell
2−cell
128−cell
1k−cell
Dome
50%−epiboly
Shield
75%−epiboly
1−4−somites
14−19−somites
20−25−somites
Prim−5
Prim−15
Prim−25
Long−pec
Protruding−mouth
Day−4
Day−5
Transcript
ENSDART00000037999
ENSDART00000170614

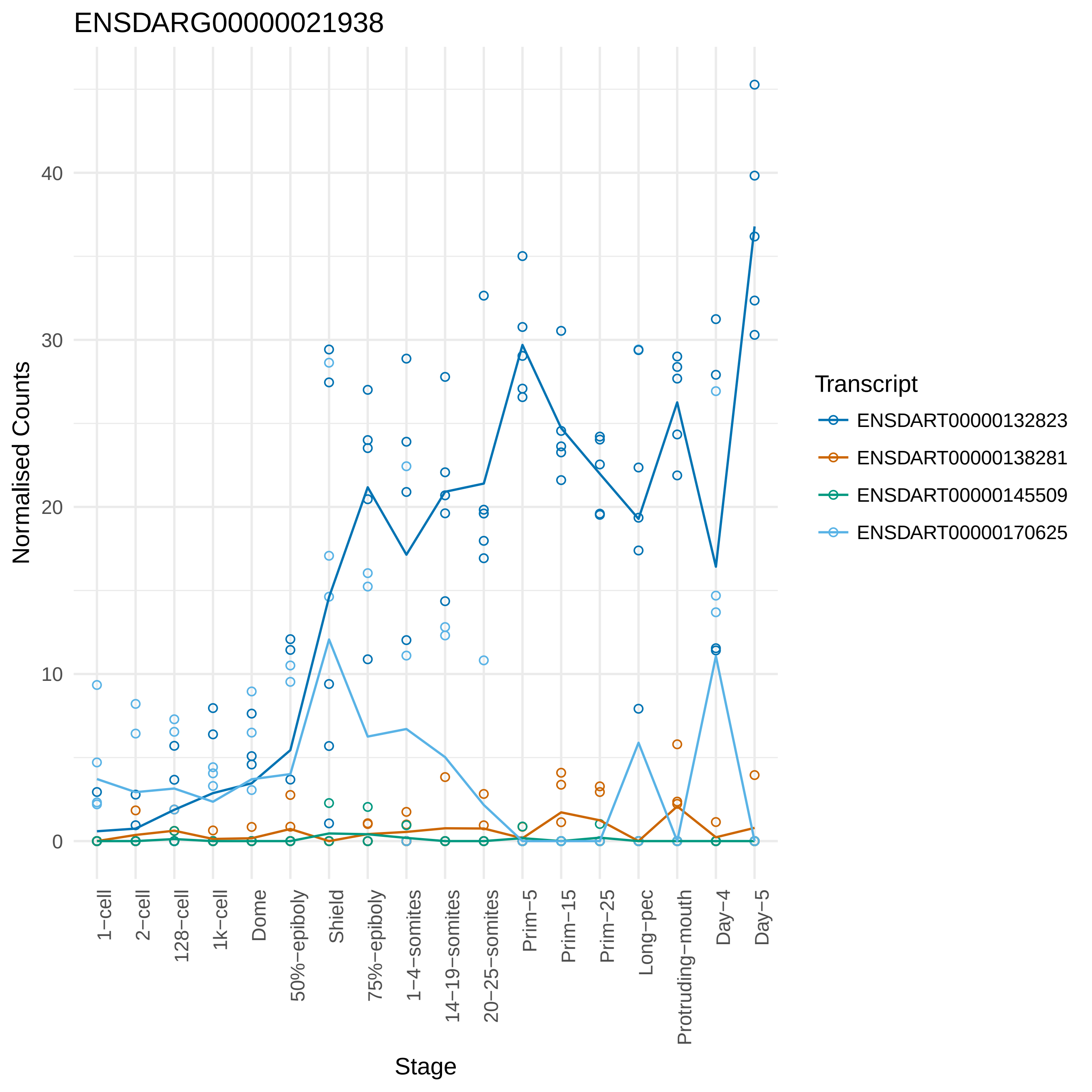
ENSDARG00000021938
Normalised Counts
0
10
20
30
40
1−cell
2−cell
128−cell
1k−cell
Dome
50%−epiboly
Shield
75%−epiboly
1−4−somites
14−19−somites
20−25−somites
Prim−5
Prim−15
Prim−25
Long−pec
Protruding−mouth
Day−4
Day−5
Stage
Transcript
ENSDART00000132823
ENSDART00000138281
ENSDART00000145509
ENSDART00000170625

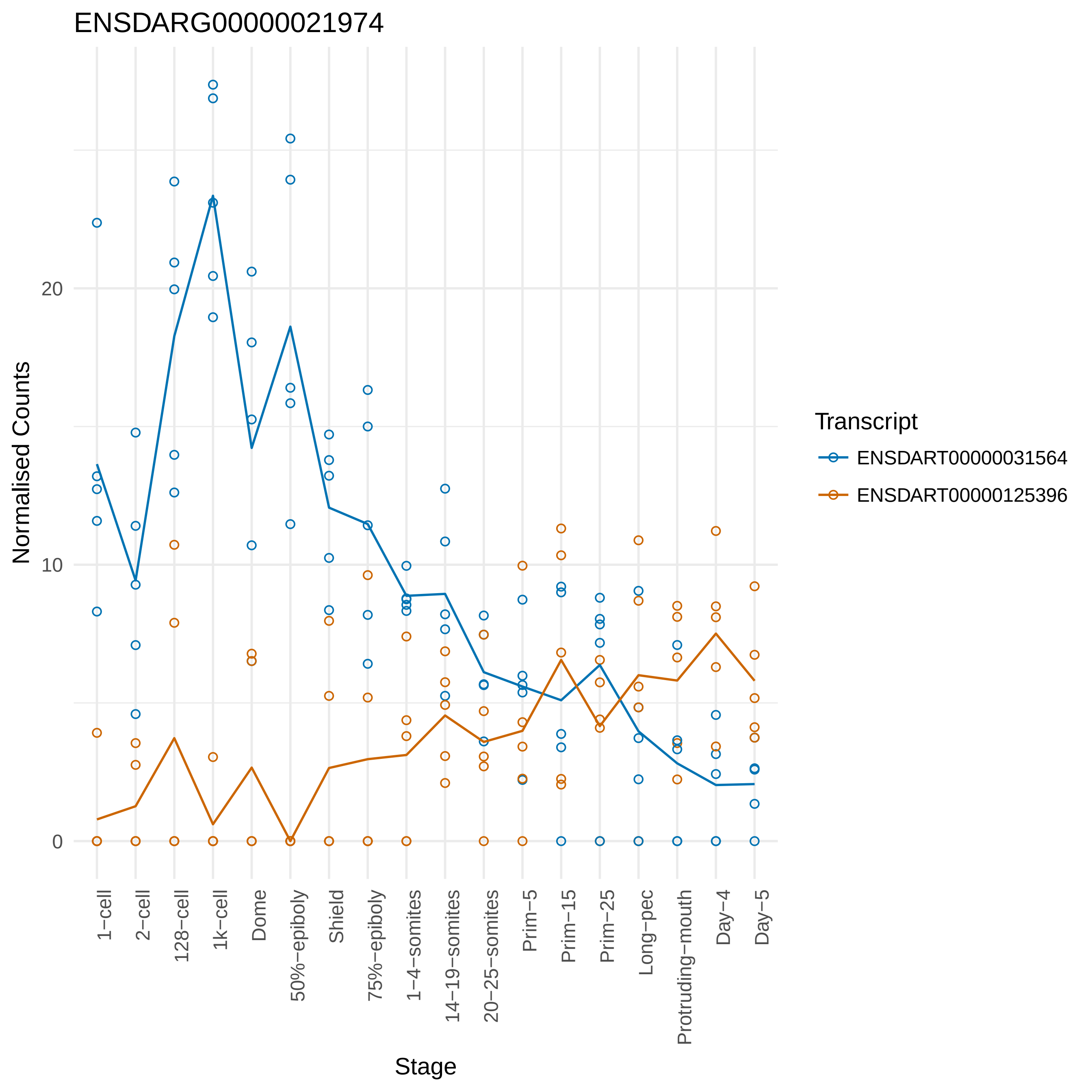
ENSDARG00000021974
Normalised Counts
0
10
20
1−cell
2−cell
128−cell
1k−cell
Dome
50%−epiboly
Shield
75%−epiboly
1−4−somites
14−19−somites
20−25−somites
Prim−5
Prim−15
Prim−25
Long−pec
Protruding−mouth
Day−4
Day−5
Stage
Transcript
ENSDART00000031564
ENSDART00000125396

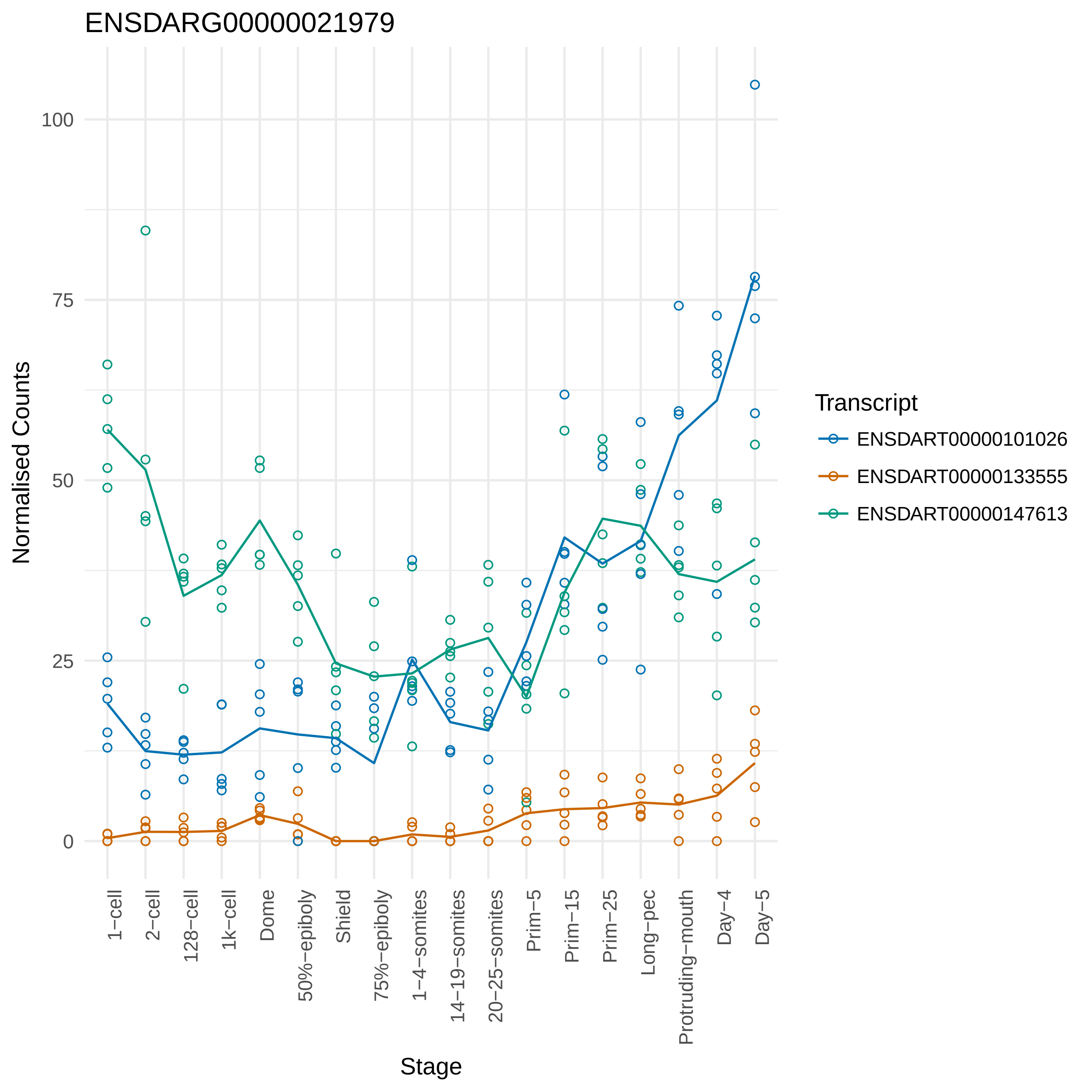
ENSDARG00000021979
Normalised Counts
100
75
50
25
0
1−cell
2−cell
128−cell
1k−cell
Dome
50%−epiboly
Shield
75%−epiboly
1−4−somites
14−19−somites
20−25−somites
Prim−5
Prim−15
Prim−25
Long−pec
Protruding−mouth
Day−4
Day−5
Stage
Transcript
ENSDART00000101026
ENSDART00000133555
ENSDART00000147613

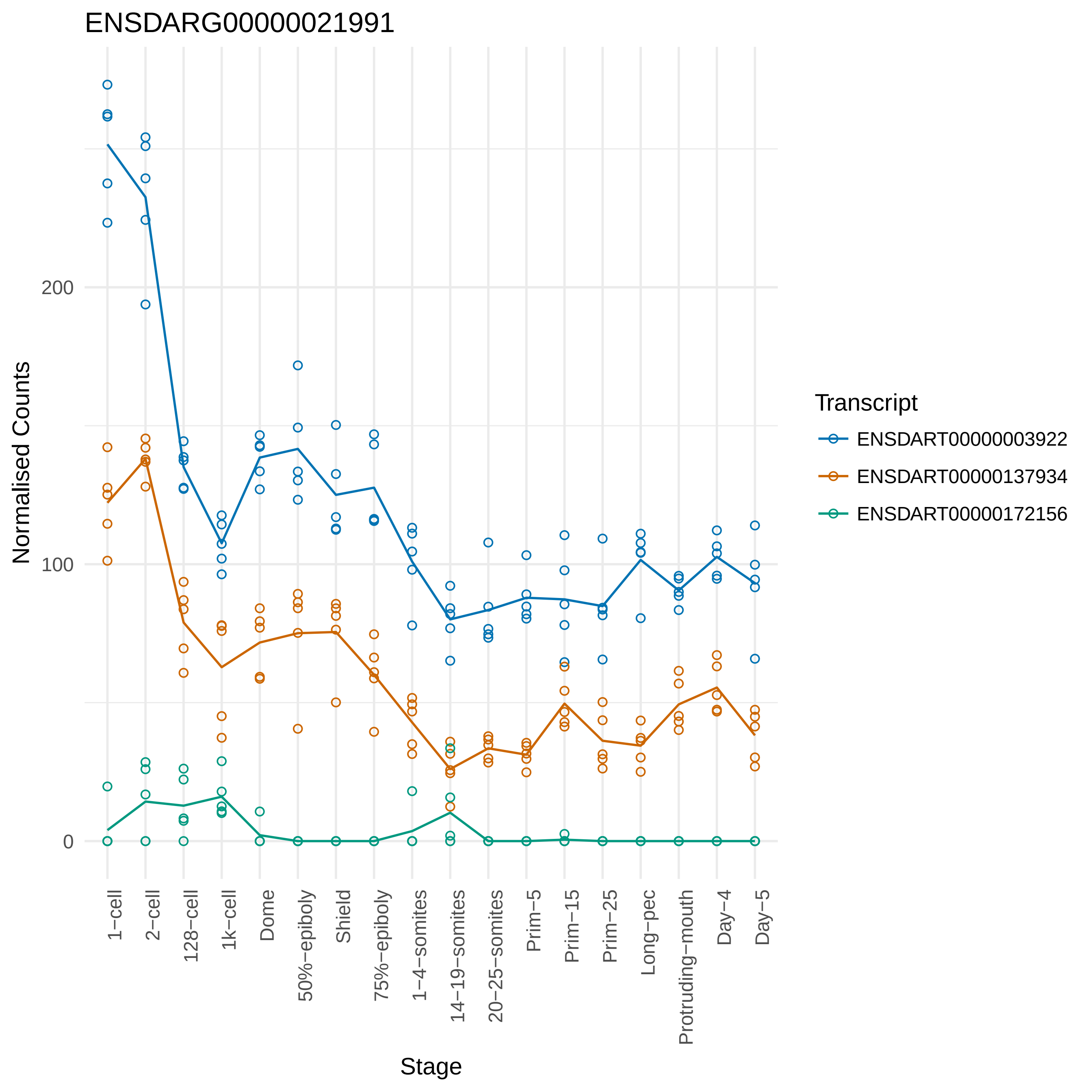
ENSDARG00000021991
Normalised Counts
200
100
0
1−cell
2−cell
128−cell
1k−cell
Dome
50%−epiboly
Shield
75%−epiboly
1−4−somites
14−19−somites
20−25−somites
Prim−5
Prim−15
Prim−25
Long−pec
Protruding−mouth
Day−4
Day−5
Stage
Transcript
ENSDART00000003922
ENSDART00000137934
ENSDART00000172156

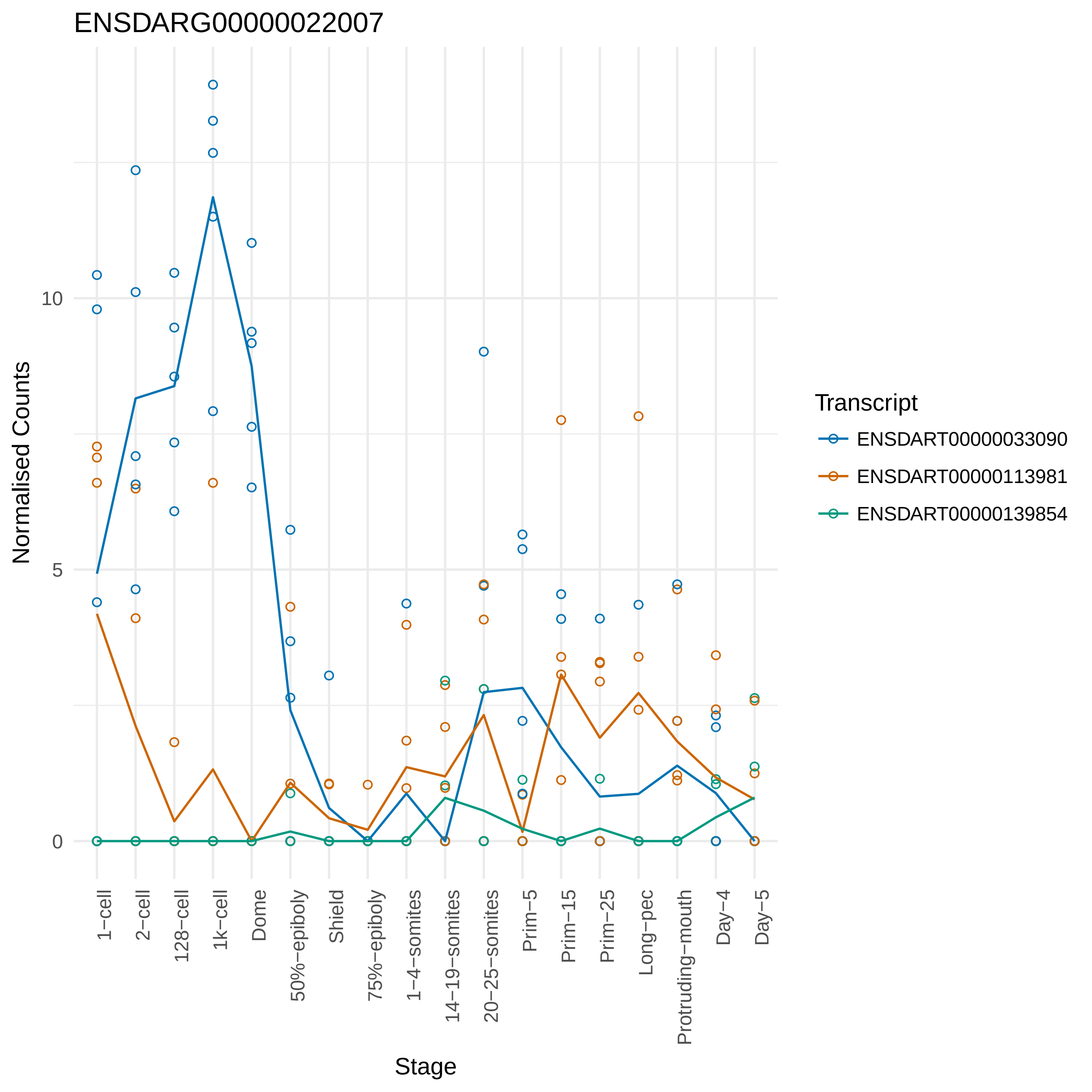

ENSDARG00000022007
Normalised Counts
0
5
10
1−cell
2−cell
128−cell
1k−cell
Dome
50%−epiboly
Shield
75%−epiboly
1−4−somites
14−19−somites
20−25−somites
Prim−5
Prim−15
Prim−25
Long−pec
Protruding−mouth
Day−4
Day−5
Stage
Transcript
ENSDART00000033090
ENSDART00000113981
ENSDART00000139854

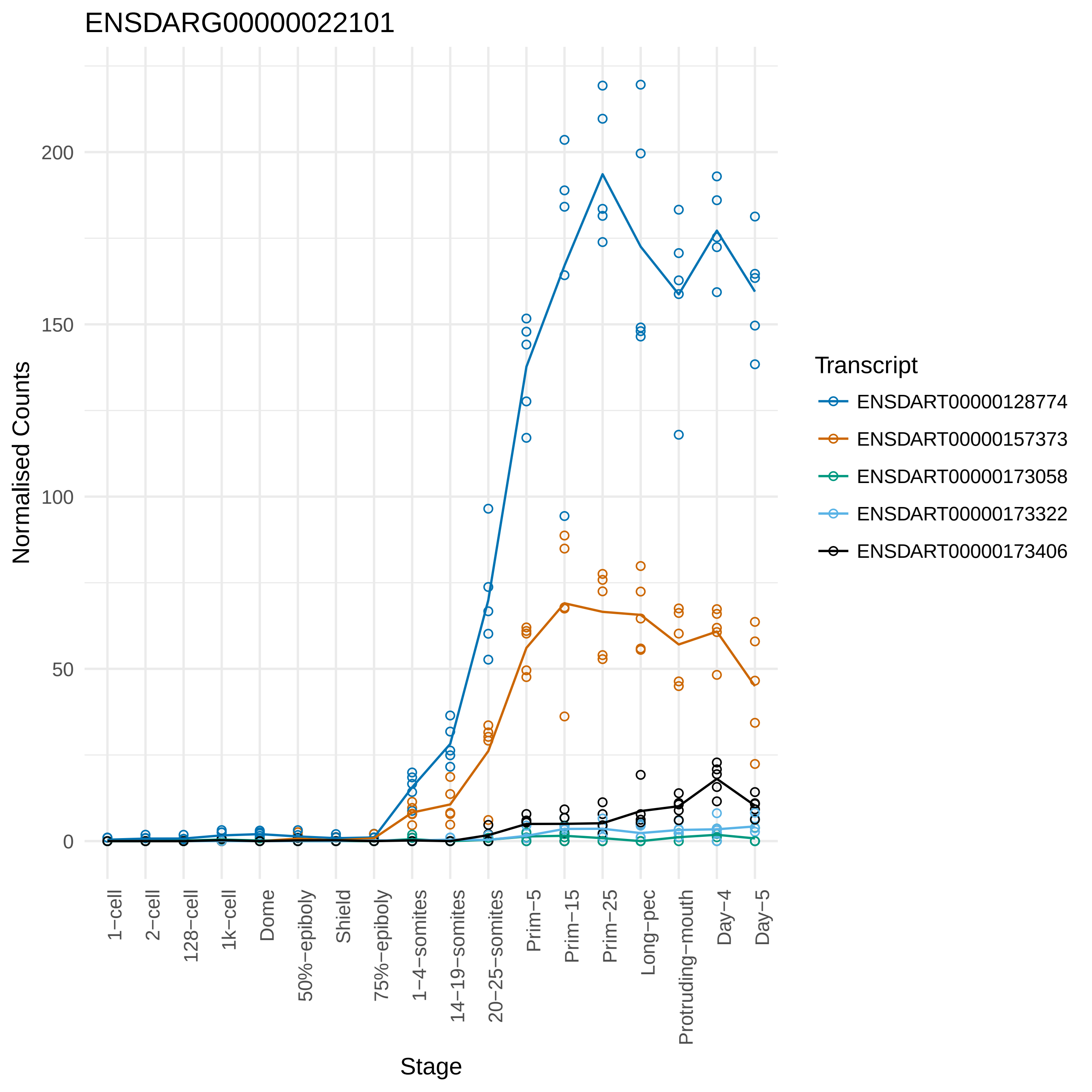
ENSDARG00000022101
Normalised Counts
0
50
100
150
200
Stage
1−cell
2−cell
128−cell
1k−cell
Dome
50%−epiboly
Shield
75%−epiboly
1−4−somites
14−19−somites
20−25−somites
Prim−5
Prim−15
Prim−25
Long−pec
Protruding−mouth
Day−4
Day−5
Transcript
ENSDART00000128774
ENSDART00000157373
ENSDART00000173058
ENSDART00000173322
ENSDART00000173406

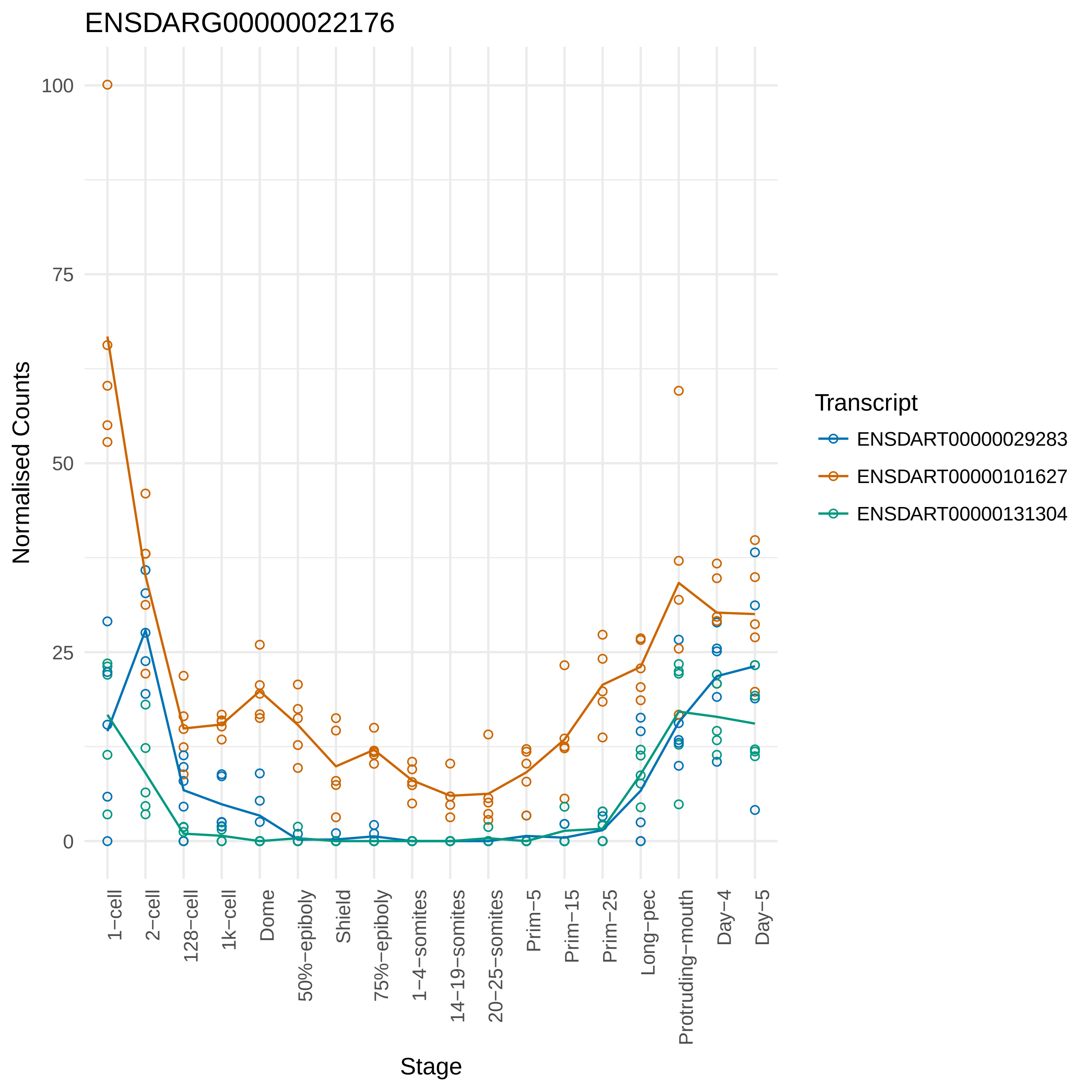

ENSDARG00000022176
Normalised Counts
100
75
50
25
0
1−cell
2−cell
128−cell
1k−cell
Dome
50%−epiboly
Shield
75%−epiboly
1−4−somites
14−19−somites
20−25−somites
Prim−5
Prim−15
Prim−25
Long−pec
Protruding−mouth
Day−4
Day−5
Stage
Transcript
ENSDART00000029283
ENSDART00000101627
ENSDART00000131304

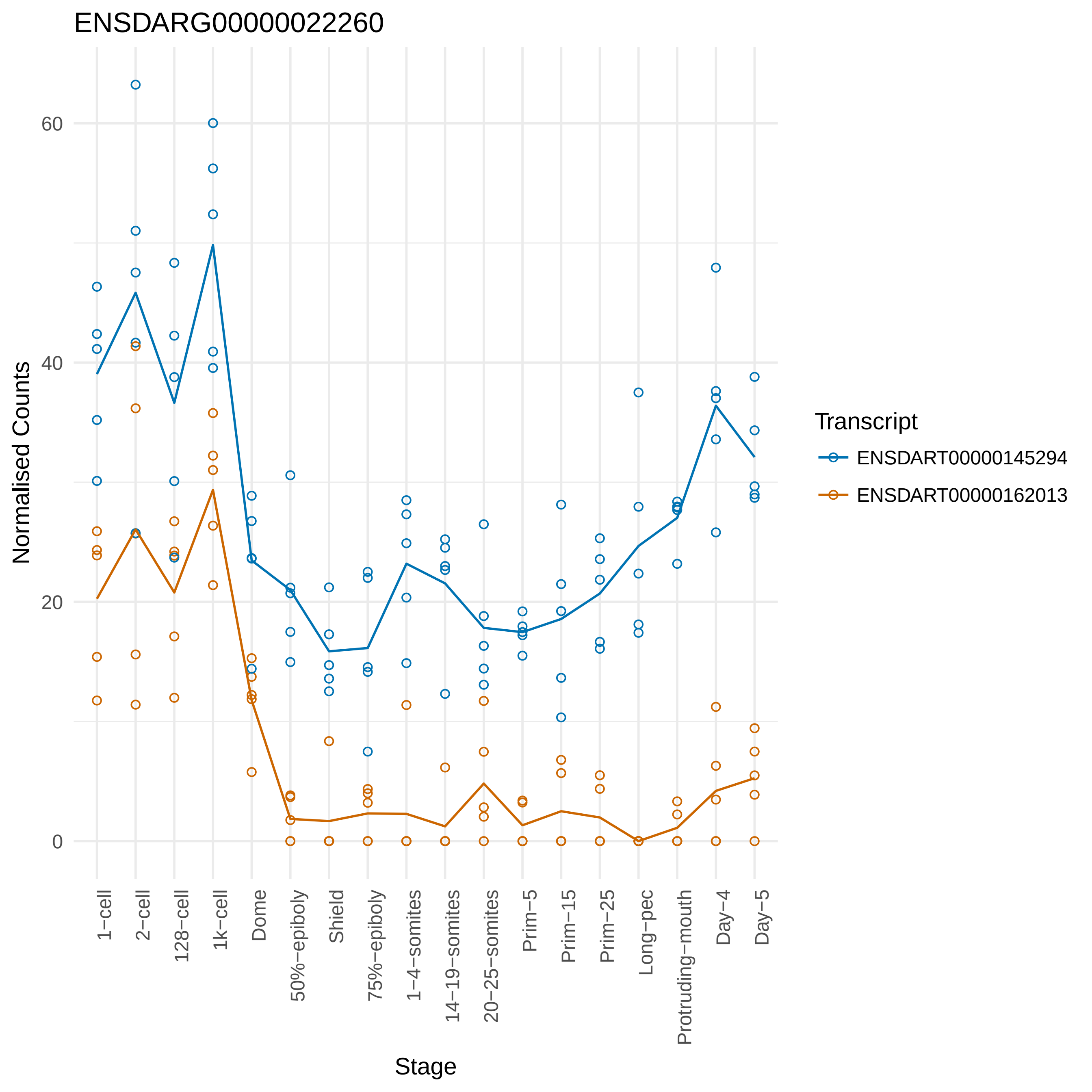

ENSDARG00000022260
Normalised Counts
60
40
20
0
1−cell
2−cell
128−cell
1k−cell
Dome
50%−epiboly
Shield
75%−epiboly
1−4−somites
14−19−somites
20−25−somites
Prim−5
Prim−15
Prim−25
Long−pec
Protruding−mouth
Day−4
Day−5
Stage
Transcript
ENSDART00000145294
ENSDART00000162013

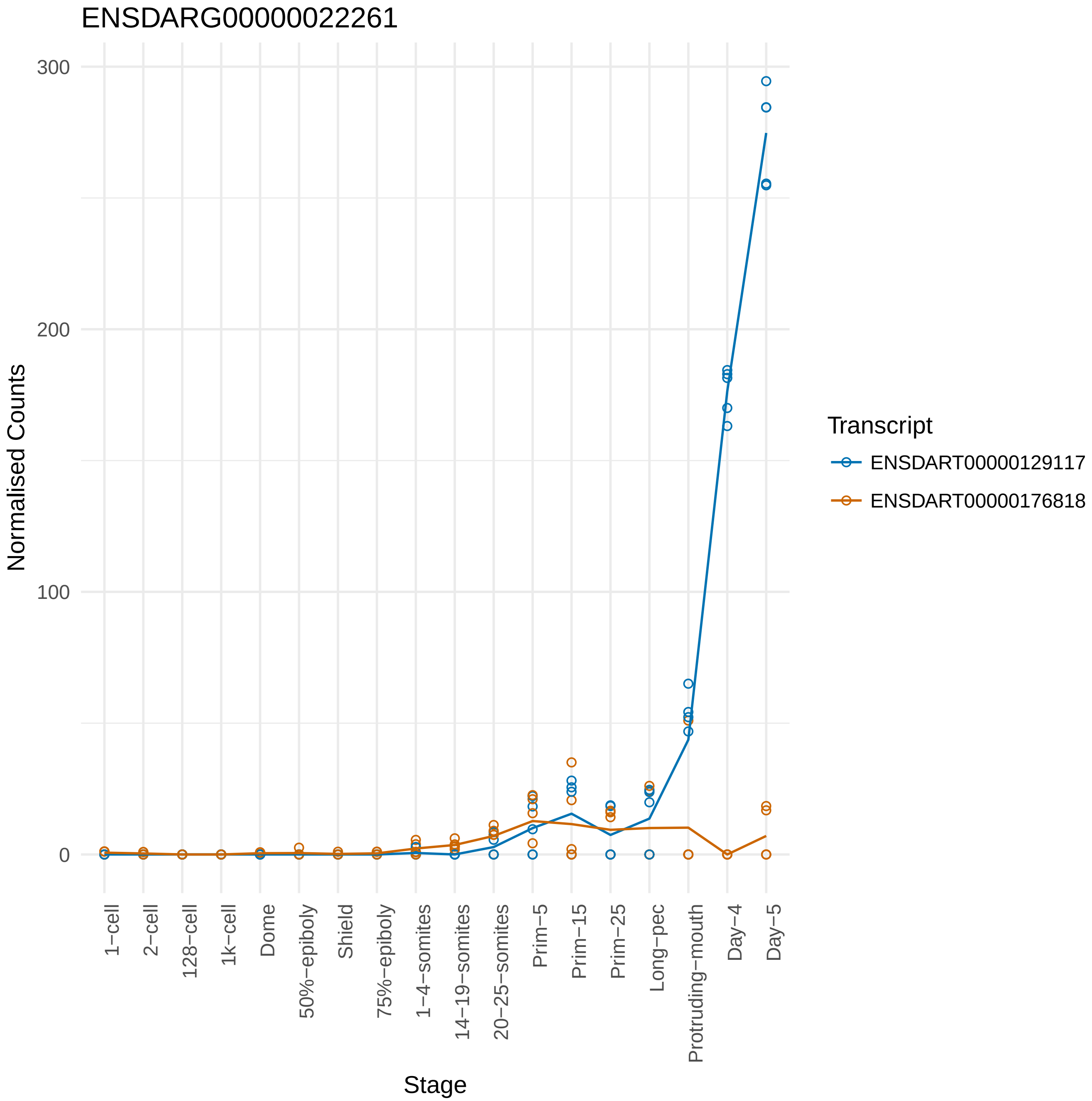

ENSDARG00000022261
Normalised Counts
300
200
100
0
1−cell
2−cell
128−cell
1k−cell
Dome
50%−epiboly
Shield
75%−epiboly
1−4−somites
14−19−somites
20−25−somites
Prim−5
Prim−15
Prim−25
Long−pec
Protruding−mouth
Day−4
Day−5
Stage
Transcript
ENSDART00000129117
ENSDART00000176818

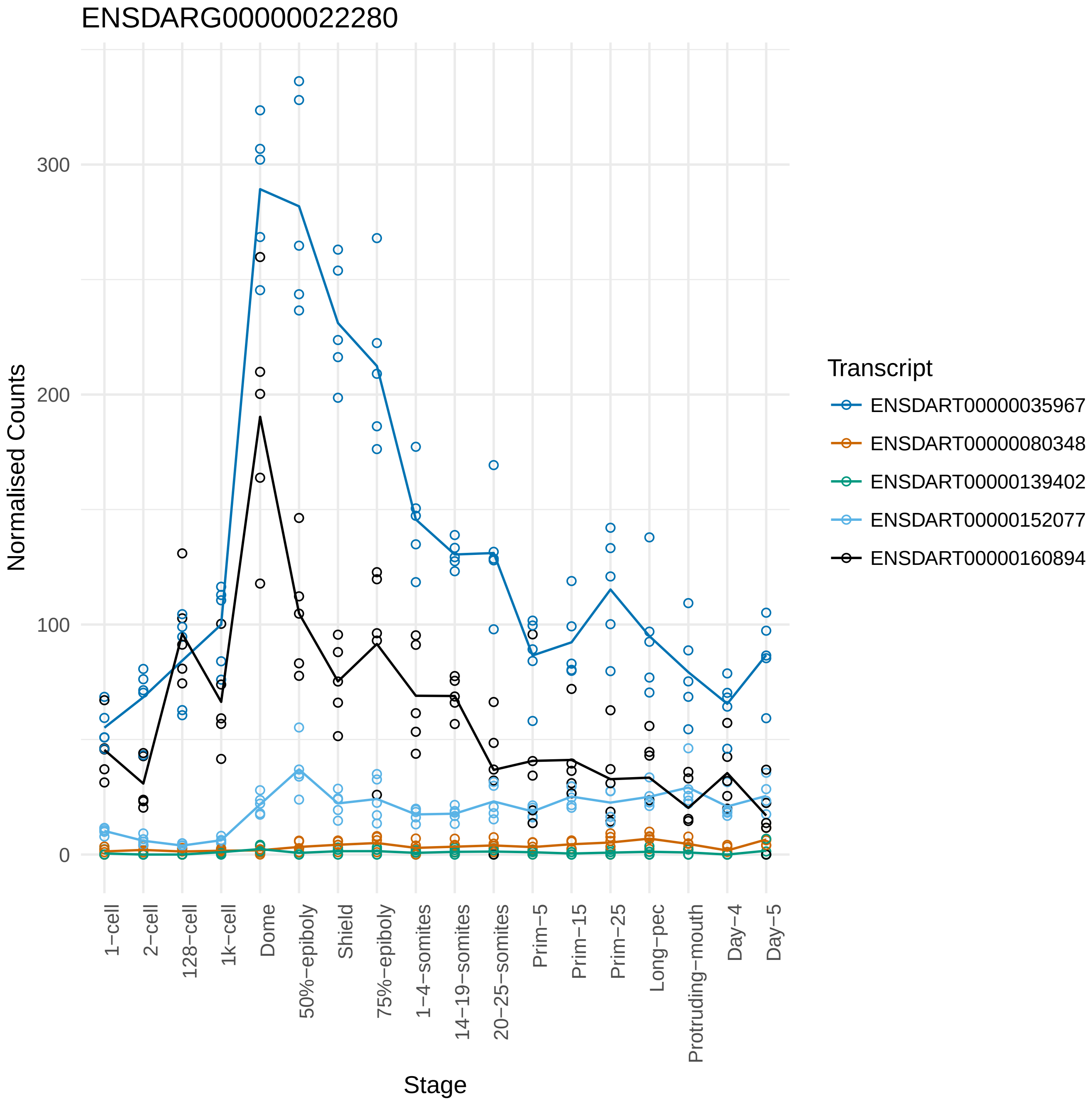
ENSDARG00000022280
Normalised Counts
300
200
100
0
1−cell
2−cell
128−cell
1k−cell
Dome
50%−epiboly
Shield
75%−epiboly
1−4−somites
14−19−somites
20−25−somites
Prim−5
Prim−15
Prim−25
Long−pec
Protruding−mouth
Day−4
Day−5
Stage
Transcript
ENSDART00000035967
ENSDART00000080348
ENSDART00000139402
ENSDART00000152077
ENSDART00000160894

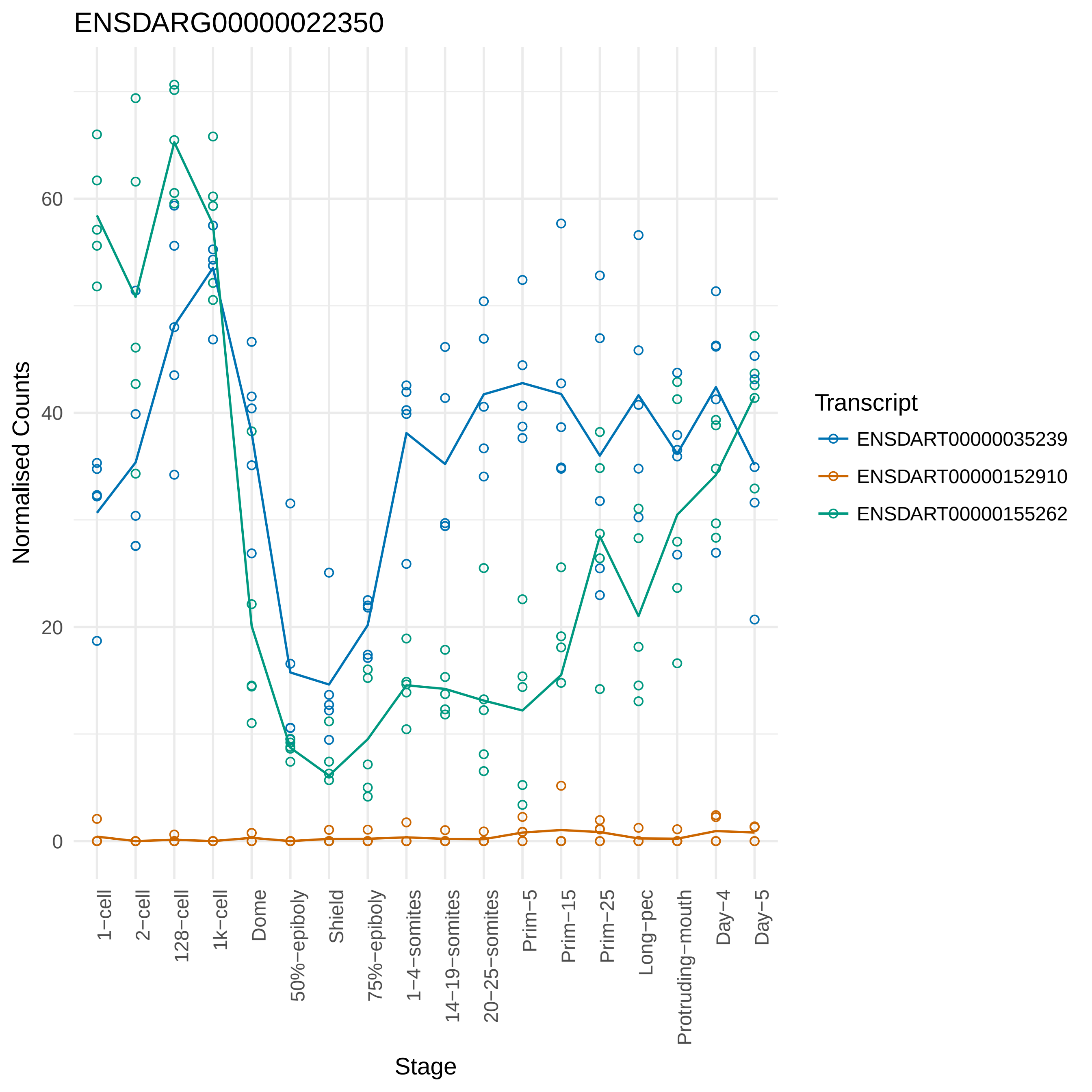
ENSDARG00000022350
Normalised Counts
0
20
40
60
Stage
1−cell
2−cell
128−cell
1k−cell
Dome
50%−epiboly
Shield
75%−epiboly
1−4−somites
14−19−somites
20−25−somites
Prim−5
Prim−15
Prim−25
Long−pec
Protruding−mouth
Day−4
Day−5
Transcript
ENSDART00000035239
ENSDART00000152910
ENSDART00000155262

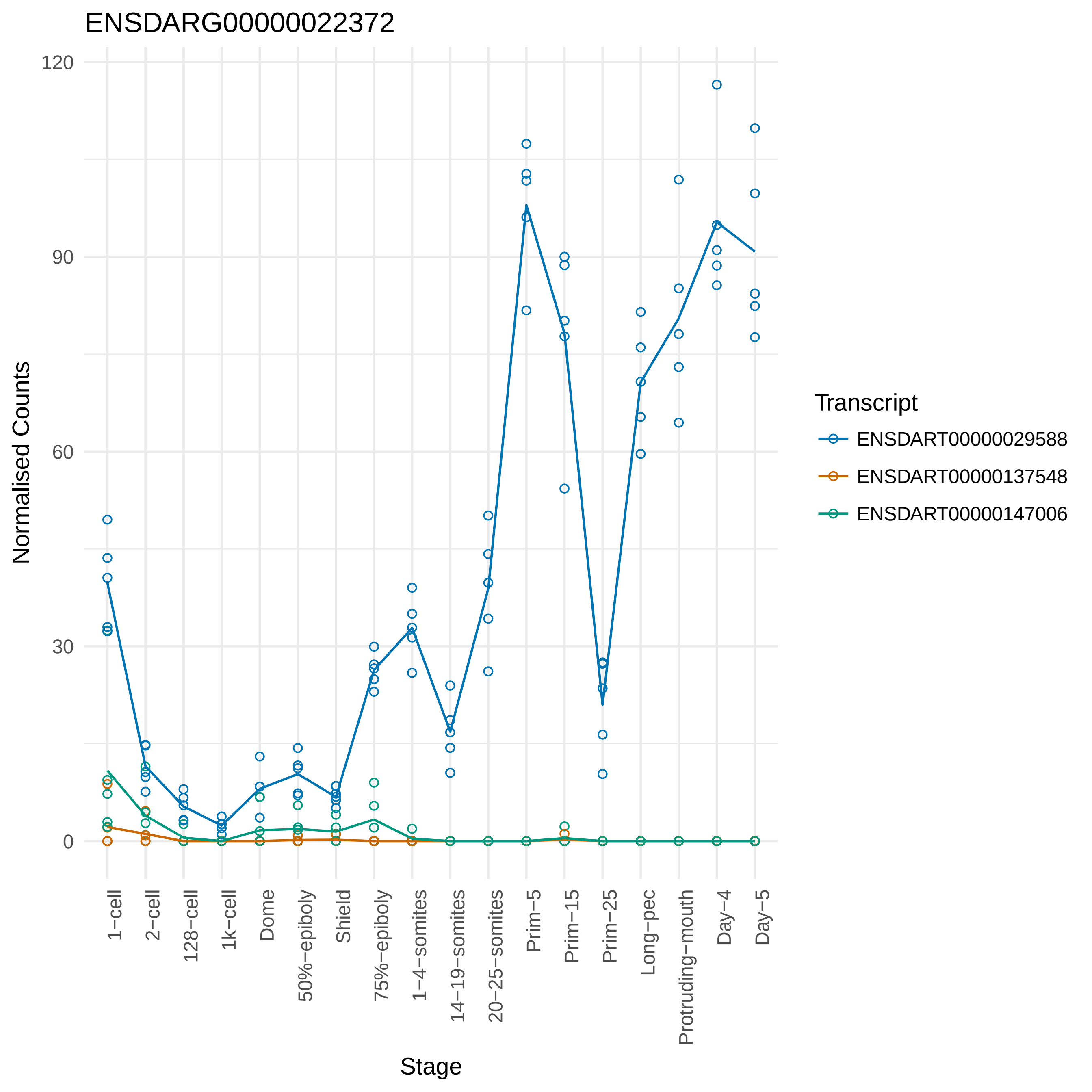
ENSDARG00000022372
Normalised Counts
120
90
60
30
0
1−cell
2−cell
128−cell
1k−cell
Dome
50%−epiboly
Shield
75%−epiboly
1−4−somites
14−19−somites
20−25−somites
Prim−5
Prim−15
Prim−25
Long−pec
Protruding−mouth
Day−4
Day−5
Stage
Transcript
ENSDART00000029588
ENSDART00000137548
ENSDART00000147006

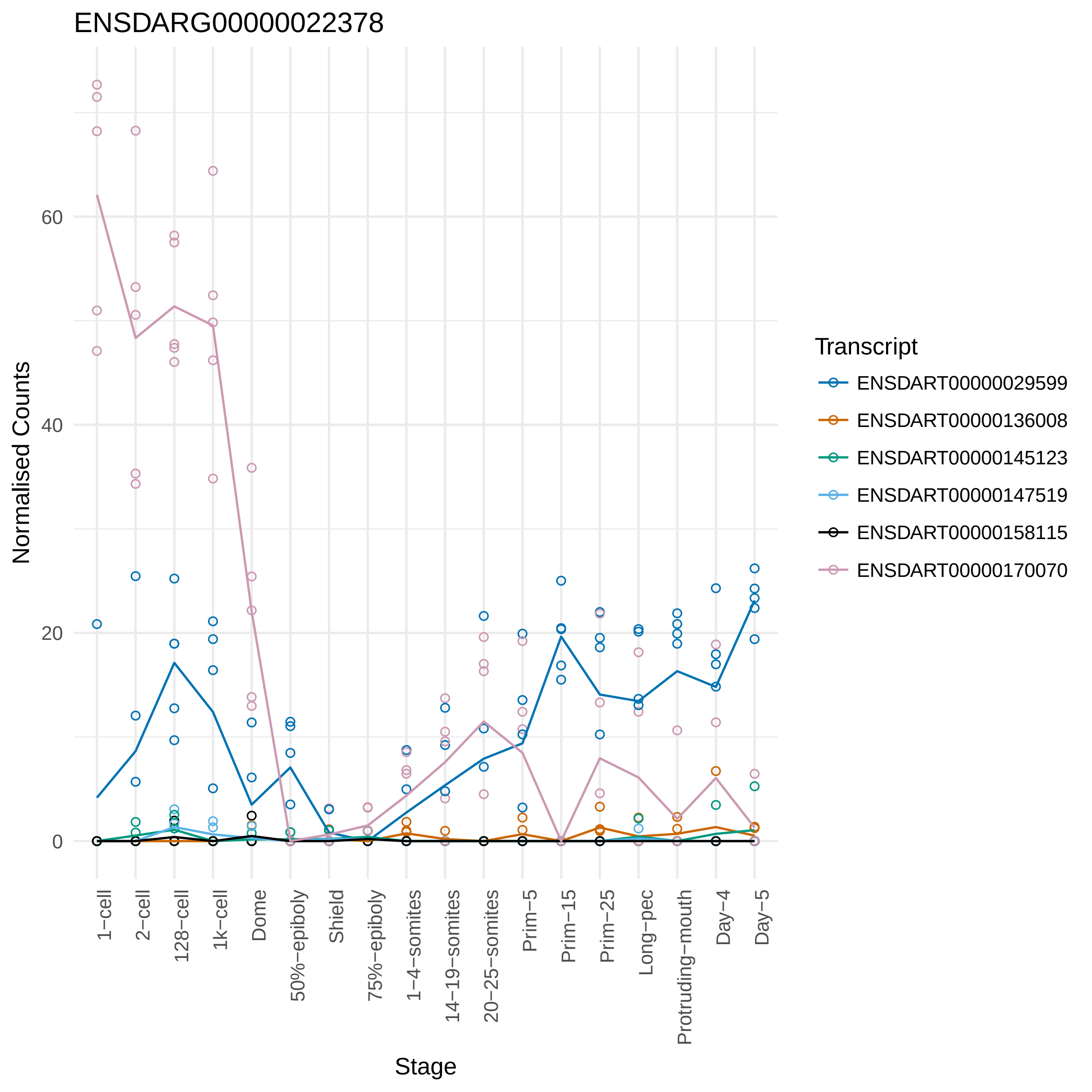
ENSDARG00000022378
Normalised Counts
0
20
40
60
Stage
1−cell
2−cell
128−cell
1k−cell
Dome
50%−epiboly
Shield
75%−epiboly
1−4−somites
14−19−somites
20−25−somites
Prim−5
Prim−15
Prim−25
Long−pec
Protruding−mouth
Day−4
Day−5
Transcript
ENSDART00000029599
ENSDART00000136008
ENSDART00000145123
ENSDART00000147519
ENSDART00000158115
ENSDART00000170070

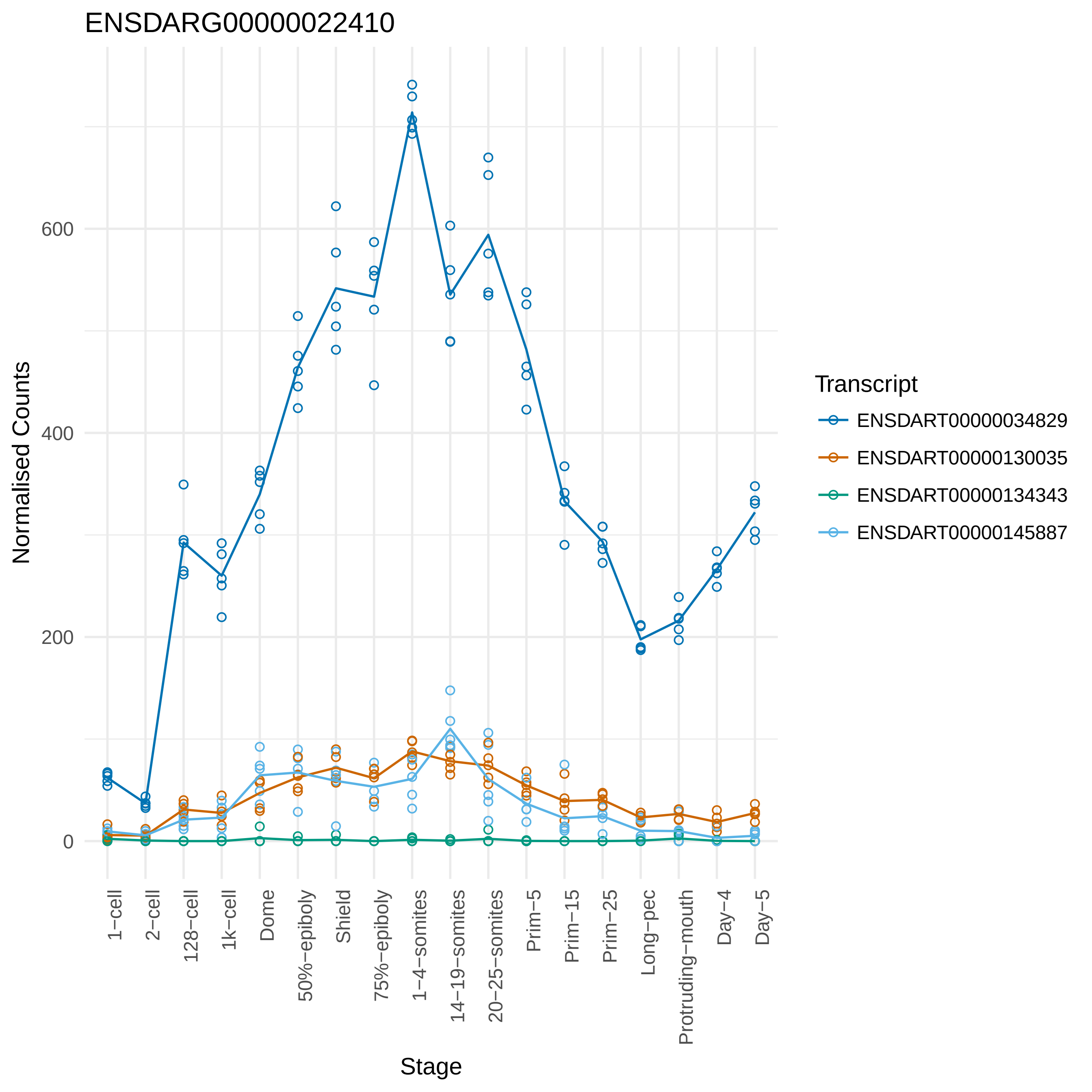

ENSDARG00000022410
Normalised Counts
600
400
200
0
1−cell
2−cell
128−cell
1k−cell
Dome
50%−epiboly
Shield
75%−epiboly
1−4−somites
14−19−somites
20−25−somites
Prim−5
Prim−15
Prim−25
Long−pec
Protruding−mouth
Day−4
Day−5
Stage
Transcript
ENSDART00000034829
ENSDART00000130035
ENSDART00000134343
ENSDART00000145887

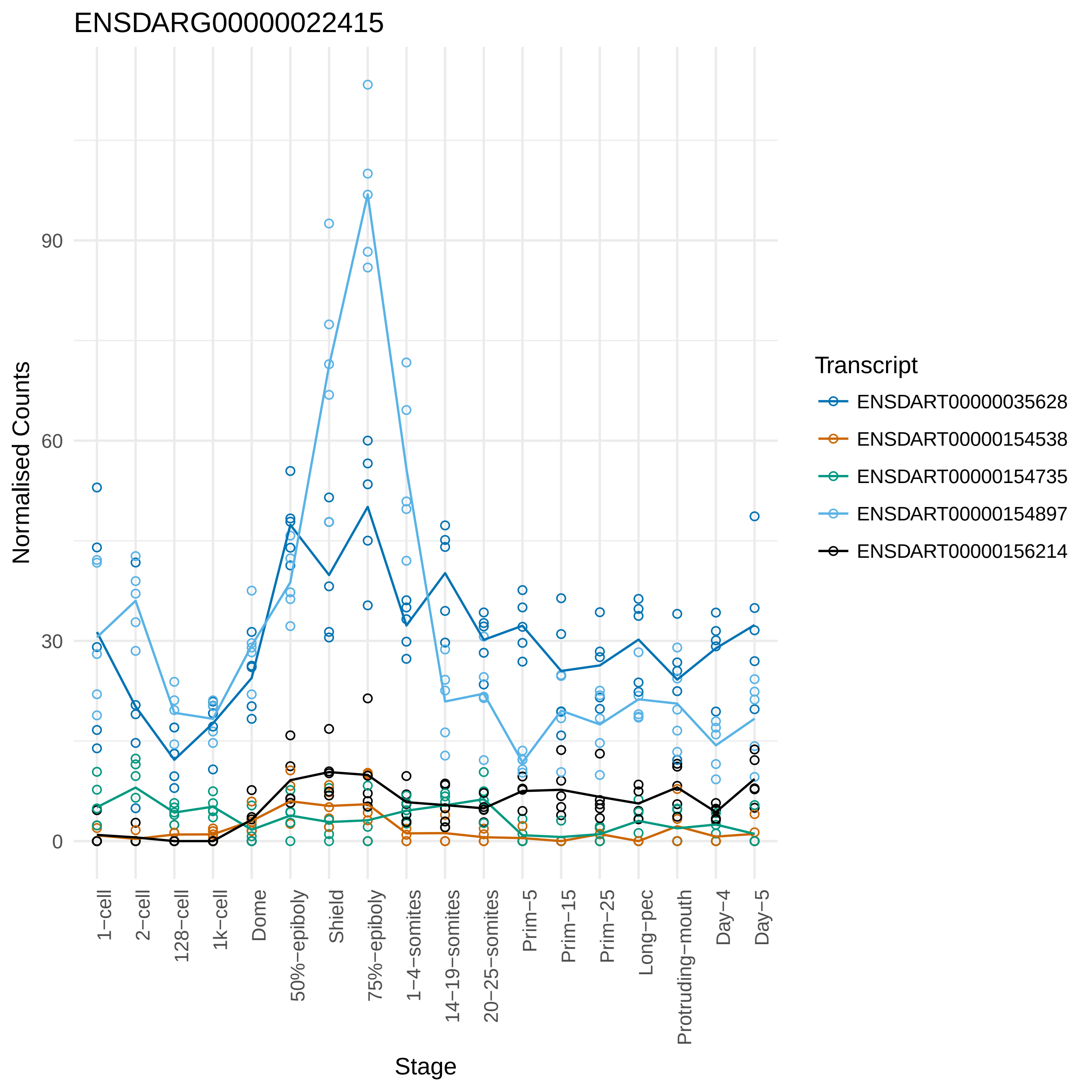

ENSDARG00000022415
Normalised Counts
0
30
60
90
1−cell
2−cell
128−cell
1k−cell
Dome
50%−epiboly
Shield
75%−epiboly
1−4−somites
14−19−somites
20−25−somites
Prim−5
Prim−15
Prim−25
Long−pec
Protruding−mouth
Day−4
Day−5
Stage
Transcript
ENSDART00000035628
ENSDART00000154538
ENSDART00000154735
ENSDART00000154897
ENSDART00000156214

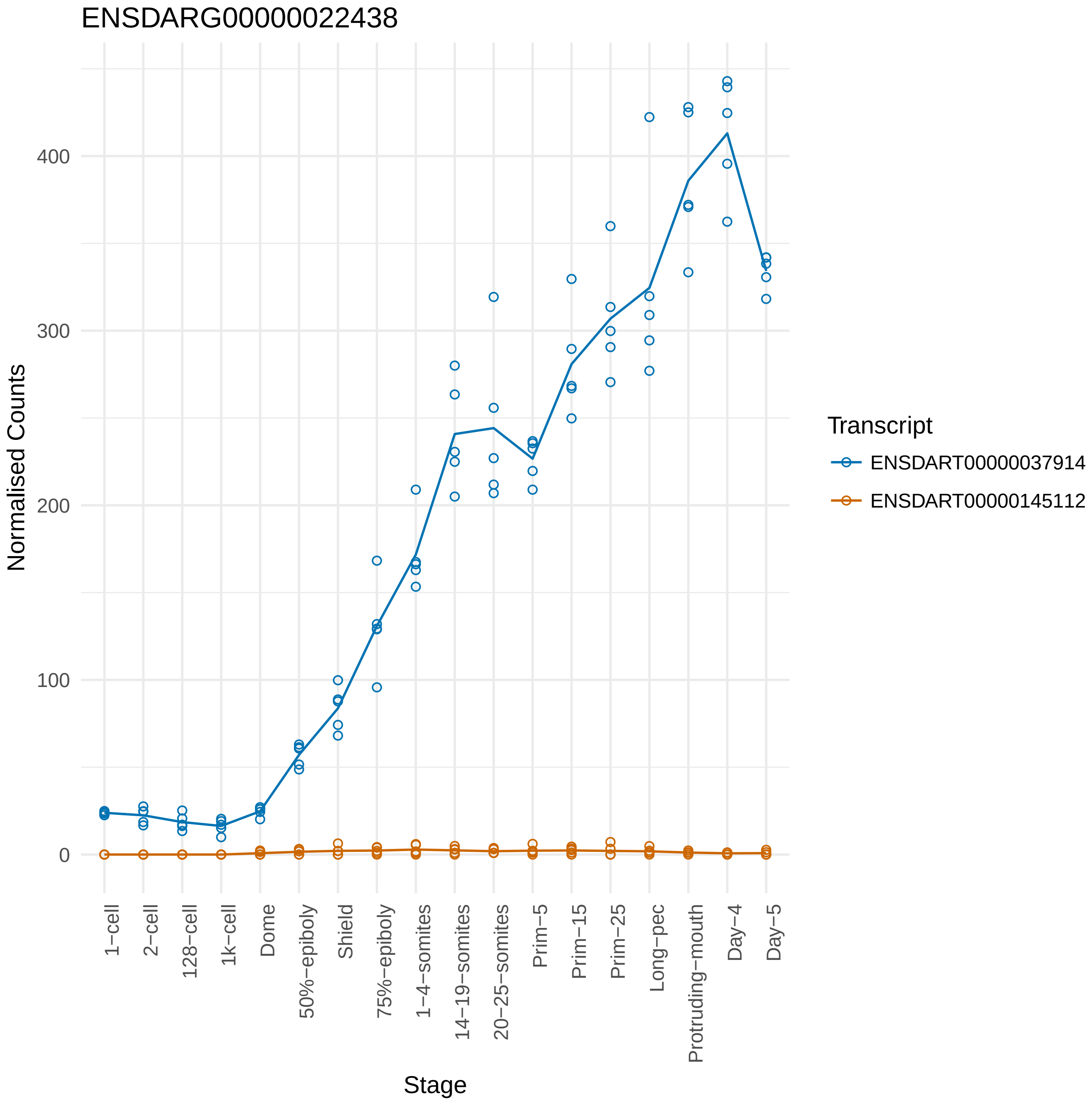

ENSDARG00000022438
Normalised Counts
0
100
200
300
400
Stage
1−cell
2−cell
128−cell
1k−cell
Dome
50%−epiboly
Shield
75%−epiboly
1−4−somites
14−19−somites
20−25−somites
Prim−5
Prim−15
Prim−25
Long−pec
Protruding−mouth
Day−4
Day−5
Transcript
ENSDART00000037914
ENSDART00000145112

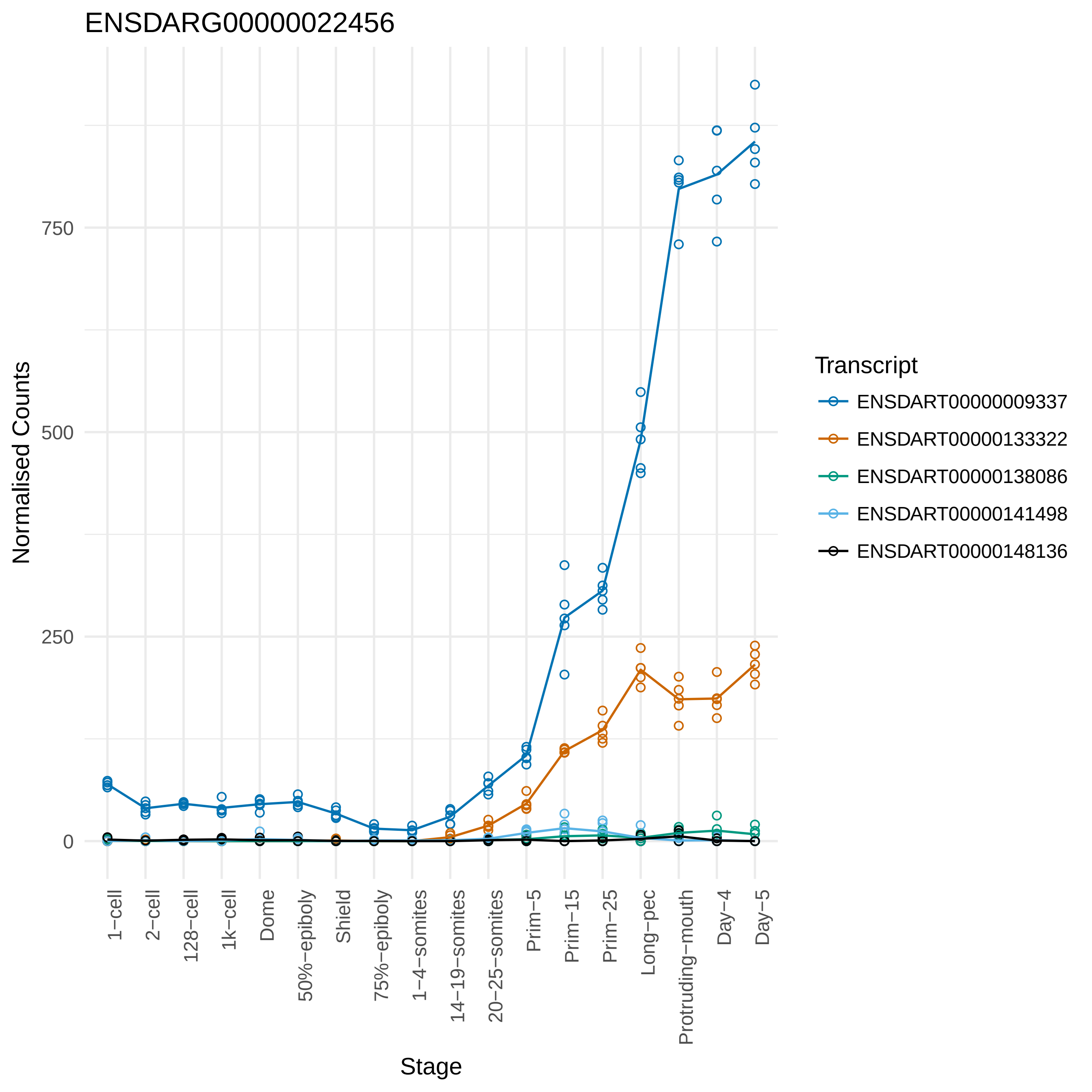
ENSDARG00000022456
Normalised Counts
750
500
250
0
1−cell
2−cell
128−cell
1k−cell
Dome
50%−epiboly
Shield
75%−epiboly
1−4−somites
14−19−somites
20−25−somites
Prim−5
Prim−15
Prim−25
Long−pec
Protruding−mouth
Day−4
Day−5
Stage
Transcript
ENSDART00000009337
ENSDART00000133322
ENSDART00000138086
ENSDART00000141498
ENSDART00000148136

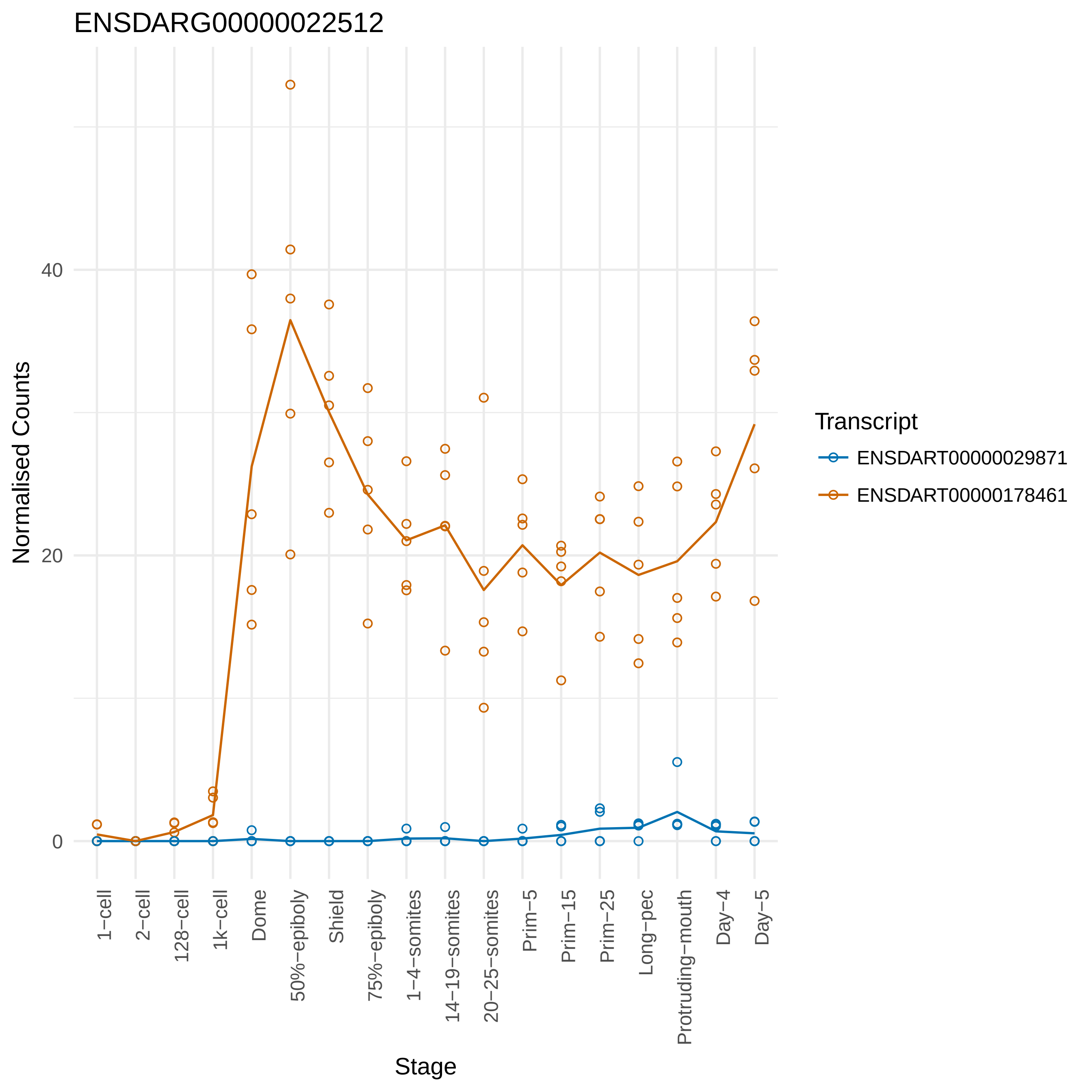

ENSDARG00000022512
Normalised Counts
40
20
0
1−cell
2−cell
128−cell
1k−cell
Dome
50%−epiboly
Shield
75%−epiboly
1−4−somites
14−19−somites
20−25−somites
Prim−5
Prim−15
Prim−25
Long−pec
Protruding−mouth
Day−4
Day−5
Stage
Transcript
ENSDART00000029871
ENSDART00000178461

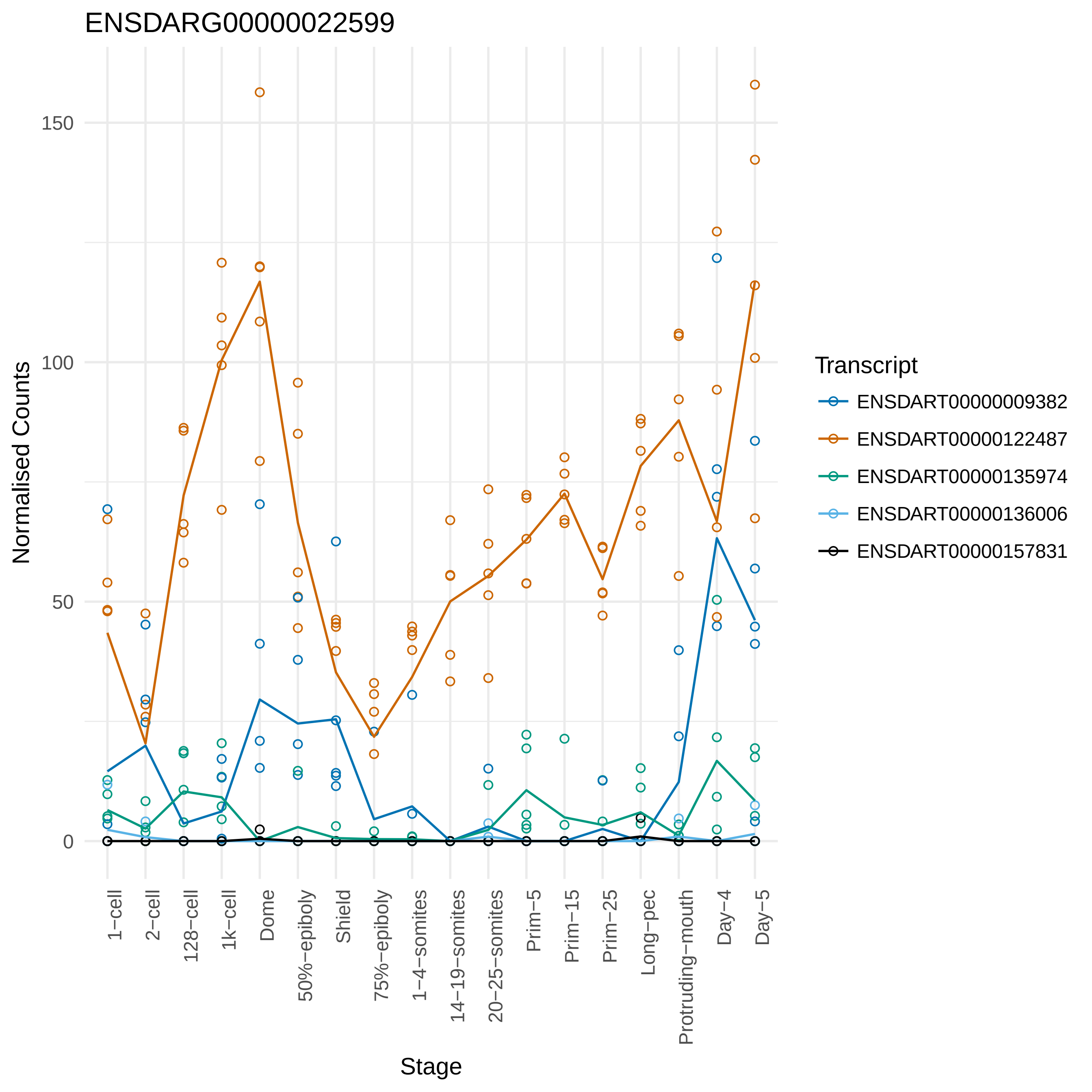

ENSDARG00000022599
Normalised Counts
150
100
50
0
1−cell
2−cell
128−cell
1k−cell
Dome
50%−epiboly
Shield
75%−epiboly
1−4−somites
14−19−somites
20−25−somites
Prim−5
Prim−15
Prim−25
Long−pec
Protruding−mouth
Day−4
Day−5
Stage
Transcript
ENSDART00000009382
ENSDART00000122487
ENSDART00000135974
ENSDART00000136006
ENSDART00000157831

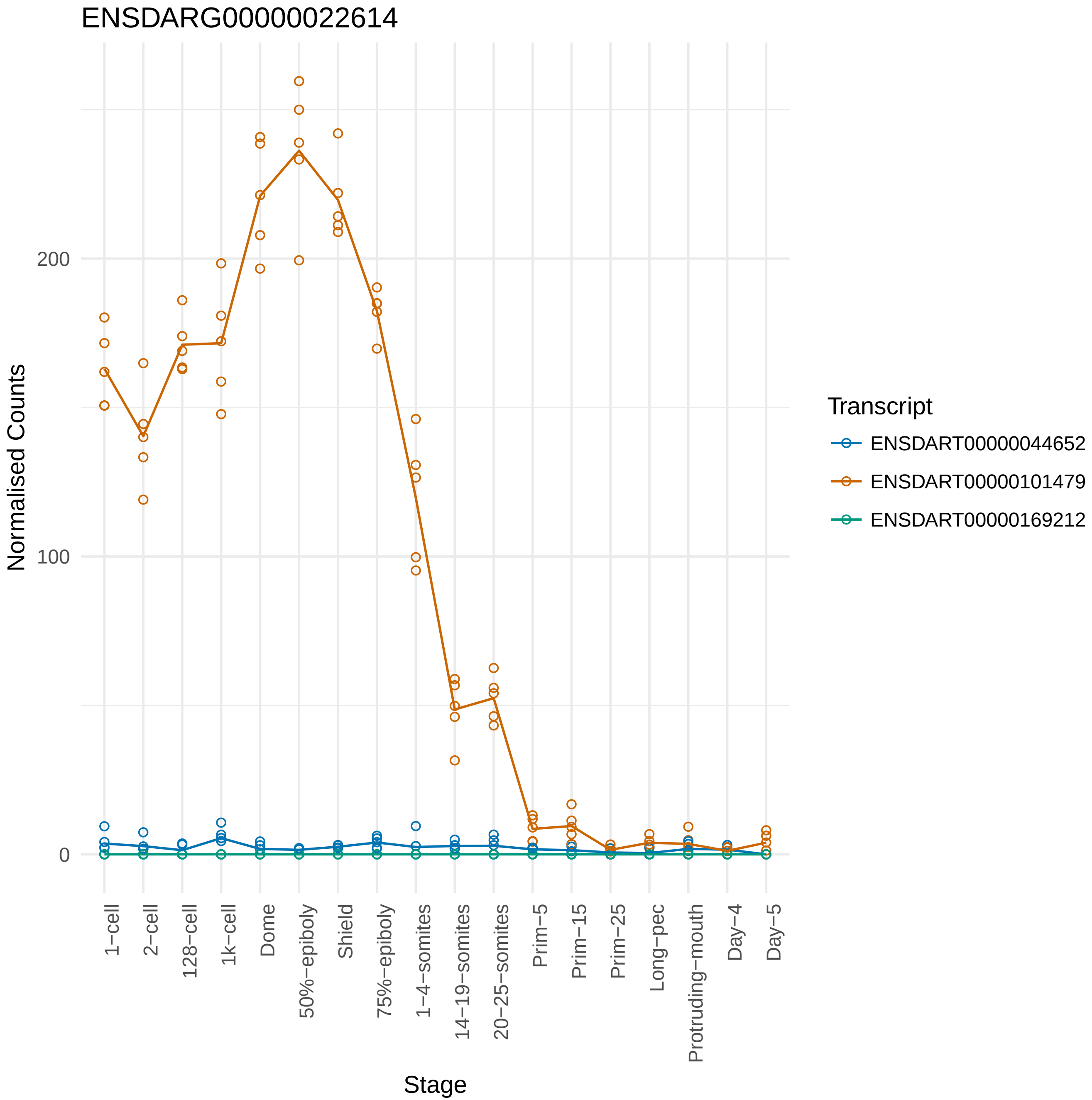
ENSDARG00000022614
Normalised Counts
200
100
0
1−cell
2−cell
128−cell
1k−cell
Dome
50%−epiboly
Shield
75%−epiboly
1−4−somites
14−19−somites
20−25−somites
Prim−5
Prim−15
Prim−25
Long−pec
Protruding−mouth
Day−4
Day−5
Stage
Transcript
ENSDART00000044652
ENSDART00000101479
ENSDART00000169212

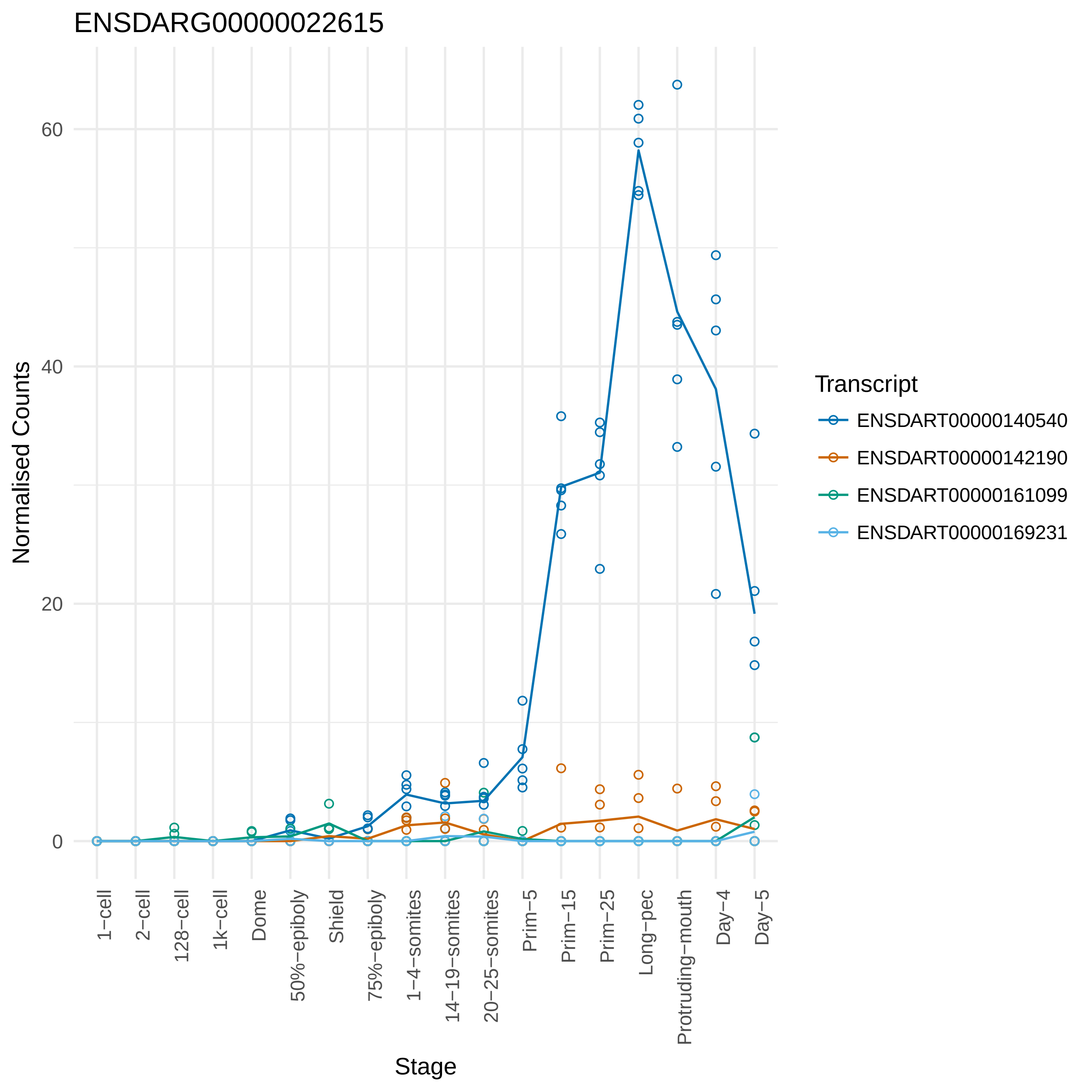

ENSDARG00000022615
Normalised Counts
0
20
40
60
1−cell
2−cell
128−cell
1k−cell
Dome
50%−epiboly
Shield
75%−epiboly
1−4−somites
14−19−somites
20−25−somites
Prim−5
Prim−15
Prim−25
Long−pec
Protruding−mouth
Day−4
Day−5
Stage
Transcript
ENSDART00000140540
ENSDART00000142190
ENSDART00000161099
ENSDART00000169231

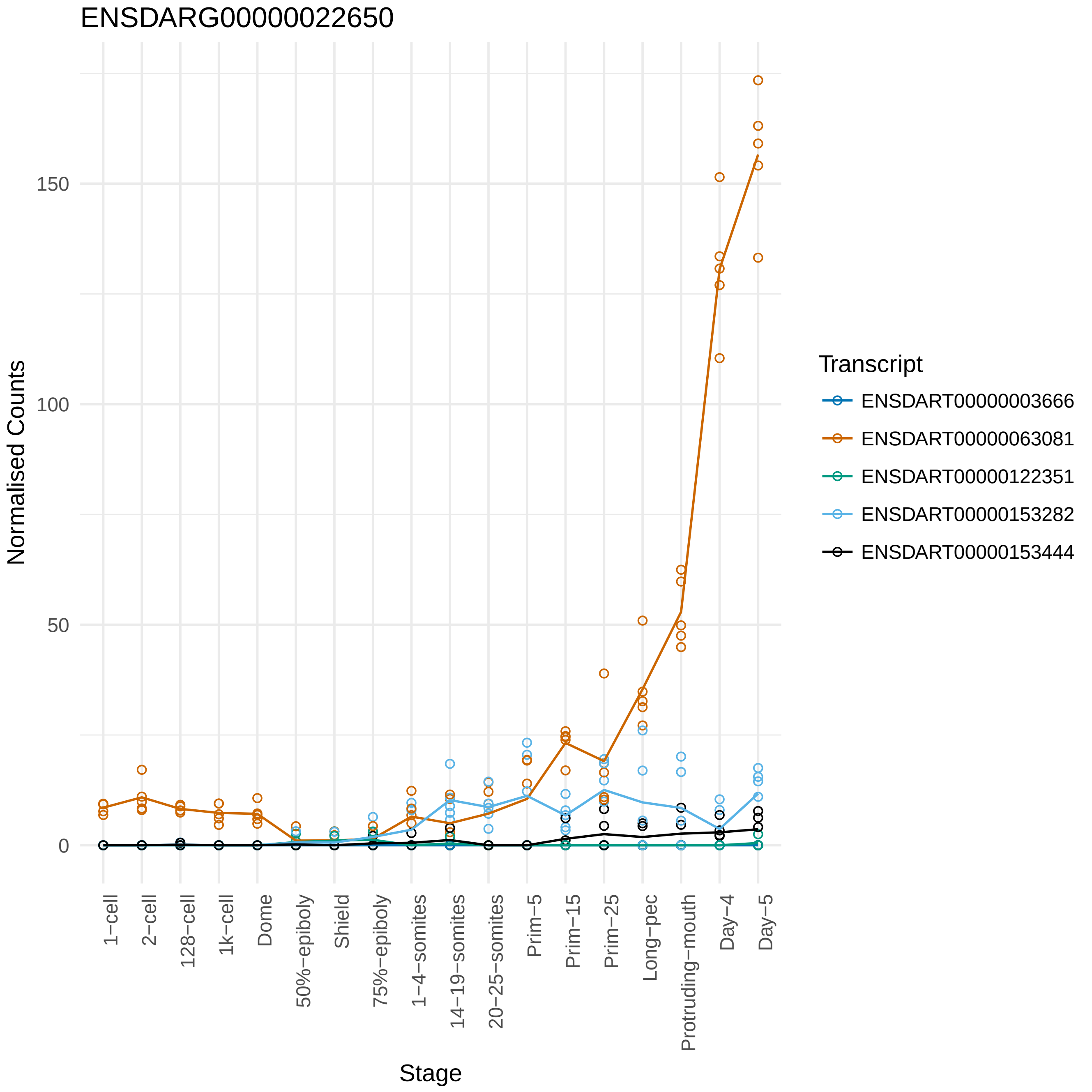
ENSDARG00000022650
Normalised Counts
150
100
50
0
1−cell
2−cell
128−cell
1k−cell
Dome
50%−epiboly
Shield
75%−epiboly
1−4−somites
14−19−somites
20−25−somites
Prim−5
Prim−15
Prim−25
Long−pec
Protruding−mouth
Day−4
Day−5
Stage
Transcript
ENSDART00000003666
ENSDART00000063081
ENSDART00000122351
ENSDART00000153282
ENSDART00000153444

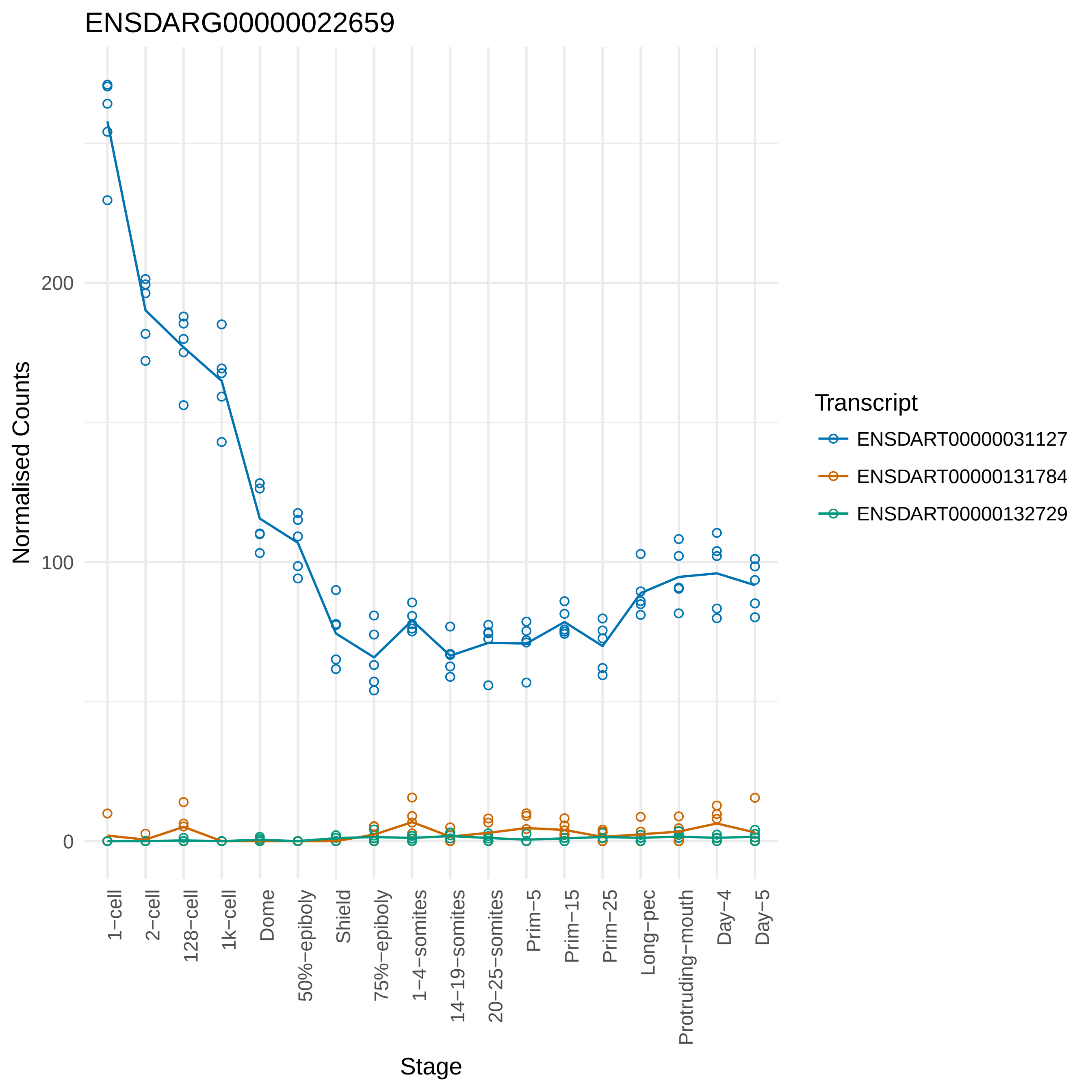

ENSDARG00000022659
Normalised Counts
200
100
0
1−cell
2−cell
128−cell
1k−cell
Dome
50%−epiboly
Shield
75%−epiboly
1−4−somites
14−19−somites
20−25−somites
Prim−5
Prim−15
Prim−25
Long−pec
Protruding−mouth
Day−4
Day−5
Stage
Transcript
ENSDART00000031127
ENSDART00000131784
ENSDART00000132729

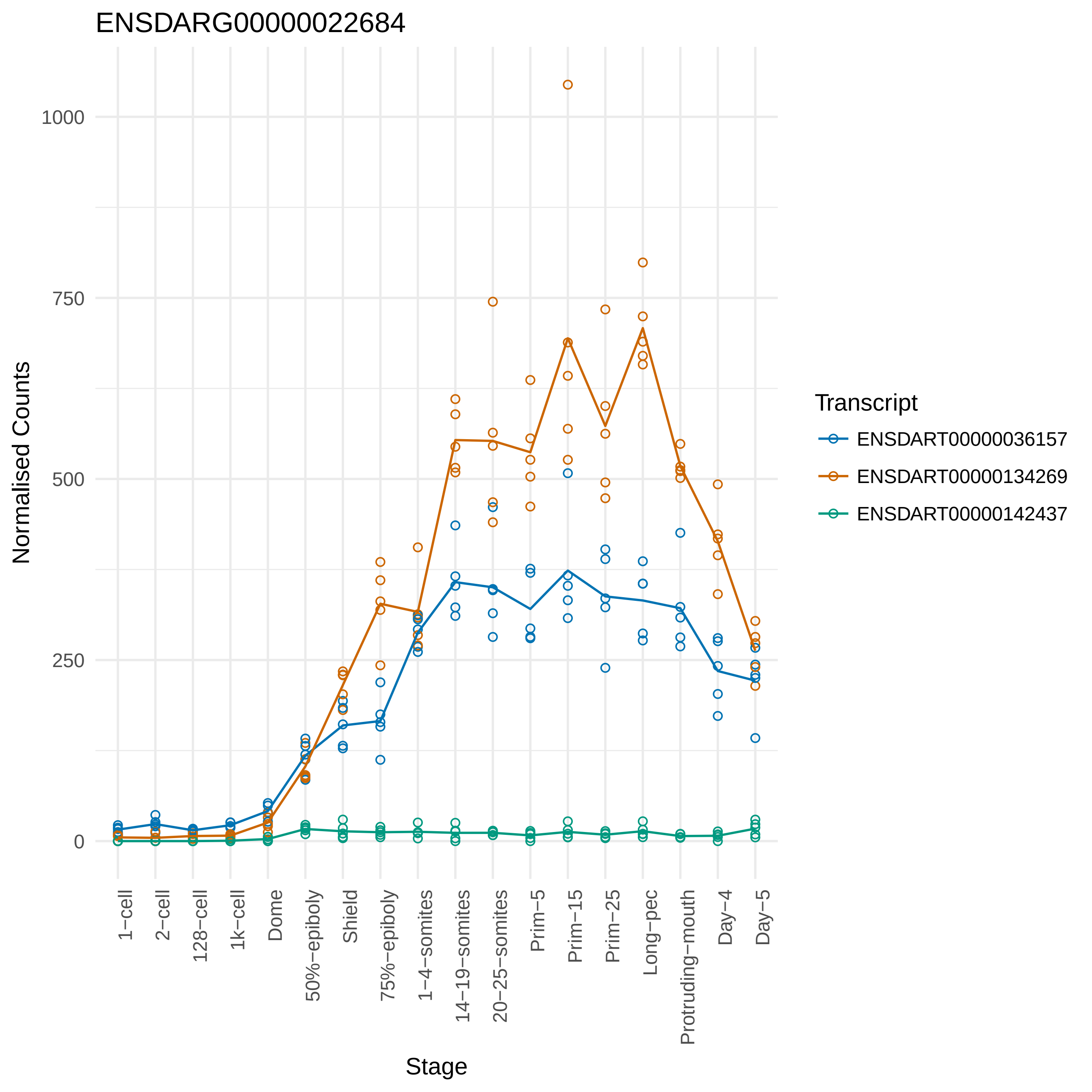

ENSDARG00000022684
Normalised Counts
1000
750
500
250
0
1−cell
2−cell
128−cell
1k−cell
Dome
50%−epiboly
Shield
75%−epiboly
1−4−somites
14−19−somites
20−25−somites
Prim−5
Prim−15
Prim−25
Long−pec
Protruding−mouth
Day−4
Day−5
Stage
Transcript
ENSDART00000036157
ENSDART00000134269
ENSDART00000142437

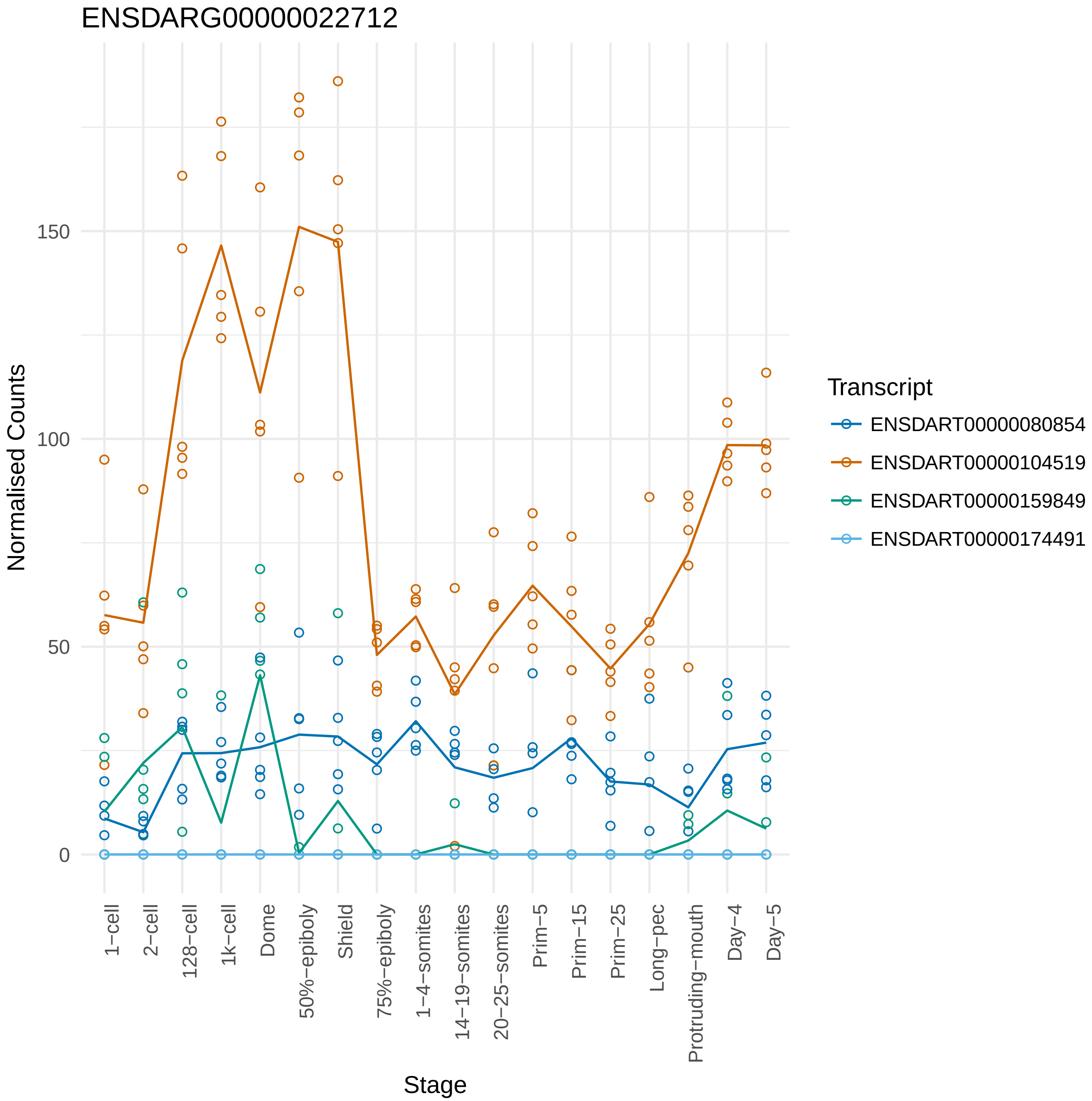
ENSDARG00000022712
Normalised Counts
0
50
100
150
Stage
1−cell
2−cell
128−cell
1k−cell
Dome
50%−epiboly
Shield
75%−epiboly
1−4−somites
14−19−somites
20−25−somites
Prim−5
Prim−15
Prim−25
Long−pec
Protruding−mouth
Day−4
Day−5
Transcript
ENSDART00000080854
ENSDART00000104519
ENSDART00000159849
ENSDART00000174491

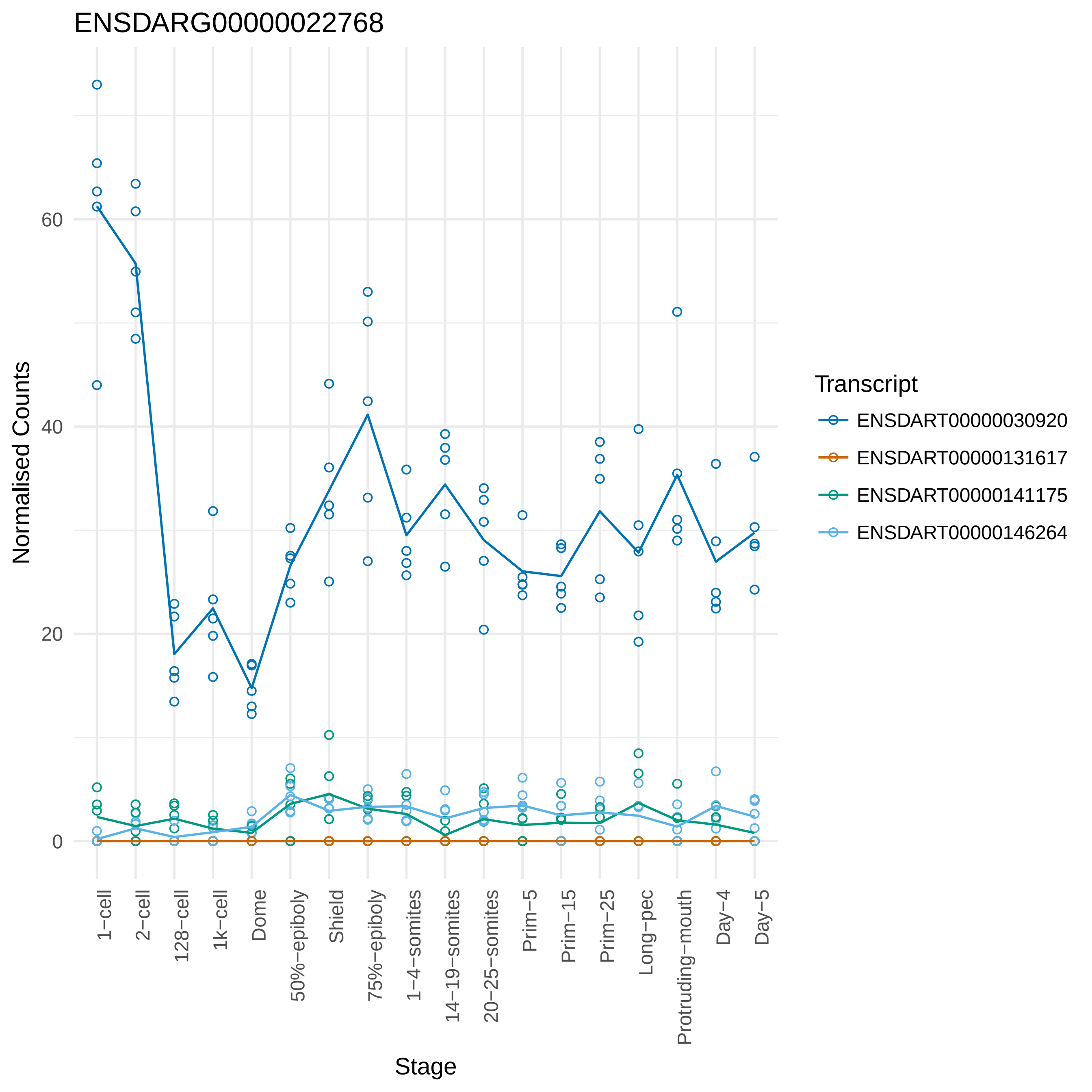
ENSDARG00000022768
Normalised Counts
0
20
40
60
Stage
1−cell
2−cell
128−cell
1k−cell
Dome
50%−epiboly
Shield
75%−epiboly
1−4−somites
14−19−somites
20−25−somites
Prim−5
Prim−15
Prim−25
Long−pec
Protruding−mouth
Day−4
Day−5
Transcript
ENSDART00000030920
ENSDART00000131617
ENSDART00000141175
ENSDART00000146264

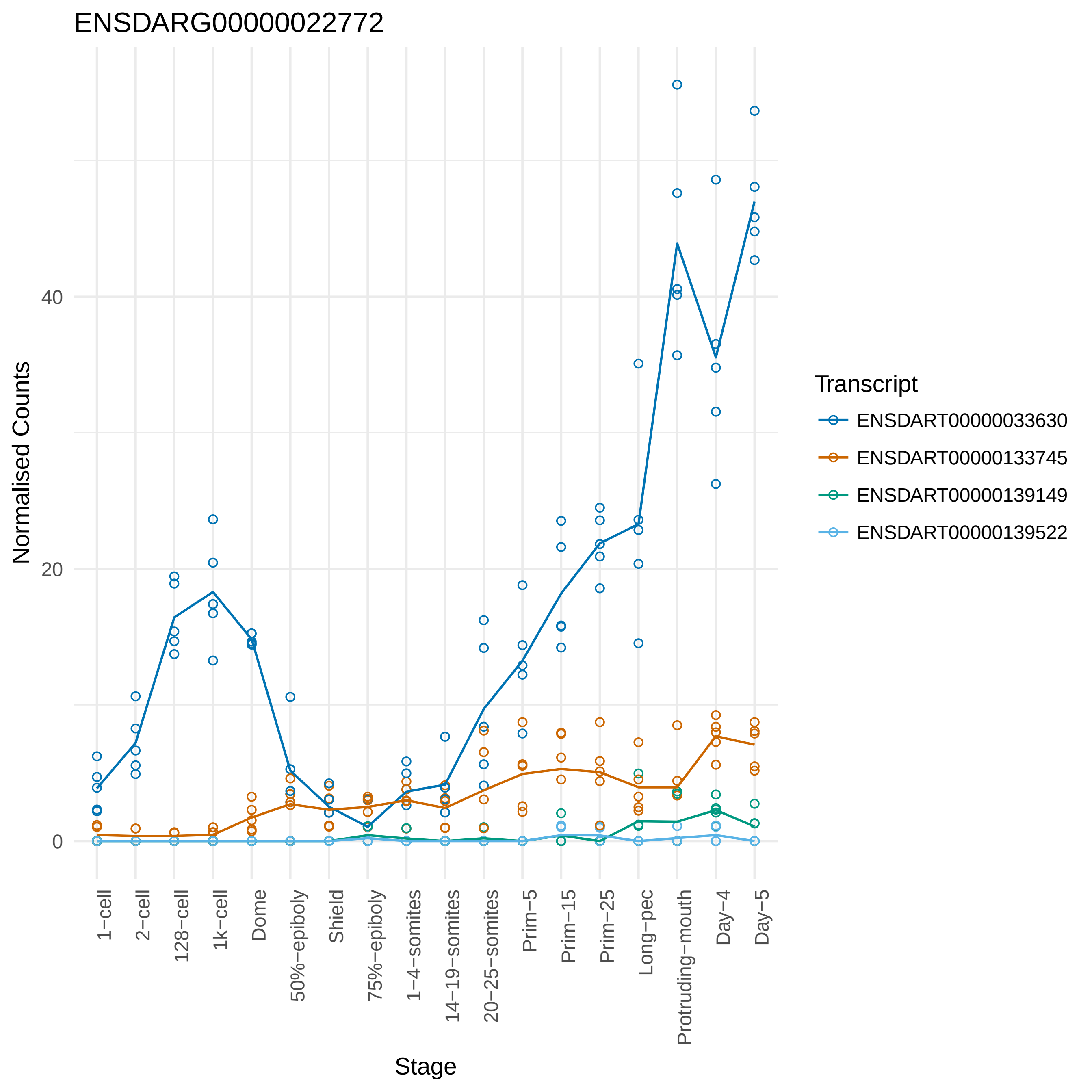
ENSDARG00000022772
Normalised Counts
0
20
40
1−cell
2−cell
128−cell
1k−cell
Dome
50%−epiboly
Shield
75%−epiboly
1−4−somites
14−19−somites
20−25−somites
Prim−5
Prim−15
Prim−25
Long−pec
Protruding−mouth
Day−4
Day−5
Stage
Transcript
ENSDART00000033630
ENSDART00000133745
ENSDART00000139149
ENSDART00000139522

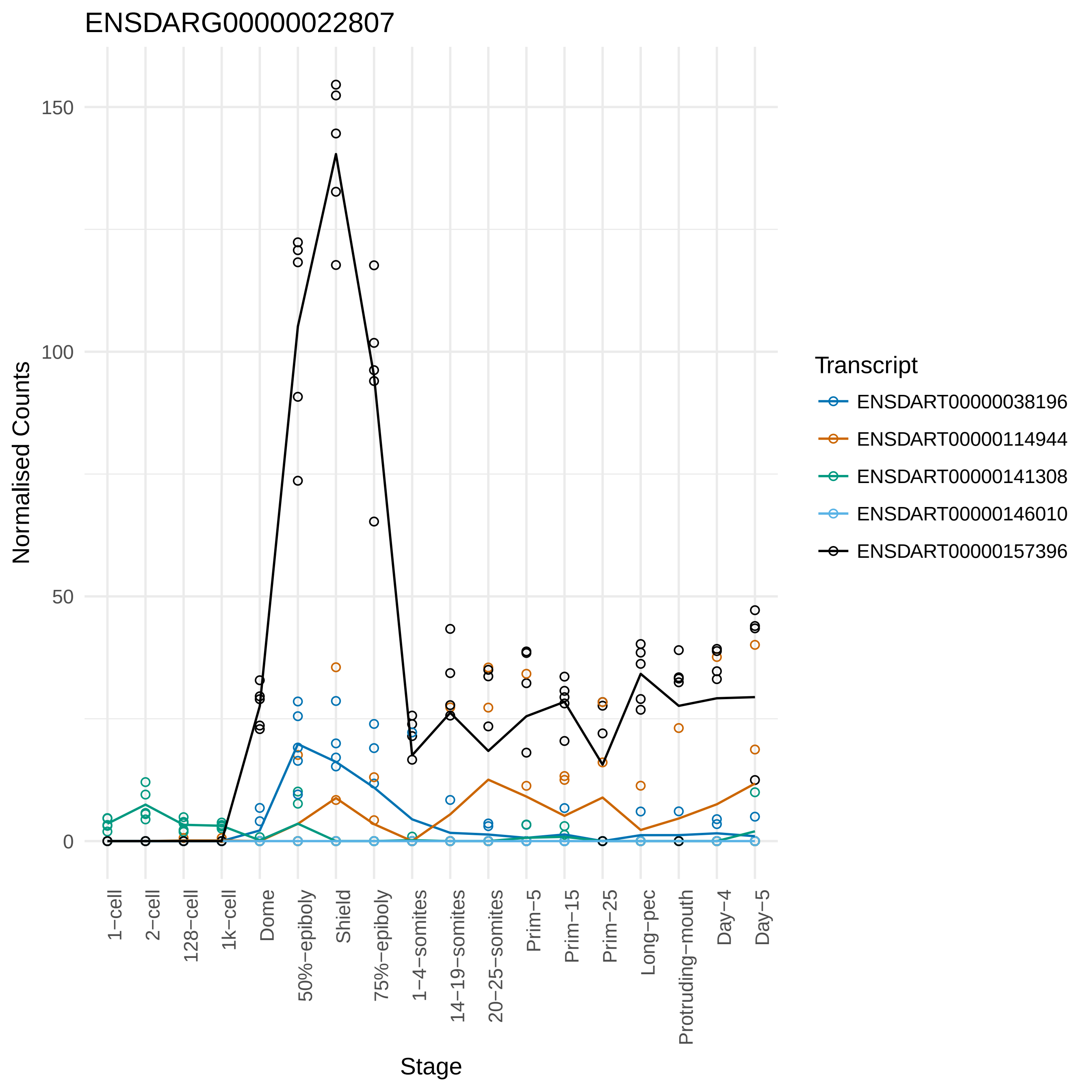
ENSDARG00000022807
Normalised Counts
150
100
50
0
Stage
1−cell
2−cell
128−cell
1k−cell
Dome
50%−epiboly
Shield
75%−epiboly
1−4−somites
14−19−somites
20−25−somites
Prim−5
Prim−15
Prim−25
Long−pec
Protruding−mouth
Day−4
Day−5
Transcript
ENSDART00000038196
ENSDART00000114944
ENSDART00000141308
ENSDART00000146010
ENSDART00000157396

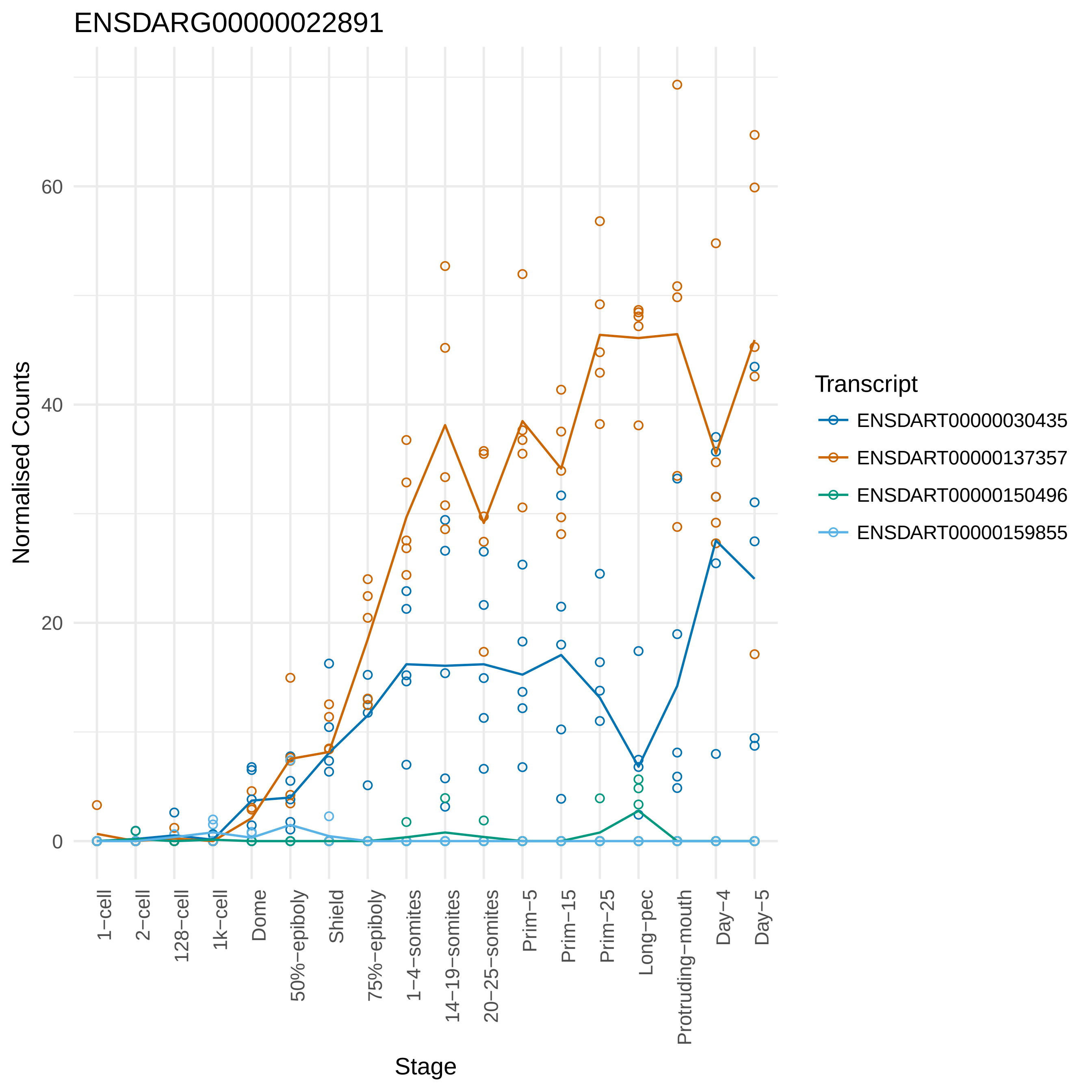
ENSDARG00000022891
Normalised Counts
0
20
40
60
Stage
1−cell
2−cell
128−cell
1k−cell
Dome
50%−epiboly
Shield
75%−epiboly
1−4−somites
14−19−somites
20−25−somites
Prim−5
Prim−15
Prim−25
Long−pec
Protruding−mouth
Day−4
Day−5
Transcript
ENSDART00000030435
ENSDART00000137357
ENSDART00000150496
ENSDART00000159855

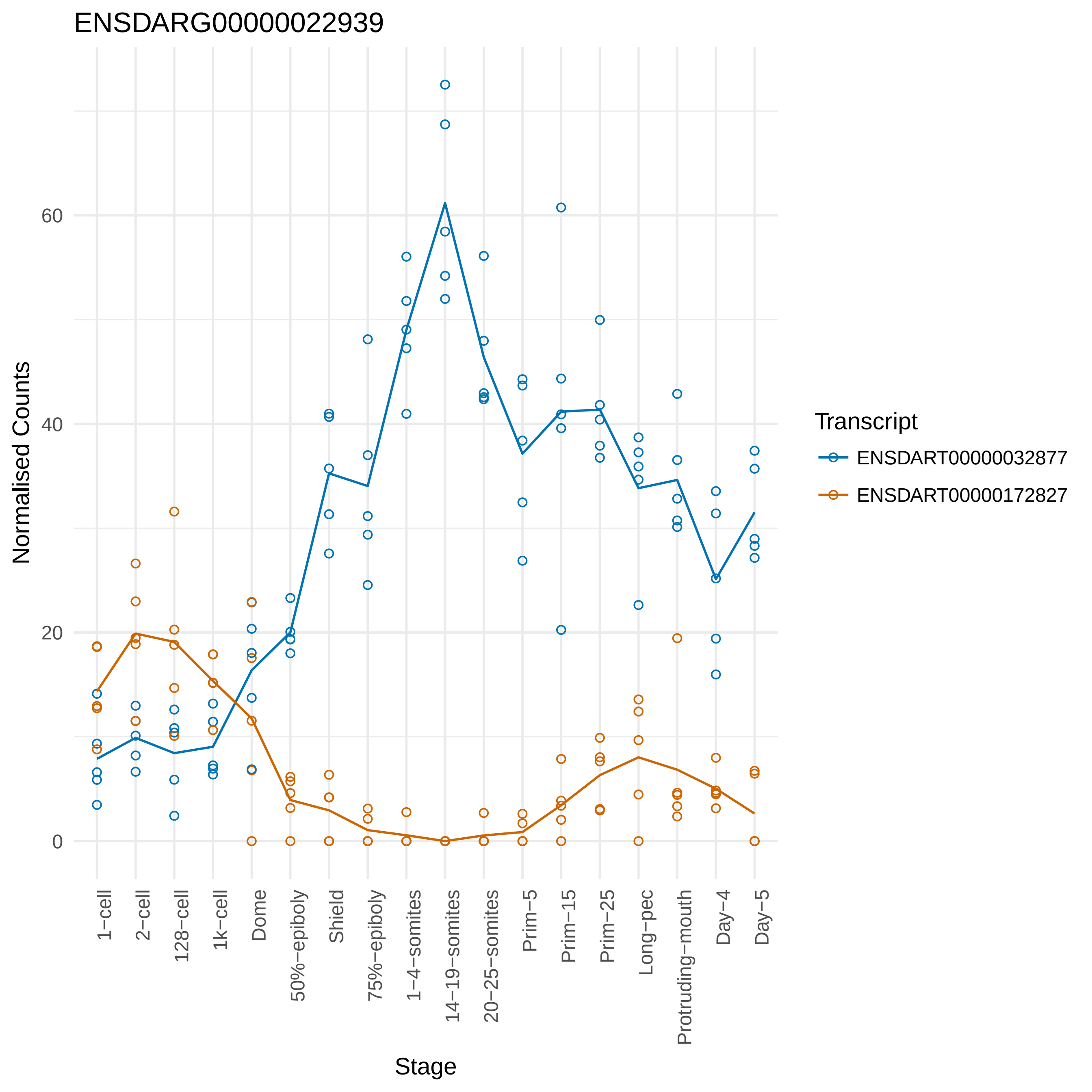
ENSDARG00000022939
Normalised Counts
0
20
40
60
Stage
1−cell
2−cell
128−cell
1k−cell
Dome
50%−epiboly
Shield
75%−epiboly
1−4−somites
14−19−somites
20−25−somites
Prim−5
Prim−15
Prim−25
Long−pec
Protruding−mouth
Day−4
Day−5
Transcript
ENSDART00000032877
ENSDART00000172827

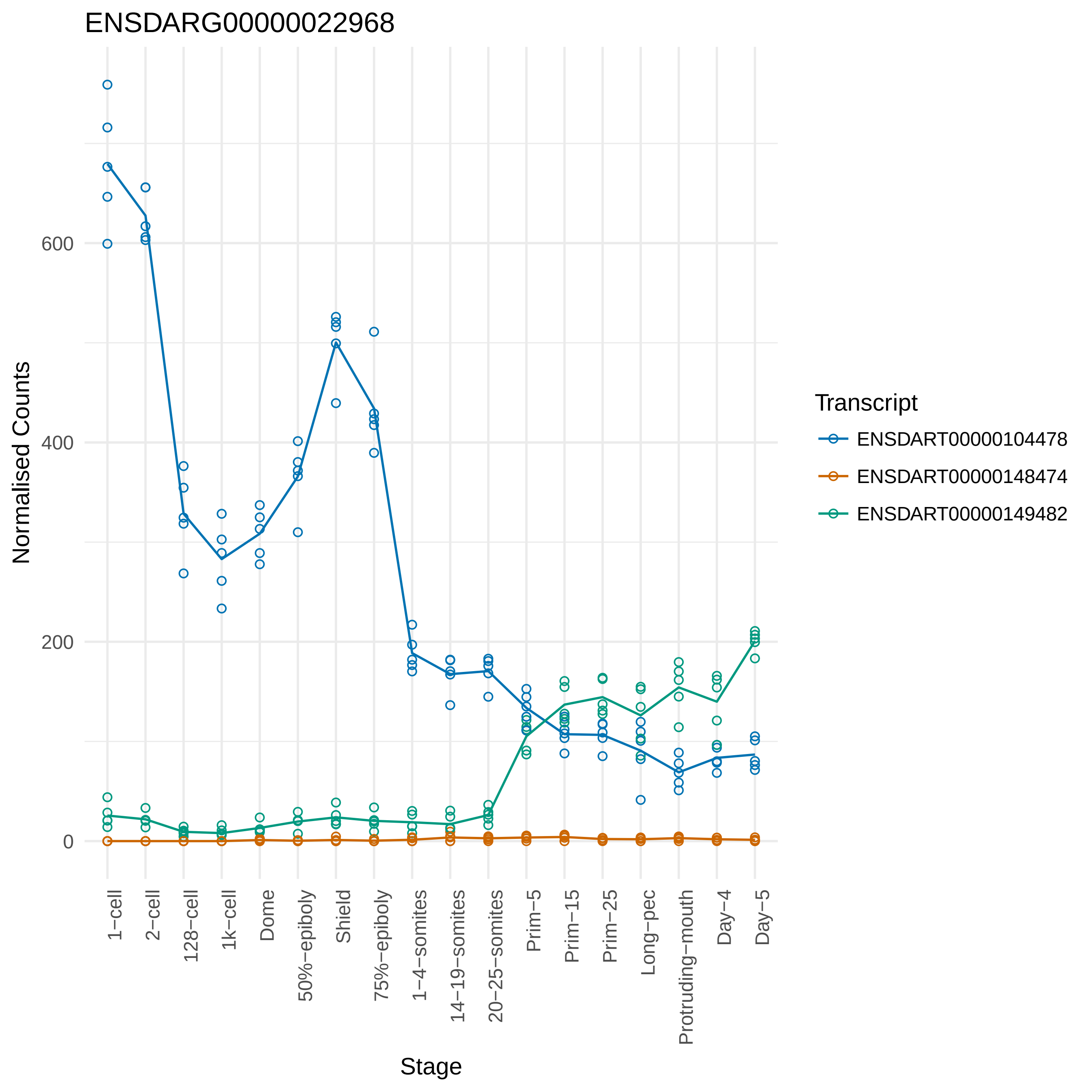

ENSDARG00000022968
Normalised Counts
600
400
200
0
1−cell
2−cell
128−cell
1k−cell
Dome
50%−epiboly
Shield
75%−epiboly
1−4−somites
14−19−somites
20−25−somites
Prim−5
Prim−15
Prim−25
Long−pec
Protruding−mouth
Day−4
Day−5
Stage
Transcript
ENSDART00000104478
ENSDART00000148474
ENSDART00000149482

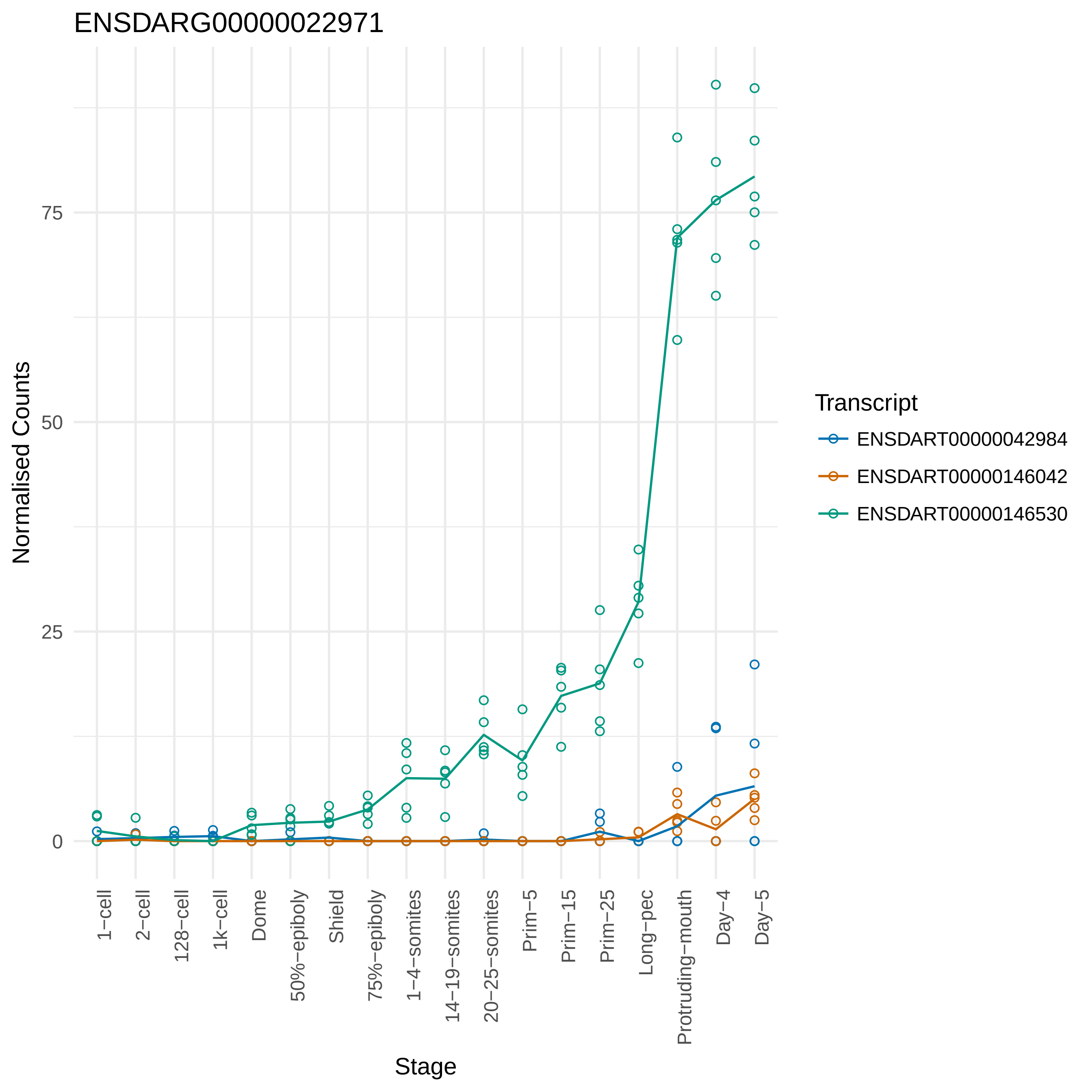

ENSDARG00000022971
Normalised Counts
0
25
50
75
Stage
1−cell
2−cell
128−cell
1k−cell
Dome
50%−epiboly
Shield
75%−epiboly
1−4−somites
14−19−somites
20−25−somites
Prim−5
Prim−15
Prim−25
Long−pec
Protruding−mouth
Day−4
Day−5
Transcript
ENSDART00000042984
ENSDART00000146042
ENSDART00000146530

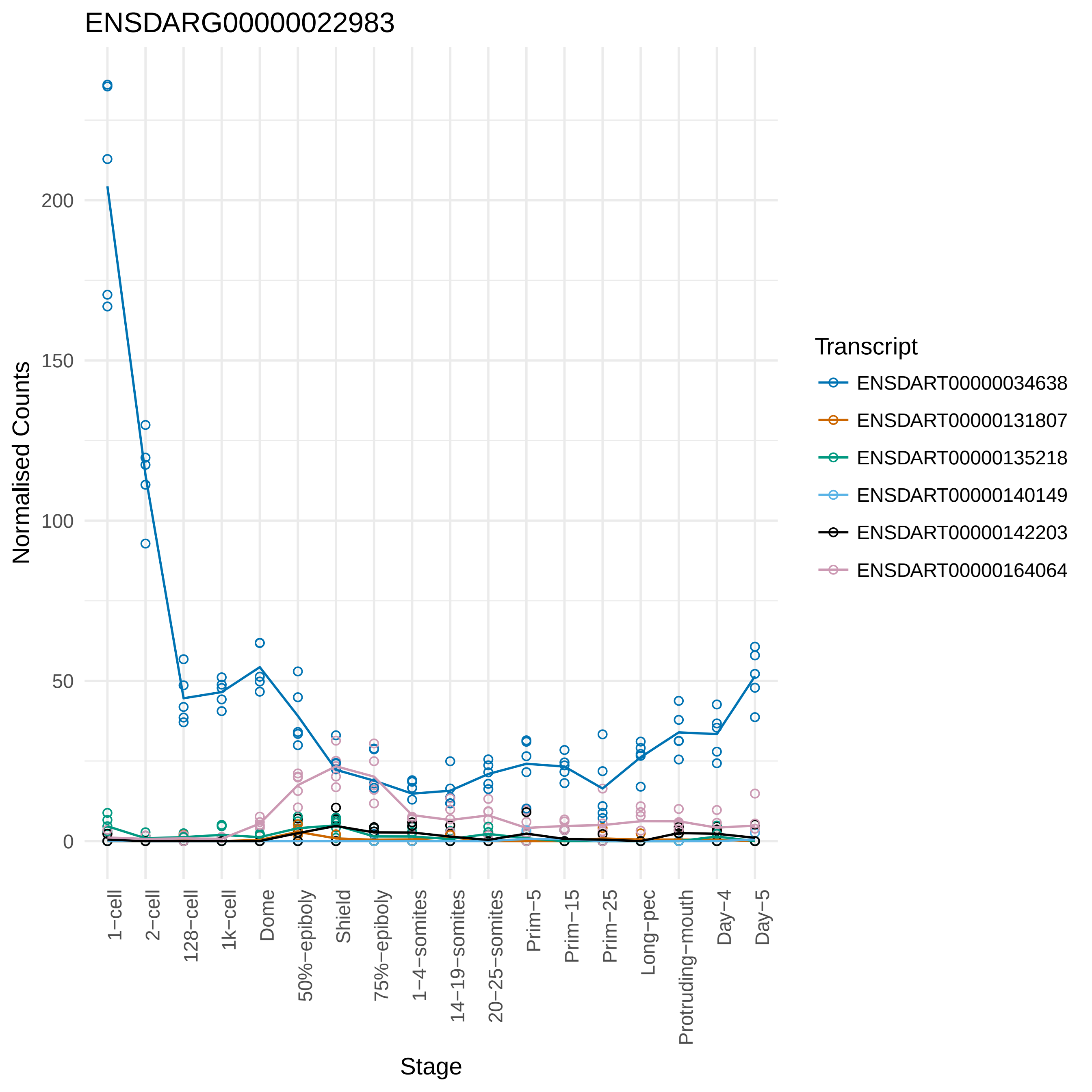
ENSDARG00000022983
Normalised Counts
200
150
100
50
0
1−cell
2−cell
128−cell
1k−cell
Dome
50%−epiboly
Shield
75%−epiboly
1−4−somites
14−19−somites
20−25−somites
Prim−5
Prim−15
Prim−25
Long−pec
Protruding−mouth
Day−4
Day−5
Stage
Transcript
ENSDART00000034638
ENSDART00000131807
ENSDART00000135218
ENSDART00000140149
ENSDART00000142203
ENSDART00000164064

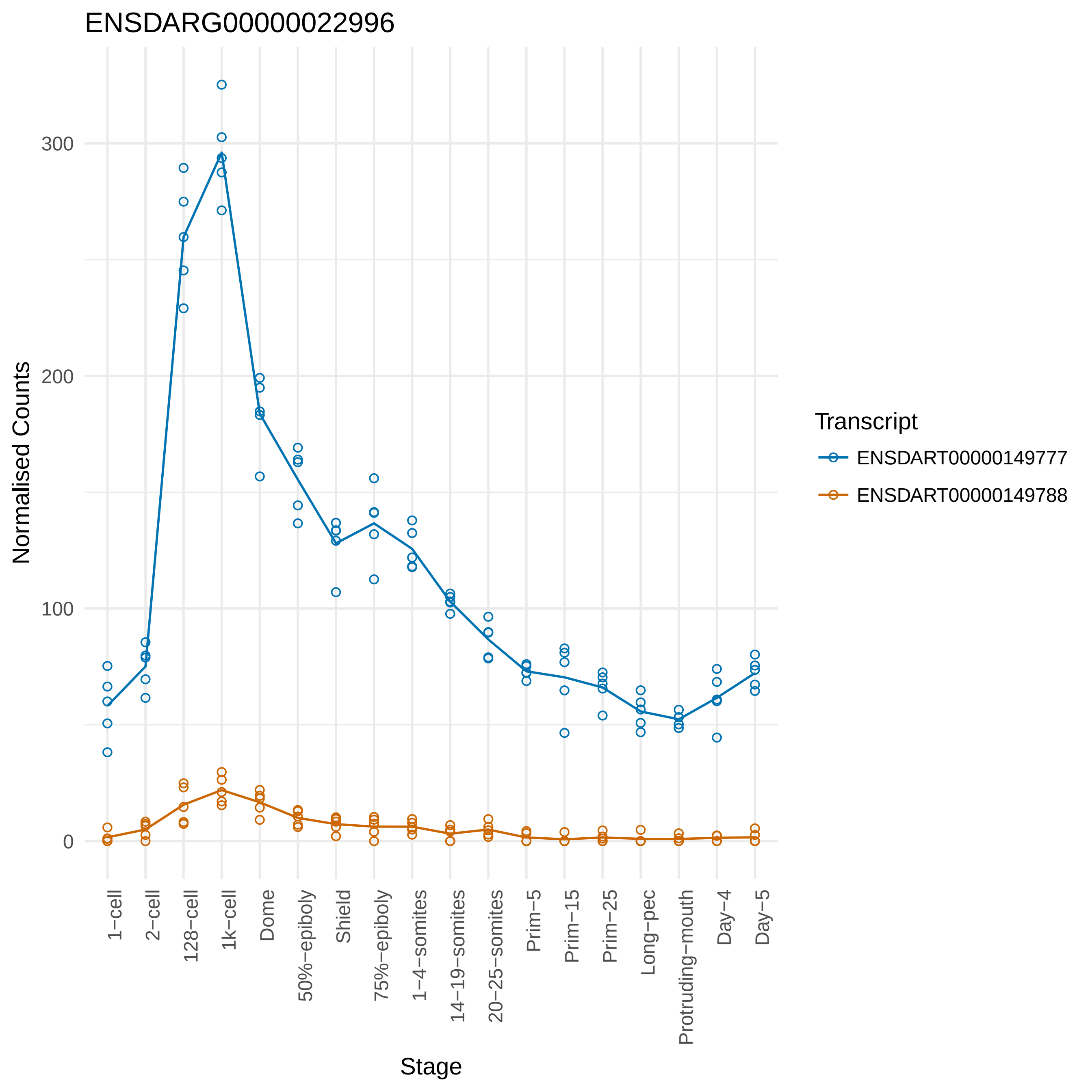
ENSDARG00000022996
Normalised Counts
300
200
100
0
1−cell
2−cell
128−cell
1k−cell
Dome
50%−epiboly
Shield
75%−epiboly
1−4−somites
14−19−somites
20−25−somites
Prim−5
Prim−15
Prim−25
Long−pec
Protruding−mouth
Day−4
Day−5
Stage
Transcript
ENSDART00000149777
ENSDART00000149788

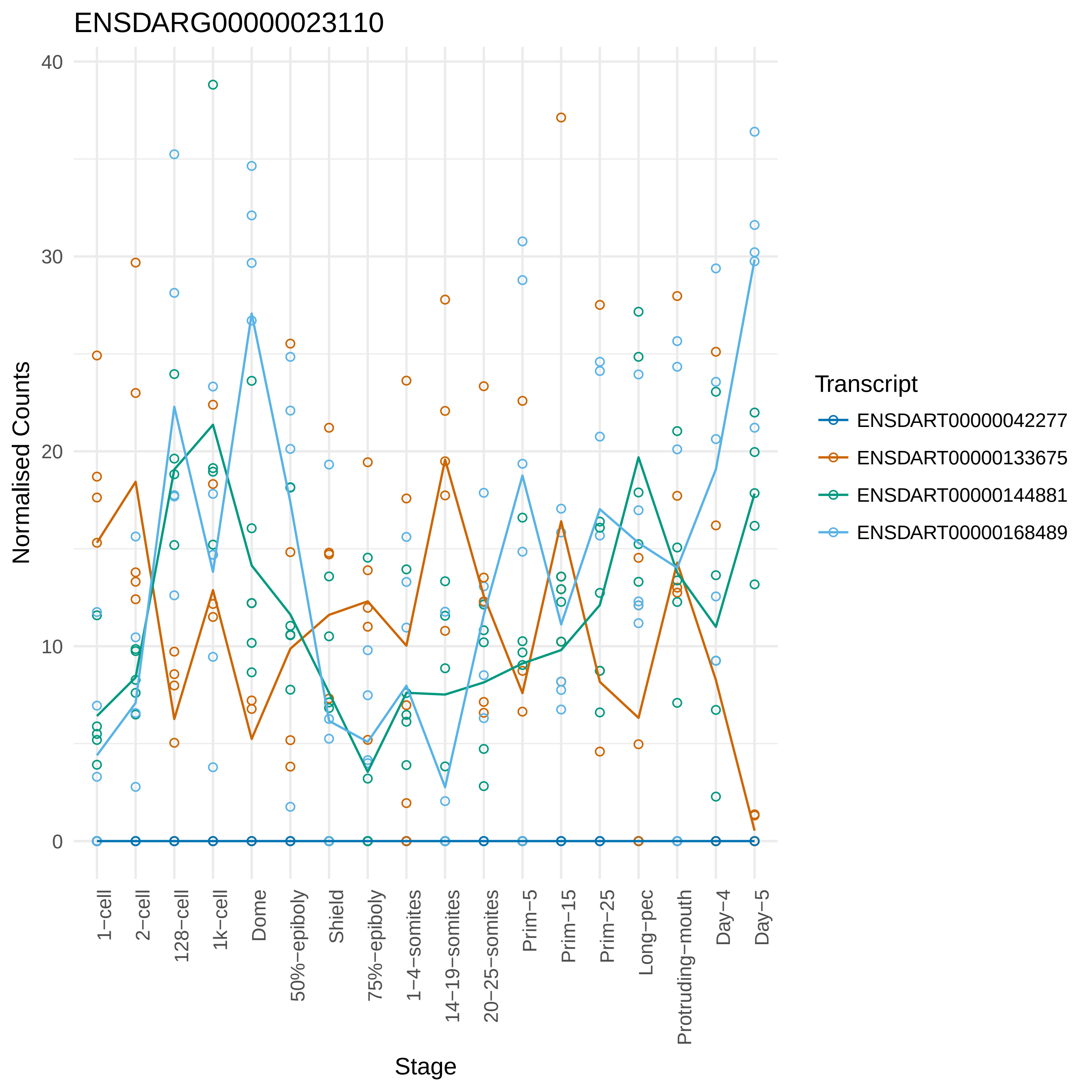

ENSDARG00000023110
Normalised Counts
40
30
20
10
0
1−cell
2−cell
128−cell
1k−cell
Dome
50%−epiboly
Shield
75%−epiboly
1−4−somites
14−19−somites
20−25−somites
Prim−5
Prim−15
Prim−25
Long−pec
Protruding−mouth
Day−4
Day−5
Stage
Transcript
ENSDART00000042277
ENSDART00000133675
ENSDART00000144881
ENSDART00000168489

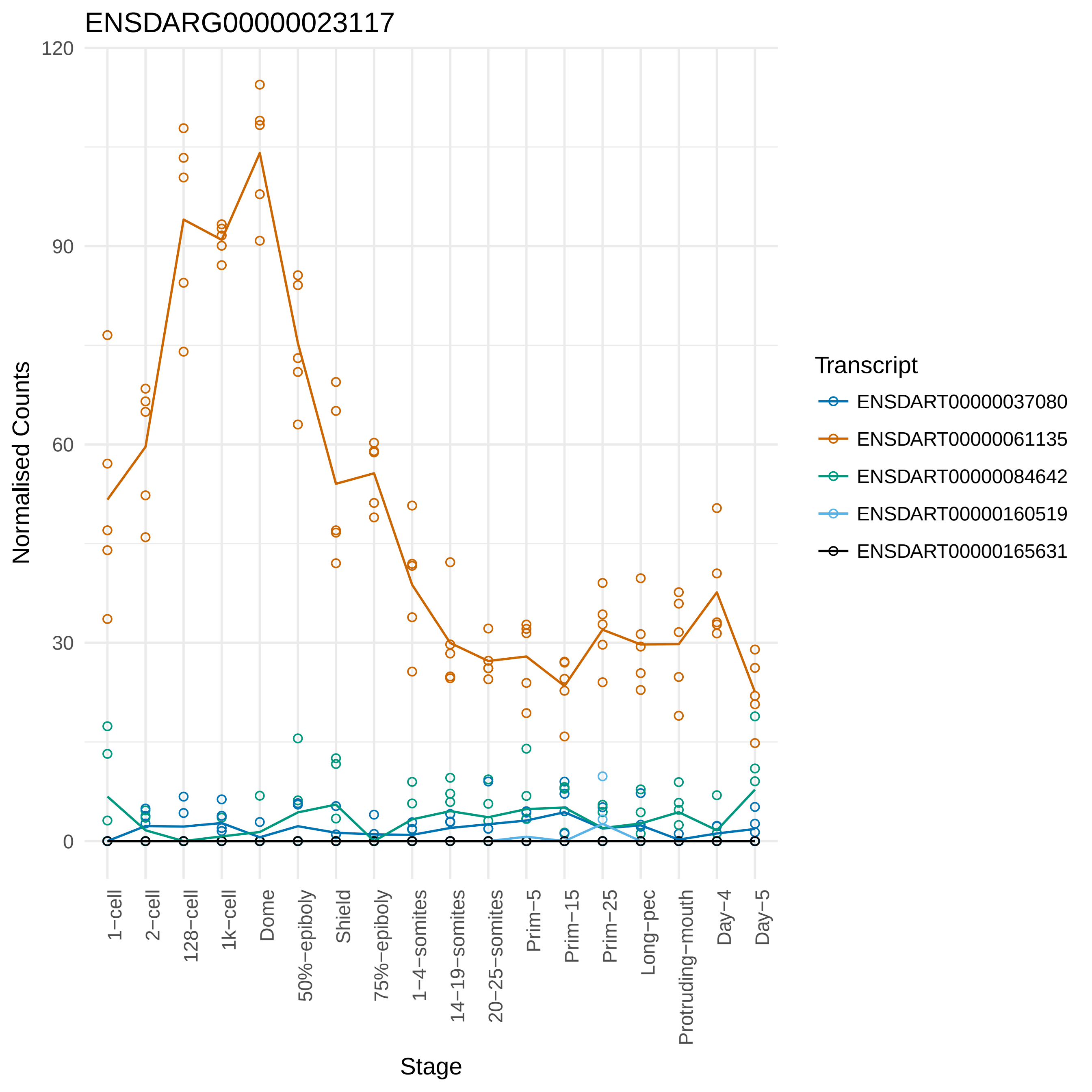
ENSDARG00000023117
Normalised Counts
120
90
60
30
0
1−cell
2−cell
128−cell
1k−cell
Dome
50%−epiboly
Shield
75%−epiboly
1−4−somites
14−19−somites
20−25−somites
Prim−5
Prim−15
Prim−25
Long−pec
Protruding−mouth
Day−4
Day−5
Stage
Transcript
ENSDART00000037080
ENSDART00000061135
ENSDART00000084642
ENSDART00000160519
ENSDART00000165631

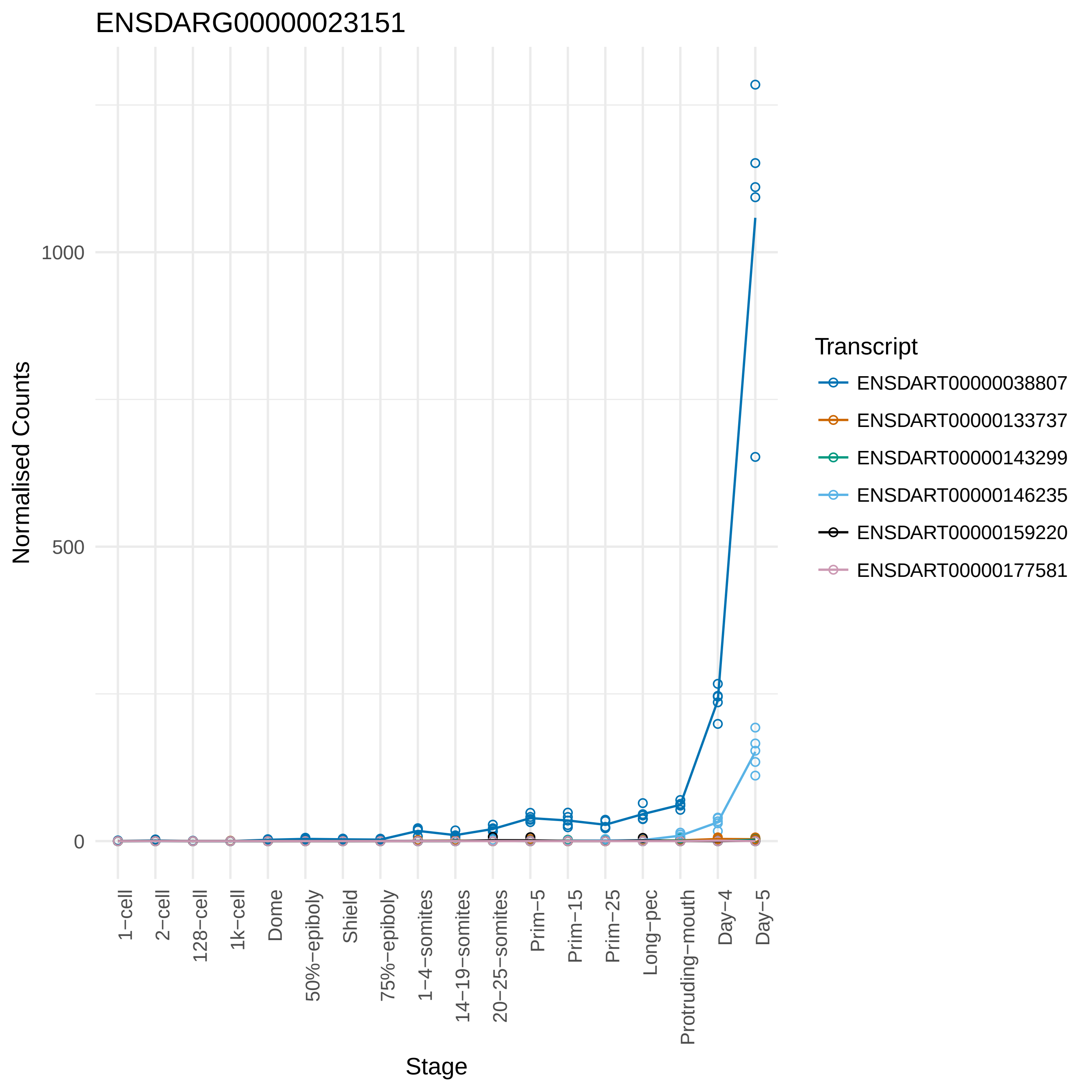

ENSDARG00000023151
Normalised Counts
1000
500
0
1−cell
2−cell
128−cell
1k−cell
Dome
50%−epiboly
Shield
75%−epiboly
1−4−somites
14−19−somites
20−25−somites
Prim−5
Prim−15
Prim−25
Long−pec
Protruding−mouth
Day−4
Day−5
Stage
Transcript
ENSDART00000038807
ENSDART00000133737
ENSDART00000143299
ENSDART00000146235
ENSDART00000159220
ENSDART00000177581

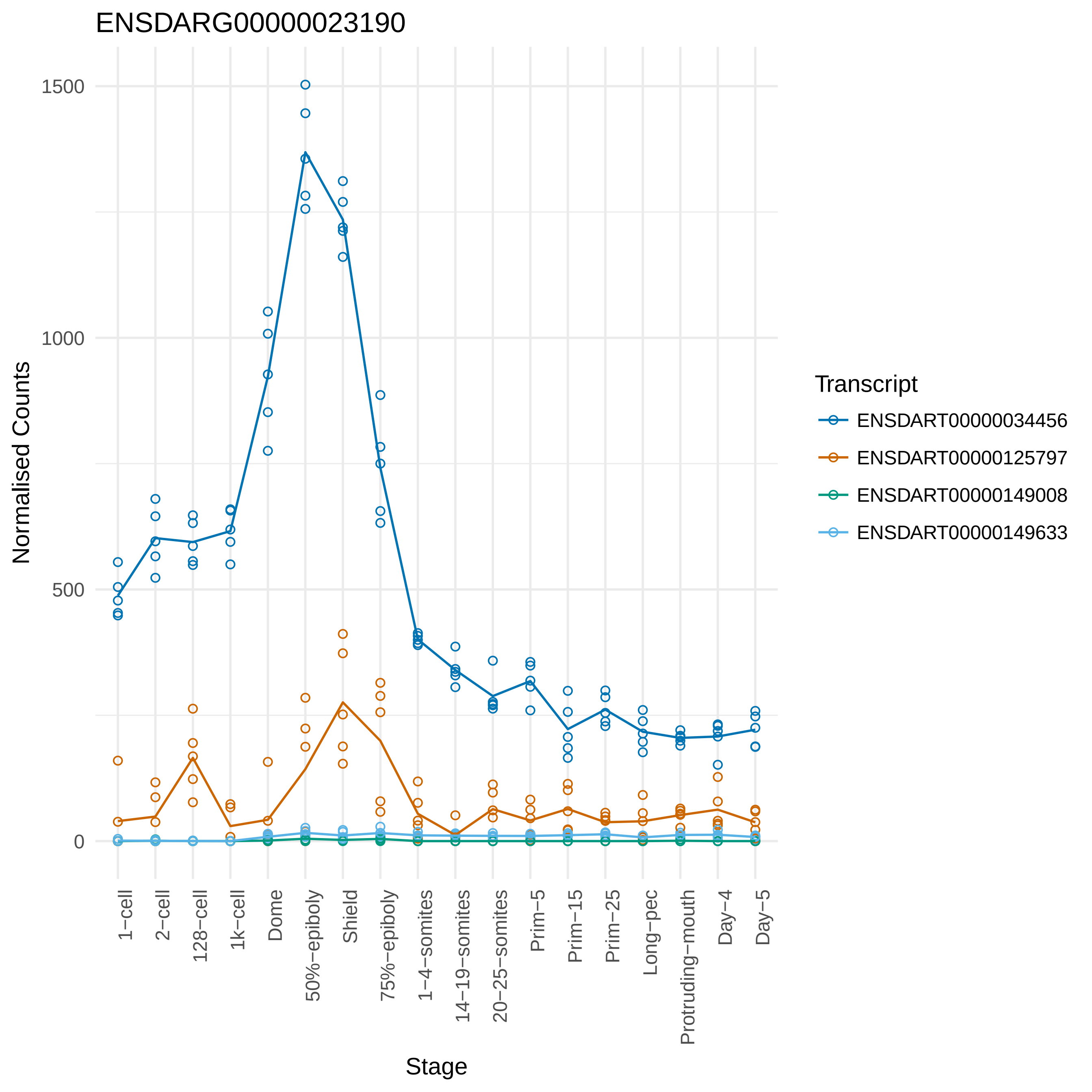

ENSDARG00000023190
Normalised Counts
1500
1000
500
0
1−cell
2−cell
128−cell
1k−cell
Dome
50%−epiboly
Shield
75%−epiboly
1−4−somites
14−19−somites
20−25−somites
Prim−5
Prim−15
Prim−25
Long−pec
Protruding−mouth
Day−4
Day−5
Stage
Transcript
ENSDART00000034456
ENSDART00000125797
ENSDART00000149008
ENSDART00000149633

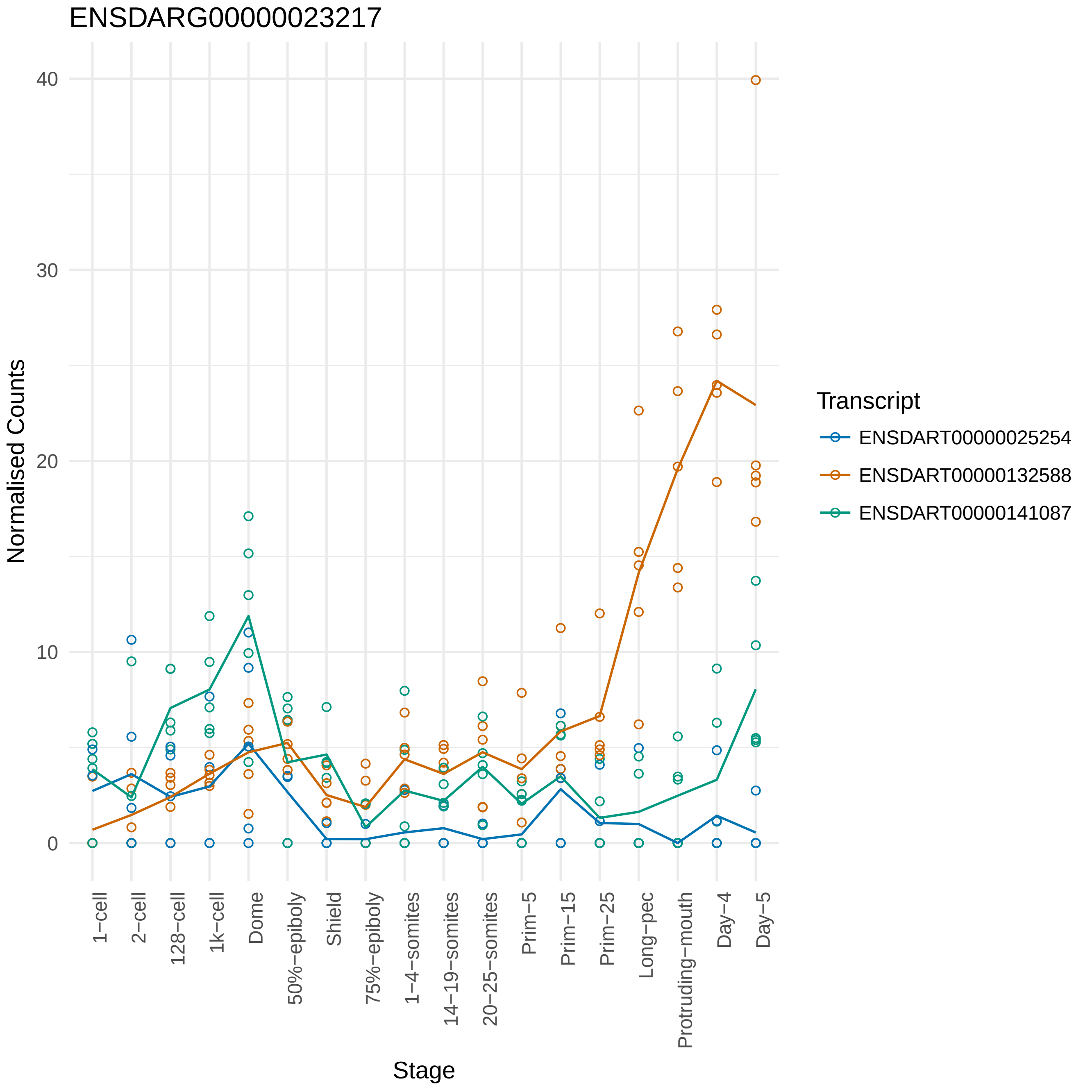

ENSDARG00000023217
Normalised Counts
0
10
20
30
40
1−cell
2−cell
128−cell
1k−cell
Dome
50%−epiboly
Shield
75%−epiboly
1−4−somites
14−19−somites
20−25−somites
Prim−5
Prim−15
Prim−25
Long−pec
Protruding−mouth
Day−4
Day−5
Stage
Transcript
ENSDART00000025254
ENSDART00000132588
ENSDART00000141087

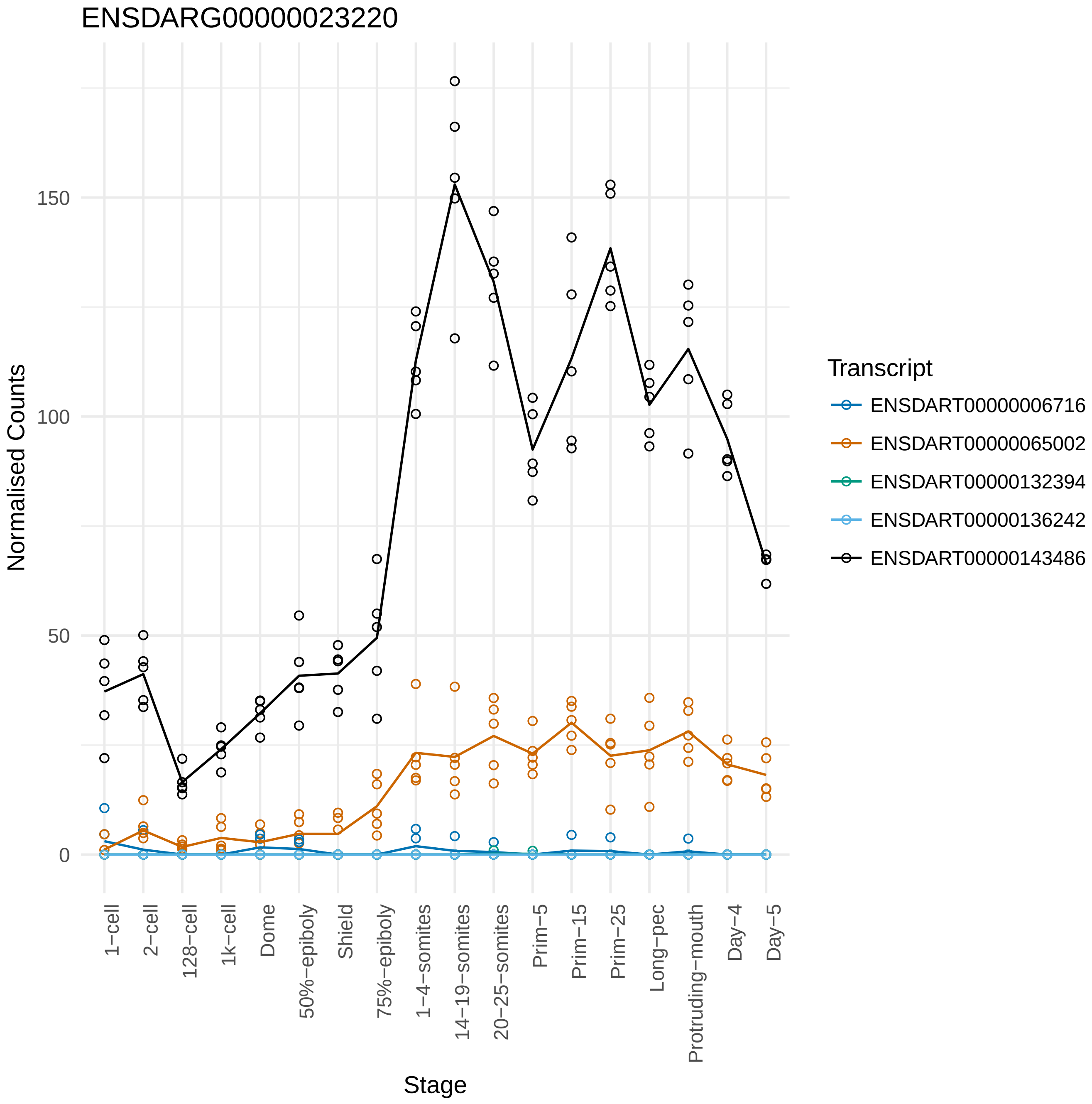
ENSDARG00000023220
Normalised Counts
0
50
100
150
1−cell
2−cell
128−cell
1k−cell
Dome
50%−epiboly
Shield
75%−epiboly
1−4−somites
14−19−somites
20−25−somites
Prim−5
Prim−15
Prim−25
Long−pec
Protruding−mouth
Day−4
Day−5
Stage
Transcript
ENSDART00000006716
ENSDART00000065002
ENSDART00000132394
ENSDART00000136242
ENSDART00000143486

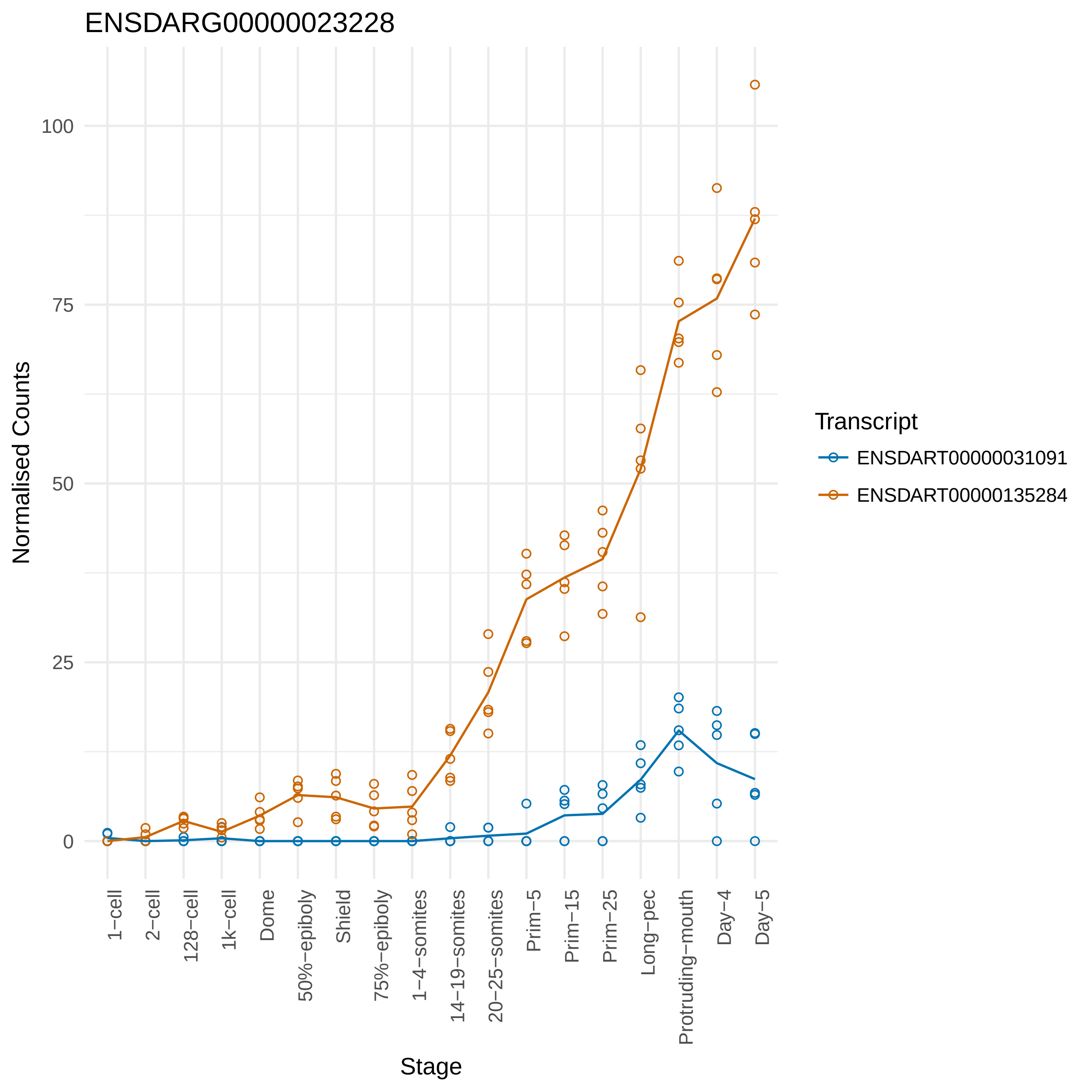
ENSDARG00000023228
Normalised Counts
100
75
50
25
0
1−cell
2−cell
128−cell
1k−cell
Dome
50%−epiboly
Shield
75%−epiboly
1−4−somites
14−19−somites
20−25−somites
Prim−5
Prim−15
Prim−25
Long−pec
Protruding−mouth
Day−4
Day−5
Stage
Transcript
ENSDART00000031091
ENSDART00000135284

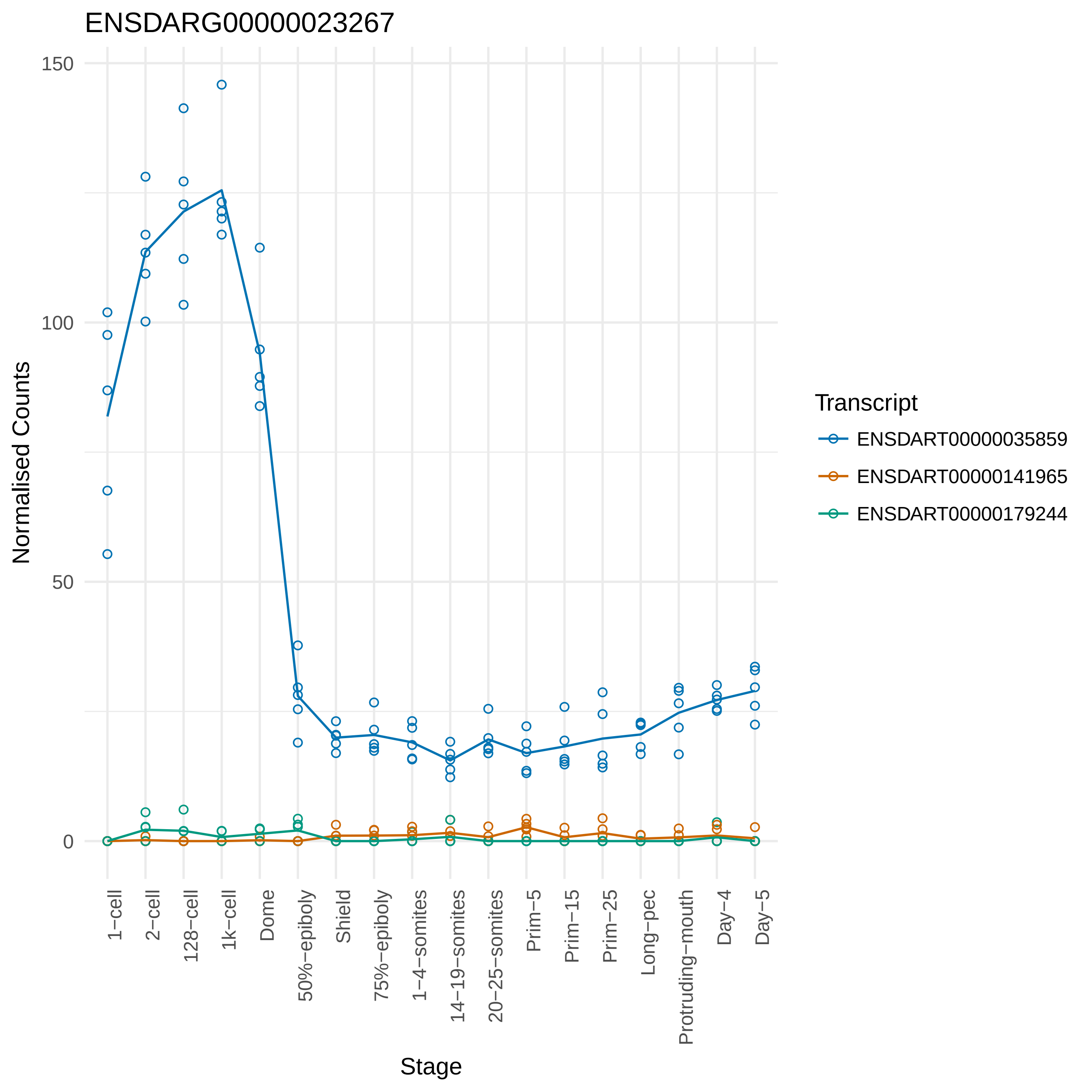

ENSDARG00000023267
Normalised Counts
150
100
50
0
1−cell
2−cell
128−cell
1k−cell
Dome
50%−epiboly
Shield
75%−epiboly
1−4−somites
14−19−somites
20−25−somites
Prim−5
Prim−15
Prim−25
Long−pec
Protruding−mouth
Day−4
Day−5
Stage
Transcript
ENSDART00000035859
ENSDART00000141965
ENSDART00000179244

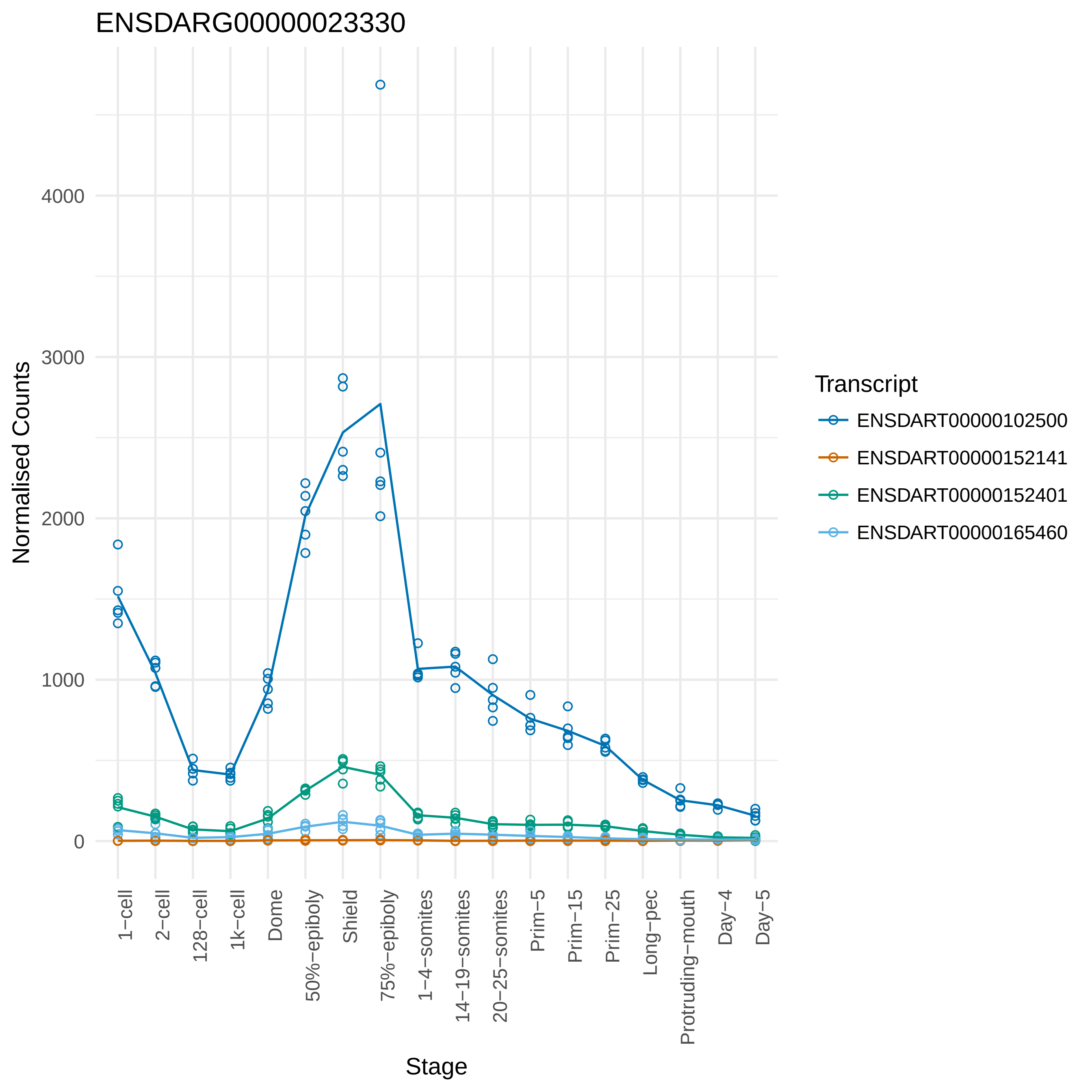
ENSDARG00000023330
Normalised Counts
0
1000
2000
3000
4000
Stage
1−cell
2−cell
128−cell
1k−cell
Dome
50%−epiboly
Shield
75%−epiboly
1−4−somites
14−19−somites
20−25−somites
Prim−5
Prim−15
Prim−25
Long−pec
Protruding−mouth
Day−4
Day−5
Transcript
ENSDART00000102500
ENSDART00000152141
ENSDART00000152401
ENSDART00000165460

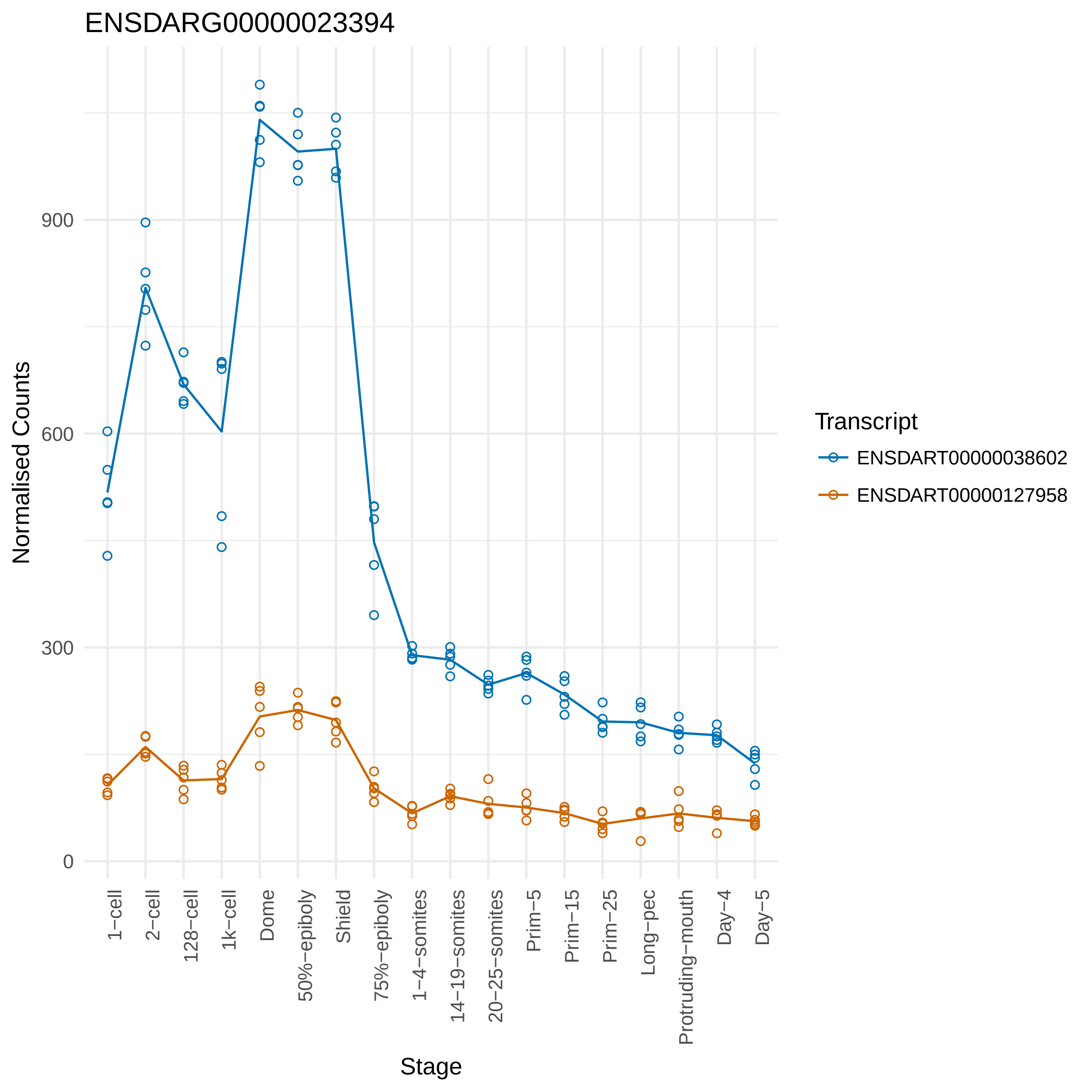

ENSDARG00000023394
Normalised Counts
900
600
300
0
1−cell
2−cell
128−cell
1k−cell
Dome
50%−epiboly
Shield
75%−epiboly
1−4−somites
14−19−somites
20−25−somites
Prim−5
Prim−15
Prim−25
Long−pec
Protruding−mouth
Day−4
Day−5
Stage
Transcript
ENSDART00000038602
ENSDART00000127958

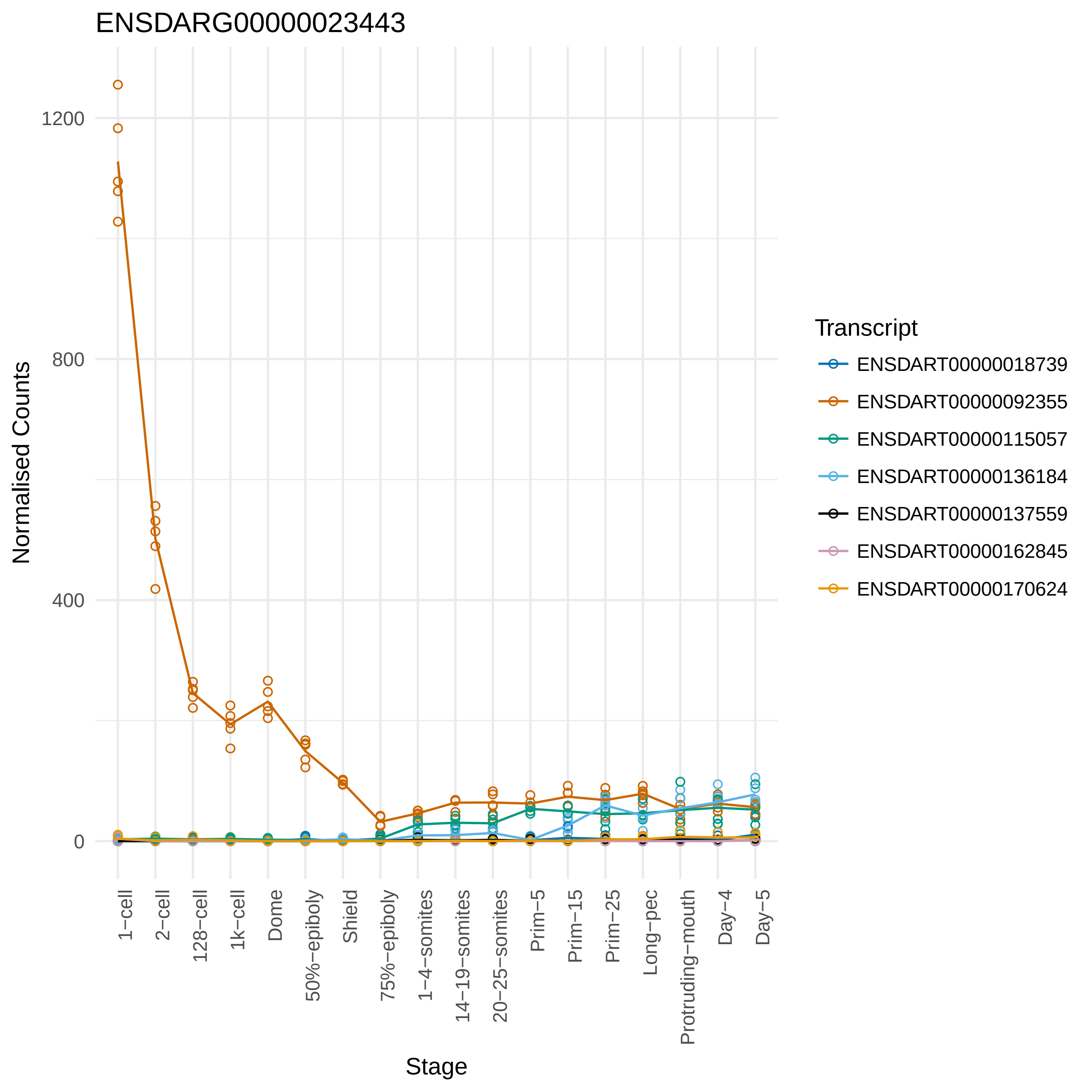

ENSDARG00000023443
Normalised Counts
1200
800
400
0
1−cell
2−cell
128−cell
1k−cell
Dome
50%−epiboly
Shield
75%−epiboly
1−4−somites
14−19−somites
20−25−somites
Prim−5
Prim−15
Prim−25
Long−pec
Protruding−mouth
Day−4
Day−5
Stage
Transcript
ENSDART00000018739
ENSDART00000092355
ENSDART00000115057
ENSDART00000136184
ENSDART00000137559
ENSDART00000162845
ENSDART00000170624

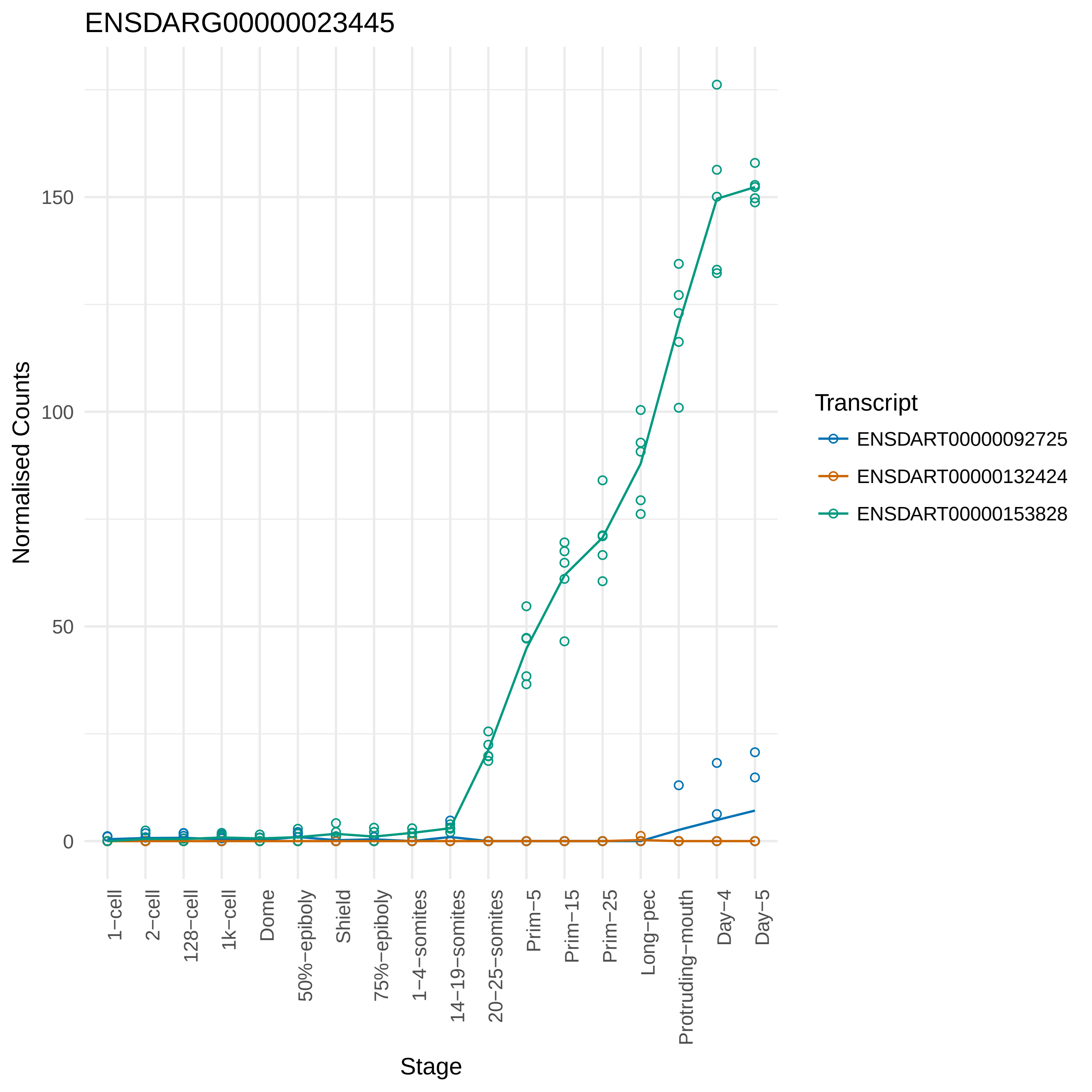

ENSDARG00000023445
Normalised Counts
150
100
50
0
1−cell
2−cell
128−cell
1k−cell
Dome
50%−epiboly
Shield
75%−epiboly
1−4−somites
14−19−somites
20−25−somites
Prim−5
Prim−15
Prim−25
Long−pec
Protruding−mouth
Day−4
Day−5
Stage
Transcript
ENSDART00000092725
ENSDART00000132424
ENSDART00000153828

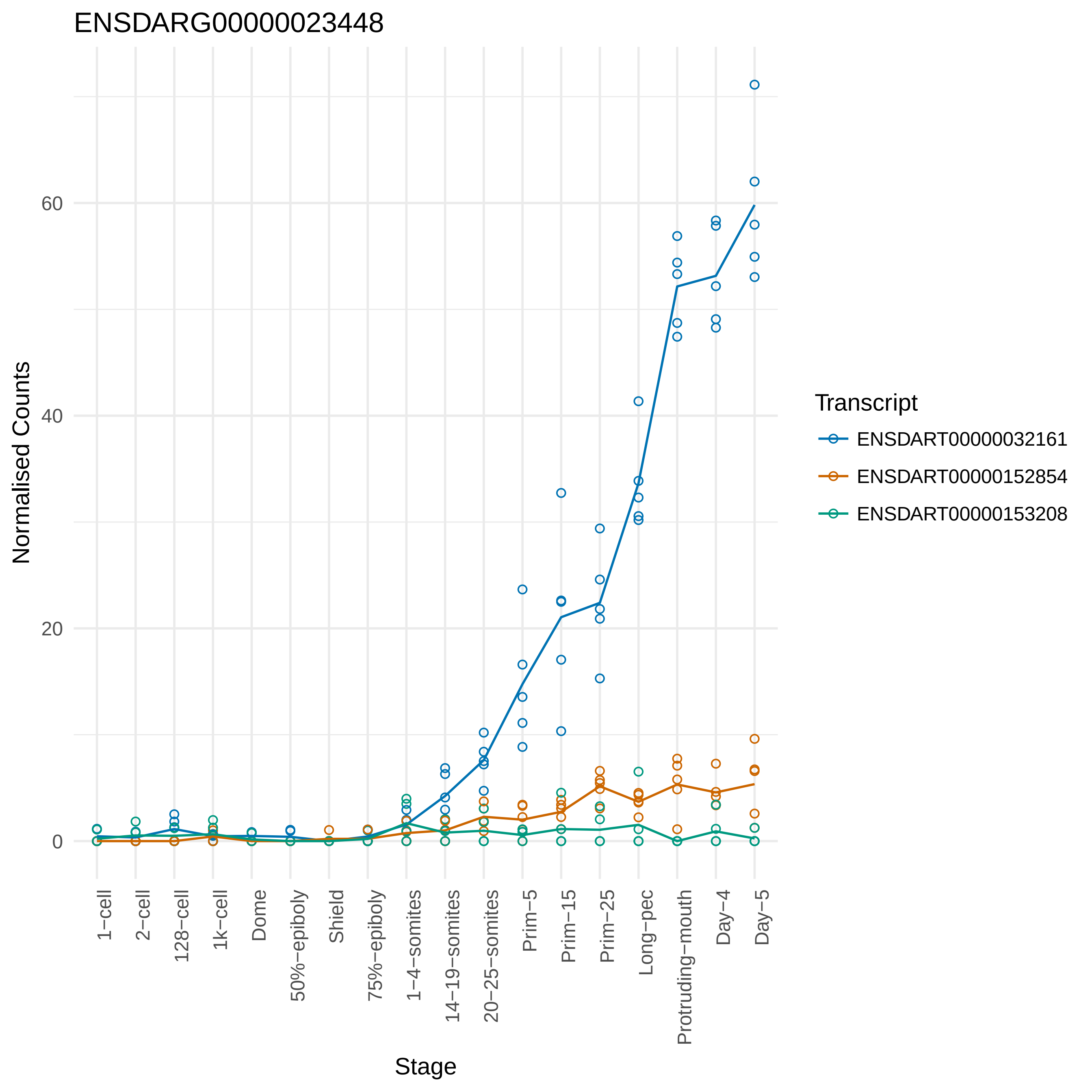

ENSDARG00000023448
Normalised Counts
0
20
40
60
Stage
1−cell
2−cell
128−cell
1k−cell
Dome
50%−epiboly
Shield
75%−epiboly
1−4−somites
14−19−somites
20−25−somites
Prim−5
Prim−15
Prim−25
Long−pec
Protruding−mouth
Day−4
Day−5
Transcript
ENSDART00000032161
ENSDART00000152854
ENSDART00000153208

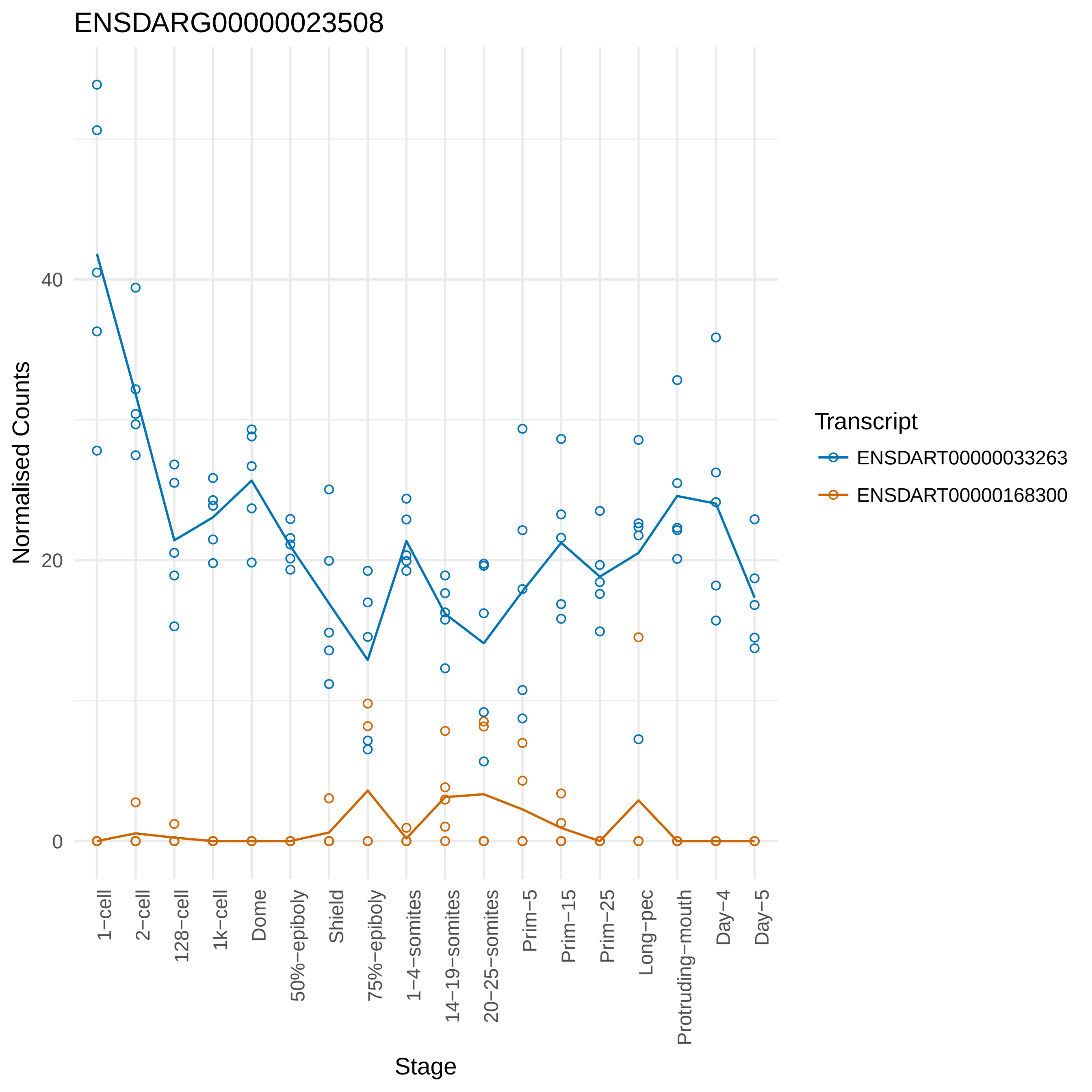
ENSDARG00000023508
Normalised Counts
40
20
0
1−cell
2−cell
128−cell
1k−cell
Dome
50%−epiboly
Shield
75%−epiboly
1−4−somites
14−19−somites
20−25−somites
Prim−5
Prim−15
Prim−25
Long−pec
Protruding−mouth
Day−4
Day−5
Stage
Transcript
ENSDART00000033263
ENSDART00000168300

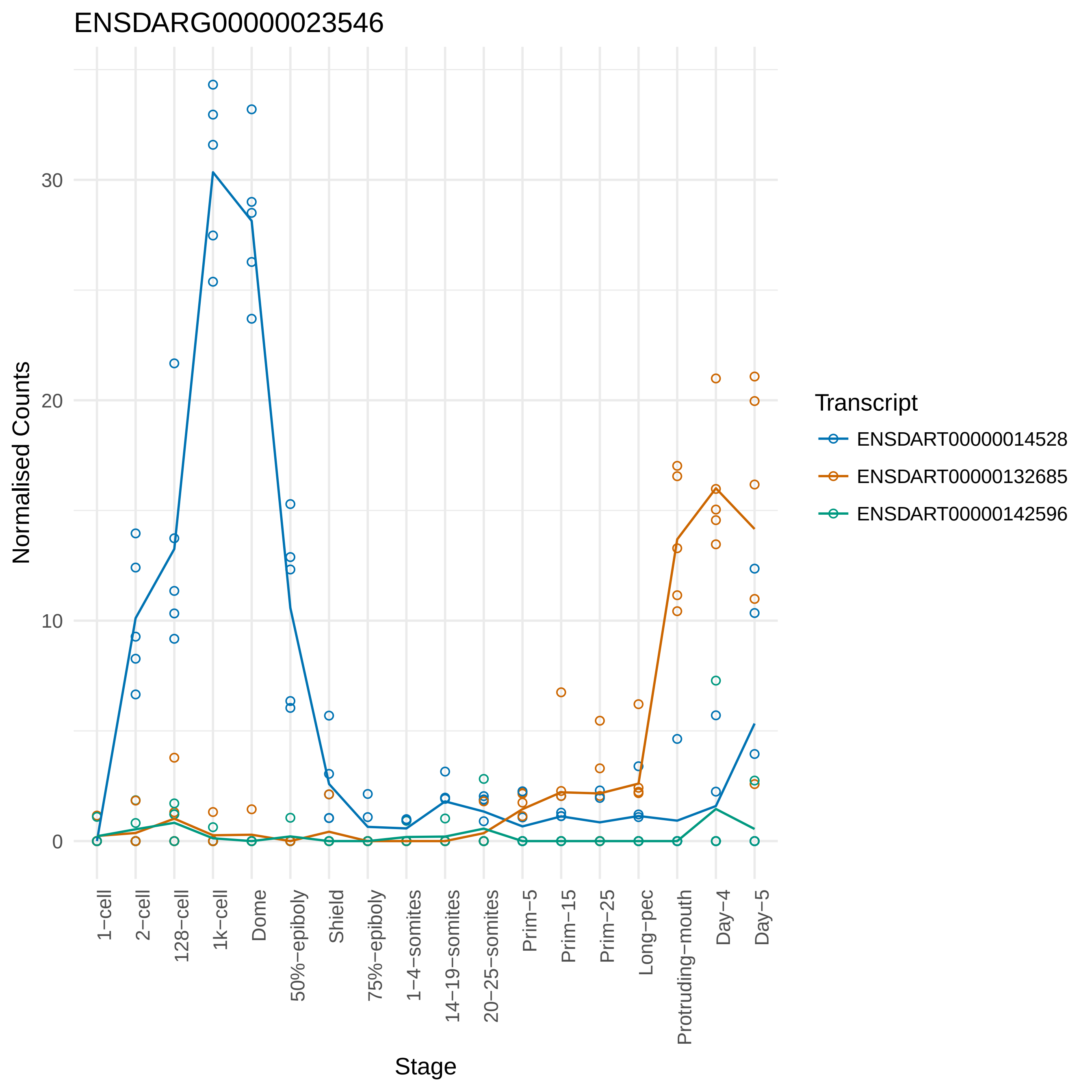
ENSDARG00000023546
Normalised Counts
0
10
20
30
1−cell
2−cell
128−cell
1k−cell
Dome
50%−epiboly
Shield
75%−epiboly
1−4−somites
14−19−somites
20−25−somites
Prim−5
Prim−15
Prim−25
Long−pec
Protruding−mouth
Day−4
Day−5
Stage
Transcript
ENSDART00000014528
ENSDART00000132685
ENSDART00000142596

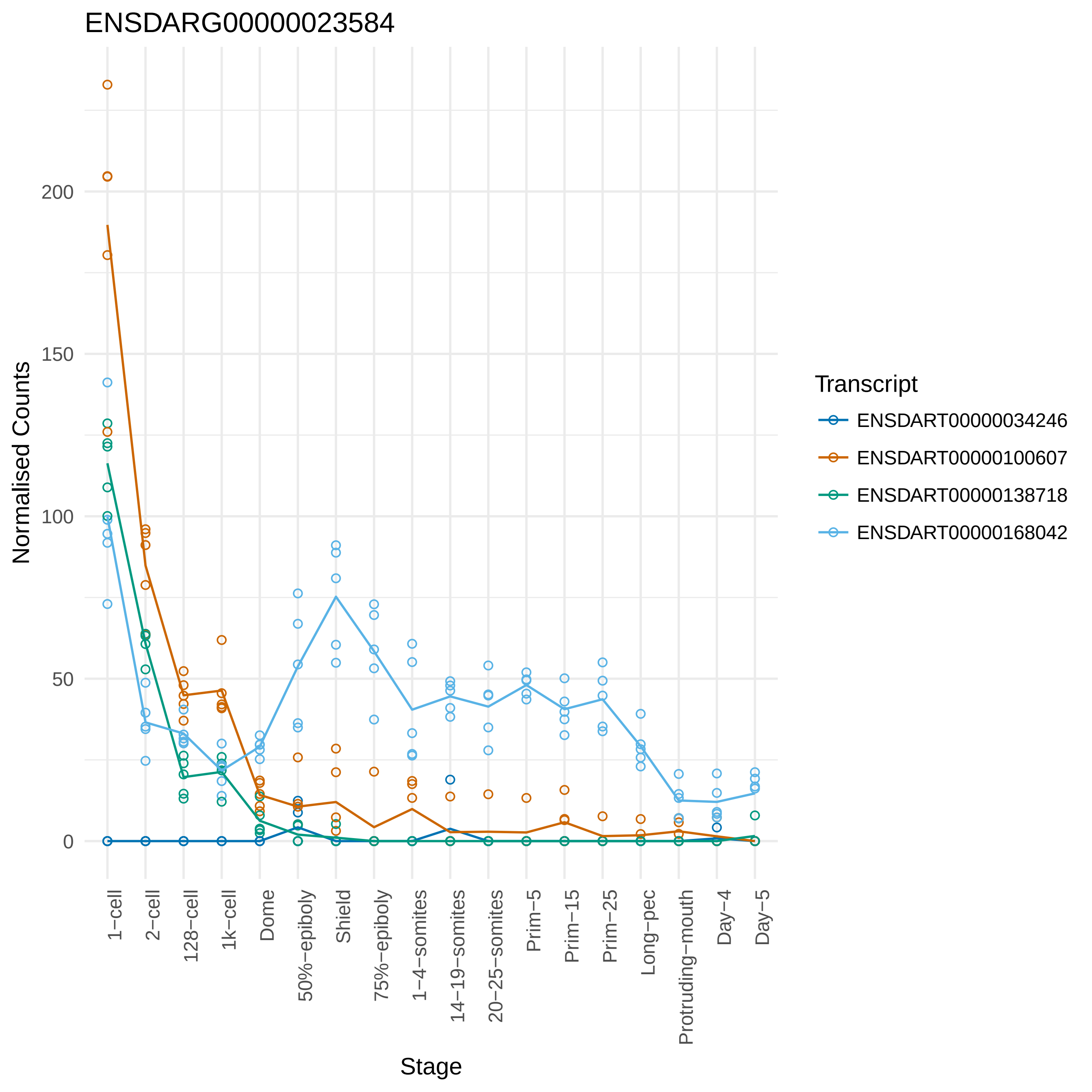

ENSDARG00000023584
Normalised Counts
0
50
100
150
200
Stage
1−cell
2−cell
128−cell
1k−cell
Dome
50%−epiboly
Shield
75%−epiboly
1−4−somites
14−19−somites
20−25−somites
Prim−5
Prim−15
Prim−25
Long−pec
Protruding−mouth
Day−4
Day−5
Transcript
ENSDART00000034246
ENSDART00000100607
ENSDART00000138718
ENSDART00000168042

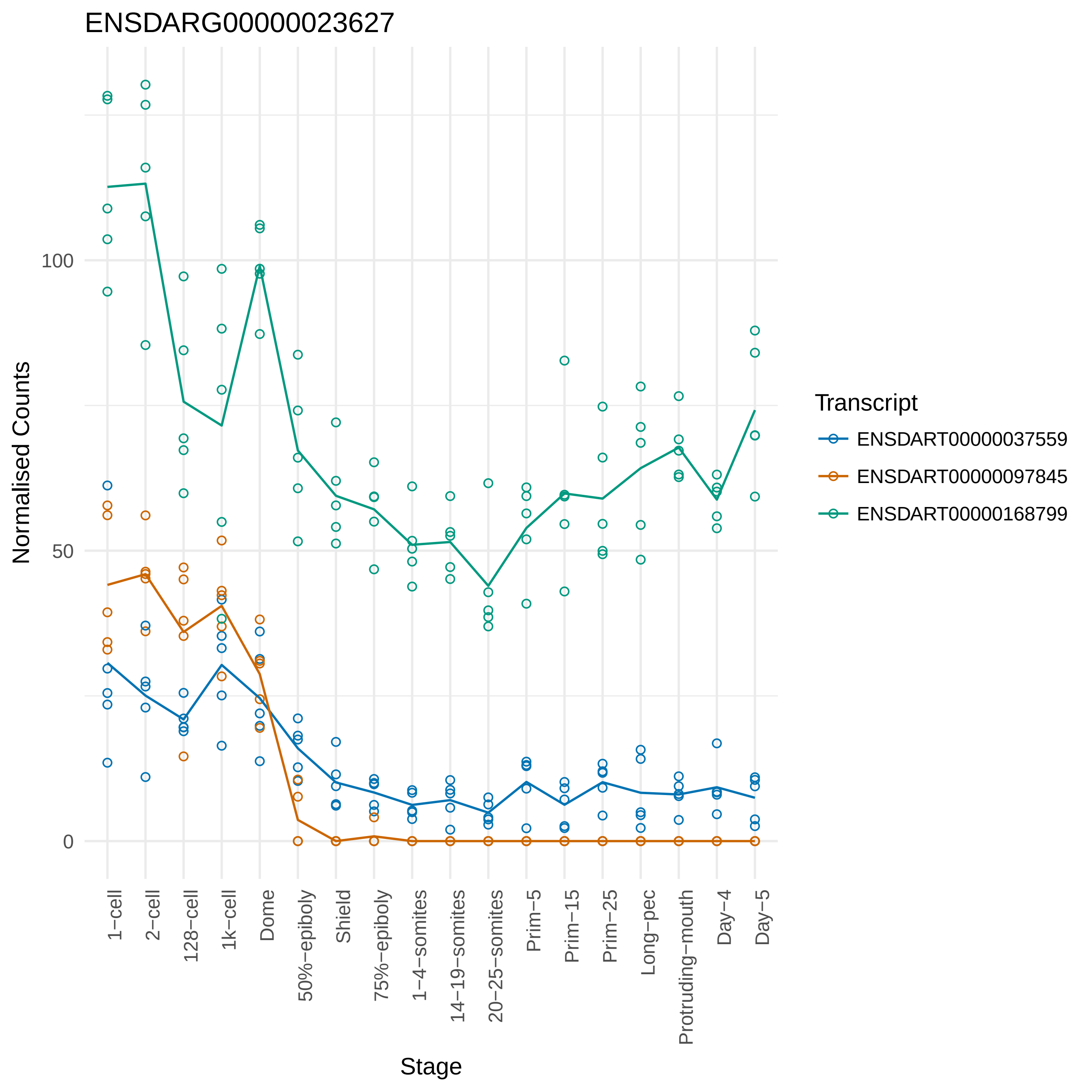

ENSDARG00000023627
Normalised Counts
100
50
0
1−cell
2−cell
128−cell
1k−cell
Dome
50%−epiboly
Shield
75%−epiboly
1−4−somites
14−19−somites
20−25−somites
Prim−5
Prim−15
Prim−25
Long−pec
Protruding−mouth
Day−4
Day−5
Stage
Transcript
ENSDART00000037559
ENSDART00000097845
ENSDART00000168799

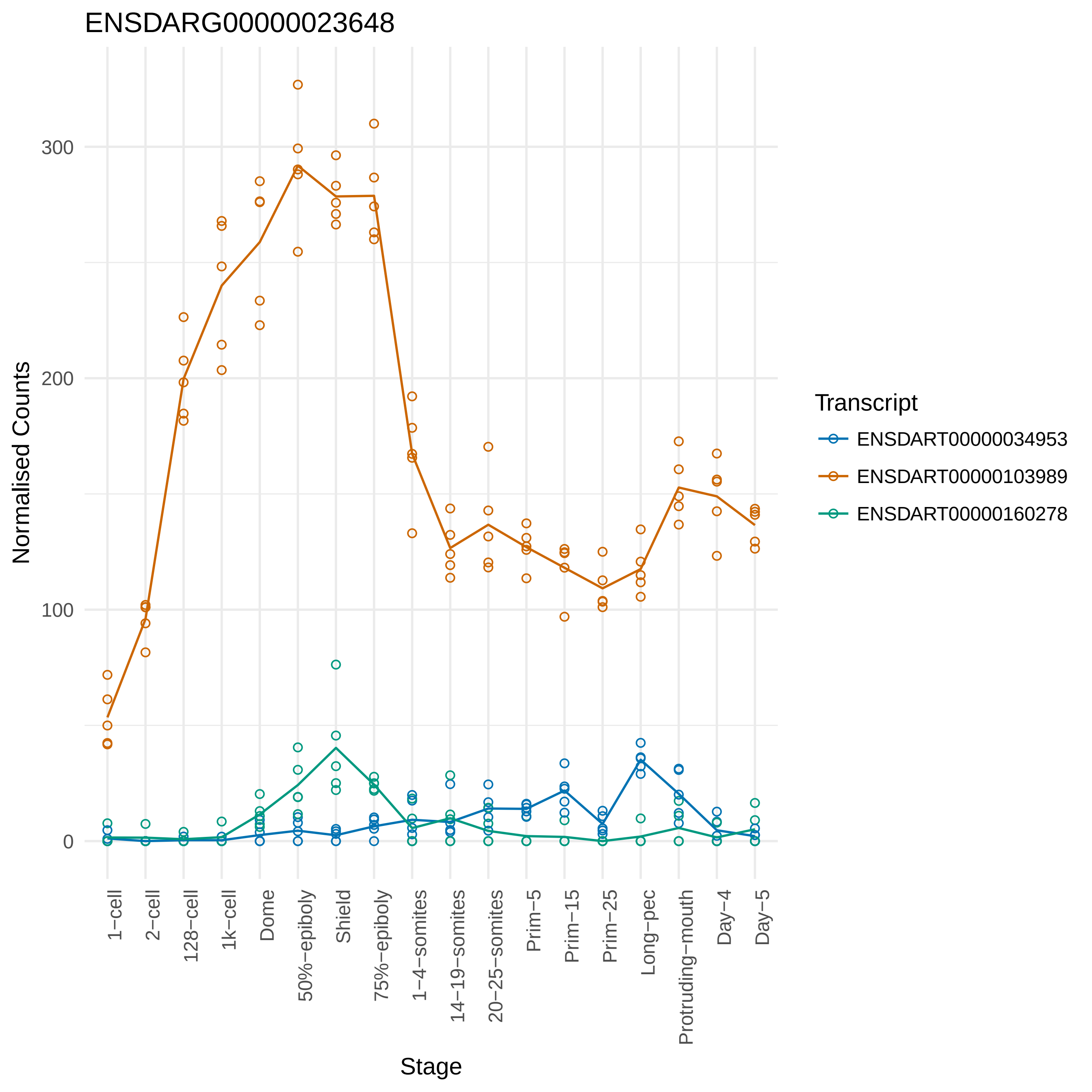
ENSDARG00000023648
Normalised Counts
300
200
100
0
Stage
1−cell
2−cell
128−cell
1k−cell
Dome
50%−epiboly
Shield
75%−epiboly
1−4−somites
14−19−somites
20−25−somites
Prim−5
Prim−15
Prim−25
Long−pec
Protruding−mouth
Day−4
Day−5
Transcript
ENSDART00000034953
ENSDART00000103989
ENSDART00000160278

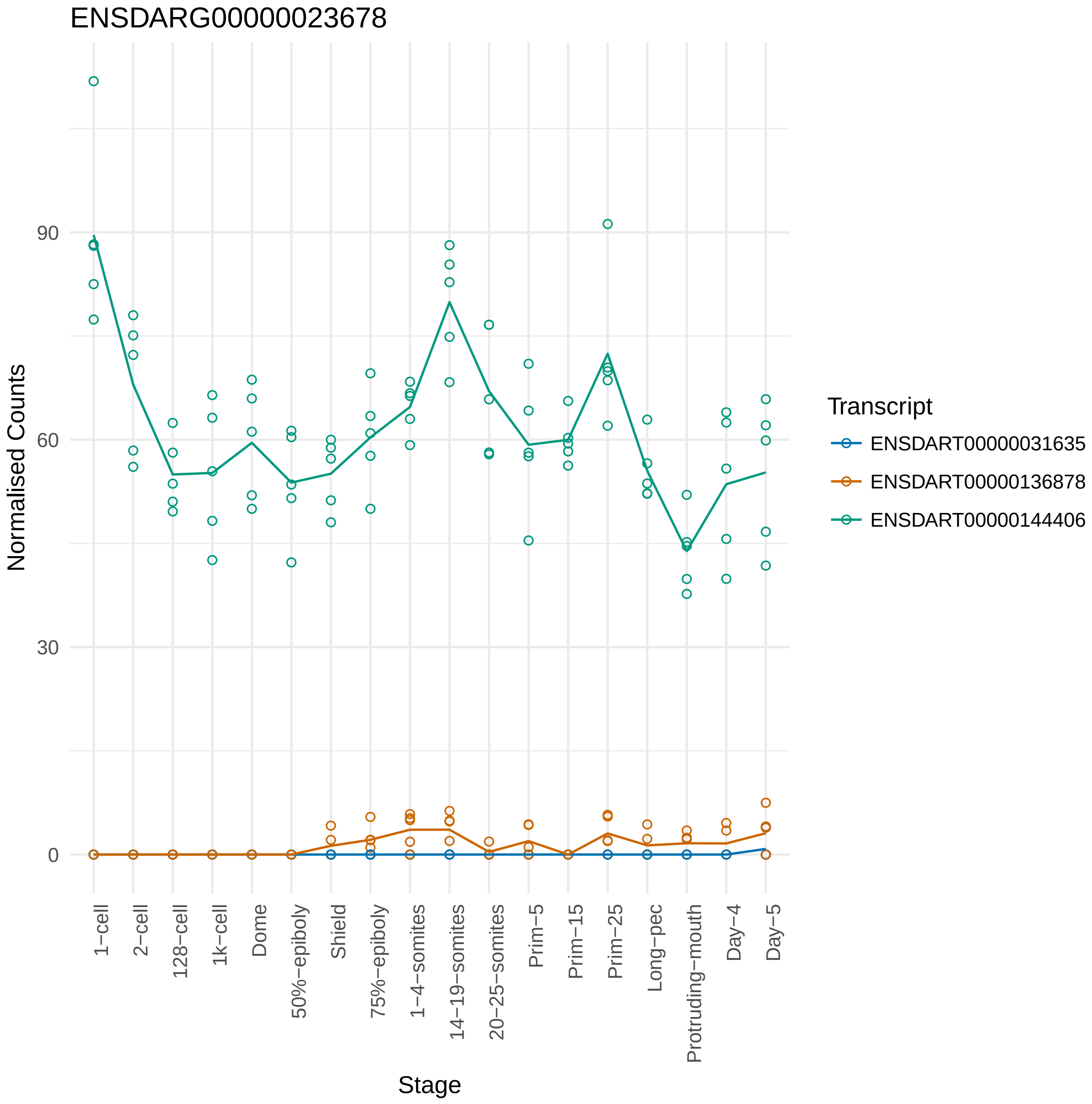
ENSDARG00000023678
Normalised Counts
0
30
60
90
Stage
1−cell
2−cell
128−cell
1k−cell
Dome
50%−epiboly
Shield
75%−epiboly
1−4−somites
14−19−somites
20−25−somites
Prim−5
Prim−15
Prim−25
Long−pec
Protruding−mouth
Day−4
Day−5
Transcript
ENSDART00000031635
ENSDART00000136878
ENSDART00000144406

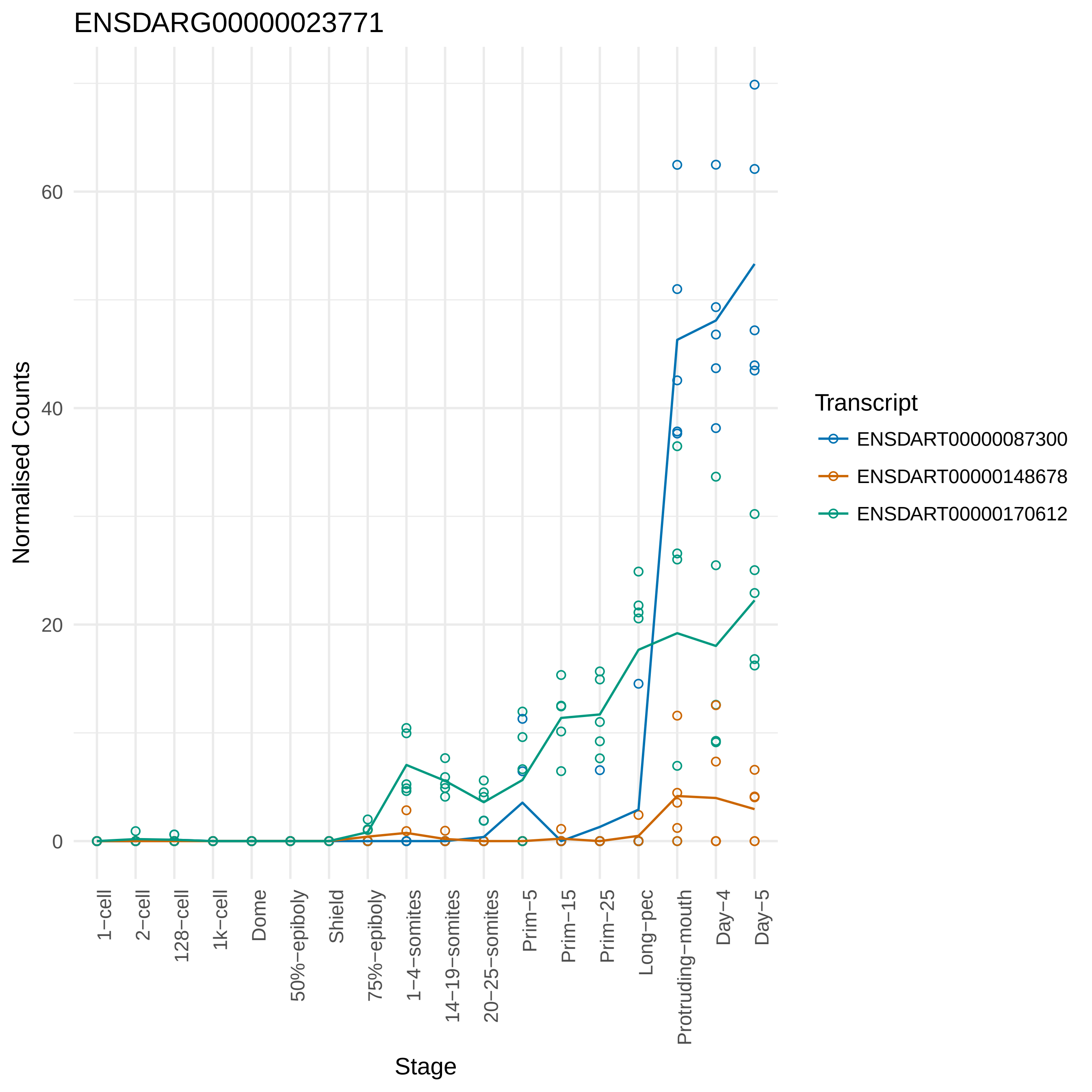
ENSDARG00000023771
Normalised Counts
0
20
40
60
Stage
1−cell
2−cell
128−cell
1k−cell
Dome
50%−epiboly
Shield
75%−epiboly
1−4−somites
14−19−somites
20−25−somites
Prim−5
Prim−15
Prim−25
Long−pec
Protruding−mouth
Day−4
Day−5
Transcript
ENSDART00000087300
ENSDART00000148678
ENSDART00000170612

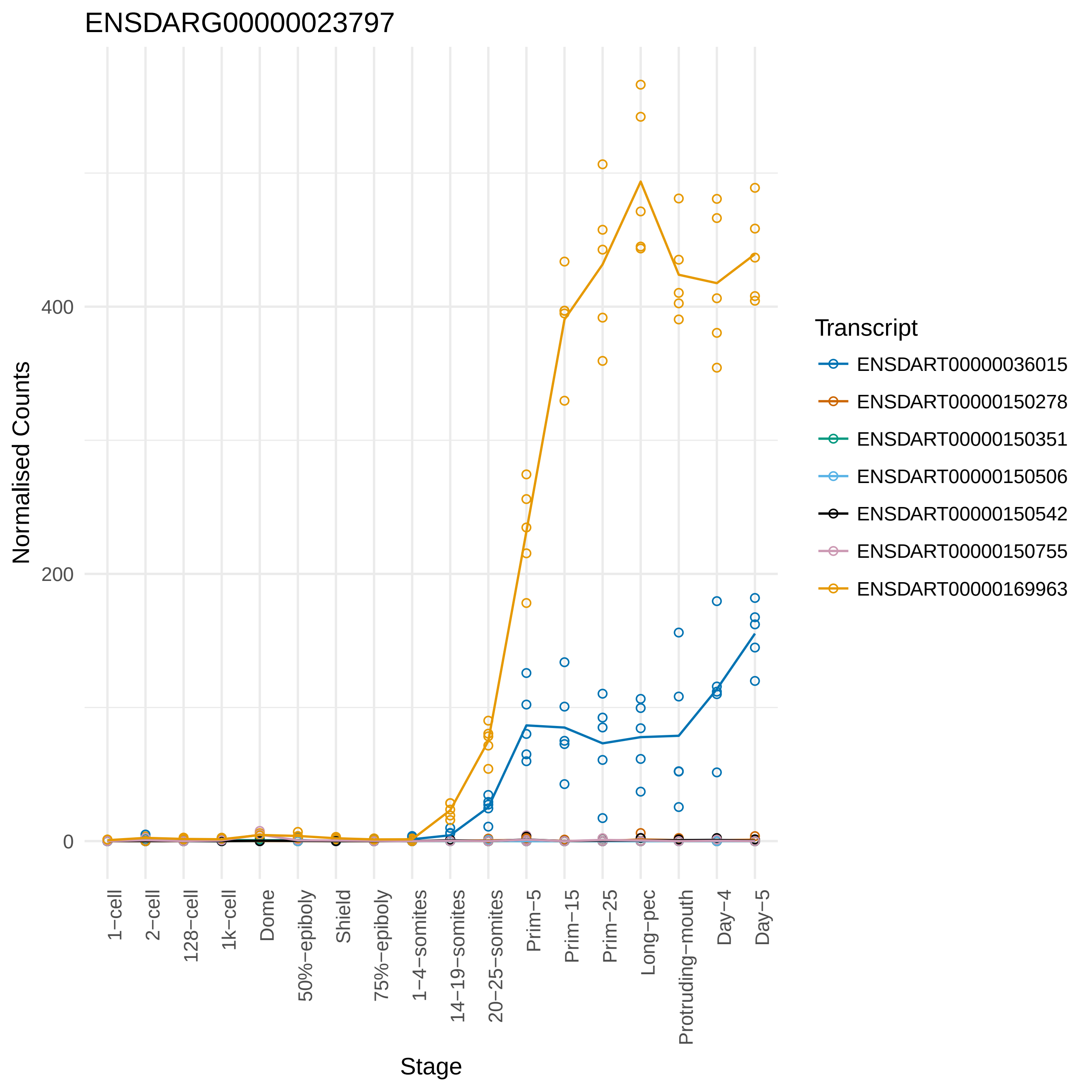

ENSDARG00000023797
Normalised Counts
400
200
0
1−cell
2−cell
128−cell
1k−cell
Dome
50%−epiboly
Shield
75%−epiboly
1−4−somites
14−19−somites
20−25−somites
Prim−5
Prim−15
Prim−25
Long−pec
Protruding−mouth
Day−4
Day−5
Stage
Transcript
ENSDART00000036015
ENSDART00000150278
ENSDART00000150351
ENSDART00000150506
ENSDART00000150542
ENSDART00000150755
ENSDART00000169963

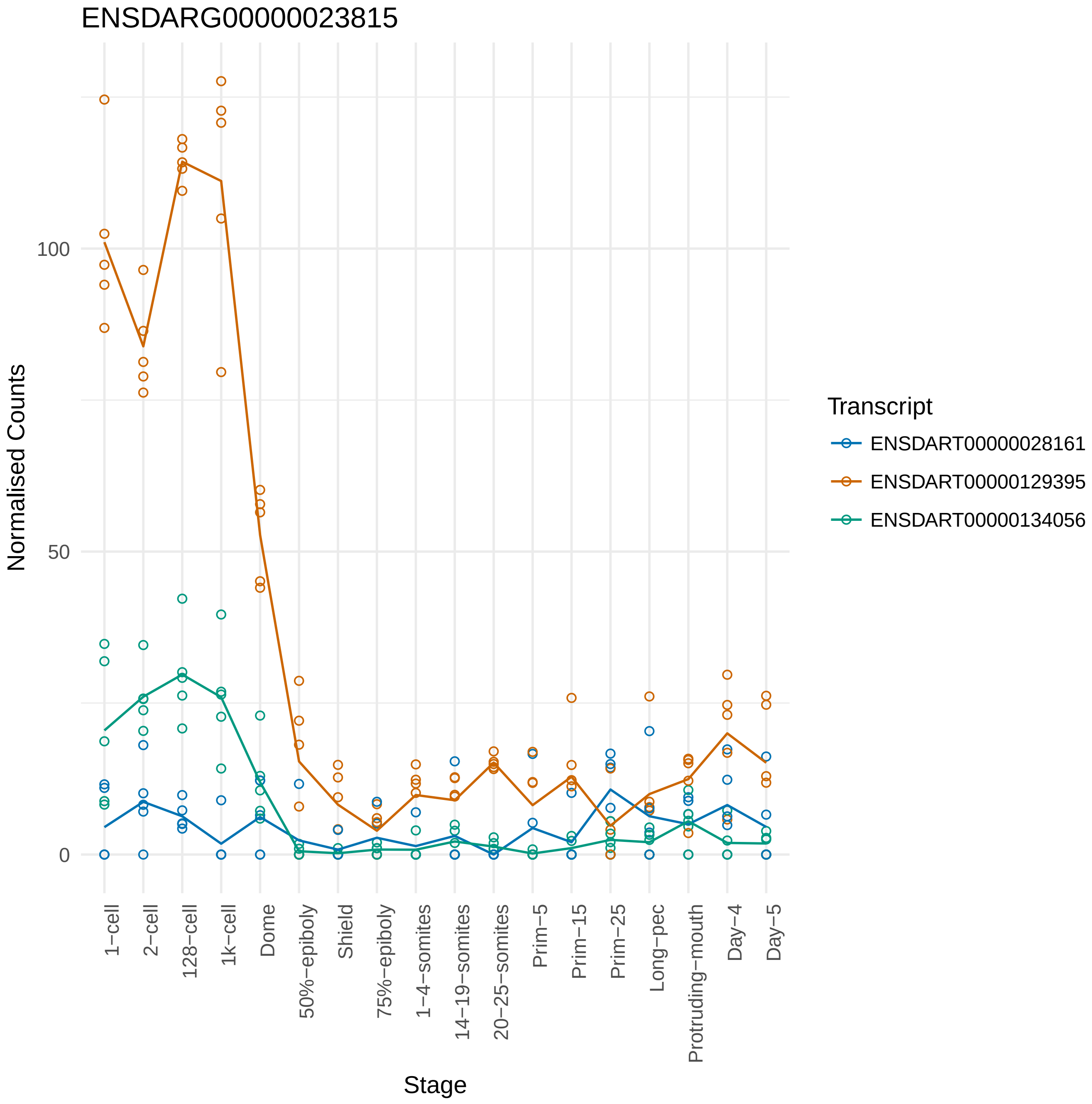
ENSDARG00000023815
Normalised Counts
100
50
0
1−cell
2−cell
128−cell
1k−cell
Dome
50%−epiboly
Shield
75%−epiboly
1−4−somites
14−19−somites
20−25−somites
Prim−5
Prim−15
Prim−25
Long−pec
Protruding−mouth
Day−4
Day−5
Stage
Transcript
ENSDART00000028161
ENSDART00000129395
ENSDART00000134056

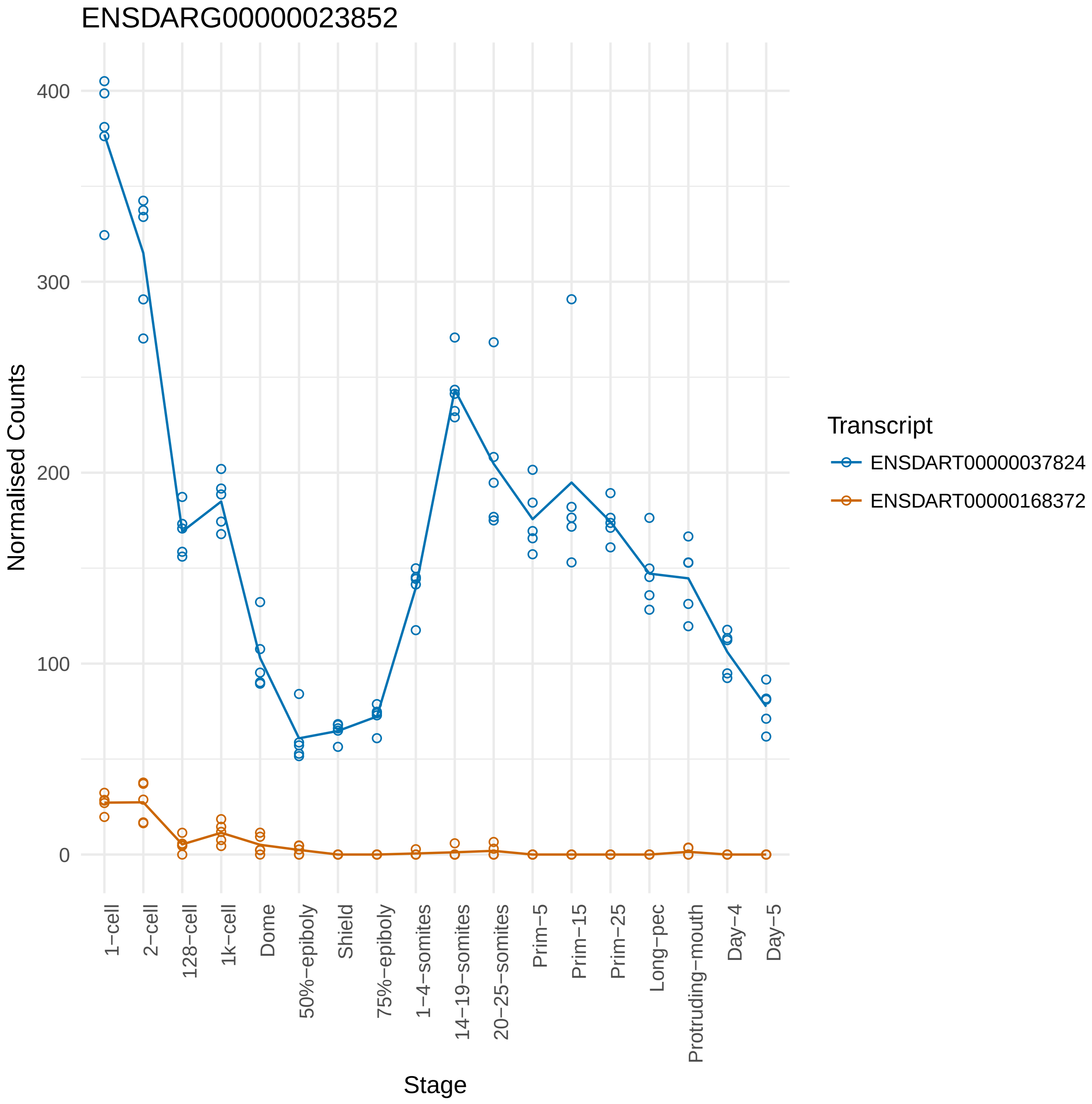
ENSDARG00000023852
Normalised Counts
400
300
200
100
0
1−cell
2−cell
128−cell
1k−cell
Dome
50%−epiboly
Shield
75%−epiboly
1−4−somites
14−19−somites
20−25−somites
Prim−5
Prim−15
Prim−25
Long−pec
Protruding−mouth
Day−4
Day−5
Stage
Transcript
ENSDART00000037824
ENSDART00000168372

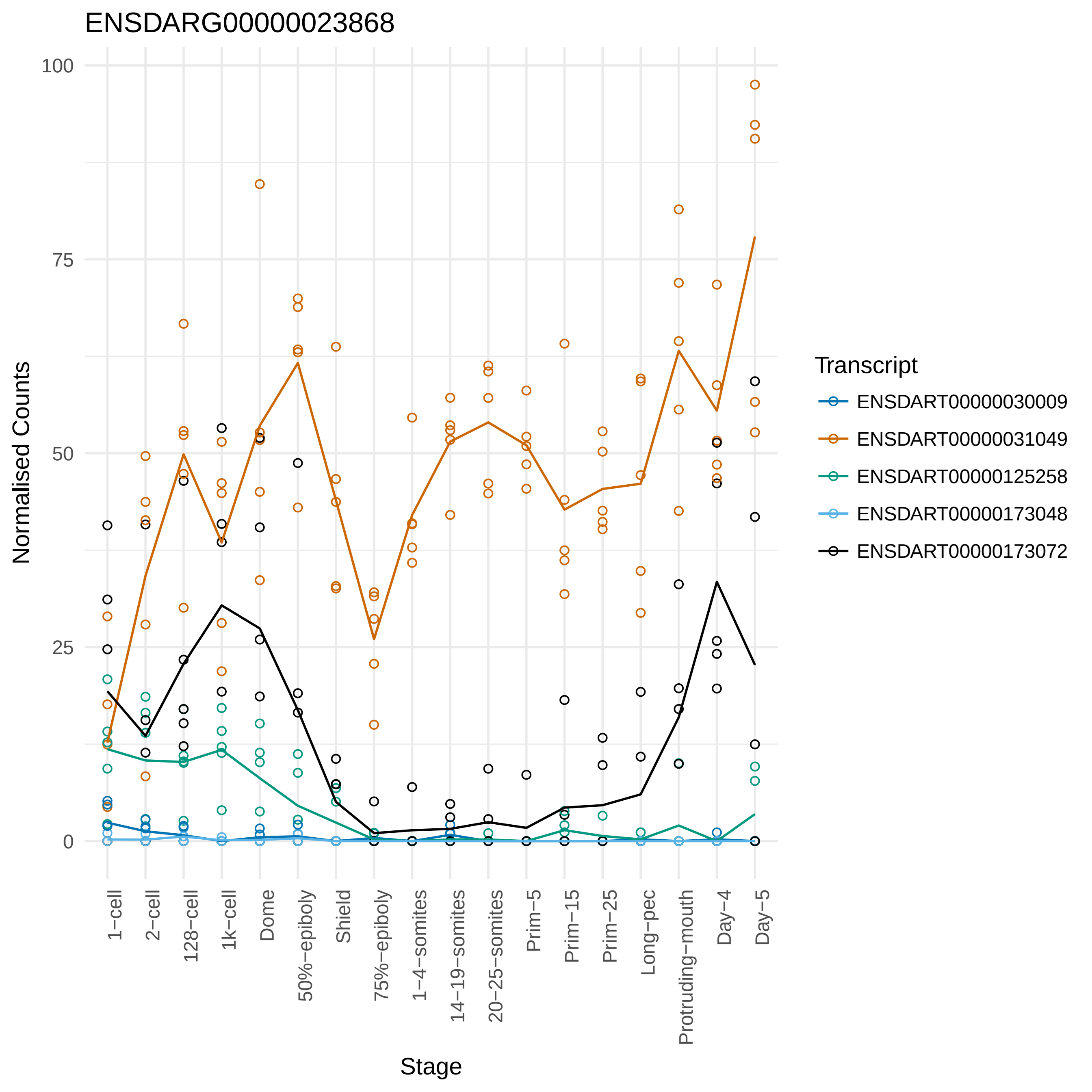

ENSDARG00000023868
Normalised Counts
100
75
50
25
0
1−cell
2−cell
128−cell
1k−cell
Dome
50%−epiboly
Shield
75%−epiboly
1−4−somites
14−19−somites
20−25−somites
Prim−5
Prim−15
Prim−25
Long−pec
Protruding−mouth
Day−4
Day−5
Stage
Transcript
ENSDART00000030009
ENSDART00000031049
ENSDART00000125258
ENSDART00000173048
ENSDART00000173072

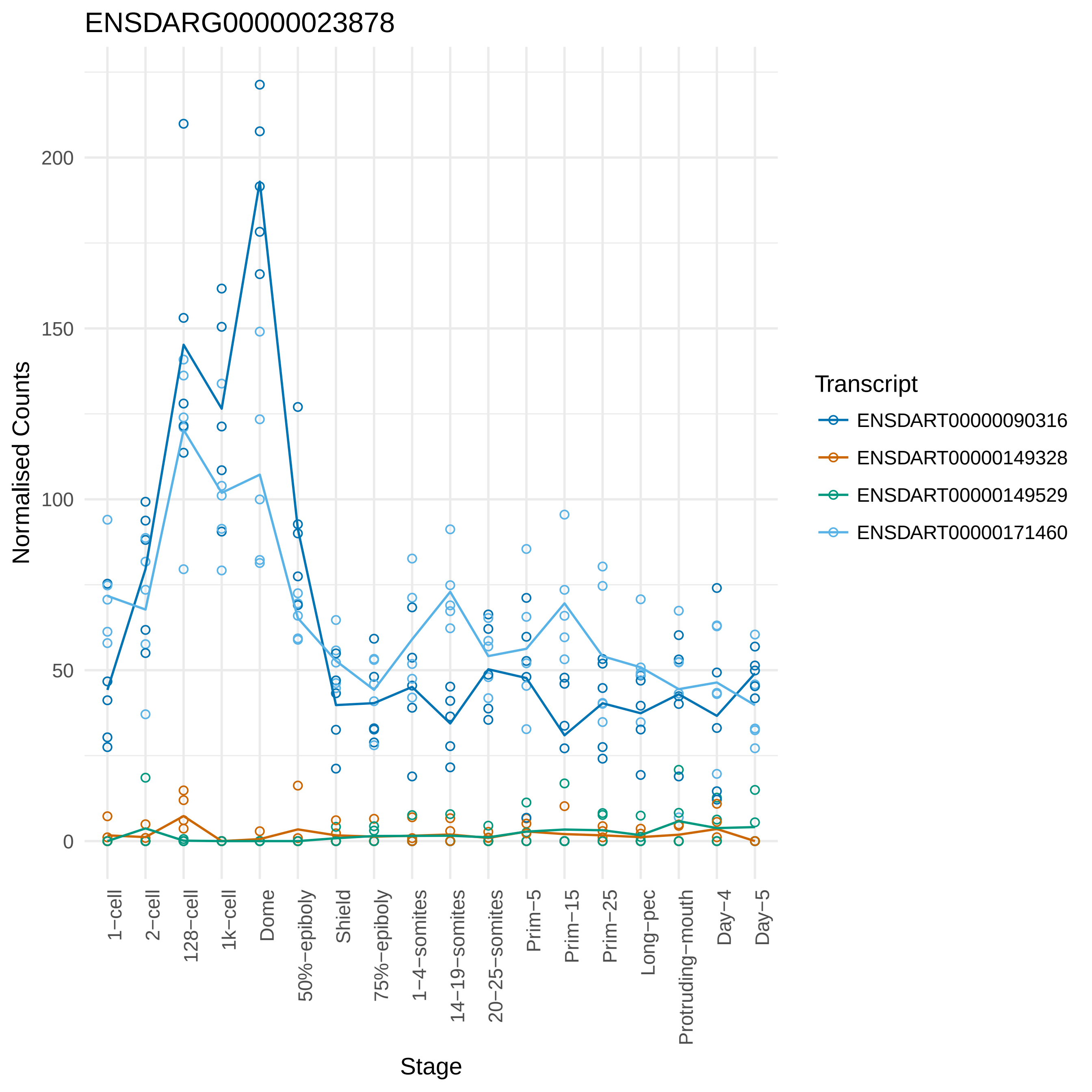

ENSDARG00000023878
Normalised Counts
0
50
100
150
200
Stage
1−cell
2−cell
128−cell
1k−cell
Dome
50%−epiboly
Shield
75%−epiboly
1−4−somites
14−19−somites
20−25−somites
Prim−5
Prim−15
Prim−25
Long−pec
Protruding−mouth
Day−4
Day−5
Transcript
ENSDART00000090316
ENSDART00000149328
ENSDART00000149529
ENSDART00000171460

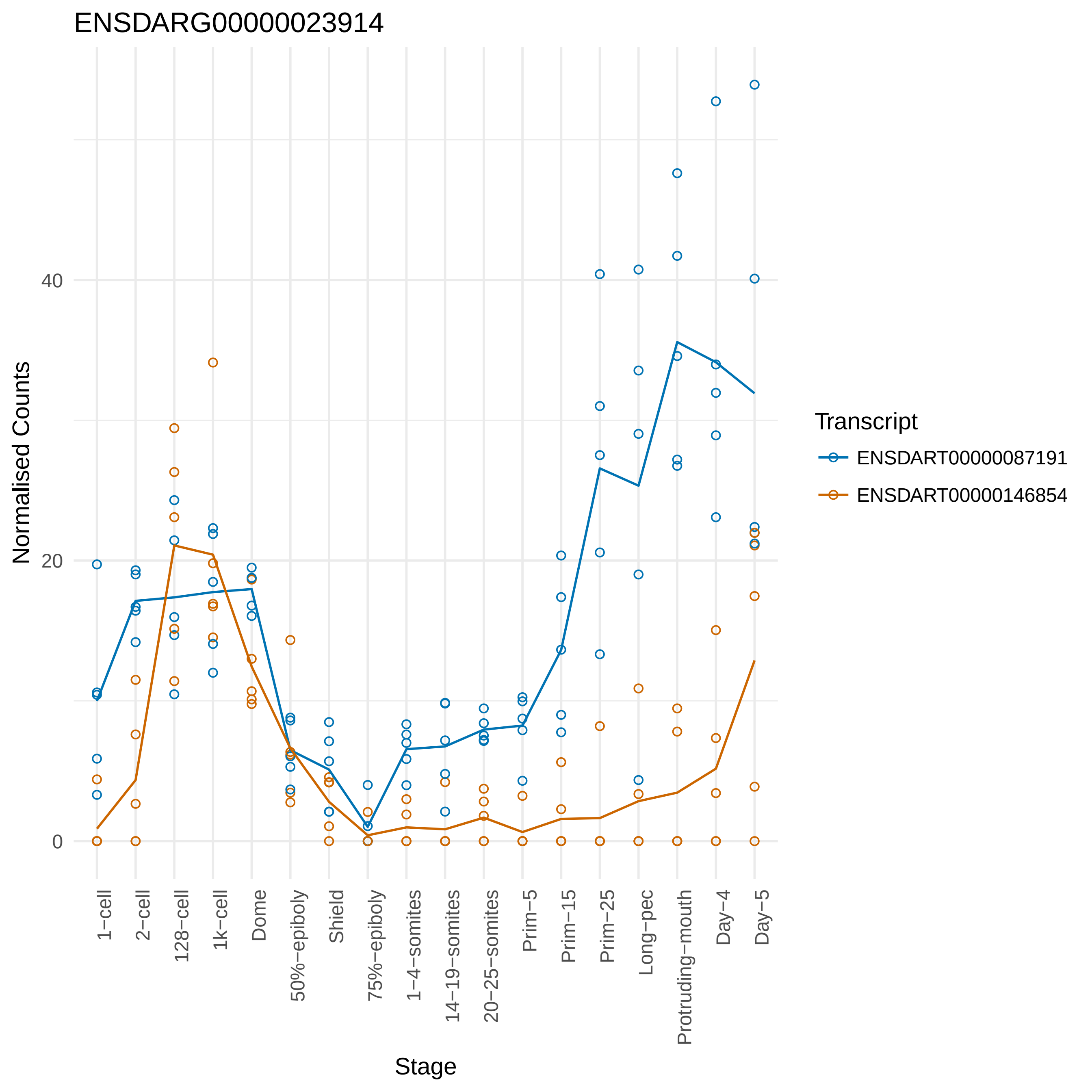
ENSDARG00000023914
Normalised Counts
0
20
40
Stage
1−cell
2−cell
128−cell
1k−cell
Dome
50%−epiboly
Shield
75%−epiboly
1−4−somites
14−19−somites
20−25−somites
Prim−5
Prim−15
Prim−25
Long−pec
Protruding−mouth
Day−4
Day−5
Transcript
ENSDART00000087191
ENSDART00000146854

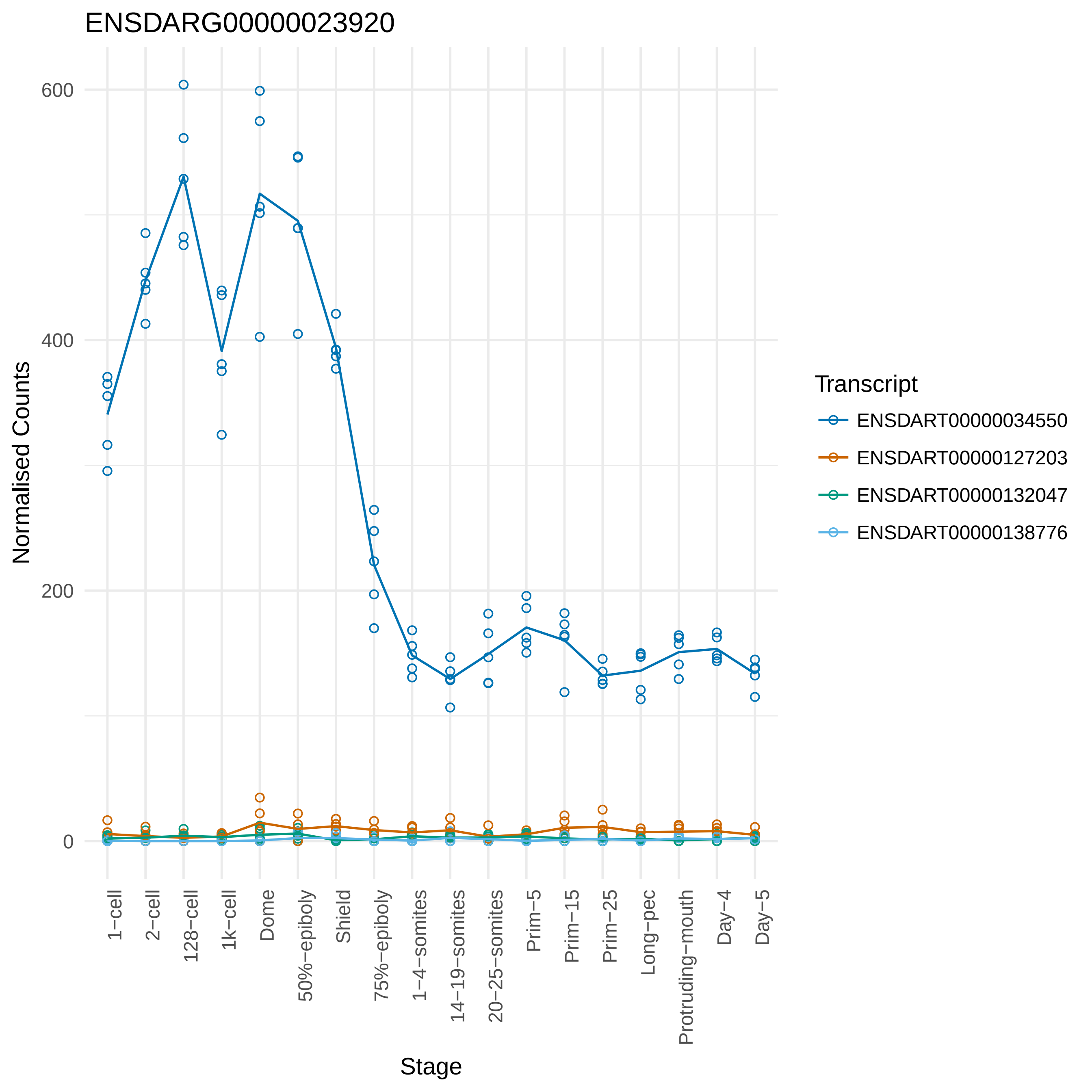

ENSDARG00000023920
Normalised Counts
600
400
200
0
Stage
1−cell
2−cell
128−cell
1k−cell
Dome
50%−epiboly
Shield
75%−epiboly
1−4−somites
14−19−somites
20−25−somites
Prim−5
Prim−15
Prim−25
Long−pec
Protruding−mouth
Day−4
Day−5
Transcript
ENSDART00000034550
ENSDART00000127203
ENSDART00000132047
ENSDART00000138776

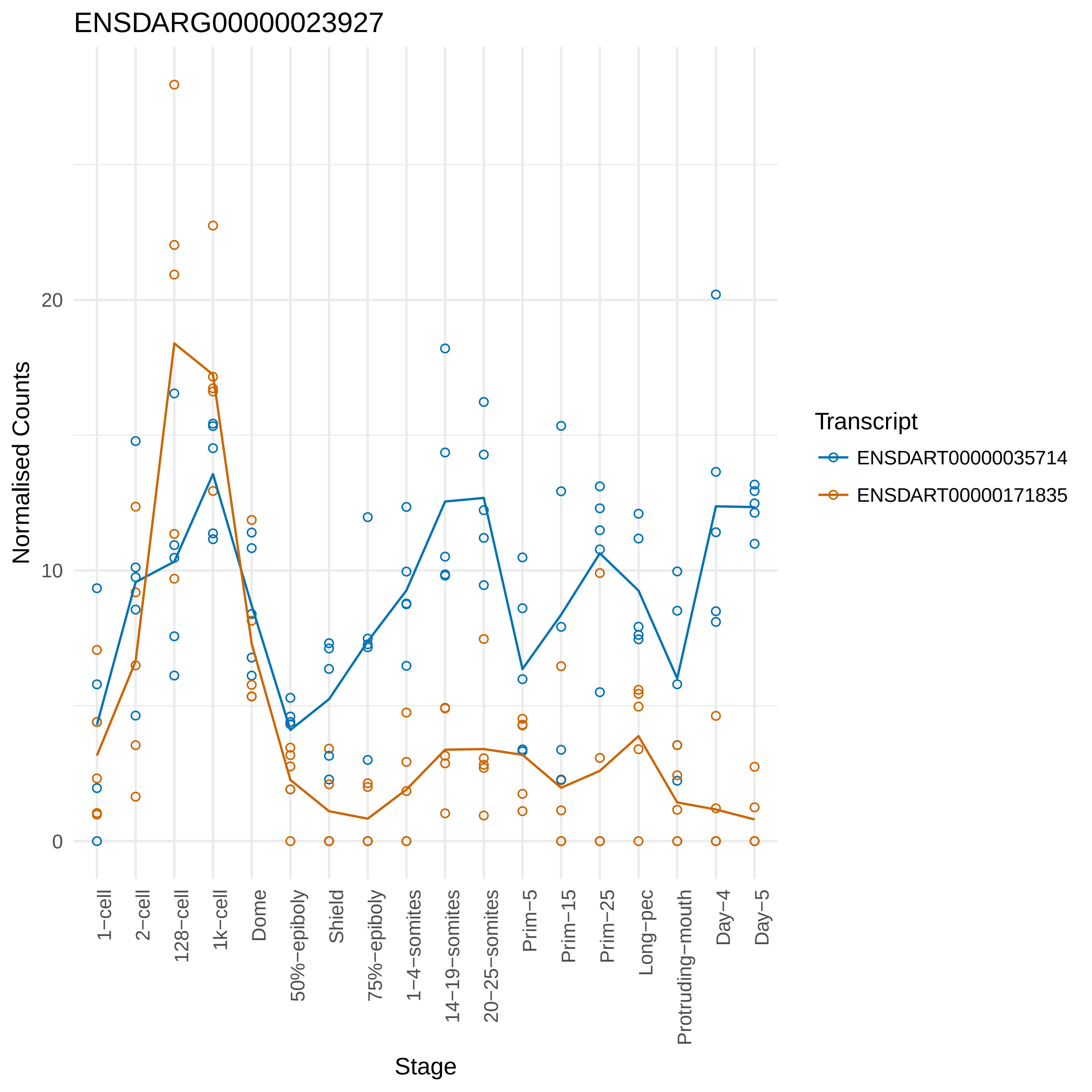
ENSDARG00000023927
Normalised Counts
20
10
0
1−cell
2−cell
128−cell
1k−cell
Dome
50%−epiboly
Shield
75%−epiboly
1−4−somites
14−19−somites
20−25−somites
Prim−5
Prim−15
Prim−25
Long−pec
Protruding−mouth
Day−4
Day−5
Stage
Transcript
ENSDART00000035714
ENSDART00000171835

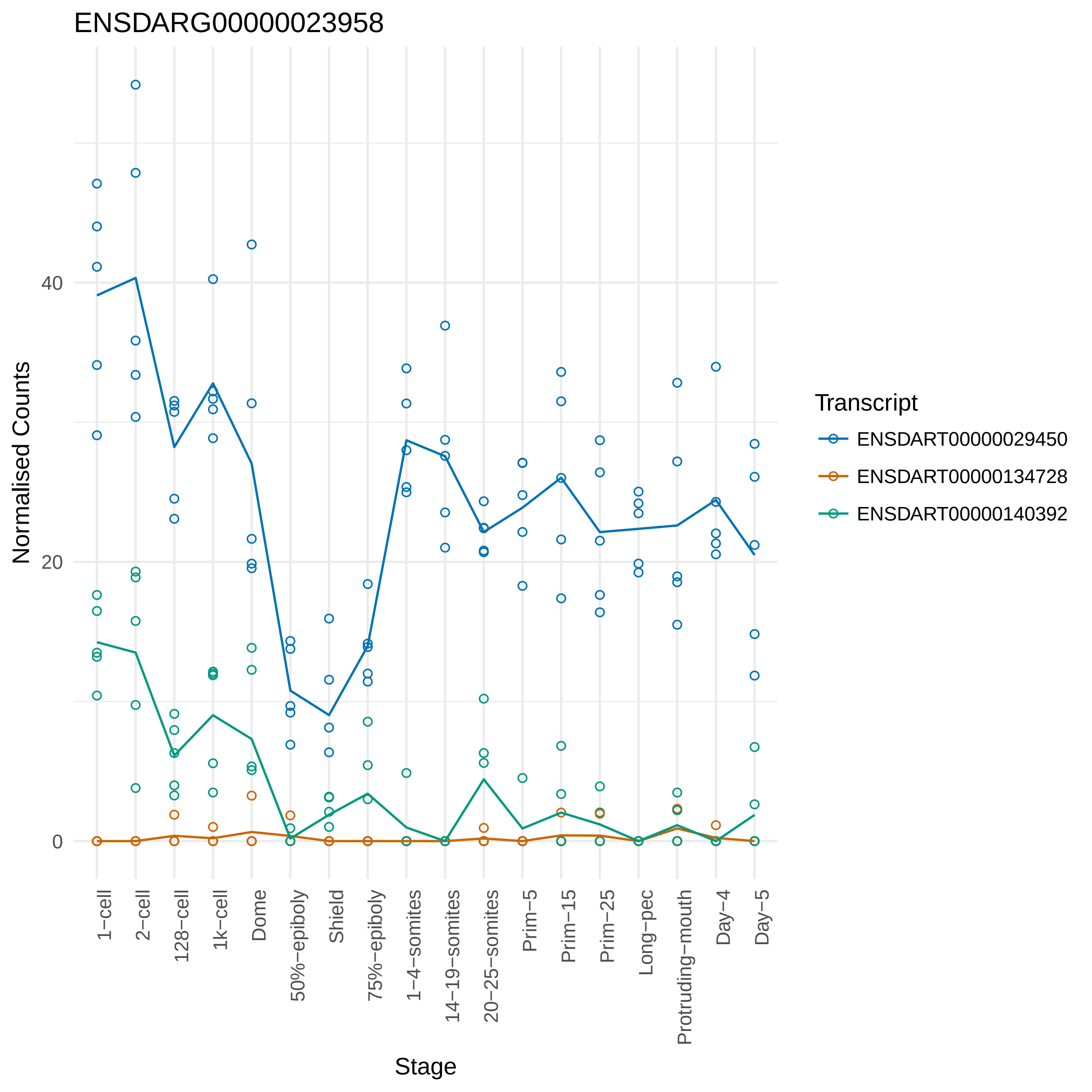

ENSDARG00000023958
Normalised Counts
0
20
40
1−cell
2−cell
128−cell
1k−cell
Dome
50%−epiboly
Shield
75%−epiboly
1−4−somites
14−19−somites
20−25−somites
Prim−5
Prim−15
Prim−25
Long−pec
Protruding−mouth
Day−4
Day−5
Stage
Transcript
ENSDART00000029450
ENSDART00000134728
ENSDART00000140392

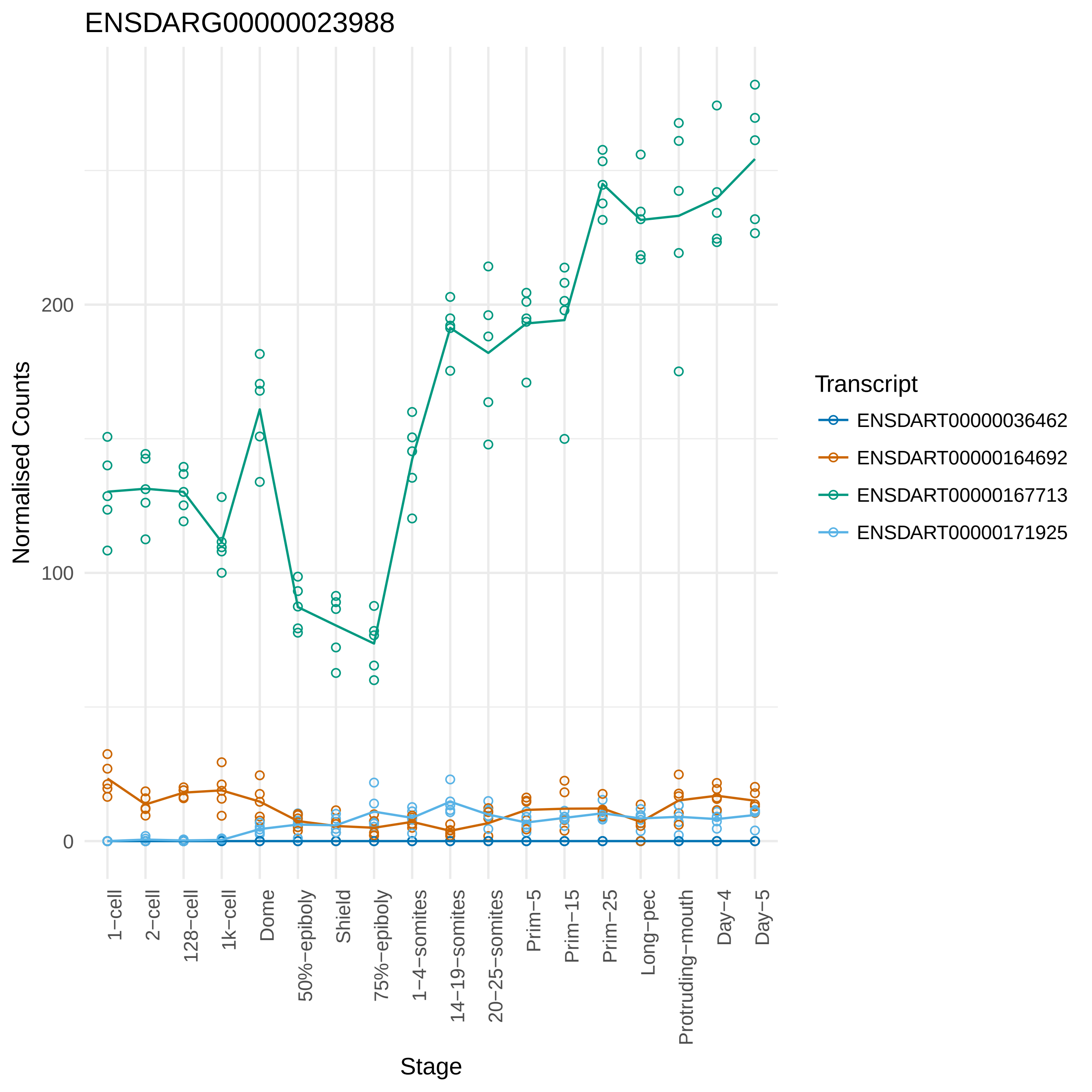
ENSDARG00000023988
Normalised Counts
200
100
0
1−cell
2−cell
128−cell
1k−cell
Dome
50%−epiboly
Shield
75%−epiboly
1−4−somites
14−19−somites
20−25−somites
Prim−5
Prim−15
Prim−25
Long−pec
Protruding−mouth
Day−4
Day−5
Stage
Transcript
ENSDART00000036462
ENSDART00000164692
ENSDART00000167713
ENSDART00000171925

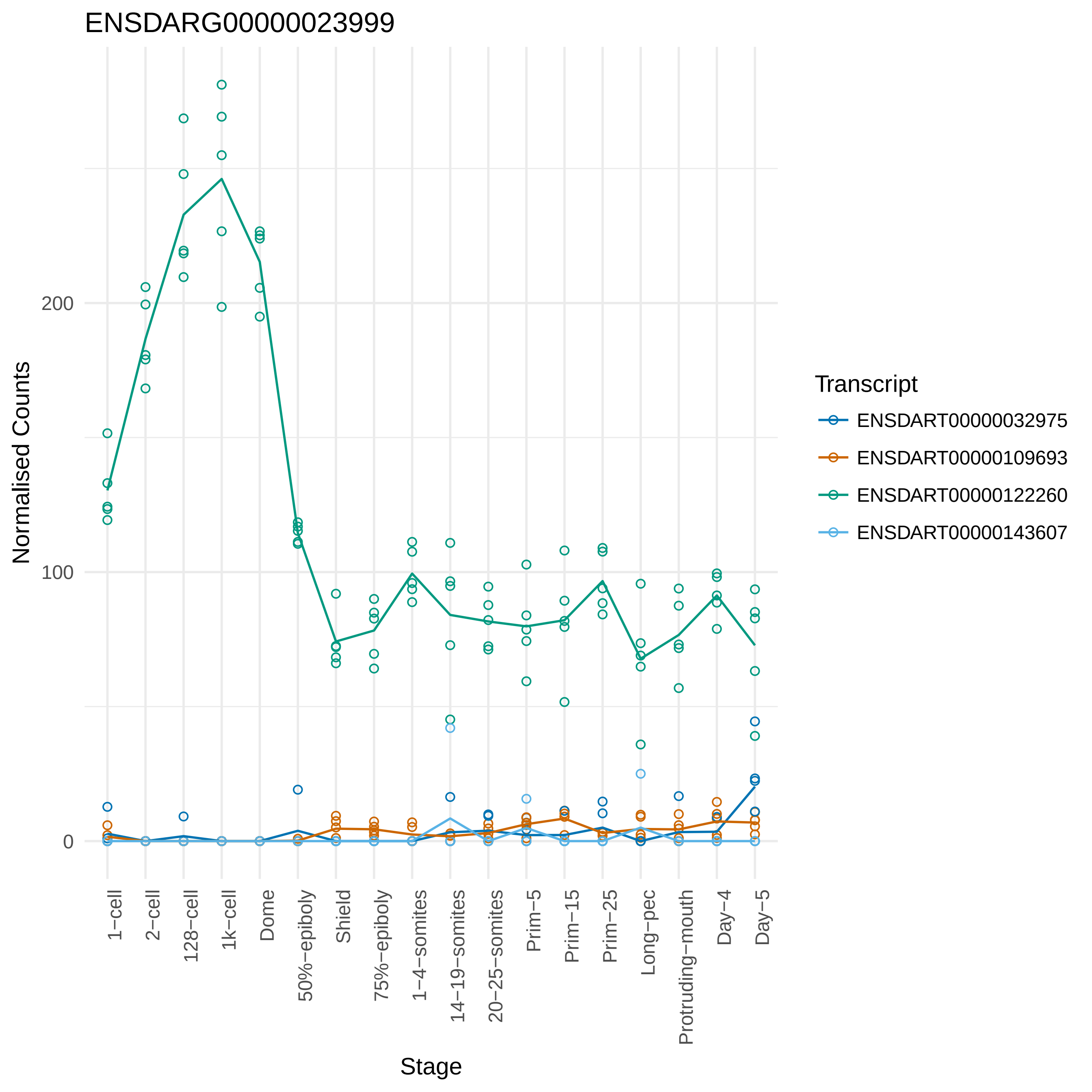

ENSDARG00000023999
Normalised Counts
200
100
0
1−cell
2−cell
128−cell
1k−cell
Dome
50%−epiboly
Shield
75%−epiboly
1−4−somites
14−19−somites
20−25−somites
Prim−5
Prim−15
Prim−25
Long−pec
Protruding−mouth
Day−4
Day−5
Stage
Transcript
ENSDART00000032975
ENSDART00000109693
ENSDART00000122260
ENSDART00000143607

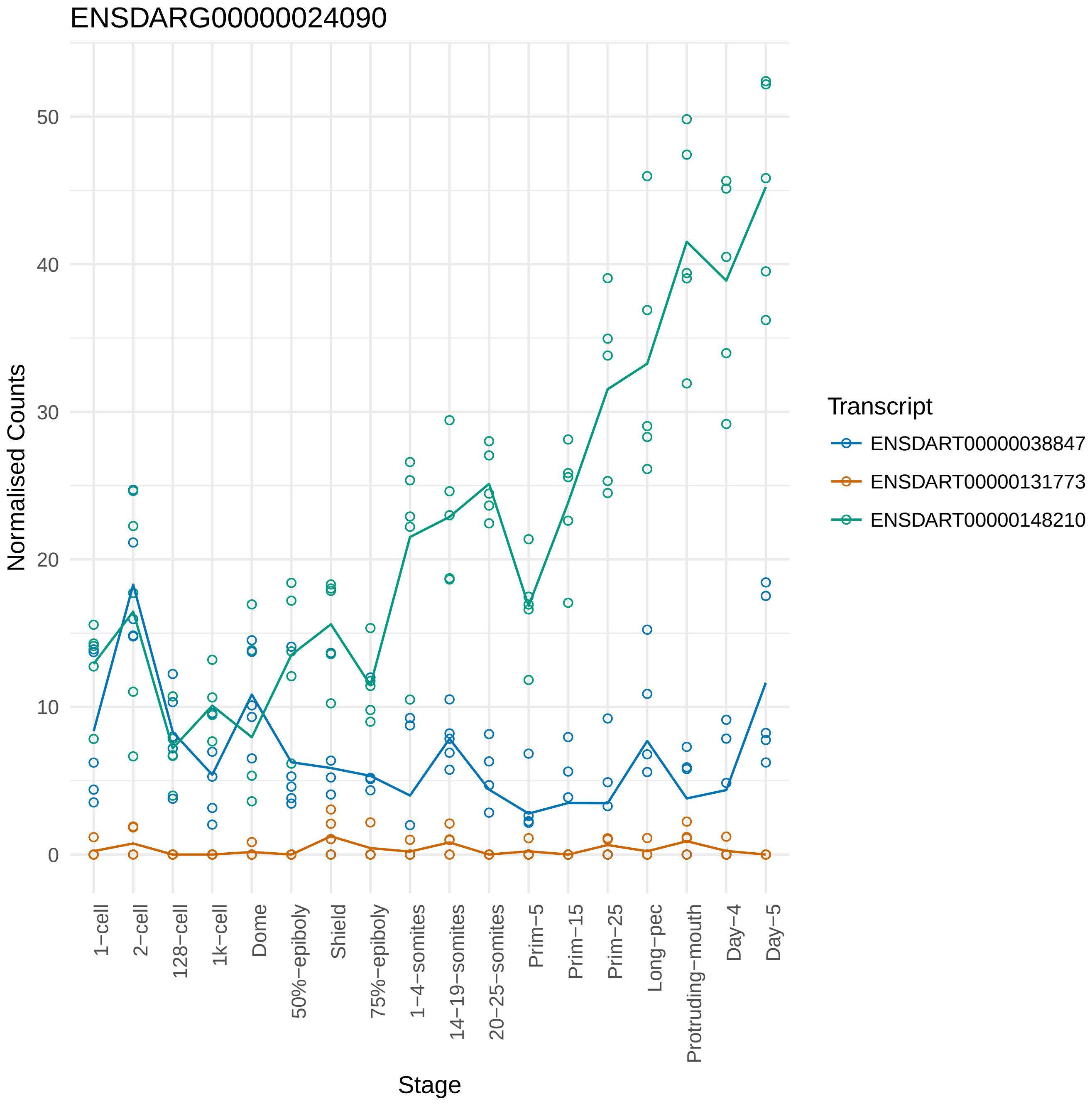

ENSDARG00000024090
Normalised Counts
0
10
20
30
40
50
1−cell
2−cell
128−cell
1k−cell
Dome
50%−epiboly
Shield
75%−epiboly
1−4−somites
14−19−somites
20−25−somites
Prim−5
Prim−15
Prim−25
Long−pec
Protruding−mouth
Day−4
Day−5
Stage
Transcript
ENSDART00000038847
ENSDART00000131773
ENSDART00000148210

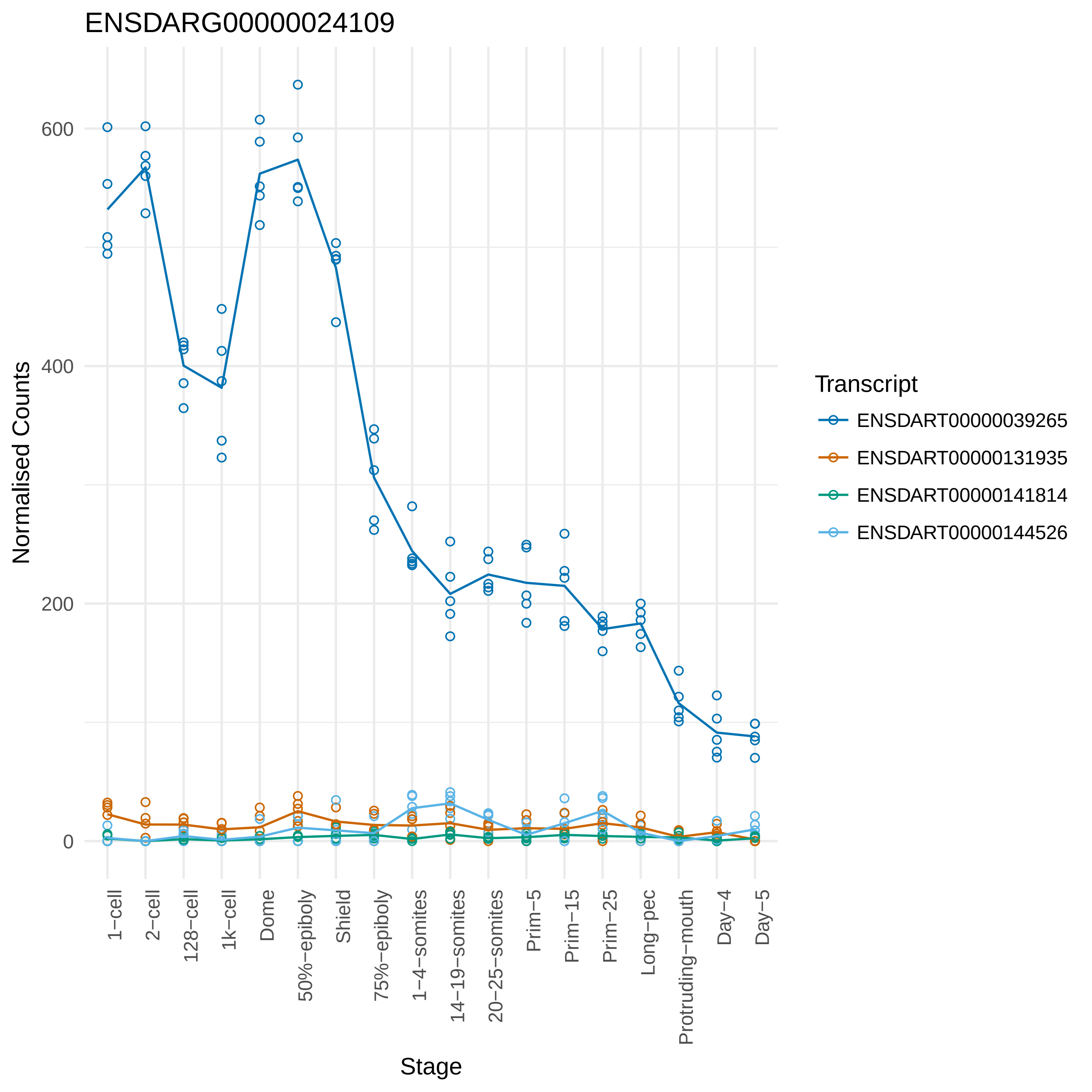
ENSDARG00000024109
Normalised Counts
600
400
200
0
1−cell
2−cell
128−cell
1k−cell
Dome
50%−epiboly
Shield
75%−epiboly
1−4−somites
14−19−somites
20−25−somites
Prim−5
Prim−15
Prim−25
Long−pec
Protruding−mouth
Day−4
Day−5
Stage
Transcript
ENSDART00000039265
ENSDART00000131935
ENSDART00000141814
ENSDART00000144526

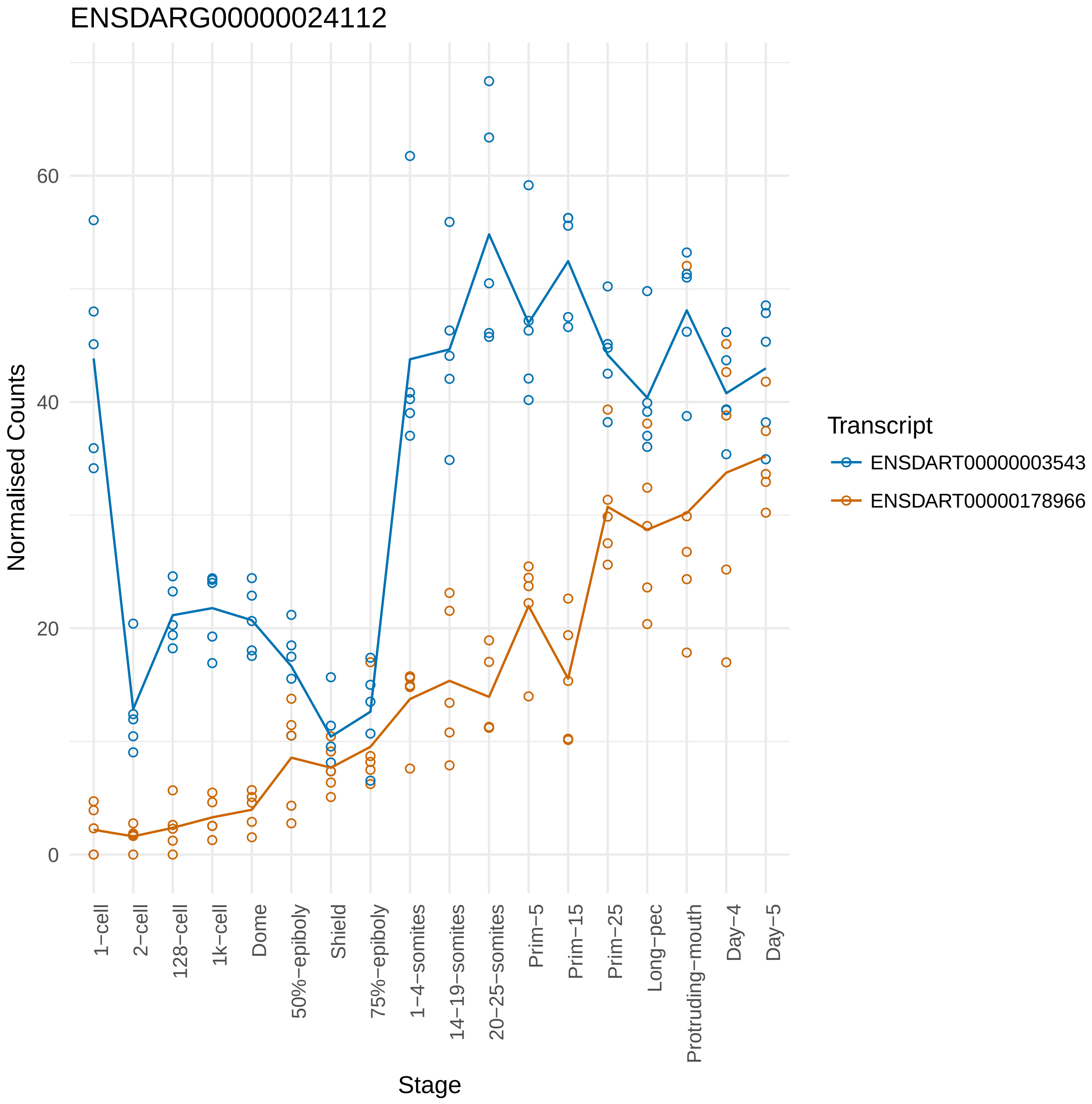
ENSDARG00000024112
Normalised Counts
0
20
40
60
1−cell
2−cell
128−cell
1k−cell
Dome
50%−epiboly
Shield
75%−epiboly
1−4−somites
14−19−somites
20−25−somites
Prim−5
Prim−15
Prim−25
Long−pec
Protruding−mouth
Day−4
Day−5
Stage
Transcript
ENSDART00000003543
ENSDART00000178966

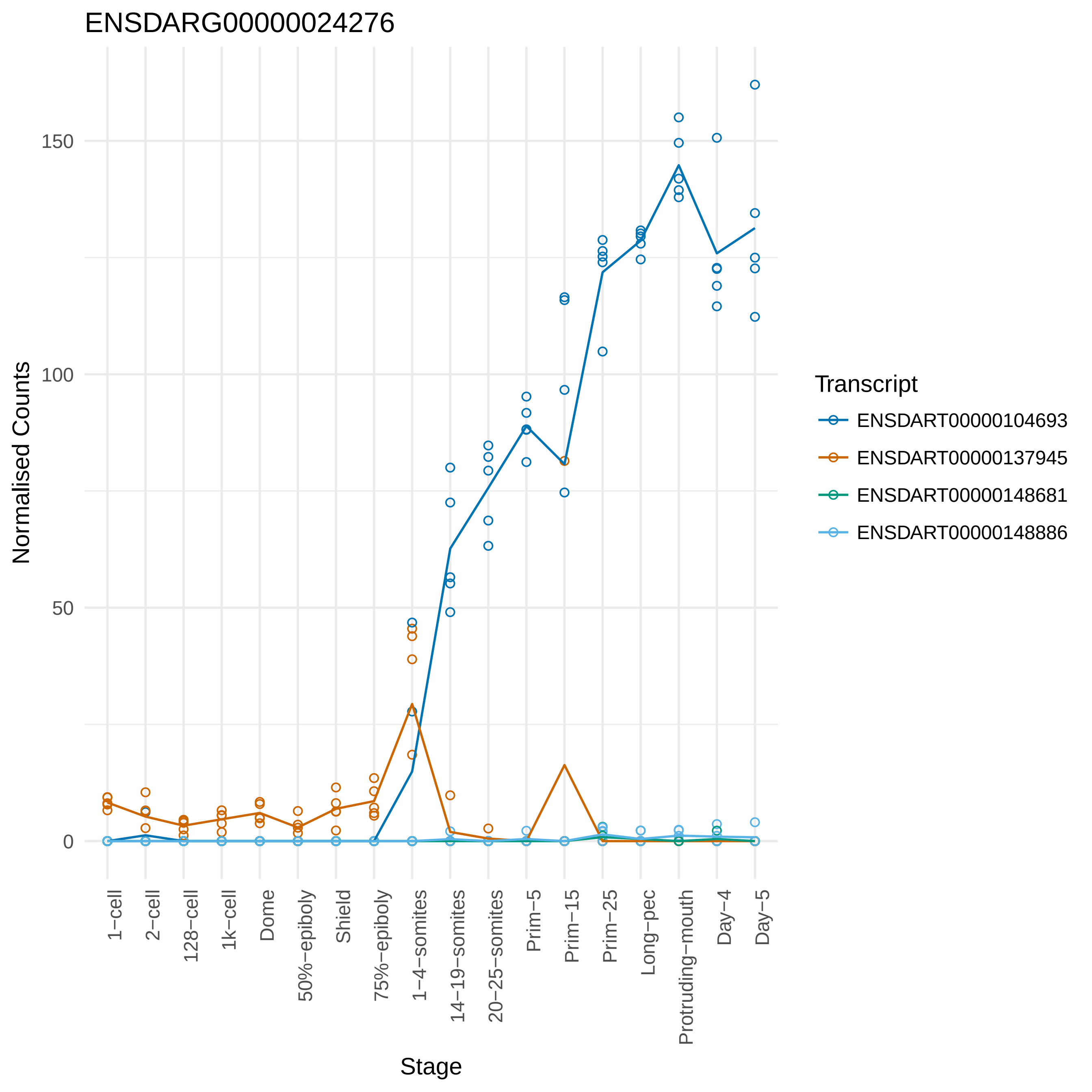

ENSDARG00000024276
Normalised Counts
150
100
50
0
1−cell
2−cell
128−cell
1k−cell
Dome
50%−epiboly
Shield
75%−epiboly
1−4−somites
14−19−somites
20−25−somites
Prim−5
Prim−15
Prim−25
Long−pec
Protruding−mouth
Day−4
Day−5
Stage
Transcript
ENSDART00000104693
ENSDART00000137945
ENSDART00000148681
ENSDART00000148886

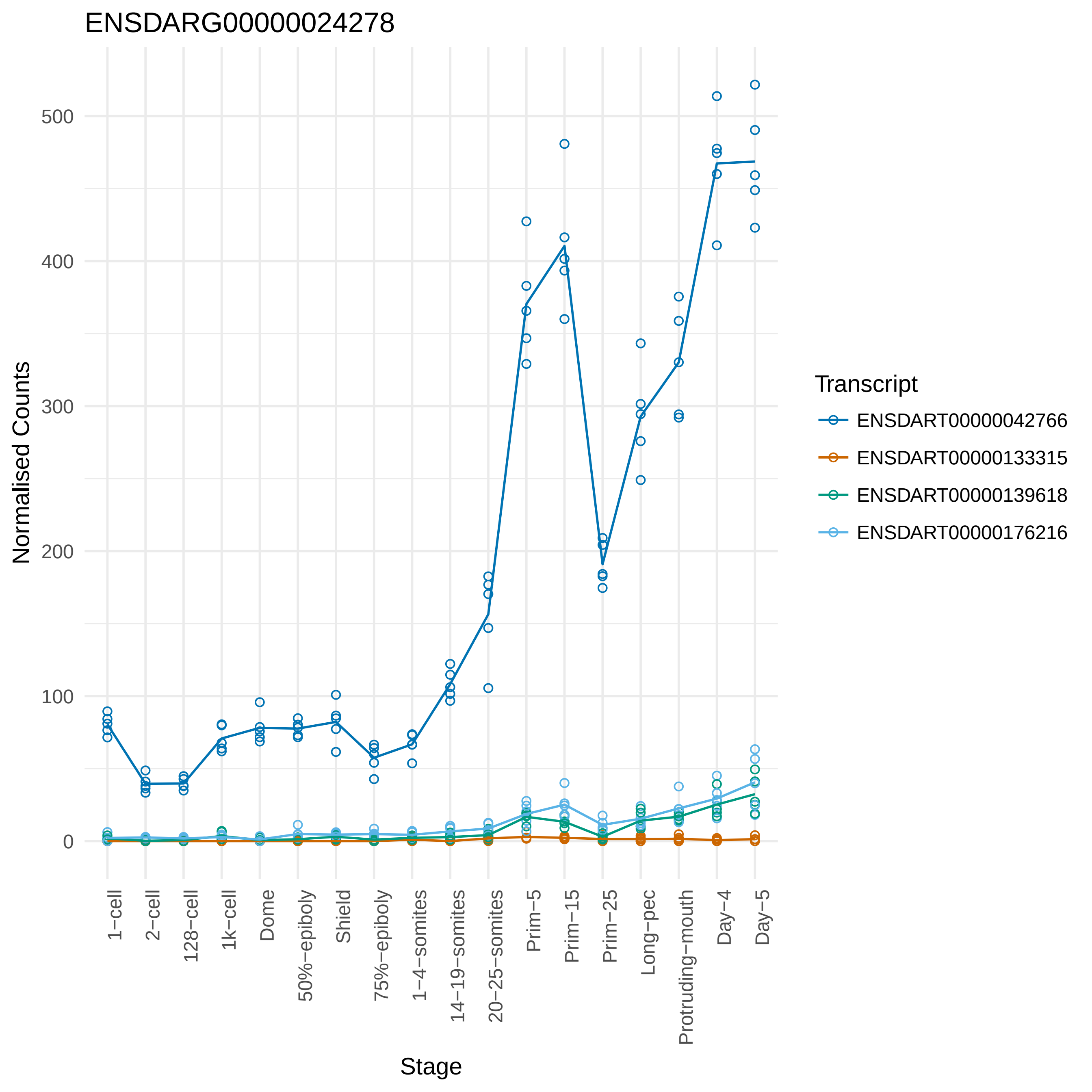
ENSDARG00000024278
Normalised Counts
0
100
200
300
400
500
Stage
1−cell
2−cell
128−cell
1k−cell
Dome
50%−epiboly
Shield
75%−epiboly
1−4−somites
14−19−somites
20−25−somites
Prim−5
Prim−15
Prim−25
Long−pec
Protruding−mouth
Day−4
Day−5
Transcript
ENSDART00000042766
ENSDART00000133315
ENSDART00000139618
ENSDART00000176216

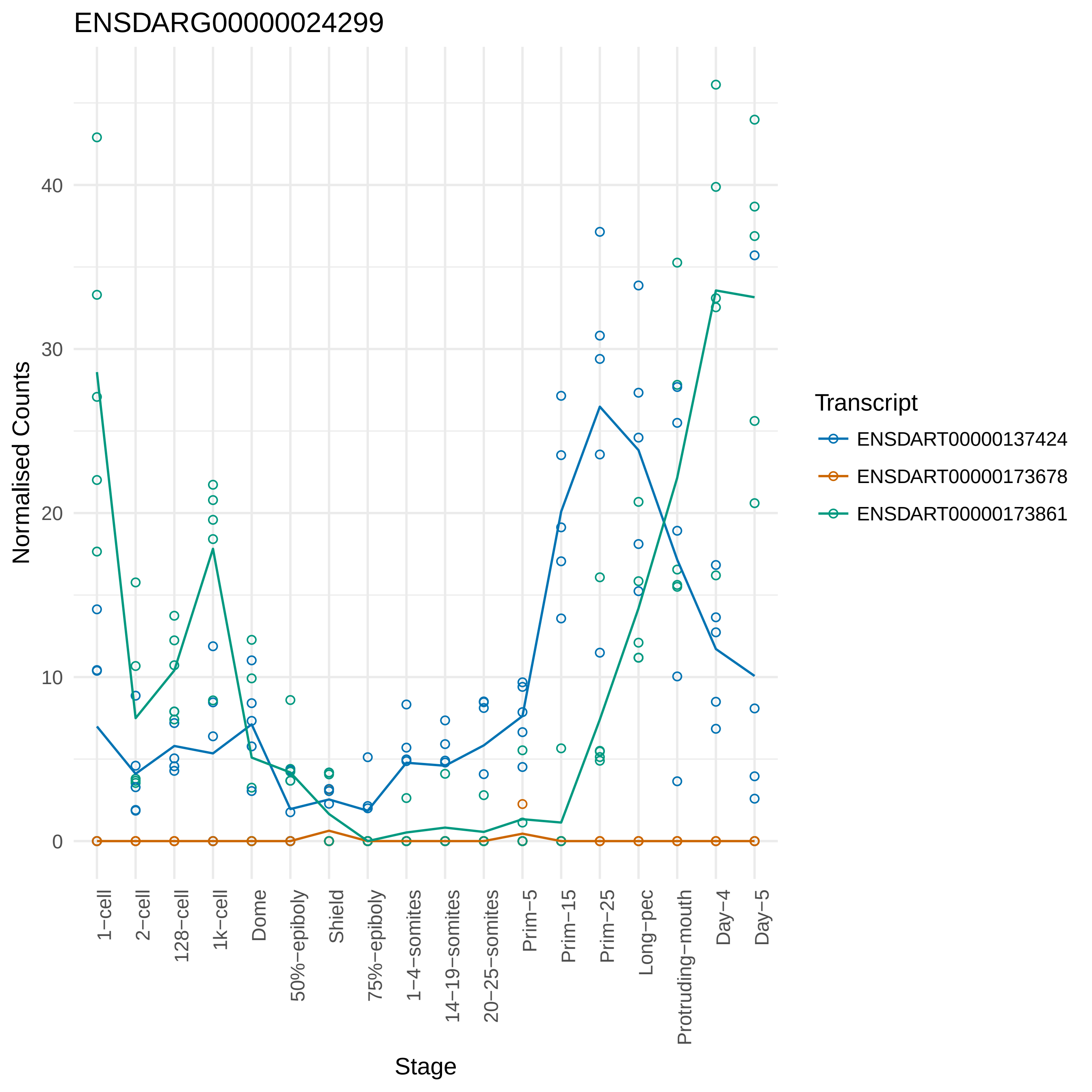

ENSDARG00000024299
Normalised Counts
0
10
20
30
40
1−cell
2−cell
128−cell
1k−cell
Dome
50%−epiboly
Shield
75%−epiboly
1−4−somites
14−19−somites
20−25−somites
Prim−5
Prim−15
Prim−25
Long−pec
Protruding−mouth
Day−4
Day−5
Stage
Transcript
ENSDART00000137424
ENSDART00000173678
ENSDART00000173861

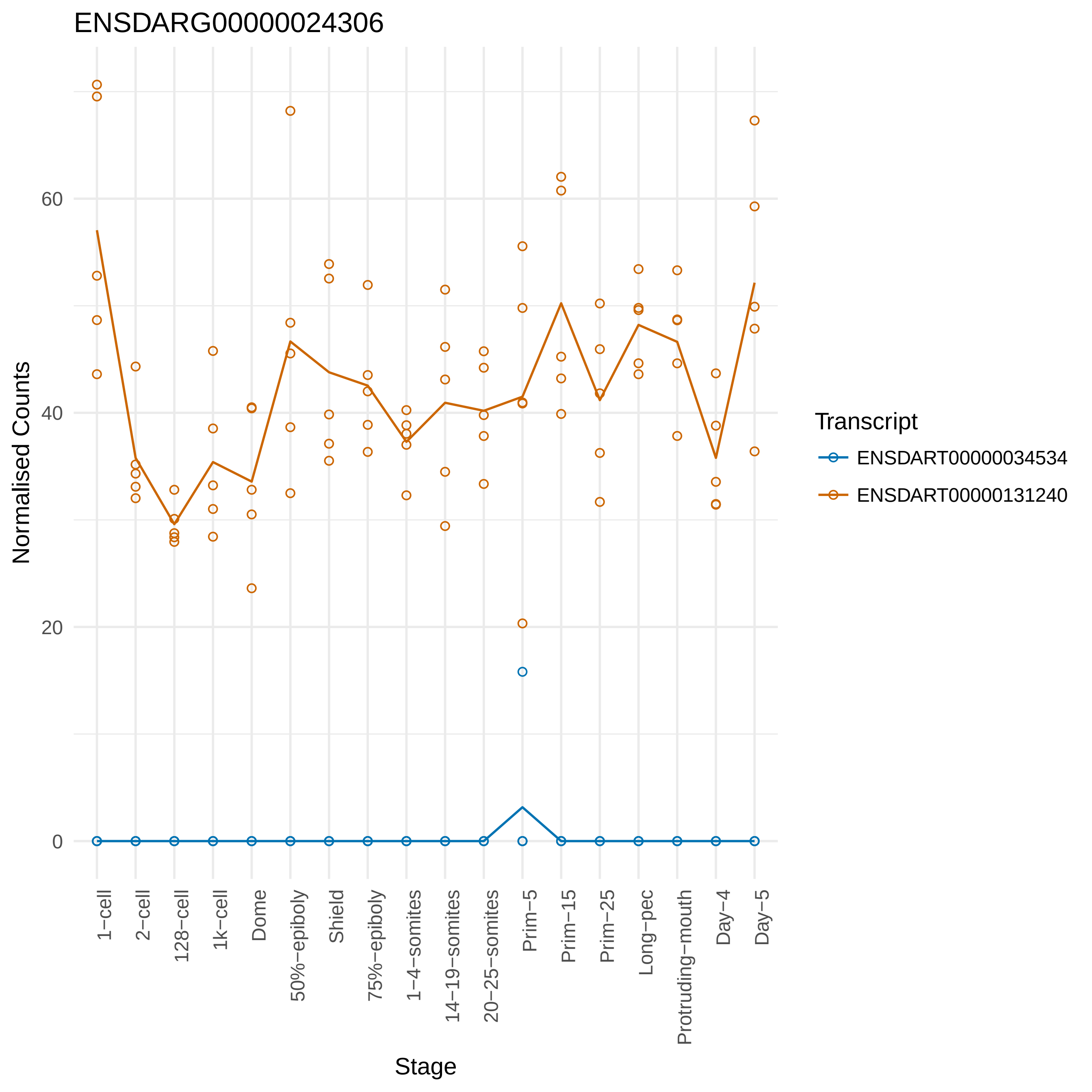
ENSDARG00000024306
Normalised Counts
0
20
40
60
Stage
1−cell
2−cell
128−cell
1k−cell
Dome
50%−epiboly
Shield
75%−epiboly
1−4−somites
14−19−somites
20−25−somites
Prim−5
Prim−15
Prim−25
Long−pec
Protruding−mouth
Day−4
Day−5
Transcript
ENSDART00000034534
ENSDART00000131240

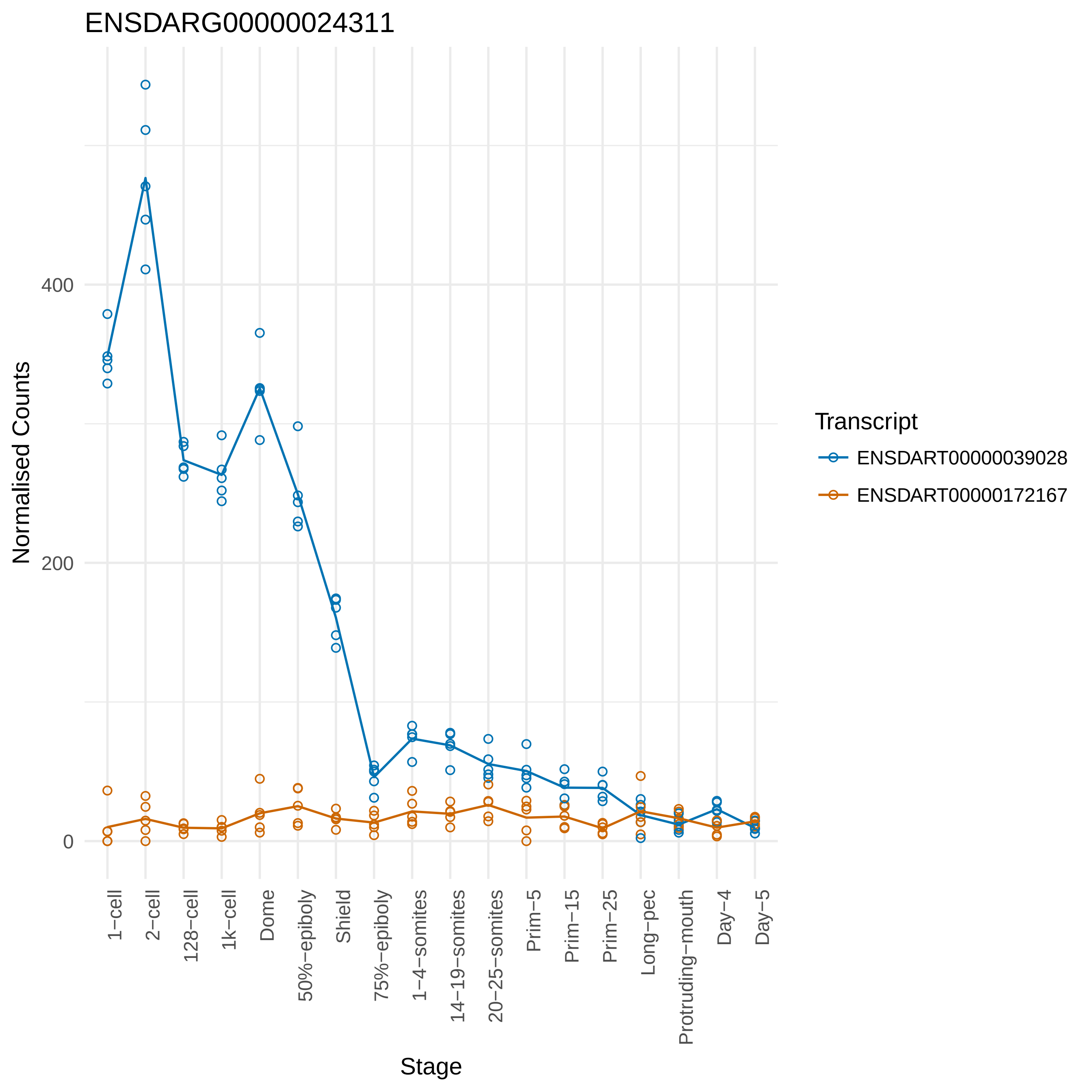

ENSDARG00000024311
Normalised Counts
400
200
0
1−cell
2−cell
128−cell
1k−cell
Dome
50%−epiboly
Shield
75%−epiboly
1−4−somites
14−19−somites
20−25−somites
Prim−5
Prim−15
Prim−25
Long−pec
Protruding−mouth
Day−4
Day−5
Stage
Transcript
ENSDART00000039028
ENSDART00000172167

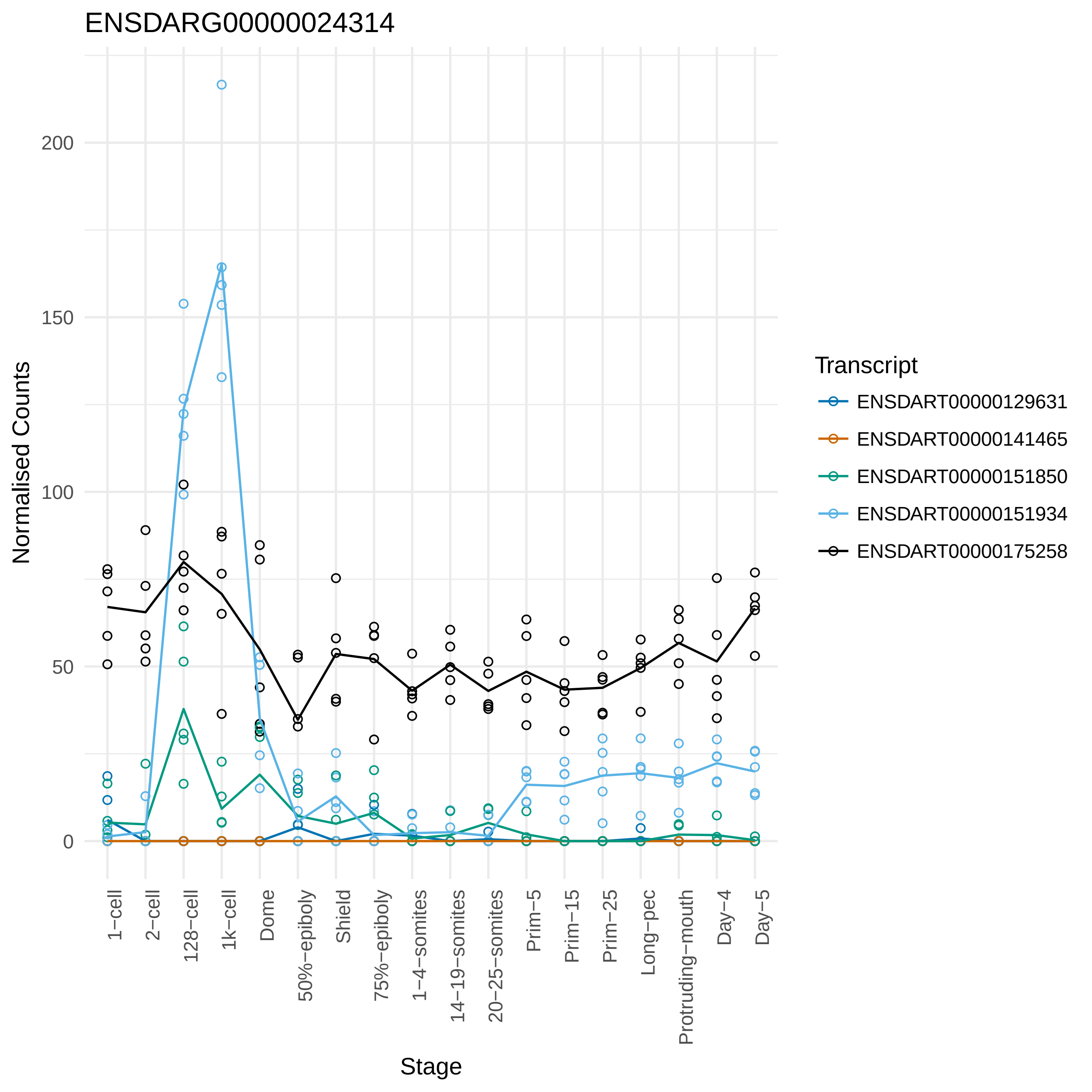

ENSDARG00000024314
Normalised Counts
0
50
100
150
200
Stage
1−cell
2−cell
128−cell
1k−cell
Dome
50%−epiboly
Shield
75%−epiboly
1−4−somites
14−19−somites
20−25−somites
Prim−5
Prim−15
Prim−25
Long−pec
Protruding−mouth
Day−4
Day−5
Transcript
ENSDART00000129631
ENSDART00000141465
ENSDART00000151850
ENSDART00000151934
ENSDART00000175258

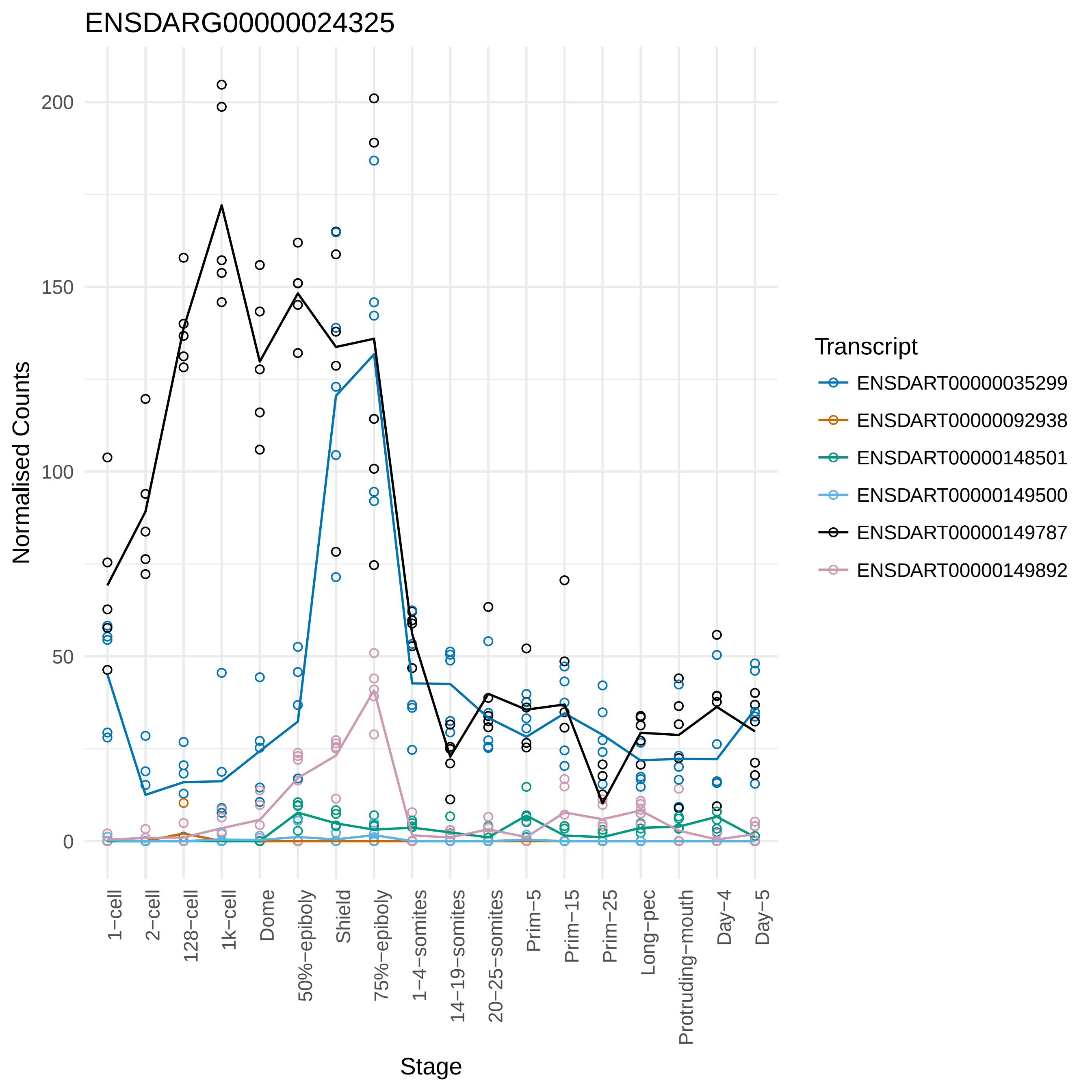
ENSDARG00000024325
Normalised Counts
200
150
100
50
0
1−cell
2−cell
128−cell
1k−cell
Dome
50%−epiboly
Shield
75%−epiboly
1−4−somites
14−19−somites
20−25−somites
Prim−5
Prim−15
Prim−25
Long−pec
Protruding−mouth
Day−4
Day−5
Stage
Transcript
ENSDART00000035299
ENSDART00000092938
ENSDART00000148501
ENSDART00000149500
ENSDART00000149787
ENSDART00000149892

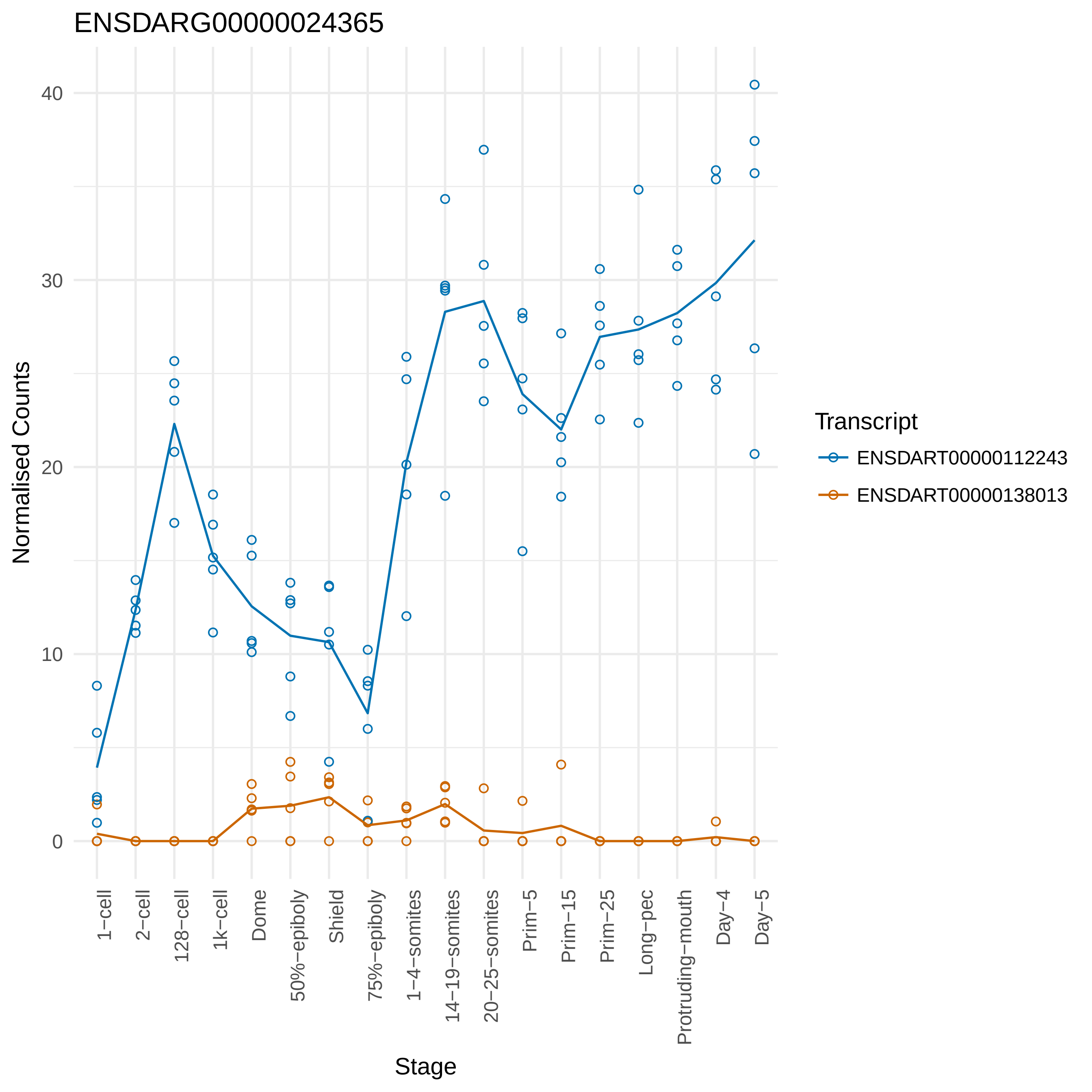

ENSDARG00000024365
Normalised Counts
0
10
20
30
40
1−cell
2−cell
128−cell
1k−cell
Dome
50%−epiboly
Shield
75%−epiboly
1−4−somites
14−19−somites
20−25−somites
Prim−5
Prim−15
Prim−25
Long−pec
Protruding−mouth
Day−4
Day−5
Stage
Transcript
ENSDART00000112243
ENSDART00000138013

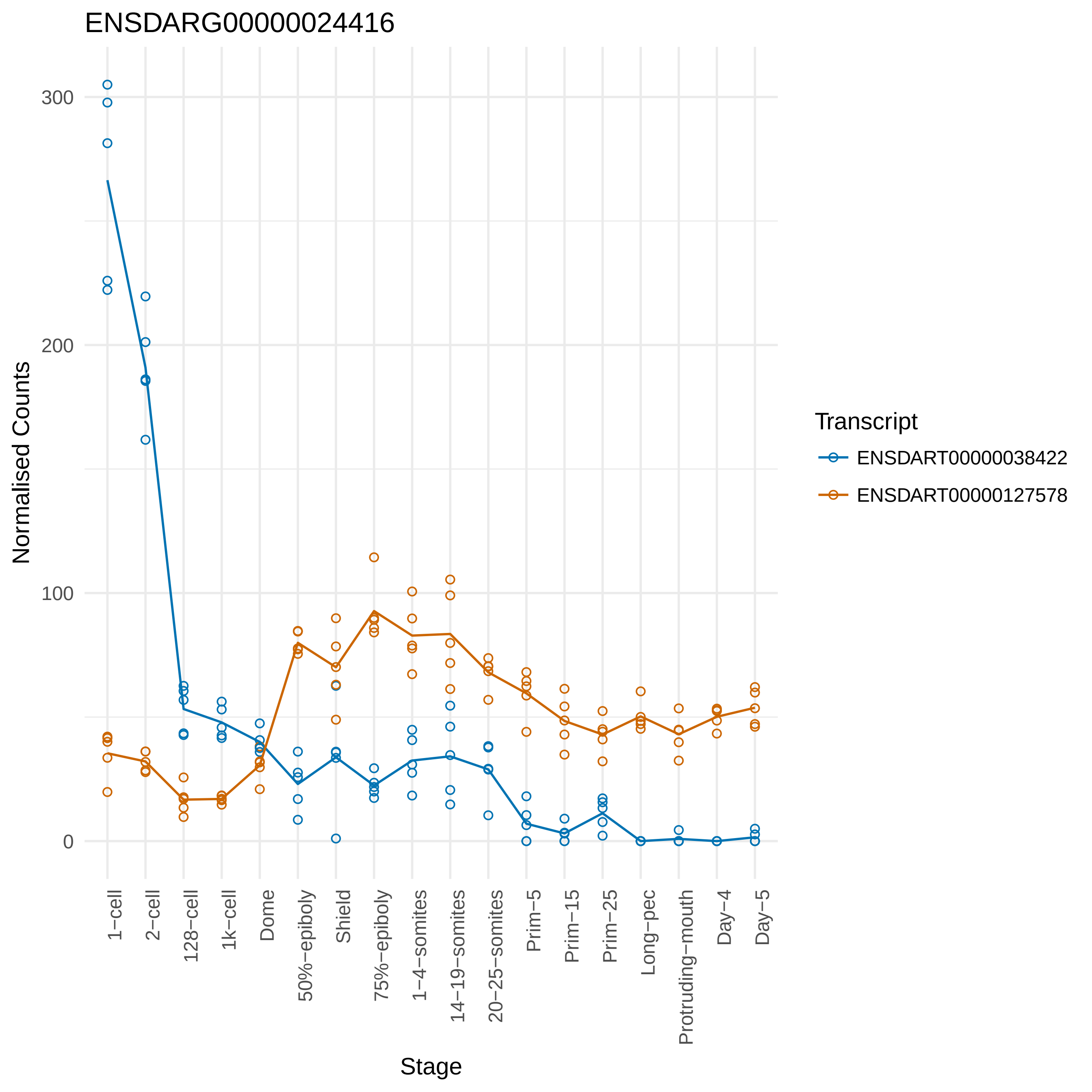
ENSDARG00000024416
Normalised Counts
300
200
100
0
1−cell
2−cell
128−cell
1k−cell
Dome
50%−epiboly
Shield
75%−epiboly
1−4−somites
14−19−somites
20−25−somites
Prim−5
Prim−15
Prim−25
Long−pec
Protruding−mouth
Day−4
Day−5
Stage
Transcript
ENSDART00000038422
ENSDART00000127578

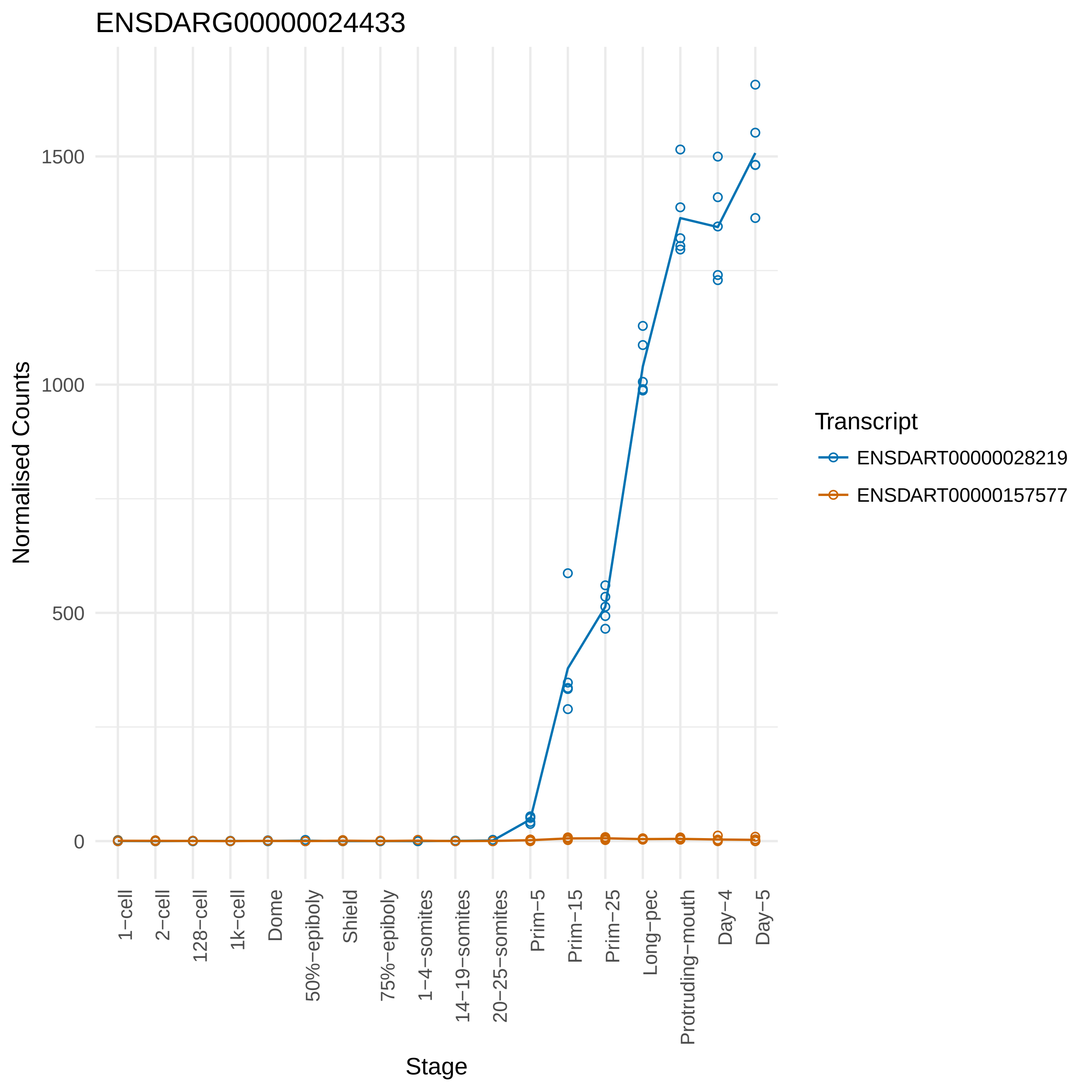

ENSDARG00000024433
Normalised Counts
1500
1000
500
0
1−cell
2−cell
128−cell
1k−cell
Dome
50%−epiboly
Shield
75%−epiboly
1−4−somites
14−19−somites
20−25−somites
Prim−5
Prim−15
Prim−25
Long−pec
Protruding−mouth
Day−4
Day−5
Stage
Transcript
ENSDART00000028219
ENSDART00000157577

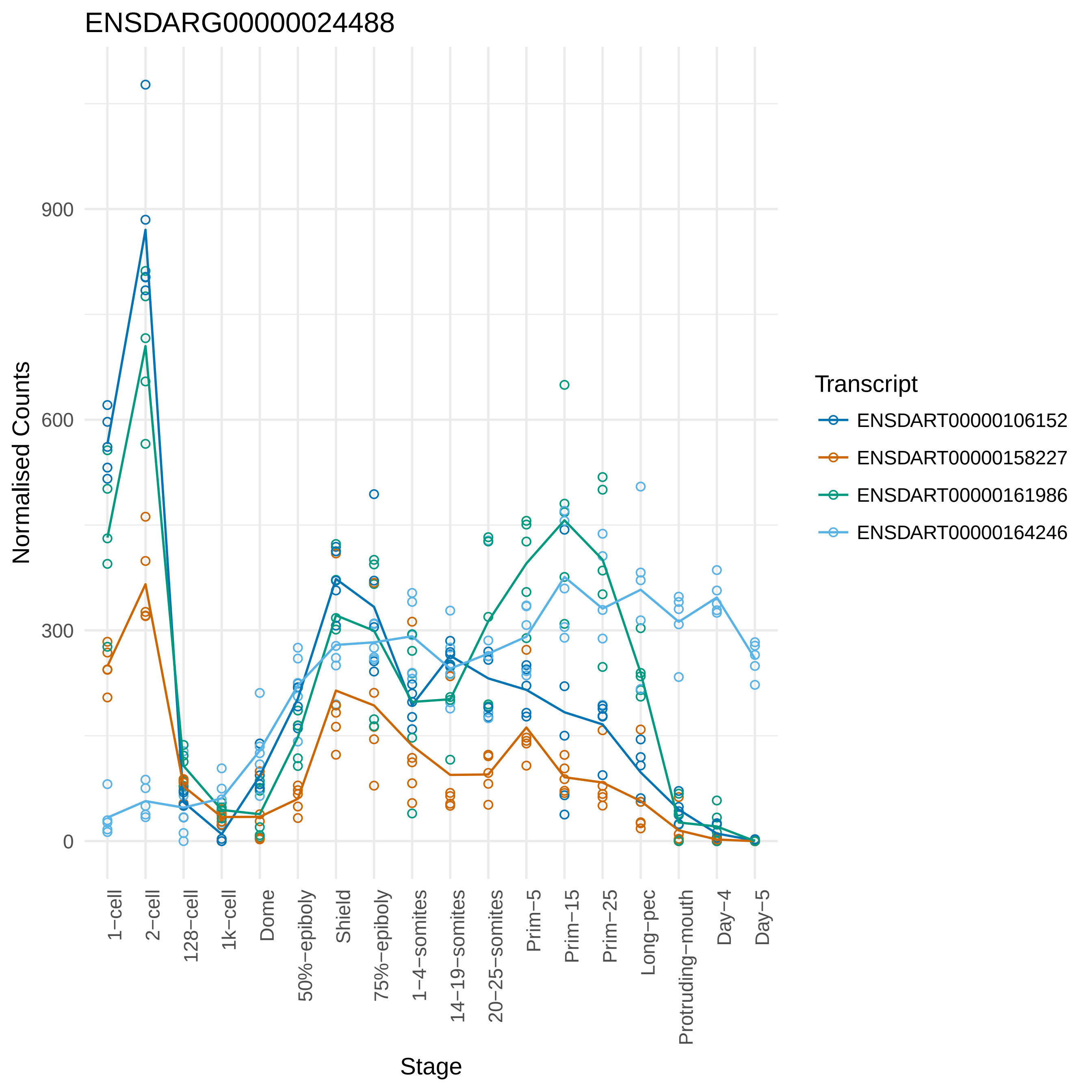

ENSDARG00000024488
Normalised Counts
900
600
300
0
1−cell
2−cell
128−cell
1k−cell
Dome
50%−epiboly
Shield
75%−epiboly
1−4−somites
14−19−somites
20−25−somites
Prim−5
Prim−15
Prim−25
Long−pec
Protruding−mouth
Day−4
Day−5
Stage
Transcript
ENSDART00000106152
ENSDART00000158227
ENSDART00000161986
ENSDART00000164246

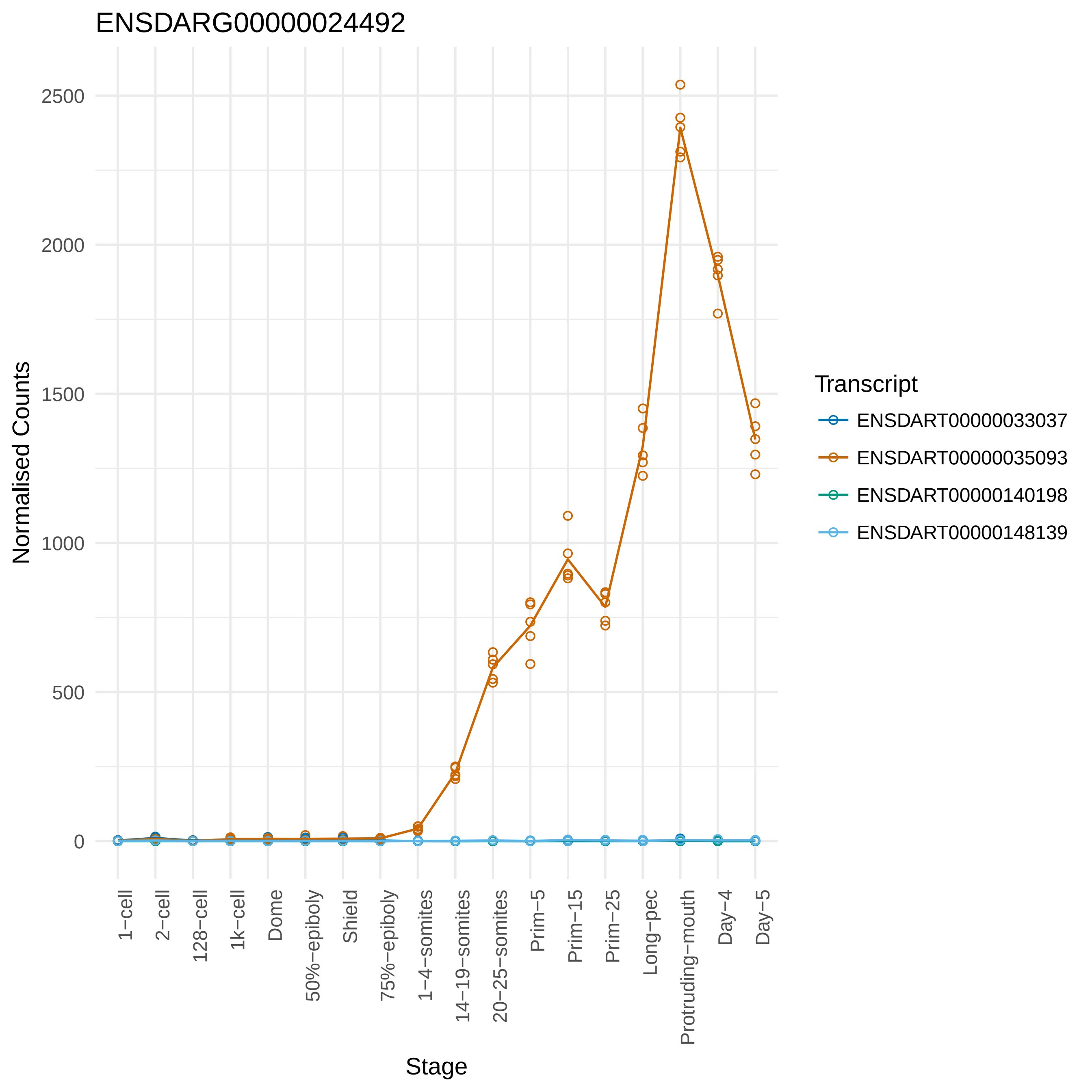

ENSDARG00000024492
Normalised Counts
0
500
1000
1500
2000
2500
Stage
1−cell
2−cell
128−cell
1k−cell
Dome
50%−epiboly
Shield
75%−epiboly
1−4−somites
14−19−somites
20−25−somites
Prim−5
Prim−15
Prim−25
Long−pec
Protruding−mouth
Day−4
Day−5
Transcript
ENSDART00000033037
ENSDART00000035093
ENSDART00000140198
ENSDART00000148139

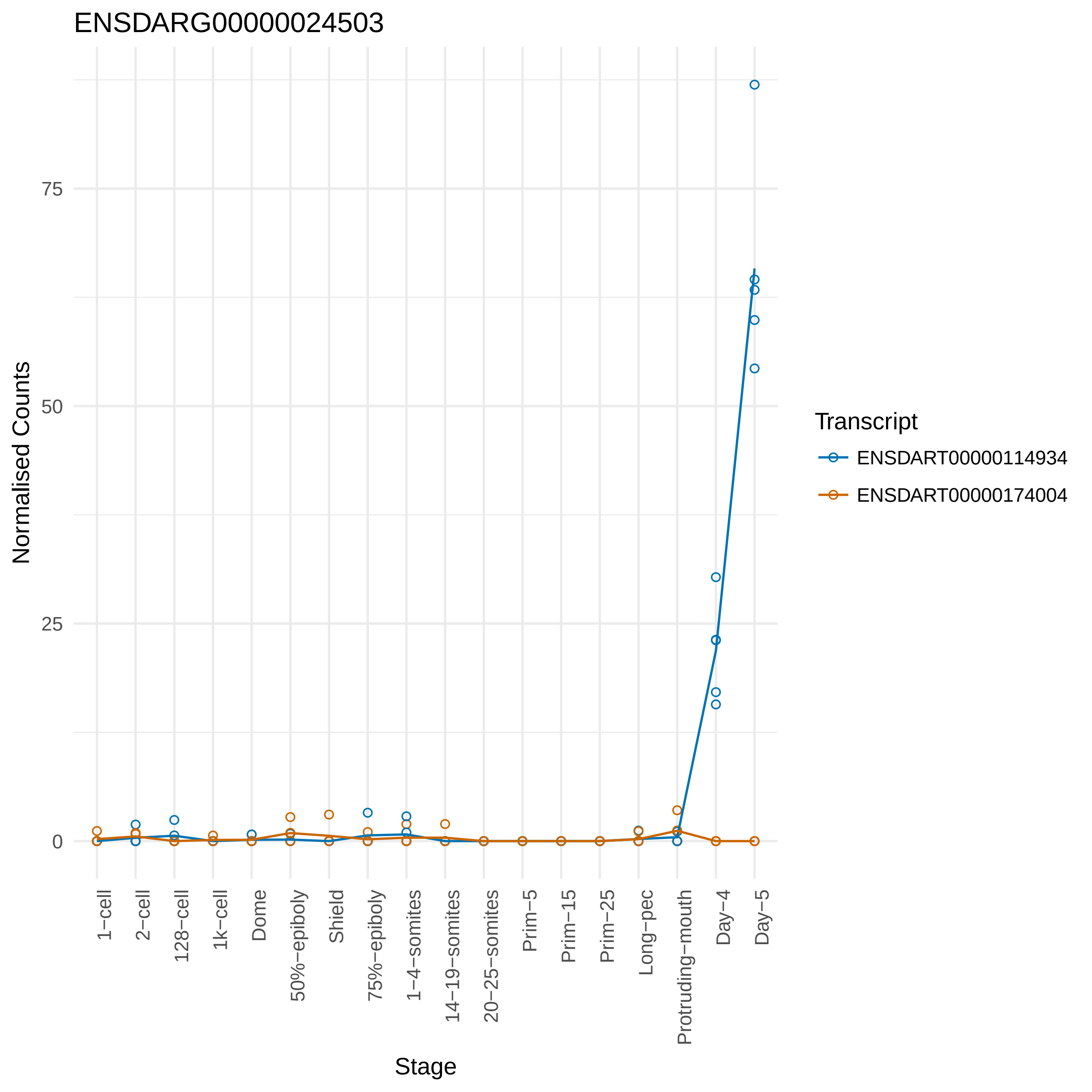

ENSDARG00000024503
Normalised Counts
0
25
50
75
Stage
1−cell
2−cell
128−cell
1k−cell
Dome
50%−epiboly
Shield
75%−epiboly
1−4−somites
14−19−somites
20−25−somites
Prim−5
Prim−15
Prim−25
Long−pec
Protruding−mouth
Day−4
Day−5
Transcript
ENSDART00000114934
ENSDART00000174004

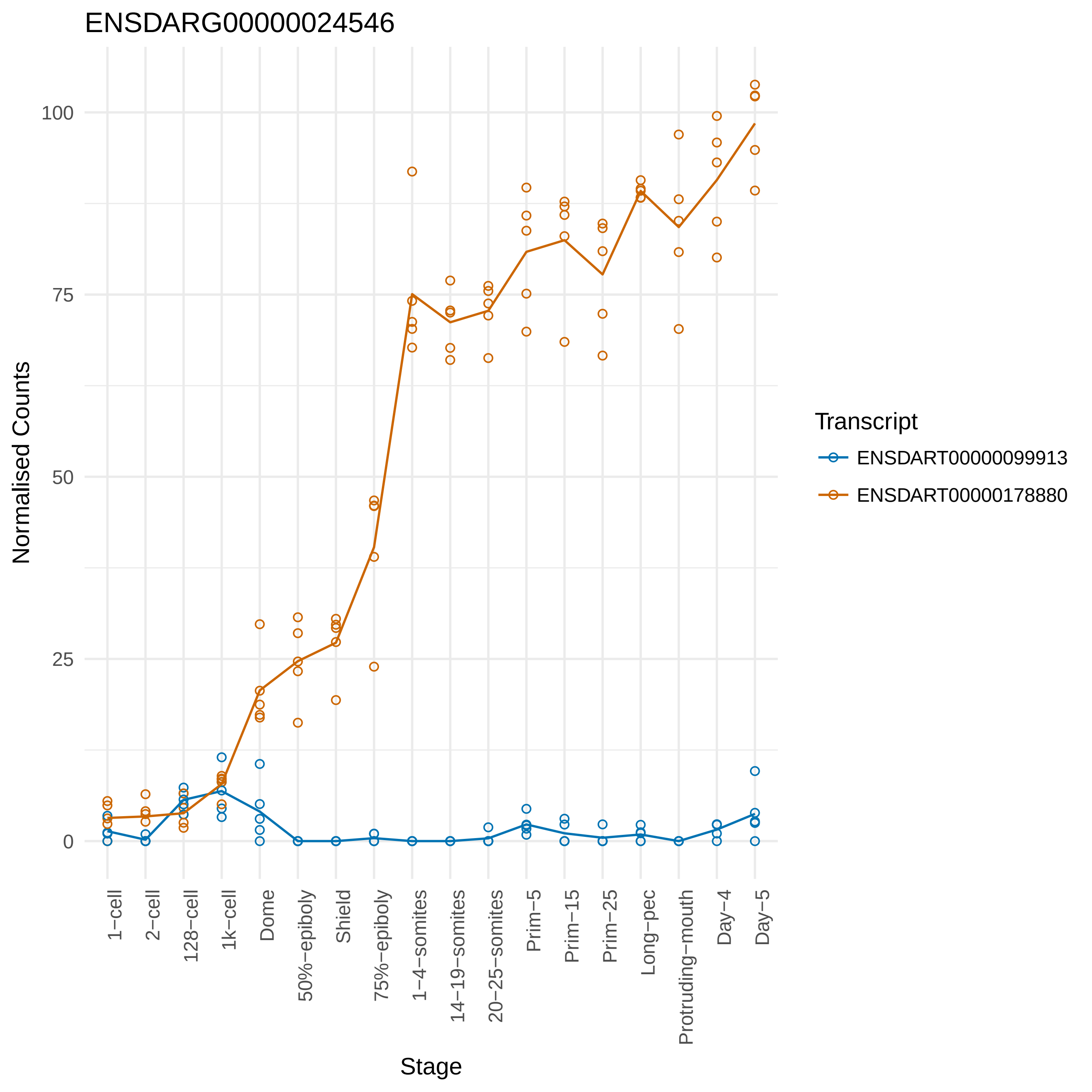
ENSDARG00000024546
Normalised Counts
100
75
50
25
0
1−cell
2−cell
128−cell
1k−cell
Dome
50%−epiboly
Shield
75%−epiboly
1−4−somites
14−19−somites
20−25−somites
Prim−5
Prim−15
Prim−25
Long−pec
Protruding−mouth
Day−4
Day−5
Stage
Transcript
ENSDART00000099913
ENSDART00000178880

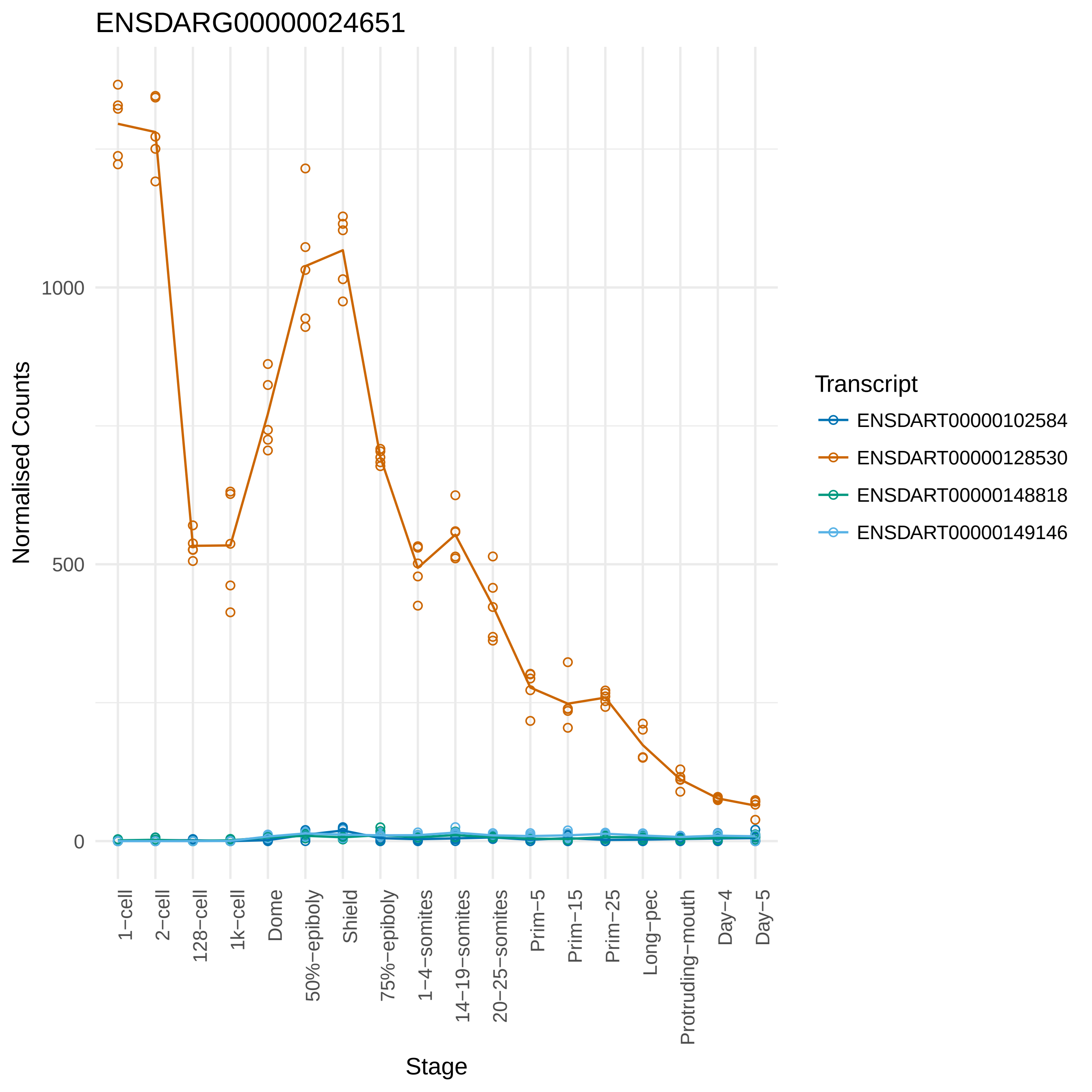

ENSDARG00000024651
Normalised Counts
1000
500
0
1−cell
2−cell
128−cell
1k−cell
Dome
50%−epiboly
Shield
75%−epiboly
1−4−somites
14−19−somites
20−25−somites
Prim−5
Prim−15
Prim−25
Long−pec
Protruding−mouth
Day−4
Day−5
Stage
Transcript
ENSDART00000102584
ENSDART00000128530
ENSDART00000148818
ENSDART00000149146

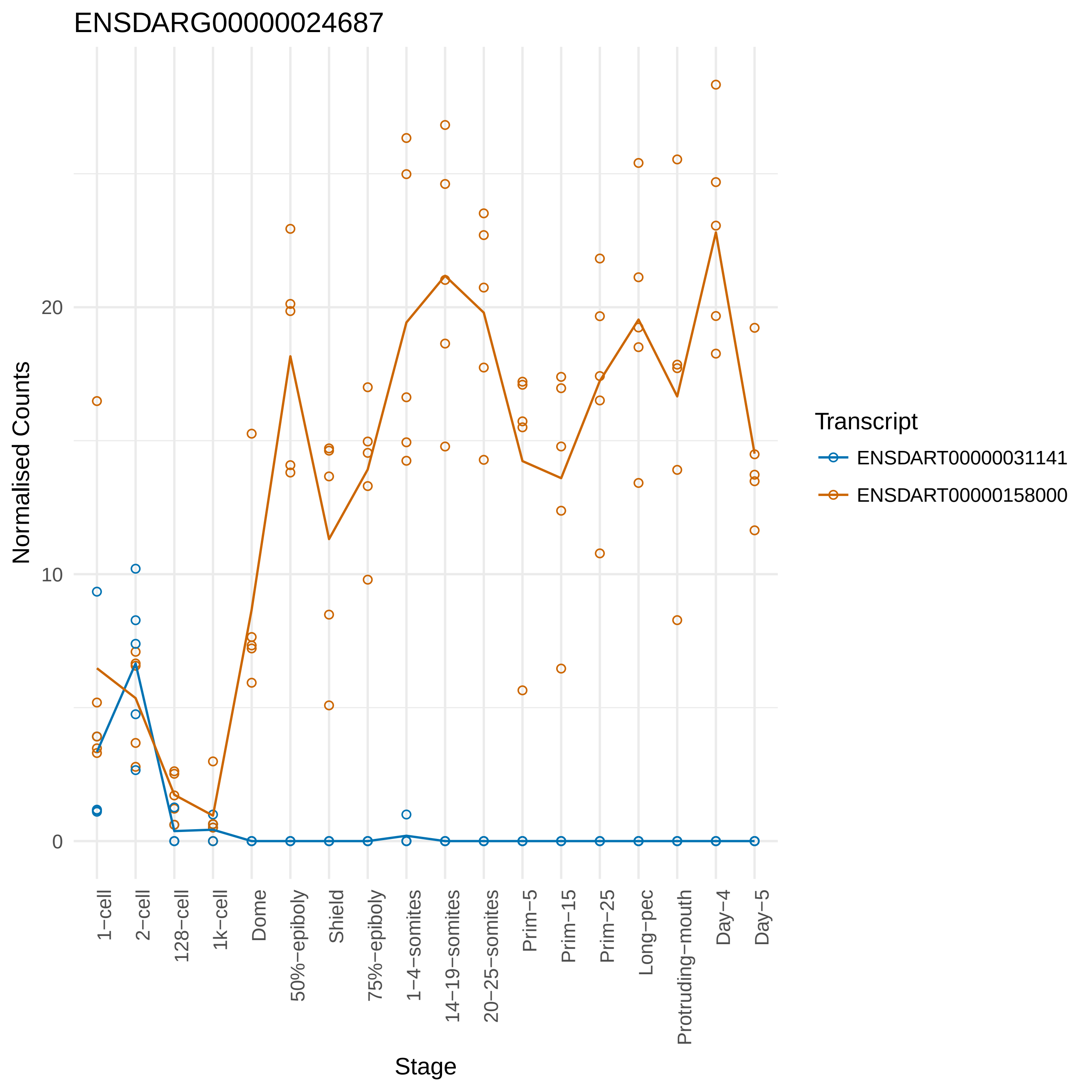

ENSDARG00000024687
Normalised Counts
20
10
0
1−cell
2−cell
128−cell
1k−cell
Dome
50%−epiboly
Shield
75%−epiboly
1−4−somites
14−19−somites
20−25−somites
Prim−5
Prim−15
Prim−25
Long−pec
Protruding−mouth
Day−4
Day−5
Stage
Transcript
ENSDART00000031141
ENSDART00000158000

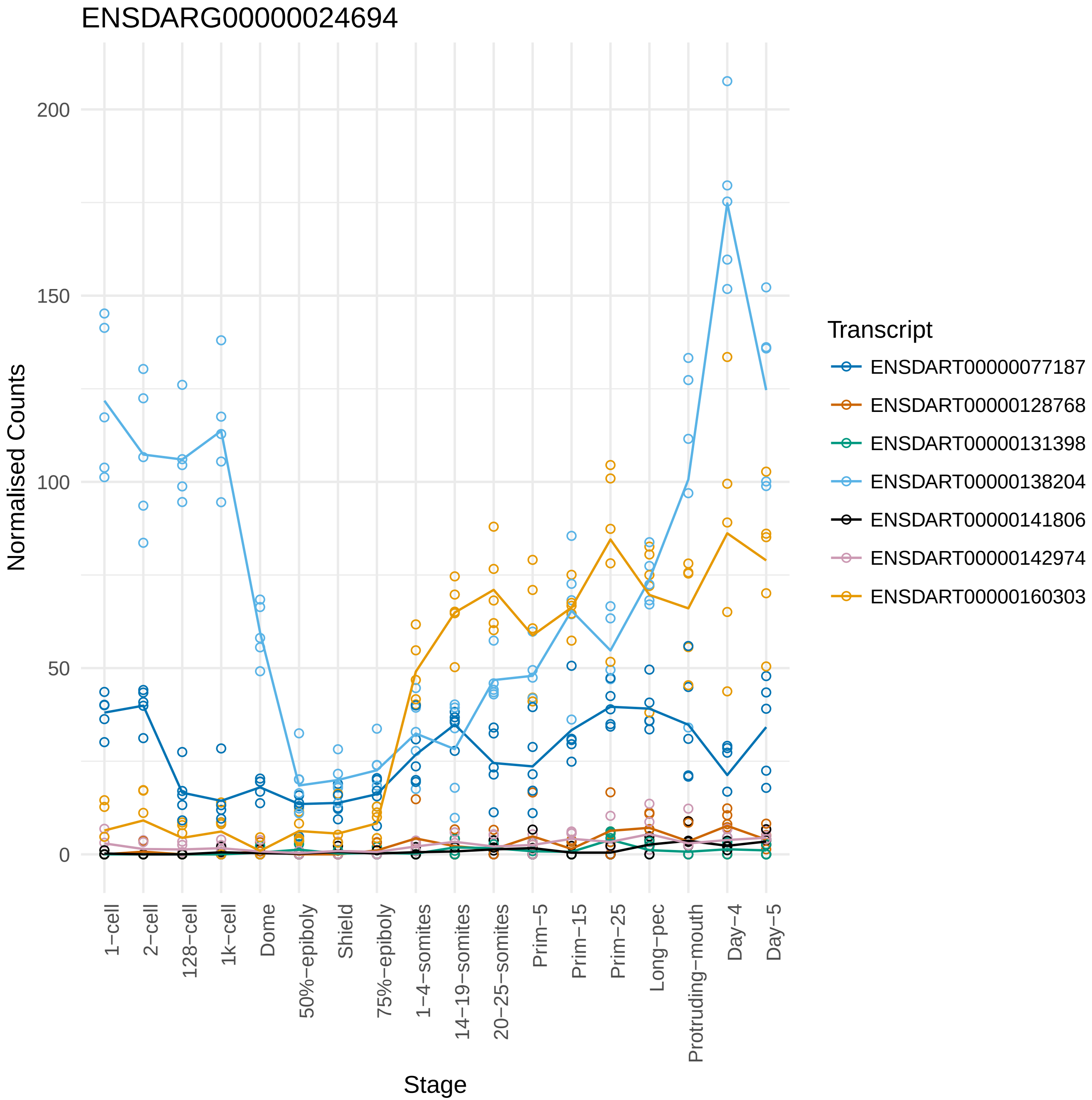

ENSDARG00000024694
Normalised Counts
200
150
100
50
0
1−cell
2−cell
128−cell
1k−cell
Dome
50%−epiboly
Shield
75%−epiboly
1−4−somites
14−19−somites
20−25−somites
Prim−5
Prim−15
Prim−25
Long−pec
Protruding−mouth
Day−4
Day−5
Stage
Transcript
ENSDART00000077187
ENSDART00000128768
ENSDART00000131398
ENSDART00000138204
ENSDART00000141806
ENSDART00000142974
ENSDART00000160303

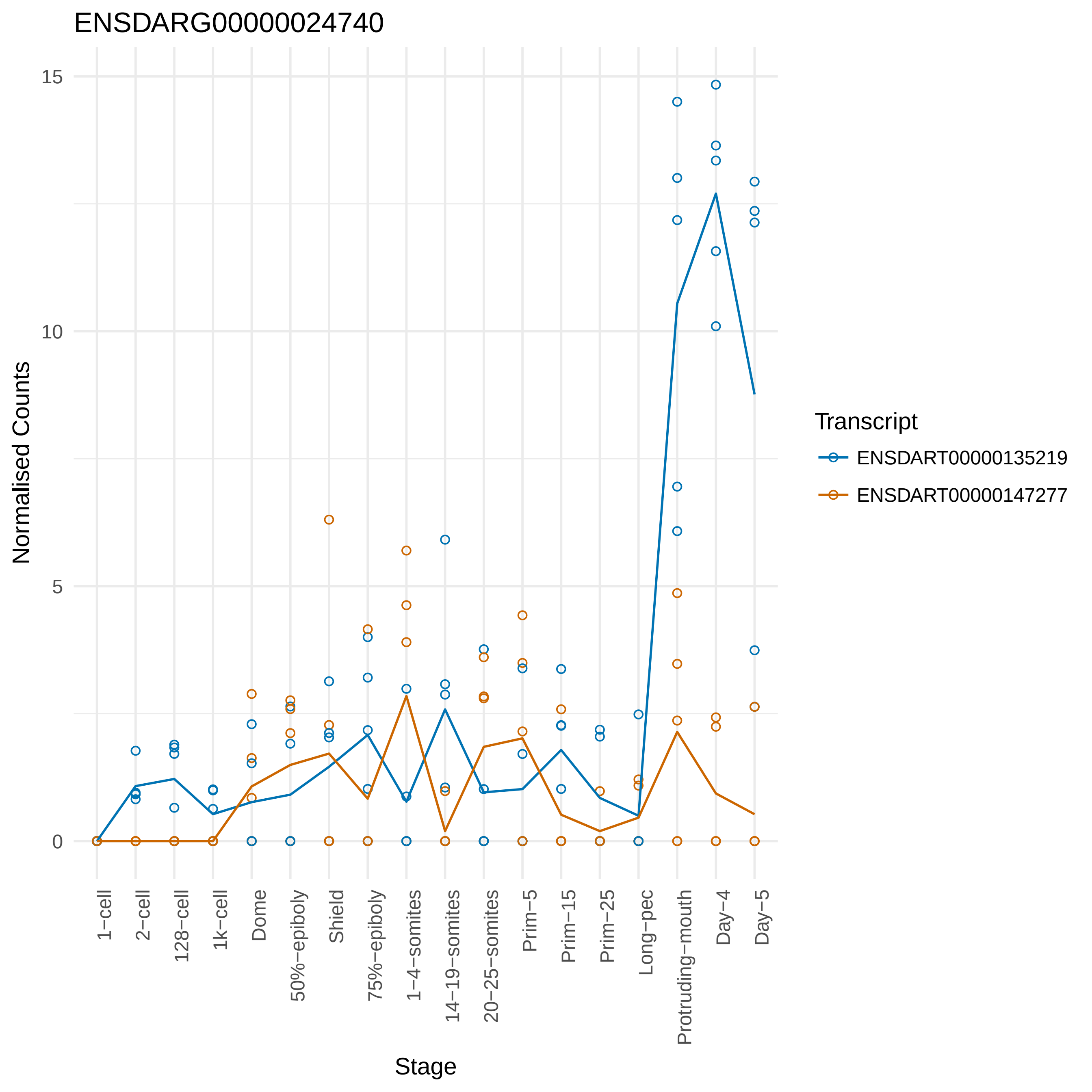

ENSDARG00000024740
Normalised Counts
15
10
5
0
Stage
1−cell
2−cell
128−cell
1k−cell
Dome
50%−epiboly
Shield
75%−epiboly
1−4−somites
14−19−somites
20−25−somites
Prim−5
Prim−15
Prim−25
Long−pec
Protruding−mouth
Day−4
Day−5
Transcript
ENSDART00000135219
ENSDART00000147277

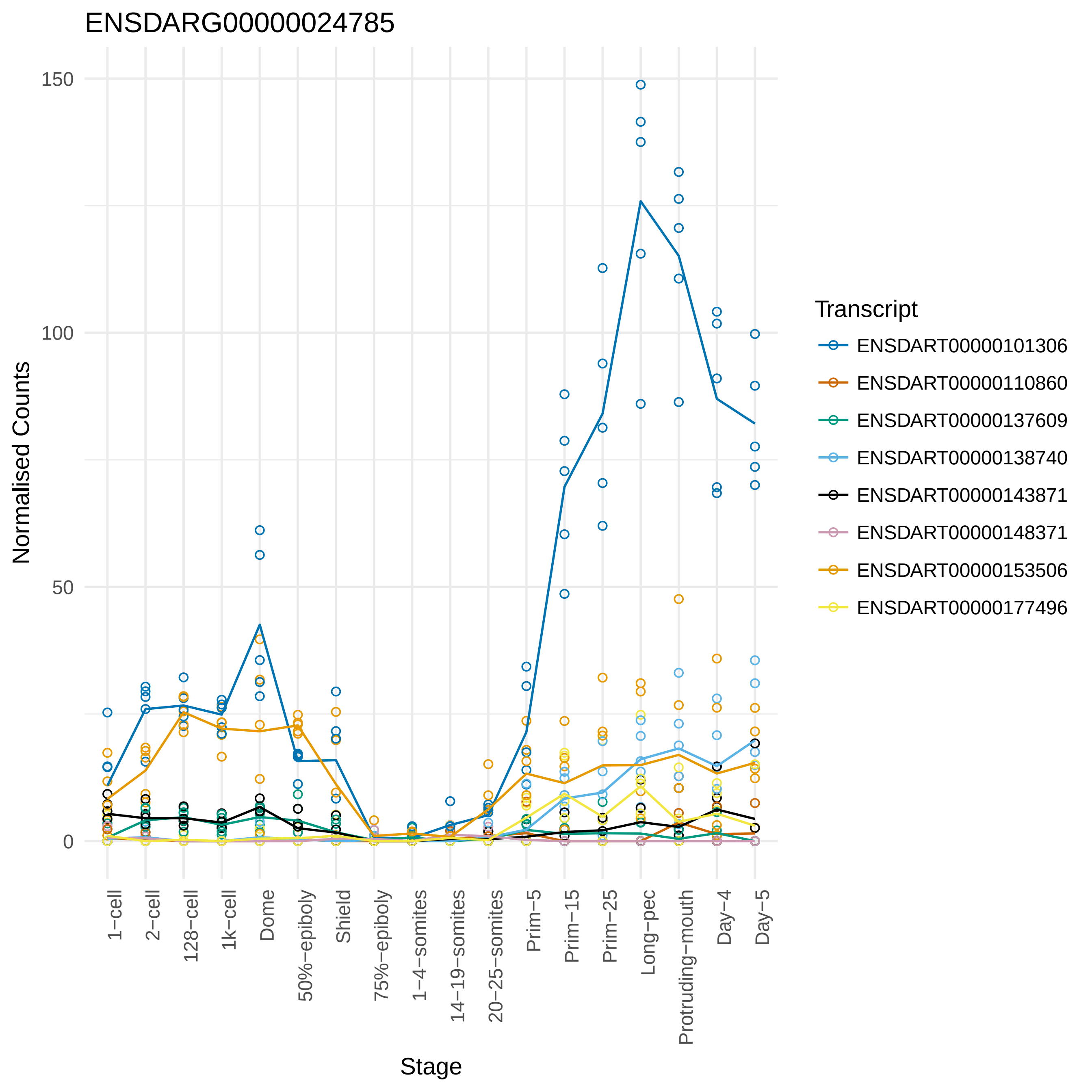

ENSDARG00000024785
Normalised Counts
150
100
50
0
1−cell
2−cell
128−cell
1k−cell
Dome
50%−epiboly
Shield
75%−epiboly
1−4−somites
14−19−somites
20−25−somites
Prim−5
Prim−15
Prim−25
Long−pec
Protruding−mouth
Day−4
Day−5
Stage
Transcript
ENSDART00000101306
ENSDART00000110860
ENSDART00000137609
ENSDART00000138740
ENSDART00000143871
ENSDART00000148371
ENSDART00000153506
ENSDART00000177496

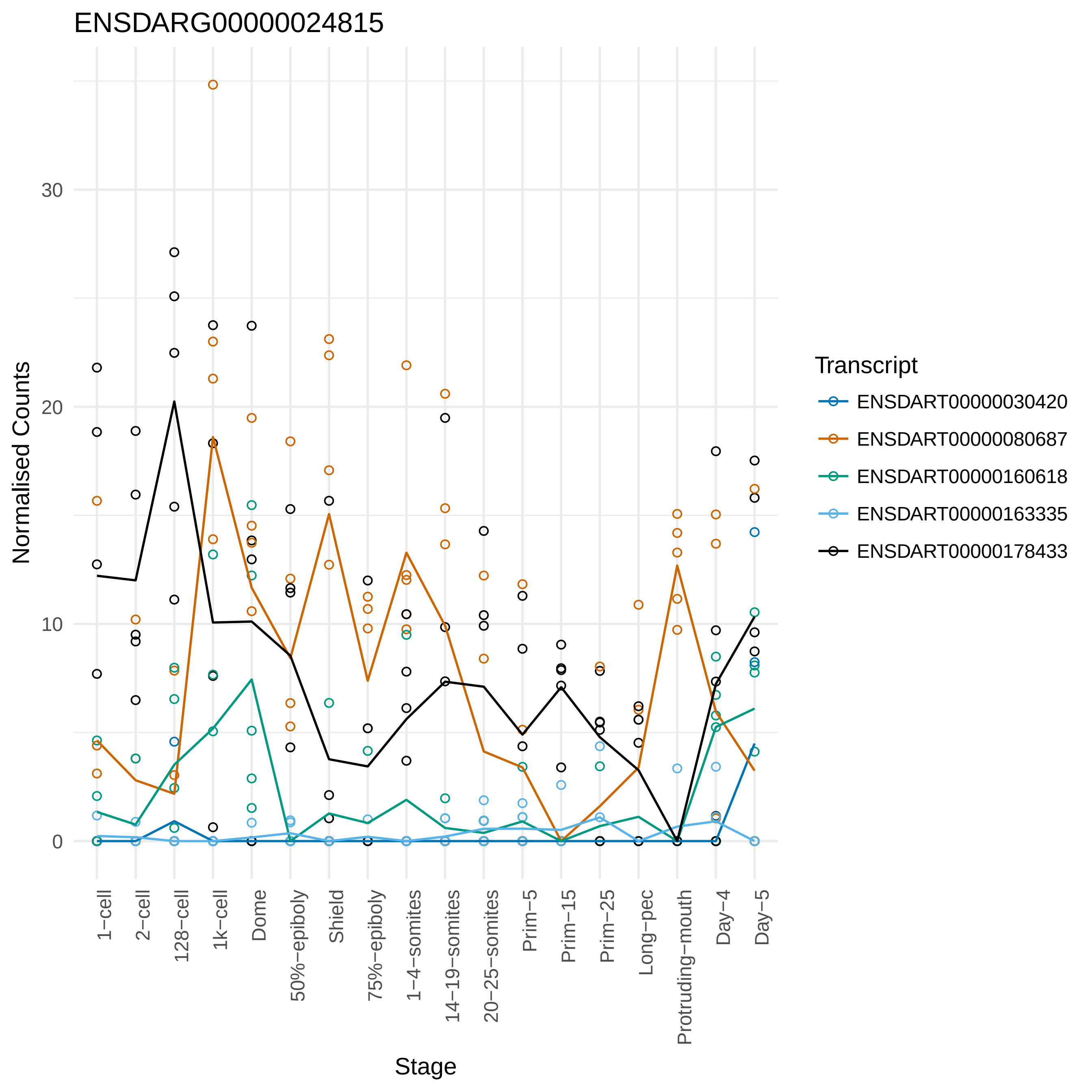

ENSDARG00000024815
Normalised Counts
0
10
20
30
Stage
1−cell
2−cell
128−cell
1k−cell
Dome
50%−epiboly
Shield
75%−epiboly
1−4−somites
14−19−somites
20−25−somites
Prim−5
Prim−15
Prim−25
Long−pec
Protruding−mouth
Day−4
Day−5
Transcript
ENSDART00000030420
ENSDART00000080687
ENSDART00000160618
ENSDART00000163335
ENSDART00000178433

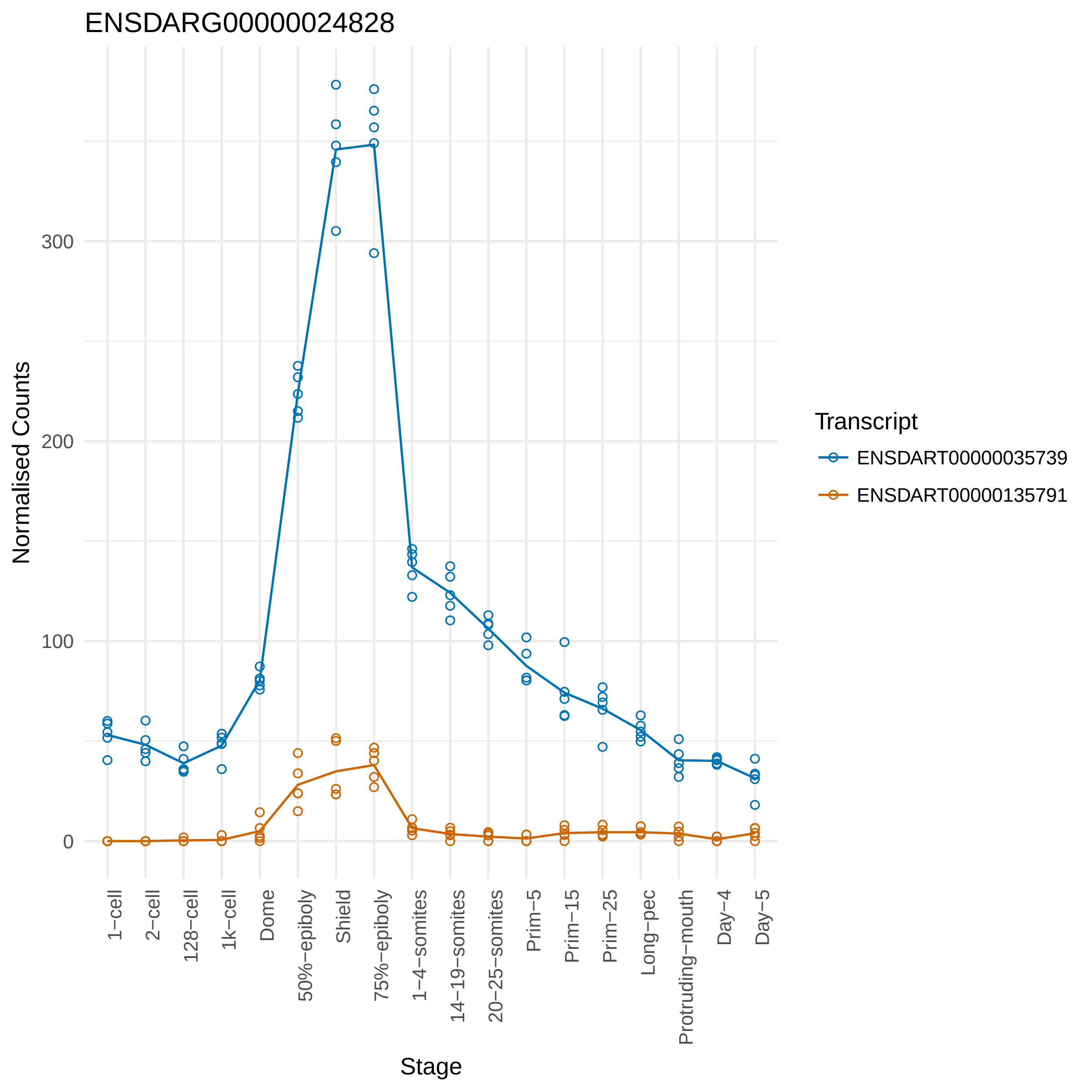
ENSDARG00000024828
Normalised Counts
300
200
100
0
1−cell
2−cell
128−cell
1k−cell
Dome
50%−epiboly
Shield
75%−epiboly
1−4−somites
14−19−somites
20−25−somites
Prim−5
Prim−15
Prim−25
Long−pec
Protruding−mouth
Day−4
Day−5
Stage
Transcript
ENSDART00000035739
ENSDART00000135791

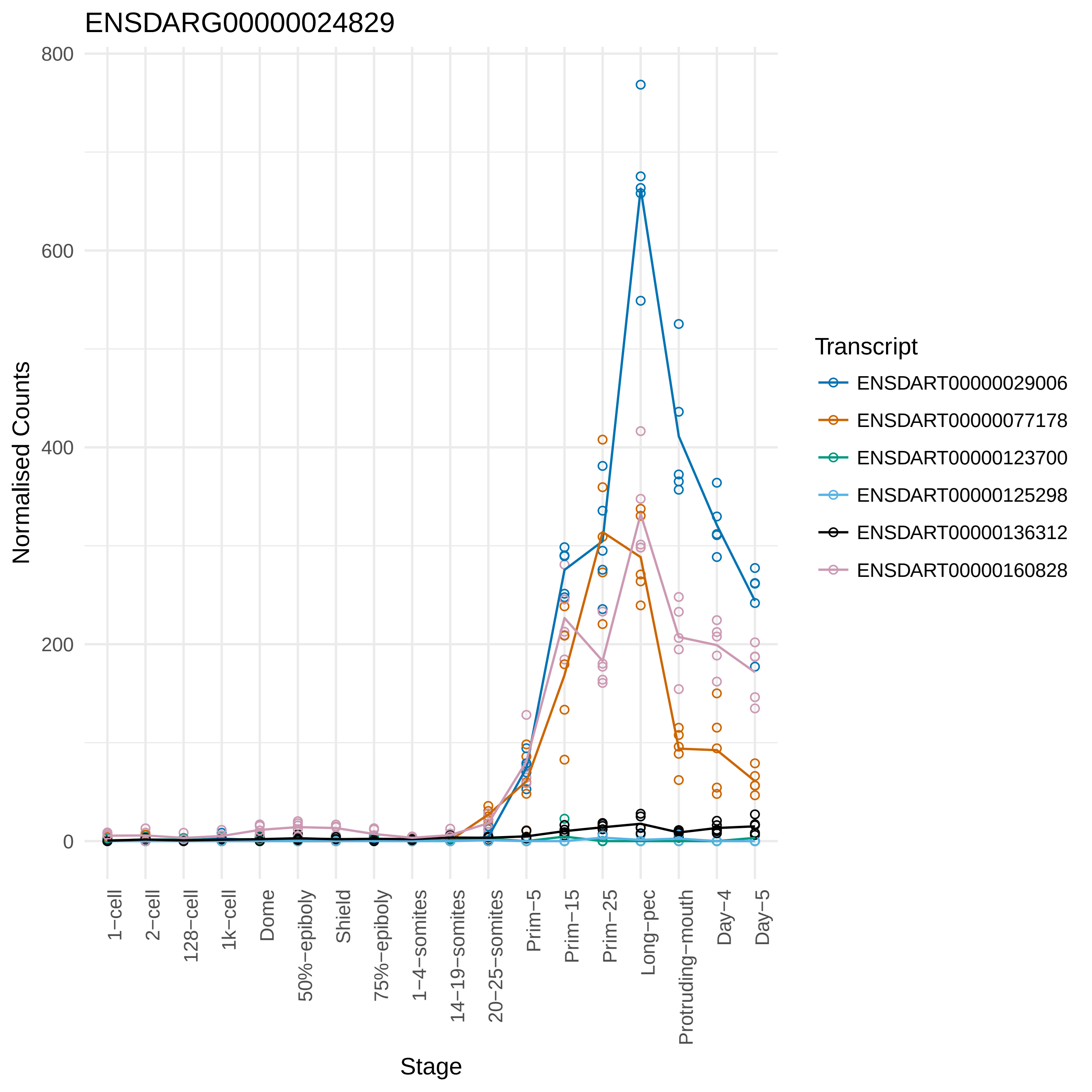
ENSDARG00000024829
Normalised Counts
800
600
400
200
0
1−cell
2−cell
128−cell
1k−cell
Dome
50%−epiboly
Shield
75%−epiboly
1−4−somites
14−19−somites
20−25−somites
Prim−5
Prim−15
Prim−25
Long−pec
Protruding−mouth
Day−4
Day−5
Stage
Transcript
ENSDART00000029006
ENSDART00000077178
ENSDART00000123700
ENSDART00000125298
ENSDART00000136312
ENSDART00000160828

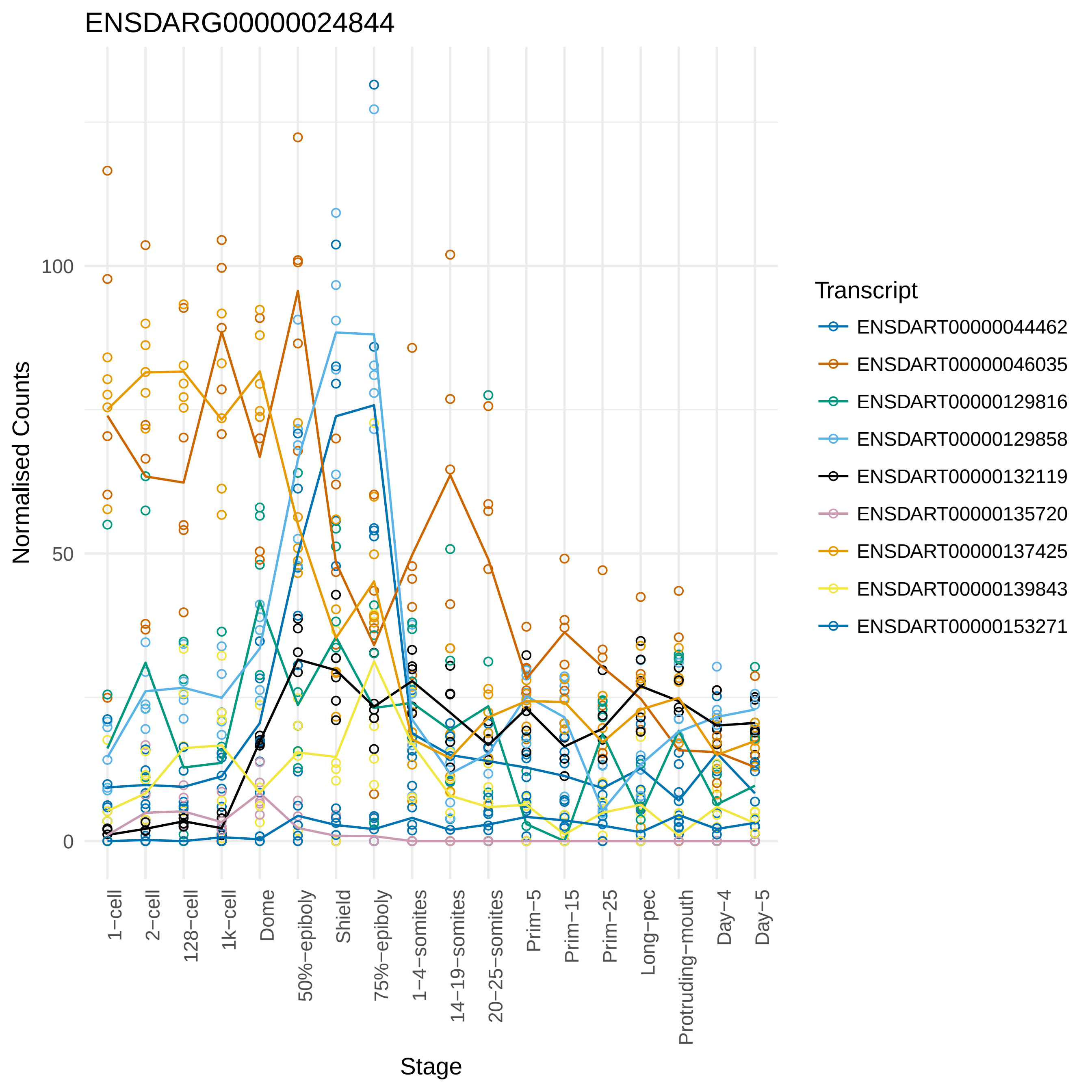

ENSDARG00000024844
Normalised Counts
100
50
0
1−cell
2−cell
128−cell
1k−cell
Dome
50%−epiboly
Shield
75%−epiboly
1−4−somites
14−19−somites
20−25−somites
Prim−5
Prim−15
Prim−25
Long−pec
Protruding−mouth
Day−4
Day−5
Stage
Transcript
ENSDART00000044462
ENSDART00000046035
ENSDART00000129816
ENSDART00000129858
ENSDART00000132119
ENSDART00000135720
ENSDART00000137425
ENSDART00000139843
ENSDART00000153271

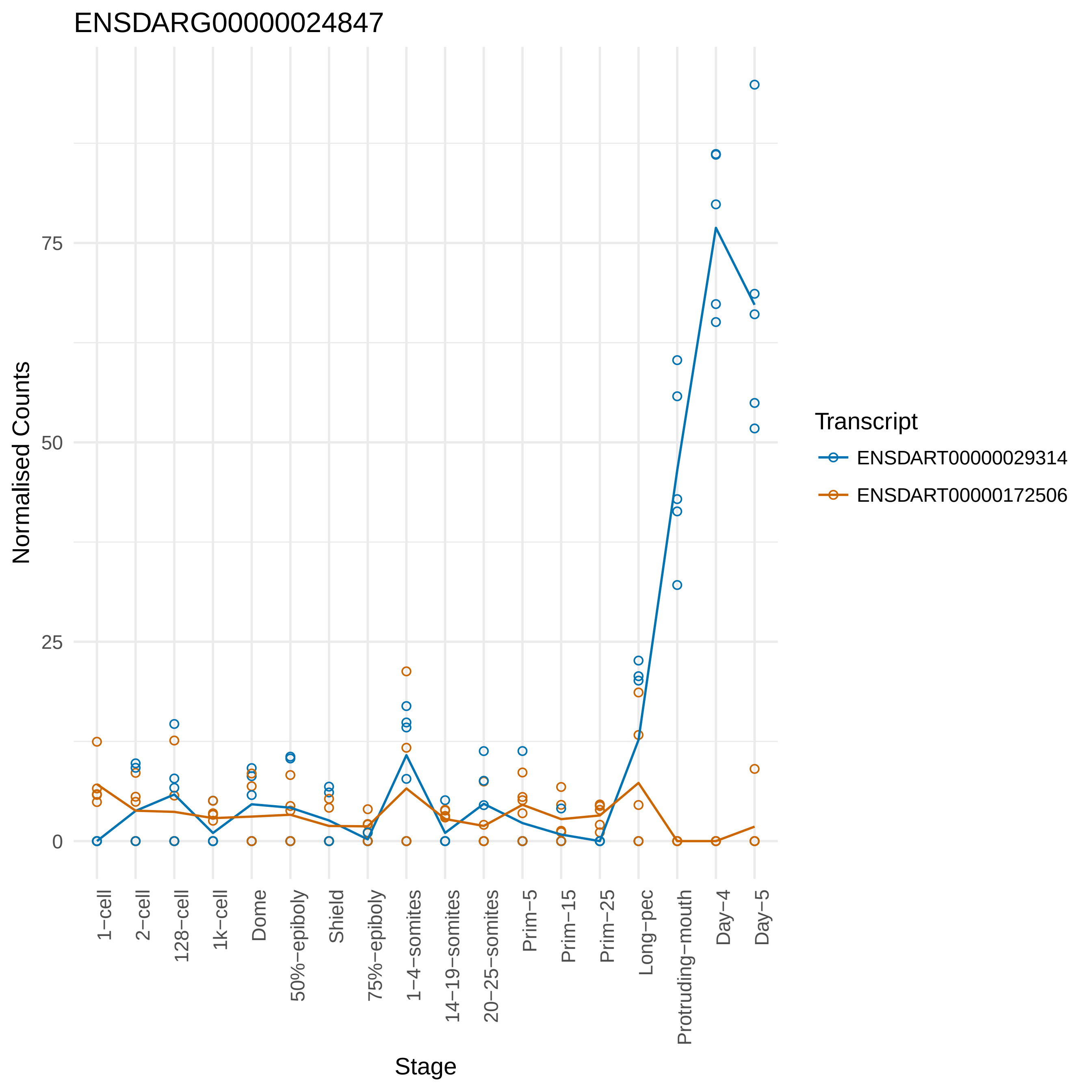

ENSDARG00000024847
Normalised Counts
0
25
50
75
Stage
1−cell
2−cell
128−cell
1k−cell
Dome
50%−epiboly
Shield
75%−epiboly
1−4−somites
14−19−somites
20−25−somites
Prim−5
Prim−15
Prim−25
Long−pec
Protruding−mouth
Day−4
Day−5
Transcript
ENSDART00000029314
ENSDART00000172506

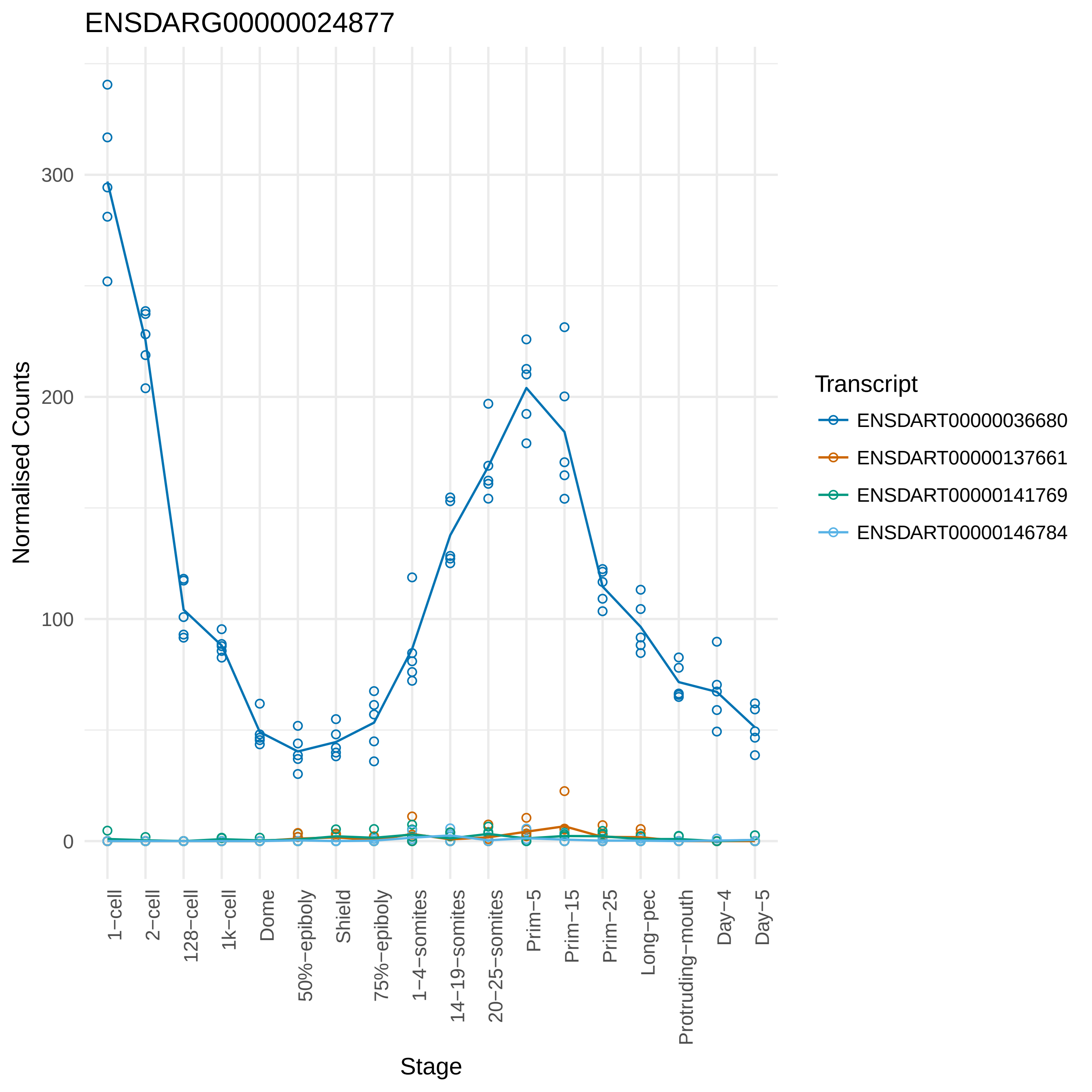

ENSDARG00000024877
Normalised Counts
300
200
100
0
1−cell
2−cell
128−cell
1k−cell
Dome
50%−epiboly
Shield
75%−epiboly
1−4−somites
14−19−somites
20−25−somites
Prim−5
Prim−15
Prim−25
Long−pec
Protruding−mouth
Day−4
Day−5
Stage
Transcript
ENSDART00000036680
ENSDART00000137661
ENSDART00000141769
ENSDART00000146784

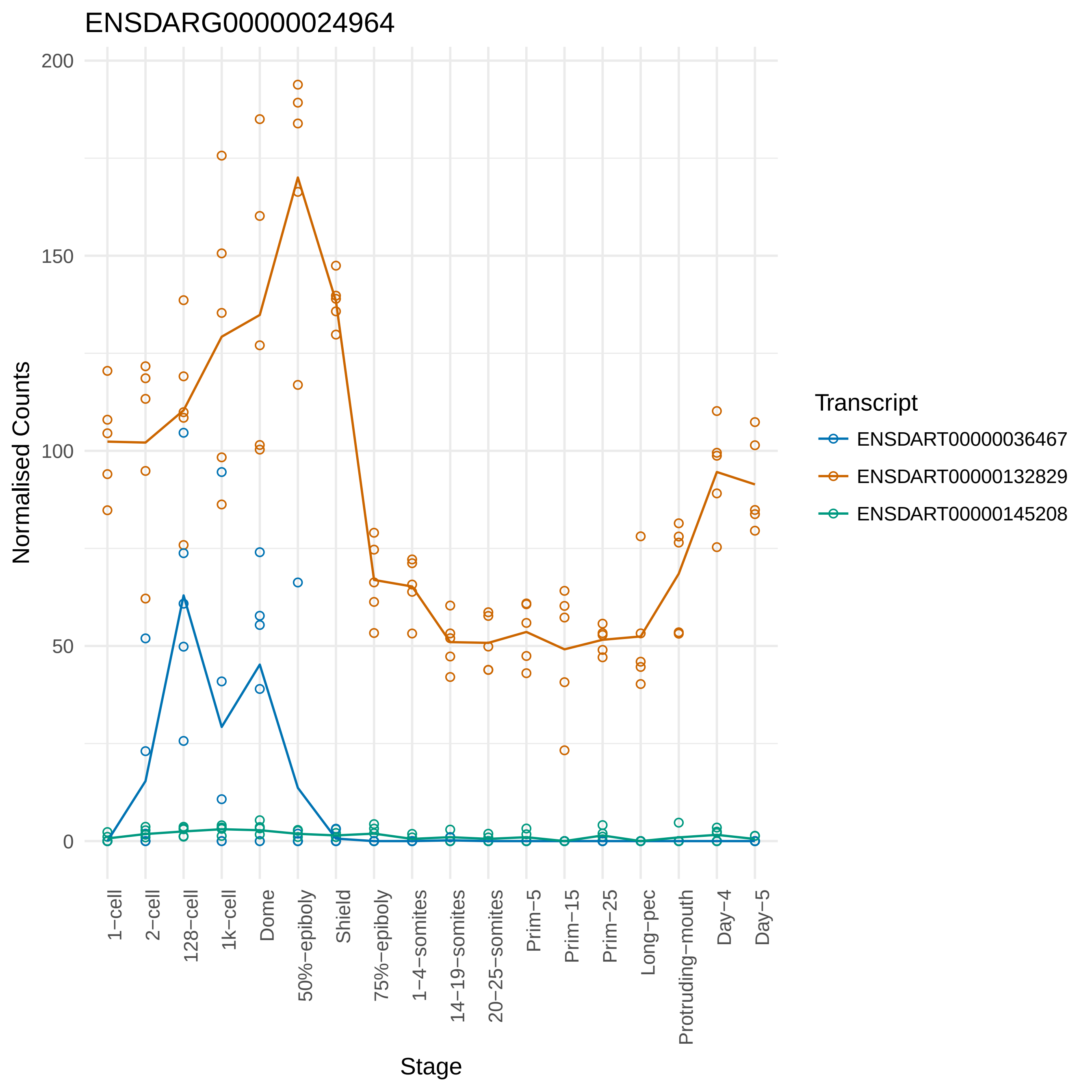

ENSDARG00000024964
Normalised Counts
0
50
100
150
200
1−cell
2−cell
128−cell
1k−cell
Dome
50%−epiboly
Shield
75%−epiboly
1−4−somites
14−19−somites
20−25−somites
Prim−5
Prim−15
Prim−25
Long−pec
Protruding−mouth
Day−4
Day−5
Stage
Transcript
ENSDART00000036467
ENSDART00000132829
ENSDART00000145208

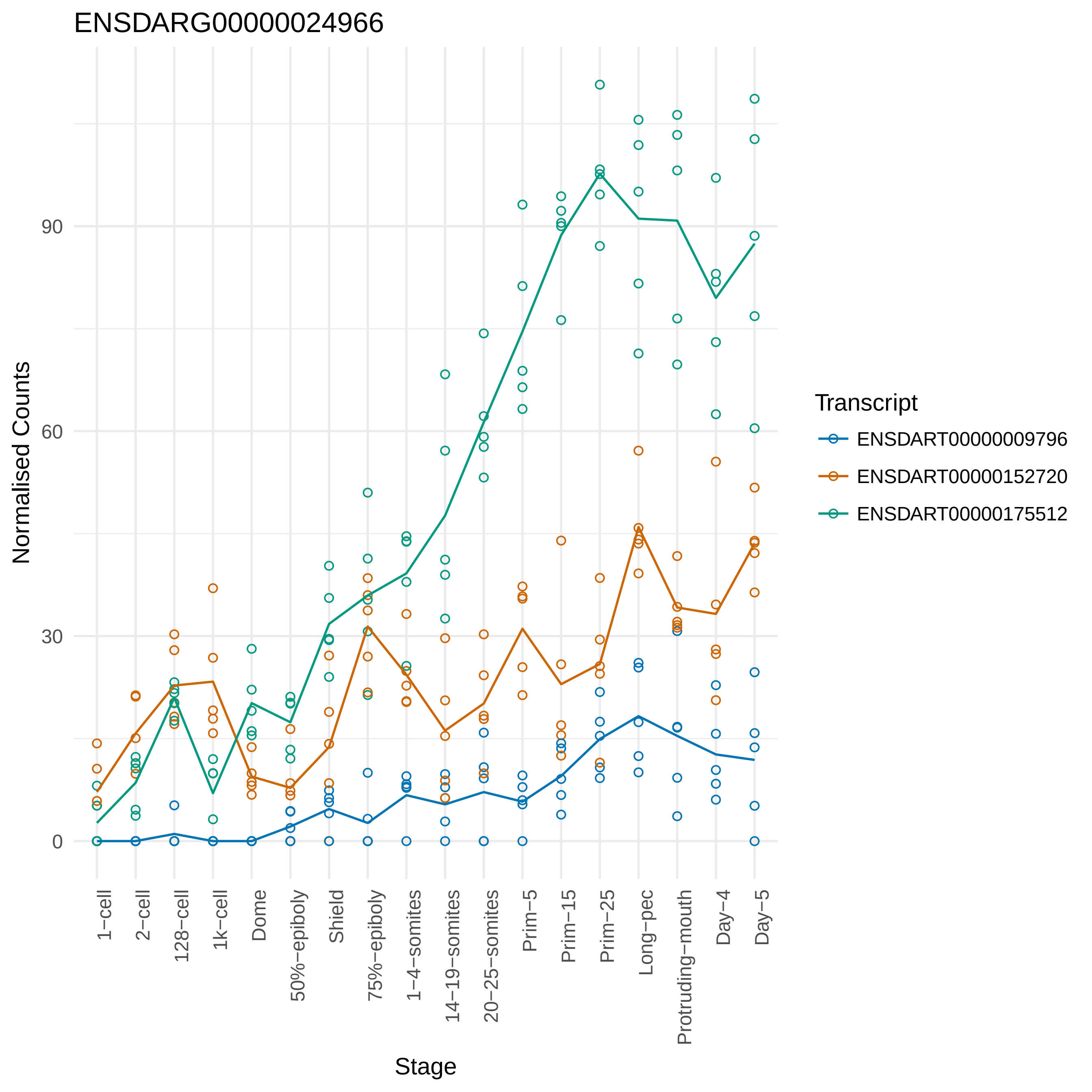

ENSDARG00000024966
Normalised Counts
0
30
60
90
Stage
1−cell
2−cell
128−cell
1k−cell
Dome
50%−epiboly
Shield
75%−epiboly
1−4−somites
14−19−somites
20−25−somites
Prim−5
Prim−15
Prim−25
Long−pec
Protruding−mouth
Day−4
Day−5
Transcript
ENSDART00000009796
ENSDART00000152720
ENSDART00000175512

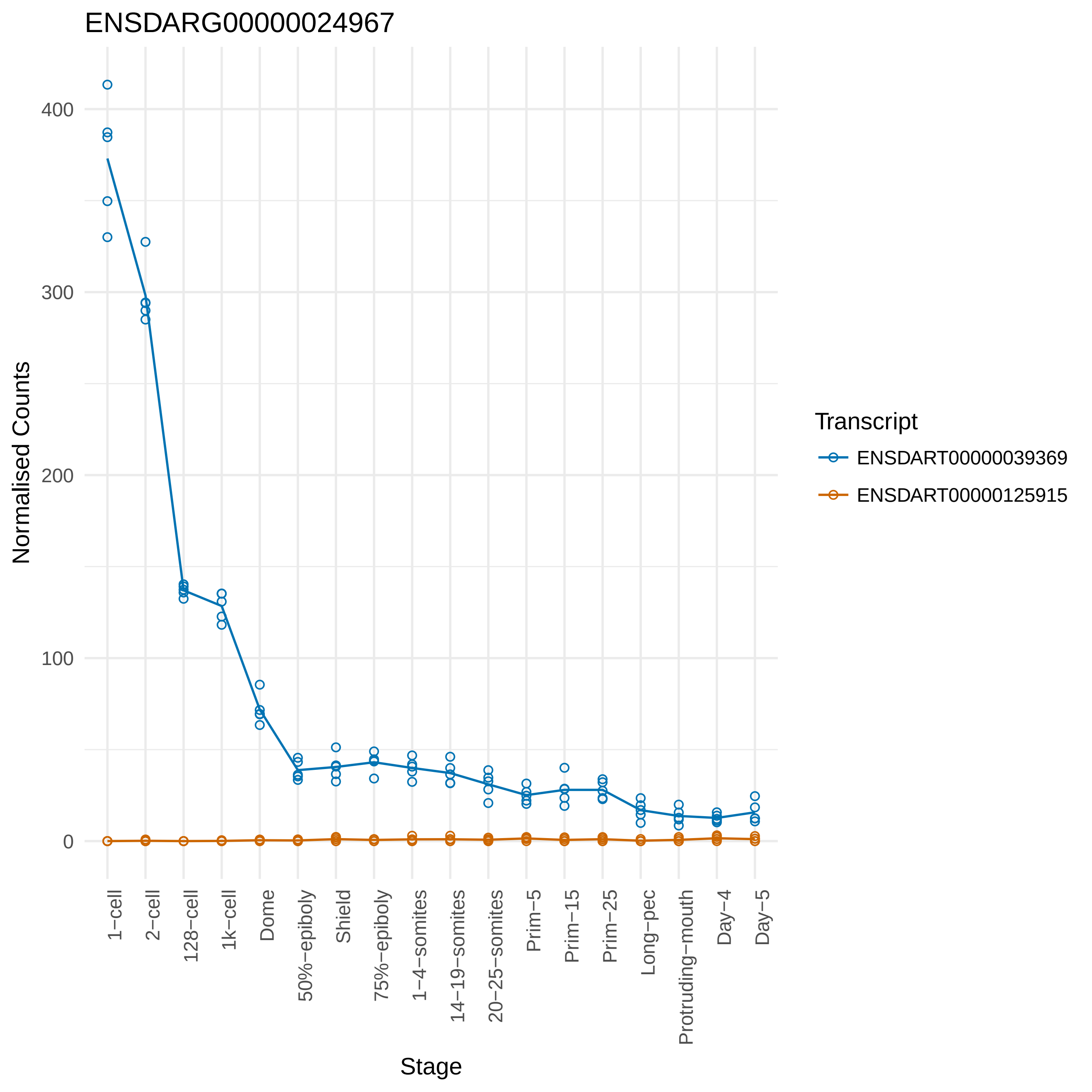

ENSDARG00000024967
Normalised Counts
0
100
200
300
400
Stage
1−cell
2−cell
128−cell
1k−cell
Dome
50%−epiboly
Shield
75%−epiboly
1−4−somites
14−19−somites
20−25−somites
Prim−5
Prim−15
Prim−25
Long−pec
Protruding−mouth
Day−4
Day−5
Transcript
ENSDART00000039369
ENSDART00000125915

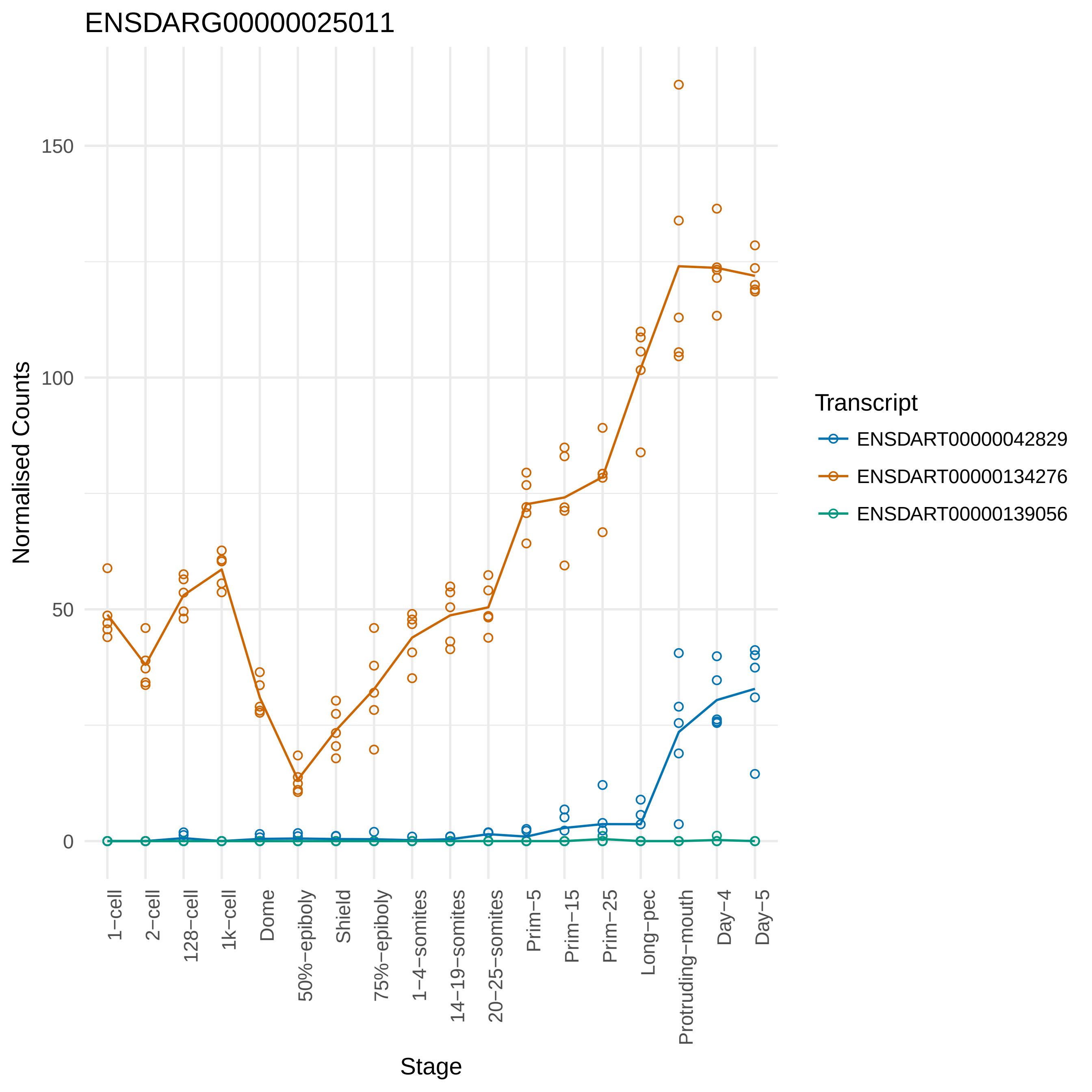

ENSDARG00000025011
Normalised Counts
150
100
50
0
1−cell
2−cell
128−cell
1k−cell
Dome
50%−epiboly
Shield
75%−epiboly
1−4−somites
14−19−somites
20−25−somites
Prim−5
Prim−15
Prim−25
Long−pec
Protruding−mouth
Day−4
Day−5
Stage
Transcript
ENSDART00000042829
ENSDART00000134276
ENSDART00000139056

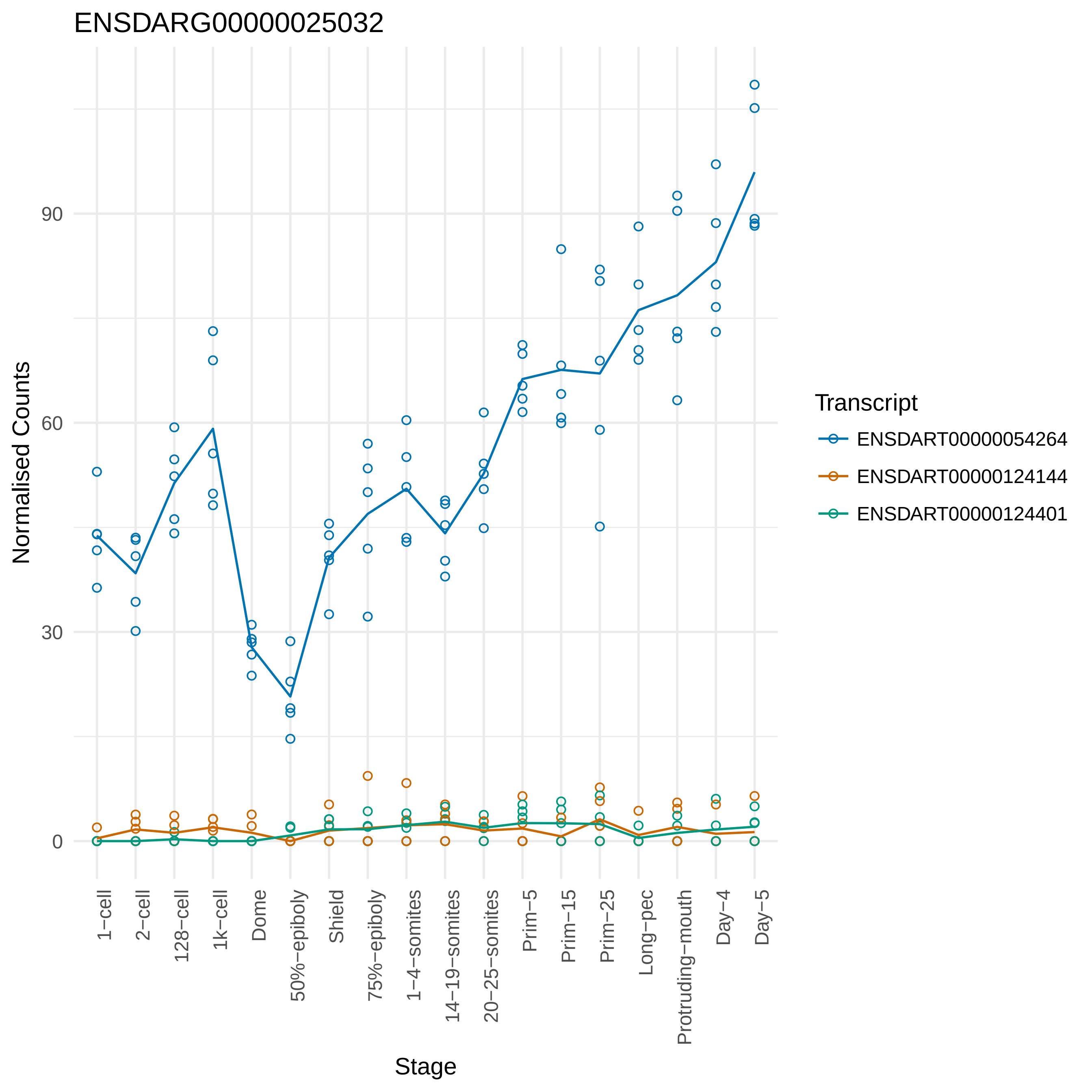
ENSDARG00000025032
Normalised Counts
0
30
60
90
1−cell
2−cell
128−cell
1k−cell
Dome
50%−epiboly
Shield
75%−epiboly
1−4−somites
14−19−somites
20−25−somites
Prim−5
Prim−15
Prim−25
Long−pec
Protruding−mouth
Day−4
Day−5
Stage
Transcript
ENSDART00000054264
ENSDART00000124144
ENSDART00000124401

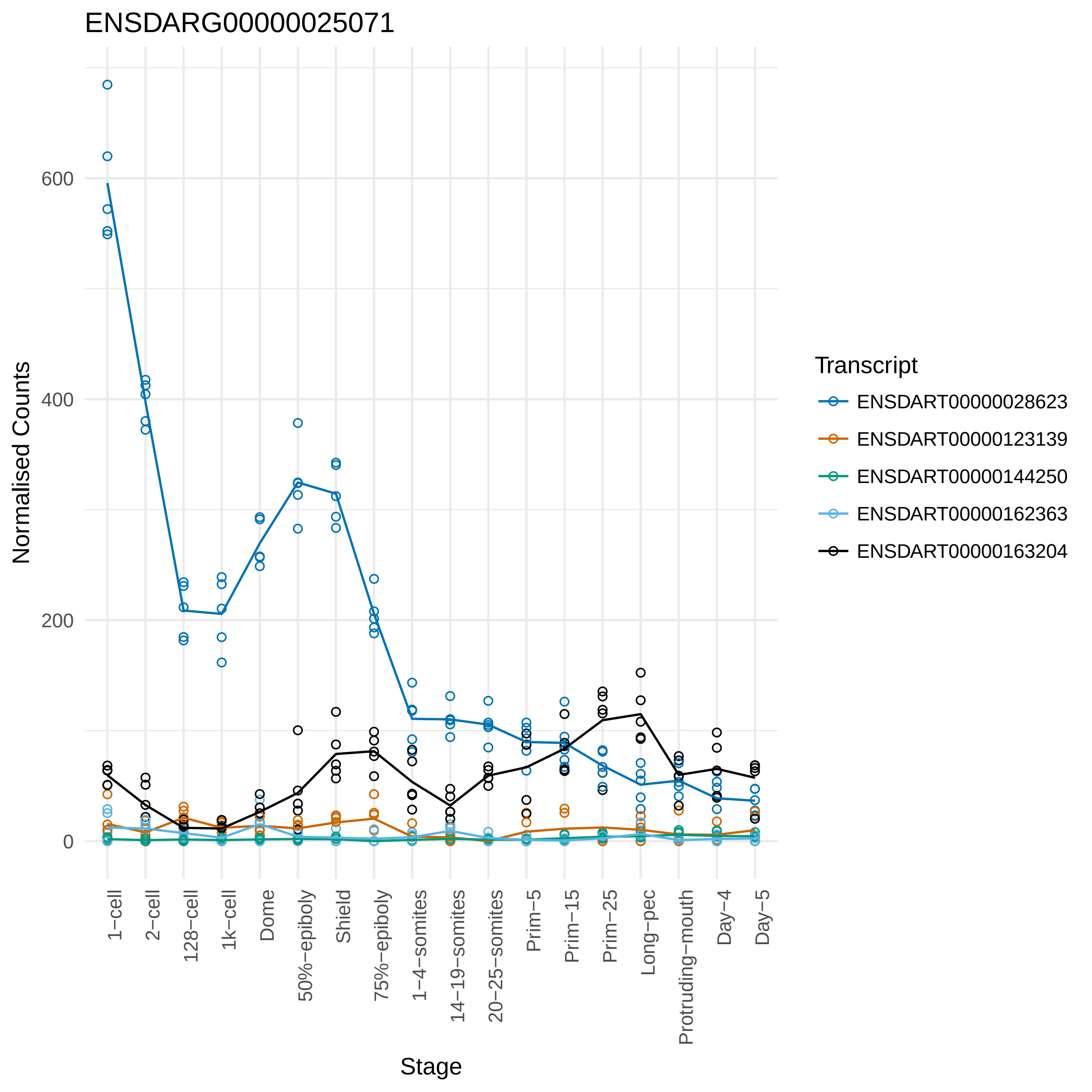
ENSDARG00000025071
Normalised Counts
0
200
400
600
Stage
1−cell
2−cell
128−cell
1k−cell
Dome
50%−epiboly
Shield
75%−epiboly
1−4−somites
14−19−somites
20−25−somites
Prim−5
Prim−15
Prim−25
Long−pec
Protruding−mouth
Day−4
Day−5
Transcript
ENSDART00000028623
ENSDART00000123139
ENSDART00000144250
ENSDART00000162363
ENSDART00000163204

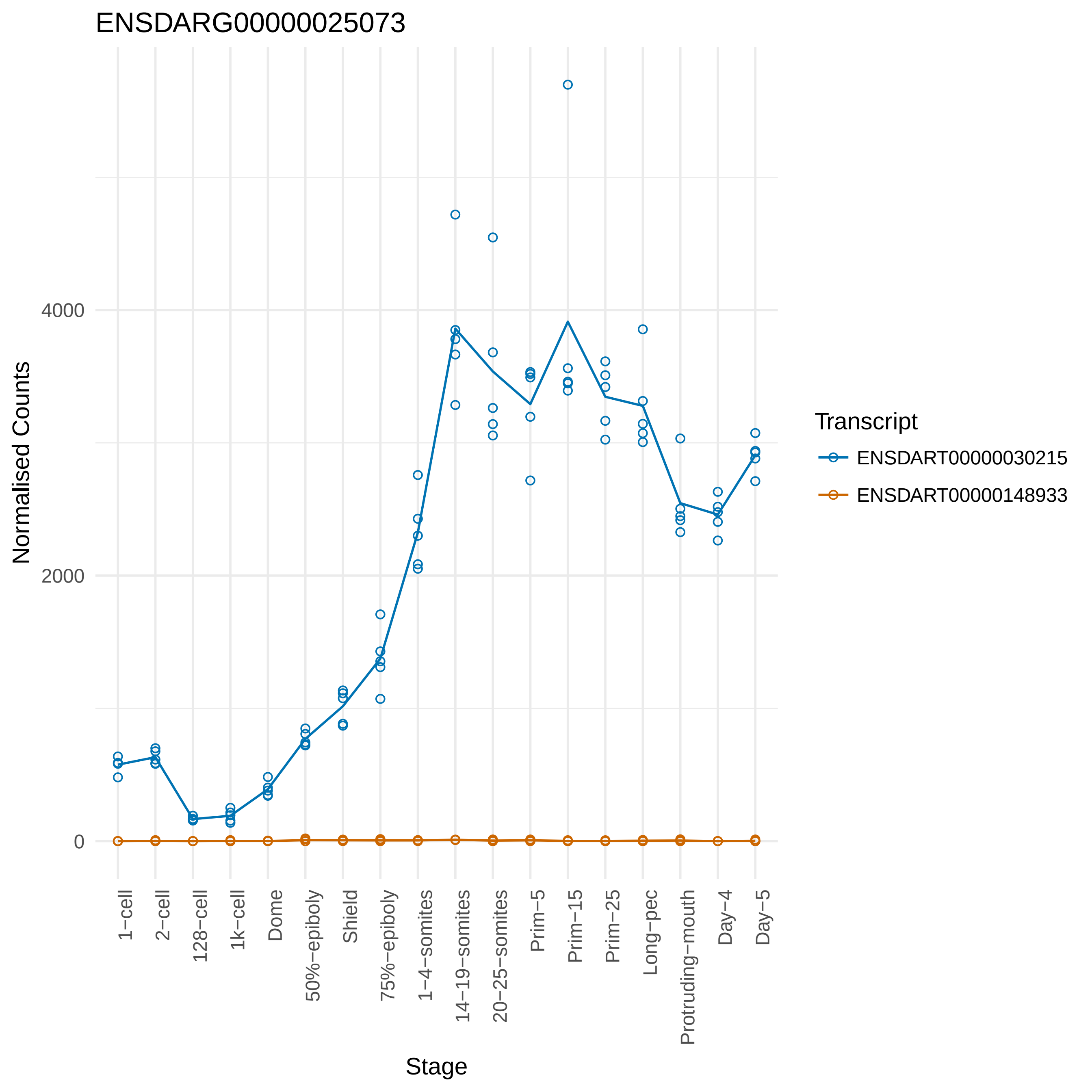
ENSDARG00000025073
Normalised Counts
4000
2000
0
1−cell
2−cell
128−cell
1k−cell
Dome
50%−epiboly
Shield
75%−epiboly
1−4−somites
14−19−somites
20−25−somites
Prim−5
Prim−15
Prim−25
Long−pec
Protruding−mouth
Day−4
Day−5
Stage
Transcript
ENSDART00000030215
ENSDART00000148933

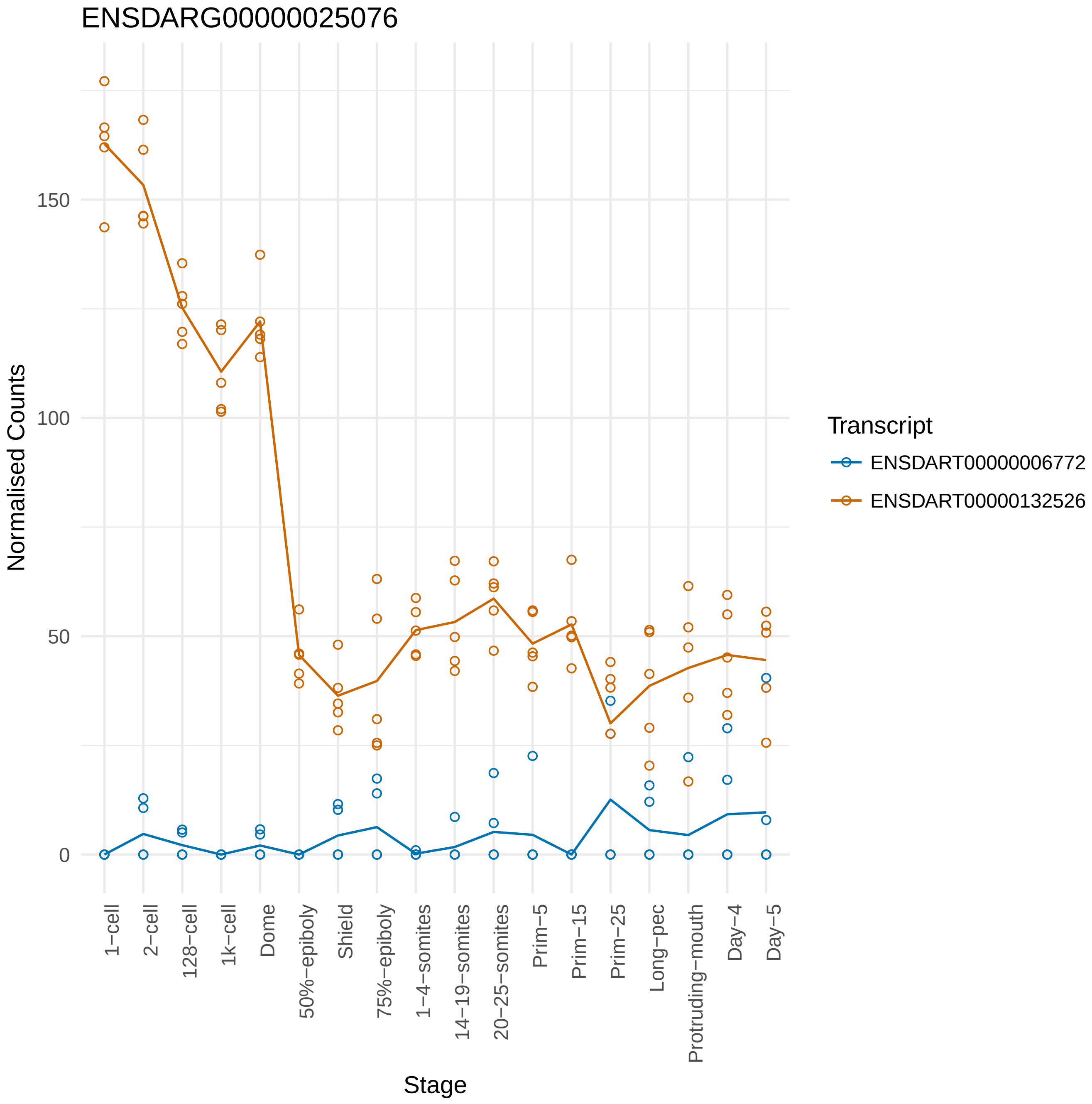

ENSDARG00000025076
Normalised Counts
150
100
50
0
1−cell
2−cell
128−cell
1k−cell
Dome
50%−epiboly
Shield
75%−epiboly
1−4−somites
14−19−somites
20−25−somites
Prim−5
Prim−15
Prim−25
Long−pec
Protruding−mouth
Day−4
Day−5
Stage
Transcript
ENSDART00000006772
ENSDART00000132526

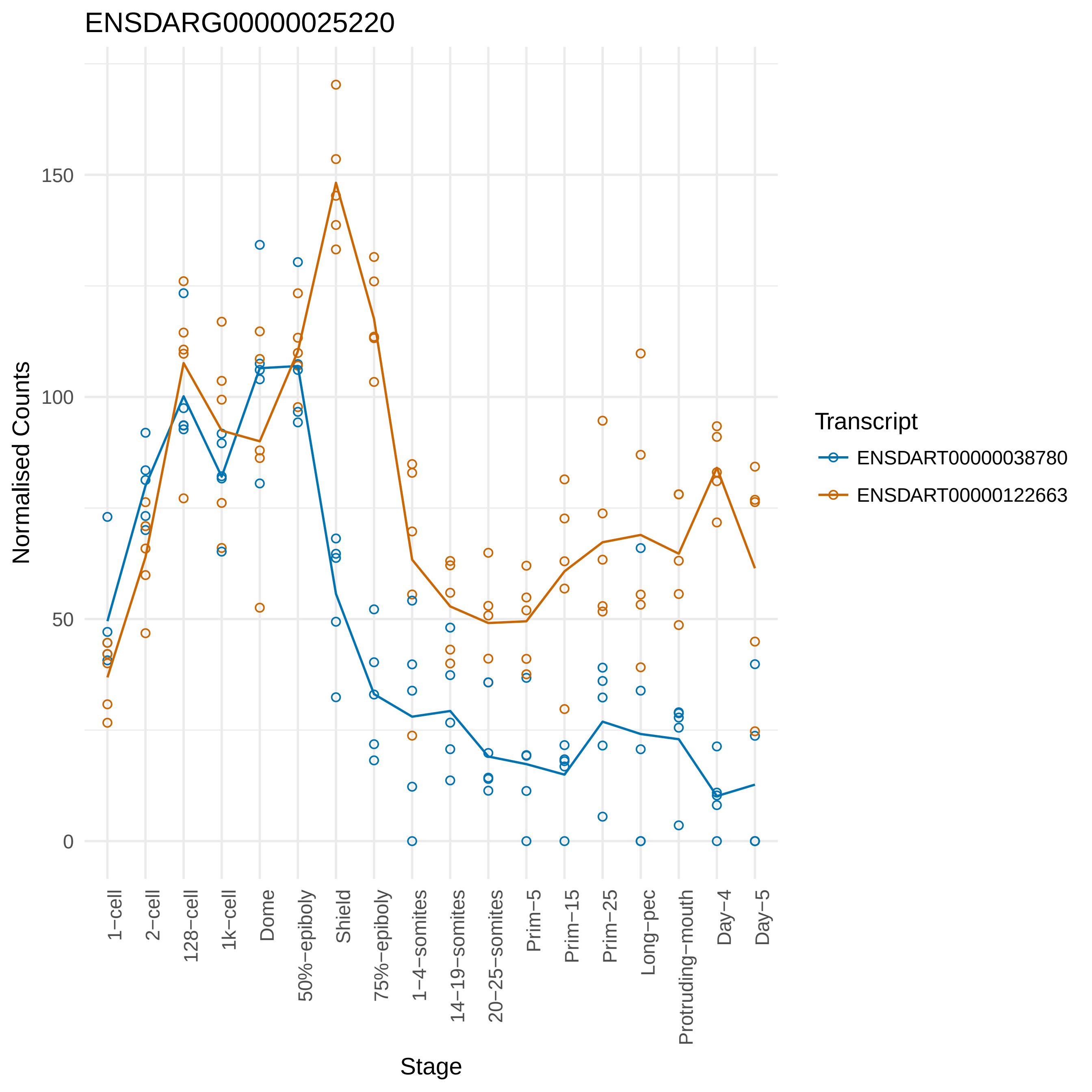

ENSDARG00000025220
Normalised Counts
0
50
100
150
1−cell
2−cell
128−cell
1k−cell
Dome
50%−epiboly
Shield
75%−epiboly
1−4−somites
14−19−somites
20−25−somites
Prim−5
Prim−15
Prim−25
Long−pec
Protruding−mouth
Day−4
Day−5
Stage
Transcript
ENSDART00000038780
ENSDART00000122663

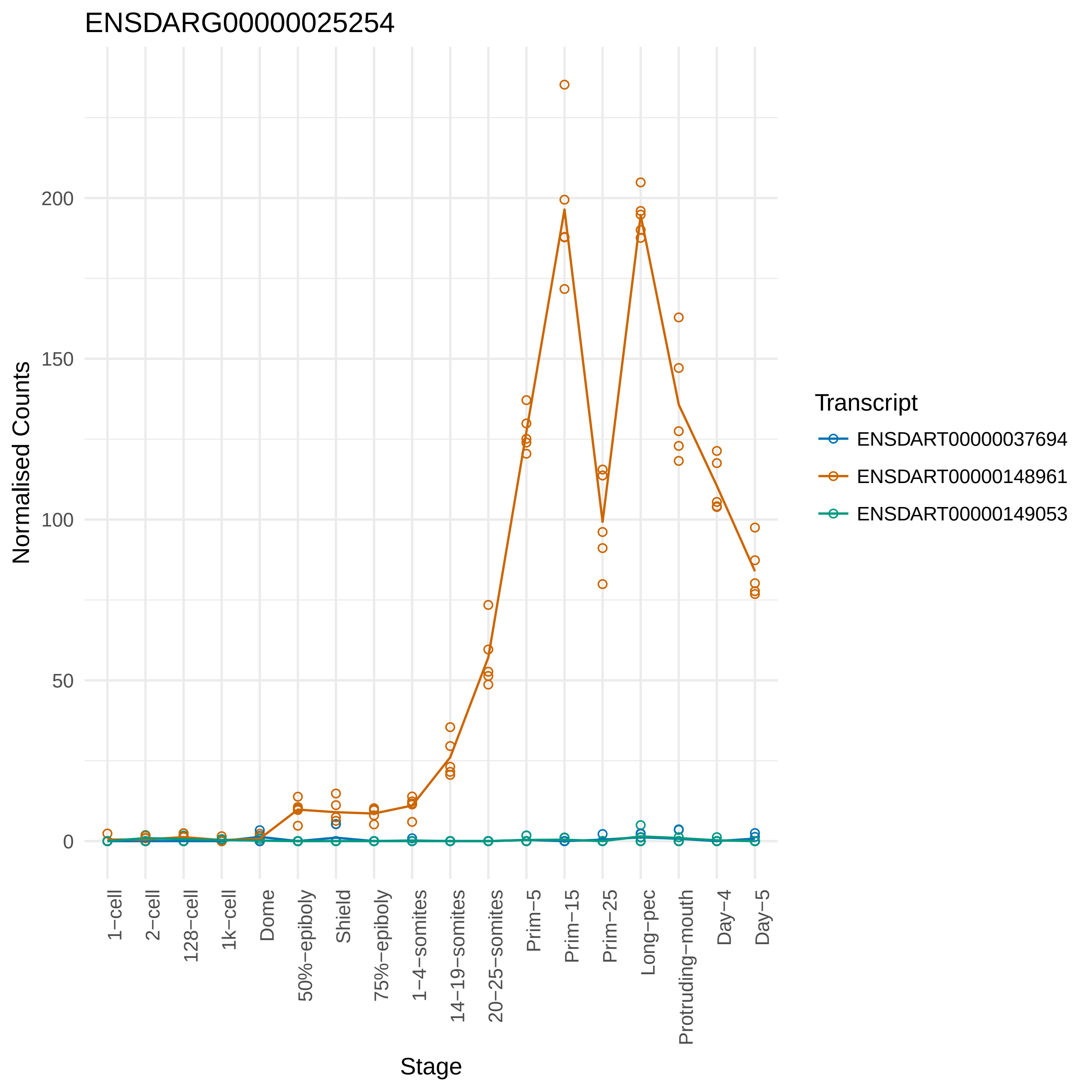

ENSDARG00000025254
Normalised Counts
200
150
100
50
0
1−cell
2−cell
128−cell
1k−cell
Dome
50%−epiboly
Shield
75%−epiboly
1−4−somites
14−19−somites
20−25−somites
Prim−5
Prim−15
Prim−25
Long−pec
Protruding−mouth
Day−4
Day−5
Stage
Transcript
ENSDART00000037694
ENSDART00000148961
ENSDART00000149053

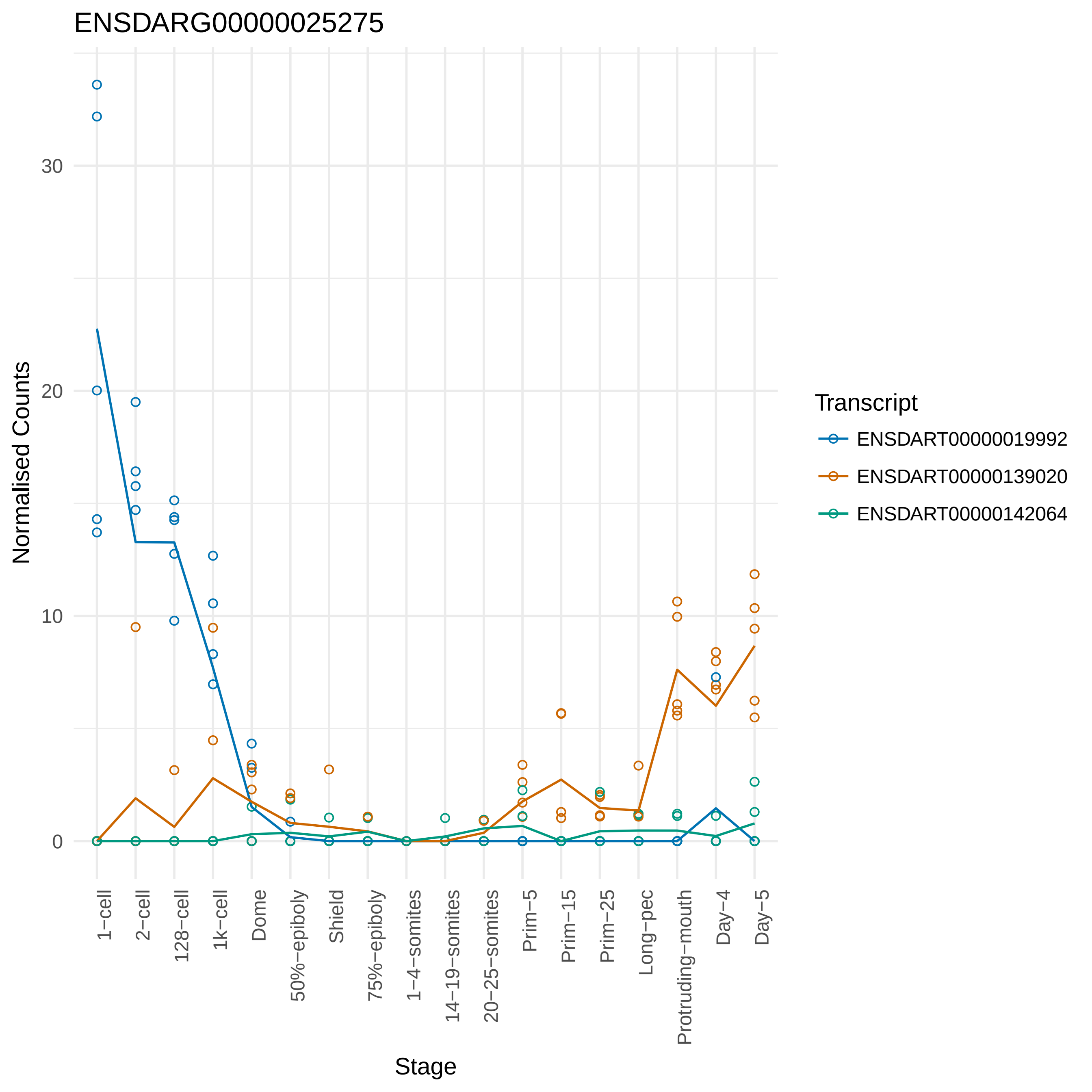
ENSDARG00000025275
Normalised Counts
30
20
10
0
1−cell
2−cell
128−cell
1k−cell
Dome
50%−epiboly
Shield
75%−epiboly
1−4−somites
14−19−somites
20−25−somites
Prim−5
Prim−15
Prim−25
Long−pec
Protruding−mouth
Day−4
Day−5
Stage
Transcript
ENSDART00000019992
ENSDART00000139020
ENSDART00000142064

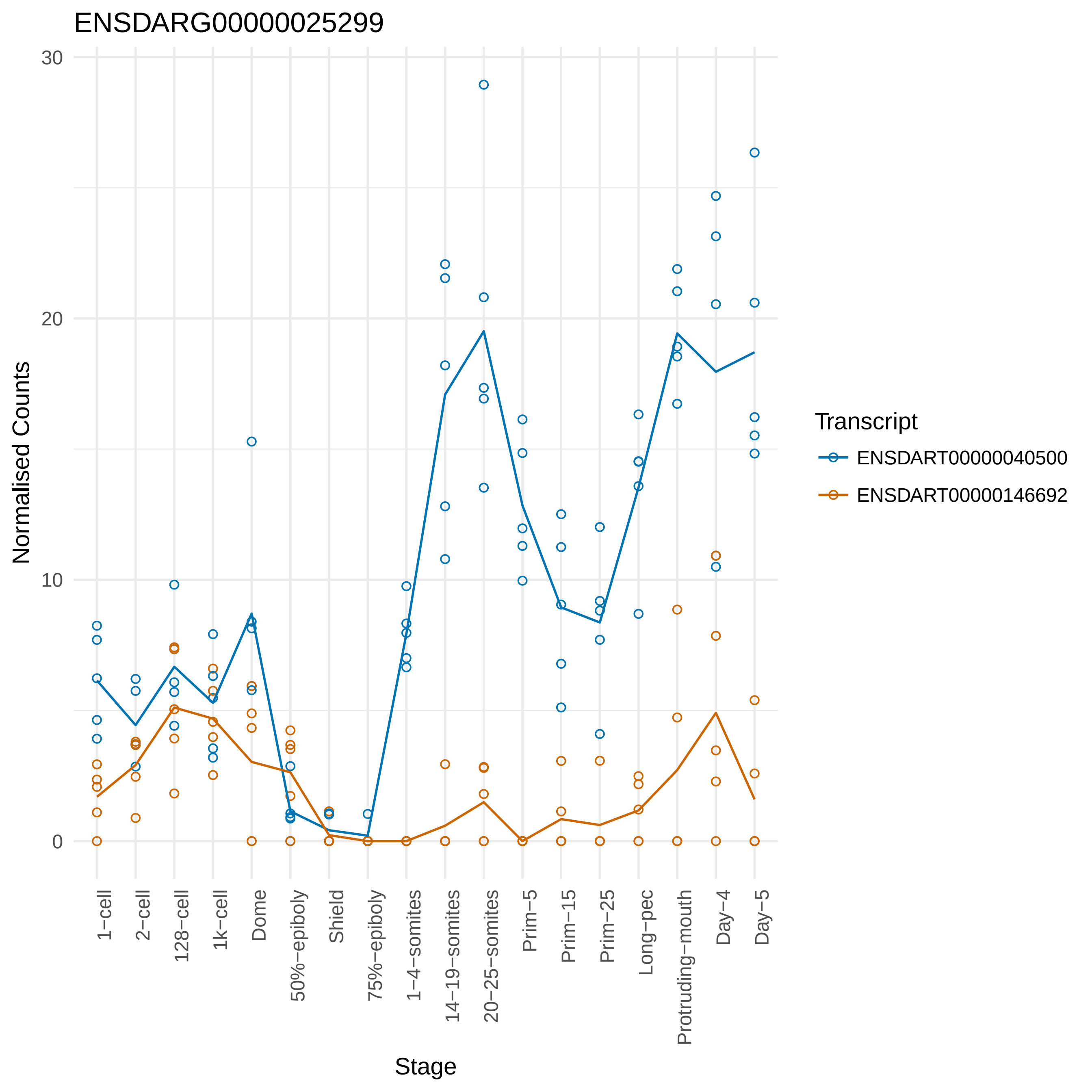
ENSDARG00000025299
Normalised Counts
30
20
10
0
Stage
1−cell
2−cell
128−cell
1k−cell
Dome
50%−epiboly
Shield
75%−epiboly
1−4−somites
14−19−somites
20−25−somites
Prim−5
Prim−15
Prim−25
Long−pec
Protruding−mouth
Day−4
Day−5
Transcript
ENSDART00000040500
ENSDART00000146692

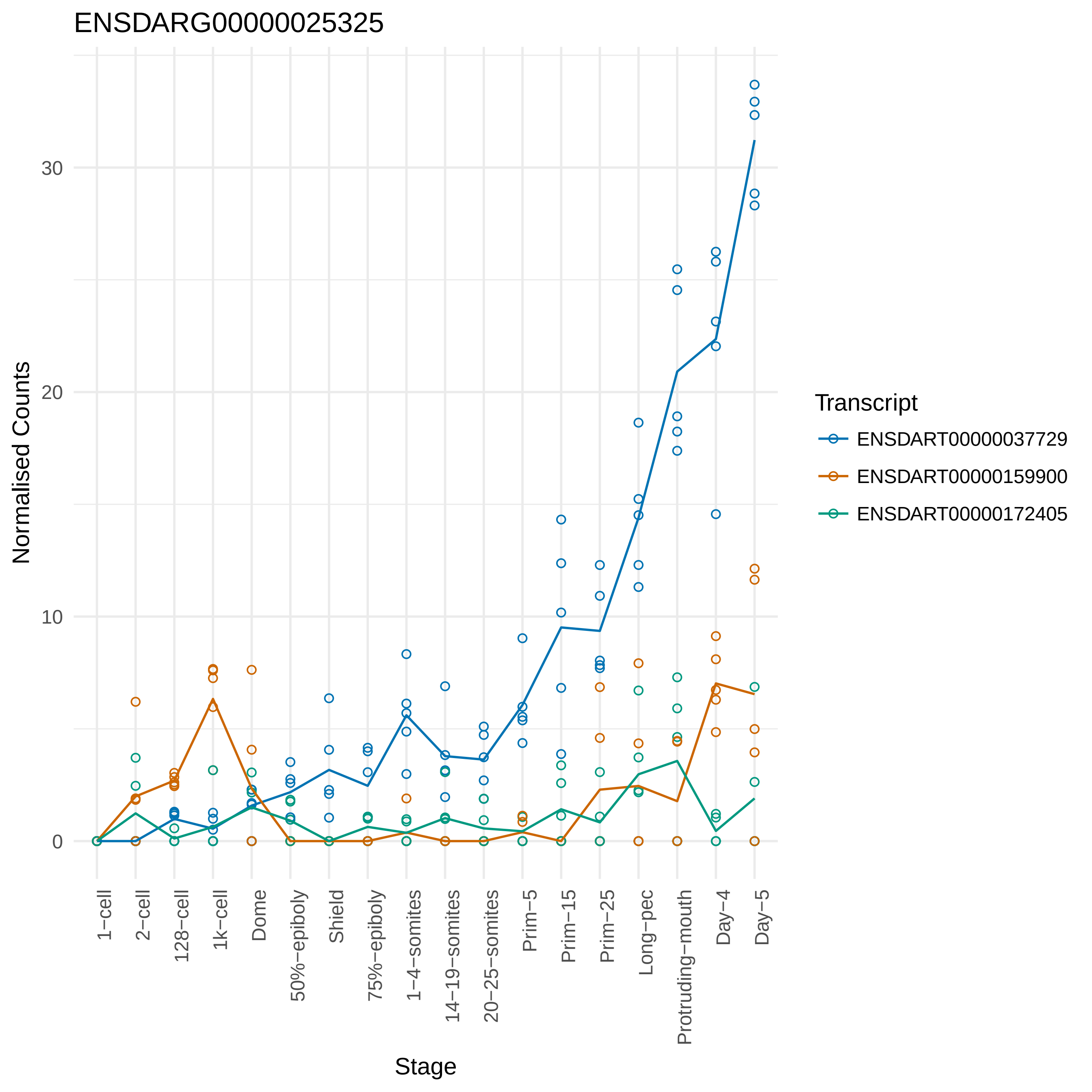

ENSDARG00000025325
Normalised Counts
0
10
20
30
Stage
1−cell
2−cell
128−cell
1k−cell
Dome
50%−epiboly
Shield
75%−epiboly
1−4−somites
14−19−somites
20−25−somites
Prim−5
Prim−15
Prim−25
Long−pec
Protruding−mouth
Day−4
Day−5
Transcript
ENSDART00000037729
ENSDART00000159900
ENSDART00000172405

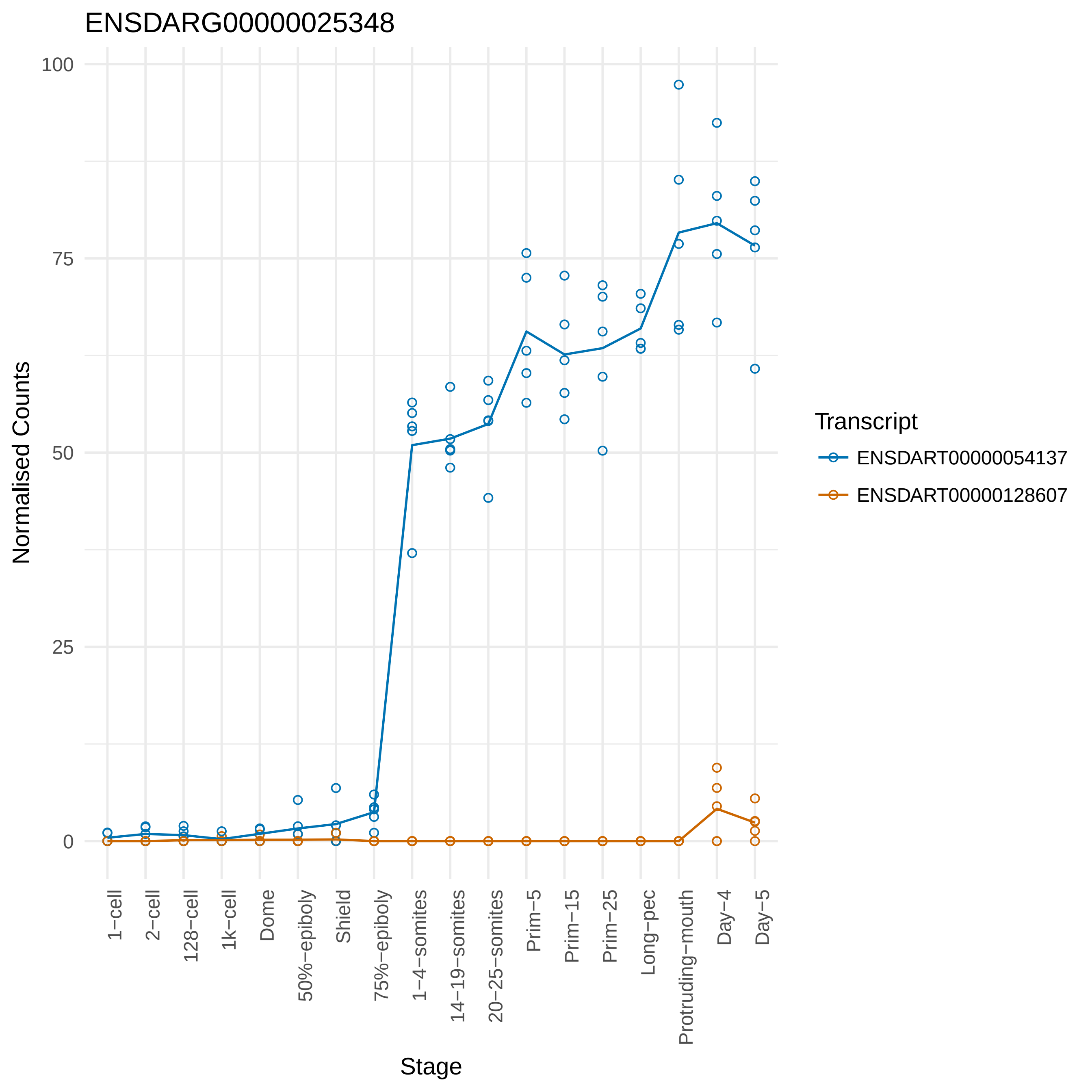

ENSDARG00000025348
Normalised Counts
100
75
50
25
0
1−cell
2−cell
128−cell
1k−cell
Dome
50%−epiboly
Shield
75%−epiboly
1−4−somites
14−19−somites
20−25−somites
Prim−5
Prim−15
Prim−25
Long−pec
Protruding−mouth
Day−4
Day−5
Stage
Transcript
ENSDART00000054137
ENSDART00000128607

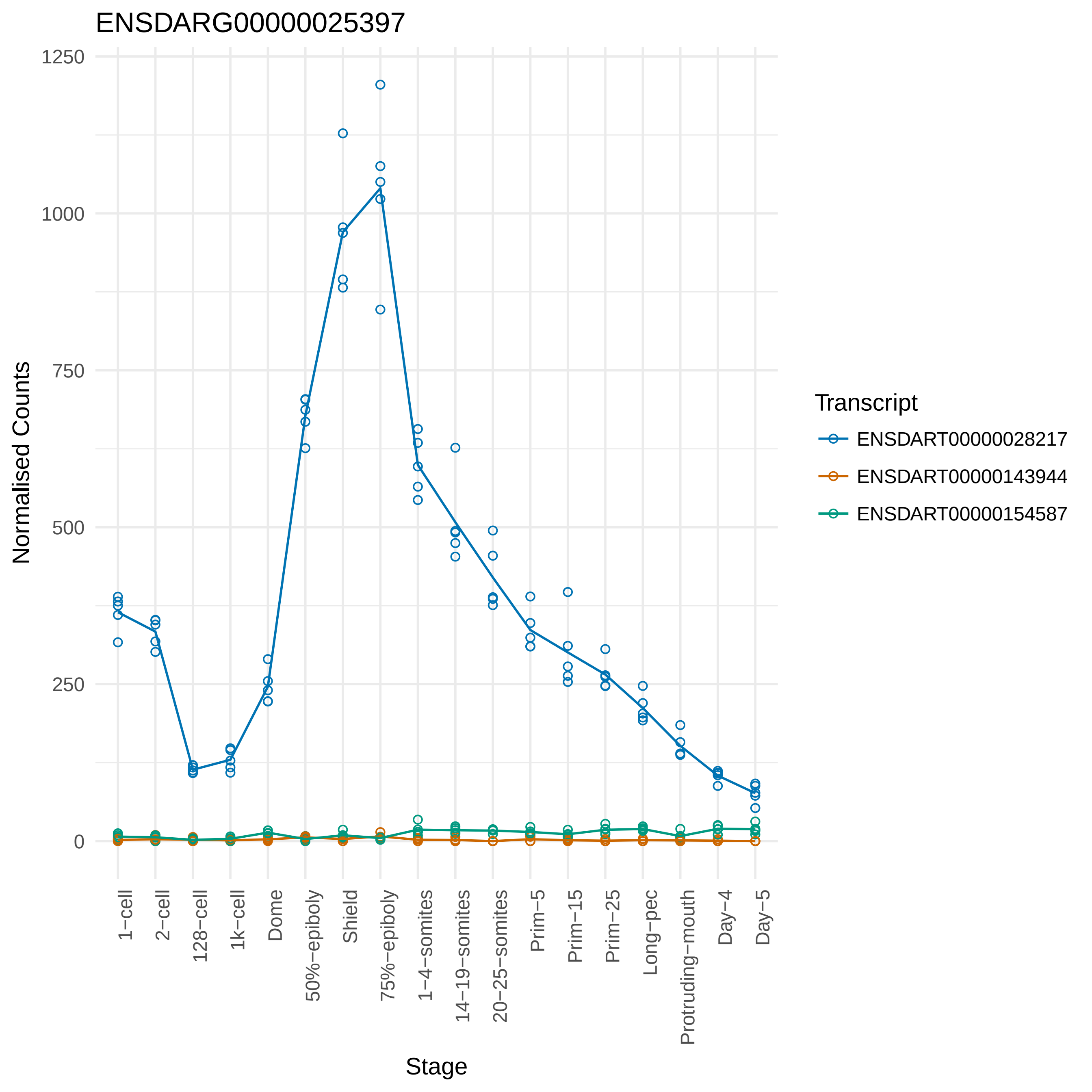
ENSDARG00000025397
Normalised Counts
1250
1000
750
500
250
0
1−cell
2−cell
128−cell
1k−cell
Dome
50%−epiboly
Shield
75%−epiboly
1−4−somites
14−19−somites
20−25−somites
Prim−5
Prim−15
Prim−25
Long−pec
Protruding−mouth
Day−4
Day−5
Stage
Transcript
ENSDART00000028217
ENSDART00000143944
ENSDART00000154587

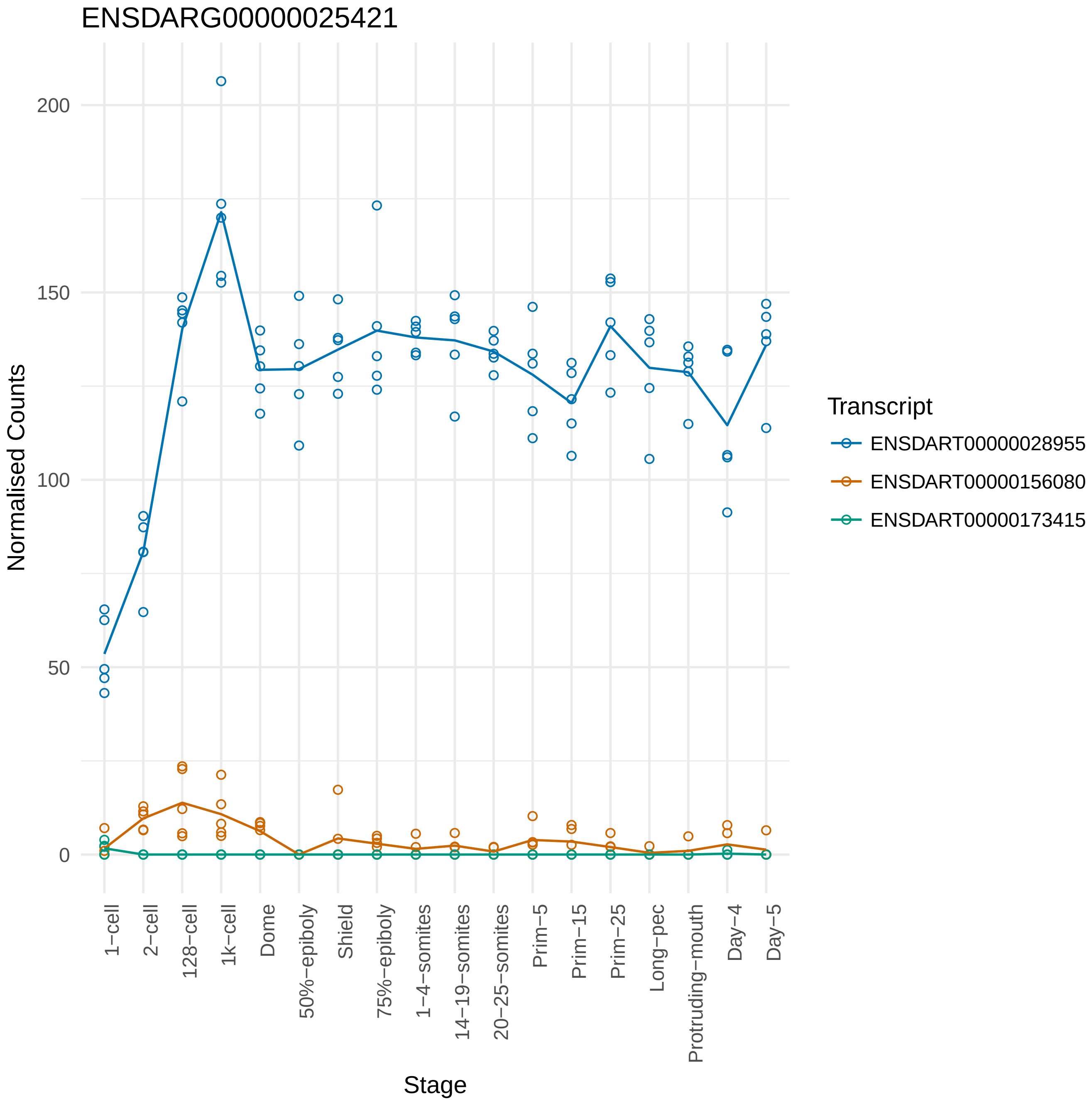
ENSDARG00000025421
Normalised Counts
0
50
100
150
200
Stage
1−cell
2−cell
128−cell
1k−cell
Dome
50%−epiboly
Shield
75%−epiboly
1−4−somites
14−19−somites
20−25−somites
Prim−5
Prim−15
Prim−25
Long−pec
Protruding−mouth
Day−4
Day−5
Transcript
ENSDART00000028955
ENSDART00000156080
ENSDART00000173415

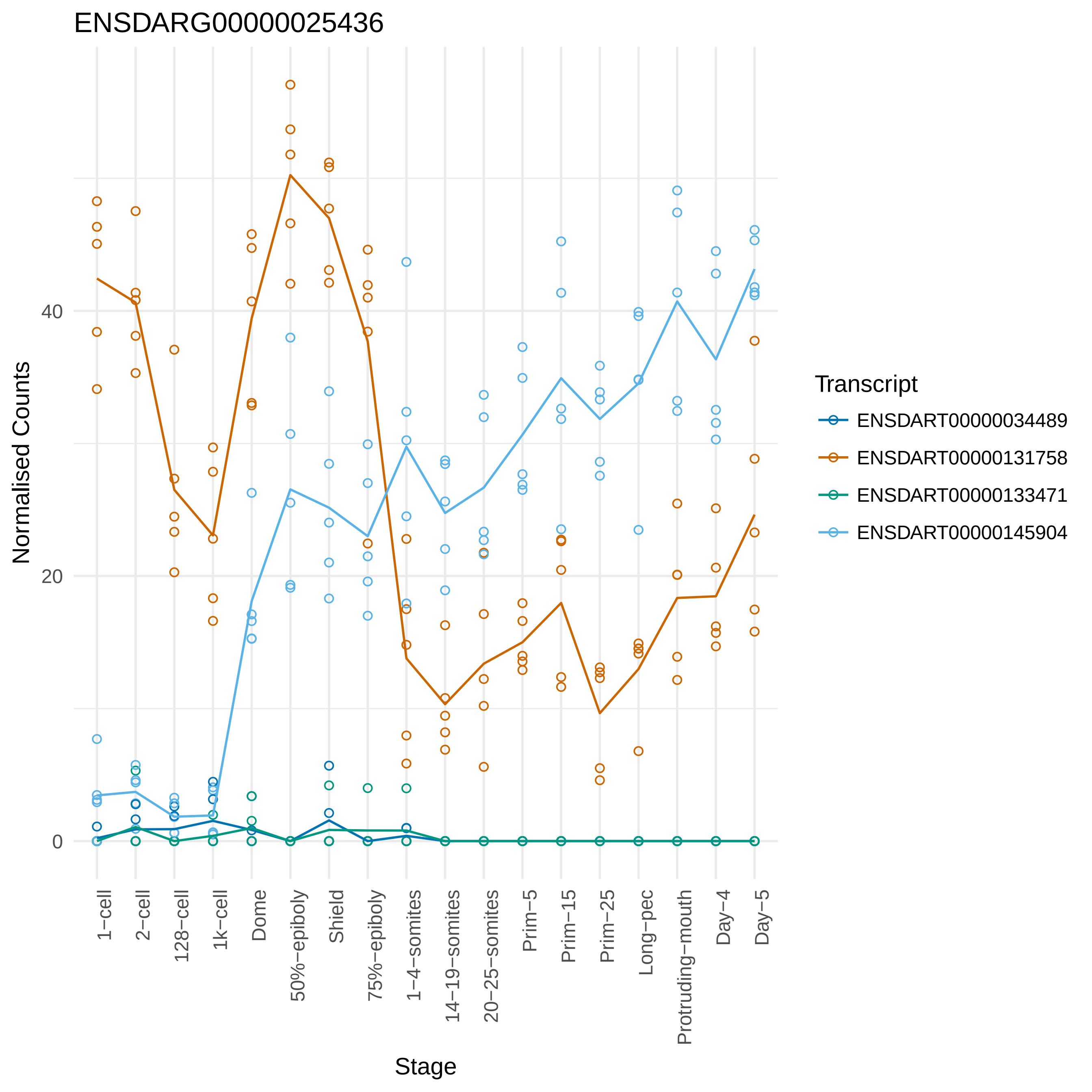

ENSDARG00000025436
Normalised Counts
0
20
40
Stage
1−cell
2−cell
128−cell
1k−cell
Dome
50%−epiboly
Shield
75%−epiboly
1−4−somites
14−19−somites
20−25−somites
Prim−5
Prim−15
Prim−25
Long−pec
Protruding−mouth
Day−4
Day−5
Transcript
ENSDART00000034489
ENSDART00000131758
ENSDART00000133471
ENSDART00000145904

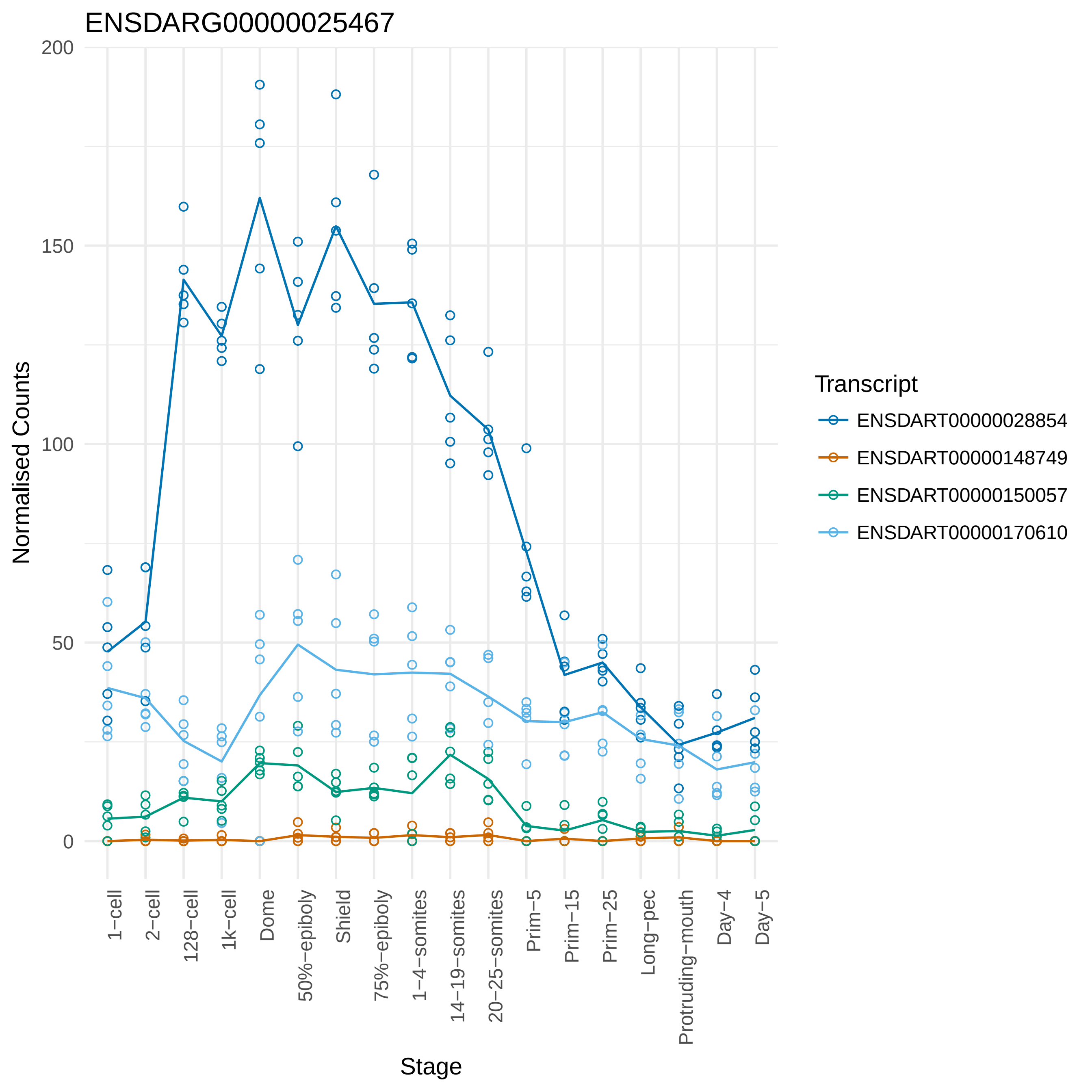

ENSDARG00000025467
Normalised Counts
0
50
100
150
200
Stage
1−cell
2−cell
128−cell
1k−cell
Dome
50%−epiboly
Shield
75%−epiboly
1−4−somites
14−19−somites
20−25−somites
Prim−5
Prim−15
Prim−25
Long−pec
Protruding−mouth
Day−4
Day−5
Transcript
ENSDART00000028854
ENSDART00000148749
ENSDART00000150057
ENSDART00000170610

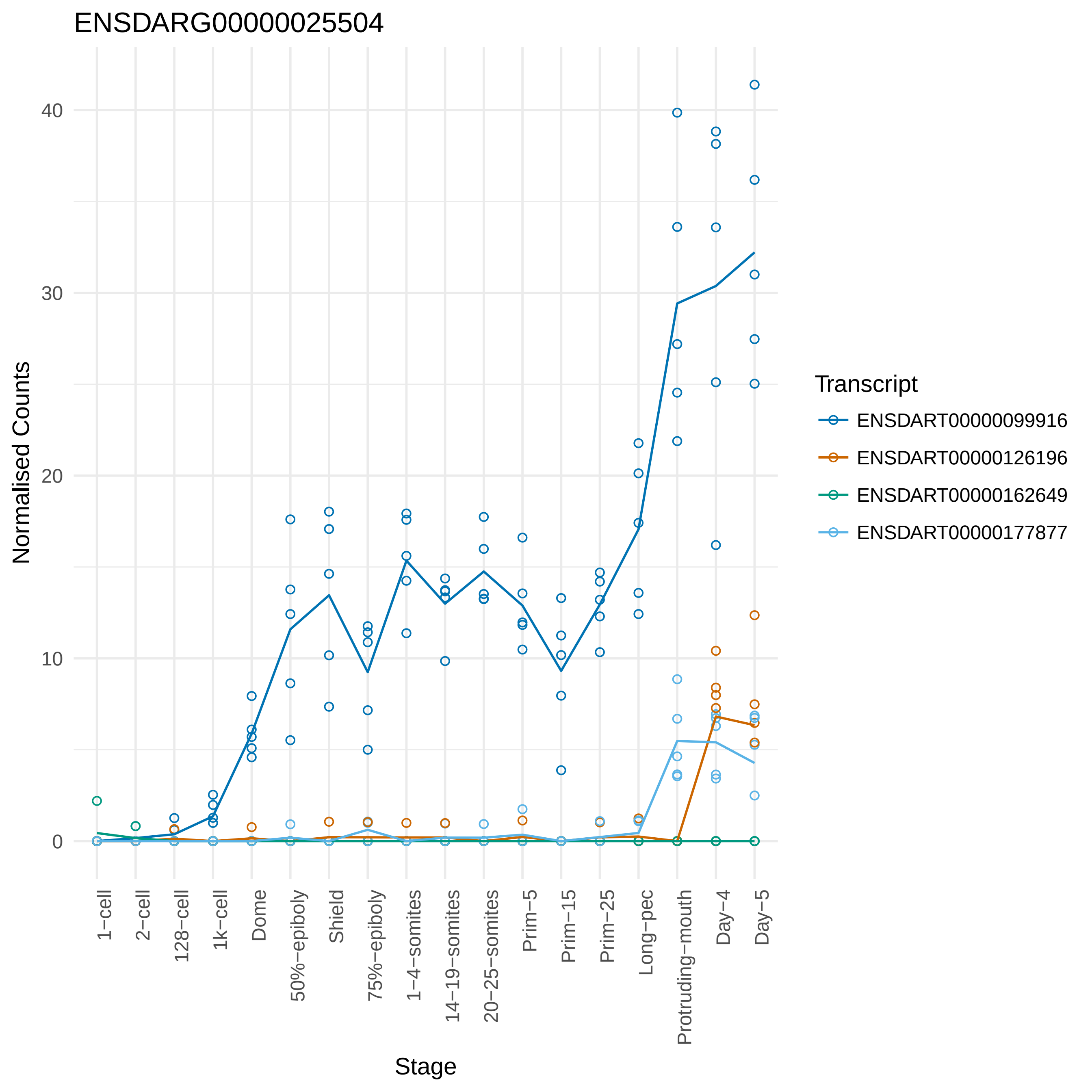

ENSDARG00000025504
Normalised Counts
0
10
20
30
40
1−cell
2−cell
128−cell
1k−cell
Dome
50%−epiboly
Shield
75%−epiboly
1−4−somites
14−19−somites
20−25−somites
Prim−5
Prim−15
Prim−25
Long−pec
Protruding−mouth
Day−4
Day−5
Stage
Transcript
ENSDART00000099916
ENSDART00000126196
ENSDART00000162649
ENSDART00000177877

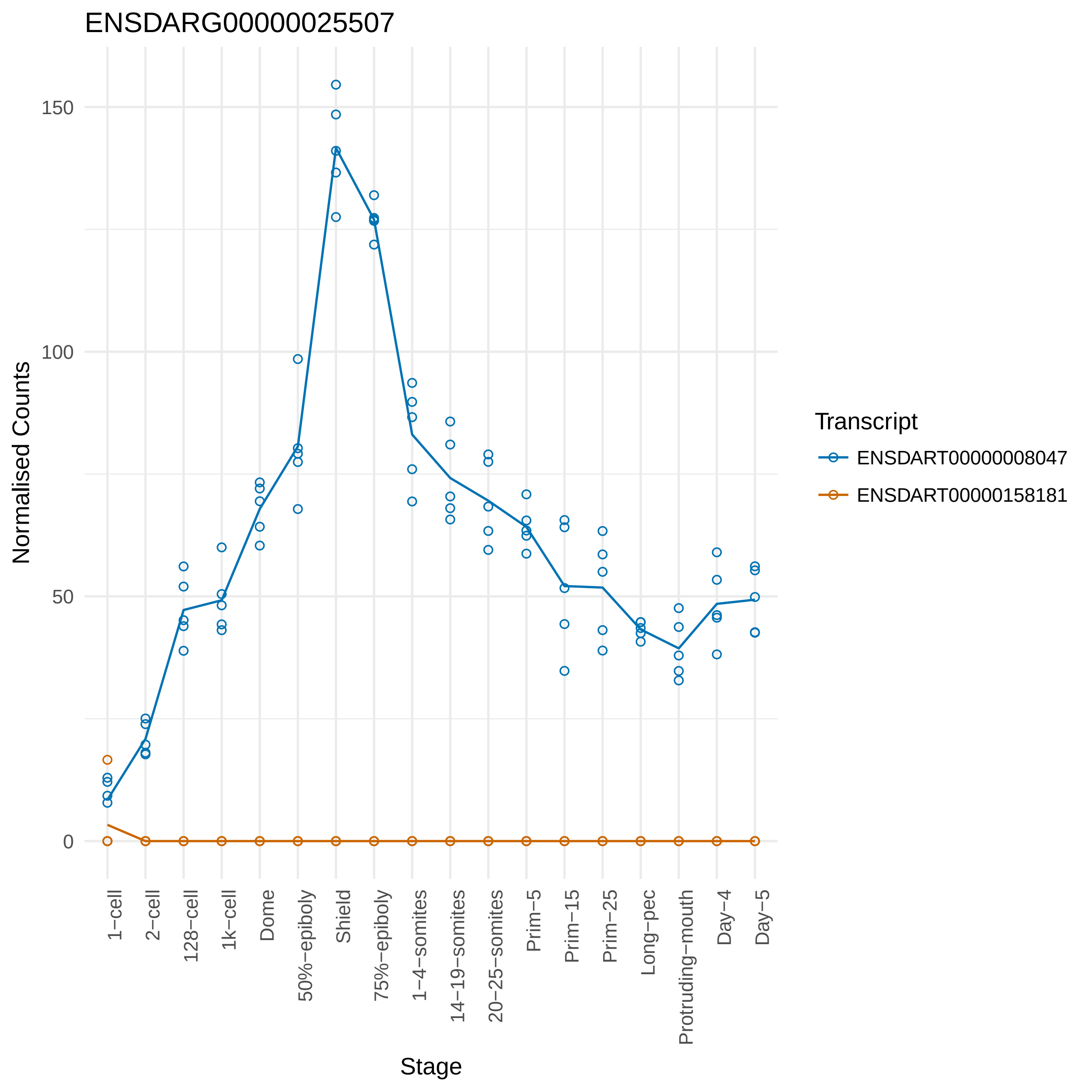
ENSDARG00000025507
Normalised Counts
150
100
50
0
1−cell
2−cell
128−cell
1k−cell
Dome
50%−epiboly
Shield
75%−epiboly
1−4−somites
14−19−somites
20−25−somites
Prim−5
Prim−15
Prim−25
Long−pec
Protruding−mouth
Day−4
Day−5
Stage
Transcript
ENSDART00000008047
ENSDART00000158181

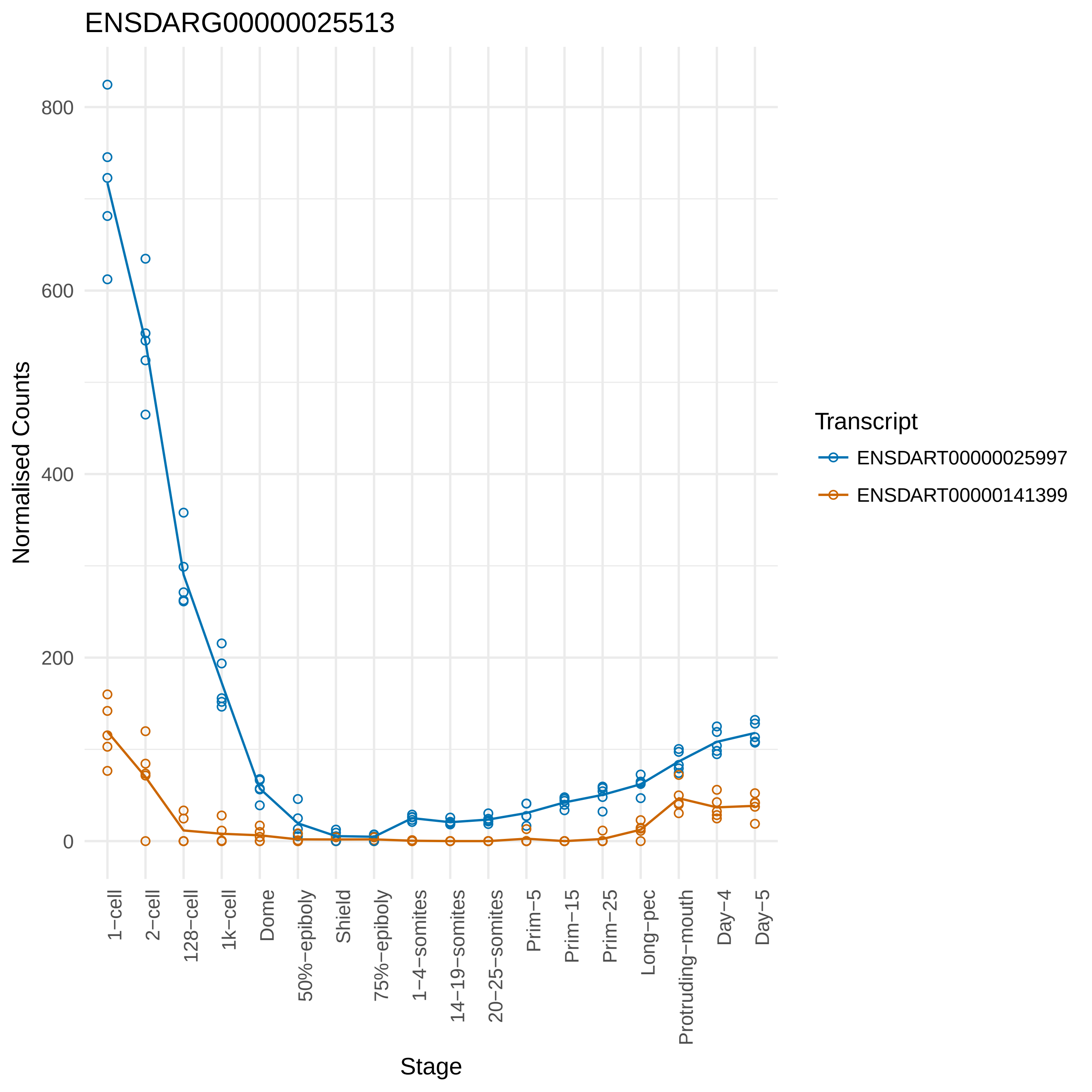

ENSDARG00000025513
Normalised Counts
0
200
400
600
800
Stage
1−cell
2−cell
128−cell
1k−cell
Dome
50%−epiboly
Shield
75%−epiboly
1−4−somites
14−19−somites
20−25−somites
Prim−5
Prim−15
Prim−25
Long−pec
Protruding−mouth
Day−4
Day−5
Transcript
ENSDART00000025997
ENSDART00000141399

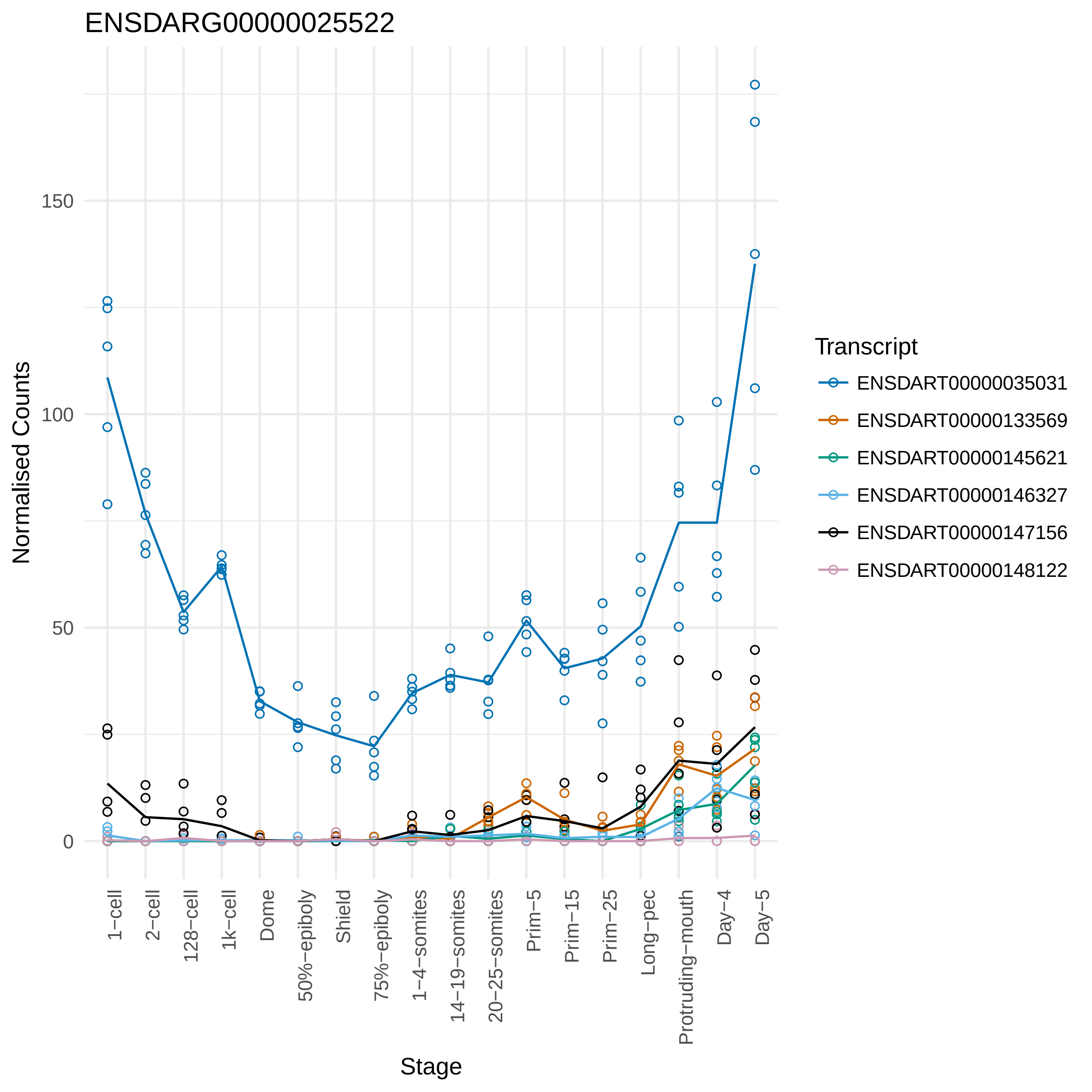
ENSDARG00000025522
Normalised Counts
150
100
50
0
1−cell
2−cell
128−cell
1k−cell
Dome
50%−epiboly
Shield
75%−epiboly
1−4−somites
14−19−somites
20−25−somites
Prim−5
Prim−15
Prim−25
Long−pec
Protruding−mouth
Day−4
Day−5
Stage
Transcript
ENSDART00000035031
ENSDART00000133569
ENSDART00000145621
ENSDART00000146327
ENSDART00000147156
ENSDART00000148122

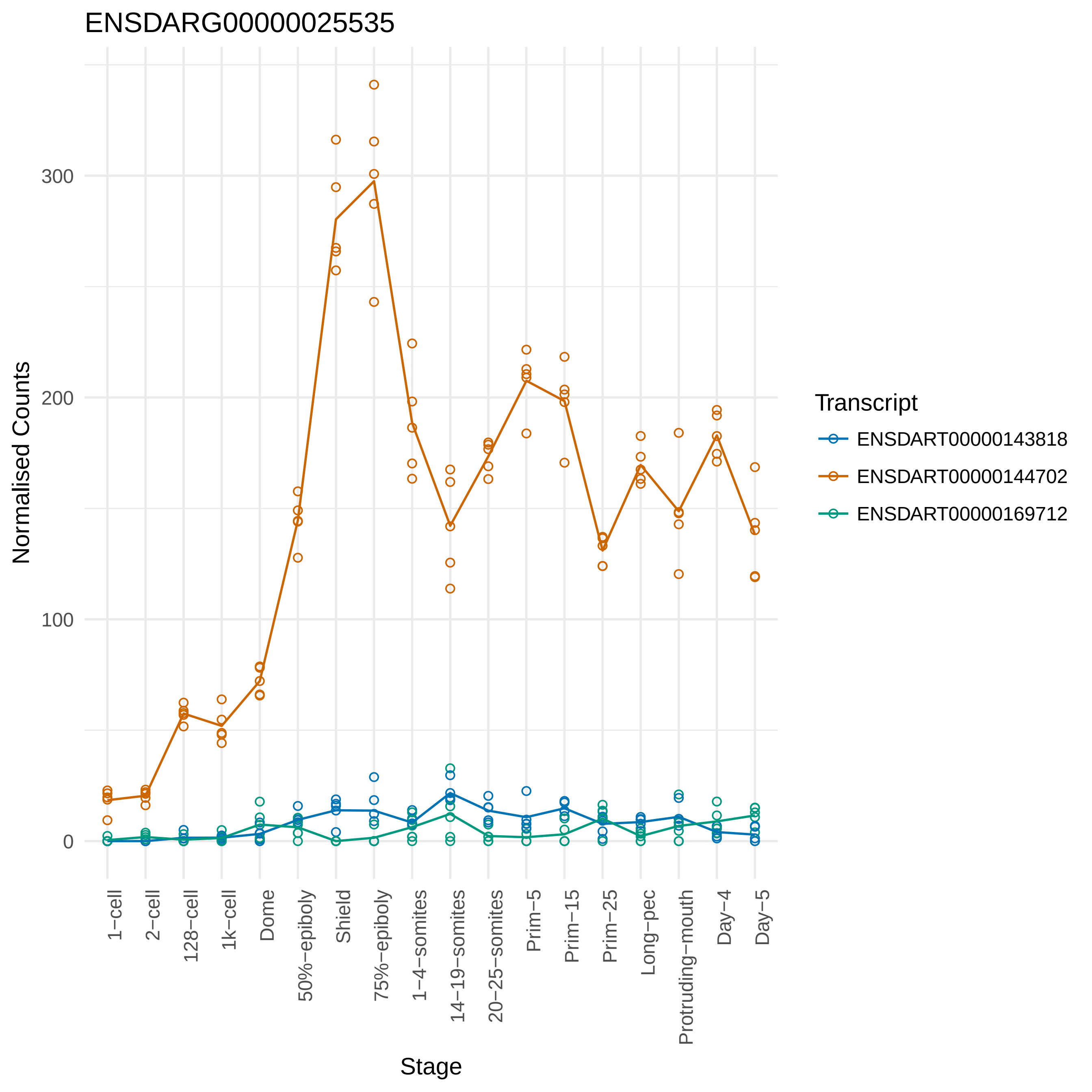

ENSDARG00000025535
Normalised Counts
300
200
100
0
1−cell
2−cell
128−cell
1k−cell
Dome
50%−epiboly
Shield
75%−epiboly
1−4−somites
14−19−somites
20−25−somites
Prim−5
Prim−15
Prim−25
Long−pec
Protruding−mouth
Day−4
Day−5
Stage
Transcript
ENSDART00000143818
ENSDART00000144702
ENSDART00000169712

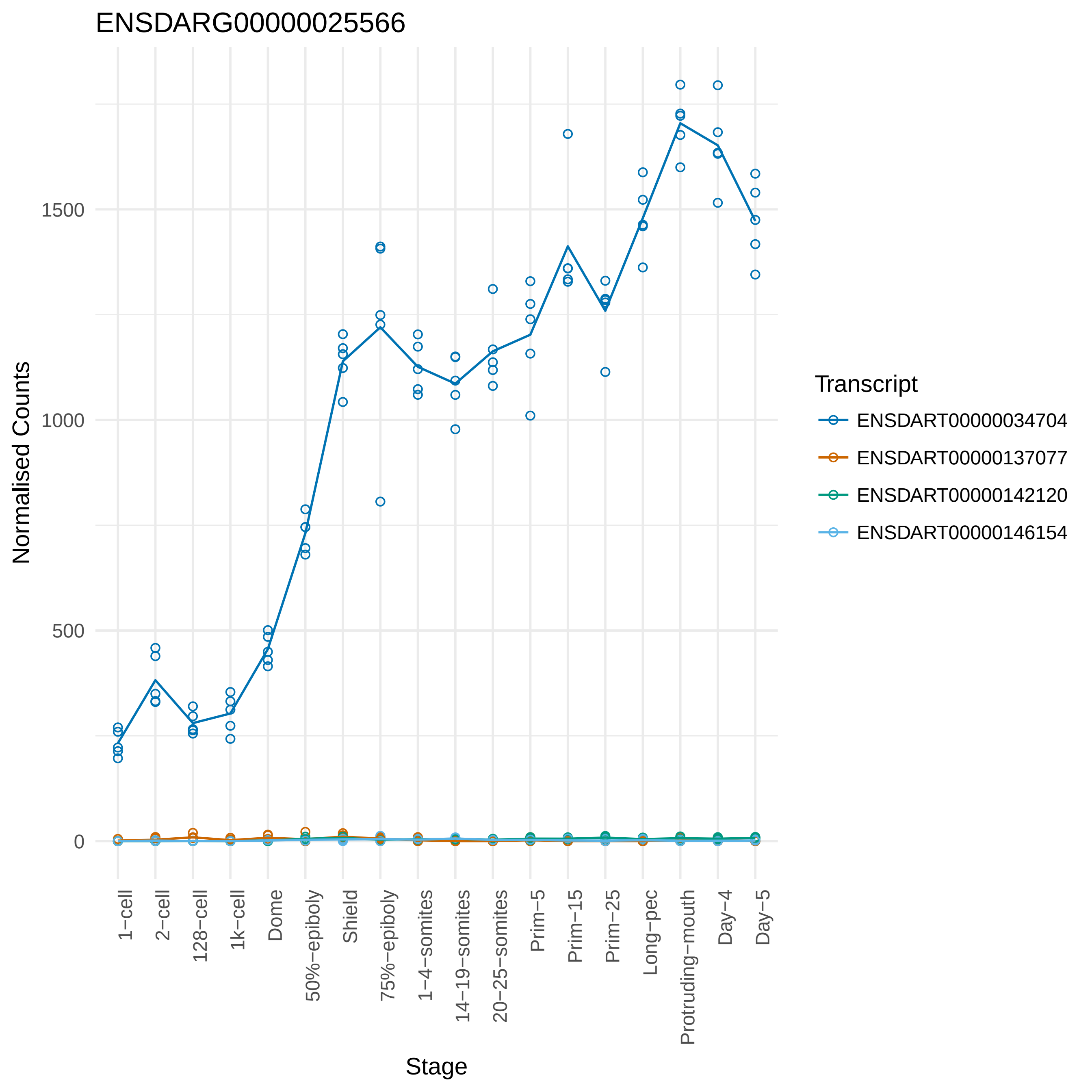
ENSDARG00000025566
Normalised Counts
0
500
1000
1500
Stage
1−cell
2−cell
128−cell
1k−cell
Dome
50%−epiboly
Shield
75%−epiboly
1−4−somites
14−19−somites
20−25−somites
Prim−5
Prim−15
Prim−25
Long−pec
Protruding−mouth
Day−4
Day−5
Transcript
ENSDART00000034704
ENSDART00000137077
ENSDART00000142120
ENSDART00000146154

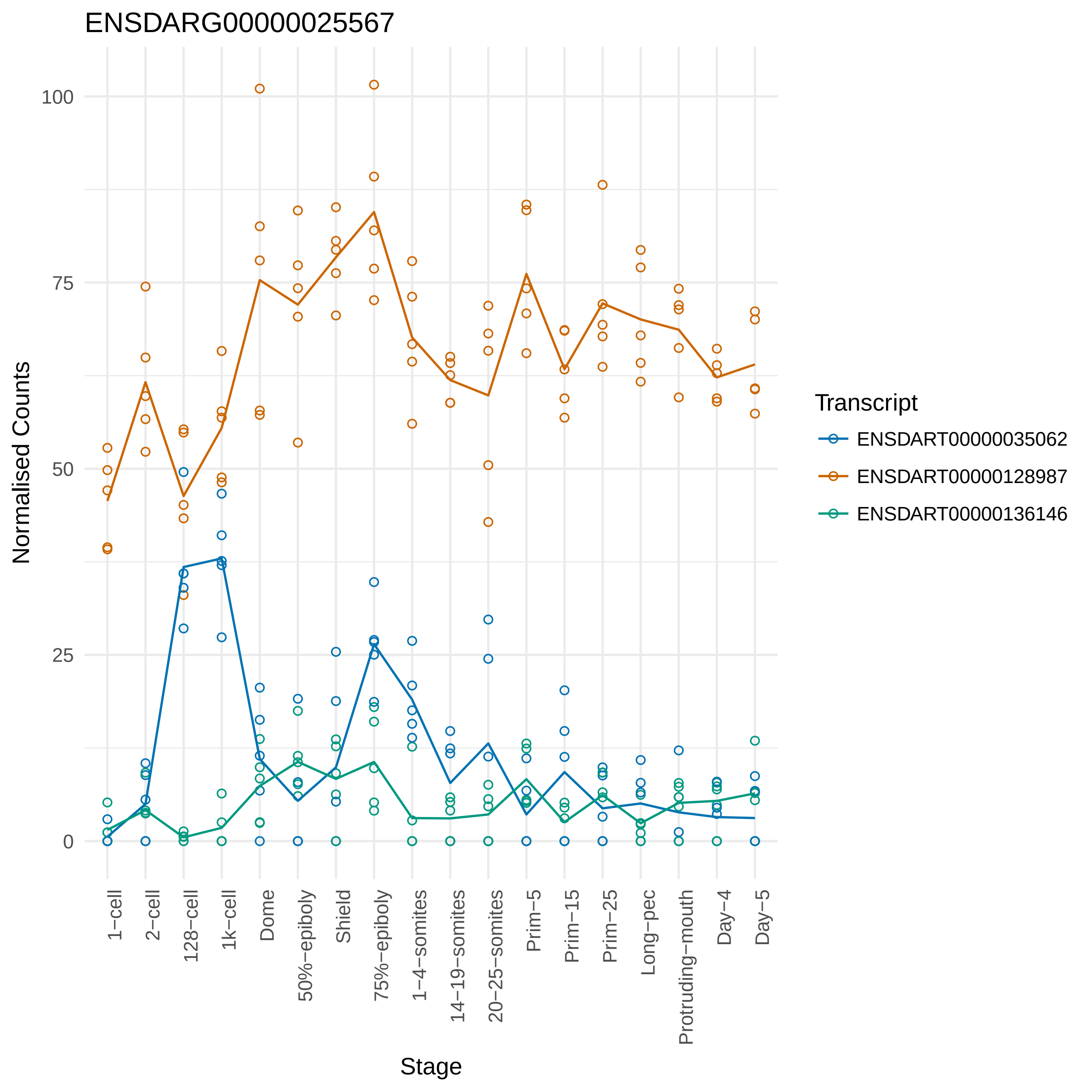
ENSDARG00000025567
Normalised Counts
100
75
50
25
0
1−cell
2−cell
128−cell
1k−cell
Dome
50%−epiboly
Shield
75%−epiboly
1−4−somites
14−19−somites
20−25−somites
Prim−5
Prim−15
Prim−25
Long−pec
Protruding−mouth
Day−4
Day−5
Stage
Transcript
ENSDART00000035062
ENSDART00000128987
ENSDART00000136146

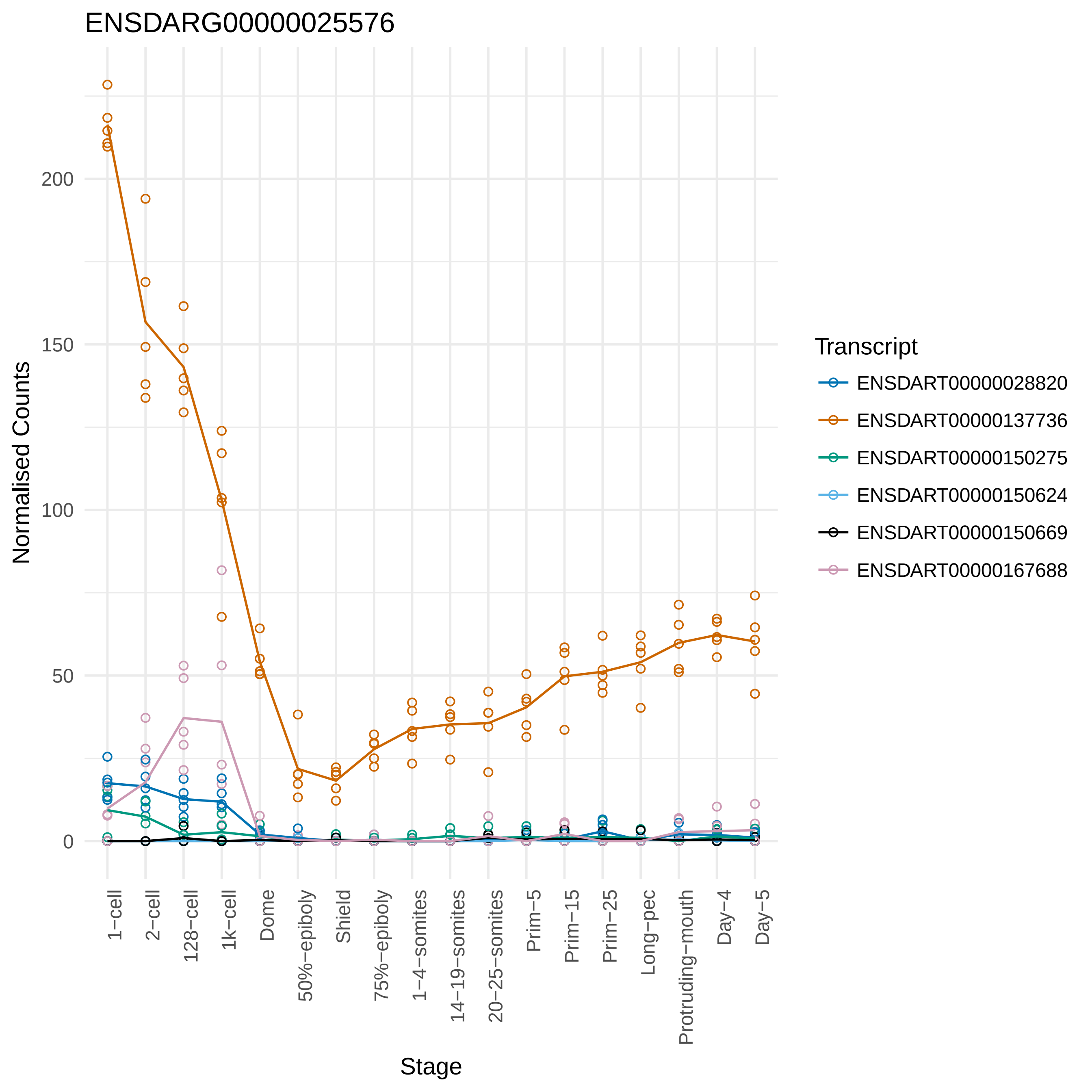

ENSDARG00000025576
Normalised Counts
0
50
100
150
200
Stage
1−cell
2−cell
128−cell
1k−cell
Dome
50%−epiboly
Shield
75%−epiboly
1−4−somites
14−19−somites
20−25−somites
Prim−5
Prim−15
Prim−25
Long−pec
Protruding−mouth
Day−4
Day−5
Transcript
ENSDART00000028820
ENSDART00000137736
ENSDART00000150275
ENSDART00000150624
ENSDART00000150669
ENSDART00000167688

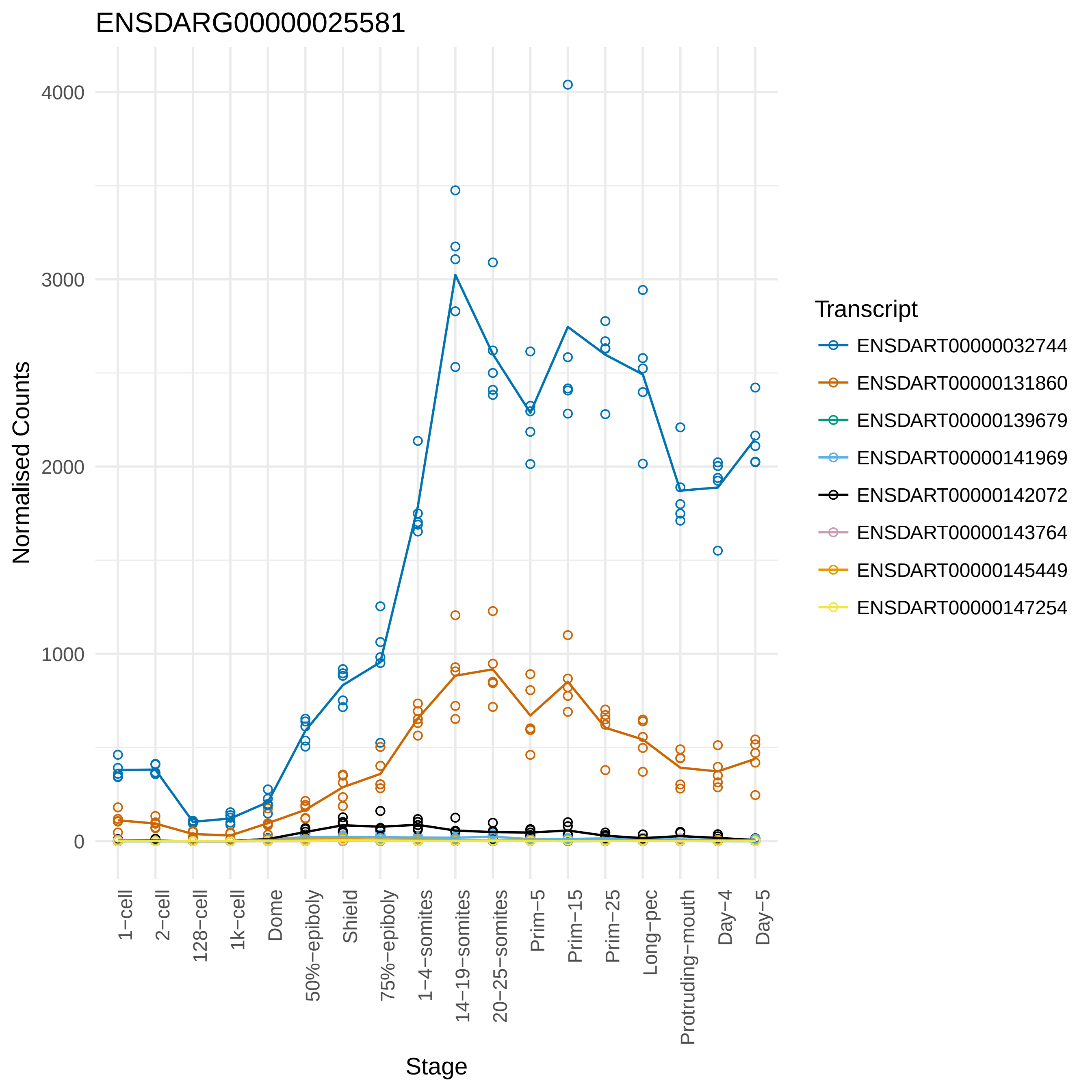
ENSDARG00000025581
Normalised Counts
4000
3000
2000
1000
0
Stage
1−cell
2−cell
128−cell
1k−cell
Dome
50%−epiboly
Shield
75%−epiboly
1−4−somites
14−19−somites
20−25−somites
Prim−5
Prim−15
Prim−25
Long−pec
Protruding−mouth
Day−4
Day−5
Transcript
ENSDART00000032744
ENSDART00000131860
ENSDART00000139679
ENSDART00000141969
ENSDART00000142072
ENSDART00000143764
ENSDART00000145449
ENSDART00000147254

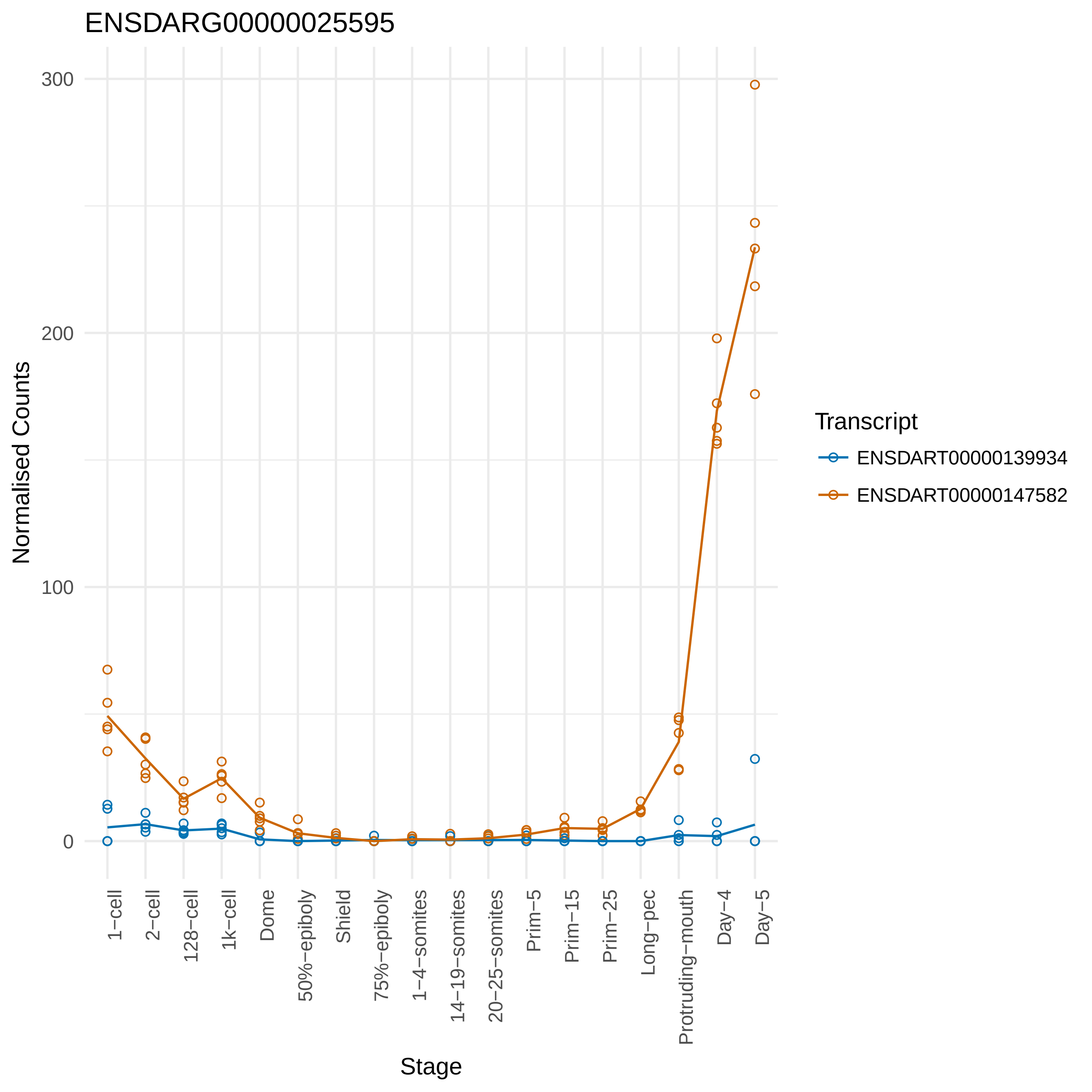

ENSDARG00000025595
Normalised Counts
300
200
100
0
1−cell
2−cell
128−cell
1k−cell
Dome
50%−epiboly
Shield
75%−epiboly
1−4−somites
14−19−somites
20−25−somites
Prim−5
Prim−15
Prim−25
Long−pec
Protruding−mouth
Day−4
Day−5
Stage
Transcript
ENSDART00000139934
ENSDART00000147582

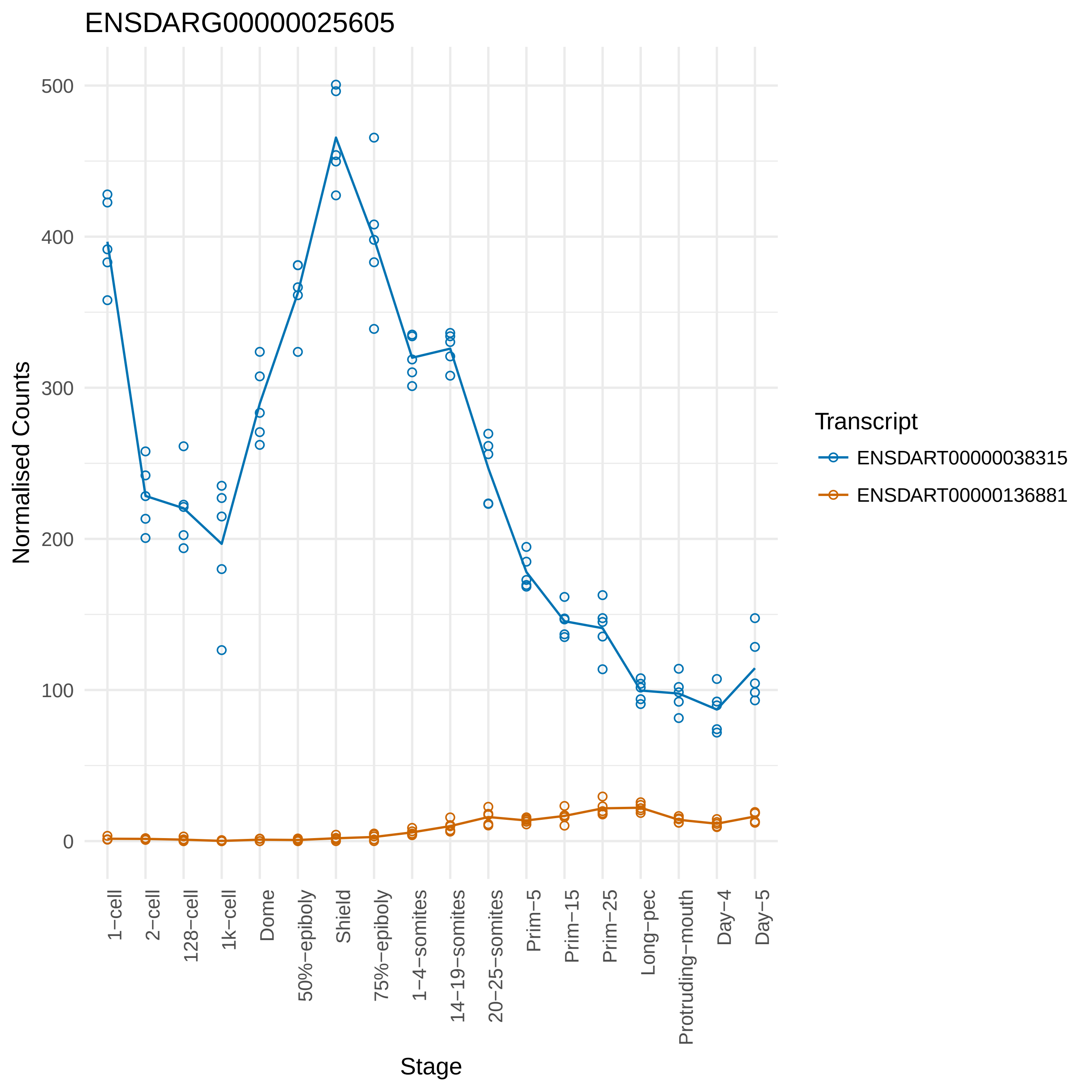

ENSDARG00000025605
Normalised Counts
500
400
300
200
100
0
1−cell
2−cell
128−cell
1k−cell
Dome
50%−epiboly
Shield
75%−epiboly
1−4−somites
14−19−somites
20−25−somites
Prim−5
Prim−15
Prim−25
Long−pec
Protruding−mouth
Day−4
Day−5
Stage
Transcript
ENSDART00000038315
ENSDART00000136881

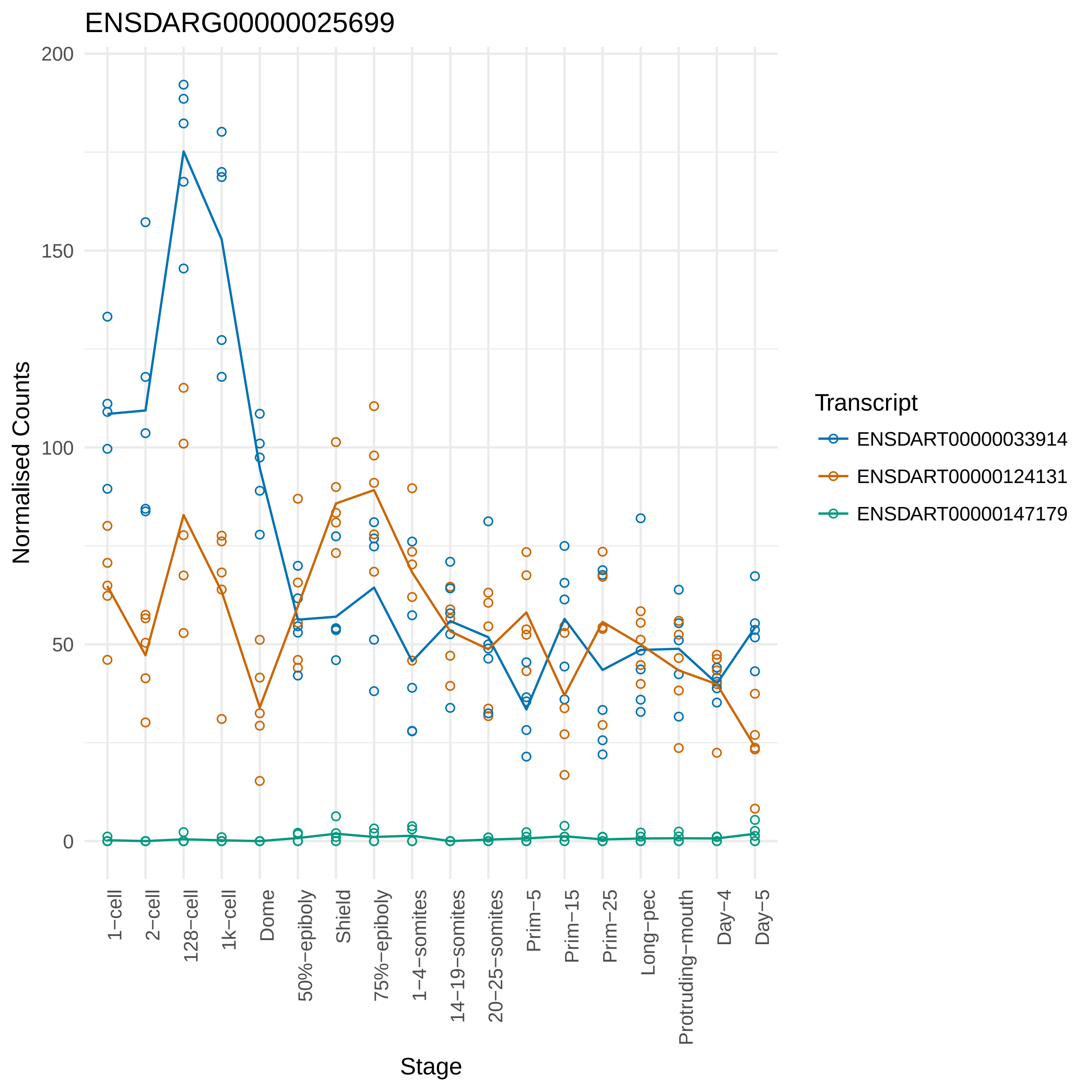

ENSDARG00000025699
Normalised Counts
200
150
100
50
0
1−cell
2−cell
128−cell
1k−cell
Dome
50%−epiboly
Shield
75%−epiboly
1−4−somites
14−19−somites
20−25−somites
Prim−5
Prim−15
Prim−25
Long−pec
Protruding−mouth
Day−4
Day−5
Stage
Transcript
ENSDART00000033914
ENSDART00000124131
ENSDART00000147179

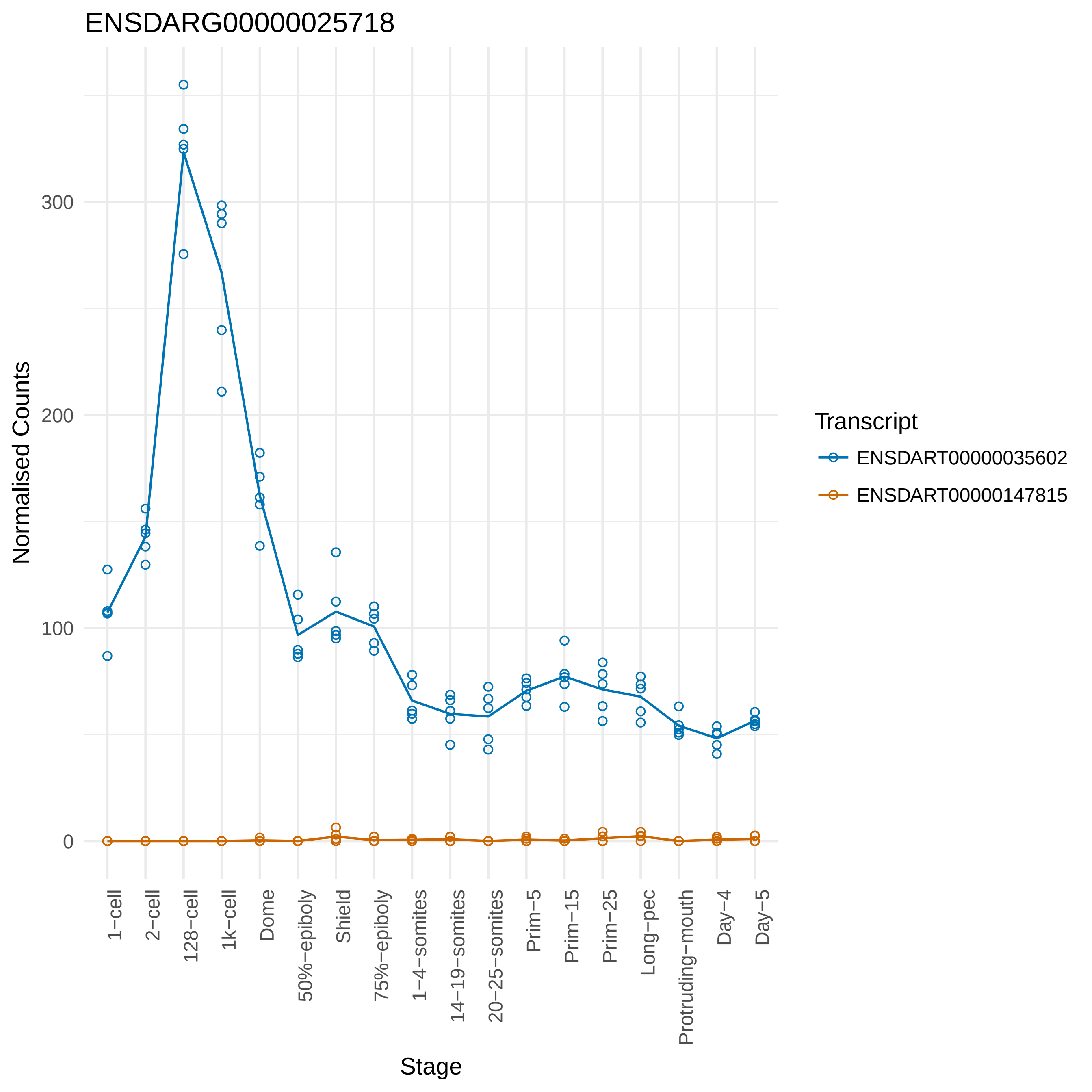
ENSDARG00000025718
Normalised Counts
300
200
100
0
Stage
1−cell
2−cell
128−cell
1k−cell
Dome
50%−epiboly
Shield
75%−epiboly
1−4−somites
14−19−somites
20−25−somites
Prim−5
Prim−15
Prim−25
Long−pec
Protruding−mouth
Day−4
Day−5
Transcript
ENSDART00000035602
ENSDART00000147815

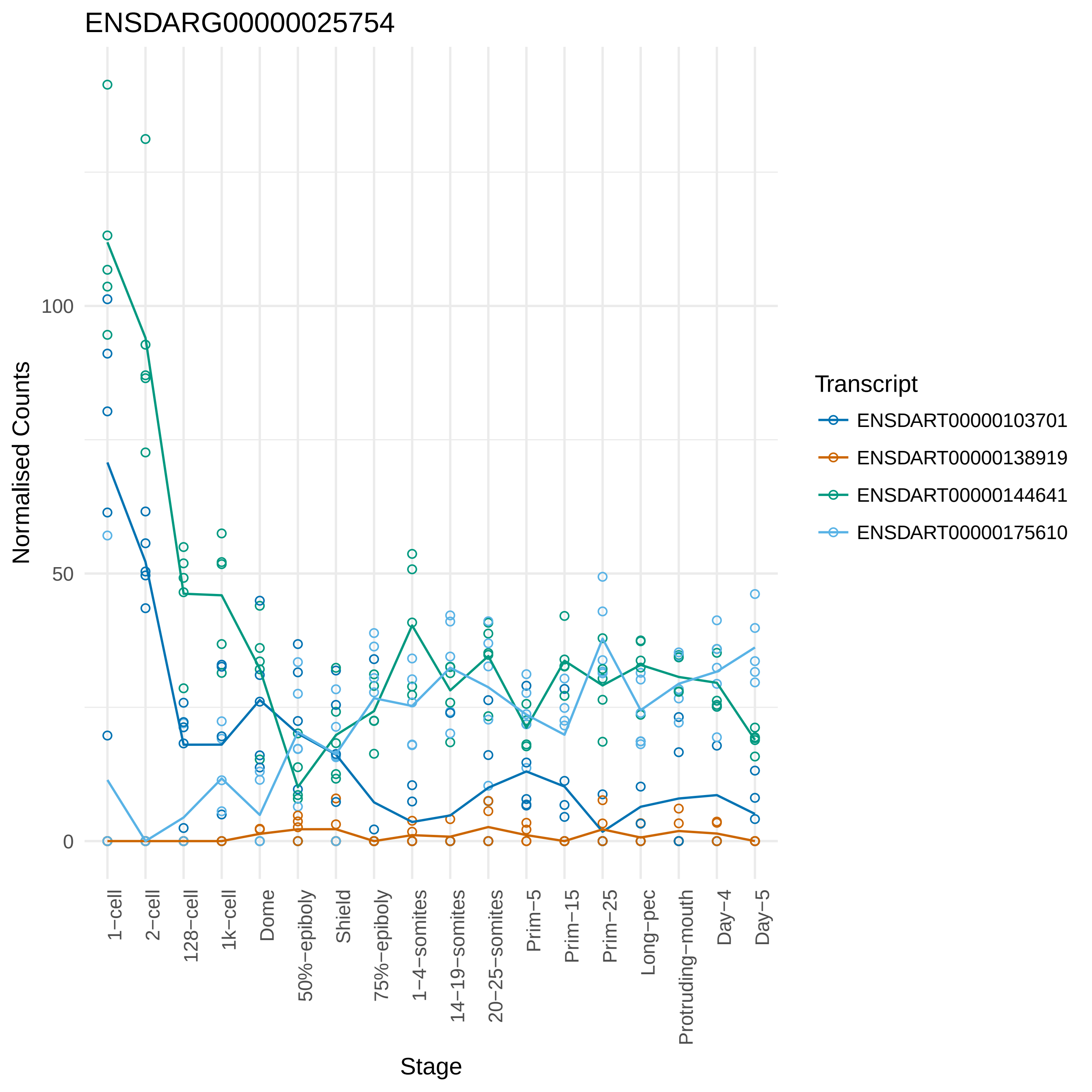

ENSDARG00000025754
Normalised Counts
100
50
0
1−cell
2−cell
128−cell
1k−cell
Dome
50%−epiboly
Shield
75%−epiboly
1−4−somites
14−19−somites
20−25−somites
Prim−5
Prim−15
Prim−25
Long−pec
Protruding−mouth
Day−4
Day−5
Stage
Transcript
ENSDART00000103701
ENSDART00000138919
ENSDART00000144641
ENSDART00000175610

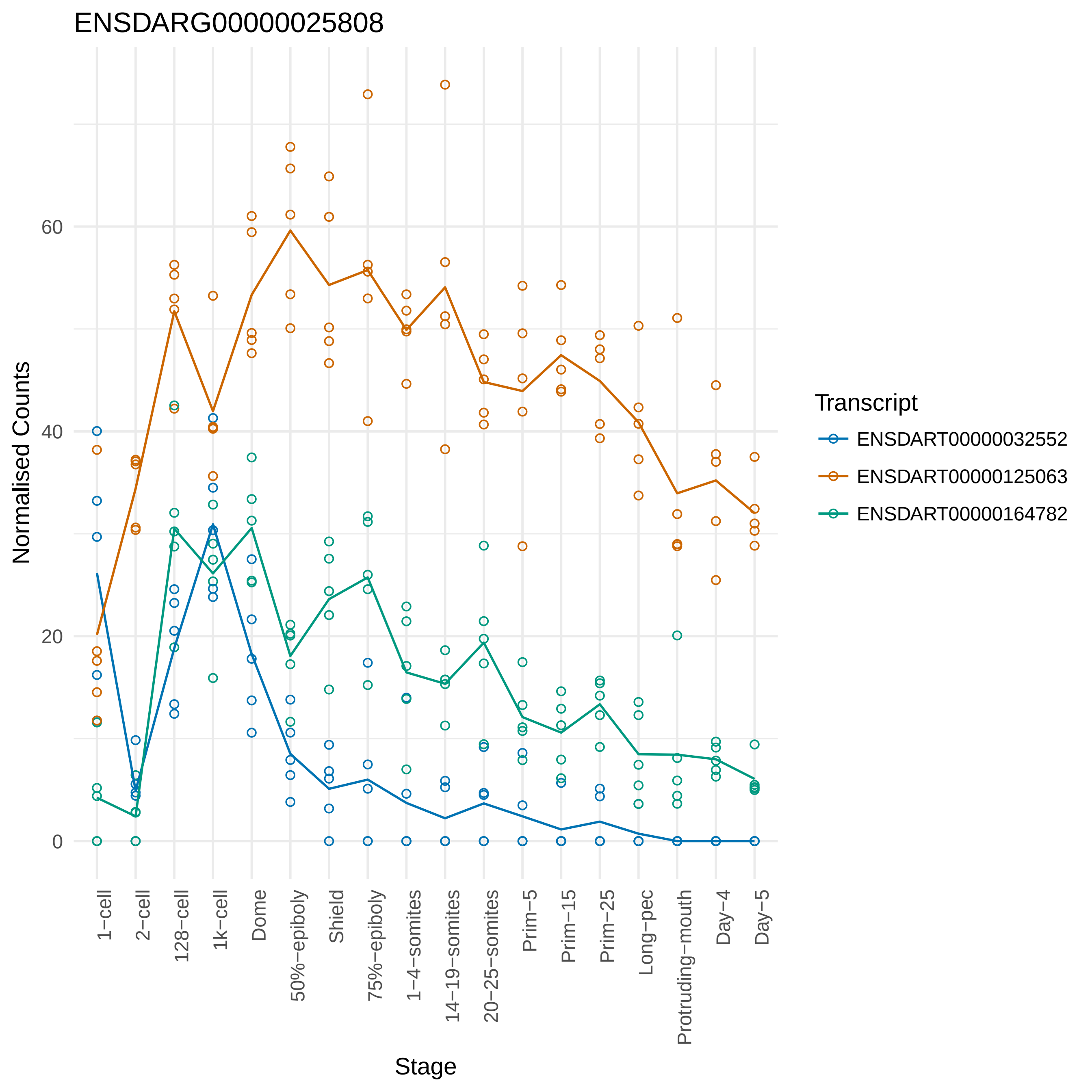
ENSDARG00000025808
Normalised Counts
0
20
40
60
Stage
1−cell
2−cell
128−cell
1k−cell
Dome
50%−epiboly
Shield
75%−epiboly
1−4−somites
14−19−somites
20−25−somites
Prim−5
Prim−15
Prim−25
Long−pec
Protruding−mouth
Day−4
Day−5
Transcript
ENSDART00000032552
ENSDART00000125063
ENSDART00000164782

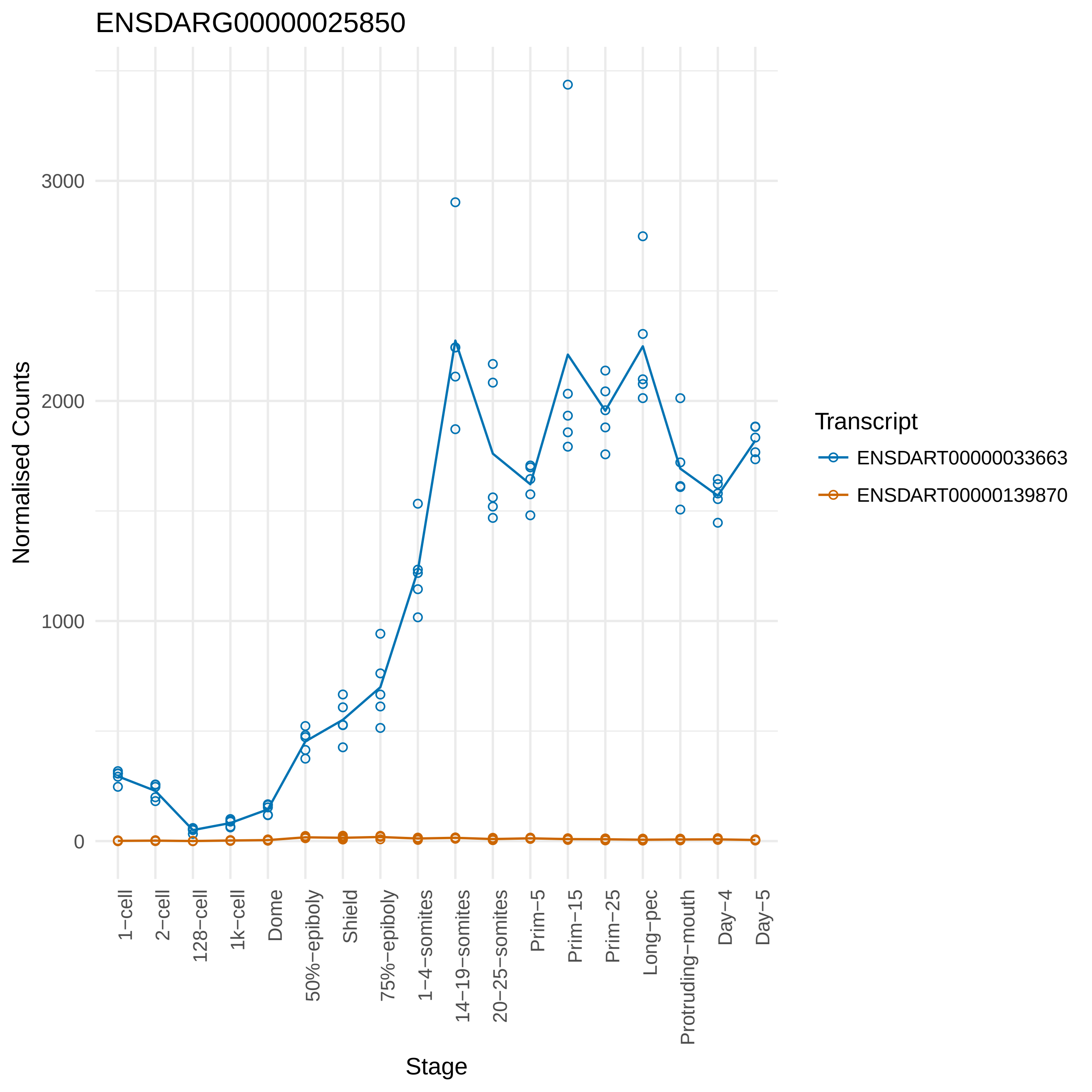

ENSDARG00000025850
Normalised Counts
0
1000
2000
3000
Stage
1−cell
2−cell
128−cell
1k−cell
Dome
50%−epiboly
Shield
75%−epiboly
1−4−somites
14−19−somites
20−25−somites
Prim−5
Prim−15
Prim−25
Long−pec
Protruding−mouth
Day−4
Day−5
Transcript
ENSDART00000033663
ENSDART00000139870

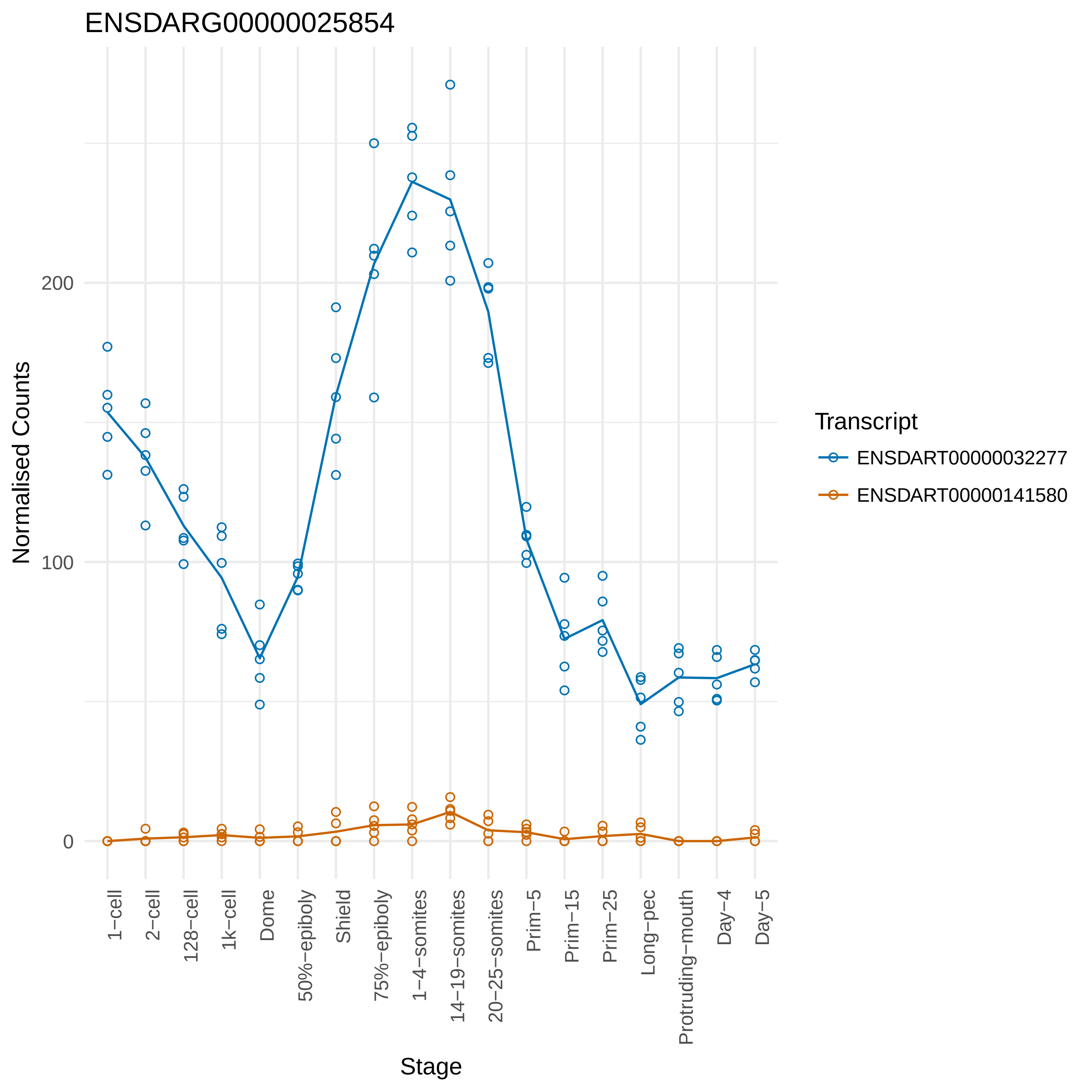

ENSDARG00000025854
Normalised Counts
200
100
0
Stage
1−cell
2−cell
128−cell
1k−cell
Dome
50%−epiboly
Shield
75%−epiboly
1−4−somites
14−19−somites
20−25−somites
Prim−5
Prim−15
Prim−25
Long−pec
Protruding−mouth
Day−4
Day−5
Transcript
ENSDART00000032277
ENSDART00000141580

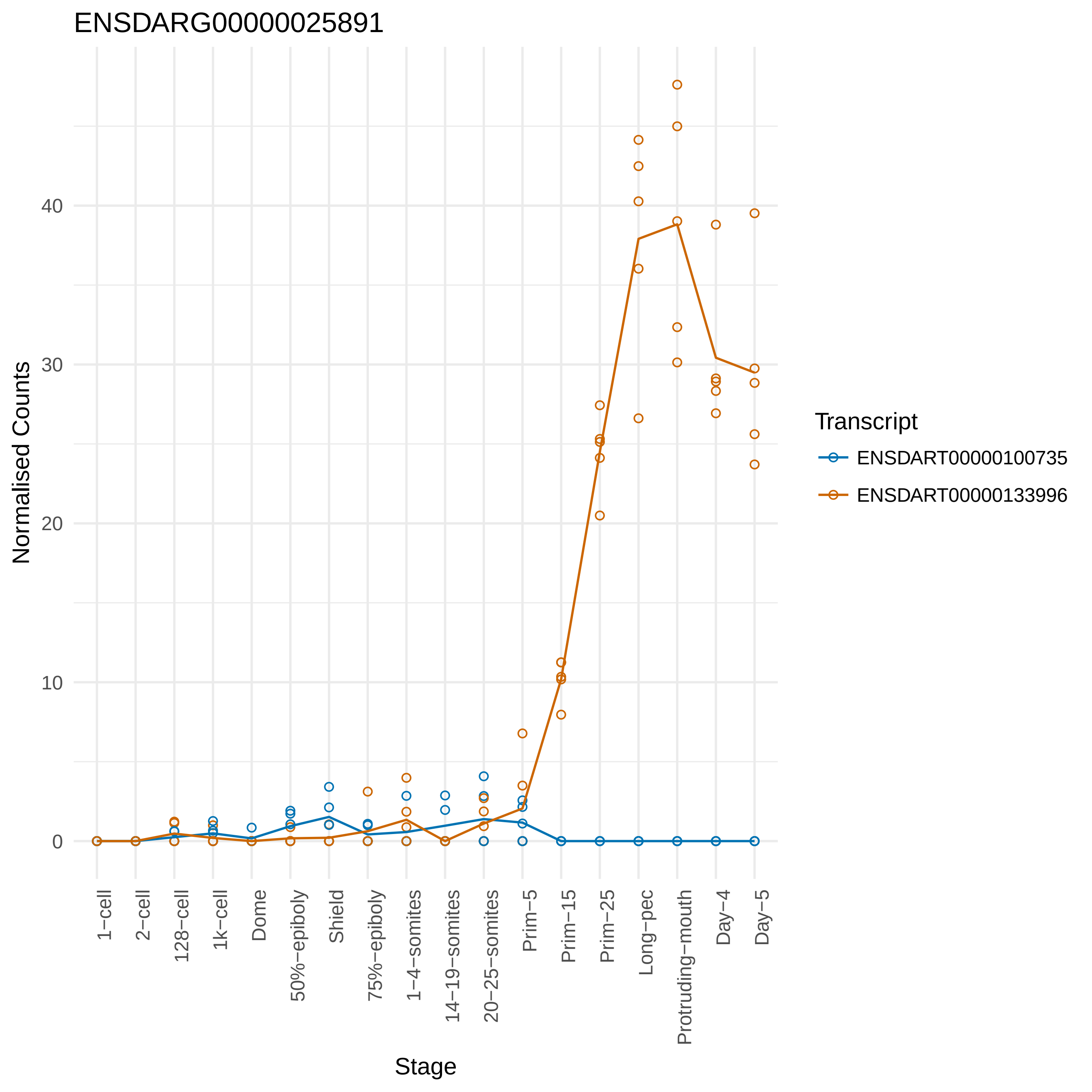

ENSDARG00000025891
Normalised Counts
0
10
20
30
40
Stage
1−cell
2−cell
128−cell
1k−cell
Dome
50%−epiboly
Shield
75%−epiboly
1−4−somites
14−19−somites
20−25−somites
Prim−5
Prim−15
Prim−25
Long−pec
Protruding−mouth
Day−4
Day−5
Transcript
ENSDART00000100735
ENSDART00000133996

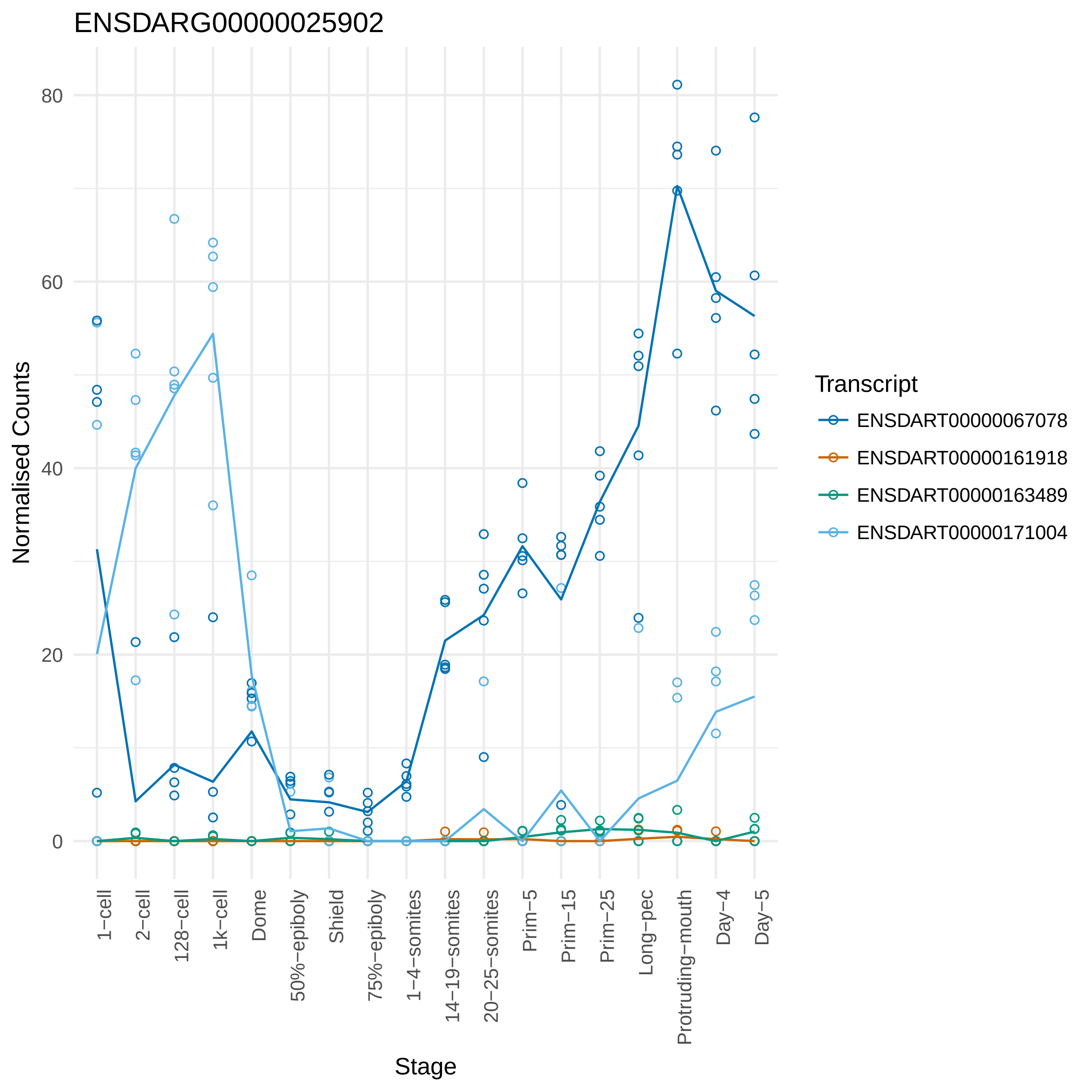

ENSDARG00000025902
Normalised Counts
0
20
40
60
80
Stage
1−cell
2−cell
128−cell
1k−cell
Dome
50%−epiboly
Shield
75%−epiboly
1−4−somites
14−19−somites
20−25−somites
Prim−5
Prim−15
Prim−25
Long−pec
Protruding−mouth
Day−4
Day−5
Transcript
ENSDART00000067078
ENSDART00000161918
ENSDART00000163489
ENSDART00000171004

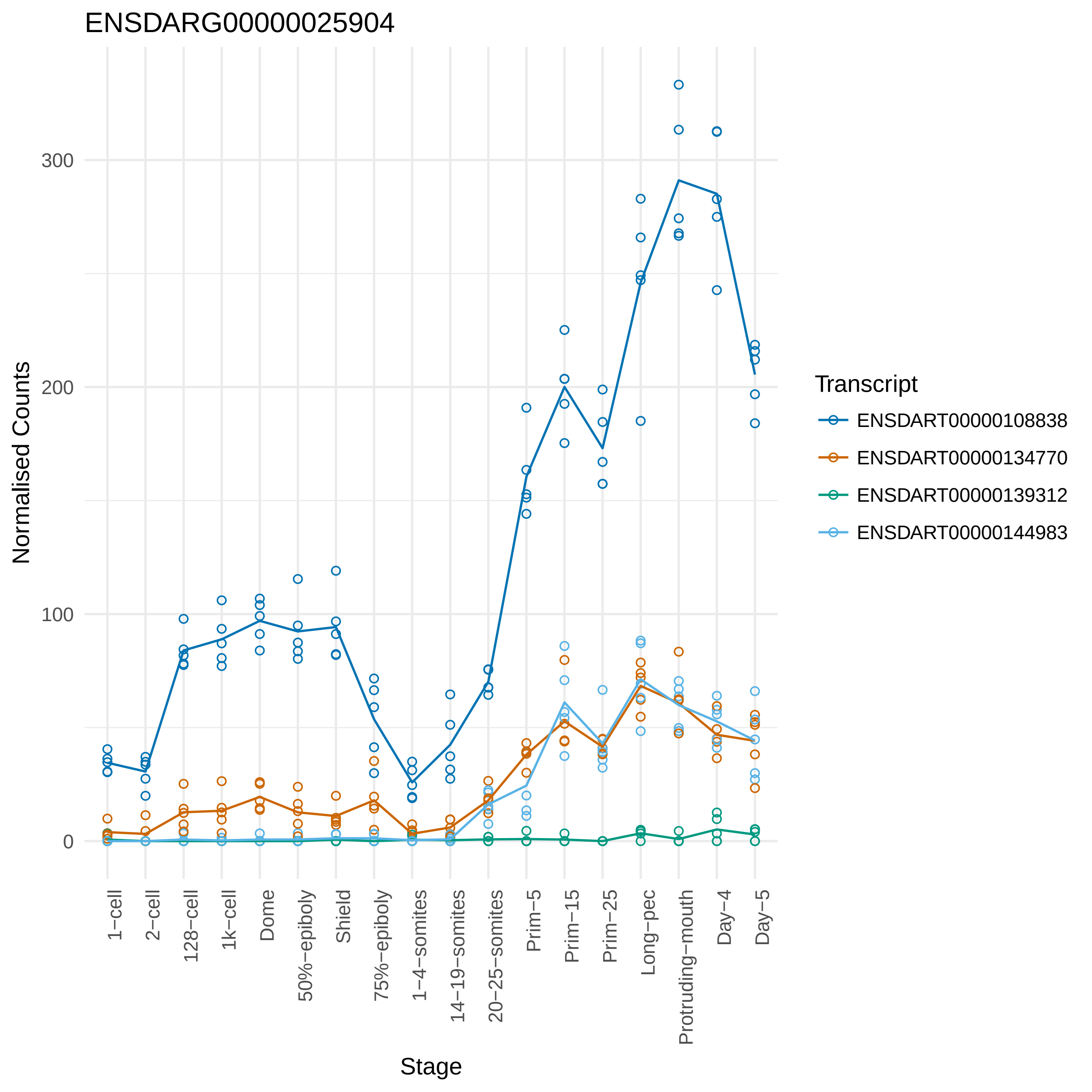

ENSDARG00000025904
Normalised Counts
300
200
100
0
1−cell
2−cell
128−cell
1k−cell
Dome
50%−epiboly
Shield
75%−epiboly
1−4−somites
14−19−somites
20−25−somites
Prim−5
Prim−15
Prim−25
Long−pec
Protruding−mouth
Day−4
Day−5
Stage
Transcript
ENSDART00000108838
ENSDART00000134770
ENSDART00000139312
ENSDART00000144983

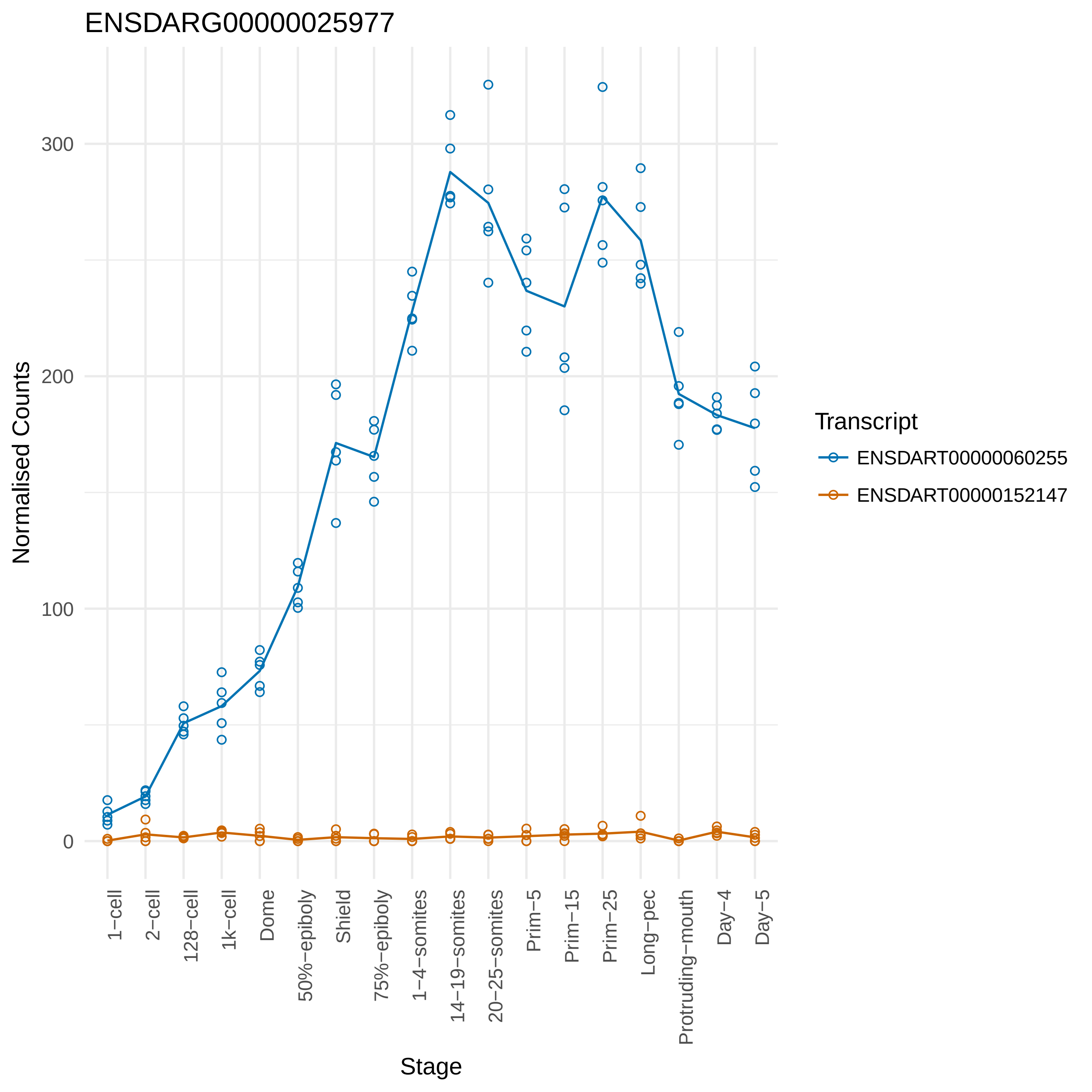

ENSDARG00000025977
Normalised Counts
300
200
100
0
1−cell
2−cell
128−cell
1k−cell
Dome
50%−epiboly
Shield
75%−epiboly
1−4−somites
14−19−somites
20−25−somites
Prim−5
Prim−15
Prim−25
Long−pec
Protruding−mouth
Day−4
Day−5
Stage
Transcript
ENSDART00000060255
ENSDART00000152147

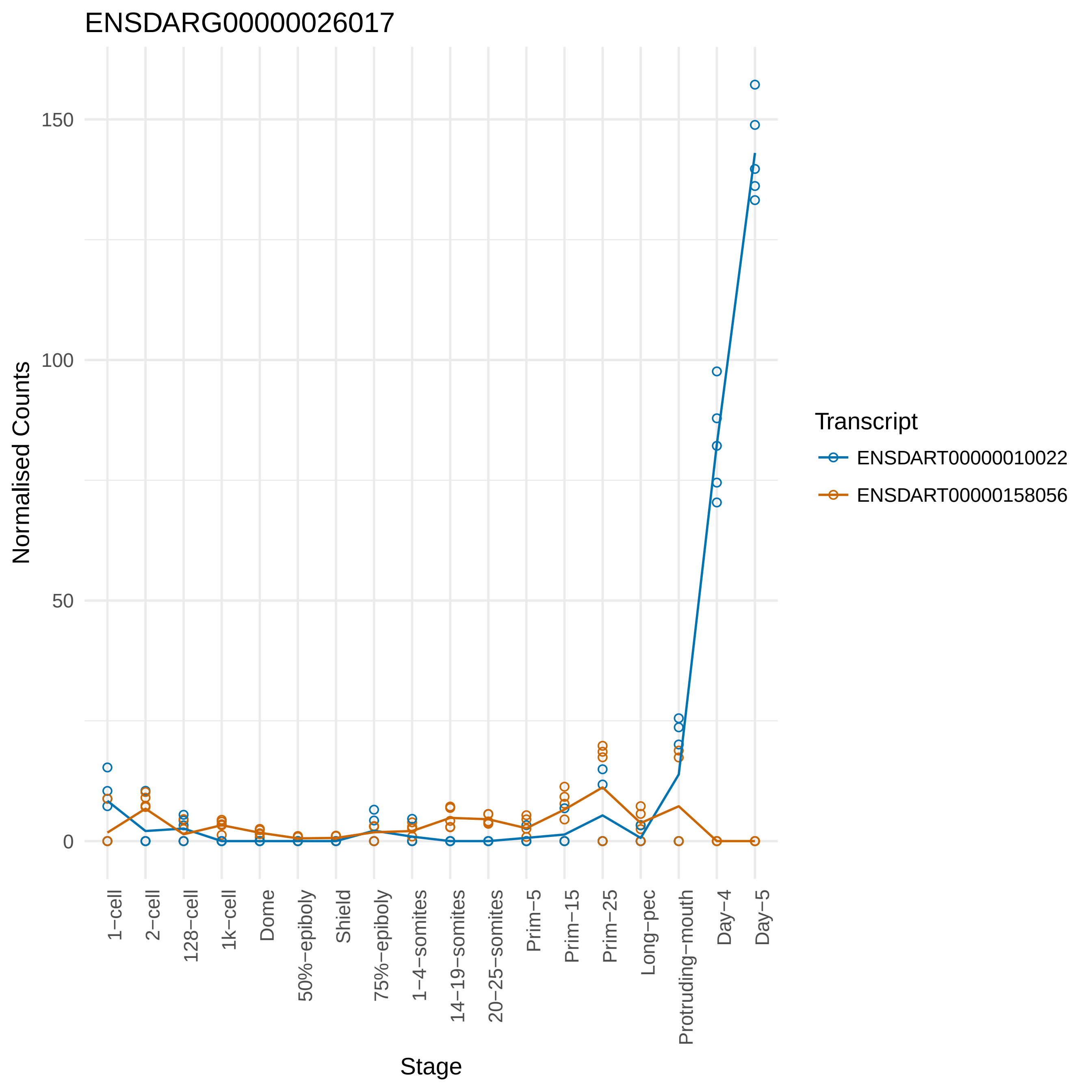

ENSDARG00000026017
Normalised Counts
150
100
50
0
1−cell
2−cell
128−cell
1k−cell
Dome
50%−epiboly
Shield
75%−epiboly
1−4−somites
14−19−somites
20−25−somites
Prim−5
Prim−15
Prim−25
Long−pec
Protruding−mouth
Day−4
Day−5
Stage
Transcript
ENSDART00000010022
ENSDART00000158056

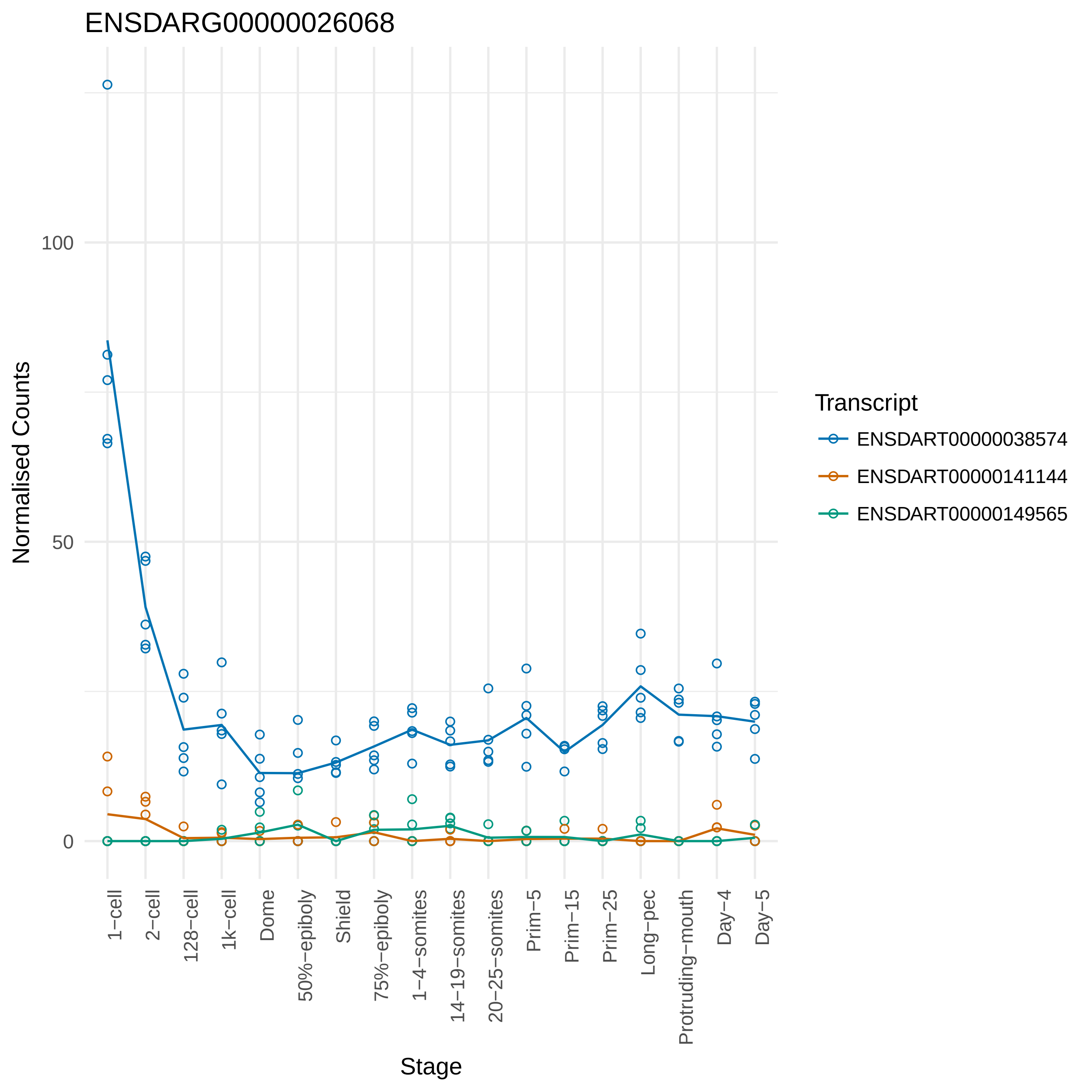

ENSDARG00000026068
Normalised Counts
100
50
0
1−cell
2−cell
128−cell
1k−cell
Dome
50%−epiboly
Shield
75%−epiboly
1−4−somites
14−19−somites
20−25−somites
Prim−5
Prim−15
Prim−25
Long−pec
Protruding−mouth
Day−4
Day−5
Stage
Transcript
ENSDART00000038574
ENSDART00000141144
ENSDART00000149565

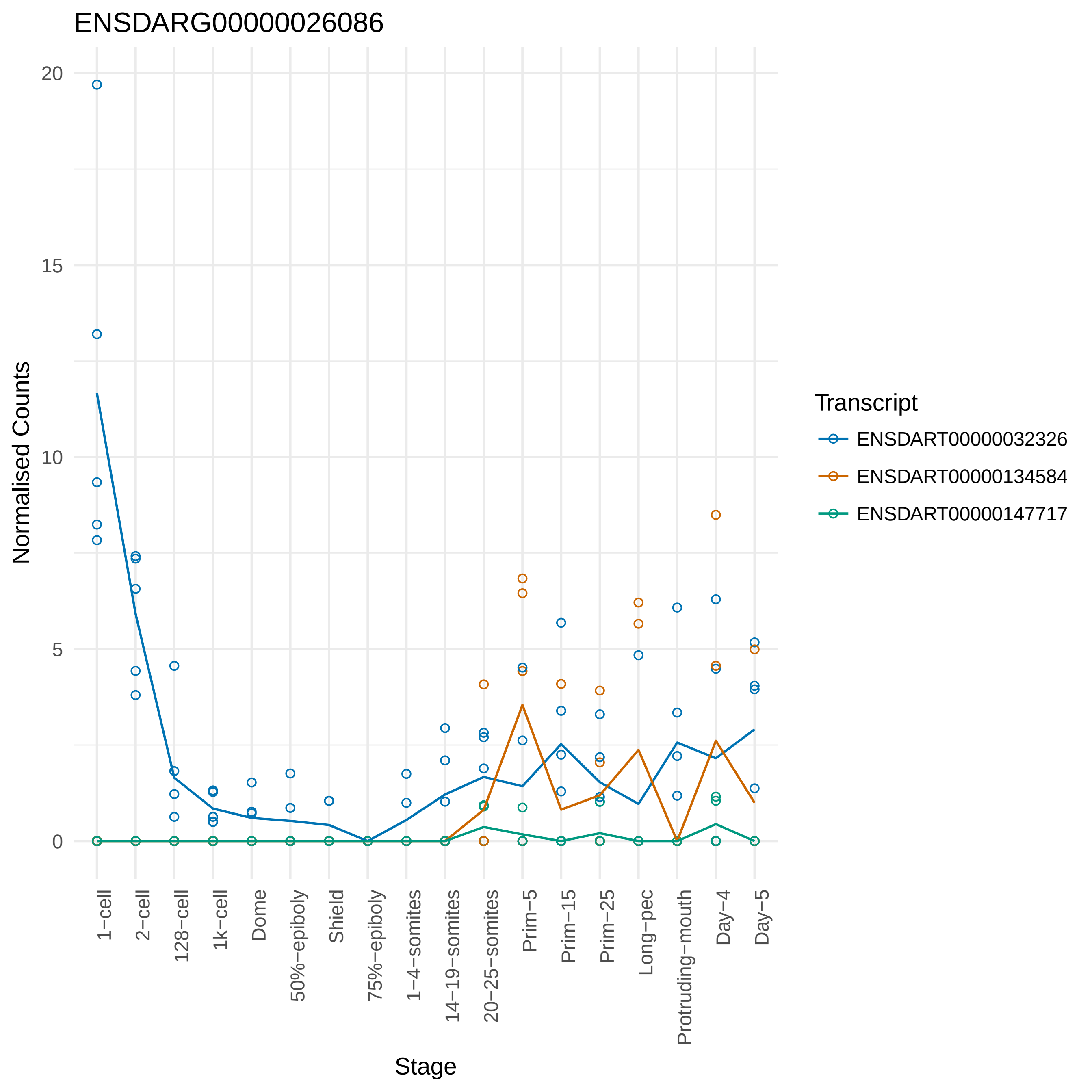

ENSDARG00000026086
Normalised Counts
20
15
10
5
0
1−cell
2−cell
128−cell
1k−cell
Dome
50%−epiboly
Shield
75%−epiboly
1−4−somites
14−19−somites
20−25−somites
Prim−5
Prim−15
Prim−25
Long−pec
Protruding−mouth
Day−4
Day−5
Stage
Transcript
ENSDART00000032326
ENSDART00000134584
ENSDART00000147717

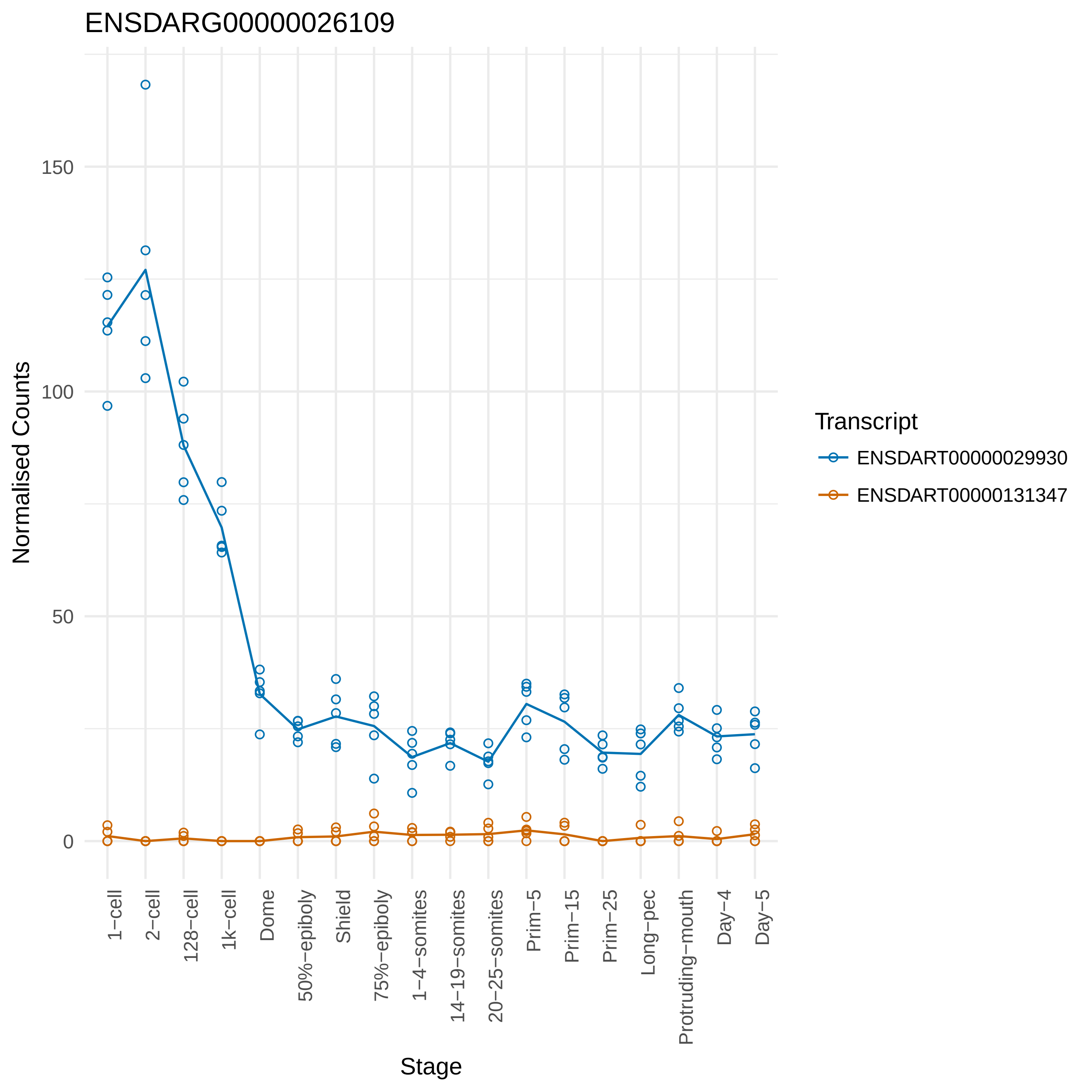
ENSDARG00000026109
Normalised Counts
150
100
50
0
1−cell
2−cell
128−cell
1k−cell
Dome
50%−epiboly
Shield
75%−epiboly
1−4−somites
14−19−somites
20−25−somites
Prim−5
Prim−15
Prim−25
Long−pec
Protruding−mouth
Day−4
Day−5
Stage
Transcript
ENSDART00000029930
ENSDART00000131347

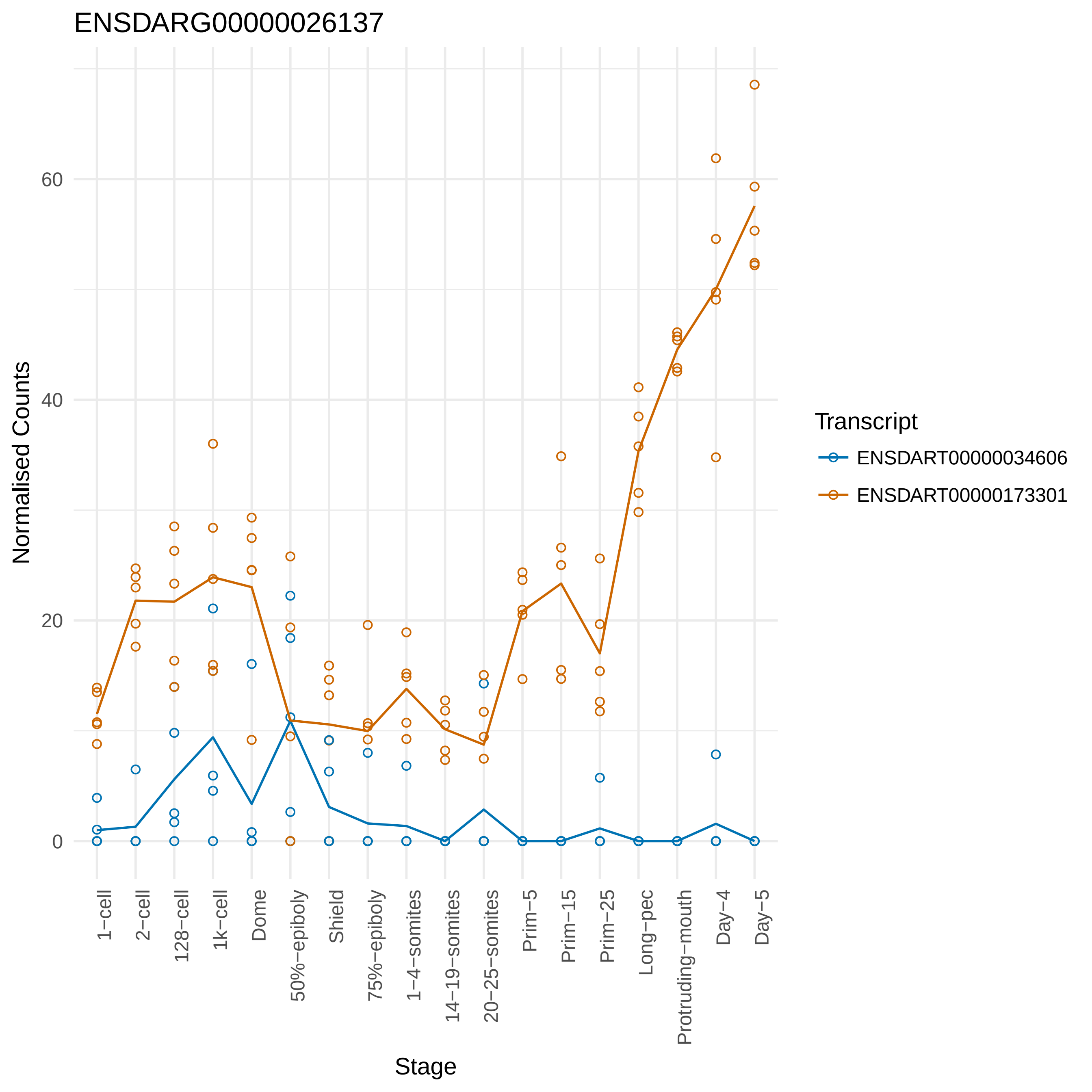

ENSDARG00000026137
Normalised Counts
60
40
20
0
1−cell
2−cell
128−cell
1k−cell
Dome
50%−epiboly
Shield
75%−epiboly
1−4−somites
14−19−somites
20−25−somites
Prim−5
Prim−15
Prim−25
Long−pec
Protruding−mouth
Day−4
Day−5
Stage
Transcript
ENSDART00000034606
ENSDART00000173301

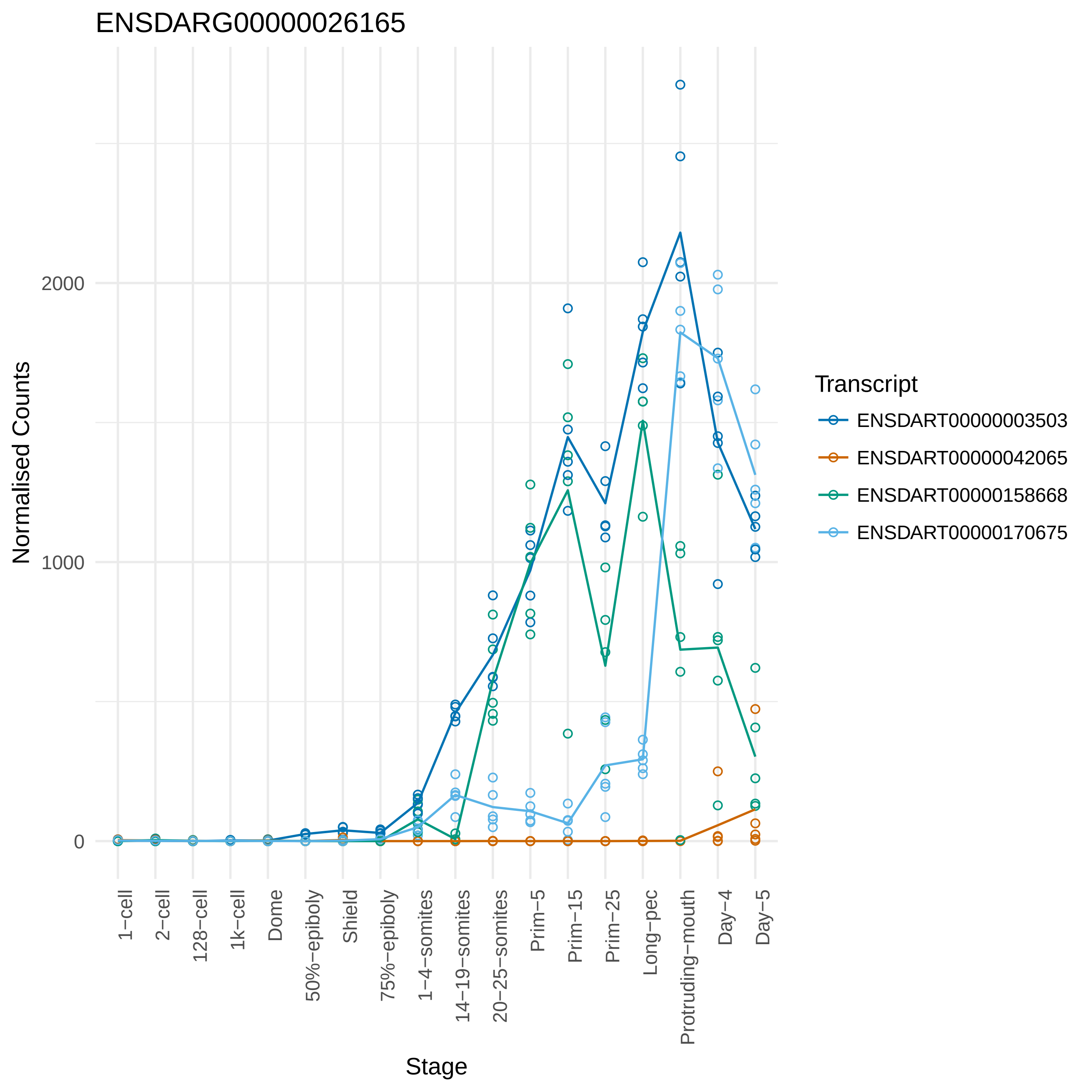

ENSDARG00000026165
Normalised Counts
2000
1000
0
1−cell
2−cell
128−cell
1k−cell
Dome
50%−epiboly
Shield
75%−epiboly
1−4−somites
14−19−somites
20−25−somites
Prim−5
Prim−15
Prim−25
Long−pec
Protruding−mouth
Day−4
Day−5
Stage
Transcript
ENSDART00000003503
ENSDART00000042065
ENSDART00000158668
ENSDART00000170675

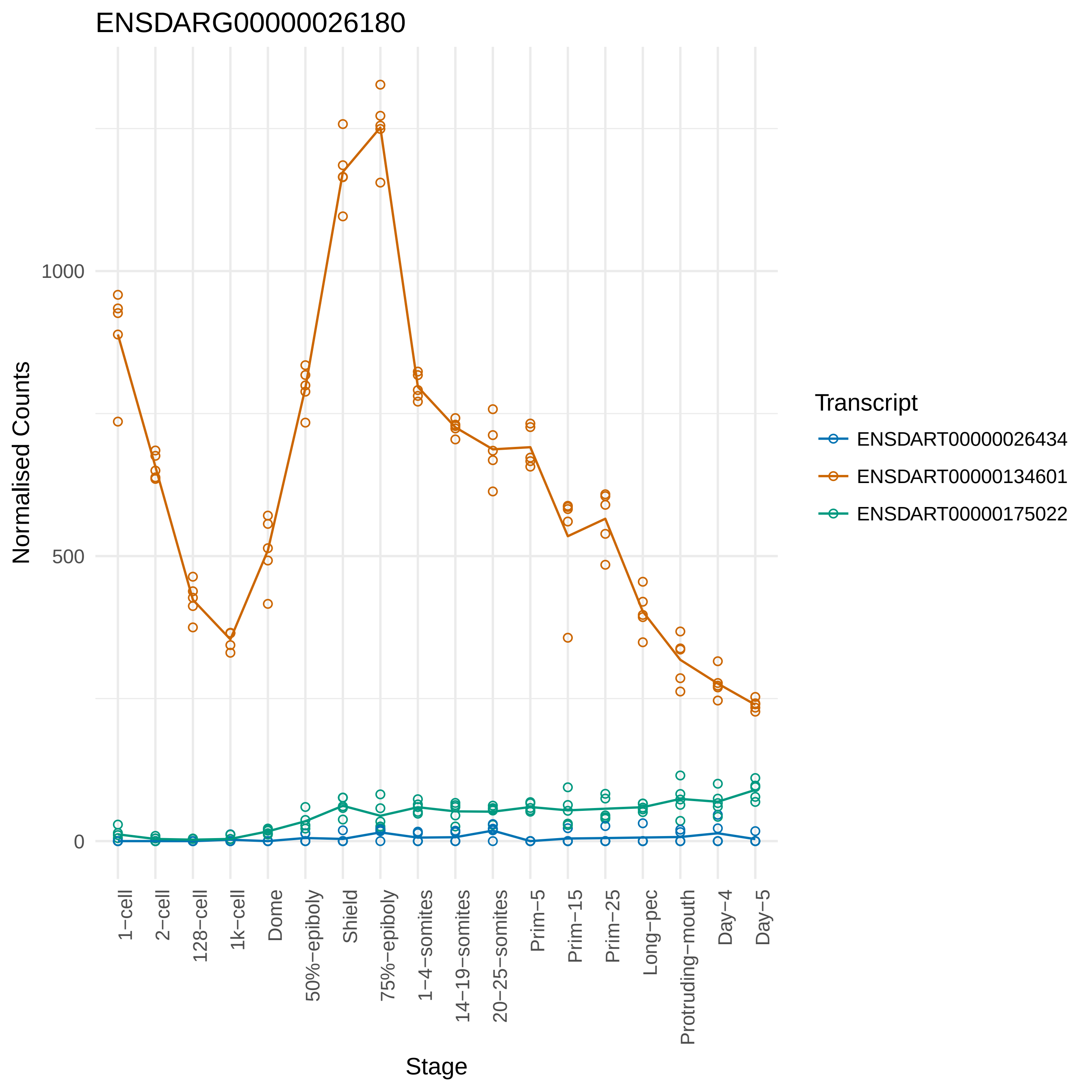

ENSDARG00000026180
Normalised Counts
1000
500
0
Stage
1−cell
2−cell
128−cell
1k−cell
Dome
50%−epiboly
Shield
75%−epiboly
1−4−somites
14−19−somites
20−25−somites
Prim−5
Prim−15
Prim−25
Long−pec
Protruding−mouth
Day−4
Day−5
Transcript
ENSDART00000026434
ENSDART00000134601
ENSDART00000175022

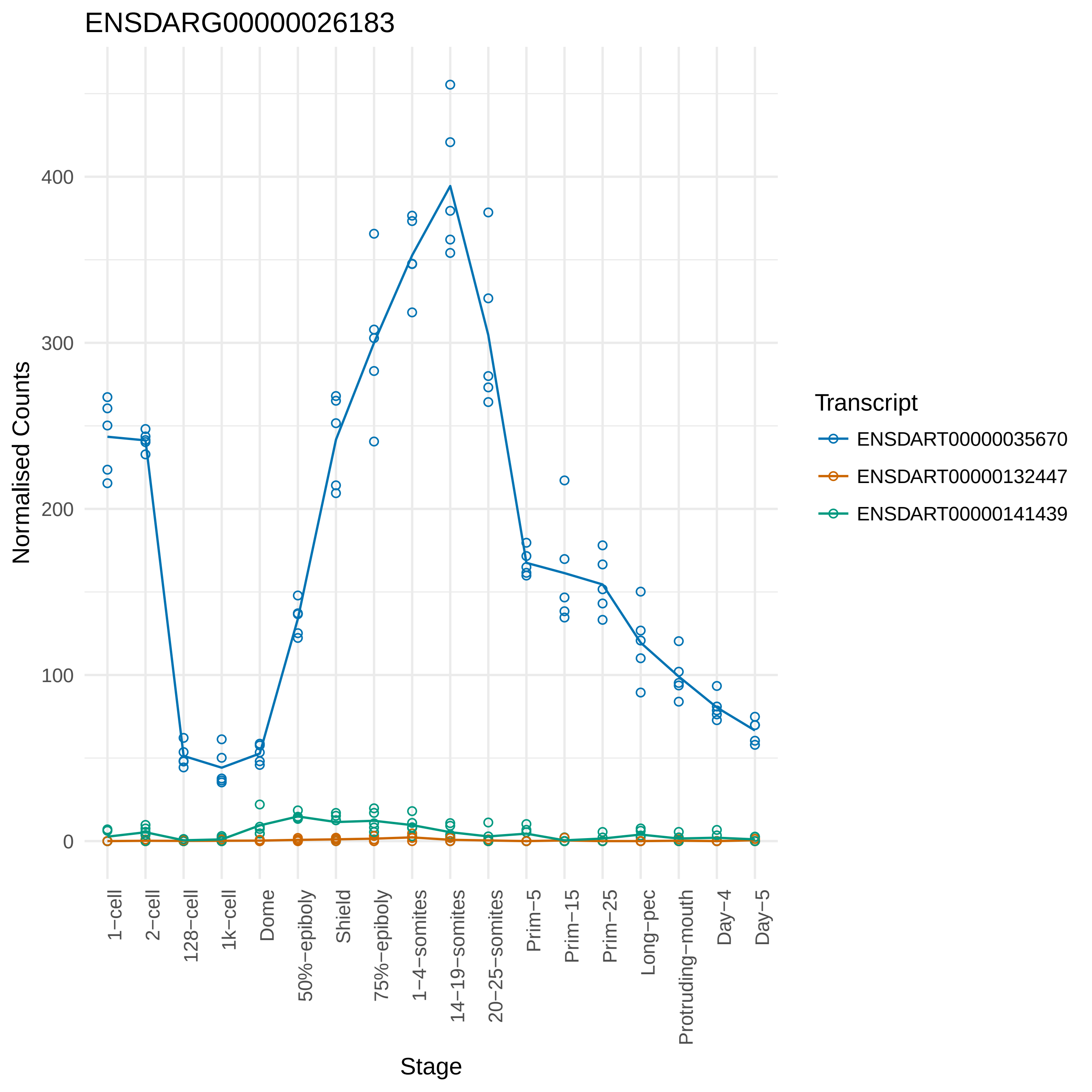
ENSDARG00000026183
Normalised Counts
0
100
200
300
400
Stage
1−cell
2−cell
128−cell
1k−cell
Dome
50%−epiboly
Shield
75%−epiboly
1−4−somites
14−19−somites
20−25−somites
Prim−5
Prim−15
Prim−25
Long−pec
Protruding−mouth
Day−4
Day−5
Transcript
ENSDART00000035670
ENSDART00000132447
ENSDART00000141439

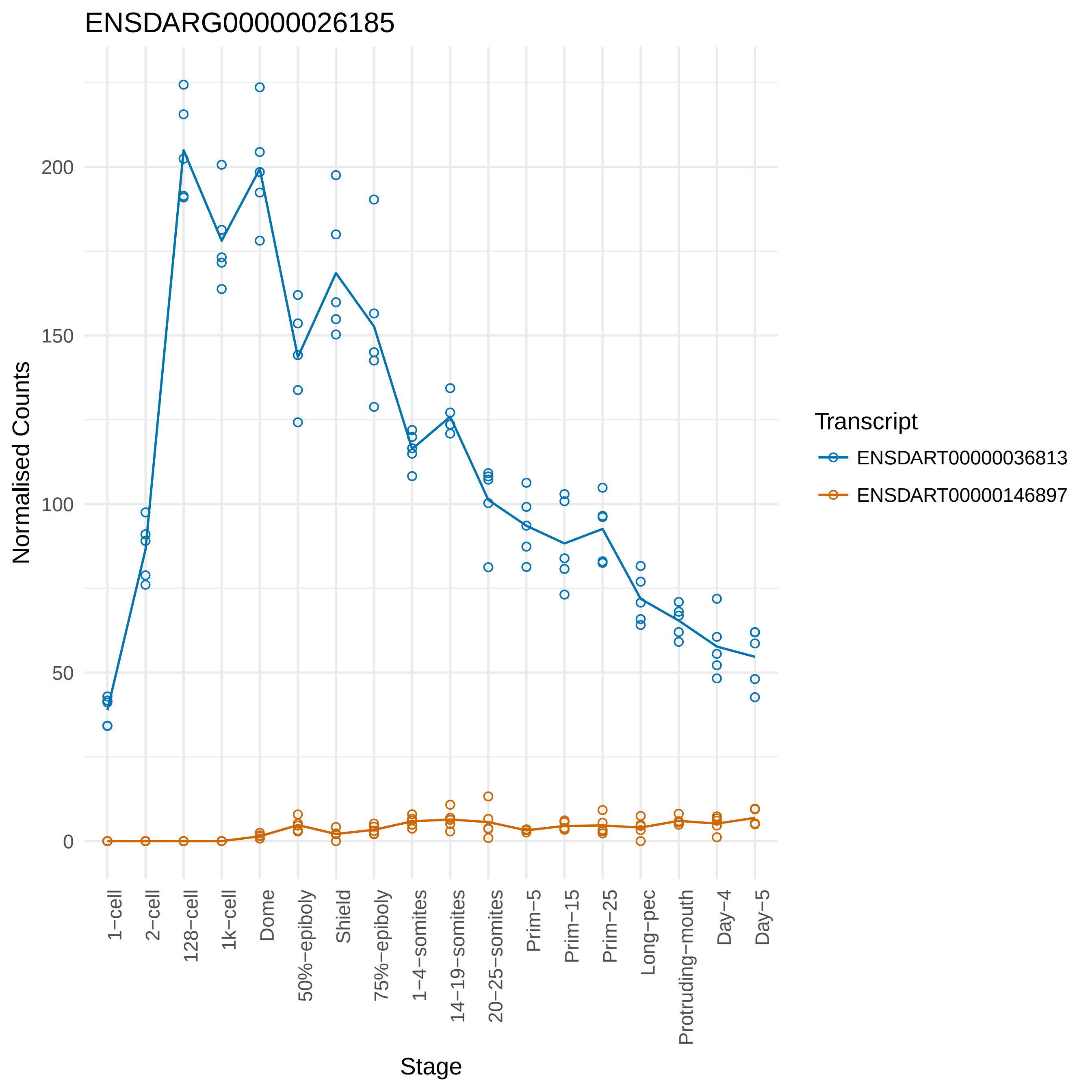
ENSDARG00000026185
Normalised Counts
200
150
100
50
0
1−cell
2−cell
128−cell
1k−cell
Dome
50%−epiboly
Shield
75%−epiboly
1−4−somites
14−19−somites
20−25−somites
Prim−5
Prim−15
Prim−25
Long−pec
Protruding−mouth
Day−4
Day−5
Stage
Transcript
ENSDART00000036813
ENSDART00000146897

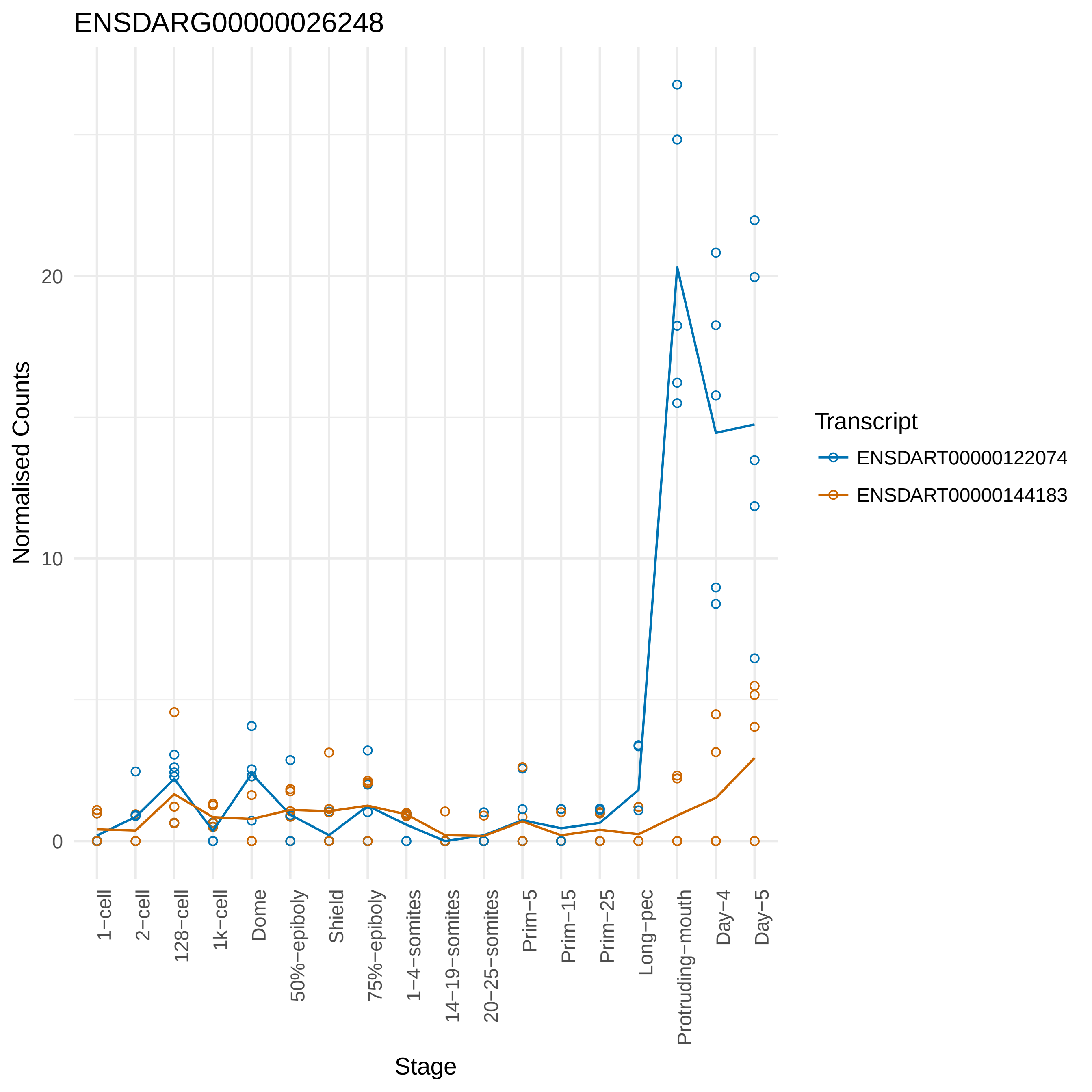
ENSDARG00000026248
Normalised Counts
0
10
20
Stage
1−cell
2−cell
128−cell
1k−cell
Dome
50%−epiboly
Shield
75%−epiboly
1−4−somites
14−19−somites
20−25−somites
Prim−5
Prim−15
Prim−25
Long−pec
Protruding−mouth
Day−4
Day−5
Transcript
ENSDART00000122074
ENSDART00000144183

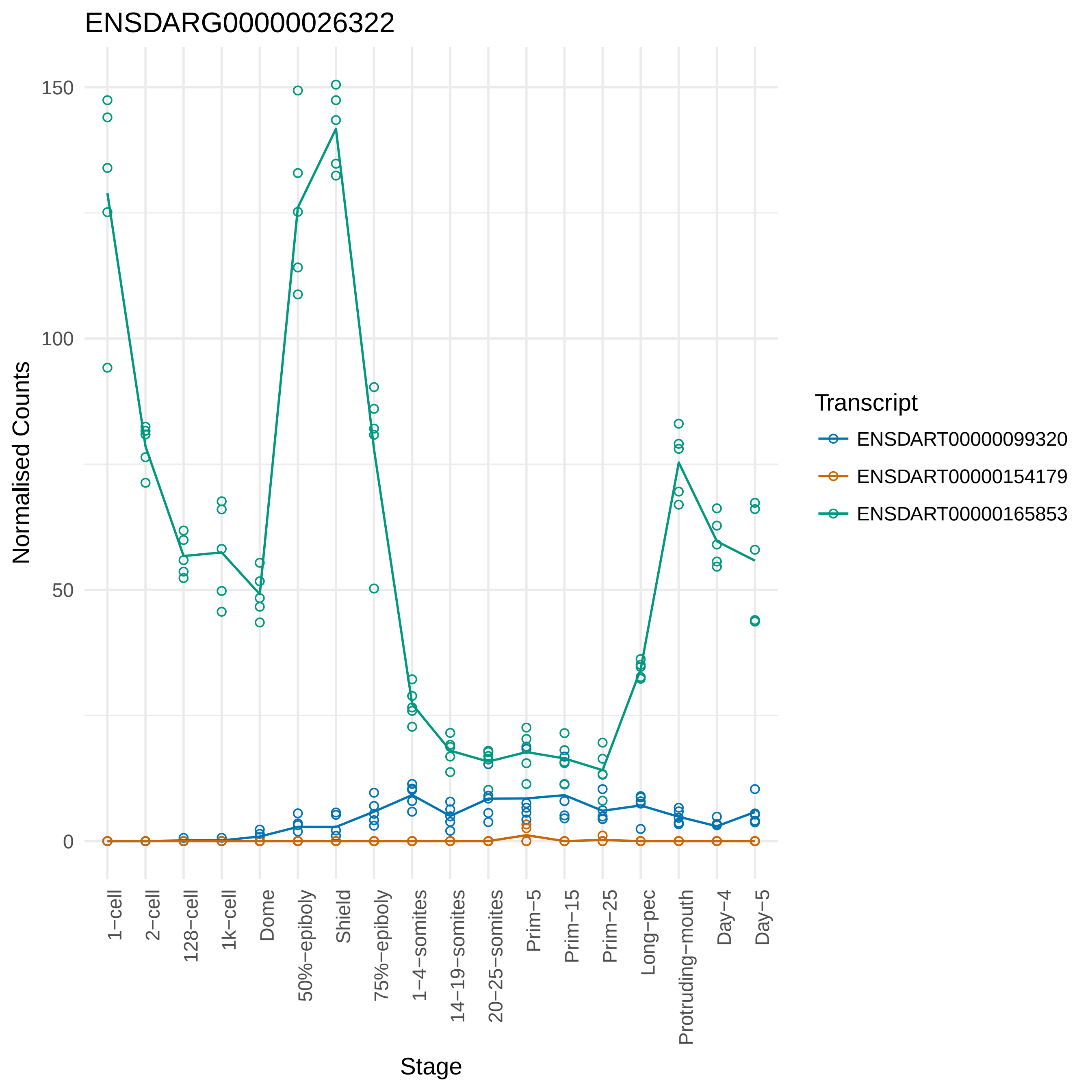
ENSDARG00000026322
Normalised Counts
150
100
50
0
1−cell
2−cell
128−cell
1k−cell
Dome
50%−epiboly
Shield
75%−epiboly
1−4−somites
14−19−somites
20−25−somites
Prim−5
Prim−15
Prim−25
Long−pec
Protruding−mouth
Day−4
Day−5
Stage
Transcript
ENSDART00000099320
ENSDART00000154179
ENSDART00000165853

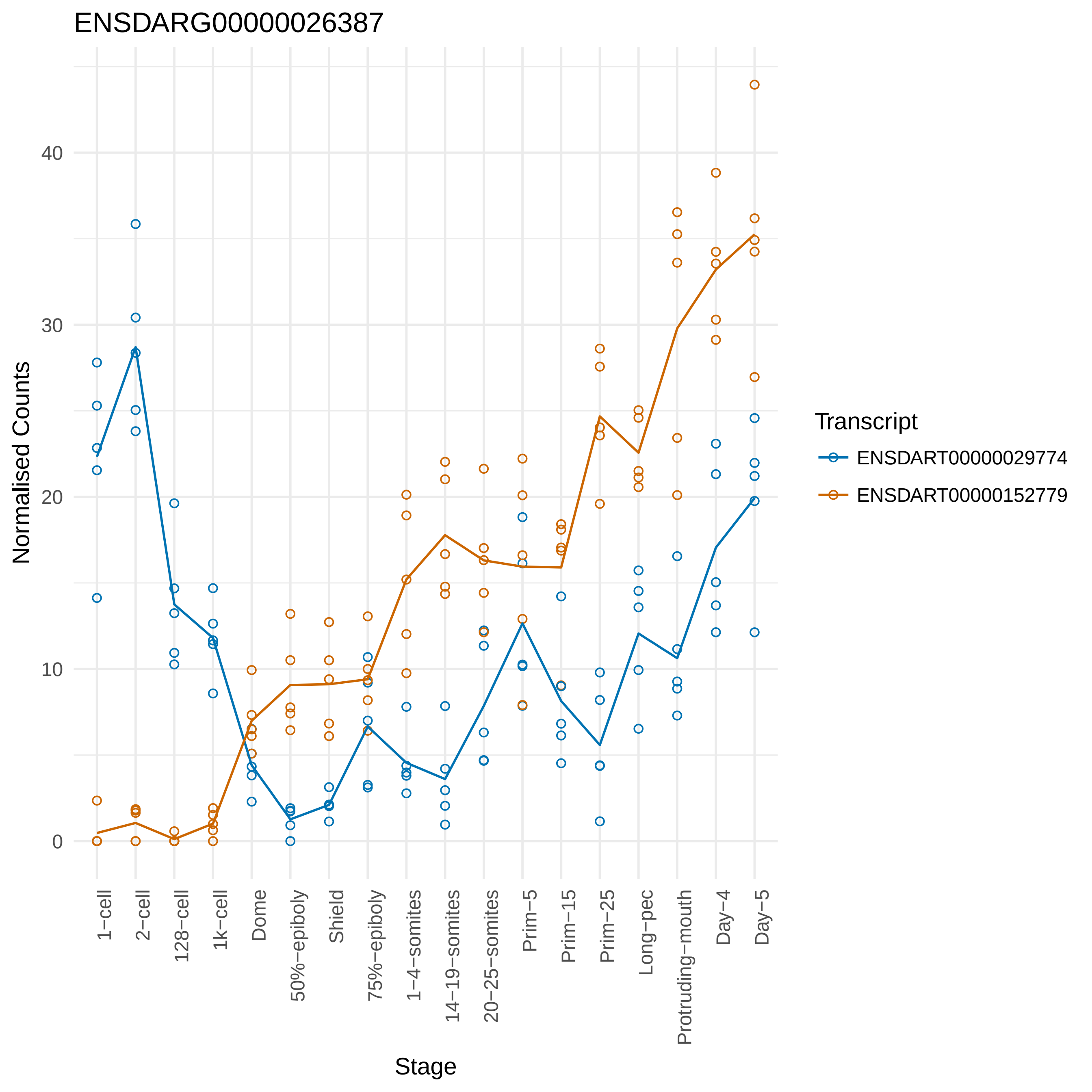
ENSDARG00000026387
Normalised Counts
0
10
20
30
40
1−cell
2−cell
128−cell
1k−cell
Dome
50%−epiboly
Shield
75%−epiboly
1−4−somites
14−19−somites
20−25−somites
Prim−5
Prim−15
Prim−25
Long−pec
Protruding−mouth
Day−4
Day−5
Stage
Transcript
ENSDART00000029774
ENSDART00000152779

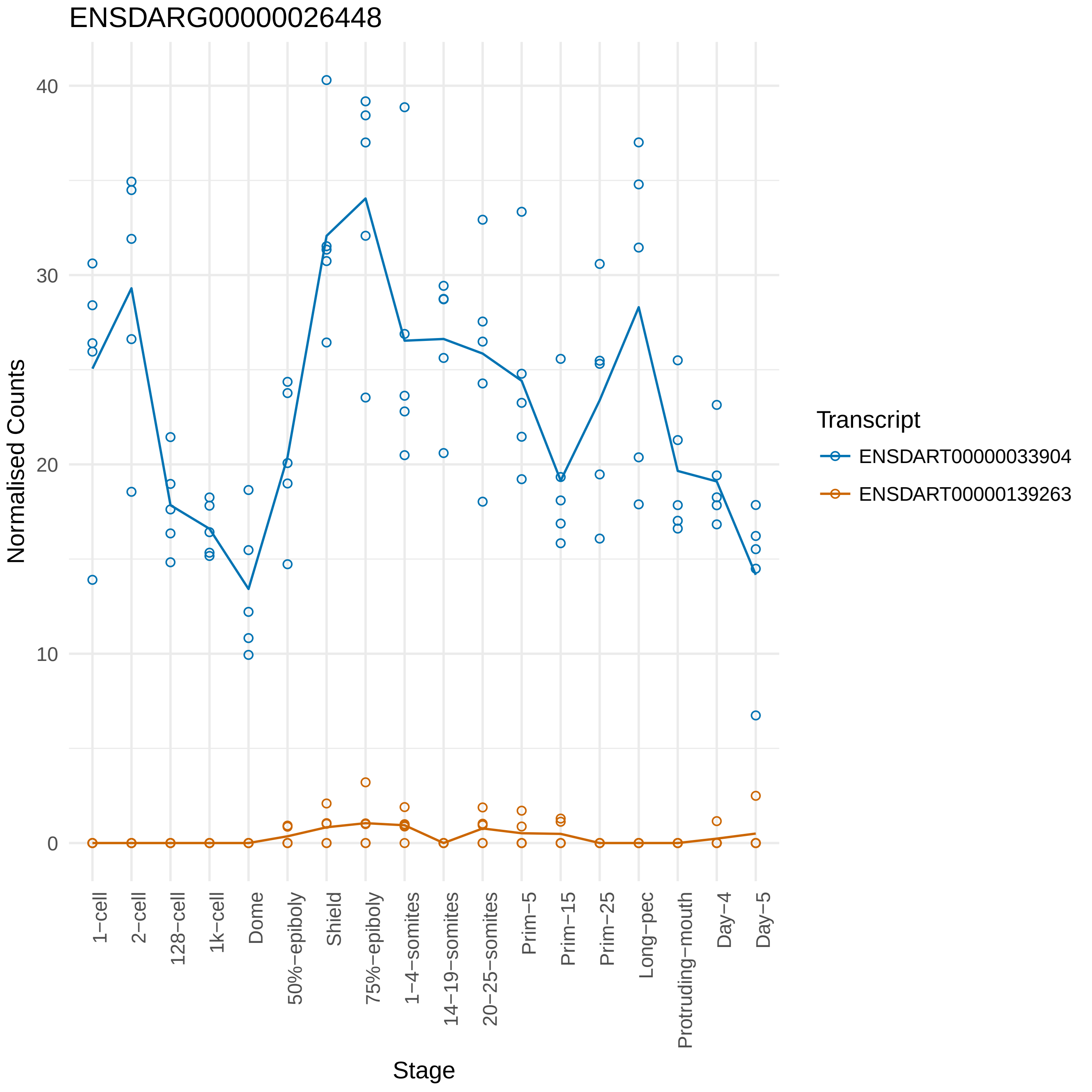

ENSDARG00000026448
Normalised Counts
40
30
20
10
0
1−cell
2−cell
128−cell
1k−cell
Dome
50%−epiboly
Shield
75%−epiboly
1−4−somites
14−19−somites
20−25−somites
Prim−5
Prim−15
Prim−25
Long−pec
Protruding−mouth
Day−4
Day−5
Stage
Transcript
ENSDART00000033904
ENSDART00000139263

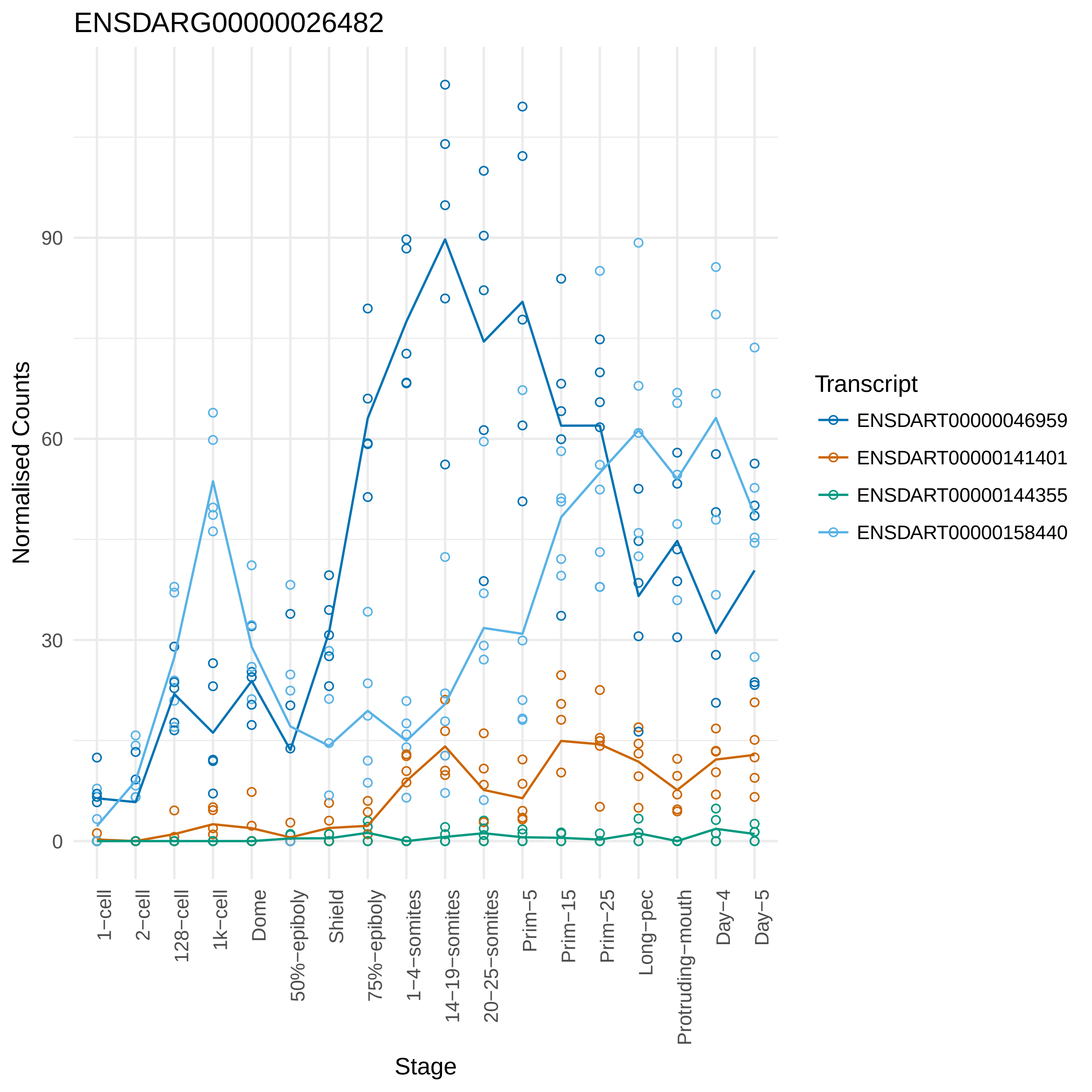

ENSDARG00000026482
Normalised Counts
0
30
60
90
1−cell
2−cell
128−cell
1k−cell
Dome
50%−epiboly
Shield
75%−epiboly
1−4−somites
14−19−somites
20−25−somites
Prim−5
Prim−15
Prim−25
Long−pec
Protruding−mouth
Day−4
Day−5
Stage
Transcript
ENSDART00000046959
ENSDART00000141401
ENSDART00000144355
ENSDART00000158440

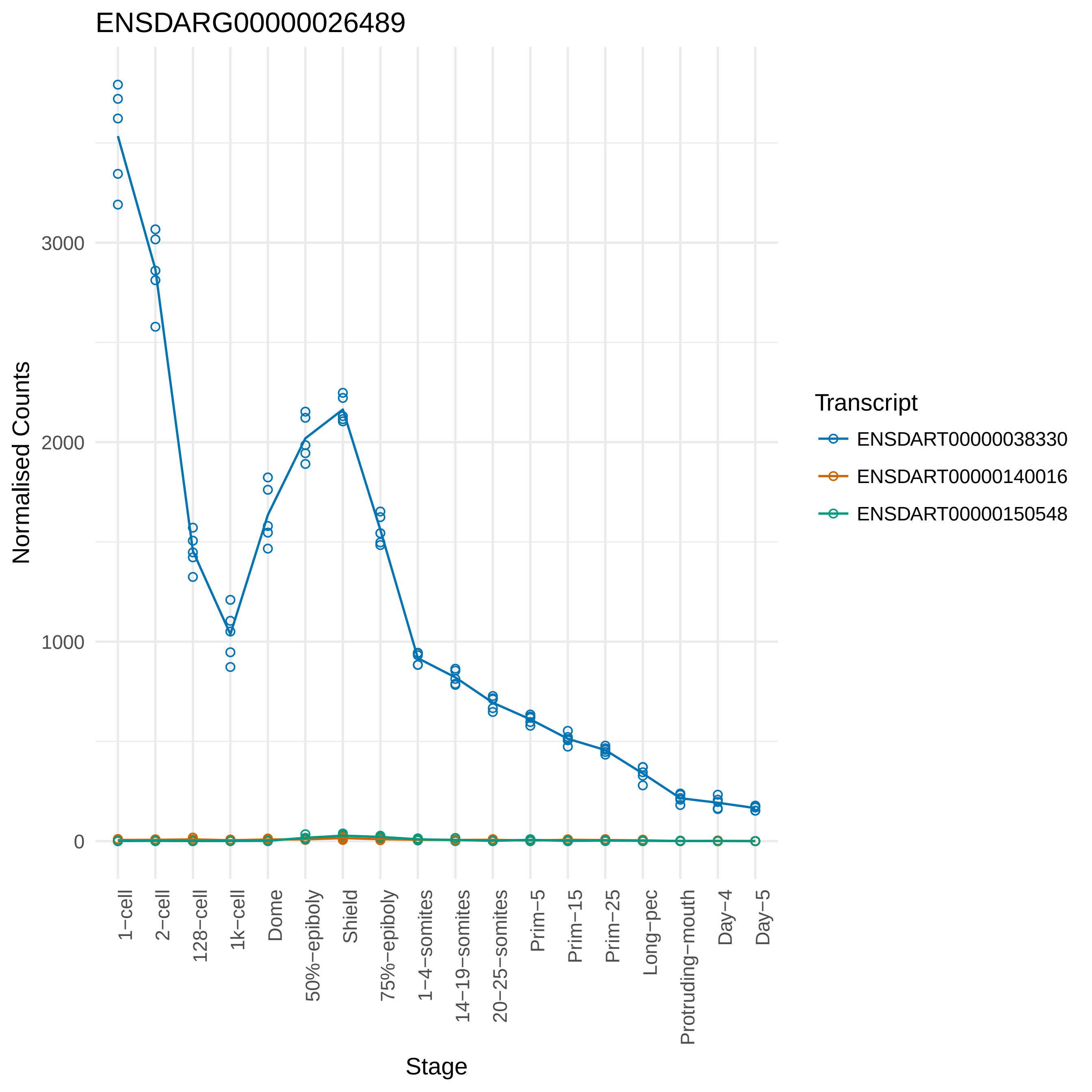

ENSDARG00000026489
Normalised Counts
3000
2000
1000
0
1−cell
2−cell
128−cell
1k−cell
Dome
50%−epiboly
Shield
75%−epiboly
1−4−somites
14−19−somites
20−25−somites
Prim−5
Prim−15
Prim−25
Long−pec
Protruding−mouth
Day−4
Day−5
Stage
Transcript
ENSDART00000038330
ENSDART00000140016
ENSDART00000150548

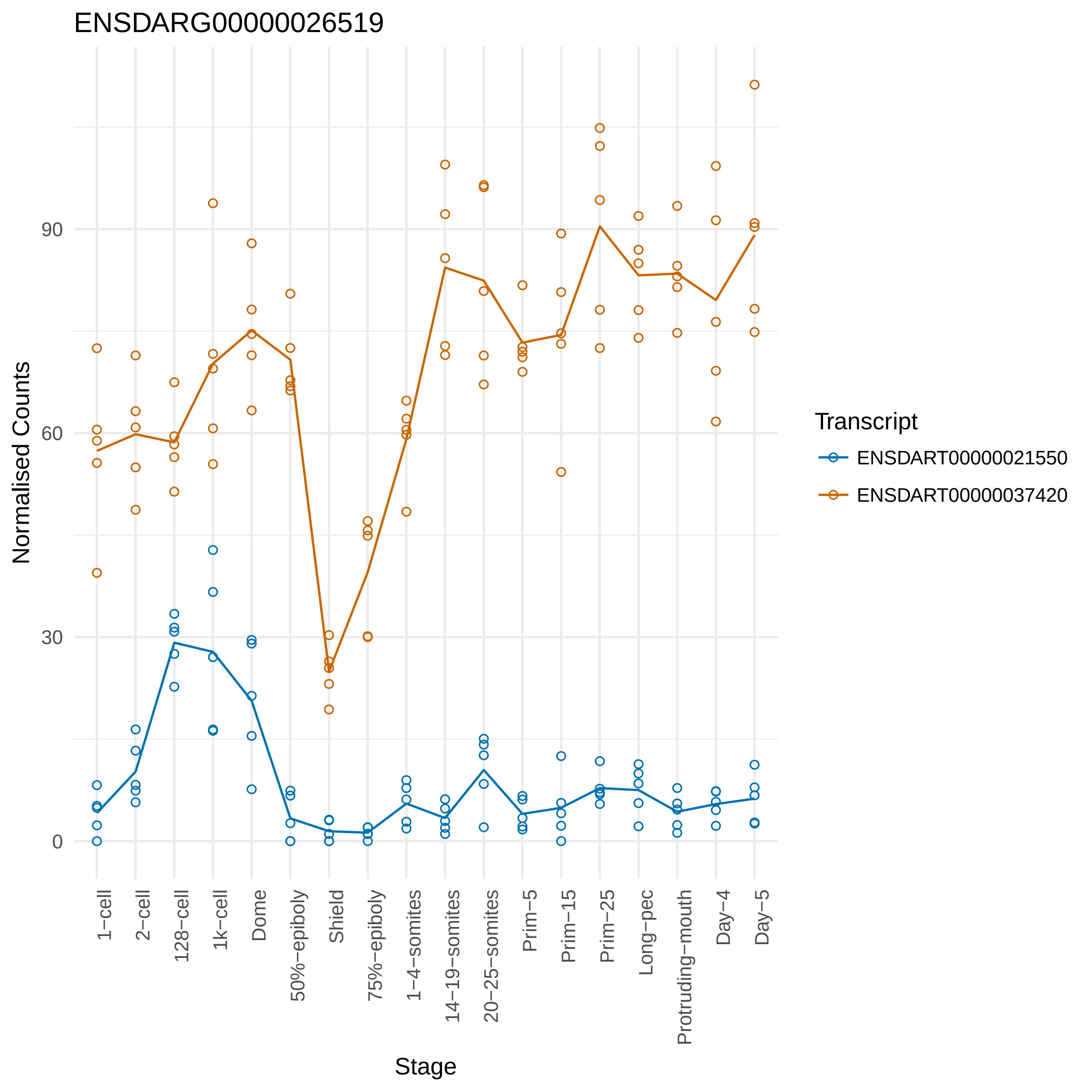
ENSDARG00000026519
Normalised Counts
0
30
60
90
1−cell
2−cell
128−cell
1k−cell
Dome
50%−epiboly
Shield
75%−epiboly
1−4−somites
14−19−somites
20−25−somites
Prim−5
Prim−15
Prim−25
Long−pec
Protruding−mouth
Day−4
Day−5
Stage
Transcript
ENSDART00000021550
ENSDART00000037420

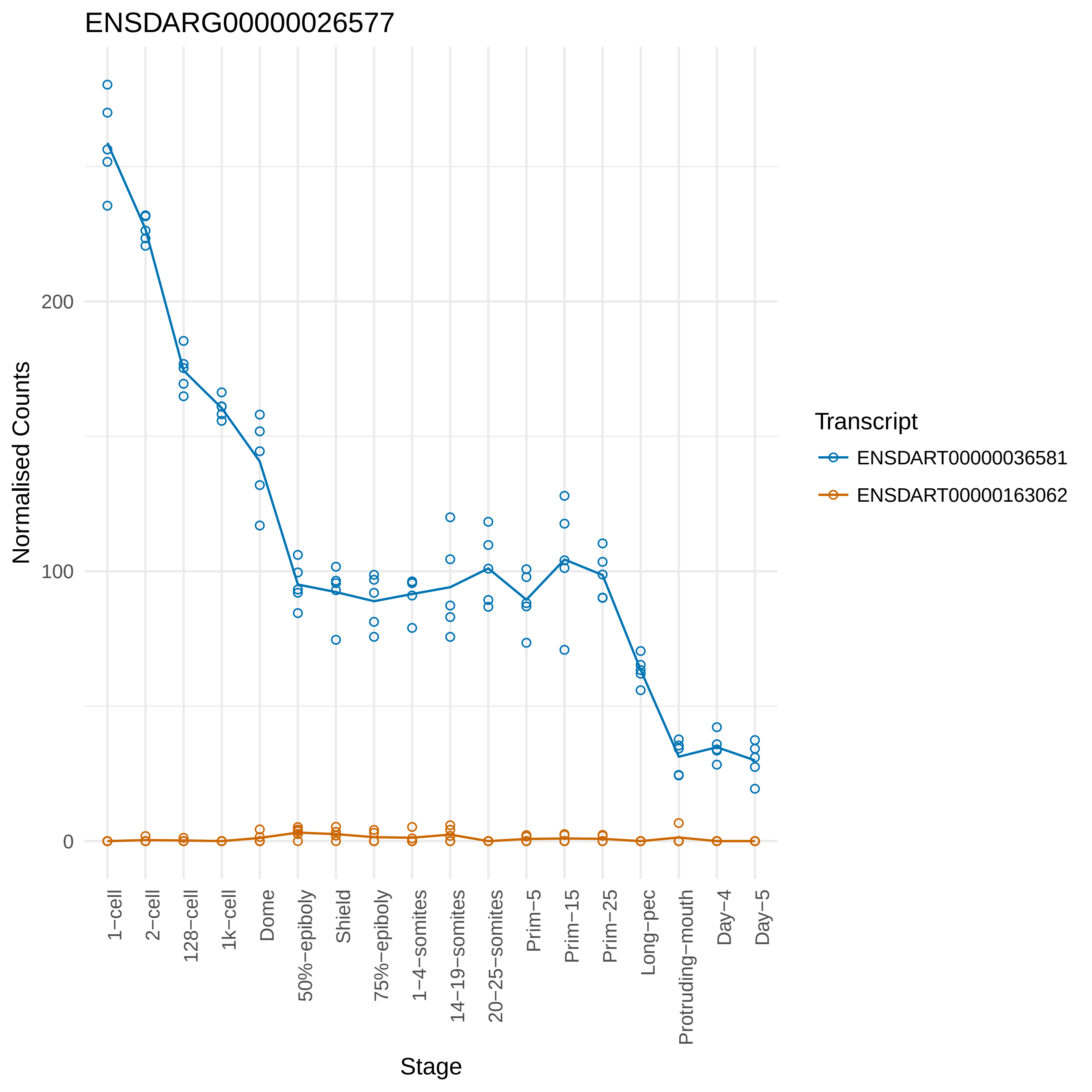

ENSDARG00000026577
Normalised Counts
200
100
0
1−cell
2−cell
128−cell
1k−cell
Dome
50%−epiboly
Shield
75%−epiboly
1−4−somites
14−19−somites
20−25−somites
Prim−5
Prim−15
Prim−25
Long−pec
Protruding−mouth
Day−4
Day−5
Stage
Transcript
ENSDART00000036581
ENSDART00000163062

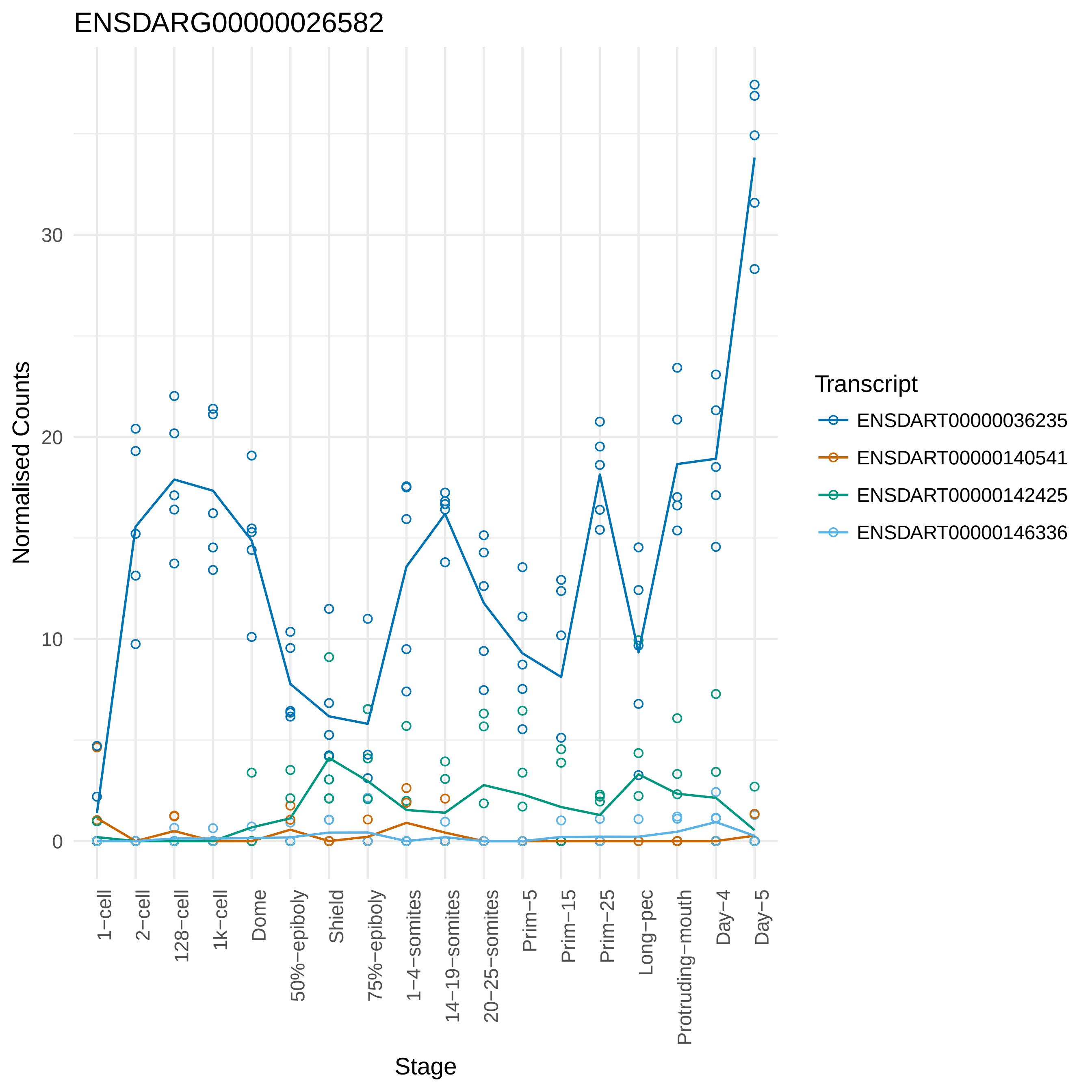
ENSDARG00000026582
Normalised Counts
0
10
20
30
Stage
1−cell
2−cell
128−cell
1k−cell
Dome
50%−epiboly
Shield
75%−epiboly
1−4−somites
14−19−somites
20−25−somites
Prim−5
Prim−15
Prim−25
Long−pec
Protruding−mouth
Day−4
Day−5
Transcript
ENSDART00000036235
ENSDART00000140541
ENSDART00000142425
ENSDART00000146336

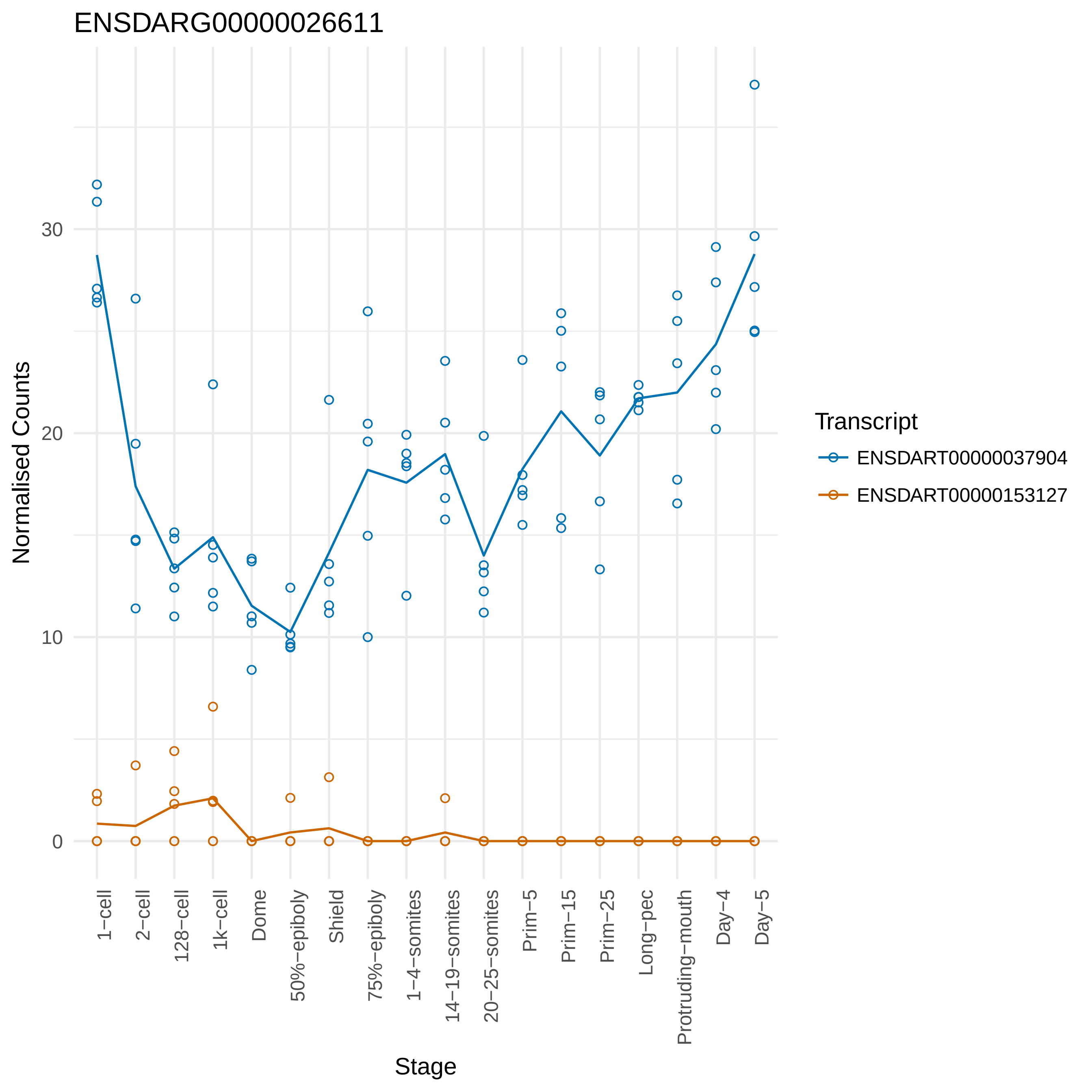

ENSDARG00000026611
Normalised Counts
30
20
10
0
1−cell
2−cell
128−cell
1k−cell
Dome
50%−epiboly
Shield
75%−epiboly
1−4−somites
14−19−somites
20−25−somites
Prim−5
Prim−15
Prim−25
Long−pec
Protruding−mouth
Day−4
Day−5
Stage
Transcript
ENSDART00000037904
ENSDART00000153127

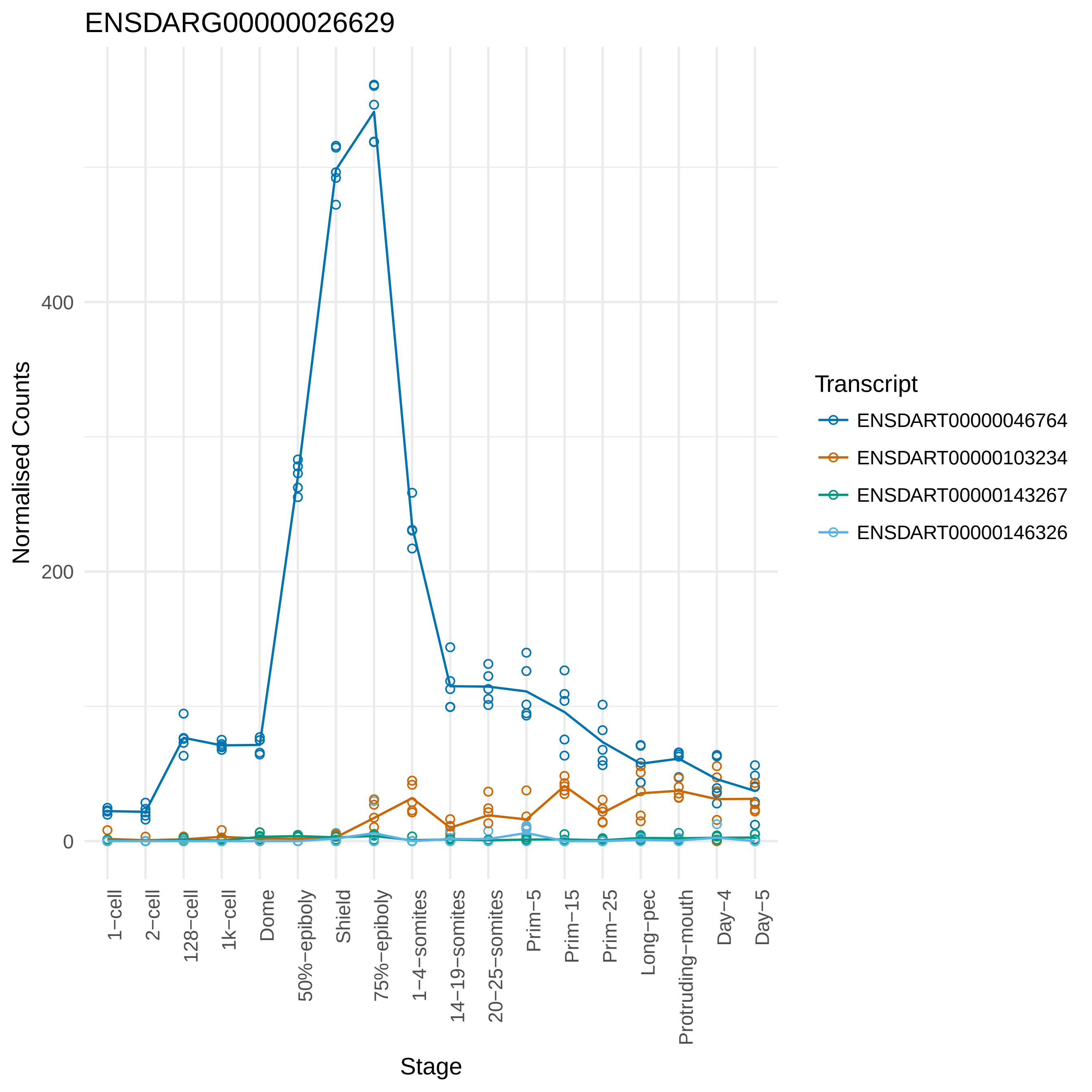
ENSDARG00000026629
Normalised Counts
0
200
400
Stage
1−cell
2−cell
128−cell
1k−cell
Dome
50%−epiboly
Shield
75%−epiboly
1−4−somites
14−19−somites
20−25−somites
Prim−5
Prim−15
Prim−25
Long−pec
Protruding−mouth
Day−4
Day−5
Transcript
ENSDART00000046764
ENSDART00000103234
ENSDART00000143267
ENSDART00000146326

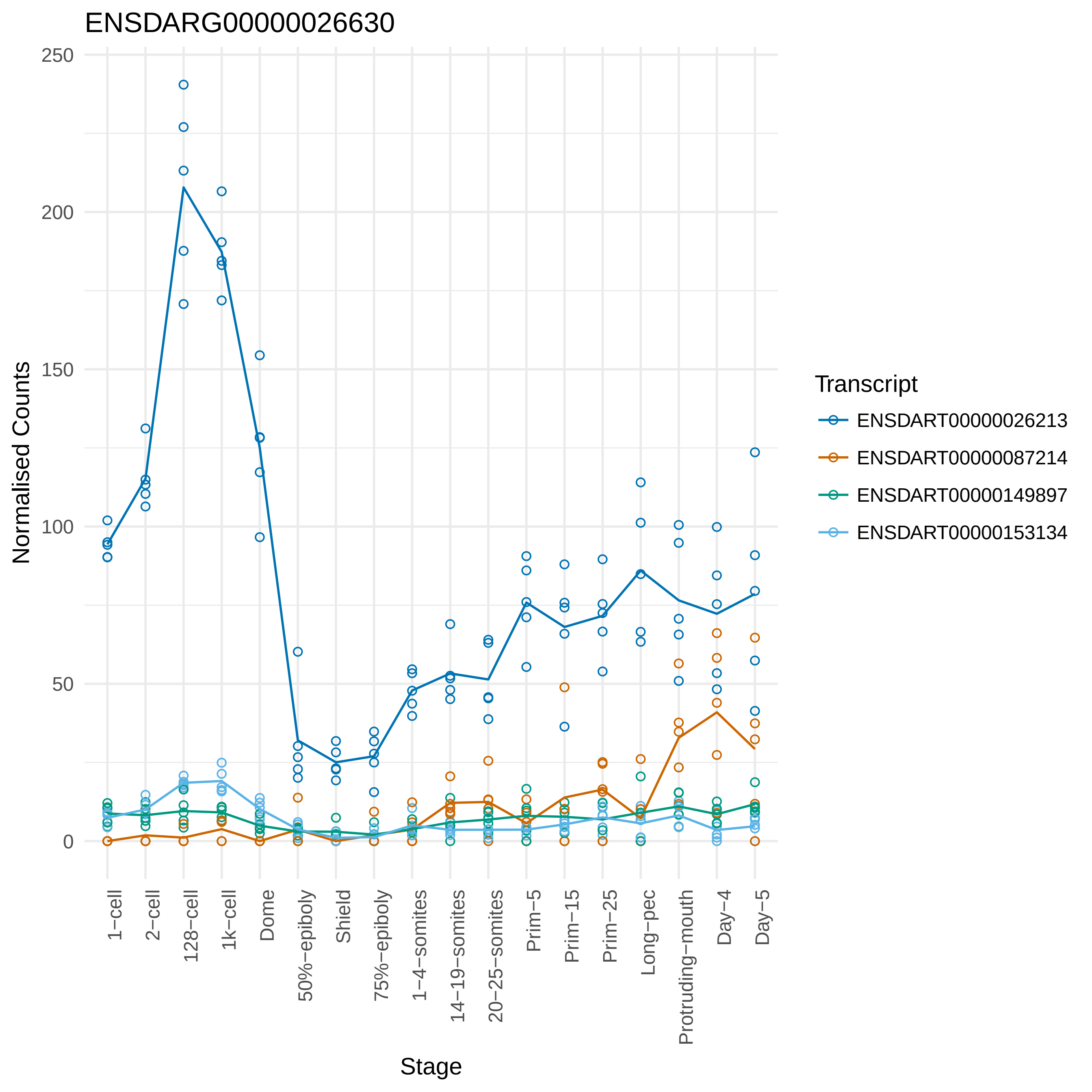

ENSDARG00000026630
Normalised Counts
250
200
150
100
50
0
1−cell
2−cell
128−cell
1k−cell
Dome
50%−epiboly
Shield
75%−epiboly
1−4−somites
14−19−somites
20−25−somites
Prim−5
Prim−15
Prim−25
Long−pec
Protruding−mouth
Day−4
Day−5
Stage
Transcript
ENSDART00000026213
ENSDART00000087214
ENSDART00000149897
ENSDART00000153134

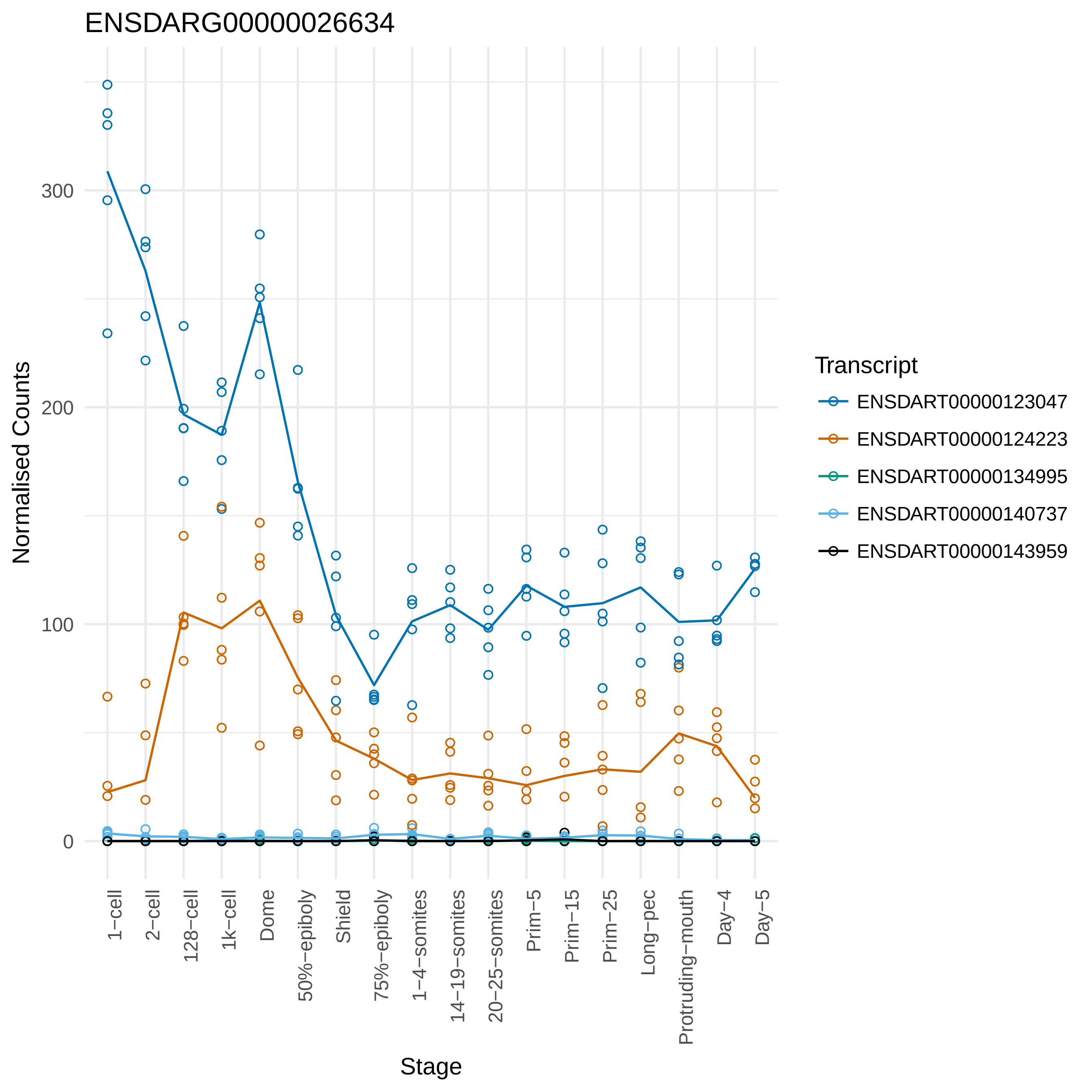
ENSDARG00000026634
Normalised Counts
300
200
100
0
1−cell
2−cell
128−cell
1k−cell
Dome
50%−epiboly
Shield
75%−epiboly
1−4−somites
14−19−somites
20−25−somites
Prim−5
Prim−15
Prim−25
Long−pec
Protruding−mouth
Day−4
Day−5
Stage
Transcript
ENSDART00000123047
ENSDART00000124223
ENSDART00000134995
ENSDART00000140737
ENSDART00000143959

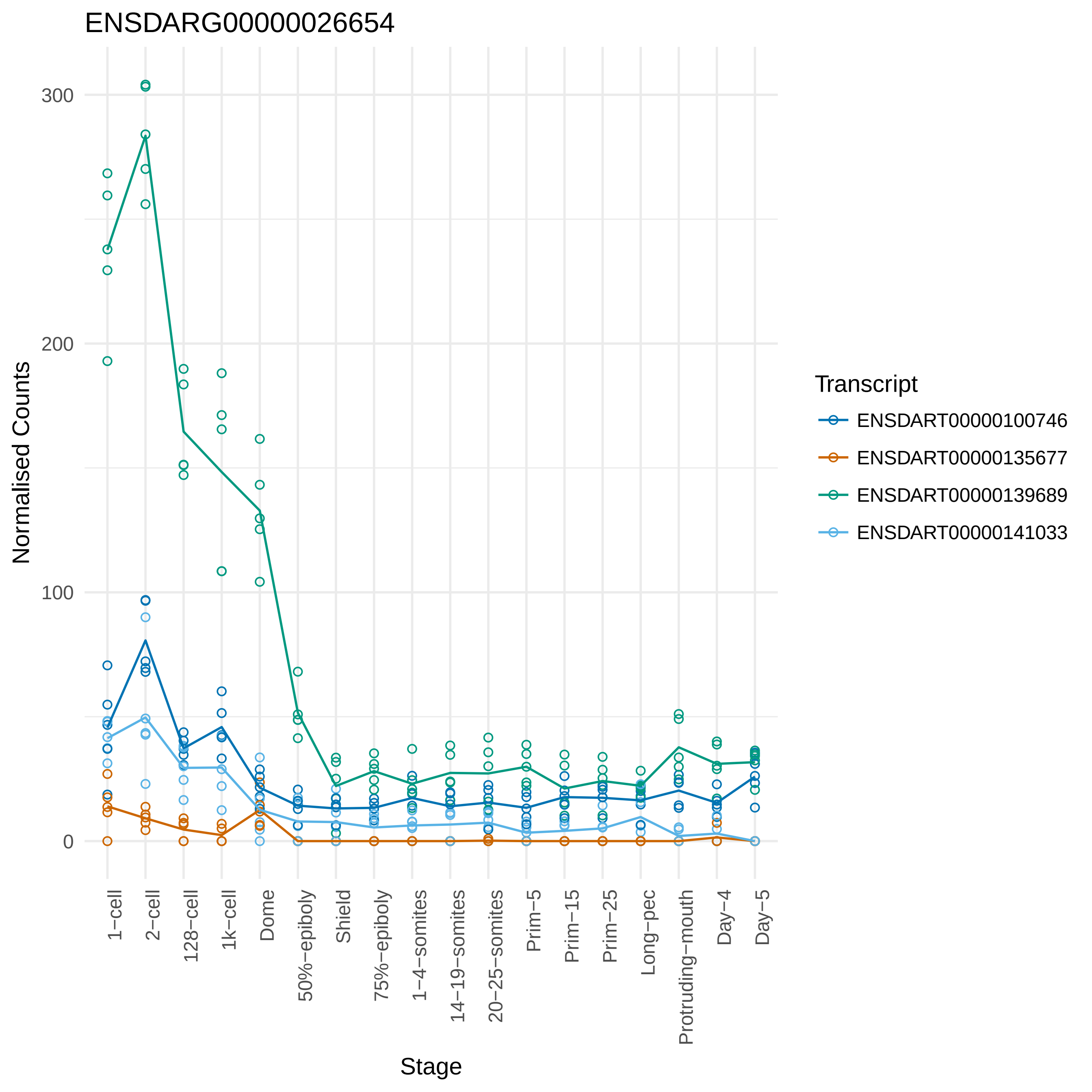

ENSDARG00000026654
Normalised Counts
300
200
100
0
1−cell
2−cell
128−cell
1k−cell
Dome
50%−epiboly
Shield
75%−epiboly
1−4−somites
14−19−somites
20−25−somites
Prim−5
Prim−15
Prim−25
Long−pec
Protruding−mouth
Day−4
Day−5
Stage
Transcript
ENSDART00000100746
ENSDART00000135677
ENSDART00000139689
ENSDART00000141033

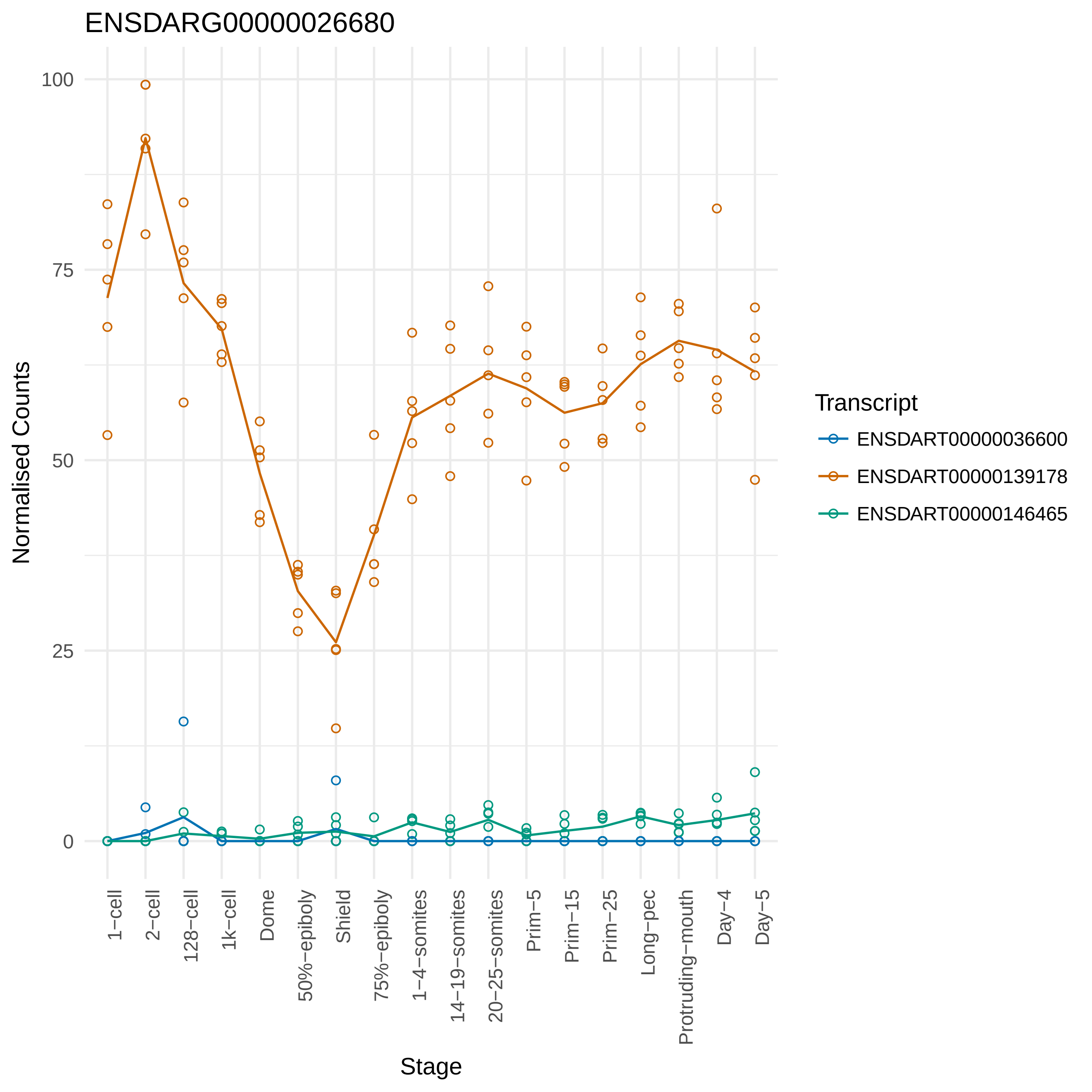

ENSDARG00000026680
Normalised Counts
100
75
50
25
0
1−cell
2−cell
128−cell
1k−cell
Dome
50%−epiboly
Shield
75%−epiboly
1−4−somites
14−19−somites
20−25−somites
Prim−5
Prim−15
Prim−25
Long−pec
Protruding−mouth
Day−4
Day−5
Stage
Transcript
ENSDART00000036600
ENSDART00000139178
ENSDART00000146465

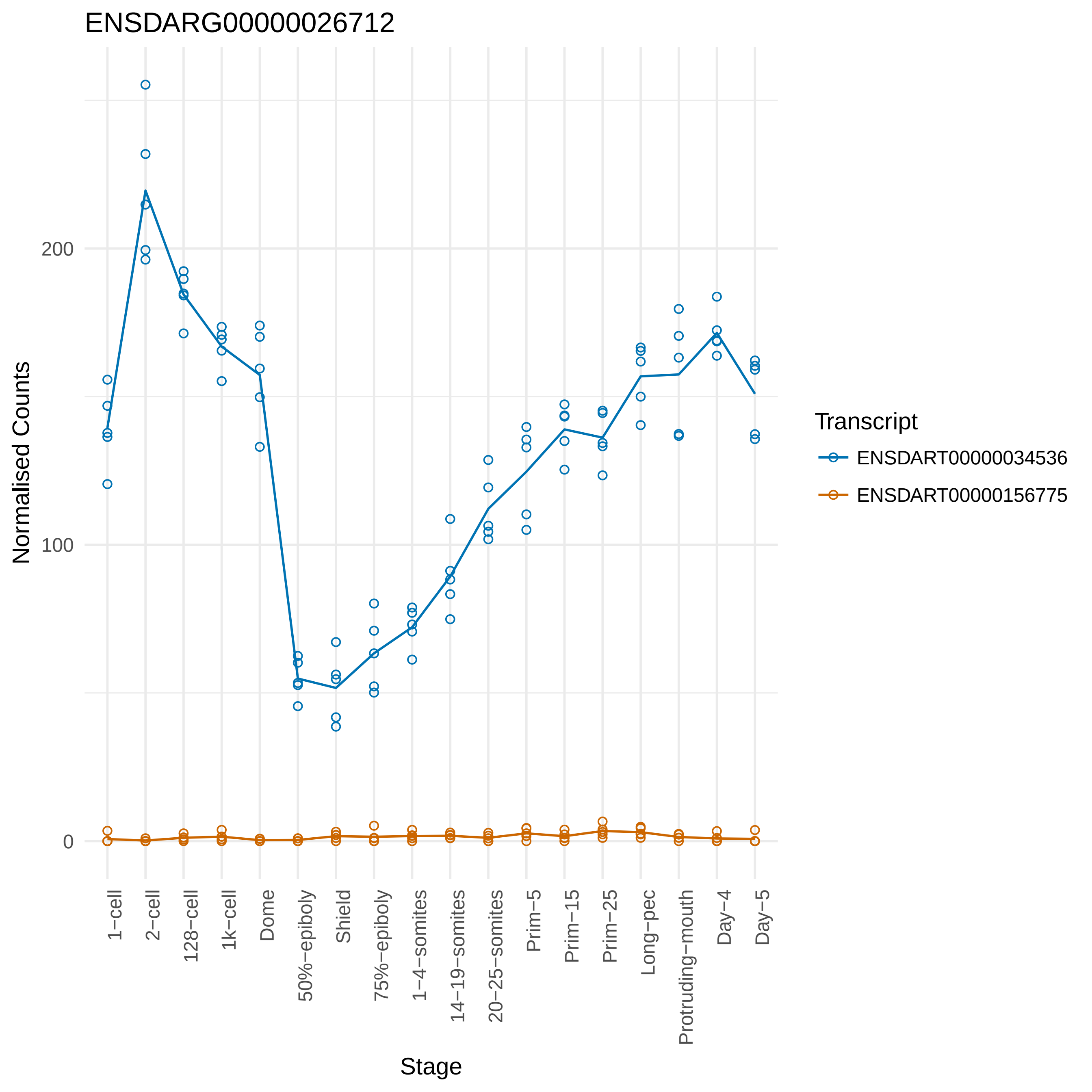

ENSDARG00000026712
Normalised Counts
200
100
0
1−cell
2−cell
128−cell
1k−cell
Dome
50%−epiboly
Shield
75%−epiboly
1−4−somites
14−19−somites
20−25−somites
Prim−5
Prim−15
Prim−25
Long−pec
Protruding−mouth
Day−4
Day−5
Stage
Transcript
ENSDART00000034536
ENSDART00000156775

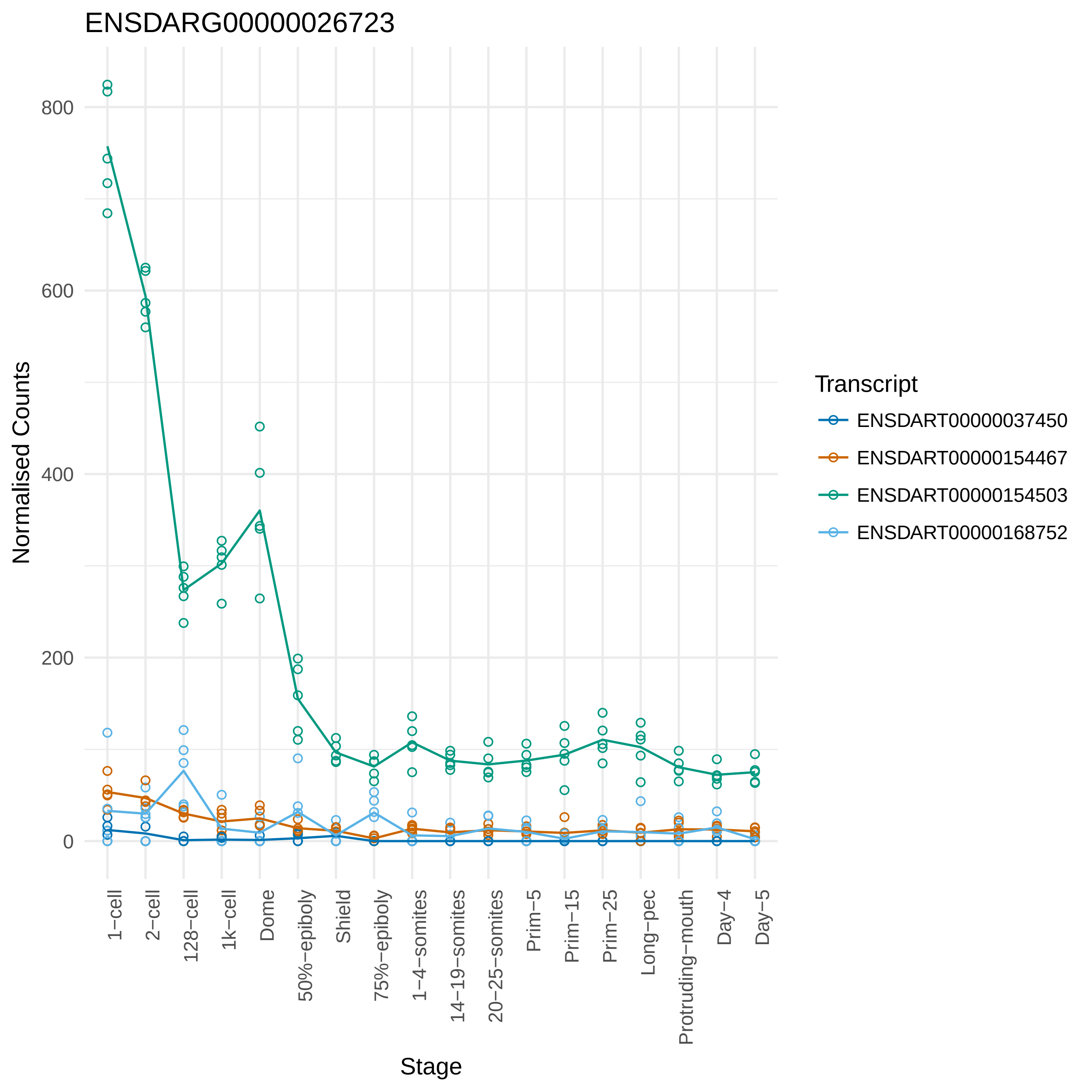

ENSDARG00000026723
Normalised Counts
800
600
400
200
0
Stage
1−cell
2−cell
128−cell
1k−cell
Dome
50%−epiboly
Shield
75%−epiboly
1−4−somites
14−19−somites
20−25−somites
Prim−5
Prim−15
Prim−25
Long−pec
Protruding−mouth
Day−4
Day−5
Transcript
ENSDART00000037450
ENSDART00000154467
ENSDART00000154503
ENSDART00000168752

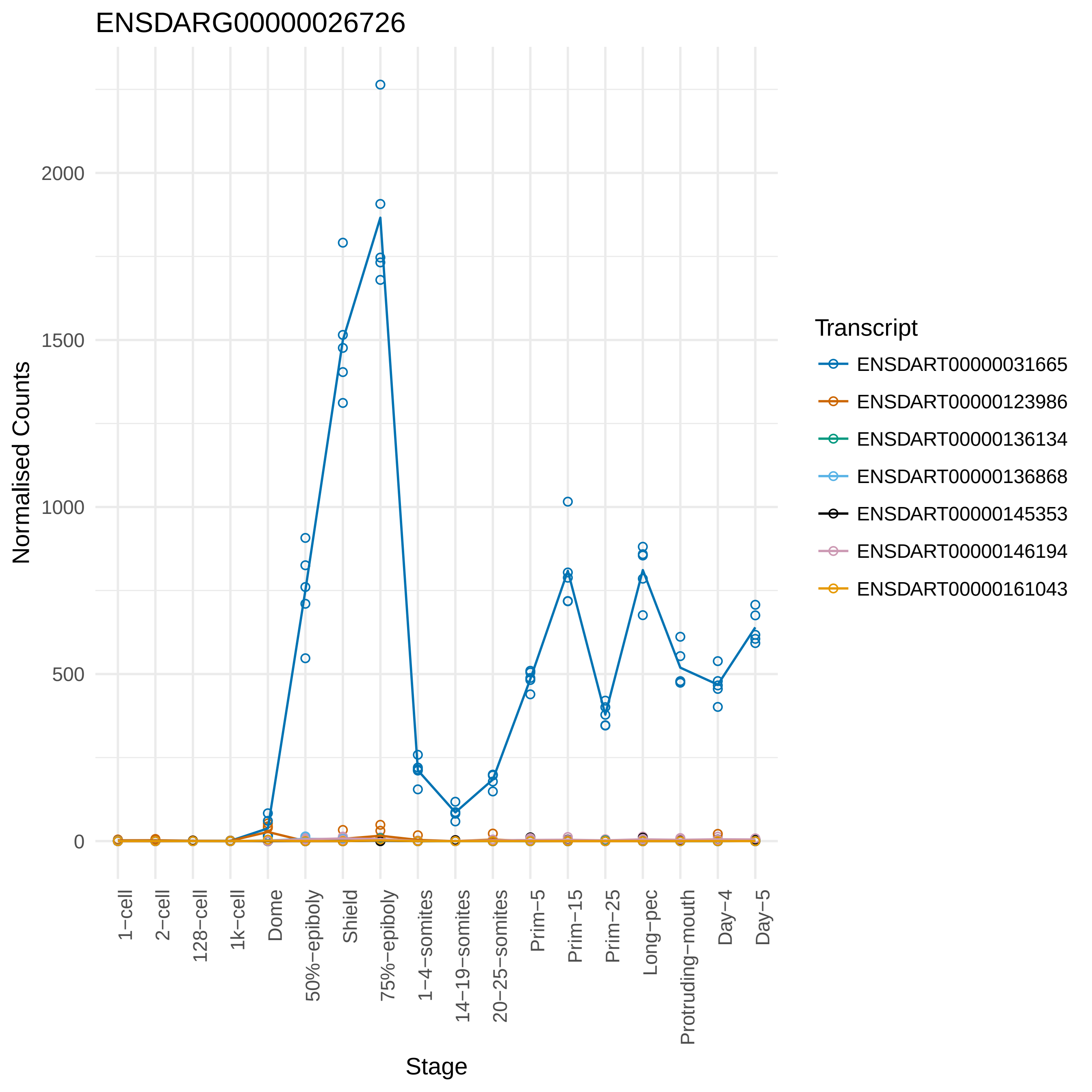

ENSDARG00000026726
Normalised Counts
2000
1500
1000
500
0
1−cell
2−cell
128−cell
1k−cell
Dome
50%−epiboly
Shield
75%−epiboly
1−4−somites
14−19−somites
20−25−somites
Prim−5
Prim−15
Prim−25
Long−pec
Protruding−mouth
Day−4
Day−5
Stage
Transcript
ENSDART00000031665
ENSDART00000123986
ENSDART00000136134
ENSDART00000136868
ENSDART00000145353
ENSDART00000146194
ENSDART00000161043

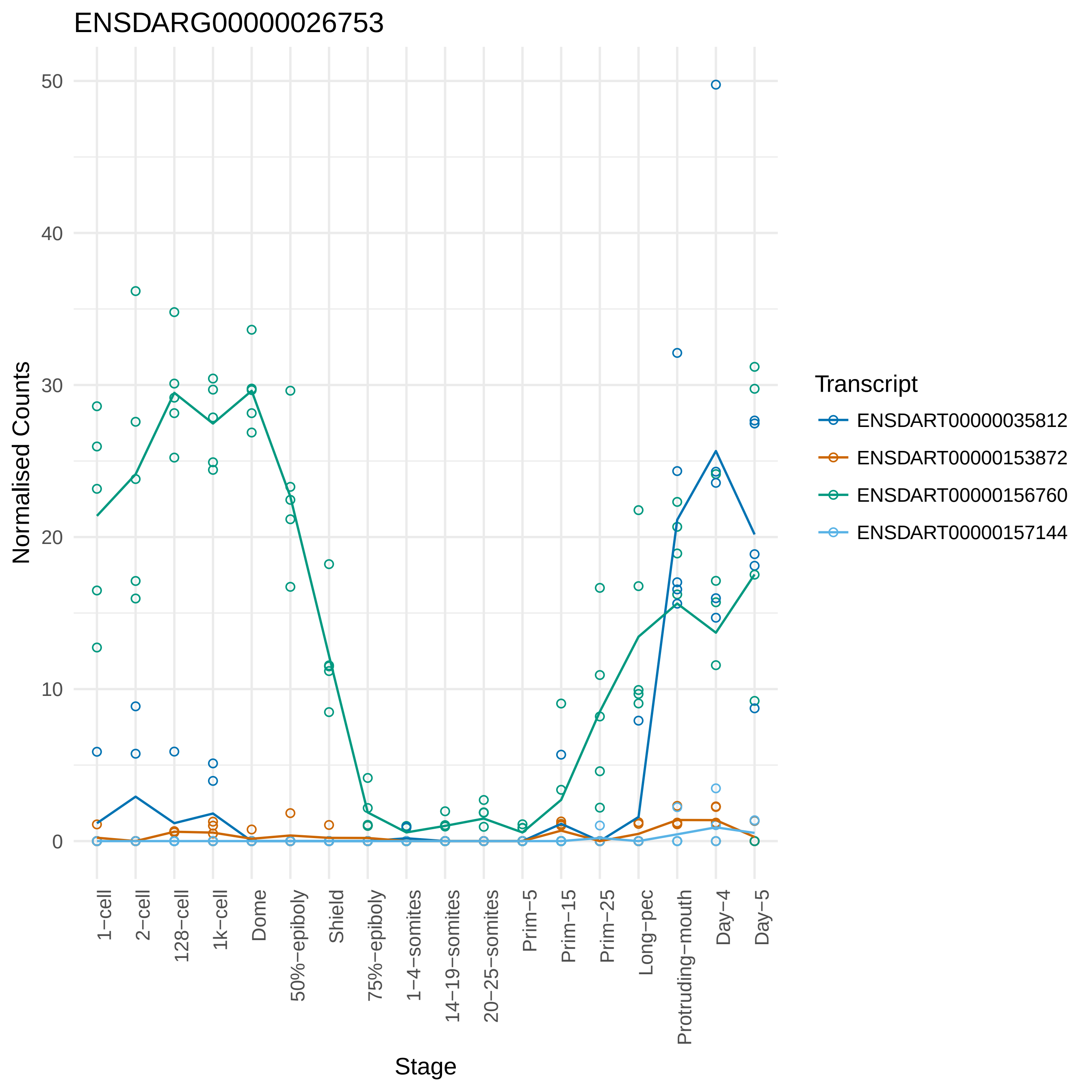

ENSDARG00000026753
Normalised Counts
0
10
20
30
40
50
1−cell
2−cell
128−cell
1k−cell
Dome
50%−epiboly
Shield
75%−epiboly
1−4−somites
14−19−somites
20−25−somites
Prim−5
Prim−15
Prim−25
Long−pec
Protruding−mouth
Day−4
Day−5
Stage
Transcript
ENSDART00000035812
ENSDART00000153872
ENSDART00000156760
ENSDART00000157144

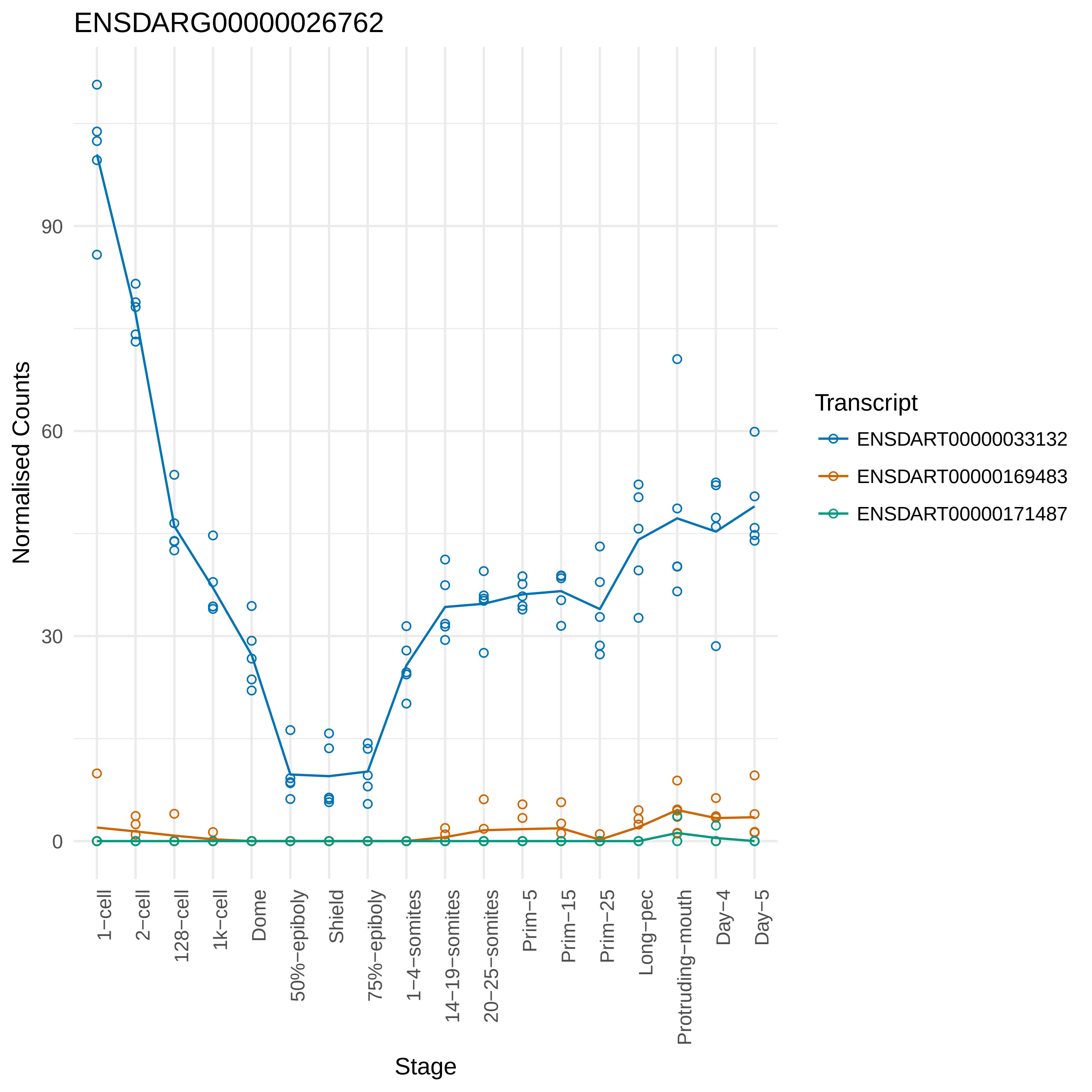
ENSDARG00000026762
Normalised Counts
90
60
30
0
1−cell
2−cell
128−cell
1k−cell
Dome
50%−epiboly
Shield
75%−epiboly
1−4−somites
14−19−somites
20−25−somites
Prim−5
Prim−15
Prim−25
Long−pec
Protruding−mouth
Day−4
Day−5
Stage
Transcript
ENSDART00000033132
ENSDART00000169483
ENSDART00000171487

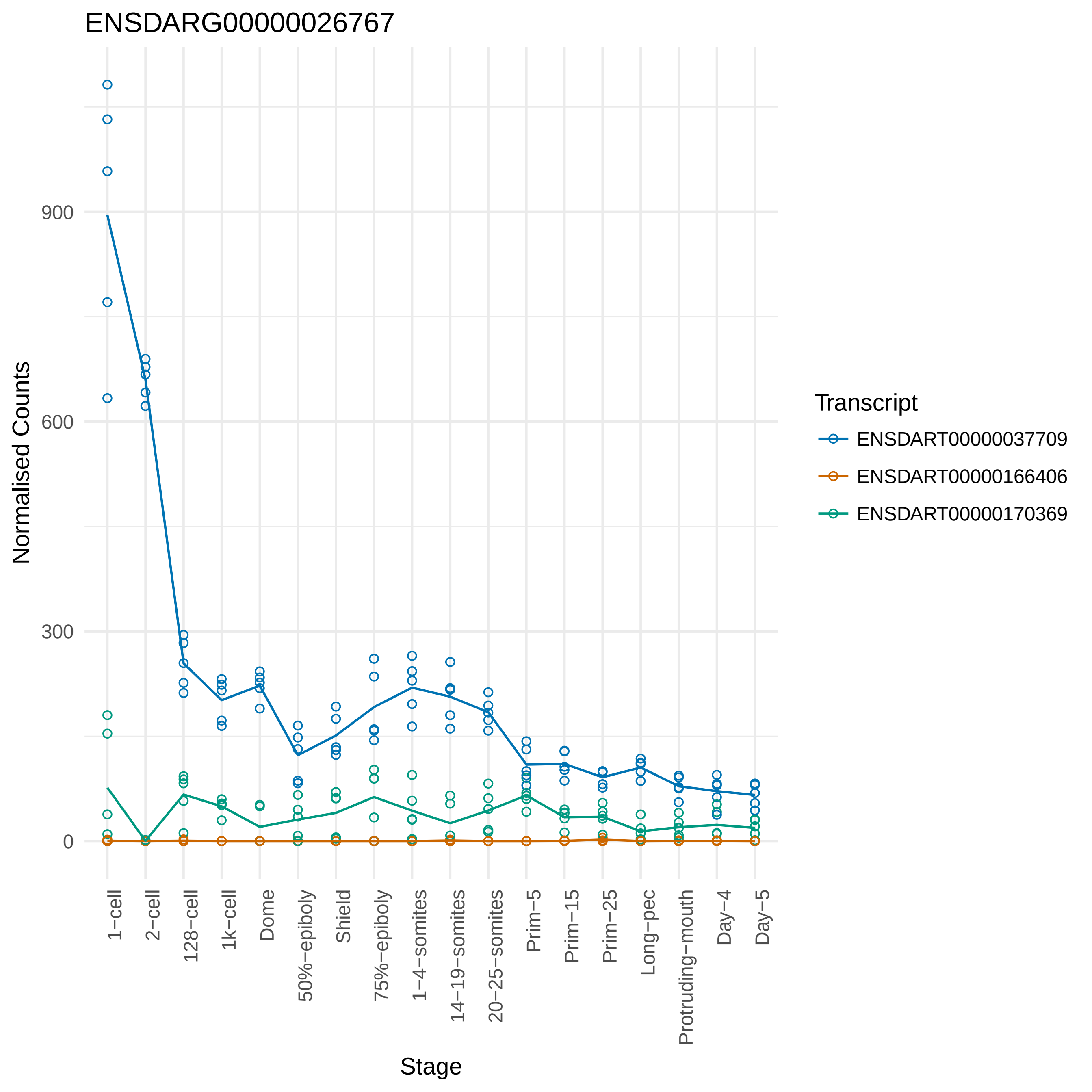

ENSDARG00000026767
Normalised Counts
900
600
300
0
1−cell
2−cell
128−cell
1k−cell
Dome
50%−epiboly
Shield
75%−epiboly
1−4−somites
14−19−somites
20−25−somites
Prim−5
Prim−15
Prim−25
Long−pec
Protruding−mouth
Day−4
Day−5
Stage
Transcript
ENSDART00000037709
ENSDART00000166406
ENSDART00000170369

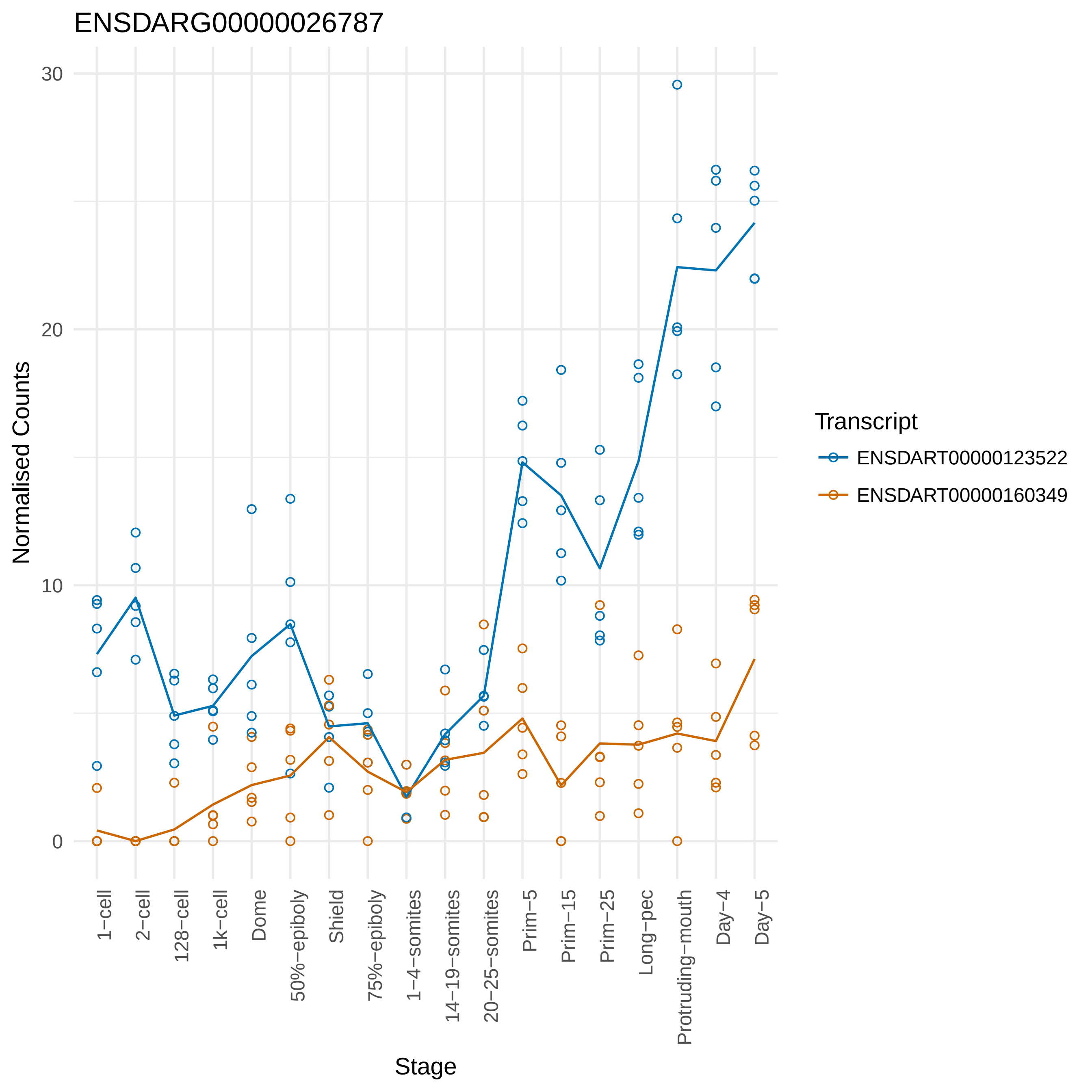
ENSDARG00000026787
Normalised Counts
30
20
10
0
Stage
1−cell
2−cell
128−cell
1k−cell
Dome
50%−epiboly
Shield
75%−epiboly
1−4−somites
14−19−somites
20−25−somites
Prim−5
Prim−15
Prim−25
Long−pec
Protruding−mouth
Day−4
Day−5
Transcript
ENSDART00000123522
ENSDART00000160349

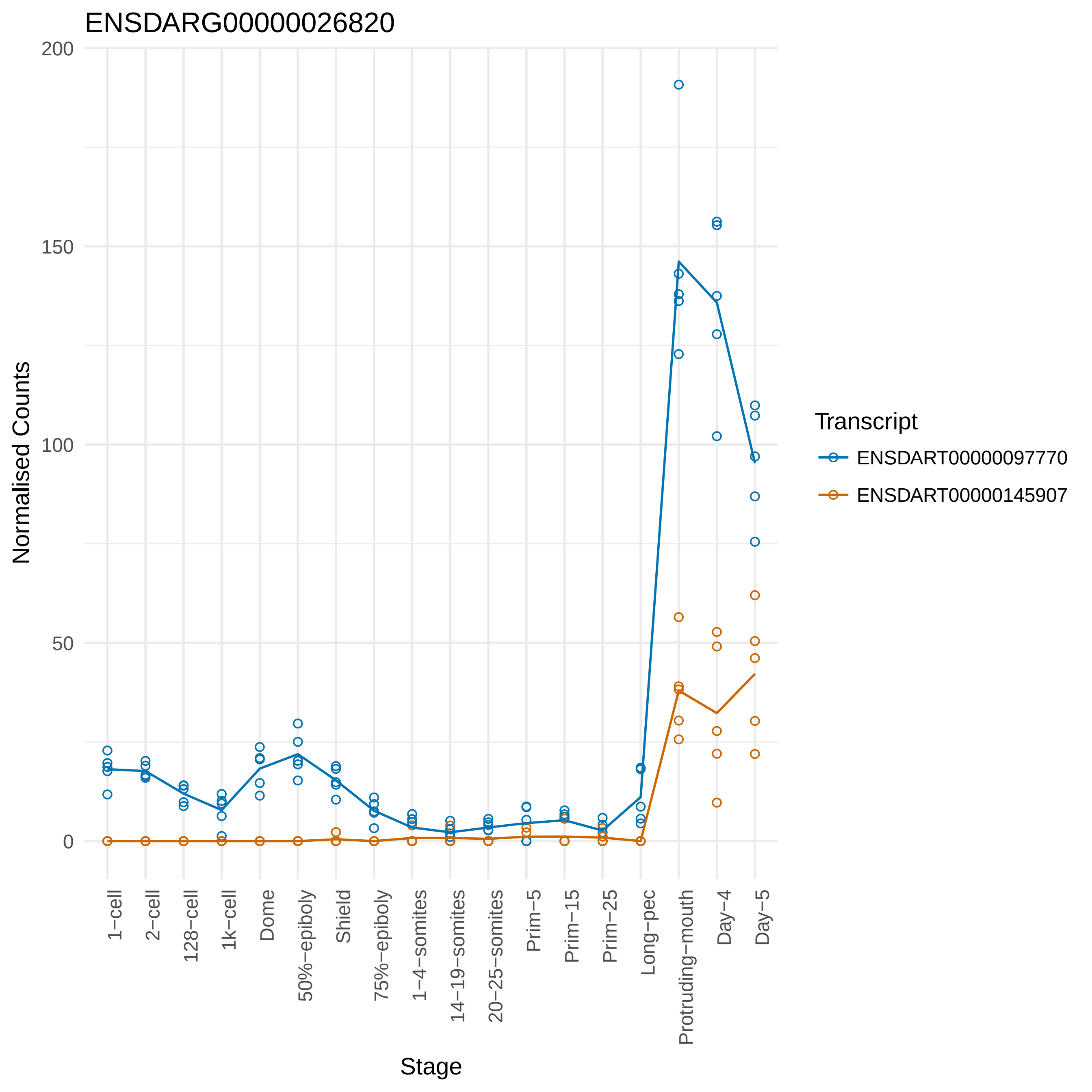

ENSDARG00000026820
Normalised Counts
200
150
100
50
0
1−cell
2−cell
128−cell
1k−cell
Dome
50%−epiboly
Shield
75%−epiboly
1−4−somites
14−19−somites
20−25−somites
Prim−5
Prim−15
Prim−25
Long−pec
Protruding−mouth
Day−4
Day−5
Stage
Transcript
ENSDART00000097770
ENSDART00000145907

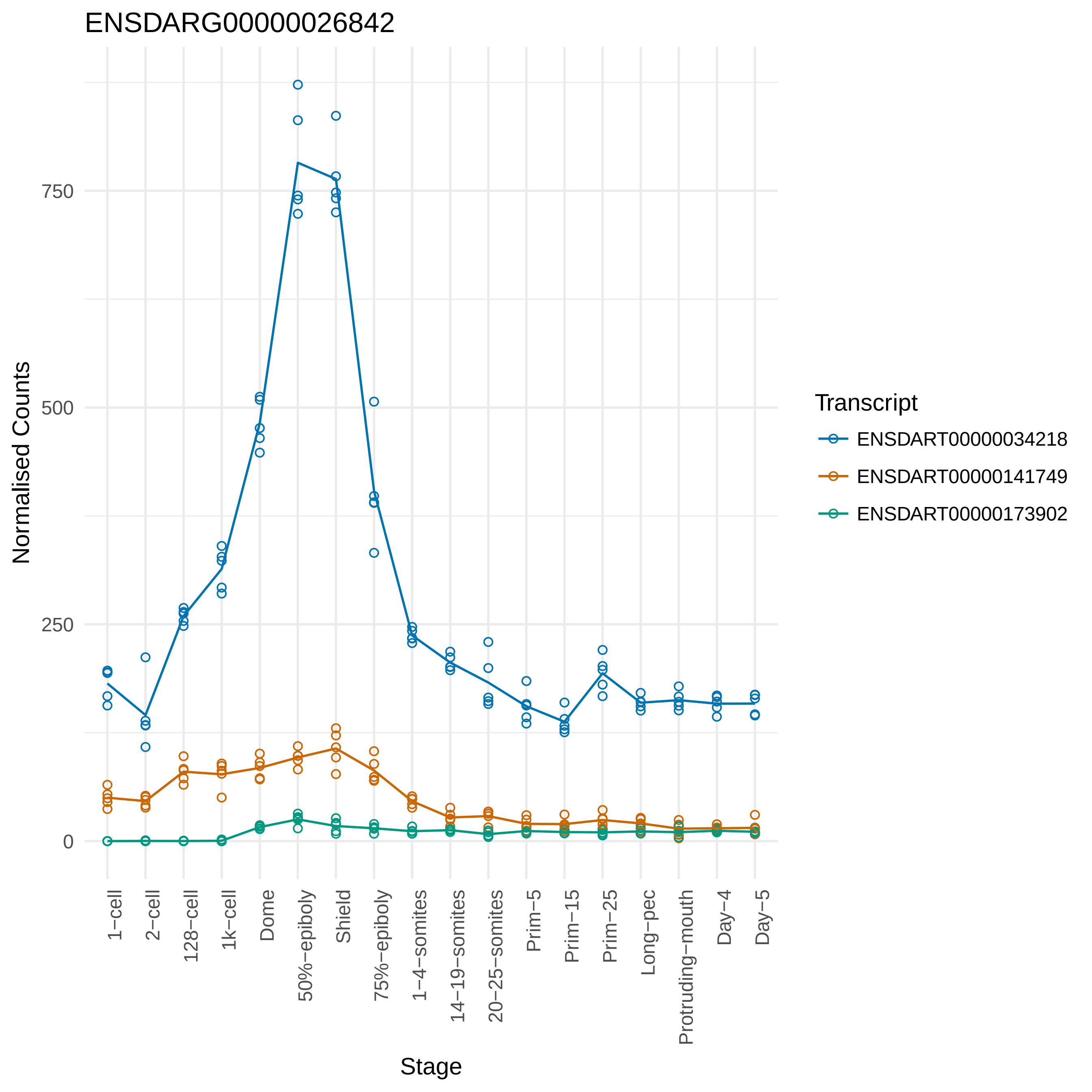
ENSDARG00000026842
Normalised Counts
750
500
250
0
1−cell
2−cell
128−cell
1k−cell
Dome
50%−epiboly
Shield
75%−epiboly
1−4−somites
14−19−somites
20−25−somites
Prim−5
Prim−15
Prim−25
Long−pec
Protruding−mouth
Day−4
Day−5
Stage
Transcript
ENSDART00000034218
ENSDART00000141749
ENSDART00000173902

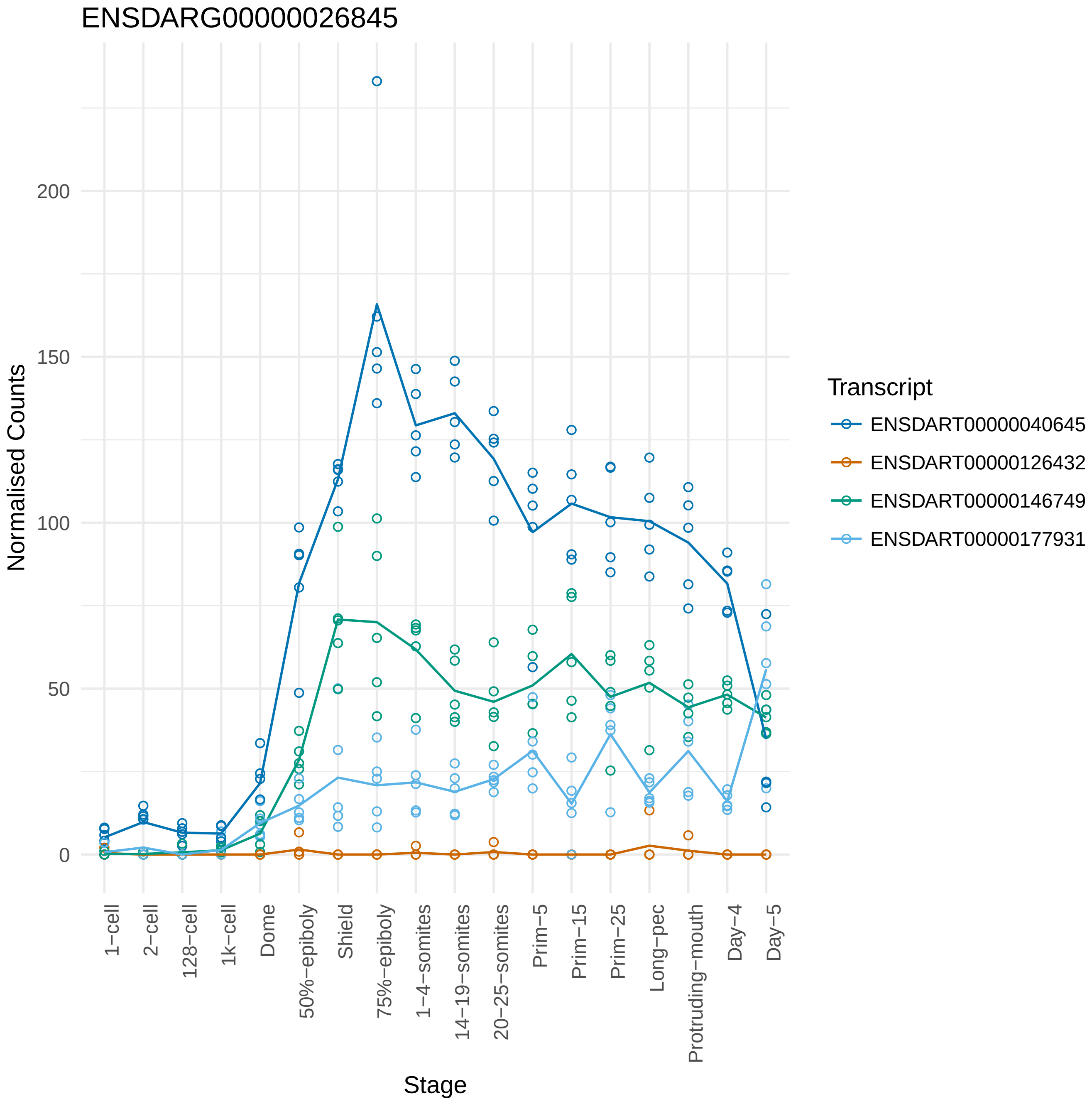
ENSDARG00000026845
Normalised Counts
0
50
100
150
200
Stage
1−cell
2−cell
128−cell
1k−cell
Dome
50%−epiboly
Shield
75%−epiboly
1−4−somites
14−19−somites
20−25−somites
Prim−5
Prim−15
Prim−25
Long−pec
Protruding−mouth
Day−4
Day−5
Transcript
ENSDART00000040645
ENSDART00000126432
ENSDART00000146749
ENSDART00000177931

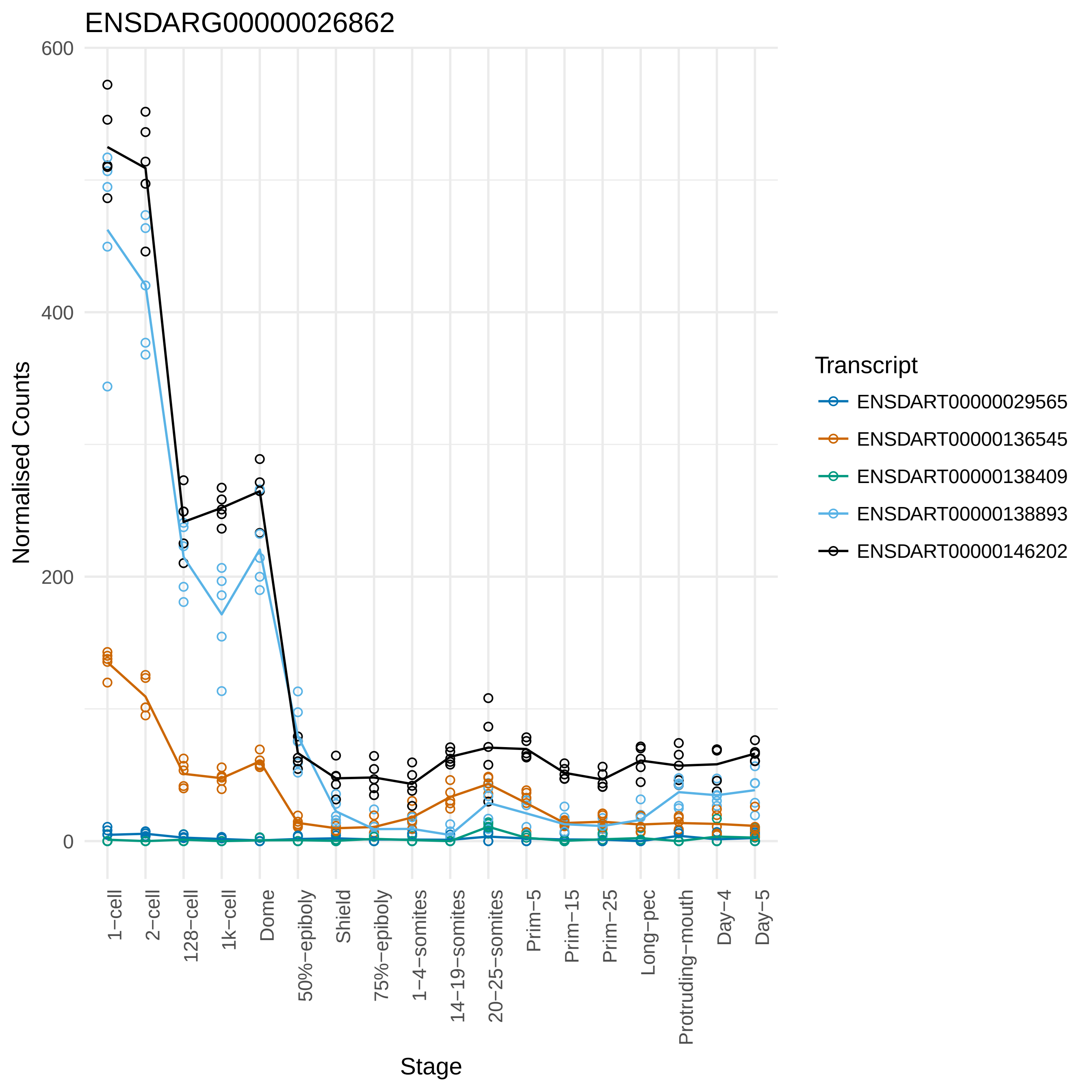

ENSDARG00000026862
600
400
200
0
Normalised Counts
1−cell
2−cell
128−cell
1k−cell
Dome
50%−epiboly
Shield
75%−epiboly
1−4−somites
14−19−somites
20−25−somites
Prim−5
Prim−15
Prim−25
Long−pec
Protruding−mouth
Day−4
Day−5
Stage
Transcript
ENSDART00000029565
ENSDART00000136545
ENSDART00000138409
ENSDART00000138893
ENSDART00000146202

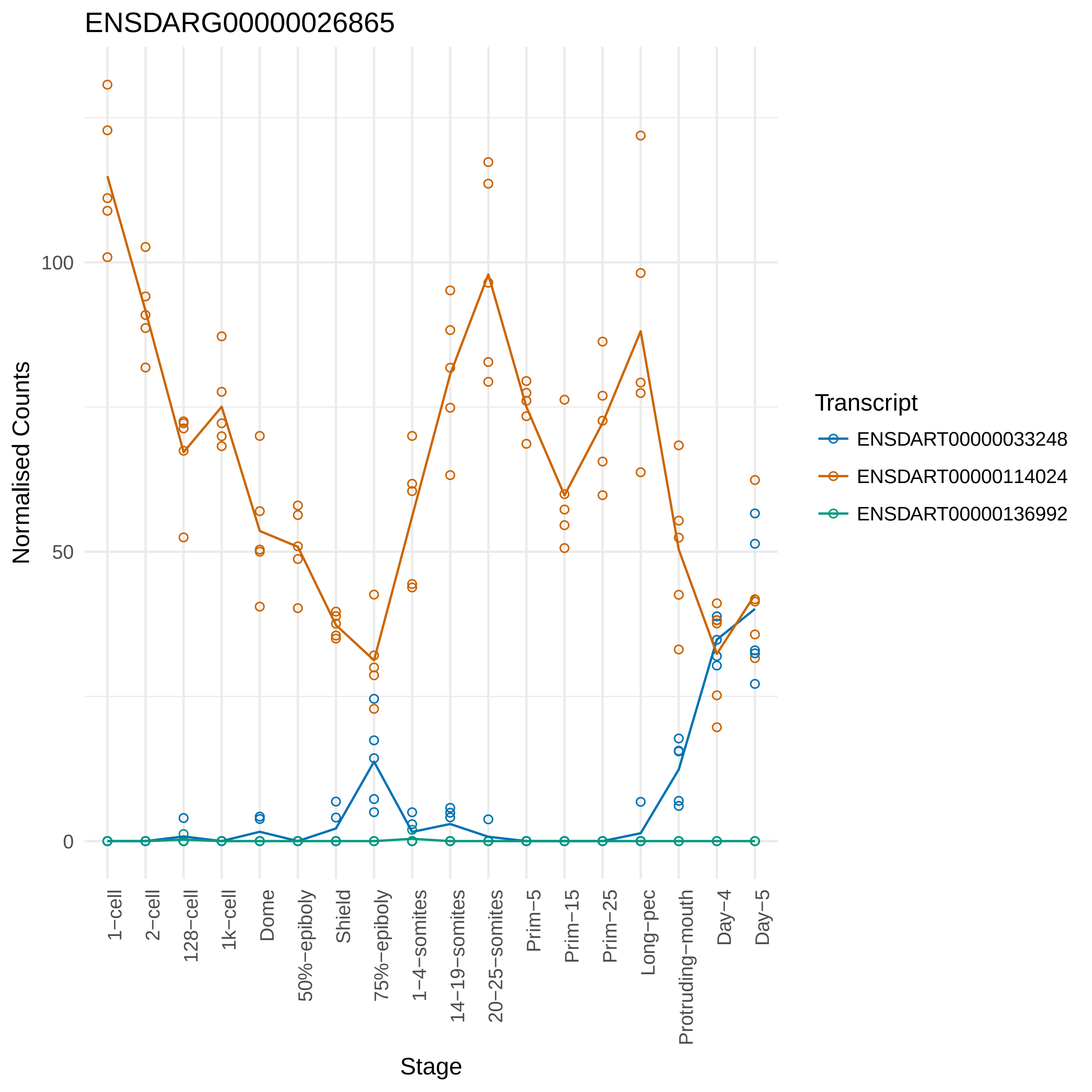
ENSDARG00000026865
Normalised Counts
100
50
0
1−cell
2−cell
128−cell
1k−cell
Dome
50%−epiboly
Shield
75%−epiboly
1−4−somites
14−19−somites
20−25−somites
Prim−5
Prim−15
Prim−25
Long−pec
Protruding−mouth
Day−4
Day−5
Stage
Transcript
ENSDART00000033248
ENSDART00000114024
ENSDART00000136992

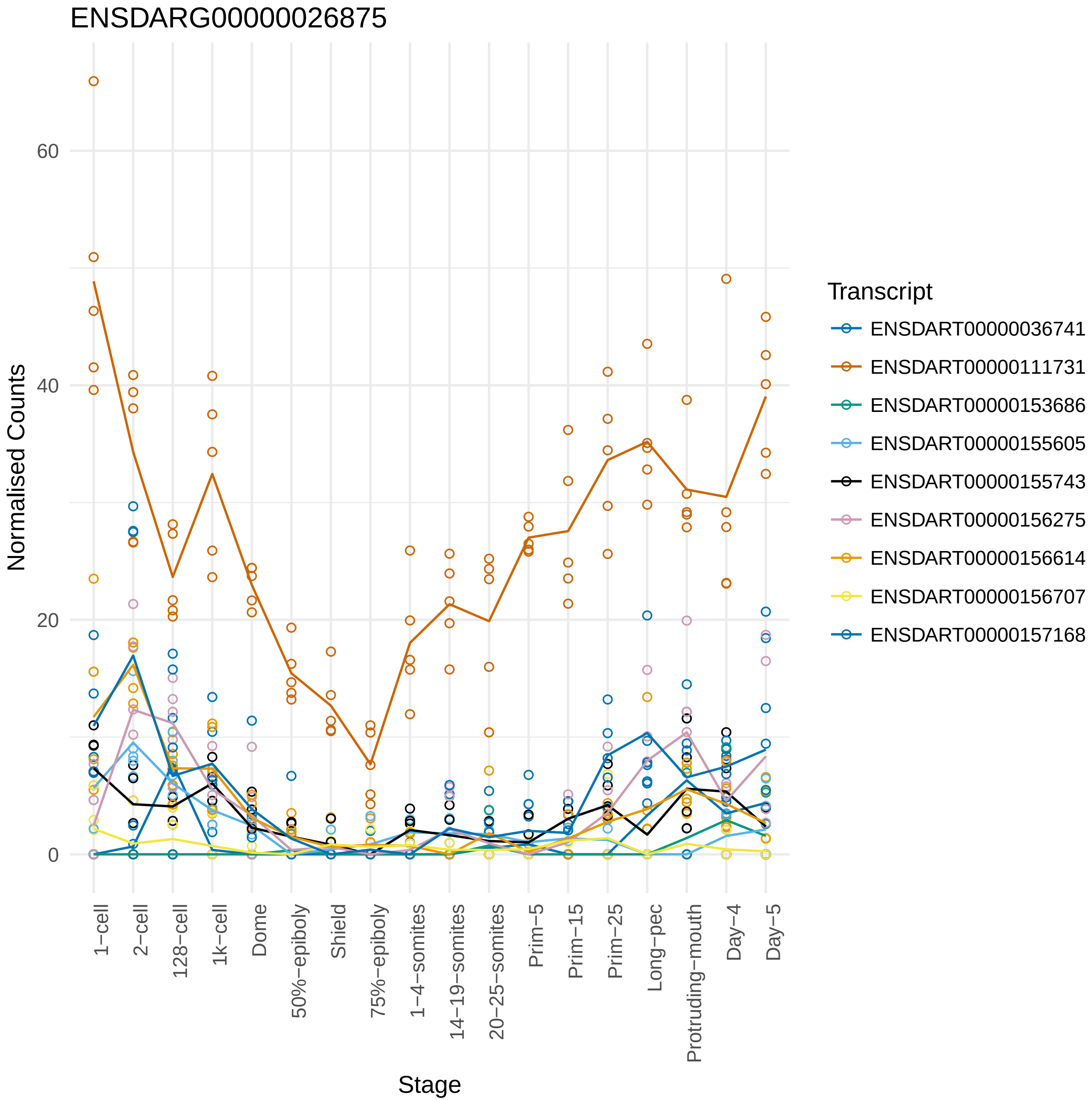
ENSDARG00000026875
Normalised Counts
0
20
40
60
1−cell
2−cell
128−cell
1k−cell
Dome
50%−epiboly
Shield
75%−epiboly
1−4−somites
14−19−somites
20−25−somites
Prim−5
Prim−15
Prim−25
Long−pec
Protruding−mouth
Day−4
Day−5
Stage
Transcript
ENSDART00000036741
ENSDART00000111731
ENSDART00000153686
ENSDART00000155605
ENSDART00000155743
ENSDART00000156275
ENSDART00000156614
ENSDART00000156707
ENSDART00000157168

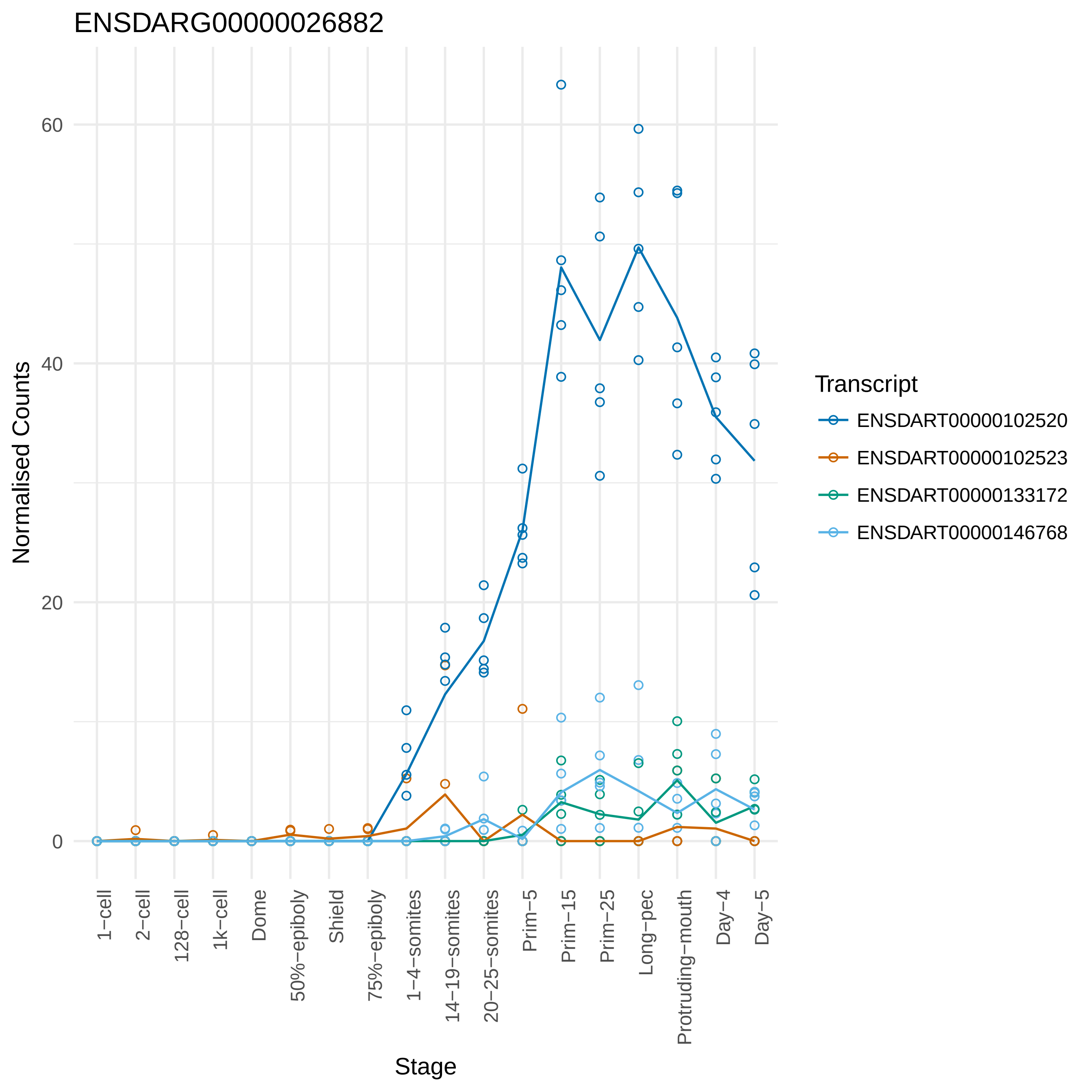

ENSDARG00000026882
Normalised Counts
60
40
20
0
1−cell
2−cell
128−cell
1k−cell
Dome
50%−epiboly
Shield
75%−epiboly
1−4−somites
14−19−somites
20−25−somites
Prim−5
Prim−15
Prim−25
Long−pec
Protruding−mouth
Day−4
Day−5
Stage
Transcript
ENSDART00000102520
ENSDART00000102523
ENSDART00000133172
ENSDART00000146768

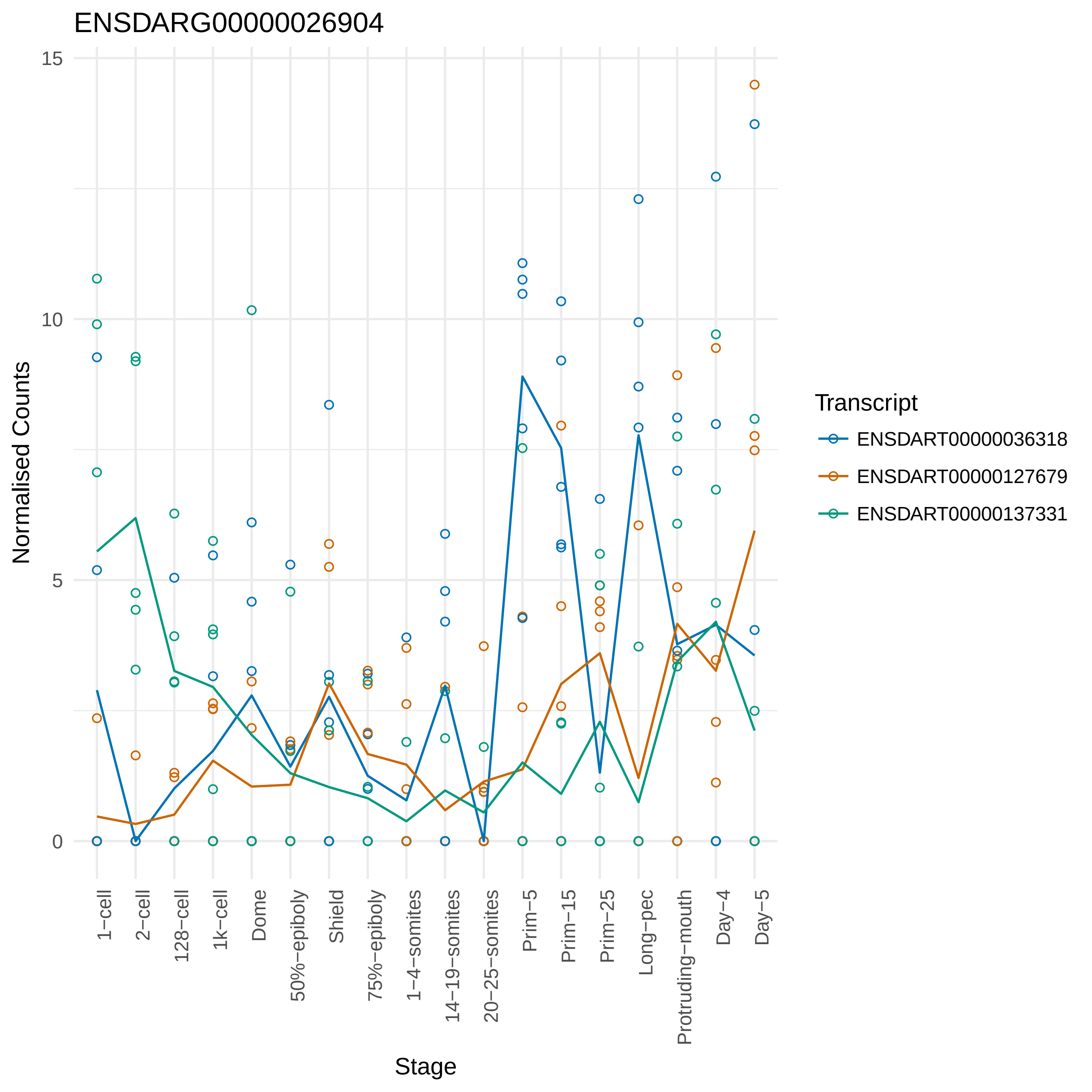
ENSDARG00000026904
Normalised Counts
0
5
10
15
Stage
1−cell
2−cell
128−cell
1k−cell
Dome
50%−epiboly
Shield
75%−epiboly
1−4−somites
14−19−somites
20−25−somites
Prim−5
Prim−15
Prim−25
Long−pec
Protruding−mouth
Day−4
Day−5
Transcript
ENSDART00000036318
ENSDART00000127679
ENSDART00000137331

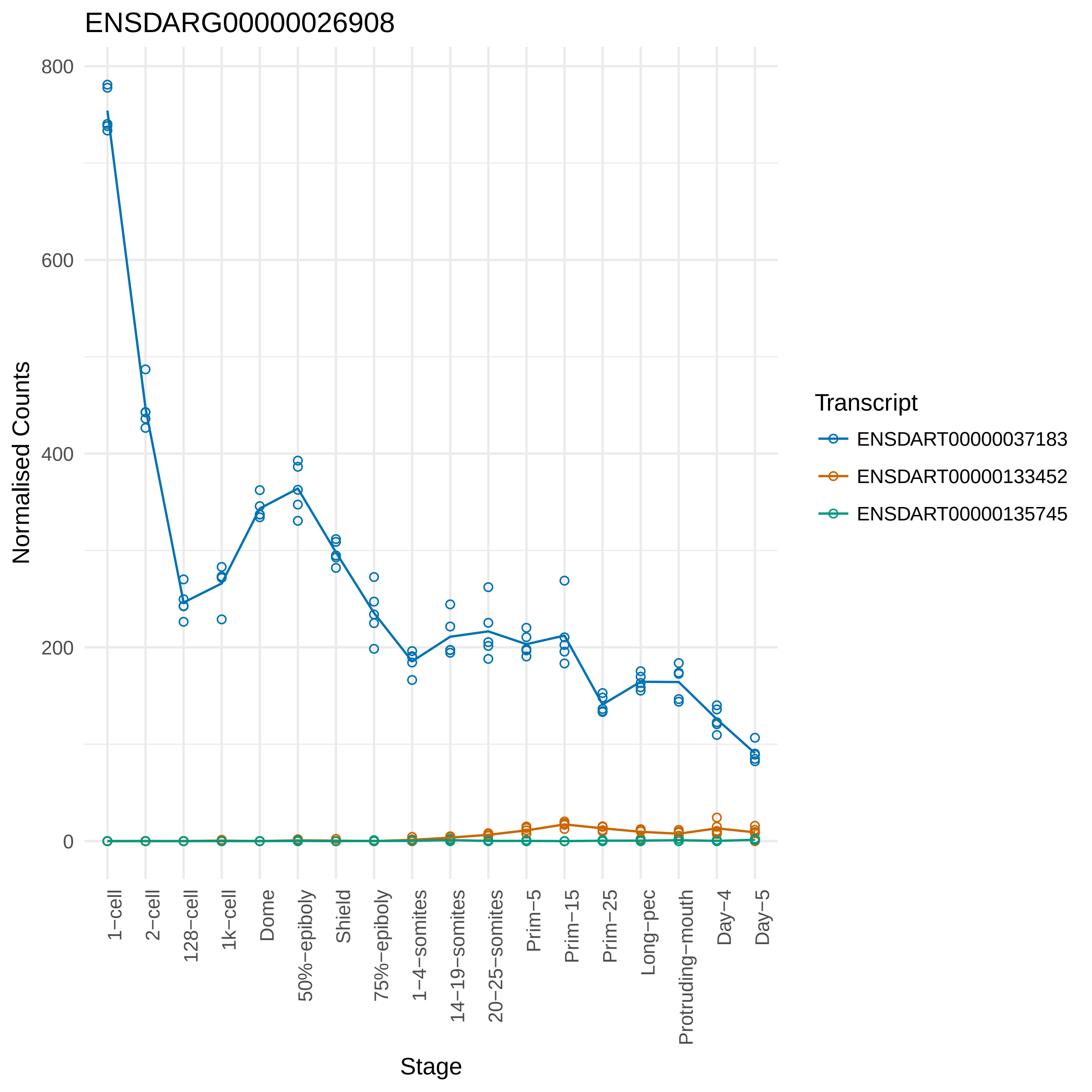
ENSDARG00000026908
Normalised Counts
800
600
400
200
0
1−cell
2−cell
128−cell
1k−cell
Dome
50%−epiboly
Shield
75%−epiboly
1−4−somites
14−19−somites
20−25−somites
Prim−5
Prim−15
Prim−25
Long−pec
Protruding−mouth
Day−4
Day−5
Stage
Transcript
ENSDART00000037183
ENSDART00000133452
ENSDART00000135745

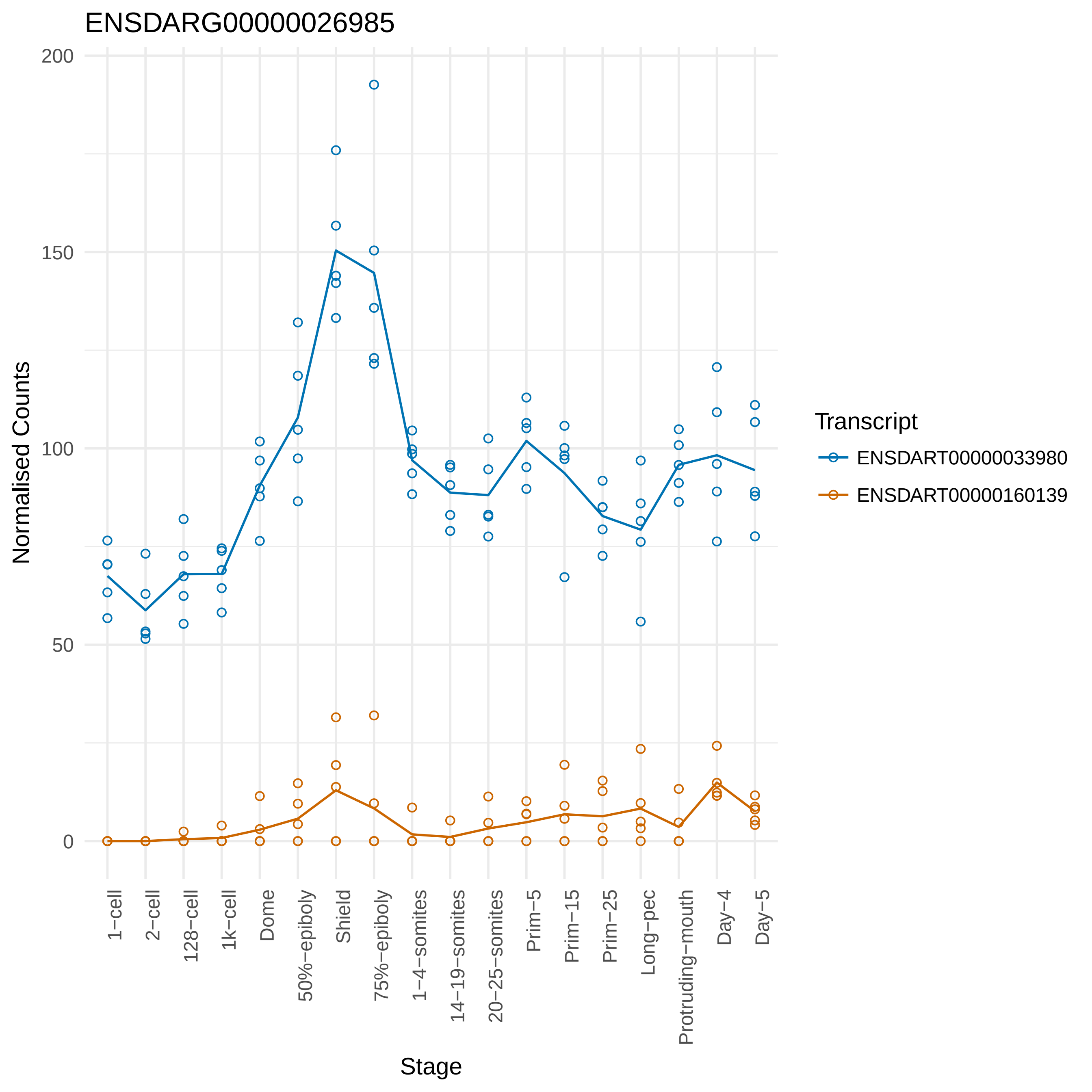

ENSDARG00000026985
Normalised Counts
200
150
100
50
0
1−cell
2−cell
128−cell
1k−cell
Dome
50%−epiboly
Shield
75%−epiboly
1−4−somites
14−19−somites
20−25−somites
Prim−5
Prim−15
Prim−25
Long−pec
Protruding−mouth
Day−4
Day−5
Stage
Transcript
ENSDART00000033980
ENSDART00000160139

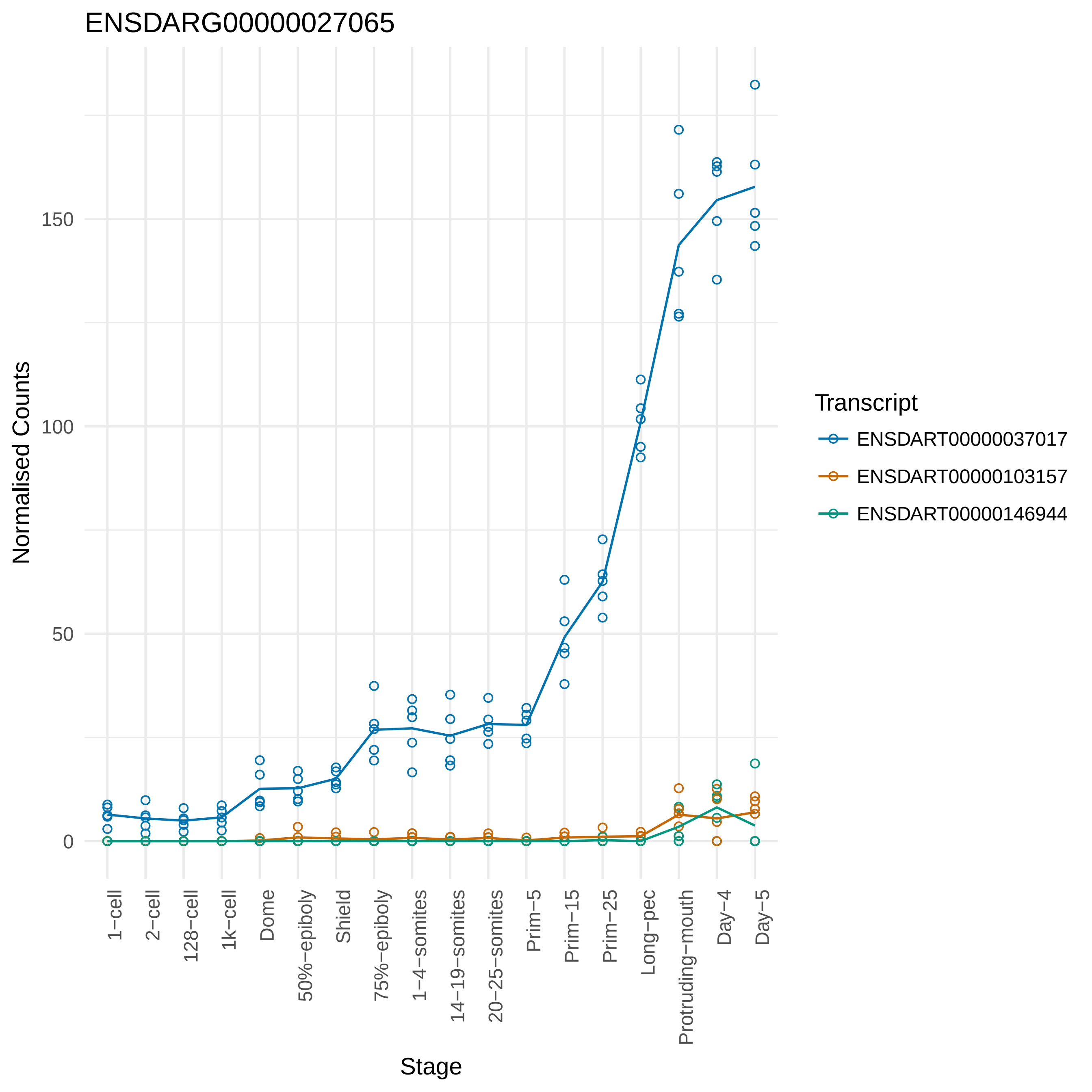
ENSDARG00000027065
Normalised Counts
0
50
100
150
Stage
1−cell
2−cell
128−cell
1k−cell
Dome
50%−epiboly
Shield
75%−epiboly
1−4−somites
14−19−somites
20−25−somites
Prim−5
Prim−15
Prim−25
Long−pec
Protruding−mouth
Day−4
Day−5
Transcript
ENSDART00000037017
ENSDART00000103157
ENSDART00000146944

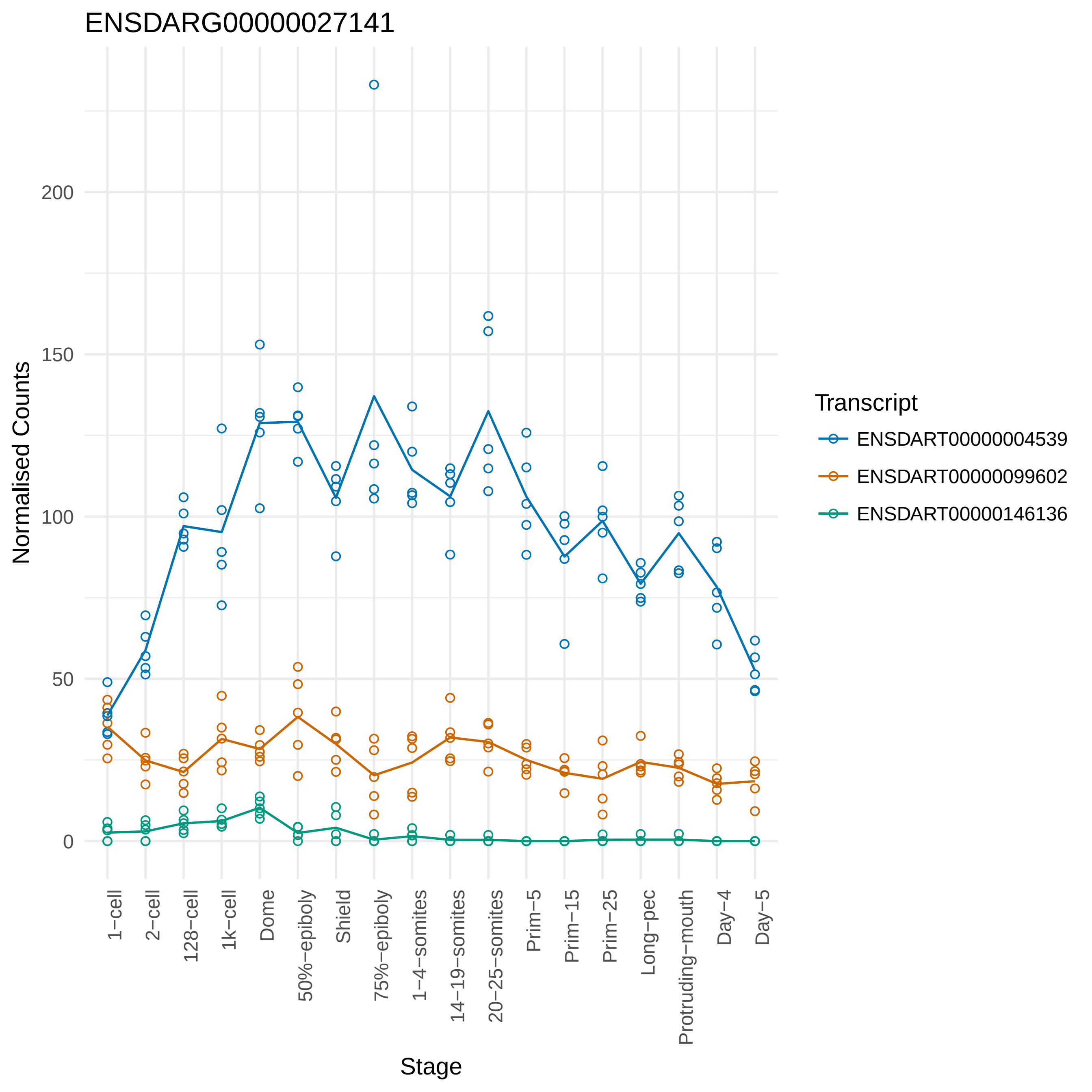
ENSDARG00000027141
Normalised Counts
0
50
100
150
200
Stage
1−cell
2−cell
128−cell
1k−cell
Dome
50%−epiboly
Shield
75%−epiboly
1−4−somites
14−19−somites
20−25−somites
Prim−5
Prim−15
Prim−25
Long−pec
Protruding−mouth
Day−4
Day−5
Transcript
ENSDART00000004539
ENSDART00000099602
ENSDART00000146136

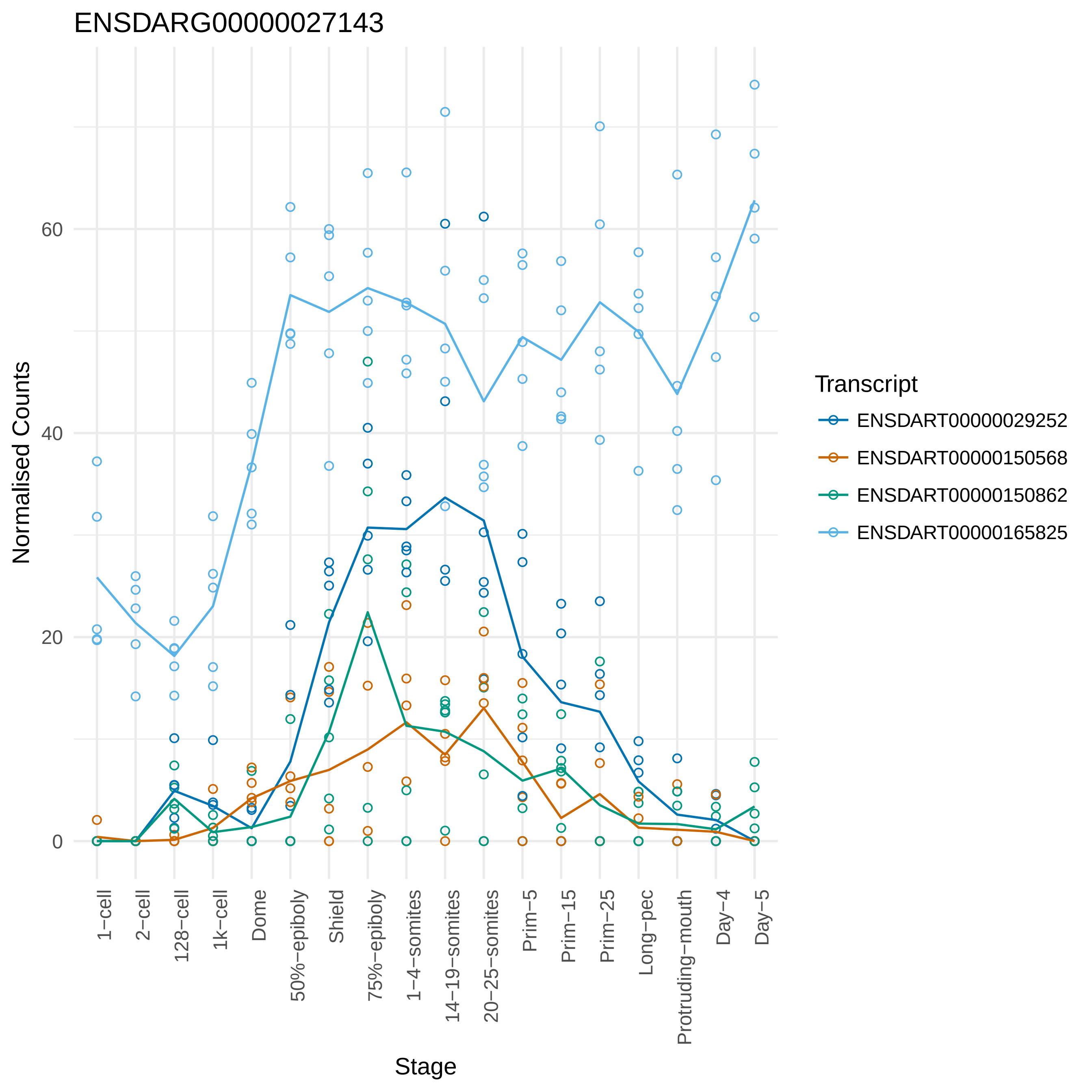

ENSDARG00000027143
Normalised Counts
0
20
40
60
Stage
1−cell
2−cell
128−cell
1k−cell
Dome
50%−epiboly
Shield
75%−epiboly
1−4−somites
14−19−somites
20−25−somites
Prim−5
Prim−15
Prim−25
Long−pec
Protruding−mouth
Day−4
Day−5
Transcript
ENSDART00000029252
ENSDART00000150568
ENSDART00000150862
ENSDART00000165825

ENSDARG00000027153

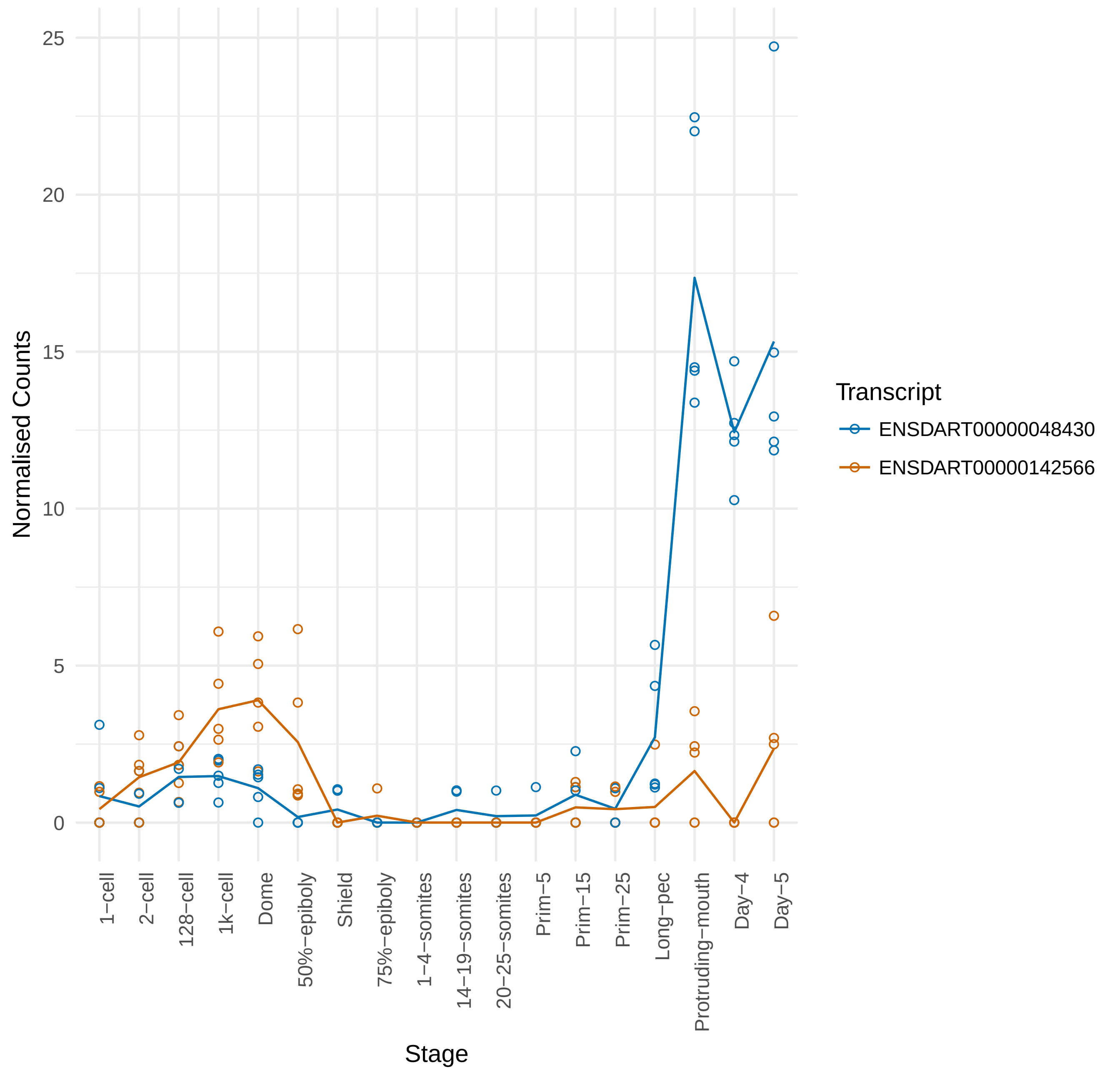

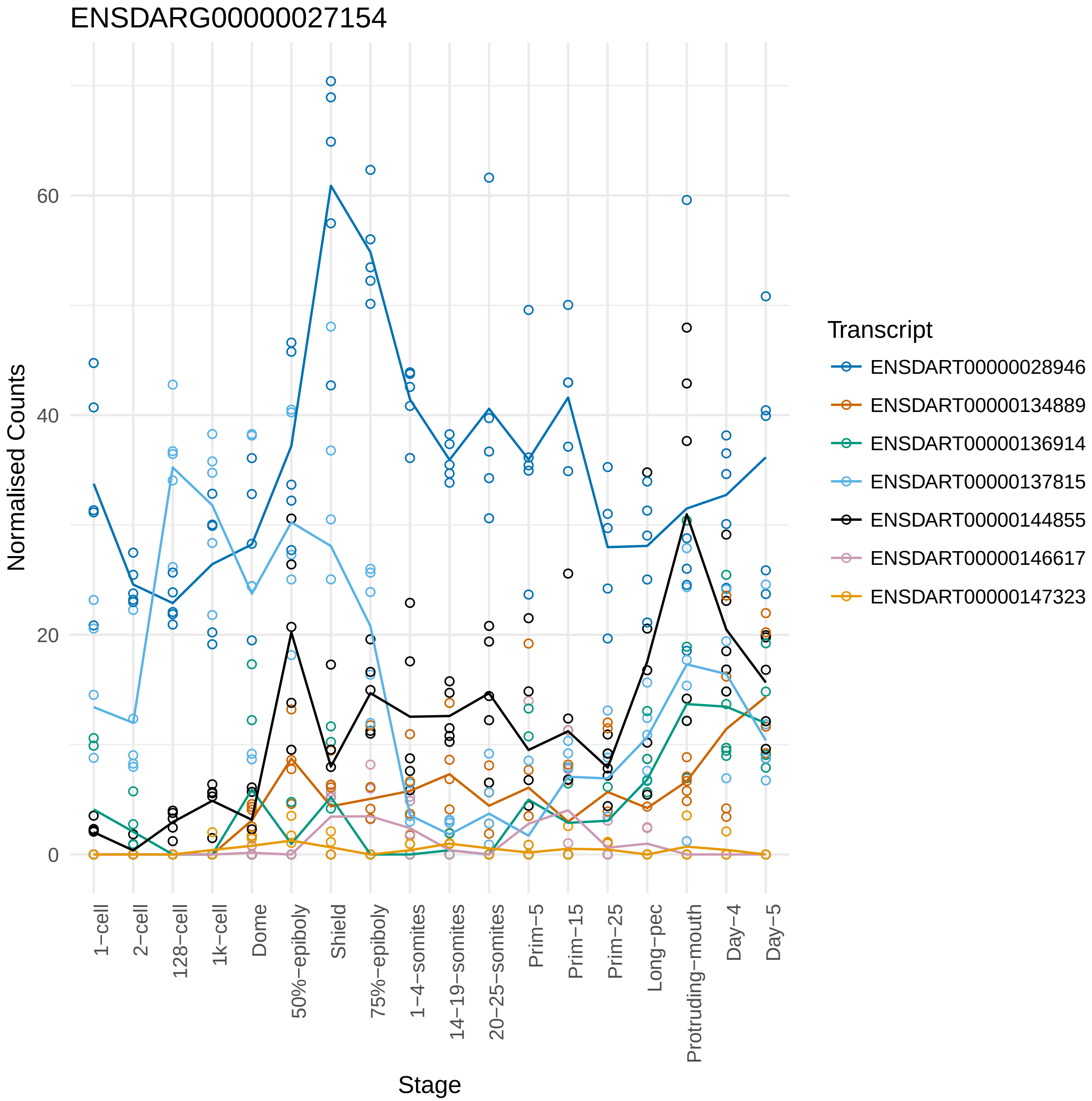
ENSDARG00000027154
Normalised Counts
0
20
40
60
Stage
1−cell
2−cell
128−cell
1k−cell
Dome
50%−epiboly
Shield
75%−epiboly
1−4−somites
14−19−somites
20−25−somites
Prim−5
Prim−15
Prim−25
Long−pec
Protruding−mouth
Day−4
Day−5
Transcript
ENSDART00000028946
ENSDART00000134889
ENSDART00000136914
ENSDART00000137815
ENSDART00000144855
ENSDART00000146617
ENSDART00000147323

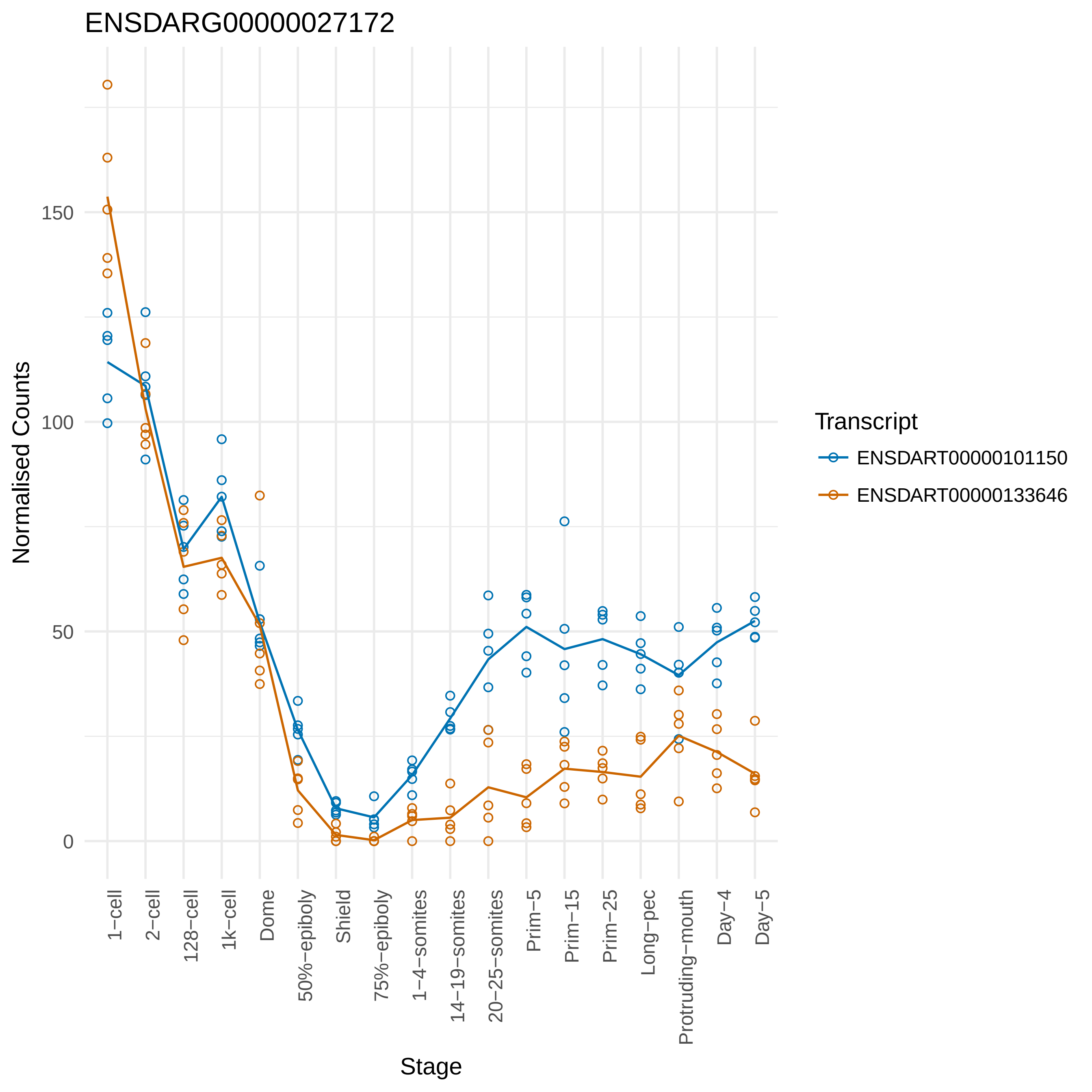

ENSDARG00000027172
Normalised Counts
150
100
50
0
1−cell
2−cell
128−cell
1k−cell
Dome
50%−epiboly
Shield
75%−epiboly
1−4−somites
14−19−somites
20−25−somites
Prim−5
Prim−15
Prim−25
Long−pec
Protruding−mouth
Day−4
Day−5
Stage
Transcript
ENSDART00000101150
ENSDART00000133646

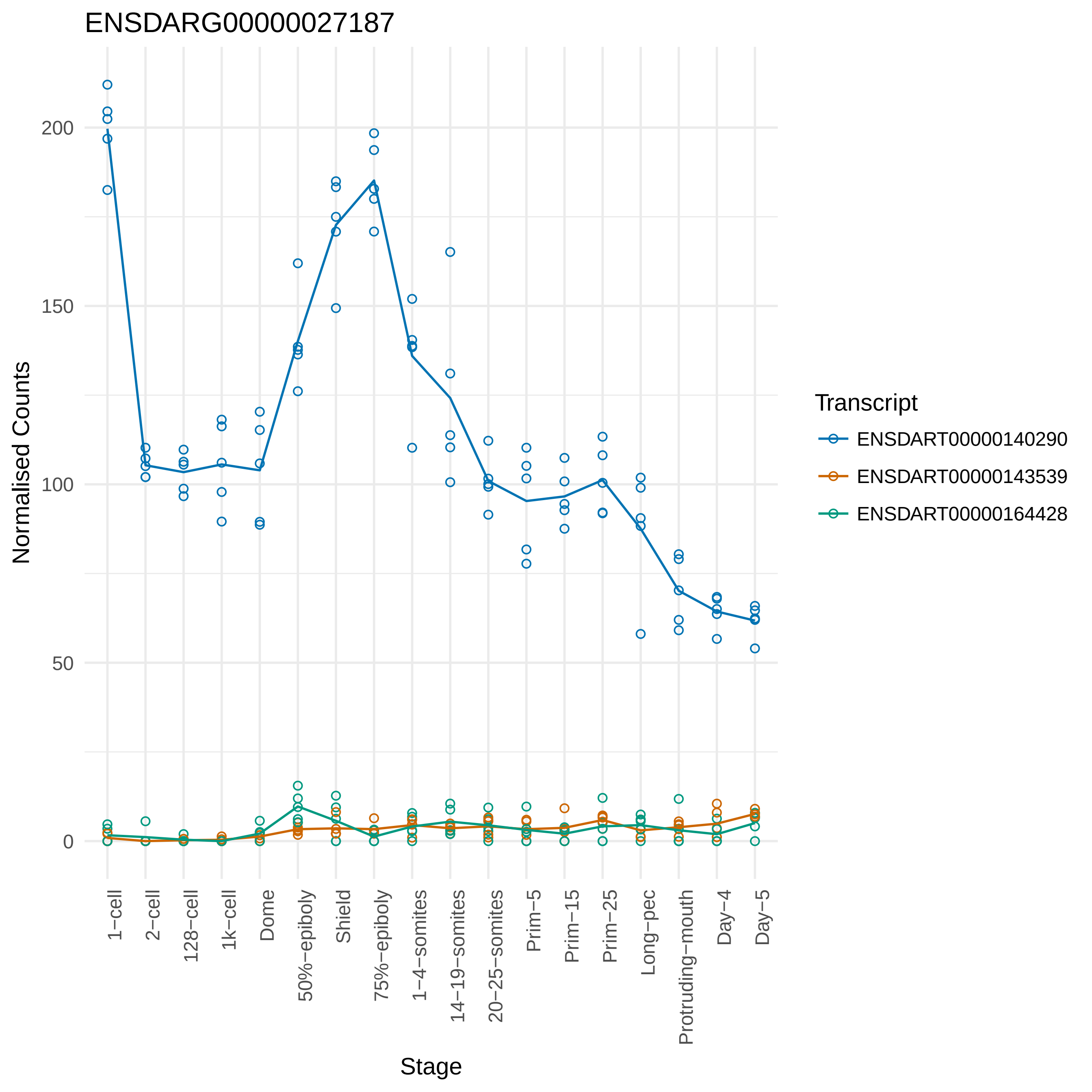

ENSDARG00000027187
Normalised Counts
200
150
100
50
0
1−cell
2−cell
128−cell
1k−cell
Dome
50%−epiboly
Shield
75%−epiboly
1−4−somites
14−19−somites
20−25−somites
Prim−5
Prim−15
Prim−25
Long−pec
Protruding−mouth
Day−4
Day−5
Stage
Transcript
ENSDART00000140290
ENSDART00000143539
ENSDART00000164428

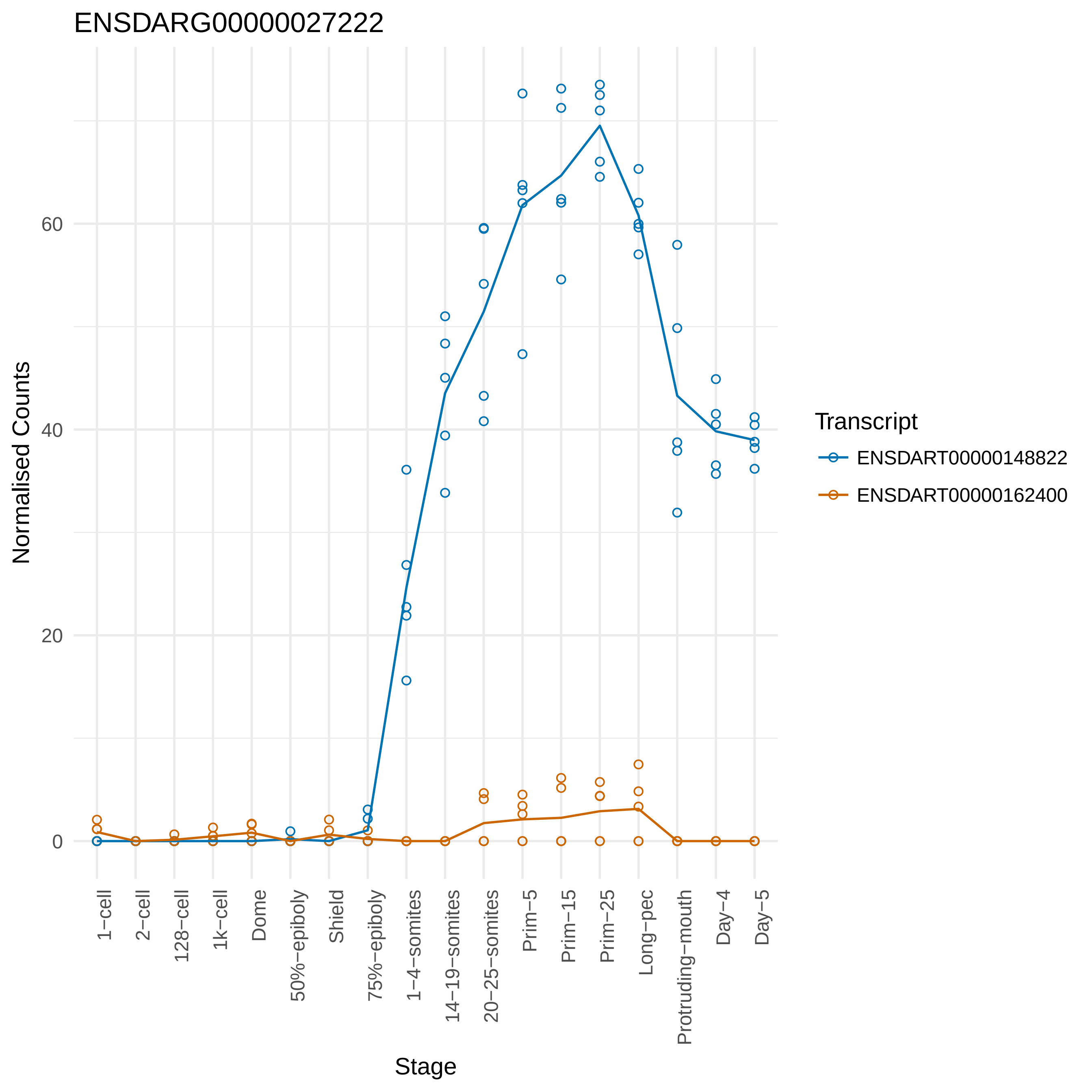

ENSDARG00000027222
Normalised Counts
0
20
40
60
1−cell
2−cell
128−cell
1k−cell
Dome
50%−epiboly
Shield
75%−epiboly
1−4−somites
14−19−somites
20−25−somites
Prim−5
Prim−15
Prim−25
Long−pec
Protruding−mouth
Day−4
Day−5
Stage
Transcript
ENSDART00000148822
ENSDART00000162400

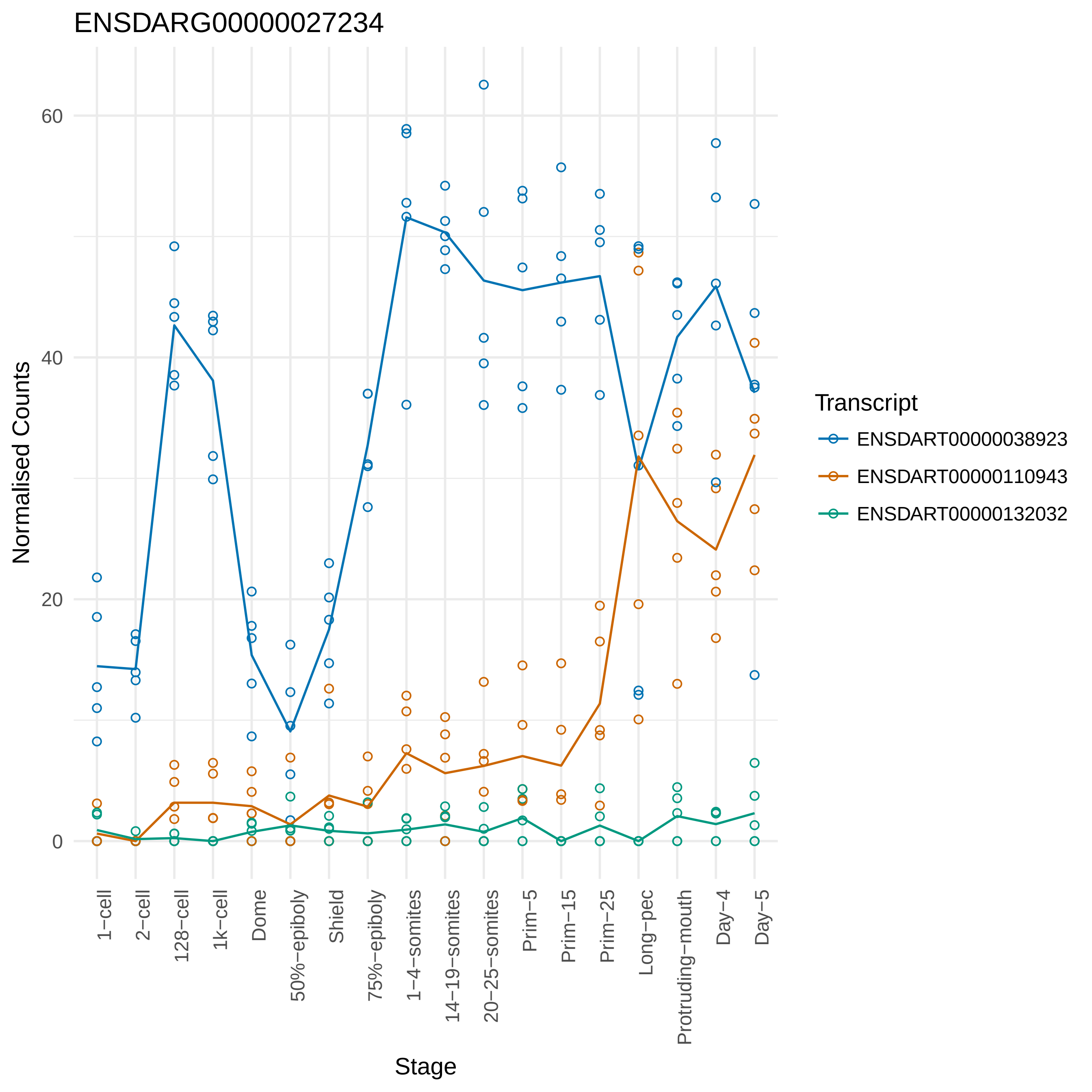
ENSDARG00000027234
Normalised Counts
0
20
40
60
1−cell
2−cell
128−cell
1k−cell
Dome
50%−epiboly
Shield
75%−epiboly
1−4−somites
14−19−somites
20−25−somites
Prim−5
Prim−15
Prim−25
Long−pec
Protruding−mouth
Day−4
Day−5
Stage
Transcript
ENSDART00000038923
ENSDART00000110943
ENSDART00000132032

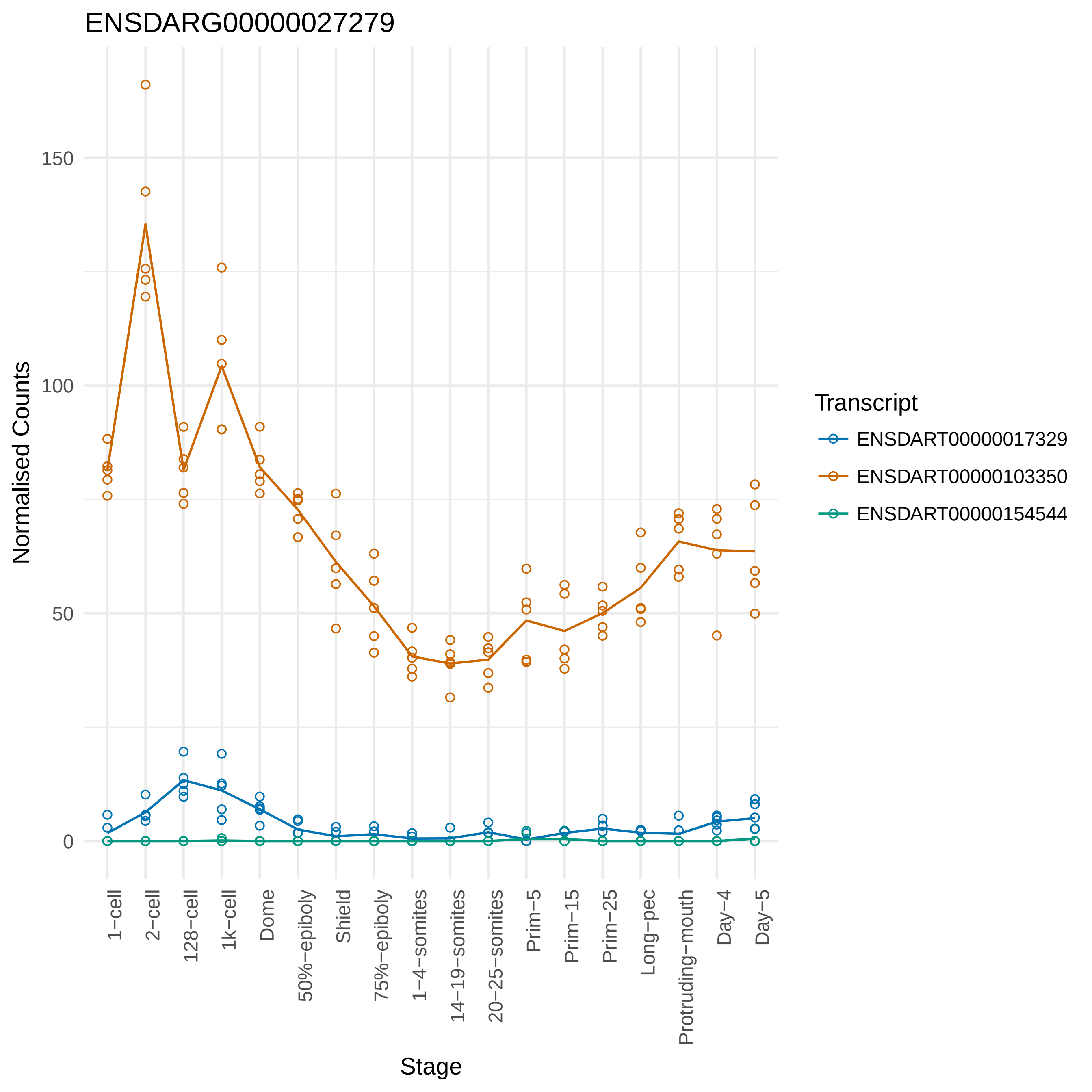

ENSDARG00000027279
Normalised Counts
150
100
50
0
1−cell
2−cell
128−cell
1k−cell
Dome
50%−epiboly
Shield
75%−epiboly
1−4−somites
14−19−somites
20−25−somites
Prim−5
Prim−15
Prim−25
Long−pec
Protruding−mouth
Day−4
Day−5
Stage
Transcript
ENSDART00000017329
ENSDART00000103350
ENSDART00000154544

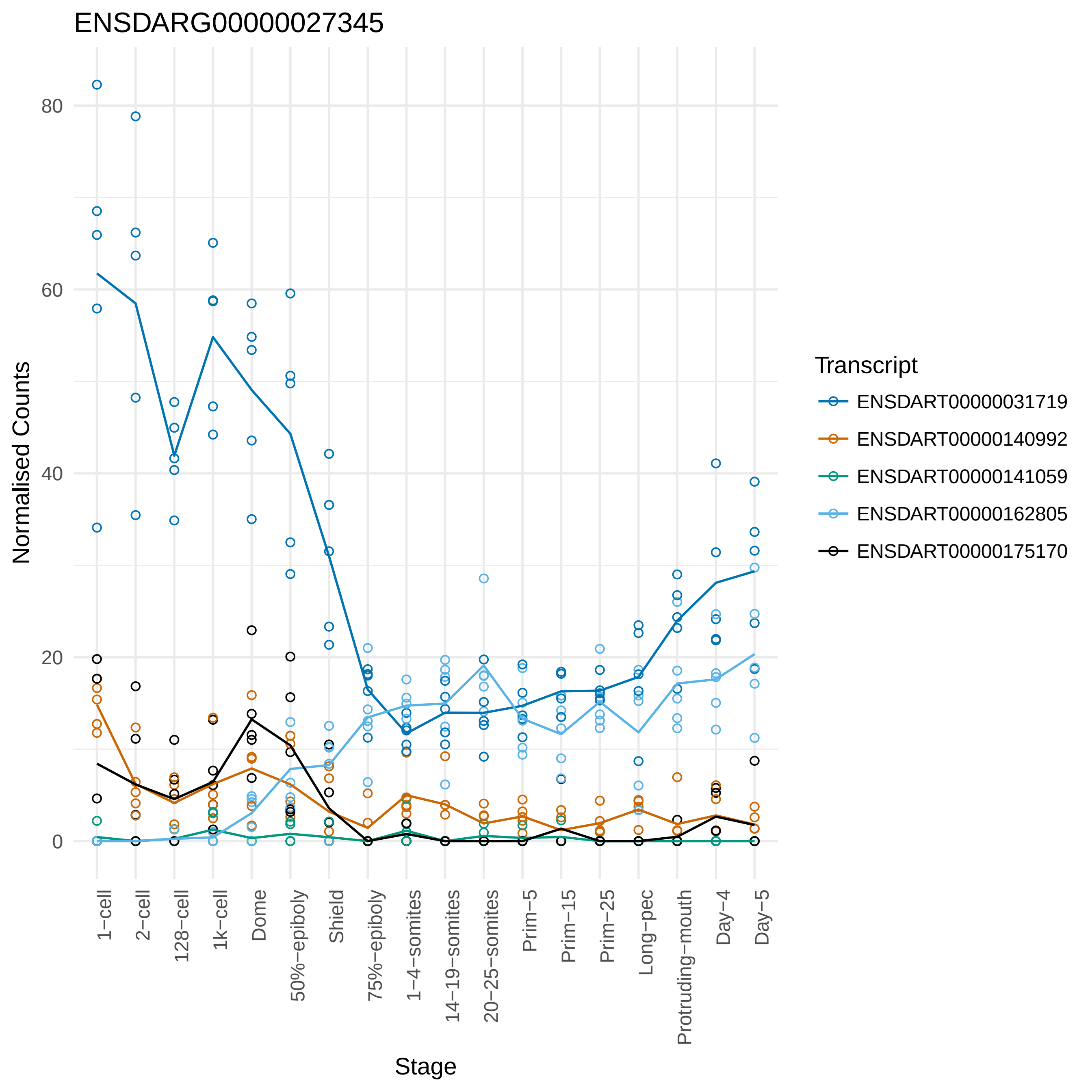
ENSDARG00000027345
Normalised Counts
0
20
40
60
80
1−cell
2−cell
128−cell
1k−cell
Dome
50%−epiboly
Shield
75%−epiboly
1−4−somites
14−19−somites
20−25−somites
Prim−5
Prim−15
Prim−25
Long−pec
Protruding−mouth
Day−4
Day−5
Stage
Transcript
ENSDART00000031719
ENSDART00000140992
ENSDART00000141059
ENSDART00000162805
ENSDART00000175170

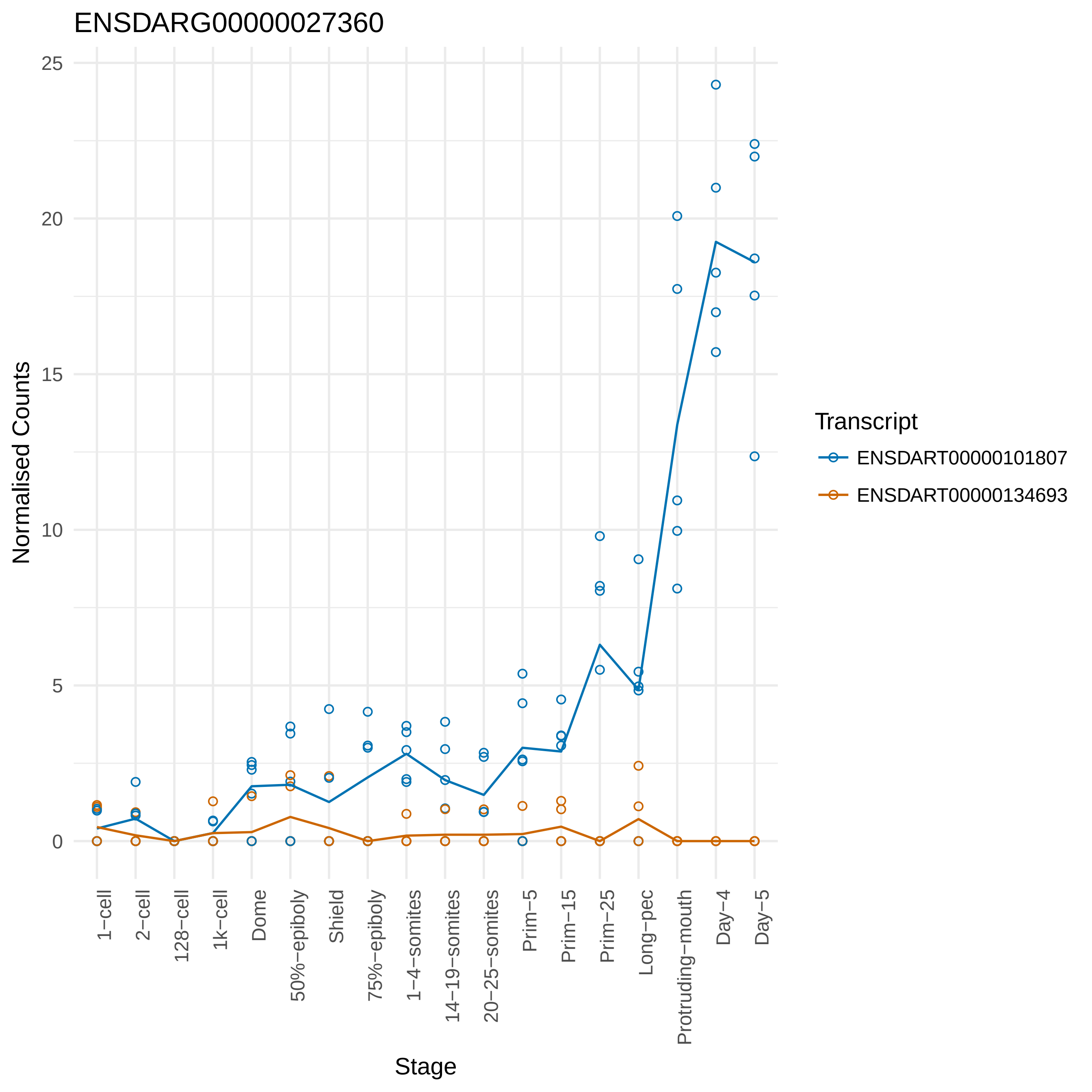

ENSDARG00000027360
Normalised Counts
0
5
10
15
20
25
1−cell
2−cell
128−cell
1k−cell
Dome
50%−epiboly
Shield
75%−epiboly
1−4−somites
14−19−somites
20−25−somites
Prim−5
Prim−15
Prim−25
Long−pec
Protruding−mouth
Day−4
Day−5
Stage
Transcript
ENSDART00000101807
ENSDART00000134693

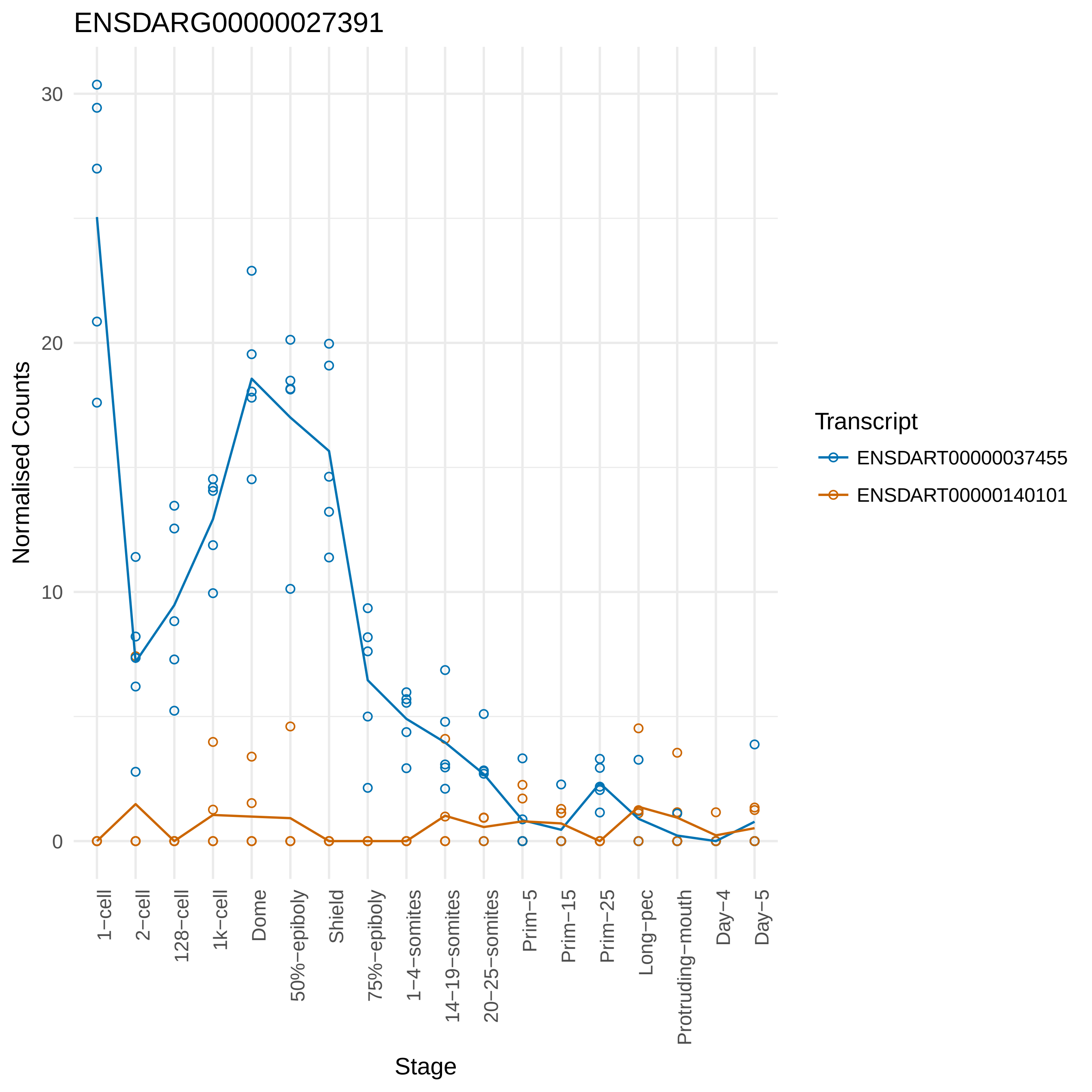

ENSDARG00000027391
Normalised Counts
30
20
10
0
1−cell
2−cell
128−cell
1k−cell
Dome
50%−epiboly
Shield
75%−epiboly
1−4−somites
14−19−somites
20−25−somites
Prim−5
Prim−15
Prim−25
Long−pec
Protruding−mouth
Day−4
Day−5
Stage
Transcript
ENSDART00000037455
ENSDART00000140101

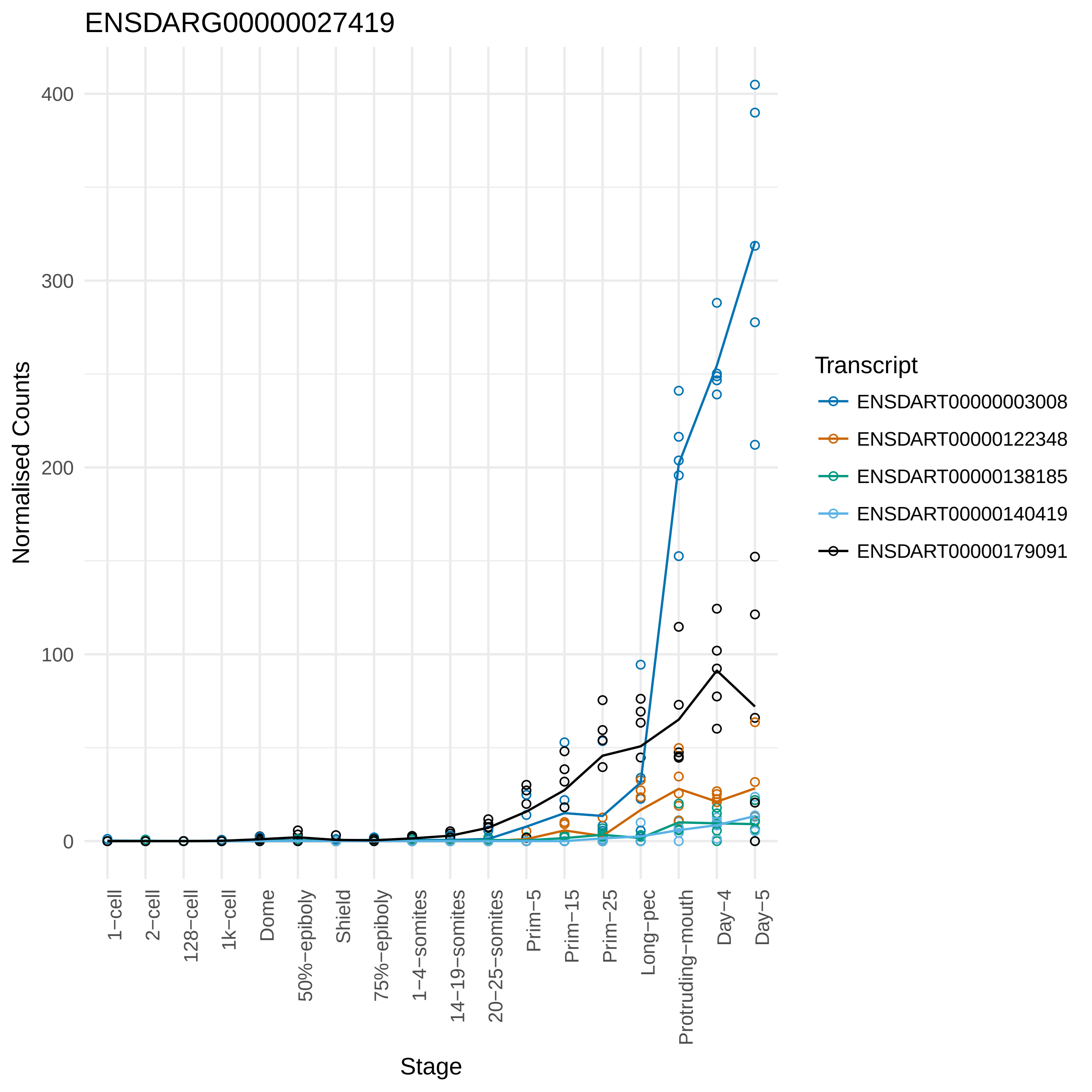

ENSDARG00000027419
Normalised Counts
400
300
200
100
0
1−cell
2−cell
128−cell
1k−cell
Dome
50%−epiboly
Shield
75%−epiboly
1−4−somites
14−19−somites
20−25−somites
Prim−5
Prim−15
Prim−25
Long−pec
Protruding−mouth
Day−4
Day−5
Stage
Transcript
ENSDART00000003008
ENSDART00000122348
ENSDART00000138185
ENSDART00000140419
ENSDART00000179091

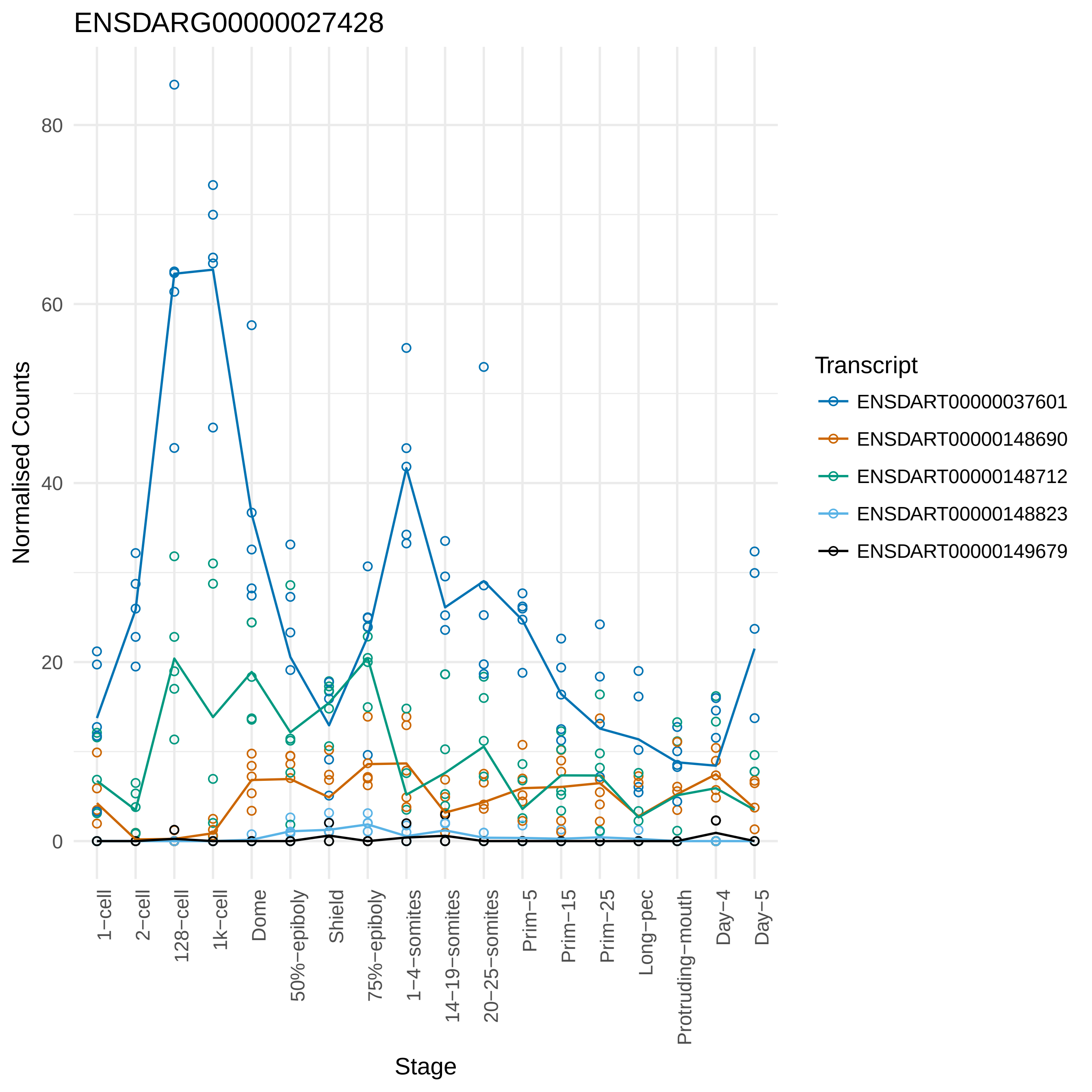
ENSDARG00000027428
Normalised Counts
Stage
Transcript
ENSDART00000037601
ENSDART00000148690
ENSDART00000148712
ENSDART00000148823
ENSDART00000149679
0
20
40
60
80
1−cell
2−cell
128−cell
1k−cell
Dome
50%−epiboly
Shield
75%−epiboly
1−4−somites
14−19−somites
20−25−somites
Prim−5
Prim−15
Prim−25
Long−pec
Protruding−mouth
Day−4
Day−5

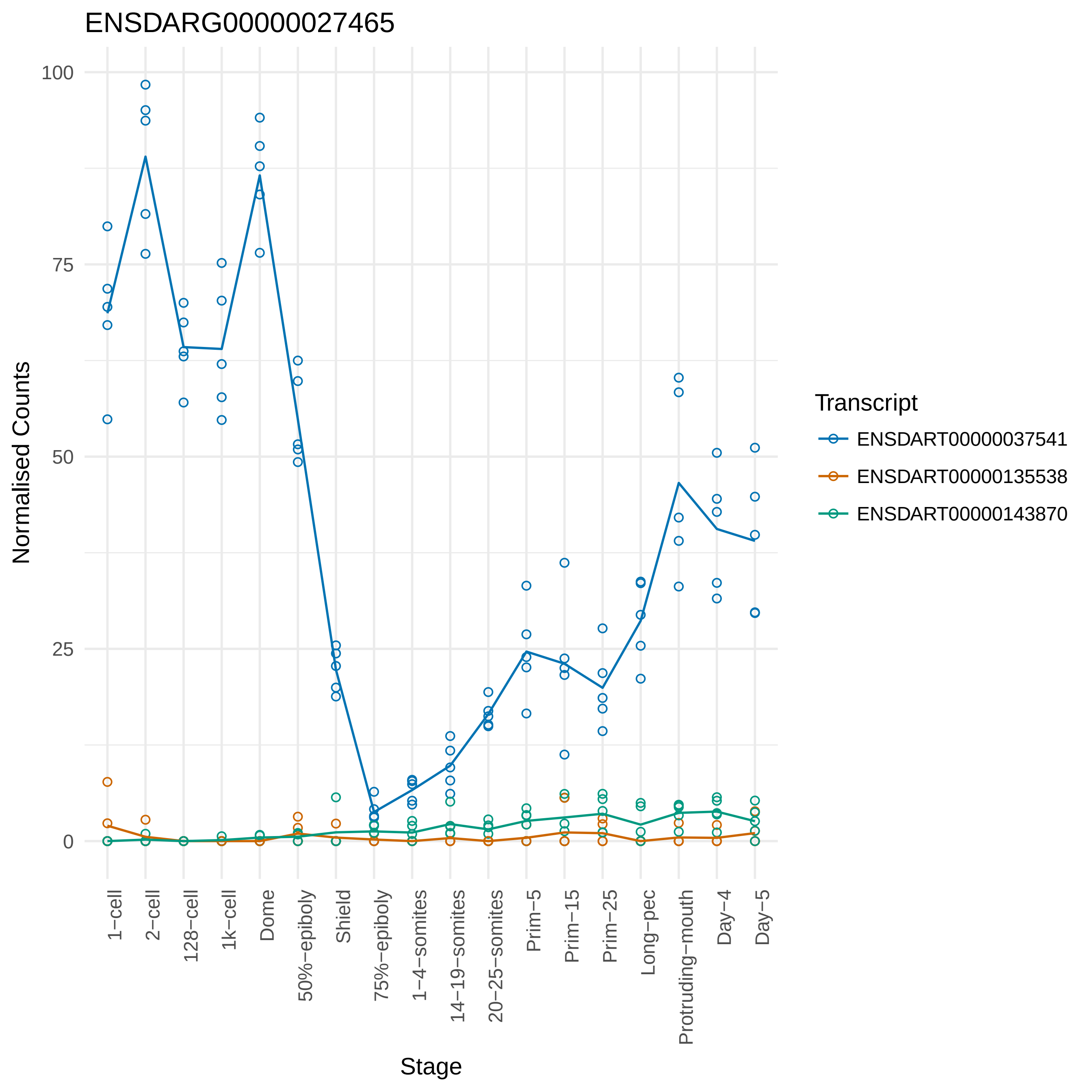

ENSDARG00000027465
Normalised Counts
100
75
50
25
0
1−cell
2−cell
128−cell
1k−cell
Dome
50%−epiboly
Shield
75%−epiboly
1−4−somites
14−19−somites
20−25−somites
Prim−5
Prim−15
Prim−25
Long−pec
Protruding−mouth
Day−4
Day−5
Stage
Transcript
ENSDART00000037541
ENSDART00000135538
ENSDART00000143870

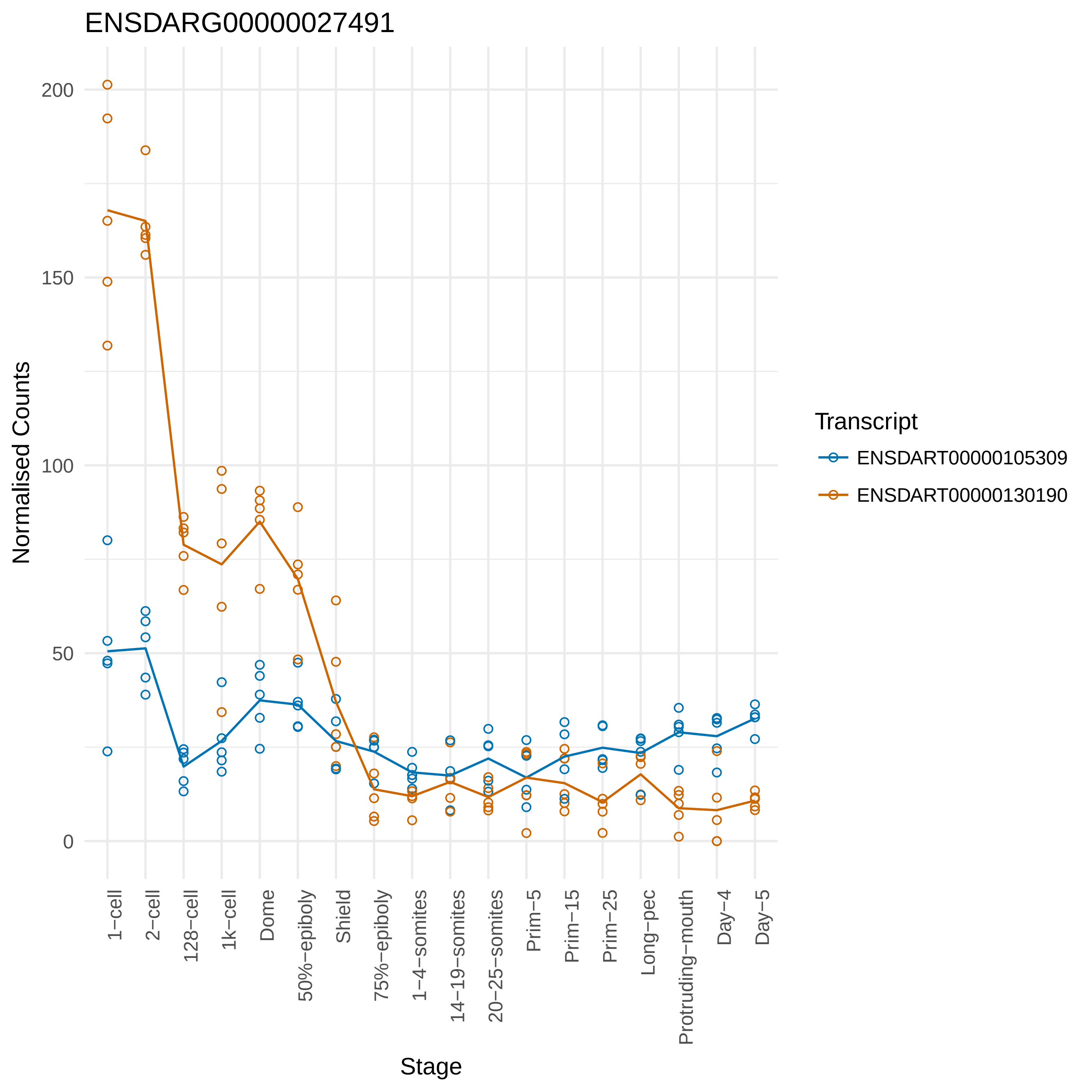
ENSDARG00000027491
Normalised Counts
0
50
100
150
200
1−cell
2−cell
128−cell
1k−cell
Dome
50%−epiboly
Shield
75%−epiboly
1−4−somites
14−19−somites
20−25−somites
Prim−5
Prim−15
Prim−25
Long−pec
Protruding−mouth
Day−4
Day−5
Stage
Transcript
ENSDART00000105309
ENSDART00000130190

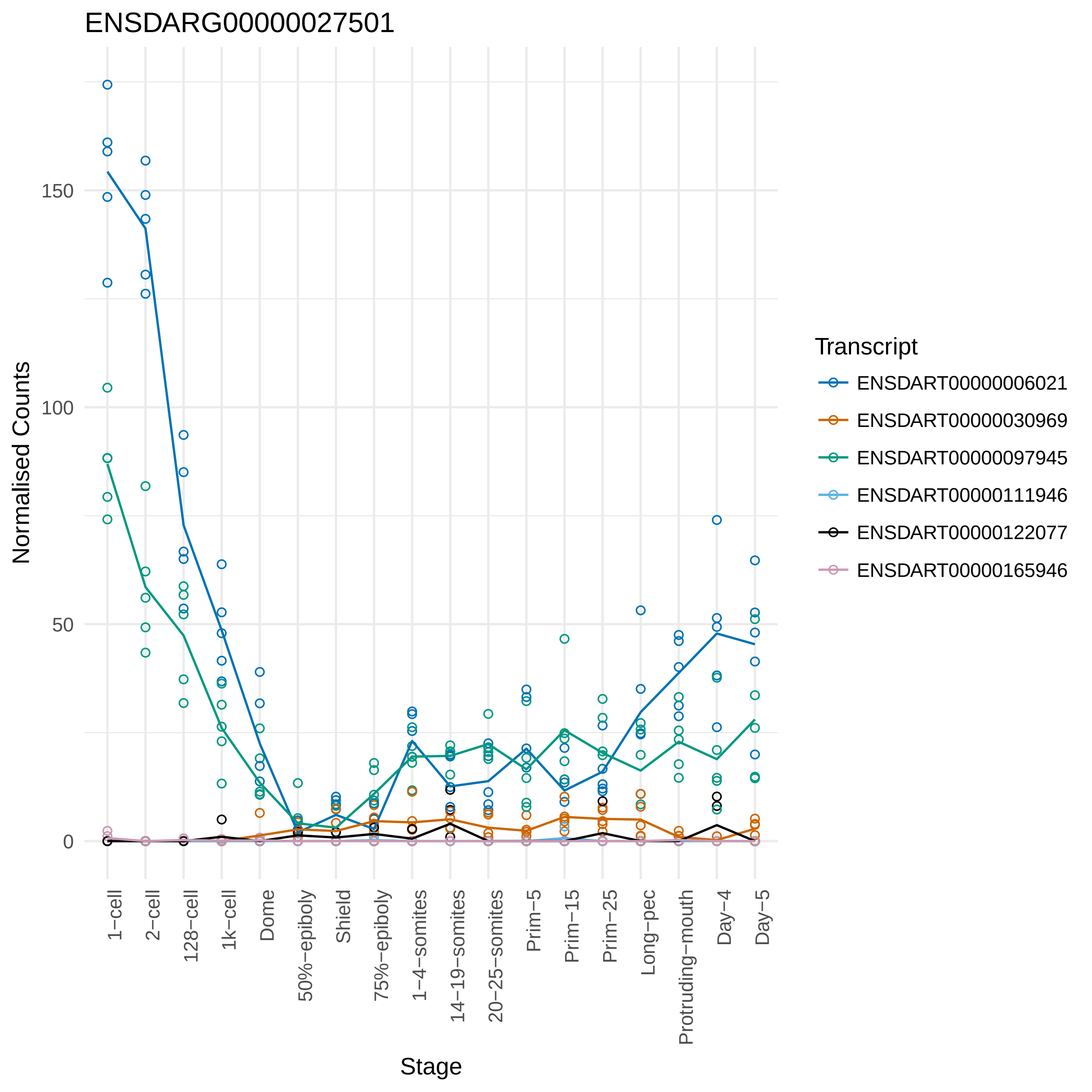

ENSDARG00000027501
Normalised Counts
0
50
100
150
Stage
1−cell
2−cell
128−cell
1k−cell
Dome
50%−epiboly
Shield
75%−epiboly
1−4−somites
14−19−somites
20−25−somites
Prim−5
Prim−15
Prim−25
Long−pec
Protruding−mouth
Day−4
Day−5
Transcript
ENSDART00000006021
ENSDART00000030969
ENSDART00000097945
ENSDART00000111946
ENSDART00000122077
ENSDART00000165946

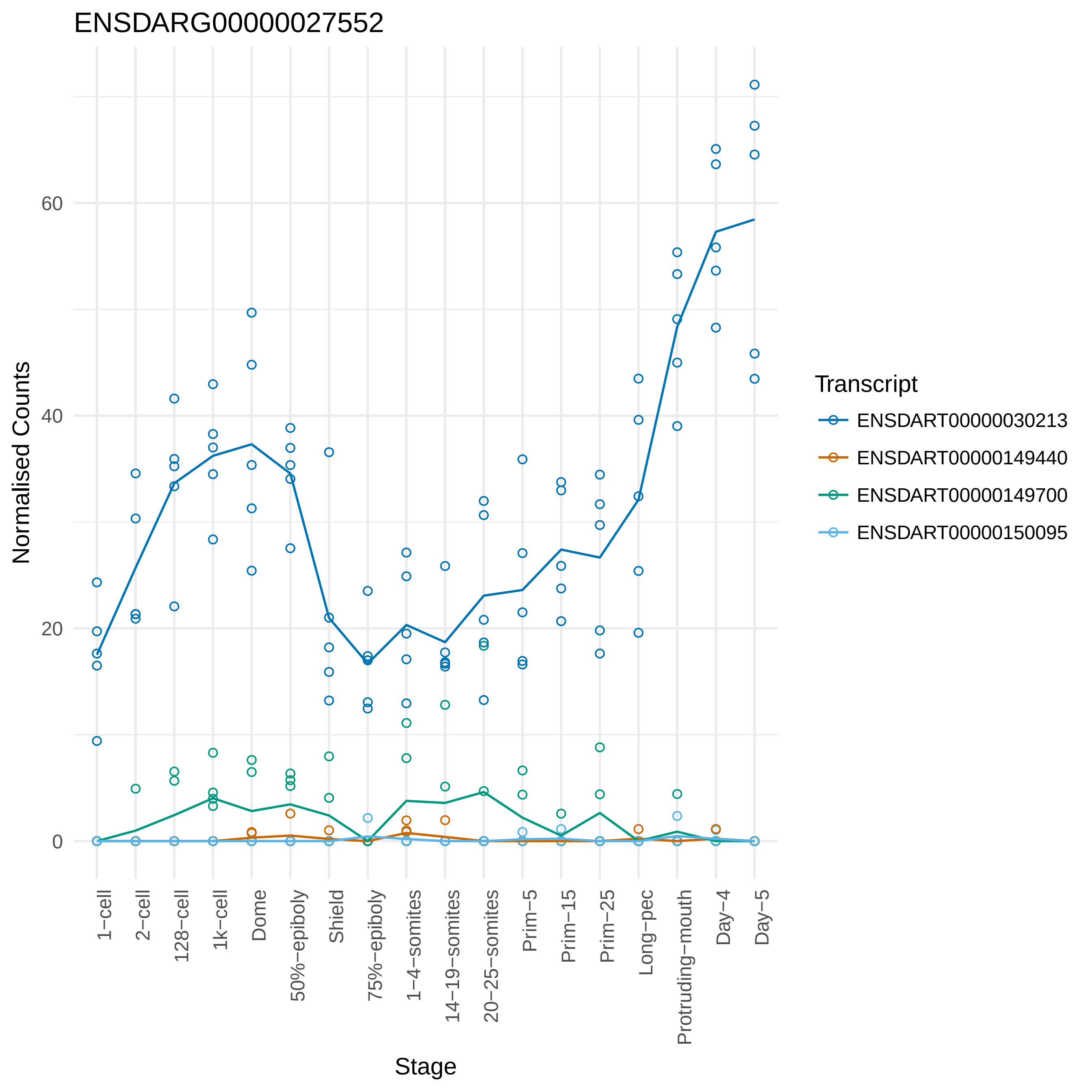
ENSDARG00000027552
Normalised Counts
60
40
20
0
1−cell
2−cell
128−cell
1k−cell
Dome
50%−epiboly
Shield
75%−epiboly
1−4−somites
14−19−somites
20−25−somites
Prim−5
Prim−15
Prim−25
Long−pec
Protruding−mouth
Day−4
Day−5
Stage
Transcript
ENSDART00000030213
ENSDART00000149440
ENSDART00000149700
ENSDART00000150095

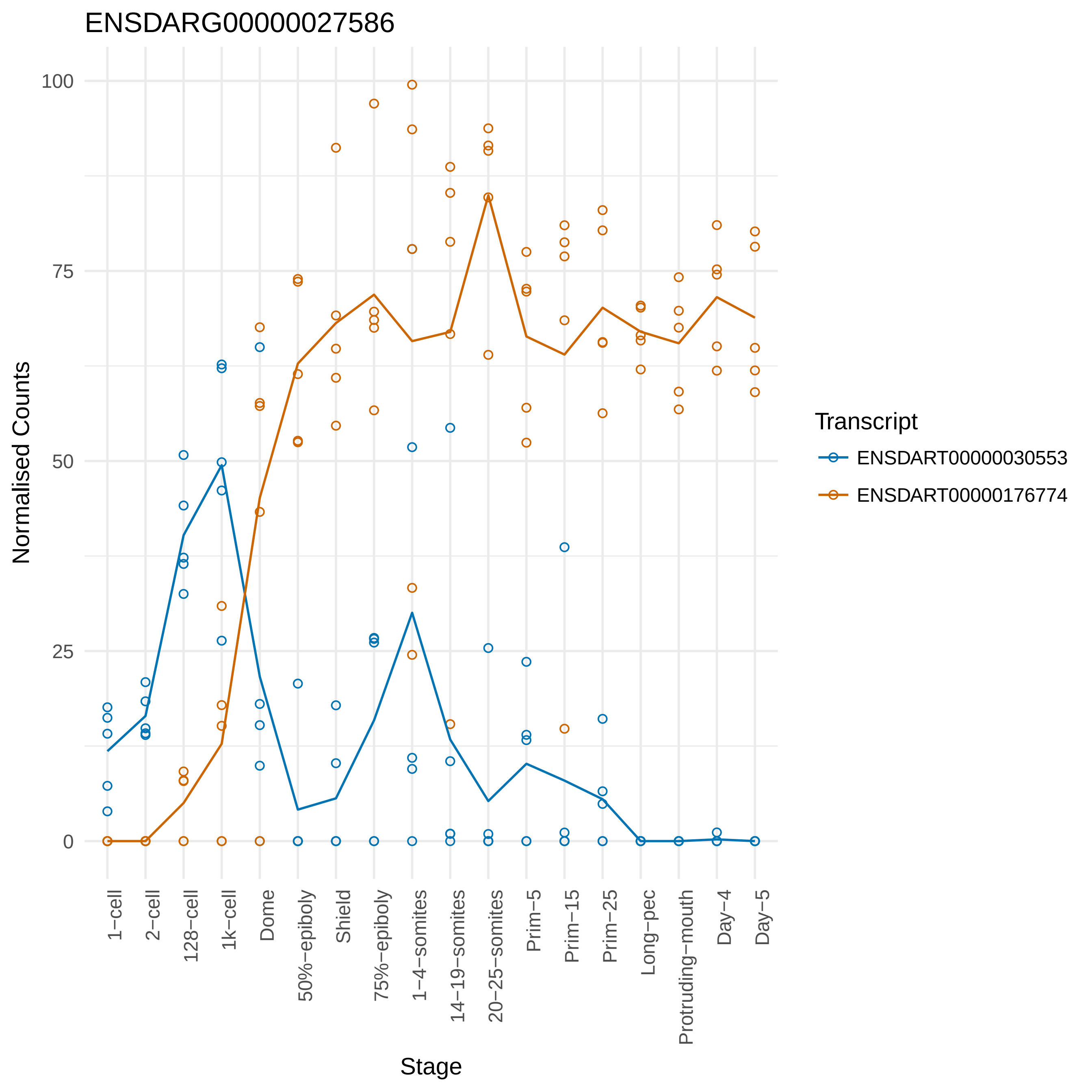

ENSDARG00000027586
Normalised Counts
100
75
50
25
0
1−cell
2−cell
128−cell
1k−cell
Dome
50%−epiboly
Shield
75%−epiboly
1−4−somites
14−19−somites
20−25−somites
Prim−5
Prim−15
Prim−25
Long−pec
Protruding−mouth
Day−4
Day−5
Stage
Transcript
ENSDART00000030553
ENSDART00000176774

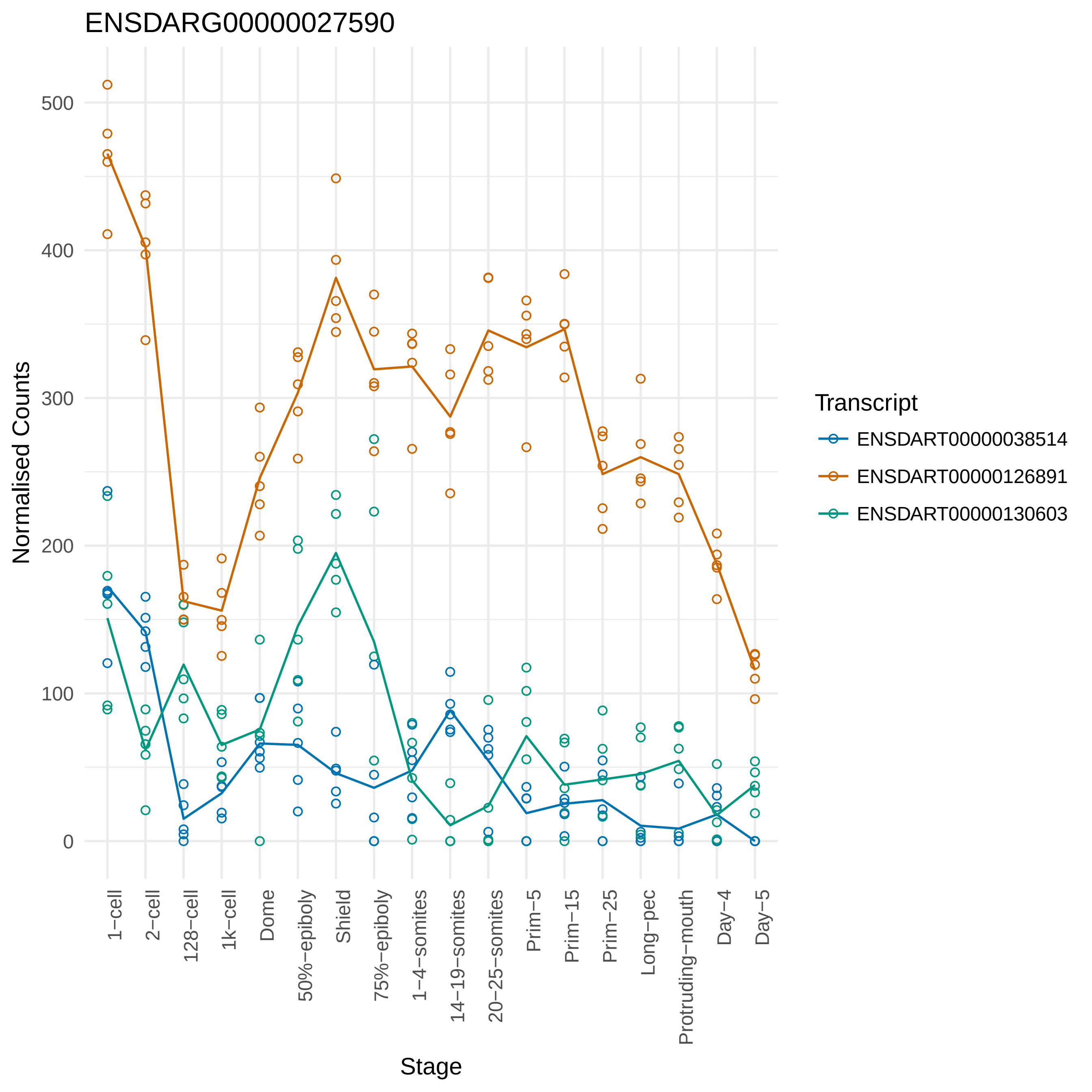

ENSDARG00000027590
Normalised Counts
0
100
200
300
400
500
1−cell
2−cell
128−cell
1k−cell
Dome
50%−epiboly
Shield
75%−epiboly
1−4−somites
14−19−somites
20−25−somites
Prim−5
Prim−15
Prim−25
Long−pec
Protruding−mouth
Day−4
Day−5
Stage
Transcript
ENSDART00000038514
ENSDART00000126891
ENSDART00000130603

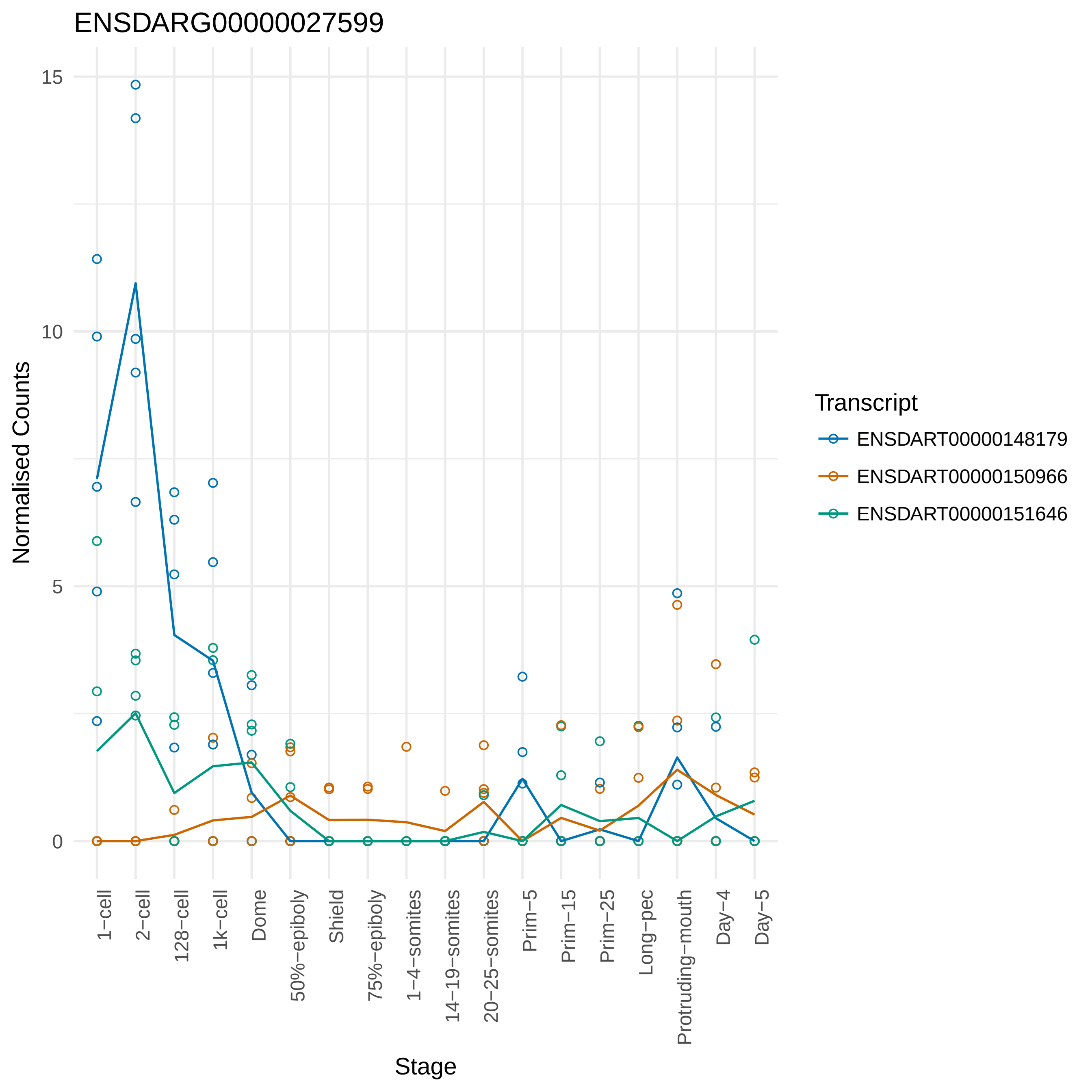
ENSDARG00000027599
Normalised Counts
15
10
5
0
1−cell
2−cell
128−cell
1k−cell
Dome
50%−epiboly
Shield
75%−epiboly
1−4−somites
14−19−somites
20−25−somites
Prim−5
Prim−15
Prim−25
Long−pec
Protruding−mouth
Day−4
Day−5
Stage
Transcript
ENSDART00000148179
ENSDART00000150966
ENSDART00000151646

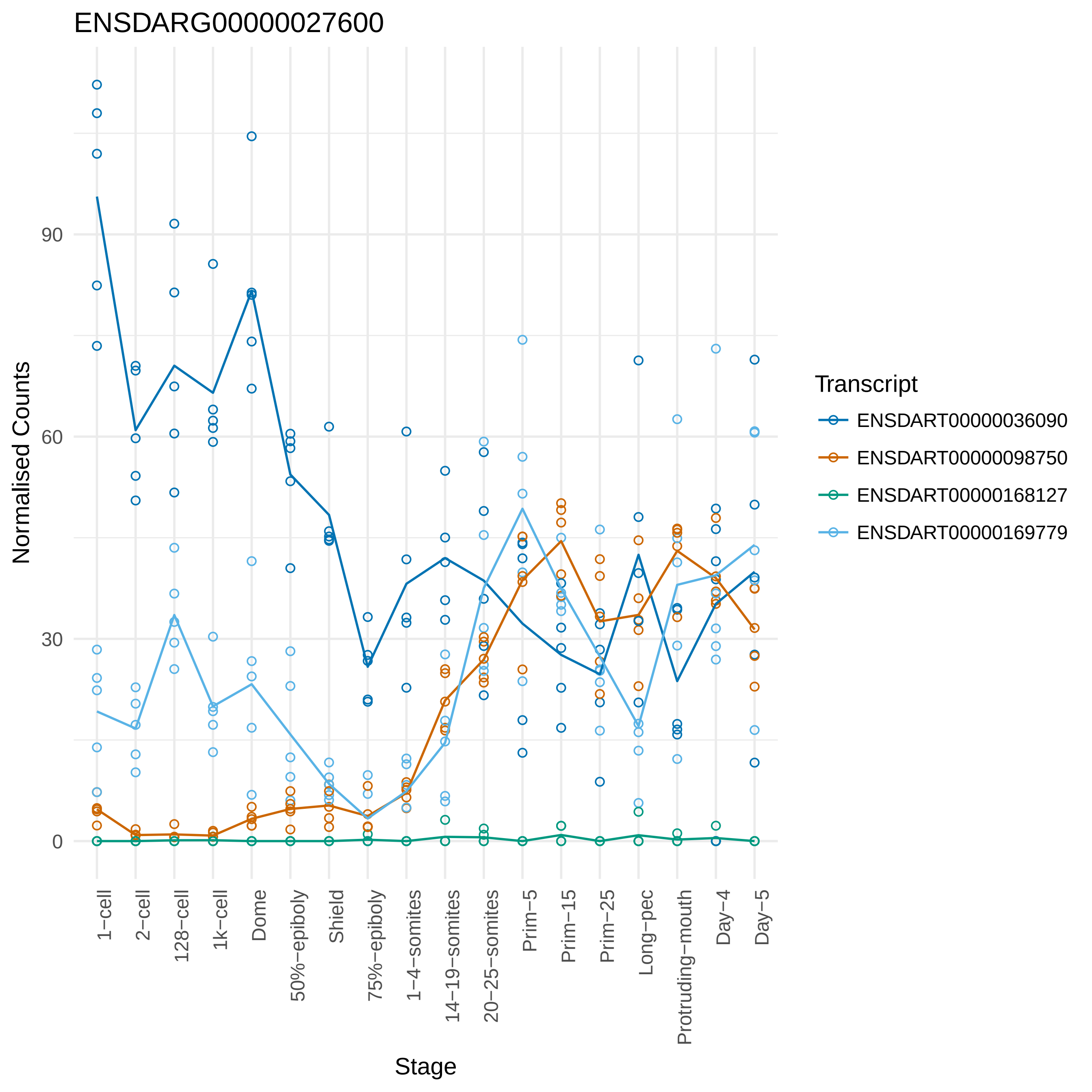

ENSDARG00000027600
Normalised Counts
0
30
60
90
Stage
1−cell
2−cell
128−cell
1k−cell
Dome
50%−epiboly
Shield
75%−epiboly
1−4−somites
14−19−somites
20−25−somites
Prim−5
Prim−15
Prim−25
Long−pec
Protruding−mouth
Day−4
Day−5
Transcript
ENSDART00000036090
ENSDART00000098750
ENSDART00000168127
ENSDART00000169779

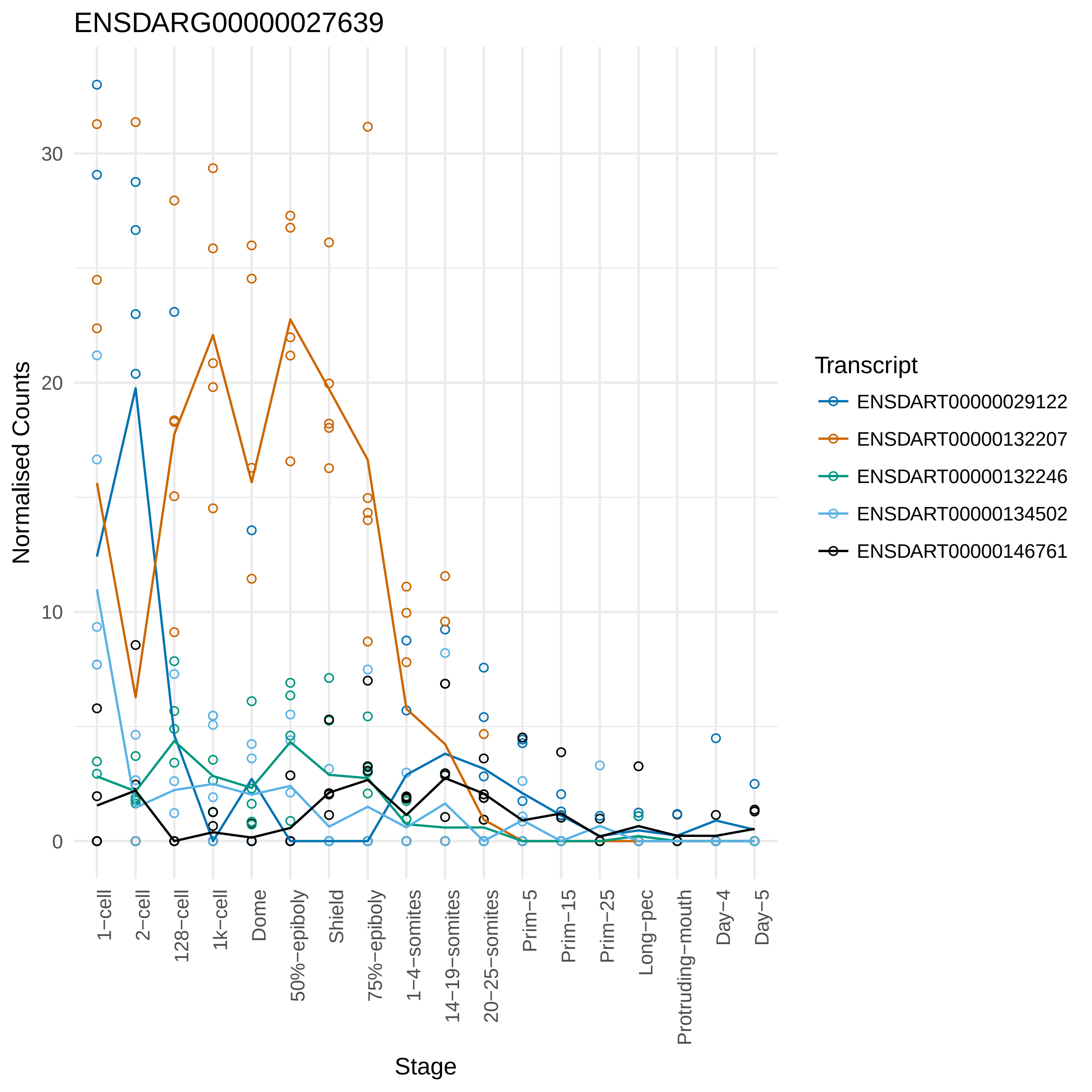

ENSDARG00000027639
Normalised Counts
30
20
10
0
1−cell
2−cell
128−cell
1k−cell
Dome
50%−epiboly
Shield
75%−epiboly
1−4−somites
14−19−somites
20−25−somites
Prim−5
Prim−15
Prim−25
Long−pec
Protruding−mouth
Day−4
Day−5
Stage
Transcript
ENSDART00000029122
ENSDART00000132207
ENSDART00000132246
ENSDART00000134502
ENSDART00000146761

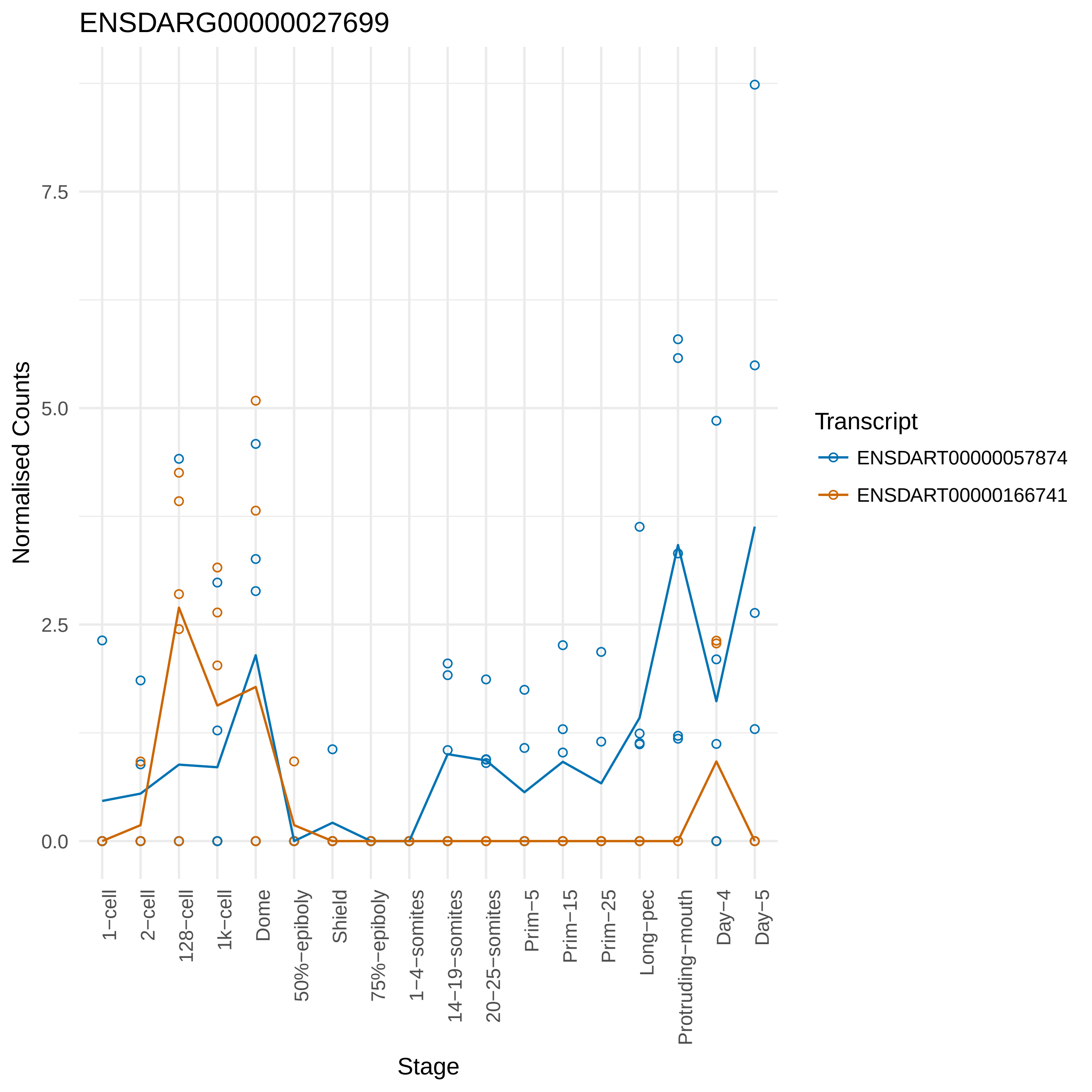

ENSDARG00000027699
Normalised Counts
0.0
2.5
5.0
7.5
1−cell
2−cell
128−cell
1k−cell
Dome
50%−epiboly
Shield
75%−epiboly
1−4−somites
14−19−somites
20−25−somites
Prim−5
Prim−15
Prim−25
Long−pec
Protruding−mouth
Day−4
Day−5
Stage
Transcript
ENSDART00000057874
ENSDART00000166741

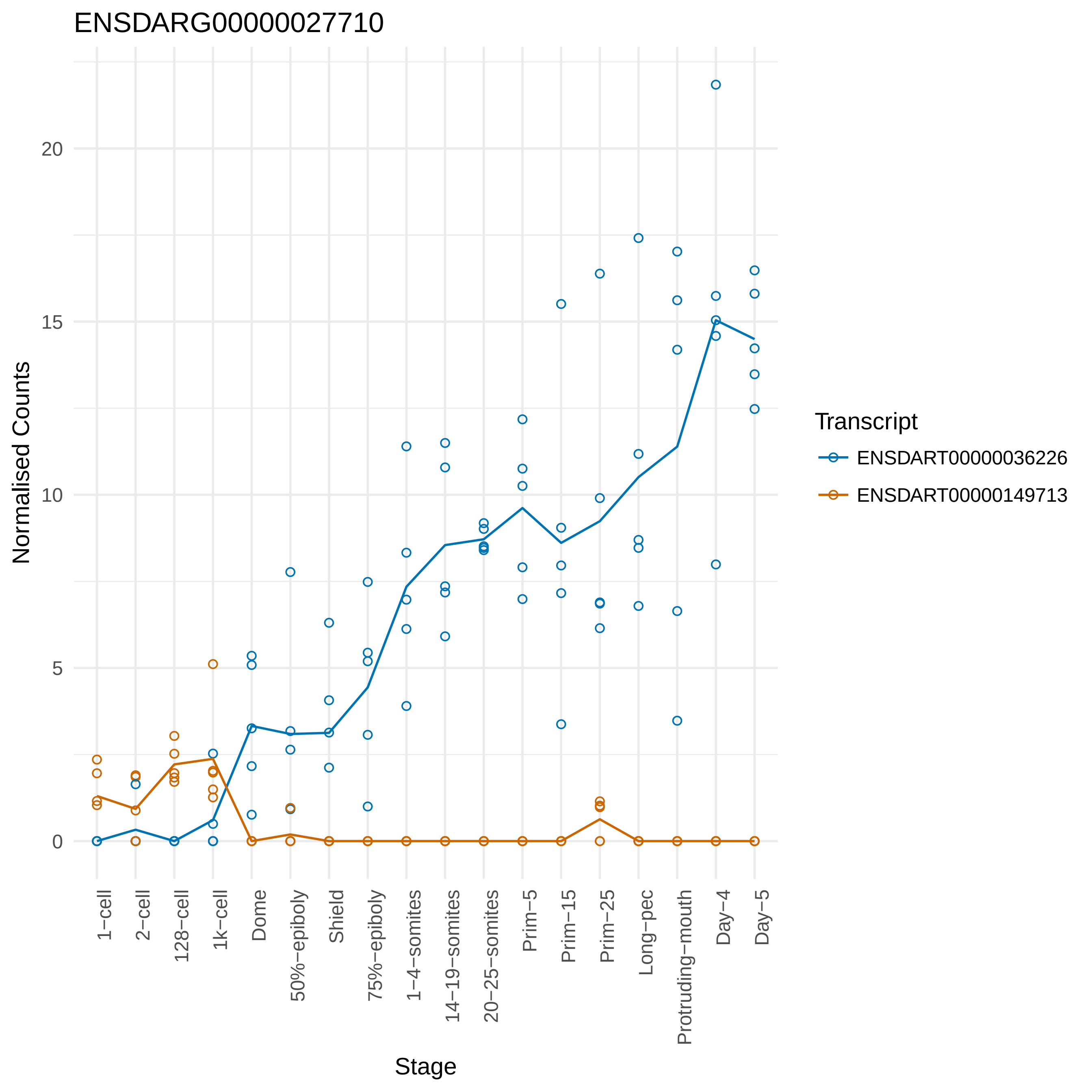

ENSDARG00000027710
Normalised Counts
0
5
10
15
20
Stage
1−cell
2−cell
128−cell
1k−cell
Dome
50%−epiboly
Shield
75%−epiboly
1−4−somites
14−19−somites
20−25−somites
Prim−5
Prim−15
Prim−25
Long−pec
Protruding−mouth
Day−4
Day−5
Transcript
ENSDART00000036226
ENSDART00000149713

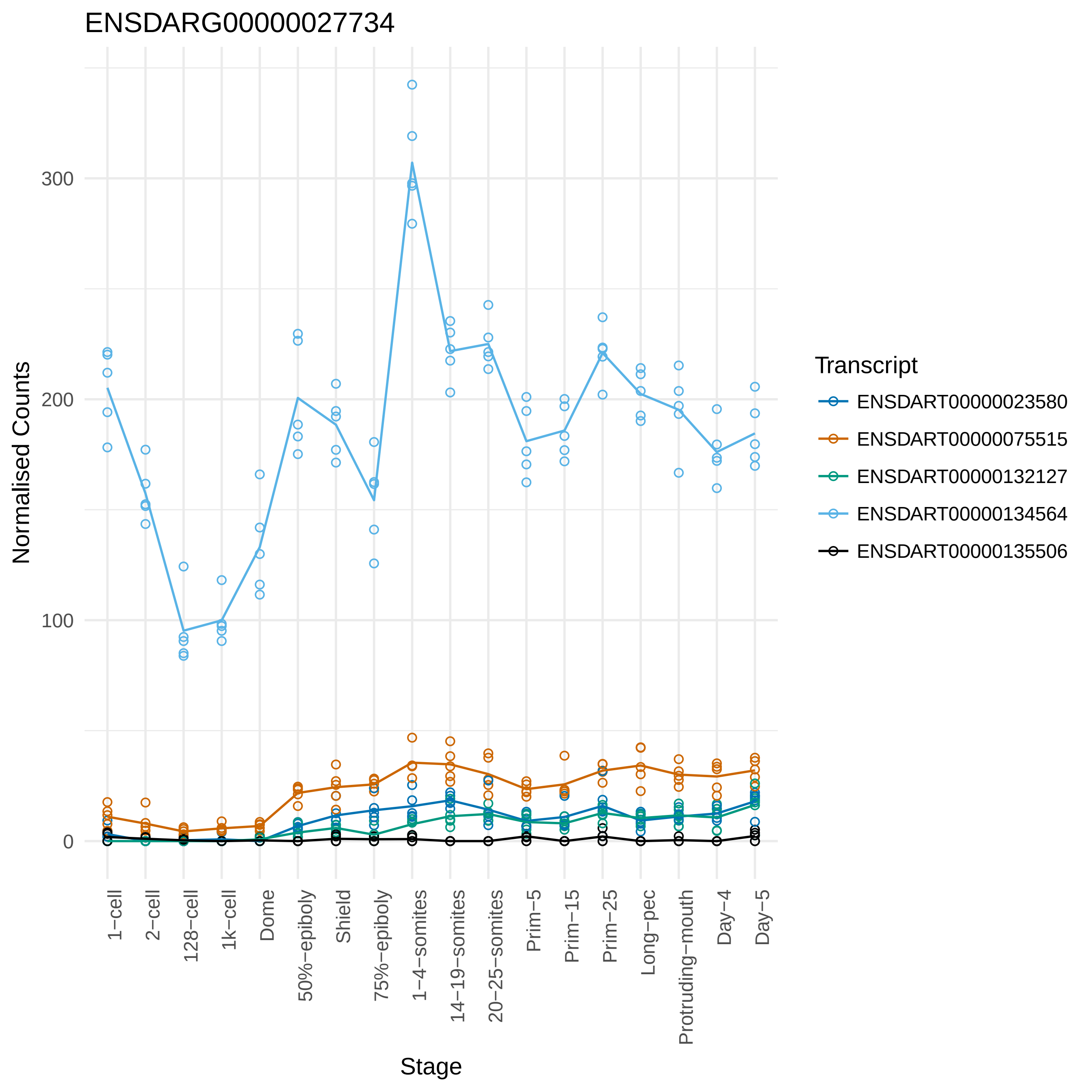

ENSDARG00000027734
Normalised Counts
300
200
100
0
1−cell
2−cell
128−cell
1k−cell
Dome
50%−epiboly
Shield
75%−epiboly
1−4−somites
14−19−somites
20−25−somites
Prim−5
Prim−15
Prim−25
Long−pec
Protruding−mouth
Day−4
Day−5
Stage
Transcript
ENSDART00000023580
ENSDART00000075515
ENSDART00000132127
ENSDART00000134564
ENSDART00000135506

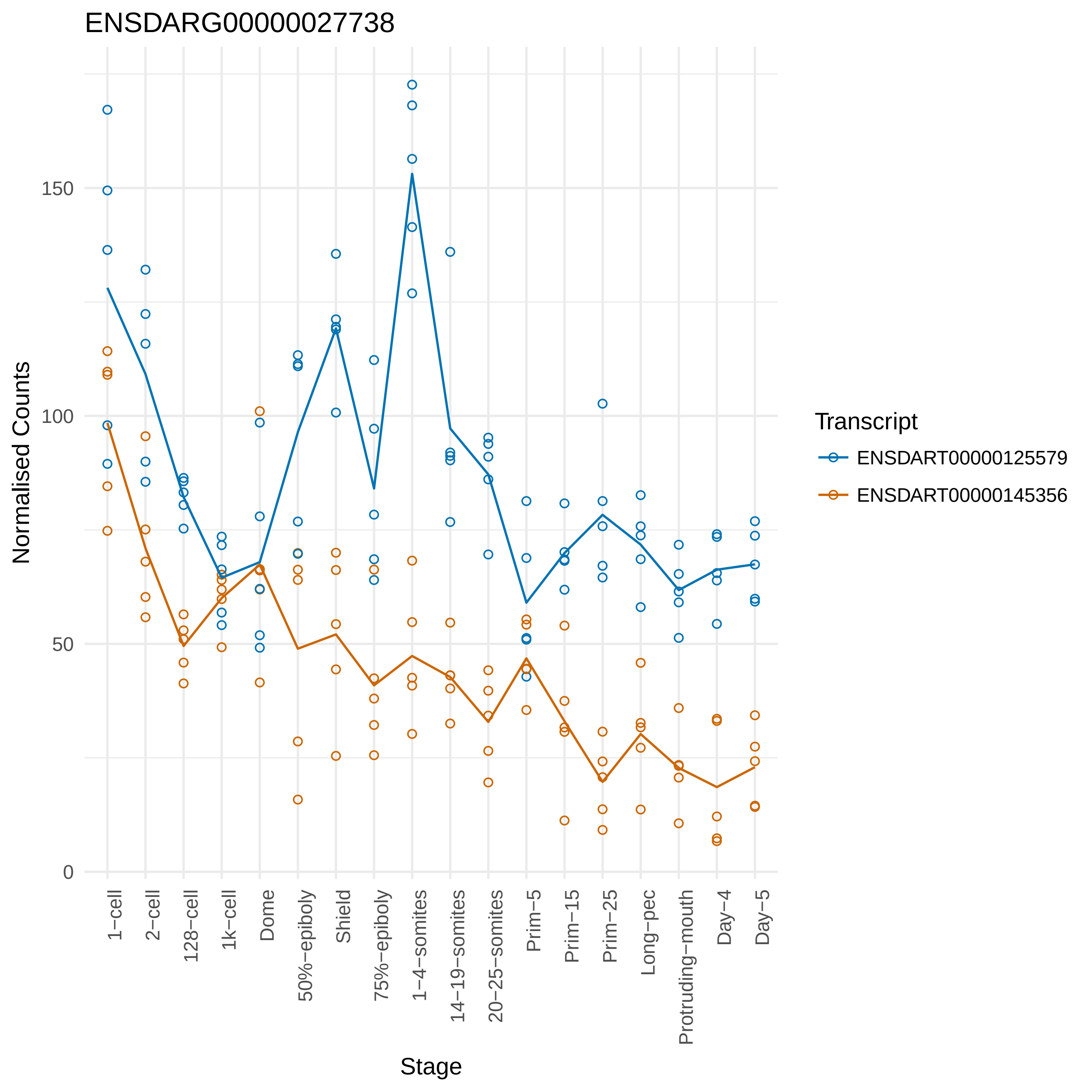

ENSDARG00000027738
Normalised Counts
150
100
50
0
1−cell
2−cell
128−cell
1k−cell
Dome
50%−epiboly
Shield
75%−epiboly
1−4−somites
14−19−somites
20−25−somites
Prim−5
Prim−15
Prim−25
Long−pec
Protruding−mouth
Day−4
Day−5
Stage
Transcript
ENSDART00000125579
ENSDART00000145356

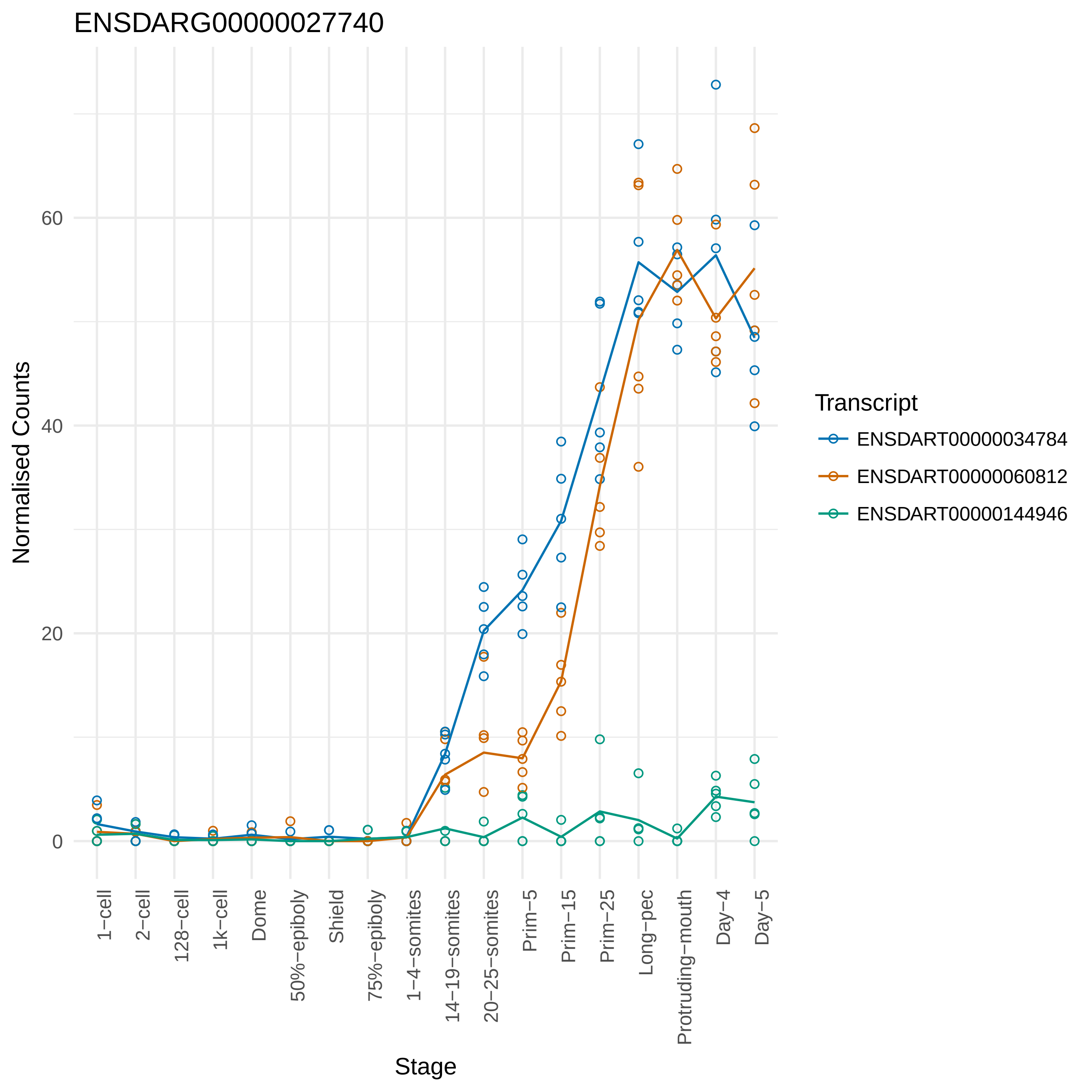

ENSDARG00000027740
Normalised Counts
0
20
40
60
1−cell
2−cell
128−cell
1k−cell
Dome
50%−epiboly
Shield
75%−epiboly
1−4−somites
14−19−somites
20−25−somites
Prim−5
Prim−15
Prim−25
Long−pec
Protruding−mouth
Day−4
Day−5
Stage
Transcript
ENSDART00000034784
ENSDART00000060812
ENSDART00000144946

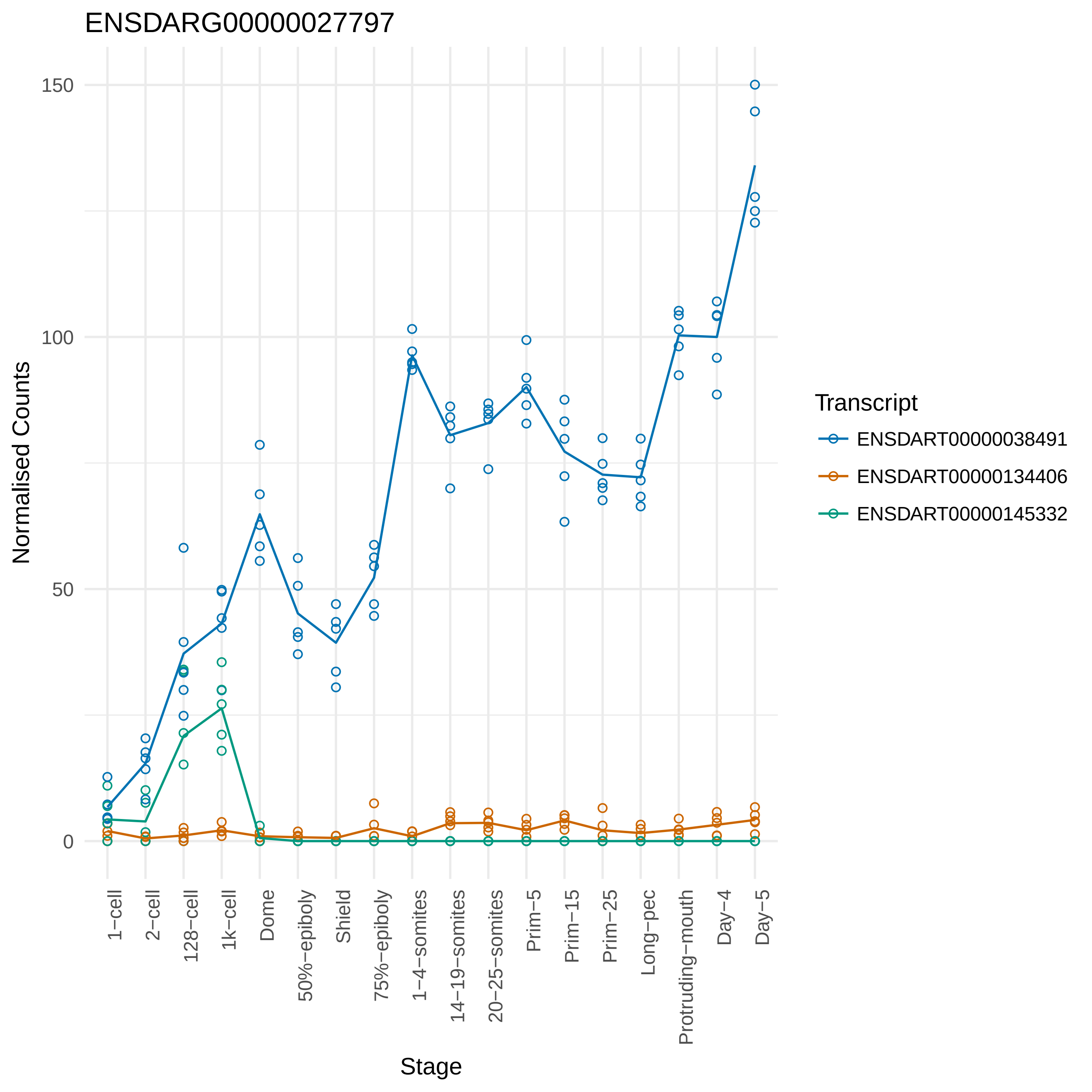

ENSDARG00000027797
Normalised Counts
150
100
50
0
1−cell
2−cell
128−cell
1k−cell
Dome
50%−epiboly
Shield
75%−epiboly
1−4−somites
14−19−somites
20−25−somites
Prim−5
Prim−15
Prim−25
Long−pec
Protruding−mouth
Day−4
Day−5
Stage
Transcript
ENSDART00000038491
ENSDART00000134406
ENSDART00000145332

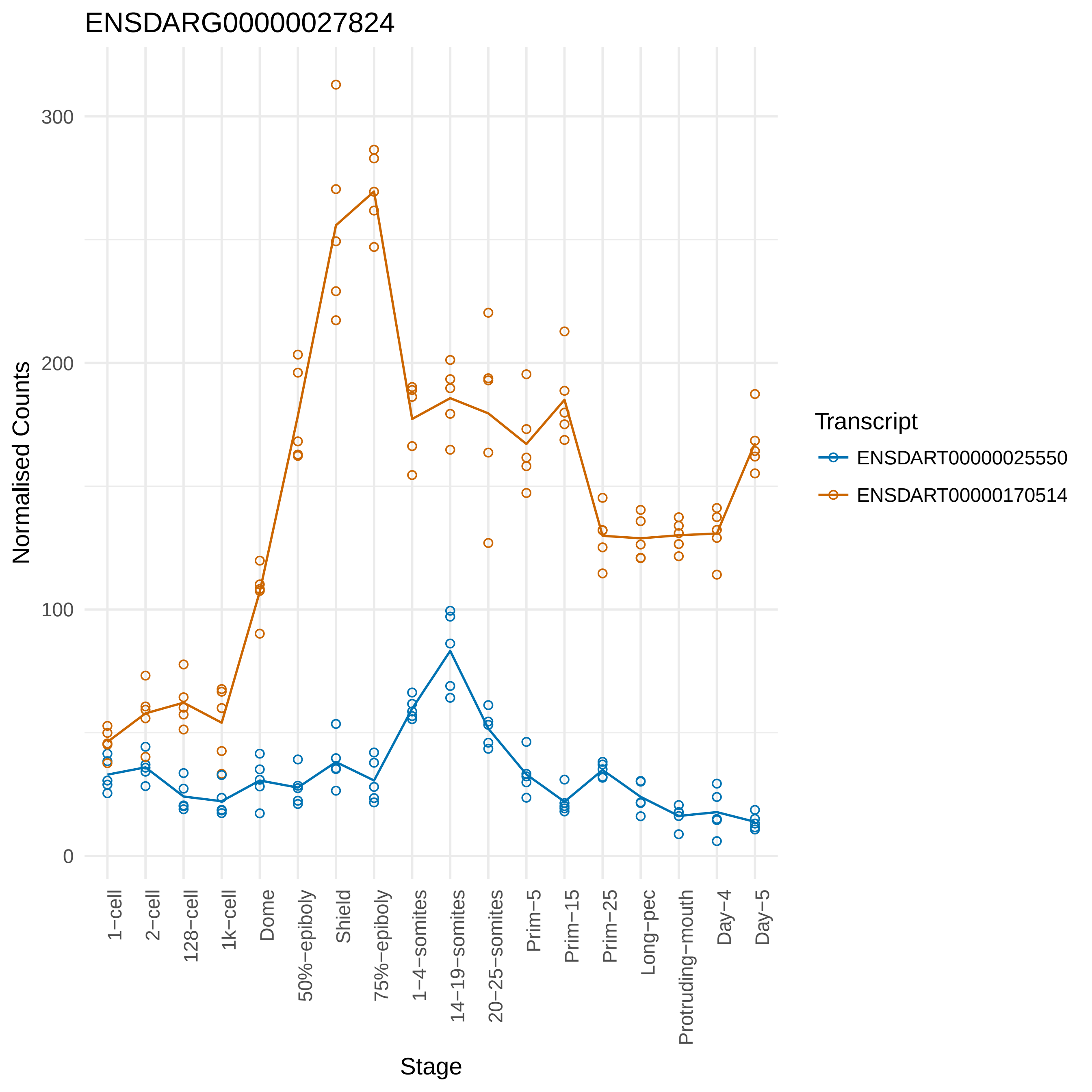

ENSDARG00000027824
Normalised Counts
300
200
100
0
1−cell
2−cell
128−cell
1k−cell
Dome
50%−epiboly
Shield
75%−epiboly
1−4−somites
14−19−somites
20−25−somites
Prim−5
Prim−15
Prim−25
Long−pec
Protruding−mouth
Day−4
Day−5
Stage
Transcript
ENSDART00000025550
ENSDART00000170514

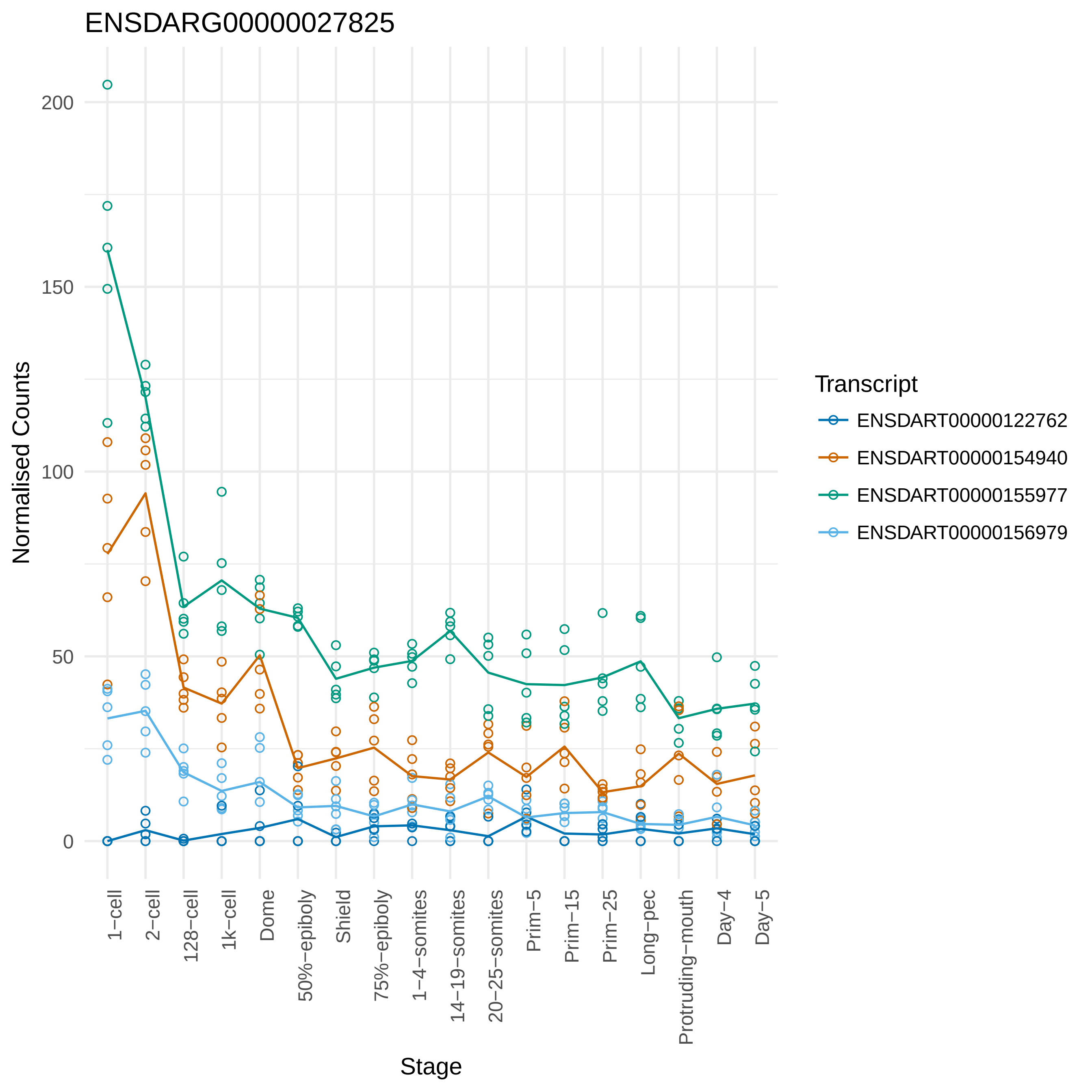

ENSDARG00000027825
Normalised Counts
200
150
100
50
0
1−cell
2−cell
128−cell
1k−cell
Dome
50%−epiboly
Shield
75%−epiboly
1−4−somites
14−19−somites
20−25−somites
Prim−5
Prim−15
Prim−25
Long−pec
Protruding−mouth
Day−4
Day−5
Stage
Transcript
ENSDART00000122762
ENSDART00000154940
ENSDART00000155977
ENSDART00000156979

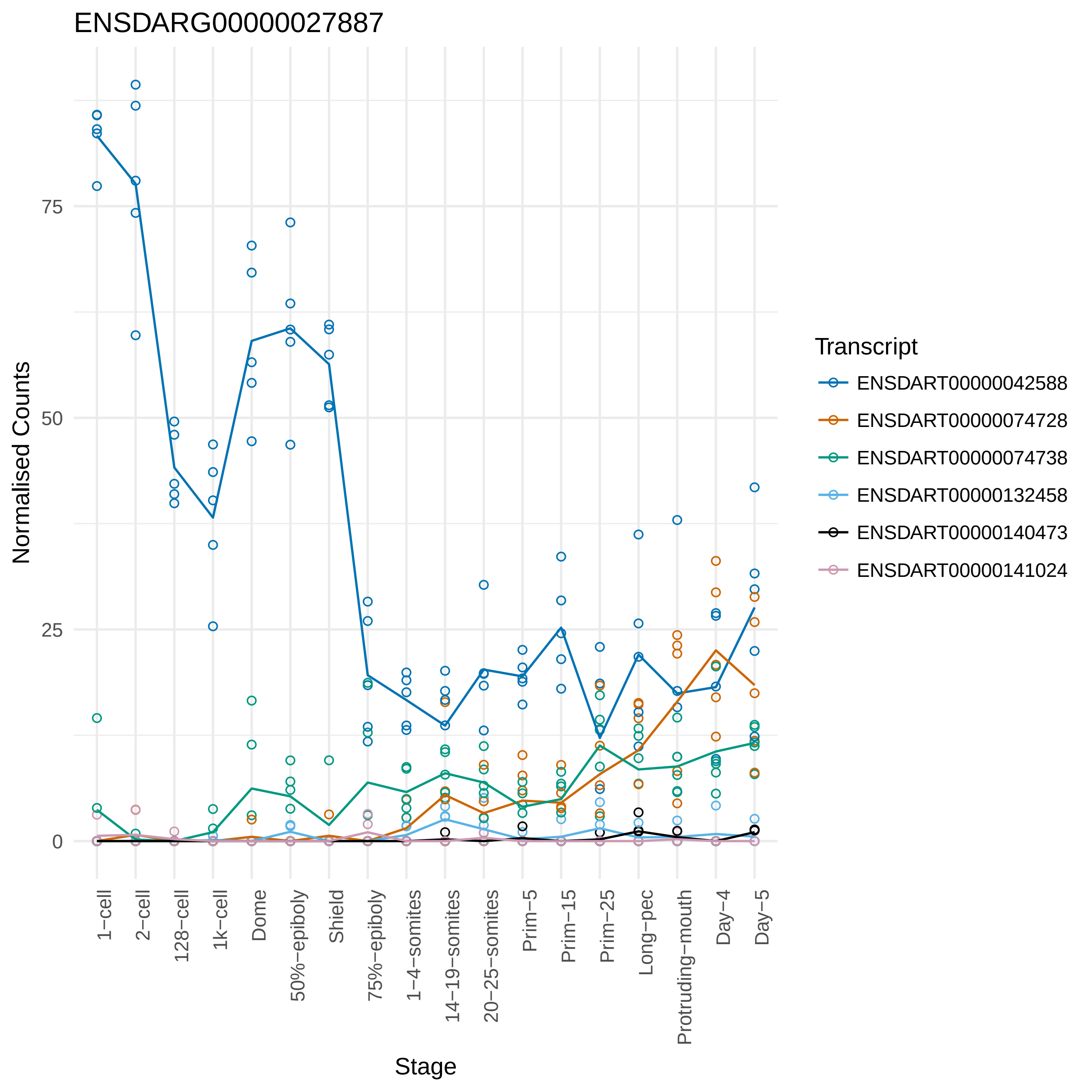

ENSDARG00000027887
Normalised Counts
75
50
25
0
1−cell
2−cell
128−cell
1k−cell
Dome
50%−epiboly
Shield
75%−epiboly
1−4−somites
14−19−somites
20−25−somites
Prim−5
Prim−15
Prim−25
Long−pec
Protruding−mouth
Day−4
Day−5
Stage
Transcript
ENSDART00000042588
ENSDART00000074728
ENSDART00000074738
ENSDART00000132458
ENSDART00000140473
ENSDART00000141024

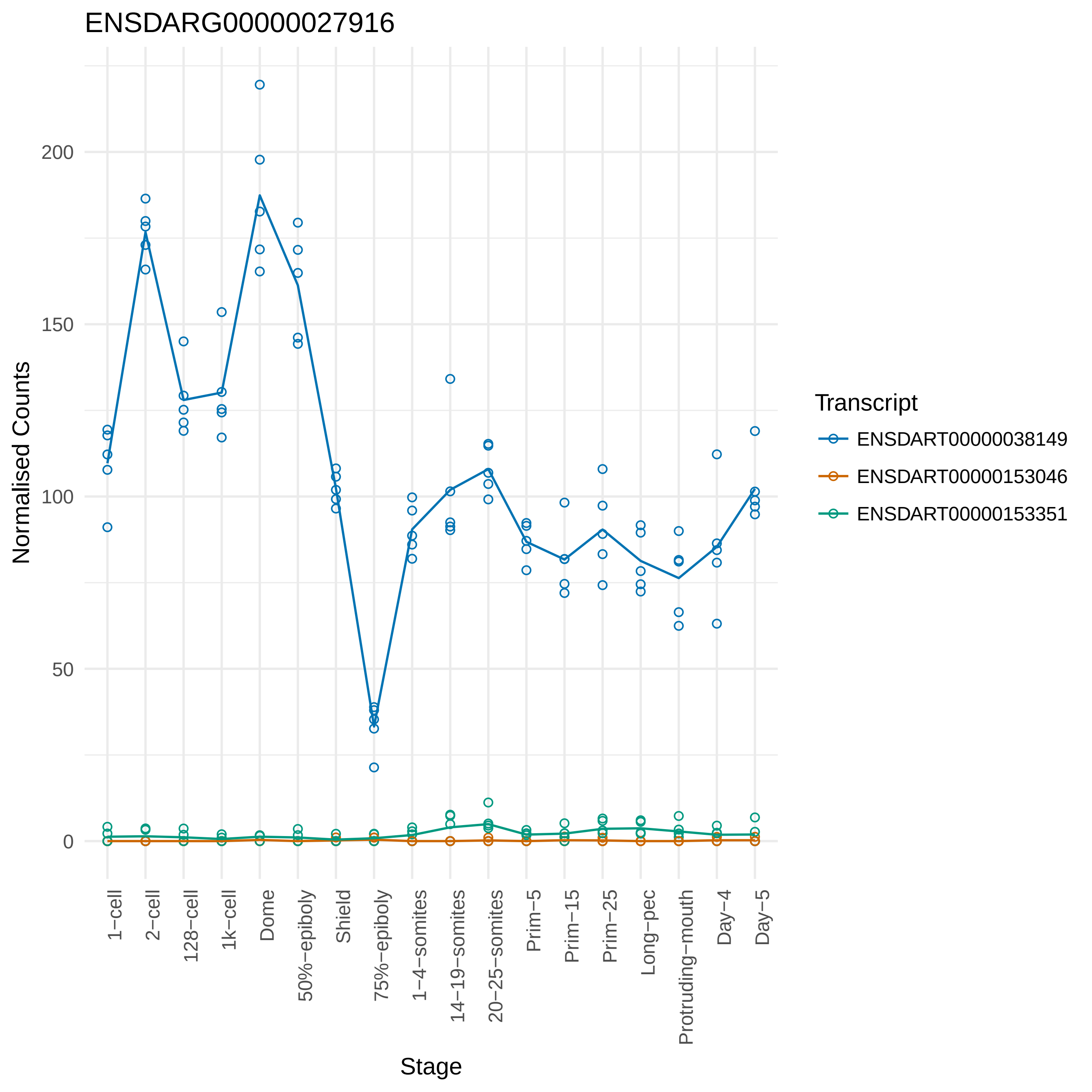
ENSDARG00000027916
Normalised Counts
200
150
100
50
0
1−cell
2−cell
128−cell
1k−cell
Dome
50%−epiboly
Shield
75%−epiboly
1−4−somites
14−19−somites
20−25−somites
Prim−5
Prim−15
Prim−25
Long−pec
Protruding−mouth
Day−4
Day−5
Stage
Transcript
ENSDART00000038149
ENSDART00000153046
ENSDART00000153351

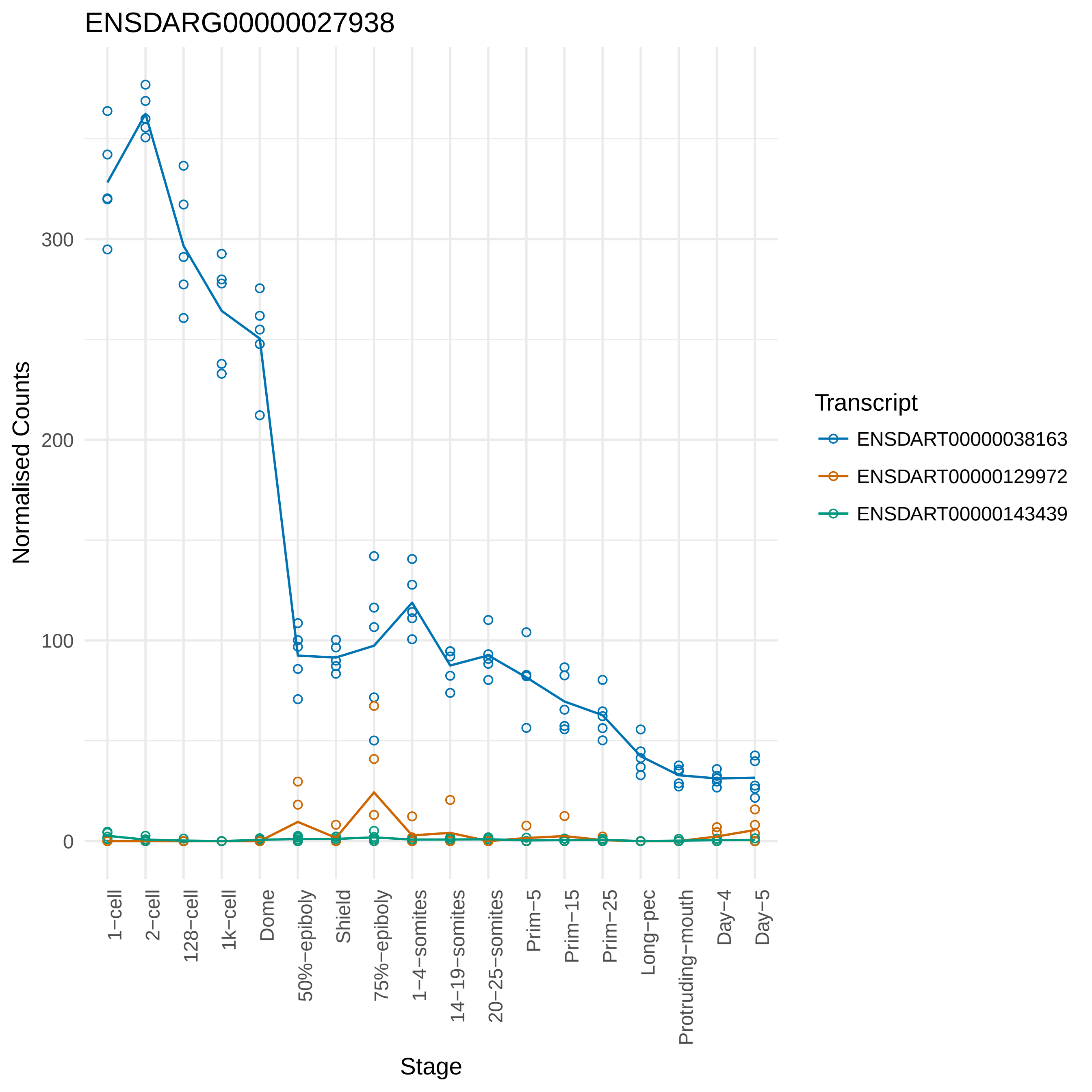
ENSDARG00000027938
Normalised Counts
0
100
200
300
Stage
1−cell
2−cell
128−cell
1k−cell
Dome
50%−epiboly
Shield
75%−epiboly
1−4−somites
14−19−somites
20−25−somites
Prim−5
Prim−15
Prim−25
Long−pec
Protruding−mouth
Day−4
Day−5
Transcript
ENSDART00000038163
ENSDART00000129972
ENSDART00000143439

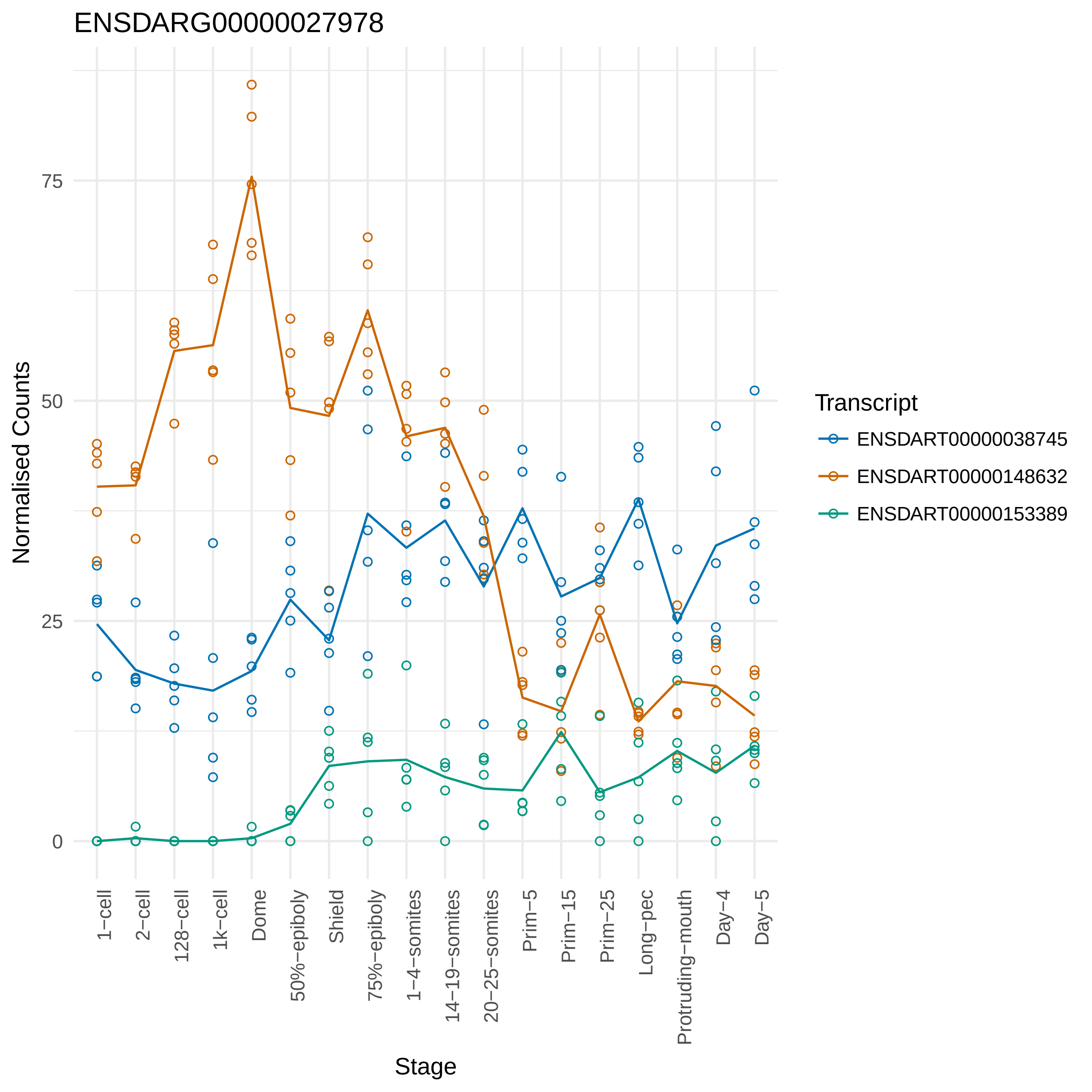

ENSDARG00000027978
Normalised Counts
0
25
50
75
1−cell
2−cell
128−cell
1k−cell
Dome
50%−epiboly
Shield
75%−epiboly
1−4−somites
14−19−somites
20−25−somites
Prim−5
Prim−15
Prim−25
Long−pec
Protruding−mouth
Day−4
Day−5
Stage
Transcript
ENSDART00000038745
ENSDART00000148632
ENSDART00000153389

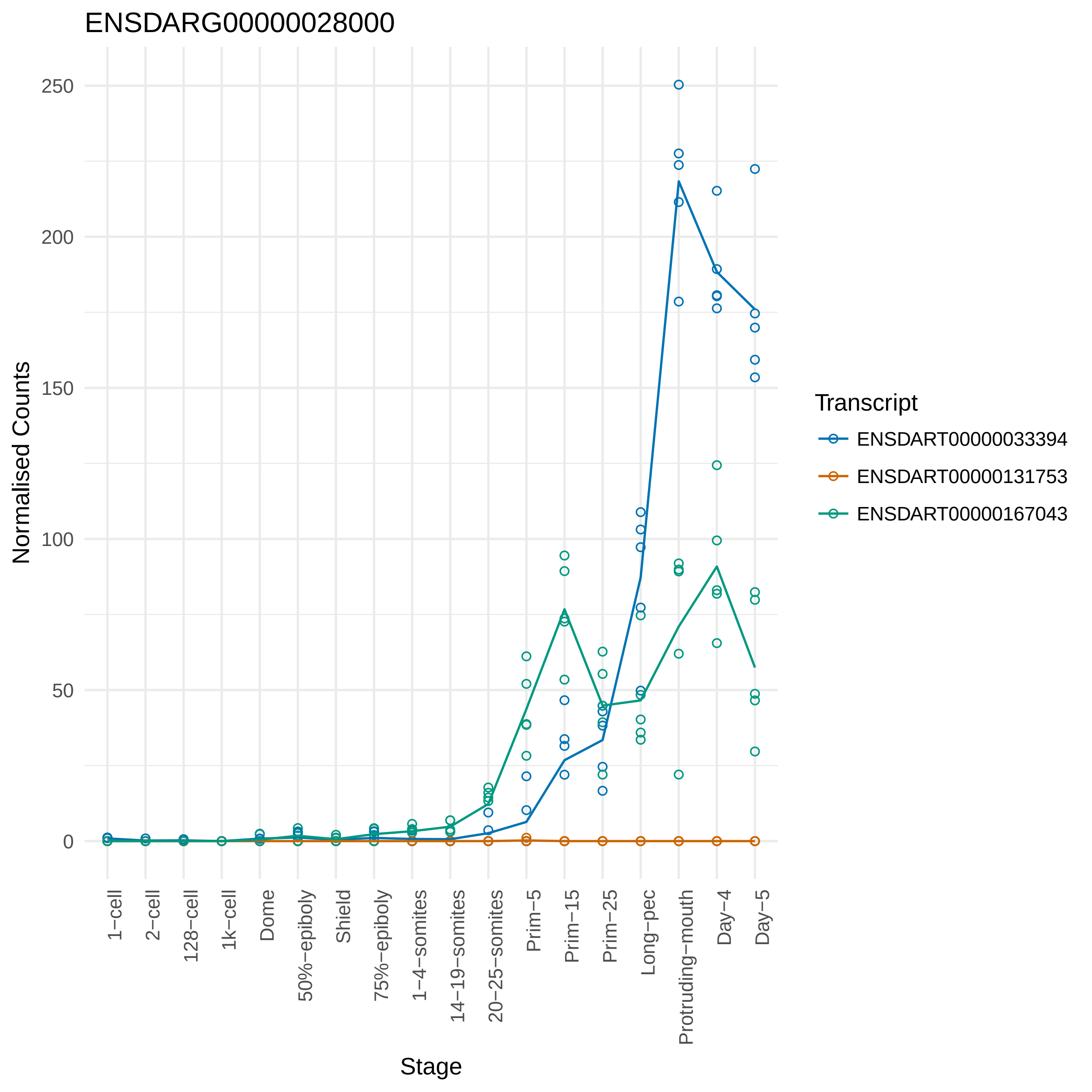

ENSDARG00000028000
Normalised Counts
250
200
150
100
50
0
1−cell
2−cell
128−cell
1k−cell
Dome
50%−epiboly
Shield
75%−epiboly
1−4−somites
14−19−somites
20−25−somites
Prim−5
Prim−15
Prim−25
Long−pec
Protruding−mouth
Day−4
Day−5
Stage
Transcript
ENSDART00000033394
ENSDART00000131753
ENSDART00000167043

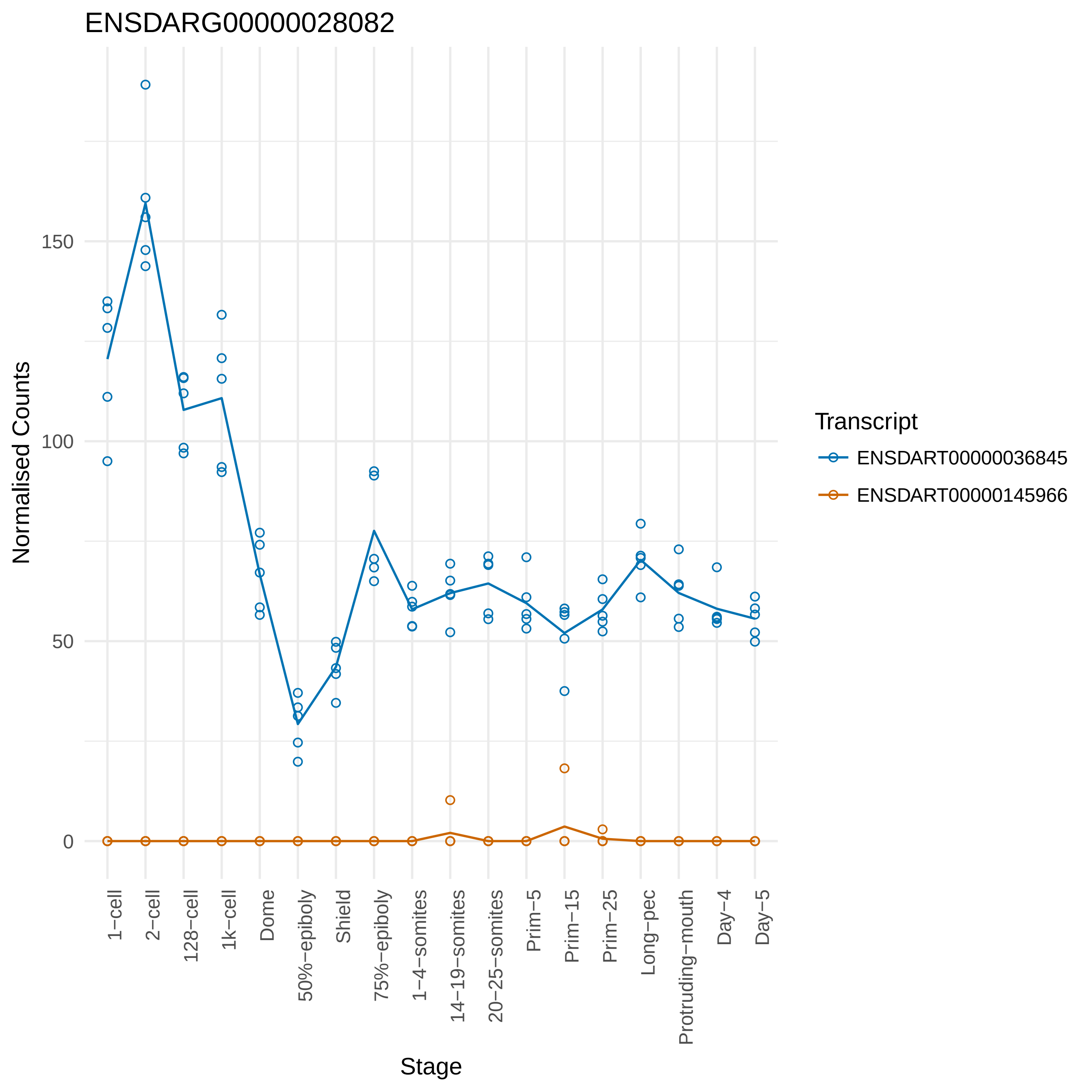
ENSDARG00000028082
Normalised Counts
150
100
50
0
1−cell
2−cell
128−cell
1k−cell
Dome
50%−epiboly
Shield
75%−epiboly
1−4−somites
14−19−somites
20−25−somites
Prim−5
Prim−15
Prim−25
Long−pec
Protruding−mouth
Day−4
Day−5
Stage
Transcript
ENSDART00000036845
ENSDART00000145966

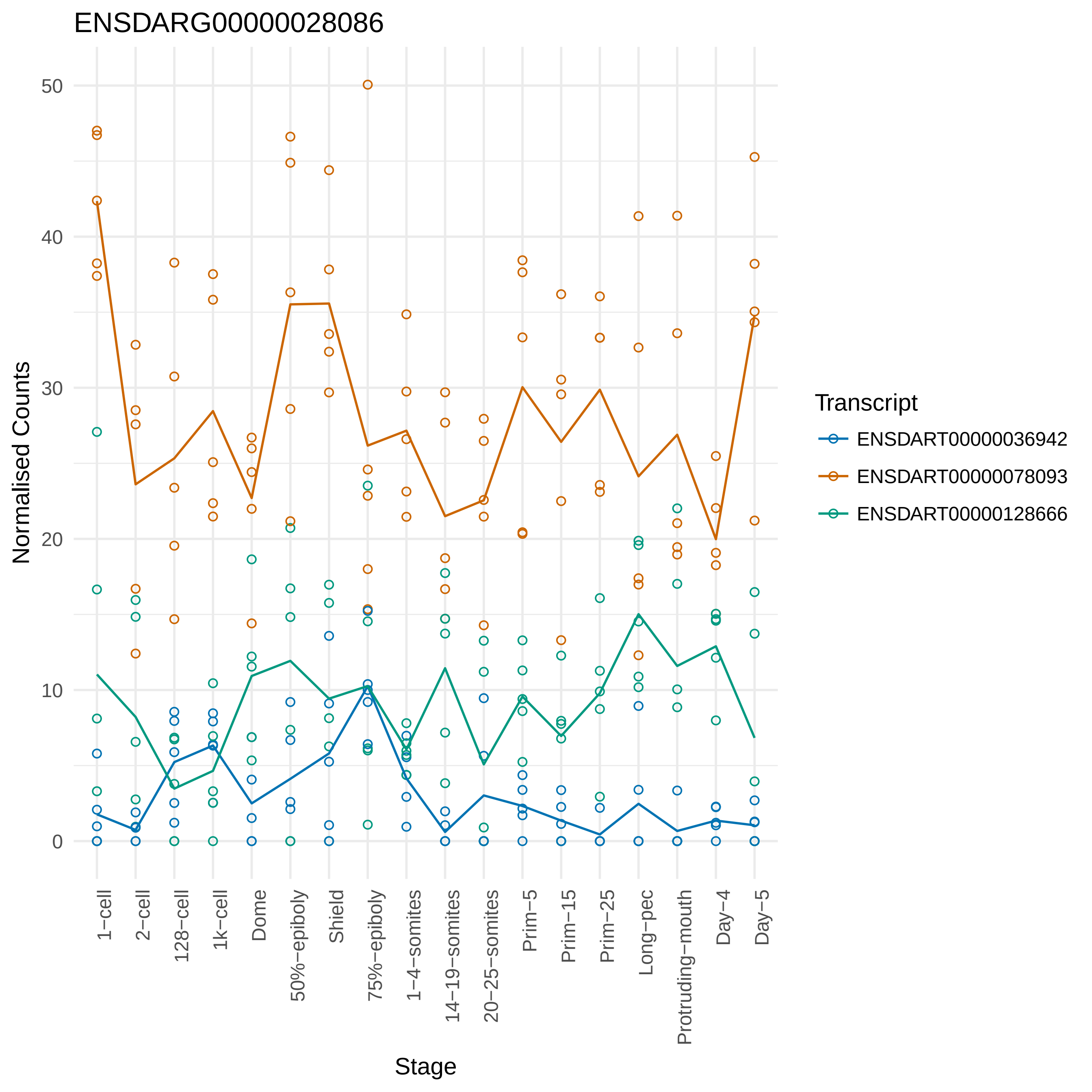
ENSDARG00000028086
Normalised Counts
50
40
30
20
10
0
1−cell
2−cell
128−cell
1k−cell
Dome
50%−epiboly
Shield
75%−epiboly
1−4−somites
14−19−somites
20−25−somites
Prim−5
Prim−15
Prim−25
Long−pec
Protruding−mouth
Day−4
Day−5
Stage
Transcript
ENSDART00000036942
ENSDART00000078093
ENSDART00000128666

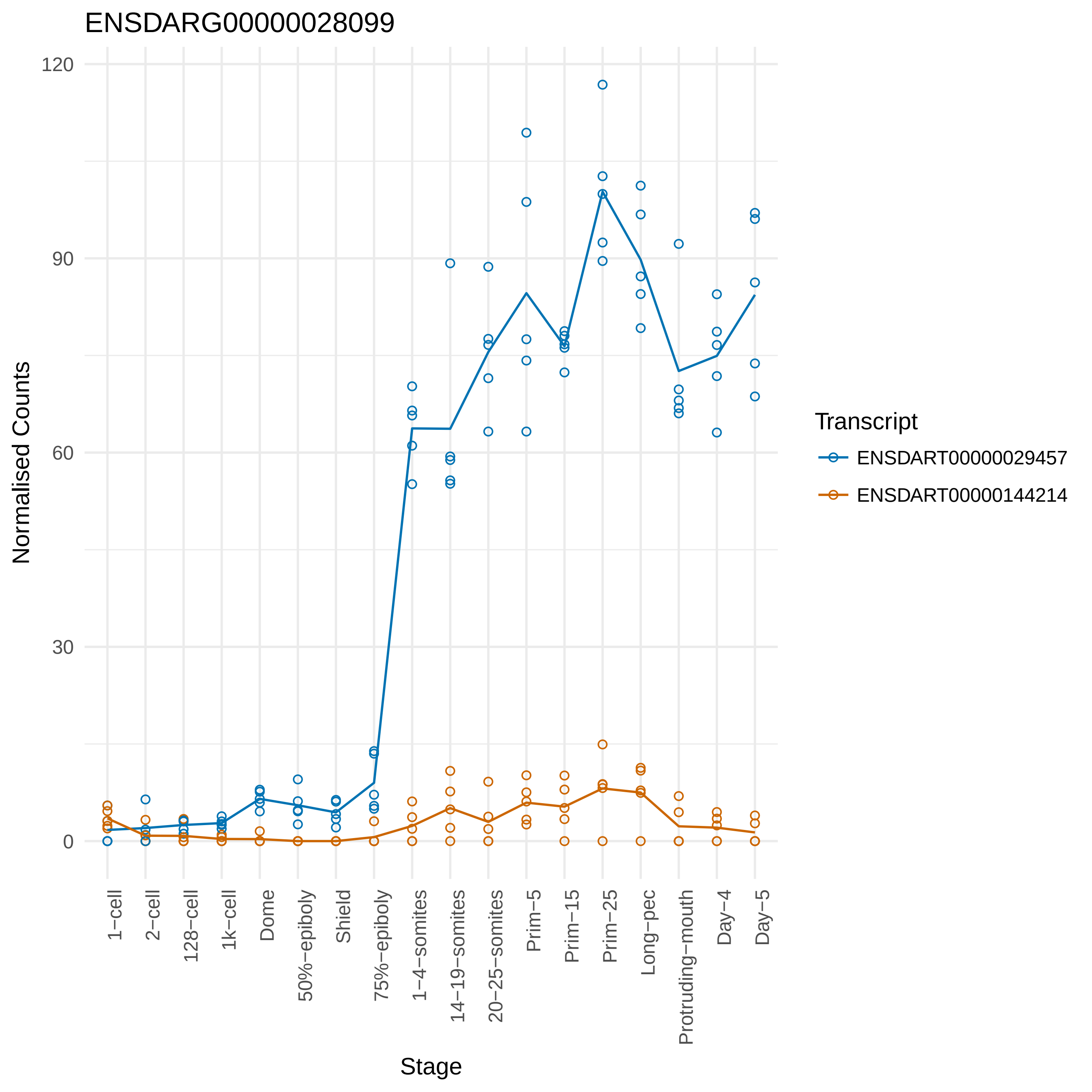

ENSDARG00000028099
Normalised Counts
120
90
60
30
0
1−cell
2−cell
128−cell
1k−cell
Dome
50%−epiboly
Shield
75%−epiboly
1−4−somites
14−19−somites
20−25−somites
Prim−5
Prim−15
Prim−25
Long−pec
Protruding−mouth
Day−4
Day−5
Stage
Transcript
ENSDART00000029457
ENSDART00000144214

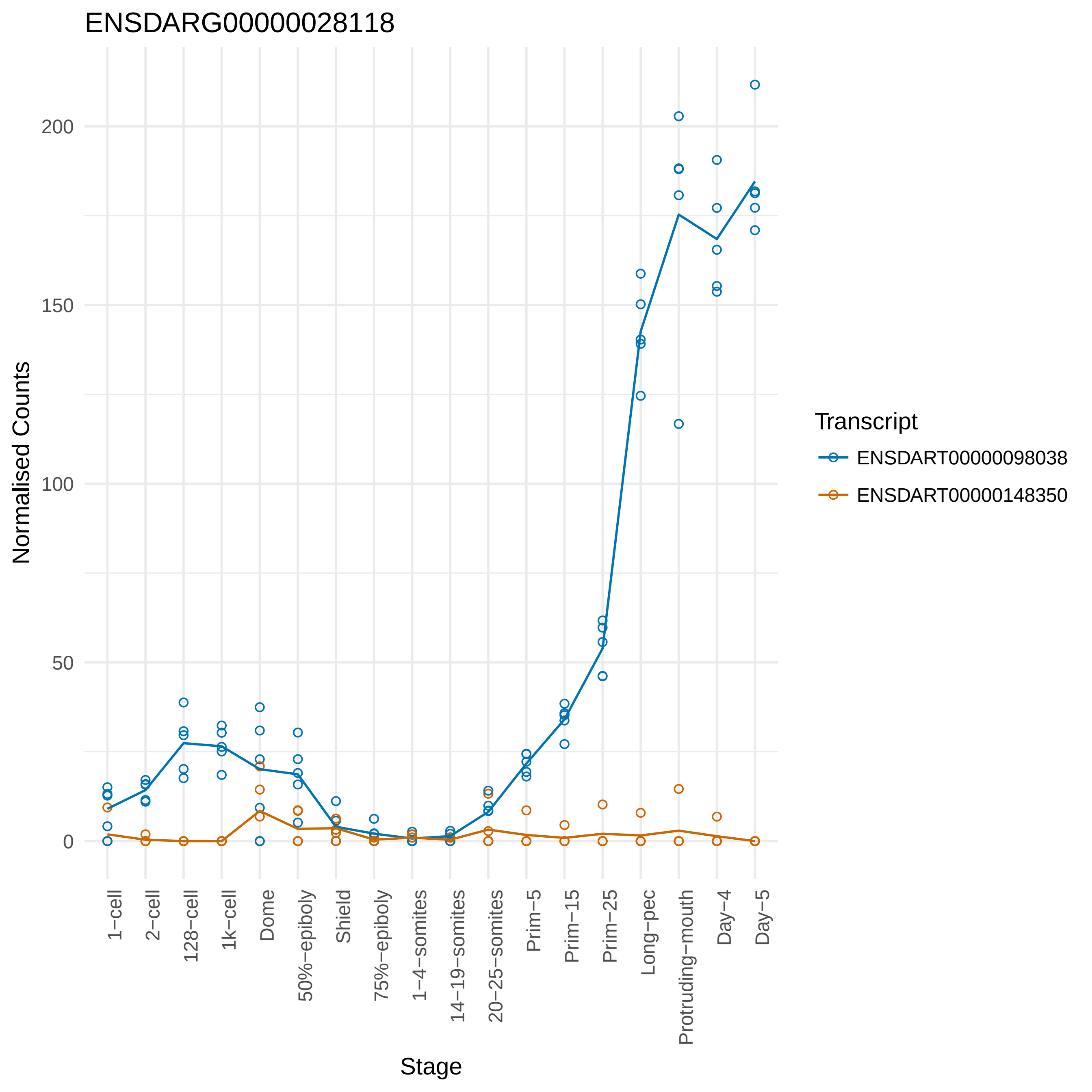
ENSDARG00000028118
Normalised Counts
0
50
100
150
200
Stage
1−cell
2−cell
128−cell
1k−cell
Dome
50%−epiboly
Shield
75%−epiboly
1−4−somites
14−19−somites
20−25−somites
Prim−5
Prim−15
Prim−25
Long−pec
Protruding−mouth
Day−4
Day−5
Transcript
ENSDART00000098038
ENSDART00000148350

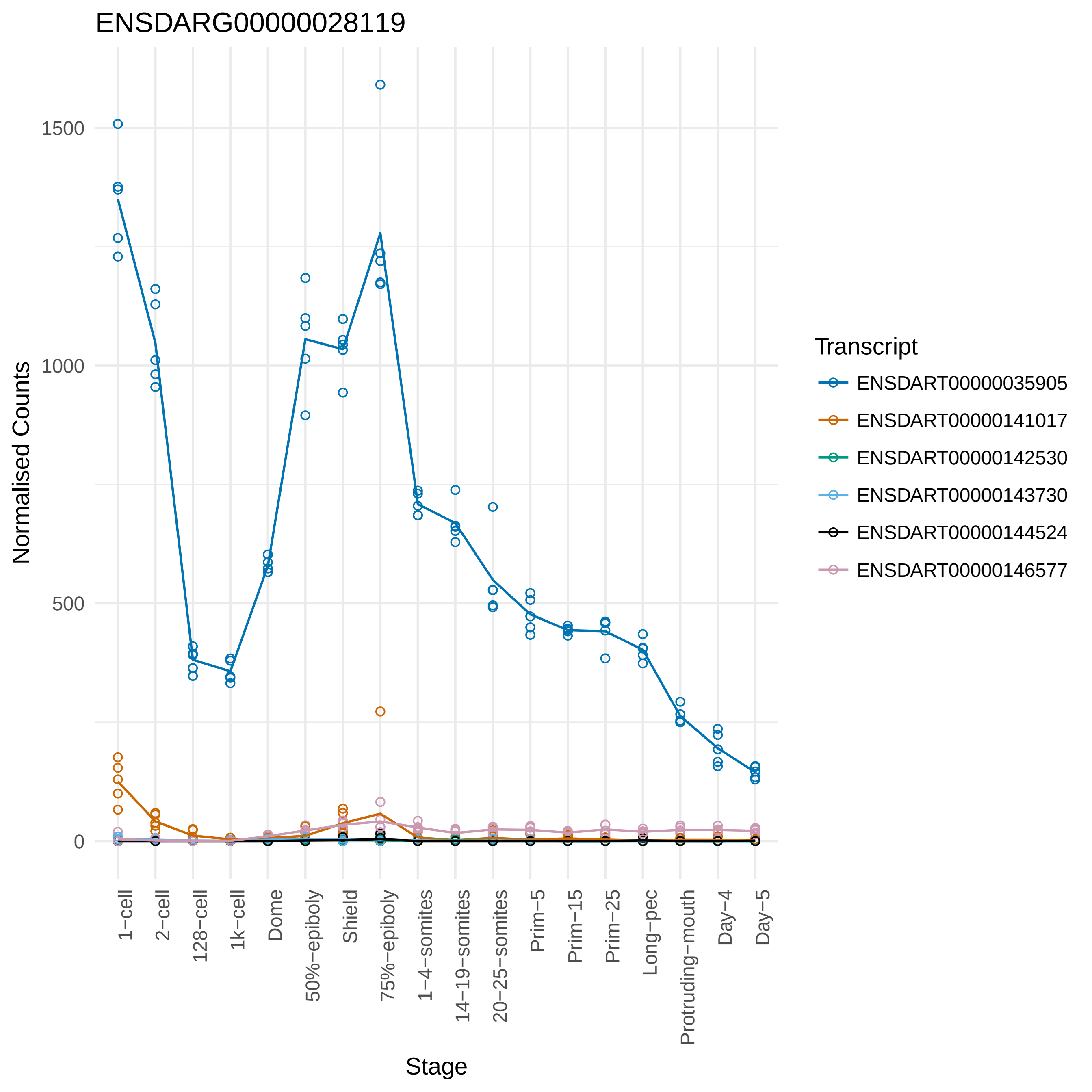
ENSDARG00000028119
Normalised Counts
1500
1000
500
0
1−cell
2−cell
128−cell
1k−cell
Dome
50%−epiboly
Shield
75%−epiboly
1−4−somites
14−19−somites
20−25−somites
Prim−5
Prim−15
Prim−25
Long−pec
Protruding−mouth
Day−4
Day−5
Stage
Transcript
ENSDART00000035905
ENSDART00000141017
ENSDART00000142530
ENSDART00000143730
ENSDART00000144524
ENSDART00000146577

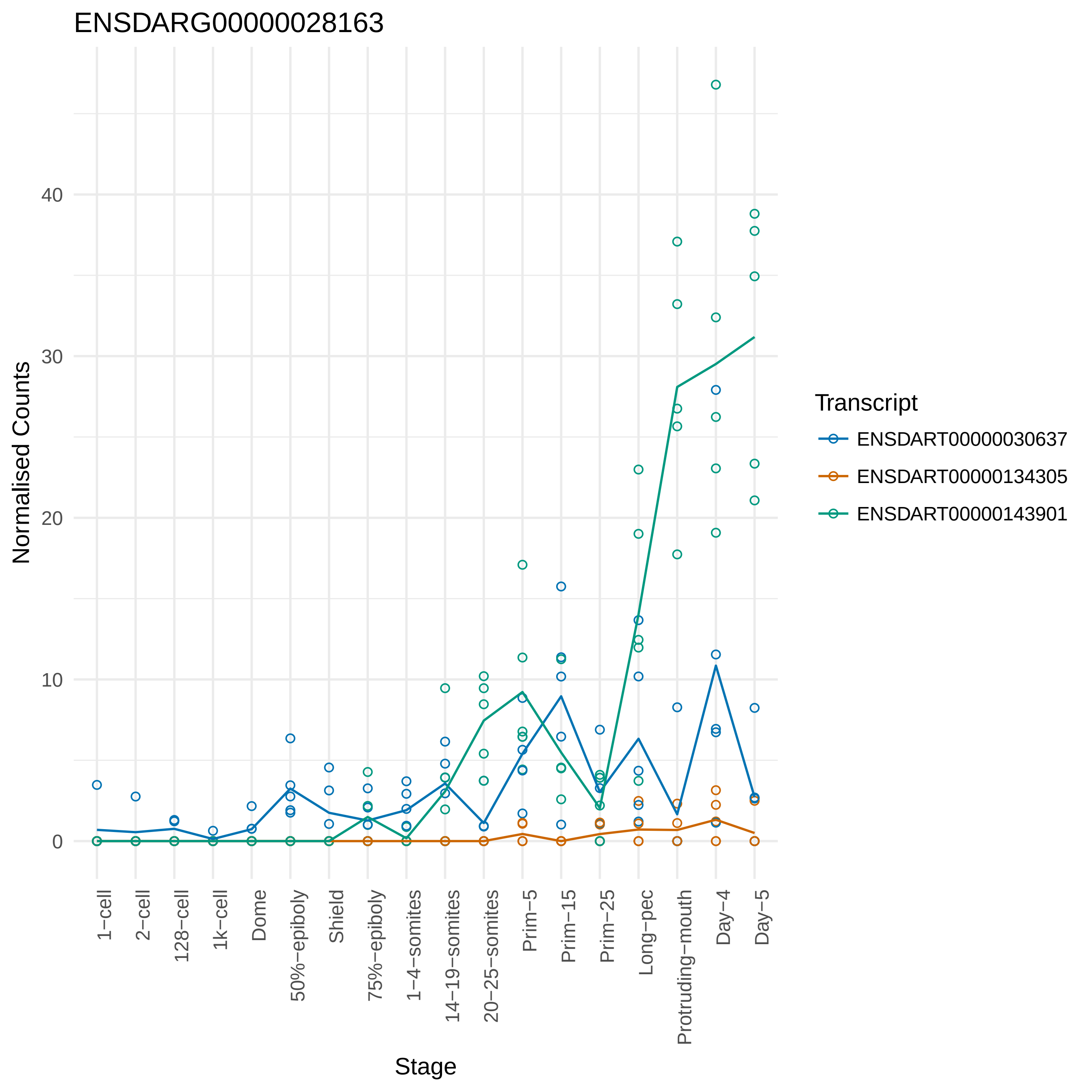
ENSDARG00000028163
Normalised Counts
0
10
20
30
40
1−cell
2−cell
128−cell
1k−cell
Dome
50%−epiboly
Shield
75%−epiboly
1−4−somites
14−19−somites
20−25−somites
Prim−5
Prim−15
Prim−25
Long−pec
Protruding−mouth
Day−4
Day−5
Stage
Transcript
ENSDART00000030637
ENSDART00000134305
ENSDART00000143901

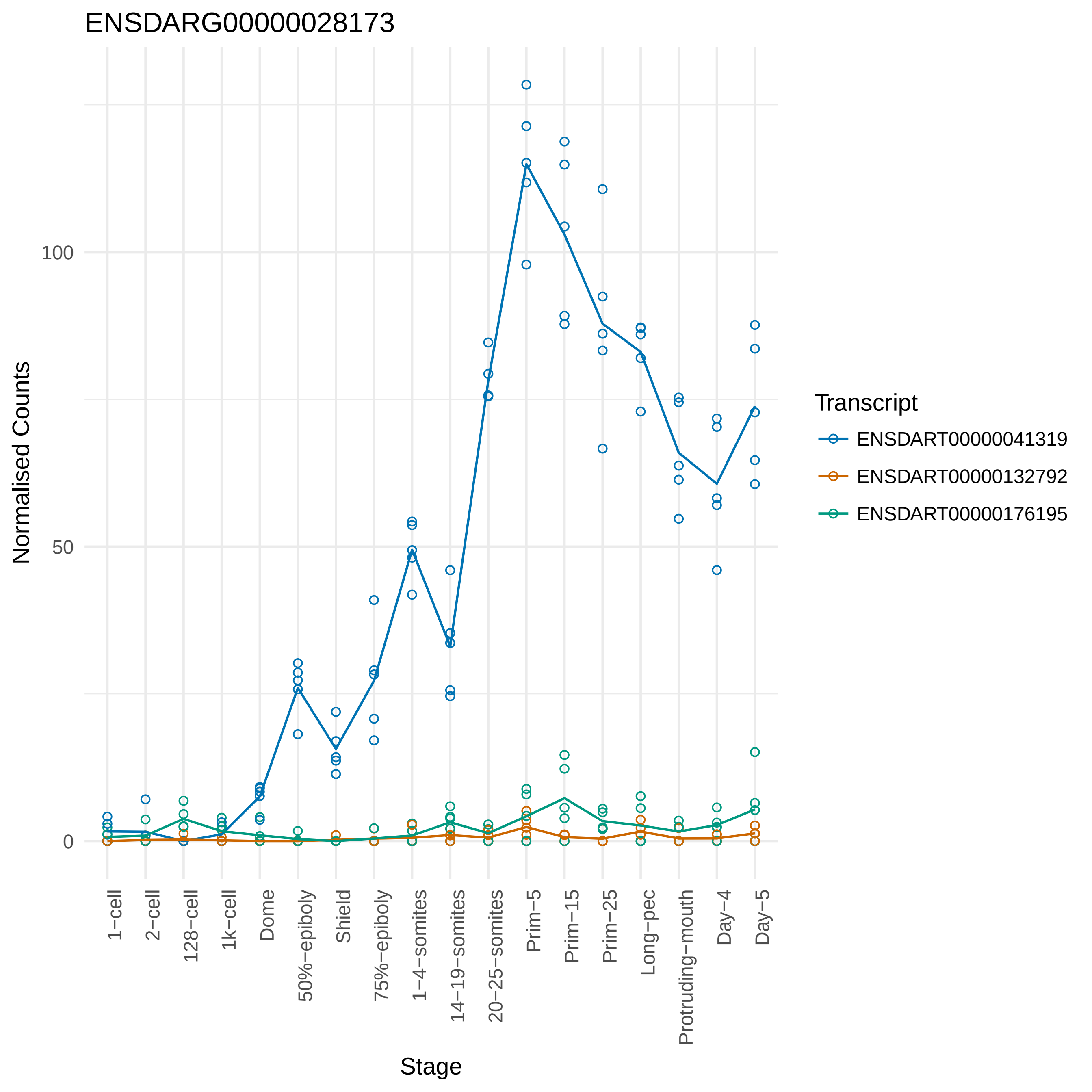
ENSDARG00000028173
Normalised Counts
100
50
0
1−cell
2−cell
128−cell
1k−cell
Dome
50%−epiboly
Shield
75%−epiboly
1−4−somites
14−19−somites
20−25−somites
Prim−5
Prim−15
Prim−25
Long−pec
Protruding−mouth
Day−4
Day−5
Stage
Transcript
ENSDART00000041319
ENSDART00000132792
ENSDART00000176195

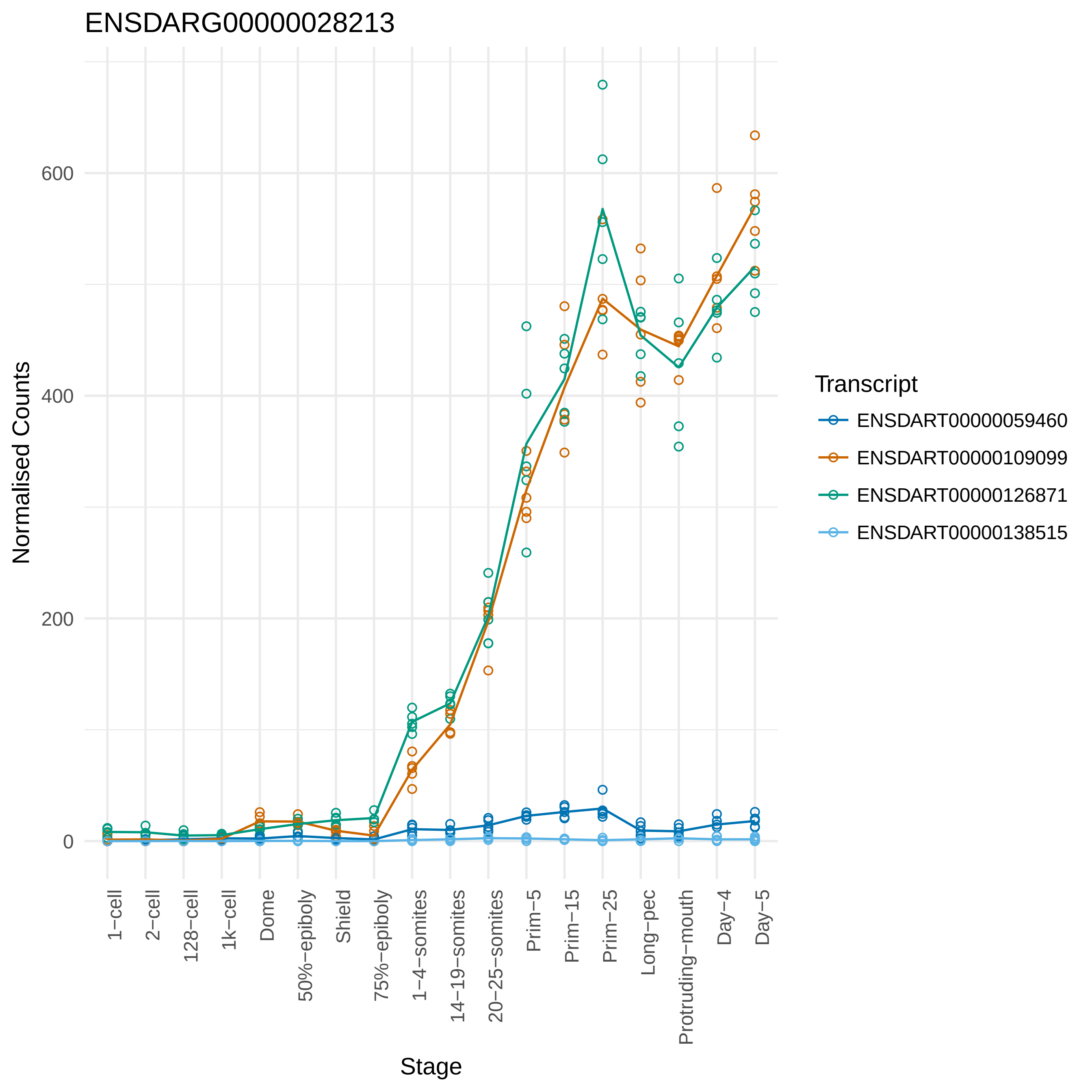

ENSDARG00000028213
Normalised Counts
600
400
200
0
1−cell
2−cell
128−cell
1k−cell
Dome
50%−epiboly
Shield
75%−epiboly
1−4−somites
14−19−somites
20−25−somites
Prim−5
Prim−15
Prim−25
Long−pec
Protruding−mouth
Day−4
Day−5
Stage
Transcript
ENSDART00000059460
ENSDART00000109099
ENSDART00000126871
ENSDART00000138515

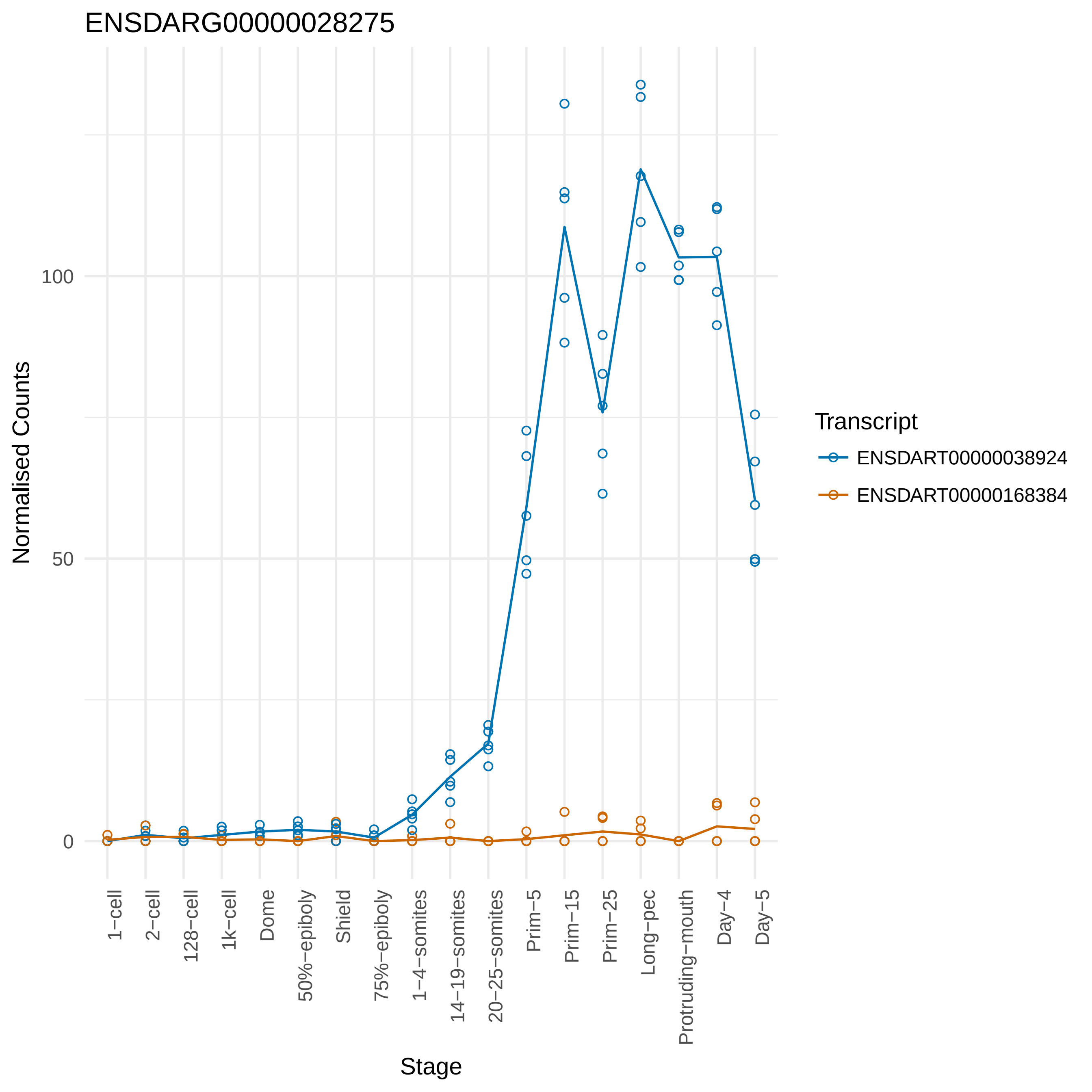

ENSDARG00000028275
Normalised Counts
100
50
0
Stage
1−cell
2−cell
128−cell
1k−cell
Dome
50%−epiboly
Shield
75%−epiboly
1−4−somites
14−19−somites
20−25−somites
Prim−5
Prim−15
Prim−25
Long−pec
Protruding−mouth
Day−4
Day−5
Transcript
ENSDART00000038924
ENSDART00000168384

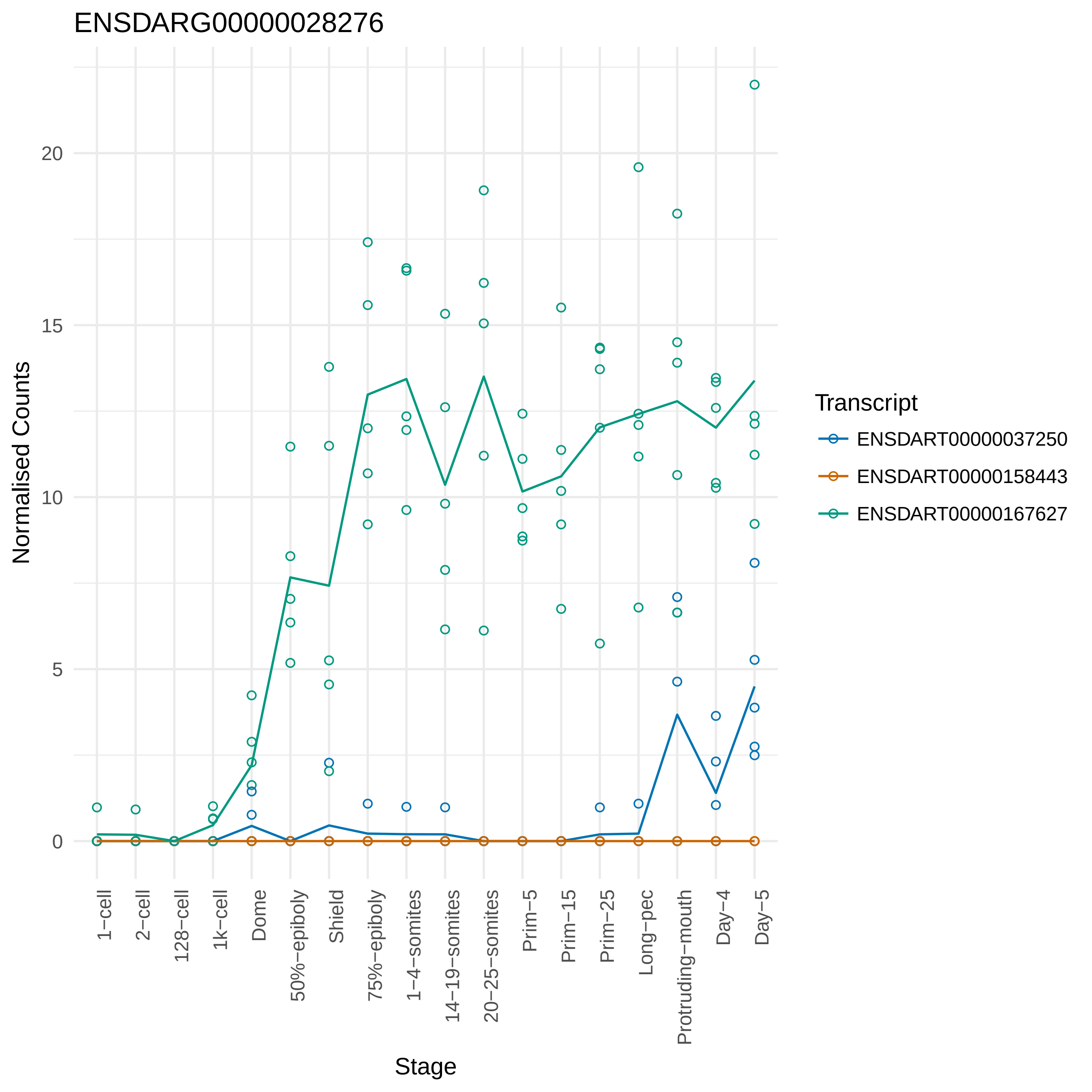

ENSDARG00000028276
Normalised Counts
0
5
10
15
20
Stage
1−cell
2−cell
128−cell
1k−cell
Dome
50%−epiboly
Shield
75%−epiboly
1−4−somites
14−19−somites
20−25−somites
Prim−5
Prim−15
Prim−25
Long−pec
Protruding−mouth
Day−4
Day−5
Transcript
ENSDART00000037250
ENSDART00000158443
ENSDART00000167627

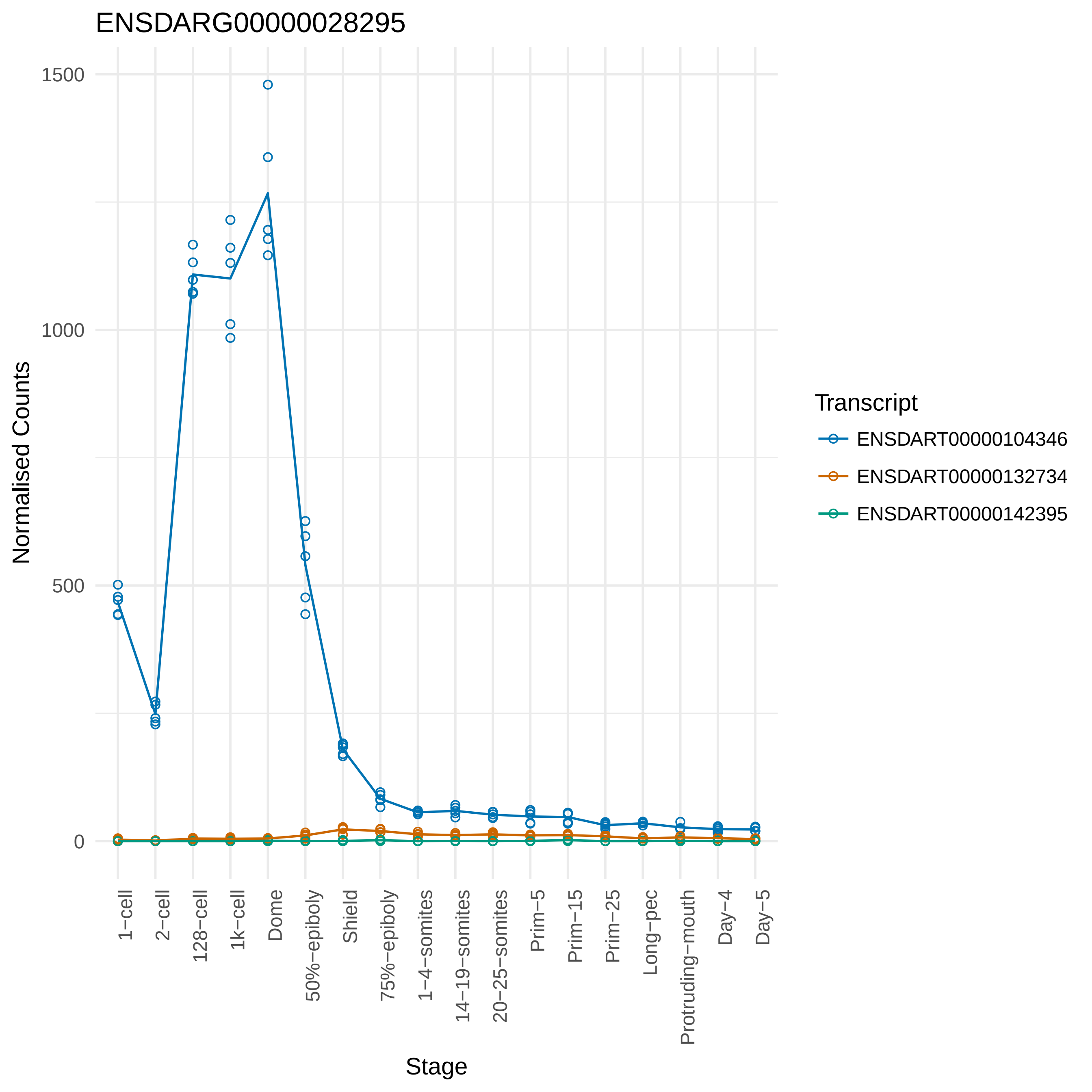
ENSDARG00000028295
Normalised Counts
1500
1000
500
0
1−cell
2−cell
128−cell
1k−cell
Dome
50%−epiboly
Shield
75%−epiboly
1−4−somites
14−19−somites
20−25−somites
Prim−5
Prim−15
Prim−25
Long−pec
Protruding−mouth
Day−4
Day−5
Stage
Transcript
ENSDART00000104346
ENSDART00000132734
ENSDART00000142395

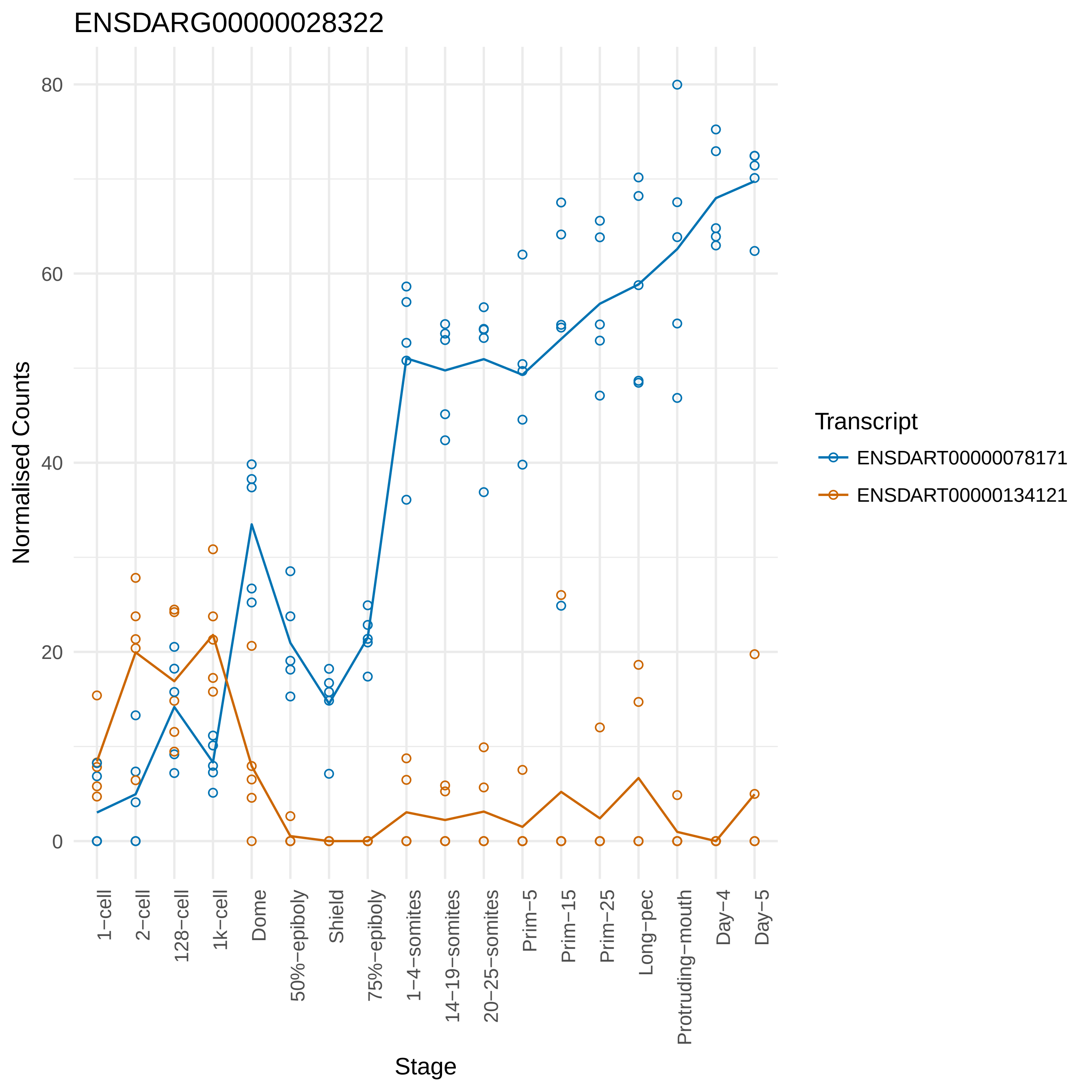
ENSDARG00000028322
Normalised Counts
0
20
40
60
80
1−cell
2−cell
128−cell
1k−cell
Dome
50%−epiboly
Shield
75%−epiboly
1−4−somites
14−19−somites
20−25−somites
Prim−5
Prim−15
Prim−25
Long−pec
Protruding−mouth
Day−4
Day−5
Stage
Transcript
ENSDART00000078171
ENSDART00000134121

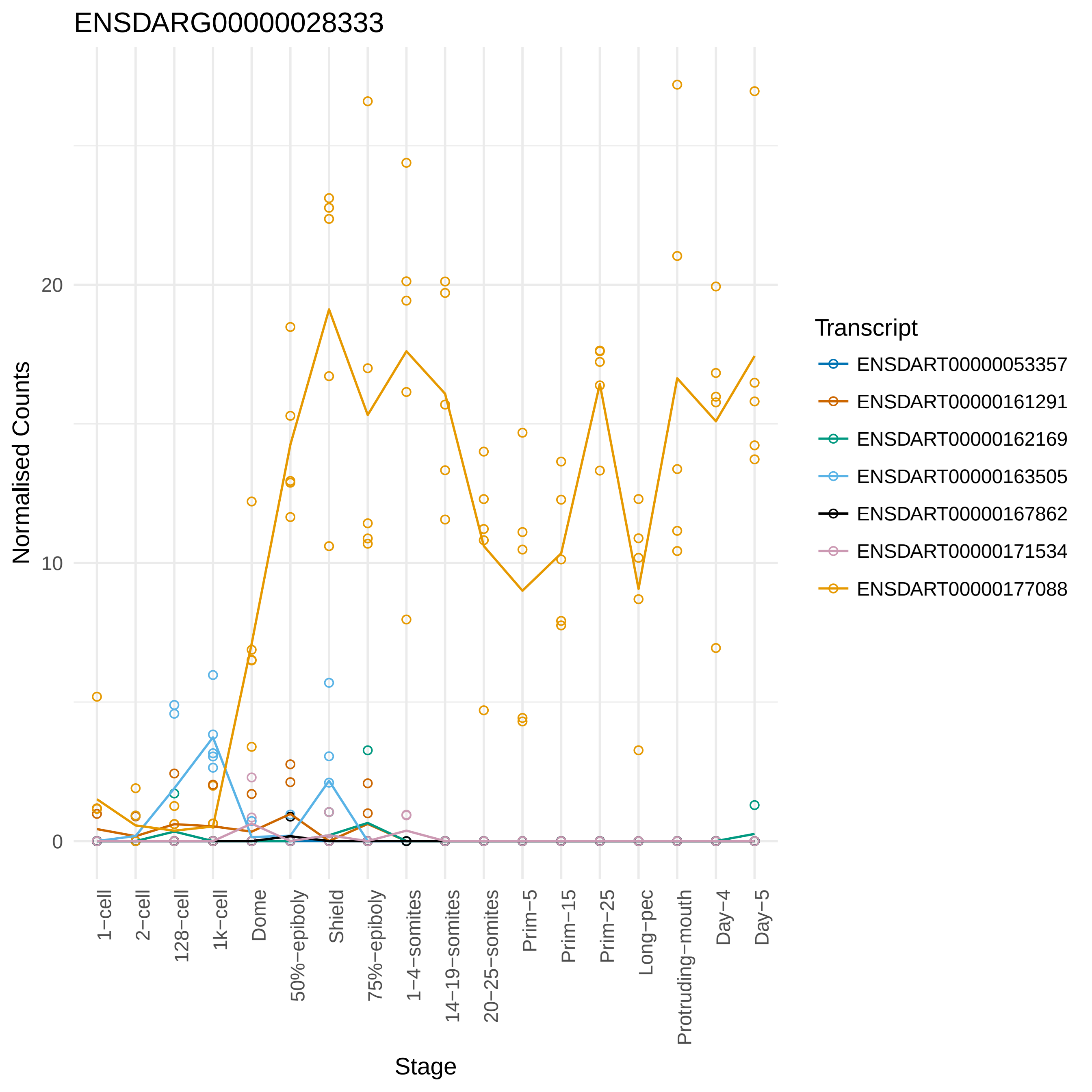
ENSDARG00000028333
Normalised Counts
0
10
20
1−cell
2−cell
128−cell
1k−cell
Dome
50%−epiboly
Shield
75%−epiboly
1−4−somites
14−19−somites
20−25−somites
Prim−5
Prim−15
Prim−25
Long−pec
Protruding−mouth
Day−4
Day−5
Stage
Transcript
ENSDART00000053357
ENSDART00000161291
ENSDART00000162169
ENSDART00000163505
ENSDART00000167862
ENSDART00000171534
ENSDART00000177088

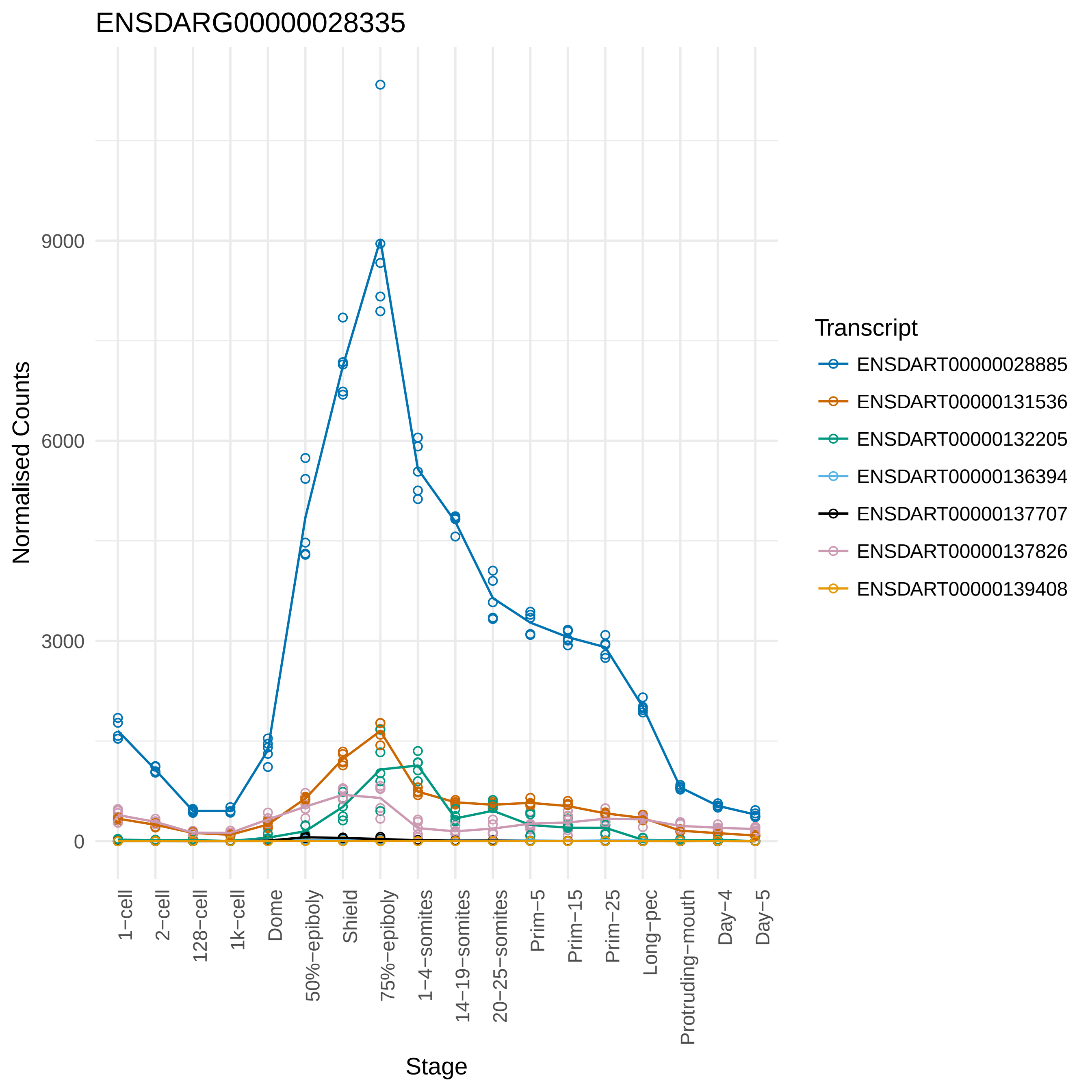

ENSDARG00000028335
Normalised Counts
9000
6000
3000
0
Stage
1−cell
2−cell
128−cell
1k−cell
Dome
50%−epiboly
Shield
75%−epiboly
1−4−somites
14−19−somites
20−25−somites
Prim−5
Prim−15
Prim−25
Long−pec
Protruding−mouth
Day−4
Day−5
Transcript
ENSDART00000028885
ENSDART00000131536
ENSDART00000132205
ENSDART00000136394
ENSDART00000137707
ENSDART00000137826
ENSDART00000139408

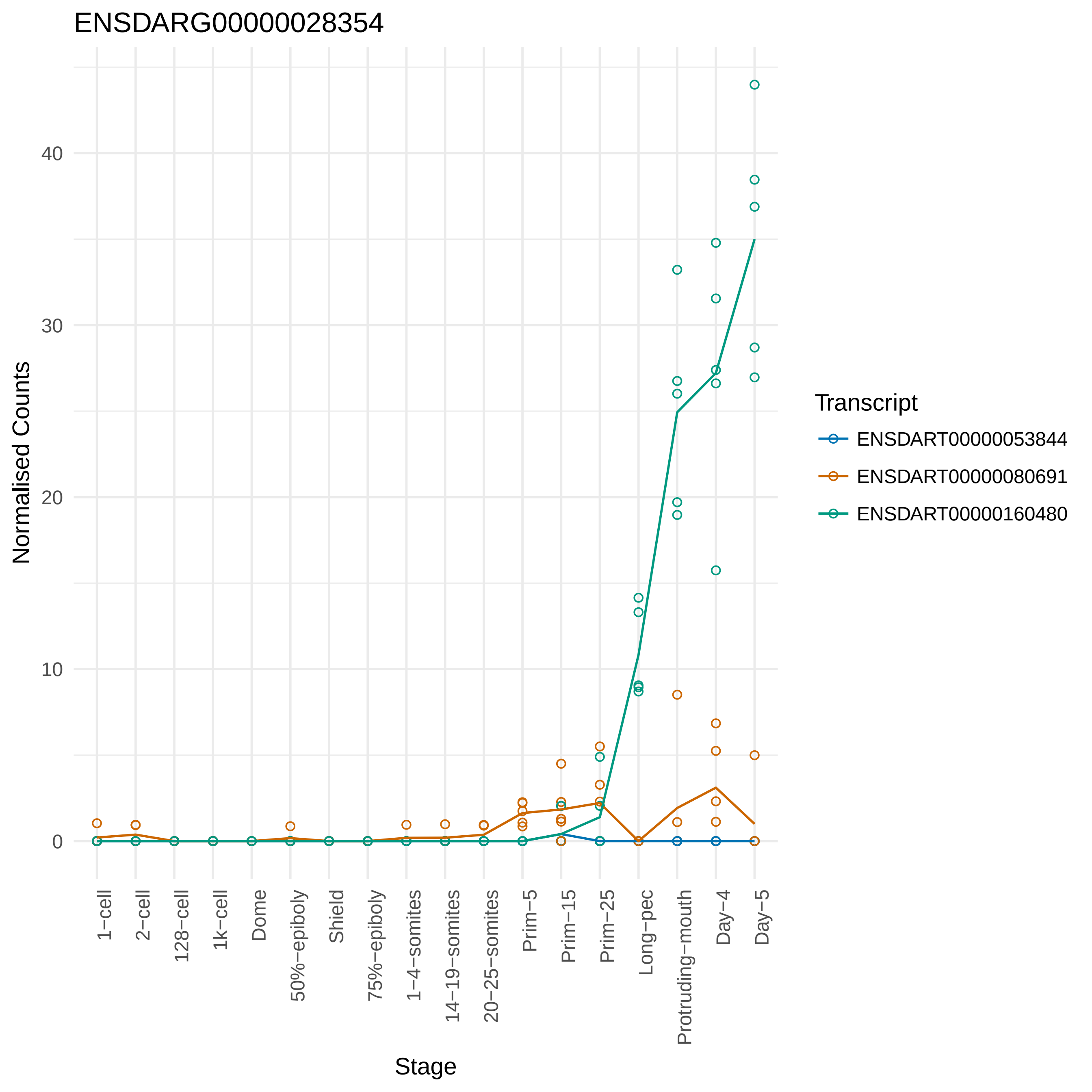

ENSDARG00000028354
Normalised Counts
0
10
20
30
40
Stage
1−cell
2−cell
128−cell
1k−cell
Dome
50%−epiboly
Shield
75%−epiboly
1−4−somites
14−19−somites
20−25−somites
Prim−5
Prim−15
Prim−25
Long−pec
Protruding−mouth
Day−4
Day−5
Transcript
ENSDART00000053844
ENSDART00000080691
ENSDART00000160480

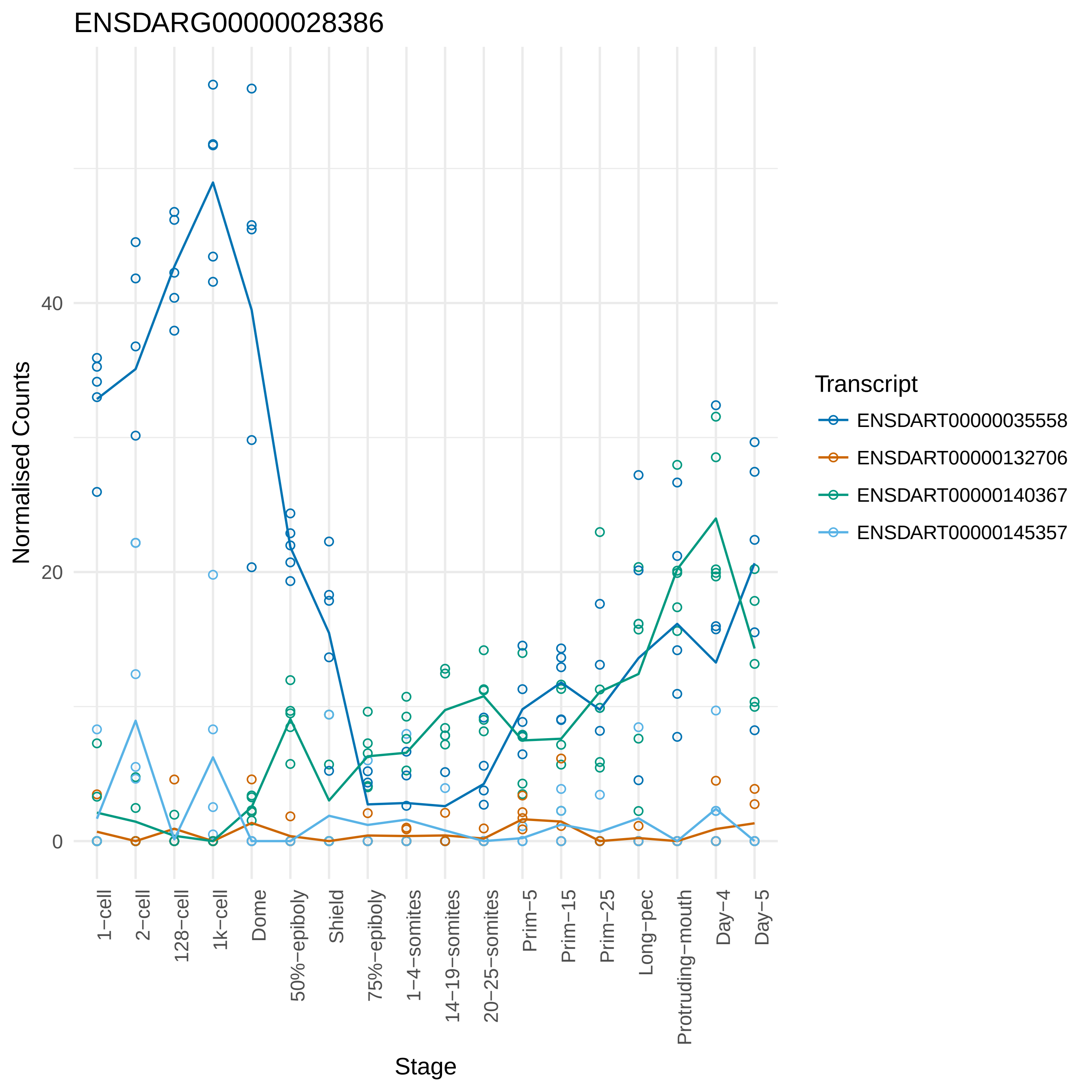

ENSDARG00000028386
Normalised Counts
40
20
0
1−cell
2−cell
128−cell
1k−cell
Dome
50%−epiboly
Shield
75%−epiboly
1−4−somites
14−19−somites
20−25−somites
Prim−5
Prim−15
Prim−25
Long−pec
Protruding−mouth
Day−4
Day−5
Stage
Transcript
ENSDART00000035558
ENSDART00000132706
ENSDART00000140367
ENSDART00000145357

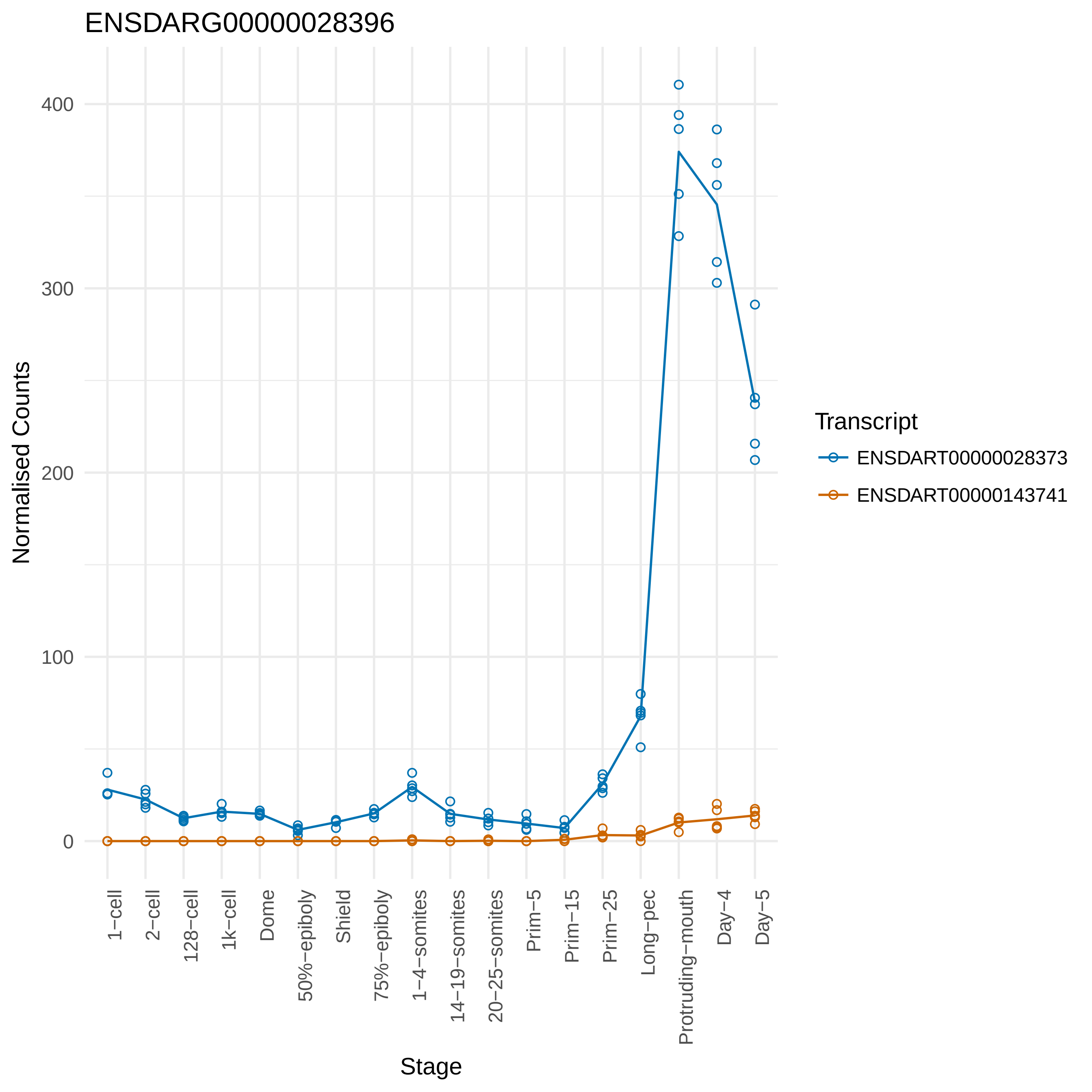

ENSDARG00000028396
Normalised Counts
400
300
200
100
0
1−cell
2−cell
128−cell
1k−cell
Dome
50%−epiboly
Shield
75%−epiboly
1−4−somites
14−19−somites
20−25−somites
Prim−5
Prim−15
Prim−25
Long−pec
Protruding−mouth
Day−4
Day−5
Stage
Transcript
ENSDART00000028373
ENSDART00000143741

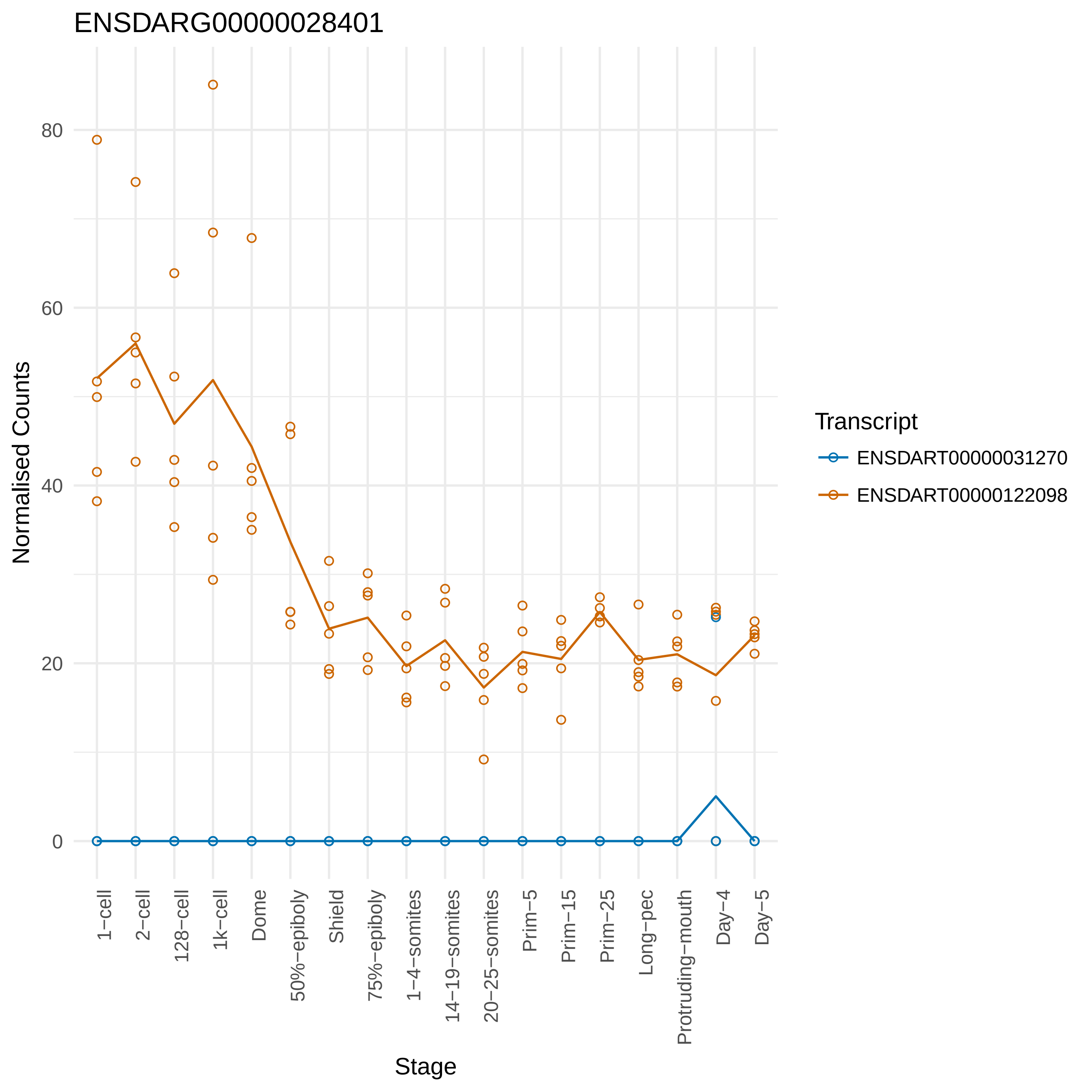

ENSDARG00000028401
Normalised Counts
0
20
40
60
80
Stage
1−cell
2−cell
128−cell
1k−cell
Dome
50%−epiboly
Shield
75%−epiboly
1−4−somites
14−19−somites
20−25−somites
Prim−5
Prim−15
Prim−25
Long−pec
Protruding−mouth
Day−4
Day−5
Transcript
ENSDART00000031270
ENSDART00000122098

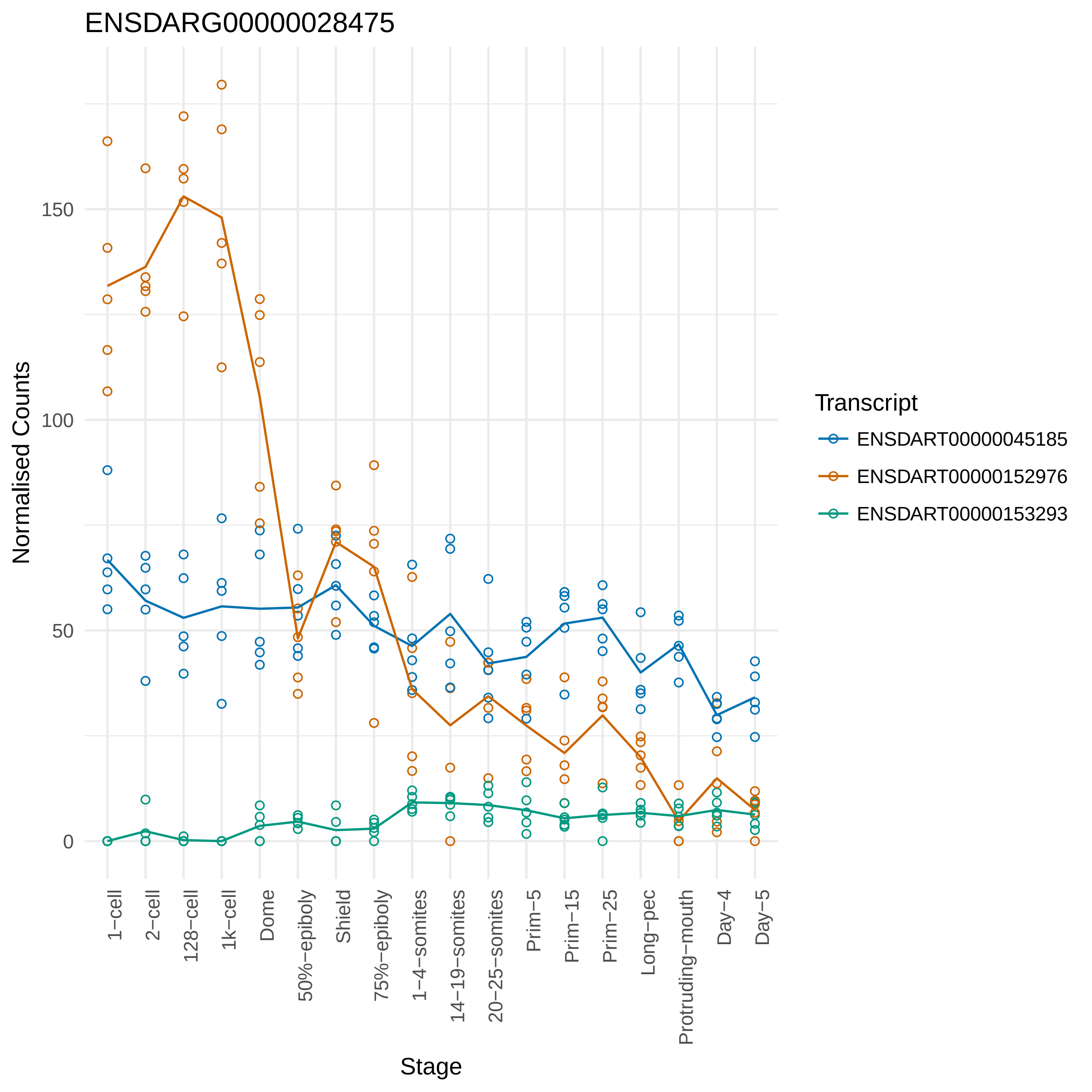

ENSDARG00000028475
Normalised Counts
150
100
50
0
1−cell
2−cell
128−cell
1k−cell
Dome
50%−epiboly
Shield
75%−epiboly
1−4−somites
14−19−somites
20−25−somites
Prim−5
Prim−15
Prim−25
Long−pec
Protruding−mouth
Day−4
Day−5
Stage
Transcript
ENSDART00000045185
ENSDART00000152976
ENSDART00000153293

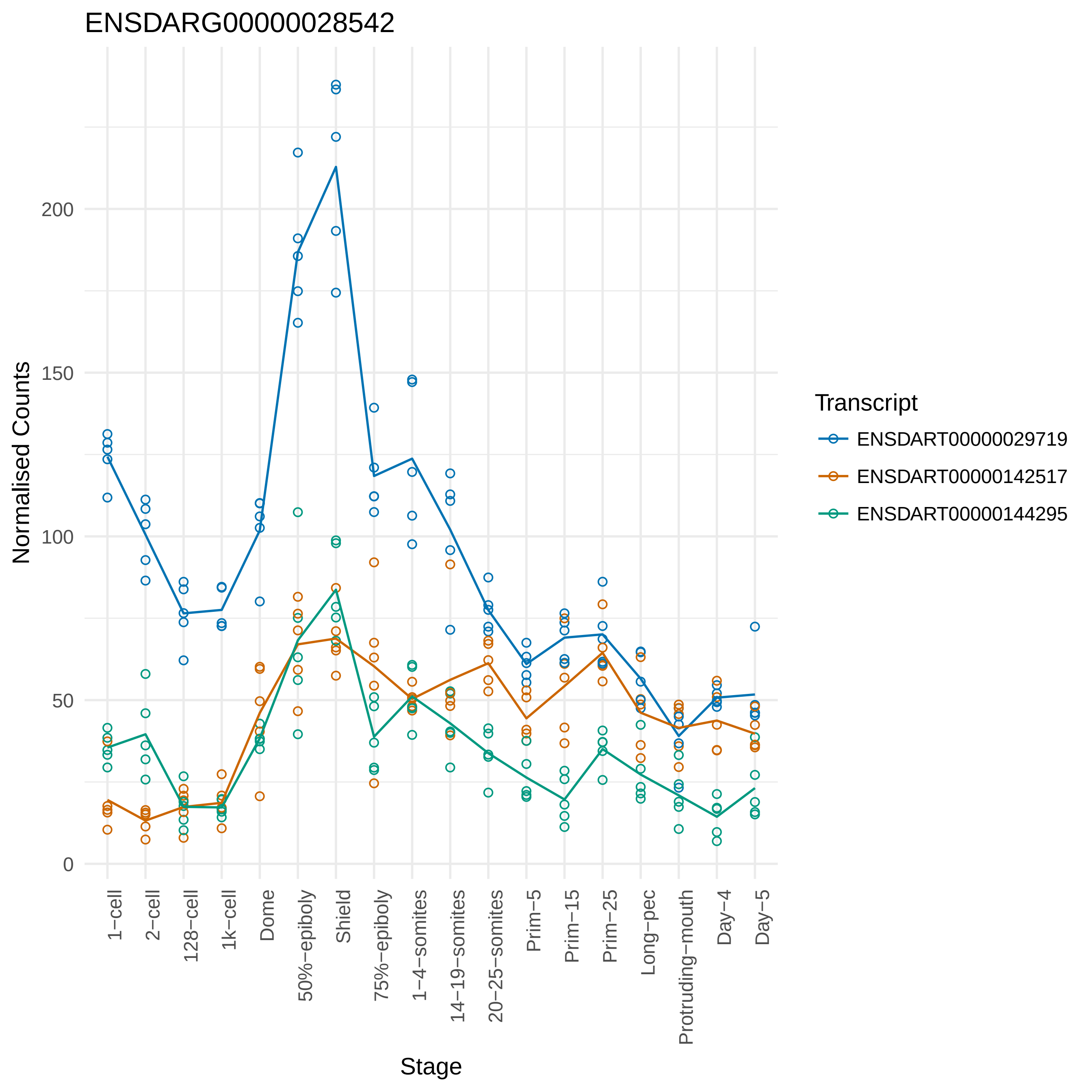
ENSDARG00000028542
Normalised Counts
0
50
100
150
200
1−cell
2−cell
128−cell
1k−cell
Dome
50%−epiboly
Shield
75%−epiboly
1−4−somites
14−19−somites
20−25−somites
Prim−5
Prim−15
Prim−25
Long−pec
Protruding−mouth
Day−4
Day−5
Stage
Transcript
ENSDART00000029719
ENSDART00000142517
ENSDART00000144295

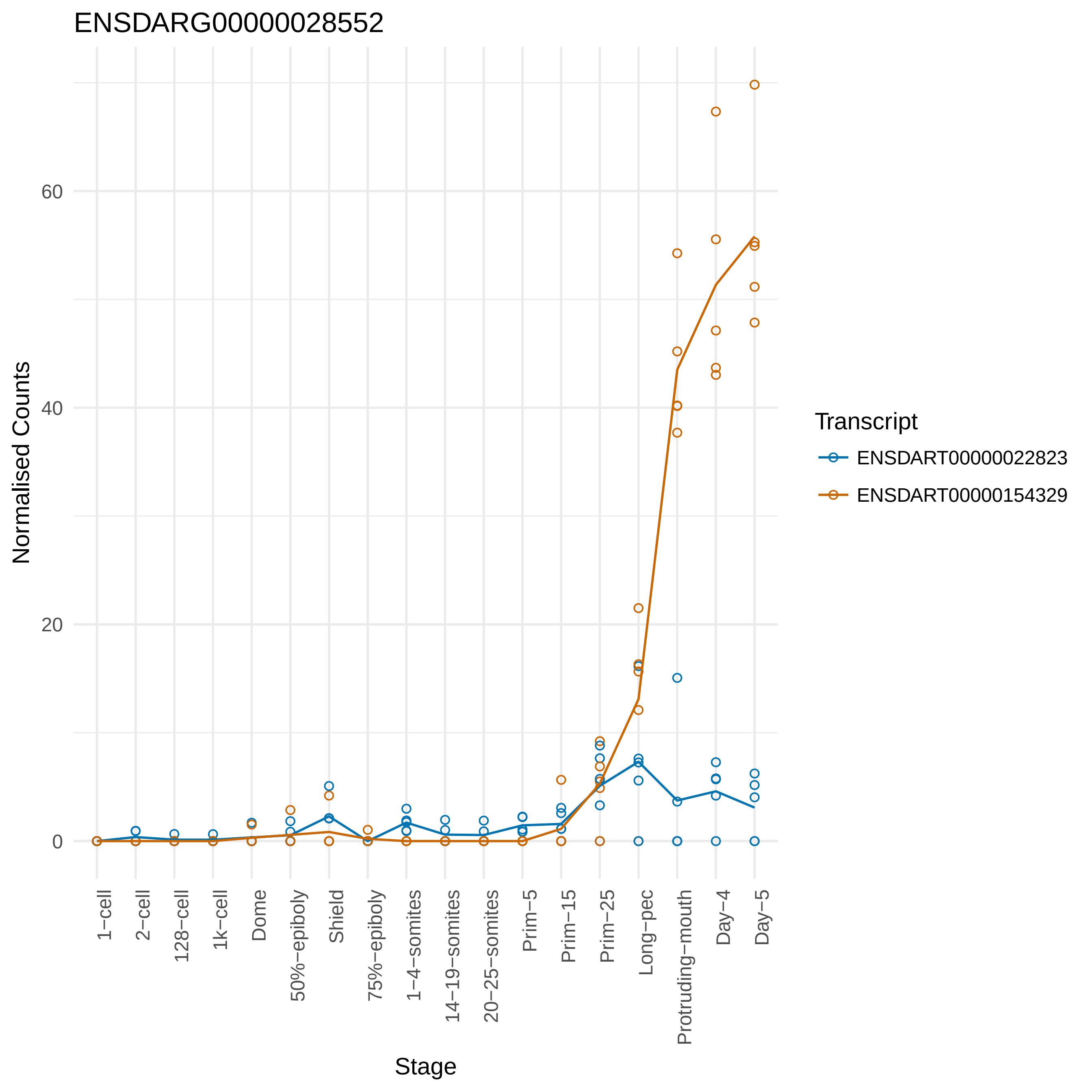
ENSDARG00000028552
Normalised Counts
0
20
40
60
Stage
1−cell
2−cell
128−cell
1k−cell
Dome
50%−epiboly
Shield
75%−epiboly
1−4−somites
14−19−somites
20−25−somites
Prim−5
Prim−15
Prim−25
Long−pec
Protruding−mouth
Day−4
Day−5
Transcript
ENSDART00000022823
ENSDART00000154329

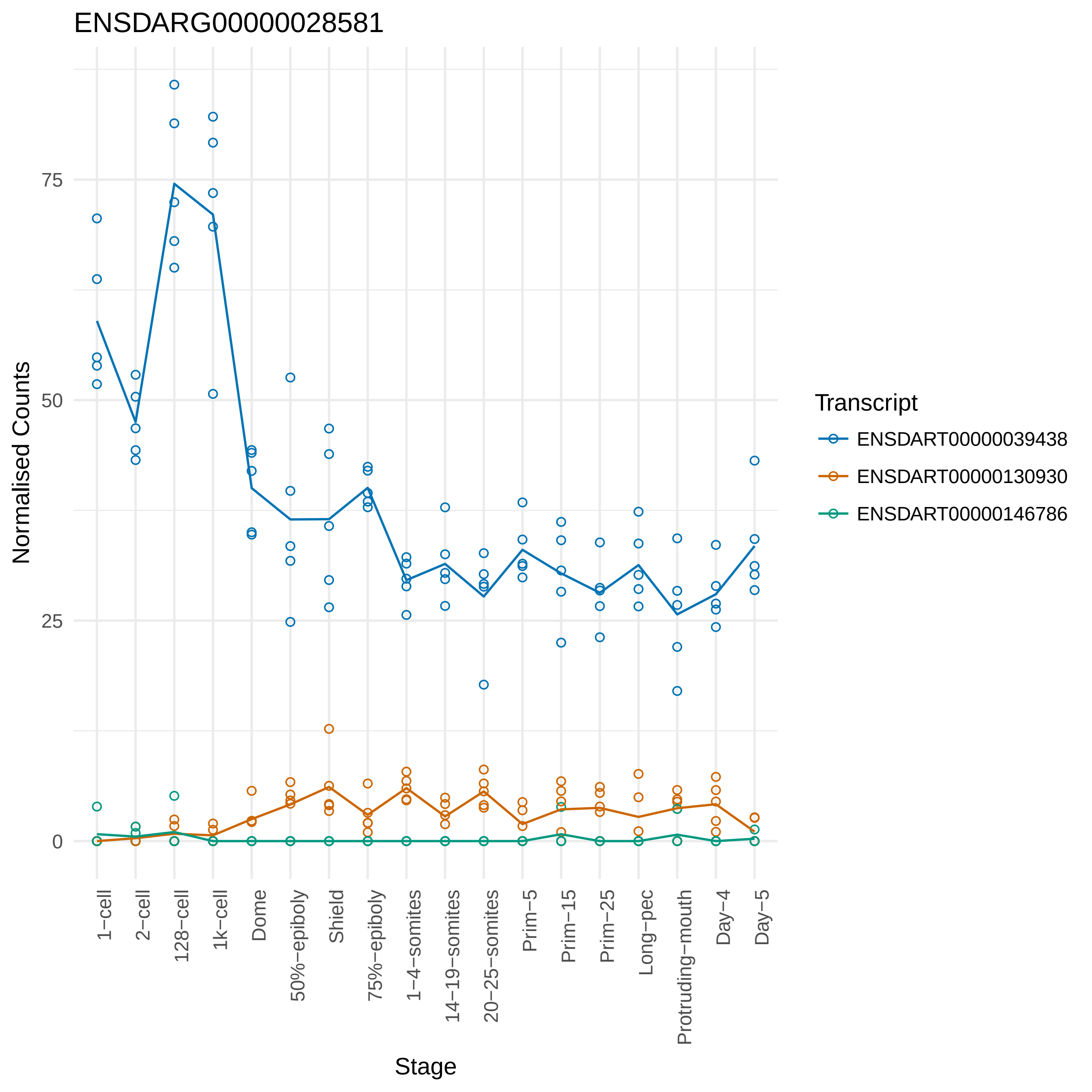

ENSDARG00000028581
Normalised Counts
75
50
25
0
1−cell
2−cell
128−cell
1k−cell
Dome
50%−epiboly
Shield
75%−epiboly
1−4−somites
14−19−somites
20−25−somites
Prim−5
Prim−15
Prim−25
Long−pec
Protruding−mouth
Day−4
Day−5
Stage
Transcript
ENSDART00000039438
ENSDART00000130930
ENSDART00000146786

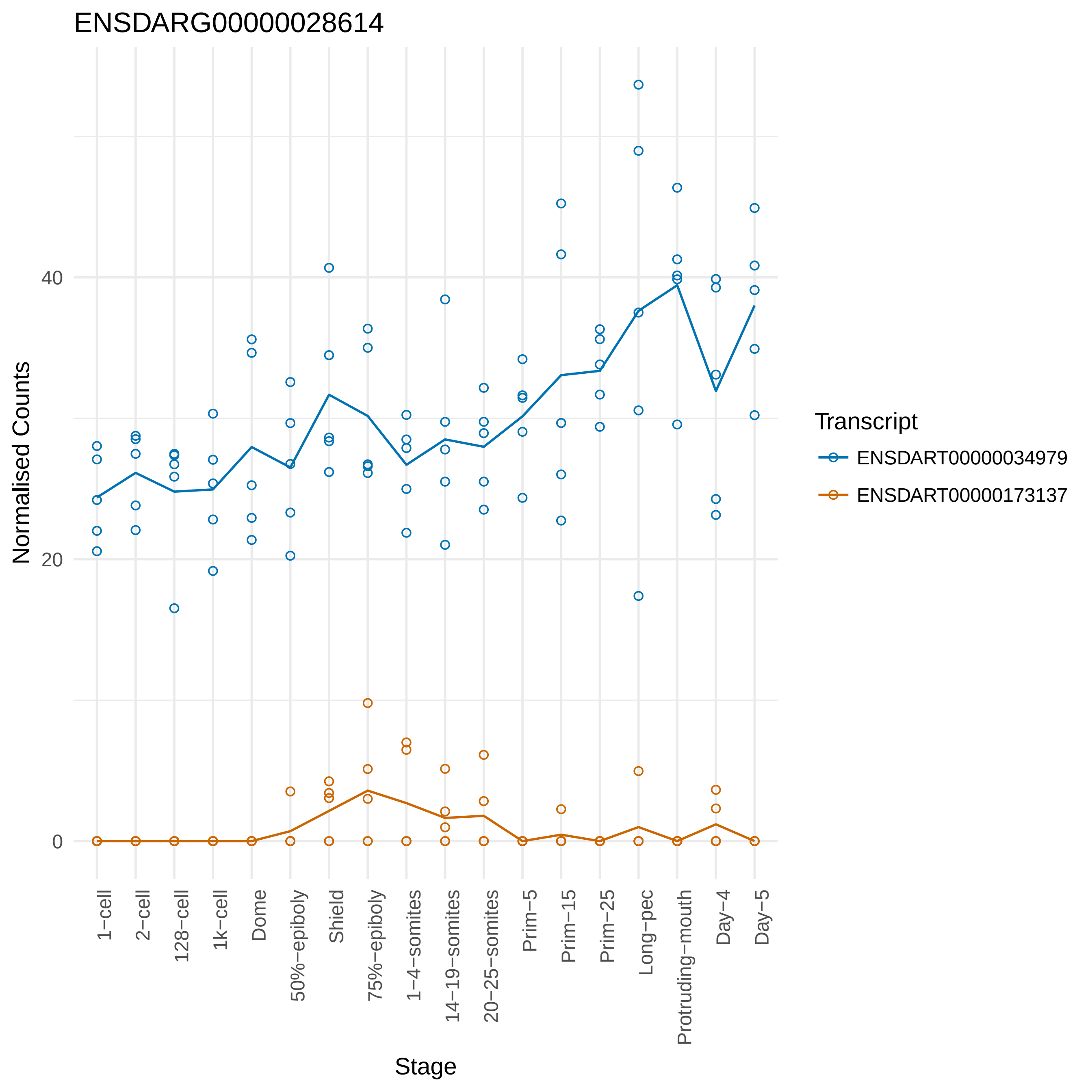

ENSDARG00000028614
Normalised Counts
40
20
0
Stage
1−cell
2−cell
128−cell
1k−cell
Dome
50%−epiboly
Shield
75%−epiboly
1−4−somites
14−19−somites
20−25−somites
Prim−5
Prim−15
Prim−25
Long−pec
Protruding−mouth
Day−4
Day−5
Transcript
ENSDART00000034979
ENSDART00000173137

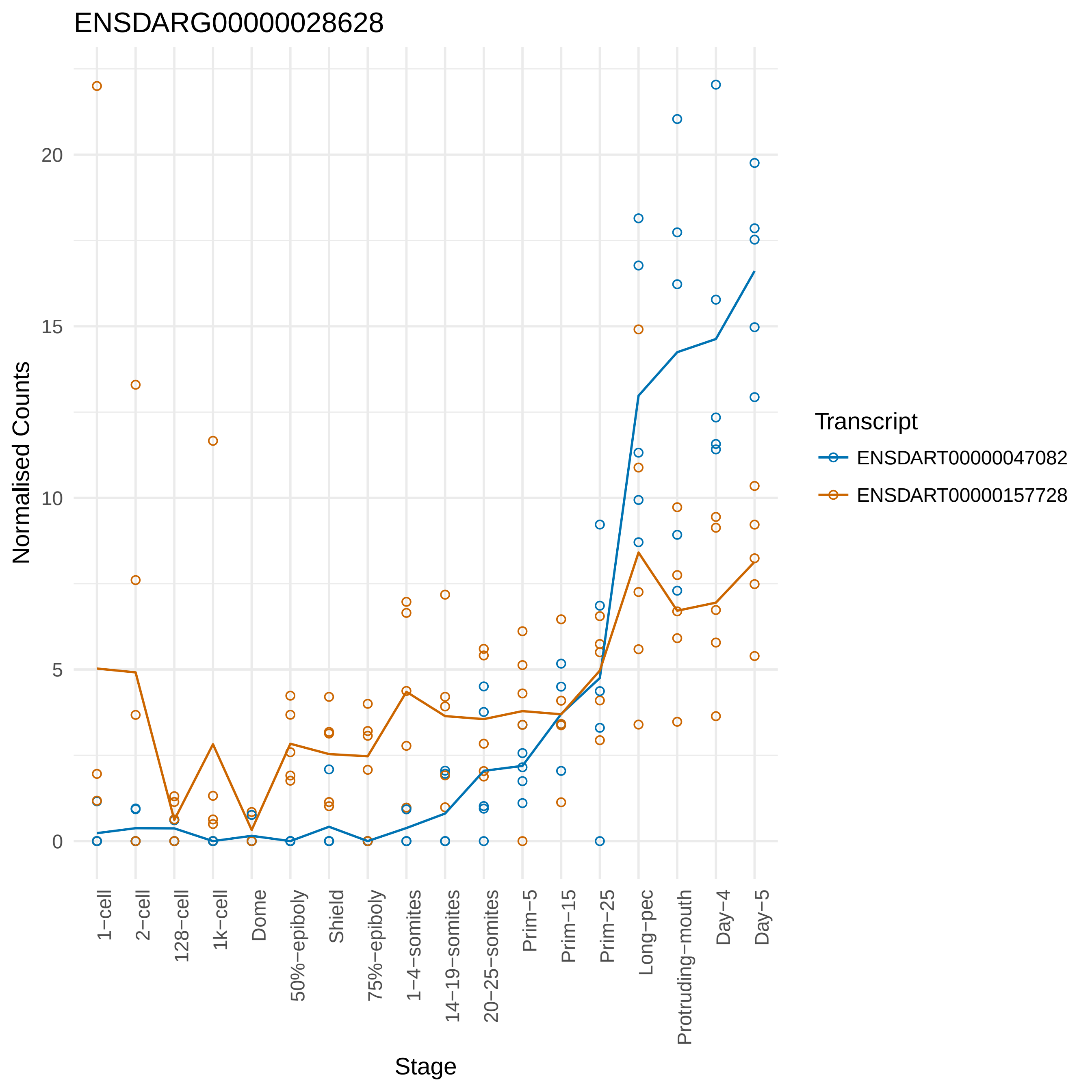

ENSDARG00000028628
Normalised Counts
0
5
10
15
20
Stage
1−cell
2−cell
128−cell
1k−cell
Dome
50%−epiboly
Shield
75%−epiboly
1−4−somites
14−19−somites
20−25−somites
Prim−5
Prim−15
Prim−25
Long−pec
Protruding−mouth
Day−4
Day−5
Transcript
ENSDART00000047082
ENSDART00000157728

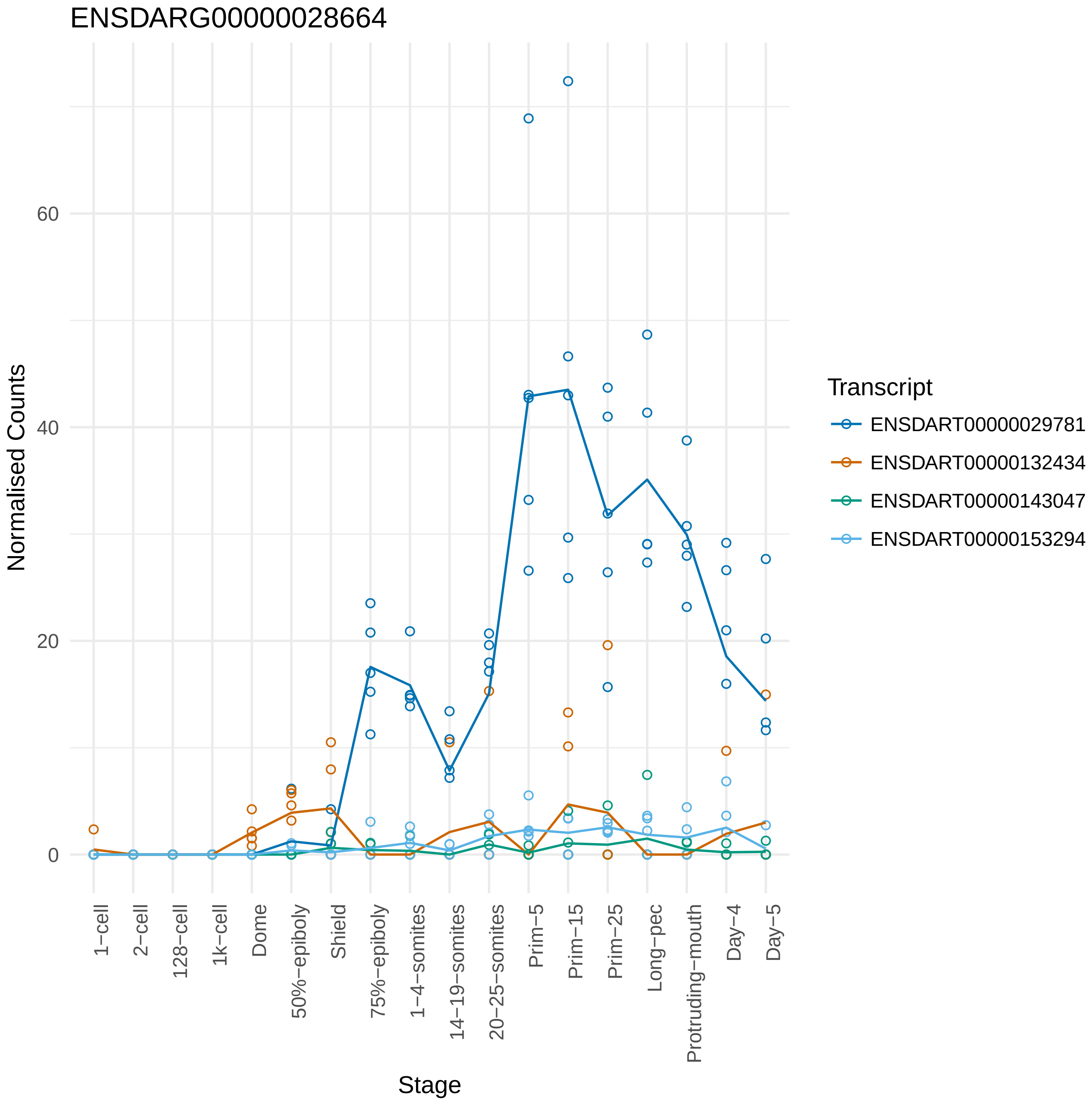

ENSDARG00000028664
Normalised Counts
0
20
40
60
1−cell
2−cell
128−cell
1k−cell
Dome
50%−epiboly
Shield
75%−epiboly
1−4−somites
14−19−somites
20−25−somites
Prim−5
Prim−15
Prim−25
Long−pec
Protruding−mouth
Day−4
Day−5
Stage
Transcript
ENSDART00000029781
ENSDART00000132434
ENSDART00000143047
ENSDART00000153294

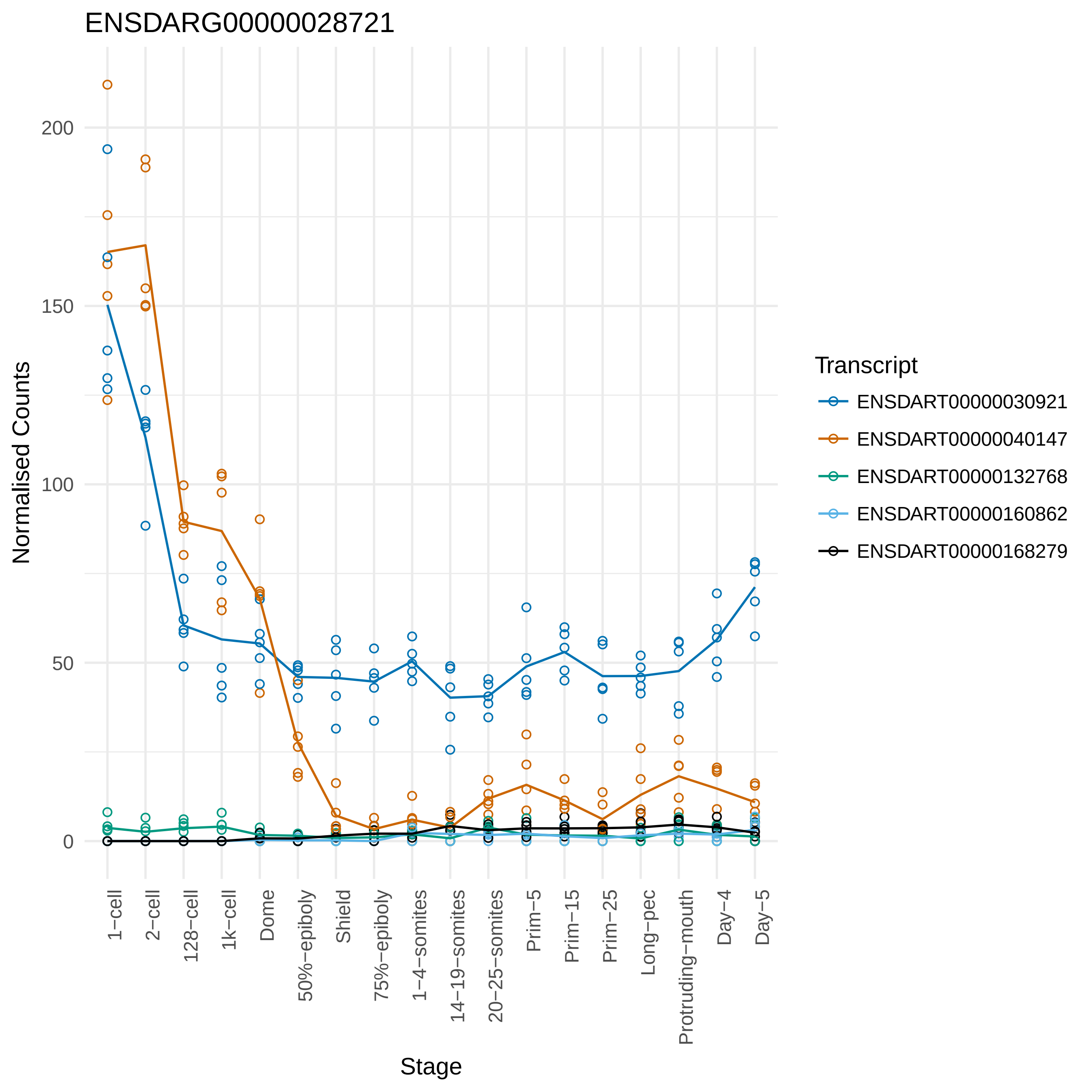

ENSDARG00000028721
Normalised Counts
200
150
100
50
0
1−cell
2−cell
128−cell
1k−cell
Dome
50%−epiboly
Shield
75%−epiboly
1−4−somites
14−19−somites
20−25−somites
Prim−5
Prim−15
Prim−25
Long−pec
Protruding−mouth
Day−4
Day−5
Stage
Transcript
ENSDART00000030921
ENSDART00000040147
ENSDART00000132768
ENSDART00000160862
ENSDART00000168279

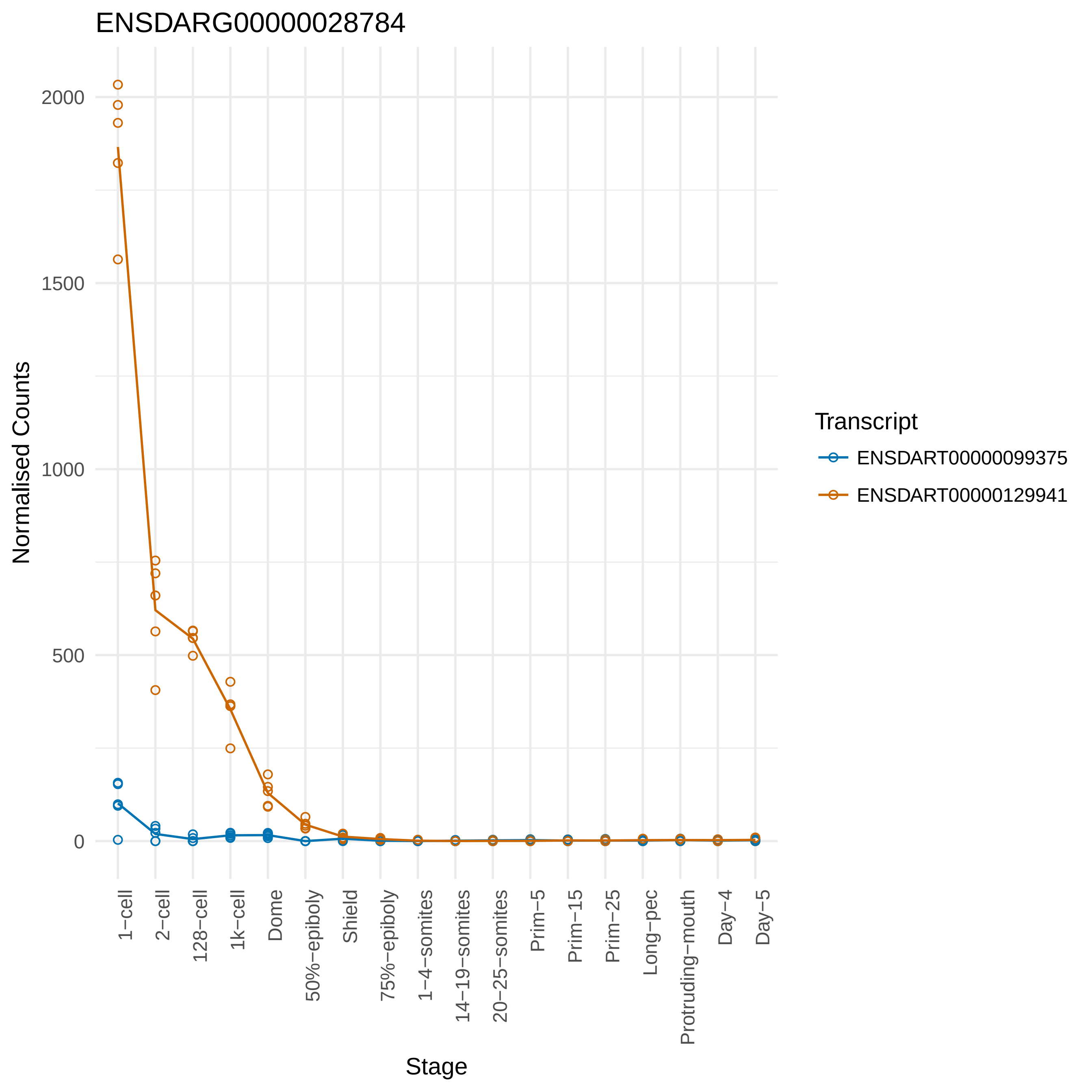

ENSDARG00000028784
Normalised Counts
2000
1500
1000
500
0
1−cell
2−cell
128−cell
1k−cell
Dome
50%−epiboly
Shield
75%−epiboly
1−4−somites
14−19−somites
20−25−somites
Prim−5
Prim−15
Prim−25
Long−pec
Protruding−mouth
Day−4
Day−5
Stage
Transcript
ENSDART00000099375
ENSDART00000129941

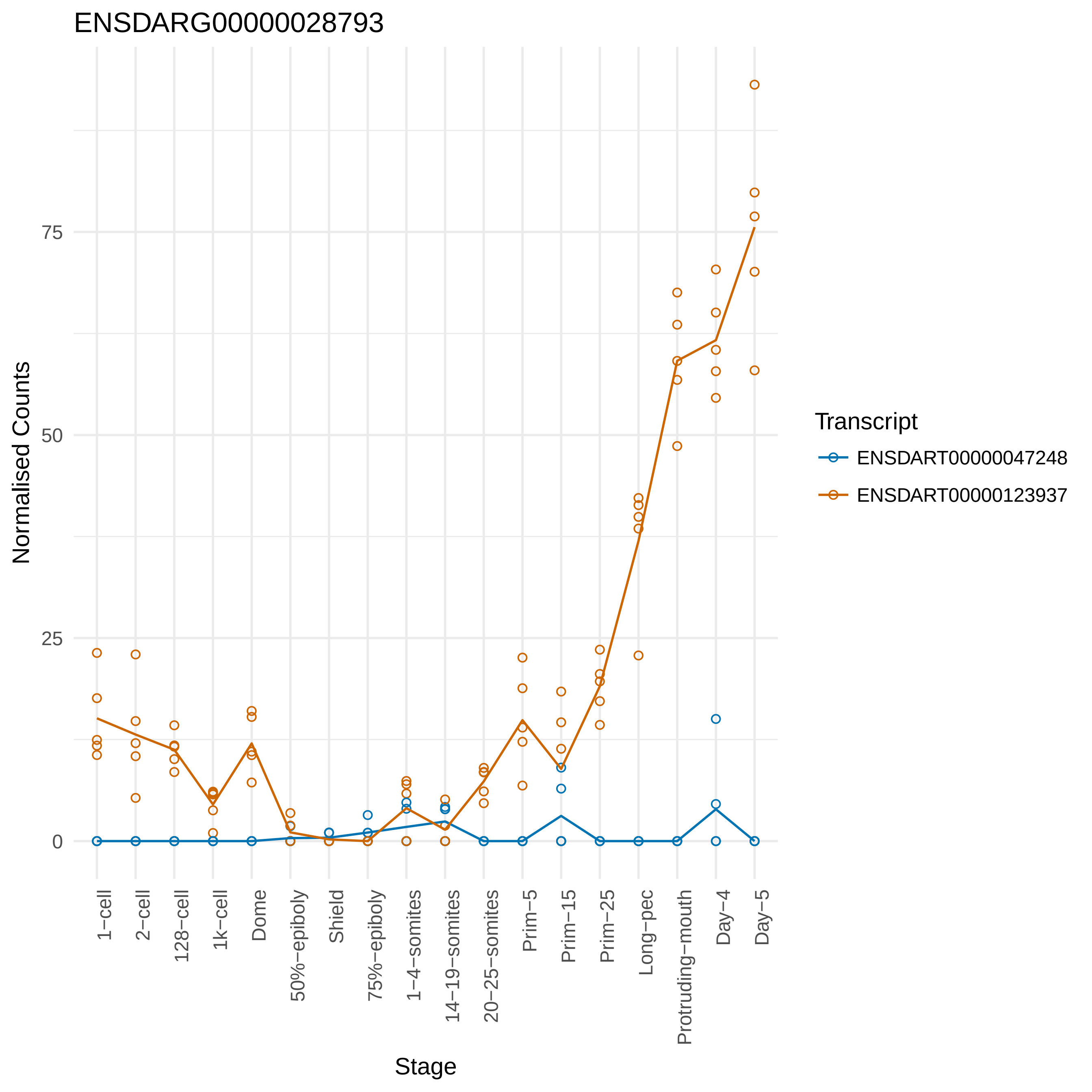
ENSDARG00000028793
Normalised Counts
75
50
25
0
1−cell
2−cell
128−cell
1k−cell
Dome
50%−epiboly
Shield
75%−epiboly
1−4−somites
14−19−somites
20−25−somites
Prim−5
Prim−15
Prim−25
Long−pec
Protruding−mouth
Day−4
Day−5
Stage
Transcript
ENSDART00000047248
ENSDART00000123937

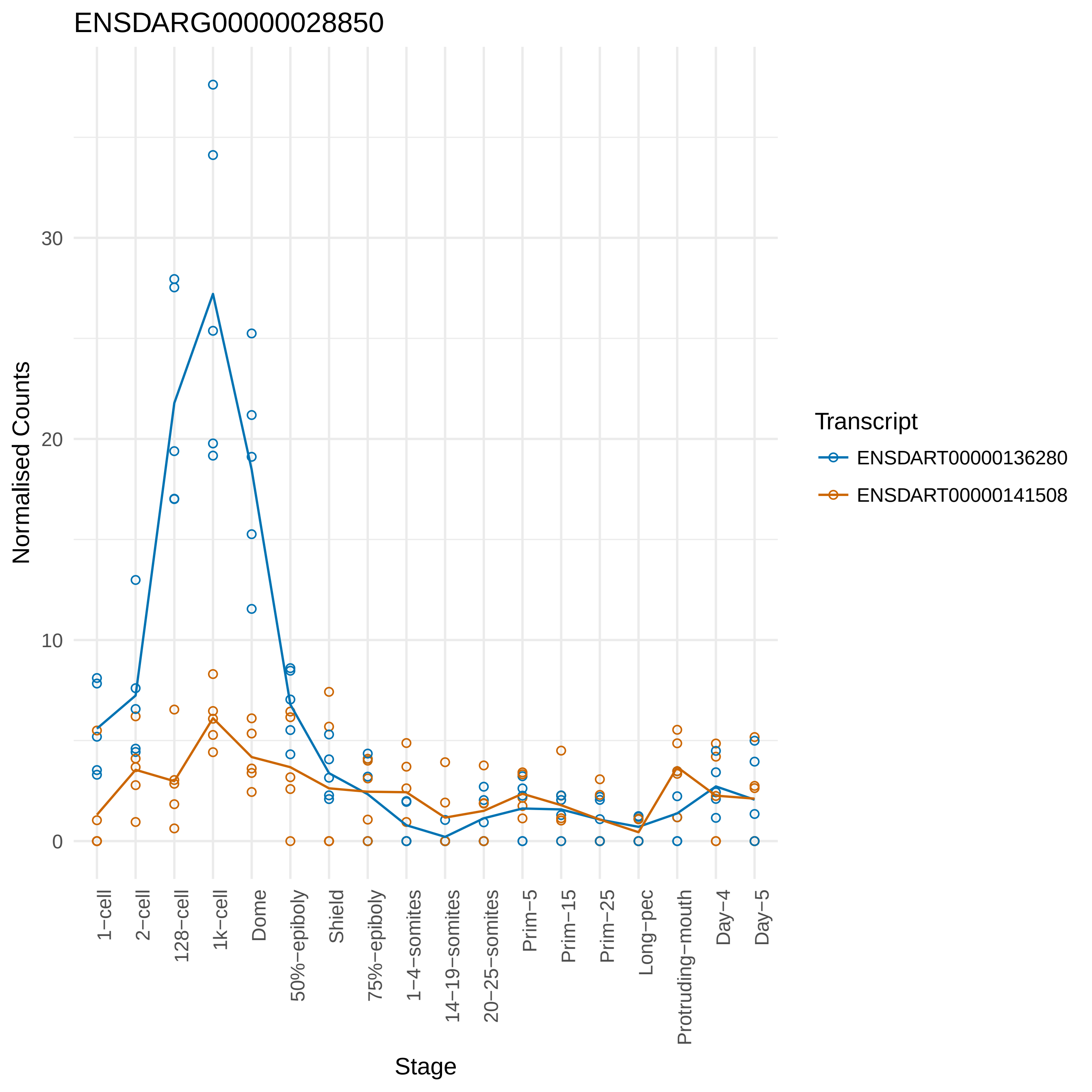

ENSDARG00000028850
Normalised Counts
30
20
10
0
1−cell
2−cell
128−cell
1k−cell
Dome
50%−epiboly
Shield
75%−epiboly
1−4−somites
14−19−somites
20−25−somites
Prim−5
Prim−15
Prim−25
Long−pec
Protruding−mouth
Day−4
Day−5
Stage
Transcript
ENSDART00000136280
ENSDART00000141508

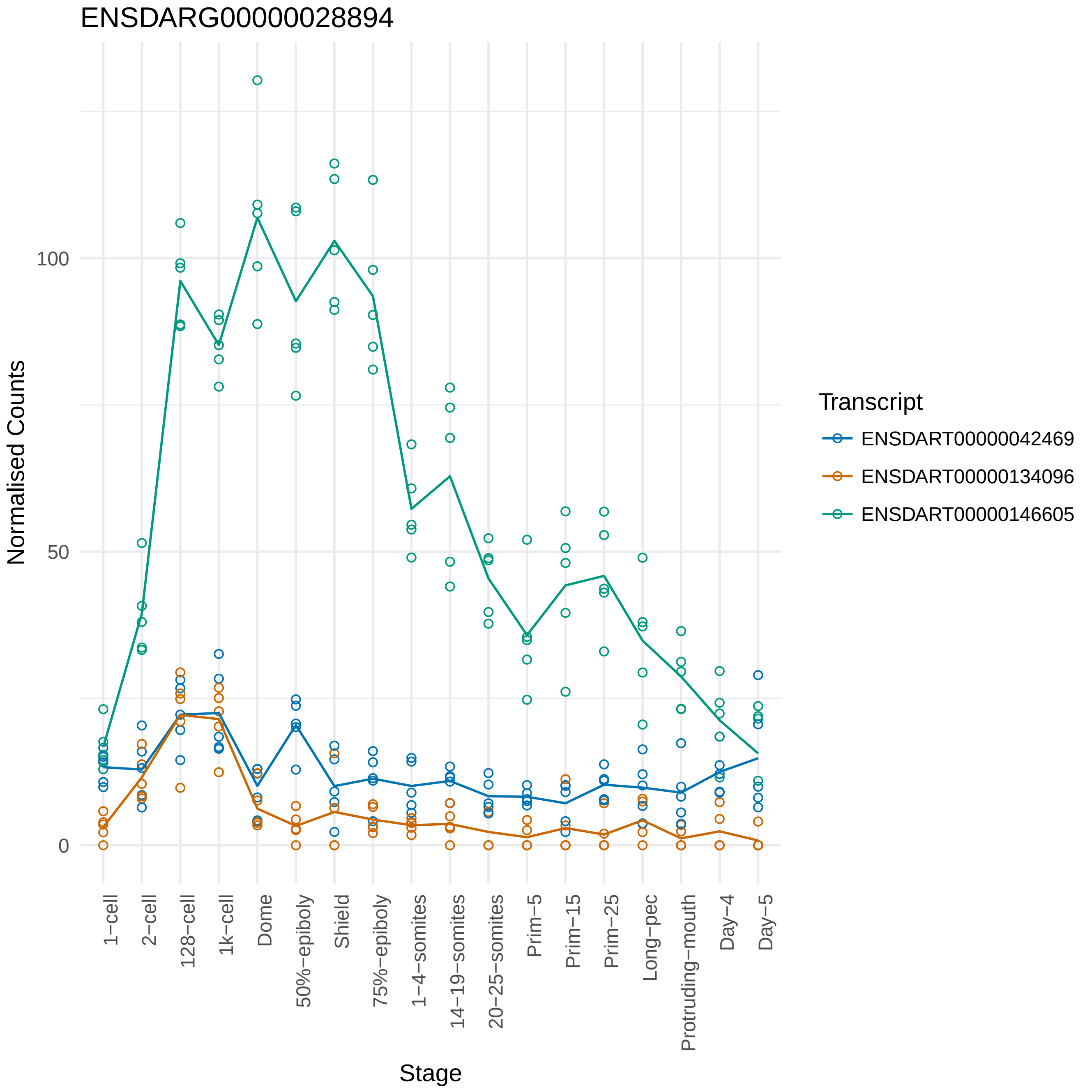

ENSDARG00000028894
Normalised Counts
100
50
0
1−cell
2−cell
128−cell
1k−cell
Dome
50%−epiboly
Shield
75%−epiboly
1−4−somites
14−19−somites
20−25−somites
Prim−5
Prim−15
Prim−25
Long−pec
Protruding−mouth
Day−4
Day−5
Stage
Transcript
ENSDART00000042469
ENSDART00000134096
ENSDART00000146605

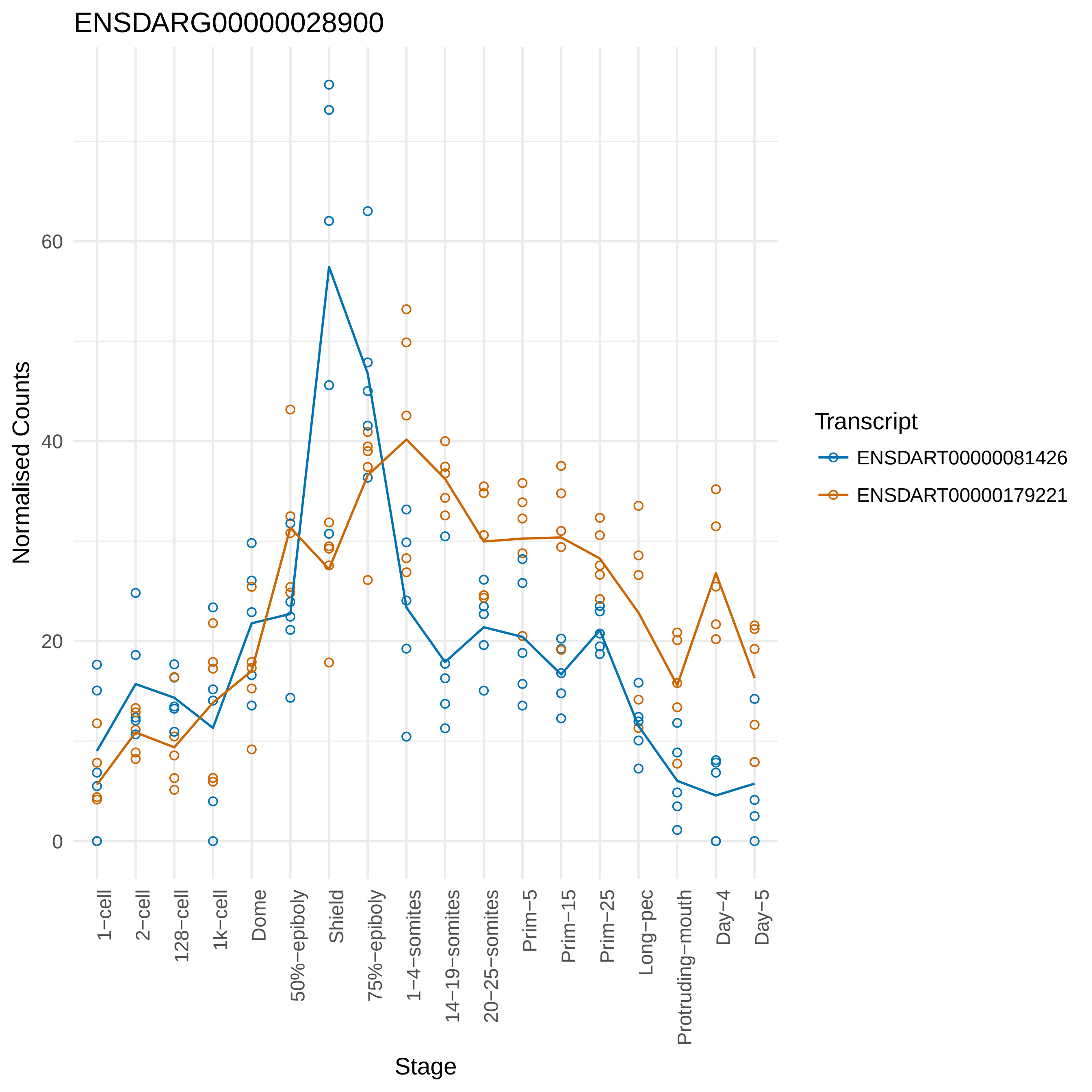

ENSDARG00000028900
Normalised Counts
0
20
40
60
Stage
1−cell
2−cell
128−cell
1k−cell
Dome
50%−epiboly
Shield
75%−epiboly
1−4−somites
14−19−somites
20−25−somites
Prim−5
Prim−15
Prim−25
Long−pec
Protruding−mouth
Day−4
Day−5
Transcript
ENSDART00000081426
ENSDART00000179221

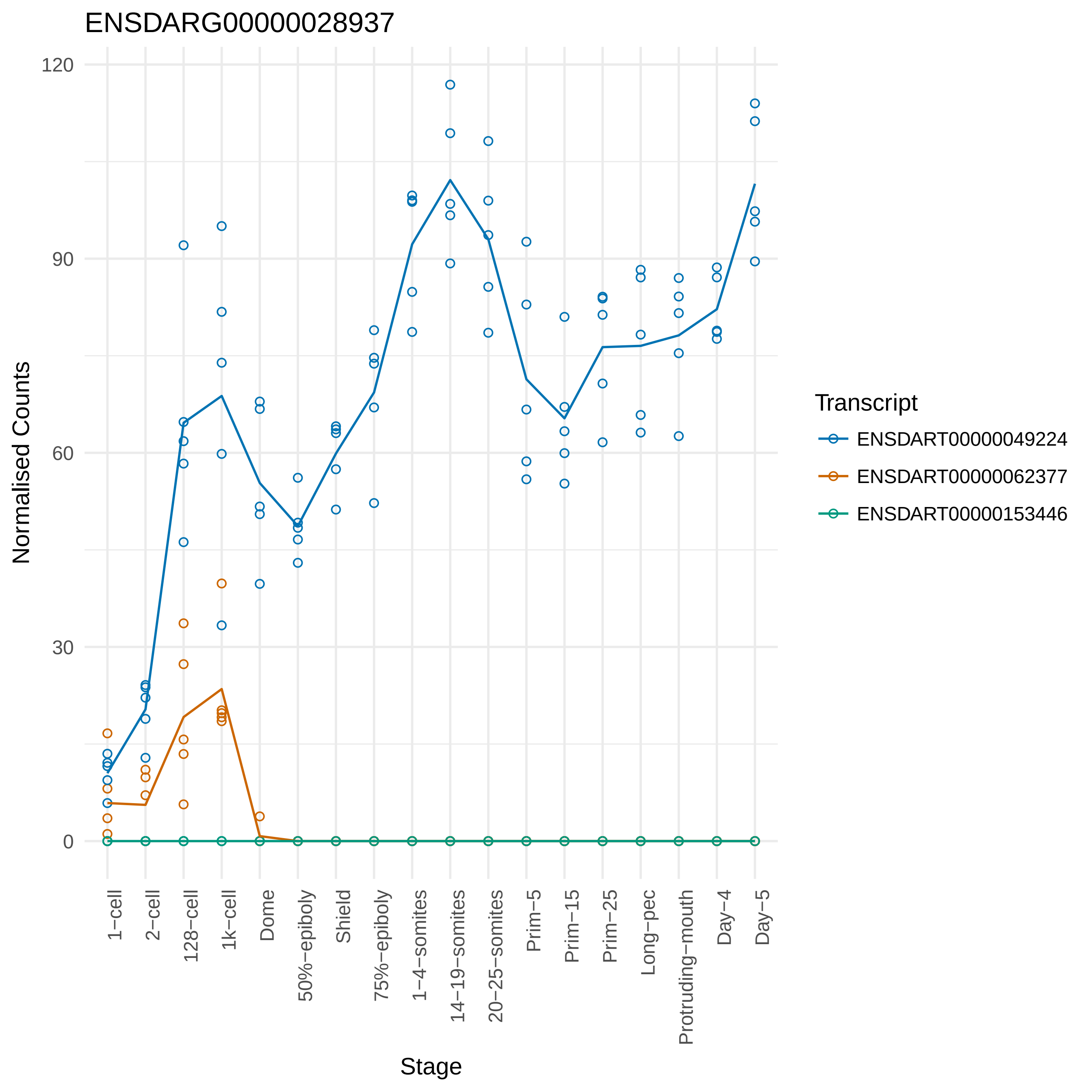

ENSDARG00000028937
Normalised Counts
120
90
60
30
0
1−cell
2−cell
128−cell
1k−cell
Dome
50%−epiboly
Shield
75%−epiboly
1−4−somites
14−19−somites
20−25−somites
Prim−5
Prim−15
Prim−25
Long−pec
Protruding−mouth
Day−4
Day−5
Stage
Transcript
ENSDART00000049224
ENSDART00000062377
ENSDART00000153446

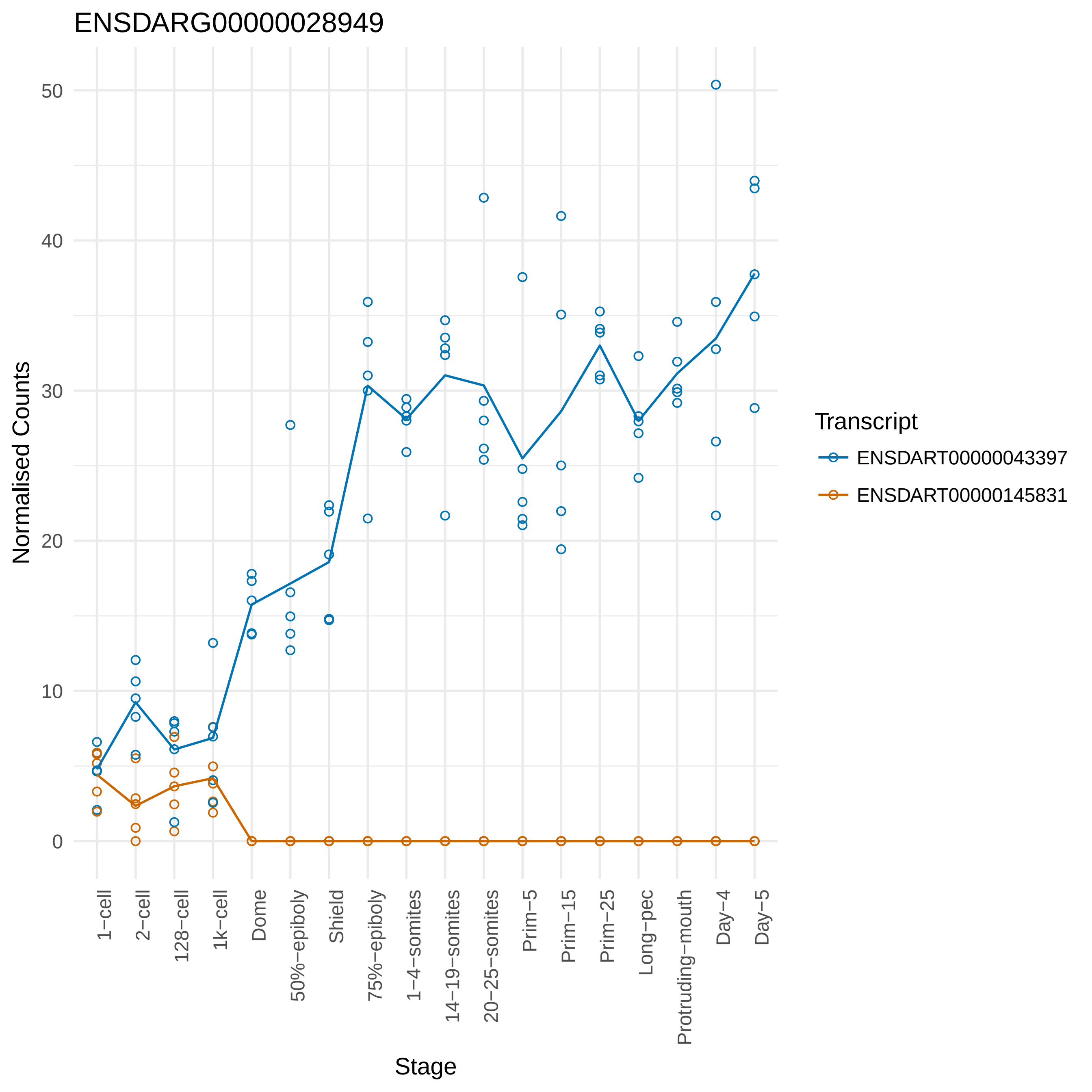

ENSDARG00000028949
Normalised Counts
50
40
30
20
10
0
1−cell
2−cell
128−cell
1k−cell
Dome
50%−epiboly
Shield
75%−epiboly
1−4−somites
14−19−somites
20−25−somites
Prim−5
Prim−15
Prim−25
Long−pec
Protruding−mouth
Day−4
Day−5
Stage
Transcript
ENSDART00000043397
ENSDART00000145831

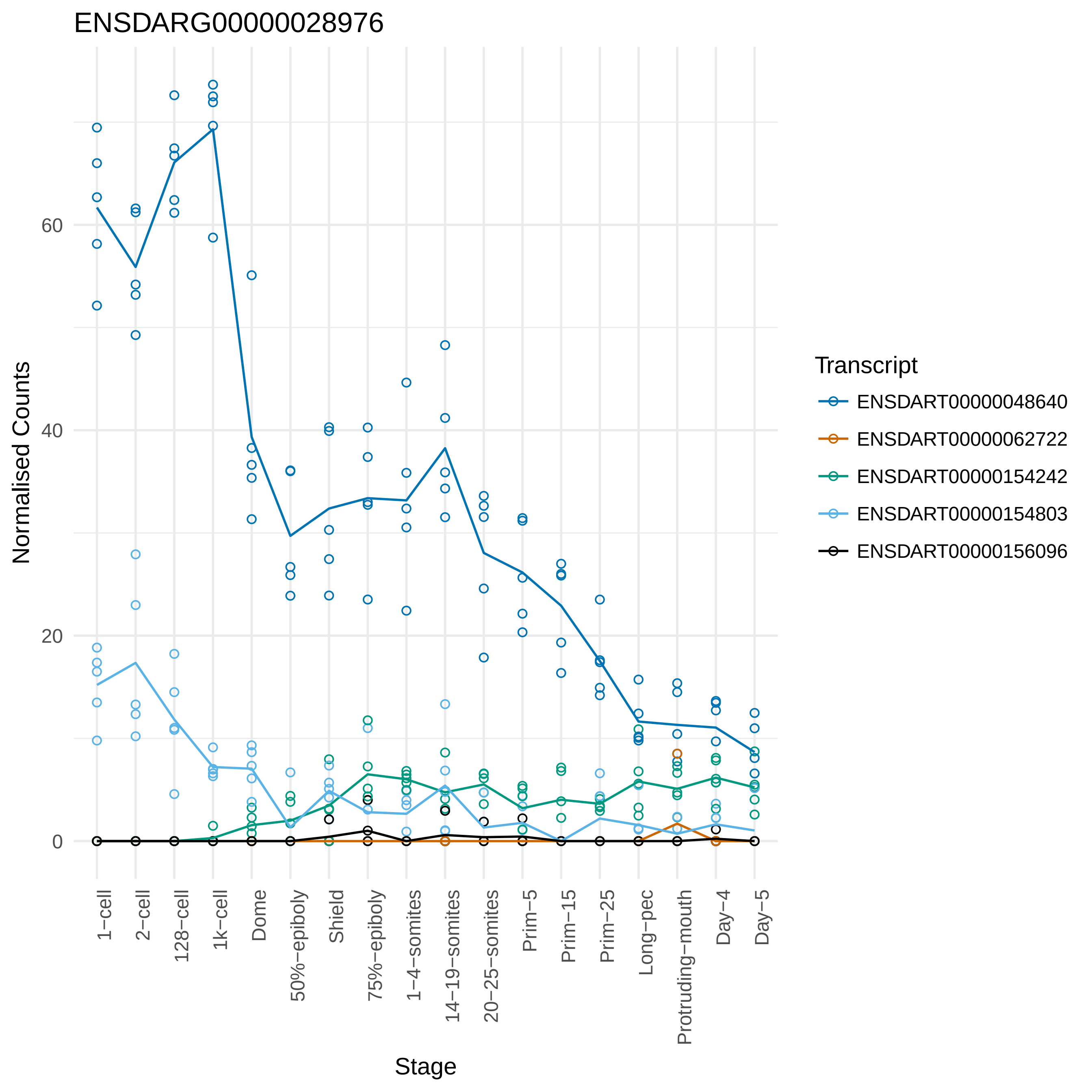
ENSDARG00000028976
Normalised Counts
0
20
40
60
Stage
1−cell
2−cell
128−cell
1k−cell
Dome
50%−epiboly
Shield
75%−epiboly
1−4−somites
14−19−somites
20−25−somites
Prim−5
Prim−15
Prim−25
Long−pec
Protruding−mouth
Day−4
Day−5
Transcript
ENSDART00000048640
ENSDART00000062722
ENSDART00000154242
ENSDART00000154803
ENSDART00000156096

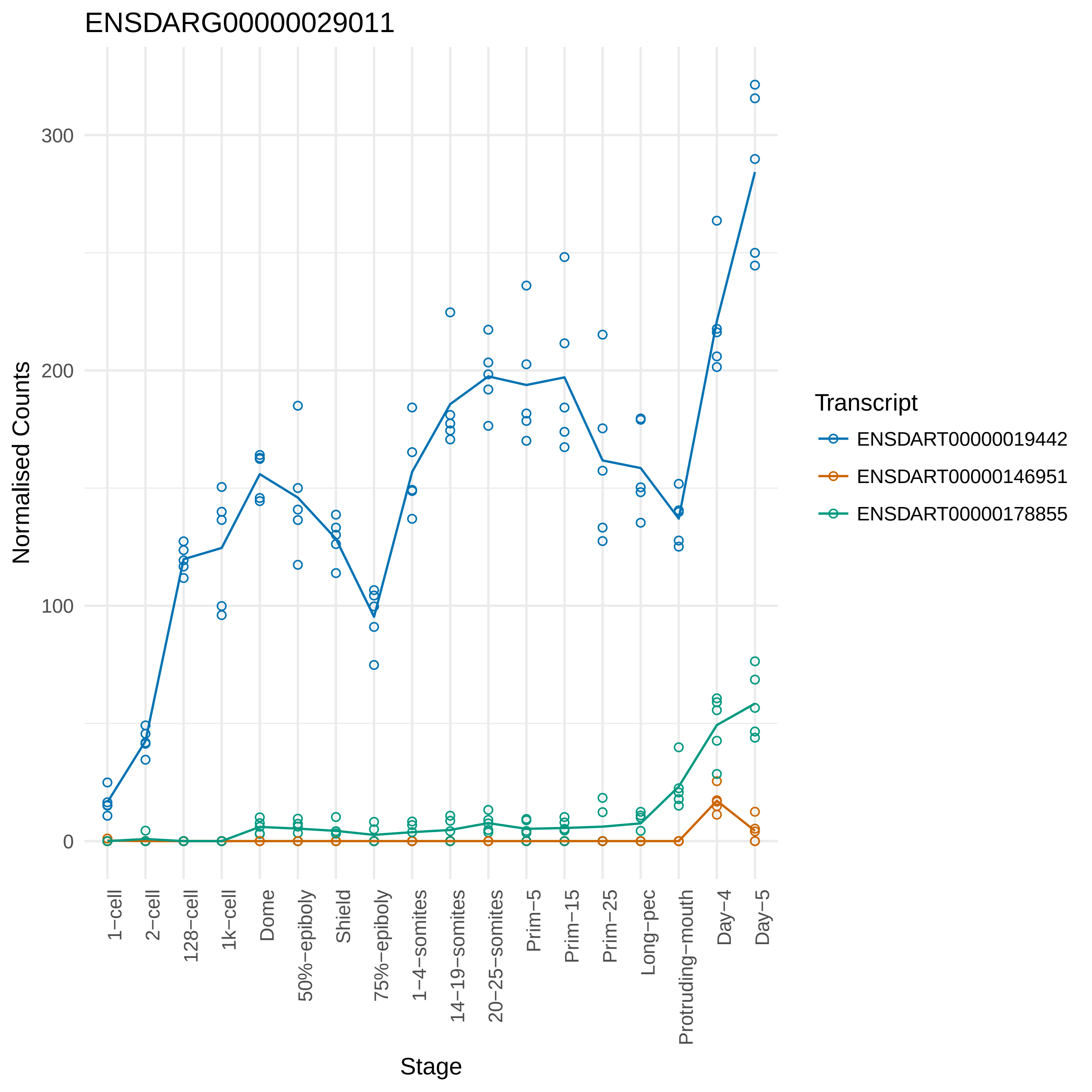

ENSDARG00000029011
Normalised Counts
0
100
200
300
1−cell
2−cell
128−cell
1k−cell
Dome
50%−epiboly
Shield
75%−epiboly
1−4−somites
14−19−somites
20−25−somites
Prim−5
Prim−15
Prim−25
Long−pec
Protruding−mouth
Day−4
Day−5
Stage
Transcript
ENSDART00000019442
ENSDART00000146951
ENSDART00000178855

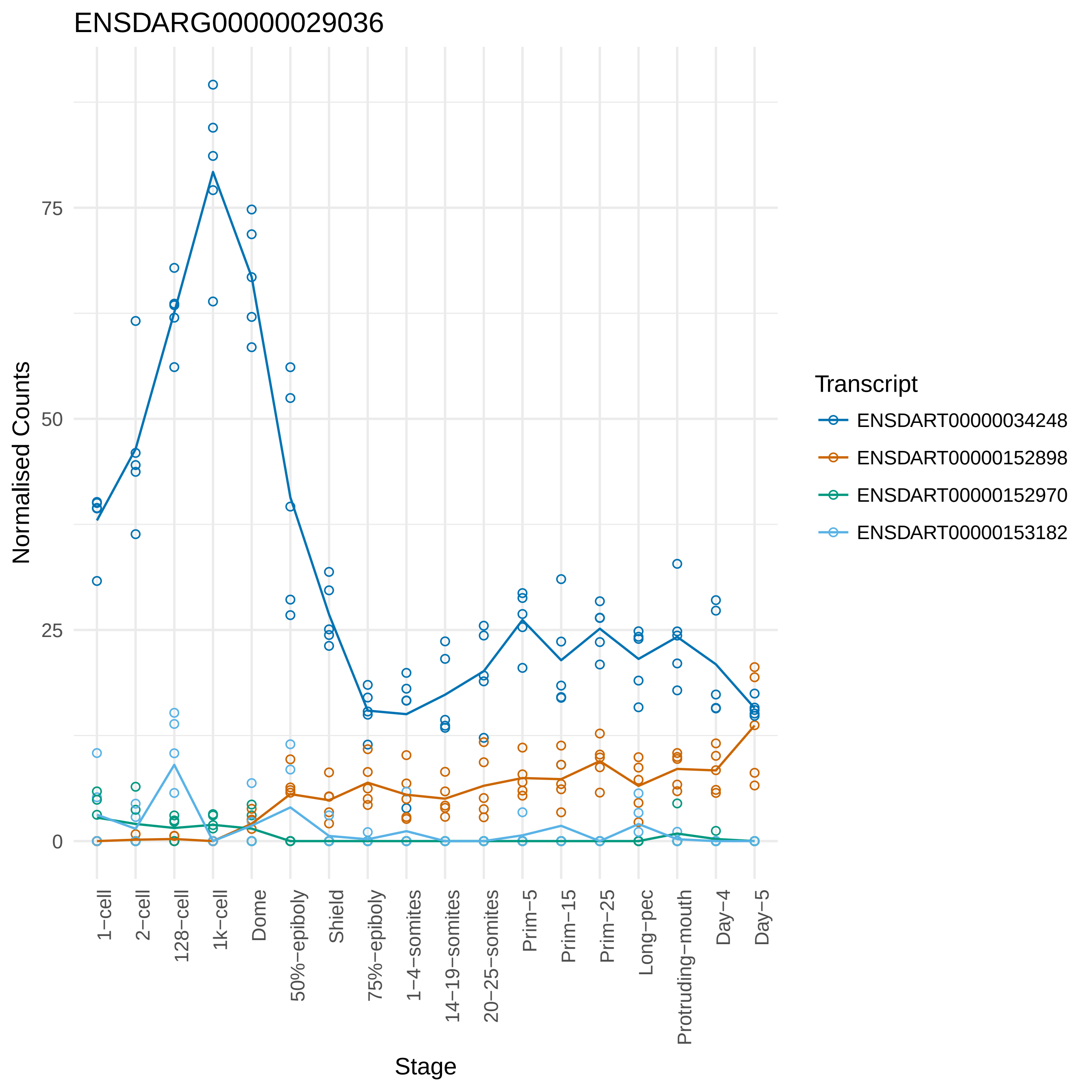
ENSDARG00000029036
Normalised Counts
75
50
25
0
1−cell
2−cell
128−cell
1k−cell
Dome
50%−epiboly
Shield
75%−epiboly
1−4−somites
14−19−somites
20−25−somites
Prim−5
Prim−15
Prim−25
Long−pec
Protruding−mouth
Day−4
Day−5
Stage
Transcript
ENSDART00000034248
ENSDART00000152898
ENSDART00000152970
ENSDART00000153182

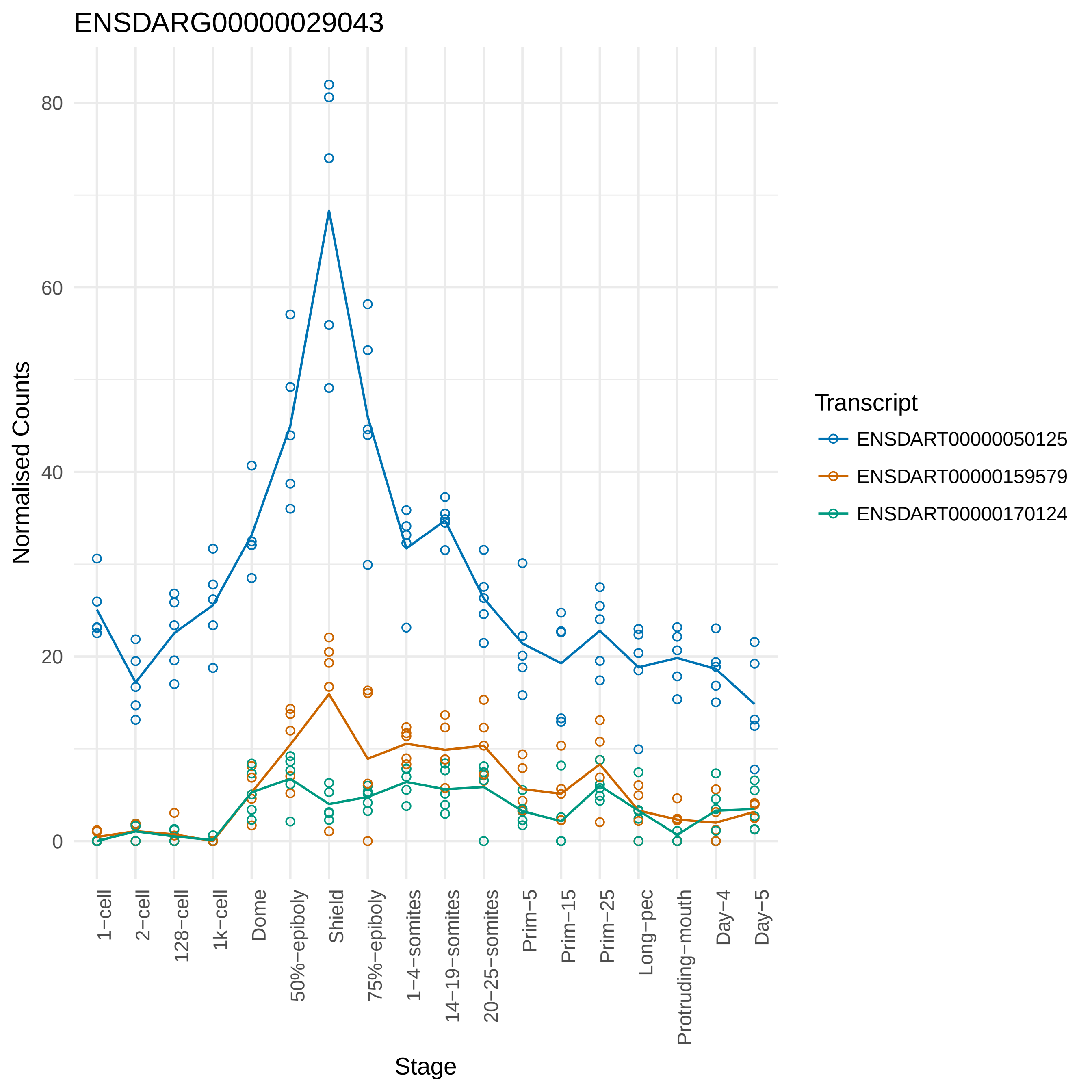
ENSDARG00000029043
Normalised Counts
0
20
40
60
80
1−cell
2−cell
128−cell
1k−cell
Dome
50%−epiboly
Shield
75%−epiboly
1−4−somites
14−19−somites
20−25−somites
Prim−5
Prim−15
Prim−25
Long−pec
Protruding−mouth
Day−4
Day−5
Stage
Transcript
ENSDART00000050125
ENSDART00000159579
ENSDART00000170124

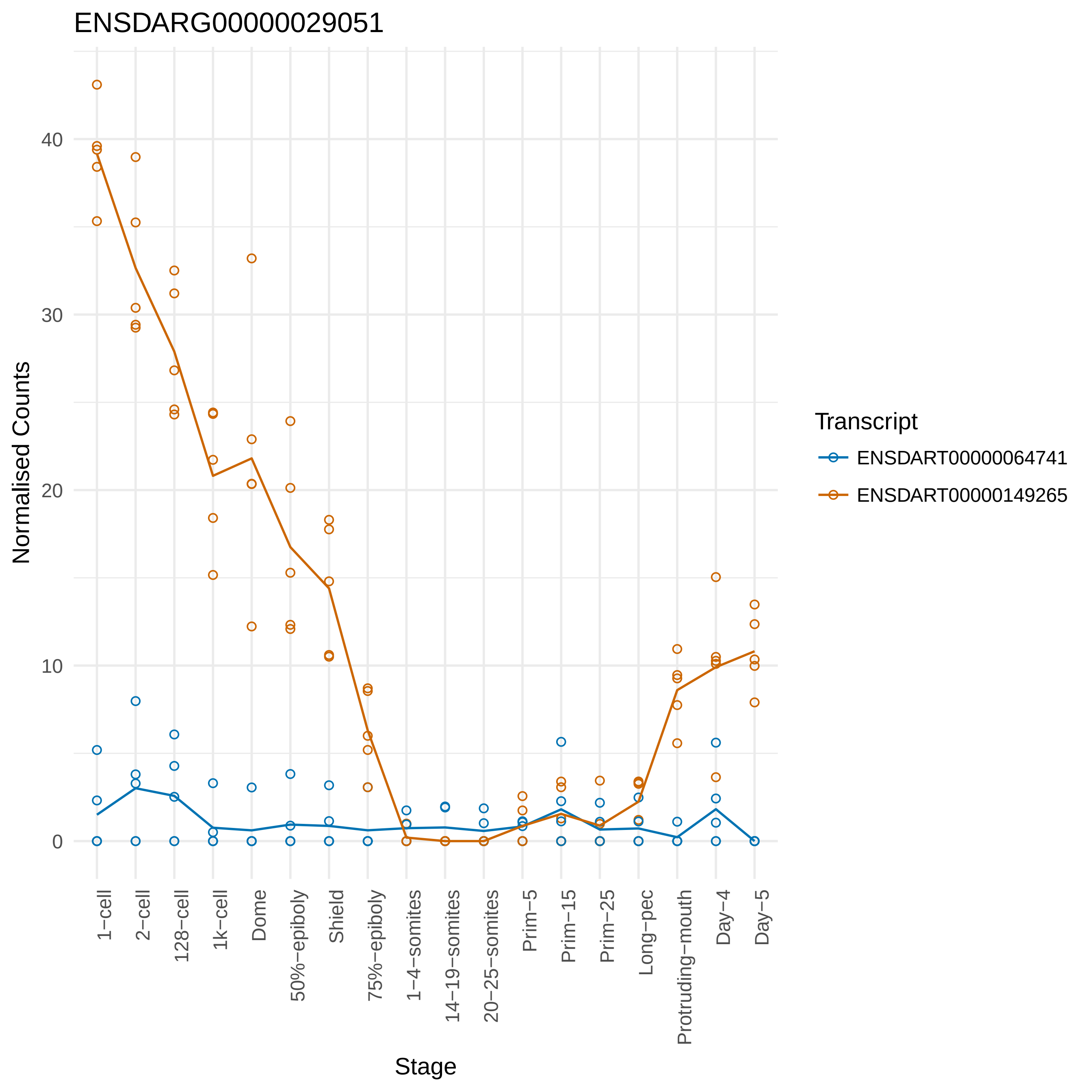
ENSDARG00000029051
Normalised Counts
0
10
20
30
40
1−cell
2−cell
128−cell
1k−cell
Dome
50%−epiboly
Shield
75%−epiboly
1−4−somites
14−19−somites
20−25−somites
Prim−5
Prim−15
Prim−25
Long−pec
Protruding−mouth
Day−4
Day−5
Stage
Transcript
ENSDART00000064741
ENSDART00000149265

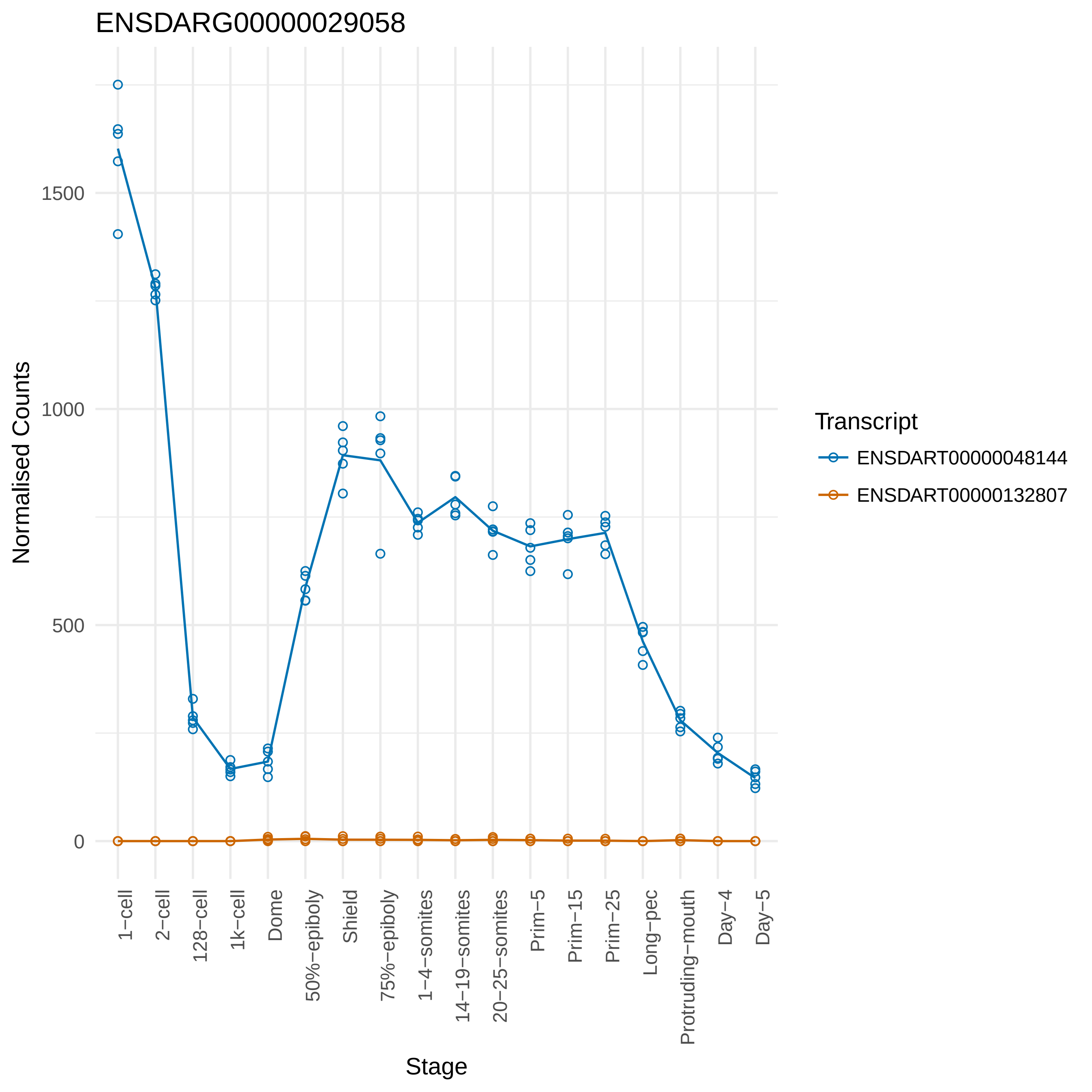
ENSDARG00000029058
Normalised Counts
1500
1000
500
0
1−cell
2−cell
128−cell
1k−cell
Dome
50%−epiboly
Shield
75%−epiboly
1−4−somites
14−19−somites
20−25−somites
Prim−5
Prim−15
Prim−25
Long−pec
Protruding−mouth
Day−4
Day−5
Stage
Transcript
ENSDART00000048144
ENSDART00000132807

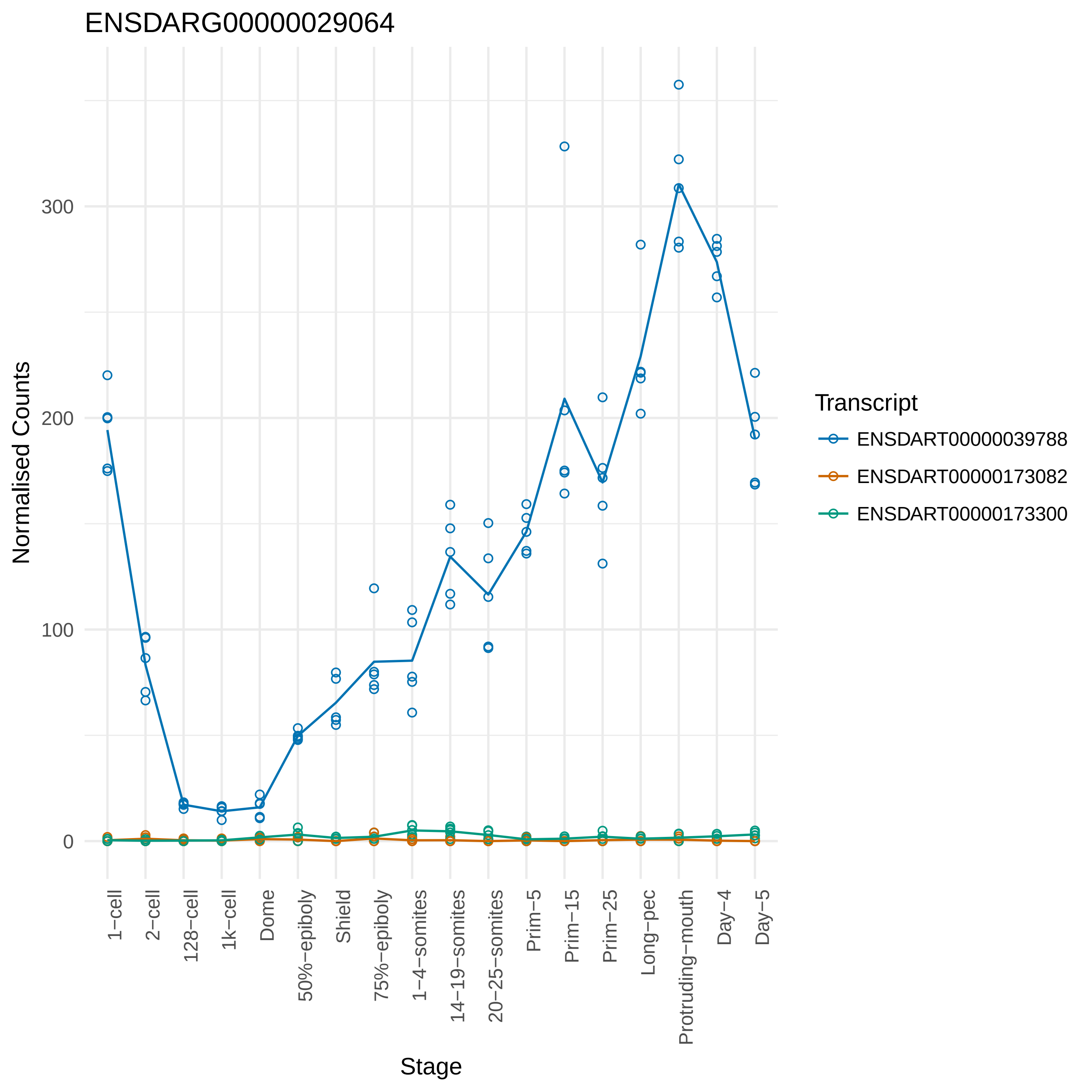
ENSDARG00000029064
Normalised Counts
300
200
100
0
1−cell
2−cell
128−cell
1k−cell
Dome
50%−epiboly
Shield
75%−epiboly
1−4−somites
14−19−somites
20−25−somites
Prim−5
Prim−15
Prim−25
Long−pec
Protruding−mouth
Day−4
Day−5
Stage
Transcript
ENSDART00000039788
ENSDART00000173082
ENSDART00000173300

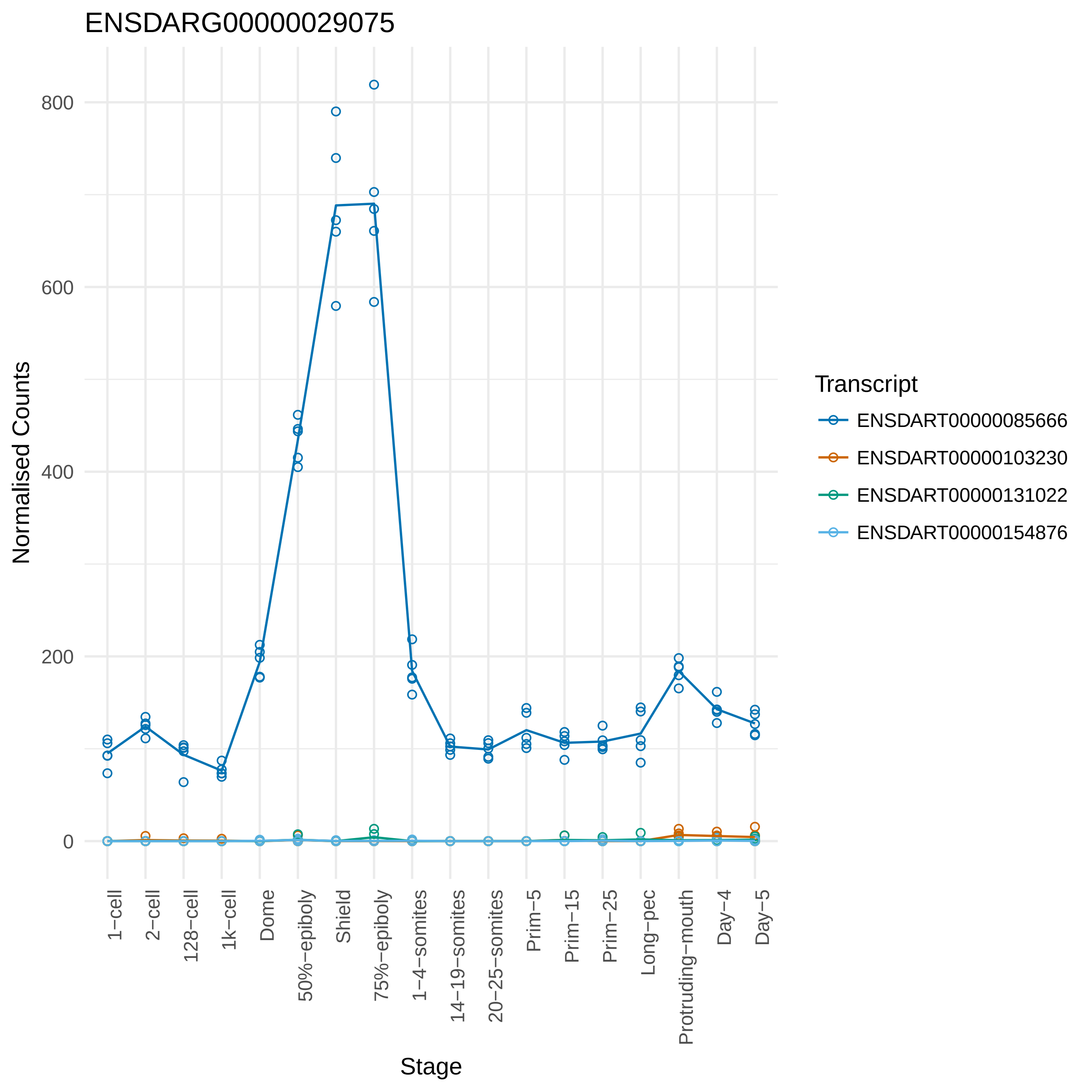

ENSDARG00000029075
Normalised Counts
800
600
400
200
0
1−cell
2−cell
128−cell
1k−cell
Dome
50%−epiboly
Shield
75%−epiboly
1−4−somites
14−19−somites
20−25−somites
Prim−5
Prim−15
Prim−25
Long−pec
Protruding−mouth
Day−4
Day−5
Stage
Transcript
ENSDART00000085666
ENSDART00000103230
ENSDART00000131022
ENSDART00000154876

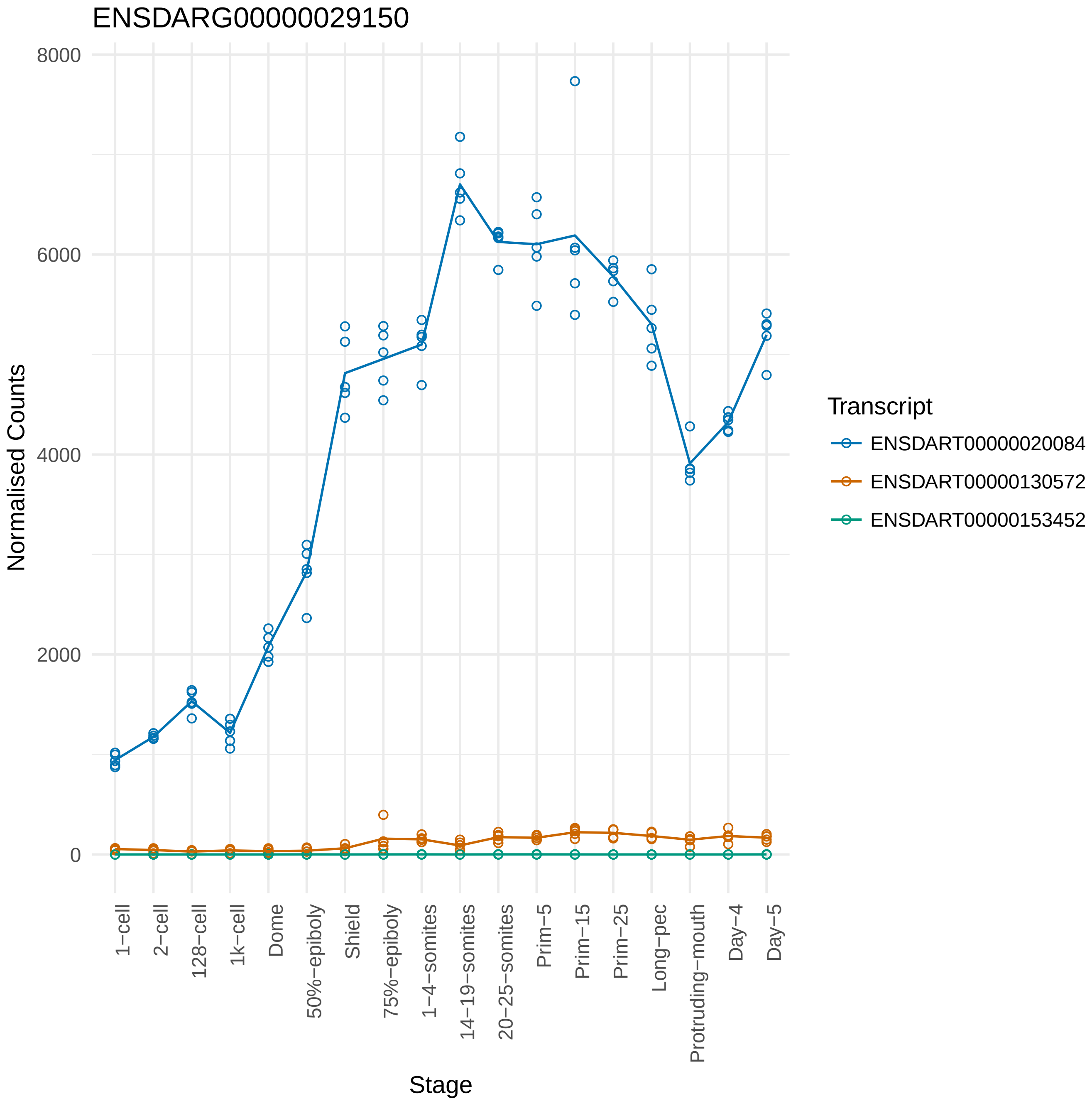
ENSDARG00000029150
Normalised Counts
8000
6000
4000
2000
0
1−cell
2−cell
128−cell
1k−cell
Dome
50%−epiboly
Shield
75%−epiboly
1−4−somites
14−19−somites
20−25−somites
Prim−5
Prim−15
Prim−25
Long−pec
Protruding−mouth
Day−4
Day−5
Stage
Transcript
ENSDART00000020084
ENSDART00000130572
ENSDART00000153452

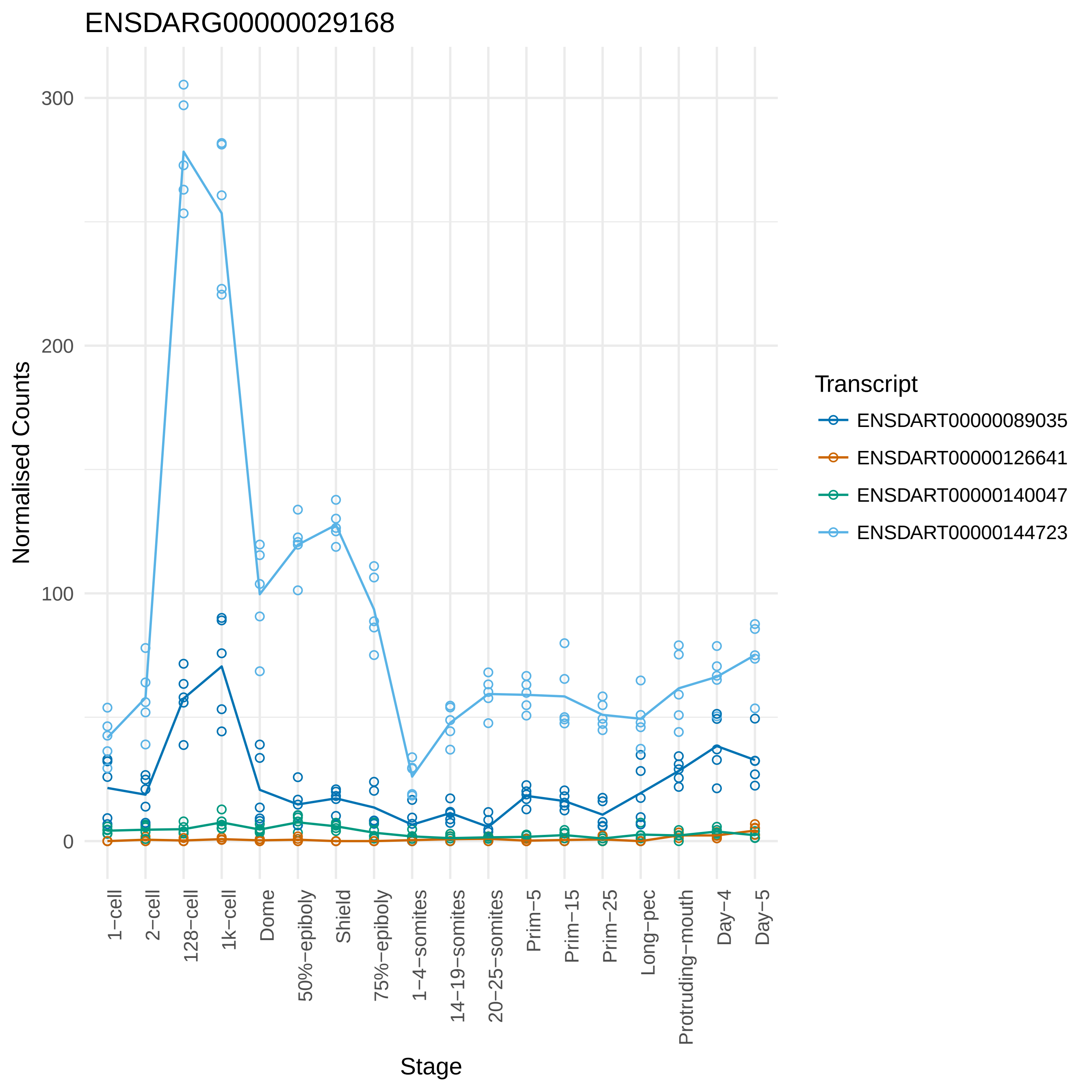
ENSDARG00000029168
Normalised Counts
300
200
100
0
1−cell
2−cell
128−cell
1k−cell
Dome
50%−epiboly
Shield
75%−epiboly
1−4−somites
14−19−somites
20−25−somites
Prim−5
Prim−15
Prim−25
Long−pec
Protruding−mouth
Day−4
Day−5
Stage
Transcript
ENSDART00000089035
ENSDART00000126641
ENSDART00000140047
ENSDART00000144723

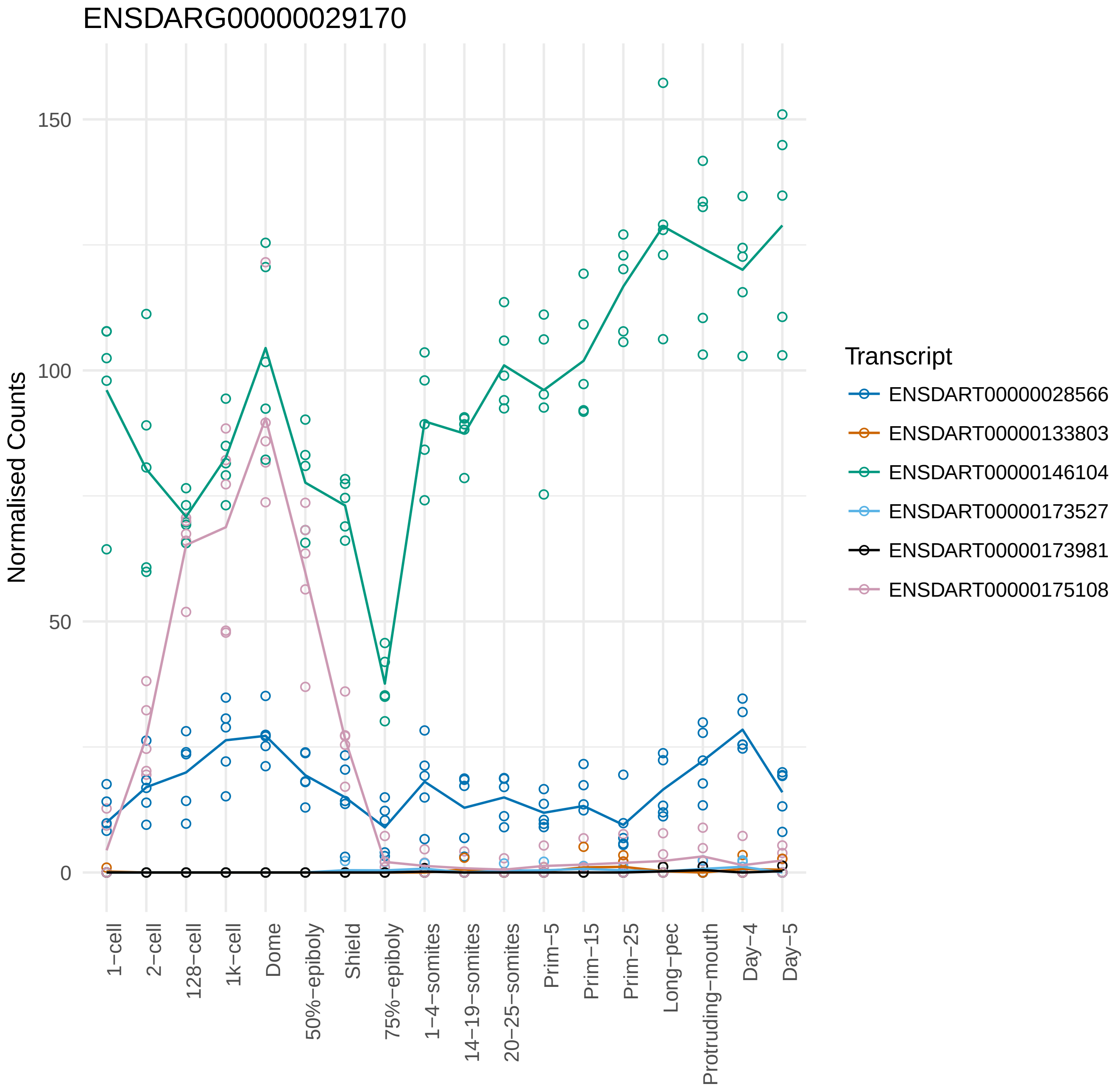

ENSDARG00000029170
Normalised Counts
150
100
50
0
1−cell
2−cell
128−cell
1k−cell
Dome
50%−epiboly
Shield
75%−epiboly
1−4−somites
14−19−somites
20−25−somites
Prim−5
Prim−15
Prim−25
Long−pec
Protruding−mouth
Day−4
Day−5
Stage
Transcript
ENSDART00000028566
ENSDART00000133803
ENSDART00000146104
ENSDART00000173527
ENSDART00000173981
ENSDART00000175108

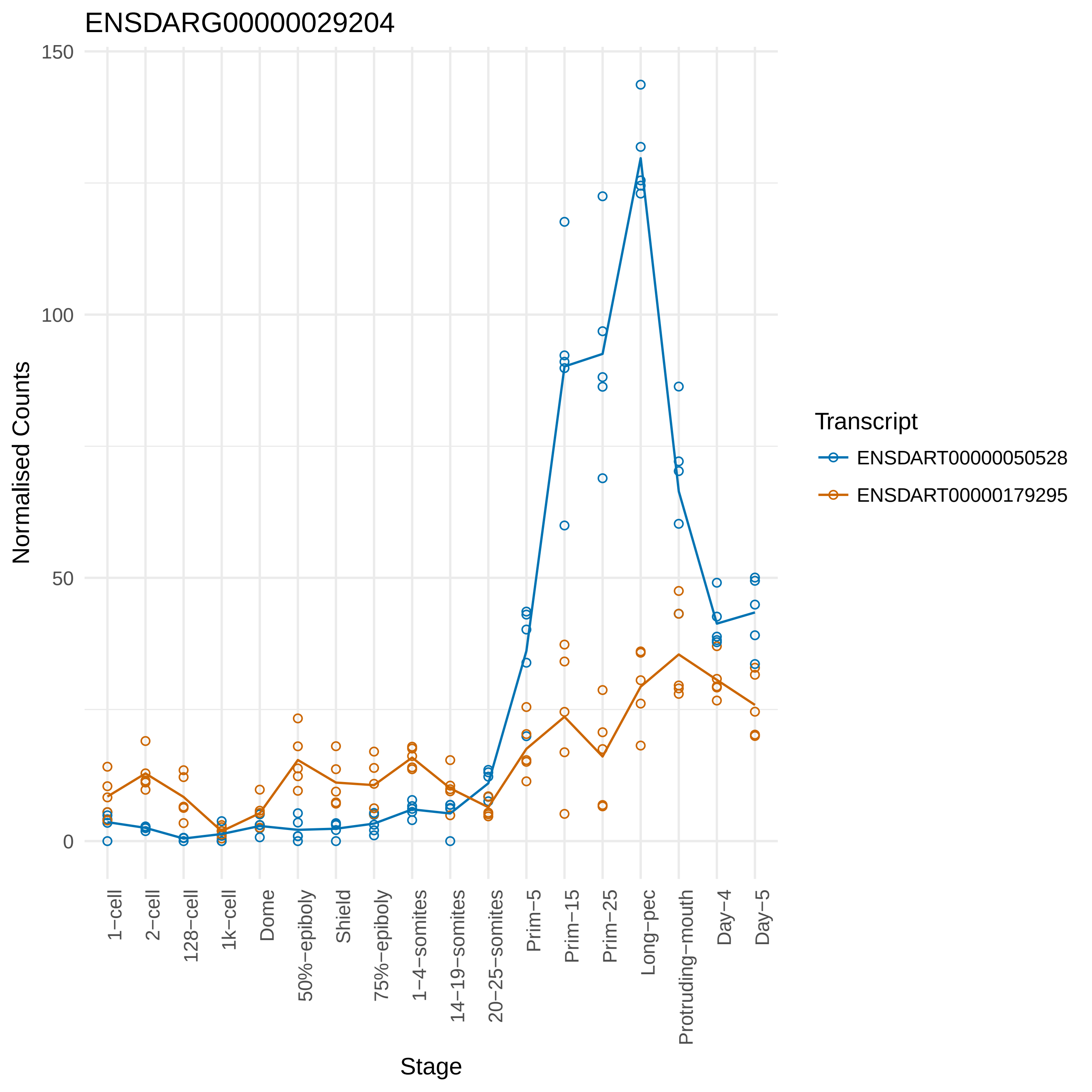
ENSDARG00000029204
150
100
50
0
Normalised Counts
1−cell
2−cell
128−cell
1k−cell
Dome
50%−epiboly
Shield
75%−epiboly
1−4−somites
14−19−somites
20−25−somites
Prim−5
Prim−15
Prim−25
Long−pec
Protruding−mouth
Day−4
Day−5
Stage
Transcript
ENSDART00000050528
ENSDART00000179295

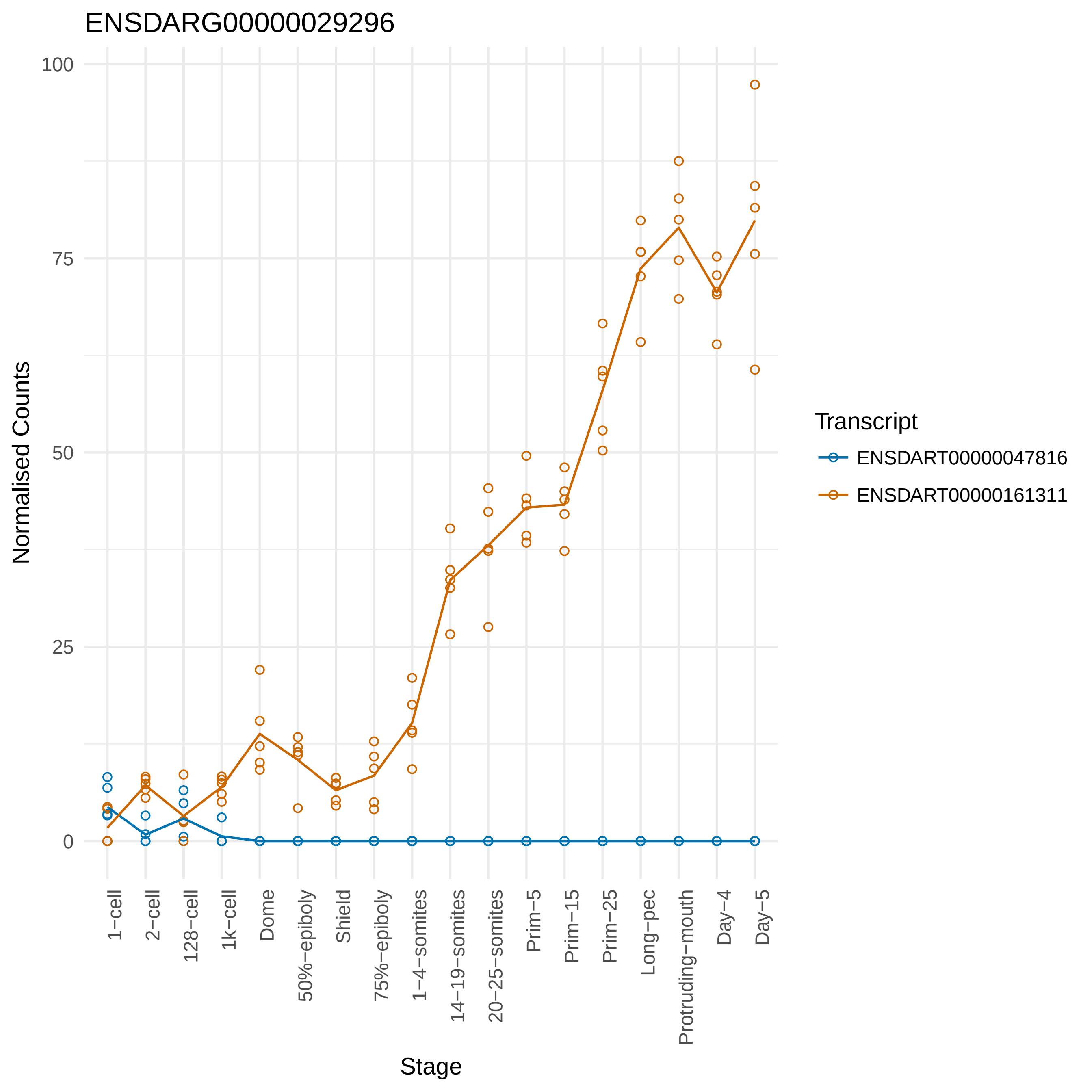
ENSDARG00000029296
Normalised Counts
100
75
50
25
0
1−cell
2−cell
128−cell
1k−cell
Dome
50%−epiboly
Shield
75%−epiboly
1−4−somites
14−19−somites
20−25−somites
Prim−5
Prim−15
Prim−25
Long−pec
Protruding−mouth
Day−4
Day−5
Stage
Transcript
ENSDART00000047816
ENSDART00000161311

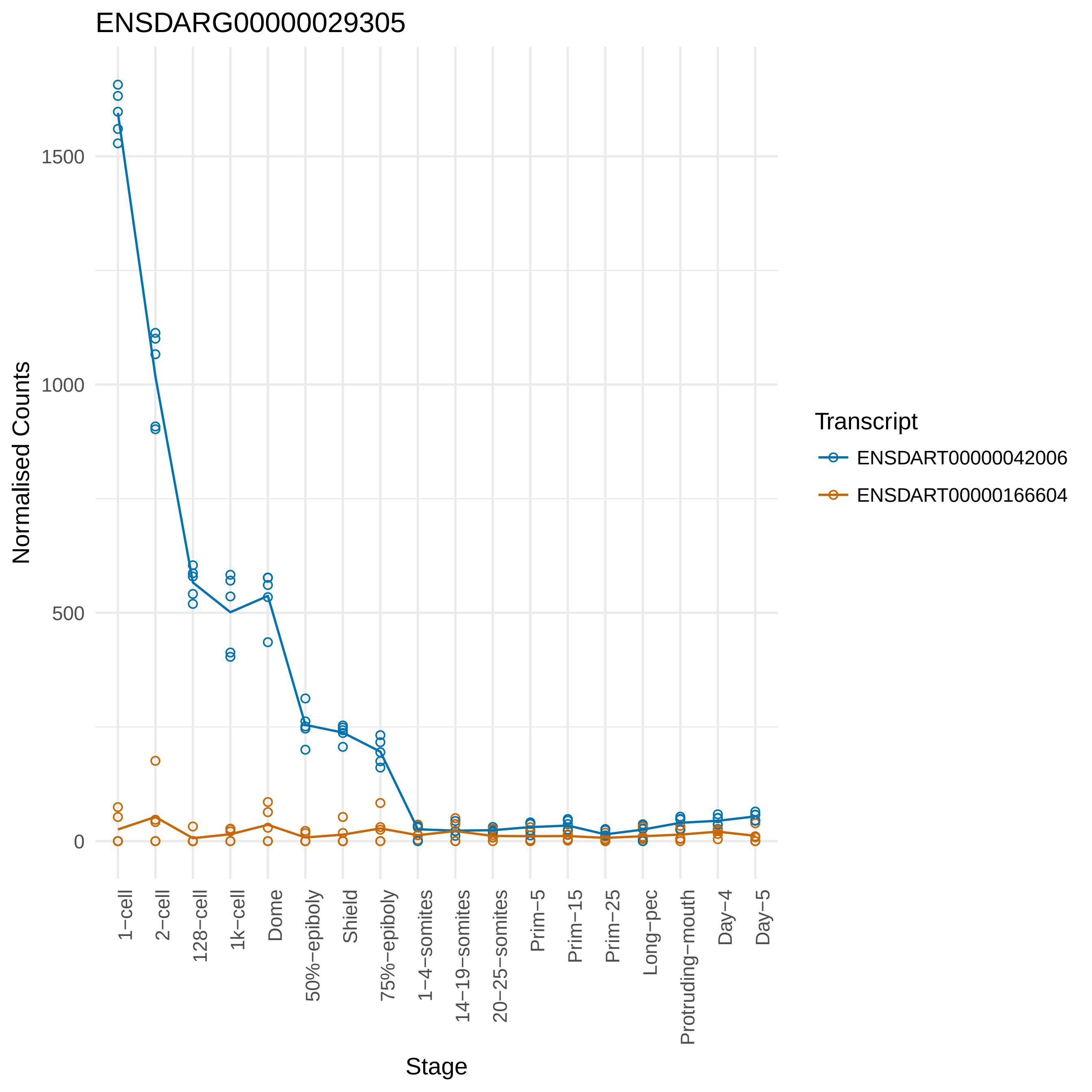

ENSDARG00000029305
Normalised Counts
1500
1000
500
0
1−cell
2−cell
128−cell
1k−cell
Dome
50%−epiboly
Shield
75%−epiboly
1−4−somites
14−19−somites
20−25−somites
Prim−5
Prim−15
Prim−25
Long−pec
Protruding−mouth
Day−4
Day−5
Stage
Transcript
ENSDART00000042006
ENSDART00000166604

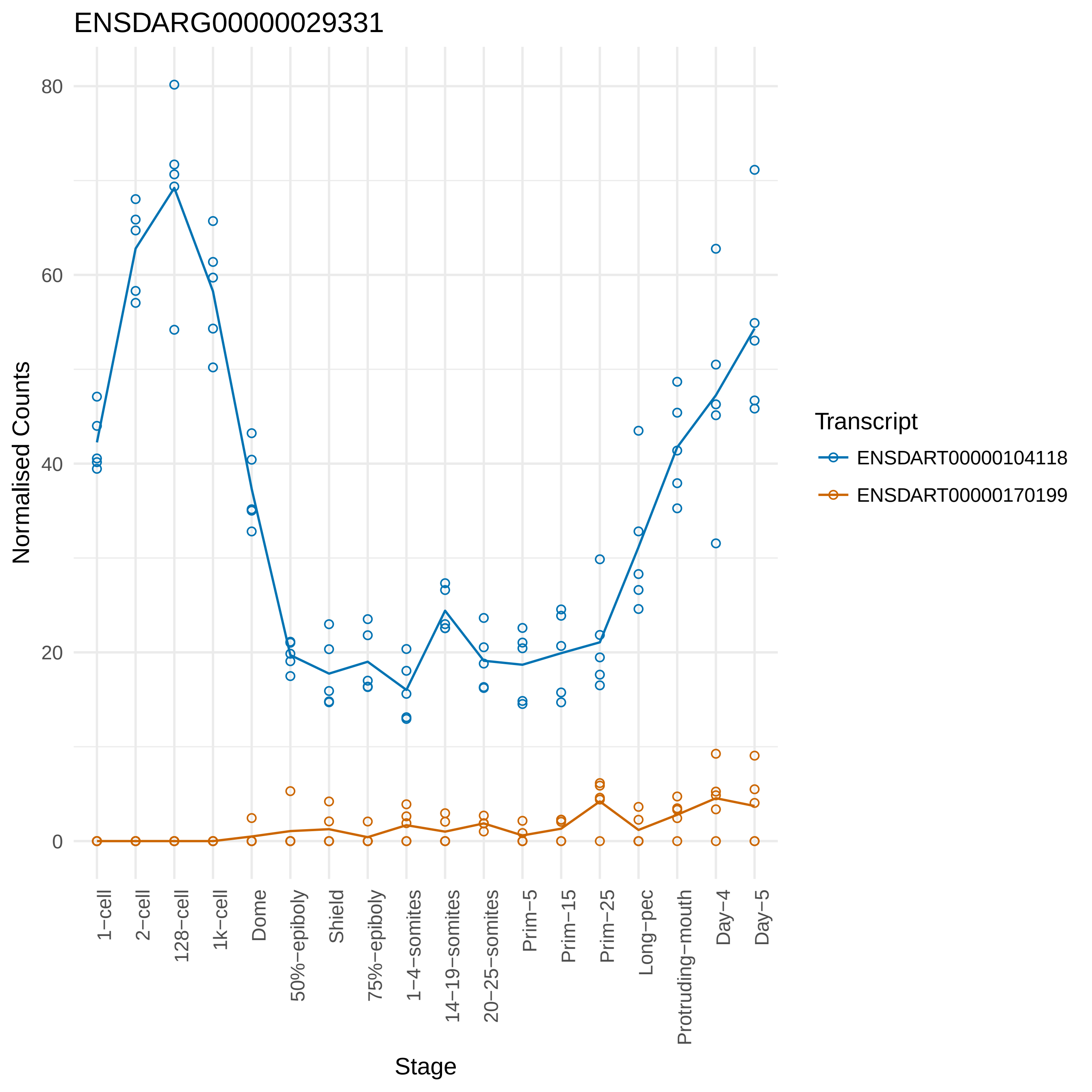
ENSDARG00000029331
Normalised Counts
80
60
40
20
0
1−cell
2−cell
128−cell
1k−cell
Dome
50%−epiboly
Shield
75%−epiboly
1−4−somites
14−19−somites
20−25−somites
Prim−5
Prim−15
Prim−25
Long−pec
Protruding−mouth
Day−4
Day−5
Stage
Transcript
ENSDART00000104118
ENSDART00000170199

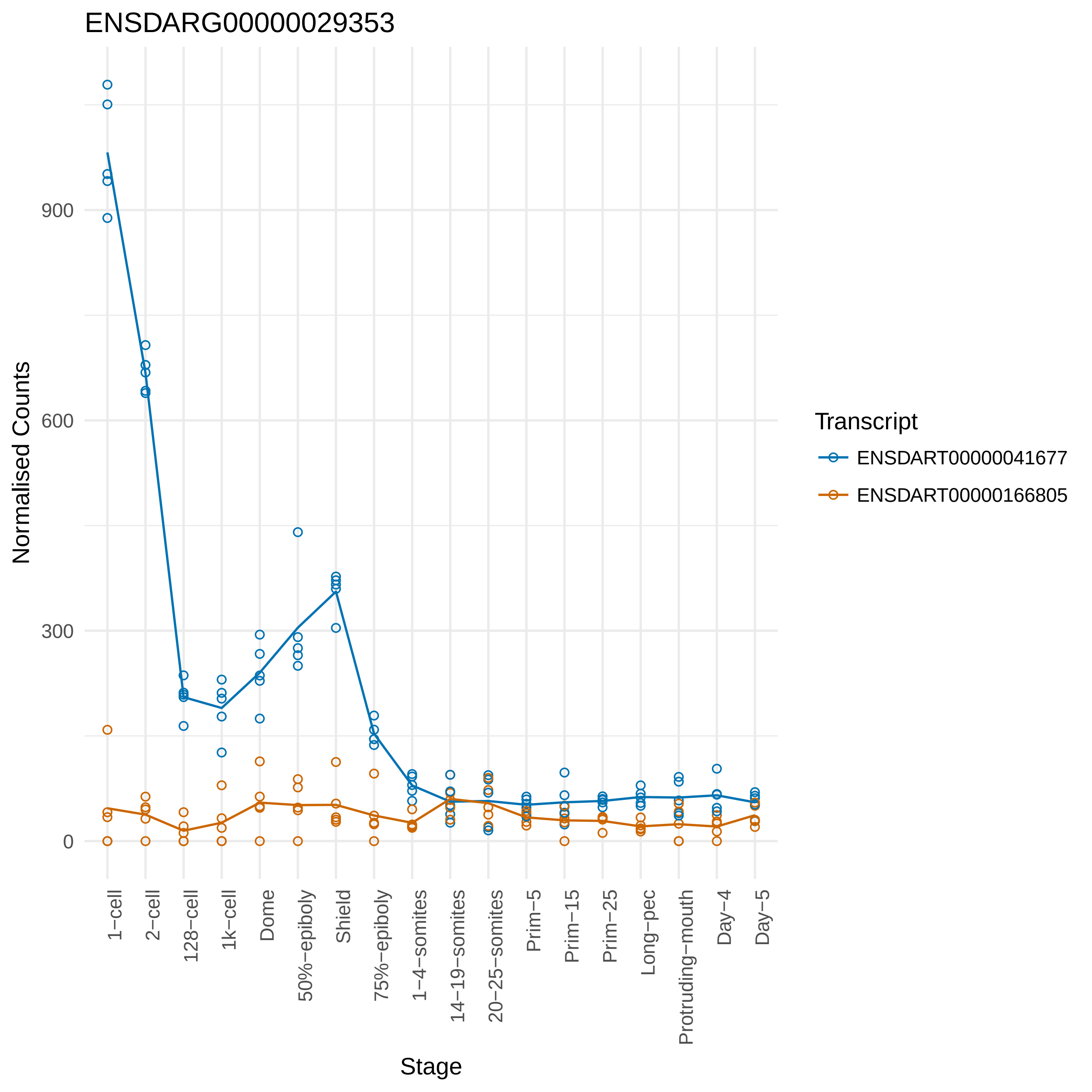

ENSDARG00000029353
Normalised Counts
900
600
300
0
1−cell
2−cell
128−cell
1k−cell
Dome
50%−epiboly
Shield
75%−epiboly
1−4−somites
14−19−somites
20−25−somites
Prim−5
Prim−15
Prim−25
Long−pec
Protruding−mouth
Day−4
Day−5
Stage
Transcript
ENSDART00000041677
ENSDART00000166805

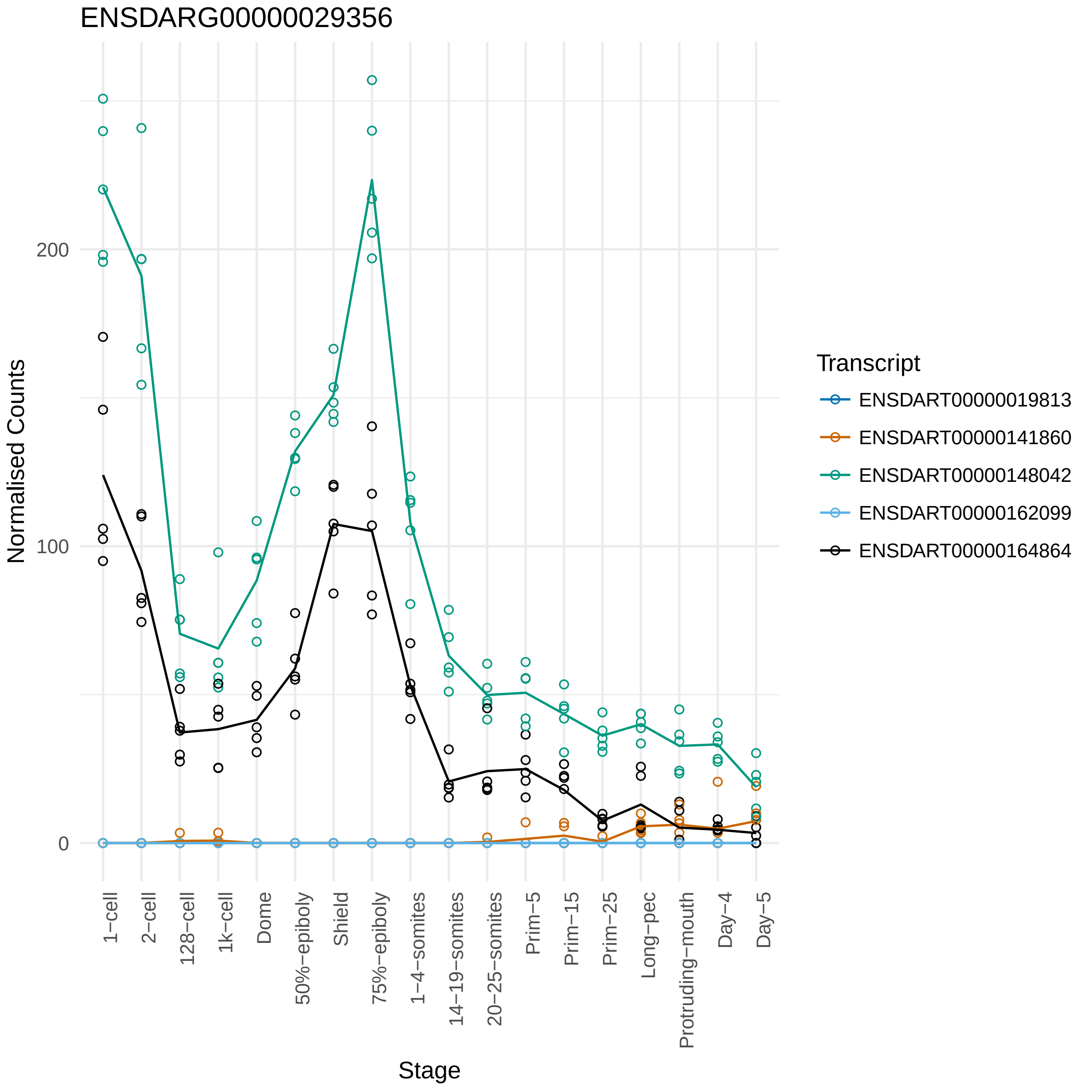
ENSDARG00000029356
Normalised Counts
0
100
200
Stage
1−cell
2−cell
128−cell
1k−cell
Dome
50%−epiboly
Shield
75%−epiboly
1−4−somites
14−19−somites
20−25−somites
Prim−5
Prim−15
Prim−25
Long−pec
Protruding−mouth
Day−4
Day−5
Transcript
ENSDART00000019813
ENSDART00000141860
ENSDART00000148042
ENSDART00000162099
ENSDART00000164864

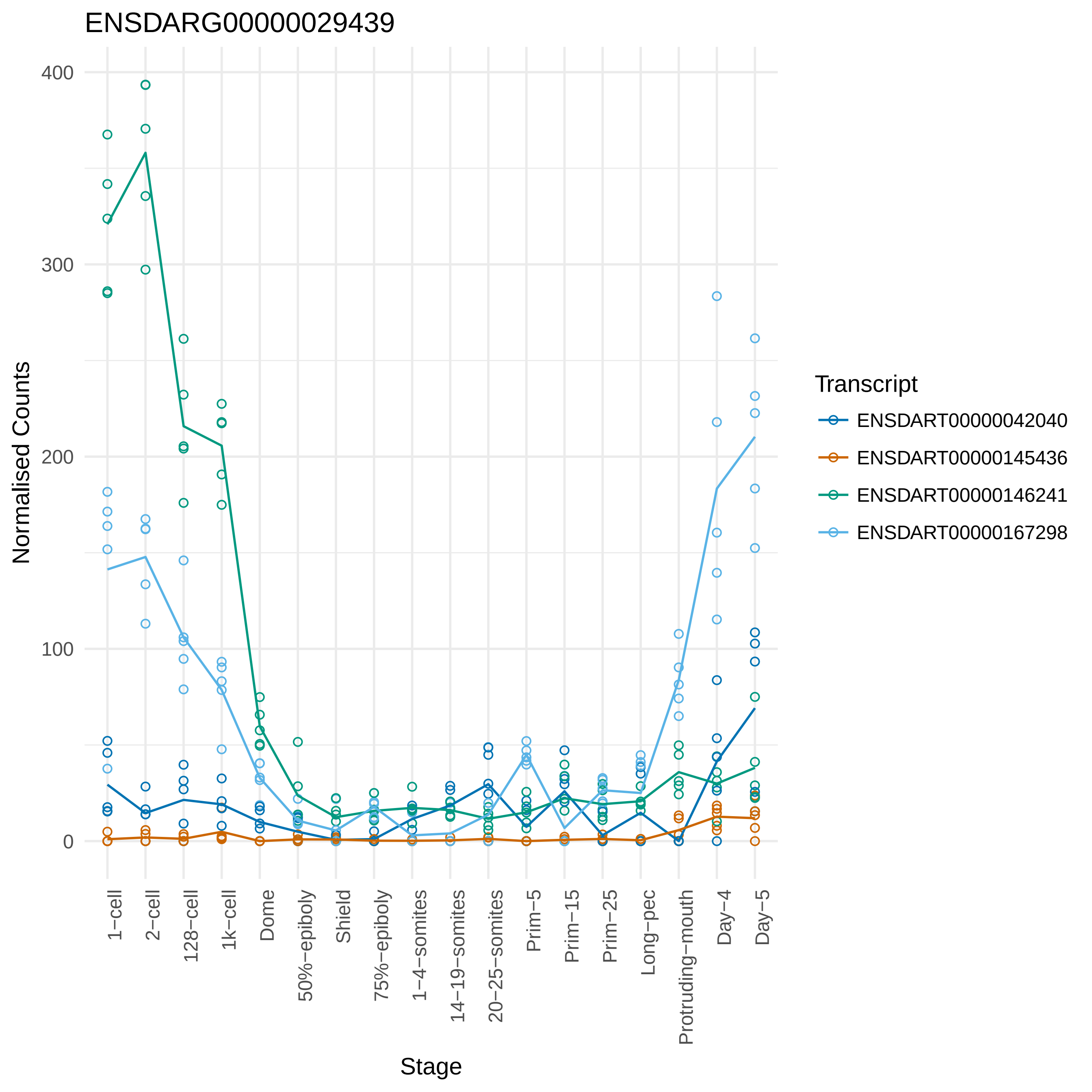

ENSDARG00000029439
Normalised Counts
400
300
200
100
0
1−cell
2−cell
128−cell
1k−cell
Dome
50%−epiboly
Shield
75%−epiboly
1−4−somites
14−19−somites
20−25−somites
Prim−5
Prim−15
Prim−25
Long−pec
Protruding−mouth
Day−4
Day−5
Stage
Transcript
ENSDART00000042040
ENSDART00000145436
ENSDART00000146241
ENSDART00000167298

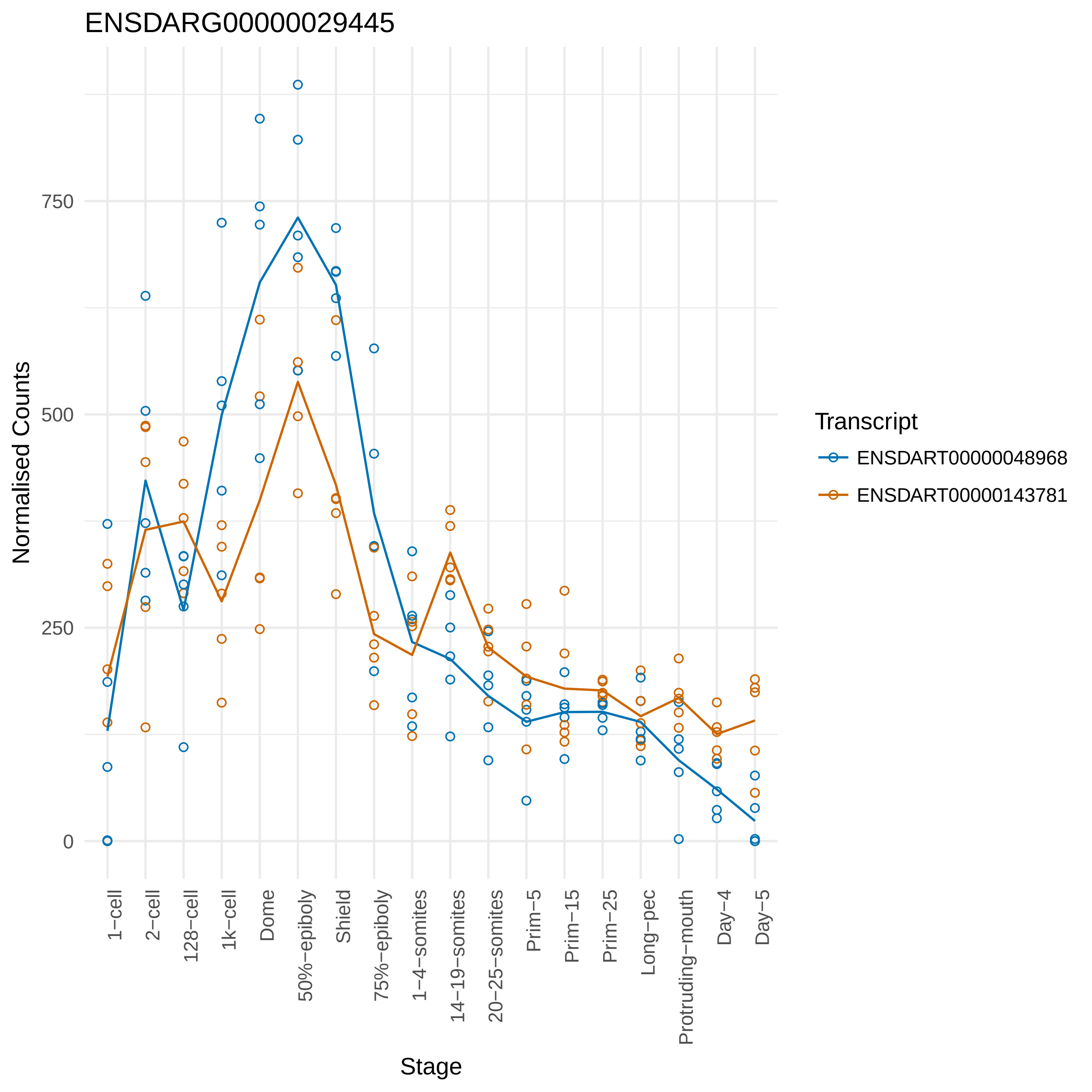
ENSDARG00000029445
Normalised Counts
750
500
250
0
1−cell
2−cell
128−cell
1k−cell
Dome
50%−epiboly
Shield
75%−epiboly
1−4−somites
14−19−somites
20−25−somites
Prim−5
Prim−15
Prim−25
Long−pec
Protruding−mouth
Day−4
Day−5
Stage
Transcript
ENSDART00000048968
ENSDART00000143781

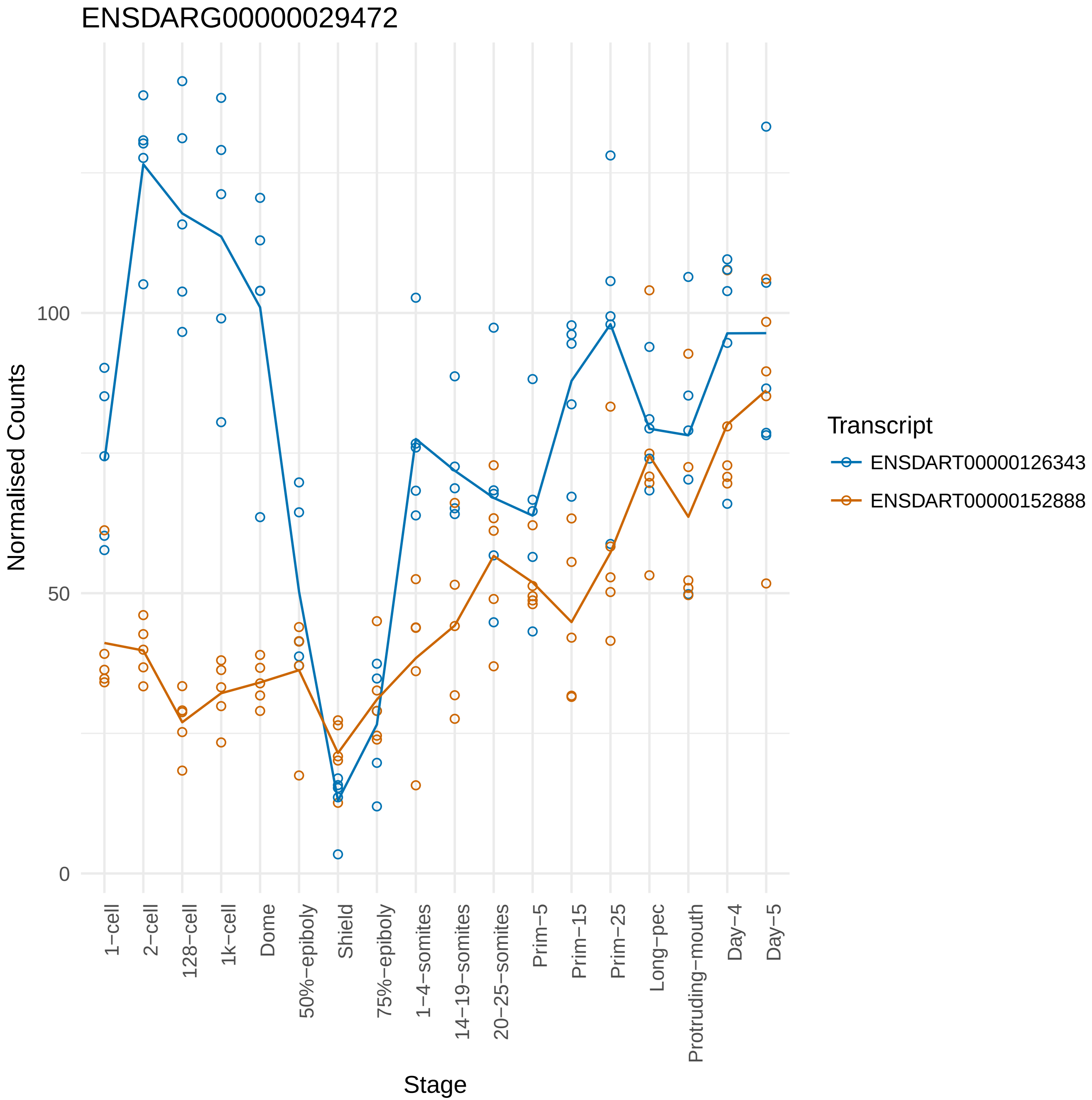

ENSDARG00000029472
Normalised Counts
100
50
0
1−cell
2−cell
128−cell
1k−cell
Dome
50%−epiboly
Shield
75%−epiboly
1−4−somites
14−19−somites
20−25−somites
Prim−5
Prim−15
Prim−25
Long−pec
Protruding−mouth
Day−4
Day−5
Stage
Transcript
ENSDART00000126343
ENSDART00000152888

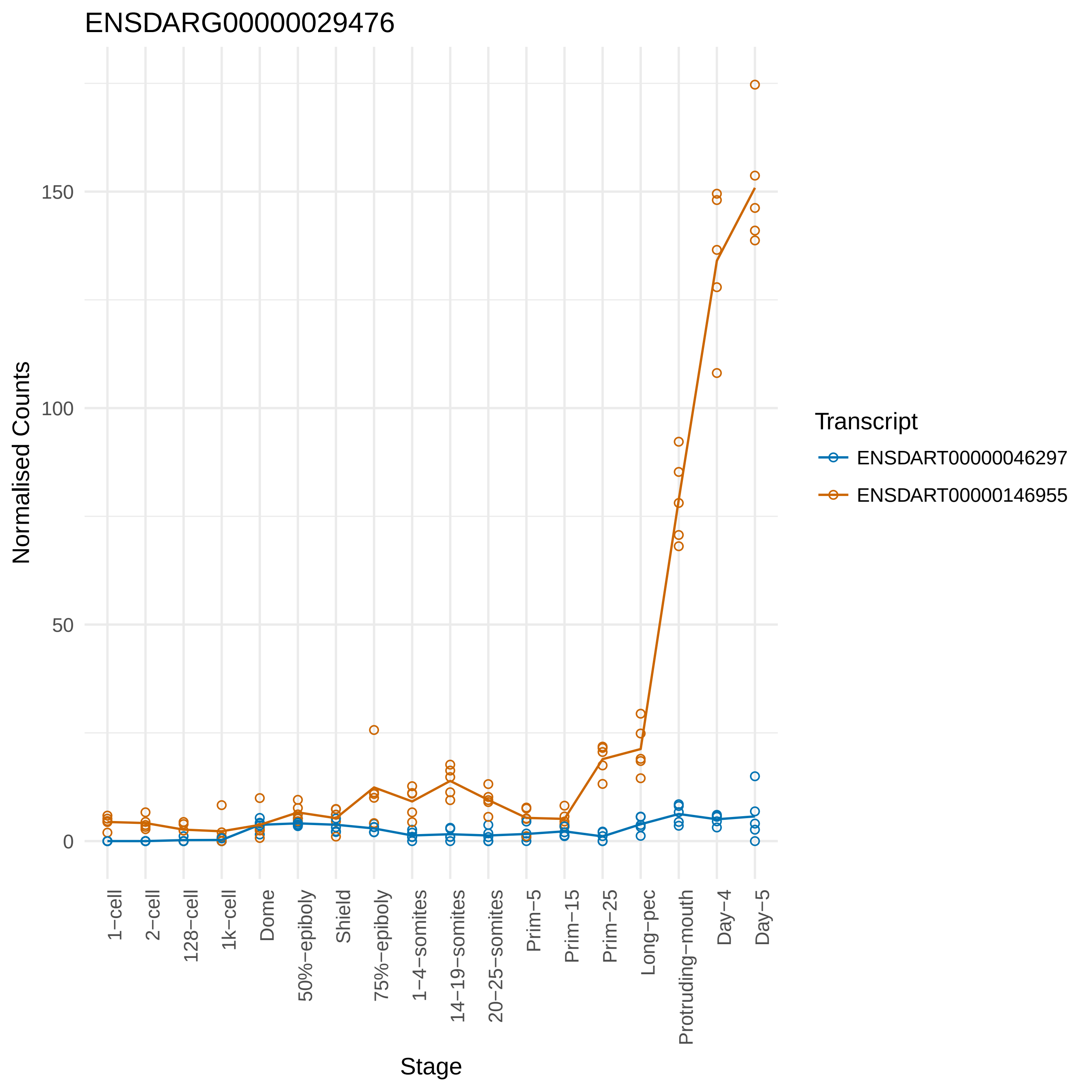
ENSDARG00000029476
Normalised Counts
150
100
50
0
1−cell
2−cell
128−cell
1k−cell
Dome
50%−epiboly
Shield
75%−epiboly
1−4−somites
14−19−somites
20−25−somites
Prim−5
Prim−15
Prim−25
Long−pec
Protruding−mouth
Day−4
Day−5
Stage
Transcript
ENSDART00000046297
ENSDART00000146955

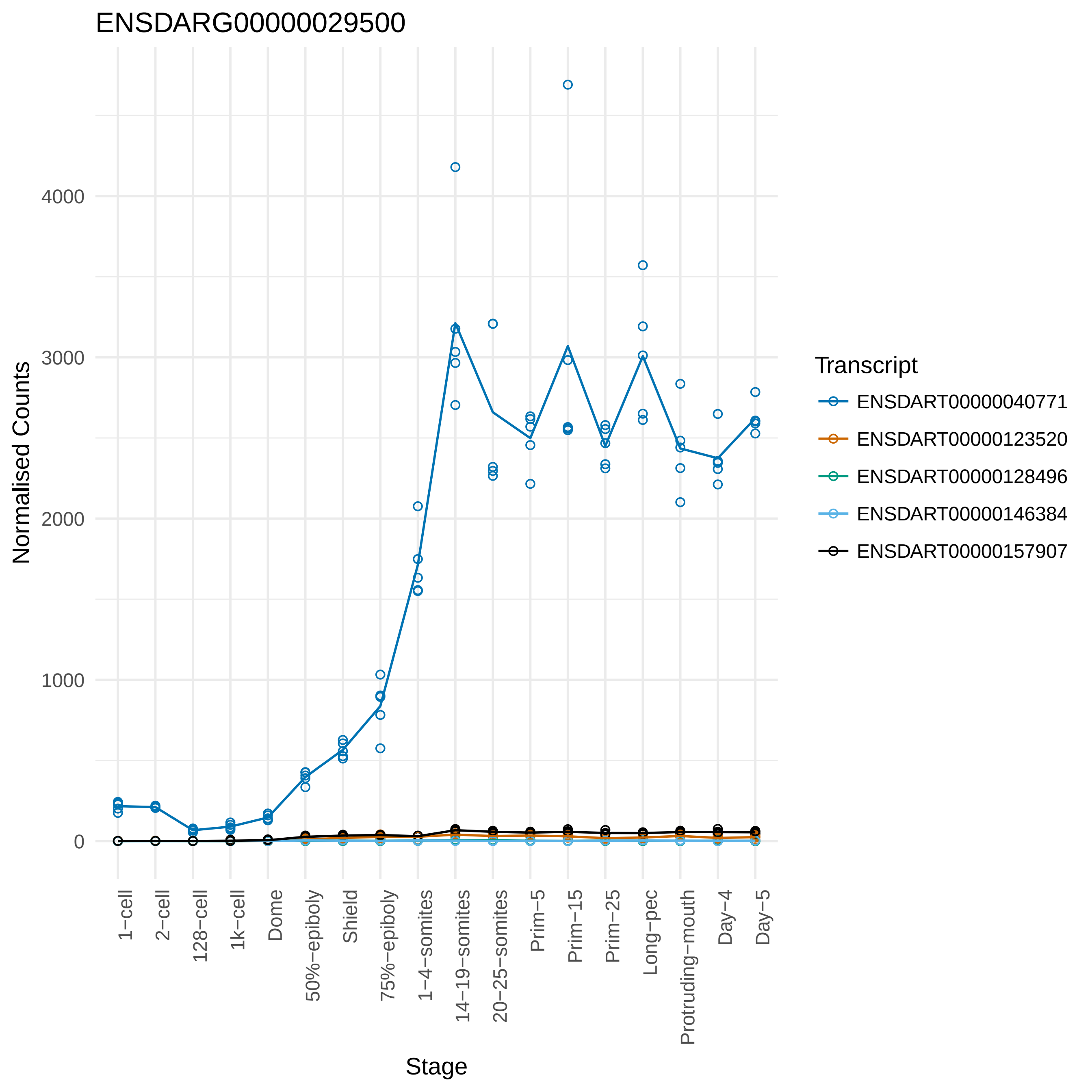
ENSDARG00000029500
Normalised Counts
4000
3000
2000
1000
0
1−cell
2−cell
128−cell
1k−cell
Dome
50%−epiboly
Shield
75%−epiboly
1−4−somites
14−19−somites
20−25−somites
Prim−5
Prim−15
Prim−25
Long−pec
Protruding−mouth
Day−4
Day−5
Stage
Transcript
ENSDART00000040771
ENSDART00000123520
ENSDART00000128496
ENSDART00000146384
ENSDART00000157907

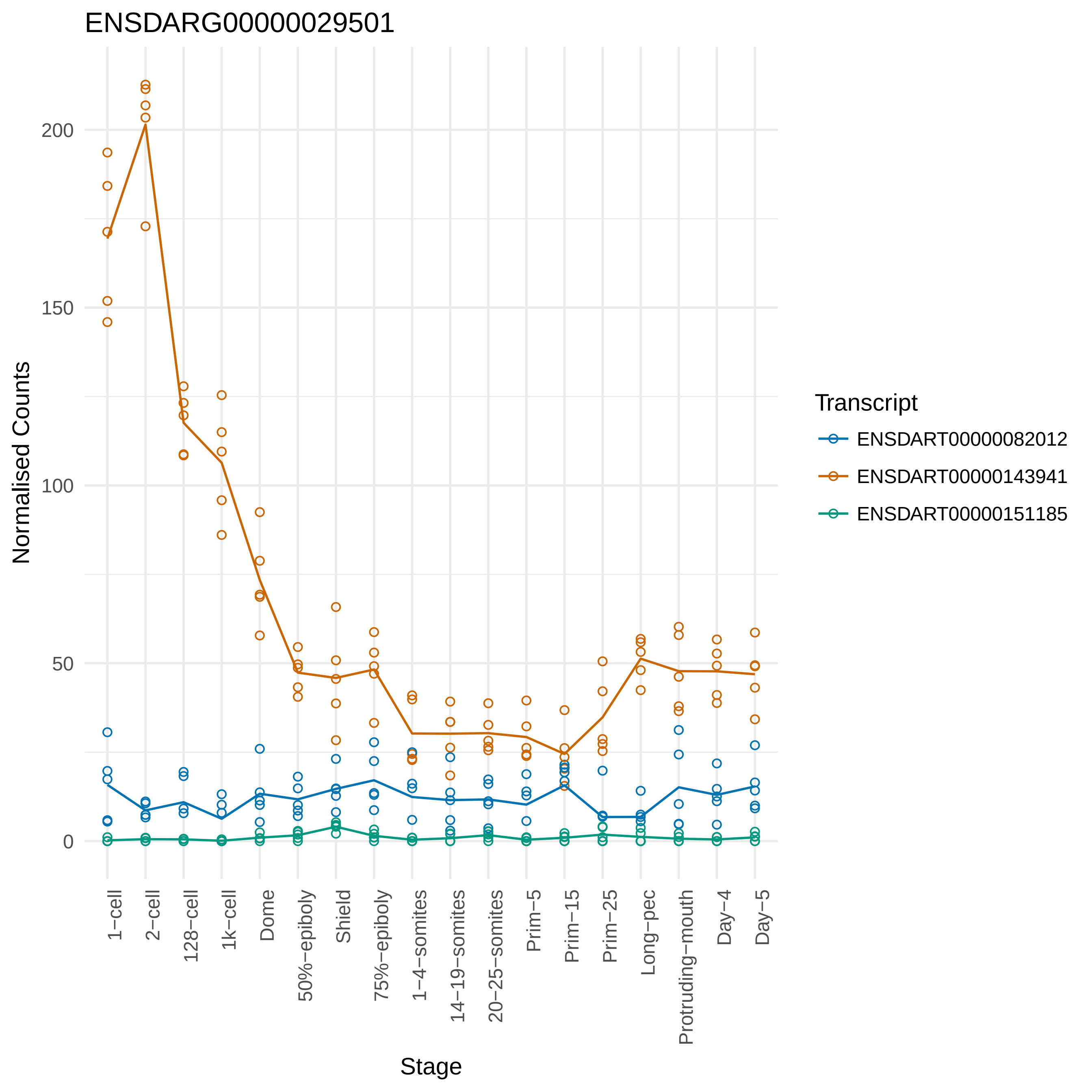
ENSDARG00000029501
Normalised Counts
0
50
100
150
200
Stage
1−cell
2−cell
128−cell
1k−cell
Dome
50%−epiboly
Shield
75%−epiboly
1−4−somites
14−19−somites
20−25−somites
Prim−5
Prim−15
Prim−25
Long−pec
Protruding−mouth
Day−4
Day−5
Transcript
ENSDART00000082012
ENSDART00000143941
ENSDART00000151185

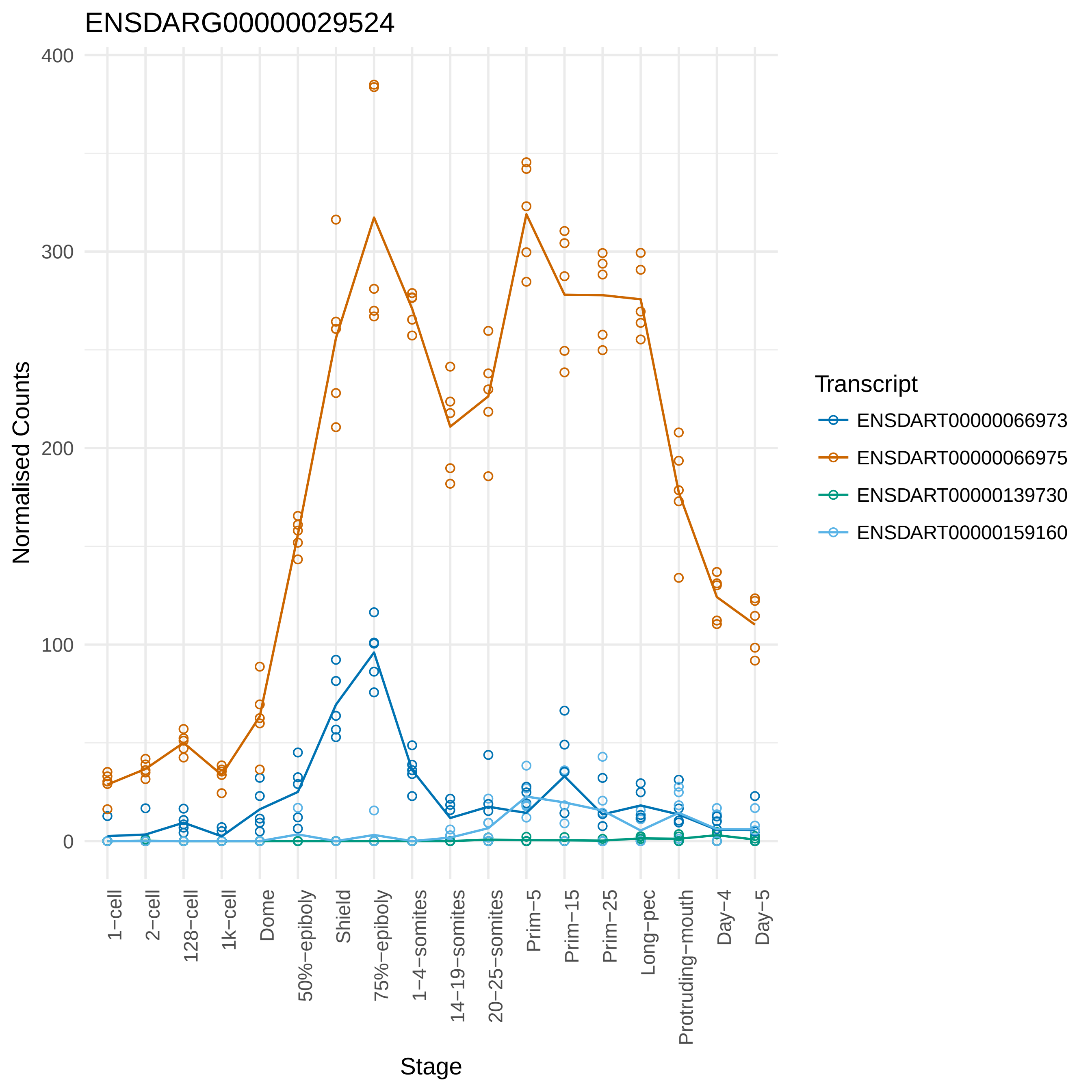
ENSDARG00000029524
400
300
200
100
0
Normalised Counts
1−cell
2−cell
128−cell
1k−cell
Dome
50%−epiboly
Shield
75%−epiboly
1−4−somites
14−19−somites
20−25−somites
Prim−5
Prim−15
Prim−25
Long−pec
Protruding−mouth
Day−4
Day−5
Stage
Transcript
ENSDART00000066973
ENSDART00000066975
ENSDART00000139730
ENSDART00000159160

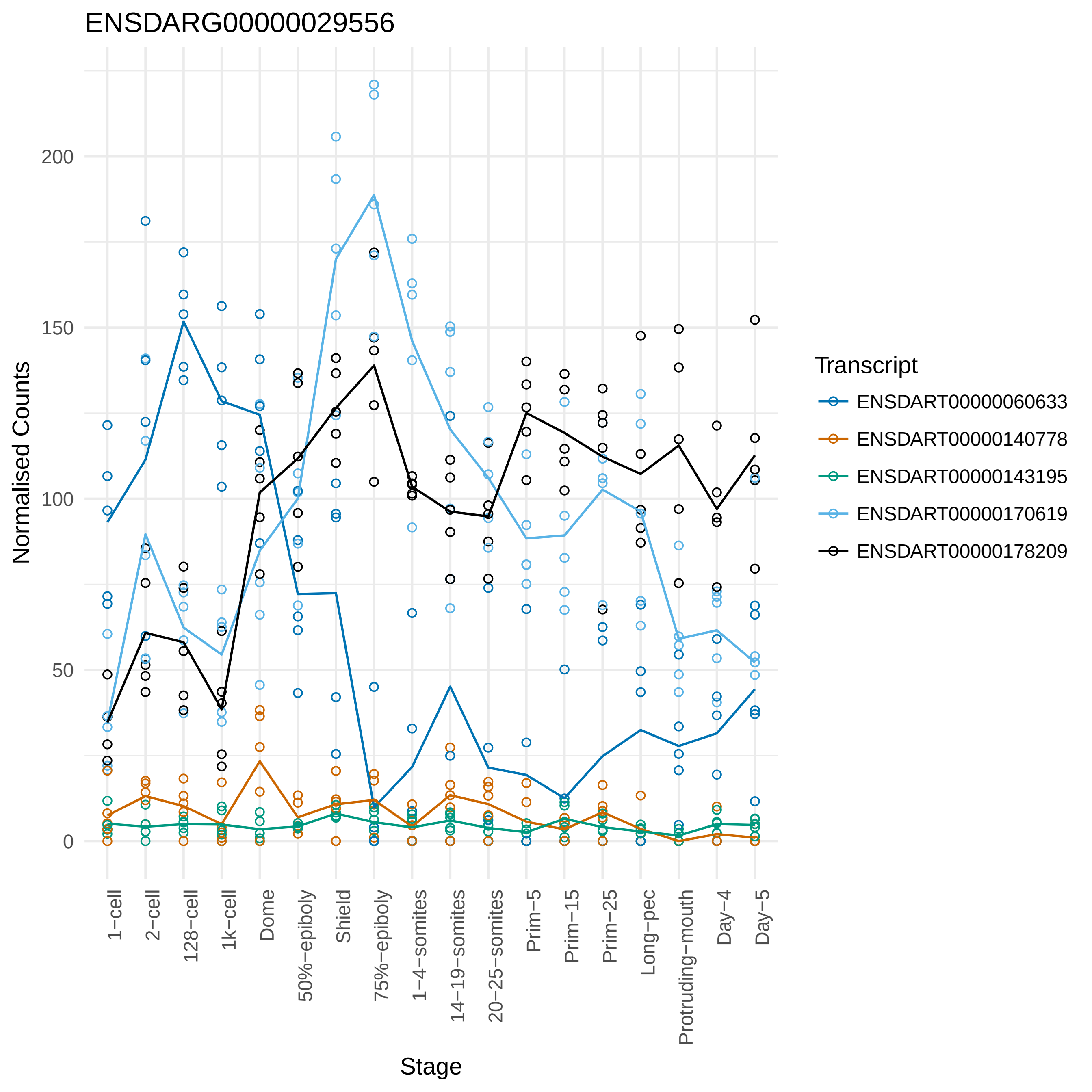

ENSDARG00000029556
Normalised Counts
0
50
100
150
200
Stage
1−cell
2−cell
128−cell
1k−cell
Dome
50%−epiboly
Shield
75%−epiboly
1−4−somites
14−19−somites
20−25−somites
Prim−5
Prim−15
Prim−25
Long−pec
Protruding−mouth
Day−4
Day−5
Transcript
ENSDART00000060633
ENSDART00000140778
ENSDART00000143195
ENSDART00000170619
ENSDART00000178209

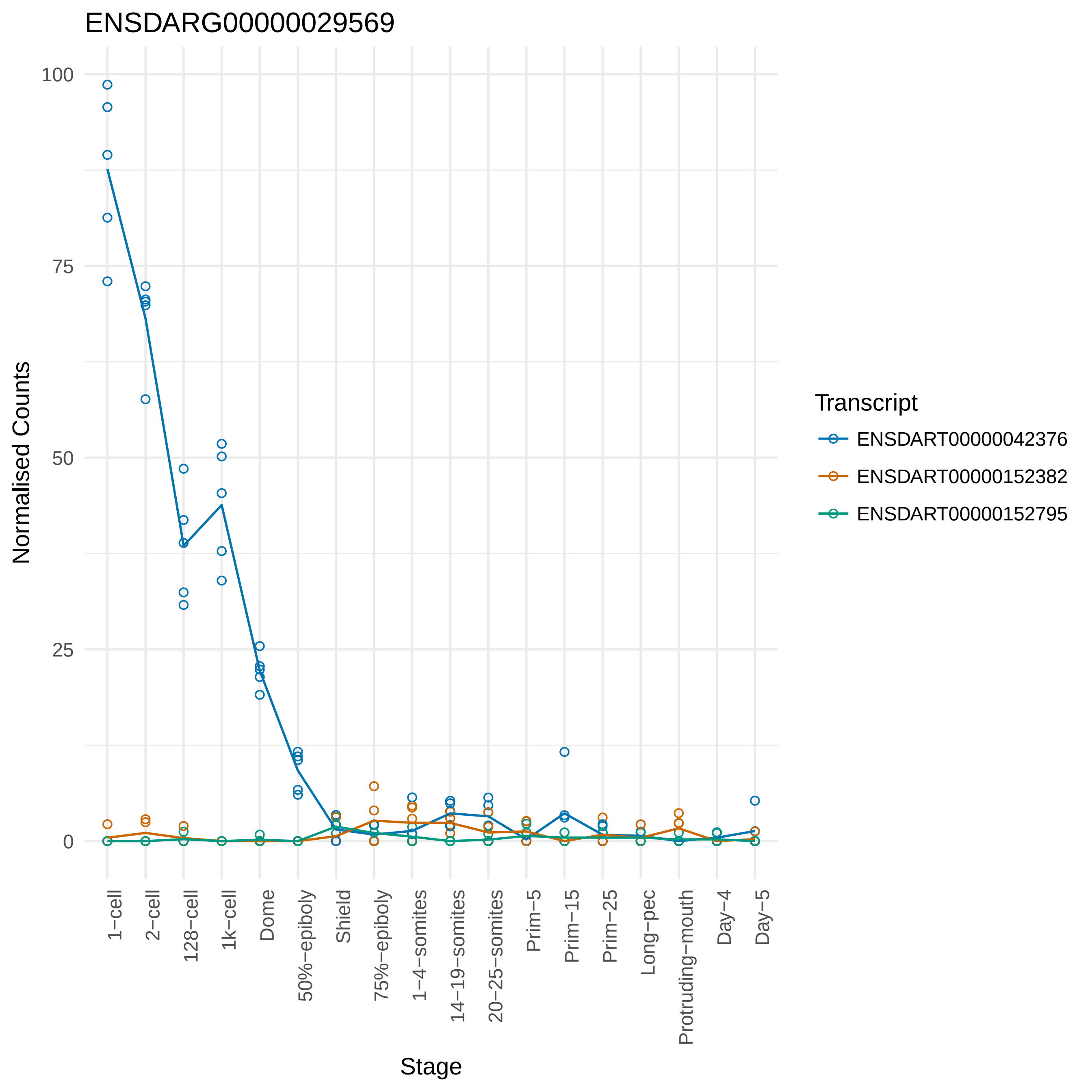

ENSDARG00000029569
Normalised Counts
100
75
50
25
0
1−cell
2−cell
128−cell
1k−cell
Dome
50%−epiboly
Shield
75%−epiboly
1−4−somites
14−19−somites
20−25−somites
Prim−5
Prim−15
Prim−25
Long−pec
Protruding−mouth
Day−4
Day−5
Stage
Transcript
ENSDART00000042376
ENSDART00000152382
ENSDART00000152795

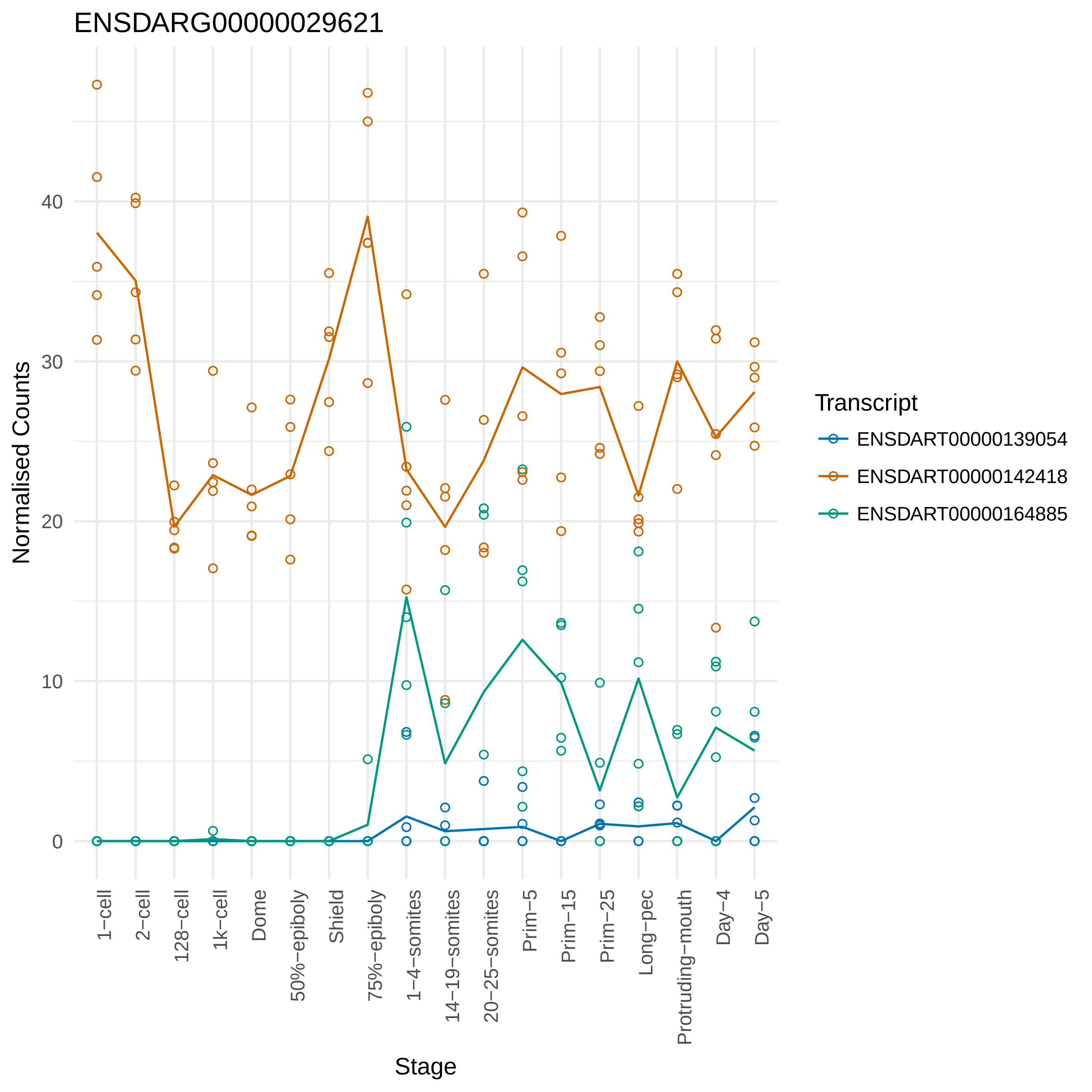
ENSDARG00000029621
Normalised Counts
40
30
20
10
0
1−cell
2−cell
128−cell
1k−cell
Dome
50%−epiboly
Shield
75%−epiboly
1−4−somites
14−19−somites
20−25−somites
Prim−5
Prim−15
Prim−25
Long−pec
Protruding−mouth
Day−4
Day−5
Stage
Transcript
ENSDART00000139054
ENSDART00000142418
ENSDART00000164885

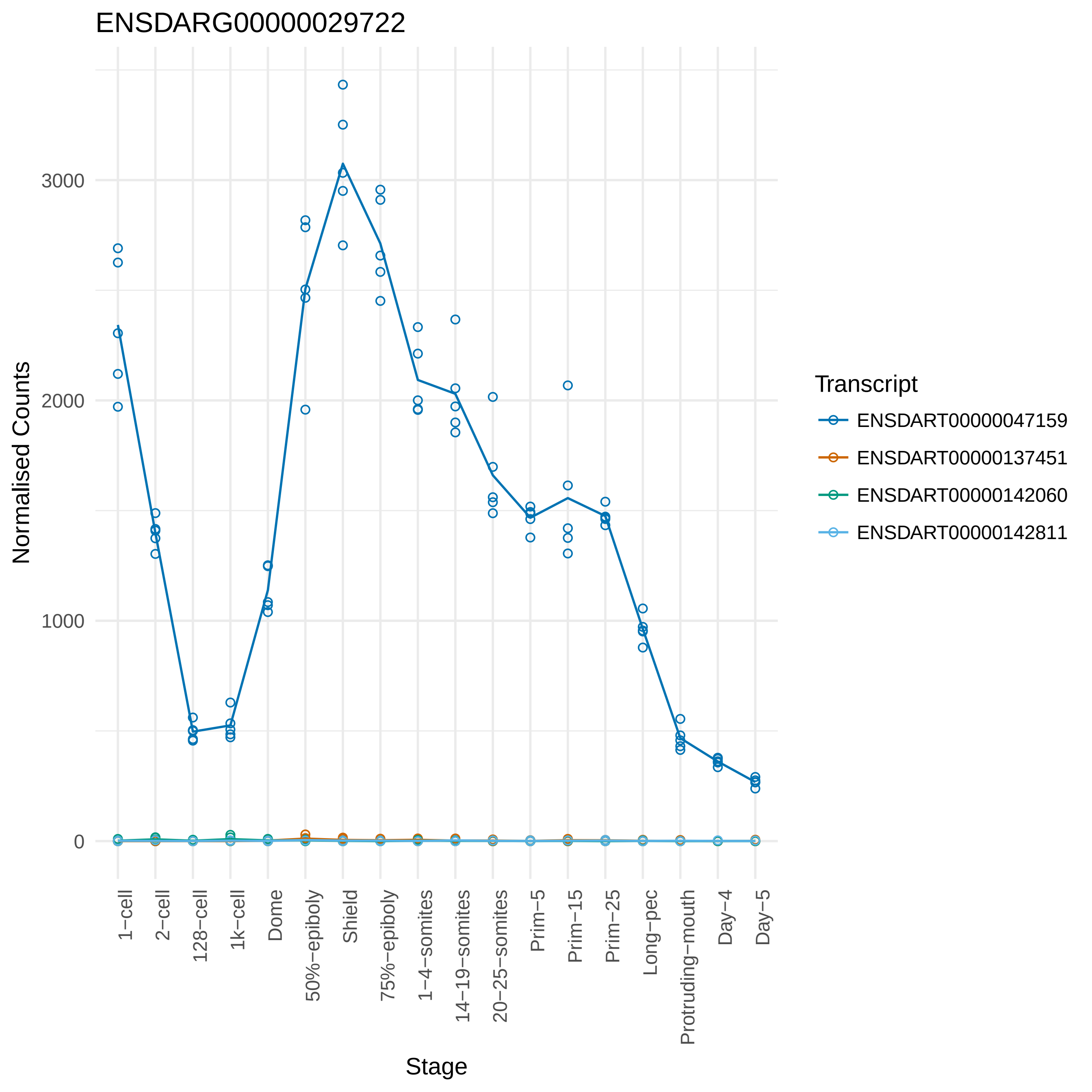

ENSDARG00000029722
Normalised Counts
3000
2000
1000
0
1−cell
2−cell
128−cell
1k−cell
Dome
50%−epiboly
Shield
75%−epiboly
1−4−somites
14−19−somites
20−25−somites
Prim−5
Prim−15
Prim−25
Long−pec
Protruding−mouth
Day−4
Day−5
Stage
Transcript
ENSDART00000047159
ENSDART00000137451
ENSDART00000142060
ENSDART00000142811

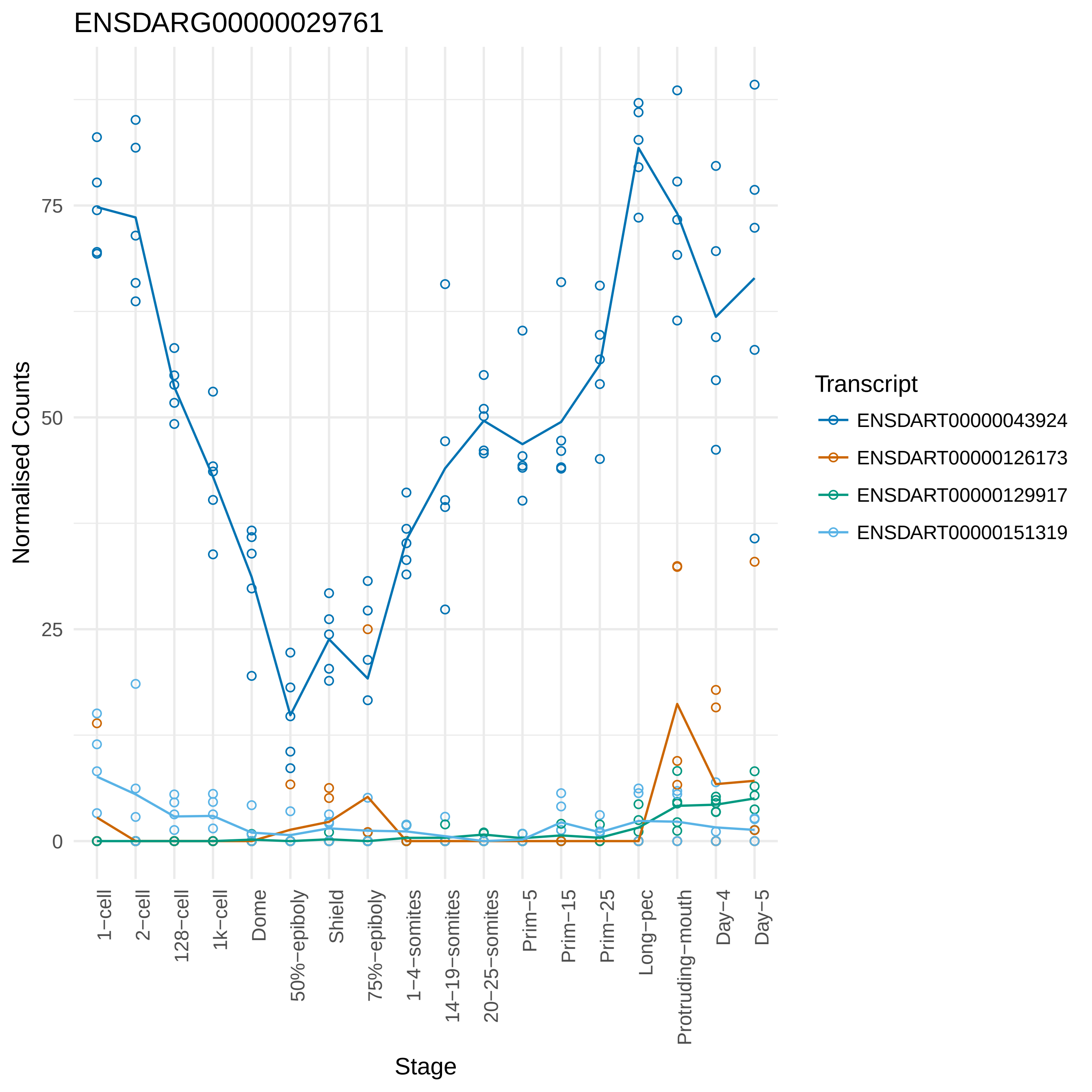
ENSDARG00000029761
Normalised Counts
75
50
25
0
1−cell
2−cell
128−cell
1k−cell
Dome
50%−epiboly
Shield
75%−epiboly
1−4−somites
14−19−somites
20−25−somites
Prim−5
Prim−15
Prim−25
Long−pec
Protruding−mouth
Day−4
Day−5
Stage
Transcript
ENSDART00000043924
ENSDART00000126173
ENSDART00000129917
ENSDART00000151319

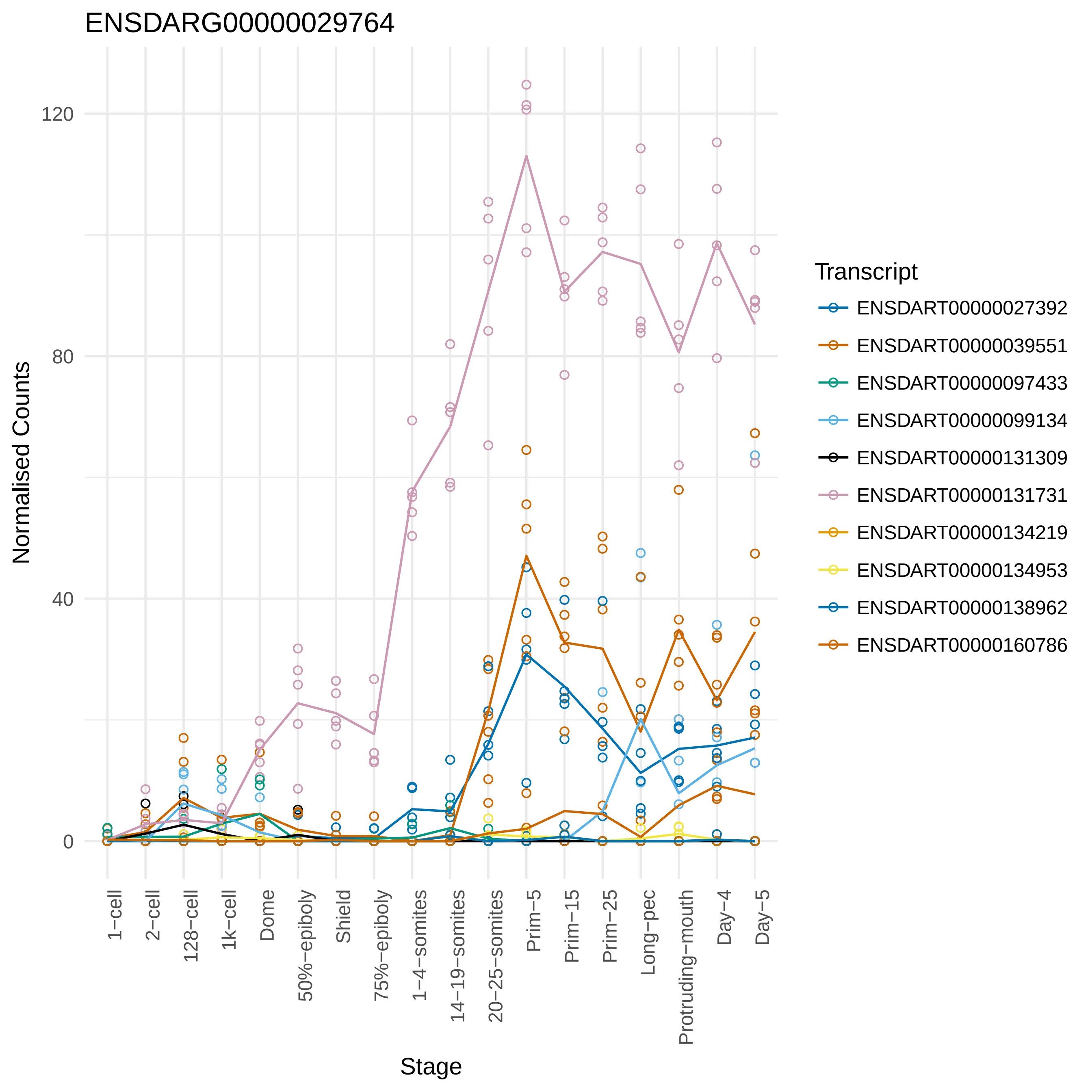

ENSDARG00000029764
Normalised Counts
120
80
40
0
Stage
1−cell
2−cell
128−cell
1k−cell
Dome
50%−epiboly
Shield
75%−epiboly
1−4−somites
14−19−somites
20−25−somites
Prim−5
Prim−15
Prim−25
Long−pec
Protruding−mouth
Day−4
Day−5
Transcript
ENSDART00000027392
ENSDART00000039551
ENSDART00000097433
ENSDART00000099134
ENSDART00000131309
ENSDART00000131731
ENSDART00000134219
ENSDART00000134953
ENSDART00000138962
ENSDART00000160786

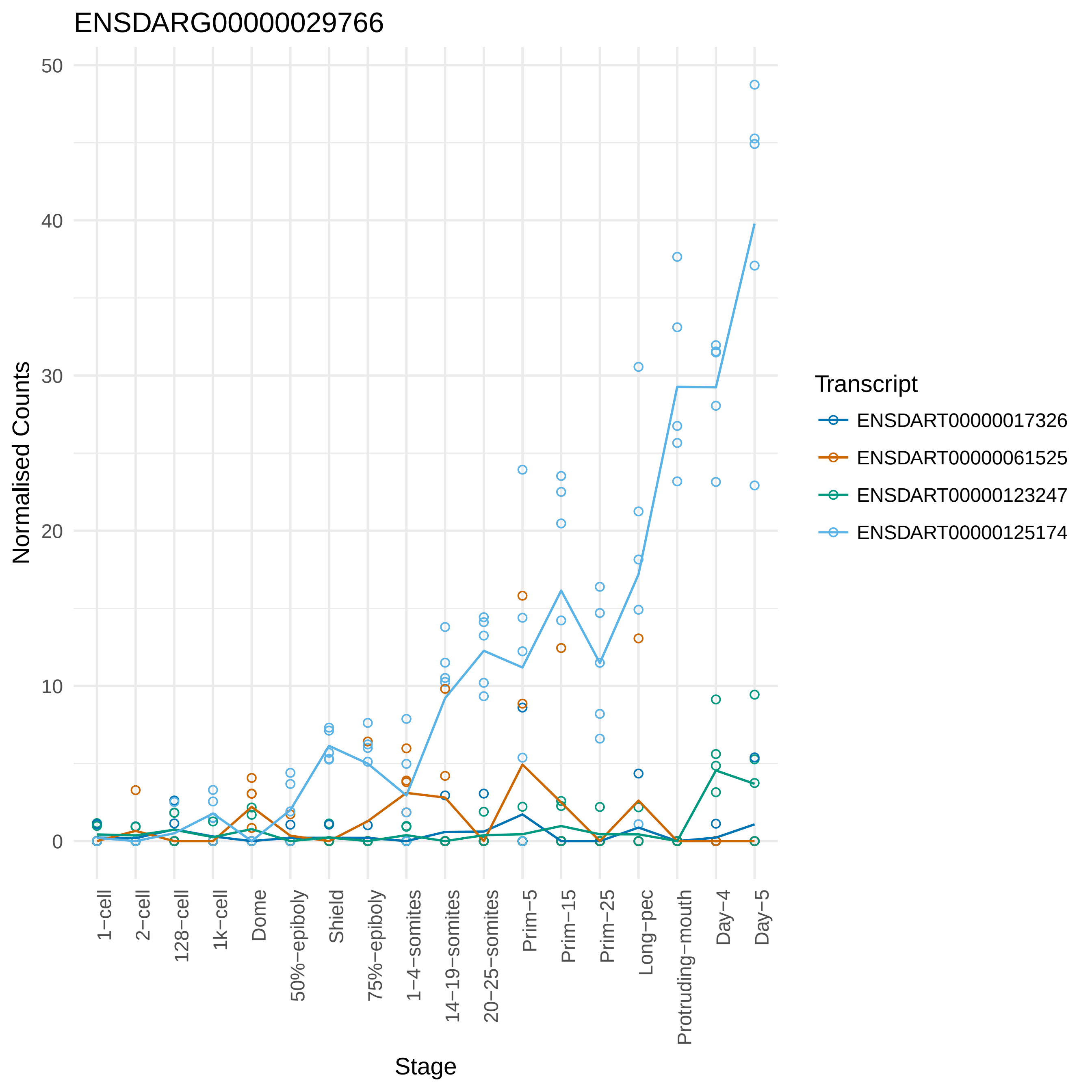

ENSDARG00000029766
Normalised Counts
0
10
20
30
40
50
1−cell
2−cell
128−cell
1k−cell
Dome
50%−epiboly
Shield
75%−epiboly
1−4−somites
14−19−somites
20−25−somites
Prim−5
Prim−15
Prim−25
Long−pec
Protruding−mouth
Day−4
Day−5
Stage
Transcript
ENSDART00000017326
ENSDART00000061525
ENSDART00000123247
ENSDART00000125174

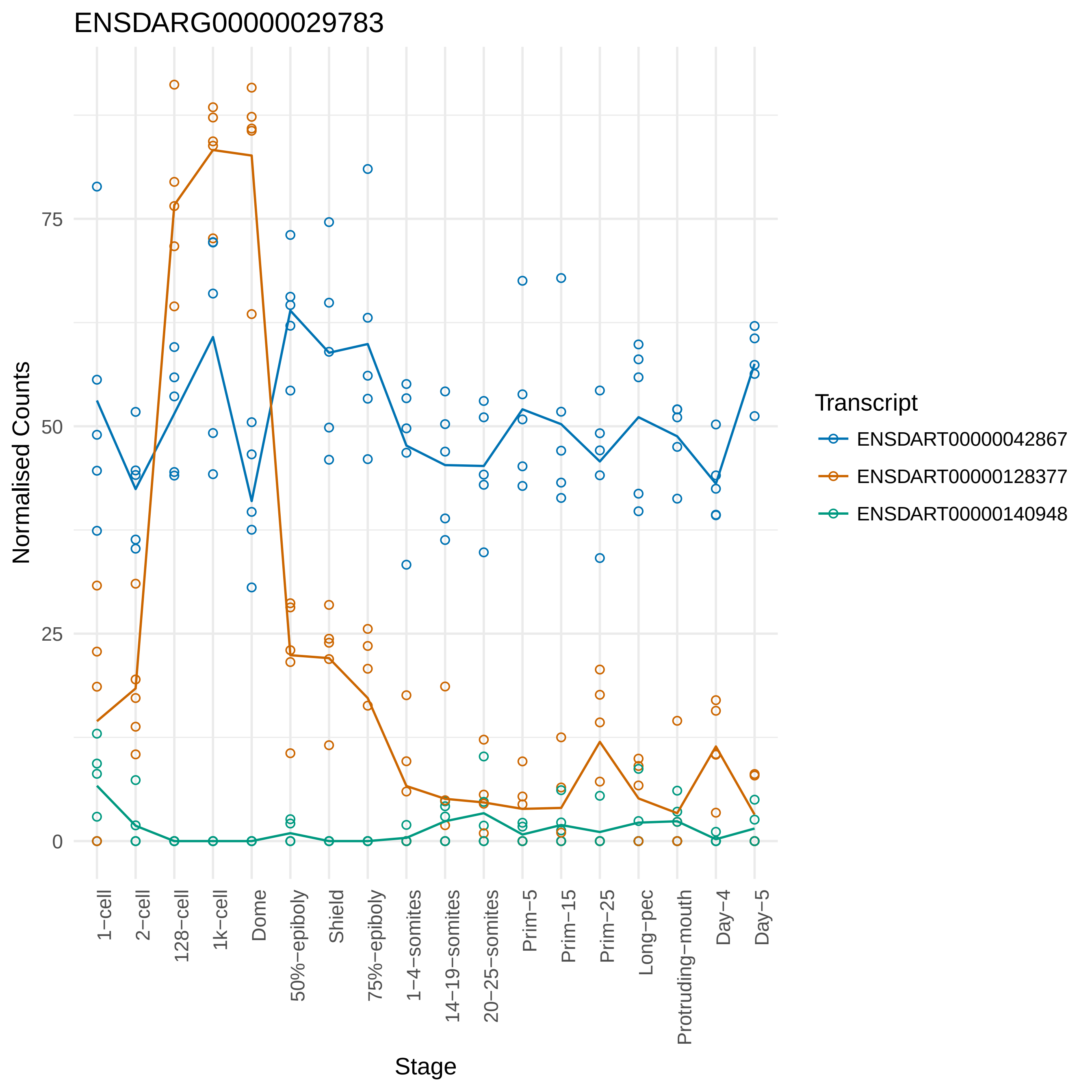

ENSDARG00000029783
Normalised Counts
75
50
25
0
1−cell
2−cell
128−cell
1k−cell
Dome
50%−epiboly
Shield
75%−epiboly
1−4−somites
14−19−somites
20−25−somites
Prim−5
Prim−15
Prim−25
Long−pec
Protruding−mouth
Day−4
Day−5
Stage
Transcript
ENSDART00000042867
ENSDART00000128377
ENSDART00000140948

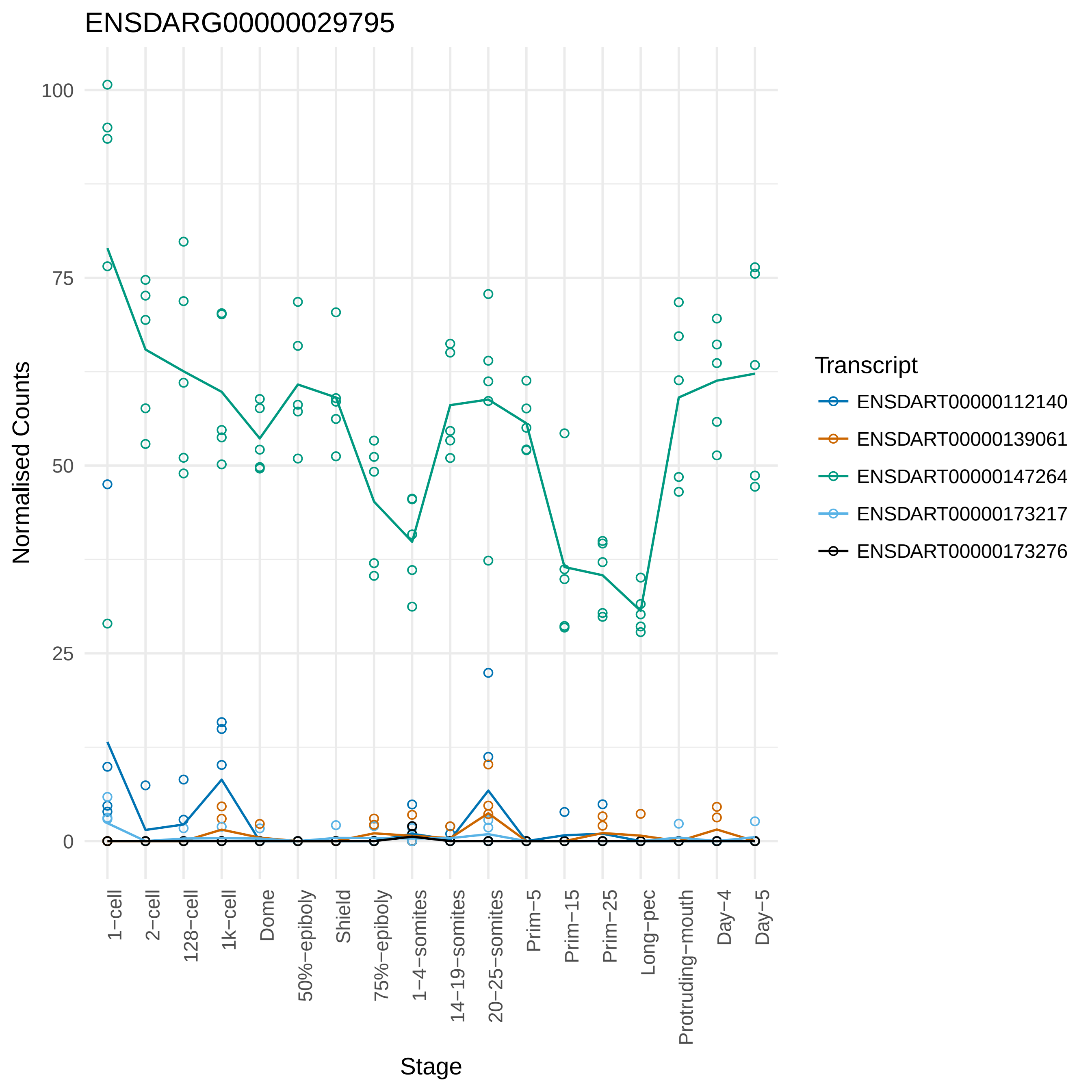

ENSDARG00000029795
Normalised Counts
100
75
50
25
0
1−cell
2−cell
128−cell
1k−cell
Dome
50%−epiboly
Shield
75%−epiboly
1−4−somites
14−19−somites
20−25−somites
Prim−5
Prim−15
Prim−25
Long−pec
Protruding−mouth
Day−4
Day−5
Stage
Transcript
ENSDART00000112140
ENSDART00000139061
ENSDART00000147264
ENSDART00000173217
ENSDART00000173276

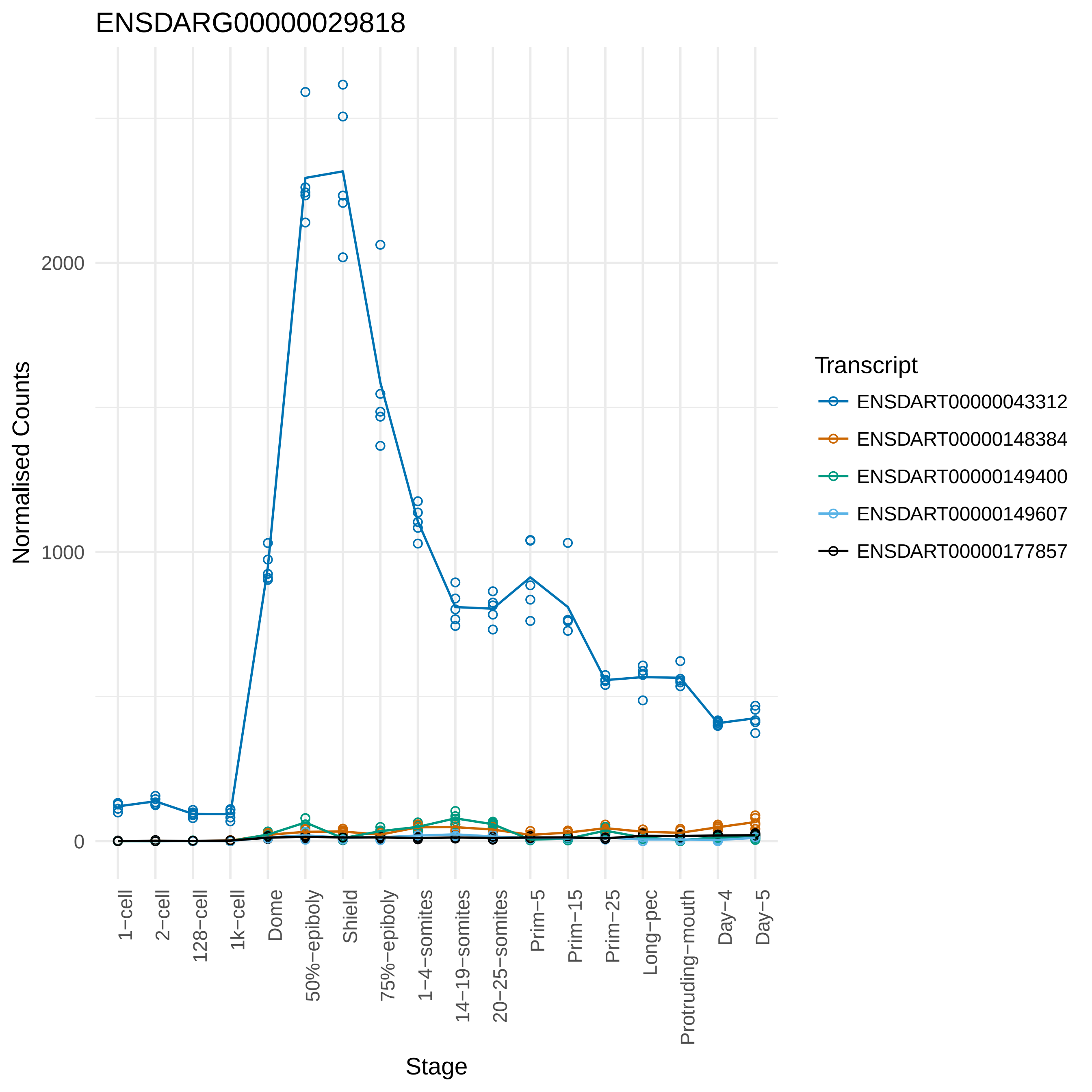

ENSDARG00000029818
Normalised Counts
2000
1000
0
1−cell
2−cell
128−cell
1k−cell
Dome
50%−epiboly
Shield
75%−epiboly
1−4−somites
14−19−somites
20−25−somites
Prim−5
Prim−15
Prim−25
Long−pec
Protruding−mouth
Day−4
Day−5
Stage
Transcript
ENSDART00000043312
ENSDART00000148384
ENSDART00000149400
ENSDART00000149607
ENSDART00000177857

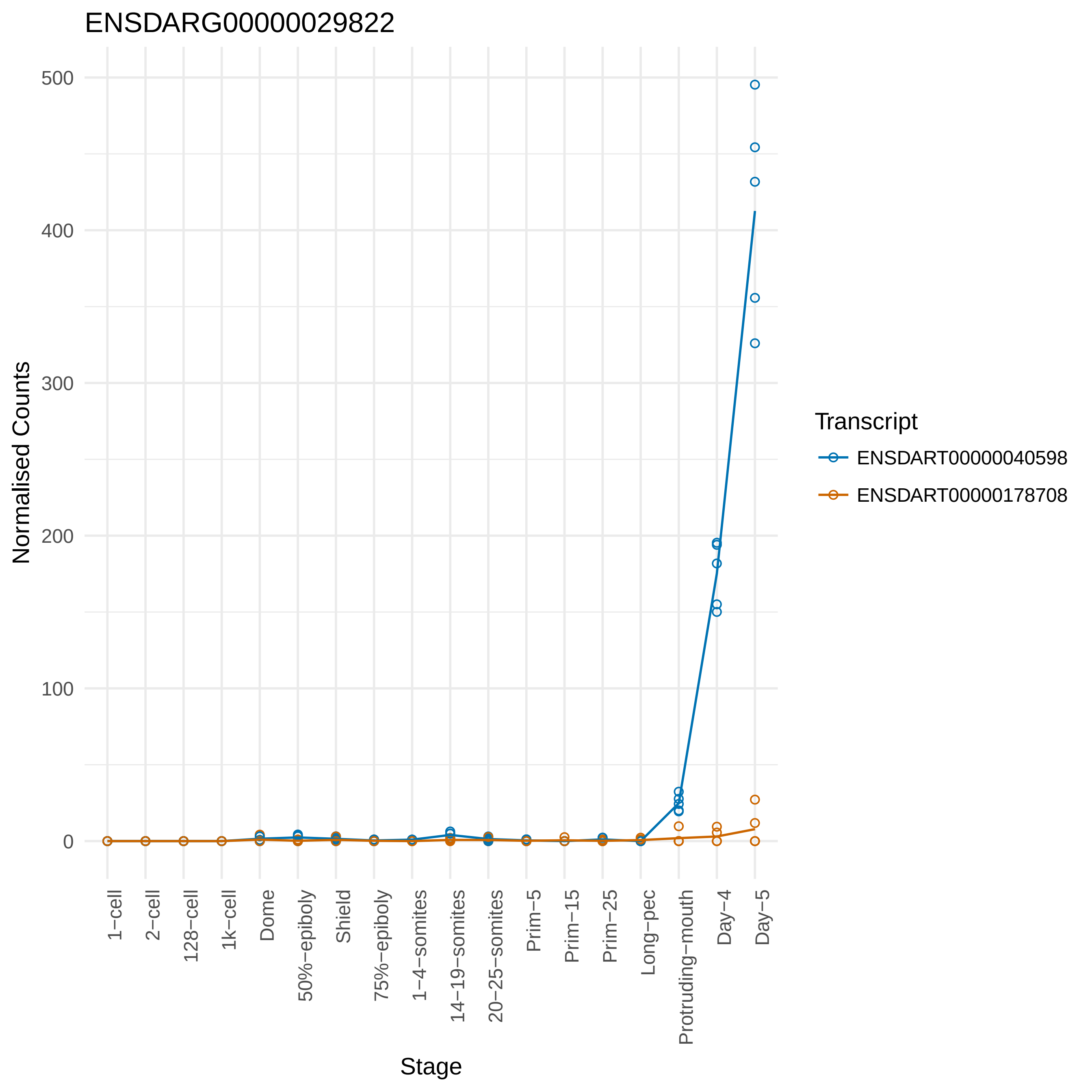

ENSDARG00000029822
Normalised Counts
500
400
300
200
100
0
1−cell
2−cell
128−cell
1k−cell
Dome
50%−epiboly
Shield
75%−epiboly
1−4−somites
14−19−somites
20−25−somites
Prim−5
Prim−15
Prim−25
Long−pec
Protruding−mouth
Day−4
Day−5
Stage
Transcript
ENSDART00000040598
ENSDART00000178708

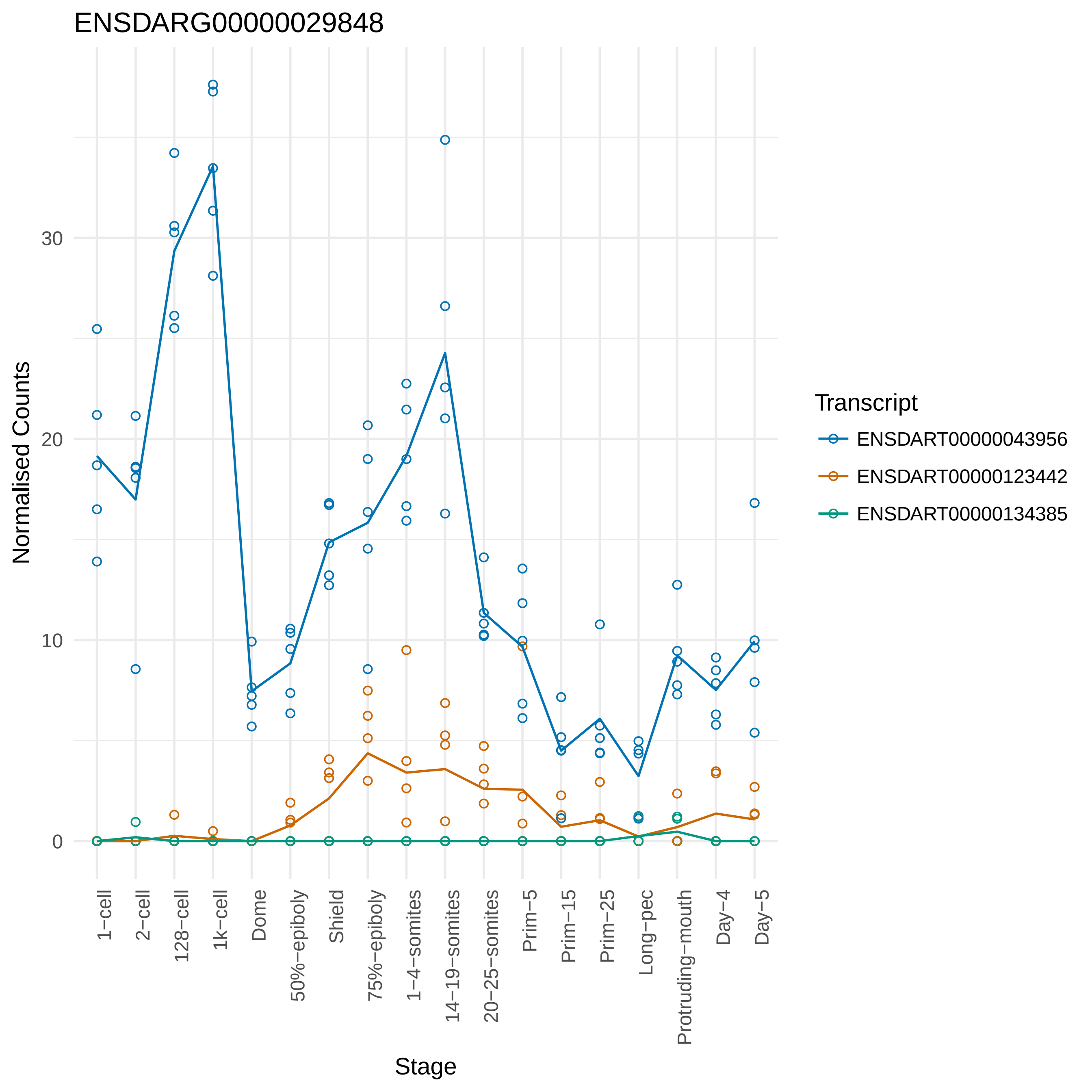

ENSDARG00000029848
Normalised Counts
30
20
10
0
1−cell
2−cell
128−cell
1k−cell
Dome
50%−epiboly
Shield
75%−epiboly
1−4−somites
14−19−somites
20−25−somites
Prim−5
Prim−15
Prim−25
Long−pec
Protruding−mouth
Day−4
Day−5
Stage
Transcript
ENSDART00000043956
ENSDART00000123442
ENSDART00000134385

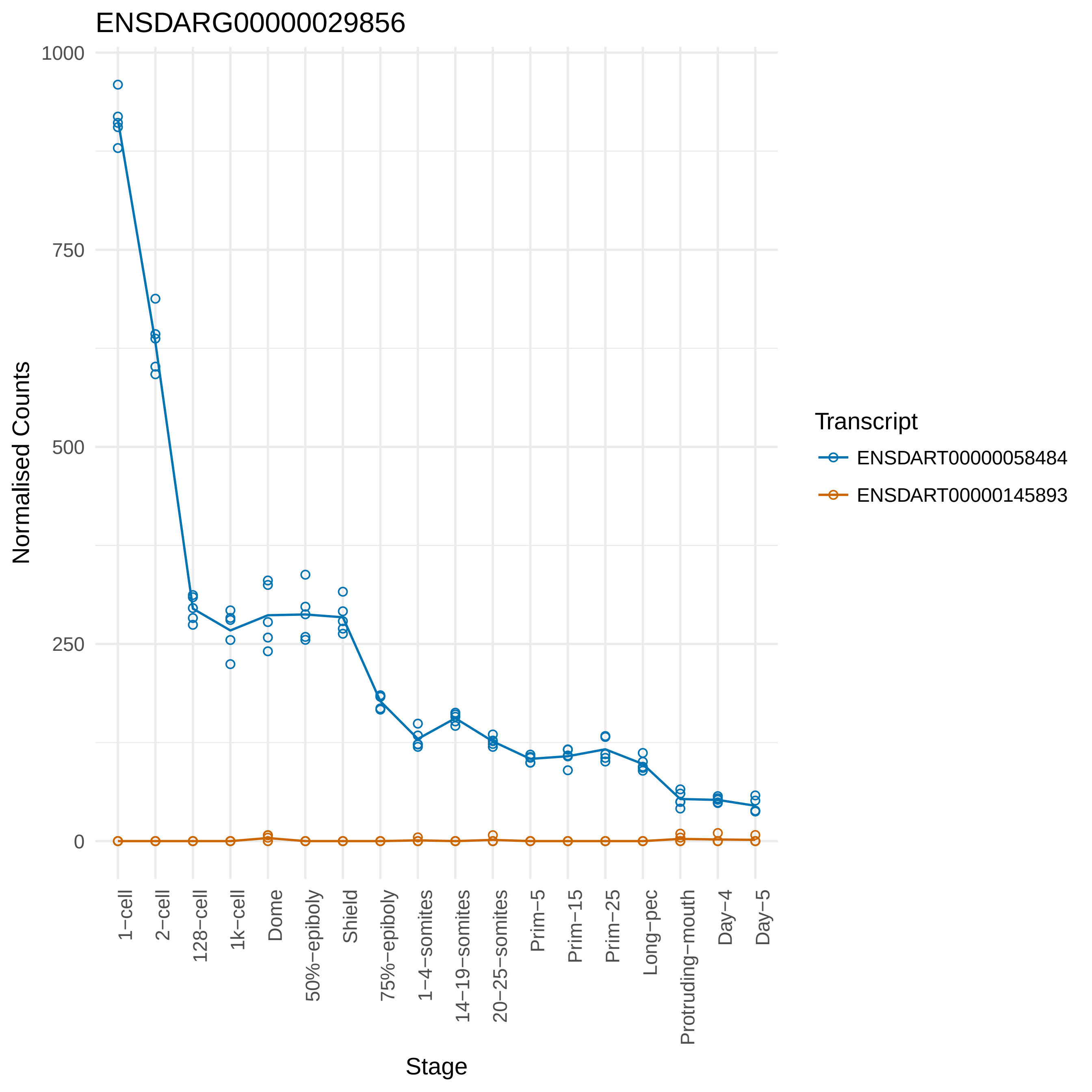

ENSDARG00000029856
Normalised Counts
1000
750
500
250
0
1−cell
2−cell
128−cell
1k−cell
Dome
50%−epiboly
Shield
75%−epiboly
1−4−somites
14−19−somites
20−25−somites
Prim−5
Prim−15
Prim−25
Long−pec
Protruding−mouth
Day−4
Day−5
Stage
Transcript
ENSDART00000058484
ENSDART00000145893

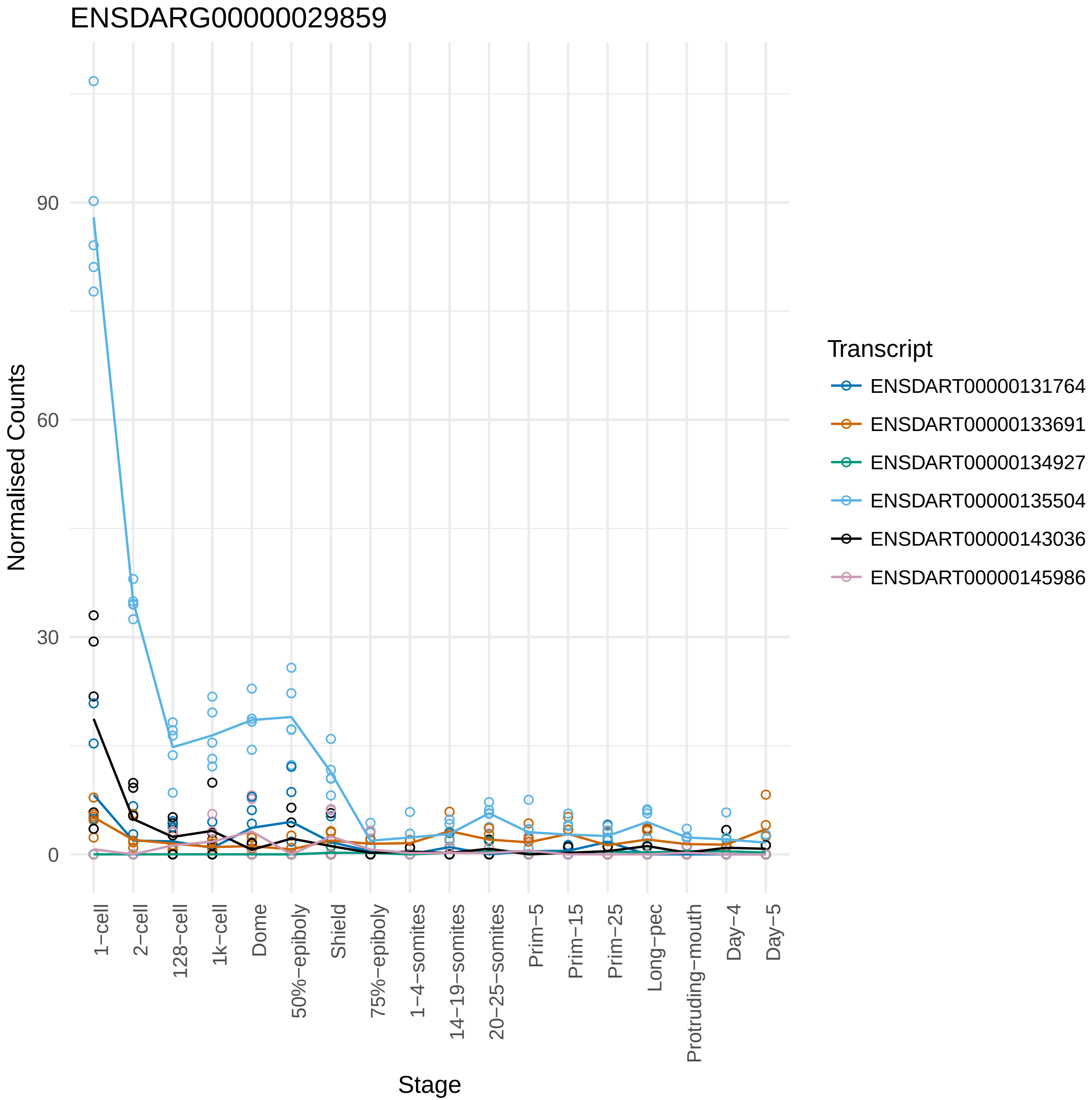

ENSDARG00000029859
Normalised Counts
0
30
60
90
1−cell
2−cell
128−cell
1k−cell
Dome
50%−epiboly
Shield
75%−epiboly
1−4−somites
14−19−somites
20−25−somites
Prim−5
Prim−15
Prim−25
Long−pec
Protruding−mouth
Day−4
Day−5
Stage
Transcript
ENSDART00000131764
ENSDART00000133691
ENSDART00000134927
ENSDART00000135504
ENSDART00000143036
ENSDART00000145986

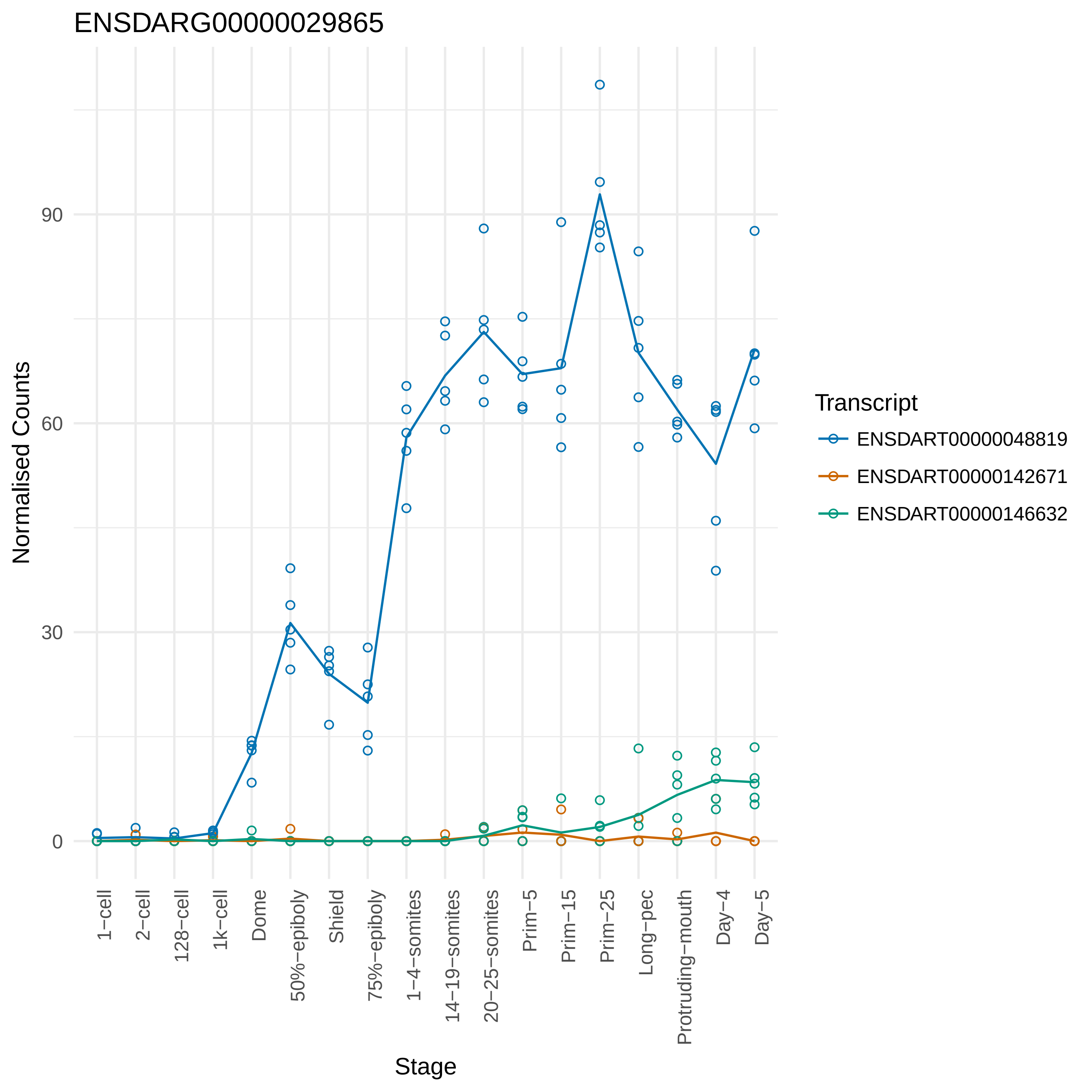

ENSDARG00000029865
Normalised Counts
90
60
30
0
1−cell
2−cell
128−cell
1k−cell
Dome
50%−epiboly
Shield
75%−epiboly
1−4−somites
14−19−somites
20−25−somites
Prim−5
Prim−15
Prim−25
Long−pec
Protruding−mouth
Day−4
Day−5
Stage
Transcript
ENSDART00000048819
ENSDART00000142671
ENSDART00000146632

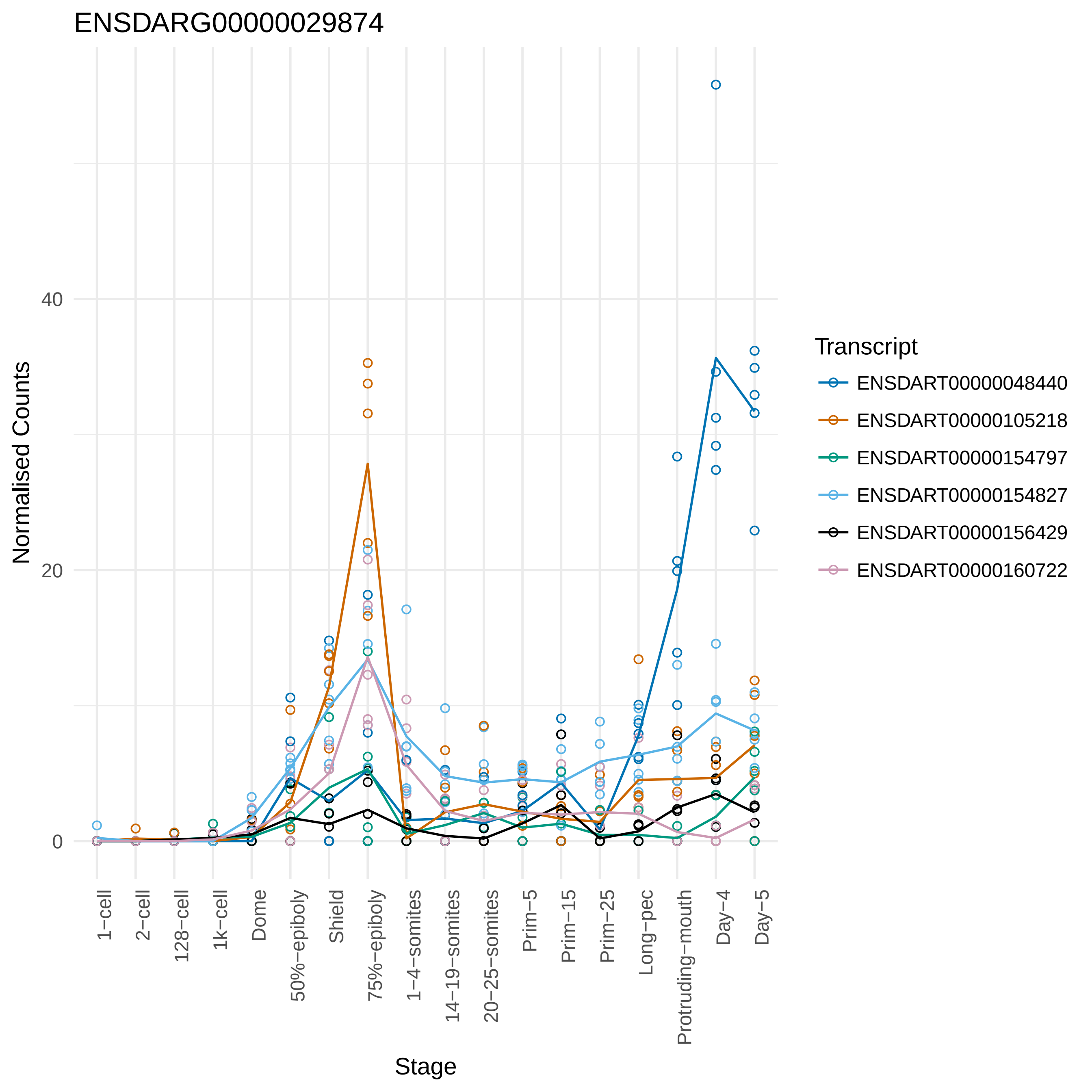

ENSDARG00000029874
Normalised Counts
0
20
40
Stage
1−cell
2−cell
128−cell
1k−cell
Dome
50%−epiboly
Shield
75%−epiboly
1−4−somites
14−19−somites
20−25−somites
Prim−5
Prim−15
Prim−25
Long−pec
Protruding−mouth
Day−4
Day−5
Transcript
ENSDART00000048440
ENSDART00000105218
ENSDART00000154797
ENSDART00000154827
ENSDART00000156429
ENSDART00000160722

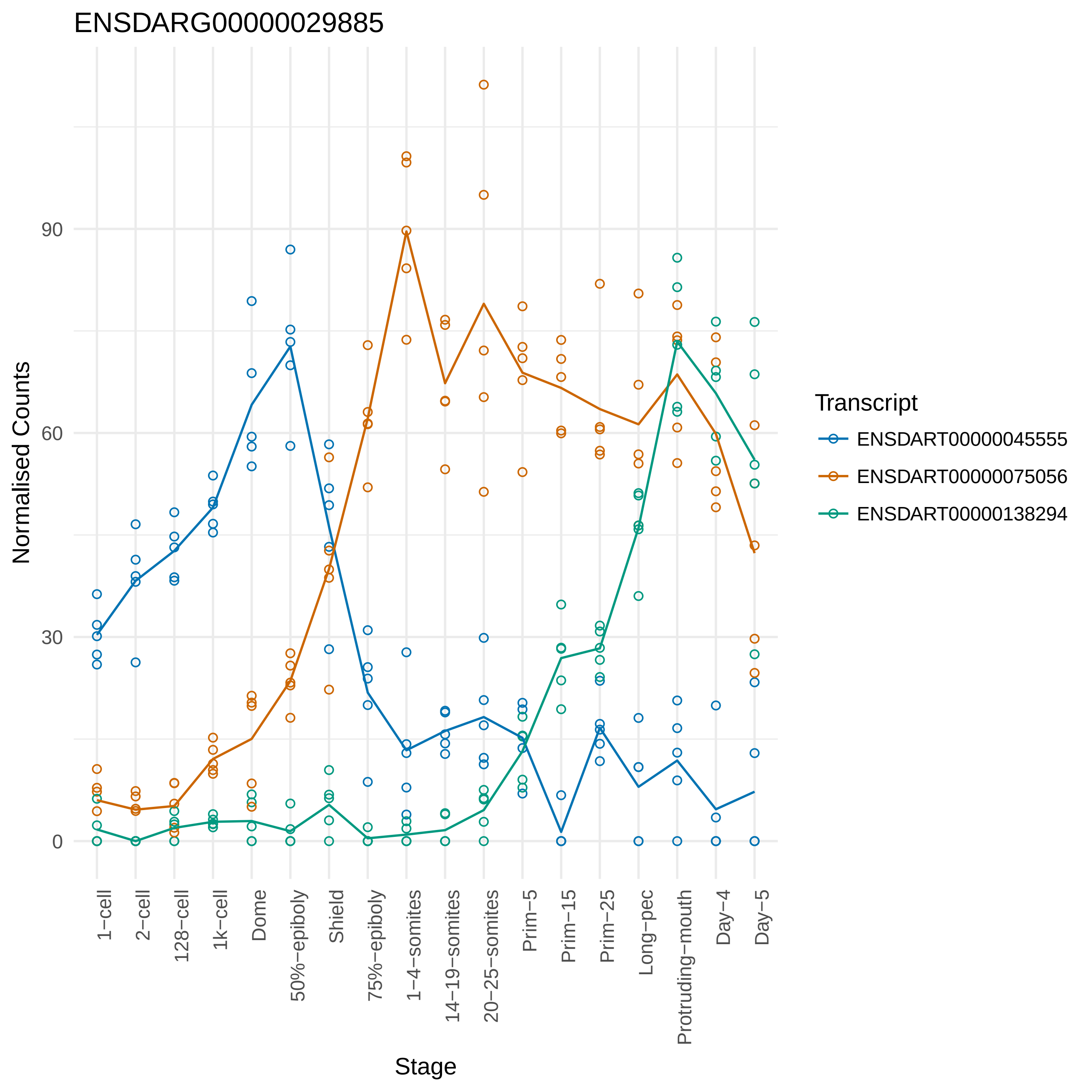

ENSDARG00000029885
Normalised Counts
0
30
60
90
1−cell
2−cell
128−cell
1k−cell
Dome
50%−epiboly
Shield
75%−epiboly
1−4−somites
14−19−somites
20−25−somites
Prim−5
Prim−15
Prim−25
Long−pec
Protruding−mouth
Day−4
Day−5
Stage
Transcript
ENSDART00000045555
ENSDART00000075056
ENSDART00000138294

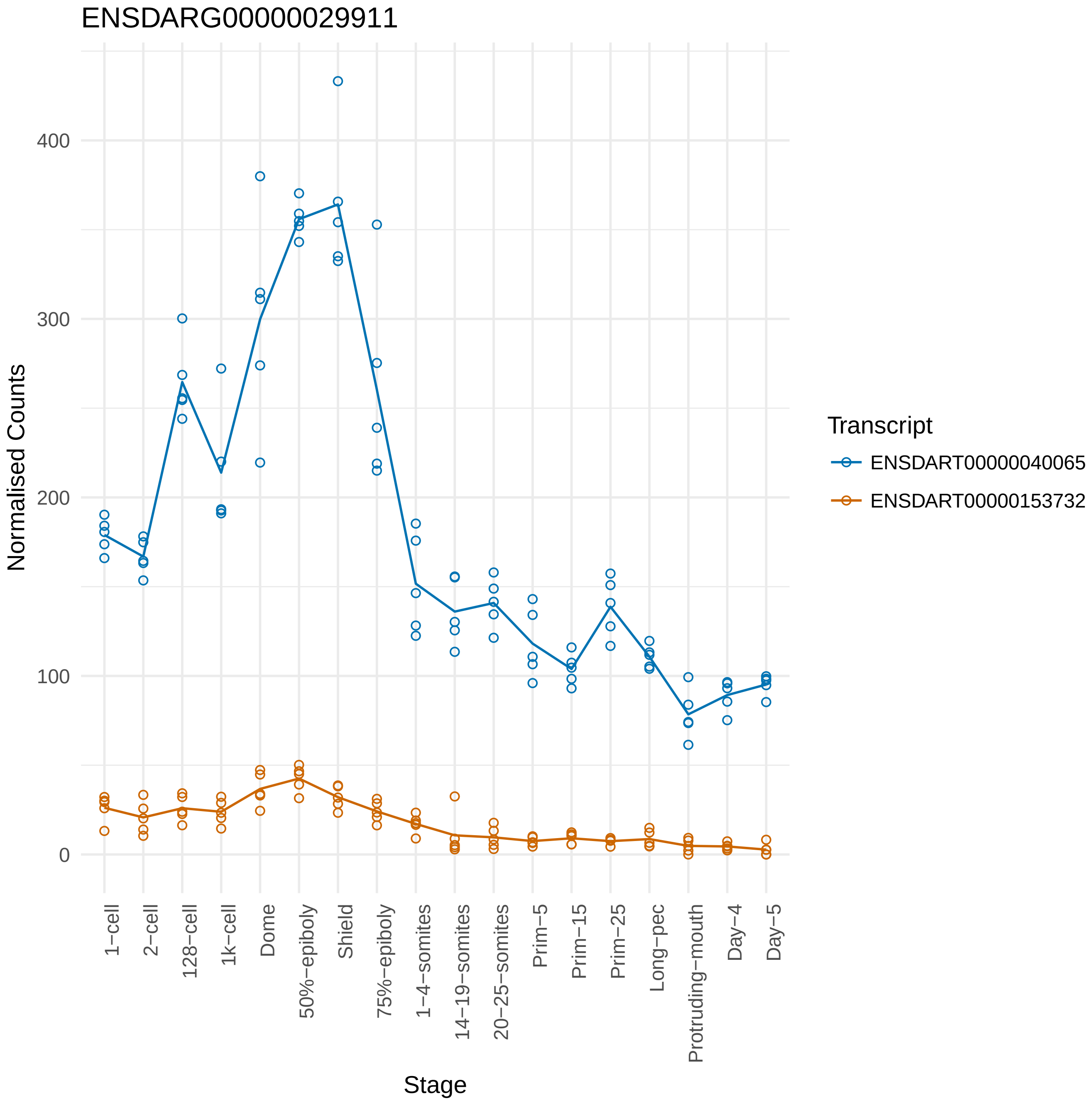
ENSDARG00000029911
Normalised Counts
400
300
200
100
0
1−cell
2−cell
128−cell
1k−cell
Dome
50%−epiboly
Shield
75%−epiboly
1−4−somites
14−19−somites
20−25−somites
Prim−5
Prim−15
Prim−25
Long−pec
Protruding−mouth
Day−4
Day−5
Stage
Transcript
ENSDART00000040065
ENSDART00000153732

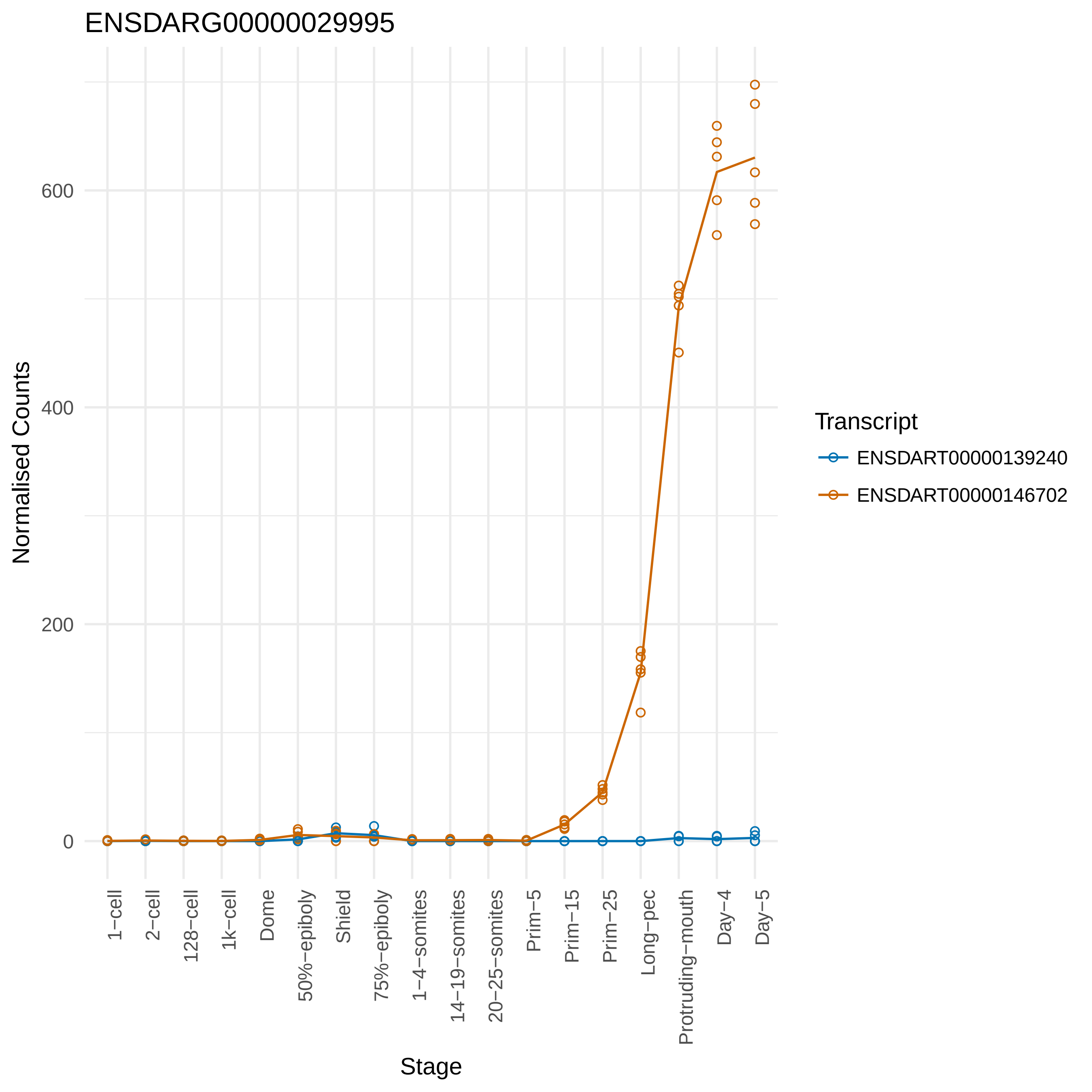
ENSDARG00000029995
Normalised Counts
0
200
400
600
Stage
1−cell
2−cell
128−cell
1k−cell
Dome
50%−epiboly
Shield
75%−epiboly
1−4−somites
14−19−somites
20−25−somites
Prim−5
Prim−15
Prim−25
Long−pec
Protruding−mouth
Day−4
Day−5
Transcript
ENSDART00000139240
ENSDART00000146702

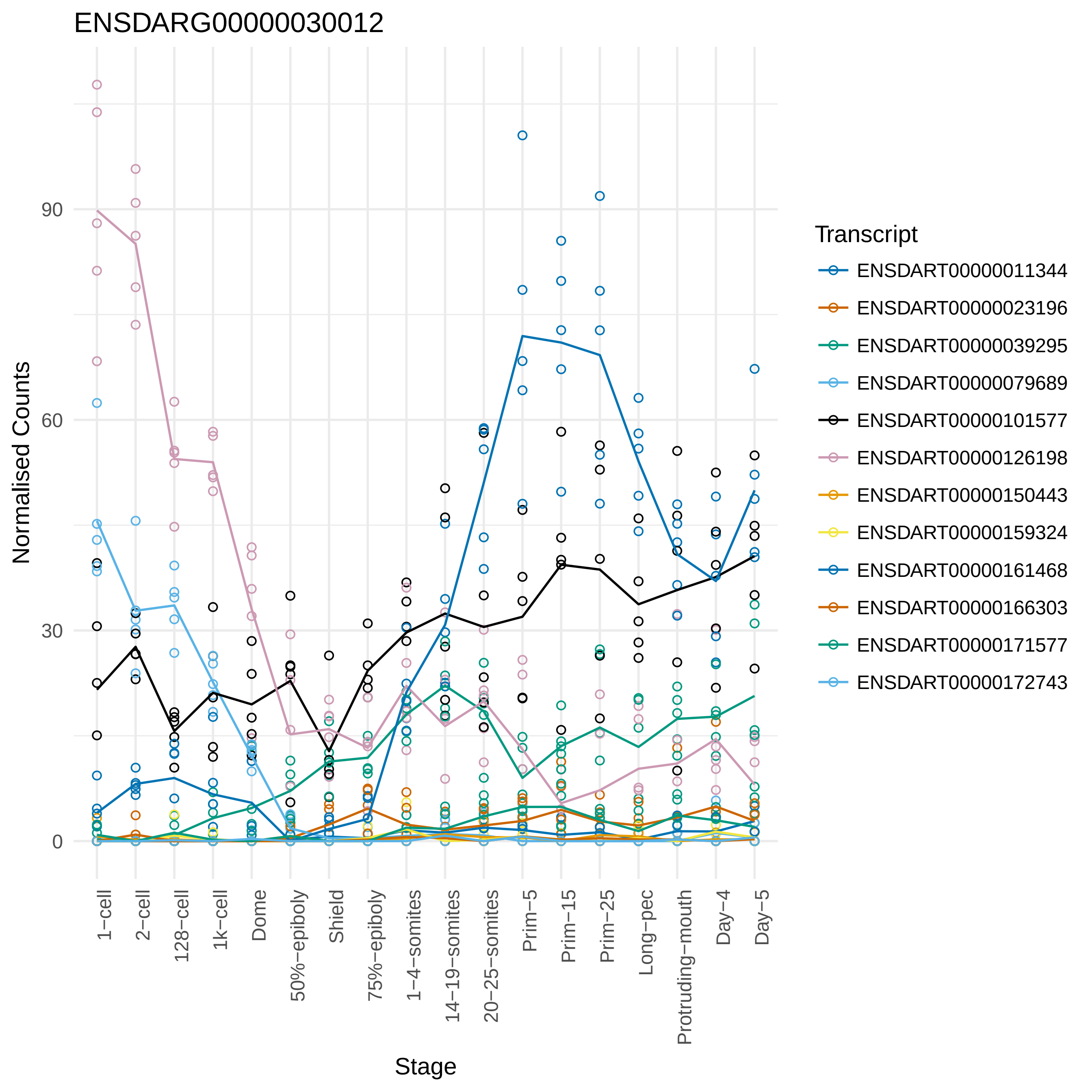

ENSDARG00000030012
Normalised Counts
0
30
60
90
Stage
1−cell
2−cell
128−cell
1k−cell
Dome
50%−epiboly
Shield
75%−epiboly
1−4−somites
14−19−somites
20−25−somites
Prim−5
Prim−15
Prim−25
Long−pec
Protruding−mouth
Day−4
Day−5
Transcript
ENSDART00000011344
ENSDART00000023196
ENSDART00000039295
ENSDART00000079689
ENSDART00000101577
ENSDART00000126198
ENSDART00000150443
ENSDART00000159324
ENSDART00000161468
ENSDART00000166303
ENSDART00000171577
ENSDART00000172743

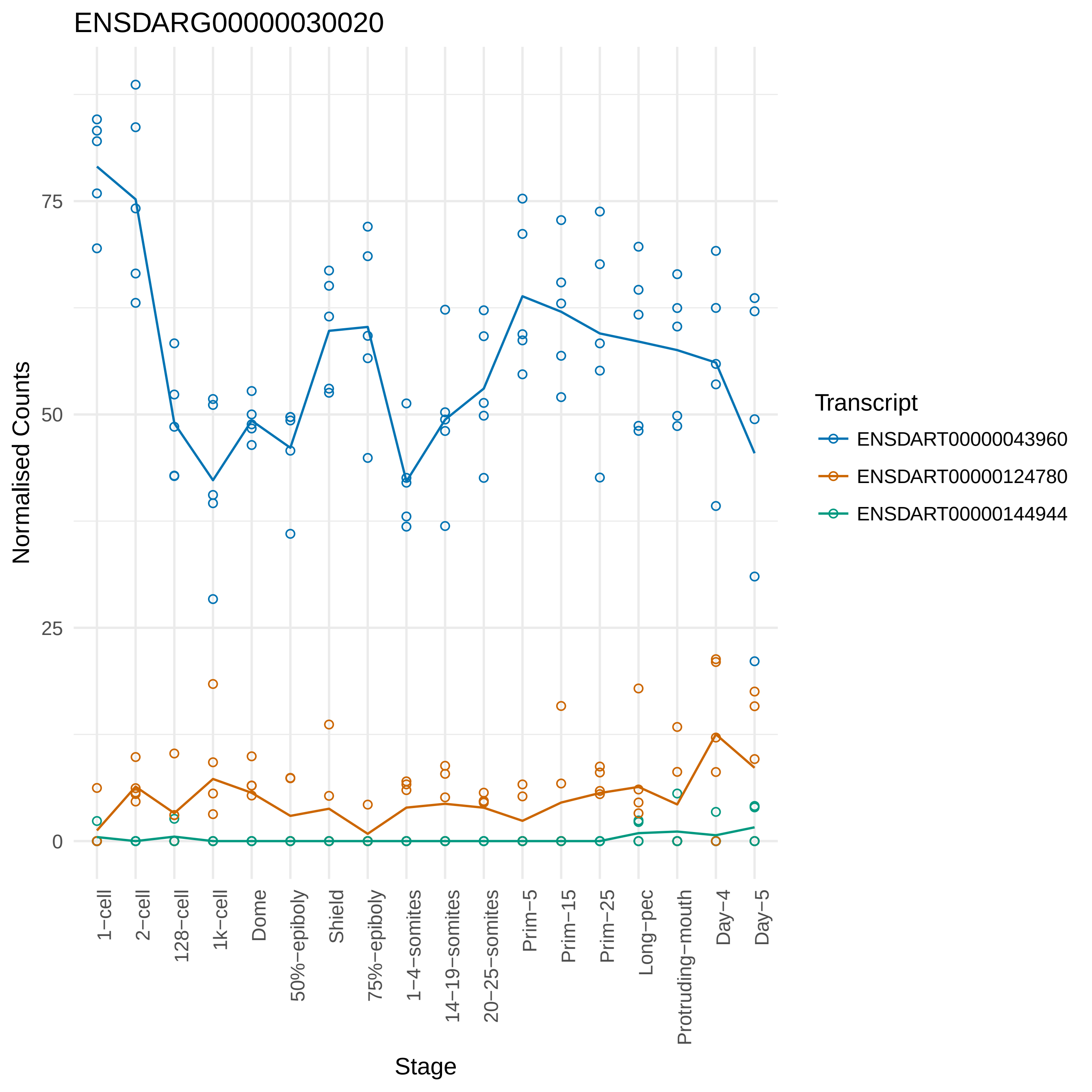

ENSDARG00000030020
Normalised Counts
0
25
50
75
1−cell
2−cell
128−cell
1k−cell
Dome
50%−epiboly
Shield
75%−epiboly
1−4−somites
14−19−somites
20−25−somites
Prim−5
Prim−15
Prim−25
Long−pec
Protruding−mouth
Day−4
Day−5
Stage
Transcript
ENSDART00000043960
ENSDART00000124780
ENSDART00000144944

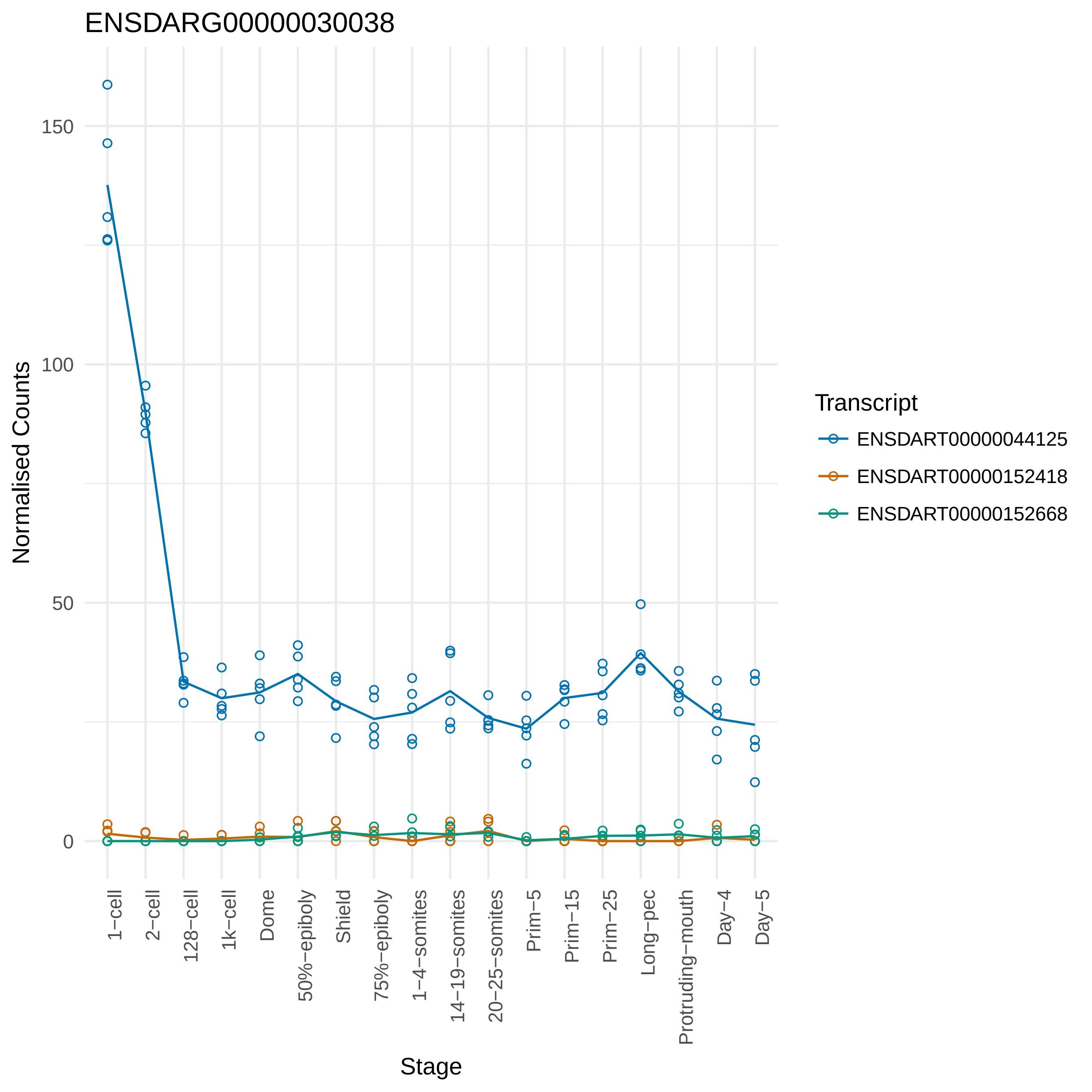

ENSDARG00000030038
Normalised Counts
150
100
50
0
Stage
1−cell
2−cell
128−cell
1k−cell
Dome
50%−epiboly
Shield
75%−epiboly
1−4−somites
14−19−somites
20−25−somites
Prim−5
Prim−15
Prim−25
Long−pec
Protruding−mouth
Day−4
Day−5
Transcript
ENSDART00000044125
ENSDART00000152418
ENSDART00000152668

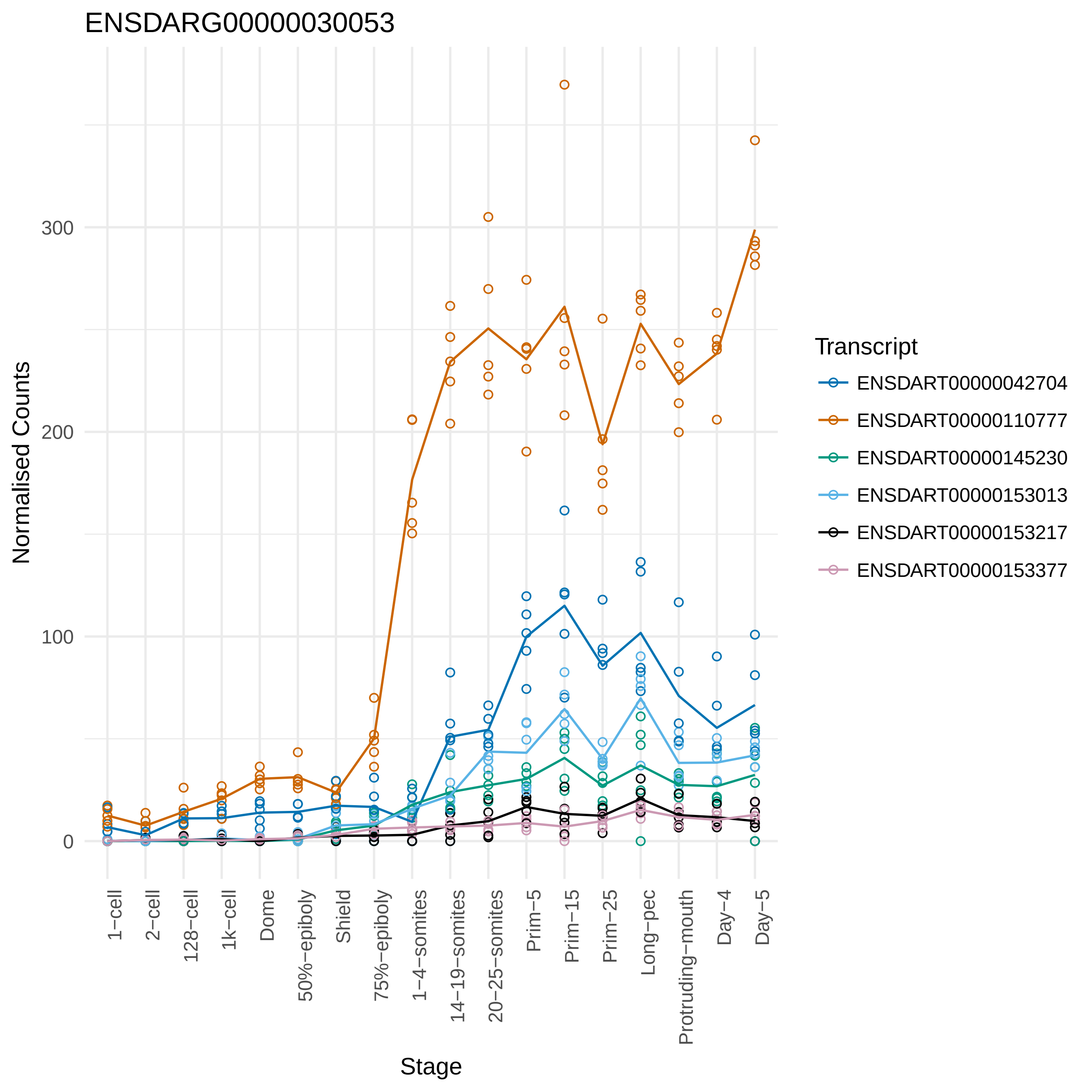

ENSDARG00000030053
Normalised Counts
300
200
100
0
1−cell
2−cell
128−cell
1k−cell
Dome
50%−epiboly
Shield
75%−epiboly
1−4−somites
14−19−somites
20−25−somites
Prim−5
Prim−15
Prim−25
Long−pec
Protruding−mouth
Day−4
Day−5
Stage
Transcript
ENSDART00000042704
ENSDART00000110777
ENSDART00000145230
ENSDART00000153013
ENSDART00000153217
ENSDART00000153377

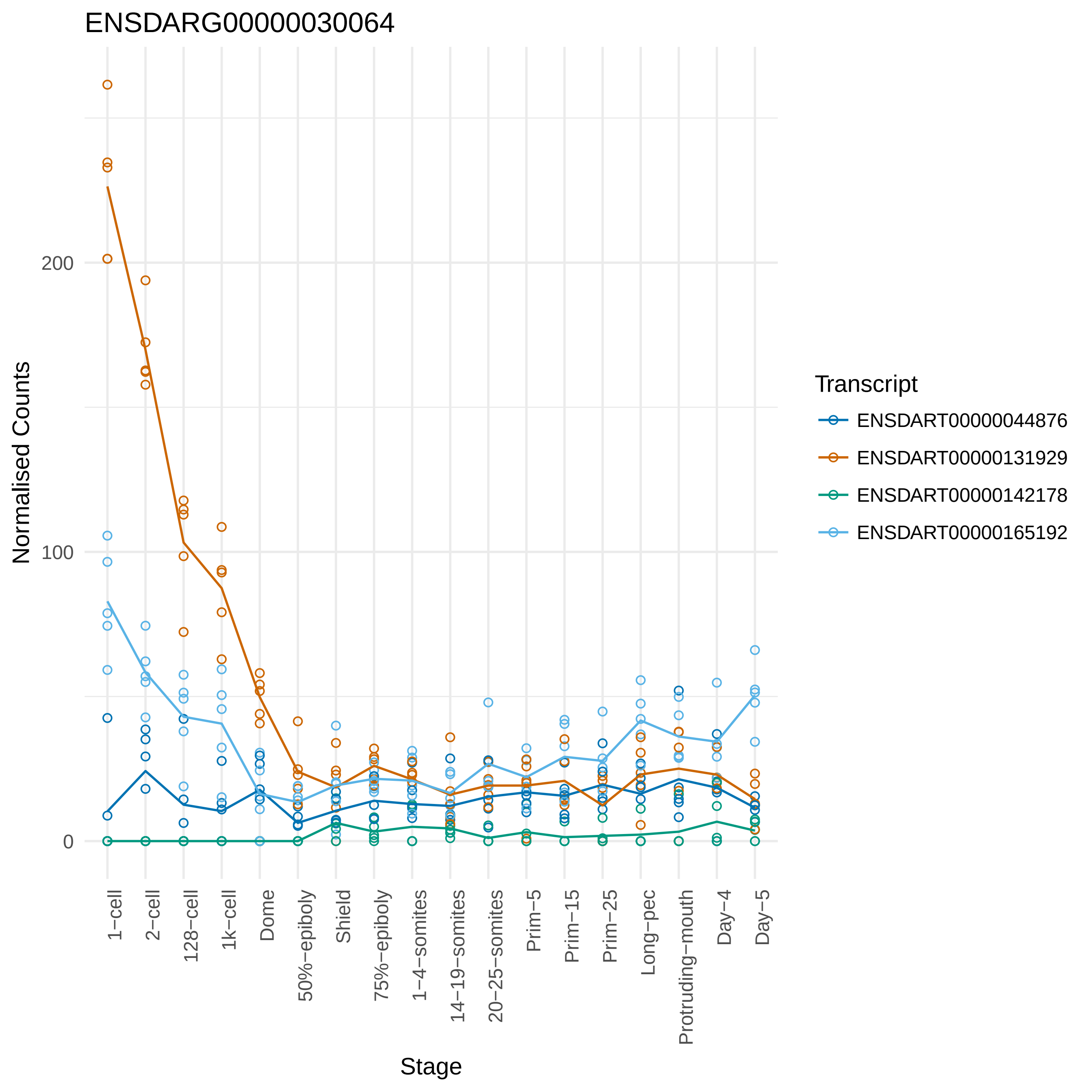
ENSDARG00000030064
Normalised Counts
200
100
0
1−cell
2−cell
128−cell
1k−cell
Dome
50%−epiboly
Shield
75%−epiboly
1−4−somites
14−19−somites
20−25−somites
Prim−5
Prim−15
Prim−25
Long−pec
Protruding−mouth
Day−4
Day−5
Stage
Transcript
ENSDART00000044876
ENSDART00000131929
ENSDART00000142178
ENSDART00000165192

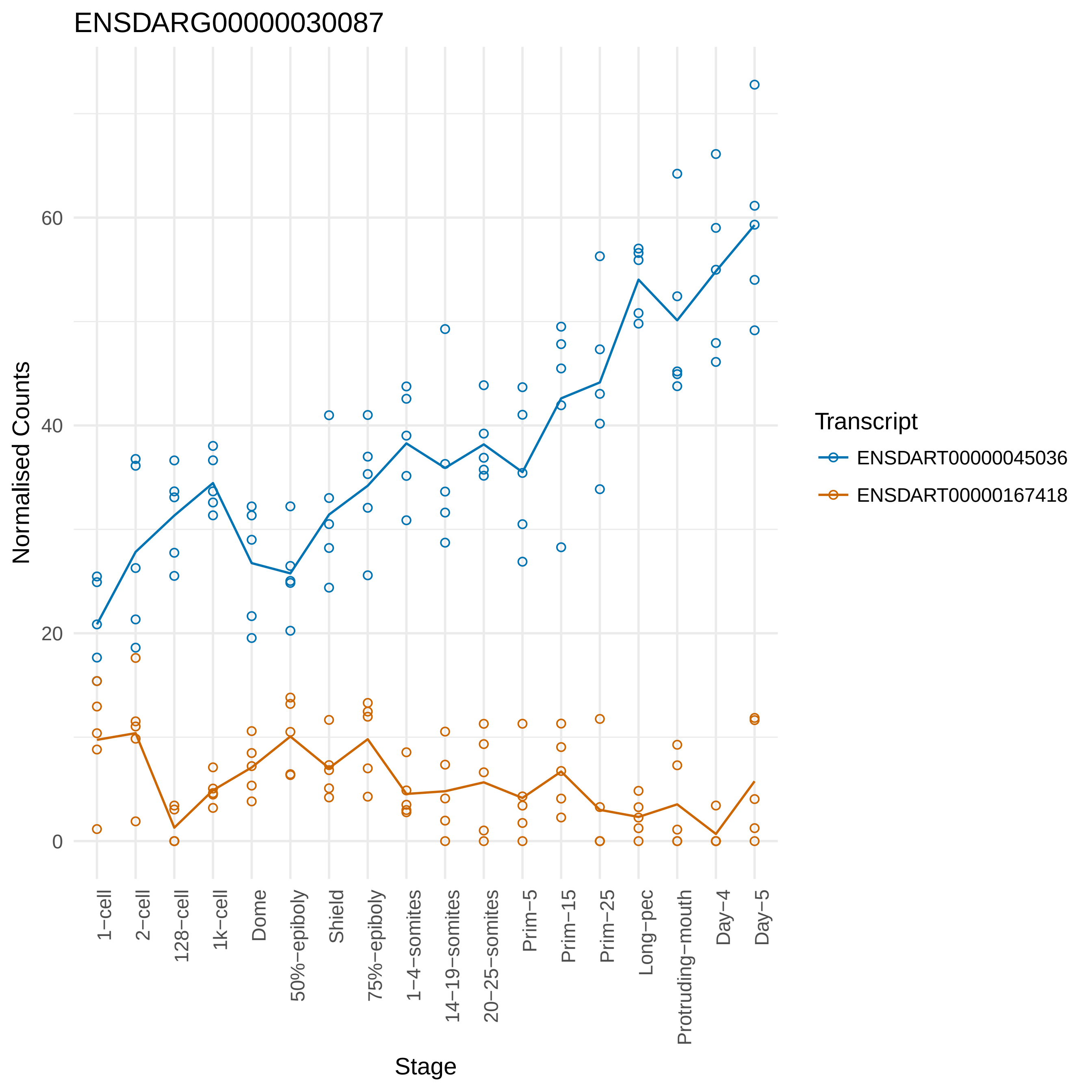
ENSDARG00000030087
Normalised Counts
0
20
40
60
Stage
1−cell
2−cell
128−cell
1k−cell
Dome
50%−epiboly
Shield
75%−epiboly
1−4−somites
14−19−somites
20−25−somites
Prim−5
Prim−15
Prim−25
Long−pec
Protruding−mouth
Day−4
Day−5
Transcript
ENSDART00000045036
ENSDART00000167418

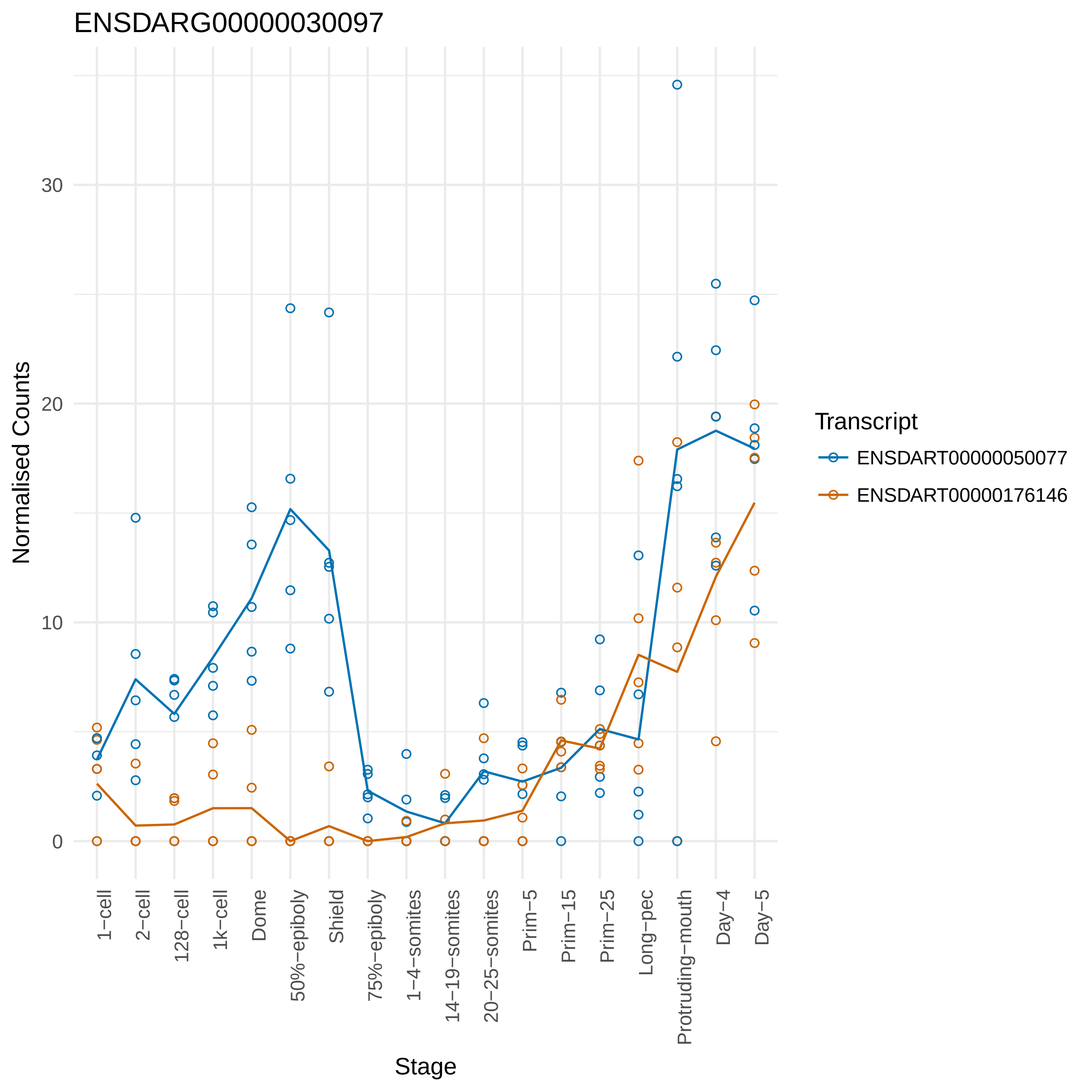

ENSDARG00000030097
Normalised Counts
0
10
20
30
Stage
1−cell
2−cell
128−cell
1k−cell
Dome
50%−epiboly
Shield
75%−epiboly
1−4−somites
14−19−somites
20−25−somites
Prim−5
Prim−15
Prim−25
Long−pec
Protruding−mouth
Day−4
Day−5
Transcript
ENSDART00000050077
ENSDART00000176146

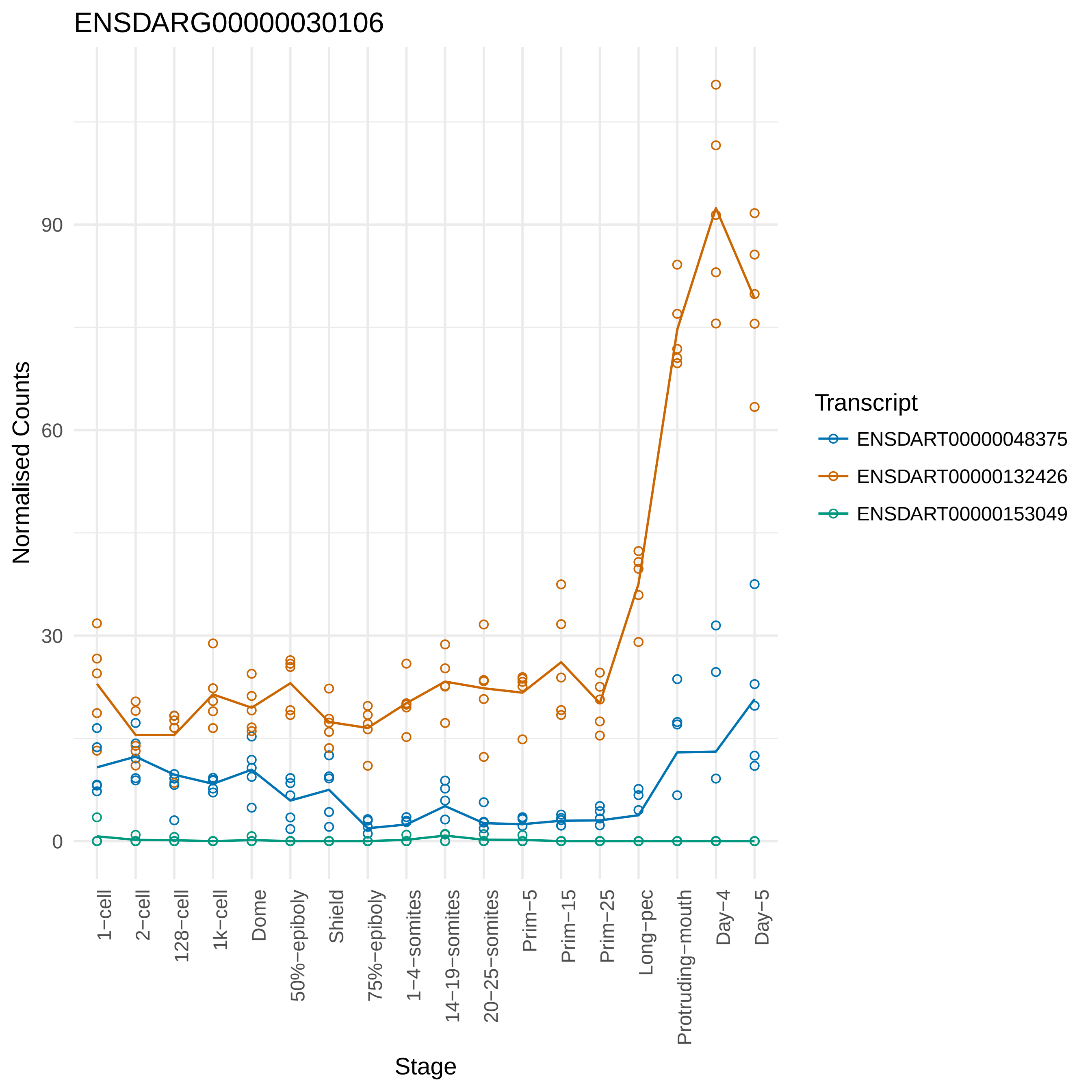
ENSDARG00000030106
Normalised Counts
90
60
30
0
1−cell
2−cell
128−cell
1k−cell
Dome
50%−epiboly
Shield
75%−epiboly
1−4−somites
14−19−somites
20−25−somites
Prim−5
Prim−15
Prim−25
Long−pec
Protruding−mouth
Day−4
Day−5
Stage
Transcript
ENSDART00000048375
ENSDART00000132426
ENSDART00000153049

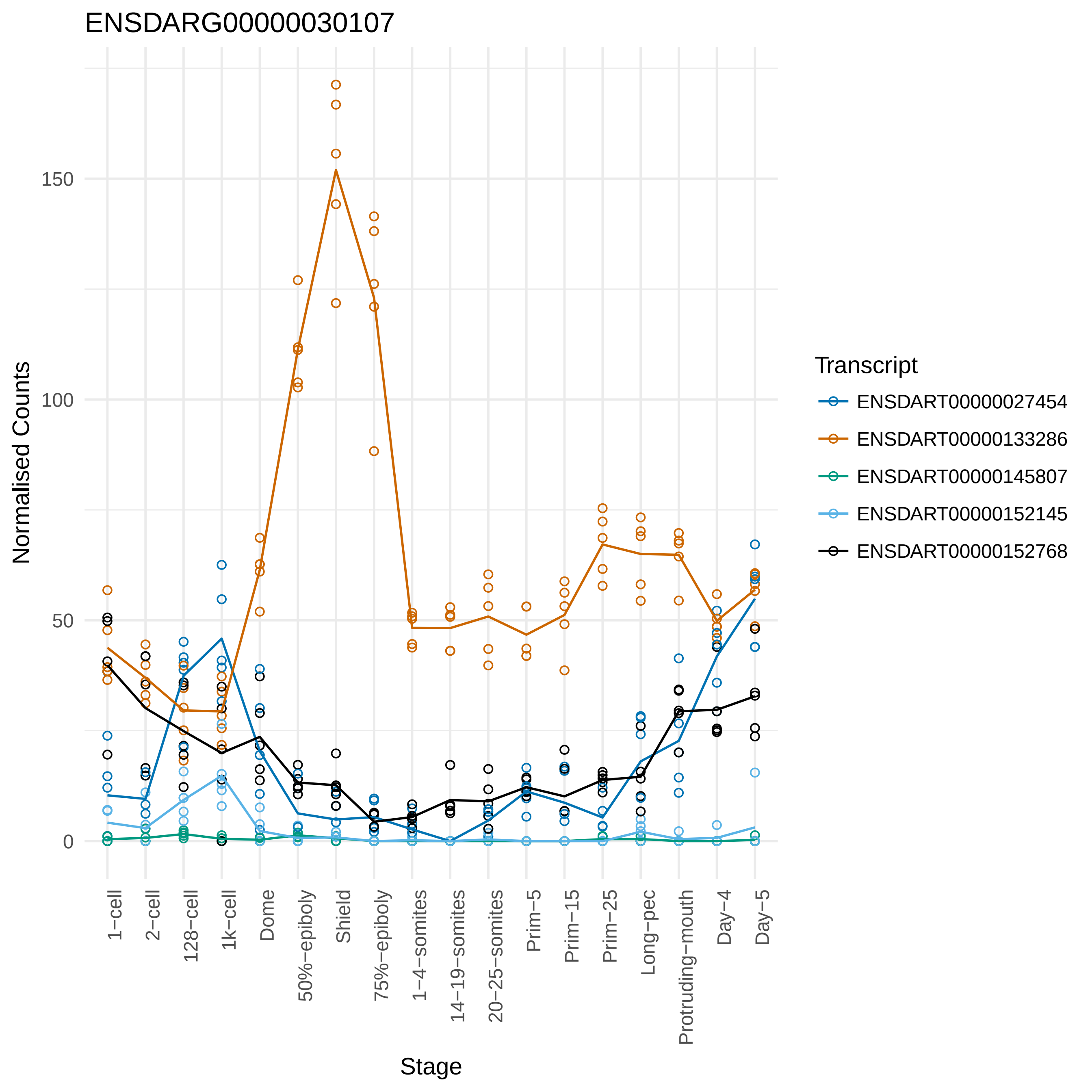
ENSDARG00000030107
Normalised Counts
150
100
50
0
1−cell
2−cell
128−cell
1k−cell
Dome
50%−epiboly
Shield
75%−epiboly
1−4−somites
14−19−somites
20−25−somites
Prim−5
Prim−15
Prim−25
Long−pec
Protruding−mouth
Day−4
Day−5
Stage
Transcript
ENSDART00000027454
ENSDART00000133286
ENSDART00000145807
ENSDART00000152145
ENSDART00000152768

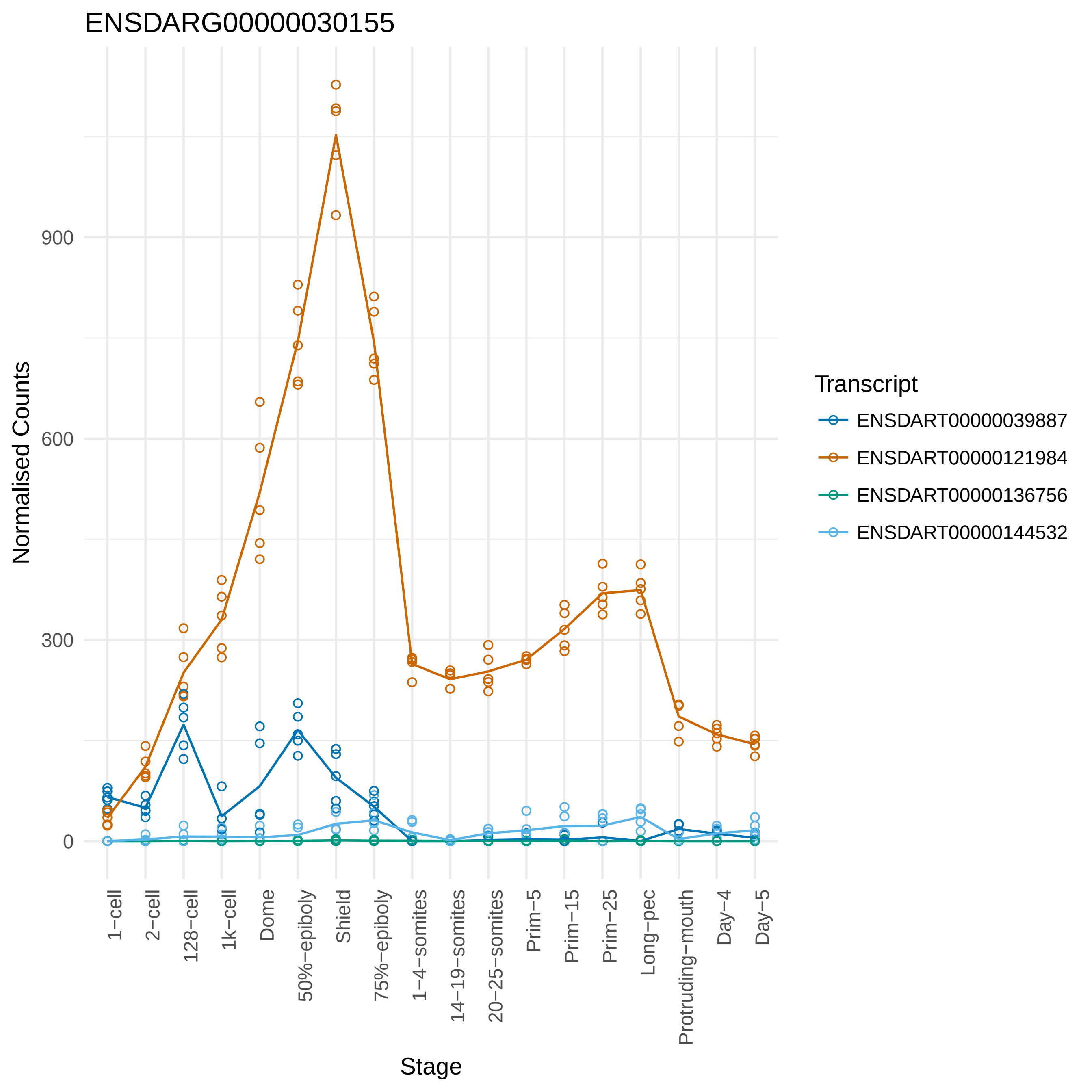
ENSDARG00000030155
Normalised Counts
0
300
600
900
Stage
1−cell
2−cell
128−cell
1k−cell
Dome
50%−epiboly
Shield
75%−epiboly
1−4−somites
14−19−somites
20−25−somites
Prim−5
Prim−15
Prim−25
Long−pec
Protruding−mouth
Day−4
Day−5
Transcript
ENSDART00000039887
ENSDART00000121984
ENSDART00000136756
ENSDART00000144532

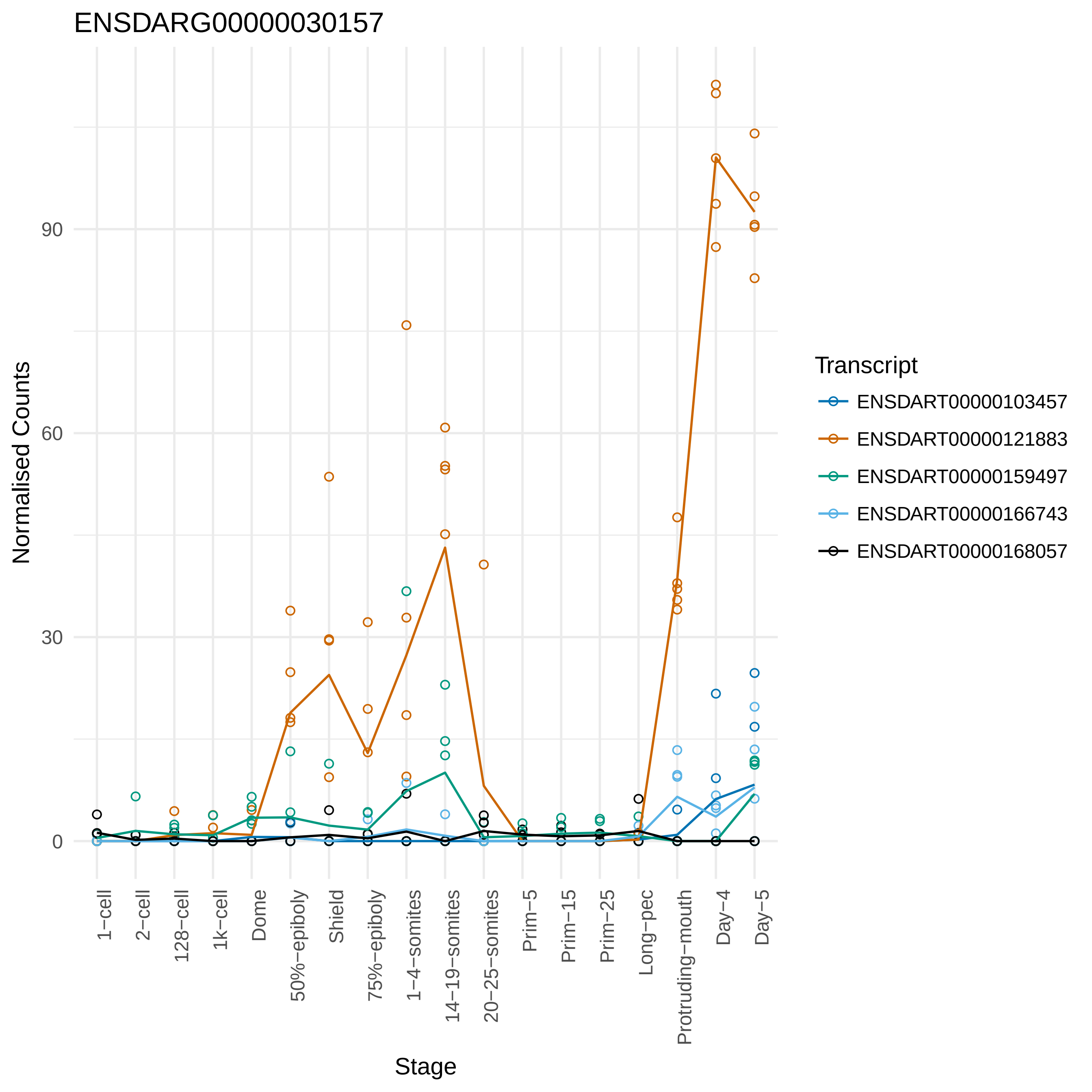
ENSDARG00000030157
Normalised Counts
0
30
60
90
Stage
1−cell
2−cell
128−cell
1k−cell
Dome
50%−epiboly
Shield
75%−epiboly
1−4−somites
14−19−somites
20−25−somites
Prim−5
Prim−15
Prim−25
Long−pec
Protruding−mouth
Day−4
Day−5
Transcript
ENSDART00000103457
ENSDART00000121883
ENSDART00000159497
ENSDART00000166743
ENSDART00000168057

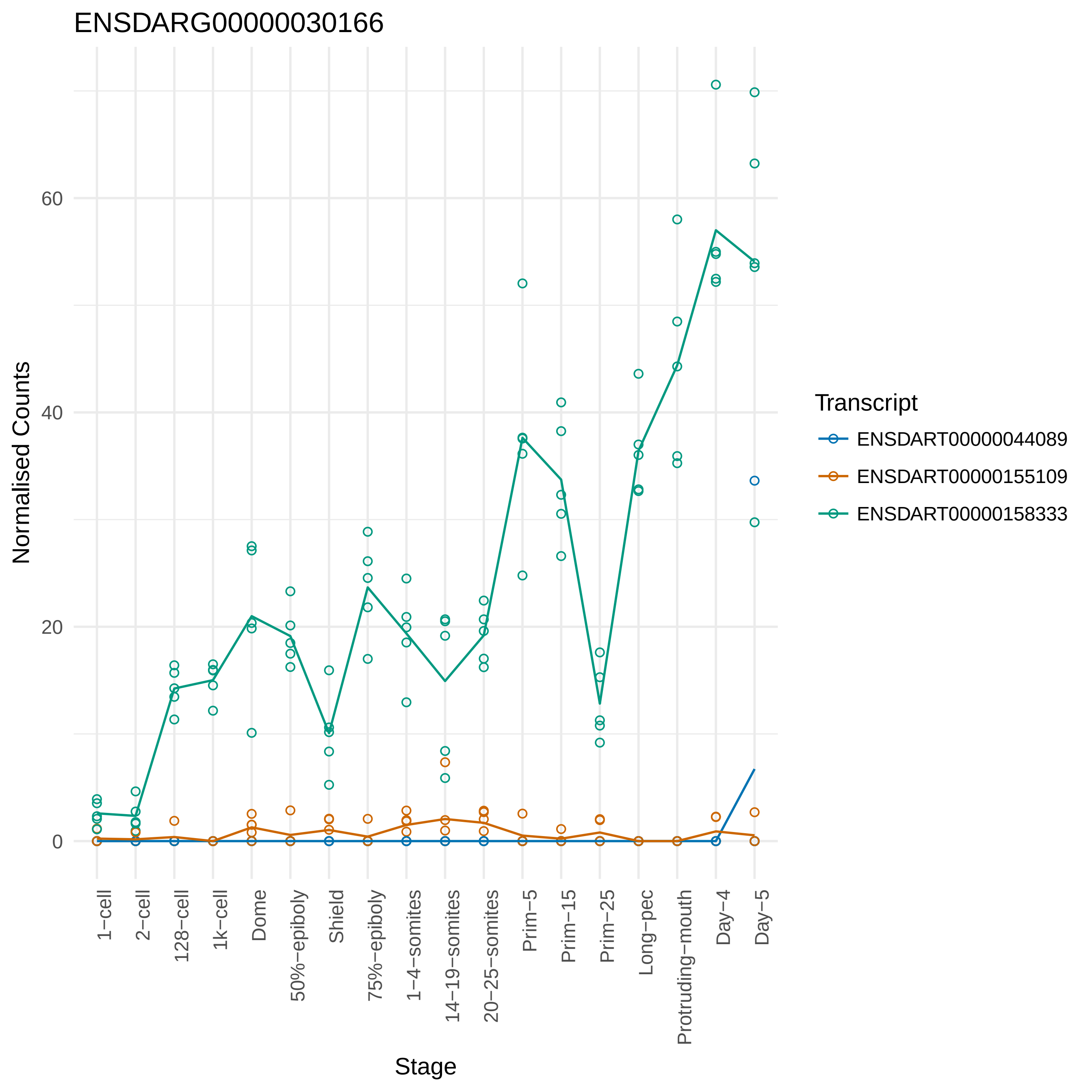

ENSDARG00000030166
Normalised Counts
60
40
20
0
1−cell
2−cell
128−cell
1k−cell
Dome
50%−epiboly
Shield
75%−epiboly
1−4−somites
14−19−somites
20−25−somites
Prim−5
Prim−15
Prim−25
Long−pec
Protruding−mouth
Day−4
Day−5
Stage
Transcript
ENSDART00000044089
ENSDART00000155109
ENSDART00000158333

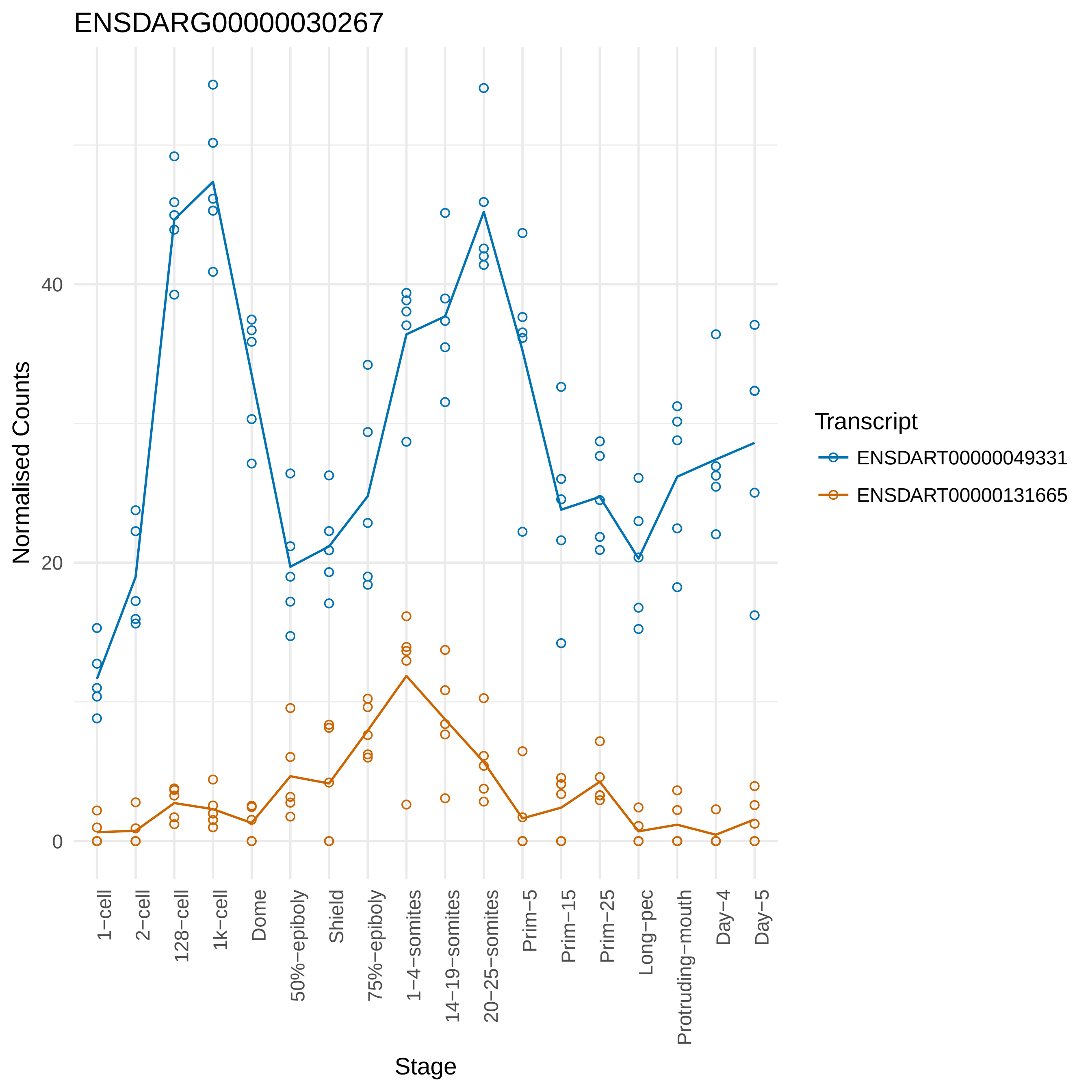
ENSDARG00000030267
Normalised Counts
0
20
40
1−cell
2−cell
128−cell
1k−cell
Dome
50%−epiboly
Shield
75%−epiboly
1−4−somites
14−19−somites
20−25−somites
Prim−5
Prim−15
Prim−25
Long−pec
Protruding−mouth
Day−4
Day−5
Stage
Transcript
ENSDART00000049331
ENSDART00000131665

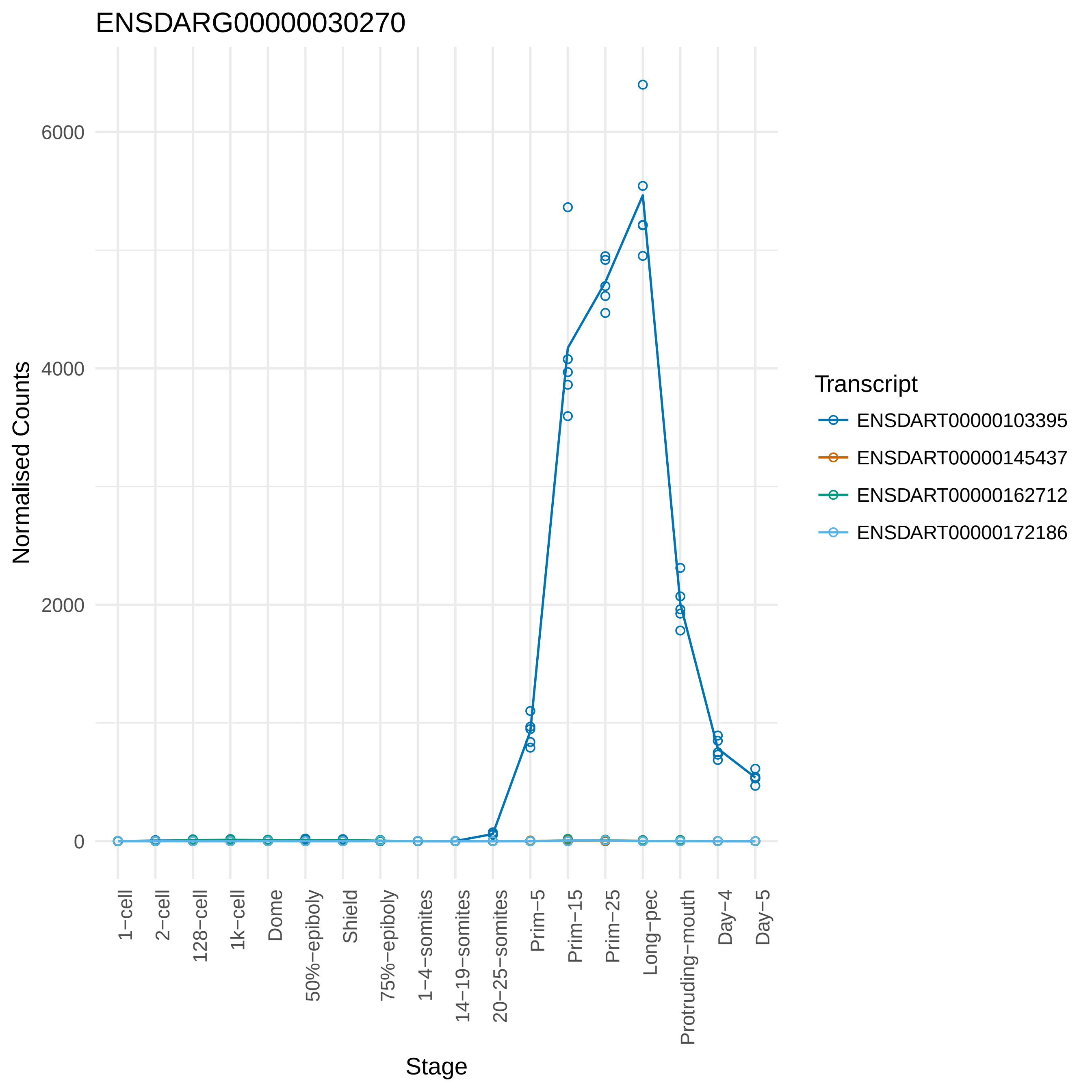

ENSDARG00000030270
Normalised Counts
6000
4000
2000
0
1−cell
2−cell
128−cell
1k−cell
Dome
50%−epiboly
Shield
75%−epiboly
1−4−somites
14−19−somites
20−25−somites
Prim−5
Prim−15
Prim−25
Long−pec
Protruding−mouth
Day−4
Day−5
Stage
Transcript
ENSDART00000103395
ENSDART00000145437
ENSDART00000162712
ENSDART00000172186

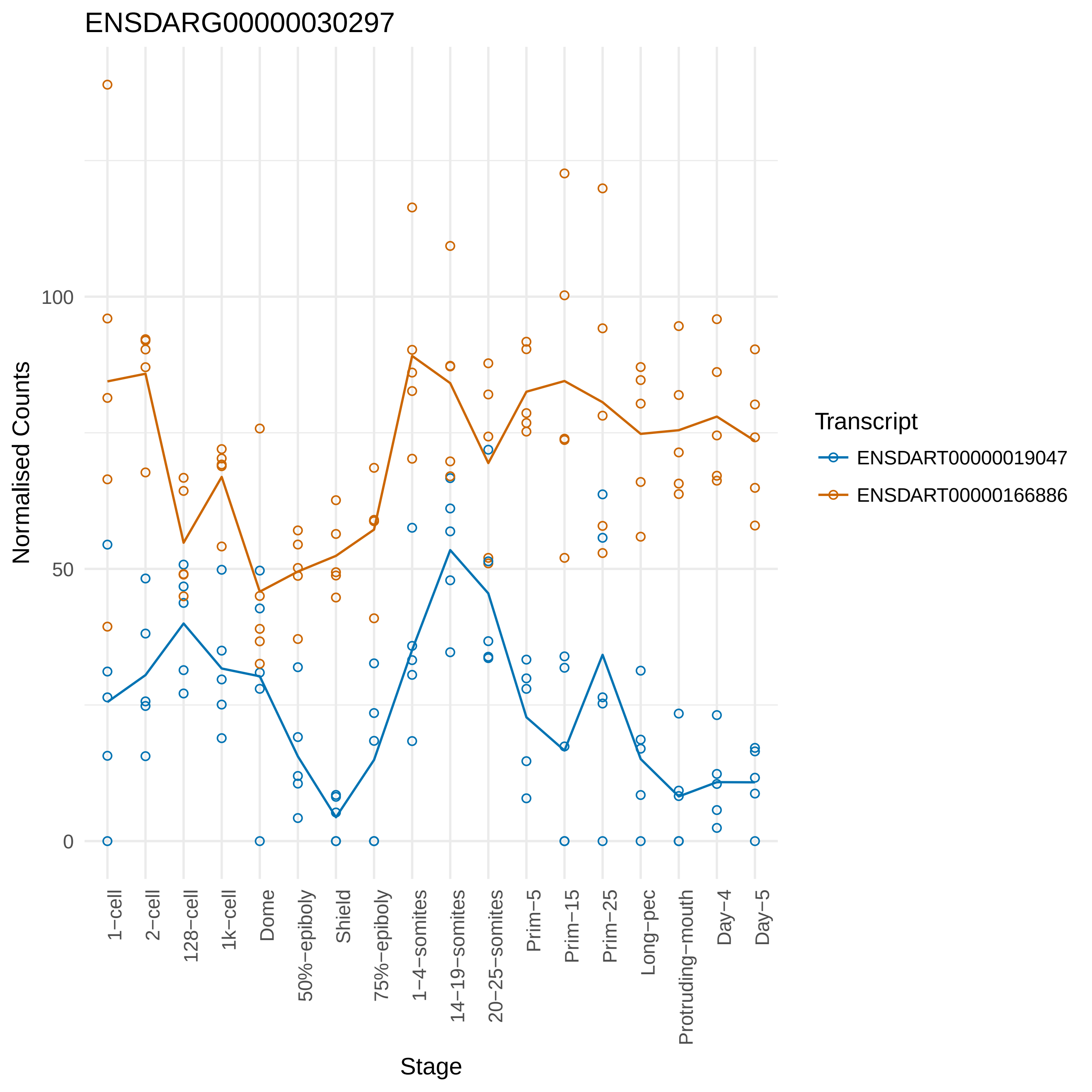
ENSDARG00000030297
Normalised Counts
100
50
0
1−cell
2−cell
128−cell
1k−cell
Dome
50%−epiboly
Shield
75%−epiboly
1−4−somites
14−19−somites
20−25−somites
Prim−5
Prim−15
Prim−25
Long−pec
Protruding−mouth
Day−4
Day−5
Stage
Transcript
ENSDART00000019047
ENSDART00000166886

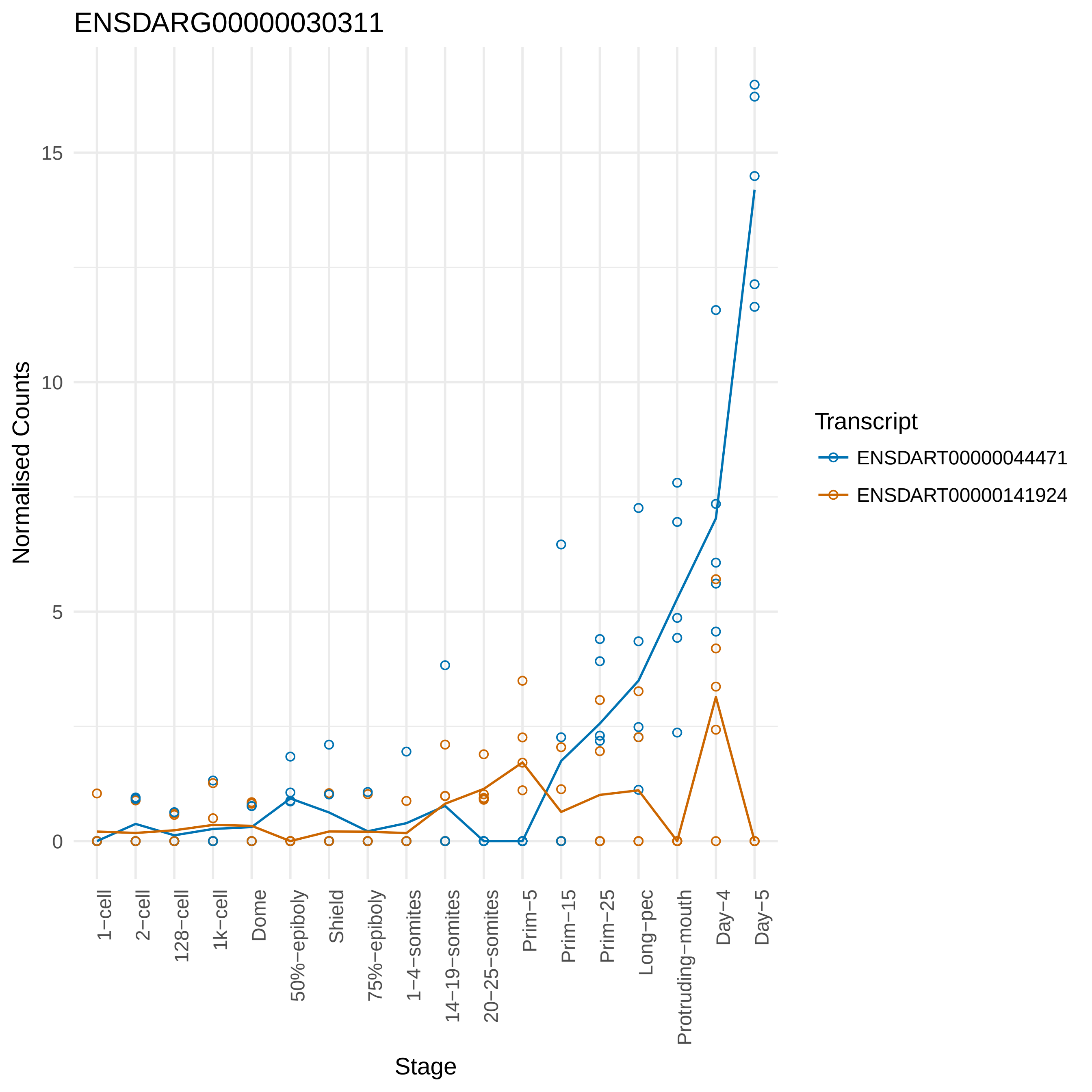

ENSDARG00000030311
Normalised Counts
0
5
10
15
Stage
1−cell
2−cell
128−cell
1k−cell
Dome
50%−epiboly
Shield
75%−epiboly
1−4−somites
14−19−somites
20−25−somites
Prim−5
Prim−15
Prim−25
Long−pec
Protruding−mouth
Day−4
Day−5
Transcript
ENSDART00000044471
ENSDART00000141924

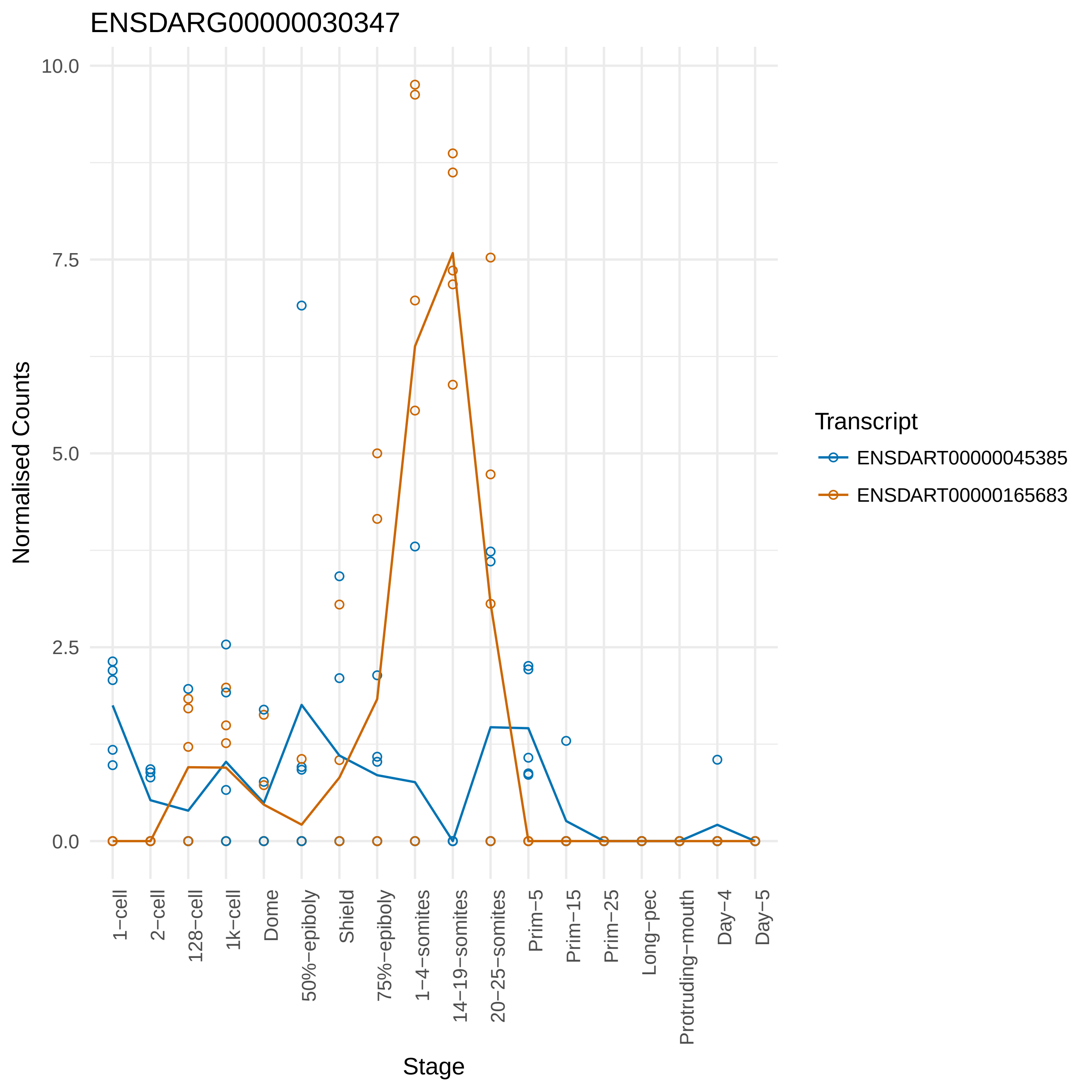

ENSDARG00000030347
Normalised Counts
10.0
7.5
5.0
2.5
0.0
1−cell
2−cell
128−cell
1k−cell
Dome
50%−epiboly
Shield
75%−epiboly
1−4−somites
14−19−somites
20−25−somites
Prim−5
Prim−15
Prim−25
Long−pec
Protruding−mouth
Day−4
Day−5
Stage
Transcript
ENSDART00000045385
ENSDART00000165683

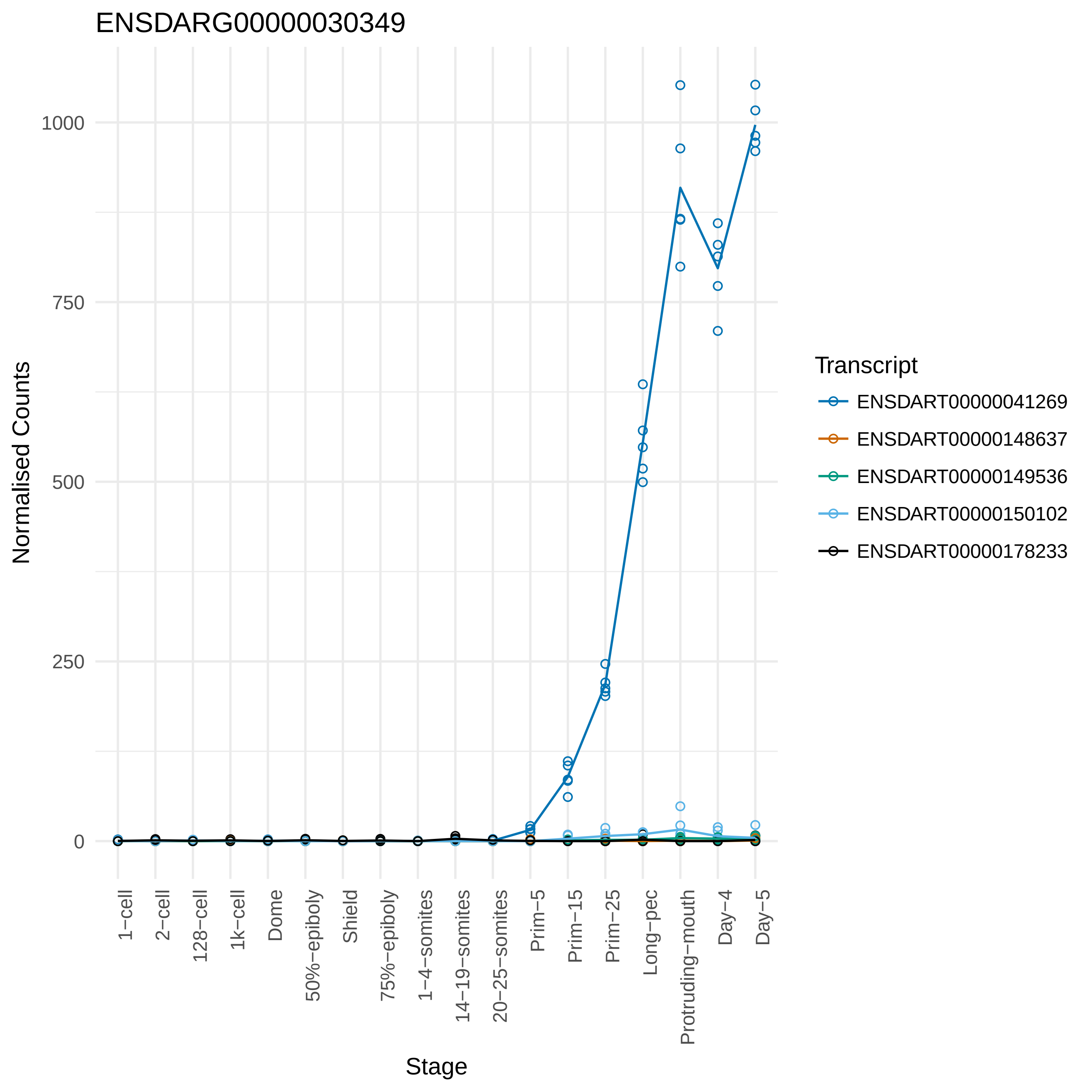

ENSDARG00000030349
Normalised Counts
1000
750
500
250
0
Stage
1−cell
2−cell
128−cell
1k−cell
Dome
50%−epiboly
Shield
75%−epiboly
1−4−somites
14−19−somites
20−25−somites
Prim−5
Prim−15
Prim−25
Long−pec
Protruding−mouth
Day−4
Day−5
Transcript
ENSDART00000041269
ENSDART00000148637
ENSDART00000149536
ENSDART00000150102
ENSDART00000178233

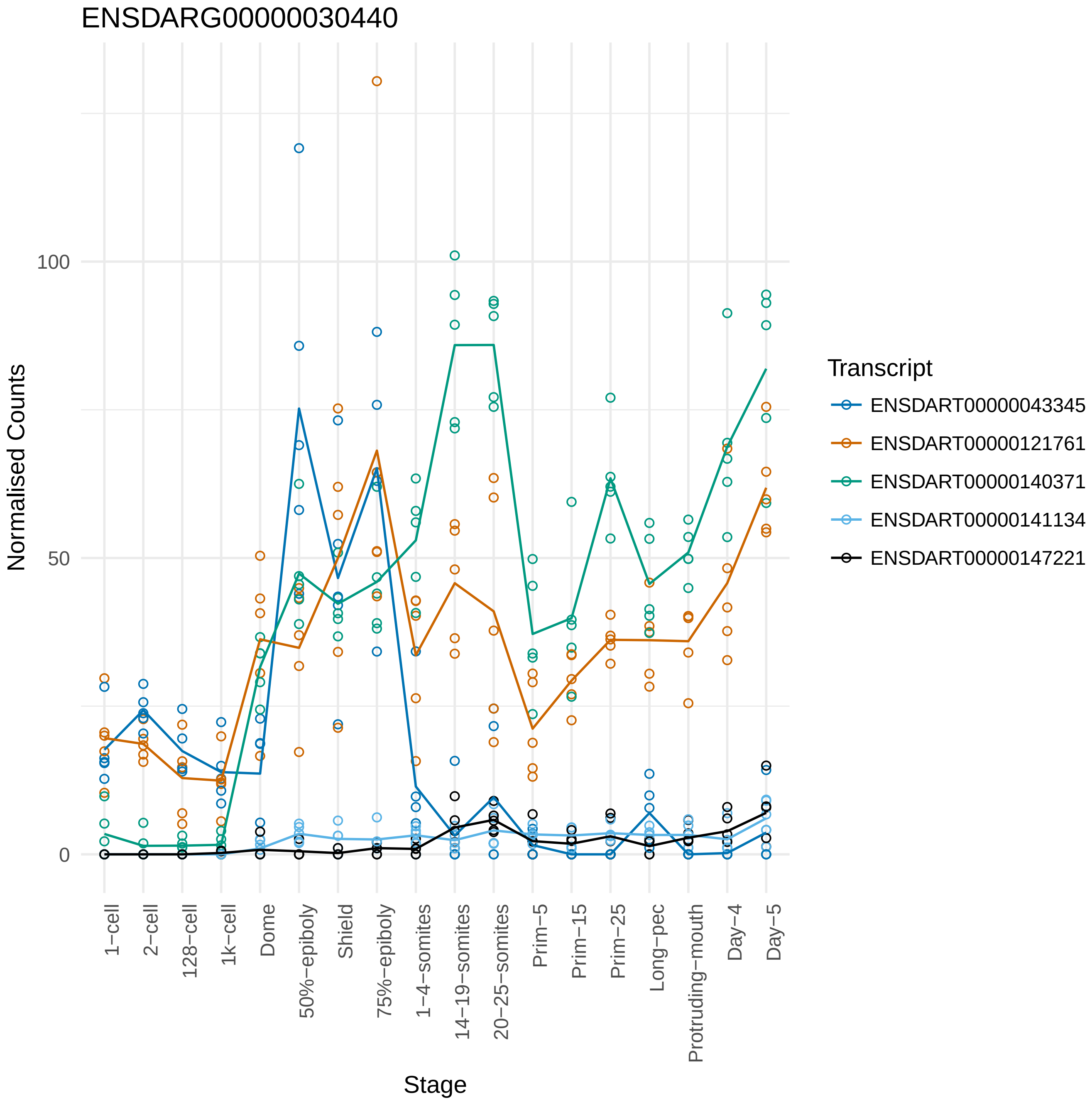

ENSDARG00000030440
Normalised Counts
100
50
0
Stage
1−cell
2−cell
128−cell
1k−cell
Dome
50%−epiboly
Shield
75%−epiboly
1−4−somites
14−19−somites
20−25−somites
Prim−5
Prim−15
Prim−25
Long−pec
Protruding−mouth
Day−4
Day−5
Transcript
ENSDART00000043345
ENSDART00000121761
ENSDART00000140371
ENSDART00000141134
ENSDART00000147221

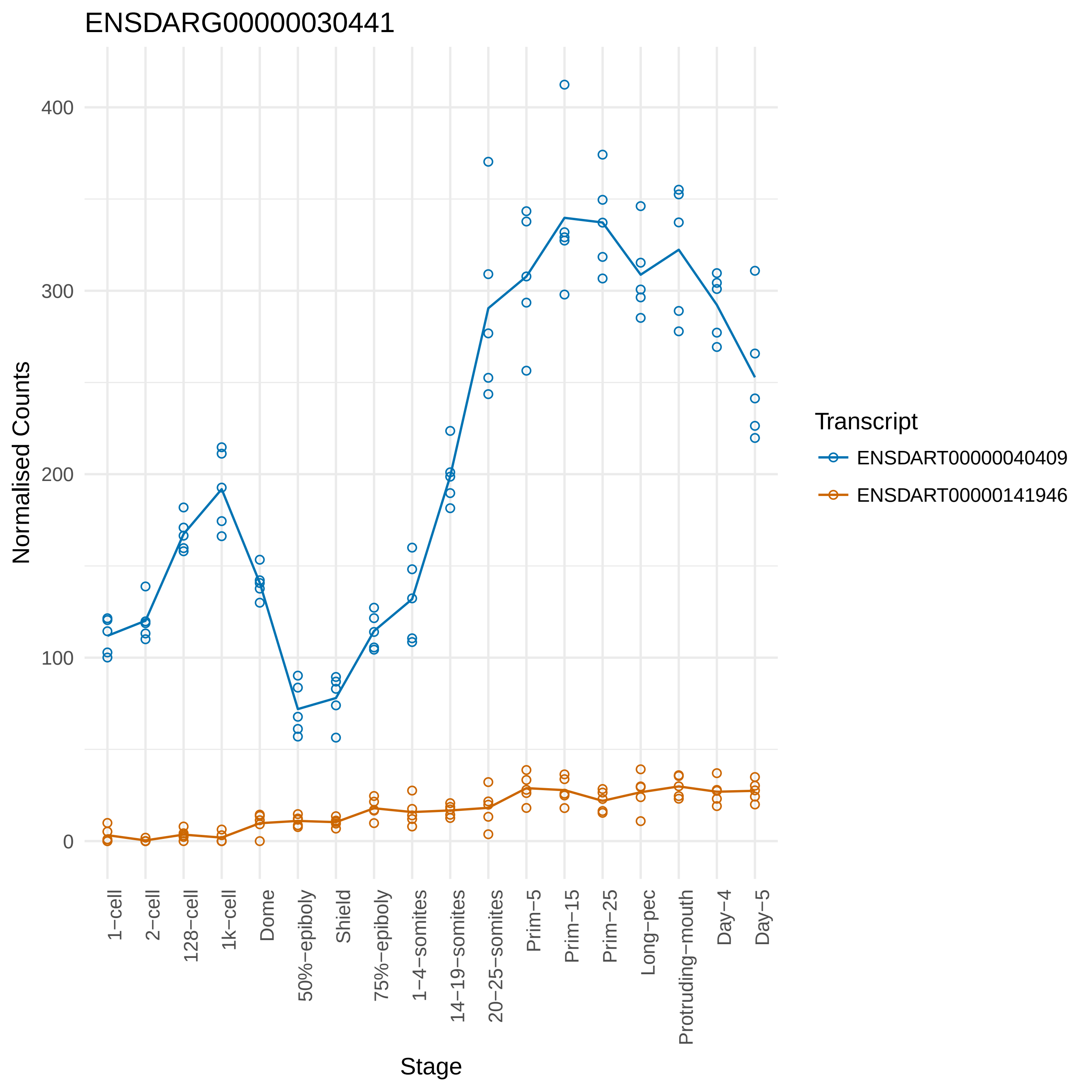

ENSDARG00000030441
Normalised Counts
400
300
200
100
0
1−cell
2−cell
128−cell
1k−cell
Dome
50%−epiboly
Shield
75%−epiboly
1−4−somites
14−19−somites
20−25−somites
Prim−5
Prim−15
Prim−25
Long−pec
Protruding−mouth
Day−4
Day−5
Stage
Transcript
ENSDART00000040409
ENSDART00000141946

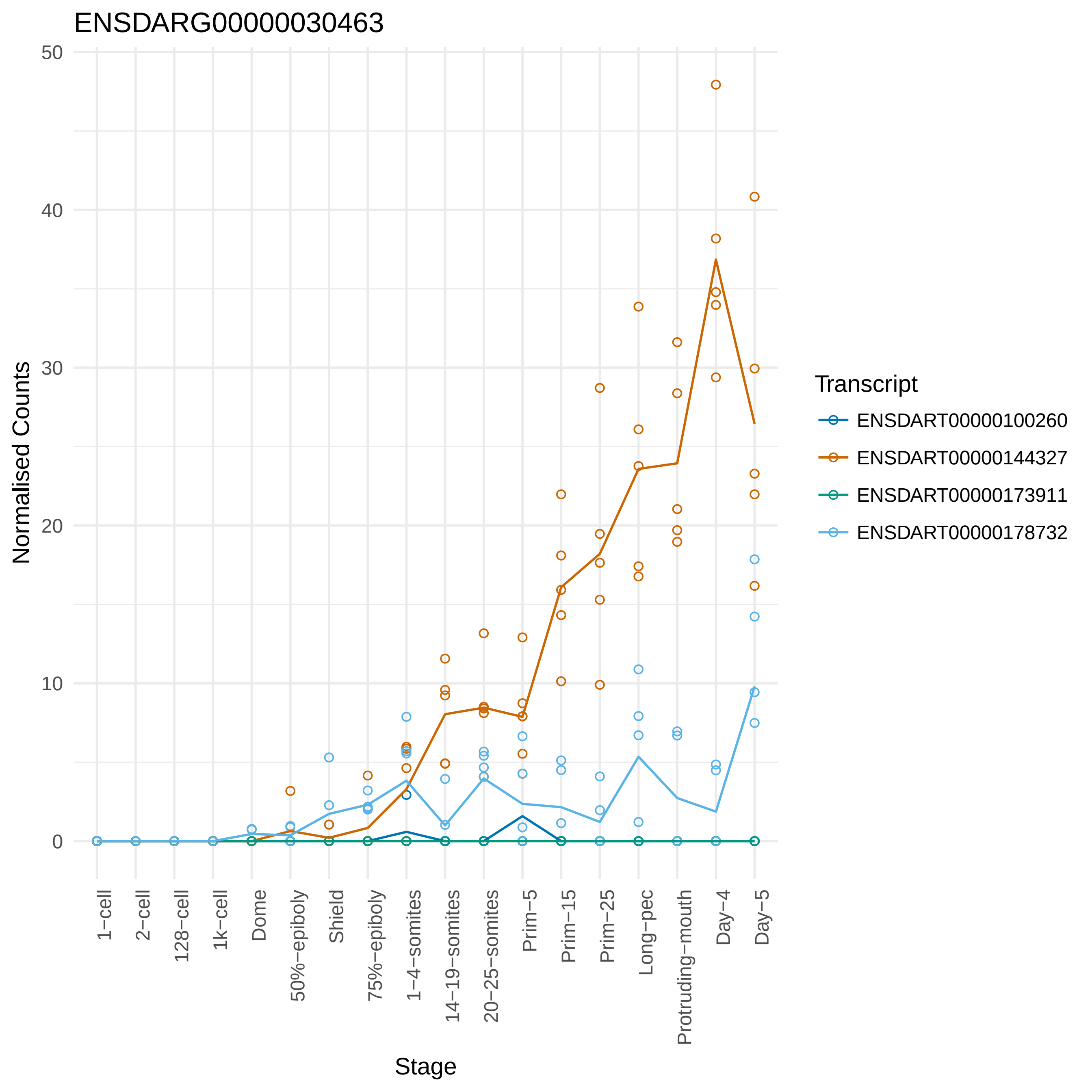

ENSDARG00000030463
Normalised Counts
50
40
30
20
10
0
1−cell
2−cell
128−cell
1k−cell
Dome
50%−epiboly
Shield
75%−epiboly
1−4−somites
14−19−somites
20−25−somites
Prim−5
Prim−15
Prim−25
Long−pec
Protruding−mouth
Day−4
Day−5
Stage
Transcript
ENSDART00000100260
ENSDART00000144327
ENSDART00000173911
ENSDART00000178732

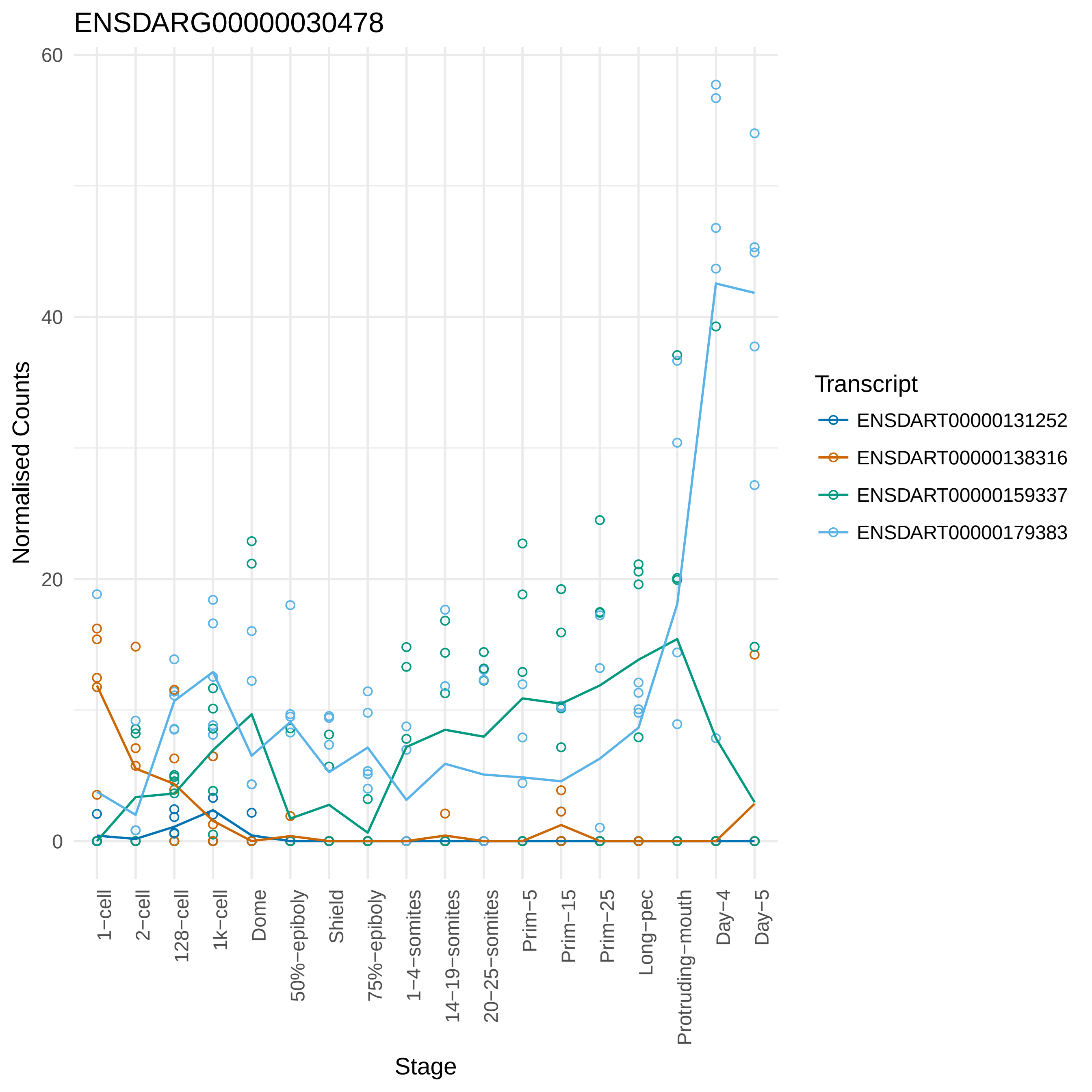

ENSDARG00000030478
Normalised Counts
0
20
40
60
1−cell
2−cell
128−cell
1k−cell
Dome
50%−epiboly
Shield
75%−epiboly
1−4−somites
14−19−somites
20−25−somites
Prim−5
Prim−15
Prim−25
Long−pec
Protruding−mouth
Day−4
Day−5
Stage
Transcript
ENSDART00000131252
ENSDART00000138316
ENSDART00000159337
ENSDART00000179383

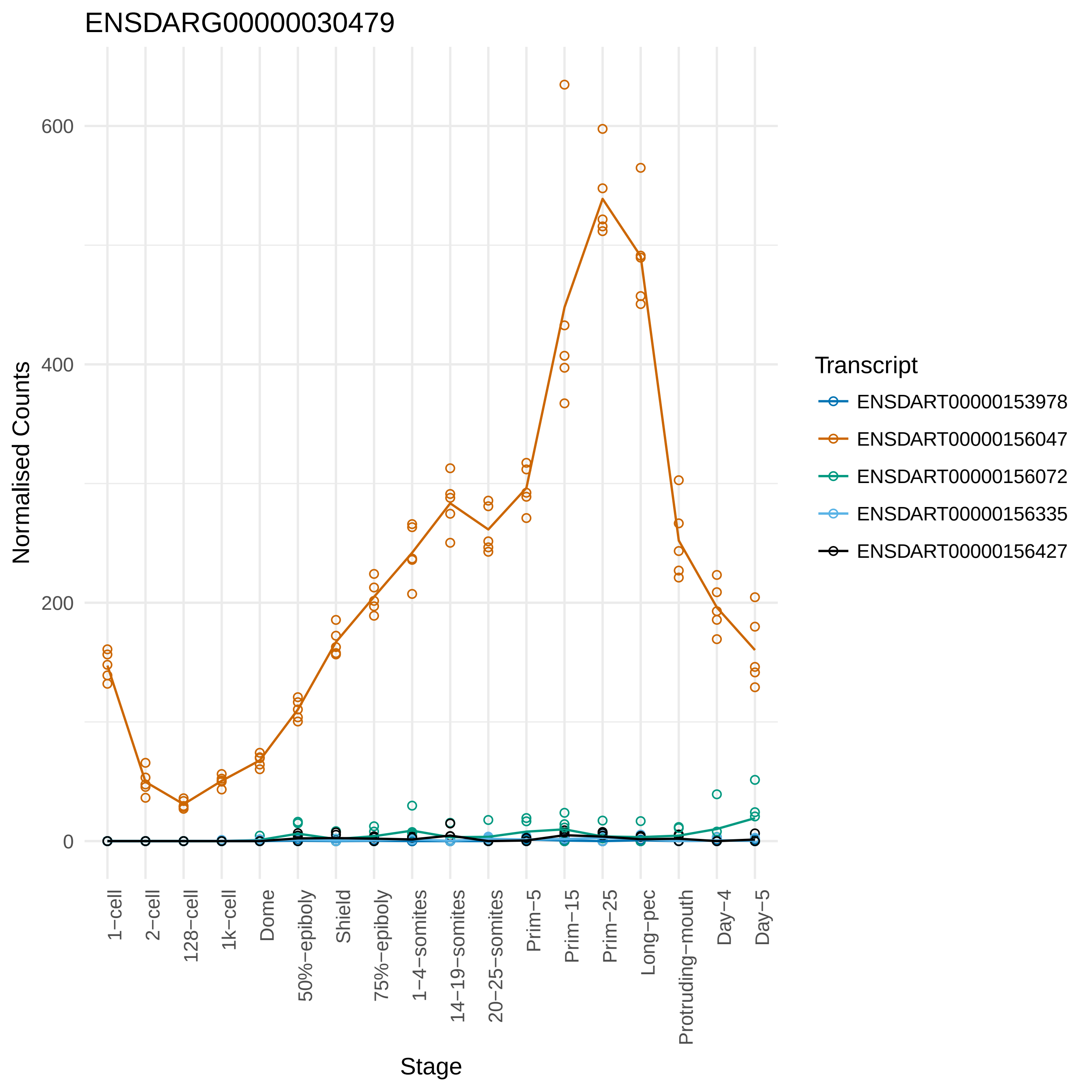

ENSDARG00000030479
Normalised Counts
600
400
200
0
1−cell
2−cell
128−cell
1k−cell
Dome
50%−epiboly
Shield
75%−epiboly
1−4−somites
14−19−somites
20−25−somites
Prim−5
Prim−15
Prim−25
Long−pec
Protruding−mouth
Day−4
Day−5
Stage
Transcript
ENSDART00000153978
ENSDART00000156047
ENSDART00000156072
ENSDART00000156335
ENSDART00000156427

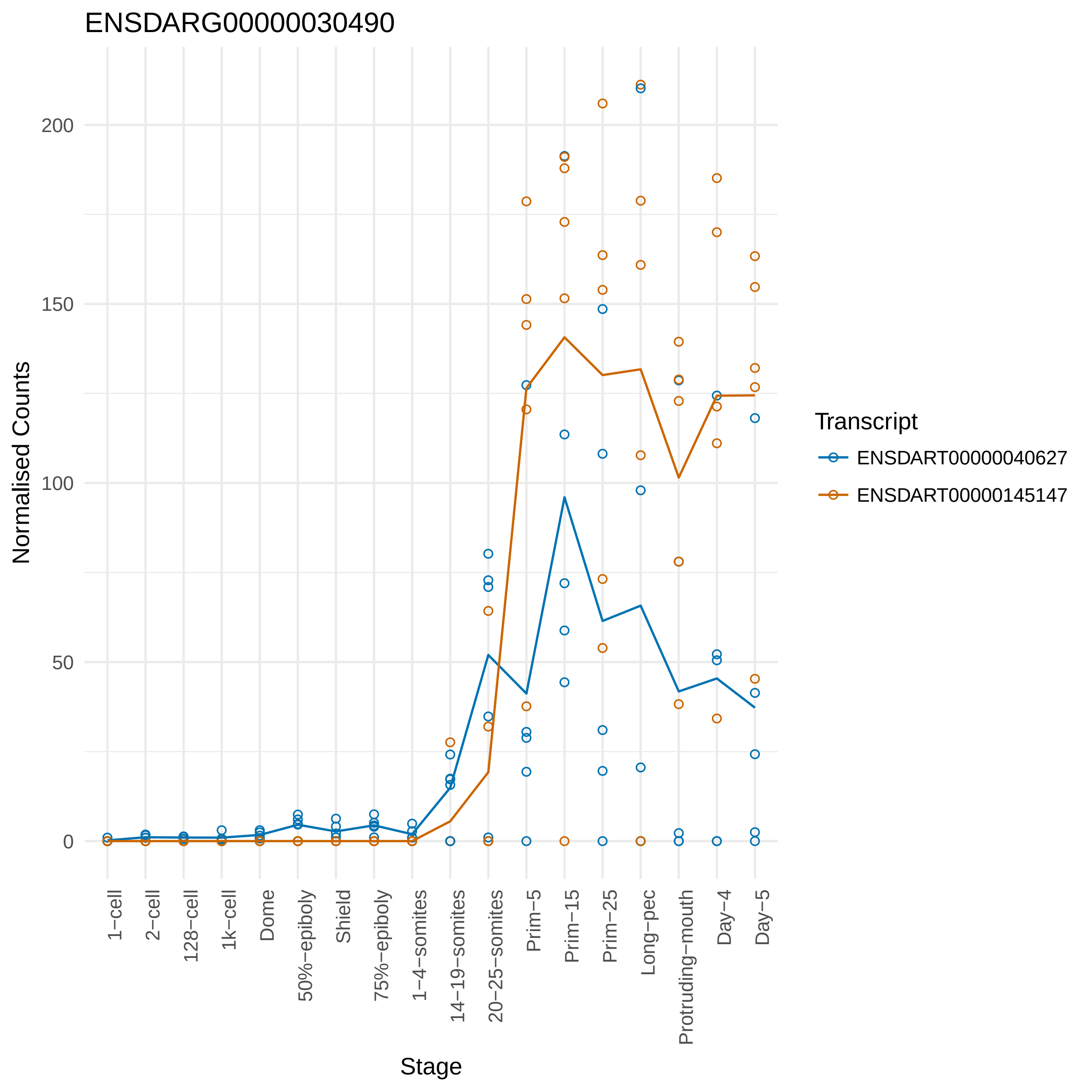

ENSDARG00000030490
Normalised Counts
200
150
100
50
0
1−cell
2−cell
128−cell
1k−cell
Dome
50%−epiboly
Shield
75%−epiboly
1−4−somites
14−19−somites
20−25−somites
Prim−5
Prim−15
Prim−25
Long−pec
Protruding−mouth
Day−4
Day−5
Stage
Transcript
ENSDART00000040627
ENSDART00000145147

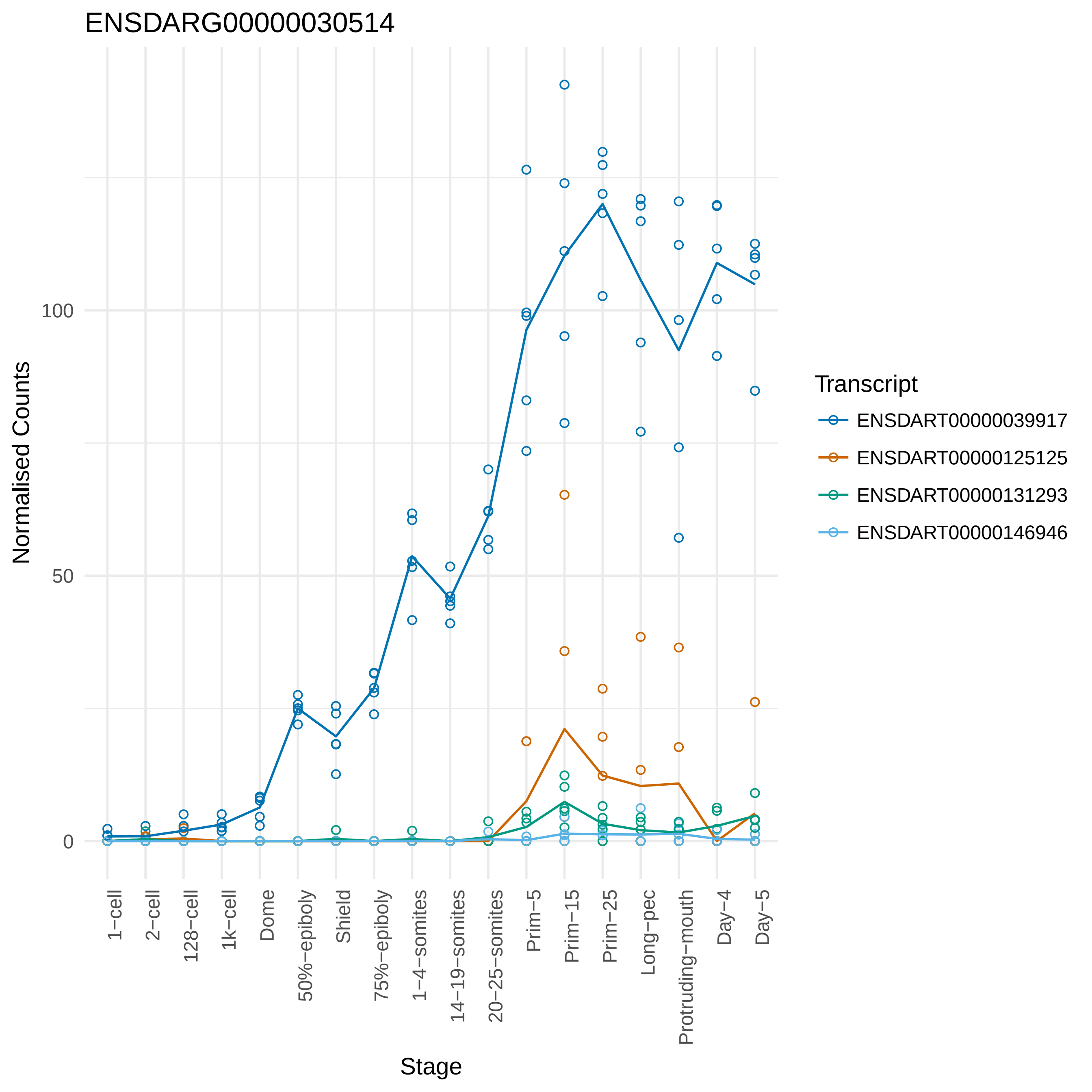
ENSDARG00000030514
Normalised Counts
100
50
0
1−cell
2−cell
128−cell
1k−cell
Dome
50%−epiboly
Shield
75%−epiboly
1−4−somites
14−19−somites
20−25−somites
Prim−5
Prim−15
Prim−25
Long−pec
Protruding−mouth
Day−4
Day−5
Stage
Transcript
ENSDART00000039917
ENSDART00000125125
ENSDART00000131293
ENSDART00000146946

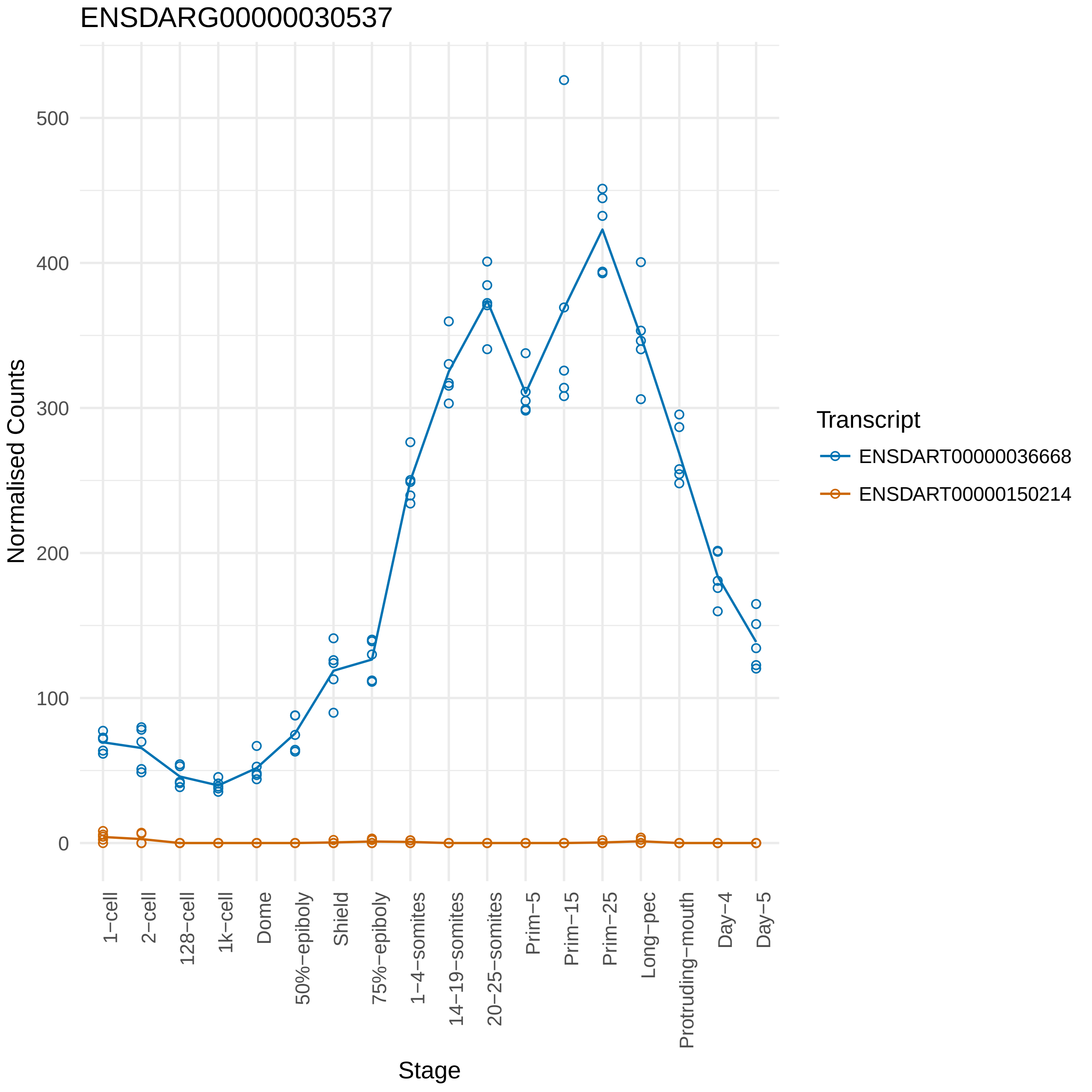
ENSDARG00000030537
Normalised Counts
500
400
300
200
100
0
1−cell
2−cell
128−cell
1k−cell
Dome
50%−epiboly
Shield
75%−epiboly
1−4−somites
14−19−somites
20−25−somites
Prim−5
Prim−15
Prim−25
Long−pec
Protruding−mouth
Day−4
Day−5
Stage
Transcript
ENSDART00000036668
ENSDART00000150214

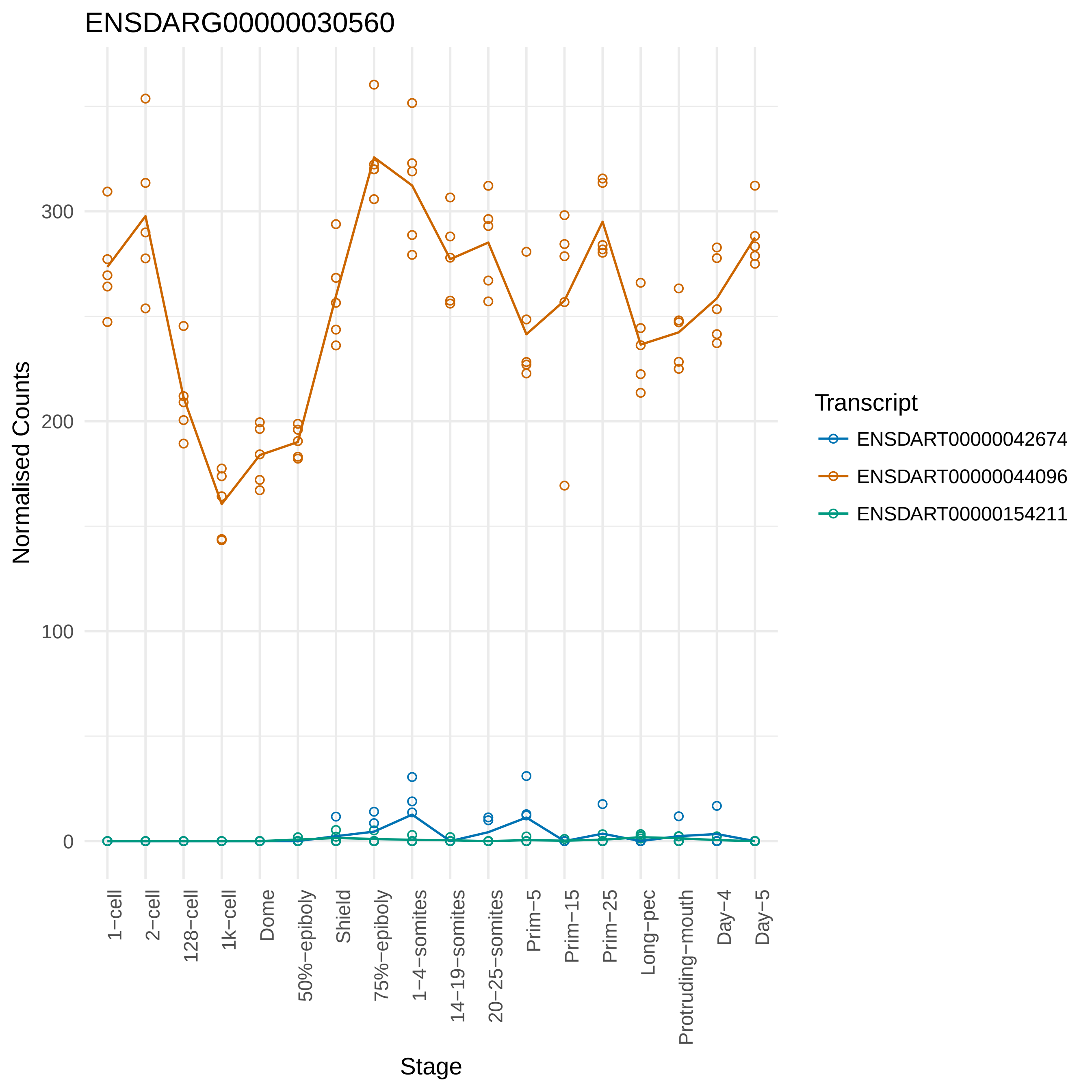

ENSDARG00000030560
Normalised Counts
300
200
100
0
1−cell
2−cell
128−cell
1k−cell
Dome
50%−epiboly
Shield
75%−epiboly
1−4−somites
14−19−somites
20−25−somites
Prim−5
Prim−15
Prim−25
Long−pec
Protruding−mouth
Day−4
Day−5
Stage
Transcript
ENSDART00000042674
ENSDART00000044096
ENSDART00000154211

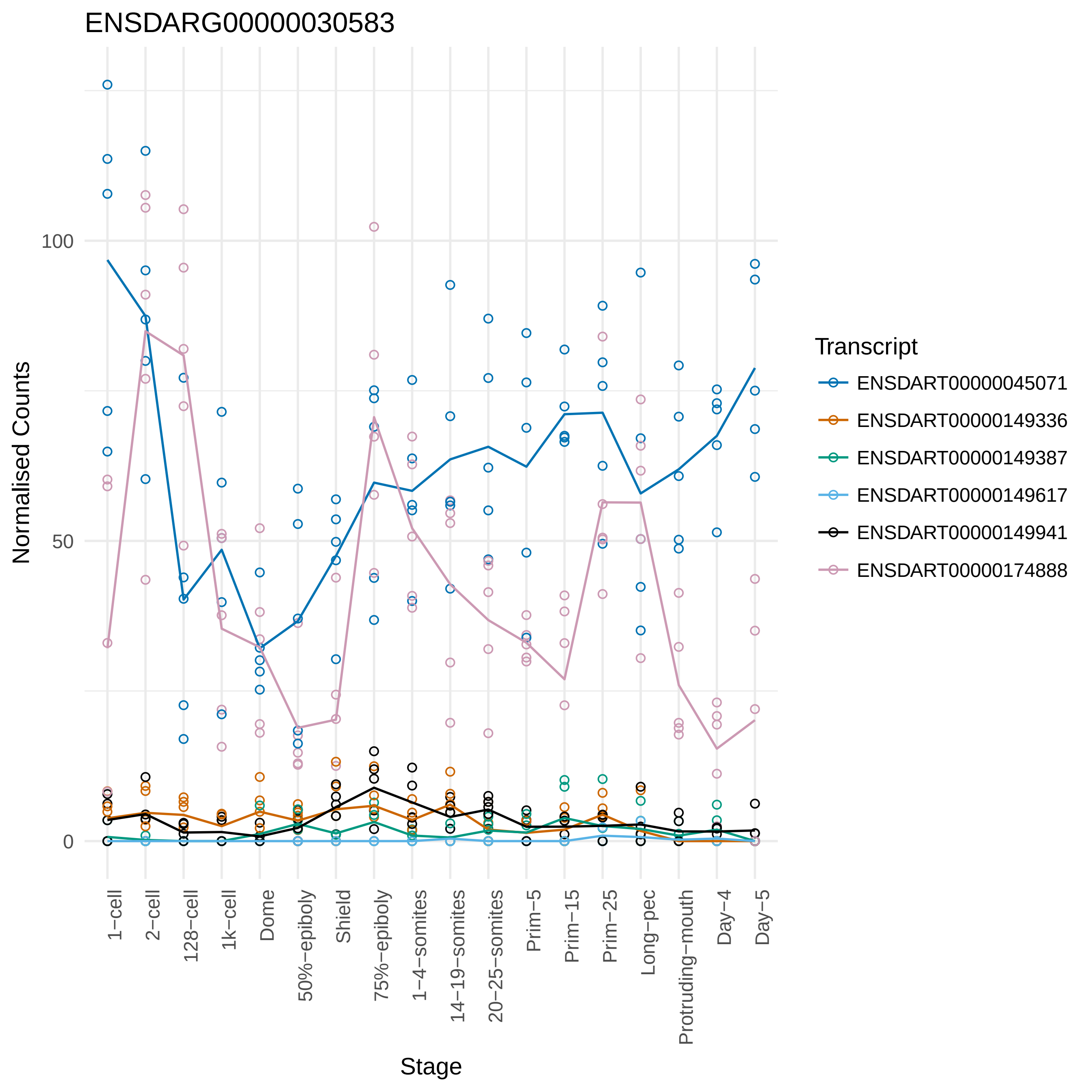
ENSDARG00000030583
Normalised Counts
100
50
0
1−cell
2−cell
128−cell
1k−cell
Dome
50%−epiboly
Shield
75%−epiboly
1−4−somites
14−19−somites
20−25−somites
Prim−5
Prim−15
Prim−25
Long−pec
Protruding−mouth
Day−4
Day−5
Stage
Transcript
ENSDART00000045071
ENSDART00000149336
ENSDART00000149387
ENSDART00000149617
ENSDART00000149941
ENSDART00000174888

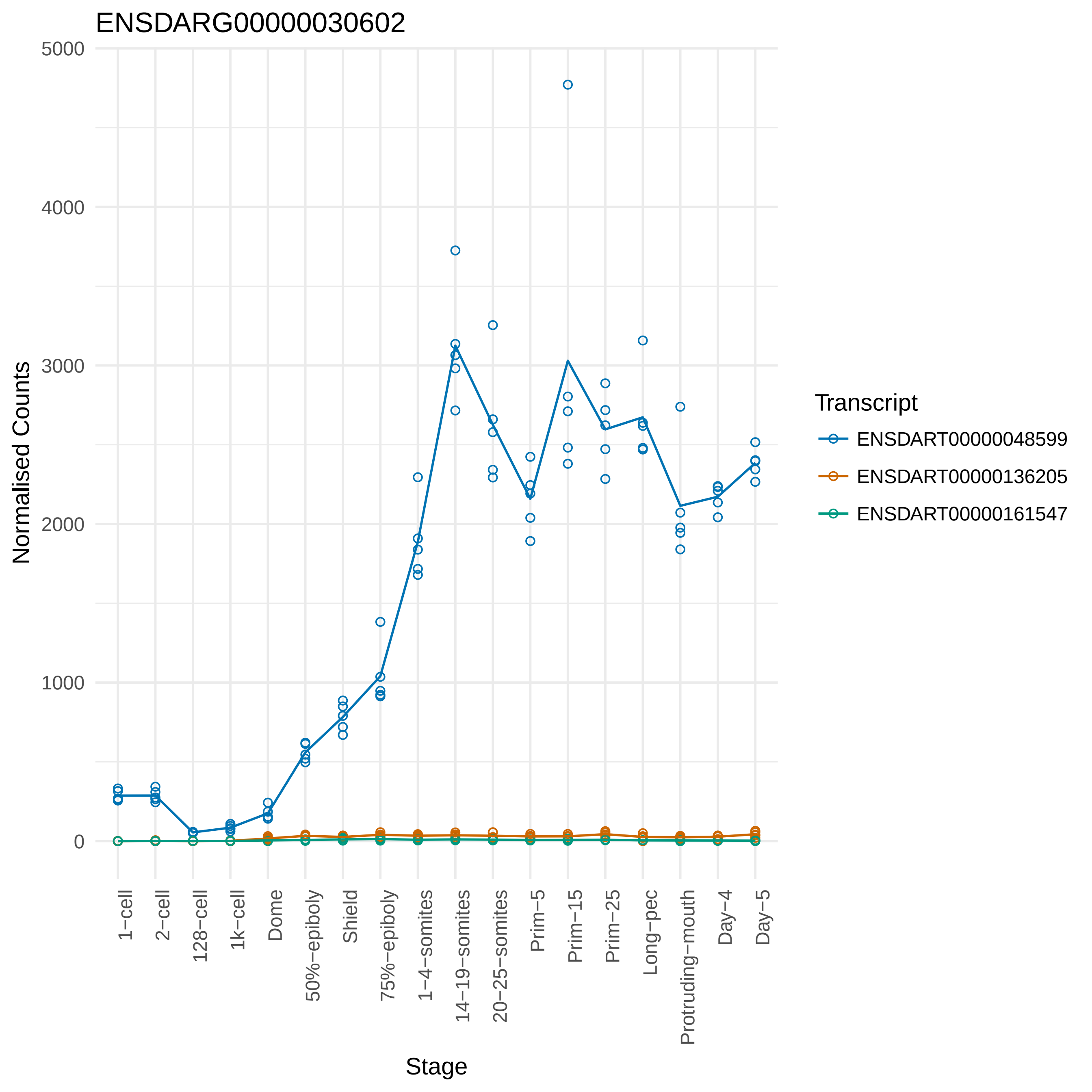

ENSDARG00000030602
Normalised Counts
5000
4000
3000
2000
1000
0
Stage
1−cell
2−cell
128−cell
1k−cell
Dome
50%−epiboly
Shield
75%−epiboly
1−4−somites
14−19−somites
20−25−somites
Prim−5
Prim−15
Prim−25
Long−pec
Protruding−mouth
Day−4
Day−5
Transcript
ENSDART00000048599
ENSDART00000136205
ENSDART00000161547

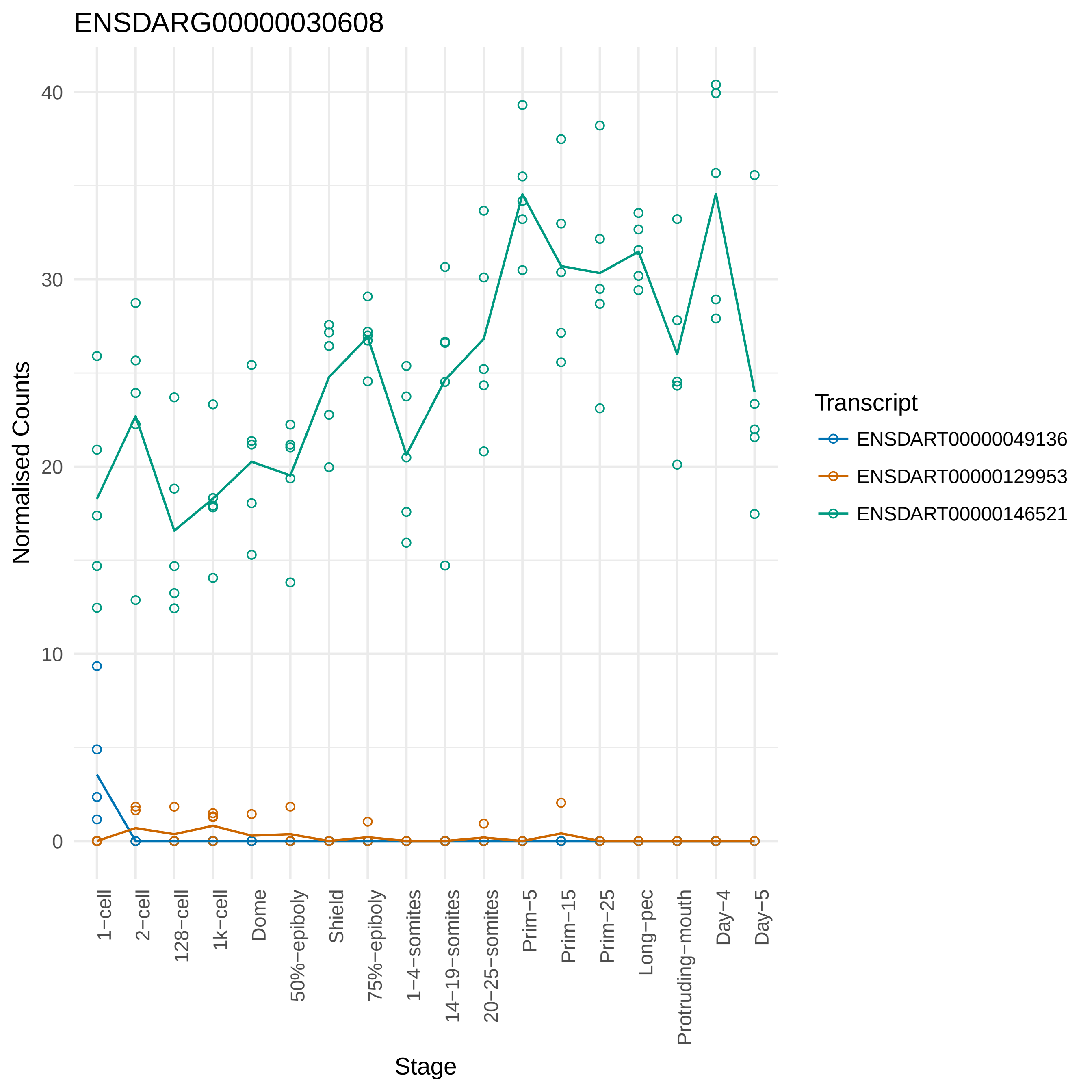
ENSDARG00000030608
Normalised Counts
0
10
20
30
40
1−cell
2−cell
128−cell
1k−cell
Dome
50%−epiboly
Shield
75%−epiboly
1−4−somites
14−19−somites
20−25−somites
Prim−5
Prim−15
Prim−25
Long−pec
Protruding−mouth
Day−4
Day−5
Stage
Transcript
ENSDART00000049136
ENSDART00000129953
ENSDART00000146521

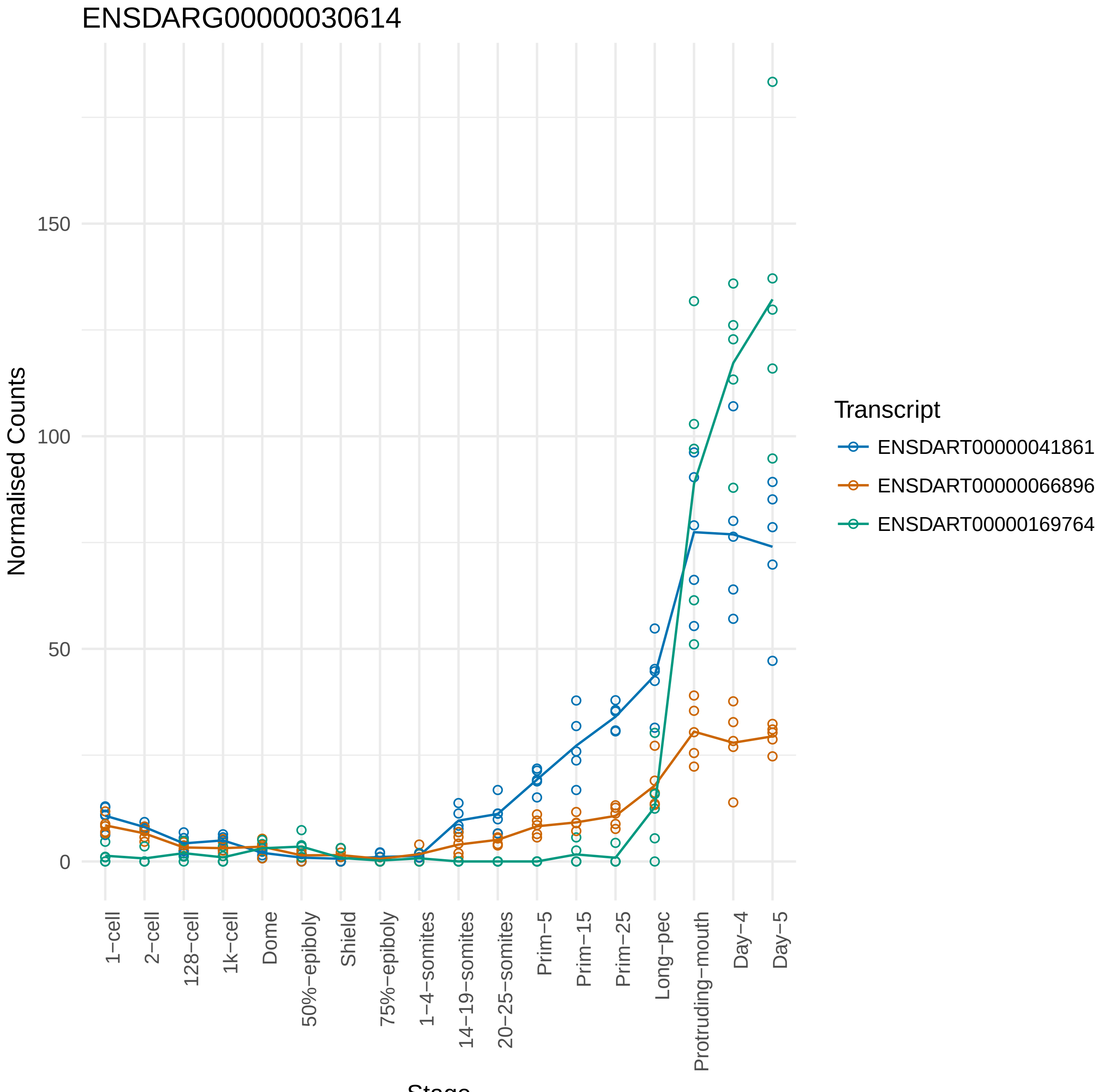

ENSDARG00000030614
Normalised Counts
150
100
50
0
1−cell
2−cell
128−cell
1k−cell
Dome
50%−epiboly
Shield
75%−epiboly
1−4−somites
14−19−somites
20−25−somites
Prim−5
Prim−15
Prim−25
Long−pec
Protruding−mouth
Day−4
Day−5
Stage
Transcript
ENSDART00000041861
ENSDART00000066896
ENSDART00000169764

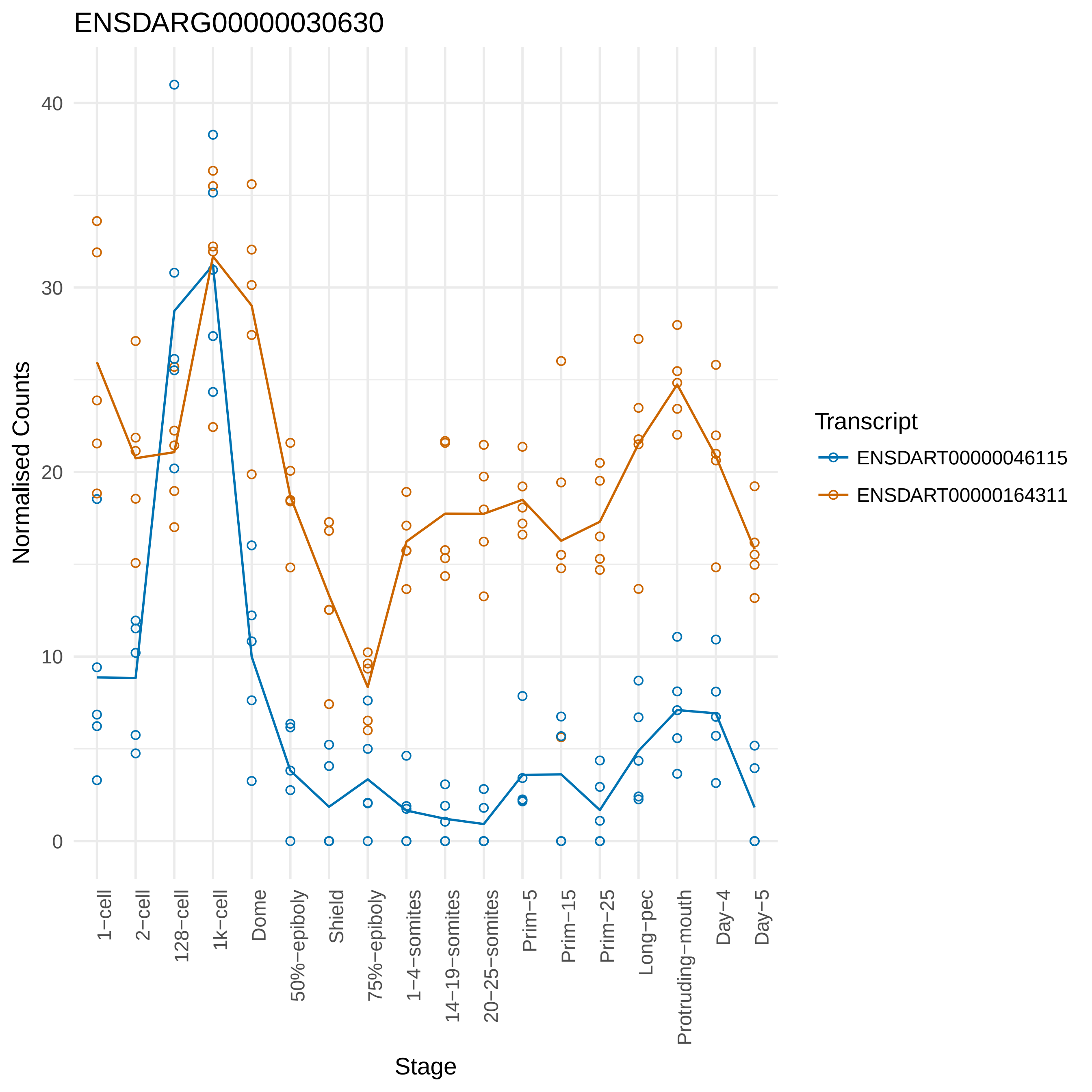

ENSDARG00000030630
Normalised Counts
0
10
20
30
40
1−cell
2−cell
128−cell
1k−cell
Dome
50%−epiboly
Shield
75%−epiboly
1−4−somites
14−19−somites
20−25−somites
Prim−5
Prim−15
Prim−25
Long−pec
Protruding−mouth
Day−4
Day−5
Stage
Transcript
ENSDART00000046115
ENSDART00000164311

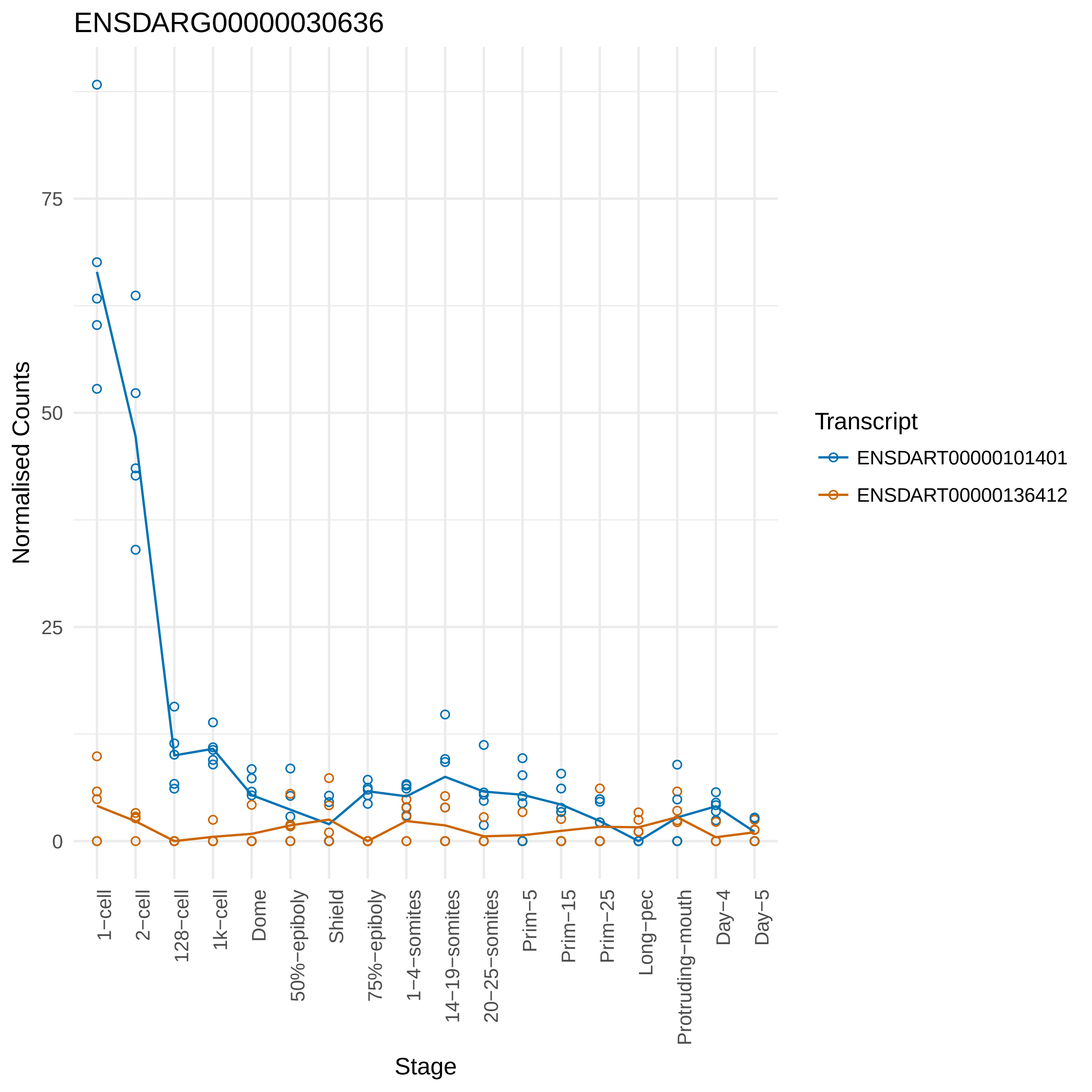

ENSDARG00000030636
Normalised Counts
75
50
25
0
1−cell
2−cell
128−cell
1k−cell
Dome
50%−epiboly
Shield
75%−epiboly
1−4−somites
14−19−somites
20−25−somites
Prim−5
Prim−15
Prim−25
Long−pec
Protruding−mouth
Day−4
Day−5
Stage
Transcript
ENSDART00000101401
ENSDART00000136412

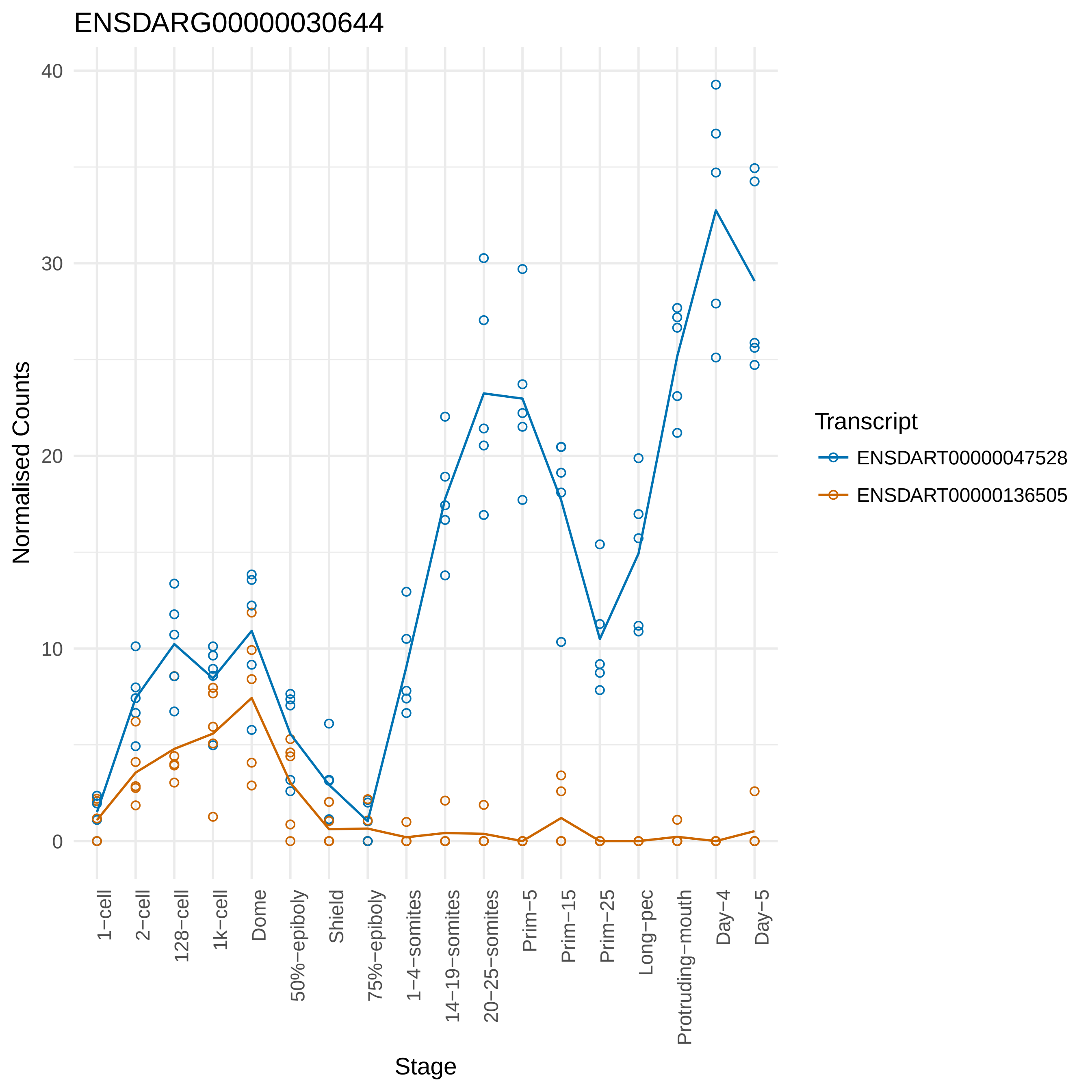
ENSDARG00000030644
Normalised Counts
0
10
20
30
40
1−cell
2−cell
128−cell
1k−cell
Dome
50%−epiboly
Shield
75%−epiboly
1−4−somites
14−19−somites
20−25−somites
Prim−5
Prim−15
Prim−25
Long−pec
Protruding−mouth
Day−4
Day−5
Stage
Transcript
ENSDART00000047528
ENSDART00000136505

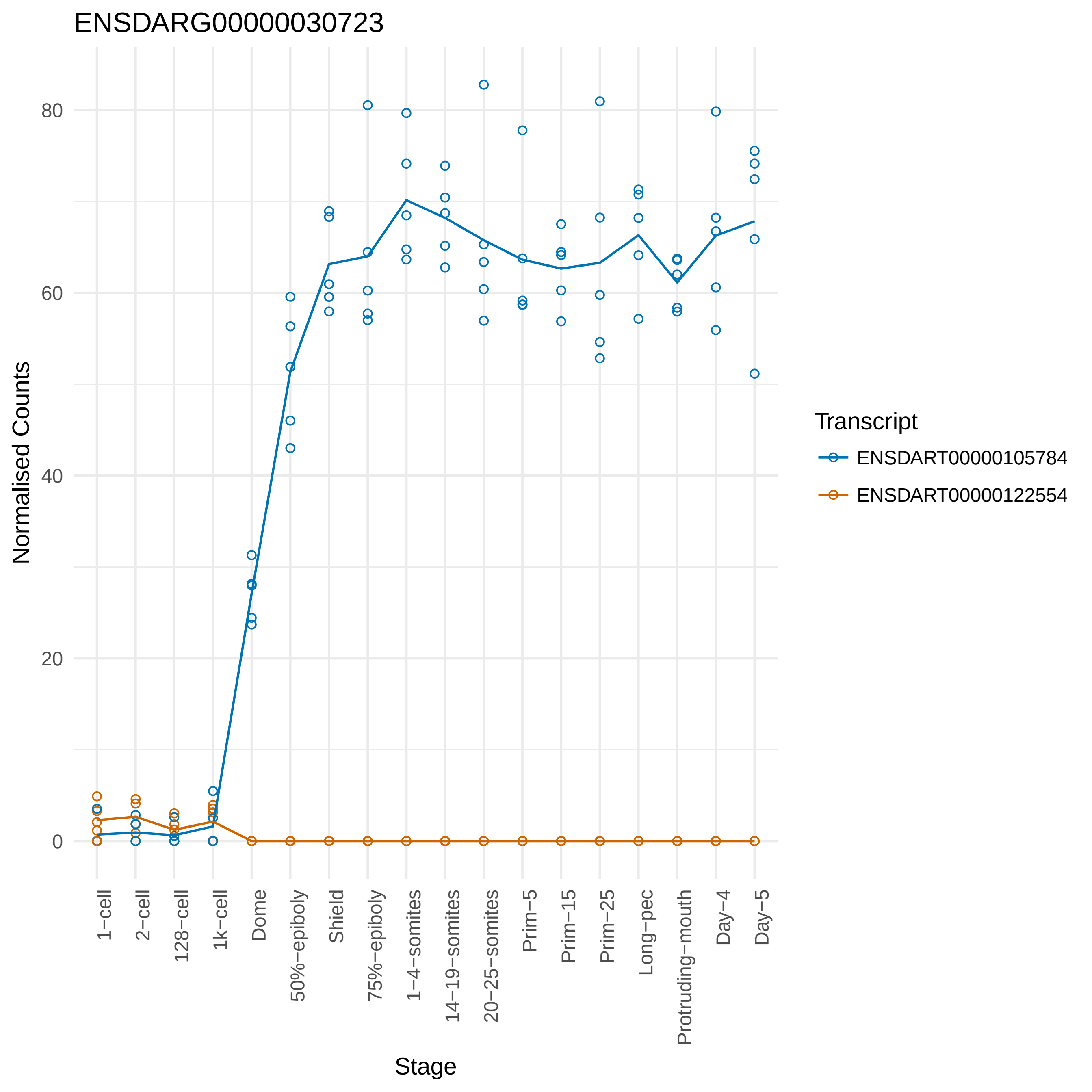
ENSDARG00000030723
Normalised Counts
80
60
40
20
0
1−cell
2−cell
128−cell
1k−cell
Dome
50%−epiboly
Shield
75%−epiboly
1−4−somites
14−19−somites
20−25−somites
Prim−5
Prim−15
Prim−25
Long−pec
Protruding−mouth
Day−4
Day−5
Stage
Transcript
ENSDART00000105784
ENSDART00000122554

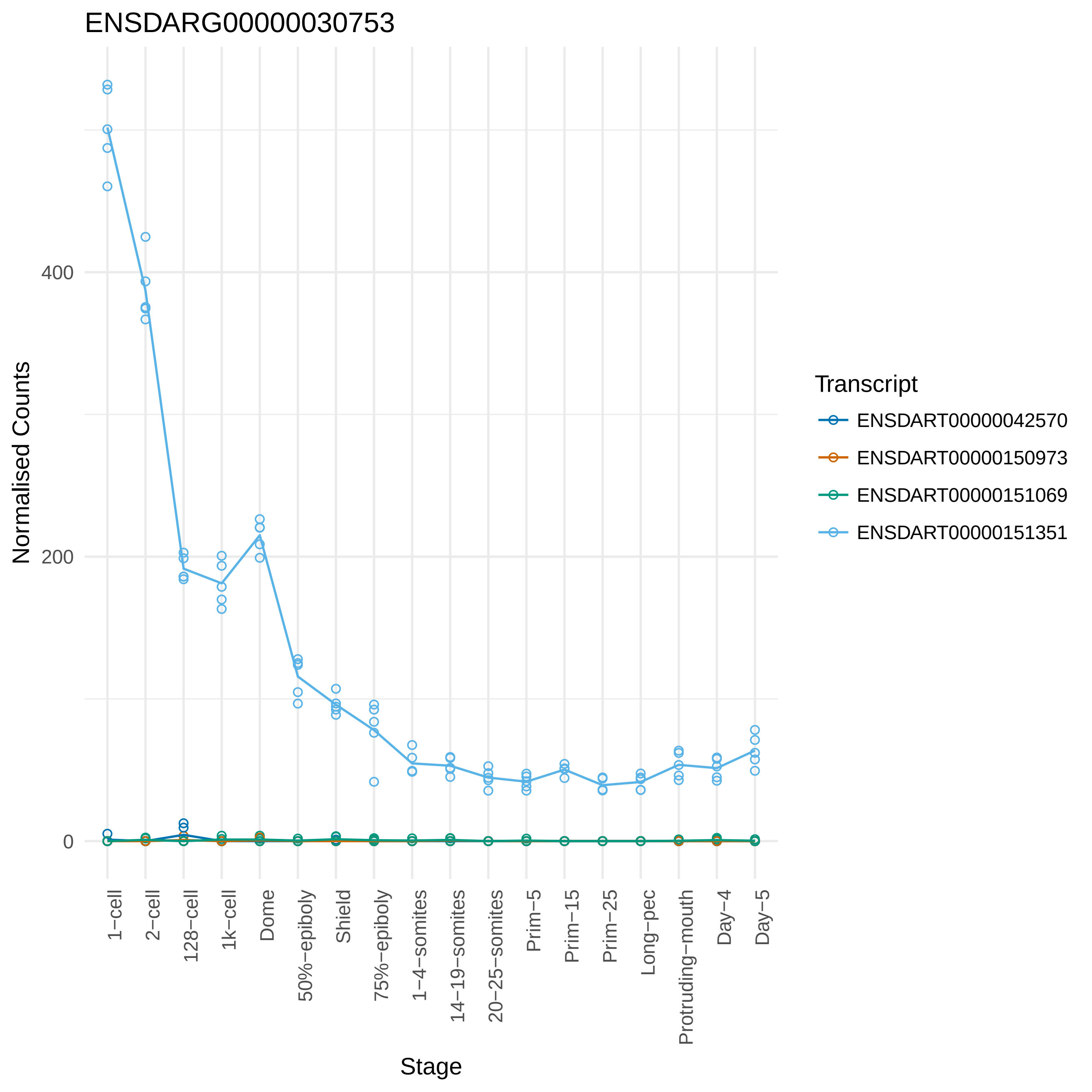

ENSDARG00000030753
Normalised Counts
400
200
0
1−cell
2−cell
128−cell
1k−cell
Dome
50%−epiboly
Shield
75%−epiboly
1−4−somites
14−19−somites
20−25−somites
Prim−5
Prim−15
Prim−25
Long−pec
Protruding−mouth
Day−4
Day−5
Stage
Transcript
ENSDART00000042570
ENSDART00000150973
ENSDART00000151069
ENSDART00000151351

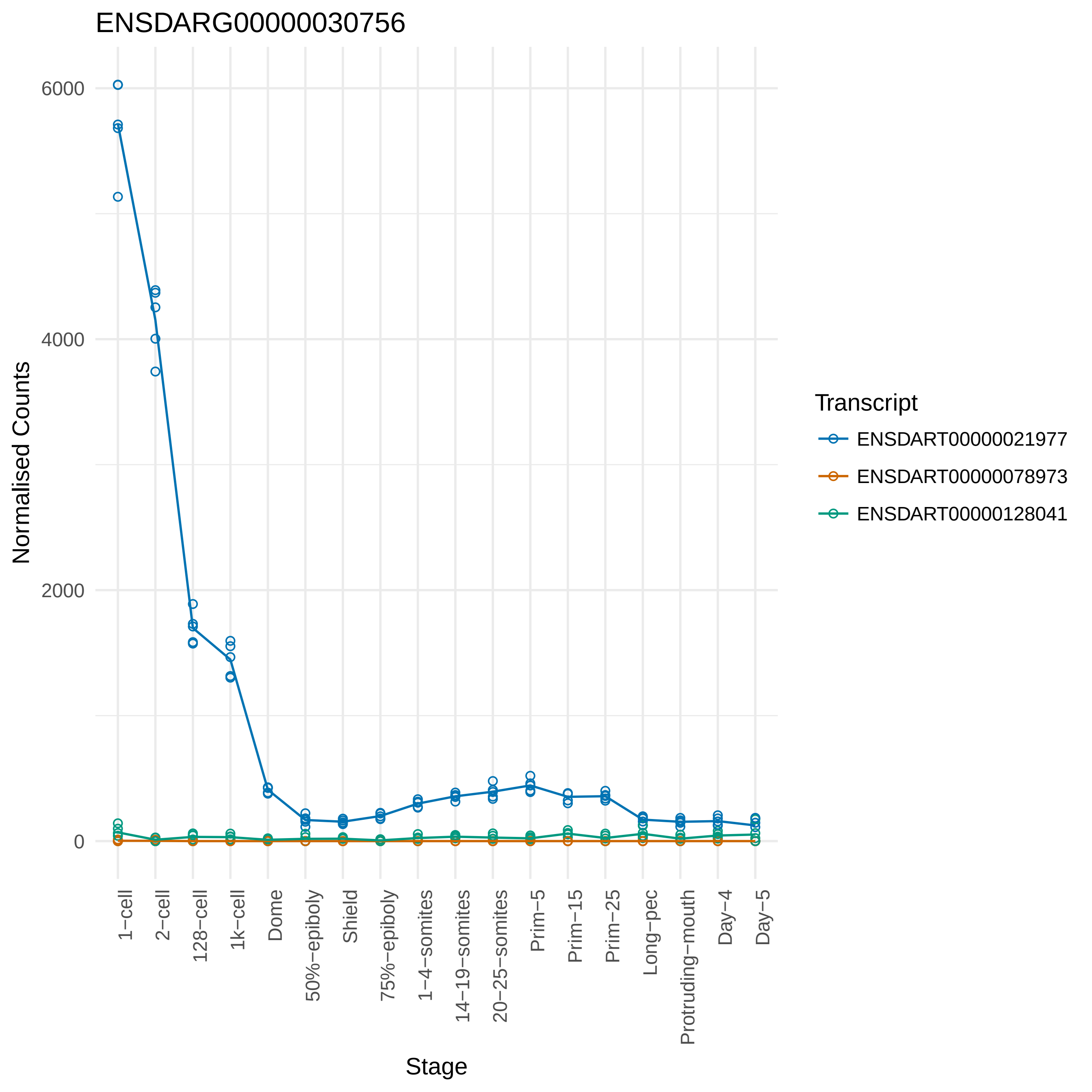
ENSDARG00000030756
Normalised Counts
6000
4000
2000
0
1−cell
2−cell
128−cell
1k−cell
Dome
50%−epiboly
Shield
75%−epiboly
1−4−somites
14−19−somites
20−25−somites
Prim−5
Prim−15
Prim−25
Long−pec
Protruding−mouth
Day−4
Day−5
Stage
Transcript
ENSDART00000021977
ENSDART00000078973
ENSDART00000128041

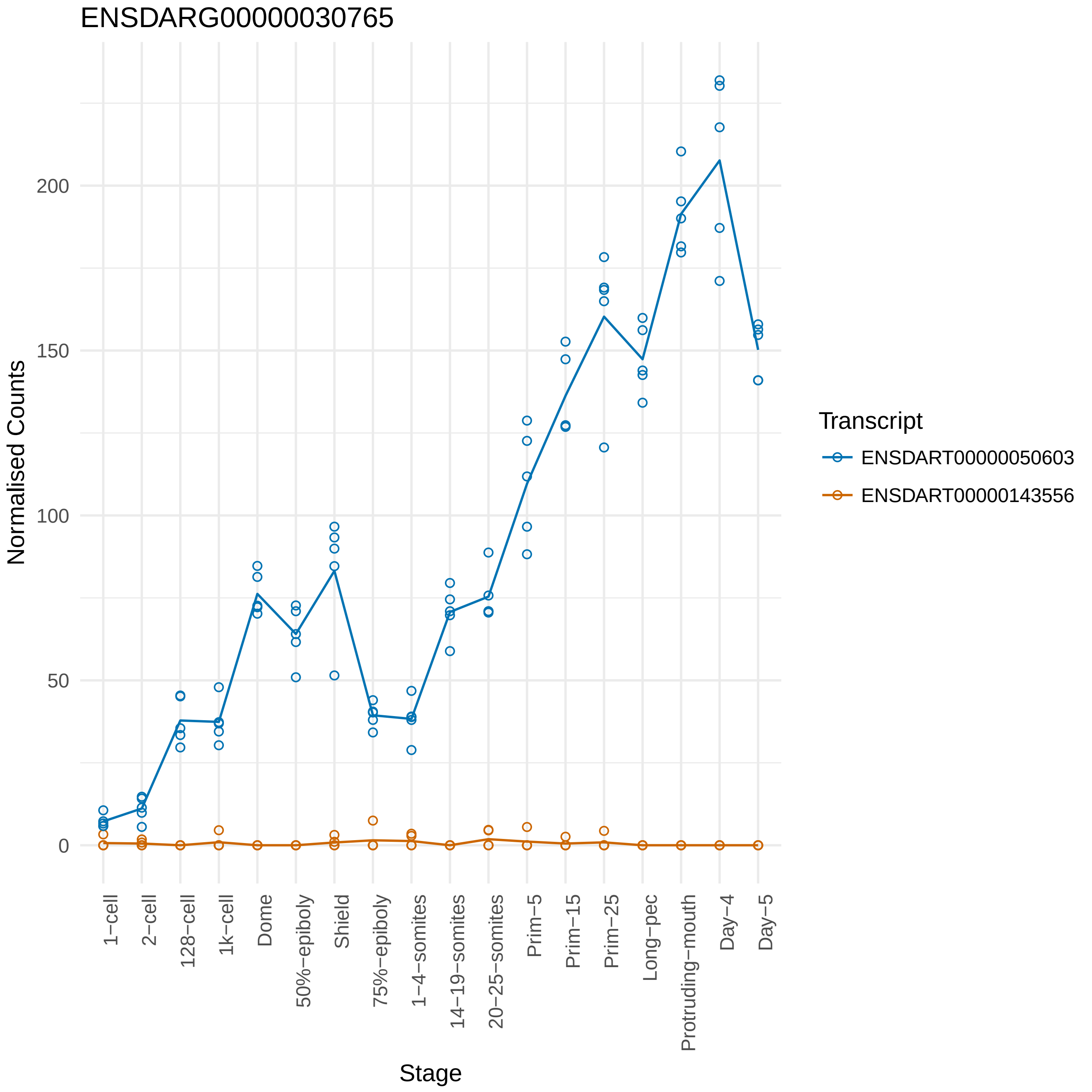
ENSDARG00000030765
Normalised Counts
0
50
100
150
200
Stage
1−cell
2−cell
128−cell
1k−cell
Dome
50%−epiboly
Shield
75%−epiboly
1−4−somites
14−19−somites
20−25−somites
Prim−5
Prim−15
Prim−25
Long−pec
Protruding−mouth
Day−4
Day−5
Transcript
ENSDART00000050603
ENSDART00000143556

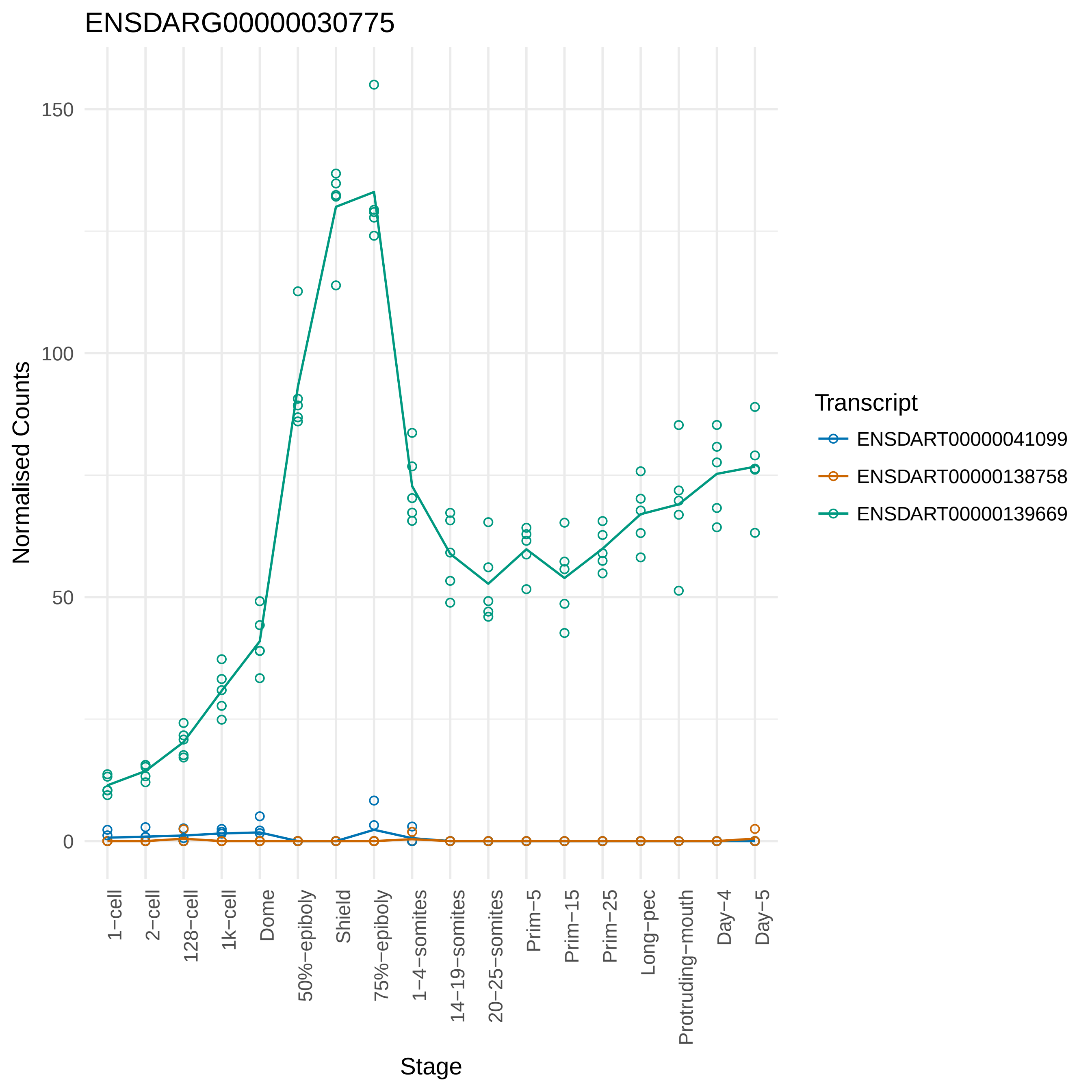
ENSDARG00000030775
Normalised Counts
150
100
50
0
1−cell
2−cell
128−cell
1k−cell
Dome
50%−epiboly
Shield
75%−epiboly
1−4−somites
14−19−somites
20−25−somites
Prim−5
Prim−15
Prim−25
Long−pec
Protruding−mouth
Day−4
Day−5
Stage
Transcript
ENSDART00000041099
ENSDART00000138758
ENSDART00000139669

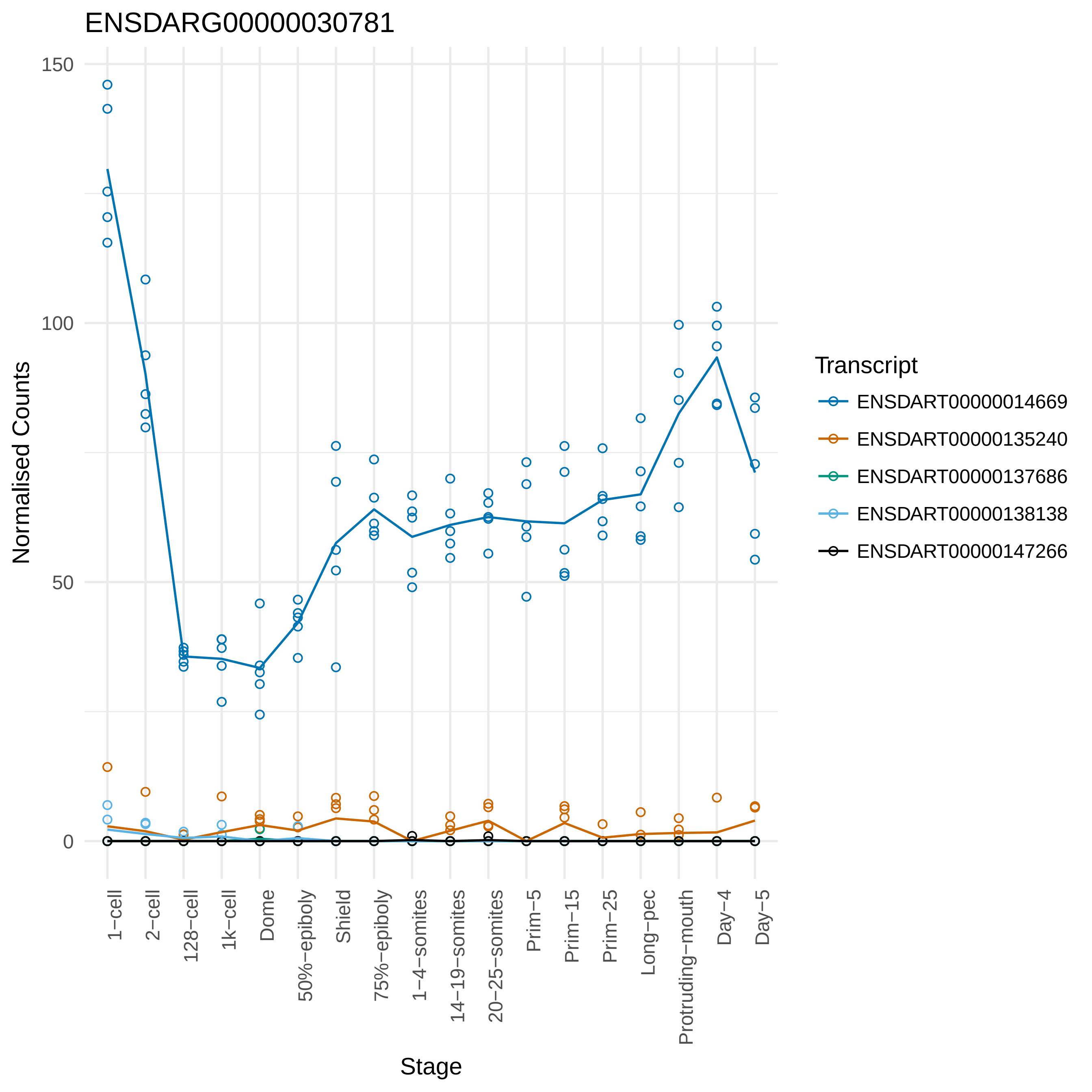

ENSDARG00000030781
Normalised Counts
150
100
50
0
Stage
1−cell
2−cell
128−cell
1k−cell
Dome
50%−epiboly
Shield
75%−epiboly
1−4−somites
14−19−somites
20−25−somites
Prim−5
Prim−15
Prim−25
Long−pec
Protruding−mouth
Day−4
Day−5
Transcript
ENSDART00000014669
ENSDART00000135240
ENSDART00000137686
ENSDART00000138138
ENSDART00000147266

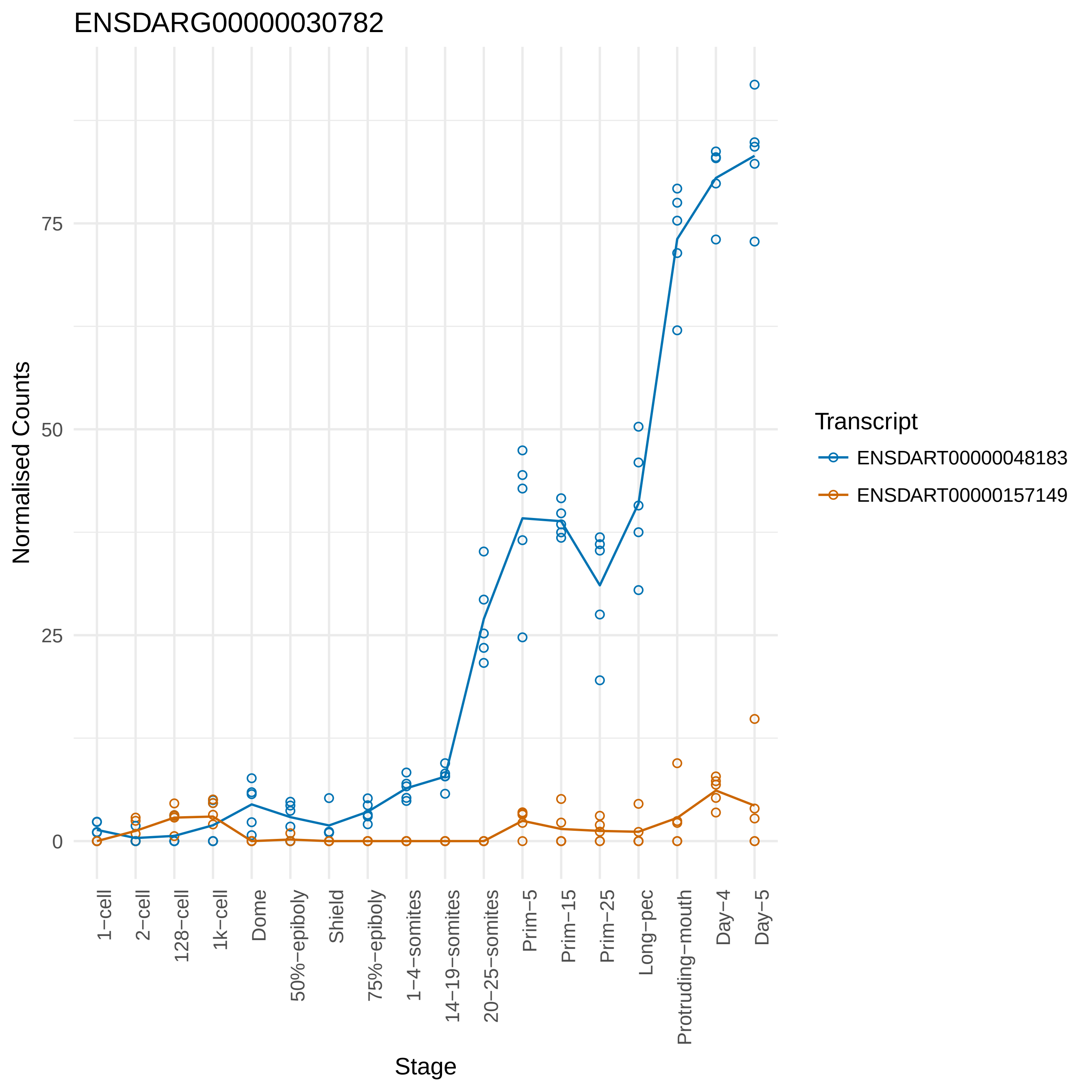

ENSDARG00000030782
Normalised Counts
75
50
25
0
1−cell
2−cell
128−cell
1k−cell
Dome
50%−epiboly
Shield
75%−epiboly
1−4−somites
14−19−somites
20−25−somites
Prim−5
Prim−15
Prim−25
Long−pec
Protruding−mouth
Day−4
Day−5
Stage
Transcript
ENSDART00000048183
ENSDART00000157149

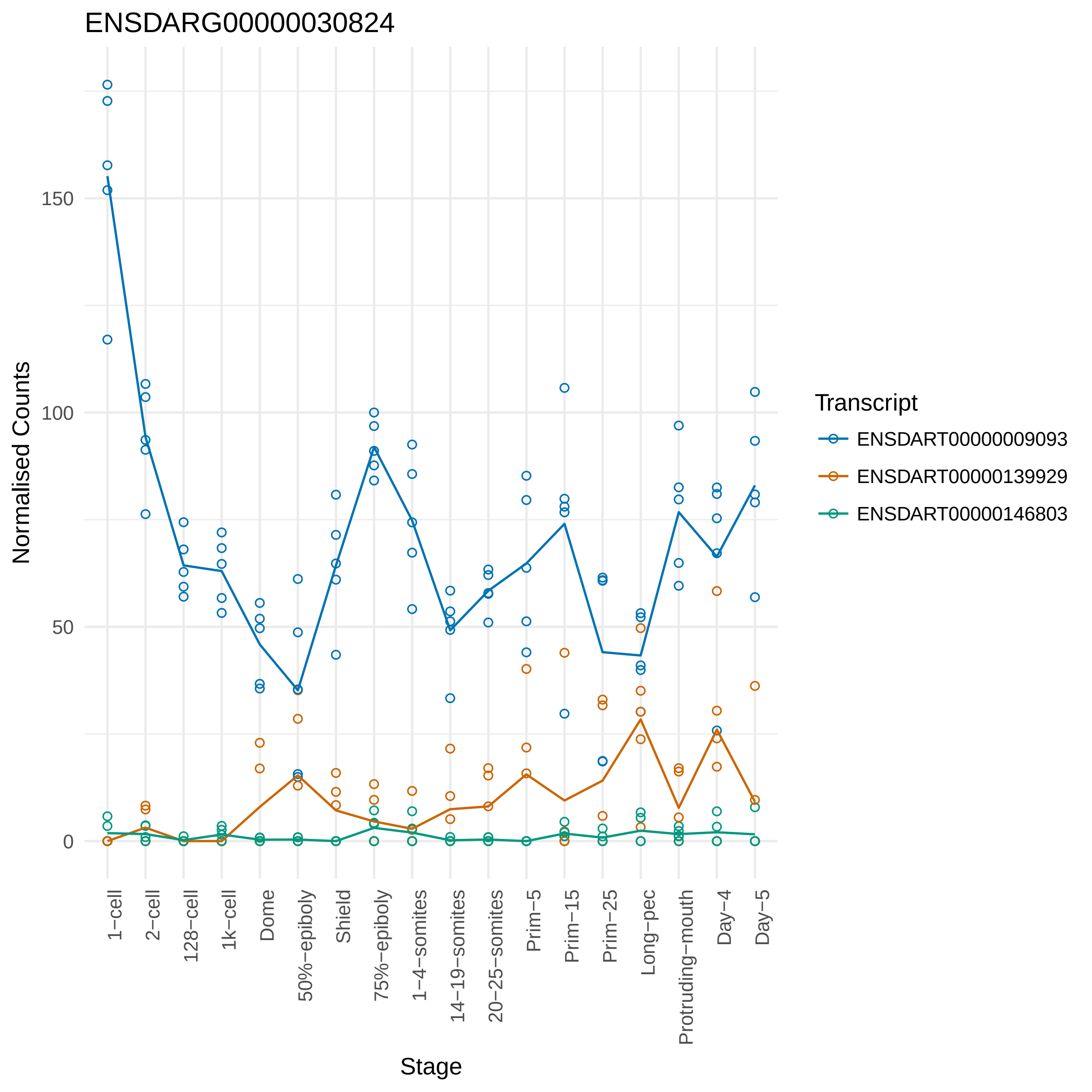

ENSDARG00000030824
Normalised Counts
150
100
50
0
1−cell
2−cell
128−cell
1k−cell
Dome
50%−epiboly
Shield
75%−epiboly
1−4−somites
14−19−somites
20−25−somites
Prim−5
Prim−15
Prim−25
Long−pec
Protruding−mouth
Day−4
Day−5
Stage
Transcript
ENSDART00000009093
ENSDART00000139929
ENSDART00000146803

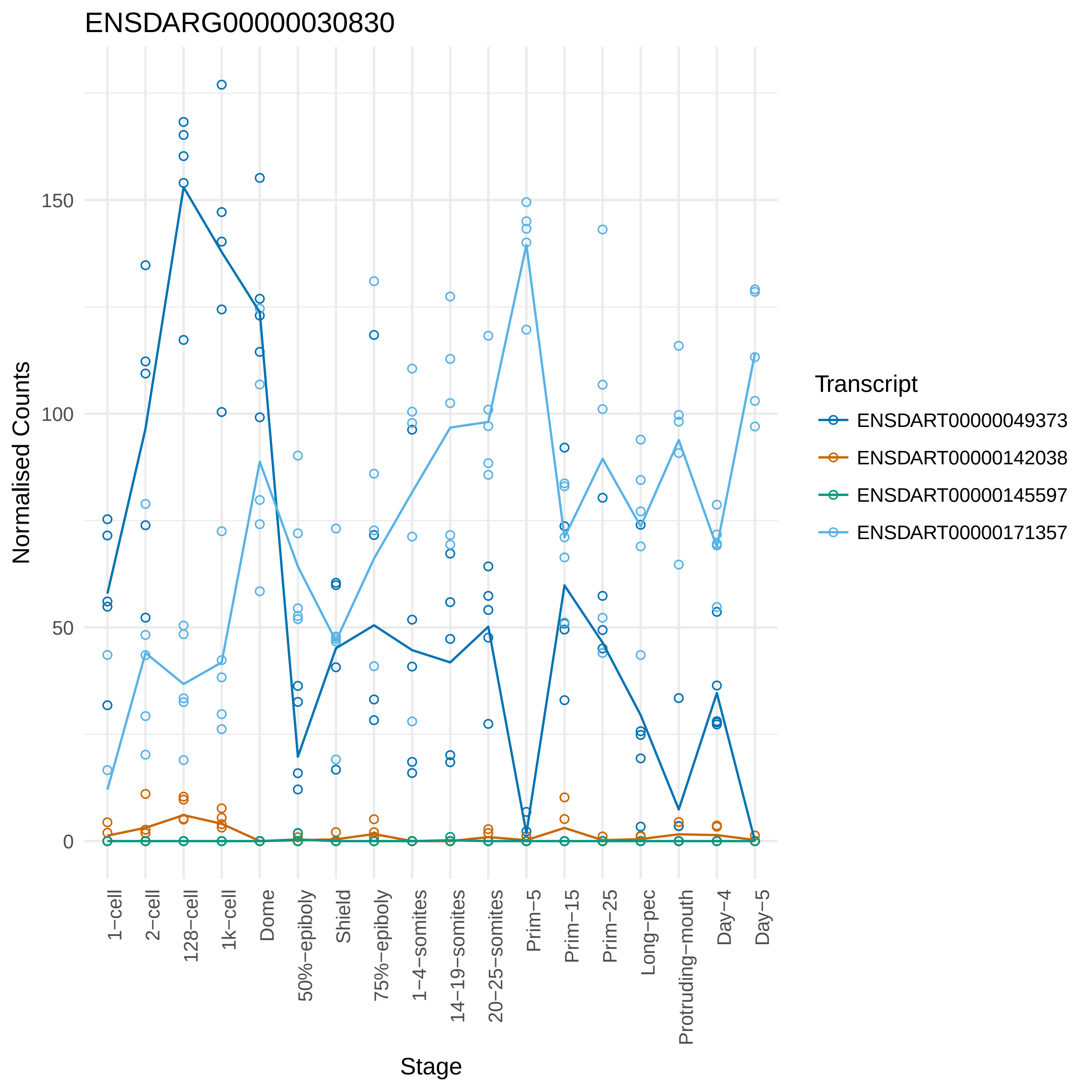
ENSDARG00000030830
Normalised Counts
0
50
100
150
1−cell
2−cell
128−cell
1k−cell
Dome
50%−epiboly
Shield
75%−epiboly
1−4−somites
14−19−somites
20−25−somites
Prim−5
Prim−15
Prim−25
Long−pec
Protruding−mouth
Day−4
Day−5
Stage
Transcript
ENSDART00000049373
ENSDART00000142038
ENSDART00000145597
ENSDART00000171357

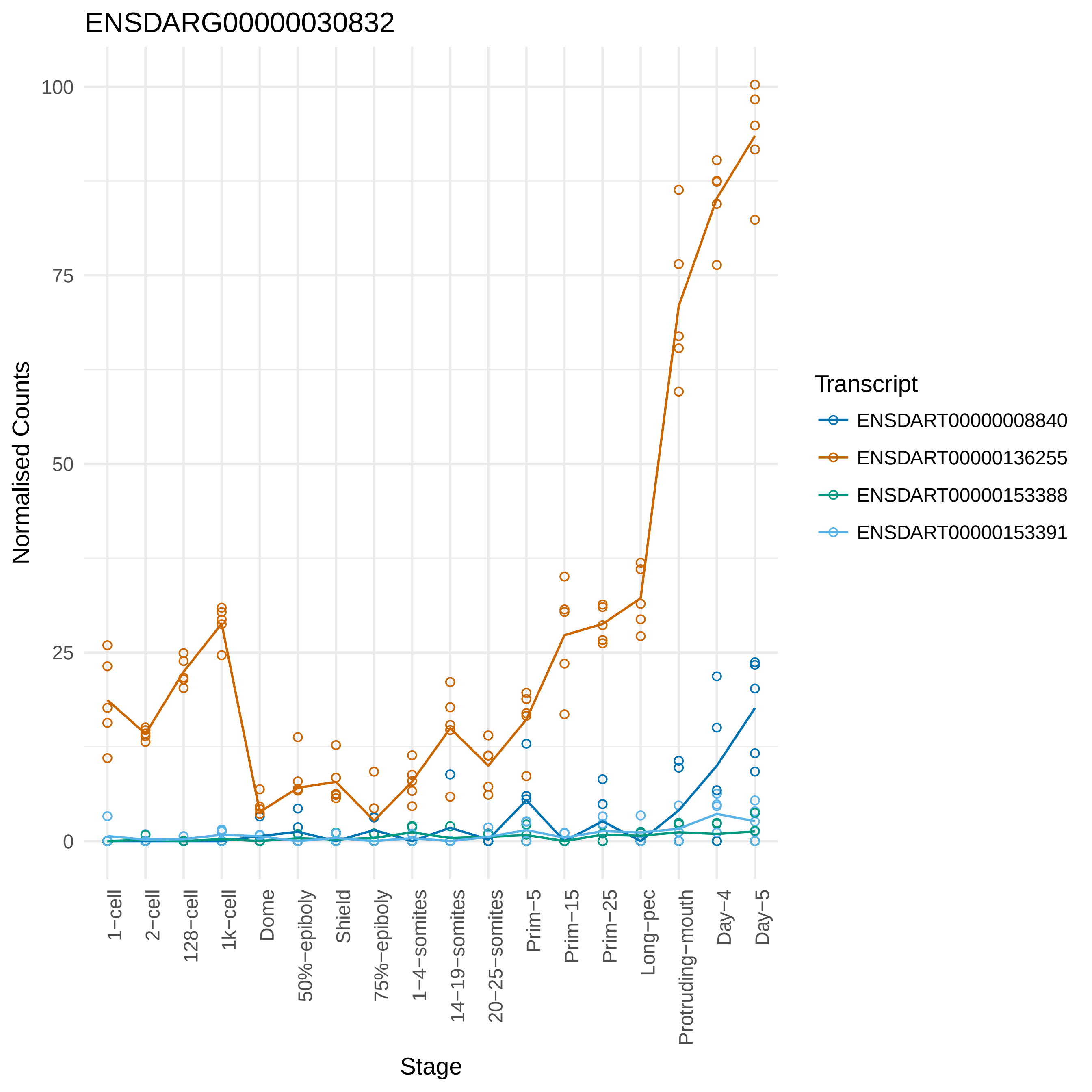

ENSDARG00000030832
Normalised Counts
100
75
50
25
0
1−cell
2−cell
128−cell
1k−cell
Dome
50%−epiboly
Shield
75%−epiboly
1−4−somites
14−19−somites
20−25−somites
Prim−5
Prim−15
Prim−25
Long−pec
Protruding−mouth
Day−4
Day−5
Stage
Transcript
ENSDART00000008840
ENSDART00000136255
ENSDART00000153388
ENSDART00000153391

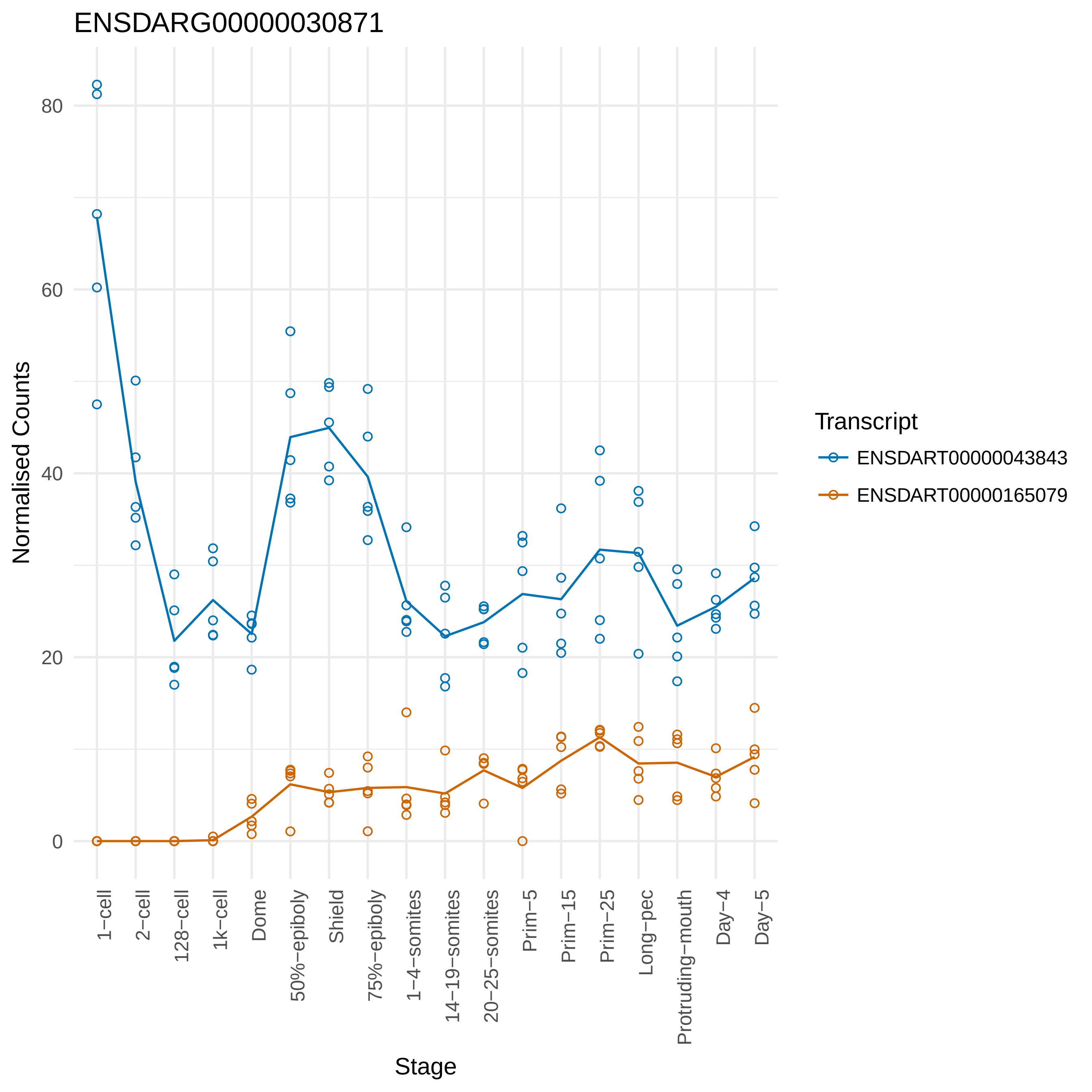
ENSDARG00000030871
Normalised Counts
0
20
40
60
80
Stage
1−cell
2−cell
128−cell
1k−cell
Dome
50%−epiboly
Shield
75%−epiboly
1−4−somites
14−19−somites
20−25−somites
Prim−5
Prim−15
Prim−25
Long−pec
Protruding−mouth
Day−4
Day−5
Transcript
ENSDART00000043843
ENSDART00000165079

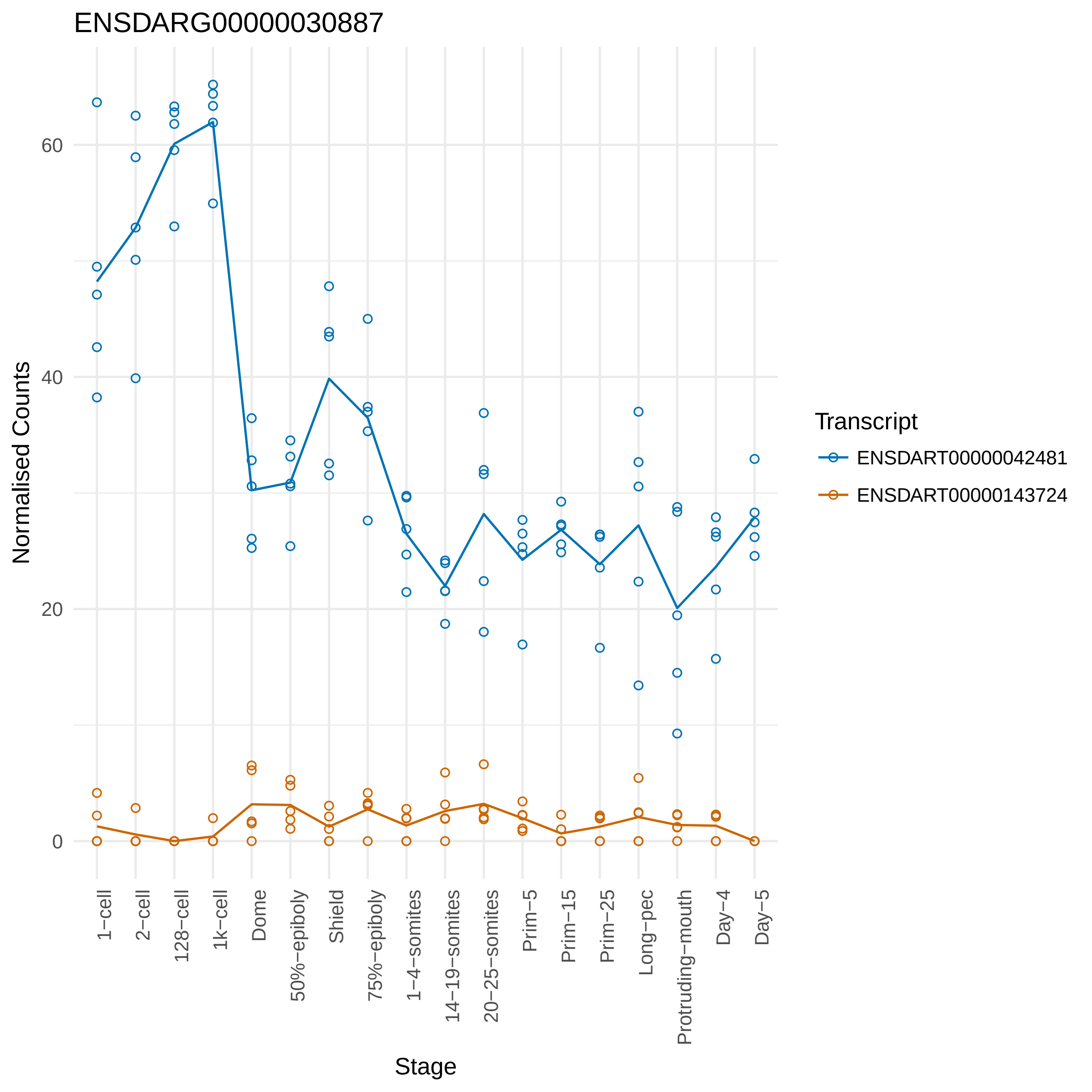

ENSDARG00000030887
Normalised Counts
Stage
0
20
40
60
1−cell
2−cell
128−cell
1k−cell
Dome
50%−epiboly
Shield
75%−epiboly
1−4−somites
14−19−somites
20−25−somites
Prim−5
Prim−15
Prim−25
Long−pec
Protruding−mouth
Day−4
Day−5
Transcript
ENSDART00000042481
ENSDART00000143724

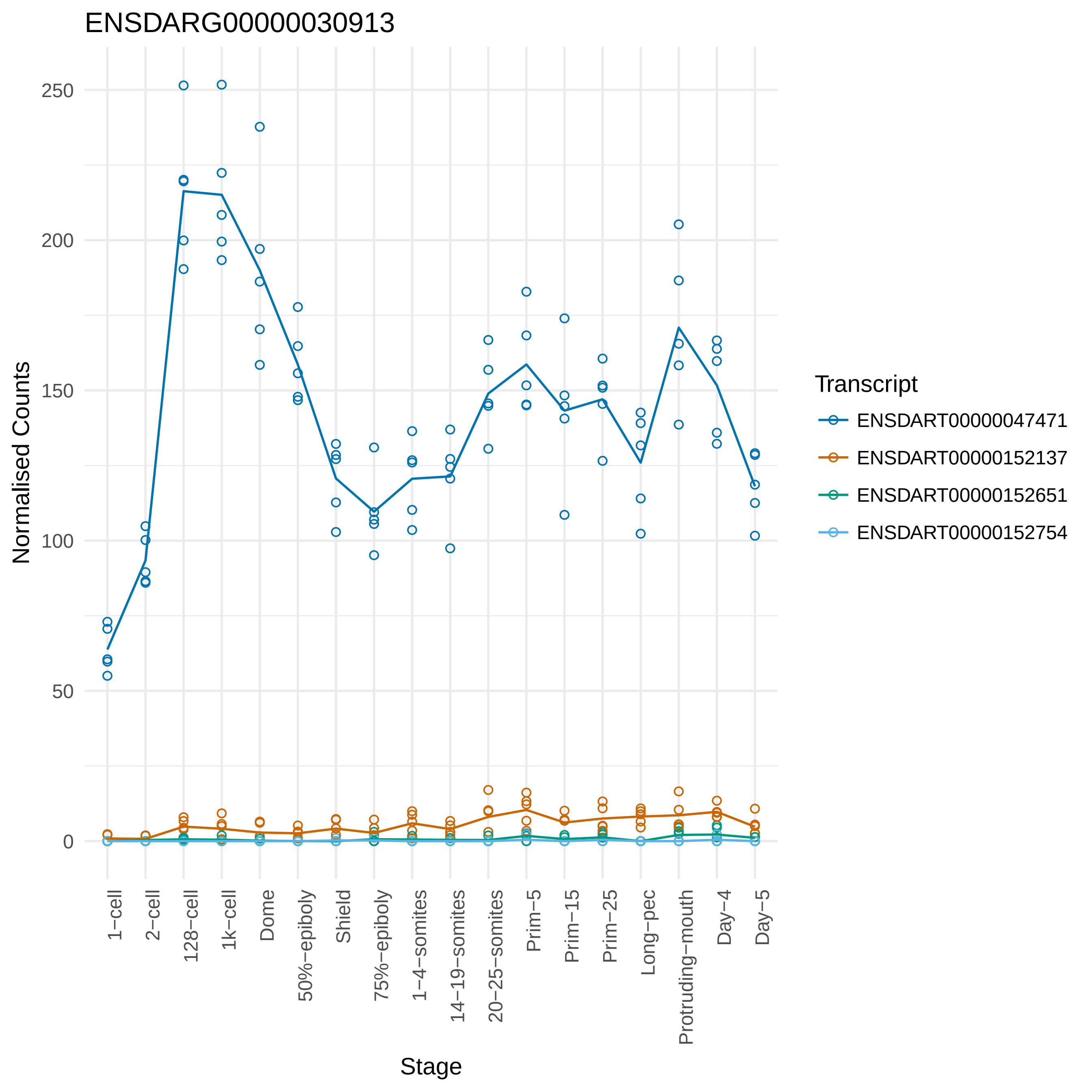

ENSDARG00000030913
Normalised Counts
250
200
150
100
50
0
1−cell
2−cell
128−cell
1k−cell
Dome
50%−epiboly
Shield
75%−epiboly
1−4−somites
14−19−somites
20−25−somites
Prim−5
Prim−15
Prim−25
Long−pec
Protruding−mouth
Day−4
Day−5
Stage
Transcript
ENSDART00000047471
ENSDART00000152137
ENSDART00000152651
ENSDART00000152754

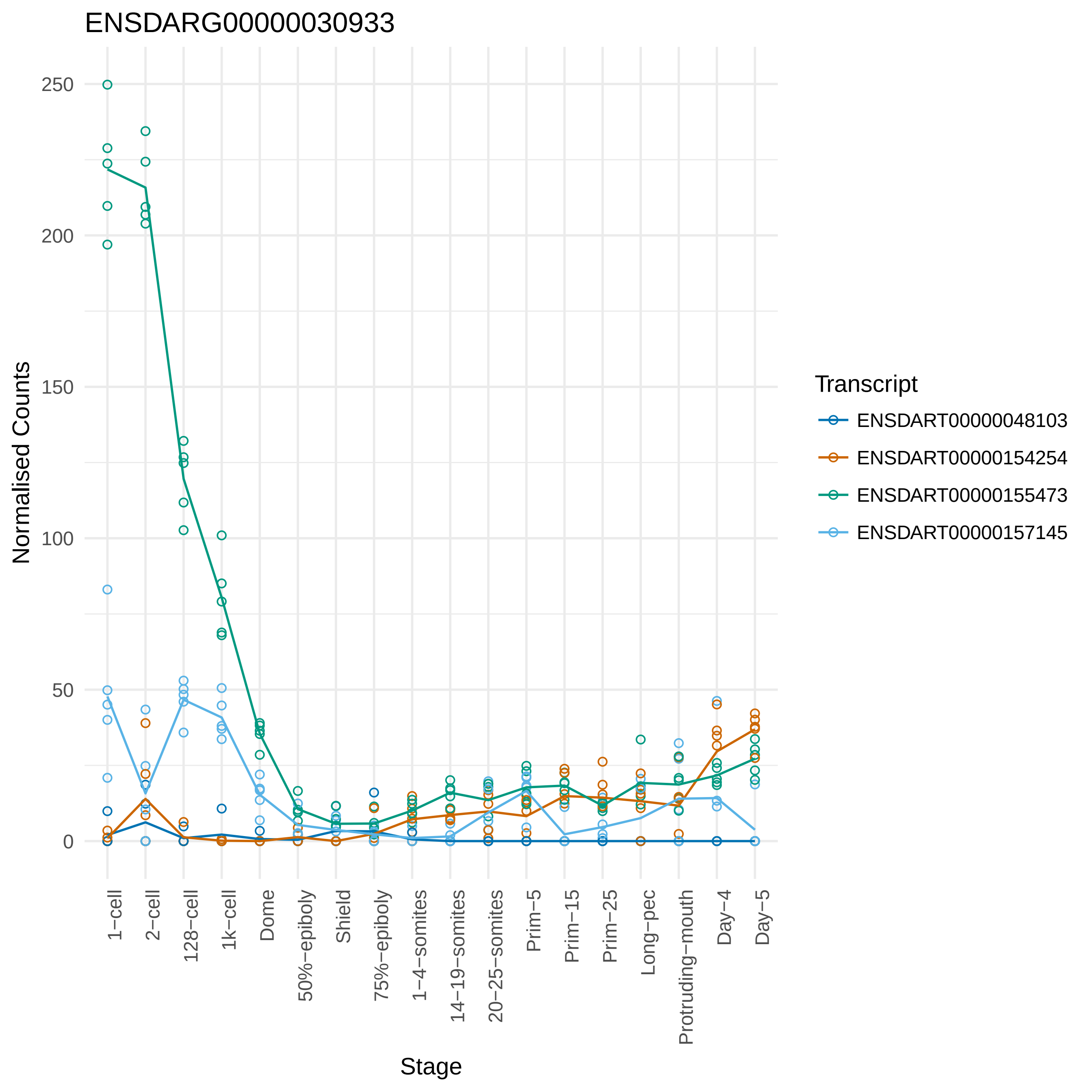

ENSDARG00000030933
Normalised Counts
250
200
150
100
50
0
1−cell
2−cell
128−cell
1k−cell
Dome
50%−epiboly
Shield
75%−epiboly
1−4−somites
14−19−somites
20−25−somites
Prim−5
Prim−15
Prim−25
Long−pec
Protruding−mouth
Day−4
Day−5
Stage
Transcript
ENSDART00000048103
ENSDART00000154254
ENSDART00000155473
ENSDART00000157145

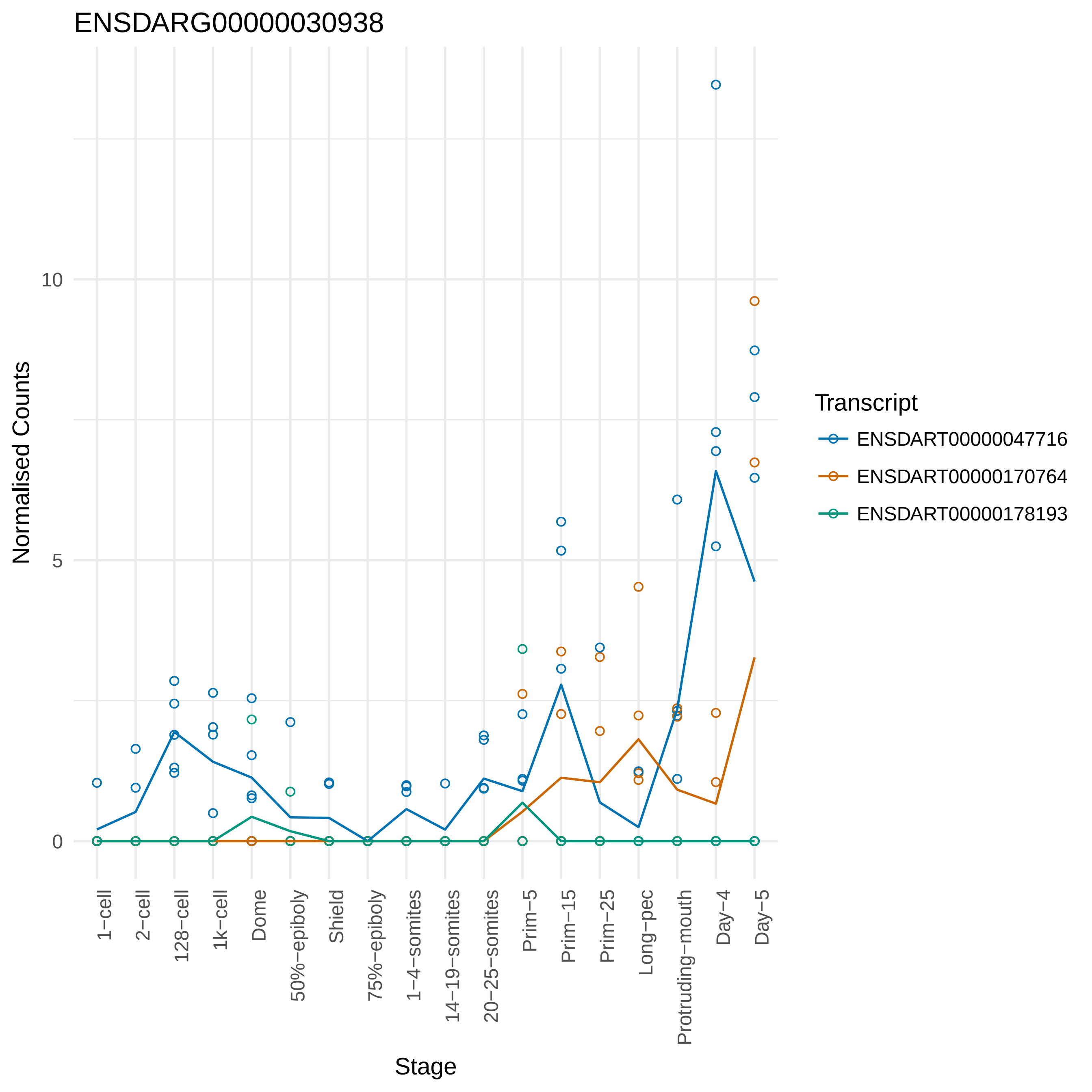

ENSDARG00000030938
Normalised Counts
10
5
0
Stage
1−cell
2−cell
128−cell
1k−cell
Dome
50%−epiboly
Shield
75%−epiboly
1−4−somites
14−19−somites
20−25−somites
Prim−5
Prim−15
Prim−25
Long−pec
Protruding−mouth
Day−4
Day−5
Transcript
ENSDART00000047716
ENSDART00000170764
ENSDART00000178193

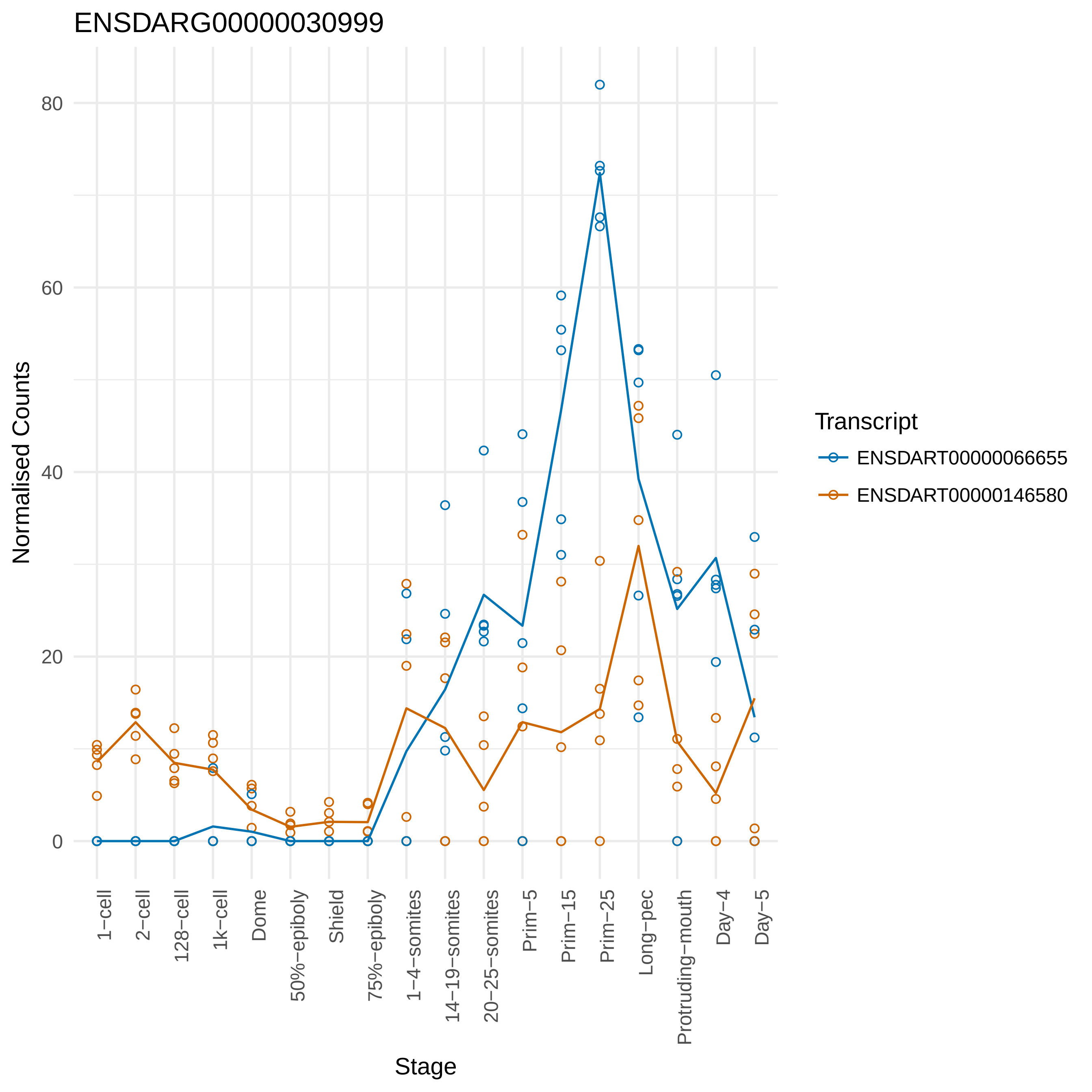

ENSDARG00000030999
Normalised Counts
0
20
40
60
80
1−cell
2−cell
128−cell
1k−cell
Dome
50%−epiboly
Shield
75%−epiboly
1−4−somites
14−19−somites
20−25−somites
Prim−5
Prim−15
Prim−25
Long−pec
Protruding−mouth
Day−4
Day−5
Stage
Transcript
ENSDART00000066655
ENSDART00000146580

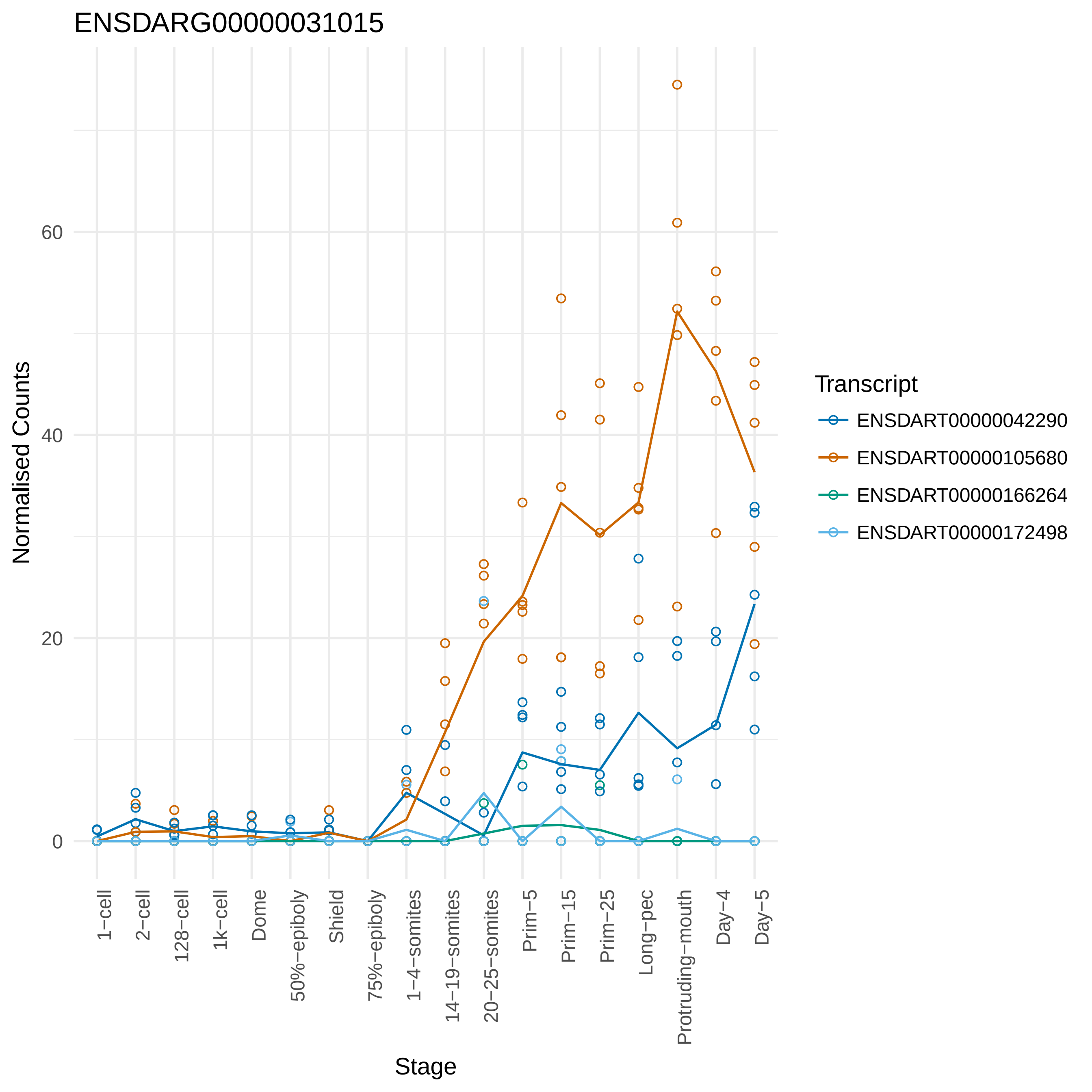

ENSDARG00000031015
Normalised Counts
0
20
40
60
Stage
1−cell
2−cell
128−cell
1k−cell
Dome
50%−epiboly
Shield
75%−epiboly
1−4−somites
14−19−somites
20−25−somites
Prim−5
Prim−15
Prim−25
Long−pec
Protruding−mouth
Day−4
Day−5
Transcript
ENSDART00000042290
ENSDART00000105680
ENSDART00000166264
ENSDART00000172498

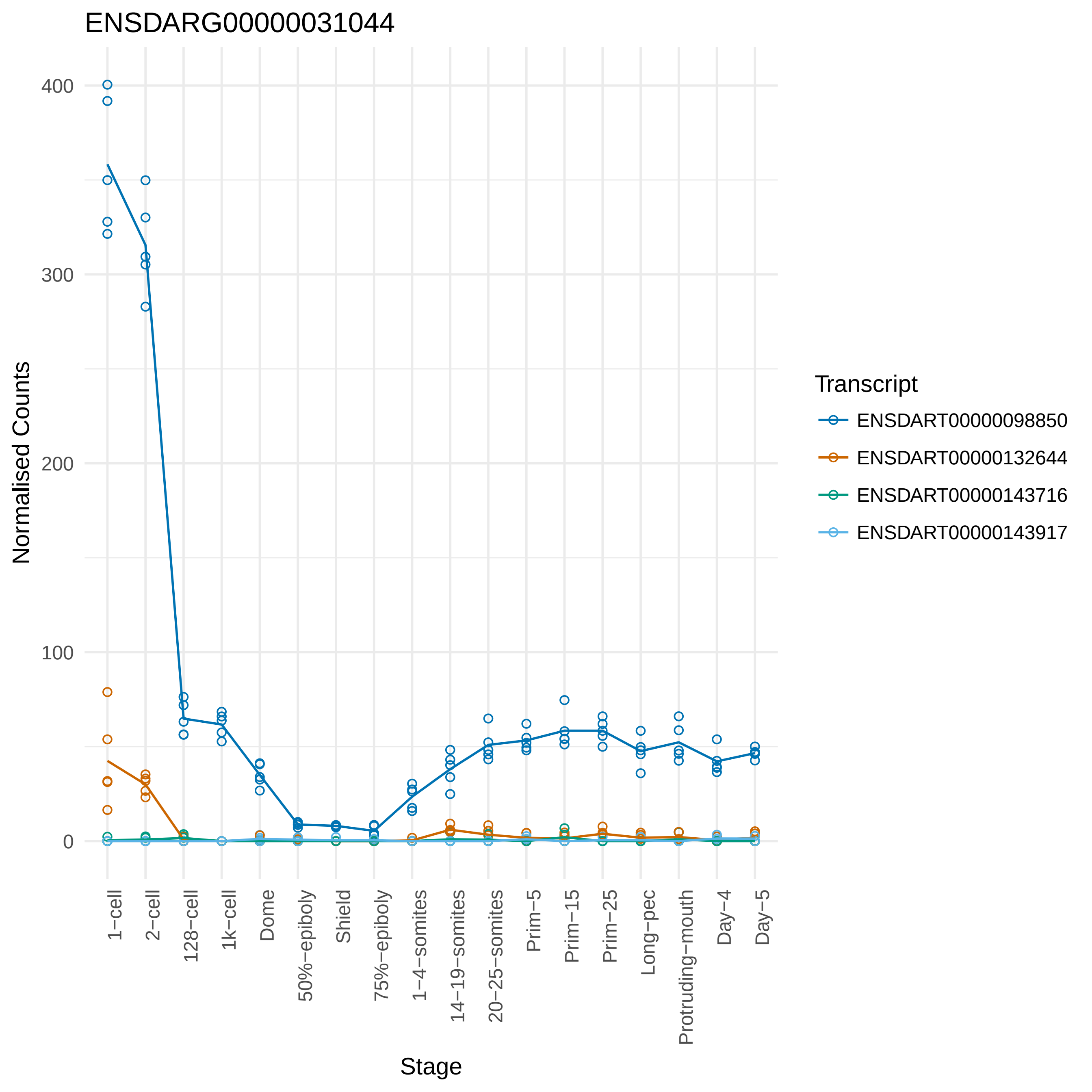
ENSDARG00000031044
Normalised Counts
400
300
200
100
0
1−cell
2−cell
128−cell
1k−cell
Dome
50%−epiboly
Shield
75%−epiboly
1−4−somites
14−19−somites
20−25−somites
Prim−5
Prim−15
Prim−25
Long−pec
Protruding−mouth
Day−4
Day−5
Stage
Transcript
ENSDART00000098850
ENSDART00000132644
ENSDART00000143716
ENSDART00000143917

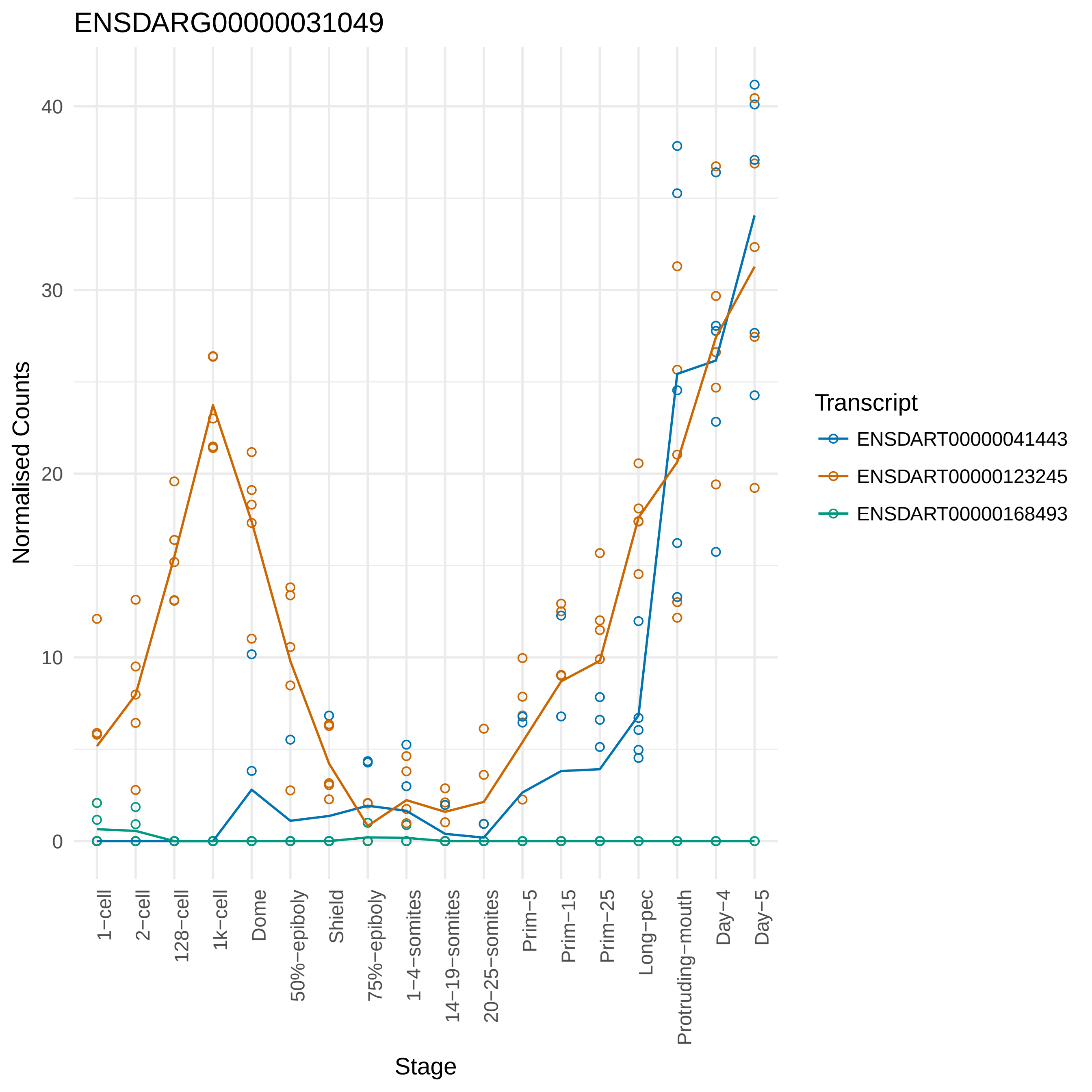
ENSDARG00000031049
Normalised Counts
0
10
20
30
40
1−cell
2−cell
128−cell
1k−cell
Dome
50%−epiboly
Shield
75%−epiboly
1−4−somites
14−19−somites
20−25−somites
Prim−5
Prim−15
Prim−25
Long−pec
Protruding−mouth
Day−4
Day−5
Stage
Transcript
ENSDART00000041443
ENSDART00000123245
ENSDART00000168493

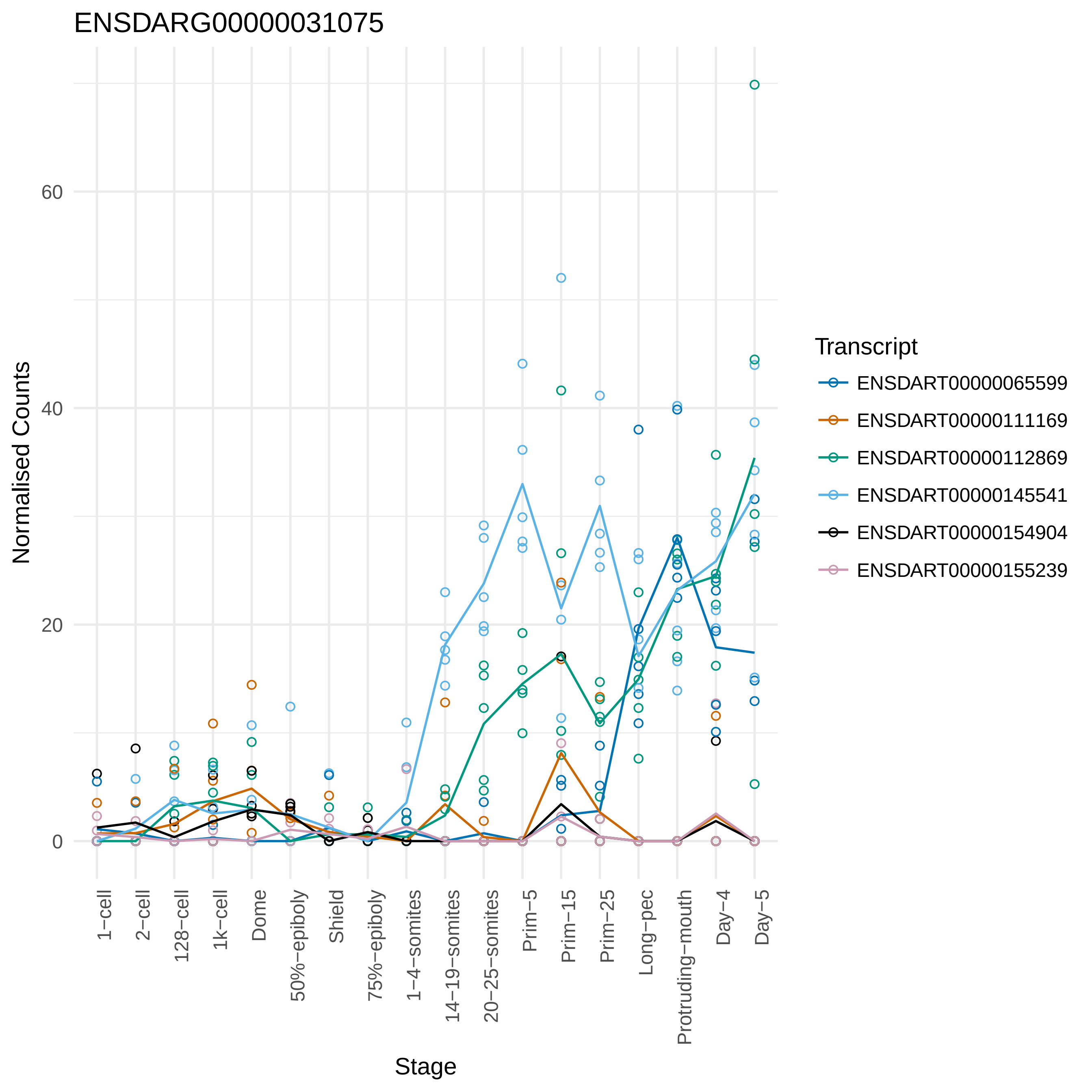

ENSDARG00000031075
Normalised Counts
0
20
40
60
Stage
1−cell
2−cell
128−cell
1k−cell
Dome
50%−epiboly
Shield
75%−epiboly
1−4−somites
14−19−somites
20−25−somites
Prim−5
Prim−15
Prim−25
Long−pec
Protruding−mouth
Day−4
Day−5
Transcript
ENSDART00000065599
ENSDART00000111169
ENSDART00000112869
ENSDART00000145541
ENSDART00000154904
ENSDART00000155239

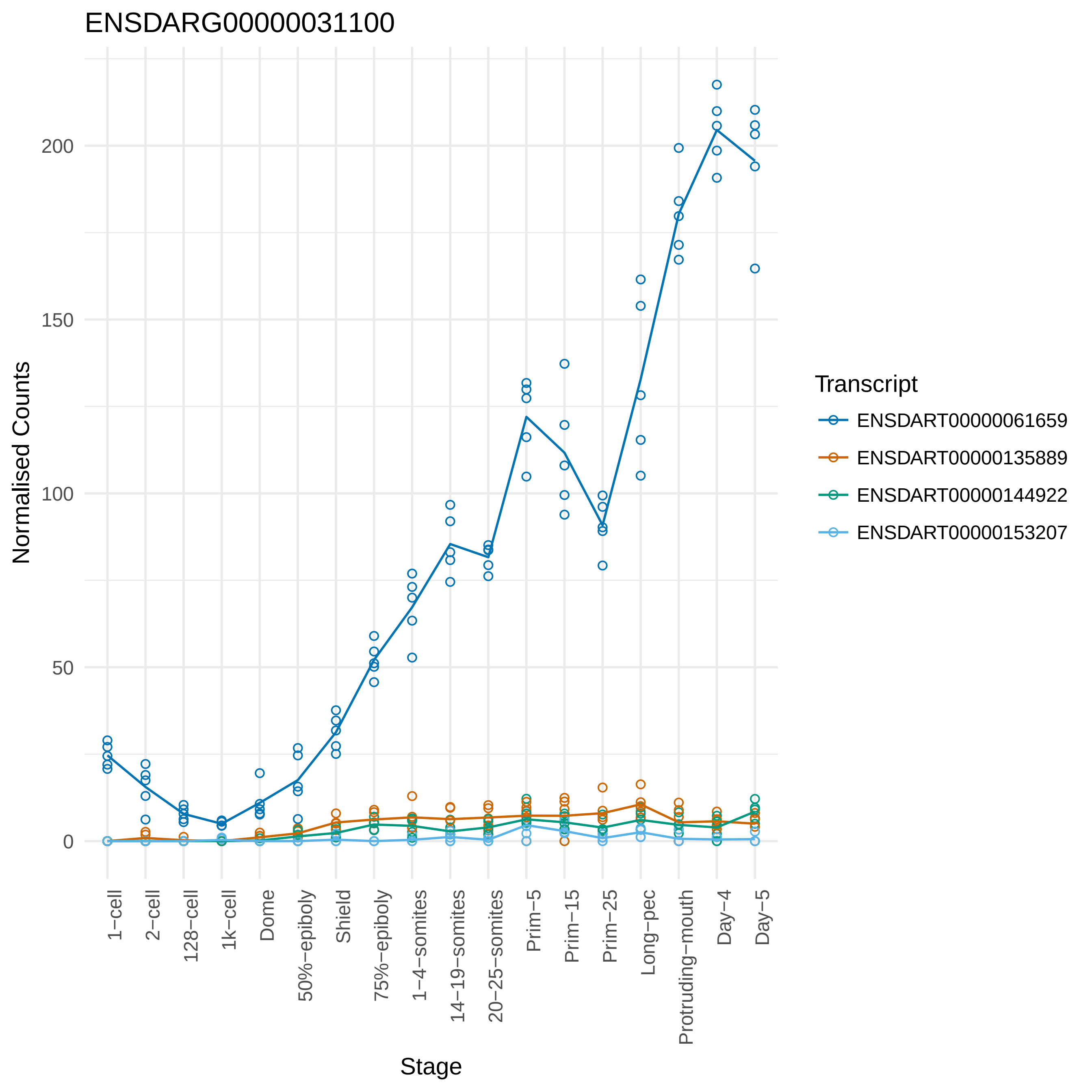

ENSDARG00000031100
Normalised Counts
0
50
100
150
200
Stage
1−cell
2−cell
128−cell
1k−cell
Dome
50%−epiboly
Shield
75%−epiboly
1−4−somites
14−19−somites
20−25−somites
Prim−5
Prim−15
Prim−25
Long−pec
Protruding−mouth
Day−4
Day−5
Transcript
ENSDART00000061659
ENSDART00000135889
ENSDART00000144922
ENSDART00000153207

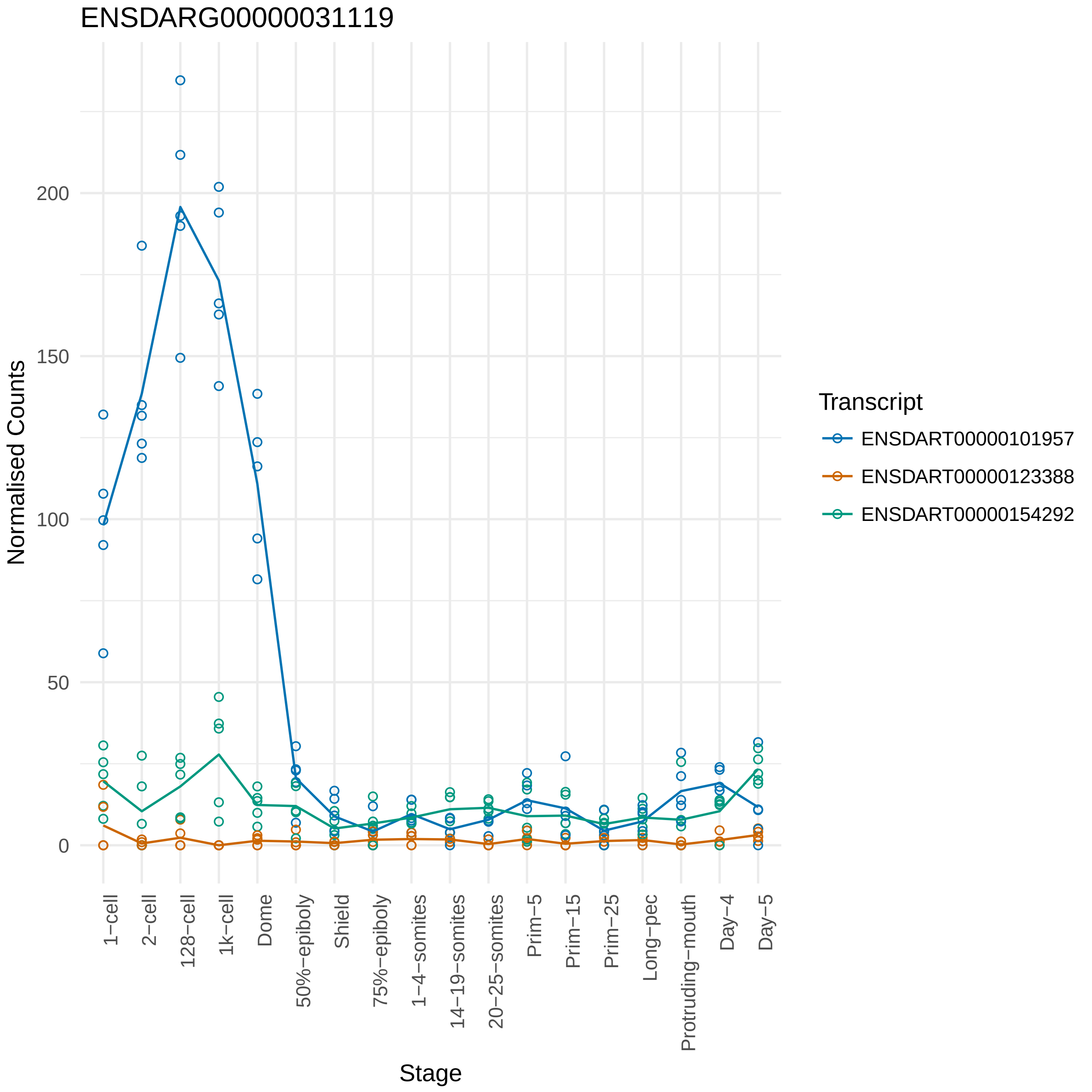

ENSDARG00000031119
Normalised Counts
200
150
100
50
0
1−cell
2−cell
128−cell
1k−cell
Dome
50%−epiboly
Shield
75%−epiboly
1−4−somites
14−19−somites
20−25−somites
Prim−5
Prim−15
Prim−25
Long−pec
Protruding−mouth
Day−4
Day−5
Stage
Transcript
ENSDART00000101957
ENSDART00000123388
ENSDART00000154292

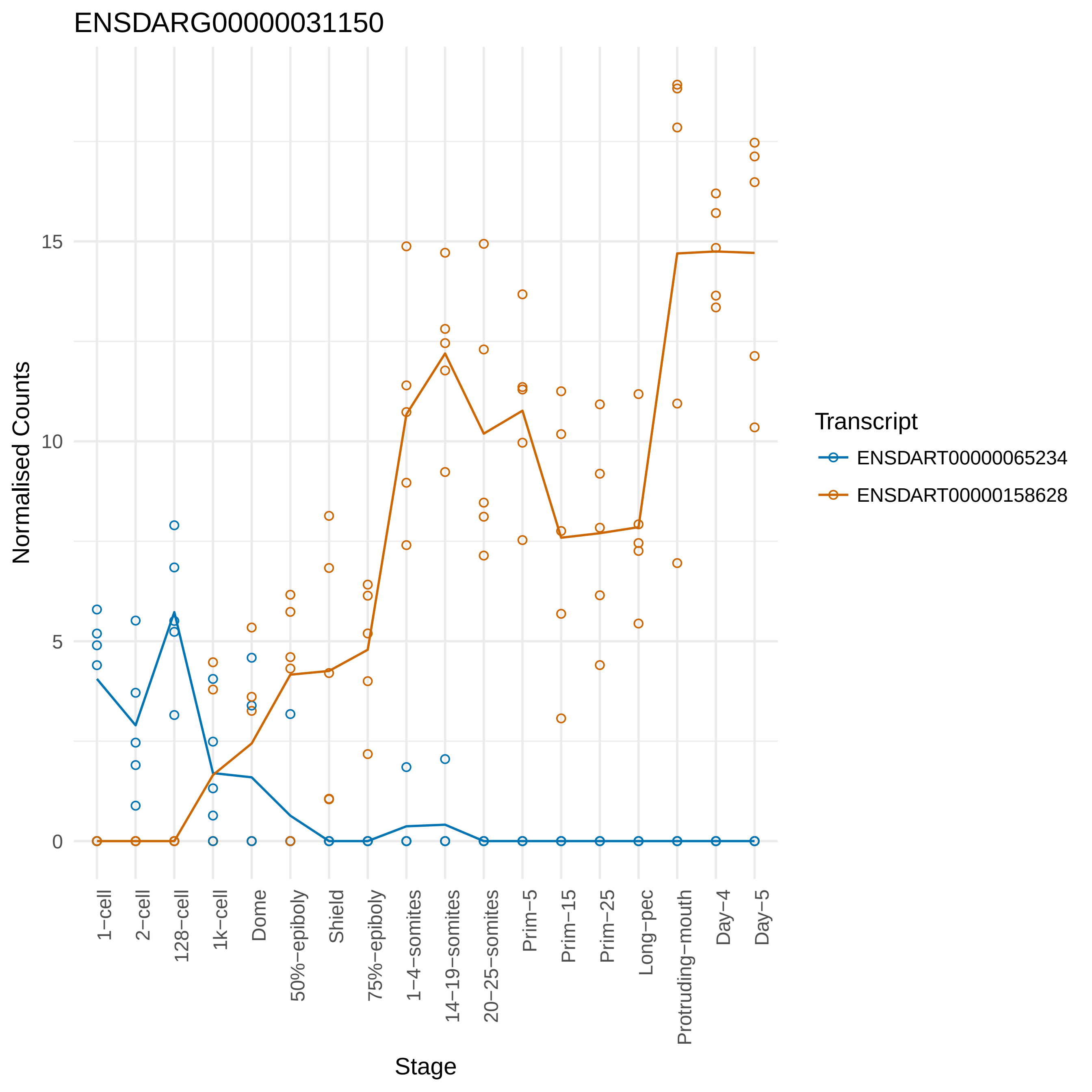

ENSDARG00000031150
Normalised Counts
0
5
10
15
Stage
1−cell
2−cell
128−cell
1k−cell
Dome
50%−epiboly
Shield
75%−epiboly
1−4−somites
14−19−somites
20−25−somites
Prim−5
Prim−15
Prim−25
Long−pec
Protruding−mouth
Day−4
Day−5
Transcript
ENSDART00000065234
ENSDART00000158628

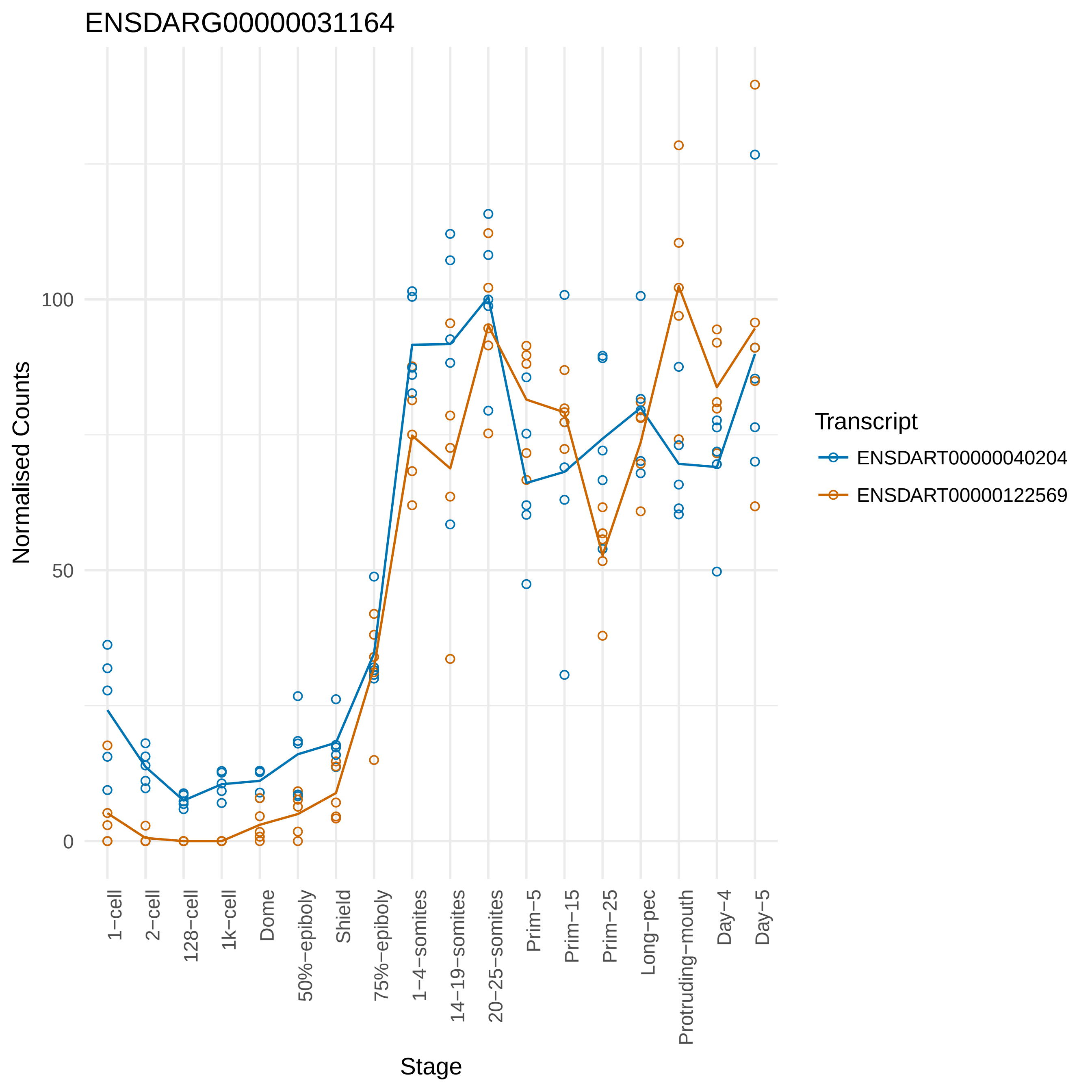
ENSDARG00000031164
Normalised Counts
0
50
100
Stage
1−cell
2−cell
128−cell
1k−cell
Dome
50%−epiboly
Shield
75%−epiboly
1−4−somites
14−19−somites
20−25−somites
Prim−5
Prim−15
Prim−25
Long−pec
Protruding−mouth
Day−4
Day−5
Transcript
ENSDART00000040204
ENSDART00000122569

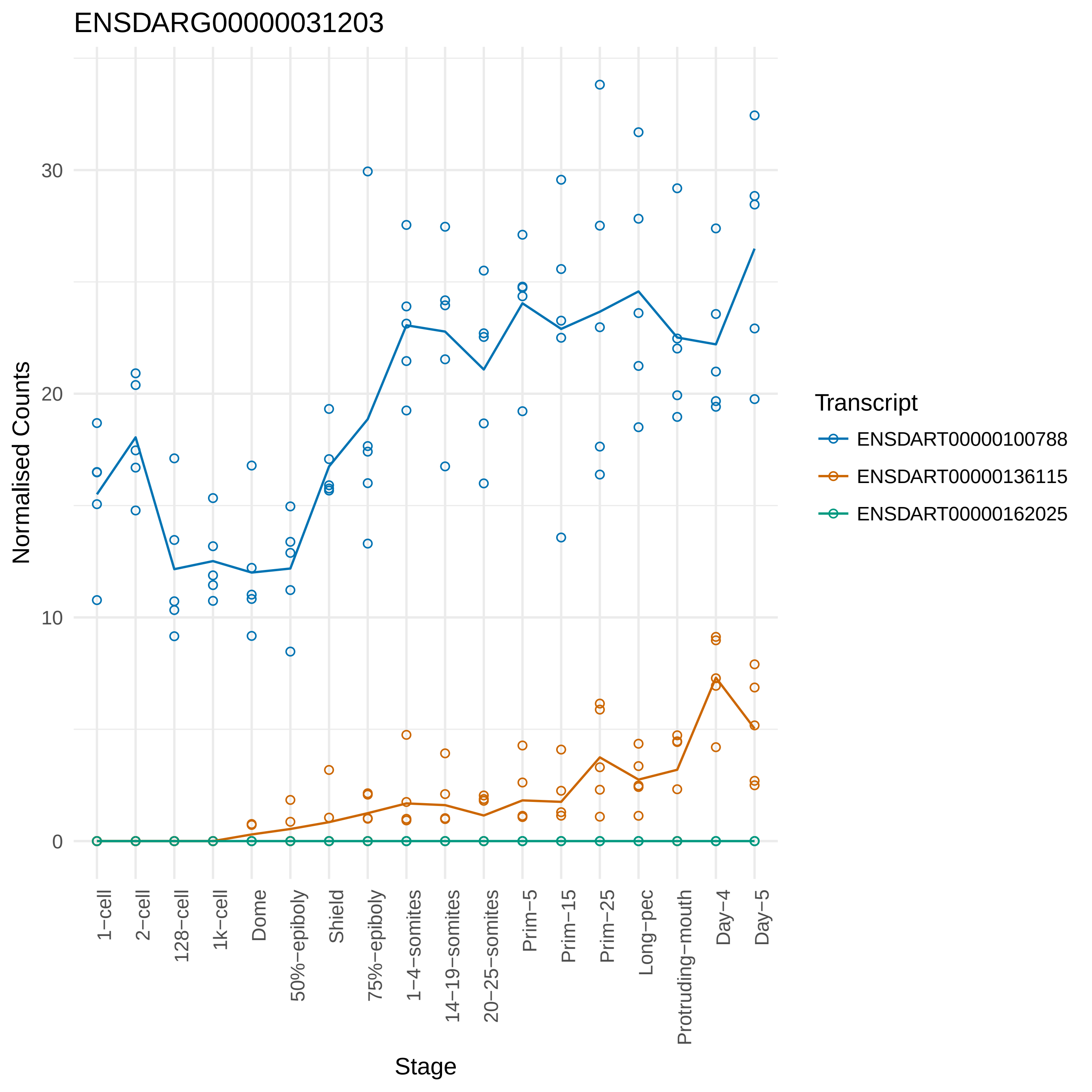

ENSDARG00000031203
Normalised Counts
0
10
20
30
Stage
1−cell
2−cell
128−cell
1k−cell
Dome
50%−epiboly
Shield
75%−epiboly
1−4−somites
14−19−somites
20−25−somites
Prim−5
Prim−15
Prim−25
Long−pec
Protruding−mouth
Day−4
Day−5
Transcript
ENSDART00000100788
ENSDART00000136115
ENSDART00000162025

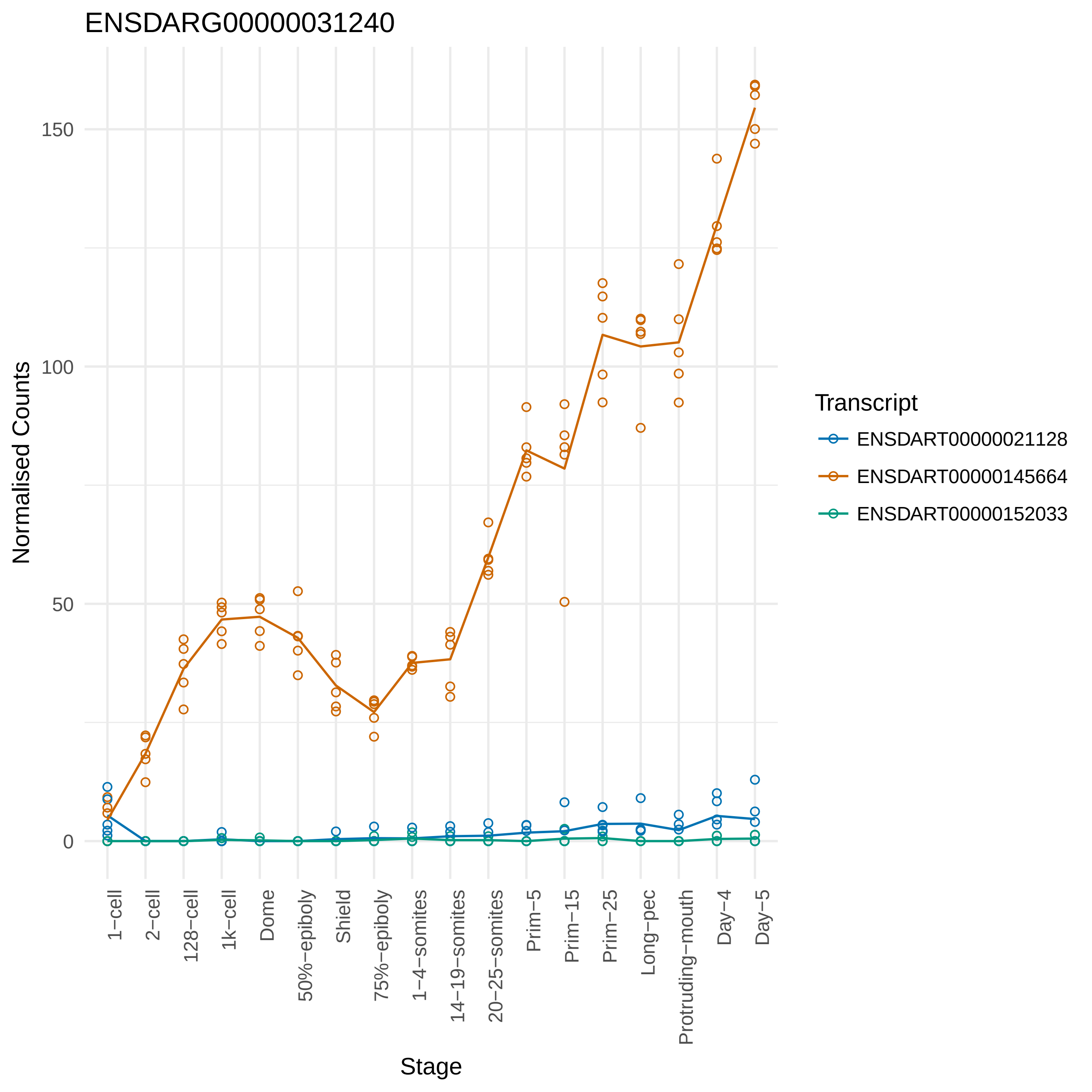

ENSDARG00000031240
Normalised Counts
150
100
50
0
1−cell
2−cell
128−cell
1k−cell
Dome
50%−epiboly
Shield
75%−epiboly
1−4−somites
14−19−somites
20−25−somites
Prim−5
Prim−15
Prim−25
Long−pec
Protruding−mouth
Day−4
Day−5
Stage
Transcript
ENSDART00000021128
ENSDART00000145664
ENSDART00000152033

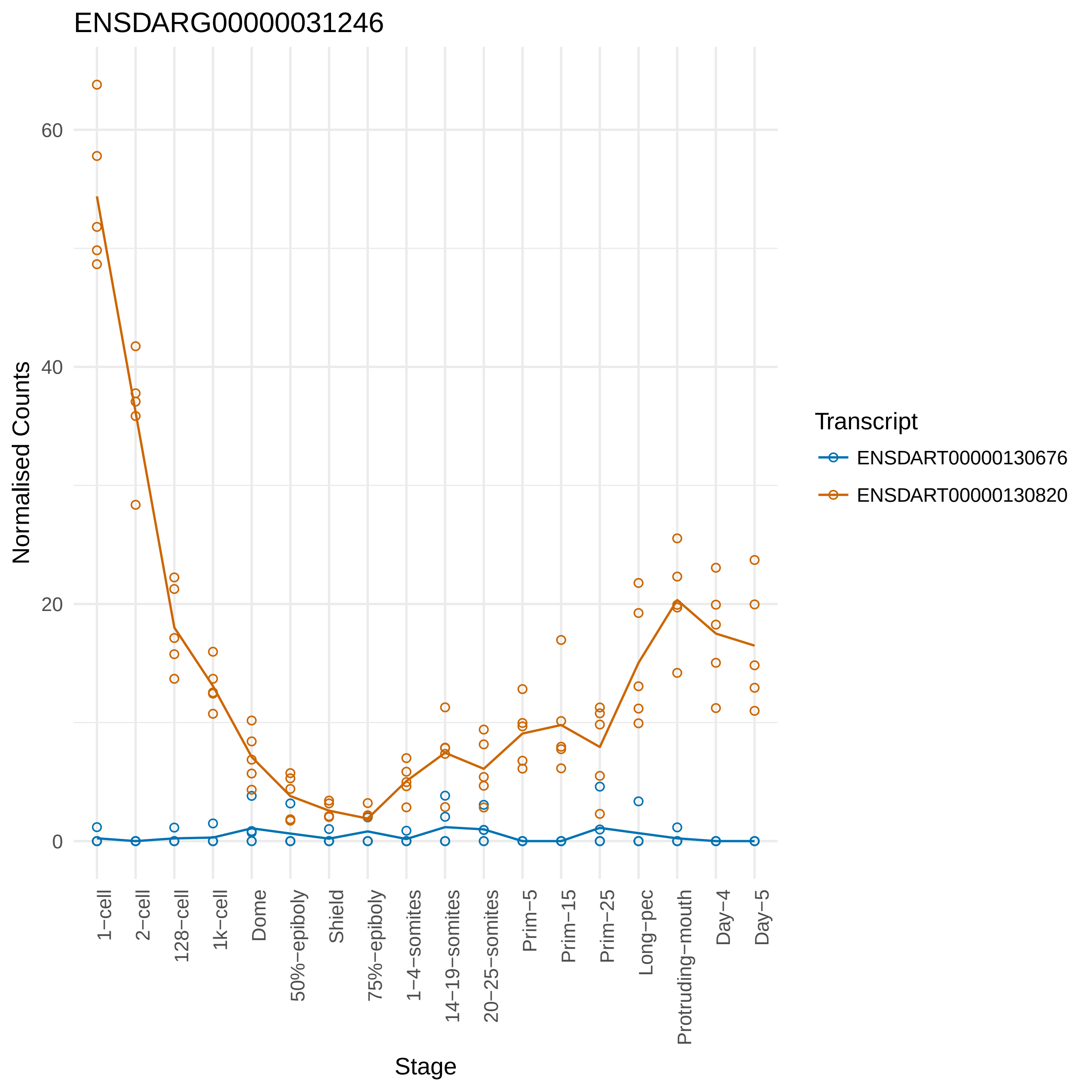
ENSDARG00000031246
Normalised Counts
60
40
20
0
1−cell
2−cell
128−cell
1k−cell
Dome
50%−epiboly
Shield
75%−epiboly
1−4−somites
14−19−somites
20−25−somites
Prim−5
Prim−15
Prim−25
Long−pec
Protruding−mouth
Day−4
Day−5
Stage
Transcript
ENSDART00000130676
ENSDART00000130820

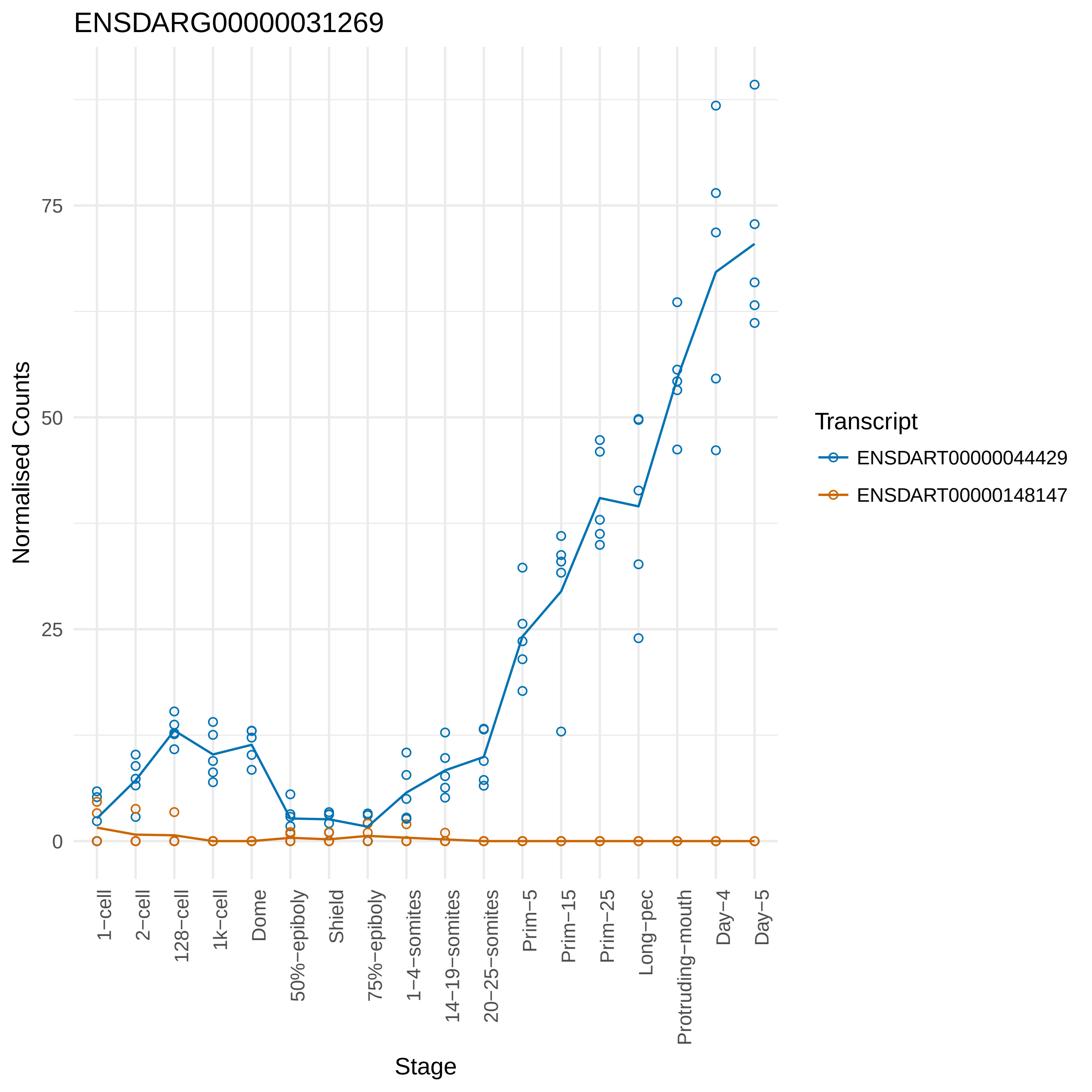

ENSDARG00000031269
Normalised Counts
75
50
25
0
1−cell
2−cell
128−cell
1k−cell
Dome
50%−epiboly
Shield
75%−epiboly
1−4−somites
14−19−somites
20−25−somites
Prim−5
Prim−15
Prim−25
Long−pec
Protruding−mouth
Day−4
Day−5
Stage
Transcript
ENSDART00000044429
ENSDART00000148147

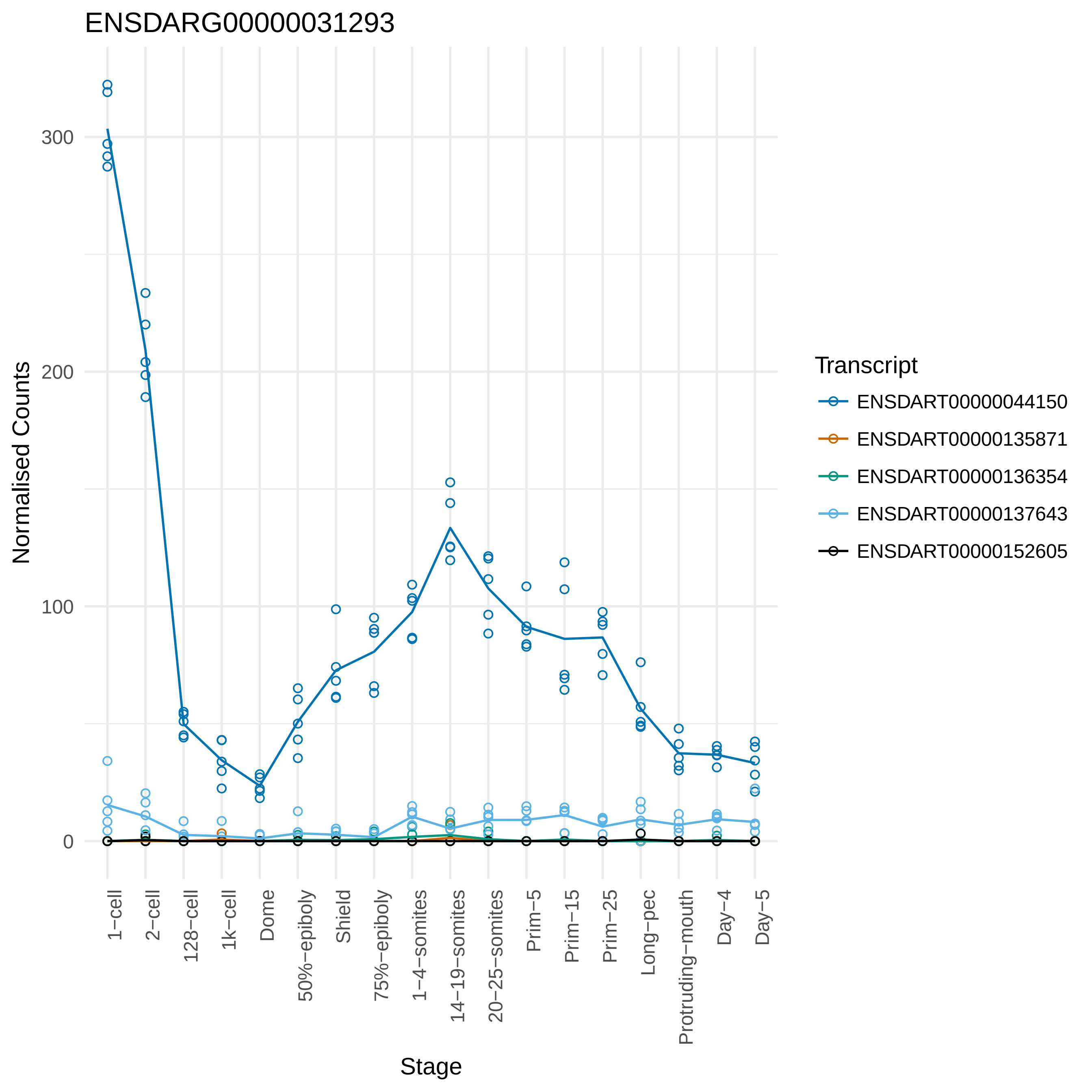
ENSDARG00000031293
Normalised Counts
300
200
100
0
1−cell
2−cell
128−cell
1k−cell
Dome
50%−epiboly
Shield
75%−epiboly
1−4−somites
14−19−somites
20−25−somites
Prim−5
Prim−15
Prim−25
Long−pec
Protruding−mouth
Day−4
Day−5
Stage
Transcript
ENSDART00000044150
ENSDART00000135871
ENSDART00000136354
ENSDART00000137643
ENSDART00000152605

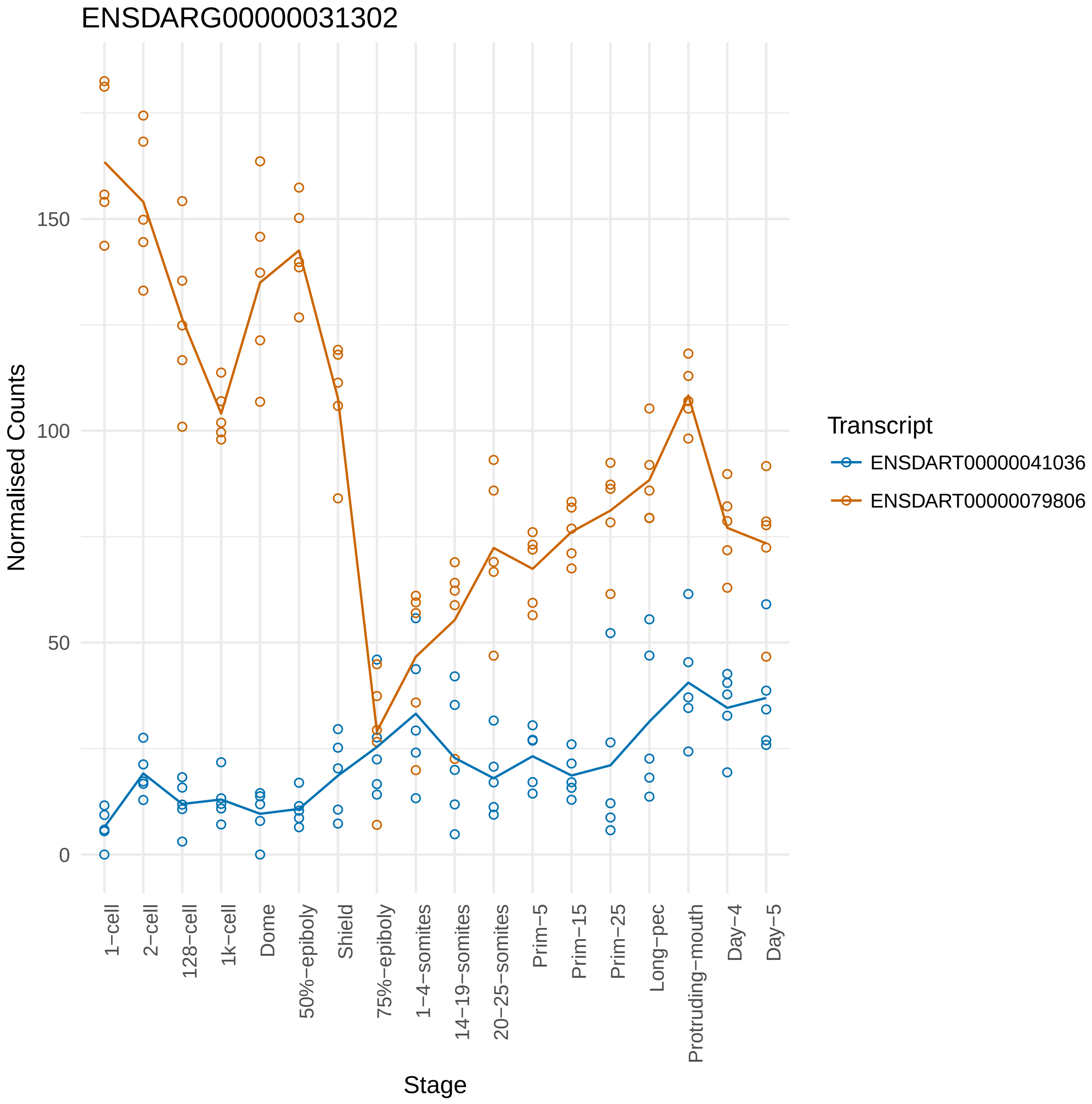
ENSDARG00000031302
Normalised Counts
0
50
100
150
1−cell
2−cell
128−cell
1k−cell
Dome
50%−epiboly
Shield
75%−epiboly
1−4−somites
14−19−somites
20−25−somites
Prim−5
Prim−15
Prim−25
Long−pec
Protruding−mouth
Day−4
Day−5
Stage
Transcript
ENSDART00000041036
ENSDART00000079806

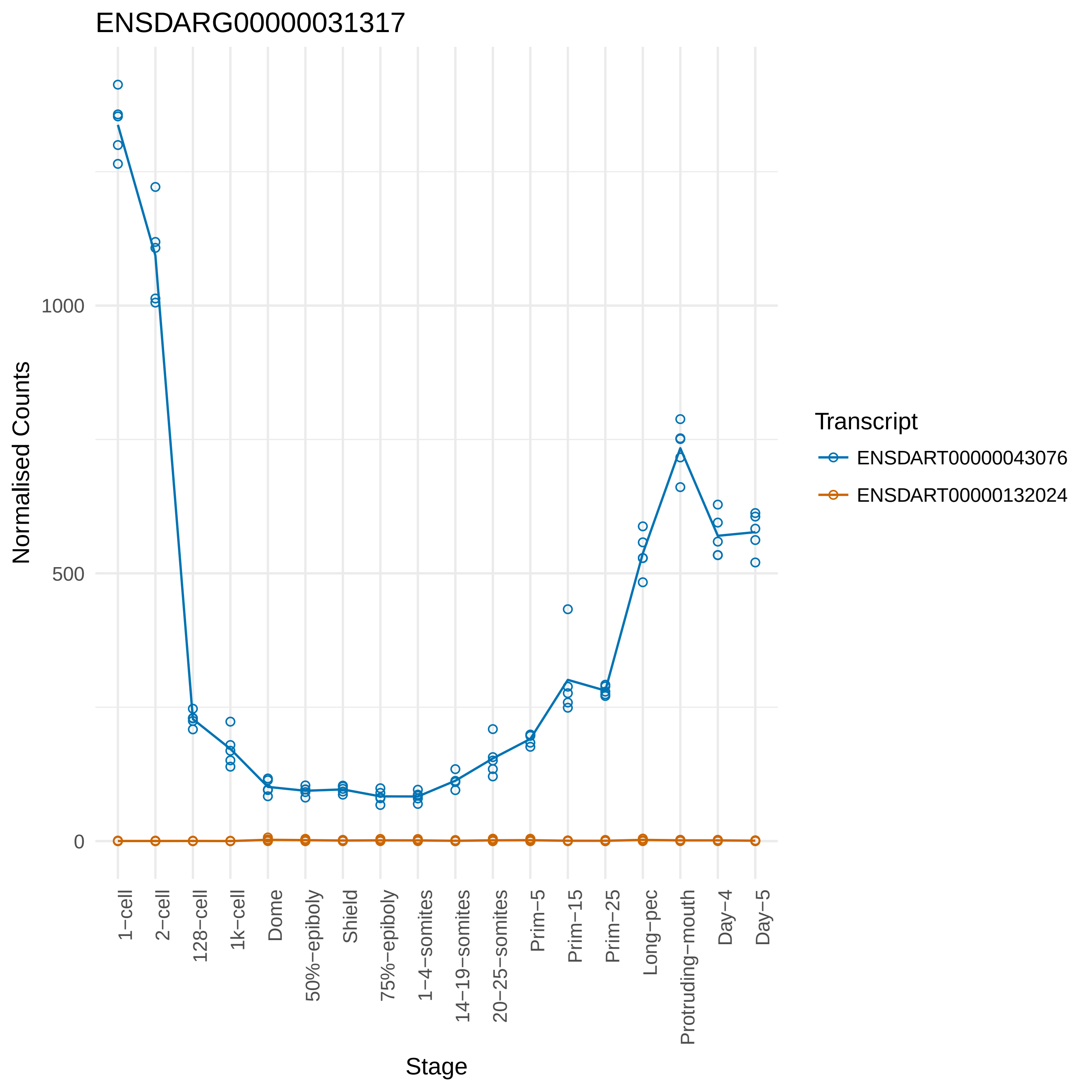
ENSDARG00000031317
Normalised Counts
1000
500
0
1−cell
2−cell
128−cell
1k−cell
Dome
50%−epiboly
Shield
75%−epiboly
1−4−somites
14−19−somites
20−25−somites
Prim−5
Prim−15
Prim−25
Long−pec
Protruding−mouth
Day−4
Day−5
Stage
Transcript
ENSDART00000043076
ENSDART00000132024

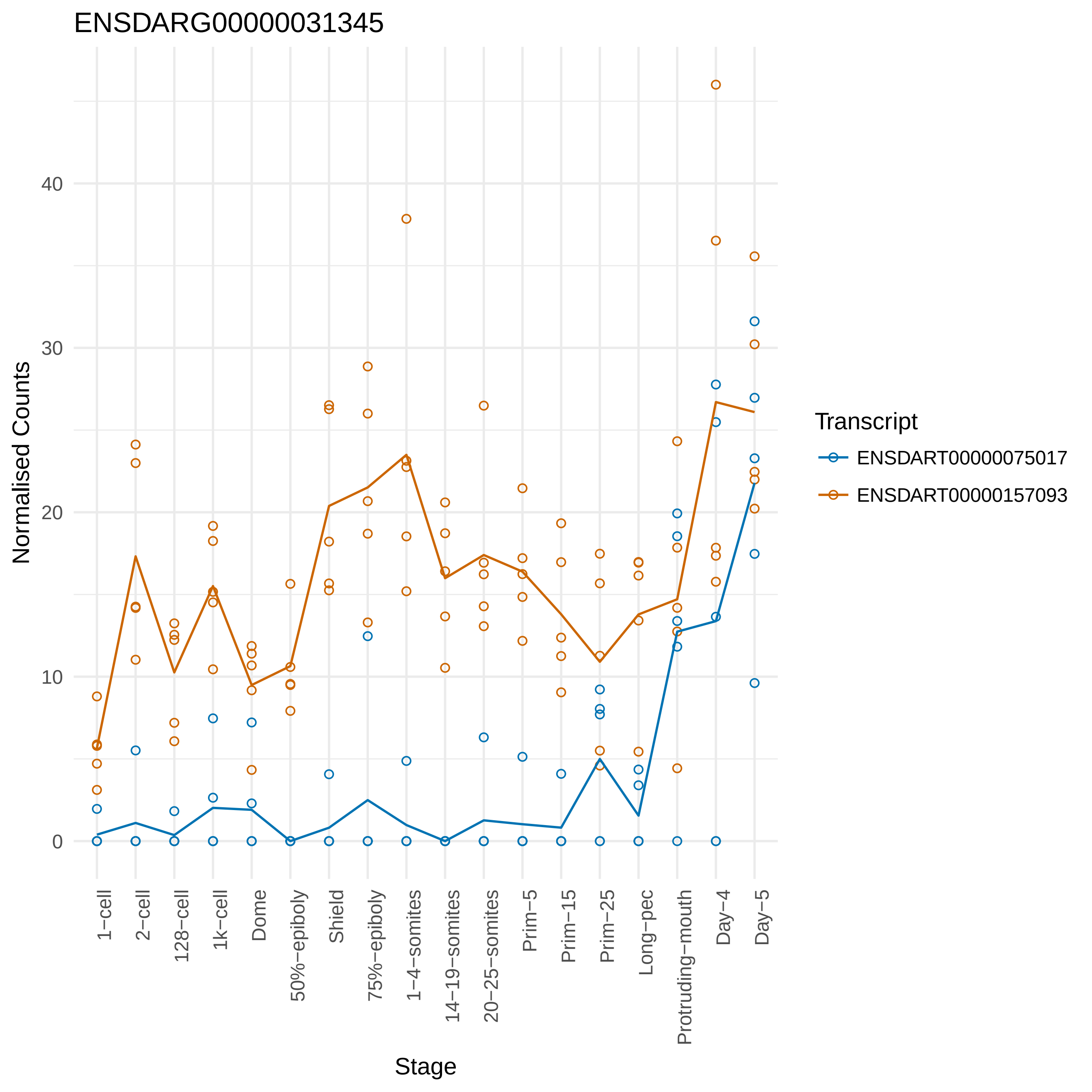

ENSDARG00000031345
Normalised Counts
0
10
20
30
40
1−cell
2−cell
128−cell
1k−cell
Dome
50%−epiboly
Shield
75%−epiboly
1−4−somites
14−19−somites
20−25−somites
Prim−5
Prim−15
Prim−25
Long−pec
Protruding−mouth
Day−4
Day−5
Stage
Transcript
ENSDART00000075017
ENSDART00000157093

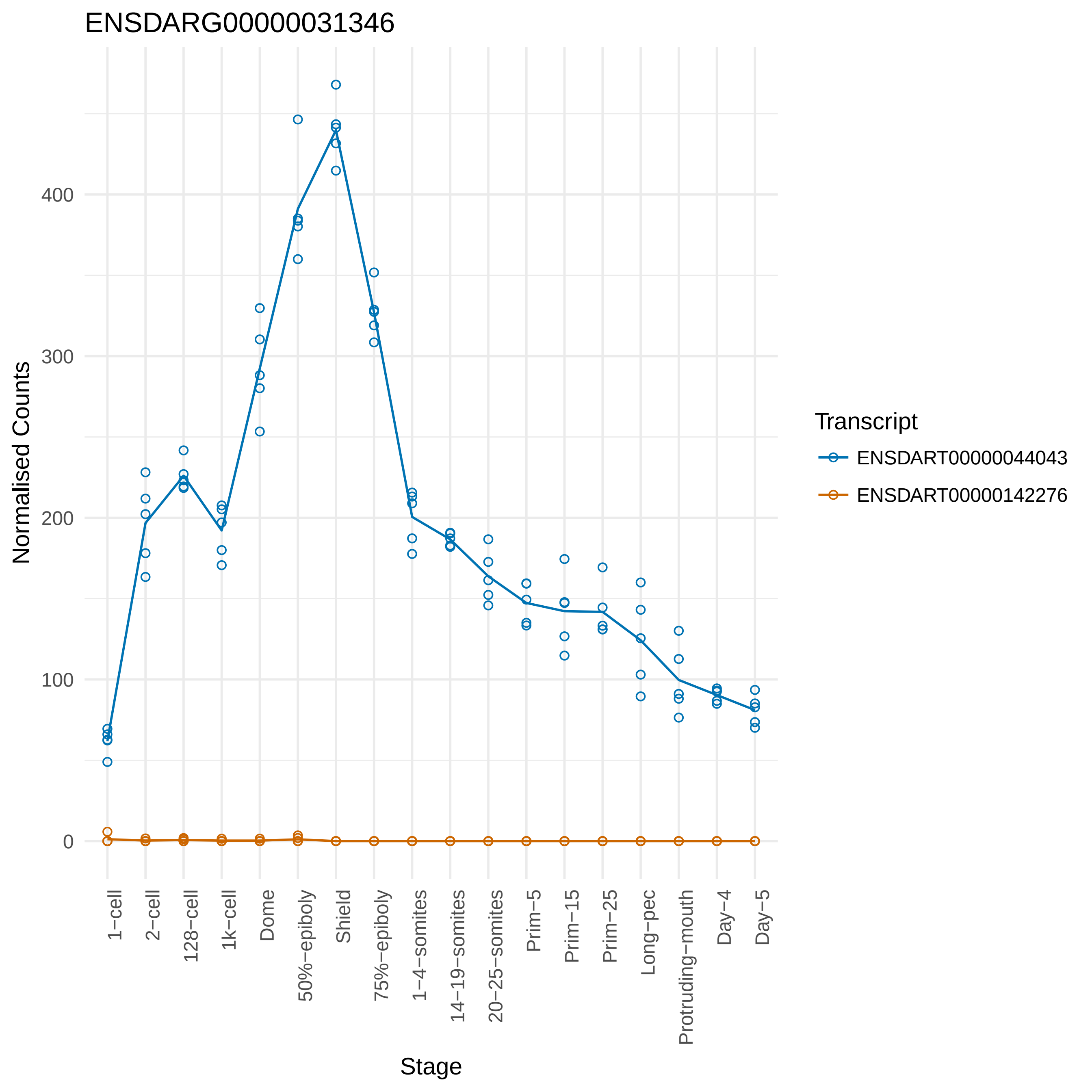
ENSDARG00000031346
Normalised Counts
400
300
200
100
0
1−cell
2−cell
128−cell
1k−cell
Dome
50%−epiboly
Shield
75%−epiboly
1−4−somites
14−19−somites
20−25−somites
Prim−5
Prim−15
Prim−25
Long−pec
Protruding−mouth
Day−4
Day−5
Stage
Transcript
ENSDART00000044043
ENSDART00000142276

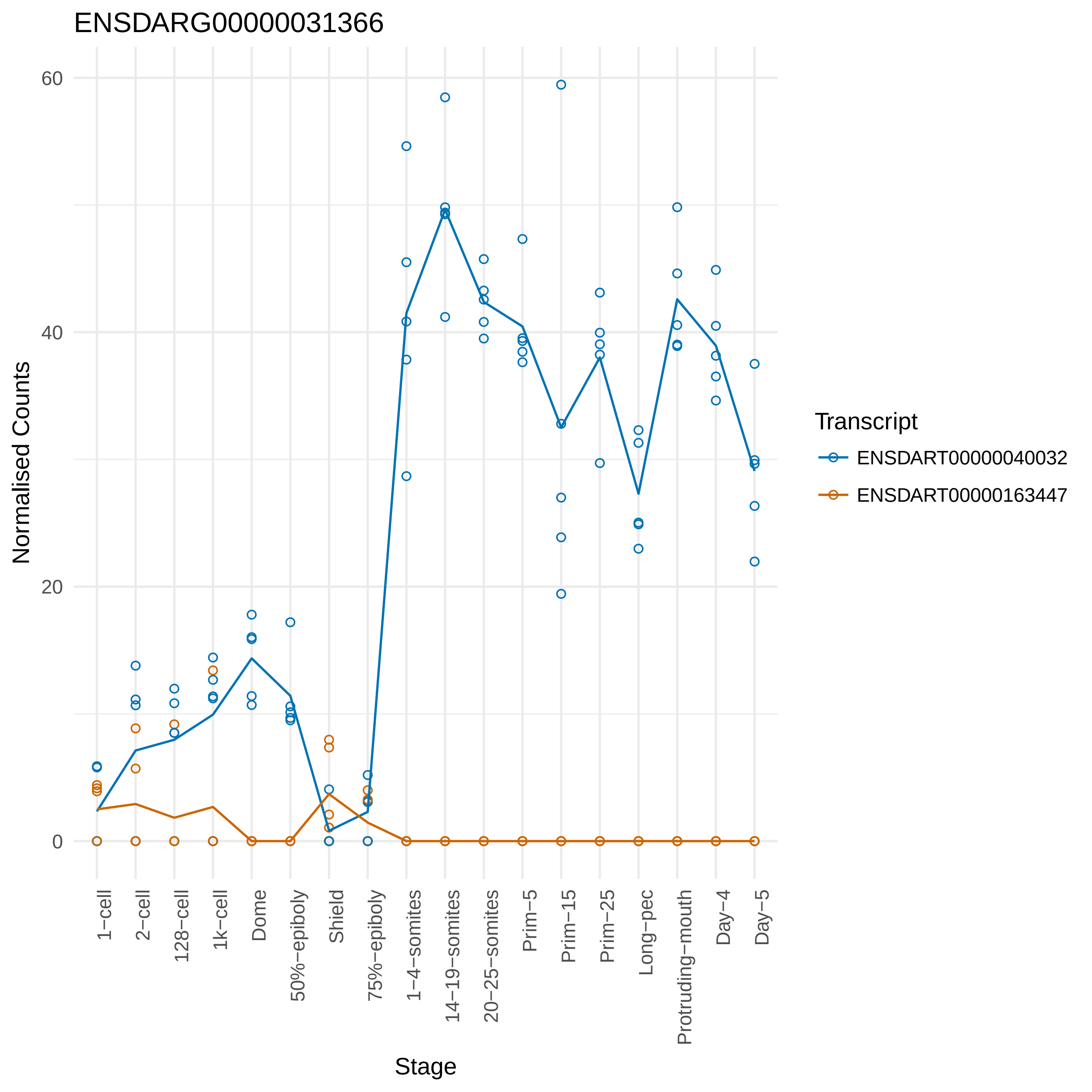

ENSDARG00000031366
Normalised Counts
60
40
20
0
1−cell
2−cell
128−cell
1k−cell
Dome
50%−epiboly
Shield
75%−epiboly
1−4−somites
14−19−somites
20−25−somites
Prim−5
Prim−15
Prim−25
Long−pec
Protruding−mouth
Day−4
Day−5
Stage
Transcript
ENSDART00000040032
ENSDART00000163447

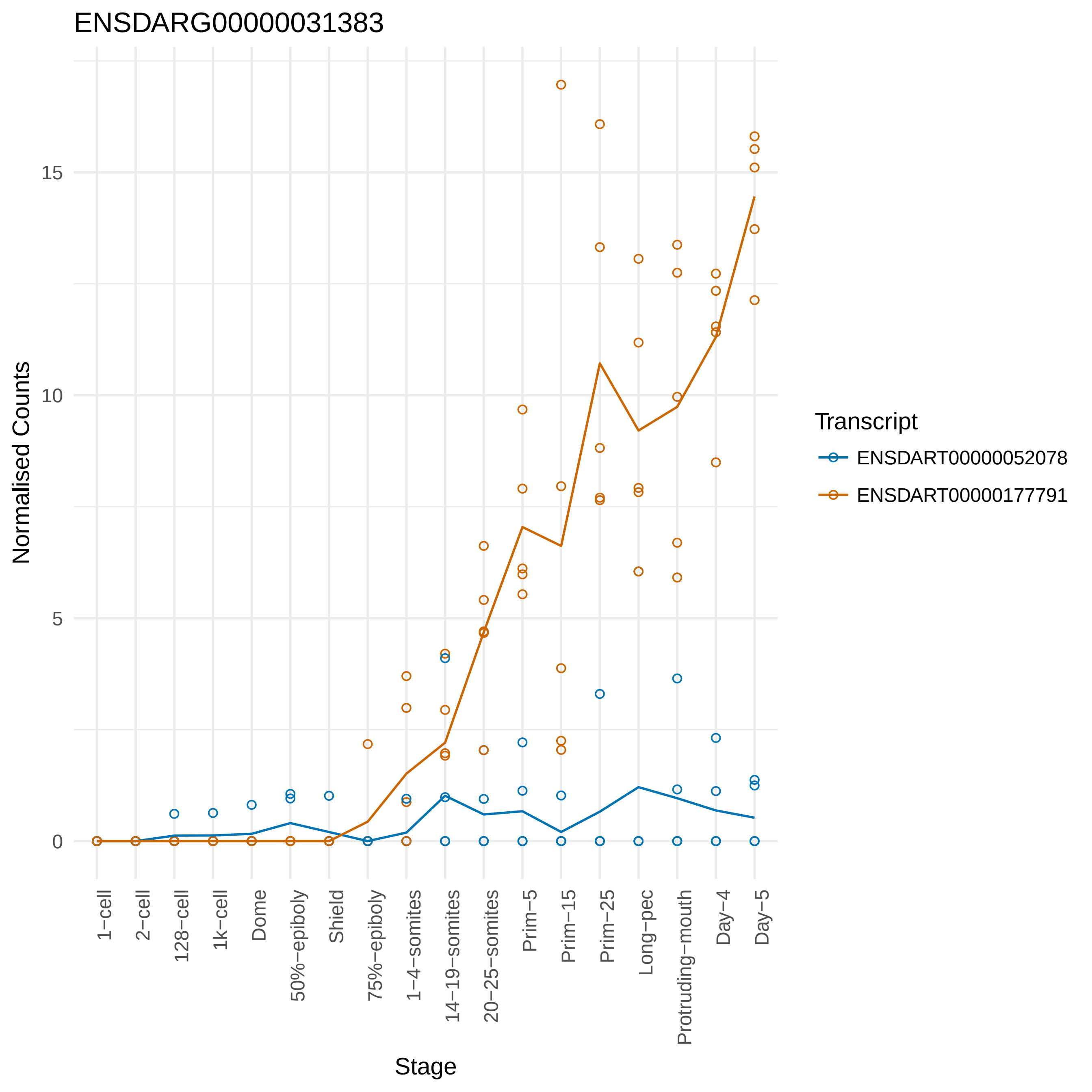

ENSDARG00000031383
Normalised Counts
15
10
5
0
1−cell
2−cell
128−cell
1k−cell
Dome
50%−epiboly
Shield
75%−epiboly
1−4−somites
14−19−somites
20−25−somites
Prim−5
Prim−15
Prim−25
Long−pec
Protruding−mouth
Day−4
Day−5
Stage
Transcript
ENSDART00000052078
ENSDART00000177791

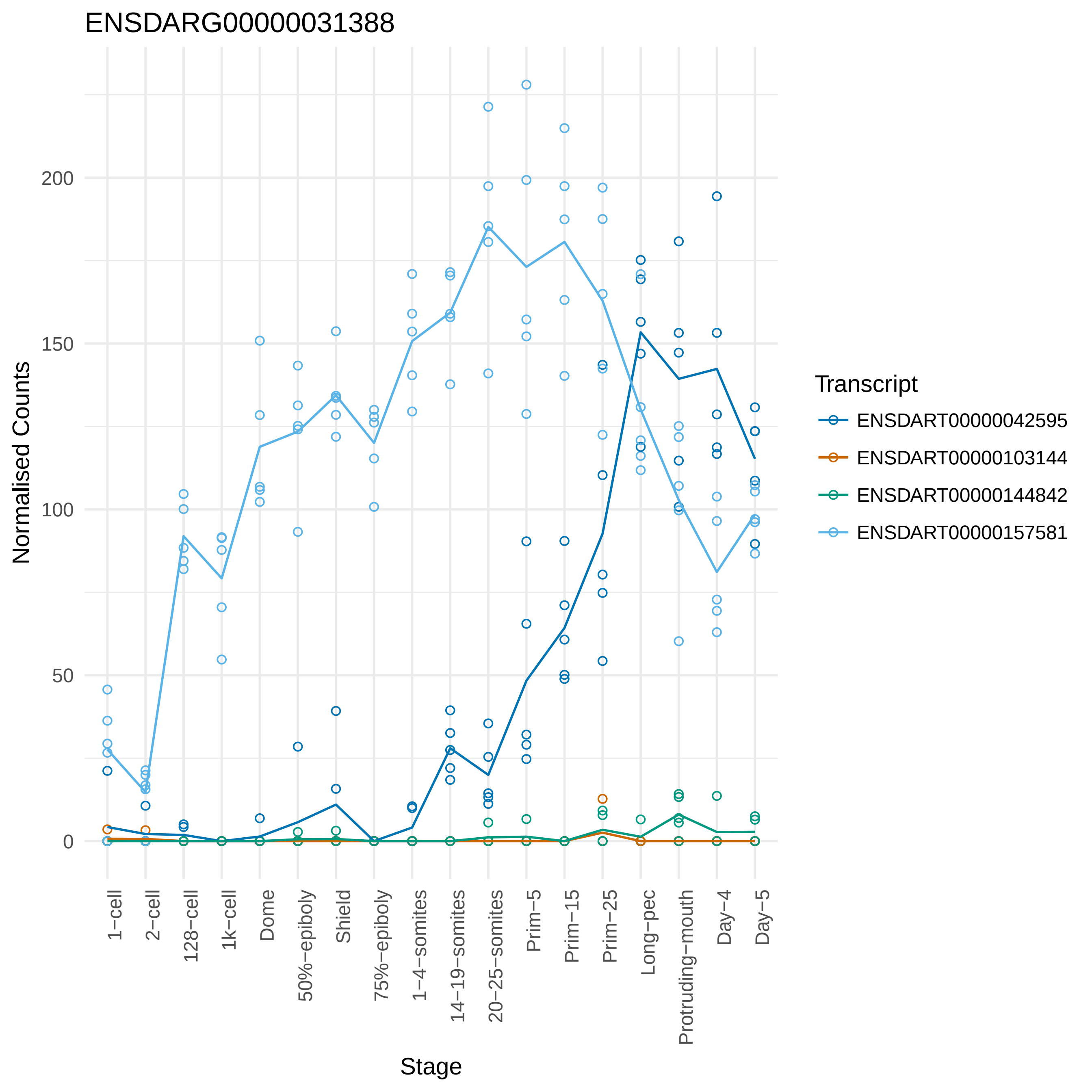
ENSDARG00000031388
Normalised Counts
0
50
100
150
200
Stage
1−cell
2−cell
128−cell
1k−cell
Dome
50%−epiboly
Shield
75%−epiboly
1−4−somites
14−19−somites
20−25−somites
Prim−5
Prim−15
Prim−25
Long−pec
Protruding−mouth
Day−4
Day−5
Transcript
ENSDART00000042595
ENSDART00000103144
ENSDART00000144842
ENSDART00000157581

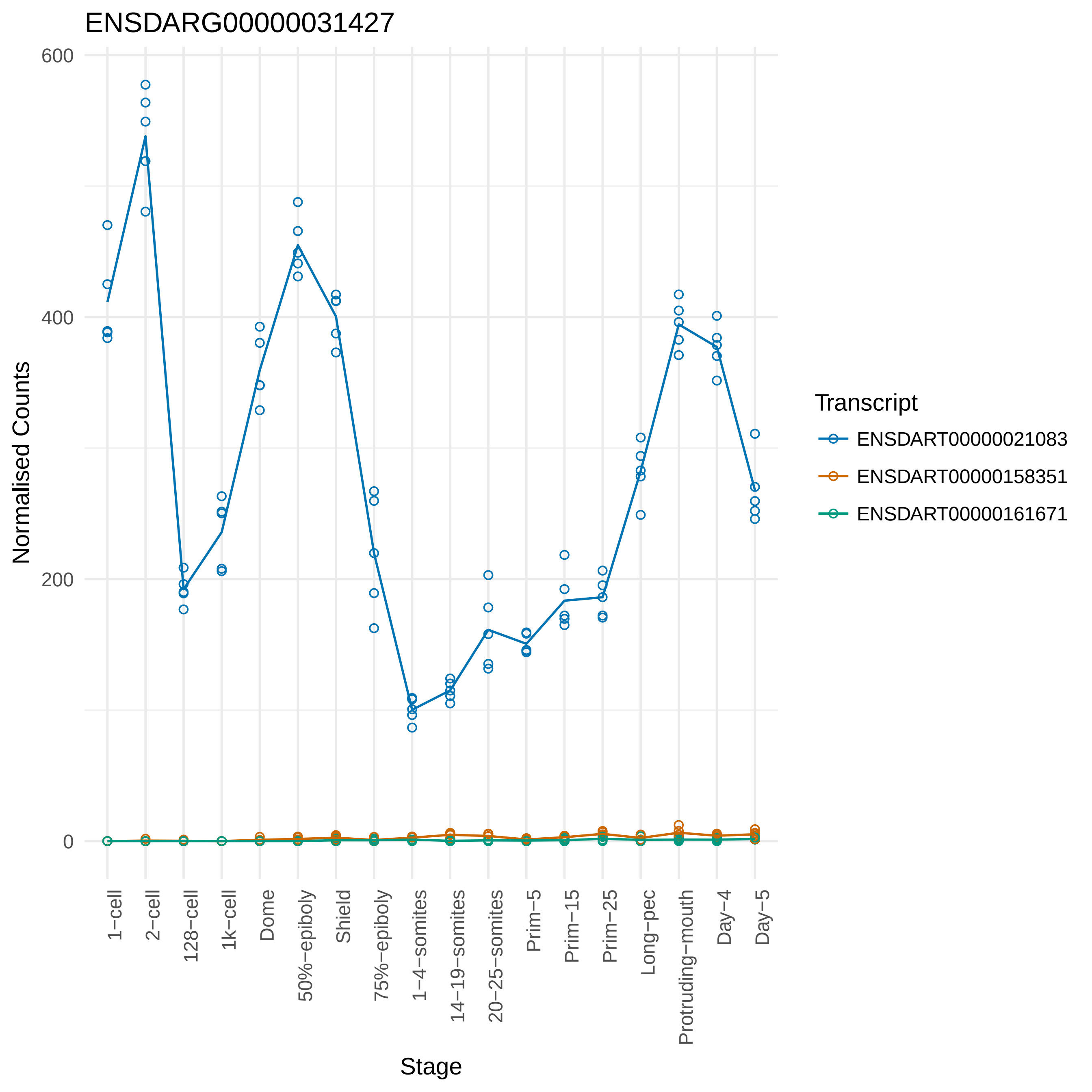
ENSDARG00000031427
Normalised Counts
600
400
200
0
1−cell
2−cell
128−cell
1k−cell
Dome
50%−epiboly
Shield
75%−epiboly
1−4−somites
14−19−somites
20−25−somites
Prim−5
Prim−15
Prim−25
Long−pec
Protruding−mouth
Day−4
Day−5
Stage
Transcript
ENSDART00000021083
ENSDART00000158351
ENSDART00000161671

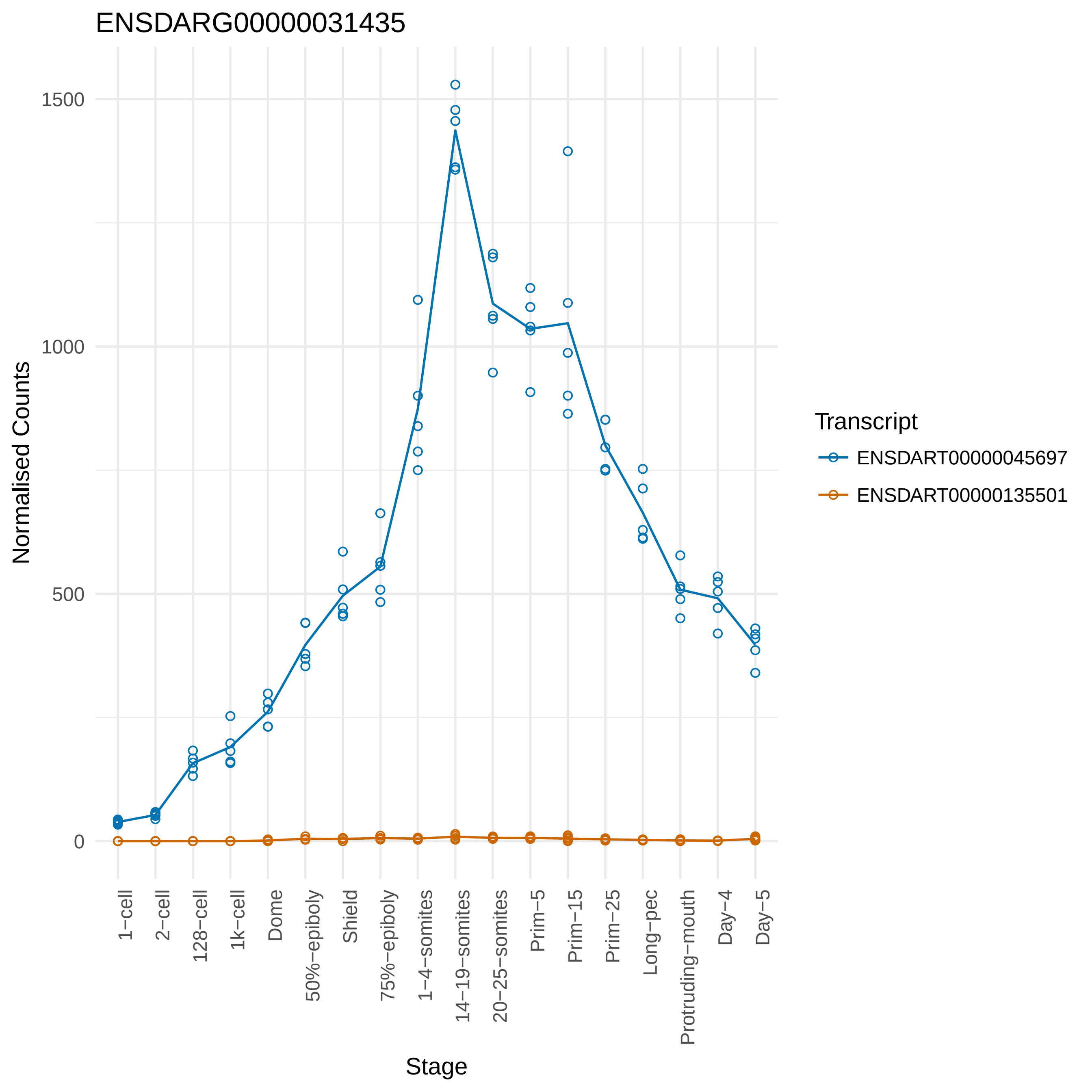

ENSDARG00000031435
Normalised Counts
0
500
1000
1500
Stage
1−cell
2−cell
128−cell
1k−cell
Dome
50%−epiboly
Shield
75%−epiboly
1−4−somites
14−19−somites
20−25−somites
Prim−5
Prim−15
Prim−25
Long−pec
Protruding−mouth
Day−4
Day−5
Transcript
ENSDART00000045697
ENSDART00000135501

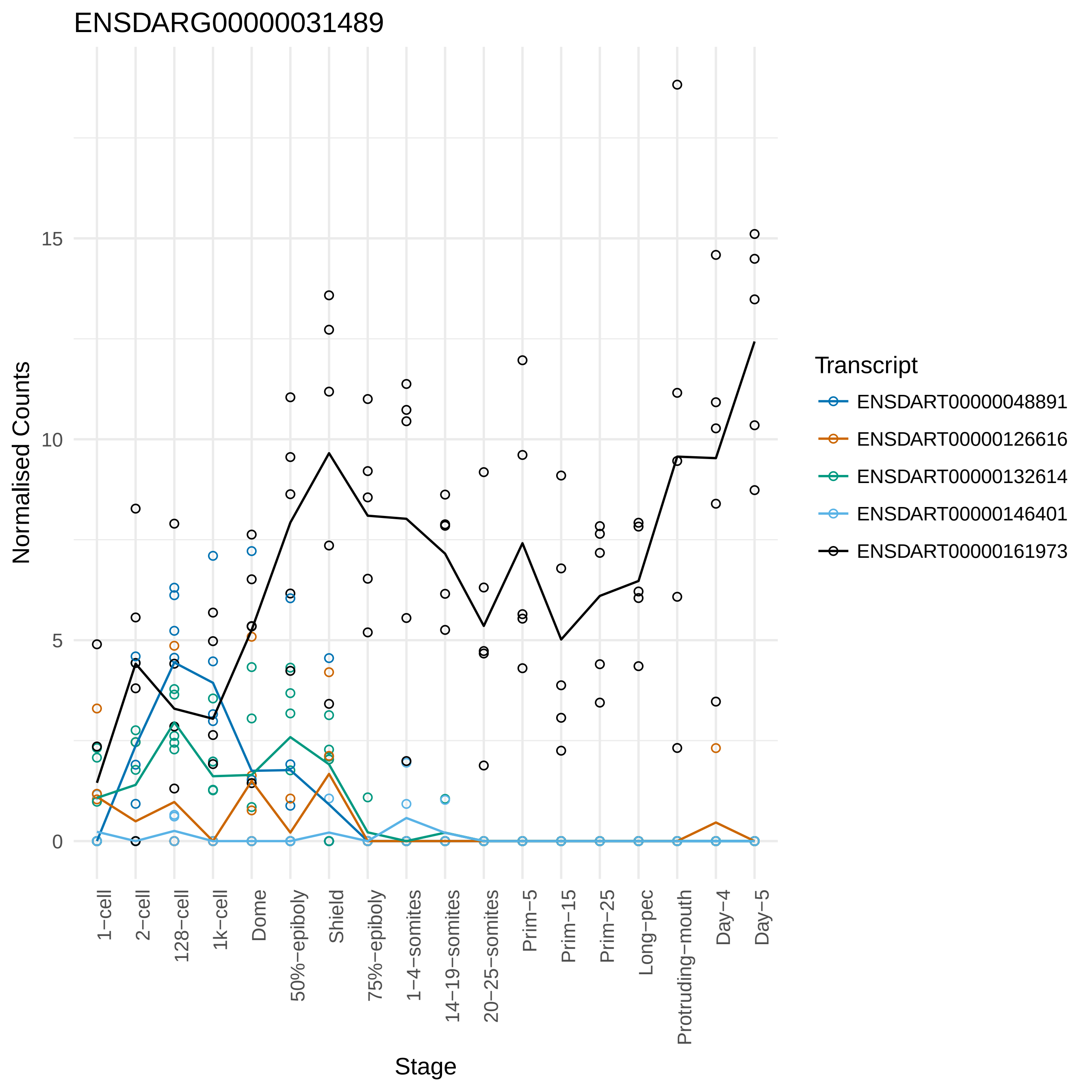
ENSDARG00000031489
Normalised Counts
0
5
10
15
1−cell
2−cell
128−cell
1k−cell
Dome
50%−epiboly
Shield
75%−epiboly
1−4−somites
14−19−somites
20−25−somites
Prim−5
Prim−15
Prim−25
Long−pec
Protruding−mouth
Day−4
Day−5
Stage
Transcript
ENSDART00000048891
ENSDART00000126616
ENSDART00000132614
ENSDART00000146401
ENSDART00000161973

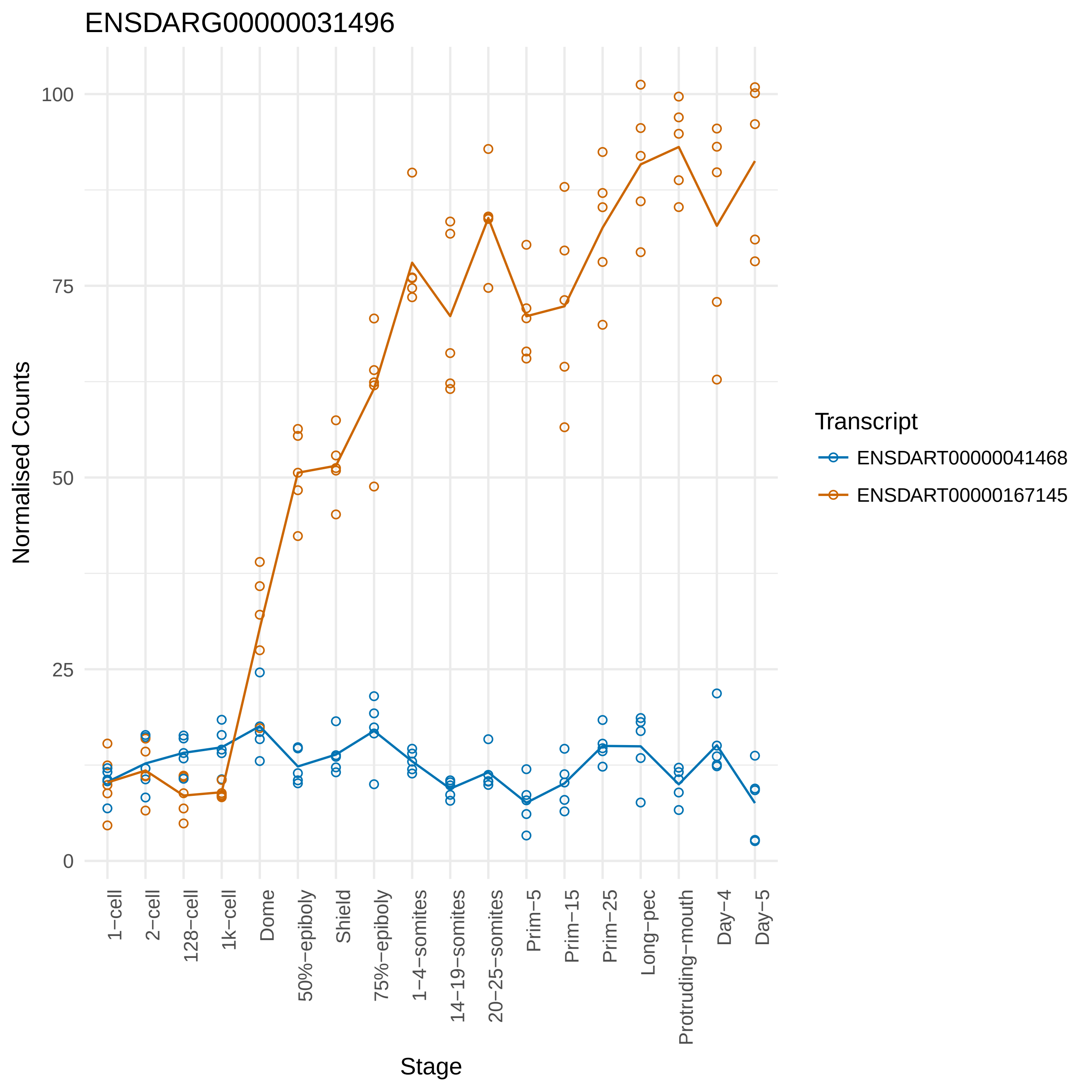
ENSDARG00000031496
Normalised Counts
100
75
50
25
0
1−cell
2−cell
128−cell
1k−cell
Dome
50%−epiboly
Shield
75%−epiboly
1−4−somites
14−19−somites
20−25−somites
Prim−5
Prim−15
Prim−25
Long−pec
Protruding−mouth
Day−4
Day−5
Stage
Transcript
ENSDART00000041468
ENSDART00000167145

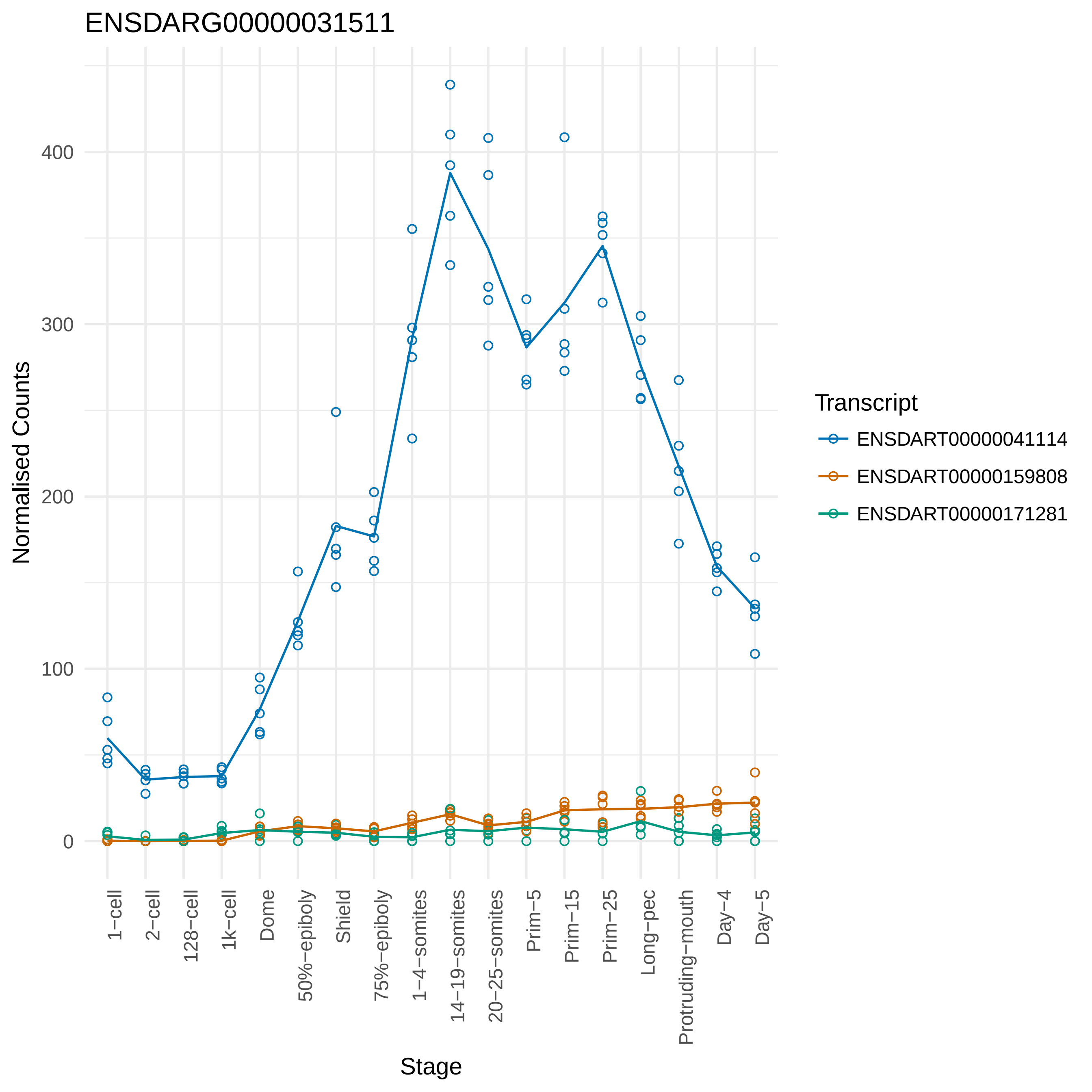

ENSDARG00000031511
Normalised Counts
0
100
200
300
400
Stage
1−cell
2−cell
128−cell
1k−cell
Dome
50%−epiboly
Shield
75%−epiboly
1−4−somites
14−19−somites
20−25−somites
Prim−5
Prim−15
Prim−25
Long−pec
Protruding−mouth
Day−4
Day−5
Transcript
ENSDART00000041114
ENSDART00000159808
ENSDART00000171281

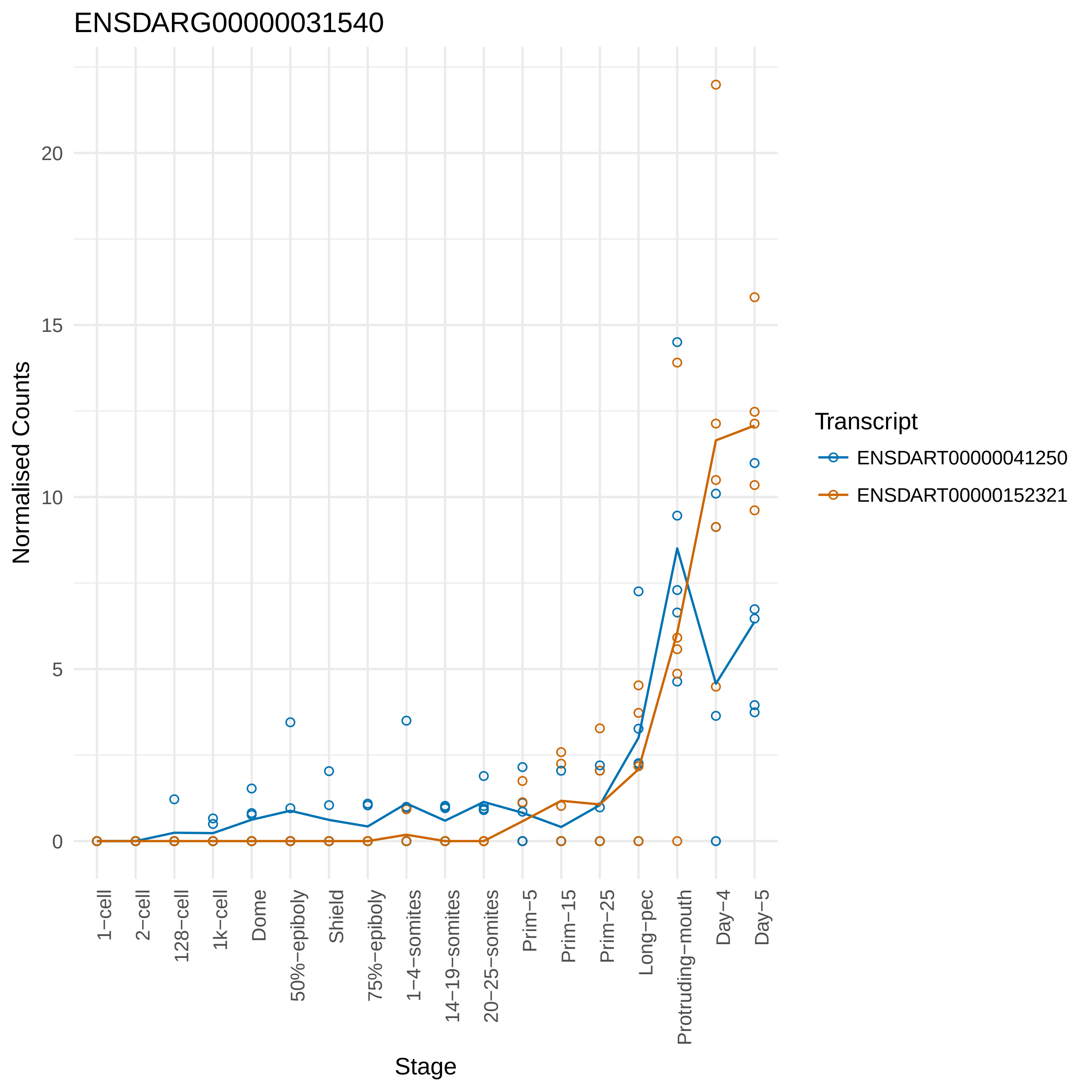

ENSDARG00000031540
Normalised Counts
0
5
10
15
20
1−cell
2−cell
128−cell
1k−cell
Dome
50%−epiboly
Shield
75%−epiboly
1−4−somites
14−19−somites
20−25−somites
Prim−5
Prim−15
Prim−25
Long−pec
Protruding−mouth
Day−4
Day−5
Stage
Transcript
ENSDART00000041250
ENSDART00000152321

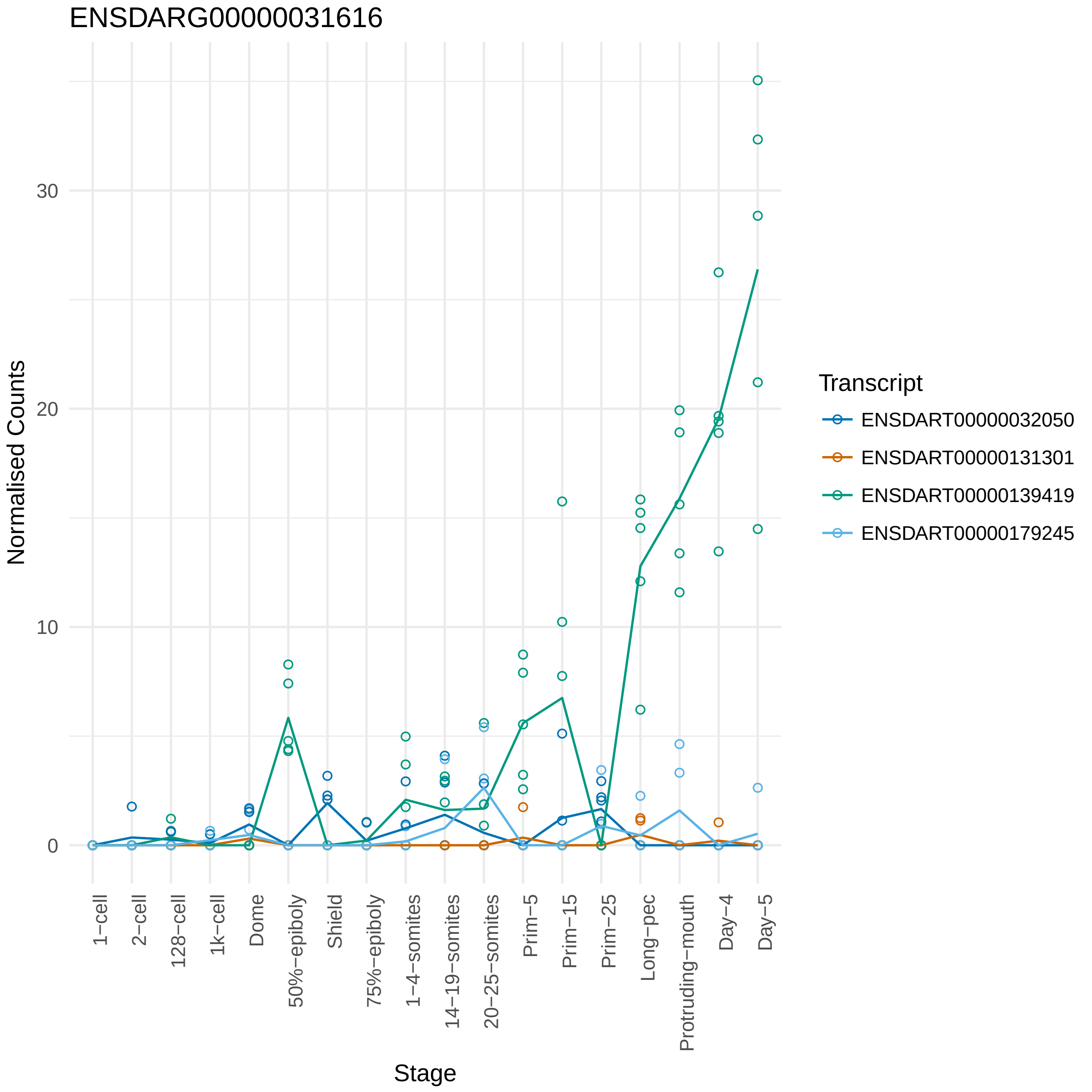

ENSDARG00000031616
Normalised Counts
0
10
20
30
Stage
1−cell
2−cell
128−cell
1k−cell
Dome
50%−epiboly
Shield
75%−epiboly
1−4−somites
14−19−somites
20−25−somites
Prim−5
Prim−15
Prim−25
Long−pec
Protruding−mouth
Day−4
Day−5
Transcript
ENSDART00000032050
ENSDART00000131301
ENSDART00000139419
ENSDART00000179245

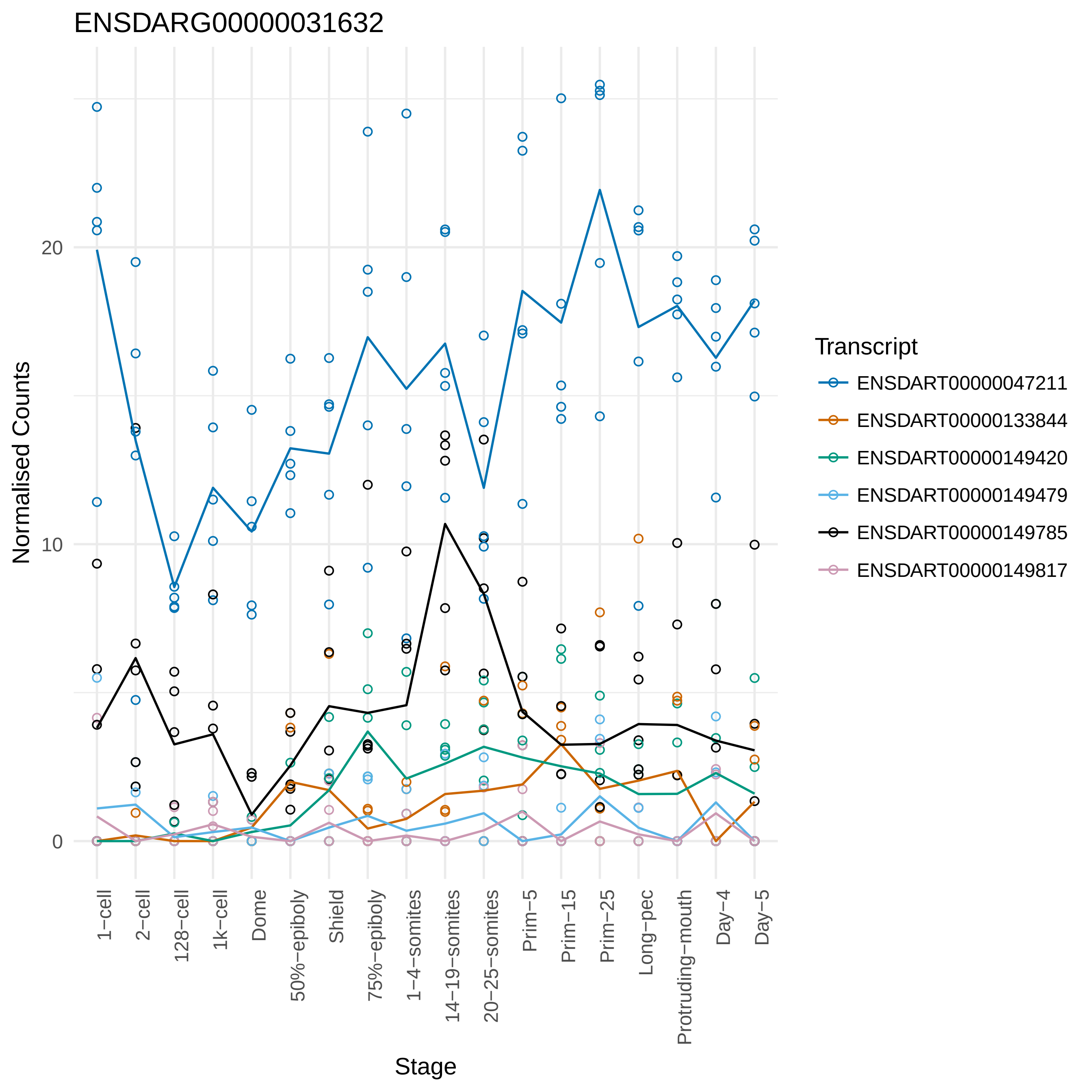

ENSDARG00000031632
Normalised Counts
0
10
20
1−cell
2−cell
128−cell
1k−cell
Dome
50%−epiboly
Shield
75%−epiboly
1−4−somites
14−19−somites
20−25−somites
Prim−5
Prim−15
Prim−25
Long−pec
Protruding−mouth
Day−4
Day−5
Stage
Transcript
ENSDART00000047211
ENSDART00000133844
ENSDART00000149420
ENSDART00000149479
ENSDART00000149785
ENSDART00000149817

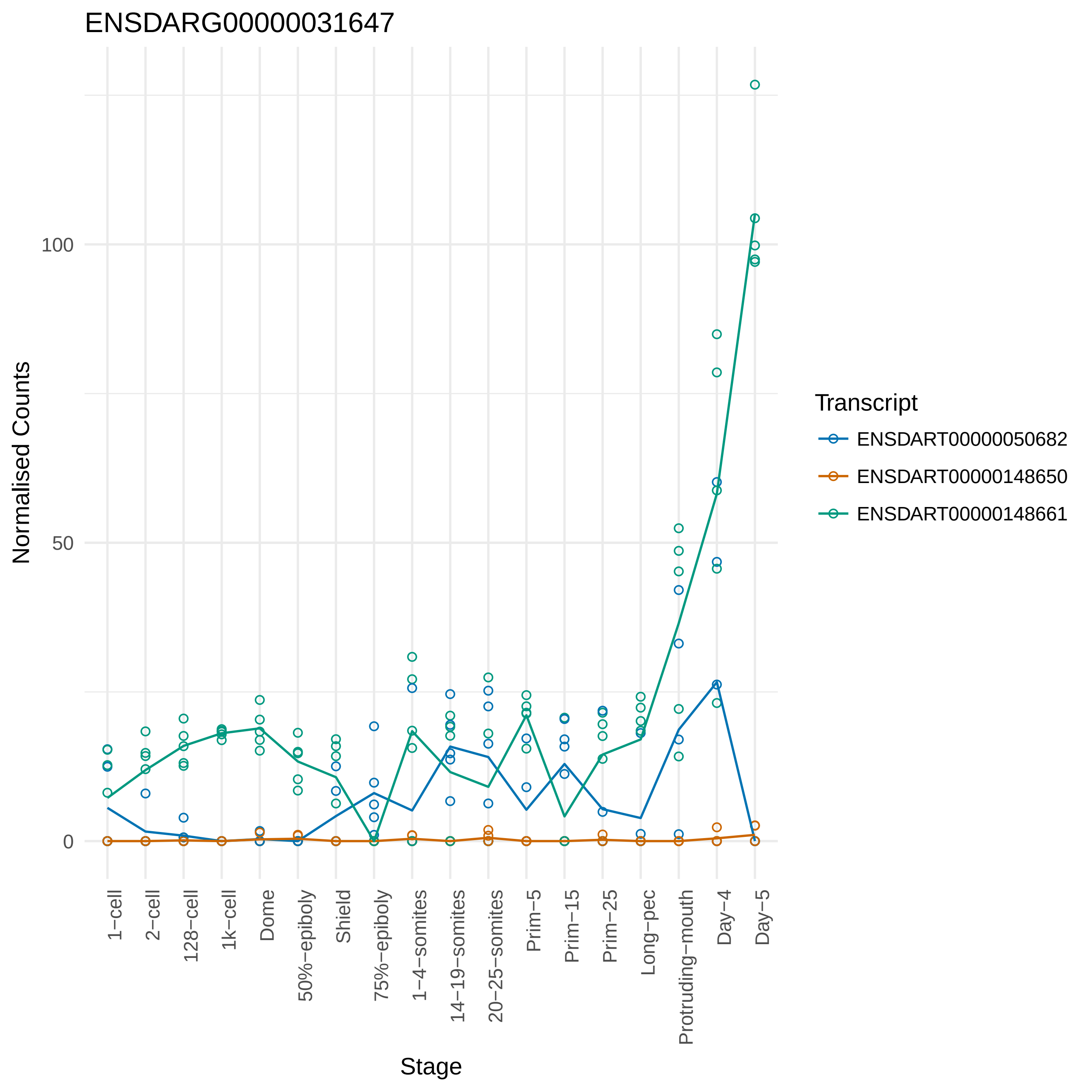
ENSDARG00000031647
Normalised Counts
100
50
0
1−cell
2−cell
128−cell
1k−cell
Dome
50%−epiboly
Shield
75%−epiboly
1−4−somites
14−19−somites
20−25−somites
Prim−5
Prim−15
Prim−25
Long−pec
Protruding−mouth
Day−4
Day−5
Stage
Transcript
ENSDART00000050682
ENSDART00000148650
ENSDART00000148661

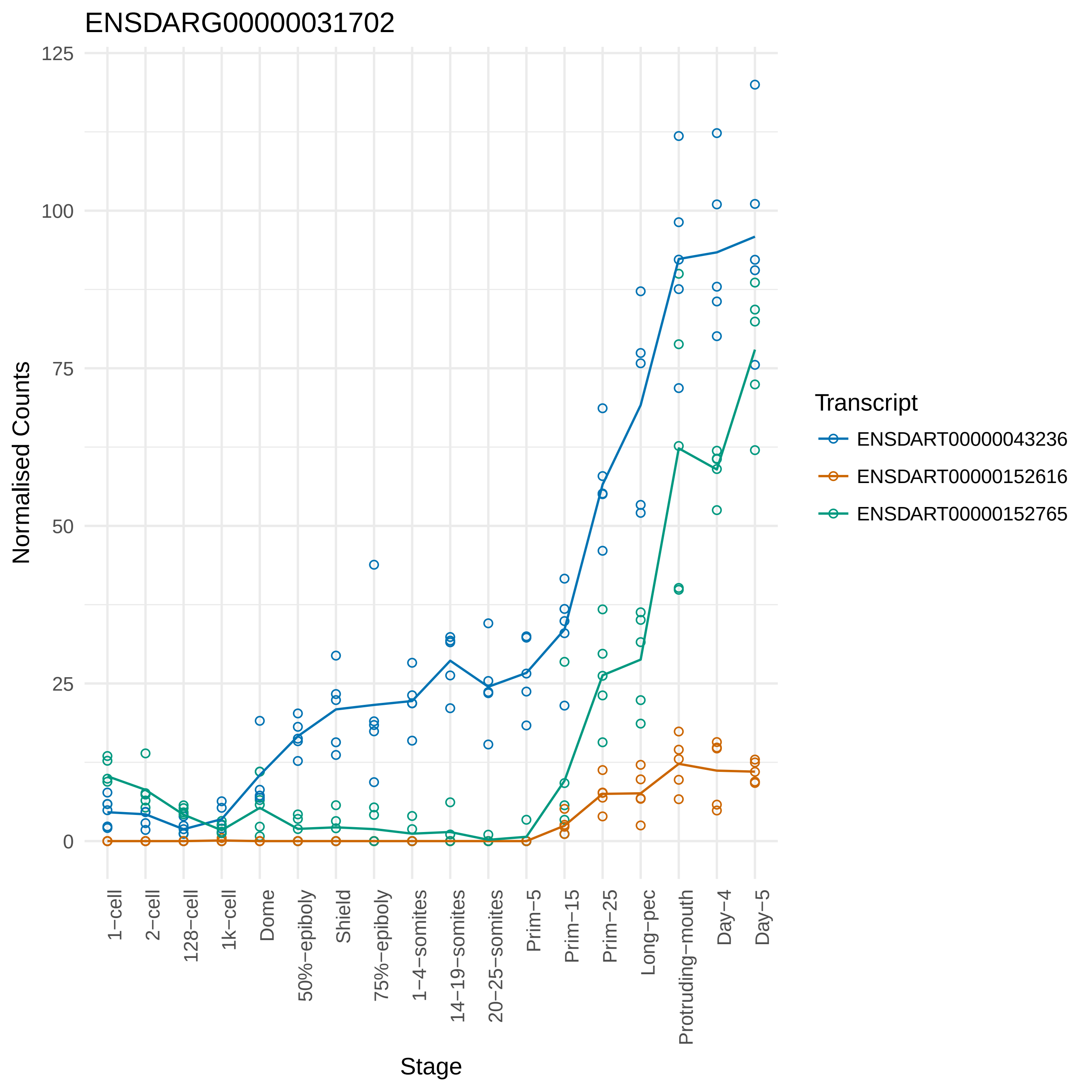

ENSDARG00000031702
Normalised Counts
125
100
75
50
25
0
1−cell
2−cell
128−cell
1k−cell
Dome
50%−epiboly
Shield
75%−epiboly
1−4−somites
14−19−somites
20−25−somites
Prim−5
Prim−15
Prim−25
Long−pec
Protruding−mouth
Day−4
Day−5
Stage
Transcript
ENSDART00000043236
ENSDART00000152616
ENSDART00000152765

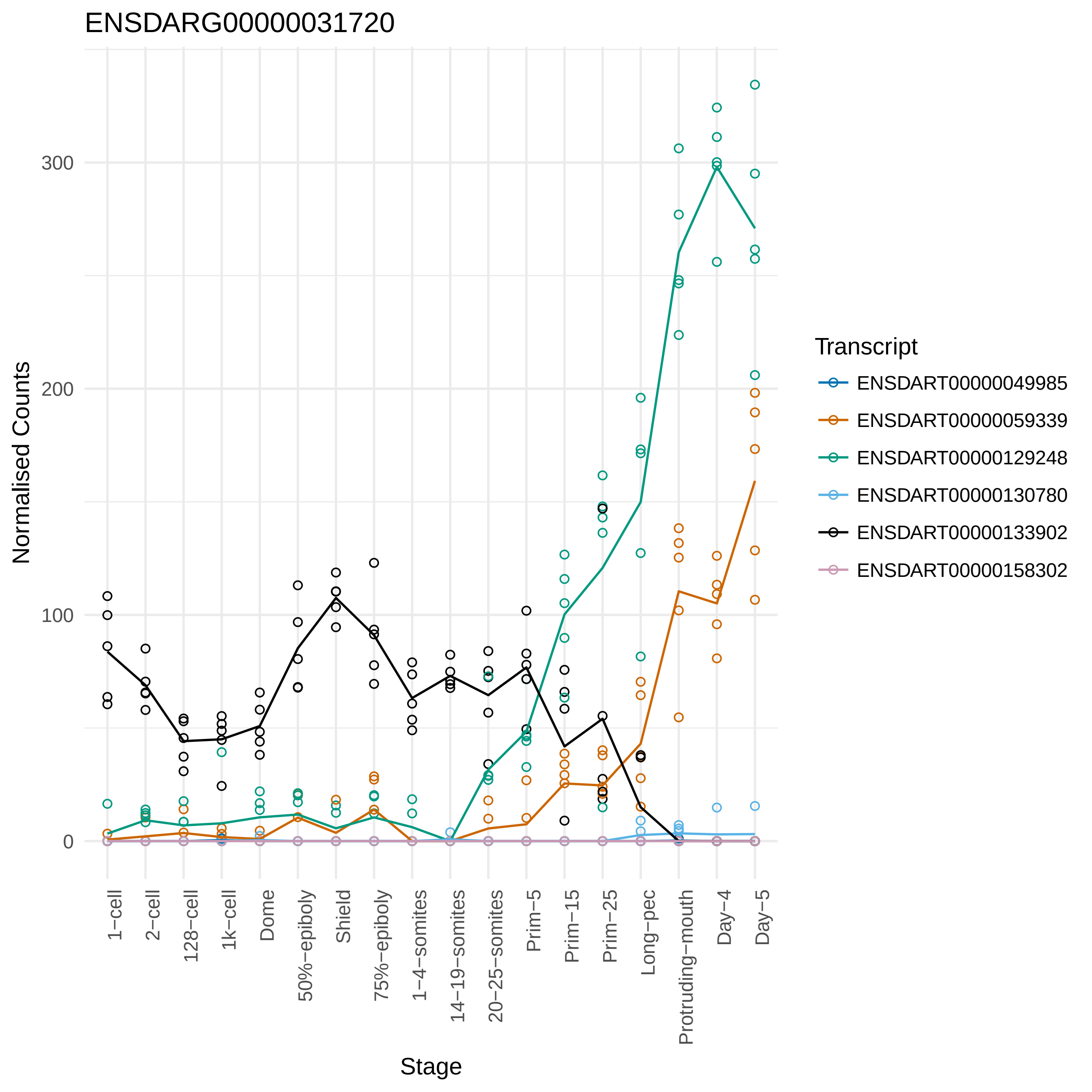

ENSDARG00000031720
Normalised Counts
300
200
100
0
1−cell
2−cell
128−cell
1k−cell
Dome
50%−epiboly
Shield
75%−epiboly
1−4−somites
14−19−somites
20−25−somites
Prim−5
Prim−15
Prim−25
Long−pec
Protruding−mouth
Day−4
Day−5
Stage
Transcript
ENSDART00000049985
ENSDART00000059339
ENSDART00000129248
ENSDART00000130780
ENSDART00000133902
ENSDART00000158302

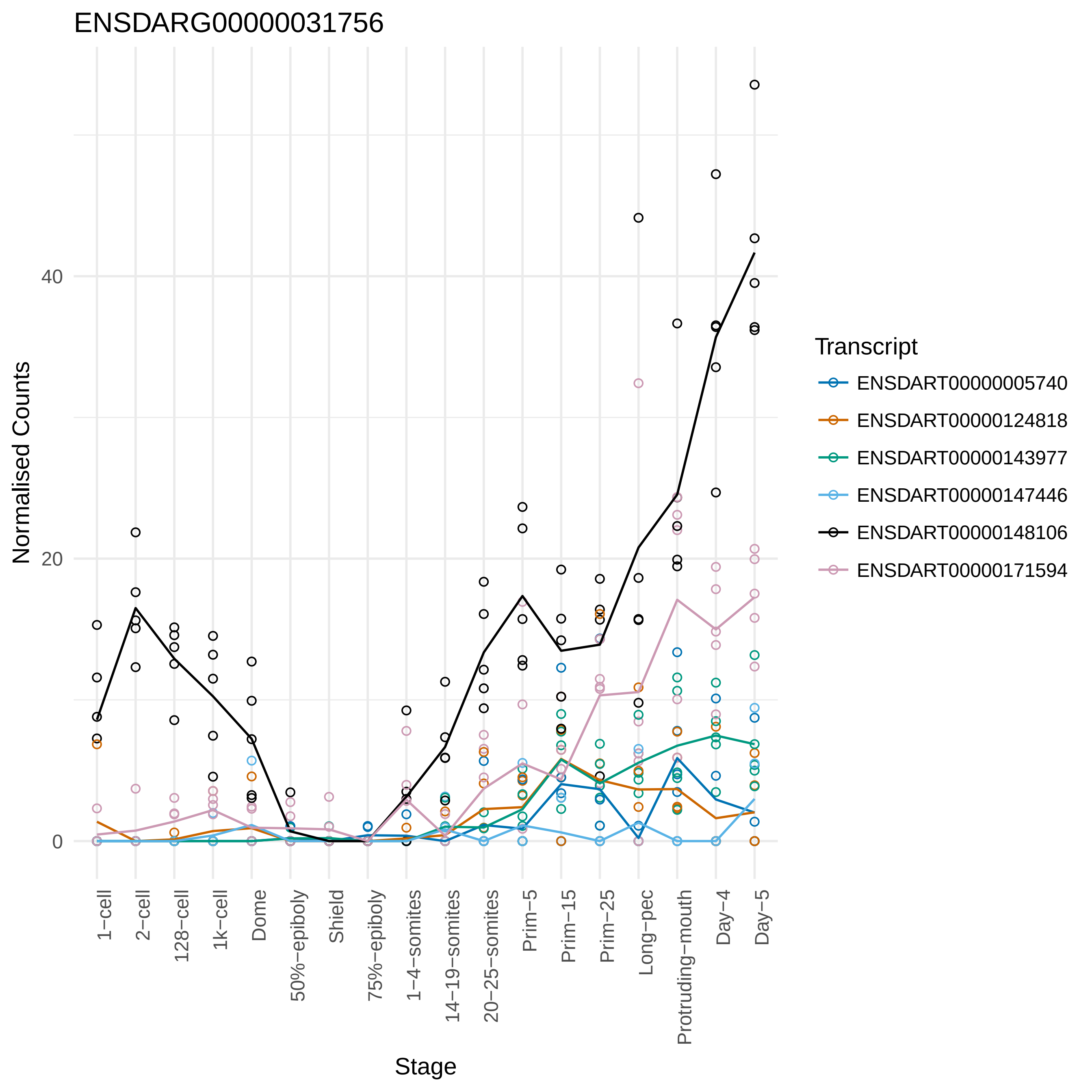

ENSDARG00000031756
Normalised Counts
40
20
0
1−cell
2−cell
128−cell
1k−cell
Dome
50%−epiboly
Shield
75%−epiboly
1−4−somites
14−19−somites
20−25−somites
Prim−5
Prim−15
Prim−25
Long−pec
Protruding−mouth
Day−4
Day−5
Stage
Transcript
ENSDART00000005740
ENSDART00000124818
ENSDART00000143977
ENSDART00000147446
ENSDART00000148106
ENSDART00000171594

ENSDARG00000031761

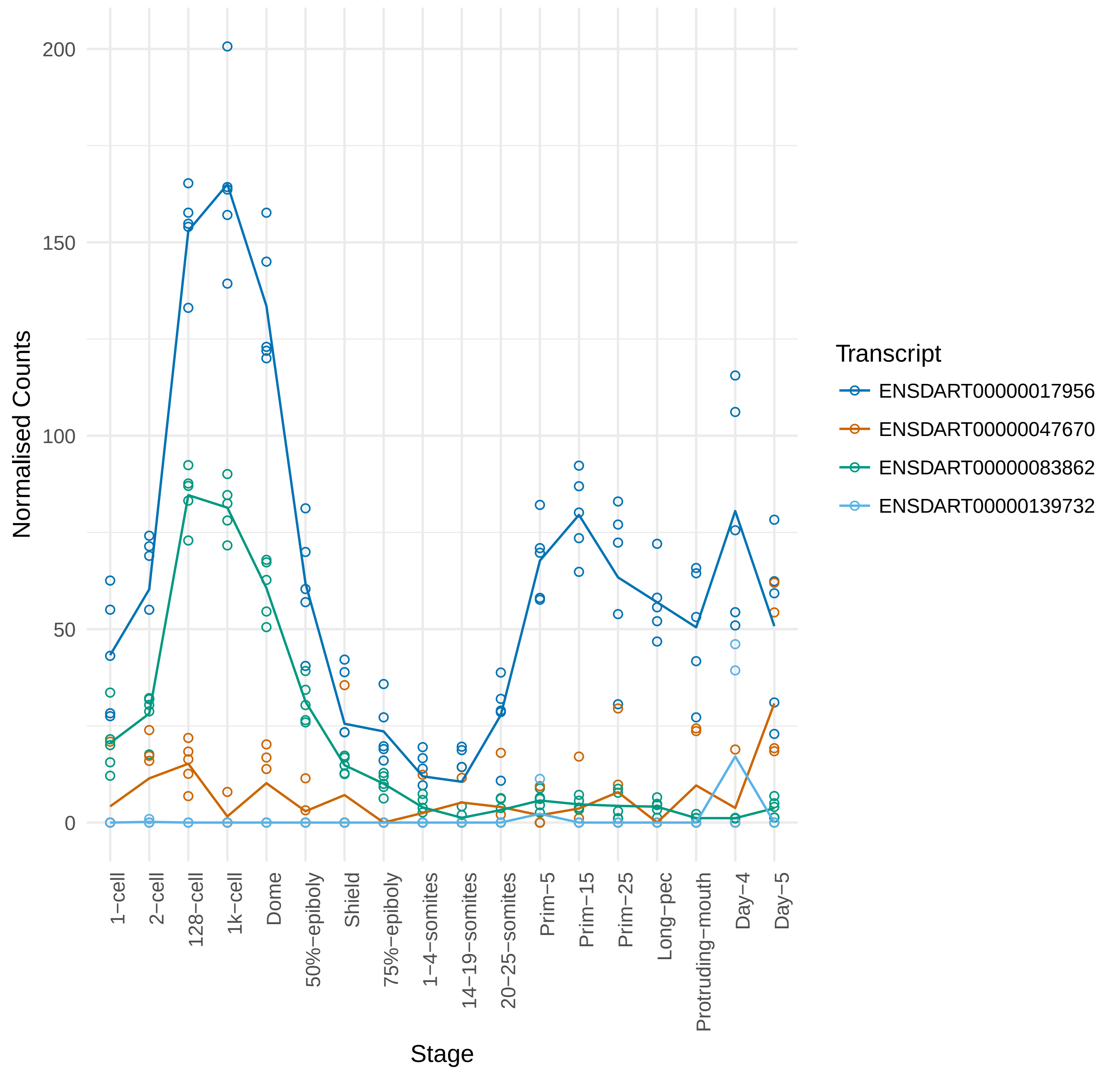

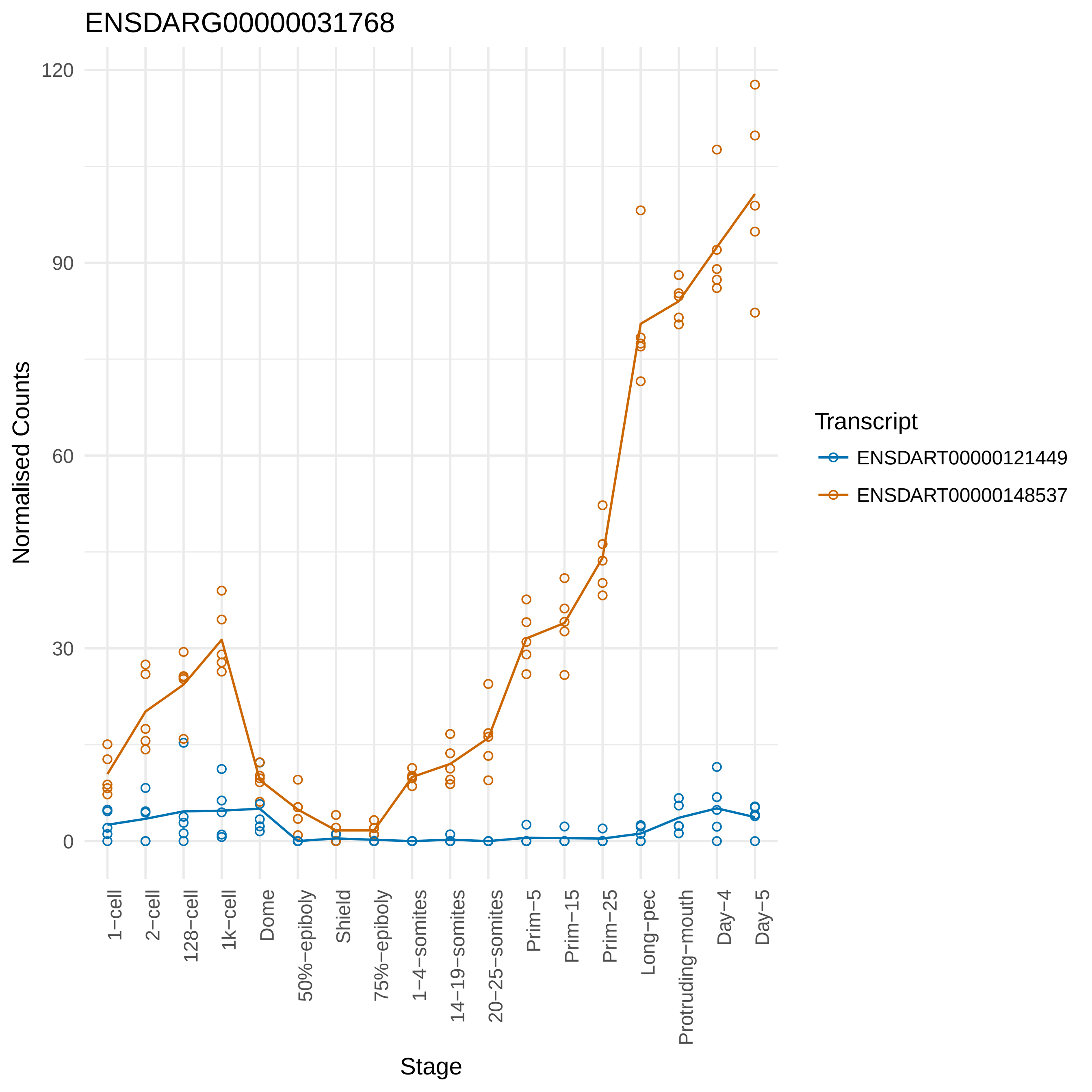

ENSDARG00000031768
Normalised Counts
120
90
60
30
0
1−cell
2−cell
128−cell
1k−cell
Dome
50%−epiboly
Shield
75%−epiboly
1−4−somites
14−19−somites
20−25−somites
Prim−5
Prim−15
Prim−25
Long−pec
Protruding−mouth
Day−4
Day−5
Stage
Transcript
ENSDART00000121449
ENSDART00000148537

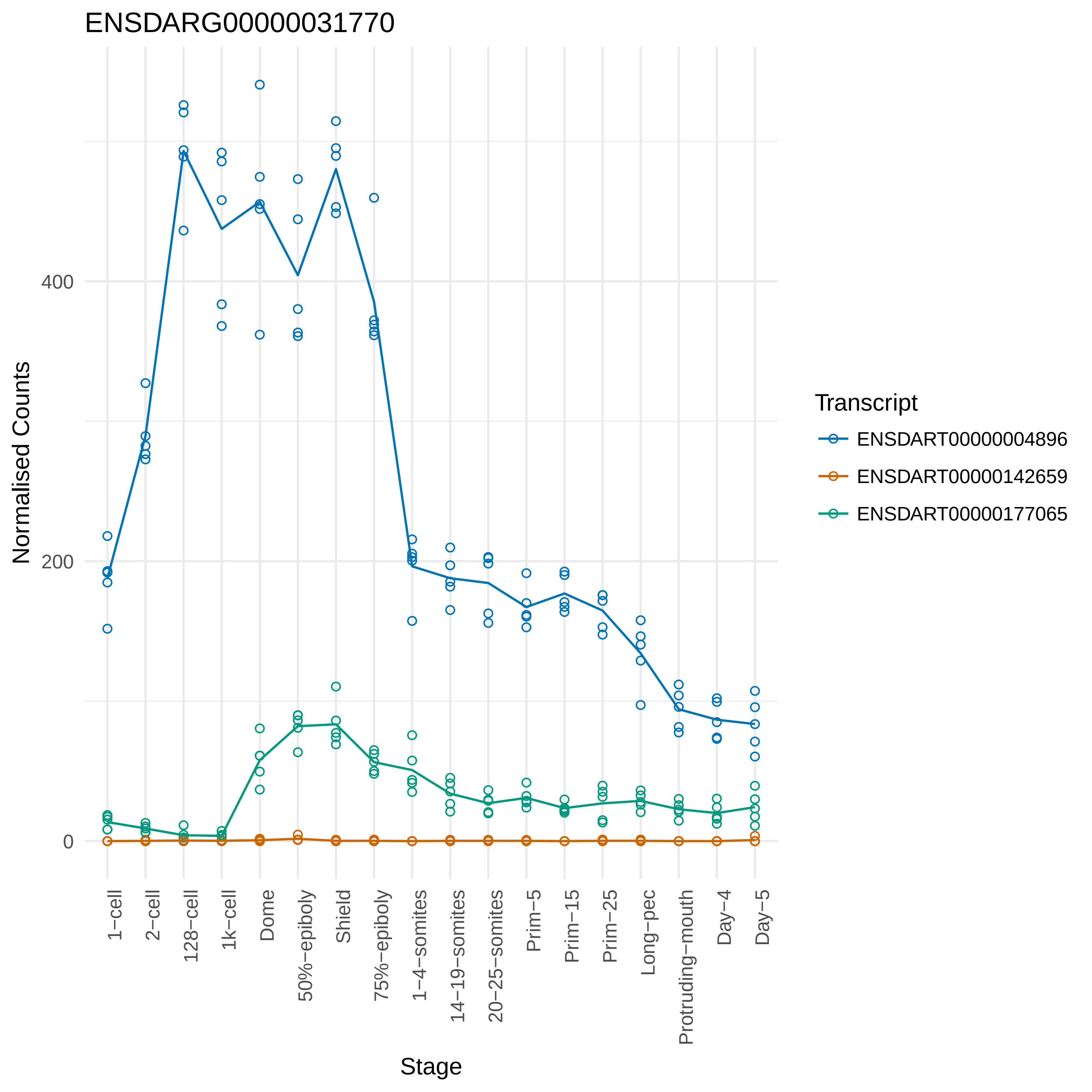

ENSDARG00000031770
Normalised Counts
400
200
0
1−cell
2−cell
128−cell
1k−cell
Dome
50%−epiboly
Shield
75%−epiboly
1−4−somites
14−19−somites
20−25−somites
Prim−5
Prim−15
Prim−25
Long−pec
Protruding−mouth
Day−4
Day−5
Stage
Transcript
ENSDART00000004896
ENSDART00000142659
ENSDART00000177065

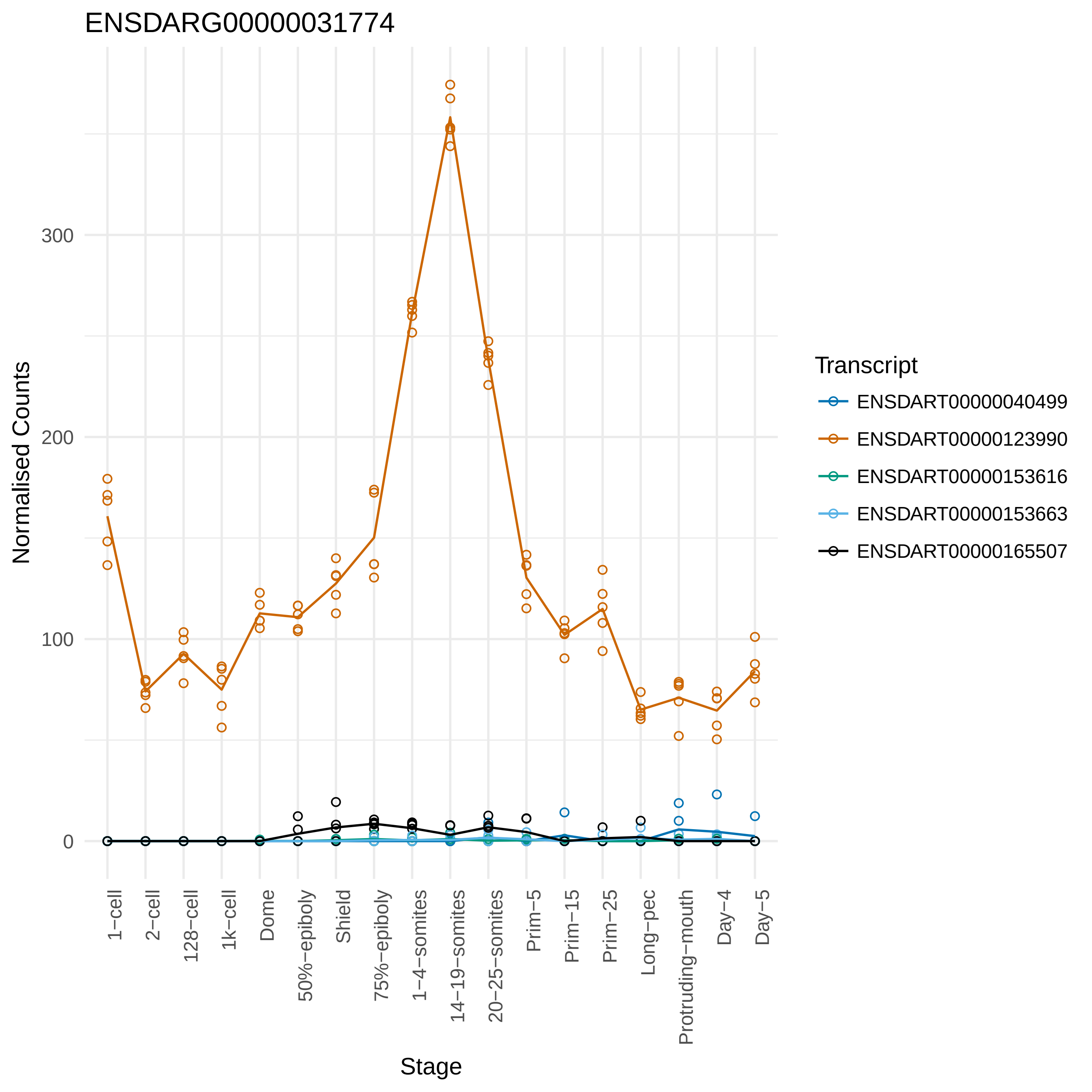

ENSDARG00000031774
Normalised Counts
300
200
100
0
1−cell
2−cell
128−cell
1k−cell
Dome
50%−epiboly
Shield
75%−epiboly
1−4−somites
14−19−somites
20−25−somites
Prim−5
Prim−15
Prim−25
Long−pec
Protruding−mouth
Day−4
Day−5
Stage
Transcript
ENSDART00000040499
ENSDART00000123990
ENSDART00000153616
ENSDART00000153663
ENSDART00000165507

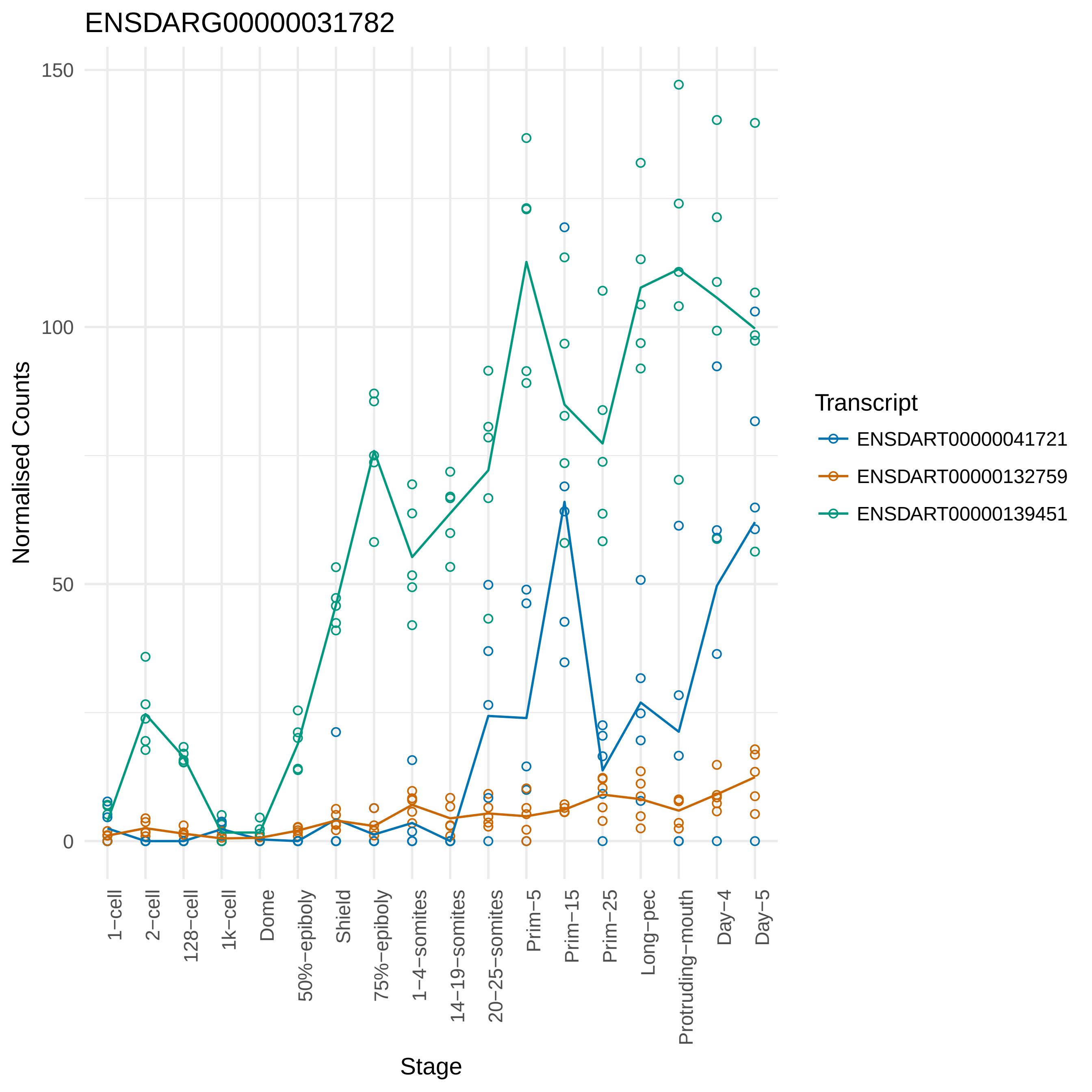

ENSDARG00000031782
Normalised Counts
150
100
50
0
1−cell
2−cell
128−cell
1k−cell
Dome
50%−epiboly
Shield
75%−epiboly
1−4−somites
14−19−somites
20−25−somites
Prim−5
Prim−15
Prim−25
Long−pec
Protruding−mouth
Day−4
Day−5
Stage
Transcript
ENSDART00000041721
ENSDART00000132759
ENSDART00000139451

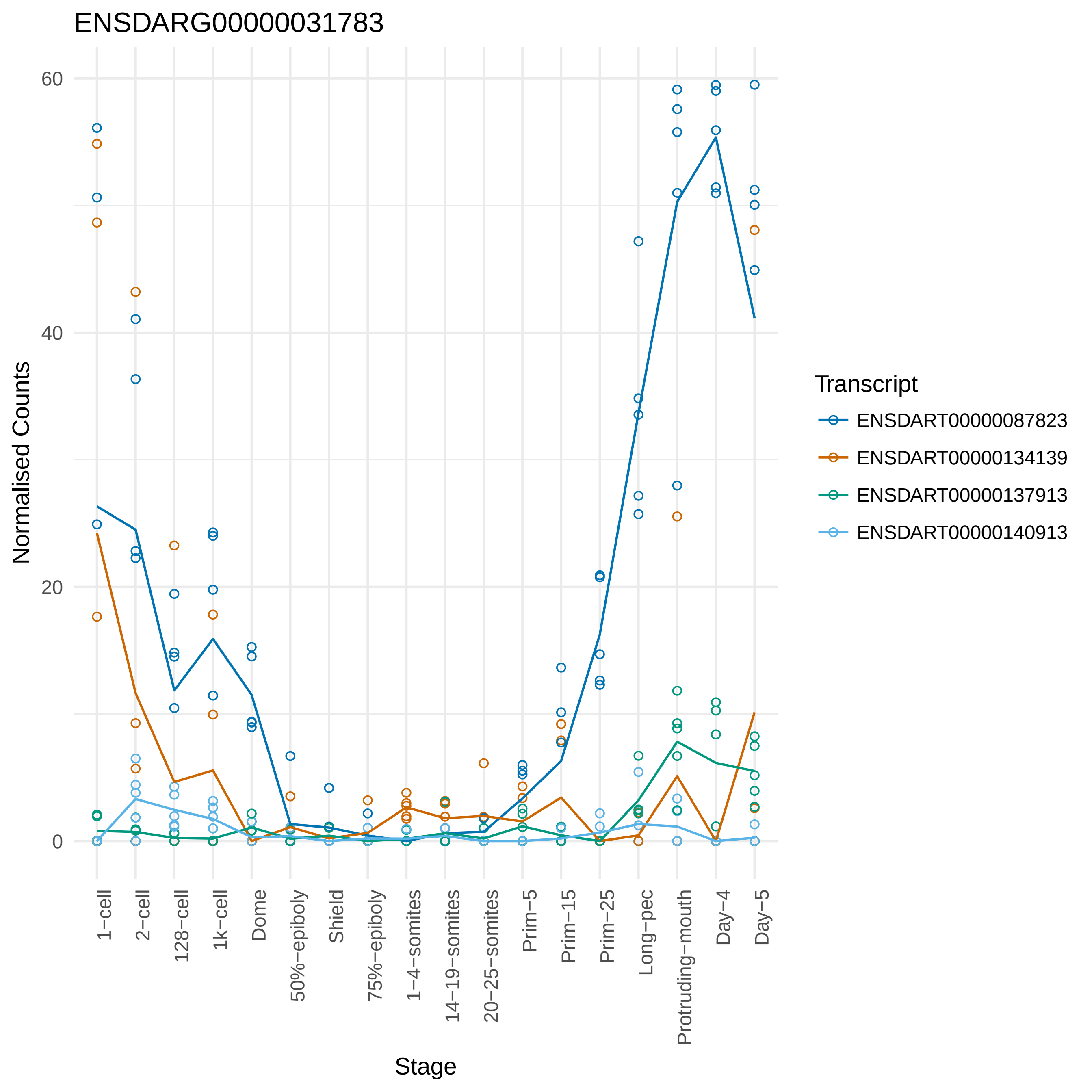
ENSDARG00000031783
Normalised Counts
0
20
40
60
Stage
1−cell
2−cell
128−cell
1k−cell
Dome
50%−epiboly
Shield
75%−epiboly
1−4−somites
14−19−somites
20−25−somites
Prim−5
Prim−15
Prim−25
Long−pec
Protruding−mouth
Day−4
Day−5
Transcript
ENSDART00000087823
ENSDART00000134139
ENSDART00000137913
ENSDART00000140913

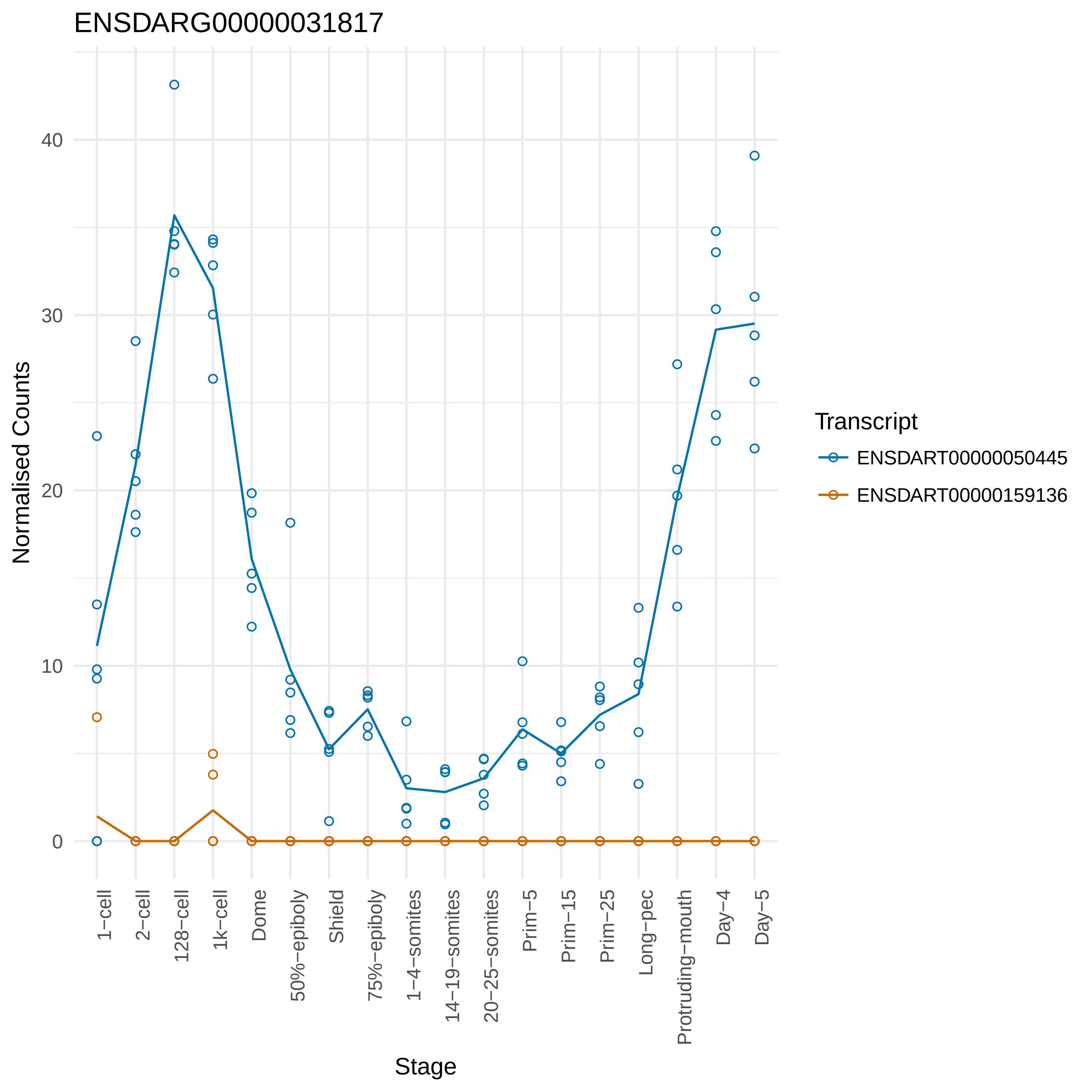
ENSDARG00000031817
Normalised Counts
0
10
20
30
40
Stage
1−cell
2−cell
128−cell
1k−cell
Dome
50%−epiboly
Shield
75%−epiboly
1−4−somites
14−19−somites
20−25−somites
Prim−5
Prim−15
Prim−25
Long−pec
Protruding−mouth
Day−4
Day−5
Transcript
ENSDART00000050445
ENSDART00000159136

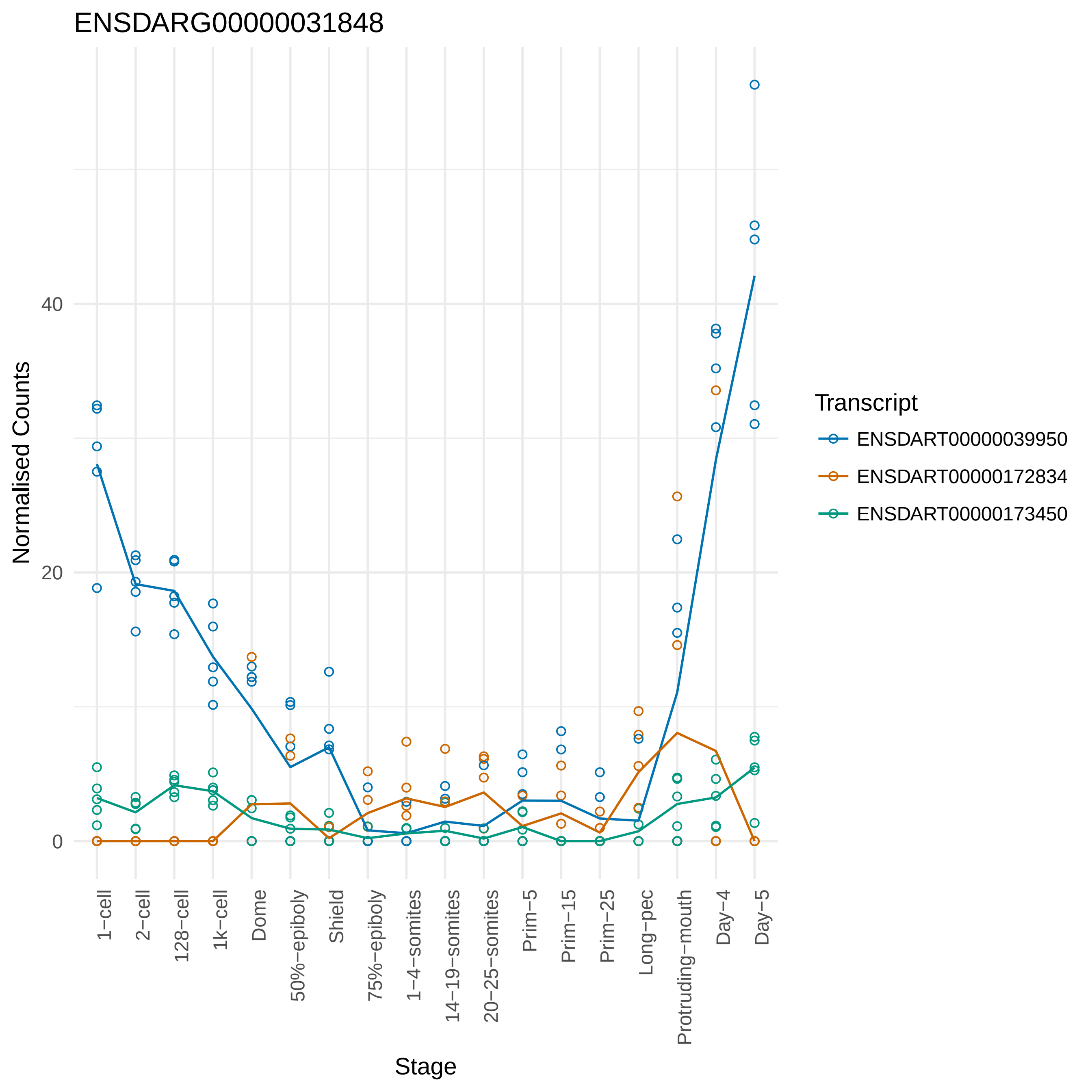

ENSDARG00000031848
Normalised Counts
0
20
40
Stage
1−cell
2−cell
128−cell
1k−cell
Dome
50%−epiboly
Shield
75%−epiboly
1−4−somites
14−19−somites
20−25−somites
Prim−5
Prim−15
Prim−25
Long−pec
Protruding−mouth
Day−4
Day−5
Transcript
ENSDART00000039950
ENSDART00000172834
ENSDART00000173450

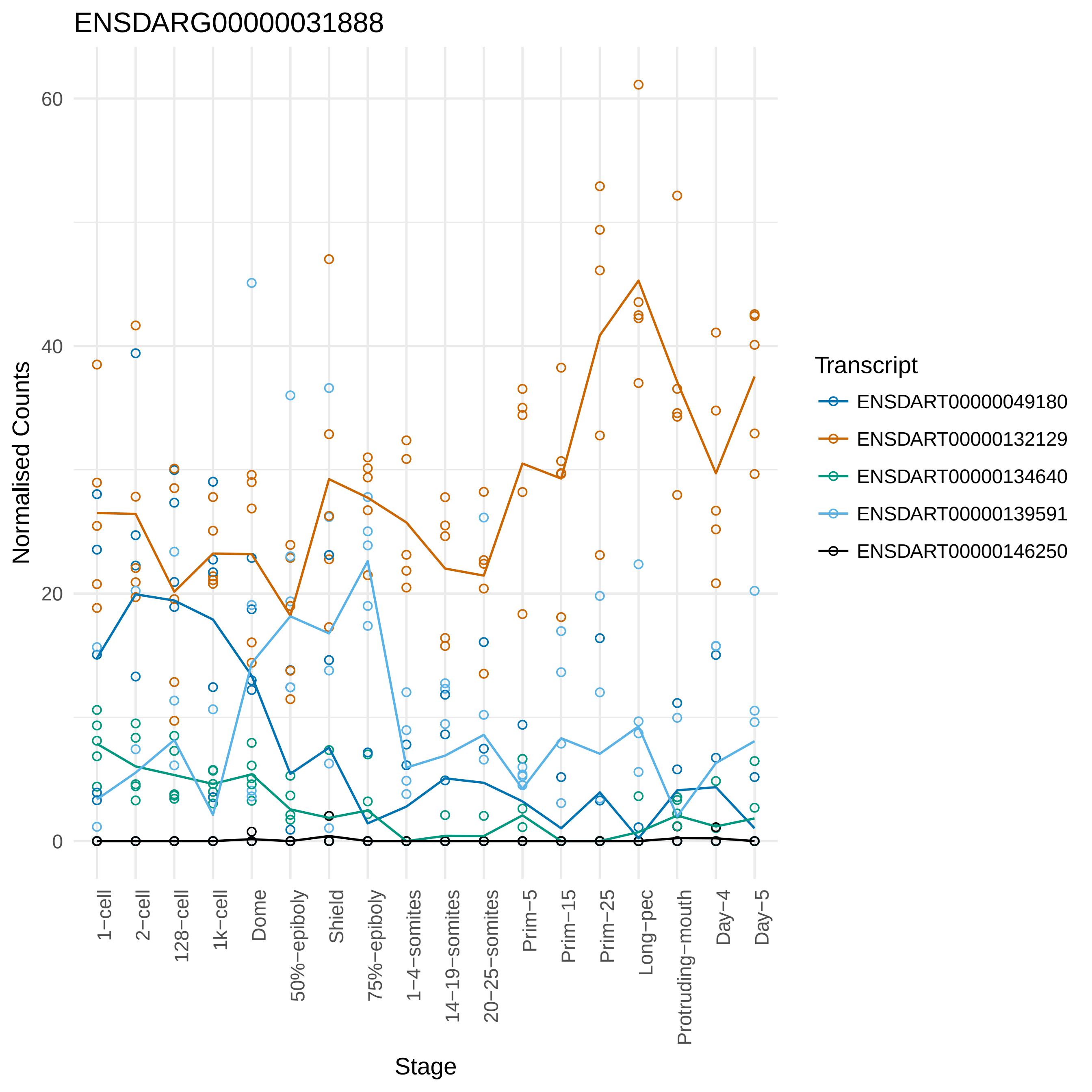

ENSDARG00000031888
Normalised Counts
0
20
40
60
Stage
1−cell
2−cell
128−cell
1k−cell
Dome
50%−epiboly
Shield
75%−epiboly
1−4−somites
14−19−somites
20−25−somites
Prim−5
Prim−15
Prim−25
Long−pec
Protruding−mouth
Day−4
Day−5
Transcript
ENSDART00000049180
ENSDART00000132129
ENSDART00000134640
ENSDART00000139591
ENSDART00000146250

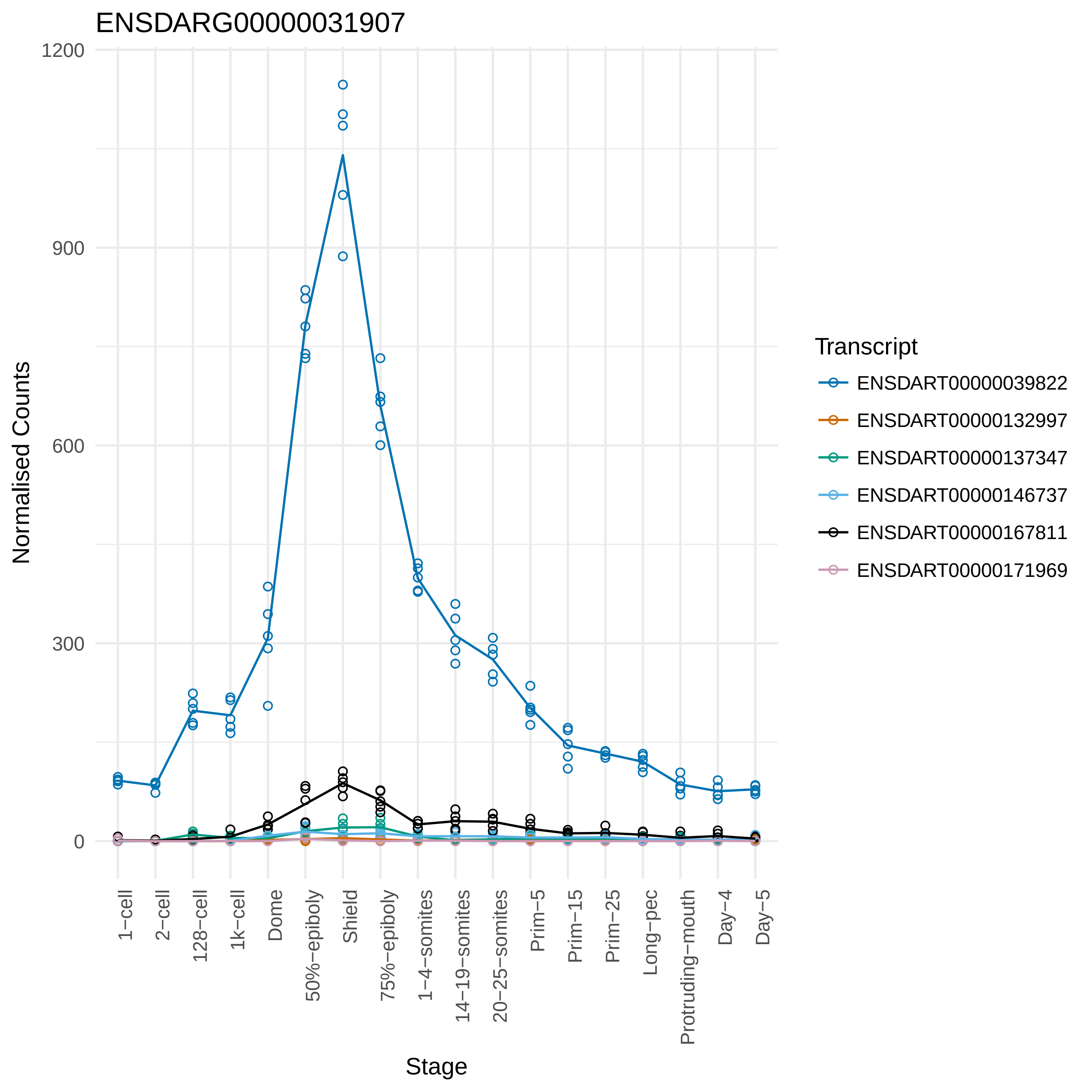
ENSDARG00000031907
Normalised Counts
1200
900
600
300
0
1−cell
2−cell
128−cell
1k−cell
Dome
50%−epiboly
Shield
75%−epiboly
1−4−somites
14−19−somites
20−25−somites
Prim−5
Prim−15
Prim−25
Long−pec
Protruding−mouth
Day−4
Day−5
Stage
Transcript
ENSDART00000039822
ENSDART00000132997
ENSDART00000137347
ENSDART00000146737
ENSDART00000167811
ENSDART00000171969

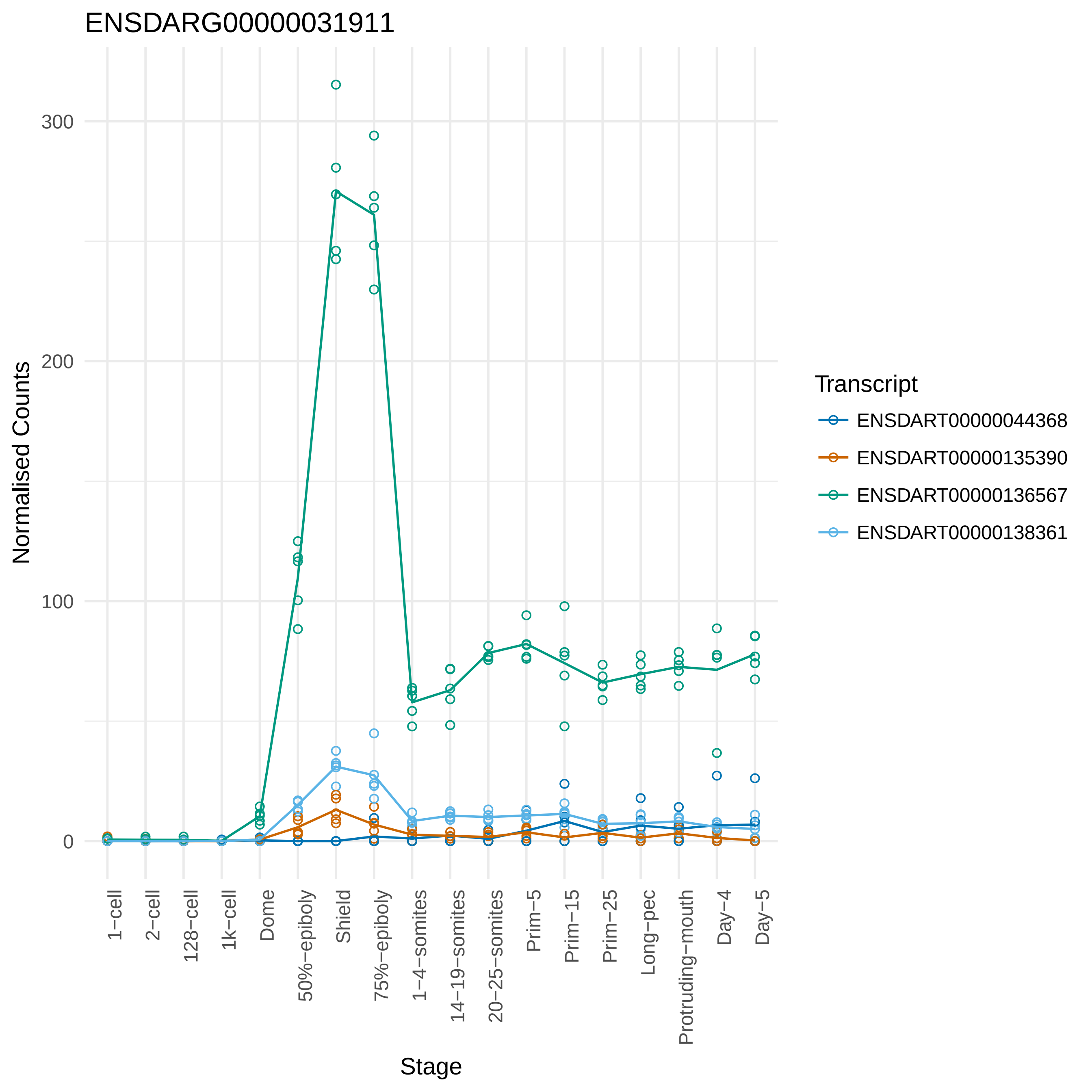
ENSDARG00000031911
Normalised Counts
300
200
100
0
1−cell
2−cell
128−cell
1k−cell
Dome
50%−epiboly
Shield
75%−epiboly
1−4−somites
14−19−somites
20−25−somites
Prim−5
Prim−15
Prim−25
Long−pec
Protruding−mouth
Day−4
Day−5
Stage
Transcript
ENSDART00000044368
ENSDART00000135390
ENSDART00000136567
ENSDART00000138361

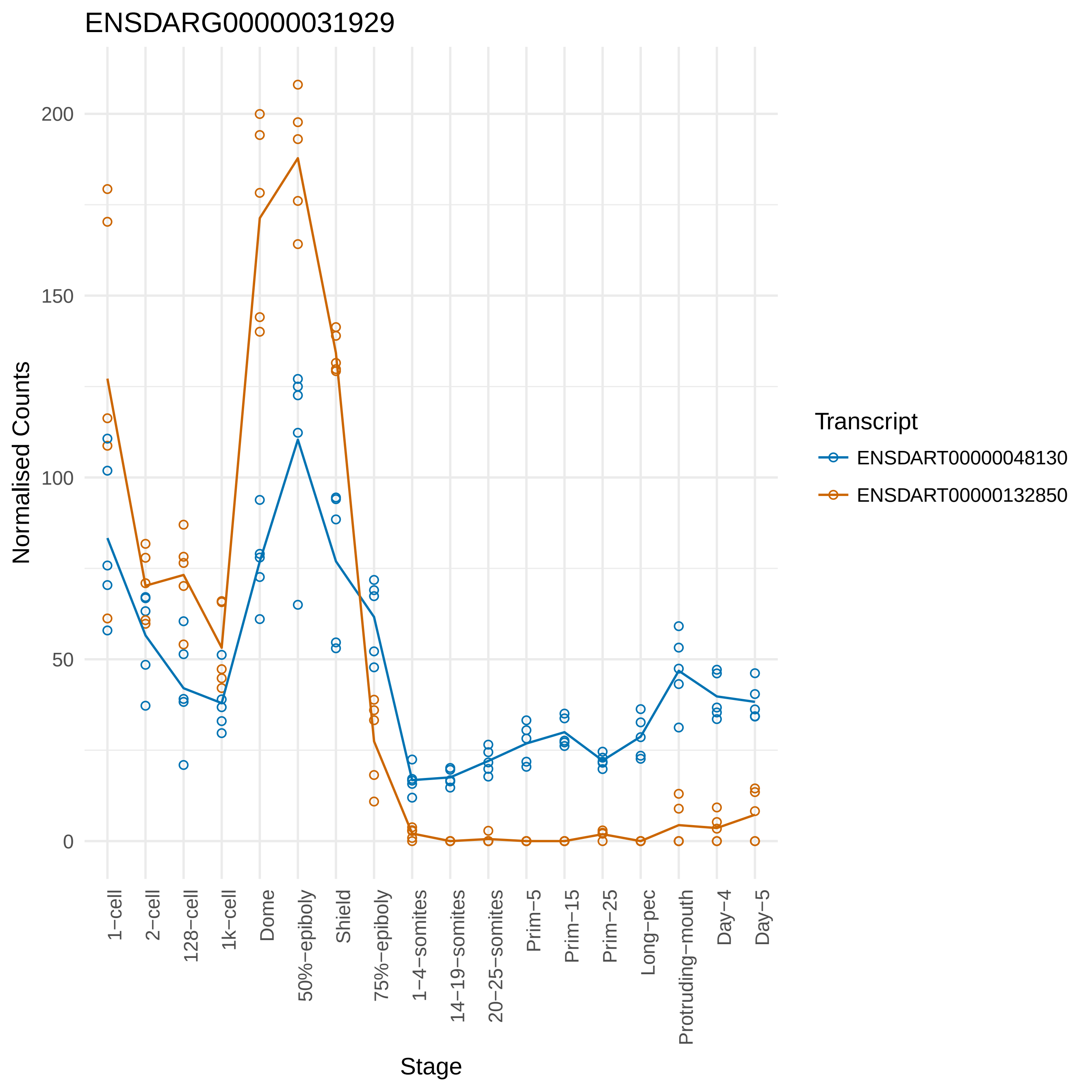

ENSDARG00000031929
Normalised Counts
200
150
100
50
0
1−cell
2−cell
128−cell
1k−cell
Dome
50%−epiboly
Shield
75%−epiboly
1−4−somites
14−19−somites
20−25−somites
Prim−5
Prim−15
Prim−25
Long−pec
Protruding−mouth
Day−4
Day−5
Stage
Transcript
ENSDART00000048130
ENSDART00000132850

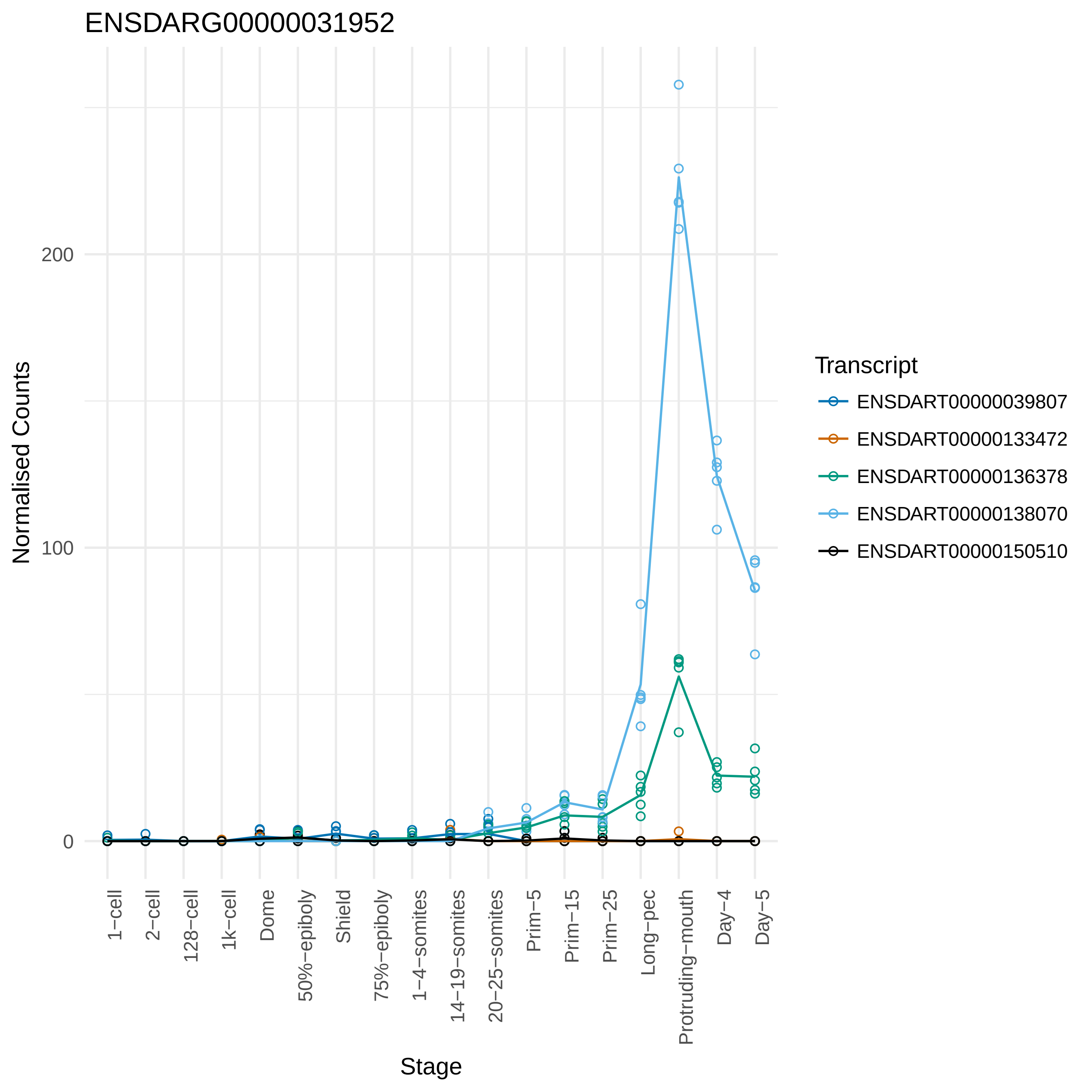

ENSDARG00000031952
Normalised Counts
200
100
0
1−cell
2−cell
128−cell
1k−cell
Dome
50%−epiboly
Shield
75%−epiboly
1−4−somites
14−19−somites
20−25−somites
Prim−5
Prim−15
Prim−25
Long−pec
Protruding−mouth
Day−4
Day−5
Stage
Transcript
ENSDART00000039807
ENSDART00000133472
ENSDART00000136378
ENSDART00000138070
ENSDART00000150510

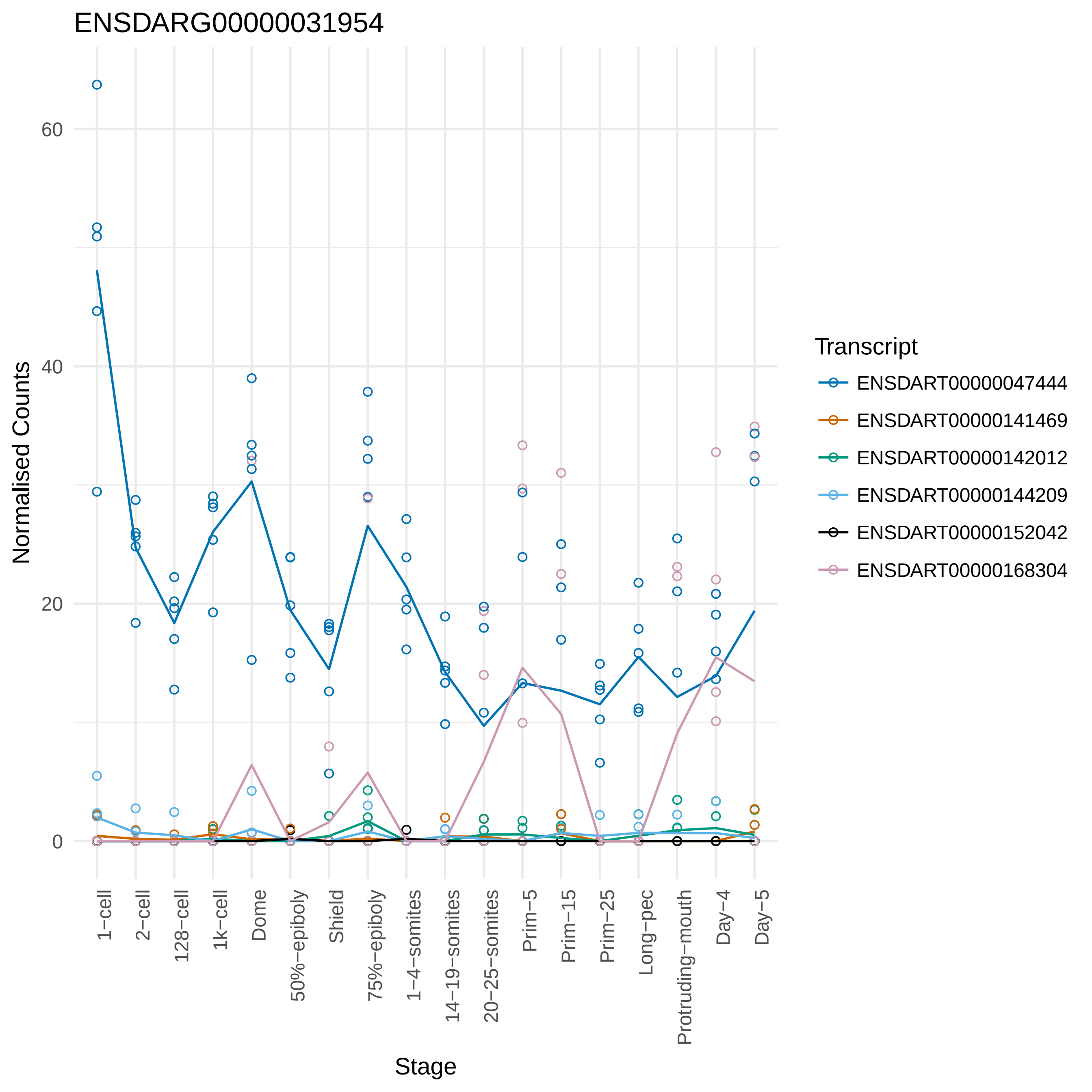

ENSDARG00000031954
Normalised Counts
60
40
20
0
1−cell
2−cell
128−cell
1k−cell
Dome
50%−epiboly
Shield
75%−epiboly
1−4−somites
14−19−somites
20−25−somites
Prim−5
Prim−15
Prim−25
Long−pec
Protruding−mouth
Day−4
Day−5
Stage
Transcript
ENSDART00000047444
ENSDART00000141469
ENSDART00000142012
ENSDART00000144209
ENSDART00000152042
ENSDART00000168304

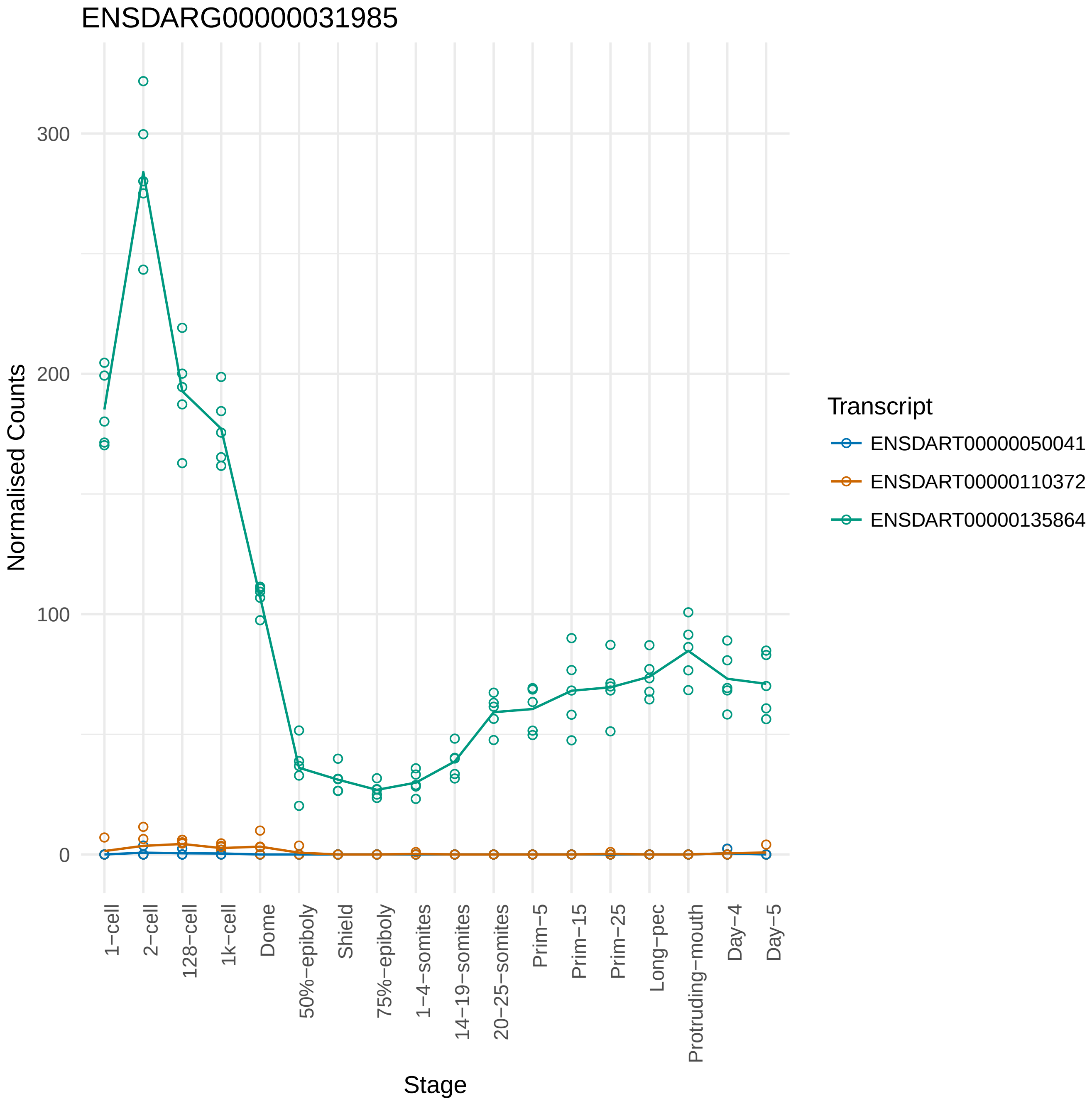

ENSDARG00000031985
Normalised Counts
300
200
100
0
1−cell
2−cell
128−cell
1k−cell
Dome
50%−epiboly
Shield
75%−epiboly
1−4−somites
14−19−somites
20−25−somites
Prim−5
Prim−15
Prim−25
Long−pec
Protruding−mouth
Day−4
Day−5
Stage
Transcript
ENSDART00000050041
ENSDART00000110372
ENSDART00000135864

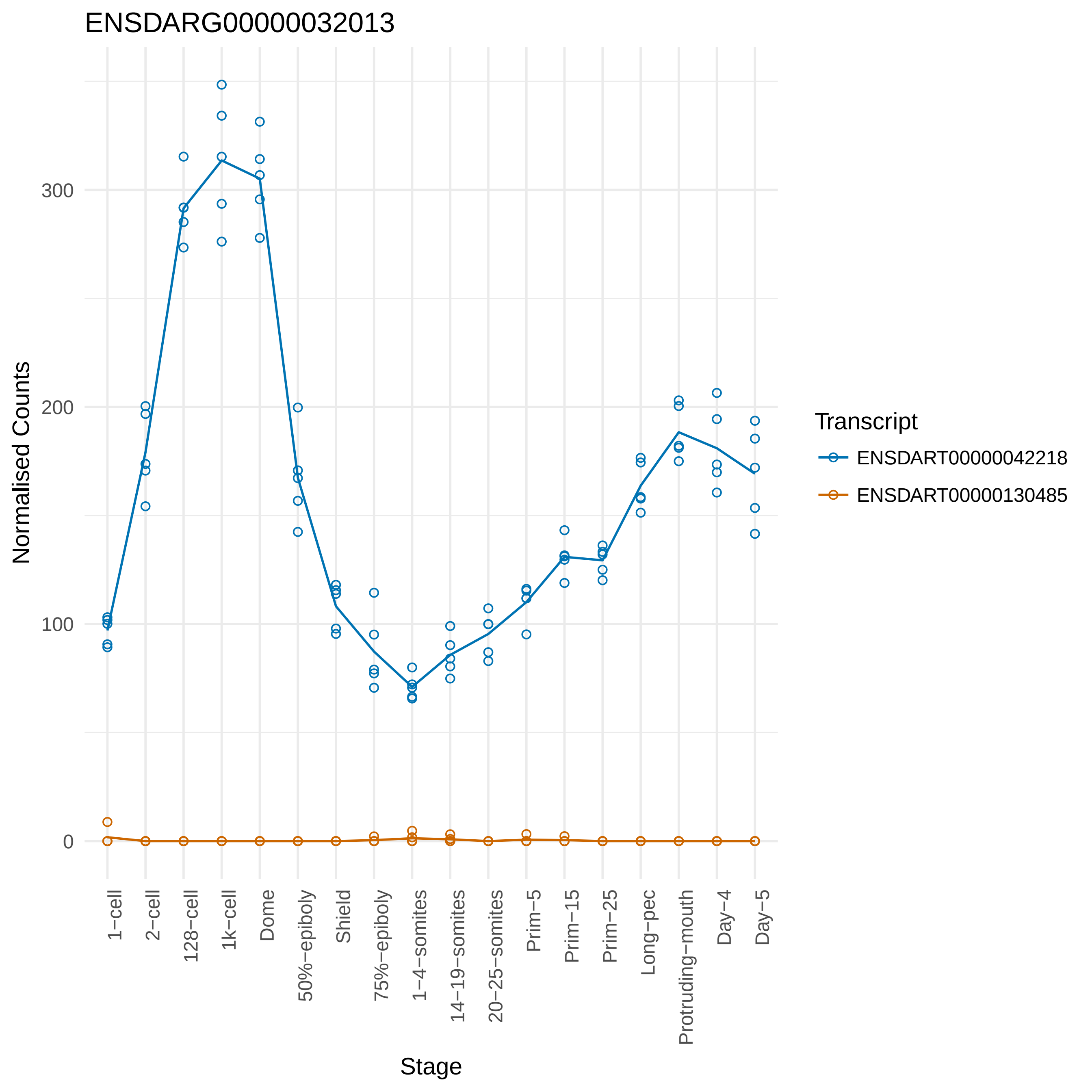
ENSDARG00000032013
Normalised Counts
300
200
100
0
Stage
1−cell
2−cell
128−cell
1k−cell
Dome
50%−epiboly
Shield
75%−epiboly
1−4−somites
14−19−somites
20−25−somites
Prim−5
Prim−15
Prim−25
Long−pec
Protruding−mouth
Day−4
Day−5
Transcript
ENSDART00000042218
ENSDART00000130485

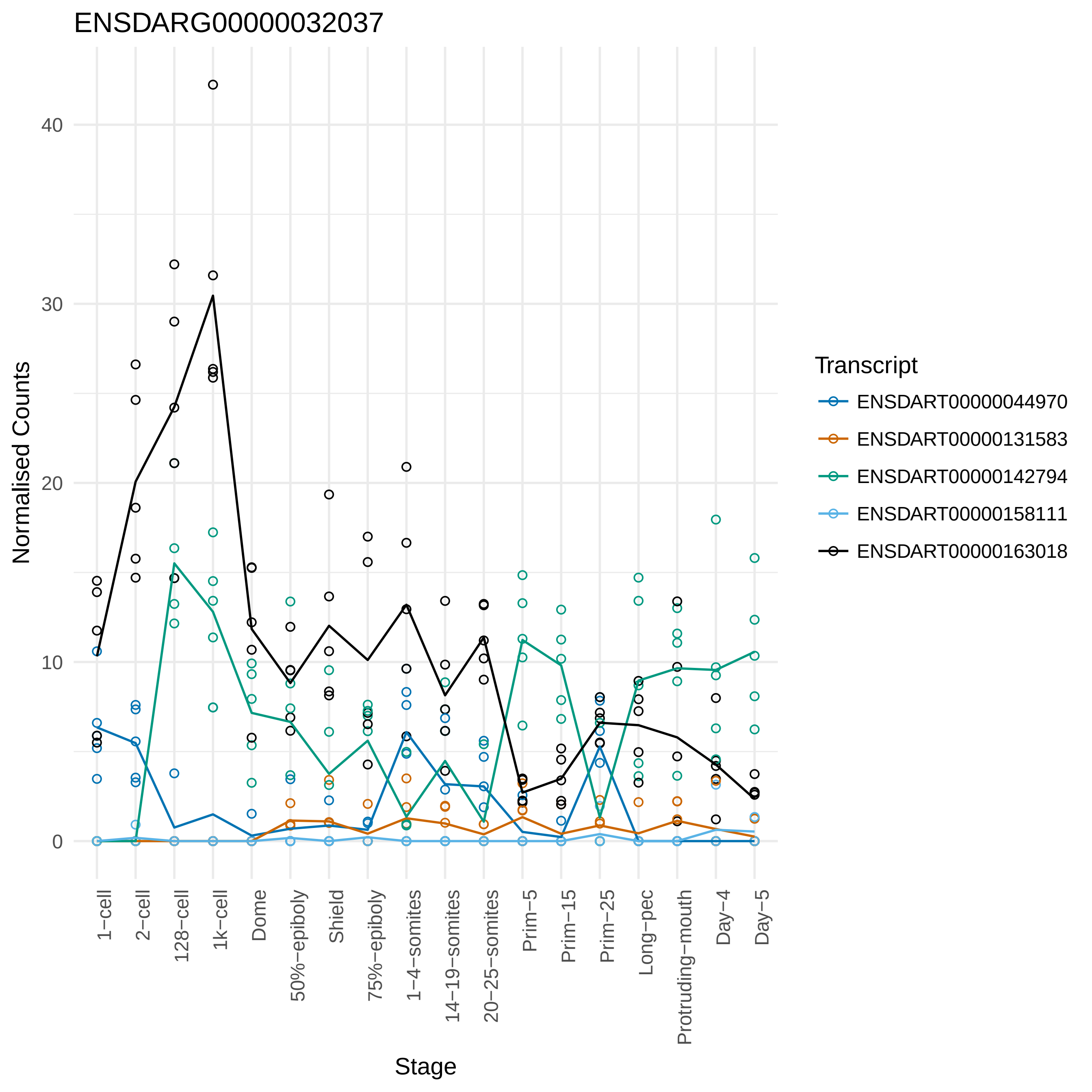
ENSDARG00000032037
Normalised Counts
0
10
20
30
40
Stage
1−cell
2−cell
128−cell
1k−cell
Dome
50%−epiboly
Shield
75%−epiboly
1−4−somites
14−19−somites
20−25−somites
Prim−5
Prim−15
Prim−25
Long−pec
Protruding−mouth
Day−4
Day−5
Transcript
ENSDART00000044970
ENSDART00000131583
ENSDART00000142794
ENSDART00000158111
ENSDART00000163018

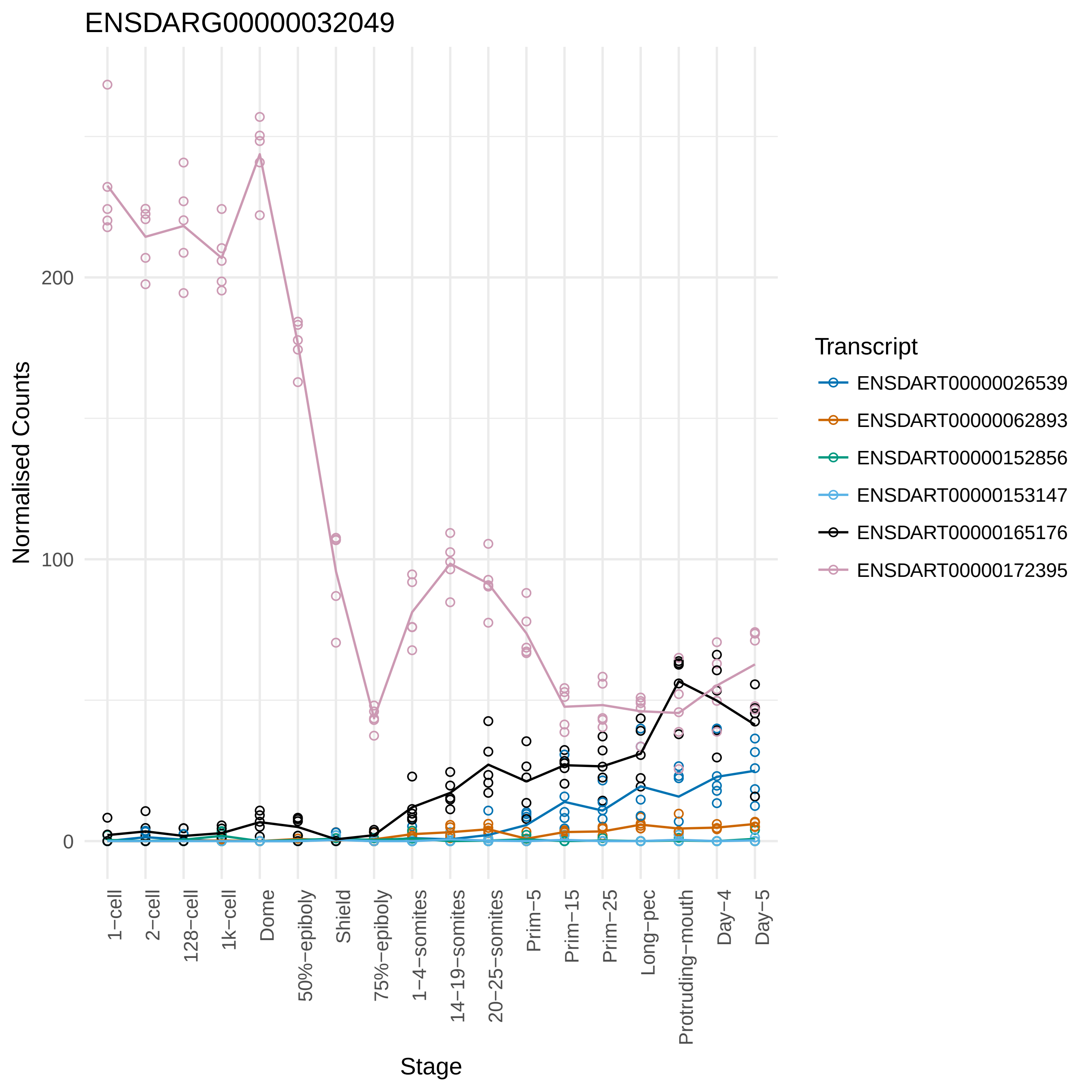
ENSDARG00000032049
Normalised Counts
200
100
0
1−cell
2−cell
128−cell
1k−cell
Dome
50%−epiboly
Shield
75%−epiboly
1−4−somites
14−19−somites
20−25−somites
Prim−5
Prim−15
Prim−25
Long−pec
Protruding−mouth
Day−4
Day−5
Stage
Transcript
ENSDART00000026539
ENSDART00000062893
ENSDART00000152856
ENSDART00000153147
ENSDART00000165176
ENSDART00000172395

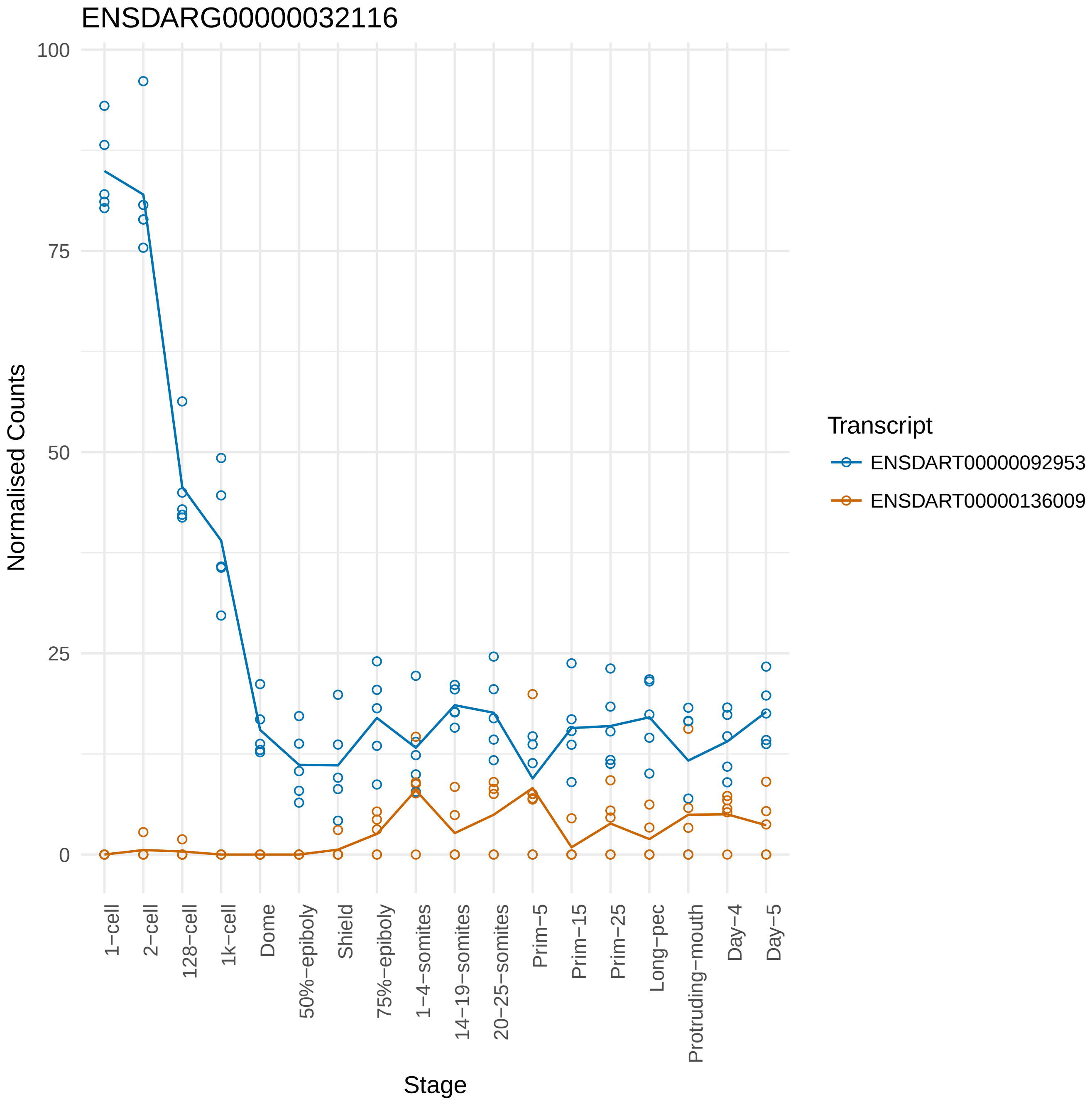

ENSDARG00000032116
Normalised Counts
100
75
50
25
0
1−cell
2−cell
128−cell
1k−cell
Dome
50%−epiboly
Shield
75%−epiboly
1−4−somites
14−19−somites
20−25−somites
Prim−5
Prim−15
Prim−25
Long−pec
Protruding−mouth
Day−4
Day−5
Stage
Transcript
ENSDART00000092953
ENSDART00000136009

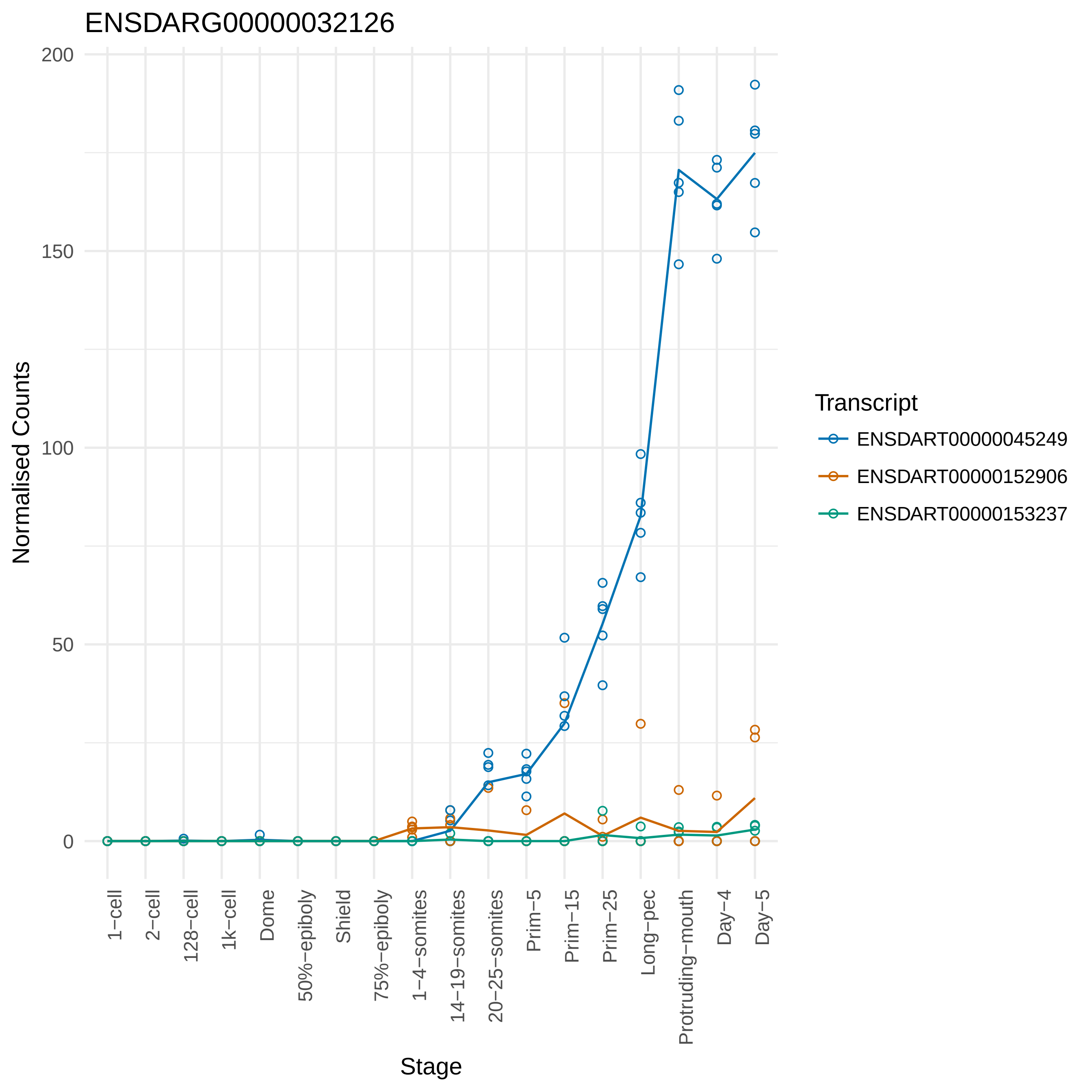
ENSDARG00000032126
Normalised Counts
200
150
100
50
0
1−cell
2−cell
128−cell
1k−cell
Dome
50%−epiboly
Shield
75%−epiboly
1−4−somites
14−19−somites
20−25−somites
Prim−5
Prim−15
Prim−25
Long−pec
Protruding−mouth
Day−4
Day−5
Stage
Transcript
ENSDART00000045249
ENSDART00000152906
ENSDART00000153237

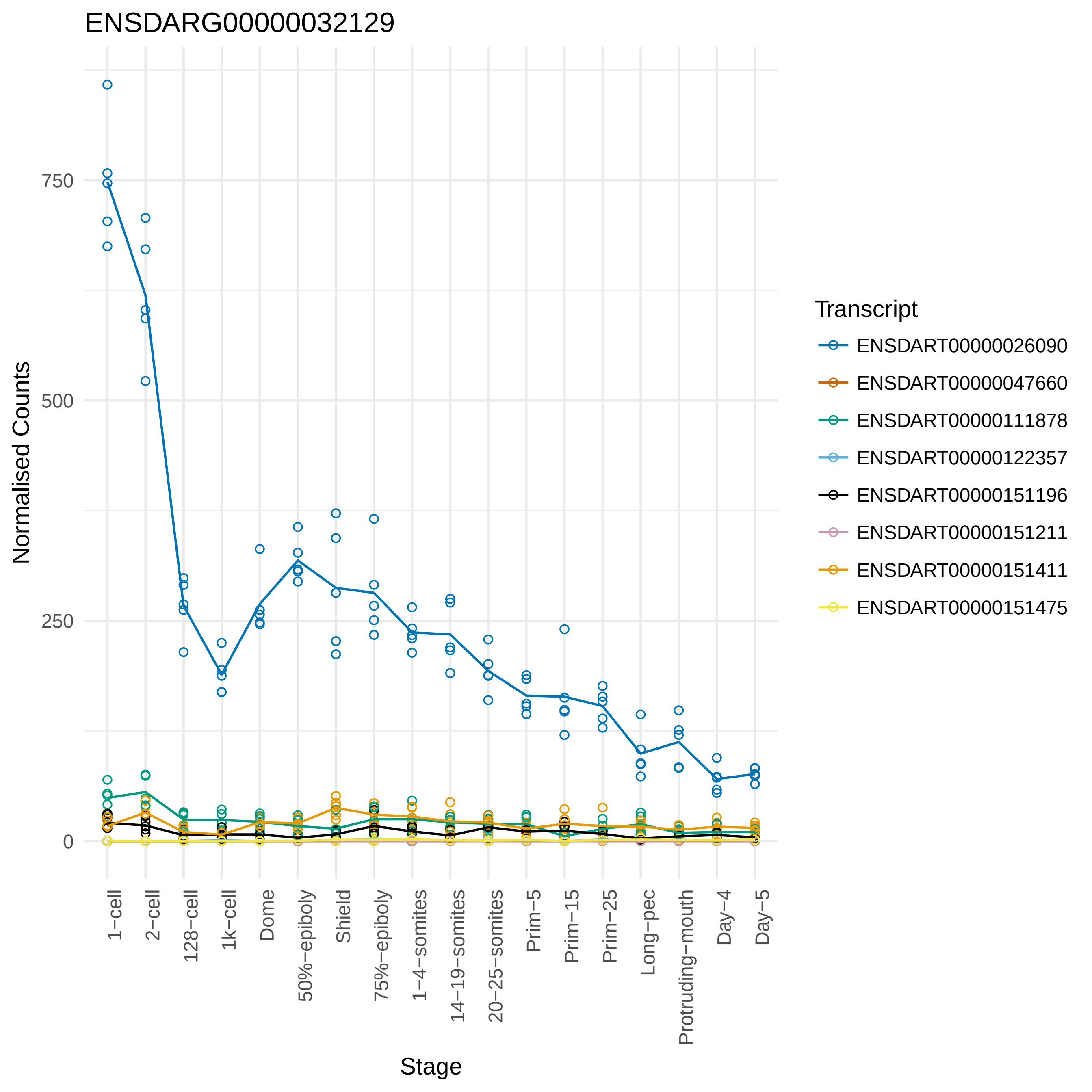
ENSDARG00000032129
Normalised Counts
0
250
500
750
Stage
1−cell
2−cell
128−cell
1k−cell
Dome
50%−epiboly
Shield
75%−epiboly
1−4−somites
14−19−somites
20−25−somites
Prim−5
Prim−15
Prim−25
Long−pec
Protruding−mouth
Day−4
Day−5
Transcript
ENSDART00000026090
ENSDART00000047660
ENSDART00000111878
ENSDART00000122357
ENSDART00000151196
ENSDART00000151211
ENSDART00000151411
ENSDART00000151475

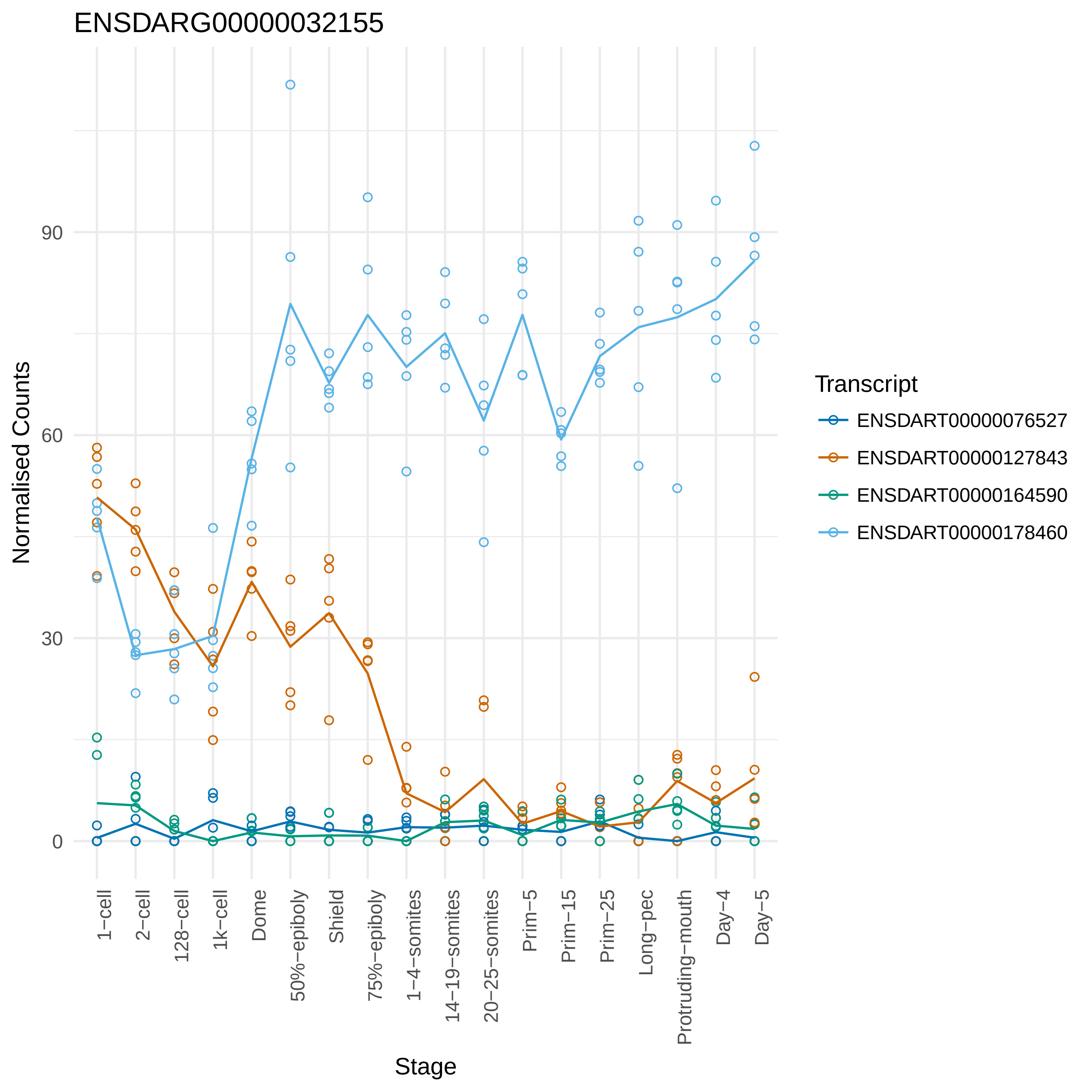
ENSDARG00000032155
Normalised Counts
0
30
60
90
Stage
1−cell
2−cell
128−cell
1k−cell
Dome
50%−epiboly
Shield
75%−epiboly
1−4−somites
14−19−somites
20−25−somites
Prim−5
Prim−15
Prim−25
Long−pec
Protruding−mouth
Day−4
Day−5
Transcript
ENSDART00000076527
ENSDART00000127843
ENSDART00000164590
ENSDART00000178460

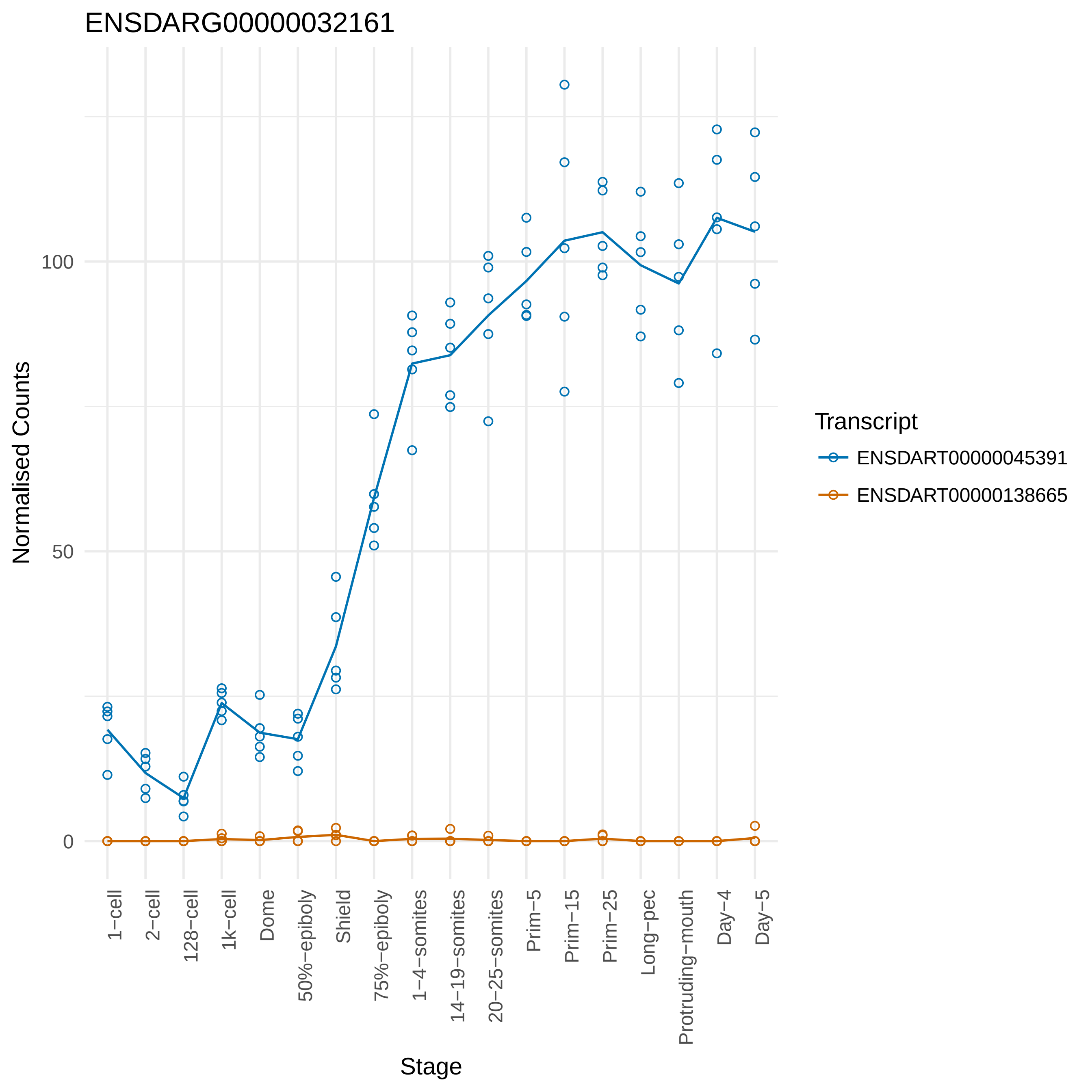
ENSDARG00000032161
Normalised Counts
100
50
0
1−cell
2−cell
128−cell
1k−cell
Dome
50%−epiboly
Shield
75%−epiboly
1−4−somites
14−19−somites
20−25−somites
Prim−5
Prim−15
Prim−25
Long−pec
Protruding−mouth
Day−4
Day−5
Stage
Transcript
ENSDART00000045391
ENSDART00000138665

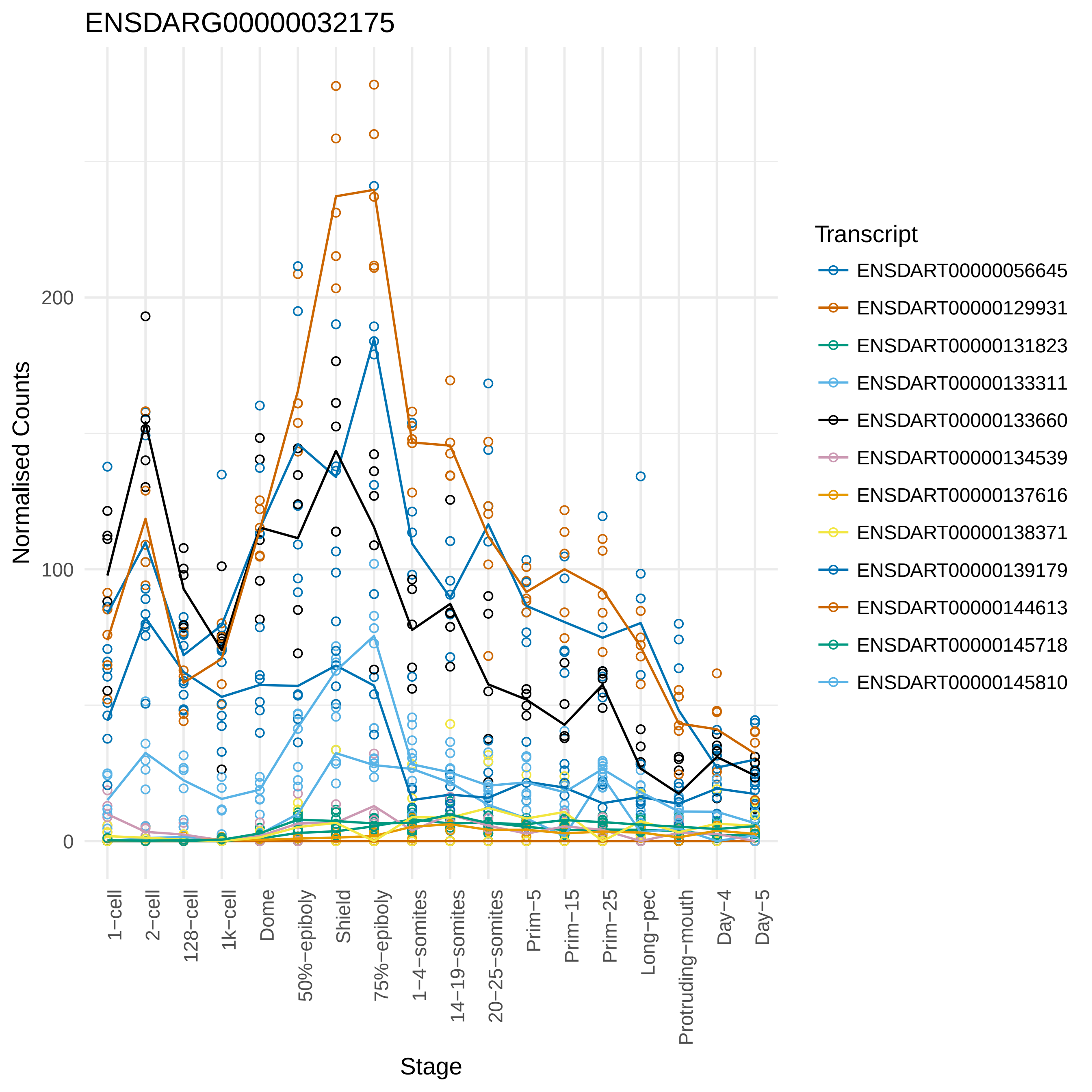
ENSDARG00000032175
Normalised Counts
0
100
200
1−cell
2−cell
128−cell
1k−cell
Dome
50%−epiboly
Shield
75%−epiboly
1−4−somites
14−19−somites
20−25−somites
Prim−5
Prim−15
Prim−25
Long−pec
Protruding−mouth
Day−4
Day−5
Stage
Transcript
ENSDART00000056645
ENSDART00000129931
ENSDART00000131823
ENSDART00000133311
ENSDART00000133660
ENSDART00000134539
ENSDART00000137616
ENSDART00000138371
ENSDART00000139179
ENSDART00000144613
ENSDART00000145718
ENSDART00000145810

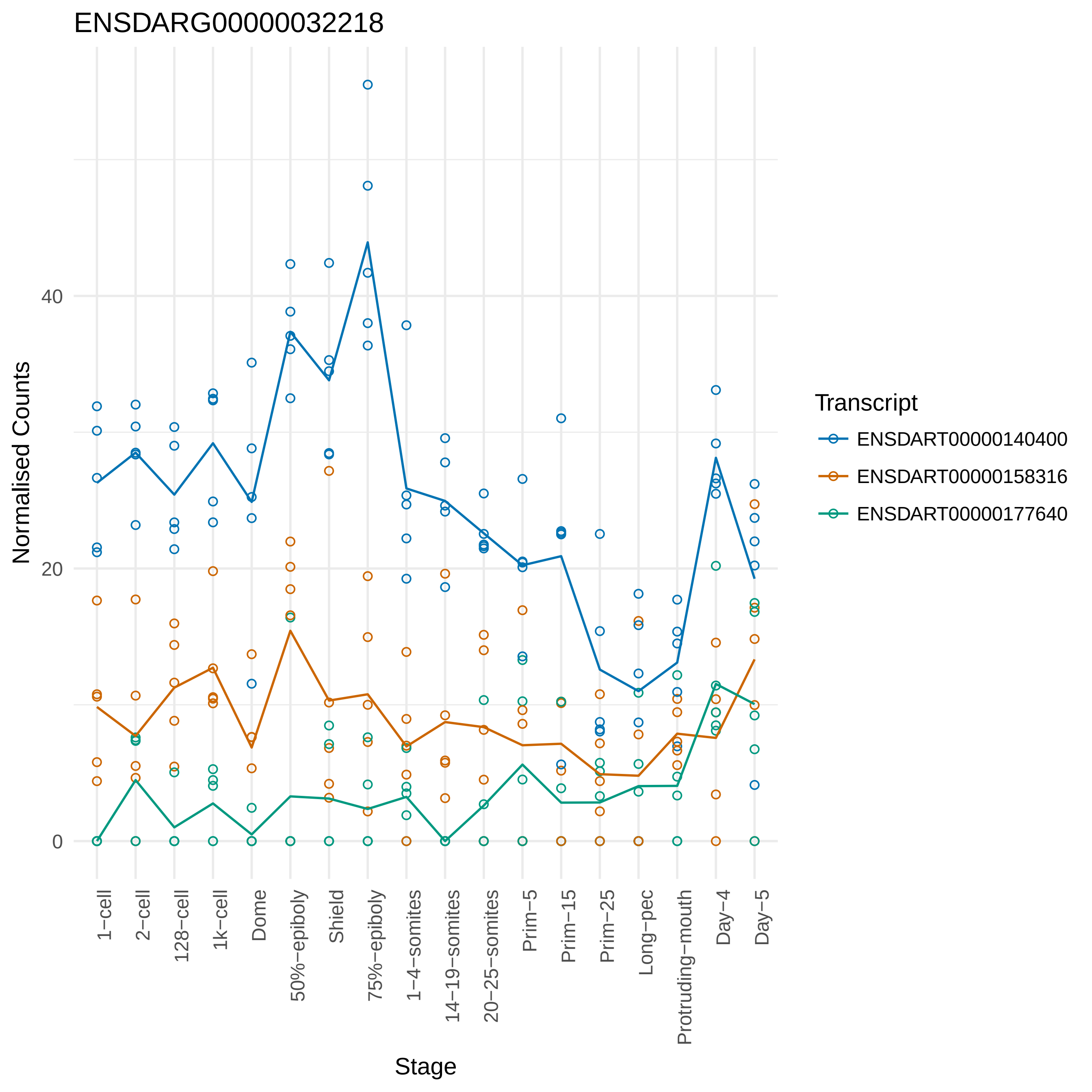

ENSDARG00000032218
Normalised Counts
0
20
40
Stage
1−cell
2−cell
128−cell
1k−cell
Dome
50%−epiboly
Shield
75%−epiboly
1−4−somites
14−19−somites
20−25−somites
Prim−5
Prim−15
Prim−25
Long−pec
Protruding−mouth
Day−4
Day−5
Transcript
ENSDART00000140400
ENSDART00000158316
ENSDART00000177640

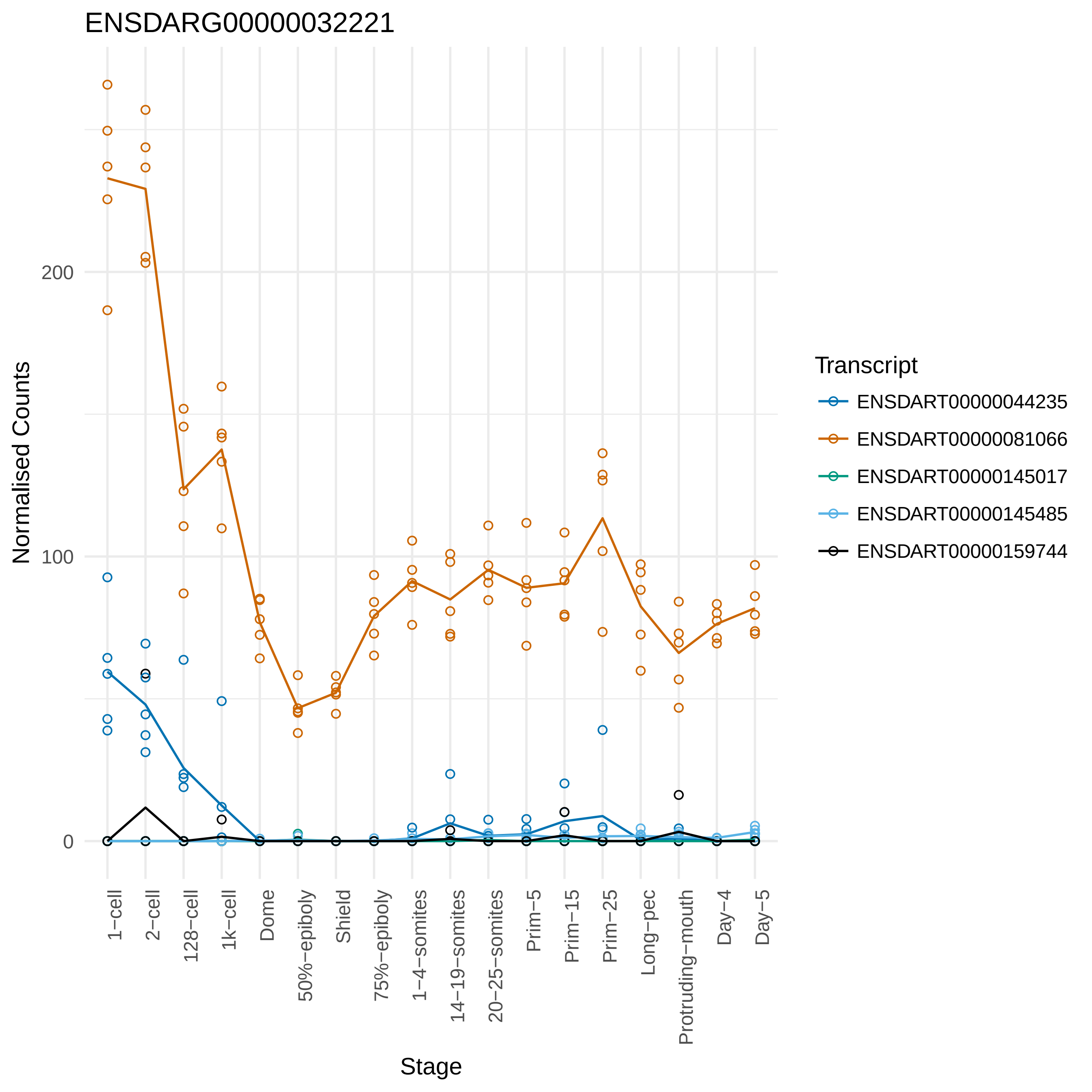

ENSDARG00000032221
Normalised Counts
200
100
0
1−cell
2−cell
128−cell
1k−cell
Dome
50%−epiboly
Shield
75%−epiboly
1−4−somites
14−19−somites
20−25−somites
Prim−5
Prim−15
Prim−25
Long−pec
Protruding−mouth
Day−4
Day−5
Stage
Transcript
ENSDART00000044235
ENSDART00000081066
ENSDART00000145017
ENSDART00000145485
ENSDART00000159744

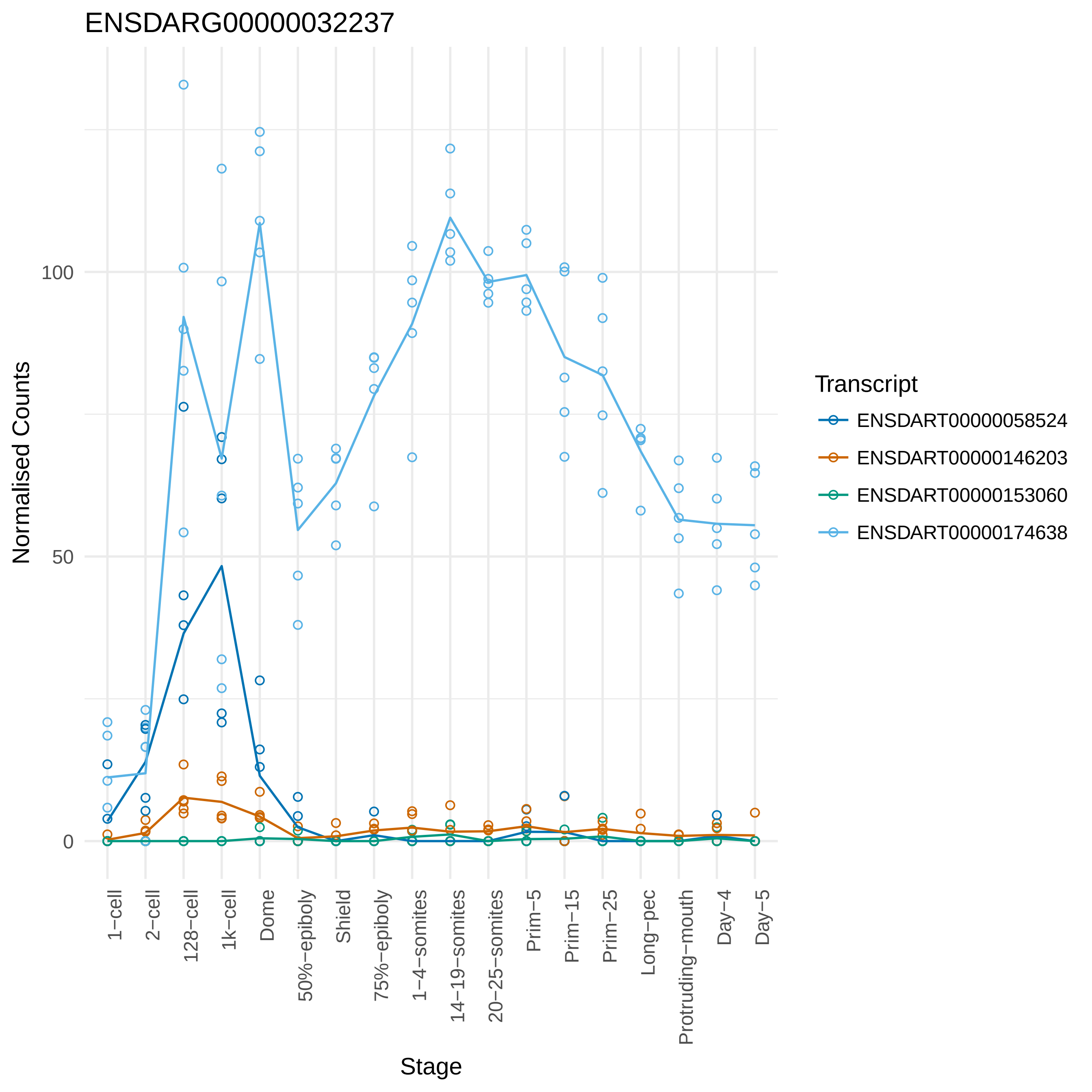

ENSDARG00000032237
Normalised Counts
100
50
0
1−cell
2−cell
128−cell
1k−cell
Dome
50%−epiboly
Shield
75%−epiboly
1−4−somites
14−19−somites
20−25−somites
Prim−5
Prim−15
Prim−25
Long−pec
Protruding−mouth
Day−4
Day−5
Stage
Transcript
ENSDART00000058524
ENSDART00000146203
ENSDART00000153060
ENSDART00000174638

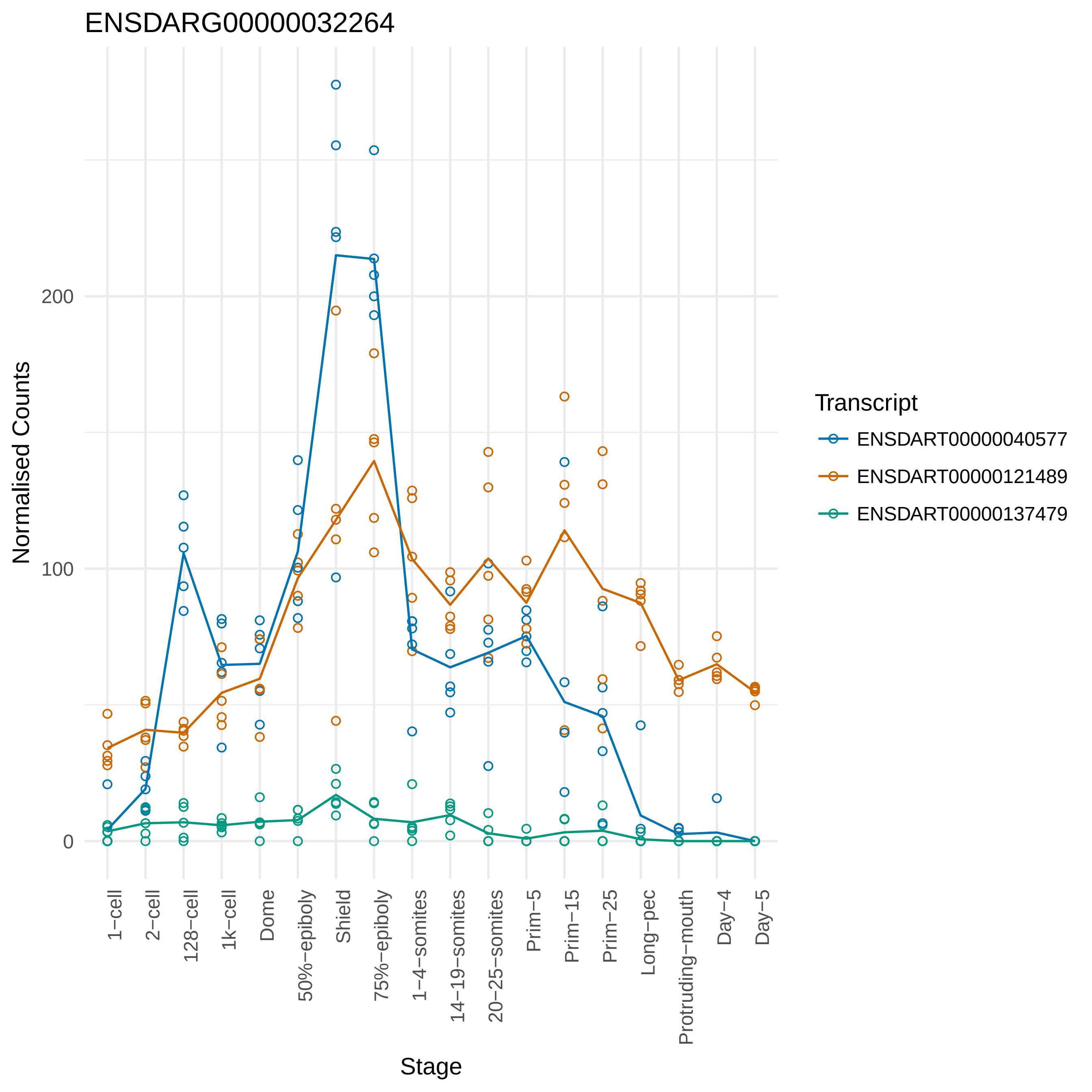

ENSDARG00000032264
Normalised Counts
0
100
200
Stage
1−cell
2−cell
128−cell
1k−cell
Dome
50%−epiboly
Shield
75%−epiboly
1−4−somites
14−19−somites
20−25−somites
Prim−5
Prim−15
Prim−25
Long−pec
Protruding−mouth
Day−4
Day−5
Transcript
ENSDART00000040577
ENSDART00000121489
ENSDART00000137479

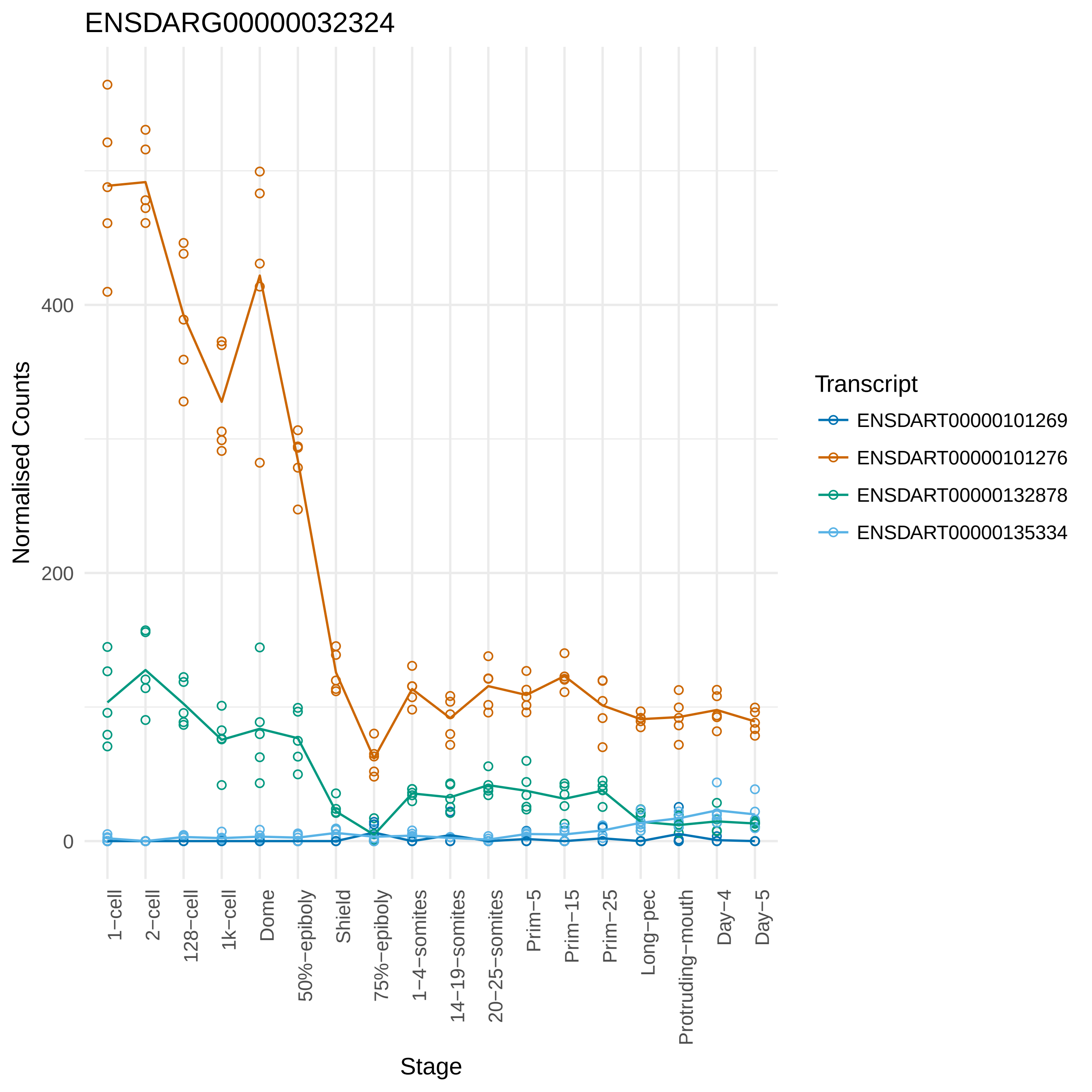

ENSDARG00000032324
Normalised Counts
0
200
400
1−cell
2−cell
128−cell
1k−cell
Dome
50%−epiboly
Shield
75%−epiboly
1−4−somites
14−19−somites
20−25−somites
Prim−5
Prim−15
Prim−25
Long−pec
Protruding−mouth
Day−4
Day−5
Stage
Transcript
ENSDART00000101269
ENSDART00000101276
ENSDART00000132878
ENSDART00000135334

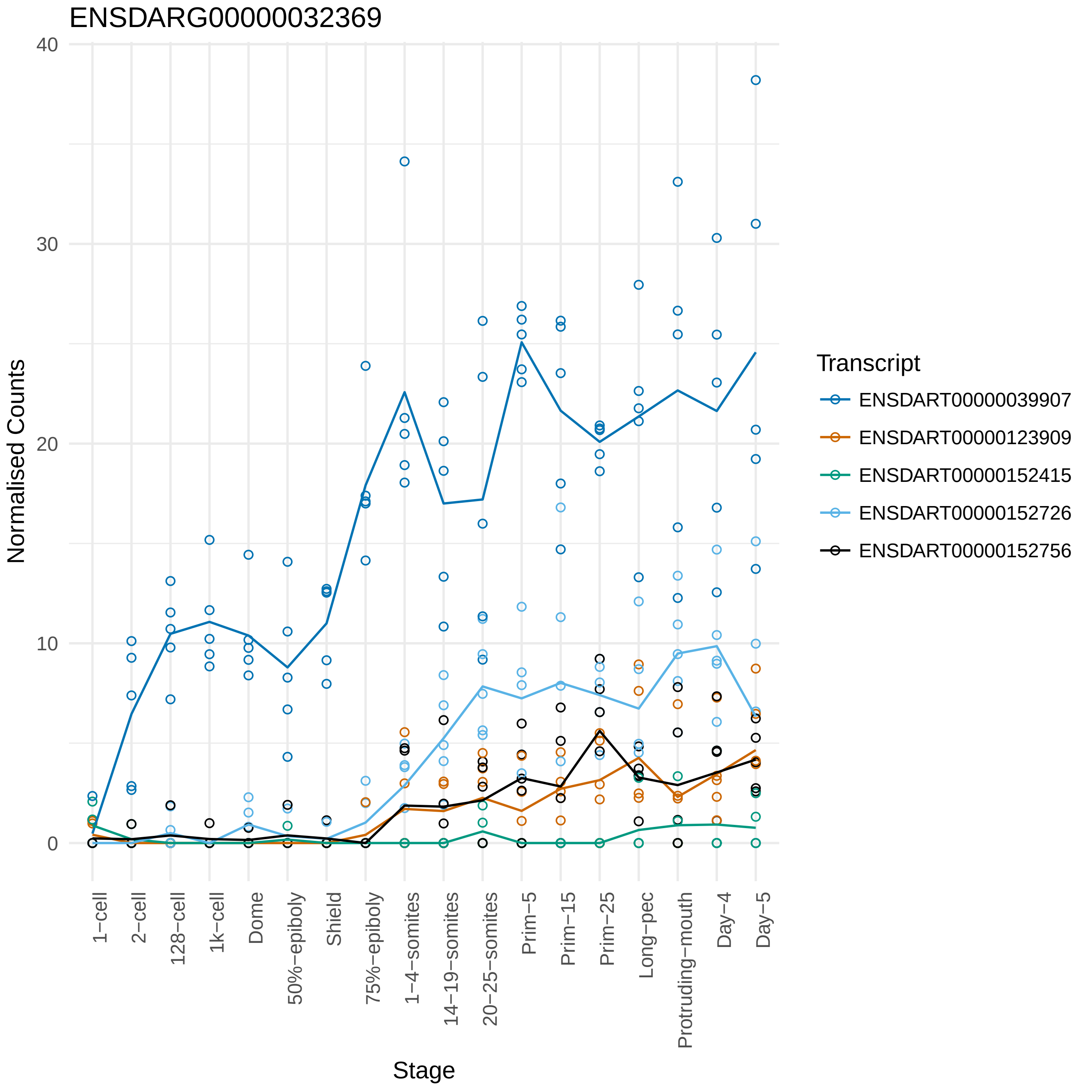

ENSDARG00000032369
Normalised Counts
0
10
20
30
40
1−cell
2−cell
128−cell
1k−cell
Dome
50%−epiboly
Shield
75%−epiboly
1−4−somites
14−19−somites
20−25−somites
Prim−5
Prim−15
Prim−25
Long−pec
Protruding−mouth
Day−4
Day−5
Stage
Transcript
ENSDART00000039907
ENSDART00000123909
ENSDART00000152415
ENSDART00000152726
ENSDART00000152756

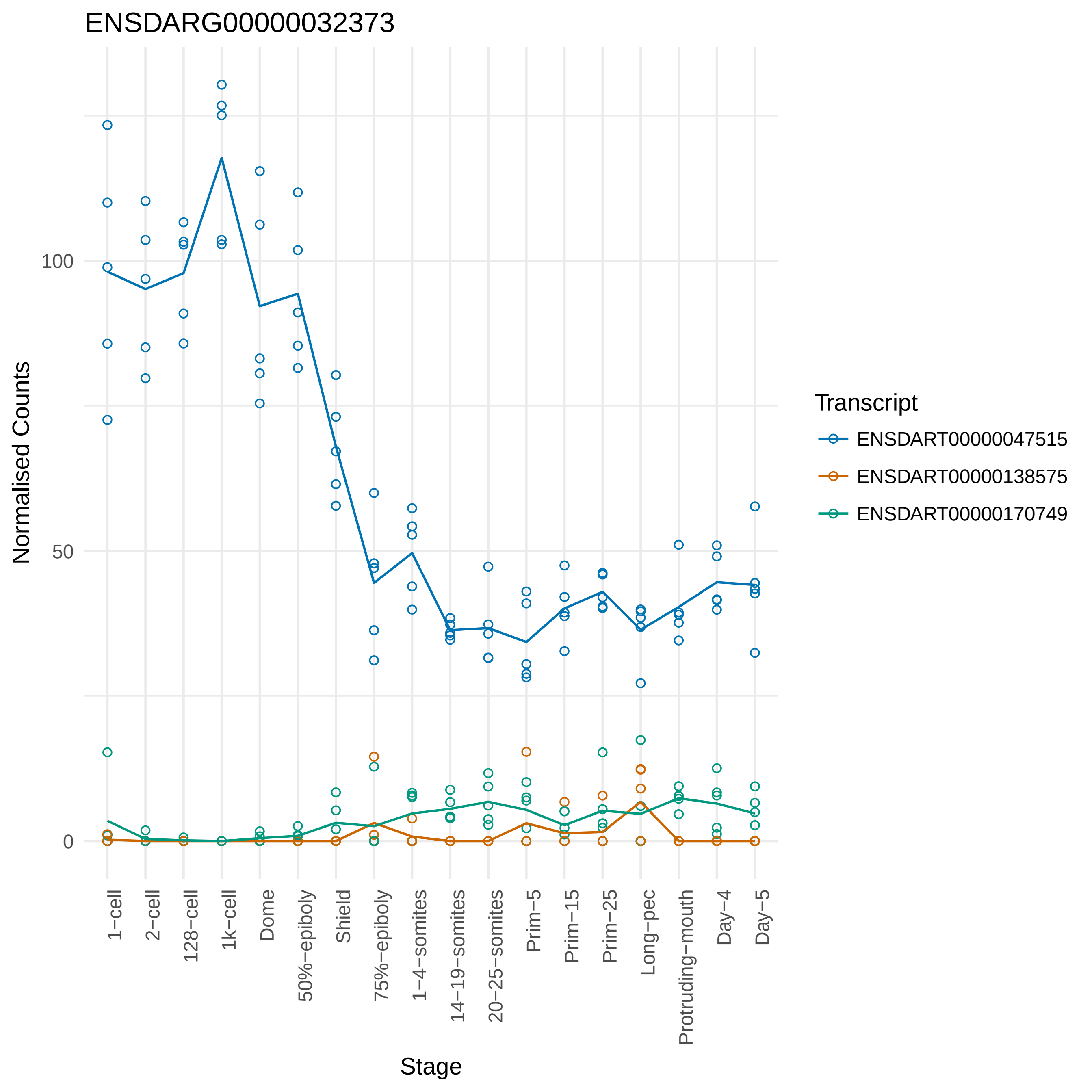

ENSDARG00000032373
Normalised Counts
100
50
0
1−cell
2−cell
128−cell
1k−cell
Dome
50%−epiboly
Shield
75%−epiboly
1−4−somites
14−19−somites
20−25−somites
Prim−5
Prim−15
Prim−25
Long−pec
Protruding−mouth
Day−4
Day−5
Stage
Transcript
ENSDART00000047515
ENSDART00000138575
ENSDART00000170749

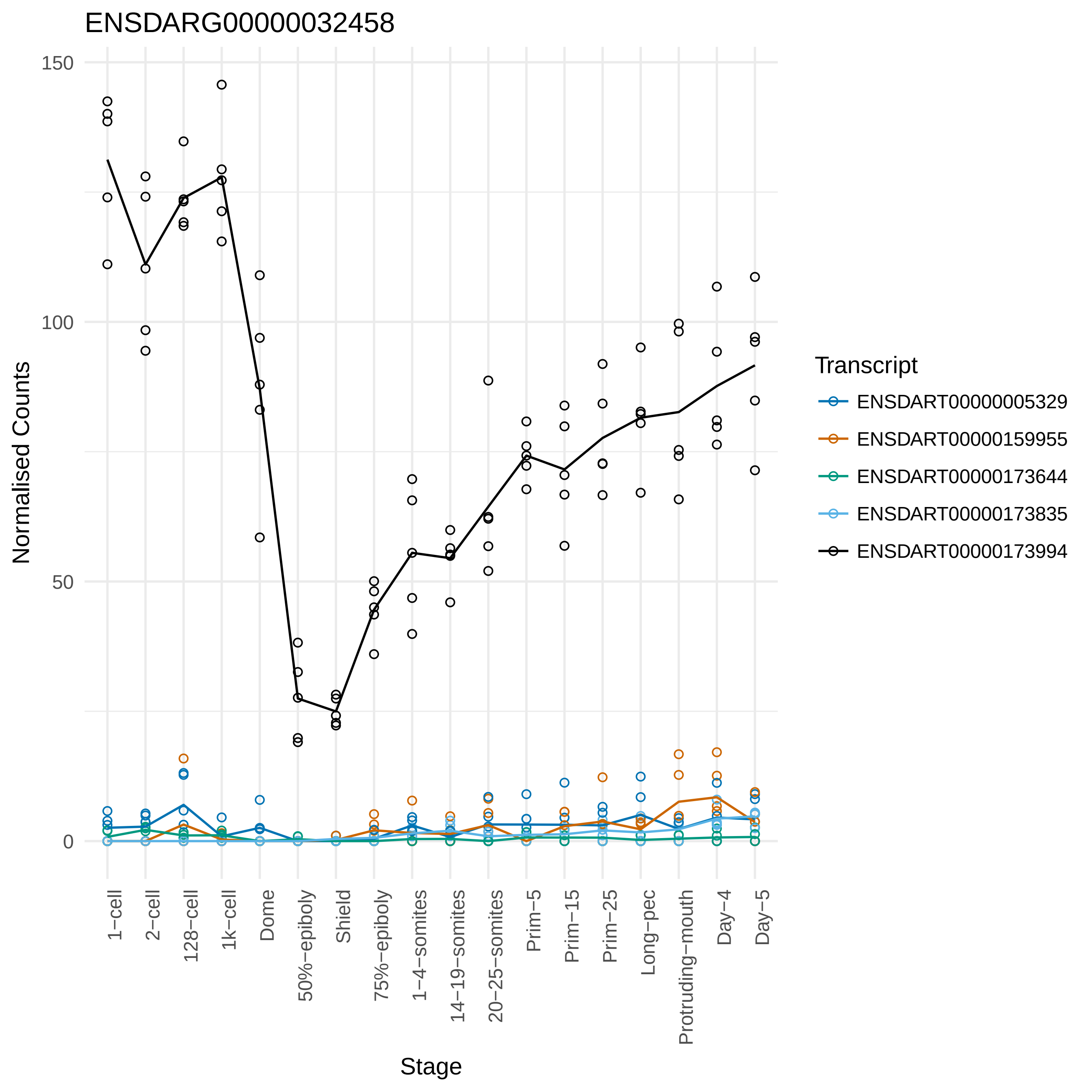
ENSDARG00000032458
Normalised Counts
150
100
50
0
Stage
1−cell
2−cell
128−cell
1k−cell
Dome
50%−epiboly
Shield
75%−epiboly
1−4−somites
14−19−somites
20−25−somites
Prim−5
Prim−15
Prim−25
Long−pec
Protruding−mouth
Day−4
Day−5
Transcript
ENSDART00000005329
ENSDART00000159955
ENSDART00000173644
ENSDART00000173835
ENSDART00000173994

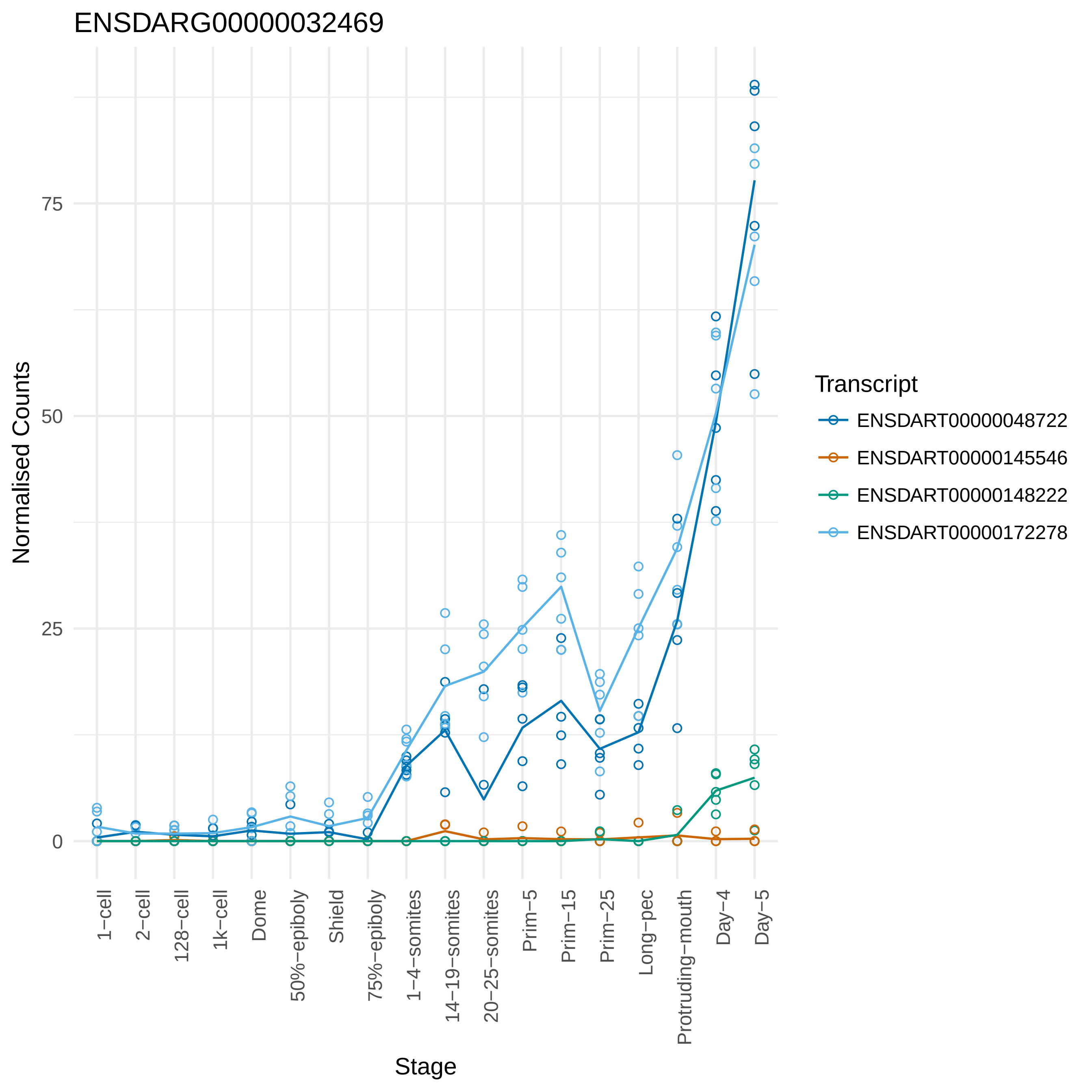

ENSDARG00000032469
Normalised Counts
0
25
50
75
Stage
1−cell
2−cell
128−cell
1k−cell
Dome
50%−epiboly
Shield
75%−epiboly
1−4−somites
14−19−somites
20−25−somites
Prim−5
Prim−15
Prim−25
Long−pec
Protruding−mouth
Day−4
Day−5
Transcript
ENSDART00000048722
ENSDART00000145546
ENSDART00000148222
ENSDART00000172278

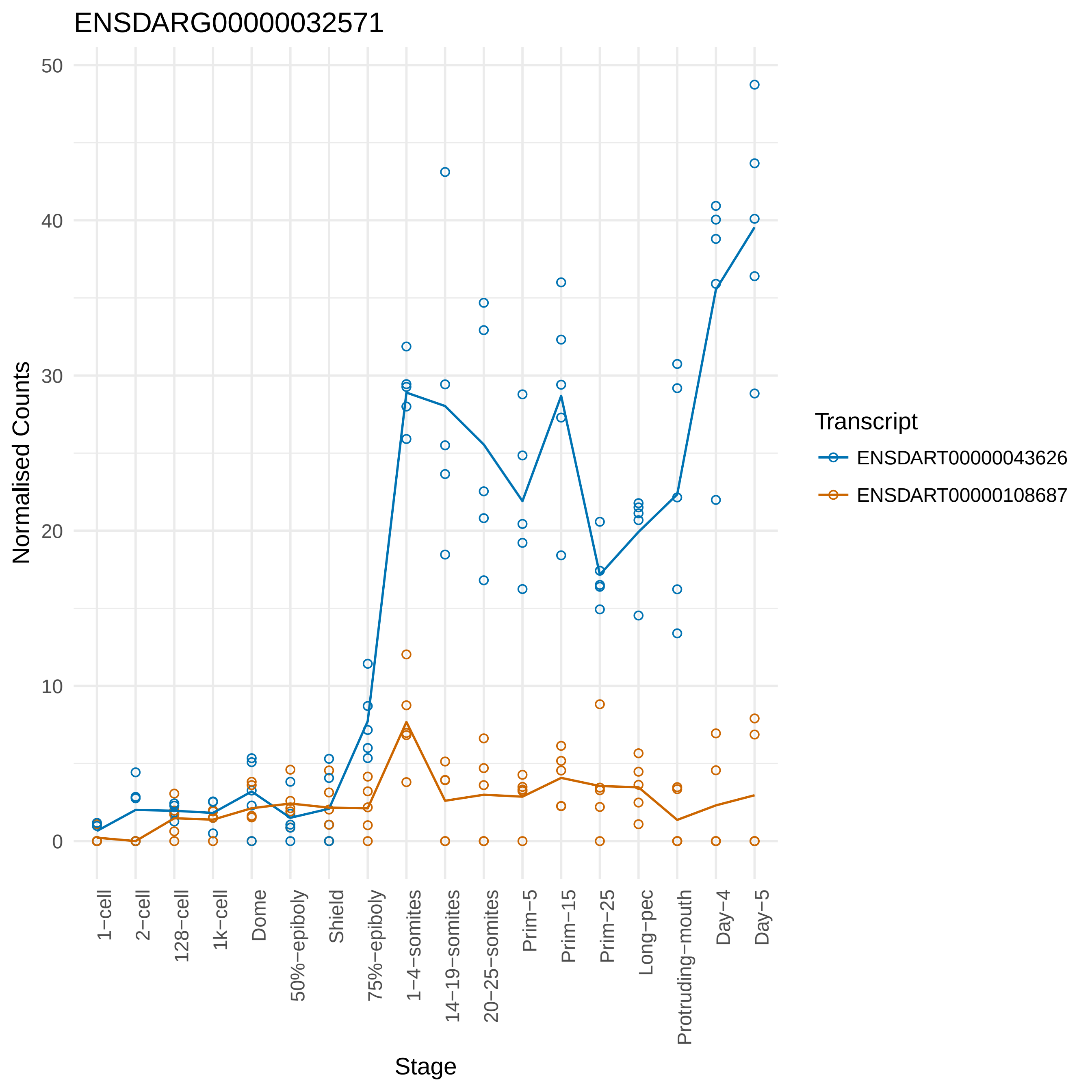

ENSDARG00000032571
Normalised Counts
0
10
20
30
40
50
1−cell
2−cell
128−cell
1k−cell
Dome
50%−epiboly
Shield
75%−epiboly
1−4−somites
14−19−somites
20−25−somites
Prim−5
Prim−15
Prim−25
Long−pec
Protruding−mouth
Day−4
Day−5
Stage
Transcript
ENSDART00000043626
ENSDART00000108687

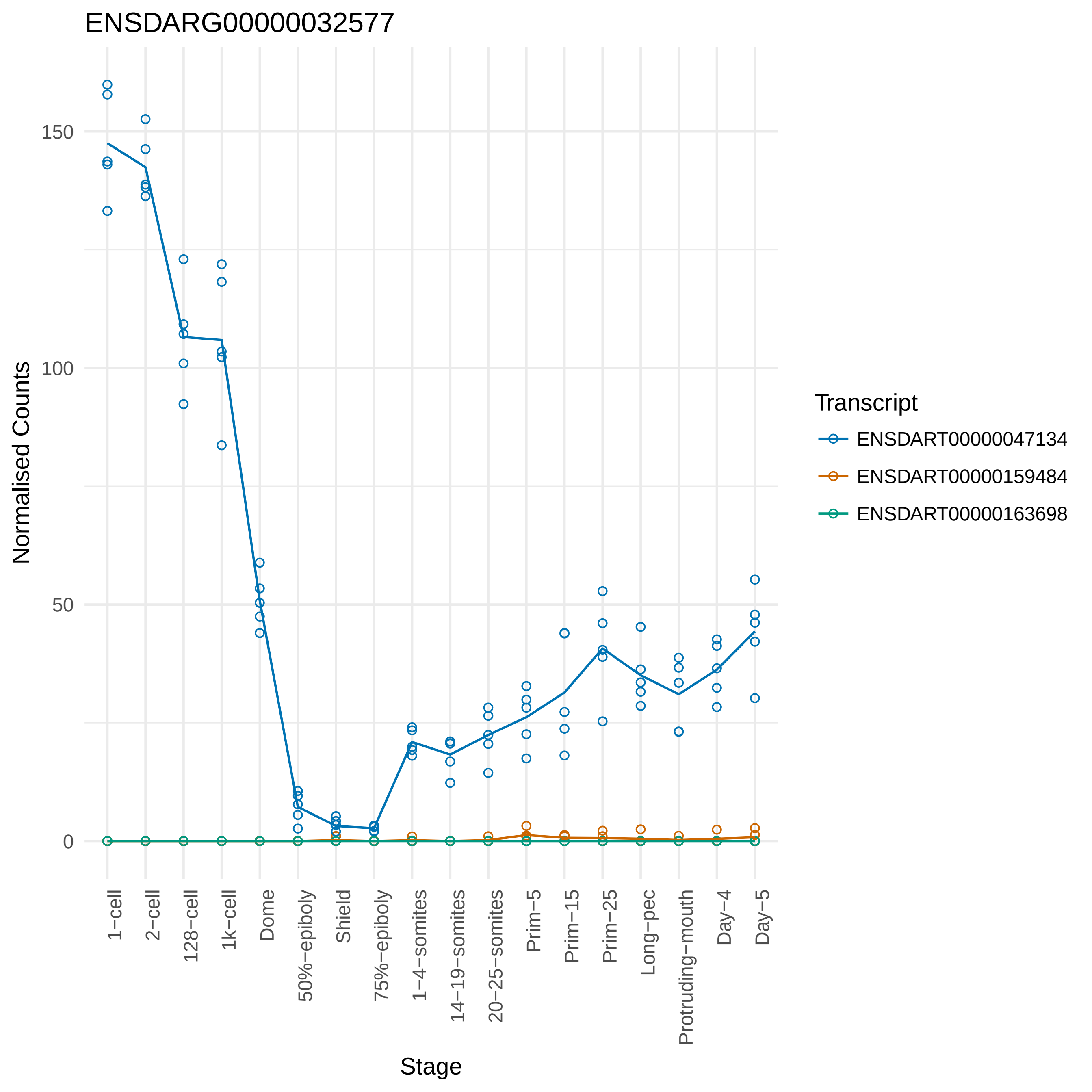

ENSDARG00000032577
Normalised Counts
150
100
50
0
1−cell
2−cell
128−cell
1k−cell
Dome
50%−epiboly
Shield
75%−epiboly
1−4−somites
14−19−somites
20−25−somites
Prim−5
Prim−15
Prim−25
Long−pec
Protruding−mouth
Day−4
Day−5
Stage
Transcript
ENSDART00000047134
ENSDART00000159484
ENSDART00000163698

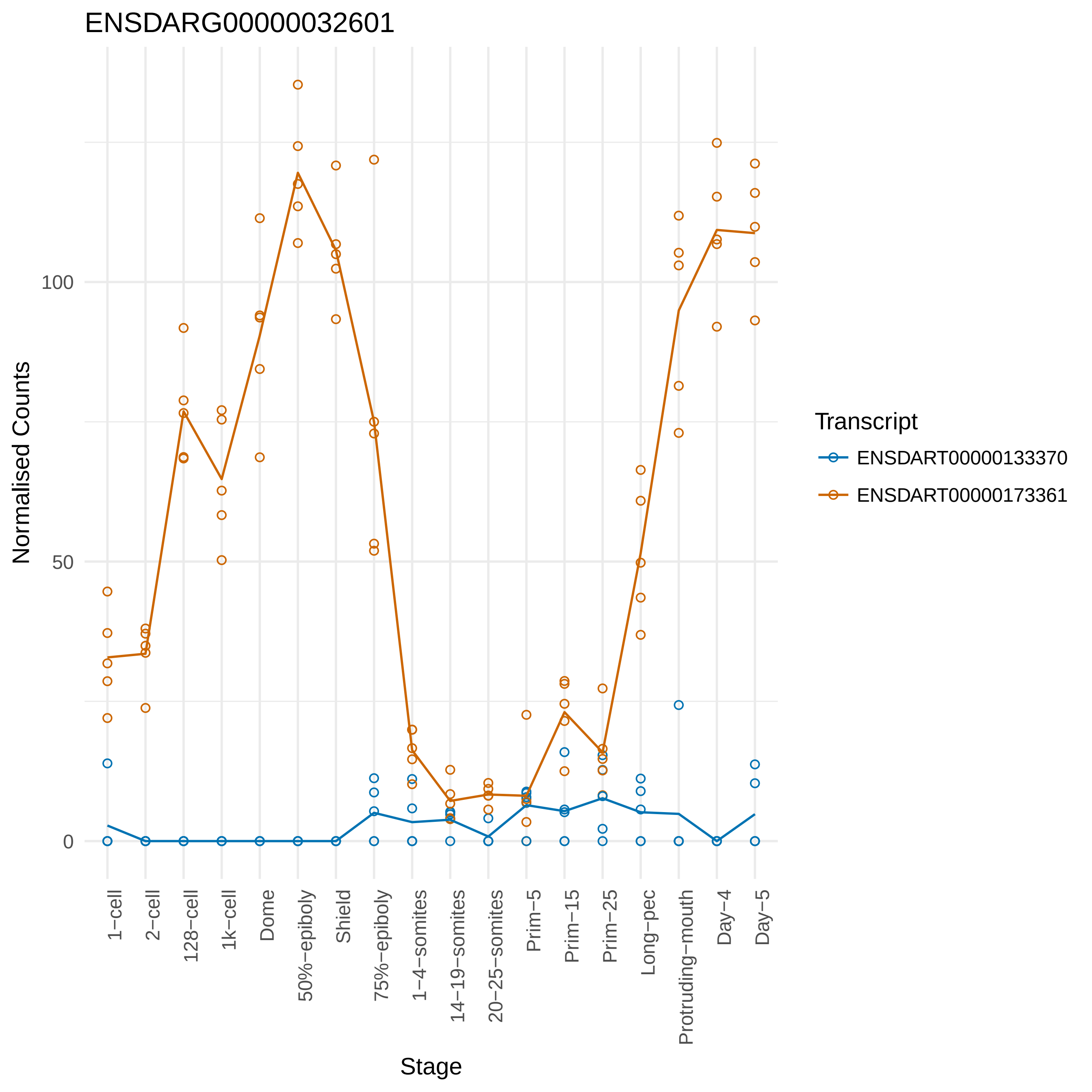
ENSDARG00000032601
Normalised Counts
0
50
100
Stage
1−cell
2−cell
128−cell
1k−cell
Dome
50%−epiboly
Shield
75%−epiboly
1−4−somites
14−19−somites
20−25−somites
Prim−5
Prim−15
Prim−25
Long−pec
Protruding−mouth
Day−4
Day−5
Transcript
ENSDART00000133370
ENSDART00000173361

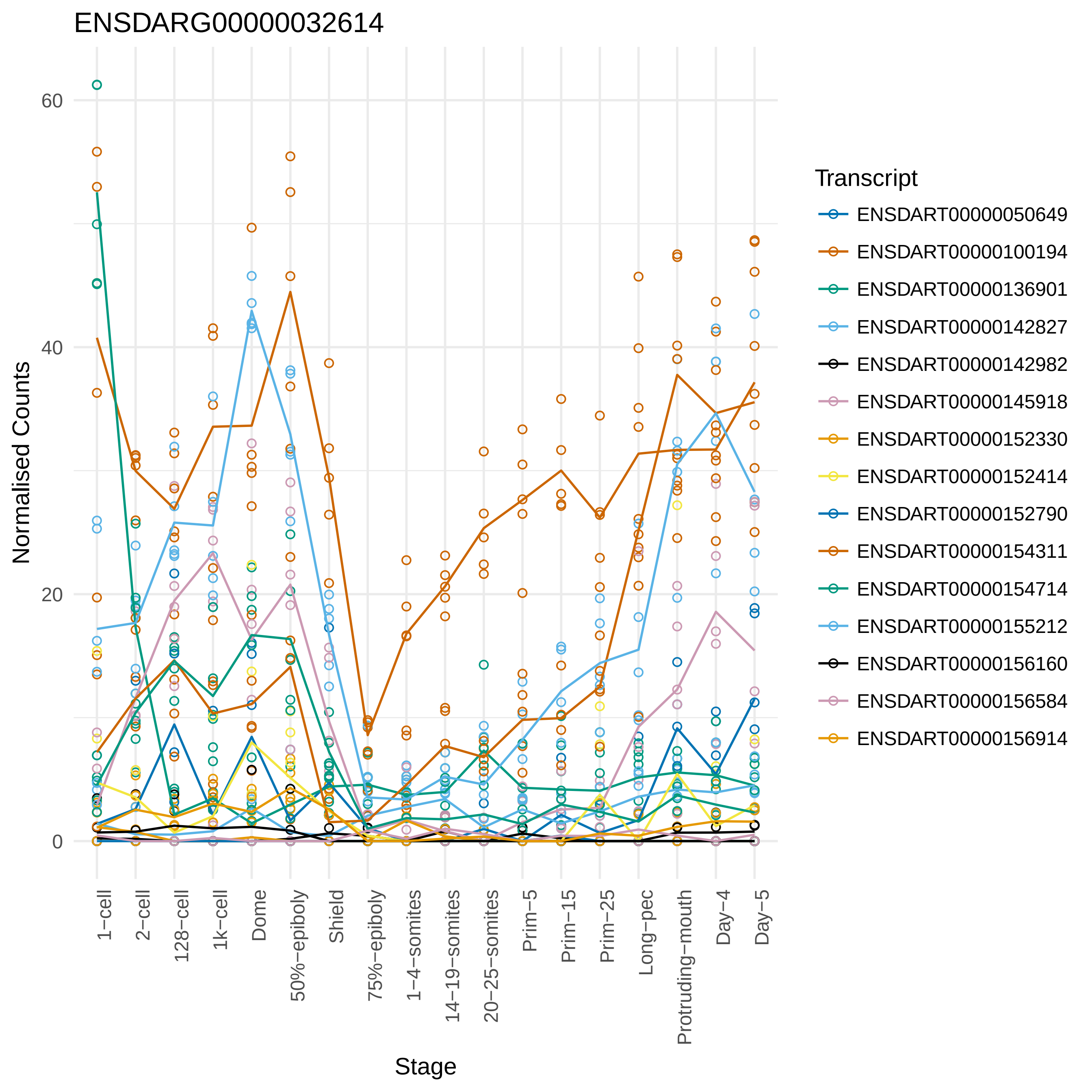
ENSDARG00000032614
Normalised Counts
0
20
40
60
1−cell
2−cell
128−cell
1k−cell
Dome
50%−epiboly
Shield
75%−epiboly
1−4−somites
14−19−somites
20−25−somites
Prim−5
Prim−15
Prim−25
Long−pec
Protruding−mouth
Day−4
Day−5
Stage
Transcript
ENSDART00000050649
ENSDART00000100194
ENSDART00000136901
ENSDART00000142827
ENSDART00000142982
ENSDART00000145918
ENSDART00000152330
ENSDART00000152414
ENSDART00000152790
ENSDART00000154311
ENSDART00000154714
ENSDART00000155212
ENSDART00000156160
ENSDART00000156584
ENSDART00000156914

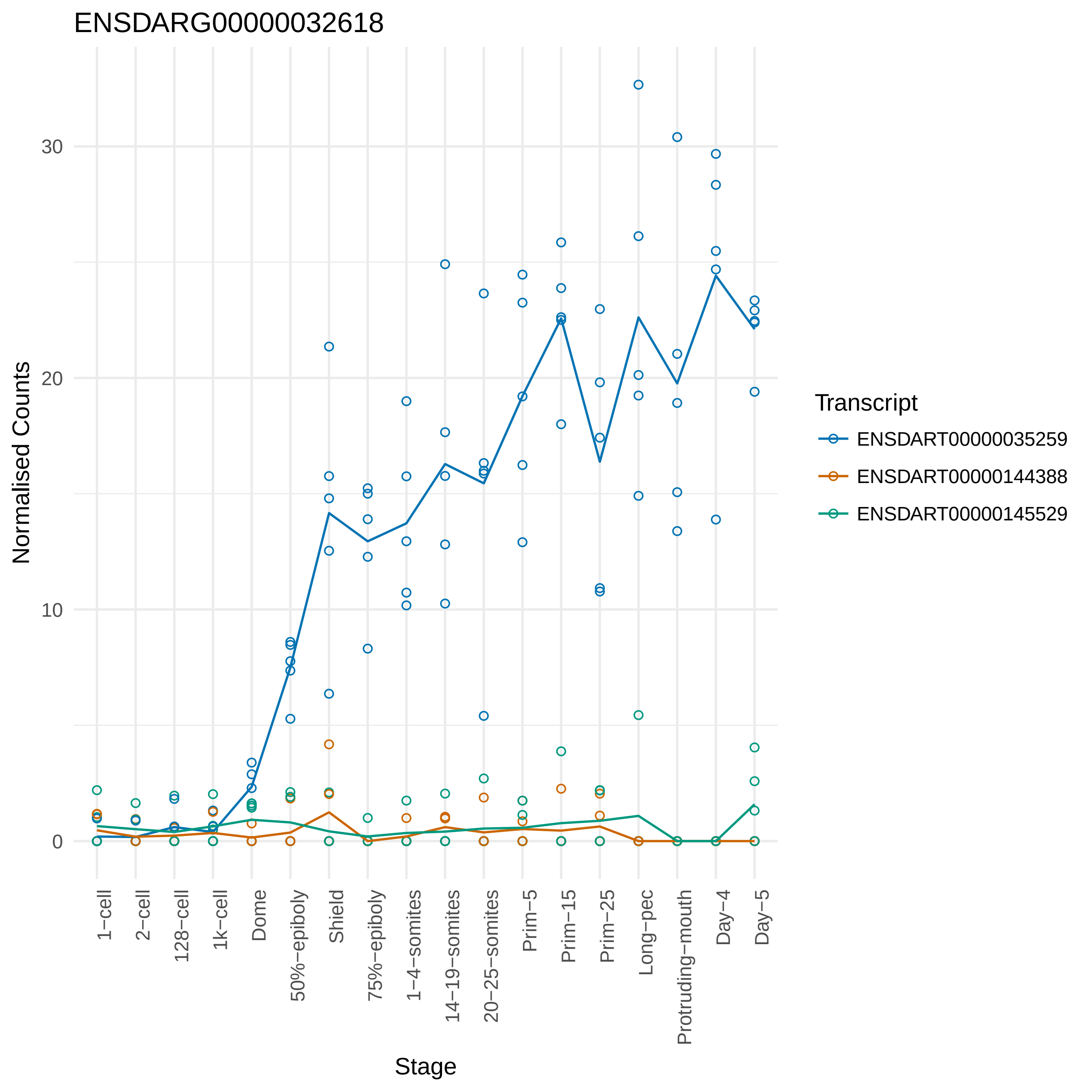
ENSDARG00000032618
Normalised Counts
30
20
10
0
1−cell
2−cell
128−cell
1k−cell
Dome
50%−epiboly
Shield
75%−epiboly
1−4−somites
14−19−somites
20−25−somites
Prim−5
Prim−15
Prim−25
Long−pec
Protruding−mouth
Day−4
Day−5
Stage
Transcript
ENSDART00000035259
ENSDART00000144388
ENSDART00000145529

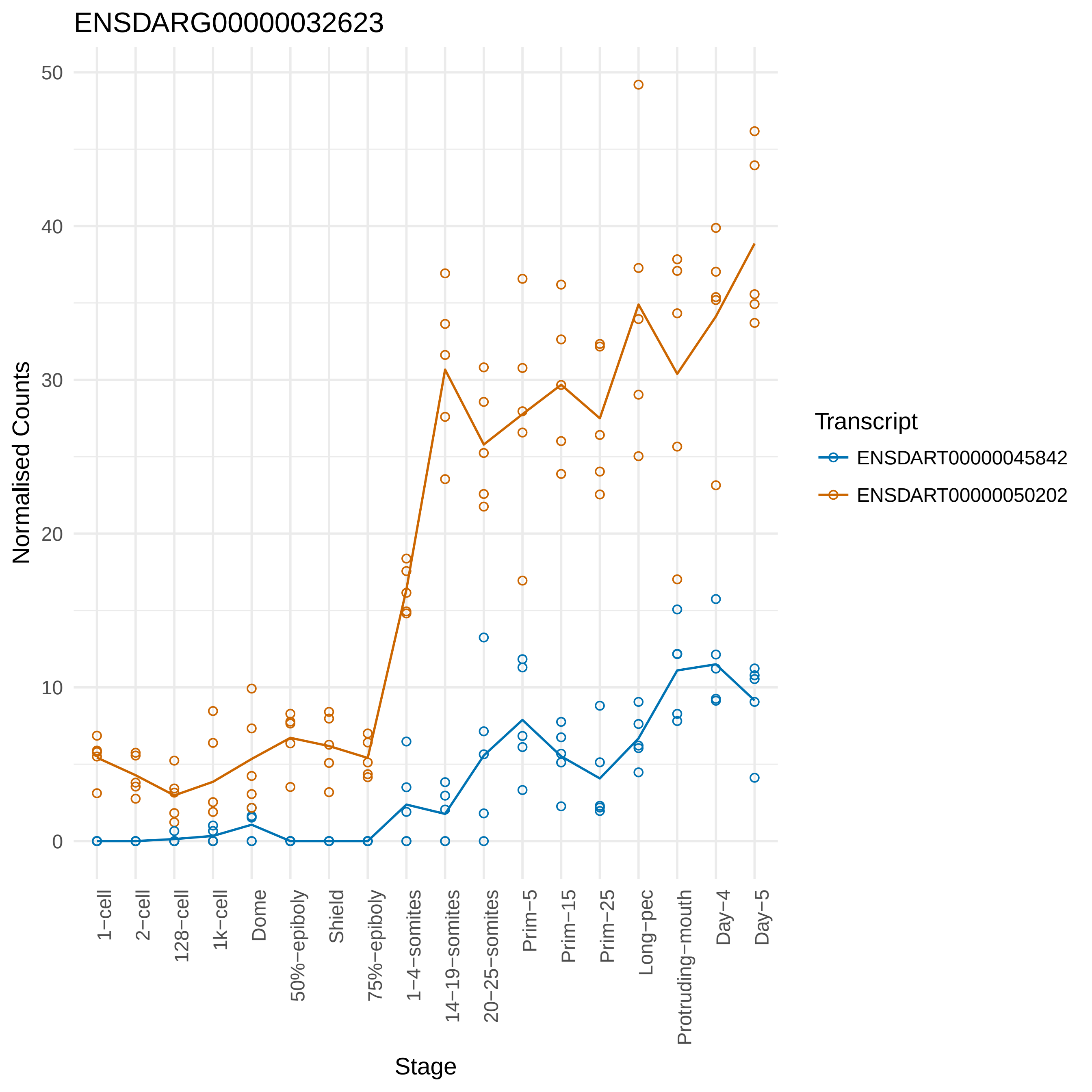

ENSDARG00000032623
Normalised Counts
0
10
20
30
40
50
1−cell
2−cell
128−cell
1k−cell
Dome
50%−epiboly
Shield
75%−epiboly
1−4−somites
14−19−somites
20−25−somites
Prim−5
Prim−15
Prim−25
Long−pec
Protruding−mouth
Day−4
Day−5
Stage
Transcript
ENSDART00000045842
ENSDART00000050202

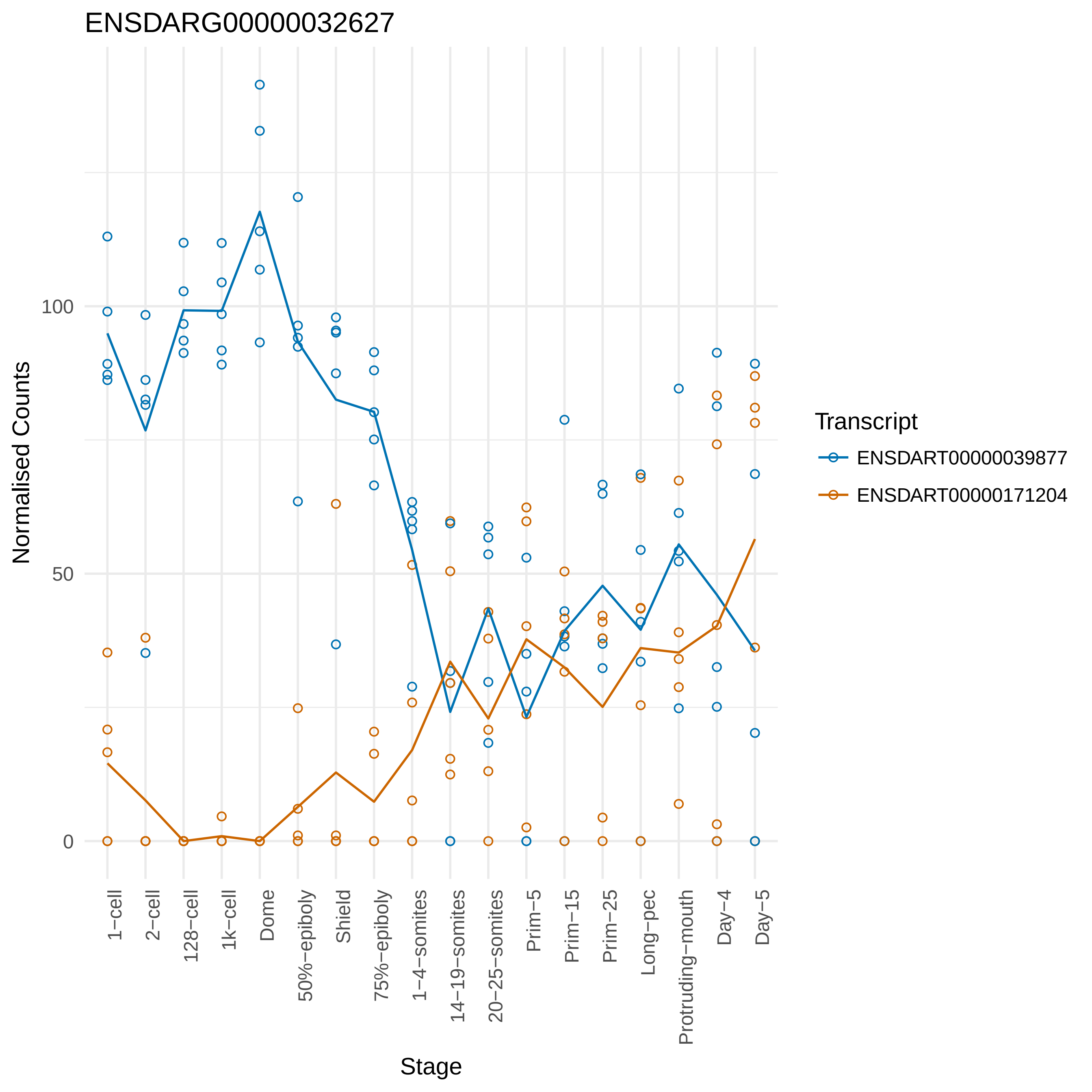

ENSDARG00000032627
Normalised Counts
100
50
0
1−cell
2−cell
128−cell
1k−cell
Dome
50%−epiboly
Shield
75%−epiboly
1−4−somites
14−19−somites
20−25−somites
Prim−5
Prim−15
Prim−25
Long−pec
Protruding−mouth
Day−4
Day−5
Stage
Transcript
ENSDART00000039877
ENSDART00000171204

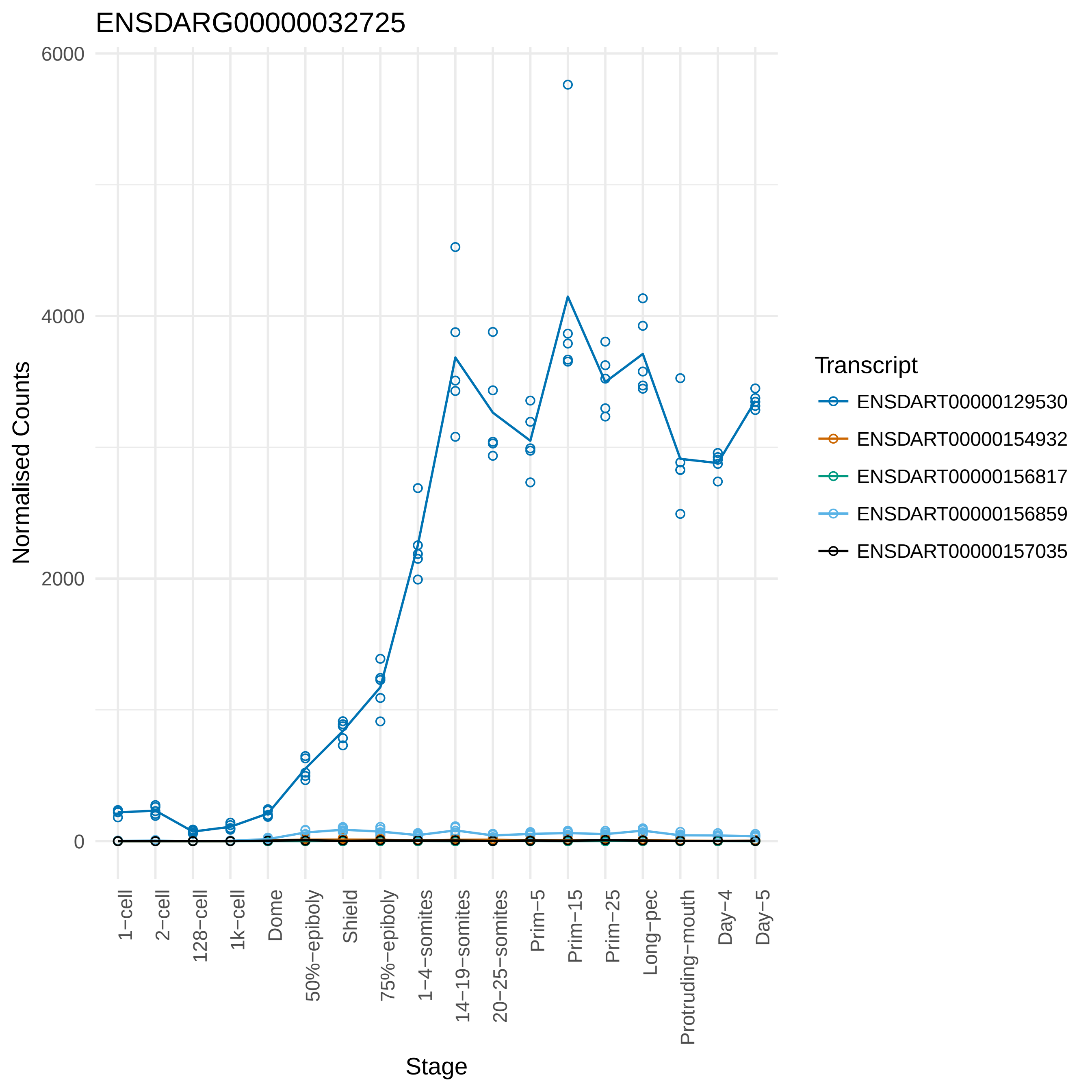
ENSDARG00000032725
6000
4000
2000
0
Normalised Counts
1−cell
2−cell
128−cell
1k−cell
Dome
50%−epiboly
Shield
75%−epiboly
1−4−somites
14−19−somites
20−25−somites
Prim−5
Prim−15
Prim−25
Long−pec
Protruding−mouth
Day−4
Day−5
Stage
Transcript
ENSDART00000129530
ENSDART00000154932
ENSDART00000156817
ENSDART00000156859
ENSDART00000157035

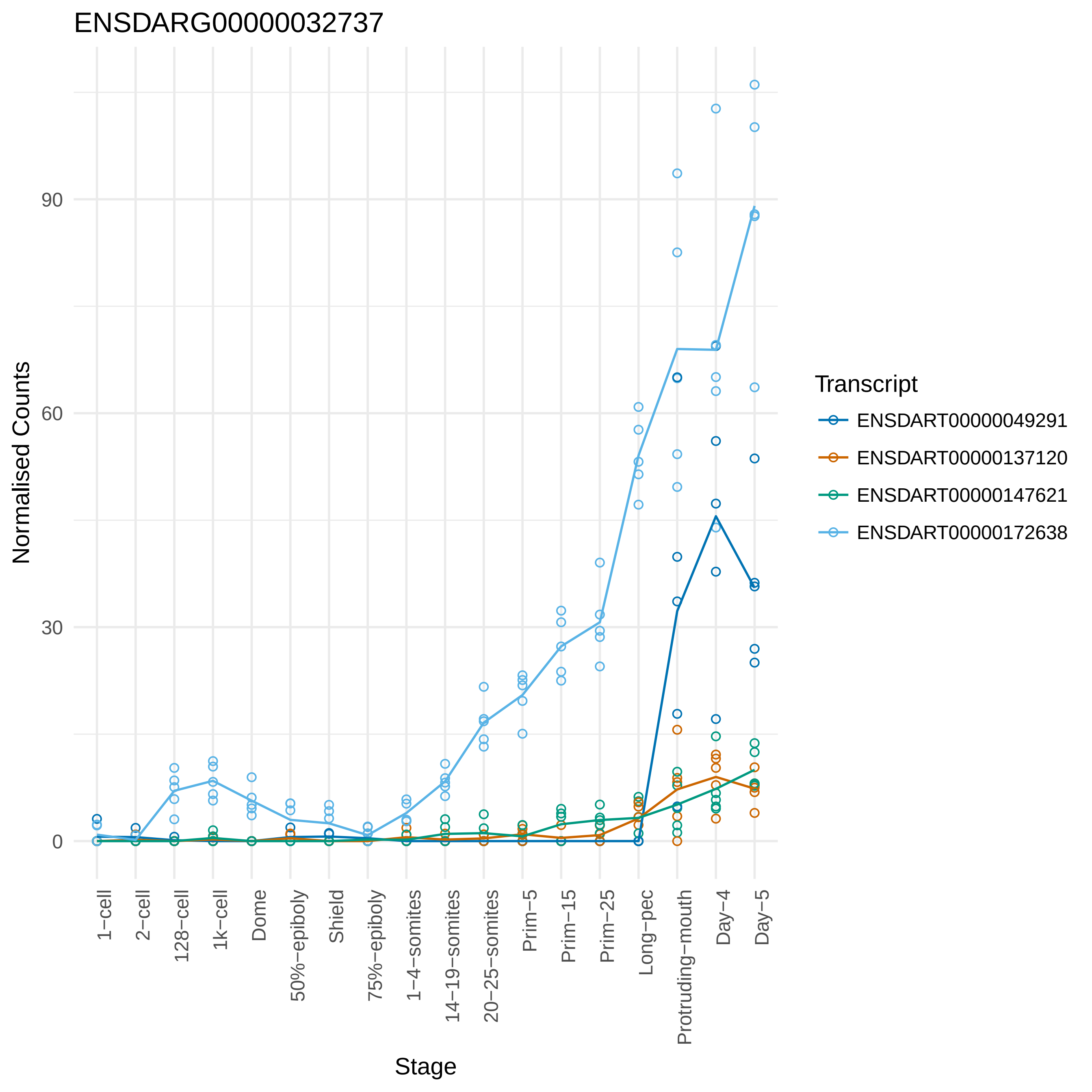
ENSDARG00000032737
Normalised Counts
90
60
30
0
1−cell
2−cell
128−cell
1k−cell
Dome
50%−epiboly
Shield
75%−epiboly
1−4−somites
14−19−somites
20−25−somites
Prim−5
Prim−15
Prim−25
Long−pec
Protruding−mouth
Day−4
Day−5
Stage
Transcript
ENSDART00000049291
ENSDART00000137120
ENSDART00000147621
ENSDART00000172638

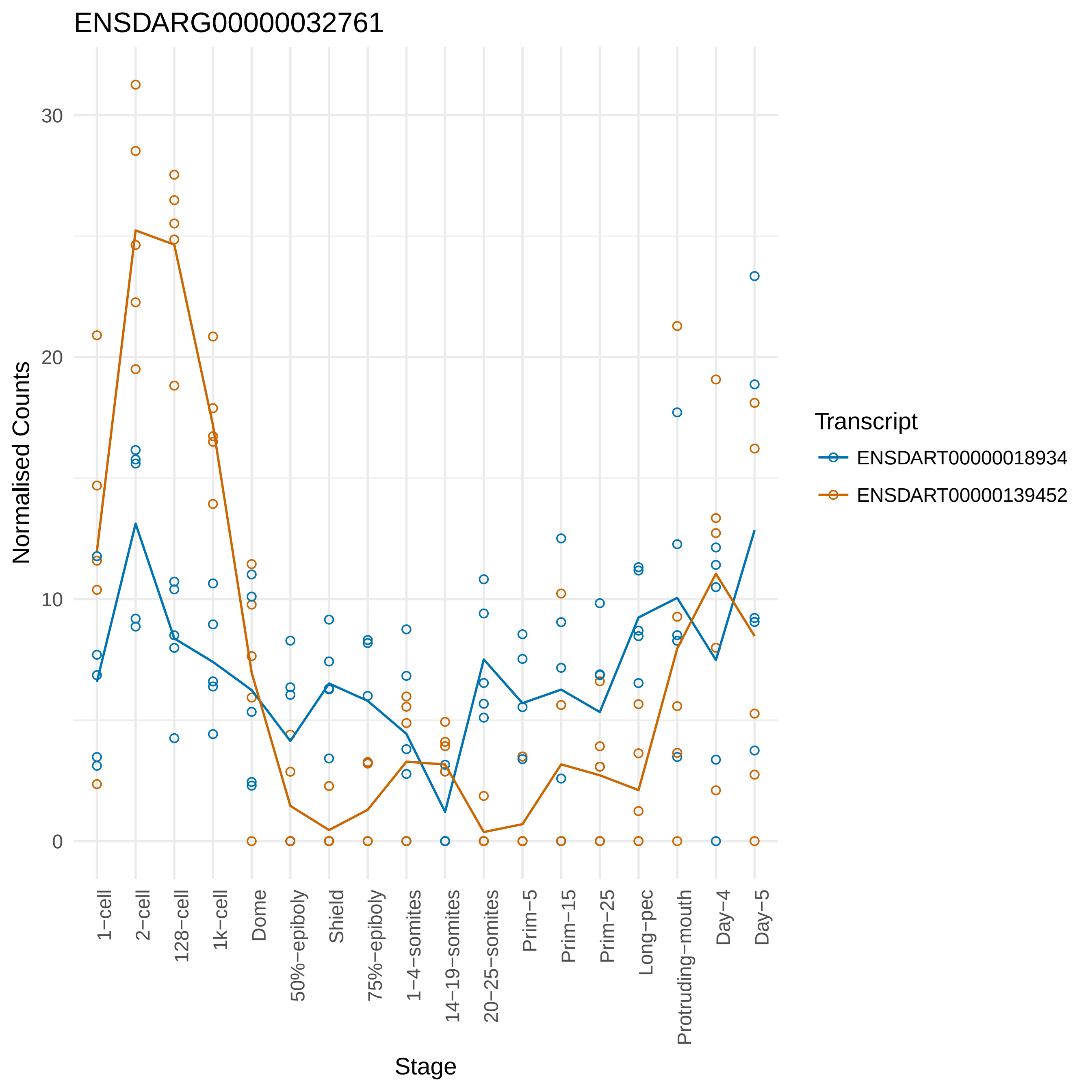

ENSDARG00000032761
Normalised Counts
0
10
20
30
Stage
1−cell
2−cell
128−cell
1k−cell
Dome
50%−epiboly
Shield
75%−epiboly
1−4−somites
14−19−somites
20−25−somites
Prim−5
Prim−15
Prim−25
Long−pec
Protruding−mouth
Day−4
Day−5
Transcript
ENSDART00000018934
ENSDART00000139452

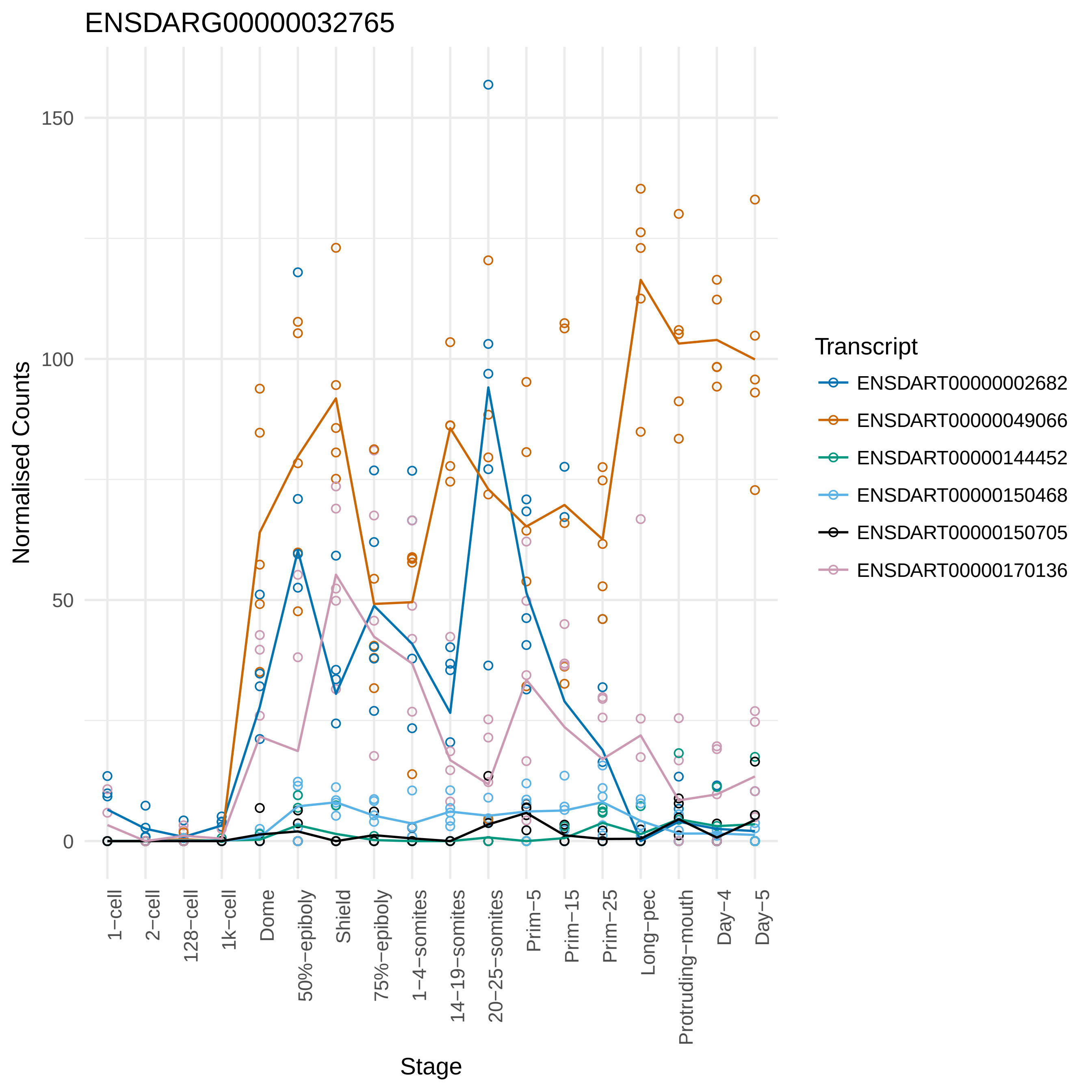
ENSDARG00000032765
Normalised Counts
150
100
50
0
1−cell
2−cell
128−cell
1k−cell
Dome
50%−epiboly
Shield
75%−epiboly
1−4−somites
14−19−somites
20−25−somites
Prim−5
Prim−15
Prim−25
Long−pec
Protruding−mouth
Day−4
Day−5
Stage
Transcript
ENSDART00000002682
ENSDART00000049066
ENSDART00000144452
ENSDART00000150468
ENSDART00000150705
ENSDART00000170136

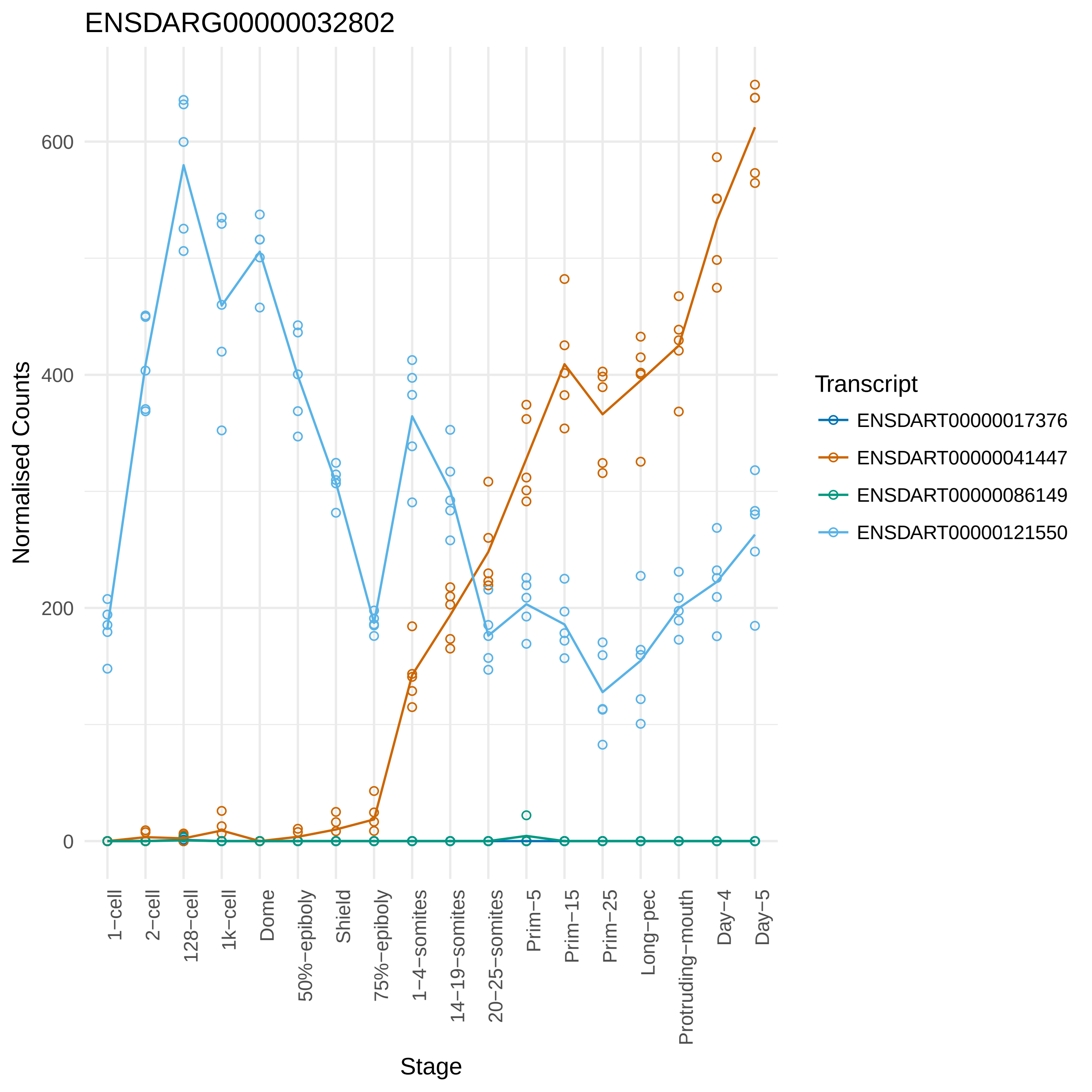
ENSDARG00000032802
Normalised Counts
600
400
200
0
1−cell
2−cell
128−cell
1k−cell
Dome
50%−epiboly
Shield
75%−epiboly
1−4−somites
14−19−somites
20−25−somites
Prim−5
Prim−15
Prim−25
Long−pec
Protruding−mouth
Day−4
Day−5
Stage
Transcript
ENSDART00000017376
ENSDART00000041447
ENSDART00000086149
ENSDART00000121550

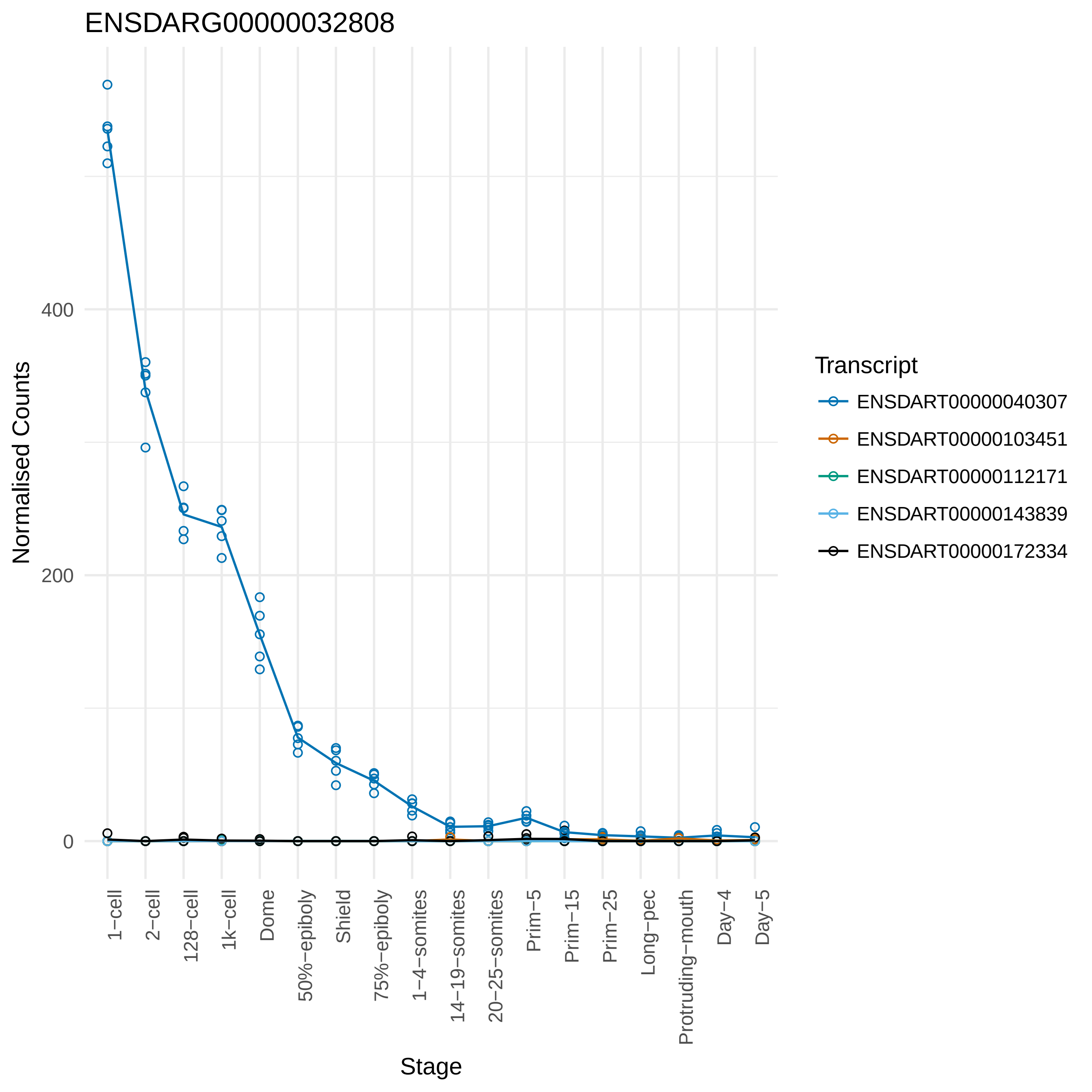

ENSDARG00000032808
Normalised Counts
400
200
0
Stage
1−cell
2−cell
128−cell
1k−cell
Dome
50%−epiboly
Shield
75%−epiboly
1−4−somites
14−19−somites
20−25−somites
Prim−5
Prim−15
Prim−25
Long−pec
Protruding−mouth
Day−4
Day−5
Transcript
ENSDART00000040307
ENSDART00000103451
ENSDART00000112171
ENSDART00000143839
ENSDART00000172334

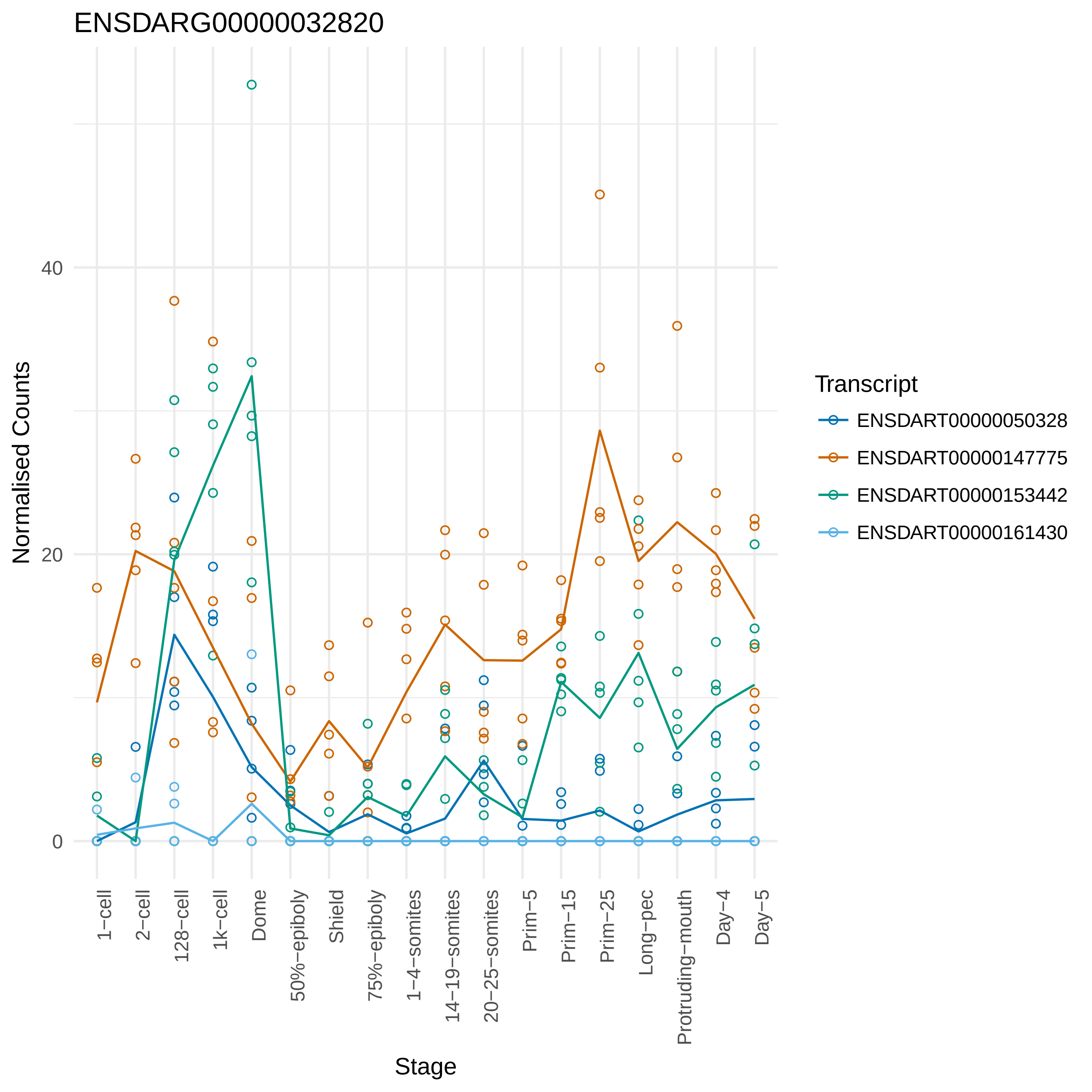
ENSDARG00000032820
Normalised Counts
40
20
0
1−cell
2−cell
128−cell
1k−cell
Dome
50%−epiboly
Shield
75%−epiboly
1−4−somites
14−19−somites
20−25−somites
Prim−5
Prim−15
Prim−25
Long−pec
Protruding−mouth
Day−4
Day−5
Stage
Transcript
ENSDART00000050328
ENSDART00000147775
ENSDART00000153442
ENSDART00000161430

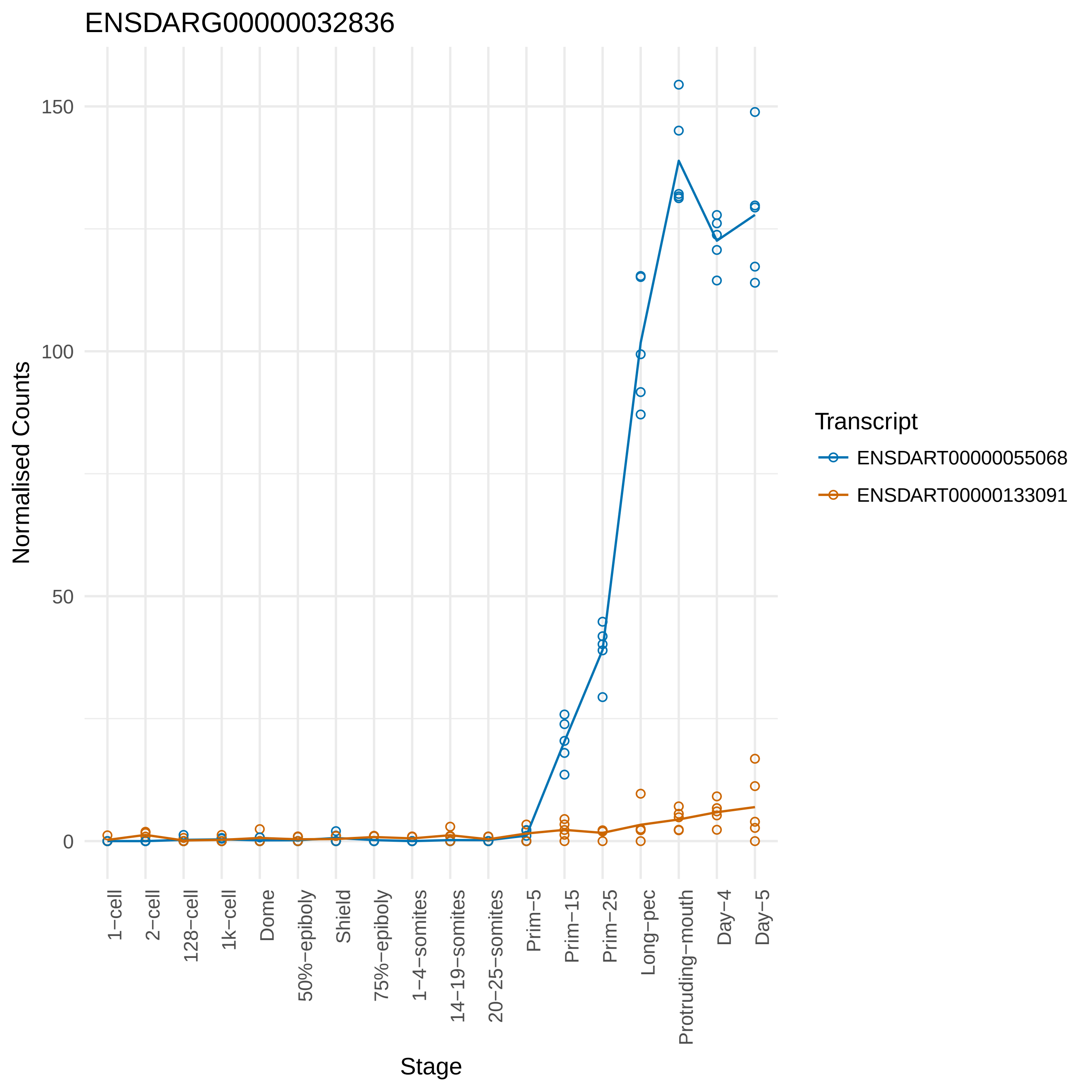
ENSDARG00000032836
Normalised Counts
150
100
50
0
1−cell
2−cell
128−cell
1k−cell
Dome
50%−epiboly
Shield
75%−epiboly
1−4−somites
14−19−somites
20−25−somites
Prim−5
Prim−15
Prim−25
Long−pec
Protruding−mouth
Day−4
Day−5
Stage
Transcript
ENSDART00000055068
ENSDART00000133091

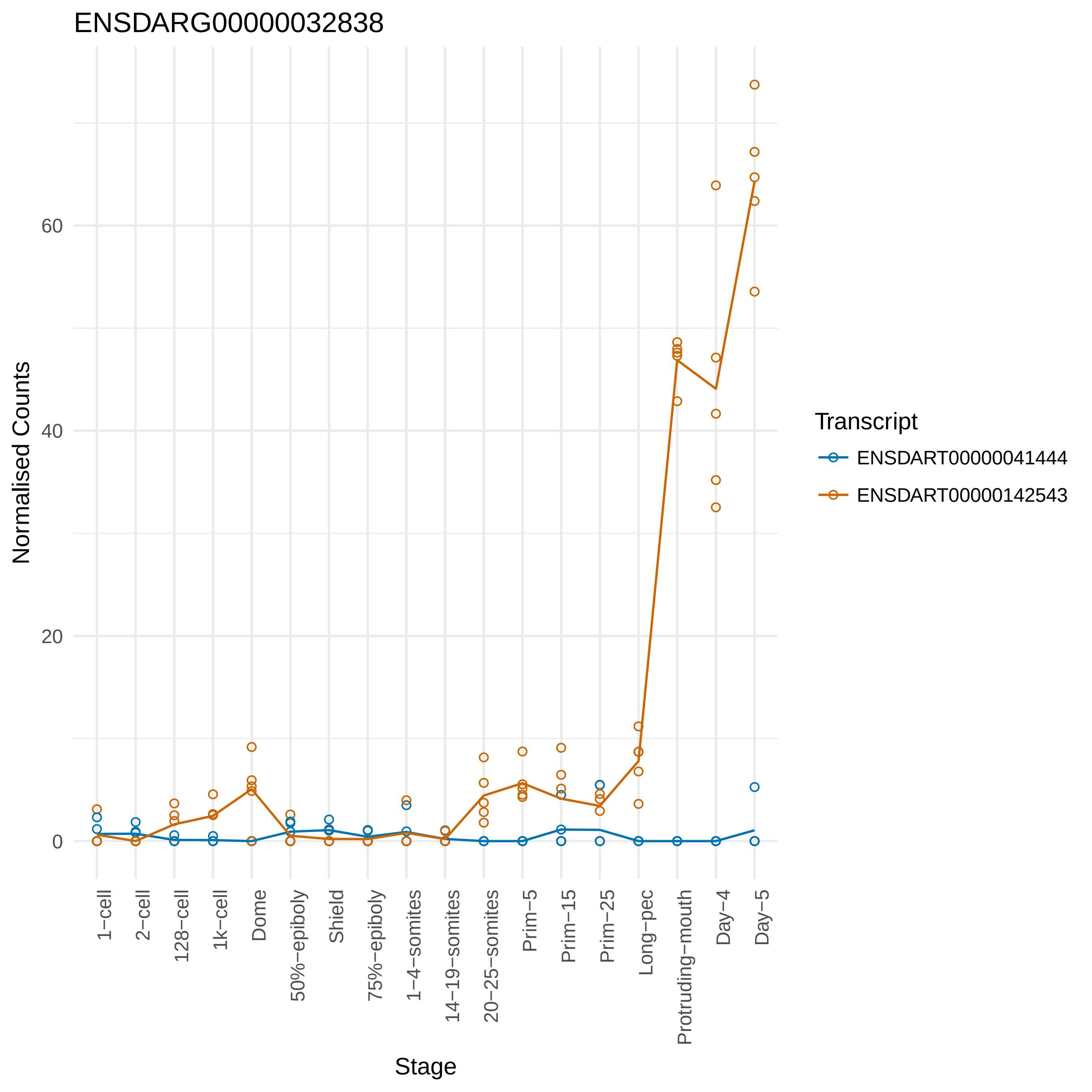
ENSDARG00000032838
Normalised Counts
0
20
40
60
Stage
1−cell
2−cell
128−cell
1k−cell
Dome
50%−epiboly
Shield
75%−epiboly
1−4−somites
14−19−somites
20−25−somites
Prim−5
Prim−15
Prim−25
Long−pec
Protruding−mouth
Day−4
Day−5
Transcript
ENSDART00000041444
ENSDART00000142543

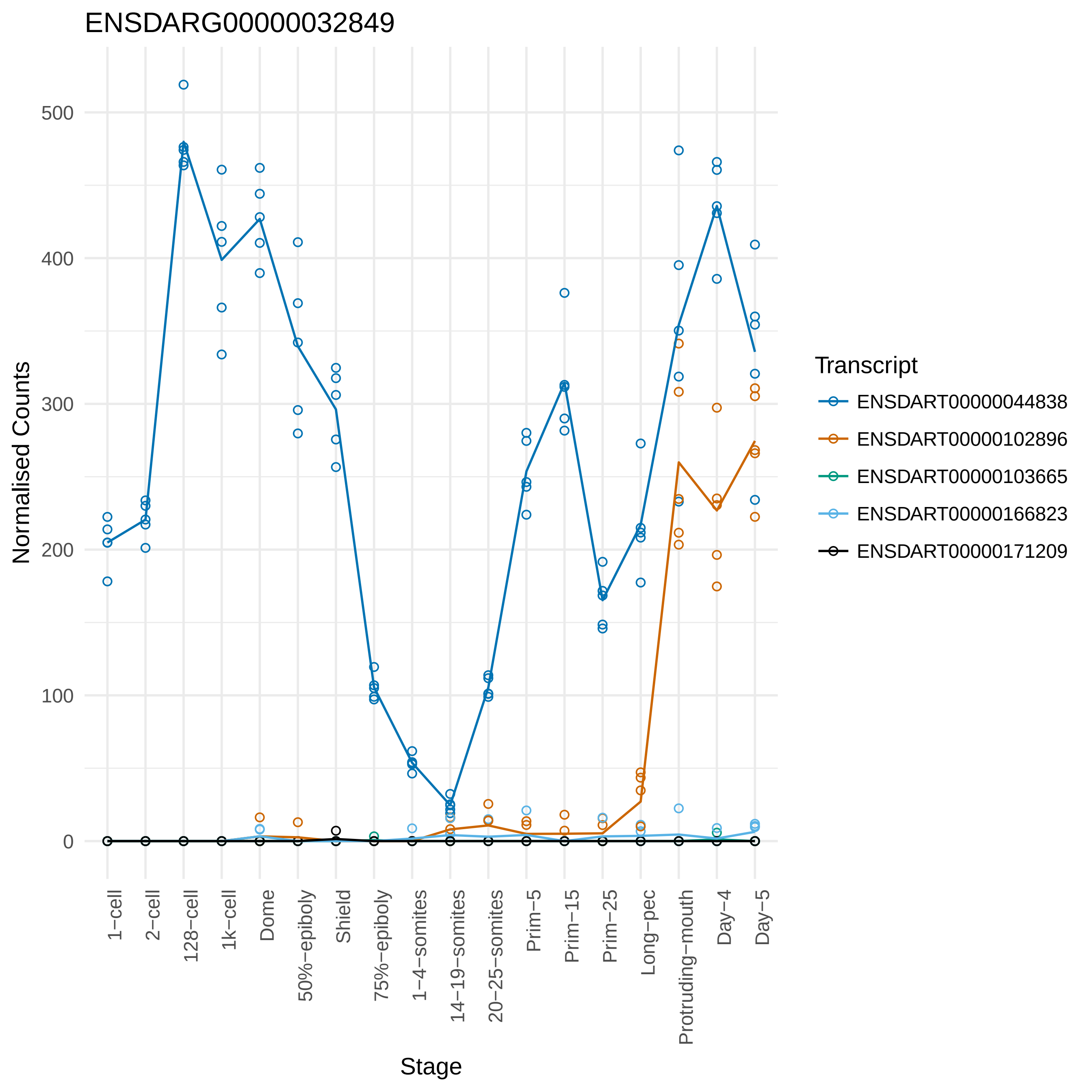

ENSDARG00000032849
Normalised Counts
0
100
200
300
400
500
1−cell
2−cell
128−cell
1k−cell
Dome
50%−epiboly
Shield
75%−epiboly
1−4−somites
14−19−somites
20−25−somites
Prim−5
Prim−15
Prim−25
Long−pec
Protruding−mouth
Day−4
Day−5
Stage
Transcript
ENSDART00000044838
ENSDART00000102896
ENSDART00000103665
ENSDART00000166823
ENSDART00000171209

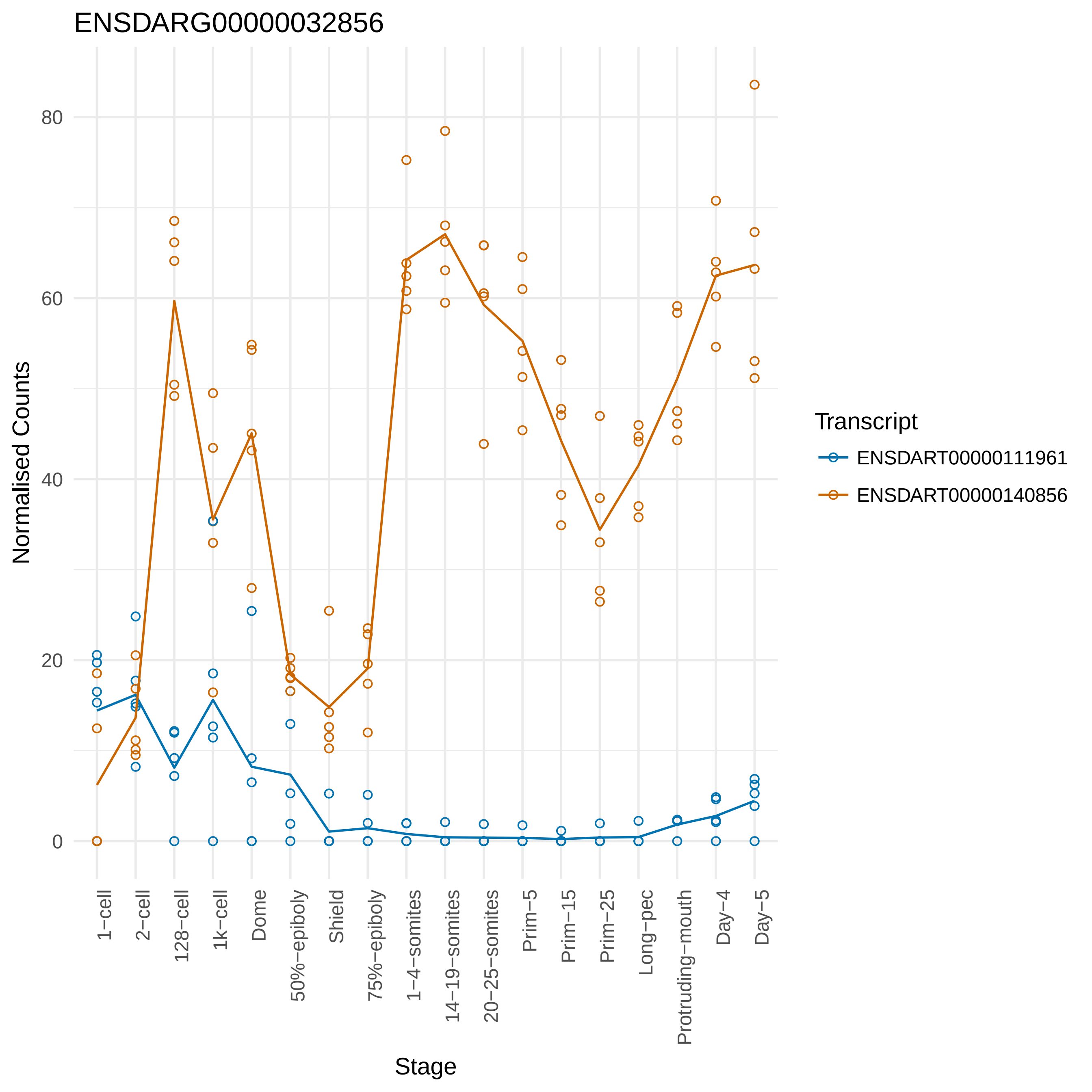

ENSDARG00000032856
Normalised Counts
0
20
40
60
80
1−cell
2−cell
128−cell
1k−cell
Dome
50%−epiboly
Shield
75%−epiboly
1−4−somites
14−19−somites
20−25−somites
Prim−5
Prim−15
Prim−25
Long−pec
Protruding−mouth
Day−4
Day−5
Stage
Transcript
ENSDART00000111961
ENSDART00000140856

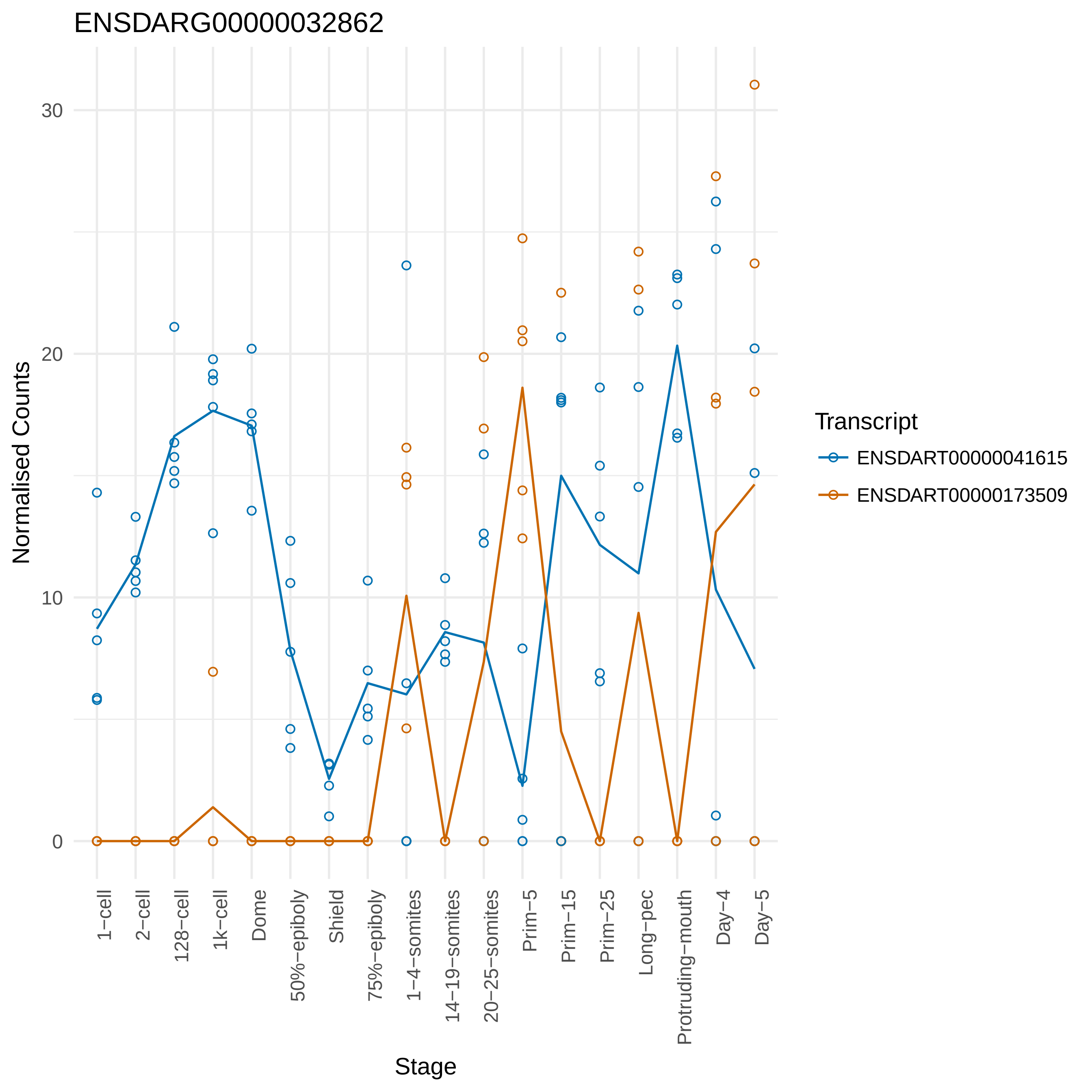

ENSDARG00000032862
Normalised Counts
30
20
10
0
1−cell
2−cell
128−cell
1k−cell
Dome
50%−epiboly
Shield
75%−epiboly
1−4−somites
14−19−somites
20−25−somites
Prim−5
Prim−15
Prim−25
Long−pec
Protruding−mouth
Day−4
Day−5
Stage
Transcript
ENSDART00000041615
ENSDART00000173509

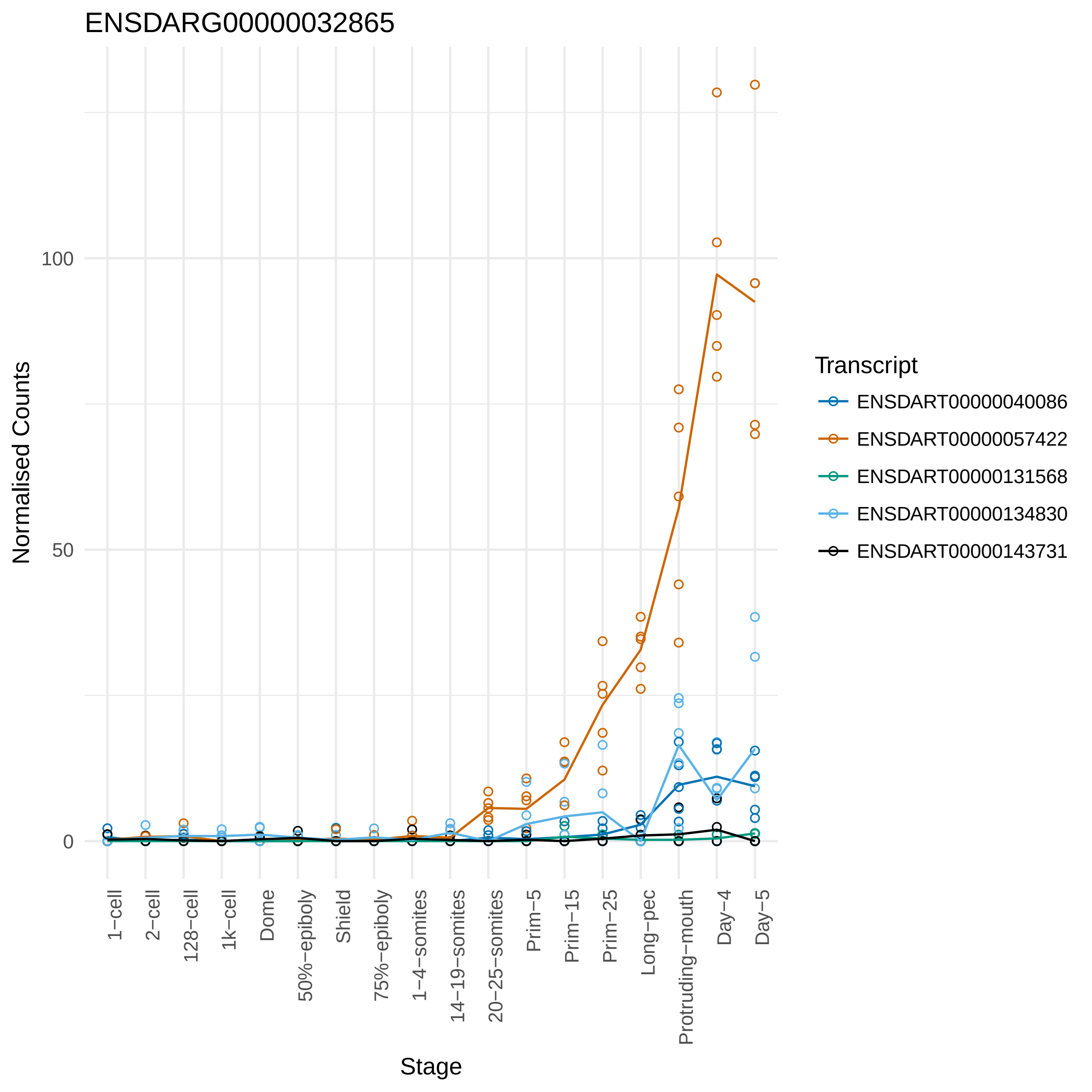
ENSDARG00000032865
Normalised Counts
0
50
100
Stage
1−cell
2−cell
128−cell
1k−cell
Dome
50%−epiboly
Shield
75%−epiboly
1−4−somites
14−19−somites
20−25−somites
Prim−5
Prim−15
Prim−25
Long−pec
Protruding−mouth
Day−4
Day−5
Transcript
ENSDART00000040086
ENSDART00000057422
ENSDART00000131568
ENSDART00000134830
ENSDART00000143731

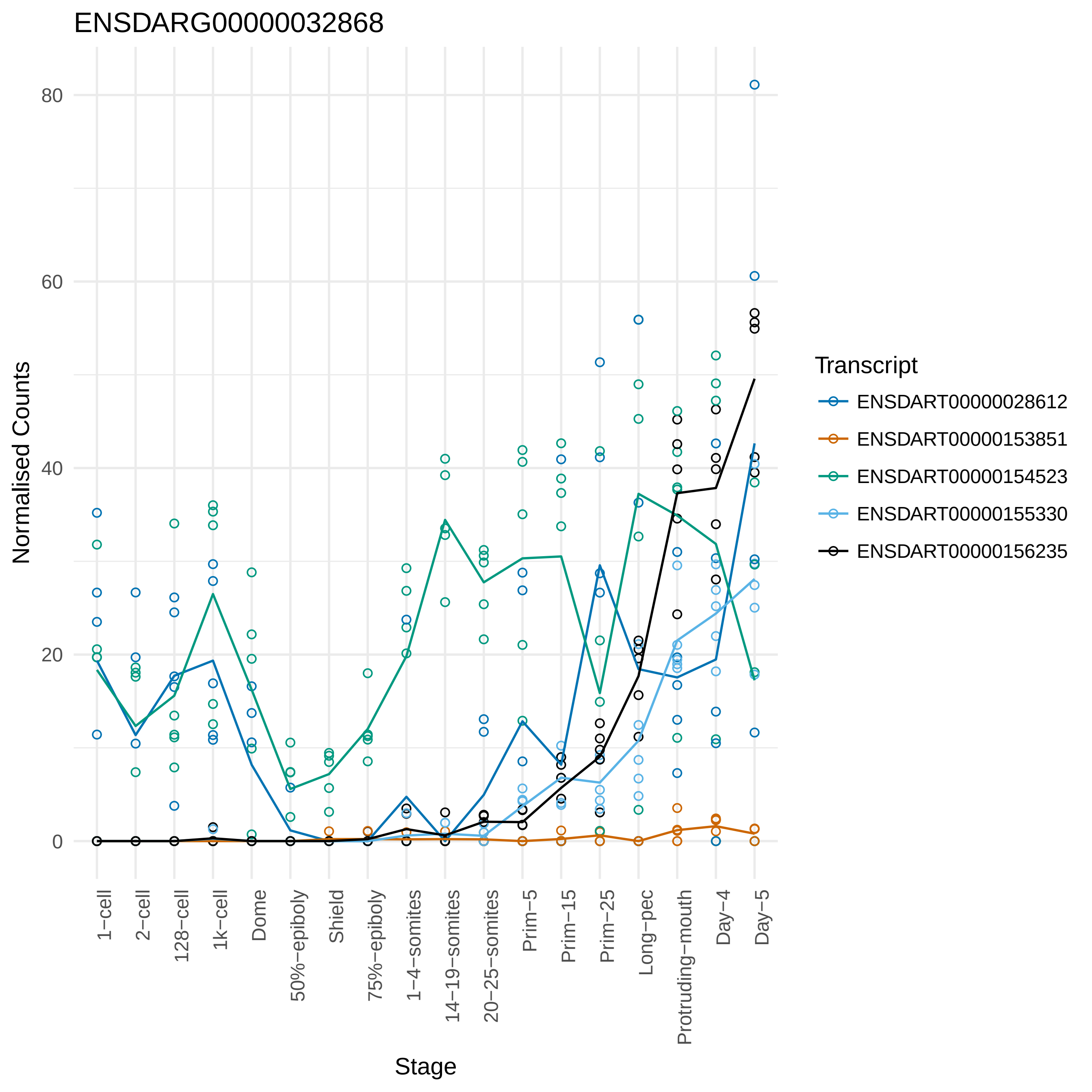

ENSDARG00000032868
Normalised Counts
80
60
40
20
0
1−cell
2−cell
128−cell
1k−cell
Dome
50%−epiboly
Shield
75%−epiboly
1−4−somites
14−19−somites
20−25−somites
Prim−5
Prim−15
Prim−25
Long−pec
Protruding−mouth
Day−4
Day−5
Stage
Transcript
ENSDART00000028612
ENSDART00000153851
ENSDART00000154523
ENSDART00000155330
ENSDART00000156235

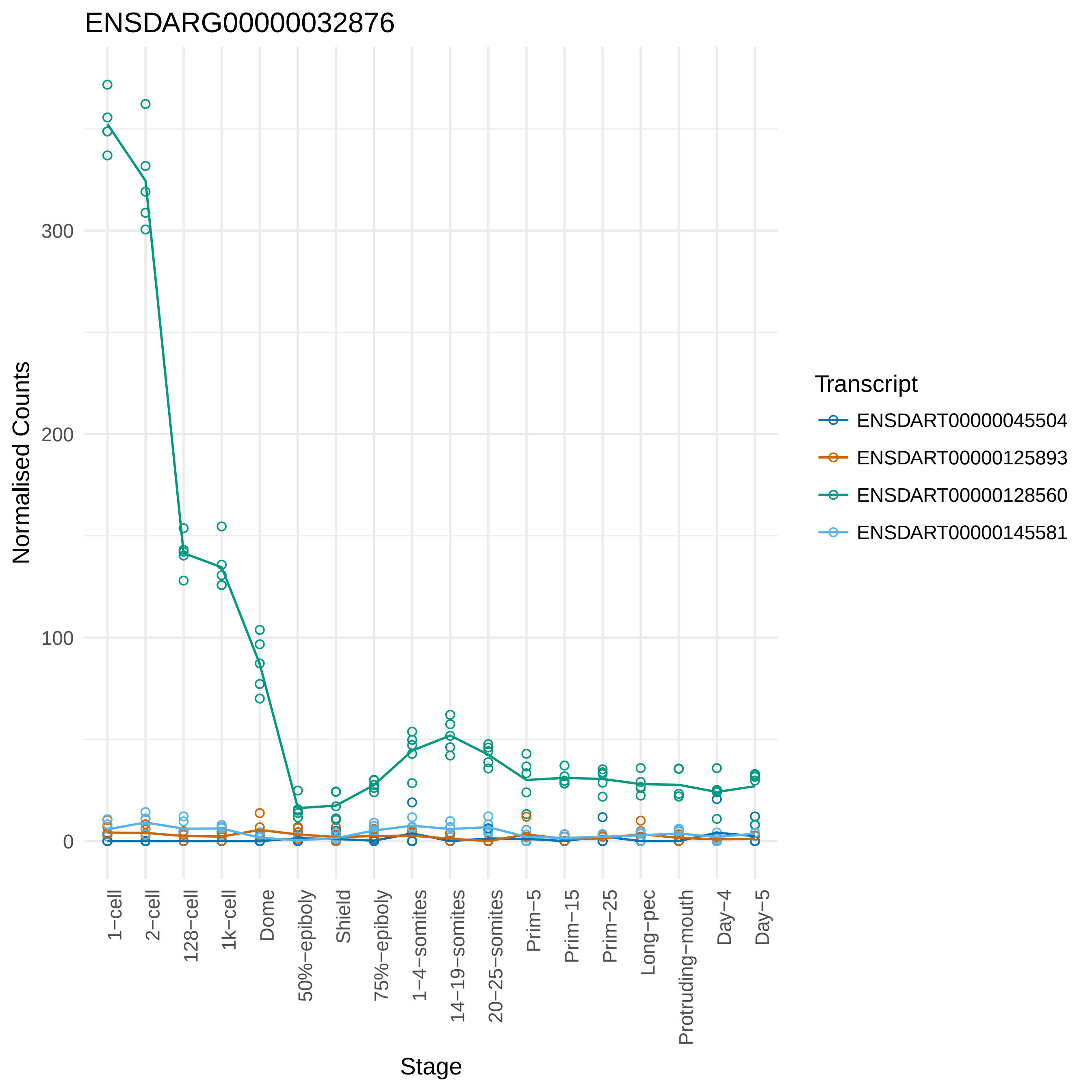
ENSDARG00000032876
Normalised Counts
300
200
100
0
1−cell
2−cell
128−cell
1k−cell
Dome
50%−epiboly
Shield
75%−epiboly
1−4−somites
14−19−somites
20−25−somites
Prim−5
Prim−15
Prim−25
Long−pec
Protruding−mouth
Day−4
Day−5
Stage
Transcript
ENSDART00000045504
ENSDART00000125893
ENSDART00000128560
ENSDART00000145581

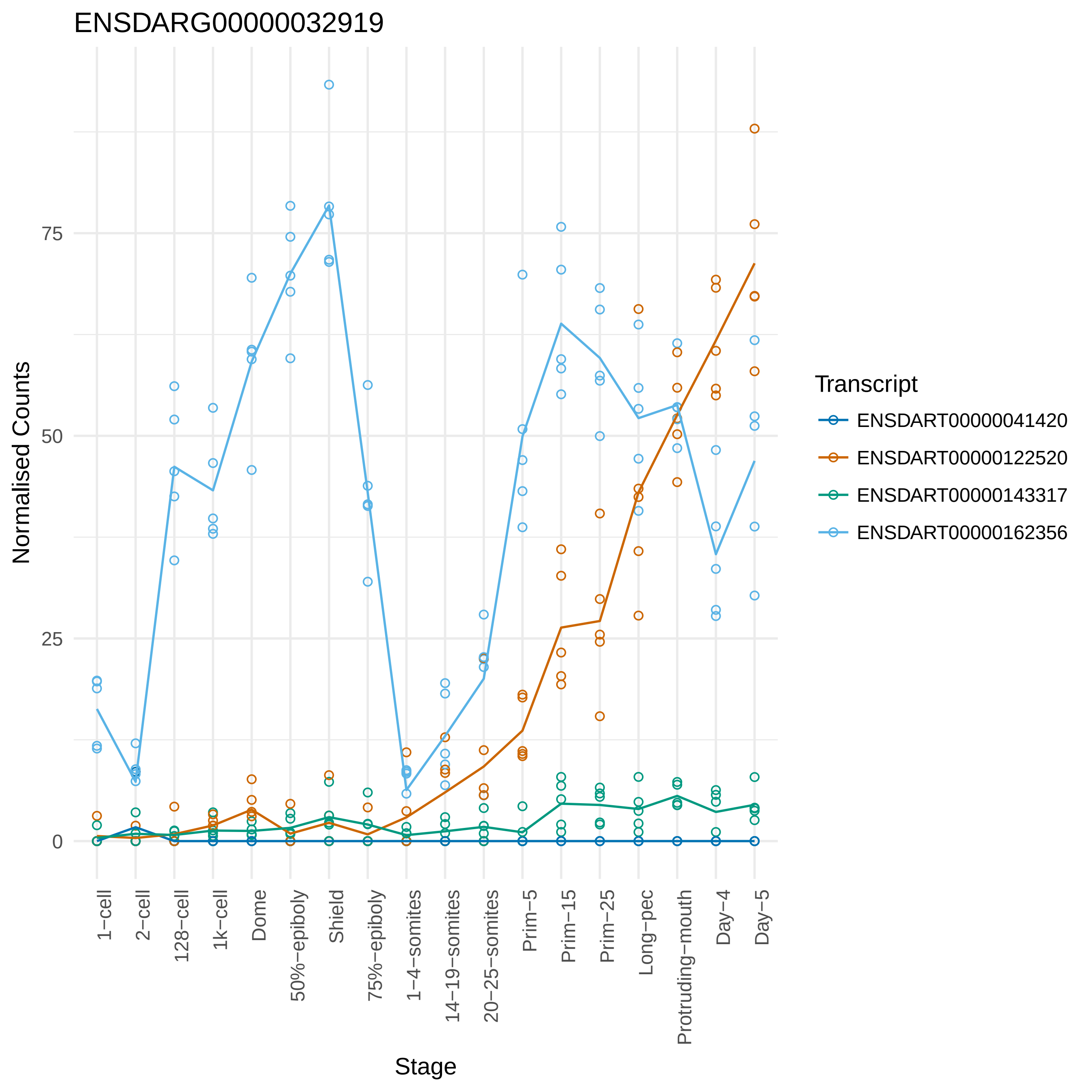

ENSDARG00000032919
Normalised Counts
0
25
50
75
1−cell
2−cell
128−cell
1k−cell
Dome
50%−epiboly
Shield
75%−epiboly
1−4−somites
14−19−somites
20−25−somites
Prim−5
Prim−15
Prim−25
Long−pec
Protruding−mouth
Day−4
Day−5
Stage
Transcript
ENSDART00000041420
ENSDART00000122520
ENSDART00000143317
ENSDART00000162356

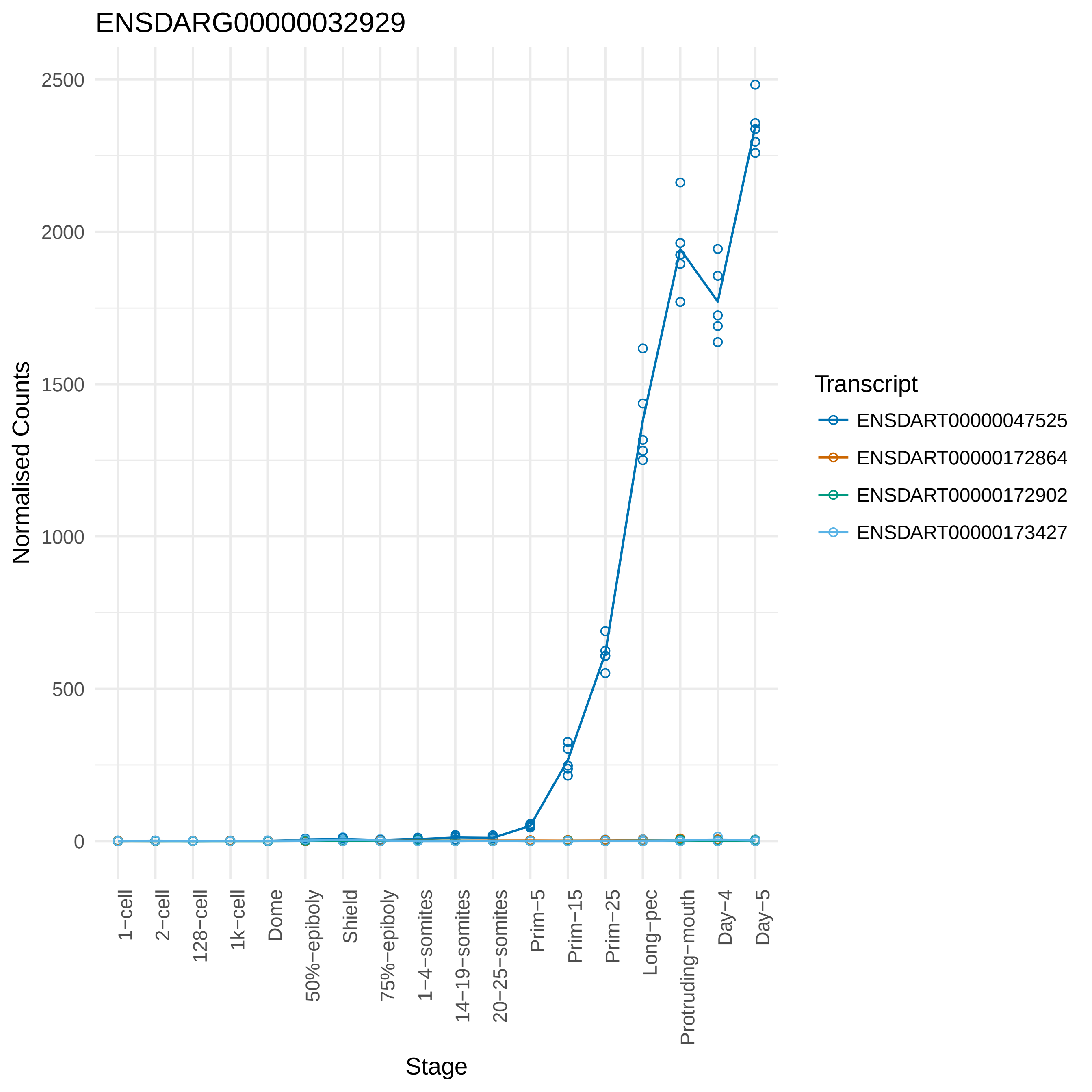
ENSDARG00000032929
Normalised Counts
2500
2000
1500
1000
500
0
Stage
1−cell
2−cell
128−cell
1k−cell
Dome
50%−epiboly
Shield
75%−epiboly
1−4−somites
14−19−somites
20−25−somites
Prim−5
Prim−15
Prim−25
Long−pec
Protruding−mouth
Day−4
Day−5
Transcript
ENSDART00000047525
ENSDART00000172864
ENSDART00000172902
ENSDART00000173427

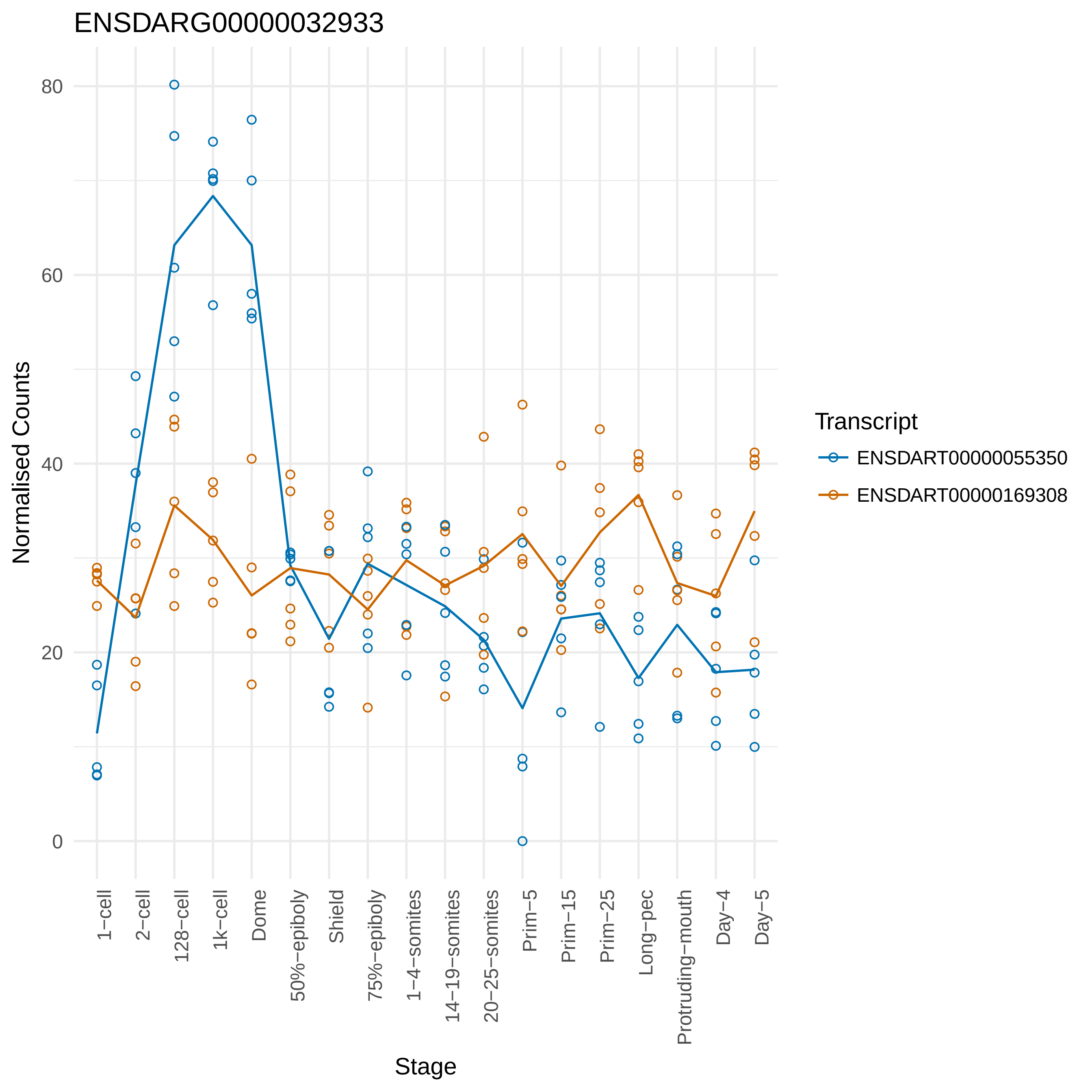
ENSDARG00000032933
Normalised Counts
0
20
40
60
80
Stage
1−cell
2−cell
128−cell
1k−cell
Dome
50%−epiboly
Shield
75%−epiboly
1−4−somites
14−19−somites
20−25−somites
Prim−5
Prim−15
Prim−25
Long−pec
Protruding−mouth
Day−4
Day−5
Transcript
ENSDART00000055350
ENSDART00000169308

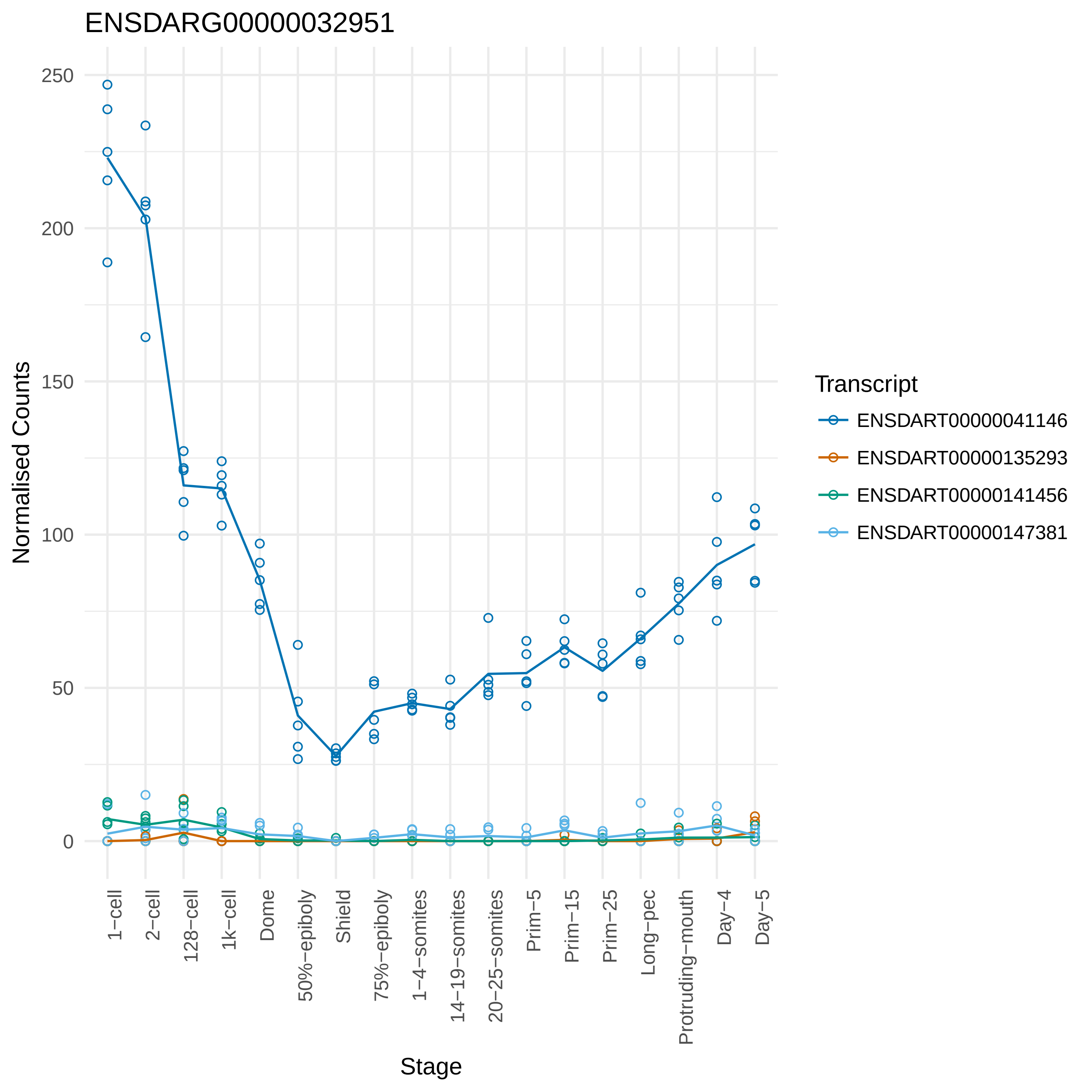

ENSDARG00000032951
Normalised Counts
250
200
150
100
50
0
1−cell
2−cell
128−cell
1k−cell
Dome
50%−epiboly
Shield
75%−epiboly
1−4−somites
14−19−somites
20−25−somites
Prim−5
Prim−15
Prim−25
Long−pec
Protruding−mouth
Day−4
Day−5
Stage
Transcript
ENSDART00000041146
ENSDART00000135293
ENSDART00000141456
ENSDART00000147381

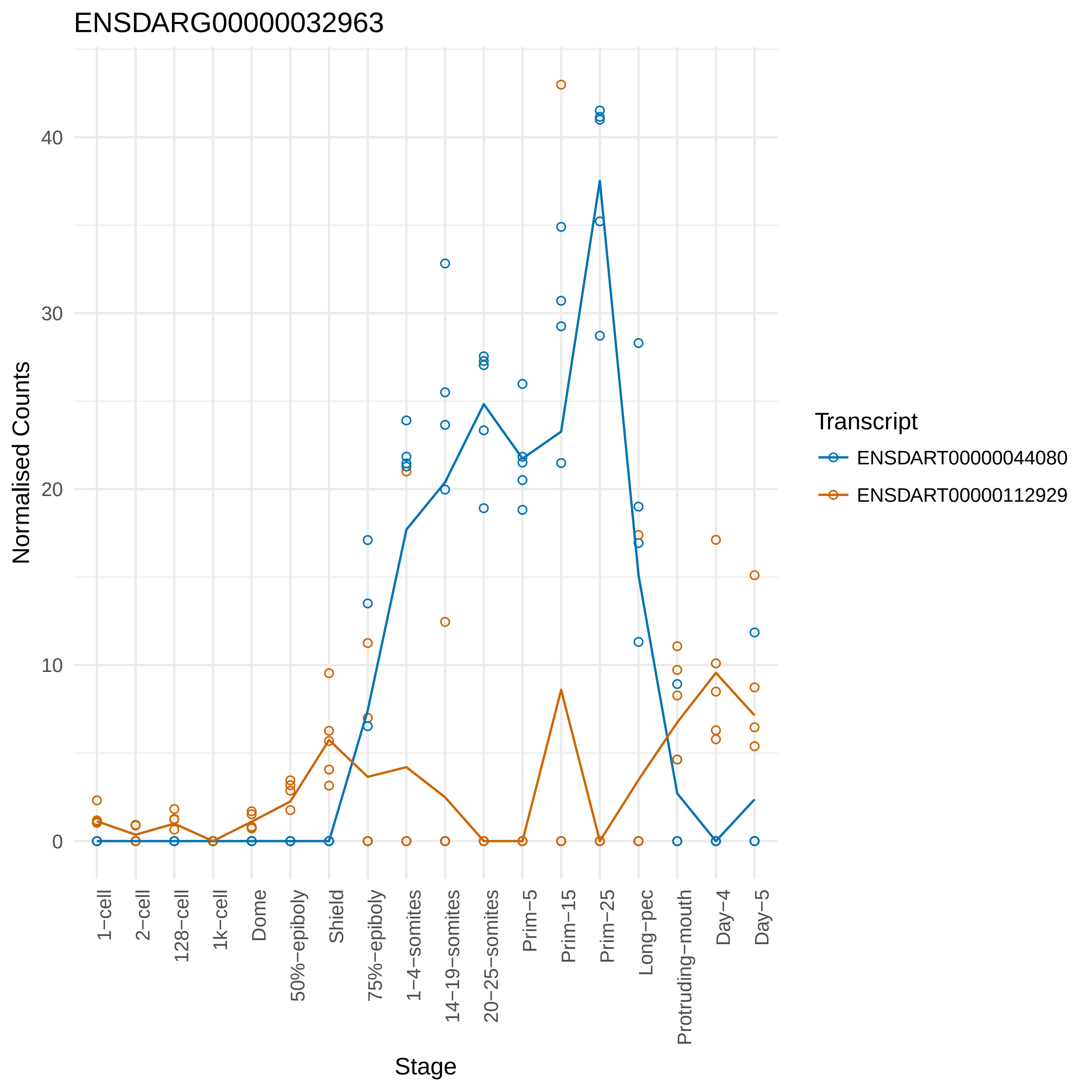

ENSDARG00000032963
Normalised Counts
0
10
20
30
40
Stage
1−cell
2−cell
128−cell
1k−cell
Dome
50%−epiboly
Shield
75%−epiboly
1−4−somites
14−19−somites
20−25−somites
Prim−5
Prim−15
Prim−25
Long−pec
Protruding−mouth
Day−4
Day−5
Transcript
ENSDART00000044080
ENSDART00000112929

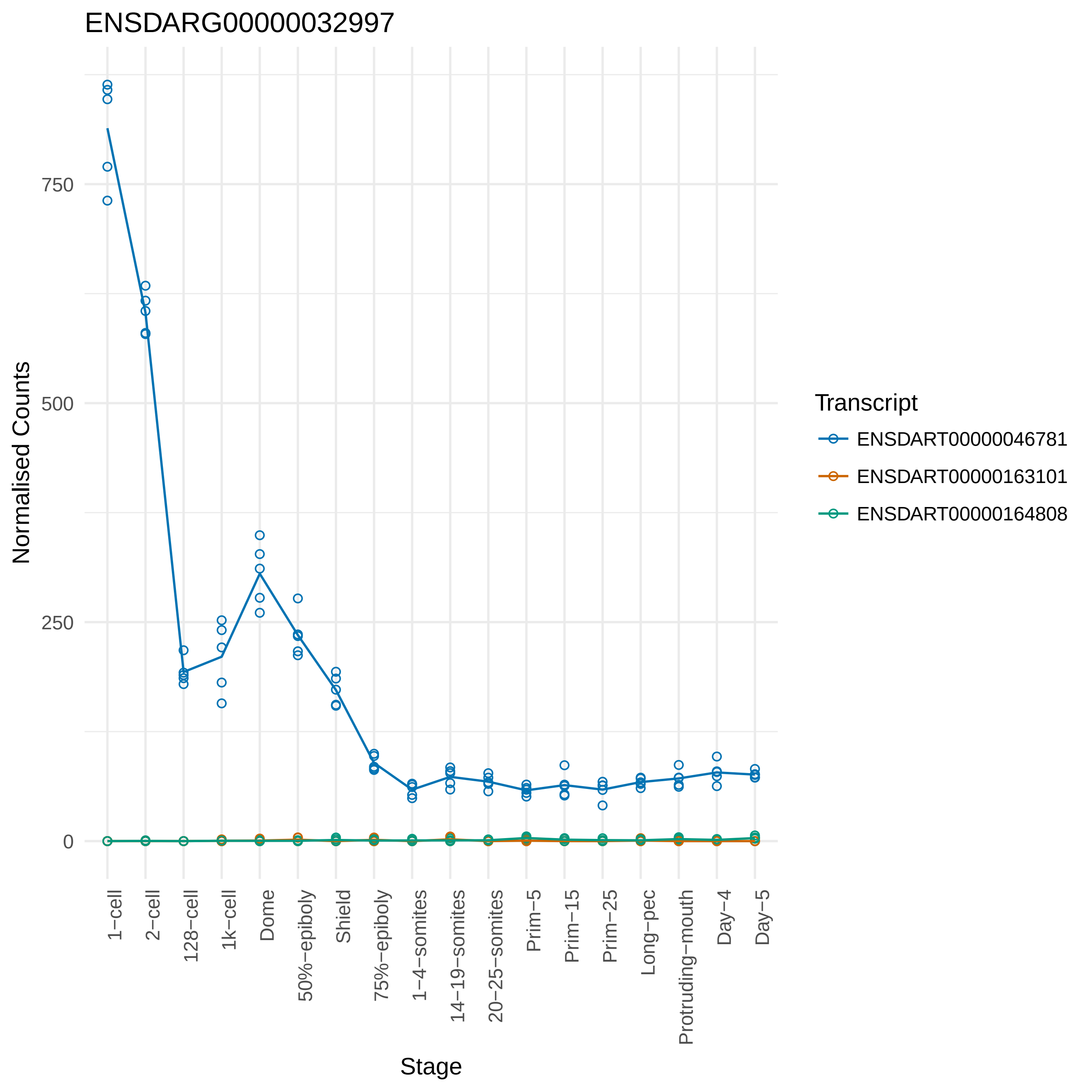

ENSDARG00000032997
Normalised Counts
750
500
250
0
1−cell
2−cell
128−cell
1k−cell
Dome
50%−epiboly
Shield
75%−epiboly
1−4−somites
14−19−somites
20−25−somites
Prim−5
Prim−15
Prim−25
Long−pec
Protruding−mouth
Day−4
Day−5
Stage
Transcript
ENSDART00000046781
ENSDART00000163101
ENSDART00000164808

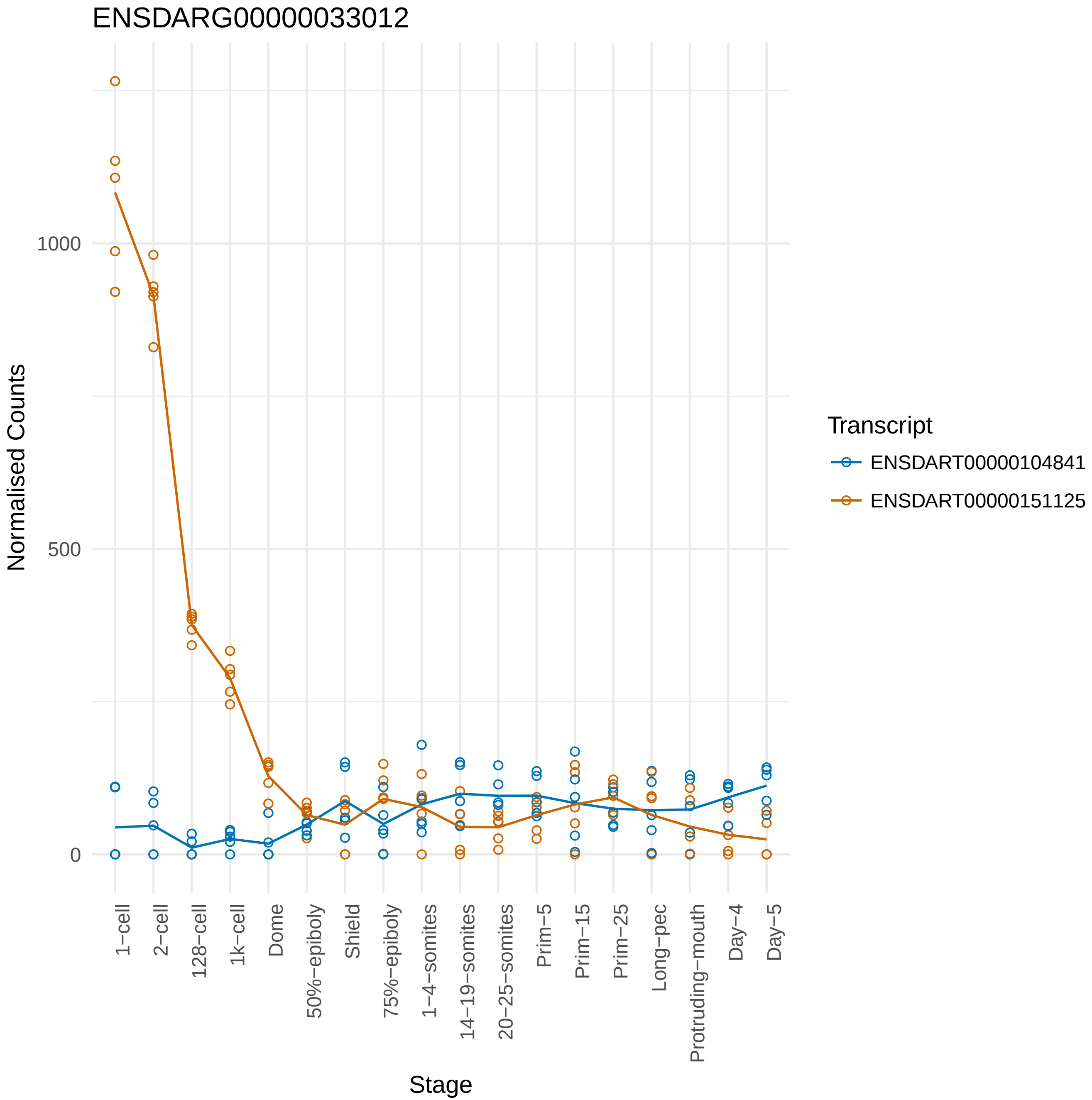
ENSDARG00000033012
Normalised Counts
1000
500
0
1−cell
2−cell
128−cell
1k−cell
Dome
50%−epiboly
Shield
75%−epiboly
1−4−somites
14−19−somites
20−25−somites
Prim−5
Prim−15
Prim−25
Long−pec
Protruding−mouth
Day−4
Day−5
Stage
Transcript
ENSDART00000104841
ENSDART00000151125

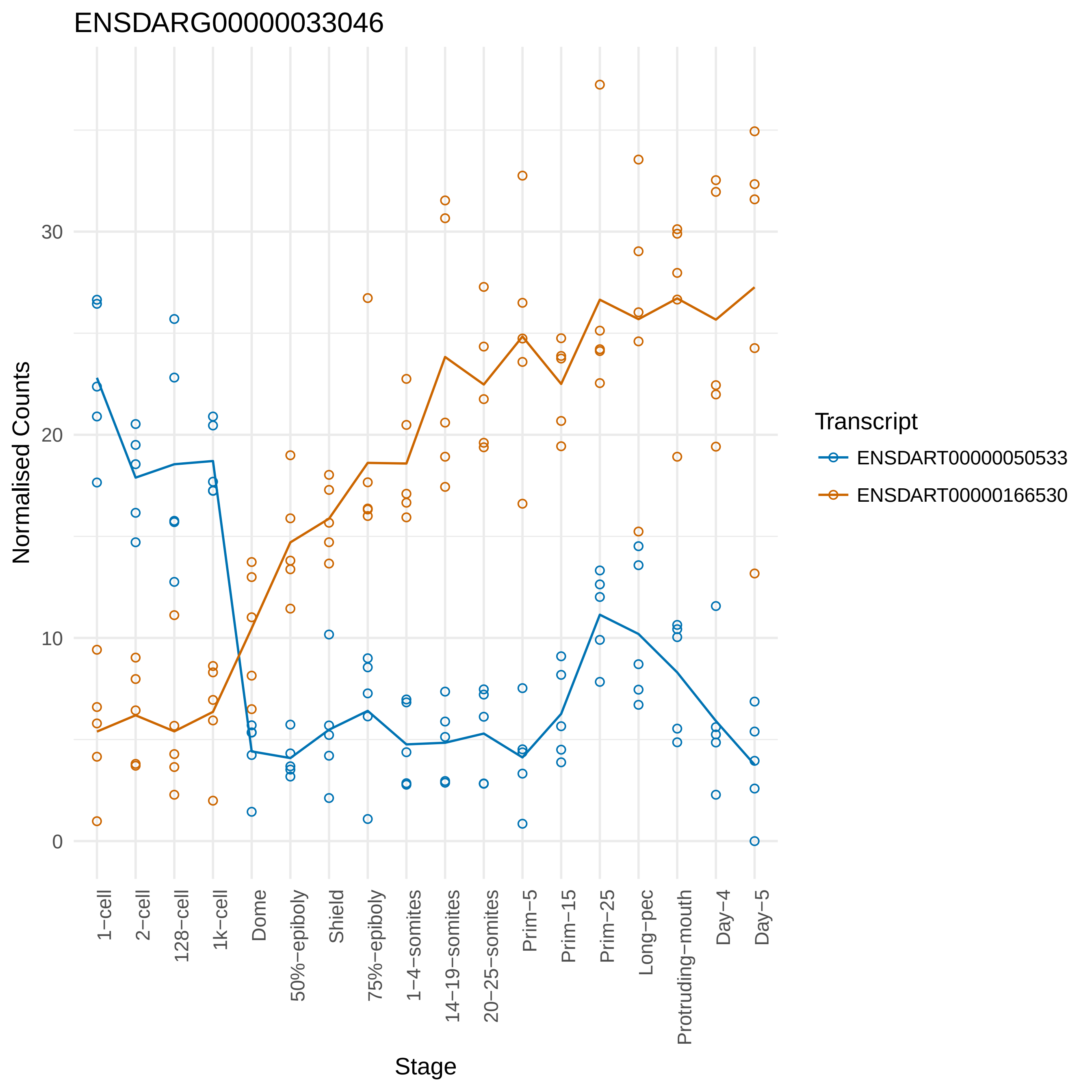

ENSDARG00000033046
Normalised Counts
0
10
20
30
1−cell
2−cell
128−cell
1k−cell
Dome
50%−epiboly
Shield
75%−epiboly
1−4−somites
14−19−somites
20−25−somites
Prim−5
Prim−15
Prim−25
Long−pec
Protruding−mouth
Day−4
Day−5
Stage
Transcript
ENSDART00000050533
ENSDART00000166530

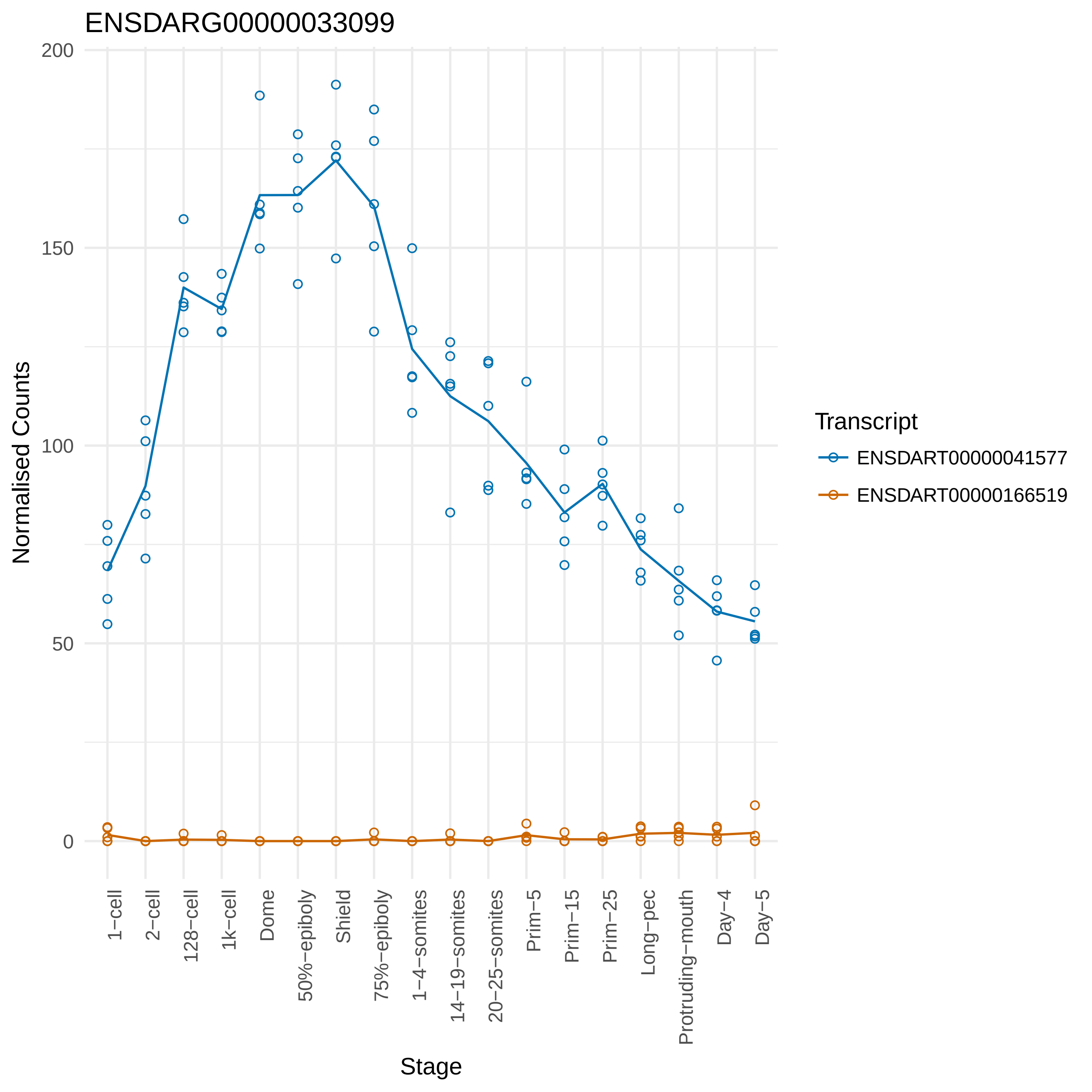
ENSDARG00000033099
Normalised Counts
200
150
100
50
0
1−cell
2−cell
128−cell
1k−cell
Dome
50%−epiboly
Shield
75%−epiboly
1−4−somites
14−19−somites
20−25−somites
Prim−5
Prim−15
Prim−25
Long−pec
Protruding−mouth
Day−4
Day−5
Stage
Transcript
ENSDART00000041577
ENSDART00000166519

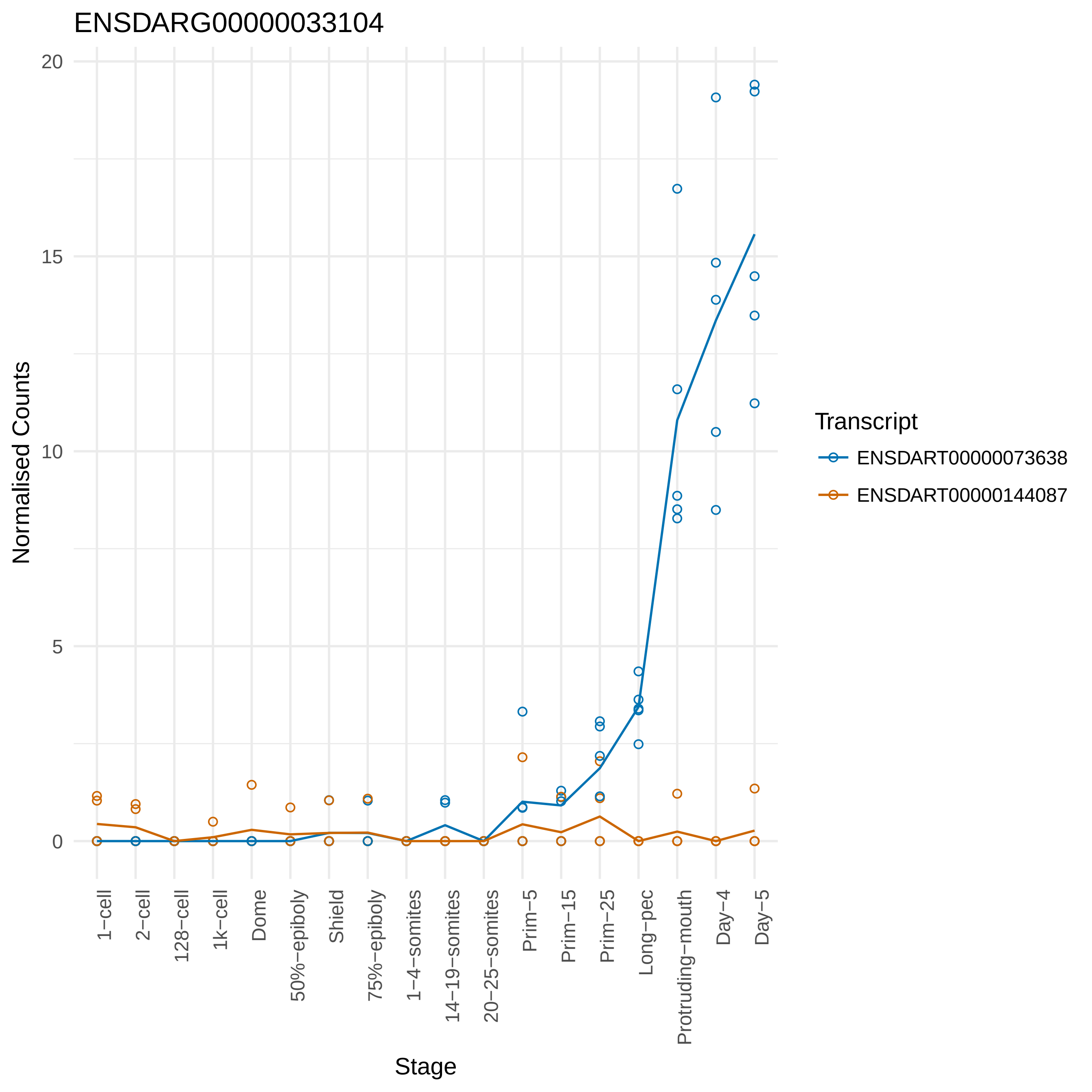
ENSDARG00000033104
Normalised Counts
20
15
10
5
0
Stage
1−cell
2−cell
128−cell
1k−cell
Dome
50%−epiboly
Shield
75%−epiboly
1−4−somites
14−19−somites
20−25−somites
Prim−5
Prim−15
Prim−25
Long−pec
Protruding−mouth
Day−4
Day−5
Transcript
ENSDART00000073638
ENSDART00000144087

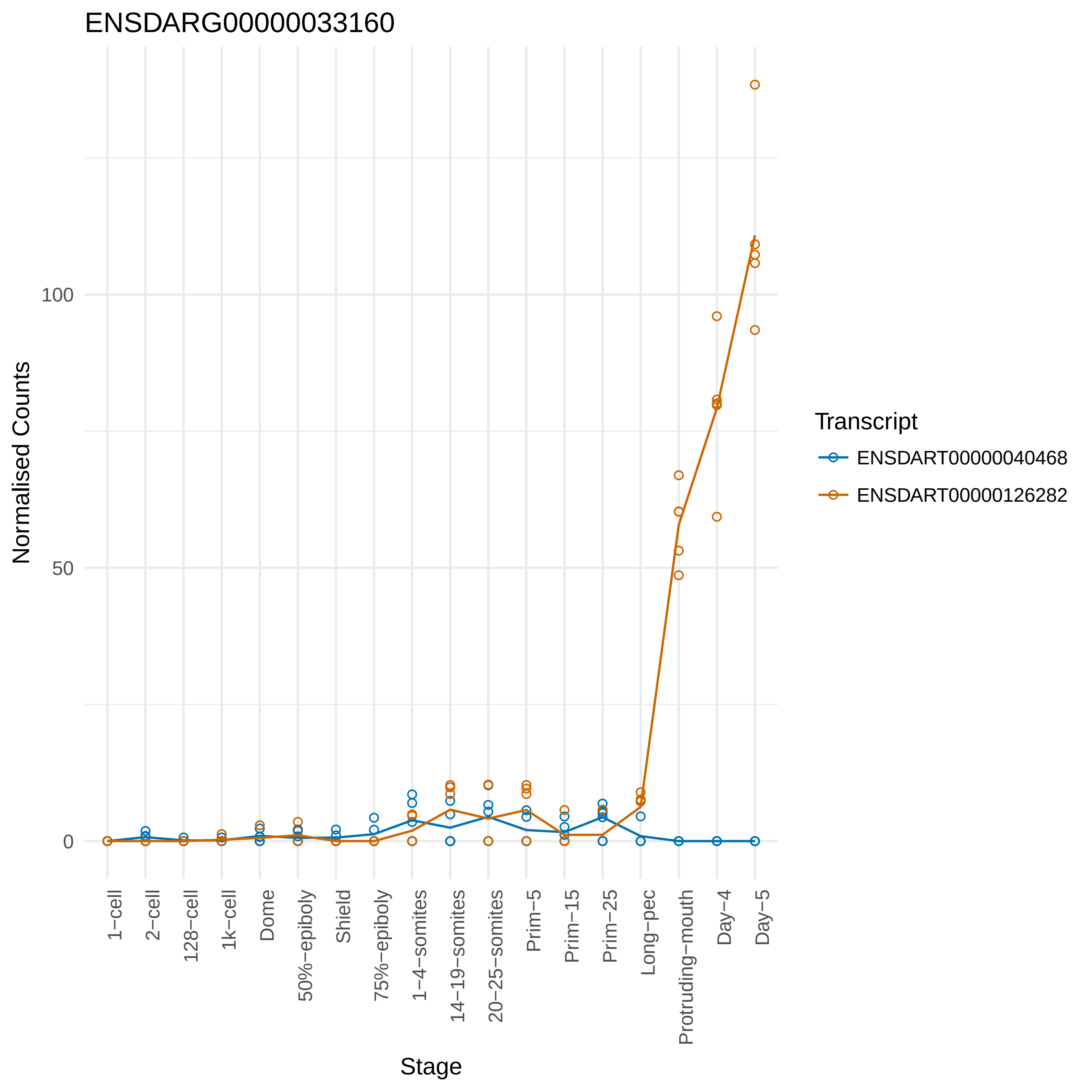

ENSDARG00000033160
Normalised Counts
100
50
0
1−cell
2−cell
128−cell
1k−cell
Dome
50%−epiboly
Shield
75%−epiboly
1−4−somites
14−19−somites
20−25−somites
Prim−5
Prim−15
Prim−25
Long−pec
Protruding−mouth
Day−4
Day−5
Stage
Transcript
ENSDART00000040468
ENSDART00000126282

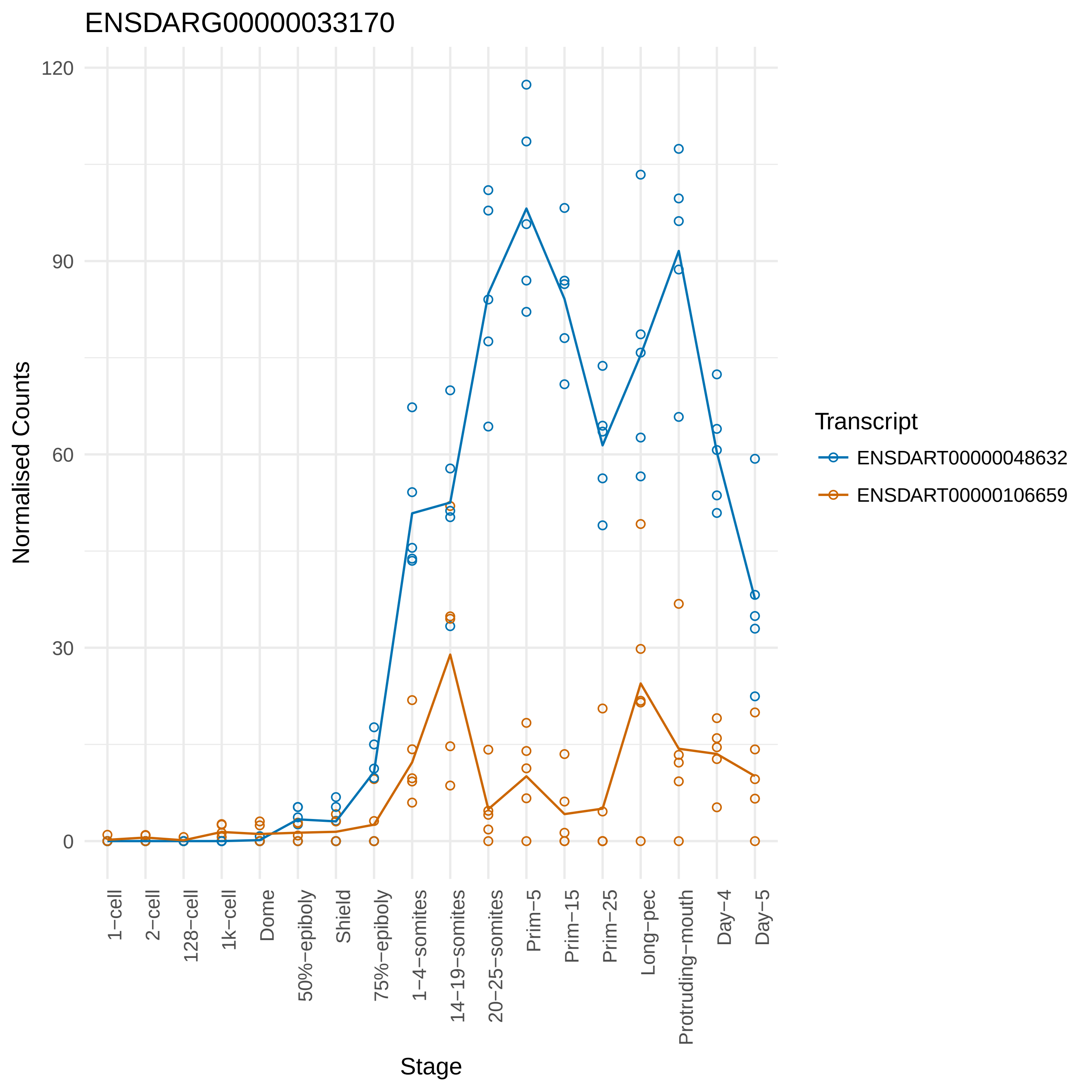

ENSDARG00000033170
Normalised Counts
120
90
60
30
0
1−cell
2−cell
128−cell
1k−cell
Dome
50%−epiboly
Shield
75%−epiboly
1−4−somites
14−19−somites
20−25−somites
Prim−5
Prim−15
Prim−25
Long−pec
Protruding−mouth
Day−4
Day−5
Stage
Transcript
ENSDART00000048632
ENSDART00000106659

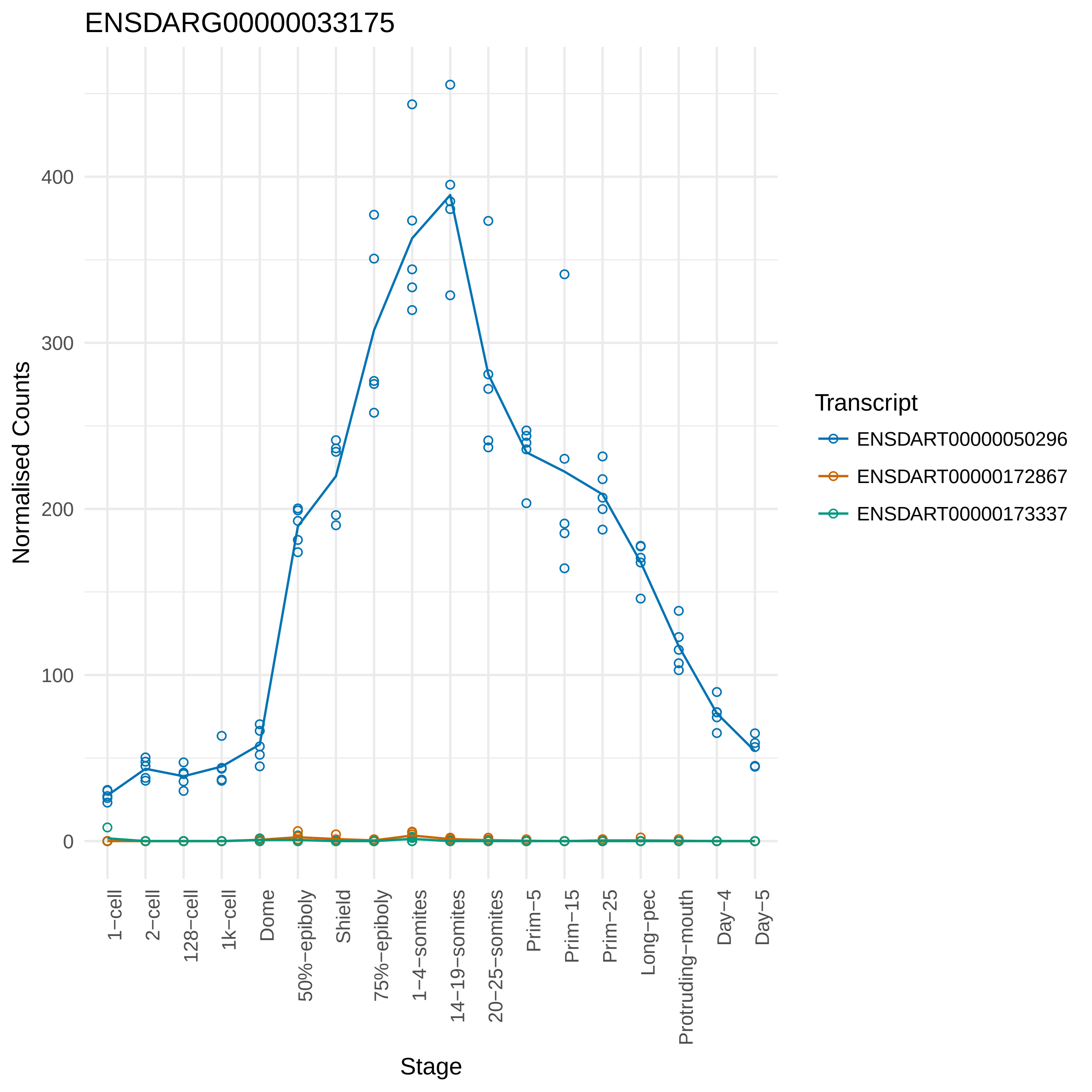
ENSDARG00000033175
Normalised Counts
400
300
200
100
0
1−cell
2−cell
128−cell
1k−cell
Dome
50%−epiboly
Shield
75%−epiboly
1−4−somites
14−19−somites
20−25−somites
Prim−5
Prim−15
Prim−25
Long−pec
Protruding−mouth
Day−4
Day−5
Stage
Transcript
ENSDART00000050296
ENSDART00000172867
ENSDART00000173337

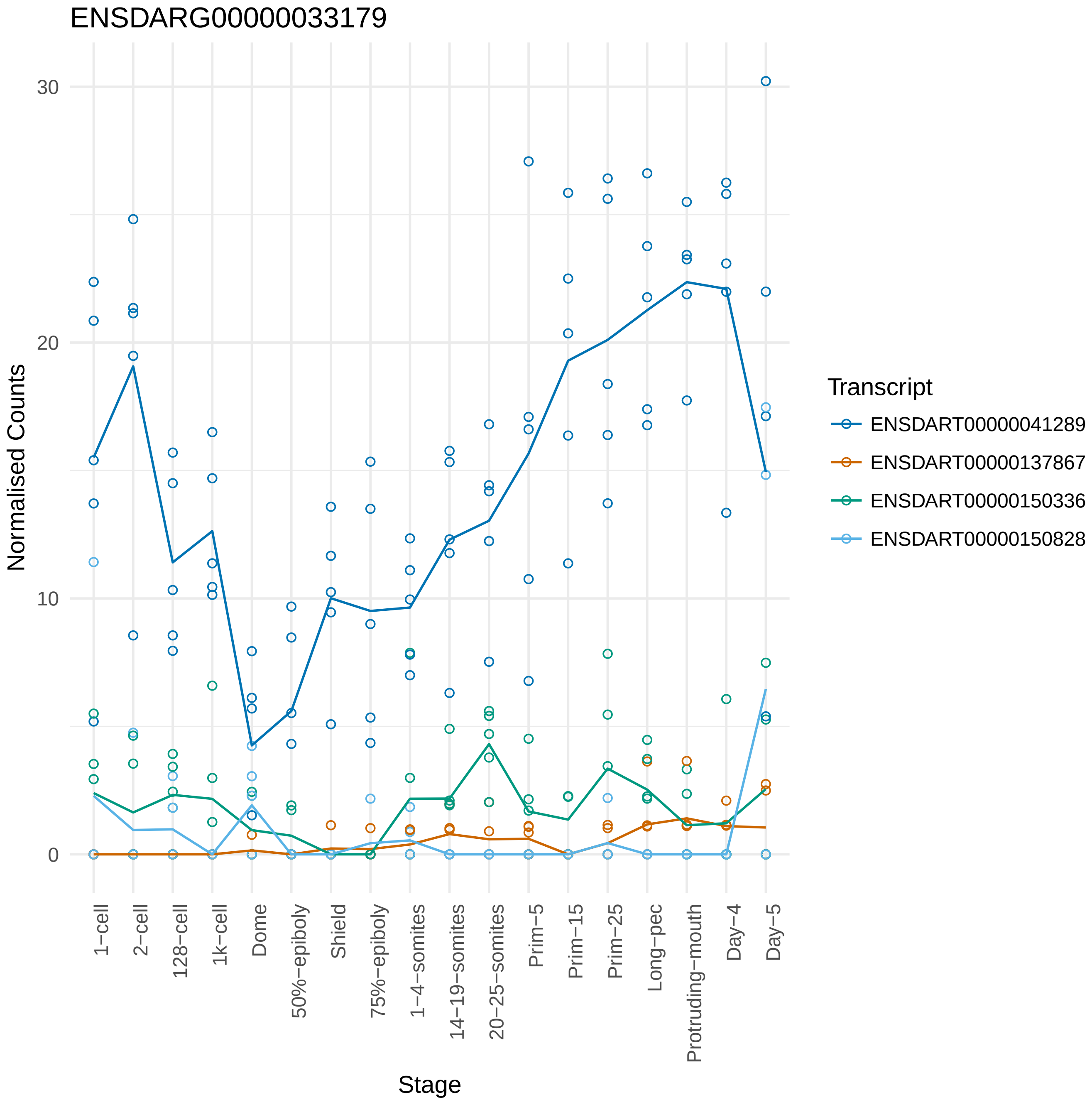

ENSDARG00000033179
Normalised Counts
30
20
10
0
1−cell
2−cell
128−cell
1k−cell
Dome
50%−epiboly
Shield
75%−epiboly
1−4−somites
14−19−somites
20−25−somites
Prim−5
Prim−15
Prim−25
Long−pec
Protruding−mouth
Day−4
Day−5
Stage
Transcript
ENSDART00000041289
ENSDART00000137867
ENSDART00000150336
ENSDART00000150828

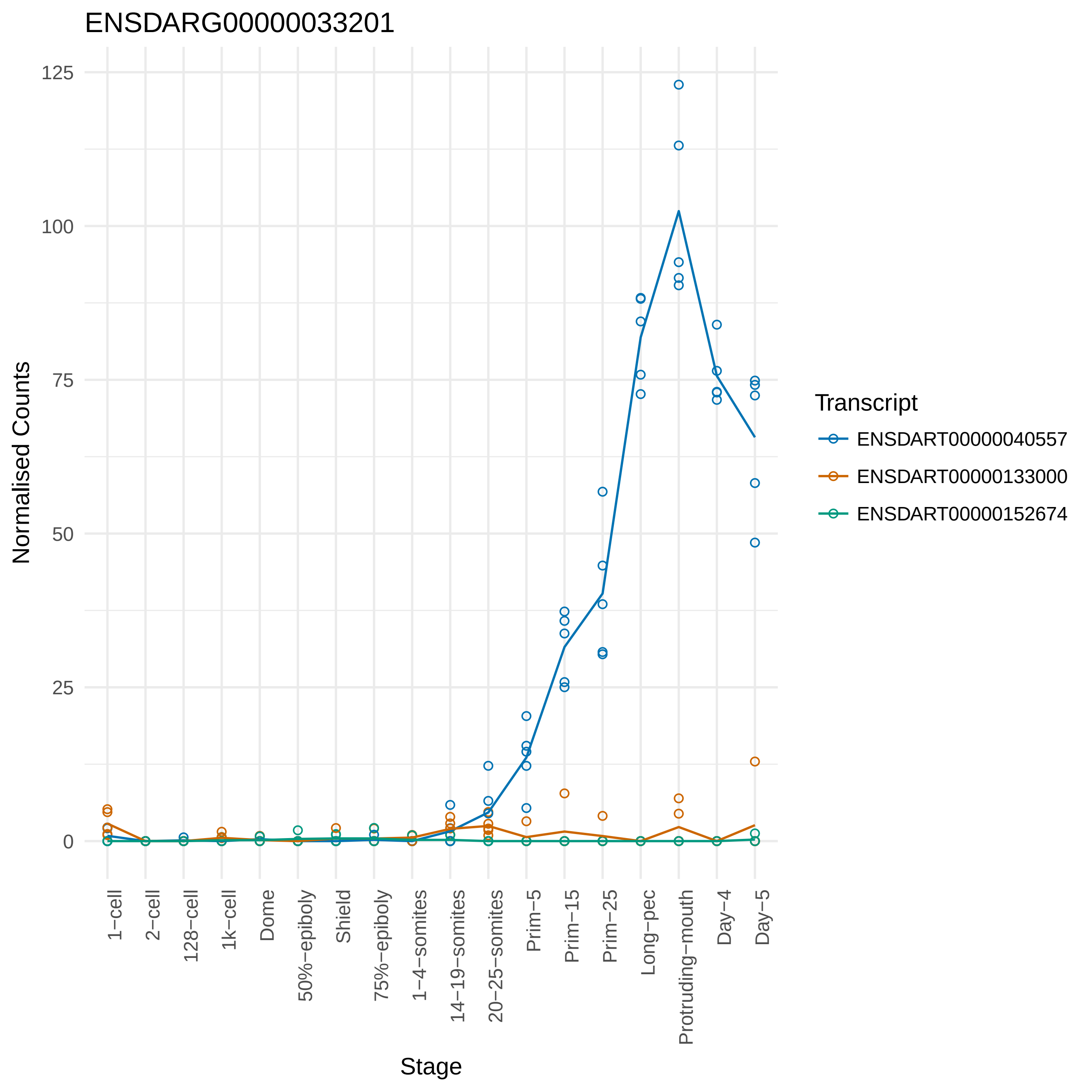
ENSDARG00000033201
Normalised Counts
125
100
75
50
25
0
1−cell
2−cell
128−cell
1k−cell
Dome
50%−epiboly
Shield
75%−epiboly
1−4−somites
14−19−somites
20−25−somites
Prim−5
Prim−15
Prim−25
Long−pec
Protruding−mouth
Day−4
Day−5
Stage
Transcript
ENSDART00000040557
ENSDART00000133000
ENSDART00000152674

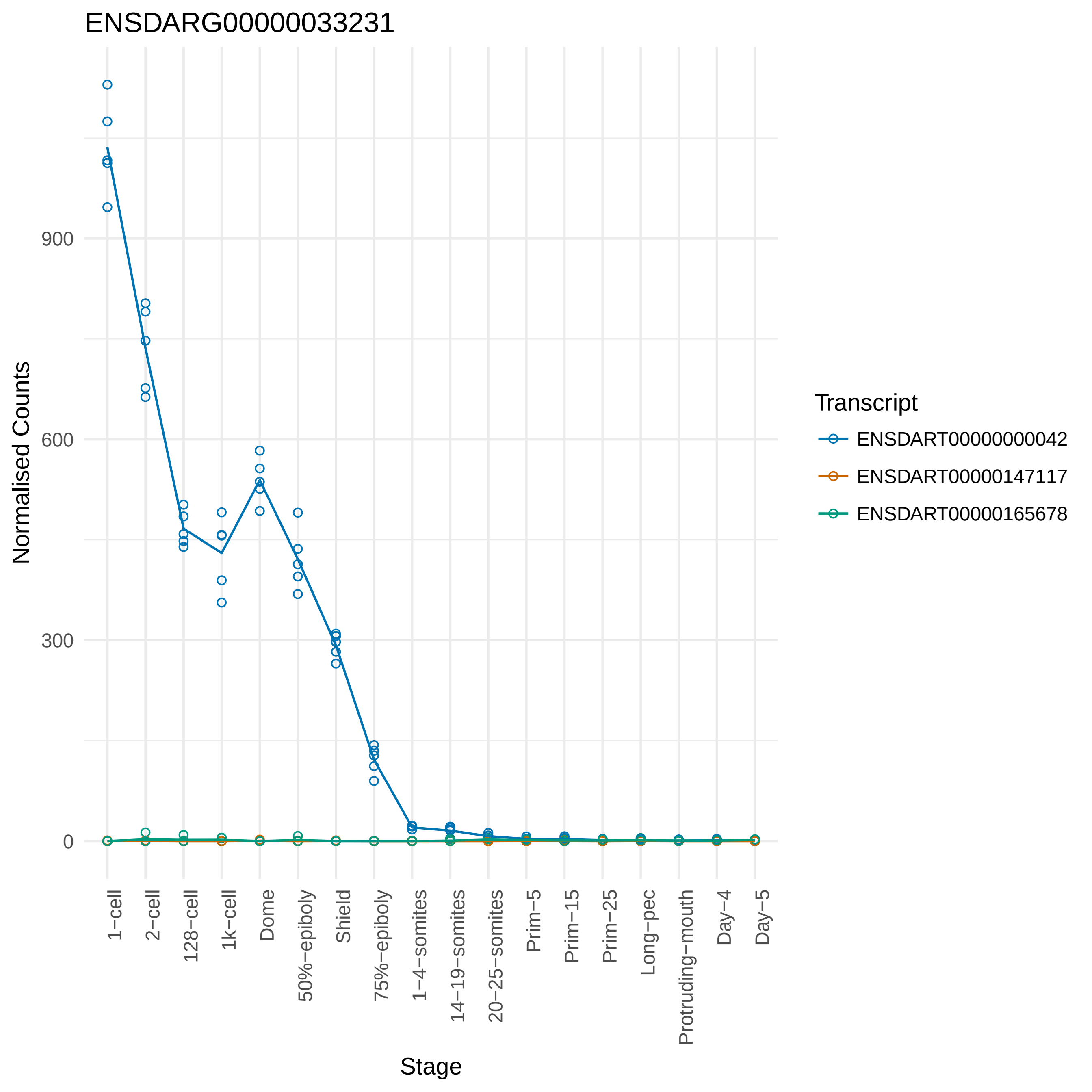

ENSDARG00000033231
Normalised Counts
900
600
300
0
1−cell
2−cell
128−cell
1k−cell
Dome
50%−epiboly
Shield
75%−epiboly
1−4−somites
14−19−somites
20−25−somites
Prim−5
Prim−15
Prim−25
Long−pec
Protruding−mouth
Day−4
Day−5
Stage
Transcript
ENSDART00000000042
ENSDART00000147117
ENSDART00000165678

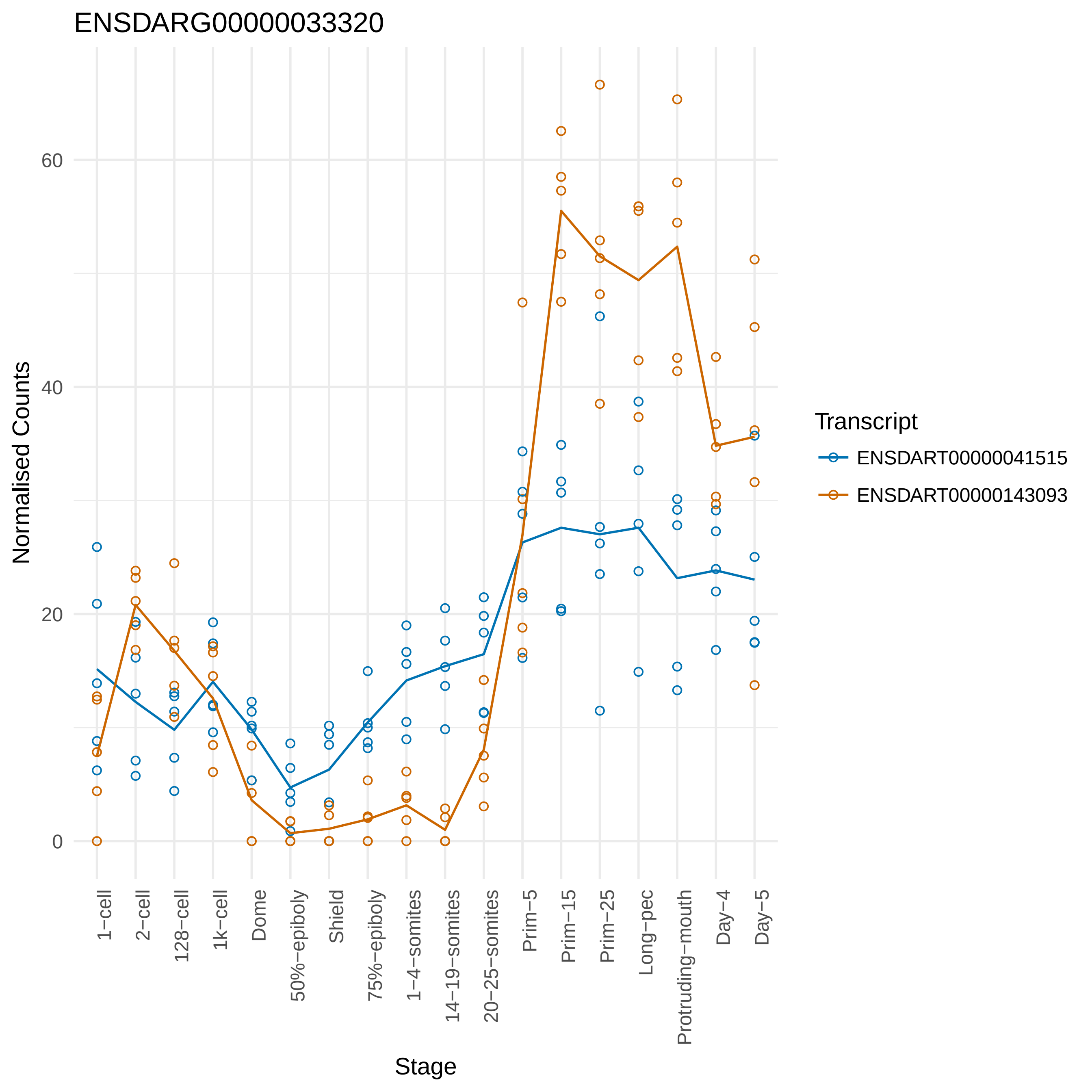

ENSDARG00000033320
Normalised Counts
60
40
20
0
1−cell
2−cell
128−cell
1k−cell
Dome
50%−epiboly
Shield
75%−epiboly
1−4−somites
14−19−somites
20−25−somites
Prim−5
Prim−15
Prim−25
Long−pec
Protruding−mouth
Day−4
Day−5
Stage
Transcript
ENSDART00000041515
ENSDART00000143093

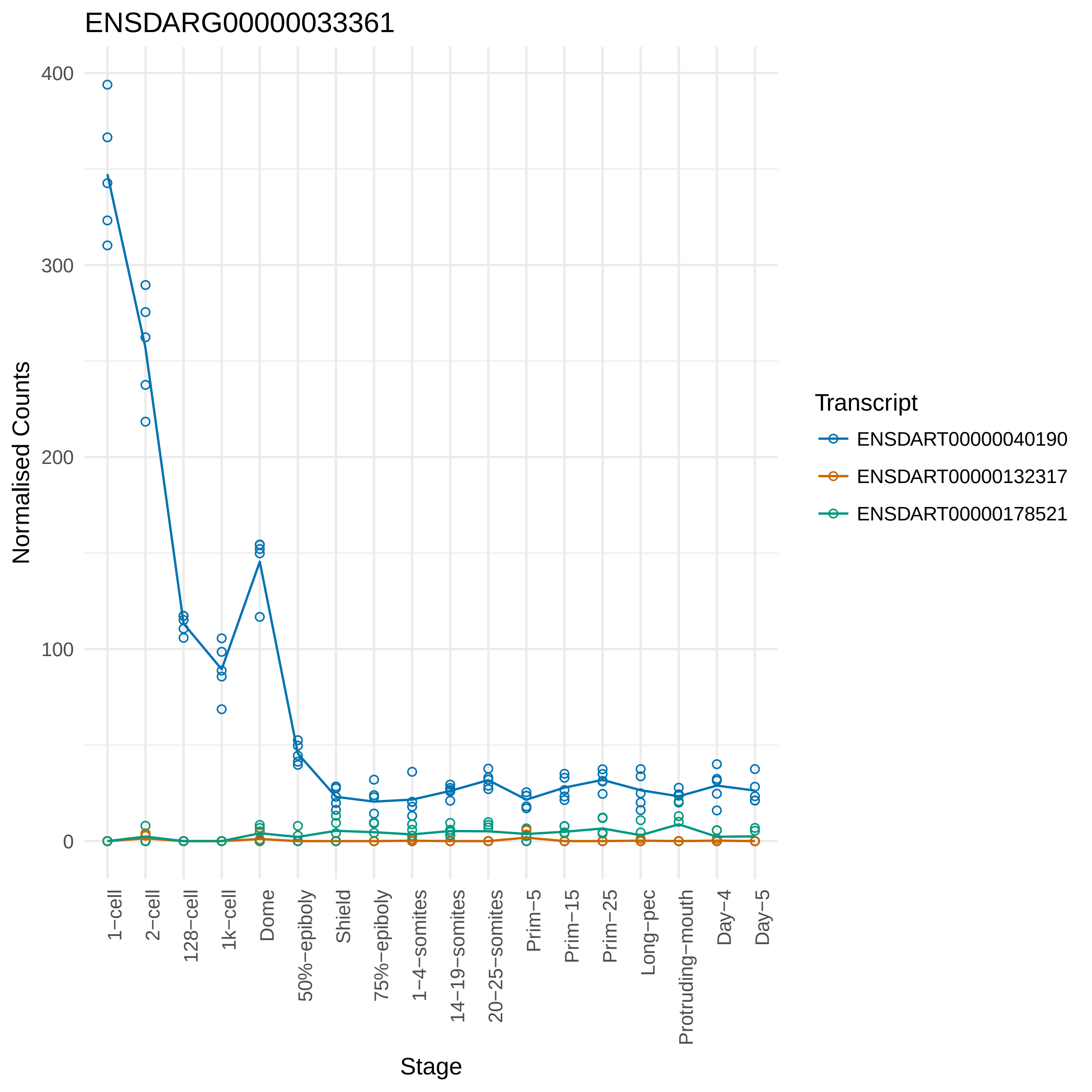

ENSDARG00000033361
Normalised Counts
400
300
200
100
0
1−cell
2−cell
128−cell
1k−cell
Dome
50%−epiboly
Shield
75%−epiboly
1−4−somites
14−19−somites
20−25−somites
Prim−5
Prim−15
Prim−25
Long−pec
Protruding−mouth
Day−4
Day−5
Stage
Transcript
ENSDART00000040190
ENSDART00000132317
ENSDART00000178521

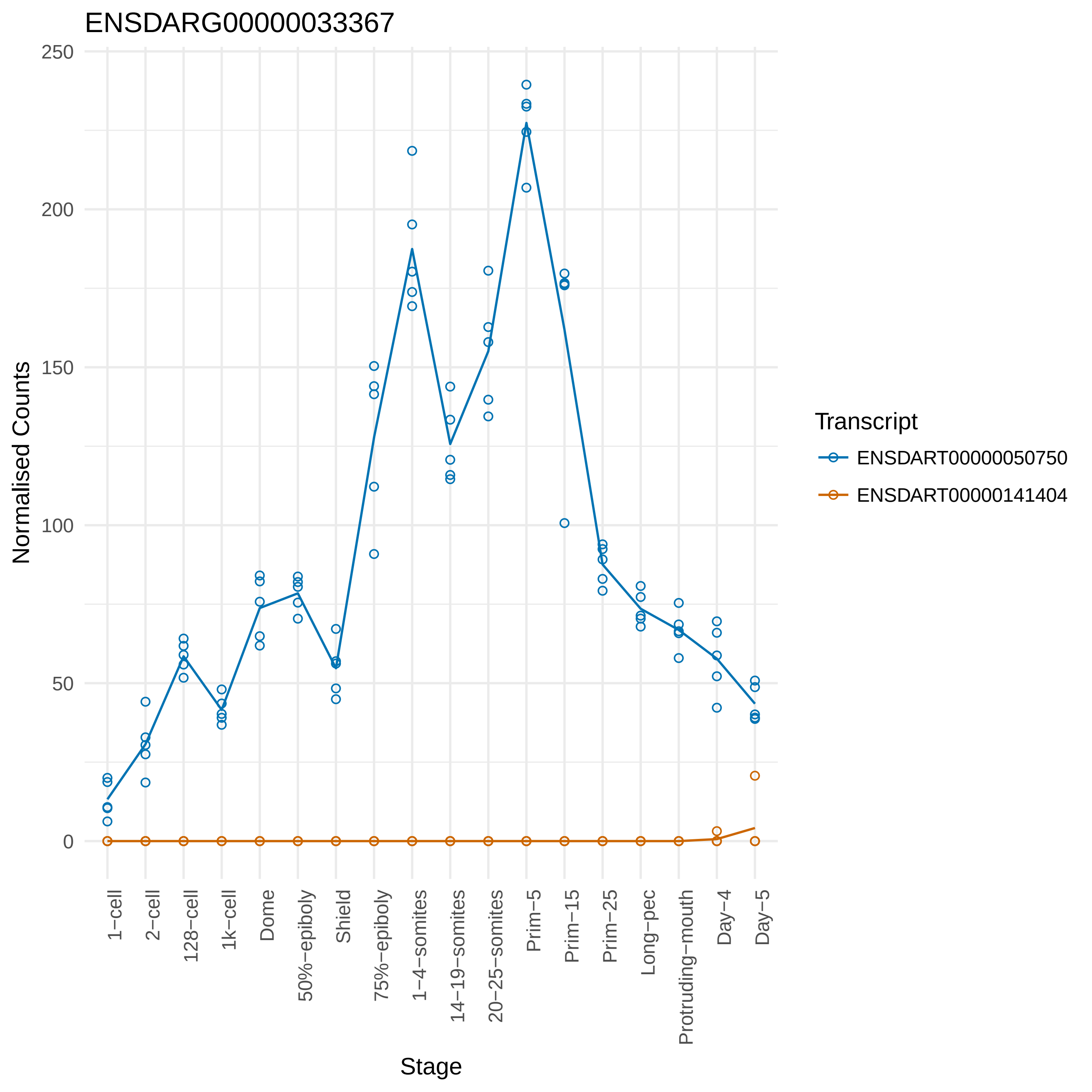

ENSDARG00000033367
Normalised Counts
250
200
150
100
50
0
1−cell
2−cell
128−cell
1k−cell
Dome
50%−epiboly
Shield
75%−epiboly
1−4−somites
14−19−somites
20−25−somites
Prim−5
Prim−15
Prim−25
Long−pec
Protruding−mouth
Day−4
Day−5
Stage
Transcript
ENSDART00000050750
ENSDART00000141404

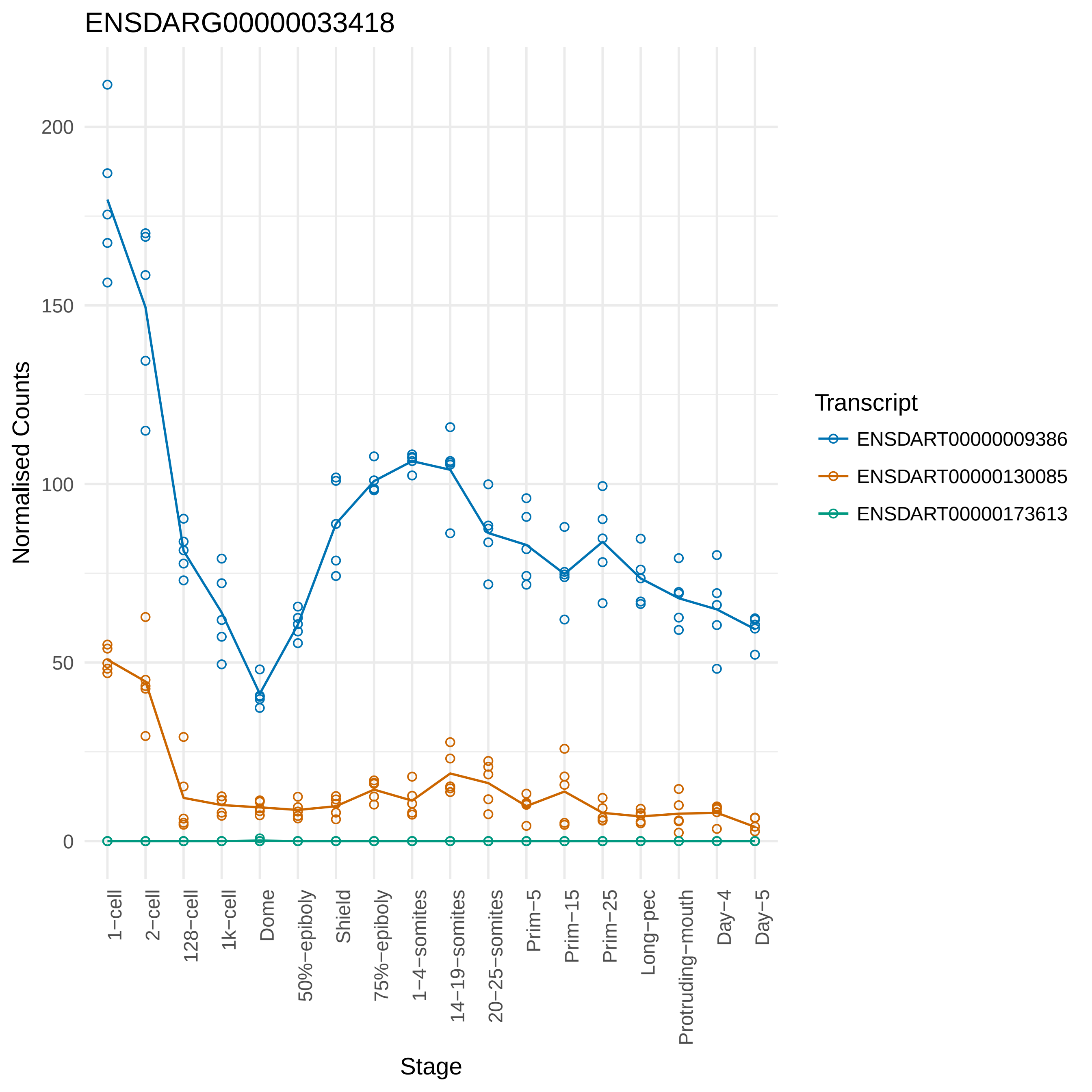

ENSDARG00000033418
Normalised Counts
200
150
100
50
0
1−cell
2−cell
128−cell
1k−cell
Dome
50%−epiboly
Shield
75%−epiboly
1−4−somites
14−19−somites
20−25−somites
Prim−5
Prim−15
Prim−25
Long−pec
Protruding−mouth
Day−4
Day−5
Stage
Transcript
ENSDART00000009386
ENSDART00000130085
ENSDART00000173613

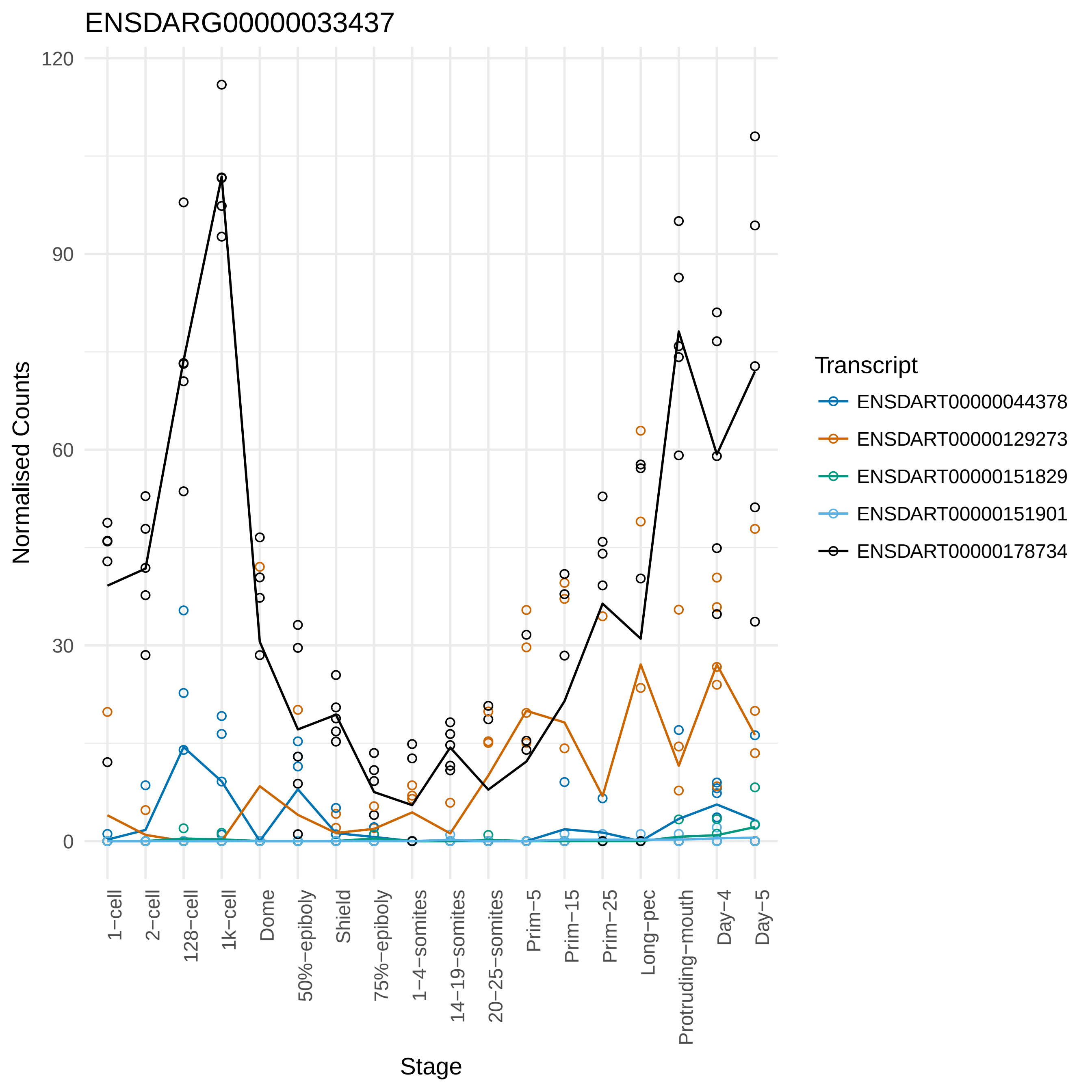
ENSDARG00000033437
Normalised Counts
120
90
60
30
0
1−cell
2−cell
128−cell
1k−cell
Dome
50%−epiboly
Shield
75%−epiboly
1−4−somites
14−19−somites
20−25−somites
Prim−5
Prim−15
Prim−25
Long−pec
Protruding−mouth
Day−4
Day−5
Stage
Transcript
ENSDART00000044378
ENSDART00000129273
ENSDART00000151829
ENSDART00000151901
ENSDART00000178734

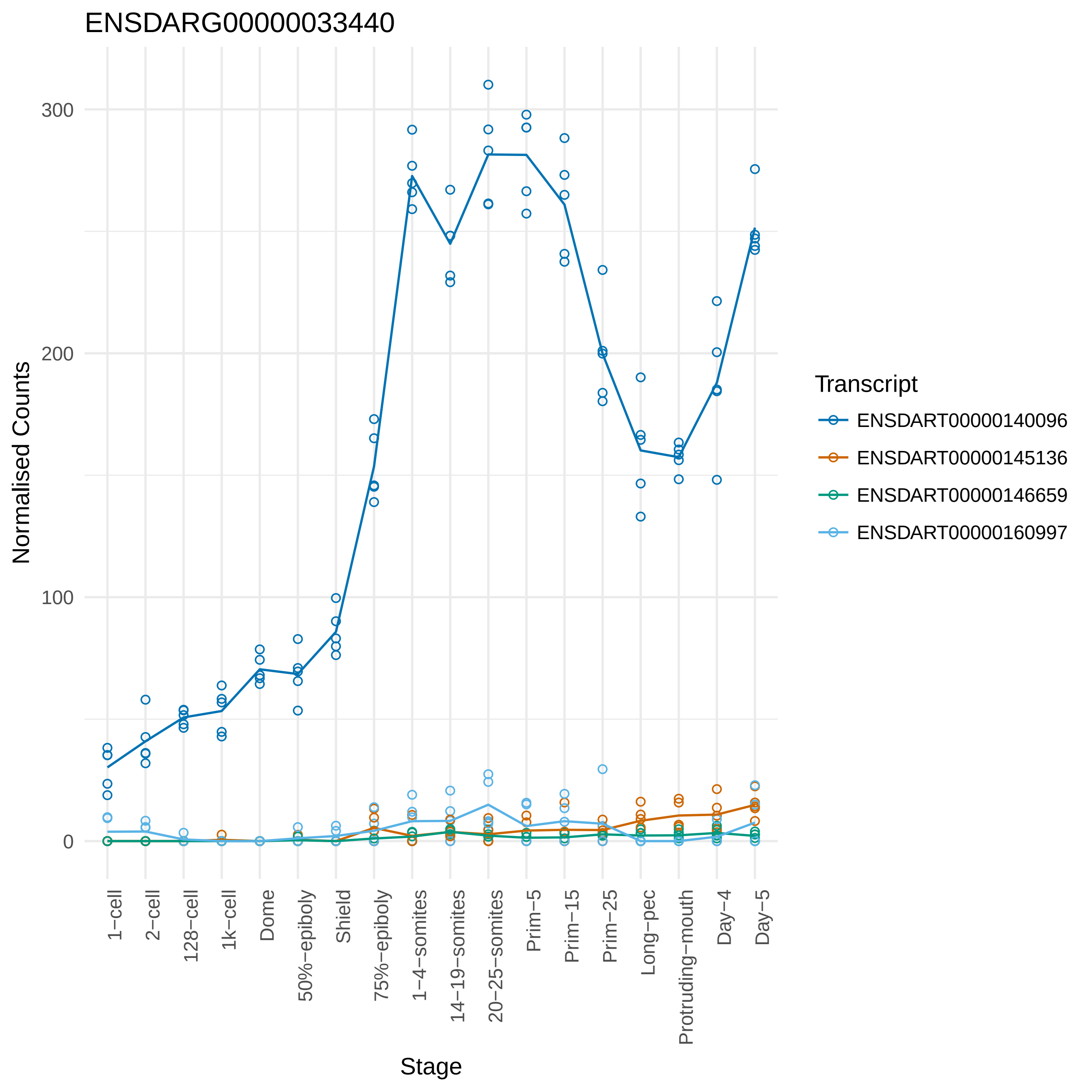

ENSDARG00000033440
Normalised Counts
300
200
100
0
1−cell
2−cell
128−cell
1k−cell
Dome
50%−epiboly
Shield
75%−epiboly
1−4−somites
14−19−somites
20−25−somites
Prim−5
Prim−15
Prim−25
Long−pec
Protruding−mouth
Day−4
Day−5
Stage
Transcript
ENSDART00000140096
ENSDART00000145136
ENSDART00000146659
ENSDART00000160997

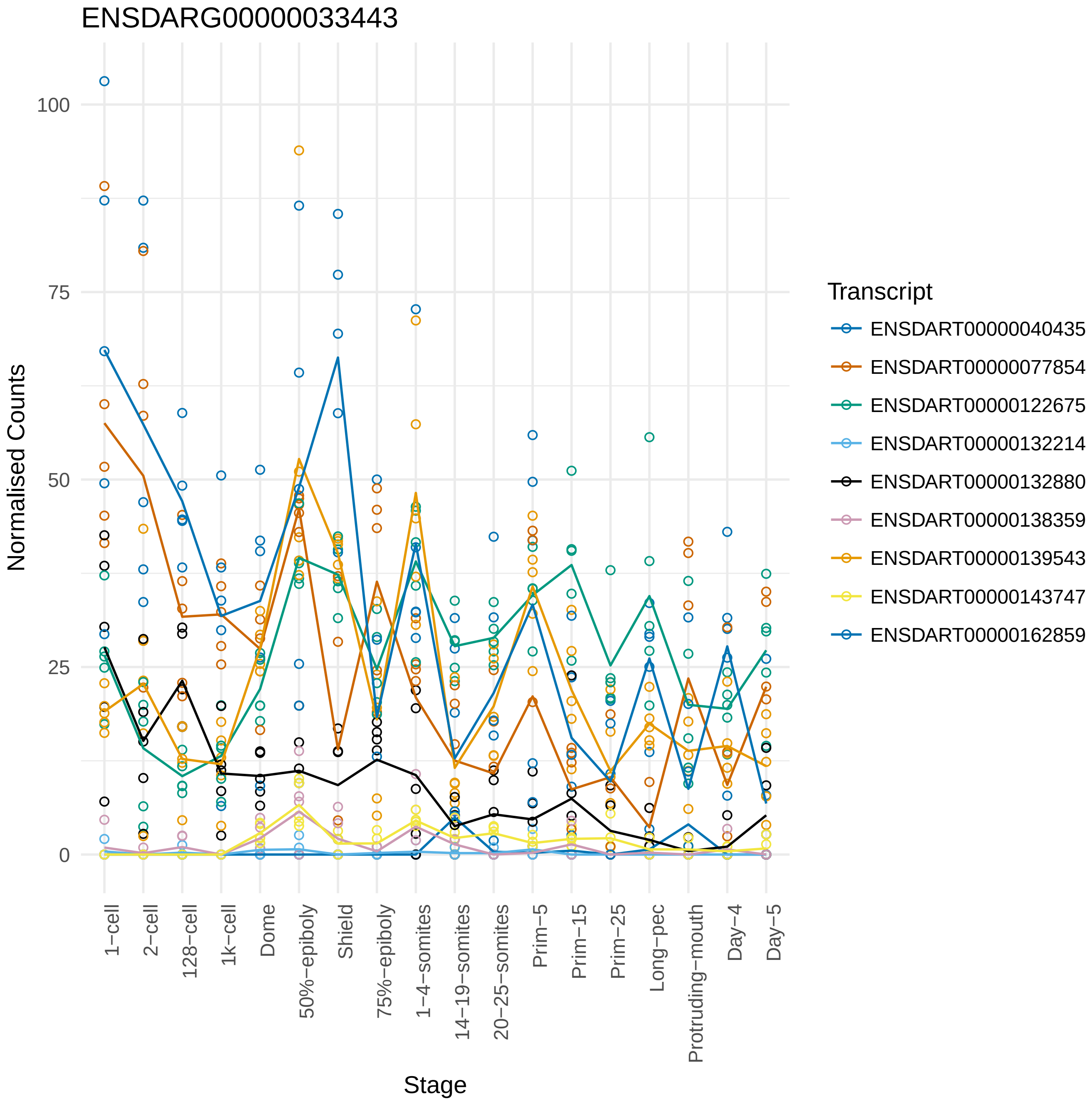

ENSDARG00000033443
Normalised Counts
0
25
50
75
100
1−cell
2−cell
128−cell
1k−cell
Dome
50%−epiboly
Shield
75%−epiboly
1−4−somites
14−19−somites
20−25−somites
Prim−5
Prim−15
Prim−25
Long−pec
Protruding−mouth
Day−4
Day−5
Stage
Transcript
ENSDART00000040435
ENSDART00000077854
ENSDART00000122675
ENSDART00000132214
ENSDART00000132880
ENSDART00000138359
ENSDART00000139543
ENSDART00000143747
ENSDART00000162859

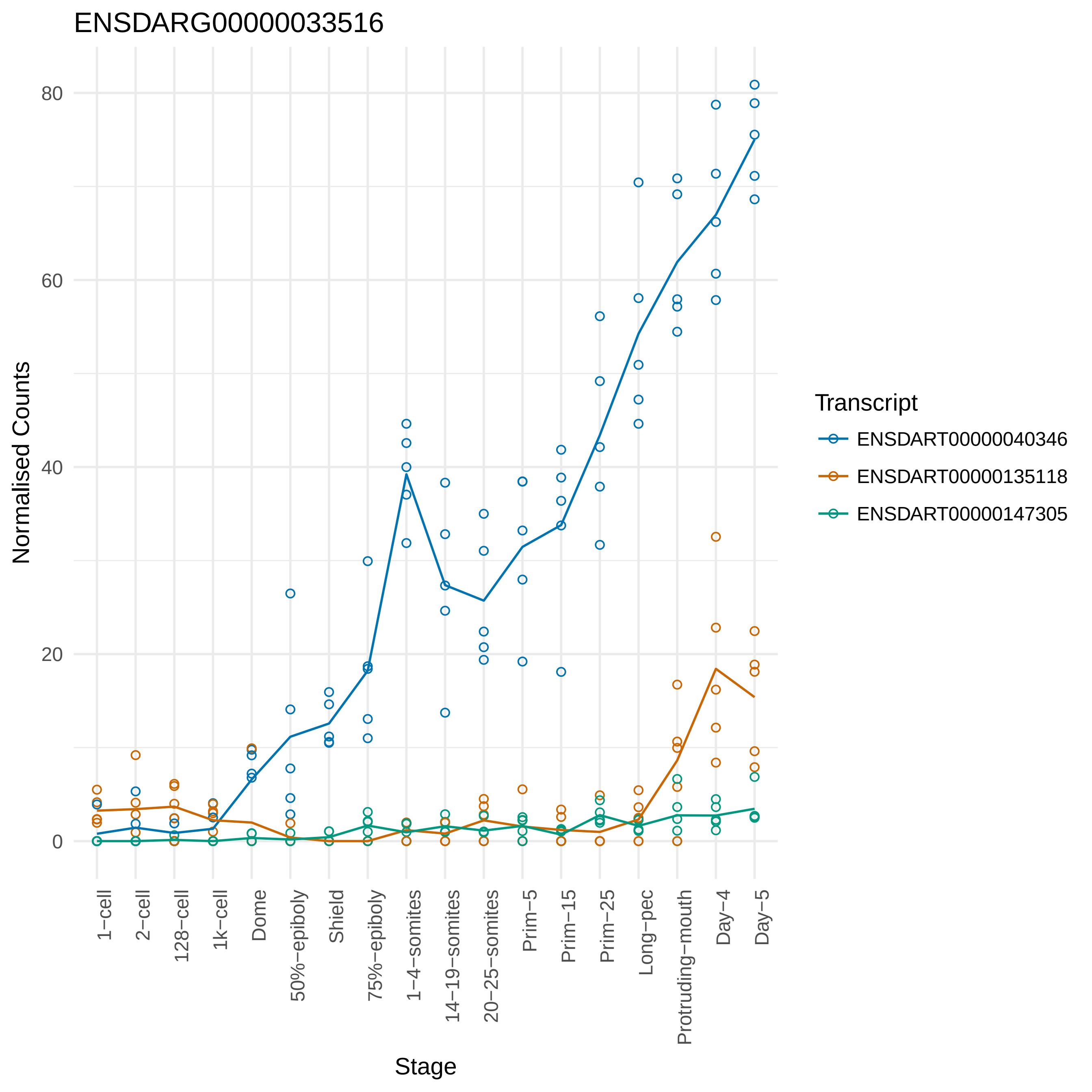
ENSDARG00000033516
Normalised Counts
0
20
40
60
80
Stage
1−cell
2−cell
128−cell
1k−cell
Dome
50%−epiboly
Shield
75%−epiboly
1−4−somites
14−19−somites
20−25−somites
Prim−5
Prim−15
Prim−25
Long−pec
Protruding−mouth
Day−4
Day−5
Transcript
ENSDART00000040346
ENSDART00000135118
ENSDART00000147305

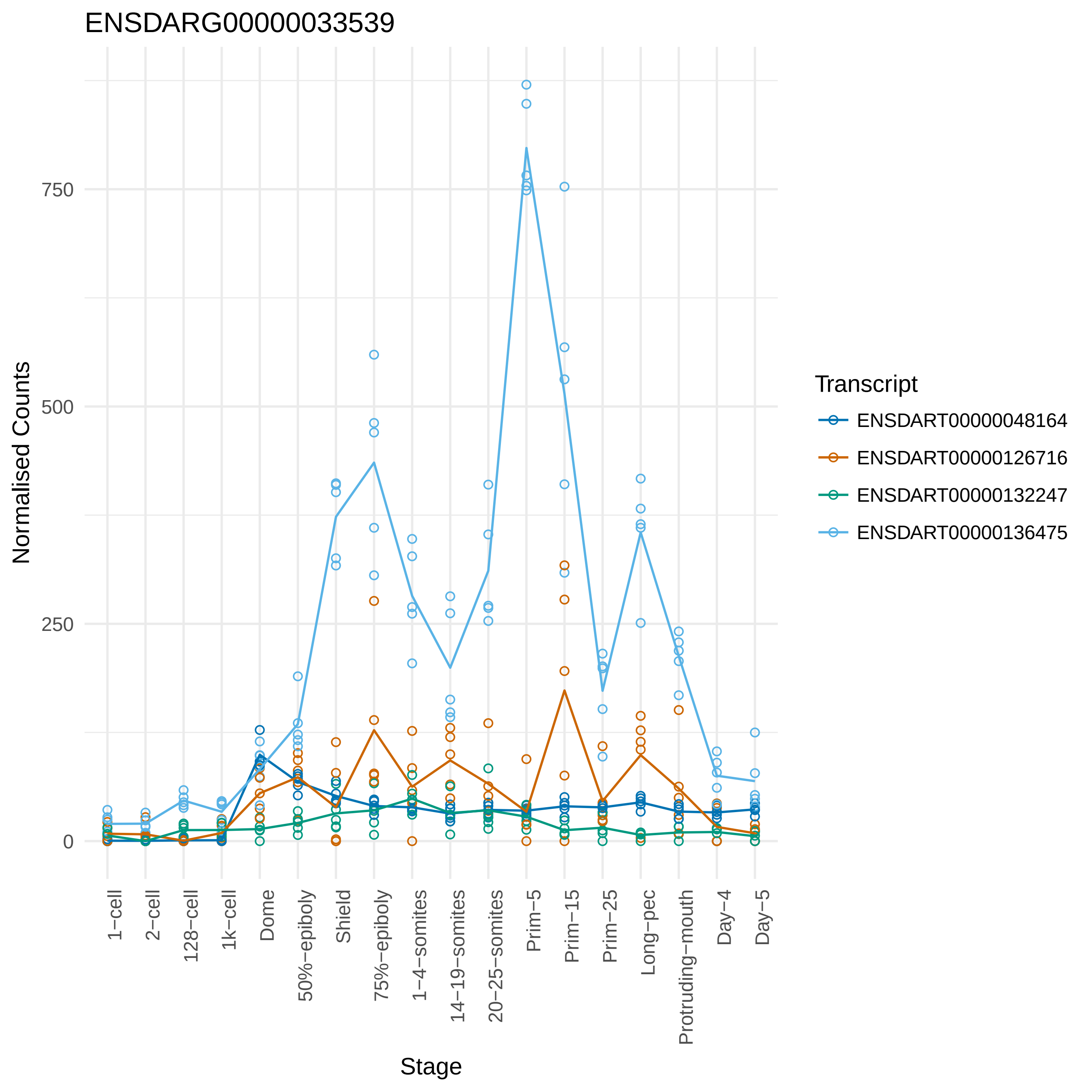

ENSDARG00000033539
Normalised Counts
0
250
500
750
Stage
1−cell
2−cell
128−cell
1k−cell
Dome
50%−epiboly
Shield
75%−epiboly
1−4−somites
14−19−somites
20−25−somites
Prim−5
Prim−15
Prim−25
Long−pec
Protruding−mouth
Day−4
Day−5
Transcript
ENSDART00000048164
ENSDART00000126716
ENSDART00000132247
ENSDART00000136475

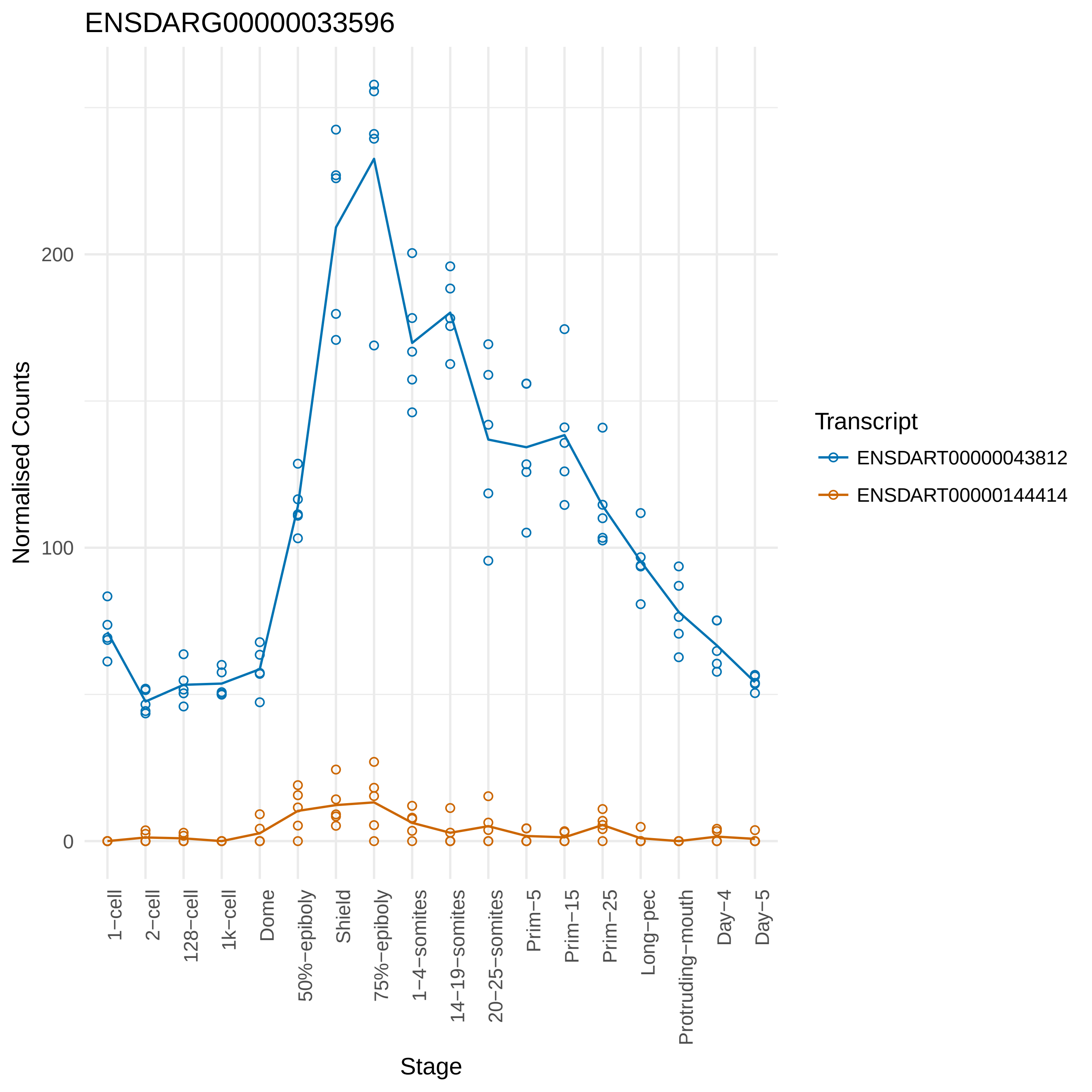

ENSDARG00000033596
Normalised Counts
200
100
0
1−cell
2−cell
128−cell
1k−cell
Dome
50%−epiboly
Shield
75%−epiboly
1−4−somites
14−19−somites
20−25−somites
Prim−5
Prim−15
Prim−25
Long−pec
Protruding−mouth
Day−4
Day−5
Stage
Transcript
ENSDART00000043812
ENSDART00000144414

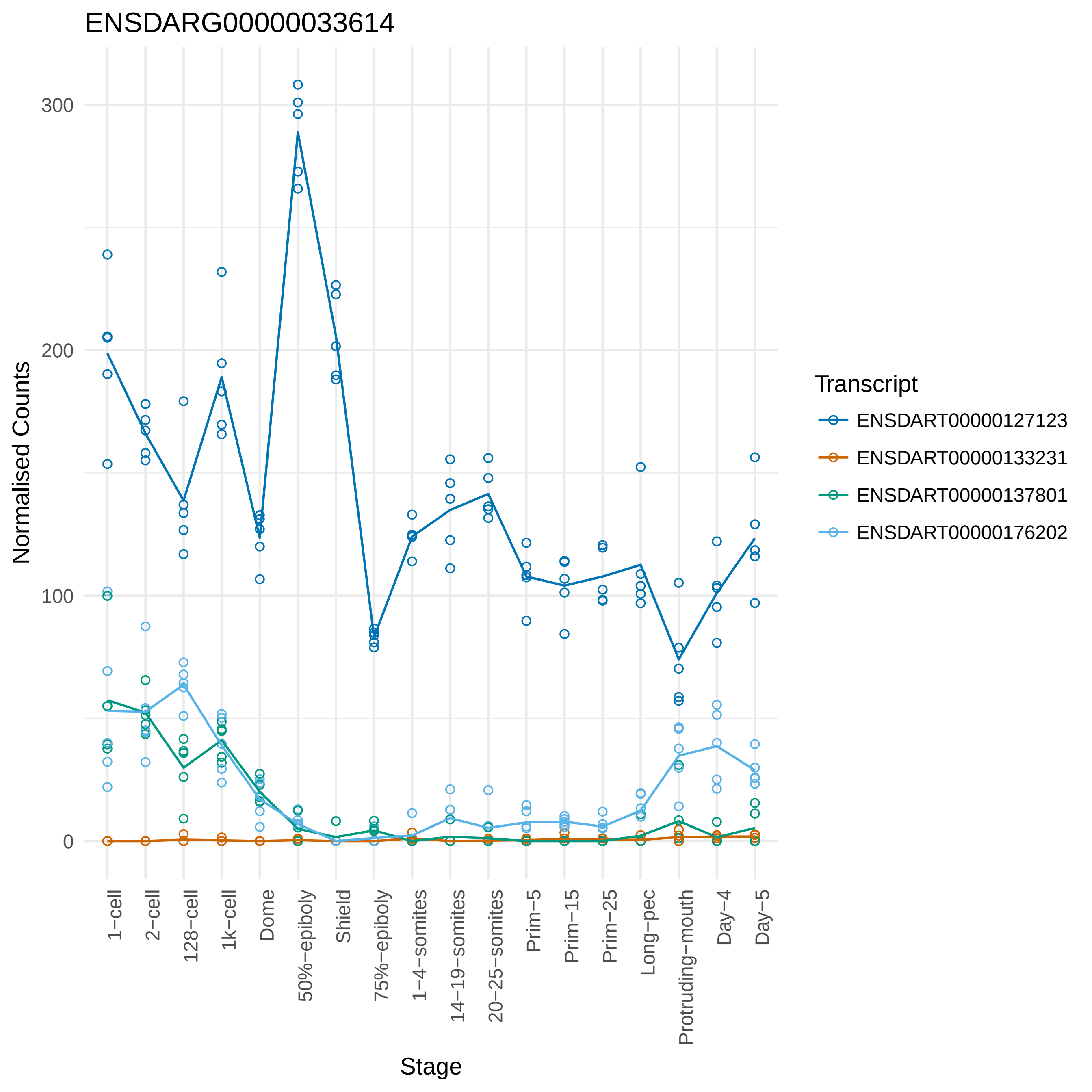

ENSDARG00000033614
Normalised Counts
300
200
100
0
1−cell
2−cell
128−cell
1k−cell
Dome
50%−epiboly
Shield
75%−epiboly
1−4−somites
14−19−somites
20−25−somites
Prim−5
Prim−15
Prim−25
Long−pec
Protruding−mouth
Day−4
Day−5
Stage
Transcript
ENSDART00000127123
ENSDART00000133231
ENSDART00000137801
ENSDART00000176202

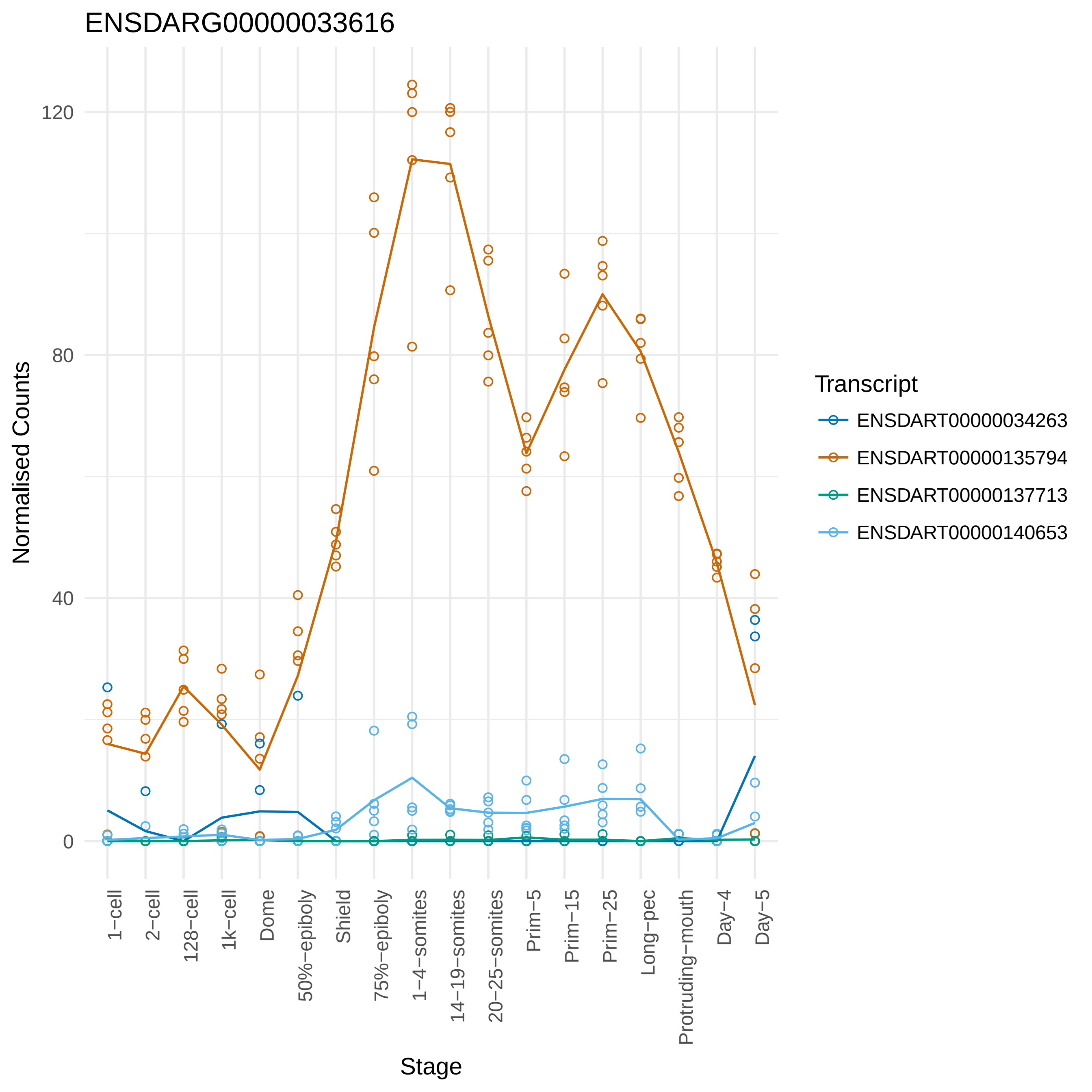

ENSDARG00000033616
Normalised Counts
120
80
40
0
1−cell
2−cell
128−cell
1k−cell
Dome
50%−epiboly
Shield
75%−epiboly
1−4−somites
14−19−somites
20−25−somites
Prim−5
Prim−15
Prim−25
Long−pec
Protruding−mouth
Day−4
Day−5
Stage
Transcript
ENSDART00000034263
ENSDART00000135794
ENSDART00000137713
ENSDART00000140653

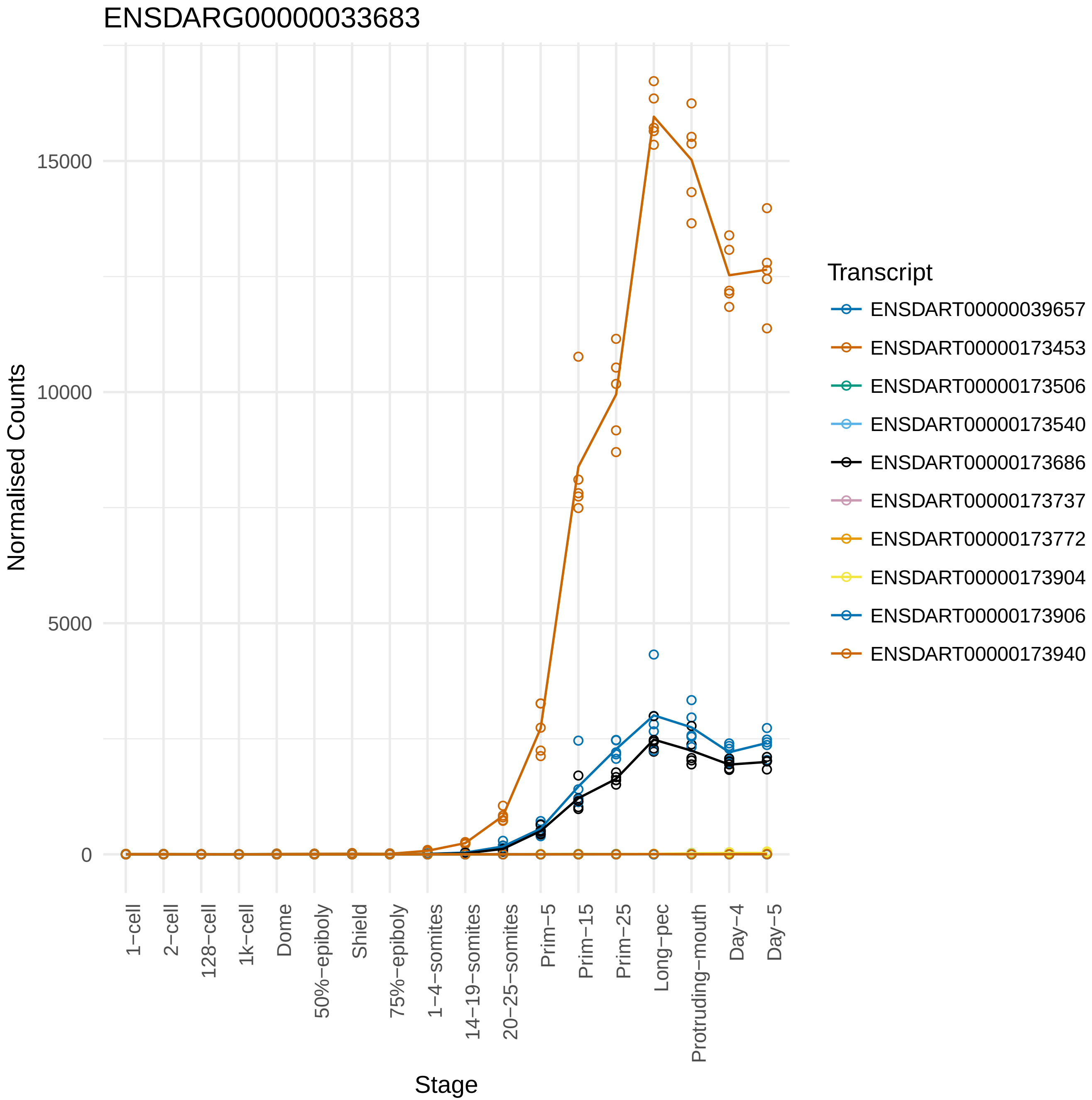

ENSDARG00000033683
Normalised Counts
15000
10000
5000
0
1−cell
2−cell
128−cell
1k−cell
Dome
50%−epiboly
Shield
75%−epiboly
1−4−somites
14−19−somites
20−25−somites
Prim−5
Prim−15
Prim−25
Long−pec
Protruding−mouth
Day−4
Day−5
Stage
Transcript
ENSDART00000039657
ENSDART00000173453
ENSDART00000173506
ENSDART00000173540
ENSDART00000173686
ENSDART00000173737
ENSDART00000173772
ENSDART00000173904
ENSDART00000173906
ENSDART00000173940

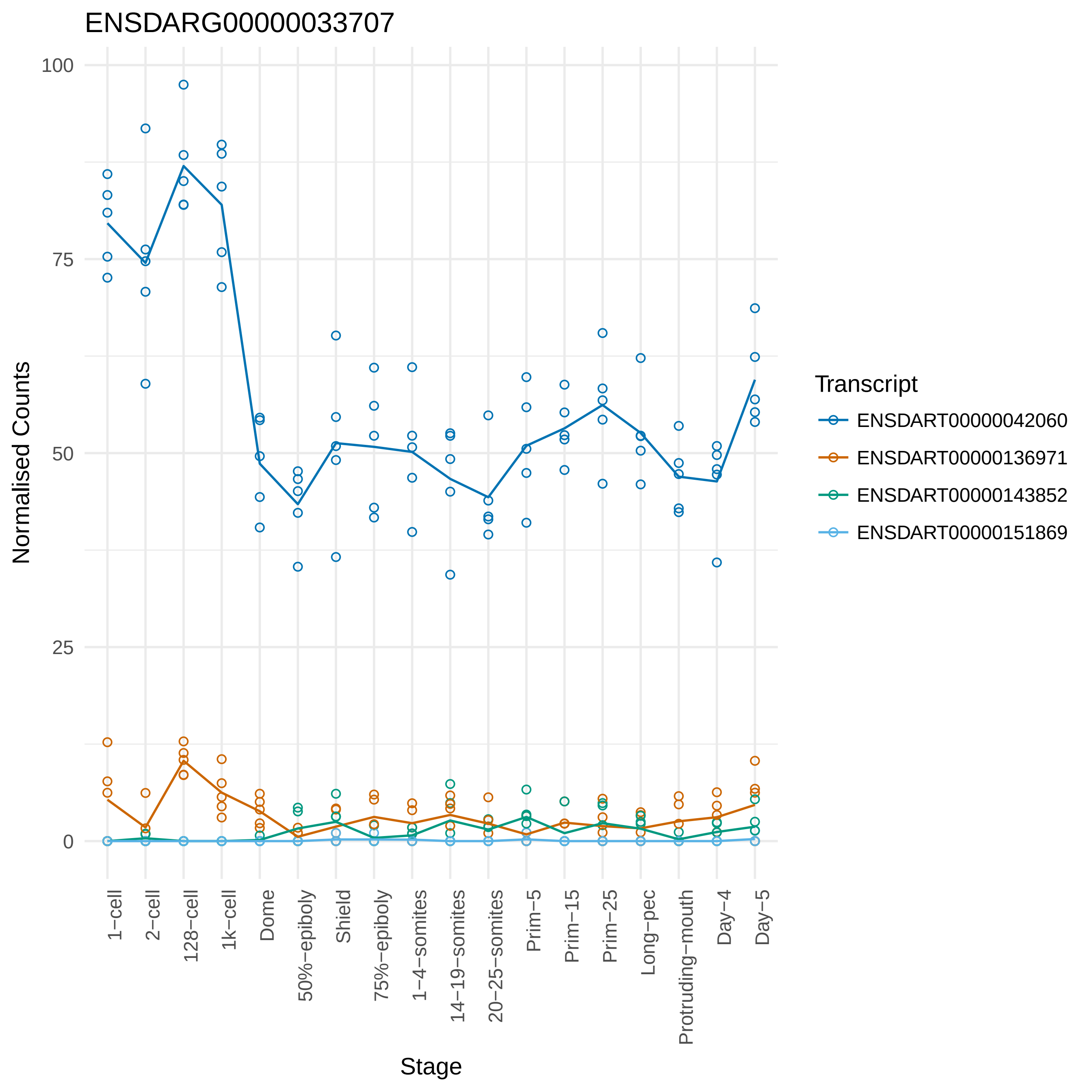
ENSDARG00000033707
Normalised Counts
100
75
50
25
0
1−cell
2−cell
128−cell
1k−cell
Dome
50%−epiboly
Shield
75%−epiboly
1−4−somites
14−19−somites
20−25−somites
Prim−5
Prim−15
Prim−25
Long−pec
Protruding−mouth
Day−4
Day−5
Stage
Transcript
ENSDART00000042060
ENSDART00000136971
ENSDART00000143852
ENSDART00000151869

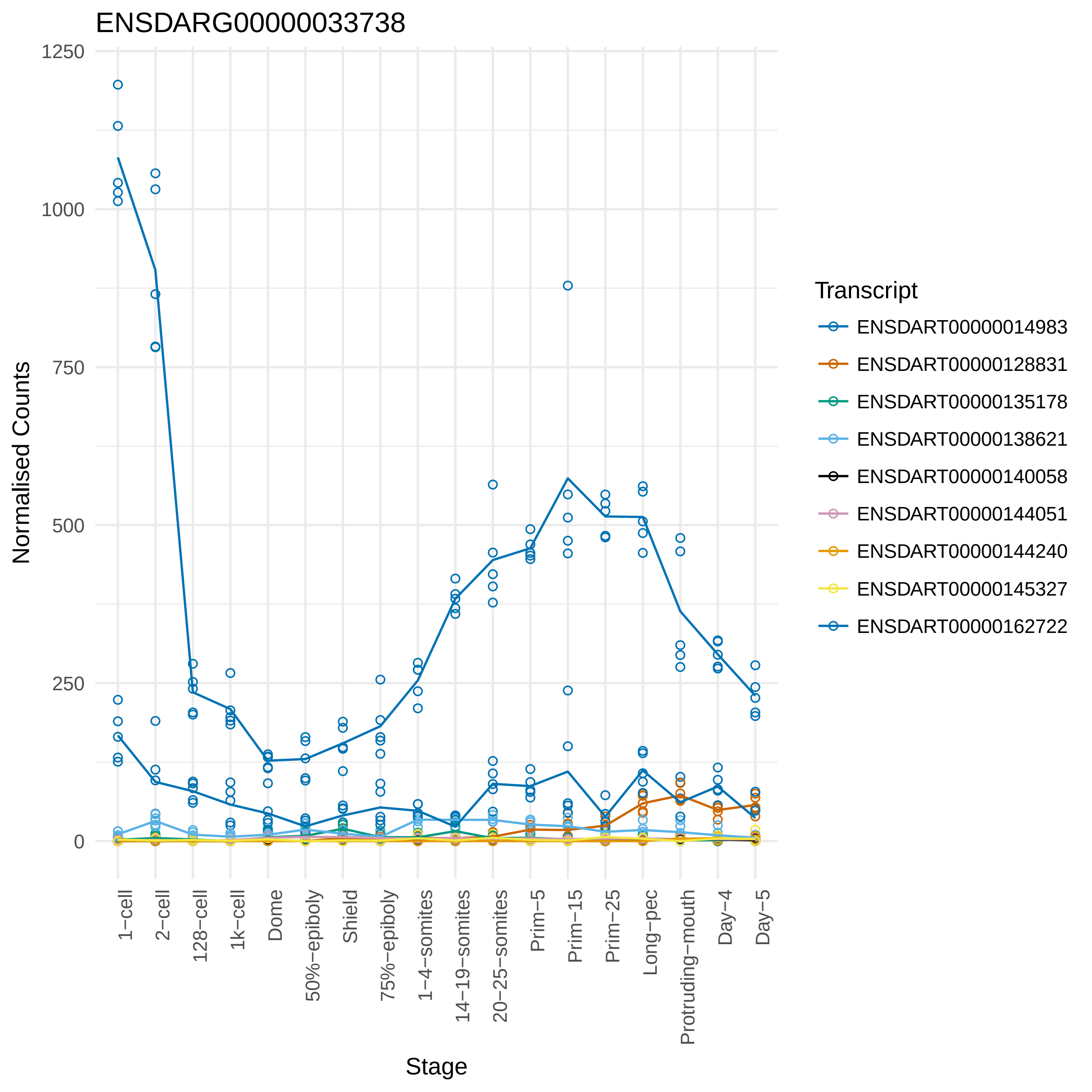

ENSDARG00000033738
Normalised Counts
1250
1000
750
500
250
0
1−cell
2−cell
128−cell
1k−cell
Dome
50%−epiboly
Shield
75%−epiboly
1−4−somites
14−19−somites
20−25−somites
Prim−5
Prim−15
Prim−25
Long−pec
Protruding−mouth
Day−4
Day−5
Stage
Transcript
ENSDART00000014983
ENSDART00000128831
ENSDART00000135178
ENSDART00000138621
ENSDART00000140058
ENSDART00000144051
ENSDART00000144240
ENSDART00000145327
ENSDART00000162722

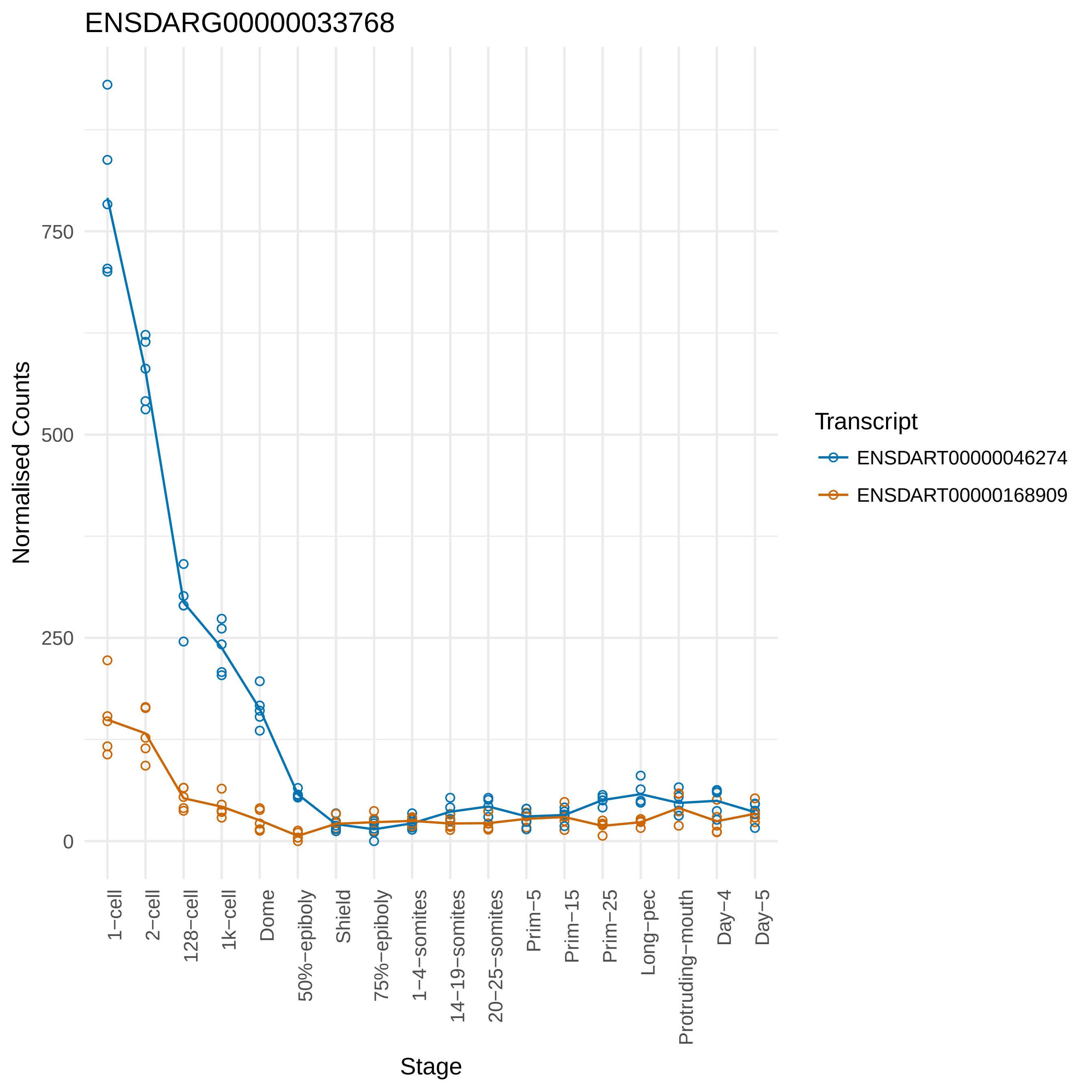
ENSDARG00000033768
Normalised Counts
750
500
250
0
1−cell
2−cell
128−cell
1k−cell
Dome
50%−epiboly
Shield
75%−epiboly
1−4−somites
14−19−somites
20−25−somites
Prim−5
Prim−15
Prim−25
Long−pec
Protruding−mouth
Day−4
Day−5
Stage
Transcript
ENSDART00000046274
ENSDART00000168909

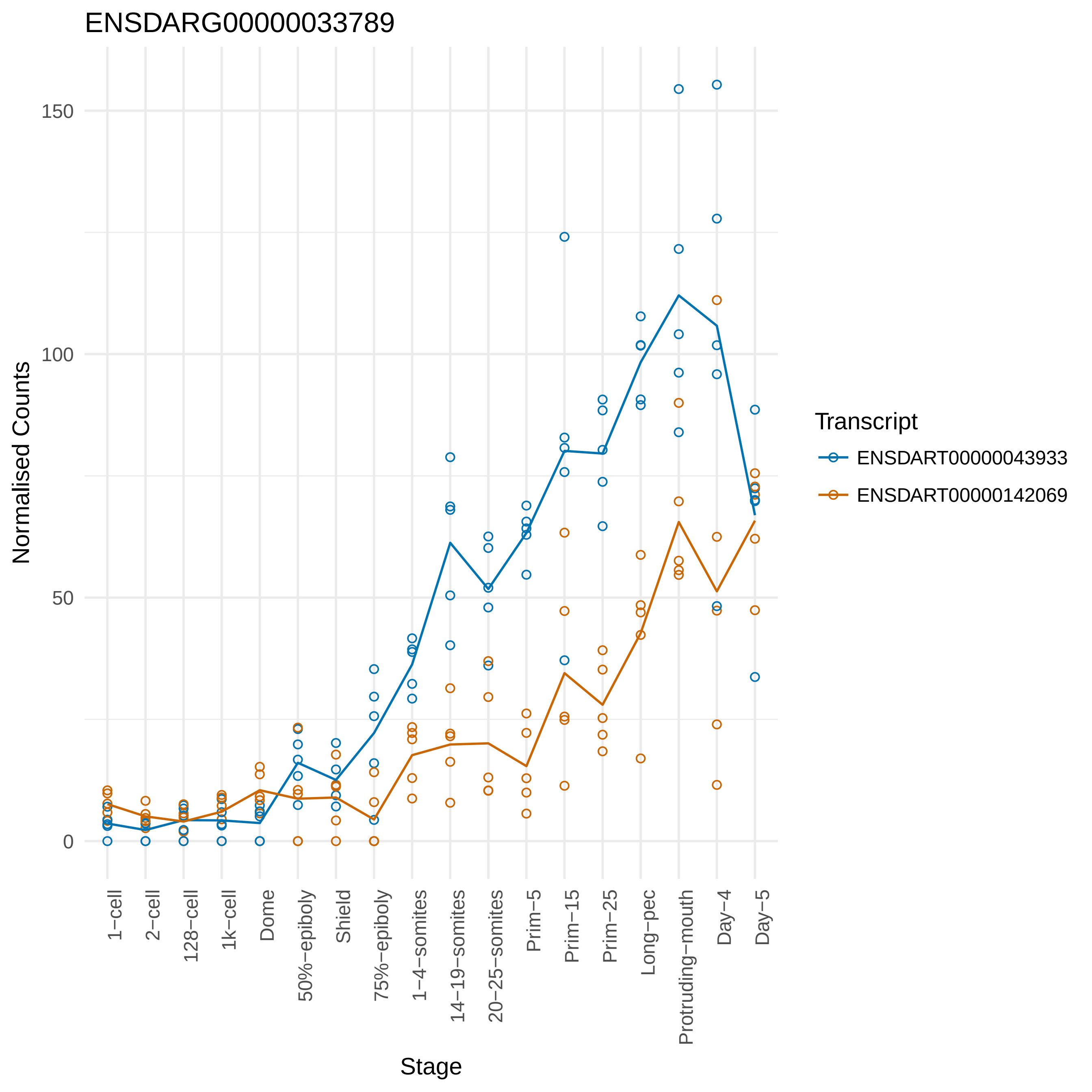

ENSDARG00000033789
Normalised Counts
0
50
100
150
Stage
1−cell
2−cell
128−cell
1k−cell
Dome
50%−epiboly
Shield
75%−epiboly
1−4−somites
14−19−somites
20−25−somites
Prim−5
Prim−15
Prim−25
Long−pec
Protruding−mouth
Day−4
Day−5
Transcript
ENSDART00000043933
ENSDART00000142069

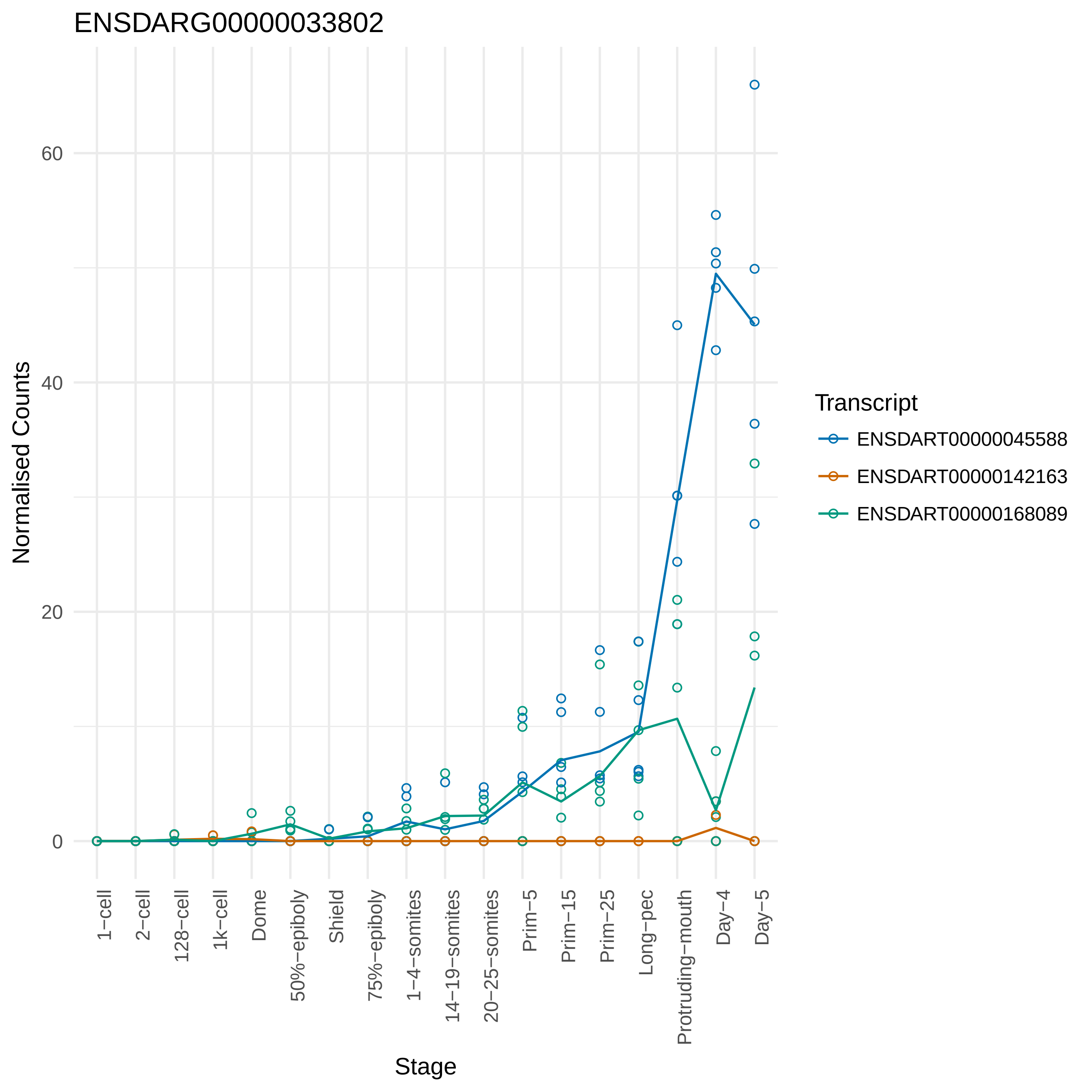
ENSDARG00000033802
Normalised Counts
0
20
40
60
Stage
1−cell
2−cell
128−cell
1k−cell
Dome
50%−epiboly
Shield
75%−epiboly
1−4−somites
14−19−somites
20−25−somites
Prim−5
Prim−15
Prim−25
Long−pec
Protruding−mouth
Day−4
Day−5
Transcript
ENSDART00000045588
ENSDART00000142163
ENSDART00000168089

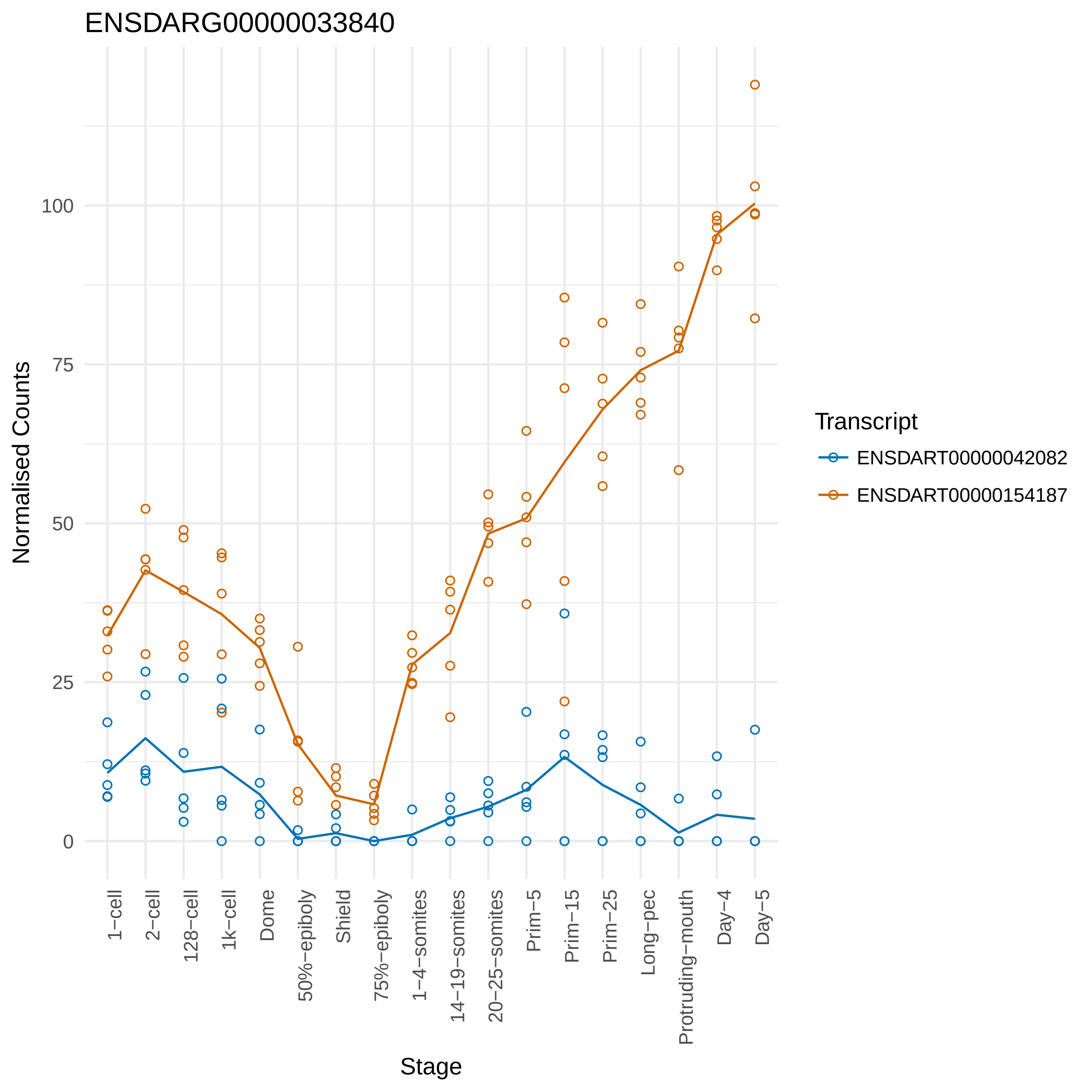

ENSDARG00000033840
Normalised Counts
100
75
50
25
0
1−cell
2−cell
128−cell
1k−cell
Dome
50%−epiboly
Shield
75%−epiboly
1−4−somites
14−19−somites
20−25−somites
Prim−5
Prim−15
Prim−25
Long−pec
Protruding−mouth
Day−4
Day−5
Stage
Transcript
ENSDART00000042082
ENSDART00000154187

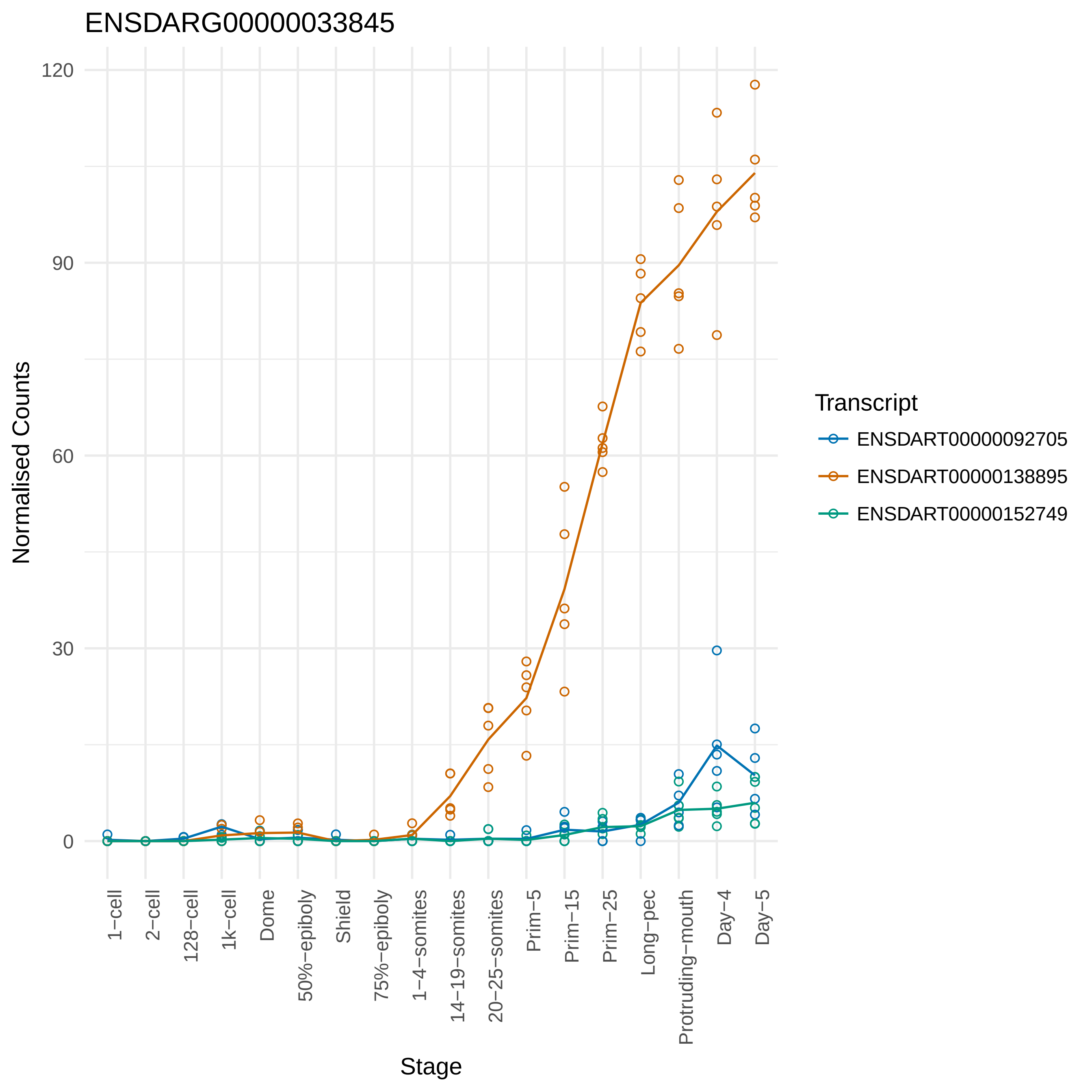

ENSDARG00000033845
Normalised Counts
120
90
60
30
0
1−cell
2−cell
128−cell
1k−cell
Dome
50%−epiboly
Shield
75%−epiboly
1−4−somites
14−19−somites
20−25−somites
Prim−5
Prim−15
Prim−25
Long−pec
Protruding−mouth
Day−4
Day−5
Stage
Transcript
ENSDART00000092705
ENSDART00000138895
ENSDART00000152749

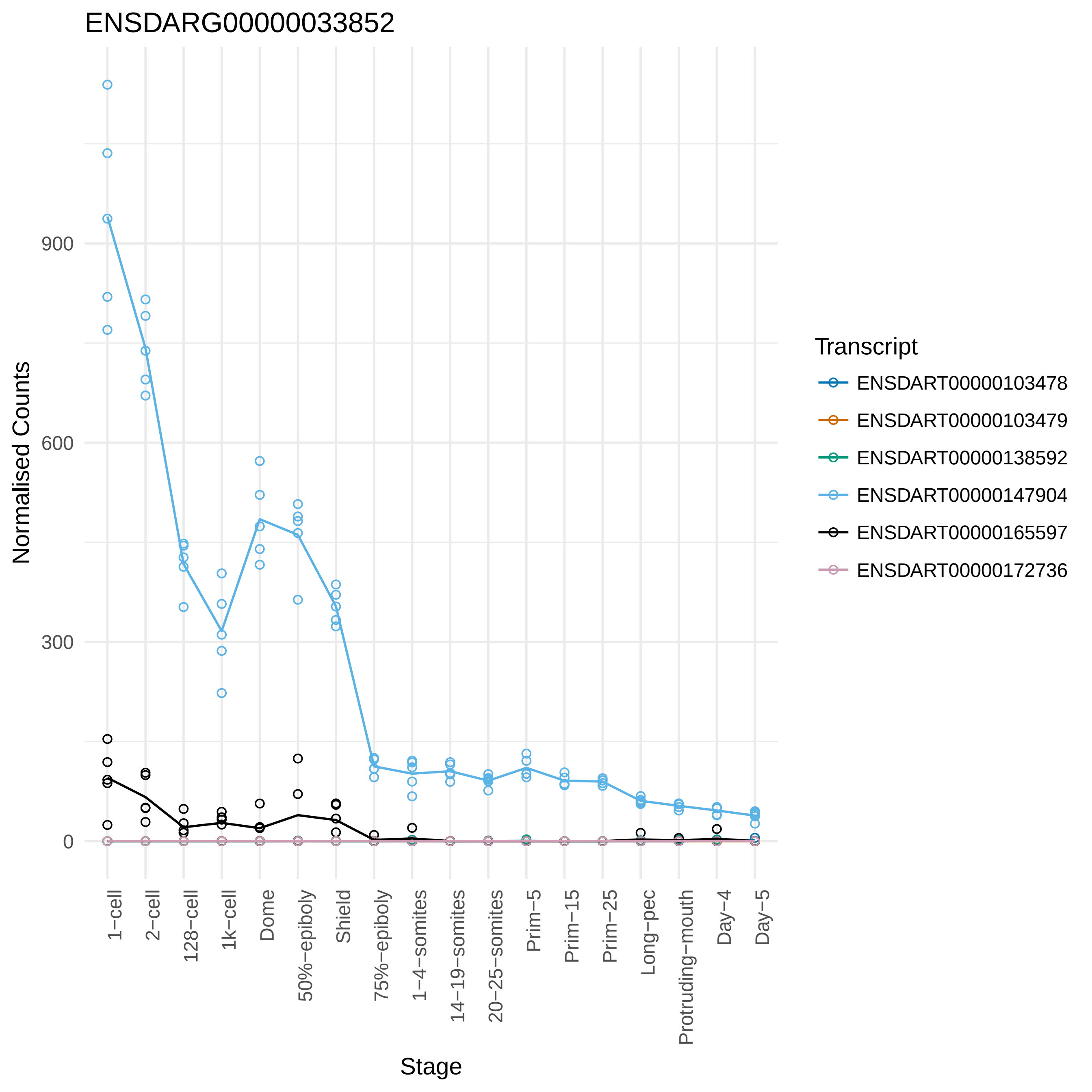

ENSDARG00000033852
Normalised Counts
900
600
300
0
1−cell
2−cell
128−cell
1k−cell
Dome
50%−epiboly
Shield
75%−epiboly
1−4−somites
14−19−somites
20−25−somites
Prim−5
Prim−15
Prim−25
Long−pec
Protruding−mouth
Day−4
Day−5
Stage
Transcript
ENSDART00000103478
ENSDART00000103479
ENSDART00000138592
ENSDART00000147904
ENSDART00000165597
ENSDART00000172736

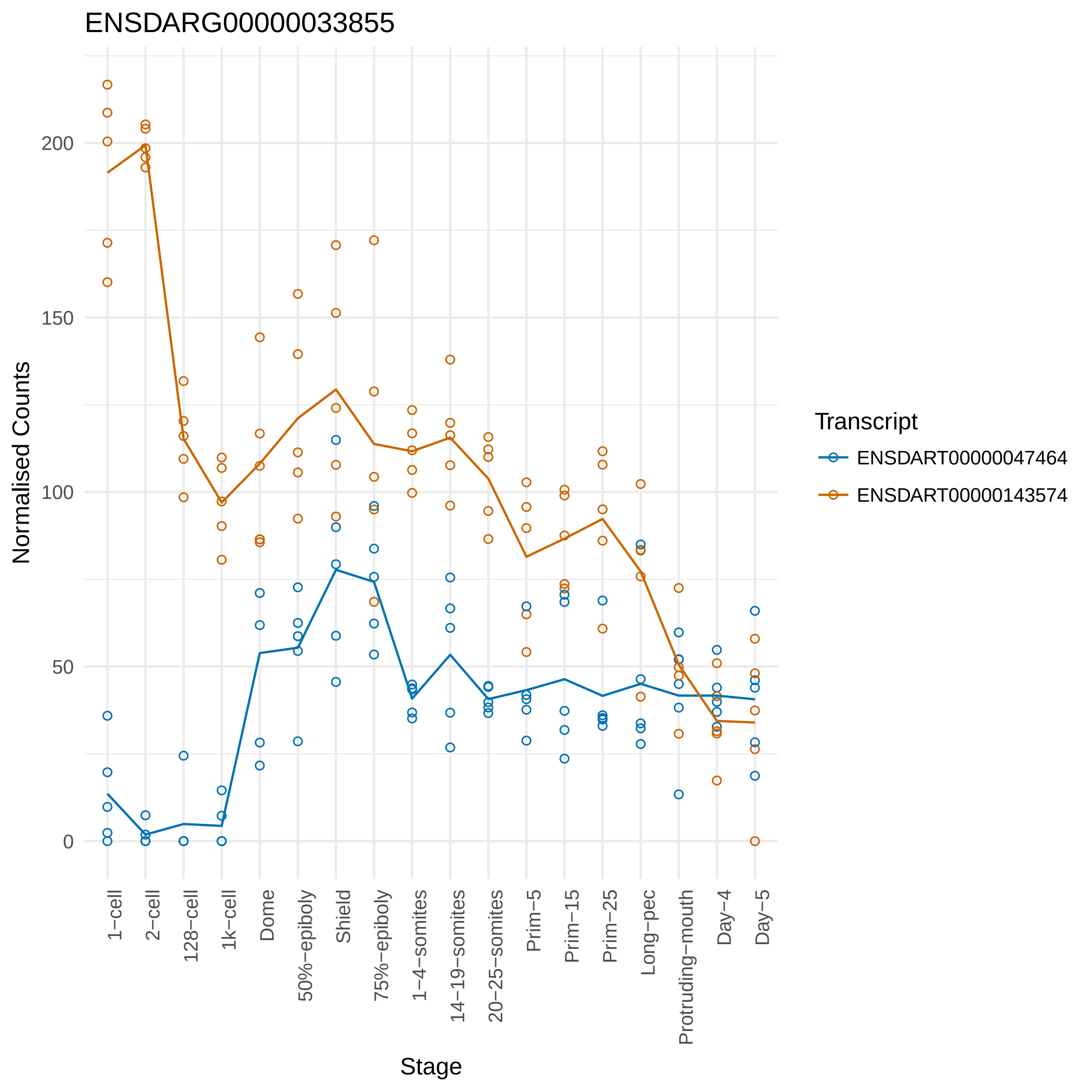

ENSDARG00000033855
Normalised Counts
0
50
100
150
200
1−cell
2−cell
128−cell
1k−cell
Dome
50%−epiboly
Shield
75%−epiboly
1−4−somites
14−19−somites
20−25−somites
Prim−5
Prim−15
Prim−25
Long−pec
Protruding−mouth
Day−4
Day−5
Stage
Transcript
ENSDART00000047464
ENSDART00000143574

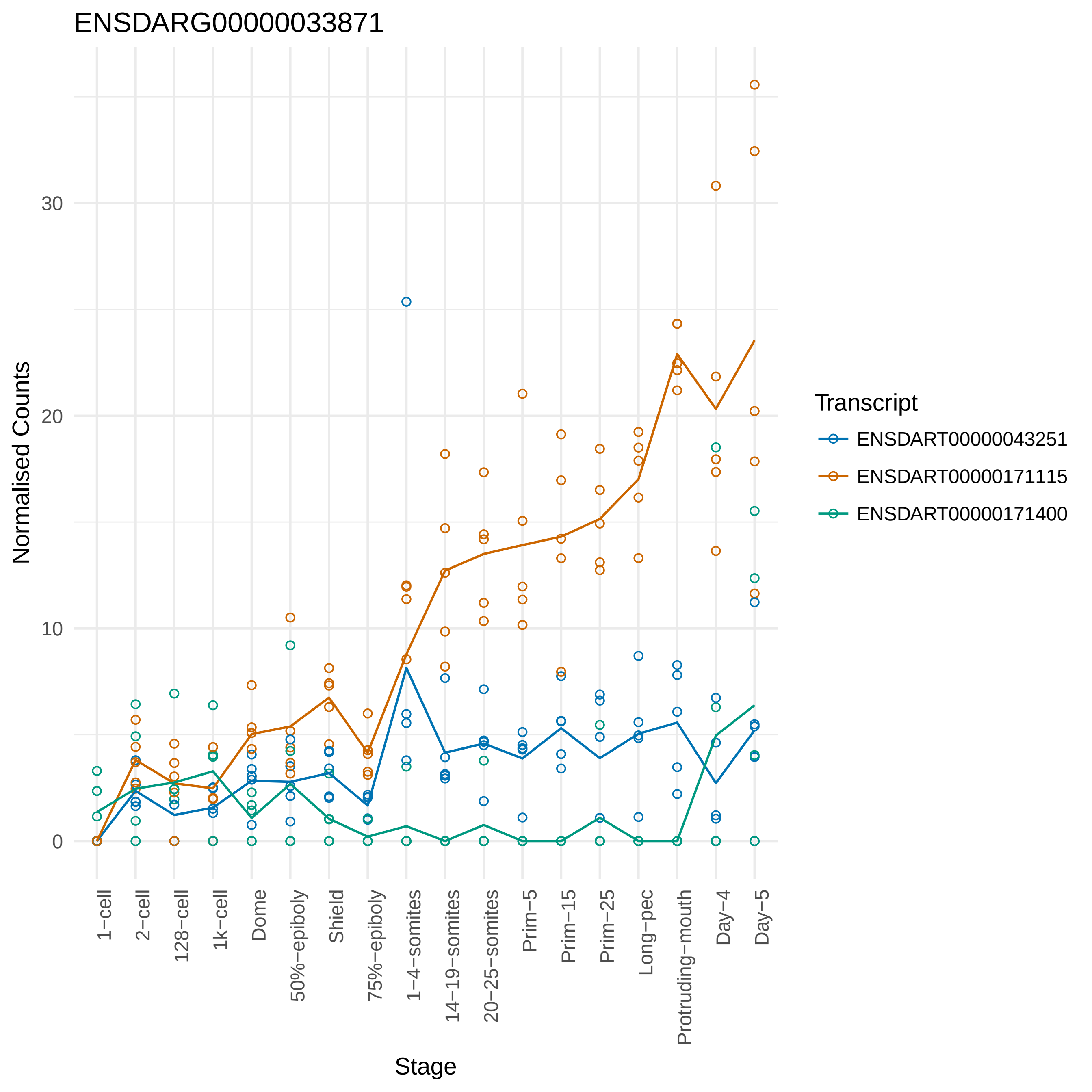

ENSDARG00000033871
Normalised Counts
0
10
20
30
1−cell
2−cell
128−cell
1k−cell
Dome
50%−epiboly
Shield
75%−epiboly
1−4−somites
14−19−somites
20−25−somites
Prim−5
Prim−15
Prim−25
Long−pec
Protruding−mouth
Day−4
Day−5
Stage
Transcript
ENSDART00000043251
ENSDART00000171115
ENSDART00000171400

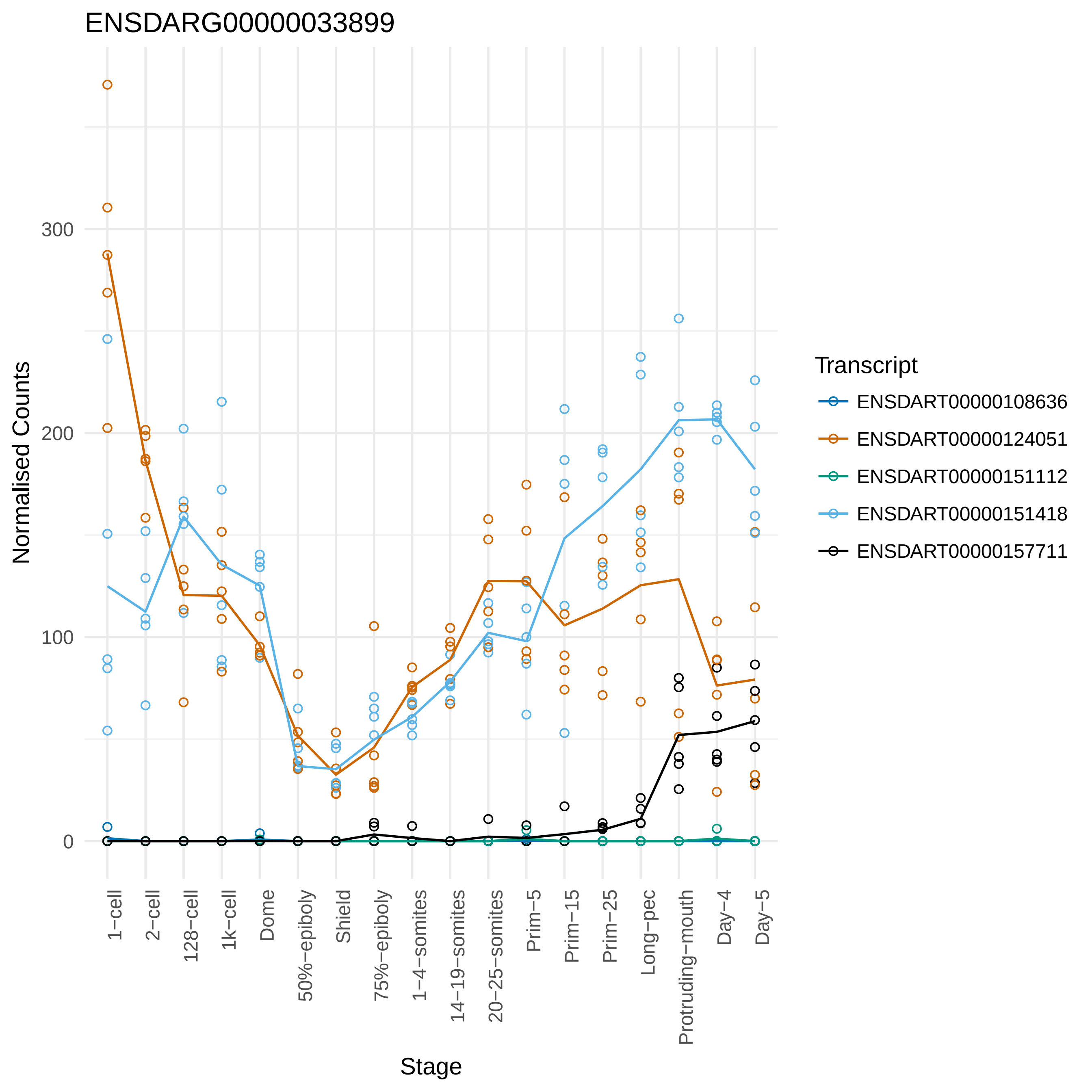

ENSDARG00000033899
Normalised Counts
0
100
200
300
1−cell
2−cell
128−cell
1k−cell
Dome
50%−epiboly
Shield
75%−epiboly
1−4−somites
14−19−somites
20−25−somites
Prim−5
Prim−15
Prim−25
Long−pec
Protruding−mouth
Day−4
Day−5
Stage
Transcript
ENSDART00000108636
ENSDART00000124051
ENSDART00000151112
ENSDART00000151418
ENSDART00000157711

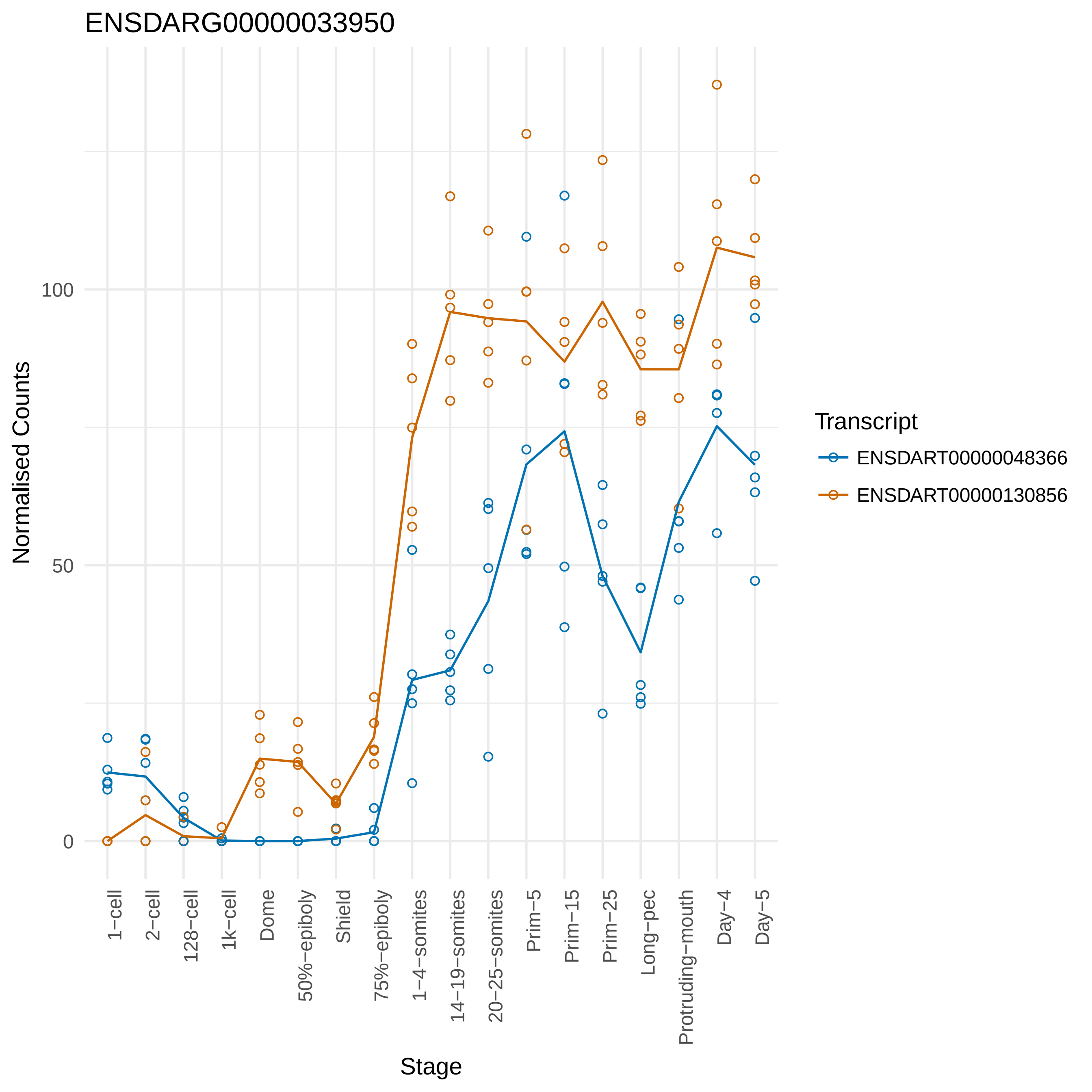

ENSDARG00000033950
Normalised Counts
100
50
0
1−cell
2−cell
128−cell
1k−cell
Dome
50%−epiboly
Shield
75%−epiboly
1−4−somites
14−19−somites
20−25−somites
Prim−5
Prim−15
Prim−25
Long−pec
Protruding−mouth
Day−4
Day−5
Stage
Transcript
ENSDART00000048366
ENSDART00000130856

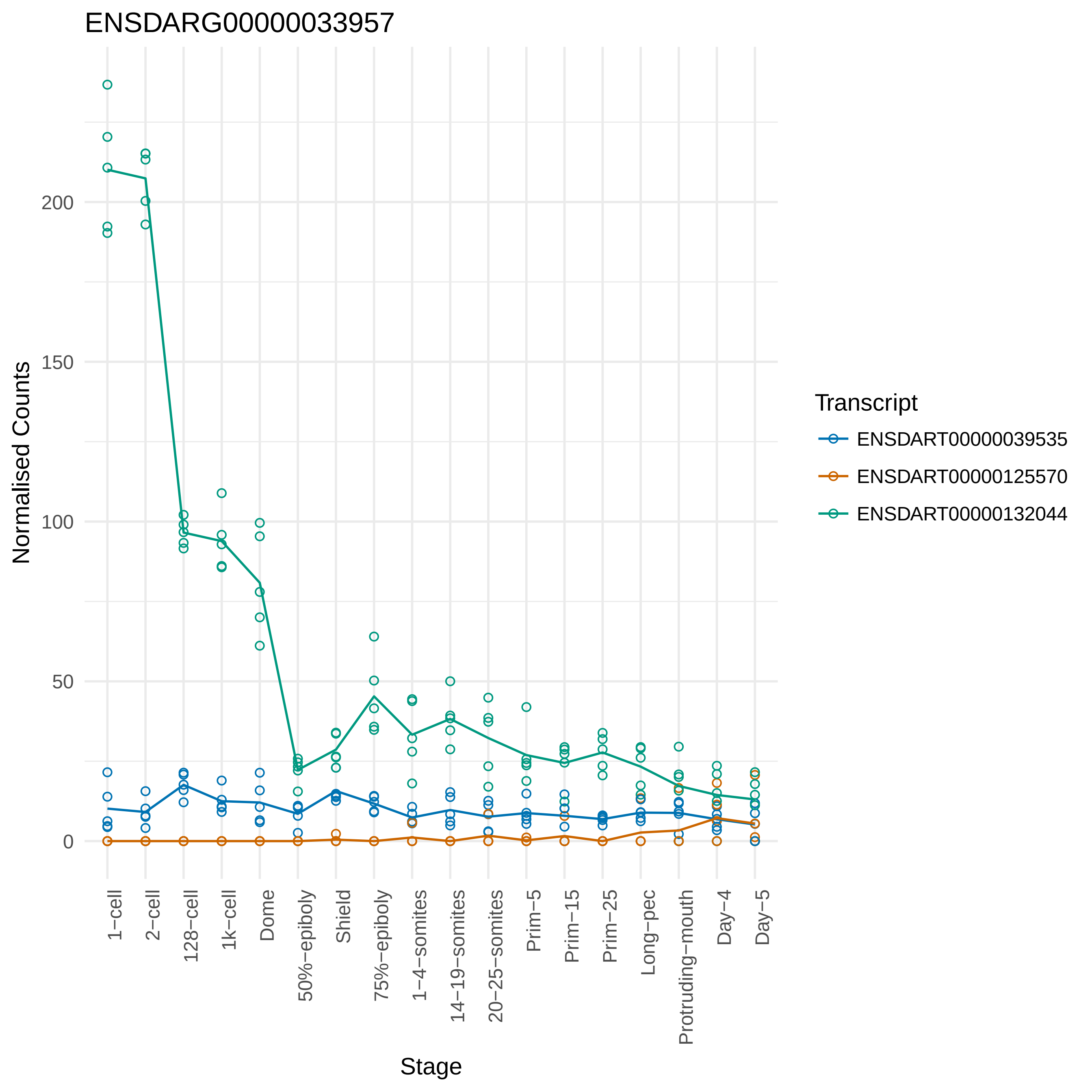

ENSDARG00000033957
Normalised Counts
200
150
100
50
0
1−cell
2−cell
128−cell
1k−cell
Dome
50%−epiboly
Shield
75%−epiboly
1−4−somites
14−19−somites
20−25−somites
Prim−5
Prim−15
Prim−25
Long−pec
Protruding−mouth
Day−4
Day−5
Stage
Transcript
ENSDART00000039535
ENSDART00000125570
ENSDART00000132044

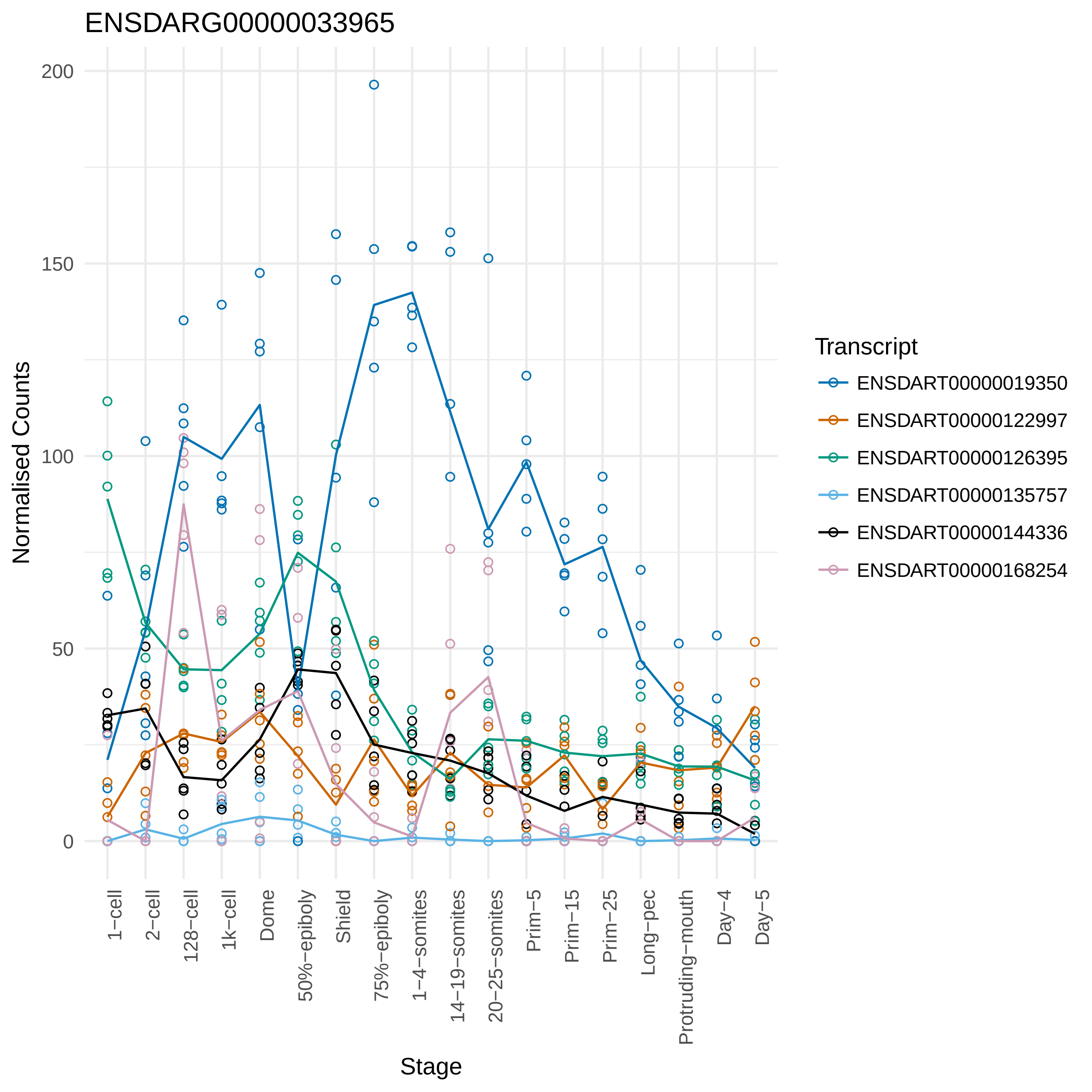

ENSDARG00000033965
Normalised Counts
200
150
100
50
0
1−cell
2−cell
128−cell
1k−cell
Dome
50%−epiboly
Shield
75%−epiboly
1−4−somites
14−19−somites
20−25−somites
Prim−5
Prim−15
Prim−25
Long−pec
Protruding−mouth
Day−4
Day−5
Stage
Transcript
ENSDART00000019350
ENSDART00000122997
ENSDART00000126395
ENSDART00000135757
ENSDART00000144336
ENSDART00000168254

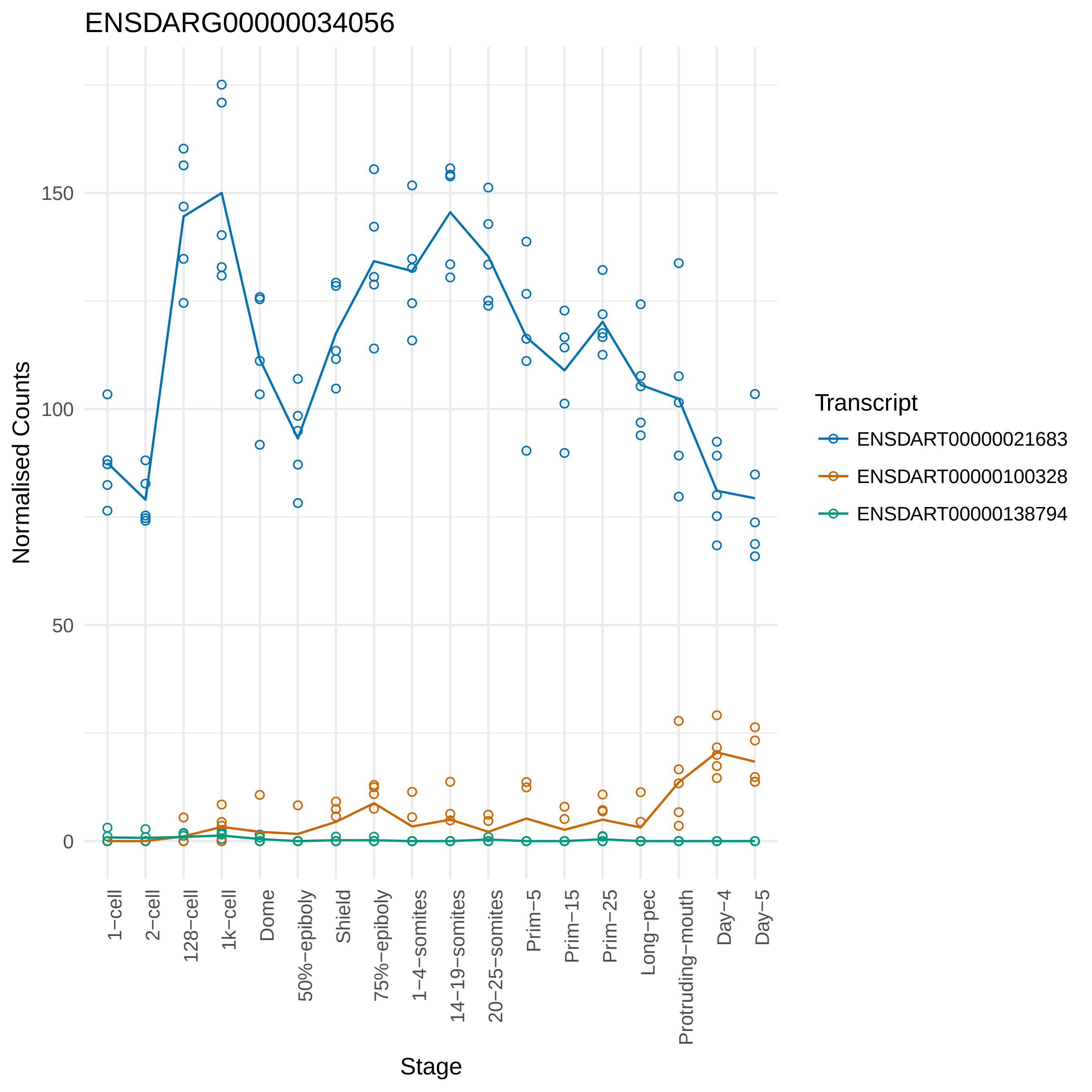

ENSDARG00000034056
Normalised Counts
0
50
100
150
1−cell
2−cell
128−cell
1k−cell
Dome
50%−epiboly
Shield
75%−epiboly
1−4−somites
14−19−somites
20−25−somites
Prim−5
Prim−15
Prim−25
Long−pec
Protruding−mouth
Day−4
Day−5
Stage
Transcript
ENSDART00000021683
ENSDART00000100328
ENSDART00000138794

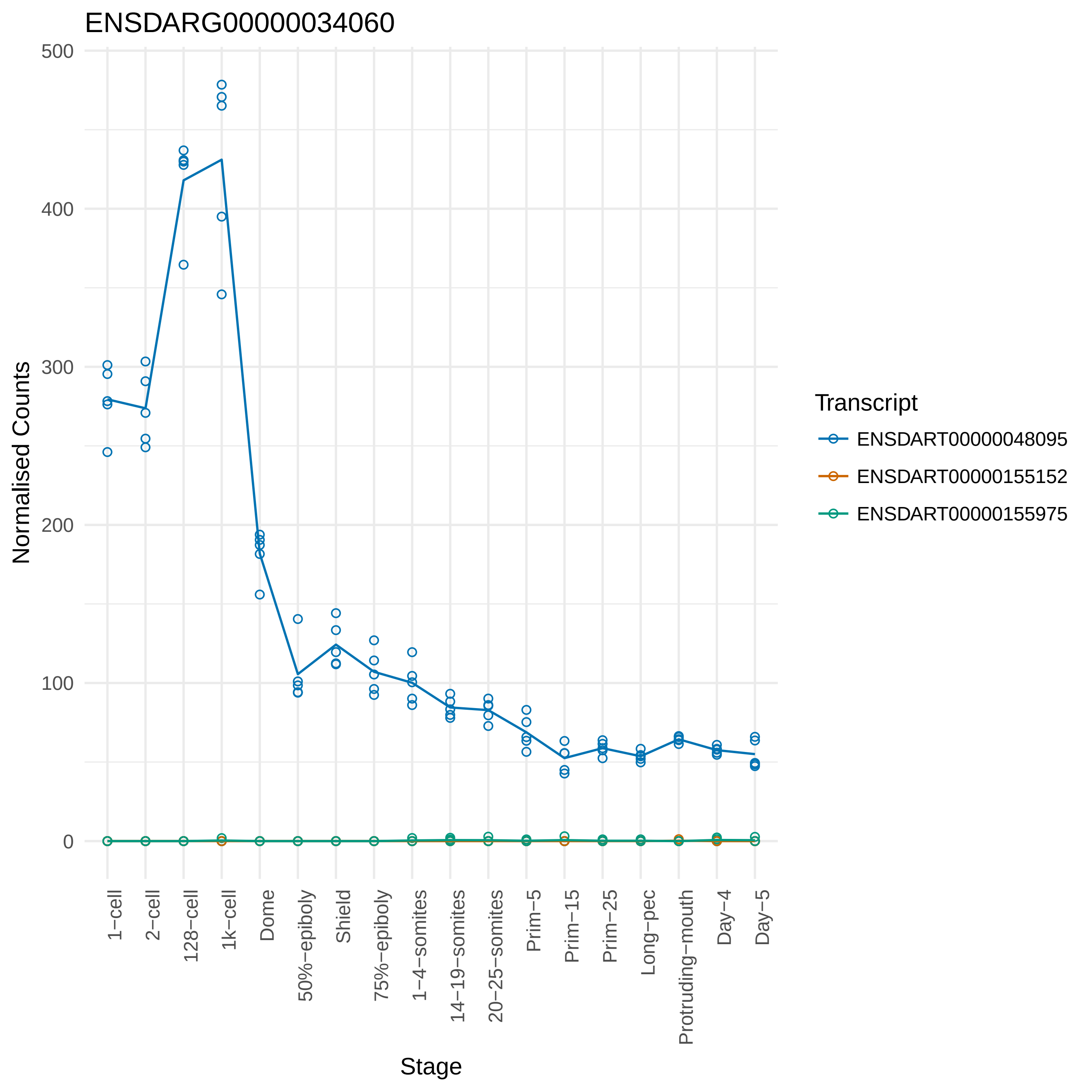

ENSDARG00000034060
Normalised Counts
500
400
300
200
100
0
1−cell
2−cell
128−cell
1k−cell
Dome
50%−epiboly
Shield
75%−epiboly
1−4−somites
14−19−somites
20−25−somites
Prim−5
Prim−15
Prim−25
Long−pec
Protruding−mouth
Day−4
Day−5
Stage
Transcript
ENSDART00000048095
ENSDART00000155152
ENSDART00000155975

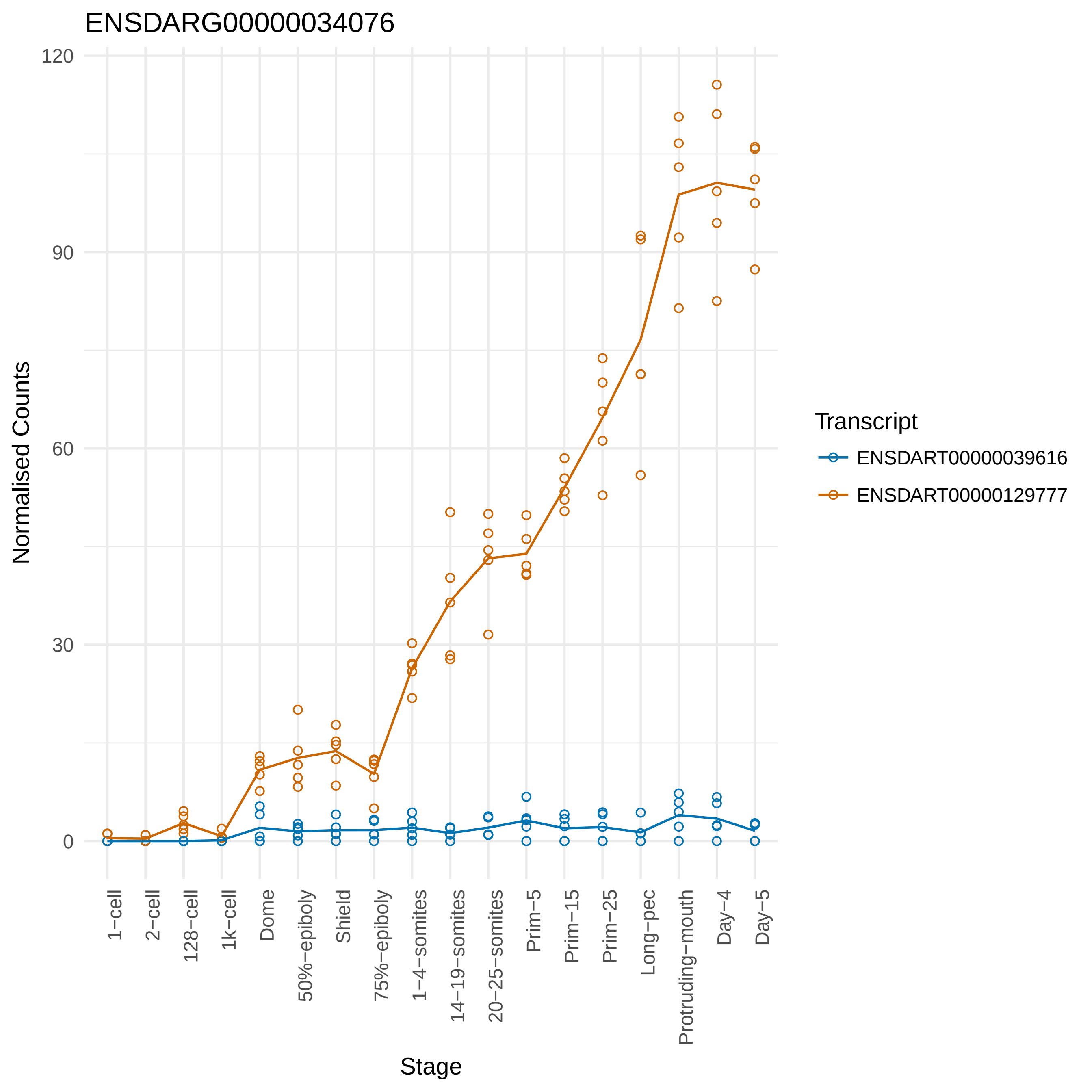

ENSDARG00000034076
Normalised Counts
120
90
60
30
0
1−cell
2−cell
128−cell
1k−cell
Dome
50%−epiboly
Shield
75%−epiboly
1−4−somites
14−19−somites
20−25−somites
Prim−5
Prim−15
Prim−25
Long−pec
Protruding−mouth
Day−4
Day−5
Stage
Transcript
ENSDART00000039616
ENSDART00000129777

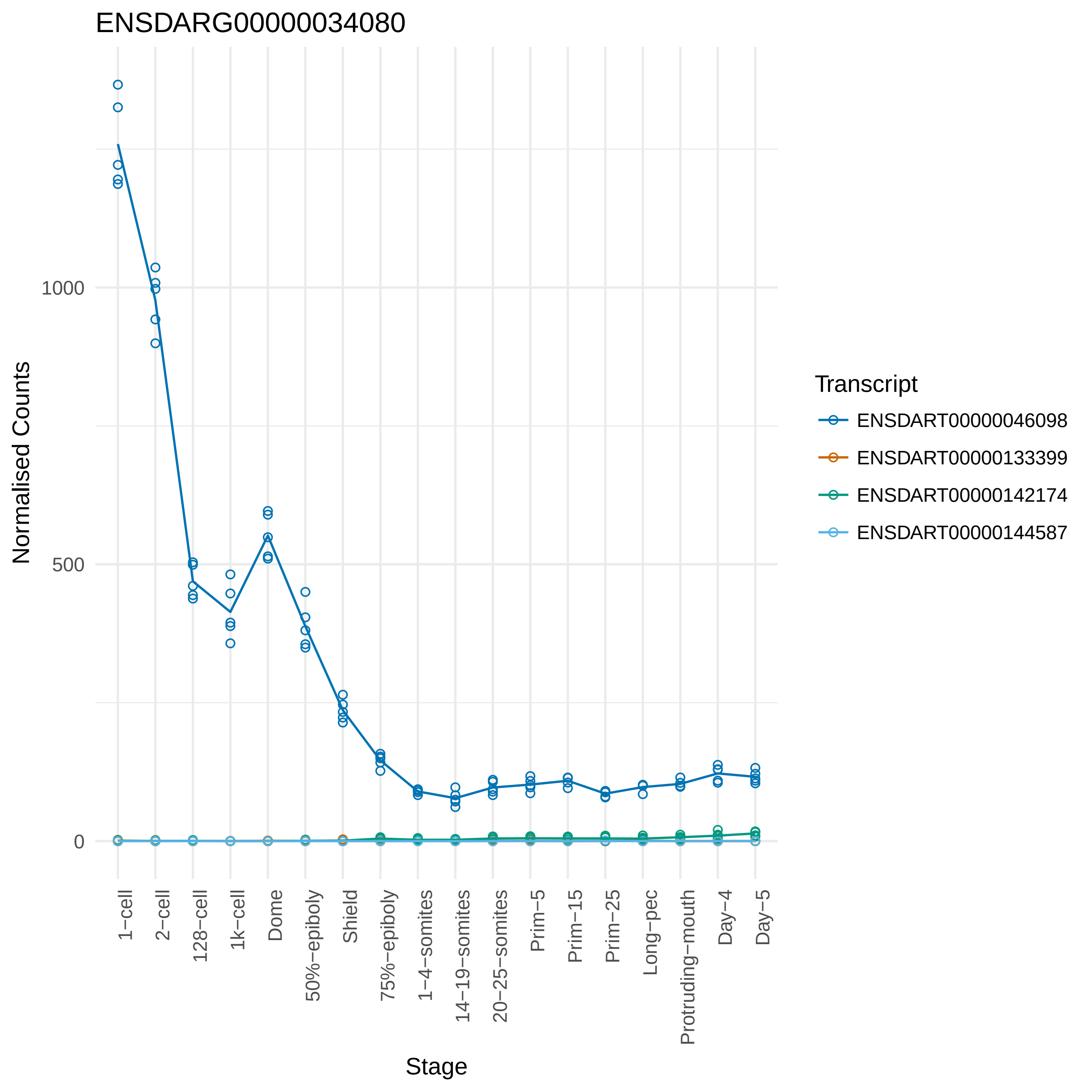

ENSDARG00000034080
Normalised Counts
1000
500
0
1−cell
2−cell
128−cell
1k−cell
Dome
50%−epiboly
Shield
75%−epiboly
1−4−somites
14−19−somites
20−25−somites
Prim−5
Prim−15
Prim−25
Long−pec
Protruding−mouth
Day−4
Day−5
Stage
Transcript
ENSDART00000046098
ENSDART00000133399
ENSDART00000142174
ENSDART00000144587

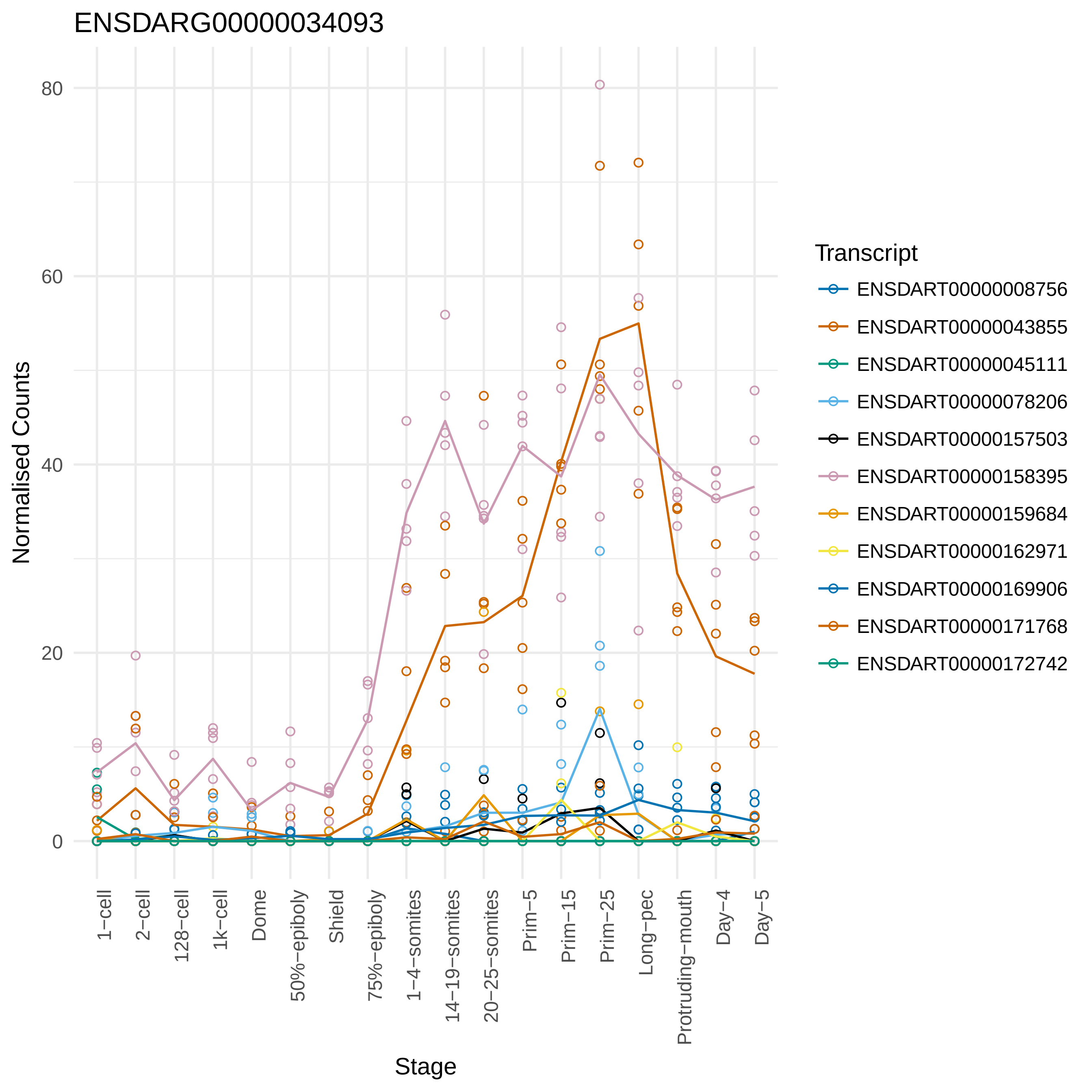

ENSDARG00000034093
Normalised Counts
80
60
40
20
0
1−cell
2−cell
128−cell
1k−cell
Dome
50%−epiboly
Shield
75%−epiboly
1−4−somites
14−19−somites
20−25−somites
Prim−5
Prim−15
Prim−25
Long−pec
Protruding−mouth
Day−4
Day−5
Stage
Transcript
ENSDART00000008756
ENSDART00000043855
ENSDART00000045111
ENSDART00000078206
ENSDART00000157503
ENSDART00000158395
ENSDART00000159684
ENSDART00000162971
ENSDART00000169906
ENSDART00000171768
ENSDART00000172742

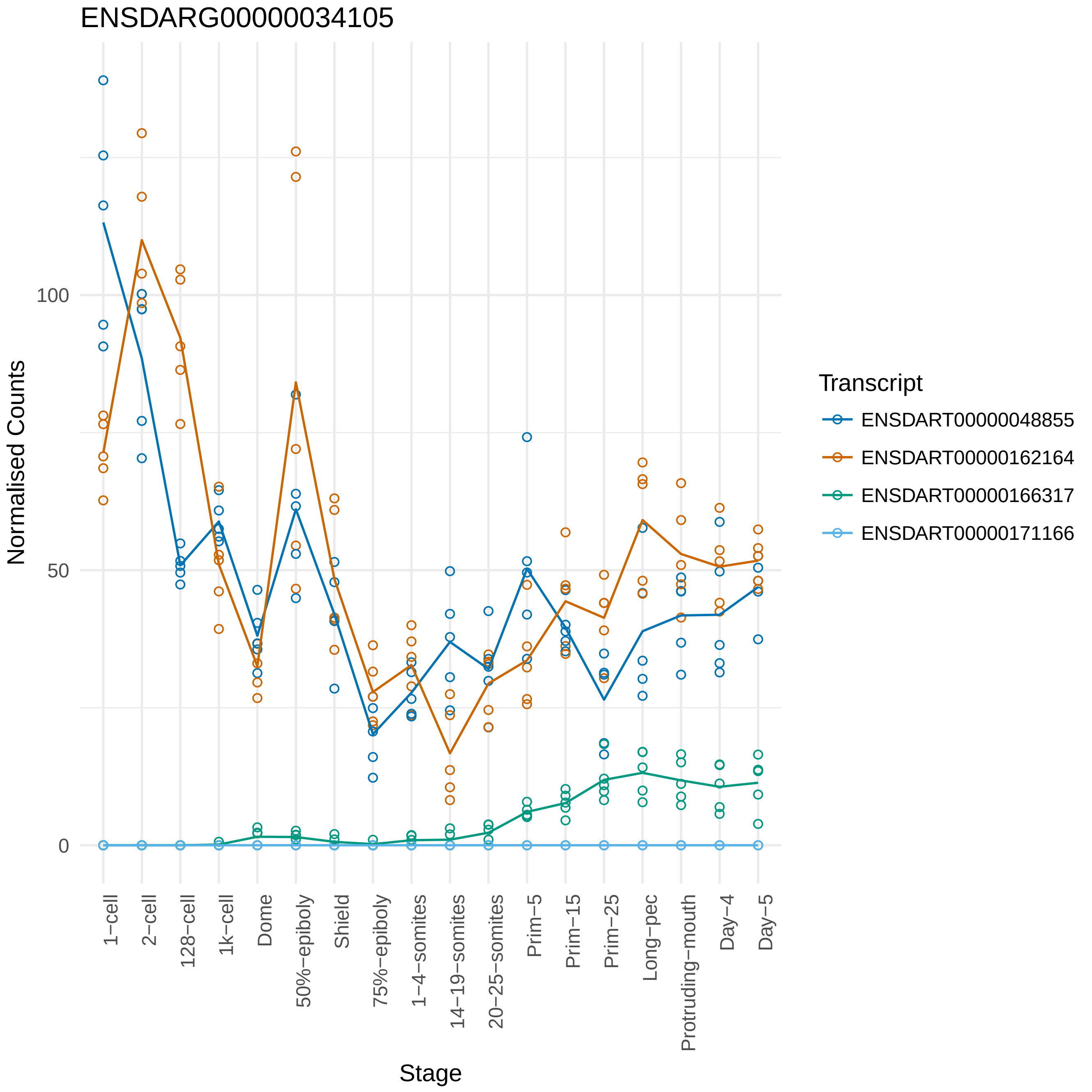

ENSDARG00000034105
Normalised Counts
100
50
0
1−cell
2−cell
128−cell
1k−cell
Dome
50%−epiboly
Shield
75%−epiboly
1−4−somites
14−19−somites
20−25−somites
Prim−5
Prim−15
Prim−25
Long−pec
Protruding−mouth
Day−4
Day−5
Stage
Transcript
ENSDART00000048855
ENSDART00000162164
ENSDART00000166317
ENSDART00000171166

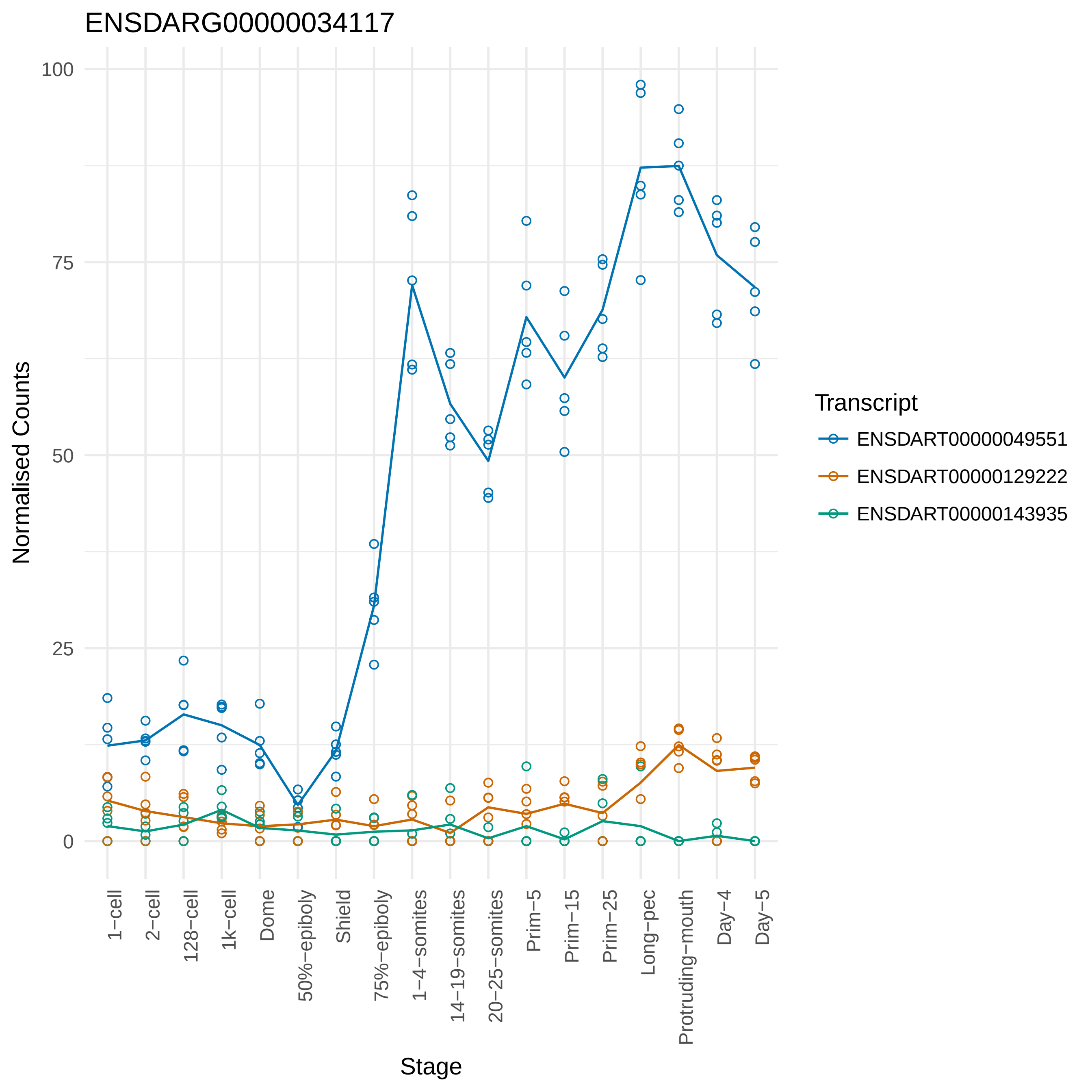

ENSDARG00000034117
Normalised Counts
100
75
50
25
0
1−cell
2−cell
128−cell
1k−cell
Dome
50%−epiboly
Shield
75%−epiboly
1−4−somites
14−19−somites
20−25−somites
Prim−5
Prim−15
Prim−25
Long−pec
Protruding−mouth
Day−4
Day−5
Stage
Transcript
ENSDART00000049551
ENSDART00000129222
ENSDART00000143935

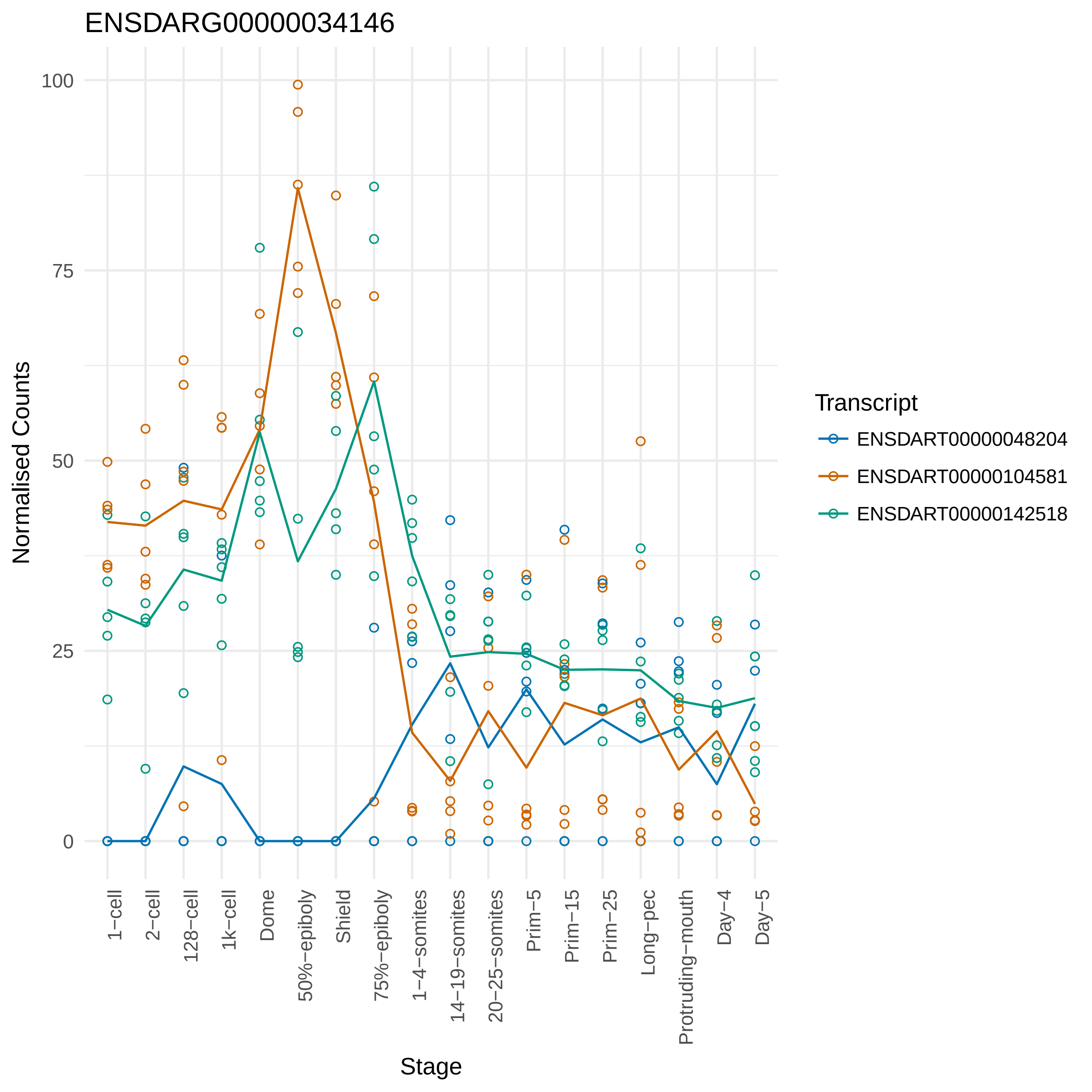
ENSDARG00000034146
Normalised Counts
100
75
50
25
0
1−cell
2−cell
128−cell
1k−cell
Dome
50%−epiboly
Shield
75%−epiboly
1−4−somites
14−19−somites
20−25−somites
Prim−5
Prim−15
Prim−25
Long−pec
Protruding−mouth
Day−4
Day−5
Stage
Transcript
ENSDART00000048204
ENSDART00000104581
ENSDART00000142518

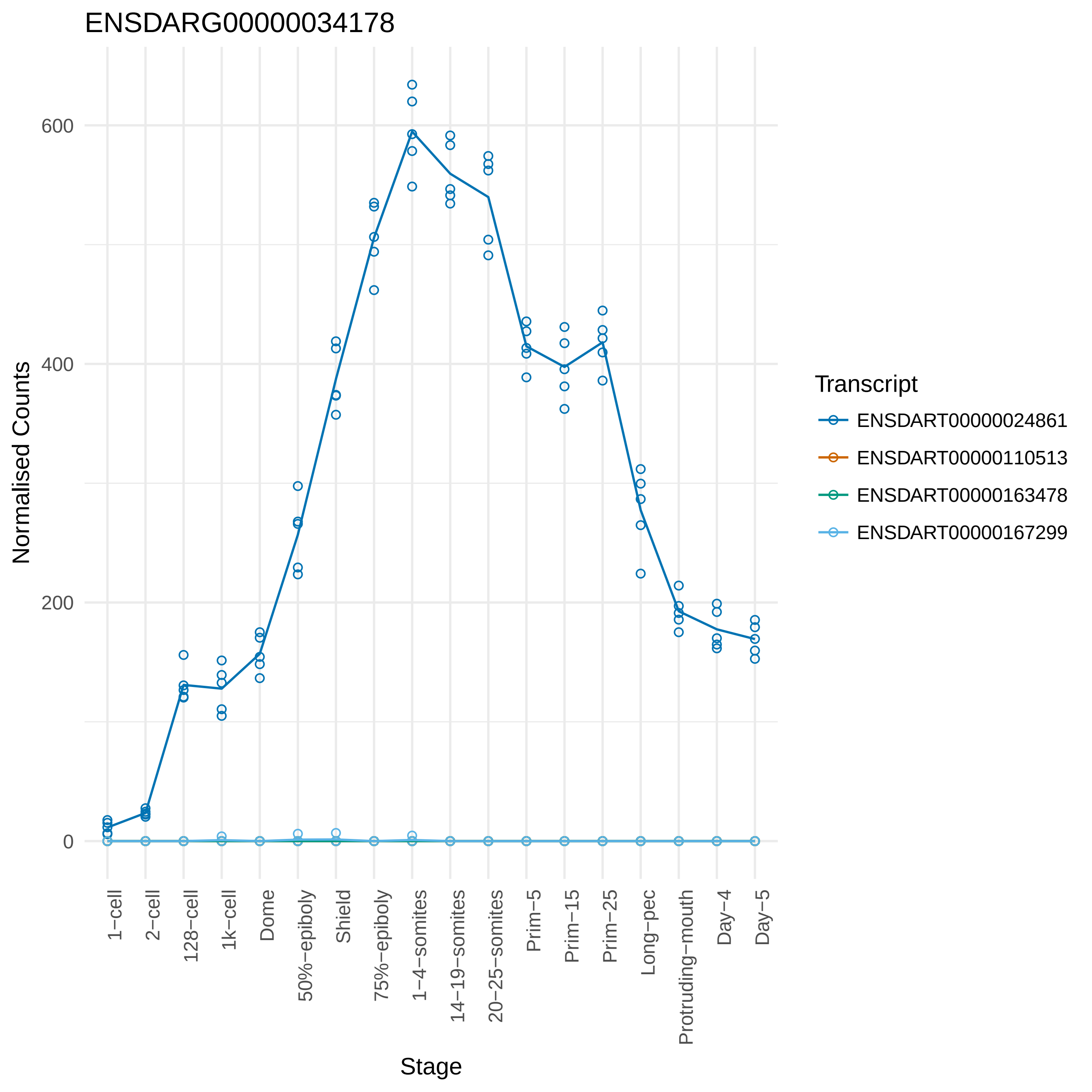

ENSDARG00000034178
Normalised Counts
600
400
200
0
Stage
1−cell
2−cell
128−cell
1k−cell
Dome
50%−epiboly
Shield
75%−epiboly
1−4−somites
14−19−somites
20−25−somites
Prim−5
Prim−15
Prim−25
Long−pec
Protruding−mouth
Day−4
Day−5
Transcript
ENSDART00000024861
ENSDART00000110513
ENSDART00000163478
ENSDART00000167299

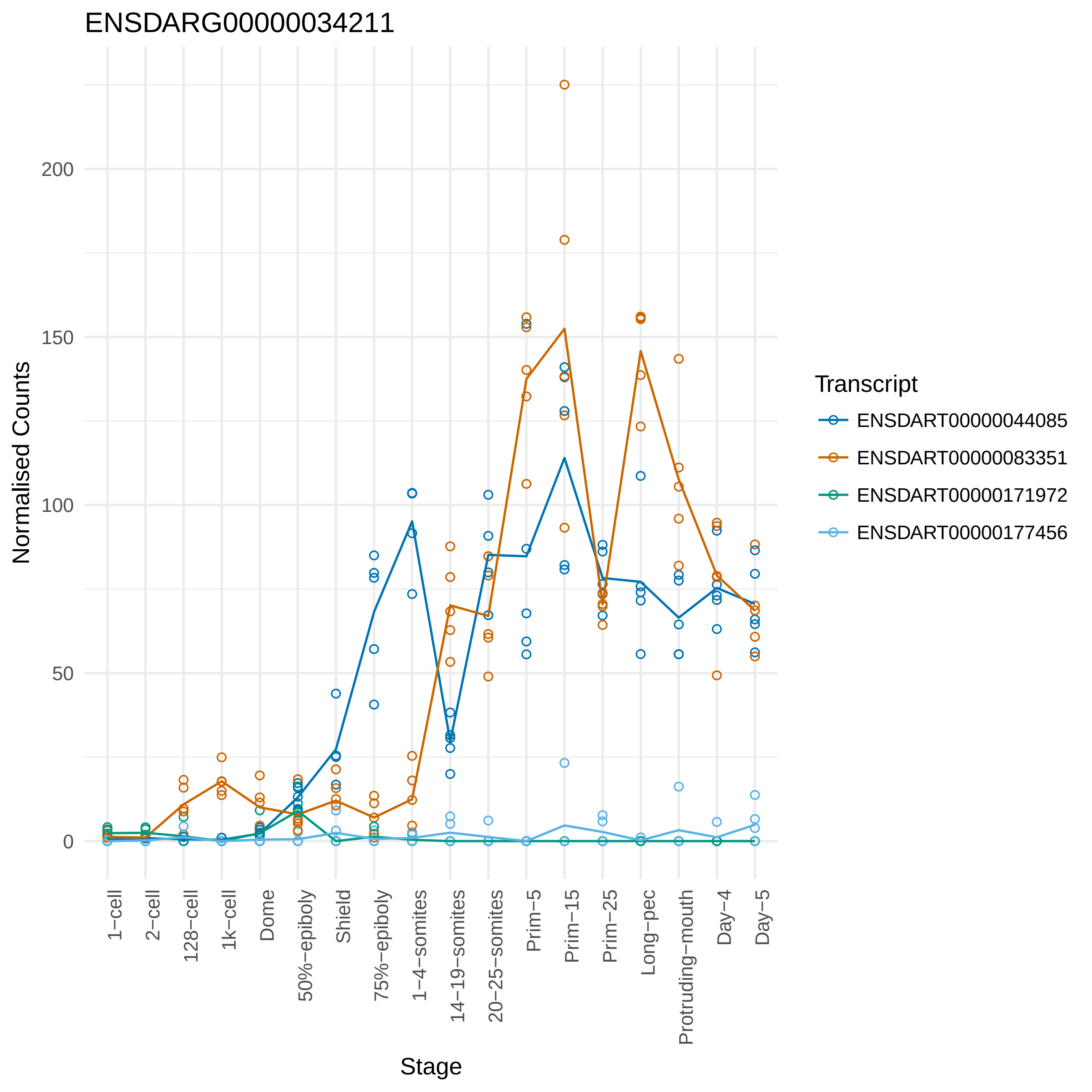

ENSDARG00000034211
Normalised Counts
0
50
100
150
200
Stage
1−cell
2−cell
128−cell
1k−cell
Dome
50%−epiboly
Shield
75%−epiboly
1−4−somites
14−19−somites
20−25−somites
Prim−5
Prim−15
Prim−25
Long−pec
Protruding−mouth
Day−4
Day−5
Transcript
ENSDART00000044085
ENSDART00000083351
ENSDART00000171972
ENSDART00000177456

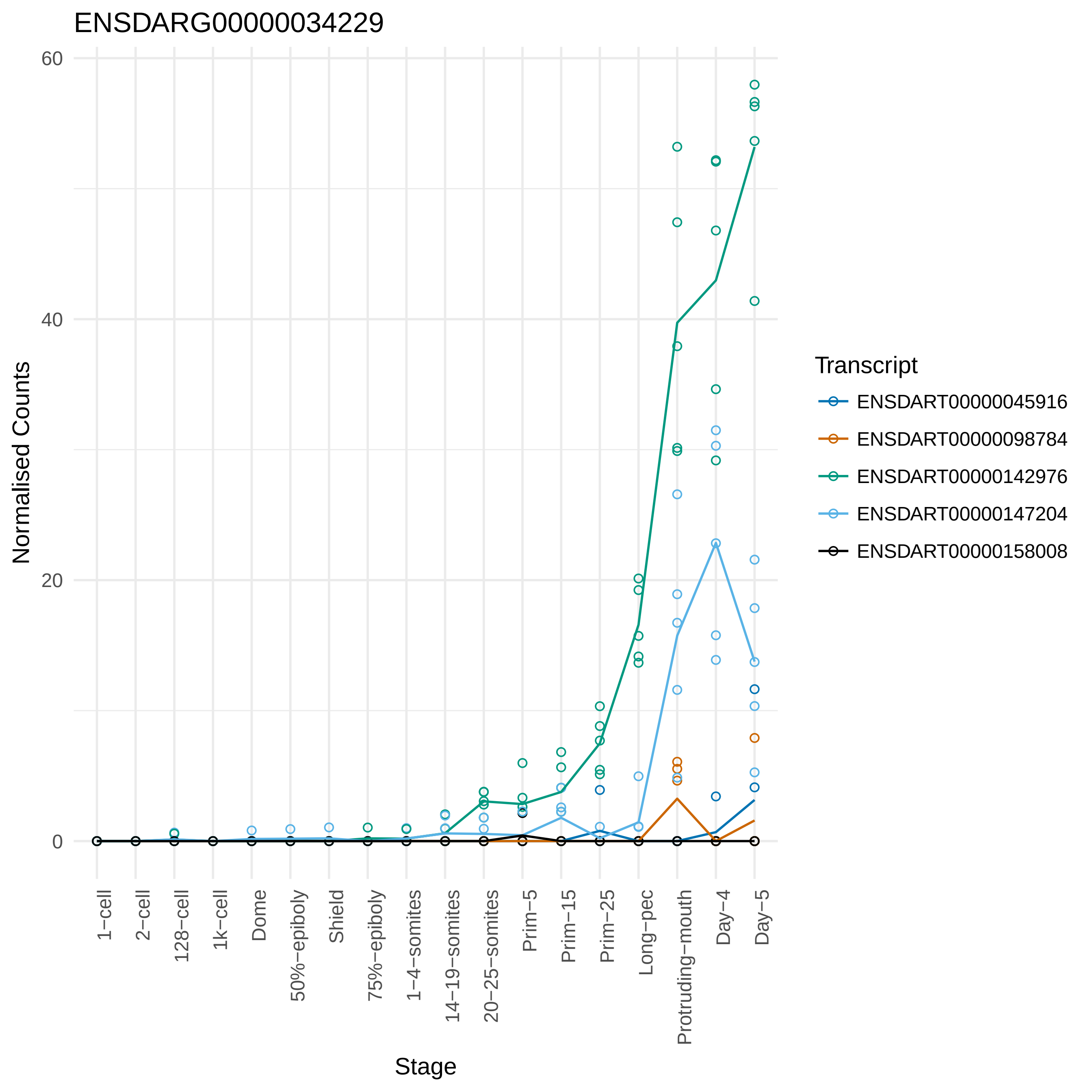

ENSDARG00000034229
Normalised Counts
0
20
40
60
Stage
1−cell
2−cell
128−cell
1k−cell
Dome
50%−epiboly
Shield
75%−epiboly
1−4−somites
14−19−somites
20−25−somites
Prim−5
Prim−15
Prim−25
Long−pec
Protruding−mouth
Day−4
Day−5
Transcript
ENSDART00000045916
ENSDART00000098784
ENSDART00000142976
ENSDART00000147204
ENSDART00000158008

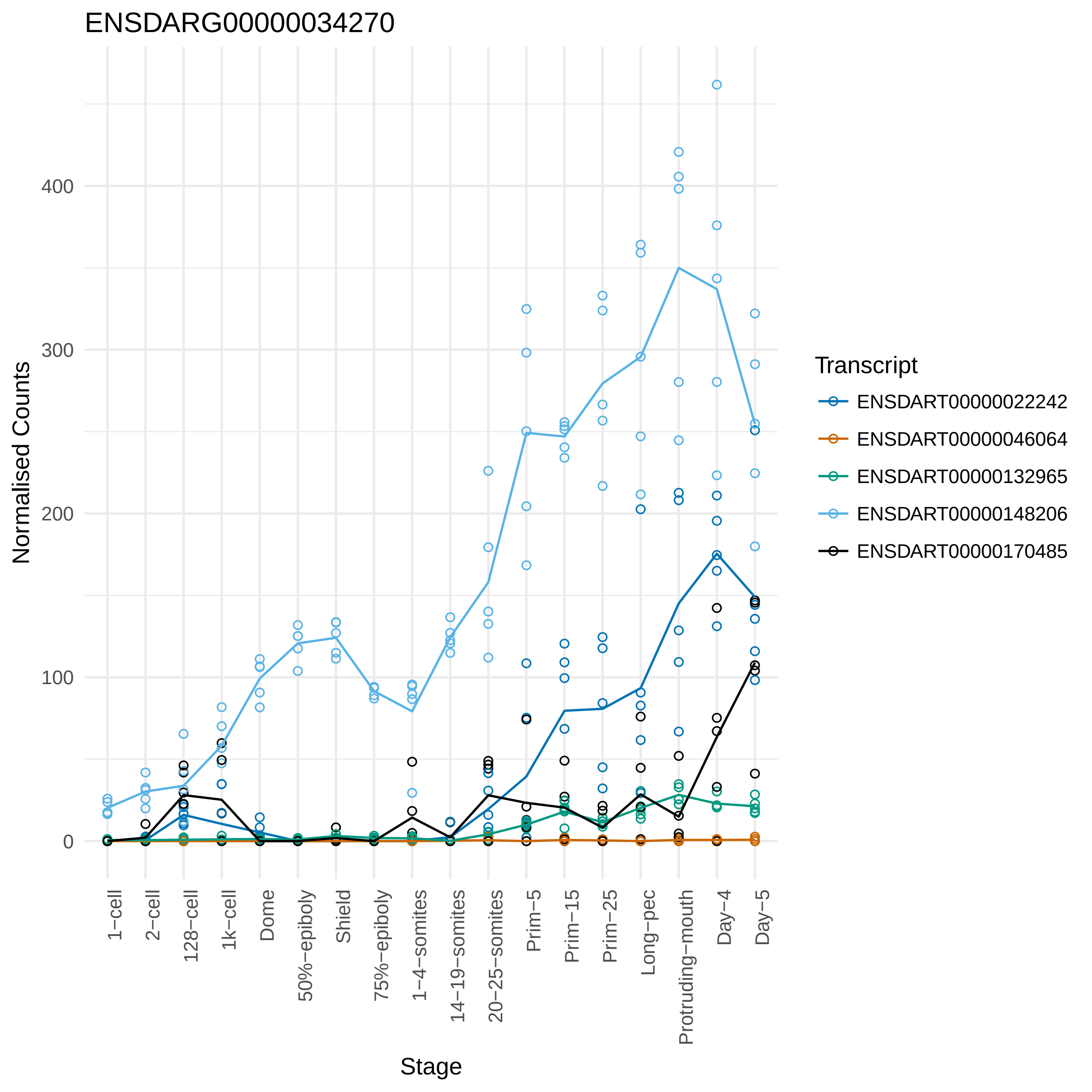

ENSDARG00000034270
Normalised Counts
0
100
200
300
400
Stage
1−cell
2−cell
128−cell
1k−cell
Dome
50%−epiboly
Shield
75%−epiboly
1−4−somites
14−19−somites
20−25−somites
Prim−5
Prim−15
Prim−25
Long−pec
Protruding−mouth
Day−4
Day−5
Transcript
ENSDART00000022242
ENSDART00000046064
ENSDART00000132965
ENSDART00000148206
ENSDART00000170485

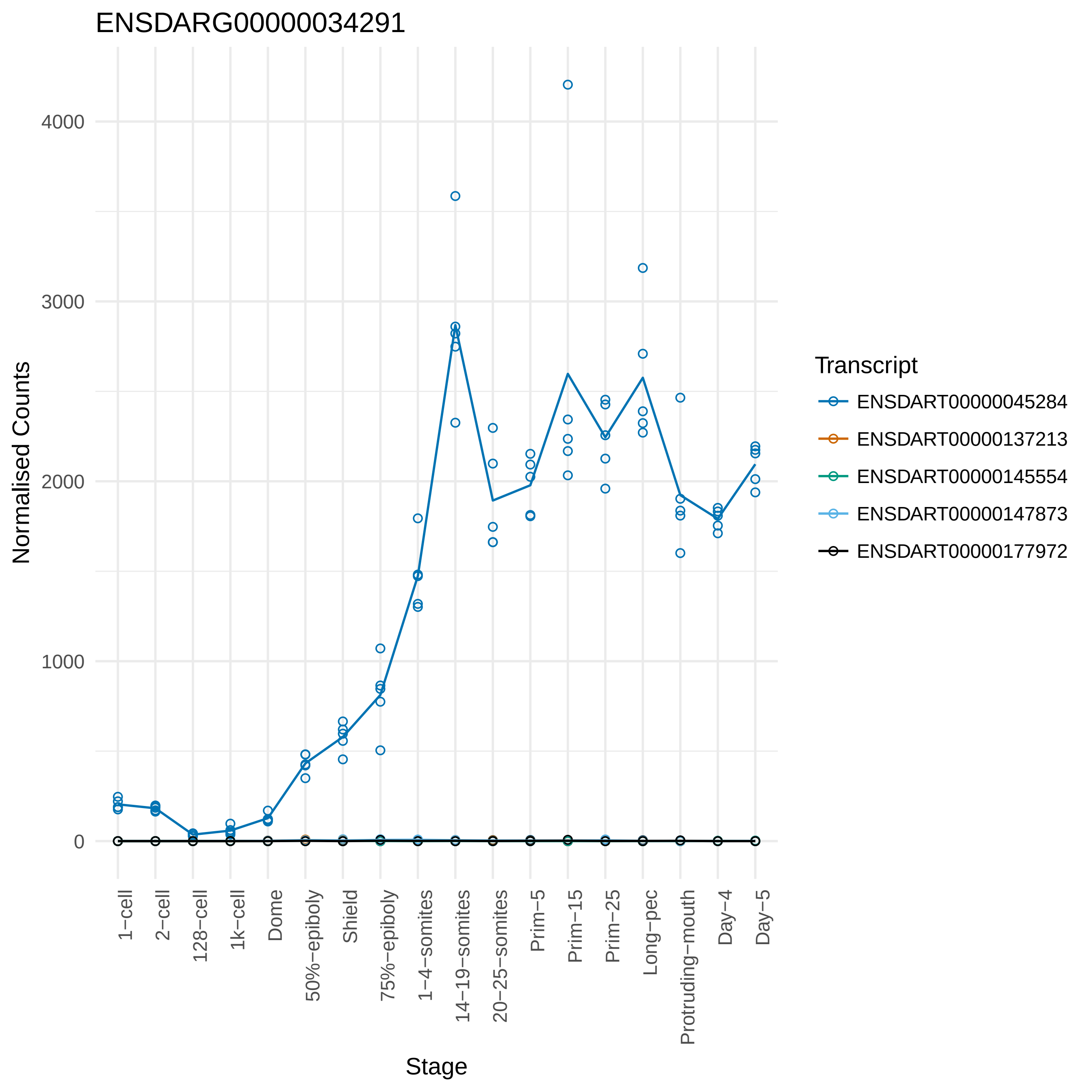

ENSDARG00000034291
Normalised Counts
4000
3000
2000
1000
0
1−cell
2−cell
128−cell
1k−cell
Dome
50%−epiboly
Shield
75%−epiboly
1−4−somites
14−19−somites
20−25−somites
Prim−5
Prim−15
Prim−25
Long−pec
Protruding−mouth
Day−4
Day−5
Stage
Transcript
ENSDART00000045284
ENSDART00000137213
ENSDART00000145554
ENSDART00000147873
ENSDART00000177972

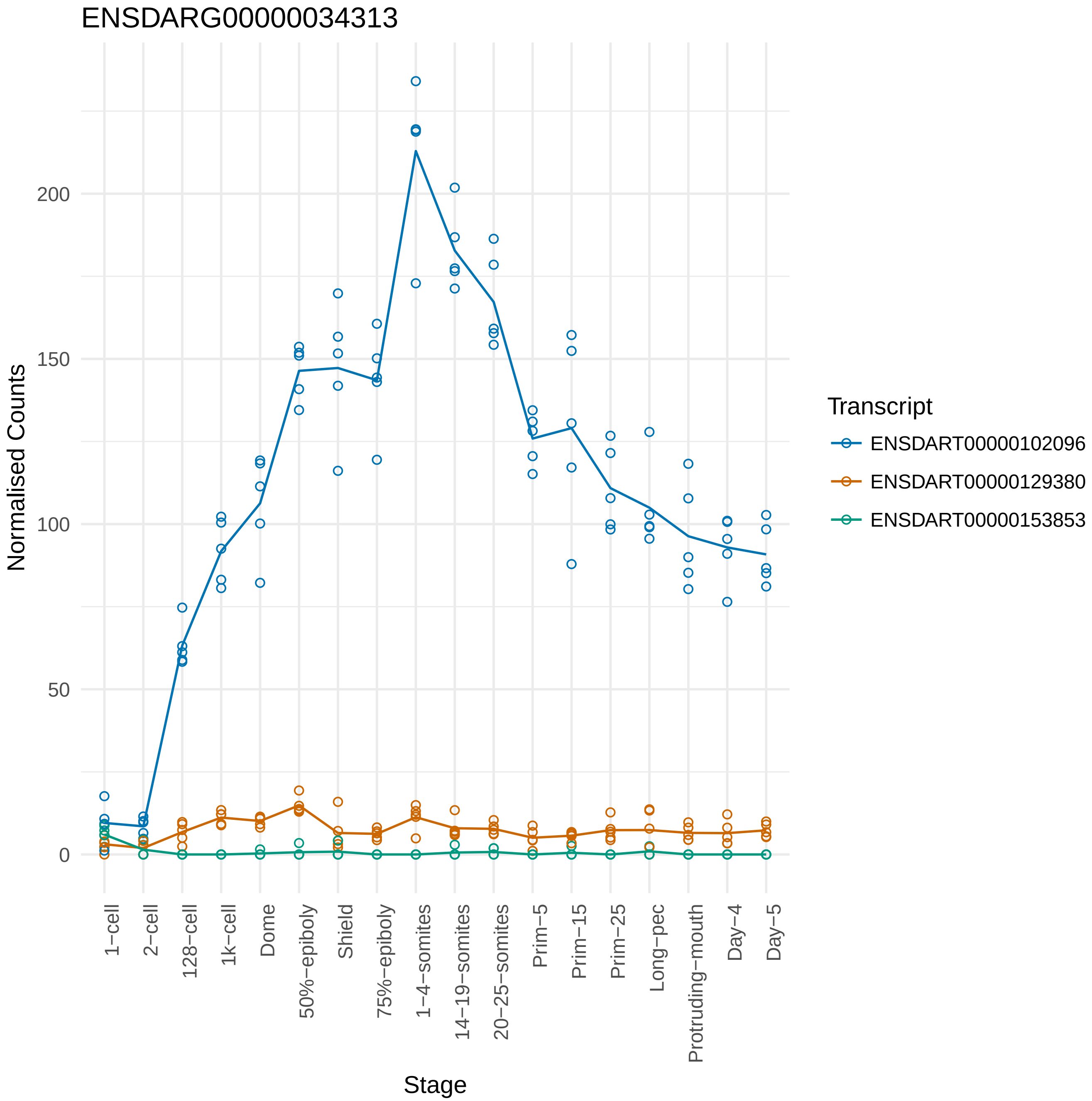
ENSDARG00000034313
Normalised Counts
0
50
100
150
200
Stage
1−cell
2−cell
128−cell
1k−cell
Dome
50%−epiboly
Shield
75%−epiboly
1−4−somites
14−19−somites
20−25−somites
Prim−5
Prim−15
Prim−25
Long−pec
Protruding−mouth
Day−4
Day−5
Transcript
ENSDART00000102096
ENSDART00000129380
ENSDART00000153853

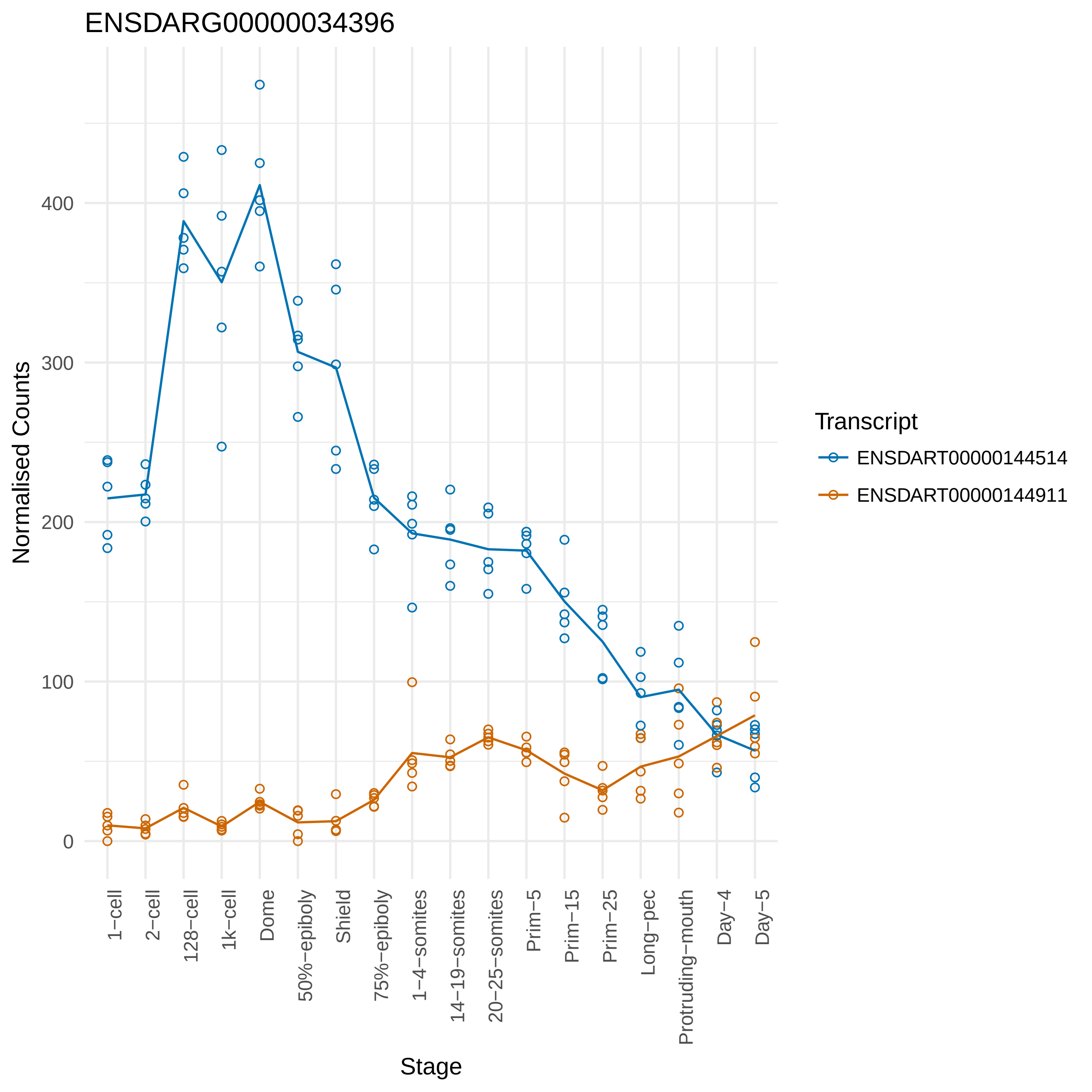
ENSDARG00000034396
Normalised Counts
0
100
200
300
400
Stage
1−cell
2−cell
128−cell
1k−cell
Dome
50%−epiboly
Shield
75%−epiboly
1−4−somites
14−19−somites
20−25−somites
Prim−5
Prim−15
Prim−25
Long−pec
Protruding−mouth
Day−4
Day−5
Transcript
ENSDART00000144514
ENSDART00000144911

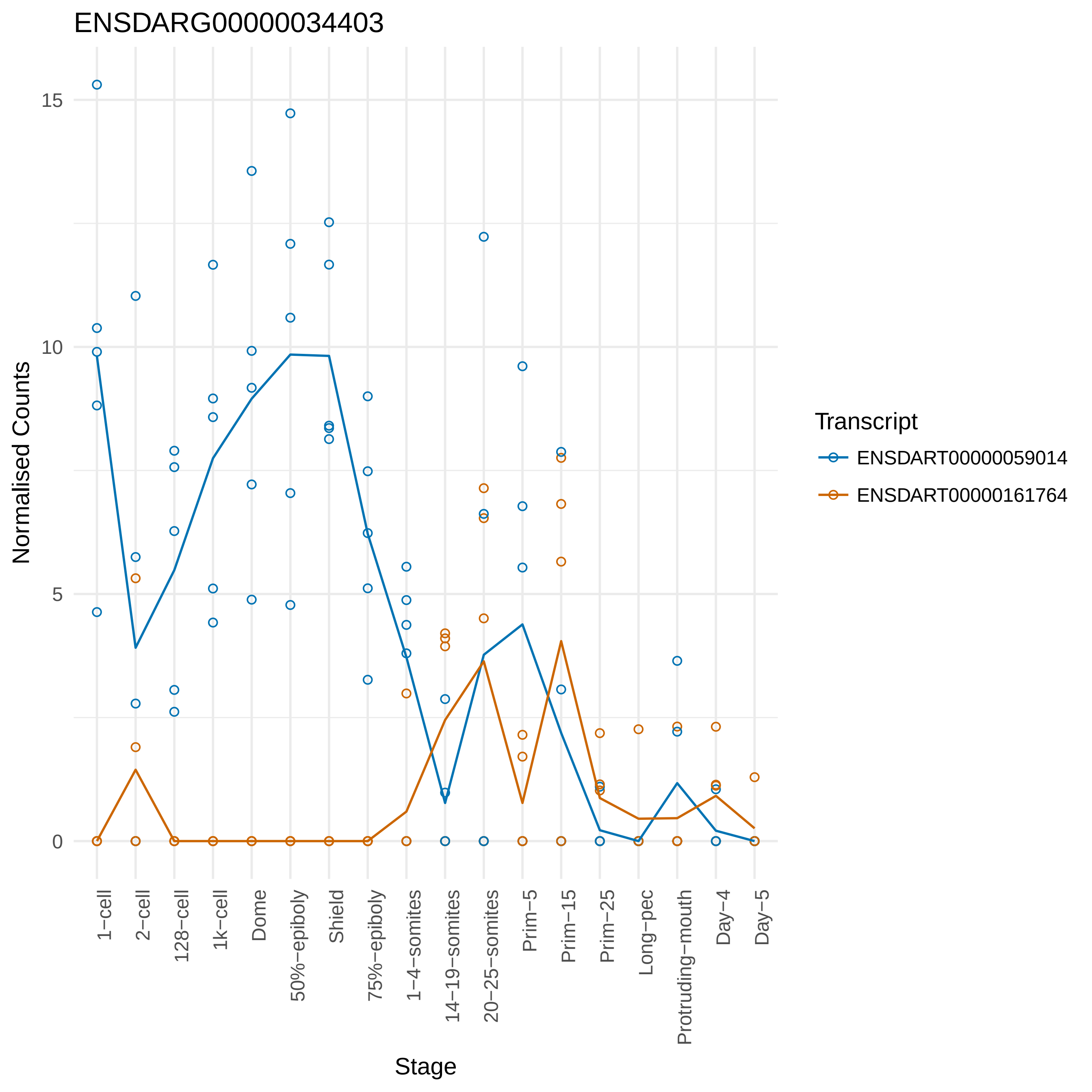

ENSDARG00000034403
Normalised Counts
0
5
10
15
Stage
1−cell
2−cell
128−cell
1k−cell
Dome
50%−epiboly
Shield
75%−epiboly
1−4−somites
14−19−somites
20−25−somites
Prim−5
Prim−15
Prim−25
Long−pec
Protruding−mouth
Day−4
Day−5
Transcript
ENSDART00000059014
ENSDART00000161764

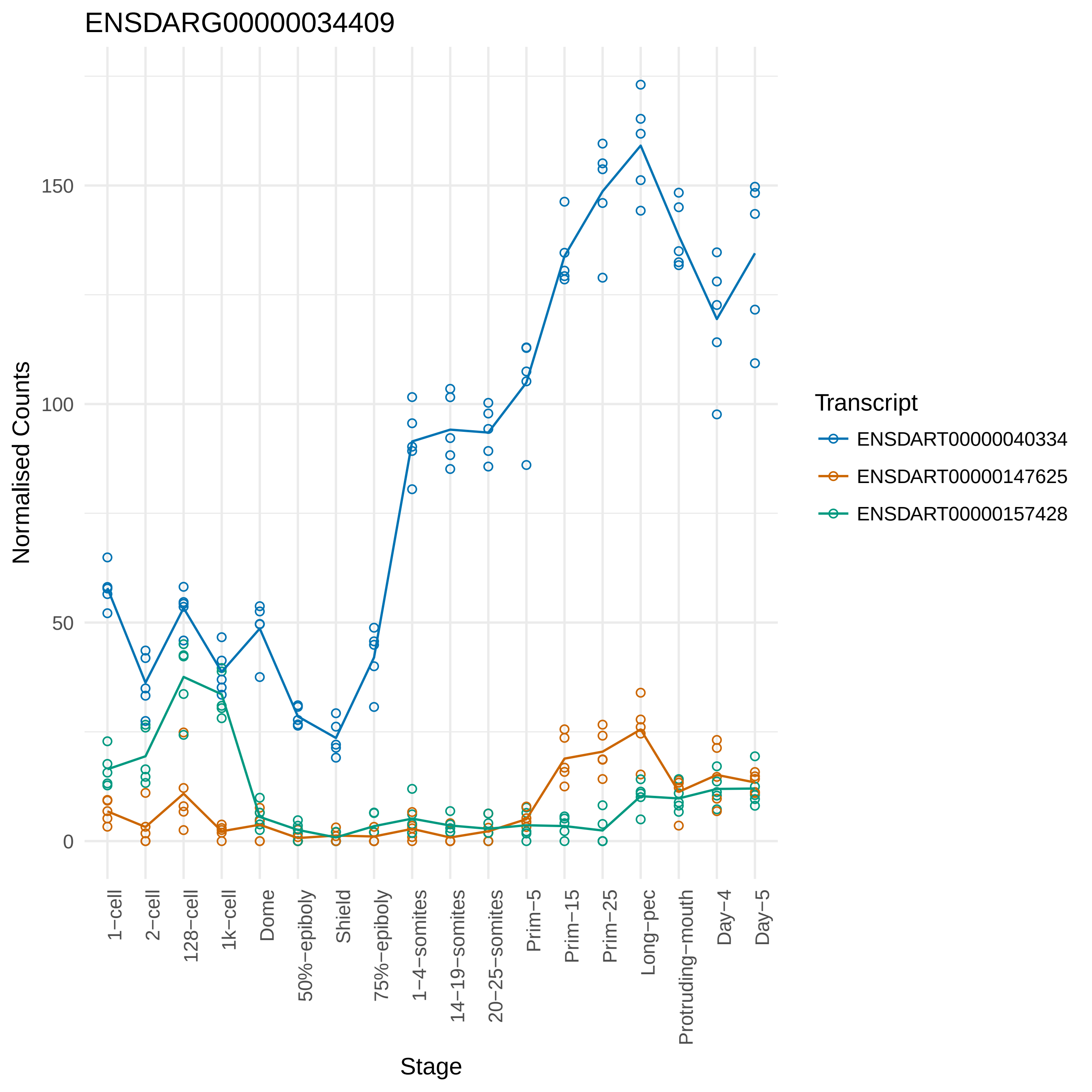
ENSDARG00000034409
Normalised Counts
0
50
100
150
1−cell
2−cell
128−cell
1k−cell
Dome
50%−epiboly
Shield
75%−epiboly
1−4−somites
14−19−somites
20−25−somites
Prim−5
Prim−15
Prim−25
Long−pec
Protruding−mouth
Day−4
Day−5
Stage
Transcript
ENSDART00000040334
ENSDART00000147625
ENSDART00000157428

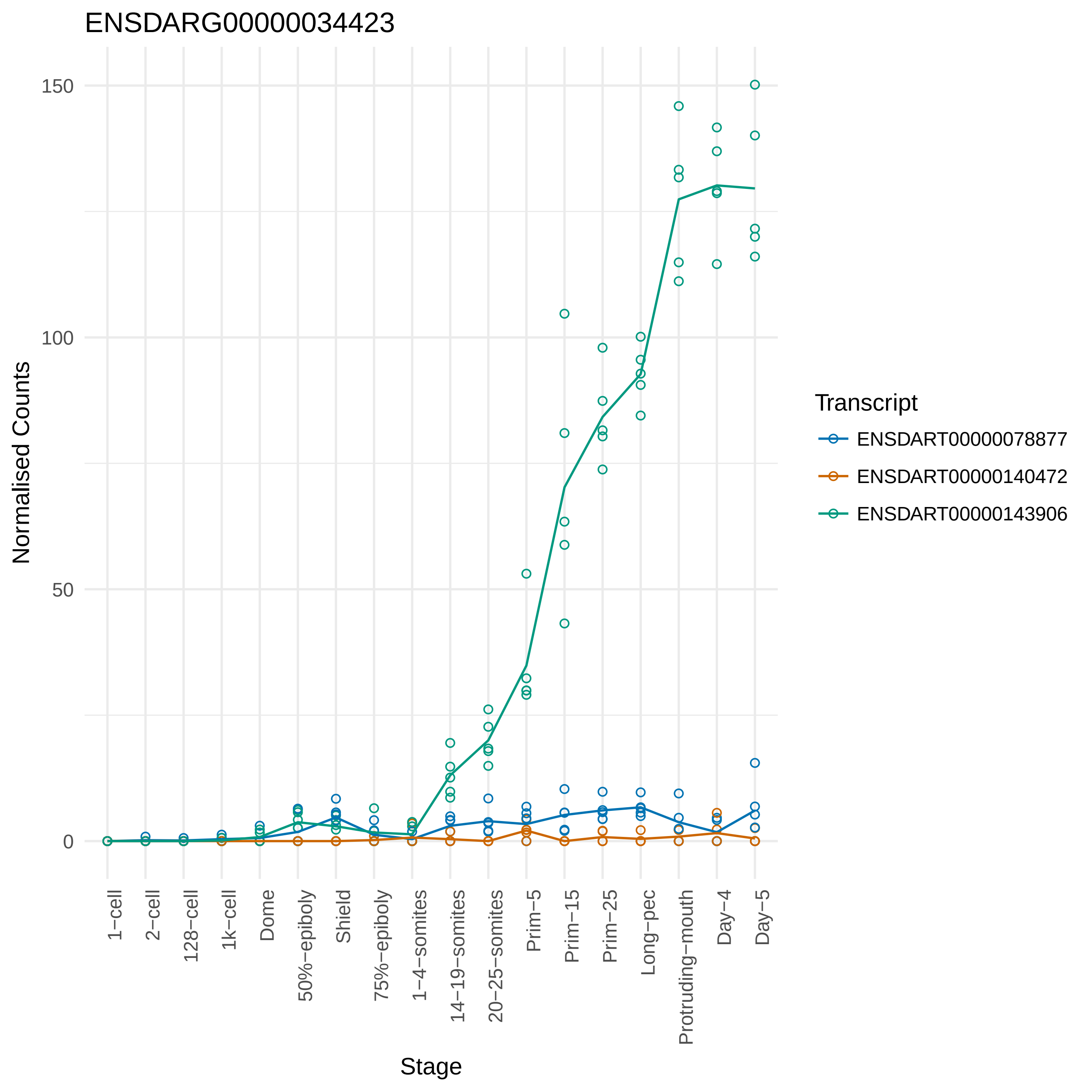

ENSDARG00000034423
Normalised Counts
150
100
50
0
1−cell
2−cell
128−cell
1k−cell
Dome
50%−epiboly
Shield
75%−epiboly
1−4−somites
14−19−somites
20−25−somites
Prim−5
Prim−15
Prim−25
Long−pec
Protruding−mouth
Day−4
Day−5
Stage
Transcript
ENSDART00000078877
ENSDART00000140472
ENSDART00000143906

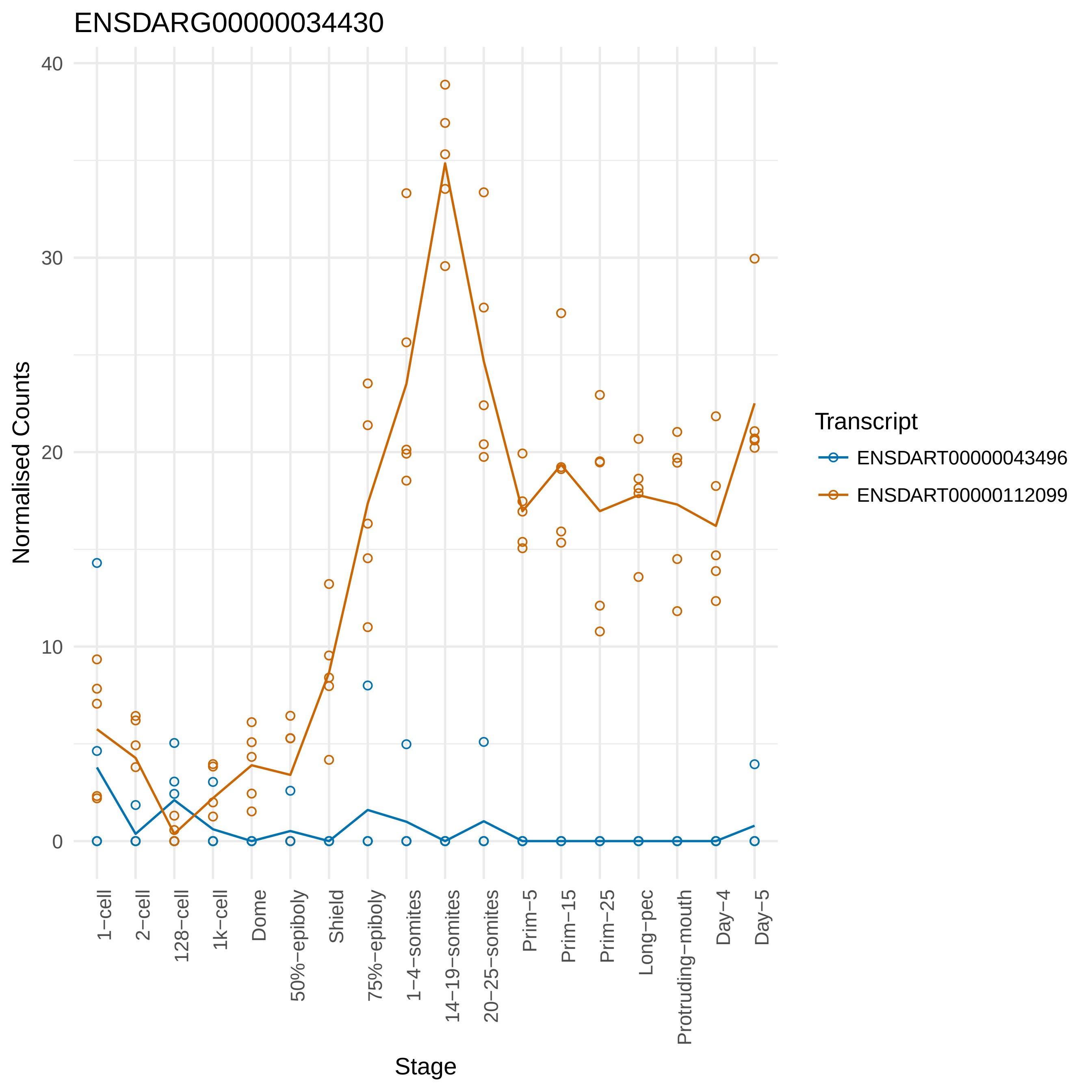

ENSDARG00000034430
Normalised Counts
Stage
0
10
20
30
40
1−cell
2−cell
128−cell
1k−cell
Dome
50%−epiboly
Shield
75%−epiboly
1−4−somites
14−19−somites
20−25−somites
Prim−5
Prim−15
Prim−25
Long−pec
Protruding−mouth
Day−4
Day−5
Transcript
ENSDART00000043496
ENSDART00000112099

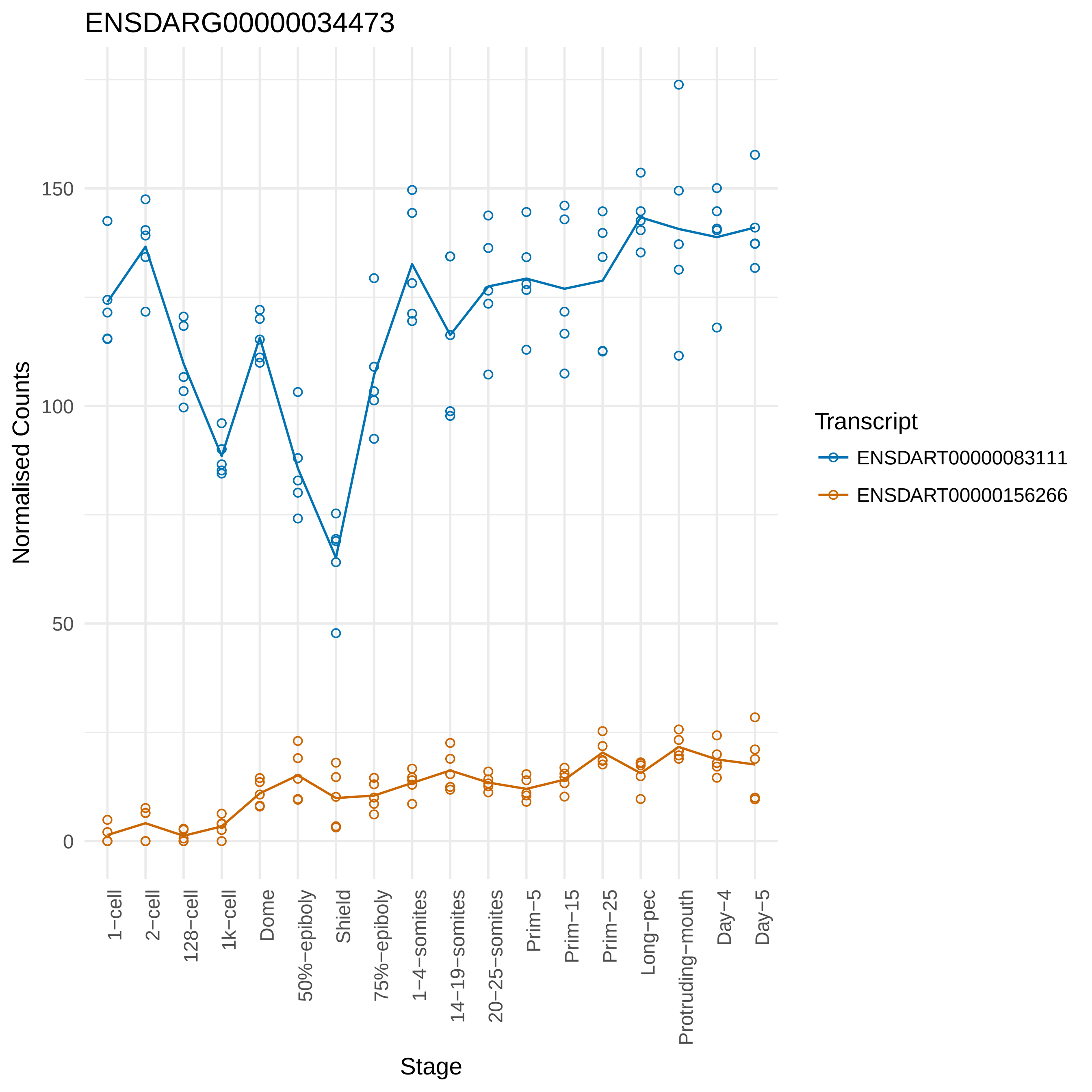

ENSDARG00000034473
Normalised Counts
150
100
50
0
1−cell
2−cell
128−cell
1k−cell
Dome
50%−epiboly
Shield
75%−epiboly
1−4−somites
14−19−somites
20−25−somites
Prim−5
Prim−15
Prim−25
Long−pec
Protruding−mouth
Day−4
Day−5
Stage
Transcript
ENSDART00000083111
ENSDART00000156266

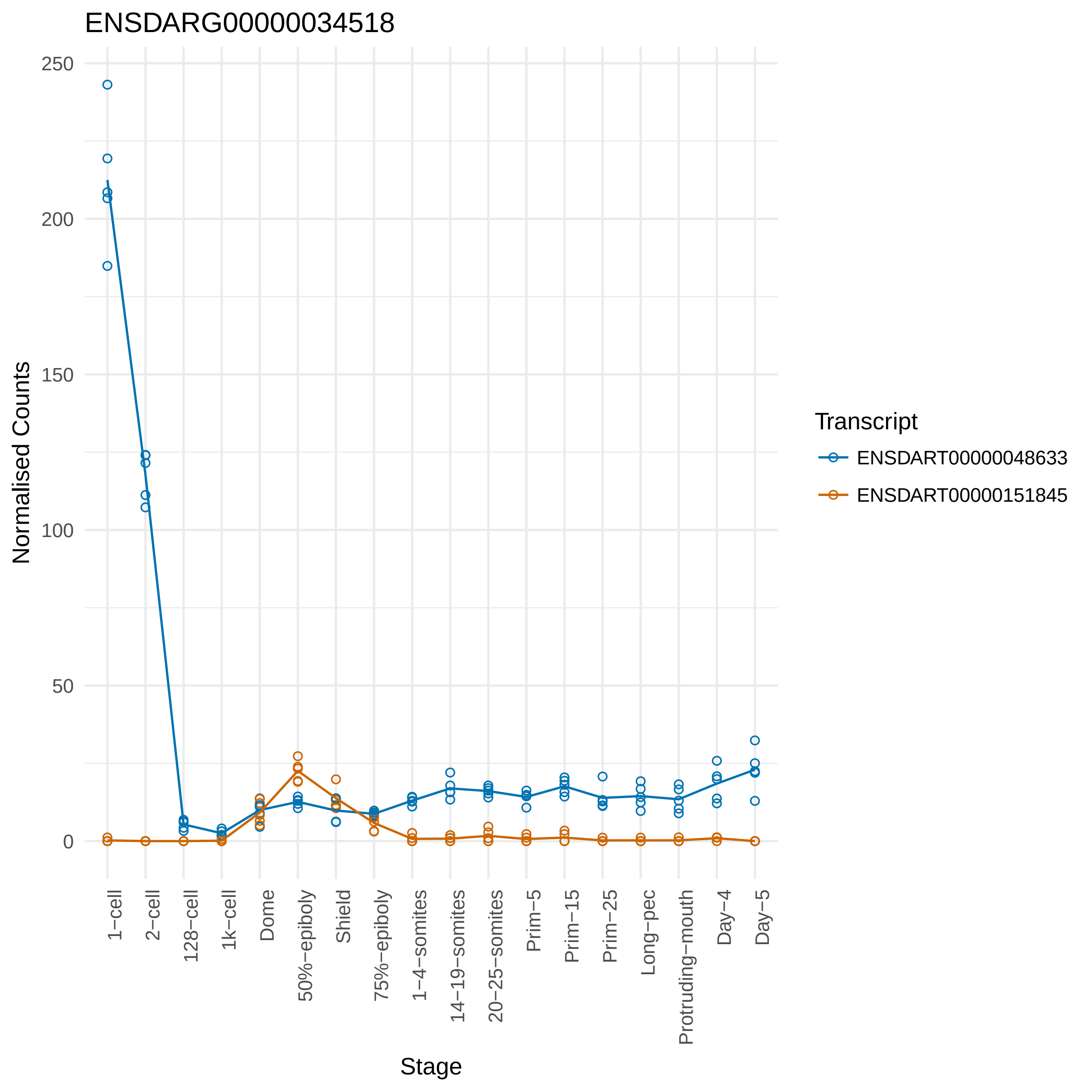

ENSDARG00000034518
Normalised Counts
250
200
150
100
50
0
1−cell
2−cell
128−cell
1k−cell
Dome
50%−epiboly
Shield
75%−epiboly
1−4−somites
14−19−somites
20−25−somites
Prim−5
Prim−15
Prim−25
Long−pec
Protruding−mouth
Day−4
Day−5
Stage
Transcript
ENSDART00000048633
ENSDART00000151845

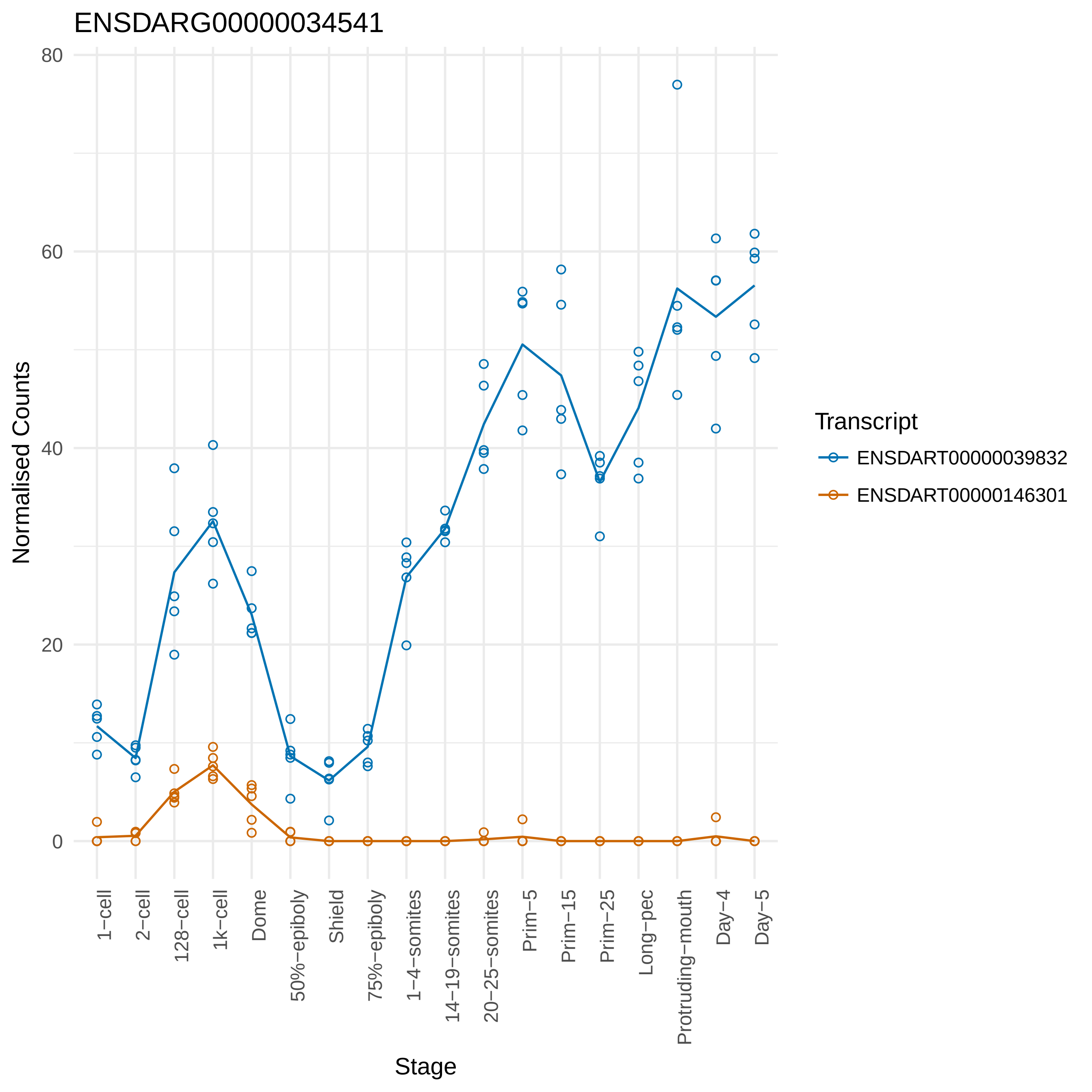
ENSDARG00000034541
Normalised Counts
80
60
40
20
0
1−cell
2−cell
128−cell
1k−cell
Dome
50%−epiboly
Shield
75%−epiboly
1−4−somites
14−19−somites
20−25−somites
Prim−5
Prim−15
Prim−25
Long−pec
Protruding−mouth
Day−4
Day−5
Stage
Transcript
ENSDART00000039832
ENSDART00000146301

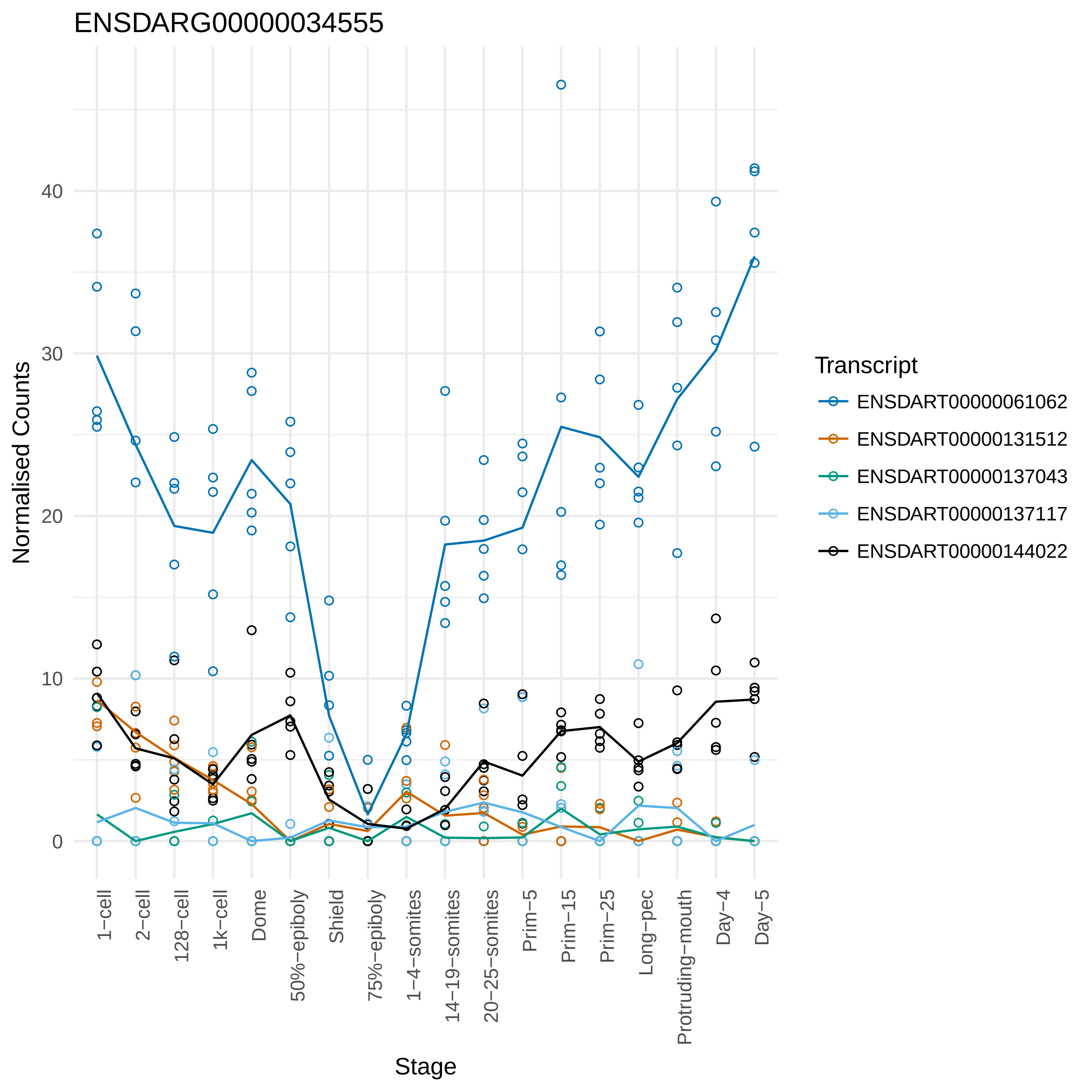

ENSDARG00000034555
Normalised Counts
0
10
20
30
40
Stage
1−cell
2−cell
128−cell
1k−cell
Dome
50%−epiboly
Shield
75%−epiboly
1−4−somites
14−19−somites
20−25−somites
Prim−5
Prim−15
Prim−25
Long−pec
Protruding−mouth
Day−4
Day−5
Transcript
ENSDART00000061062
ENSDART00000131512
ENSDART00000137043
ENSDART00000137117
ENSDART00000144022

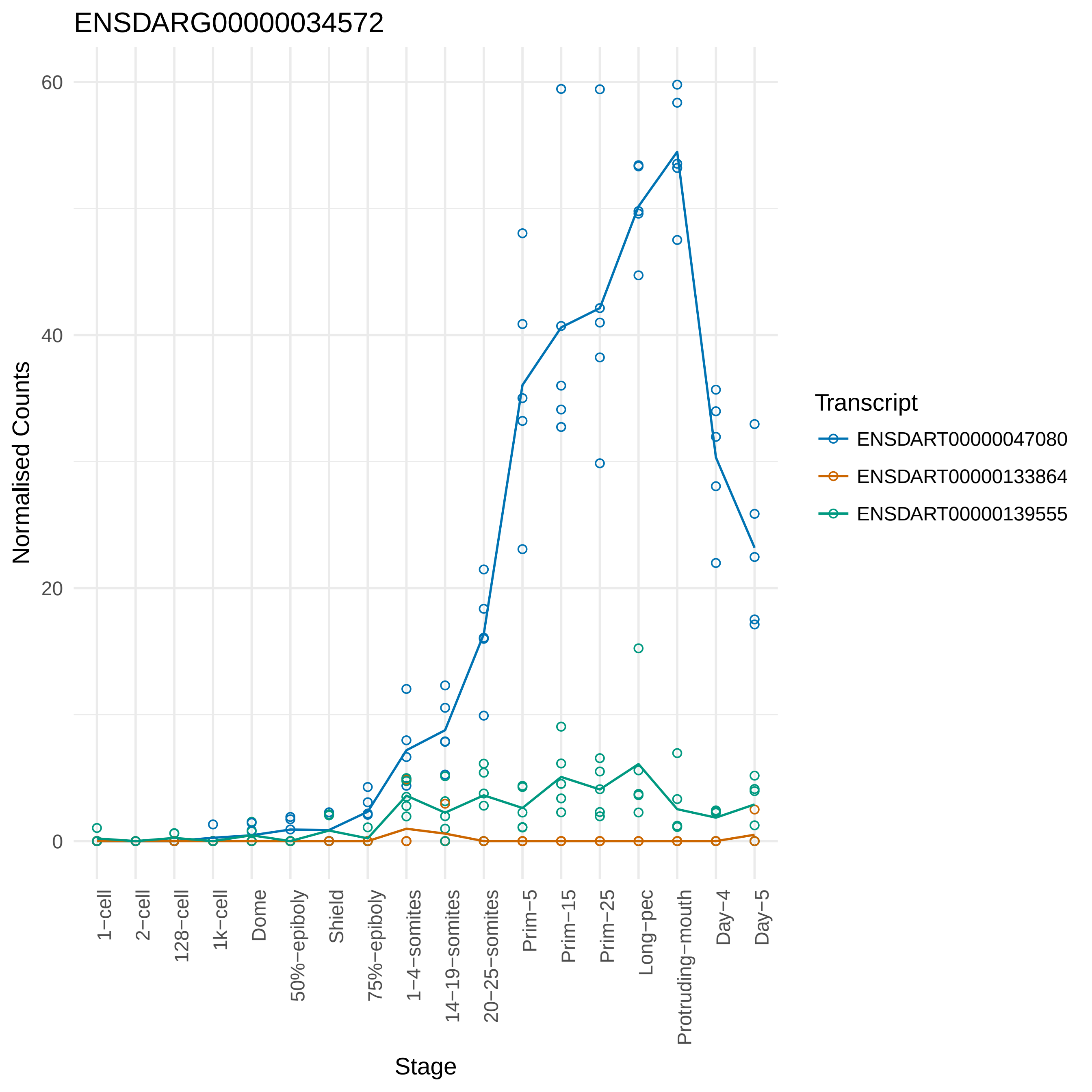
ENSDARG00000034572
Normalised Counts
0
20
40
60
Stage
1−cell
2−cell
128−cell
1k−cell
Dome
50%−epiboly
Shield
75%−epiboly
1−4−somites
14−19−somites
20−25−somites
Prim−5
Prim−15
Prim−25
Long−pec
Protruding−mouth
Day−4
Day−5
Transcript
ENSDART00000047080
ENSDART00000133864
ENSDART00000139555

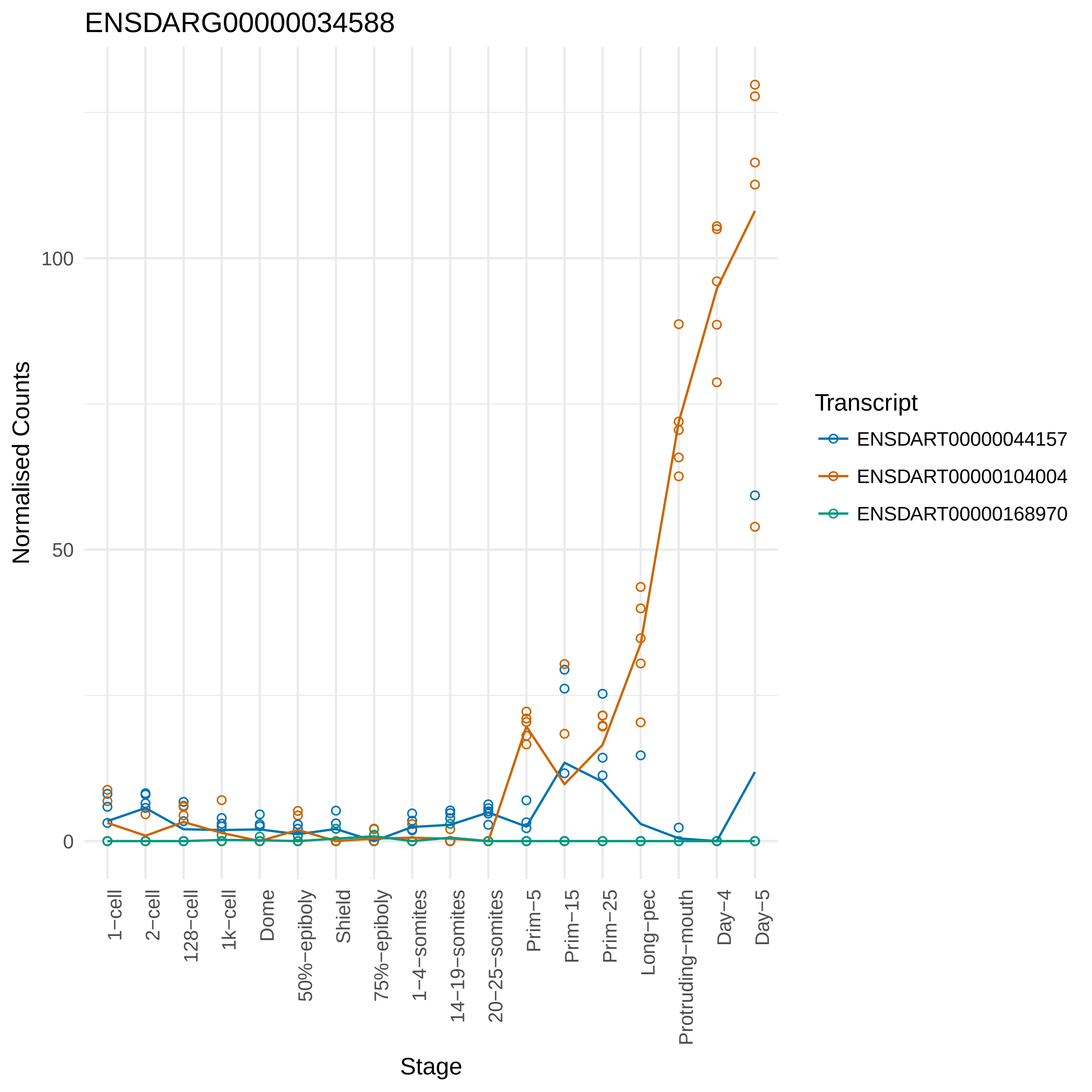

ENSDARG00000034588
Normalised Counts
100
50
0
1−cell
2−cell
128−cell
1k−cell
Dome
50%−epiboly
Shield
75%−epiboly
1−4−somites
14−19−somites
20−25−somites
Prim−5
Prim−15
Prim−25
Long−pec
Protruding−mouth
Day−4
Day−5
Stage
Transcript
ENSDART00000044157
ENSDART00000104004
ENSDART00000168970

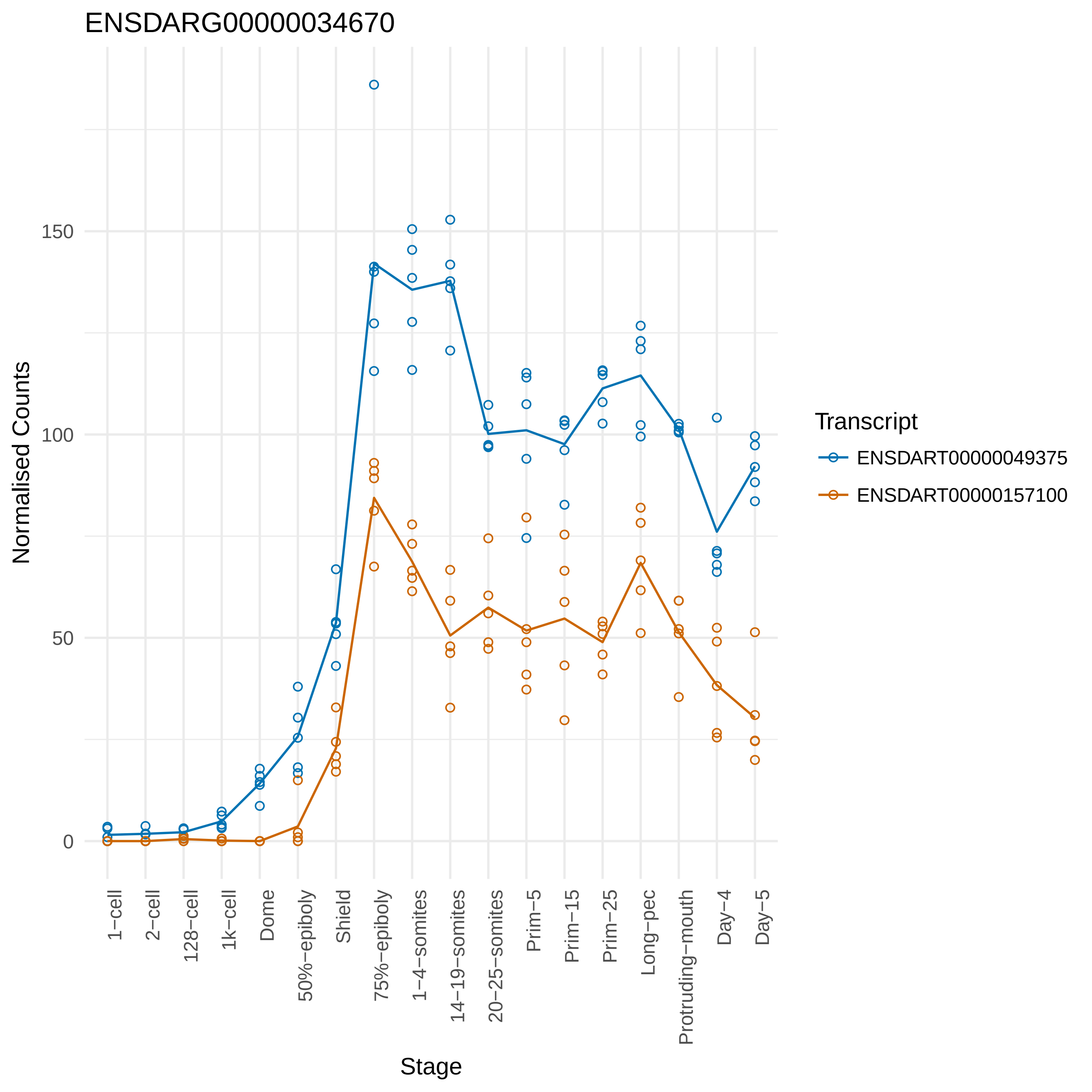

ENSDARG00000034670
Normalised Counts
150
100
50
0
1−cell
2−cell
128−cell
1k−cell
Dome
50%−epiboly
Shield
75%−epiboly
1−4−somites
14−19−somites
20−25−somites
Prim−5
Prim−15
Prim−25
Long−pec
Protruding−mouth
Day−4
Day−5
Stage
Transcript
ENSDART00000049375
ENSDART00000157100

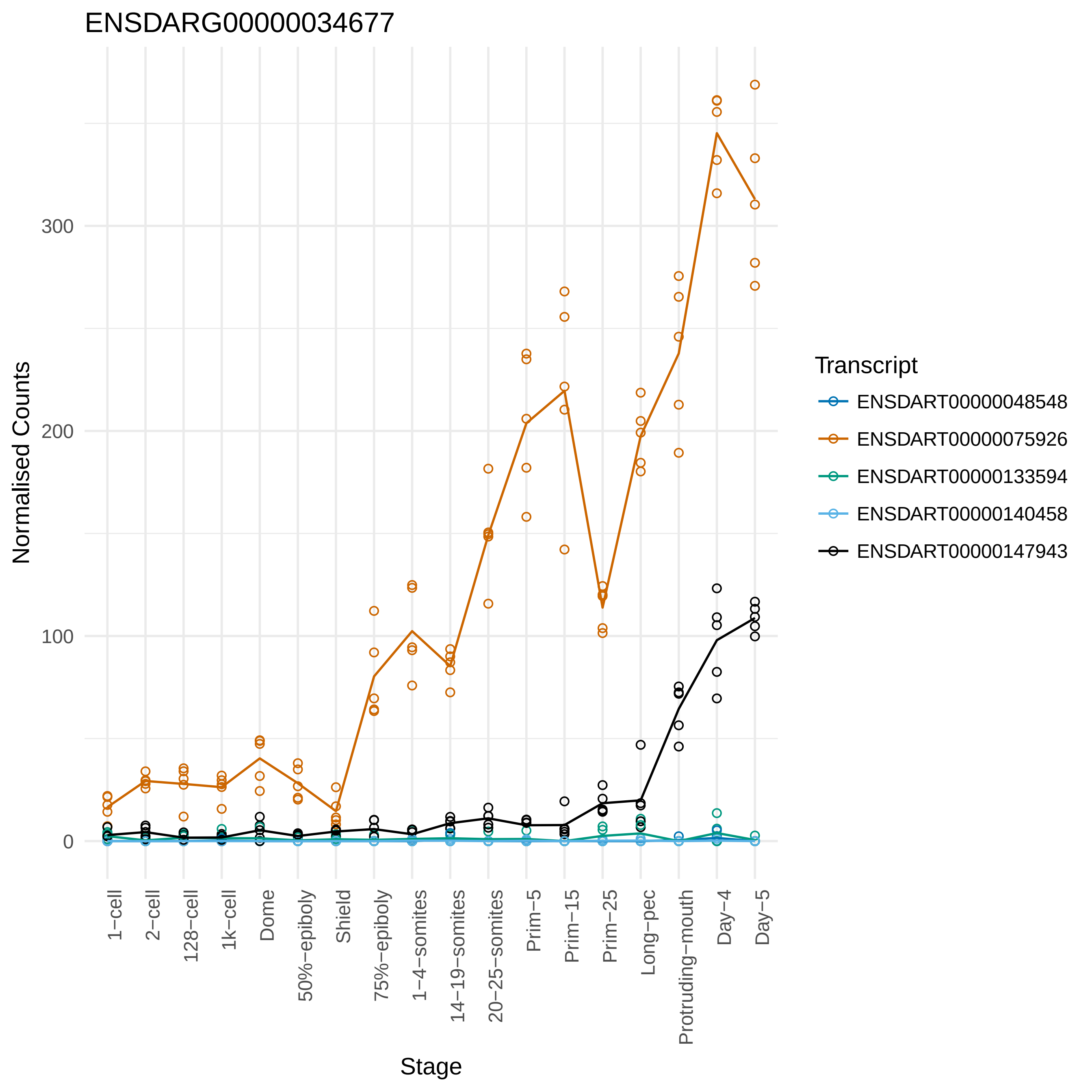

ENSDARG00000034677
Normalised Counts
0
100
200
300
Stage
1−cell
2−cell
128−cell
1k−cell
Dome
50%−epiboly
Shield
75%−epiboly
1−4−somites
14−19−somites
20−25−somites
Prim−5
Prim−15
Prim−25
Long−pec
Protruding−mouth
Day−4
Day−5
Transcript
ENSDART00000048548
ENSDART00000075926
ENSDART00000133594
ENSDART00000140458
ENSDART00000147943

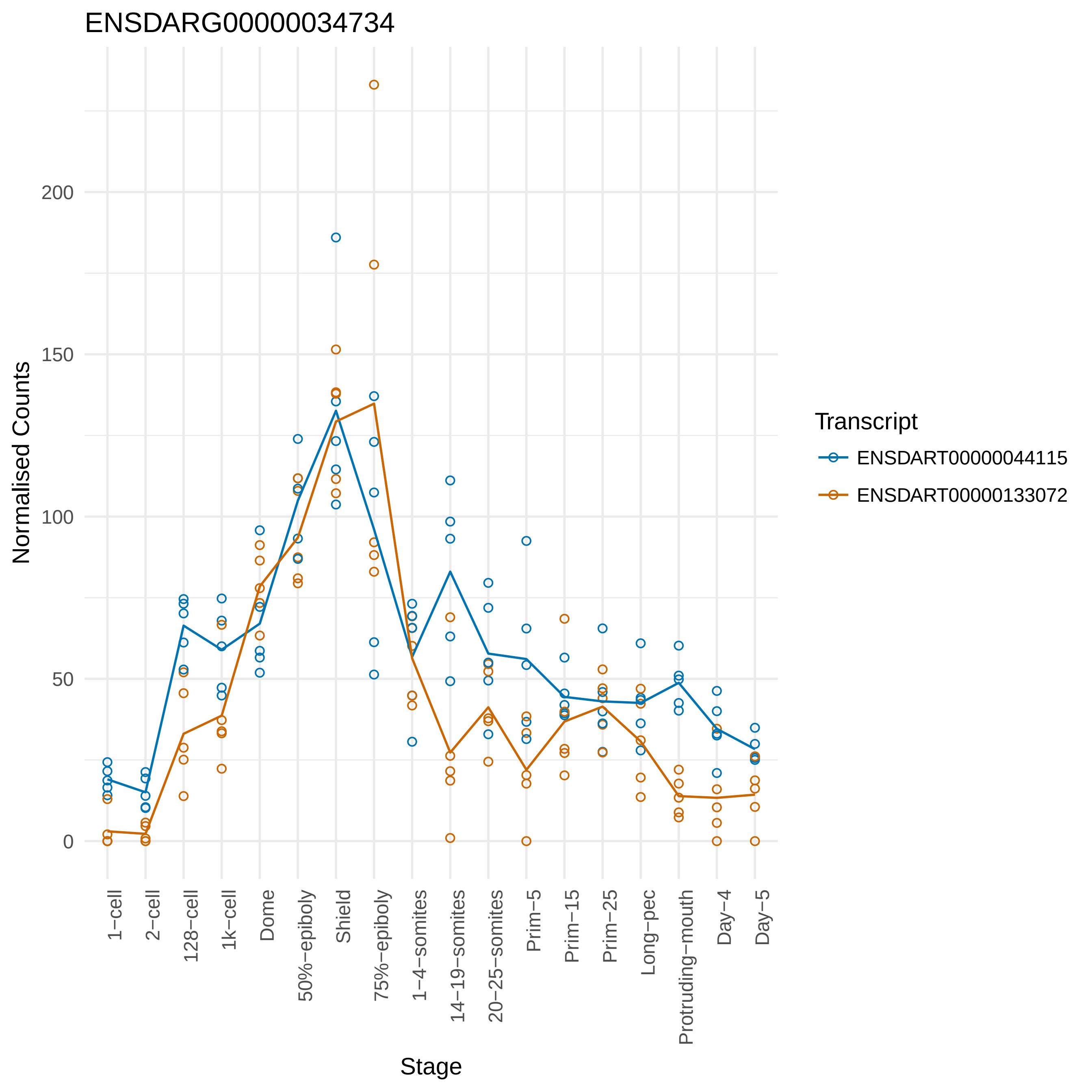

ENSDARG00000034734
Normalised Counts
0
50
100
150
200
Stage
1−cell
2−cell
128−cell
1k−cell
Dome
50%−epiboly
Shield
75%−epiboly
1−4−somites
14−19−somites
20−25−somites
Prim−5
Prim−15
Prim−25
Long−pec
Protruding−mouth
Day−4
Day−5
Transcript
ENSDART00000044115
ENSDART00000133072

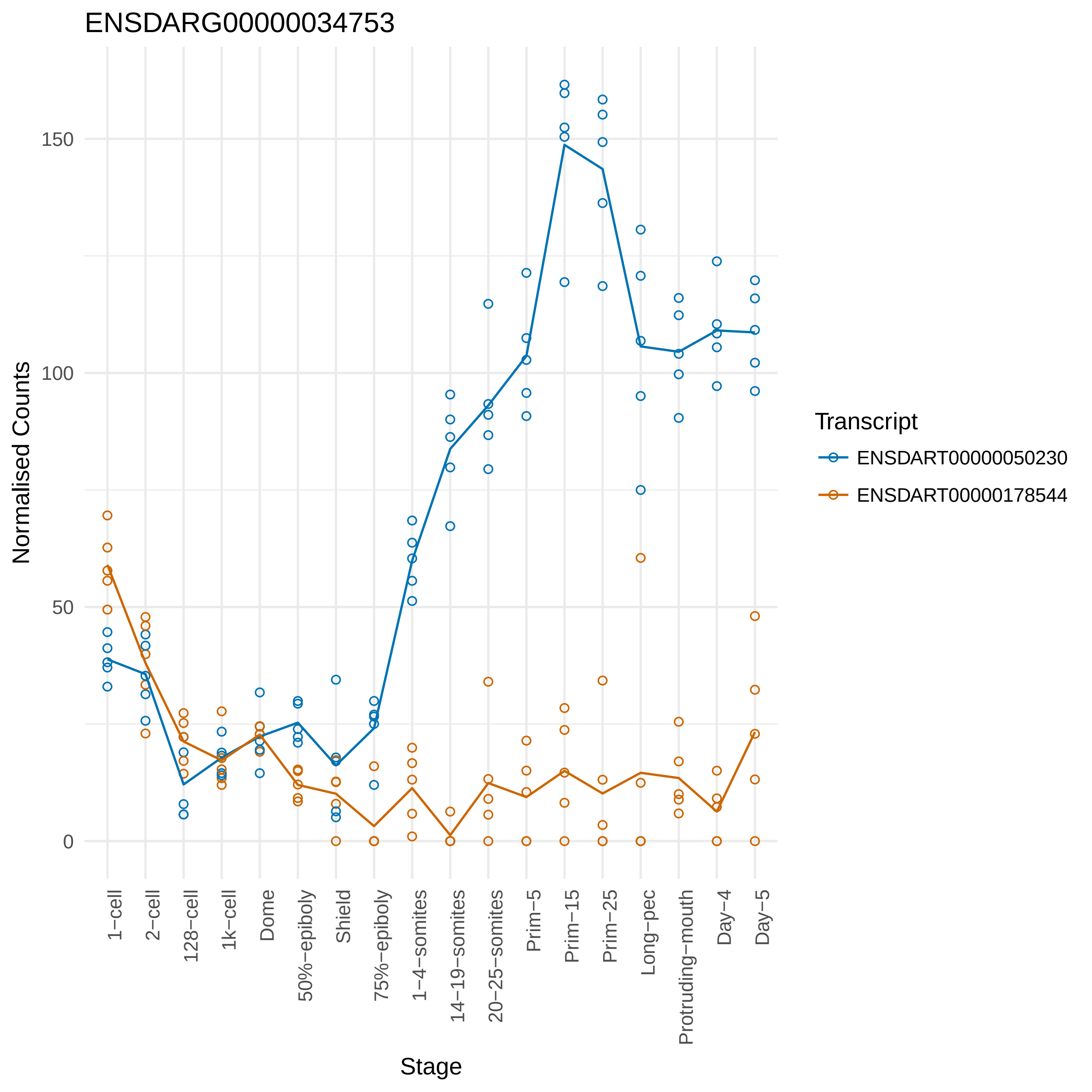
ENSDARG00000034753
Normalised Counts
0
50
100
150
Stage
1−cell
2−cell
128−cell
1k−cell
Dome
50%−epiboly
Shield
75%−epiboly
1−4−somites
14−19−somites
20−25−somites
Prim−5
Prim−15
Prim−25
Long−pec
Protruding−mouth
Day−4
Day−5
Transcript
ENSDART00000050230
ENSDART00000178544

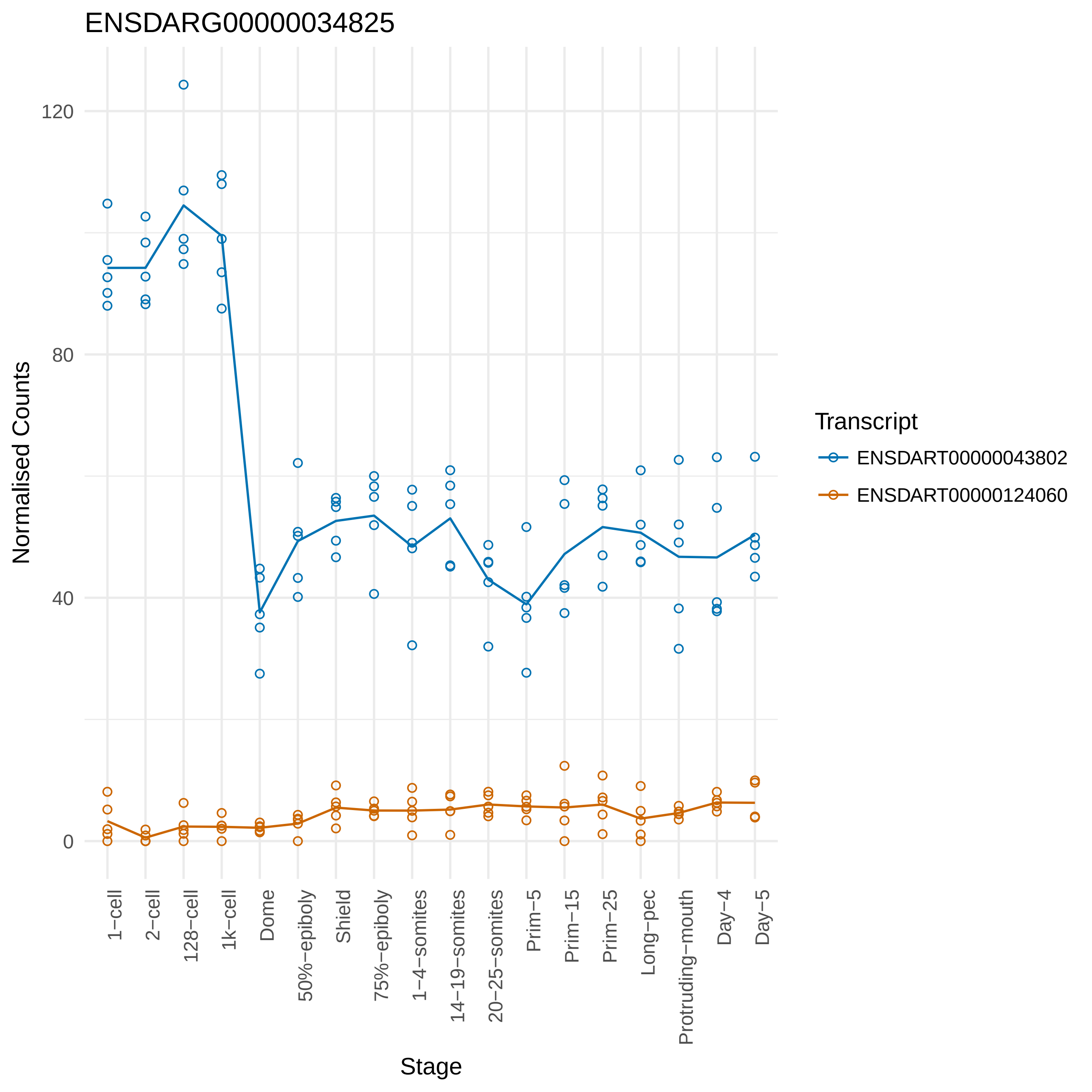

ENSDARG00000034825
Normalised Counts
120
80
40
0
Stage
1−cell
2−cell
128−cell
1k−cell
Dome
50%−epiboly
Shield
75%−epiboly
1−4−somites
14−19−somites
20−25−somites
Prim−5
Prim−15
Prim−25
Long−pec
Protruding−mouth
Day−4
Day−5
Transcript
ENSDART00000043802
ENSDART00000124060

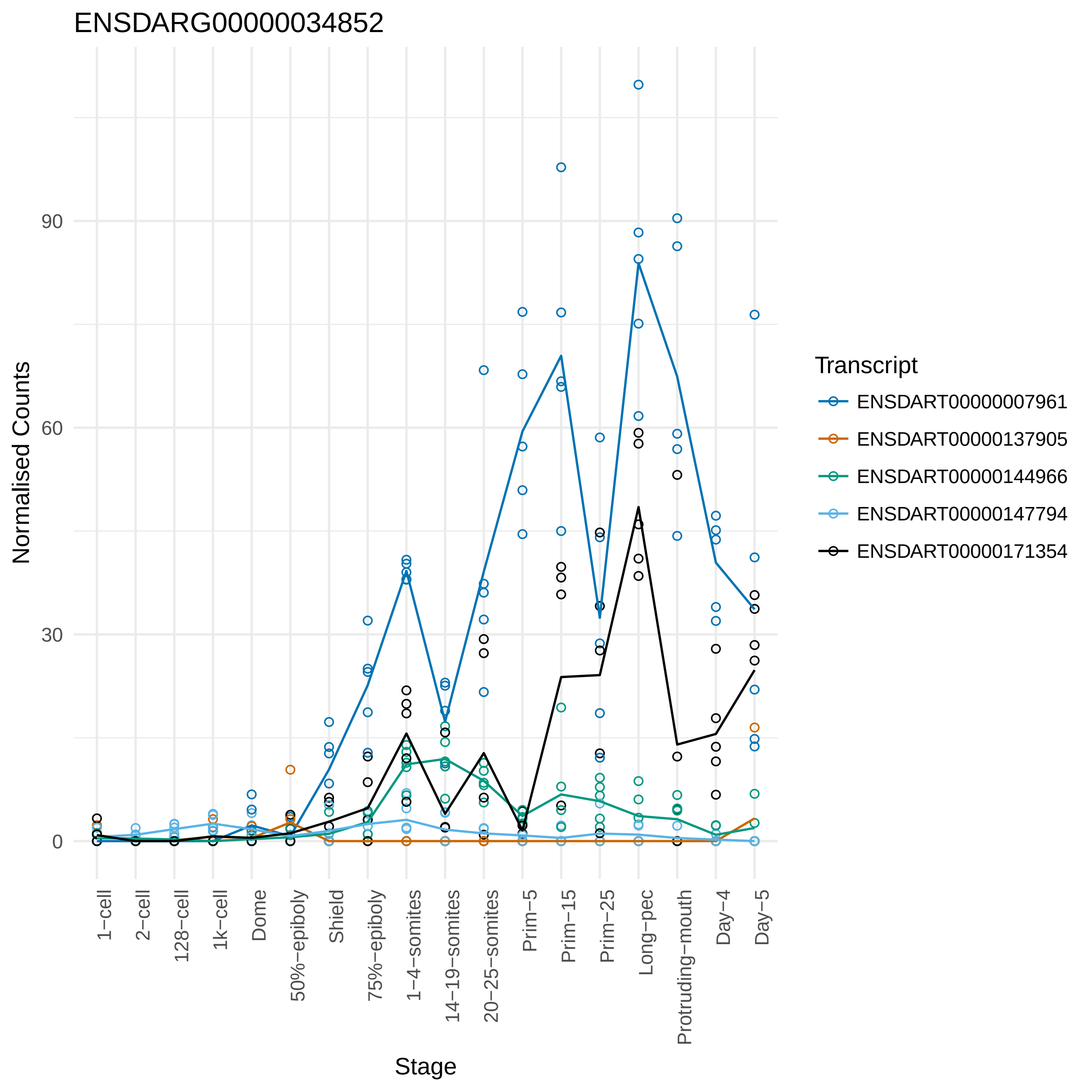
ENSDARG00000034852
Normalised Counts
0
30
60
90
Stage
1−cell
2−cell
128−cell
1k−cell
Dome
50%−epiboly
Shield
75%−epiboly
1−4−somites
14−19−somites
20−25−somites
Prim−5
Prim−15
Prim−25
Long−pec
Protruding−mouth
Day−4
Day−5
Transcript
ENSDART00000007961
ENSDART00000137905
ENSDART00000144966
ENSDART00000147794
ENSDART00000171354

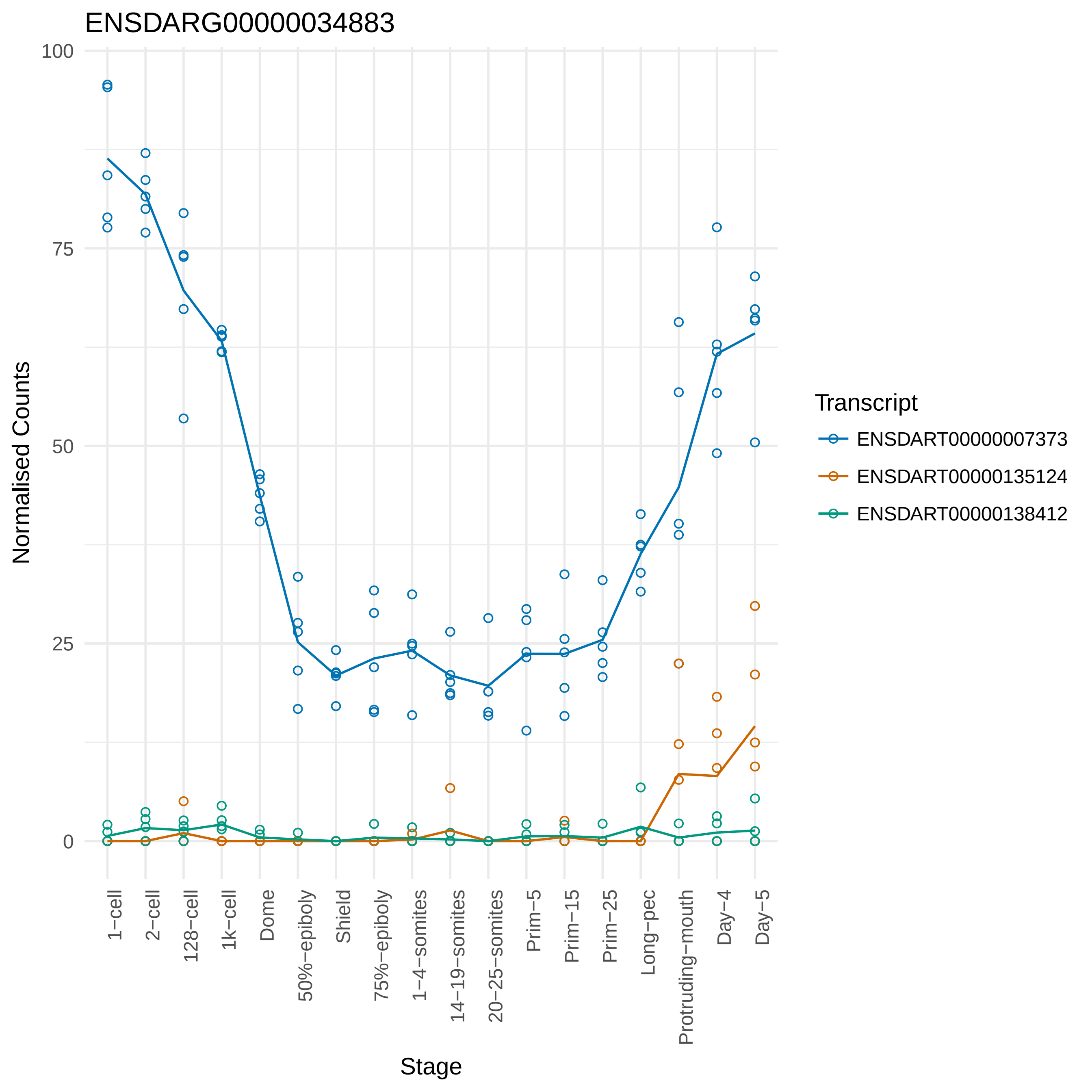

ENSDARG00000034883
Normalised Counts
100
75
50
25
0
1−cell
2−cell
128−cell
1k−cell
Dome
50%−epiboly
Shield
75%−epiboly
1−4−somites
14−19−somites
20−25−somites
Prim−5
Prim−15
Prim−25
Long−pec
Protruding−mouth
Day−4
Day−5
Stage
Transcript
ENSDART00000007373
ENSDART00000135124
ENSDART00000138412

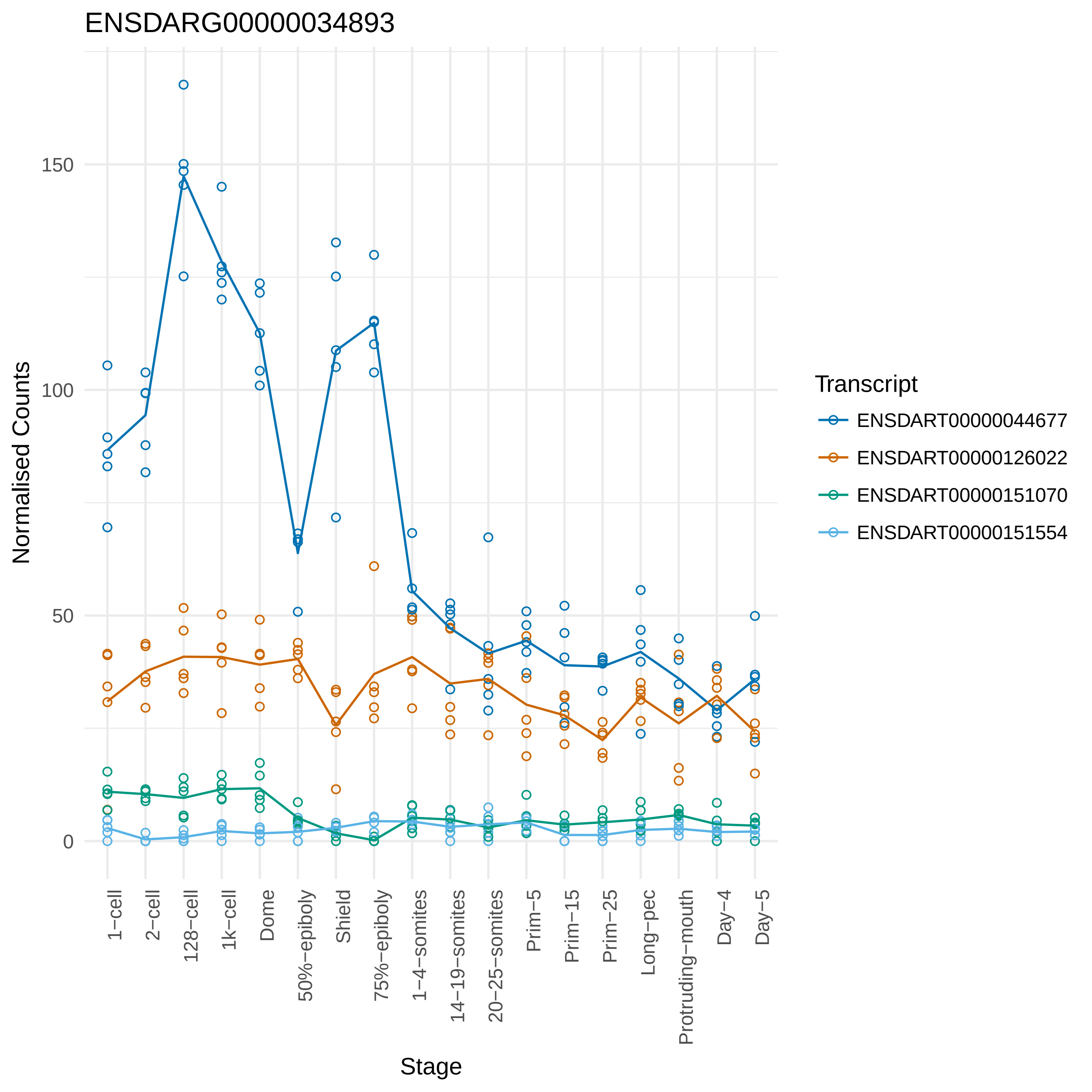

ENSDARG00000034893
Normalised Counts
150
100
50
0
1−cell
2−cell
128−cell
1k−cell
Dome
50%−epiboly
Shield
75%−epiboly
1−4−somites
14−19−somites
20−25−somites
Prim−5
Prim−15
Prim−25
Long−pec
Protruding−mouth
Day−4
Day−5
Stage
Transcript
ENSDART00000044677
ENSDART00000126022
ENSDART00000151070
ENSDART00000151554

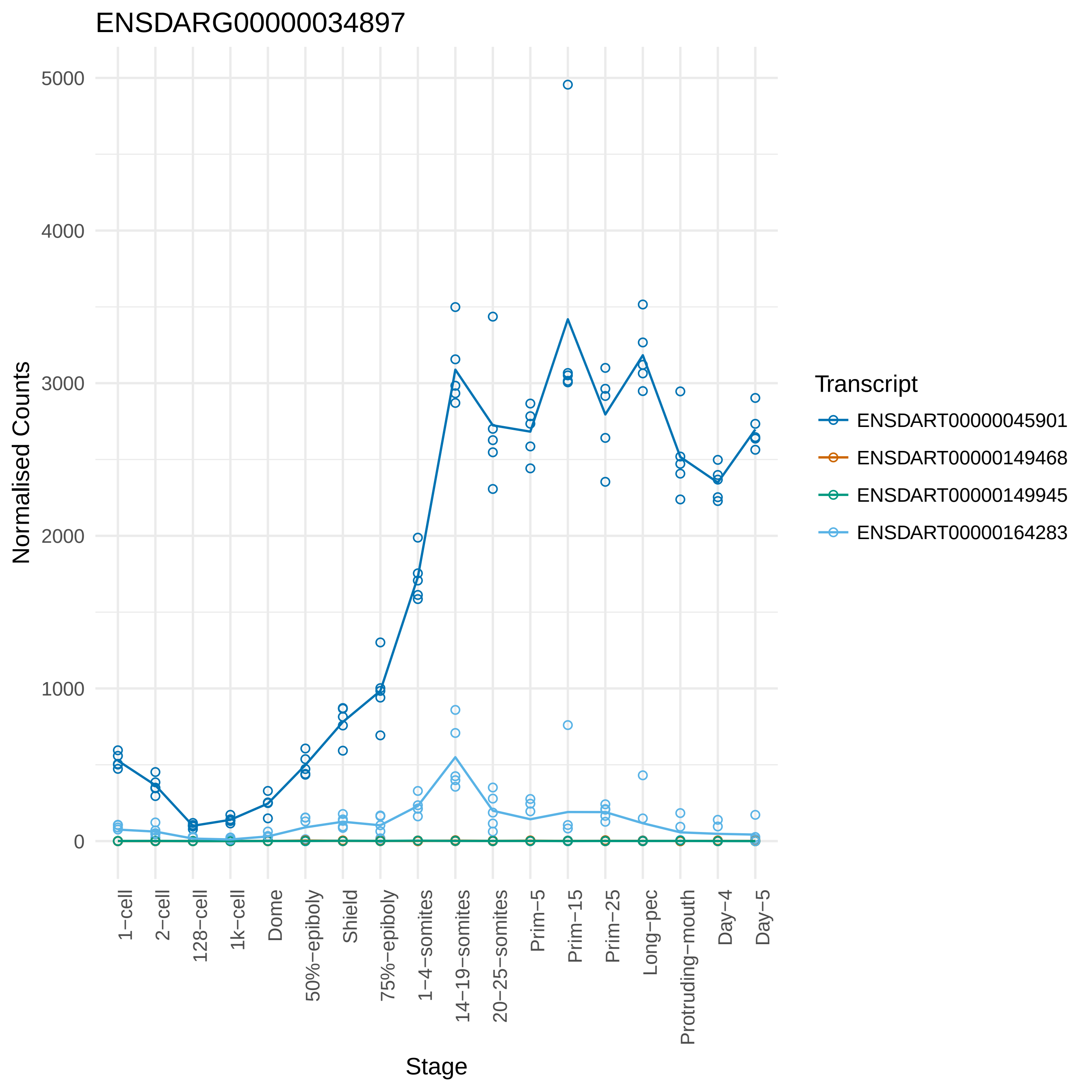

ENSDARG00000034897
Normalised Counts
0
1000
2000
3000
4000
5000
1−cell
2−cell
128−cell
1k−cell
Dome
50%−epiboly
Shield
75%−epiboly
1−4−somites
14−19−somites
20−25−somites
Prim−5
Prim−15
Prim−25
Long−pec
Protruding−mouth
Day−4
Day−5
Stage
Transcript
ENSDART00000045901
ENSDART00000149468
ENSDART00000149945
ENSDART00000164283

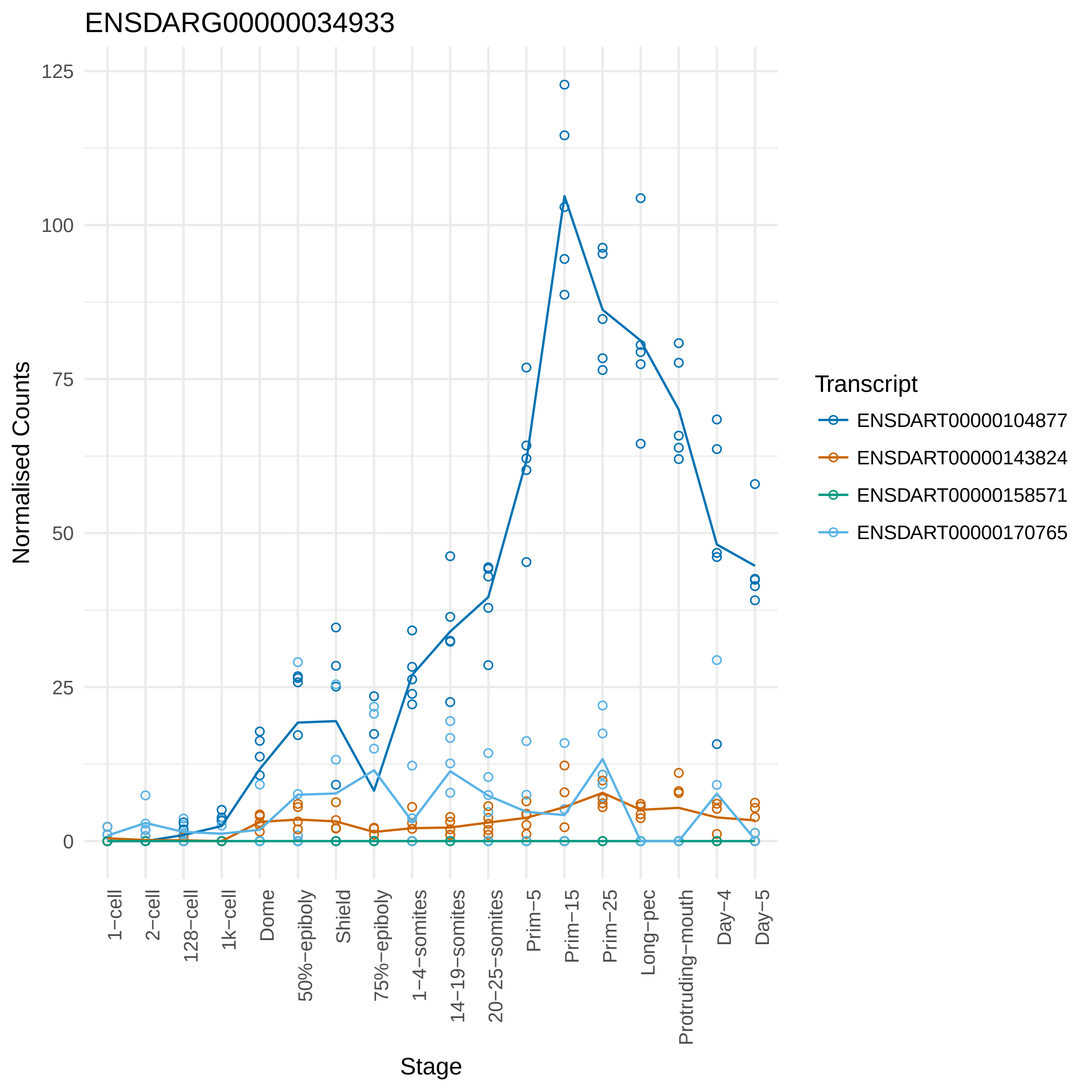

ENSDARG00000034933
Normalised Counts
125
100
75
50
25
0
1−cell
2−cell
128−cell
1k−cell
Dome
50%−epiboly
Shield
75%−epiboly
1−4−somites
14−19−somites
20−25−somites
Prim−5
Prim−15
Prim−25
Long−pec
Protruding−mouth
Day−4
Day−5
Stage
Transcript
ENSDART00000104877
ENSDART00000143824
ENSDART00000158571
ENSDART00000170765

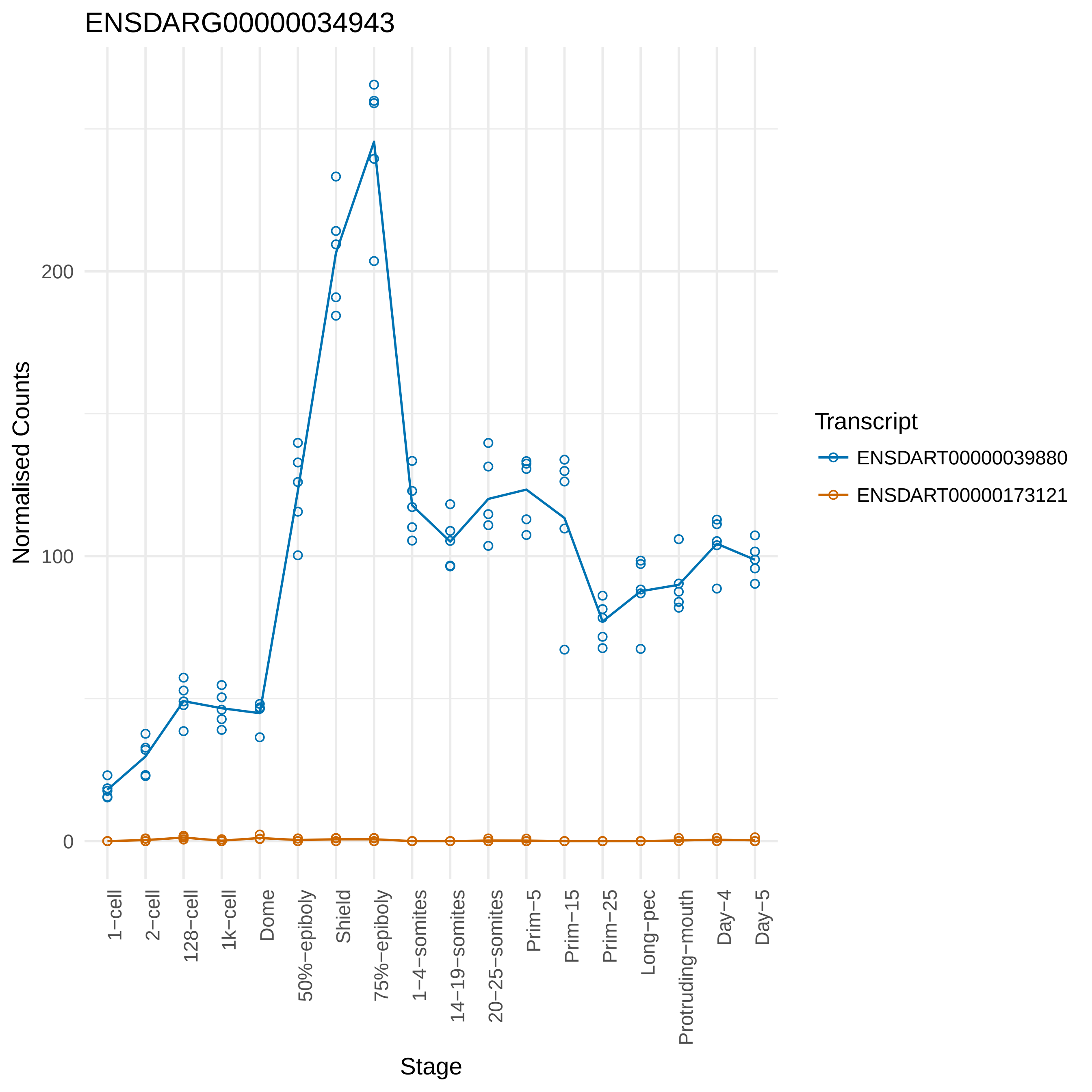

ENSDARG00000034943
Normalised Counts
200
100
0
1−cell
2−cell
128−cell
1k−cell
Dome
50%−epiboly
Shield
75%−epiboly
1−4−somites
14−19−somites
20−25−somites
Prim−5
Prim−15
Prim−25
Long−pec
Protruding−mouth
Day−4
Day−5
Stage
Transcript
ENSDART00000039880
ENSDART00000173121

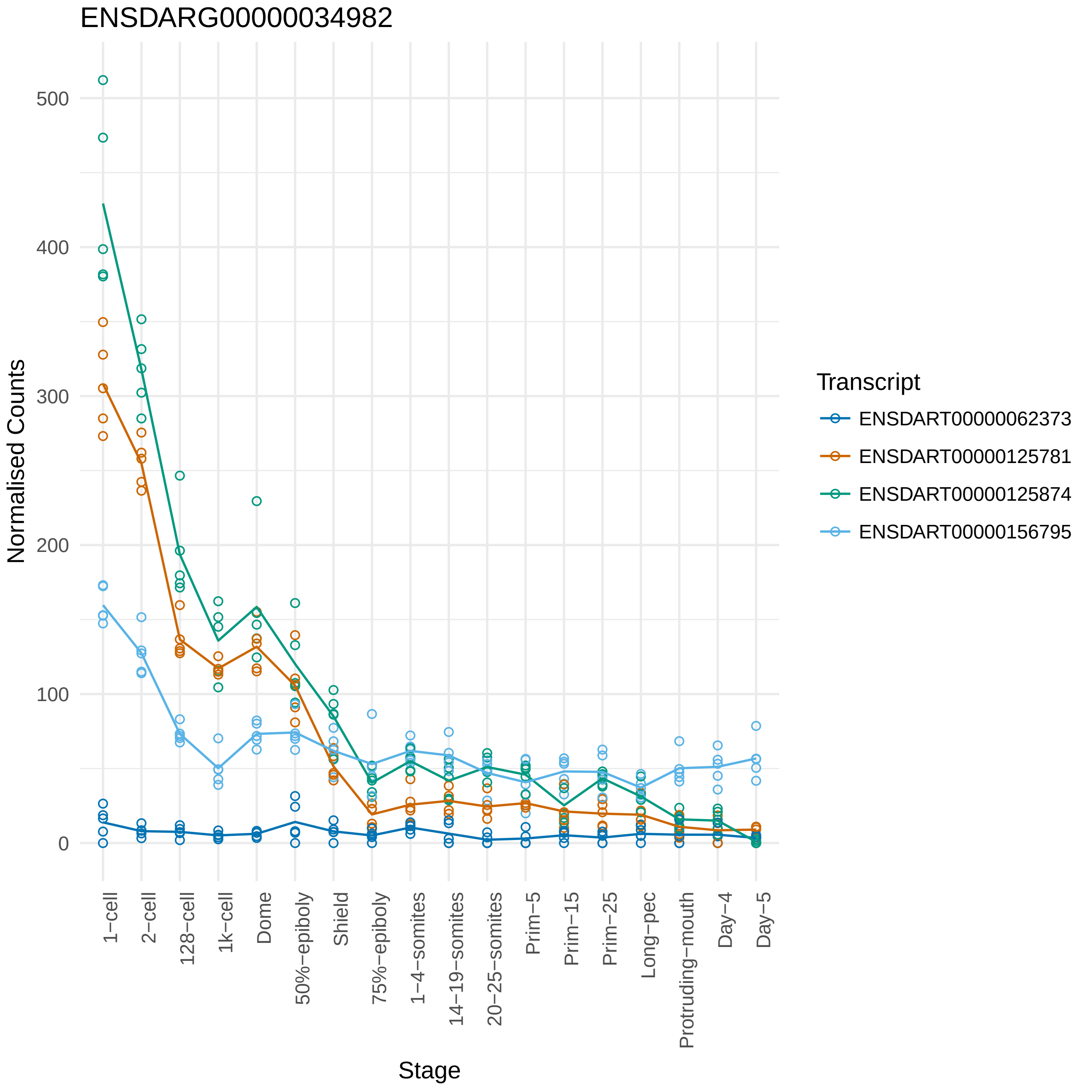

ENSDARG00000034982
Normalised Counts
500
400
300
200
100
0
1−cell
2−cell
128−cell
1k−cell
Dome
50%−epiboly
Shield
75%−epiboly
1−4−somites
14−19−somites
20−25−somites
Prim−5
Prim−15
Prim−25
Long−pec
Protruding−mouth
Day−4
Day−5
Stage
Transcript
ENSDART00000062373
ENSDART00000125781
ENSDART00000125874
ENSDART00000156795

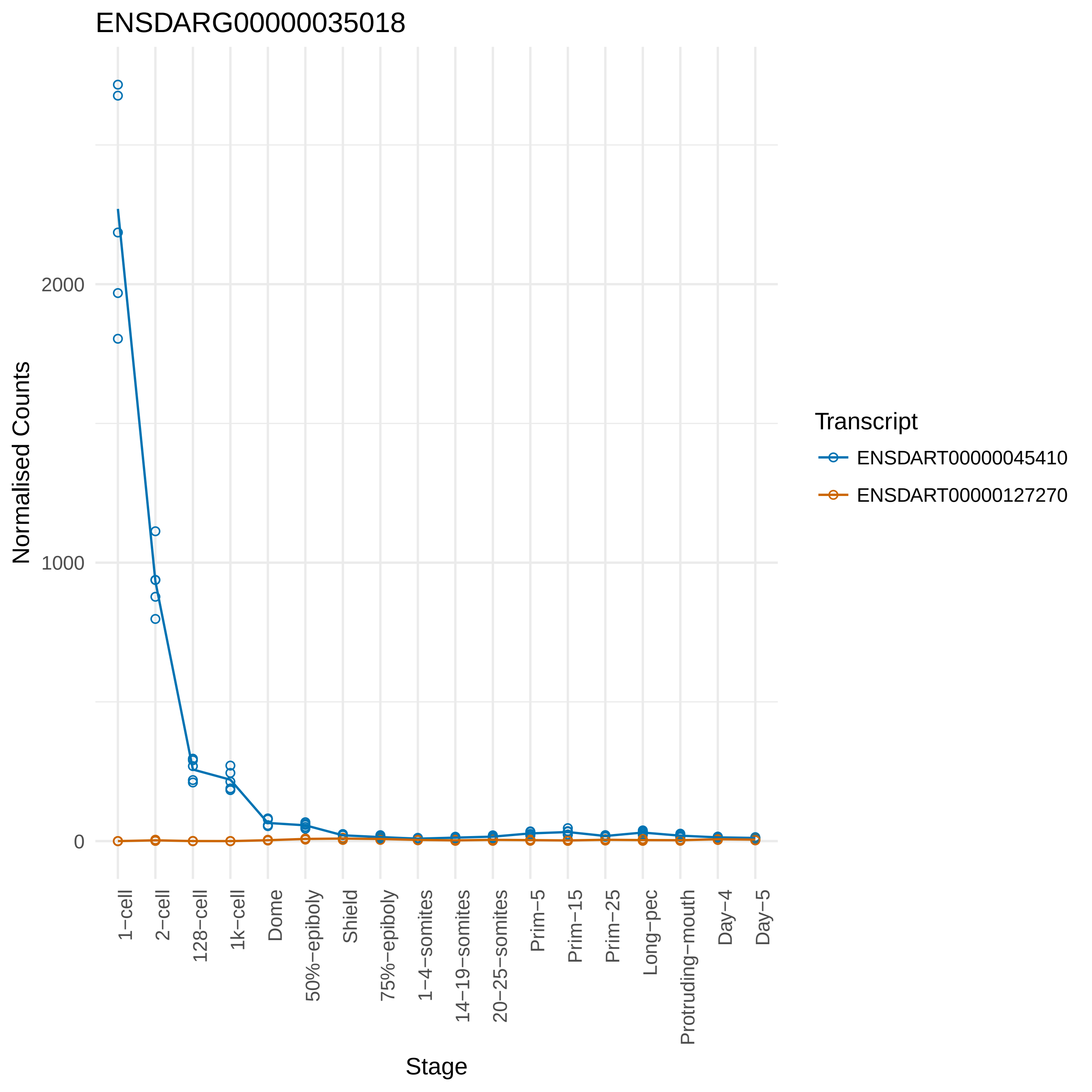
ENSDARG00000035018
Normalised Counts
2000
1000
0
Stage
1−cell
2−cell
128−cell
1k−cell
Dome
50%−epiboly
Shield
75%−epiboly
1−4−somites
14−19−somites
20−25−somites
Prim−5
Prim−15
Prim−25
Long−pec
Protruding−mouth
Day−4
Day−5
Transcript
ENSDART00000045410
ENSDART00000127270

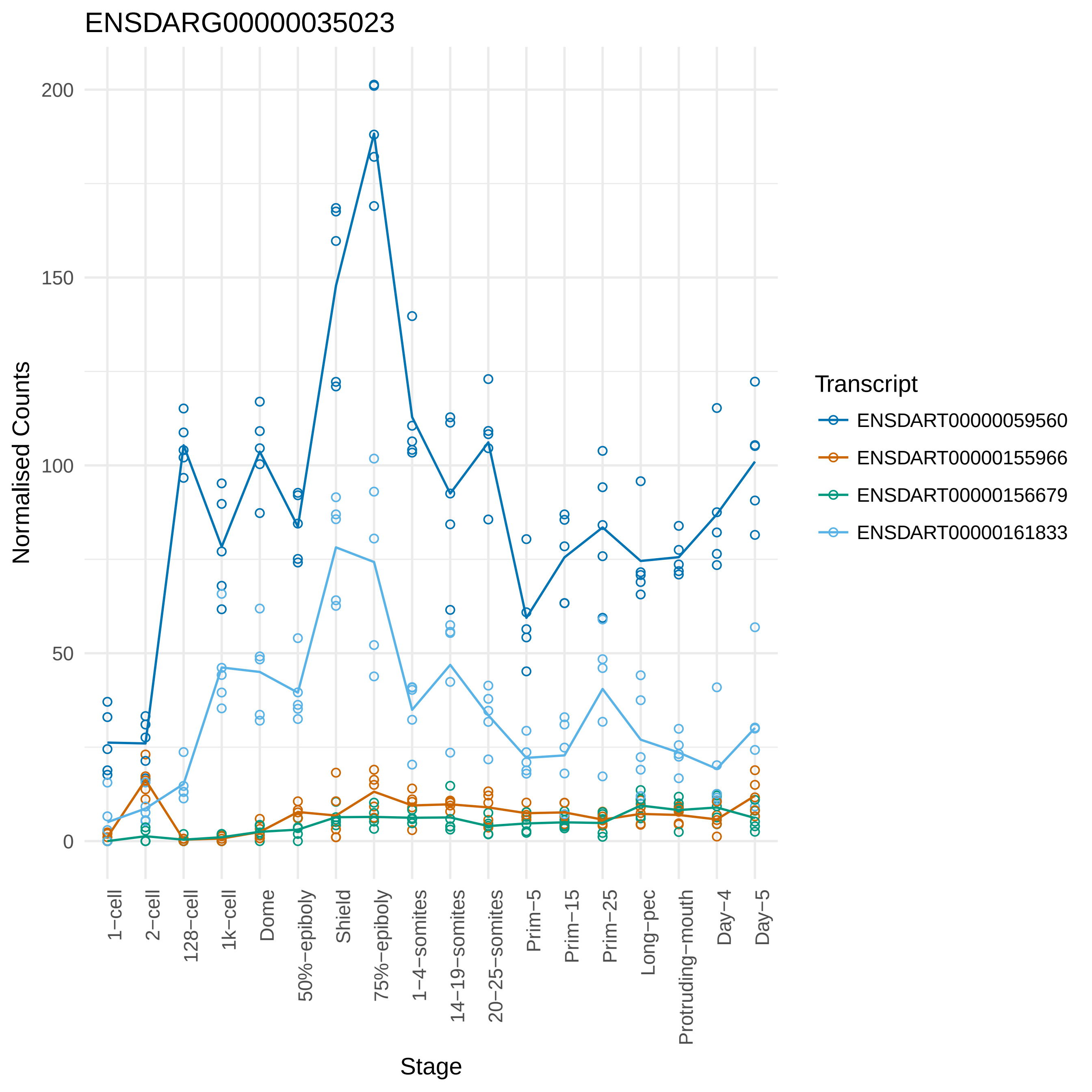

ENSDARG00000035023
Normalised Counts
200
150
100
50
0
1−cell
2−cell
128−cell
1k−cell
Dome
50%−epiboly
Shield
75%−epiboly
1−4−somites
14−19−somites
20−25−somites
Prim−5
Prim−15
Prim−25
Long−pec
Protruding−mouth
Day−4
Day−5
Stage
Transcript
ENSDART00000059560
ENSDART00000155966
ENSDART00000156679
ENSDART00000161833

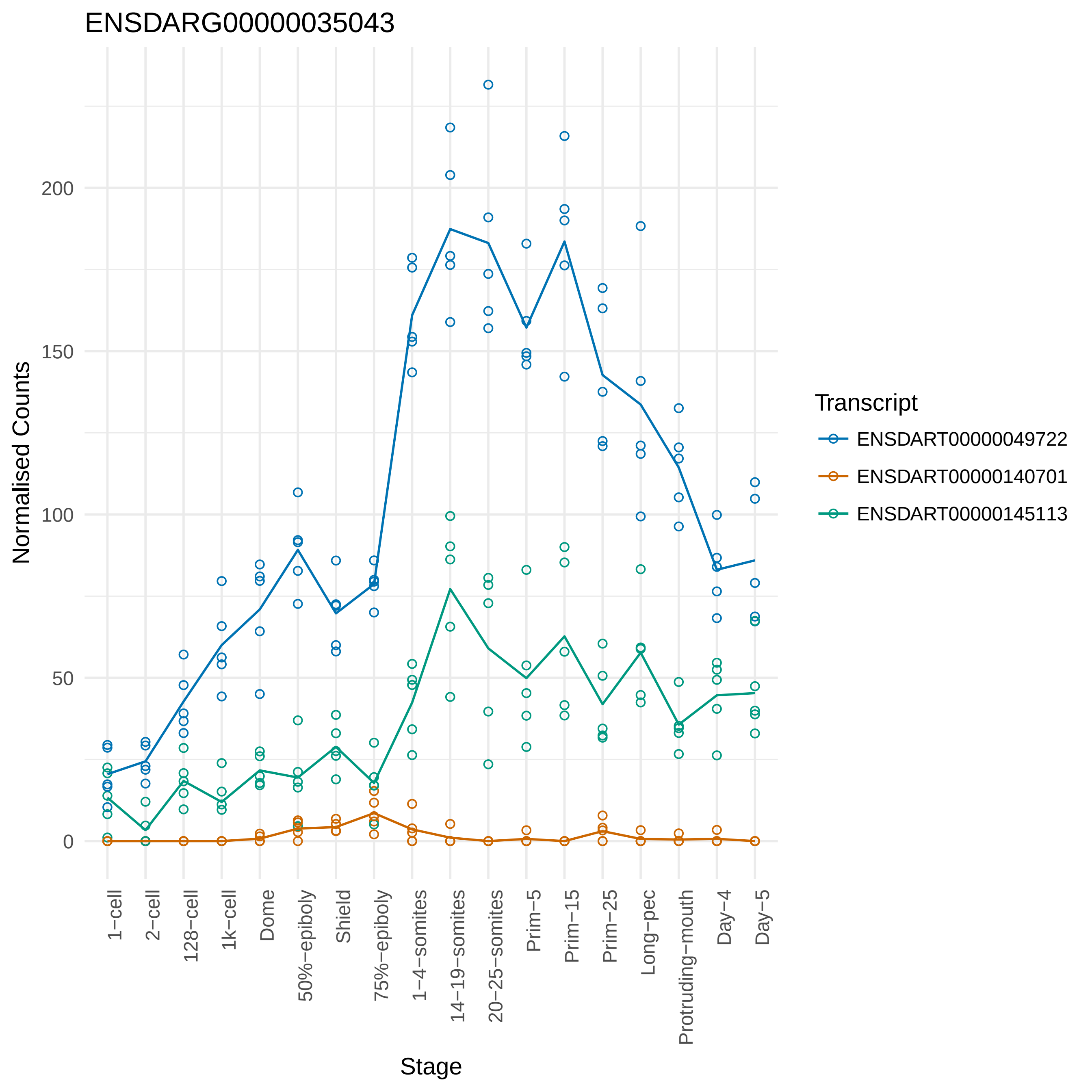

ENSDARG00000035043
Normalised Counts
200
150
100
50
0
1−cell
2−cell
128−cell
1k−cell
Dome
50%−epiboly
Shield
75%−epiboly
1−4−somites
14−19−somites
20−25−somites
Prim−5
Prim−15
Prim−25
Long−pec
Protruding−mouth
Day−4
Day−5
Stage
Transcript
ENSDART00000049722
ENSDART00000140701
ENSDART00000145113

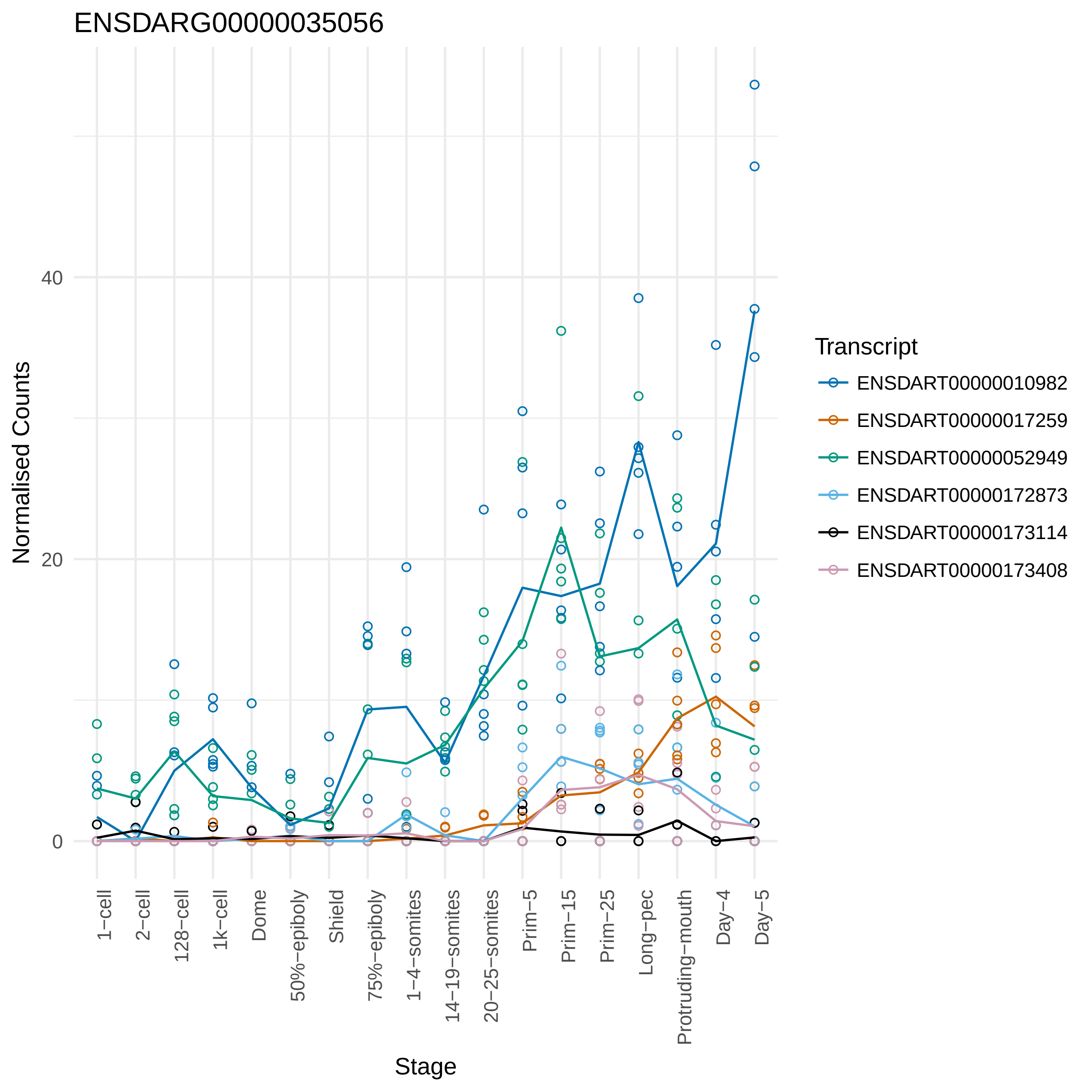
ENSDARG00000035056
Normalised Counts
0
20
40
Stage
1−cell
2−cell
128−cell
1k−cell
Dome
50%−epiboly
Shield
75%−epiboly
1−4−somites
14−19−somites
20−25−somites
Prim−5
Prim−15
Prim−25
Long−pec
Protruding−mouth
Day−4
Day−5
Transcript
ENSDART00000010982
ENSDART00000017259
ENSDART00000052949
ENSDART00000172873
ENSDART00000173114
ENSDART00000173408

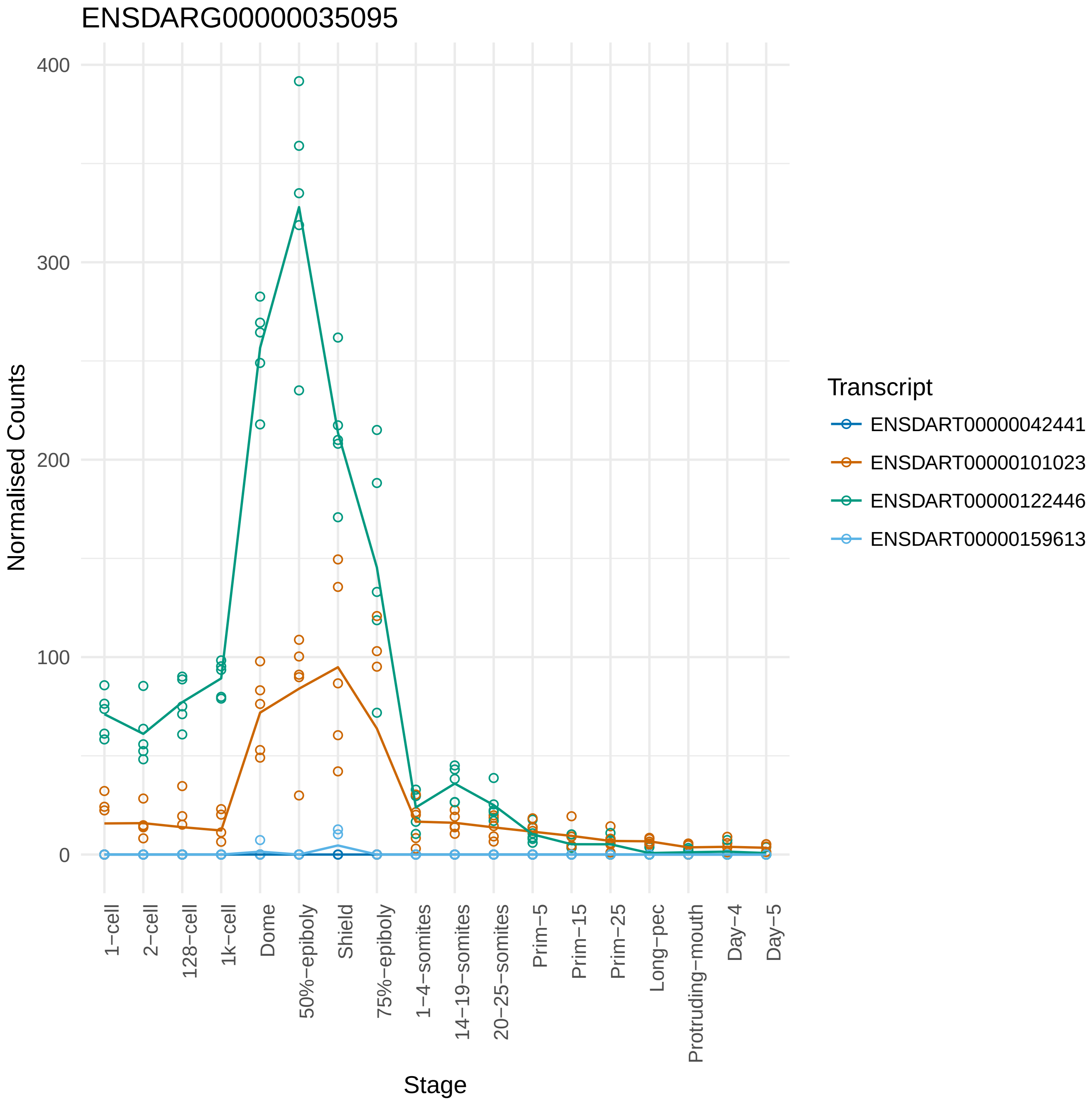
ENSDARG00000035095
Normalised Counts
400
300
200
100
0
1−cell
2−cell
128−cell
1k−cell
Dome
50%−epiboly
Shield
75%−epiboly
1−4−somites
14−19−somites
20−25−somites
Prim−5
Prim−15
Prim−25
Long−pec
Protruding−mouth
Day−4
Day−5
Stage
Transcript
ENSDART00000042441
ENSDART00000101023
ENSDART00000122446
ENSDART00000159613

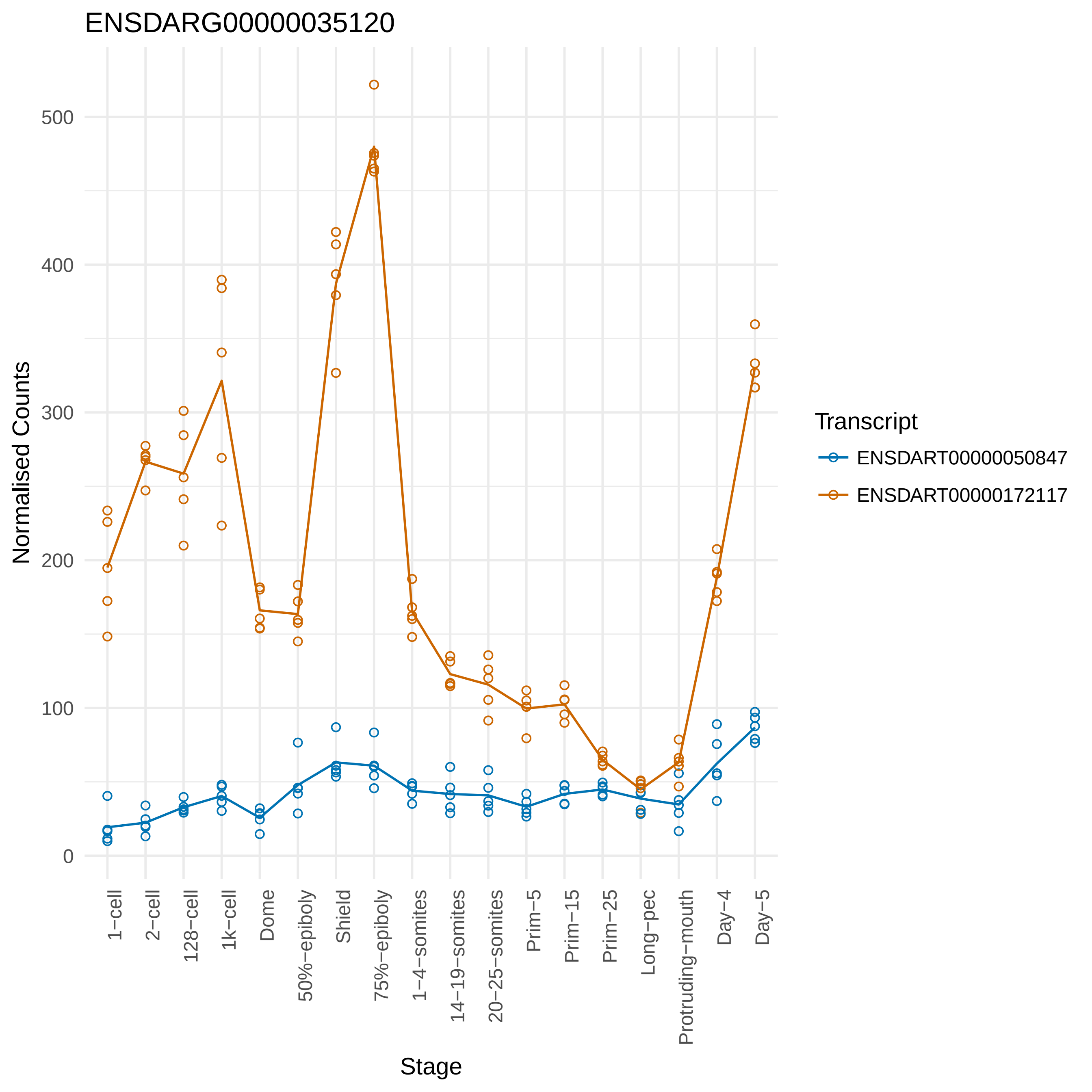

ENSDARG00000035120
Normalised Counts
500
400
300
200
100
0
1−cell
2−cell
128−cell
1k−cell
Dome
50%−epiboly
Shield
75%−epiboly
1−4−somites
14−19−somites
20−25−somites
Prim−5
Prim−15
Prim−25
Long−pec
Protruding−mouth
Day−4
Day−5
Stage
Transcript
ENSDART00000050847
ENSDART00000172117

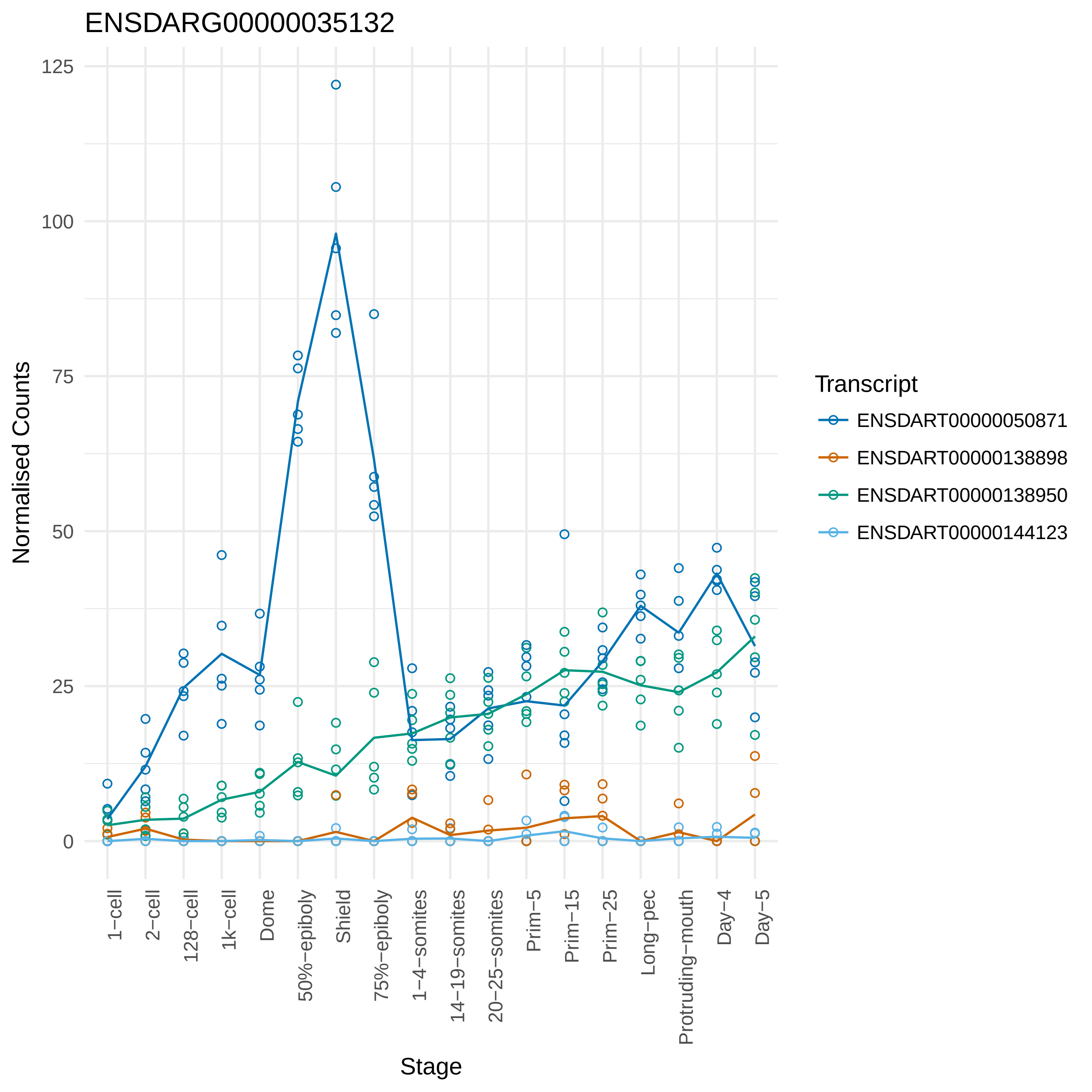

ENSDARG00000035132
Normalised Counts
125
100
75
50
25
0
1−cell
2−cell
128−cell
1k−cell
Dome
50%−epiboly
Shield
75%−epiboly
1−4−somites
14−19−somites
20−25−somites
Prim−5
Prim−15
Prim−25
Long−pec
Protruding−mouth
Day−4
Day−5
Stage
Transcript
ENSDART00000050871
ENSDART00000138898
ENSDART00000138950
ENSDART00000144123

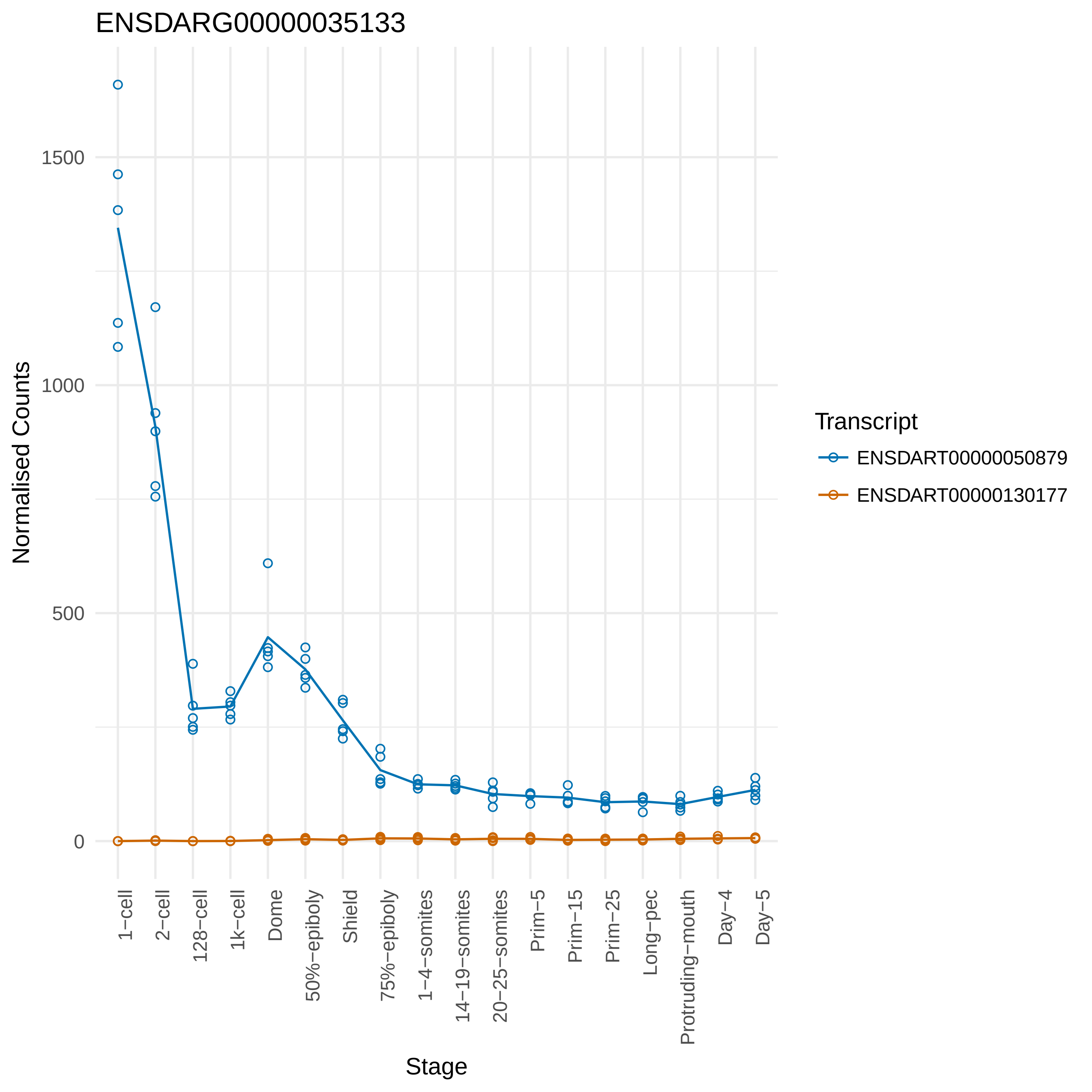
ENSDARG00000035133
Normalised Counts
1500
1000
500
0
1−cell
2−cell
128−cell
1k−cell
Dome
50%−epiboly
Shield
75%−epiboly
1−4−somites
14−19−somites
20−25−somites
Prim−5
Prim−15
Prim−25
Long−pec
Protruding−mouth
Day−4
Day−5
Stage
Transcript
ENSDART00000050879
ENSDART00000130177

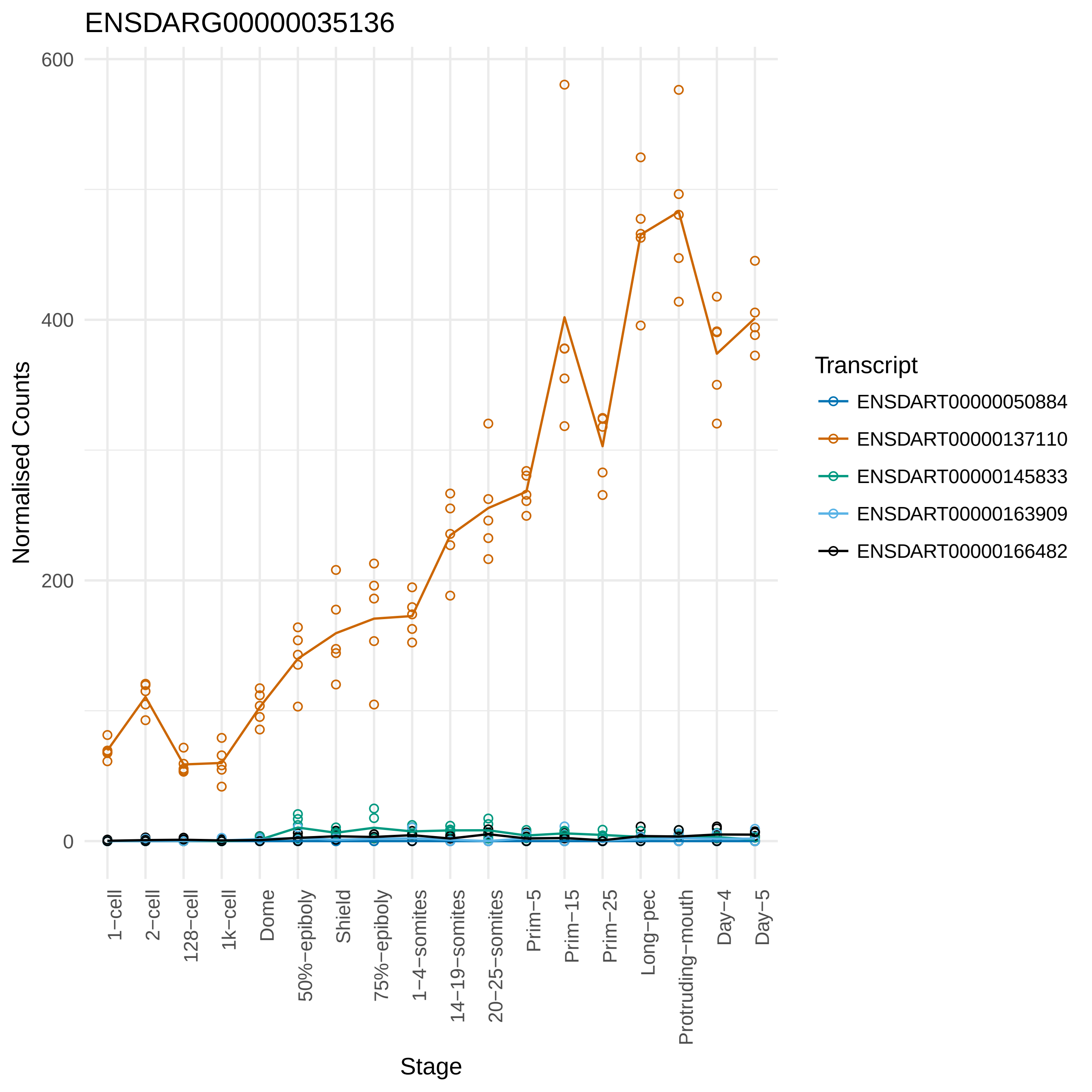

ENSDARG00000035136
Normalised Counts
0
200
400
600
Stage
1−cell
2−cell
128−cell
1k−cell
Dome
50%−epiboly
Shield
75%−epiboly
1−4−somites
14−19−somites
20−25−somites
Prim−5
Prim−15
Prim−25
Long−pec
Protruding−mouth
Day−4
Day−5
Transcript
ENSDART00000050884
ENSDART00000137110
ENSDART00000145833
ENSDART00000163909
ENSDART00000166482

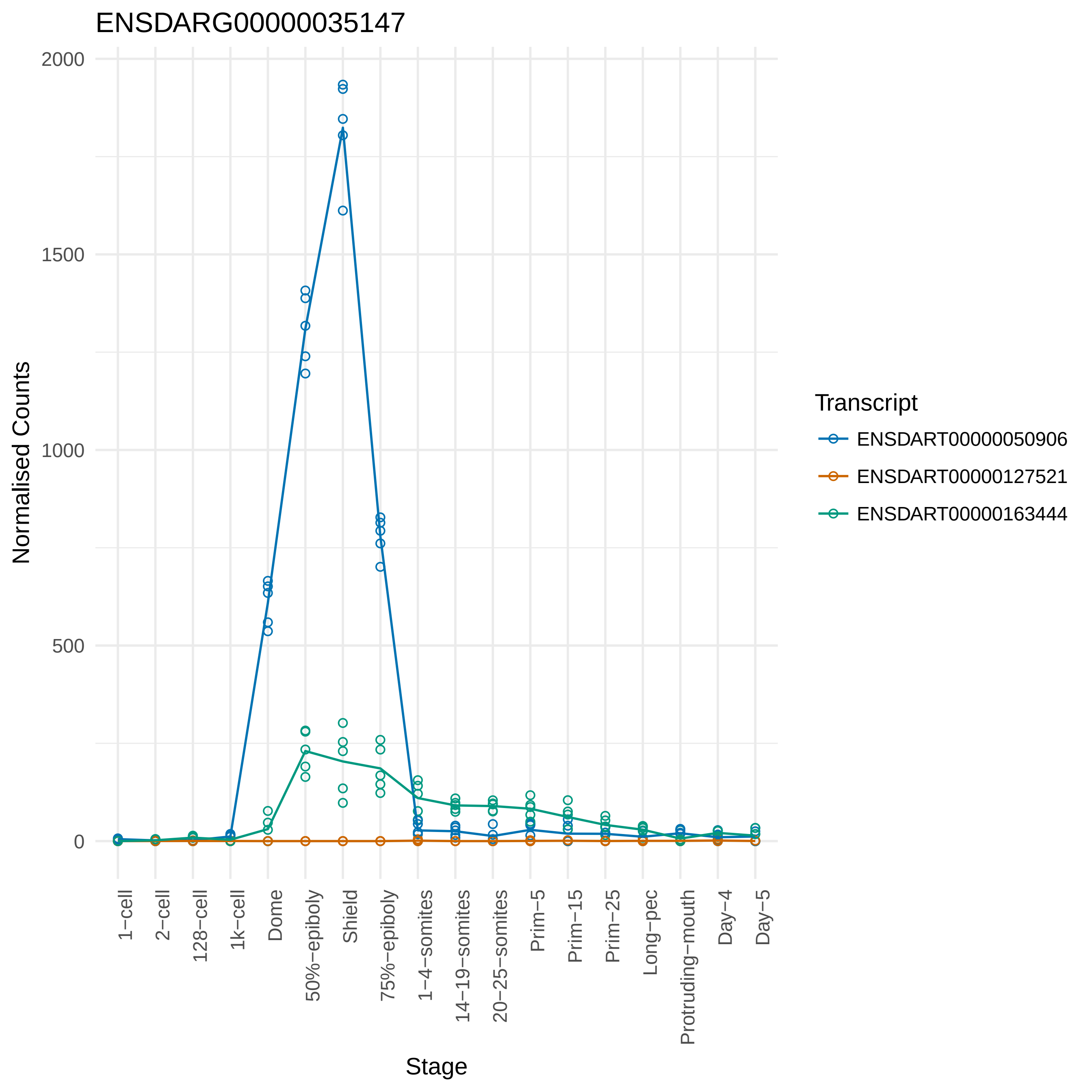

ENSDARG00000035147
Normalised Counts
2000
1500
1000
500
0
1−cell
2−cell
128−cell
1k−cell
Dome
50%−epiboly
Shield
75%−epiboly
1−4−somites
14−19−somites
20−25−somites
Prim−5
Prim−15
Prim−25
Long−pec
Protruding−mouth
Day−4
Day−5
Stage
Transcript
ENSDART00000050906
ENSDART00000127521
ENSDART00000163444

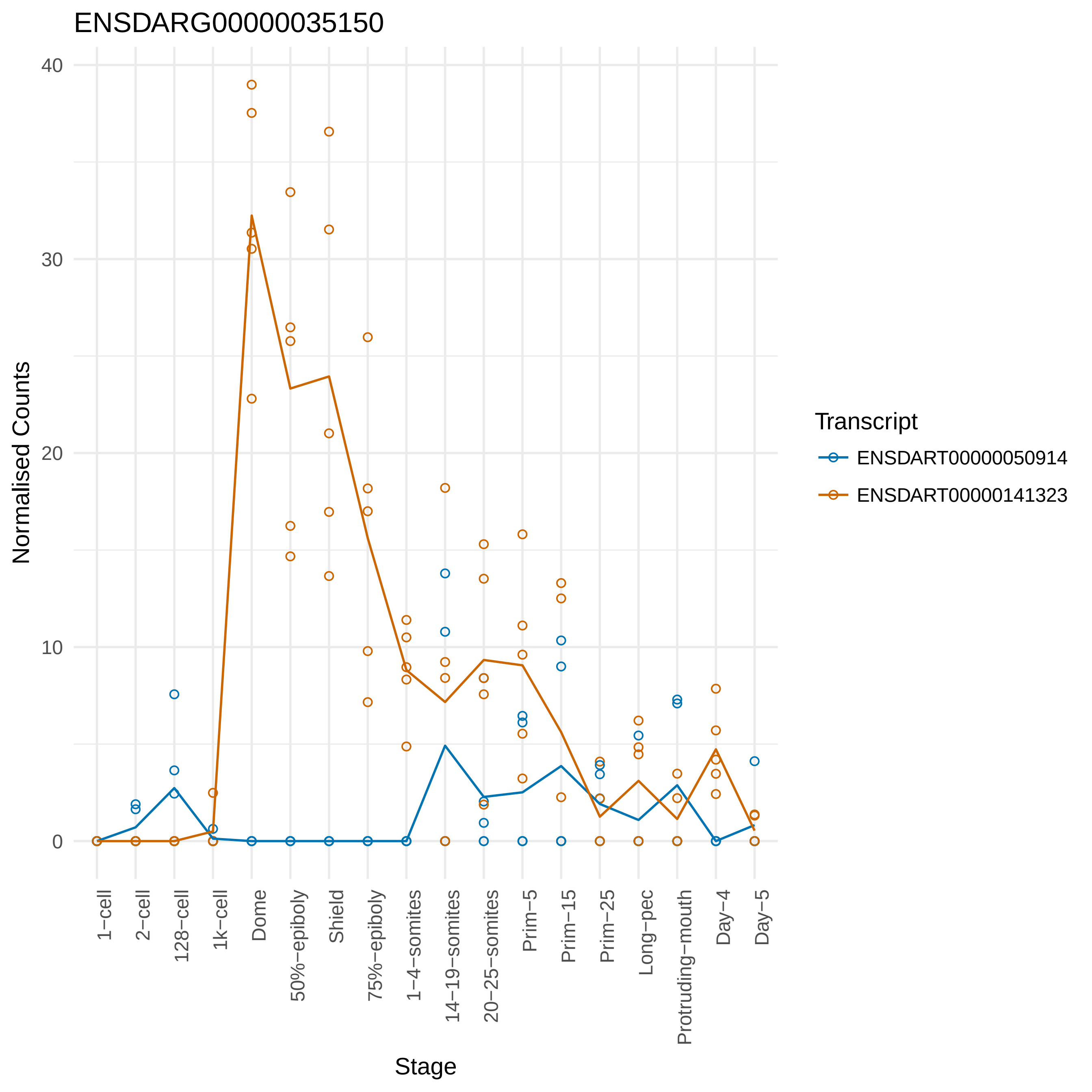

ENSDARG00000035150
Normalised Counts
0
10
20
30
40
1−cell
2−cell
128−cell
1k−cell
Dome
50%−epiboly
Shield
75%−epiboly
1−4−somites
14−19−somites
20−25−somites
Prim−5
Prim−15
Prim−25
Long−pec
Protruding−mouth
Day−4
Day−5
Stage
Transcript
ENSDART00000050914
ENSDART00000141323

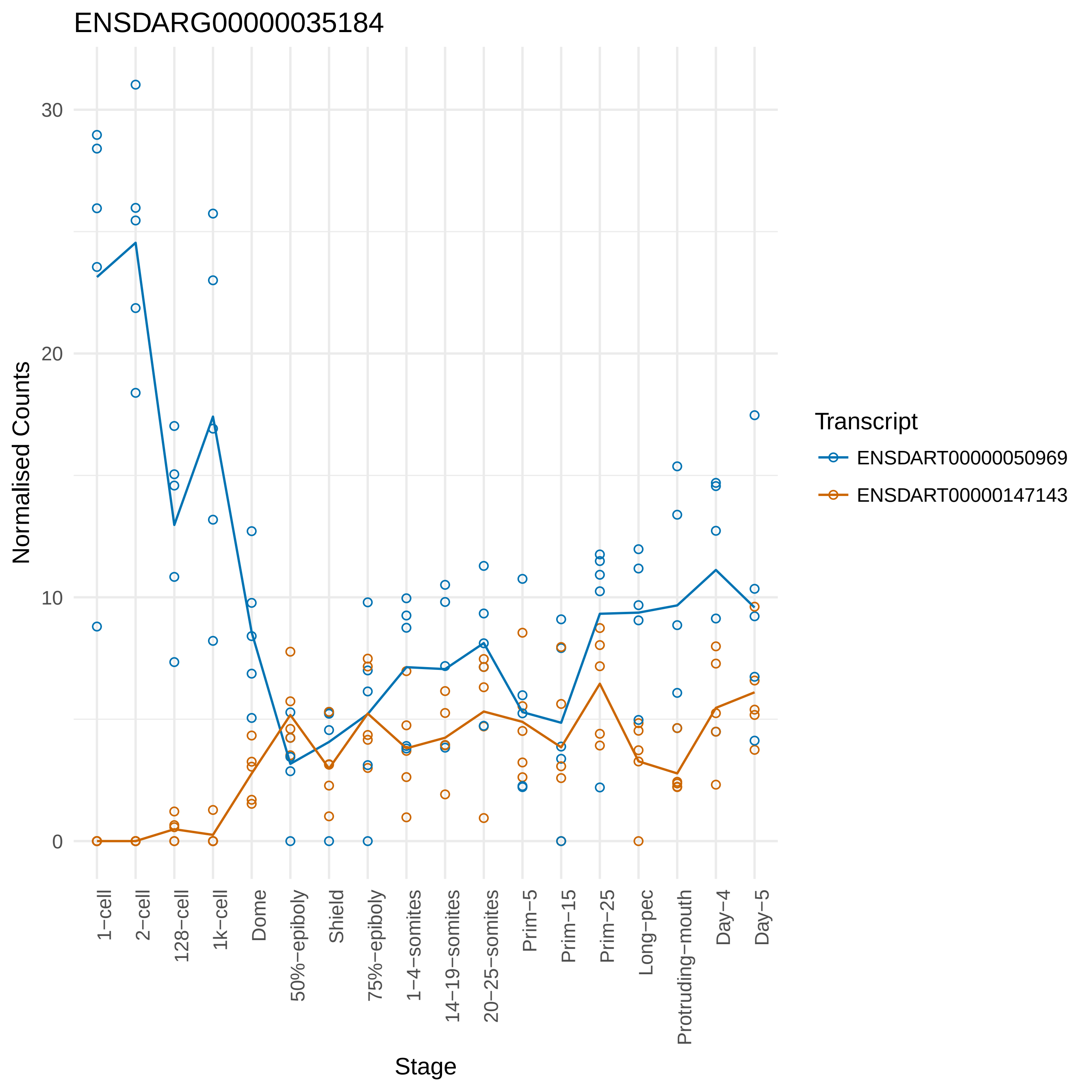
ENSDARG00000035184
Normalised Counts
30
20
10
0
1−cell
2−cell
128−cell
1k−cell
Dome
50%−epiboly
Shield
75%−epiboly
1−4−somites
14−19−somites
20−25−somites
Prim−5
Prim−15
Prim−25
Long−pec
Protruding−mouth
Day−4
Day−5
Stage
Transcript
ENSDART00000050969
ENSDART00000147143

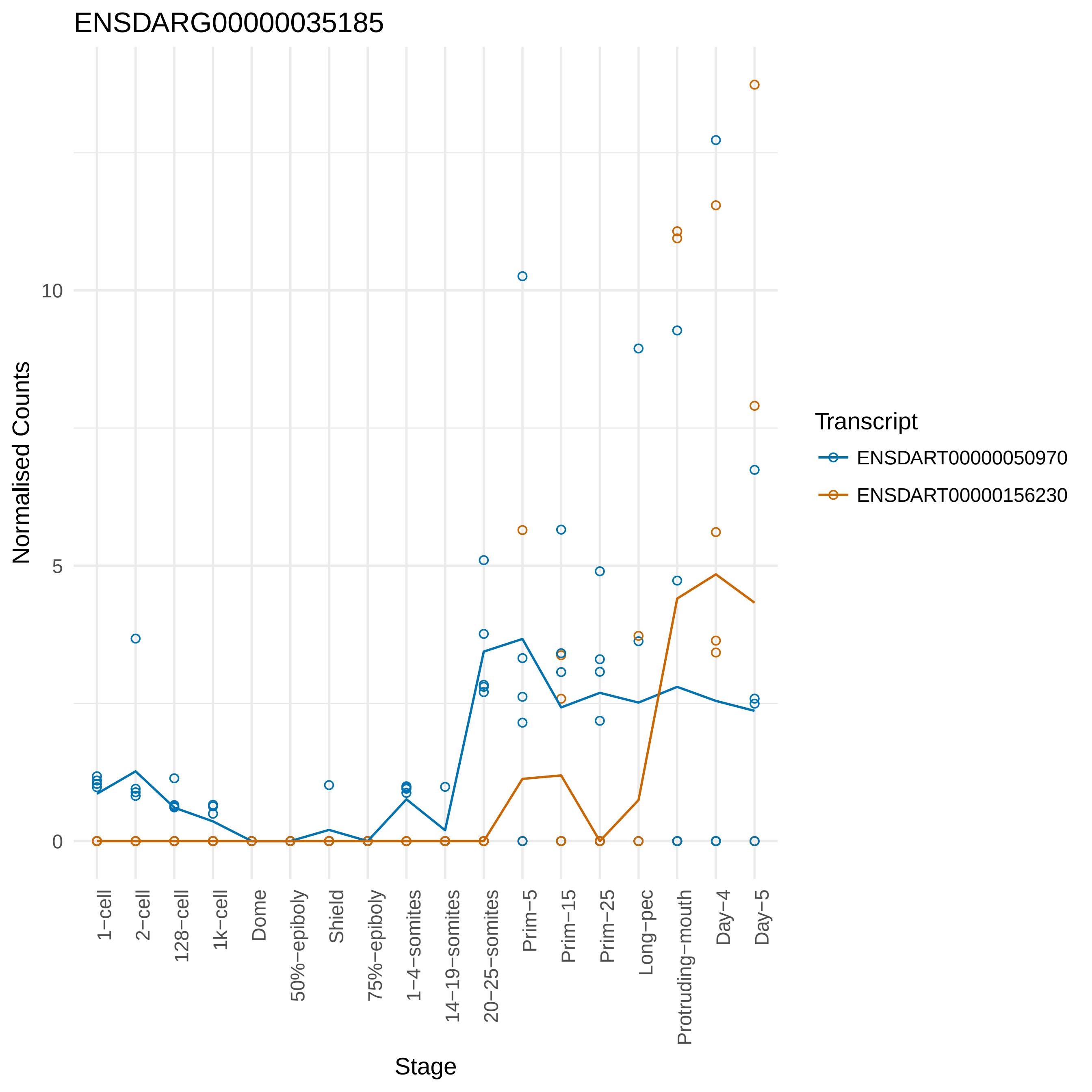
ENSDARG00000035185
Normalised Counts
0
5
10
1−cell
2−cell
128−cell
1k−cell
Dome
50%−epiboly
Shield
75%−epiboly
1−4−somites
14−19−somites
20−25−somites
Prim−5
Prim−15
Prim−25
Long−pec
Protruding−mouth
Day−4
Day−5
Stage
Transcript
ENSDART00000050970
ENSDART00000156230

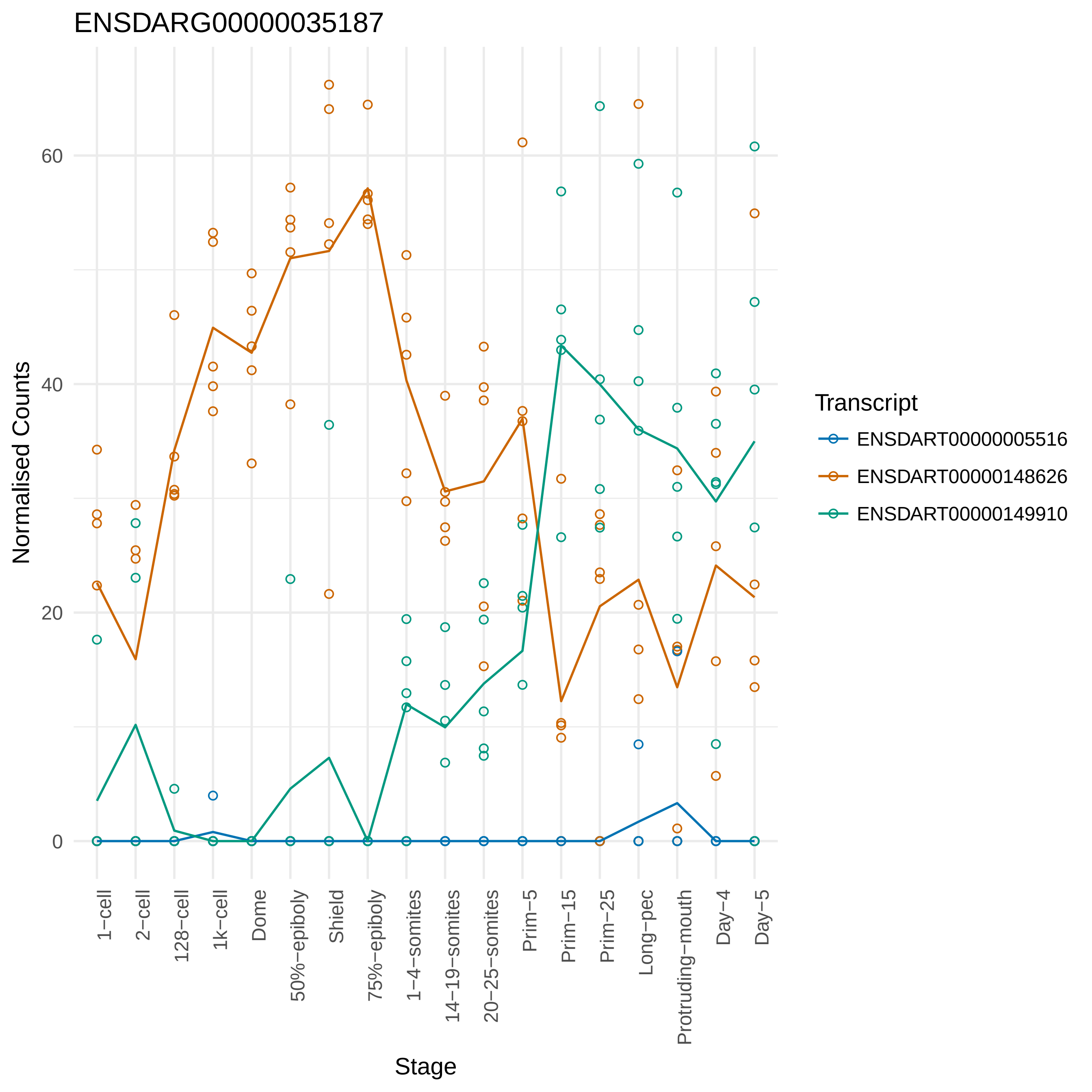

ENSDARG00000035187
Normalised Counts
60
40
20
0
1−cell
2−cell
128−cell
1k−cell
Dome
50%−epiboly
Shield
75%−epiboly
1−4−somites
14−19−somites
20−25−somites
Prim−5
Prim−15
Prim−25
Long−pec
Protruding−mouth
Day−4
Day−5
Stage
Transcript
ENSDART00000005516
ENSDART00000148626
ENSDART00000149910

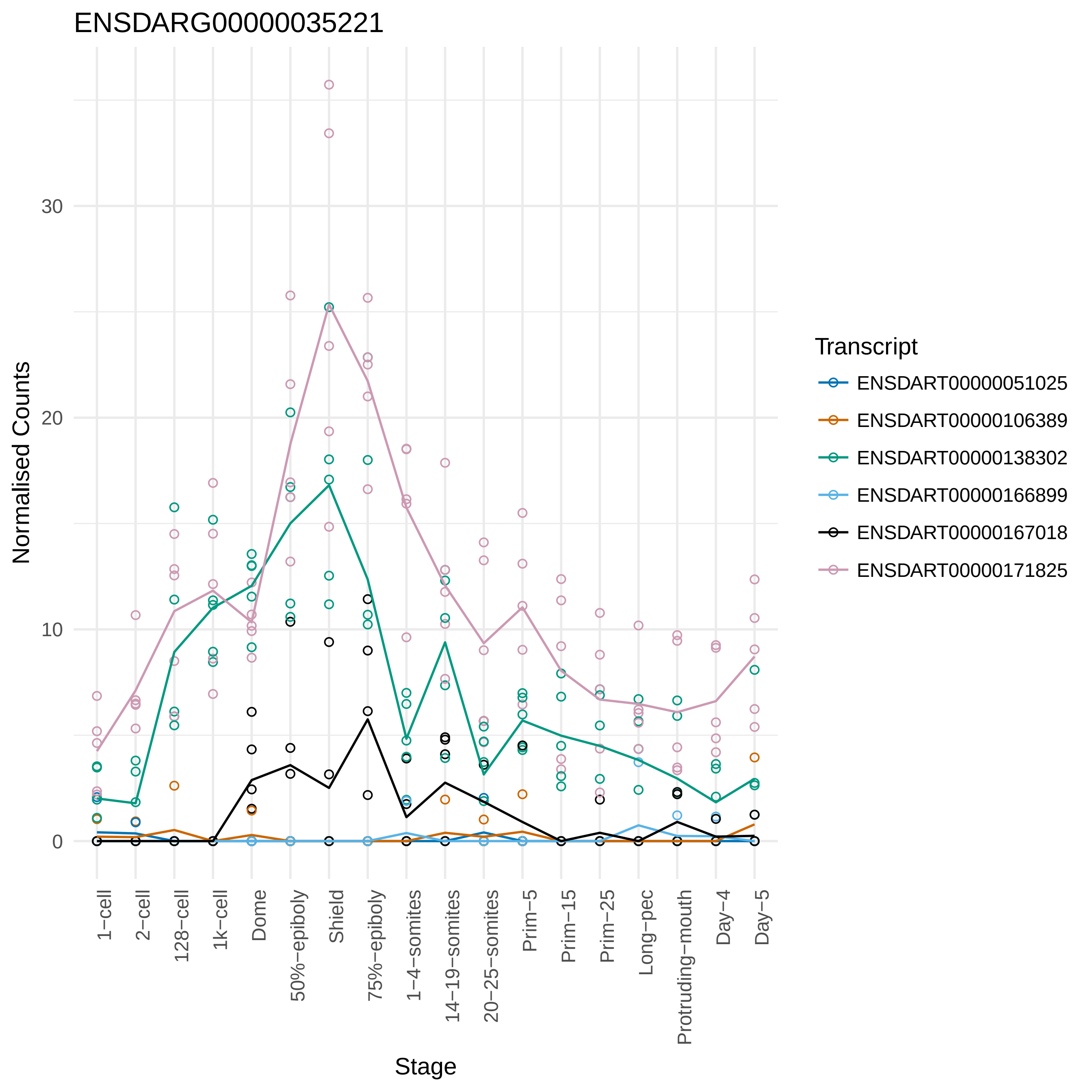
ENSDARG00000035221
Normalised Counts
30
20
10
0
1−cell
2−cell
128−cell
1k−cell
Dome
50%−epiboly
Shield
75%−epiboly
1−4−somites
14−19−somites
20−25−somites
Prim−5
Prim−15
Prim−25
Long−pec
Protruding−mouth
Day−4
Day−5
Stage
Transcript
ENSDART00000051025
ENSDART00000106389
ENSDART00000138302
ENSDART00000166899
ENSDART00000167018
ENSDART00000171825

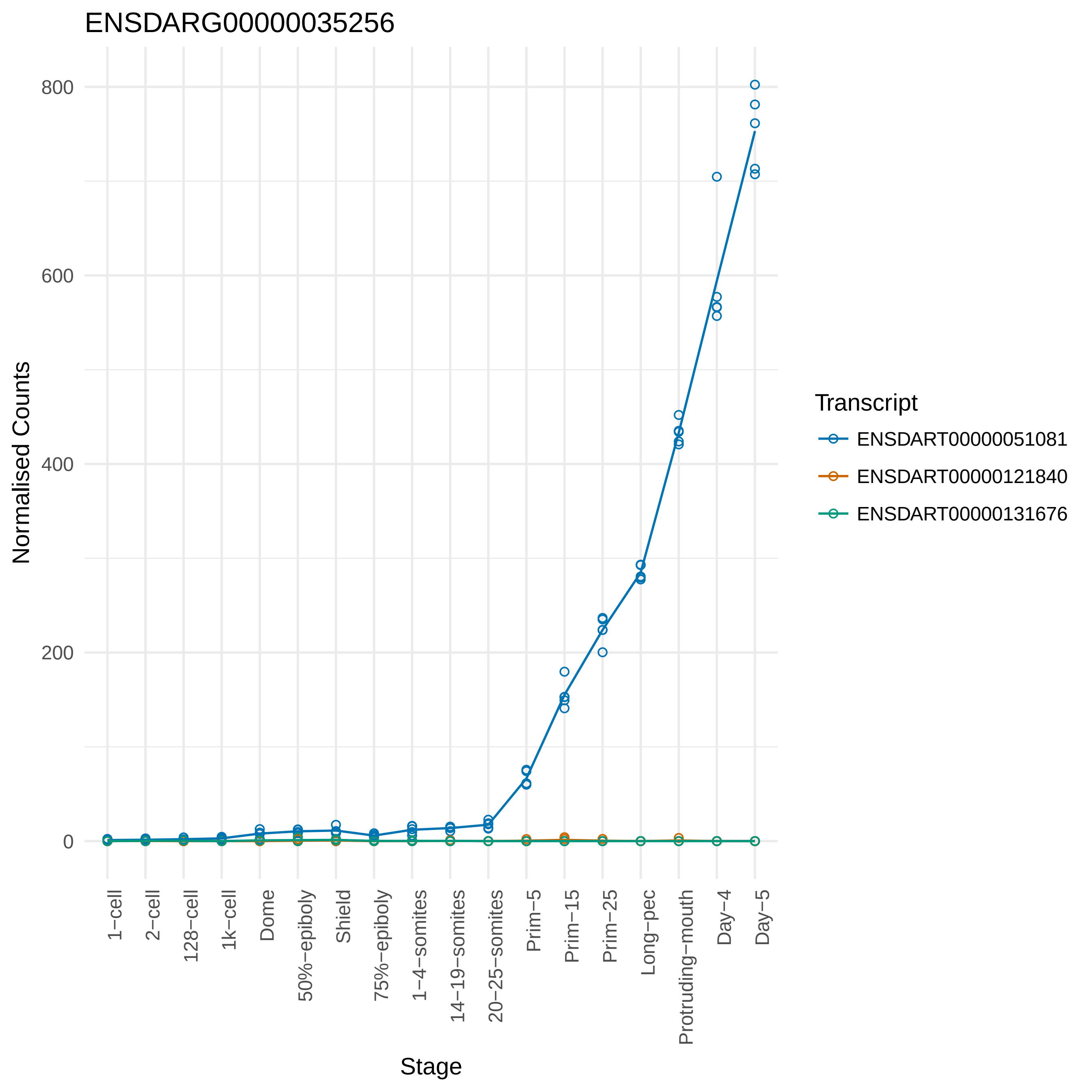

ENSDARG00000035256
Normalised Counts
800
600
400
200
0
1−cell
2−cell
128−cell
1k−cell
Dome
50%−epiboly
Shield
75%−epiboly
1−4−somites
14−19−somites
20−25−somites
Prim−5
Prim−15
Prim−25
Long−pec
Protruding−mouth
Day−4
Day−5
Stage
Transcript
ENSDART00000051081
ENSDART00000121840
ENSDART00000131676

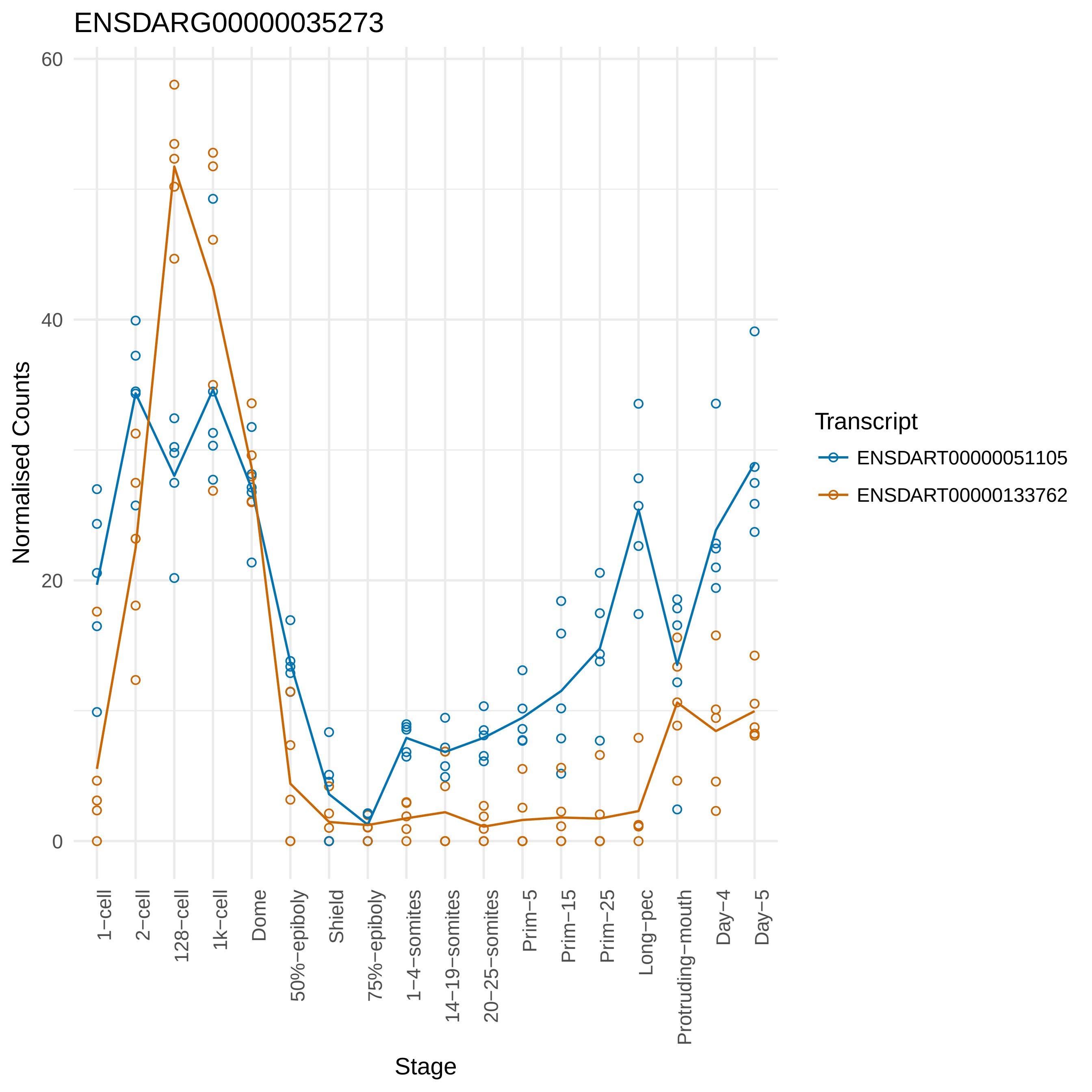
ENSDARG00000035273
Normalised Counts
60
40
20
0
1−cell
2−cell
128−cell
1k−cell
Dome
50%−epiboly
Shield
75%−epiboly
1−4−somites
14−19−somites
20−25−somites
Prim−5
Prim−15
Prim−25
Long−pec
Protruding−mouth
Day−4
Day−5
Stage
Transcript
ENSDART00000051105
ENSDART00000133762

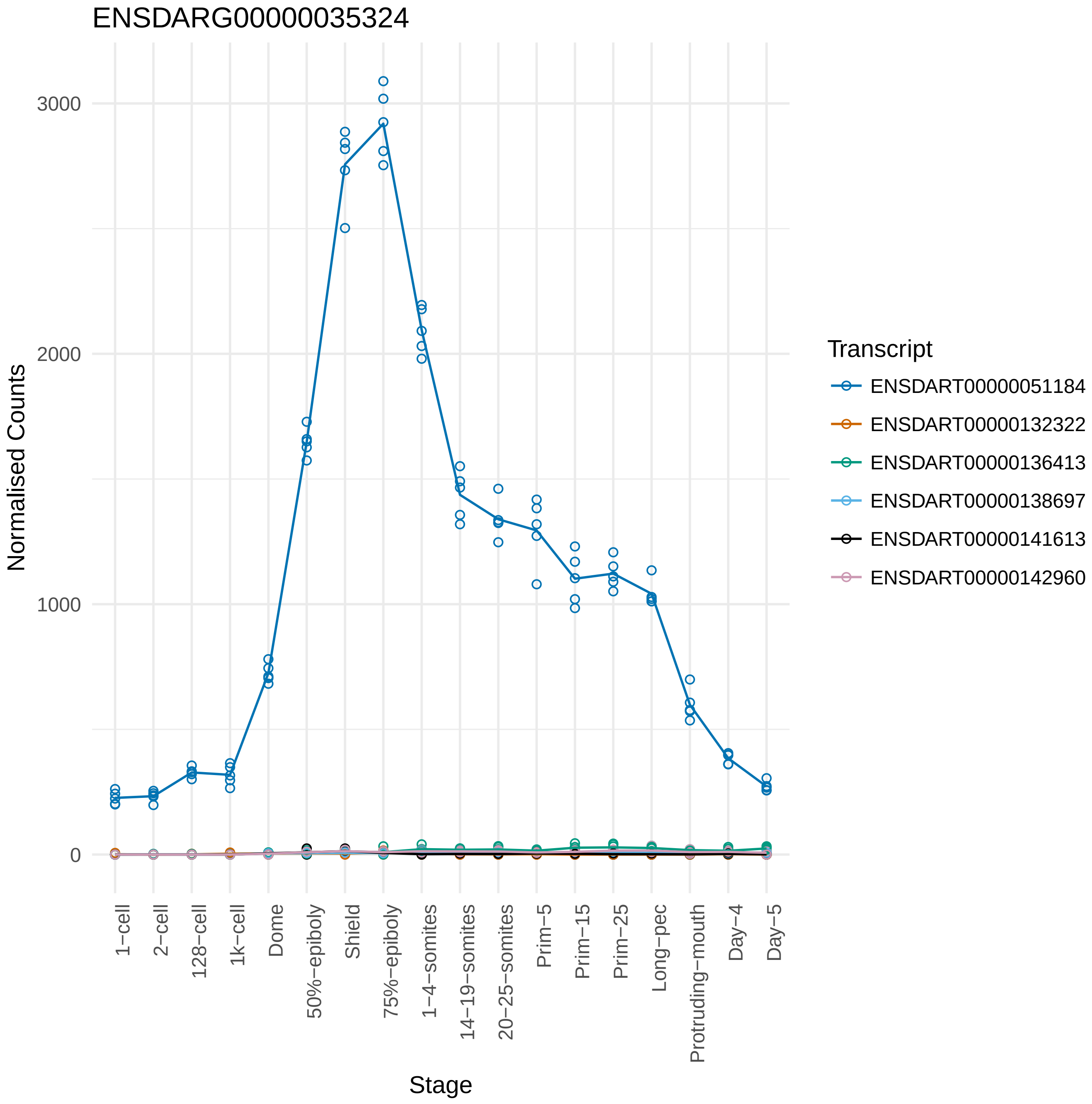

ENSDARG00000035324
Normalised Counts
3000
2000
1000
0
Stage
1−cell
2−cell
128−cell
1k−cell
Dome
50%−epiboly
Shield
75%−epiboly
1−4−somites
14−19−somites
20−25−somites
Prim−5
Prim−15
Prim−25
Long−pec
Protruding−mouth
Day−4
Day−5
Transcript
ENSDART00000051184
ENSDART00000132322
ENSDART00000136413
ENSDART00000138697
ENSDART00000141613
ENSDART00000142960

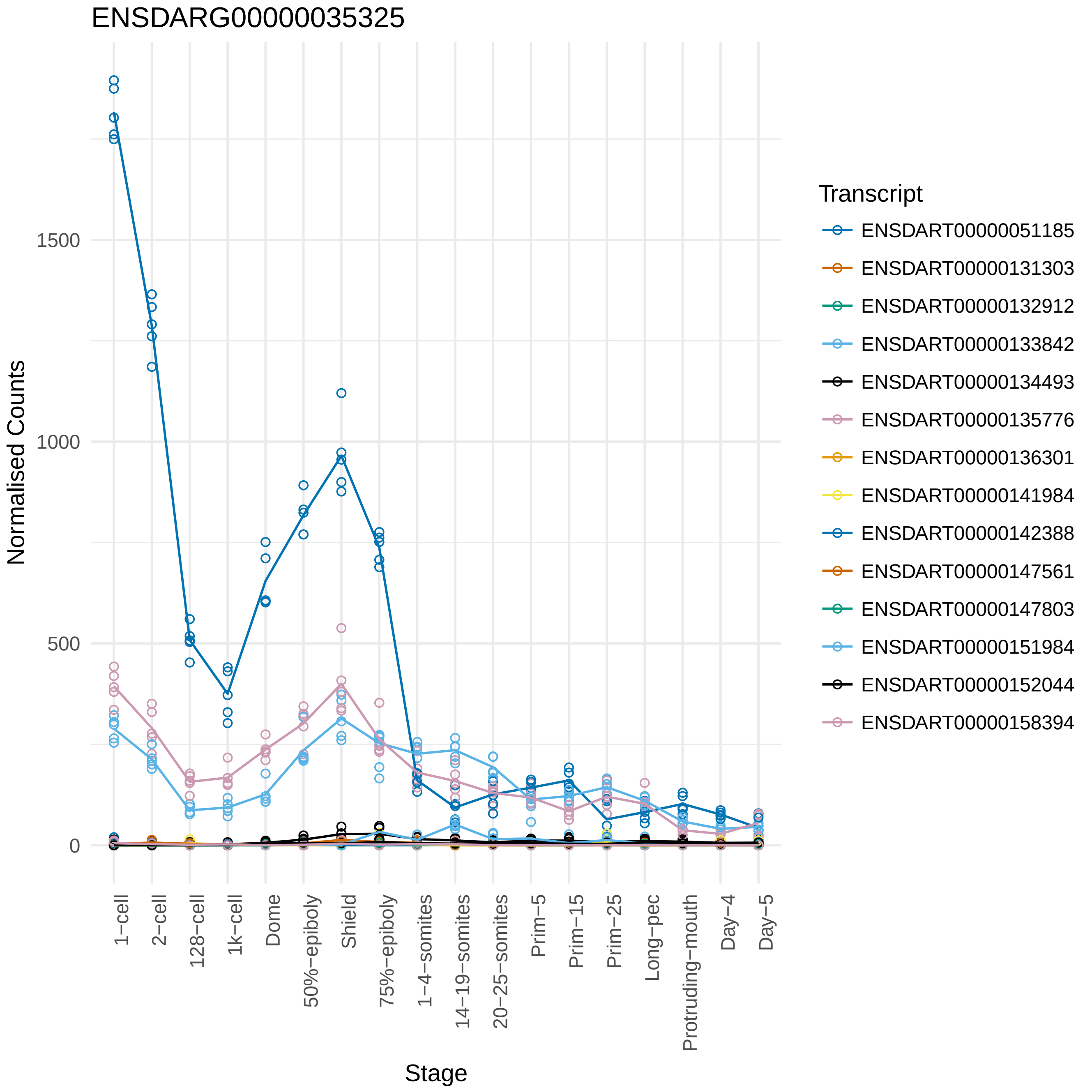
ENSDARG00000035325
Normalised Counts
0
500
1000
1500
Stage
1−cell
2−cell
128−cell
1k−cell
Dome
50%−epiboly
Shield
75%−epiboly
1−4−somites
14−19−somites
20−25−somites
Prim−5
Prim−15
Prim−25
Long−pec
Protruding−mouth
Day−4
Day−5
Transcript
ENSDART00000051185
ENSDART00000131303
ENSDART00000132912
ENSDART00000133842
ENSDART00000134493
ENSDART00000135776
ENSDART00000136301
ENSDART00000141984
ENSDART00000142388
ENSDART00000147561
ENSDART00000147803
ENSDART00000151984
ENSDART00000152044
ENSDART00000158394

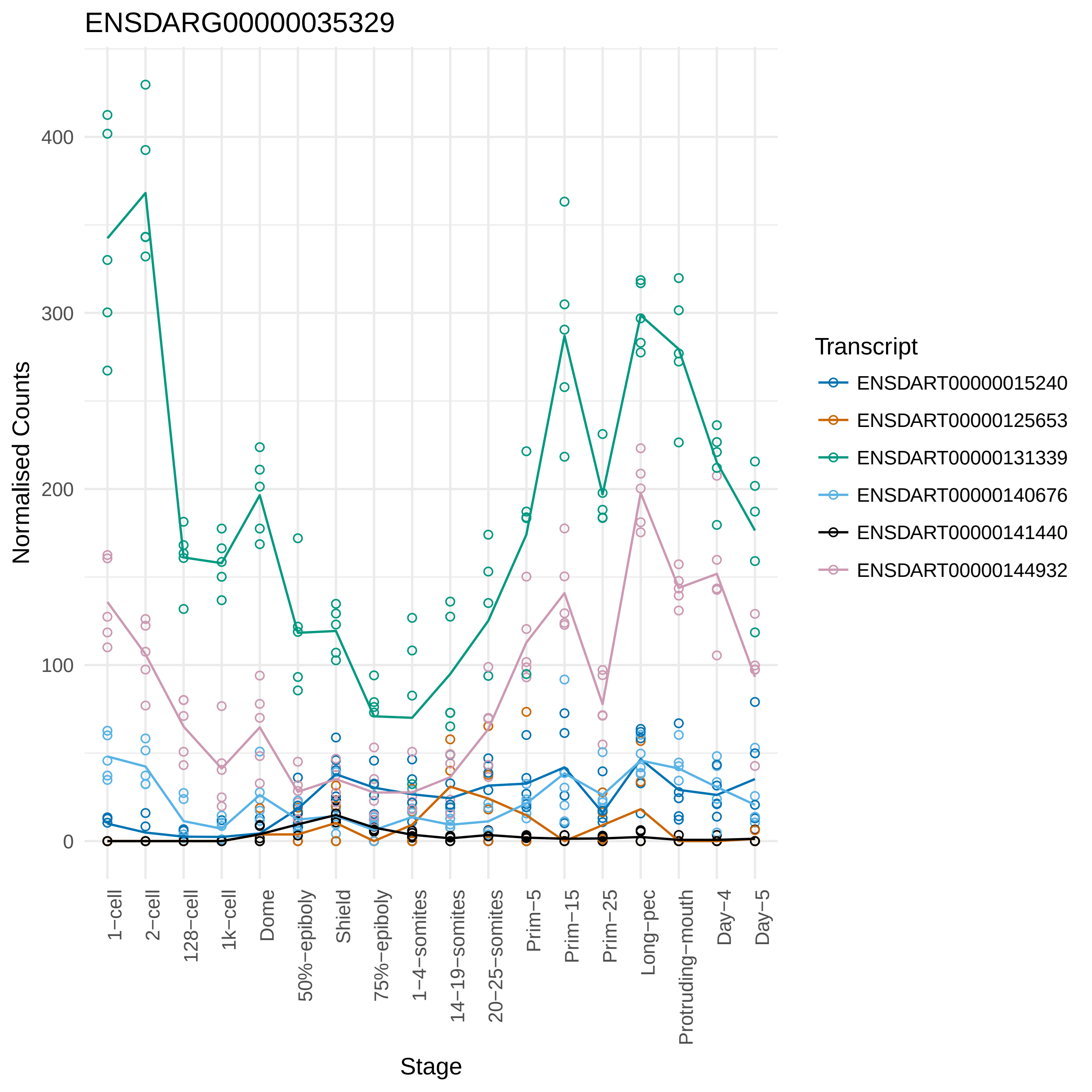

ENSDARG00000035329
Normalised Counts
0
100
200
300
400
Stage
1−cell
2−cell
128−cell
1k−cell
Dome
50%−epiboly
Shield
75%−epiboly
1−4−somites
14−19−somites
20−25−somites
Prim−5
Prim−15
Prim−25
Long−pec
Protruding−mouth
Day−4
Day−5
Transcript
ENSDART00000015240
ENSDART00000125653
ENSDART00000131339
ENSDART00000140676
ENSDART00000141440
ENSDART00000144932

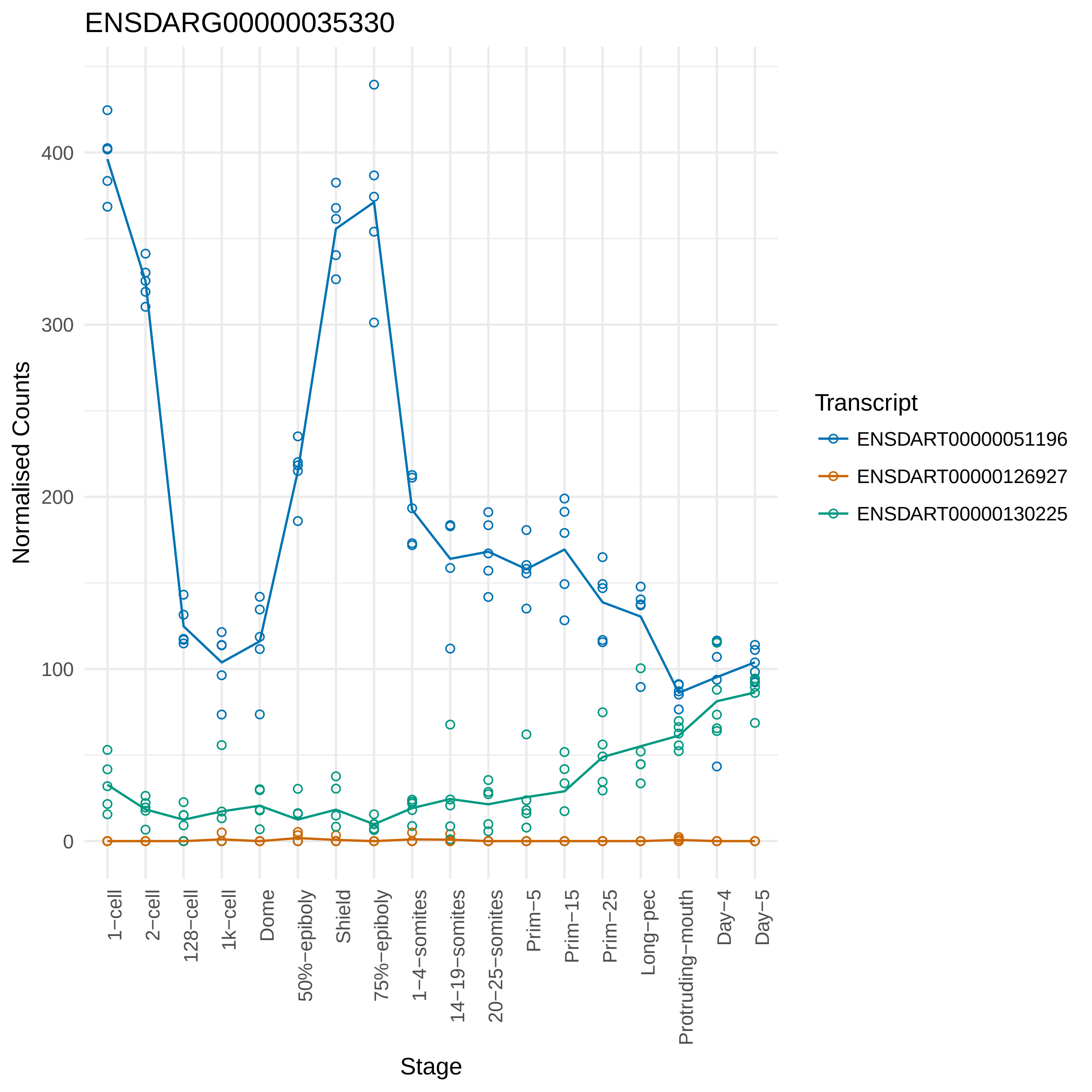

ENSDARG00000035330
Normalised Counts
0
100
200
300
400
Stage
1−cell
2−cell
128−cell
1k−cell
Dome
50%−epiboly
Shield
75%−epiboly
1−4−somites
14−19−somites
20−25−somites
Prim−5
Prim−15
Prim−25
Long−pec
Protruding−mouth
Day−4
Day−5
Transcript
ENSDART00000051196
ENSDART00000126927
ENSDART00000130225

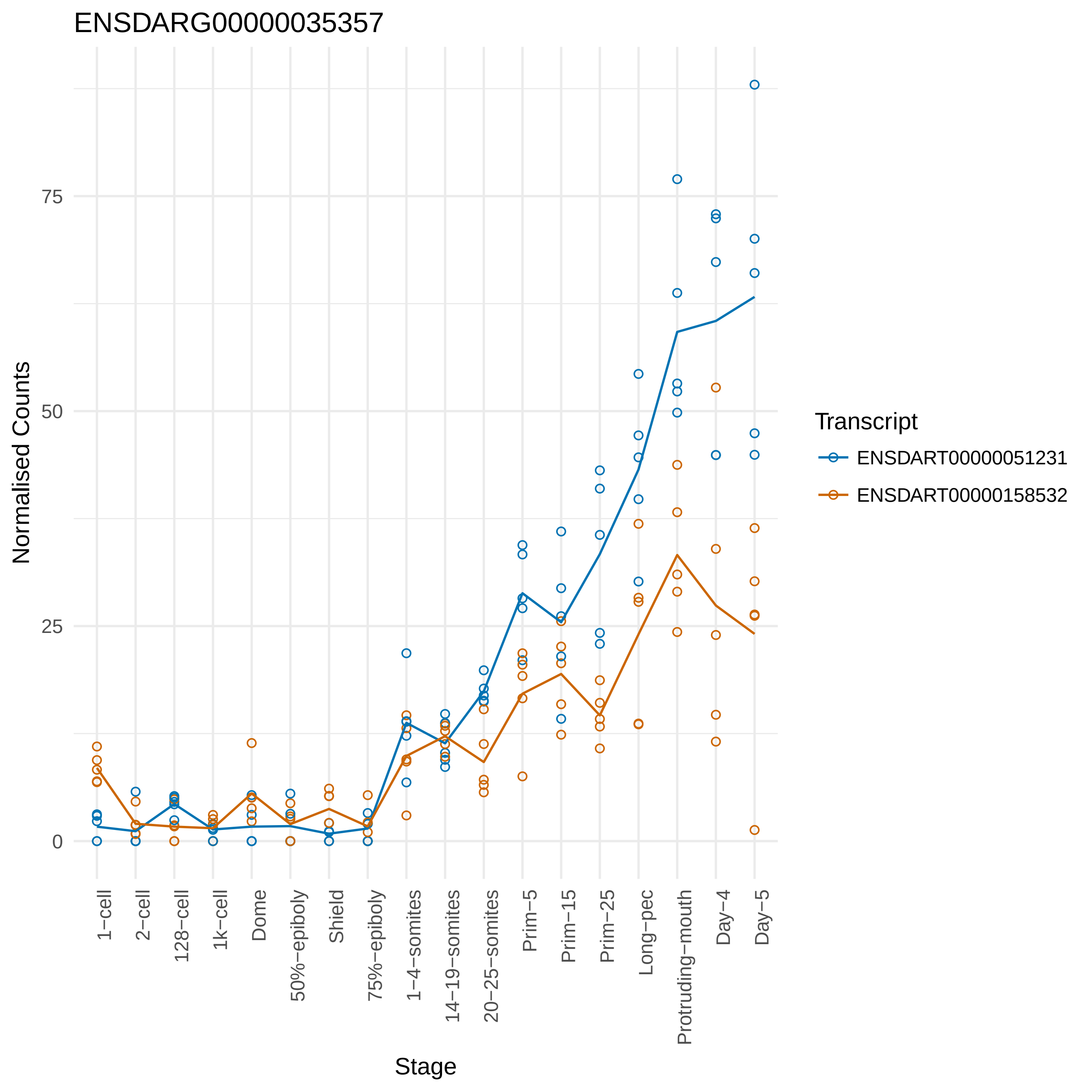

ENSDARG00000035357
Normalised Counts
75
50
25
0
1−cell
2−cell
128−cell
1k−cell
Dome
50%−epiboly
Shield
75%−epiboly
1−4−somites
14−19−somites
20−25−somites
Prim−5
Prim−15
Prim−25
Long−pec
Protruding−mouth
Day−4
Day−5
Stage
Transcript
ENSDART00000051231
ENSDART00000158532

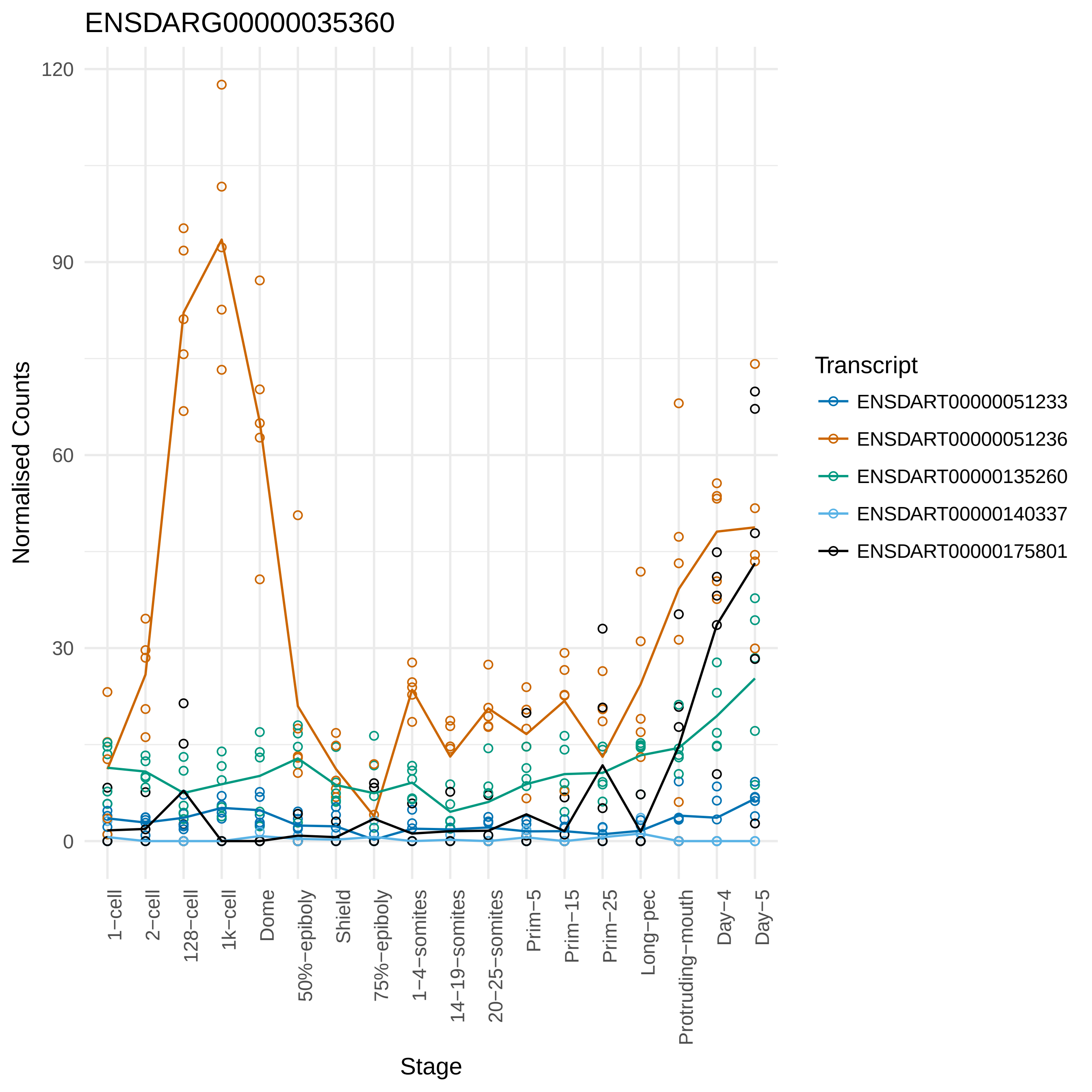

ENSDARG00000035360
Normalised Counts
120
90
60
30
0
1−cell
2−cell
128−cell
1k−cell
Dome
50%−epiboly
Shield
75%−epiboly
1−4−somites
14−19−somites
20−25−somites
Prim−5
Prim−15
Prim−25
Long−pec
Protruding−mouth
Day−4
Day−5
Stage
Transcript
ENSDART00000051233
ENSDART00000051236
ENSDART00000135260
ENSDART00000140337
ENSDART00000175801

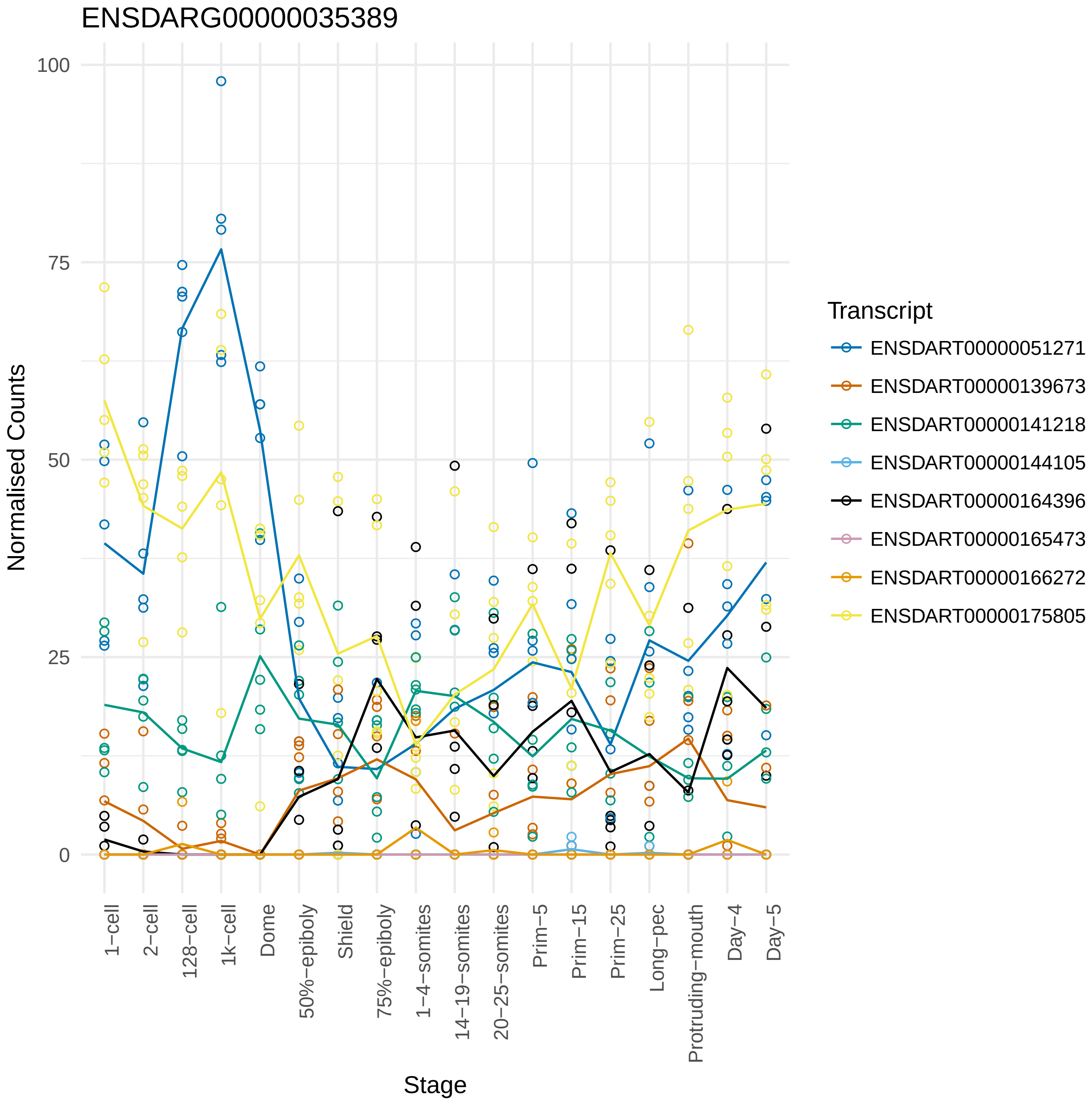
ENSDARG00000035389
Normalised Counts
100
75
50
25
0
1−cell
2−cell
128−cell
1k−cell
Dome
50%−epiboly
Shield
75%−epiboly
1−4−somites
14−19−somites
20−25−somites
Prim−5
Prim−15
Prim−25
Long−pec
Protruding−mouth
Day−4
Day−5
Stage
Transcript
ENSDART00000051271
ENSDART00000139673
ENSDART00000141218
ENSDART00000144105
ENSDART00000164396
ENSDART00000165473
ENSDART00000166272
ENSDART00000175805

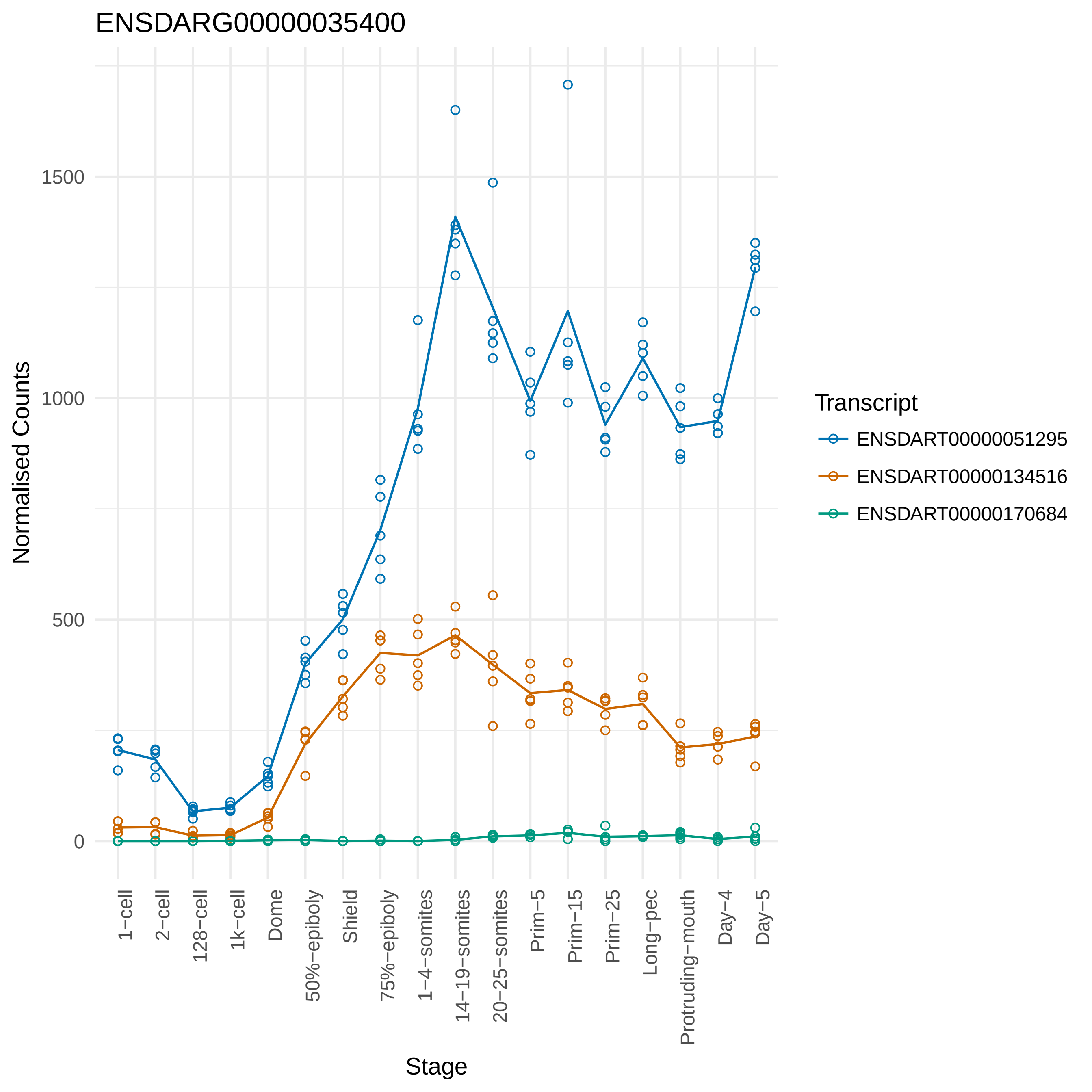

ENSDARG00000035400
Normalised Counts
1500
1000
500
0
Stage
1−cell
2−cell
128−cell
1k−cell
Dome
50%−epiboly
Shield
75%−epiboly
1−4−somites
14−19−somites
20−25−somites
Prim−5
Prim−15
Prim−25
Long−pec
Protruding−mouth
Day−4
Day−5
Transcript
ENSDART00000051295
ENSDART00000134516
ENSDART00000170684

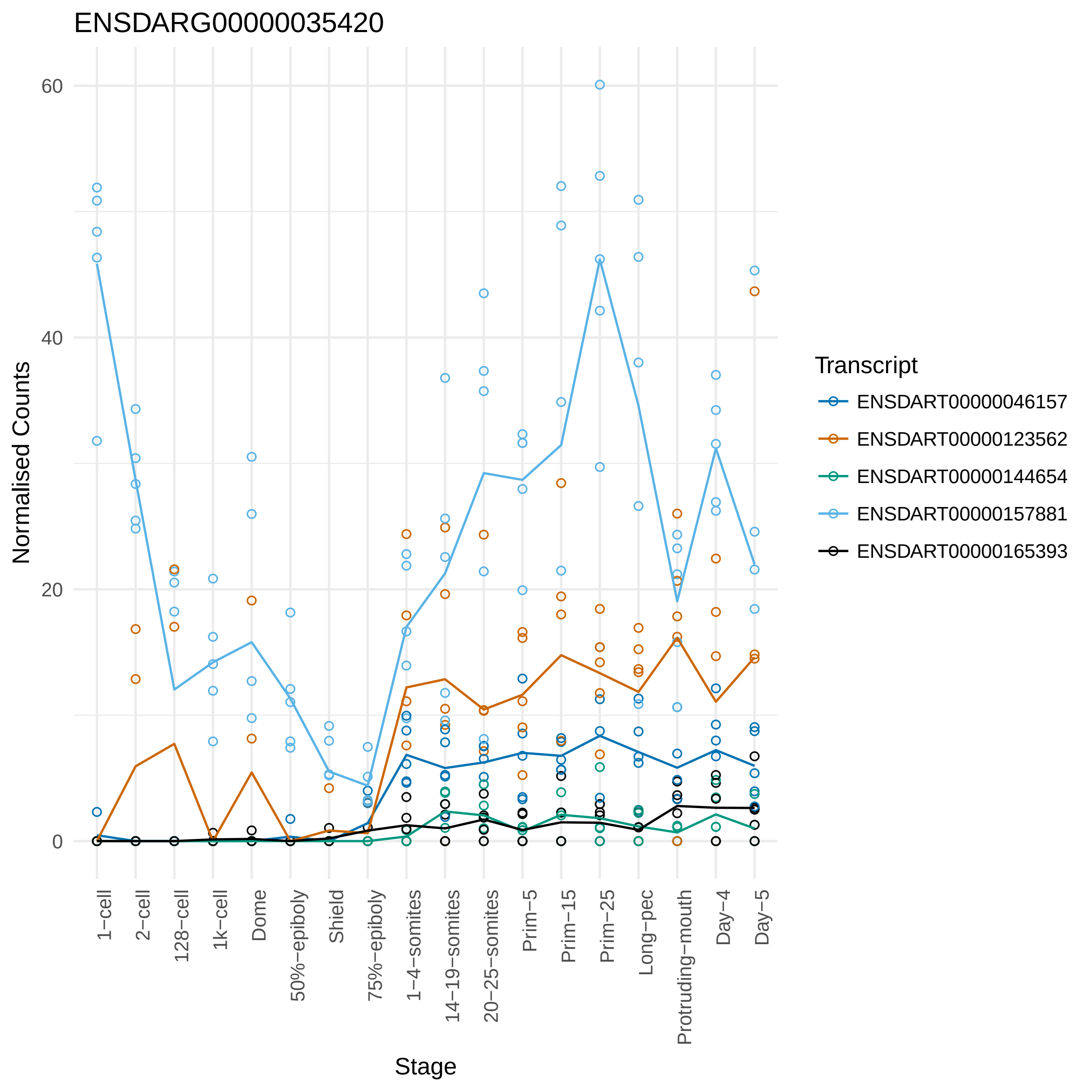

ENSDARG00000035420
Normalised Counts
60
40
20
0
1−cell
2−cell
128−cell
1k−cell
Dome
50%−epiboly
Shield
75%−epiboly
1−4−somites
14−19−somites
20−25−somites
Prim−5
Prim−15
Prim−25
Long−pec
Protruding−mouth
Day−4
Day−5
Stage
Transcript
ENSDART00000046157
ENSDART00000123562
ENSDART00000144654
ENSDART00000157881
ENSDART00000165393

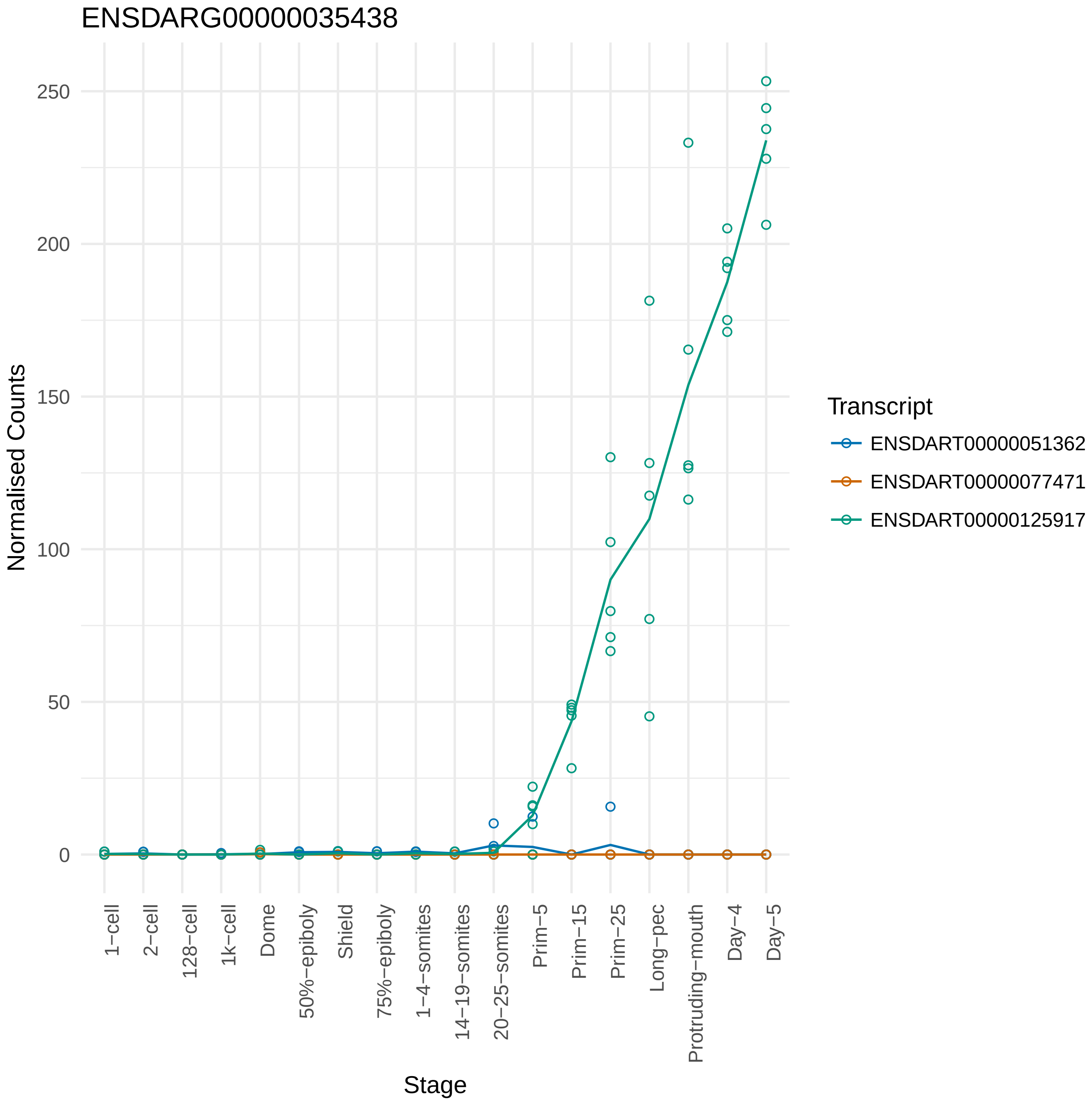

ENSDARG00000035438
Normalised Counts
250
200
150
100
50
0
1−cell
2−cell
128−cell
1k−cell
Dome
50%−epiboly
Shield
75%−epiboly
1−4−somites
14−19−somites
20−25−somites
Prim−5
Prim−15
Prim−25
Long−pec
Protruding−mouth
Day−4
Day−5
Stage
Transcript
ENSDART00000051362
ENSDART00000077471
ENSDART00000125917

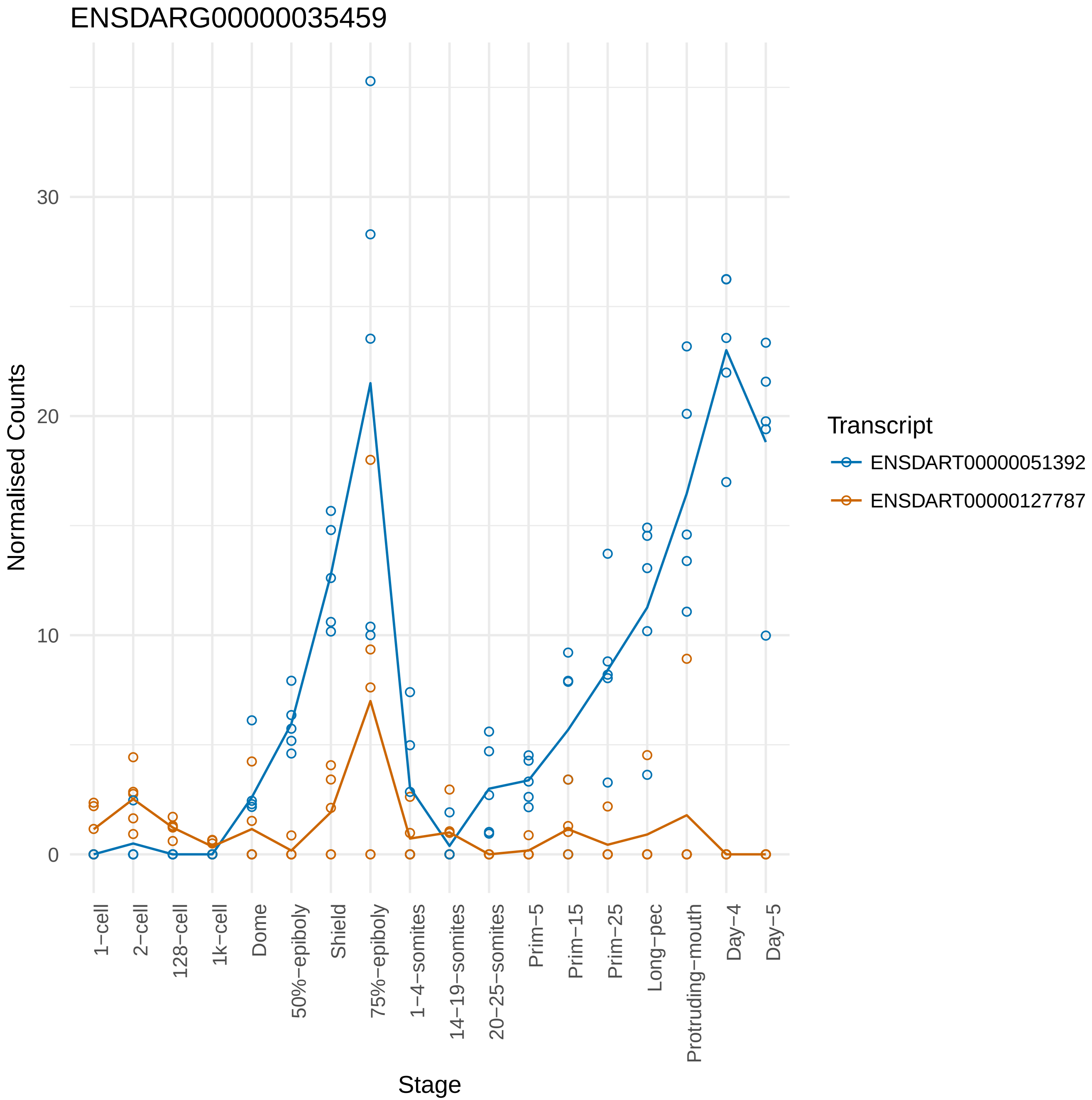

ENSDARG00000035459
Normalised Counts
0
10
20
30
Stage
1−cell
2−cell
128−cell
1k−cell
Dome
50%−epiboly
Shield
75%−epiboly
1−4−somites
14−19−somites
20−25−somites
Prim−5
Prim−15
Prim−25
Long−pec
Protruding−mouth
Day−4
Day−5
Transcript
ENSDART00000051392
ENSDART00000127787

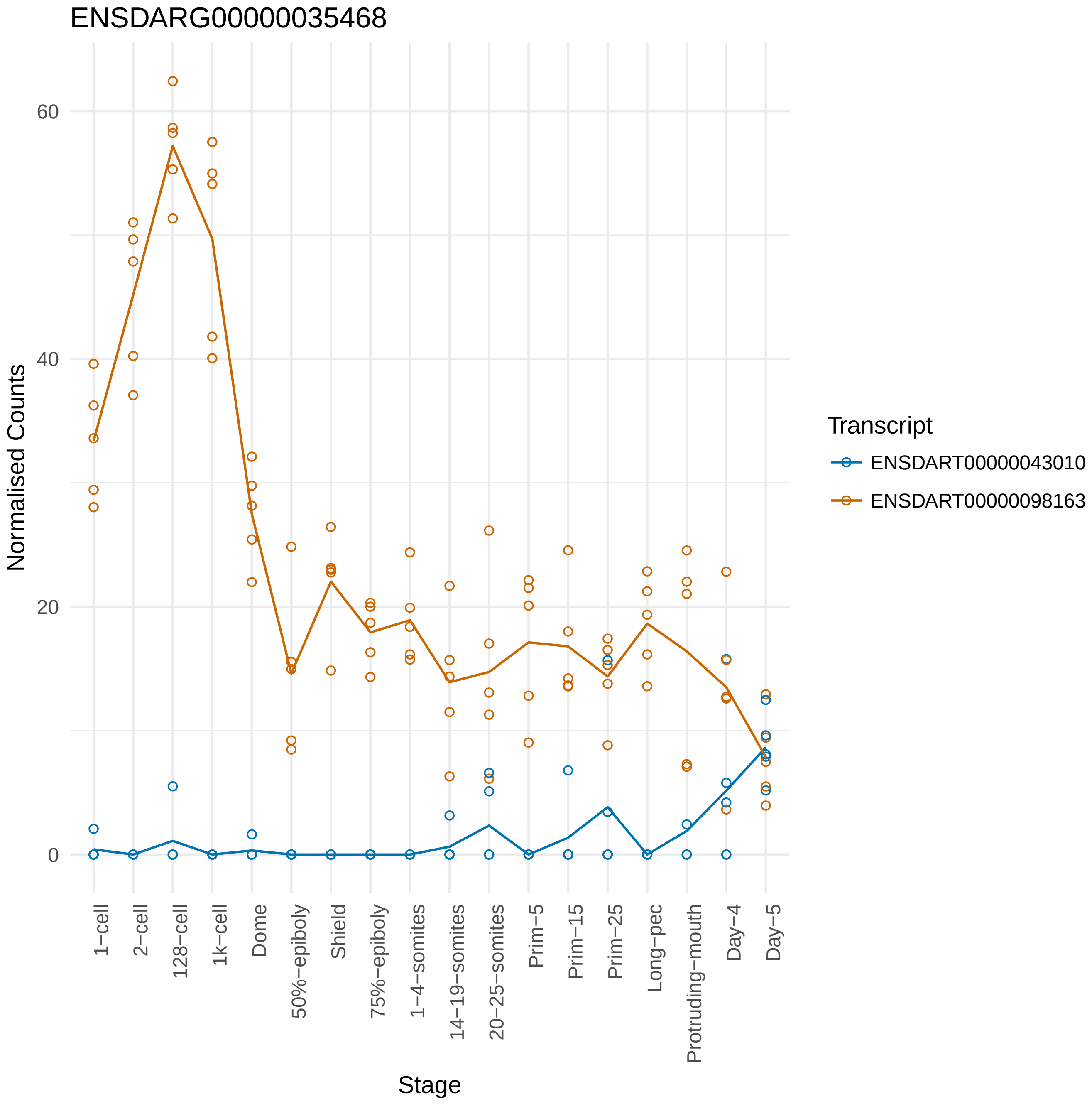
ENSDARG00000035468
Normalised Counts
60
40
20
0
1−cell
2−cell
128−cell
1k−cell
Dome
50%−epiboly
Shield
75%−epiboly
1−4−somites
14−19−somites
20−25−somites
Prim−5
Prim−15
Prim−25
Long−pec
Protruding−mouth
Day−4
Day−5
Stage
Transcript
ENSDART00000043010
ENSDART00000098163

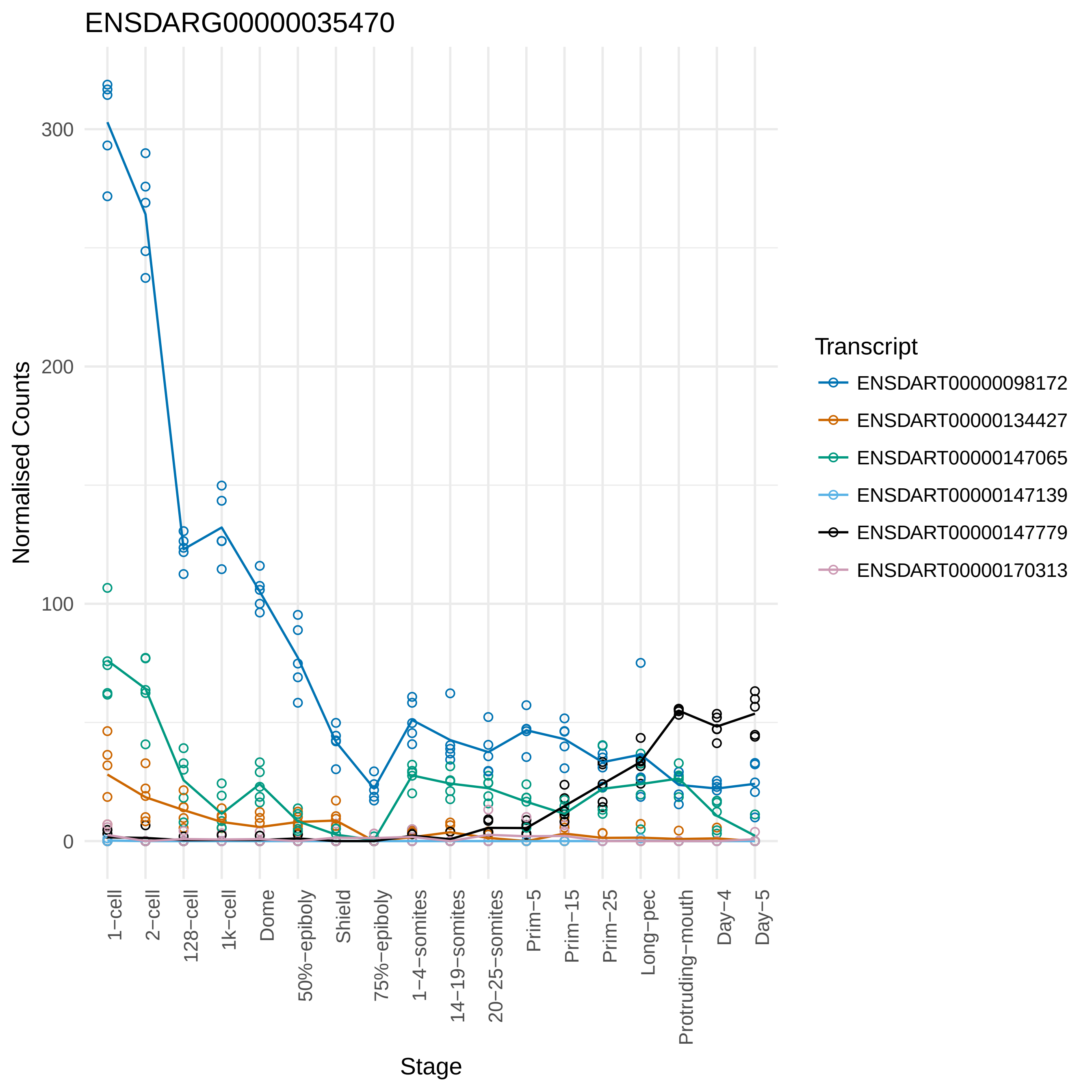

ENSDARG00000035470
Normalised Counts
300
200
100
0
1−cell
2−cell
128−cell
1k−cell
Dome
50%−epiboly
Shield
75%−epiboly
1−4−somites
14−19−somites
20−25−somites
Prim−5
Prim−15
Prim−25
Long−pec
Protruding−mouth
Day−4
Day−5
Stage
Transcript
ENSDART00000098172
ENSDART00000134427
ENSDART00000147065
ENSDART00000147139
ENSDART00000147779
ENSDART00000170313

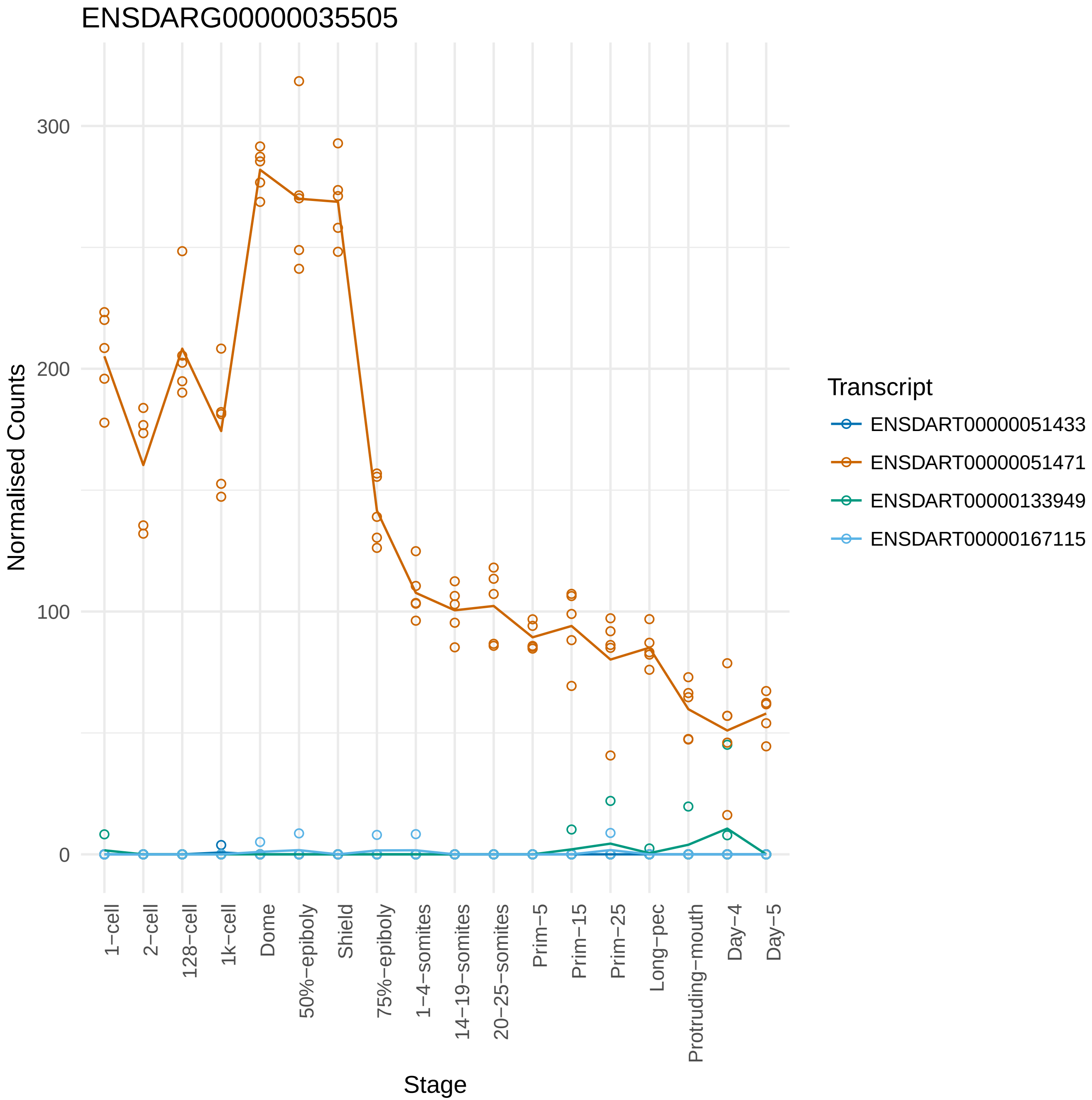

ENSDARG00000035505
Normalised Counts
300
200
100
0
1−cell
2−cell
128−cell
1k−cell
Dome
50%−epiboly
Shield
75%−epiboly
1−4−somites
14−19−somites
20−25−somites
Prim−5
Prim−15
Prim−25
Long−pec
Protruding−mouth
Day−4
Day−5
Stage
Transcript
ENSDART00000051433
ENSDART00000051471
ENSDART00000133949
ENSDART00000167115

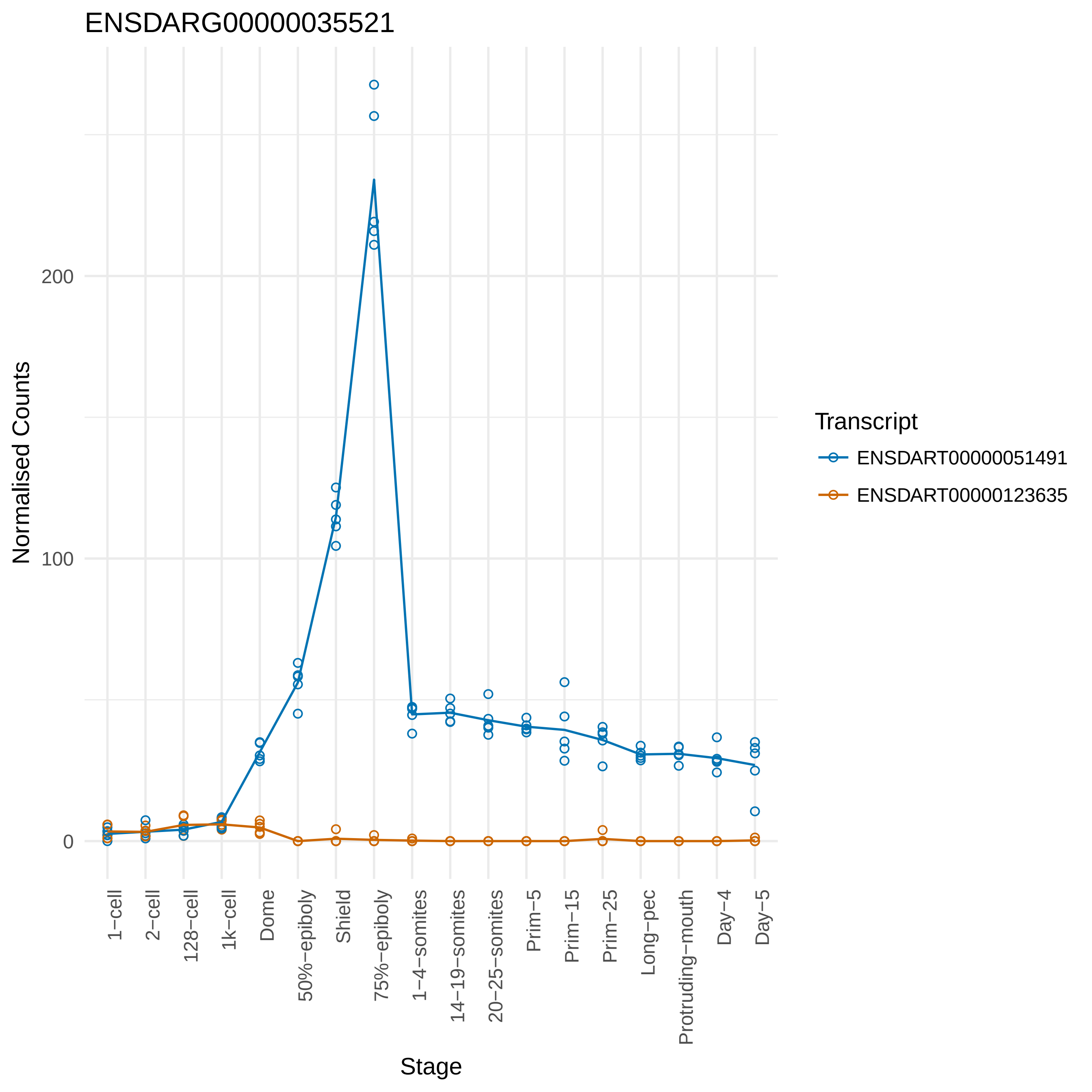

ENSDARG00000035521
Normalised Counts
200
100
0
1−cell
2−cell
128−cell
1k−cell
Dome
50%−epiboly
Shield
75%−epiboly
1−4−somites
14−19−somites
20−25−somites
Prim−5
Prim−15
Prim−25
Long−pec
Protruding−mouth
Day−4
Day−5
Stage
Transcript
ENSDART00000051491
ENSDART00000123635

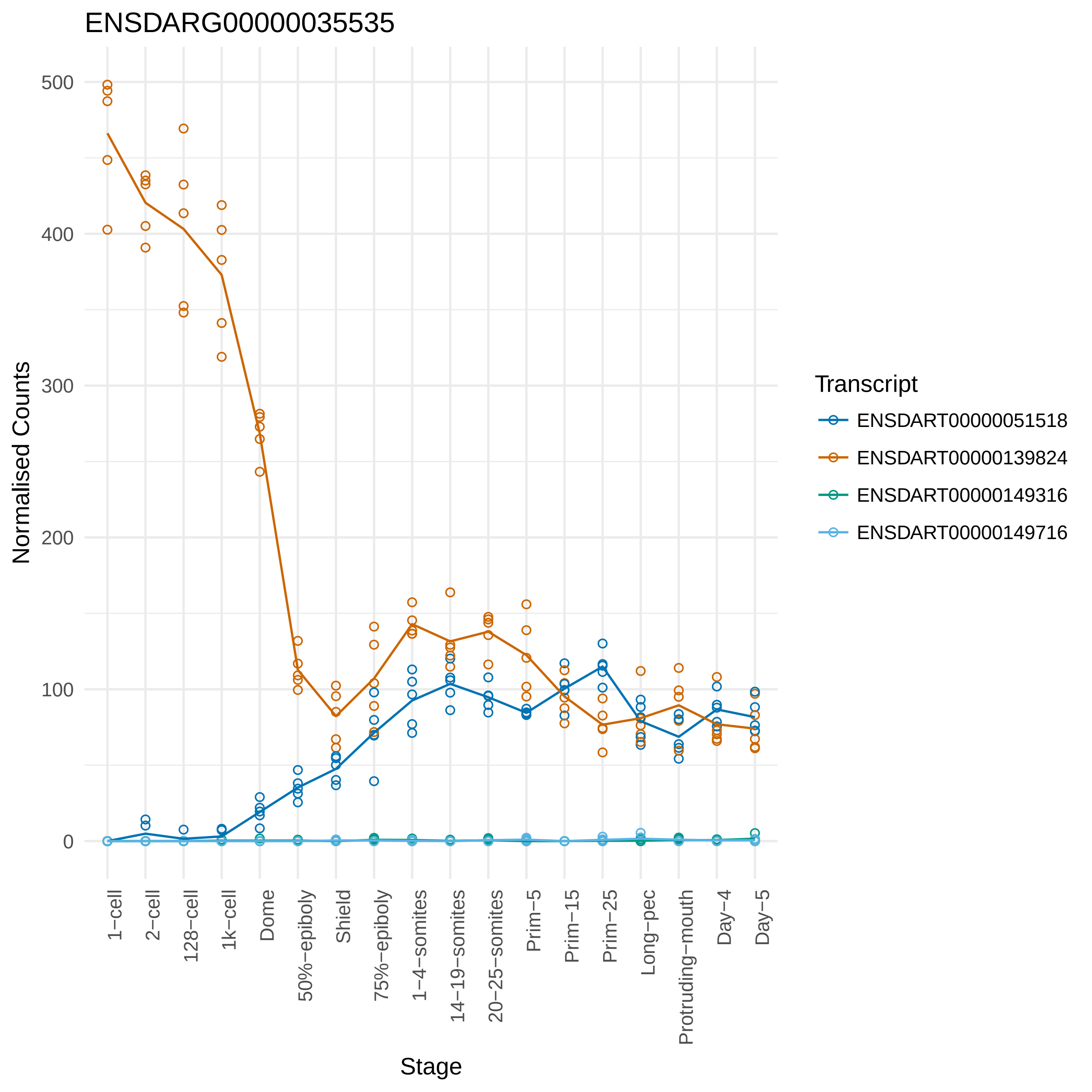

ENSDARG00000035535
Normalised Counts
500
400
300
200
100
0
1−cell
2−cell
128−cell
1k−cell
Dome
50%−epiboly
Shield
75%−epiboly
1−4−somites
14−19−somites
20−25−somites
Prim−5
Prim−15
Prim−25
Long−pec
Protruding−mouth
Day−4
Day−5
Stage
Transcript
ENSDART00000051518
ENSDART00000139824
ENSDART00000149316
ENSDART00000149716

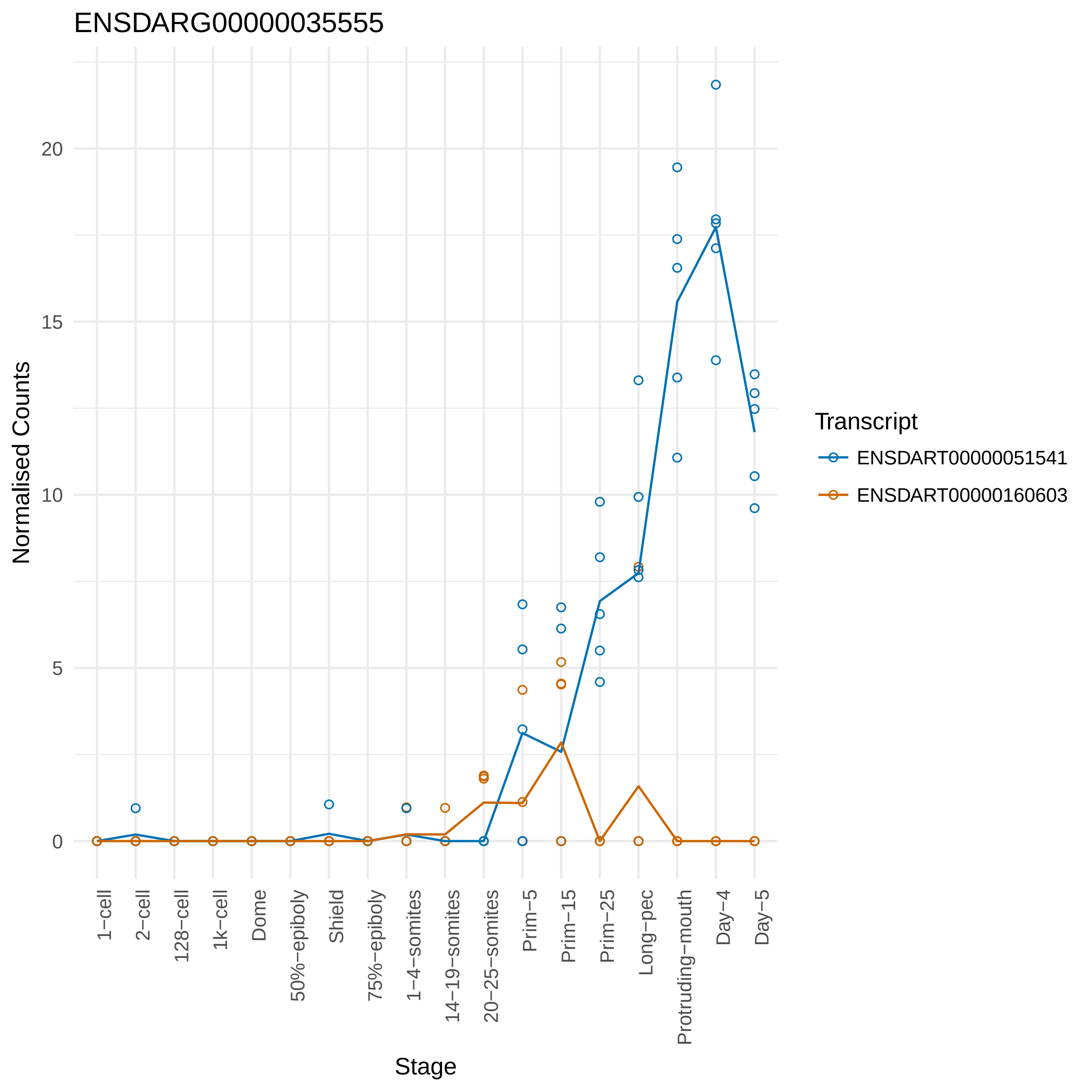
ENSDARG00000035555
Normalised Counts
0
5
10
15
20
Stage
1−cell
2−cell
128−cell
1k−cell
Dome
50%−epiboly
Shield
75%−epiboly
1−4−somites
14−19−somites
20−25−somites
Prim−5
Prim−15
Prim−25
Long−pec
Protruding−mouth
Day−4
Day−5
Transcript
ENSDART00000051541
ENSDART00000160603

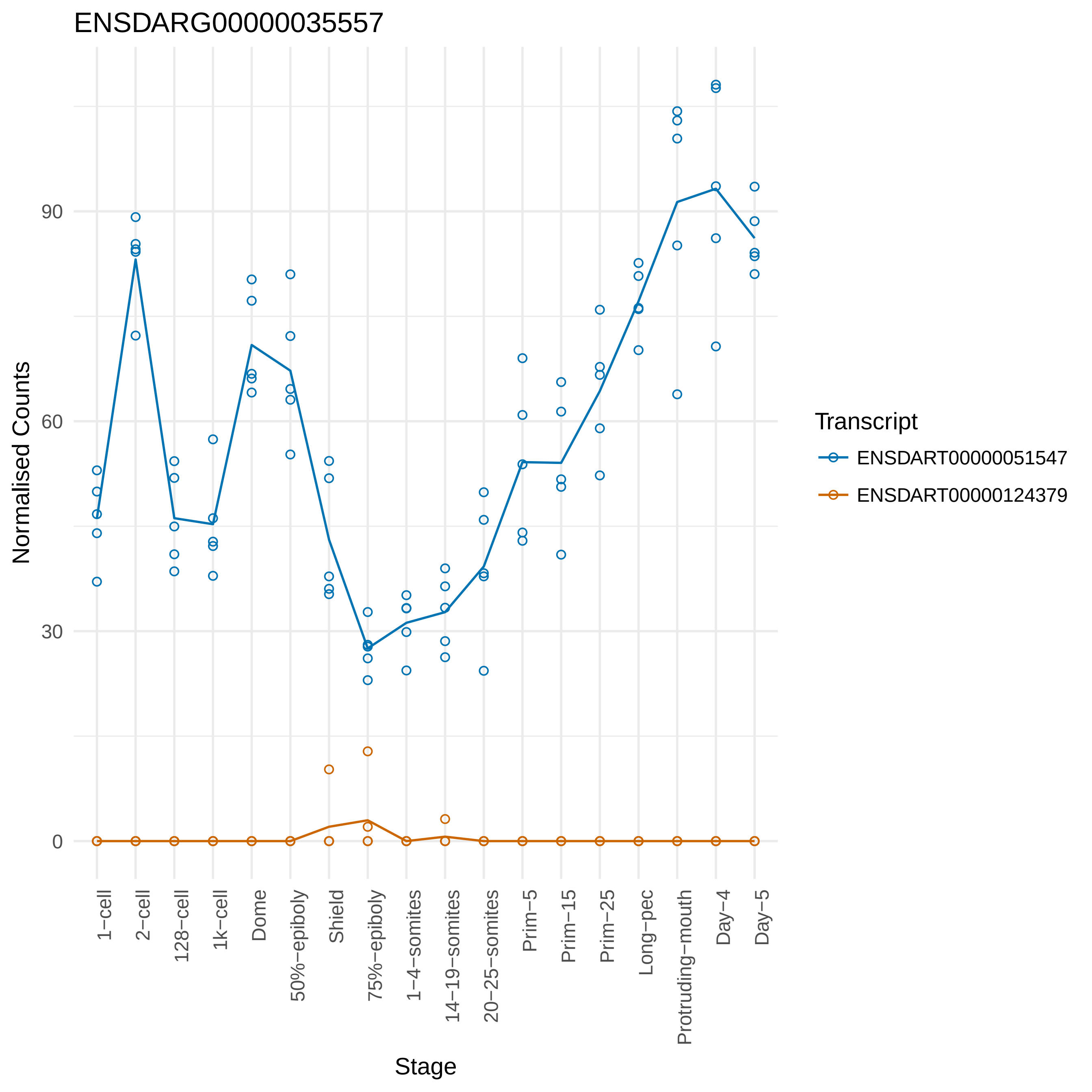

ENSDARG00000035557
Normalised Counts
90
60
30
0
1−cell
2−cell
128−cell
1k−cell
Dome
50%−epiboly
Shield
75%−epiboly
1−4−somites
14−19−somites
20−25−somites
Prim−5
Prim−15
Prim−25
Long−pec
Protruding−mouth
Day−4
Day−5
Stage
Transcript
ENSDART00000051547
ENSDART00000124379

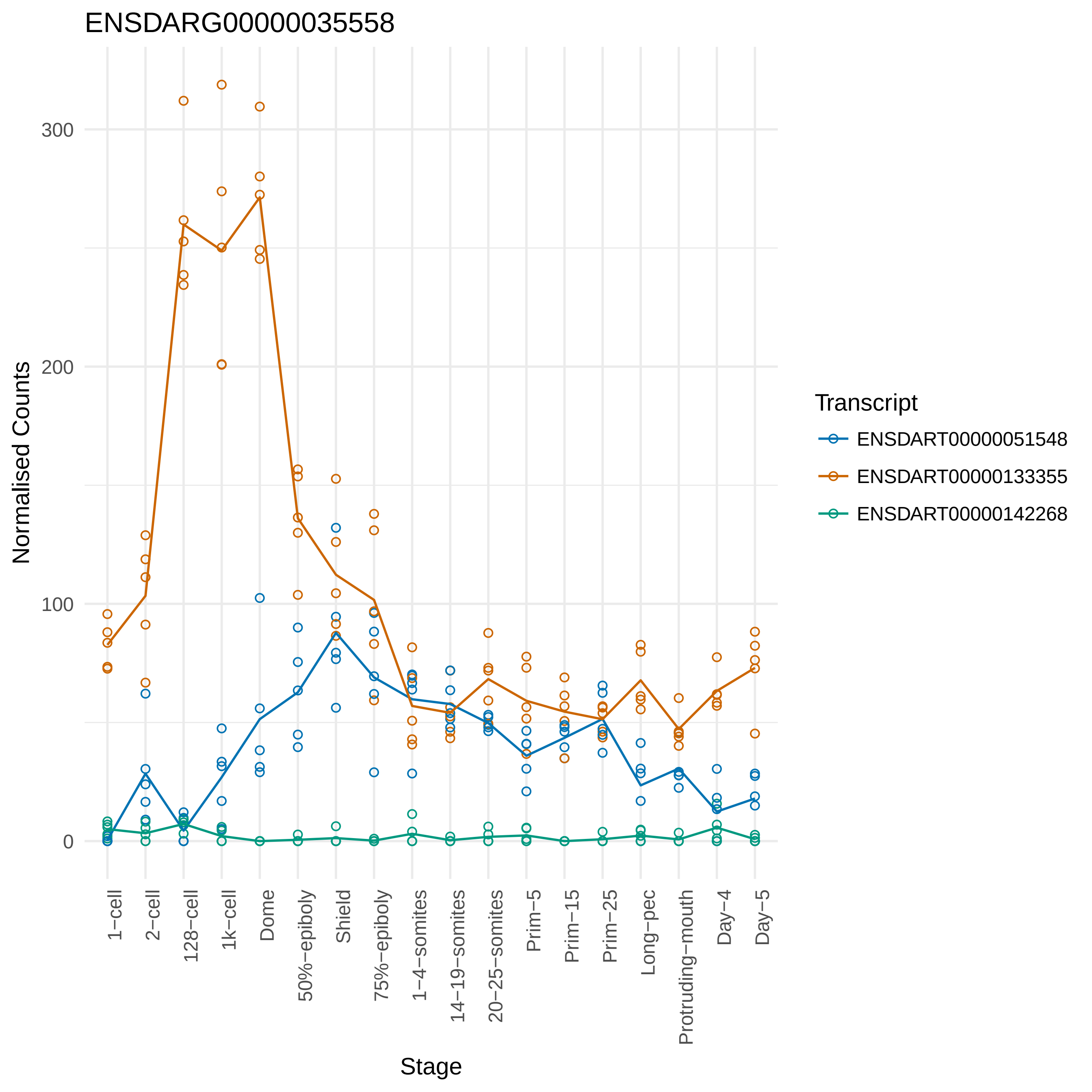
ENSDARG00000035558
Normalised Counts
300
200
100
0
1−cell
2−cell
128−cell
1k−cell
Dome
50%−epiboly
Shield
75%−epiboly
1−4−somites
14−19−somites
20−25−somites
Prim−5
Prim−15
Prim−25
Long−pec
Protruding−mouth
Day−4
Day−5
Stage
Transcript
ENSDART00000051548
ENSDART00000133355
ENSDART00000142268

ENSDARG00000035559

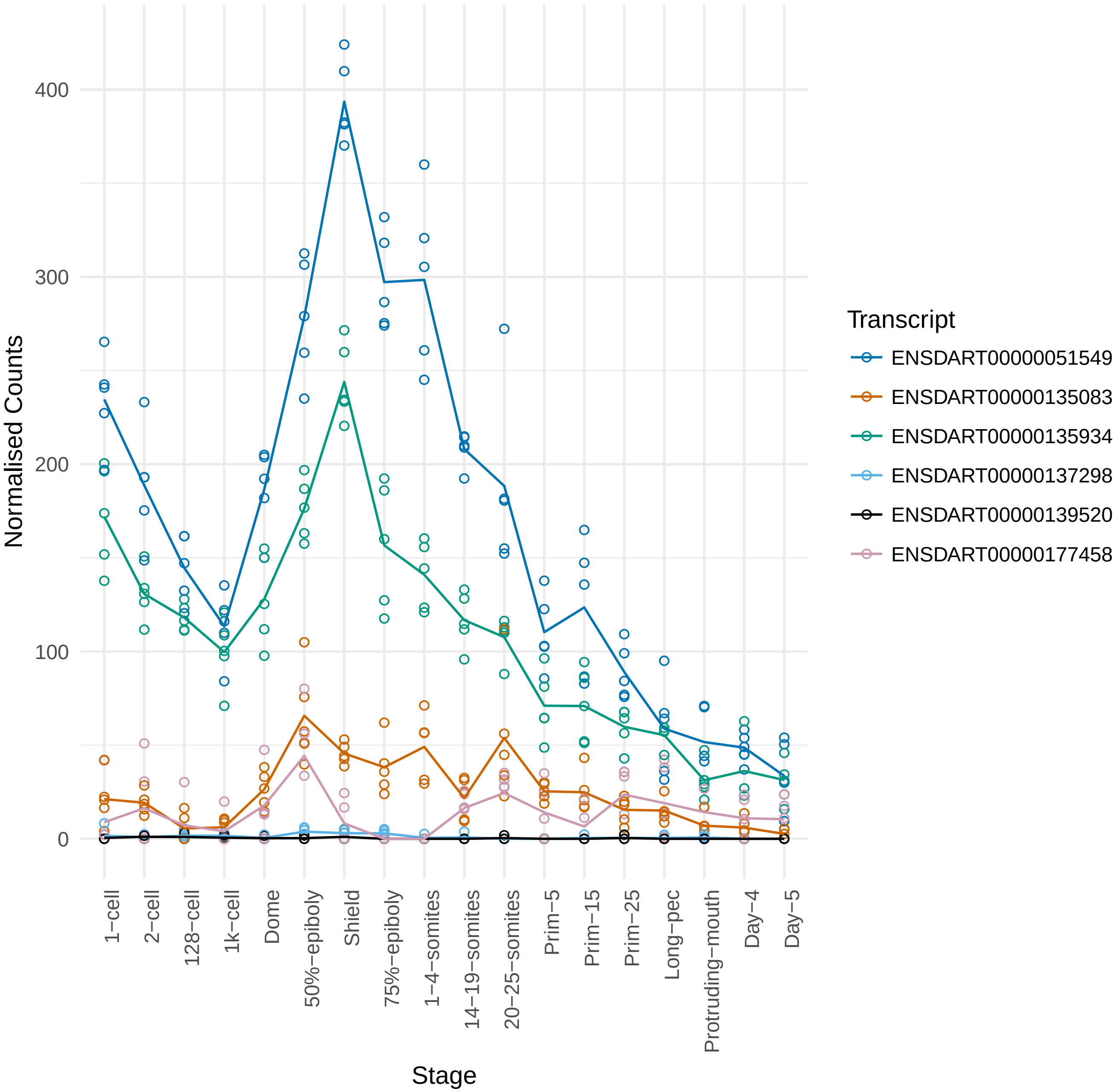

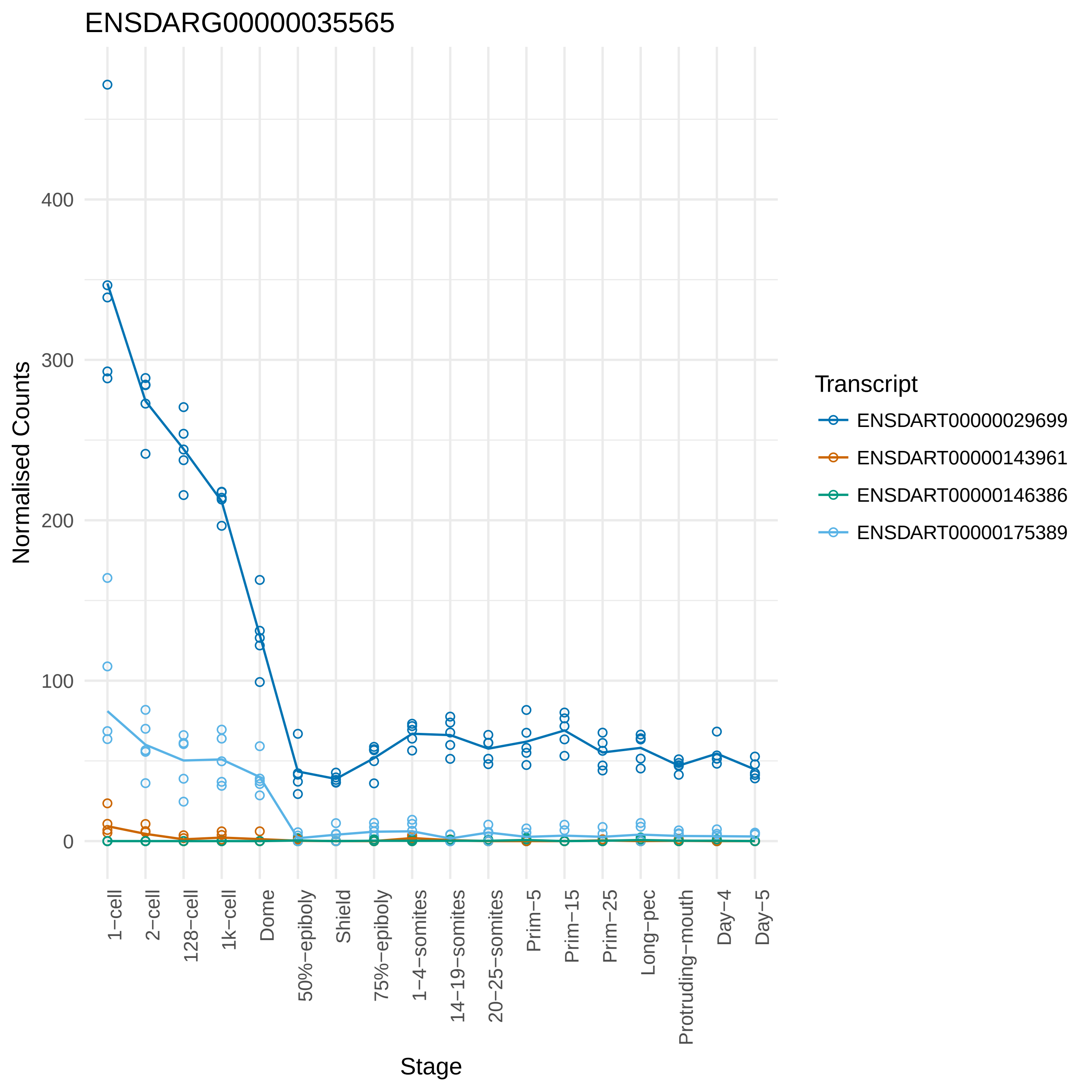
ENSDARG00000035565
Normalised Counts
0
100
200
300
400
Stage
1−cell
2−cell
128−cell
1k−cell
Dome
50%−epiboly
Shield
75%−epiboly
1−4−somites
14−19−somites
20−25−somites
Prim−5
Prim−15
Prim−25
Long−pec
Protruding−mouth
Day−4
Day−5
Transcript
ENSDART00000029699
ENSDART00000143961
ENSDART00000146386
ENSDART00000175389

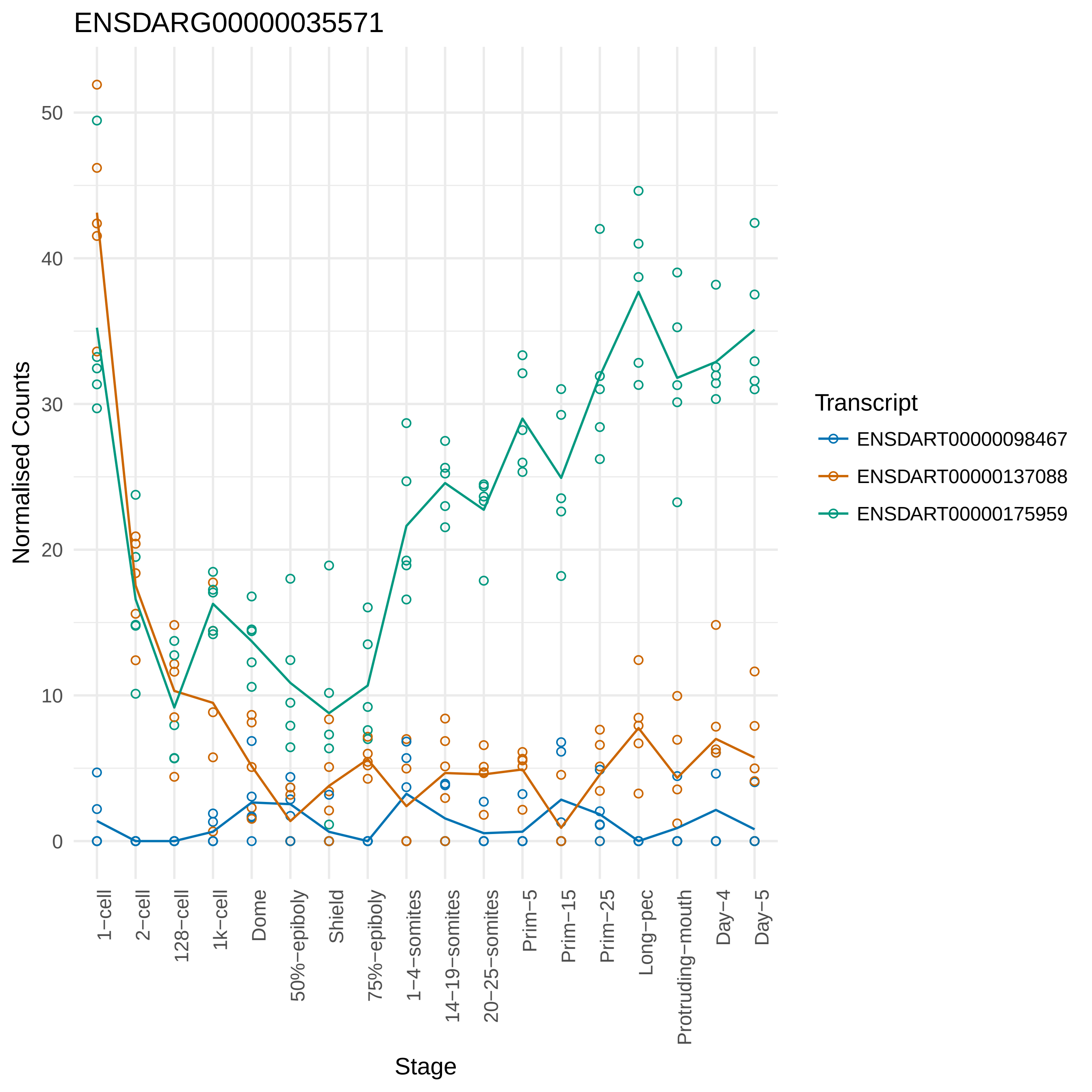

ENSDARG00000035571
Normalised Counts
0
10
20
30
40
50
1−cell
2−cell
128−cell
1k−cell
Dome
50%−epiboly
Shield
75%−epiboly
1−4−somites
14−19−somites
20−25−somites
Prim−5
Prim−15
Prim−25
Long−pec
Protruding−mouth
Day−4
Day−5
Stage
Transcript
ENSDART00000098467
ENSDART00000137088
ENSDART00000175959

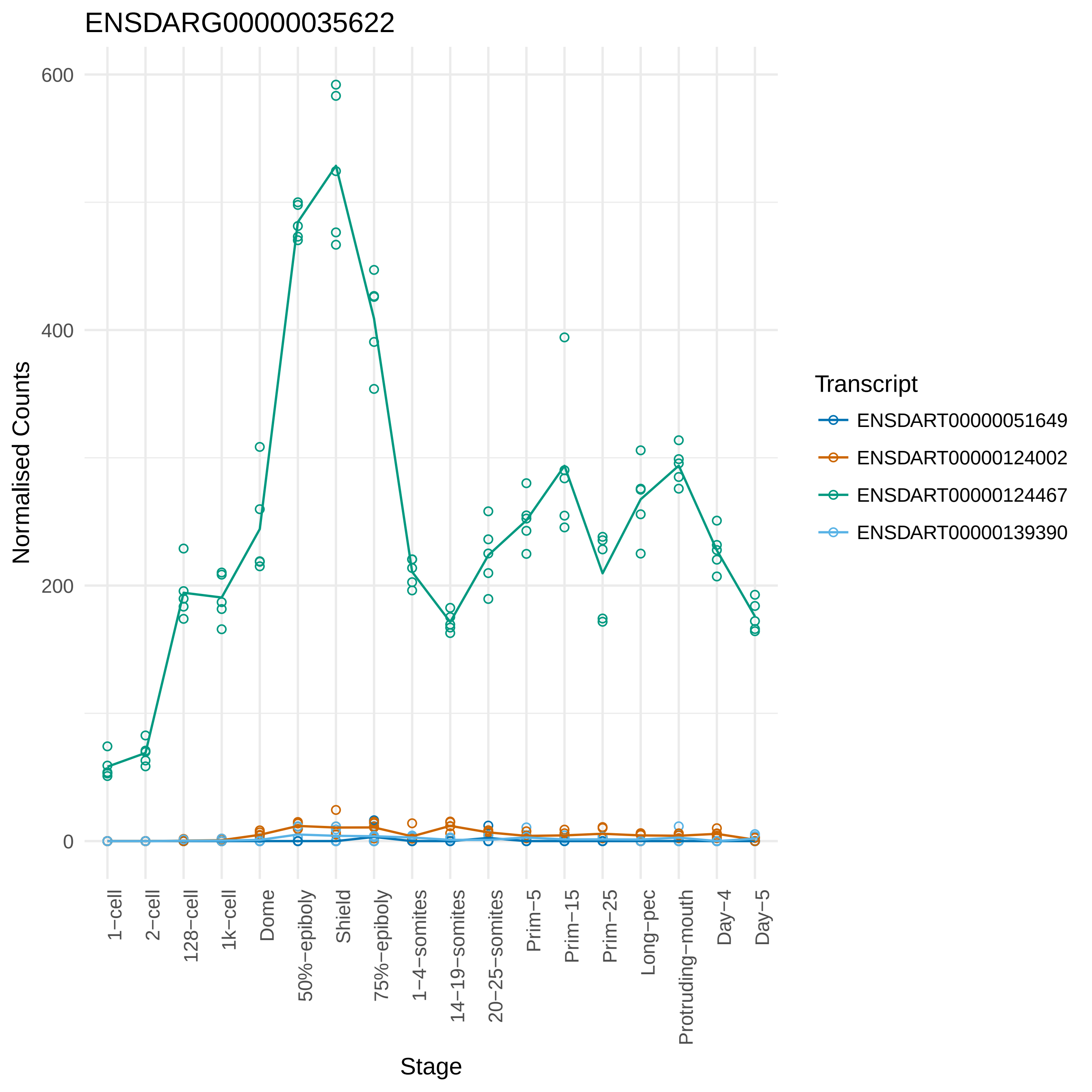

ENSDARG00000035622
Normalised Counts
600
400
200
0
Stage
1−cell
2−cell
128−cell
1k−cell
Dome
50%−epiboly
Shield
75%−epiboly
1−4−somites
14−19−somites
20−25−somites
Prim−5
Prim−15
Prim−25
Long−pec
Protruding−mouth
Day−4
Day−5
Transcript
ENSDART00000051649
ENSDART00000124002
ENSDART00000124467
ENSDART00000139390

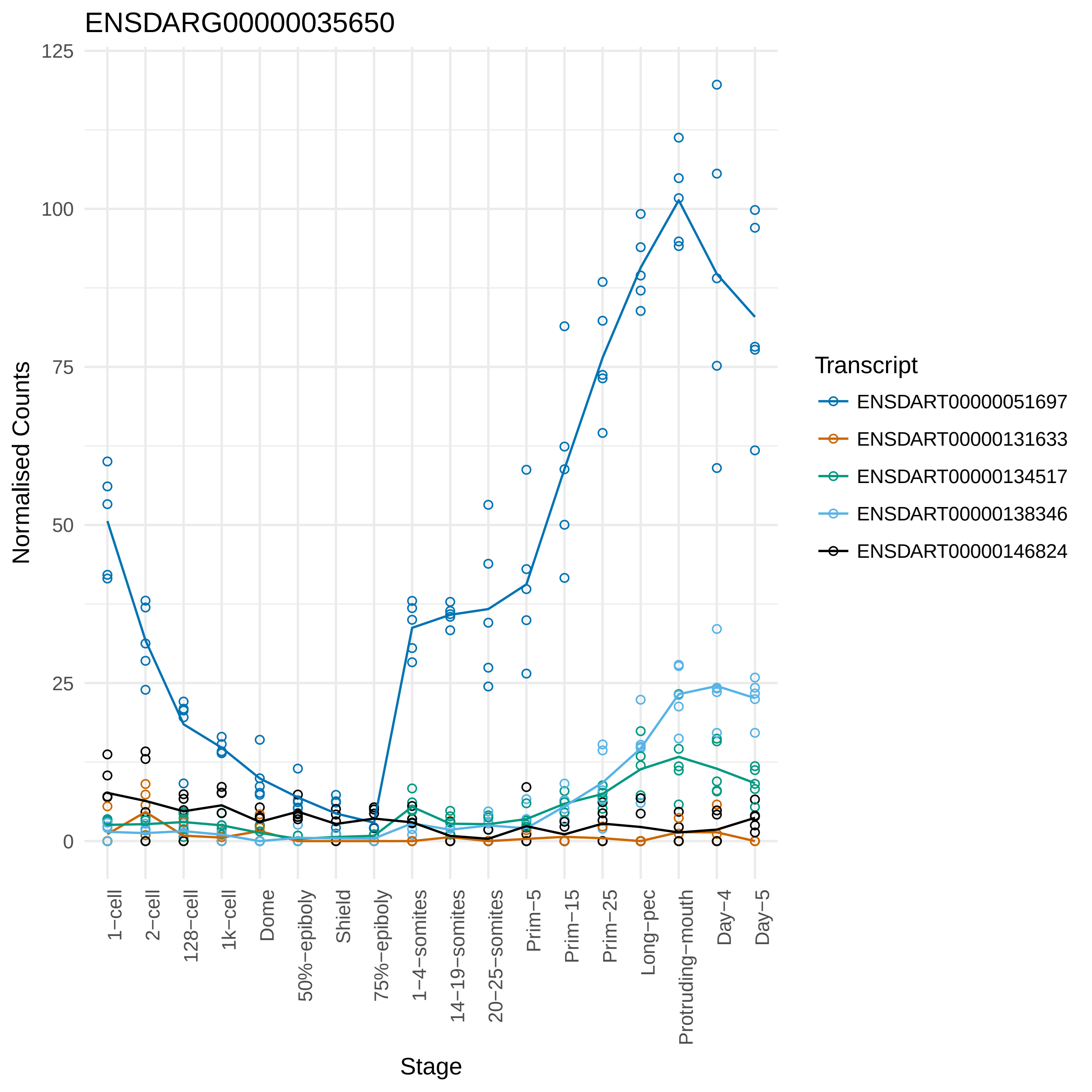

ENSDARG00000035650
125
100
75
50
25
0
Normalised Counts
1−cell
2−cell
128−cell
1k−cell
Dome
50%−epiboly
Shield
75%−epiboly
1−4−somites
14−19−somites
20−25−somites
Prim−5
Prim−15
Prim−25
Long−pec
Protruding−mouth
Day−4
Day−5
Stage
Transcript
ENSDART00000051697
ENSDART00000131633
ENSDART00000134517
ENSDART00000138346
ENSDART00000146824

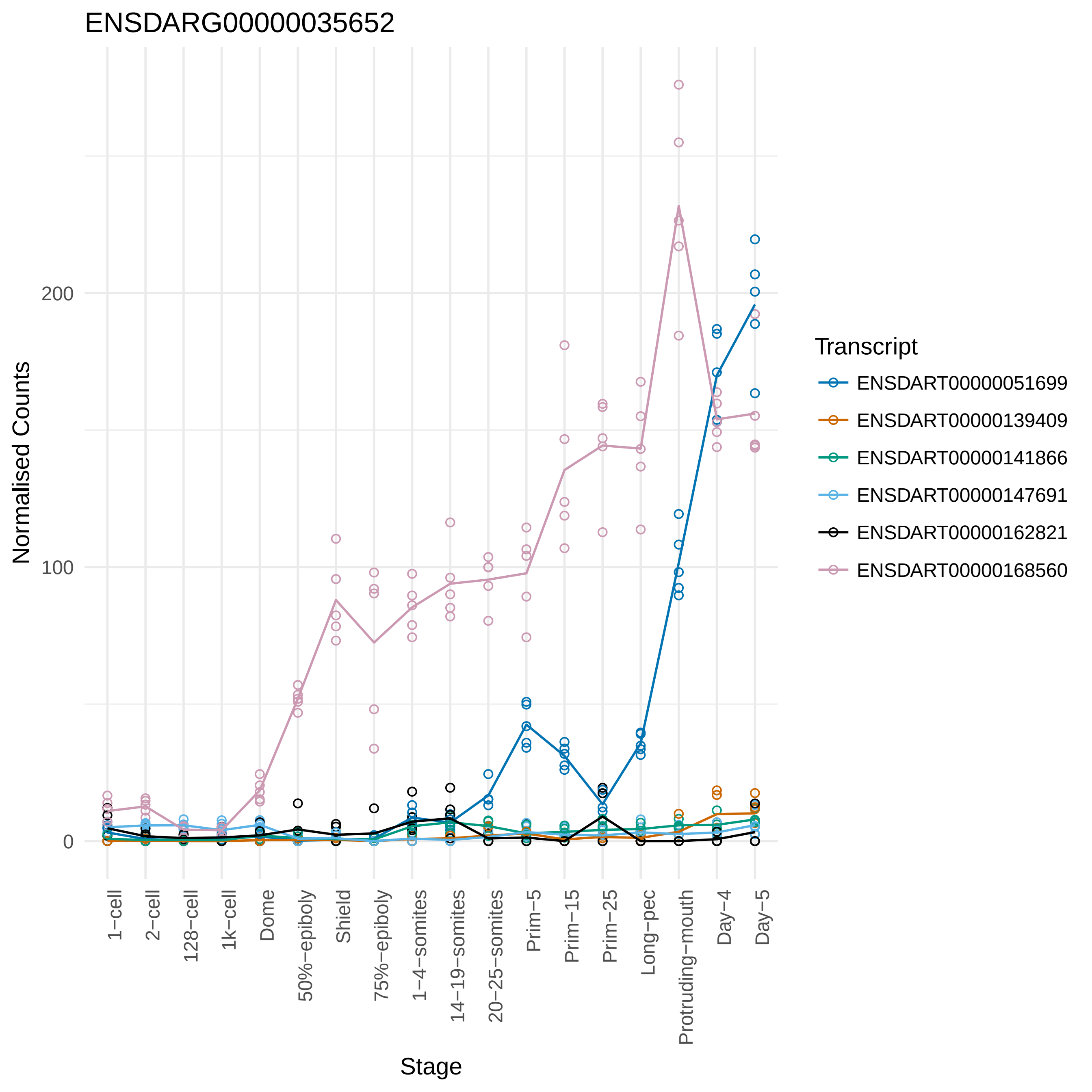

ENSDARG00000035652
Normalised Counts
200
100
0
1−cell
2−cell
128−cell
1k−cell
Dome
50%−epiboly
Shield
75%−epiboly
1−4−somites
14−19−somites
20−25−somites
Prim−5
Prim−15
Prim−25
Long−pec
Protruding−mouth
Day−4
Day−5
Stage
Transcript
ENSDART00000051699
ENSDART00000139409
ENSDART00000141866
ENSDART00000147691
ENSDART00000162821
ENSDART00000168560

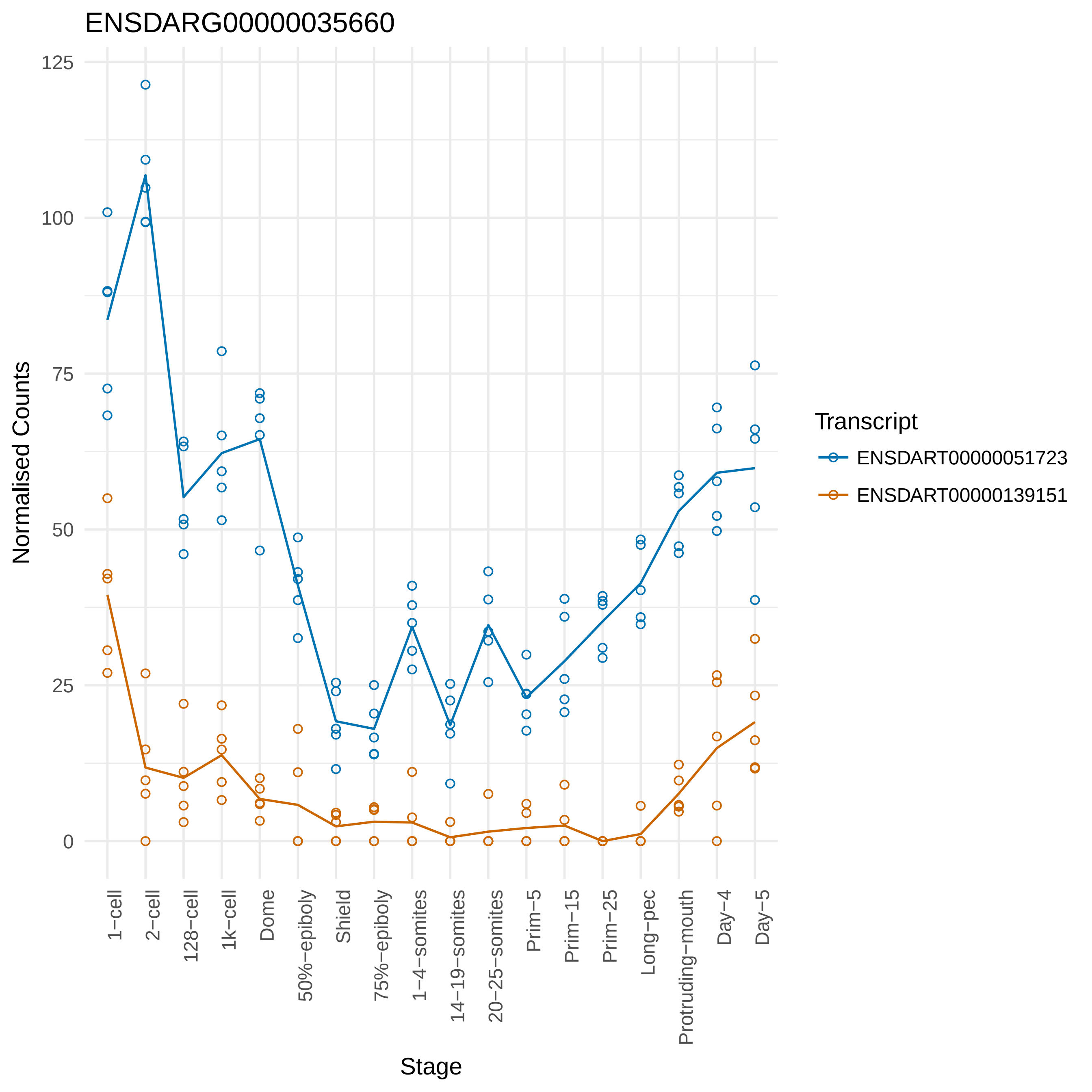
ENSDARG00000035660
Normalised Counts
125
100
75
50
25
0
1−cell
2−cell
128−cell
1k−cell
Dome
50%−epiboly
Shield
75%−epiboly
1−4−somites
14−19−somites
20−25−somites
Prim−5
Prim−15
Prim−25
Long−pec
Protruding−mouth
Day−4
Day−5
Stage
Transcript
ENSDART00000051723
ENSDART00000139151

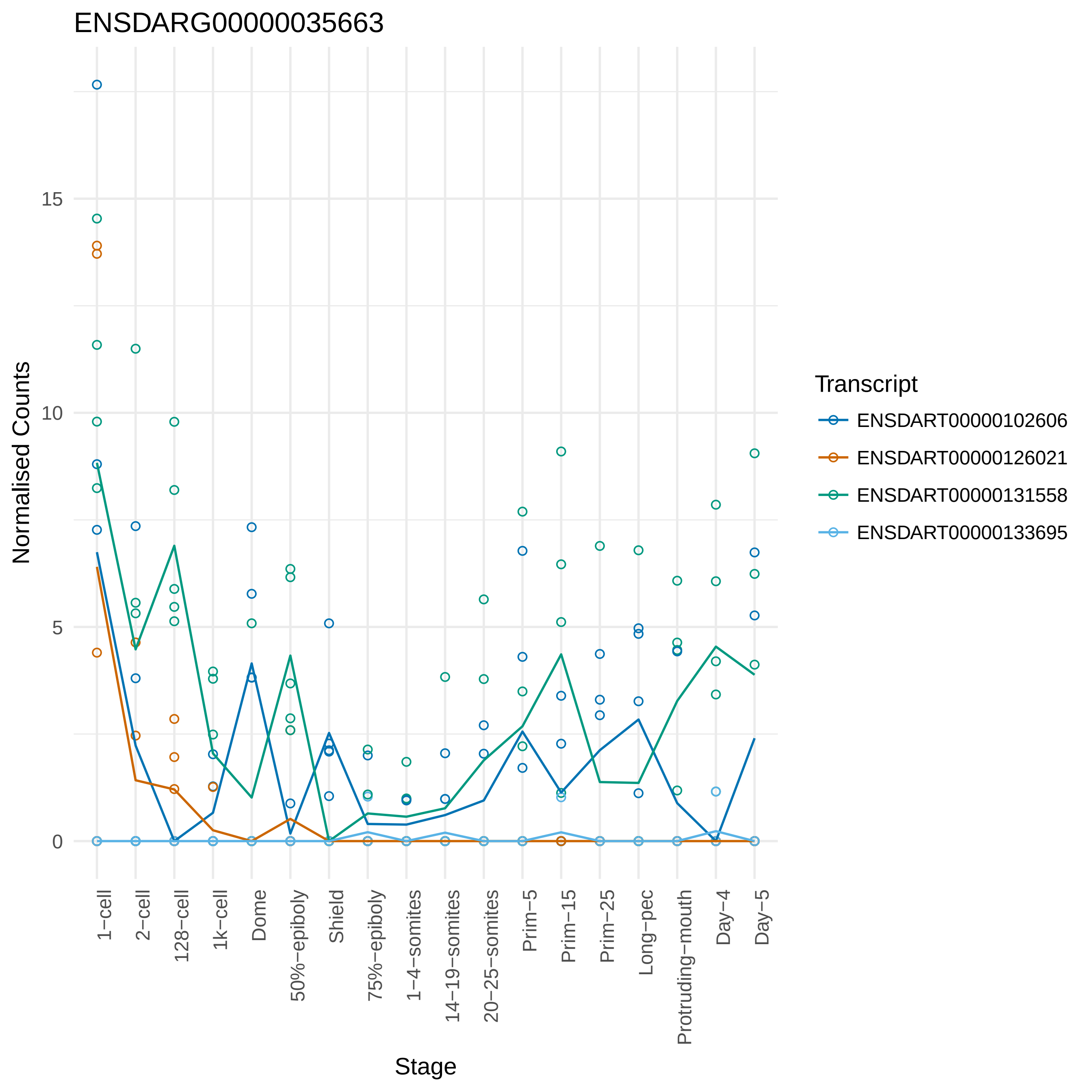

ENSDARG00000035663
Normalised Counts
15
10
5
0
1−cell
2−cell
128−cell
1k−cell
Dome
50%−epiboly
Shield
75%−epiboly
1−4−somites
14−19−somites
20−25−somites
Prim−5
Prim−15
Prim−25
Long−pec
Protruding−mouth
Day−4
Day−5
Stage
Transcript
ENSDART00000102606
ENSDART00000126021
ENSDART00000131558
ENSDART00000133695

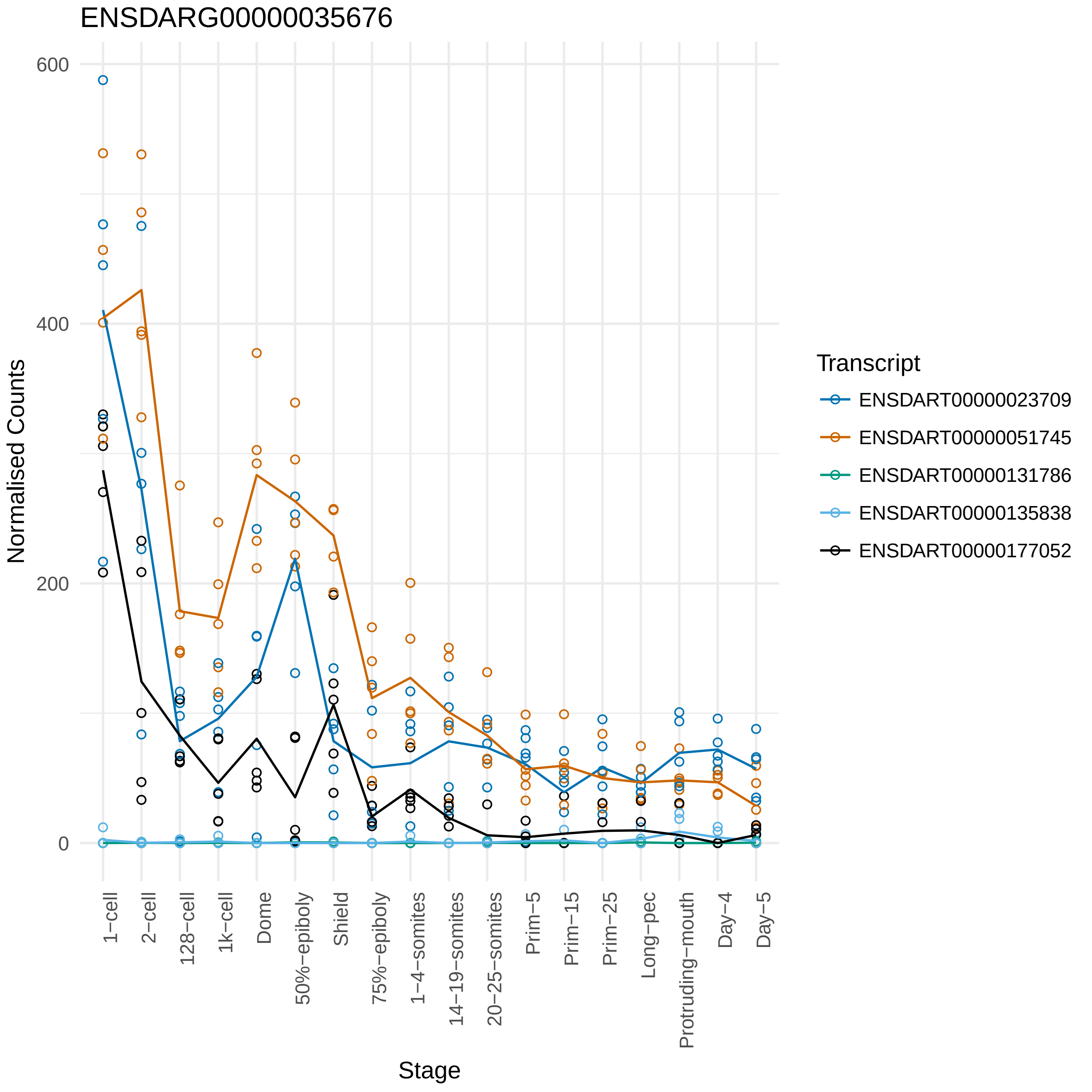
ENSDARG00000035676
Normalised Counts
600
400
200
0
Stage
1−cell
2−cell
128−cell
1k−cell
Dome
50%−epiboly
Shield
75%−epiboly
1−4−somites
14−19−somites
20−25−somites
Prim−5
Prim−15
Prim−25
Long−pec
Protruding−mouth
Day−4
Day−5
Transcript
ENSDART00000023709
ENSDART00000051745
ENSDART00000131786
ENSDART00000135838
ENSDART00000177052

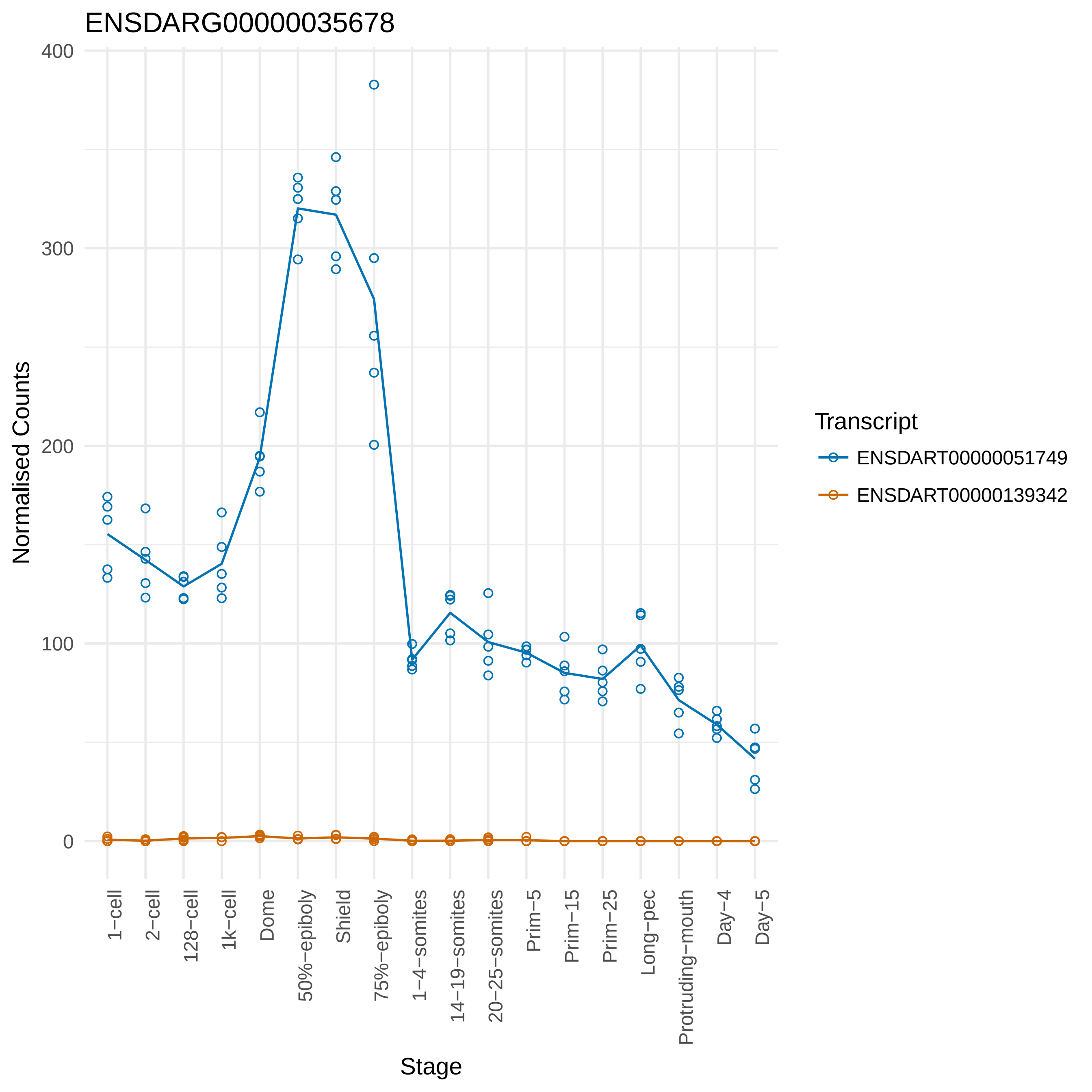

ENSDARG00000035678
Normalised Counts
400
300
200
100
0
1−cell
2−cell
128−cell
1k−cell
Dome
50%−epiboly
Shield
75%−epiboly
1−4−somites
14−19−somites
20−25−somites
Prim−5
Prim−15
Prim−25
Long−pec
Protruding−mouth
Day−4
Day−5
Stage
Transcript
ENSDART00000051749
ENSDART00000139342

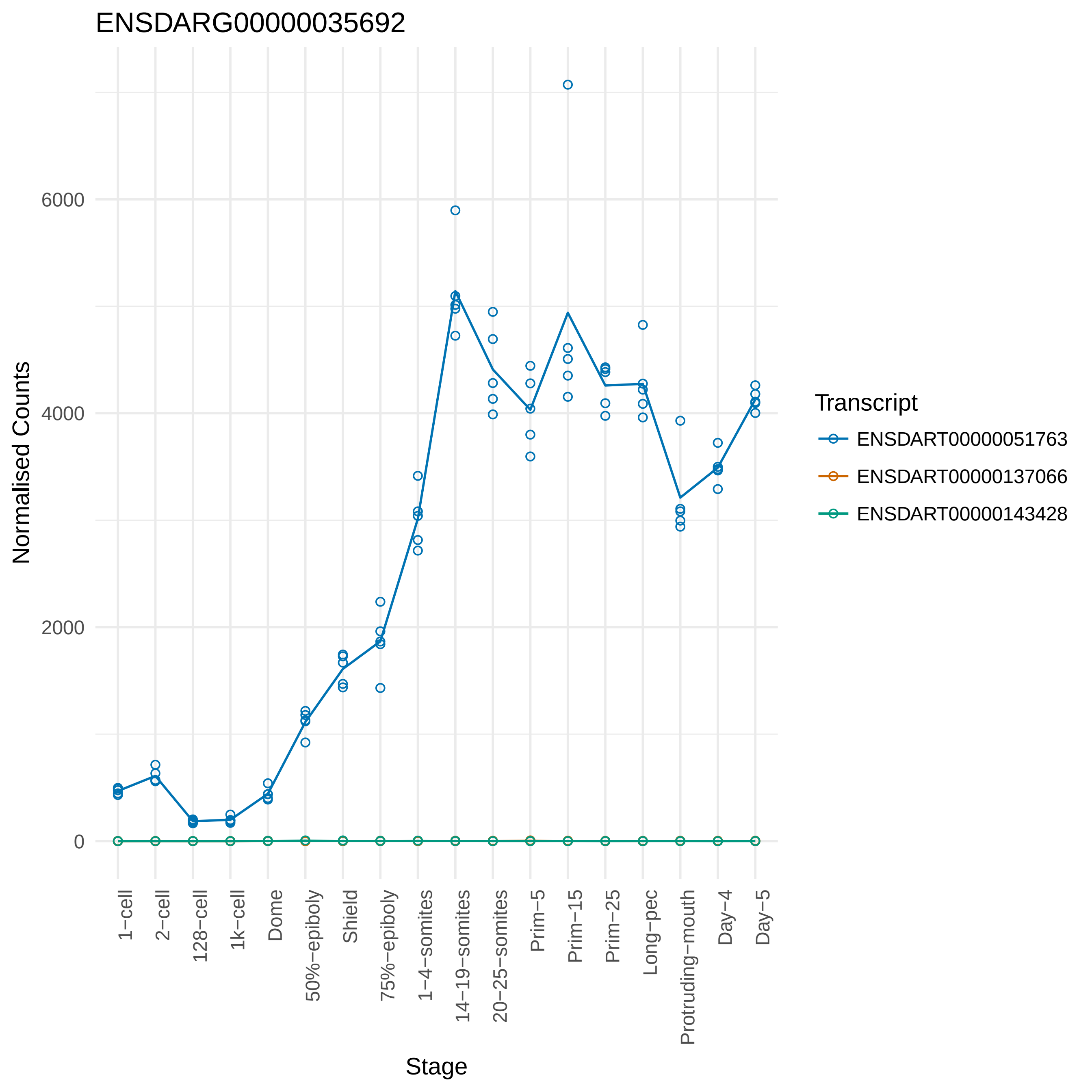

ENSDARG00000035692
Normalised Counts
6000
4000
2000
0
Stage
1−cell
2−cell
128−cell
1k−cell
Dome
50%−epiboly
Shield
75%−epiboly
1−4−somites
14−19−somites
20−25−somites
Prim−5
Prim−15
Prim−25
Long−pec
Protruding−mouth
Day−4
Day−5
Transcript
ENSDART00000051763
ENSDART00000137066
ENSDART00000143428

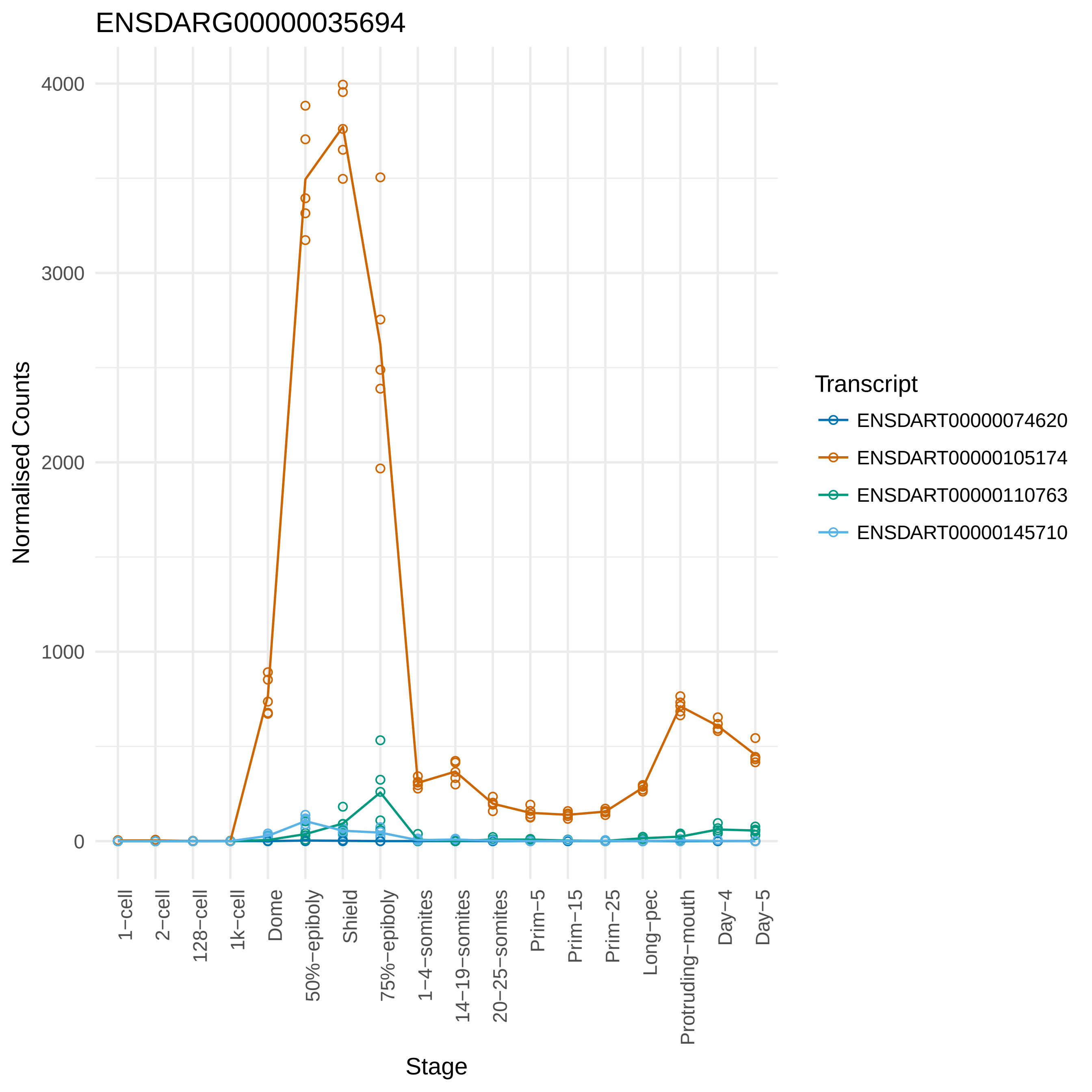

ENSDARG00000035694
Normalised Counts
4000
3000
2000
1000
0
1−cell
2−cell
128−cell
1k−cell
Dome
50%−epiboly
Shield
75%−epiboly
1−4−somites
14−19−somites
20−25−somites
Prim−5
Prim−15
Prim−25
Long−pec
Protruding−mouth
Day−4
Day−5
Stage
Transcript
ENSDART00000074620
ENSDART00000105174
ENSDART00000110763
ENSDART00000145710

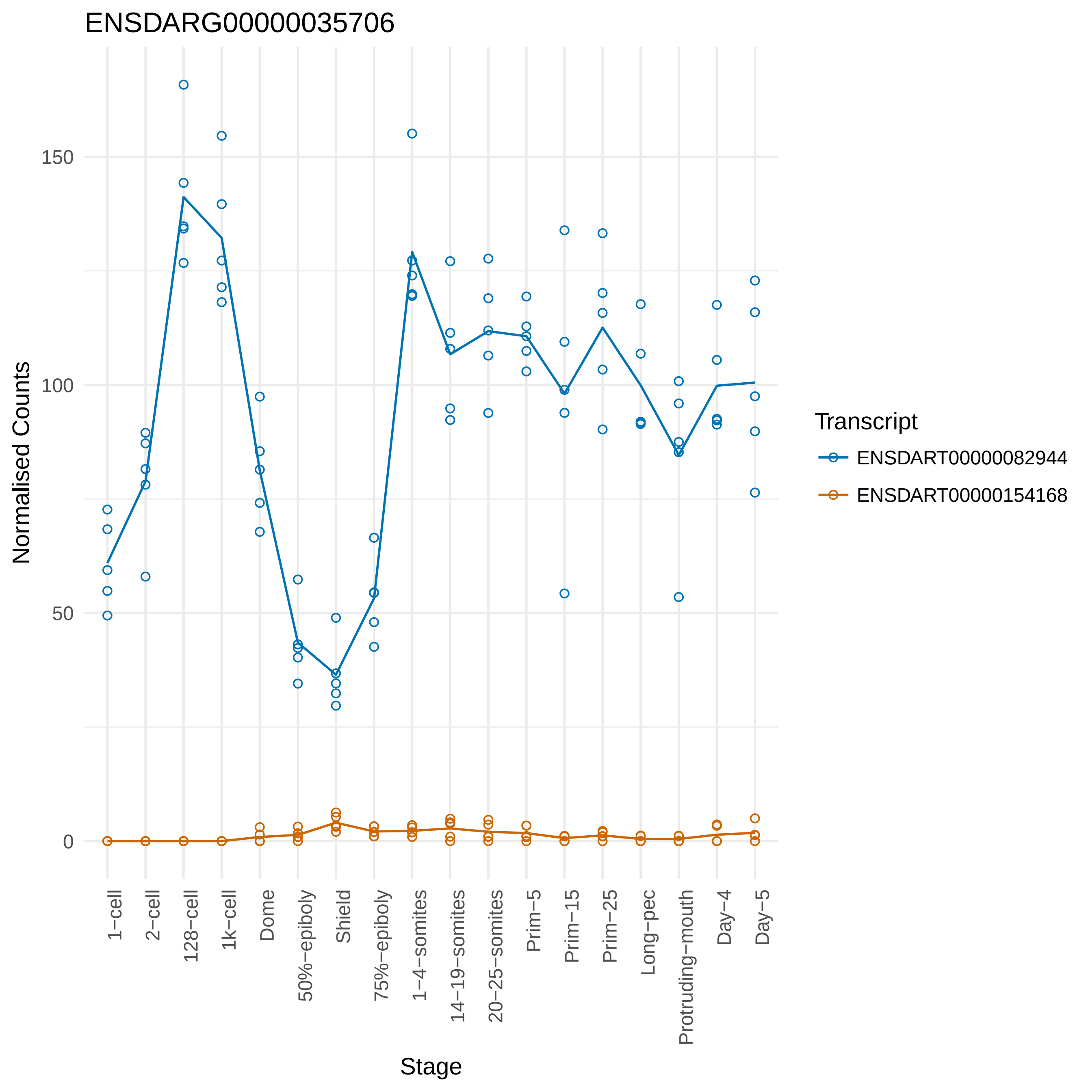
ENSDARG00000035706
Normalised Counts
150
100
50
0
1−cell
2−cell
128−cell
1k−cell
Dome
50%−epiboly
Shield
75%−epiboly
1−4−somites
14−19−somites
20−25−somites
Prim−5
Prim−15
Prim−25
Long−pec
Protruding−mouth
Day−4
Day−5
Stage
Transcript
ENSDART00000082944
ENSDART00000154168

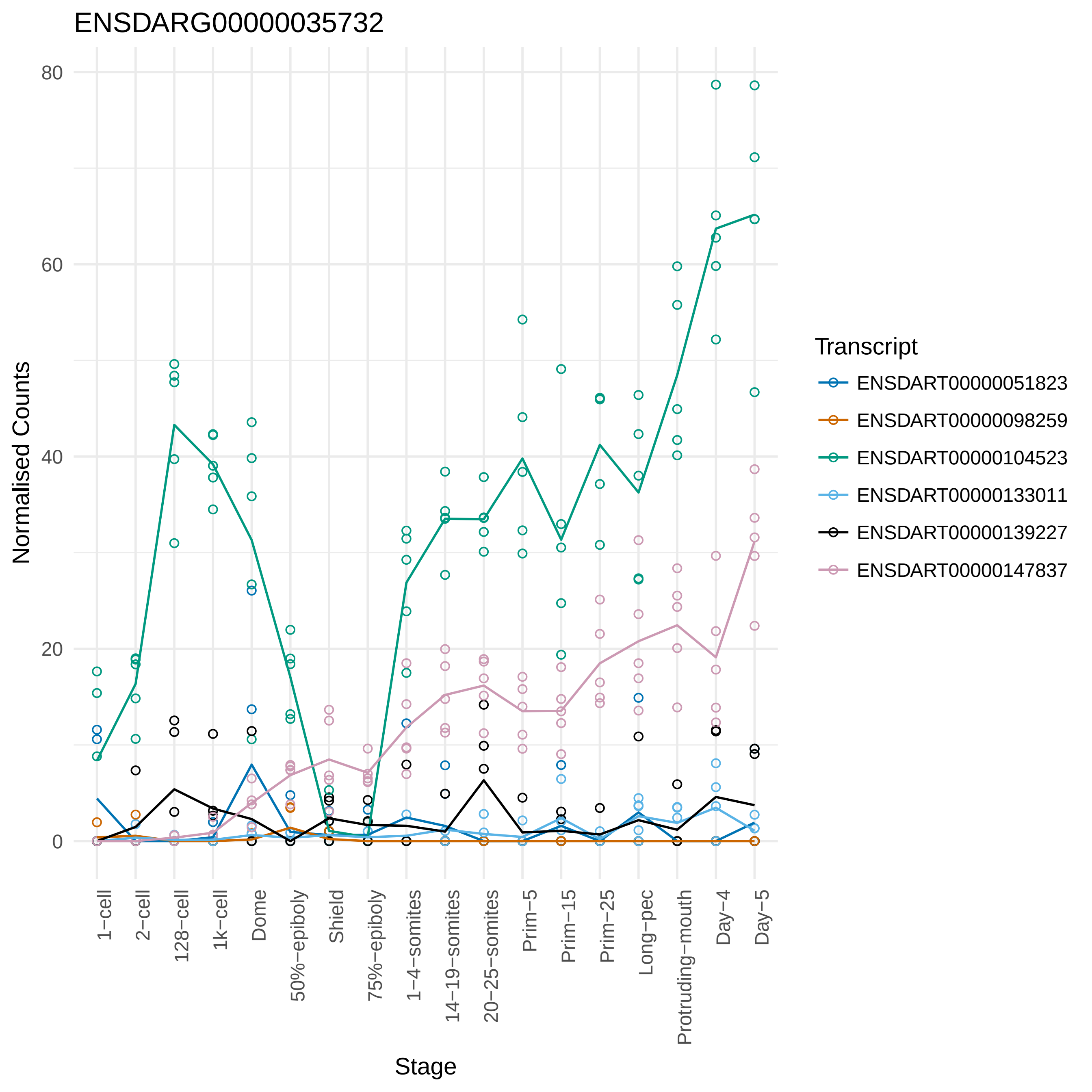

ENSDARG00000035732
Normalised Counts
0
20
40
60
80
1−cell
2−cell
128−cell
1k−cell
Dome
50%−epiboly
Shield
75%−epiboly
1−4−somites
14−19−somites
20−25−somites
Prim−5
Prim−15
Prim−25
Long−pec
Protruding−mouth
Day−4
Day−5
Stage
Transcript
ENSDART00000051823
ENSDART00000098259
ENSDART00000104523
ENSDART00000133011
ENSDART00000139227
ENSDART00000147837

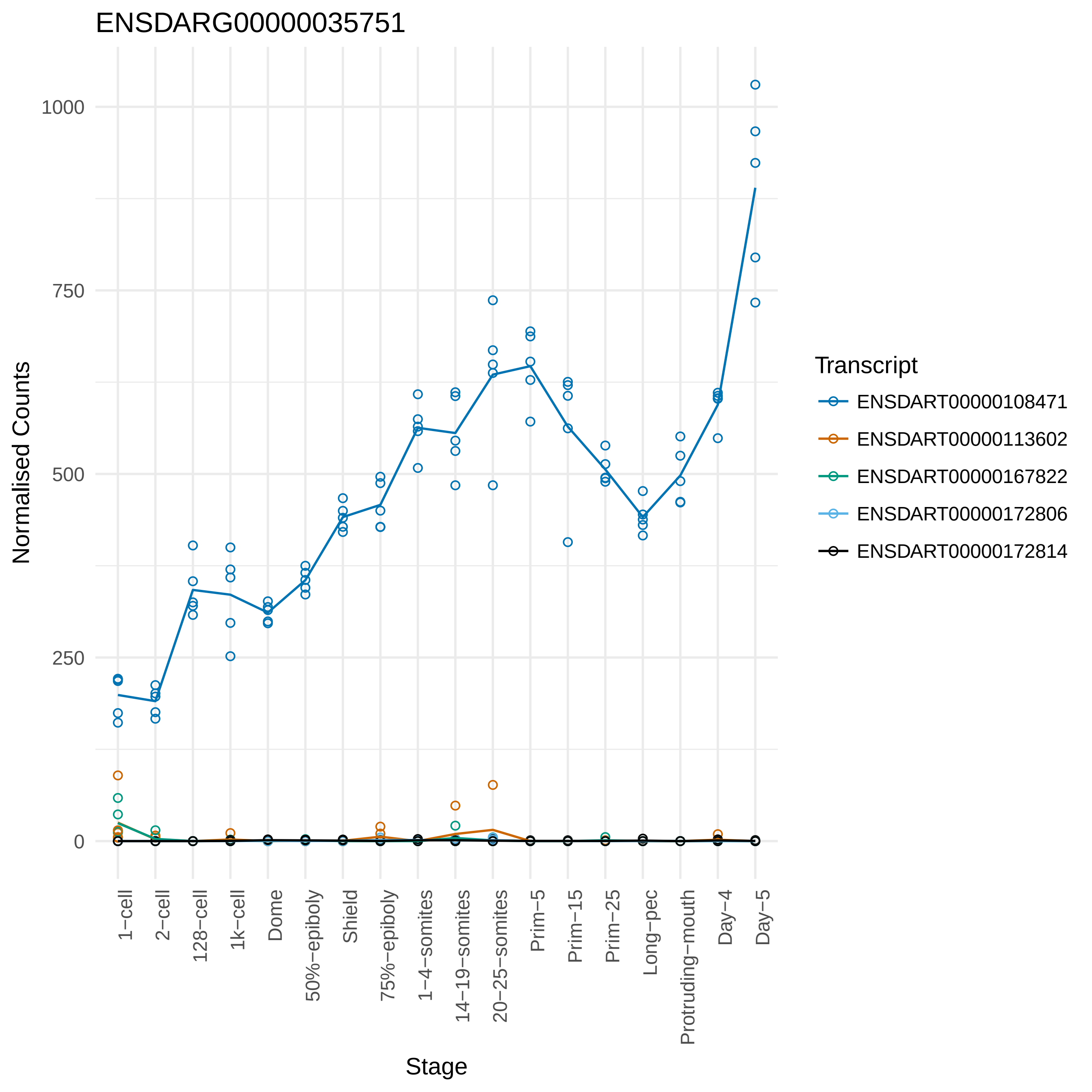

ENSDARG00000035751
Normalised Counts
1000
750
500
250
0
1−cell
2−cell
128−cell
1k−cell
Dome
50%−epiboly
Shield
75%−epiboly
1−4−somites
14−19−somites
20−25−somites
Prim−5
Prim−15
Prim−25
Long−pec
Protruding−mouth
Day−4
Day−5
Stage
Transcript
ENSDART00000108471
ENSDART00000113602
ENSDART00000167822
ENSDART00000172806
ENSDART00000172814

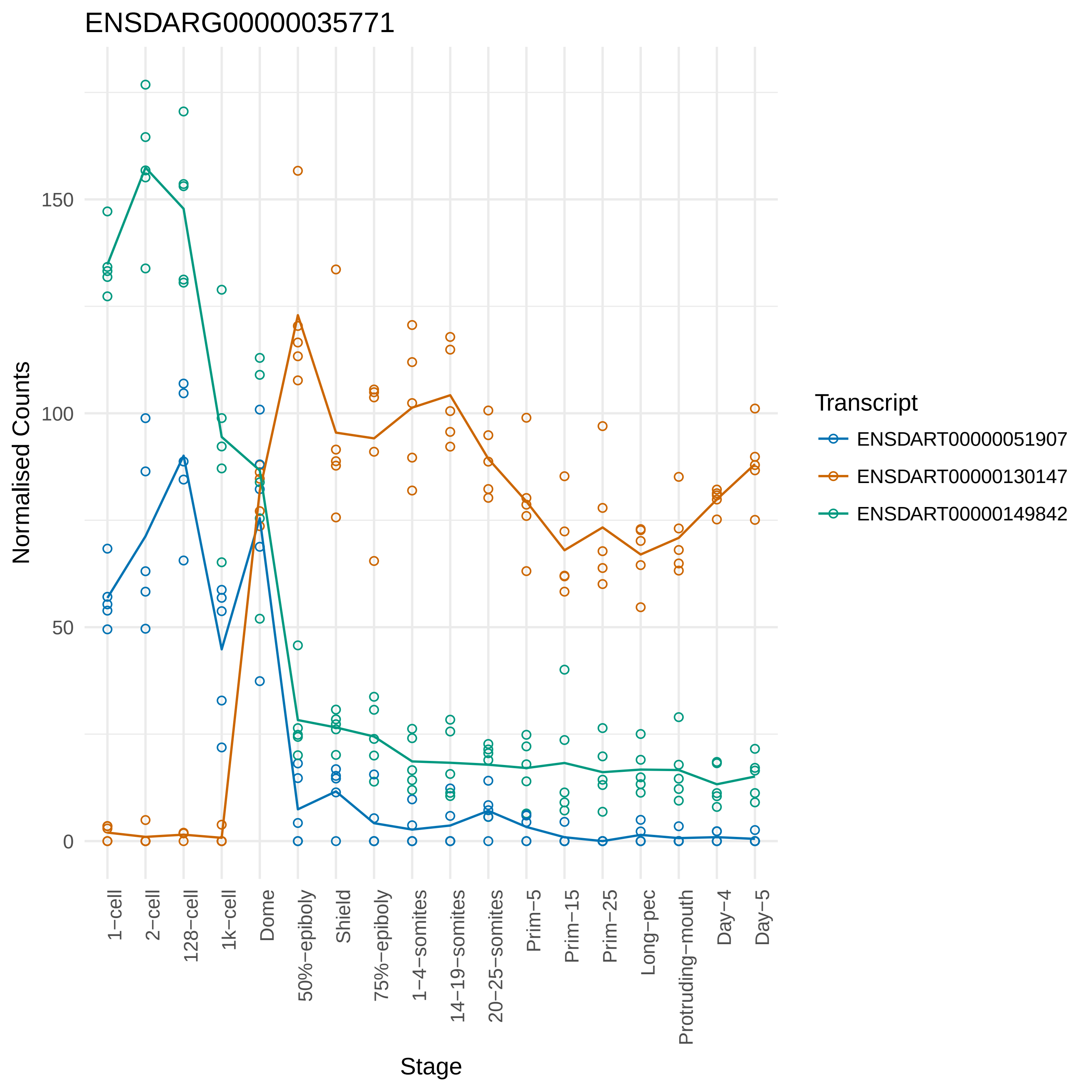
ENSDARG00000035771
Normalised Counts
0
50
100
150
Stage
1−cell
2−cell
128−cell
1k−cell
Dome
50%−epiboly
Shield
75%−epiboly
1−4−somites
14−19−somites
20−25−somites
Prim−5
Prim−15
Prim−25
Long−pec
Protruding−mouth
Day−4
Day−5
Transcript
ENSDART00000051907
ENSDART00000130147
ENSDART00000149842

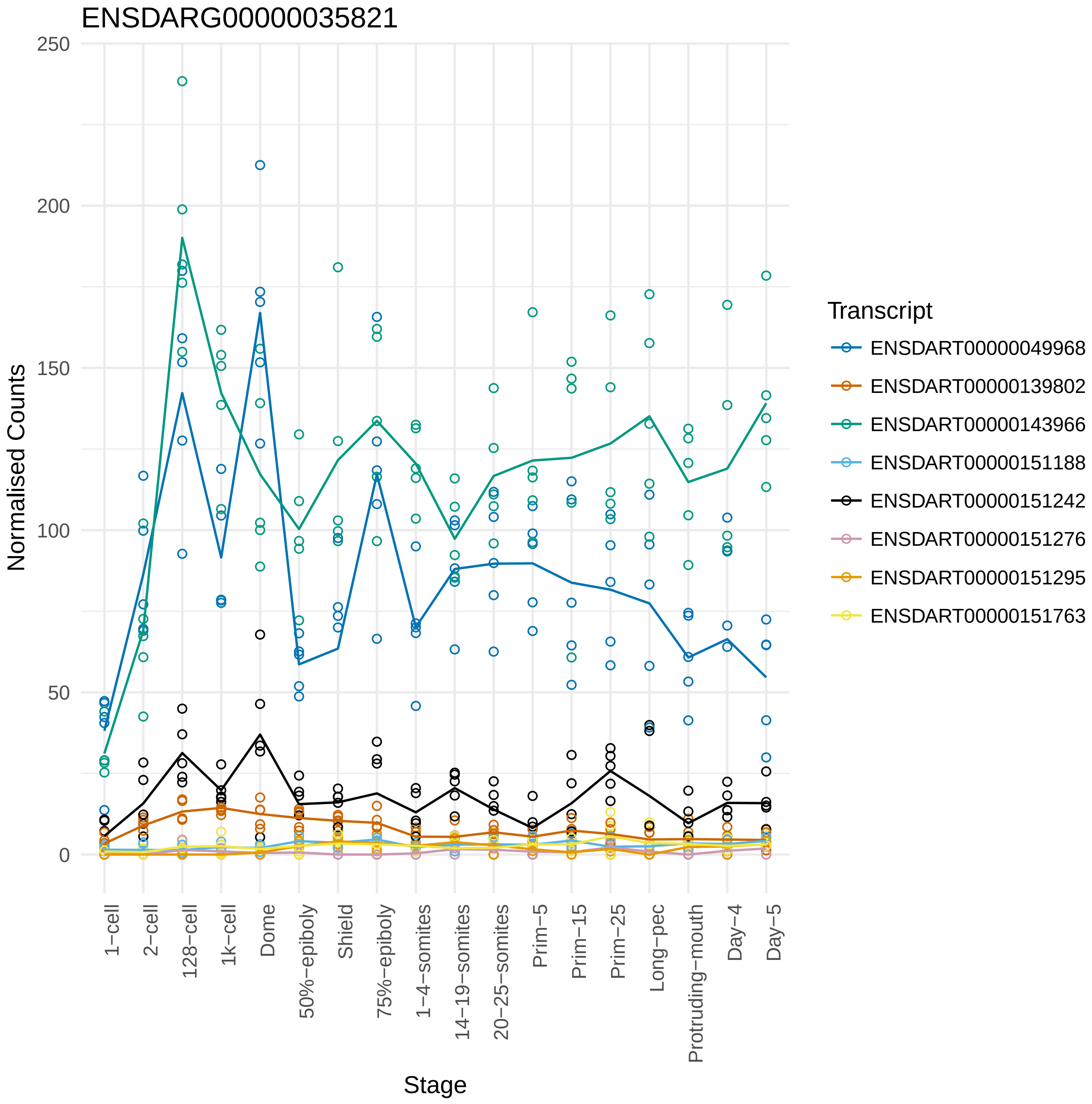
ENSDARG00000035821
Normalised Counts
250
200
150
100
50
0
1−cell
2−cell
128−cell
1k−cell
Dome
50%−epiboly
Shield
75%−epiboly
1−4−somites
14−19−somites
20−25−somites
Prim−5
Prim−15
Prim−25
Long−pec
Protruding−mouth
Day−4
Day−5
Stage
Transcript
ENSDART00000049968
ENSDART00000139802
ENSDART00000143966
ENSDART00000151188
ENSDART00000151242
ENSDART00000151276
ENSDART00000151295
ENSDART00000151763

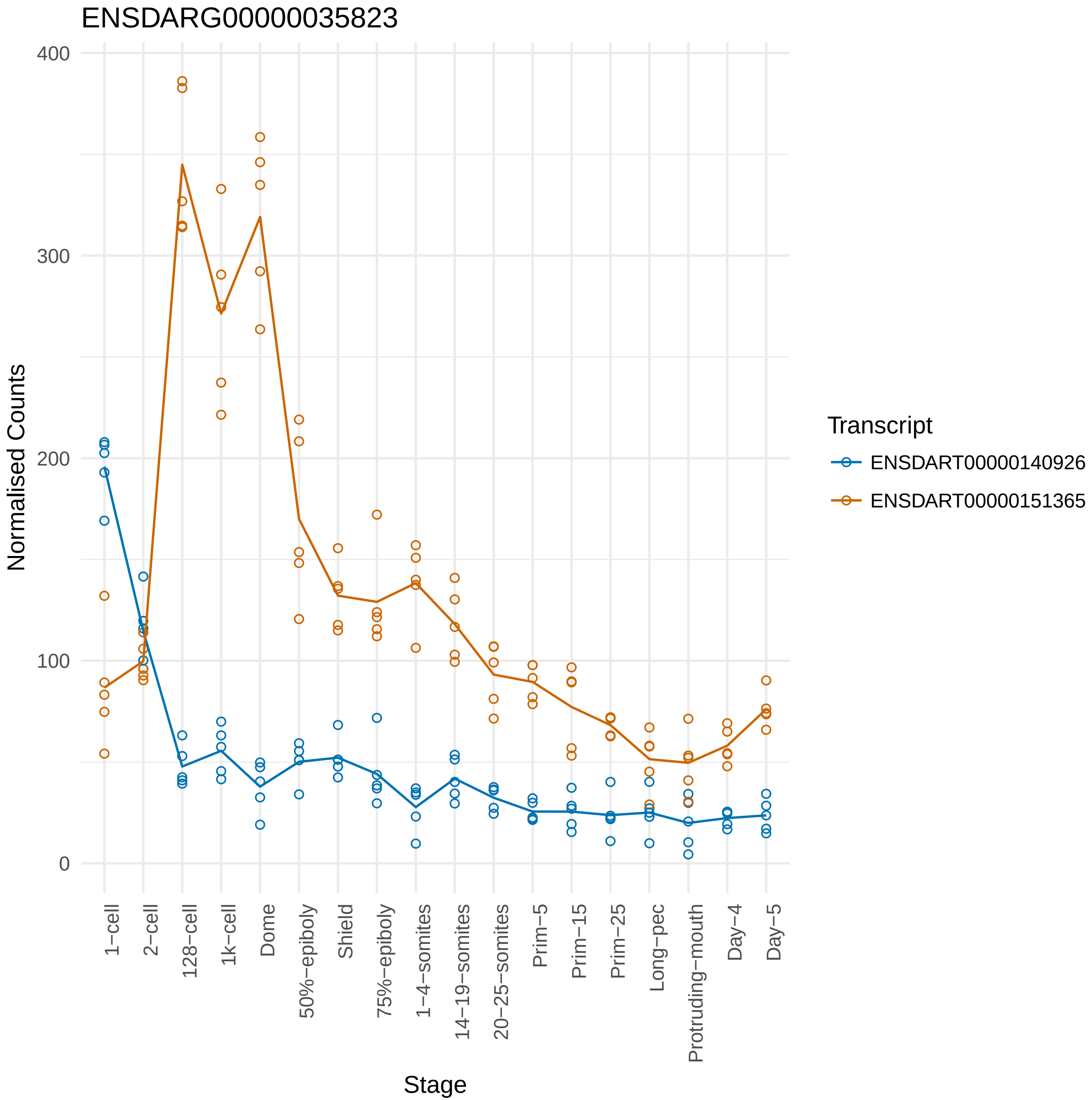
ENSDARG00000035823
Normalised Counts
400
300
200
100
0
Stage
1−cell
2−cell
128−cell
1k−cell
Dome
50%−epiboly
Shield
75%−epiboly
1−4−somites
14−19−somites
20−25−somites
Prim−5
Prim−15
Prim−25
Long−pec
Protruding−mouth
Day−4
Day−5
Transcript
ENSDART00000140926
ENSDART00000151365

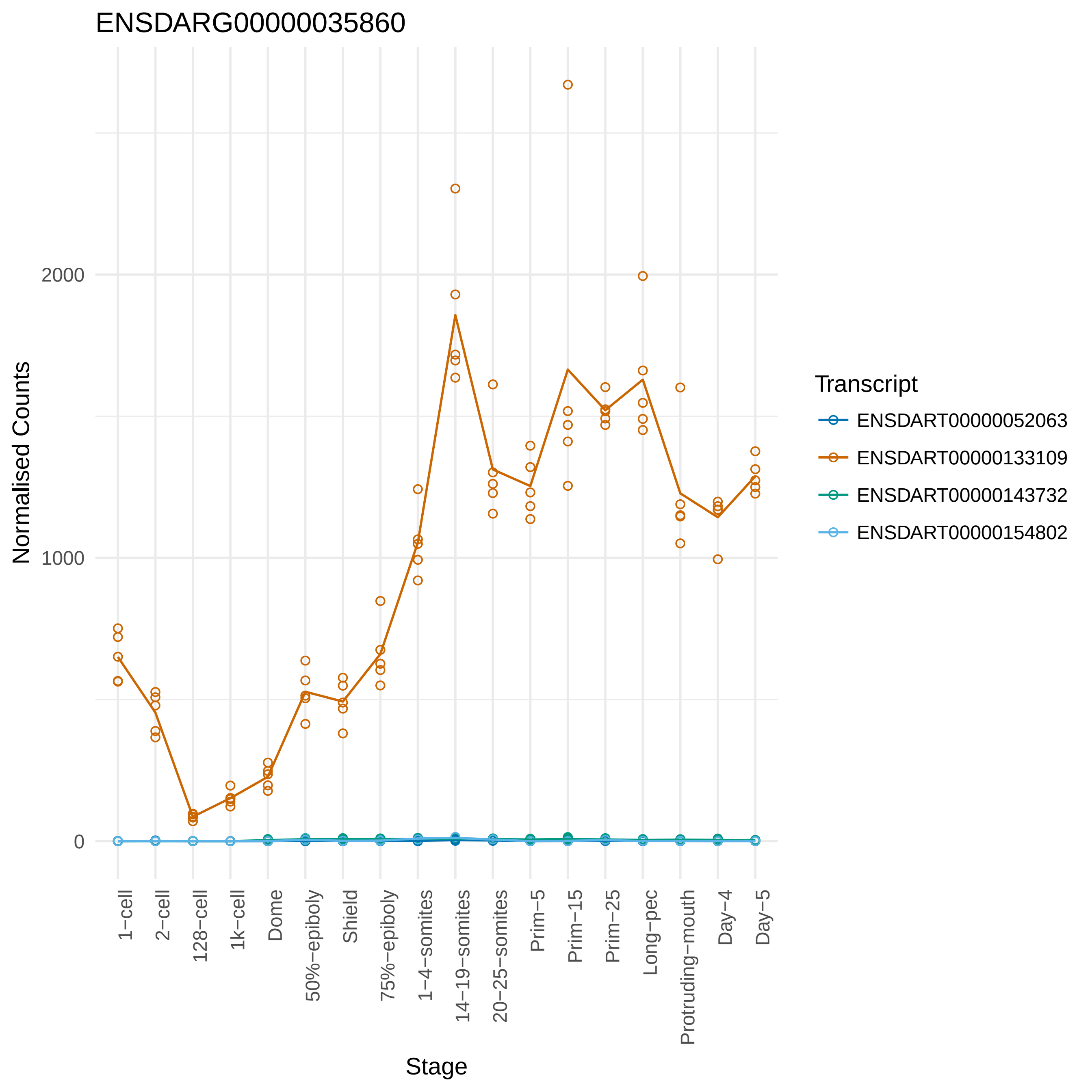

ENSDARG00000035860
Normalised Counts
2000
1000
0
1−cell
2−cell
128−cell
1k−cell
Dome
50%−epiboly
Shield
75%−epiboly
1−4−somites
14−19−somites
20−25−somites
Prim−5
Prim−15
Prim−25
Long−pec
Protruding−mouth
Day−4
Day−5
Stage
Transcript
ENSDART00000052063
ENSDART00000133109
ENSDART00000143732
ENSDART00000154802

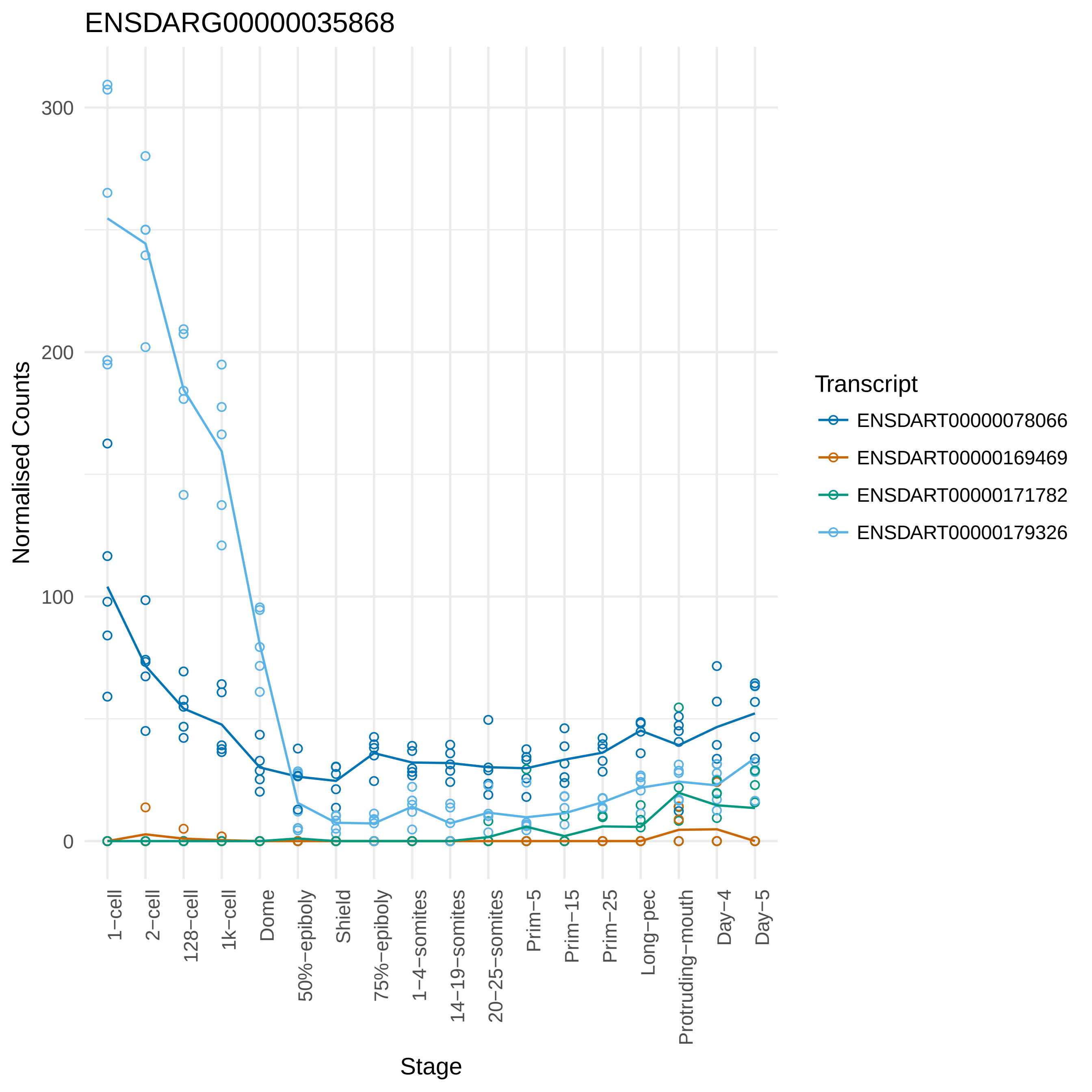

ENSDARG00000035868
Normalised Counts
300
200
100
0
1−cell
2−cell
128−cell
1k−cell
Dome
50%−epiboly
Shield
75%−epiboly
1−4−somites
14−19−somites
20−25−somites
Prim−5
Prim−15
Prim−25
Long−pec
Protruding−mouth
Day−4
Day−5
Stage
Transcript
ENSDART00000078066
ENSDART00000169469
ENSDART00000171782
ENSDART00000179326

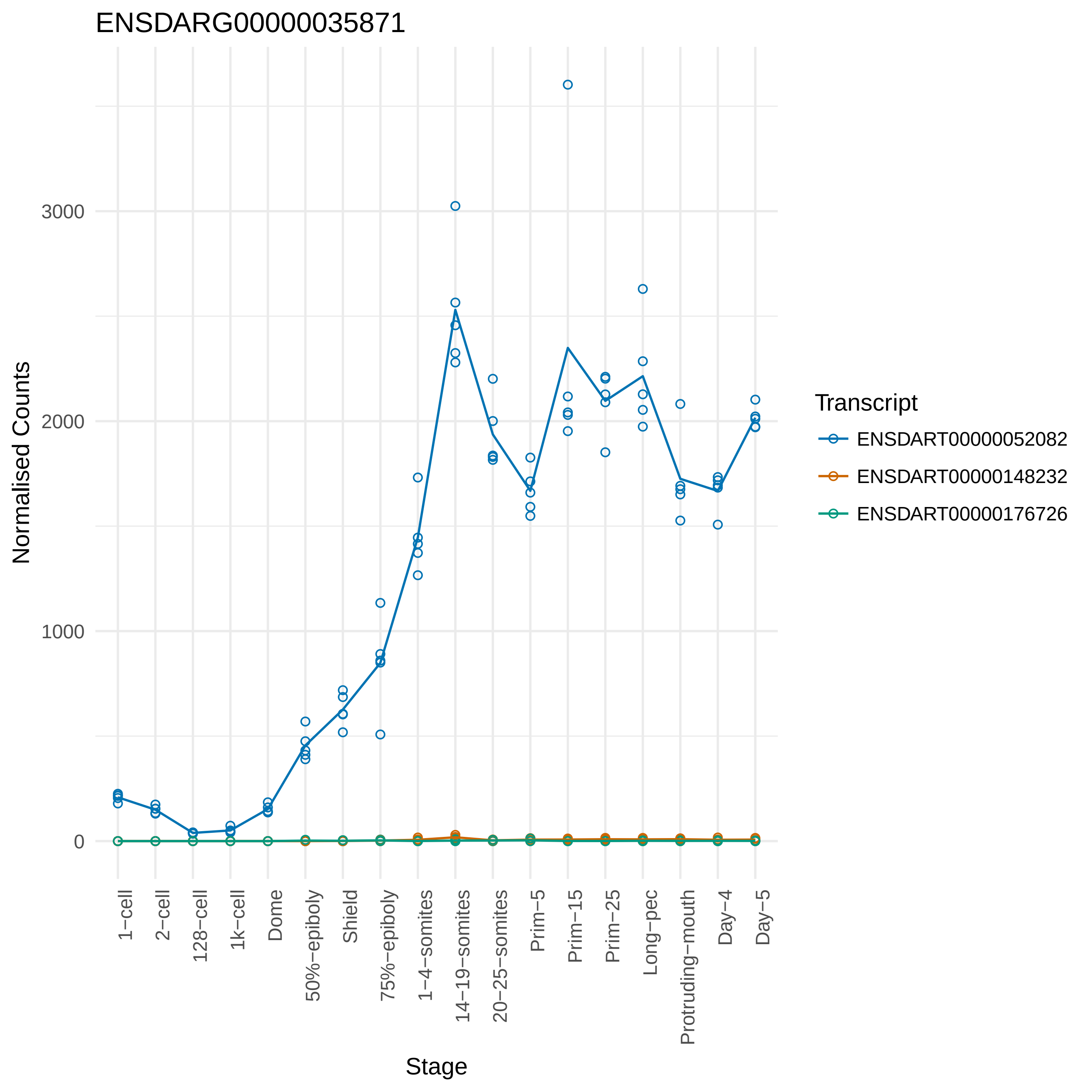

ENSDARG00000035871
Normalised Counts
0
1000
2000
3000
Stage
1−cell
2−cell
128−cell
1k−cell
Dome
50%−epiboly
Shield
75%−epiboly
1−4−somites
14−19−somites
20−25−somites
Prim−5
Prim−15
Prim−25
Long−pec
Protruding−mouth
Day−4
Day−5
Transcript
ENSDART00000052082
ENSDART00000148232
ENSDART00000176726

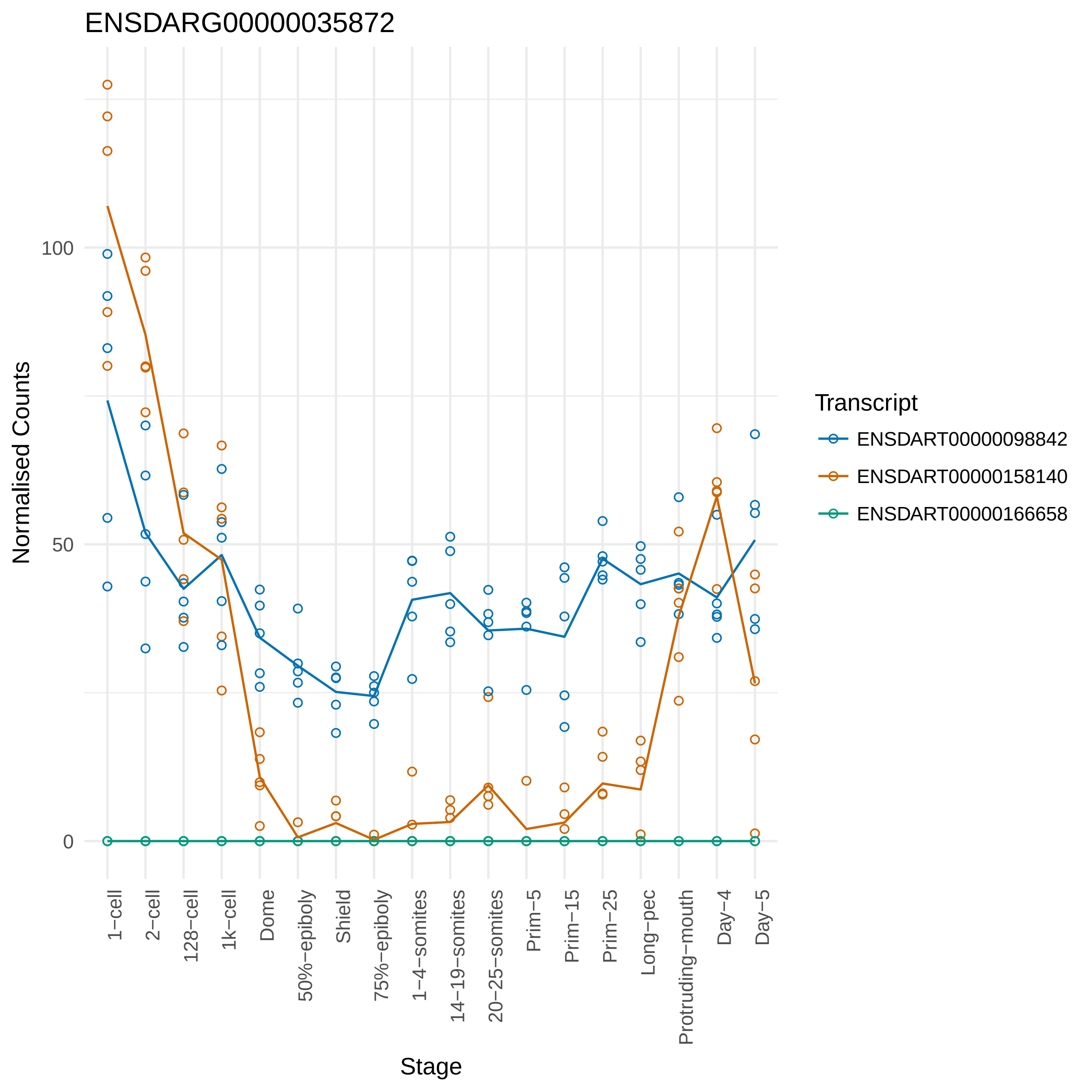

ENSDARG00000035872
Normalised Counts
100
50
0
1−cell
2−cell
128−cell
1k−cell
Dome
50%−epiboly
Shield
75%−epiboly
1−4−somites
14−19−somites
20−25−somites
Prim−5
Prim−15
Prim−25
Long−pec
Protruding−mouth
Day−4
Day−5
Stage
Transcript
ENSDART00000098842
ENSDART00000158140
ENSDART00000166658

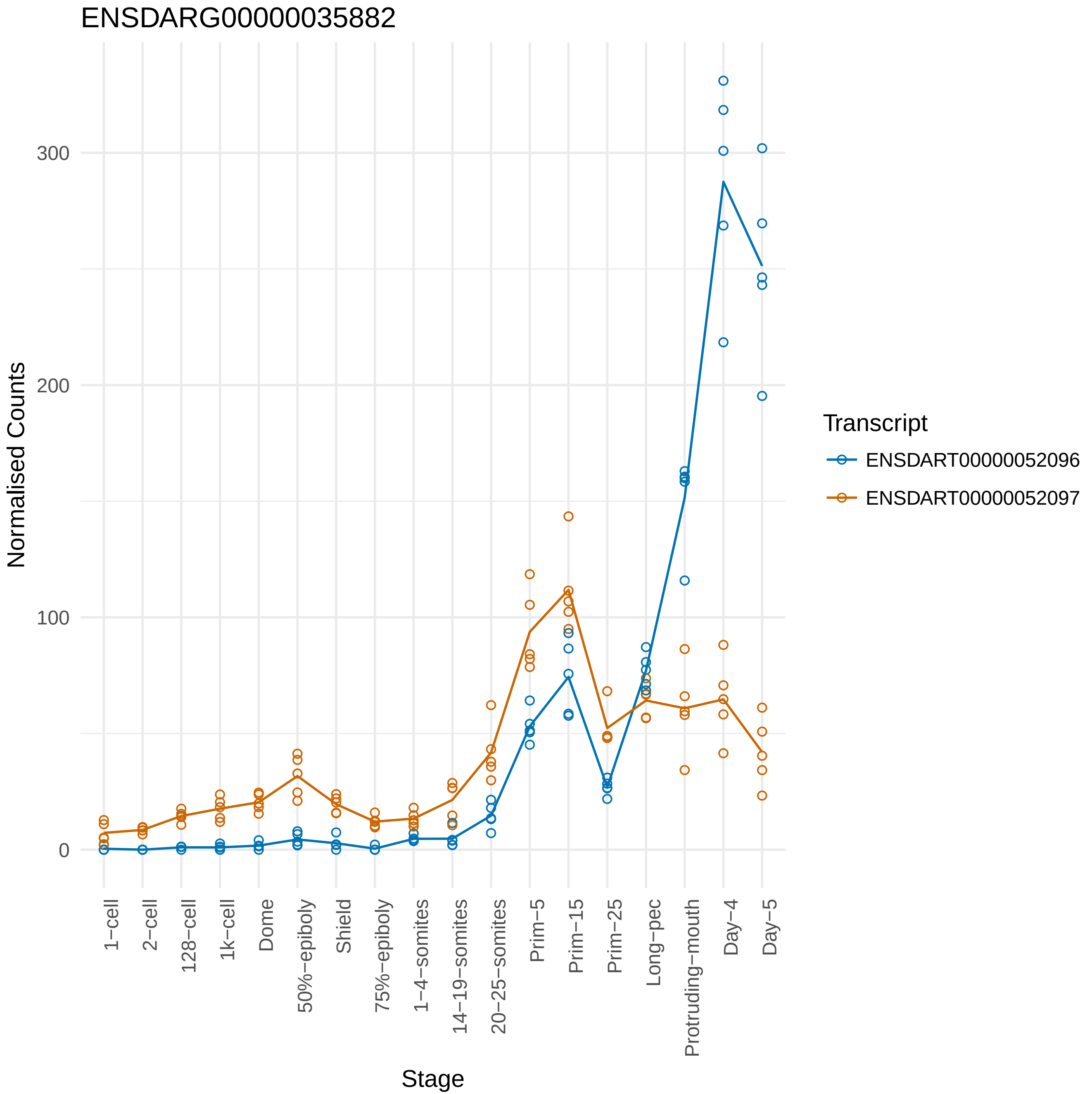

ENSDARG00000035882
Normalised Counts
300
200
100
0
1−cell
2−cell
128−cell
1k−cell
Dome
50%−epiboly
Shield
75%−epiboly
1−4−somites
14−19−somites
20−25−somites
Prim−5
Prim−15
Prim−25
Long−pec
Protruding−mouth
Day−4
Day−5
Stage
Transcript
ENSDART00000052096
ENSDART00000052097

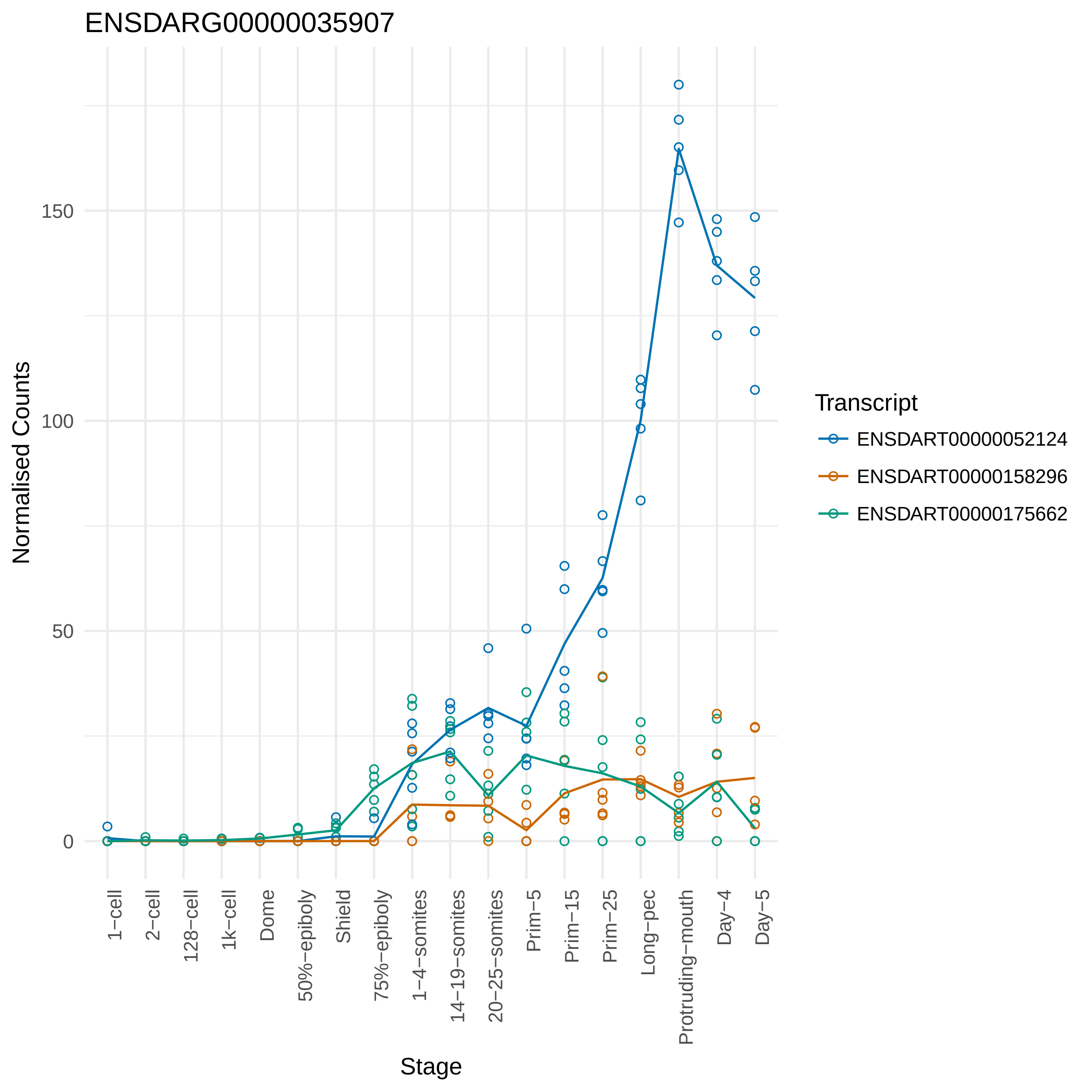

ENSDARG00000035907
Normalised Counts
150
100
50
0
1−cell
2−cell
128−cell
1k−cell
Dome
50%−epiboly
Shield
75%−epiboly
1−4−somites
14−19−somites
20−25−somites
Prim−5
Prim−15
Prim−25
Long−pec
Protruding−mouth
Day−4
Day−5
Stage
Transcript
ENSDART00000052124
ENSDART00000158296
ENSDART00000175662

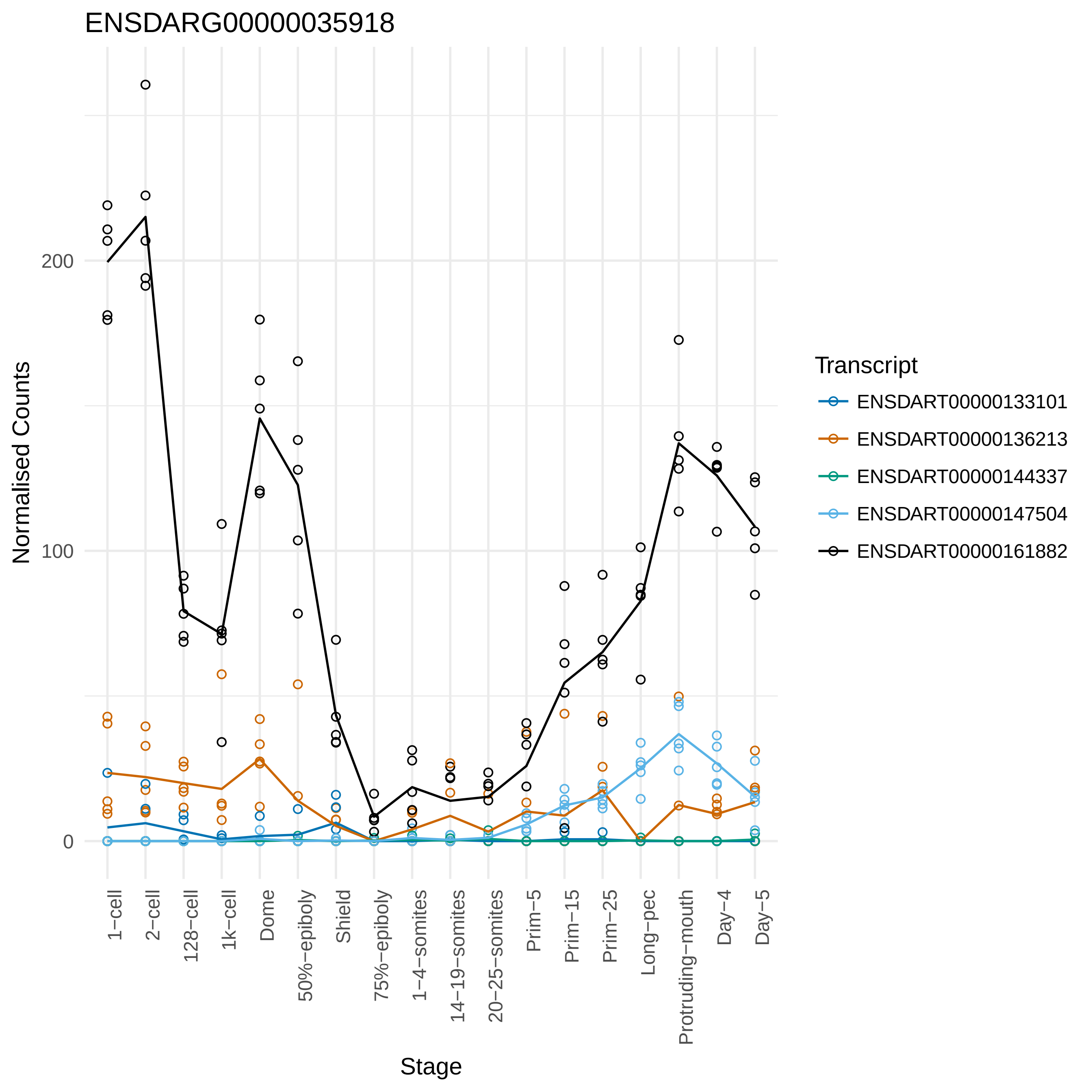
ENSDARG00000035918
Normalised Counts
200
100
0
Stage
1−cell
2−cell
128−cell
1k−cell
Dome
50%−epiboly
Shield
75%−epiboly
1−4−somites
14−19−somites
20−25−somites
Prim−5
Prim−15
Prim−25
Long−pec
Protruding−mouth
Day−4
Day−5
Transcript
ENSDART00000133101
ENSDART00000136213
ENSDART00000144337
ENSDART00000147504
ENSDART00000161882

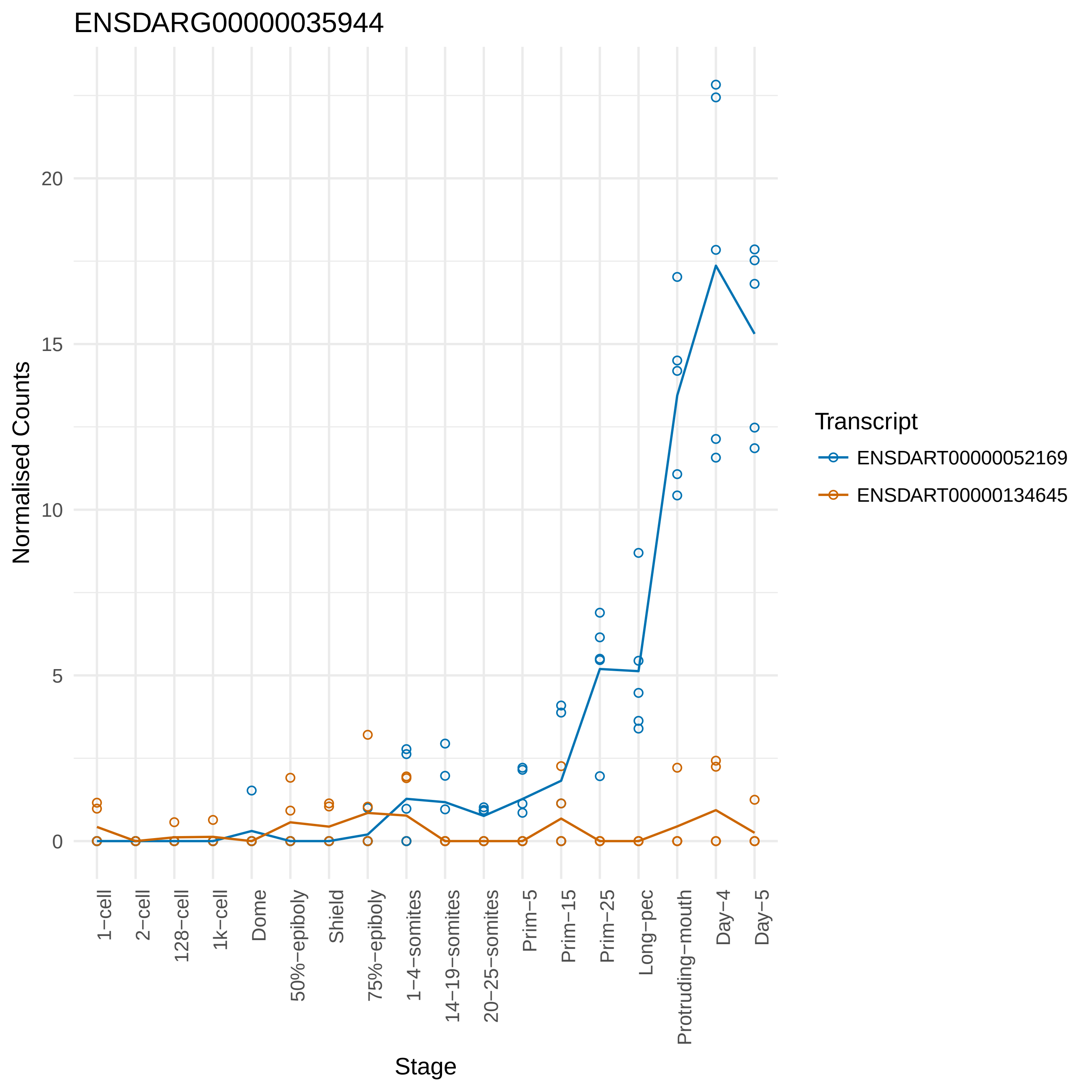

ENSDARG00000035944
Normalised Counts
0
5
10
15
20
Stage
1−cell
2−cell
128−cell
1k−cell
Dome
50%−epiboly
Shield
75%−epiboly
1−4−somites
14−19−somites
20−25−somites
Prim−5
Prim−15
Prim−25
Long−pec
Protruding−mouth
Day−4
Day−5
Transcript
ENSDART00000052169
ENSDART00000134645

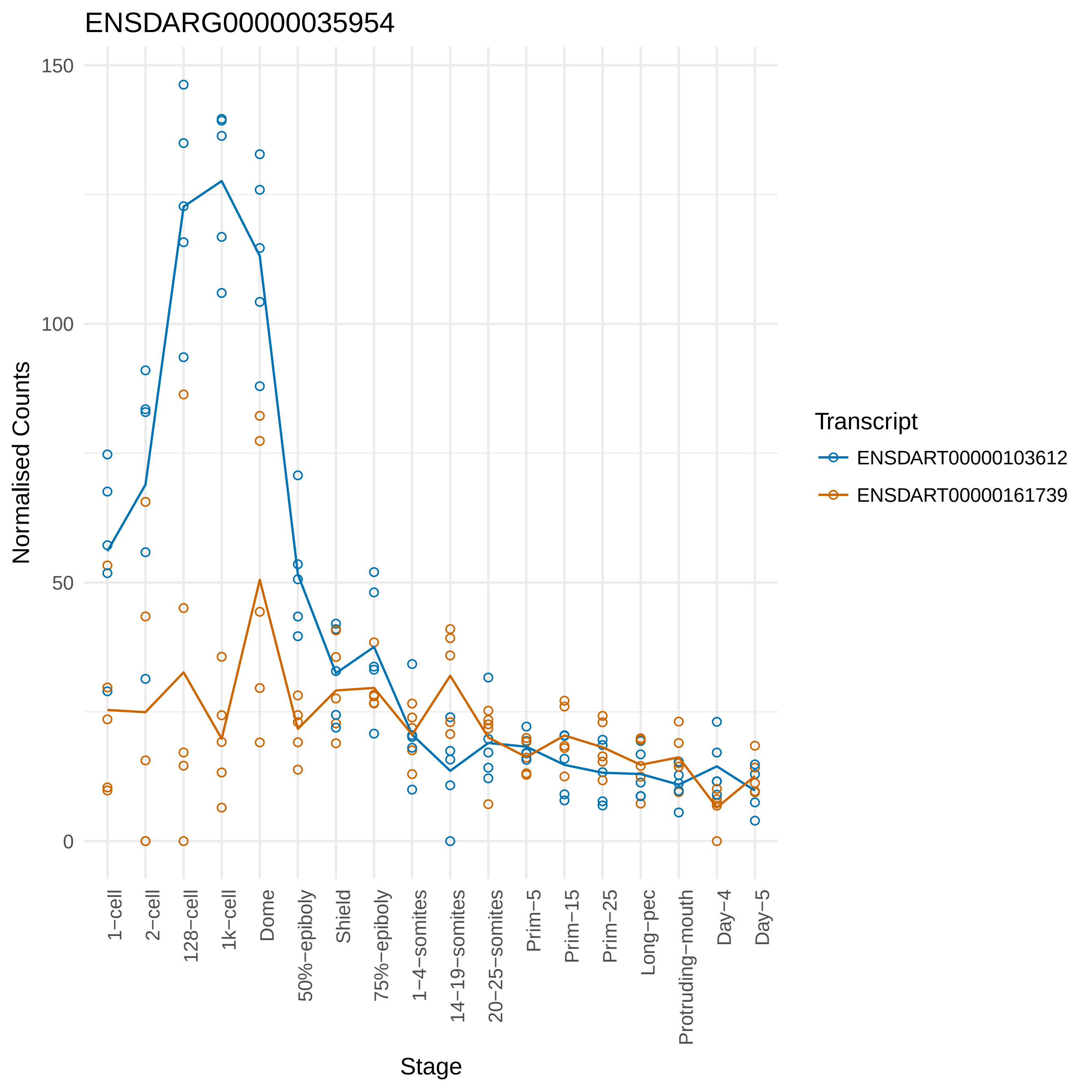

ENSDARG00000035954
Normalised Counts
150
100
50
0
1−cell
2−cell
128−cell
1k−cell
Dome
50%−epiboly
Shield
75%−epiboly
1−4−somites
14−19−somites
20−25−somites
Prim−5
Prim−15
Prim−25
Long−pec
Protruding−mouth
Day−4
Day−5
Stage
Transcript
ENSDART00000103612
ENSDART00000161739

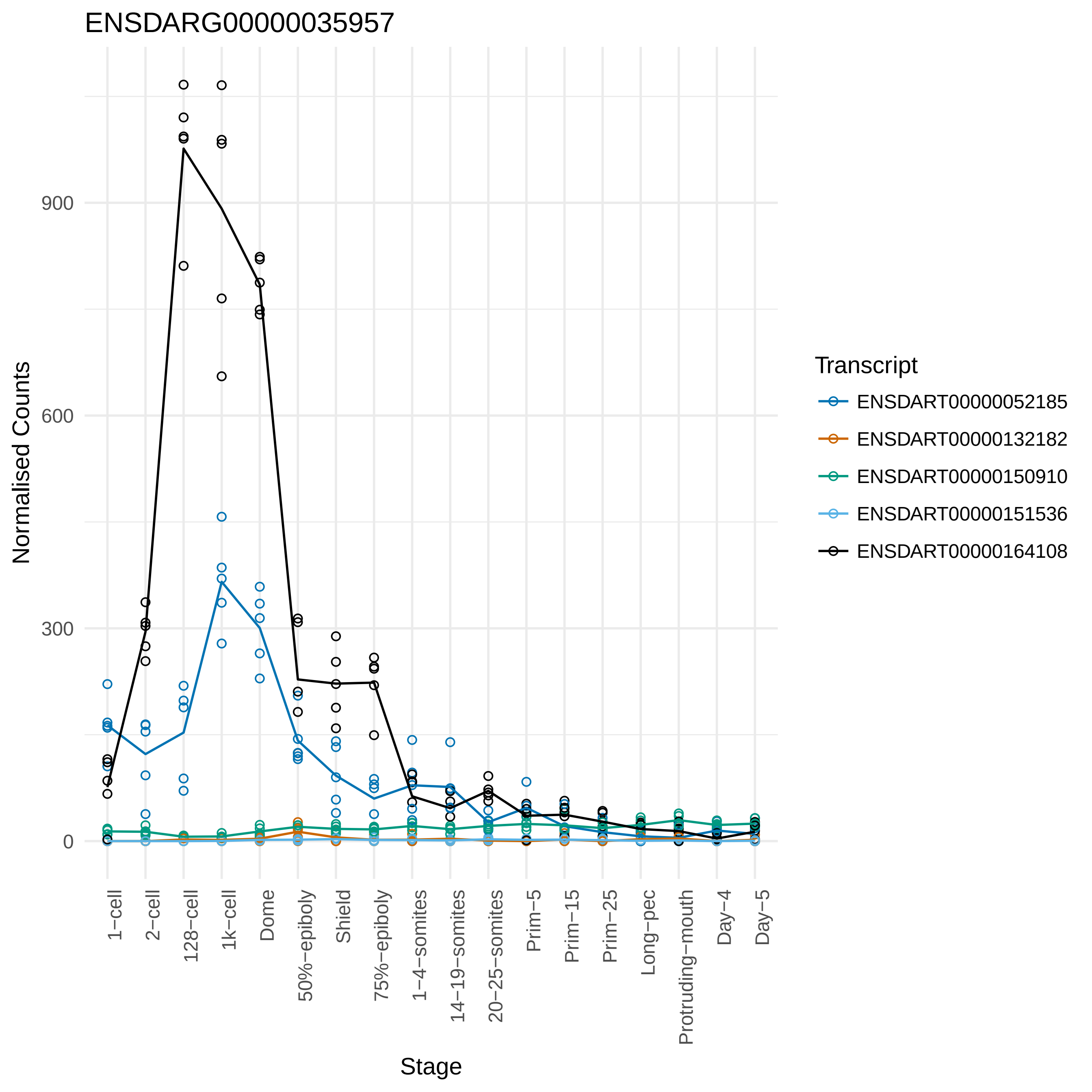

ENSDARG00000035957
Normalised Counts
900
600
300
0
1−cell
2−cell
128−cell
1k−cell
Dome
50%−epiboly
Shield
75%−epiboly
1−4−somites
14−19−somites
20−25−somites
Prim−5
Prim−15
Prim−25
Long−pec
Protruding−mouth
Day−4
Day−5
Stage
Transcript
ENSDART00000052185
ENSDART00000132182
ENSDART00000150910
ENSDART00000151536
ENSDART00000164108

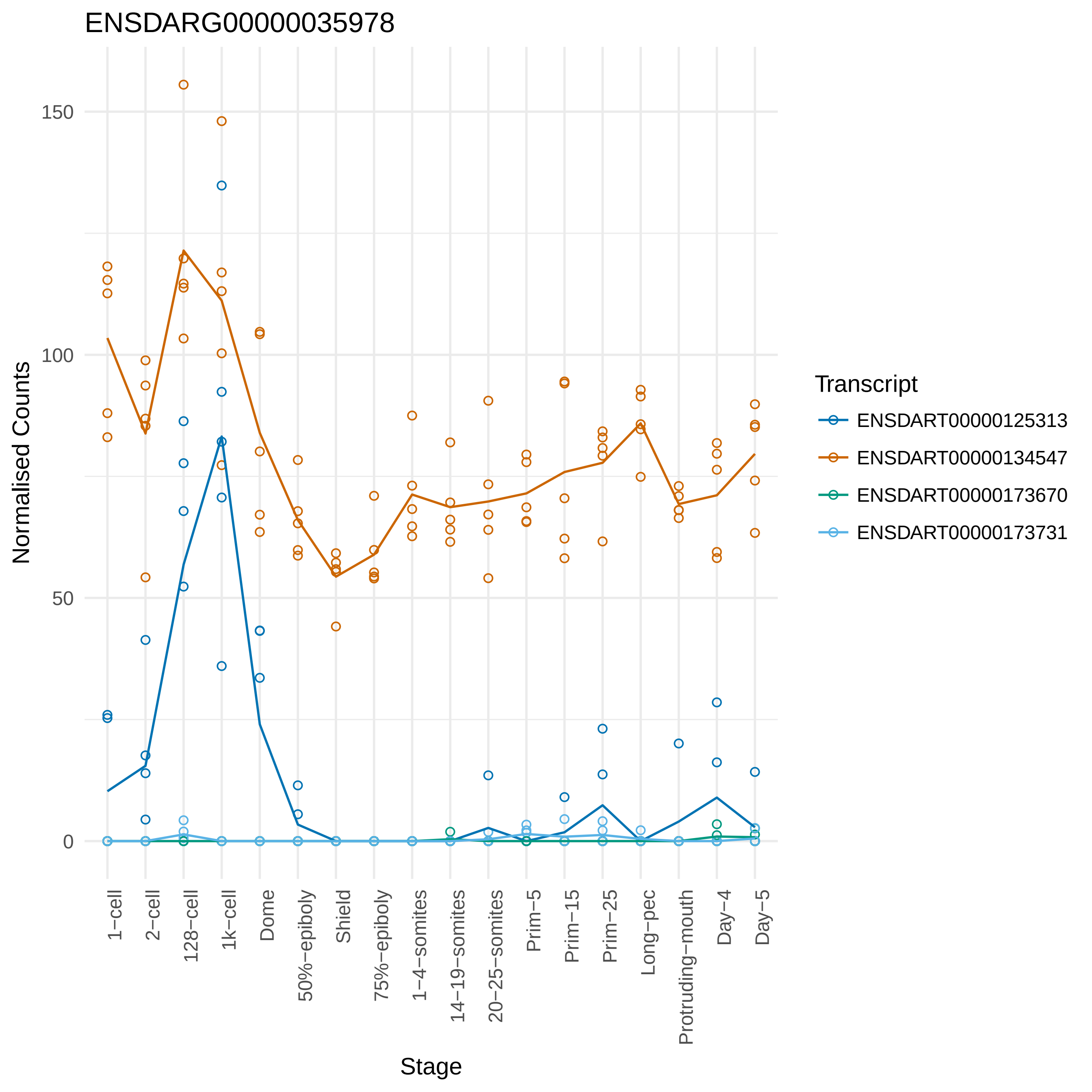
ENSDARG00000035978
Normalised Counts
150
100
50
0
1−cell
2−cell
128−cell
1k−cell
Dome
50%−epiboly
Shield
75%−epiboly
1−4−somites
14−19−somites
20−25−somites
Prim−5
Prim−15
Prim−25
Long−pec
Protruding−mouth
Day−4
Day−5
Stage
Transcript
ENSDART00000125313
ENSDART00000134547
ENSDART00000173670
ENSDART00000173731

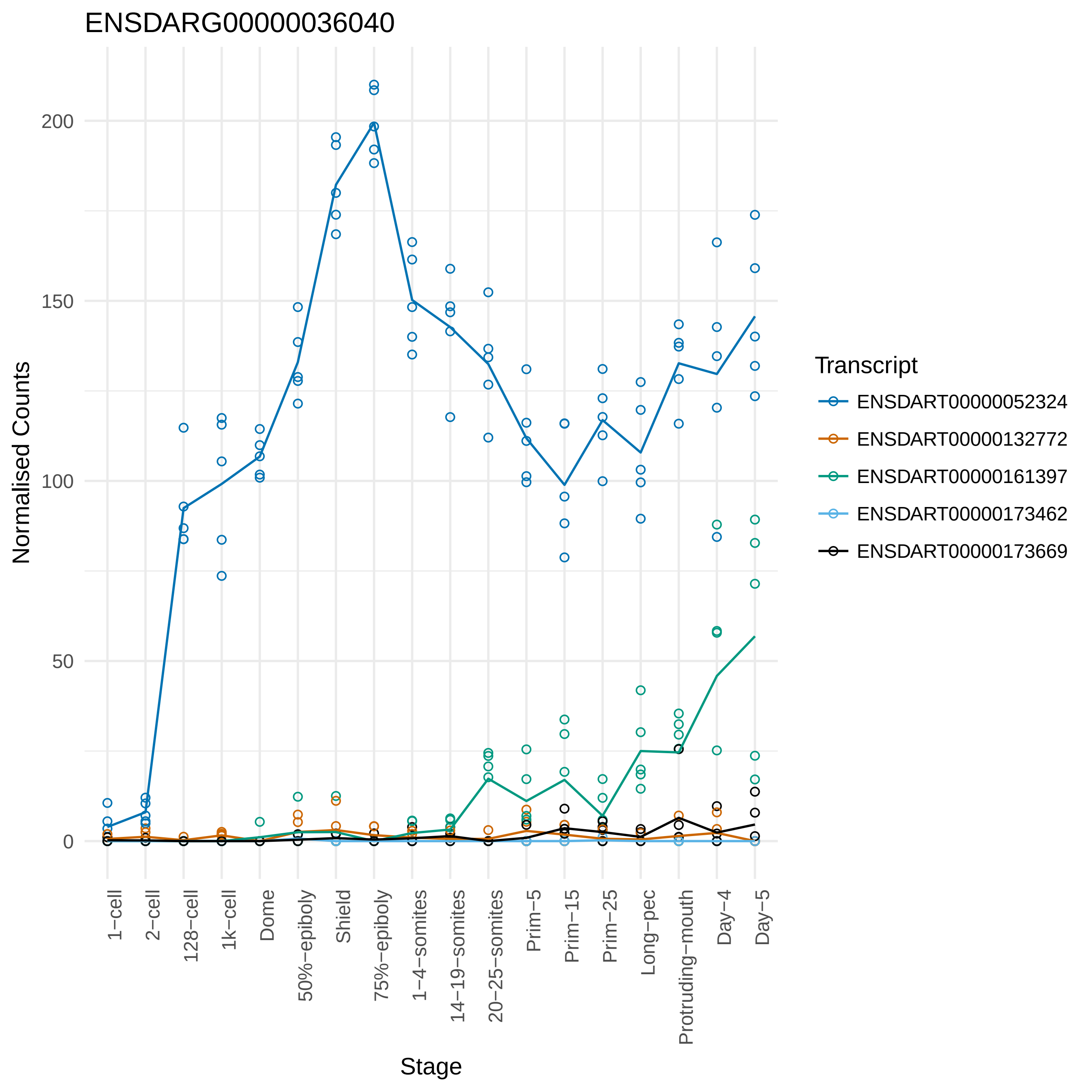
ENSDARG00000036040
Normalised Counts
200
150
100
50
0
1−cell
2−cell
128−cell
1k−cell
Dome
50%−epiboly
Shield
75%−epiboly
1−4−somites
14−19−somites
20−25−somites
Prim−5
Prim−15
Prim−25
Long−pec
Protruding−mouth
Day−4
Day−5
Stage
Transcript
ENSDART00000052324
ENSDART00000132772
ENSDART00000161397
ENSDART00000173462
ENSDART00000173669

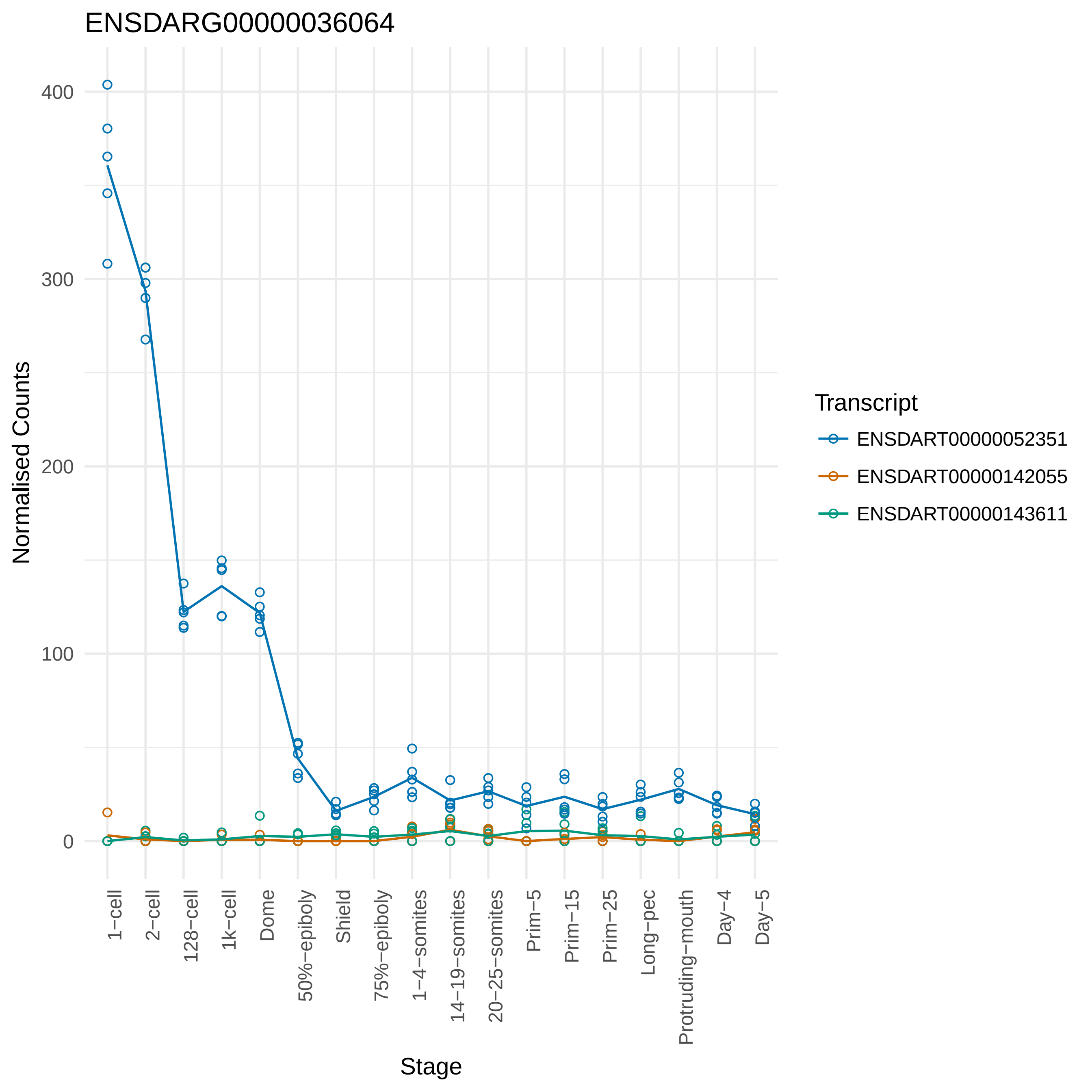
ENSDARG00000036064
Normalised Counts
400
300
200
100
0
1−cell
2−cell
128−cell
1k−cell
Dome
50%−epiboly
Shield
75%−epiboly
1−4−somites
14−19−somites
20−25−somites
Prim−5
Prim−15
Prim−25
Long−pec
Protruding−mouth
Day−4
Day−5
Stage
Transcript
ENSDART00000052351
ENSDART00000142055
ENSDART00000143611

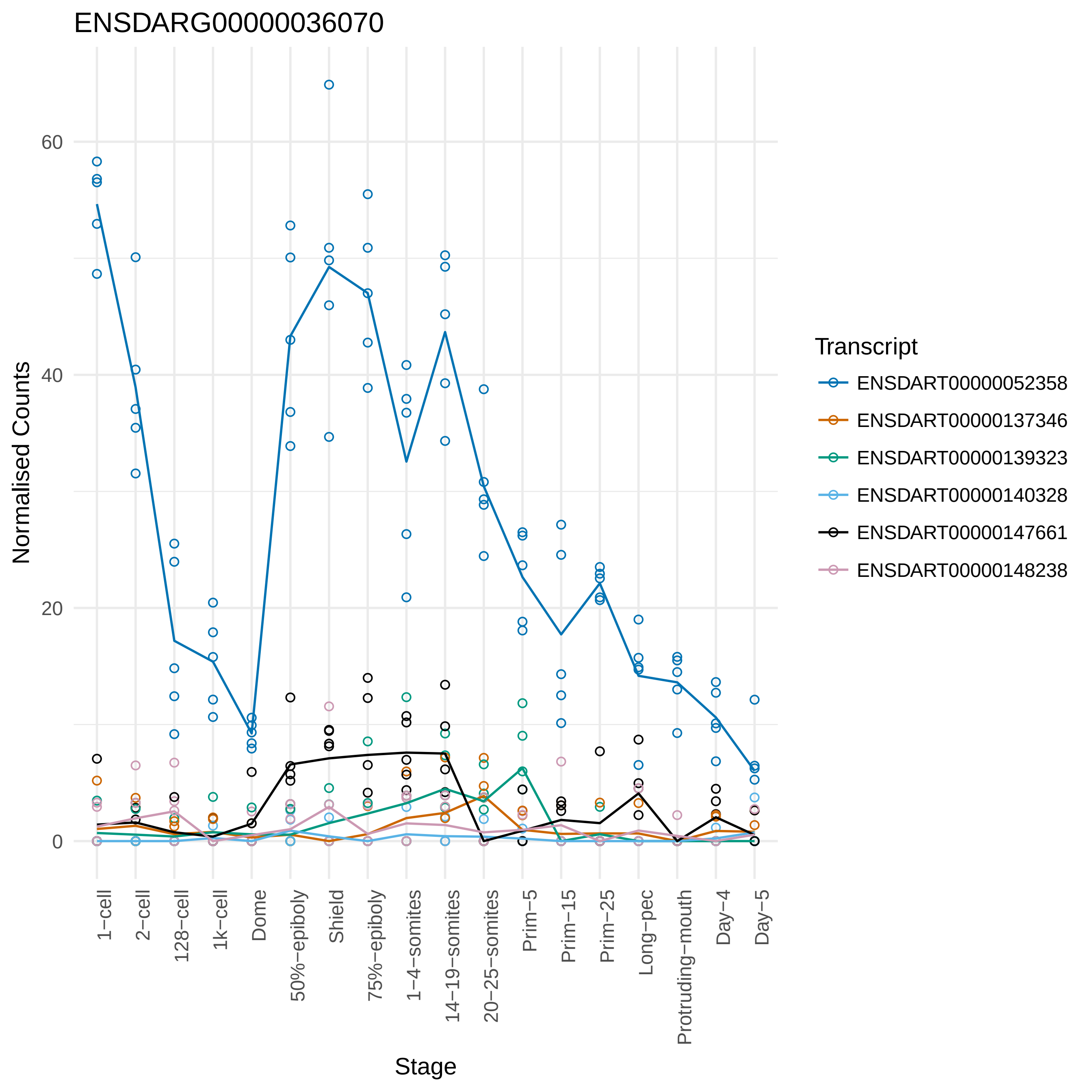

ENSDARG00000036070
Normalised Counts
60
40
20
0
Stage
1−cell
2−cell
128−cell
1k−cell
Dome
50%−epiboly
Shield
75%−epiboly
1−4−somites
14−19−somites
20−25−somites
Prim−5
Prim−15
Prim−25
Long−pec
Protruding−mouth
Day−4
Day−5
Transcript
ENSDART00000052358
ENSDART00000137346
ENSDART00000139323
ENSDART00000140328
ENSDART00000147661
ENSDART00000148238

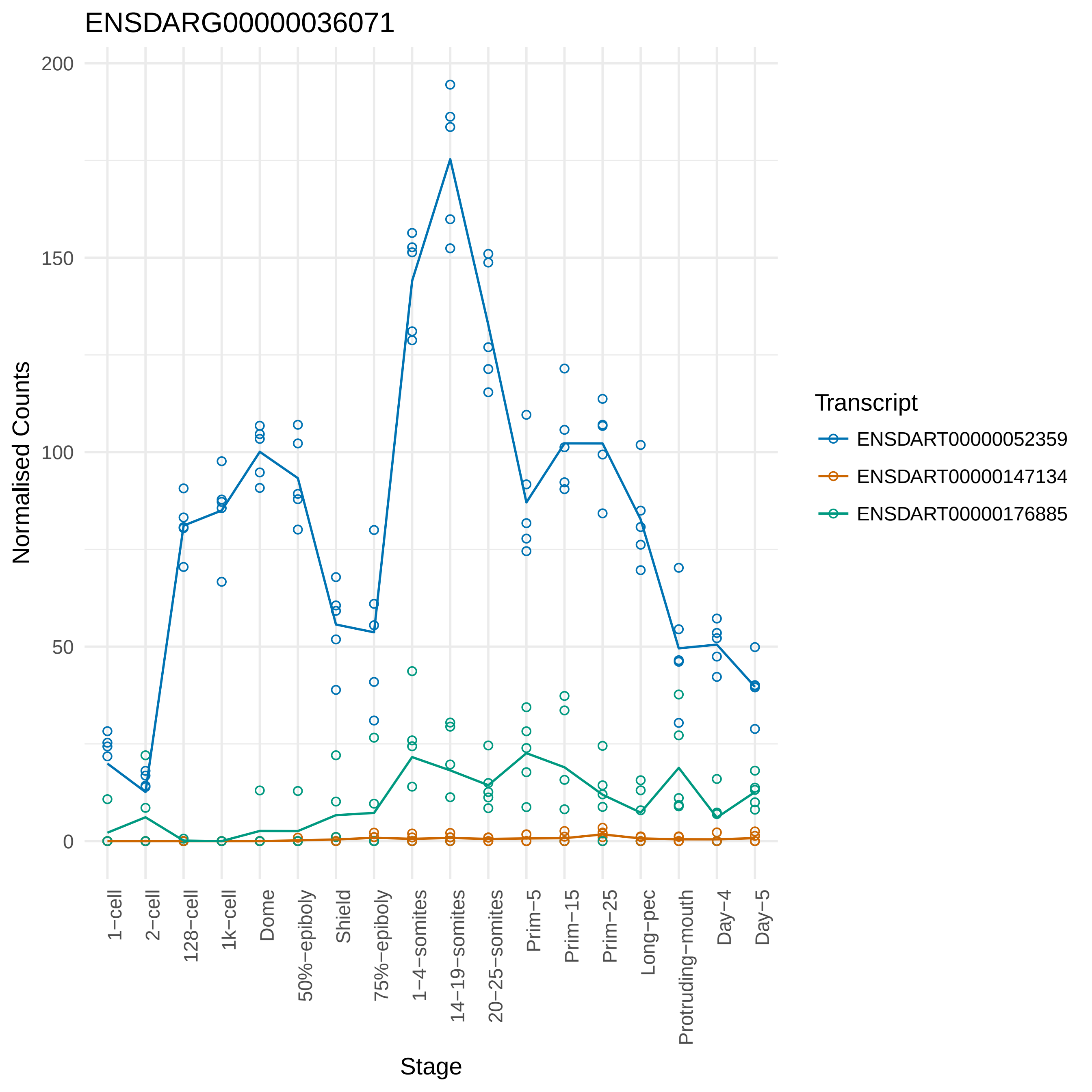
ENSDARG00000036071
Normalised Counts
200
150
100
50
0
1−cell
2−cell
128−cell
1k−cell
Dome
50%−epiboly
Shield
75%−epiboly
1−4−somites
14−19−somites
20−25−somites
Prim−5
Prim−15
Prim−25
Long−pec
Protruding−mouth
Day−4
Day−5
Stage
Transcript
ENSDART00000052359
ENSDART00000147134
ENSDART00000176885

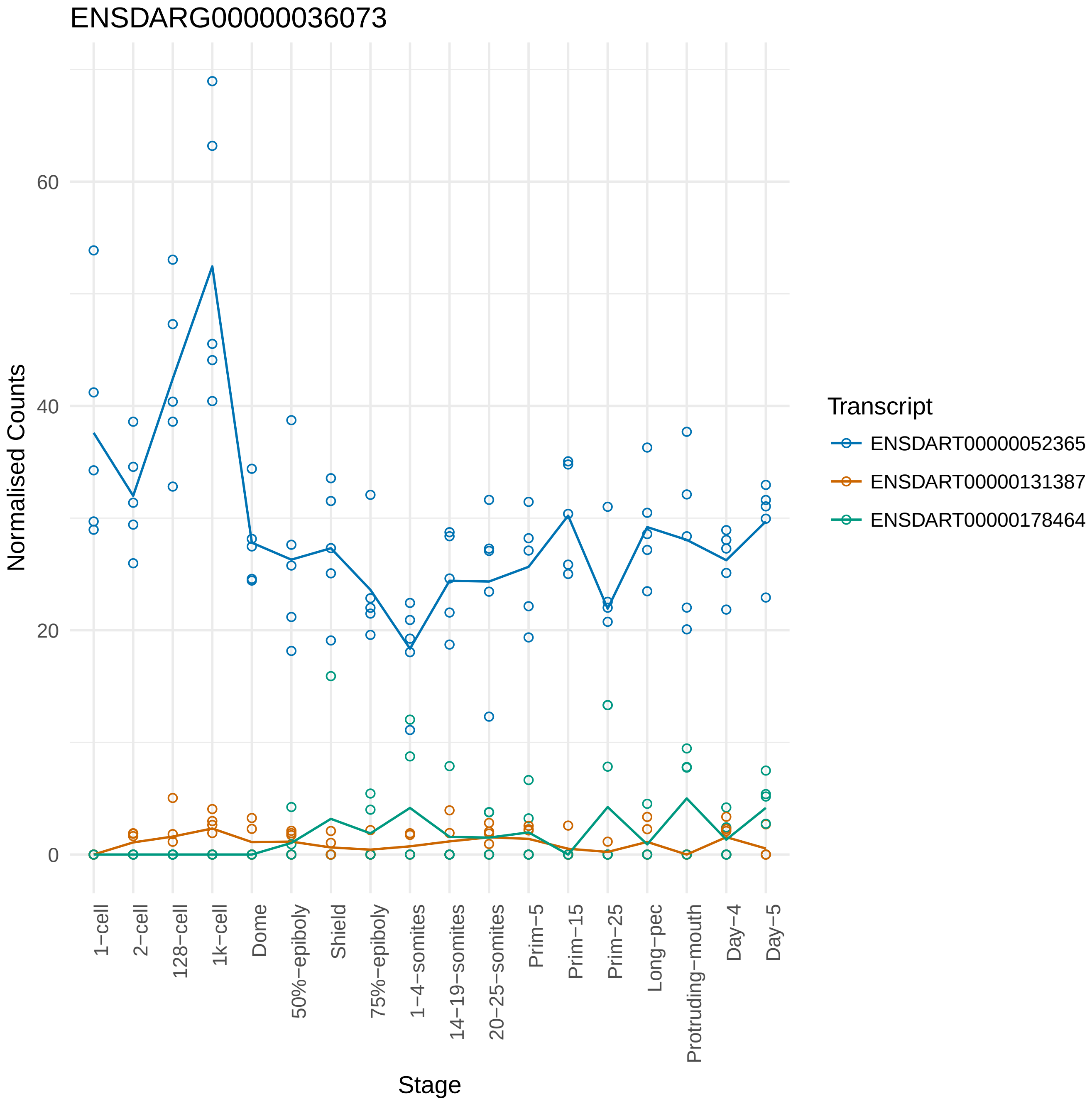
ENSDARG00000036073
Normalised Counts
0
20
40
60
Stage
1−cell
2−cell
128−cell
1k−cell
Dome
50%−epiboly
Shield
75%−epiboly
1−4−somites
14−19−somites
20−25−somites
Prim−5
Prim−15
Prim−25
Long−pec
Protruding−mouth
Day−4
Day−5
Transcript
ENSDART00000052365
ENSDART00000131387
ENSDART00000178464

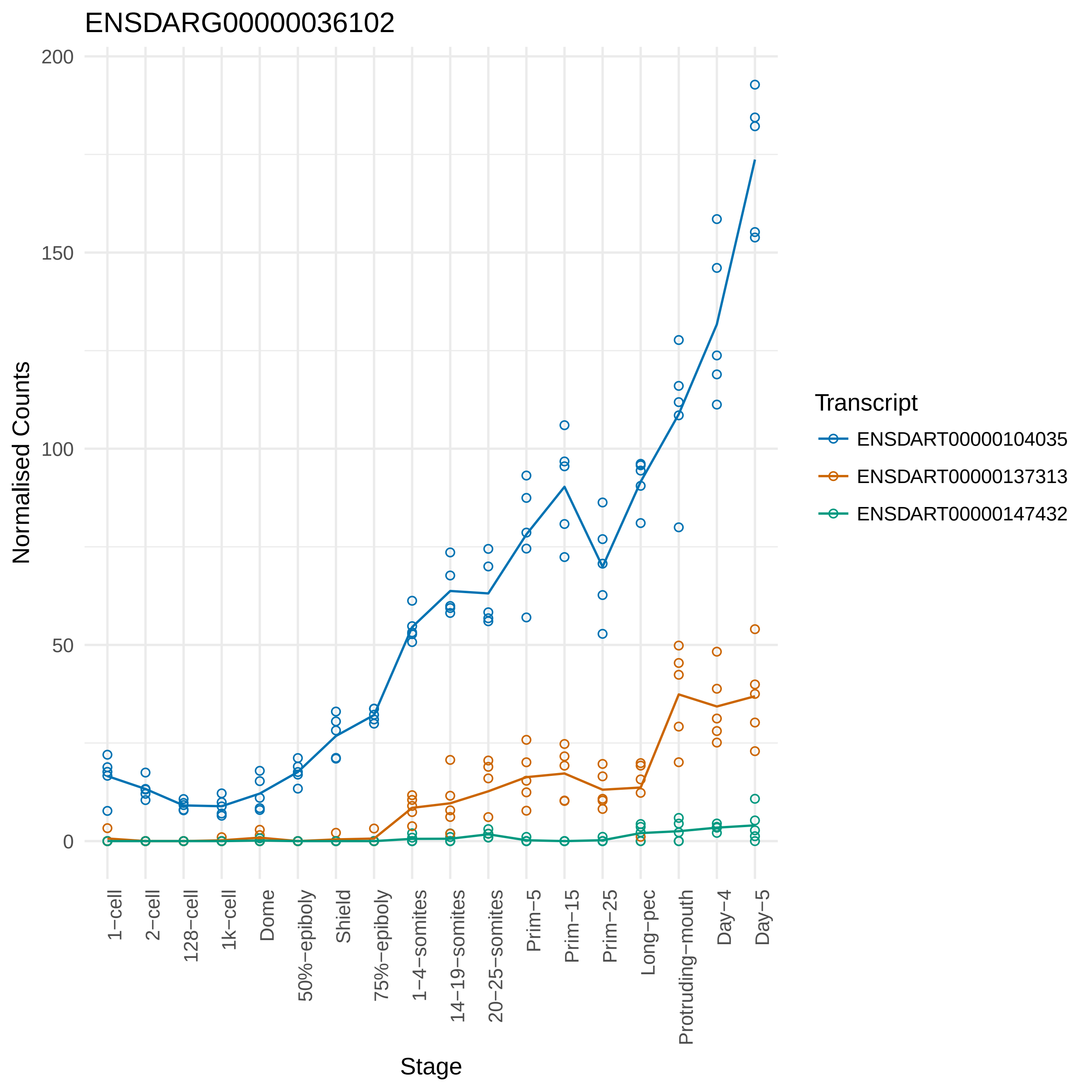

ENSDARG00000036102
Normalised Counts
200
150
100
50
0
1−cell
2−cell
128−cell
1k−cell
Dome
50%−epiboly
Shield
75%−epiboly
1−4−somites
14−19−somites
20−25−somites
Prim−5
Prim−15
Prim−25
Long−pec
Protruding−mouth
Day−4
Day−5
Stage
Transcript
ENSDART00000104035
ENSDART00000137313
ENSDART00000147432

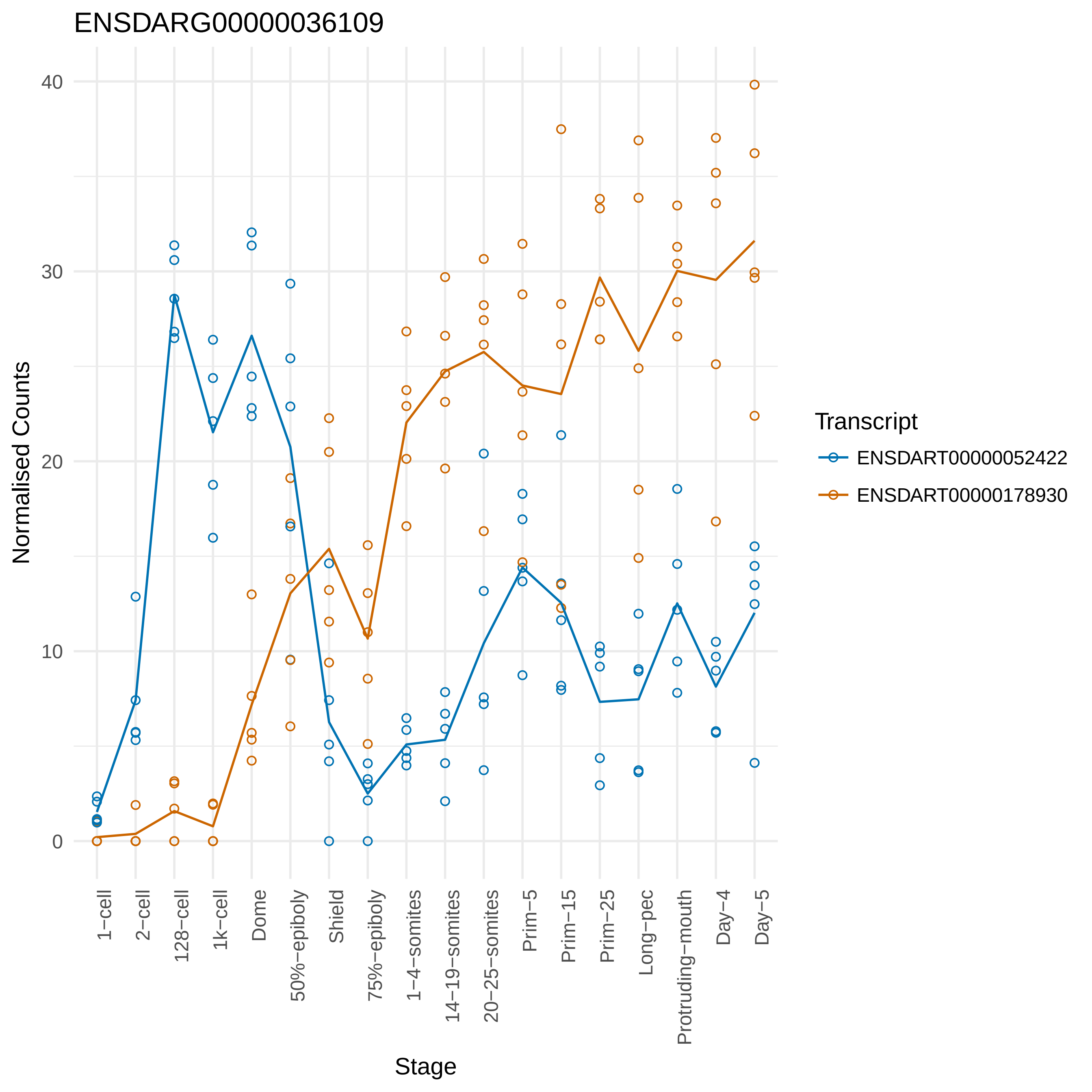
ENSDARG00000036109
Normalised Counts
0
10
20
30
40
1−cell
2−cell
128−cell
1k−cell
Dome
50%−epiboly
Shield
75%−epiboly
1−4−somites
14−19−somites
20−25−somites
Prim−5
Prim−15
Prim−25
Long−pec
Protruding−mouth
Day−4
Day−5
Stage
Transcript
ENSDART00000052422
ENSDART00000178930

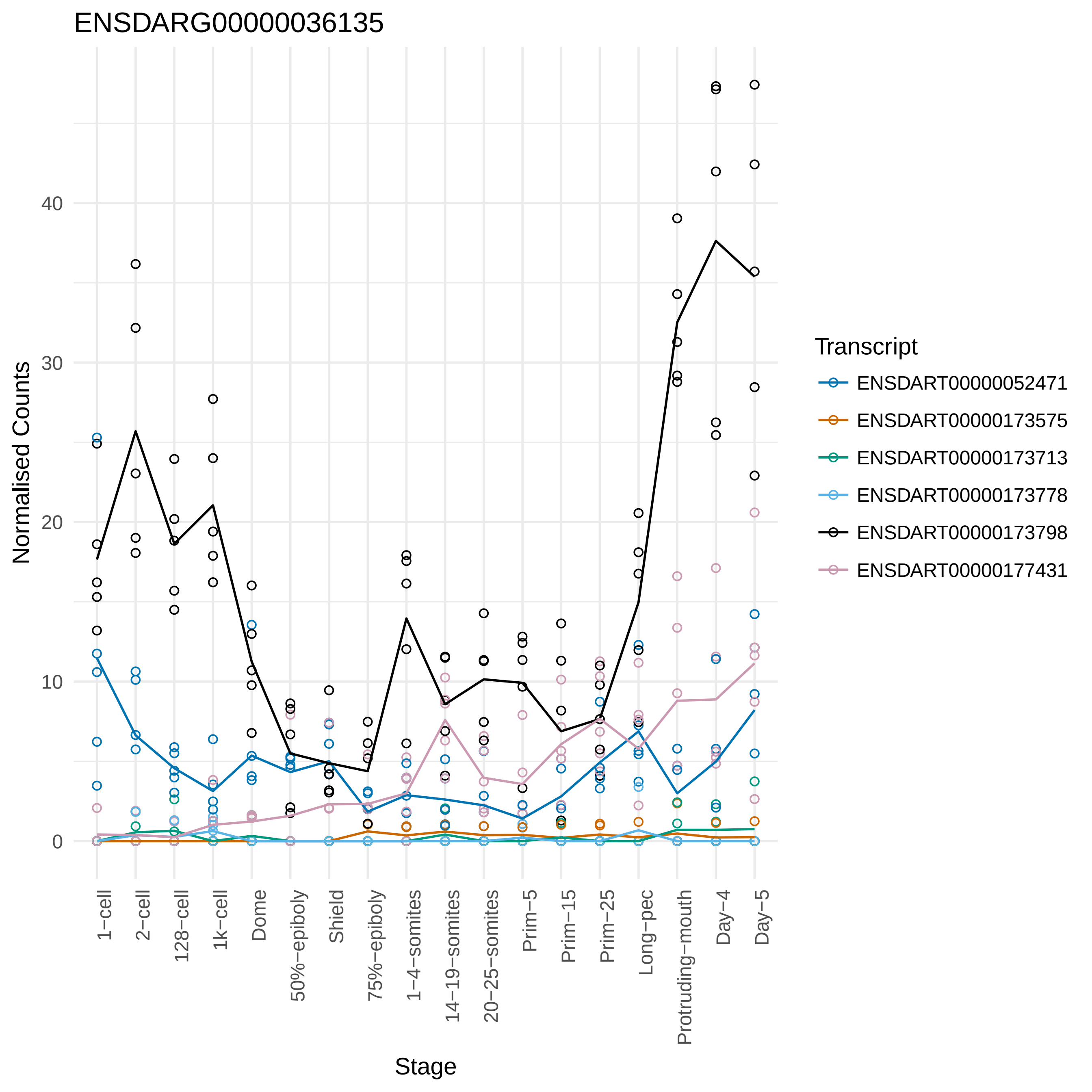

ENSDARG00000036135
Normalised Counts
0
10
20
30
40
Stage
1−cell
2−cell
128−cell
1k−cell
Dome
50%−epiboly
Shield
75%−epiboly
1−4−somites
14−19−somites
20−25−somites
Prim−5
Prim−15
Prim−25
Long−pec
Protruding−mouth
Day−4
Day−5
Transcript
ENSDART00000052471
ENSDART00000173575
ENSDART00000173713
ENSDART00000173778
ENSDART00000173798
ENSDART00000177431

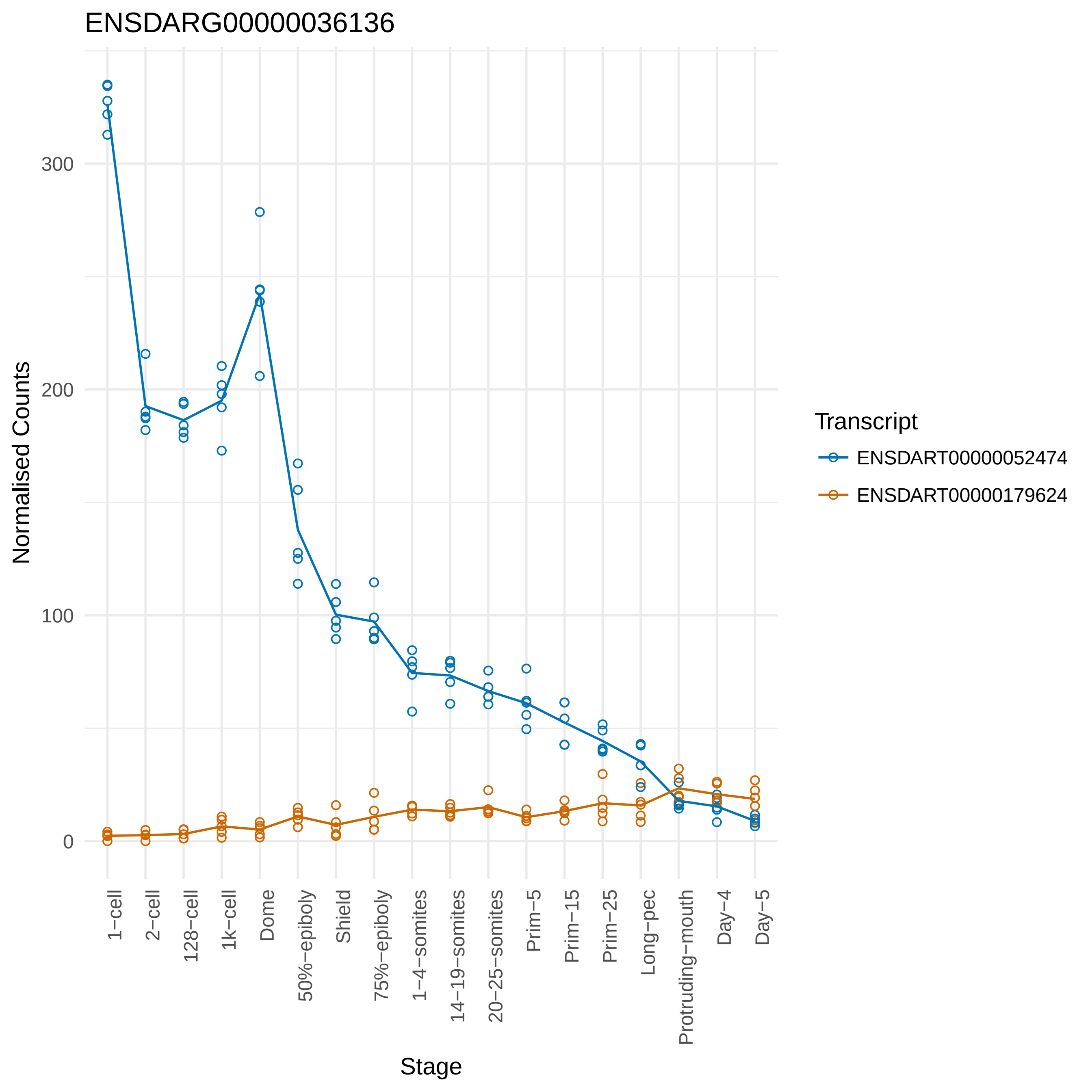
ENSDARG00000036136
Normalised Counts
300
200
100
0
1−cell
2−cell
128−cell
1k−cell
Dome
50%−epiboly
Shield
75%−epiboly
1−4−somites
14−19−somites
20−25−somites
Prim−5
Prim−15
Prim−25
Long−pec
Protruding−mouth
Day−4
Day−5
Stage
Transcript
ENSDART00000052474
ENSDART00000179624

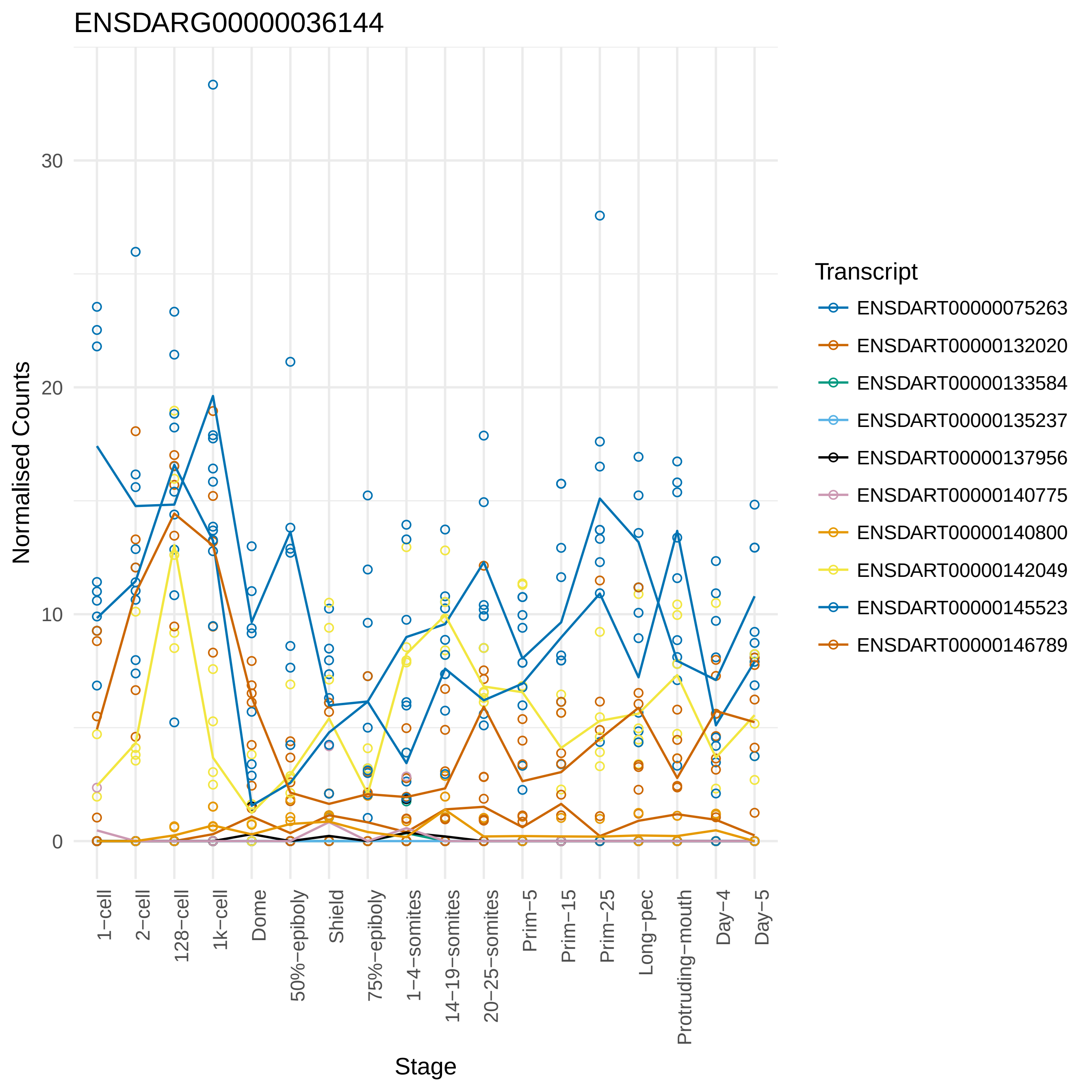

ENSDARG00000036144
Normalised Counts
30
20
10
0
1−cell
2−cell
128−cell
1k−cell
Dome
50%−epiboly
Shield
75%−epiboly
1−4−somites
14−19−somites
20−25−somites
Prim−5
Prim−15
Prim−25
Long−pec
Protruding−mouth
Day−4
Day−5
Stage
Transcript
ENSDART00000075263
ENSDART00000132020
ENSDART00000133584
ENSDART00000135237
ENSDART00000137956
ENSDART00000140775
ENSDART00000140800
ENSDART00000142049
ENSDART00000145523
ENSDART00000146789

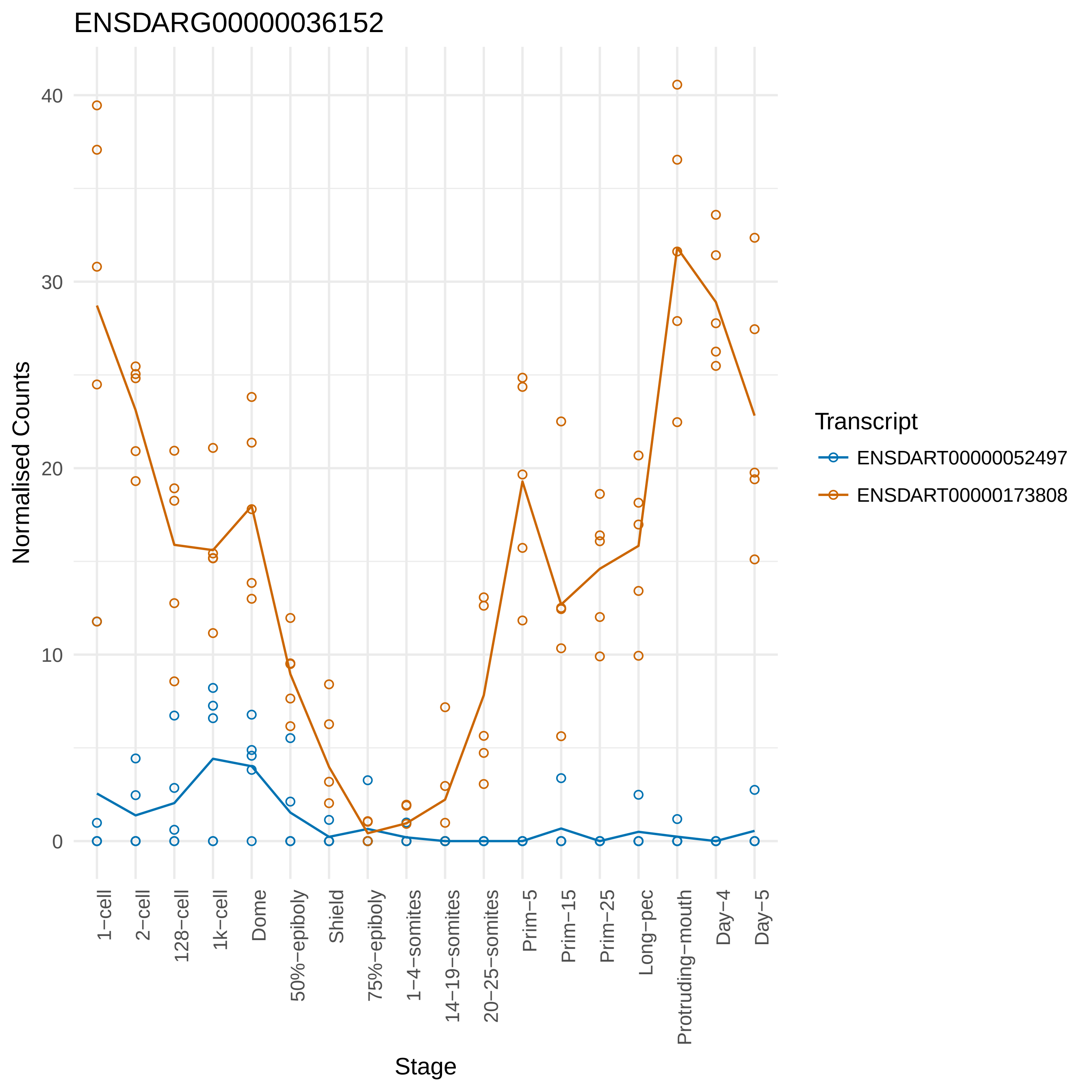

ENSDARG00000036152
Normalised Counts
40
30
20
10
0
1−cell
2−cell
128−cell
1k−cell
Dome
50%−epiboly
Shield
75%−epiboly
1−4−somites
14−19−somites
20−25−somites
Prim−5
Prim−15
Prim−25
Long−pec
Protruding−mouth
Day−4
Day−5
Stage
Transcript
ENSDART00000052497
ENSDART00000173808

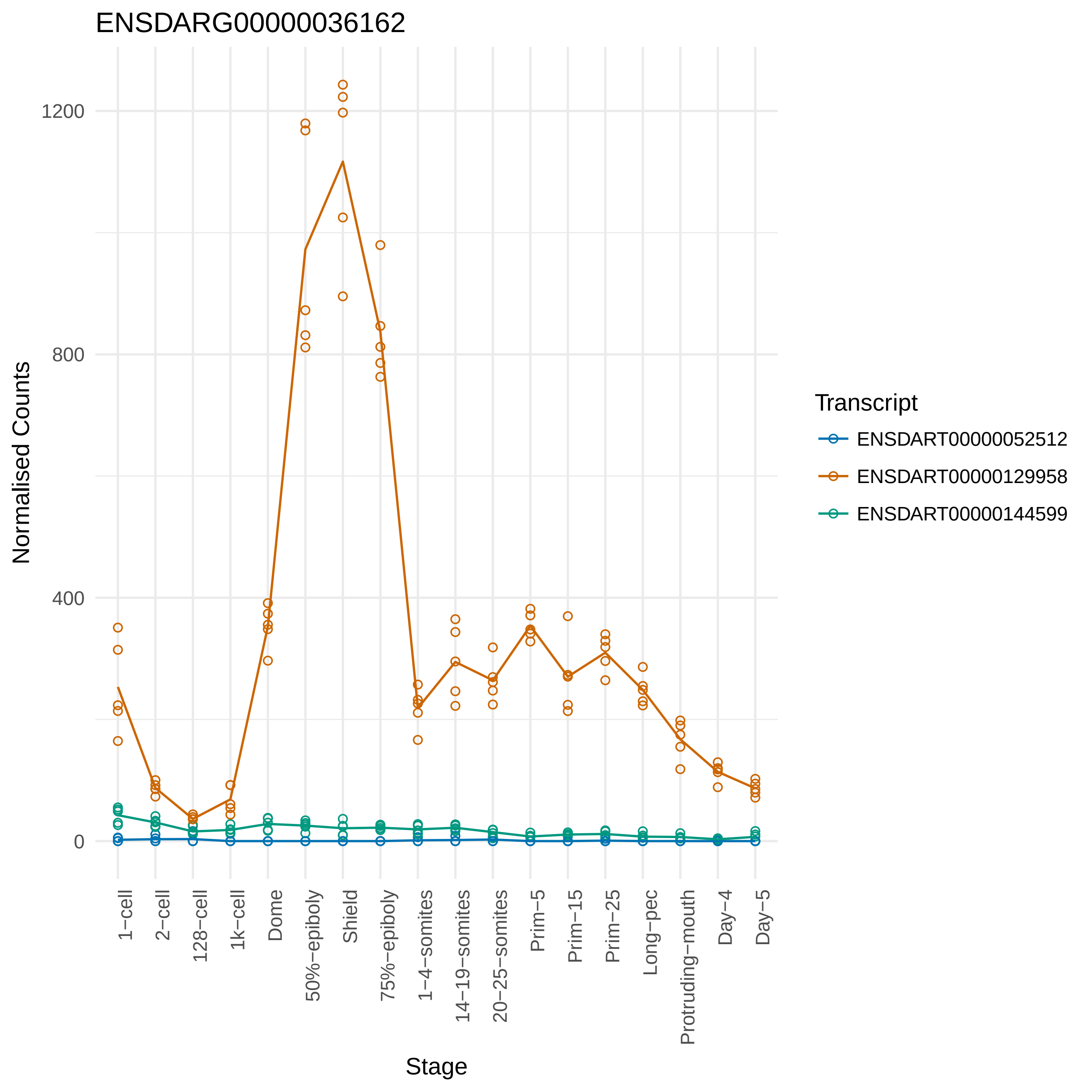
ENSDARG00000036162
Normalised Counts
1200
800
400
0
1−cell
2−cell
128−cell
1k−cell
Dome
50%−epiboly
Shield
75%−epiboly
1−4−somites
14−19−somites
20−25−somites
Prim−5
Prim−15
Prim−25
Long−pec
Protruding−mouth
Day−4
Day−5
Stage
Transcript
ENSDART00000052512
ENSDART00000129958
ENSDART00000144599

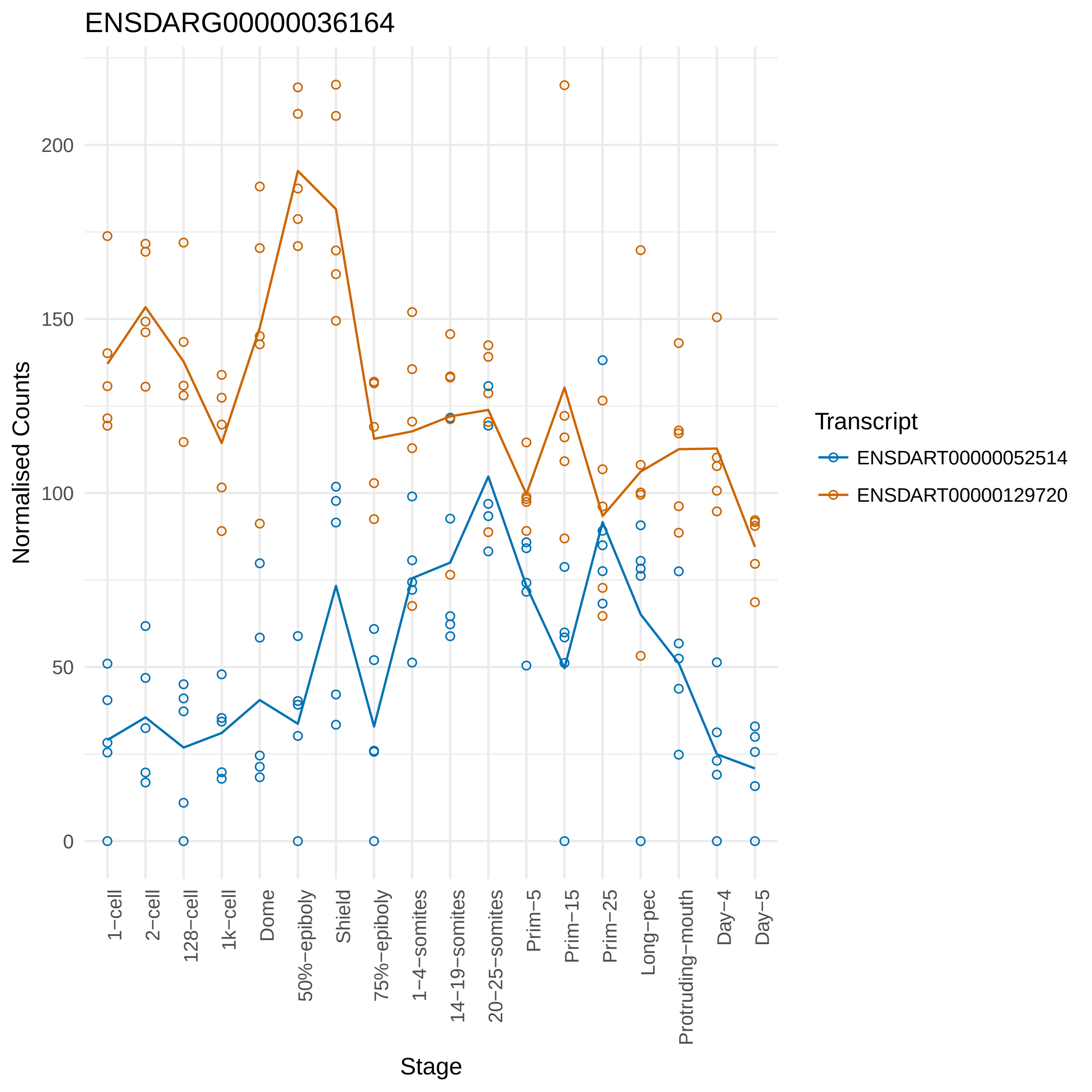

ENSDARG00000036164
Normalised Counts
0
50
100
150
200
Stage
1−cell
2−cell
128−cell
1k−cell
Dome
50%−epiboly
Shield
75%−epiboly
1−4−somites
14−19−somites
20−25−somites
Prim−5
Prim−15
Prim−25
Long−pec
Protruding−mouth
Day−4
Day−5
Transcript
ENSDART00000052514
ENSDART00000129720

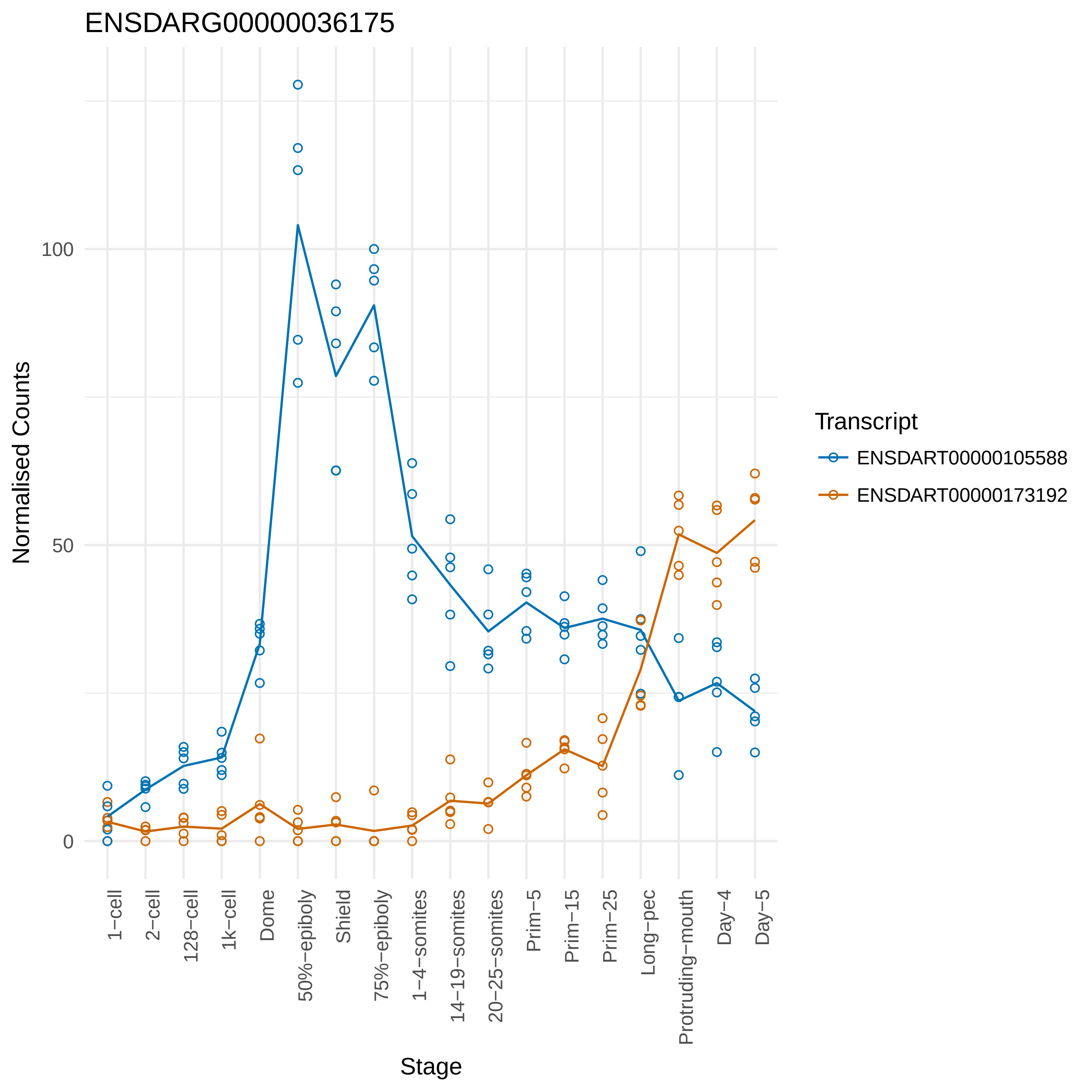
ENSDARG00000036175
Normalised Counts
100
50
0
1−cell
2−cell
128−cell
1k−cell
Dome
50%−epiboly
Shield
75%−epiboly
1−4−somites
14−19−somites
20−25−somites
Prim−5
Prim−15
Prim−25
Long−pec
Protruding−mouth
Day−4
Day−5
Stage
Transcript
ENSDART00000105588
ENSDART00000173192

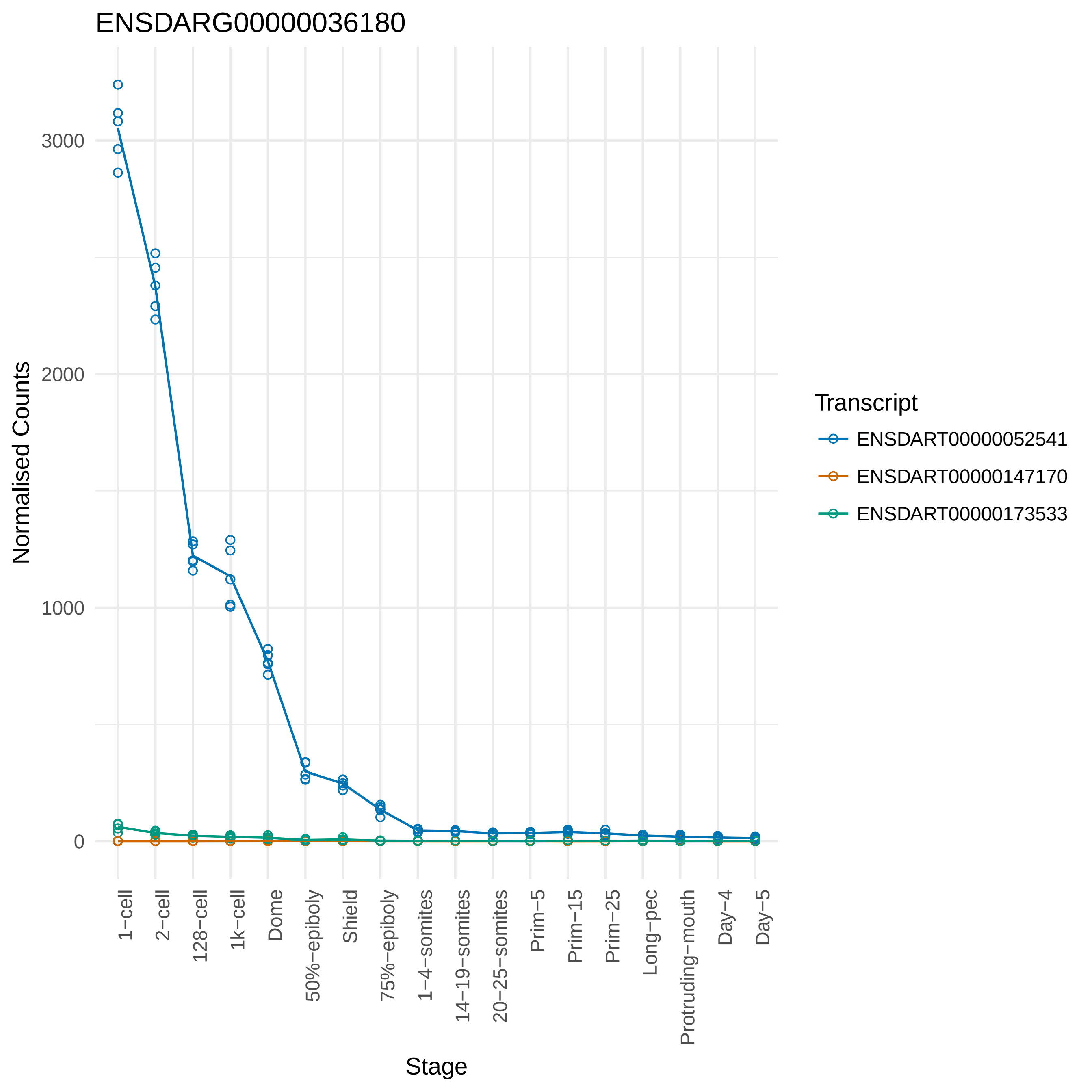
ENSDARG00000036180
Normalised Counts
3000
2000
1000
0
Stage
1−cell
2−cell
128−cell
1k−cell
Dome
50%−epiboly
Shield
75%−epiboly
1−4−somites
14−19−somites
20−25−somites
Prim−5
Prim−15
Prim−25
Long−pec
Protruding−mouth
Day−4
Day−5
Transcript
ENSDART00000052541
ENSDART00000147170
ENSDART00000173533

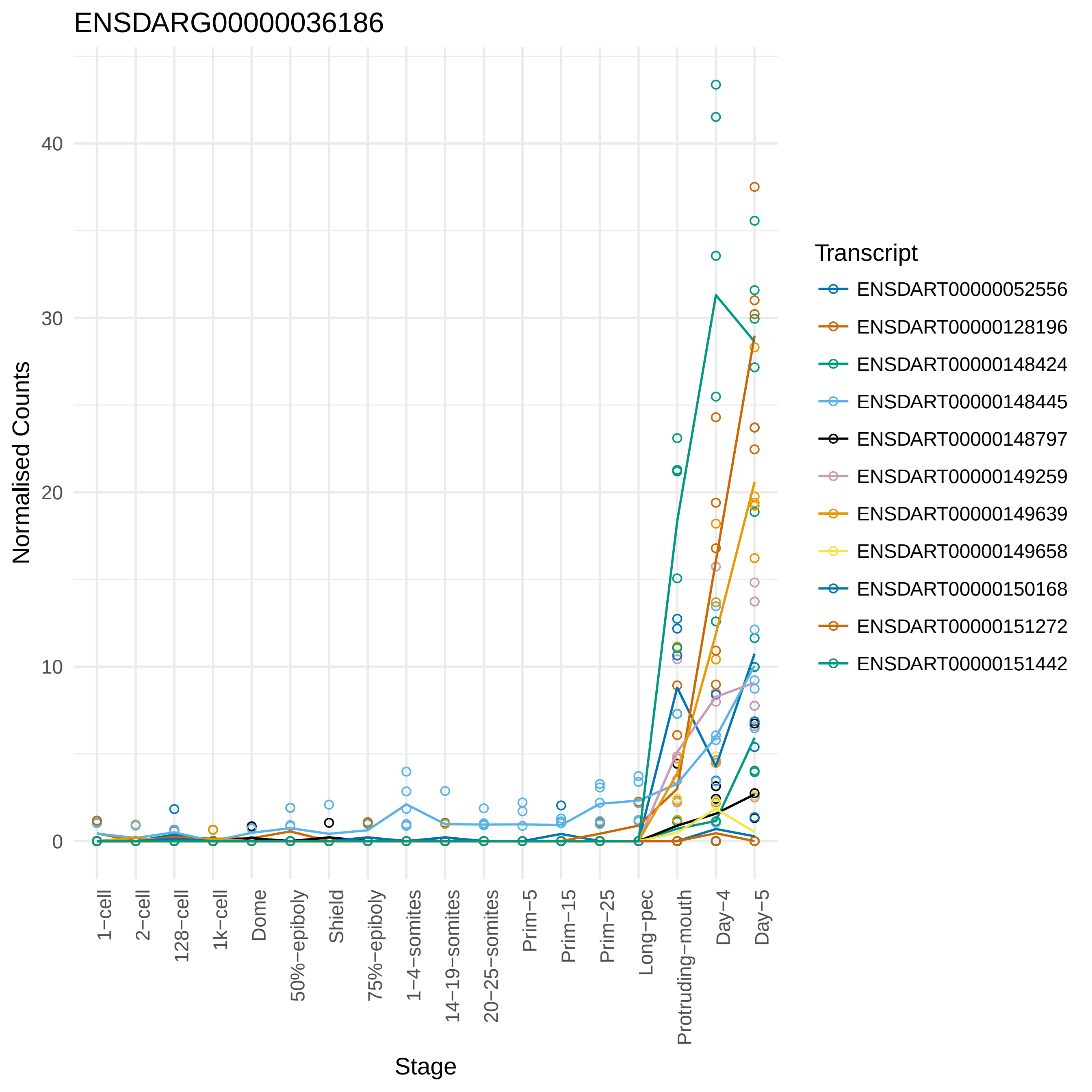
ENSDARG00000036186
Normalised Counts
0
10
20
30
40
Stage
1−cell
2−cell
128−cell
1k−cell
Dome
50%−epiboly
Shield
75%−epiboly
1−4−somites
14−19−somites
20−25−somites
Prim−5
Prim−15
Prim−25
Long−pec
Protruding−mouth
Day−4
Day−5
Transcript
ENSDART00000052556
ENSDART00000128196
ENSDART00000148424
ENSDART00000148445
ENSDART00000148797
ENSDART00000149259
ENSDART00000149639
ENSDART00000149658
ENSDART00000150168
ENSDART00000151272
ENSDART00000151442

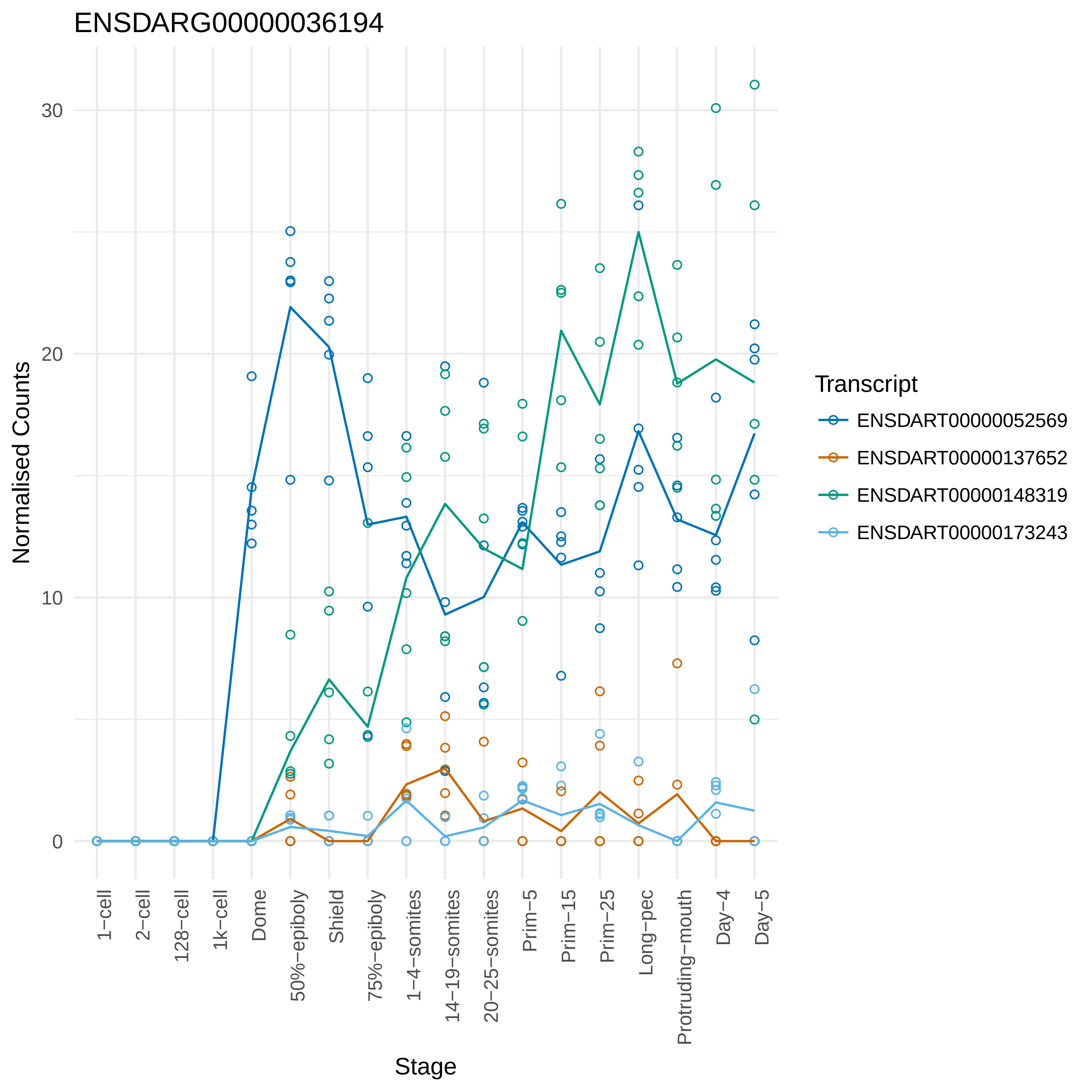

ENSDARG00000036194
Normalised Counts
30
20
10
0
1−cell
2−cell
128−cell
1k−cell
Dome
50%−epiboly
Shield
75%−epiboly
1−4−somites
14−19−somites
20−25−somites
Prim−5
Prim−15
Prim−25
Long−pec
Protruding−mouth
Day−4
Day−5
Stage
Transcript
ENSDART00000052569
ENSDART00000137652
ENSDART00000148319
ENSDART00000173243

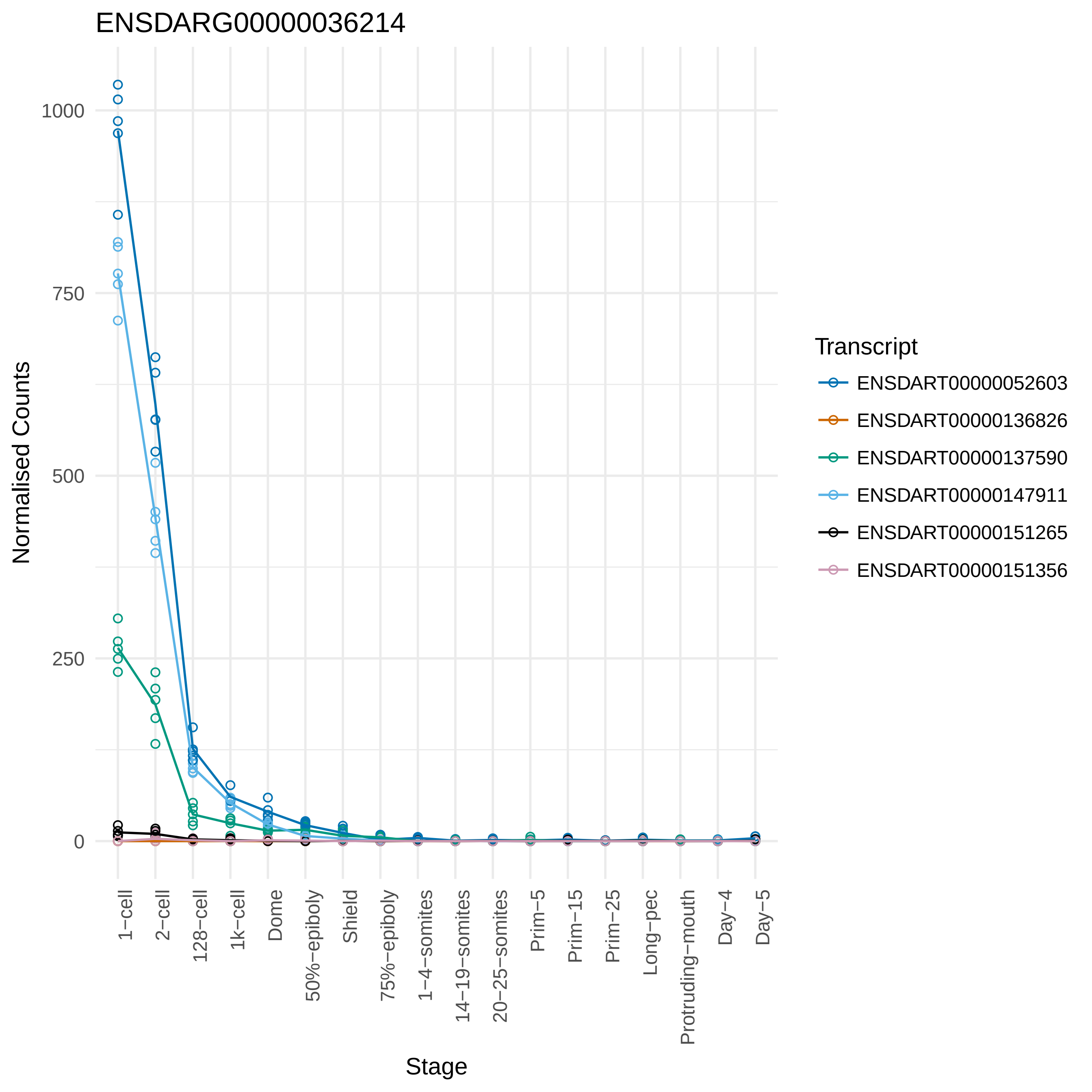

ENSDARG00000036214
Normalised Counts
1000
750
500
250
0
Stage
1−cell
2−cell
128−cell
1k−cell
Dome
50%−epiboly
Shield
75%−epiboly
1−4−somites
14−19−somites
20−25−somites
Prim−5
Prim−15
Prim−25
Long−pec
Protruding−mouth
Day−4
Day−5
Transcript
ENSDART00000052603
ENSDART00000136826
ENSDART00000137590
ENSDART00000147911
ENSDART00000151265
ENSDART00000151356

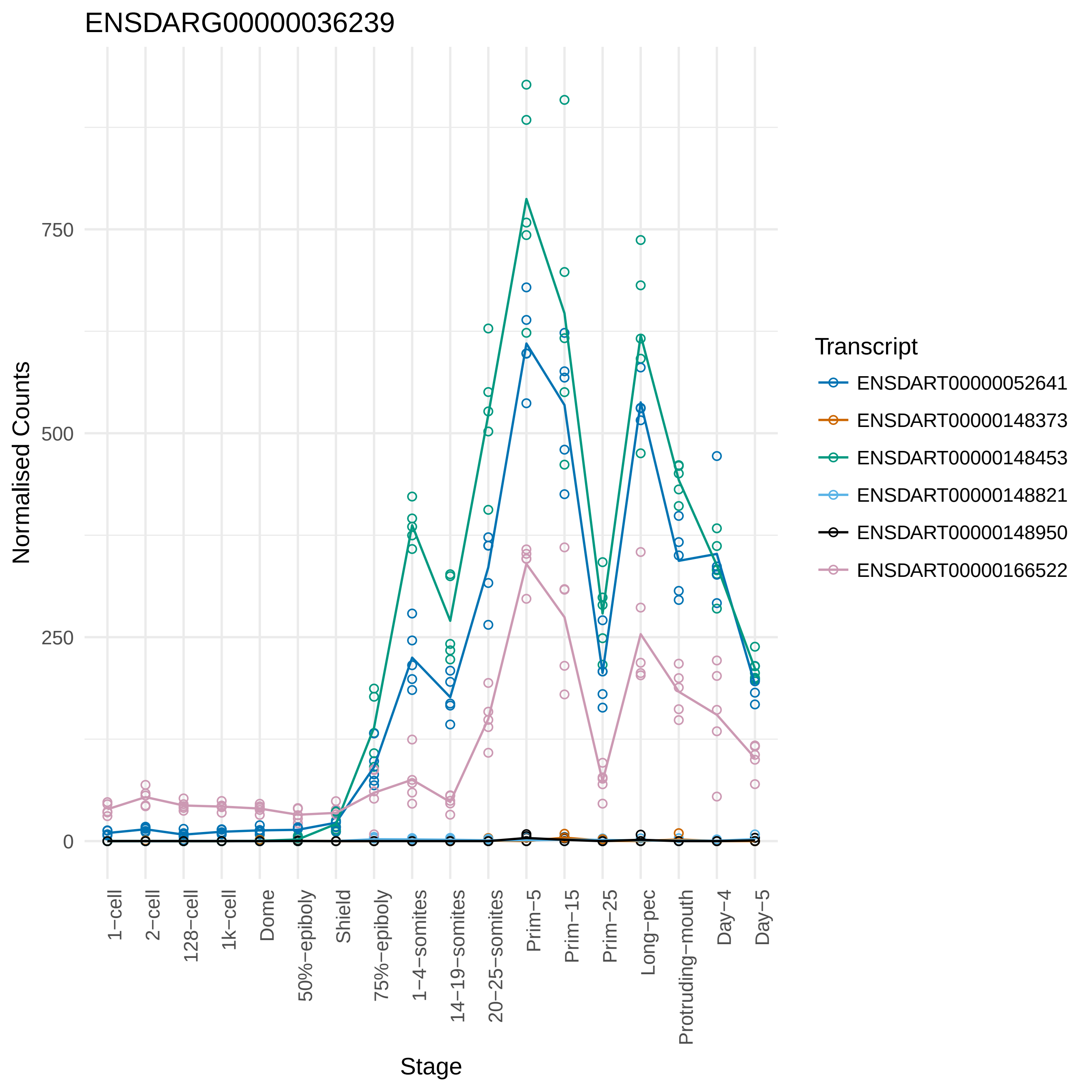

ENSDARG00000036239
Normalised Counts
750
500
250
0
1−cell
2−cell
128−cell
1k−cell
Dome
50%−epiboly
Shield
75%−epiboly
1−4−somites
14−19−somites
20−25−somites
Prim−5
Prim−15
Prim−25
Long−pec
Protruding−mouth
Day−4
Day−5
Stage
Transcript
ENSDART00000052641
ENSDART00000148373
ENSDART00000148453
ENSDART00000148821
ENSDART00000148950
ENSDART00000166522

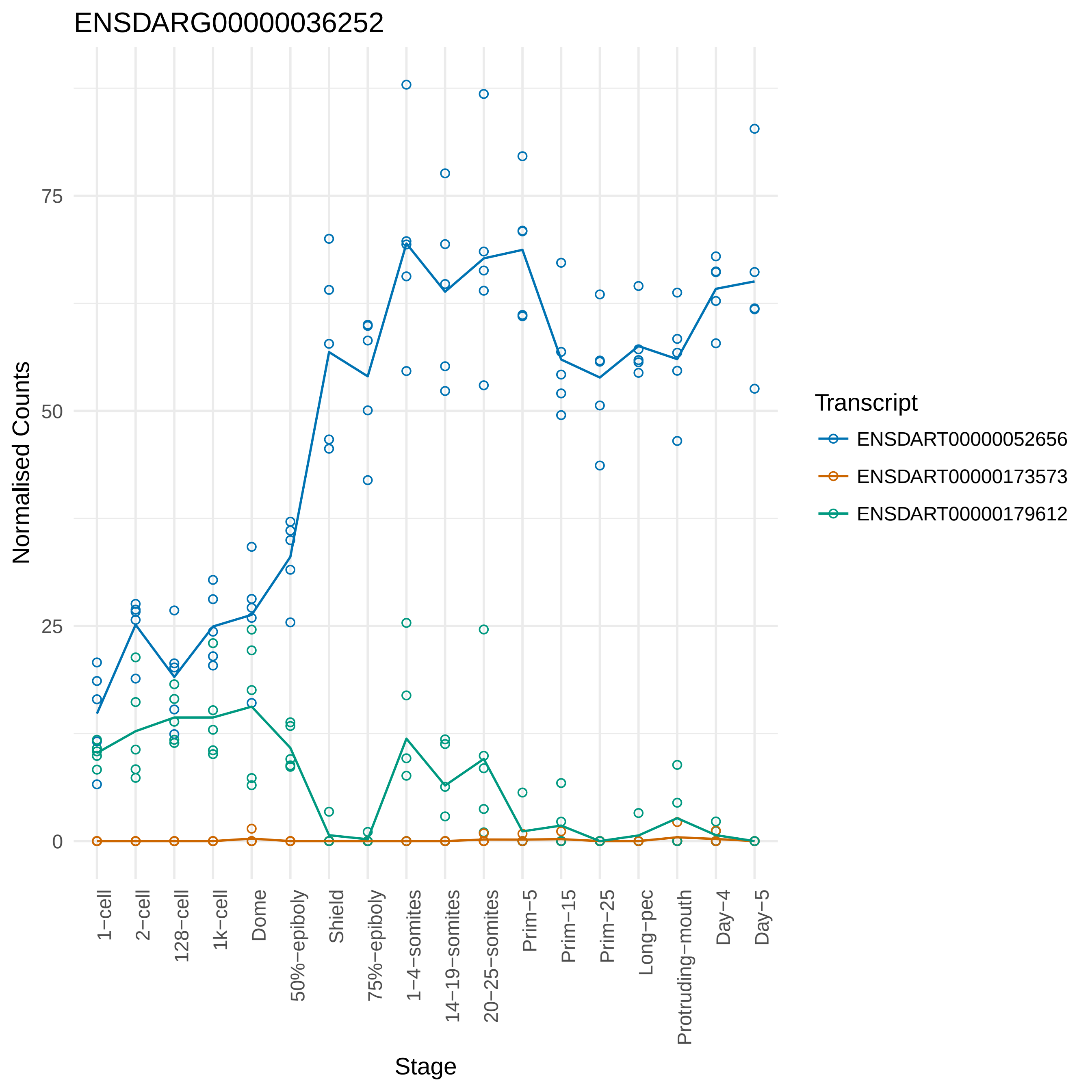
ENSDARG00000036252
Normalised Counts
75
50
25
0
1−cell
2−cell
128−cell
1k−cell
Dome
50%−epiboly
Shield
75%−epiboly
1−4−somites
14−19−somites
20−25−somites
Prim−5
Prim−15
Prim−25
Long−pec
Protruding−mouth
Day−4
Day−5
Stage
Transcript
ENSDART00000052656
ENSDART00000173573
ENSDART00000179612

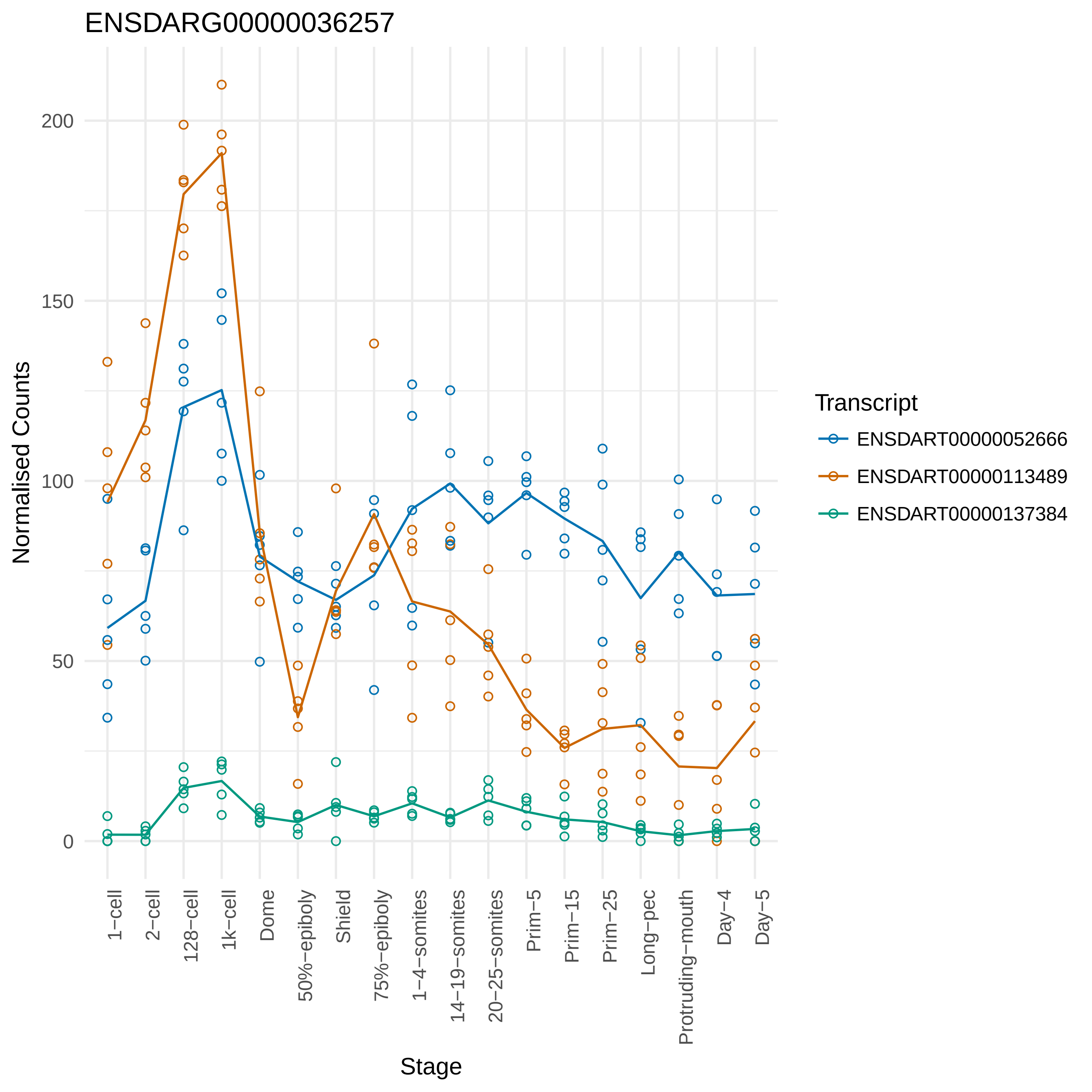

ENSDARG00000036257
Normalised Counts
0
50
100
150
200
1−cell
2−cell
128−cell
1k−cell
Dome
50%−epiboly
Shield
75%−epiboly
1−4−somites
14−19−somites
20−25−somites
Prim−5
Prim−15
Prim−25
Long−pec
Protruding−mouth
Day−4
Day−5
Stage
Transcript
ENSDART00000052666
ENSDART00000113489
ENSDART00000137384

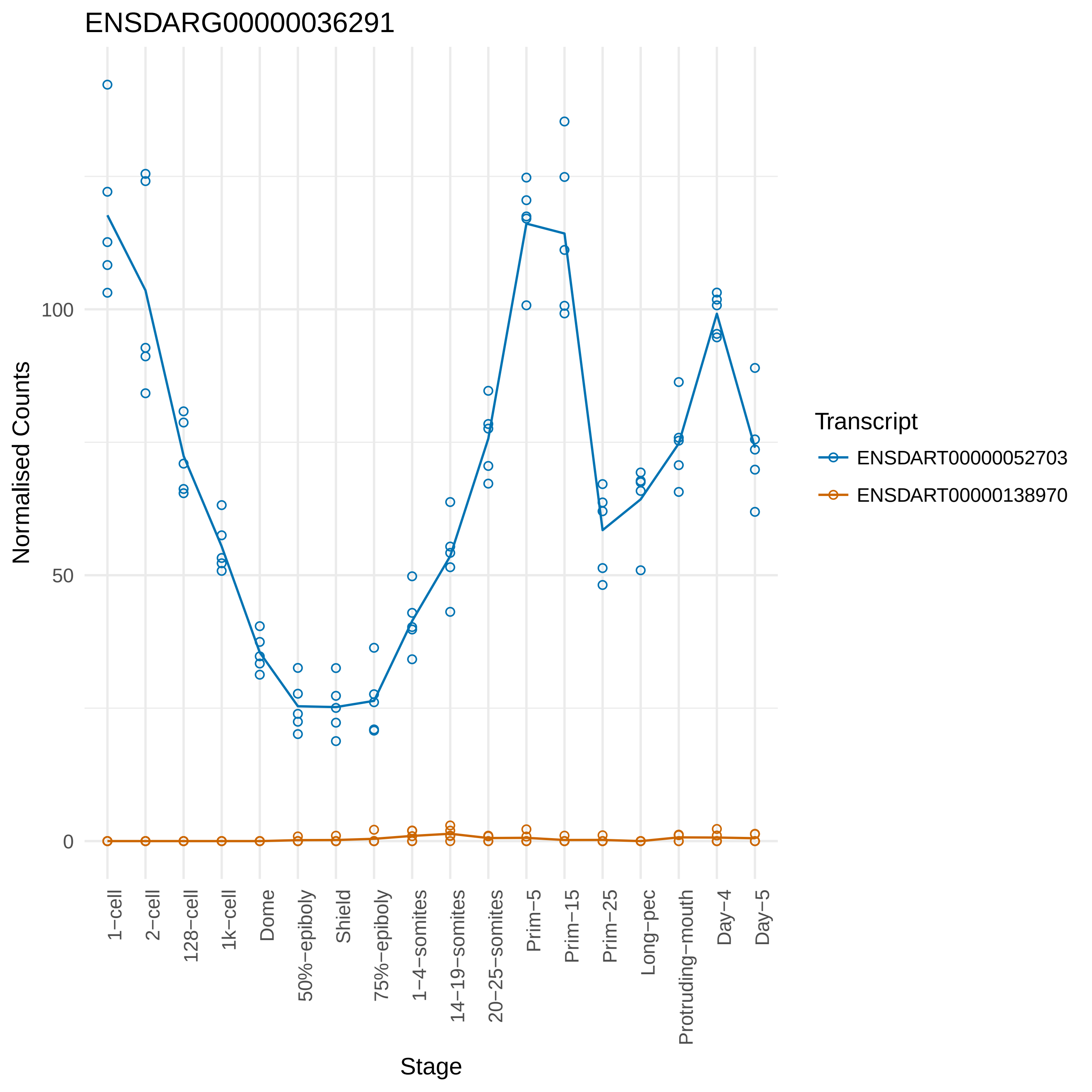

ENSDARG00000036291
Normalised Counts
100
50
0
1−cell
2−cell
128−cell
1k−cell
Dome
50%−epiboly
Shield
75%−epiboly
1−4−somites
14−19−somites
20−25−somites
Prim−5
Prim−15
Prim−25
Long−pec
Protruding−mouth
Day−4
Day−5
Stage
Transcript
ENSDART00000052703
ENSDART00000138970

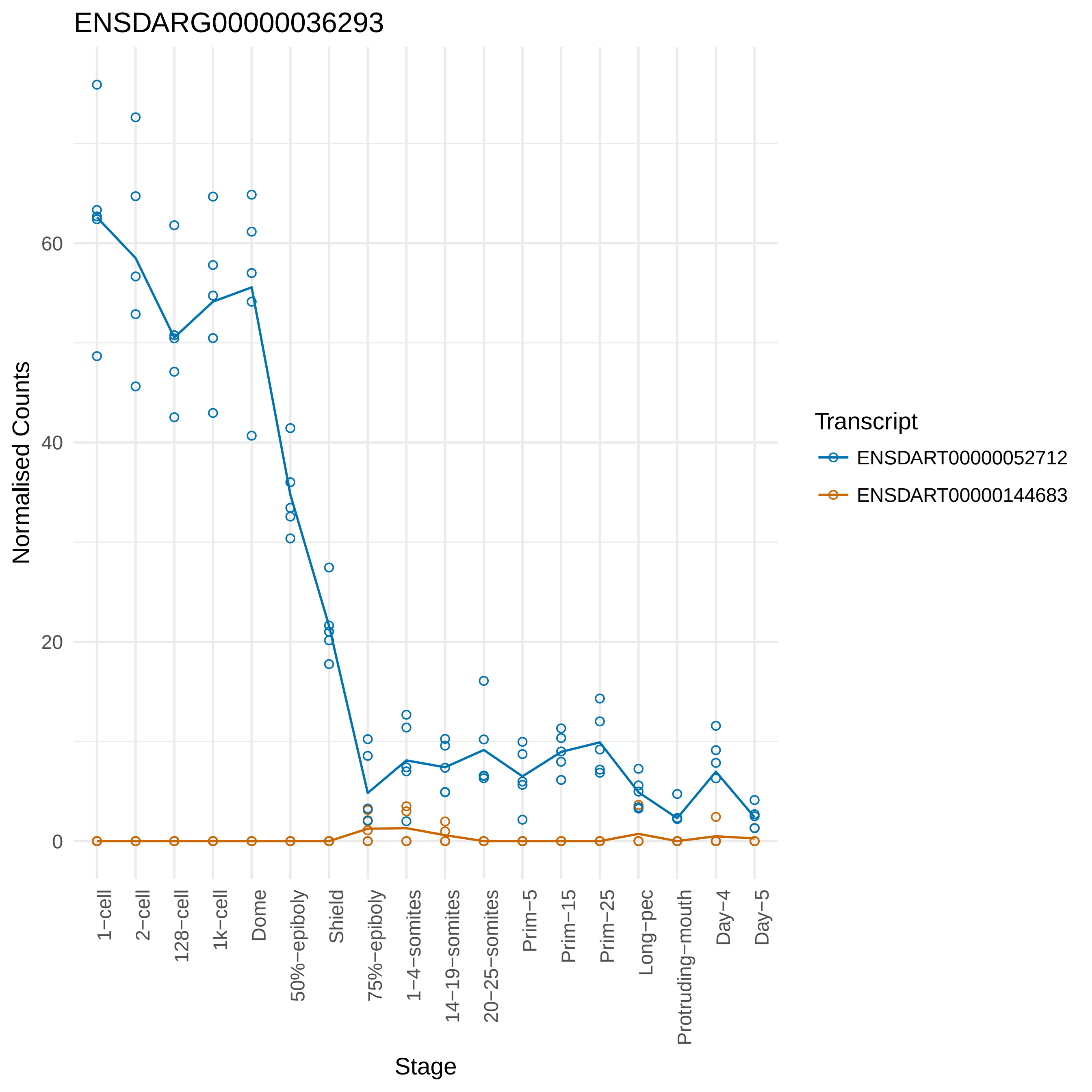
ENSDARG00000036293
Normalised Counts
0
20
40
60
Stage
1−cell
2−cell
128−cell
1k−cell
Dome
50%−epiboly
Shield
75%−epiboly
1−4−somites
14−19−somites
20−25−somites
Prim−5
Prim−15
Prim−25
Long−pec
Protruding−mouth
Day−4
Day−5
Transcript
ENSDART00000052712
ENSDART00000144683

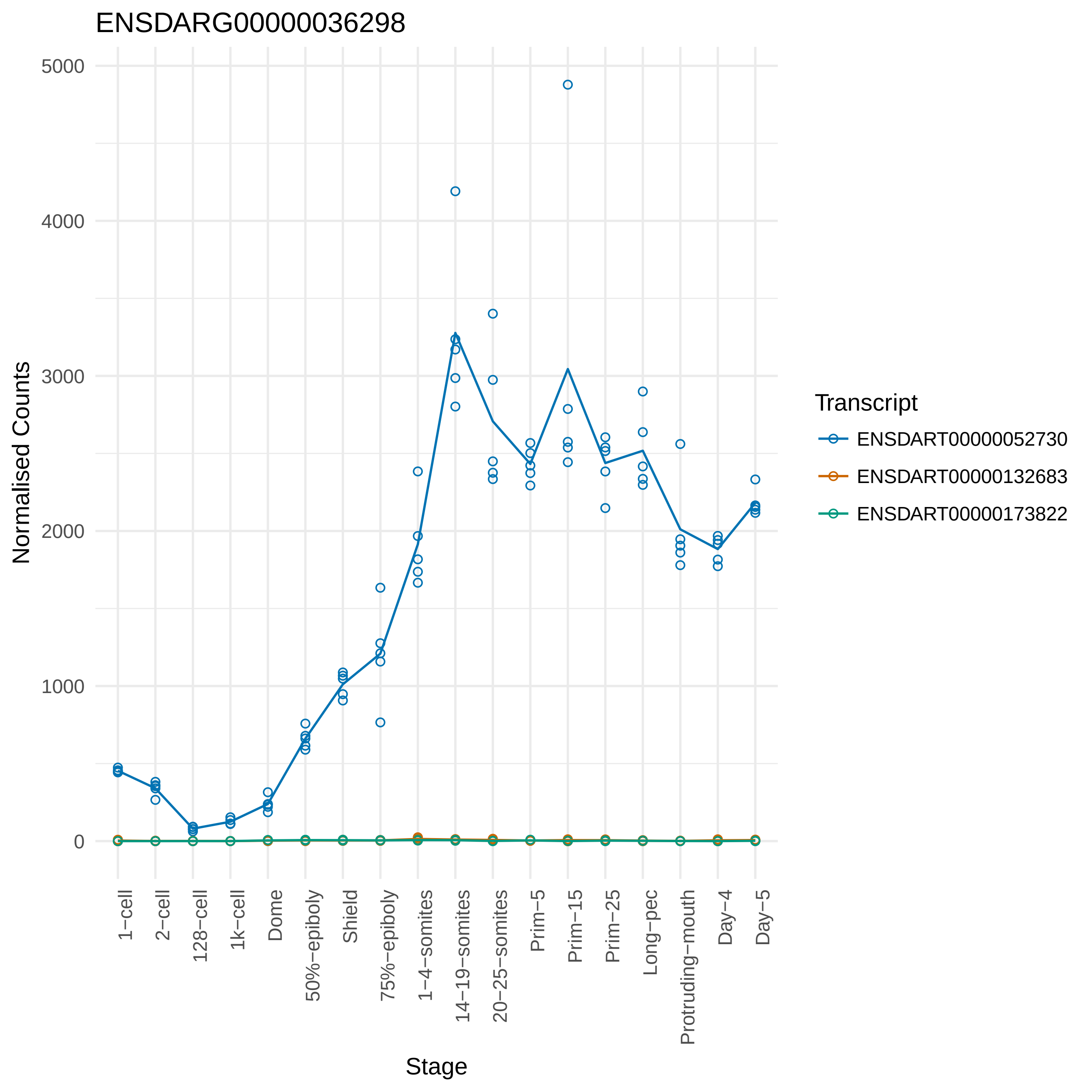

ENSDARG00000036298
Normalised Counts
5000
4000
3000
2000
1000
0
1−cell
2−cell
128−cell
1k−cell
Dome
50%−epiboly
Shield
75%−epiboly
1−4−somites
14−19−somites
20−25−somites
Prim−5
Prim−15
Prim−25
Long−pec
Protruding−mouth
Day−4
Day−5
Stage
Transcript
ENSDART00000052730
ENSDART00000132683
ENSDART00000173822

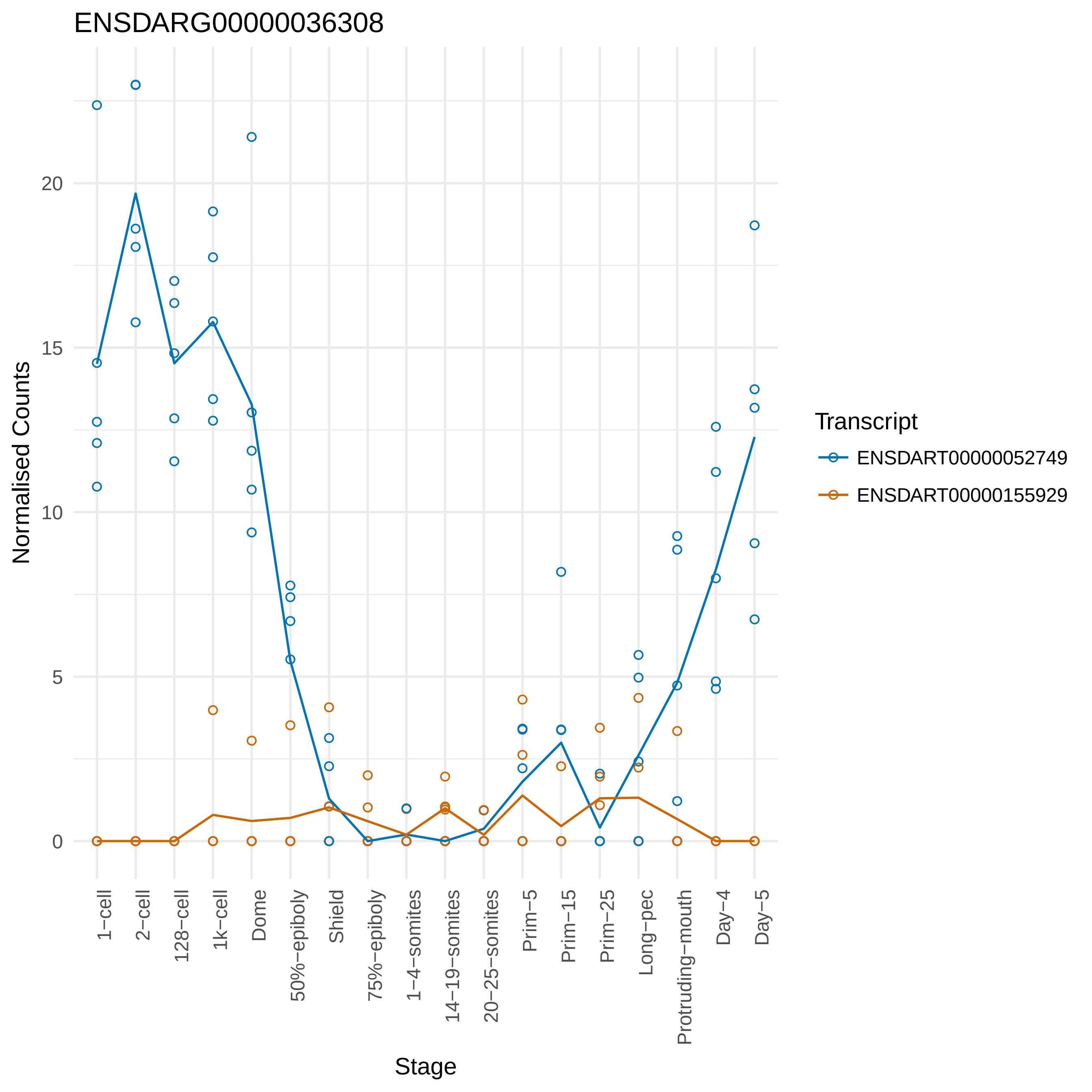

ENSDARG00000036308
Normalised Counts
0
5
10
15
20
Stage
1−cell
2−cell
128−cell
1k−cell
Dome
50%−epiboly
Shield
75%−epiboly
1−4−somites
14−19−somites
20−25−somites
Prim−5
Prim−15
Prim−25
Long−pec
Protruding−mouth
Day−4
Day−5
Transcript
ENSDART00000052749
ENSDART00000155929

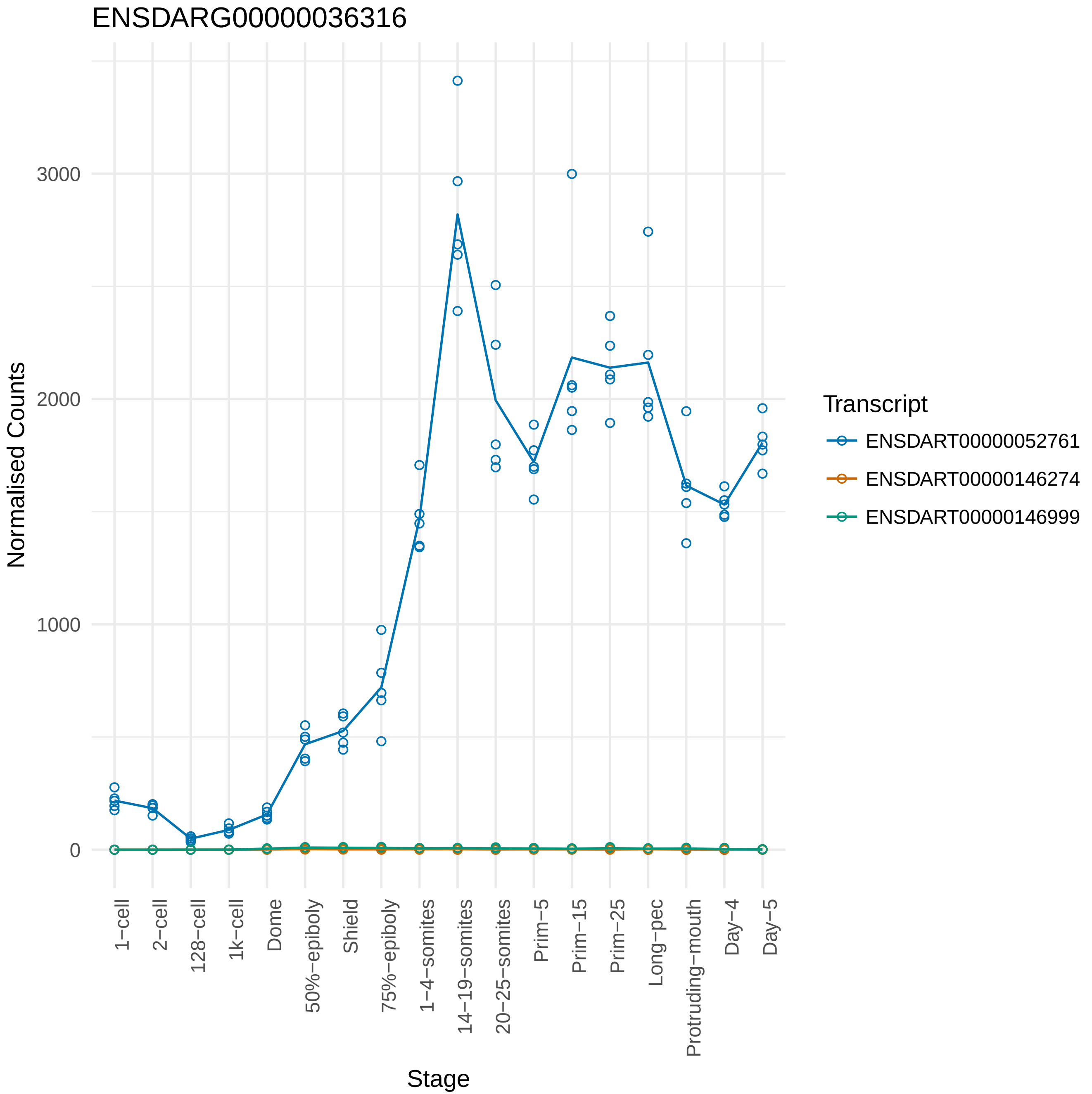
ENSDARG00000036316
Normalised Counts
3000
2000
1000
0
Stage
1−cell
2−cell
128−cell
1k−cell
Dome
50%−epiboly
Shield
75%−epiboly
1−4−somites
14−19−somites
20−25−somites
Prim−5
Prim−15
Prim−25
Long−pec
Protruding−mouth
Day−4
Day−5
Transcript
ENSDART00000052761
ENSDART00000146274
ENSDART00000146999

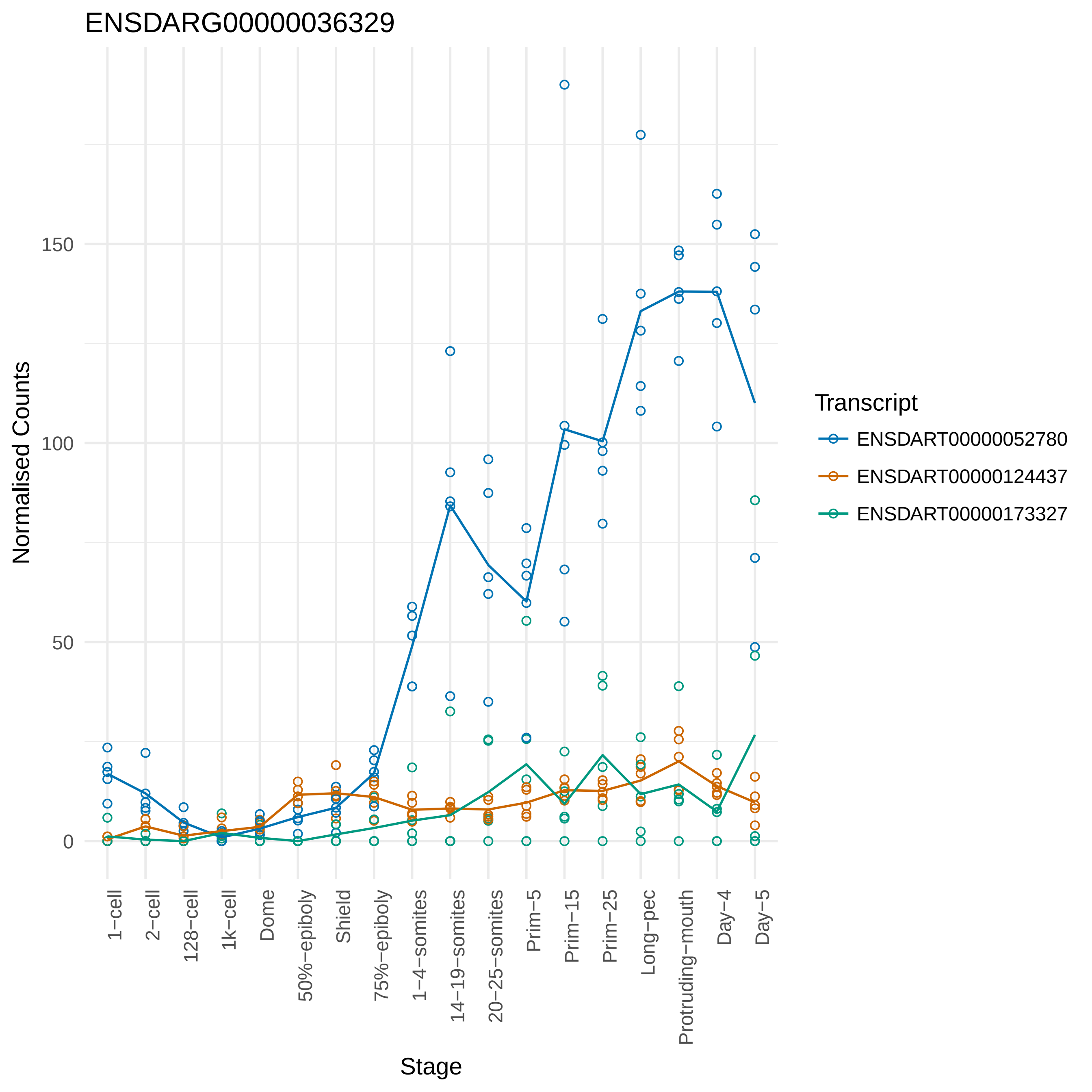

ENSDARG00000036329
Normalised Counts
150
100
50
0
1−cell
2−cell
128−cell
1k−cell
Dome
50%−epiboly
Shield
75%−epiboly
1−4−somites
14−19−somites
20−25−somites
Prim−5
Prim−15
Prim−25
Long−pec
Protruding−mouth
Day−4
Day−5
Stage
Transcript
ENSDART00000052780
ENSDART00000124437
ENSDART00000173327

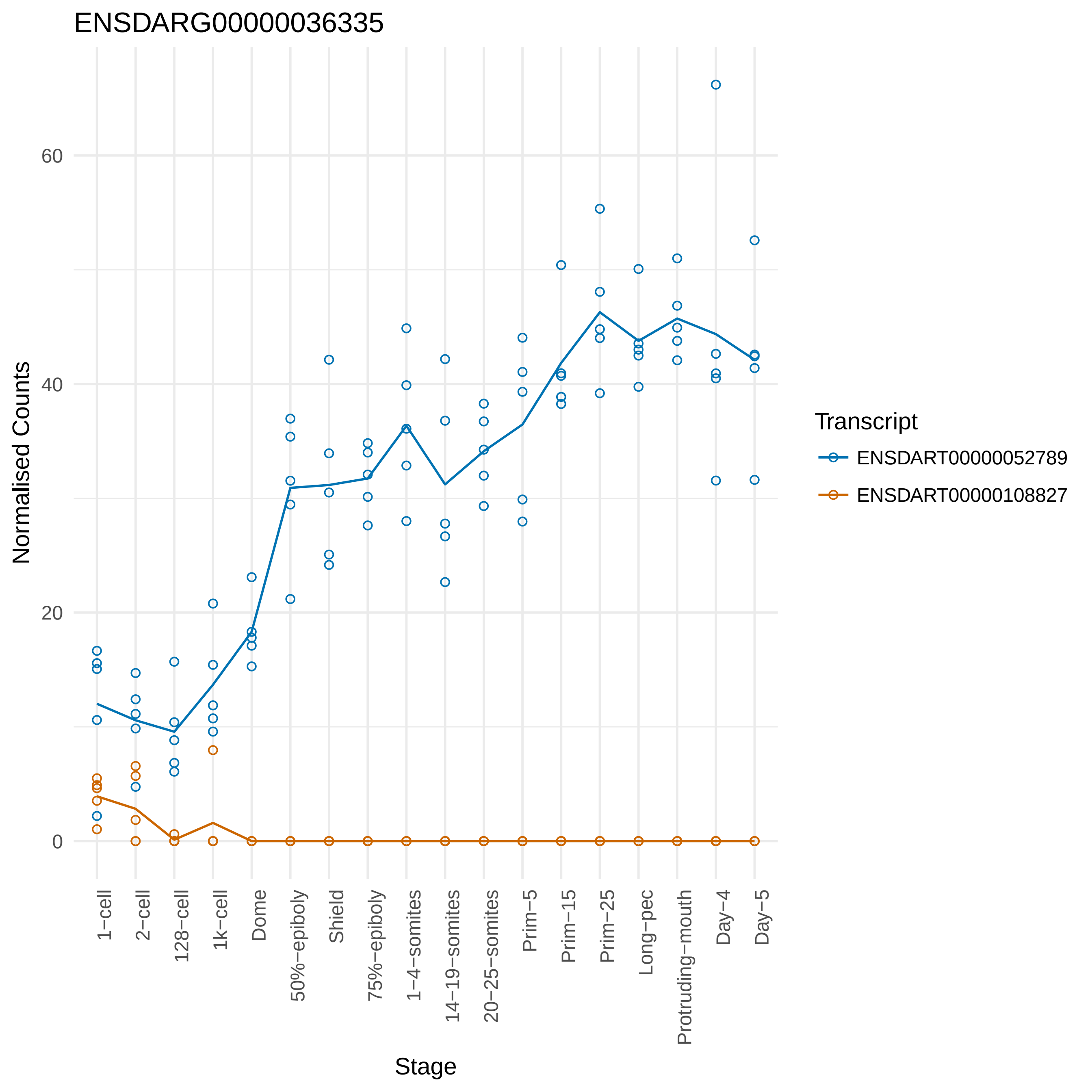
ENSDARG00000036335
Normalised Counts
0
20
40
60
1−cell
2−cell
128−cell
1k−cell
Dome
50%−epiboly
Shield
75%−epiboly
1−4−somites
14−19−somites
20−25−somites
Prim−5
Prim−15
Prim−25
Long−pec
Protruding−mouth
Day−4
Day−5
Stage
Transcript
ENSDART00000052789
ENSDART00000108827

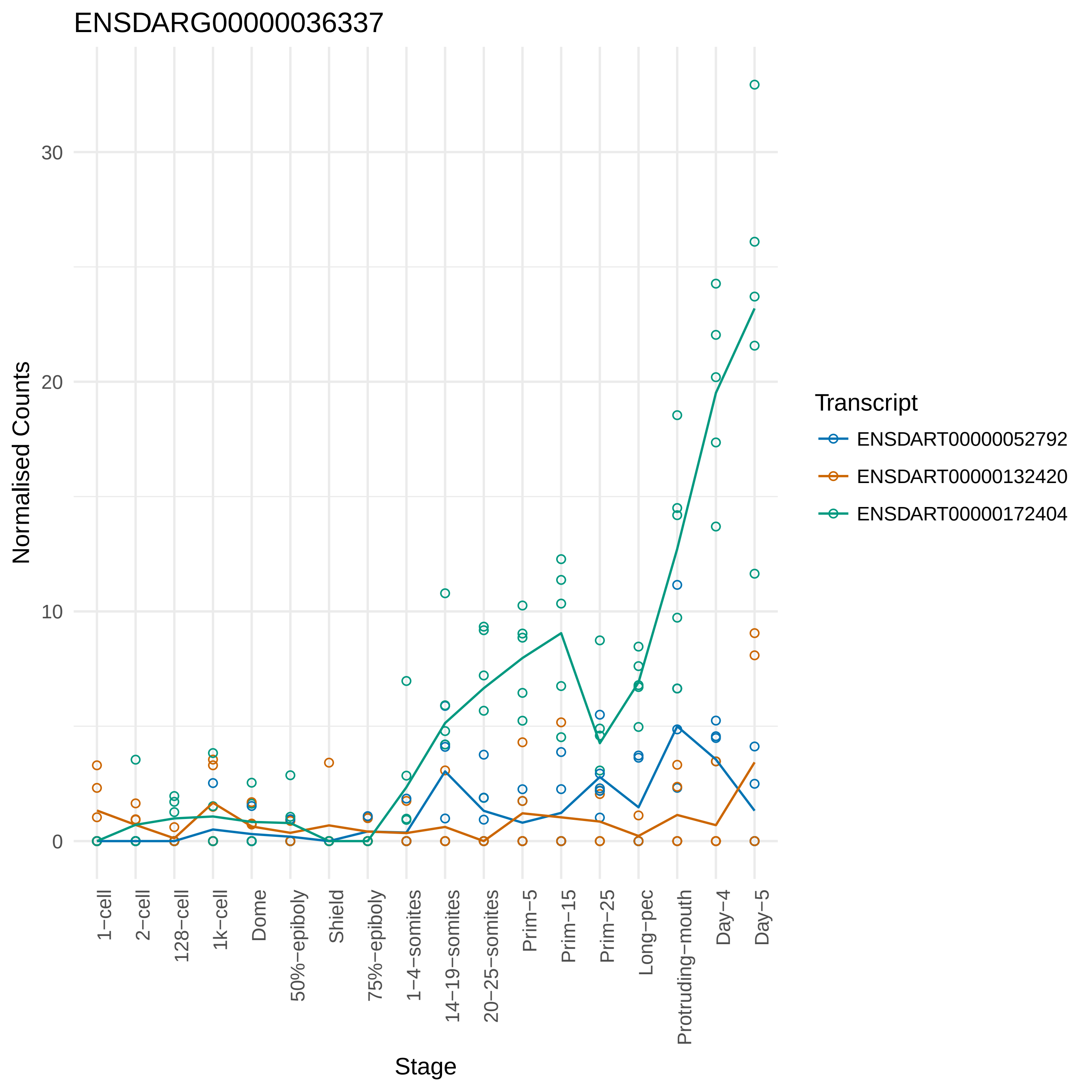

ENSDARG00000036337
Normalised Counts
30
20
10
0
1−cell
2−cell
128−cell
1k−cell
Dome
50%−epiboly
Shield
75%−epiboly
1−4−somites
14−19−somites
20−25−somites
Prim−5
Prim−15
Prim−25
Long−pec
Protruding−mouth
Day−4
Day−5
Stage
Transcript
ENSDART00000052792
ENSDART00000132420
ENSDART00000172404

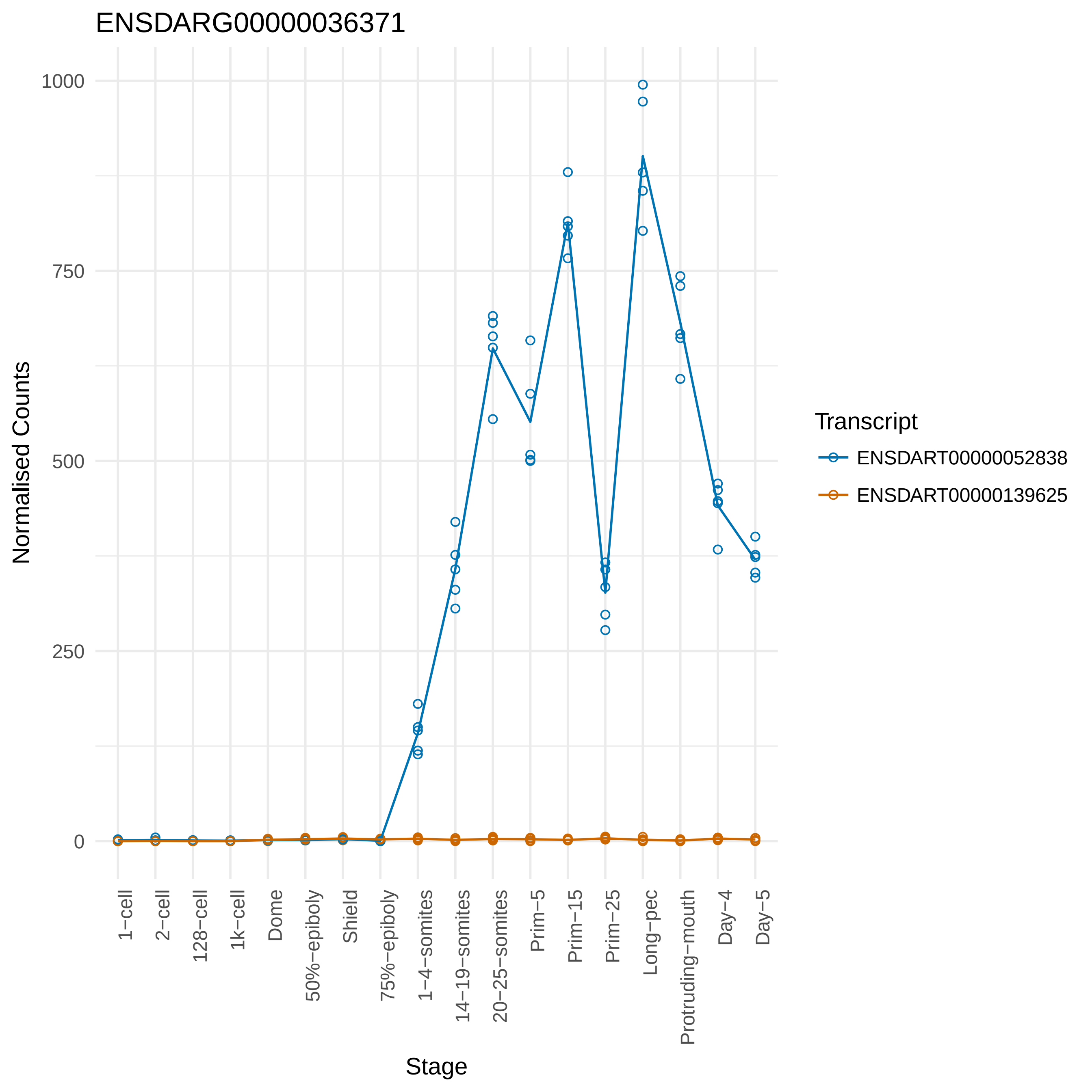

ENSDARG00000036371
Normalised Counts
1000
750
500
250
0
1−cell
2−cell
128−cell
1k−cell
Dome
50%−epiboly
Shield
75%−epiboly
1−4−somites
14−19−somites
20−25−somites
Prim−5
Prim−15
Prim−25
Long−pec
Protruding−mouth
Day−4
Day−5
Stage
Transcript
ENSDART00000052838
ENSDART00000139625

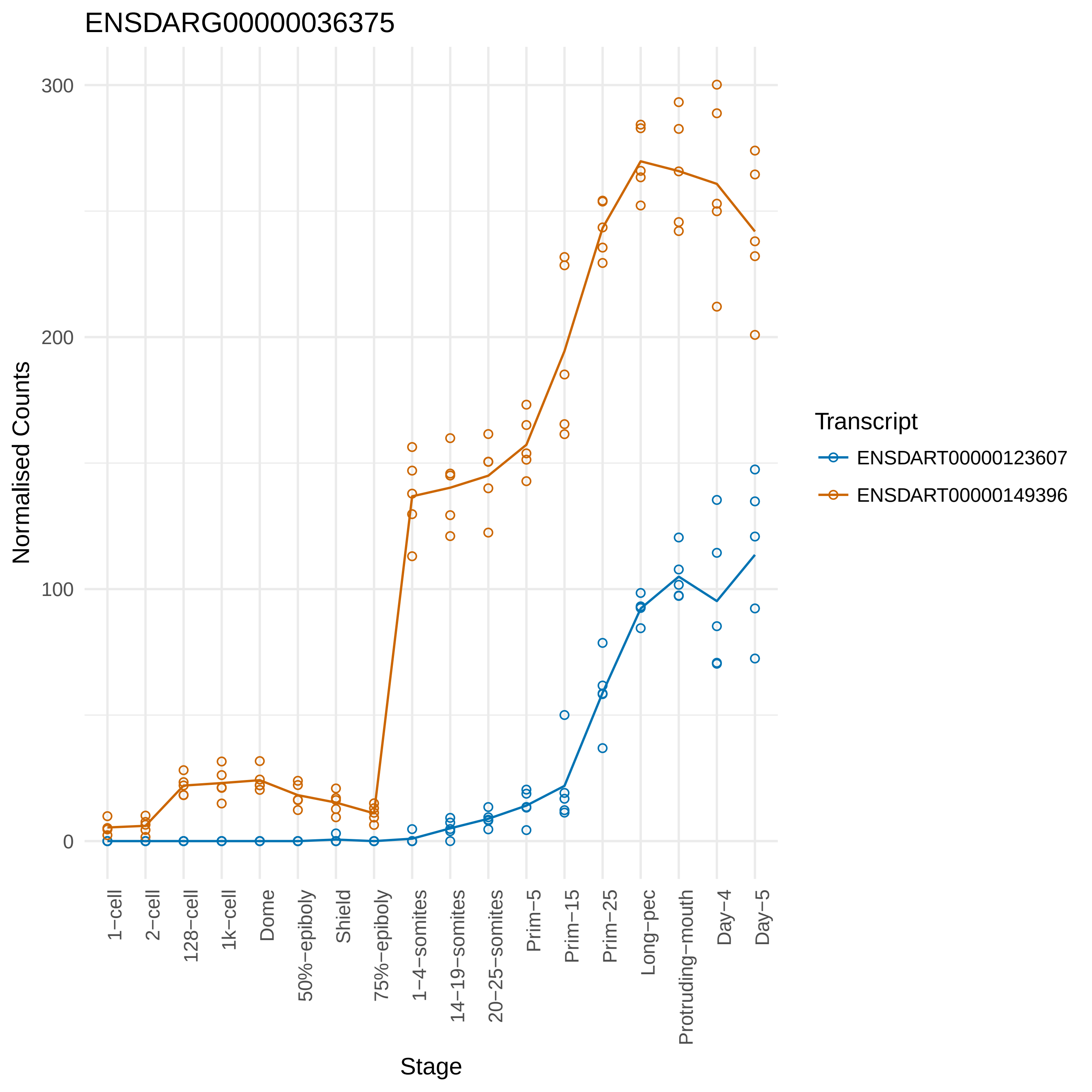
ENSDARG00000036375
Normalised Counts
300
200
100
0
1−cell
2−cell
128−cell
1k−cell
Dome
50%−epiboly
Shield
75%−epiboly
1−4−somites
14−19−somites
20−25−somites
Prim−5
Prim−15
Prim−25
Long−pec
Protruding−mouth
Day−4
Day−5
Stage
Transcript
ENSDART00000123607
ENSDART00000149396

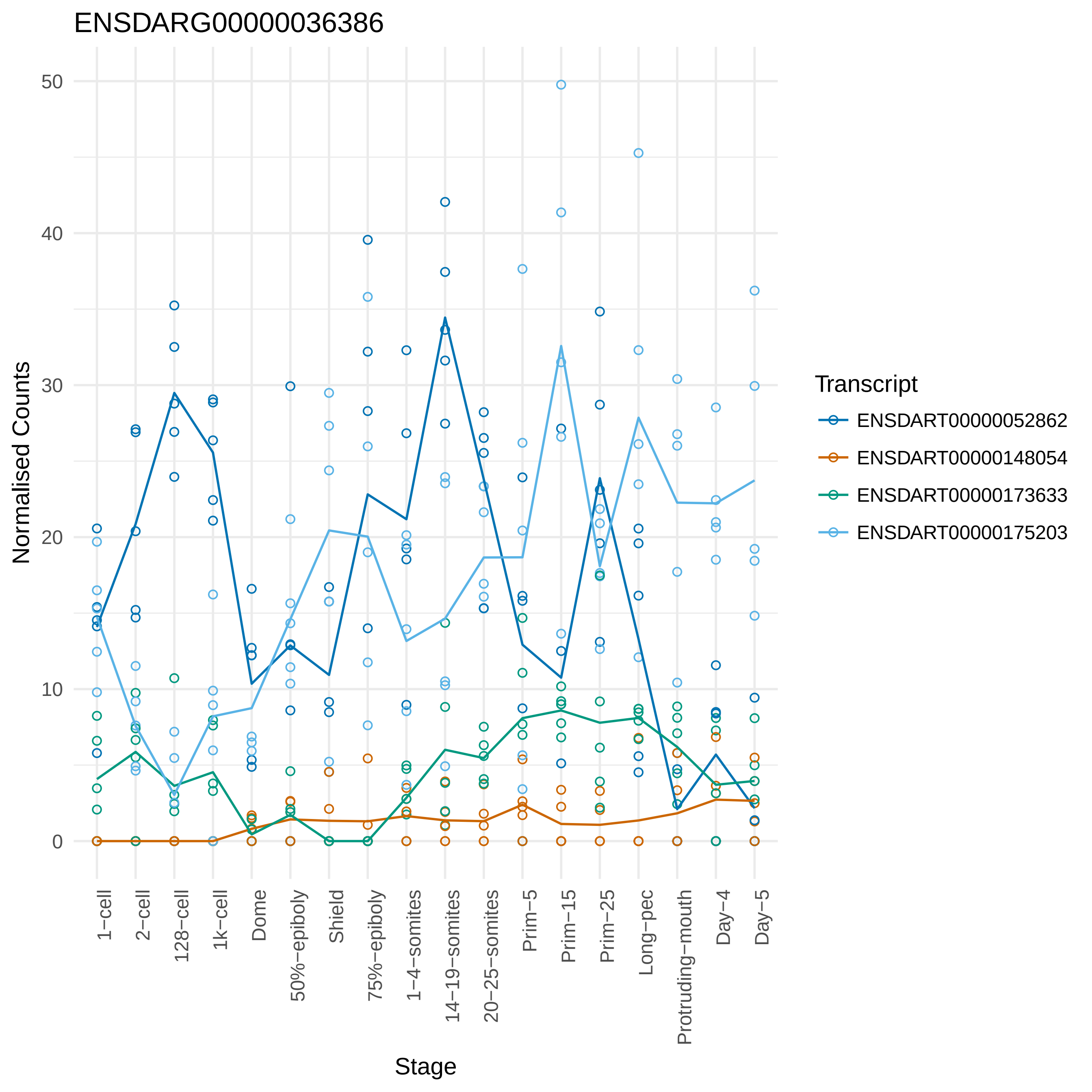
ENSDARG00000036386
Normalised Counts
0
10
20
30
40
50
1−cell
2−cell
128−cell
1k−cell
Dome
50%−epiboly
Shield
75%−epiboly
1−4−somites
14−19−somites
20−25−somites
Prim−5
Prim−15
Prim−25
Long−pec
Protruding−mouth
Day−4
Day−5
Stage
Transcript
ENSDART00000052862
ENSDART00000148054
ENSDART00000173633
ENSDART00000175203

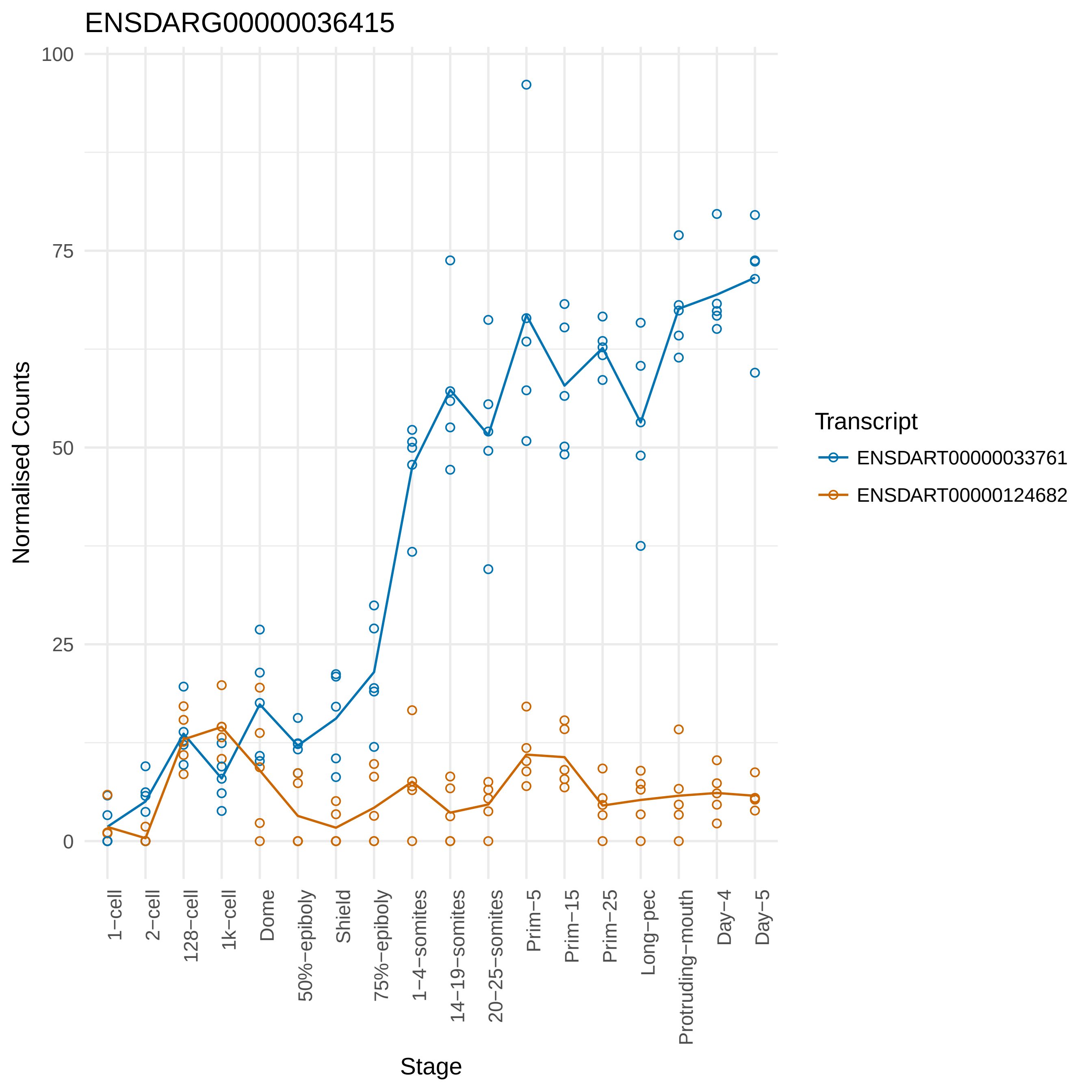
ENSDARG00000036415
Normalised Counts
100
75
50
25
0
1−cell
2−cell
128−cell
1k−cell
Dome
50%−epiboly
Shield
75%−epiboly
1−4−somites
14−19−somites
20−25−somites
Prim−5
Prim−15
Prim−25
Long−pec
Protruding−mouth
Day−4
Day−5
Stage
Transcript
ENSDART00000033761
ENSDART00000124682

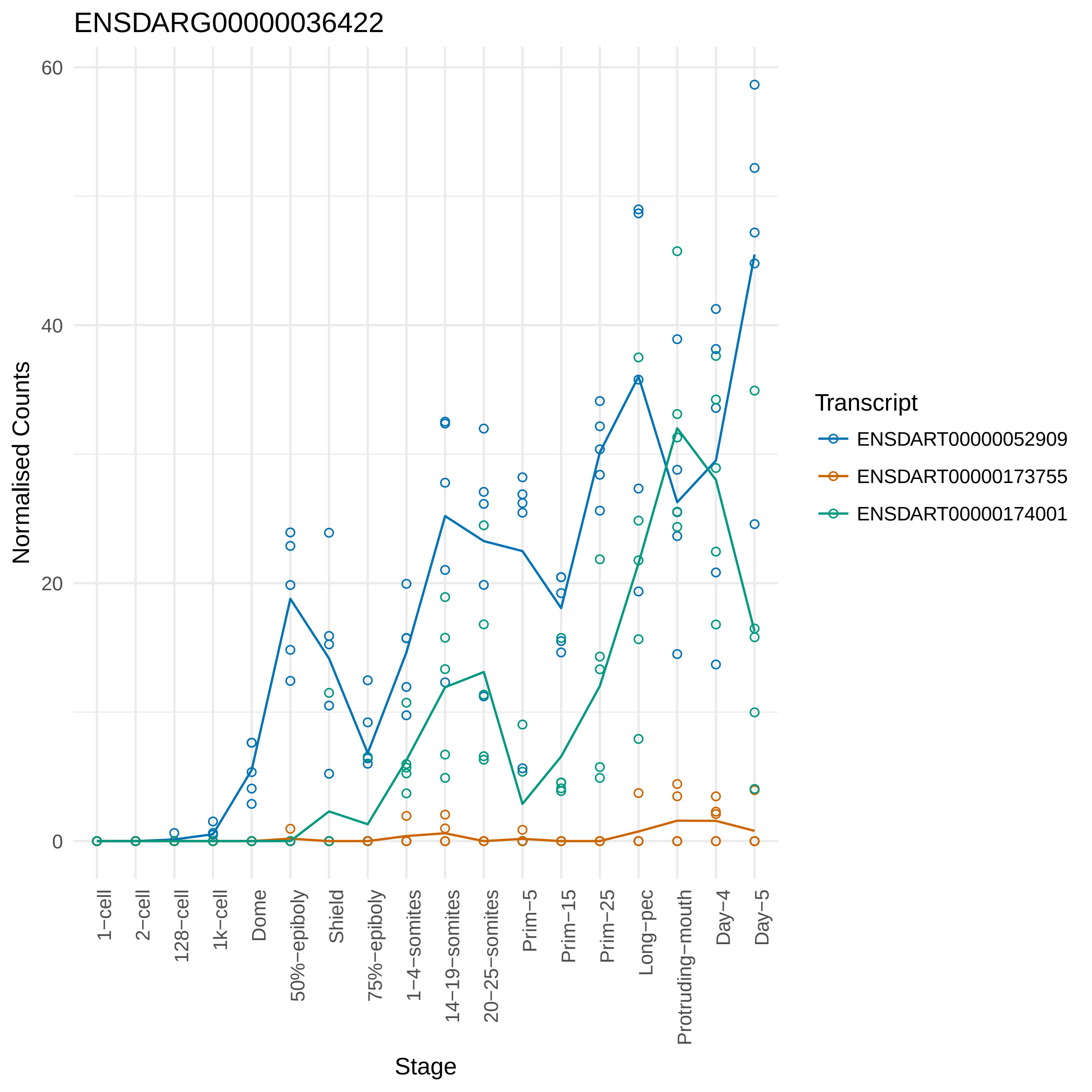

ENSDARG00000036422
Normalised Counts
0
20
40
60
Stage
1−cell
2−cell
128−cell
1k−cell
Dome
50%−epiboly
Shield
75%−epiboly
1−4−somites
14−19−somites
20−25−somites
Prim−5
Prim−15
Prim−25
Long−pec
Protruding−mouth
Day−4
Day−5
Transcript
ENSDART00000052909
ENSDART00000173755
ENSDART00000174001

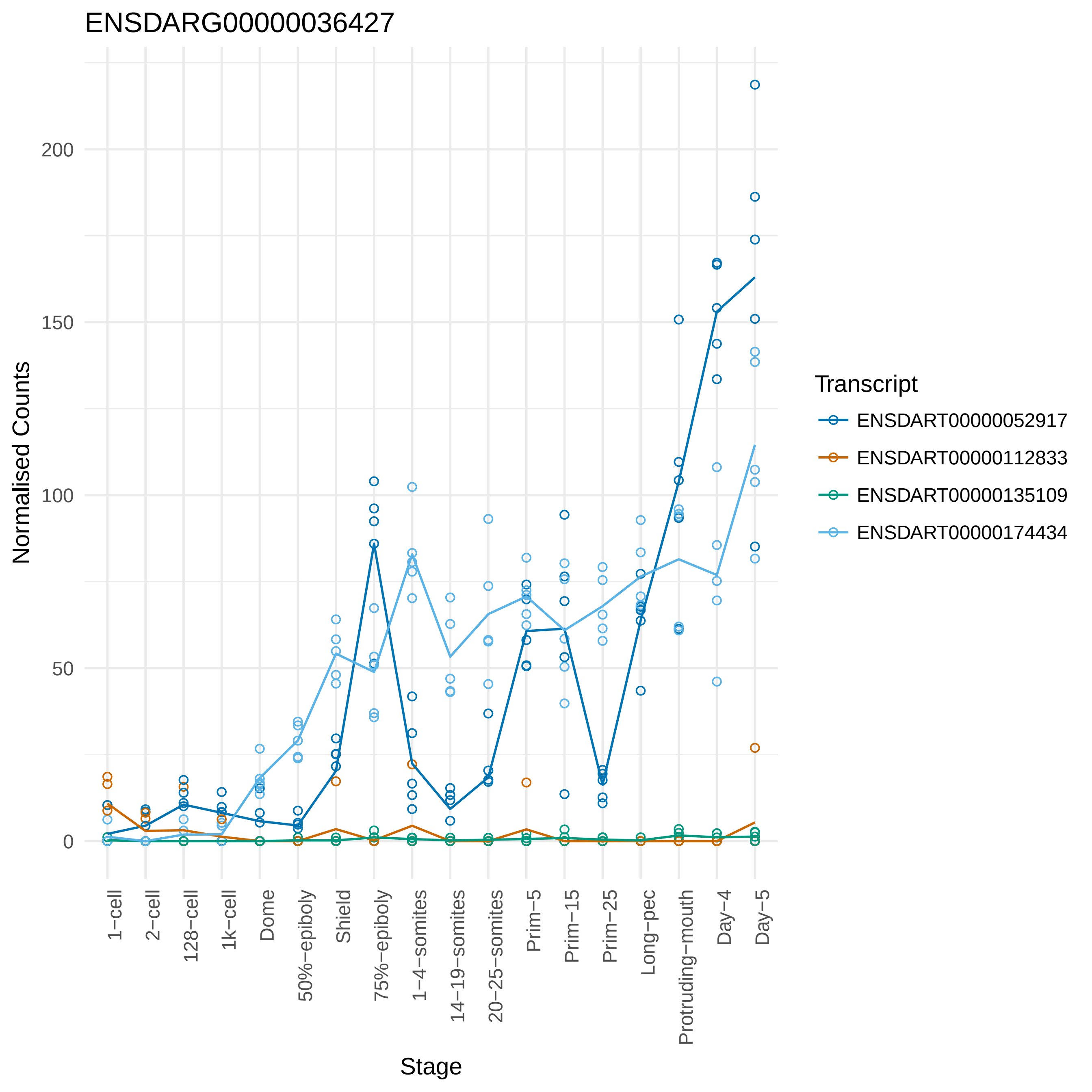

ENSDARG00000036427
Normalised Counts
0
50
100
150
200
Stage
1−cell
2−cell
128−cell
1k−cell
Dome
50%−epiboly
Shield
75%−epiboly
1−4−somites
14−19−somites
20−25−somites
Prim−5
Prim−15
Prim−25
Long−pec
Protruding−mouth
Day−4
Day−5
Transcript
ENSDART00000052917
ENSDART00000112833
ENSDART00000135109
ENSDART00000174434

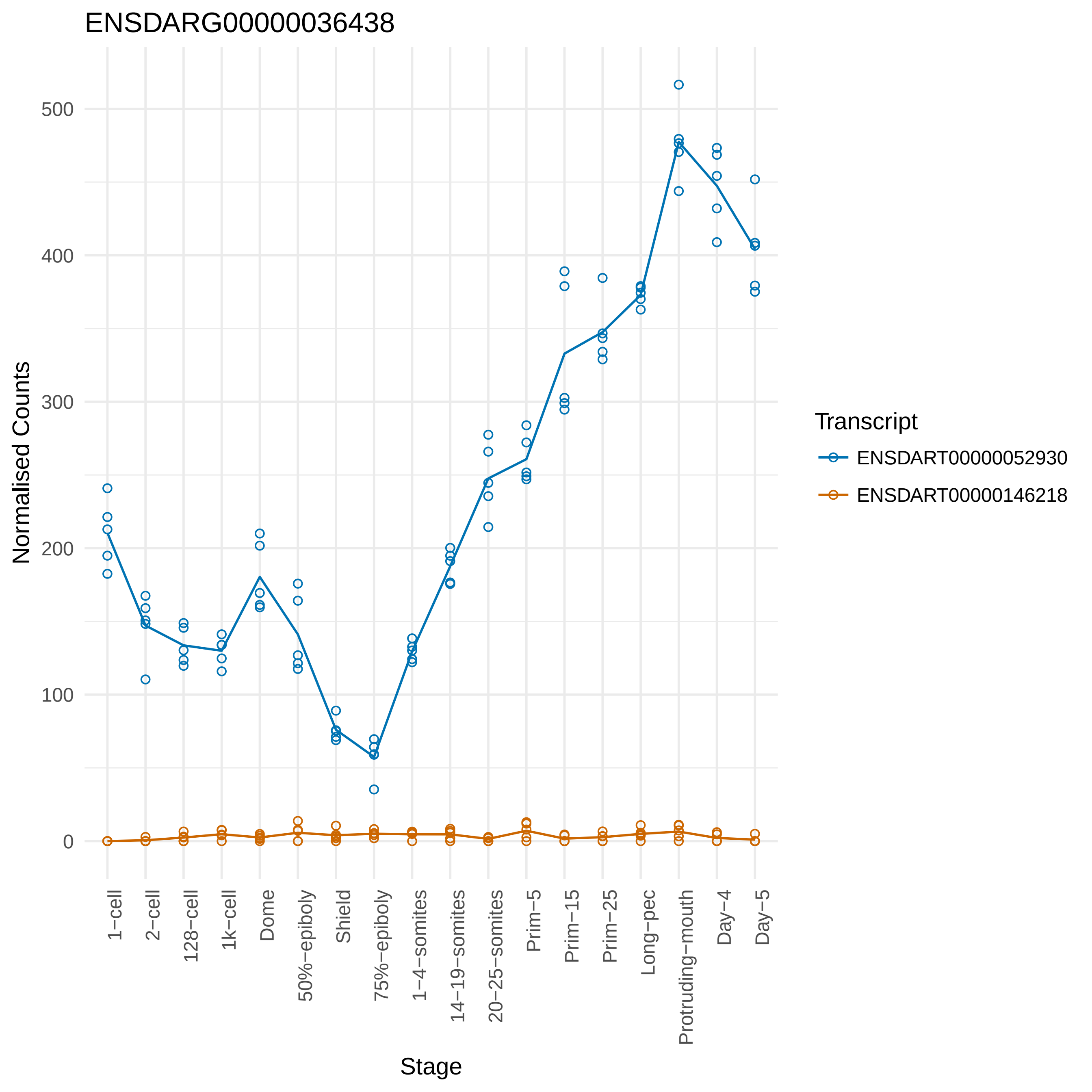

ENSDARG00000036438
Normalised Counts
0
100
200
300
400
500
1−cell
2−cell
128−cell
1k−cell
Dome
50%−epiboly
Shield
75%−epiboly
1−4−somites
14−19−somites
20−25−somites
Prim−5
Prim−15
Prim−25
Long−pec
Protruding−mouth
Day−4
Day−5
Stage
Transcript
ENSDART00000052930
ENSDART00000146218

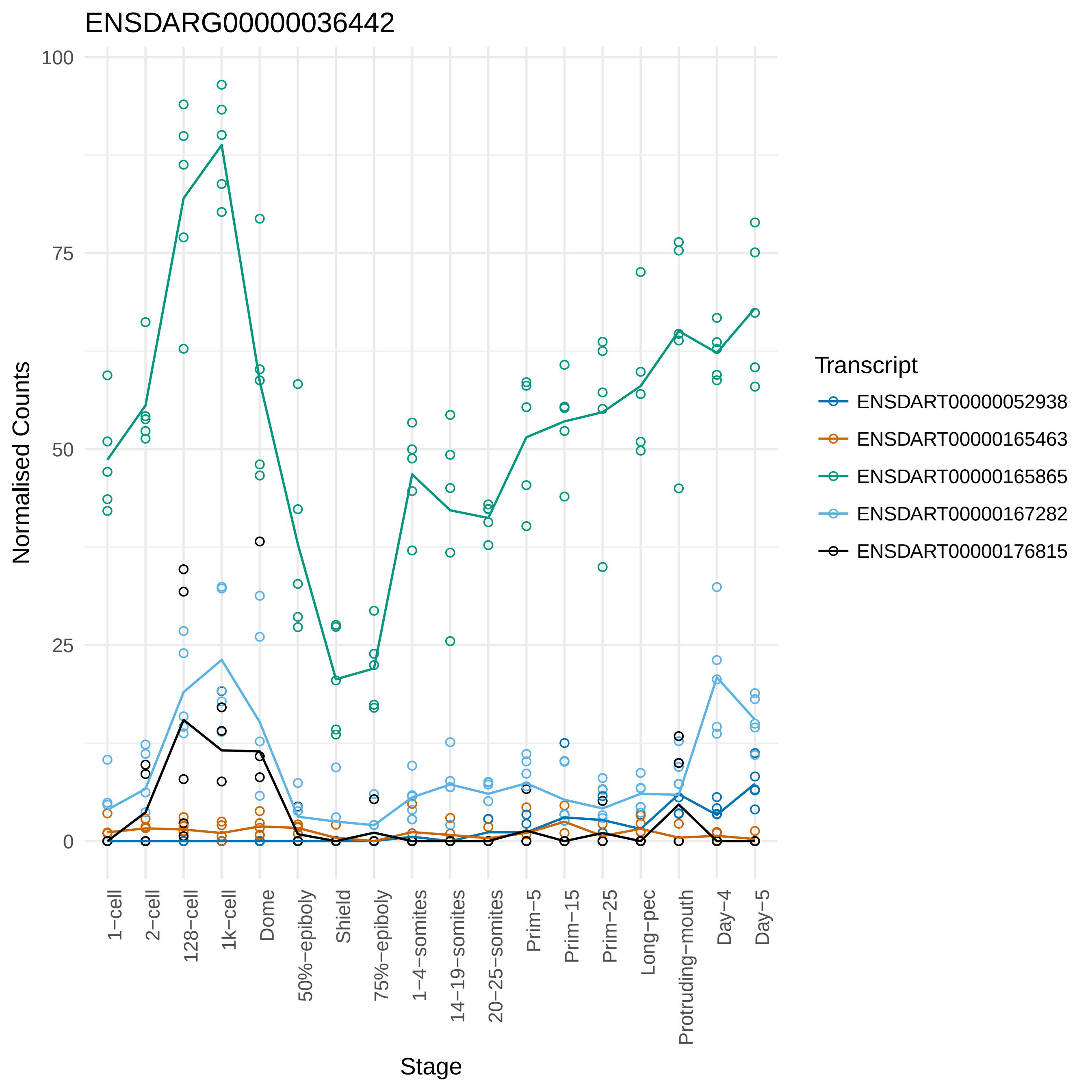

ENSDARG00000036442
Normalised Counts
100
75
50
25
0
1−cell
2−cell
128−cell
1k−cell
Dome
50%−epiboly
Shield
75%−epiboly
1−4−somites
14−19−somites
20−25−somites
Prim−5
Prim−15
Prim−25
Long−pec
Protruding−mouth
Day−4
Day−5
Stage
Transcript
ENSDART00000052938
ENSDART00000165463
ENSDART00000165865
ENSDART00000167282
ENSDART00000176815

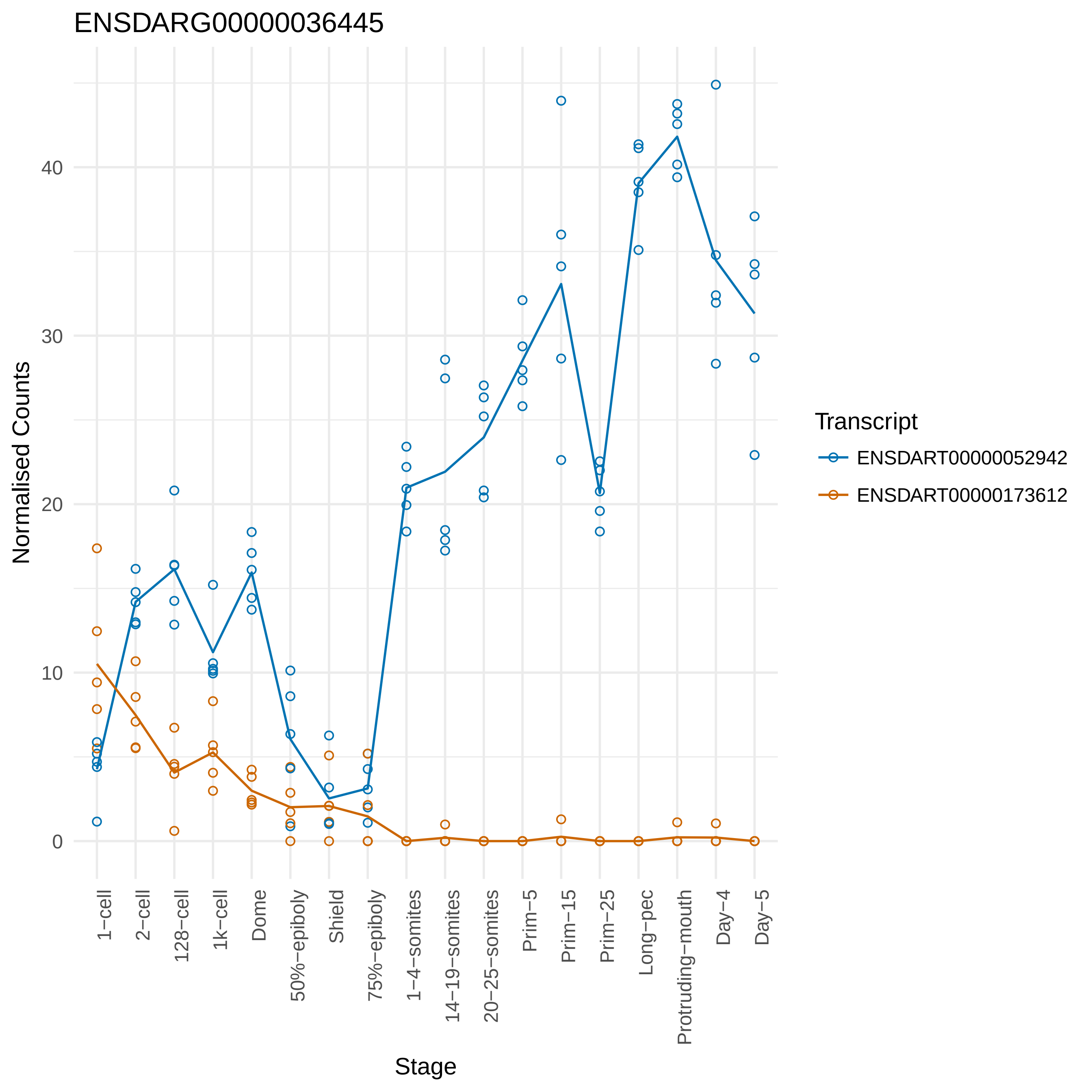
ENSDARG00000036445
Normalised Counts
0
10
20
30
40
Stage
1−cell
2−cell
128−cell
1k−cell
Dome
50%−epiboly
Shield
75%−epiboly
1−4−somites
14−19−somites
20−25−somites
Prim−5
Prim−15
Prim−25
Long−pec
Protruding−mouth
Day−4
Day−5
Transcript
ENSDART00000052942
ENSDART00000173612

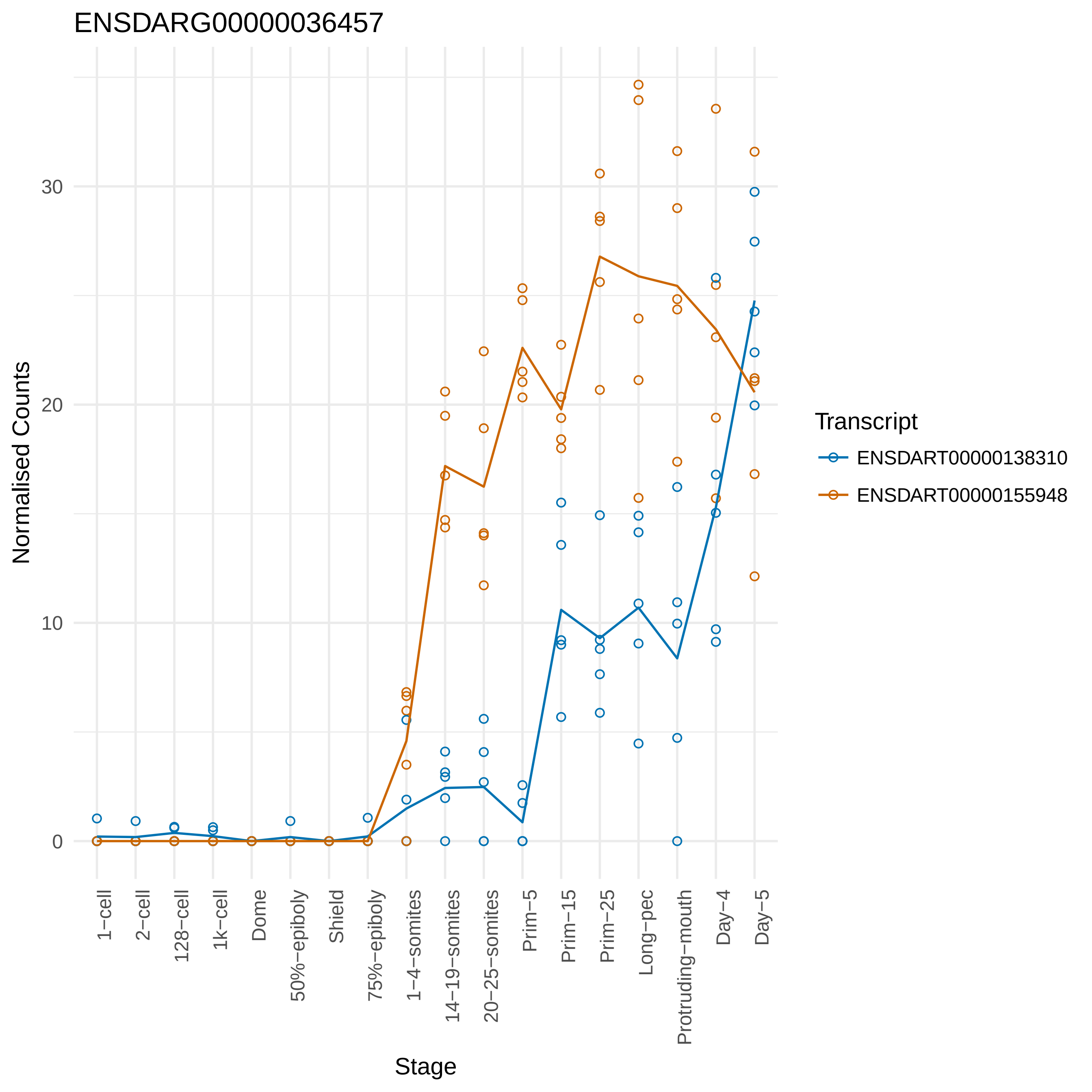

ENSDARG00000036457
Normalised Counts
0
10
20
30
Stage
1−cell
2−cell
128−cell
1k−cell
Dome
50%−epiboly
Shield
75%−epiboly
1−4−somites
14−19−somites
20−25−somites
Prim−5
Prim−15
Prim−25
Long−pec
Protruding−mouth
Day−4
Day−5
Transcript
ENSDART00000138310
ENSDART00000155948

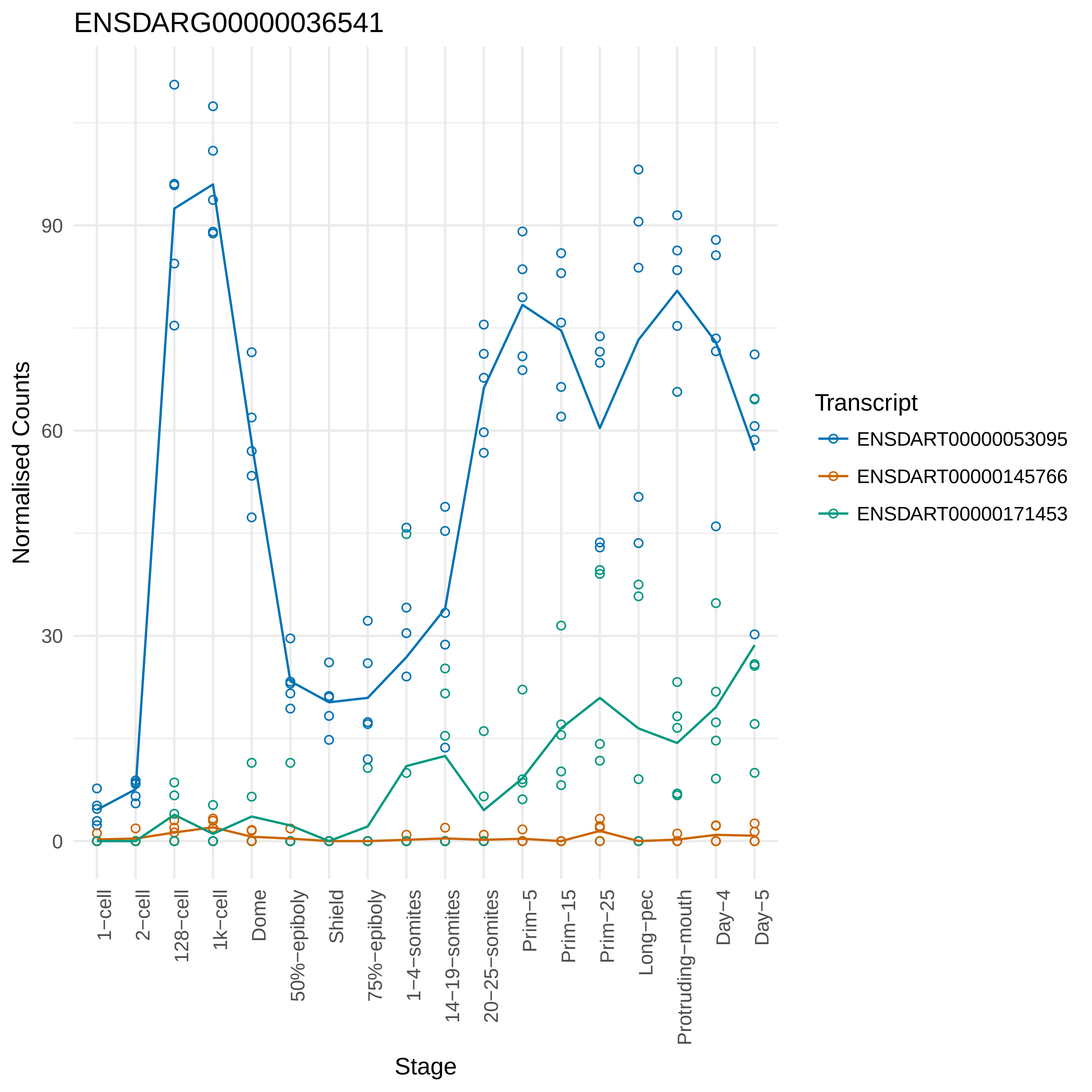
ENSDARG00000036541
Normalised Counts
90
60
30
0
1−cell
2−cell
128−cell
1k−cell
Dome
50%−epiboly
Shield
75%−epiboly
1−4−somites
14−19−somites
20−25−somites
Prim−5
Prim−15
Prim−25
Long−pec
Protruding−mouth
Day−4
Day−5
Stage
Transcript
ENSDART00000053095
ENSDART00000145766
ENSDART00000171453

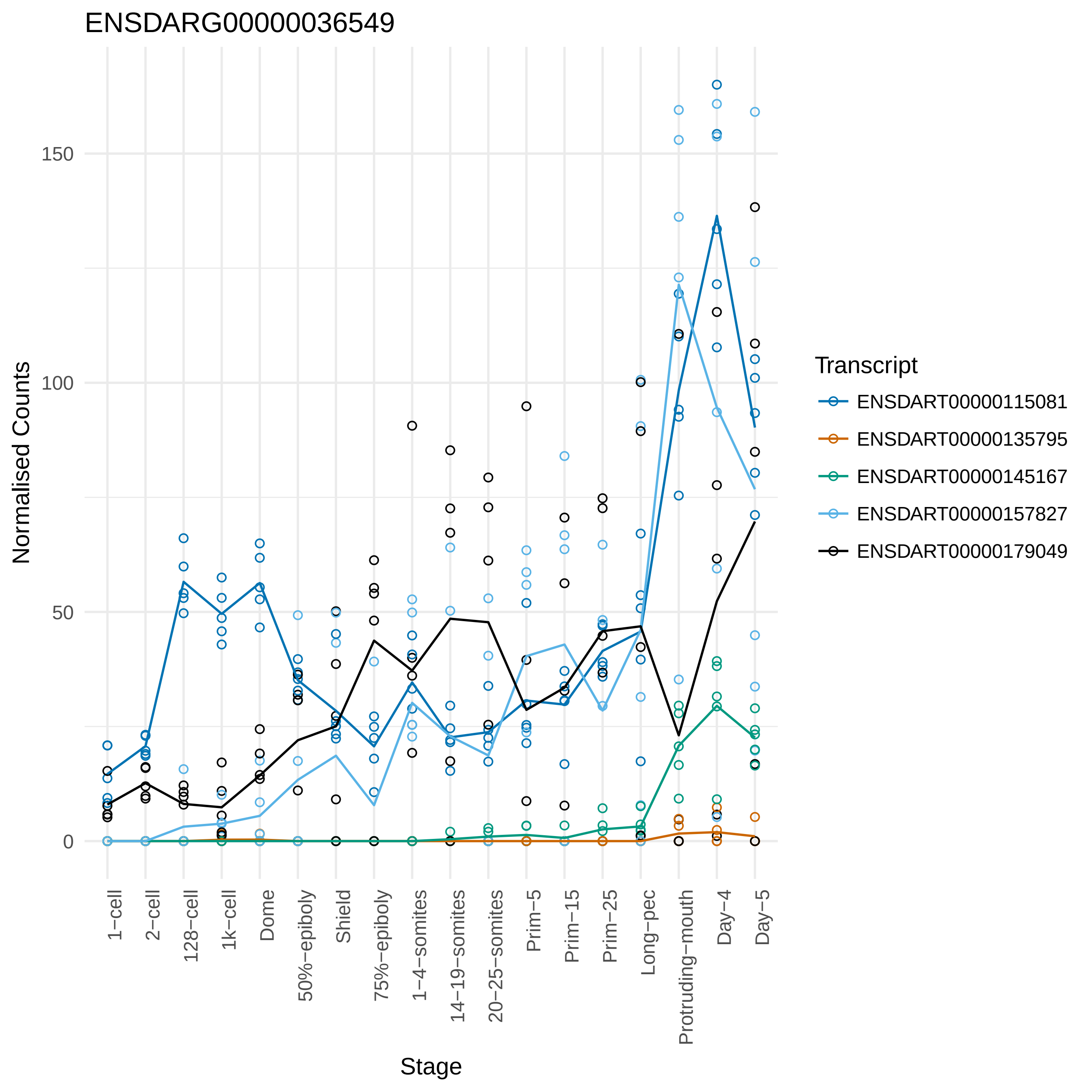

ENSDARG00000036549
Normalised Counts
150
100
50
0
1−cell
2−cell
128−cell
1k−cell
Dome
50%−epiboly
Shield
75%−epiboly
1−4−somites
14−19−somites
20−25−somites
Prim−5
Prim−15
Prim−25
Long−pec
Protruding−mouth
Day−4
Day−5
Stage
Transcript
ENSDART00000115081
ENSDART00000135795
ENSDART00000145167
ENSDART00000157827
ENSDART00000179049

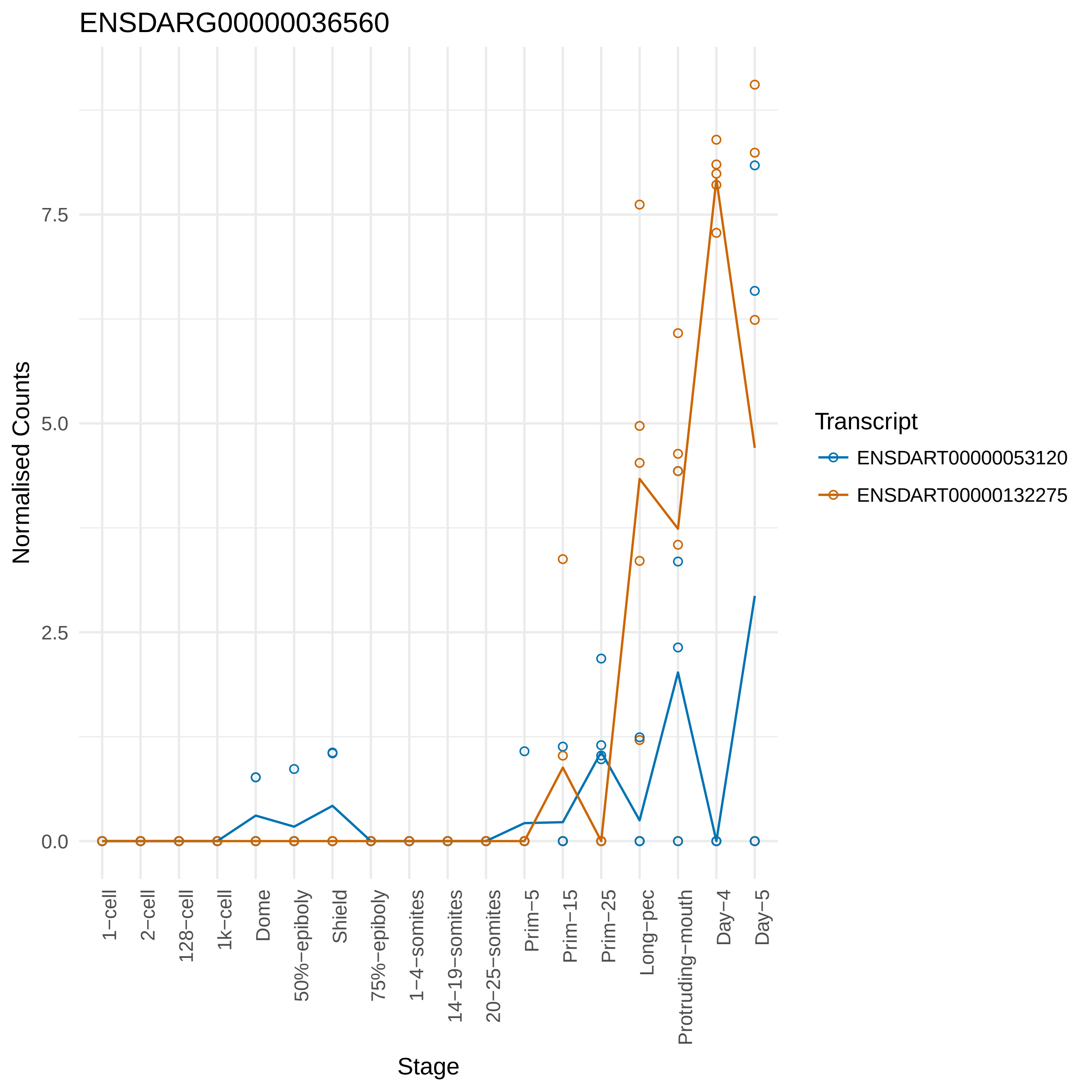

ENSDARG00000036560
Normalised Counts
7.5
5.0
2.5
0.0
1−cell
2−cell
128−cell
1k−cell
Dome
50%−epiboly
Shield
75%−epiboly
1−4−somites
14−19−somites
20−25−somites
Prim−5
Prim−15
Prim−25
Long−pec
Protruding−mouth
Day−4
Day−5
Stage
Transcript
ENSDART00000053120
ENSDART00000132275

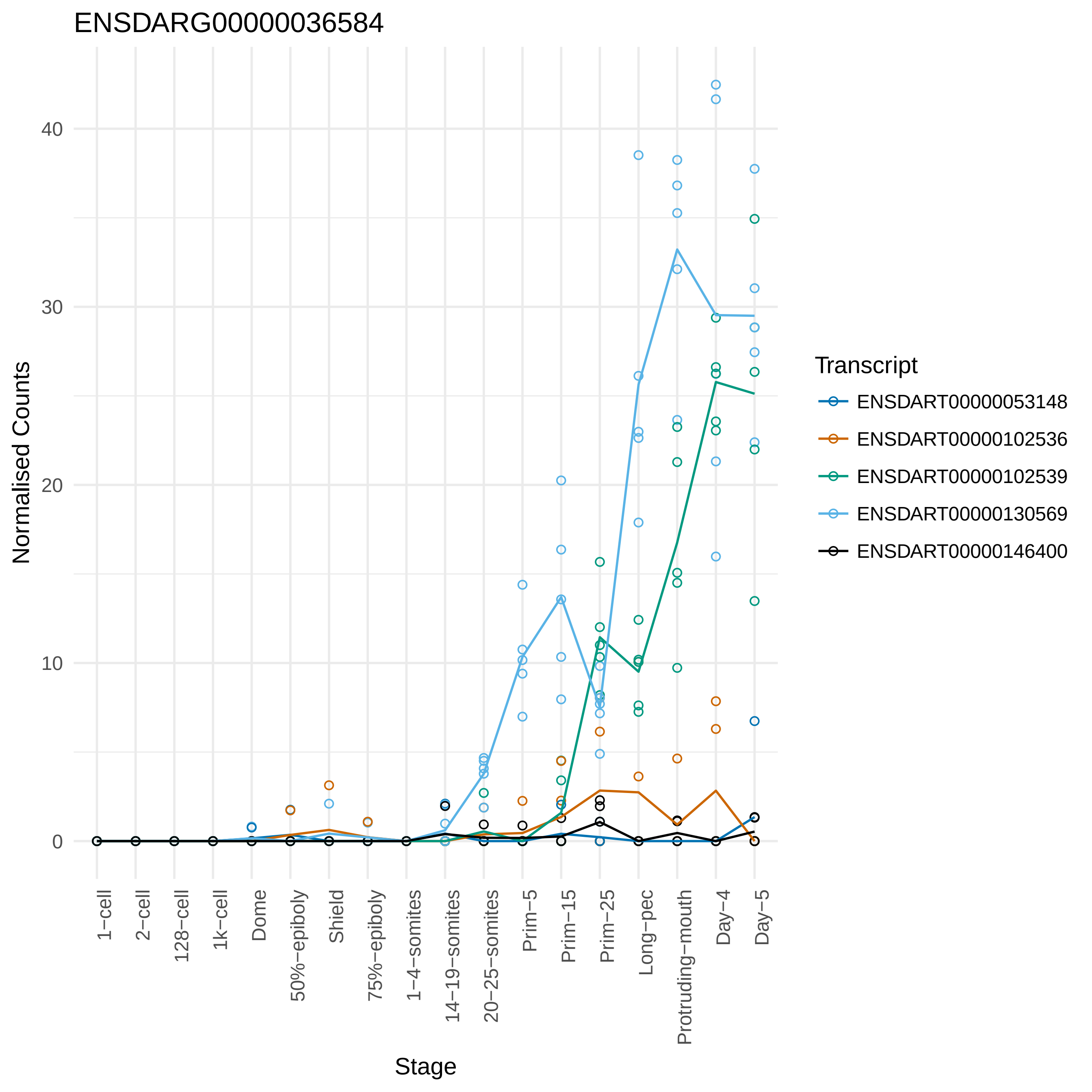

ENSDARG00000036584
Normalised Counts
0
10
20
30
40
Stage
1−cell
2−cell
128−cell
1k−cell
Dome
50%−epiboly
Shield
75%−epiboly
1−4−somites
14−19−somites
20−25−somites
Prim−5
Prim−15
Prim−25
Long−pec
Protruding−mouth
Day−4
Day−5
Transcript
ENSDART00000053148
ENSDART00000102536
ENSDART00000102539
ENSDART00000130569
ENSDART00000146400

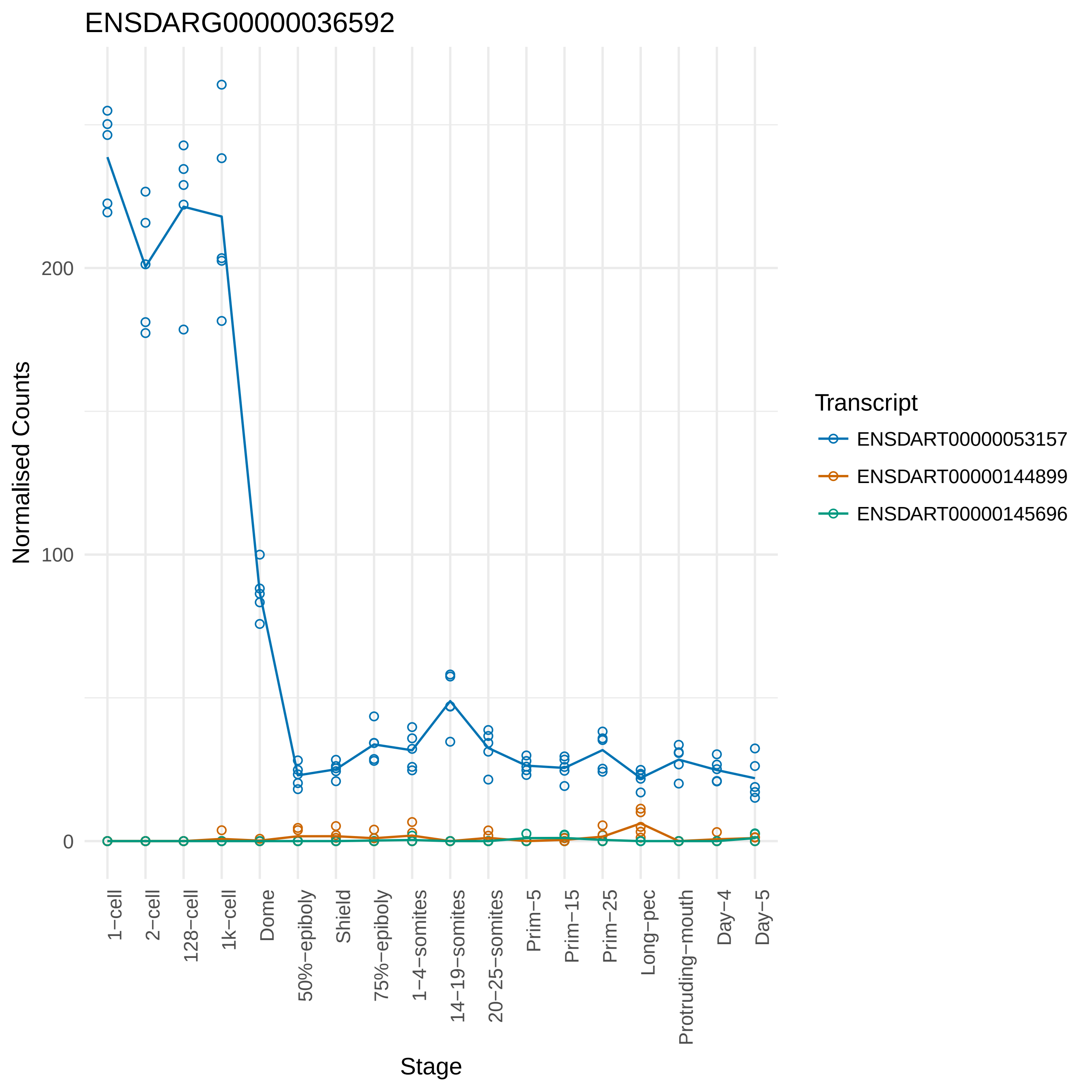
ENSDARG00000036592
Normalised Counts
200
100
0
1−cell
2−cell
128−cell
1k−cell
Dome
50%−epiboly
Shield
75%−epiboly
1−4−somites
14−19−somites
20−25−somites
Prim−5
Prim−15
Prim−25
Long−pec
Protruding−mouth
Day−4
Day−5
Stage
Transcript
ENSDART00000053157
ENSDART00000144899
ENSDART00000145696

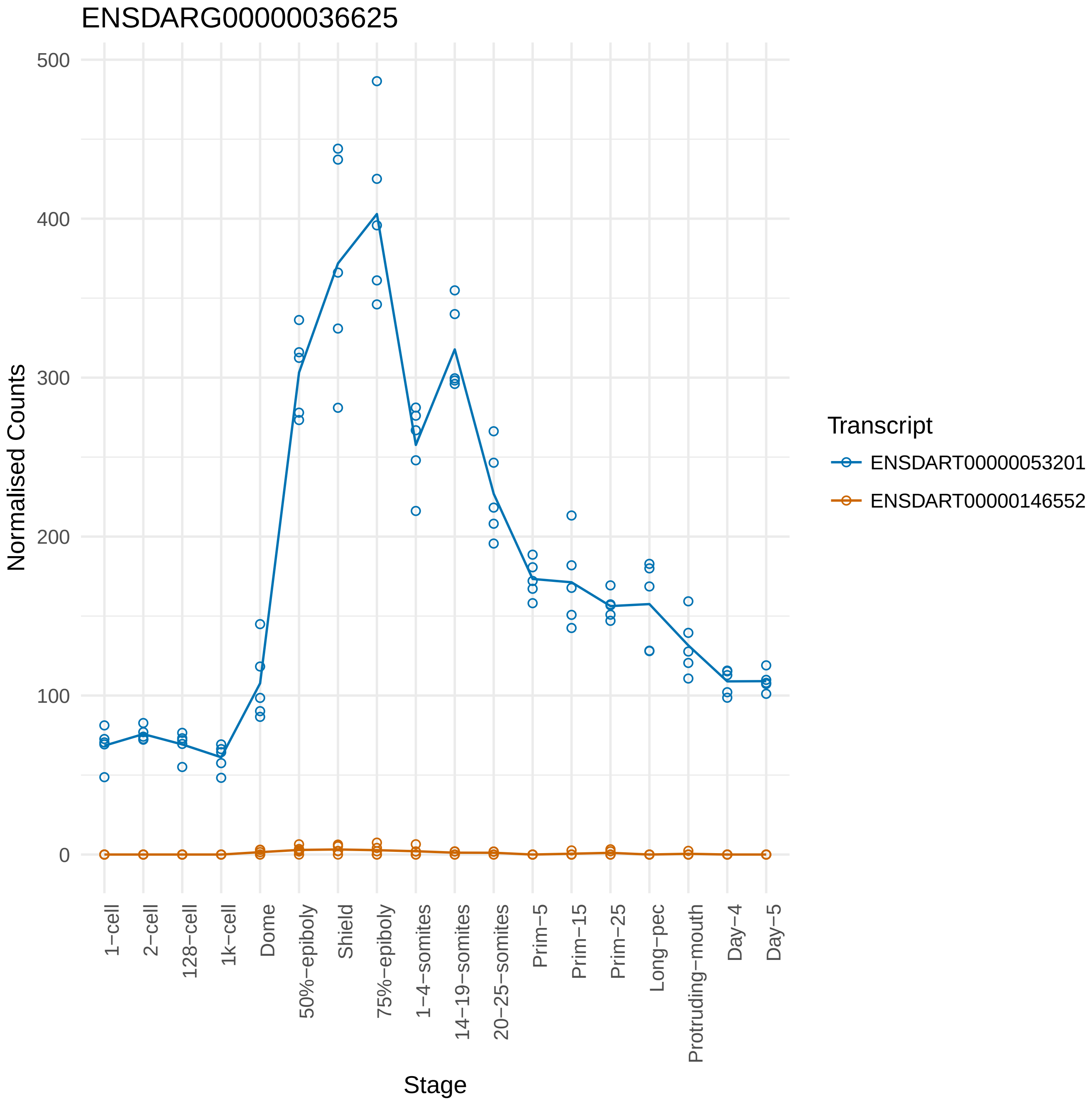
ENSDARG00000036625
Normalised Counts
500
400
300
200
100
0
1−cell
2−cell
128−cell
1k−cell
Dome
50%−epiboly
Shield
75%−epiboly
1−4−somites
14−19−somites
20−25−somites
Prim−5
Prim−15
Prim−25
Long−pec
Protruding−mouth
Day−4
Day−5
Stage
Transcript
ENSDART00000053201
ENSDART00000146552

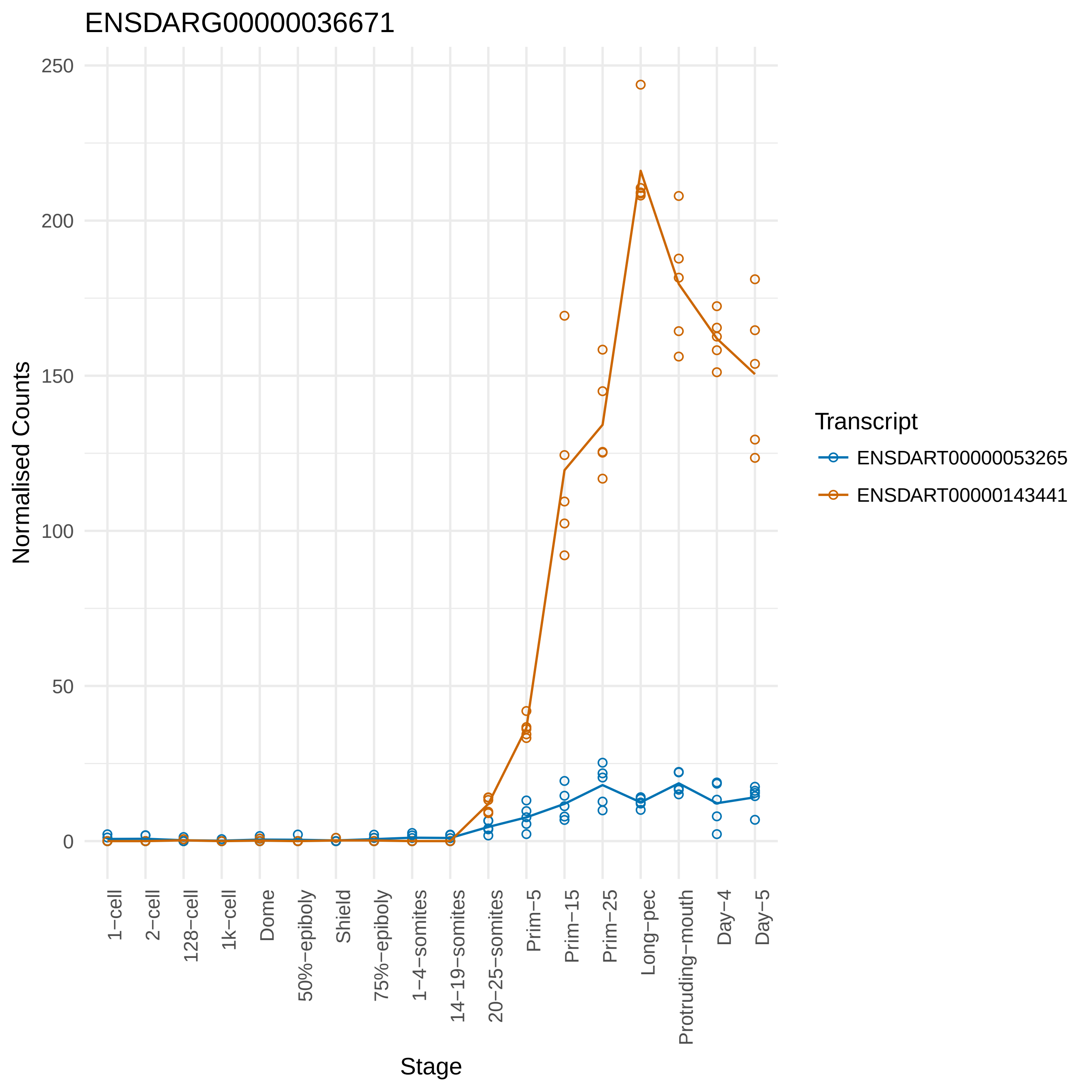
ENSDARG00000036671
Normalised Counts
0
50
100
150
200
250
Stage
1−cell
2−cell
128−cell
1k−cell
Dome
50%−epiboly
Shield
75%−epiboly
1−4−somites
14−19−somites
20−25−somites
Prim−5
Prim−15
Prim−25
Long−pec
Protruding−mouth
Day−4
Day−5
Transcript
ENSDART00000053265
ENSDART00000143441

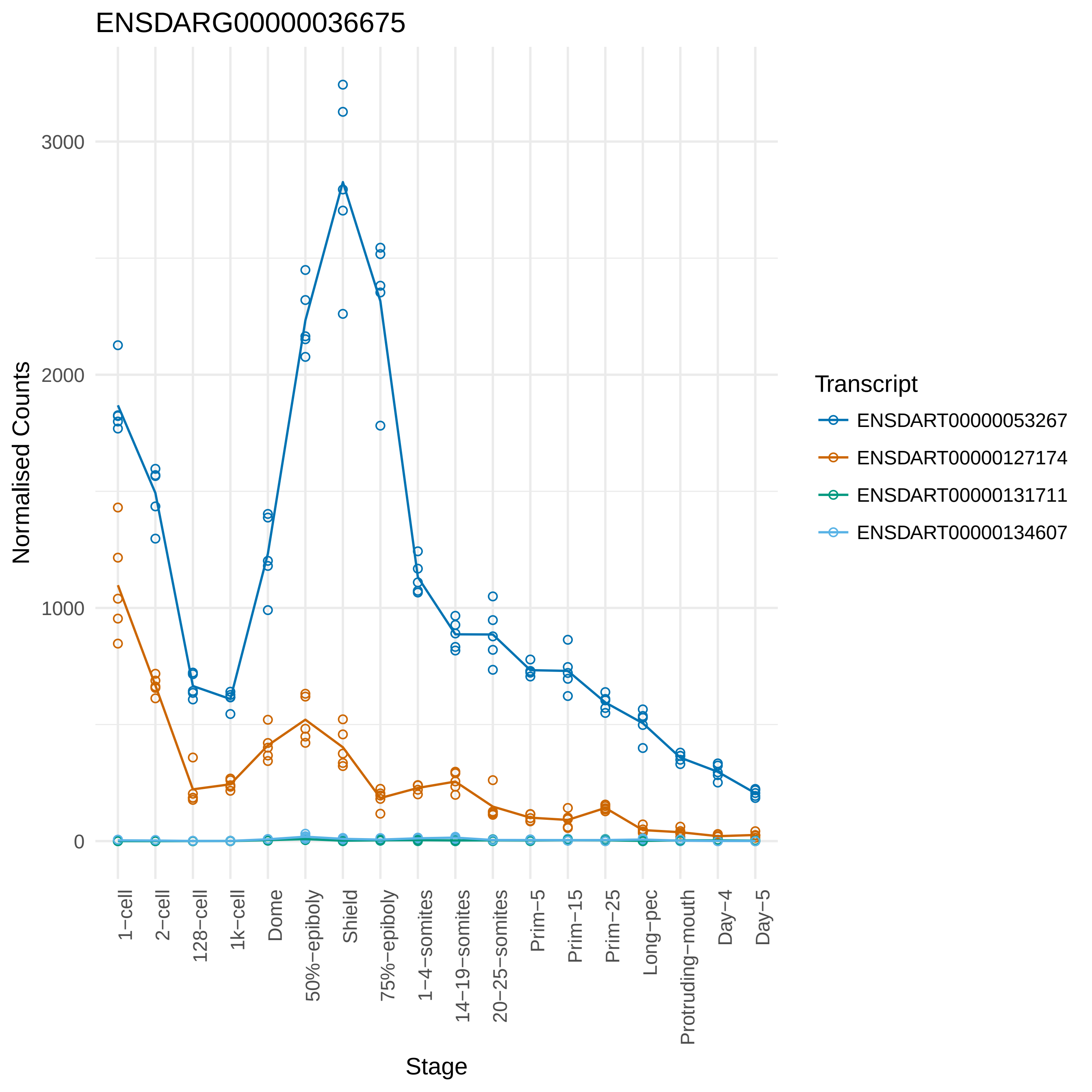
ENSDARG00000036675
Normalised Counts
3000
2000
1000
0
1−cell
2−cell
128−cell
1k−cell
Dome
50%−epiboly
Shield
75%−epiboly
1−4−somites
14−19−somites
20−25−somites
Prim−5
Prim−15
Prim−25
Long−pec
Protruding−mouth
Day−4
Day−5
Stage
Transcript
ENSDART00000053267
ENSDART00000127174
ENSDART00000131711
ENSDART00000134607

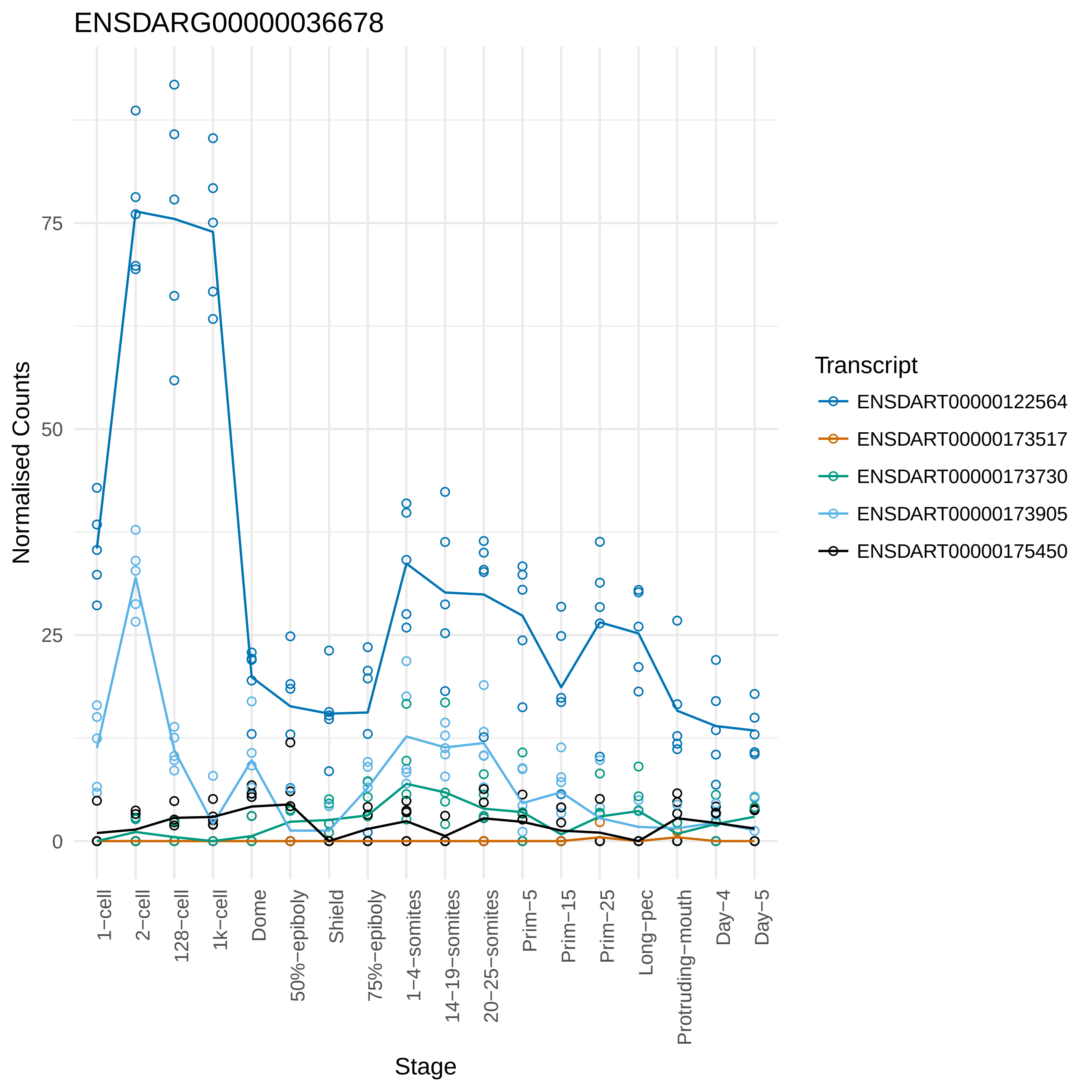

ENSDARG00000036678
Normalised Counts
0
25
50
75
Stage
1−cell
2−cell
128−cell
1k−cell
Dome
50%−epiboly
Shield
75%−epiboly
1−4−somites
14−19−somites
20−25−somites
Prim−5
Prim−15
Prim−25
Long−pec
Protruding−mouth
Day−4
Day−5
Transcript
ENSDART00000122564
ENSDART00000173517
ENSDART00000173730
ENSDART00000173905
ENSDART00000175450

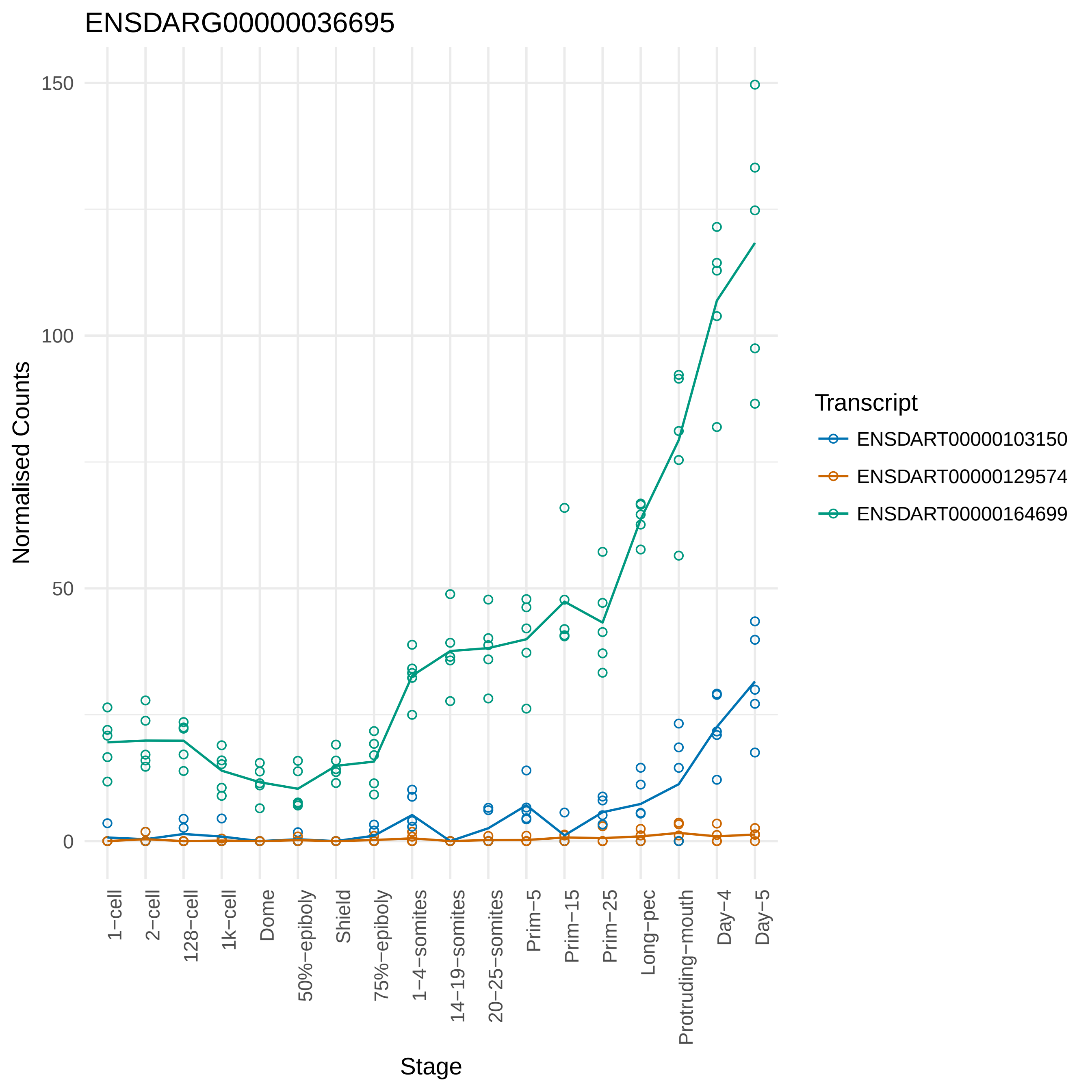
ENSDARG00000036695
Normalised Counts
150
100
50
0
1−cell
2−cell
128−cell
1k−cell
Dome
50%−epiboly
Shield
75%−epiboly
1−4−somites
14−19−somites
20−25−somites
Prim−5
Prim−15
Prim−25
Long−pec
Protruding−mouth
Day−4
Day−5
Stage
Transcript
ENSDART00000103150
ENSDART00000129574
ENSDART00000164699

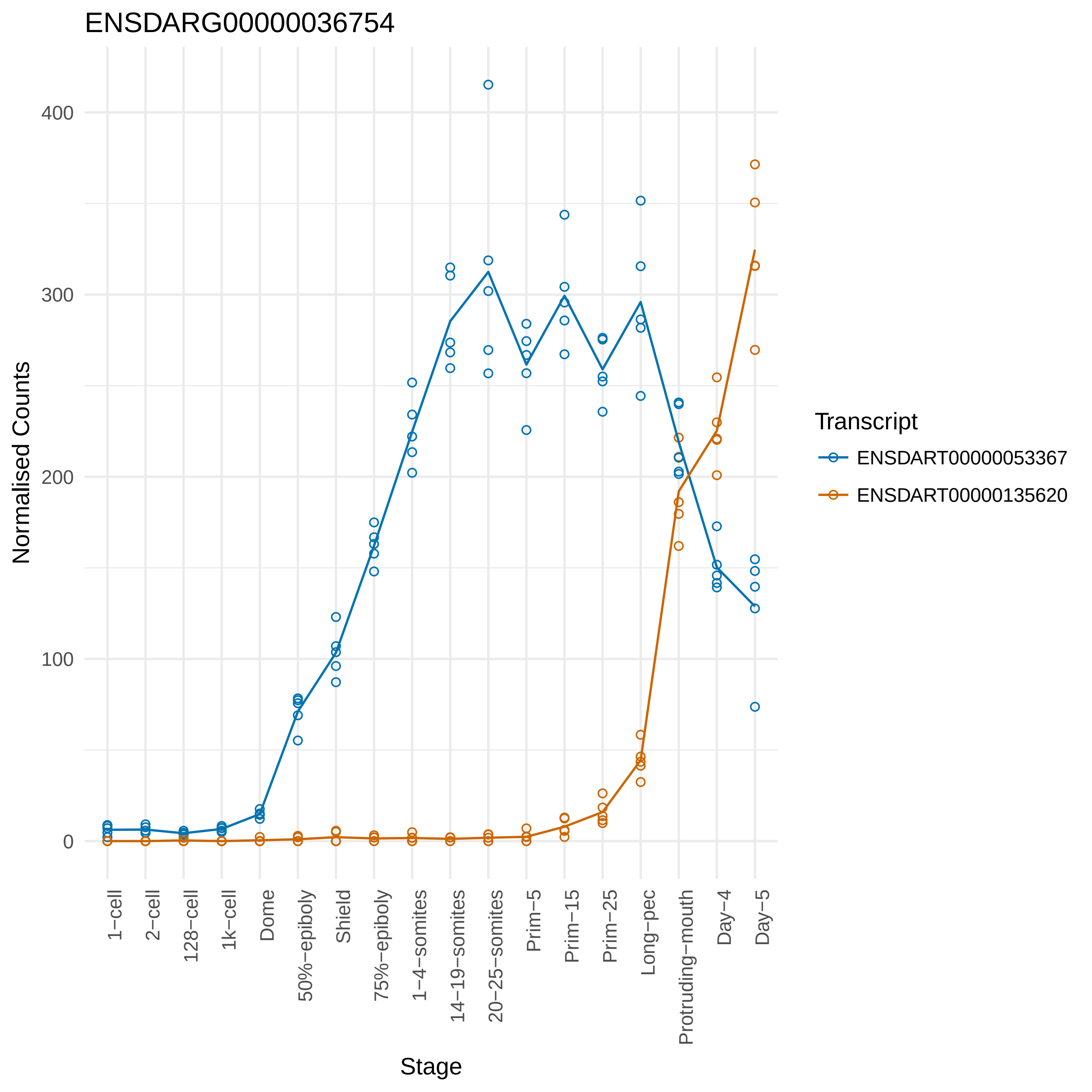

ENSDARG00000036754
Normalised Counts
400
300
200
100
0
1−cell
2−cell
128−cell
1k−cell
Dome
50%−epiboly
Shield
75%−epiboly
1−4−somites
14−19−somites
20−25−somites
Prim−5
Prim−15
Prim−25
Long−pec
Protruding−mouth
Day−4
Day−5
Stage
Transcript
ENSDART00000053367
ENSDART00000135620

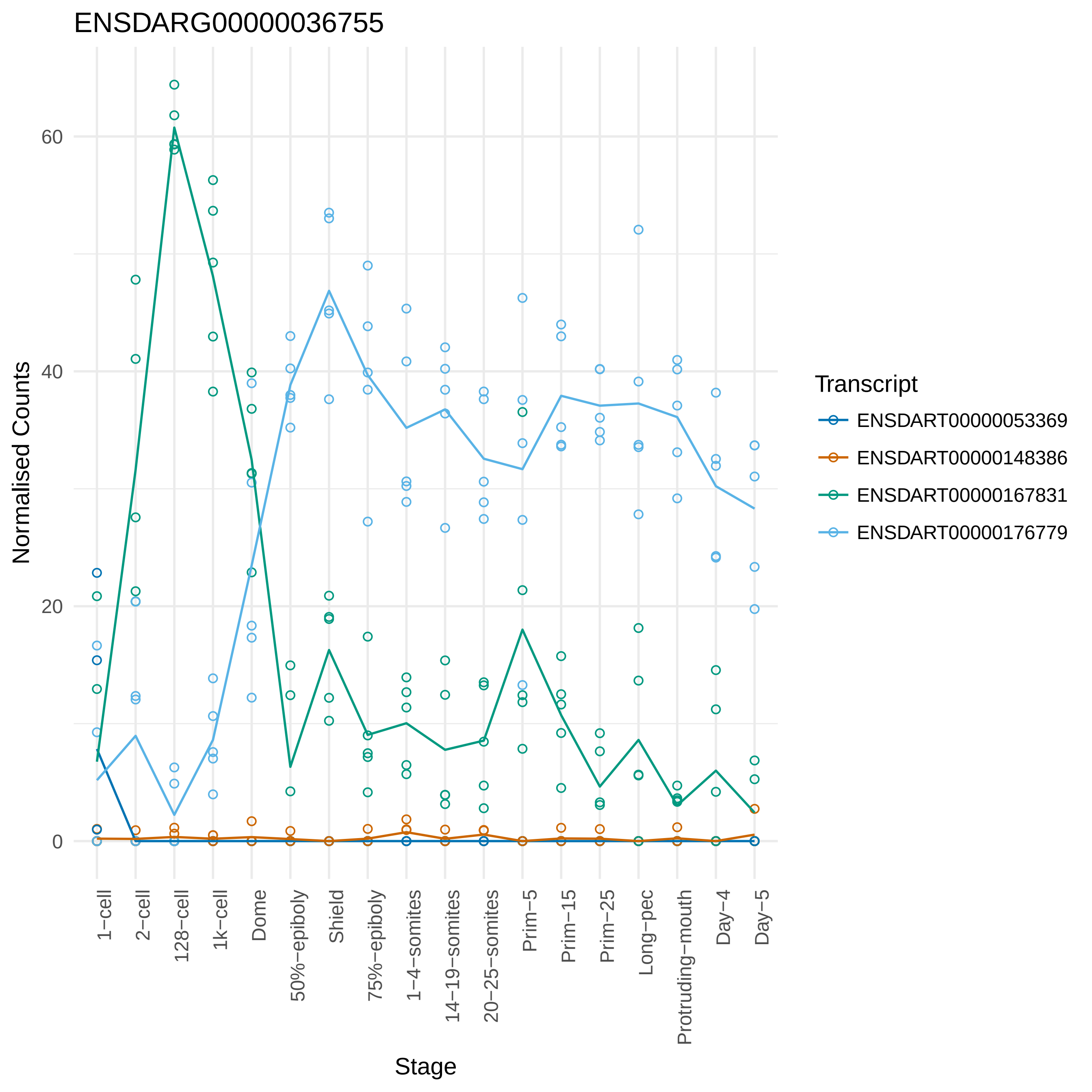

ENSDARG00000036755
Normalised Counts
0
20
40
60
1−cell
2−cell
128−cell
1k−cell
Dome
50%−epiboly
Shield
75%−epiboly
1−4−somites
14−19−somites
20−25−somites
Prim−5
Prim−15
Prim−25
Long−pec
Protruding−mouth
Day−4
Day−5
Stage
Transcript
ENSDART00000053369
ENSDART00000148386
ENSDART00000167831
ENSDART00000176779

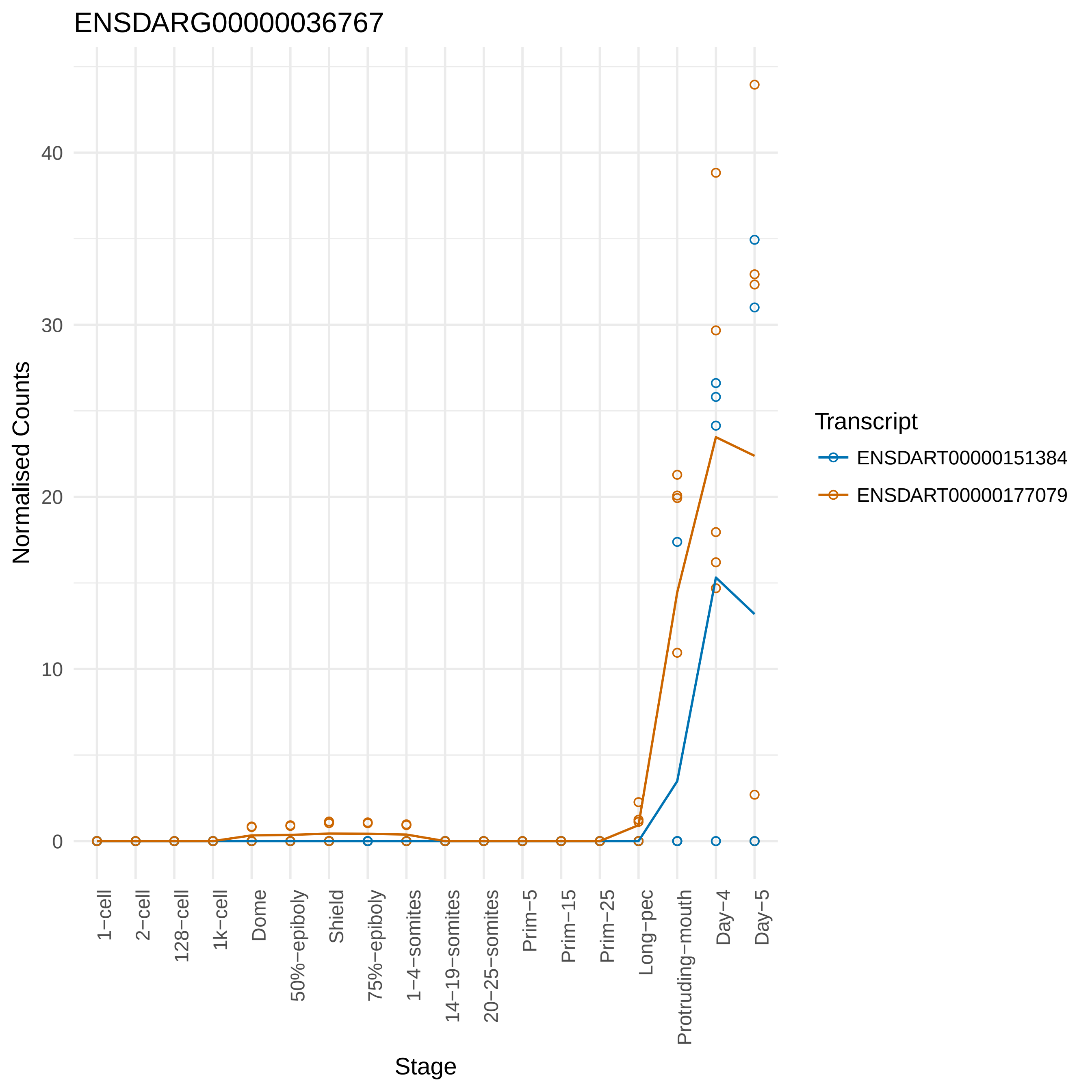
ENSDARG00000036767
Normalised Counts
0
10
20
30
40
1−cell
2−cell
128−cell
1k−cell
Dome
50%−epiboly
Shield
75%−epiboly
1−4−somites
14−19−somites
20−25−somites
Prim−5
Prim−15
Prim−25
Long−pec
Protruding−mouth
Day−4
Day−5
Stage
Transcript
ENSDART00000151384
ENSDART00000177079

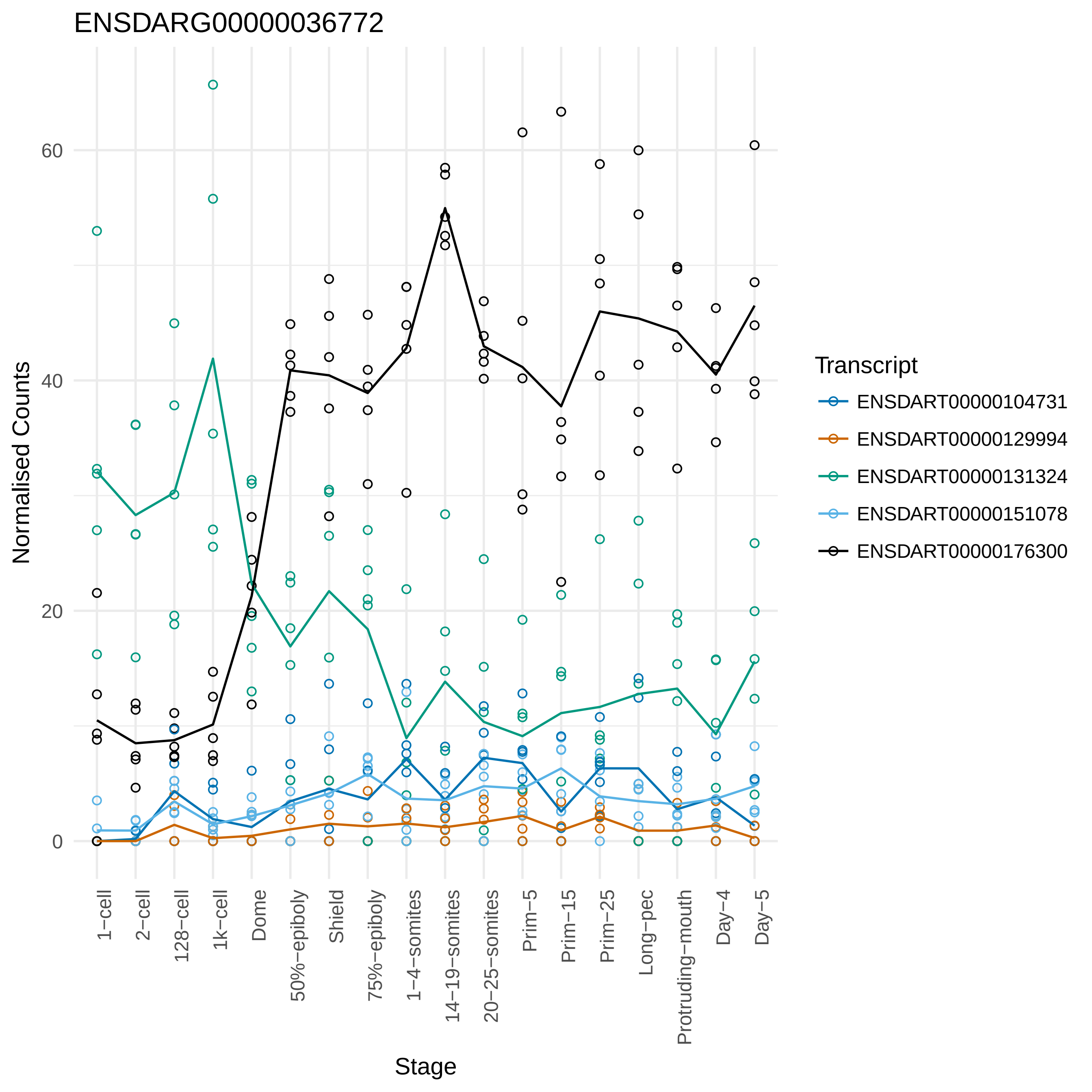

ENSDARG00000036772
Normalised Counts
60
40
20
0
1−cell
2−cell
128−cell
1k−cell
Dome
50%−epiboly
Shield
75%−epiboly
1−4−somites
14−19−somites
20−25−somites
Prim−5
Prim−15
Prim−25
Long−pec
Protruding−mouth
Day−4
Day−5
Stage
Transcript
ENSDART00000104731
ENSDART00000129994
ENSDART00000131324
ENSDART00000151078
ENSDART00000176300

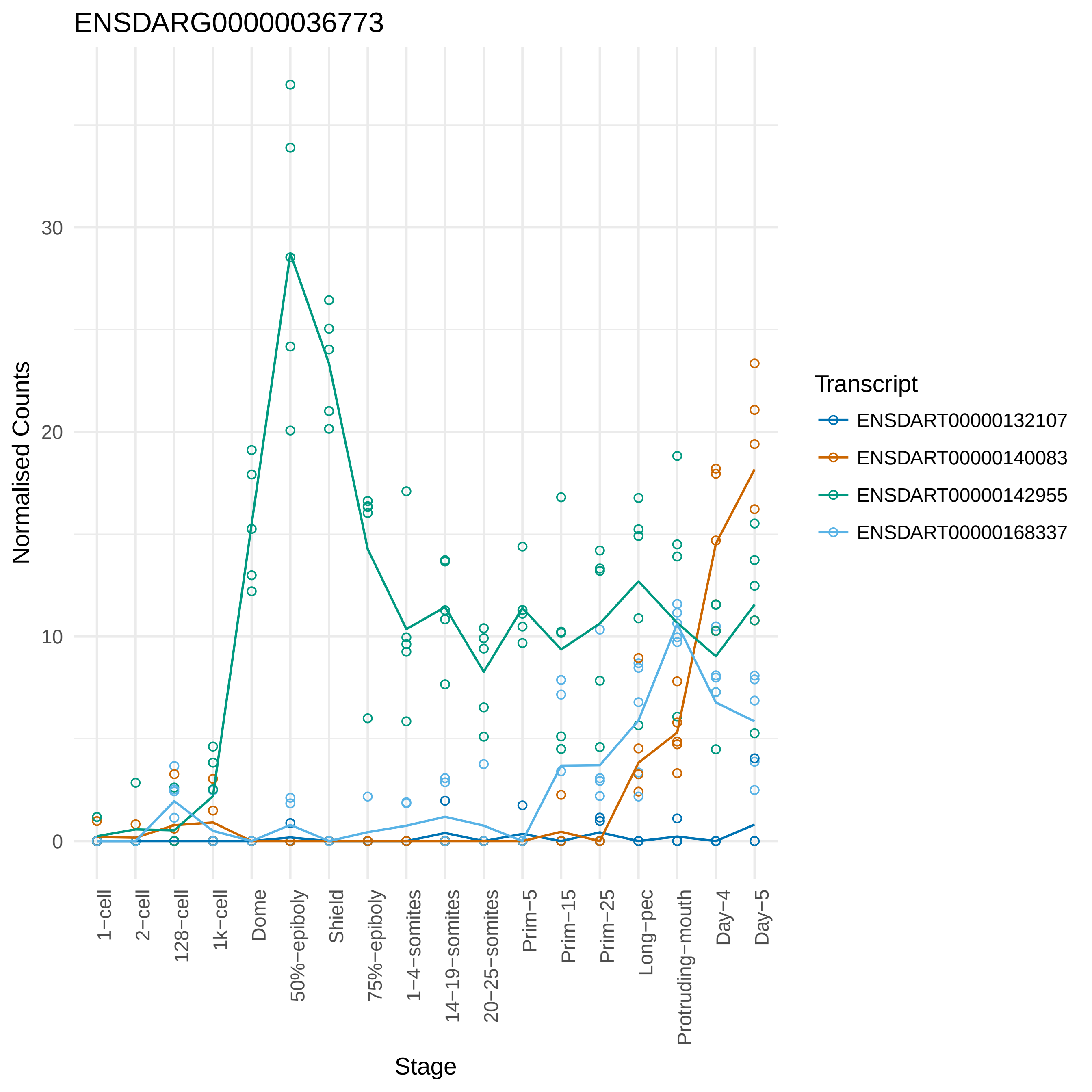
ENSDARG00000036773
Normalised Counts
0
10
20
30
Stage
1−cell
2−cell
128−cell
1k−cell
Dome
50%−epiboly
Shield
75%−epiboly
1−4−somites
14−19−somites
20−25−somites
Prim−5
Prim−15
Prim−25
Long−pec
Protruding−mouth
Day−4
Day−5
Transcript
ENSDART00000132107
ENSDART00000140083
ENSDART00000142955
ENSDART00000168337

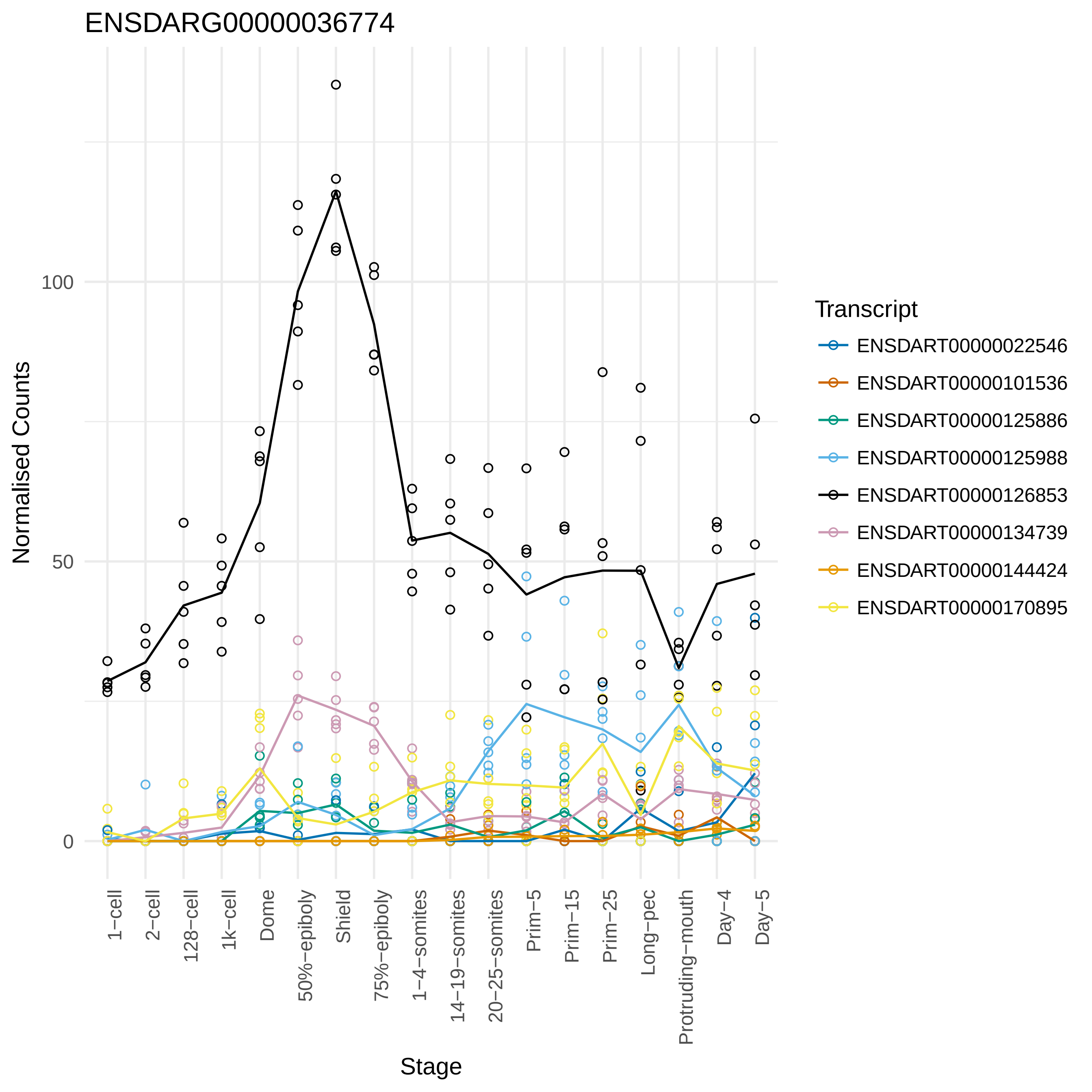

ENSDARG00000036774
Normalised Counts
0
50
100
Stage
1−cell
2−cell
128−cell
1k−cell
Dome
50%−epiboly
Shield
75%−epiboly
1−4−somites
14−19−somites
20−25−somites
Prim−5
Prim−15
Prim−25
Long−pec
Protruding−mouth
Day−4
Day−5
Transcript
ENSDART00000022546
ENSDART00000101536
ENSDART00000125886
ENSDART00000125988
ENSDART00000126853
ENSDART00000134739
ENSDART00000144424
ENSDART00000170895

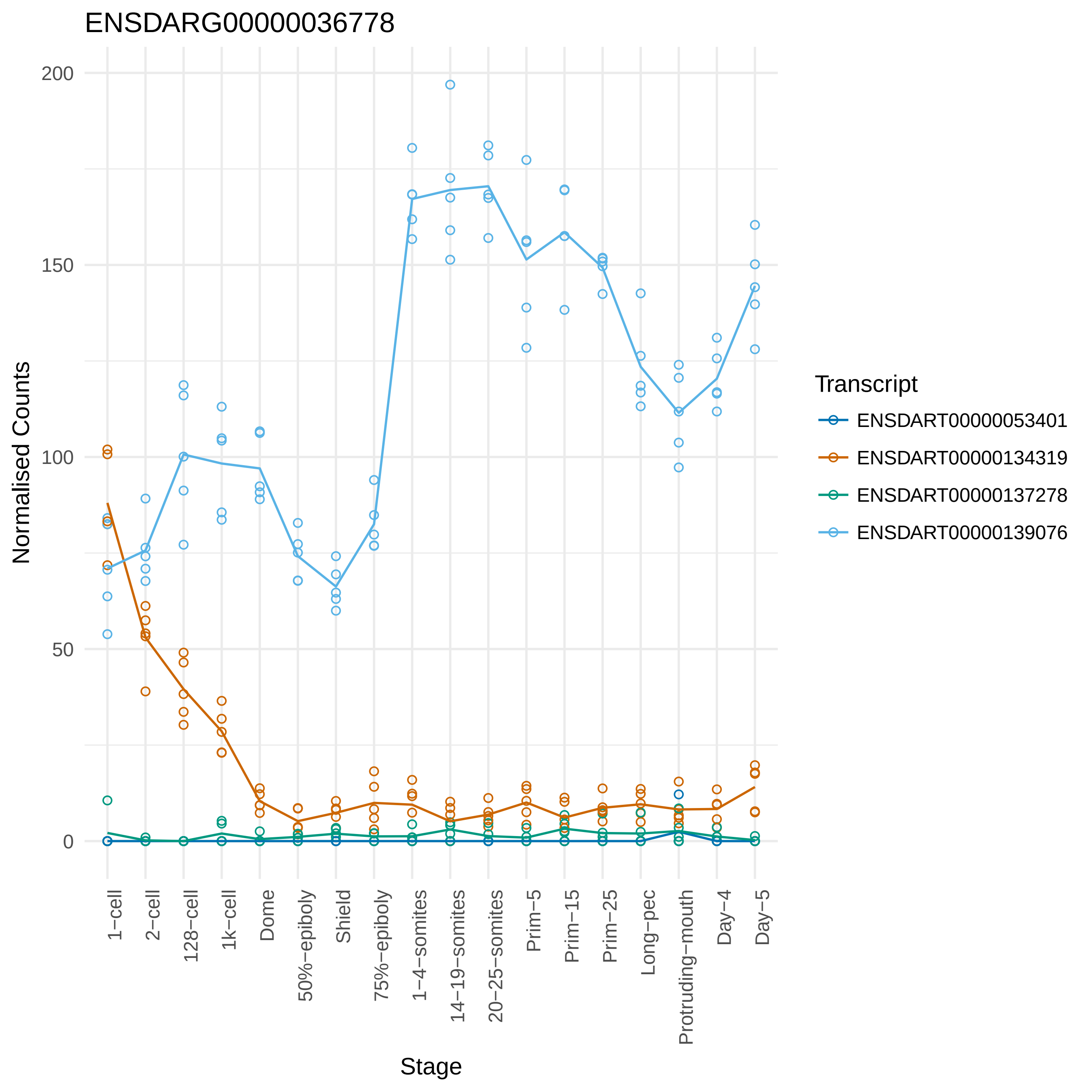

ENSDARG00000036778
Normalised Counts
0
50
100
150
200
Stage
1−cell
2−cell
128−cell
1k−cell
Dome
50%−epiboly
Shield
75%−epiboly
1−4−somites
14−19−somites
20−25−somites
Prim−5
Prim−15
Prim−25
Long−pec
Protruding−mouth
Day−4
Day−5
Transcript
ENSDART00000053401
ENSDART00000134319
ENSDART00000137278
ENSDART00000139076

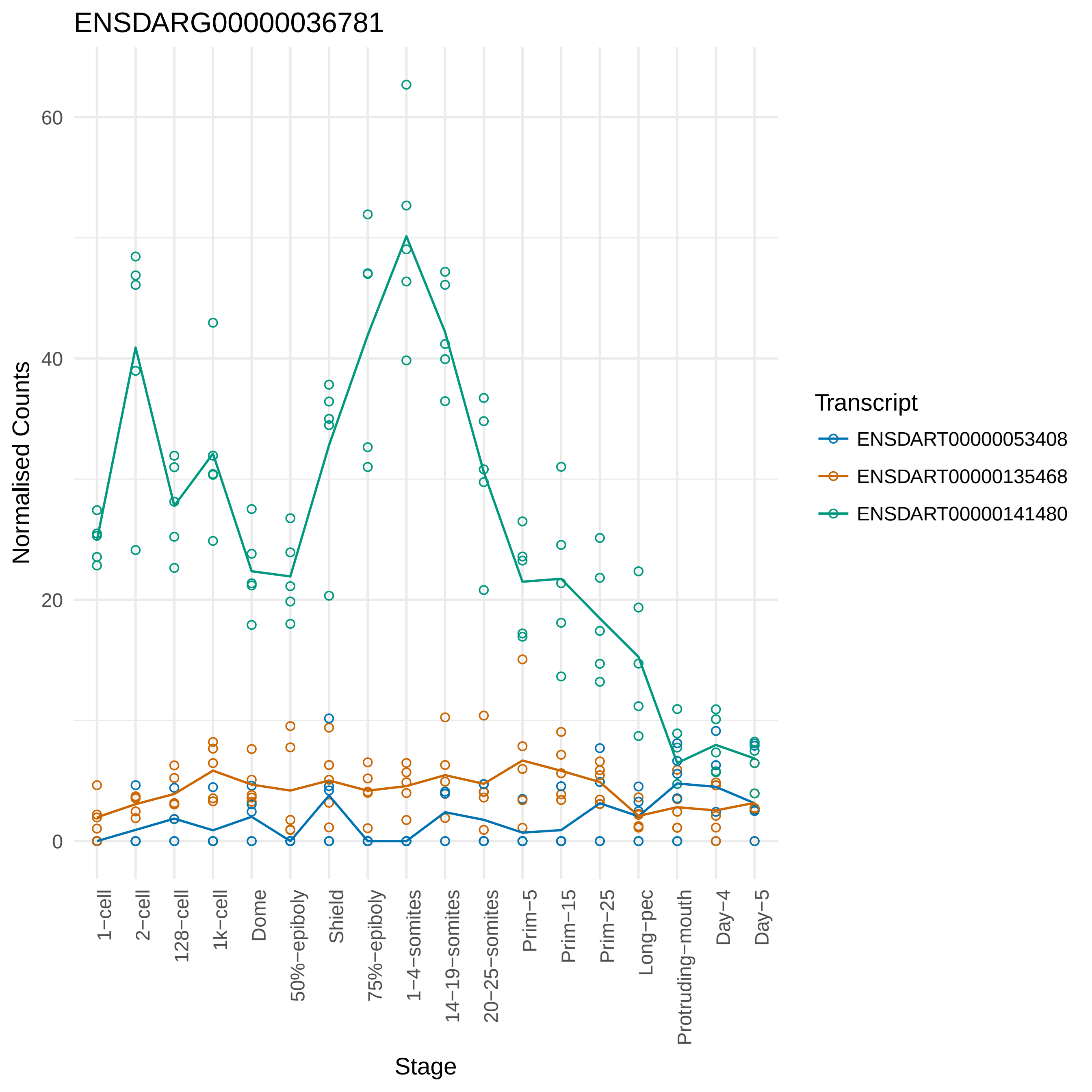

ENSDARG00000036781
Normalised Counts
0
20
40
60
Stage
1−cell
2−cell
128−cell
1k−cell
Dome
50%−epiboly
Shield
75%−epiboly
1−4−somites
14−19−somites
20−25−somites
Prim−5
Prim−15
Prim−25
Long−pec
Protruding−mouth
Day−4
Day−5
Transcript
ENSDART00000053408
ENSDART00000135468
ENSDART00000141480

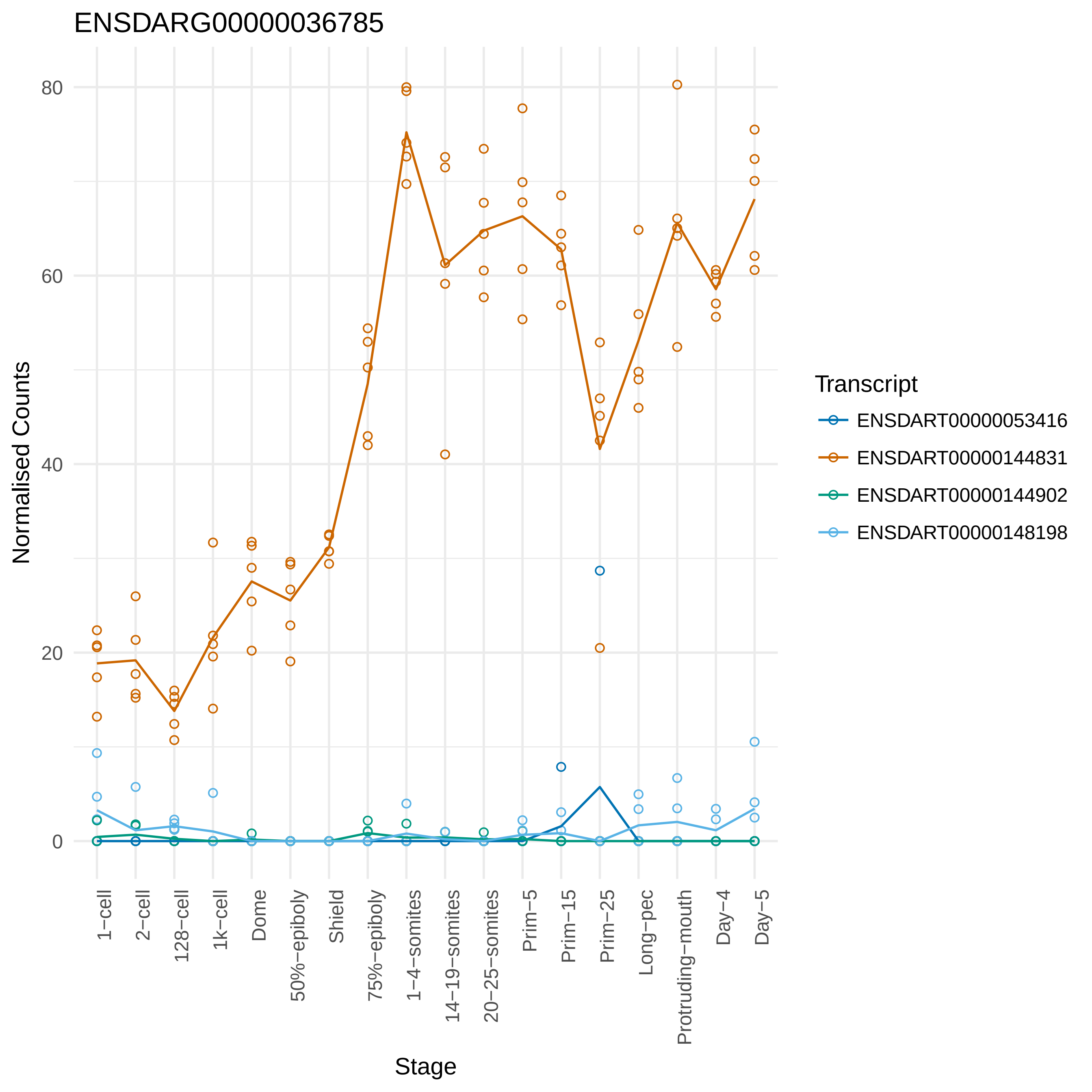

ENSDARG00000036785
Normalised Counts
0
20
40
60
80
1−cell
2−cell
128−cell
1k−cell
Dome
50%−epiboly
Shield
75%−epiboly
1−4−somites
14−19−somites
20−25−somites
Prim−5
Prim−15
Prim−25
Long−pec
Protruding−mouth
Day−4
Day−5
Stage
Transcript
ENSDART00000053416
ENSDART00000144831
ENSDART00000144902
ENSDART00000148198

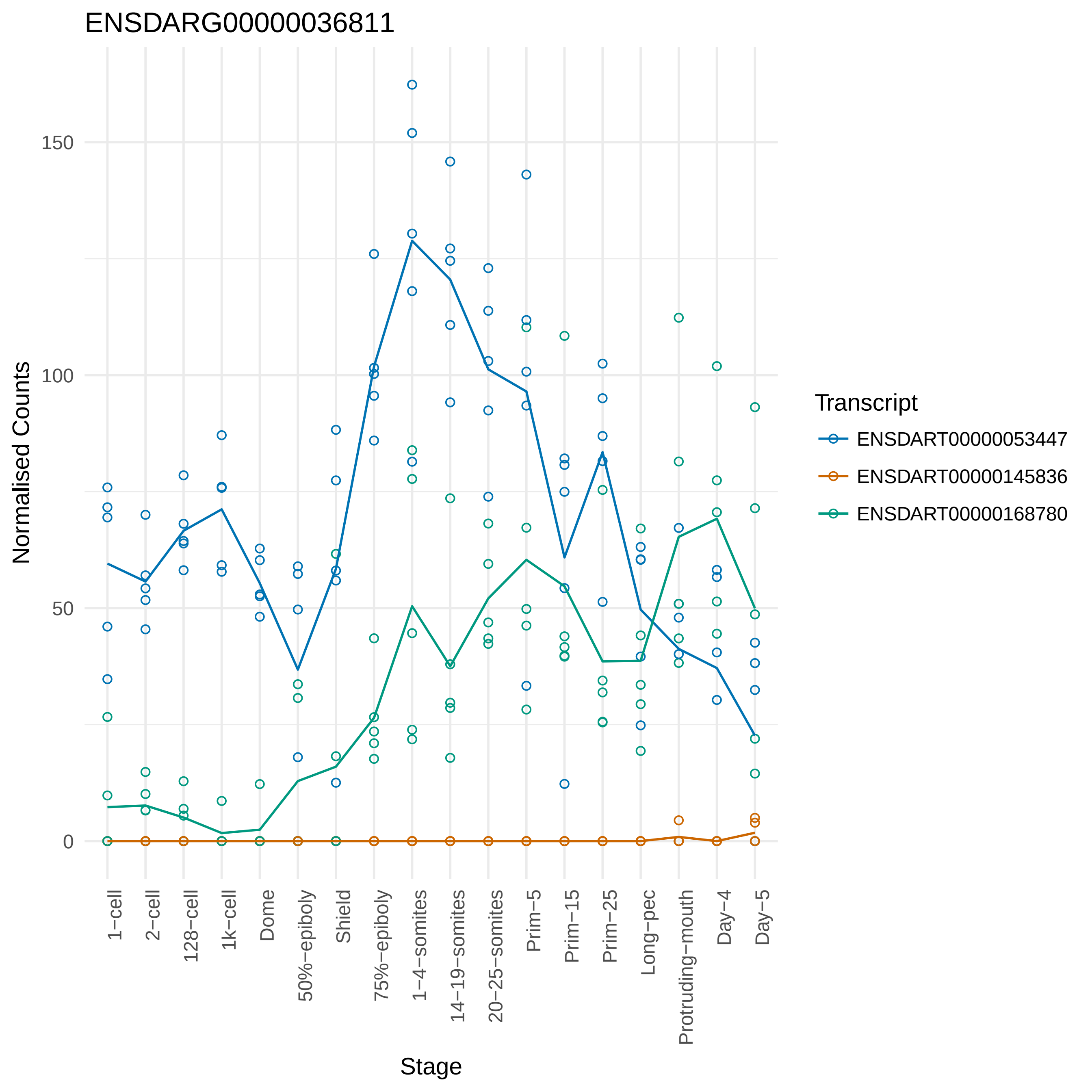
ENSDARG00000036811
Normalised Counts
150
100
50
0
1−cell
2−cell
128−cell
1k−cell
Dome
50%−epiboly
Shield
75%−epiboly
1−4−somites
14−19−somites
20−25−somites
Prim−5
Prim−15
Prim−25
Long−pec
Protruding−mouth
Day−4
Day−5
Stage
Transcript
ENSDART00000053447
ENSDART00000145836
ENSDART00000168780

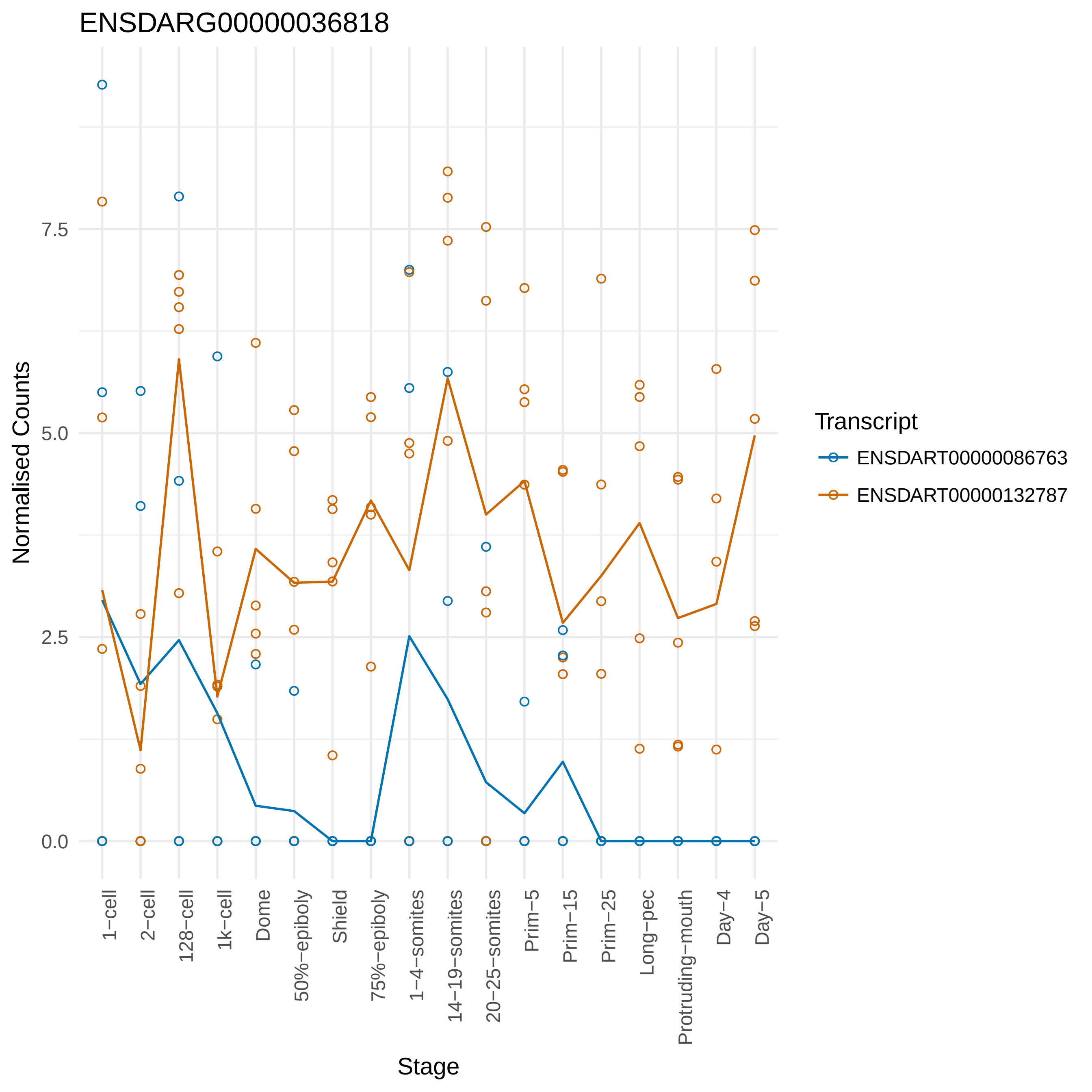

ENSDARG00000036818
Normalised Counts
0.0
2.5
5.0
7.5
Stage
1−cell
2−cell
128−cell
1k−cell
Dome
50%−epiboly
Shield
75%−epiboly
1−4−somites
14−19−somites
20−25−somites
Prim−5
Prim−15
Prim−25
Long−pec
Protruding−mouth
Day−4
Day−5
Transcript
ENSDART00000086763
ENSDART00000132787

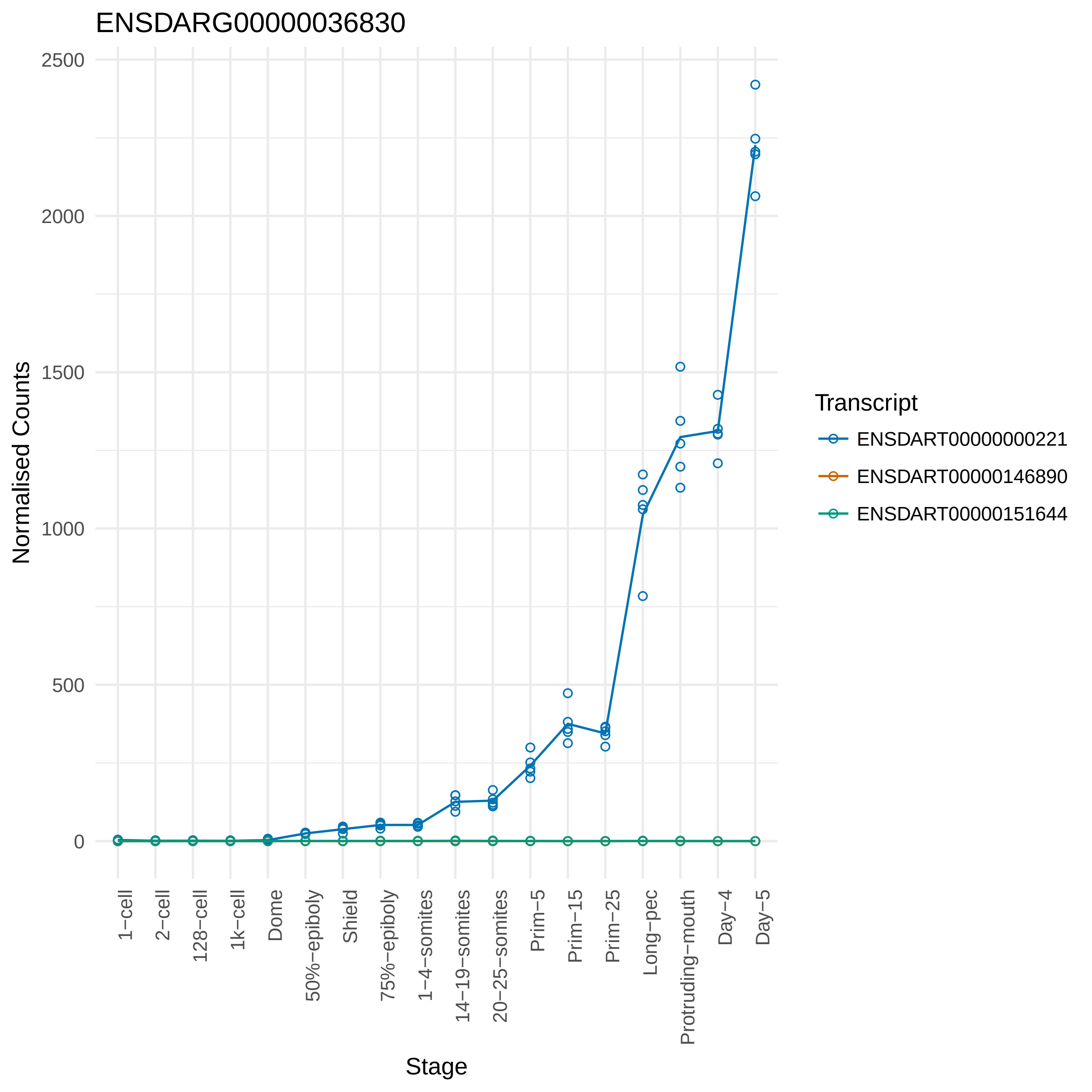

ENSDARG00000036830
Normalised Counts
2500
2000
1500
1000
500
0
Stage
1−cell
2−cell
128−cell
1k−cell
Dome
50%−epiboly
Shield
75%−epiboly
1−4−somites
14−19−somites
20−25−somites
Prim−5
Prim−15
Prim−25
Long−pec
Protruding−mouth
Day−4
Day−5
Transcript
ENSDART00000000221
ENSDART00000146890
ENSDART00000151644

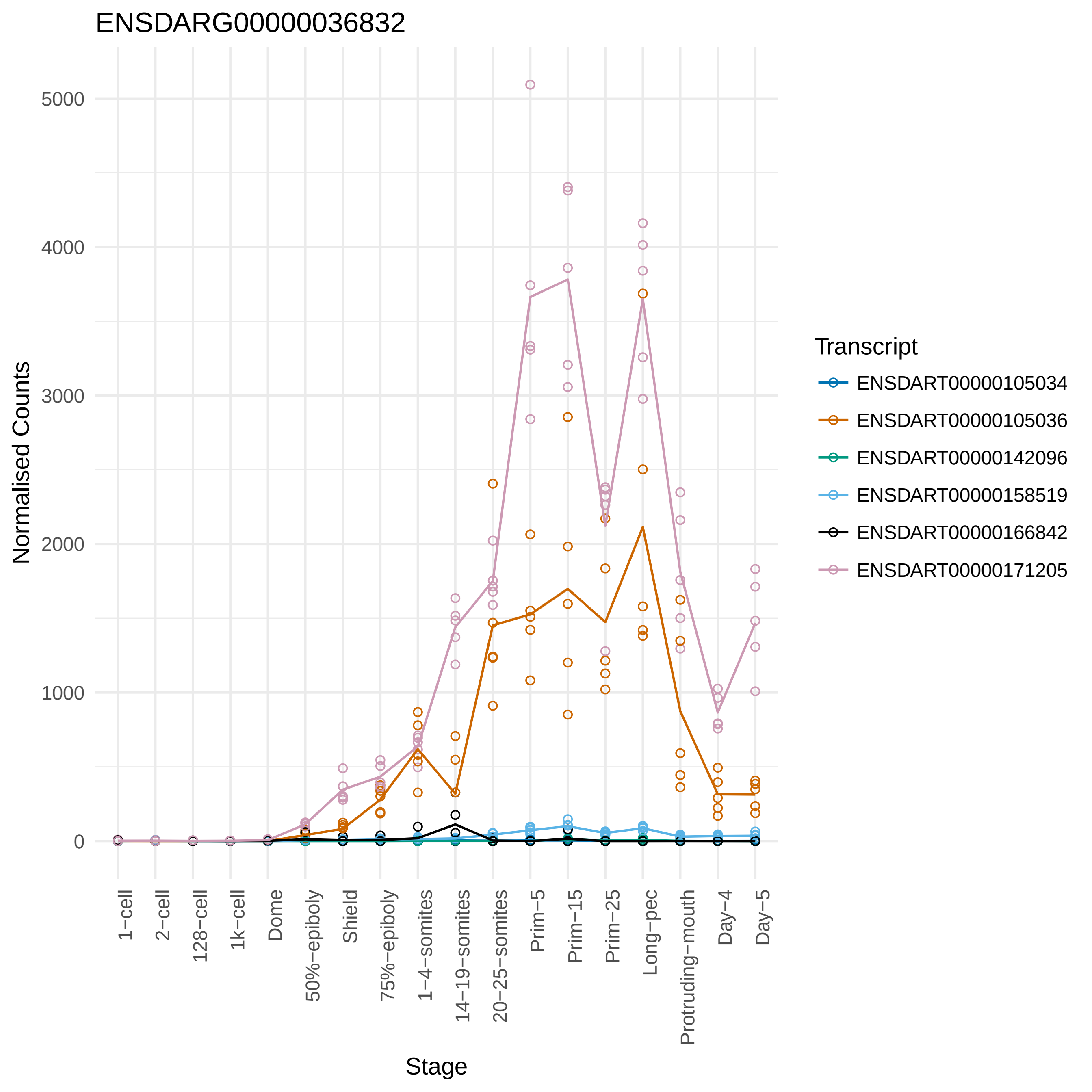
ENSDARG00000036832
Normalised Counts
5000
4000
3000
2000
1000
0
1−cell
2−cell
128−cell
1k−cell
Dome
50%−epiboly
Shield
75%−epiboly
1−4−somites
14−19−somites
20−25−somites
Prim−5
Prim−15
Prim−25
Long−pec
Protruding−mouth
Day−4
Day−5
Stage
Transcript
ENSDART00000105034
ENSDART00000105036
ENSDART00000142096
ENSDART00000158519
ENSDART00000166842
ENSDART00000171205

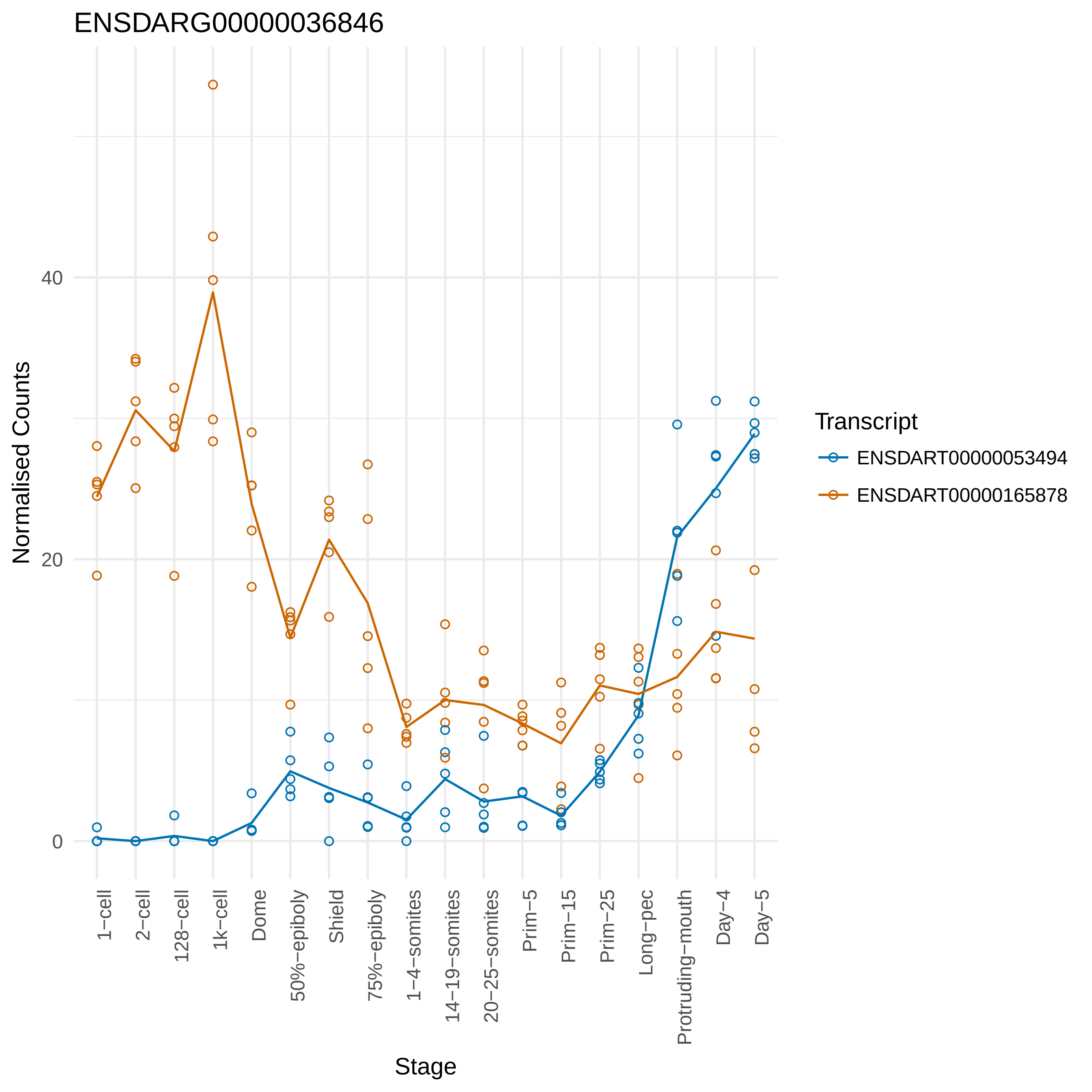
ENSDARG00000036846
Normalised Counts
0
20
40
1−cell
2−cell
128−cell
1k−cell
Dome
50%−epiboly
Shield
75%−epiboly
1−4−somites
14−19−somites
20−25−somites
Prim−5
Prim−15
Prim−25
Long−pec
Protruding−mouth
Day−4
Day−5
Stage
Transcript
ENSDART00000053494
ENSDART00000165878

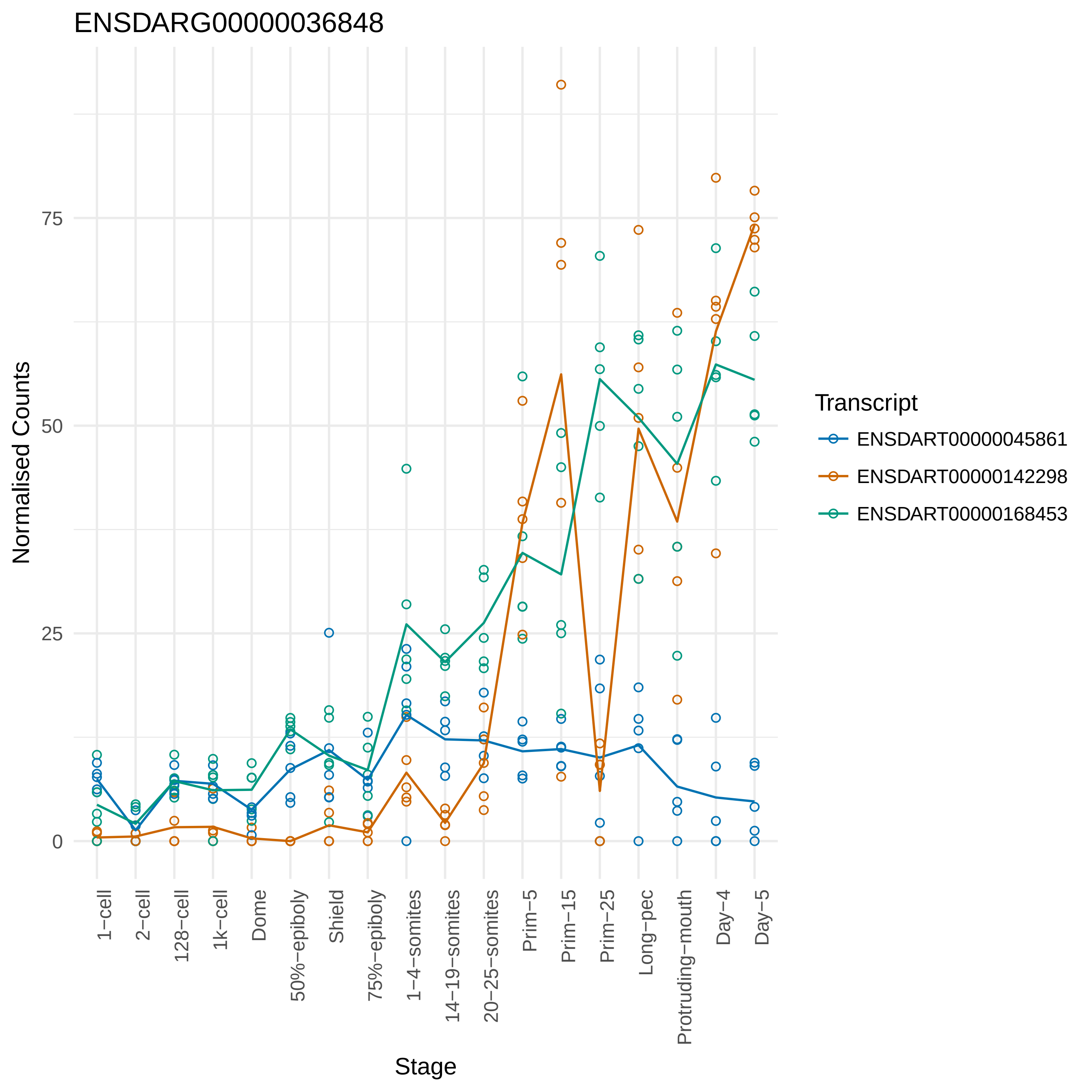

ENSDARG00000036848
Normalised Counts
0
25
50
75
Stage
1−cell
2−cell
128−cell
1k−cell
Dome
50%−epiboly
Shield
75%−epiboly
1−4−somites
14−19−somites
20−25−somites
Prim−5
Prim−15
Prim−25
Long−pec
Protruding−mouth
Day−4
Day−5
Transcript
ENSDART00000045861
ENSDART00000142298
ENSDART00000168453

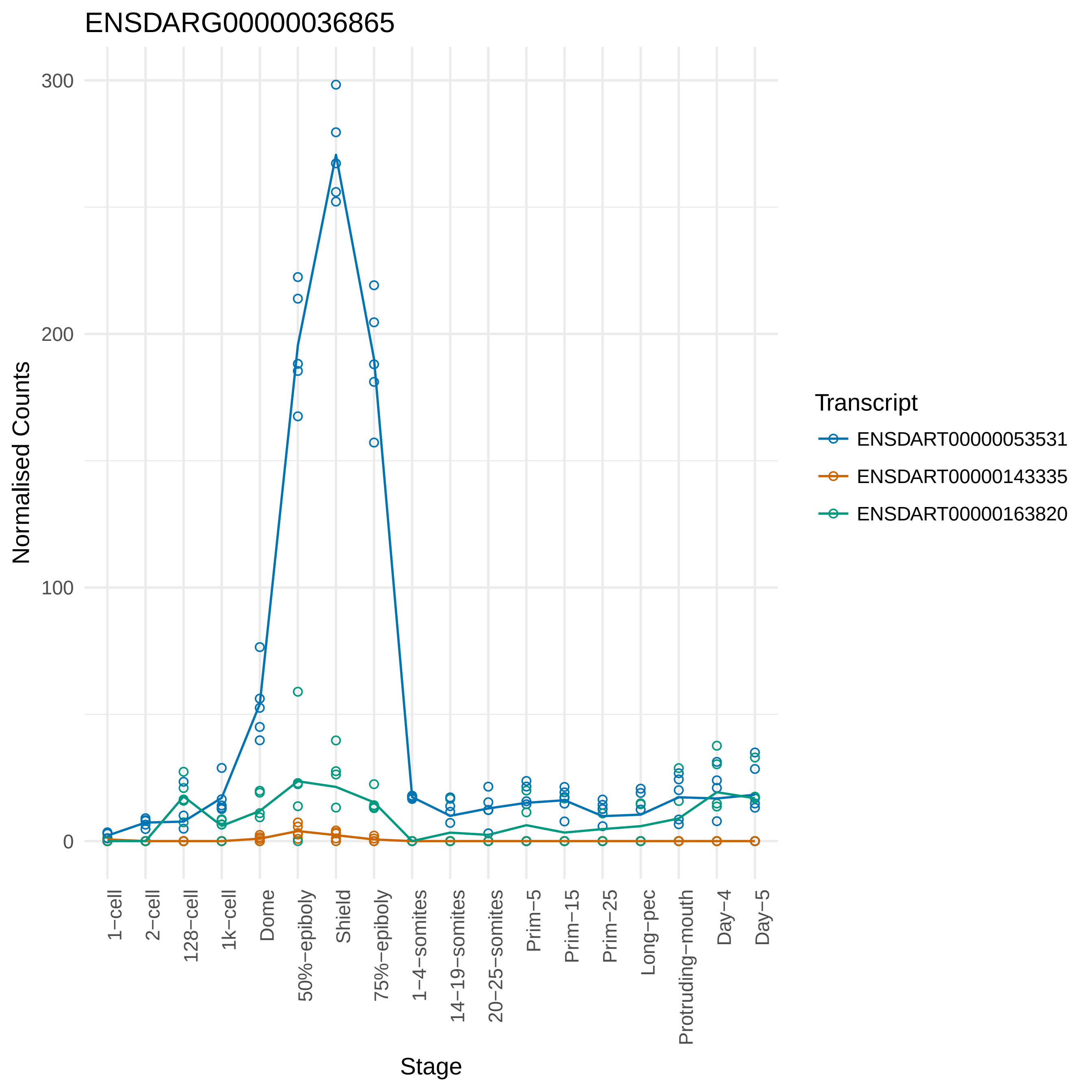
ENSDARG00000036865
Normalised Counts
300
200
100
0
1−cell
2−cell
128−cell
1k−cell
Dome
50%−epiboly
Shield
75%−epiboly
1−4−somites
14−19−somites
20−25−somites
Prim−5
Prim−15
Prim−25
Long−pec
Protruding−mouth
Day−4
Day−5
Stage
Transcript
ENSDART00000053531
ENSDART00000143335
ENSDART00000163820

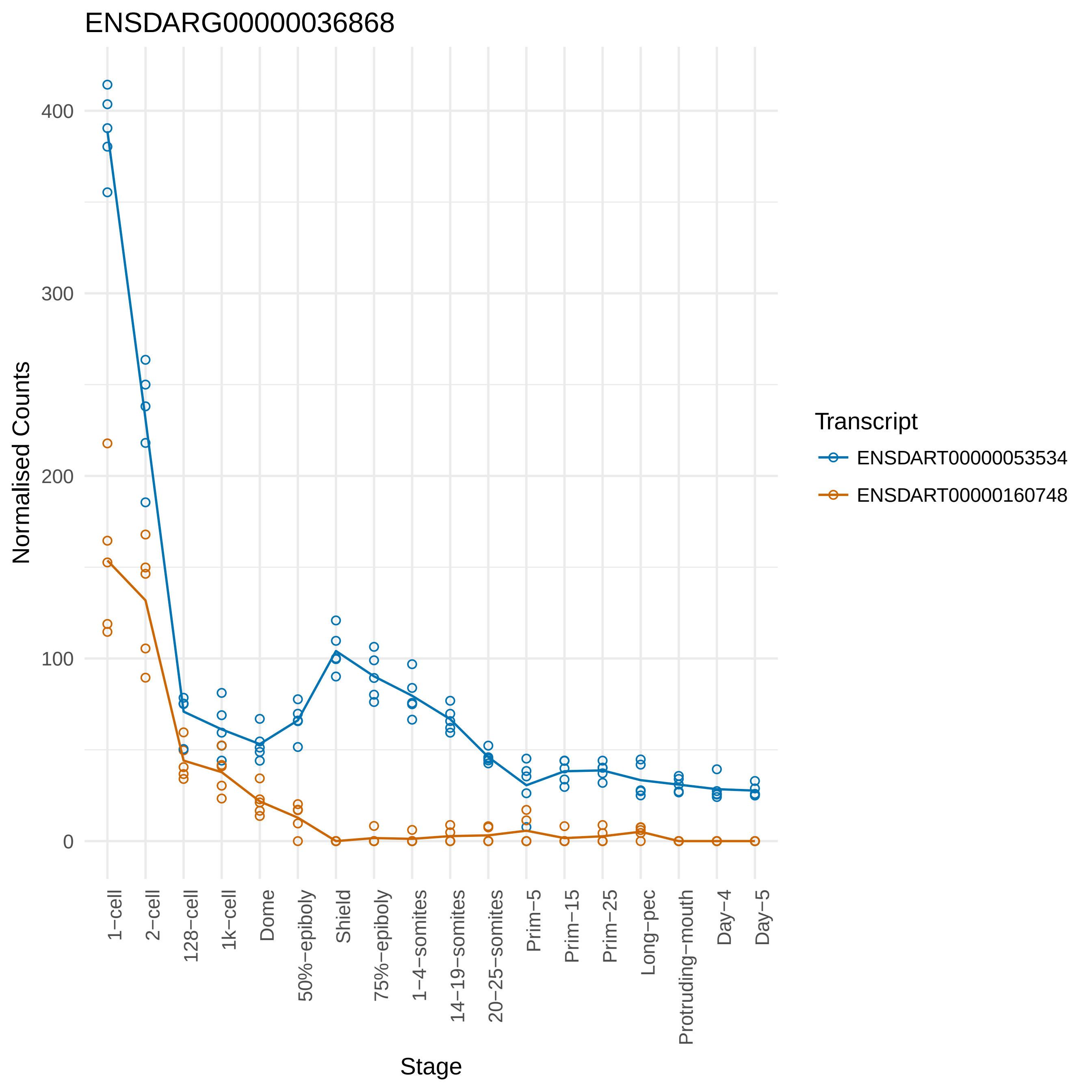

ENSDARG00000036868
Normalised Counts
0
100
200
300
400
Stage
1−cell
2−cell
128−cell
1k−cell
Dome
50%−epiboly
Shield
75%−epiboly
1−4−somites
14−19−somites
20−25−somites
Prim−5
Prim−15
Prim−25
Long−pec
Protruding−mouth
Day−4
Day−5
Transcript
ENSDART00000053534
ENSDART00000160748

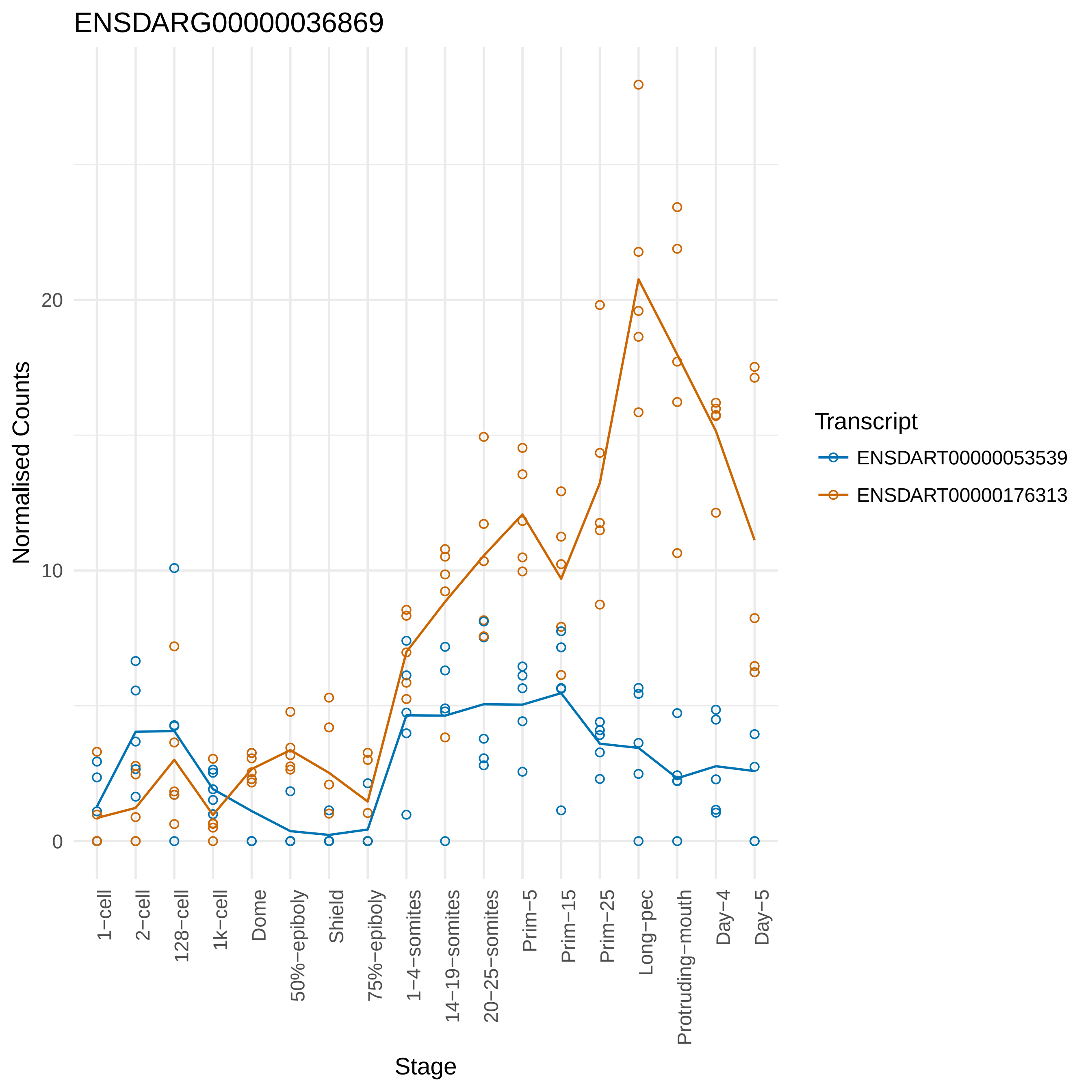

ENSDARG00000036869
Normalised Counts
0
10
20
1−cell
2−cell
128−cell
1k−cell
Dome
50%−epiboly
Shield
75%−epiboly
1−4−somites
14−19−somites
20−25−somites
Prim−5
Prim−15
Prim−25
Long−pec
Protruding−mouth
Day−4
Day−5
Stage
Transcript
ENSDART00000053539
ENSDART00000176313

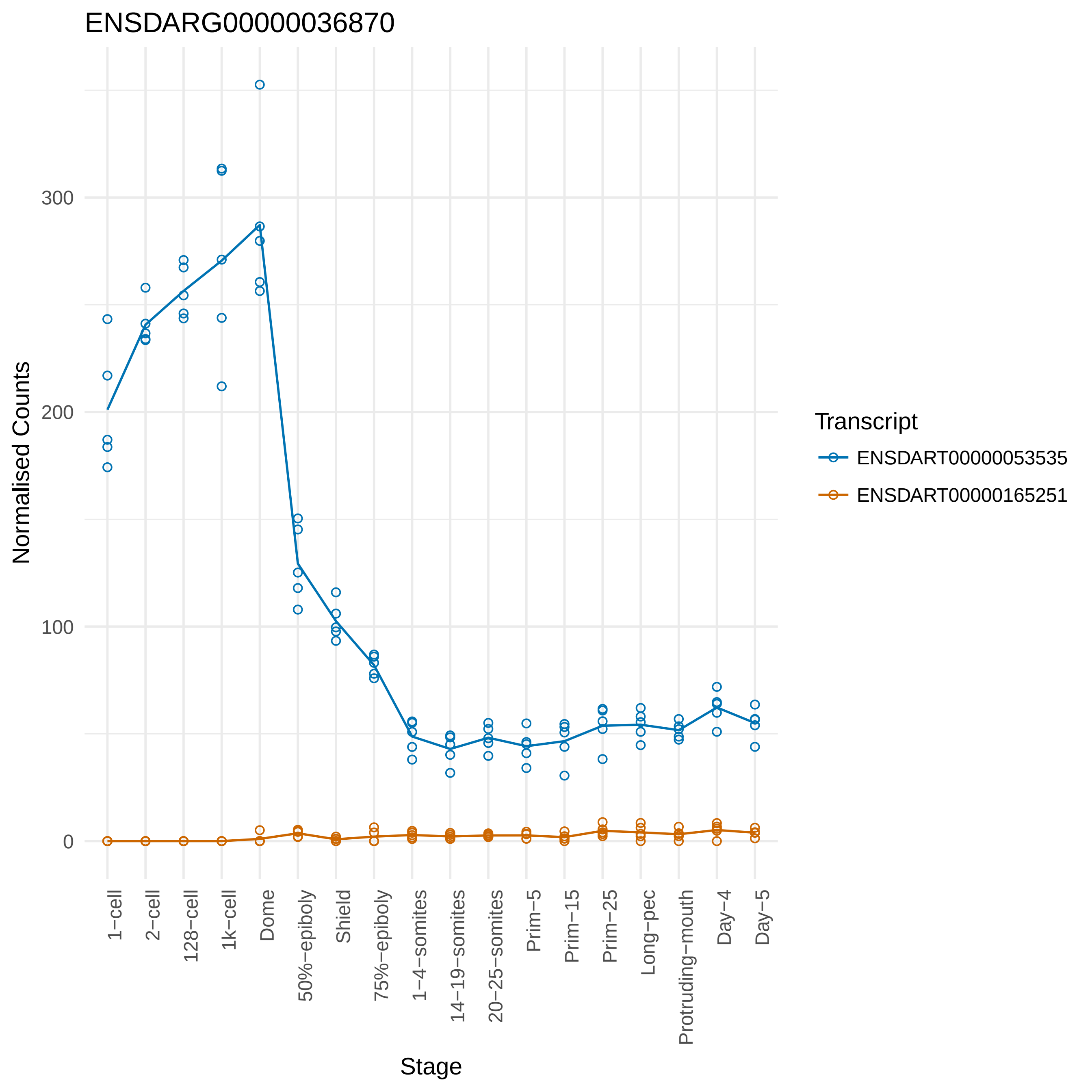

ENSDARG00000036870
Normalised Counts
300
200
100
0
1−cell
2−cell
128−cell
1k−cell
Dome
50%−epiboly
Shield
75%−epiboly
1−4−somites
14−19−somites
20−25−somites
Prim−5
Prim−15
Prim−25
Long−pec
Protruding−mouth
Day−4
Day−5
Stage
Transcript
ENSDART00000053535
ENSDART00000165251

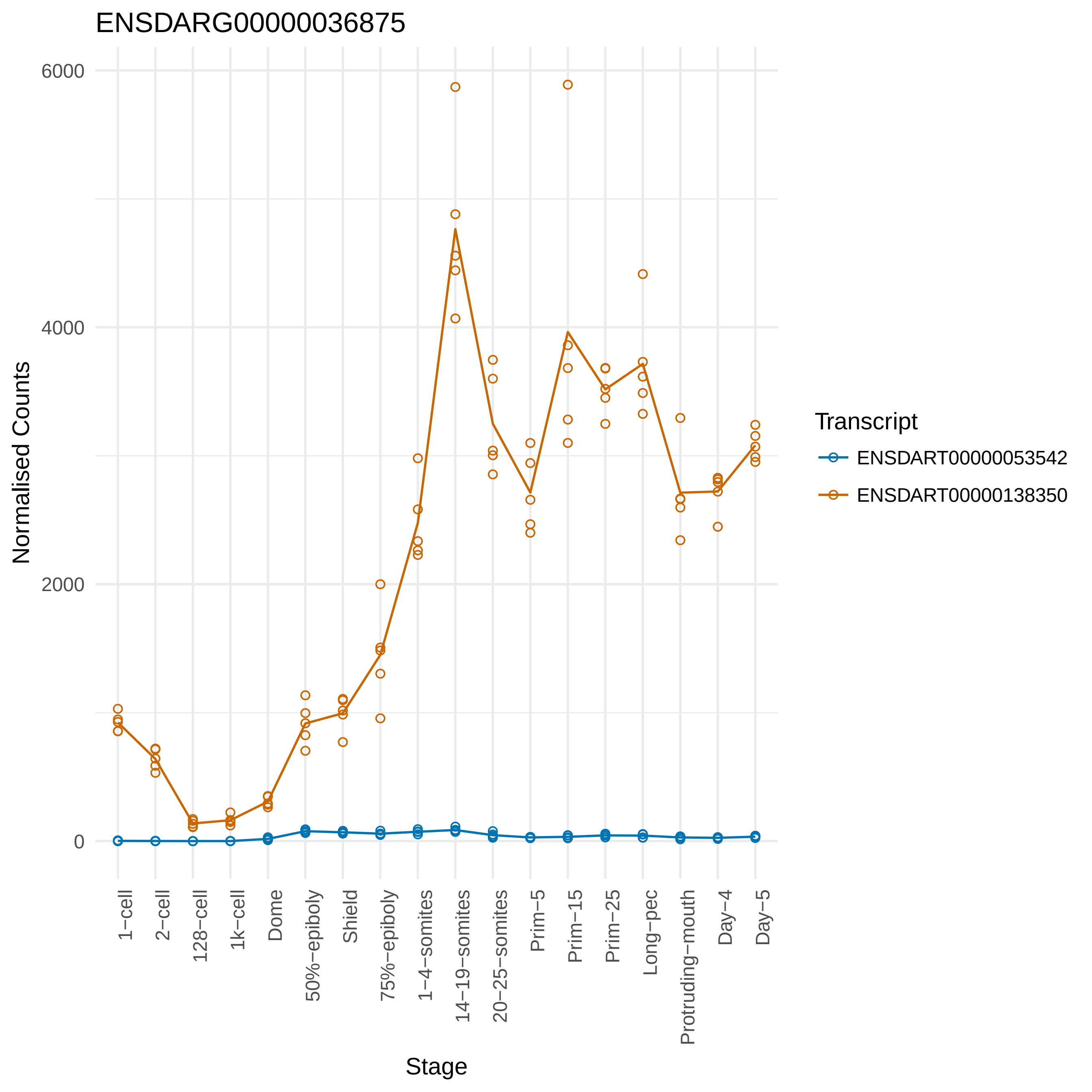

ENSDARG00000036875
Normalised Counts
6000
4000
2000
0
1−cell
2−cell
128−cell
1k−cell
Dome
50%−epiboly
Shield
75%−epiboly
1−4−somites
14−19−somites
20−25−somites
Prim−5
Prim−15
Prim−25
Long−pec
Protruding−mouth
Day−4
Day−5
Stage
Transcript
ENSDART00000053542
ENSDART00000138350

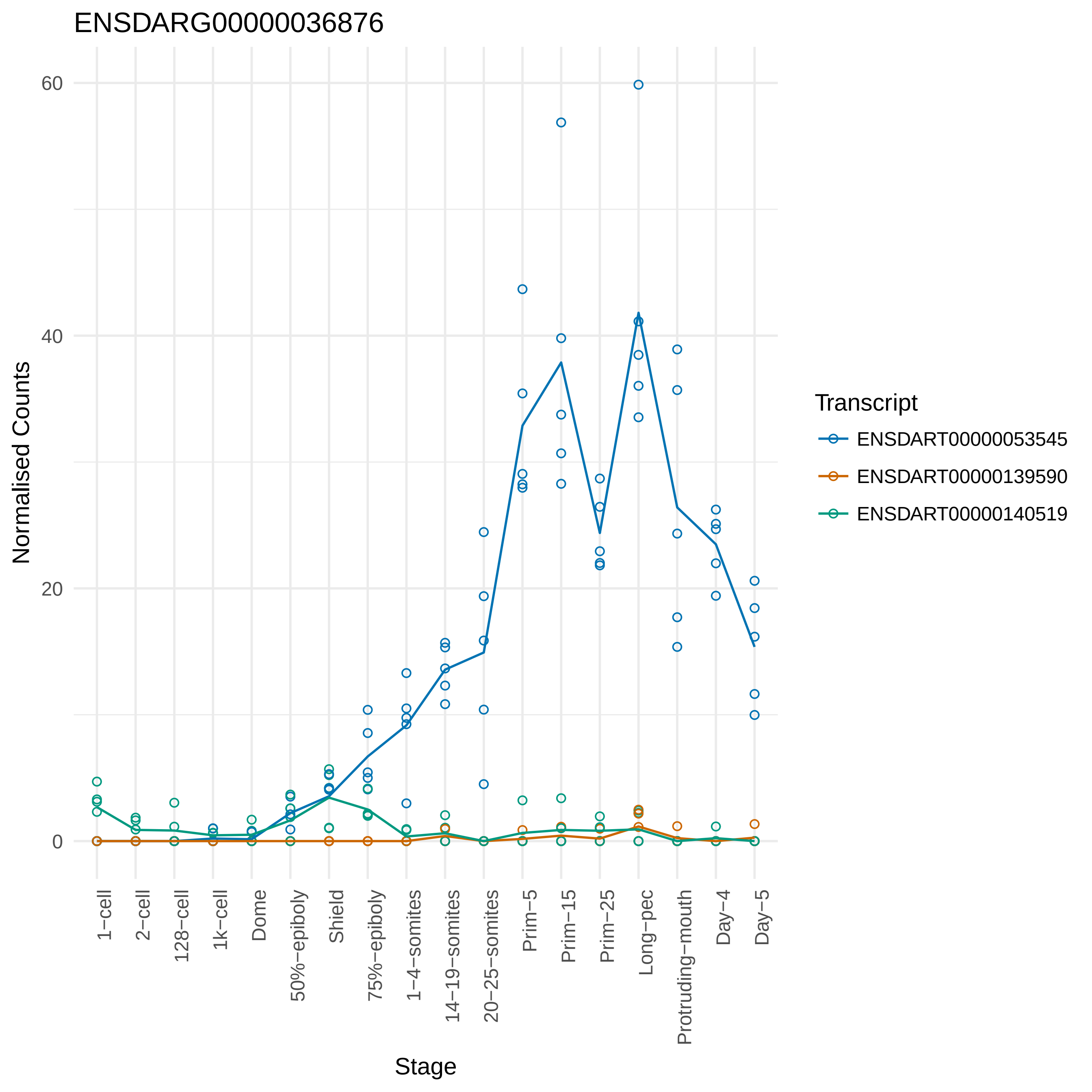
ENSDARG00000036876
Normalised Counts
60
40
20
0
Stage
1−cell
2−cell
128−cell
1k−cell
Dome
50%−epiboly
Shield
75%−epiboly
1−4−somites
14−19−somites
20−25−somites
Prim−5
Prim−15
Prim−25
Long−pec
Protruding−mouth
Day−4
Day−5
Transcript
ENSDART00000053545
ENSDART00000139590
ENSDART00000140519

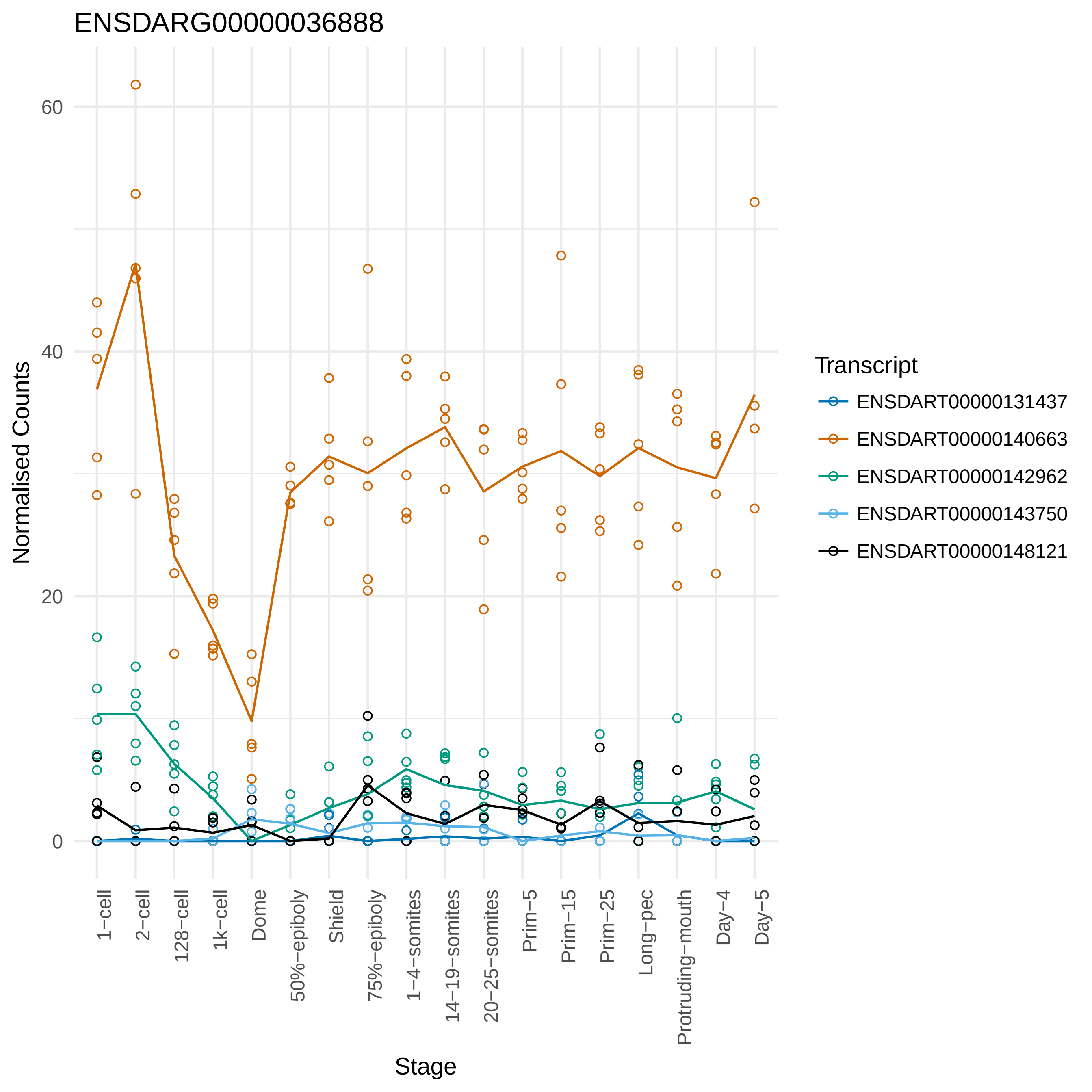

ENSDARG00000036888
Normalised Counts
0
20
40
60
Stage
1−cell
2−cell
128−cell
1k−cell
Dome
50%−epiboly
Shield
75%−epiboly
1−4−somites
14−19−somites
20−25−somites
Prim−5
Prim−15
Prim−25
Long−pec
Protruding−mouth
Day−4
Day−5
Transcript
ENSDART00000131437
ENSDART00000140663
ENSDART00000142962
ENSDART00000143750
ENSDART00000148121

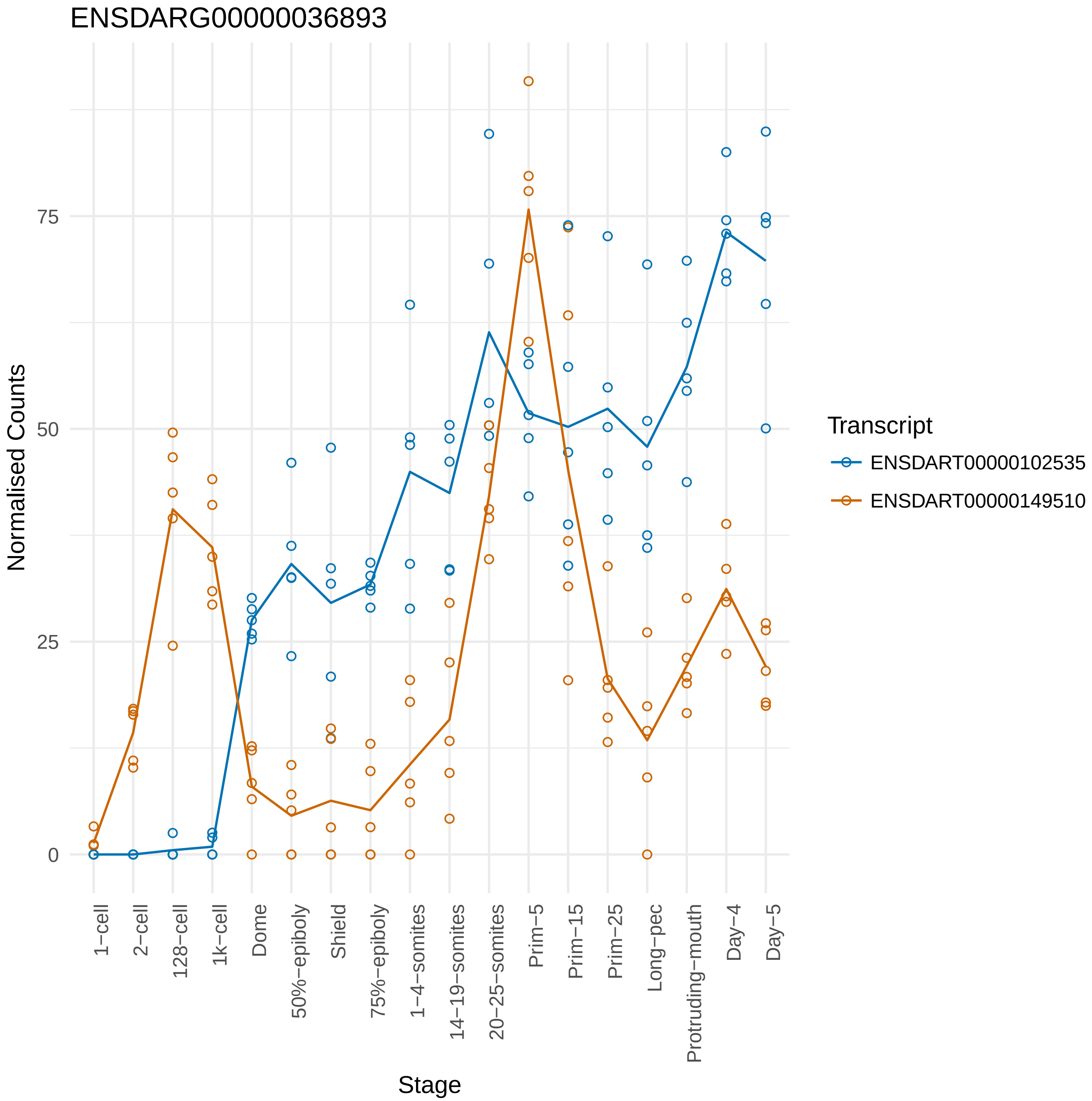
ENSDARG00000036893
Normalised Counts
0
25
50
75
Stage
1−cell
2−cell
128−cell
1k−cell
Dome
50%−epiboly
Shield
75%−epiboly
1−4−somites
14−19−somites
20−25−somites
Prim−5
Prim−15
Prim−25
Long−pec
Protruding−mouth
Day−4
Day−5
Transcript
ENSDART00000102535
ENSDART00000149510

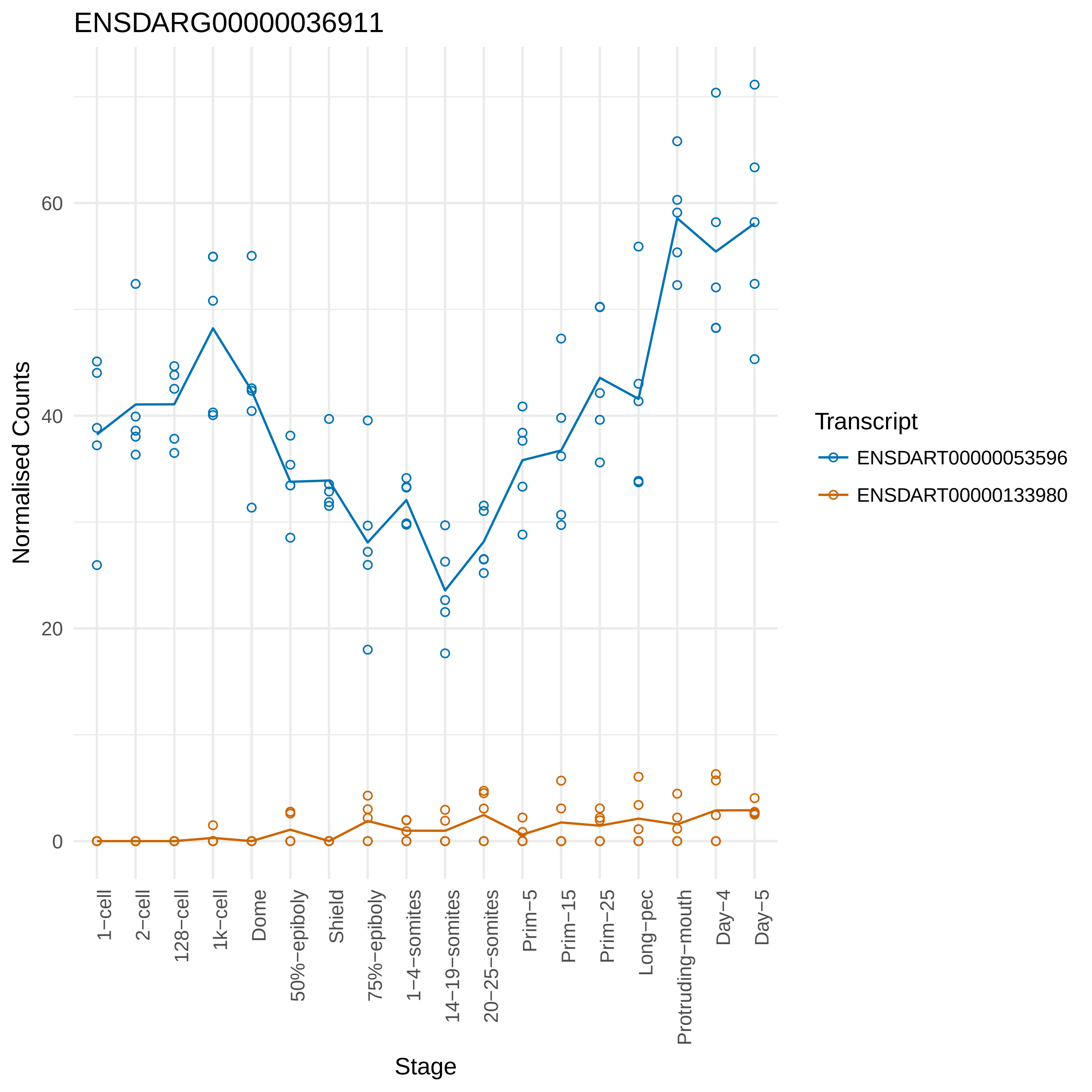

ENSDARG00000036911
Normalised Counts
60
40
20
0
1−cell
2−cell
128−cell
1k−cell
Dome
50%−epiboly
Shield
75%−epiboly
1−4−somites
14−19−somites
20−25−somites
Prim−5
Prim−15
Prim−25
Long−pec
Protruding−mouth
Day−4
Day−5
Stage
Transcript
ENSDART00000053596
ENSDART00000133980

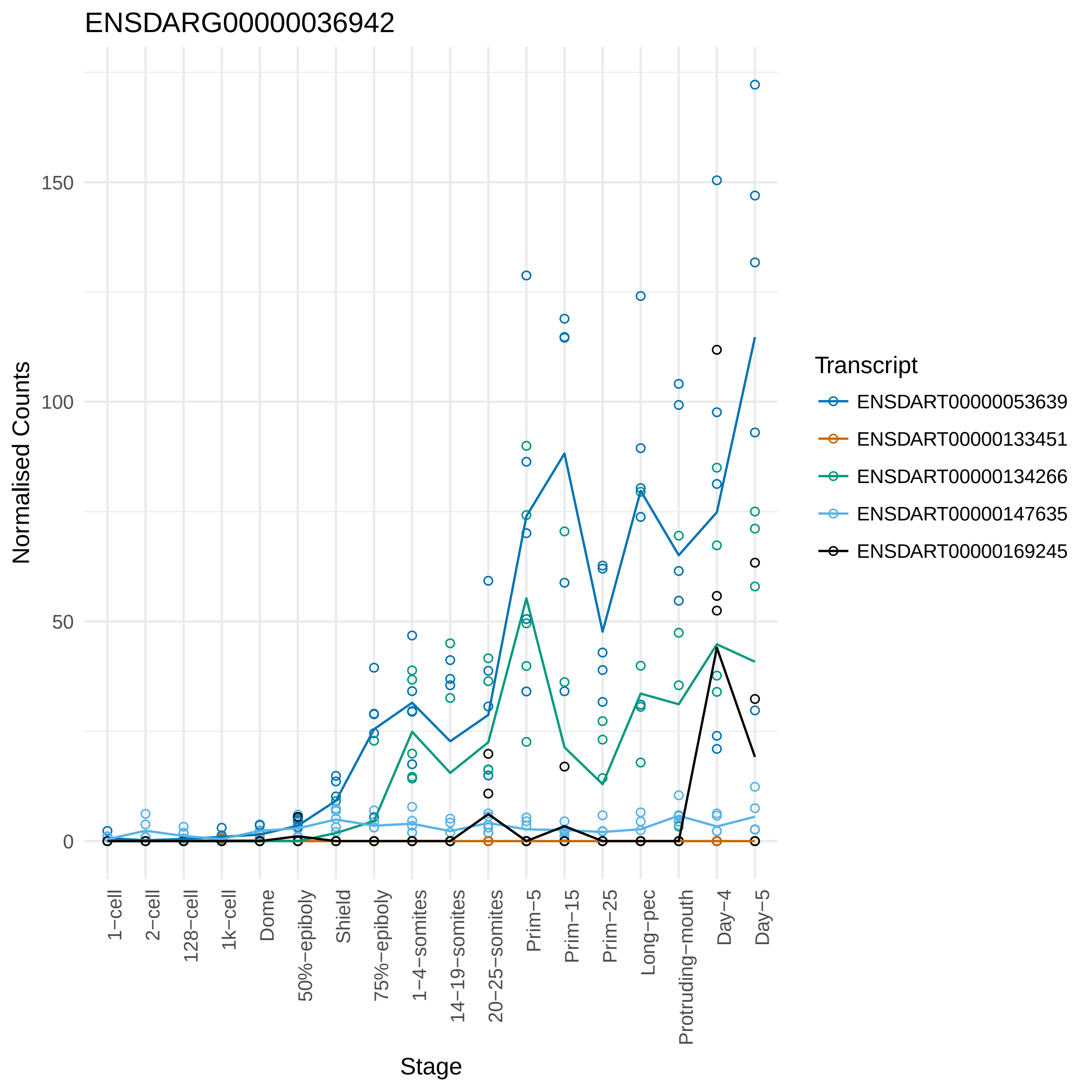

ENSDARG00000036942
Normalised Counts
150
100
50
0
1−cell
2−cell
128−cell
1k−cell
Dome
50%−epiboly
Shield
75%−epiboly
1−4−somites
14−19−somites
20−25−somites
Prim−5
Prim−15
Prim−25
Long−pec
Protruding−mouth
Day−4
Day−5
Stage
Transcript
ENSDART00000053639
ENSDART00000133451
ENSDART00000134266
ENSDART00000147635
ENSDART00000169245

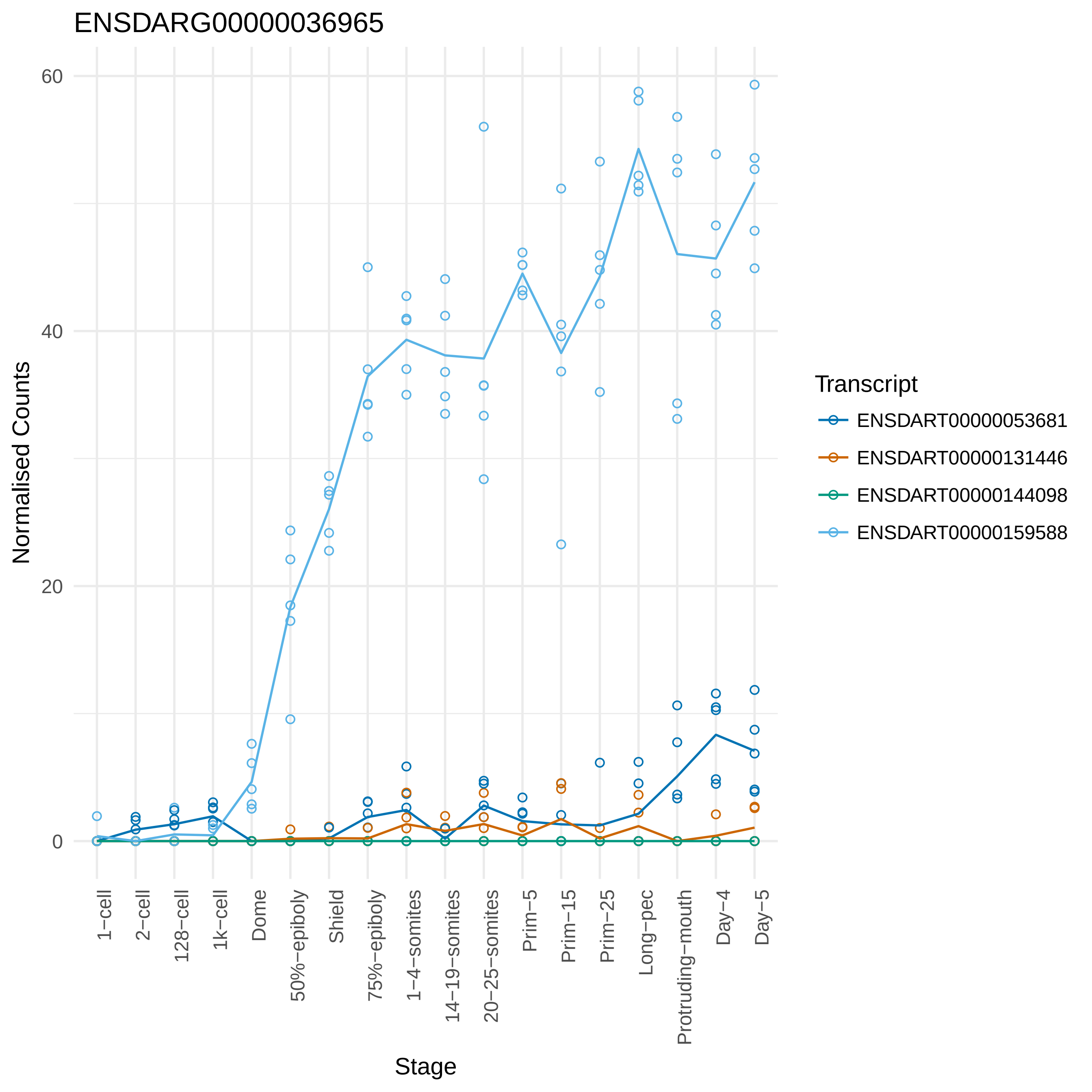

ENSDARG00000036965
Normalised Counts
60
40
20
0
1−cell
2−cell
128−cell
1k−cell
Dome
50%−epiboly
Shield
75%−epiboly
1−4−somites
14−19−somites
20−25−somites
Prim−5
Prim−15
Prim−25
Long−pec
Protruding−mouth
Day−4
Day−5
Stage
Transcript
ENSDART00000053681
ENSDART00000131446
ENSDART00000144098
ENSDART00000159588

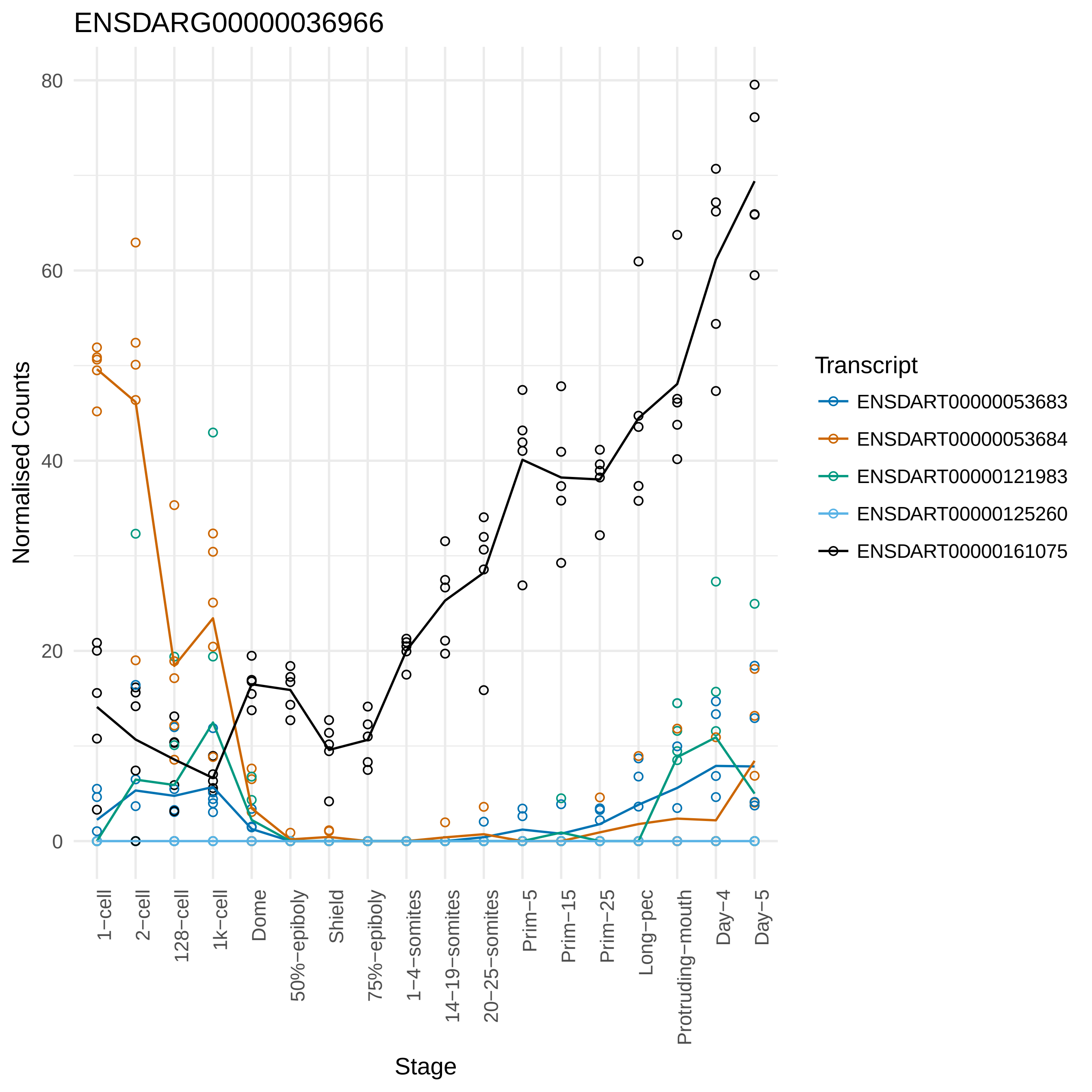

ENSDARG00000036966
Normalised Counts
80
60
40
20
0
Stage
1−cell
2−cell
128−cell
1k−cell
Dome
50%−epiboly
Shield
75%−epiboly
1−4−somites
14−19−somites
20−25−somites
Prim−5
Prim−15
Prim−25
Long−pec
Protruding−mouth
Day−4
Day−5
Transcript
ENSDART00000053683
ENSDART00000053684
ENSDART00000121983
ENSDART00000125260
ENSDART00000161075

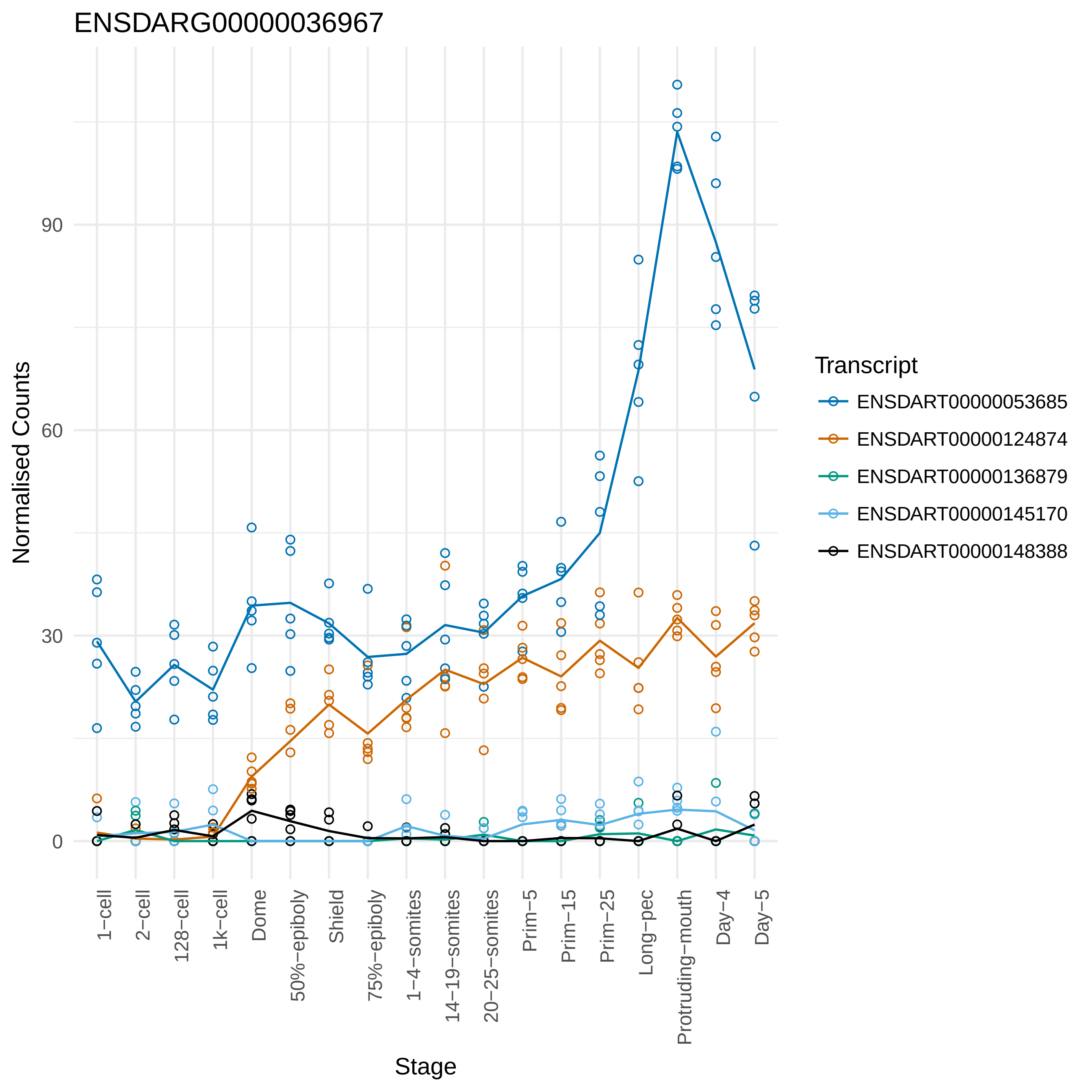
ENSDARG00000036967
Normalised Counts
90
60
30
0
1−cell
2−cell
128−cell
1k−cell
Dome
50%−epiboly
Shield
75%−epiboly
1−4−somites
14−19−somites
20−25−somites
Prim−5
Prim−15
Prim−25
Long−pec
Protruding−mouth
Day−4
Day−5
Stage
Transcript
ENSDART00000053685
ENSDART00000124874
ENSDART00000136879
ENSDART00000145170
ENSDART00000148388

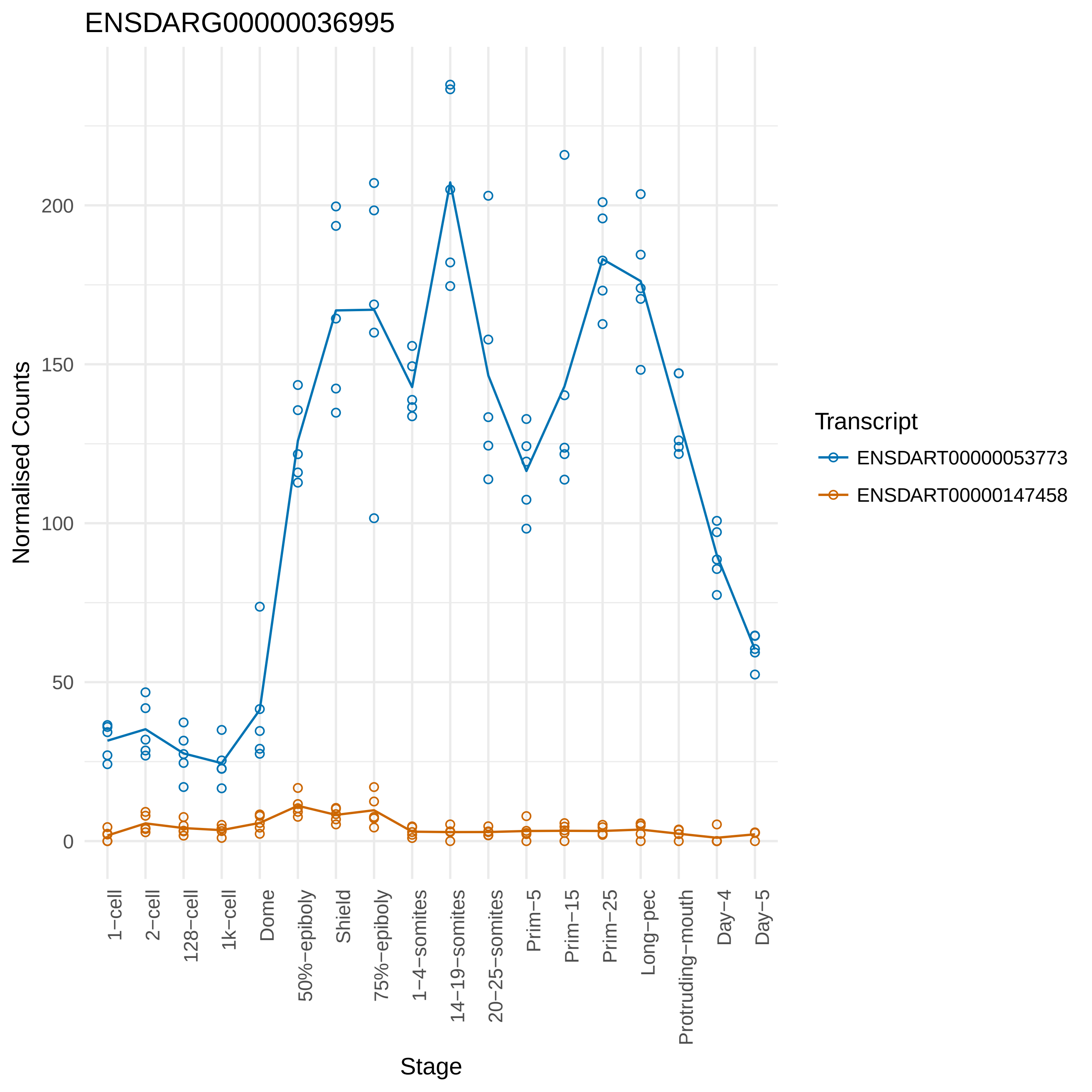

ENSDARG00000036995
Normalised Counts
0
50
100
150
200
Stage
1−cell
2−cell
128−cell
1k−cell
Dome
50%−epiboly
Shield
75%−epiboly
1−4−somites
14−19−somites
20−25−somites
Prim−5
Prim−15
Prim−25
Long−pec
Protruding−mouth
Day−4
Day−5
Transcript
ENSDART00000053773
ENSDART00000147458

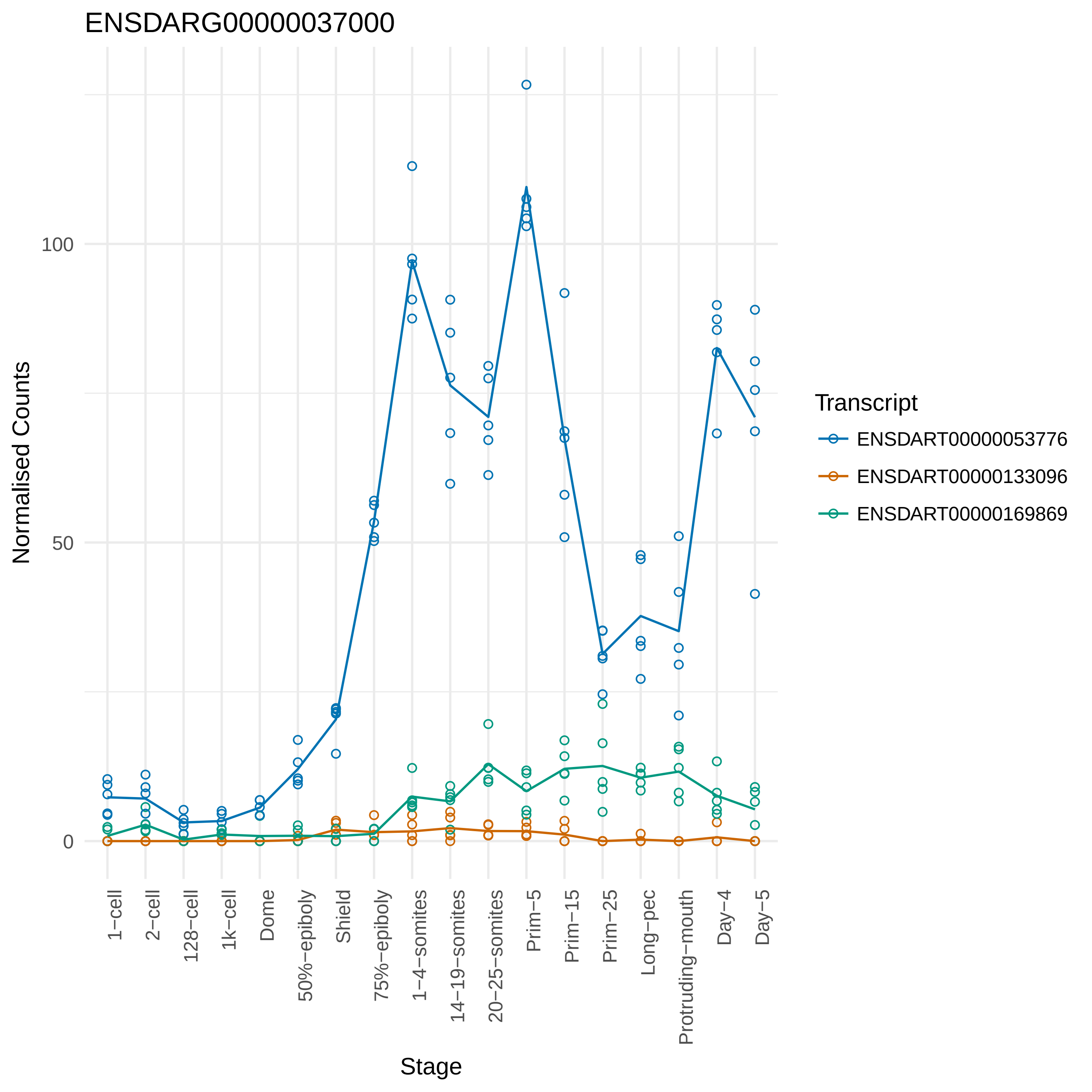

ENSDARG00000037000
Normalised Counts
100
50
0
1−cell
2−cell
128−cell
1k−cell
Dome
50%−epiboly
Shield
75%−epiboly
1−4−somites
14−19−somites
20−25−somites
Prim−5
Prim−15
Prim−25
Long−pec
Protruding−mouth
Day−4
Day−5
Stage
Transcript
ENSDART00000053776
ENSDART00000133096
ENSDART00000169869

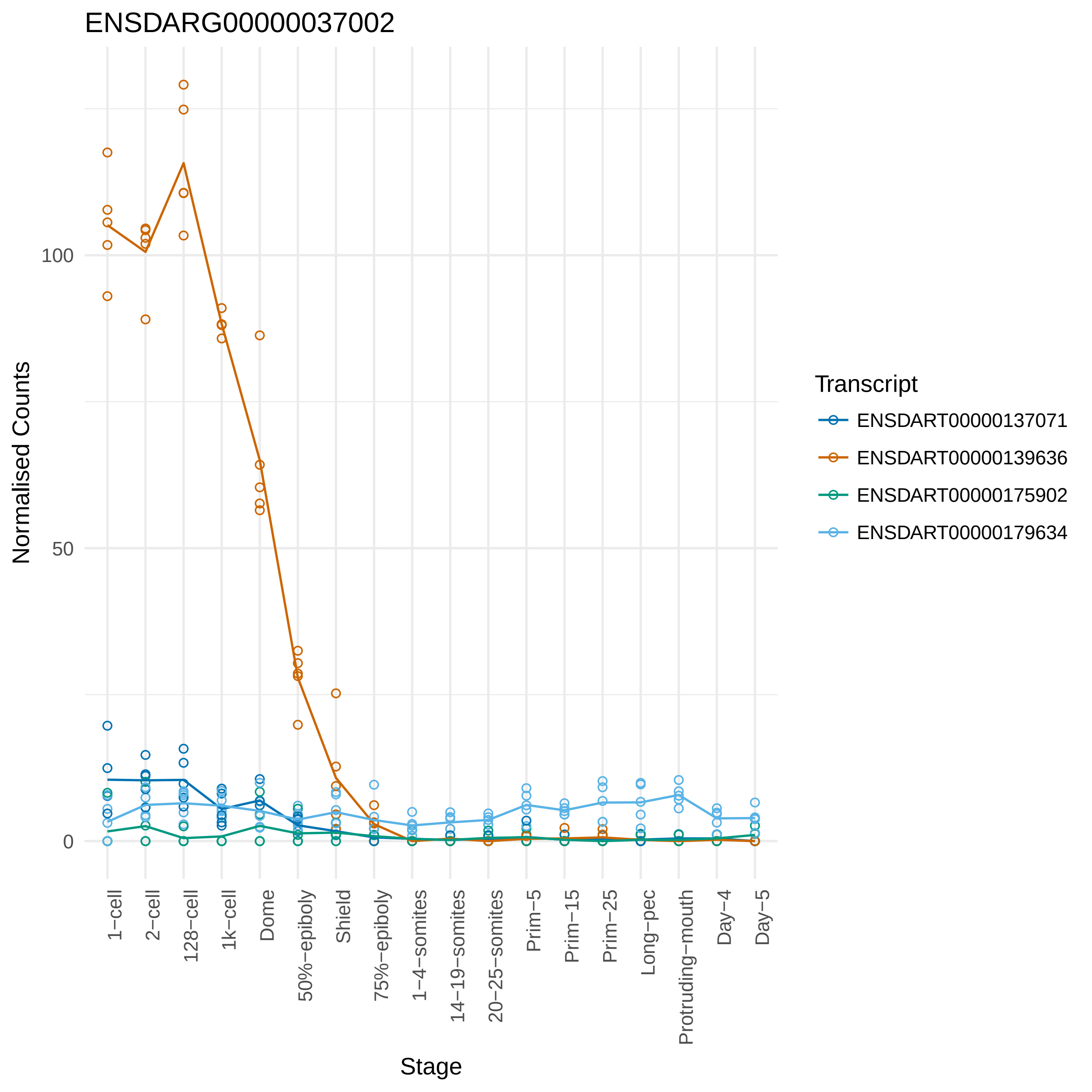
ENSDARG00000037002
Normalised Counts
100
50
0
1−cell
2−cell
128−cell
1k−cell
Dome
50%−epiboly
Shield
75%−epiboly
1−4−somites
14−19−somites
20−25−somites
Prim−5
Prim−15
Prim−25
Long−pec
Protruding−mouth
Day−4
Day−5
Stage
Transcript
ENSDART00000137071
ENSDART00000139636
ENSDART00000175902
ENSDART00000179634

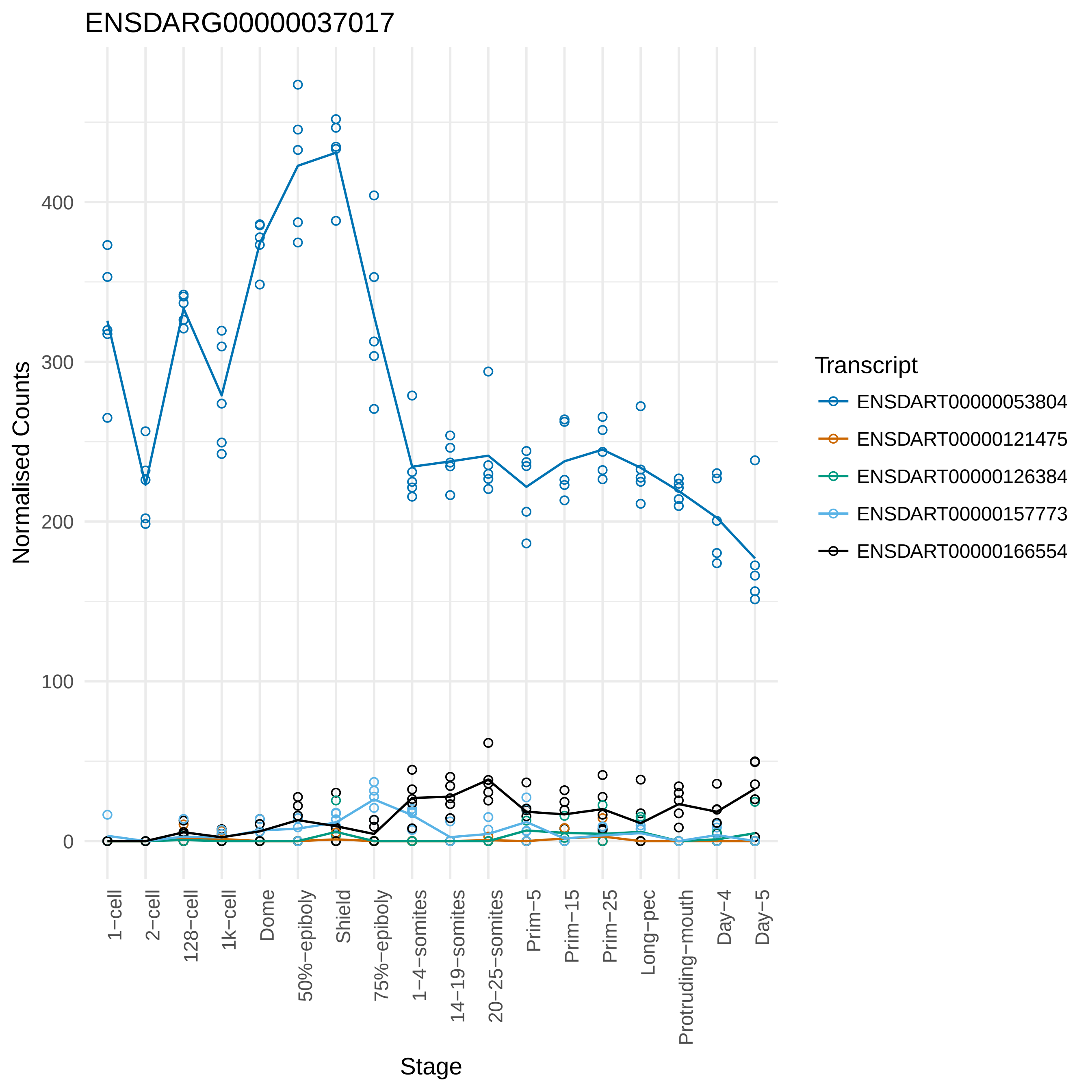

ENSDARG00000037017
Normalised Counts
400
300
200
100
0
1−cell
2−cell
128−cell
1k−cell
Dome
50%−epiboly
Shield
75%−epiboly
1−4−somites
14−19−somites
20−25−somites
Prim−5
Prim−15
Prim−25
Long−pec
Protruding−mouth
Day−4
Day−5
Stage
Transcript
ENSDART00000053804
ENSDART00000121475
ENSDART00000126384
ENSDART00000157773
ENSDART00000166554

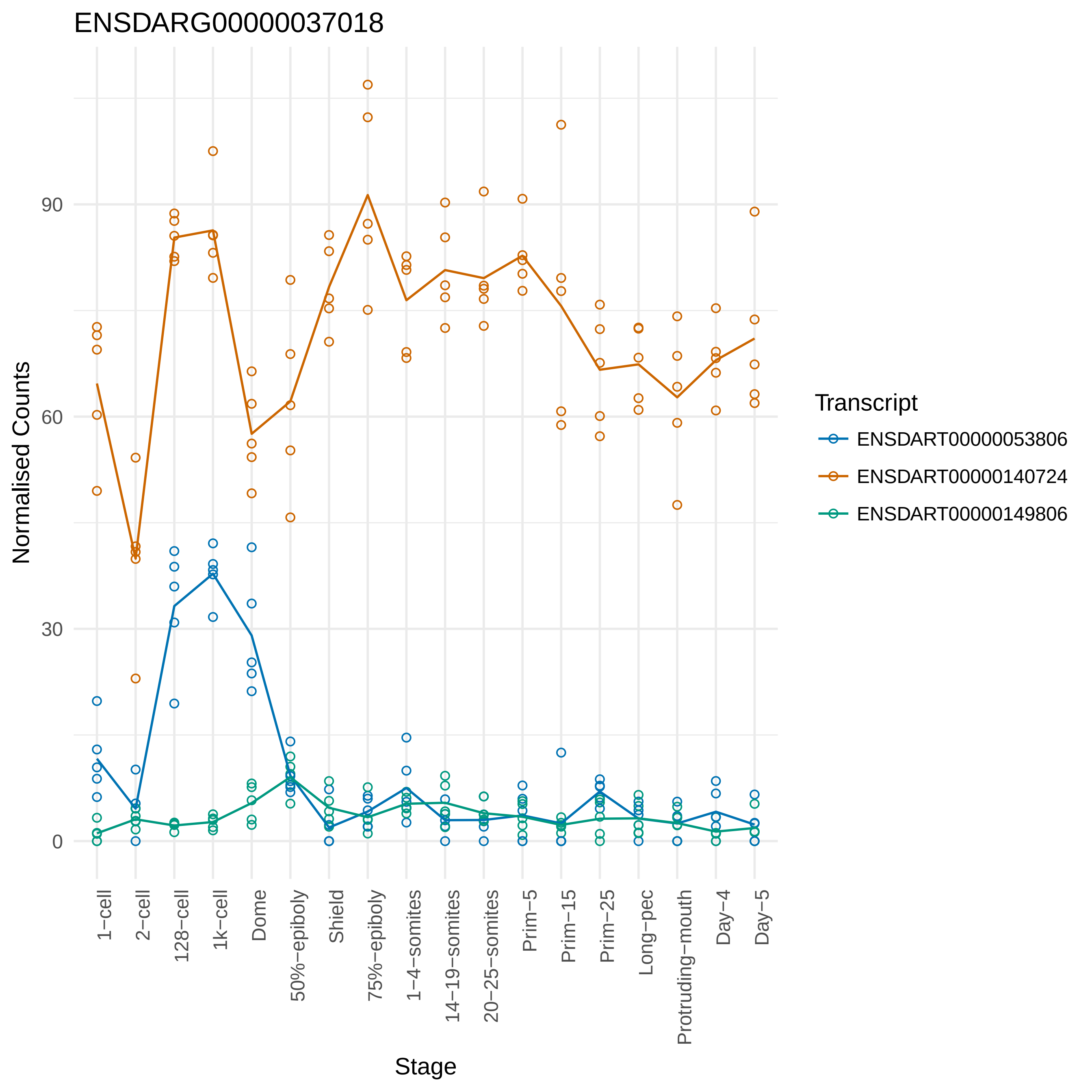
ENSDARG00000037018
Normalised Counts
90
60
30
0
1−cell
2−cell
128−cell
1k−cell
Dome
50%−epiboly
Shield
75%−epiboly
1−4−somites
14−19−somites
20−25−somites
Prim−5
Prim−15
Prim−25
Long−pec
Protruding−mouth
Day−4
Day−5
Stage
Transcript
ENSDART00000053806
ENSDART00000140724
ENSDART00000149806

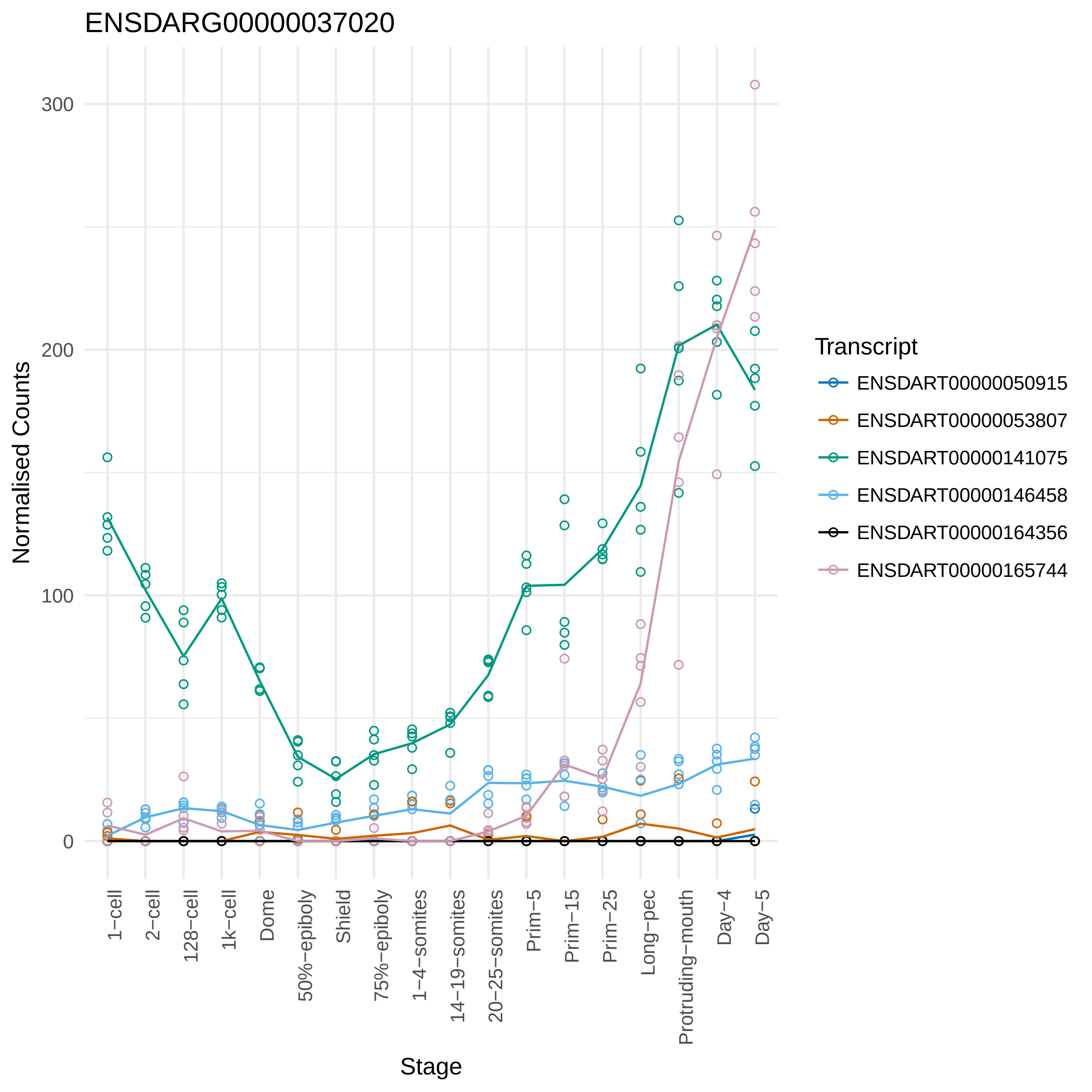

ENSDARG00000037020
Normalised Counts
300
200
100
0
1−cell
2−cell
128−cell
1k−cell
Dome
50%−epiboly
Shield
75%−epiboly
1−4−somites
14−19−somites
20−25−somites
Prim−5
Prim−15
Prim−25
Long−pec
Protruding−mouth
Day−4
Day−5
Stage
Transcript
ENSDART00000050915
ENSDART00000053807
ENSDART00000141075
ENSDART00000146458
ENSDART00000164356
ENSDART00000165744

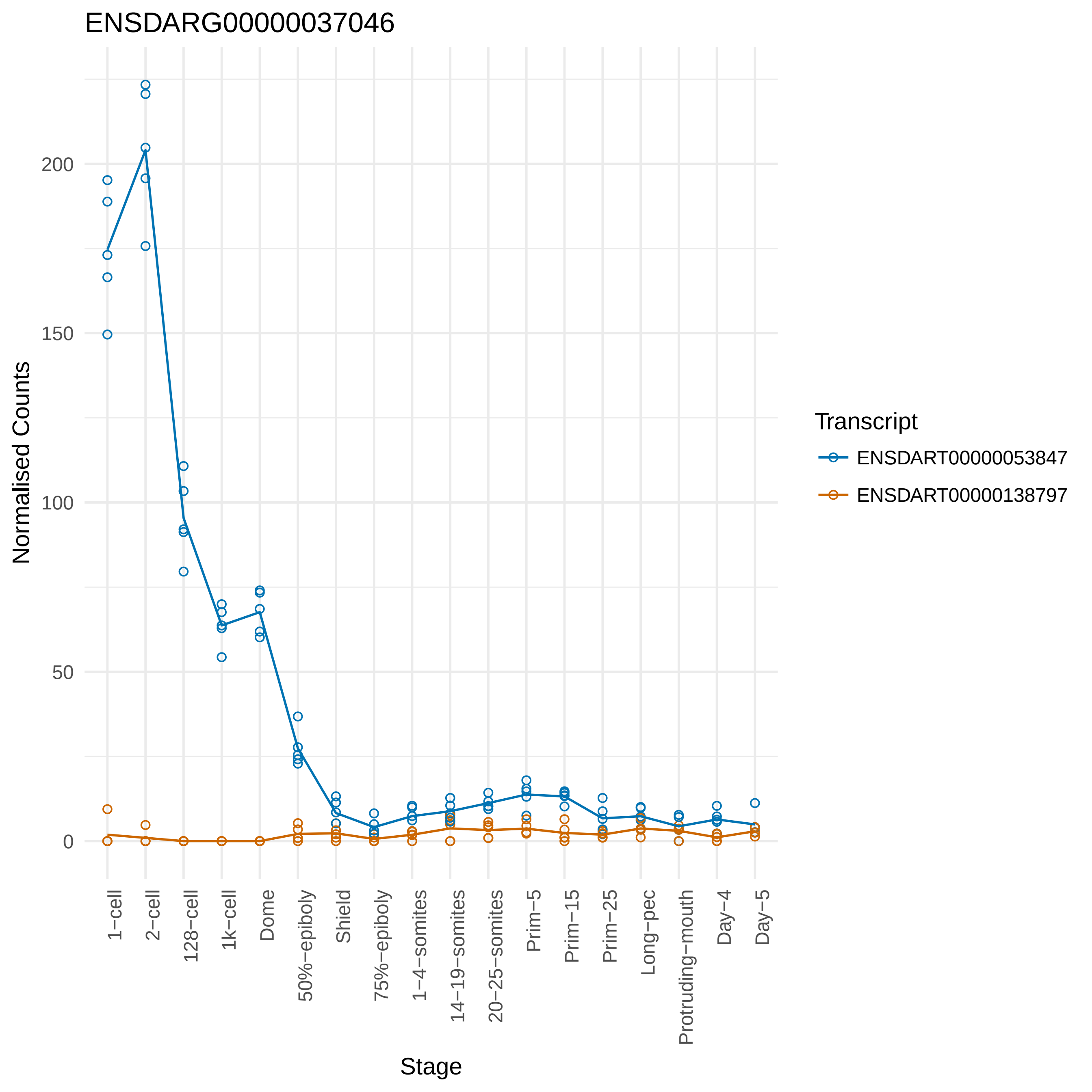
ENSDARG00000037046
Normalised Counts
0
50
100
150
200
Stage
1−cell
2−cell
128−cell
1k−cell
Dome
50%−epiboly
Shield
75%−epiboly
1−4−somites
14−19−somites
20−25−somites
Prim−5
Prim−15
Prim−25
Long−pec
Protruding−mouth
Day−4
Day−5
Transcript
ENSDART00000053847
ENSDART00000138797

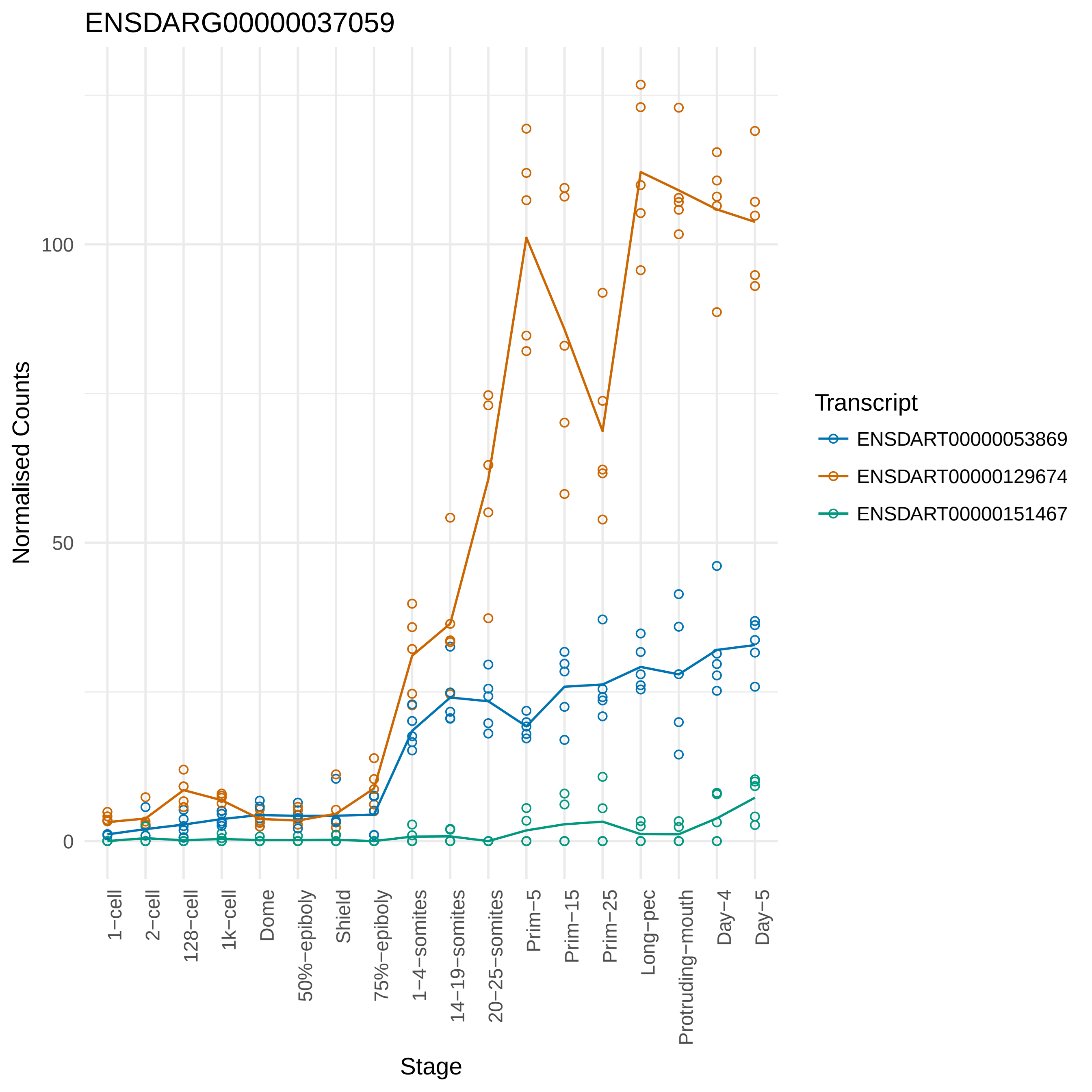

ENSDARG00000037059
Normalised Counts
100
50
0
1−cell
2−cell
128−cell
1k−cell
Dome
50%−epiboly
Shield
75%−epiboly
1−4−somites
14−19−somites
20−25−somites
Prim−5
Prim−15
Prim−25
Long−pec
Protruding−mouth
Day−4
Day−5
Stage
Transcript
ENSDART00000053869
ENSDART00000129674
ENSDART00000151467

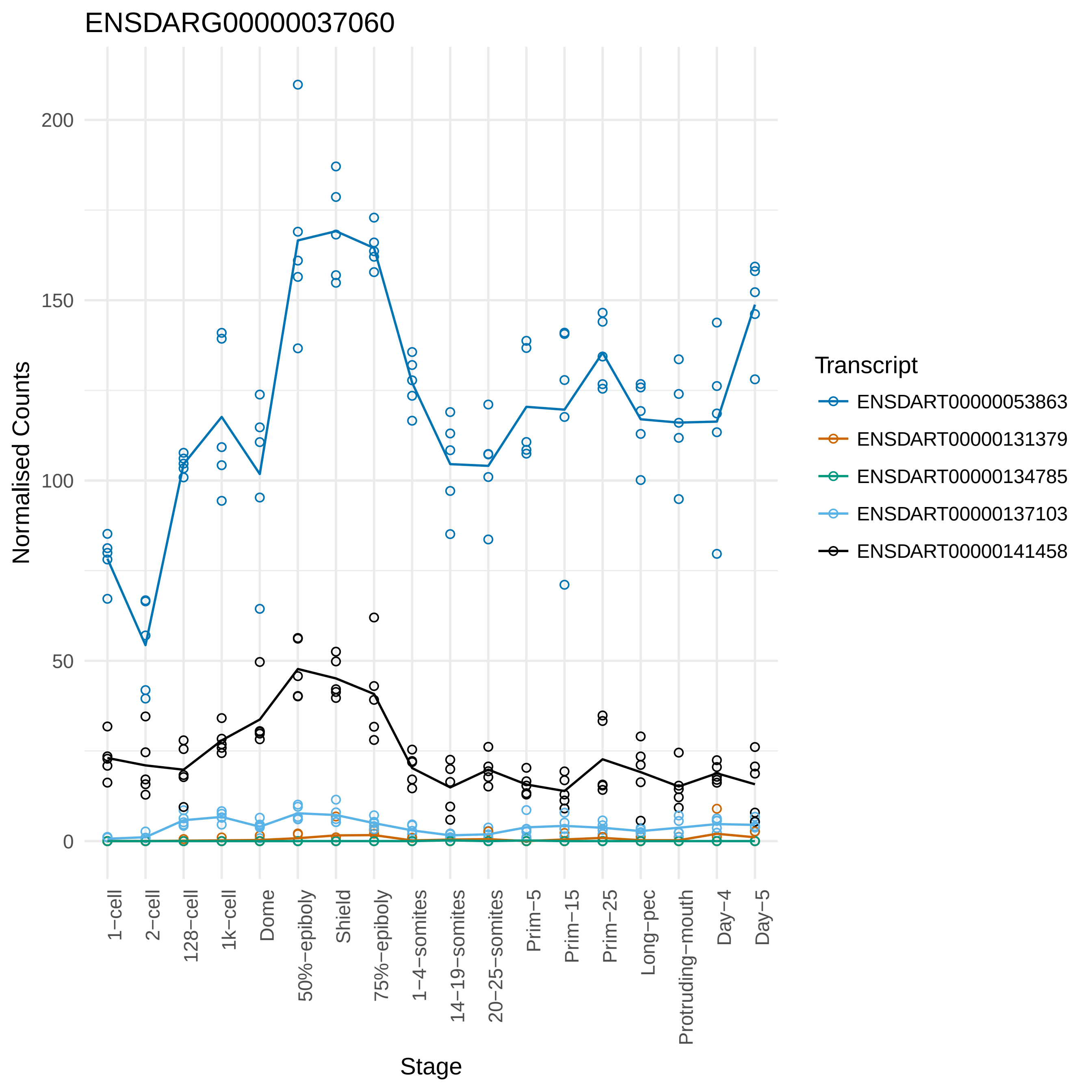

ENSDARG00000037060
Normalised Counts
200
150
100
50
0
1−cell
2−cell
128−cell
1k−cell
Dome
50%−epiboly
Shield
75%−epiboly
1−4−somites
14−19−somites
20−25−somites
Prim−5
Prim−15
Prim−25
Long−pec
Protruding−mouth
Day−4
Day−5
Stage
Transcript
ENSDART00000053863
ENSDART00000131379
ENSDART00000134785
ENSDART00000137103
ENSDART00000141458

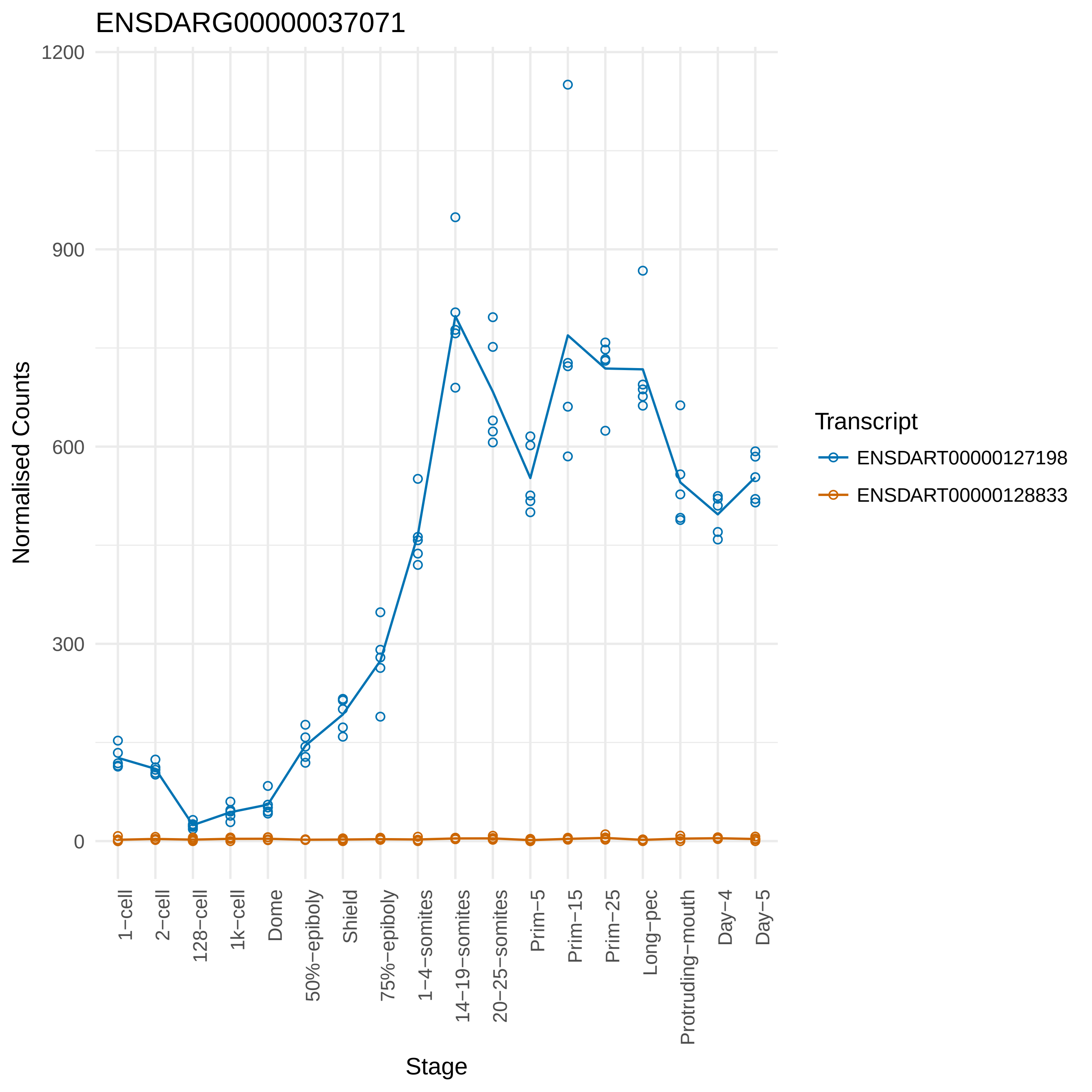

ENSDARG00000037071
Normalised Counts
1200
900
600
300
0
1−cell
2−cell
128−cell
1k−cell
Dome
50%−epiboly
Shield
75%−epiboly
1−4−somites
14−19−somites
20−25−somites
Prim−5
Prim−15
Prim−25
Long−pec
Protruding−mouth
Day−4
Day−5
Stage
Transcript
ENSDART00000127198
ENSDART00000128833

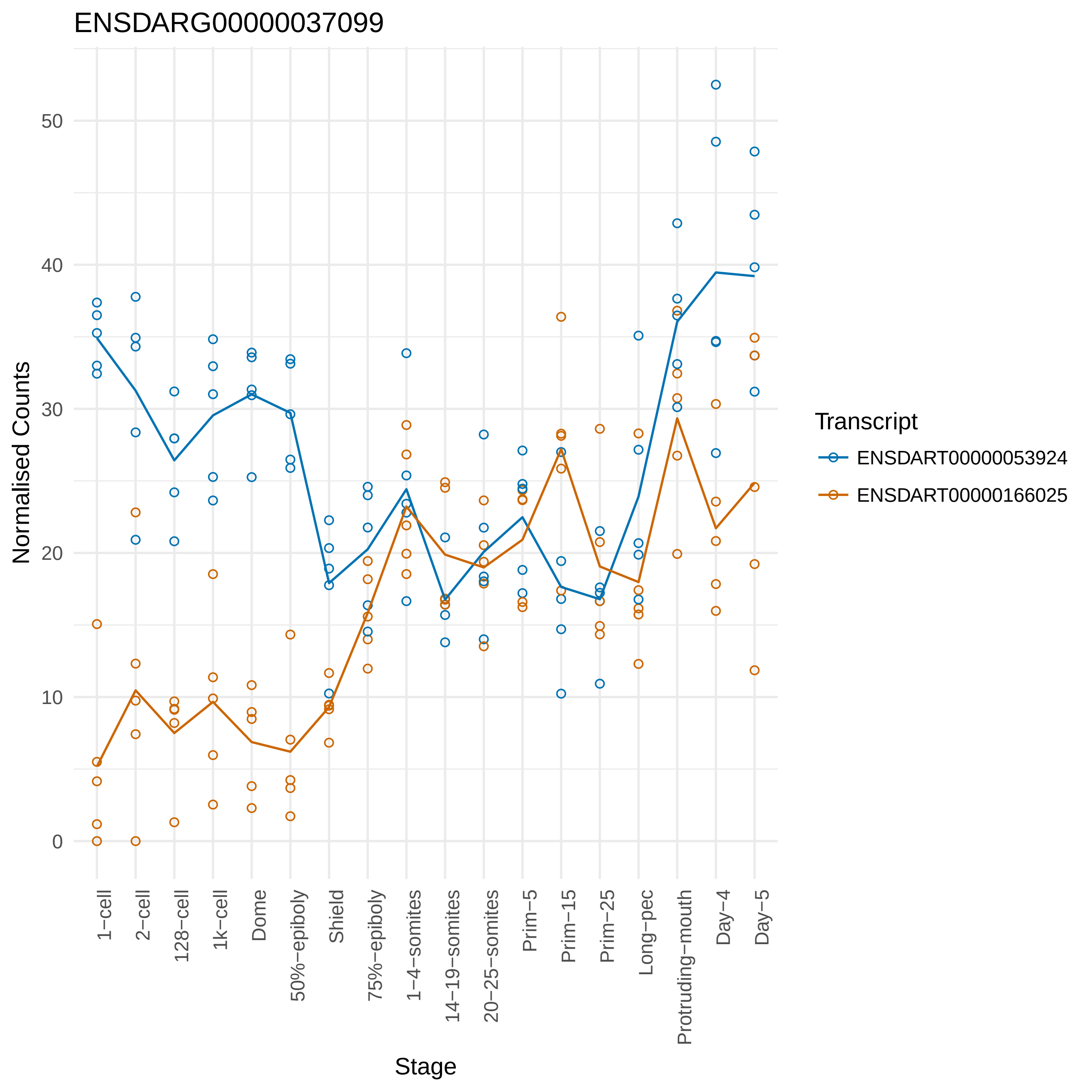

ENSDARG00000037099
Normalised Counts
0
10
20
30
40
50
1−cell
2−cell
128−cell
1k−cell
Dome
50%−epiboly
Shield
75%−epiboly
1−4−somites
14−19−somites
20−25−somites
Prim−5
Prim−15
Prim−25
Long−pec
Protruding−mouth
Day−4
Day−5
Stage
Transcript
ENSDART00000053924
ENSDART00000166025

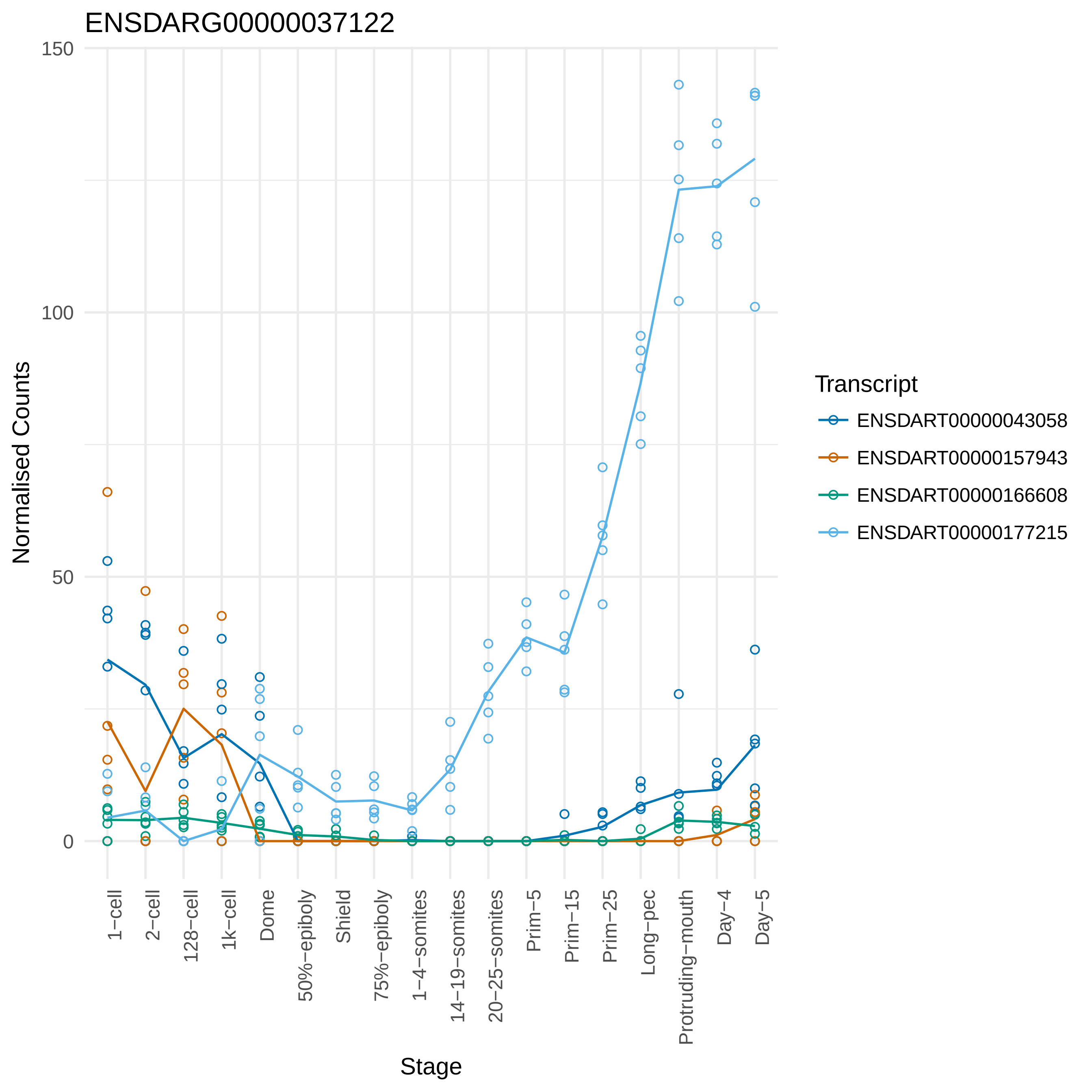

ENSDARG00000037122
Normalised Counts
150
100
50
0
1−cell
2−cell
128−cell
1k−cell
Dome
50%−epiboly
Shield
75%−epiboly
1−4−somites
14−19−somites
20−25−somites
Prim−5
Prim−15
Prim−25
Long−pec
Protruding−mouth
Day−4
Day−5
Stage
Transcript
ENSDART00000043058
ENSDART00000157943
ENSDART00000166608
ENSDART00000177215

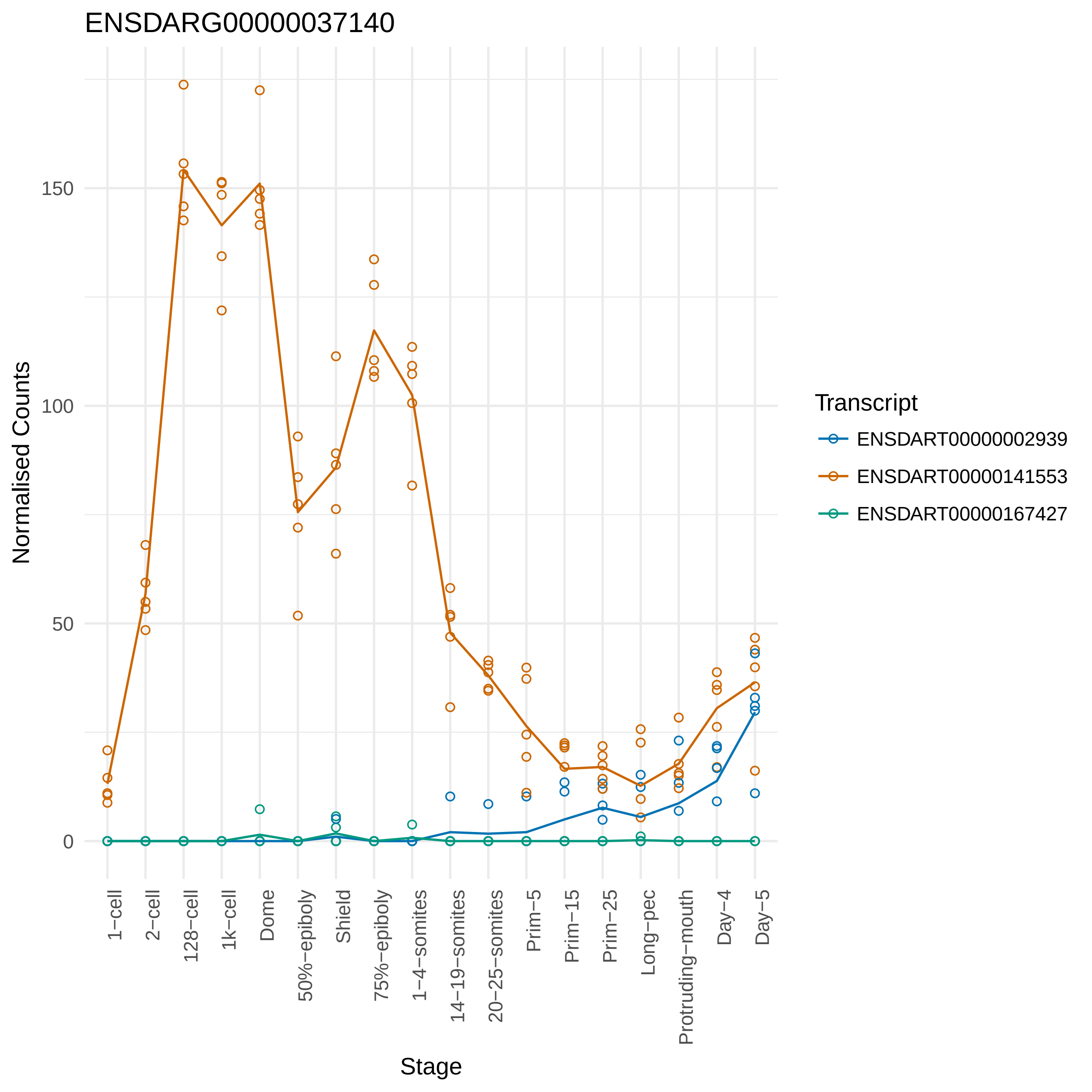

ENSDARG00000037140
Normalised Counts
150
100
50
0
1−cell
2−cell
128−cell
1k−cell
Dome
50%−epiboly
Shield
75%−epiboly
1−4−somites
14−19−somites
20−25−somites
Prim−5
Prim−15
Prim−25
Long−pec
Protruding−mouth
Day−4
Day−5
Stage
Transcript
ENSDART00000002939
ENSDART00000141553
ENSDART00000167427

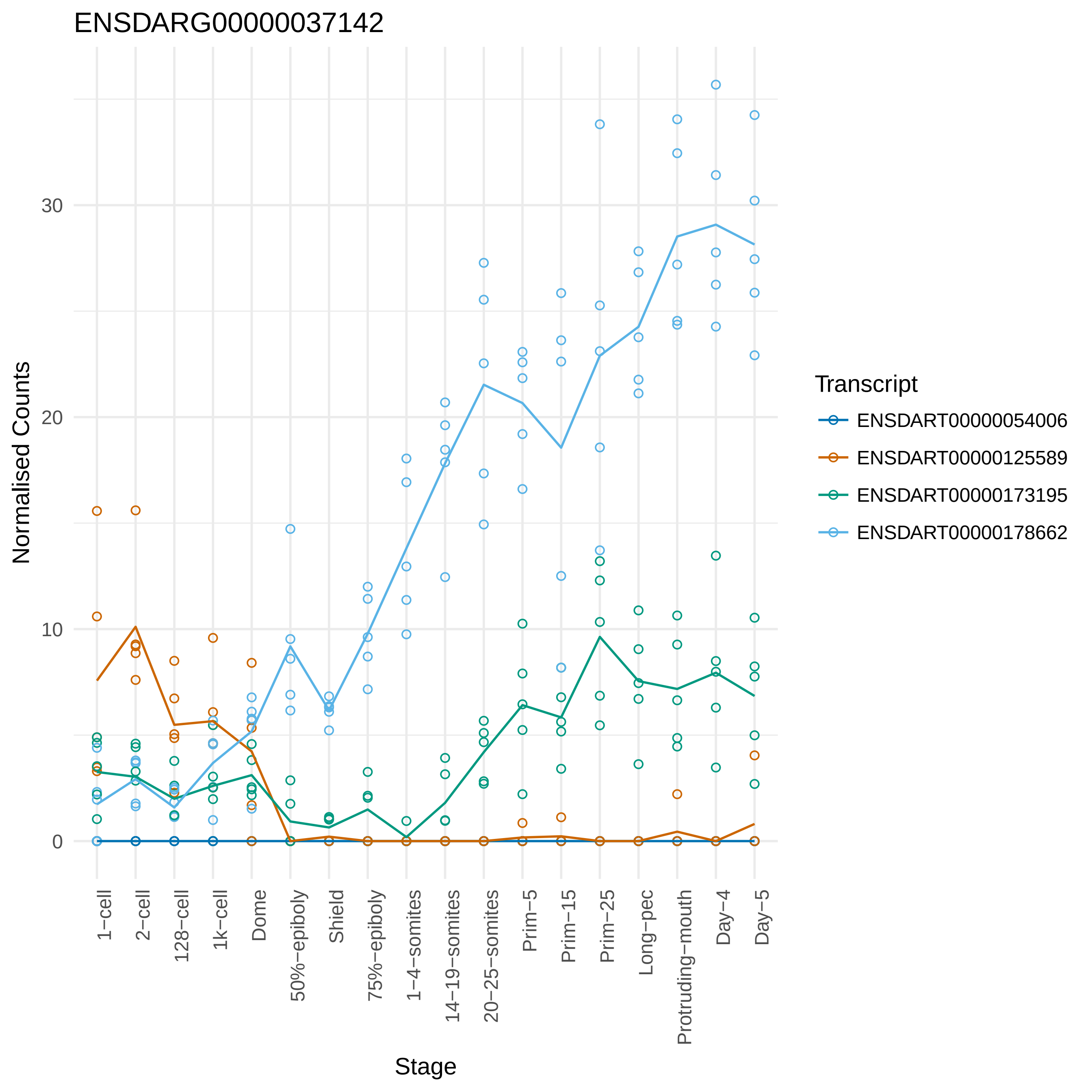

ENSDARG00000037142
Normalised Counts
30
20
10
0
1−cell
2−cell
128−cell
1k−cell
Dome
50%−epiboly
Shield
75%−epiboly
1−4−somites
14−19−somites
20−25−somites
Prim−5
Prim−15
Prim−25
Long−pec
Protruding−mouth
Day−4
Day−5
Stage
Transcript
ENSDART00000054006
ENSDART00000125589
ENSDART00000173195
ENSDART00000178662

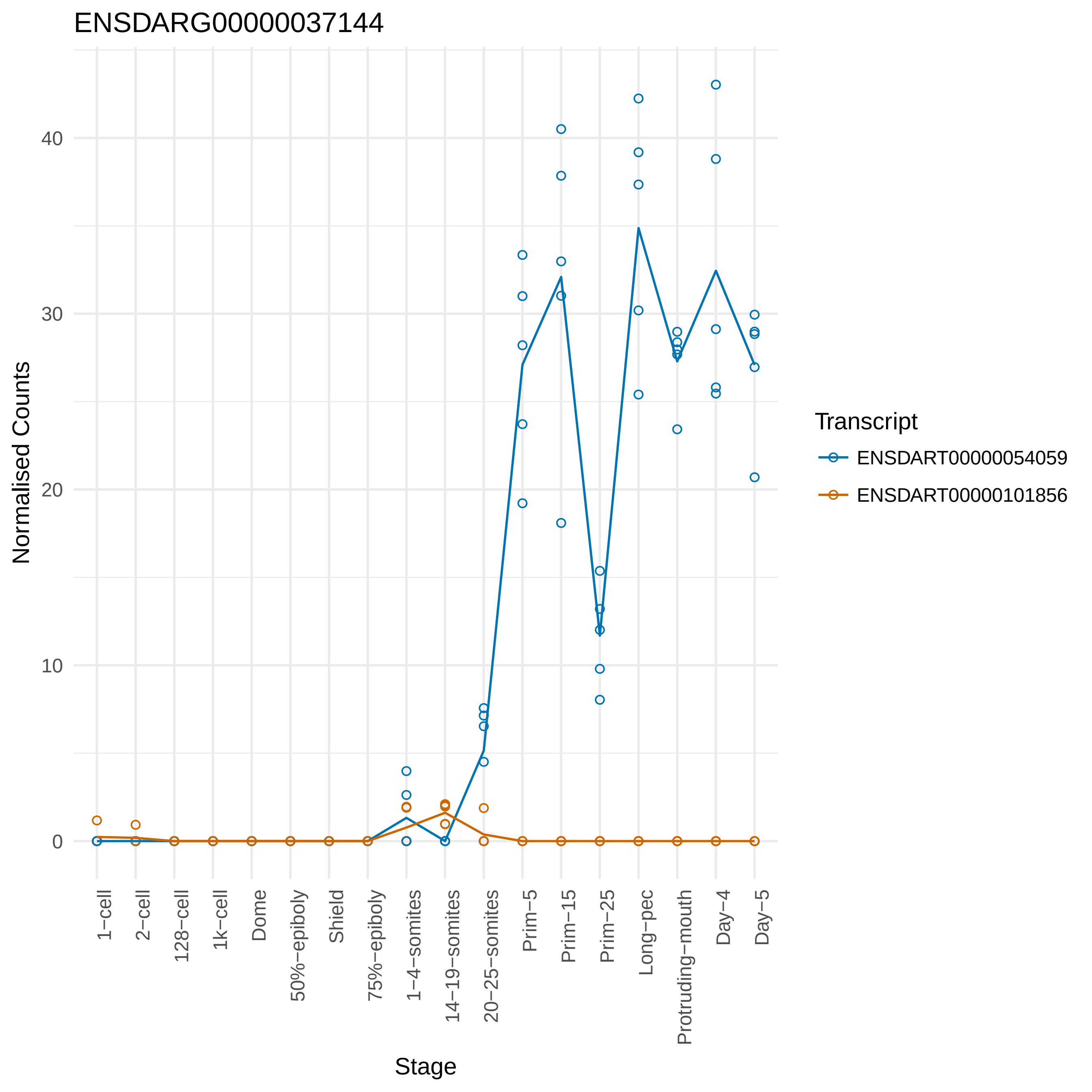
ENSDARG00000037144
Normalised Counts
0
10
20
30
40
1−cell
2−cell
128−cell
1k−cell
Dome
50%−epiboly
Shield
75%−epiboly
1−4−somites
14−19−somites
20−25−somites
Prim−5
Prim−15
Prim−25
Long−pec
Protruding−mouth
Day−4
Day−5
Stage
Transcript
ENSDART00000054059
ENSDART00000101856

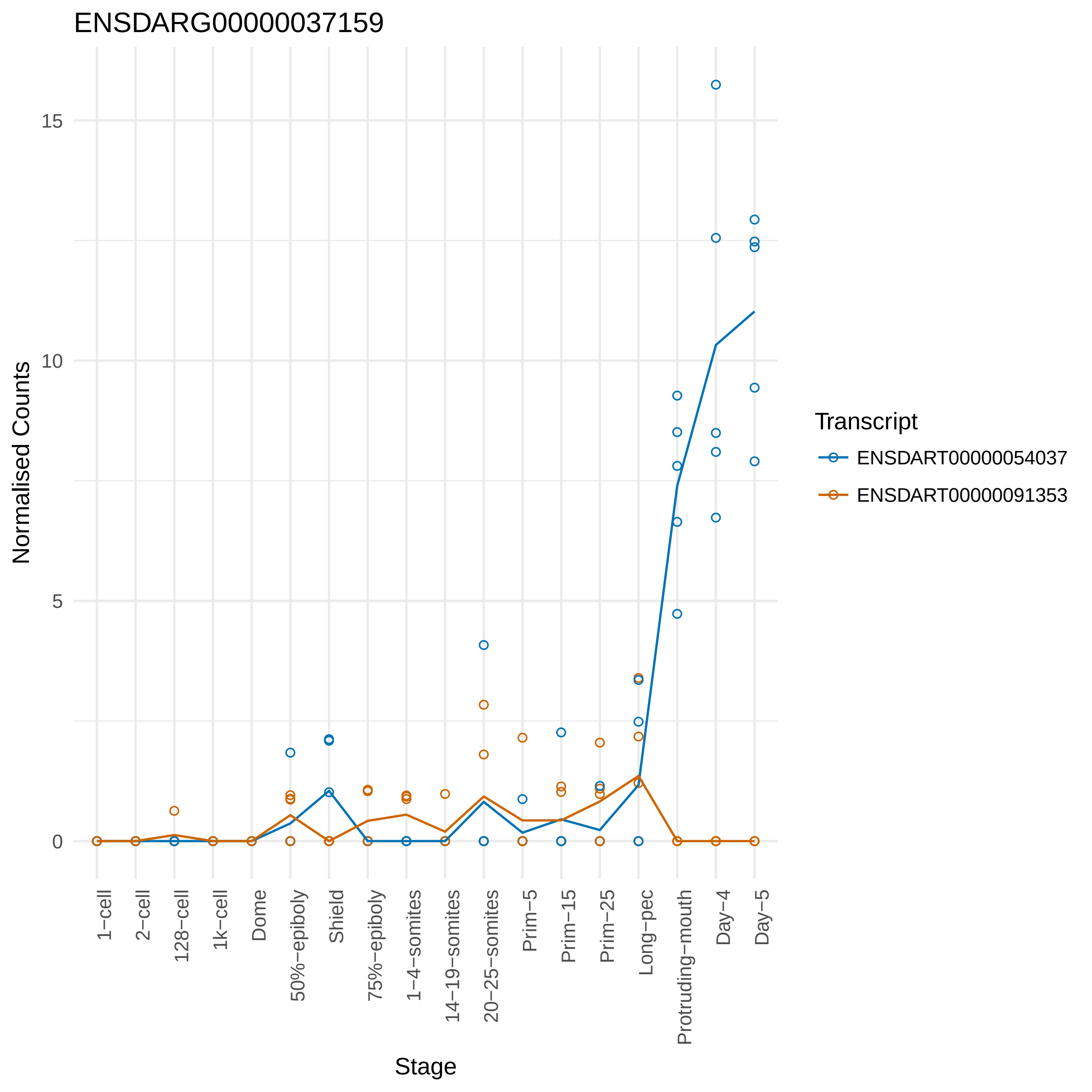

ENSDARG00000037159
Normalised Counts
0
5
10
15
1−cell
2−cell
128−cell
1k−cell
Dome
50%−epiboly
Shield
75%−epiboly
1−4−somites
14−19−somites
20−25−somites
Prim−5
Prim−15
Prim−25
Long−pec
Protruding−mouth
Day−4
Day−5
Stage
Transcript
ENSDART00000054037
ENSDART00000091353

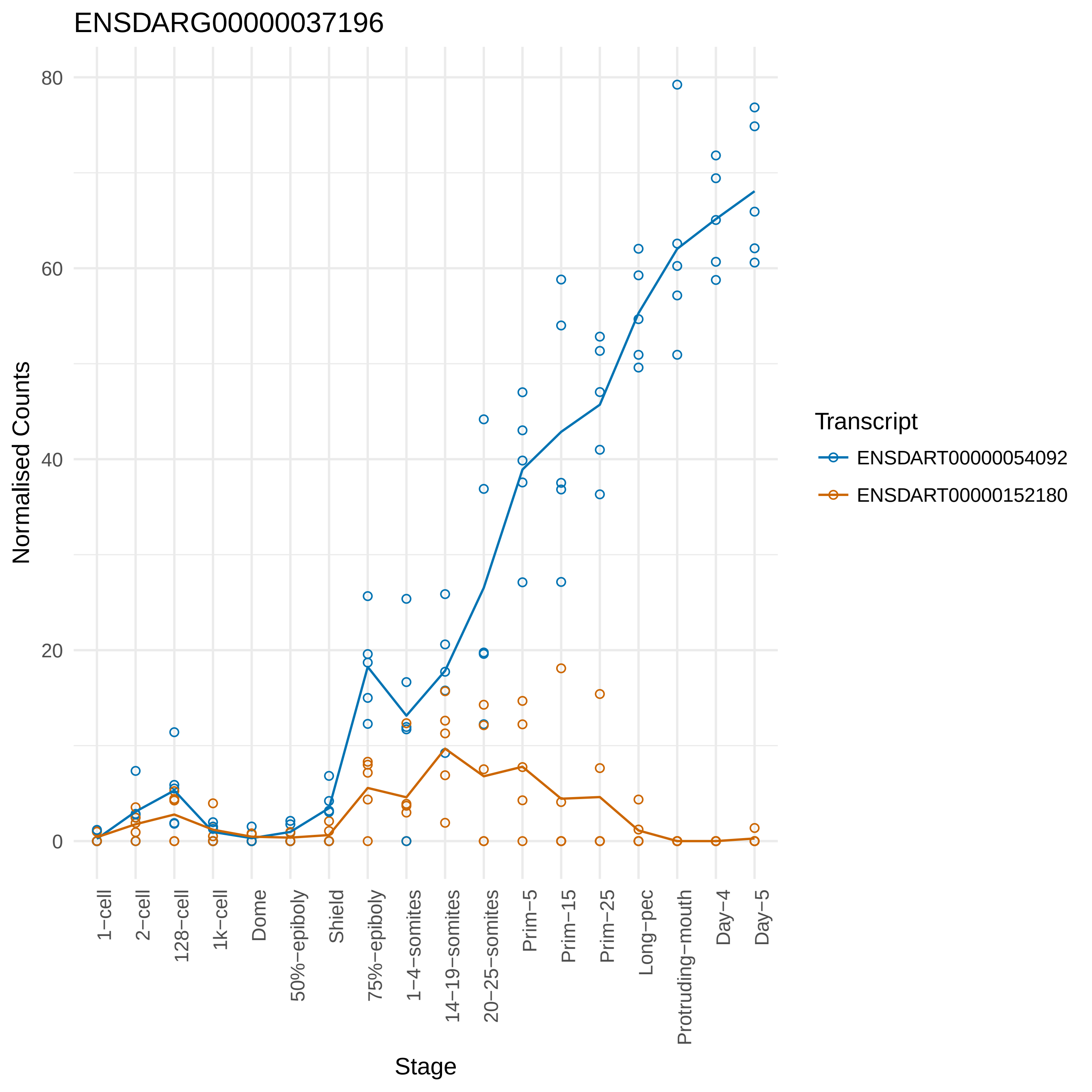

ENSDARG00000037196
Normalised Counts
80
60
40
20
0
1−cell
2−cell
128−cell
1k−cell
Dome
50%−epiboly
Shield
75%−epiboly
1−4−somites
14−19−somites
20−25−somites
Prim−5
Prim−15
Prim−25
Long−pec
Protruding−mouth
Day−4
Day−5
Stage
Transcript
ENSDART00000054092
ENSDART00000152180

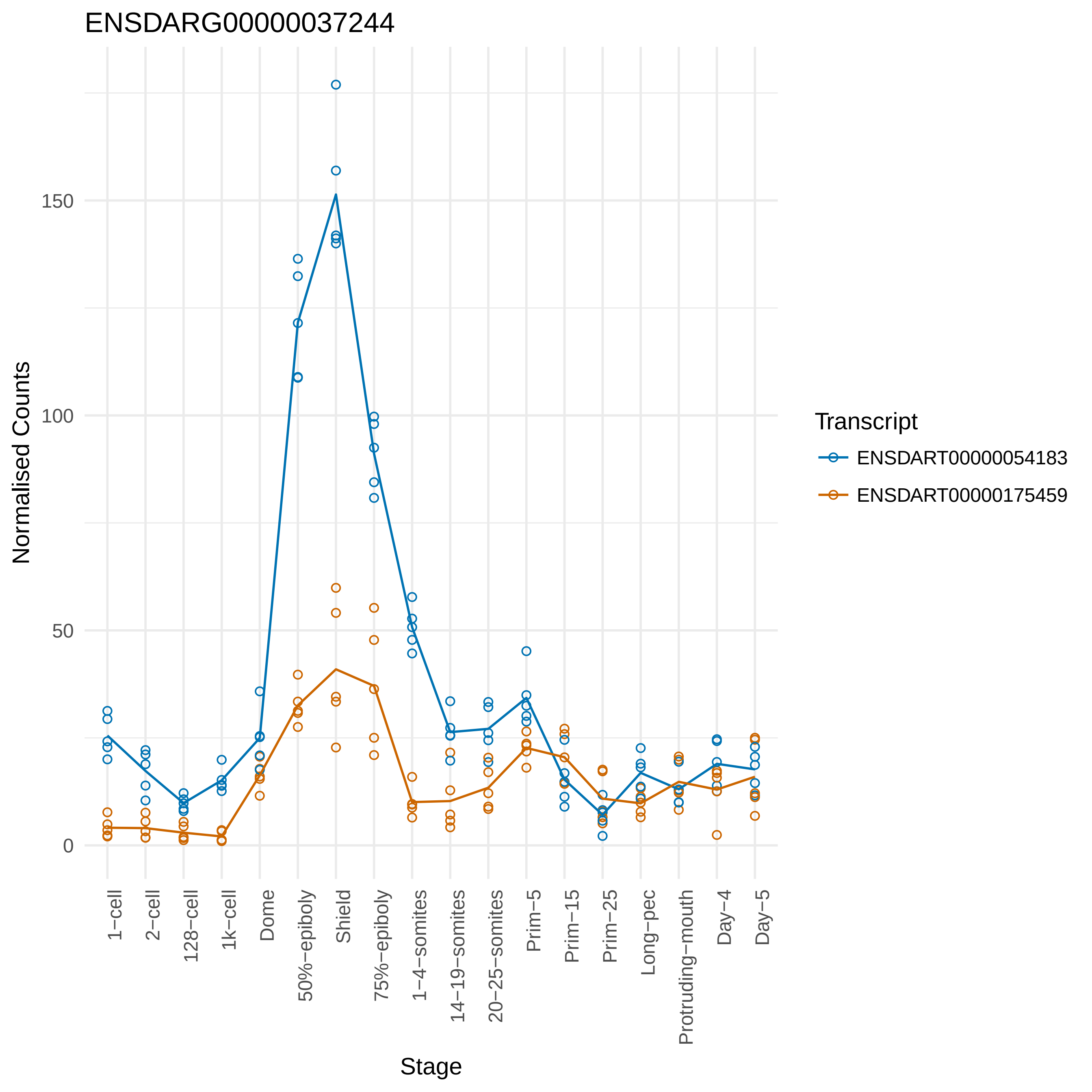

ENSDARG00000037244
Normalised Counts
150
100
50
0
1−cell
2−cell
128−cell
1k−cell
Dome
50%−epiboly
Shield
75%−epiboly
1−4−somites
14−19−somites
20−25−somites
Prim−5
Prim−15
Prim−25
Long−pec
Protruding−mouth
Day−4
Day−5
Stage
Transcript
ENSDART00000054183
ENSDART00000175459

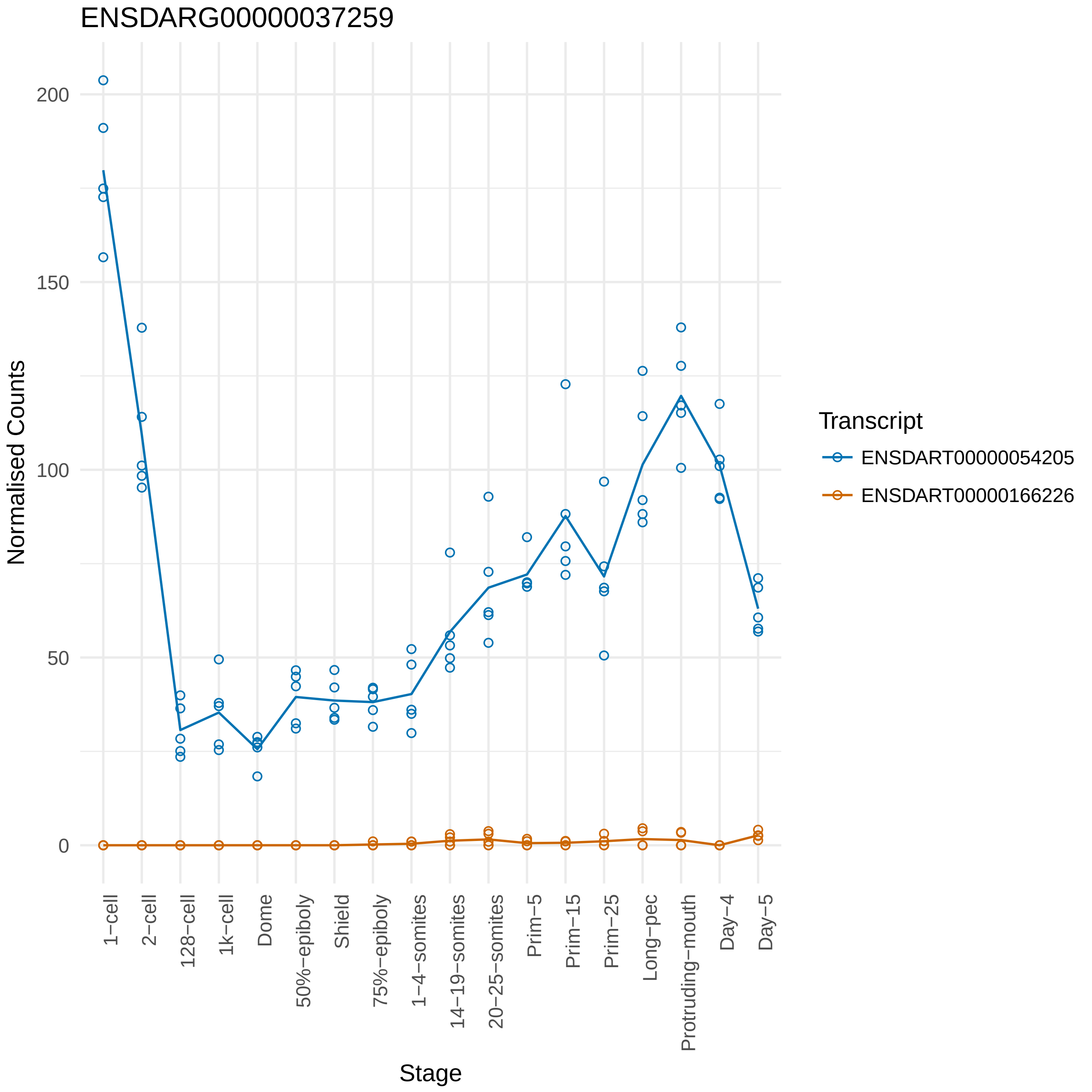
ENSDARG00000037259
Normalised Counts
200
150
100
50
0
1−cell
2−cell
128−cell
1k−cell
Dome
50%−epiboly
Shield
75%−epiboly
1−4−somites
14−19−somites
20−25−somites
Prim−5
Prim−15
Prim−25
Long−pec
Protruding−mouth
Day−4
Day−5
Stage
Transcript
ENSDART00000054205
ENSDART00000166226

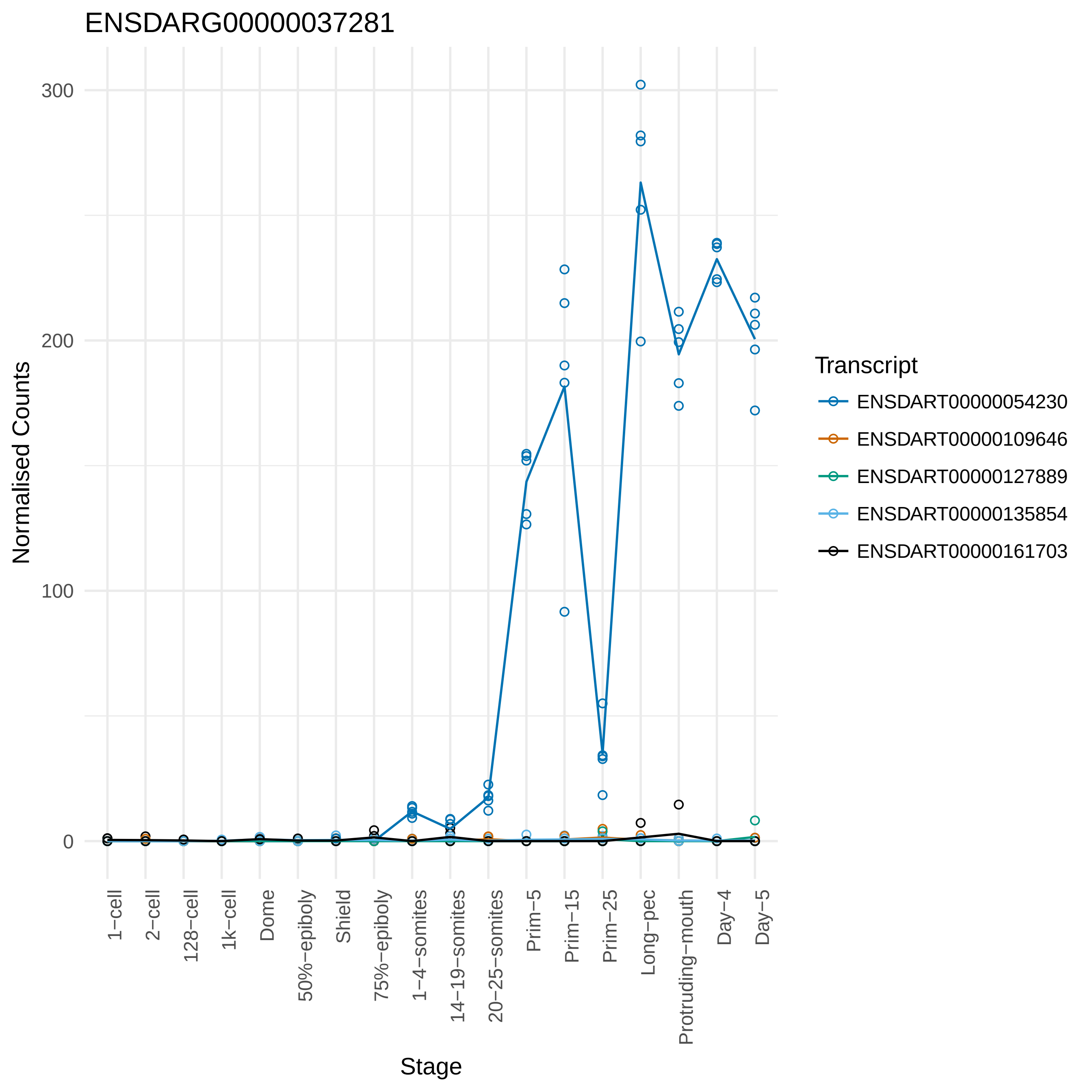

ENSDARG00000037281
Normalised Counts
300
200
100
0
1−cell
2−cell
128−cell
1k−cell
Dome
50%−epiboly
Shield
75%−epiboly
1−4−somites
14−19−somites
20−25−somites
Prim−5
Prim−15
Prim−25
Long−pec
Protruding−mouth
Day−4
Day−5
Stage
Transcript
ENSDART00000054230
ENSDART00000109646
ENSDART00000127889
ENSDART00000135854
ENSDART00000161703

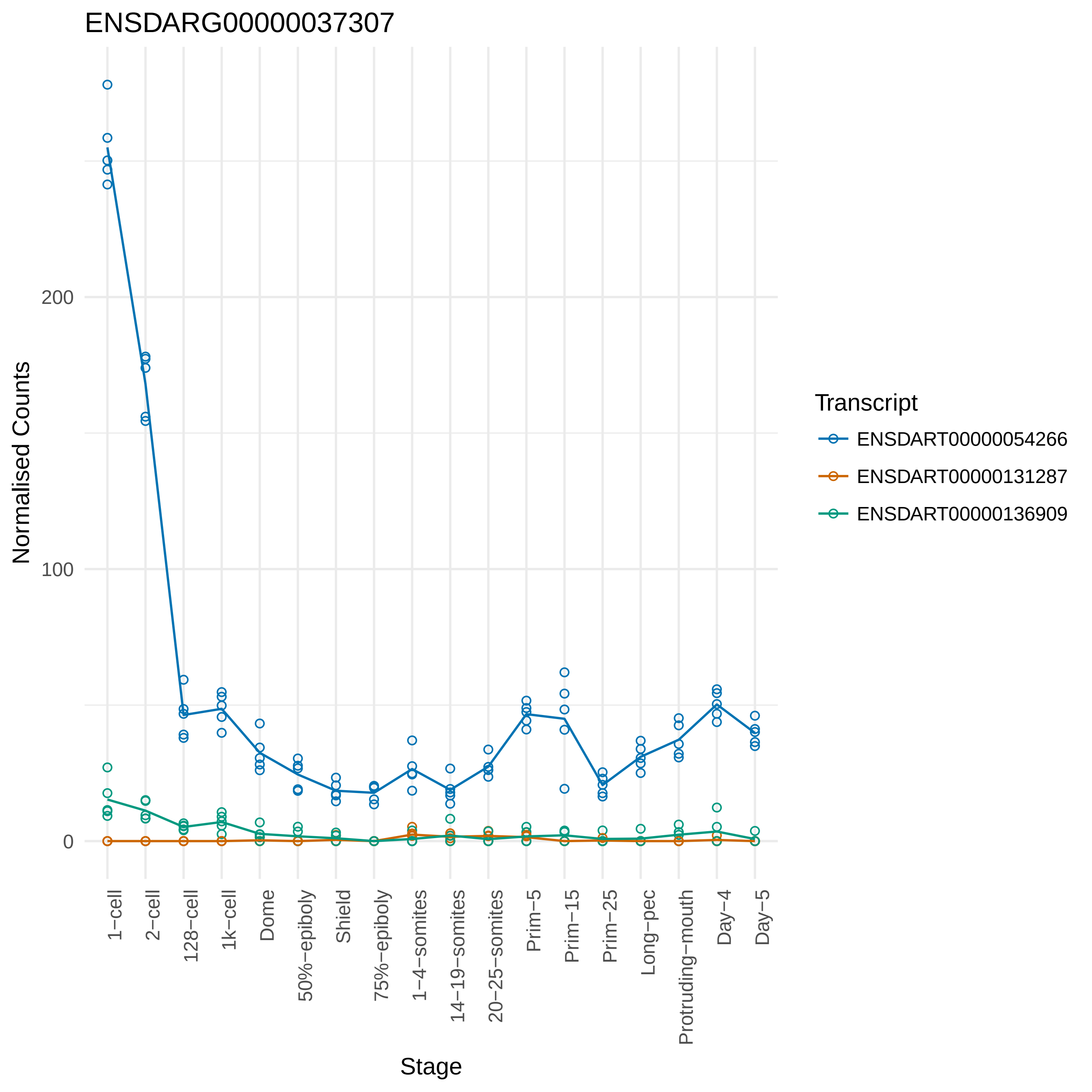
ENSDARG00000037307
Normalised Counts
200
100
0
1−cell
2−cell
128−cell
1k−cell
Dome
50%−epiboly
Shield
75%−epiboly
1−4−somites
14−19−somites
20−25−somites
Prim−5
Prim−15
Prim−25
Long−pec
Protruding−mouth
Day−4
Day−5
Stage
Transcript
ENSDART00000054266
ENSDART00000131287
ENSDART00000136909

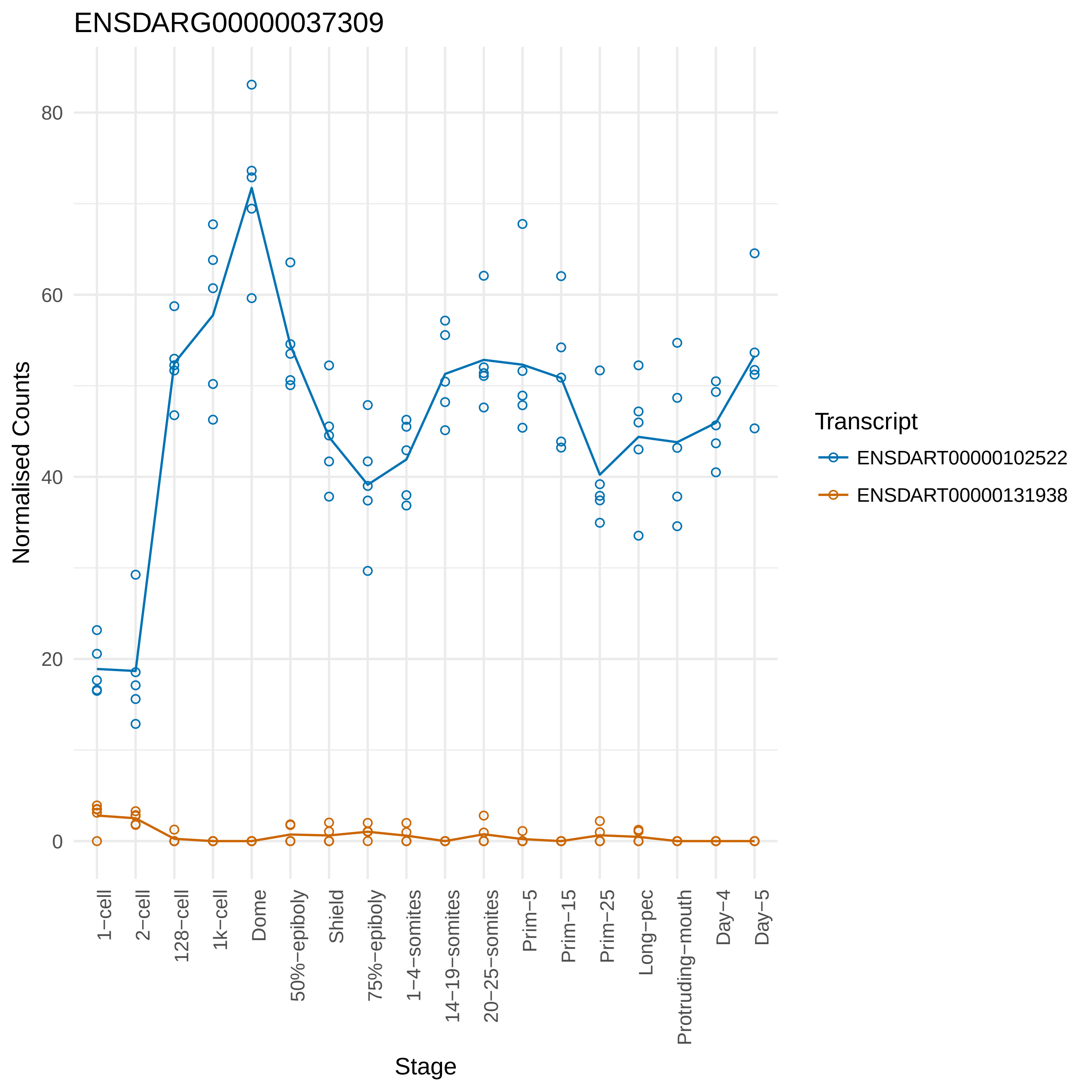

ENSDARG00000037309
Normalised Counts
80
60
40
20
0
1−cell
2−cell
128−cell
1k−cell
Dome
50%−epiboly
Shield
75%−epiboly
1−4−somites
14−19−somites
20−25−somites
Prim−5
Prim−15
Prim−25
Long−pec
Protruding−mouth
Day−4
Day−5
Stage
Transcript
ENSDART00000102522
ENSDART00000131938

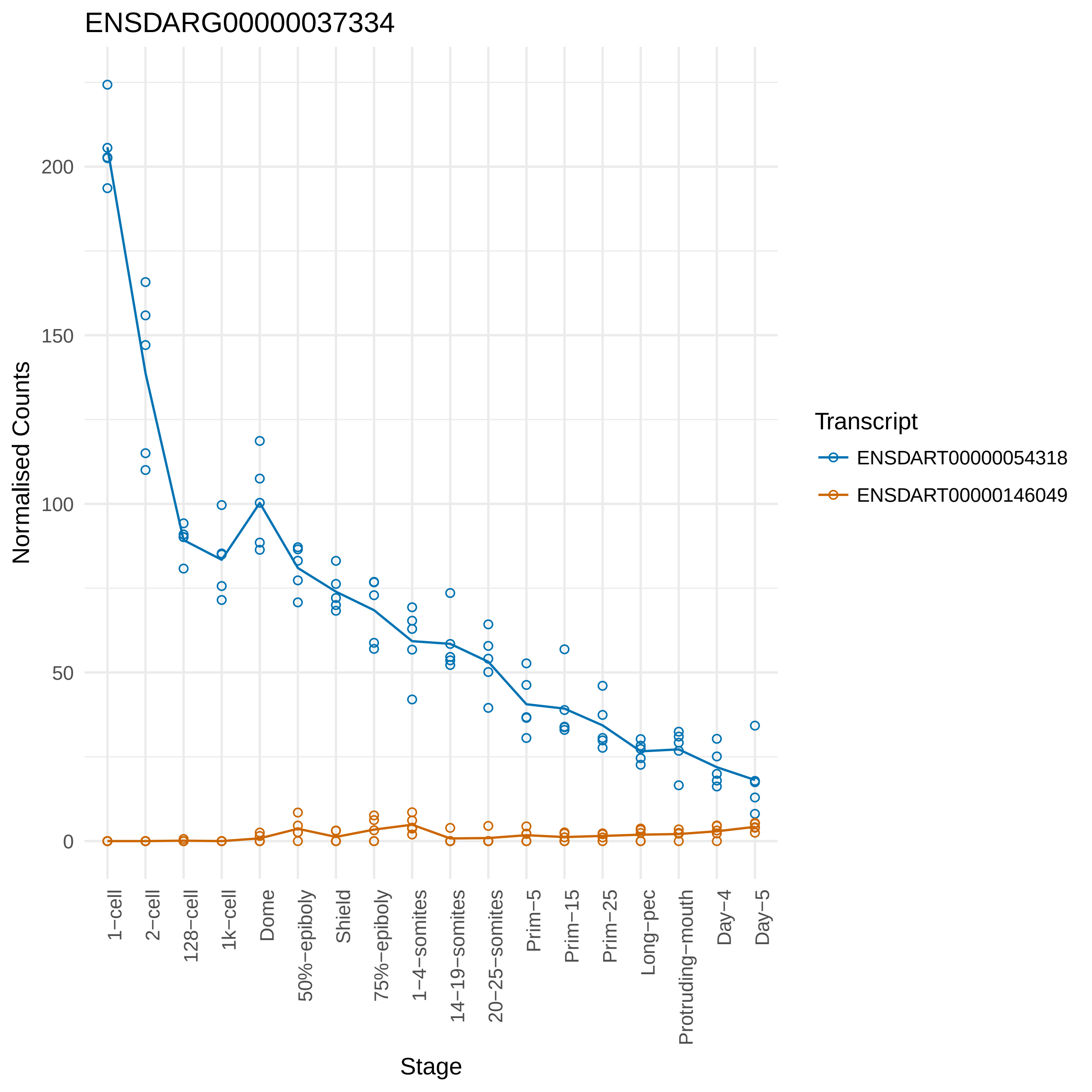

ENSDARG00000037334
Normalised Counts
200
150
100
50
0
1−cell
2−cell
128−cell
1k−cell
Dome
50%−epiboly
Shield
75%−epiboly
1−4−somites
14−19−somites
20−25−somites
Prim−5
Prim−15
Prim−25
Long−pec
Protruding−mouth
Day−4
Day−5
Stage
Transcript
ENSDART00000054318
ENSDART00000146049

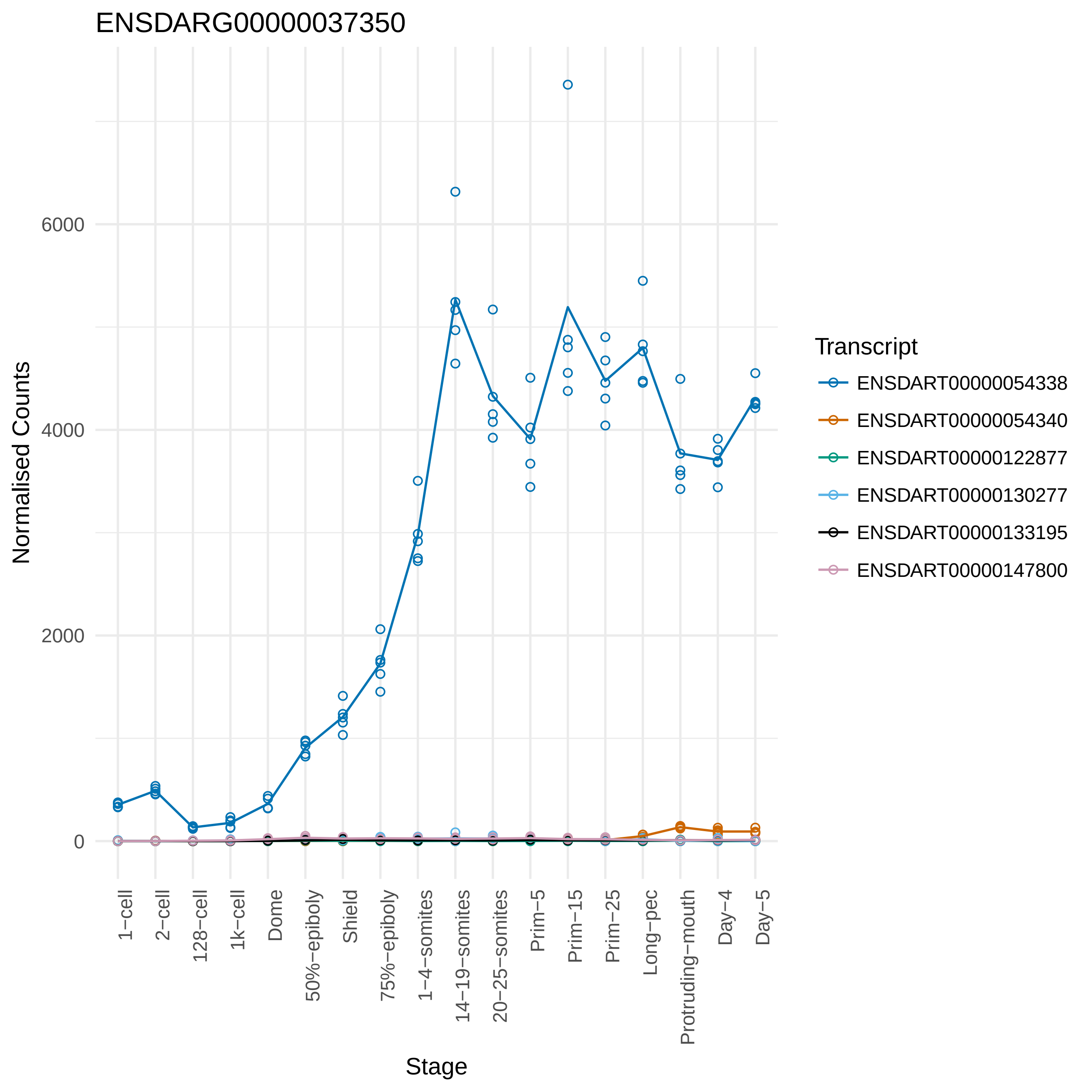
ENSDARG00000037350
Normalised Counts
0
2000
4000
6000
Stage
1−cell
2−cell
128−cell
1k−cell
Dome
50%−epiboly
Shield
75%−epiboly
1−4−somites
14−19−somites
20−25−somites
Prim−5
Prim−15
Prim−25
Long−pec
Protruding−mouth
Day−4
Day−5
Transcript
ENSDART00000054338
ENSDART00000054340
ENSDART00000122877
ENSDART00000130277
ENSDART00000133195
ENSDART00000147800

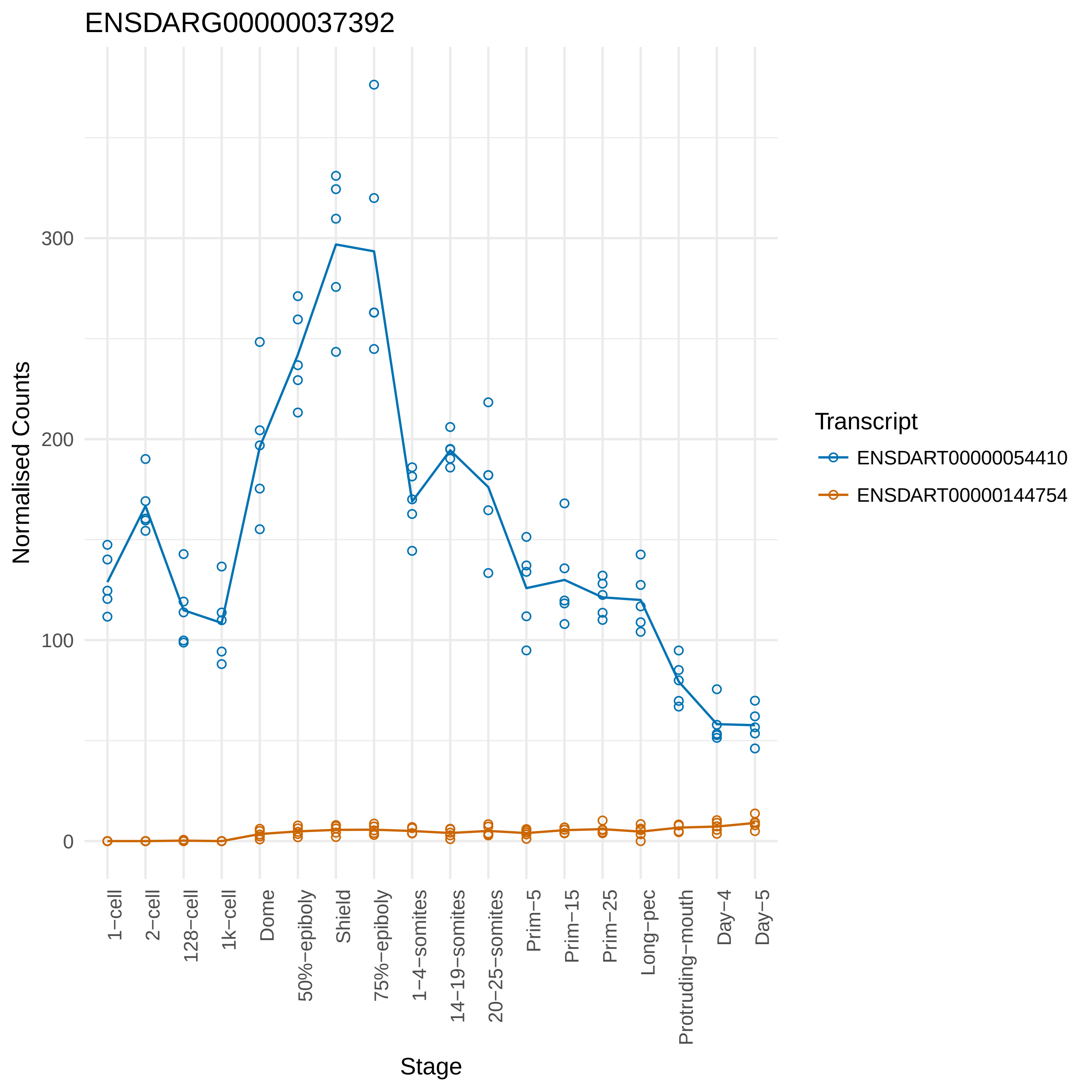

ENSDARG00000037392
Normalised Counts
300
200
100
0
1−cell
2−cell
128−cell
1k−cell
Dome
50%−epiboly
Shield
75%−epiboly
1−4−somites
14−19−somites
20−25−somites
Prim−5
Prim−15
Prim−25
Long−pec
Protruding−mouth
Day−4
Day−5
Stage
Transcript
ENSDART00000054410
ENSDART00000144754

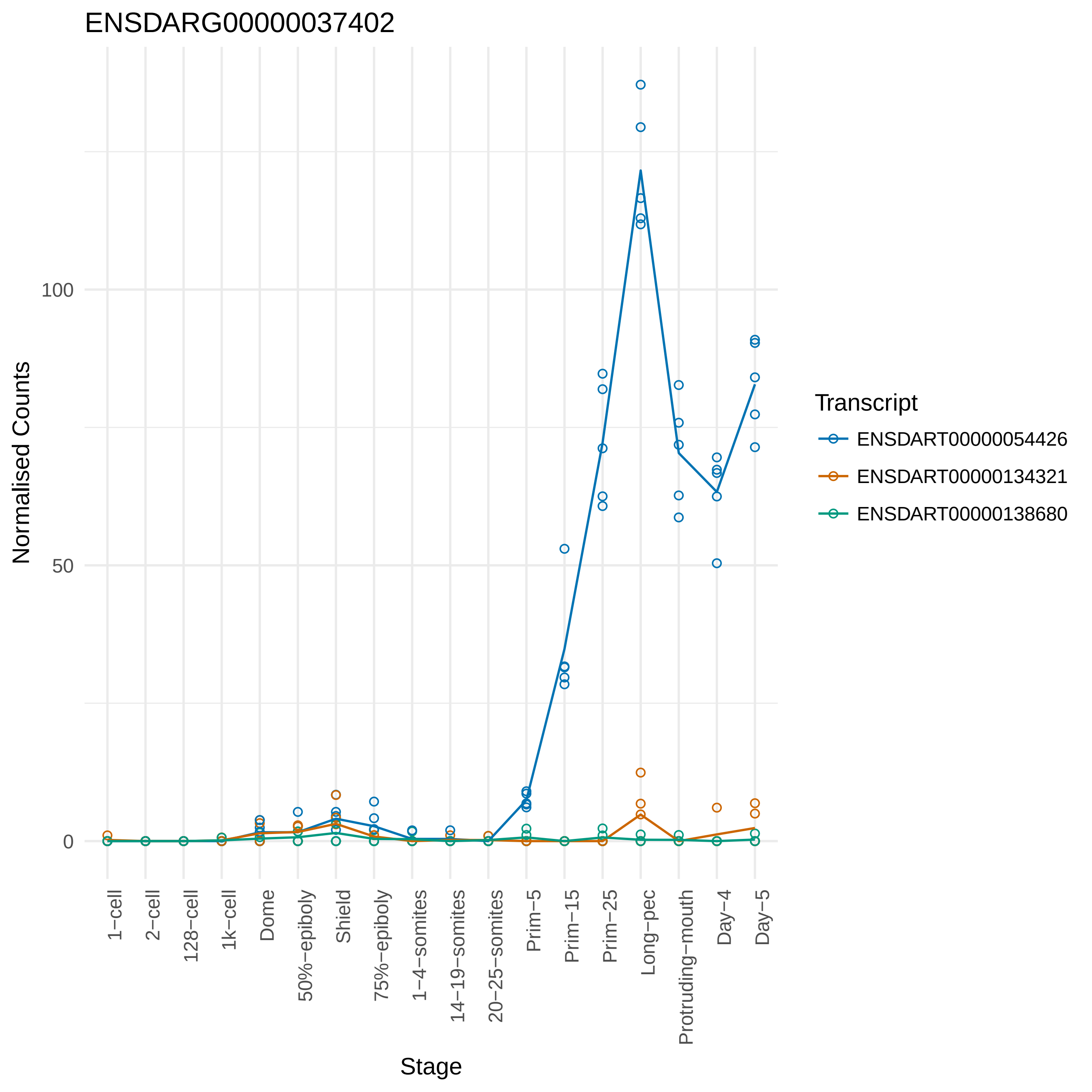

ENSDARG00000037402
Normalised Counts
100
50
0
1−cell
2−cell
128−cell
1k−cell
Dome
50%−epiboly
Shield
75%−epiboly
1−4−somites
14−19−somites
20−25−somites
Prim−5
Prim−15
Prim−25
Long−pec
Protruding−mouth
Day−4
Day−5
Stage
Transcript
ENSDART00000054426
ENSDART00000134321
ENSDART00000138680

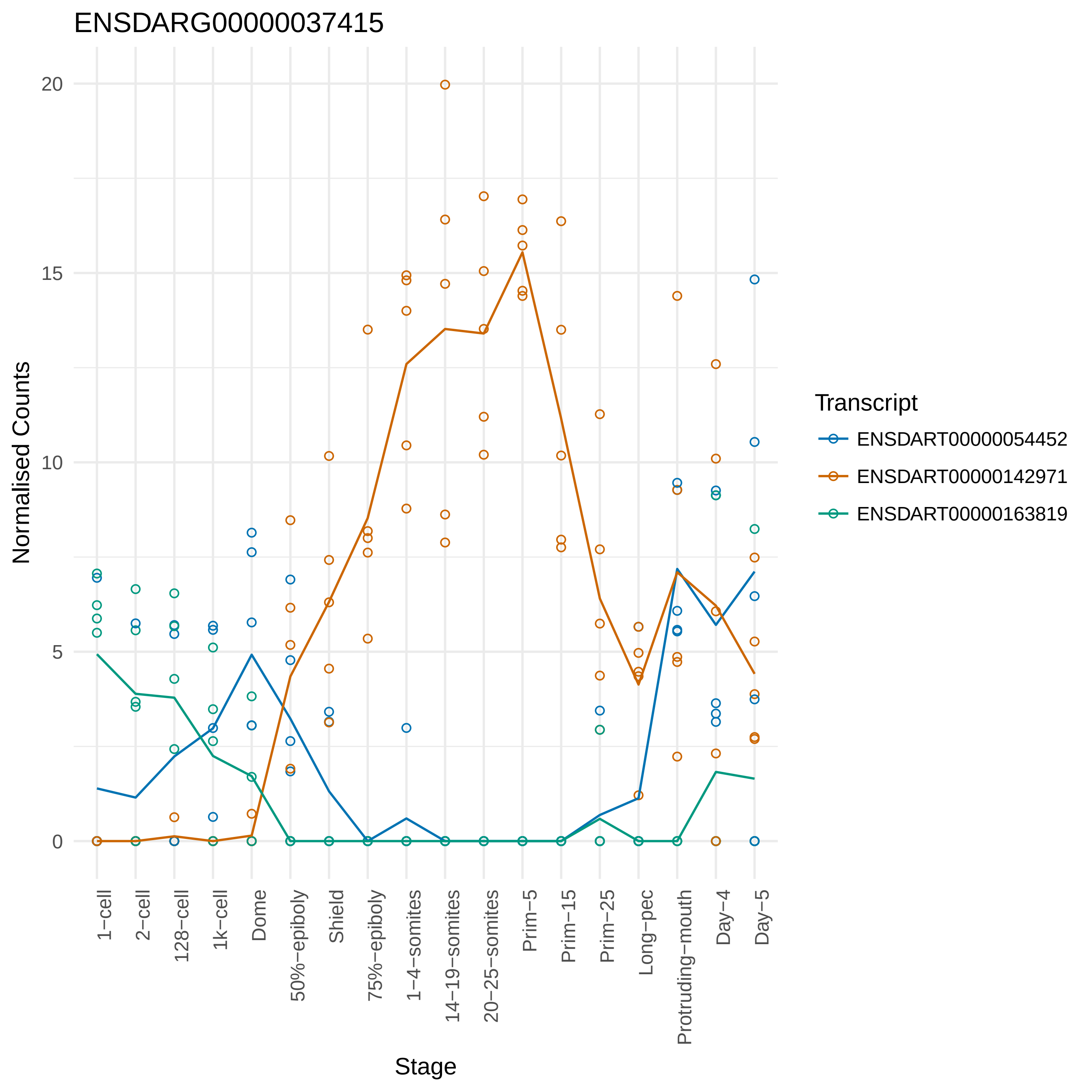

ENSDARG00000037415
Normalised Counts
0
5
10
15
20
Stage
1−cell
2−cell
128−cell
1k−cell
Dome
50%−epiboly
Shield
75%−epiboly
1−4−somites
14−19−somites
20−25−somites
Prim−5
Prim−15
Prim−25
Long−pec
Protruding−mouth
Day−4
Day−5
Transcript
ENSDART00000054452
ENSDART00000142971
ENSDART00000163819

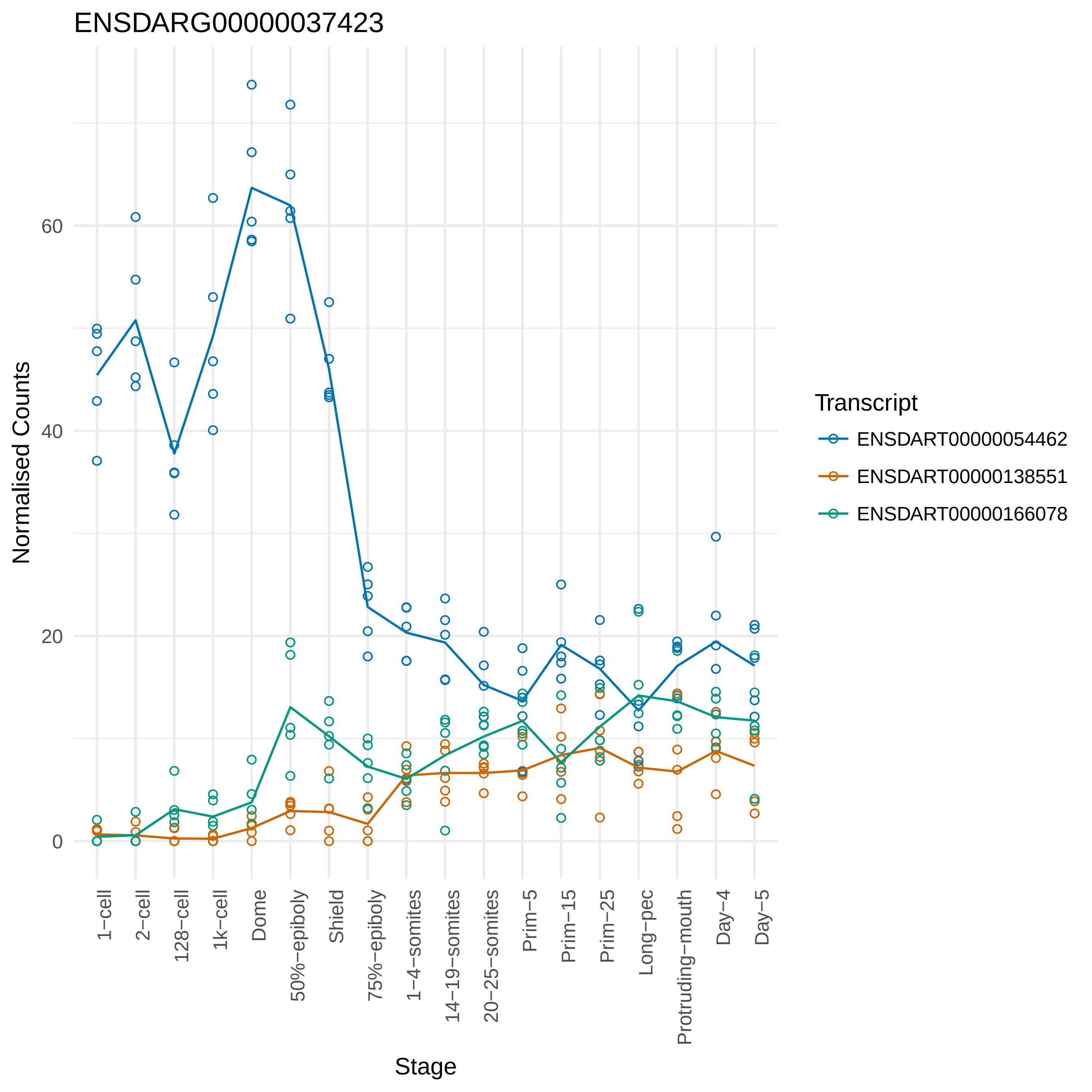

ENSDARG00000037423
Normalised Counts
0
20
40
60
1−cell
2−cell
128−cell
1k−cell
Dome
50%−epiboly
Shield
75%−epiboly
1−4−somites
14−19−somites
20−25−somites
Prim−5
Prim−15
Prim−25
Long−pec
Protruding−mouth
Day−4
Day−5
Stage
Transcript
ENSDART00000054462
ENSDART00000138551
ENSDART00000166078

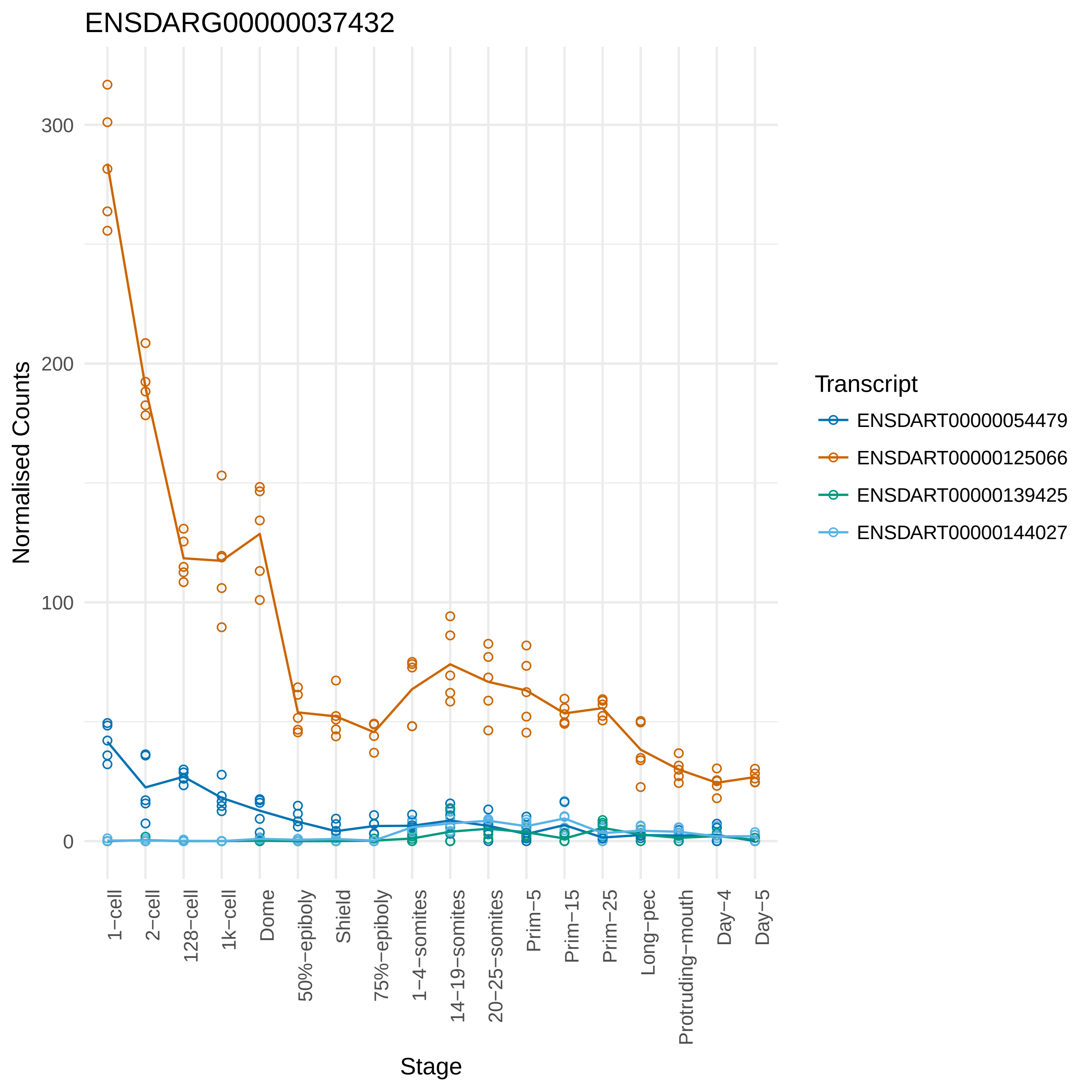

ENSDARG00000037432
Normalised Counts
300
200
100
0
1−cell
2−cell
128−cell
1k−cell
Dome
50%−epiboly
Shield
75%−epiboly
1−4−somites
14−19−somites
20−25−somites
Prim−5
Prim−15
Prim−25
Long−pec
Protruding−mouth
Day−4
Day−5
Stage
Transcript
ENSDART00000054479
ENSDART00000125066
ENSDART00000139425
ENSDART00000144027

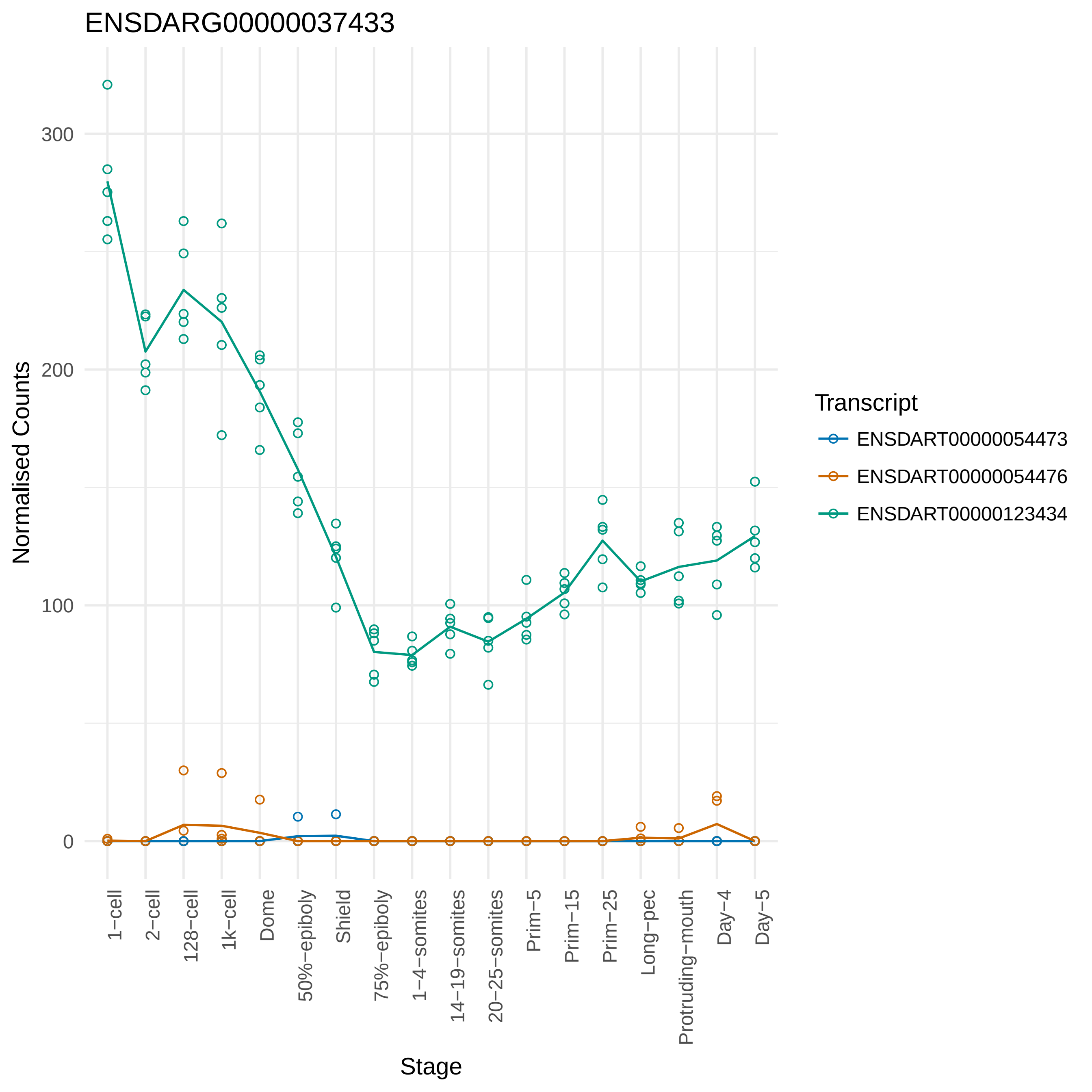

ENSDARG00000037433
Normalised Counts
300
200
100
0
1−cell
2−cell
128−cell
1k−cell
Dome
50%−epiboly
Shield
75%−epiboly
1−4−somites
14−19−somites
20−25−somites
Prim−5
Prim−15
Prim−25
Long−pec
Protruding−mouth
Day−4
Day−5
Stage
Transcript
ENSDART00000054473
ENSDART00000054476
ENSDART00000123434

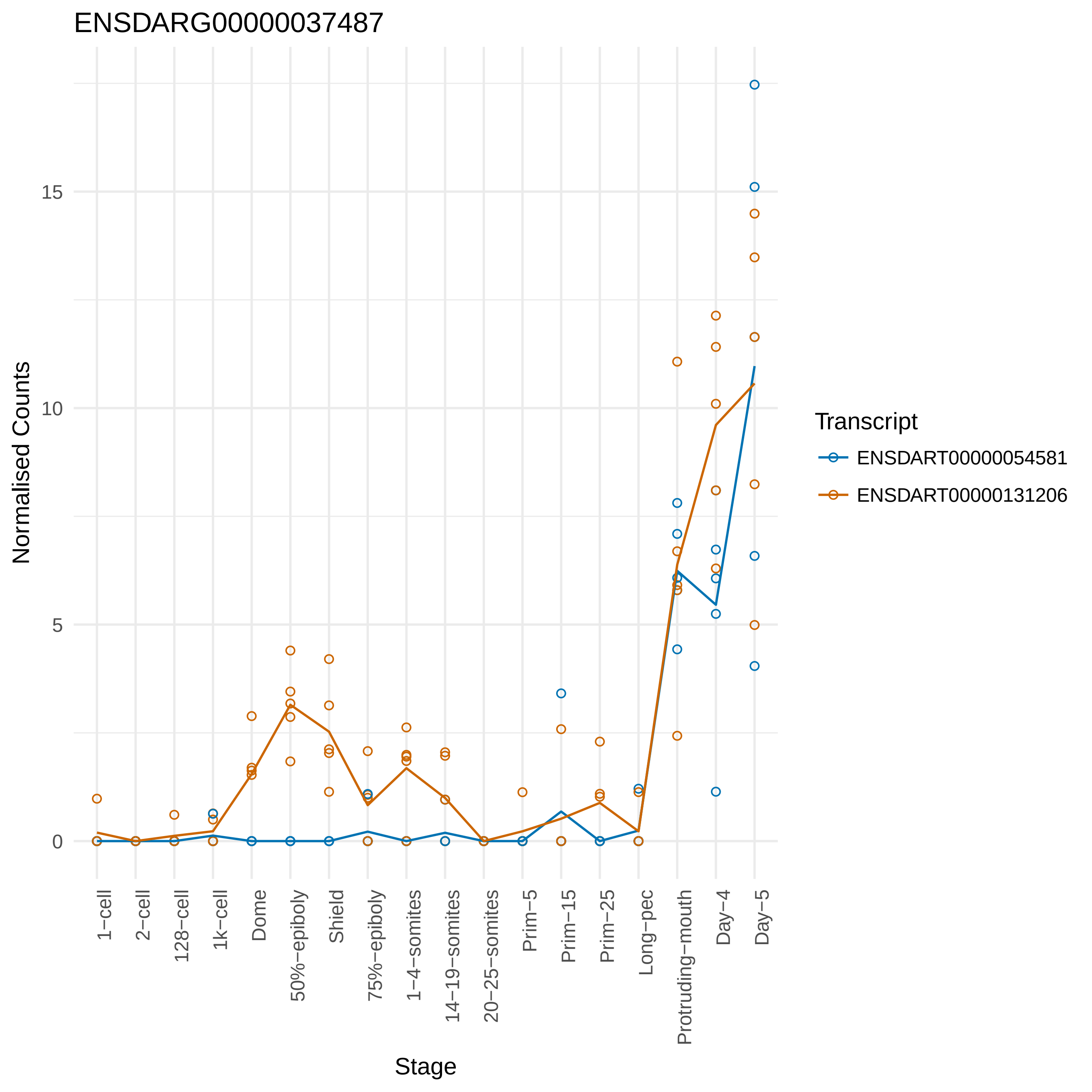

ENSDARG00000037487
Normalised Counts
0
5
10
15
Stage
1−cell
2−cell
128−cell
1k−cell
Dome
50%−epiboly
Shield
75%−epiboly
1−4−somites
14−19−somites
20−25−somites
Prim−5
Prim−15
Prim−25
Long−pec
Protruding−mouth
Day−4
Day−5
Transcript
ENSDART00000054581
ENSDART00000131206

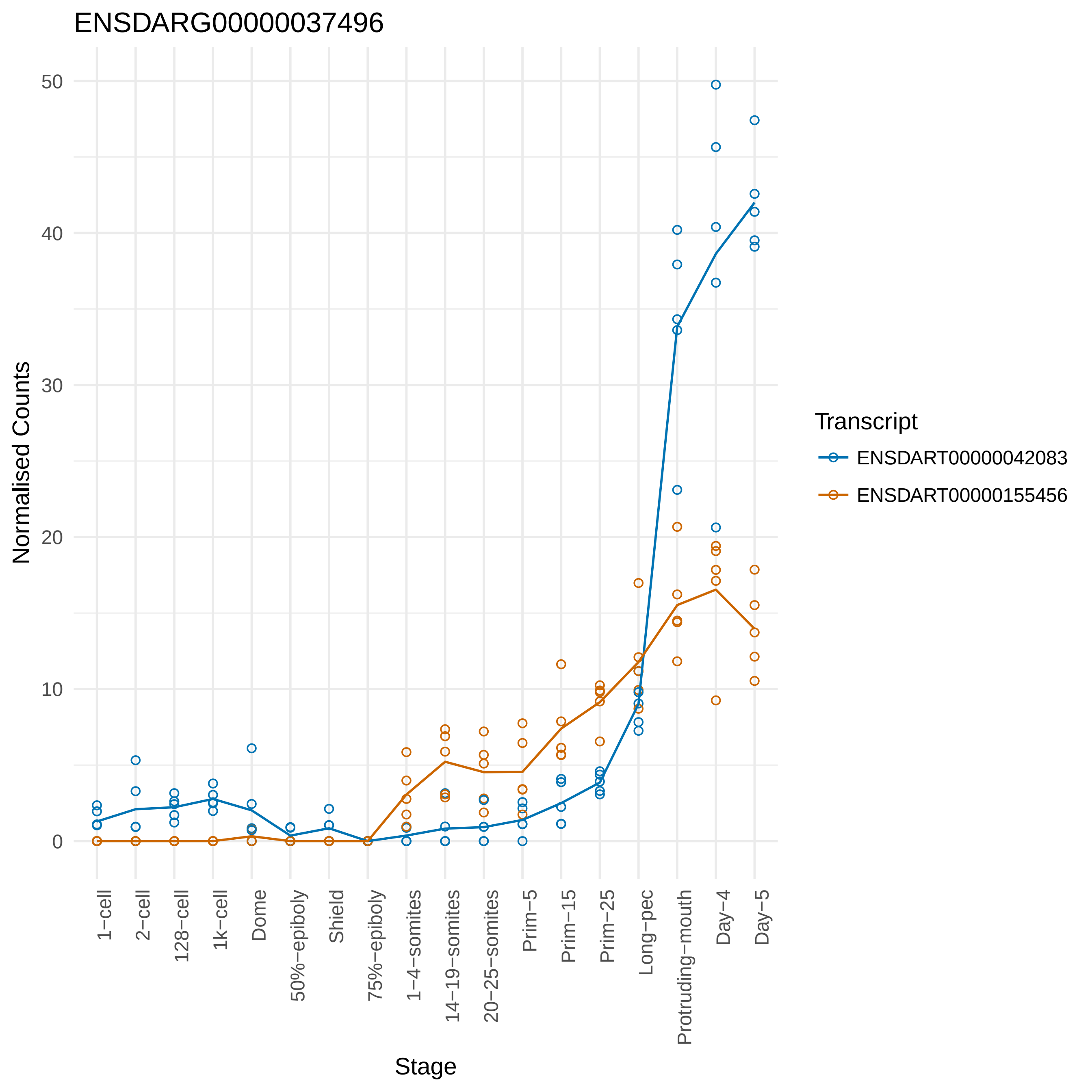
ENSDARG00000037496
Normalised Counts
0
10
20
30
40
50
1−cell
2−cell
128−cell
1k−cell
Dome
50%−epiboly
Shield
75%−epiboly
1−4−somites
14−19−somites
20−25−somites
Prim−5
Prim−15
Prim−25
Long−pec
Protruding−mouth
Day−4
Day−5
Stage
Transcript
ENSDART00000042083
ENSDART00000155456

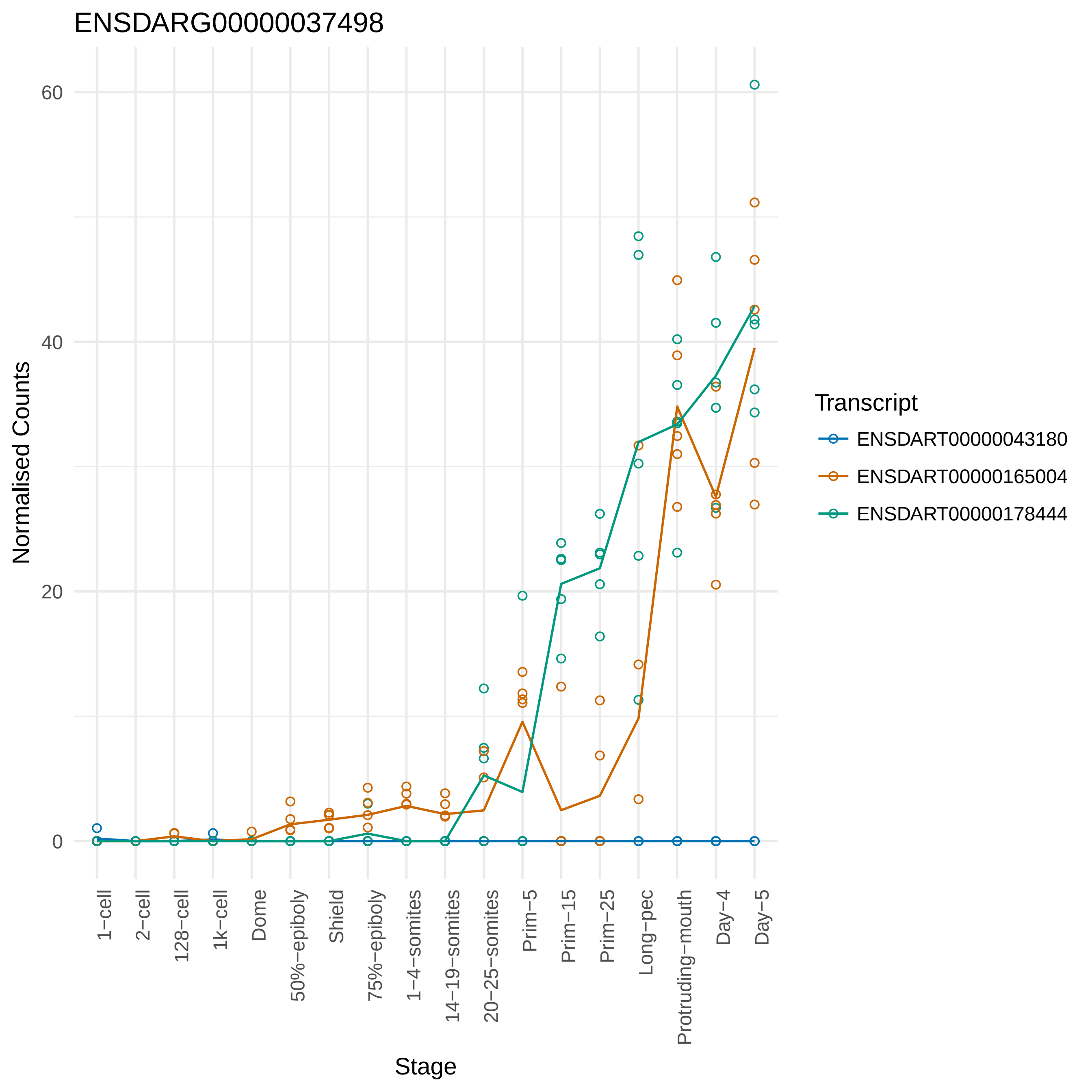
ENSDARG00000037498
Normalised Counts
60
40
20
0
1−cell
2−cell
128−cell
1k−cell
Dome
50%−epiboly
Shield
75%−epiboly
1−4−somites
14−19−somites
20−25−somites
Prim−5
Prim−15
Prim−25
Long−pec
Protruding−mouth
Day−4
Day−5
Stage
Transcript
ENSDART00000043180
ENSDART00000165004
ENSDART00000178444

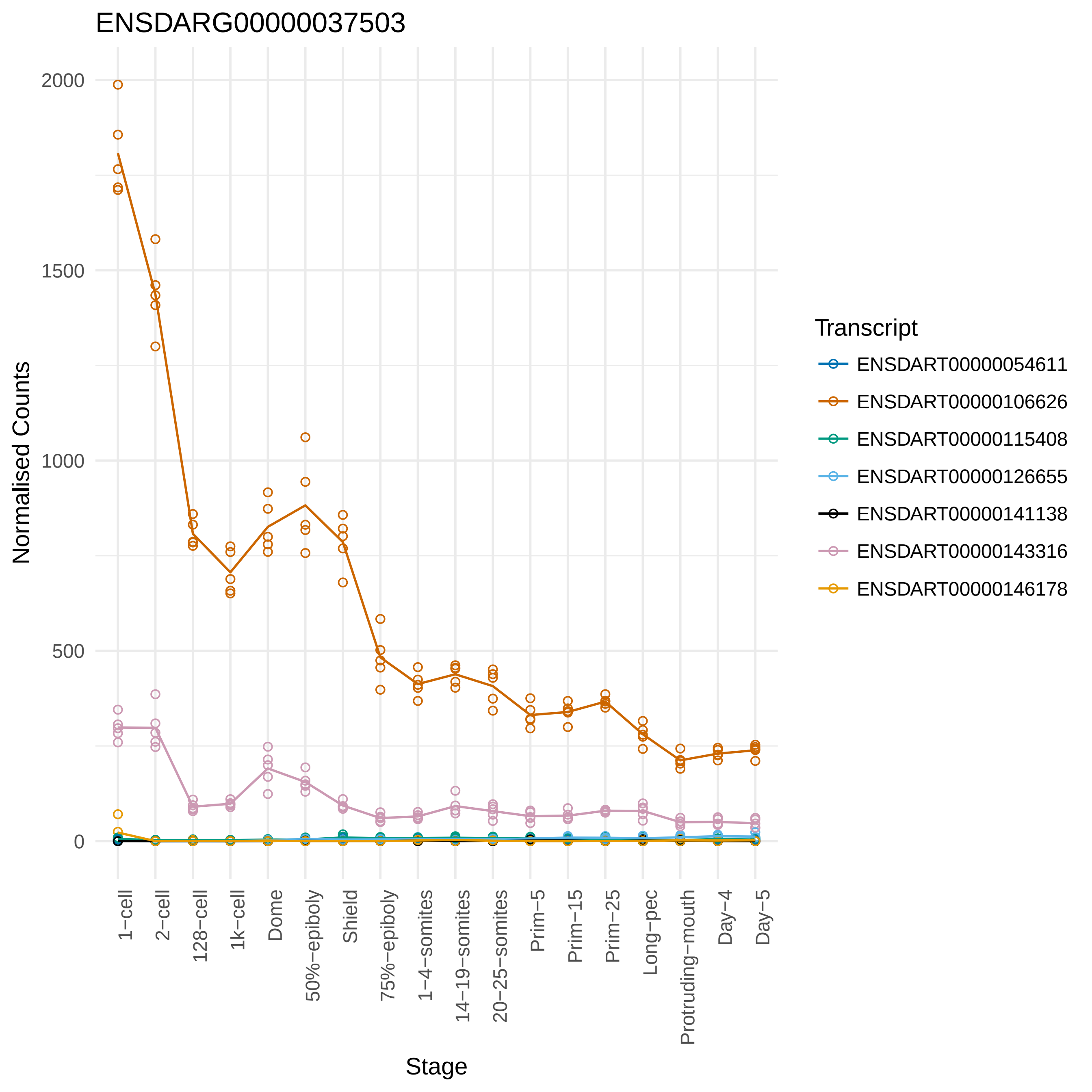

ENSDARG00000037503
Normalised Counts
2000
1500
1000
500
0
1−cell
2−cell
128−cell
1k−cell
Dome
50%−epiboly
Shield
75%−epiboly
1−4−somites
14−19−somites
20−25−somites
Prim−5
Prim−15
Prim−25
Long−pec
Protruding−mouth
Day−4
Day−5
Stage
Transcript
ENSDART00000054611
ENSDART00000106626
ENSDART00000115408
ENSDART00000126655
ENSDART00000141138
ENSDART00000143316
ENSDART00000146178

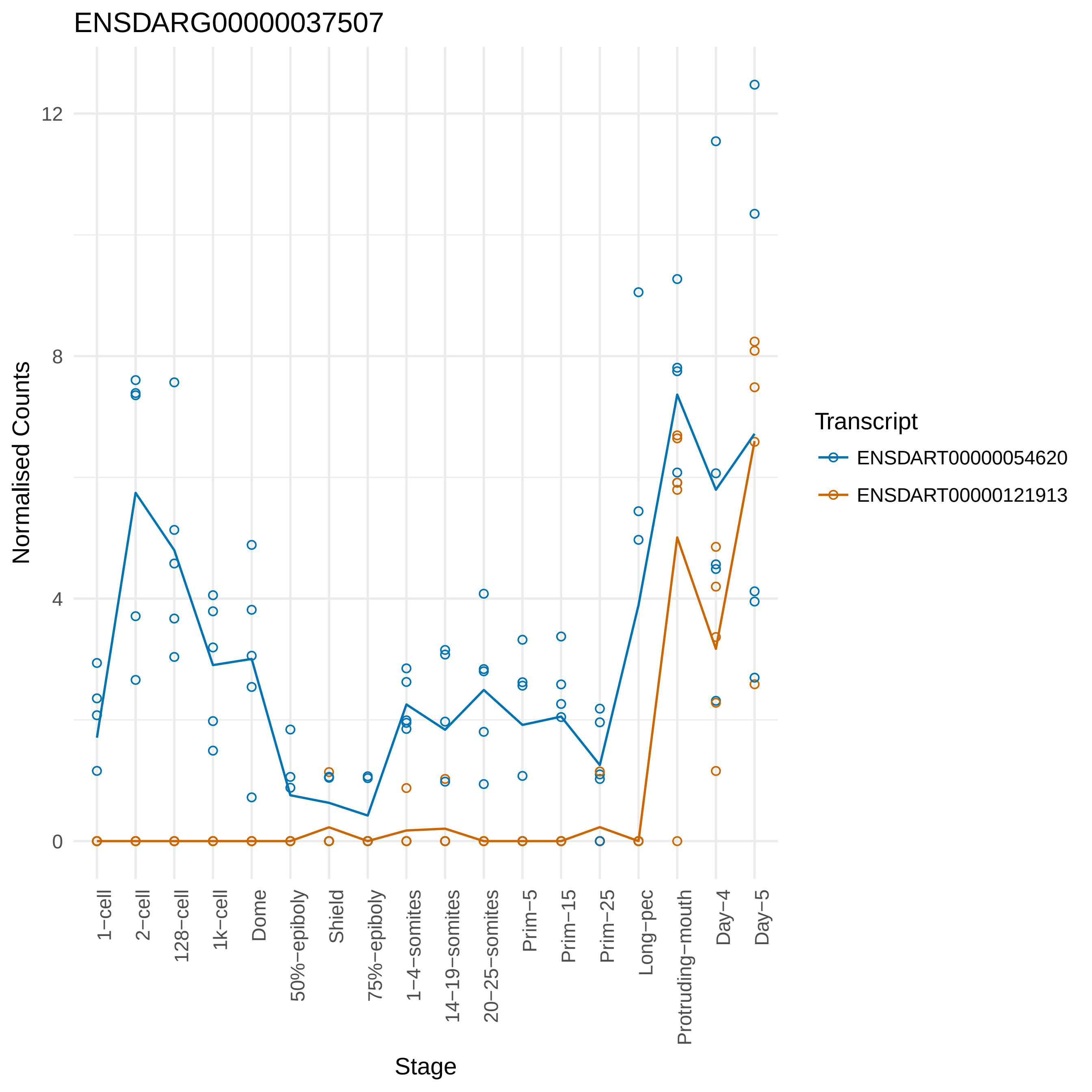

ENSDARG00000037507
Normalised Counts
12
8
4
0
1−cell
2−cell
128−cell
1k−cell
Dome
50%−epiboly
Shield
75%−epiboly
1−4−somites
14−19−somites
20−25−somites
Prim−5
Prim−15
Prim−25
Long−pec
Protruding−mouth
Day−4
Day−5
Stage
Transcript
ENSDART00000054620
ENSDART00000121913

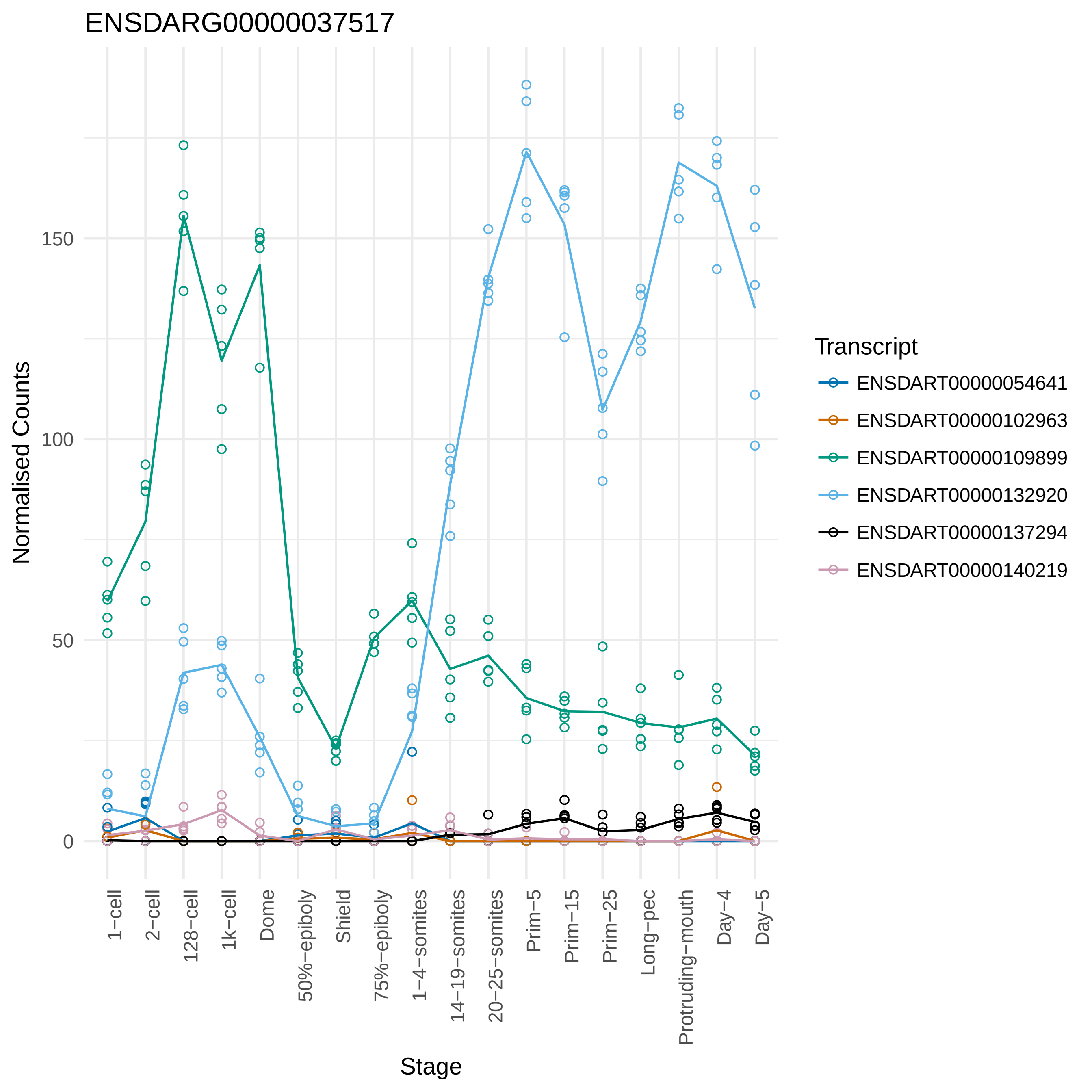
ENSDARG00000037517
Normalised Counts
0
50
100
150
Stage
1−cell
2−cell
128−cell
1k−cell
Dome
50%−epiboly
Shield
75%−epiboly
1−4−somites
14−19−somites
20−25−somites
Prim−5
Prim−15
Prim−25
Long−pec
Protruding−mouth
Day−4
Day−5
Transcript
ENSDART00000054641
ENSDART00000102963
ENSDART00000109899
ENSDART00000132920
ENSDART00000137294
ENSDART00000140219

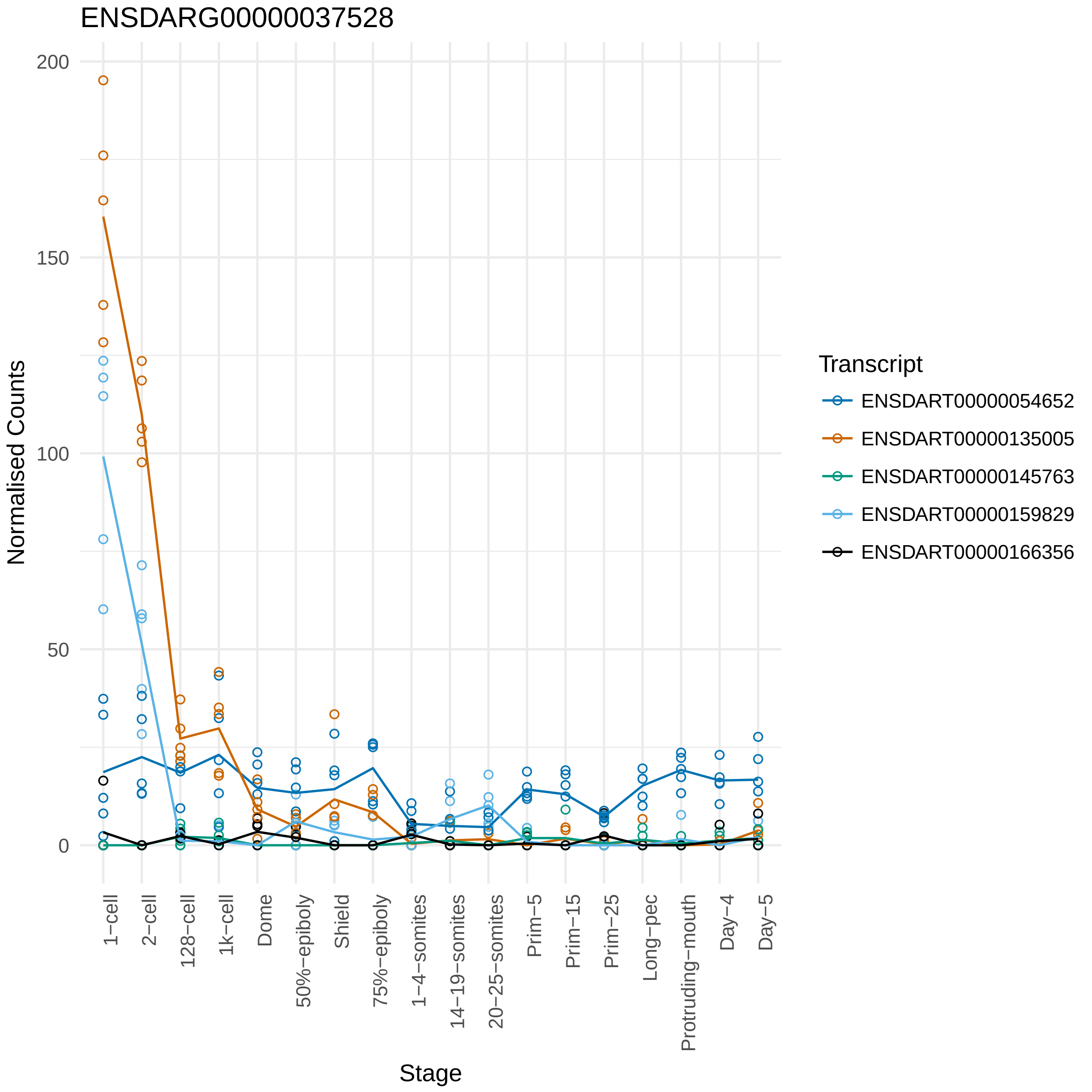
ENSDARG00000037528
Normalised Counts
200
150
100
50
0
1−cell
2−cell
128−cell
1k−cell
Dome
50%−epiboly
Shield
75%−epiboly
1−4−somites
14−19−somites
20−25−somites
Prim−5
Prim−15
Prim−25
Long−pec
Protruding−mouth
Day−4
Day−5
Stage
Transcript
ENSDART00000054652
ENSDART00000135005
ENSDART00000145763
ENSDART00000159829
ENSDART00000166356

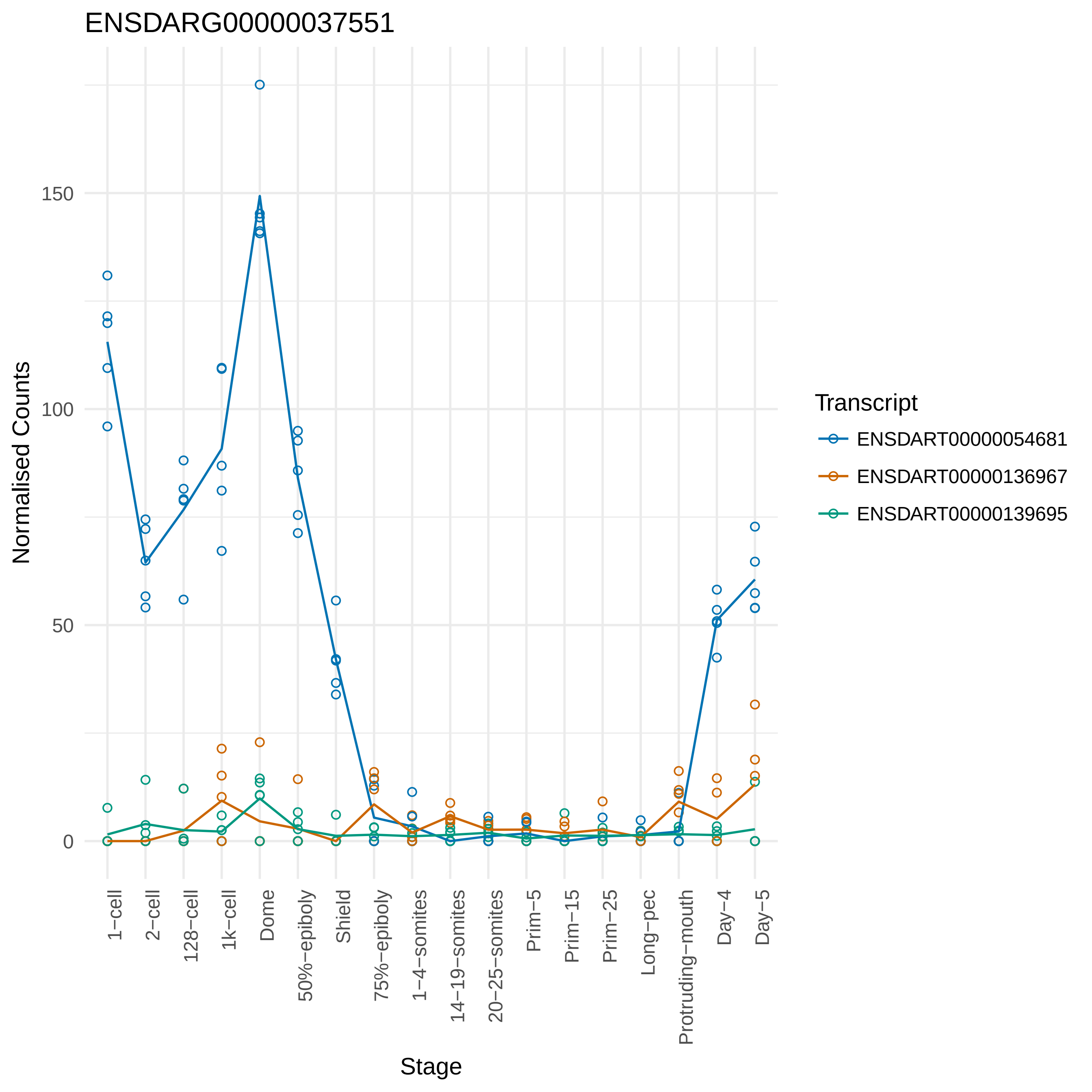

ENSDARG00000037551
Normalised Counts
150
100
50
0
1−cell
2−cell
128−cell
1k−cell
Dome
50%−epiboly
Shield
75%−epiboly
1−4−somites
14−19−somites
20−25−somites
Prim−5
Prim−15
Prim−25
Long−pec
Protruding−mouth
Day−4
Day−5
Stage
Transcript
ENSDART00000054681
ENSDART00000136967
ENSDART00000139695

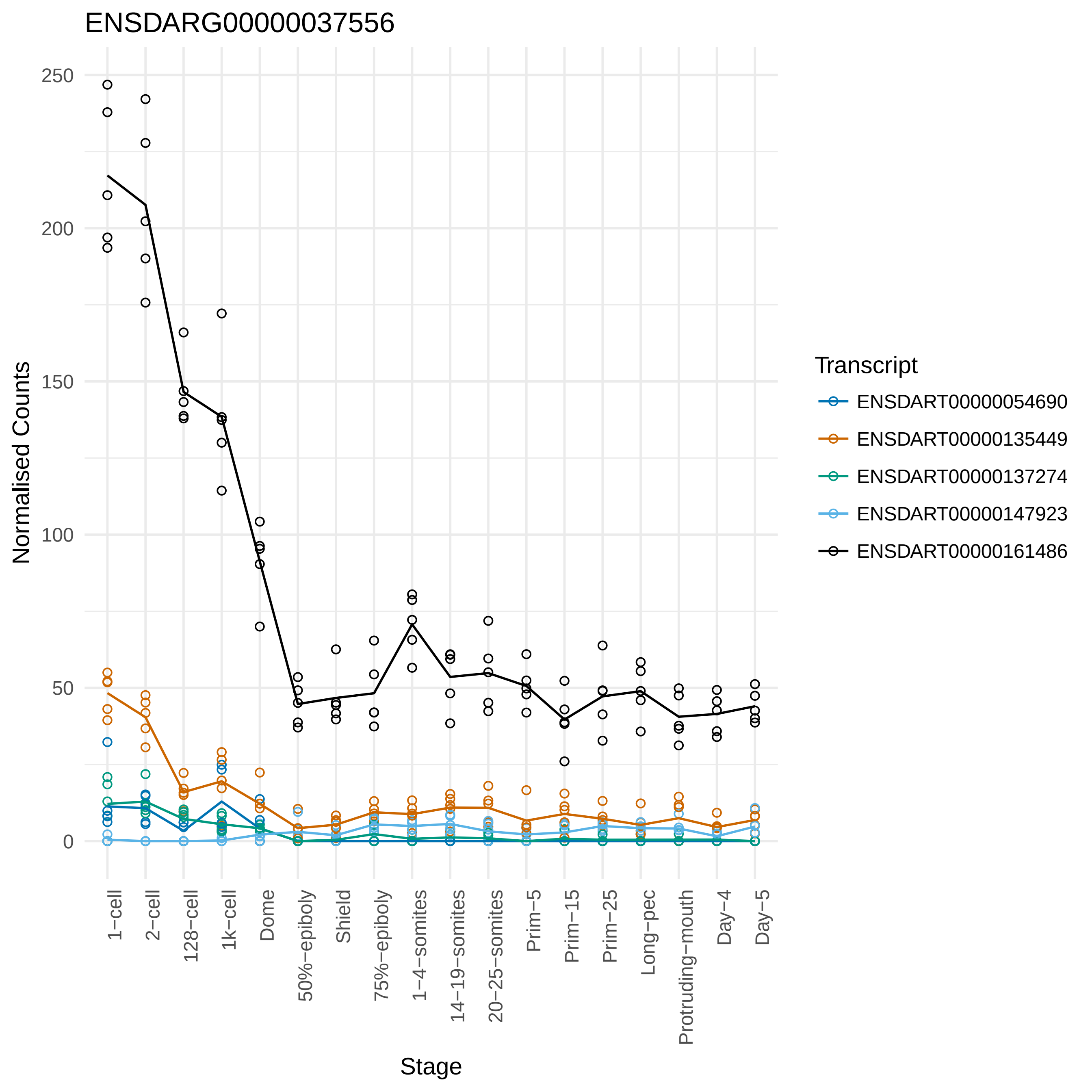

ENSDARG00000037556
Normalised Counts
250
200
150
100
50
0
1−cell
2−cell
128−cell
1k−cell
Dome
50%−epiboly
Shield
75%−epiboly
1−4−somites
14−19−somites
20−25−somites
Prim−5
Prim−15
Prim−25
Long−pec
Protruding−mouth
Day−4
Day−5
Stage
Transcript
ENSDART00000054690
ENSDART00000135449
ENSDART00000137274
ENSDART00000147923
ENSDART00000161486

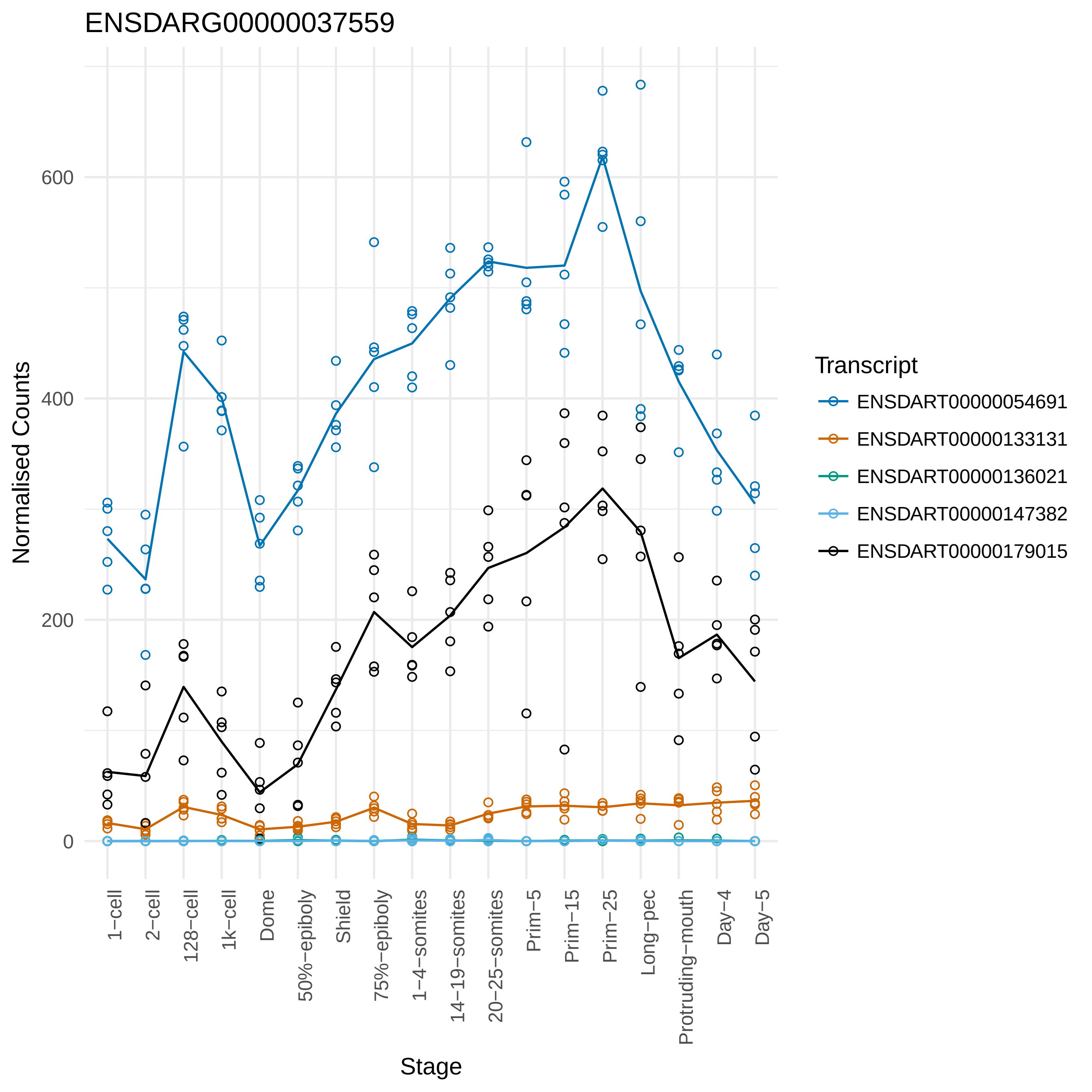
ENSDARG00000037559
Normalised Counts
600
400
200
0
1−cell
2−cell
128−cell
1k−cell
Dome
50%−epiboly
Shield
75%−epiboly
1−4−somites
14−19−somites
20−25−somites
Prim−5
Prim−15
Prim−25
Long−pec
Protruding−mouth
Day−4
Day−5
Stage
Transcript
ENSDART00000054691
ENSDART00000133131
ENSDART00000136021
ENSDART00000147382
ENSDART00000179015

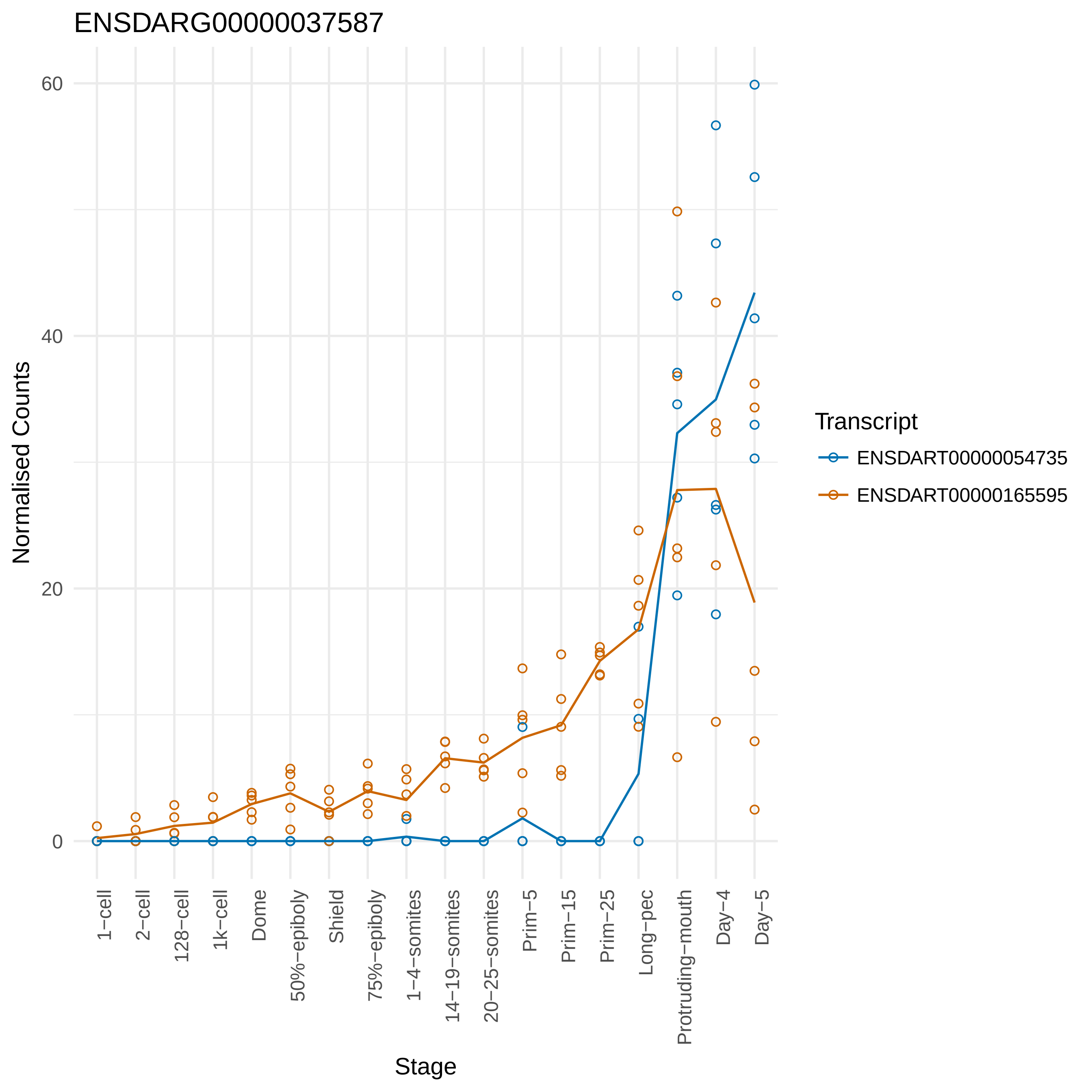

ENSDARG00000037587
Normalised Counts
60
40
20
0
1−cell
2−cell
128−cell
1k−cell
Dome
50%−epiboly
Shield
75%−epiboly
1−4−somites
14−19−somites
20−25−somites
Prim−5
Prim−15
Prim−25
Long−pec
Protruding−mouth
Day−4
Day−5
Stage
Transcript
ENSDART00000054735
ENSDART00000165595

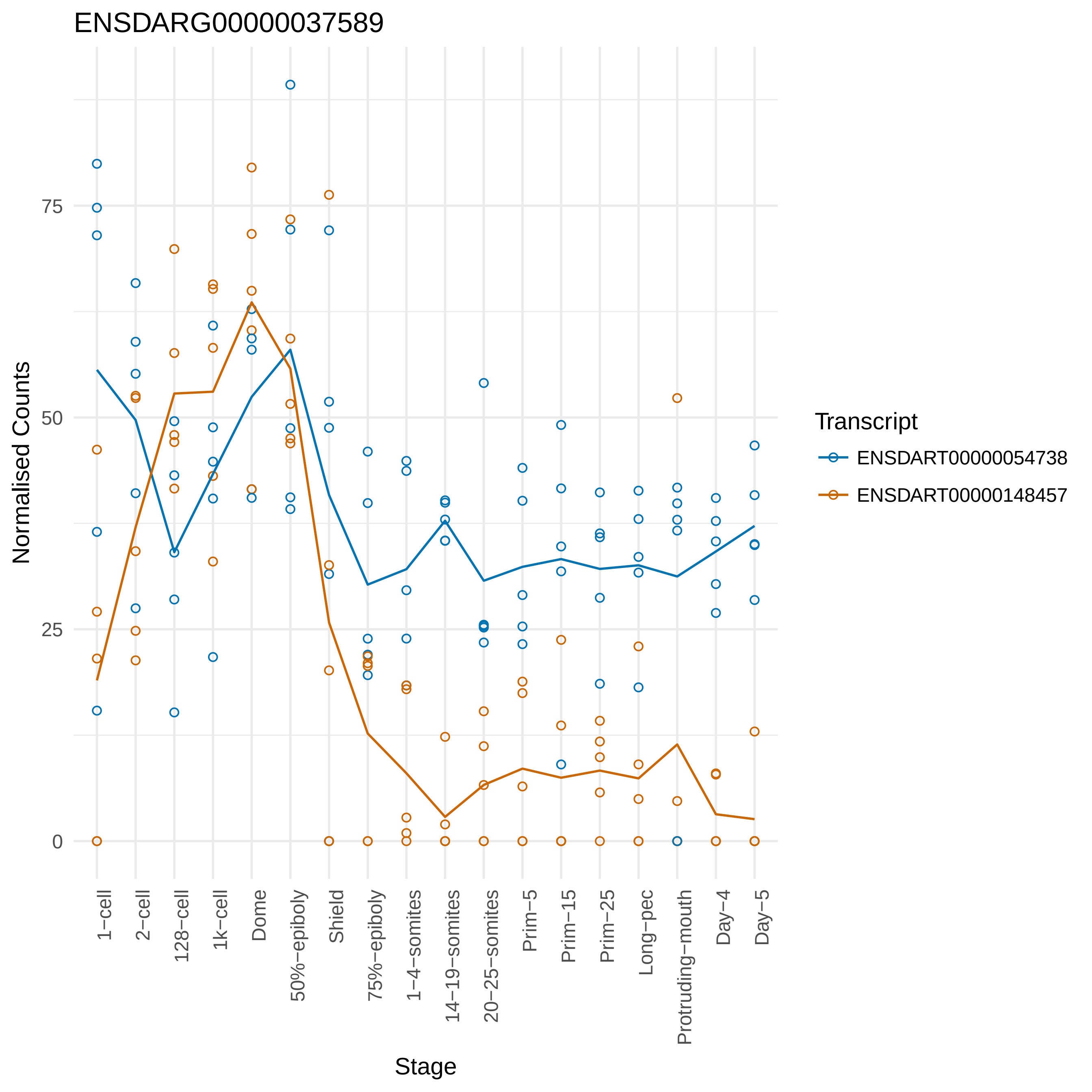

ENSDARG00000037589
Normalised Counts
75
50
25
0
1−cell
2−cell
128−cell
1k−cell
Dome
50%−epiboly
Shield
75%−epiboly
1−4−somites
14−19−somites
20−25−somites
Prim−5
Prim−15
Prim−25
Long−pec
Protruding−mouth
Day−4
Day−5
Stage
Transcript
ENSDART00000054738
ENSDART00000148457

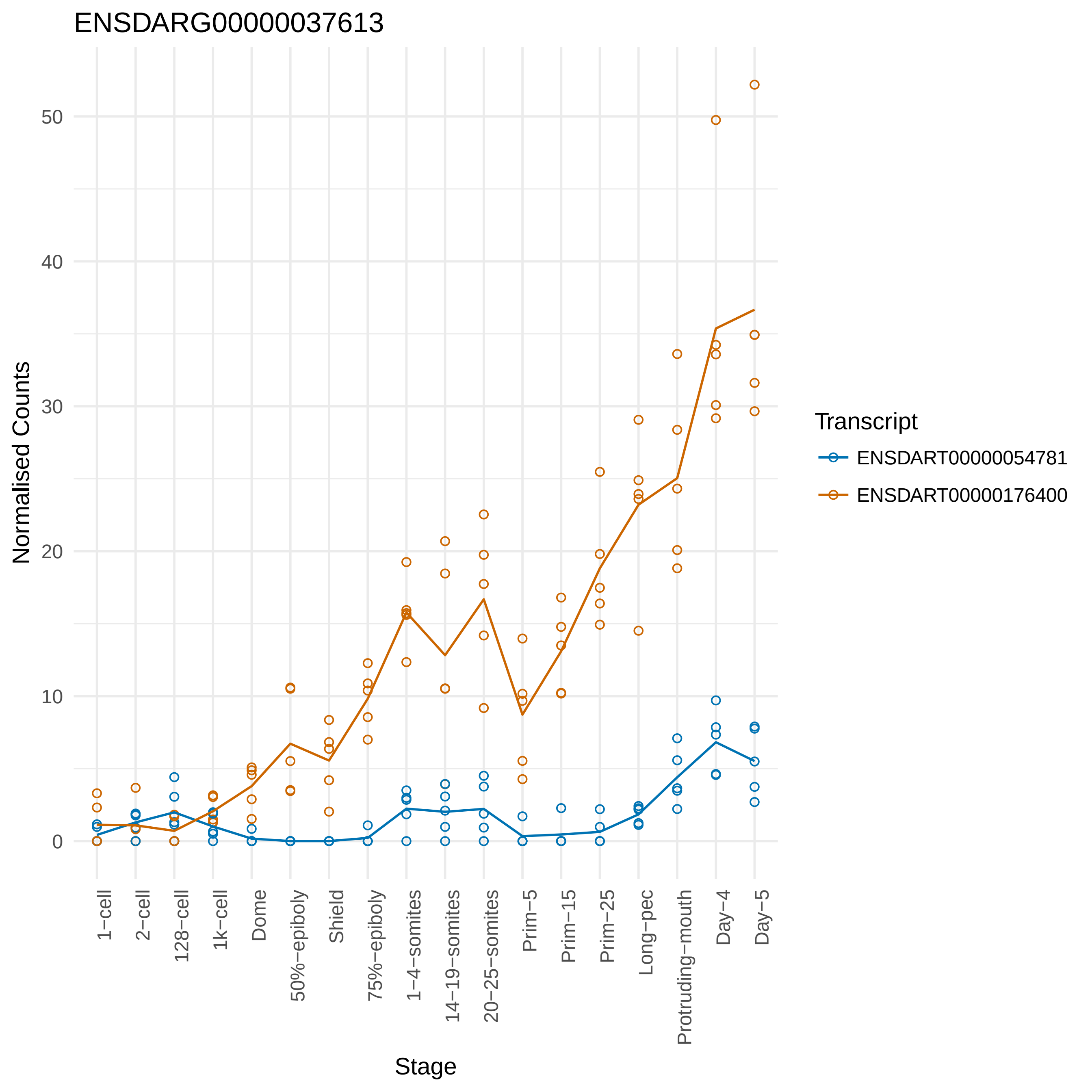

ENSDARG00000037613
Normalised Counts
0
10
20
30
40
50
1−cell
2−cell
128−cell
1k−cell
Dome
50%−epiboly
Shield
75%−epiboly
1−4−somites
14−19−somites
20−25−somites
Prim−5
Prim−15
Prim−25
Long−pec
Protruding−mouth
Day−4
Day−5
Stage
Transcript
ENSDART00000054781
ENSDART00000176400

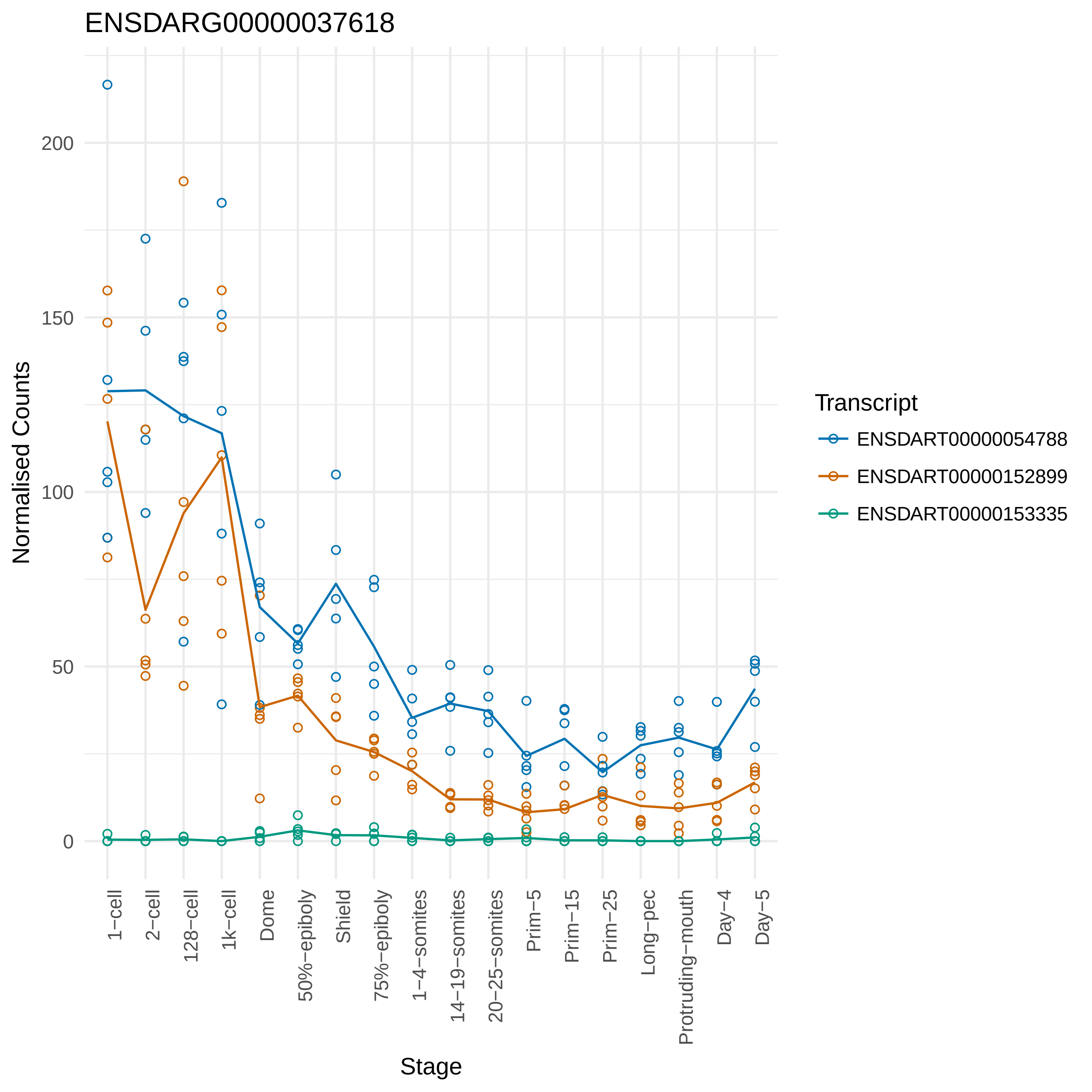

ENSDARG00000037618
Normalised Counts
0
50
100
150
200
Stage
1−cell
2−cell
128−cell
1k−cell
Dome
50%−epiboly
Shield
75%−epiboly
1−4−somites
14−19−somites
20−25−somites
Prim−5
Prim−15
Prim−25
Long−pec
Protruding−mouth
Day−4
Day−5
Transcript
ENSDART00000054788
ENSDART00000152899
ENSDART00000153335

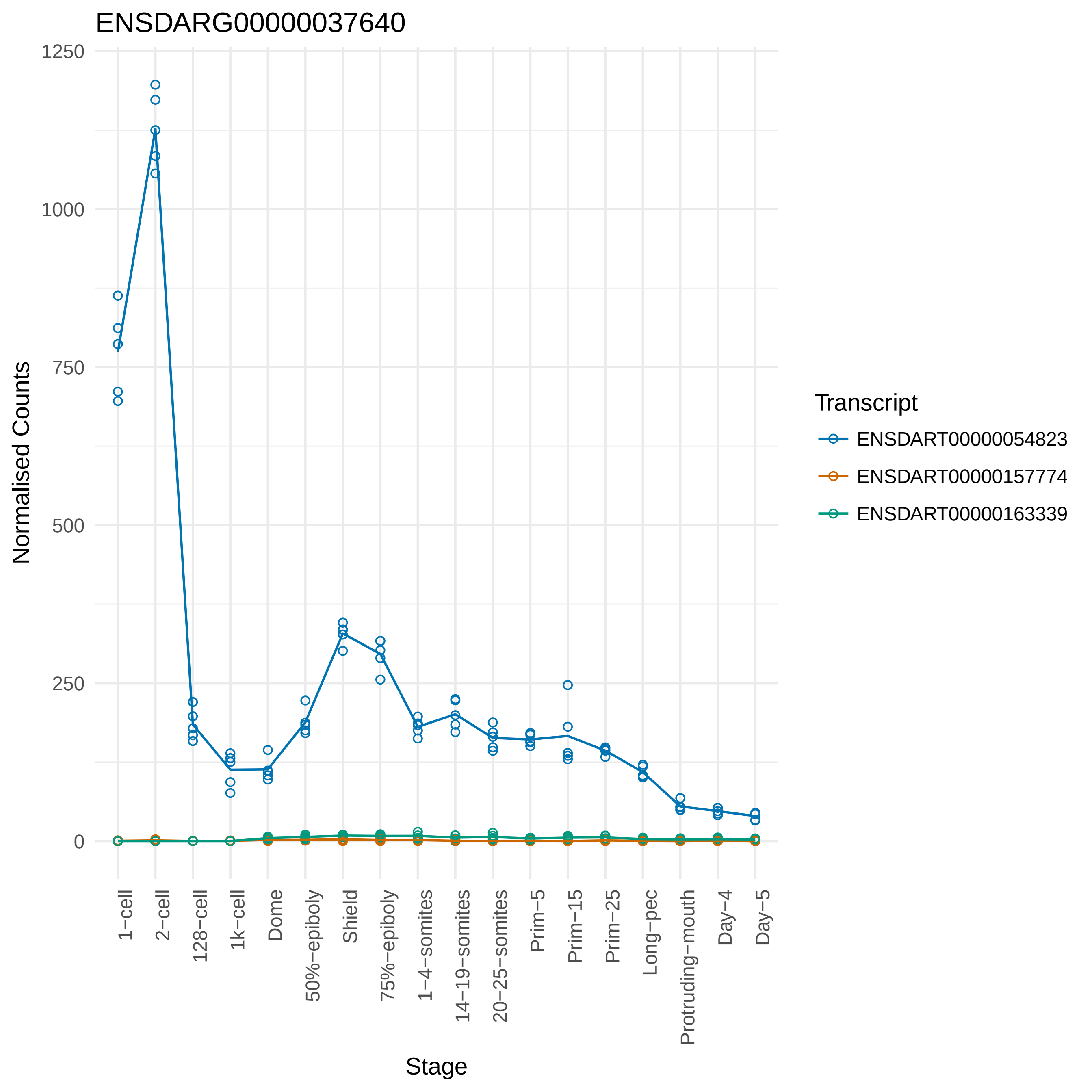

ENSDARG00000037640
Normalised Counts
1250
1000
750
500
250
0
1−cell
2−cell
128−cell
1k−cell
Dome
50%−epiboly
Shield
75%−epiboly
1−4−somites
14−19−somites
20−25−somites
Prim−5
Prim−15
Prim−25
Long−pec
Protruding−mouth
Day−4
Day−5
Stage
Transcript
ENSDART00000054823
ENSDART00000157774
ENSDART00000163339

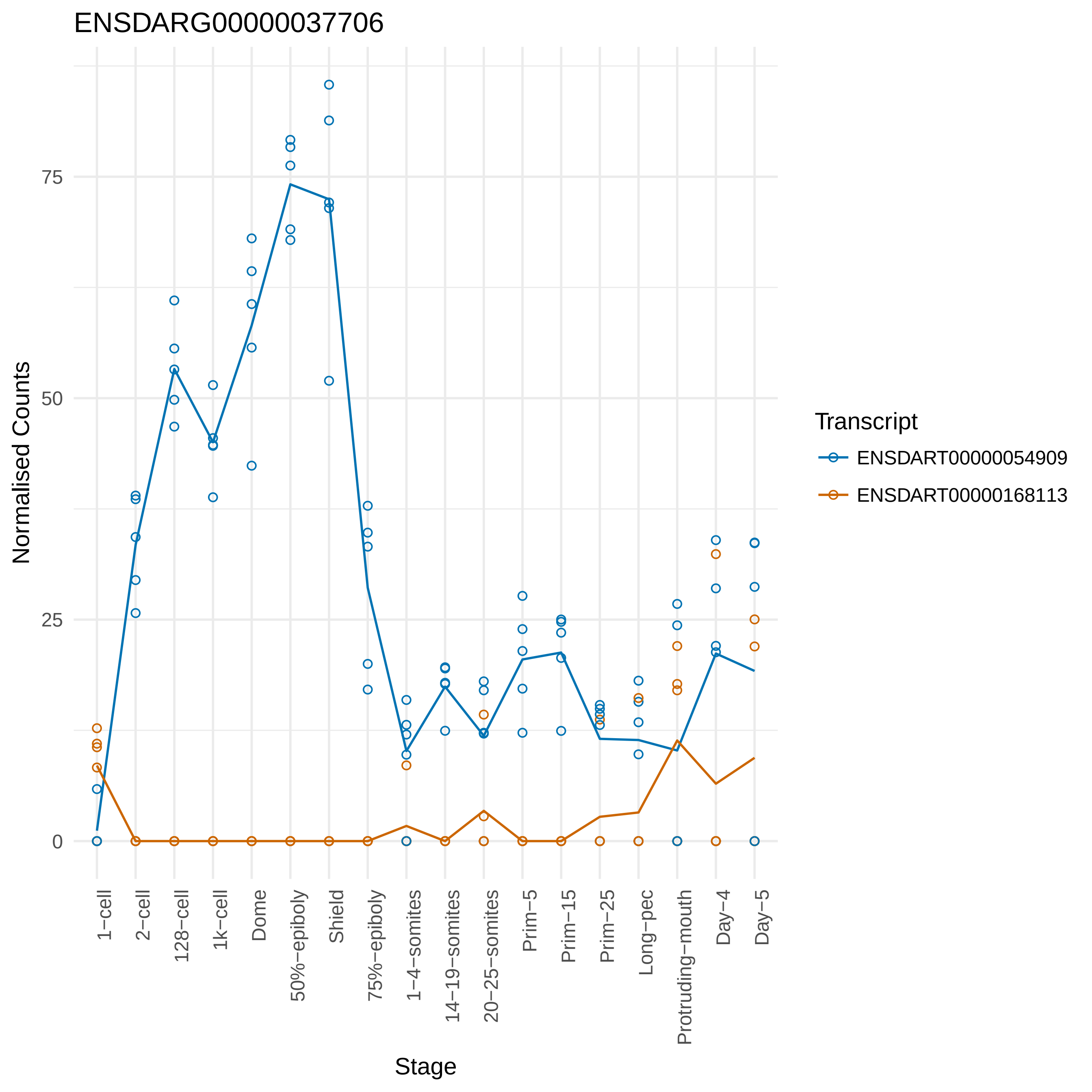

ENSDARG00000037706
Normalised Counts
75
50
25
0
1−cell
2−cell
128−cell
1k−cell
Dome
50%−epiboly
Shield
75%−epiboly
1−4−somites
14−19−somites
20−25−somites
Prim−5
Prim−15
Prim−25
Long−pec
Protruding−mouth
Day−4
Day−5
Stage
Transcript
ENSDART00000054909
ENSDART00000168113

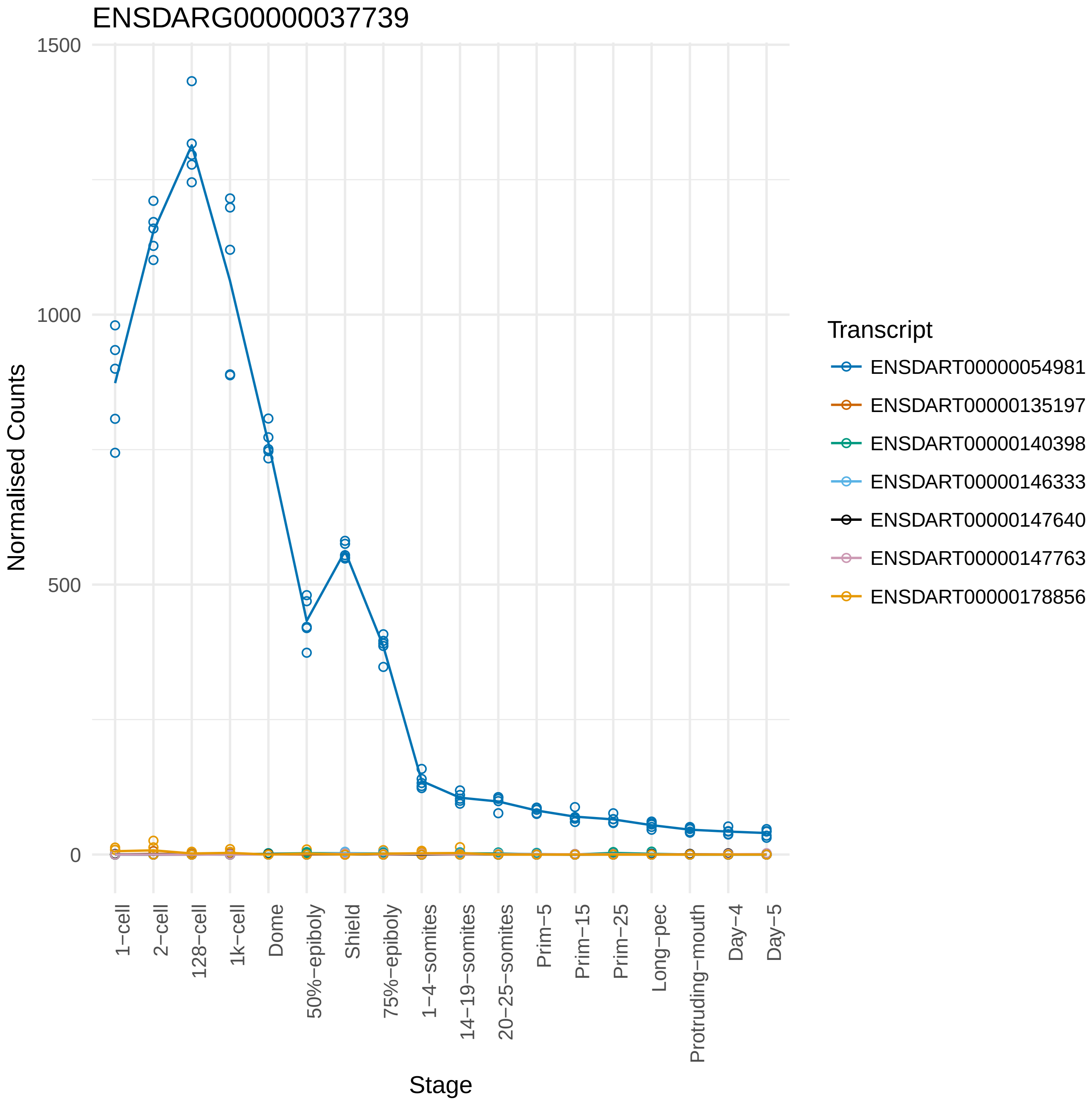
ENSDARG00000037739
1500
1000
500
0
Normalised Counts
1−cell
2−cell
128−cell
1k−cell
Dome
50%−epiboly
Shield
75%−epiboly
1−4−somites
14−19−somites
20−25−somites
Prim−5
Prim−15
Prim−25
Long−pec
Protruding−mouth
Day−4
Day−5
Stage
Transcript
ENSDART00000054981
ENSDART00000135197
ENSDART00000140398
ENSDART00000146333
ENSDART00000147640
ENSDART00000147763
ENSDART00000178856

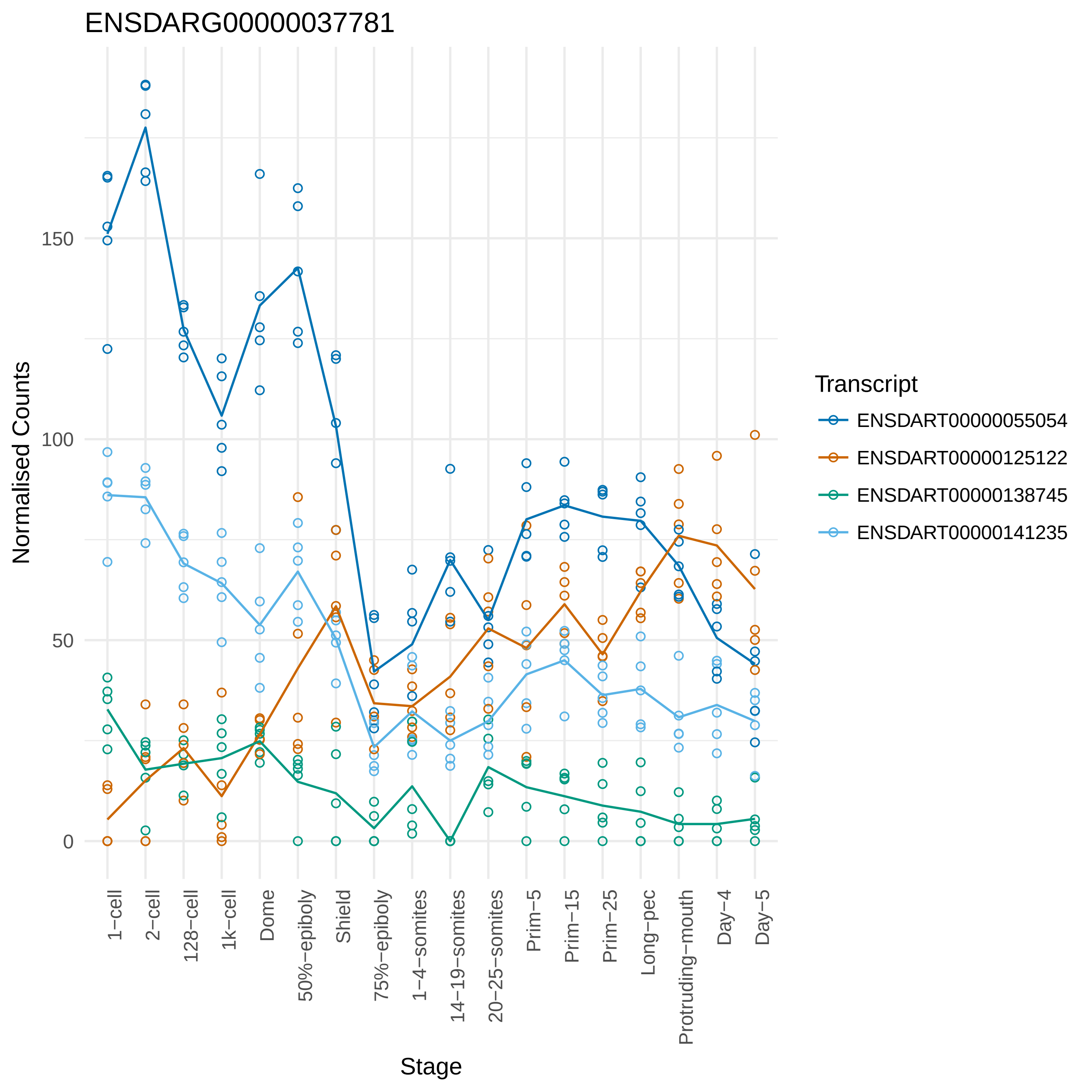

ENSDARG00000037781
Normalised Counts
150
100
50
0
1−cell
2−cell
128−cell
1k−cell
Dome
50%−epiboly
Shield
75%−epiboly
1−4−somites
14−19−somites
20−25−somites
Prim−5
Prim−15
Prim−25
Long−pec
Protruding−mouth
Day−4
Day−5
Stage
Transcript
ENSDART00000055054
ENSDART00000125122
ENSDART00000138745
ENSDART00000141235

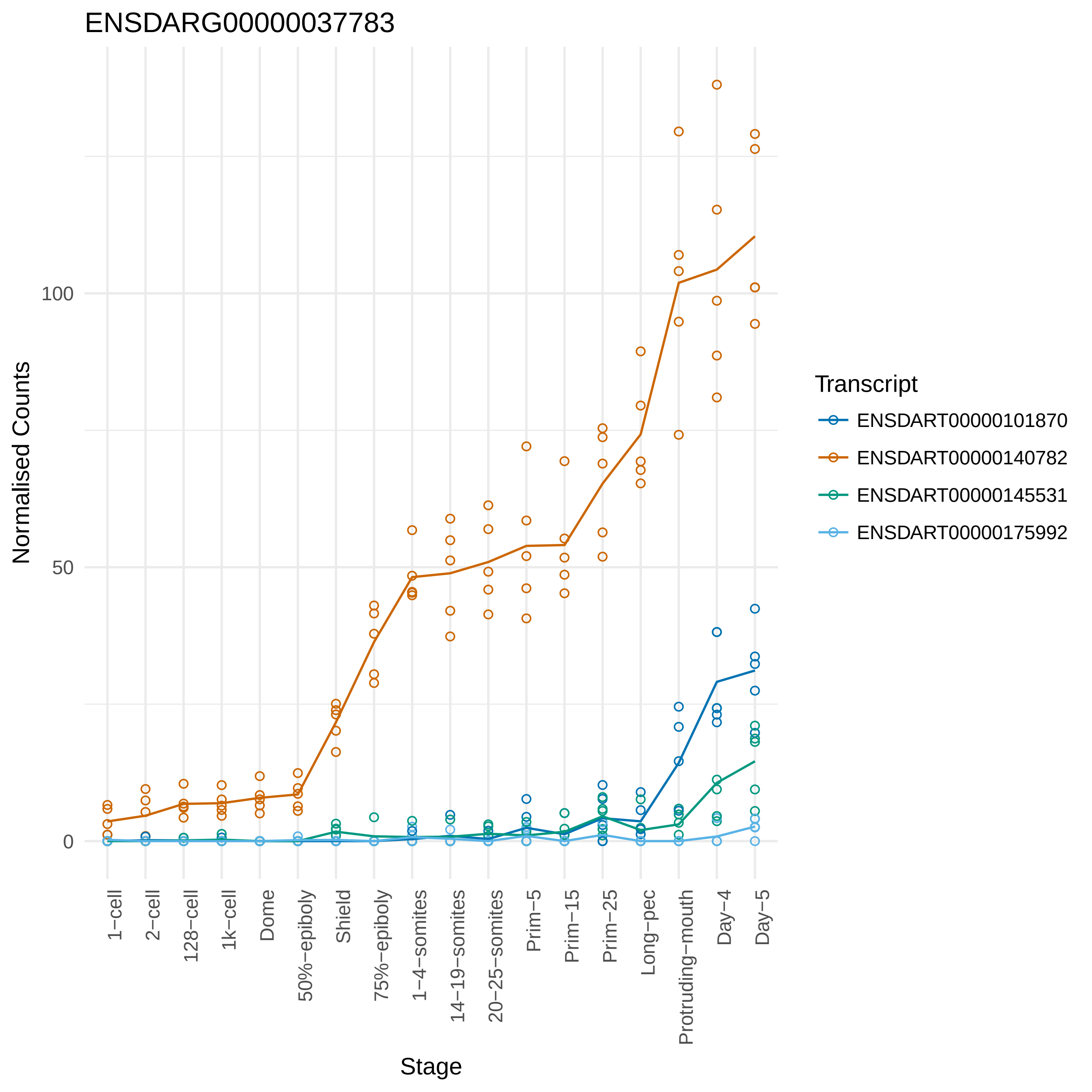
ENSDARG00000037783
Normalised Counts
0
50
100
1−cell
2−cell
128−cell
1k−cell
Dome
50%−epiboly
Shield
75%−epiboly
1−4−somites
14−19−somites
20−25−somites
Prim−5
Prim−15
Prim−25
Long−pec
Protruding−mouth
Day−4
Day−5
Stage
Transcript
ENSDART00000101870
ENSDART00000140782
ENSDART00000145531
ENSDART00000175992

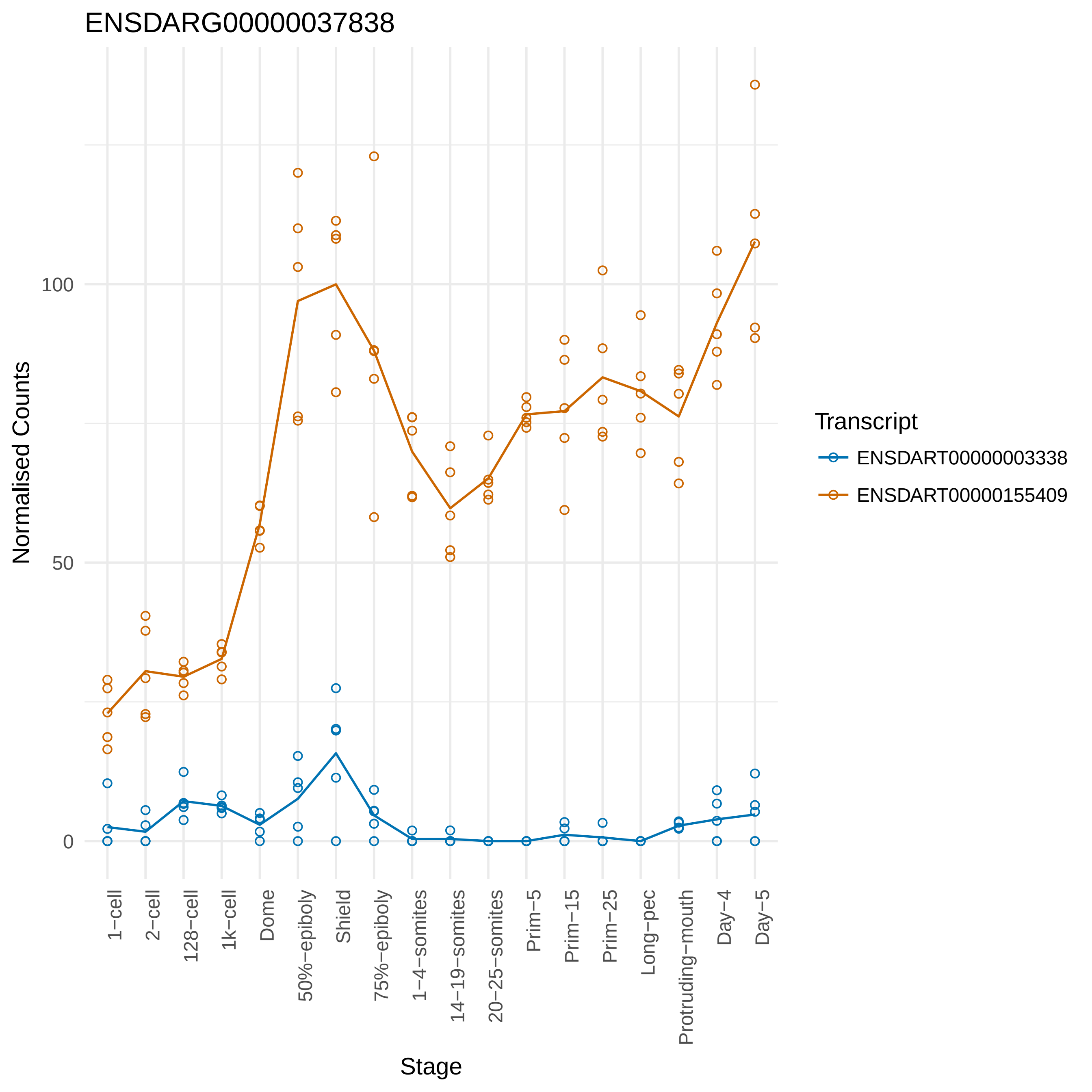

ENSDARG00000037838
Normalised Counts
100
50
0
1−cell
2−cell
128−cell
1k−cell
Dome
50%−epiboly
Shield
75%−epiboly
1−4−somites
14−19−somites
20−25−somites
Prim−5
Prim−15
Prim−25
Long−pec
Protruding−mouth
Day−4
Day−5
Stage
Transcript
ENSDART00000003338
ENSDART00000155409

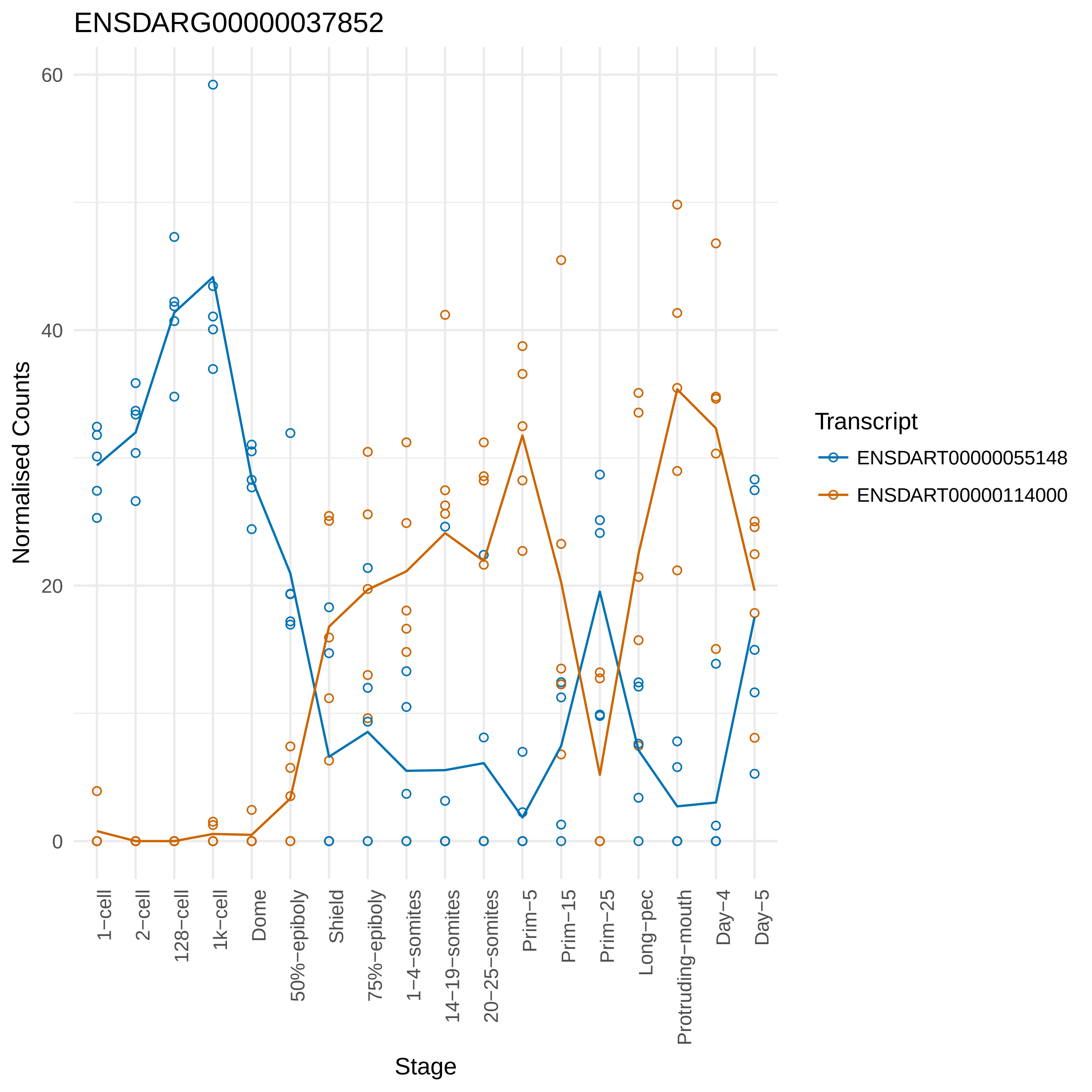
ENSDARG00000037852
Normalised Counts
60
40
20
0
1−cell
2−cell
128−cell
1k−cell
Dome
50%−epiboly
Shield
75%−epiboly
1−4−somites
14−19−somites
20−25−somites
Prim−5
Prim−15
Prim−25
Long−pec
Protruding−mouth
Day−4
Day−5
Stage
Transcript
ENSDART00000055148
ENSDART00000114000

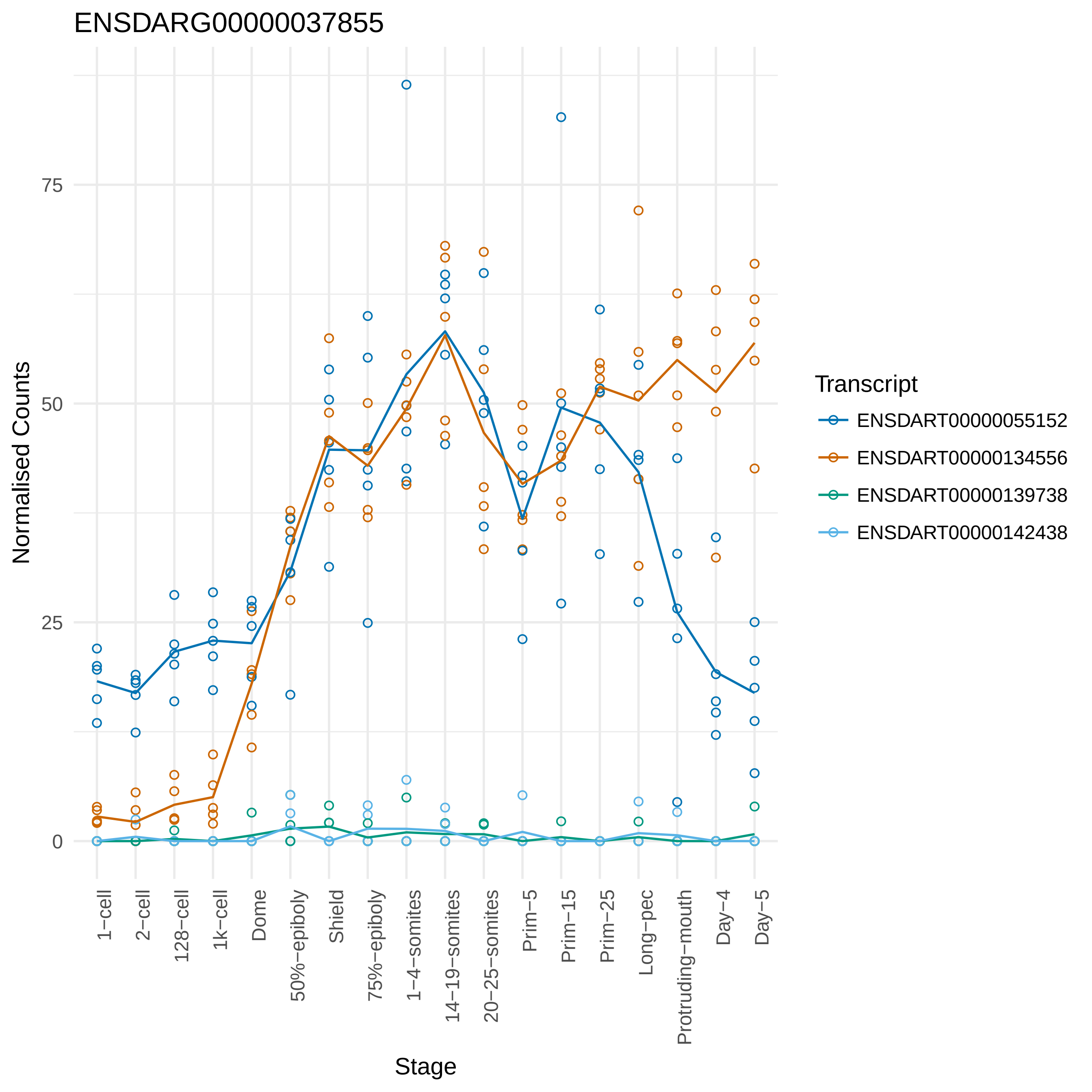

ENSDARG00000037855
Normalised Counts
0
25
50
75
Stage
1−cell
2−cell
128−cell
1k−cell
Dome
50%−epiboly
Shield
75%−epiboly
1−4−somites
14−19−somites
20−25−somites
Prim−5
Prim−15
Prim−25
Long−pec
Protruding−mouth
Day−4
Day−5
Transcript
ENSDART00000055152
ENSDART00000134556
ENSDART00000139738
ENSDART00000142438

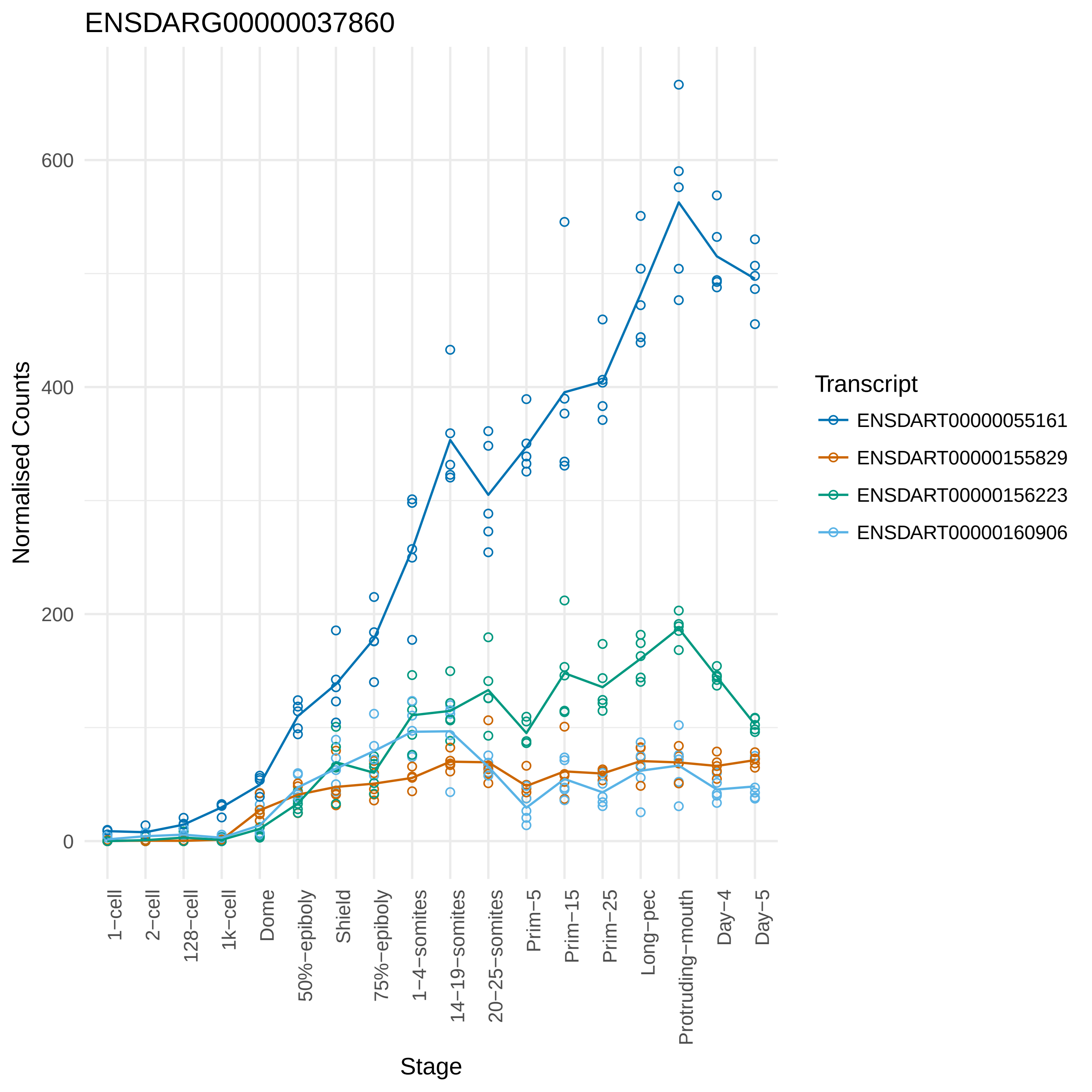

ENSDARG00000037860
Normalised Counts
0
200
400
600
Stage
1−cell
2−cell
128−cell
1k−cell
Dome
50%−epiboly
Shield
75%−epiboly
1−4−somites
14−19−somites
20−25−somites
Prim−5
Prim−15
Prim−25
Long−pec
Protruding−mouth
Day−4
Day−5
Transcript
ENSDART00000055161
ENSDART00000155829
ENSDART00000156223
ENSDART00000160906

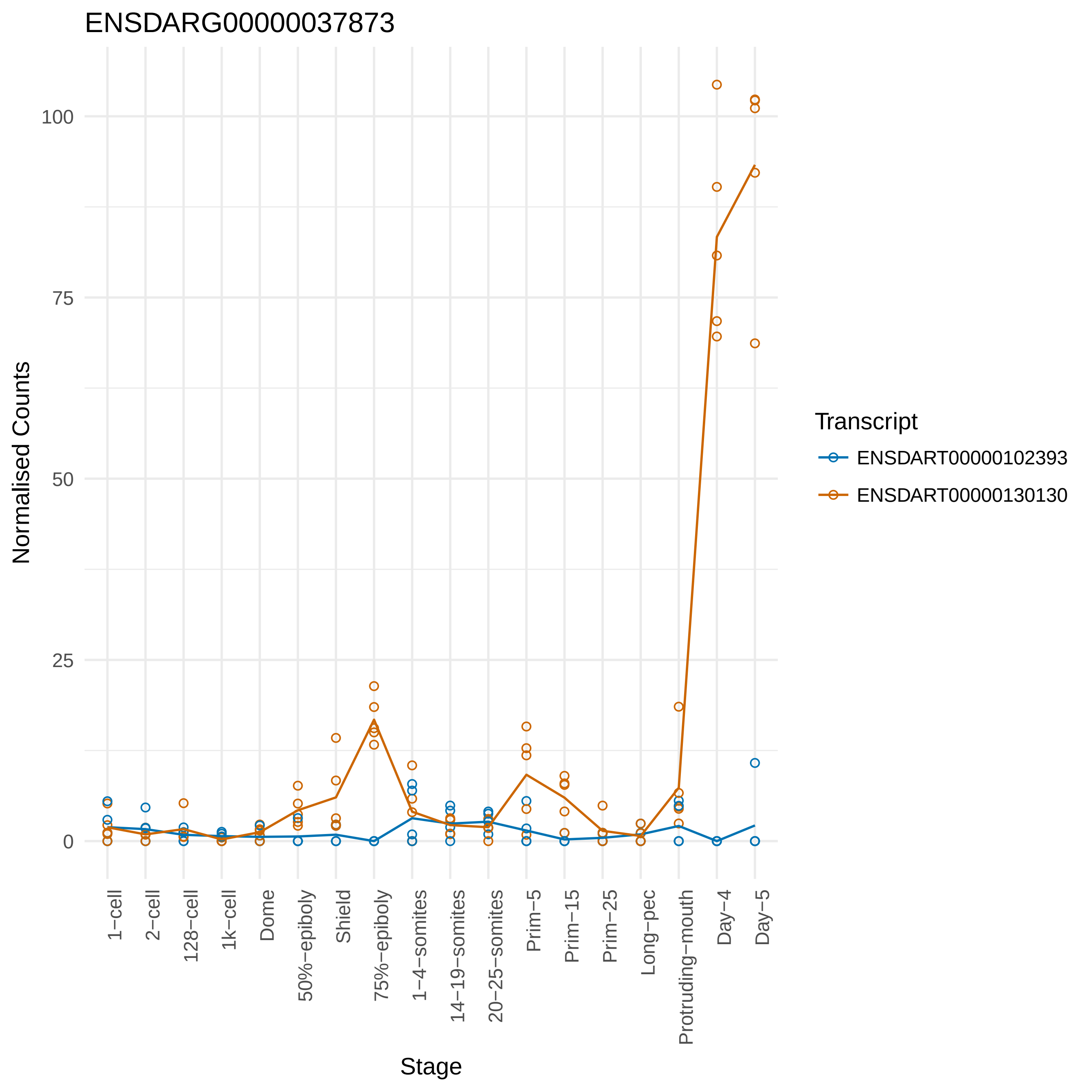
ENSDARG00000037873
Normalised Counts
100
75
50
25
0
1−cell
2−cell
128−cell
1k−cell
Dome
50%−epiboly
Shield
75%−epiboly
1−4−somites
14−19−somites
20−25−somites
Prim−5
Prim−15
Prim−25
Long−pec
Protruding−mouth
Day−4
Day−5
Stage
Transcript
ENSDART00000102393
ENSDART00000130130

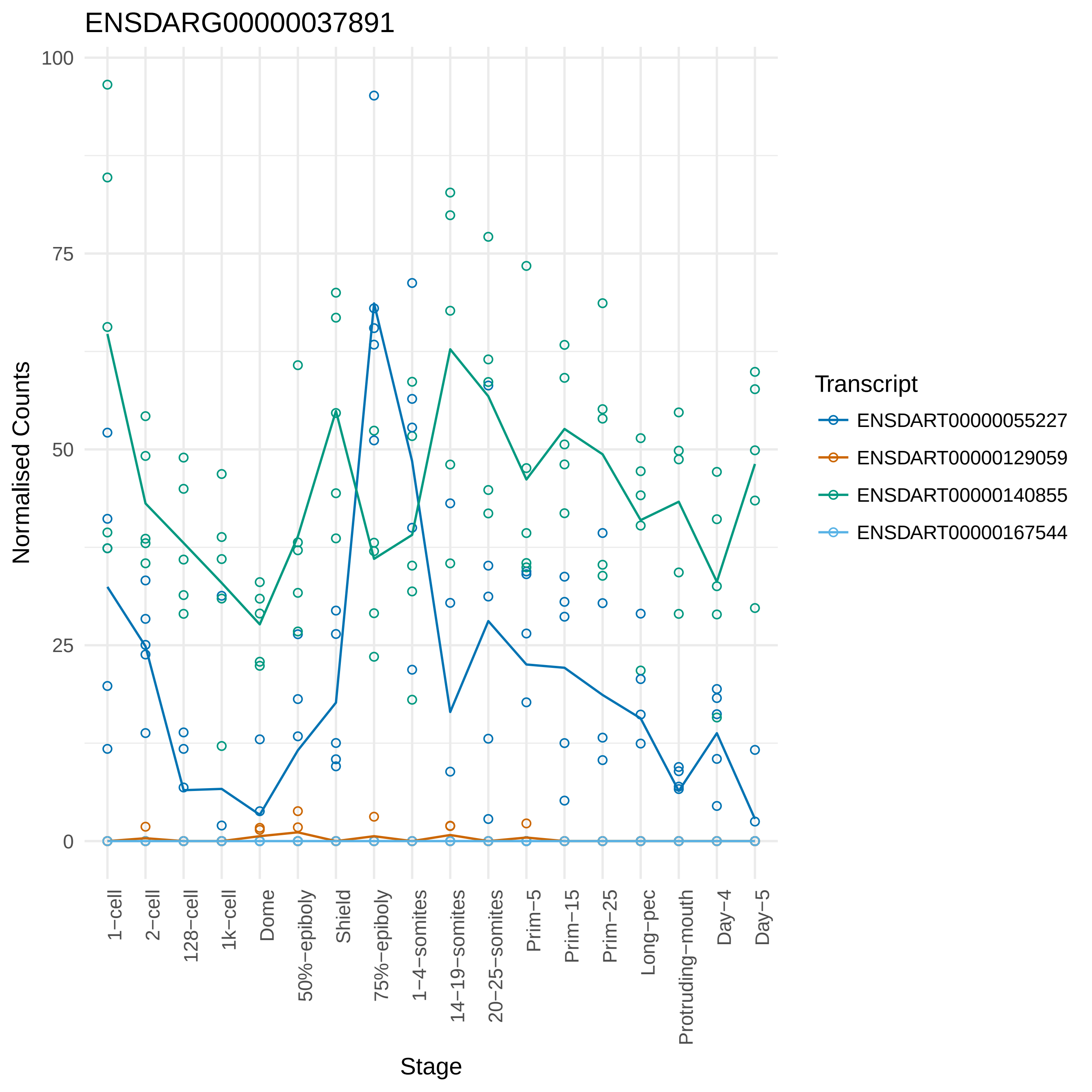

ENSDARG00000037891
Normalised Counts
100
75
50
25
0
Stage
1−cell
2−cell
128−cell
1k−cell
Dome
50%−epiboly
Shield
75%−epiboly
1−4−somites
14−19−somites
20−25−somites
Prim−5
Prim−15
Prim−25
Long−pec
Protruding−mouth
Day−4
Day−5
Transcript
ENSDART00000055227
ENSDART00000129059
ENSDART00000140855
ENSDART00000167544

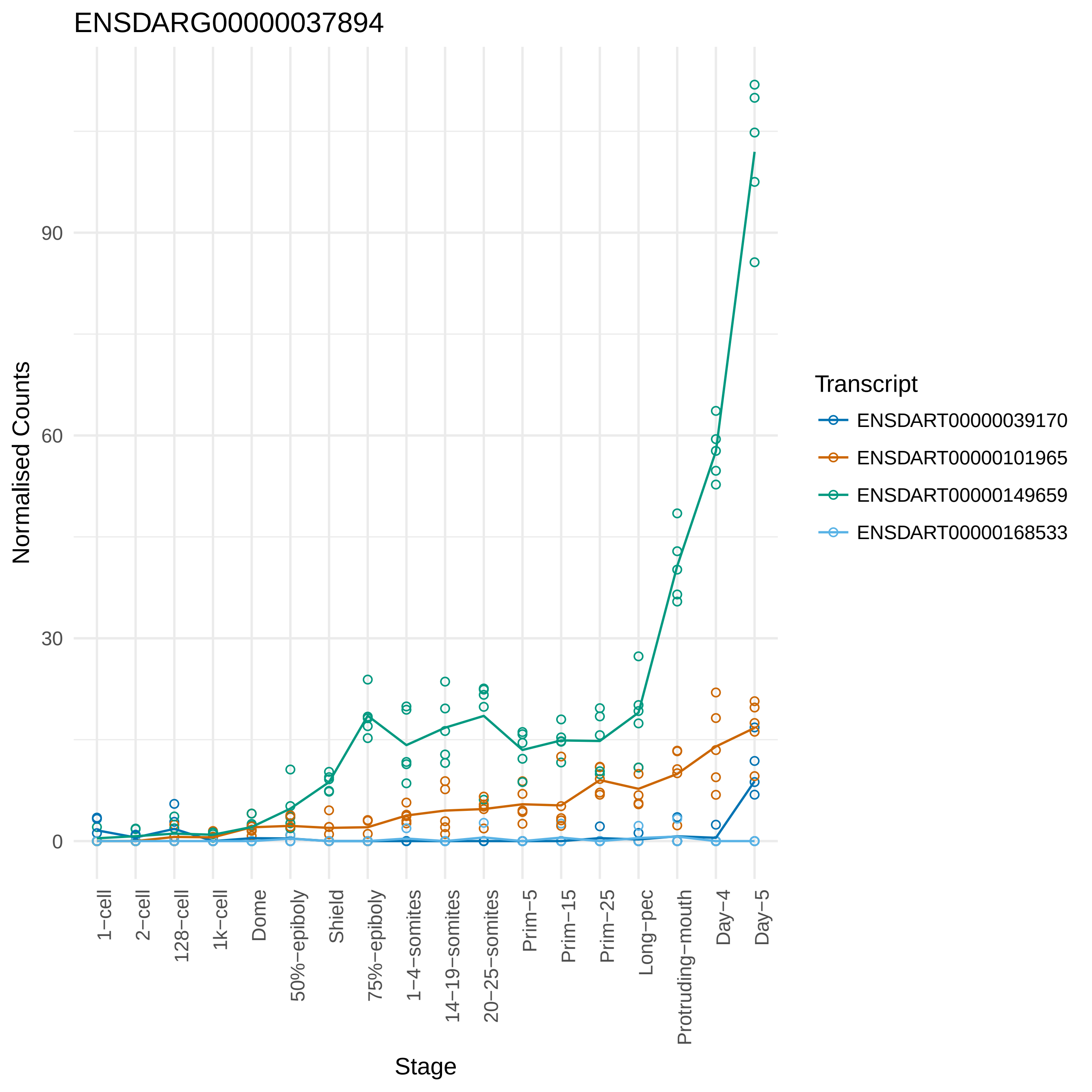

ENSDARG00000037894
Normalised Counts
0
30
60
90
Stage
1−cell
2−cell
128−cell
1k−cell
Dome
50%−epiboly
Shield
75%−epiboly
1−4−somites
14−19−somites
20−25−somites
Prim−5
Prim−15
Prim−25
Long−pec
Protruding−mouth
Day−4
Day−5
Transcript
ENSDART00000039170
ENSDART00000101965
ENSDART00000149659
ENSDART00000168533

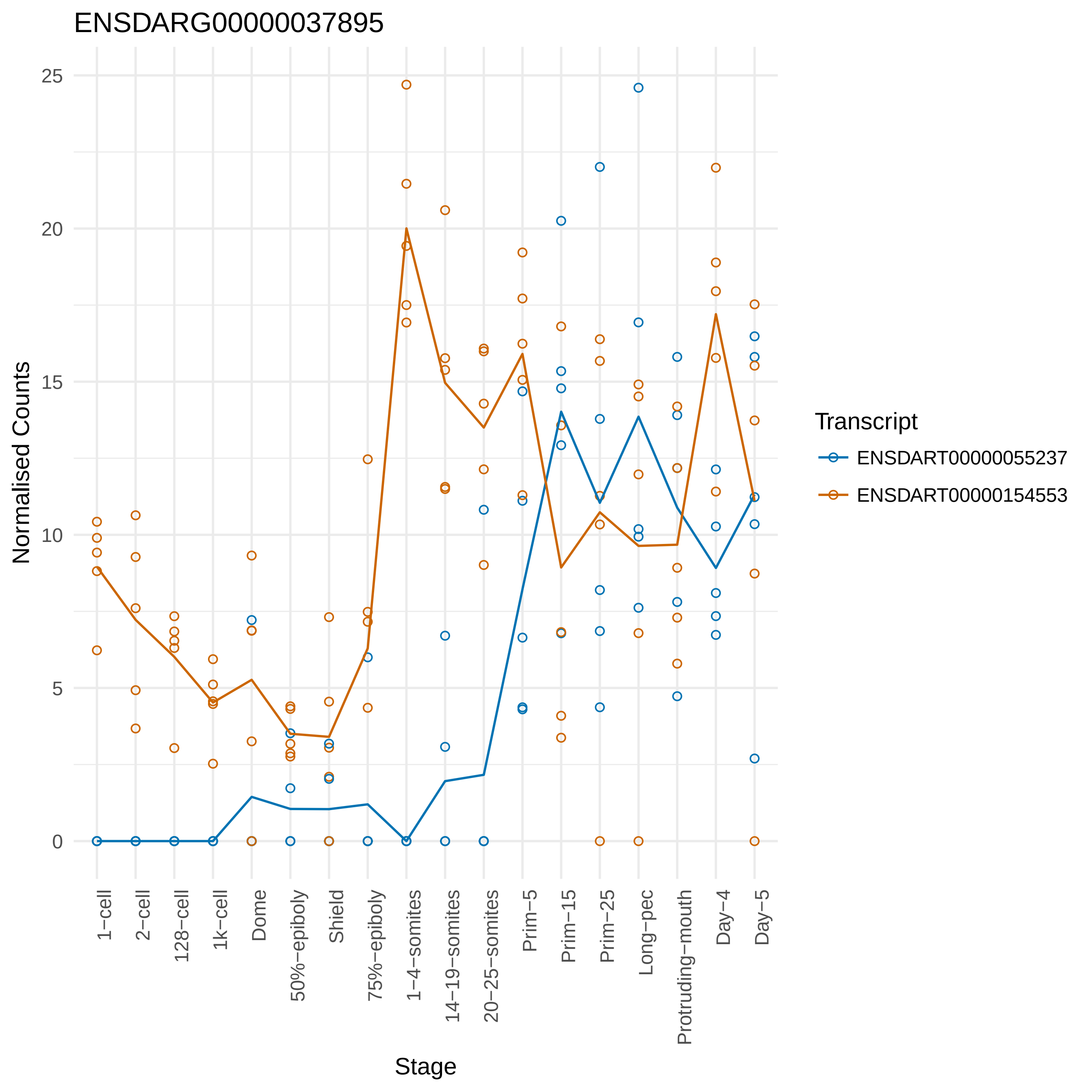

ENSDARG00000037895
Normalised Counts
0
5
10
15
20
25
1−cell
2−cell
128−cell
1k−cell
Dome
50%−epiboly
Shield
75%−epiboly
1−4−somites
14−19−somites
20−25−somites
Prim−5
Prim−15
Prim−25
Long−pec
Protruding−mouth
Day−4
Day−5
Stage
Transcript
ENSDART00000055237
ENSDART00000154553

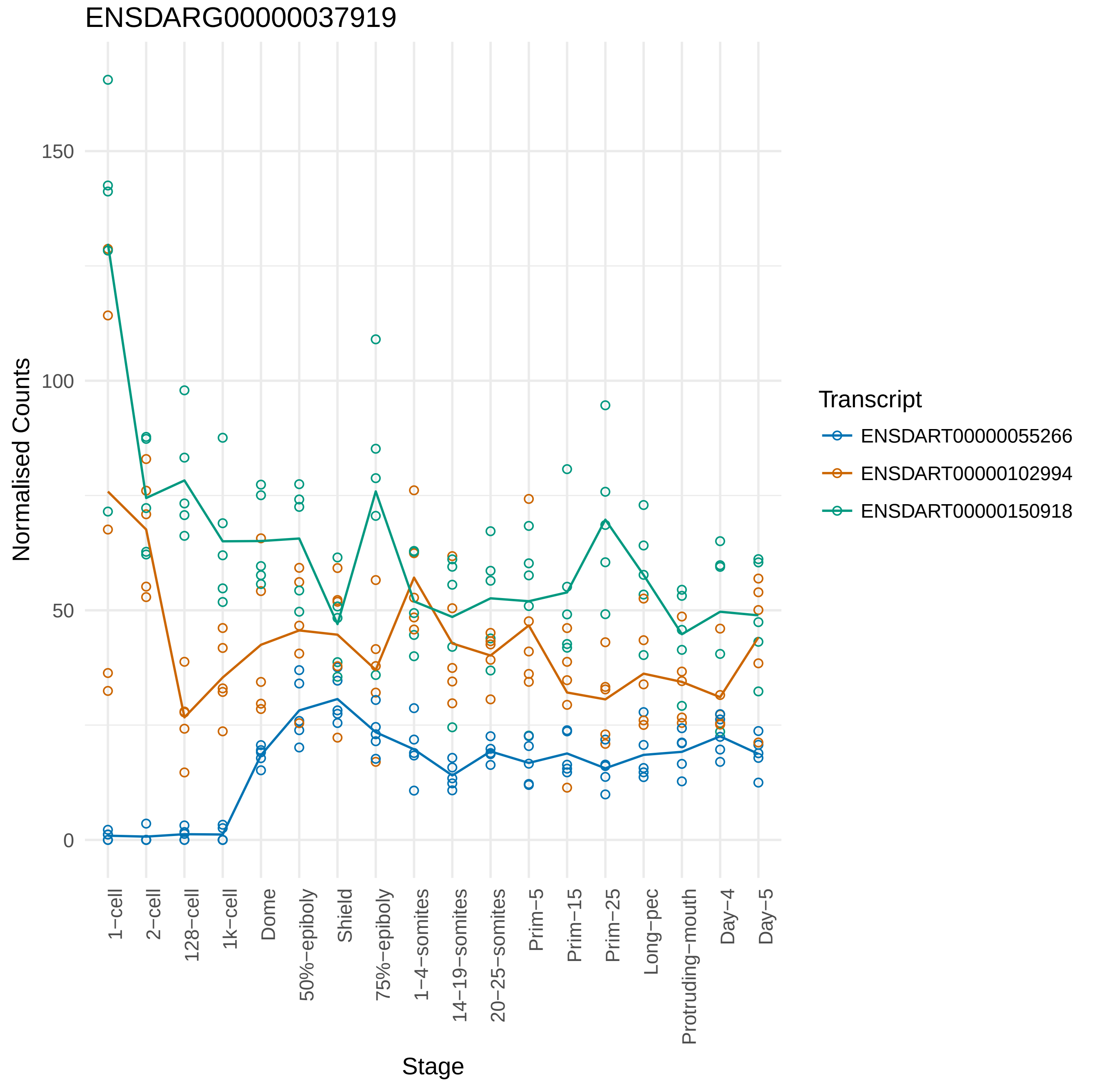
ENSDARG00000037919
Normalised Counts
150
100
50
0
Stage
1−cell
2−cell
128−cell
1k−cell
Dome
50%−epiboly
Shield
75%−epiboly
1−4−somites
14−19−somites
20−25−somites
Prim−5
Prim−15
Prim−25
Long−pec
Protruding−mouth
Day−4
Day−5
Transcript
ENSDART00000055266
ENSDART00000102994
ENSDART00000150918

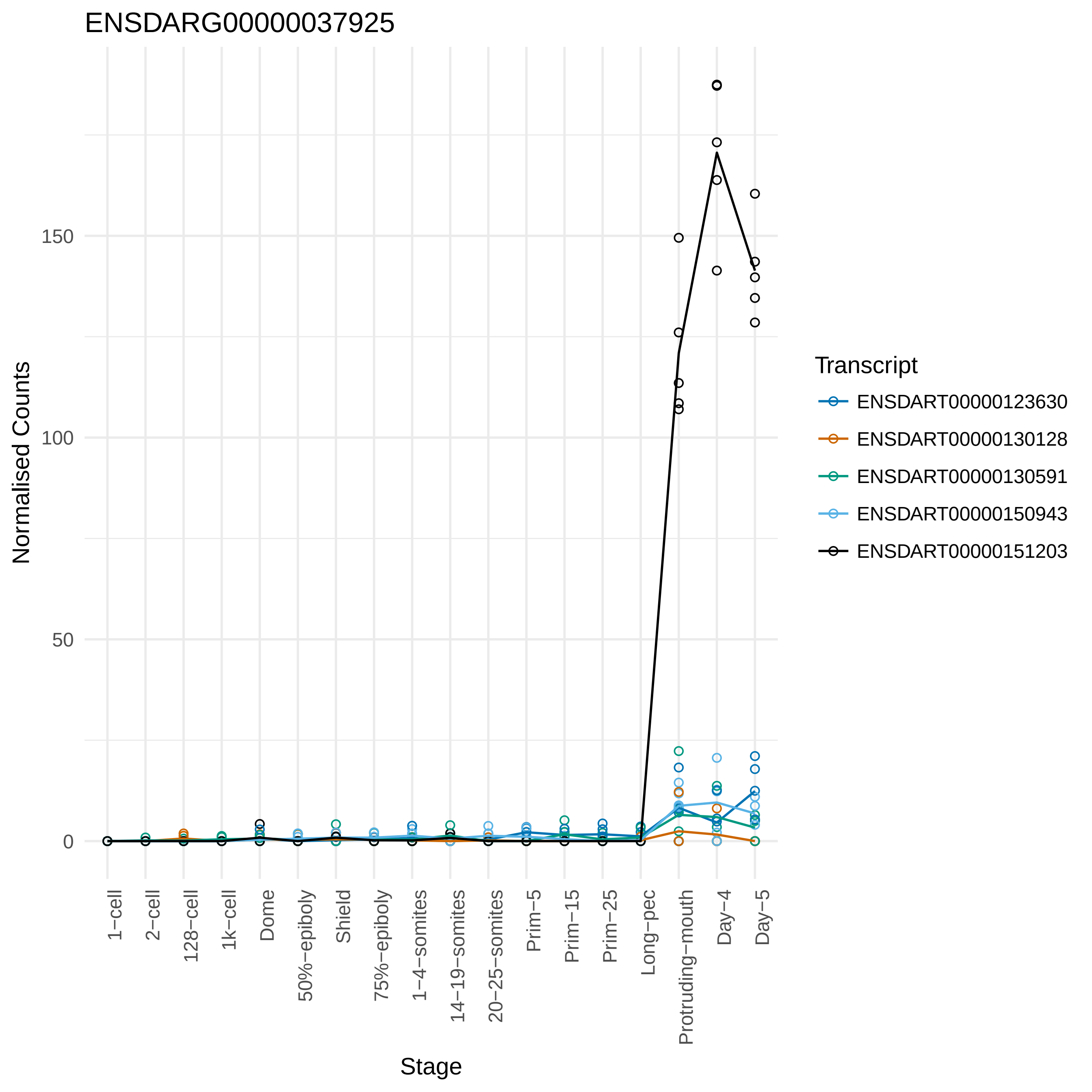
ENSDARG00000037925
Normalised Counts
0
50
100
150
Stage
1−cell
2−cell
128−cell
1k−cell
Dome
50%−epiboly
Shield
75%−epiboly
1−4−somites
14−19−somites
20−25−somites
Prim−5
Prim−15
Prim−25
Long−pec
Protruding−mouth
Day−4
Day−5
Transcript
ENSDART00000123630
ENSDART00000130128
ENSDART00000130591
ENSDART00000150943
ENSDART00000151203

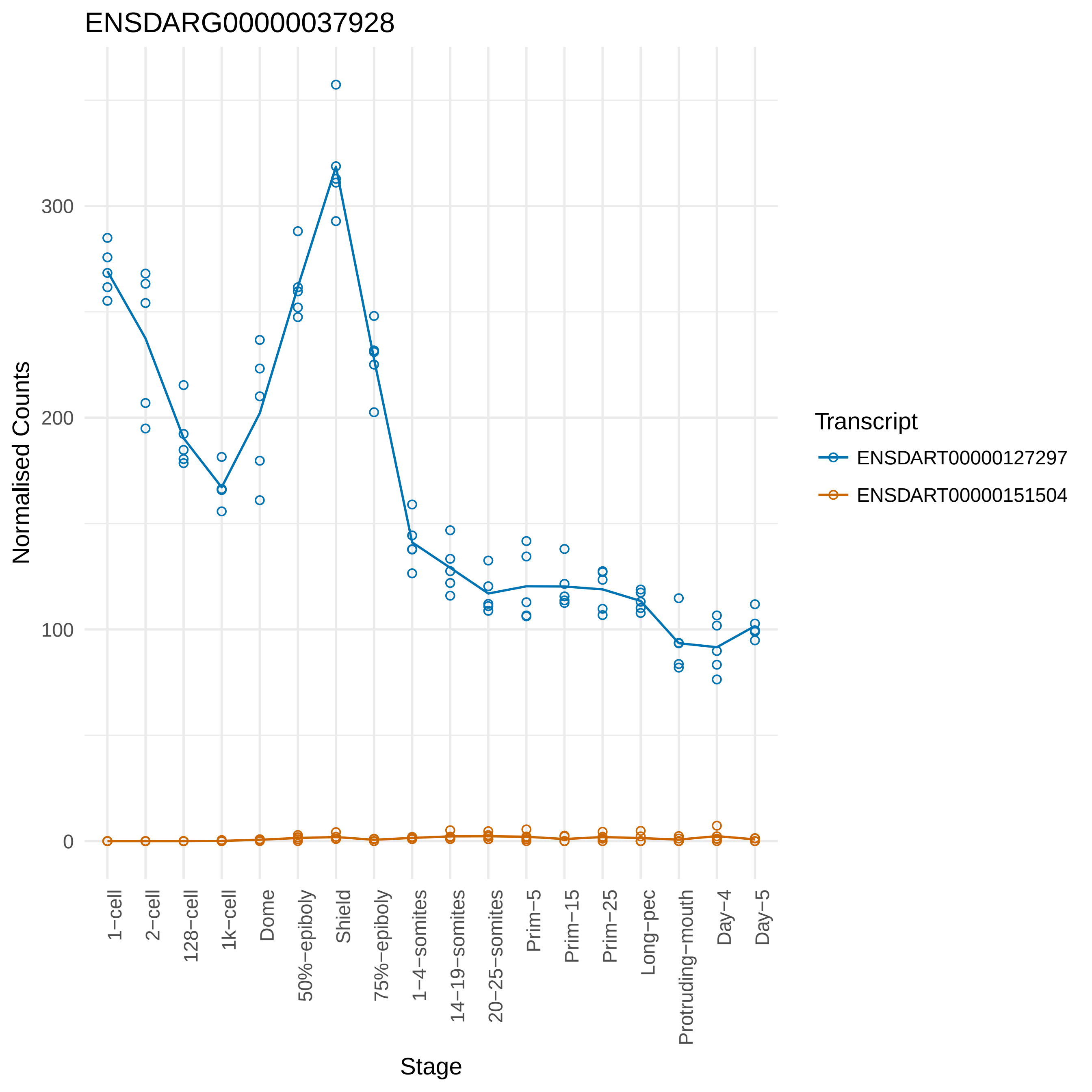

ENSDARG00000037928
Normalised Counts
300
200
100
0
1−cell
2−cell
128−cell
1k−cell
Dome
50%−epiboly
Shield
75%−epiboly
1−4−somites
14−19−somites
20−25−somites
Prim−5
Prim−15
Prim−25
Long−pec
Protruding−mouth
Day−4
Day−5
Stage
Transcript
ENSDART00000127297
ENSDART00000151504

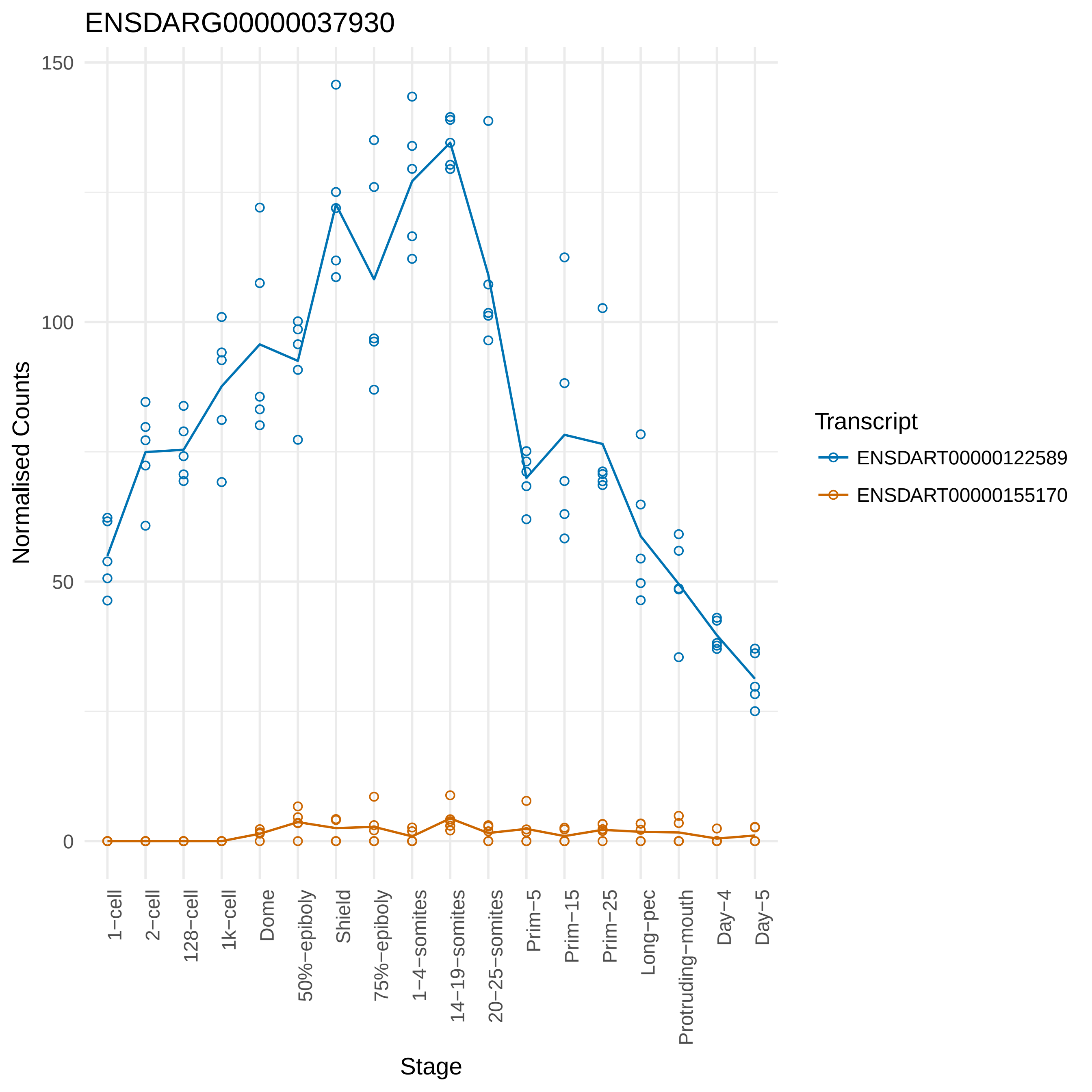
ENSDARG00000037930
Normalised Counts
150
100
50
0
1−cell
2−cell
128−cell
1k−cell
Dome
50%−epiboly
Shield
75%−epiboly
1−4−somites
14−19−somites
20−25−somites
Prim−5
Prim−15
Prim−25
Long−pec
Protruding−mouth
Day−4
Day−5
Stage
Transcript
ENSDART00000122589
ENSDART00000155170

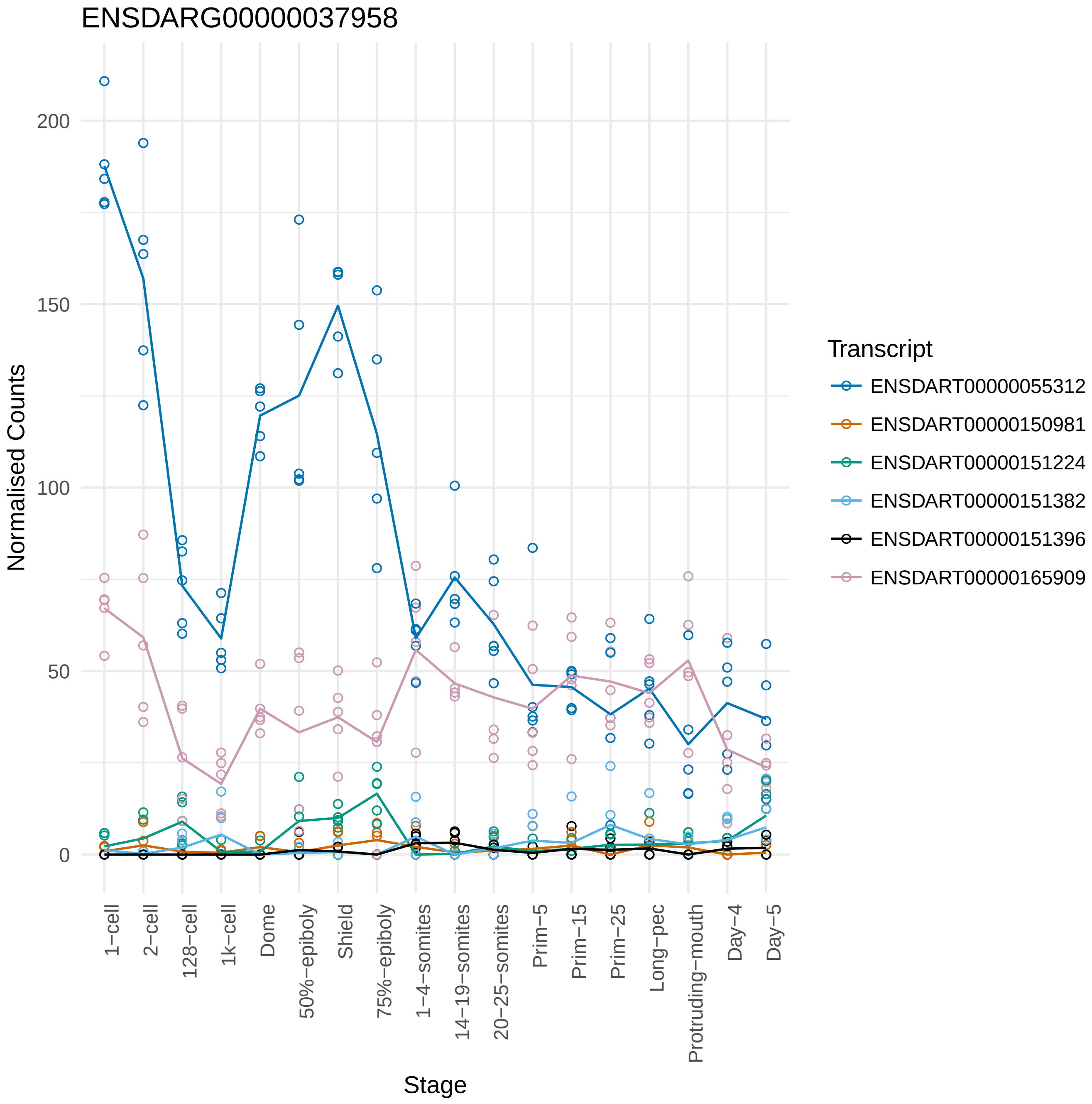
ENSDARG00000037958
Normalised Counts
200
150
100
50
0
1−cell
2−cell
128−cell
1k−cell
Dome
50%−epiboly
Shield
75%−epiboly
1−4−somites
14−19−somites
20−25−somites
Prim−5
Prim−15
Prim−25
Long−pec
Protruding−mouth
Day−4
Day−5
Stage
Transcript
ENSDART00000055312
ENSDART00000150981
ENSDART00000151224
ENSDART00000151382
ENSDART00000151396
ENSDART00000165909

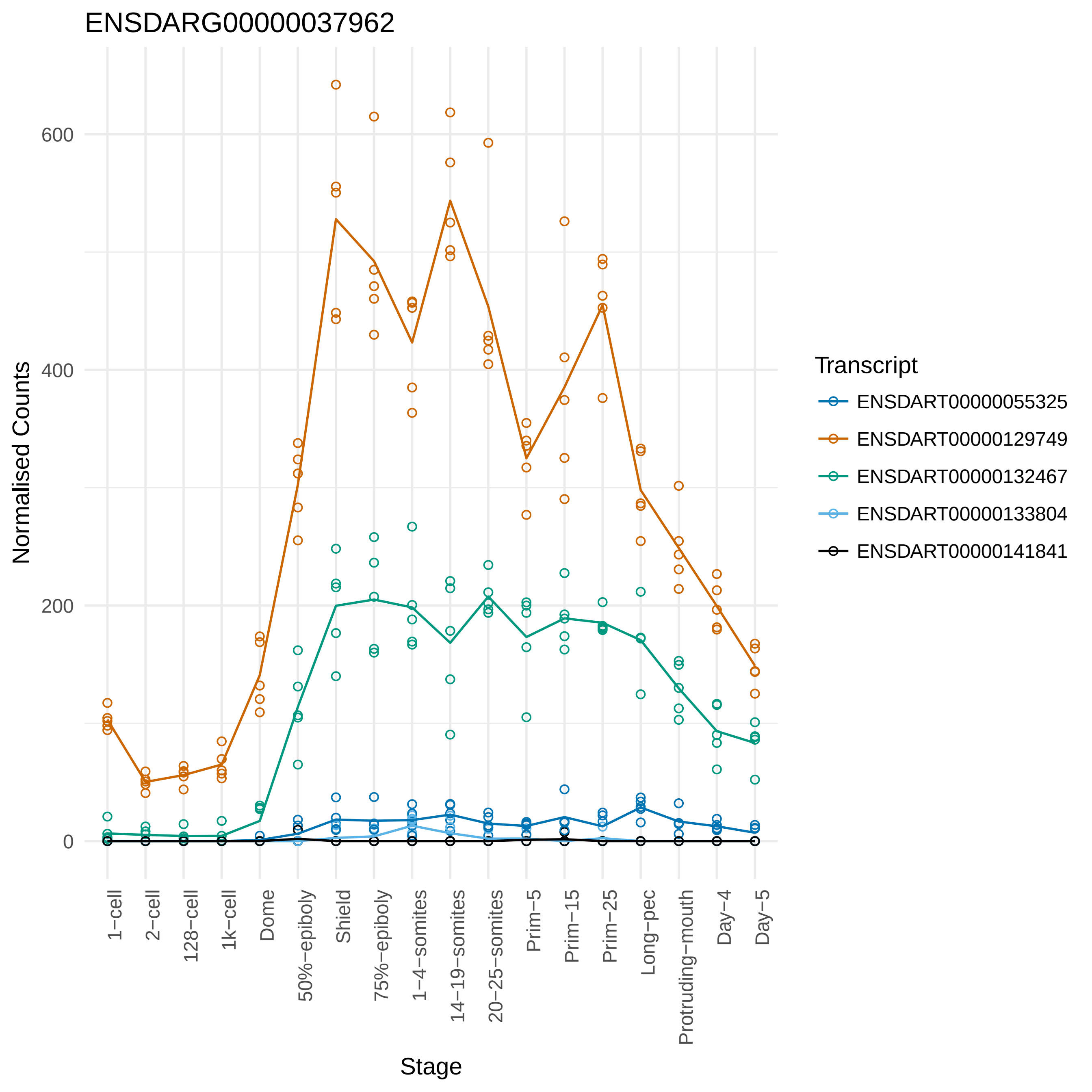

ENSDARG00000037962
Normalised Counts
600
400
200
0
1−cell
2−cell
128−cell
1k−cell
Dome
50%−epiboly
Shield
75%−epiboly
1−4−somites
14−19−somites
20−25−somites
Prim−5
Prim−15
Prim−25
Long−pec
Protruding−mouth
Day−4
Day−5
Stage
Transcript
ENSDART00000055325
ENSDART00000129749
ENSDART00000132467
ENSDART00000133804
ENSDART00000141841

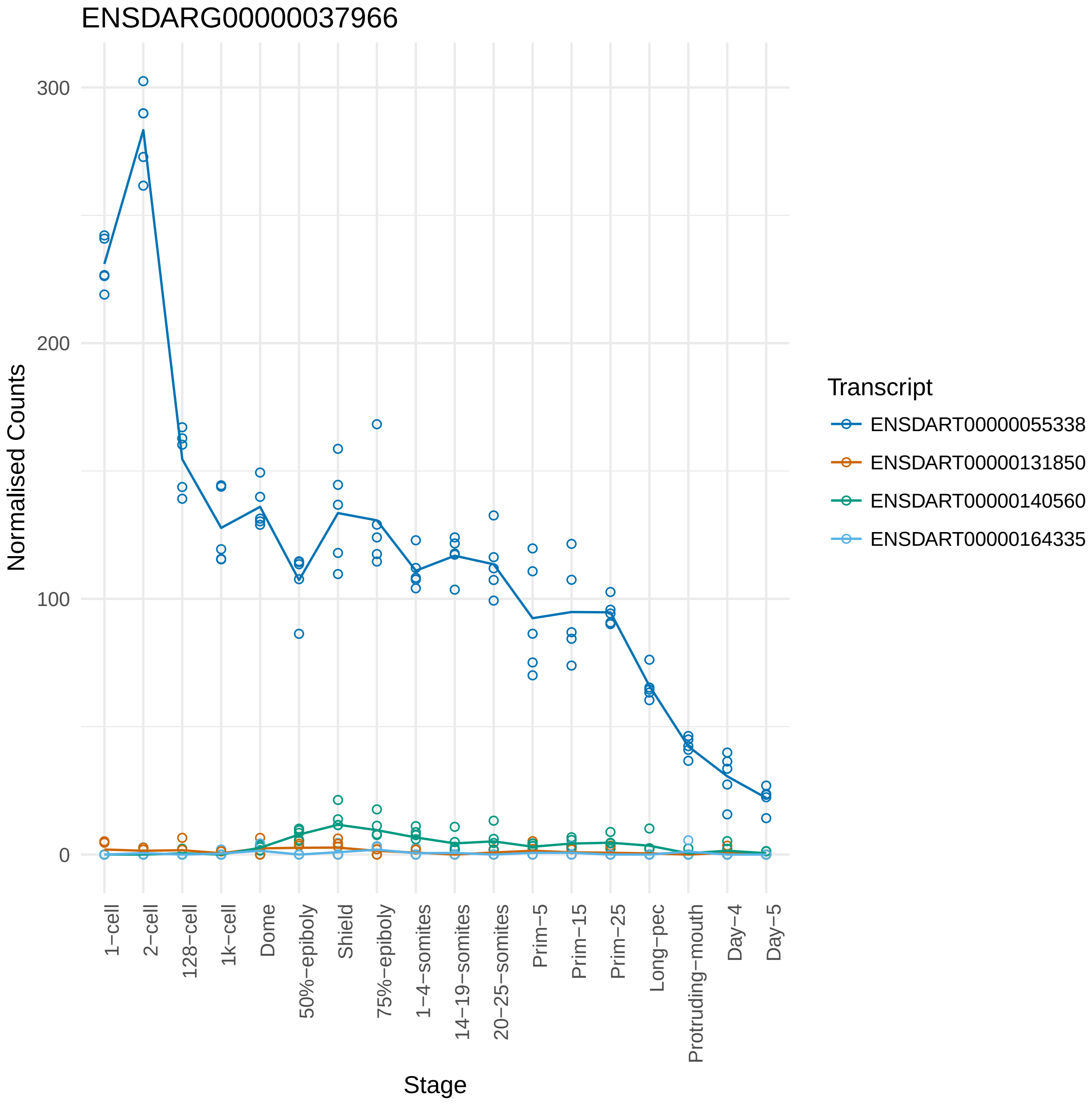

ENSDARG00000037966
Normalised Counts
300
200
100
0
1−cell
2−cell
128−cell
1k−cell
Dome
50%−epiboly
Shield
75%−epiboly
1−4−somites
14−19−somites
20−25−somites
Prim−5
Prim−15
Prim−25
Long−pec
Protruding−mouth
Day−4
Day−5
Stage
Transcript
ENSDART00000055338
ENSDART00000131850
ENSDART00000140560
ENSDART00000164335

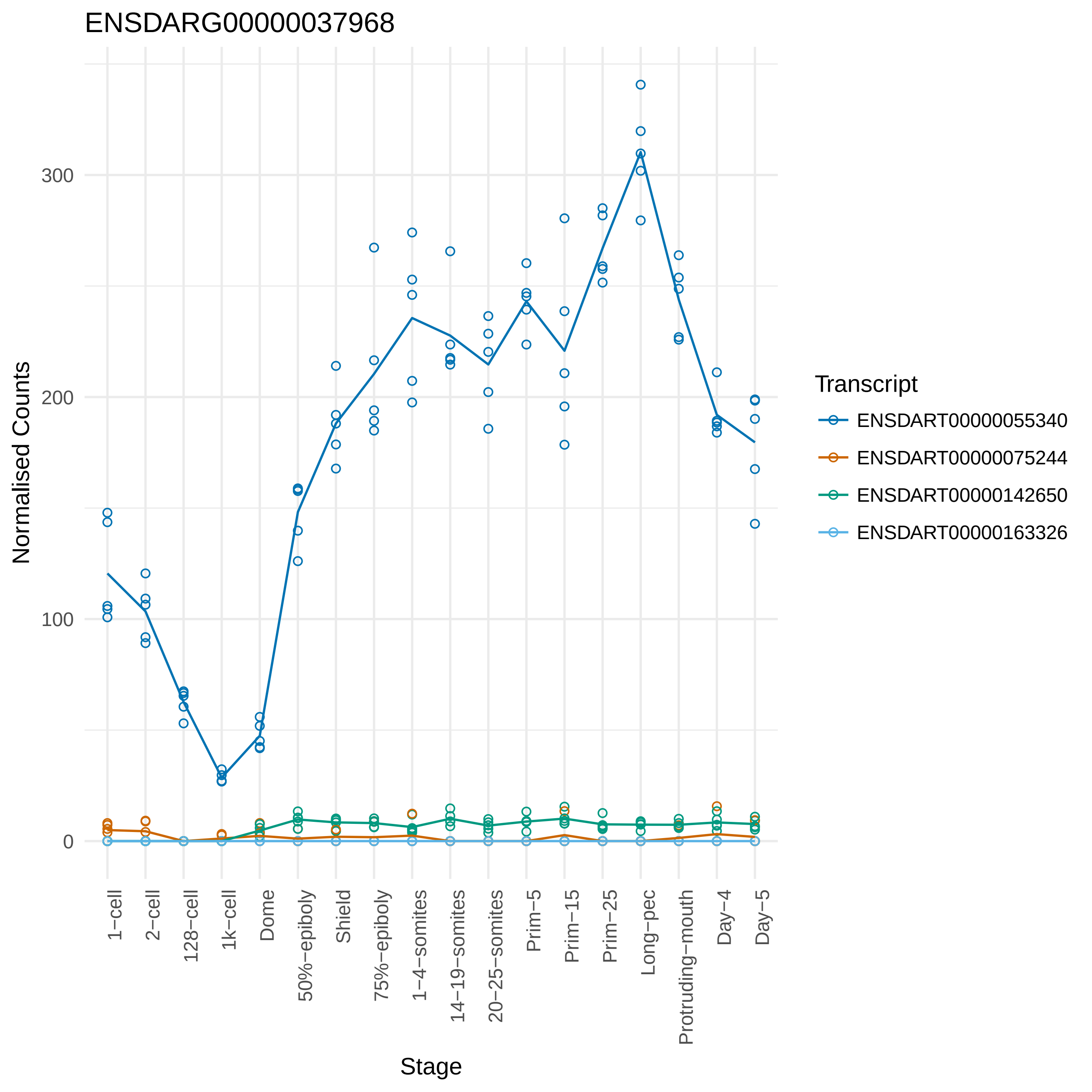
ENSDARG00000037968
Normalised Counts
300
200
100
0
1−cell
2−cell
128−cell
1k−cell
Dome
50%−epiboly
Shield
75%−epiboly
1−4−somites
14−19−somites
20−25−somites
Prim−5
Prim−15
Prim−25
Long−pec
Protruding−mouth
Day−4
Day−5
Stage
Transcript
ENSDART00000055340
ENSDART00000075244
ENSDART00000142650
ENSDART00000163326

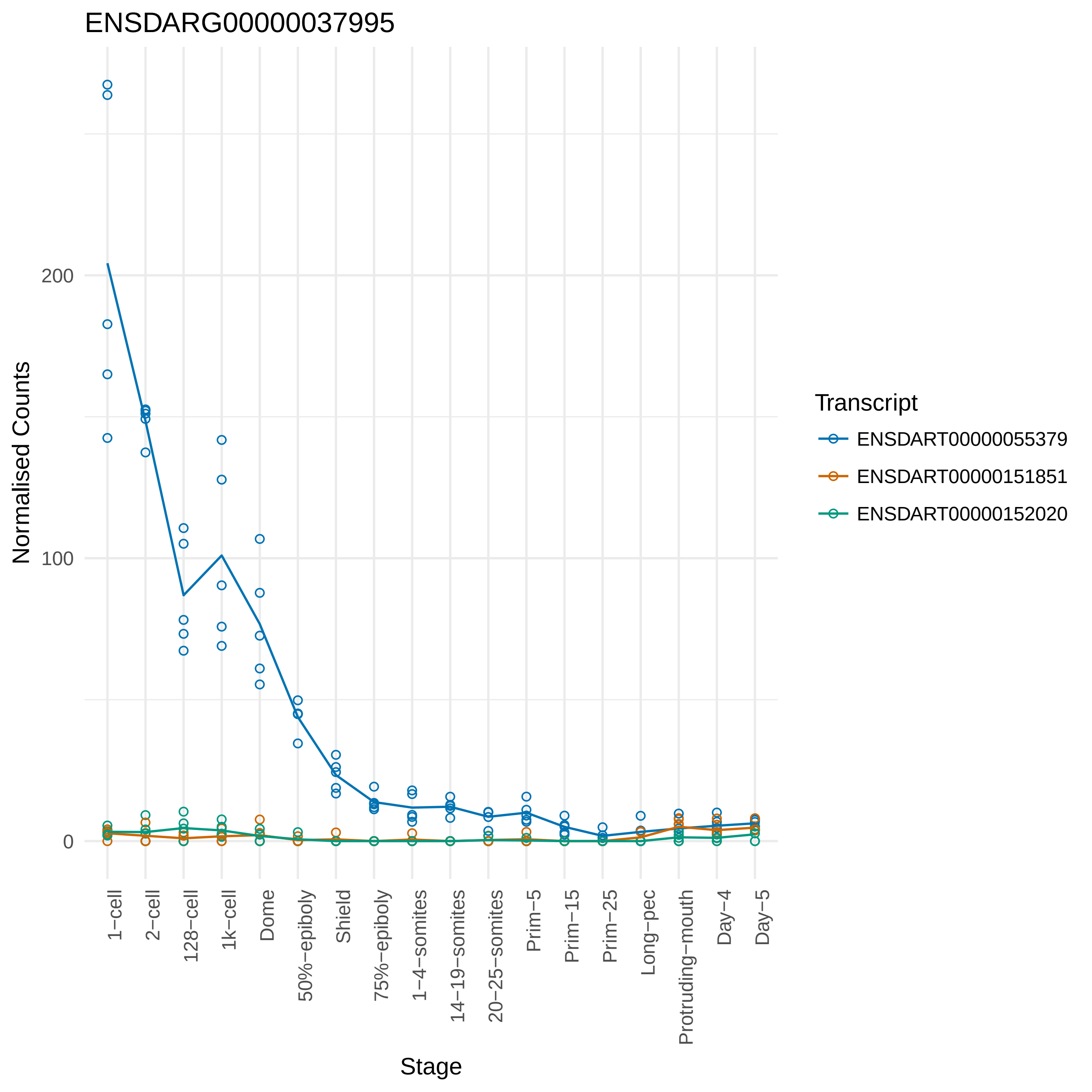

ENSDARG00000037995
Normalised Counts
200
100
0
Stage
1−cell
2−cell
128−cell
1k−cell
Dome
50%−epiboly
Shield
75%−epiboly
1−4−somites
14−19−somites
20−25−somites
Prim−5
Prim−15
Prim−25
Long−pec
Protruding−mouth
Day−4
Day−5
Transcript
ENSDART00000055379
ENSDART00000151851
ENSDART00000152020

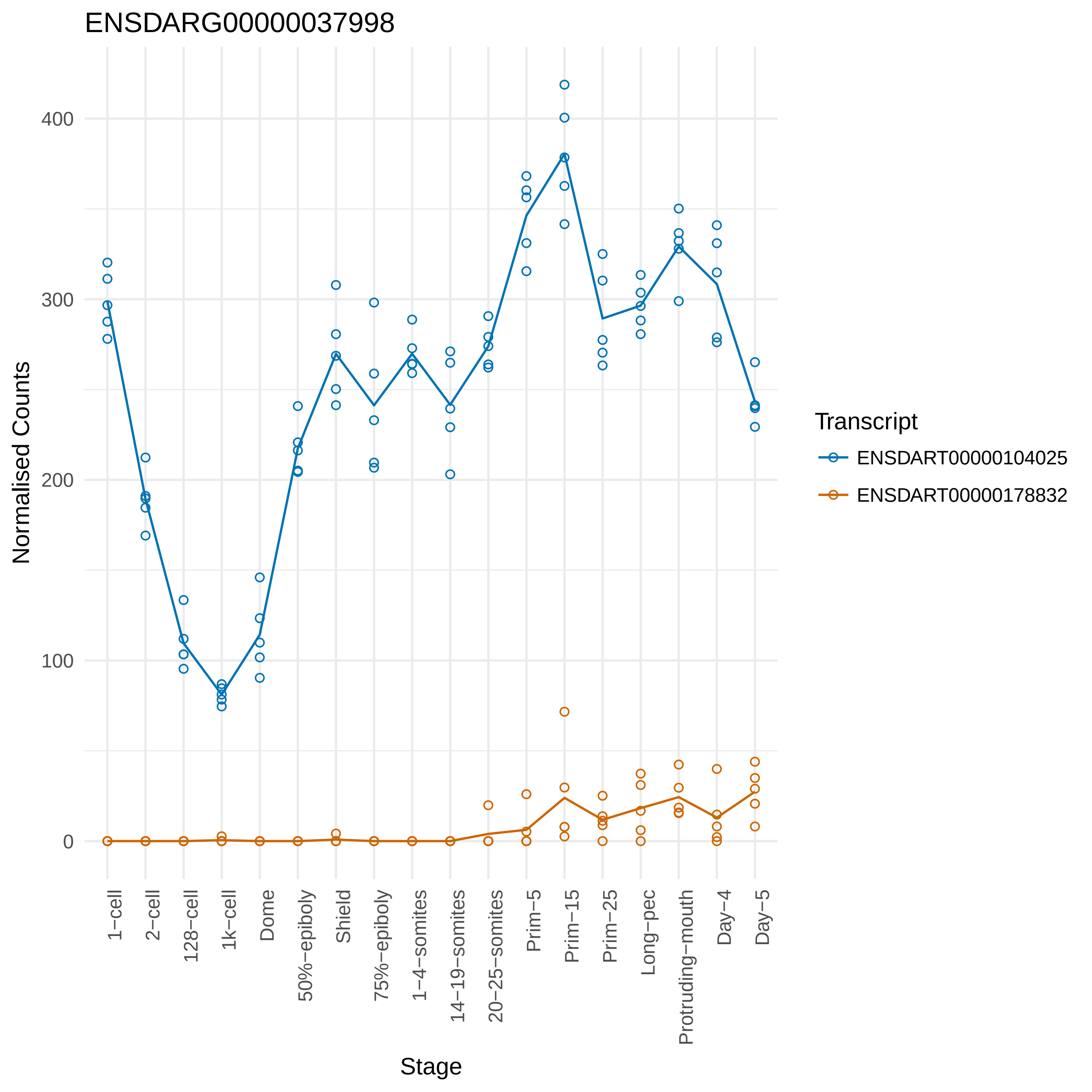

ENSDARG00000037998
Normalised Counts
400
300
200
100
0
1−cell
2−cell
128−cell
1k−cell
Dome
50%−epiboly
Shield
75%−epiboly
1−4−somites
14−19−somites
20−25−somites
Prim−5
Prim−15
Prim−25
Long−pec
Protruding−mouth
Day−4
Day−5
Stage
Transcript
ENSDART00000104025
ENSDART00000178832

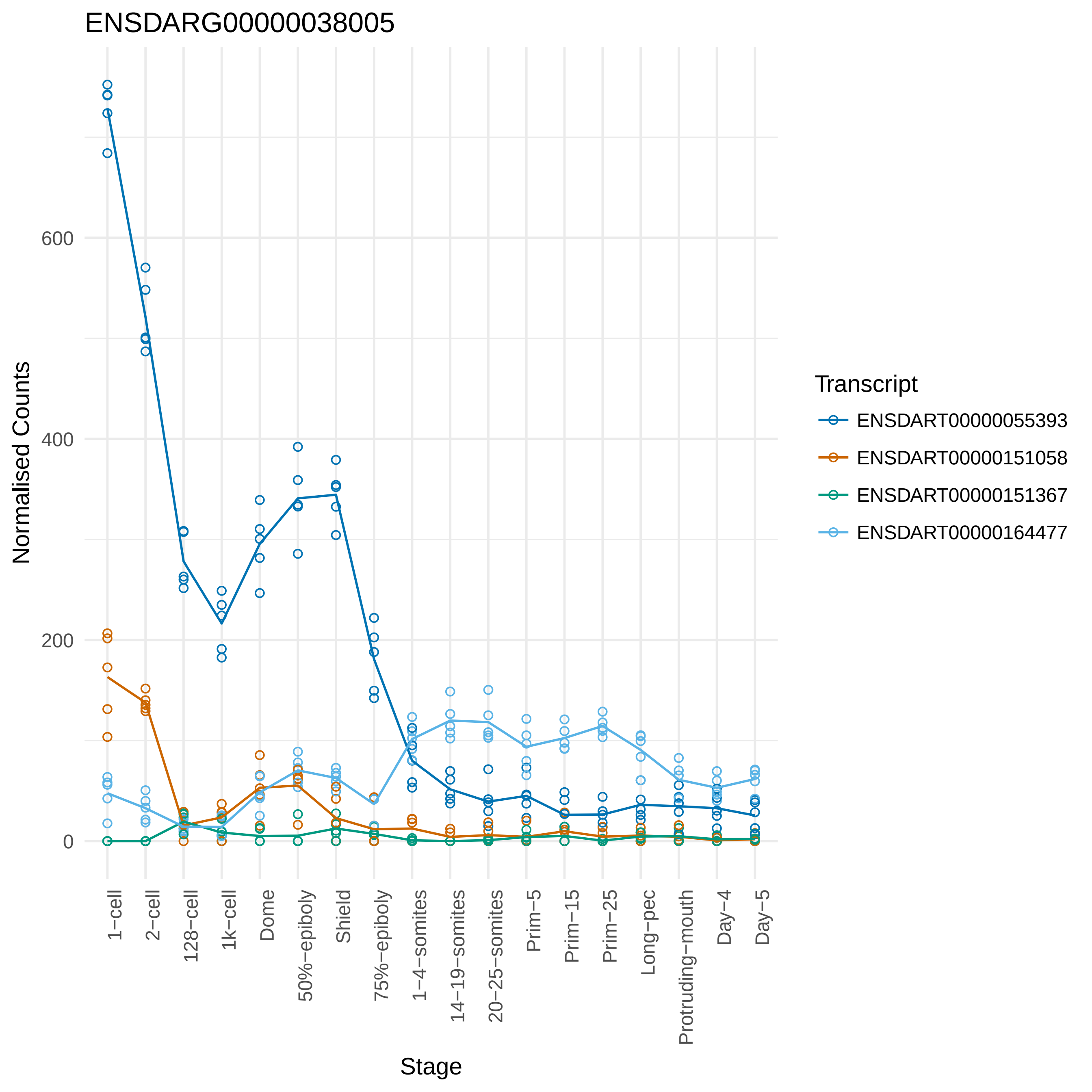

ENSDARG00000038005
Normalised Counts
600
400
200
0
1−cell
2−cell
128−cell
1k−cell
Dome
50%−epiboly
Shield
75%−epiboly
1−4−somites
14−19−somites
20−25−somites
Prim−5
Prim−15
Prim−25
Long−pec
Protruding−mouth
Day−4
Day−5
Stage
Transcript
ENSDART00000055393
ENSDART00000151058
ENSDART00000151367
ENSDART00000164477

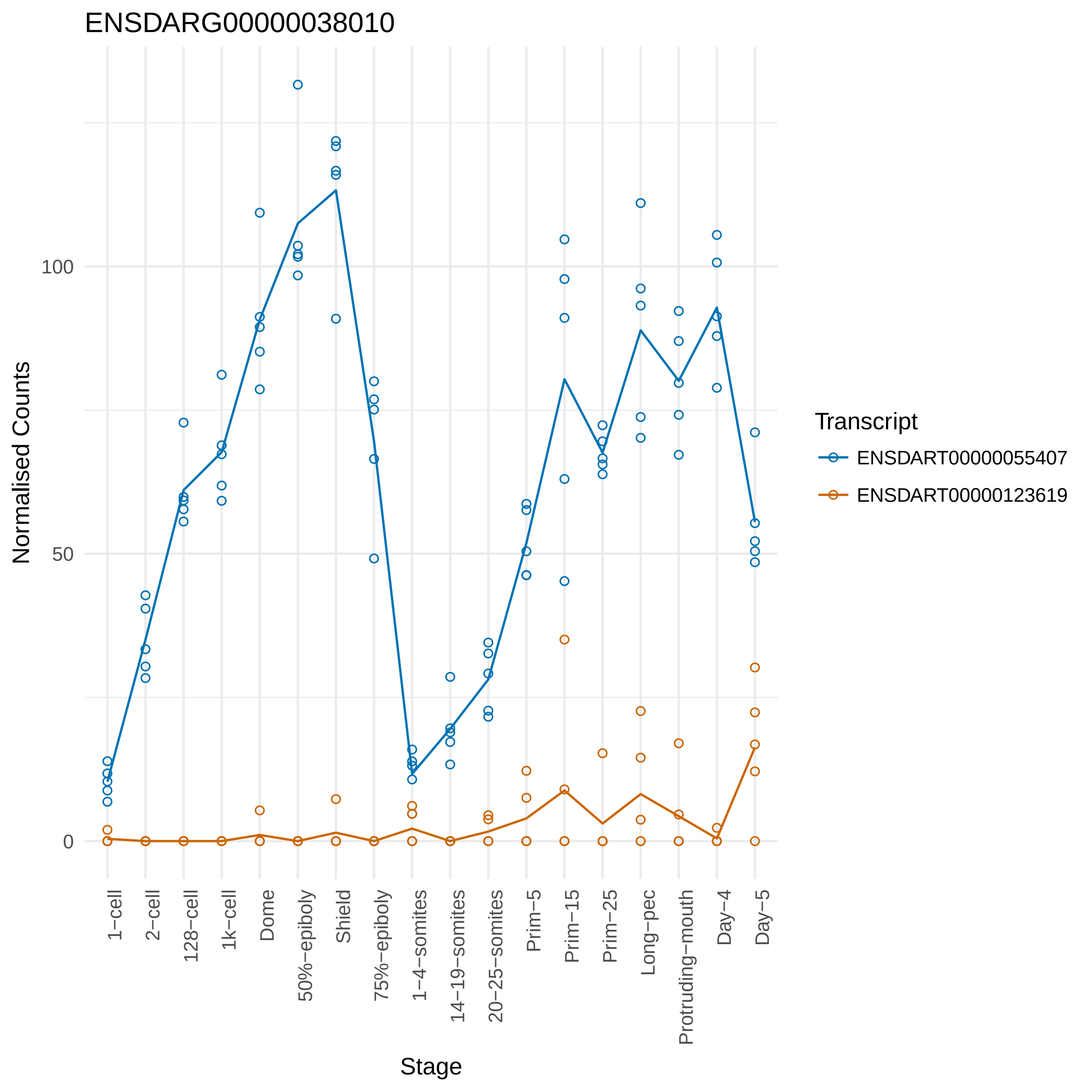

ENSDARG00000038010
Normalised Counts
0
50
100
1−cell
2−cell
128−cell
1k−cell
Dome
50%−epiboly
Shield
75%−epiboly
1−4−somites
14−19−somites
20−25−somites
Prim−5
Prim−15
Prim−25
Long−pec
Protruding−mouth
Day−4
Day−5
Stage
Transcript
ENSDART00000055407
ENSDART00000123619

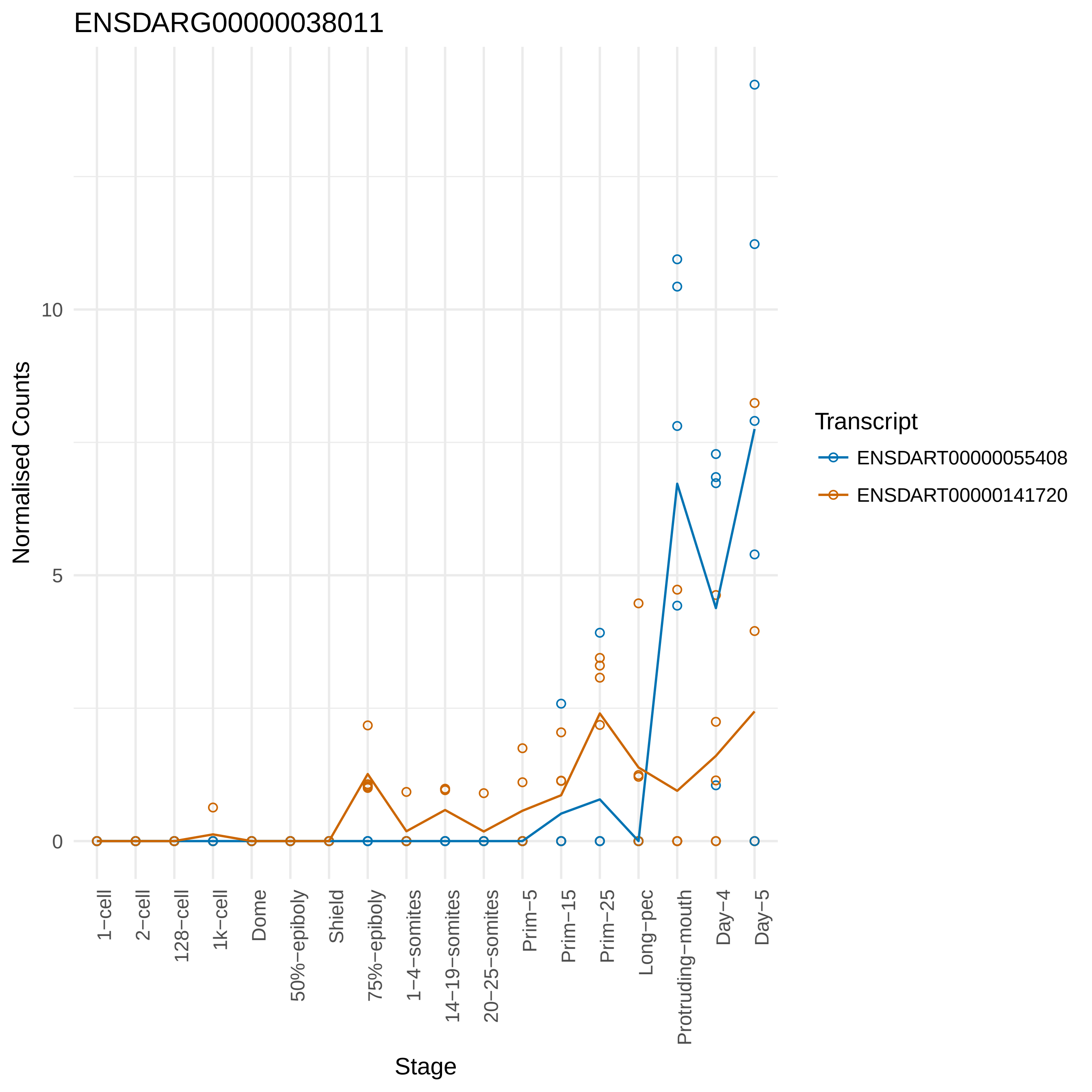
ENSDARG00000038011
Normalised Counts
0
5
10
1−cell
2−cell
128−cell
1k−cell
Dome
50%−epiboly
Shield
75%−epiboly
1−4−somites
14−19−somites
20−25−somites
Prim−5
Prim−15
Prim−25
Long−pec
Protruding−mouth
Day−4
Day−5
Stage
Transcript
ENSDART00000055408
ENSDART00000141720

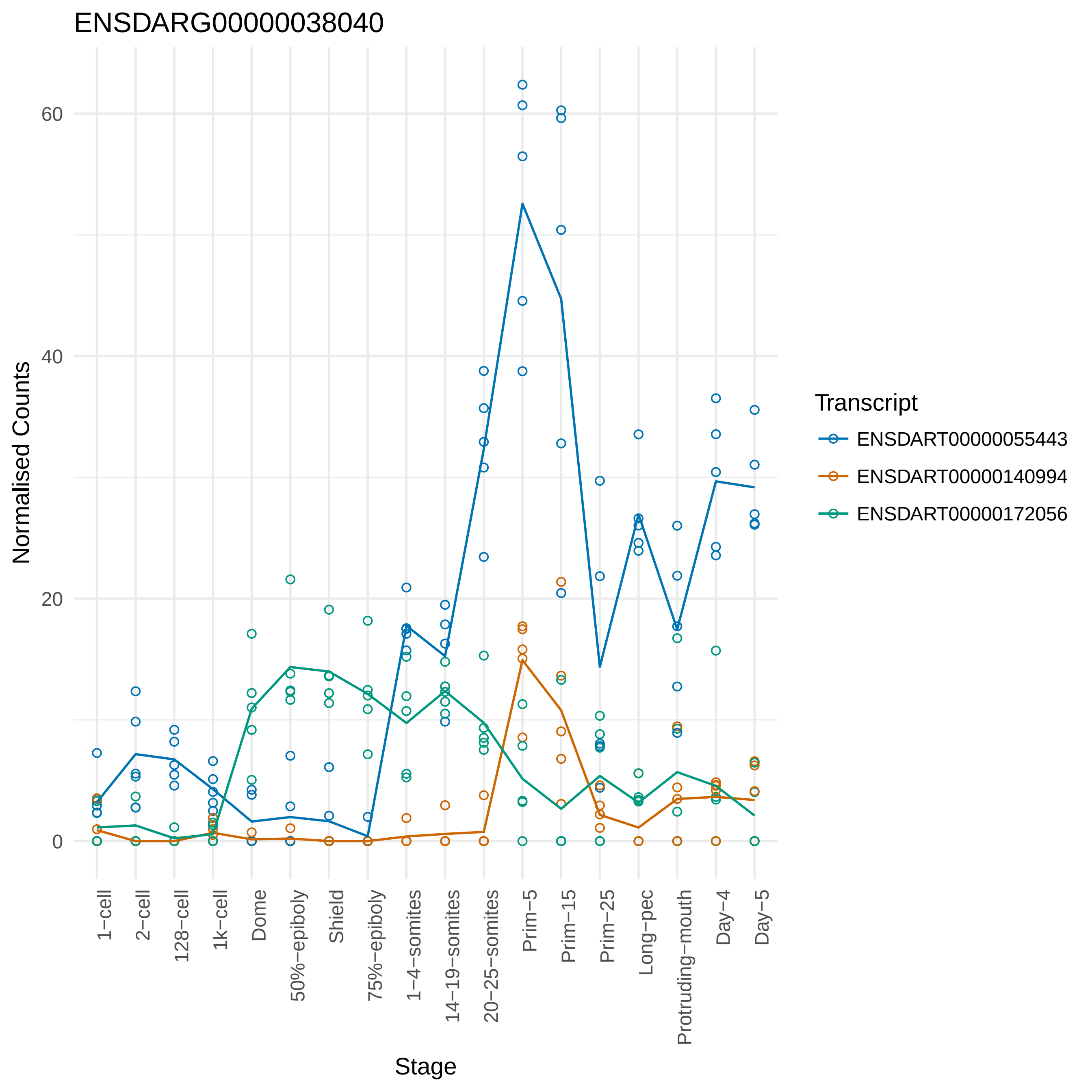

ENSDARG00000038040
Normalised Counts
0
20
40
60
Stage
1−cell
2−cell
128−cell
1k−cell
Dome
50%−epiboly
Shield
75%−epiboly
1−4−somites
14−19−somites
20−25−somites
Prim−5
Prim−15
Prim−25
Long−pec
Protruding−mouth
Day−4
Day−5
Transcript
ENSDART00000055443
ENSDART00000140994
ENSDART00000172056

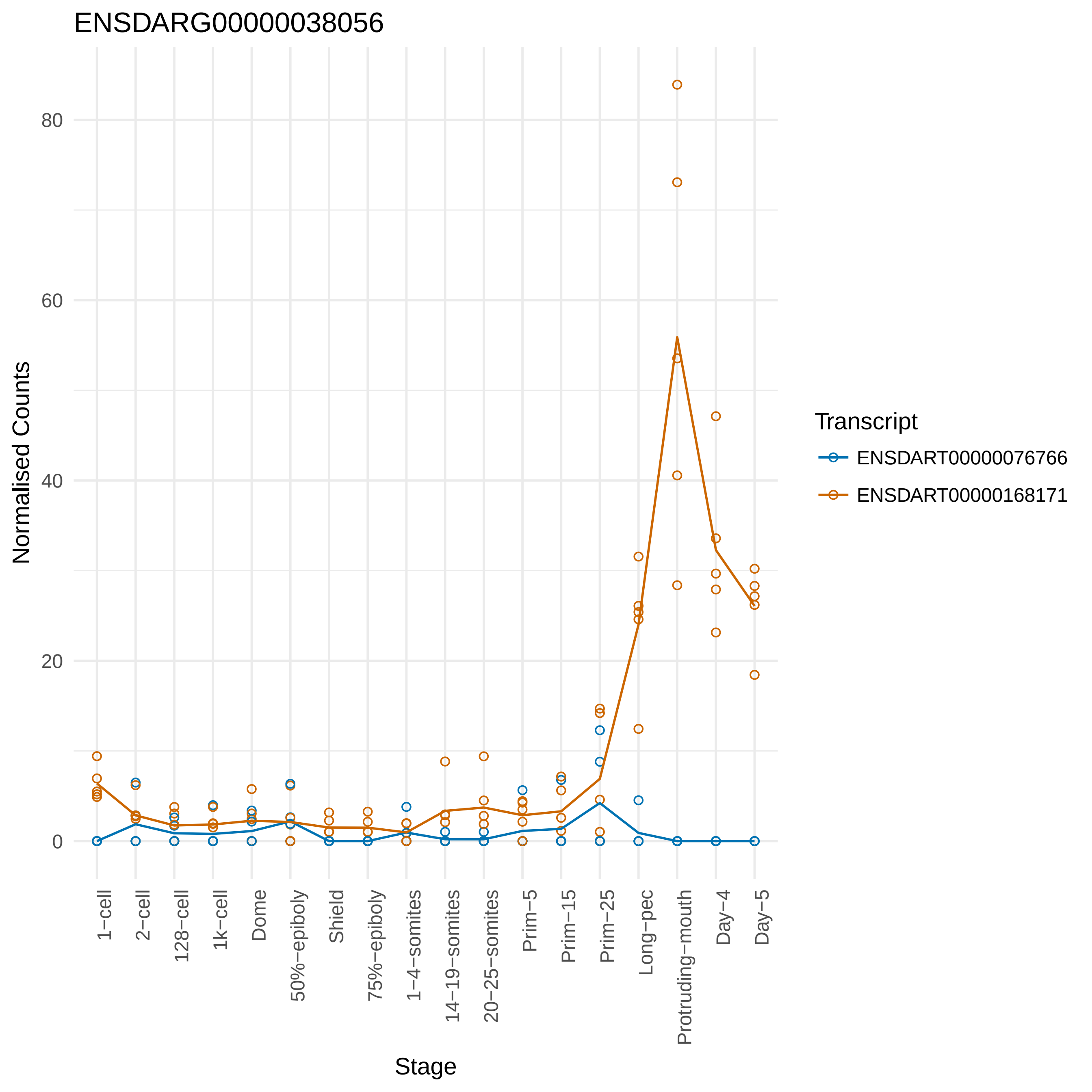

ENSDARG00000038056
Normalised Counts
0
20
40
60
80
Stage
1−cell
2−cell
128−cell
1k−cell
Dome
50%−epiboly
Shield
75%−epiboly
1−4−somites
14−19−somites
20−25−somites
Prim−5
Prim−15
Prim−25
Long−pec
Protruding−mouth
Day−4
Day−5
Transcript
ENSDART00000076766
ENSDART00000168171

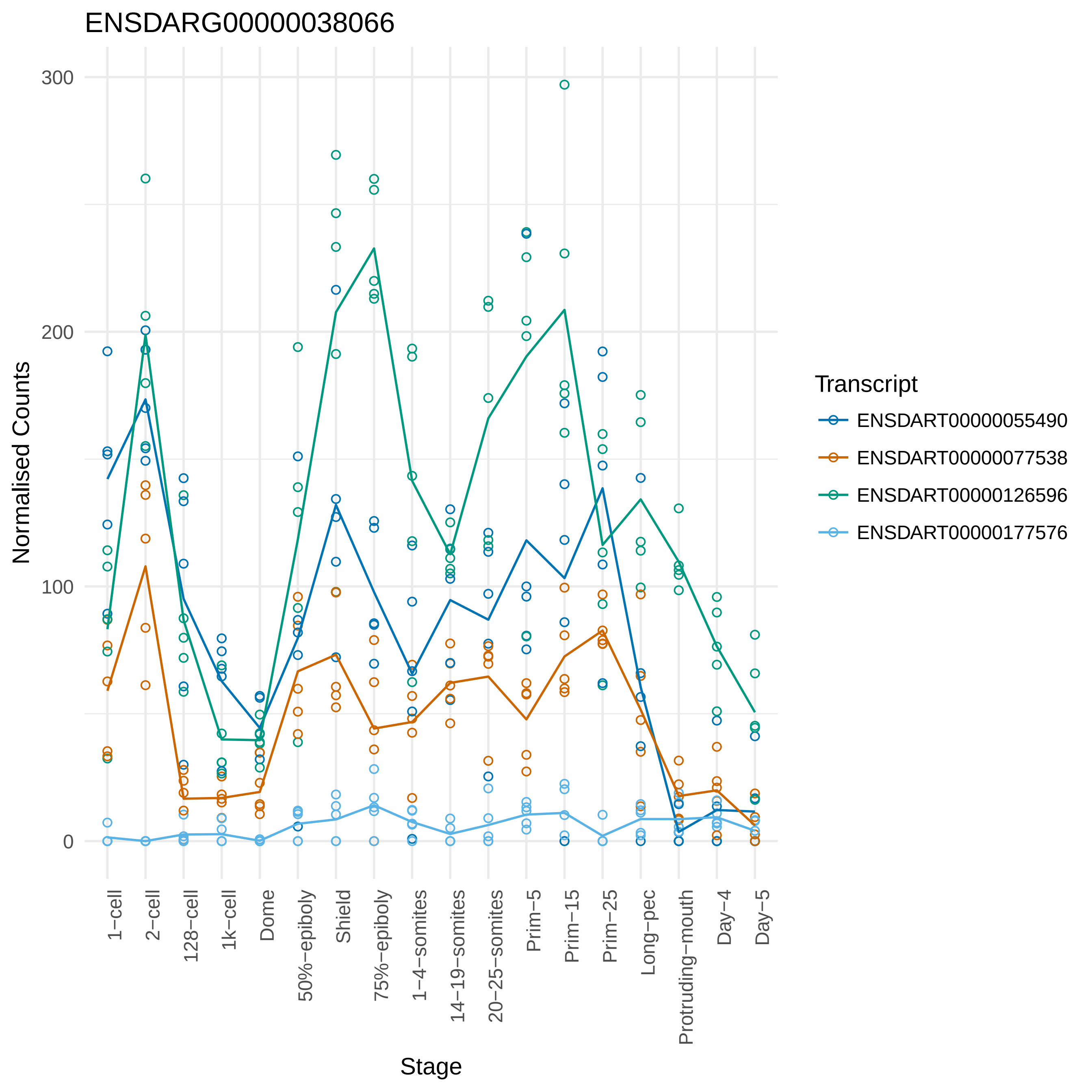

ENSDARG00000038066
Normalised Counts
300
200
100
0
1−cell
2−cell
128−cell
1k−cell
Dome
50%−epiboly
Shield
75%−epiboly
1−4−somites
14−19−somites
20−25−somites
Prim−5
Prim−15
Prim−25
Long−pec
Protruding−mouth
Day−4
Day−5
Stage
Transcript
ENSDART00000055490
ENSDART00000077538
ENSDART00000126596
ENSDART00000177576

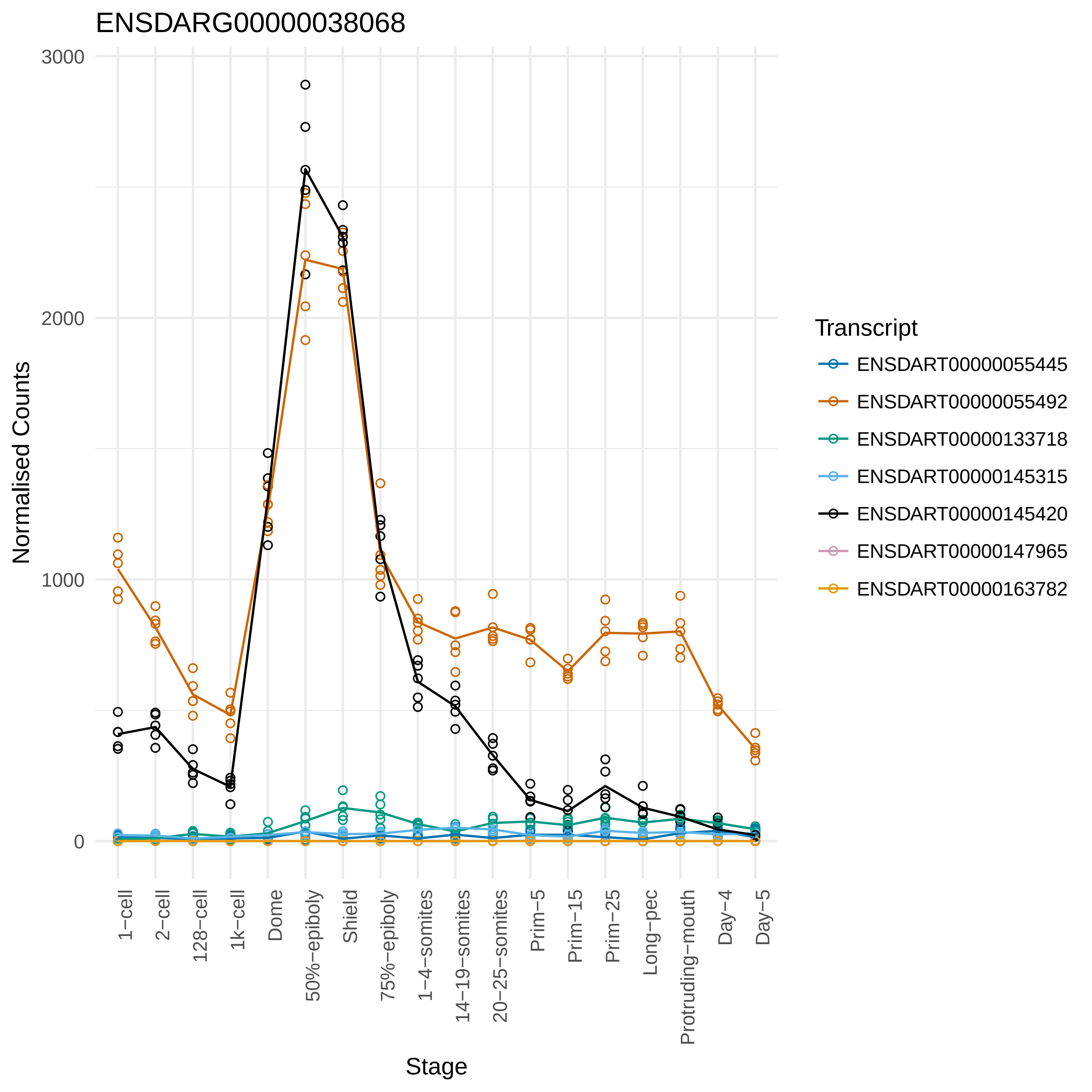

ENSDARG00000038068
Normalised Counts
3000
2000
1000
0
Stage
1−cell
2−cell
128−cell
1k−cell
Dome
50%−epiboly
Shield
75%−epiboly
1−4−somites
14−19−somites
20−25−somites
Prim−5
Prim−15
Prim−25
Long−pec
Protruding−mouth
Day−4
Day−5
Transcript
ENSDART00000055445
ENSDART00000055492
ENSDART00000133718
ENSDART00000145315
ENSDART00000145420
ENSDART00000147965
ENSDART00000163782

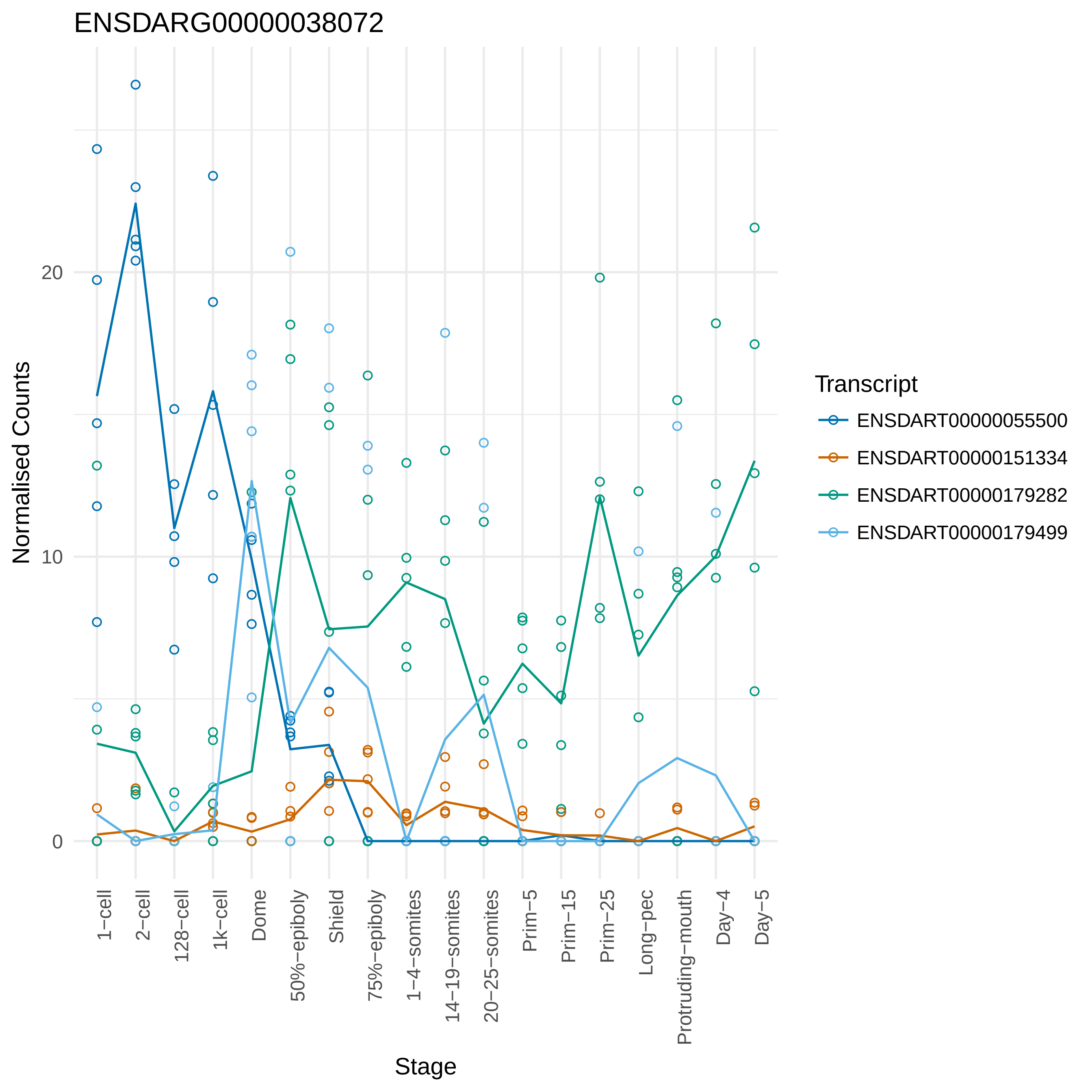

ENSDARG00000038072
Normalised Counts
20
10
0
1−cell
2−cell
128−cell
1k−cell
Dome
50%−epiboly
Shield
75%−epiboly
1−4−somites
14−19−somites
20−25−somites
Prim−5
Prim−15
Prim−25
Long−pec
Protruding−mouth
Day−4
Day−5
Stage
Transcript
ENSDART00000055500
ENSDART00000151334
ENSDART00000179282
ENSDART00000179499

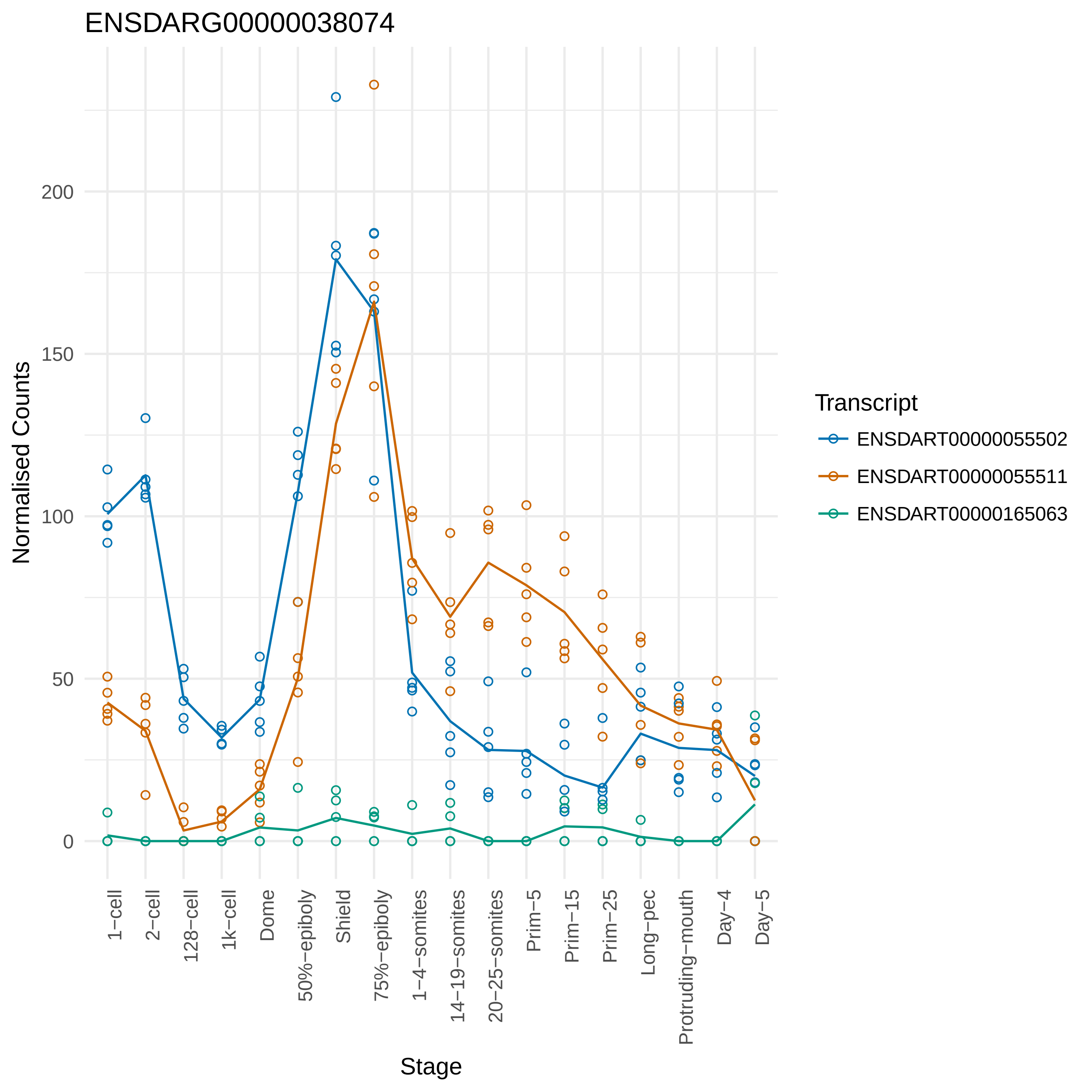

ENSDARG00000038074
Normalised Counts
0
50
100
150
200
Stage
1−cell
2−cell
128−cell
1k−cell
Dome
50%−epiboly
Shield
75%−epiboly
1−4−somites
14−19−somites
20−25−somites
Prim−5
Prim−15
Prim−25
Long−pec
Protruding−mouth
Day−4
Day−5
Transcript
ENSDART00000055502
ENSDART00000055511
ENSDART00000165063

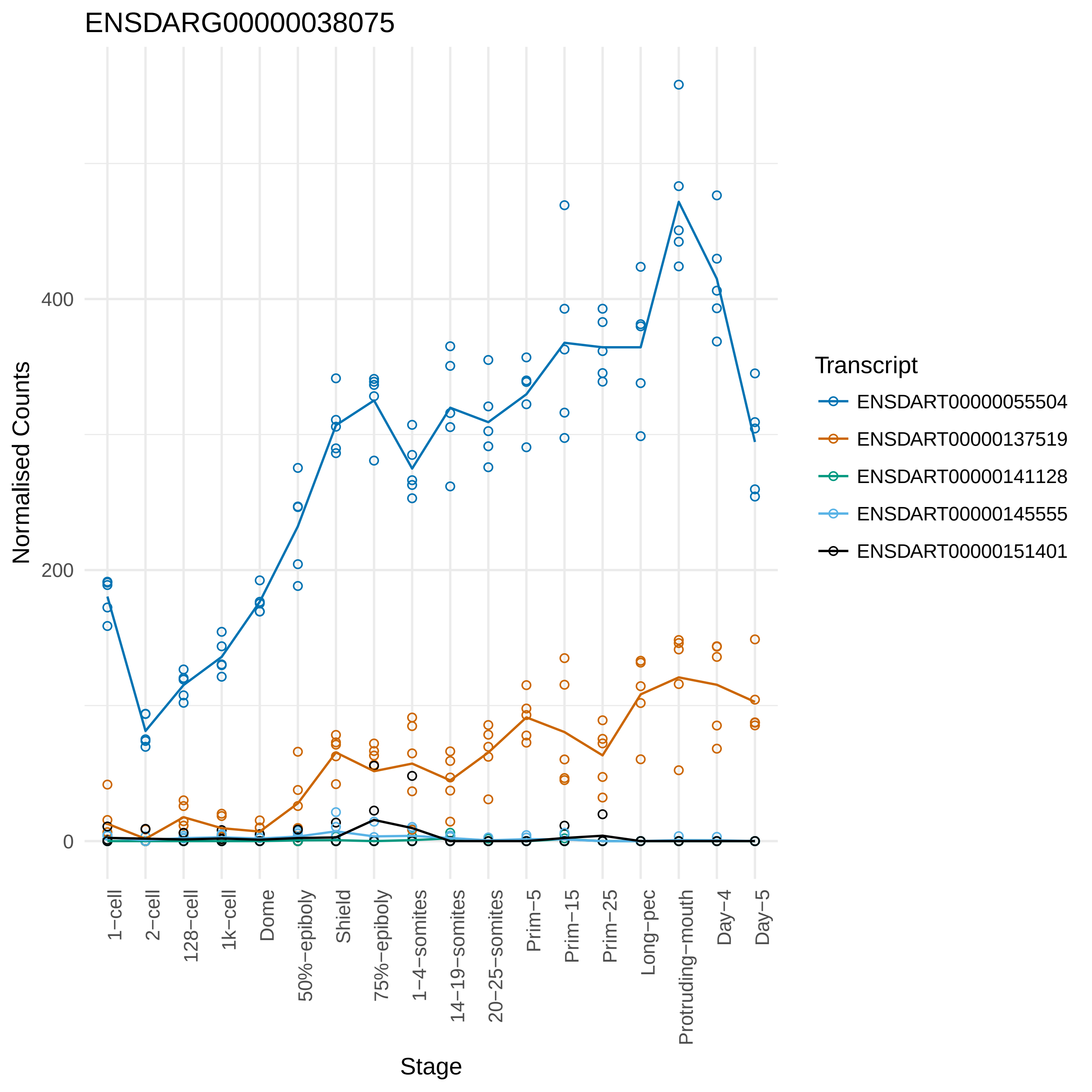

ENSDARG00000038075
Normalised Counts
0
200
400
Stage
1−cell
2−cell
128−cell
1k−cell
Dome
50%−epiboly
Shield
75%−epiboly
1−4−somites
14−19−somites
20−25−somites
Prim−5
Prim−15
Prim−25
Long−pec
Protruding−mouth
Day−4
Day−5
Transcript
ENSDART00000055504
ENSDART00000137519
ENSDART00000141128
ENSDART00000145555
ENSDART00000151401

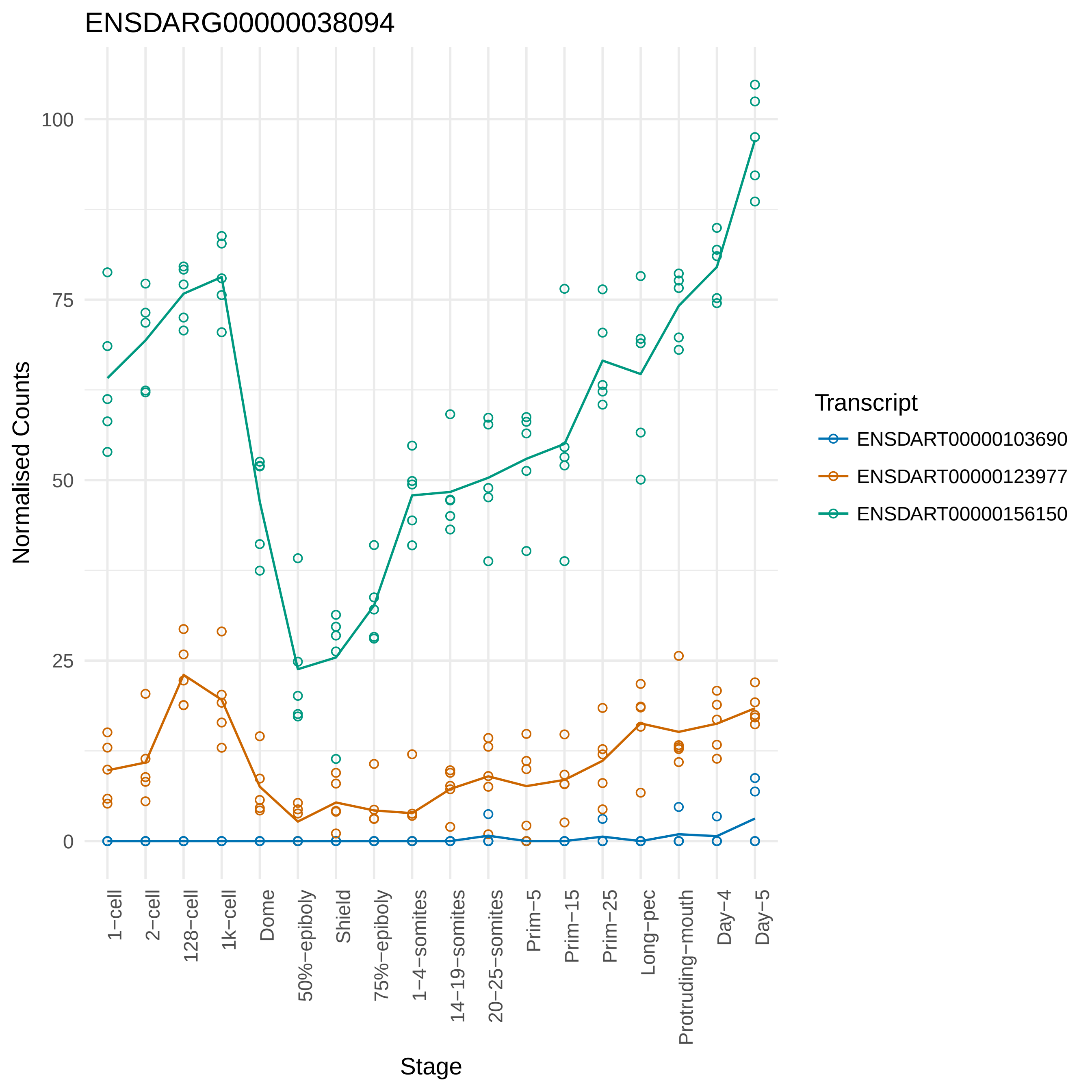

ENSDARG00000038094
Normalised Counts
100
75
50
25
0
1−cell
2−cell
128−cell
1k−cell
Dome
50%−epiboly
Shield
75%−epiboly
1−4−somites
14−19−somites
20−25−somites
Prim−5
Prim−15
Prim−25
Long−pec
Protruding−mouth
Day−4
Day−5
Stage
Transcript
ENSDART00000103690
ENSDART00000123977
ENSDART00000156150

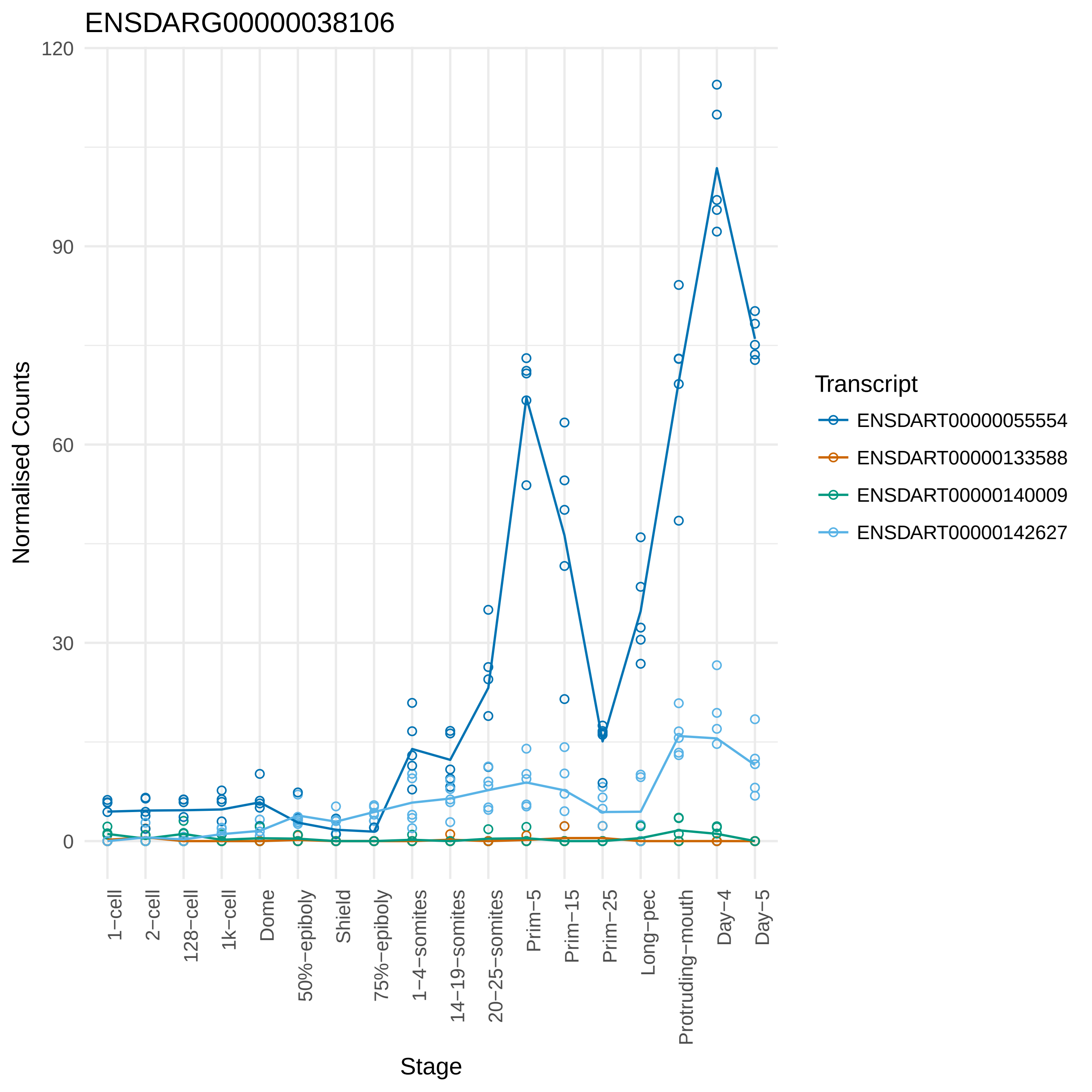

ENSDARG00000038106
Normalised Counts
0
30
60
90
120
Stage
1−cell
2−cell
128−cell
1k−cell
Dome
50%−epiboly
Shield
75%−epiboly
1−4−somites
14−19−somites
20−25−somites
Prim−5
Prim−15
Prim−25
Long−pec
Protruding−mouth
Day−4
Day−5
Transcript
ENSDART00000055554
ENSDART00000133588
ENSDART00000140009
ENSDART00000142627

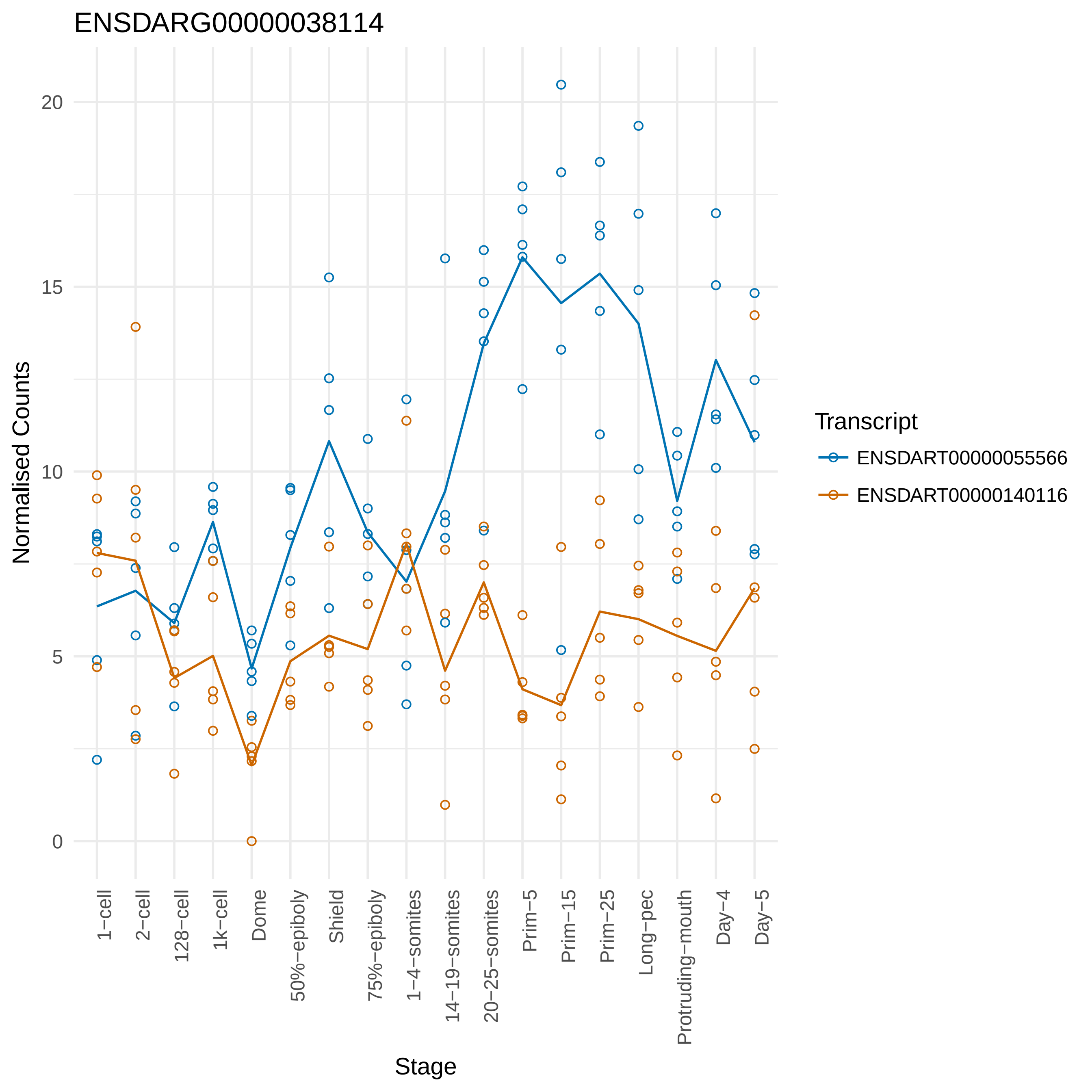

ENSDARG00000038114
Normalised Counts
0
5
10
15
20
1−cell
2−cell
128−cell
1k−cell
Dome
50%−epiboly
Shield
75%−epiboly
1−4−somites
14−19−somites
20−25−somites
Prim−5
Prim−15
Prim−25
Long−pec
Protruding−mouth
Day−4
Day−5
Stage
Transcript
ENSDART00000055566
ENSDART00000140116

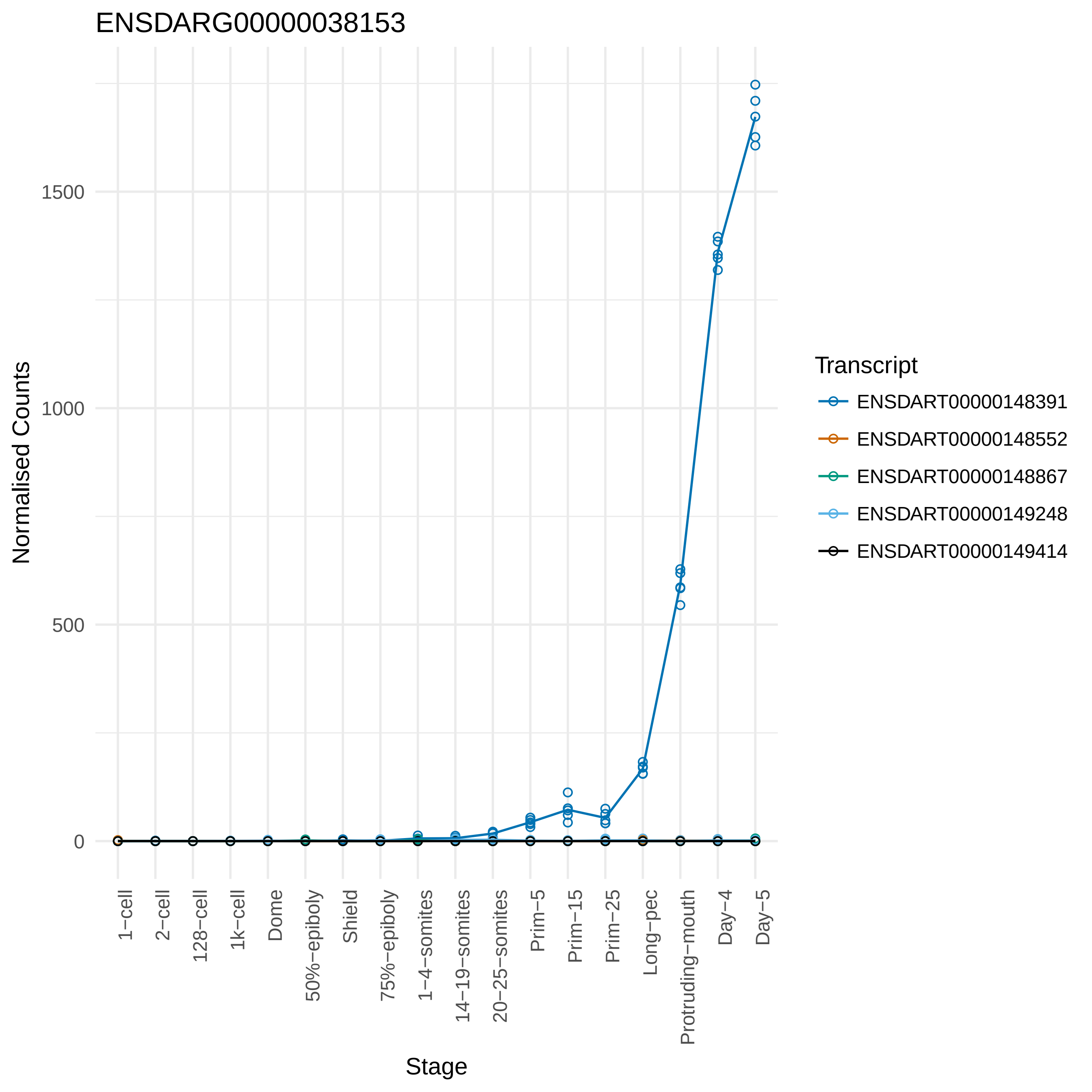
ENSDARG00000038153
Normalised Counts
0
500
1000
1500
Stage
1−cell
2−cell
128−cell
1k−cell
Dome
50%−epiboly
Shield
75%−epiboly
1−4−somites
14−19−somites
20−25−somites
Prim−5
Prim−15
Prim−25
Long−pec
Protruding−mouth
Day−4
Day−5
Transcript
ENSDART00000148391
ENSDART00000148552
ENSDART00000148867
ENSDART00000149248
ENSDART00000149414

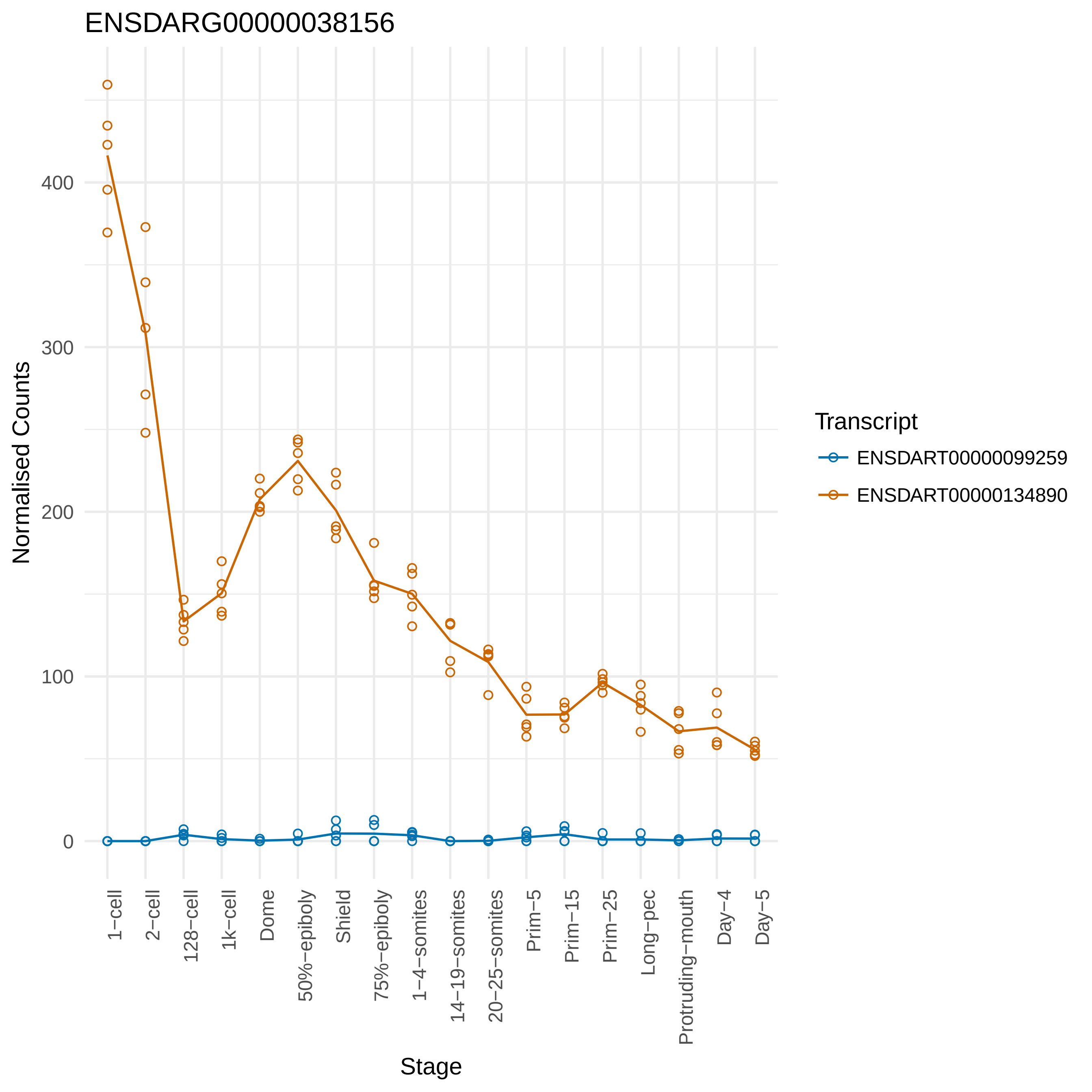

ENSDARG00000038156
Normalised Counts
400
300
200
100
0
1−cell
2−cell
128−cell
1k−cell
Dome
50%−epiboly
Shield
75%−epiboly
1−4−somites
14−19−somites
20−25−somites
Prim−5
Prim−15
Prim−25
Long−pec
Protruding−mouth
Day−4
Day−5
Stage
Transcript
ENSDART00000099259
ENSDART00000134890

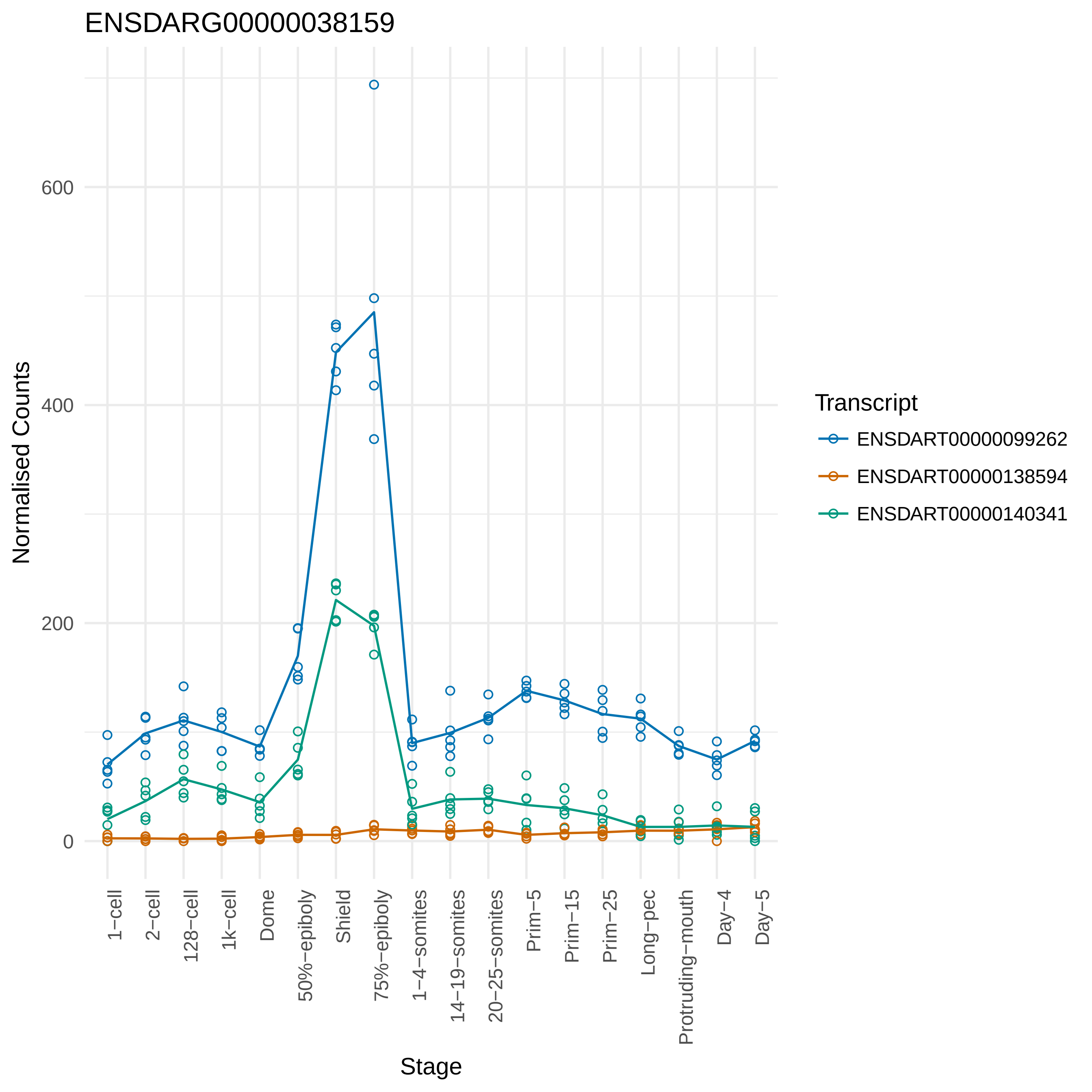

ENSDARG00000038159
Normalised Counts
0
200
400
600
1−cell
2−cell
128−cell
1k−cell
Dome
50%−epiboly
Shield
75%−epiboly
1−4−somites
14−19−somites
20−25−somites
Prim−5
Prim−15
Prim−25
Long−pec
Protruding−mouth
Day−4
Day−5
Stage
Transcript
ENSDART00000099262
ENSDART00000138594
ENSDART00000140341

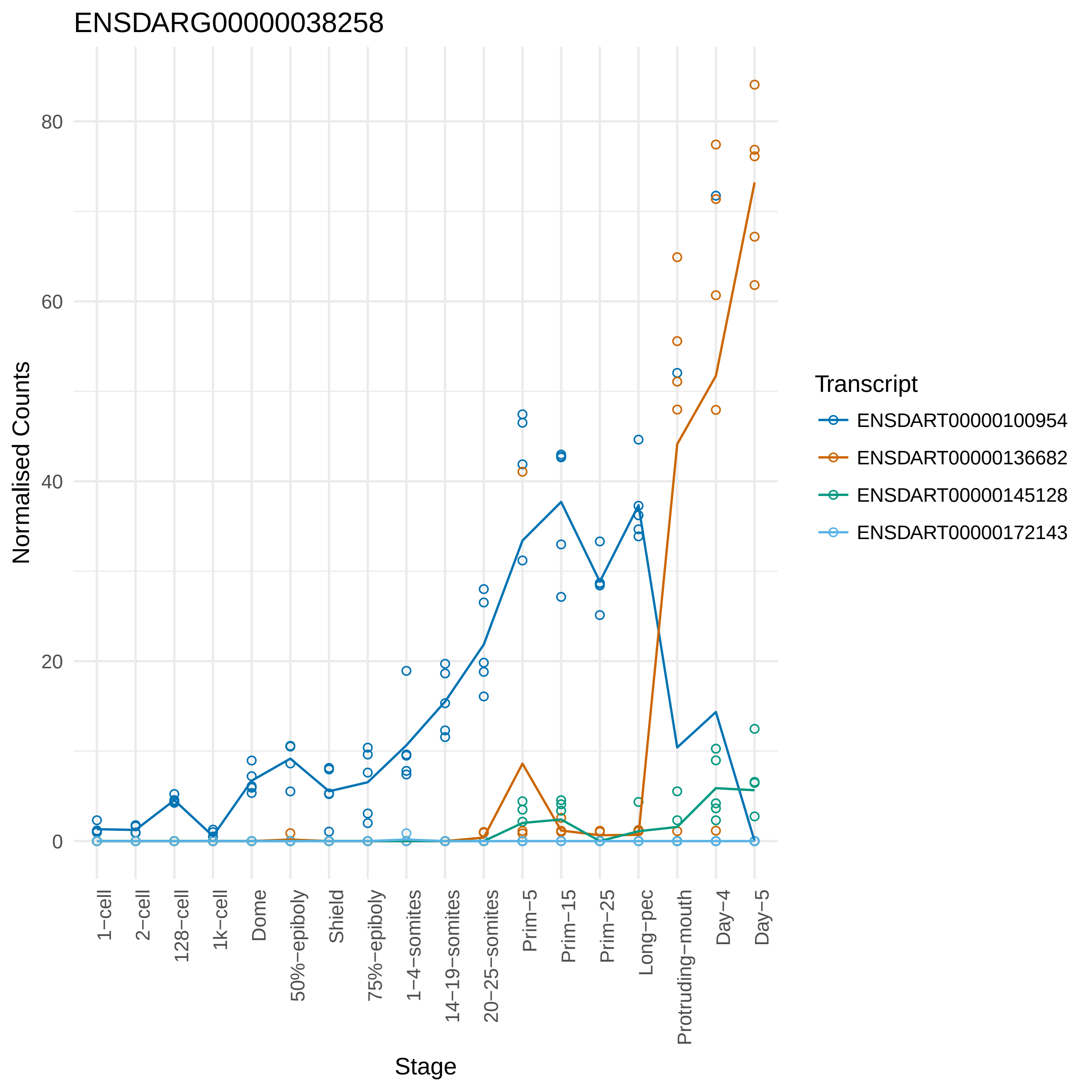

ENSDARG00000038258
Normalised Counts
0
20
40
60
80
1−cell
2−cell
128−cell
1k−cell
Dome
50%−epiboly
Shield
75%−epiboly
1−4−somites
14−19−somites
20−25−somites
Prim−5
Prim−15
Prim−25
Long−pec
Protruding−mouth
Day−4
Day−5
Stage
Transcript
ENSDART00000100954
ENSDART00000136682
ENSDART00000145128
ENSDART00000172143

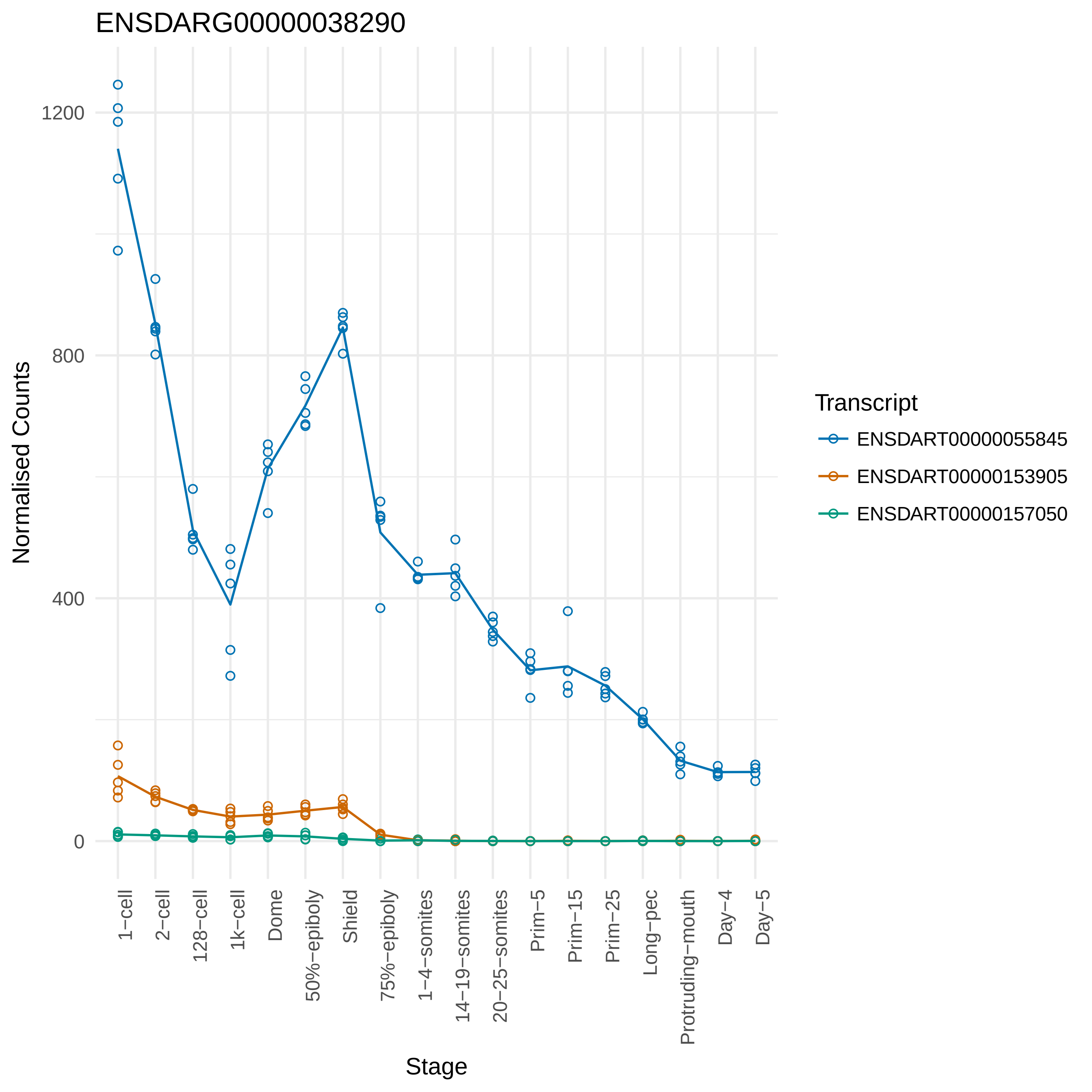
ENSDARG00000038290
Normalised Counts
1200
800
400
0
1−cell
2−cell
128−cell
1k−cell
Dome
50%−epiboly
Shield
75%−epiboly
1−4−somites
14−19−somites
20−25−somites
Prim−5
Prim−15
Prim−25
Long−pec
Protruding−mouth
Day−4
Day−5
Stage
Transcript
ENSDART00000055845
ENSDART00000153905
ENSDART00000157050

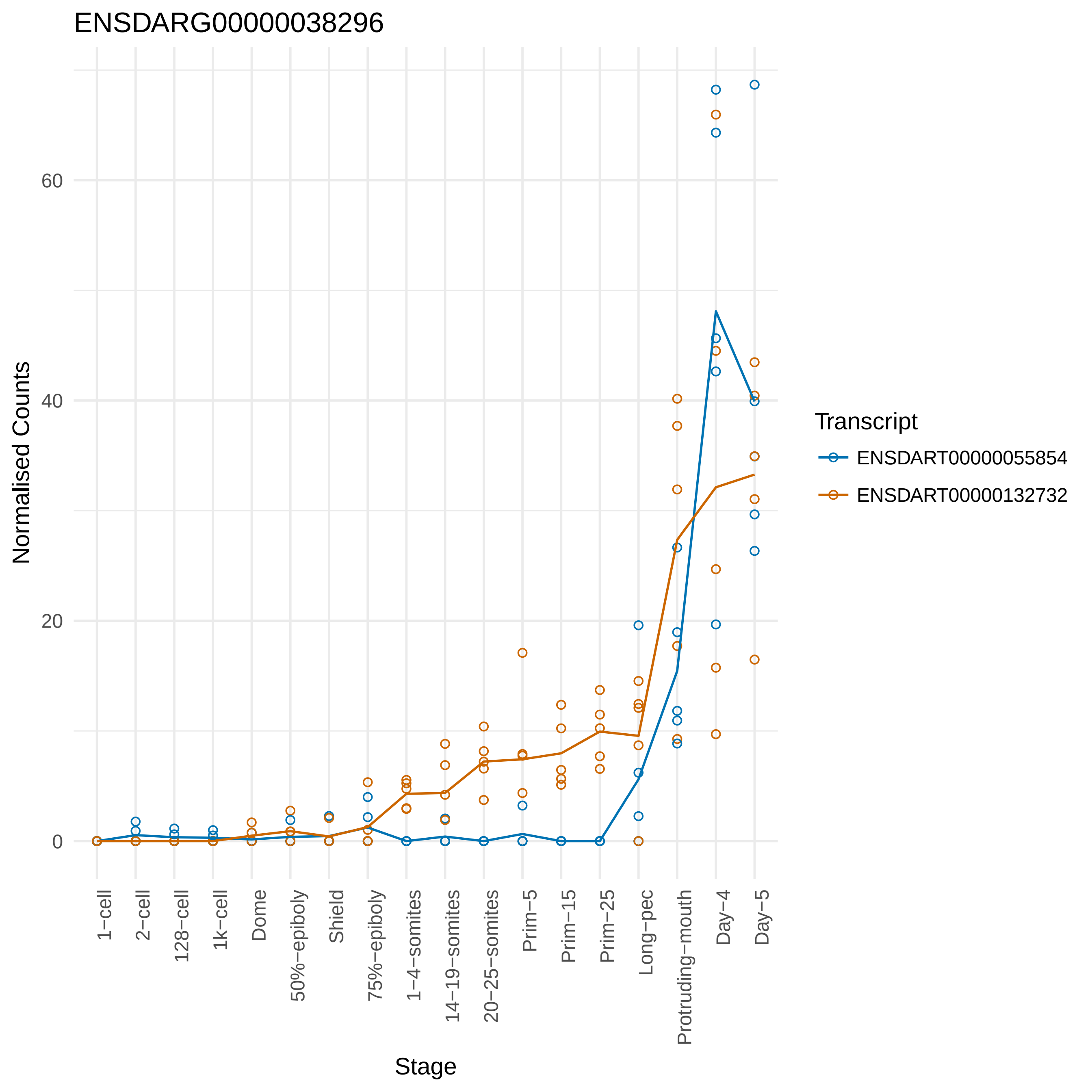
ENSDARG00000038296
Normalised Counts
0
20
40
60
Stage
1−cell
2−cell
128−cell
1k−cell
Dome
50%−epiboly
Shield
75%−epiboly
1−4−somites
14−19−somites
20−25−somites
Prim−5
Prim−15
Prim−25
Long−pec
Protruding−mouth
Day−4
Day−5
Transcript
ENSDART00000055854
ENSDART00000132732

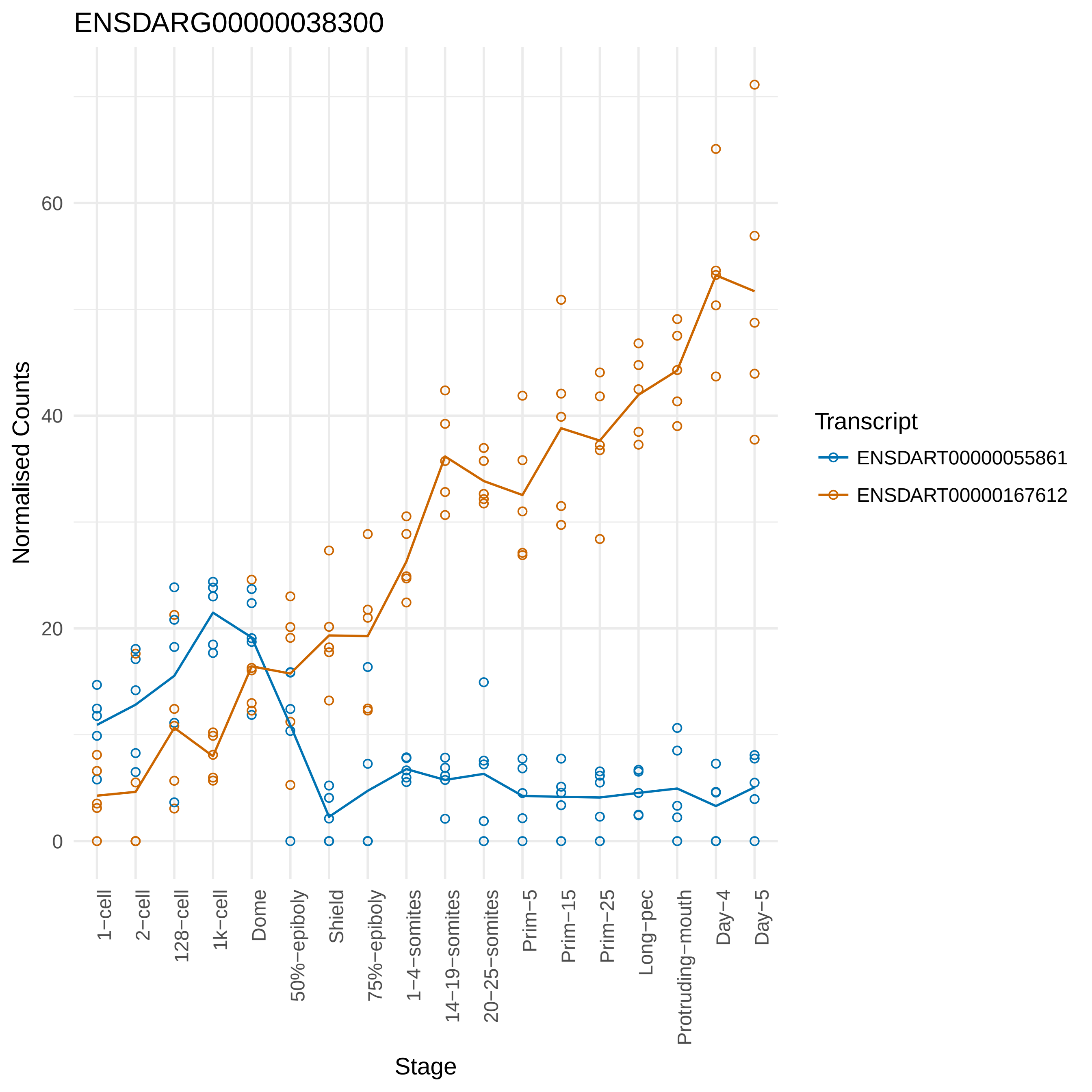
ENSDARG00000038300
Normalised Counts
0
20
40
60
Stage
1−cell
2−cell
128−cell
1k−cell
Dome
50%−epiboly
Shield
75%−epiboly
1−4−somites
14−19−somites
20−25−somites
Prim−5
Prim−15
Prim−25
Long−pec
Protruding−mouth
Day−4
Day−5
Transcript
ENSDART00000055861
ENSDART00000167612

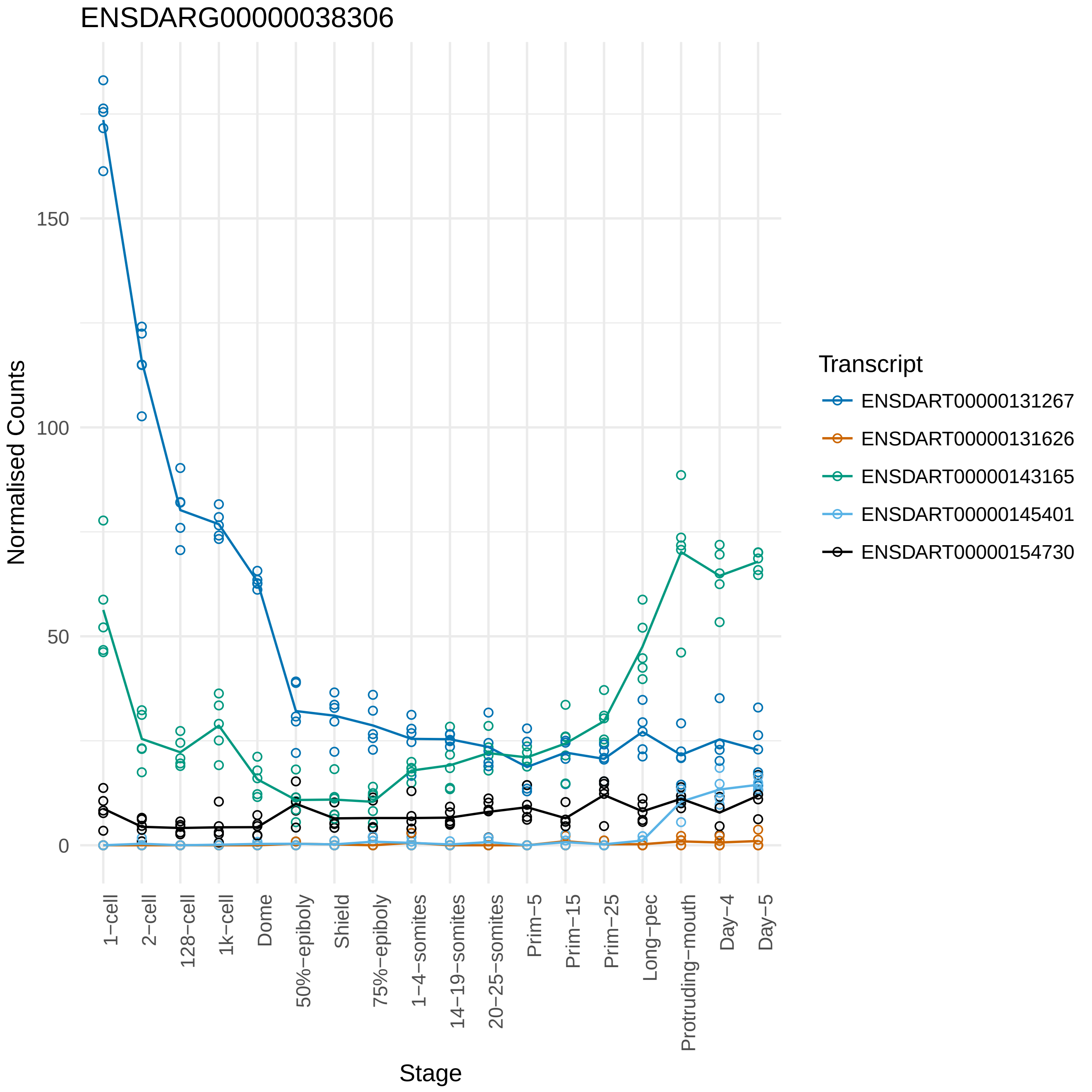
ENSDARG00000038306
Normalised Counts
150
100
50
0
Stage
1−cell
2−cell
128−cell
1k−cell
Dome
50%−epiboly
Shield
75%−epiboly
1−4−somites
14−19−somites
20−25−somites
Prim−5
Prim−15
Prim−25
Long−pec
Protruding−mouth
Day−4
Day−5
Transcript
ENSDART00000131267
ENSDART00000131626
ENSDART00000143165
ENSDART00000145401
ENSDART00000154730

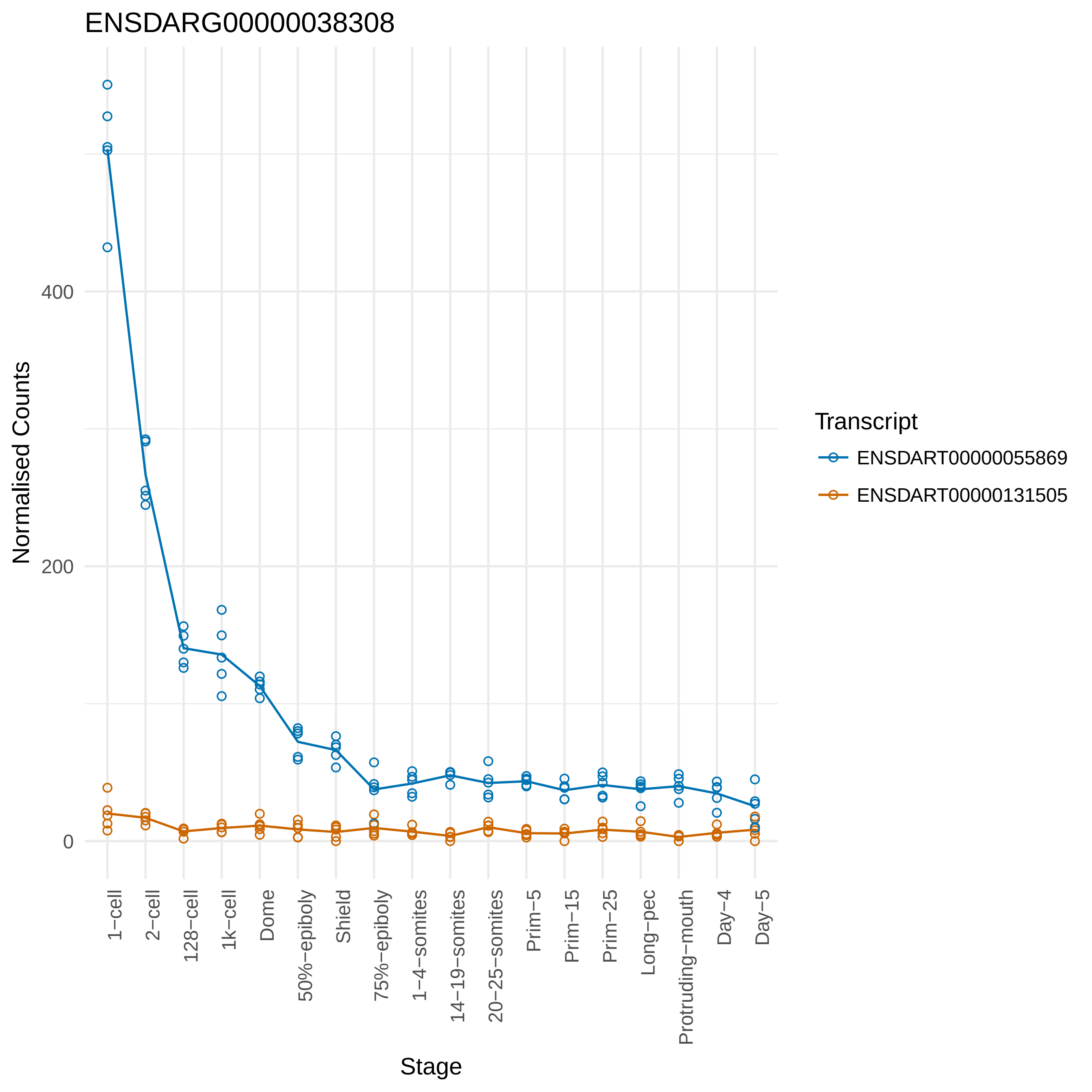

ENSDARG00000038308
Normalised Counts
400
200
0
Stage
1−cell
2−cell
128−cell
1k−cell
Dome
50%−epiboly
Shield
75%−epiboly
1−4−somites
14−19−somites
20−25−somites
Prim−5
Prim−15
Prim−25
Long−pec
Protruding−mouth
Day−4
Day−5
Transcript
ENSDART00000055869
ENSDART00000131505

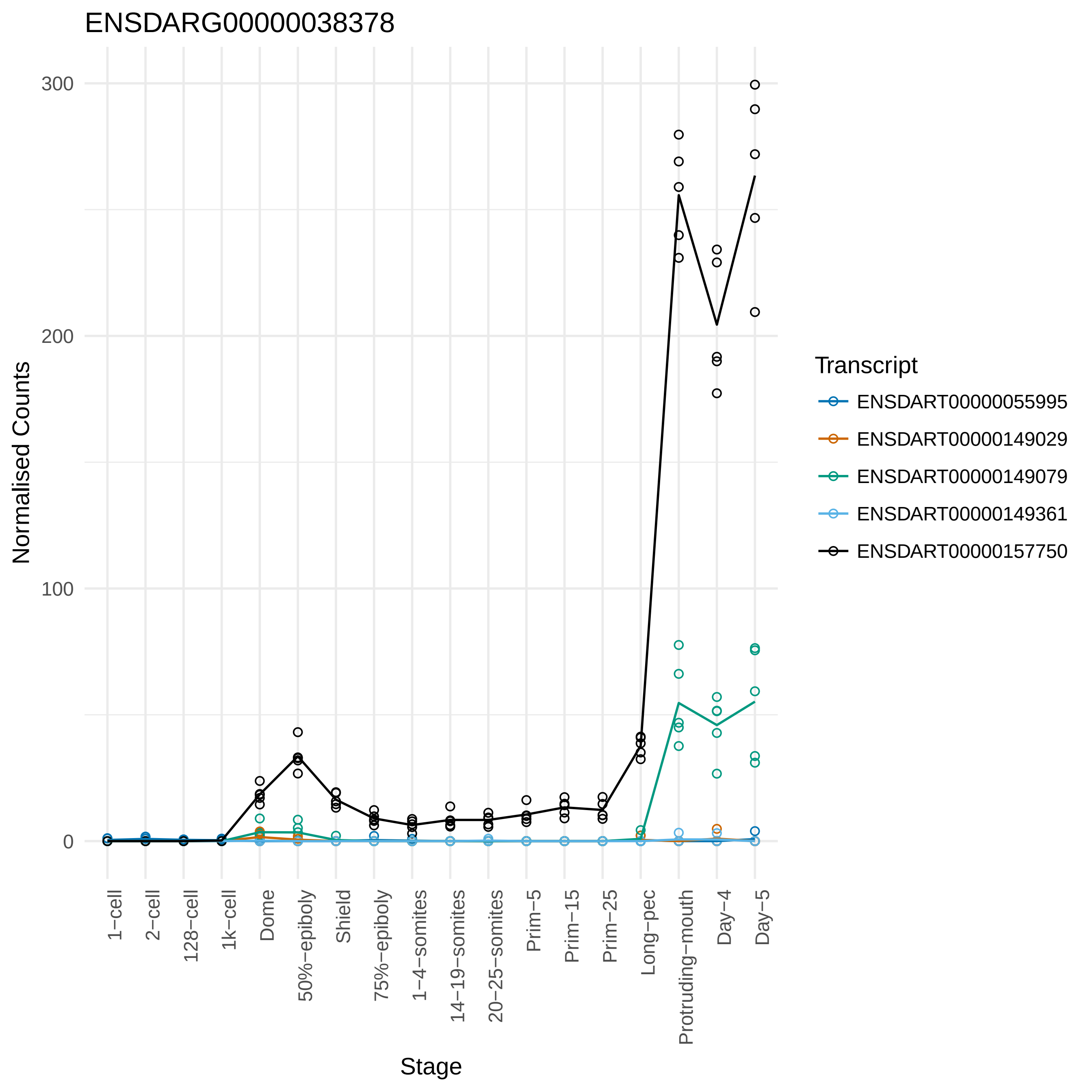

ENSDARG00000038378
Normalised Counts
300
200
100
0
1−cell
2−cell
128−cell
1k−cell
Dome
50%−epiboly
Shield
75%−epiboly
1−4−somites
14−19−somites
20−25−somites
Prim−5
Prim−15
Prim−25
Long−pec
Protruding−mouth
Day−4
Day−5
Stage
Transcript
ENSDART00000055995
ENSDART00000149029
ENSDART00000149079
ENSDART00000149361
ENSDART00000157750

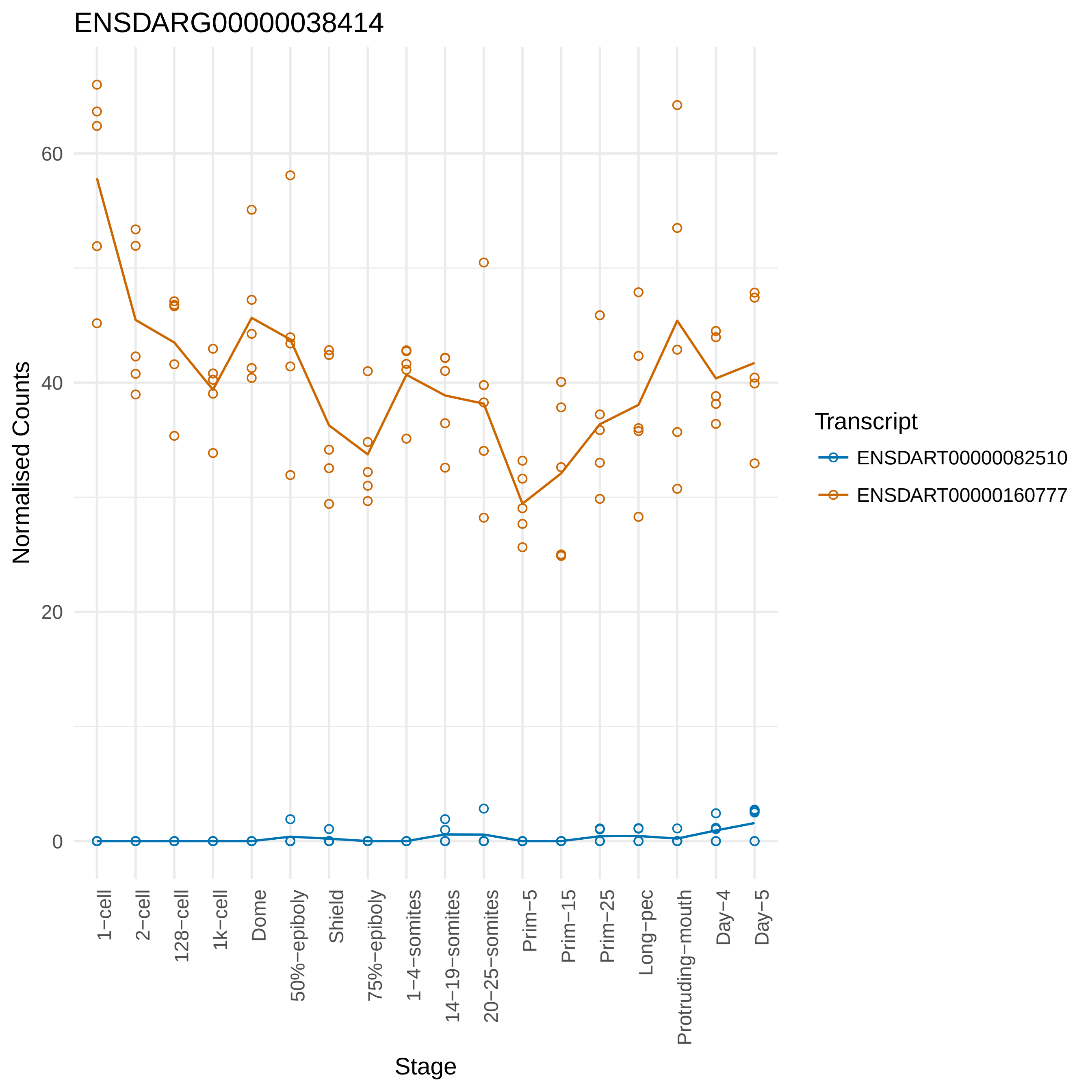
ENSDARG00000038414
Normalised Counts
60
40
20
0
1−cell
2−cell
128−cell
1k−cell
Dome
50%−epiboly
Shield
75%−epiboly
1−4−somites
14−19−somites
20−25−somites
Prim−5
Prim−15
Prim−25
Long−pec
Protruding−mouth
Day−4
Day−5
Stage
Transcript
ENSDART00000082510
ENSDART00000160777

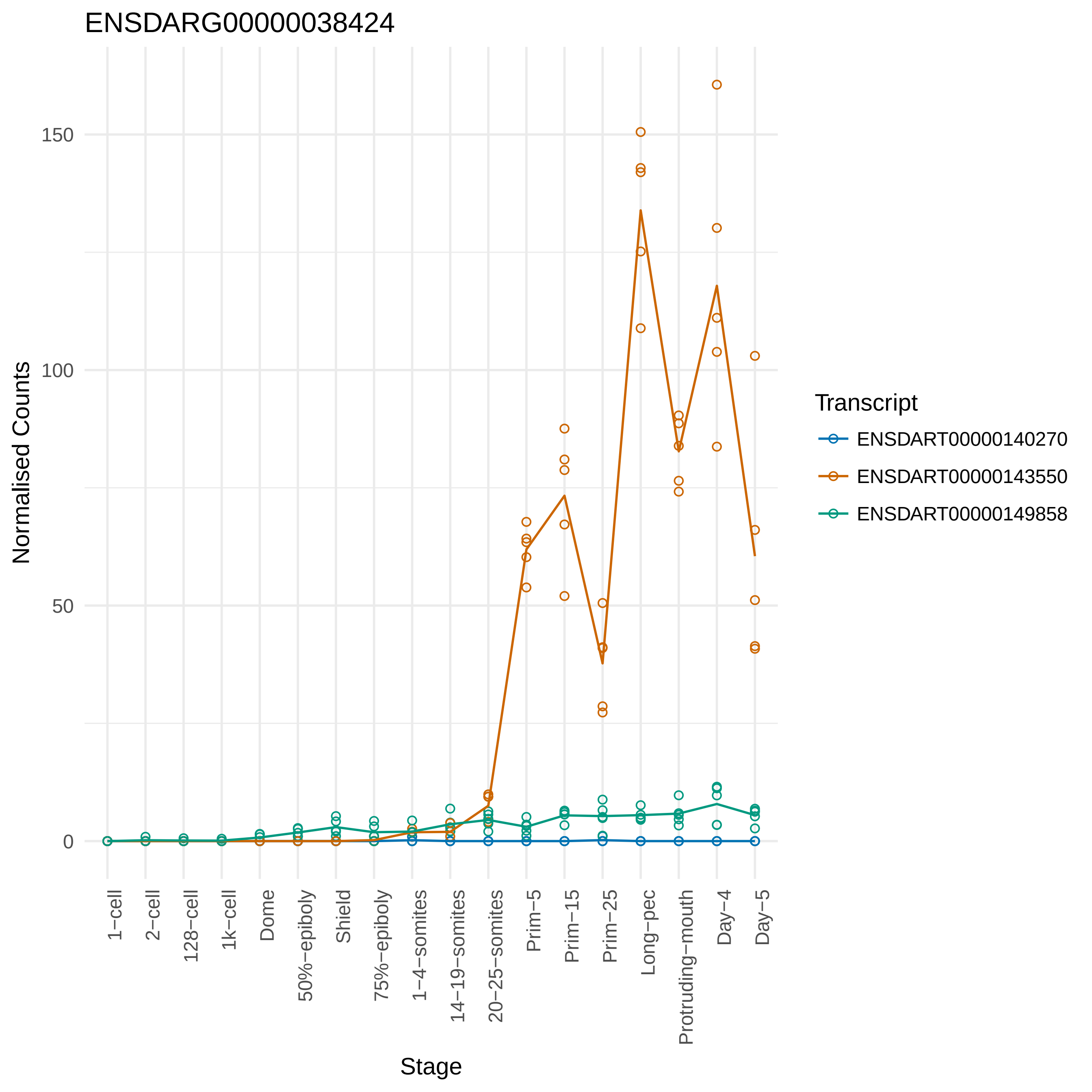
ENSDARG00000038424
Normalised Counts
150
100
50
0
1−cell
2−cell
128−cell
1k−cell
Dome
50%−epiboly
Shield
75%−epiboly
1−4−somites
14−19−somites
20−25−somites
Prim−5
Prim−15
Prim−25
Long−pec
Protruding−mouth
Day−4
Day−5
Stage
Transcript
ENSDART00000140270
ENSDART00000143550
ENSDART00000149858

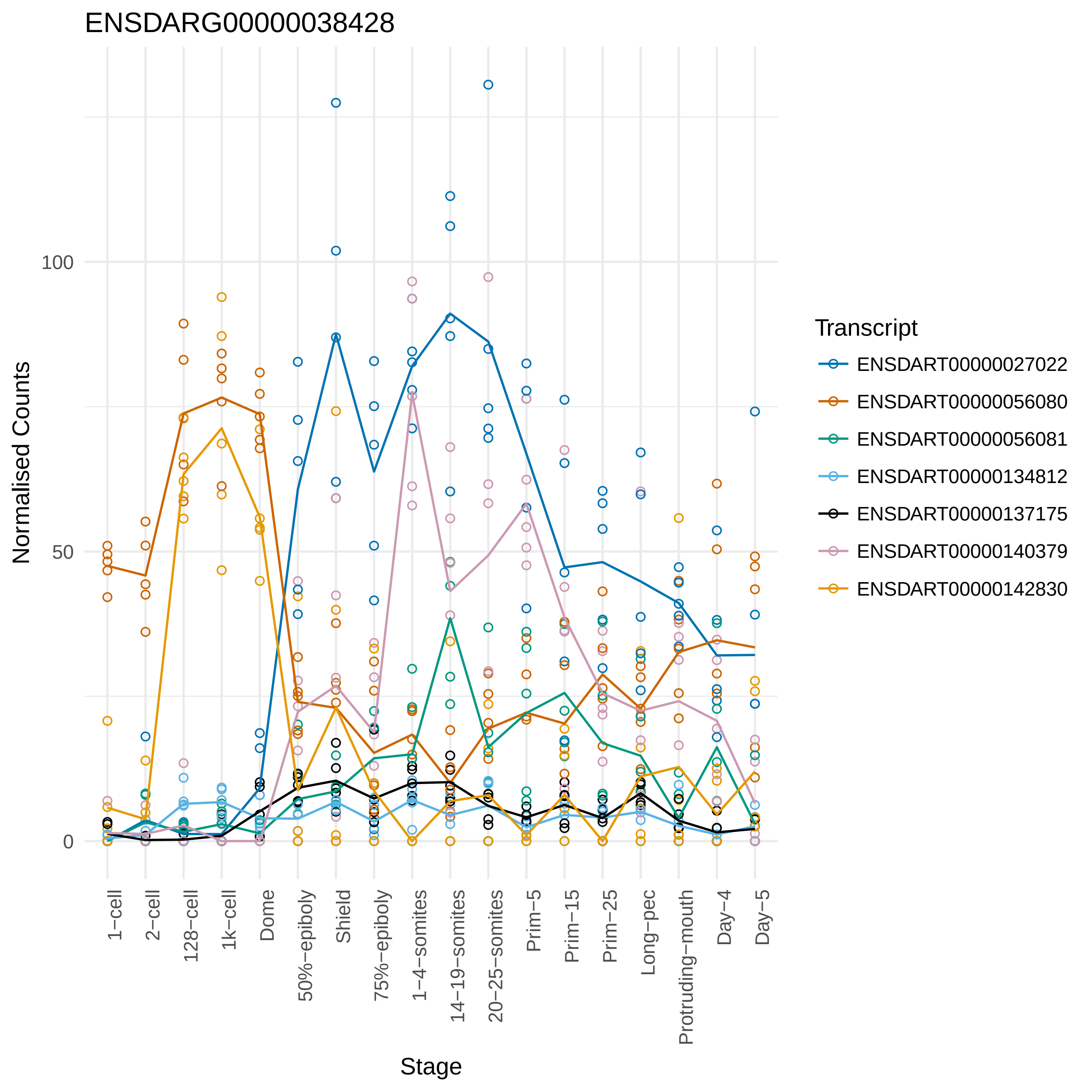
ENSDARG00000038428
Normalised Counts
0
50
100
Stage
1−cell
2−cell
128−cell
1k−cell
Dome
50%−epiboly
Shield
75%−epiboly
1−4−somites
14−19−somites
20−25−somites
Prim−5
Prim−15
Prim−25
Long−pec
Protruding−mouth
Day−4
Day−5
Transcript
ENSDART00000027022
ENSDART00000056080
ENSDART00000056081
ENSDART00000134812
ENSDART00000137175
ENSDART00000140379
ENSDART00000142830

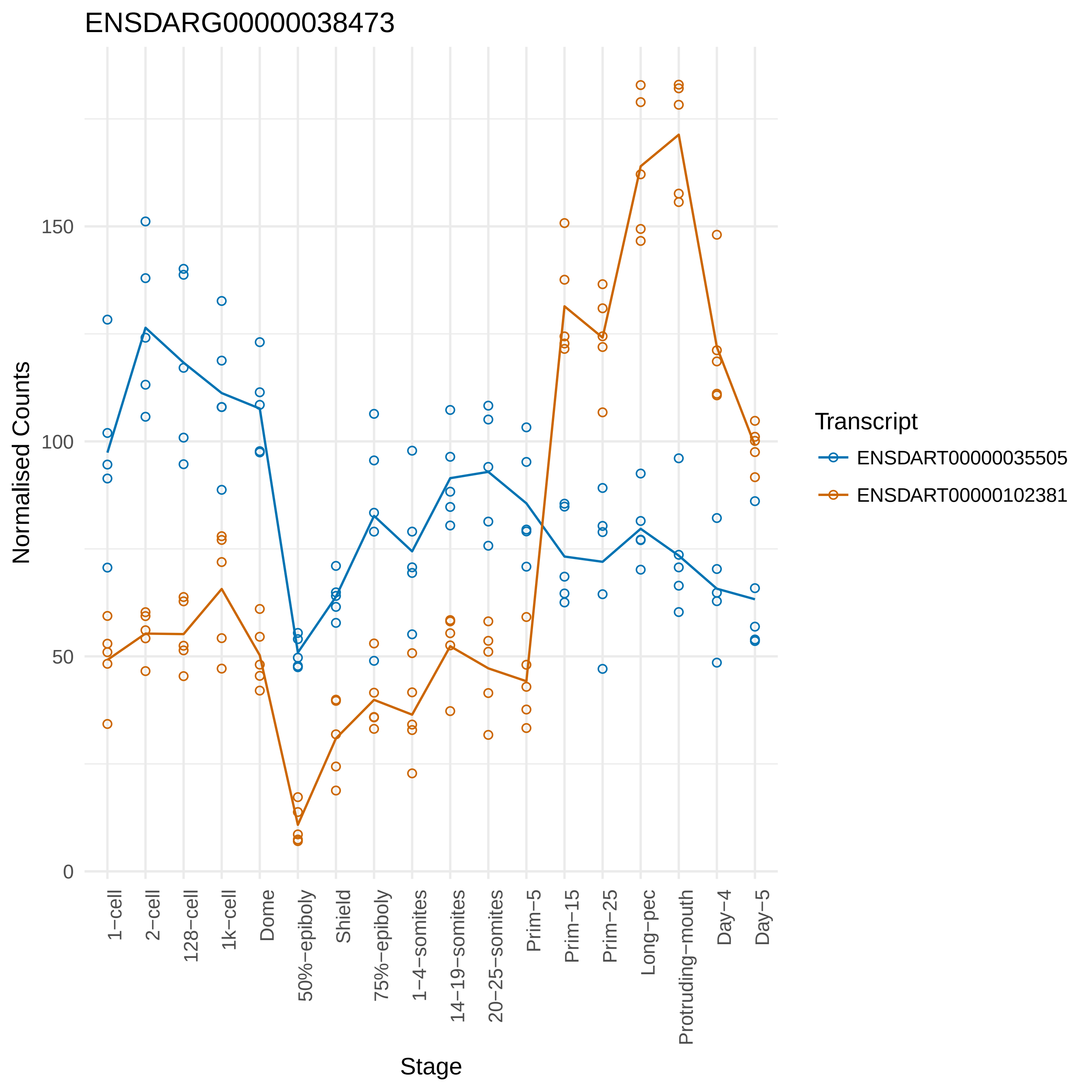

ENSDARG00000038473
Normalised Counts
0
50
100
150
1−cell
2−cell
128−cell
1k−cell
Dome
50%−epiboly
Shield
75%−epiboly
1−4−somites
14−19−somites
20−25−somites
Prim−5
Prim−15
Prim−25
Long−pec
Protruding−mouth
Day−4
Day−5
Stage
Transcript
ENSDART00000035505
ENSDART00000102381

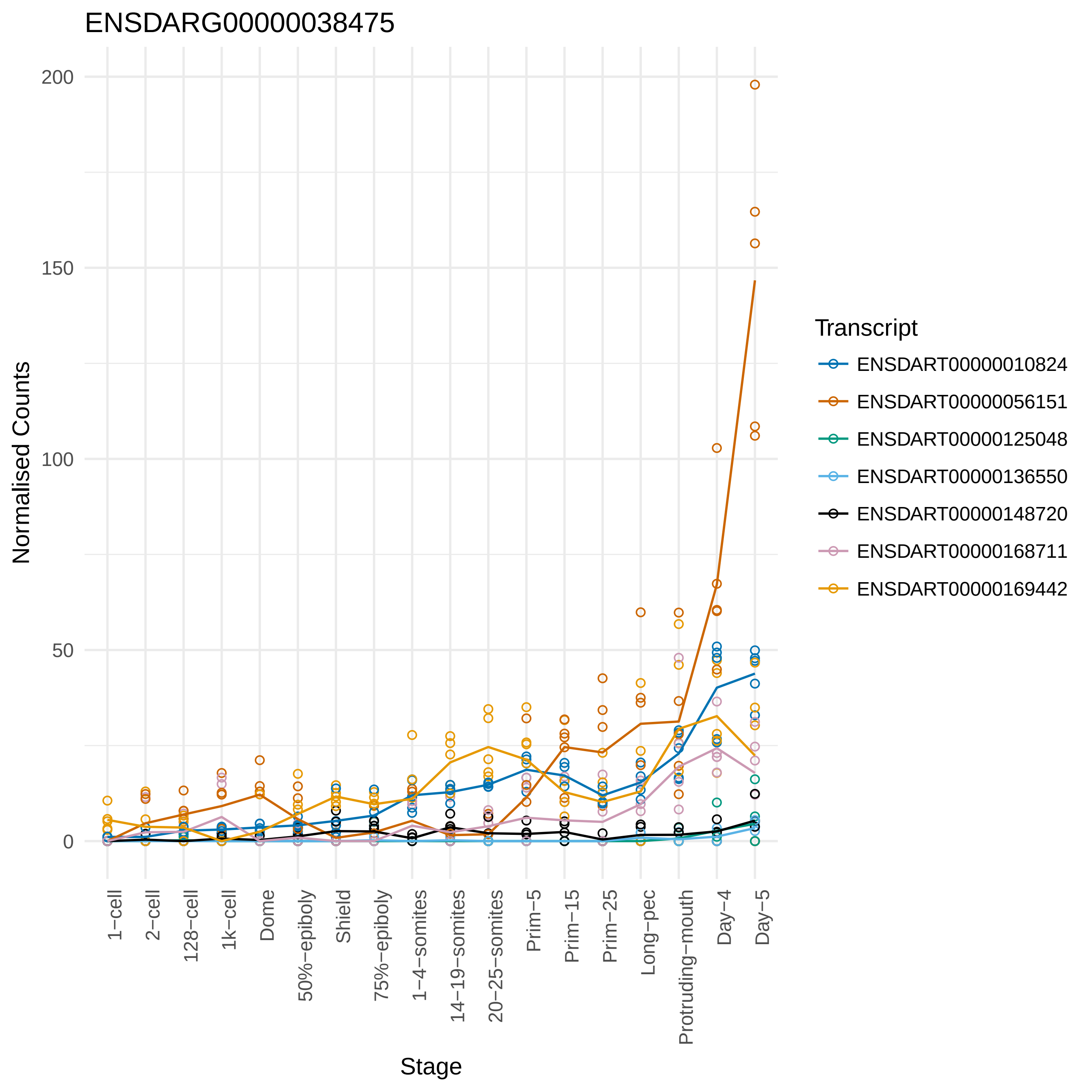

ENSDARG00000038475
Normalised Counts
200
150
100
50
0
1−cell
2−cell
128−cell
1k−cell
Dome
50%−epiboly
Shield
75%−epiboly
1−4−somites
14−19−somites
20−25−somites
Prim−5
Prim−15
Prim−25
Long−pec
Protruding−mouth
Day−4
Day−5
Stage
Transcript
ENSDART00000010824
ENSDART00000056151
ENSDART00000125048
ENSDART00000136550
ENSDART00000148720
ENSDART00000168711
ENSDART00000169442

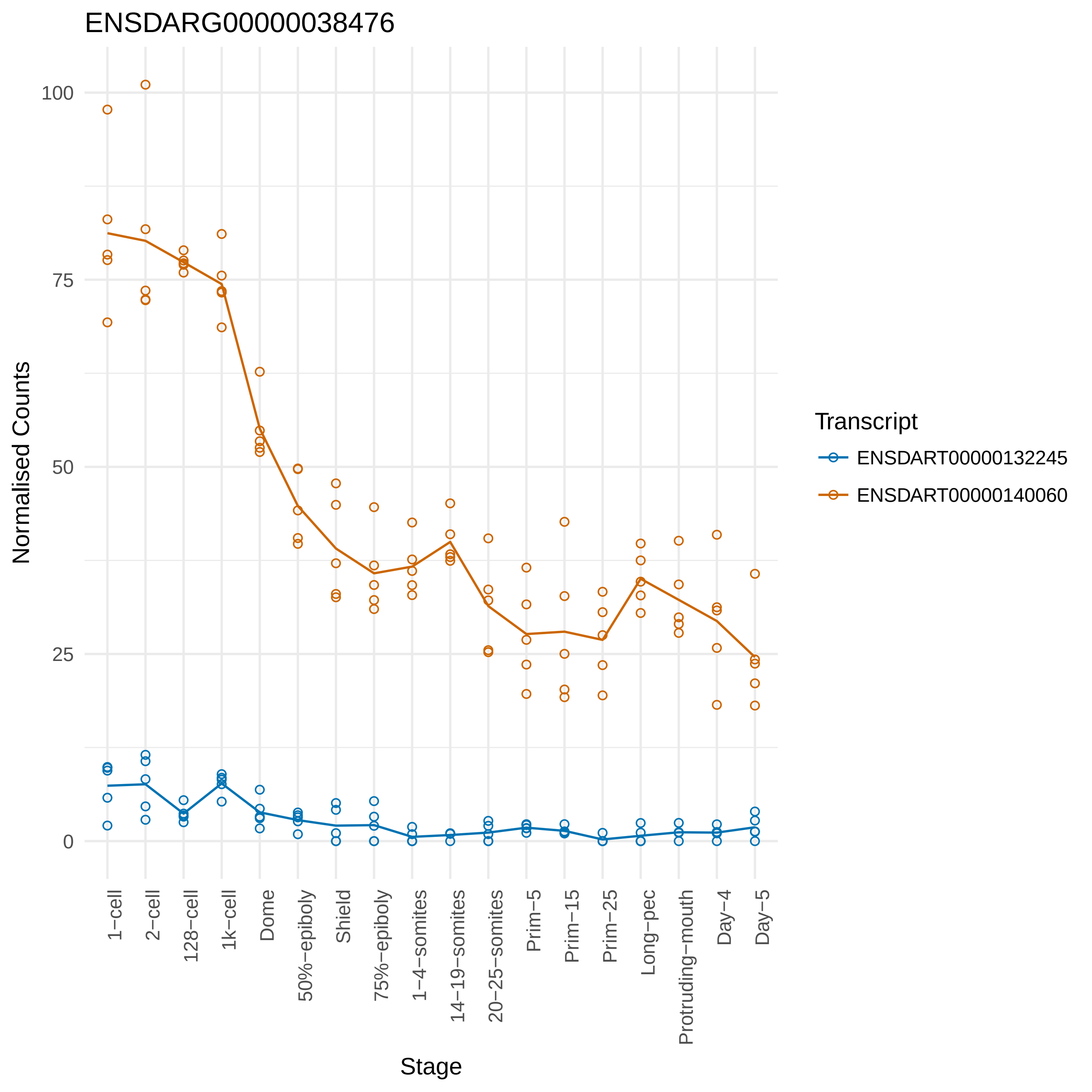

ENSDARG00000038476
Normalised Counts
100
75
50
25
0
1−cell
2−cell
128−cell
1k−cell
Dome
50%−epiboly
Shield
75%−epiboly
1−4−somites
14−19−somites
20−25−somites
Prim−5
Prim−15
Prim−25
Long−pec
Protruding−mouth
Day−4
Day−5
Stage
Transcript
ENSDART00000132245
ENSDART00000140060

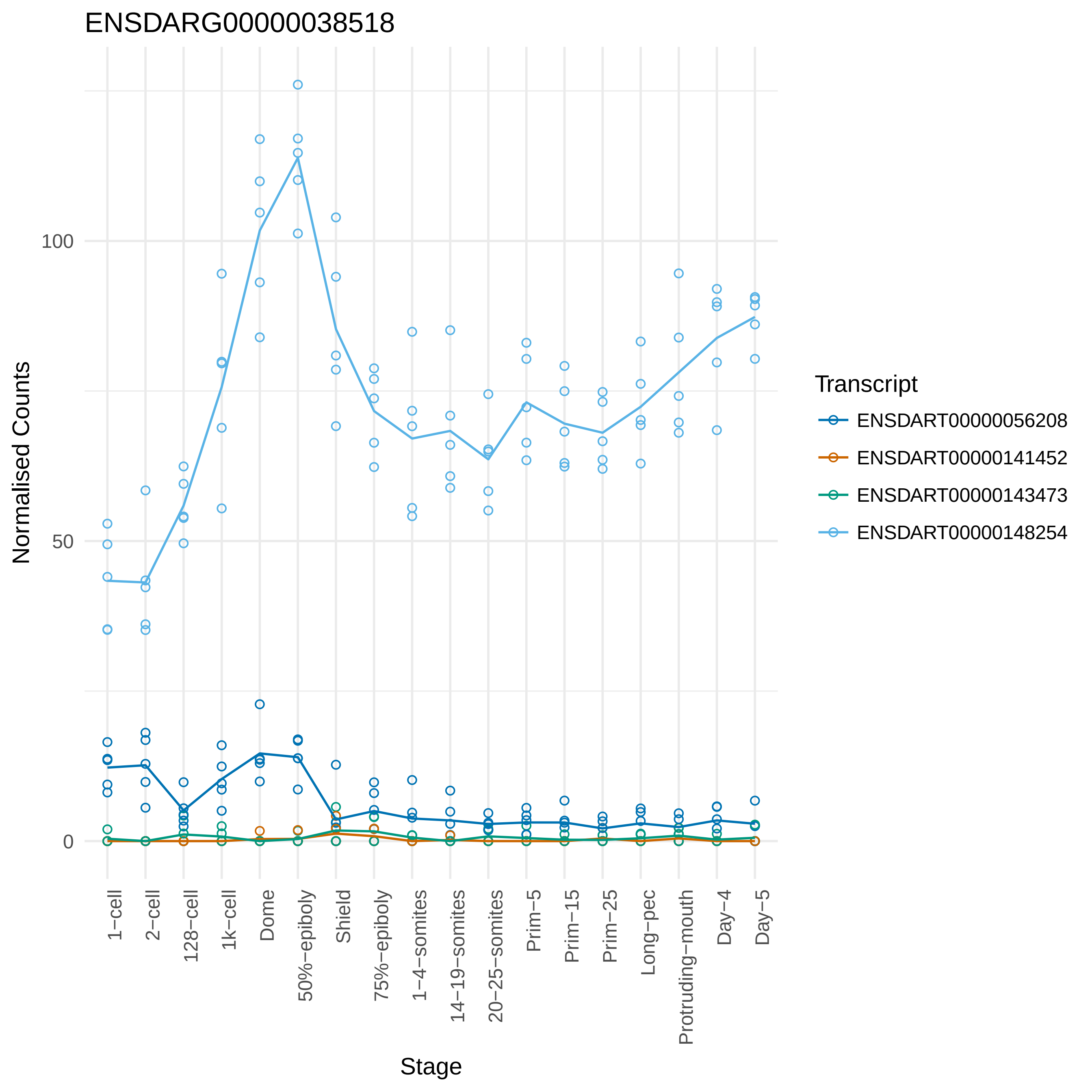

ENSDARG00000038518
Normalised Counts
100
50
0
1−cell
2−cell
128−cell
1k−cell
Dome
50%−epiboly
Shield
75%−epiboly
1−4−somites
14−19−somites
20−25−somites
Prim−5
Prim−15
Prim−25
Long−pec
Protruding−mouth
Day−4
Day−5
Stage
Transcript
ENSDART00000056208
ENSDART00000141452
ENSDART00000143473
ENSDART00000148254

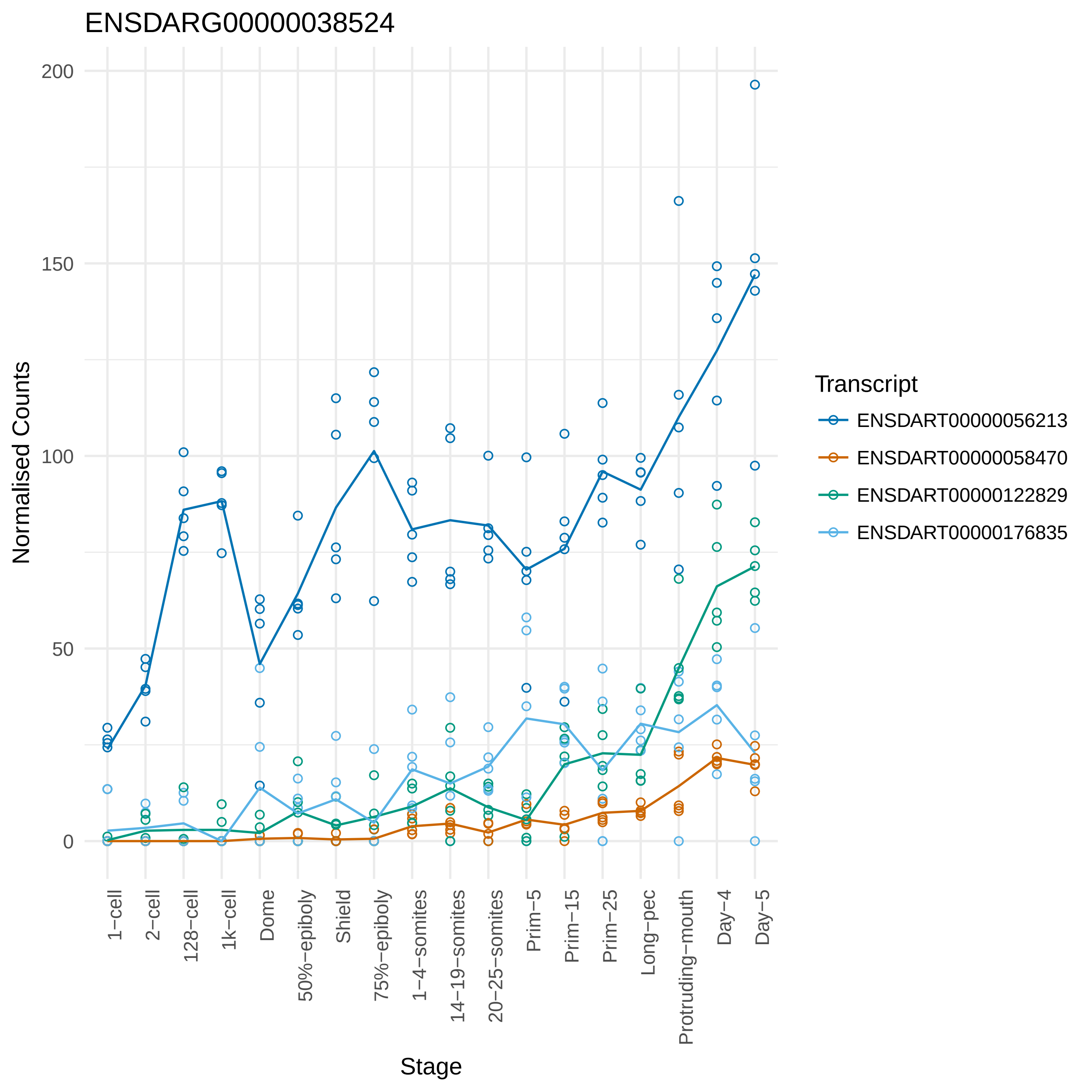

ENSDARG00000038524
Normalised Counts
0
50
100
150
200
Stage
1−cell
2−cell
128−cell
1k−cell
Dome
50%−epiboly
Shield
75%−epiboly
1−4−somites
14−19−somites
20−25−somites
Prim−5
Prim−15
Prim−25
Long−pec
Protruding−mouth
Day−4
Day−5
Transcript
ENSDART00000056213
ENSDART00000058470
ENSDART00000122829
ENSDART00000176835

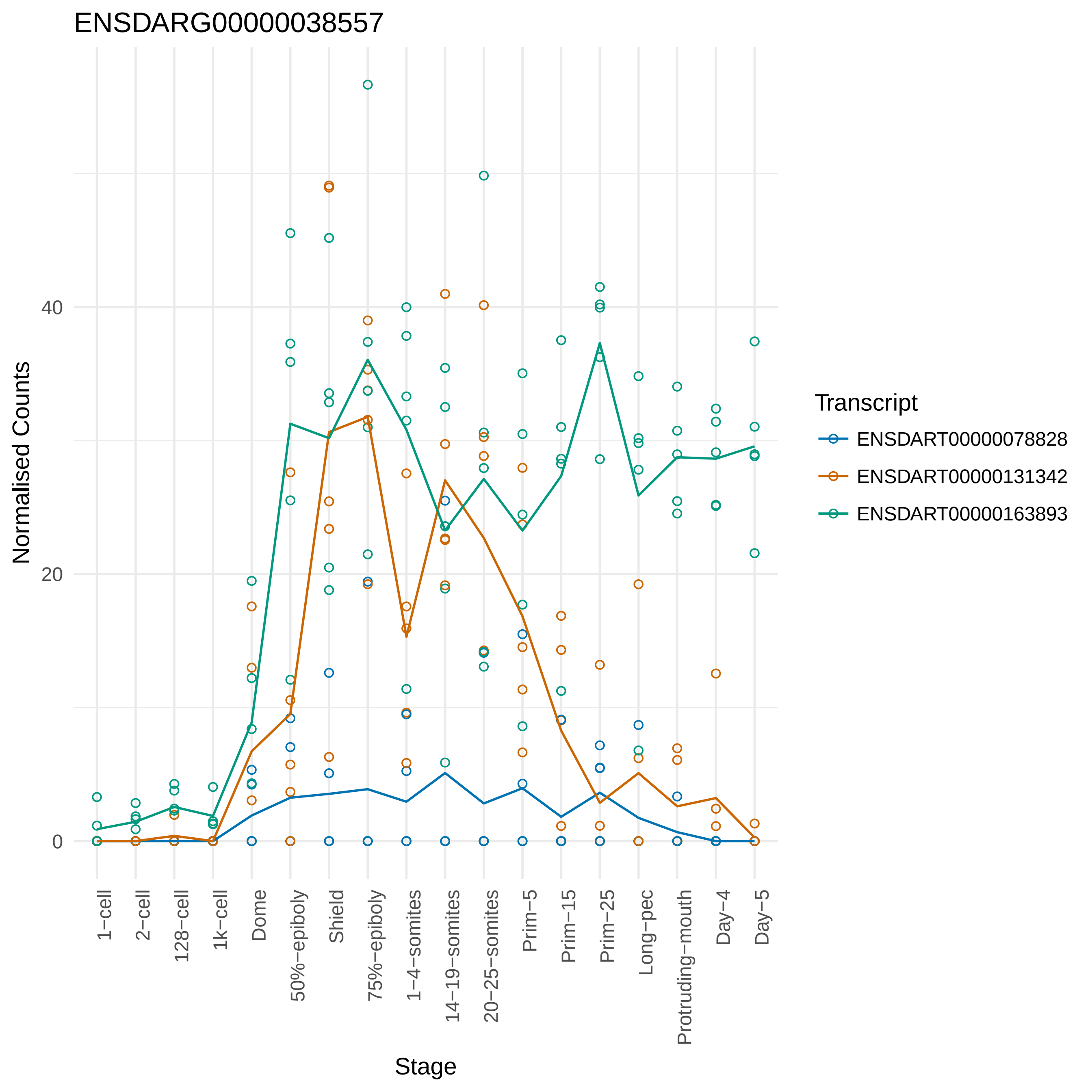
ENSDARG00000038557
Normalised Counts
0
20
40
Stage
1−cell
2−cell
128−cell
1k−cell
Dome
50%−epiboly
Shield
75%−epiboly
1−4−somites
14−19−somites
20−25−somites
Prim−5
Prim−15
Prim−25
Long−pec
Protruding−mouth
Day−4
Day−5
Transcript
ENSDART00000078828
ENSDART00000131342
ENSDART00000163893

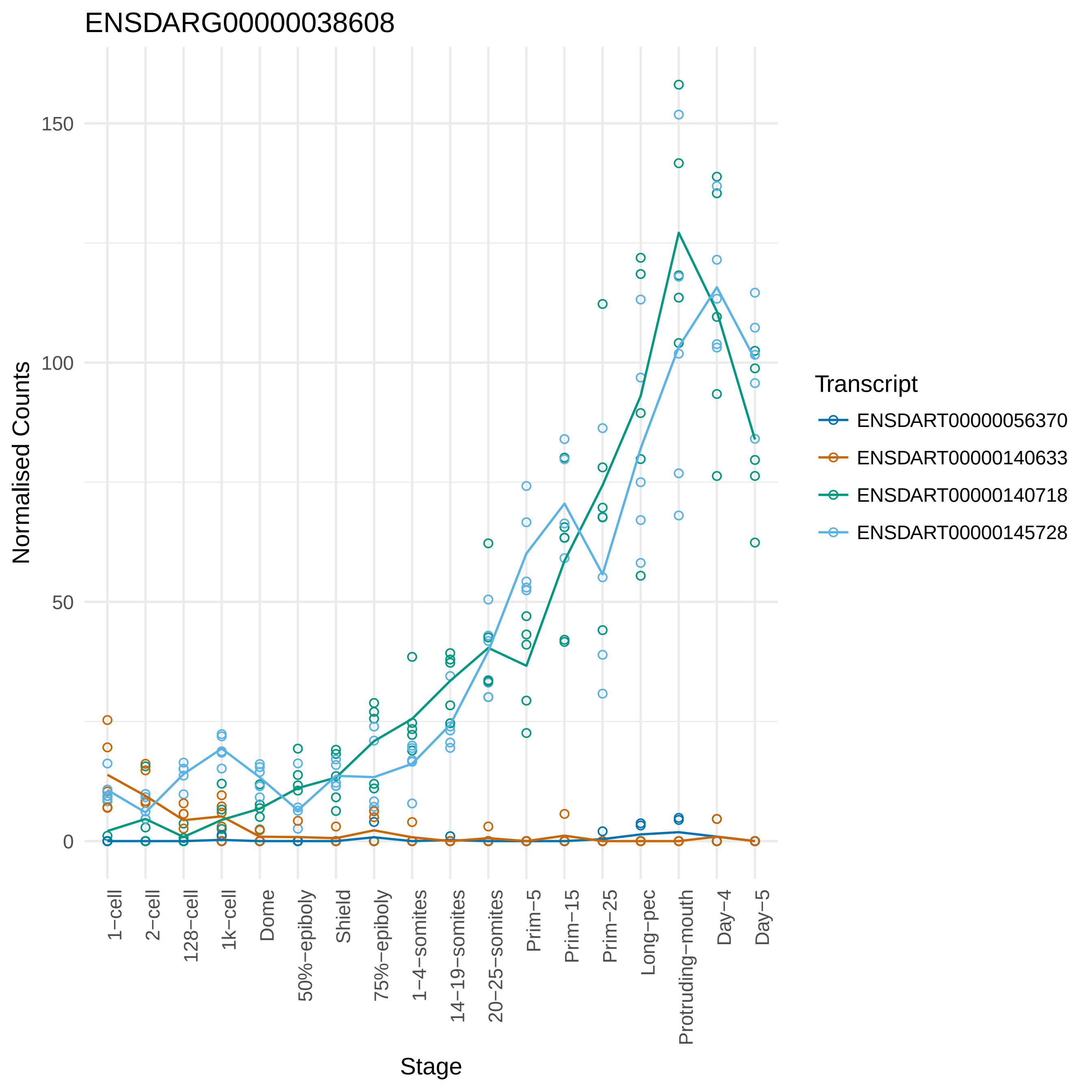

ENSDARG00000038608
Normalised Counts
150
100
50
0
Stage
1−cell
2−cell
128−cell
1k−cell
Dome
50%−epiboly
Shield
75%−epiboly
1−4−somites
14−19−somites
20−25−somites
Prim−5
Prim−15
Prim−25
Long−pec
Protruding−mouth
Day−4
Day−5
Transcript
ENSDART00000056370
ENSDART00000140633
ENSDART00000140718
ENSDART00000145728

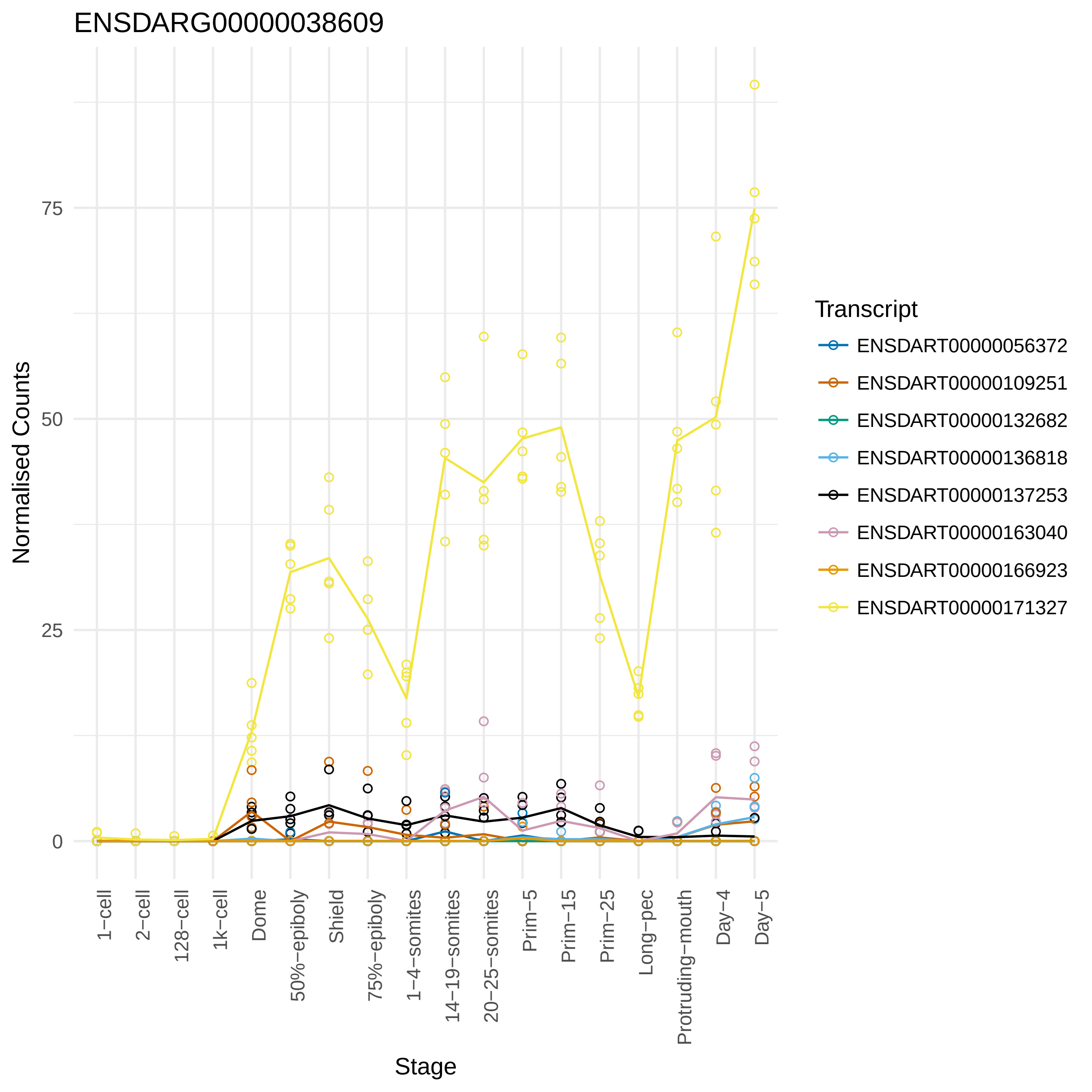

ENSDARG00000038609
Normalised Counts
0
25
50
75
Stage
1−cell
2−cell
128−cell
1k−cell
Dome
50%−epiboly
Shield
75%−epiboly
1−4−somites
14−19−somites
20−25−somites
Prim−5
Prim−15
Prim−25
Long−pec
Protruding−mouth
Day−4
Day−5
Transcript
ENSDART00000056372
ENSDART00000109251
ENSDART00000132682
ENSDART00000136818
ENSDART00000137253
ENSDART00000163040
ENSDART00000166923
ENSDART00000171327

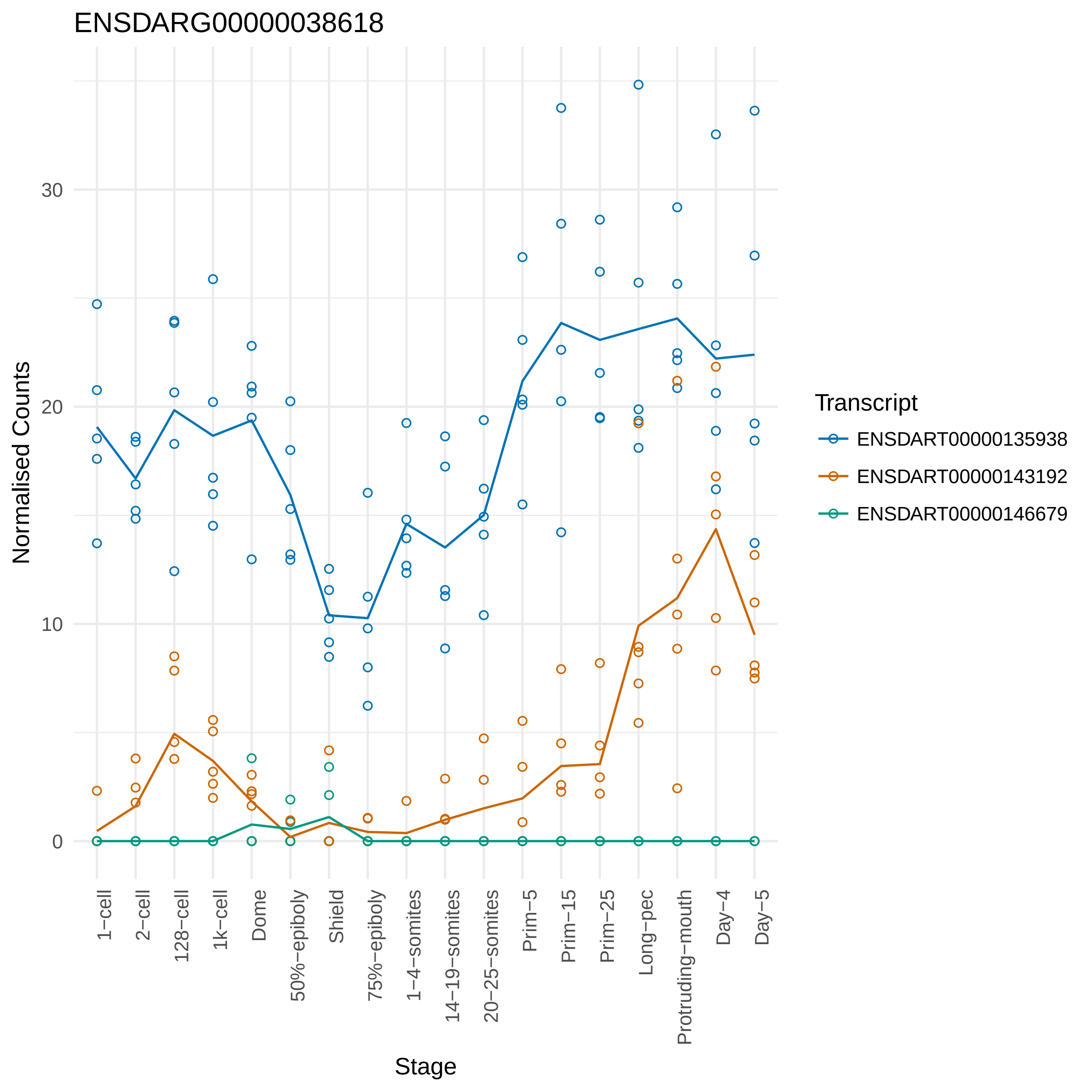
ENSDARG00000038618
Normalised Counts
30
20
10
0
1−cell
2−cell
128−cell
1k−cell
Dome
50%−epiboly
Shield
75%−epiboly
1−4−somites
14−19−somites
20−25−somites
Prim−5
Prim−15
Prim−25
Long−pec
Protruding−mouth
Day−4
Day−5
Stage
Transcript
ENSDART00000135938
ENSDART00000143192
ENSDART00000146679

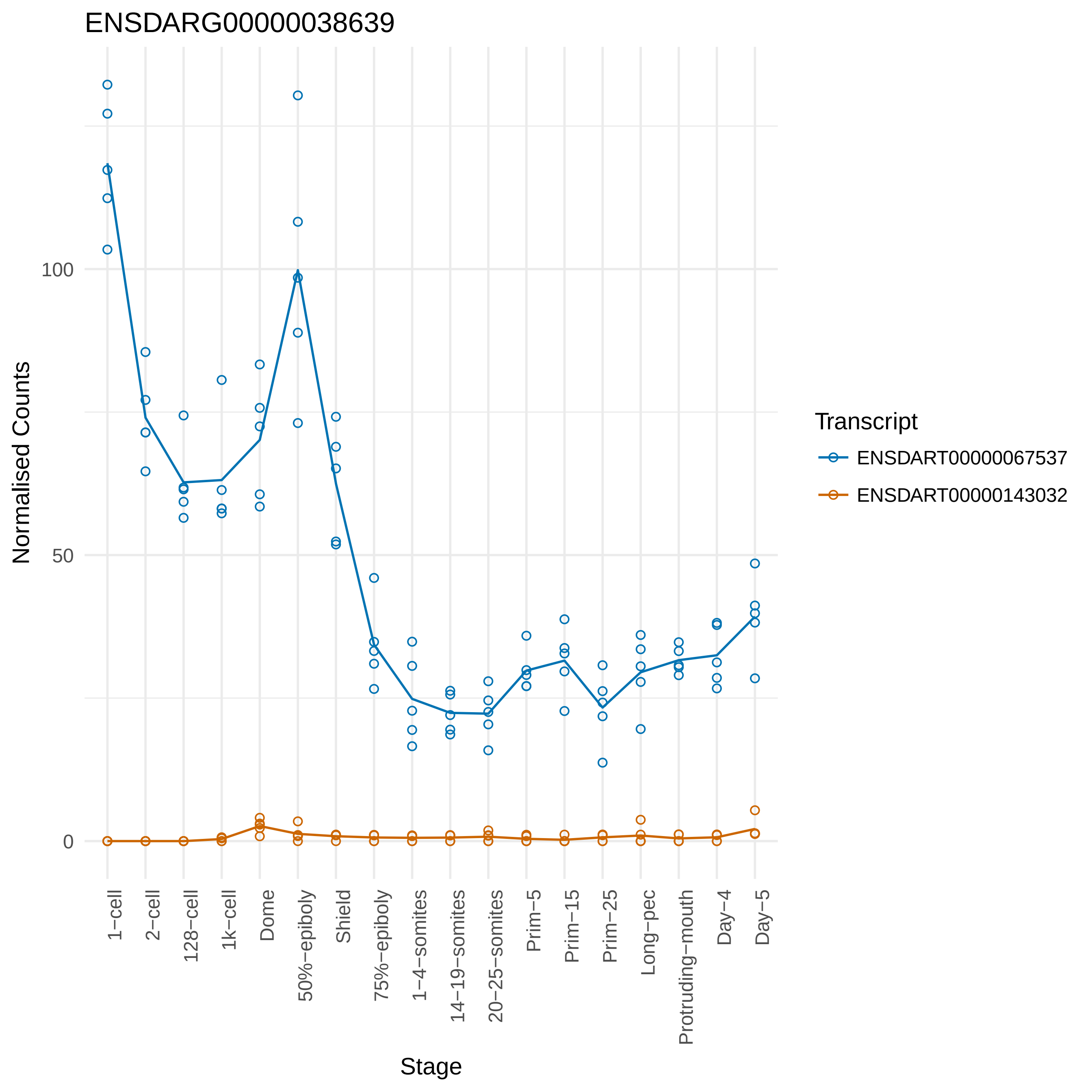

ENSDARG00000038639
Normalised Counts
100
50
0
1−cell
2−cell
128−cell
1k−cell
Dome
50%−epiboly
Shield
75%−epiboly
1−4−somites
14−19−somites
20−25−somites
Prim−5
Prim−15
Prim−25
Long−pec
Protruding−mouth
Day−4
Day−5
Stage
Transcript
ENSDART00000067537
ENSDART00000143032

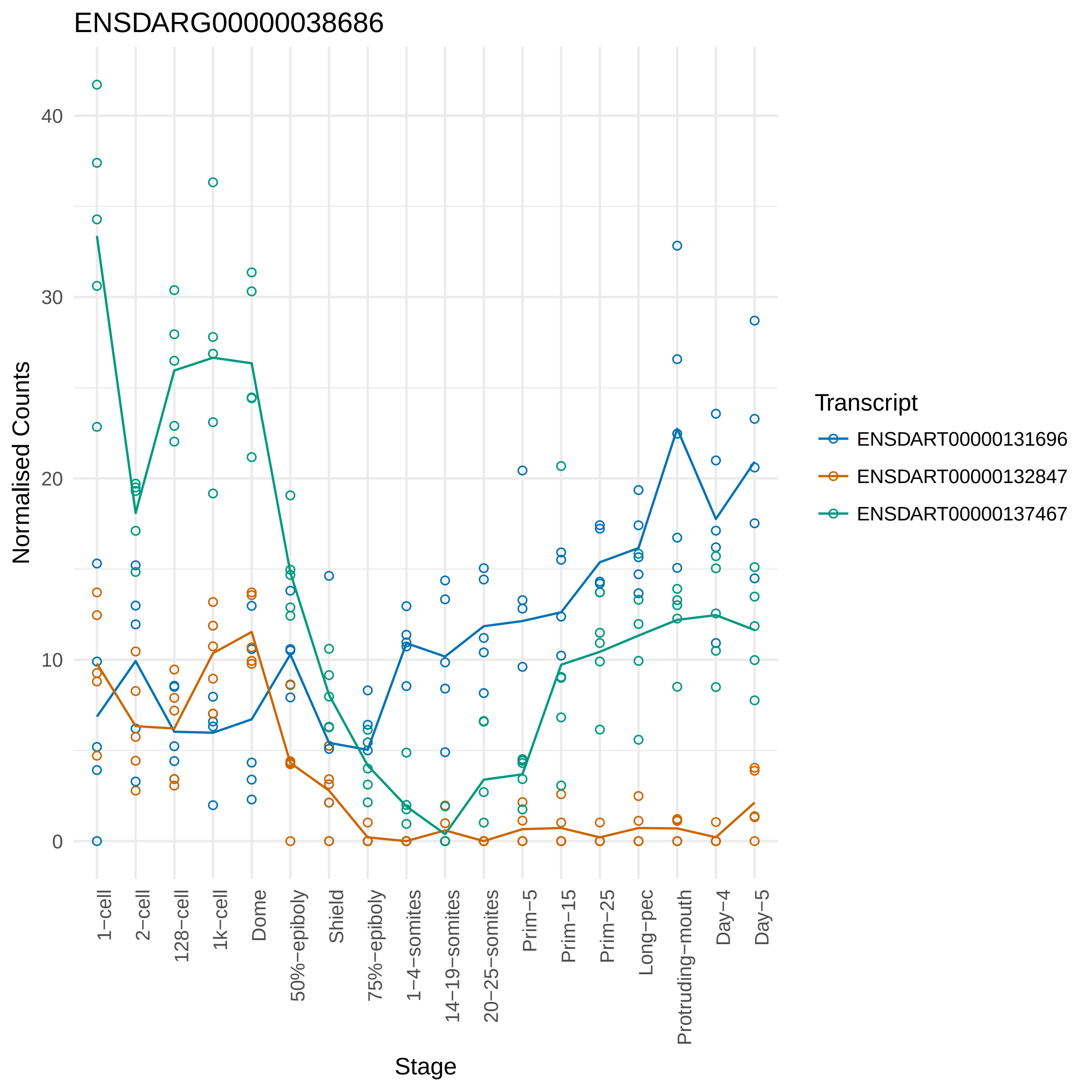
ENSDARG00000038686
Normalised Counts
0
10
20
30
40
Stage
1−cell
2−cell
128−cell
1k−cell
Dome
50%−epiboly
Shield
75%−epiboly
1−4−somites
14−19−somites
20−25−somites
Prim−5
Prim−15
Prim−25
Long−pec
Protruding−mouth
Day−4
Day−5
Transcript
ENSDART00000131696
ENSDART00000132847
ENSDART00000137467

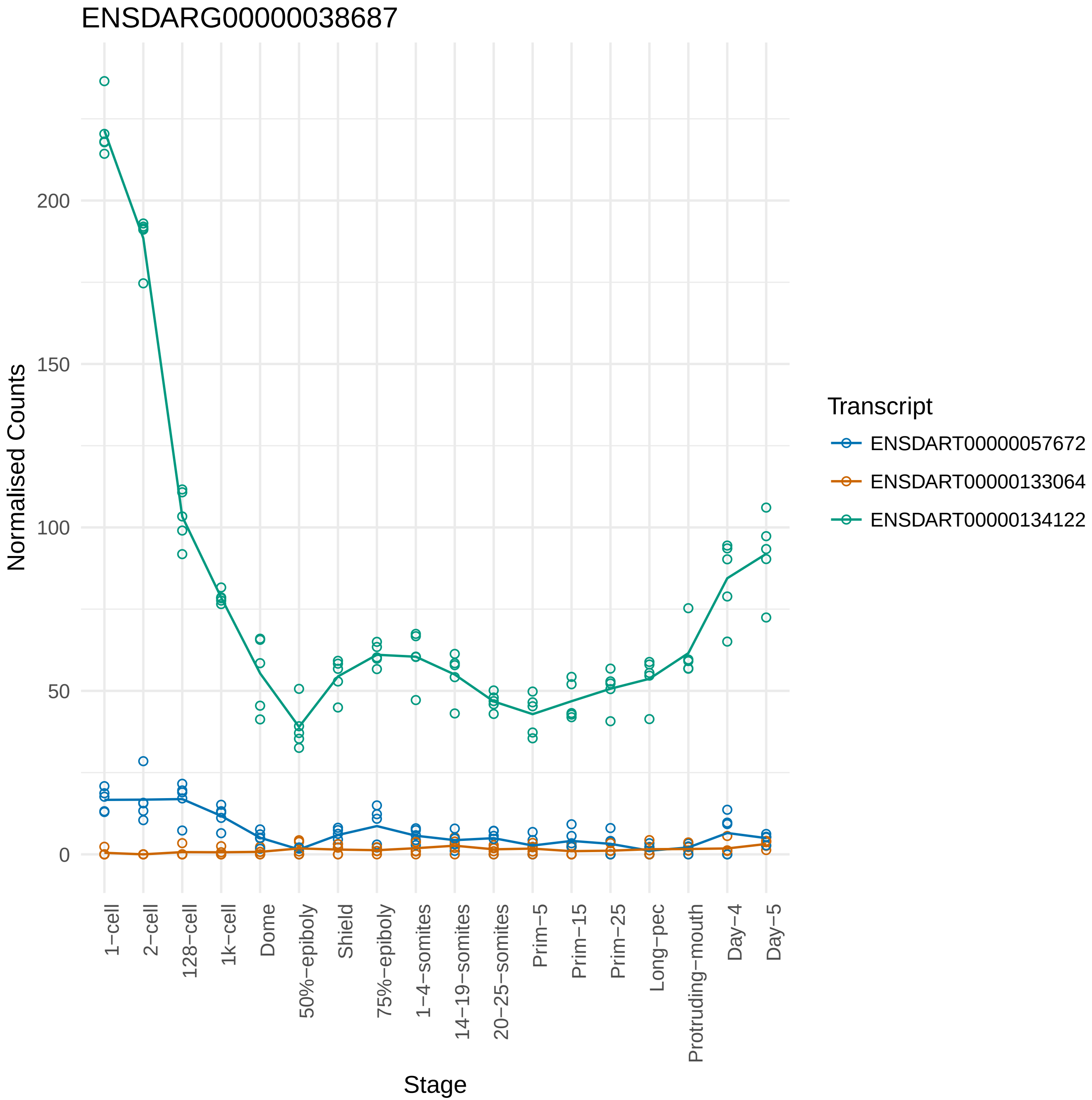

ENSDARG00000038687
Normalised Counts
200
150
100
50
0
Stage
1−cell
2−cell
128−cell
1k−cell
Dome
50%−epiboly
Shield
75%−epiboly
1−4−somites
14−19−somites
20−25−somites
Prim−5
Prim−15
Prim−25
Long−pec
Protruding−mouth
Day−4
Day−5
Transcript
ENSDART00000057672
ENSDART00000133064
ENSDART00000134122

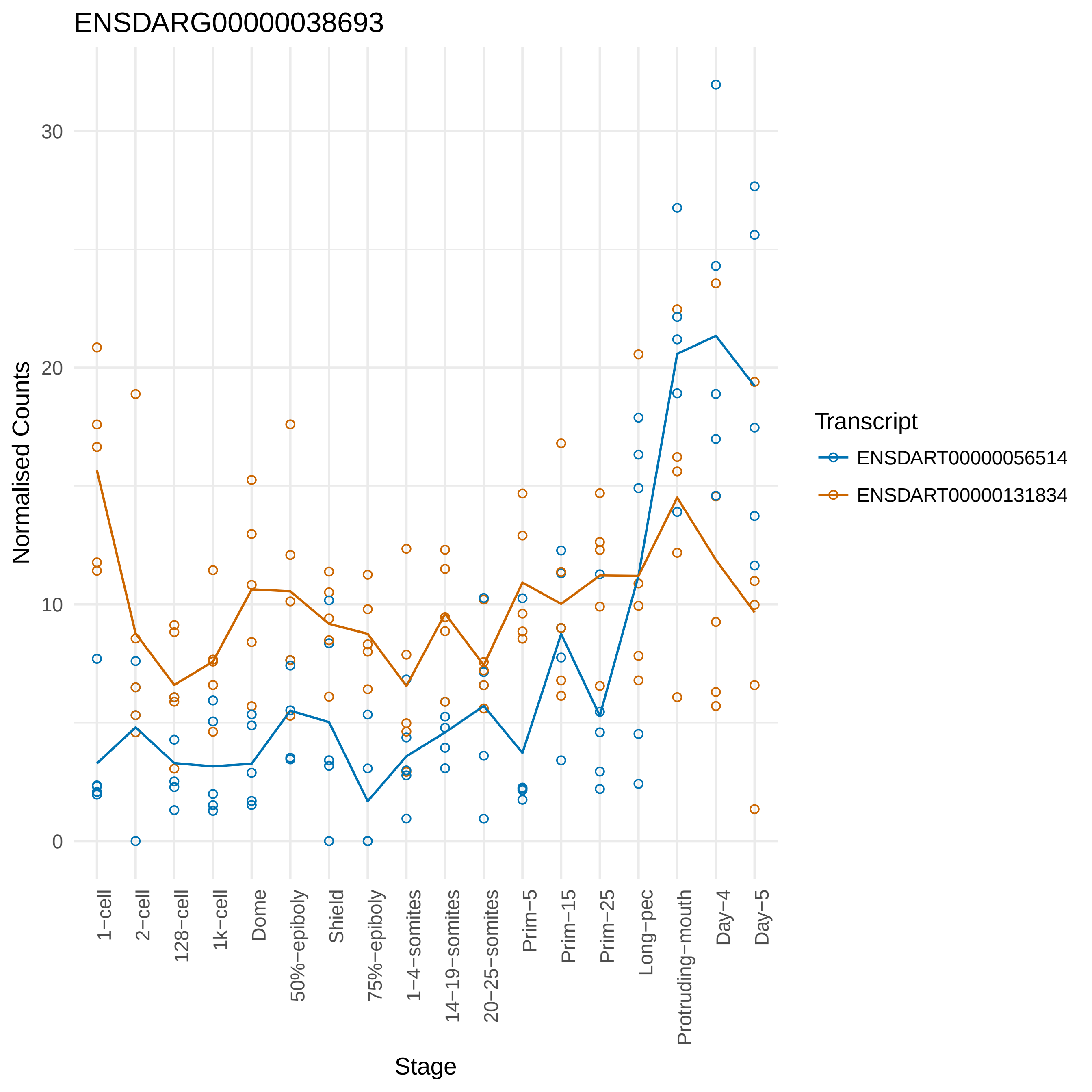

ENSDARG00000038693
Normalised Counts
0
10
20
30
Stage
1−cell
2−cell
128−cell
1k−cell
Dome
50%−epiboly
Shield
75%−epiboly
1−4−somites
14−19−somites
20−25−somites
Prim−5
Prim−15
Prim−25
Long−pec
Protruding−mouth
Day−4
Day−5
Transcript
ENSDART00000056514
ENSDART00000131834

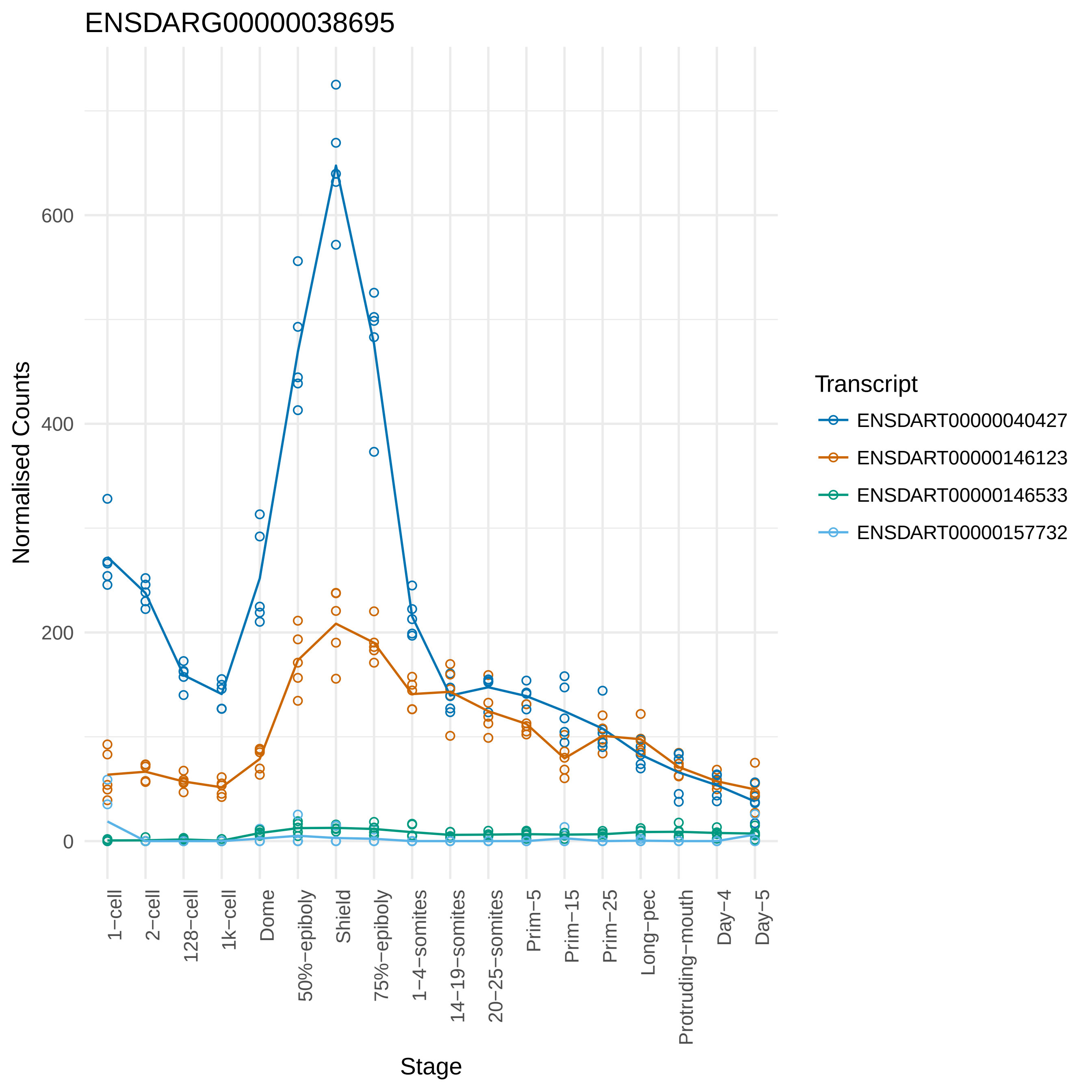

ENSDARG00000038695
Normalised Counts
600
400
200
0
1−cell
2−cell
128−cell
1k−cell
Dome
50%−epiboly
Shield
75%−epiboly
1−4−somites
14−19−somites
20−25−somites
Prim−5
Prim−15
Prim−25
Long−pec
Protruding−mouth
Day−4
Day−5
Stage
Transcript
ENSDART00000040427
ENSDART00000146123
ENSDART00000146533
ENSDART00000157732

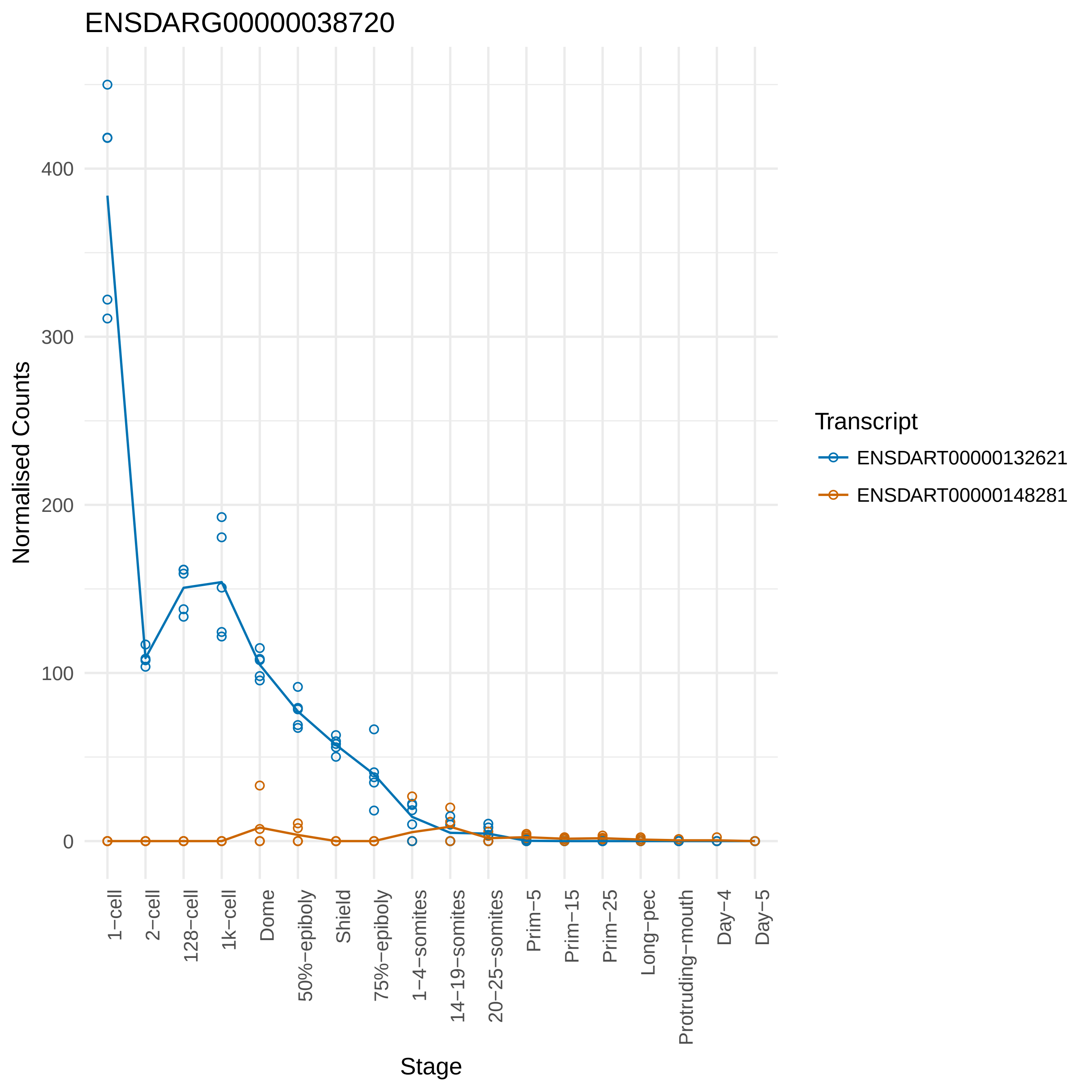
ENSDARG00000038720
Normalised Counts
0
100
200
300
400
1−cell
2−cell
128−cell
1k−cell
Dome
50%−epiboly
Shield
75%−epiboly
1−4−somites
14−19−somites
20−25−somites
Prim−5
Prim−15
Prim−25
Long−pec
Protruding−mouth
Day−4
Day−5
Stage
Transcript
ENSDART00000132621
ENSDART00000148281

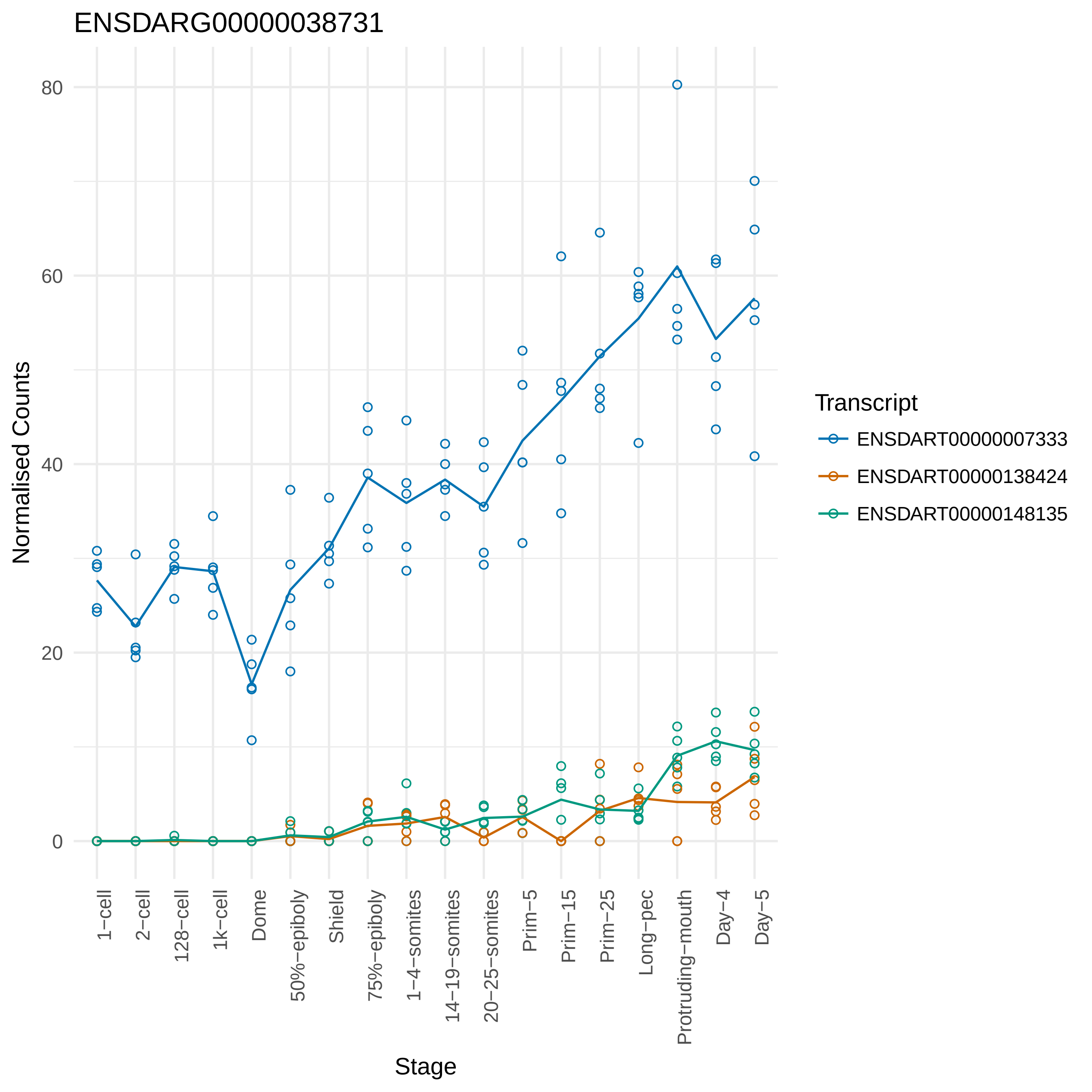
ENSDARG00000038731
Normalised Counts
0
20
40
60
80
Stage
1−cell
2−cell
128−cell
1k−cell
Dome
50%−epiboly
Shield
75%−epiboly
1−4−somites
14−19−somites
20−25−somites
Prim−5
Prim−15
Prim−25
Long−pec
Protruding−mouth
Day−4
Day−5
Transcript
ENSDART00000007333
ENSDART00000138424
ENSDART00000148135

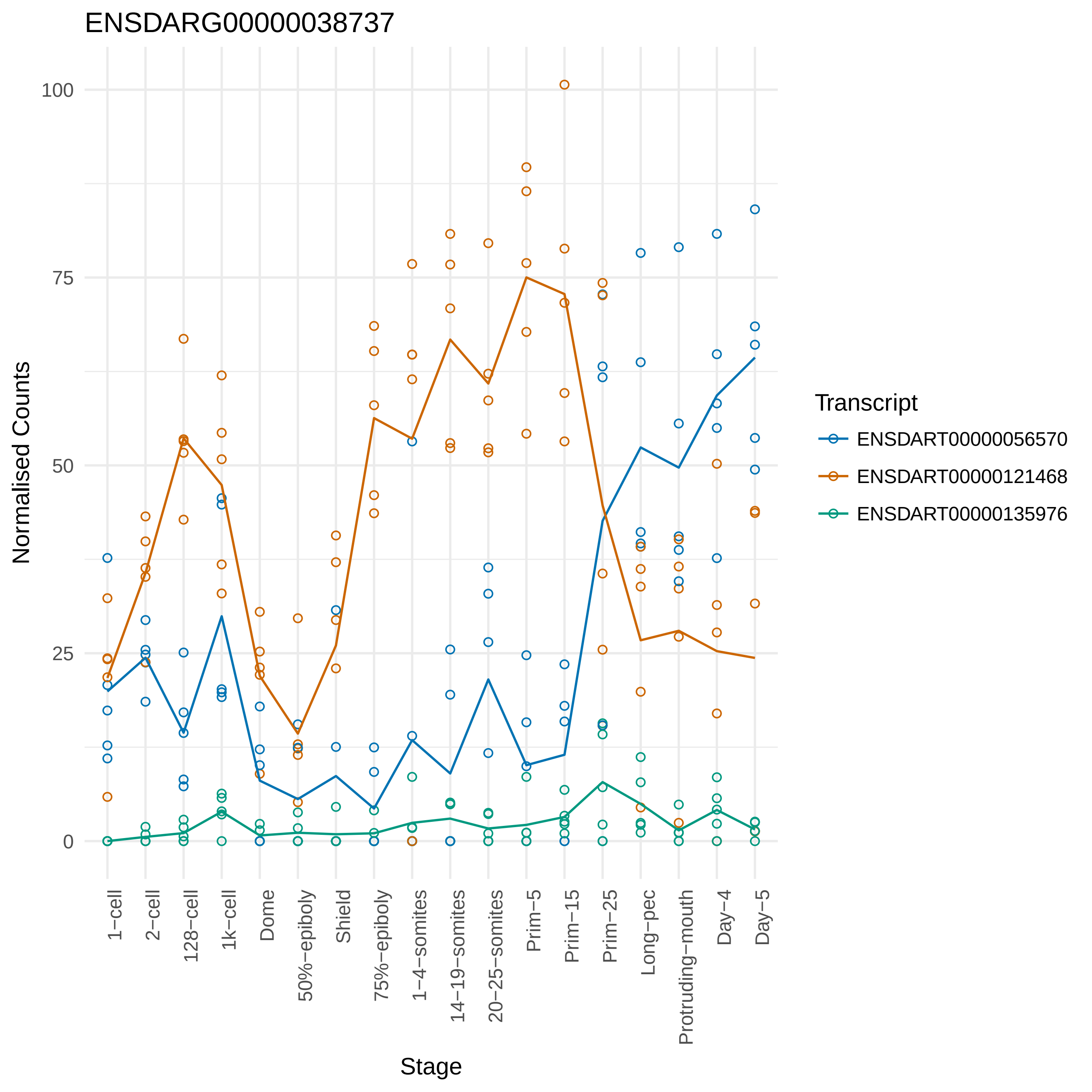

ENSDARG00000038737
Normalised Counts
0
25
50
75
100
Stage
1−cell
2−cell
128−cell
1k−cell
Dome
50%−epiboly
Shield
75%−epiboly
1−4−somites
14−19−somites
20−25−somites
Prim−5
Prim−15
Prim−25
Long−pec
Protruding−mouth
Day−4
Day−5
Transcript
ENSDART00000056570
ENSDART00000121468
ENSDART00000135976

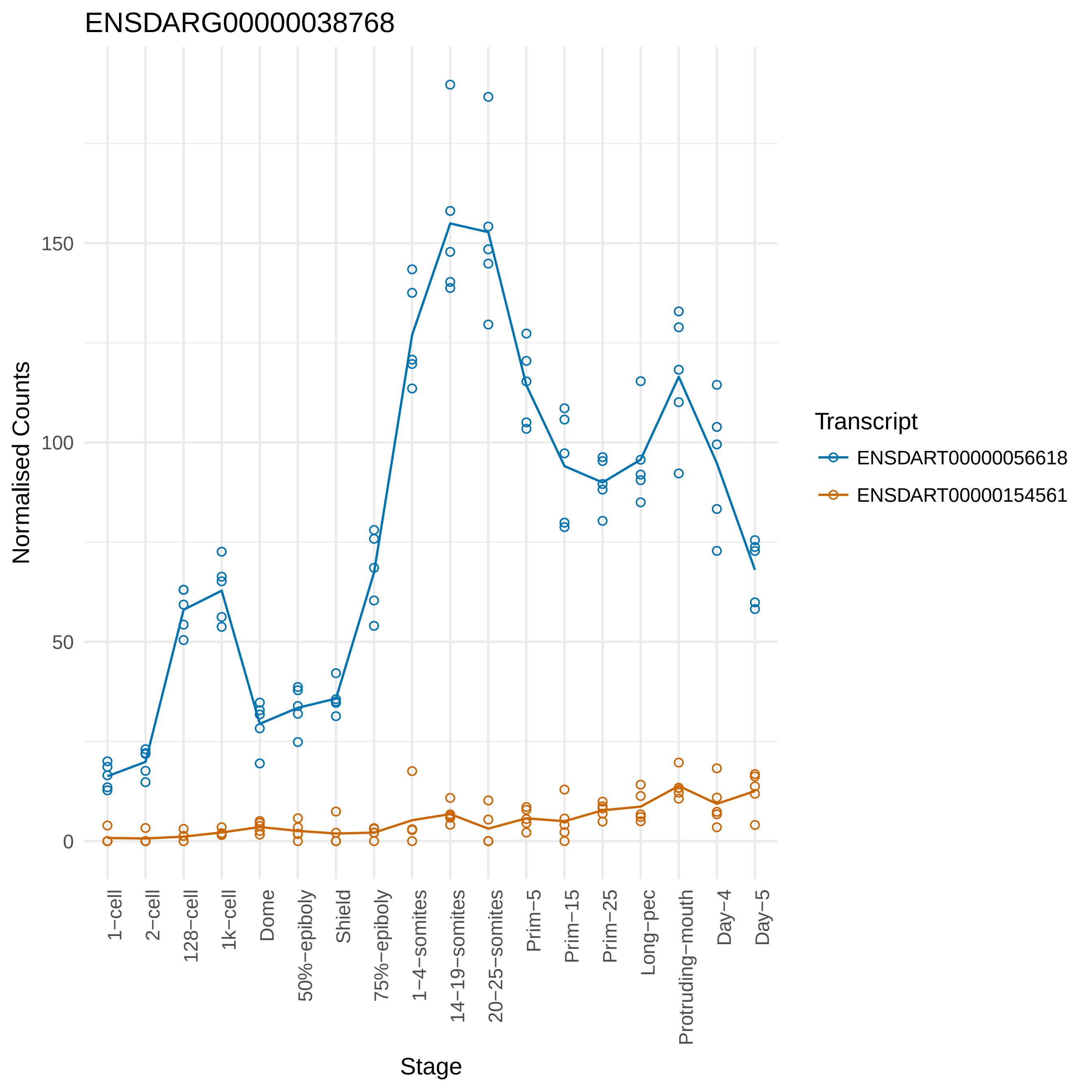
ENSDARG00000038768
Normalised Counts
0
50
100
150
Stage
1−cell
2−cell
128−cell
1k−cell
Dome
50%−epiboly
Shield
75%−epiboly
1−4−somites
14−19−somites
20−25−somites
Prim−5
Prim−15
Prim−25
Long−pec
Protruding−mouth
Day−4
Day−5
Transcript
ENSDART00000056618
ENSDART00000154561

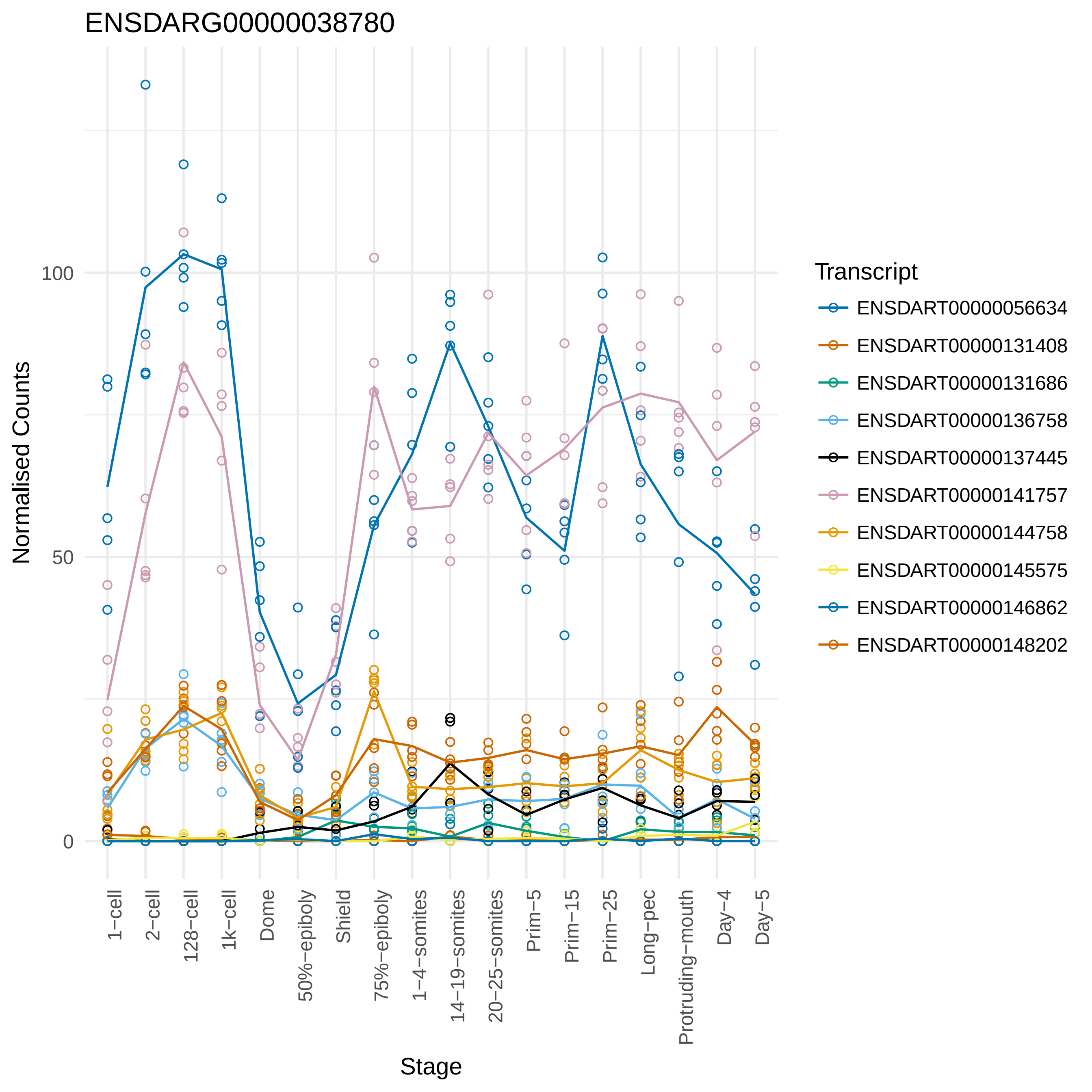

ENSDARG00000038780
Normalised Counts
100
50
0
1−cell
2−cell
128−cell
1k−cell
Dome
50%−epiboly
Shield
75%−epiboly
1−4−somites
14−19−somites
20−25−somites
Prim−5
Prim−15
Prim−25
Long−pec
Protruding−mouth
Day−4
Day−5
Stage
Transcript
ENSDART00000056634
ENSDART00000131408
ENSDART00000131686
ENSDART00000136758
ENSDART00000137445
ENSDART00000141757
ENSDART00000144758
ENSDART00000145575
ENSDART00000146862
ENSDART00000148202

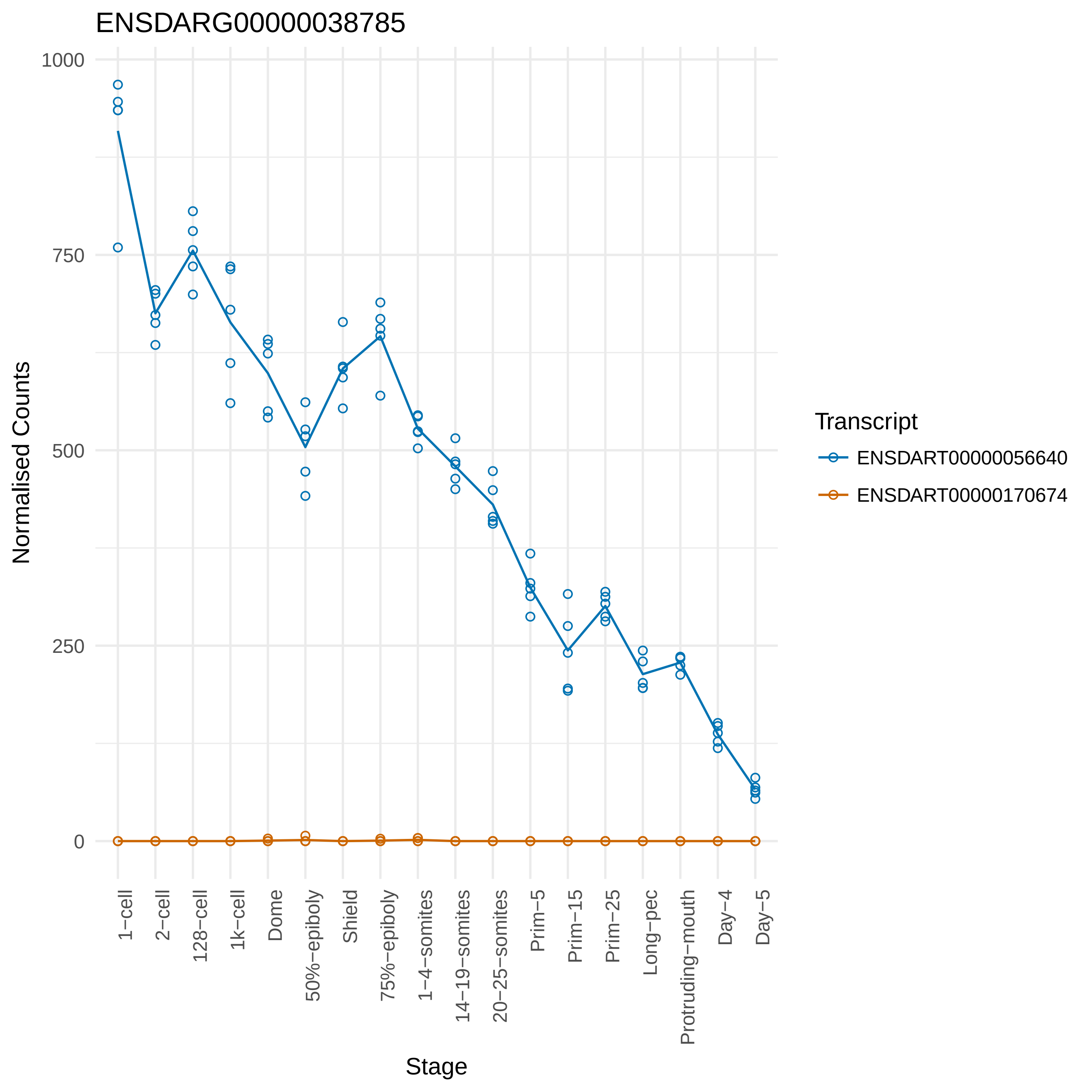
ENSDARG00000038785
Normalised Counts
1000
750
500
250
0
1−cell
2−cell
128−cell
1k−cell
Dome
50%−epiboly
Shield
75%−epiboly
1−4−somites
14−19−somites
20−25−somites
Prim−5
Prim−15
Prim−25
Long−pec
Protruding−mouth
Day−4
Day−5
Stage
Transcript
ENSDART00000056640
ENSDART00000170674

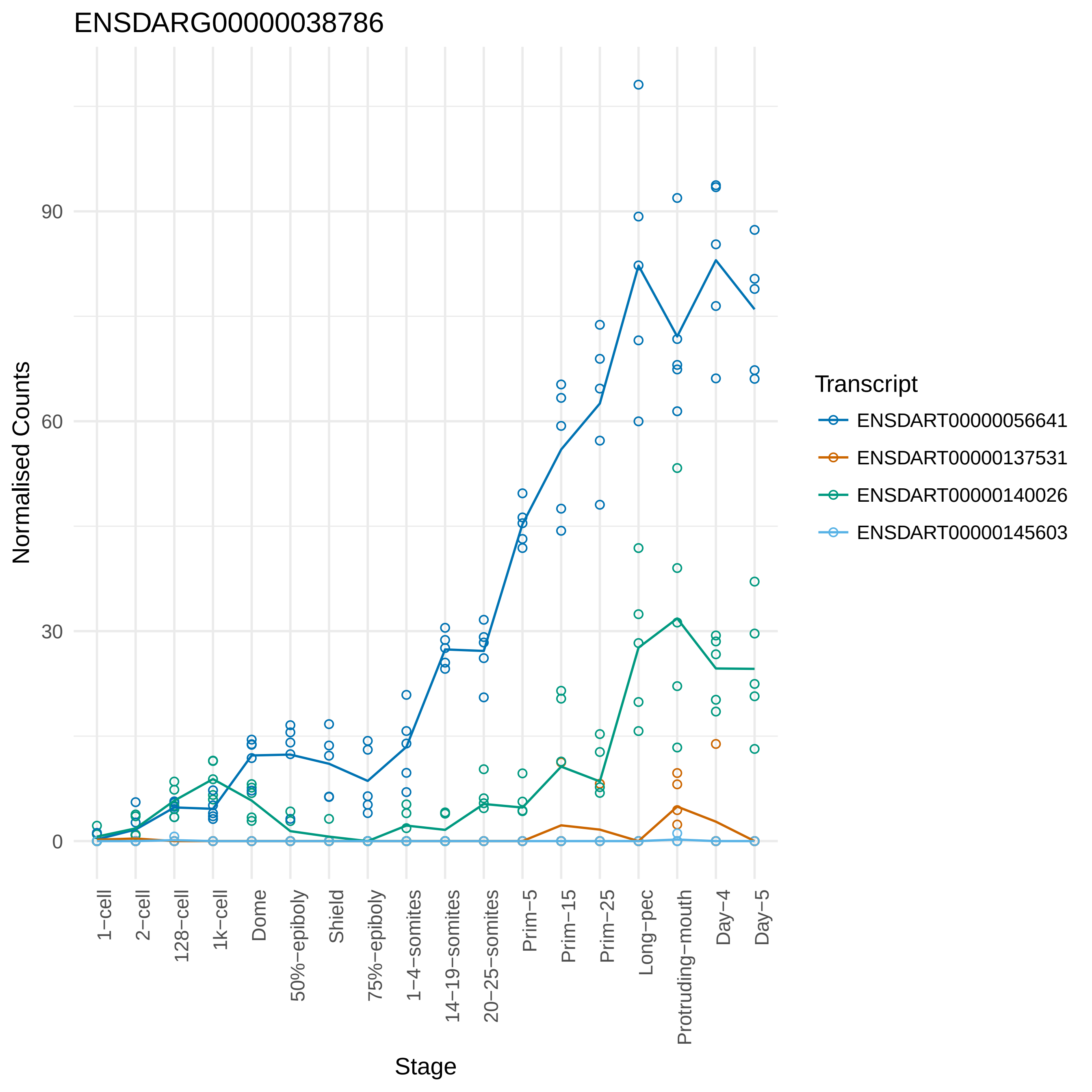
ENSDARG00000038786
Normalised Counts
0
30
60
90
Stage
1−cell
2−cell
128−cell
1k−cell
Dome
50%−epiboly
Shield
75%−epiboly
1−4−somites
14−19−somites
20−25−somites
Prim−5
Prim−15
Prim−25
Long−pec
Protruding−mouth
Day−4
Day−5
Transcript
ENSDART00000056641
ENSDART00000137531
ENSDART00000140026
ENSDART00000145603

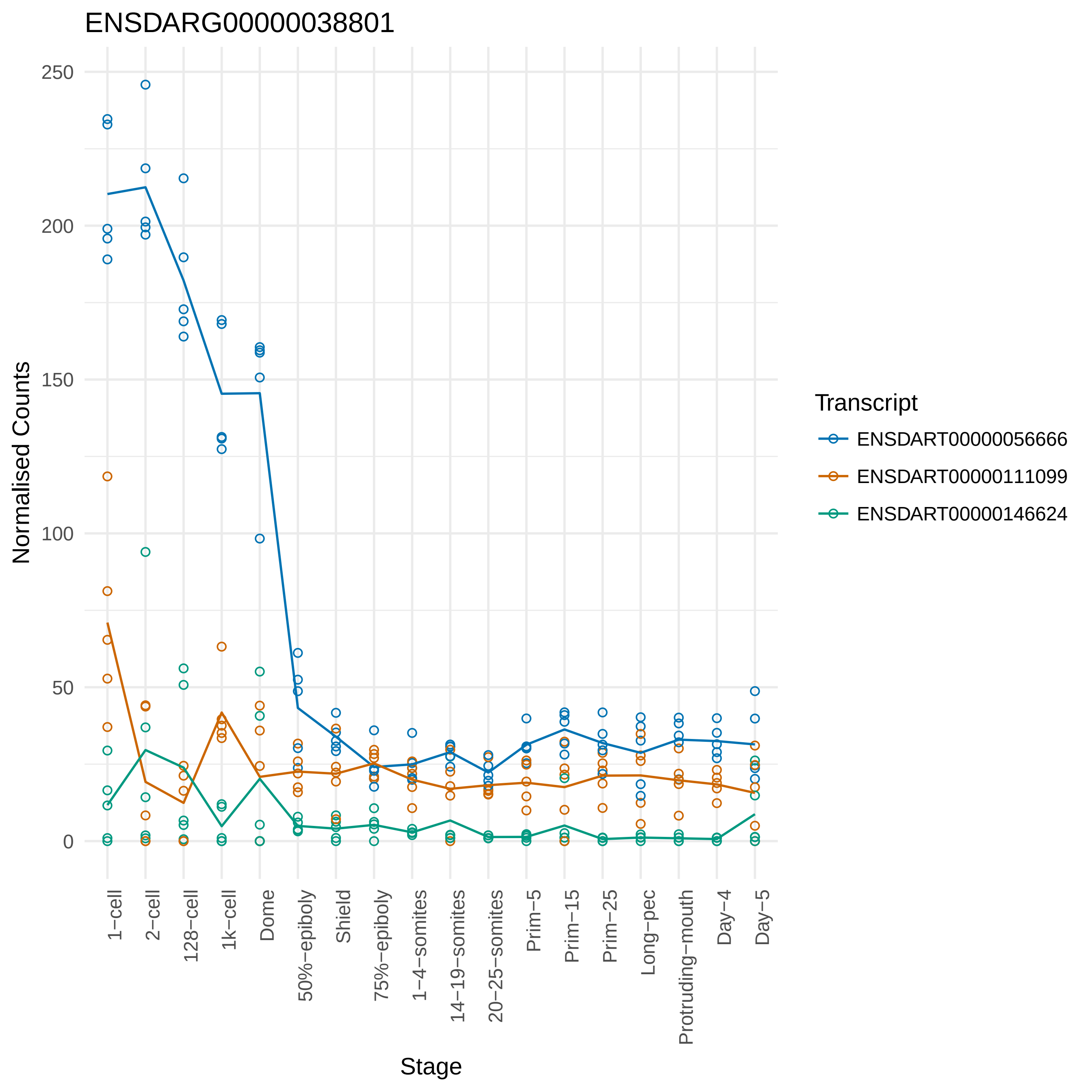
ENSDARG00000038801
Normalised Counts
250
200
150
100
50
0
1−cell
2−cell
128−cell
1k−cell
Dome
50%−epiboly
Shield
75%−epiboly
1−4−somites
14−19−somites
20−25−somites
Prim−5
Prim−15
Prim−25
Long−pec
Protruding−mouth
Day−4
Day−5
Stage
Transcript
ENSDART00000056666
ENSDART00000111099
ENSDART00000146624

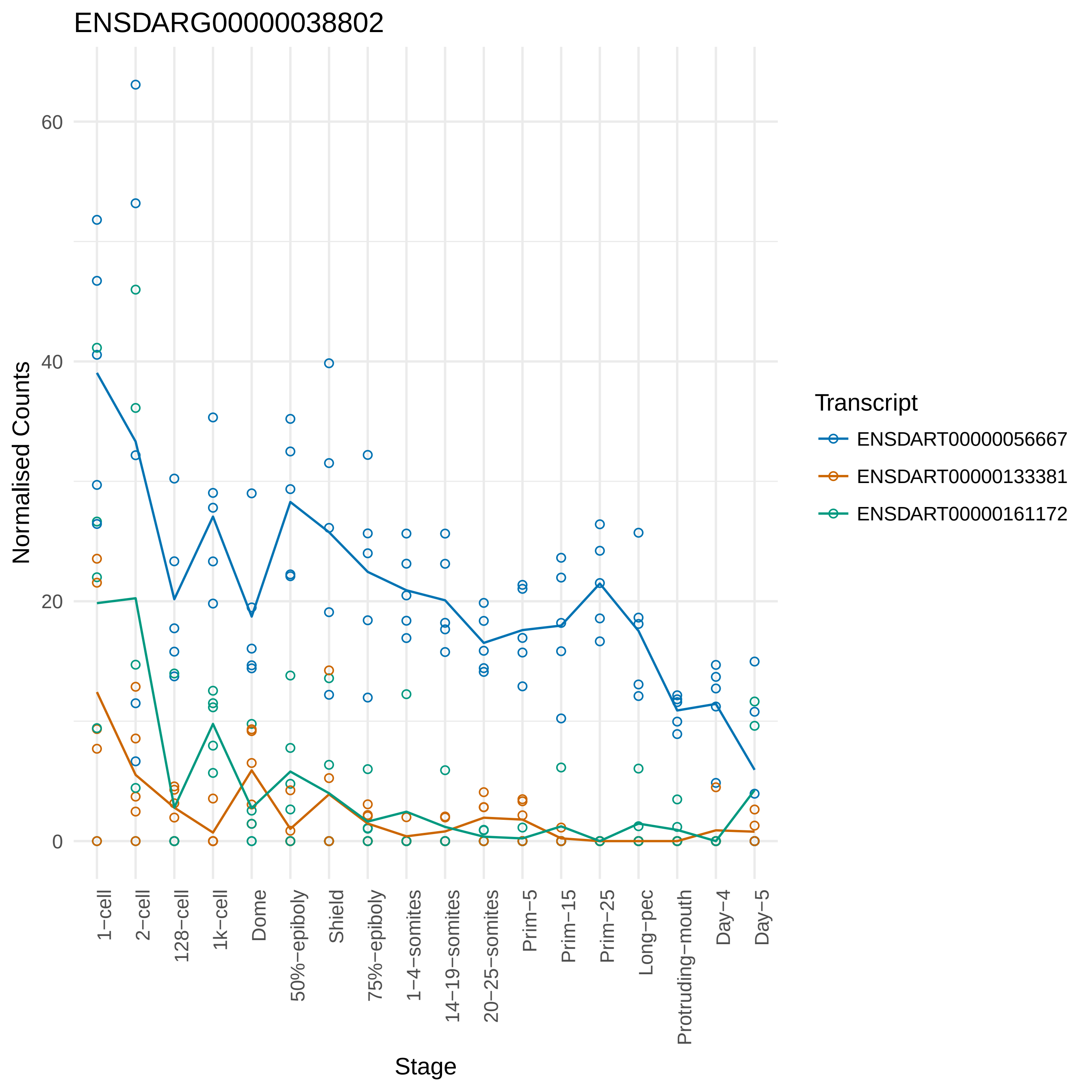
ENSDARG00000038802
Normalised Counts
0
20
40
60
1−cell
2−cell
128−cell
1k−cell
Dome
50%−epiboly
Shield
75%−epiboly
1−4−somites
14−19−somites
20−25−somites
Prim−5
Prim−15
Prim−25
Long−pec
Protruding−mouth
Day−4
Day−5
Stage
Transcript
ENSDART00000056667
ENSDART00000133381
ENSDART00000161172

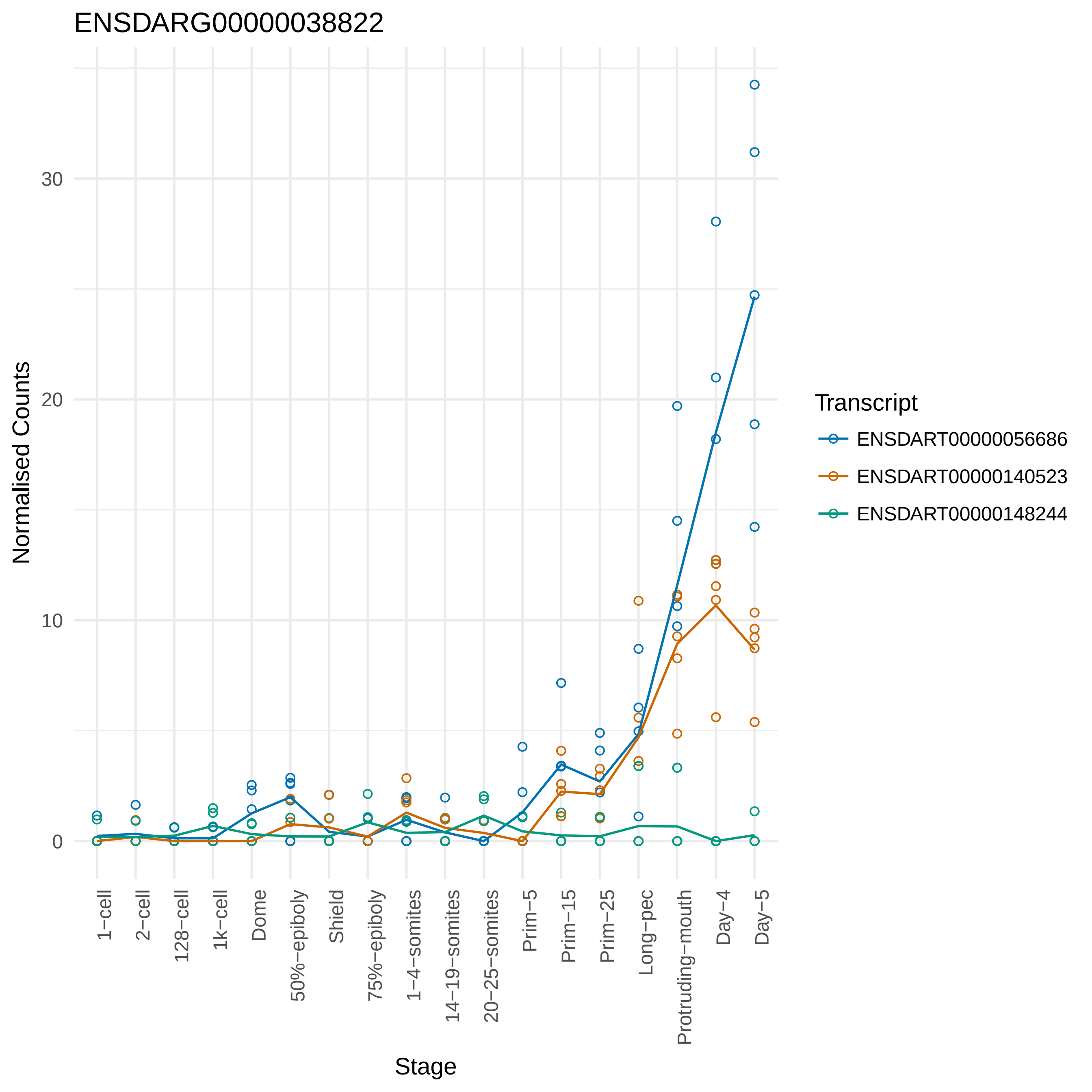

ENSDARG00000038822
Normalised Counts
0
10
20
30
1−cell
2−cell
128−cell
1k−cell
Dome
50%−epiboly
Shield
75%−epiboly
1−4−somites
14−19−somites
20−25−somites
Prim−5
Prim−15
Prim−25
Long−pec
Protruding−mouth
Day−4
Day−5
Stage
Transcript
ENSDART00000056686
ENSDART00000140523
ENSDART00000148244

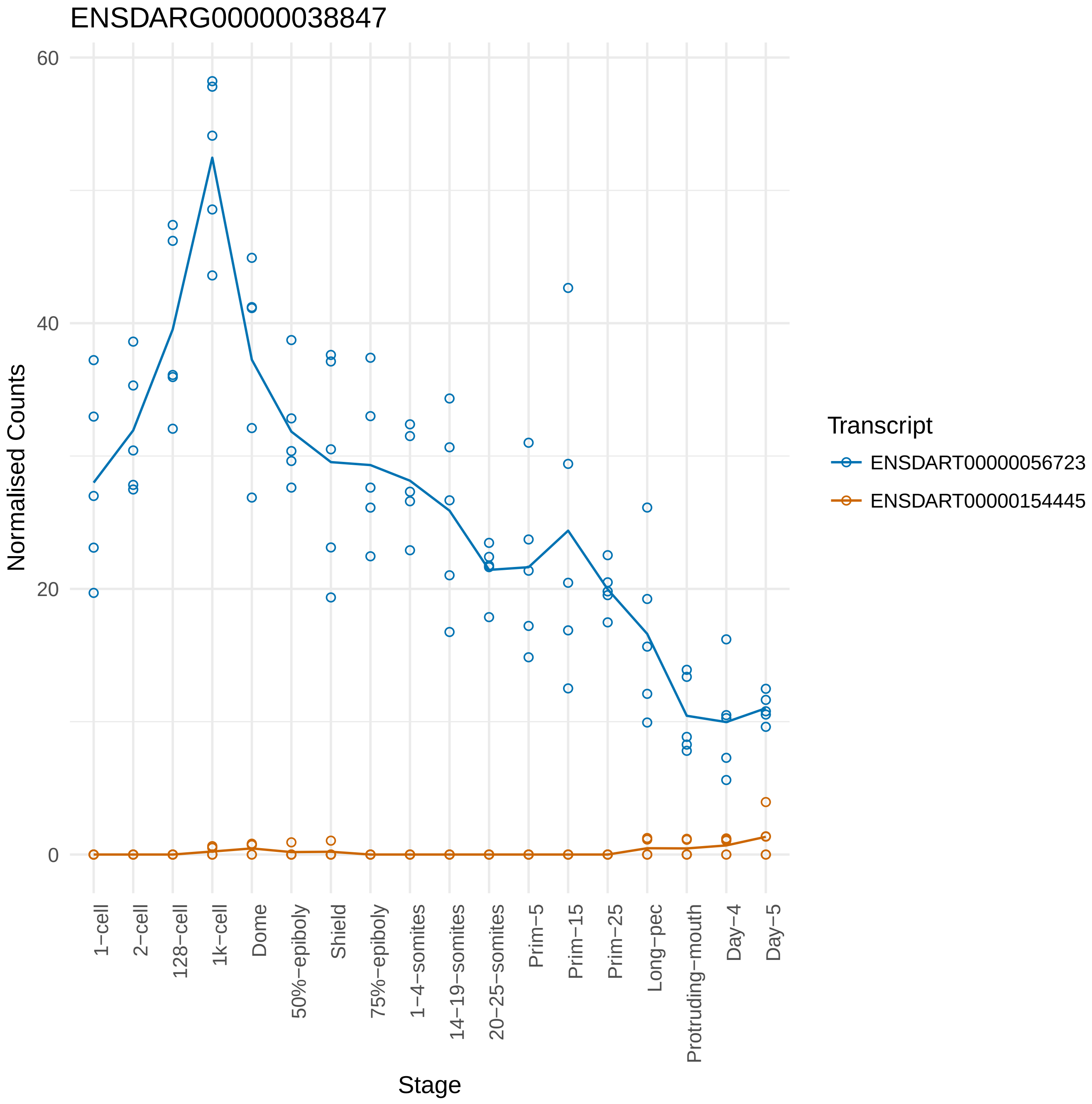
ENSDARG00000038847
Normalised Counts
60
40
20
0
1−cell
2−cell
128−cell
1k−cell
Dome
50%−epiboly
Shield
75%−epiboly
1−4−somites
14−19−somites
20−25−somites
Prim−5
Prim−15
Prim−25
Long−pec
Protruding−mouth
Day−4
Day−5
Stage
Transcript
ENSDART00000056723
ENSDART00000154445

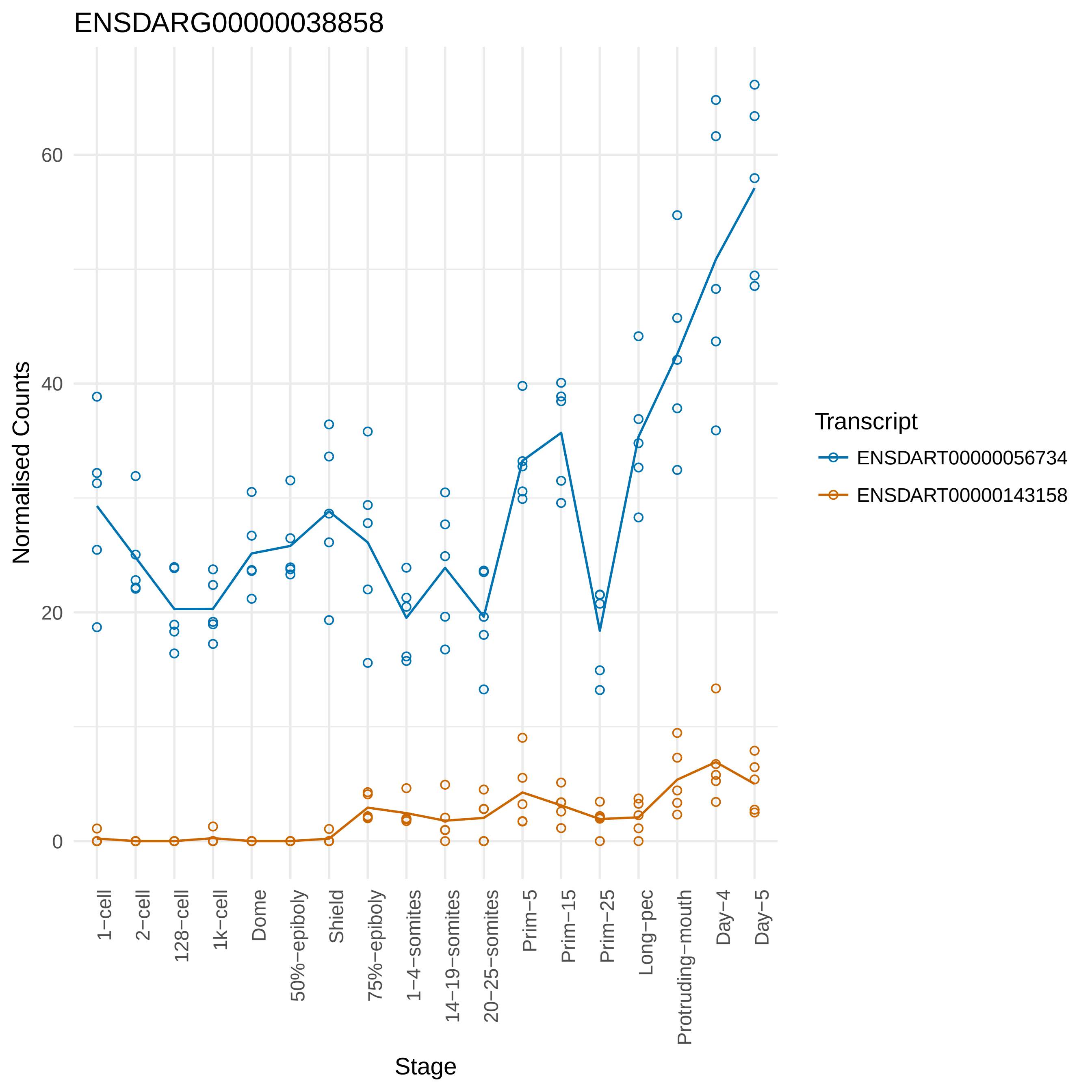

ENSDARG00000038858
Normalised Counts
0
20
40
60
Stage
1−cell
2−cell
128−cell
1k−cell
Dome
50%−epiboly
Shield
75%−epiboly
1−4−somites
14−19−somites
20−25−somites
Prim−5
Prim−15
Prim−25
Long−pec
Protruding−mouth
Day−4
Day−5
Transcript
ENSDART00000056734
ENSDART00000143158

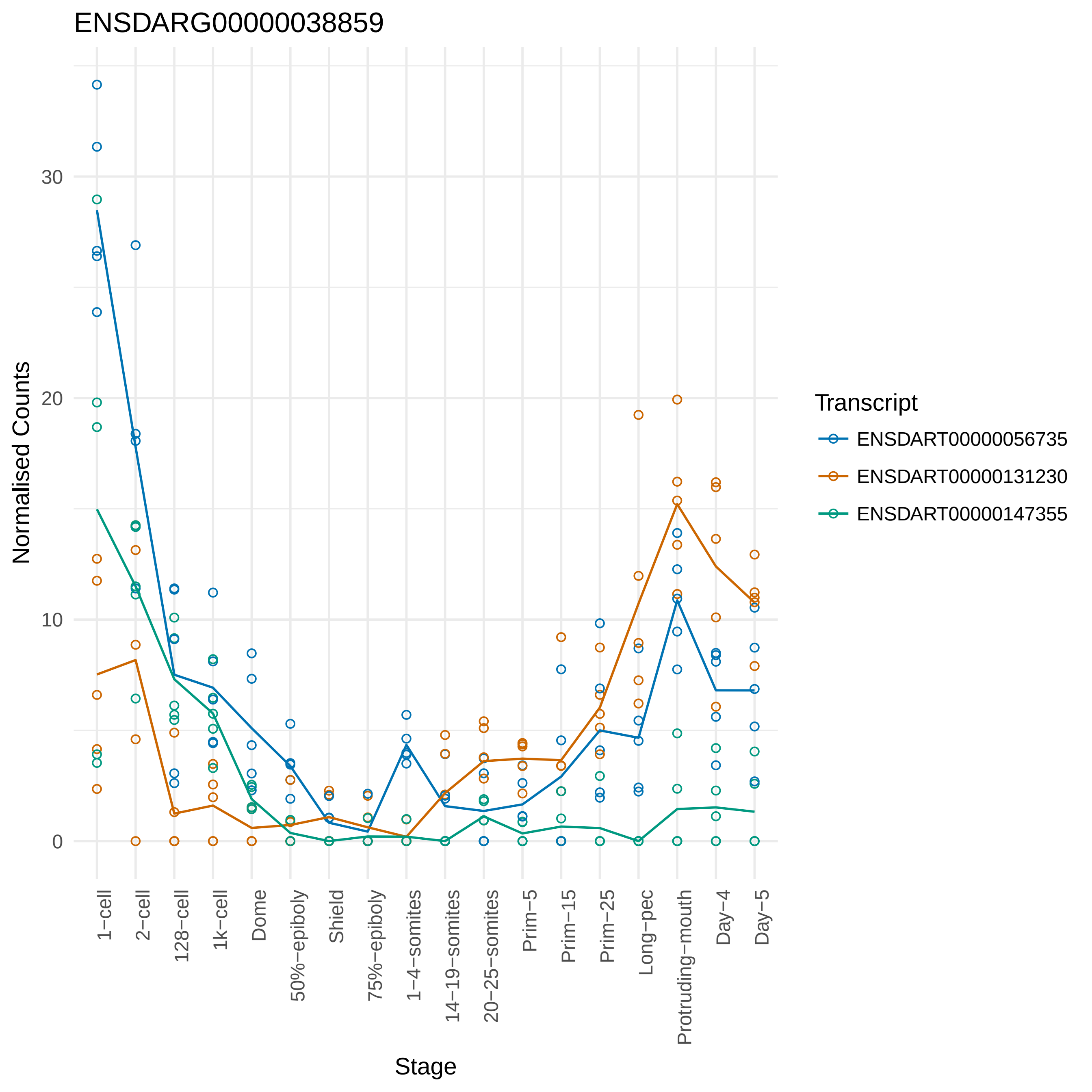

ENSDARG00000038859
Normalised Counts
30
20
10
0
1−cell
2−cell
128−cell
1k−cell
Dome
50%−epiboly
Shield
75%−epiboly
1−4−somites
14−19−somites
20−25−somites
Prim−5
Prim−15
Prim−25
Long−pec
Protruding−mouth
Day−4
Day−5
Stage
Transcript
ENSDART00000056735
ENSDART00000131230
ENSDART00000147355

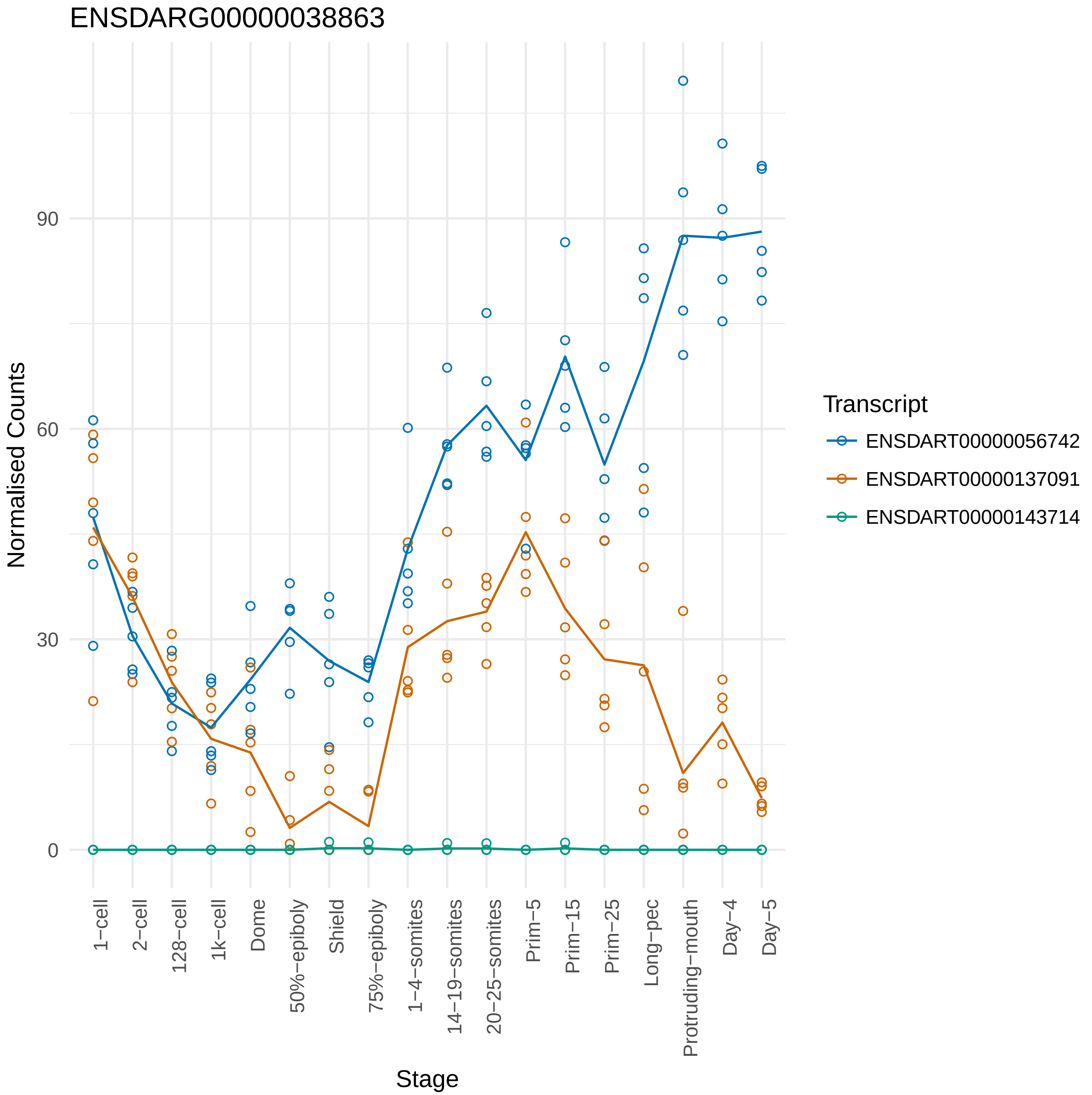
ENSDARG00000038863
Normalised Counts
0
30
60
90
1−cell
2−cell
128−cell
1k−cell
Dome
50%−epiboly
Shield
75%−epiboly
1−4−somites
14−19−somites
20−25−somites
Prim−5
Prim−15
Prim−25
Long−pec
Protruding−mouth
Day−4
Day−5
Stage
Transcript
ENSDART00000056742
ENSDART00000137091
ENSDART00000143714

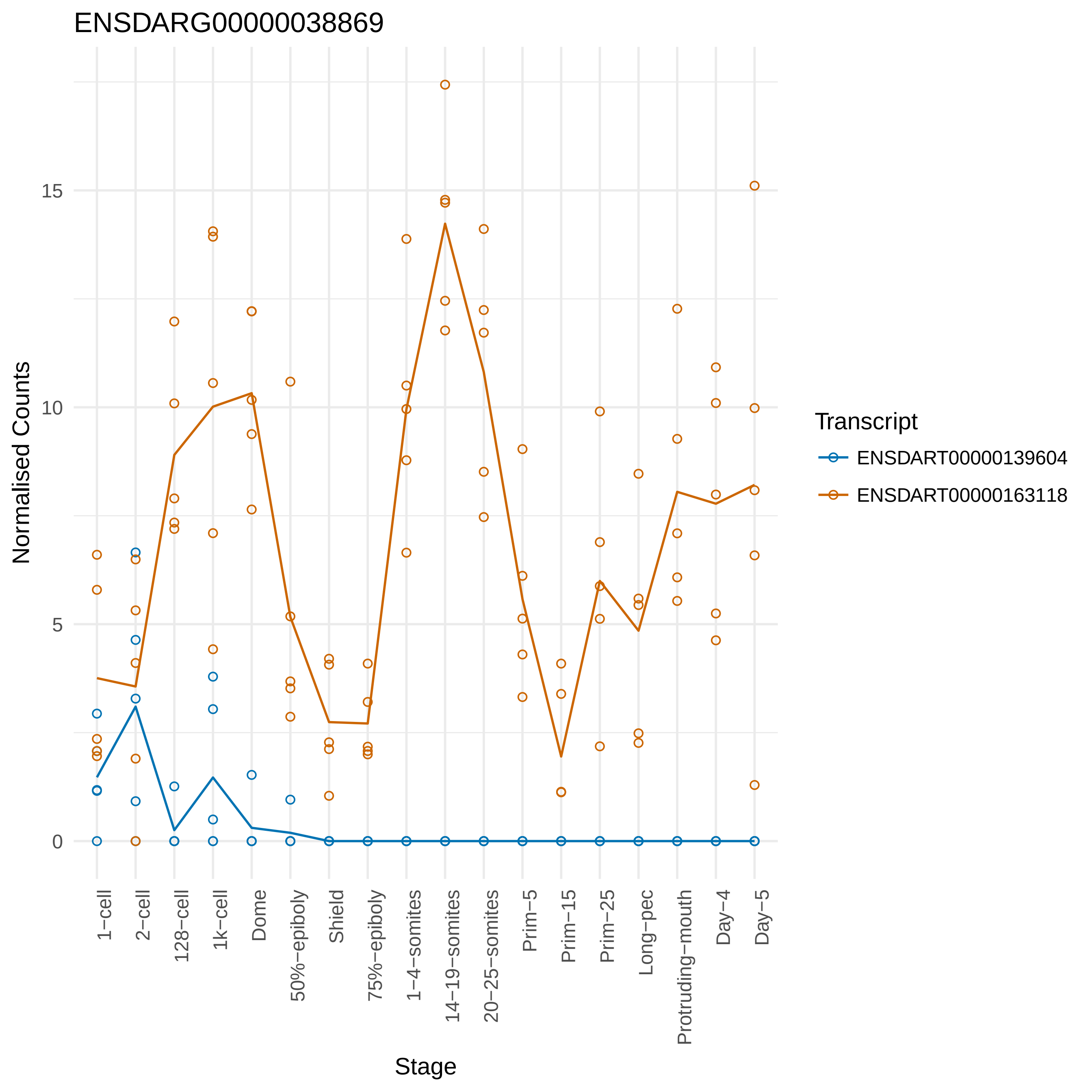

ENSDARG00000038869
Normalised Counts
15
10
5
0
1−cell
2−cell
128−cell
1k−cell
Dome
50%−epiboly
Shield
75%−epiboly
1−4−somites
14−19−somites
20−25−somites
Prim−5
Prim−15
Prim−25
Long−pec
Protruding−mouth
Day−4
Day−5
Stage
Transcript
ENSDART00000139604
ENSDART00000163118

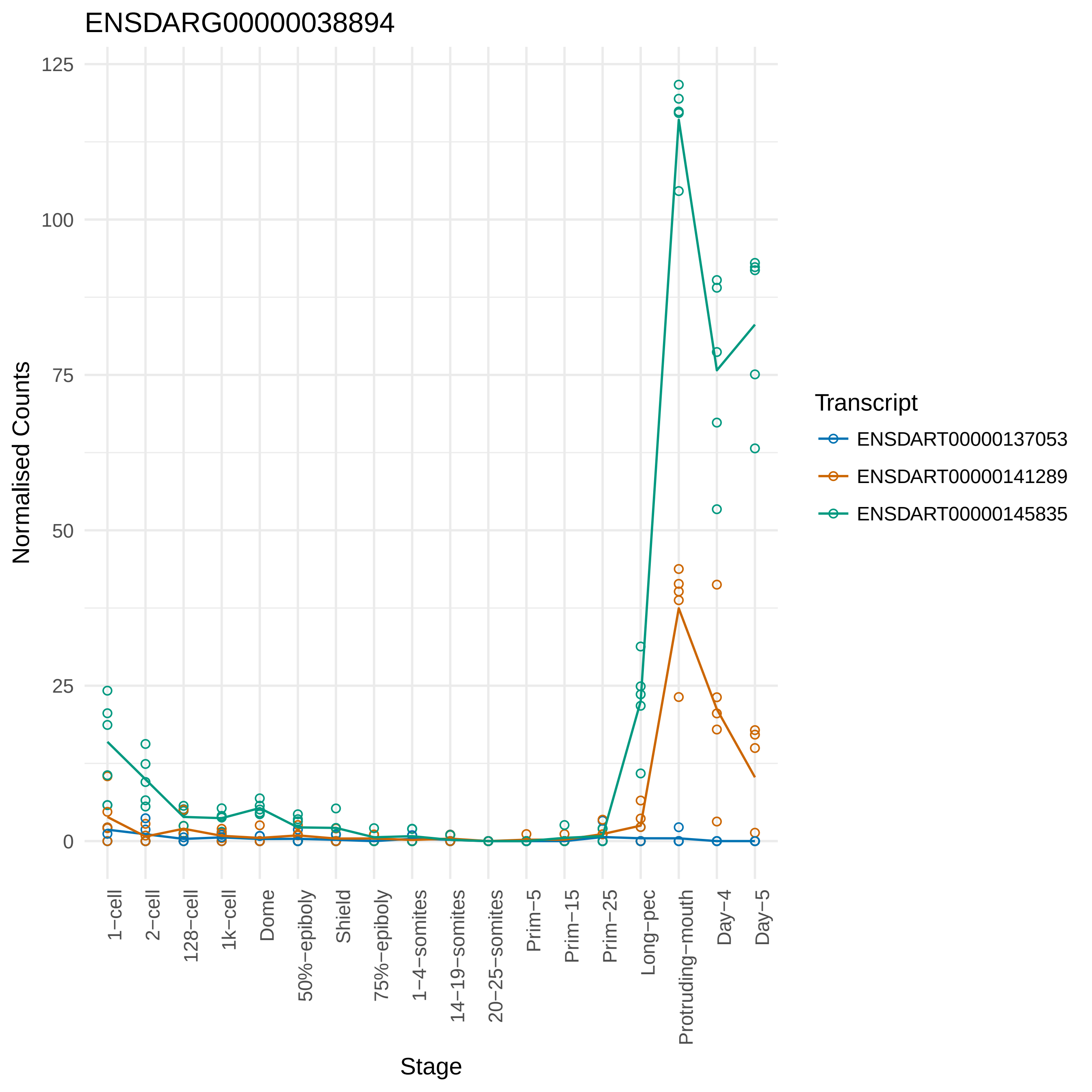

ENSDARG00000038894
Normalised Counts
125
100
75
50
25
0
1−cell
2−cell
128−cell
1k−cell
Dome
50%−epiboly
Shield
75%−epiboly
1−4−somites
14−19−somites
20−25−somites
Prim−5
Prim−15
Prim−25
Long−pec
Protruding−mouth
Day−4
Day−5
Stage
Transcript
ENSDART00000137053
ENSDART00000141289
ENSDART00000145835

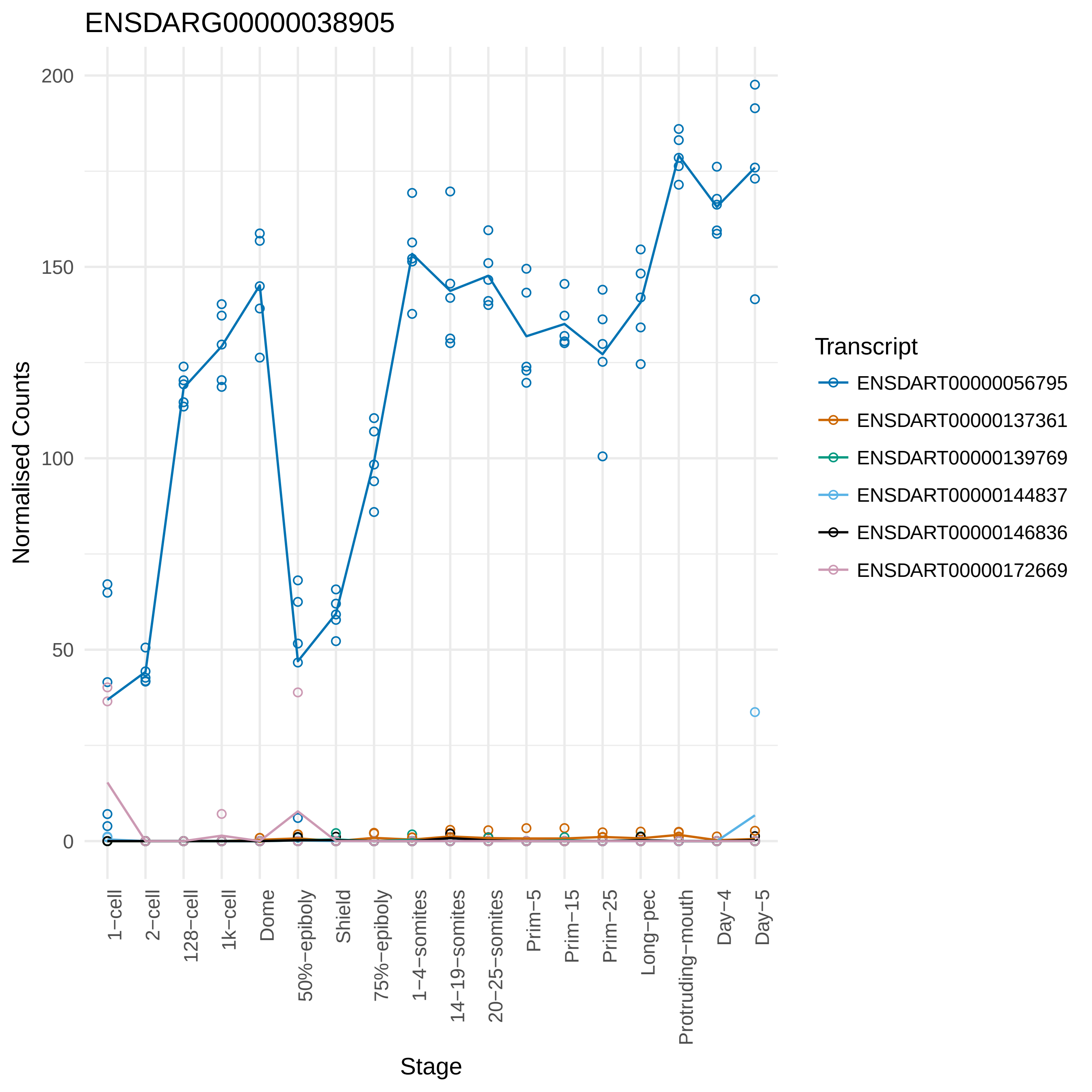

ENSDARG00000038905
Normalised Counts
200
150
100
50
0
1−cell
2−cell
128−cell
1k−cell
Dome
50%−epiboly
Shield
75%−epiboly
1−4−somites
14−19−somites
20−25−somites
Prim−5
Prim−15
Prim−25
Long−pec
Protruding−mouth
Day−4
Day−5
Stage
Transcript
ENSDART00000056795
ENSDART00000137361
ENSDART00000139769
ENSDART00000144837
ENSDART00000146836
ENSDART00000172669

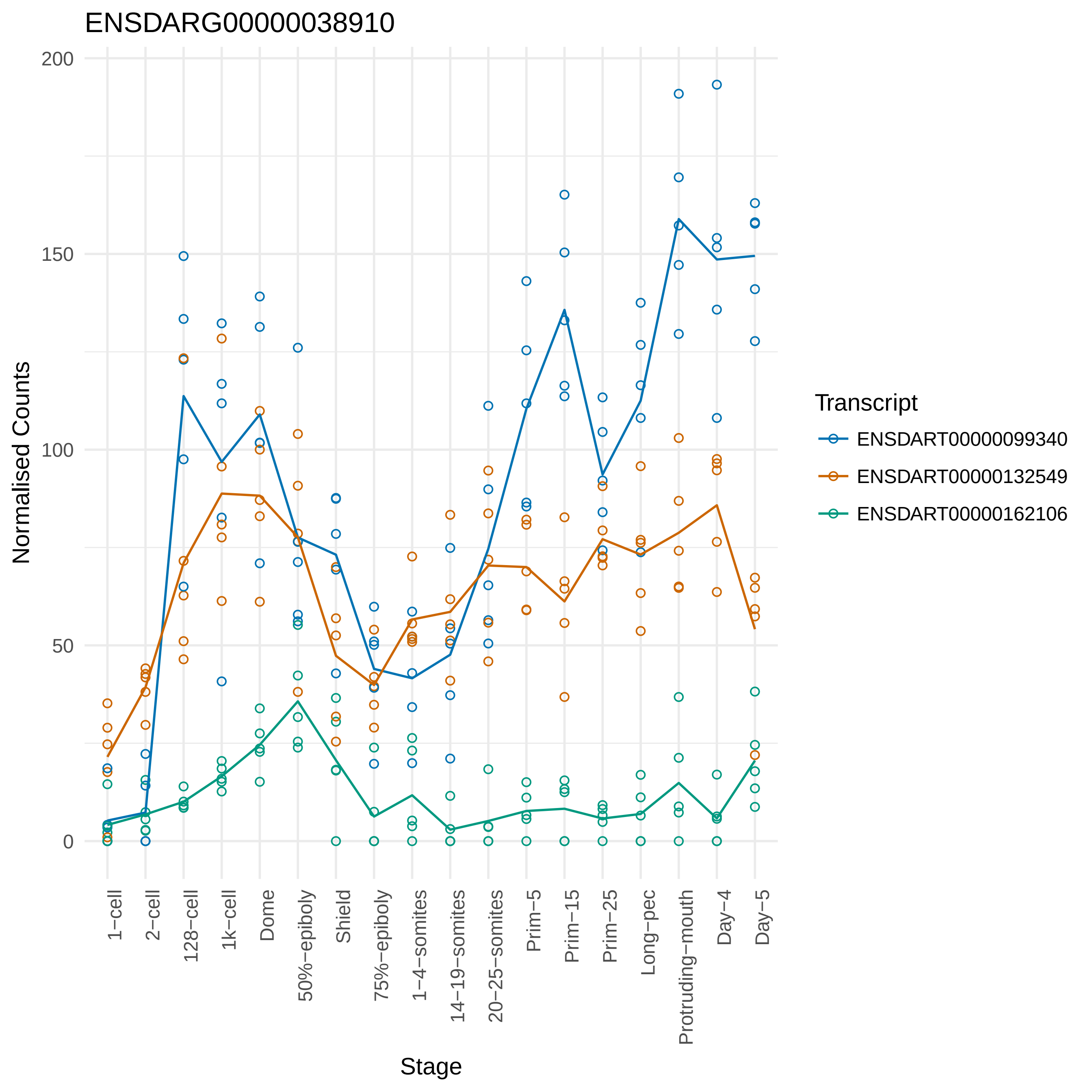
ENSDARG00000038910
Normalised Counts
200
150
100
50
0
1−cell
2−cell
128−cell
1k−cell
Dome
50%−epiboly
Shield
75%−epiboly
1−4−somites
14−19−somites
20−25−somites
Prim−5
Prim−15
Prim−25
Long−pec
Protruding−mouth
Day−4
Day−5
Stage
Transcript
ENSDART00000099340
ENSDART00000132549
ENSDART00000162106

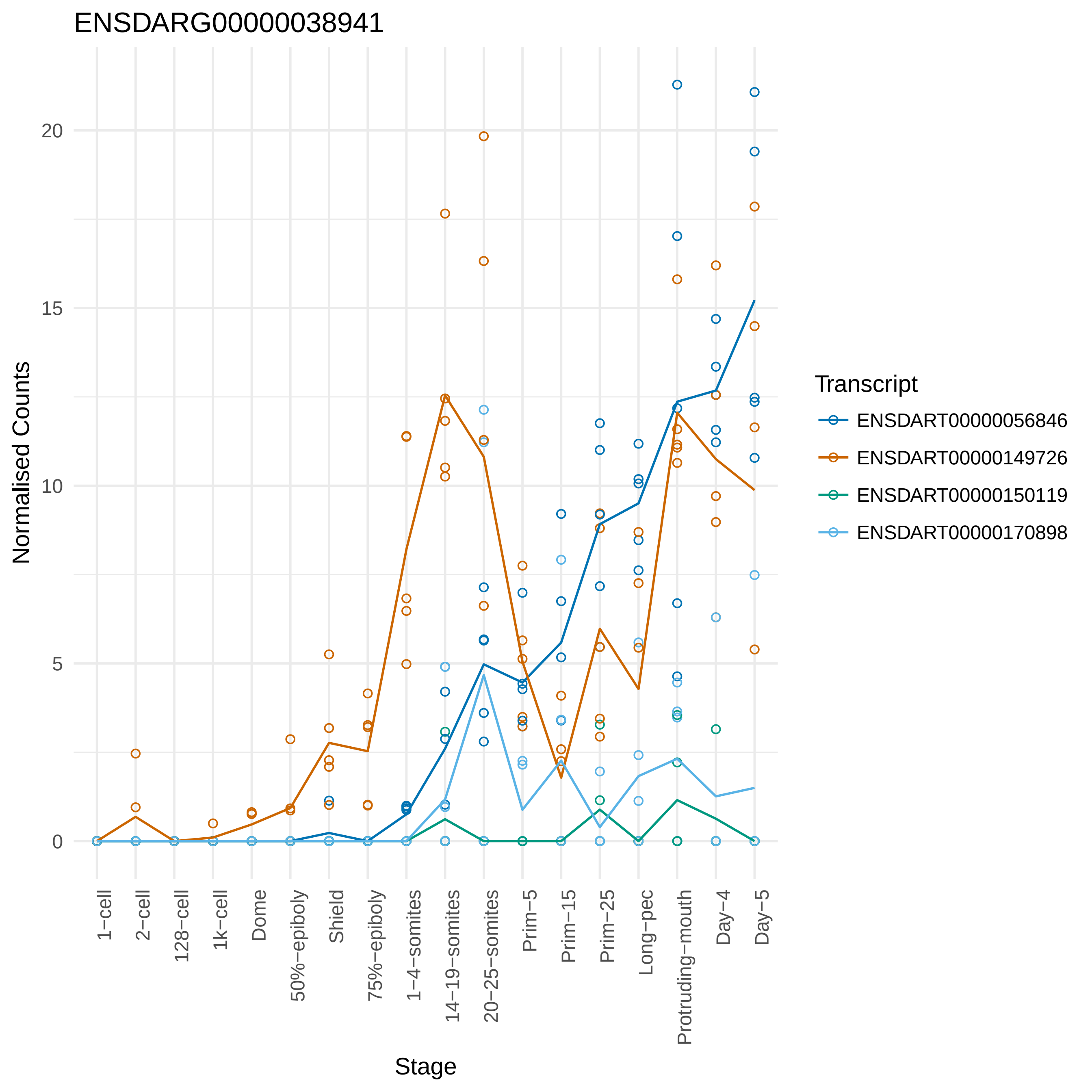

ENSDARG00000038941
Normalised Counts
0
5
10
15
20
Stage
1−cell
2−cell
128−cell
1k−cell
Dome
50%−epiboly
Shield
75%−epiboly
1−4−somites
14−19−somites
20−25−somites
Prim−5
Prim−15
Prim−25
Long−pec
Protruding−mouth
Day−4
Day−5
Transcript
ENSDART00000056846
ENSDART00000149726
ENSDART00000150119
ENSDART00000170898

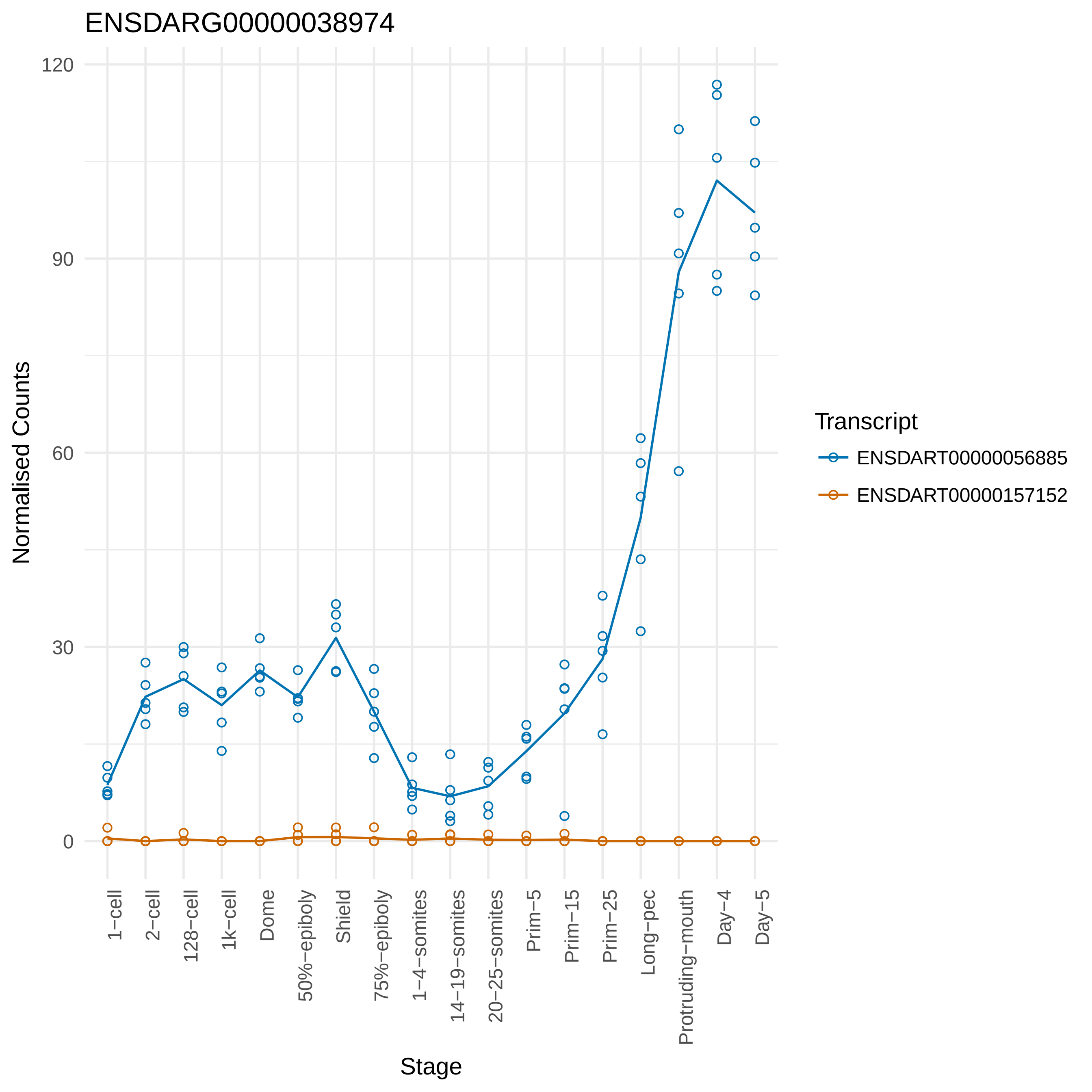
ENSDARG00000038974
Normalised Counts
120
90
60
30
0
1−cell
2−cell
128−cell
1k−cell
Dome
50%−epiboly
Shield
75%−epiboly
1−4−somites
14−19−somites
20−25−somites
Prim−5
Prim−15
Prim−25
Long−pec
Protruding−mouth
Day−4
Day−5
Stage
Transcript
ENSDART00000056885
ENSDART00000157152

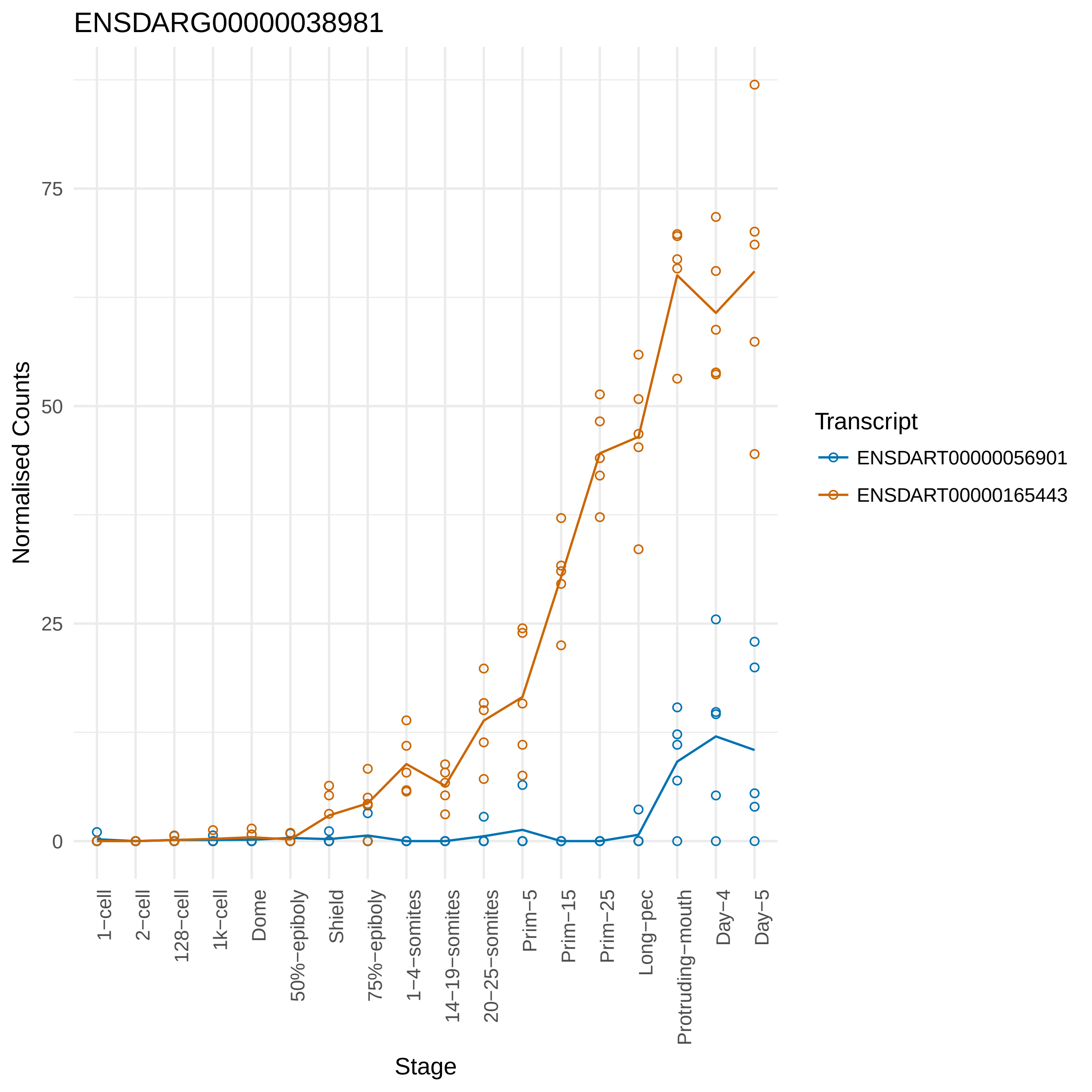
ENSDARG00000038981
Normalised Counts
75
50
25
0
1−cell
2−cell
128−cell
1k−cell
Dome
50%−epiboly
Shield
75%−epiboly
1−4−somites
14−19−somites
20−25−somites
Prim−5
Prim−15
Prim−25
Long−pec
Protruding−mouth
Day−4
Day−5
Stage
Transcript
ENSDART00000056901
ENSDART00000165443

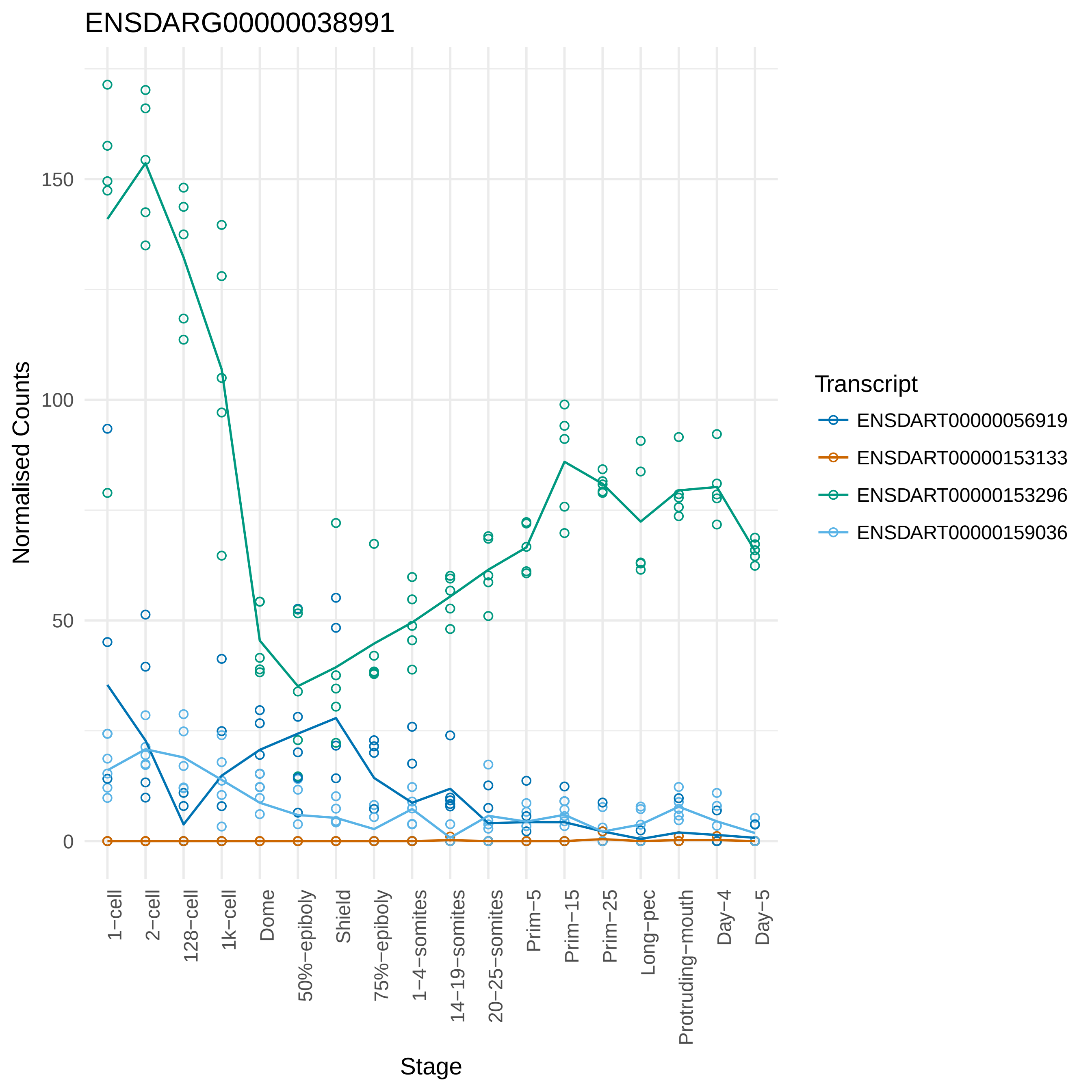
ENSDARG00000038991
Normalised Counts
150
100
50
0
1−cell
2−cell
128−cell
1k−cell
Dome
50%−epiboly
Shield
75%−epiboly
1−4−somites
14−19−somites
20−25−somites
Prim−5
Prim−15
Prim−25
Long−pec
Protruding−mouth
Day−4
Day−5
Stage
Transcript
ENSDART00000056919
ENSDART00000153133
ENSDART00000153296
ENSDART00000159036

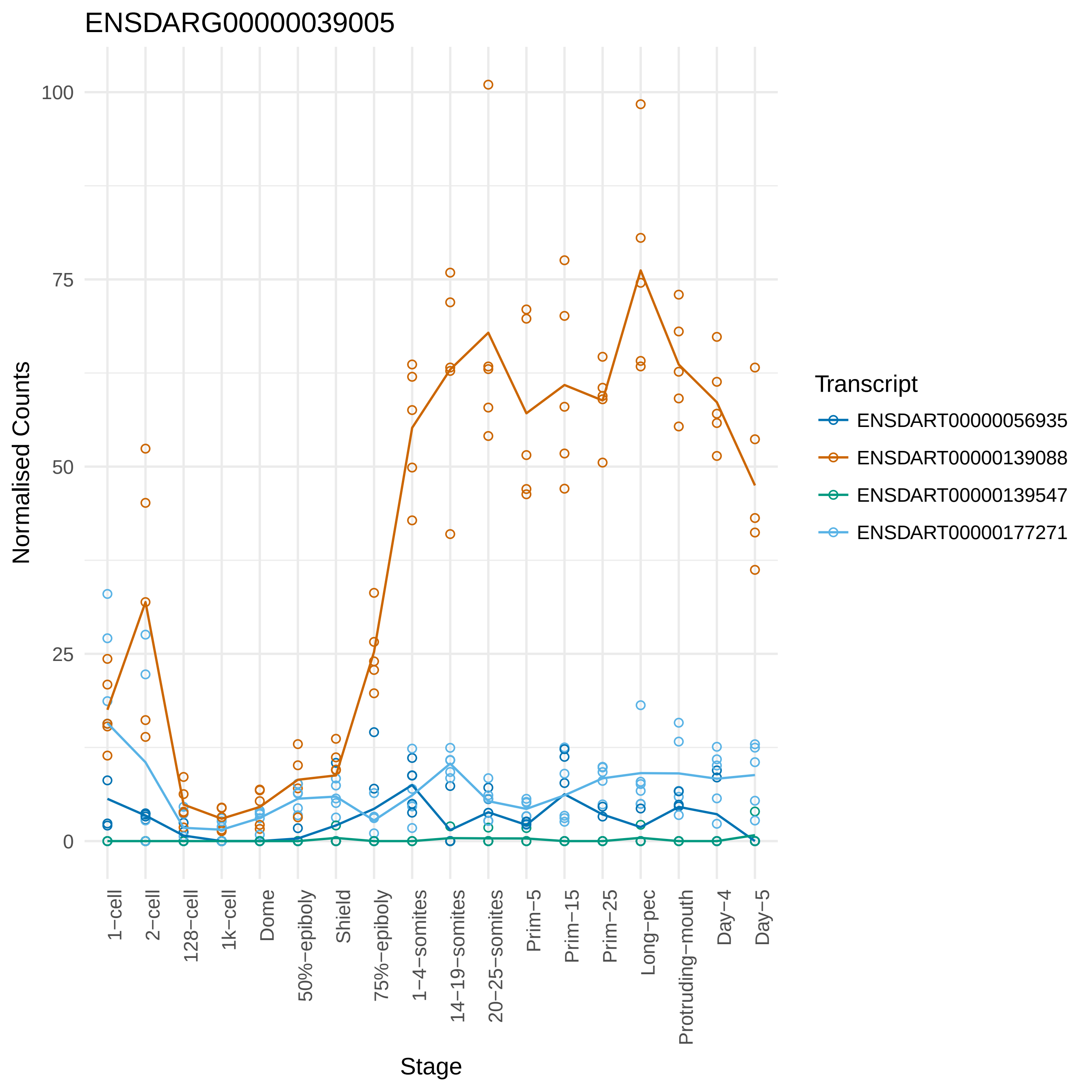

ENSDARG00000039005
Normalised Counts
100
75
50
25
0
1−cell
2−cell
128−cell
1k−cell
Dome
50%−epiboly
Shield
75%−epiboly
1−4−somites
14−19−somites
20−25−somites
Prim−5
Prim−15
Prim−25
Long−pec
Protruding−mouth
Day−4
Day−5
Stage
Transcript
ENSDART00000056935
ENSDART00000139088
ENSDART00000139547
ENSDART00000177271

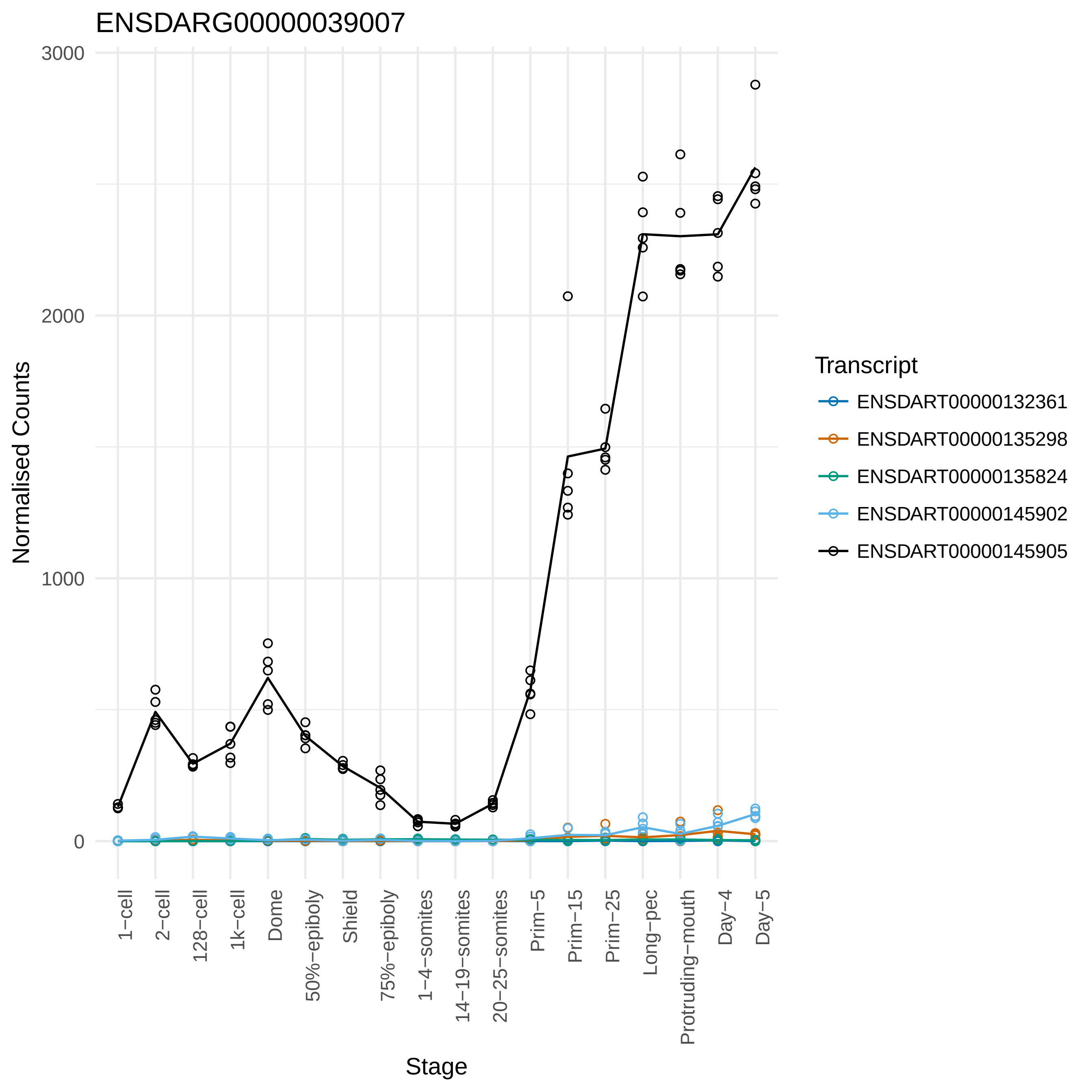

ENSDARG00000039007
Normalised Counts
3000
2000
1000
0
Stage
1−cell
2−cell
128−cell
1k−cell
Dome
50%−epiboly
Shield
75%−epiboly
1−4−somites
14−19−somites
20−25−somites
Prim−5
Prim−15
Prim−25
Long−pec
Protruding−mouth
Day−4
Day−5
Transcript
ENSDART00000132361
ENSDART00000135298
ENSDART00000135824
ENSDART00000145902
ENSDART00000145905

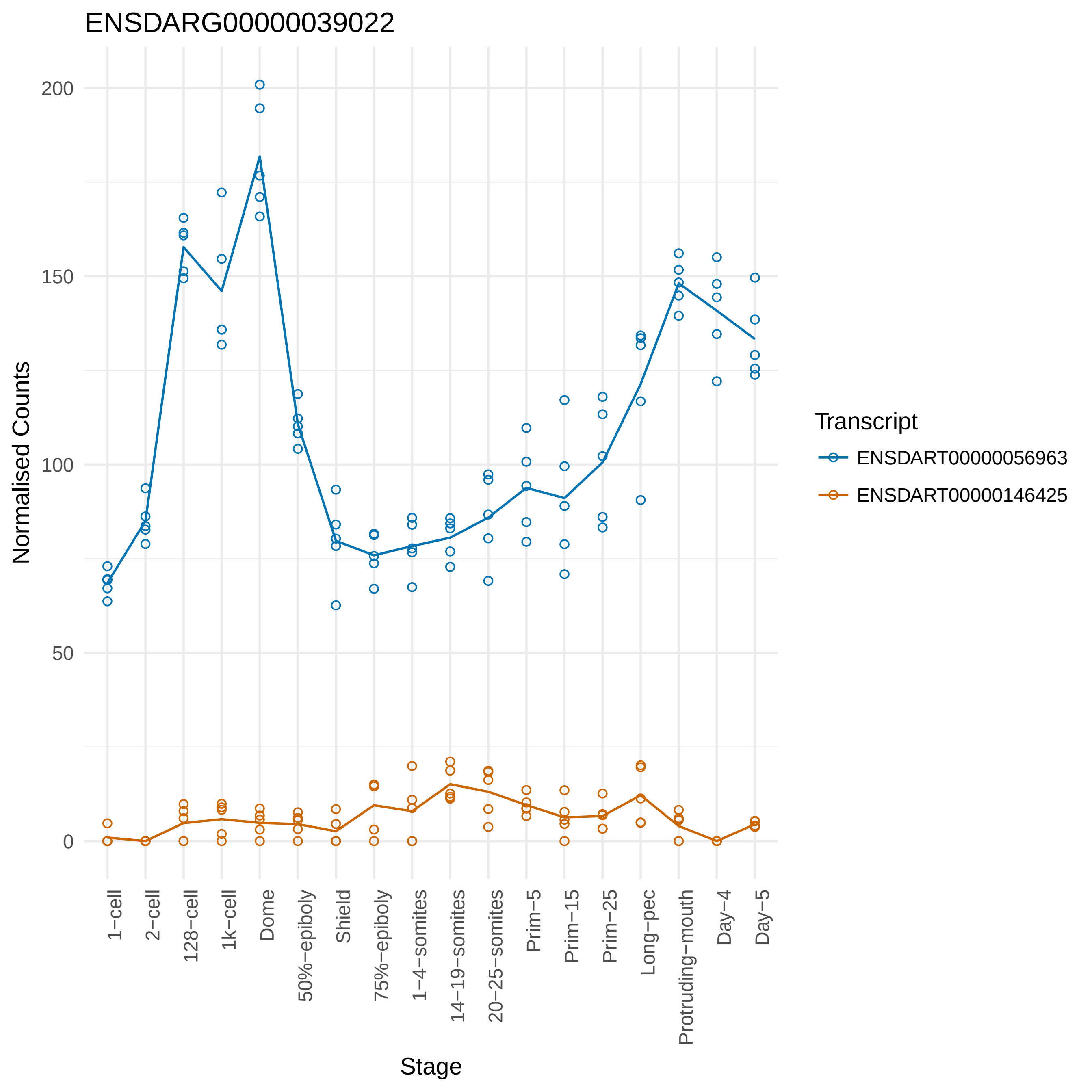
ENSDARG00000039022
Normalised Counts
200
150
100
50
0
1−cell
2−cell
128−cell
1k−cell
Dome
50%−epiboly
Shield
75%−epiboly
1−4−somites
14−19−somites
20−25−somites
Prim−5
Prim−15
Prim−25
Long−pec
Protruding−mouth
Day−4
Day−5
Stage
Transcript
ENSDART00000056963
ENSDART00000146425

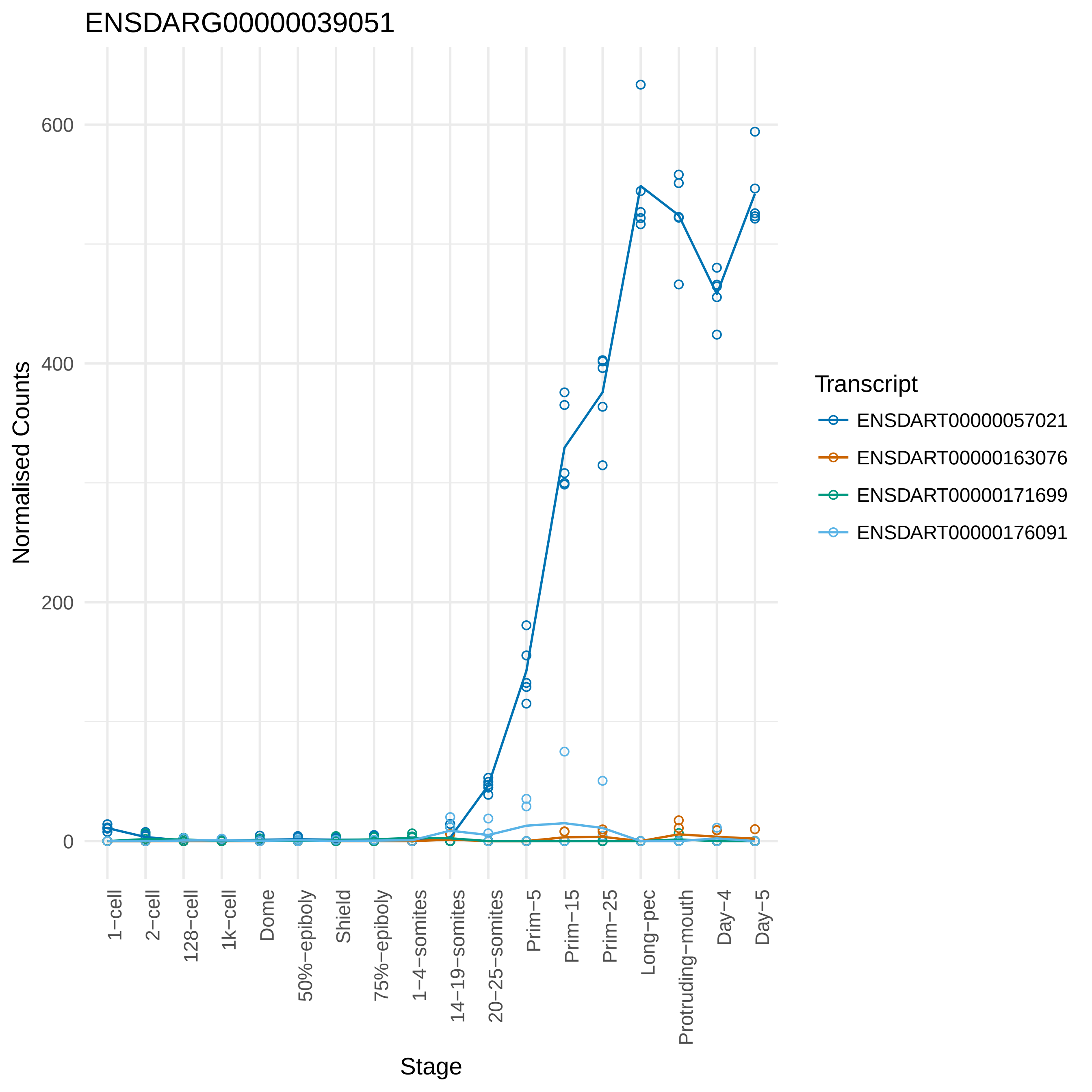

ENSDARG00000039051
Normalised Counts
600
400
200
0
1−cell
2−cell
128−cell
1k−cell
Dome
50%−epiboly
Shield
75%−epiboly
1−4−somites
14−19−somites
20−25−somites
Prim−5
Prim−15
Prim−25
Long−pec
Protruding−mouth
Day−4
Day−5
Stage
Transcript
ENSDART00000057021
ENSDART00000163076
ENSDART00000171699
ENSDART00000176091

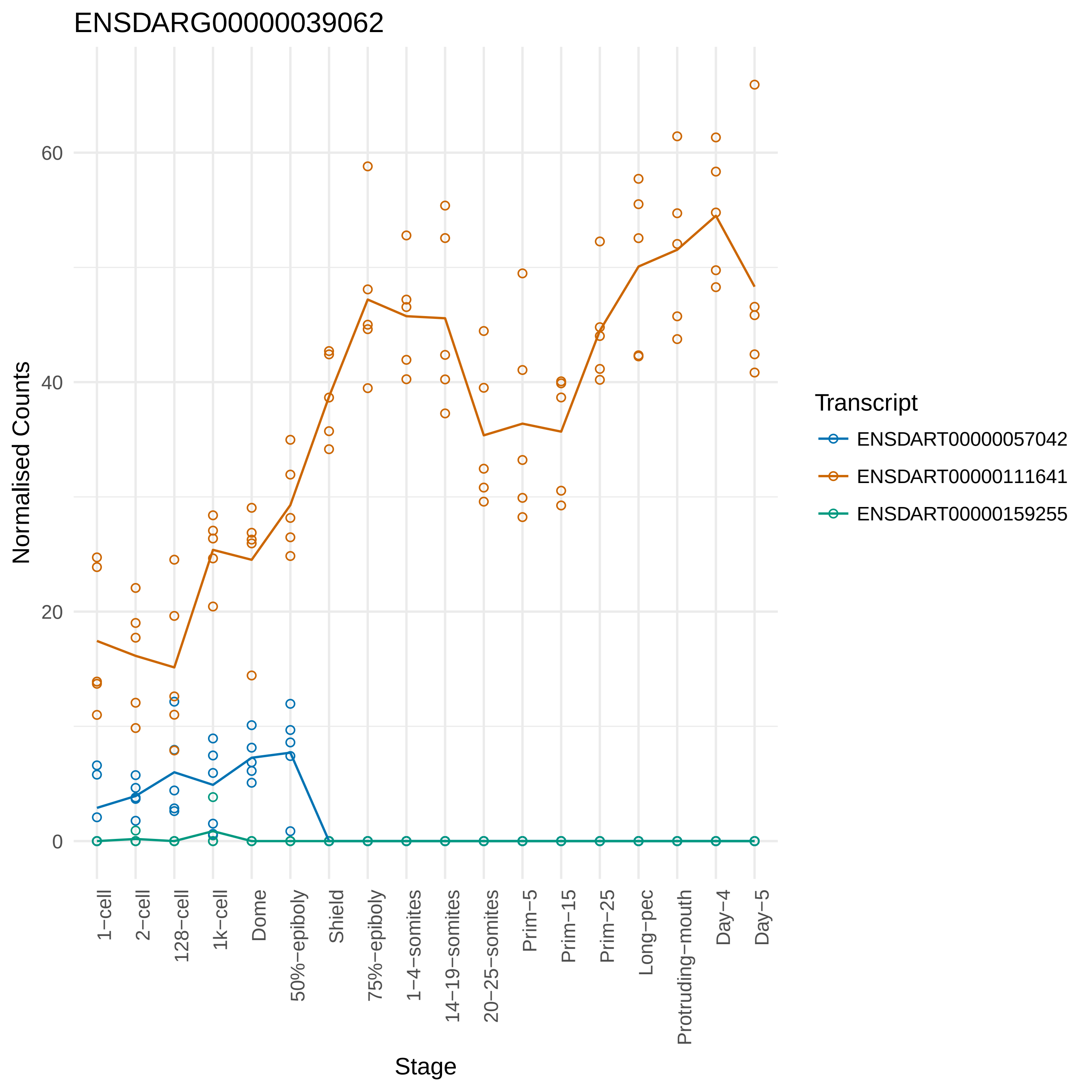
ENSDARG00000039062
Normalised Counts
60
40
20
0
1−cell
2−cell
128−cell
1k−cell
Dome
50%−epiboly
Shield
75%−epiboly
1−4−somites
14−19−somites
20−25−somites
Prim−5
Prim−15
Prim−25
Long−pec
Protruding−mouth
Day−4
Day−5
Stage
Transcript
ENSDART00000057042
ENSDART00000111641
ENSDART00000159255

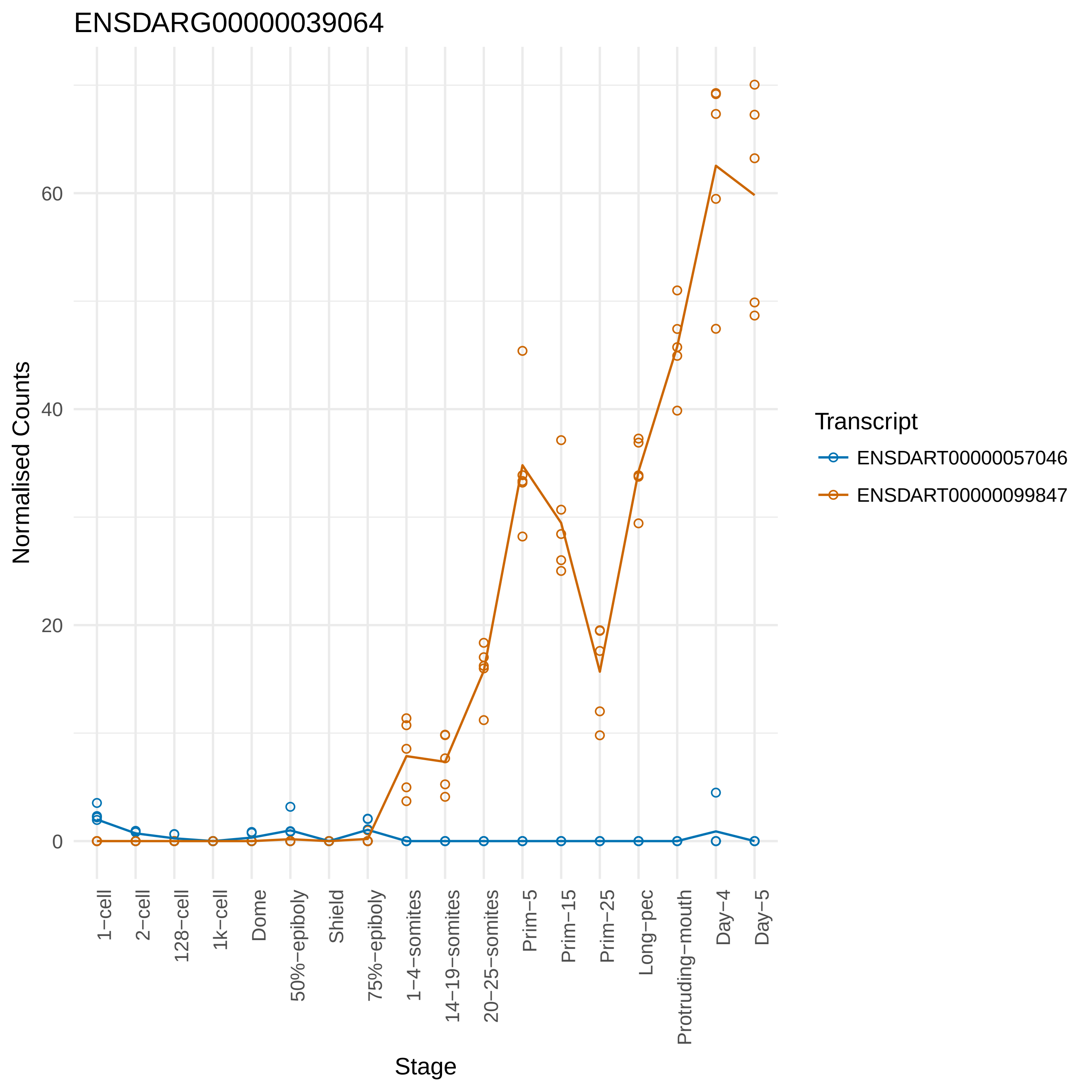

ENSDARG00000039064
Normalised Counts
60
40
20
0
1−cell
2−cell
128−cell
1k−cell
Dome
50%−epiboly
Shield
75%−epiboly
1−4−somites
14−19−somites
20−25−somites
Prim−5
Prim−15
Prim−25
Long−pec
Protruding−mouth
Day−4
Day−5
Stage
Transcript
ENSDART00000057046
ENSDART00000099847

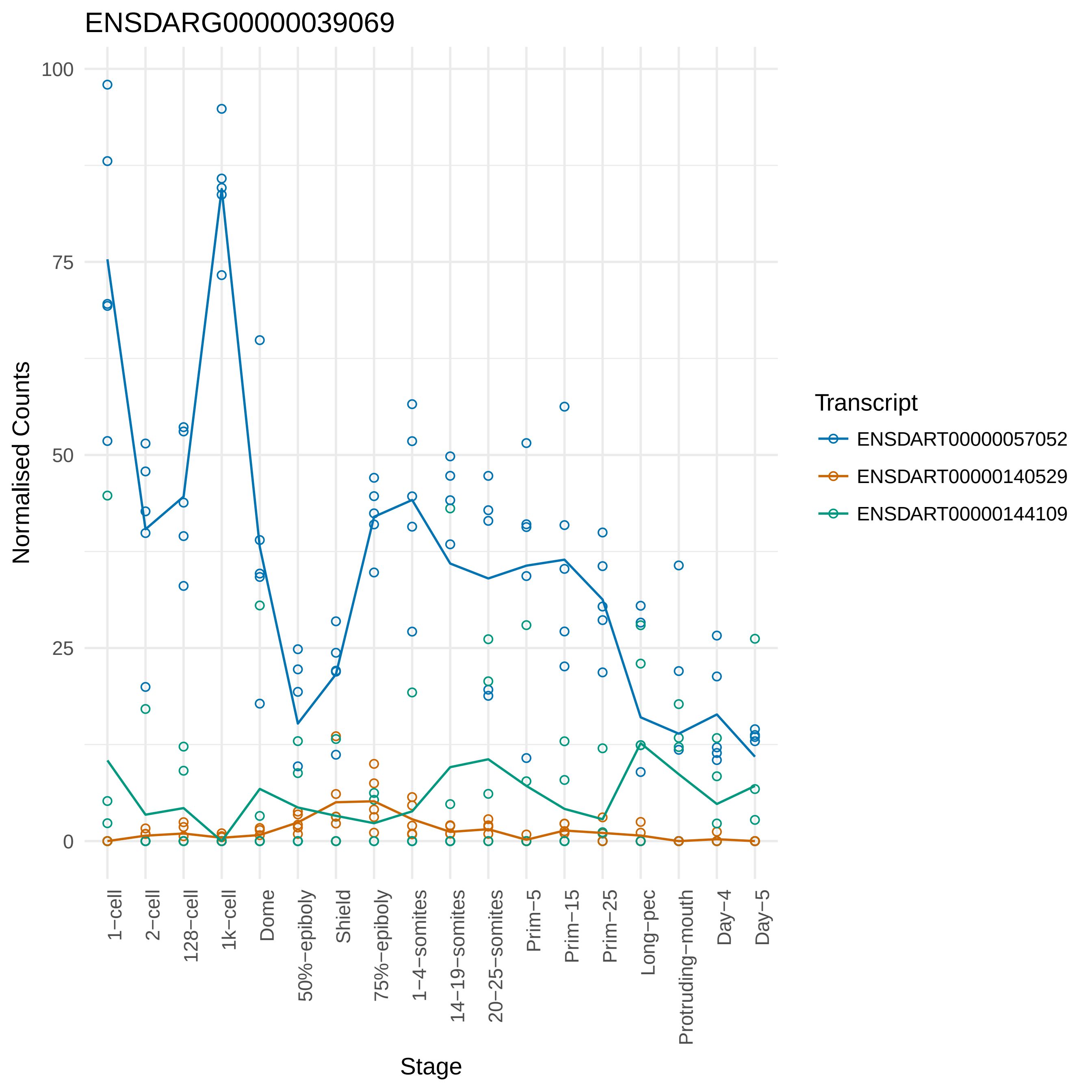

ENSDARG00000039069
Normalised Counts
100
75
50
25
0
1−cell
2−cell
128−cell
1k−cell
Dome
50%−epiboly
Shield
75%−epiboly
1−4−somites
14−19−somites
20−25−somites
Prim−5
Prim−15
Prim−25
Long−pec
Protruding−mouth
Day−4
Day−5
Stage
Transcript
ENSDART00000057052
ENSDART00000140529
ENSDART00000144109

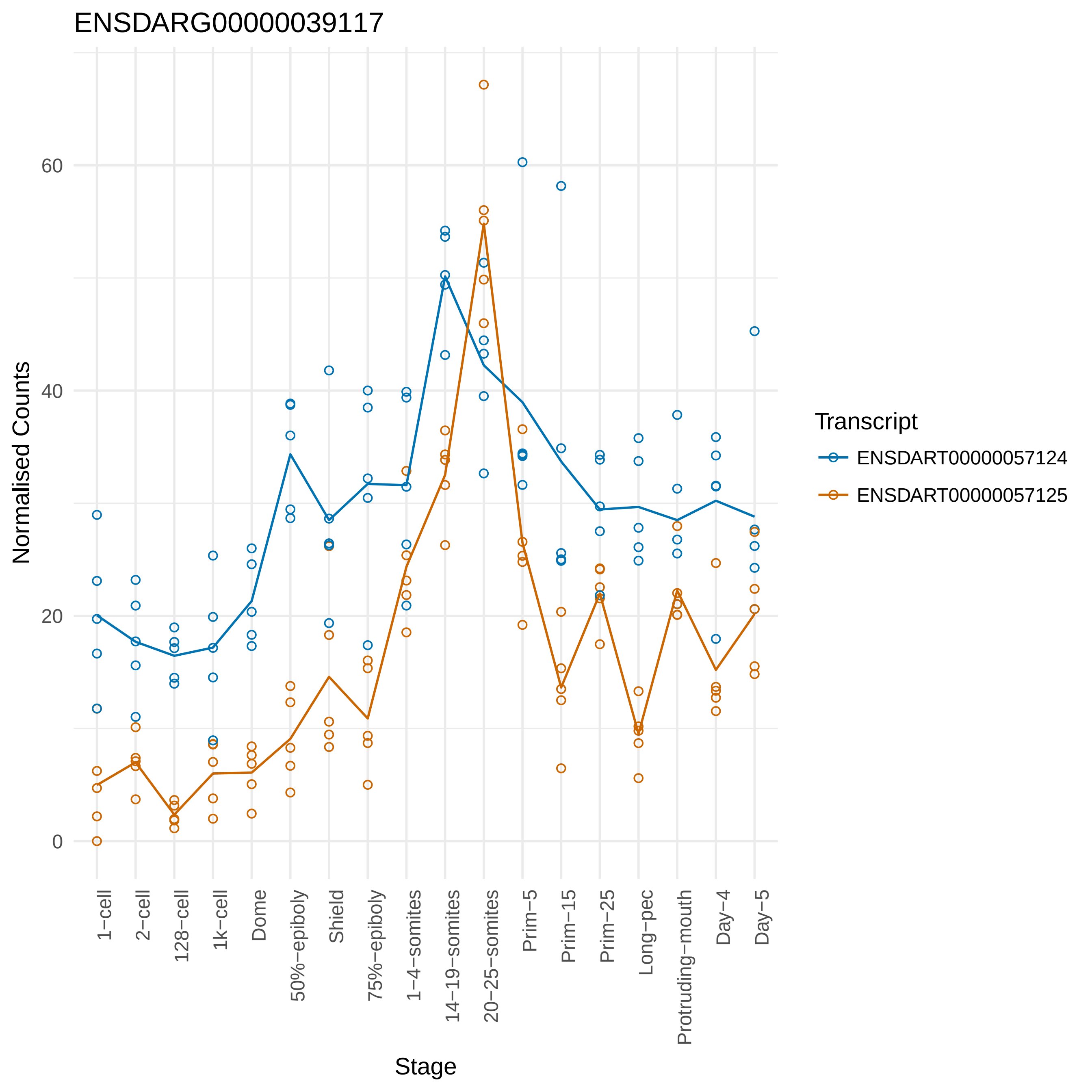
ENSDARG00000039117
Normalised Counts
60
40
20
0
Stage
1−cell
2−cell
128−cell
1k−cell
Dome
50%−epiboly
Shield
75%−epiboly
1−4−somites
14−19−somites
20−25−somites
Prim−5
Prim−15
Prim−25
Long−pec
Protruding−mouth
Day−4
Day−5
Transcript
ENSDART00000057124
ENSDART00000057125

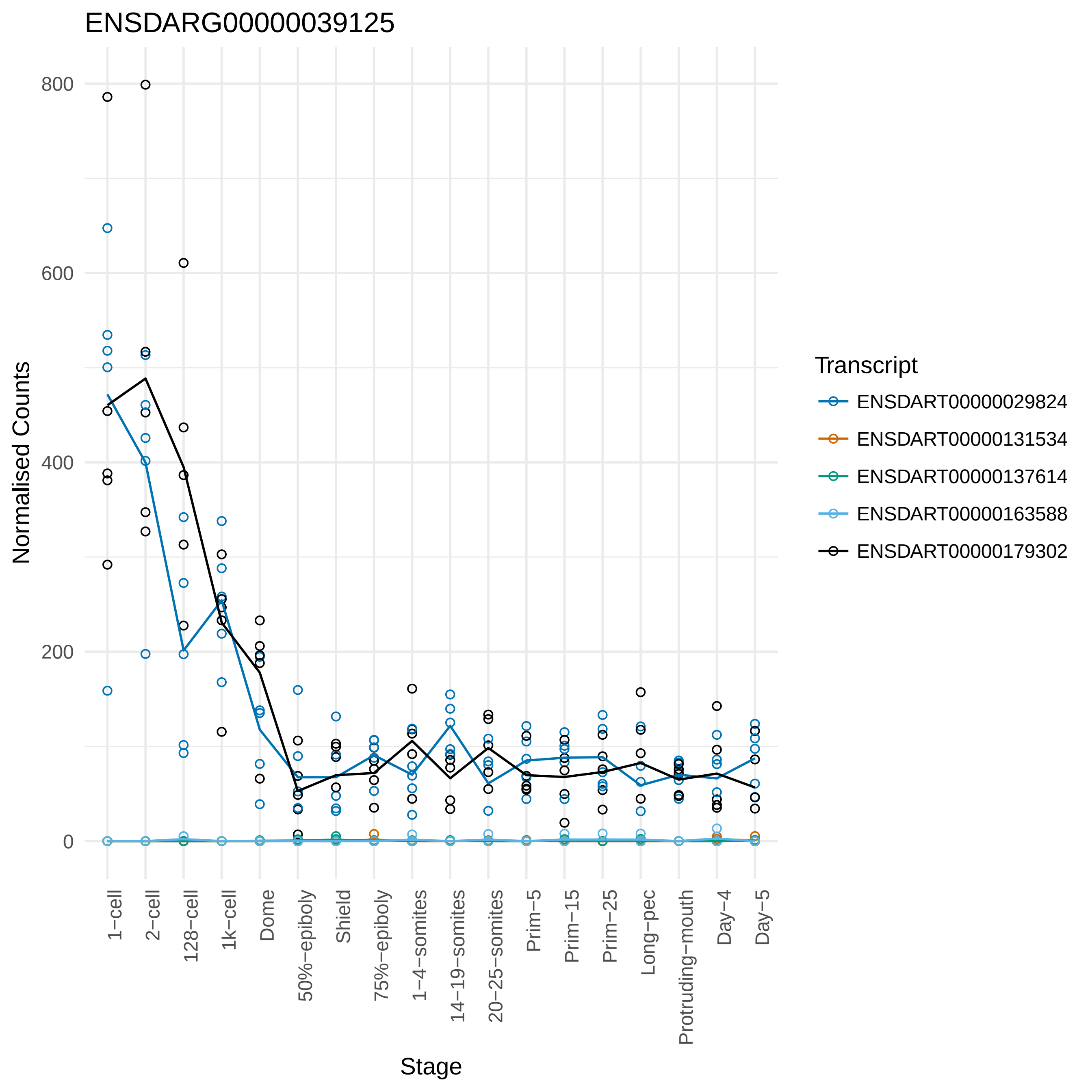

ENSDARG00000039125
Normalised Counts
800
600
400
200
0
1−cell
2−cell
128−cell
1k−cell
Dome
50%−epiboly
Shield
75%−epiboly
1−4−somites
14−19−somites
20−25−somites
Prim−5
Prim−15
Prim−25
Long−pec
Protruding−mouth
Day−4
Day−5
Stage
Transcript
ENSDART00000029824
ENSDART00000131534
ENSDART00000137614
ENSDART00000163588
ENSDART00000179302

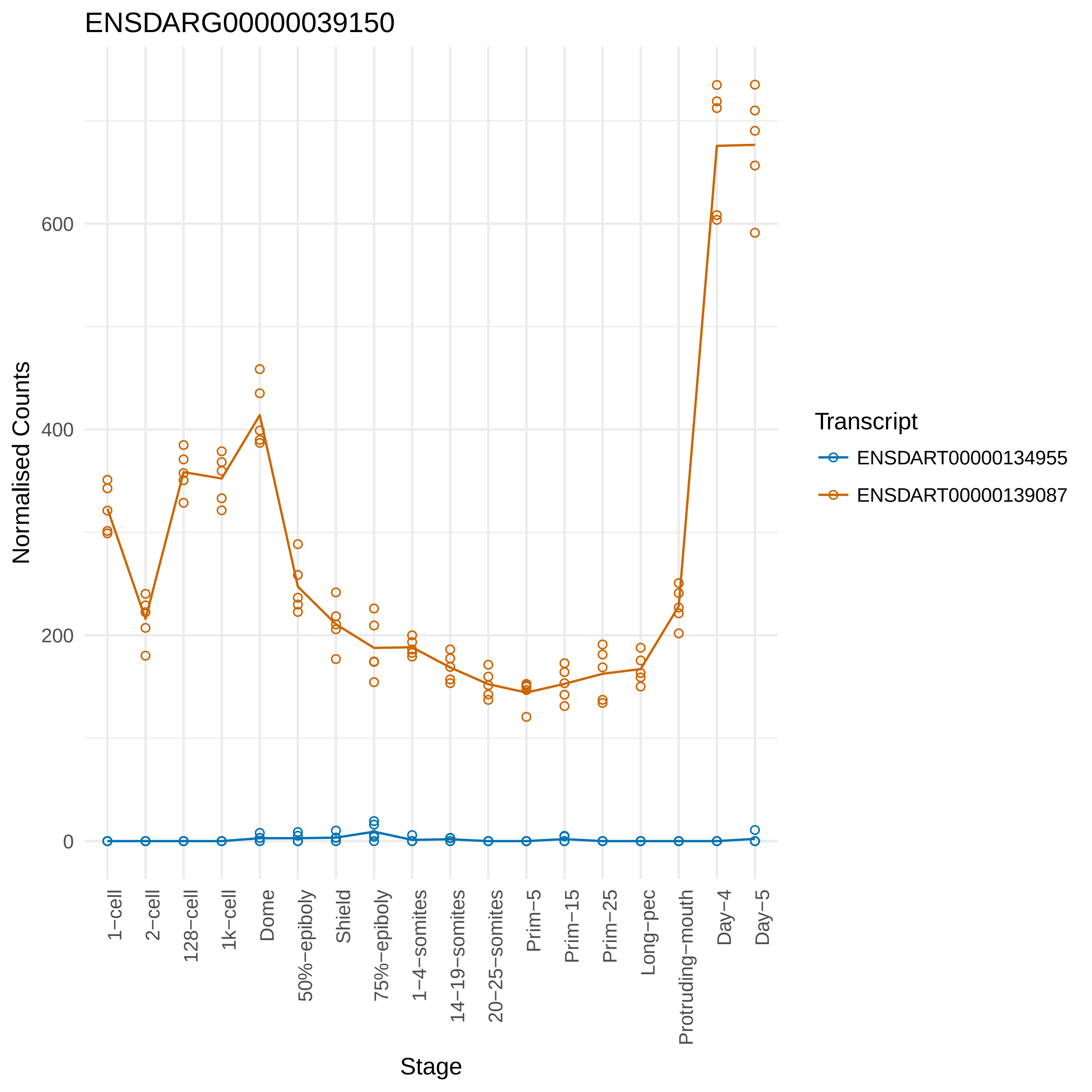
ENSDARG00000039150
Normalised Counts
0
200
400
600
Stage
1−cell
2−cell
128−cell
1k−cell
Dome
50%−epiboly
Shield
75%−epiboly
1−4−somites
14−19−somites
20−25−somites
Prim−5
Prim−15
Prim−25
Long−pec
Protruding−mouth
Day−4
Day−5
Transcript
ENSDART00000134955
ENSDART00000139087

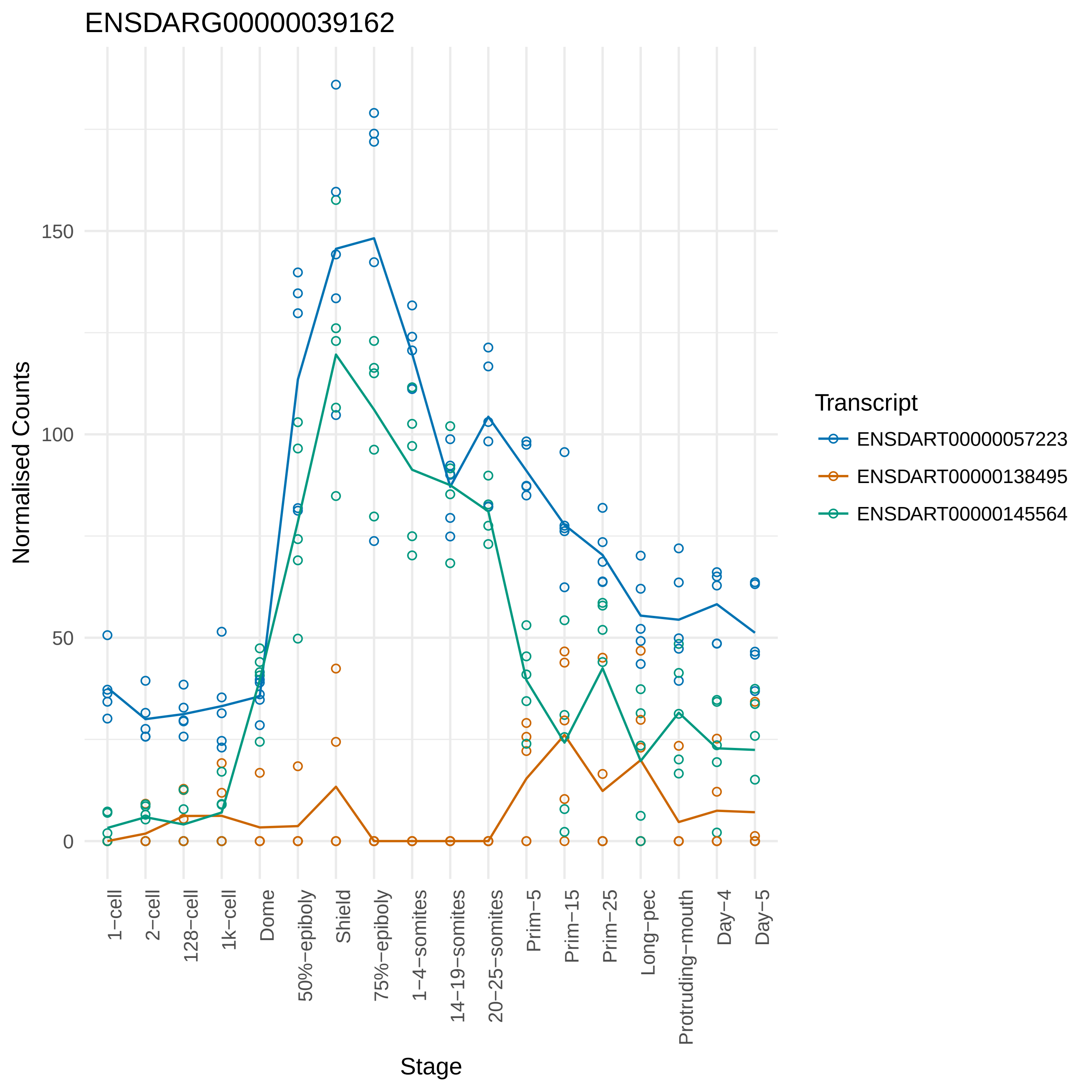

ENSDARG00000039162
Normalised Counts
0
50
100
150
1−cell
2−cell
128−cell
1k−cell
Dome
50%−epiboly
Shield
75%−epiboly
1−4−somites
14−19−somites
20−25−somites
Prim−5
Prim−15
Prim−25
Long−pec
Protruding−mouth
Day−4
Day−5
Stage
Transcript
ENSDART00000057223
ENSDART00000138495
ENSDART00000145564

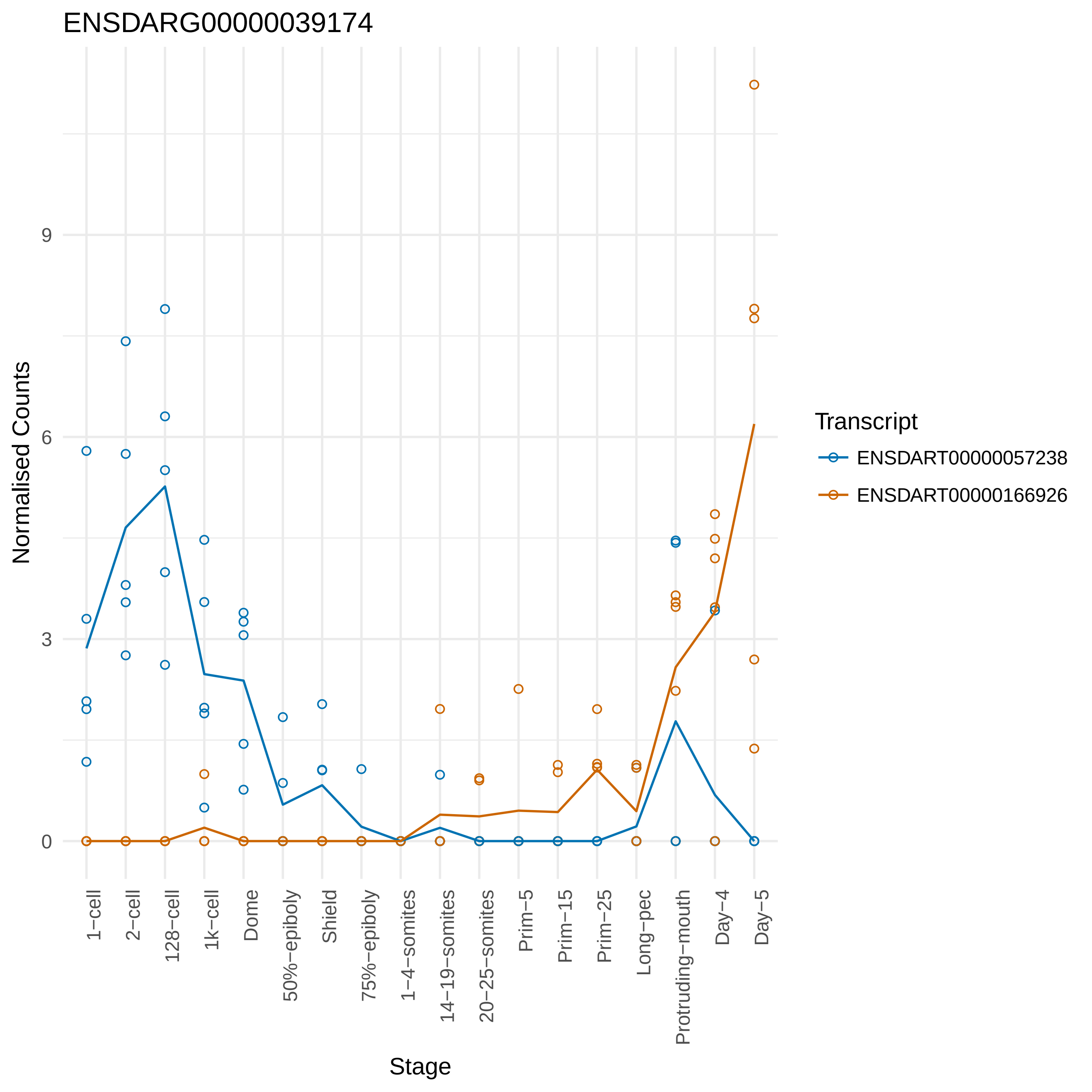

ENSDARG00000039174
Normalised Counts
0
3
6
9
1−cell
2−cell
128−cell
1k−cell
Dome
50%−epiboly
Shield
75%−epiboly
1−4−somites
14−19−somites
20−25−somites
Prim−5
Prim−15
Prim−25
Long−pec
Protruding−mouth
Day−4
Day−5
Stage
Transcript
ENSDART00000057238
ENSDART00000166926

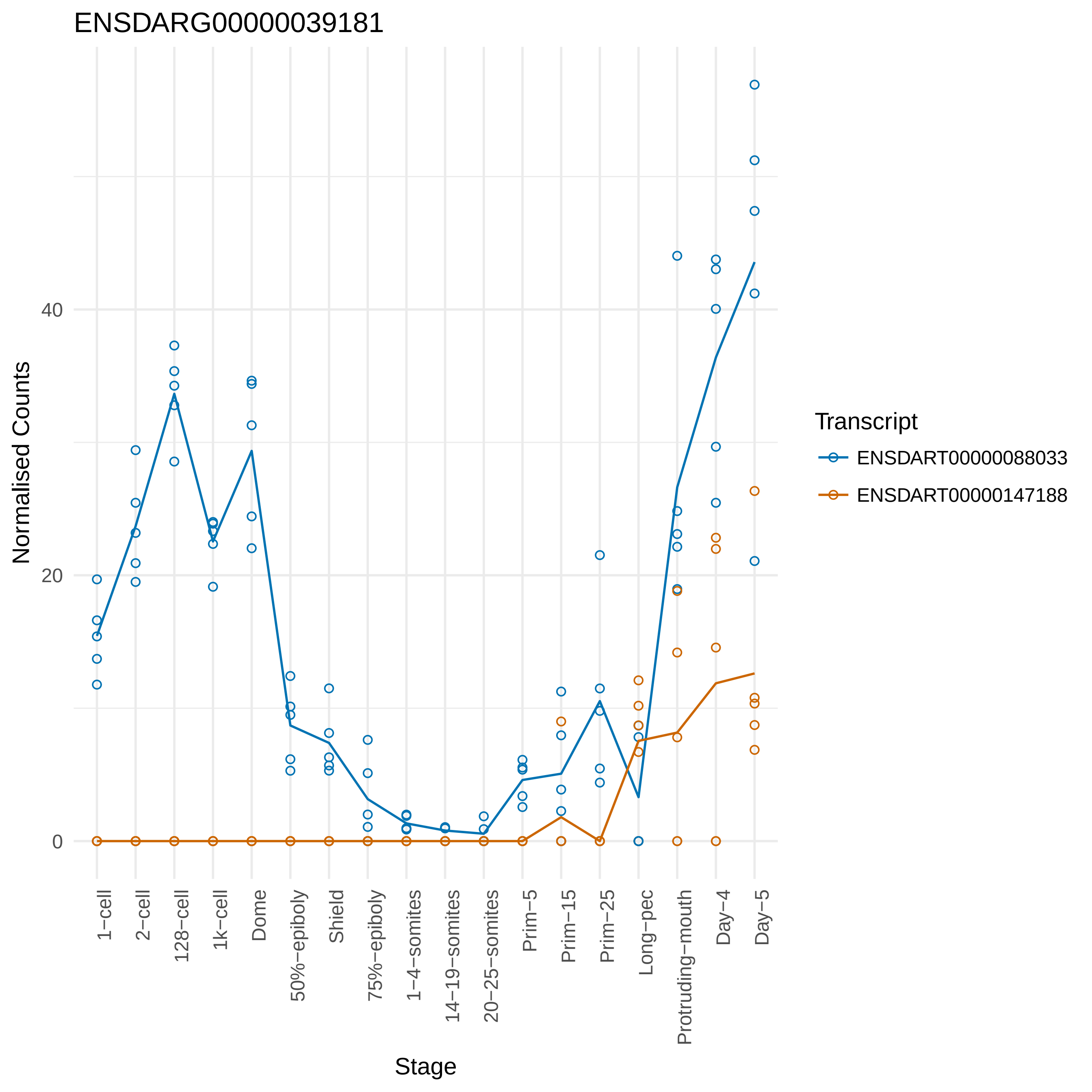
ENSDARG00000039181
Normalised Counts
40
20
0
1−cell
2−cell
128−cell
1k−cell
Dome
50%−epiboly
Shield
75%−epiboly
1−4−somites
14−19−somites
20−25−somites
Prim−5
Prim−15
Prim−25
Long−pec
Protruding−mouth
Day−4
Day−5
Stage
Transcript
ENSDART00000088033
ENSDART00000147188

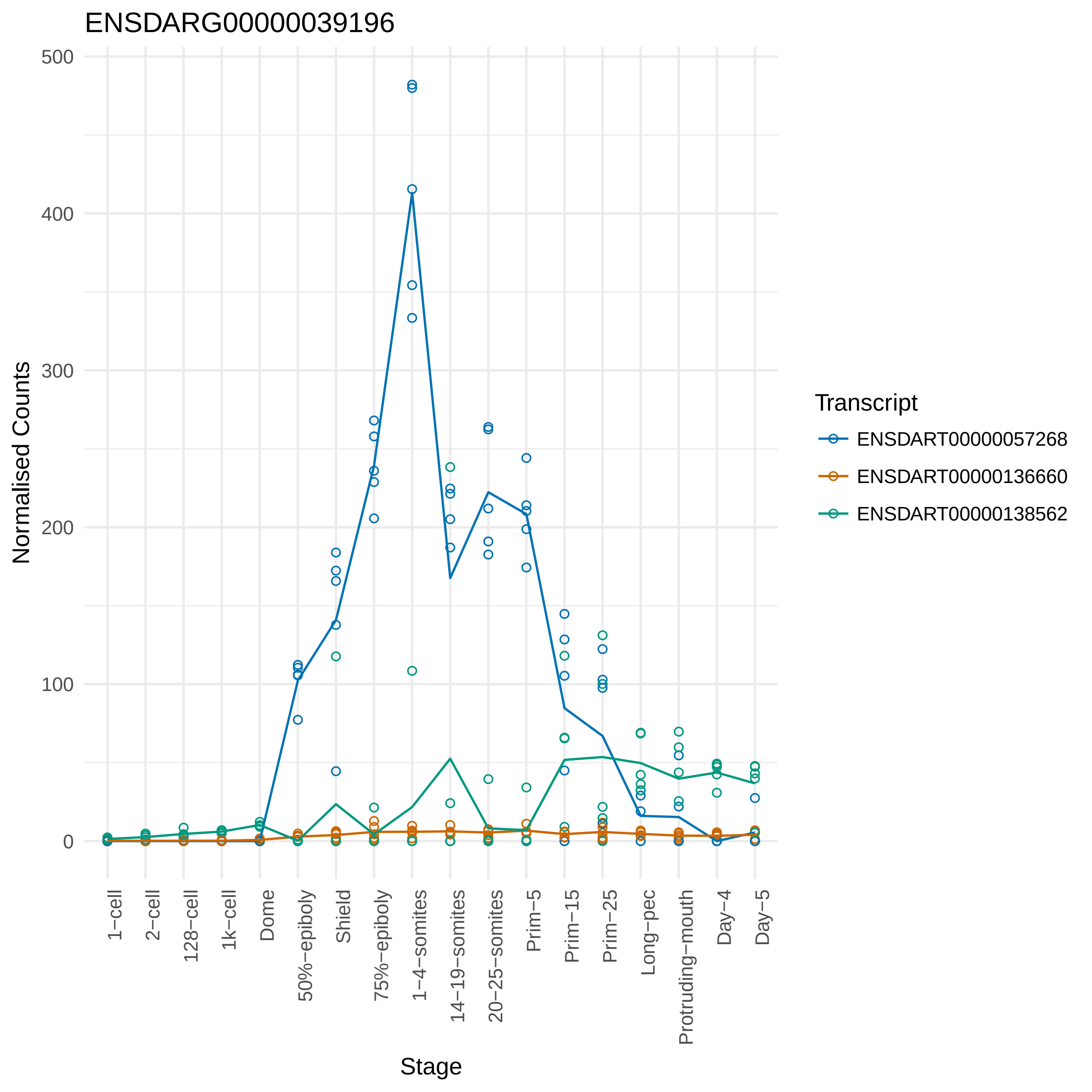
ENSDARG00000039196
Normalised Counts
500
400
300
200
100
0
1−cell
2−cell
128−cell
1k−cell
Dome
50%−epiboly
Shield
75%−epiboly
1−4−somites
14−19−somites
20−25−somites
Prim−5
Prim−15
Prim−25
Long−pec
Protruding−mouth
Day−4
Day−5
Stage
Transcript
ENSDART00000057268
ENSDART00000136660
ENSDART00000138562

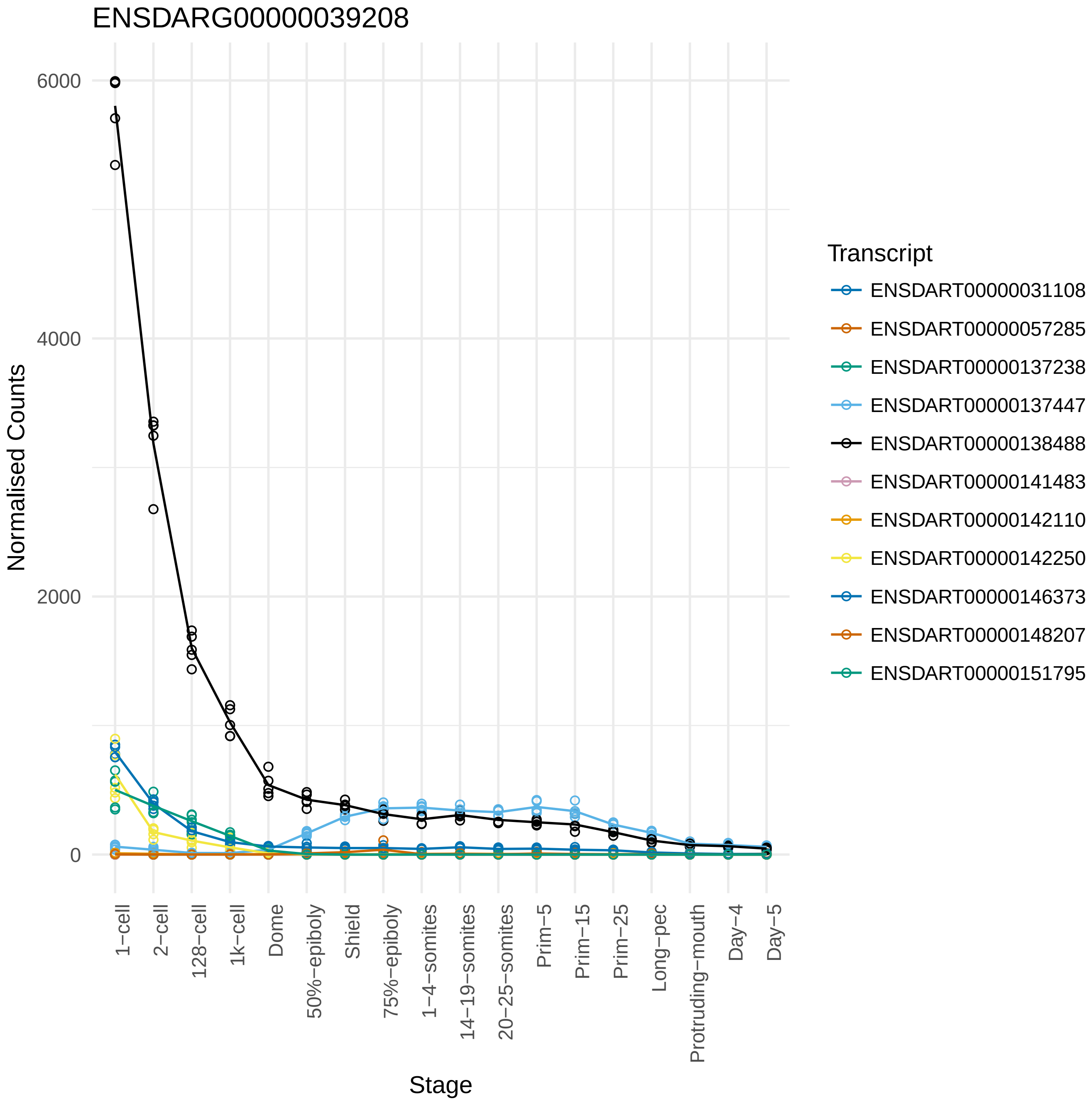

ENSDARG00000039208
Normalised Counts
6000
4000
2000
0
1−cell
2−cell
128−cell
1k−cell
Dome
50%−epiboly
Shield
75%−epiboly
1−4−somites
14−19−somites
20−25−somites
Prim−5
Prim−15
Prim−25
Long−pec
Protruding−mouth
Day−4
Day−5
Stage
Transcript
ENSDART00000031108
ENSDART00000057285
ENSDART00000137238
ENSDART00000137447
ENSDART00000138488
ENSDART00000141483
ENSDART00000142110
ENSDART00000142250
ENSDART00000146373
ENSDART00000148207
ENSDART00000151795

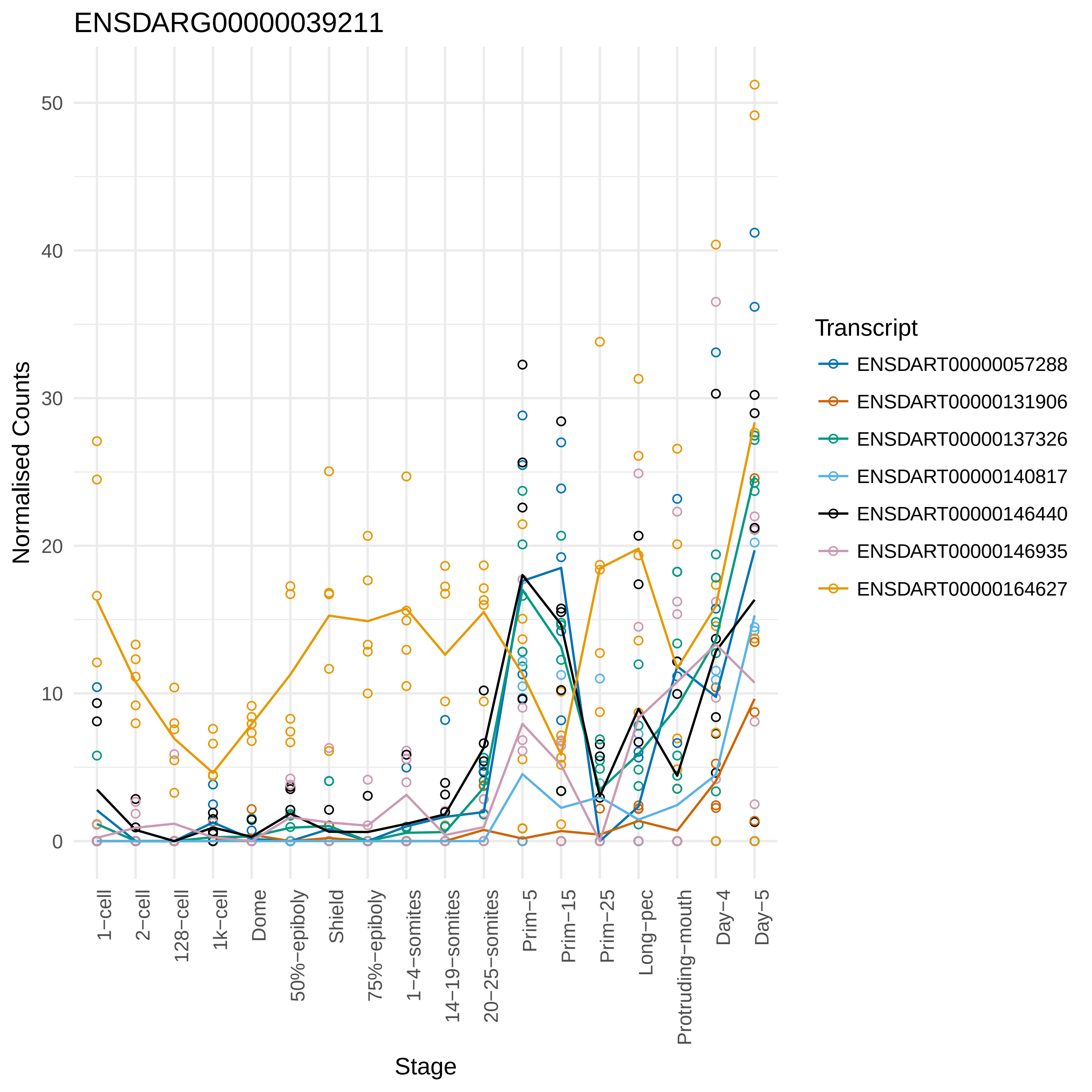
ENSDARG00000039211
Normalised Counts
0
10
20
30
40
50
1−cell
2−cell
128−cell
1k−cell
Dome
50%−epiboly
Shield
75%−epiboly
1−4−somites
14−19−somites
20−25−somites
Prim−5
Prim−15
Prim−25
Long−pec
Protruding−mouth
Day−4
Day−5
Stage
Transcript
ENSDART00000057288
ENSDART00000131906
ENSDART00000137326
ENSDART00000140817
ENSDART00000146440
ENSDART00000146935
ENSDART00000164627

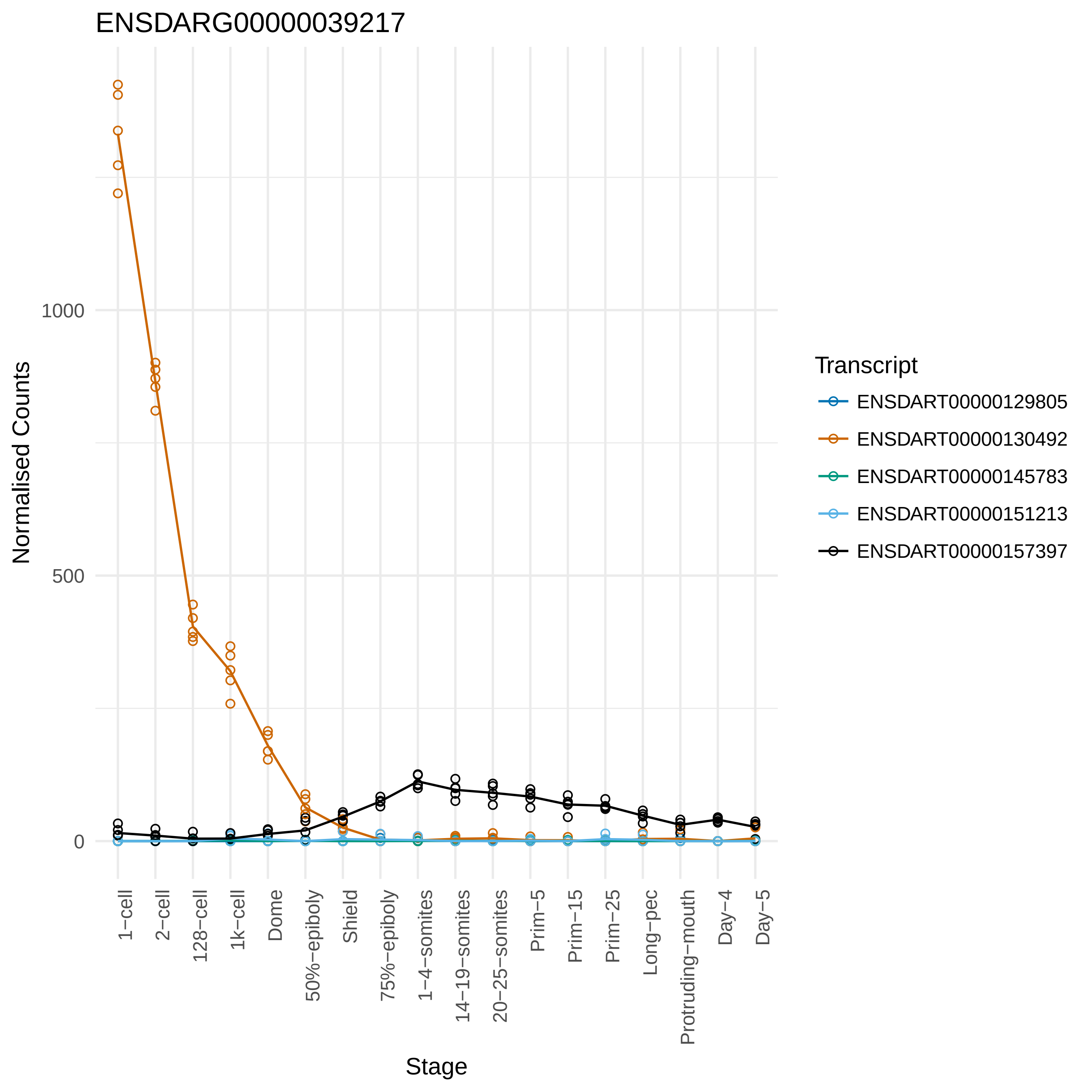

ENSDARG00000039217
Normalised Counts
1000
500
0
1−cell
2−cell
128−cell
1k−cell
Dome
50%−epiboly
Shield
75%−epiboly
1−4−somites
14−19−somites
20−25−somites
Prim−5
Prim−15
Prim−25
Long−pec
Protruding−mouth
Day−4
Day−5
Stage
Transcript
ENSDART00000129805
ENSDART00000130492
ENSDART00000145783
ENSDART00000151213
ENSDART00000157397

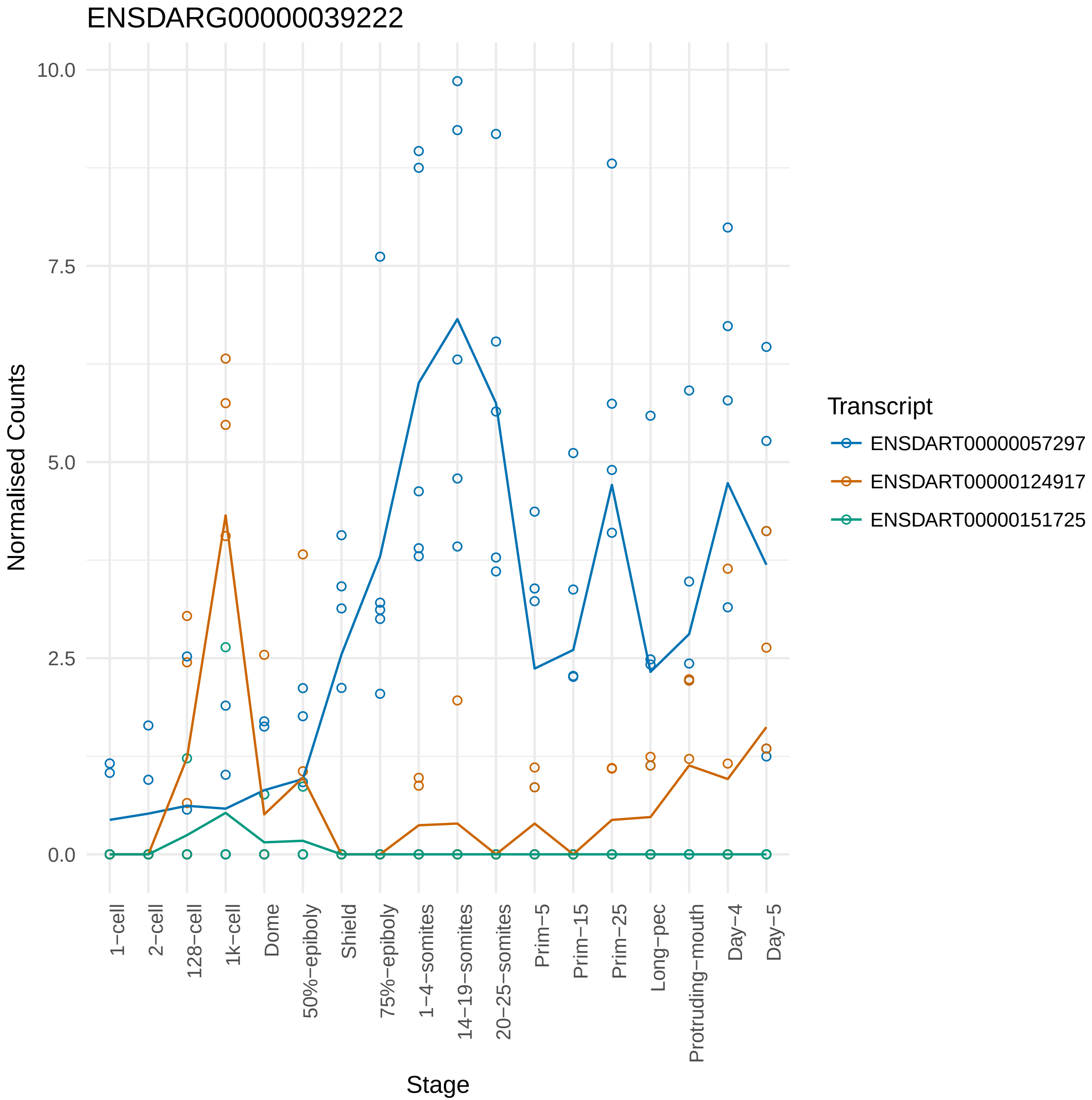

ENSDARG00000039222
Normalised Counts
10.0
7.5
5.0
2.5
0.0
1−cell
2−cell
128−cell
1k−cell
Dome
50%−epiboly
Shield
75%−epiboly
1−4−somites
14−19−somites
20−25−somites
Prim−5
Prim−15
Prim−25
Long−pec
Protruding−mouth
Day−4
Day−5
Stage
Transcript
ENSDART00000057297
ENSDART00000124917
ENSDART00000151725

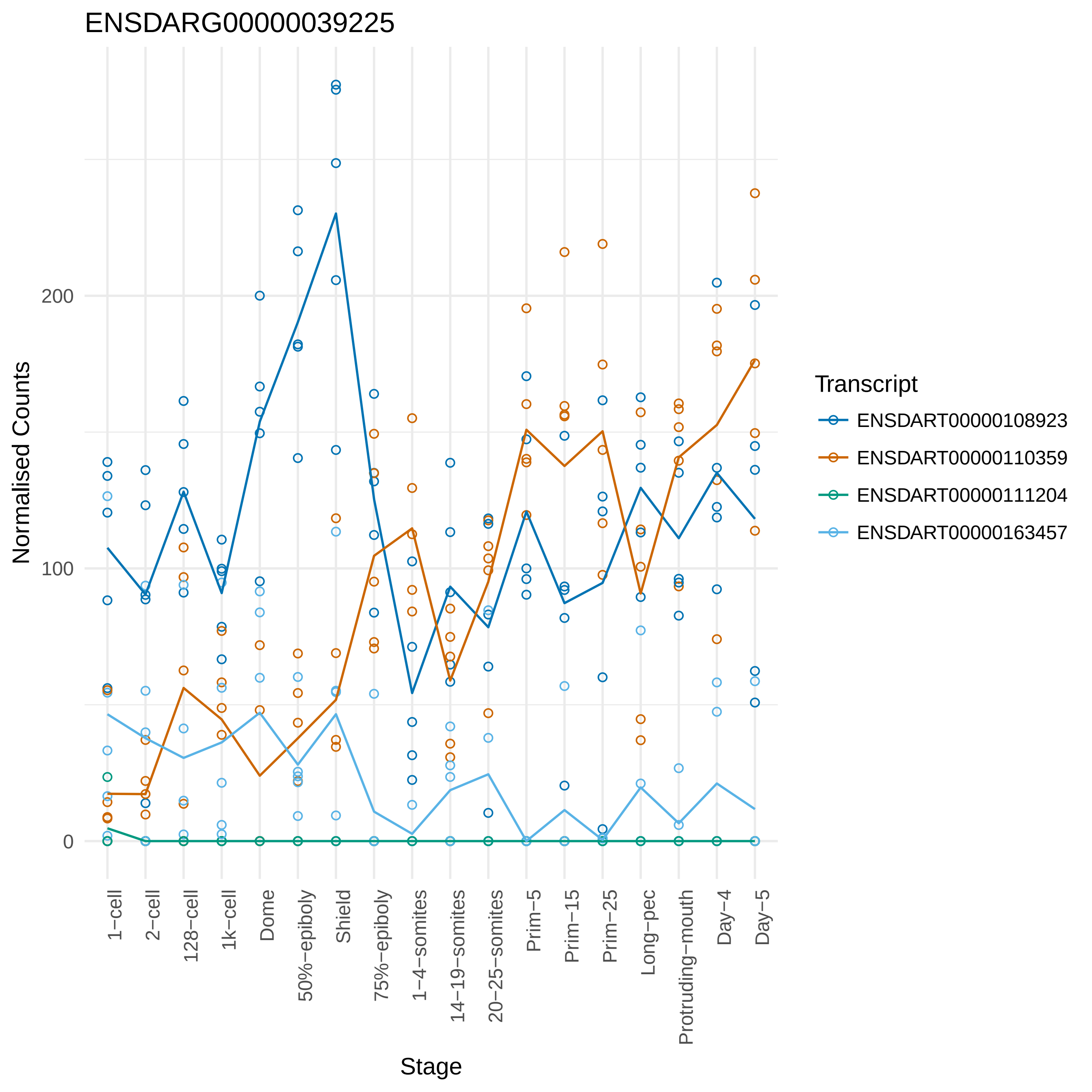
ENSDARG00000039225
Normalised Counts
0
100
200
1−cell
2−cell
128−cell
1k−cell
Dome
50%−epiboly
Shield
75%−epiboly
1−4−somites
14−19−somites
20−25−somites
Prim−5
Prim−15
Prim−25
Long−pec
Protruding−mouth
Day−4
Day−5
Stage
Transcript
ENSDART00000108923
ENSDART00000110359
ENSDART00000111204
ENSDART00000163457

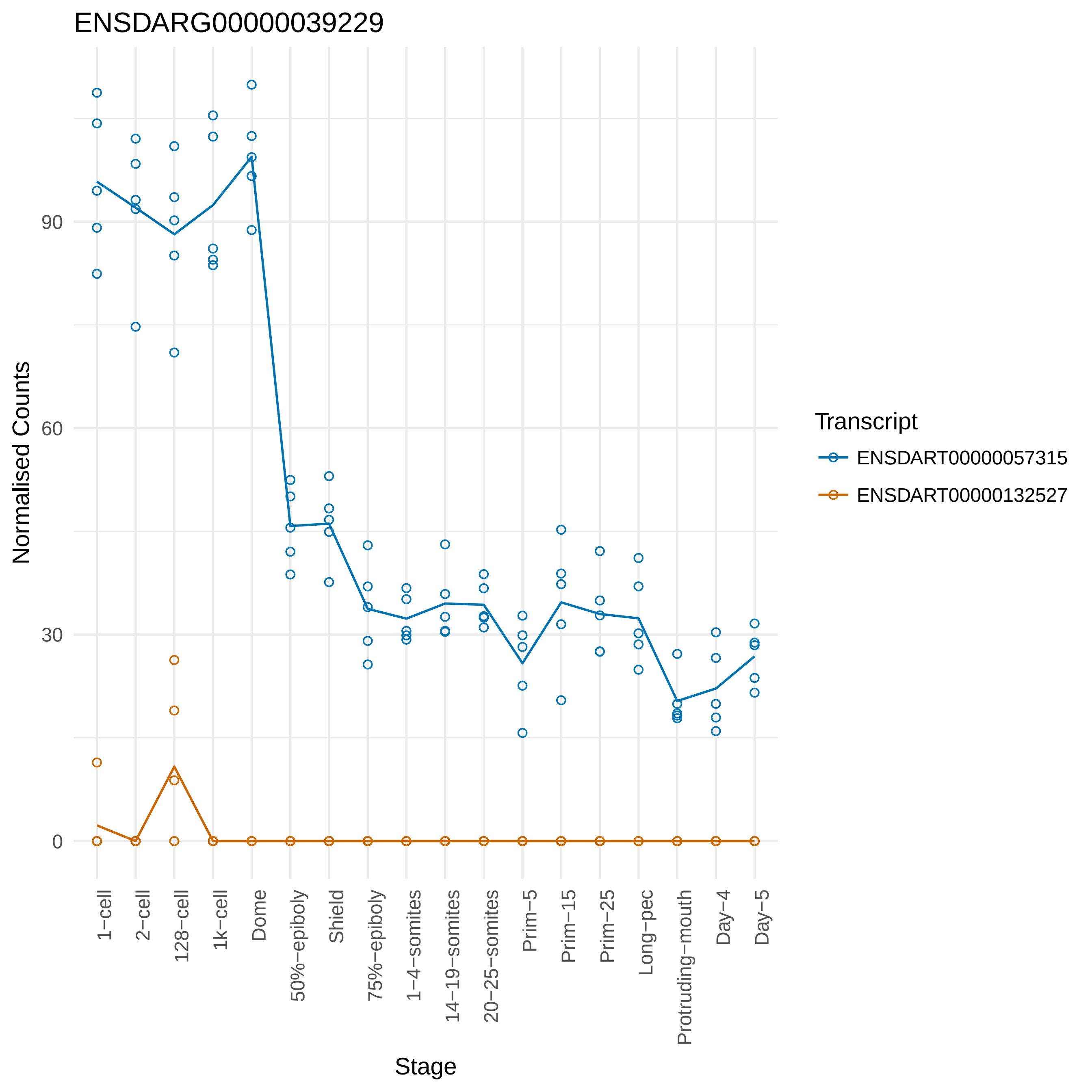

ENSDARG00000039229
Normalised Counts
0
30
60
90
Stage
1−cell
2−cell
128−cell
1k−cell
Dome
50%−epiboly
Shield
75%−epiboly
1−4−somites
14−19−somites
20−25−somites
Prim−5
Prim−15
Prim−25
Long−pec
Protruding−mouth
Day−4
Day−5
Transcript
ENSDART00000057315
ENSDART00000132527

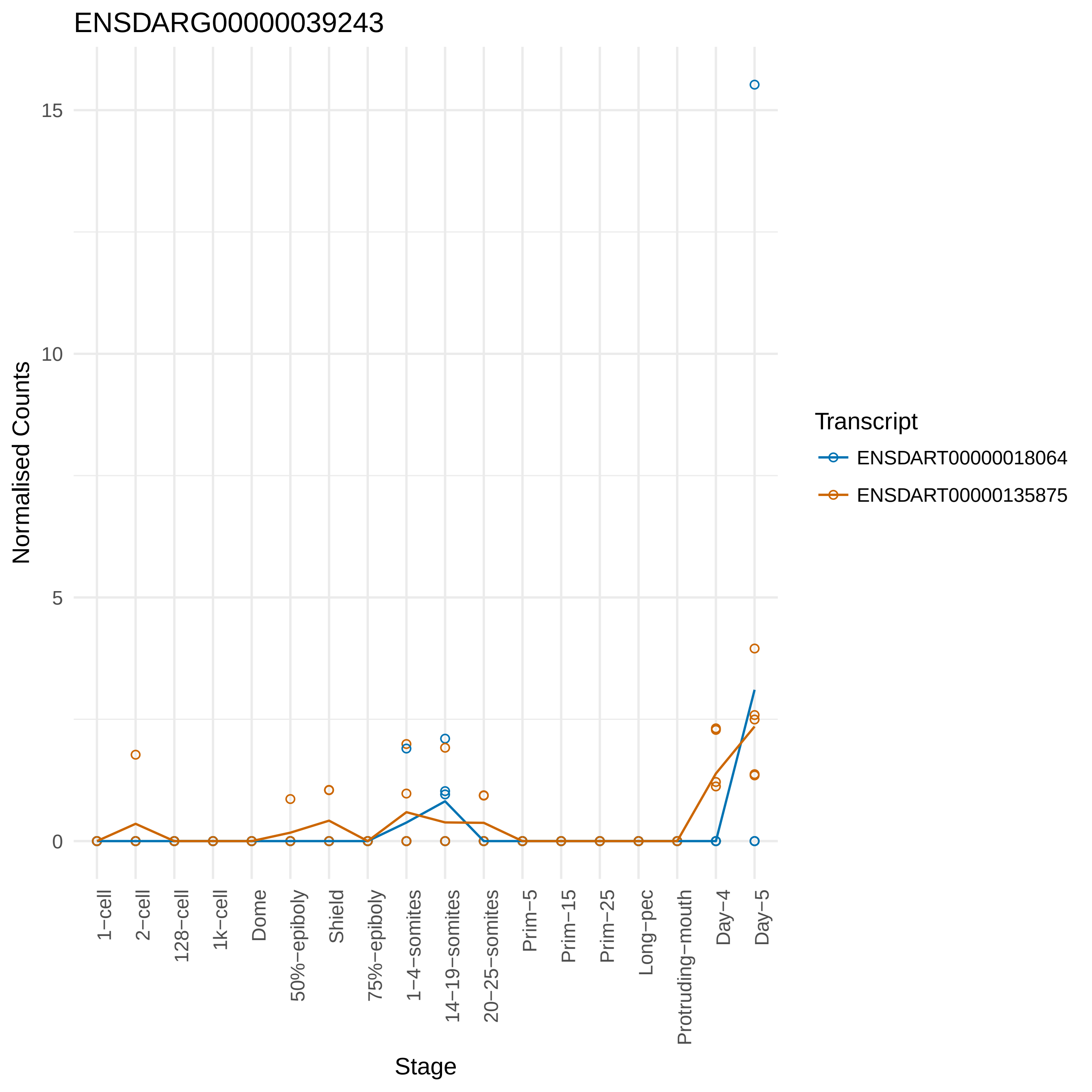
ENSDARG00000039243
Normalised Counts
15
10
5
0
1−cell
2−cell
128−cell
1k−cell
Dome
50%−epiboly
Shield
75%−epiboly
1−4−somites
14−19−somites
20−25−somites
Prim−5
Prim−15
Prim−25
Long−pec
Protruding−mouth
Day−4
Day−5
Stage
Transcript
ENSDART00000018064
ENSDART00000135875

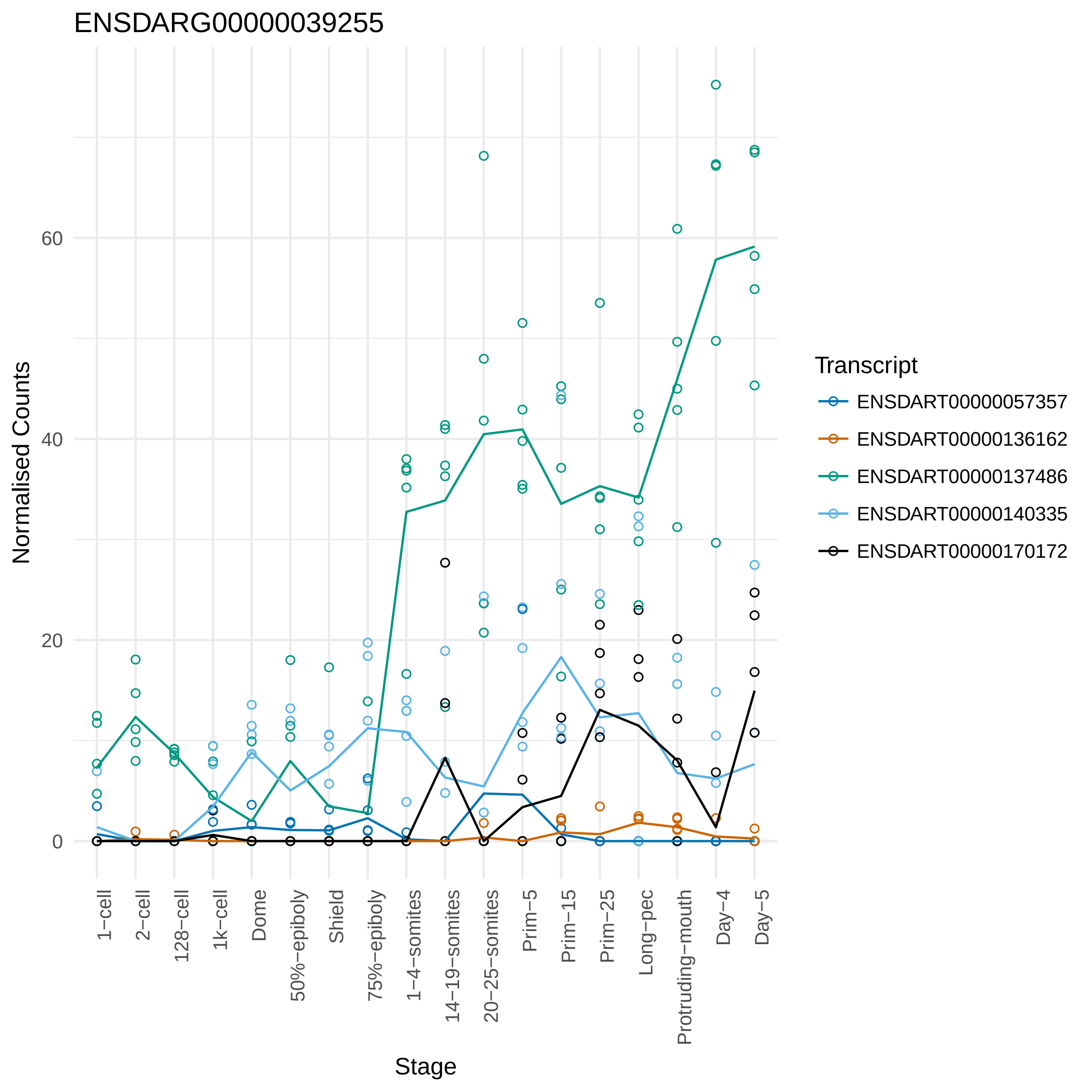
ENSDARG00000039255
Normalised Counts
0
20
40
60
Stage
1−cell
2−cell
128−cell
1k−cell
Dome
50%−epiboly
Shield
75%−epiboly
1−4−somites
14−19−somites
20−25−somites
Prim−5
Prim−15
Prim−25
Long−pec
Protruding−mouth
Day−4
Day−5
Transcript
ENSDART00000057357
ENSDART00000136162
ENSDART00000137486
ENSDART00000140335
ENSDART00000170172

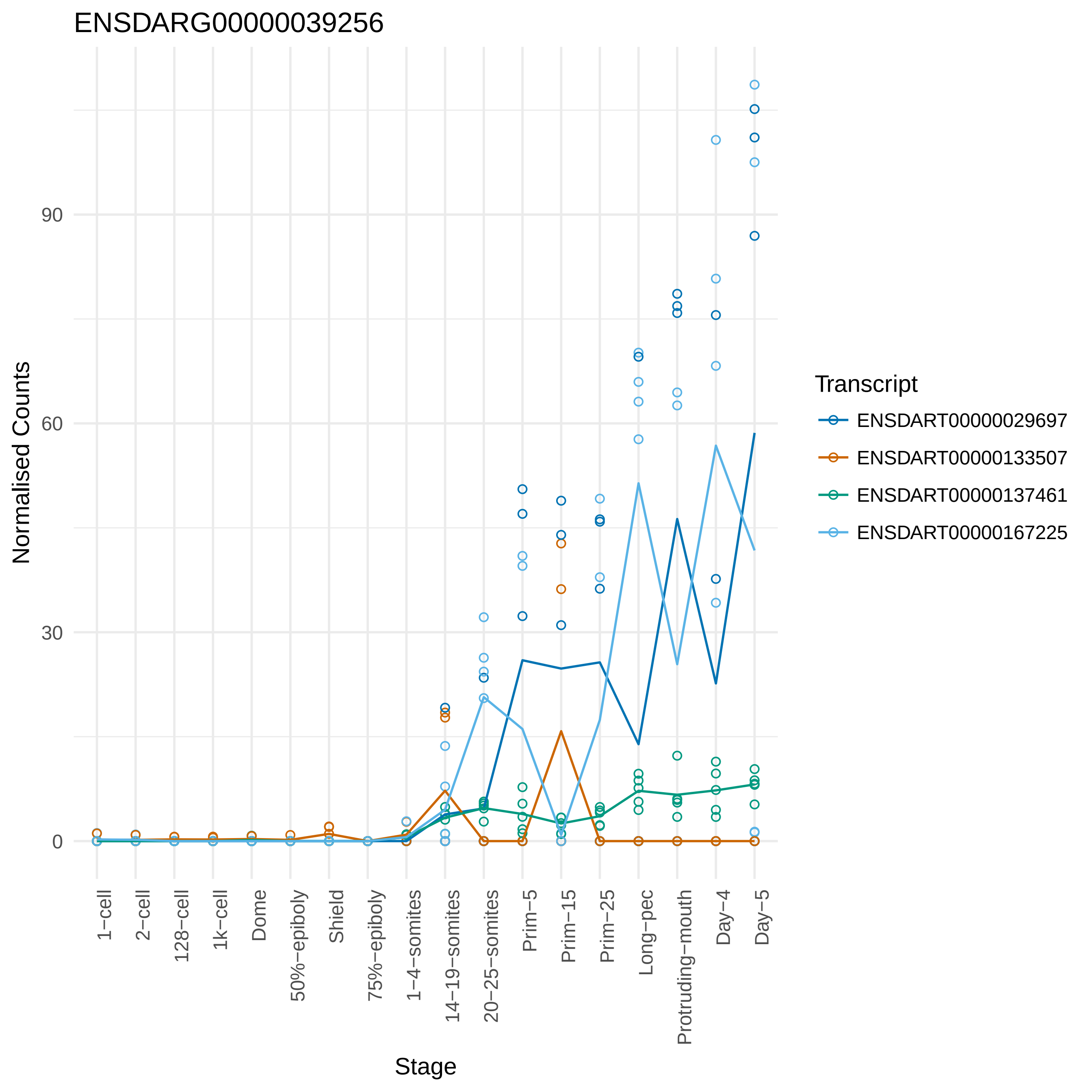

ENSDARG00000039256
Normalised Counts
0
30
60
90
Stage
1−cell
2−cell
128−cell
1k−cell
Dome
50%−epiboly
Shield
75%−epiboly
1−4−somites
14−19−somites
20−25−somites
Prim−5
Prim−15
Prim−25
Long−pec
Protruding−mouth
Day−4
Day−5
Transcript
ENSDART00000029697
ENSDART00000133507
ENSDART00000137461
ENSDART00000167225

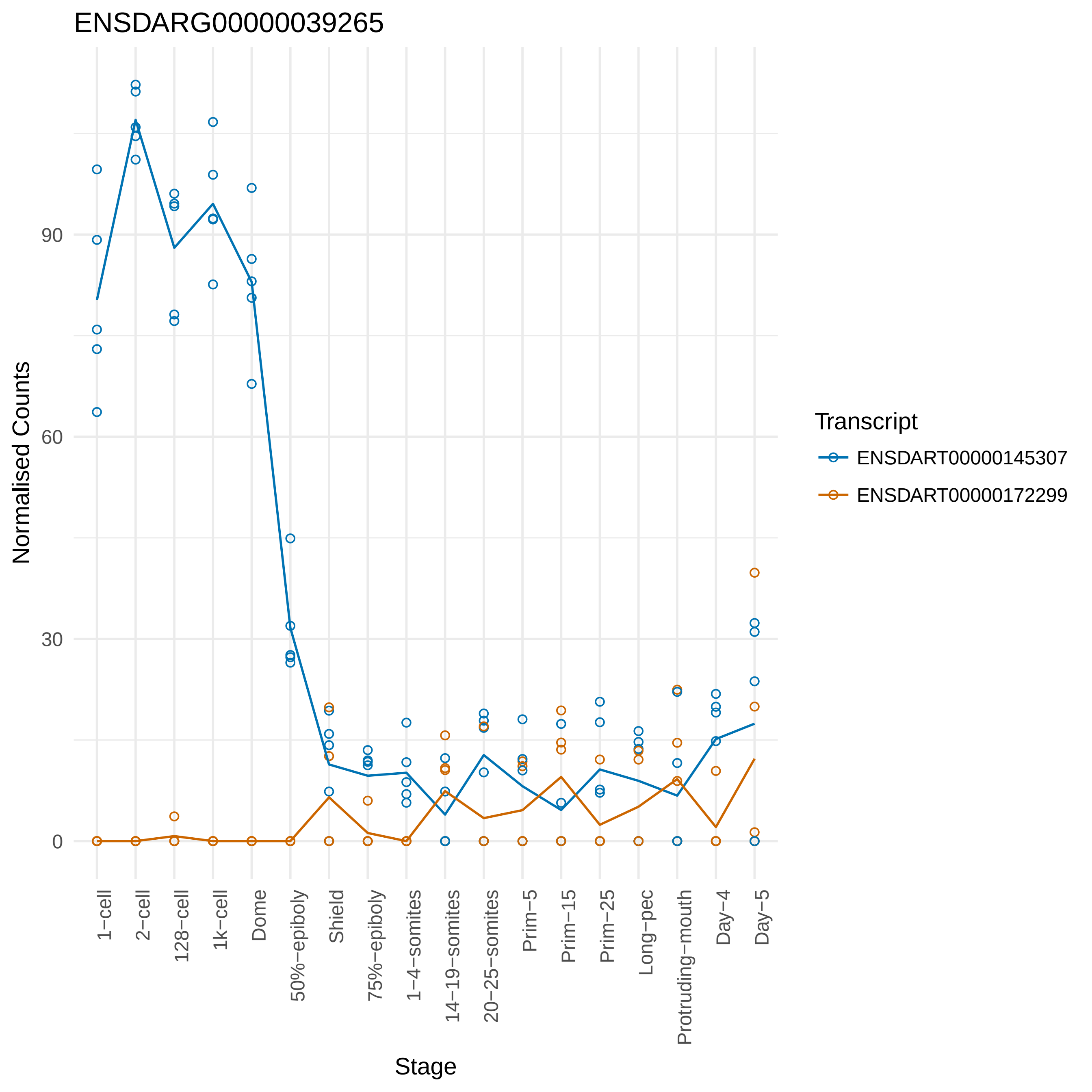

ENSDARG00000039265
Normalised Counts
90
60
30
0
1−cell
2−cell
128−cell
1k−cell
Dome
50%−epiboly
Shield
75%−epiboly
1−4−somites
14−19−somites
20−25−somites
Prim−5
Prim−15
Prim−25
Long−pec
Protruding−mouth
Day−4
Day−5
Stage
Transcript
ENSDART00000145307
ENSDART00000172299

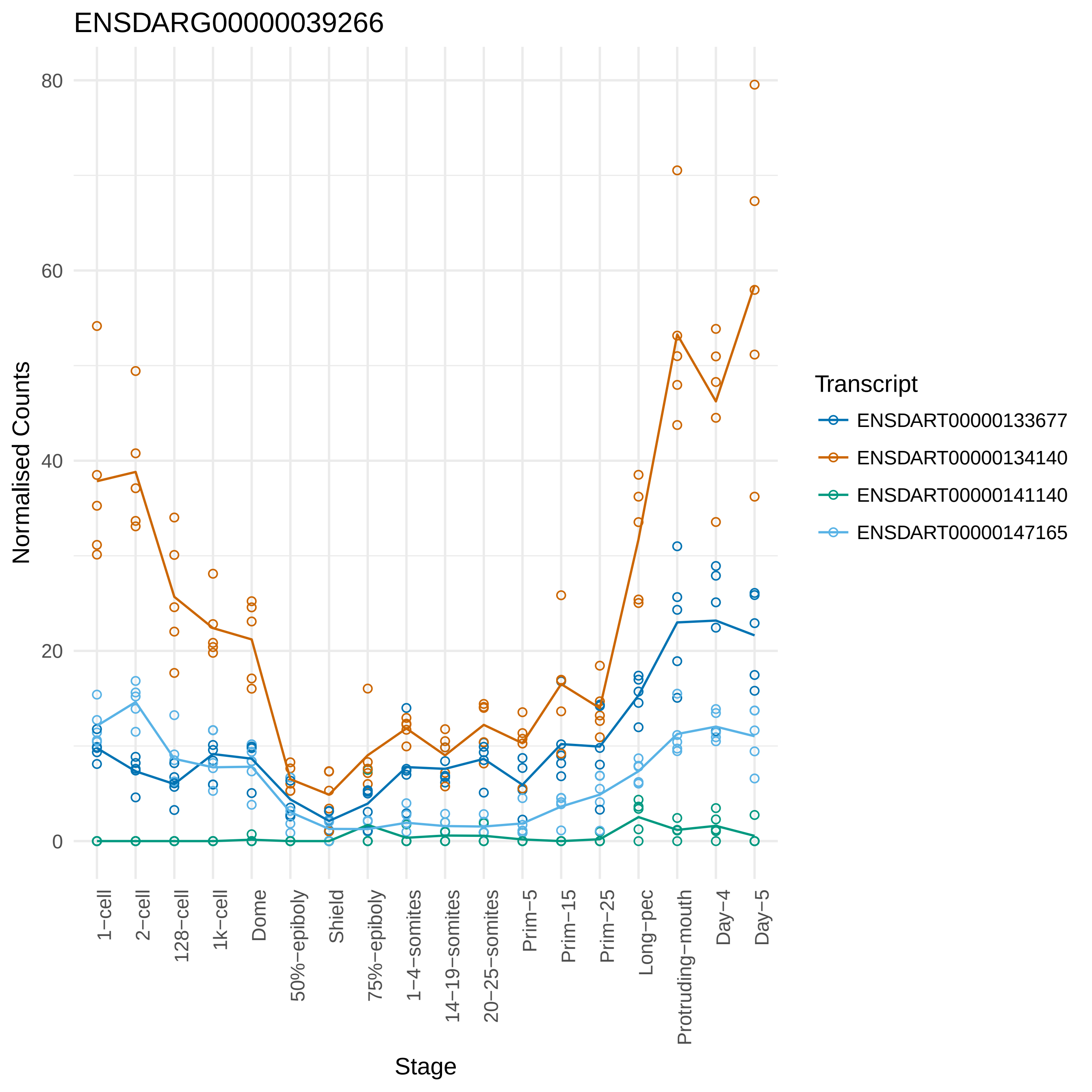
ENSDARG00000039266
Normalised Counts
80
60
40
20
0
1−cell
2−cell
128−cell
1k−cell
Dome
50%−epiboly
Shield
75%−epiboly
1−4−somites
14−19−somites
20−25−somites
Prim−5
Prim−15
Prim−25
Long−pec
Protruding−mouth
Day−4
Day−5
Stage
Transcript
ENSDART00000133677
ENSDART00000134140
ENSDART00000141140
ENSDART00000147165

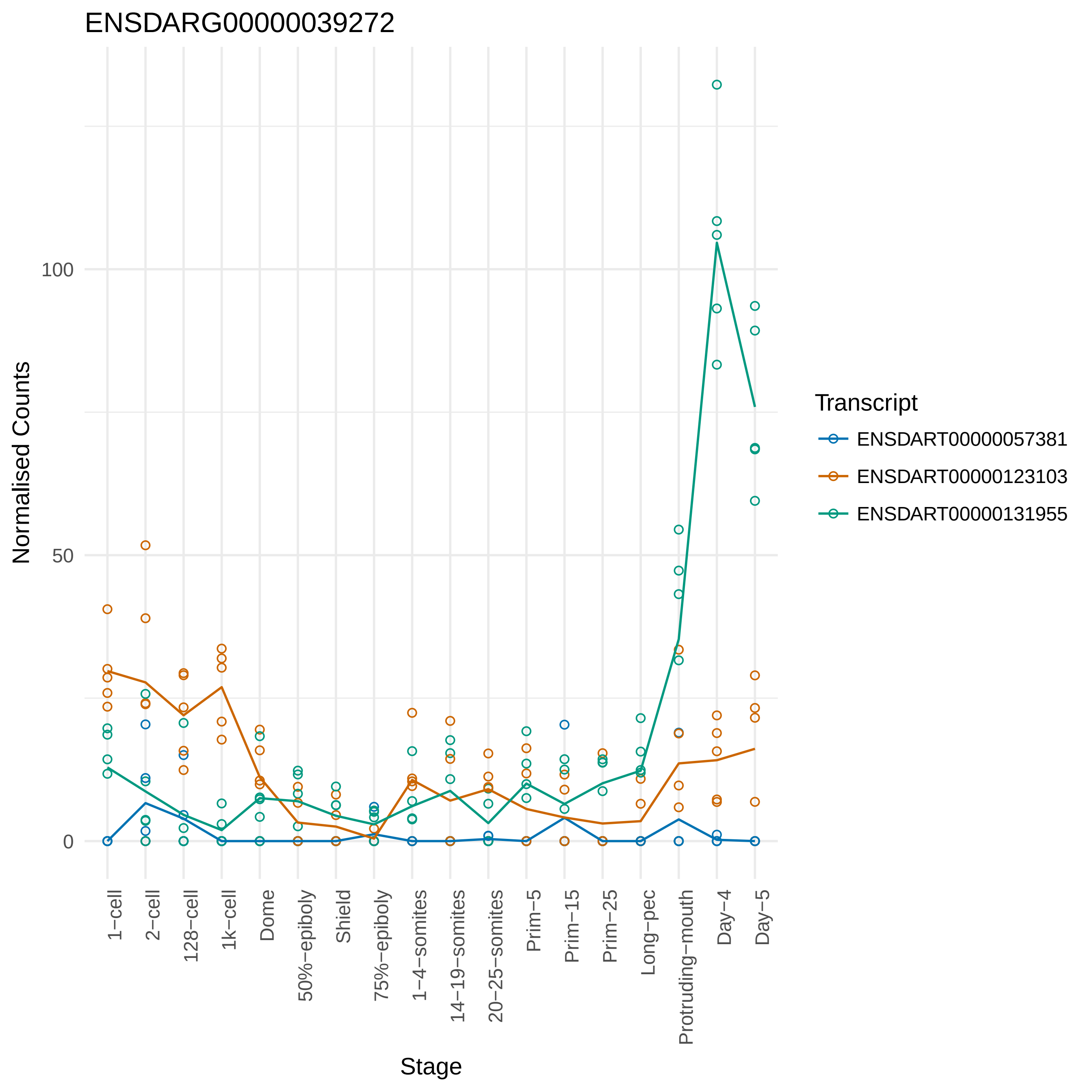
ENSDARG00000039272
Normalised Counts
100
50
0
1−cell
2−cell
128−cell
1k−cell
Dome
50%−epiboly
Shield
75%−epiboly
1−4−somites
14−19−somites
20−25−somites
Prim−5
Prim−15
Prim−25
Long−pec
Protruding−mouth
Day−4
Day−5
Stage
Transcript
ENSDART00000057381
ENSDART00000123103
ENSDART00000131955

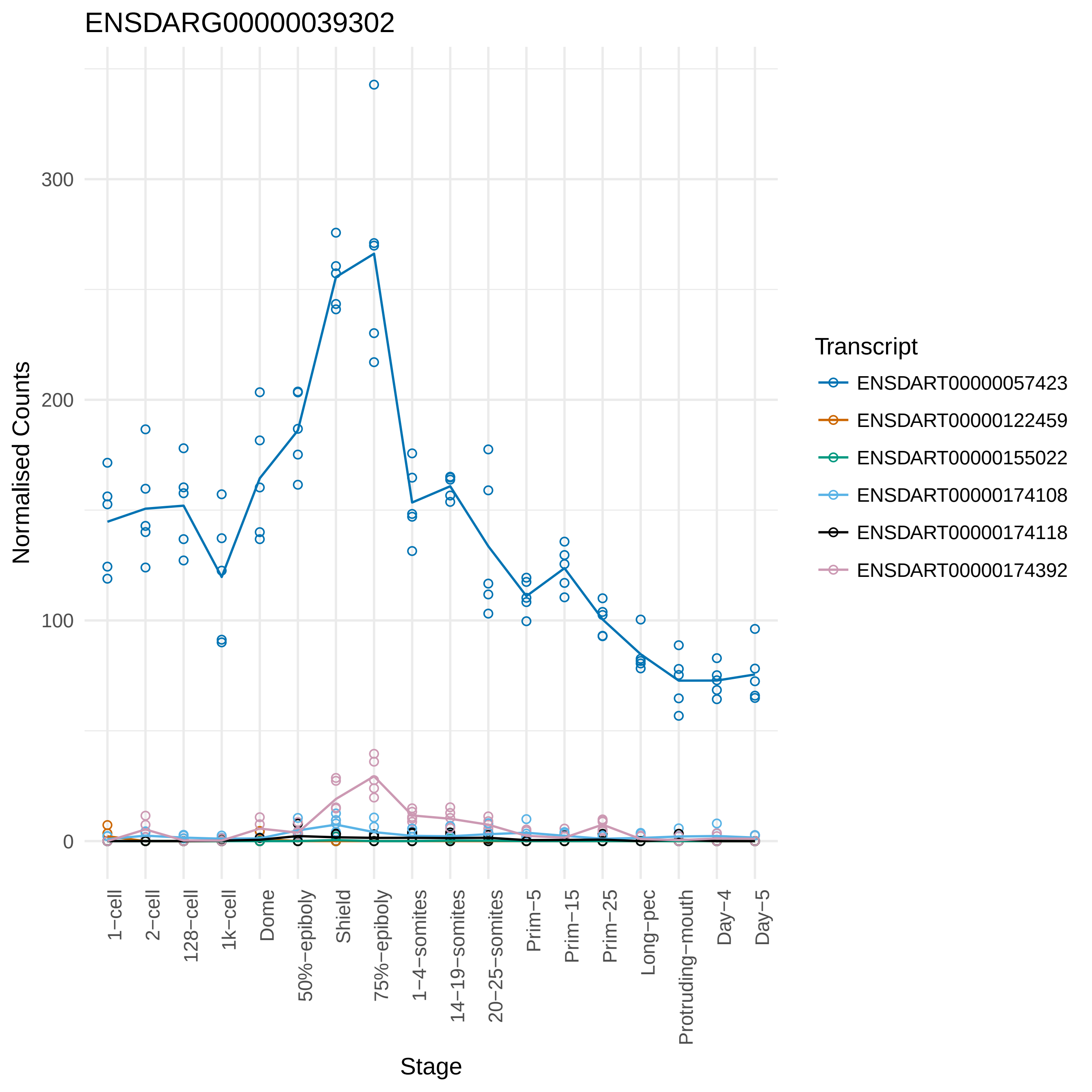
ENSDARG00000039302
Normalised Counts
0
100
200
300
Stage
1−cell
2−cell
128−cell
1k−cell
Dome
50%−epiboly
Shield
75%−epiboly
1−4−somites
14−19−somites
20−25−somites
Prim−5
Prim−15
Prim−25
Long−pec
Protruding−mouth
Day−4
Day−5
Transcript
ENSDART00000057423
ENSDART00000122459
ENSDART00000155022
ENSDART00000174108
ENSDART00000174118
ENSDART00000174392

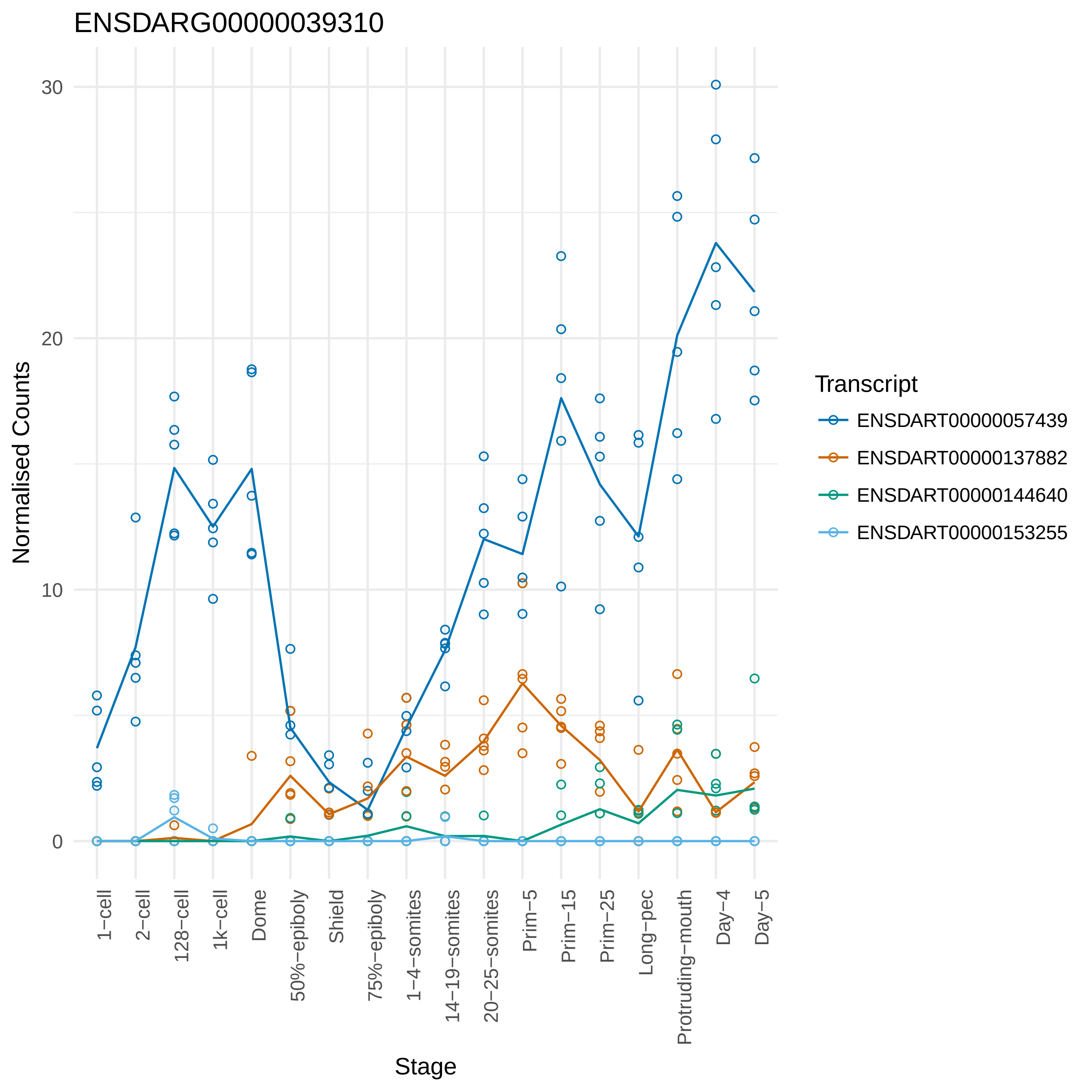
ENSDARG00000039310
Normalised Counts
30
20
10
0
1−cell
2−cell
128−cell
1k−cell
Dome
50%−epiboly
Shield
75%−epiboly
1−4−somites
14−19−somites
20−25−somites
Prim−5
Prim−15
Prim−25
Long−pec
Protruding−mouth
Day−4
Day−5
Stage
Transcript
ENSDART00000057439
ENSDART00000137882
ENSDART00000144640
ENSDART00000153255

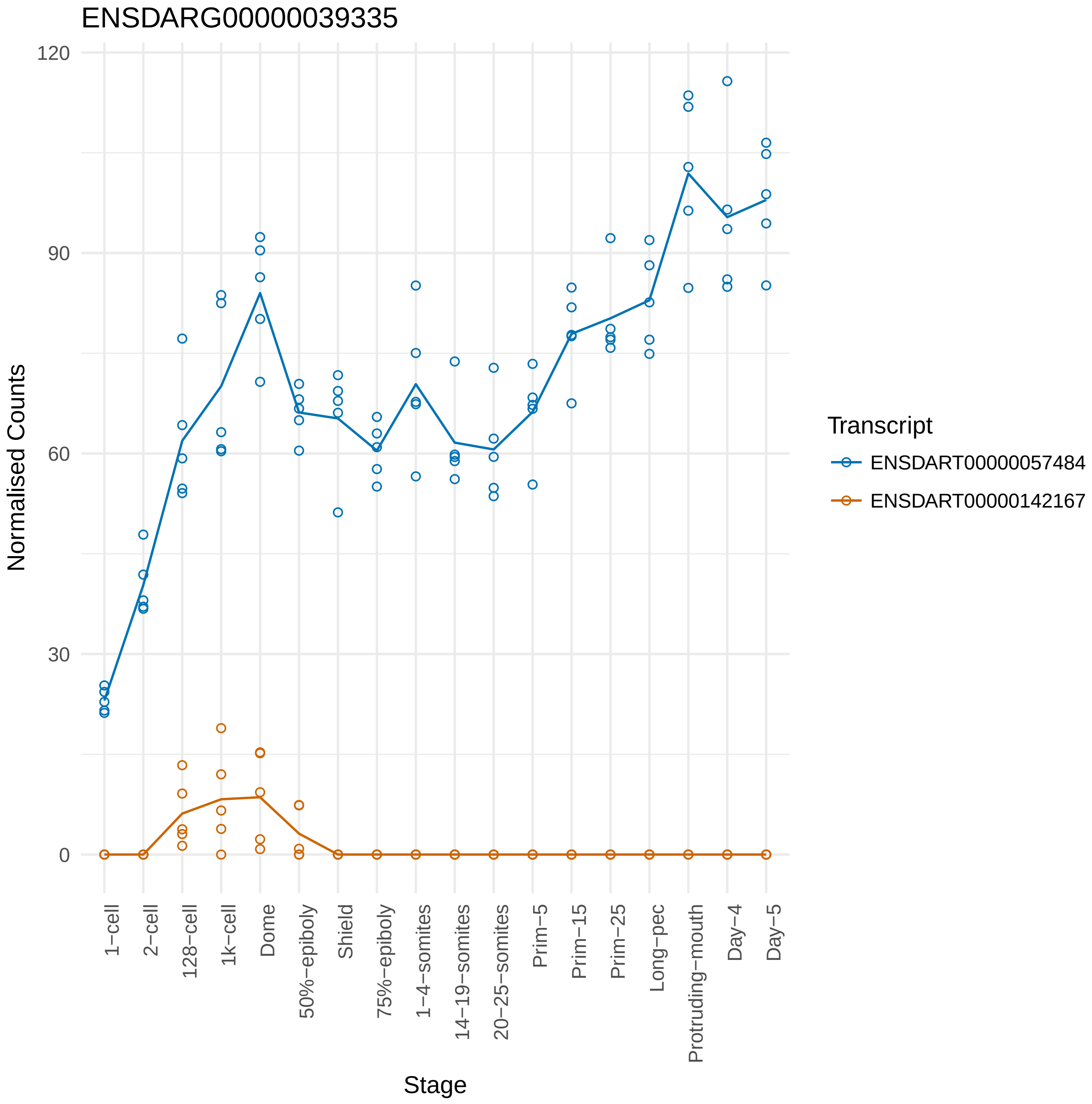

ENSDARG00000039335
Normalised Counts
120
90
60
30
0
1−cell
2−cell
128−cell
1k−cell
Dome
50%−epiboly
Shield
75%−epiboly
1−4−somites
14−19−somites
20−25−somites
Prim−5
Prim−15
Prim−25
Long−pec
Protruding−mouth
Day−4
Day−5
Stage
Transcript
ENSDART00000057484
ENSDART00000142167

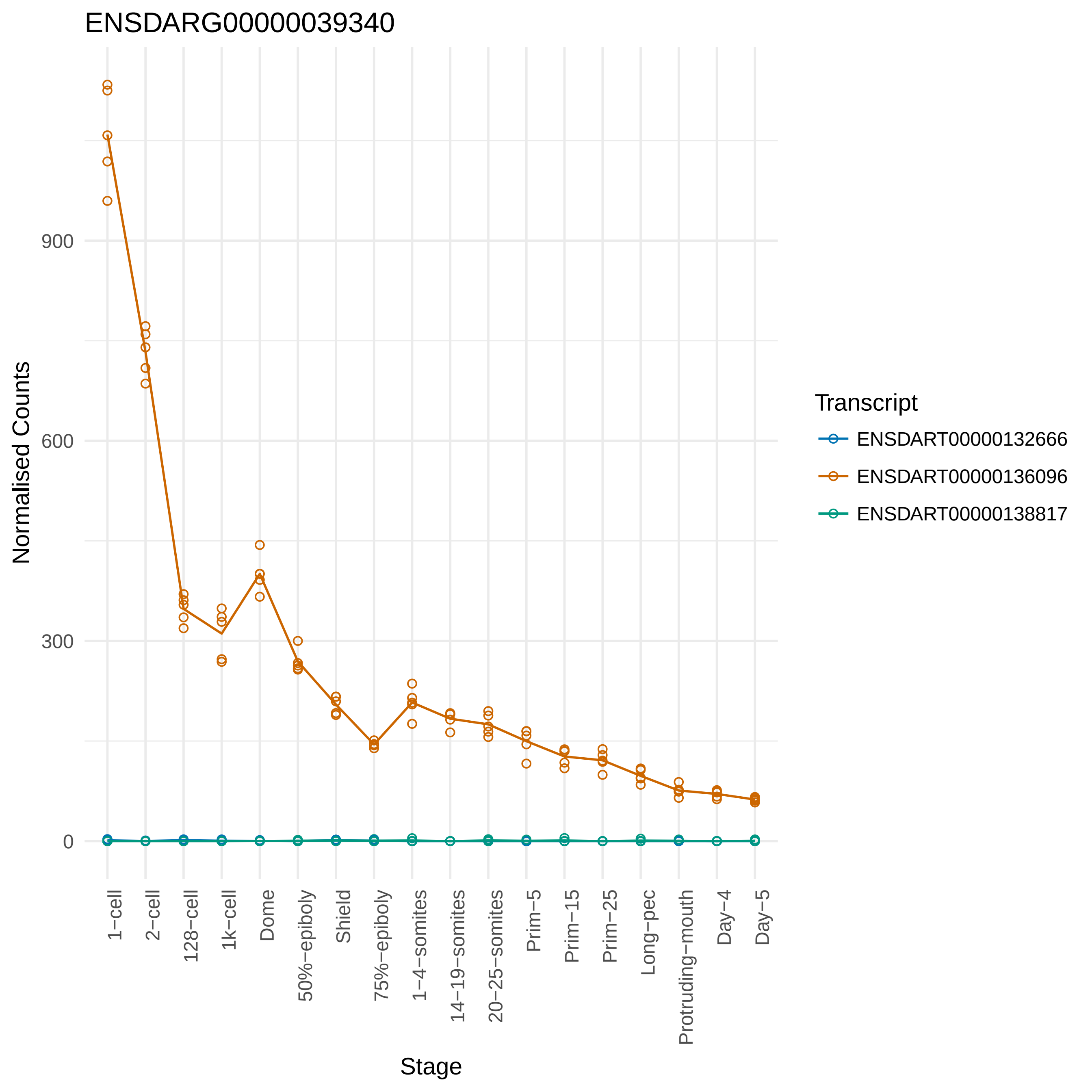

ENSDARG00000039340
Normalised Counts
900
600
300
0
1−cell
2−cell
128−cell
1k−cell
Dome
50%−epiboly
Shield
75%−epiboly
1−4−somites
14−19−somites
20−25−somites
Prim−5
Prim−15
Prim−25
Long−pec
Protruding−mouth
Day−4
Day−5
Stage
Transcript
ENSDART00000132666
ENSDART00000136096
ENSDART00000138817

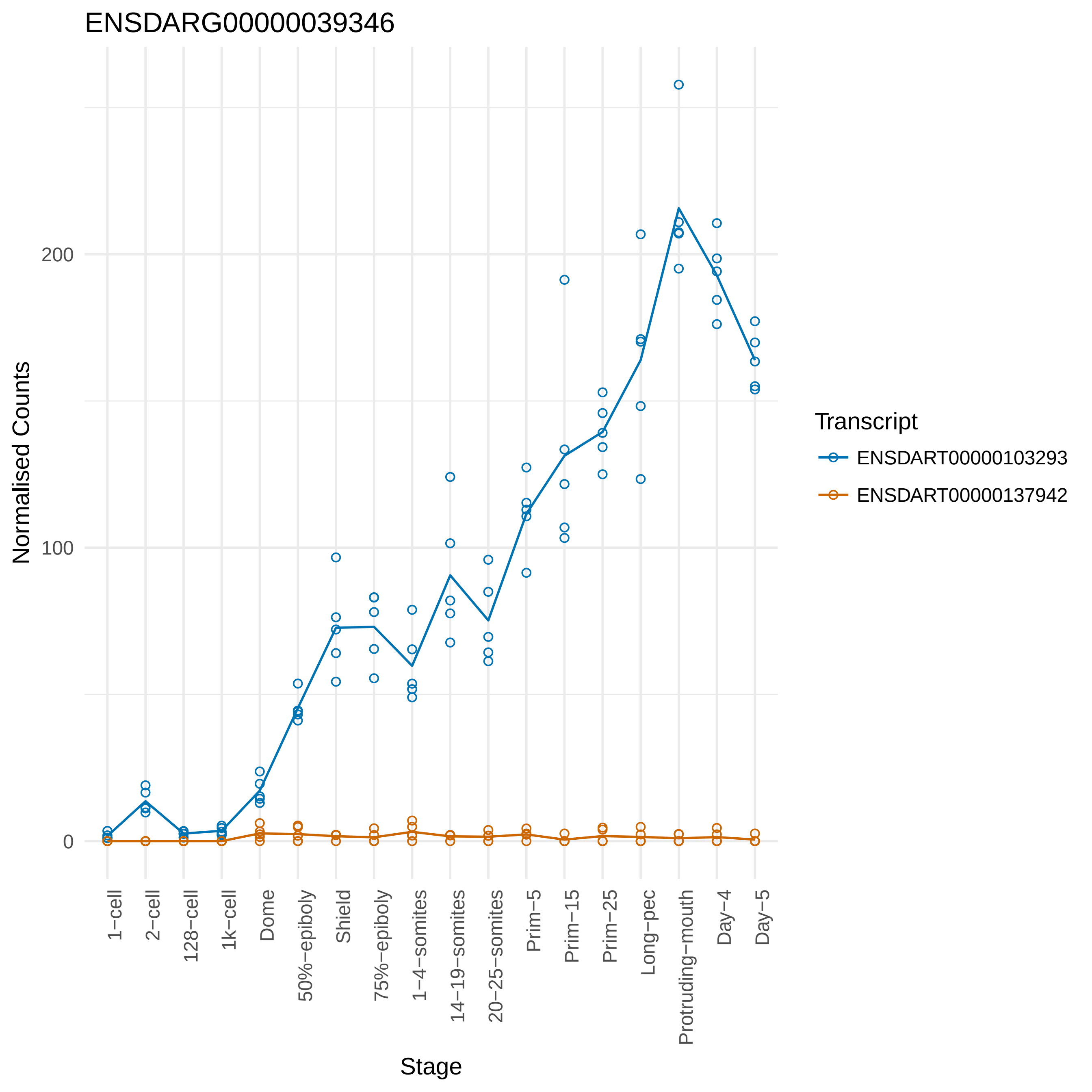

ENSDARG00000039346
Normalised Counts
200
100
0
1−cell
2−cell
128−cell
1k−cell
Dome
50%−epiboly
Shield
75%−epiboly
1−4−somites
14−19−somites
20−25−somites
Prim−5
Prim−15
Prim−25
Long−pec
Protruding−mouth
Day−4
Day−5
Stage
Transcript
ENSDART00000103293
ENSDART00000137942

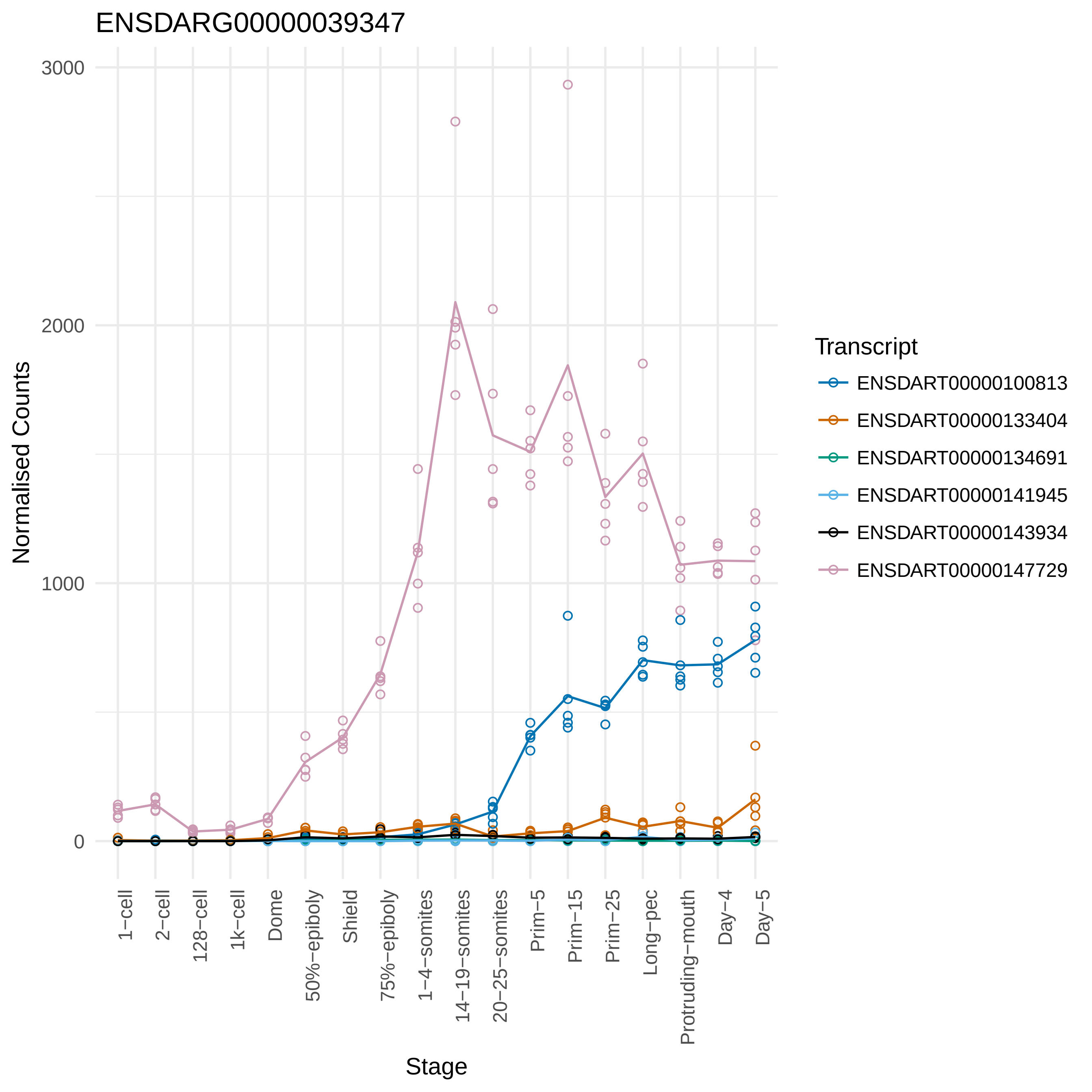
ENSDARG00000039347
Normalised Counts
3000
2000
1000
0
Stage
1−cell
2−cell
128−cell
1k−cell
Dome
50%−epiboly
Shield
75%−epiboly
1−4−somites
14−19−somites
20−25−somites
Prim−5
Prim−15
Prim−25
Long−pec
Protruding−mouth
Day−4
Day−5
Transcript
ENSDART00000100813
ENSDART00000133404
ENSDART00000134691
ENSDART00000141945
ENSDART00000143934
ENSDART00000147729

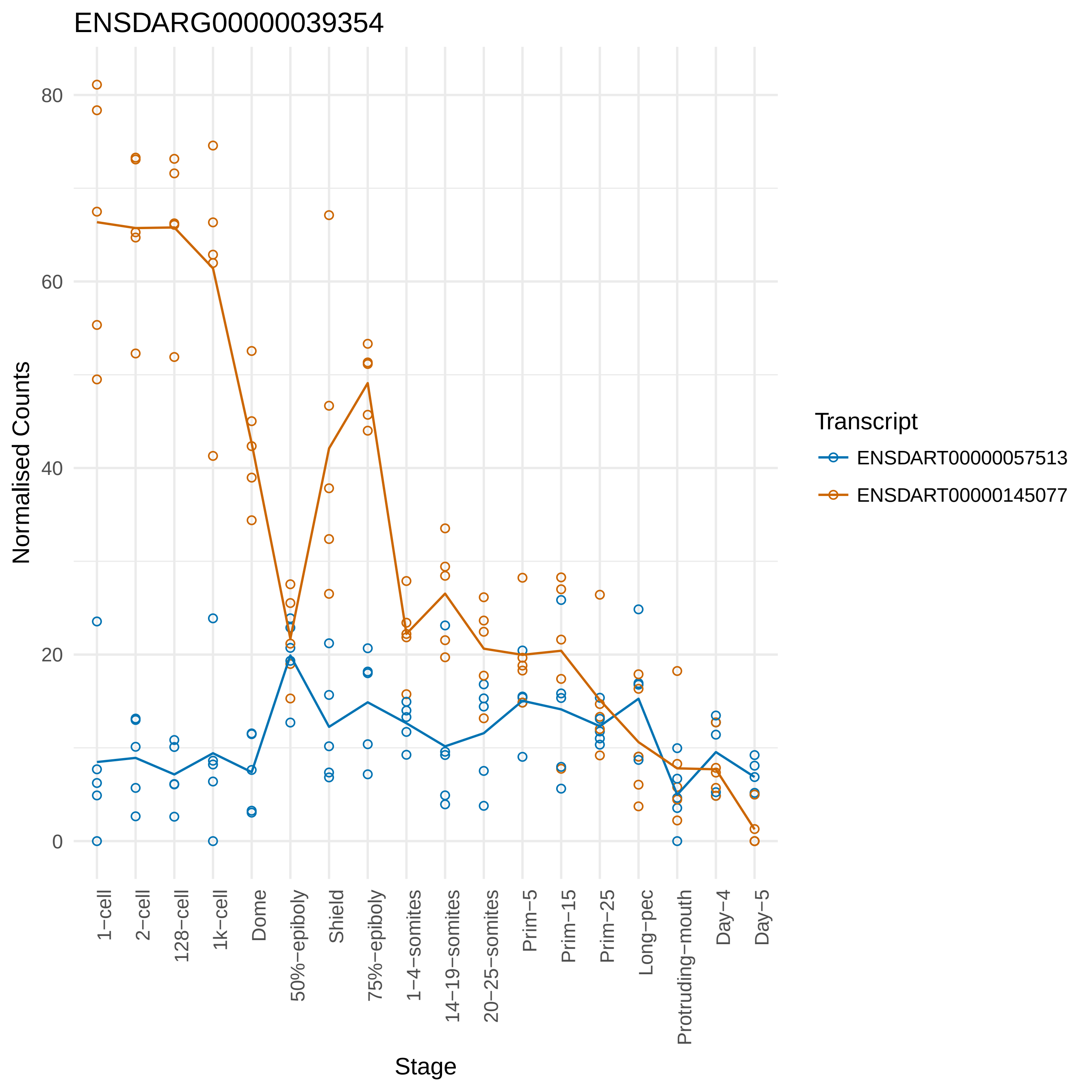

ENSDARG00000039354
Normalised Counts
0
20
40
60
80
1−cell
2−cell
128−cell
1k−cell
Dome
50%−epiboly
Shield
75%−epiboly
1−4−somites
14−19−somites
20−25−somites
Prim−5
Prim−15
Prim−25
Long−pec
Protruding−mouth
Day−4
Day−5
Stage
Transcript
ENSDART00000057513
ENSDART00000145077

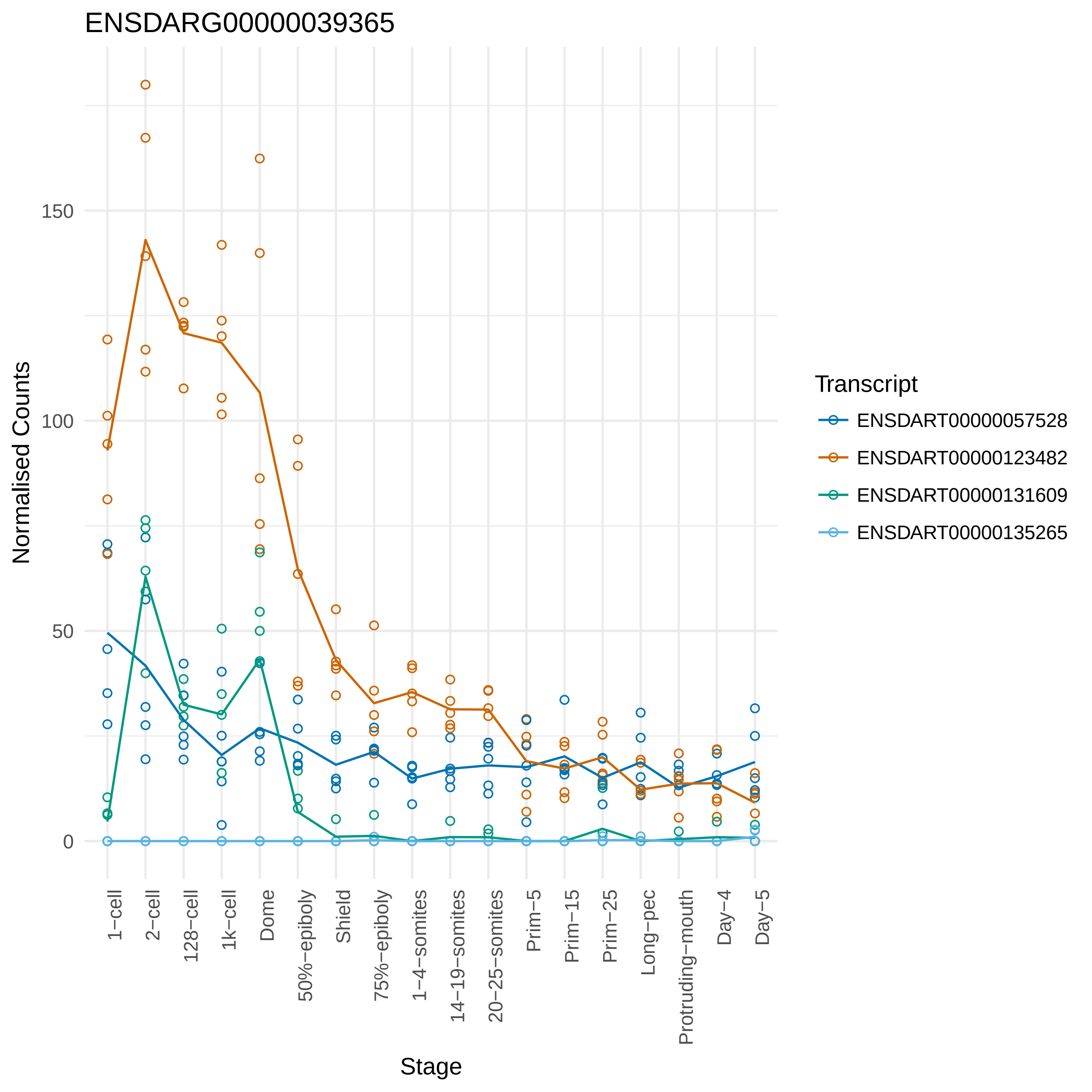

ENSDARG00000039365
Normalised Counts
0
50
100
150
1−cell
2−cell
128−cell
1k−cell
Dome
50%−epiboly
Shield
75%−epiboly
1−4−somites
14−19−somites
20−25−somites
Prim−5
Prim−15
Prim−25
Long−pec
Protruding−mouth
Day−4
Day−5
Stage
Transcript
ENSDART00000057528
ENSDART00000123482
ENSDART00000131609
ENSDART00000135265

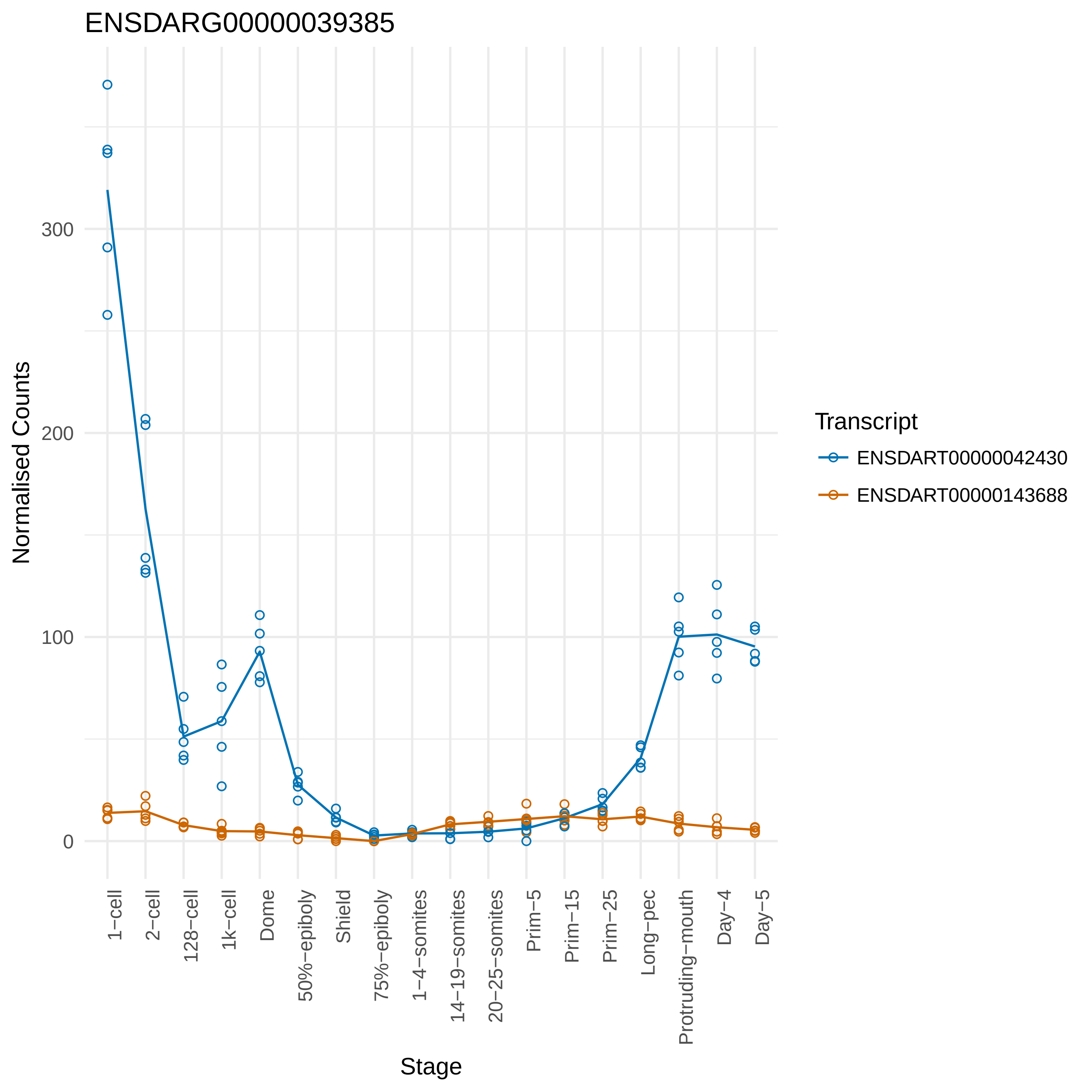

ENSDARG00000039385
Normalised Counts
300
200
100
0
1−cell
2−cell
128−cell
1k−cell
Dome
50%−epiboly
Shield
75%−epiboly
1−4−somites
14−19−somites
20−25−somites
Prim−5
Prim−15
Prim−25
Long−pec
Protruding−mouth
Day−4
Day−5
Stage
Transcript
ENSDART00000042430
ENSDART00000143688

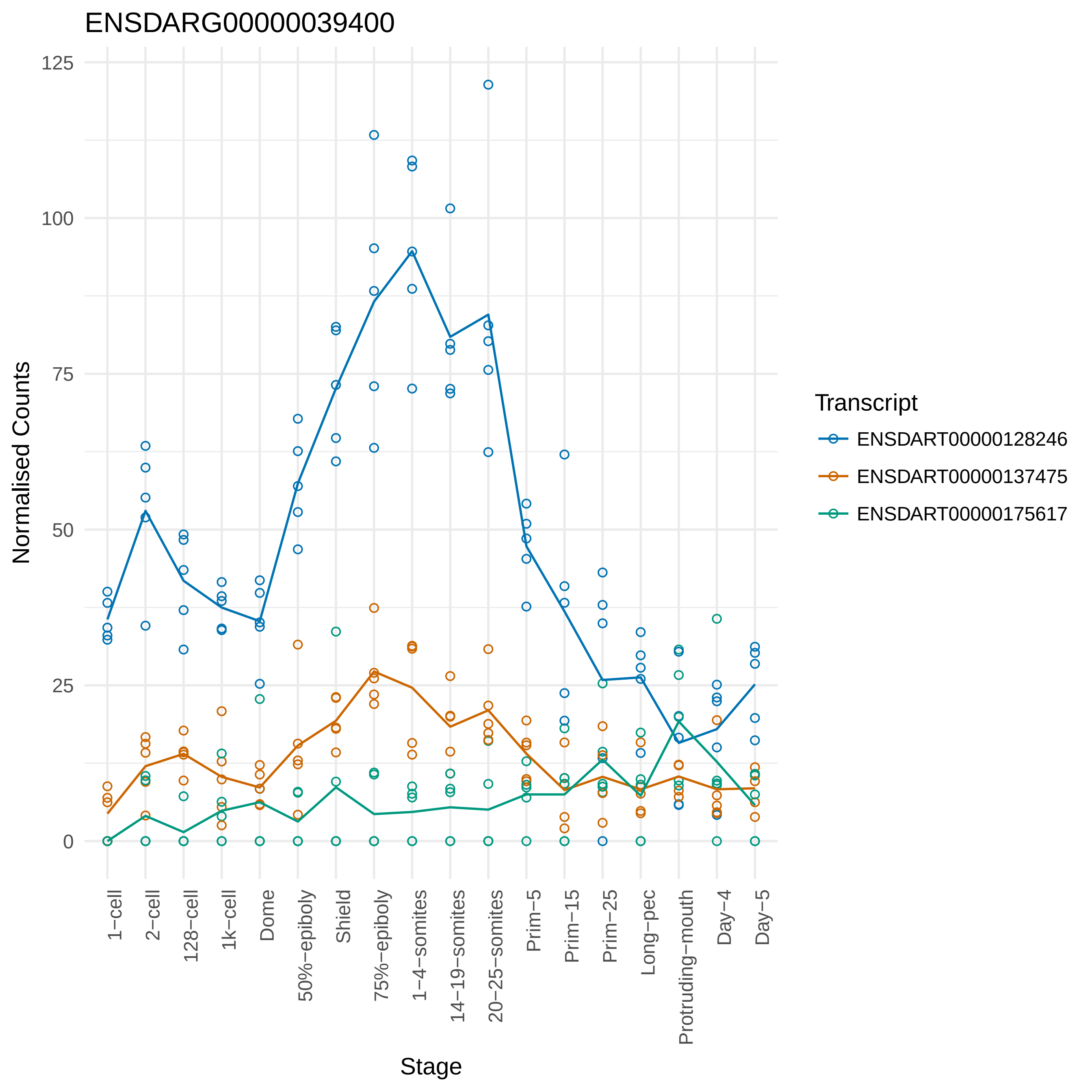
ENSDARG00000039400
Normalised Counts
125
100
75
50
25
0
1−cell
2−cell
128−cell
1k−cell
Dome
50%−epiboly
Shield
75%−epiboly
1−4−somites
14−19−somites
20−25−somites
Prim−5
Prim−15
Prim−25
Long−pec
Protruding−mouth
Day−4
Day−5
Stage
Transcript
ENSDART00000128246
ENSDART00000137475
ENSDART00000175617

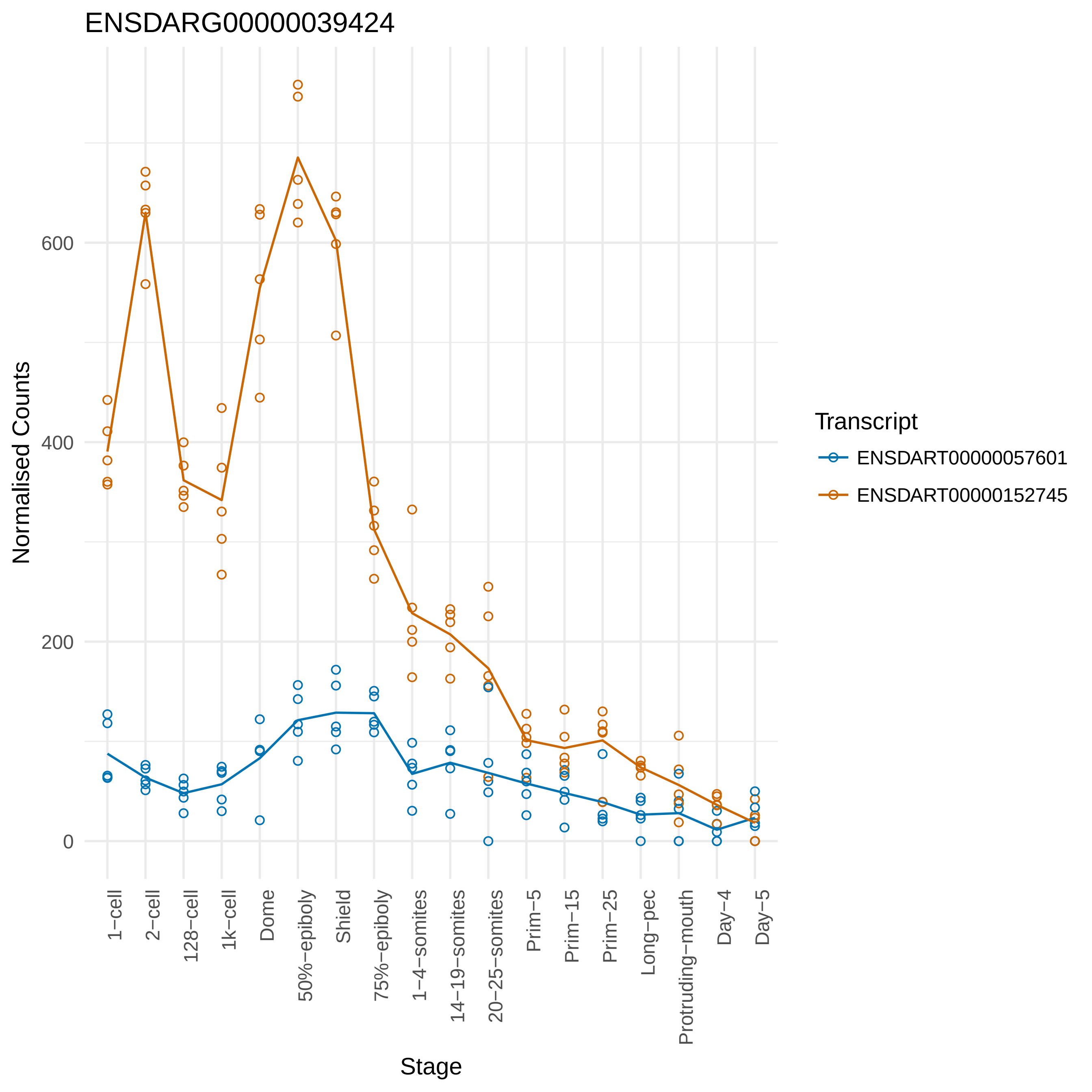

ENSDARG00000039424
Normalised Counts
0
200
400
600
Stage
1−cell
2−cell
128−cell
1k−cell
Dome
50%−epiboly
Shield
75%−epiboly
1−4−somites
14−19−somites
20−25−somites
Prim−5
Prim−15
Prim−25
Long−pec
Protruding−mouth
Day−4
Day−5
Transcript
ENSDART00000057601
ENSDART00000152745

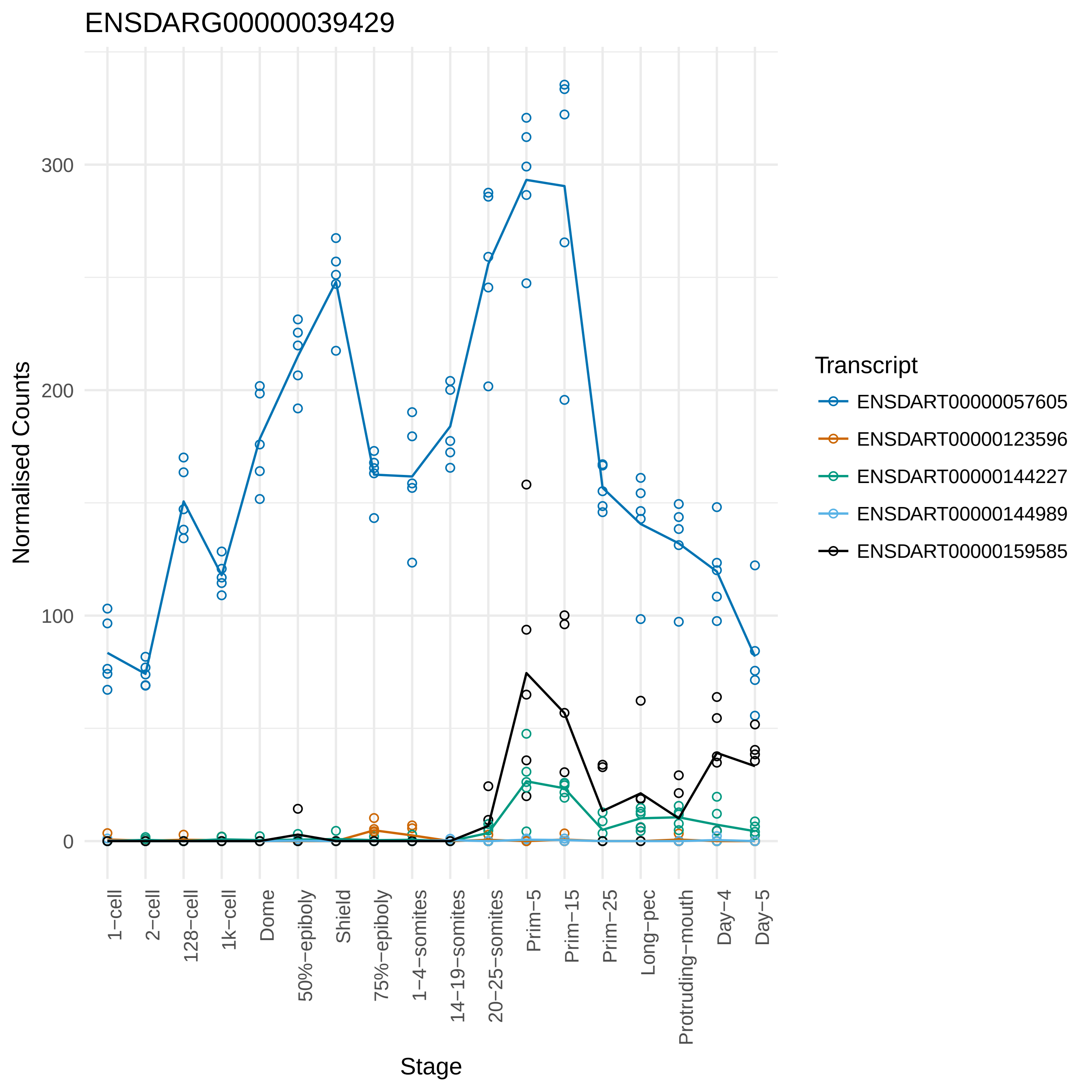
ENSDARG00000039429
Normalised Counts
300
200
100
0
1−cell
2−cell
128−cell
1k−cell
Dome
50%−epiboly
Shield
75%−epiboly
1−4−somites
14−19−somites
20−25−somites
Prim−5
Prim−15
Prim−25
Long−pec
Protruding−mouth
Day−4
Day−5
Stage
Transcript
ENSDART00000057605
ENSDART00000123596
ENSDART00000144227
ENSDART00000144989
ENSDART00000159585

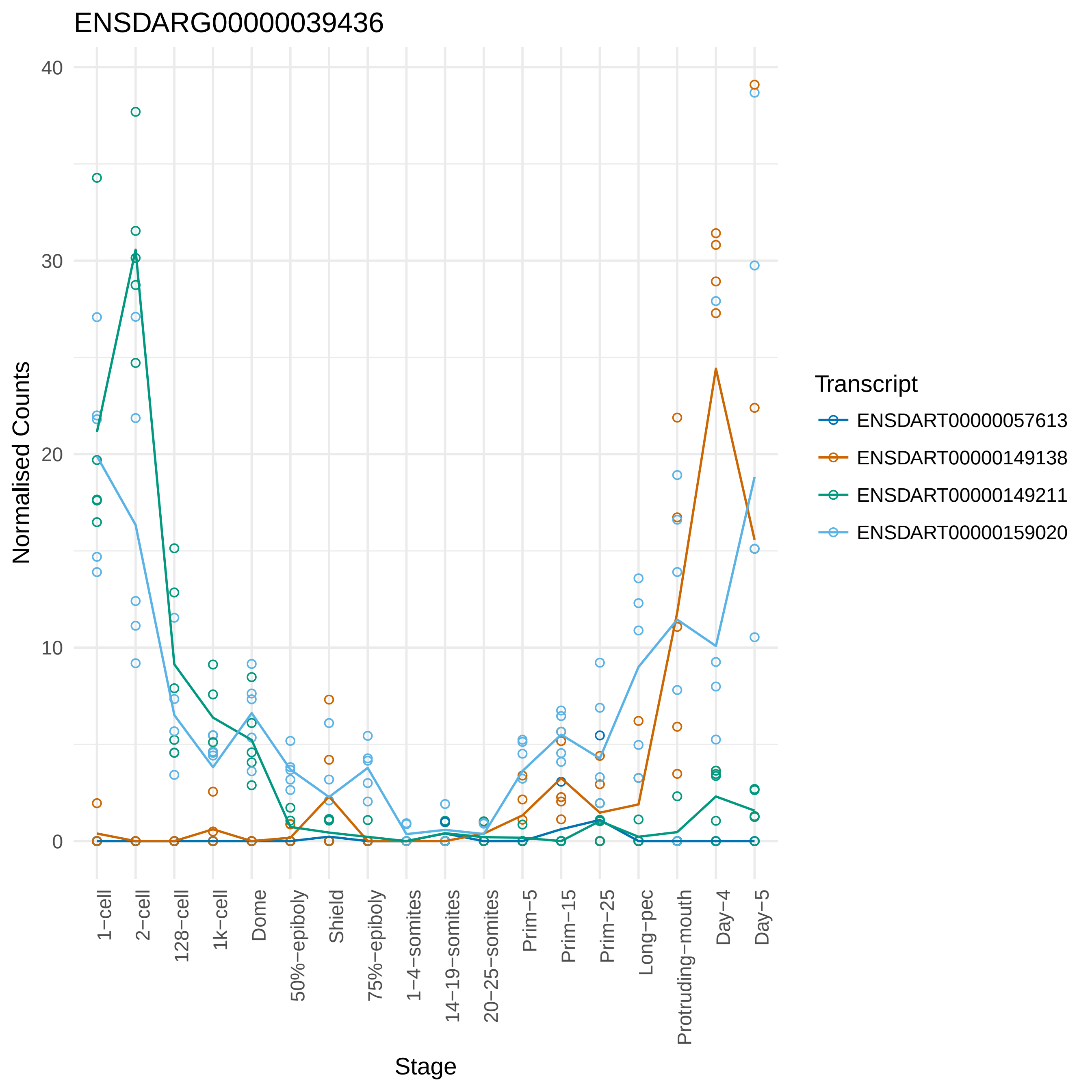

ENSDARG00000039436
Normalised Counts
40
30
20
10
0
1−cell
2−cell
128−cell
1k−cell
Dome
50%−epiboly
Shield
75%−epiboly
1−4−somites
14−19−somites
20−25−somites
Prim−5
Prim−15
Prim−25
Long−pec
Protruding−mouth
Day−4
Day−5
Stage
Transcript
ENSDART00000057613
ENSDART00000149138
ENSDART00000149211
ENSDART00000159020

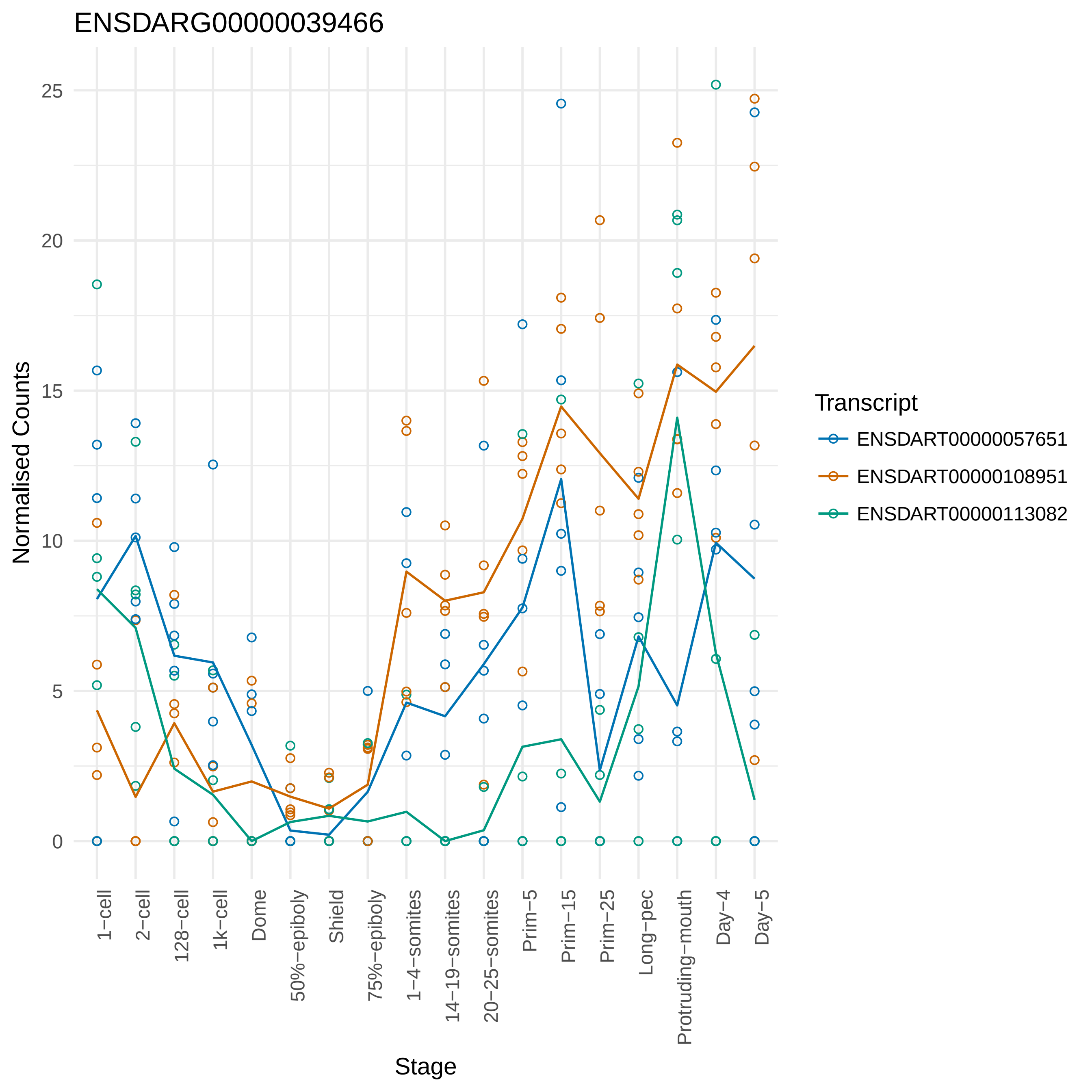

ENSDARG00000039466
Normalised Counts
0
5
10
15
20
25
Stage
1−cell
2−cell
128−cell
1k−cell
Dome
50%−epiboly
Shield
75%−epiboly
1−4−somites
14−19−somites
20−25−somites
Prim−5
Prim−15
Prim−25
Long−pec
Protruding−mouth
Day−4
Day−5
Transcript
ENSDART00000057651
ENSDART00000108951
ENSDART00000113082

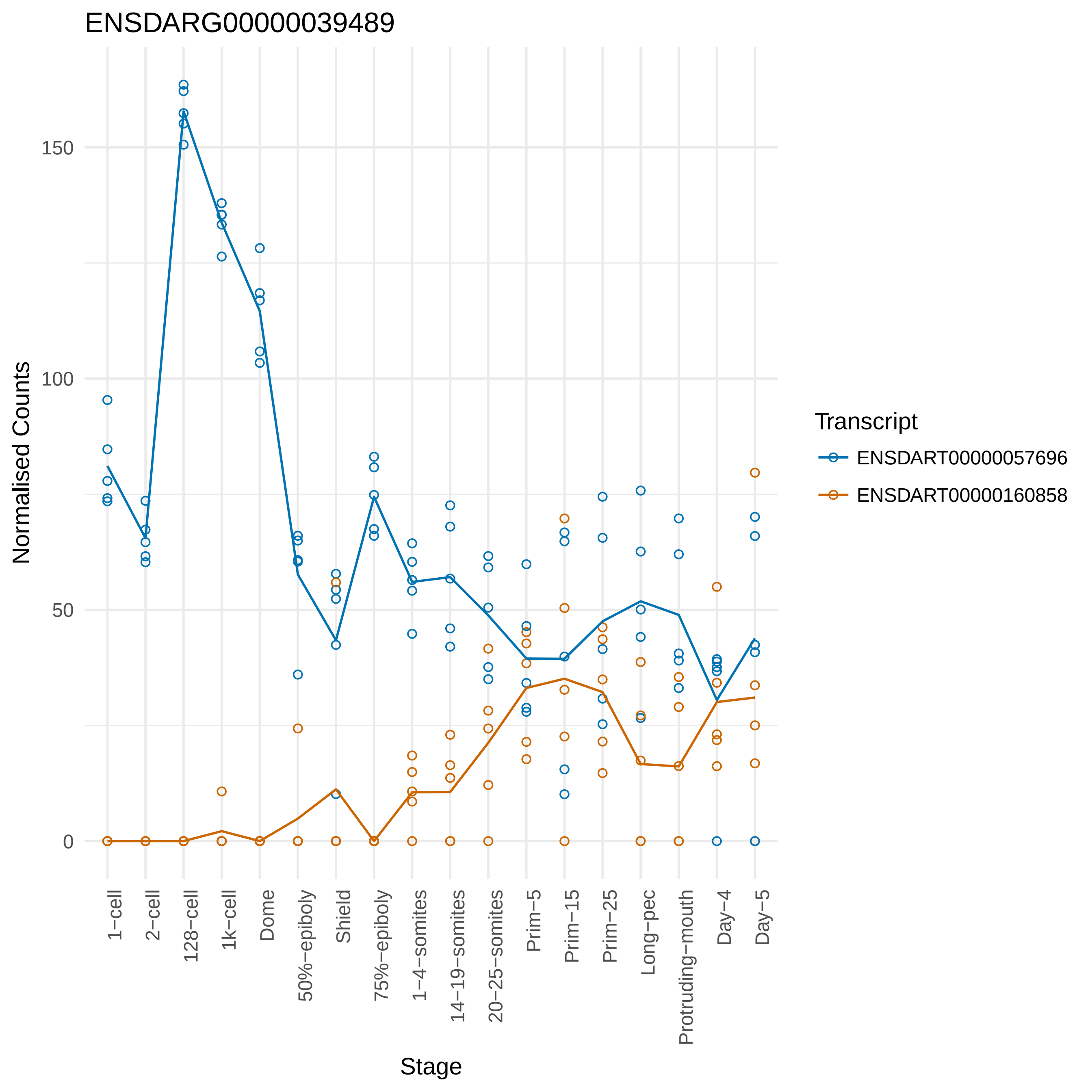
ENSDARG00000039489
Normalised Counts
150
100
50
0
1−cell
2−cell
128−cell
1k−cell
Dome
50%−epiboly
Shield
75%−epiboly
1−4−somites
14−19−somites
20−25−somites
Prim−5
Prim−15
Prim−25
Long−pec
Protruding−mouth
Day−4
Day−5
Stage
Transcript
ENSDART00000057696
ENSDART00000160858

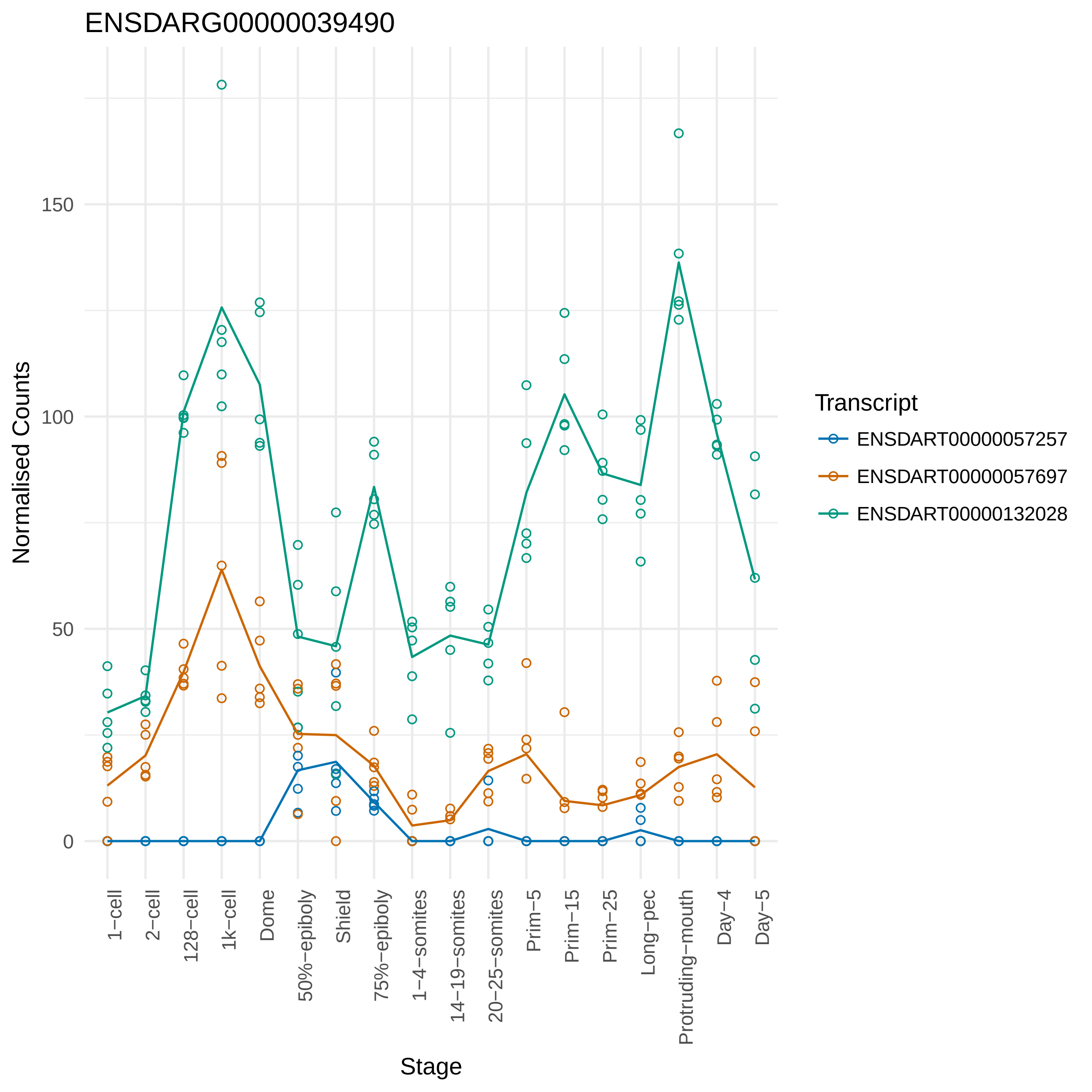

ENSDARG00000039490
Normalised Counts
150
100
50
0
1−cell
2−cell
128−cell
1k−cell
Dome
50%−epiboly
Shield
75%−epiboly
1−4−somites
14−19−somites
20−25−somites
Prim−5
Prim−15
Prim−25
Long−pec
Protruding−mouth
Day−4
Day−5
Stage
Transcript
ENSDART00000057257
ENSDART00000057697
ENSDART00000132028

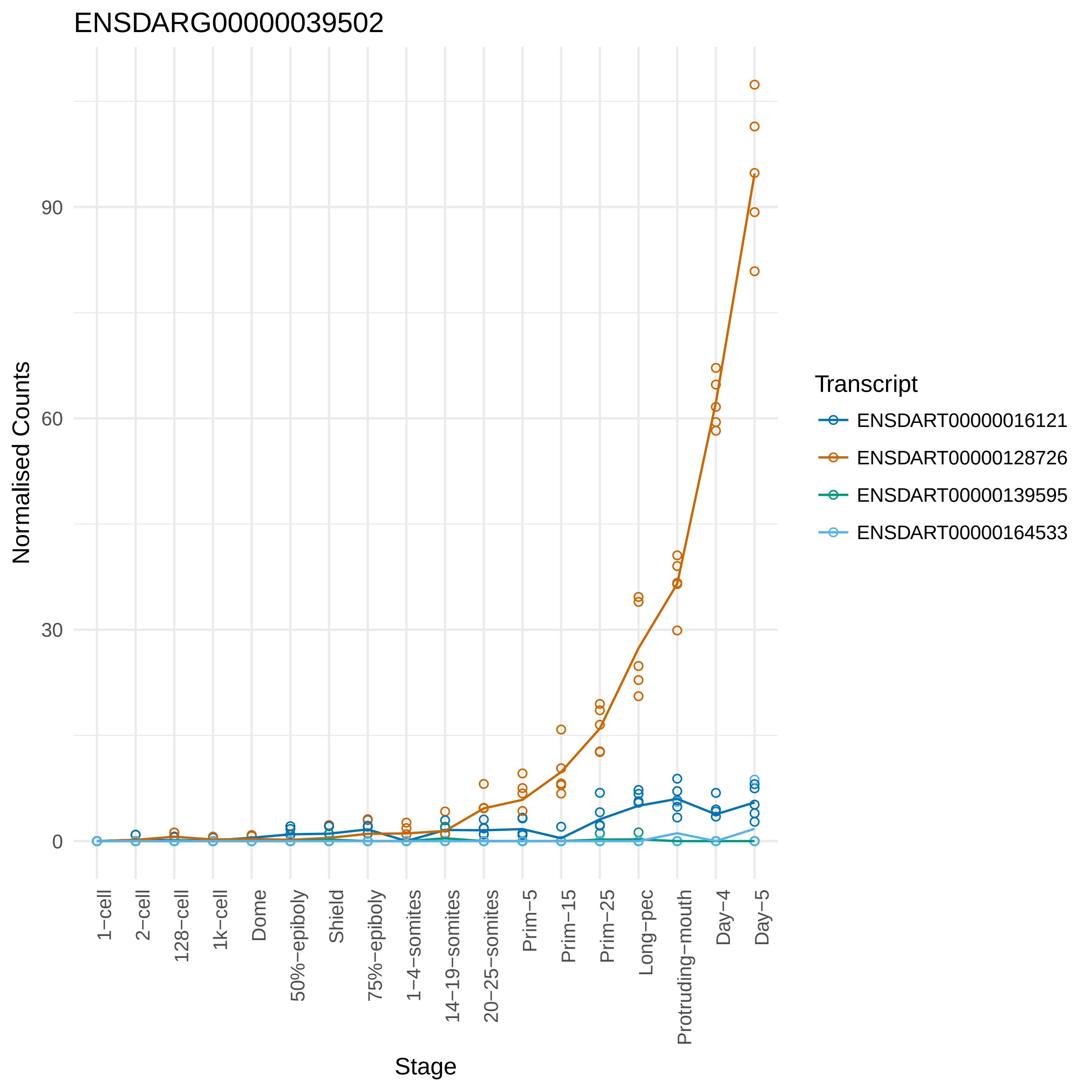
ENSDARG00000039502
Normalised Counts
0
30
60
90
1−cell
2−cell
128−cell
1k−cell
Dome
50%−epiboly
Shield
75%−epiboly
1−4−somites
14−19−somites
20−25−somites
Prim−5
Prim−15
Prim−25
Long−pec
Protruding−mouth
Day−4
Day−5
Stage
Transcript
ENSDART00000016121
ENSDART00000128726
ENSDART00000139595
ENSDART00000164533

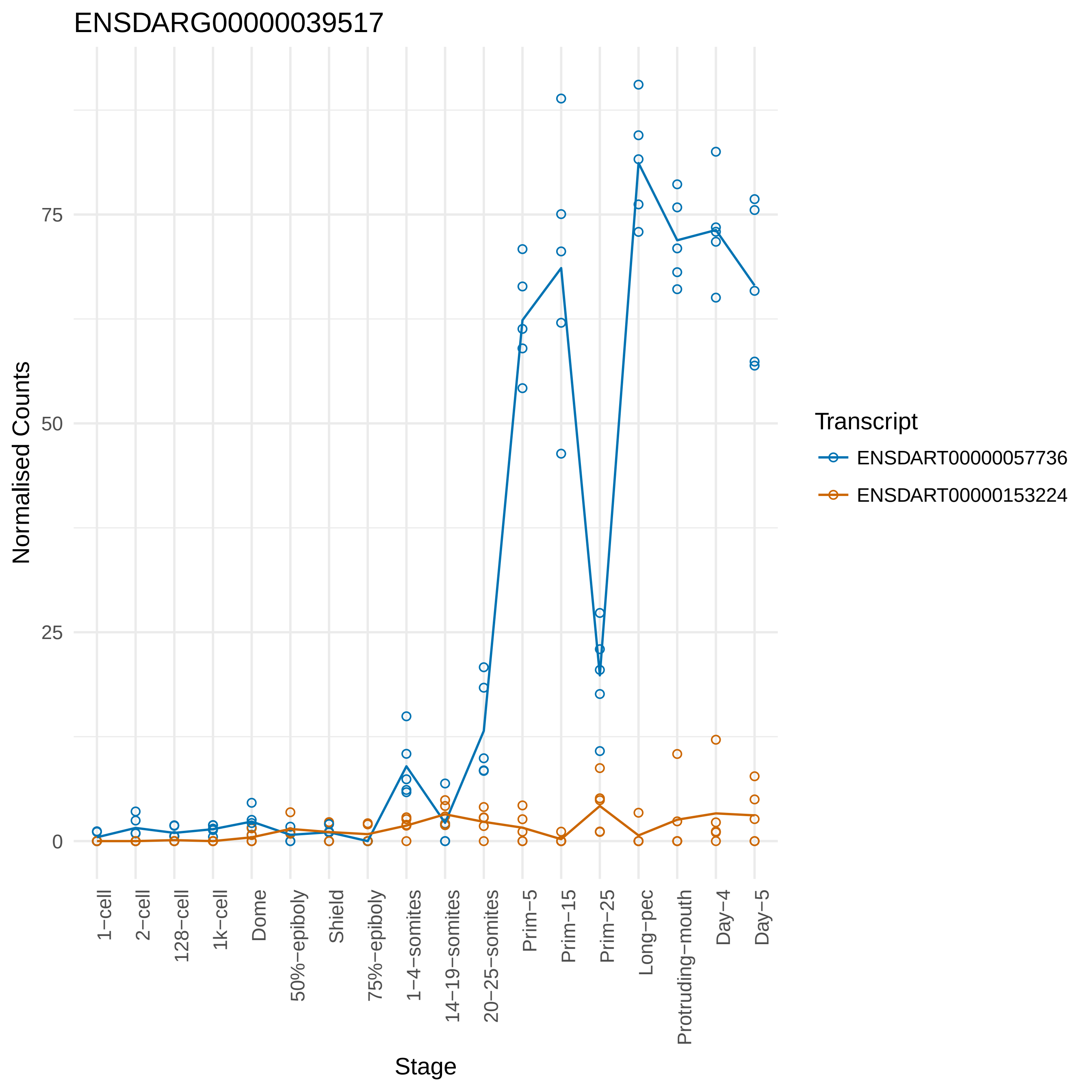

ENSDARG00000039517
Normalised Counts
75
50
25
0
1−cell
2−cell
128−cell
1k−cell
Dome
50%−epiboly
Shield
75%−epiboly
1−4−somites
14−19−somites
20−25−somites
Prim−5
Prim−15
Prim−25
Long−pec
Protruding−mouth
Day−4
Day−5
Stage
Transcript
ENSDART00000057736
ENSDART00000153224

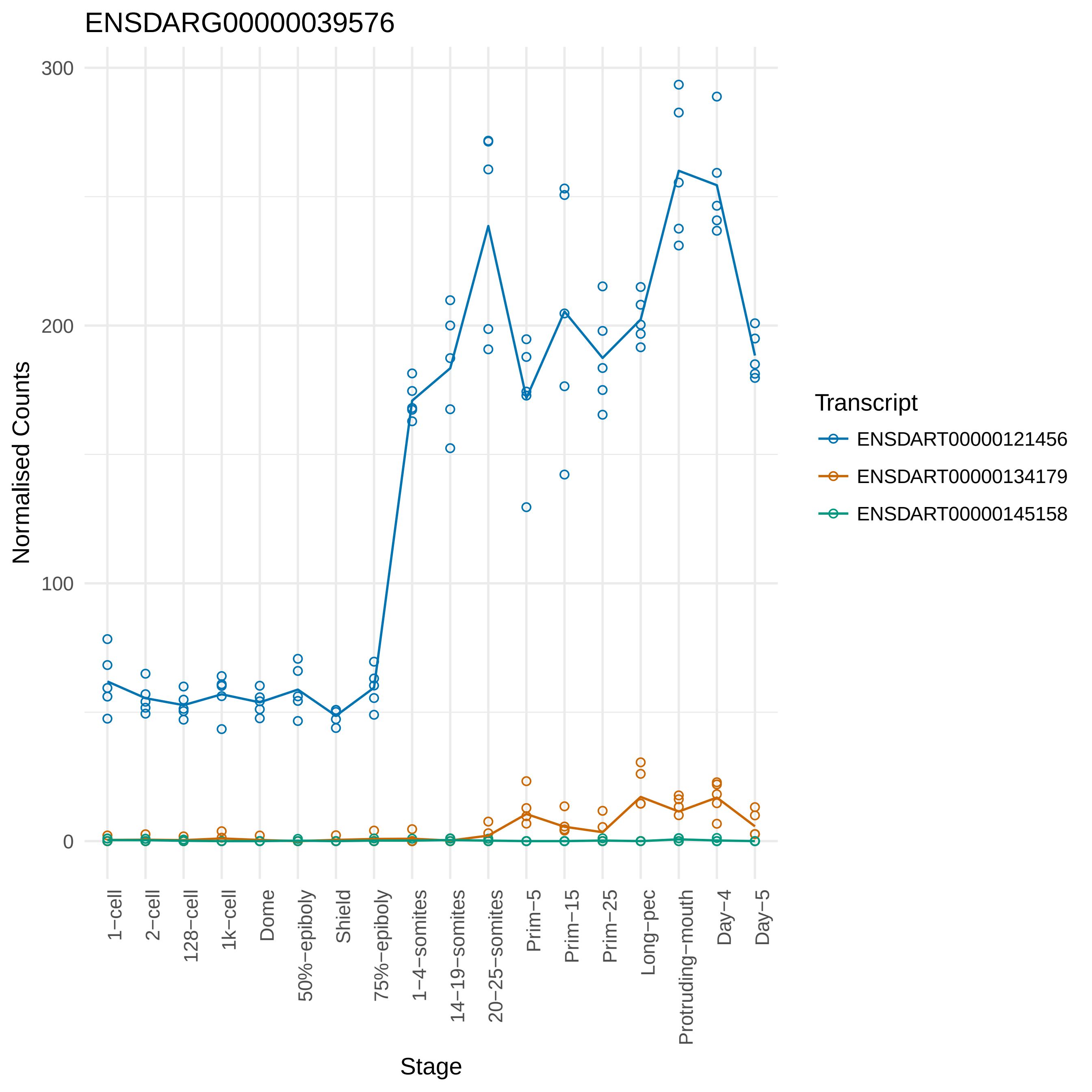

ENSDARG00000039576
Normalised Counts
300
200
100
0
1−cell
2−cell
128−cell
1k−cell
Dome
50%−epiboly
Shield
75%−epiboly
1−4−somites
14−19−somites
20−25−somites
Prim−5
Prim−15
Prim−25
Long−pec
Protruding−mouth
Day−4
Day−5
Stage
Transcript
ENSDART00000121456
ENSDART00000134179
ENSDART00000145158

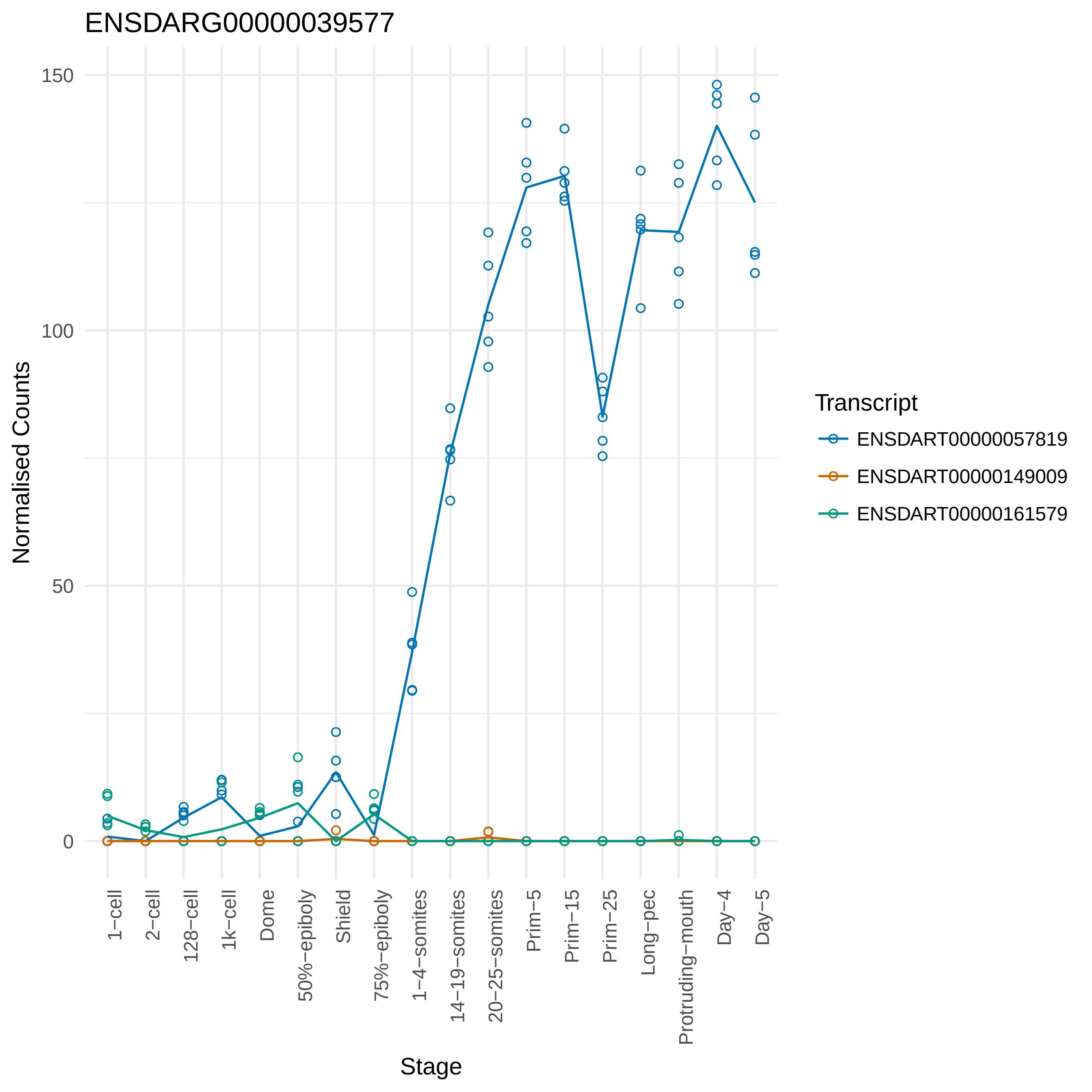

ENSDARG00000039577
Normalised Counts
150
100
50
0
1−cell
2−cell
128−cell
1k−cell
Dome
50%−epiboly
Shield
75%−epiboly
1−4−somites
14−19−somites
20−25−somites
Prim−5
Prim−15
Prim−25
Long−pec
Protruding−mouth
Day−4
Day−5
Stage
Transcript
ENSDART00000057819
ENSDART00000149009
ENSDART00000161579

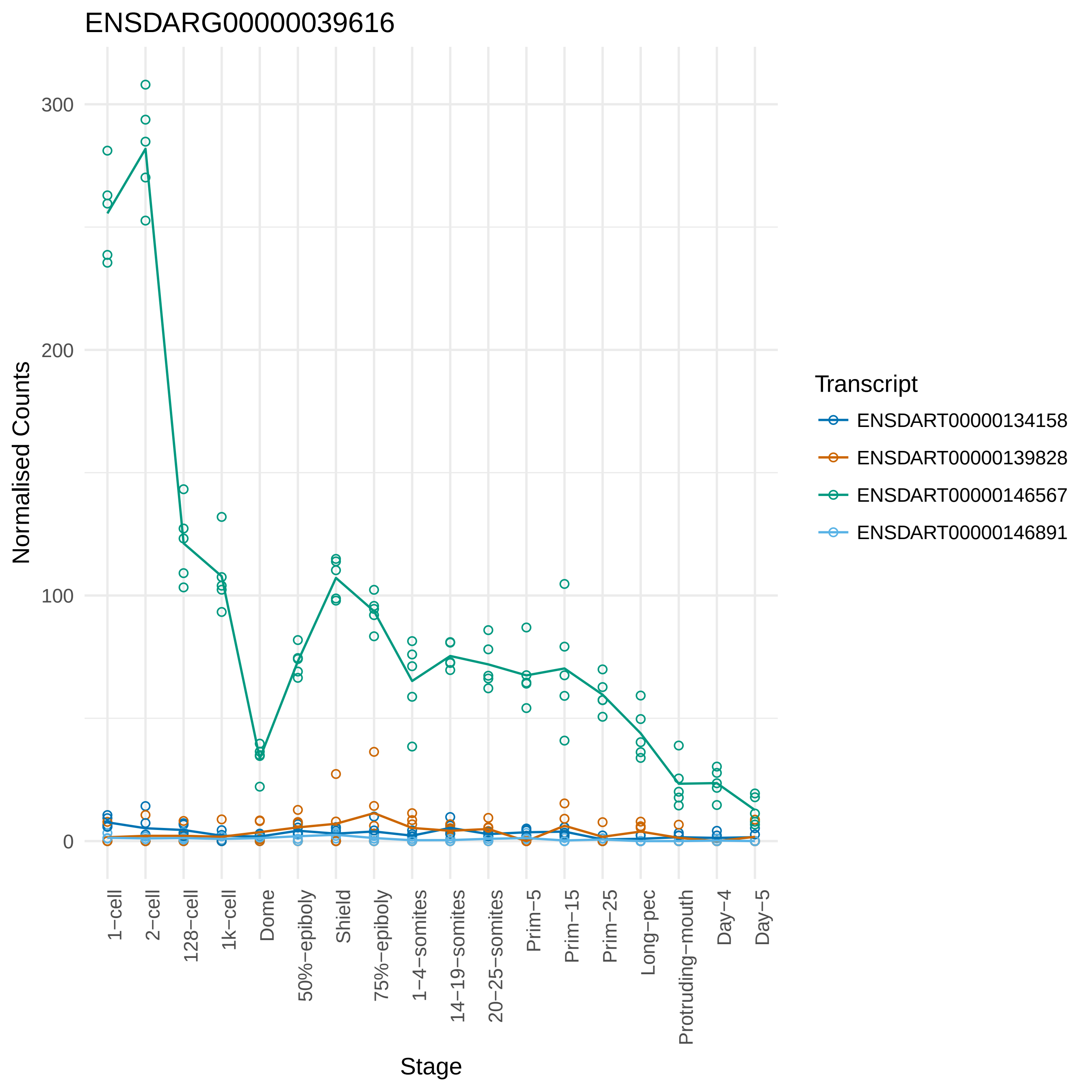
ENSDARG00000039616
Normalised Counts
300
200
100
0
Stage
1−cell
2−cell
128−cell
1k−cell
Dome
50%−epiboly
Shield
75%−epiboly
1−4−somites
14−19−somites
20−25−somites
Prim−5
Prim−15
Prim−25
Long−pec
Protruding−mouth
Day−4
Day−5
Transcript
ENSDART00000134158
ENSDART00000139828
ENSDART00000146567
ENSDART00000146891

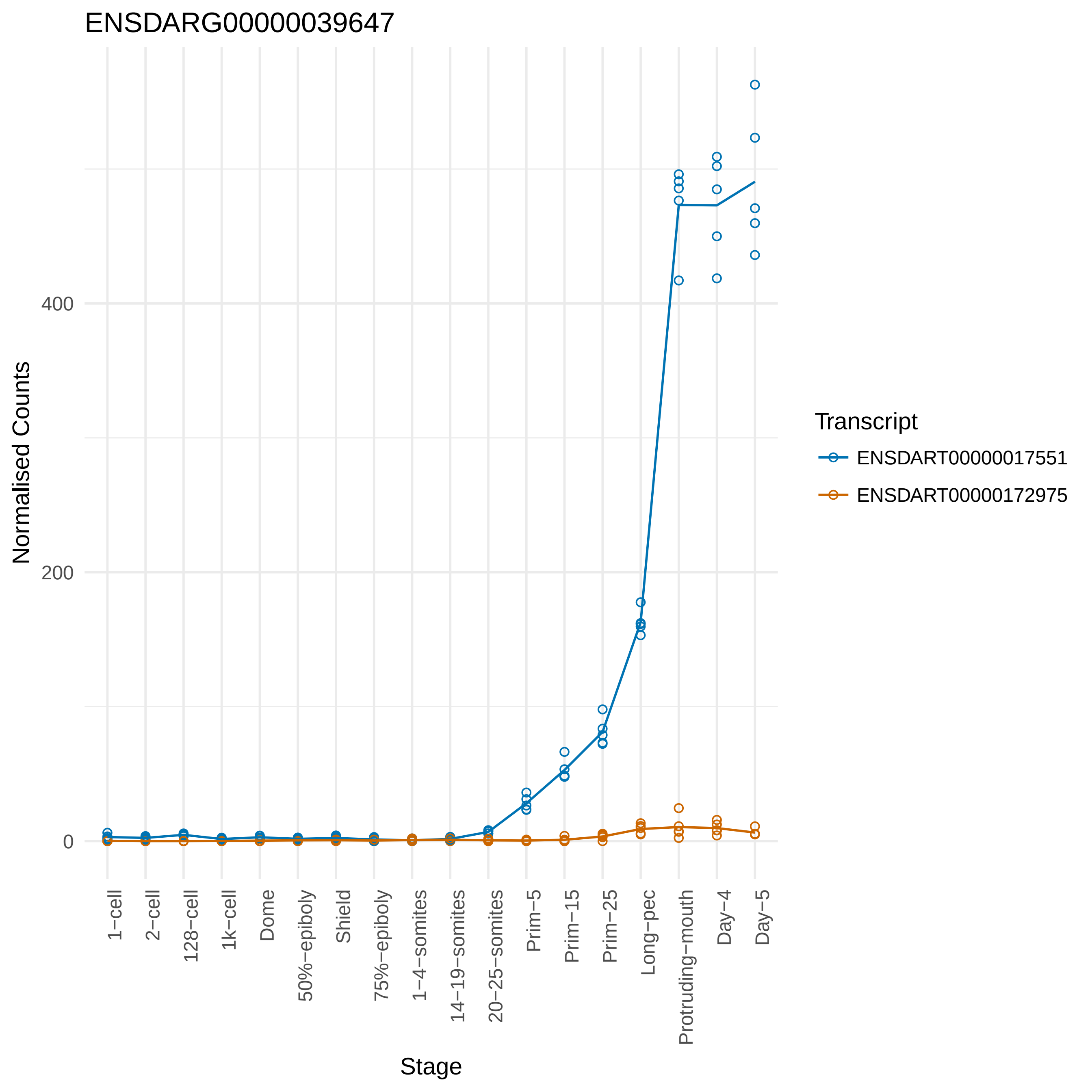

ENSDARG00000039647
Normalised Counts
0
200
400
Stage
1−cell
2−cell
128−cell
1k−cell
Dome
50%−epiboly
Shield
75%−epiboly
1−4−somites
14−19−somites
20−25−somites
Prim−5
Prim−15
Prim−25
Long−pec
Protruding−mouth
Day−4
Day−5
Transcript
ENSDART00000017551
ENSDART00000172975

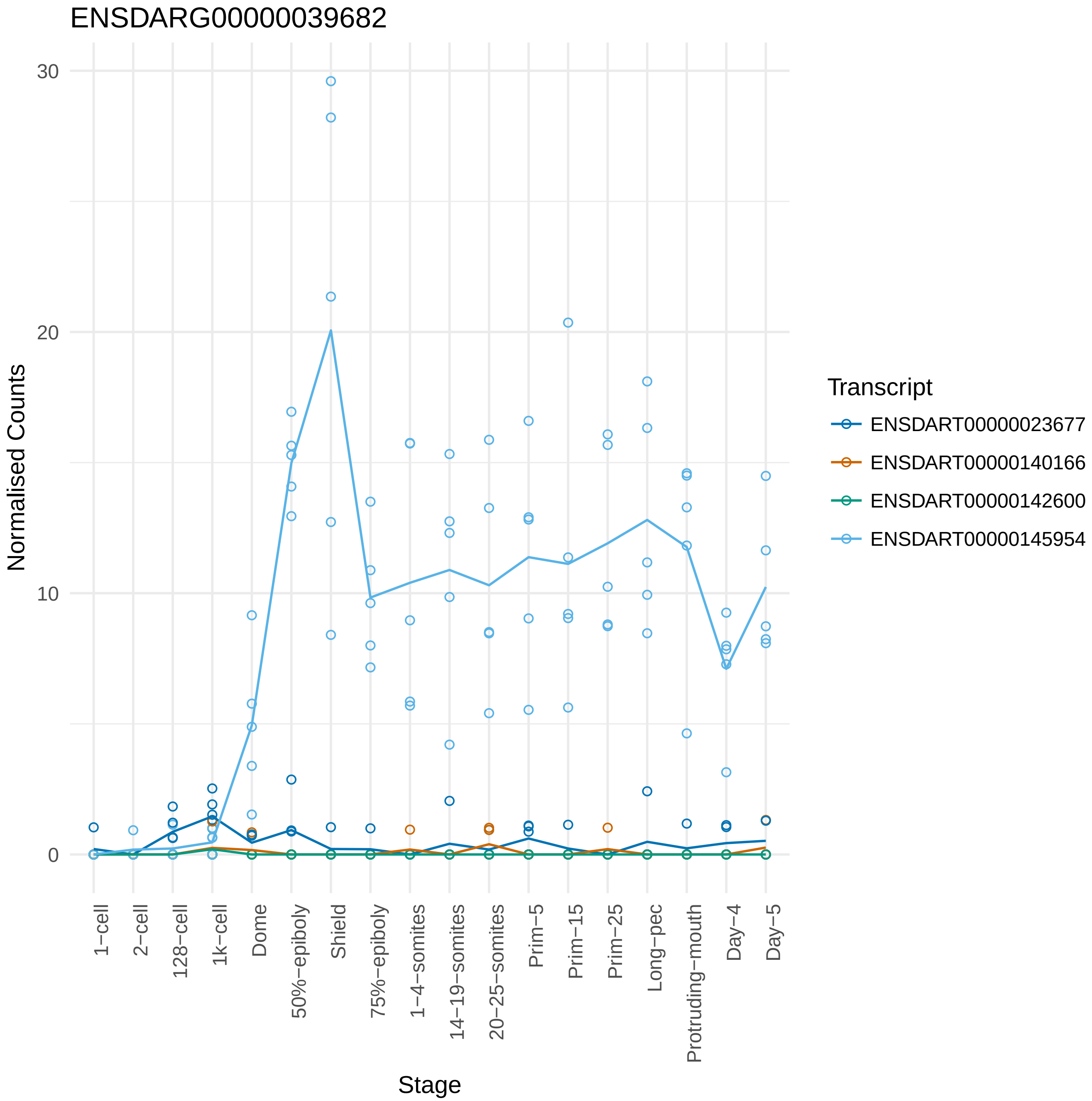
ENSDARG00000039682
Normalised Counts
30
20
10
0
1−cell
2−cell
128−cell
1k−cell
Dome
50%−epiboly
Shield
75%−epiboly
1−4−somites
14−19−somites
20−25−somites
Prim−5
Prim−15
Prim−25
Long−pec
Protruding−mouth
Day−4
Day−5
Stage
Transcript
ENSDART00000023677
ENSDART00000140166
ENSDART00000142600
ENSDART00000145954

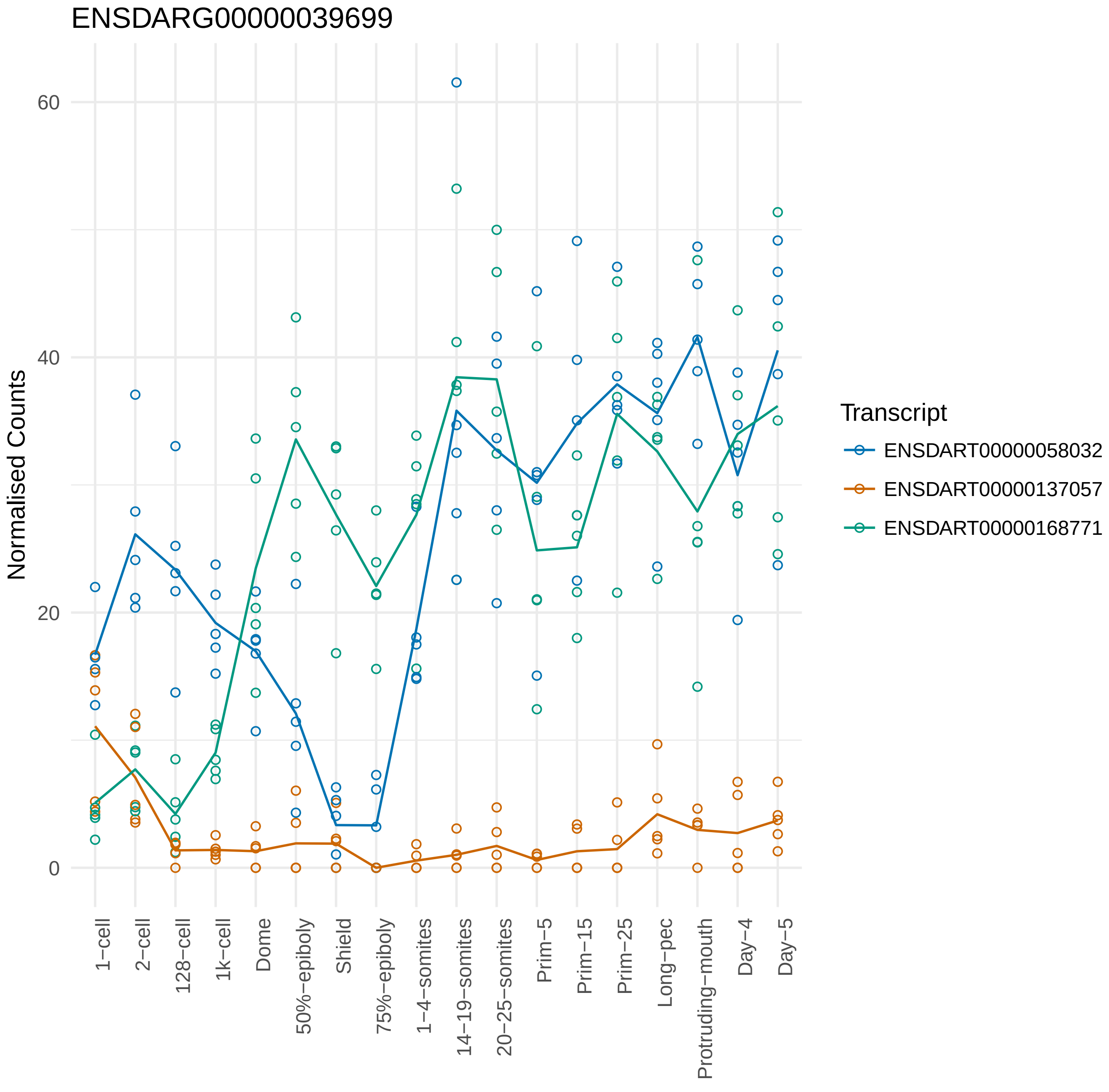
ENSDARG00000039699
Normalised Counts
0
20
40
60
1−cell
2−cell
128−cell
1k−cell
Dome
50%−epiboly
Shield
75%−epiboly
1−4−somites
14−19−somites
20−25−somites
Prim−5
Prim−15
Prim−25
Long−pec
Protruding−mouth
Day−4
Day−5
Stage
Transcript
ENSDART00000058032
ENSDART00000137057
ENSDART00000168771

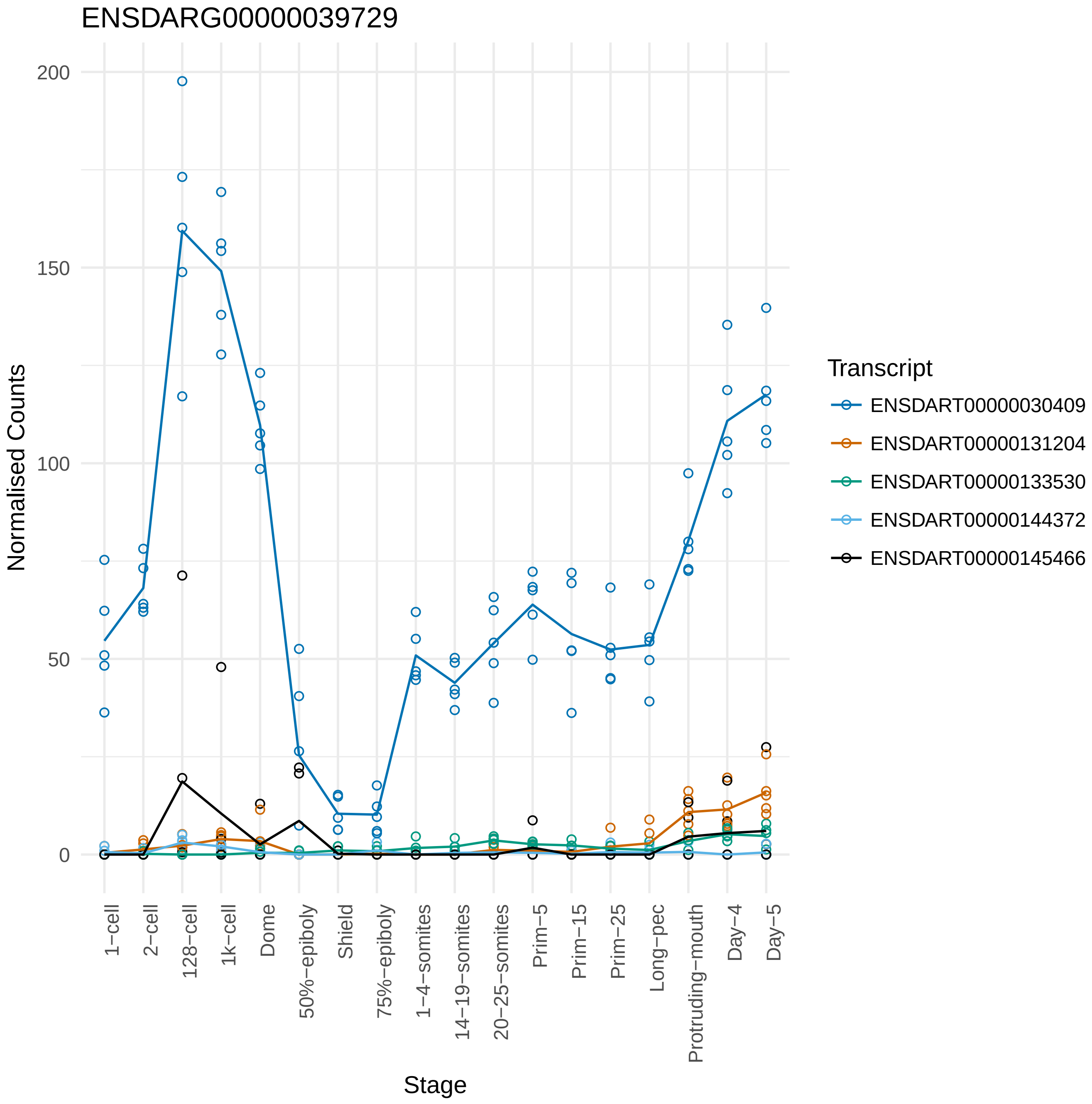
ENSDARG00000039729
Normalised Counts
200
150
100
50
0
1−cell
2−cell
128−cell
1k−cell
Dome
50%−epiboly
Shield
75%−epiboly
1−4−somites
14−19−somites
20−25−somites
Prim−5
Prim−15
Prim−25
Long−pec
Protruding−mouth
Day−4
Day−5
Stage
Transcript
ENSDART00000030409
ENSDART00000131204
ENSDART00000133530
ENSDART00000144372
ENSDART00000145466

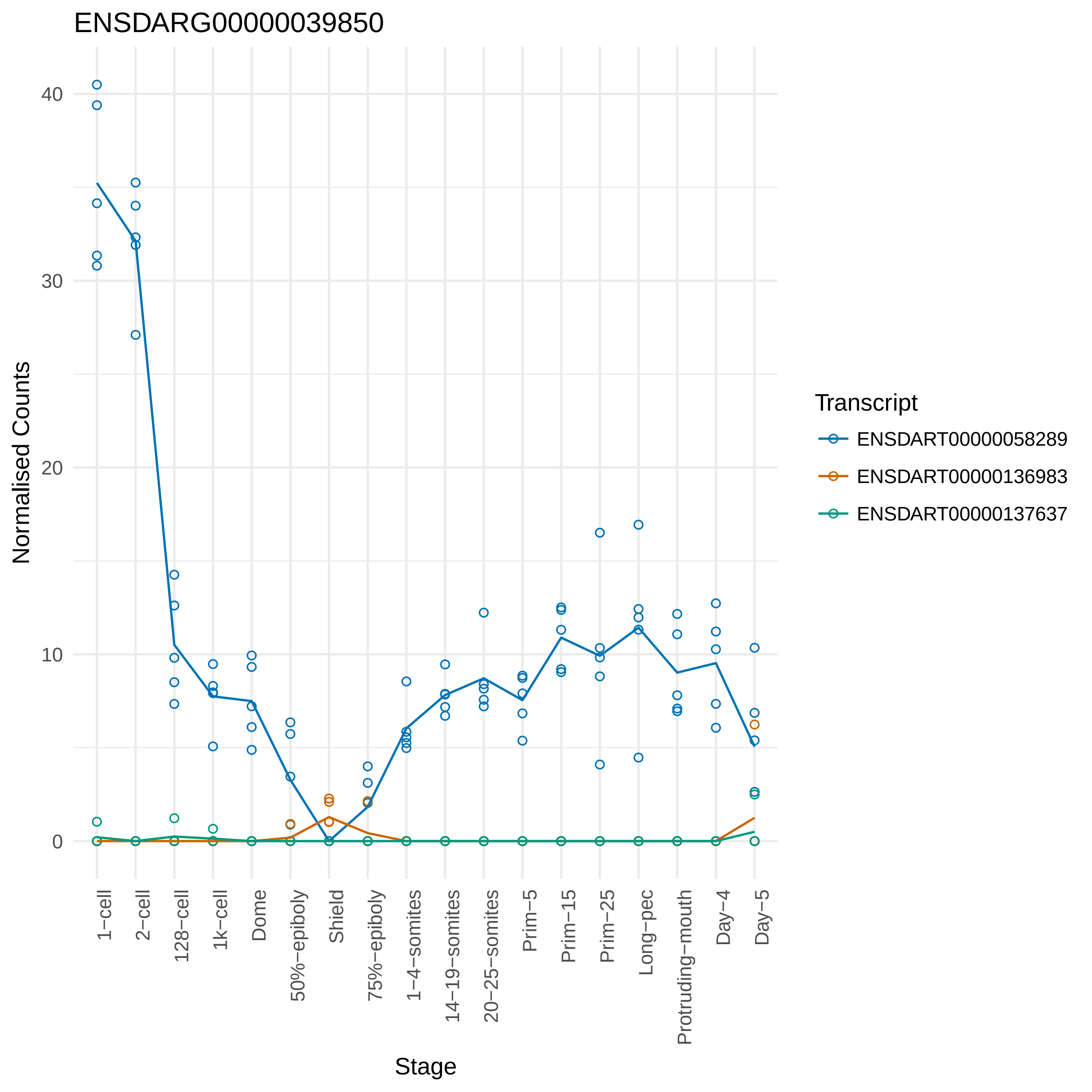

ENSDARG00000039850
Normalised Counts
40
30
20
10
0
1−cell
2−cell
128−cell
1k−cell
Dome
50%−epiboly
Shield
75%−epiboly
1−4−somites
14−19−somites
20−25−somites
Prim−5
Prim−15
Prim−25
Long−pec
Protruding−mouth
Day−4
Day−5
Stage
Transcript
ENSDART00000058289
ENSDART00000136983
ENSDART00000137637

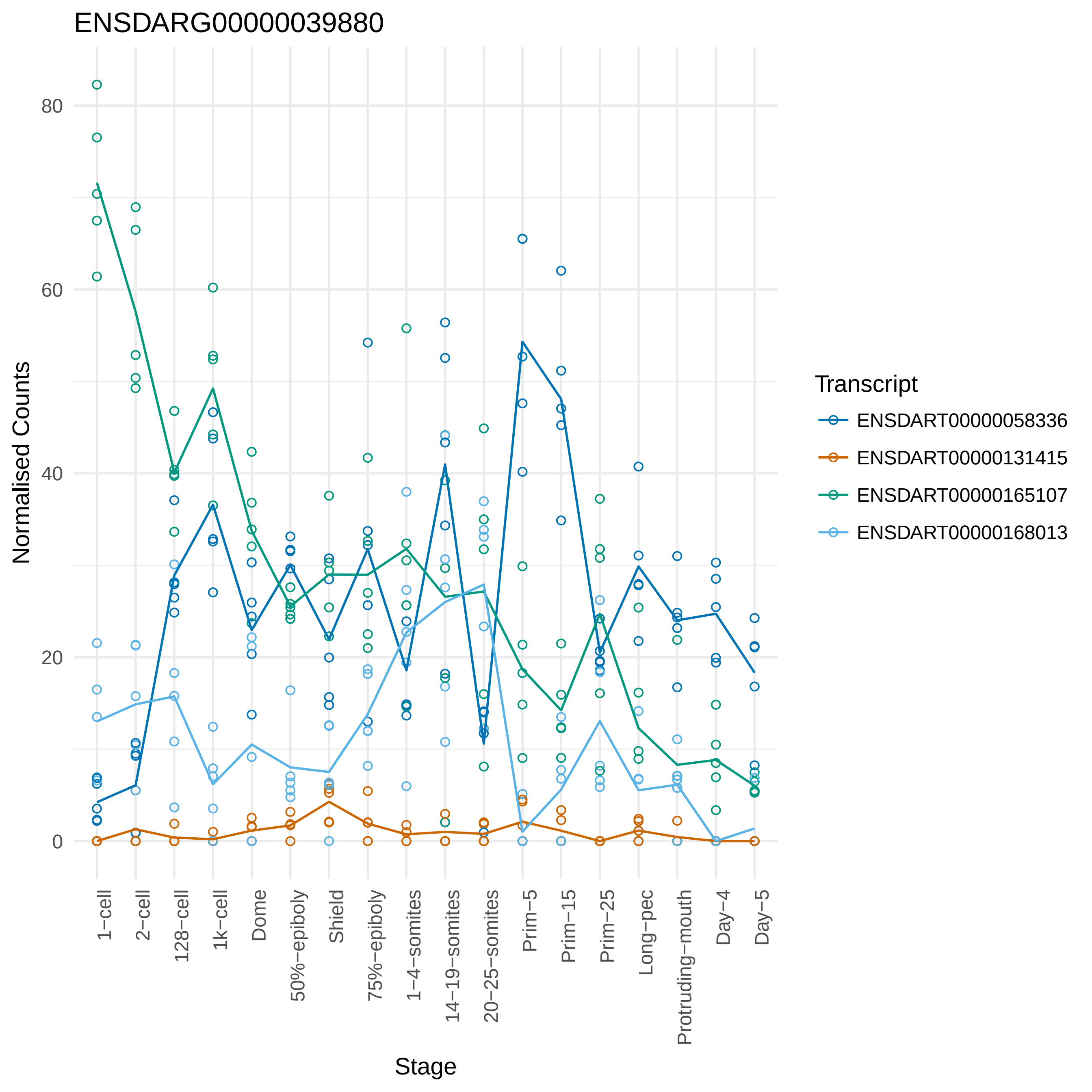
ENSDARG00000039880
Normalised Counts
0
20
40
60
80
Stage
1−cell
2−cell
128−cell
1k−cell
Dome
50%−epiboly
Shield
75%−epiboly
1−4−somites
14−19−somites
20−25−somites
Prim−5
Prim−15
Prim−25
Long−pec
Protruding−mouth
Day−4
Day−5
Transcript
ENSDART00000058336
ENSDART00000131415
ENSDART00000165107
ENSDART00000168013

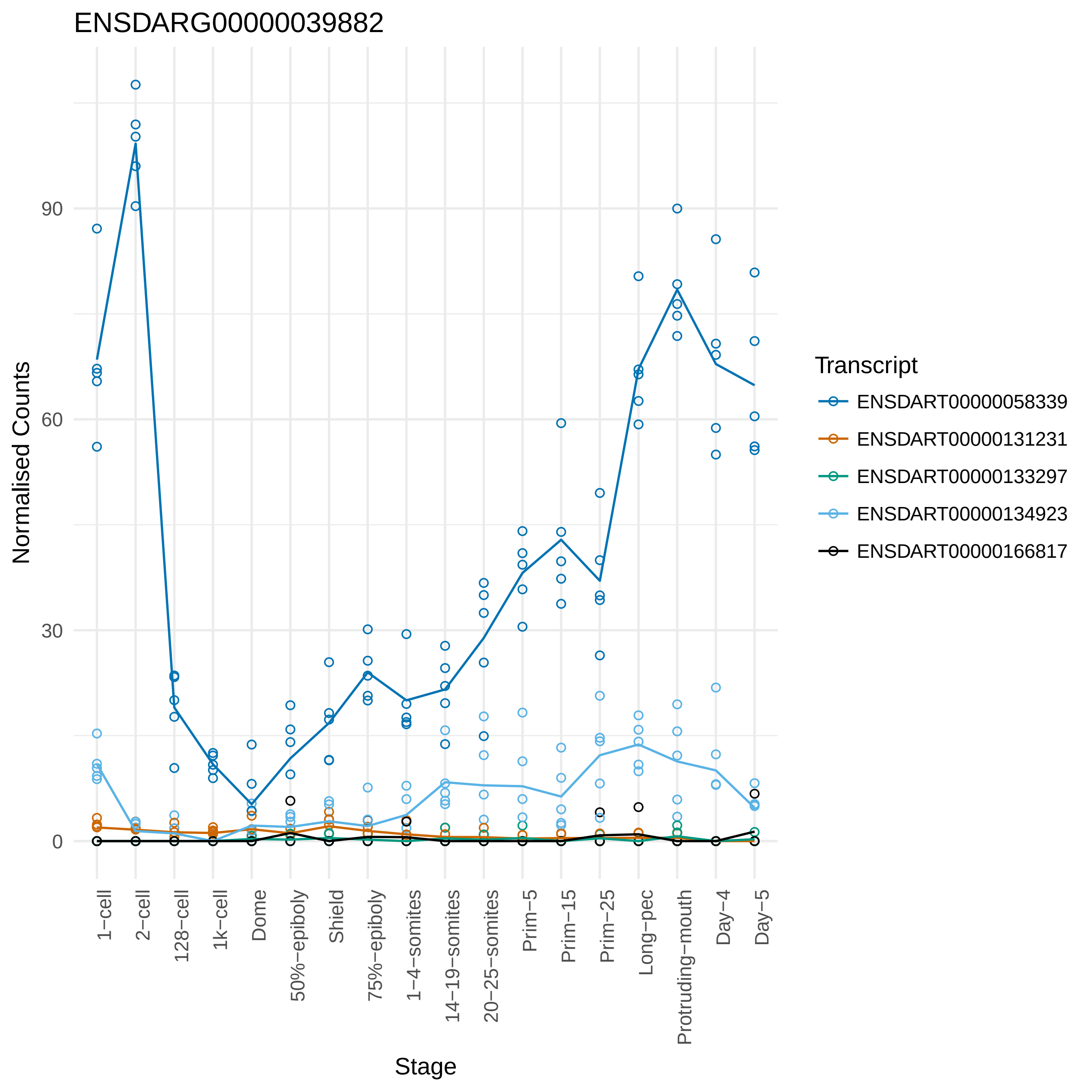
ENSDARG00000039882
Normalised Counts
0
30
60
90
Stage
1−cell
2−cell
128−cell
1k−cell
Dome
50%−epiboly
Shield
75%−epiboly
1−4−somites
14−19−somites
20−25−somites
Prim−5
Prim−15
Prim−25
Long−pec
Protruding−mouth
Day−4
Day−5
Transcript
ENSDART00000058339
ENSDART00000131231
ENSDART00000133297
ENSDART00000134923
ENSDART00000166817

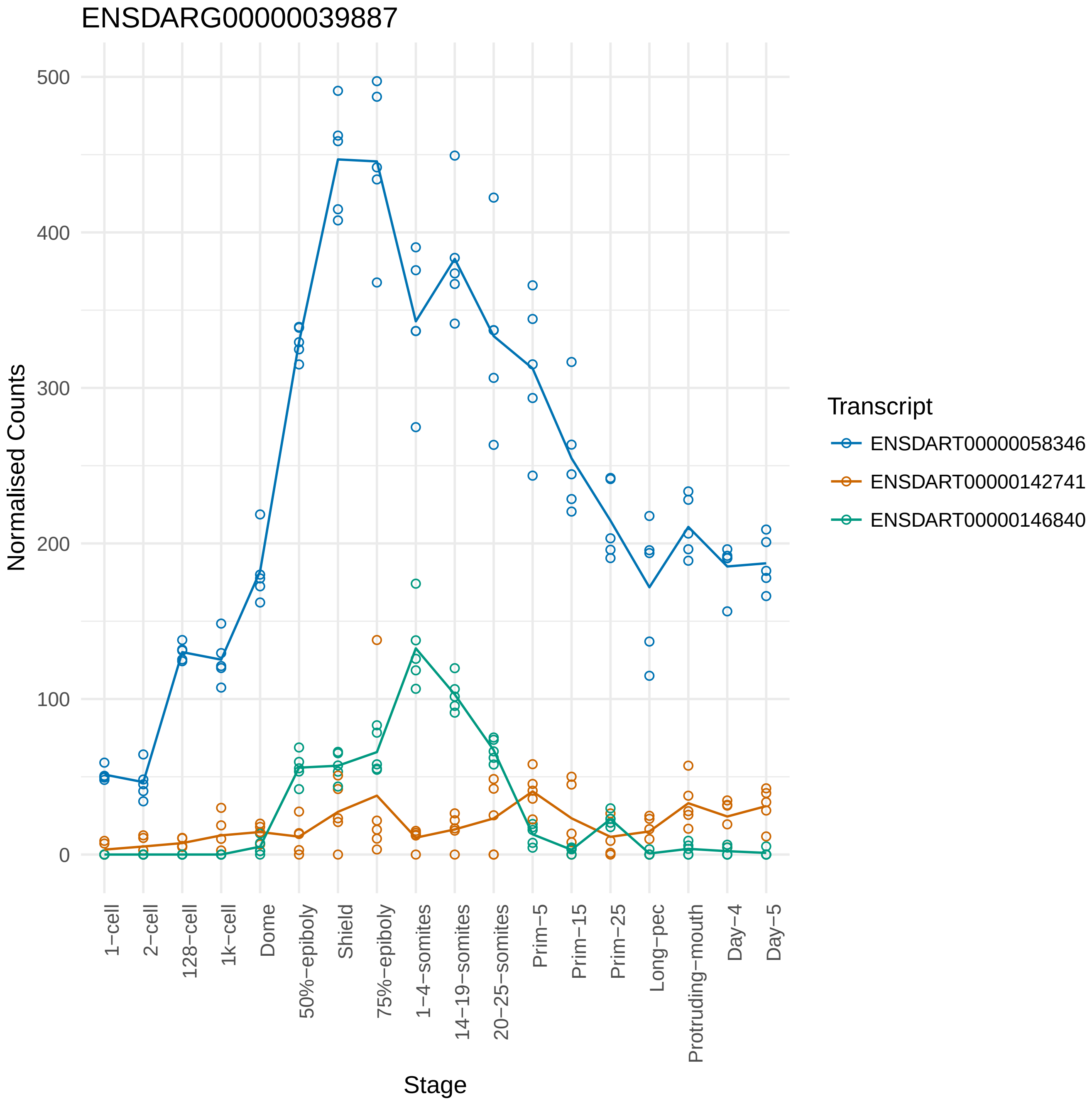
ENSDARG00000039887
Normalised Counts
500
400
300
200
100
0
1−cell
2−cell
128−cell
1k−cell
Dome
50%−epiboly
Shield
75%−epiboly
1−4−somites
14−19−somites
20−25−somites
Prim−5
Prim−15
Prim−25
Long−pec
Protruding−mouth
Day−4
Day−5
Stage
Transcript
ENSDART00000058346
ENSDART00000142741
ENSDART00000146840

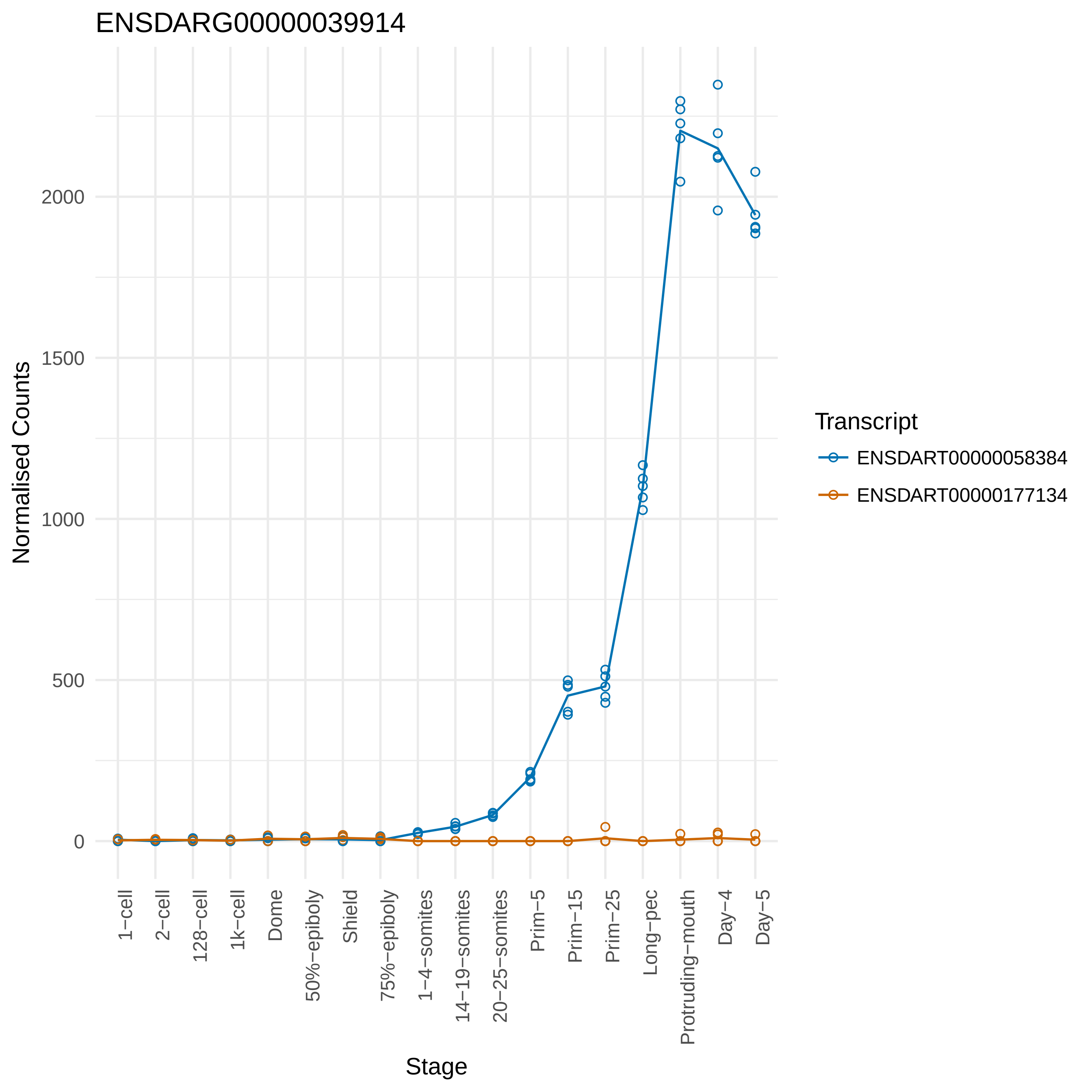
ENSDARG00000039914
Normalised Counts
0
500
1000
1500
2000
1−cell
2−cell
128−cell
1k−cell
Dome
50%−epiboly
Shield
75%−epiboly
1−4−somites
14−19−somites
20−25−somites
Prim−5
Prim−15
Prim−25
Long−pec
Protruding−mouth
Day−4
Day−5
Stage
Transcript
ENSDART00000058384
ENSDART00000177134

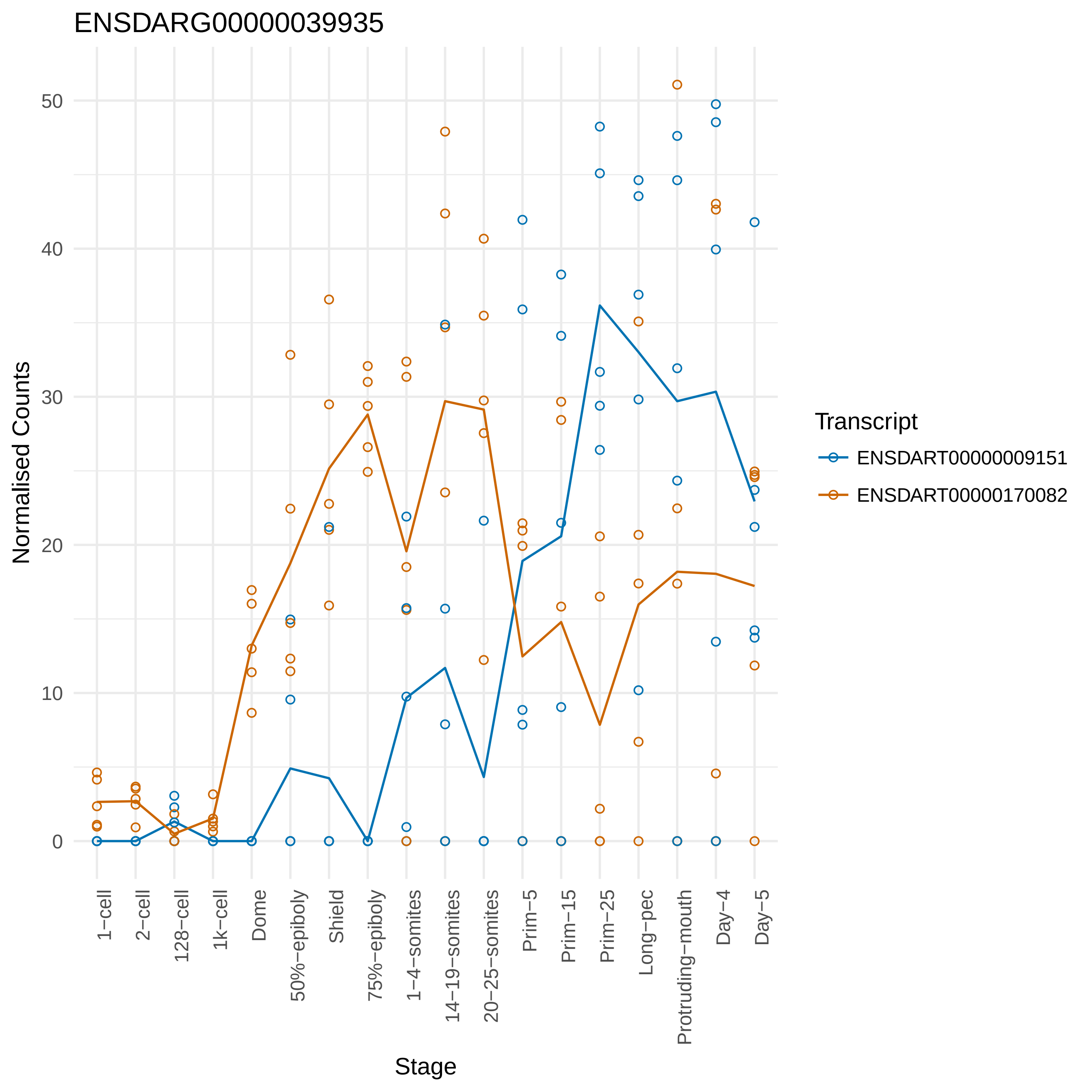

ENSDARG00000039935
Normalised Counts
50
40
30
20
10
0
1−cell
2−cell
128−cell
1k−cell
Dome
50%−epiboly
Shield
75%−epiboly
1−4−somites
14−19−somites
20−25−somites
Prim−5
Prim−15
Prim−25
Long−pec
Protruding−mouth
Day−4
Day−5
Stage
Transcript
ENSDART00000009151
ENSDART00000170082

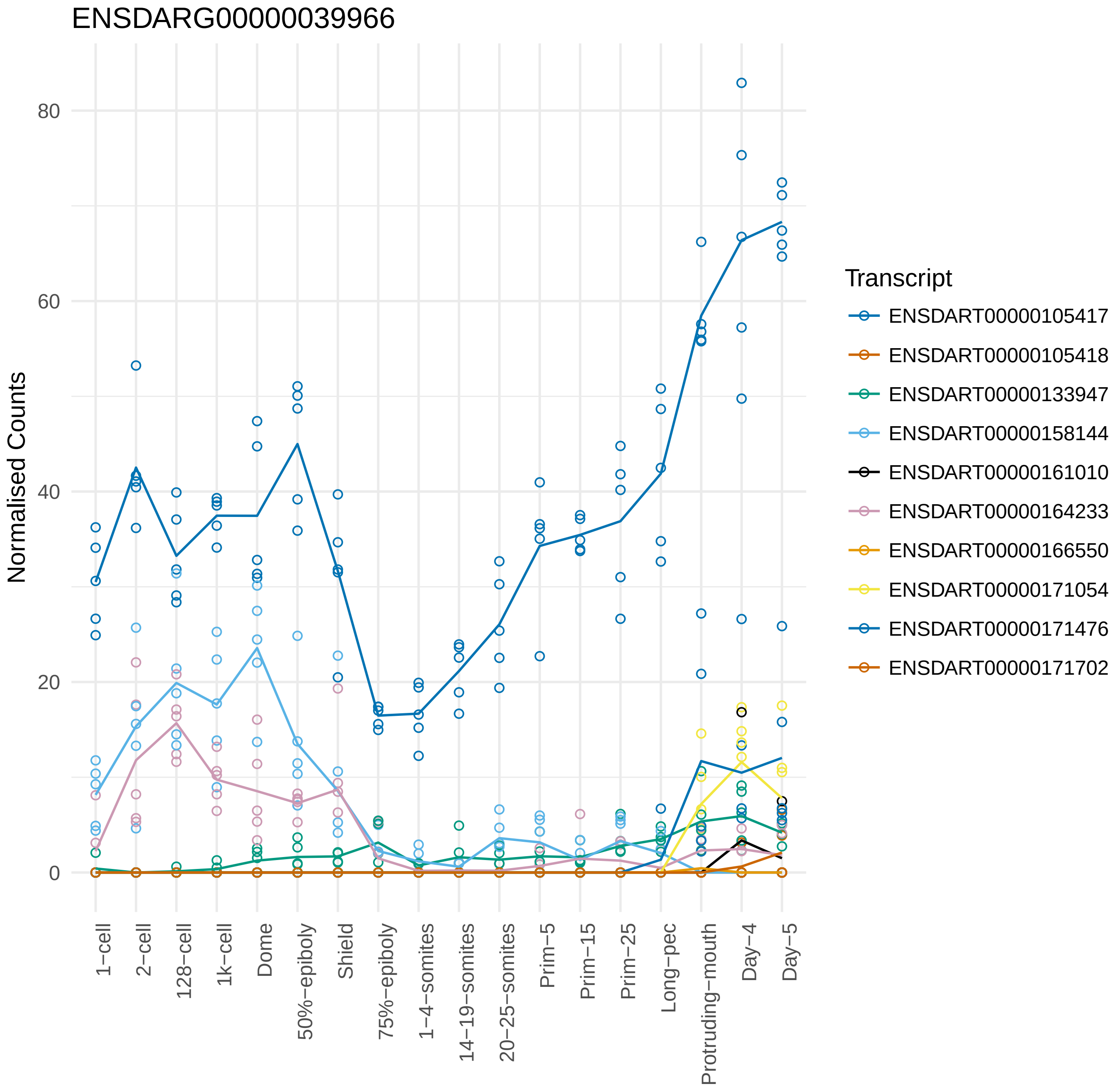
ENSDARG00000039966
Normalised Counts
0
20
40
60
80
1−cell
2−cell
128−cell
1k−cell
Dome
50%−epiboly
Shield
75%−epiboly
1−4−somites
14−19−somites
20−25−somites
Prim−5
Prim−15
Prim−25
Long−pec
Protruding−mouth
Day−4
Day−5
Stage
Transcript
ENSDART00000105417
ENSDART00000105418
ENSDART00000133947
ENSDART00000158144
ENSDART00000161010
ENSDART00000164233
ENSDART00000166550
ENSDART00000171054
ENSDART00000171476
ENSDART00000171702

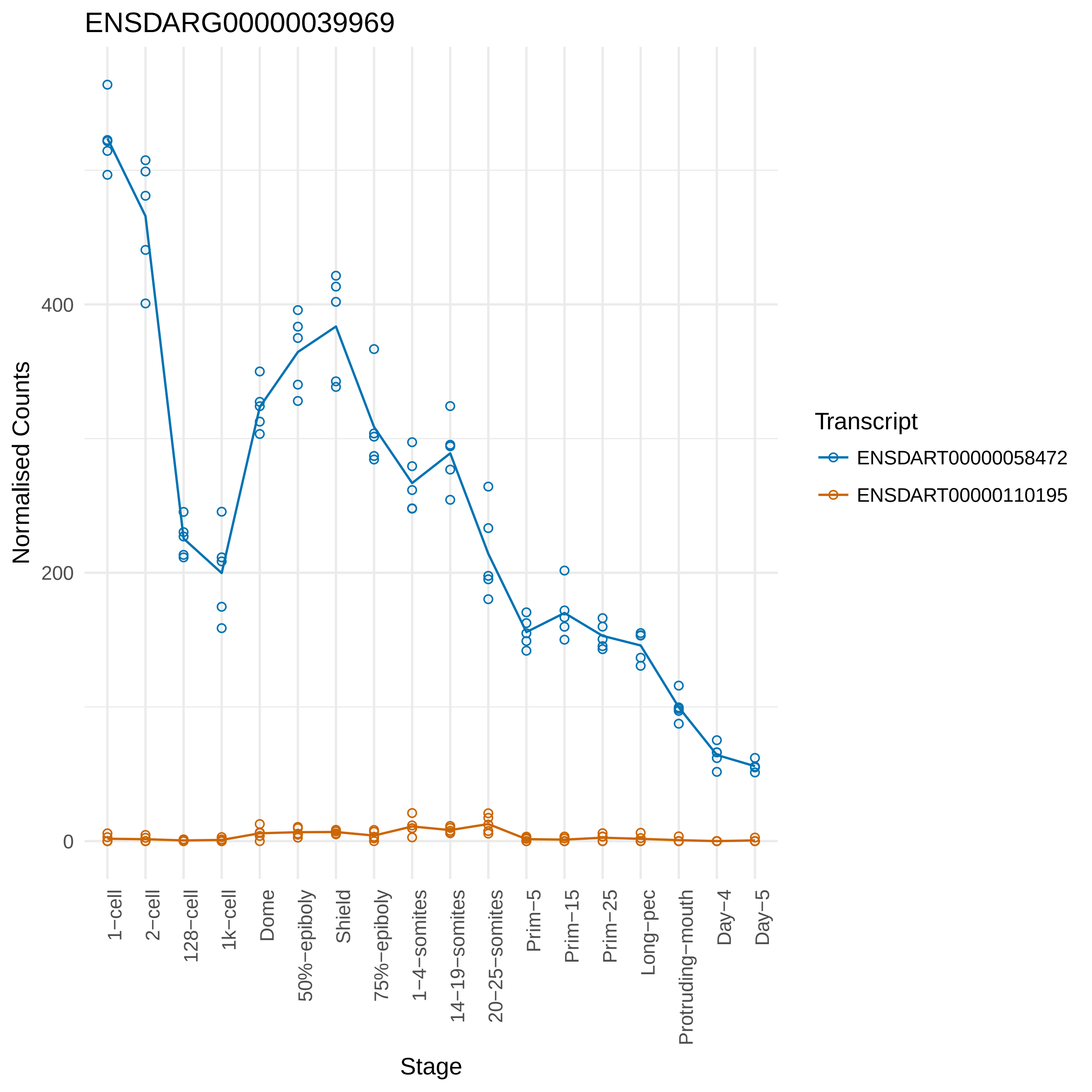
ENSDARG00000039969
Normalised Counts
400
200
0
1−cell
2−cell
128−cell
1k−cell
Dome
50%−epiboly
Shield
75%−epiboly
1−4−somites
14−19−somites
20−25−somites
Prim−5
Prim−15
Prim−25
Long−pec
Protruding−mouth
Day−4
Day−5
Stage
Transcript
ENSDART00000058472
ENSDART00000110195

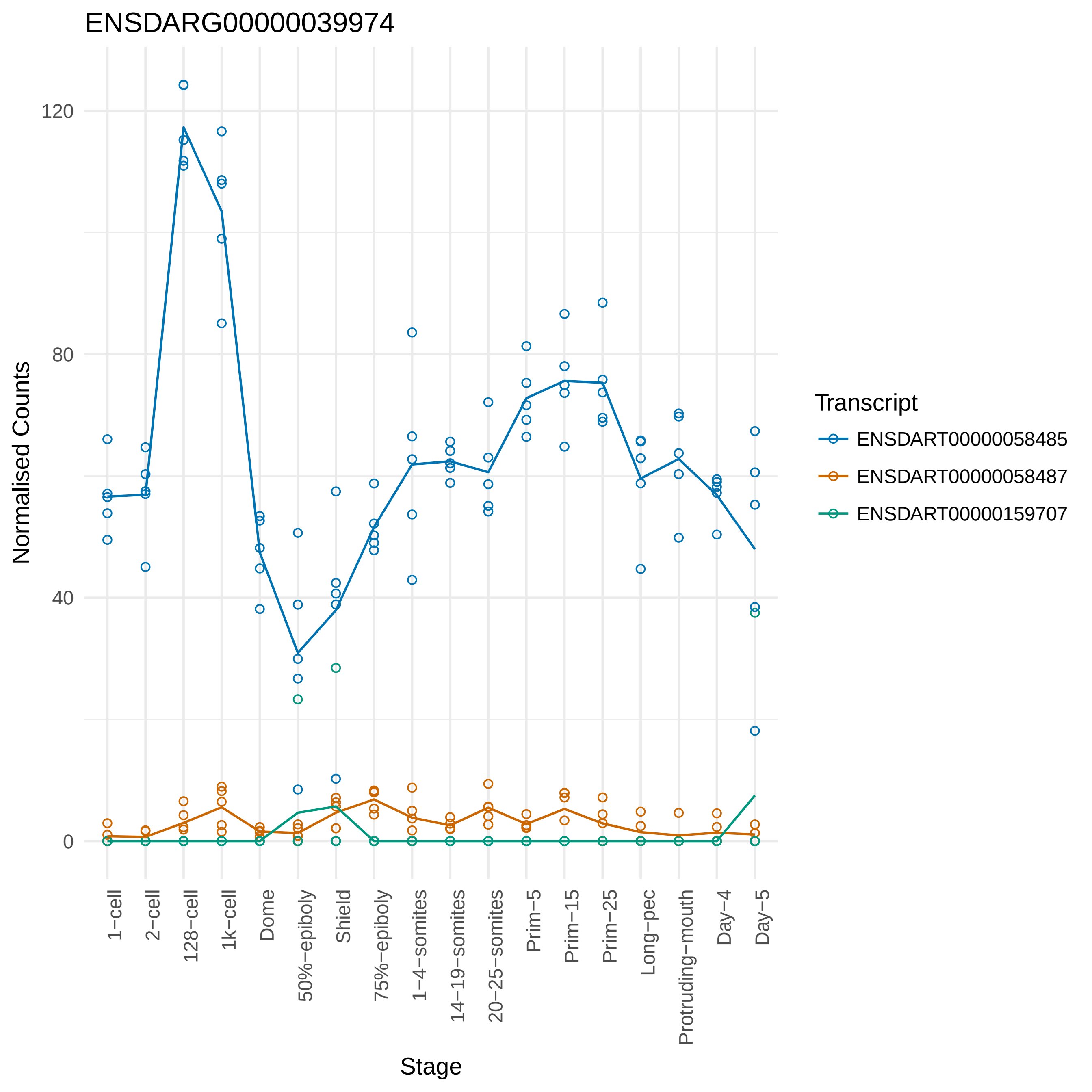

ENSDARG00000039974
Normalised Counts
120
80
40
0
1−cell
2−cell
128−cell
1k−cell
Dome
50%−epiboly
Shield
75%−epiboly
1−4−somites
14−19−somites
20−25−somites
Prim−5
Prim−15
Prim−25
Long−pec
Protruding−mouth
Day−4
Day−5
Stage
Transcript
ENSDART00000058485
ENSDART00000058487
ENSDART00000159707

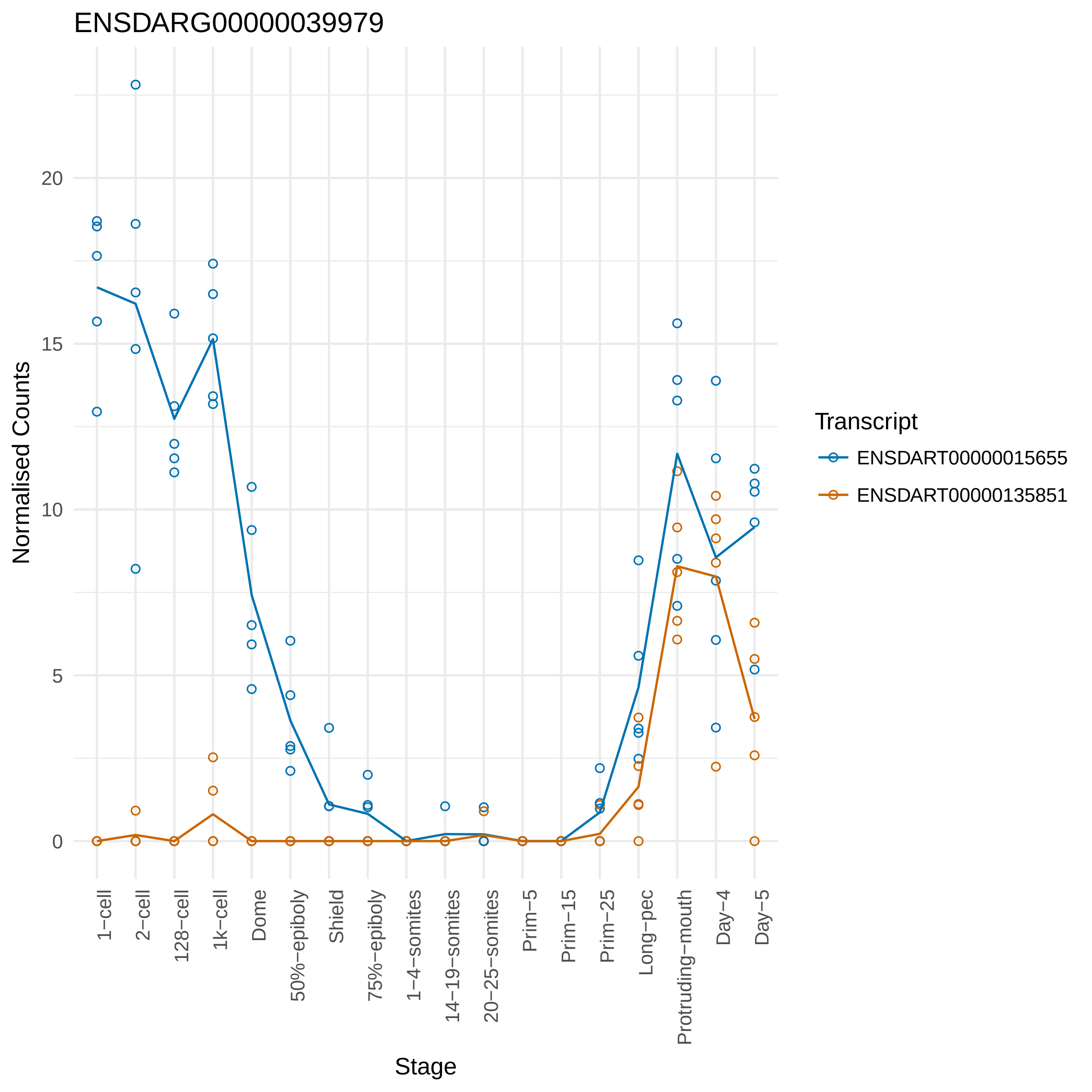

ENSDARG00000039979
Normalised Counts
0
5
10
15
20
Stage
1−cell
2−cell
128−cell
1k−cell
Dome
50%−epiboly
Shield
75%−epiboly
1−4−somites
14−19−somites
20−25−somites
Prim−5
Prim−15
Prim−25
Long−pec
Protruding−mouth
Day−4
Day−5
Transcript
ENSDART00000015655
ENSDART00000135851

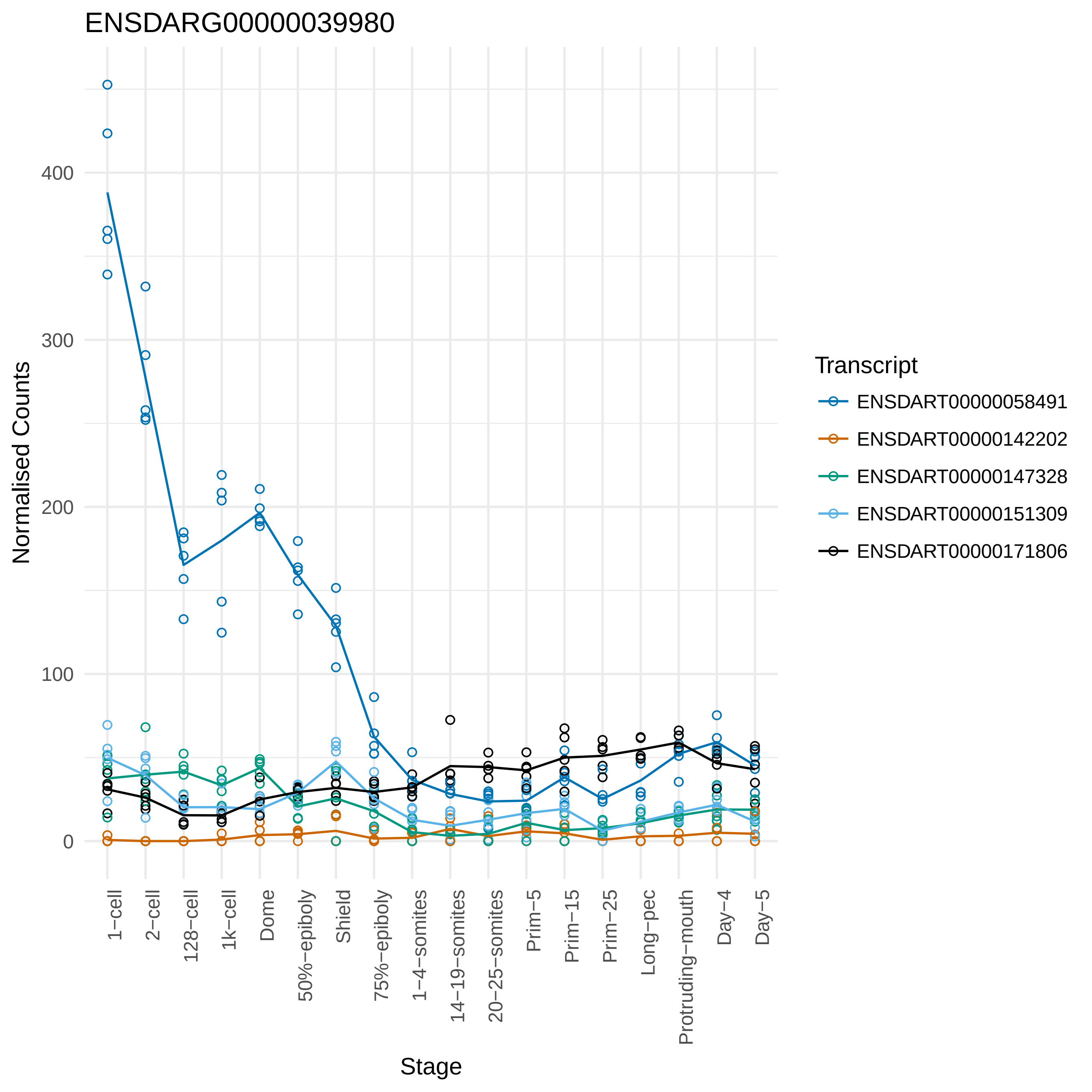

ENSDARG00000039980
Normalised Counts
400
300
200
100
0
1−cell
2−cell
128−cell
1k−cell
Dome
50%−epiboly
Shield
75%−epiboly
1−4−somites
14−19−somites
20−25−somites
Prim−5
Prim−15
Prim−25
Long−pec
Protruding−mouth
Day−4
Day−5
Stage
Transcript
ENSDART00000058491
ENSDART00000142202
ENSDART00000147328
ENSDART00000151309
ENSDART00000171806

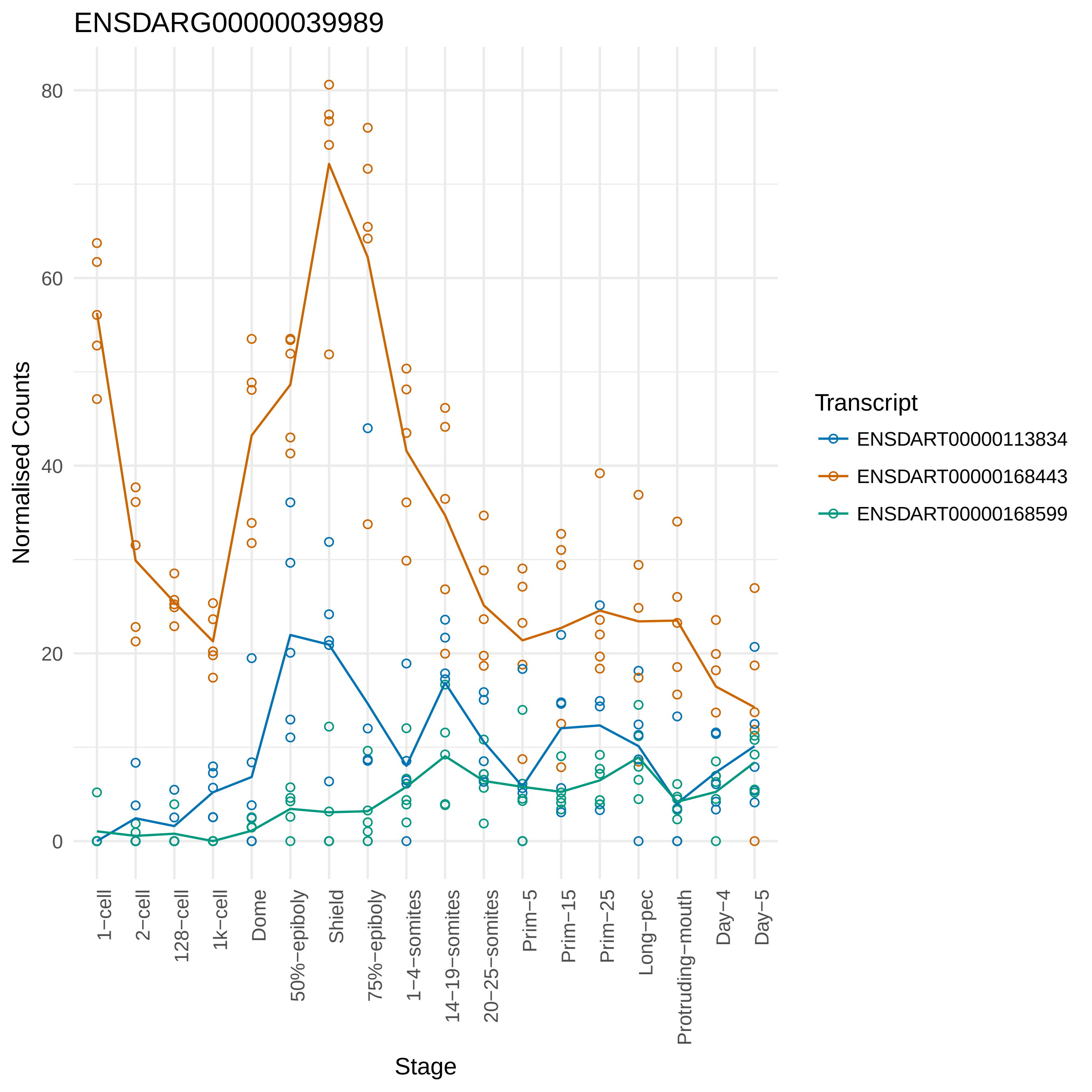
ENSDARG00000039989
Normalised Counts
0
20
40
60
80
1−cell
2−cell
128−cell
1k−cell
Dome
50%−epiboly
Shield
75%−epiboly
1−4−somites
14−19−somites
20−25−somites
Prim−5
Prim−15
Prim−25
Long−pec
Protruding−mouth
Day−4
Day−5
Stage
Transcript
ENSDART00000113834
ENSDART00000168443
ENSDART00000168599

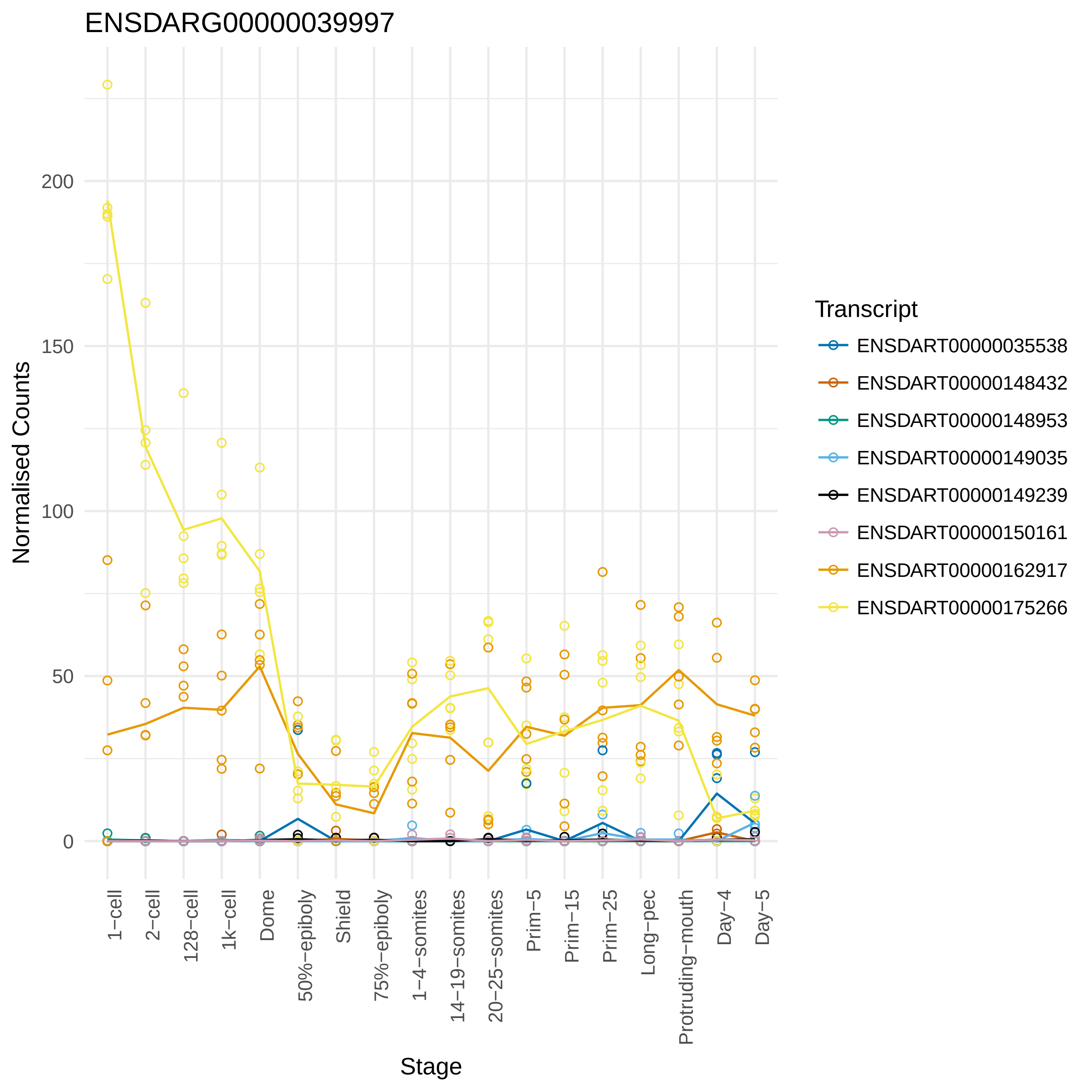

ENSDARG00000039997
Normalised Counts
0
50
100
150
200
Stage
1−cell
2−cell
128−cell
1k−cell
Dome
50%−epiboly
Shield
75%−epiboly
1−4−somites
14−19−somites
20−25−somites
Prim−5
Prim−15
Prim−25
Long−pec
Protruding−mouth
Day−4
Day−5
Transcript
ENSDART00000035538
ENSDART00000148432
ENSDART00000148953
ENSDART00000149035
ENSDART00000149239
ENSDART00000150161
ENSDART00000162917
ENSDART00000175266

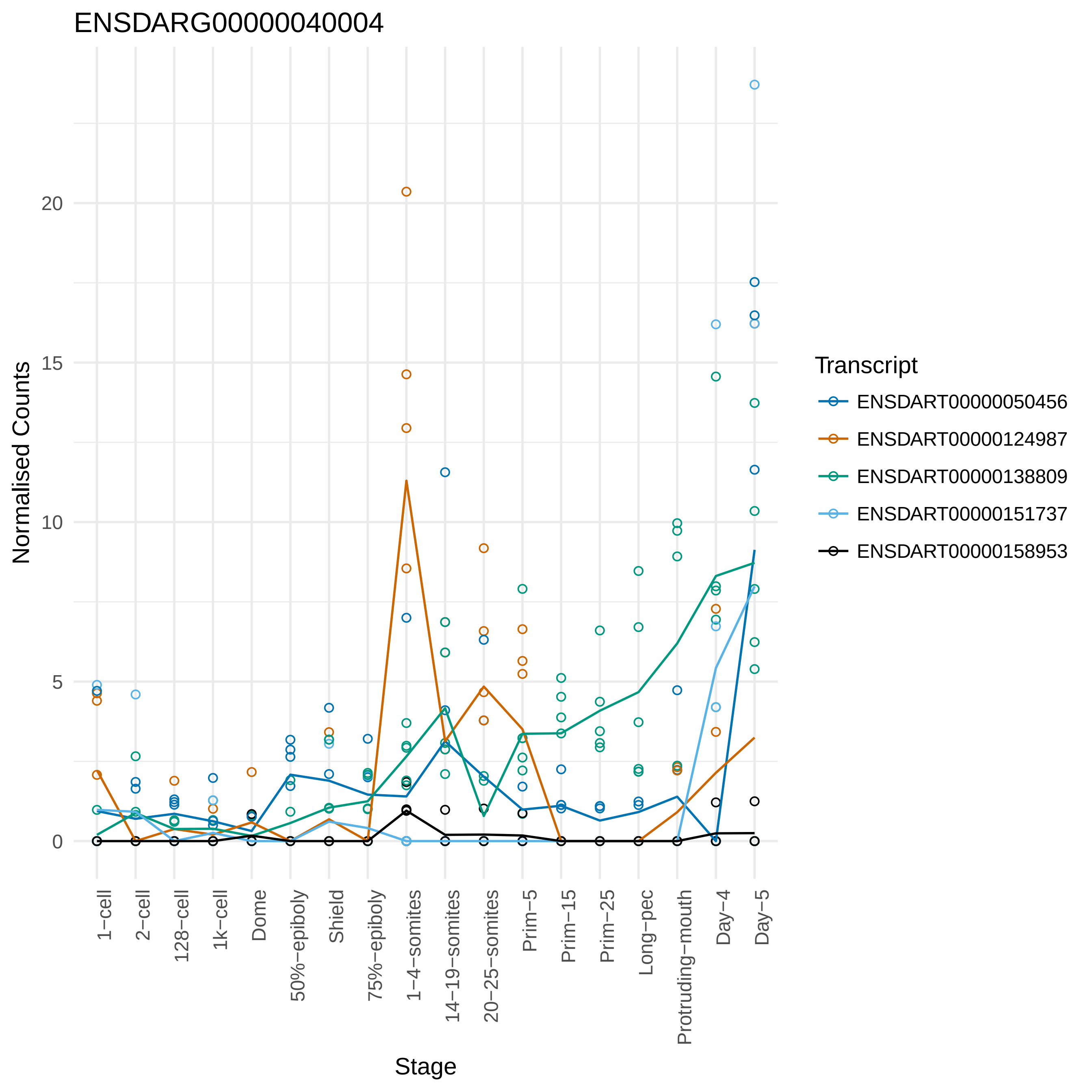
ENSDARG00000040004
Normalised Counts
Stage
0
5
10
15
20
1−cell
2−cell
128−cell
1k−cell
Dome
50%−epiboly
Shield
75%−epiboly
1−4−somites
14−19−somites
20−25−somites
Prim−5
Prim−15
Prim−25
Long−pec
Protruding−mouth
Day−4
Day−5
Transcript
ENSDART00000050456
ENSDART00000124987
ENSDART00000138809
ENSDART00000151737
ENSDART00000158953

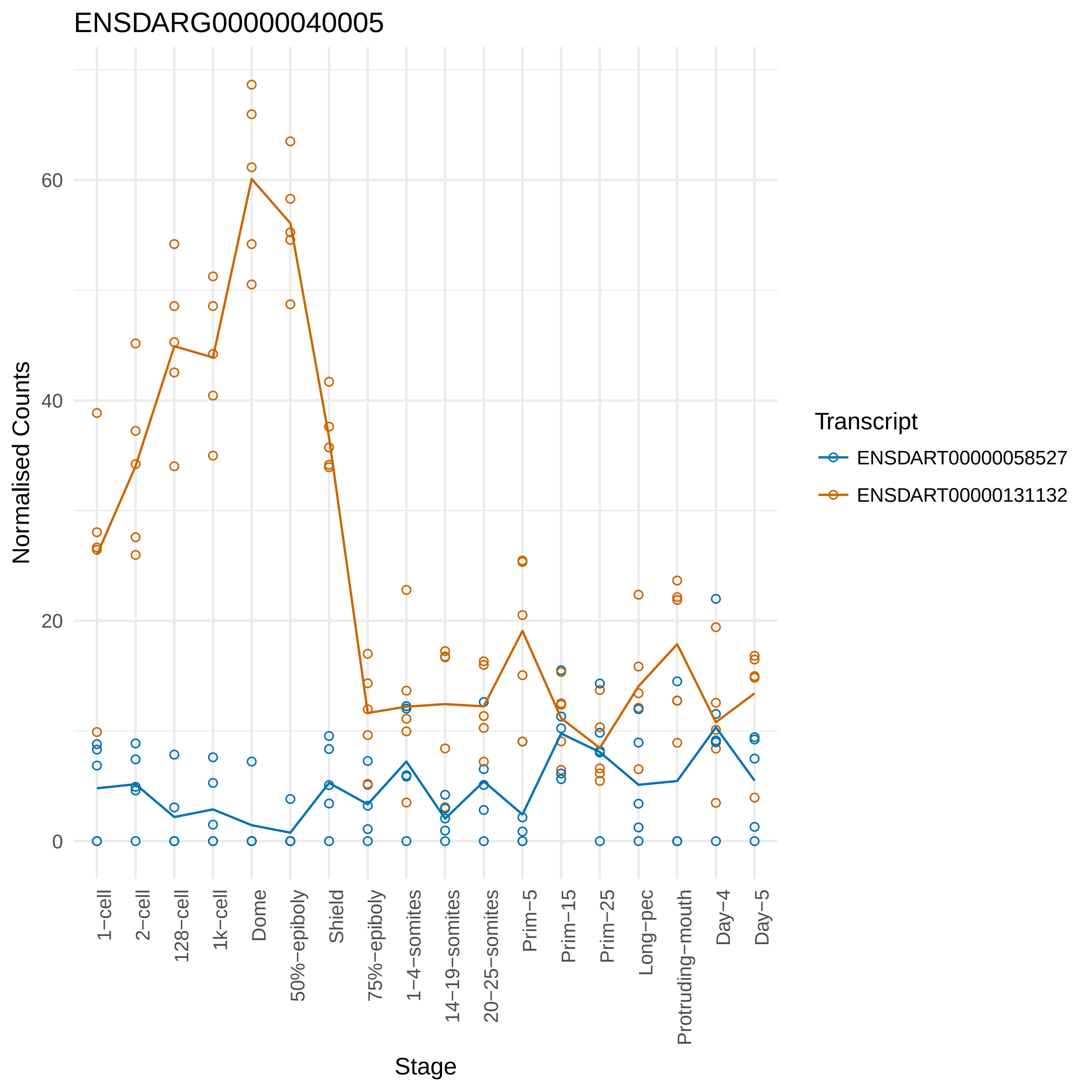

ENSDARG00000040005
Normalised Counts
0
20
40
60
1−cell
2−cell
128−cell
1k−cell
Dome
50%−epiboly
Shield
75%−epiboly
1−4−somites
14−19−somites
20−25−somites
Prim−5
Prim−15
Prim−25
Long−pec
Protruding−mouth
Day−4
Day−5
Stage
Transcript
ENSDART00000058527
ENSDART00000131132

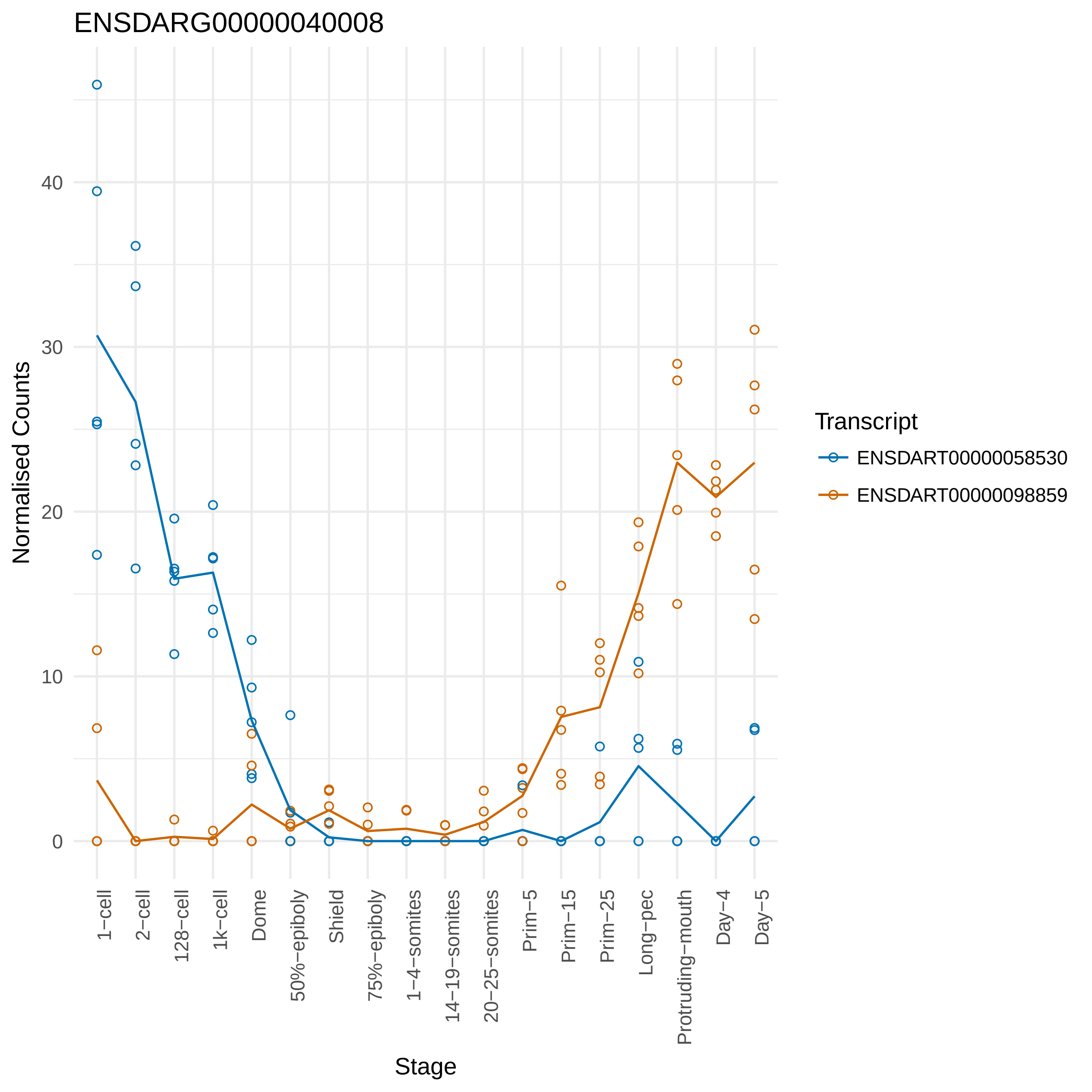

ENSDARG00000040008
Normalised Counts
0
10
20
30
40
1−cell
2−cell
128−cell
1k−cell
Dome
50%−epiboly
Shield
75%−epiboly
1−4−somites
14−19−somites
20−25−somites
Prim−5
Prim−15
Prim−25
Long−pec
Protruding−mouth
Day−4
Day−5
Stage
Transcript
ENSDART00000058530
ENSDART00000098859

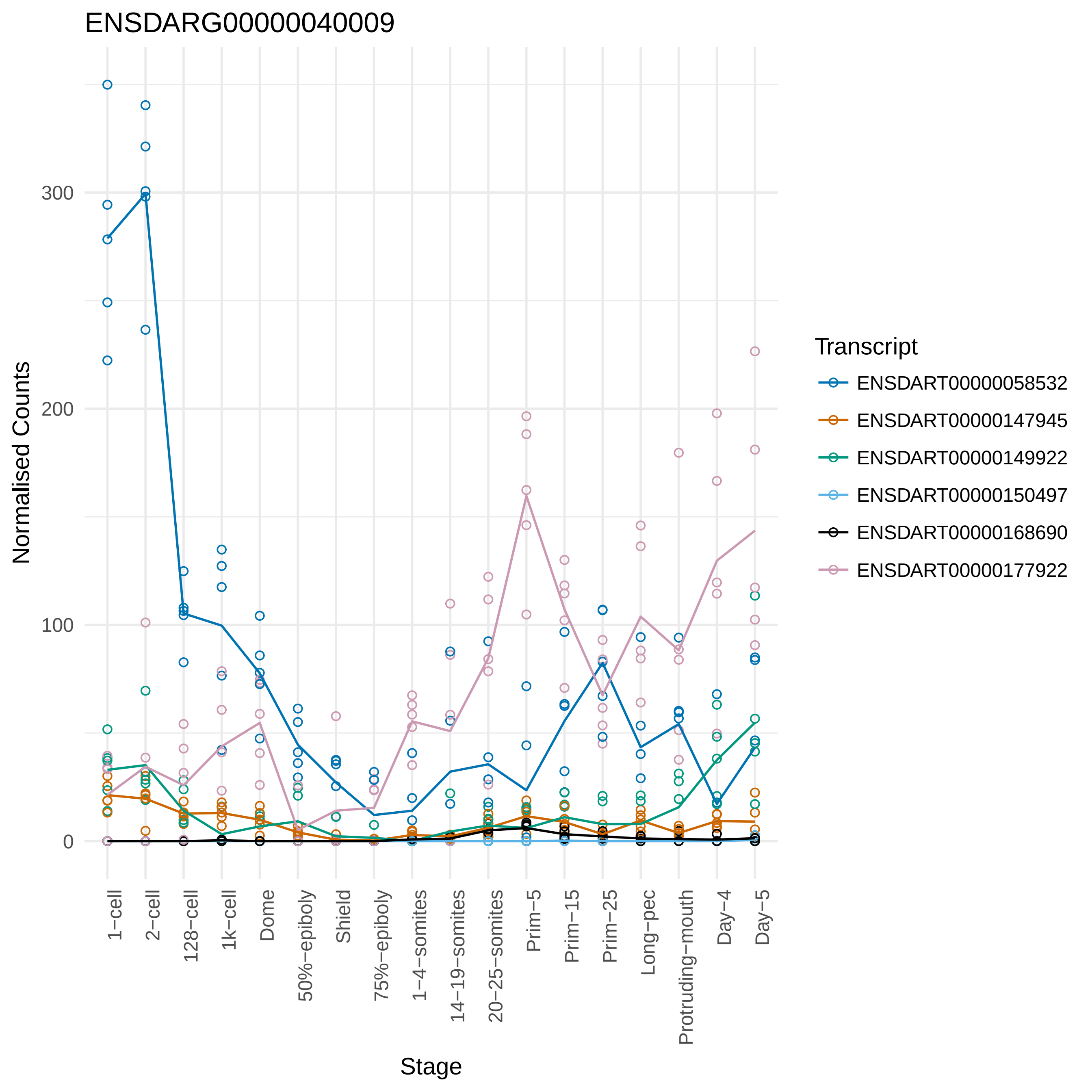

ENSDARG00000040009
Normalised Counts
300
200
100
0
Stage
1−cell
2−cell
128−cell
1k−cell
Dome
50%−epiboly
Shield
75%−epiboly
1−4−somites
14−19−somites
20−25−somites
Prim−5
Prim−15
Prim−25
Long−pec
Protruding−mouth
Day−4
Day−5
Transcript
ENSDART00000058532
ENSDART00000147945
ENSDART00000149922
ENSDART00000150497
ENSDART00000168690
ENSDART00000177922

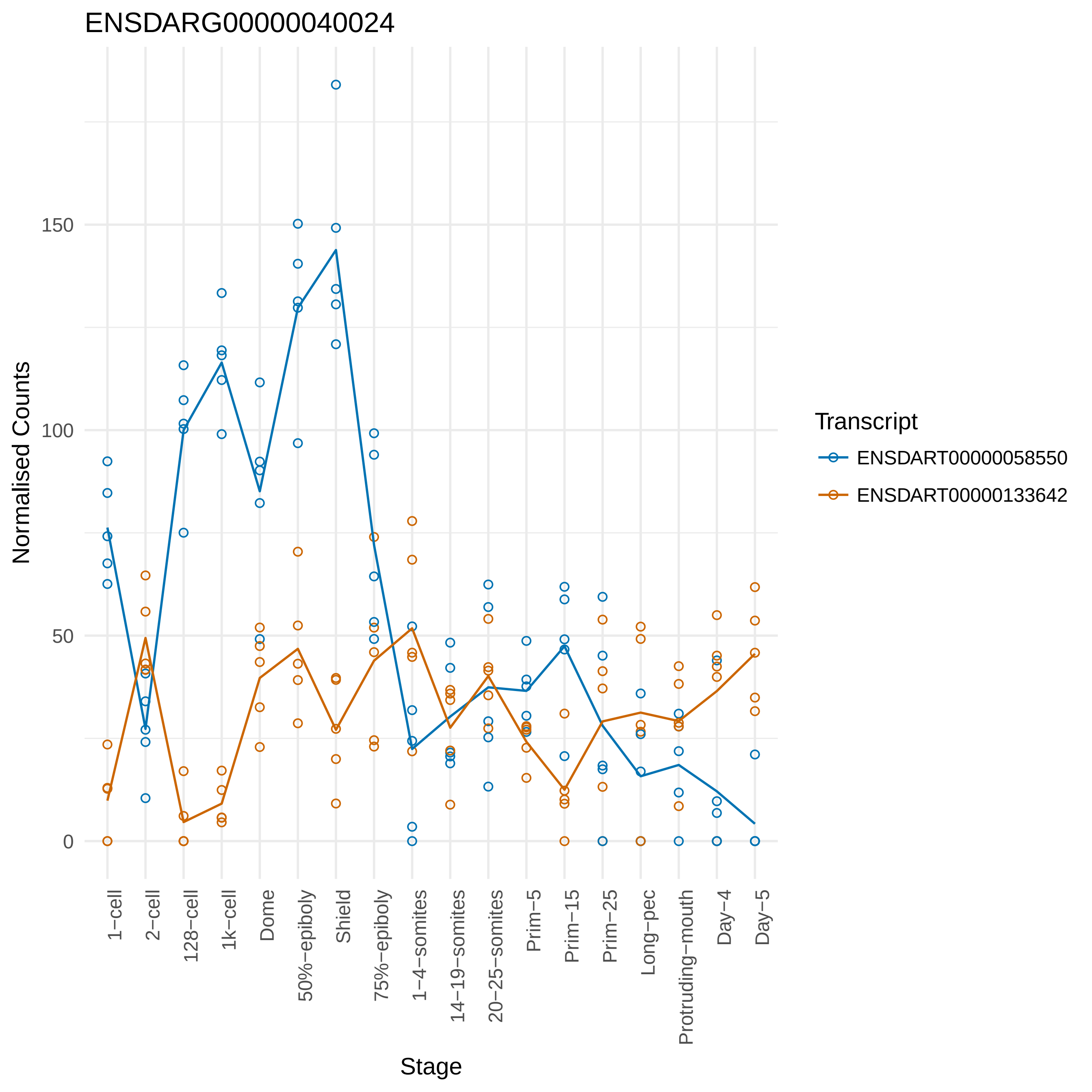

ENSDARG00000040024
Normalised Counts
150
100
50
0
1−cell
2−cell
128−cell
1k−cell
Dome
50%−epiboly
Shield
75%−epiboly
1−4−somites
14−19−somites
20−25−somites
Prim−5
Prim−15
Prim−25
Long−pec
Protruding−mouth
Day−4
Day−5
Stage
Transcript
ENSDART00000058550
ENSDART00000133642

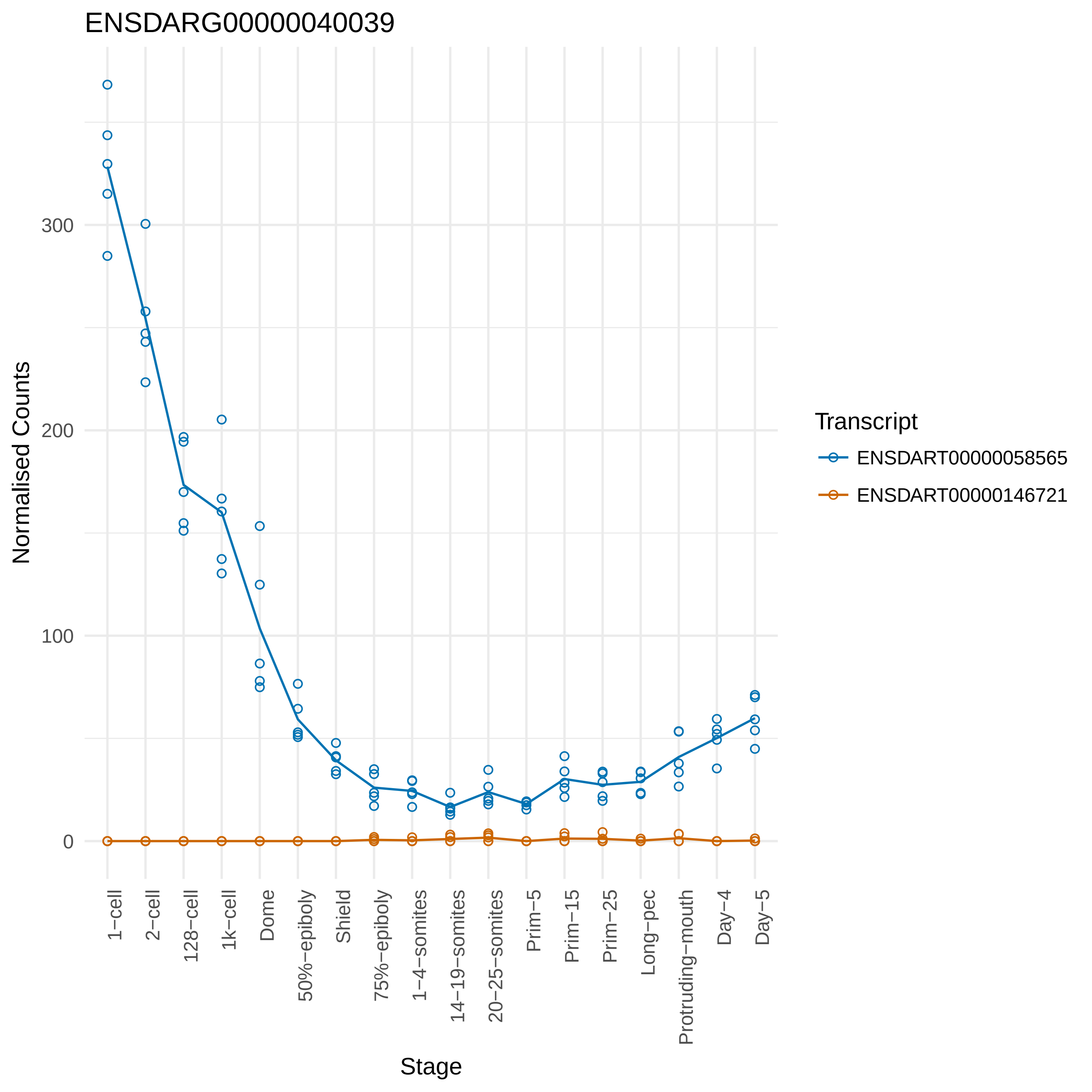

ENSDARG00000040039
Normalised Counts
300
200
100
0
1−cell
2−cell
128−cell
1k−cell
Dome
50%−epiboly
Shield
75%−epiboly
1−4−somites
14−19−somites
20−25−somites
Prim−5
Prim−15
Prim−25
Long−pec
Protruding−mouth
Day−4
Day−5
Stage
Transcript
ENSDART00000058565
ENSDART00000146721

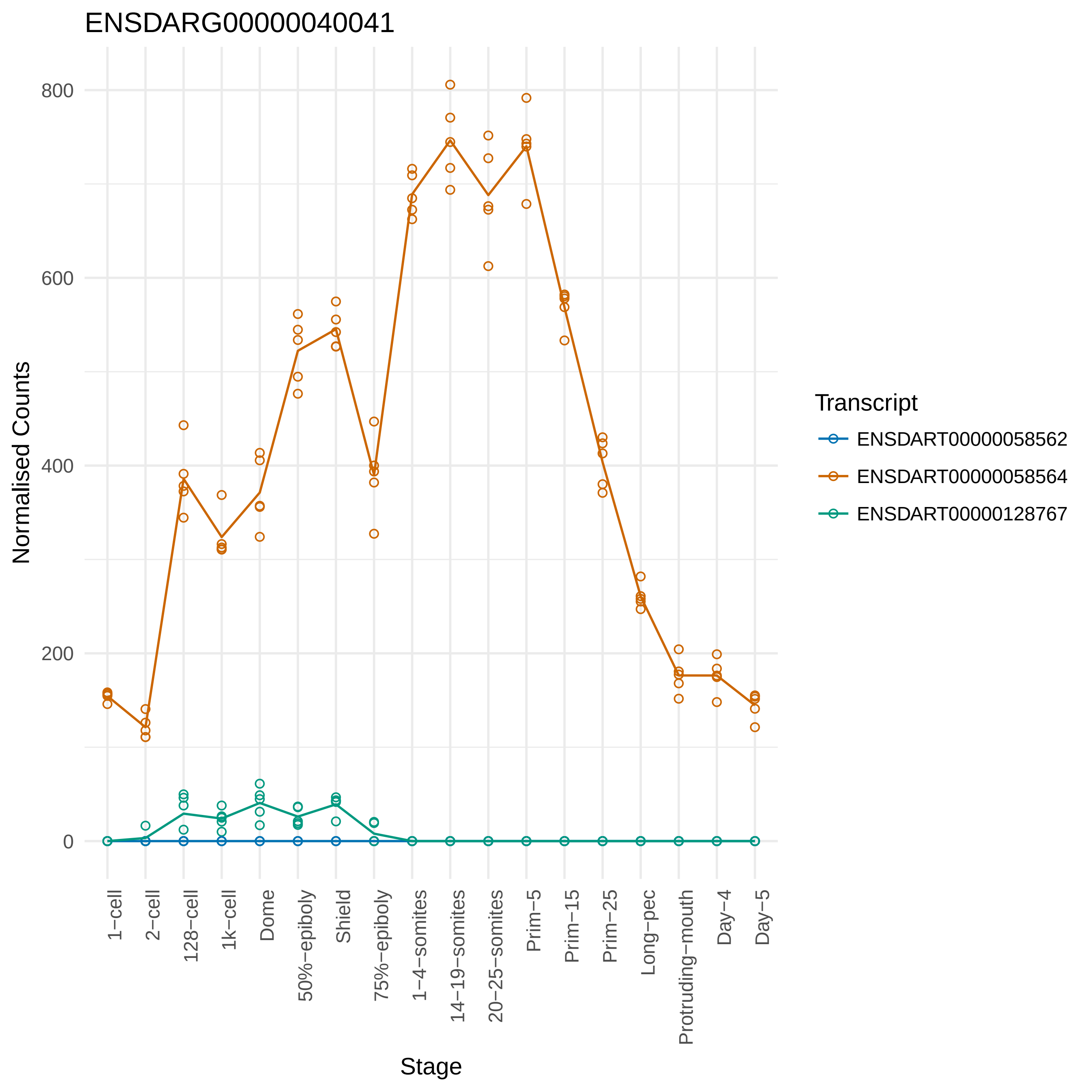
ENSDARG00000040041
Normalised Counts
800
600
400
200
0
1−cell
2−cell
128−cell
1k−cell
Dome
50%−epiboly
Shield
75%−epiboly
1−4−somites
14−19−somites
20−25−somites
Prim−5
Prim−15
Prim−25
Long−pec
Protruding−mouth
Day−4
Day−5
Stage
Transcript
ENSDART00000058562
ENSDART00000058564
ENSDART00000128767

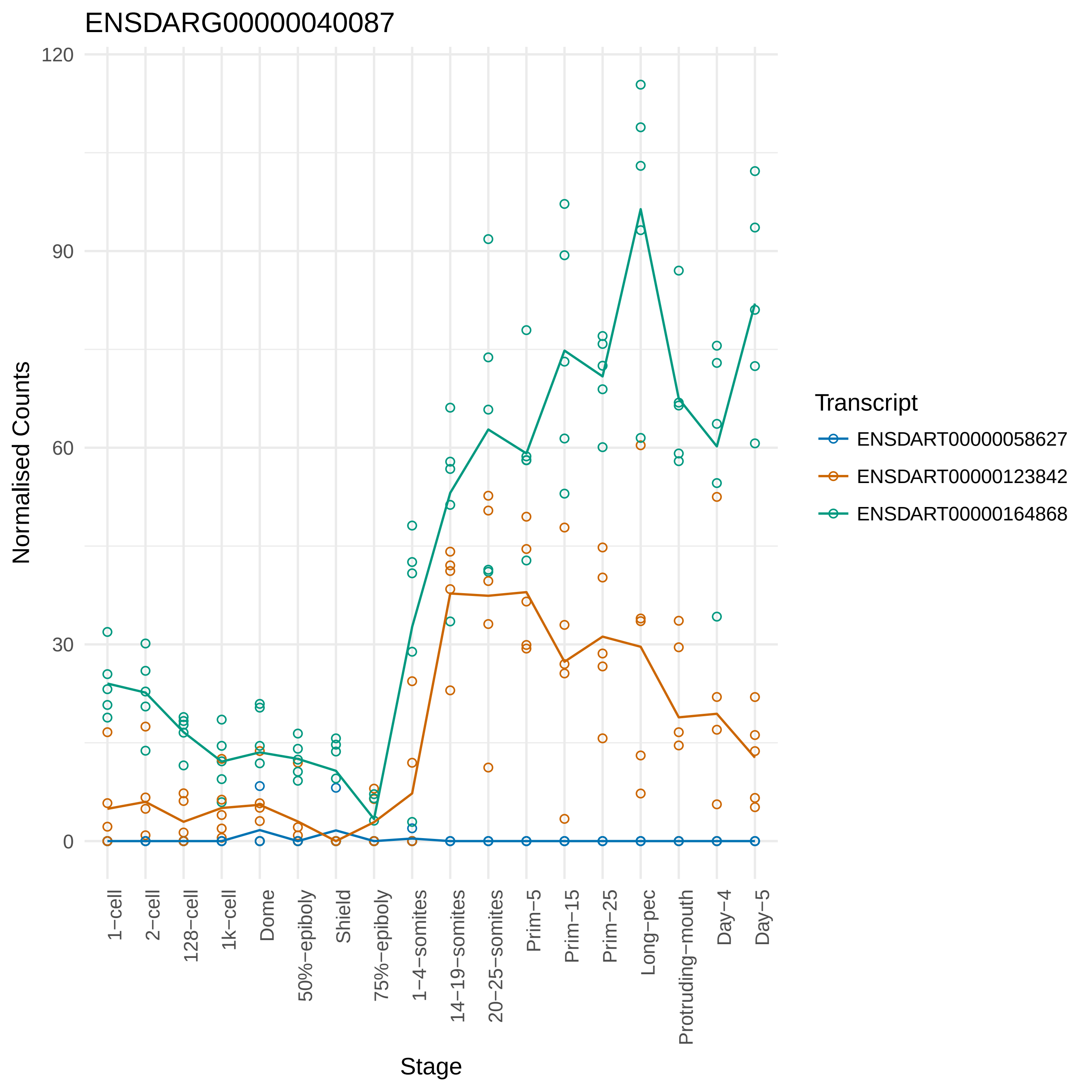

ENSDARG00000040087
Normalised Counts
120
90
60
30
0
1−cell
2−cell
128−cell
1k−cell
Dome
50%−epiboly
Shield
75%−epiboly
1−4−somites
14−19−somites
20−25−somites
Prim−5
Prim−15
Prim−25
Long−pec
Protruding−mouth
Day−4
Day−5
Stage
Transcript
ENSDART00000058627
ENSDART00000123842
ENSDART00000164868

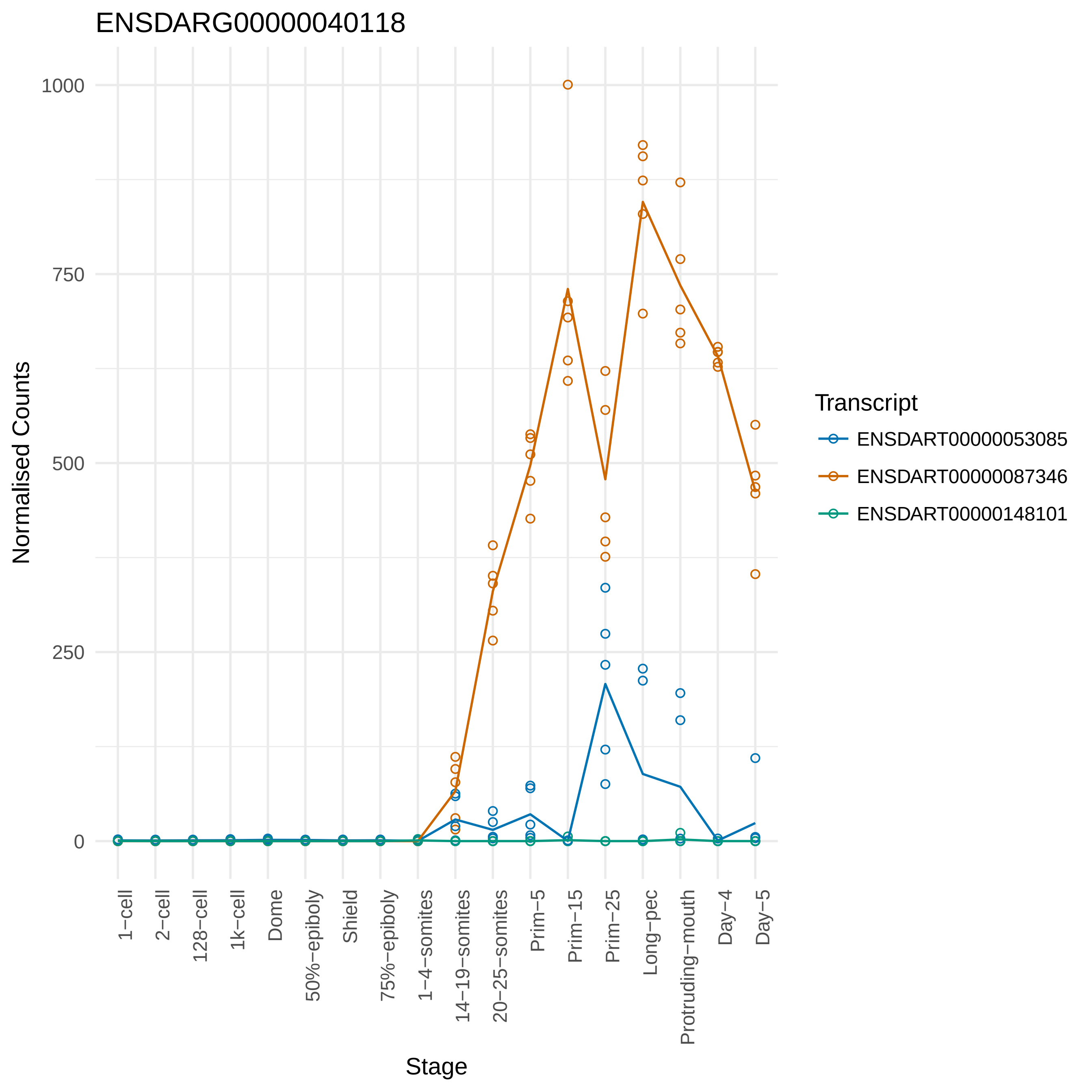

ENSDARG00000040118
Normalised Counts
1000
750
500
250
0
Stage
1−cell
2−cell
128−cell
1k−cell
Dome
50%−epiboly
Shield
75%−epiboly
1−4−somites
14−19−somites
20−25−somites
Prim−5
Prim−15
Prim−25
Long−pec
Protruding−mouth
Day−4
Day−5
Transcript
ENSDART00000053085
ENSDART00000087346
ENSDART00000148101

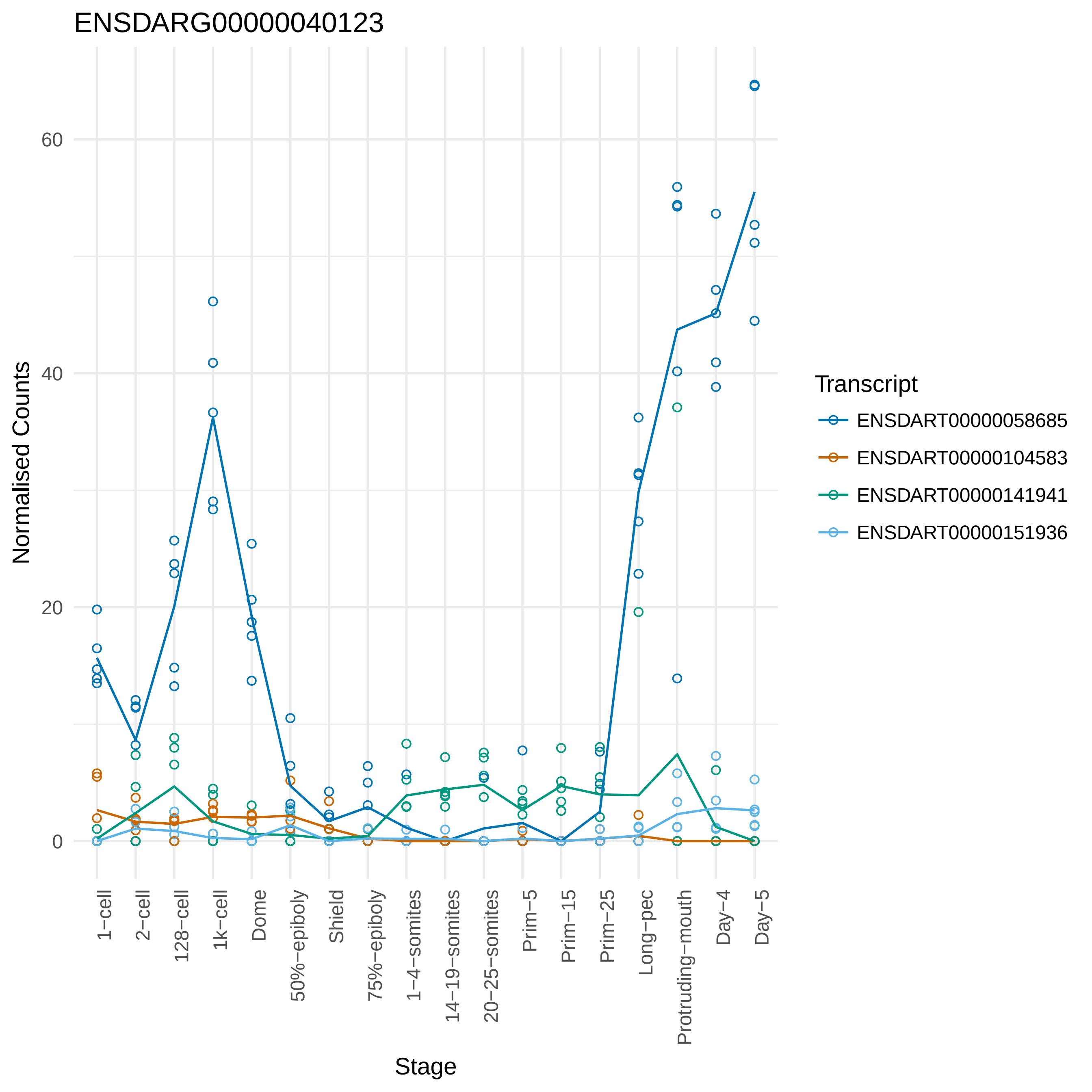

ENSDARG00000040123
Normalised Counts
0
20
40
60
1−cell
2−cell
128−cell
1k−cell
Dome
50%−epiboly
Shield
75%−epiboly
1−4−somites
14−19−somites
20−25−somites
Prim−5
Prim−15
Prim−25
Long−pec
Protruding−mouth
Day−4
Day−5
Stage
Transcript
ENSDART00000058685
ENSDART00000104583
ENSDART00000141941
ENSDART00000151936

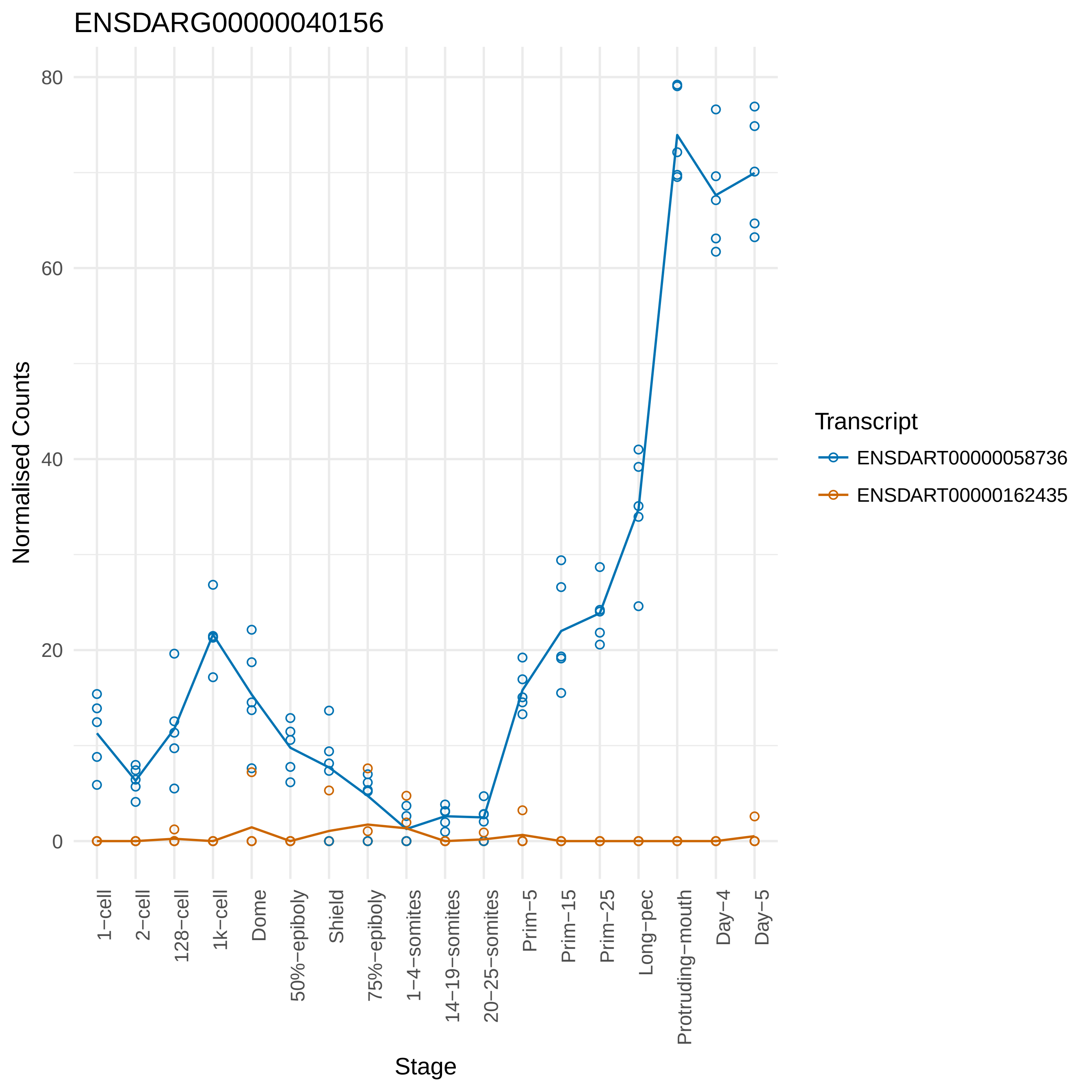
ENSDARG00000040156
Normalised Counts
0
20
40
60
80
1−cell
2−cell
128−cell
1k−cell
Dome
50%−epiboly
Shield
75%−epiboly
1−4−somites
14−19−somites
20−25−somites
Prim−5
Prim−15
Prim−25
Long−pec
Protruding−mouth
Day−4
Day−5
Stage
Transcript
ENSDART00000058736
ENSDART00000162435

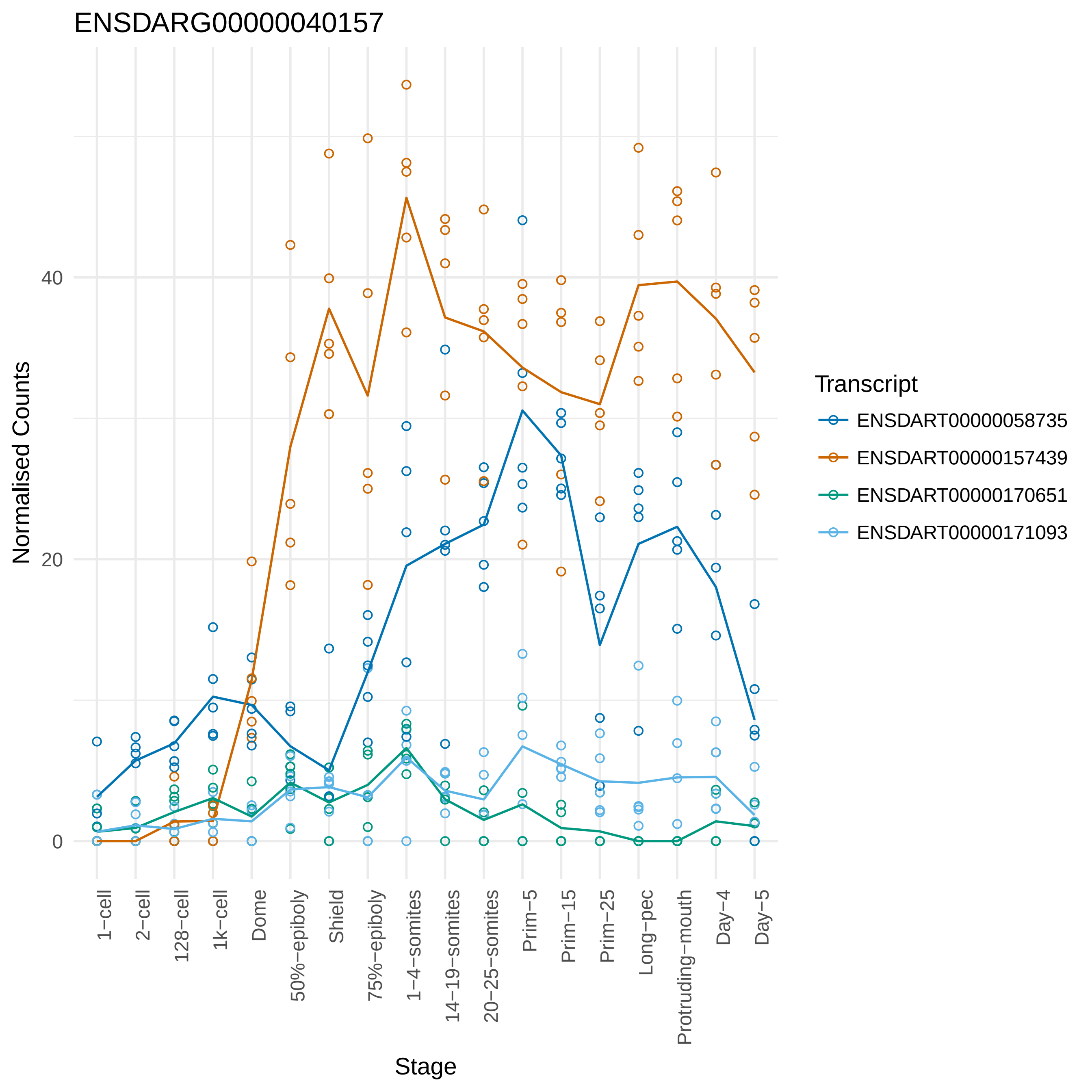

ENSDARG00000040157
Normalised Counts
0
20
40
Stage
1−cell
2−cell
128−cell
1k−cell
Dome
50%−epiboly
Shield
75%−epiboly
1−4−somites
14−19−somites
20−25−somites
Prim−5
Prim−15
Prim−25
Long−pec
Protruding−mouth
Day−4
Day−5
Transcript
ENSDART00000058735
ENSDART00000157439
ENSDART00000170651
ENSDART00000171093

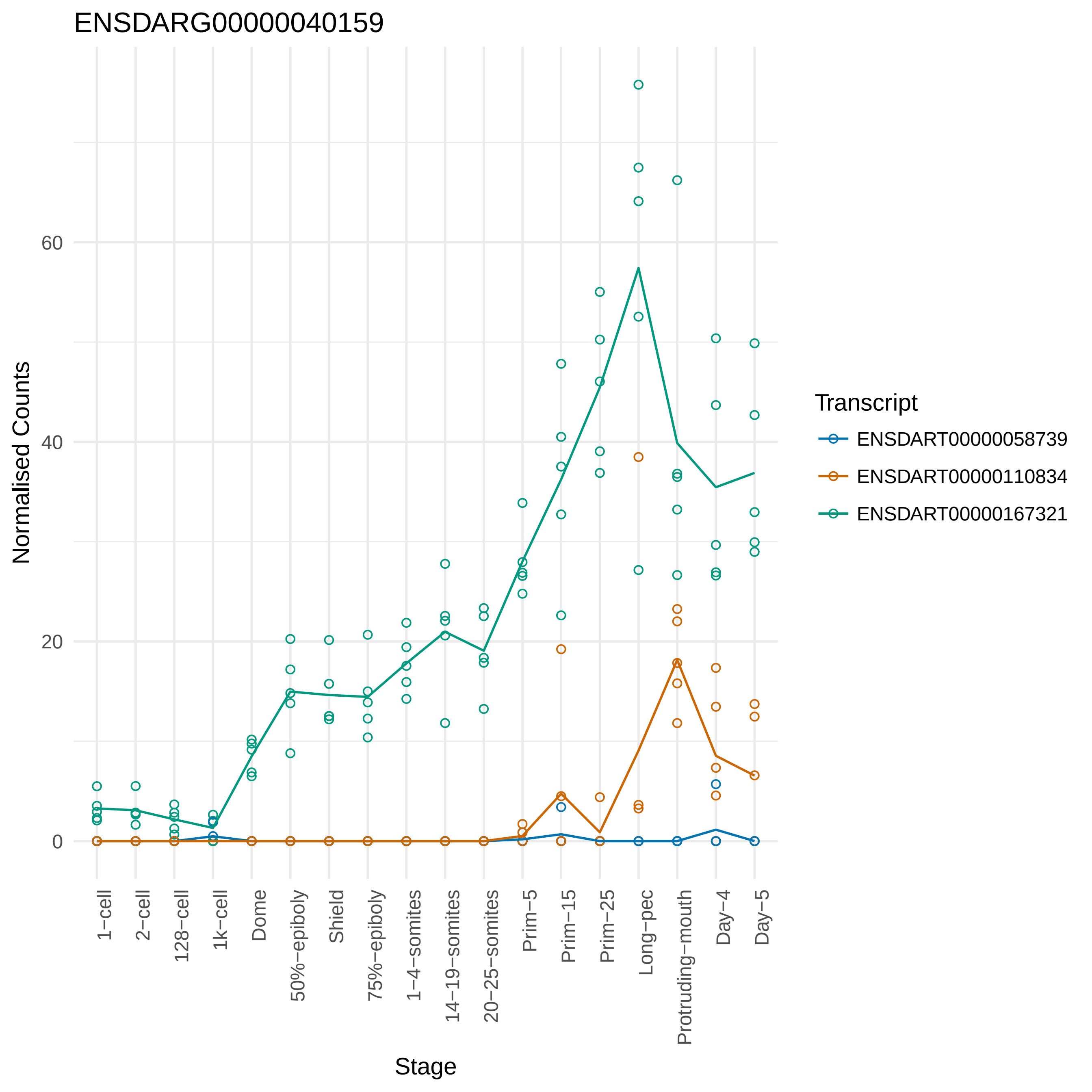

ENSDARG00000040159
Normalised Counts
0
20
40
60
Stage
1−cell
2−cell
128−cell
1k−cell
Dome
50%−epiboly
Shield
75%−epiboly
1−4−somites
14−19−somites
20−25−somites
Prim−5
Prim−15
Prim−25
Long−pec
Protruding−mouth
Day−4
Day−5
Transcript
ENSDART00000058739
ENSDART00000110834
ENSDART00000167321

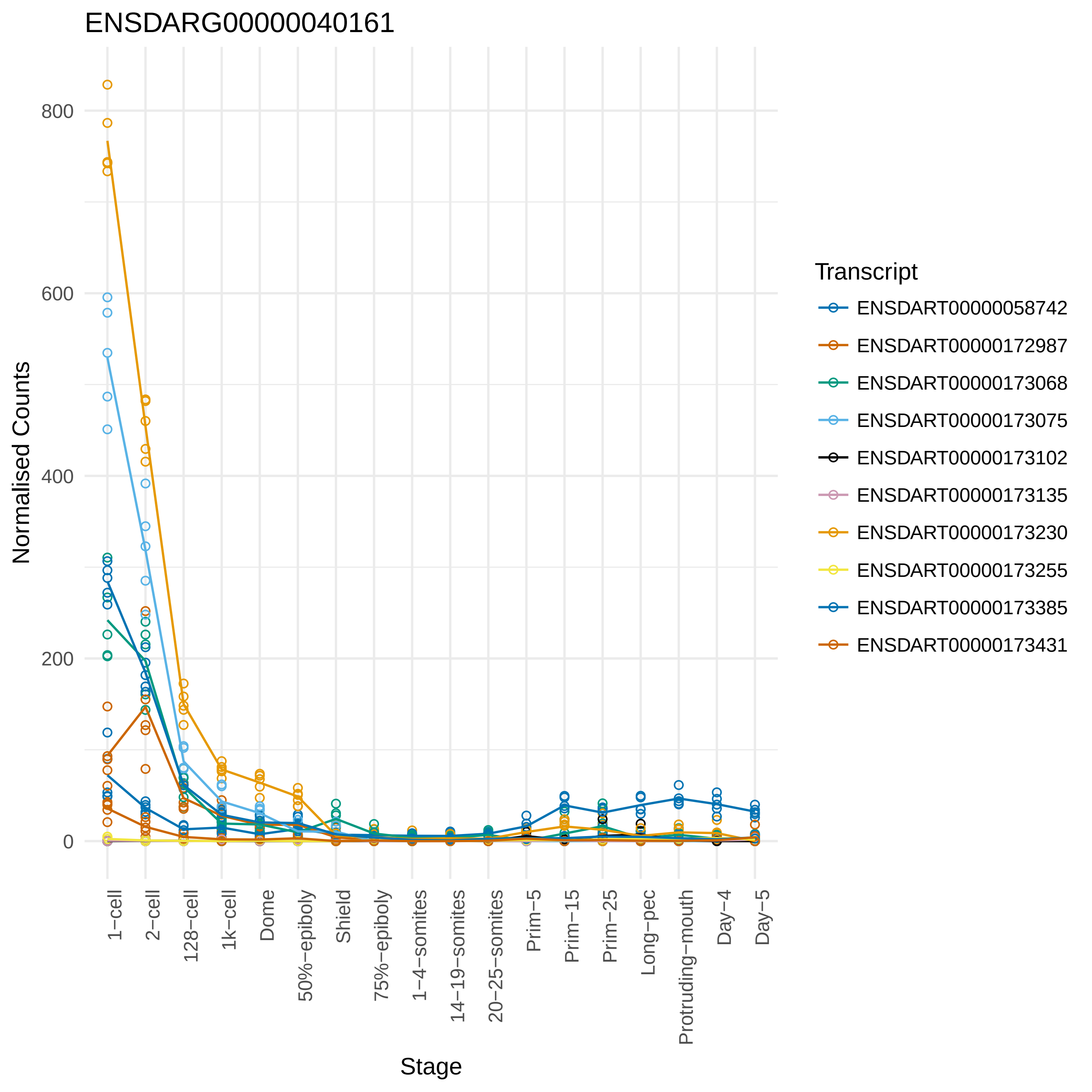
ENSDARG00000040161
Normalised Counts
0
200
400
600
800
Stage
1−cell
2−cell
128−cell
1k−cell
Dome
50%−epiboly
Shield
75%−epiboly
1−4−somites
14−19−somites
20−25−somites
Prim−5
Prim−15
Prim−25
Long−pec
Protruding−mouth
Day−4
Day−5
Transcript
ENSDART00000058742
ENSDART00000172987
ENSDART00000173068
ENSDART00000173075
ENSDART00000173102
ENSDART00000173135
ENSDART00000173230
ENSDART00000173255
ENSDART00000173385
ENSDART00000173431

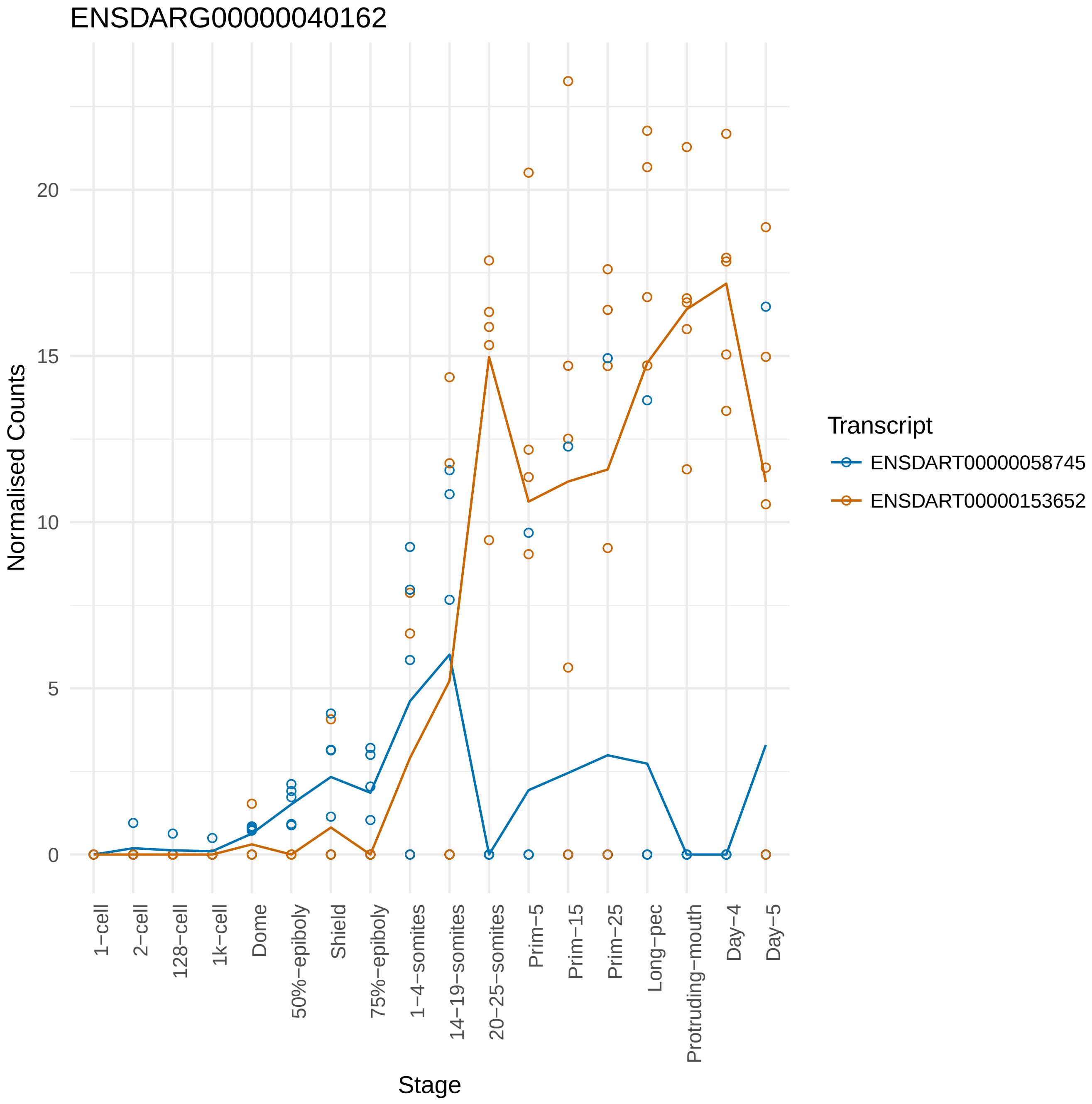

ENSDARG00000040162
Normalised Counts
0
5
10
15
20
1−cell
2−cell
128−cell
1k−cell
Dome
50%−epiboly
Shield
75%−epiboly
1−4−somites
14−19−somites
20−25−somites
Prim−5
Prim−15
Prim−25
Long−pec
Protruding−mouth
Day−4
Day−5
Stage
Transcript
ENSDART00000058745
ENSDART00000153652

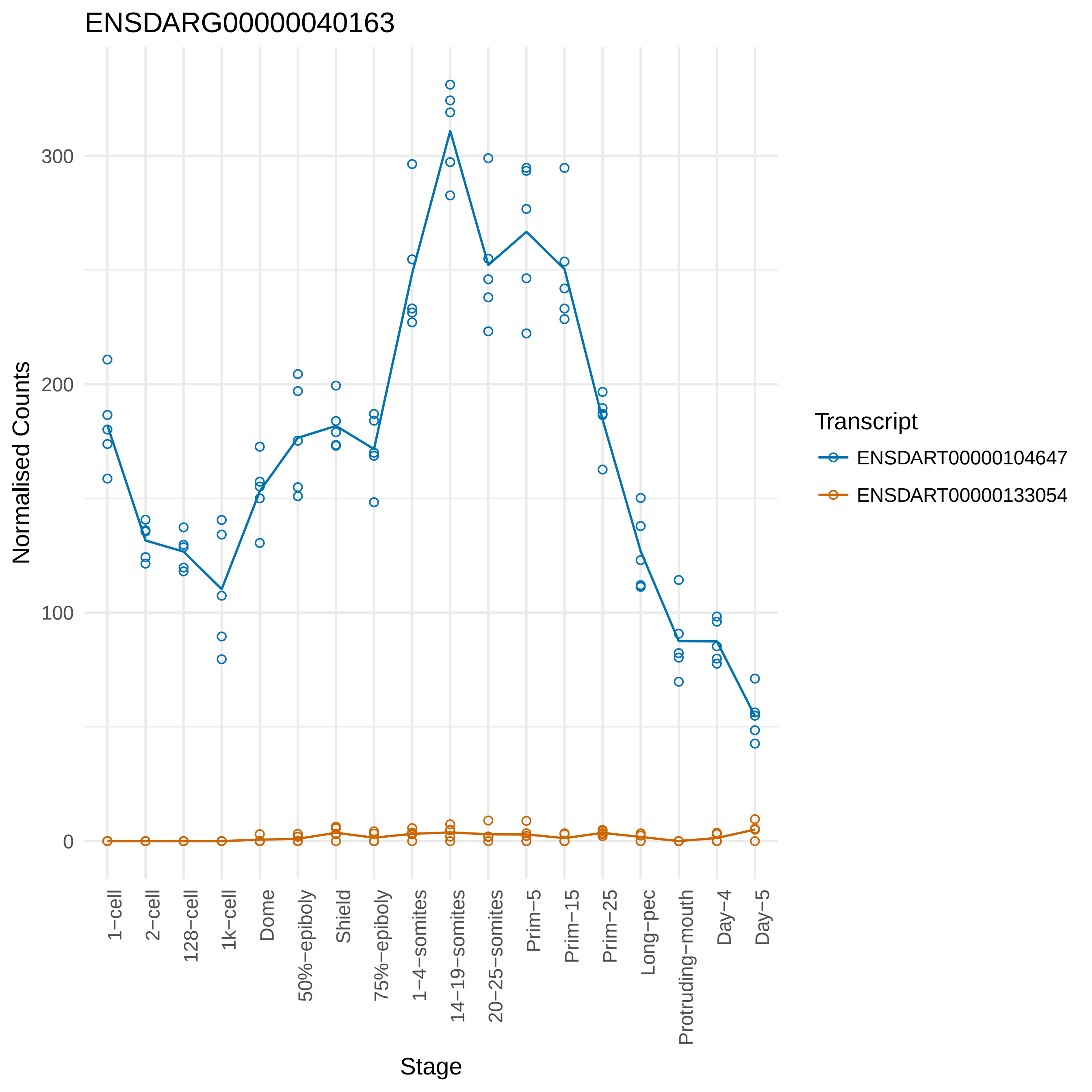

ENSDARG00000040163
Normalised Counts
300
200
100
0
1−cell
2−cell
128−cell
1k−cell
Dome
50%−epiboly
Shield
75%−epiboly
1−4−somites
14−19−somites
20−25−somites
Prim−5
Prim−15
Prim−25
Long−pec
Protruding−mouth
Day−4
Day−5
Stage
Transcript
ENSDART00000104647
ENSDART00000133054

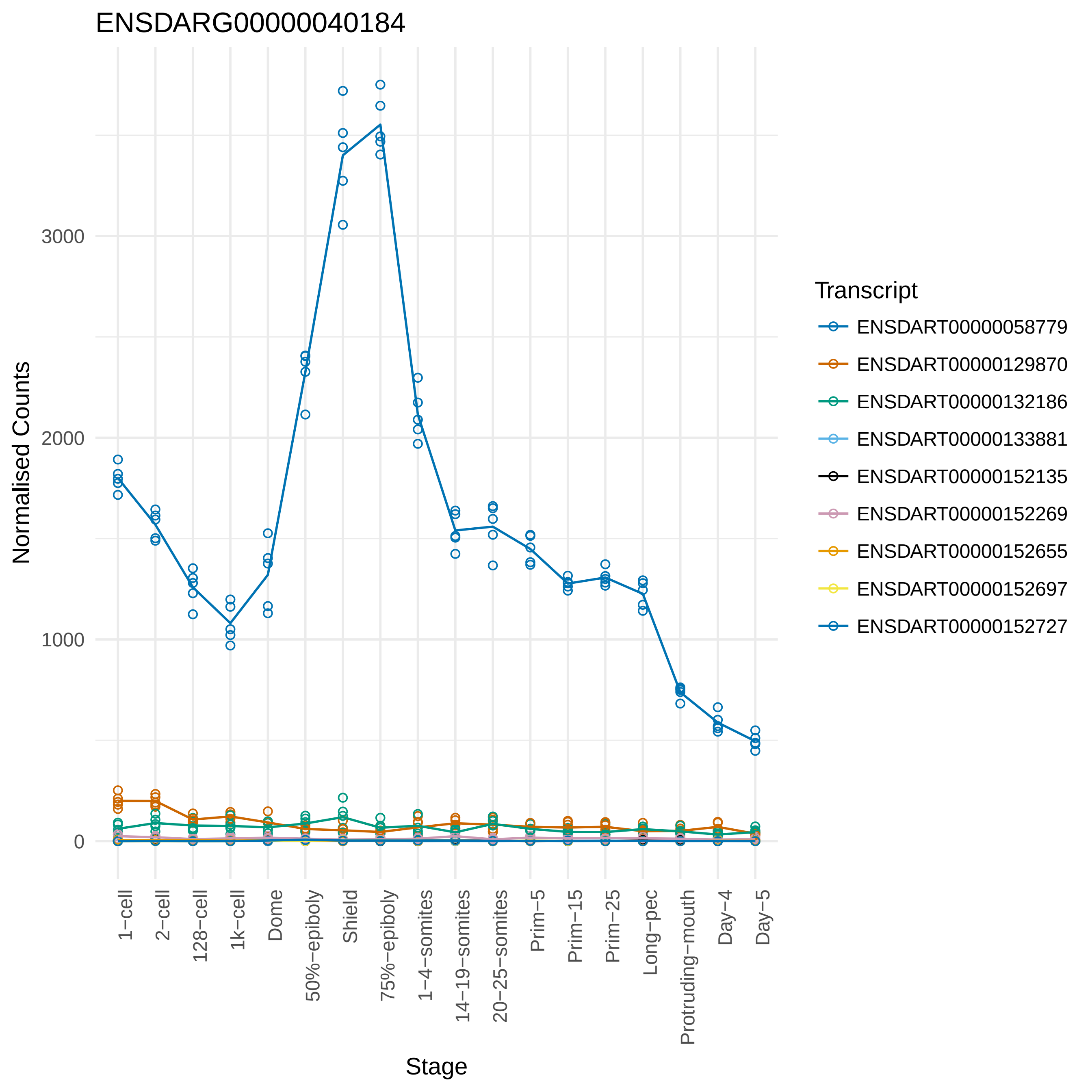

ENSDARG00000040184
Normalised Counts
3000
2000
1000
0
Stage
1−cell
2−cell
128−cell
1k−cell
Dome
50%−epiboly
Shield
75%−epiboly
1−4−somites
14−19−somites
20−25−somites
Prim−5
Prim−15
Prim−25
Long−pec
Protruding−mouth
Day−4
Day−5
Transcript
ENSDART00000058779
ENSDART00000129870
ENSDART00000132186
ENSDART00000133881
ENSDART00000152135
ENSDART00000152269
ENSDART00000152655
ENSDART00000152697
ENSDART00000152727

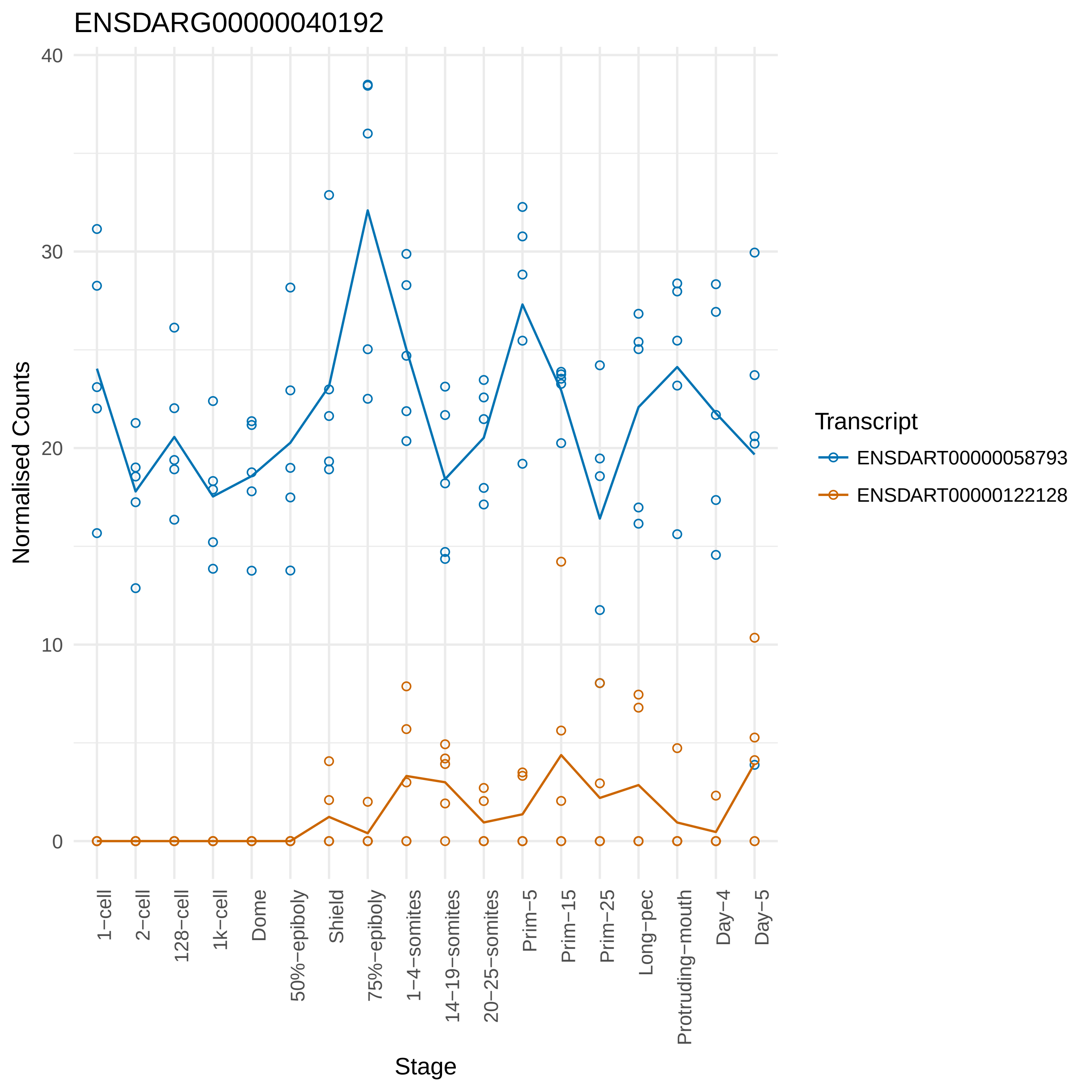

ENSDARG00000040192
Normalised Counts
40
30
20
10
0
1−cell
2−cell
128−cell
1k−cell
Dome
50%−epiboly
Shield
75%−epiboly
1−4−somites
14−19−somites
20−25−somites
Prim−5
Prim−15
Prim−25
Long−pec
Protruding−mouth
Day−4
Day−5
Stage
Transcript
ENSDART00000058793
ENSDART00000122128

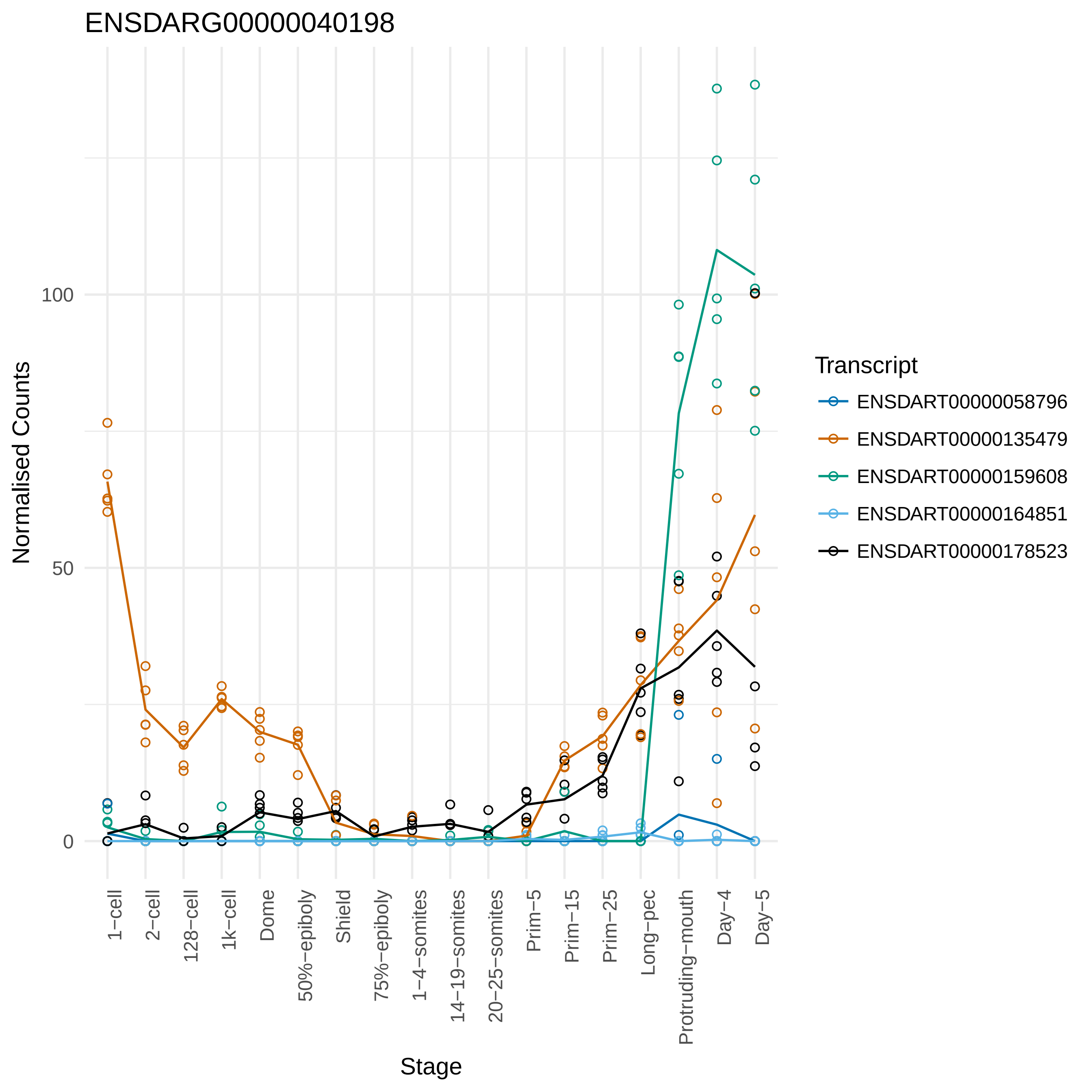

ENSDARG00000040198
Normalised Counts
0
50
100
1−cell
2−cell
128−cell
1k−cell
Dome
50%−epiboly
Shield
75%−epiboly
1−4−somites
14−19−somites
20−25−somites
Prim−5
Prim−15
Prim−25
Long−pec
Protruding−mouth
Day−4
Day−5
Stage
Transcript
ENSDART00000058796
ENSDART00000135479
ENSDART00000159608
ENSDART00000164851
ENSDART00000178523

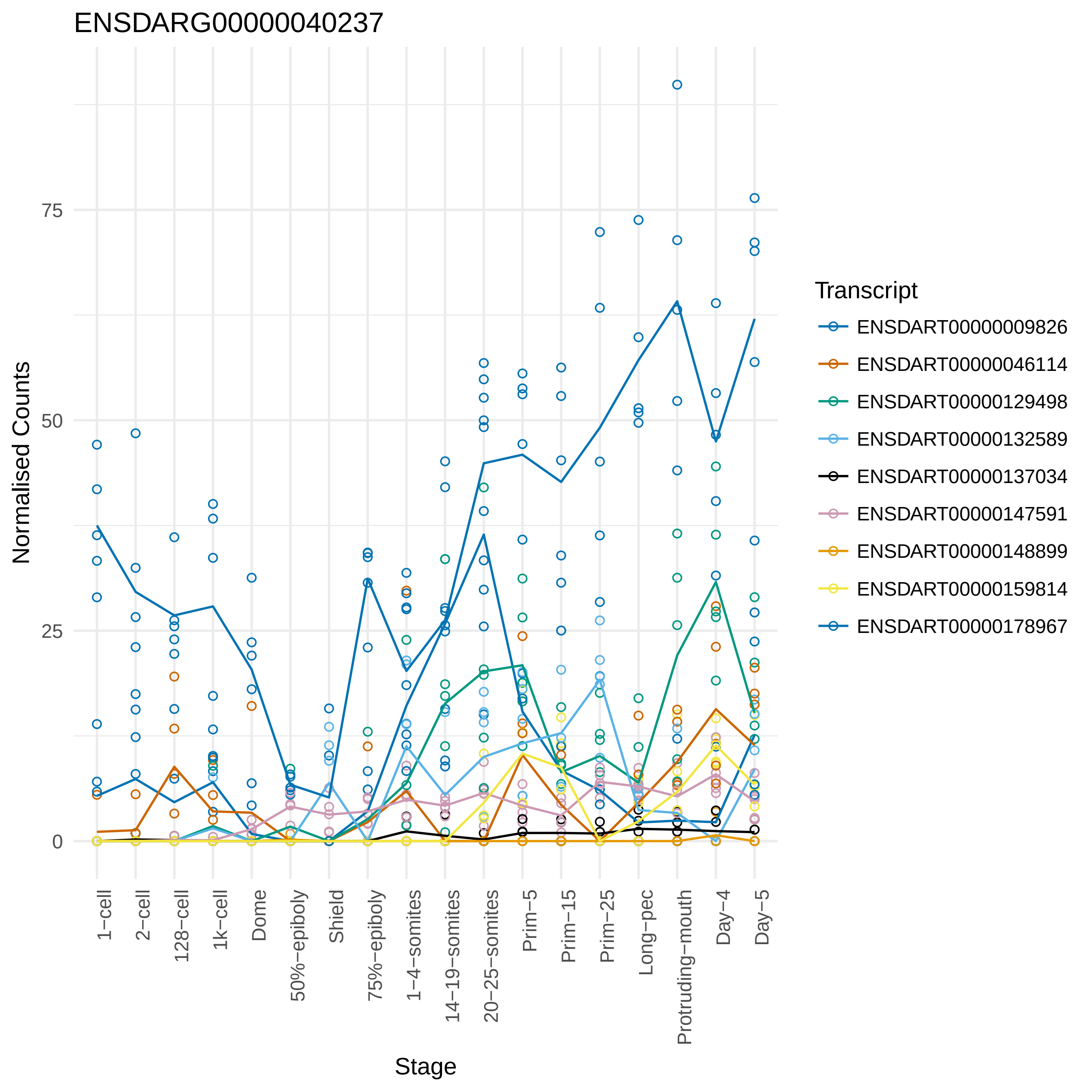
ENSDARG00000040237
Normalised Counts
0
25
50
75
Stage
1−cell
2−cell
128−cell
1k−cell
Dome
50%−epiboly
Shield
75%−epiboly
1−4−somites
14−19−somites
20−25−somites
Prim−5
Prim−15
Prim−25
Long−pec
Protruding−mouth
Day−4
Day−5
Transcript
ENSDART00000009826
ENSDART00000046114
ENSDART00000129498
ENSDART00000132589
ENSDART00000137034
ENSDART00000147591
ENSDART00000148899
ENSDART00000159814
ENSDART00000178967

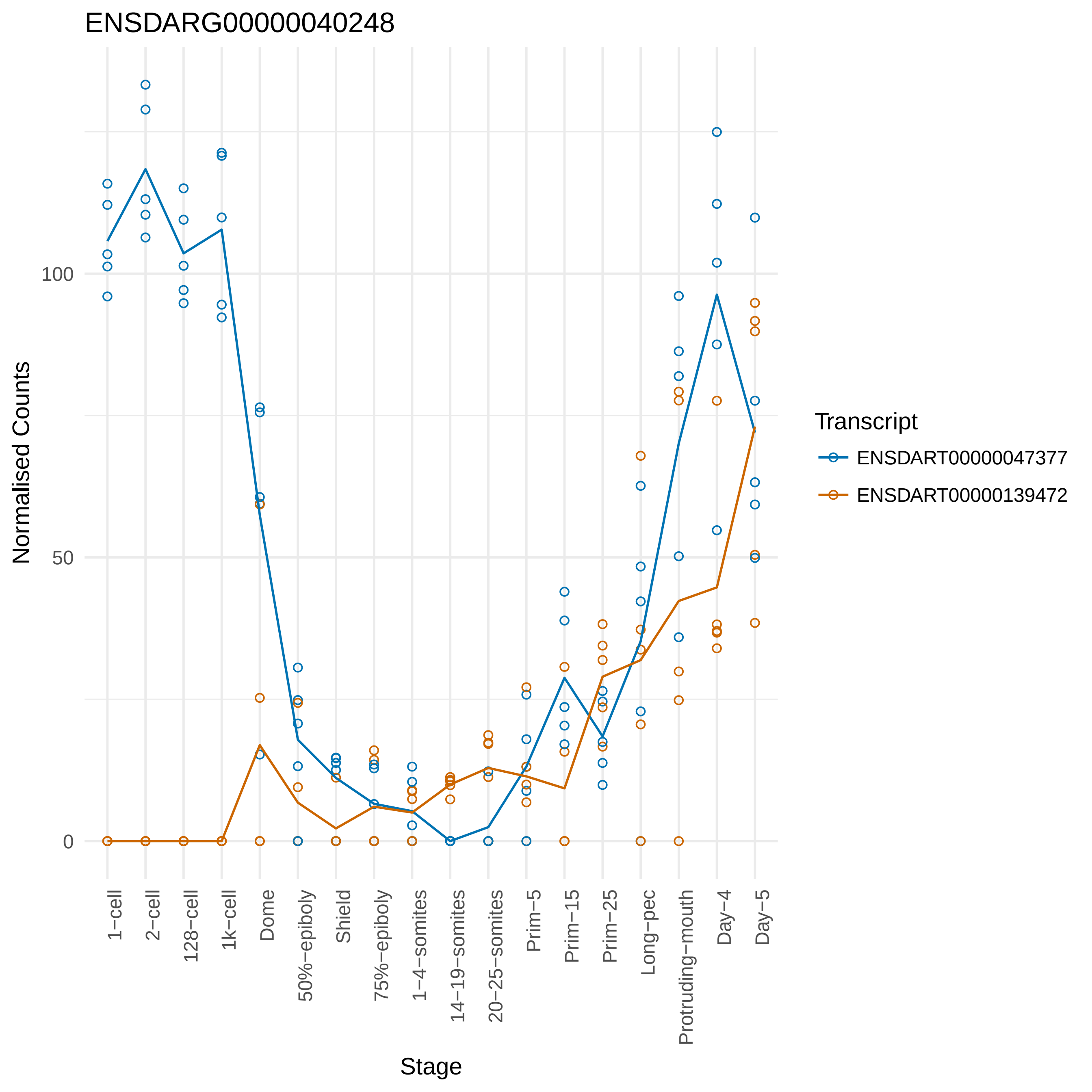
ENSDARG00000040248
Normalised Counts
100
50
0
1−cell
2−cell
128−cell
1k−cell
Dome
50%−epiboly
Shield
75%−epiboly
1−4−somites
14−19−somites
20−25−somites
Prim−5
Prim−15
Prim−25
Long−pec
Protruding−mouth
Day−4
Day−5
Stage
Transcript
ENSDART00000047377
ENSDART00000139472

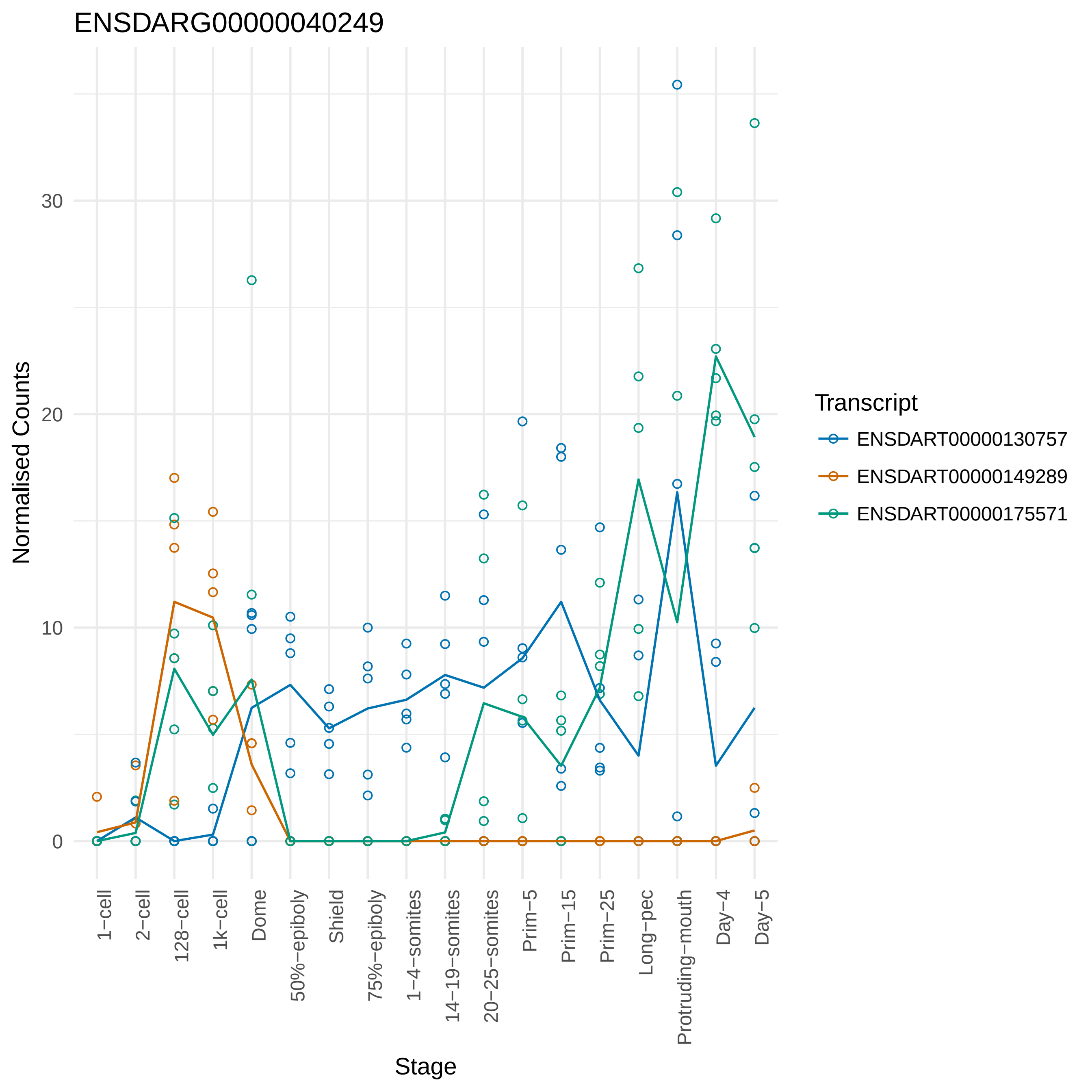

ENSDARG00000040249
Normalised Counts
30
20
10
0
1−cell
2−cell
128−cell
1k−cell
Dome
50%−epiboly
Shield
75%−epiboly
1−4−somites
14−19−somites
20−25−somites
Prim−5
Prim−15
Prim−25
Long−pec
Protruding−mouth
Day−4
Day−5
Stage
Transcript
ENSDART00000130757
ENSDART00000149289
ENSDART00000175571

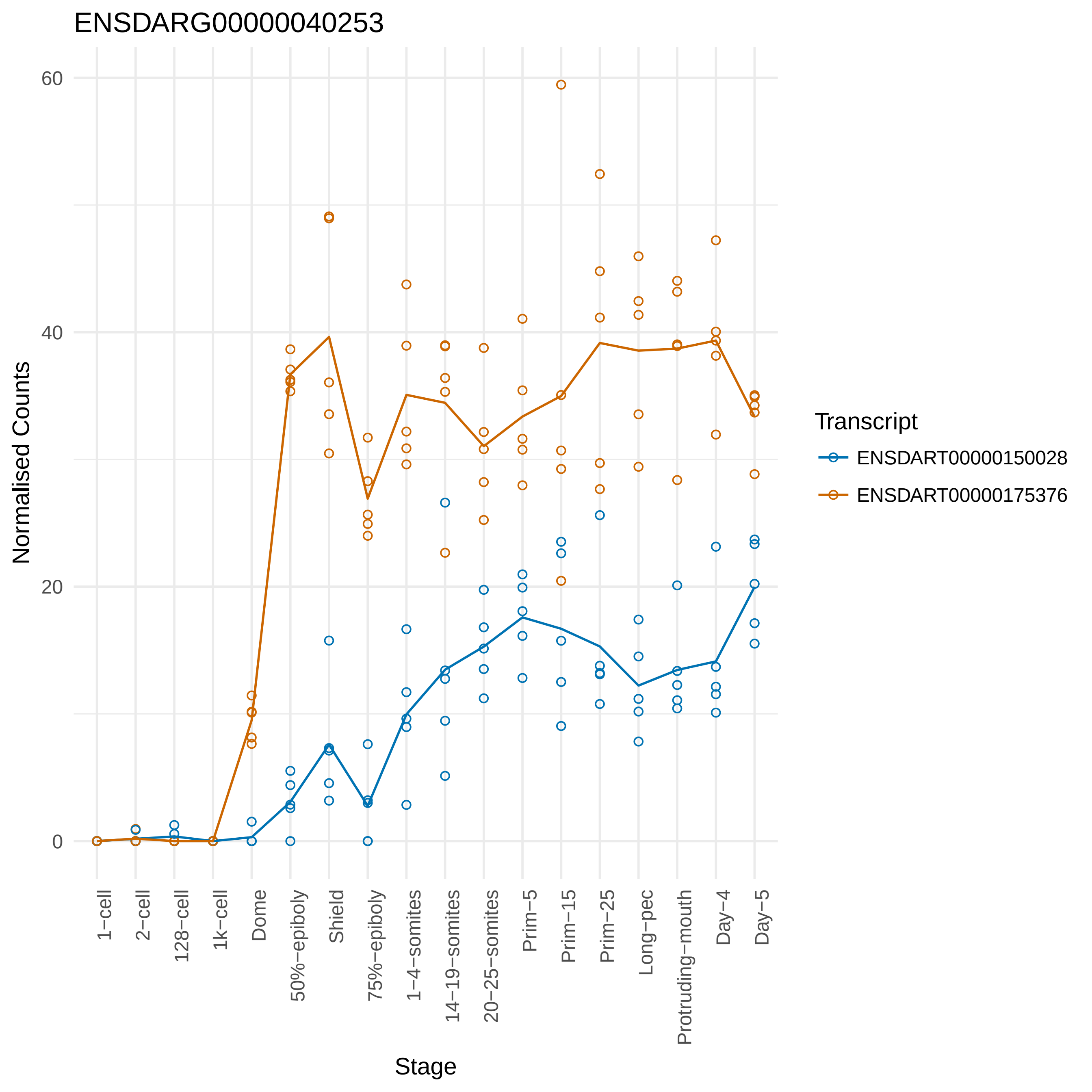

ENSDARG00000040253
Normalised Counts
60
40
20
0
1−cell
2−cell
128−cell
1k−cell
Dome
50%−epiboly
Shield
75%−epiboly
1−4−somites
14−19−somites
20−25−somites
Prim−5
Prim−15
Prim−25
Long−pec
Protruding−mouth
Day−4
Day−5
Stage
Transcript
ENSDART00000150028
ENSDART00000175376

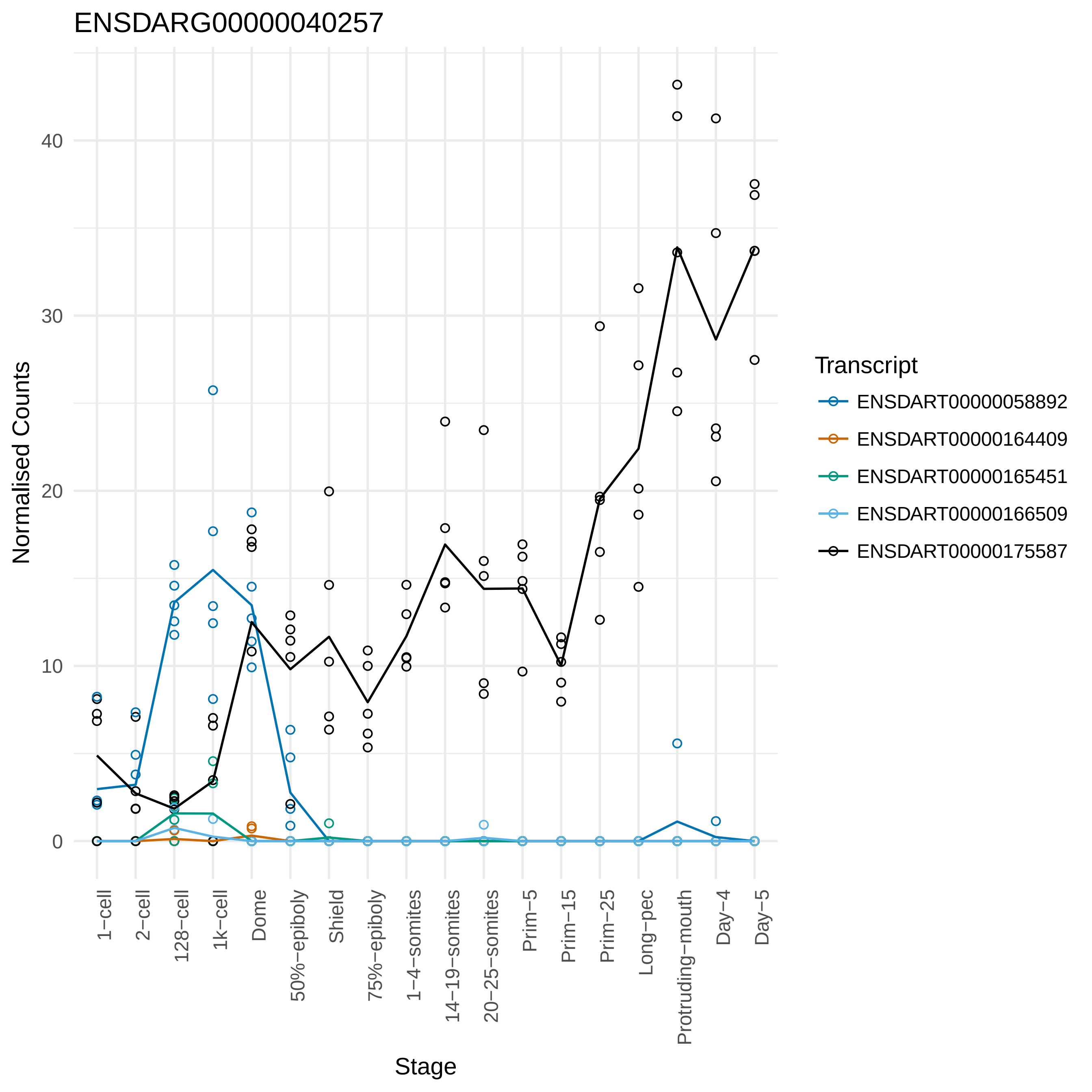

ENSDARG00000040257
Normalised Counts
0
10
20
30
40
Stage
1−cell
2−cell
128−cell
1k−cell
Dome
50%−epiboly
Shield
75%−epiboly
1−4−somites
14−19−somites
20−25−somites
Prim−5
Prim−15
Prim−25
Long−pec
Protruding−mouth
Day−4
Day−5
Transcript
ENSDART00000058892
ENSDART00000164409
ENSDART00000165451
ENSDART00000166509
ENSDART00000175587

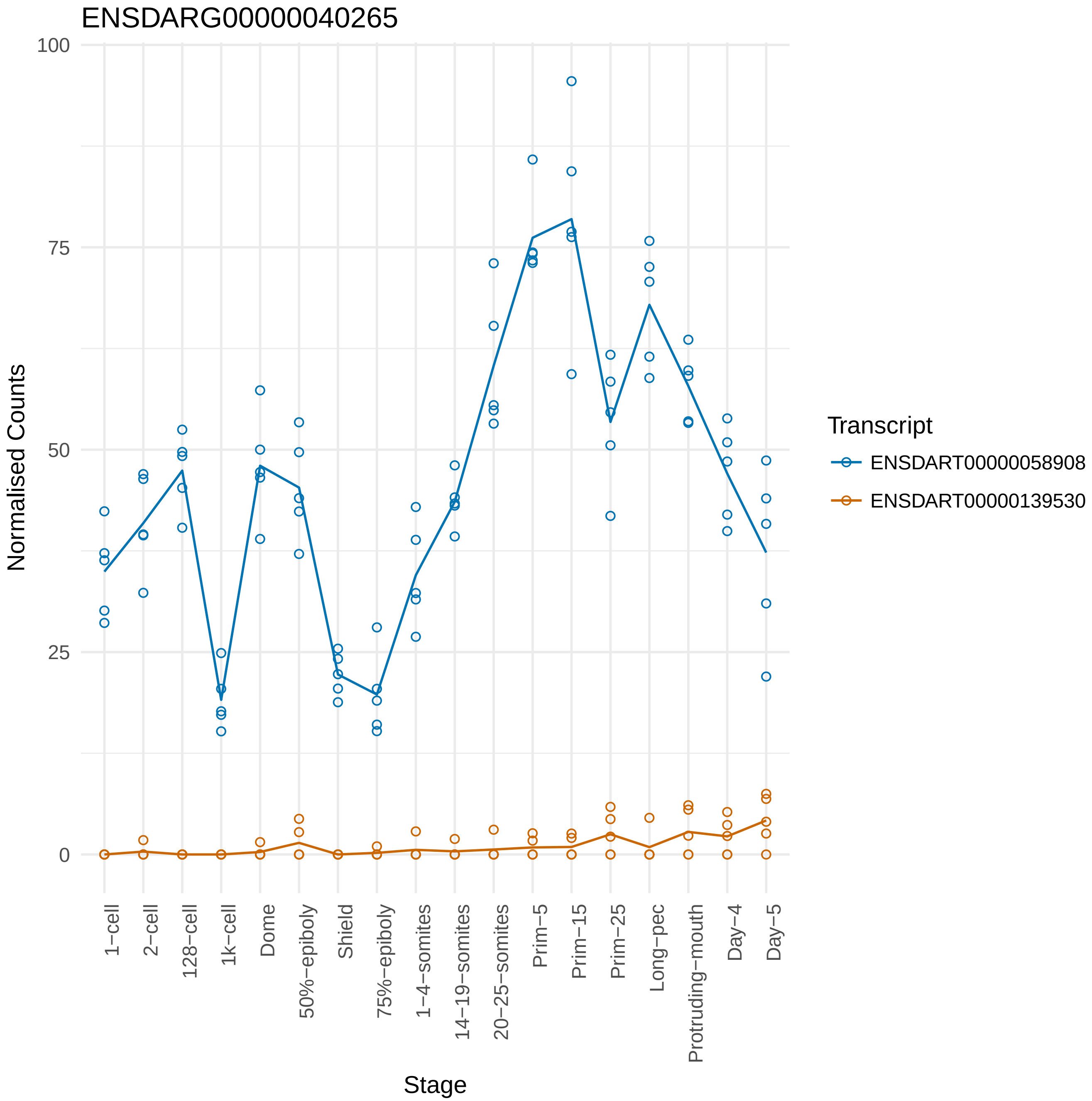

ENSDARG00000040265
Normalised Counts
100
75
50
25
0
1−cell
2−cell
128−cell
1k−cell
Dome
50%−epiboly
Shield
75%−epiboly
1−4−somites
14−19−somites
20−25−somites
Prim−5
Prim−15
Prim−25
Long−pec
Protruding−mouth
Day−4
Day−5
Stage
Transcript
ENSDART00000058908
ENSDART00000139530

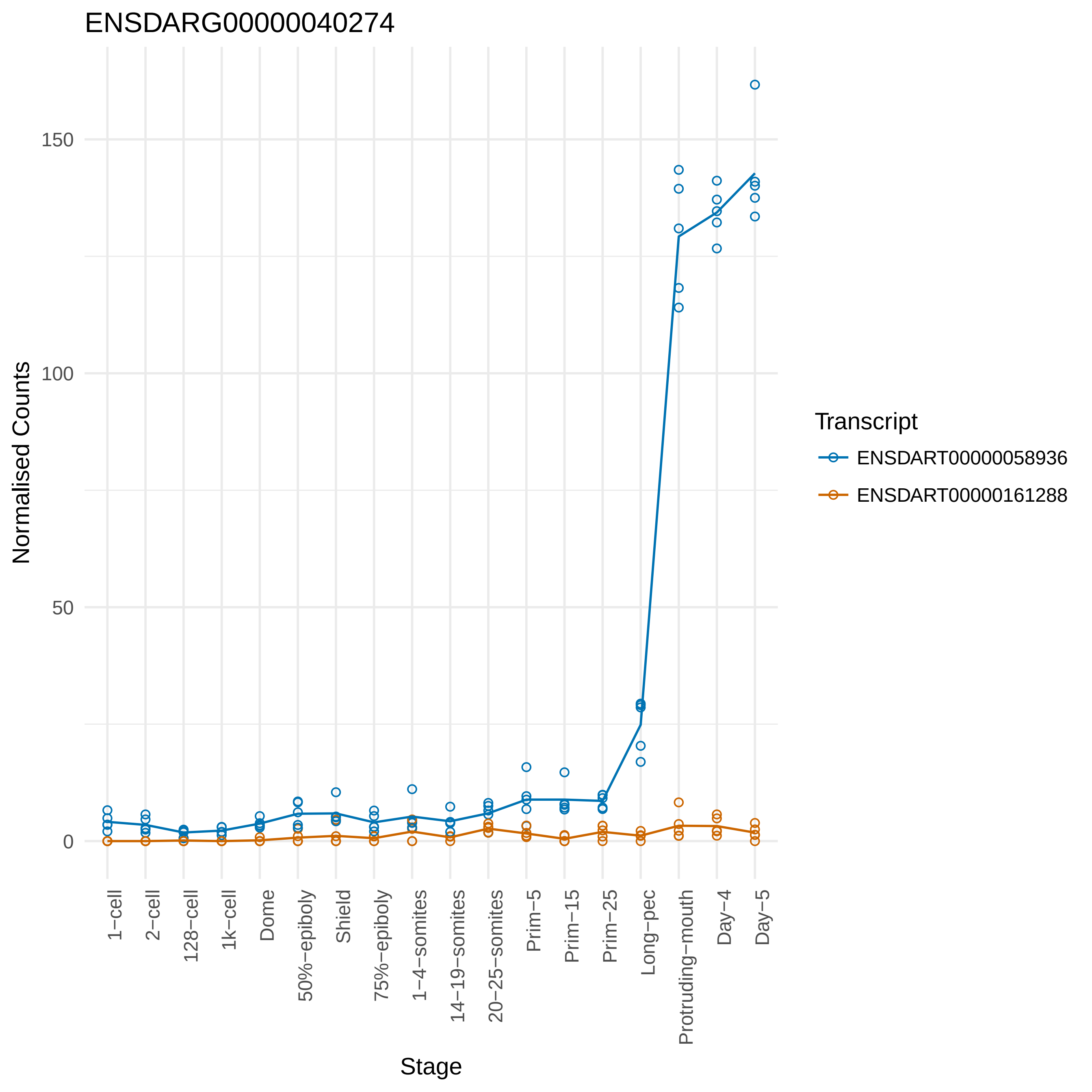

ENSDARG00000040274
Normalised Counts
150
100
50
0
1−cell
2−cell
128−cell
1k−cell
Dome
50%−epiboly
Shield
75%−epiboly
1−4−somites
14−19−somites
20−25−somites
Prim−5
Prim−15
Prim−25
Long−pec
Protruding−mouth
Day−4
Day−5
Stage
Transcript
ENSDART00000058936
ENSDART00000161288

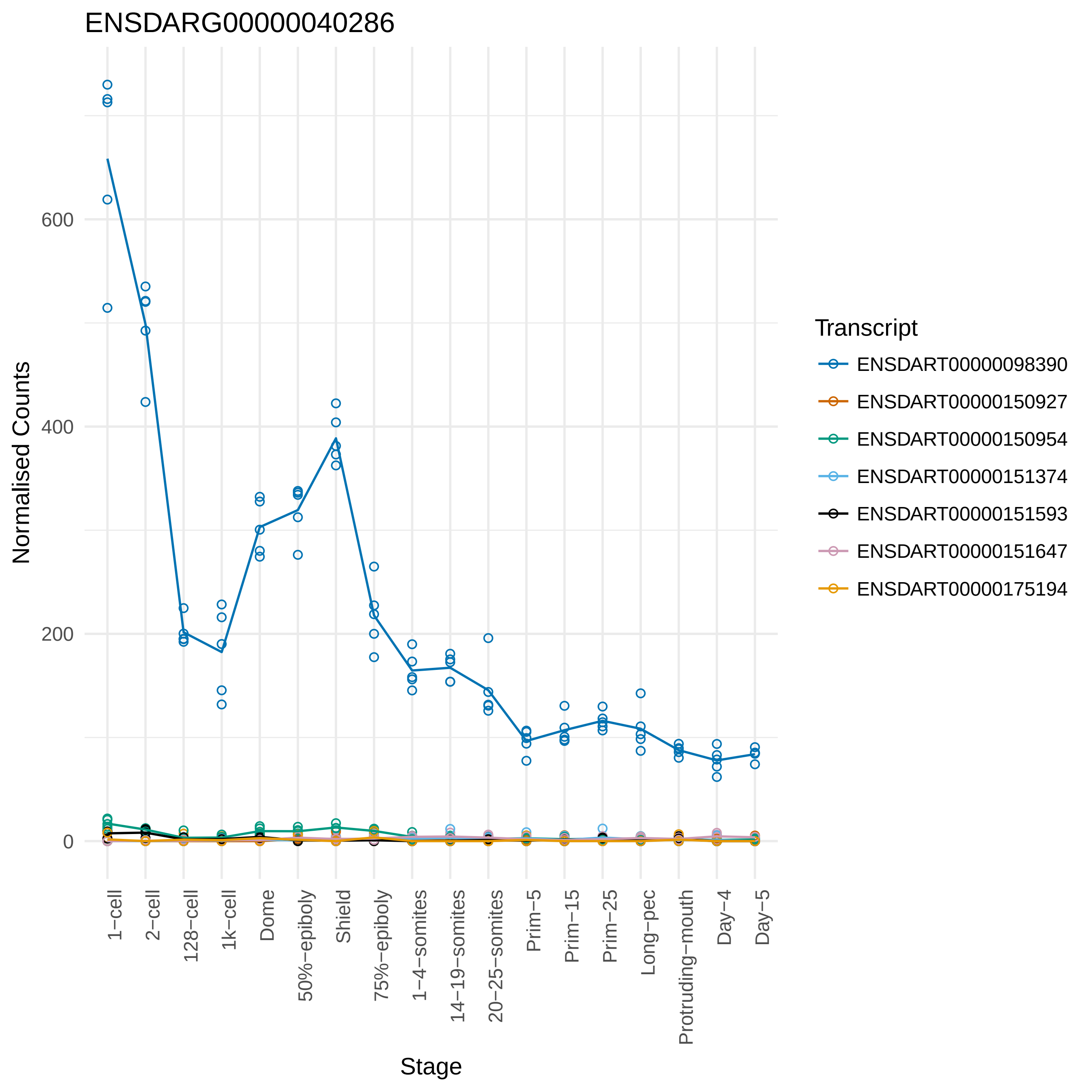

ENSDARG00000040286
Normalised Counts
600
400
200
0
1−cell
2−cell
128−cell
1k−cell
Dome
50%−epiboly
Shield
75%−epiboly
1−4−somites
14−19−somites
20−25−somites
Prim−5
Prim−15
Prim−25
Long−pec
Protruding−mouth
Day−4
Day−5
Stage
Transcript
ENSDART00000098390
ENSDART00000150927
ENSDART00000150954
ENSDART00000151374
ENSDART00000151593
ENSDART00000151647
ENSDART00000175194

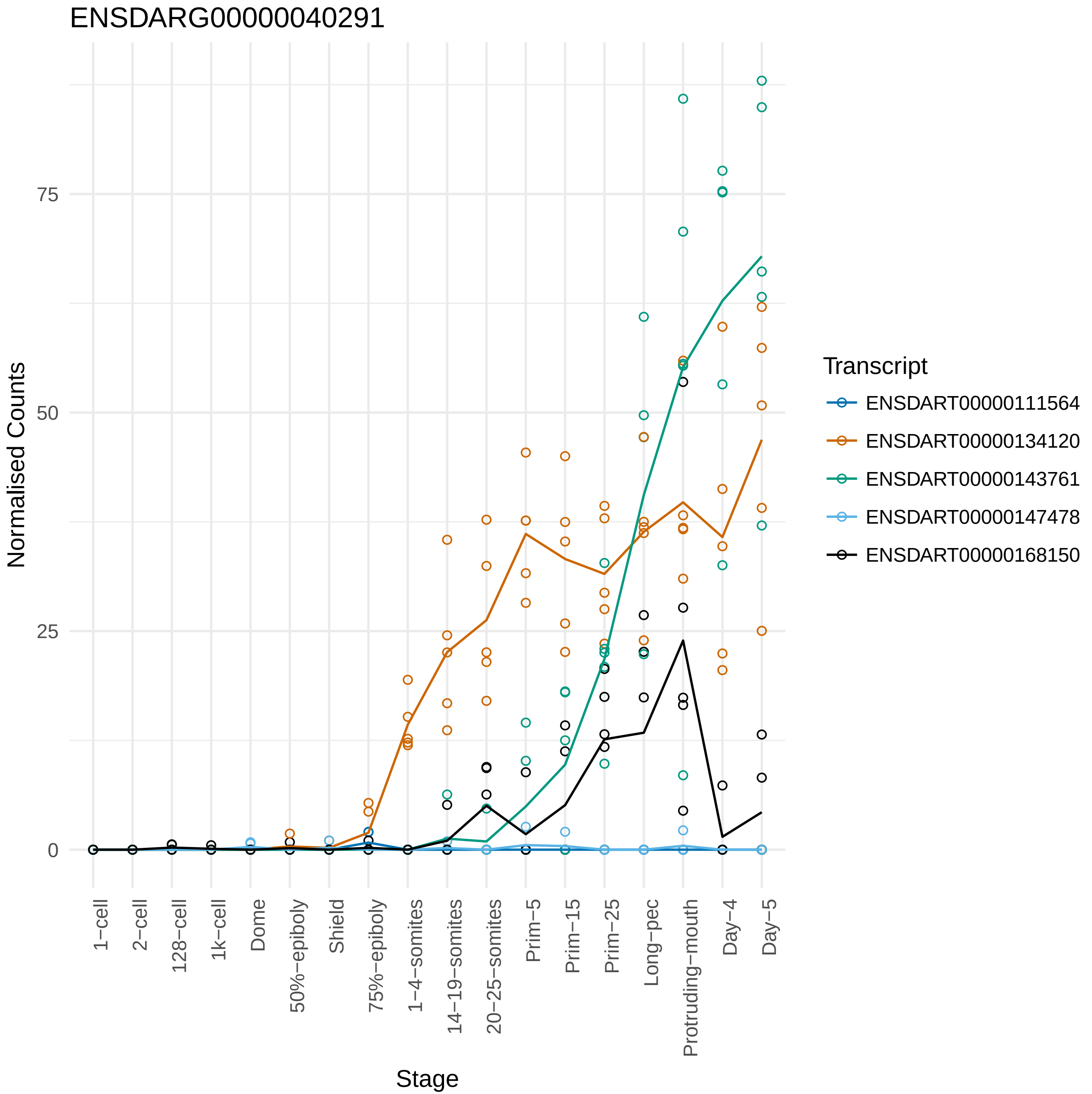

ENSDARG00000040291
Normalised Counts
75
50
25
0
1−cell
2−cell
128−cell
1k−cell
Dome
50%−epiboly
Shield
75%−epiboly
1−4−somites
14−19−somites
20−25−somites
Prim−5
Prim−15
Prim−25
Long−pec
Protruding−mouth
Day−4
Day−5
Stage
Transcript
ENSDART00000111564
ENSDART00000134120
ENSDART00000143761
ENSDART00000147478
ENSDART00000168150

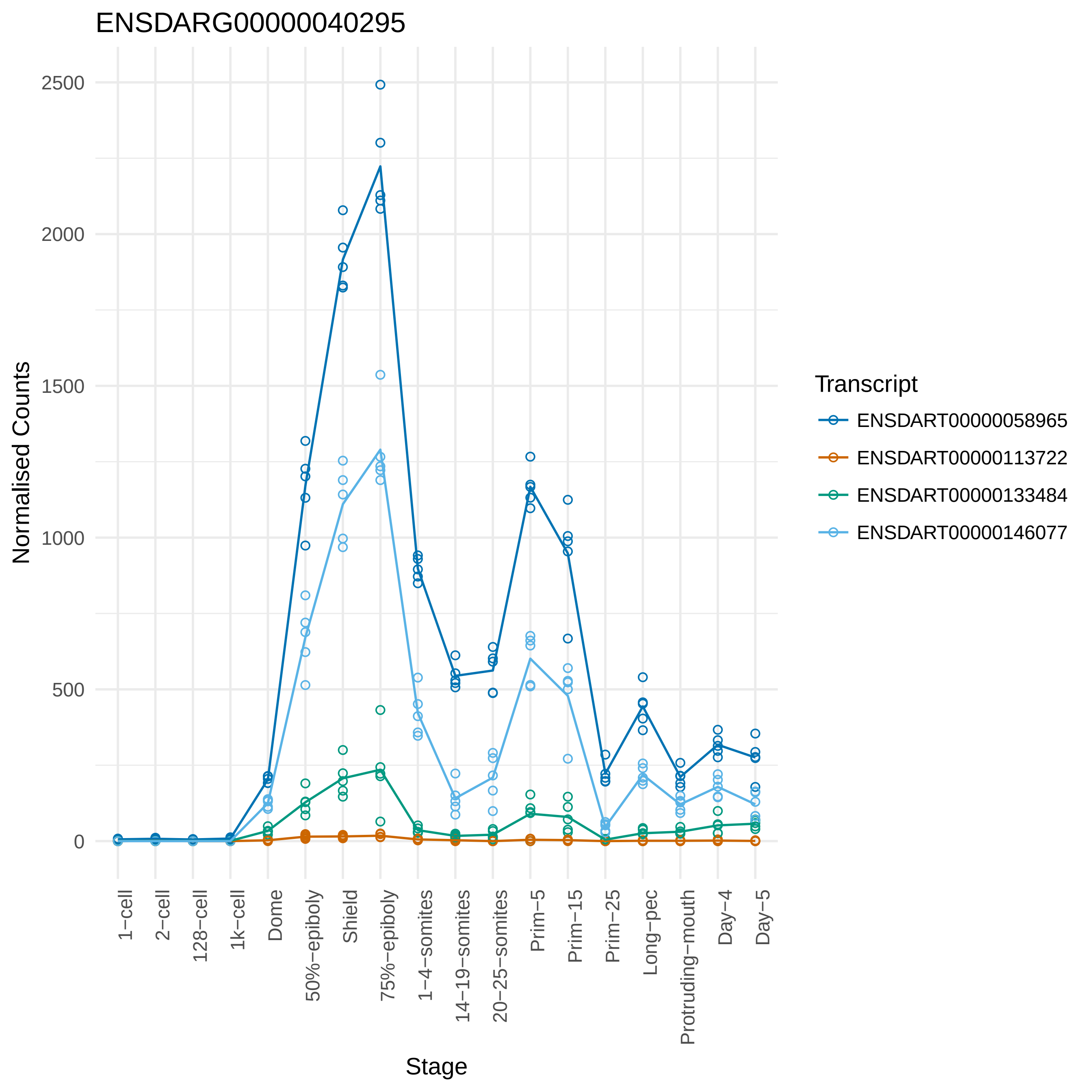

ENSDARG00000040295
Normalised Counts
2500
2000
1500
1000
500
0
1−cell
2−cell
128−cell
1k−cell
Dome
50%−epiboly
Shield
75%−epiboly
1−4−somites
14−19−somites
20−25−somites
Prim−5
Prim−15
Prim−25
Long−pec
Protruding−mouth
Day−4
Day−5
Stage
Transcript
ENSDART00000058965
ENSDART00000113722
ENSDART00000133484
ENSDART00000146077

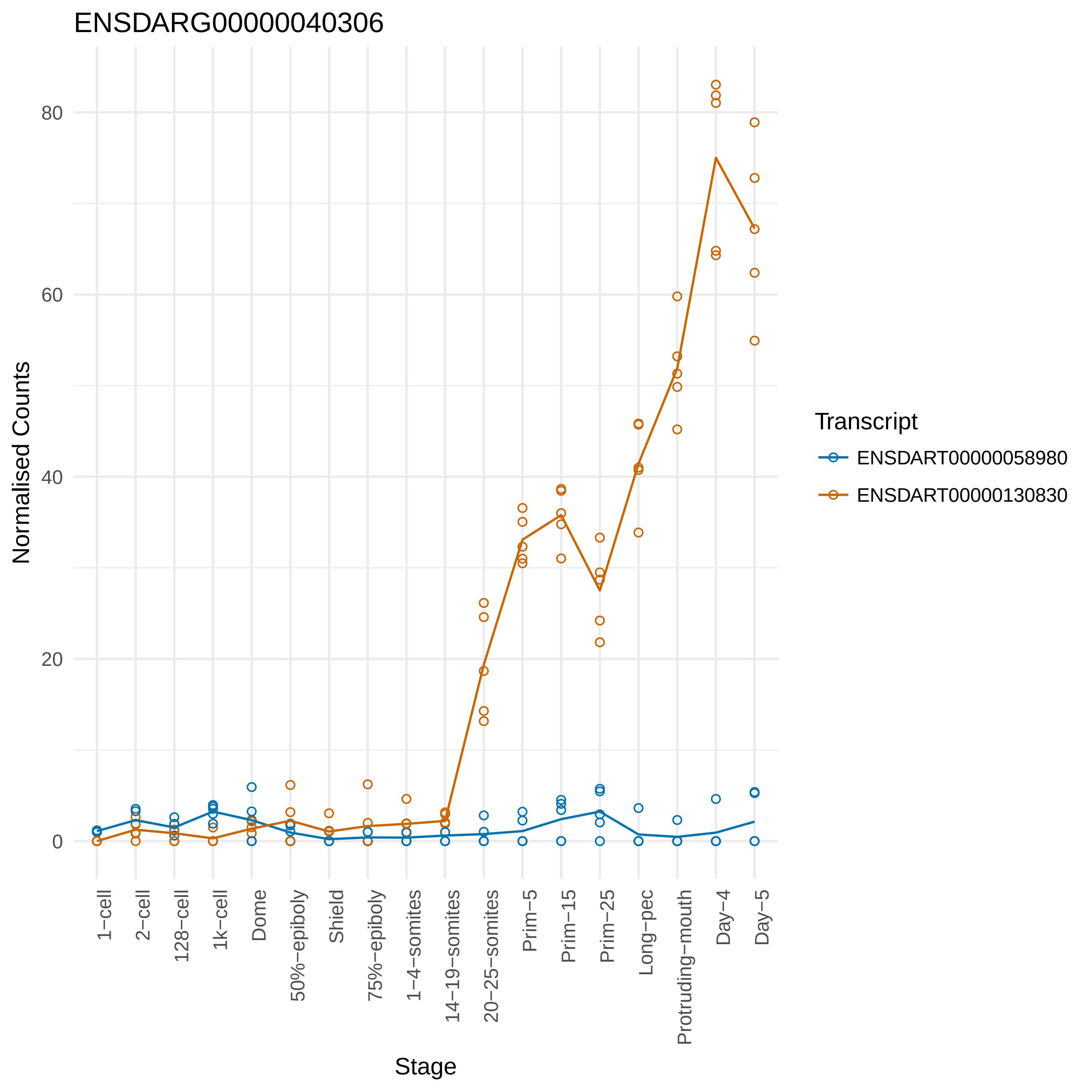

ENSDARG00000040306
Normalised Counts
0
20
40
60
80
1−cell
2−cell
128−cell
1k−cell
Dome
50%−epiboly
Shield
75%−epiboly
1−4−somites
14−19−somites
20−25−somites
Prim−5
Prim−15
Prim−25
Long−pec
Protruding−mouth
Day−4
Day−5
Stage
Transcript
ENSDART00000058980
ENSDART00000130830

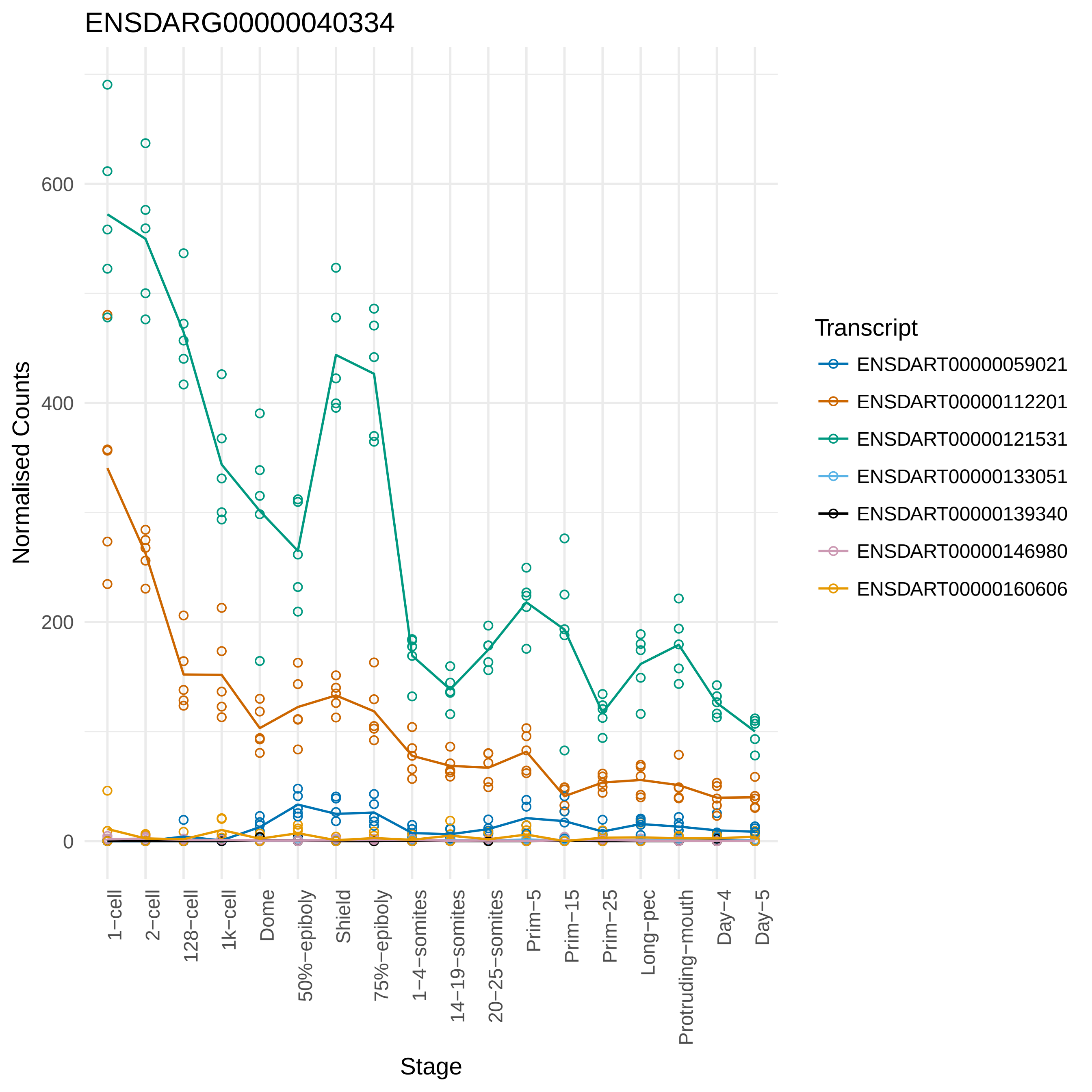
ENSDARG00000040334
Normalised Counts
0
200
400
600
Stage
1−cell
2−cell
128−cell
1k−cell
Dome
50%−epiboly
Shield
75%−epiboly
1−4−somites
14−19−somites
20−25−somites
Prim−5
Prim−15
Prim−25
Long−pec
Protruding−mouth
Day−4
Day−5
Transcript
ENSDART00000059021
ENSDART00000112201
ENSDART00000121531
ENSDART00000133051
ENSDART00000139340
ENSDART00000146980
ENSDART00000160606

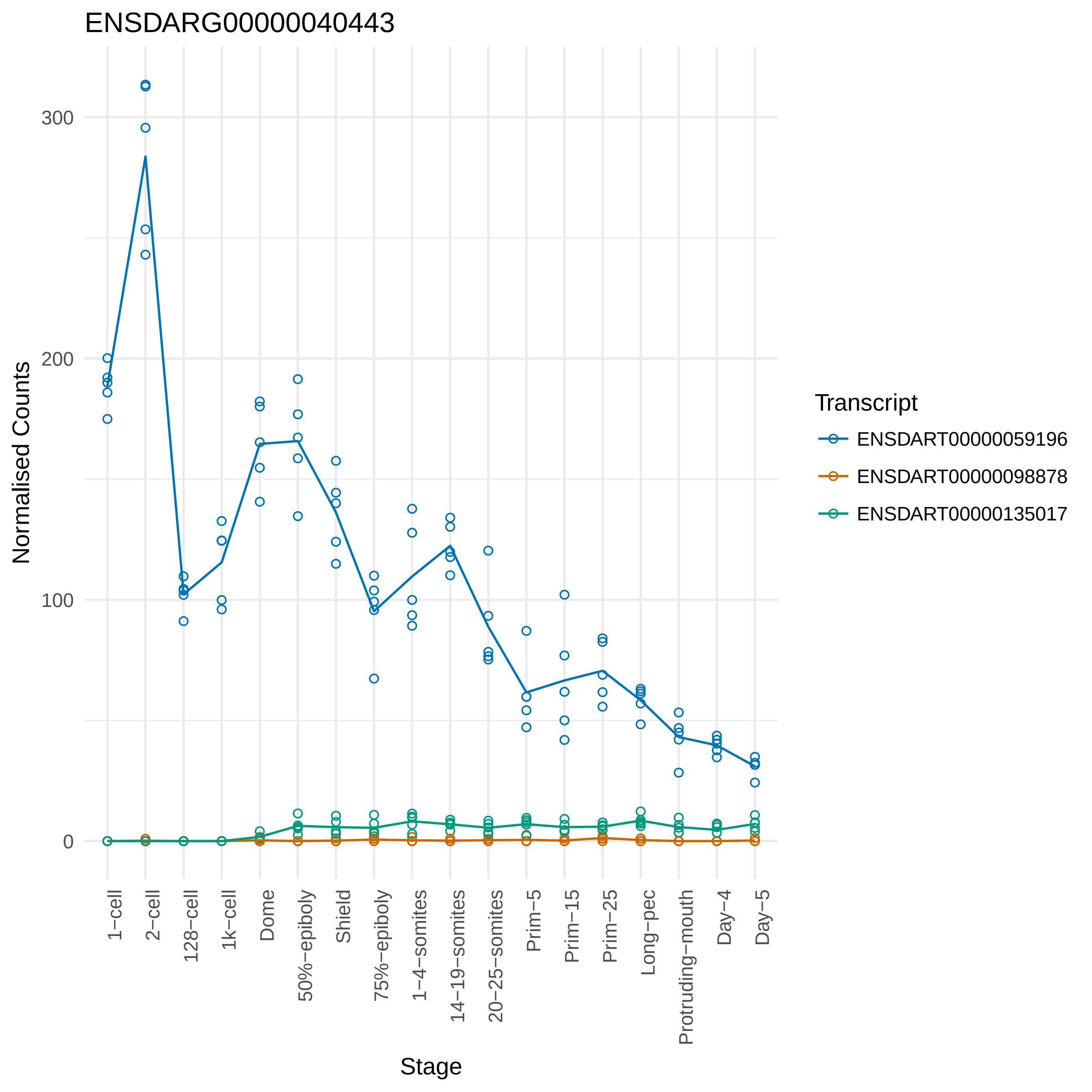

ENSDARG00000040443
Normalised Counts
300
200
100
0
1−cell
2−cell
128−cell
1k−cell
Dome
50%−epiboly
Shield
75%−epiboly
1−4−somites
14−19−somites
20−25−somites
Prim−5
Prim−15
Prim−25
Long−pec
Protruding−mouth
Day−4
Day−5
Stage
Transcript
ENSDART00000059196
ENSDART00000098878
ENSDART00000135017

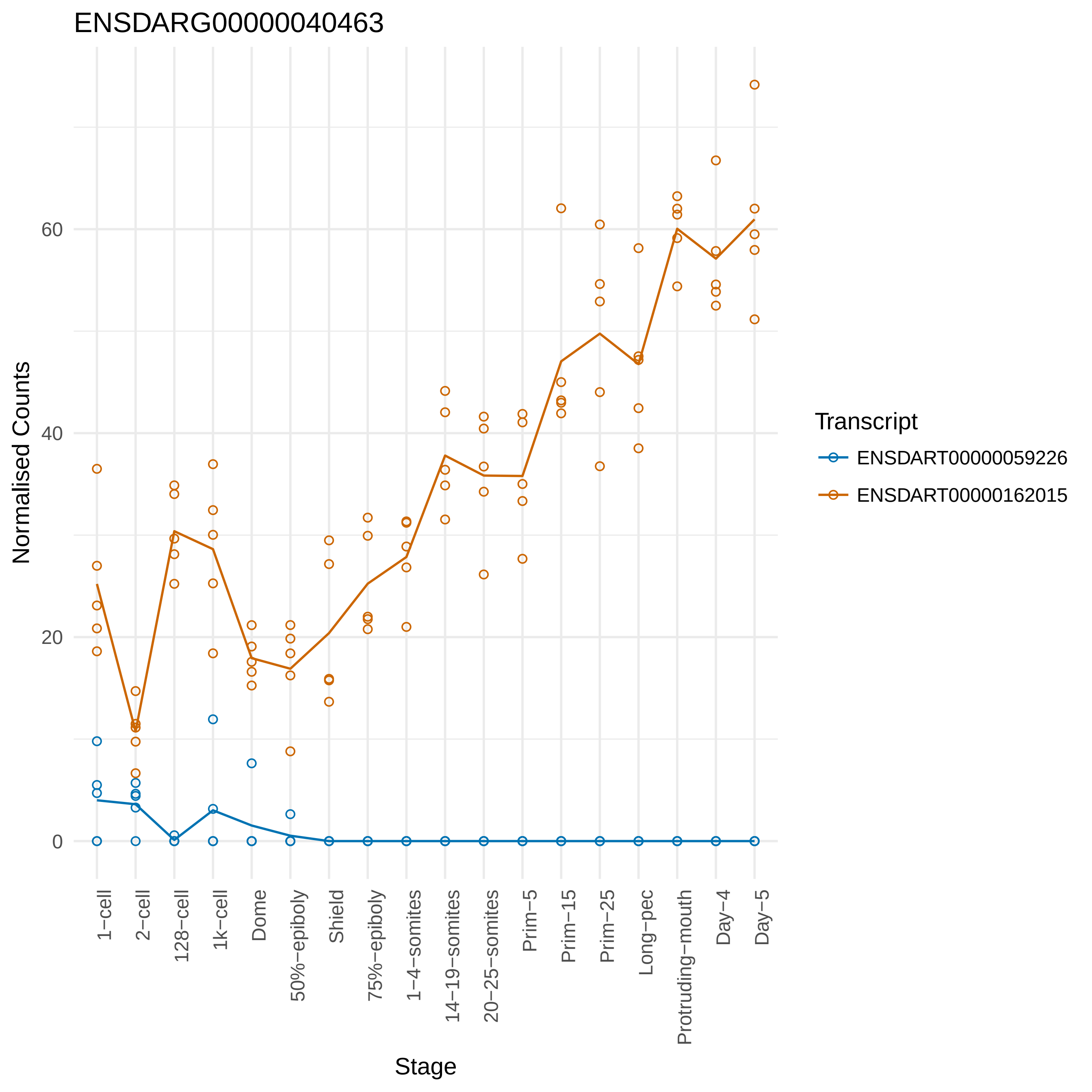
ENSDARG00000040463
Normalised Counts
0
20
40
60
Stage
1−cell
2−cell
128−cell
1k−cell
Dome
50%−epiboly
Shield
75%−epiboly
1−4−somites
14−19−somites
20−25−somites
Prim−5
Prim−15
Prim−25
Long−pec
Protruding−mouth
Day−4
Day−5
Transcript
ENSDART00000059226
ENSDART00000162015

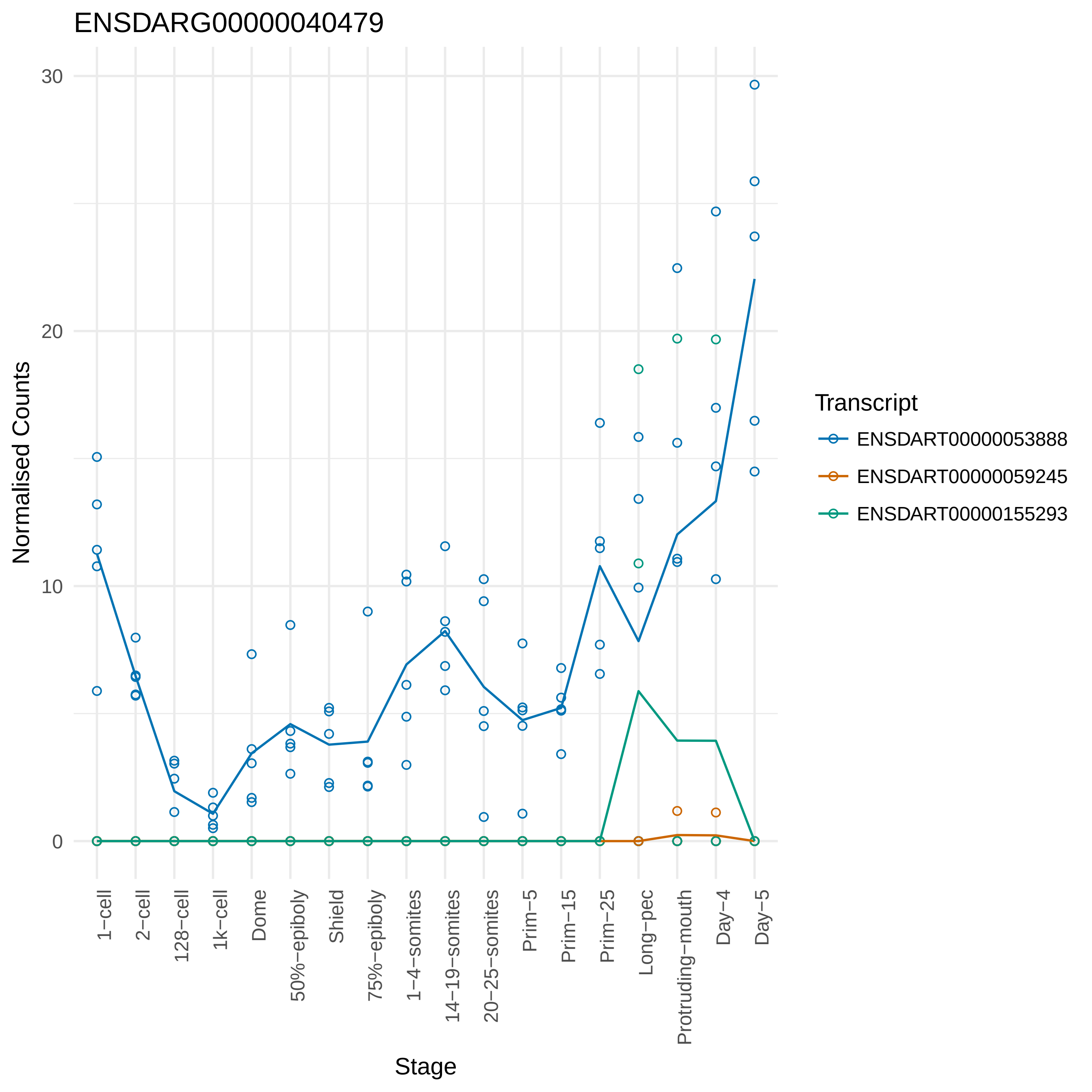

ENSDARG00000040479
Normalised Counts
0
10
20
30
Stage
1−cell
2−cell
128−cell
1k−cell
Dome
50%−epiboly
Shield
75%−epiboly
1−4−somites
14−19−somites
20−25−somites
Prim−5
Prim−15
Prim−25
Long−pec
Protruding−mouth
Day−4
Day−5
Transcript
ENSDART00000053888
ENSDART00000059245
ENSDART00000155293

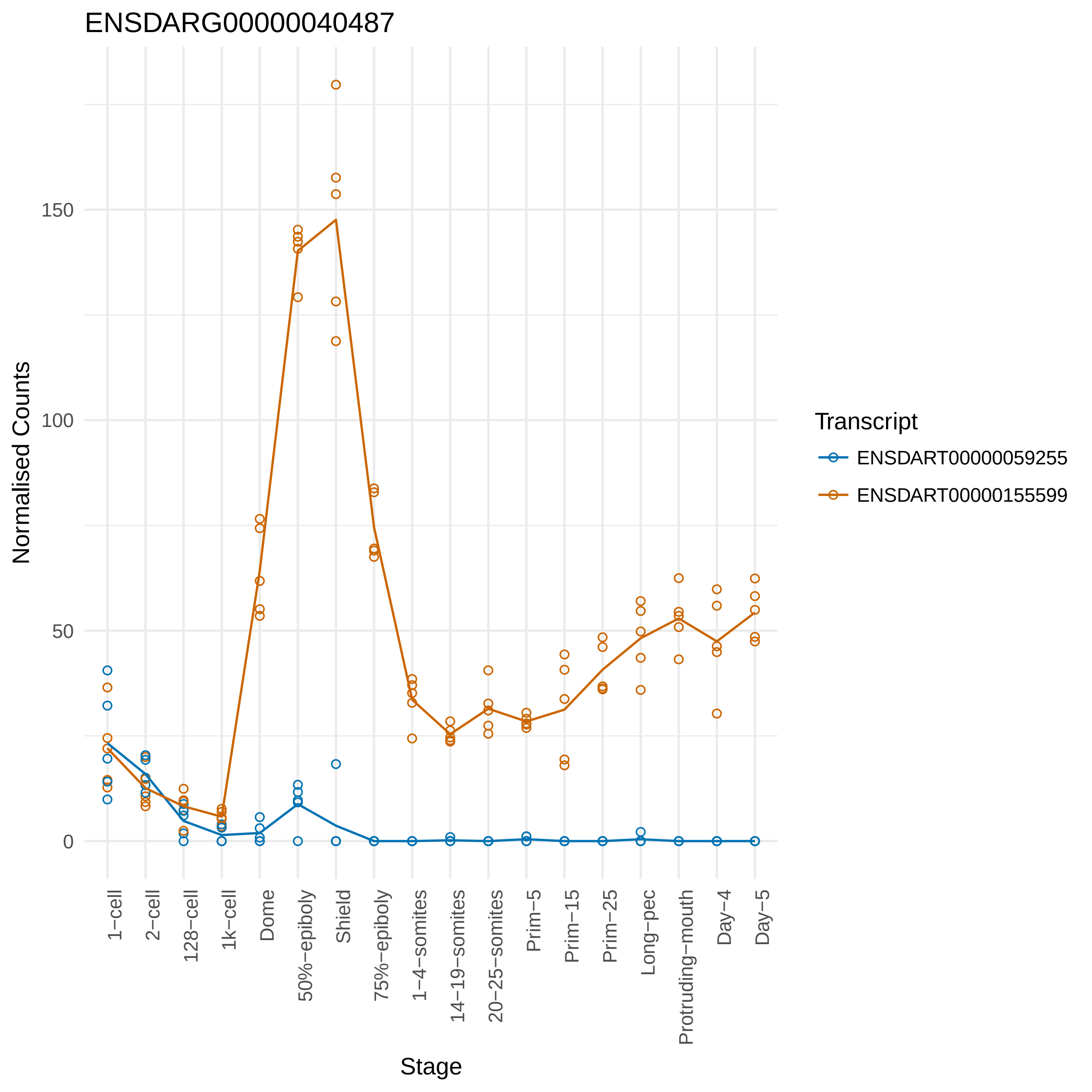

ENSDARG00000040487
Normalised Counts
150
100
50
0
1−cell
2−cell
128−cell
1k−cell
Dome
50%−epiboly
Shield
75%−epiboly
1−4−somites
14−19−somites
20−25−somites
Prim−5
Prim−15
Prim−25
Long−pec
Protruding−mouth
Day−4
Day−5
Stage
Transcript
ENSDART00000059255
ENSDART00000155599

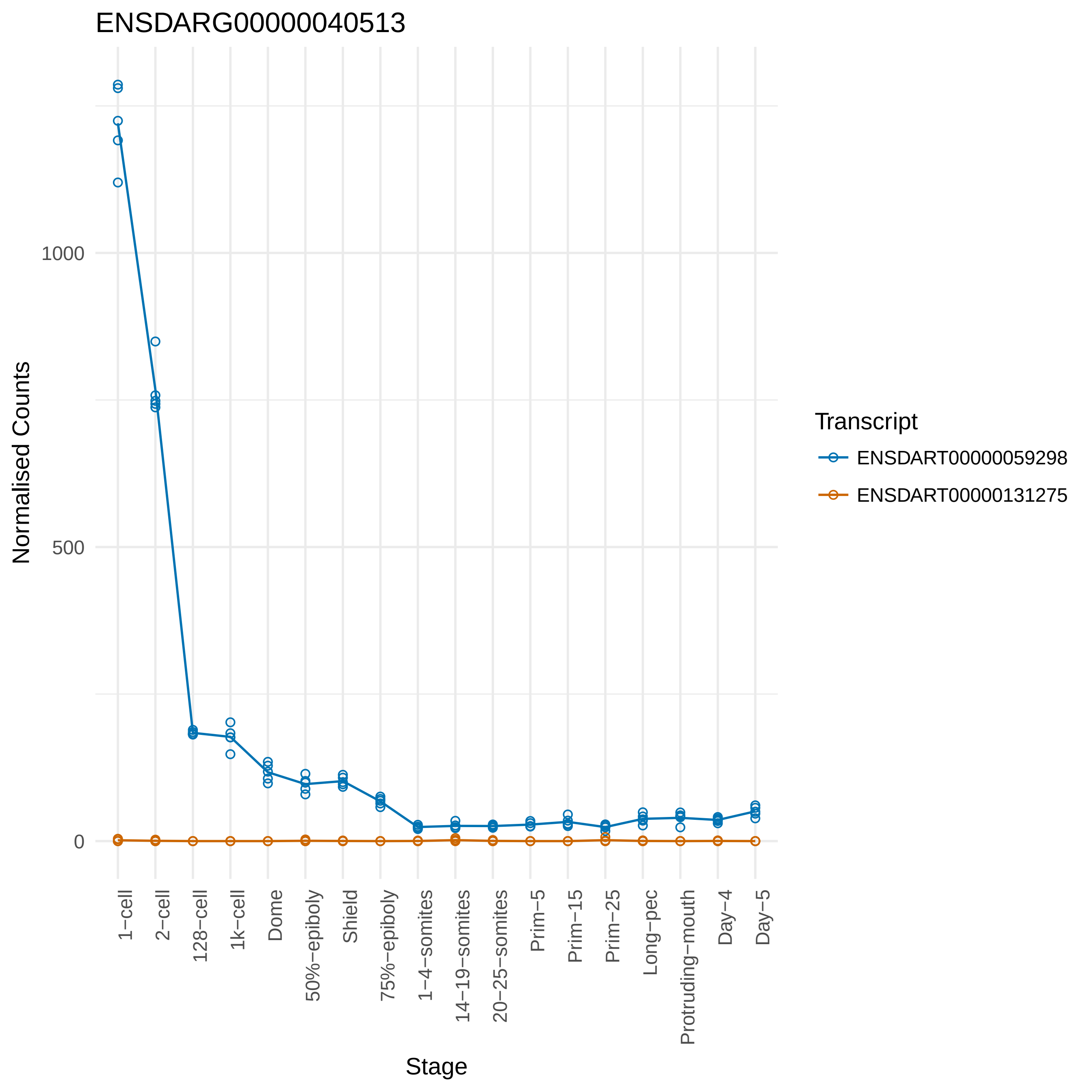

ENSDARG00000040513
Normalised Counts
1000
500
0
Stage
1−cell
2−cell
128−cell
1k−cell
Dome
50%−epiboly
Shield
75%−epiboly
1−4−somites
14−19−somites
20−25−somites
Prim−5
Prim−15
Prim−25
Long−pec
Protruding−mouth
Day−4
Day−5
Transcript
ENSDART00000059298
ENSDART00000131275

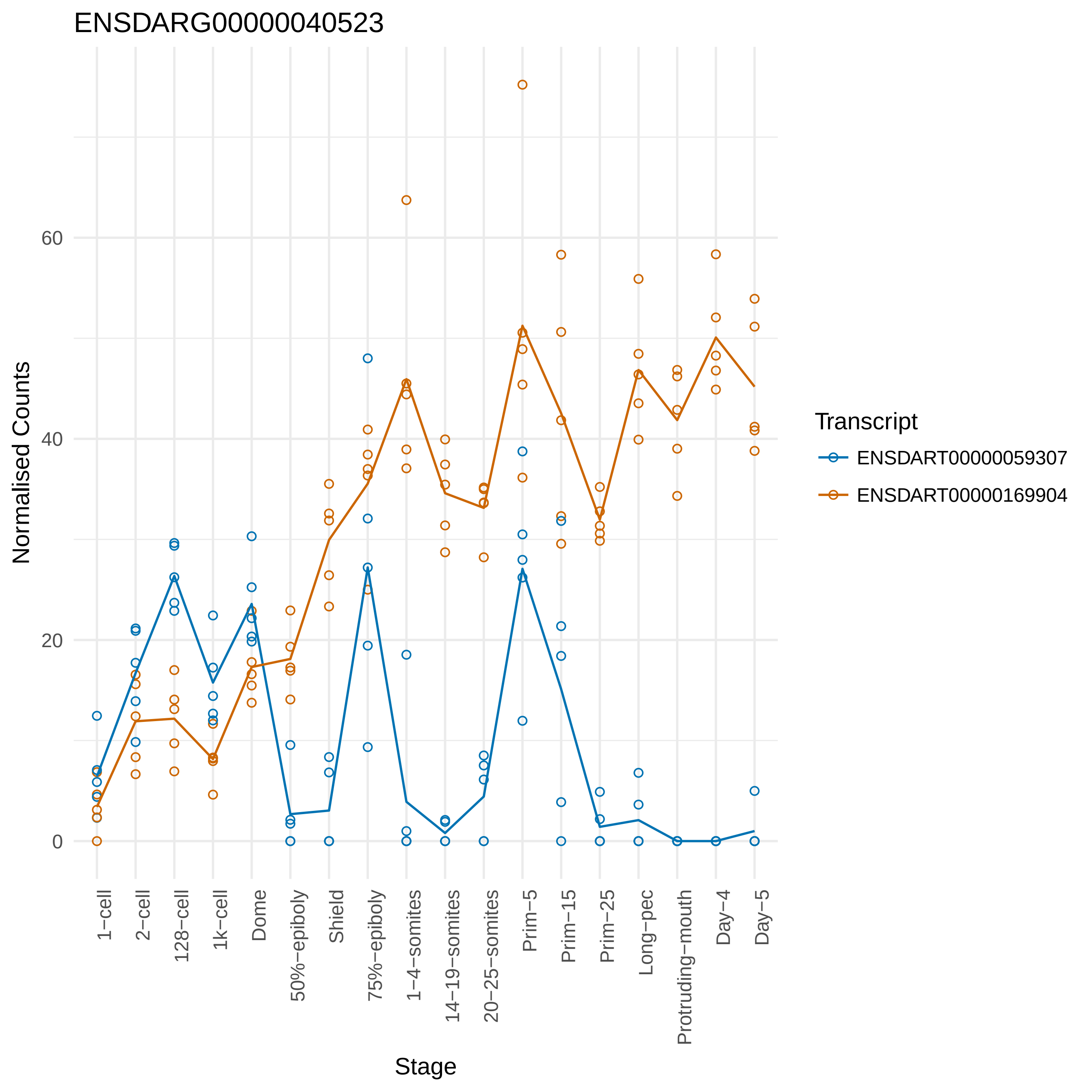

ENSDARG00000040523
Normalised Counts
0
20
40
60
Stage
1−cell
2−cell
128−cell
1k−cell
Dome
50%−epiboly
Shield
75%−epiboly
1−4−somites
14−19−somites
20−25−somites
Prim−5
Prim−15
Prim−25
Long−pec
Protruding−mouth
Day−4
Day−5
Transcript
ENSDART00000059307
ENSDART00000169904

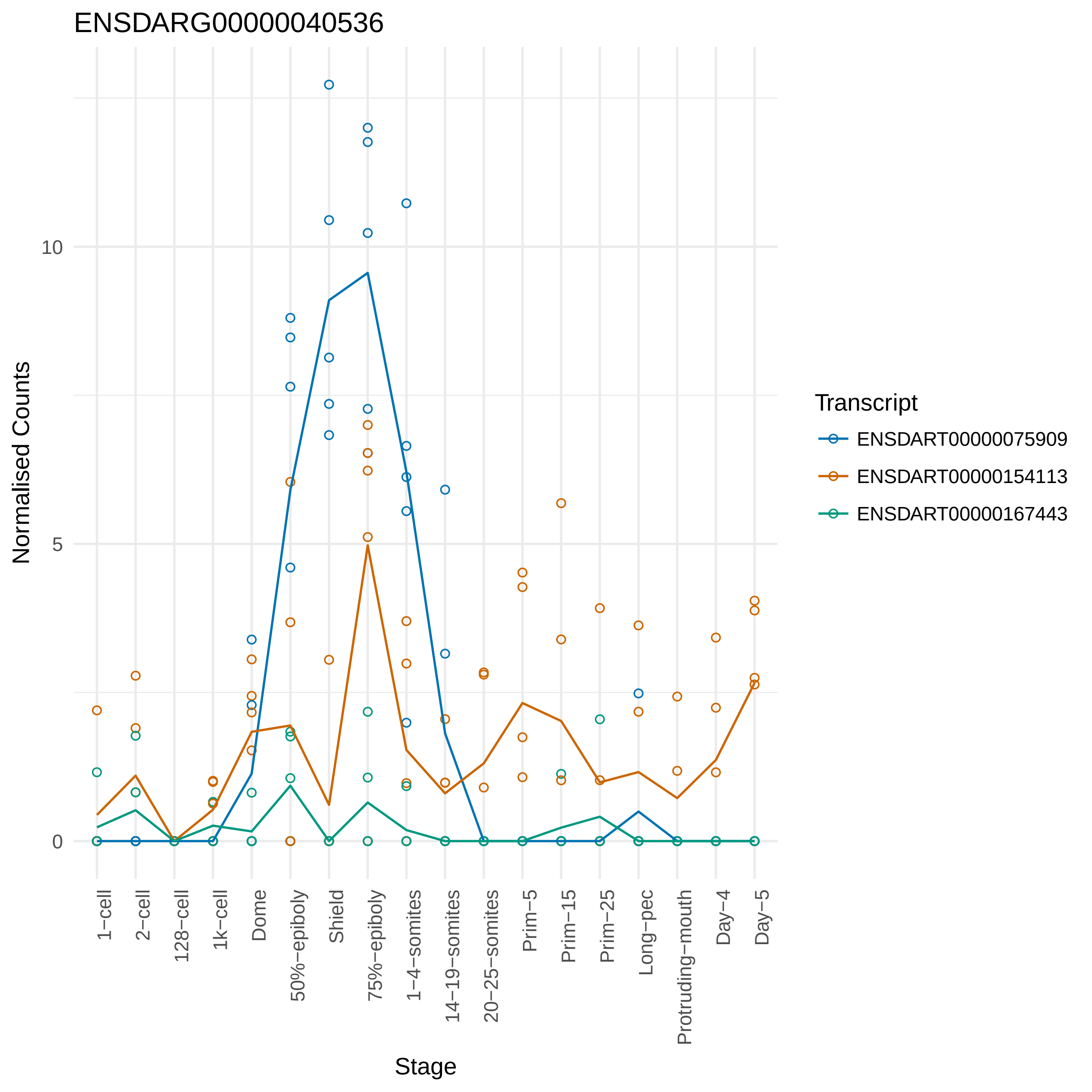

ENSDARG00000040536
Normalised Counts
10
5
0
1−cell
2−cell
128−cell
1k−cell
Dome
50%−epiboly
Shield
75%−epiboly
1−4−somites
14−19−somites
20−25−somites
Prim−5
Prim−15
Prim−25
Long−pec
Protruding−mouth
Day−4
Day−5
Stage
Transcript
ENSDART00000075909
ENSDART00000154113
ENSDART00000167443

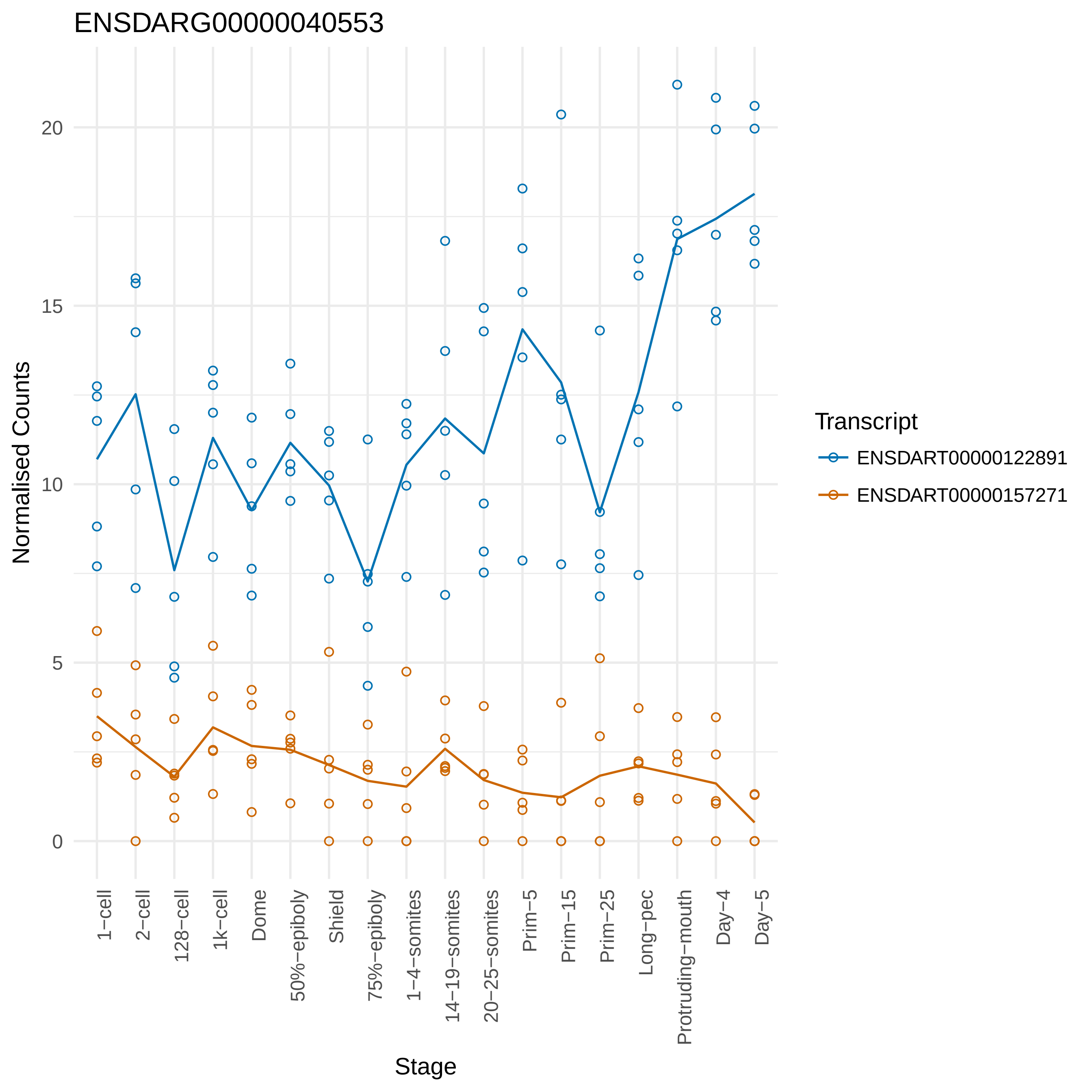

ENSDARG00000040553
Normalised Counts
0
5
10
15
20
1−cell
2−cell
128−cell
1k−cell
Dome
50%−epiboly
Shield
75%−epiboly
1−4−somites
14−19−somites
20−25−somites
Prim−5
Prim−15
Prim−25
Long−pec
Protruding−mouth
Day−4
Day−5
Stage
Transcript
ENSDART00000122891
ENSDART00000157271

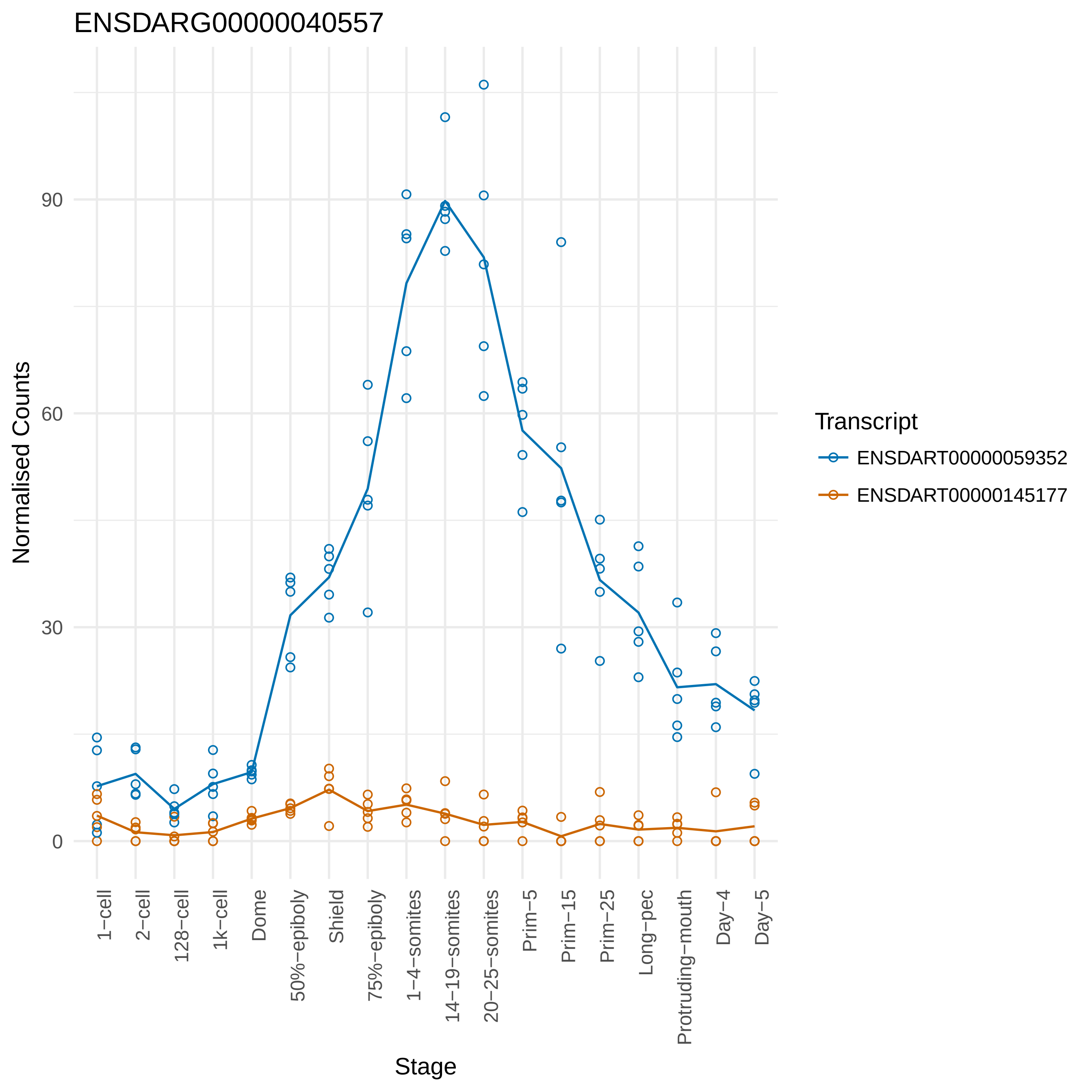

ENSDARG00000040557
Normalised Counts
90
60
30
0
1−cell
2−cell
128−cell
1k−cell
Dome
50%−epiboly
Shield
75%−epiboly
1−4−somites
14−19−somites
20−25−somites
Prim−5
Prim−15
Prim−25
Long−pec
Protruding−mouth
Day−4
Day−5
Stage
Transcript
ENSDART00000059352
ENSDART00000145177

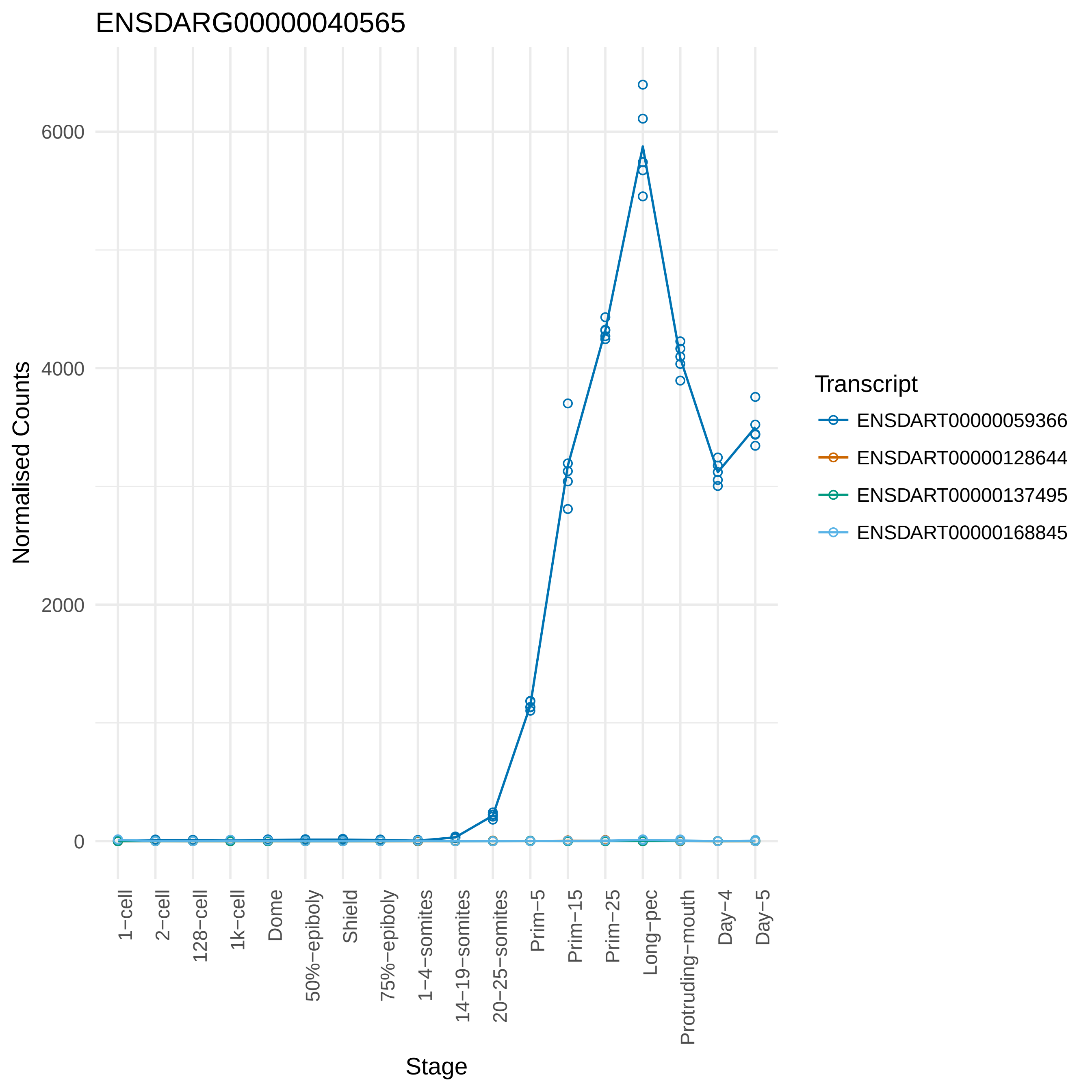

ENSDARG00000040565
Normalised Counts
6000
4000
2000
0
1−cell
2−cell
128−cell
1k−cell
Dome
50%−epiboly
Shield
75%−epiboly
1−4−somites
14−19−somites
20−25−somites
Prim−5
Prim−15
Prim−25
Long−pec
Protruding−mouth
Day−4
Day−5
Stage
Transcript
ENSDART00000059366
ENSDART00000128644
ENSDART00000137495
ENSDART00000168845

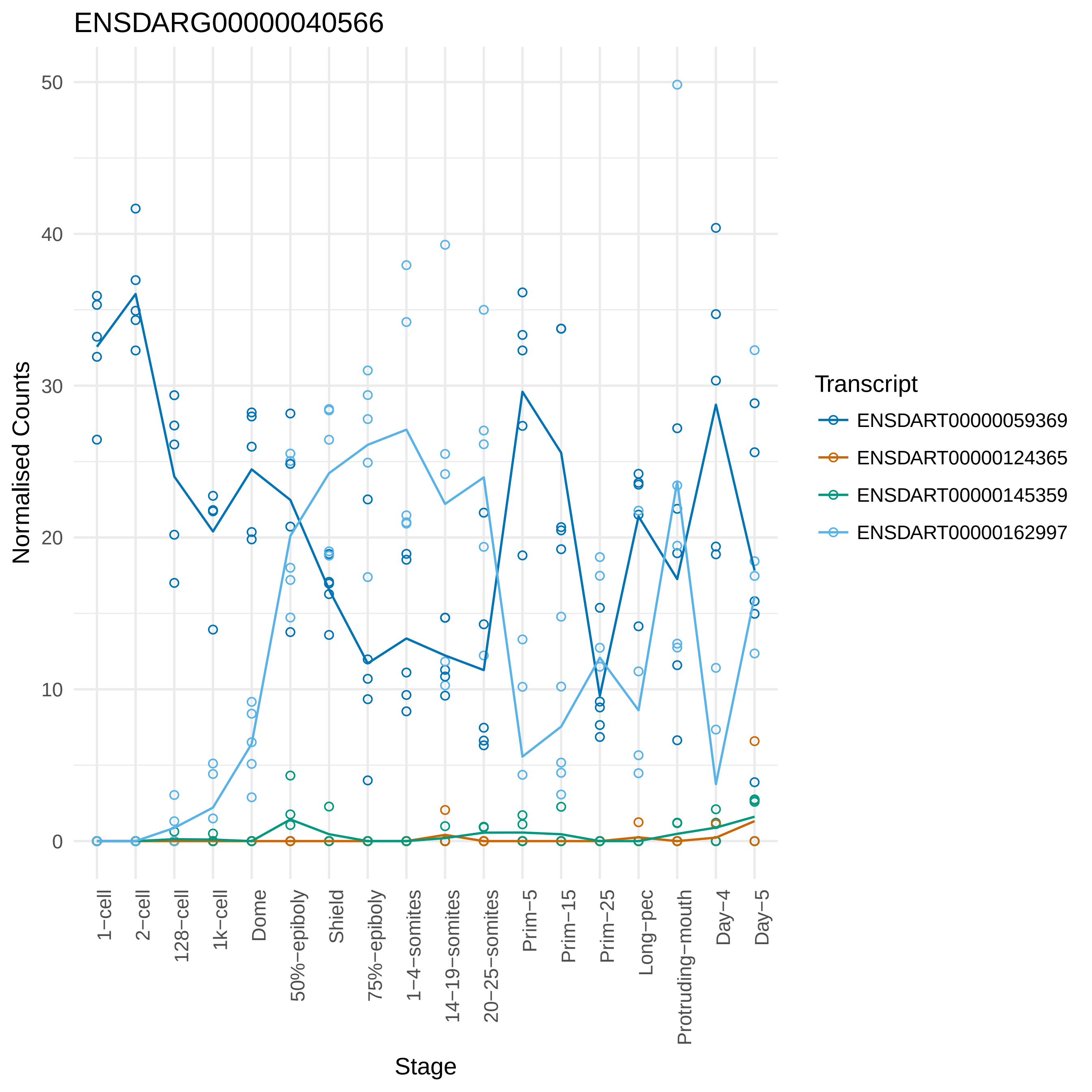
ENSDARG00000040566
Normalised Counts
0
10
20
30
40
50
Stage
1−cell
2−cell
128−cell
1k−cell
Dome
50%−epiboly
Shield
75%−epiboly
1−4−somites
14−19−somites
20−25−somites
Prim−5
Prim−15
Prim−25
Long−pec
Protruding−mouth
Day−4
Day−5
Transcript
ENSDART00000059369
ENSDART00000124365
ENSDART00000145359
ENSDART00000162997

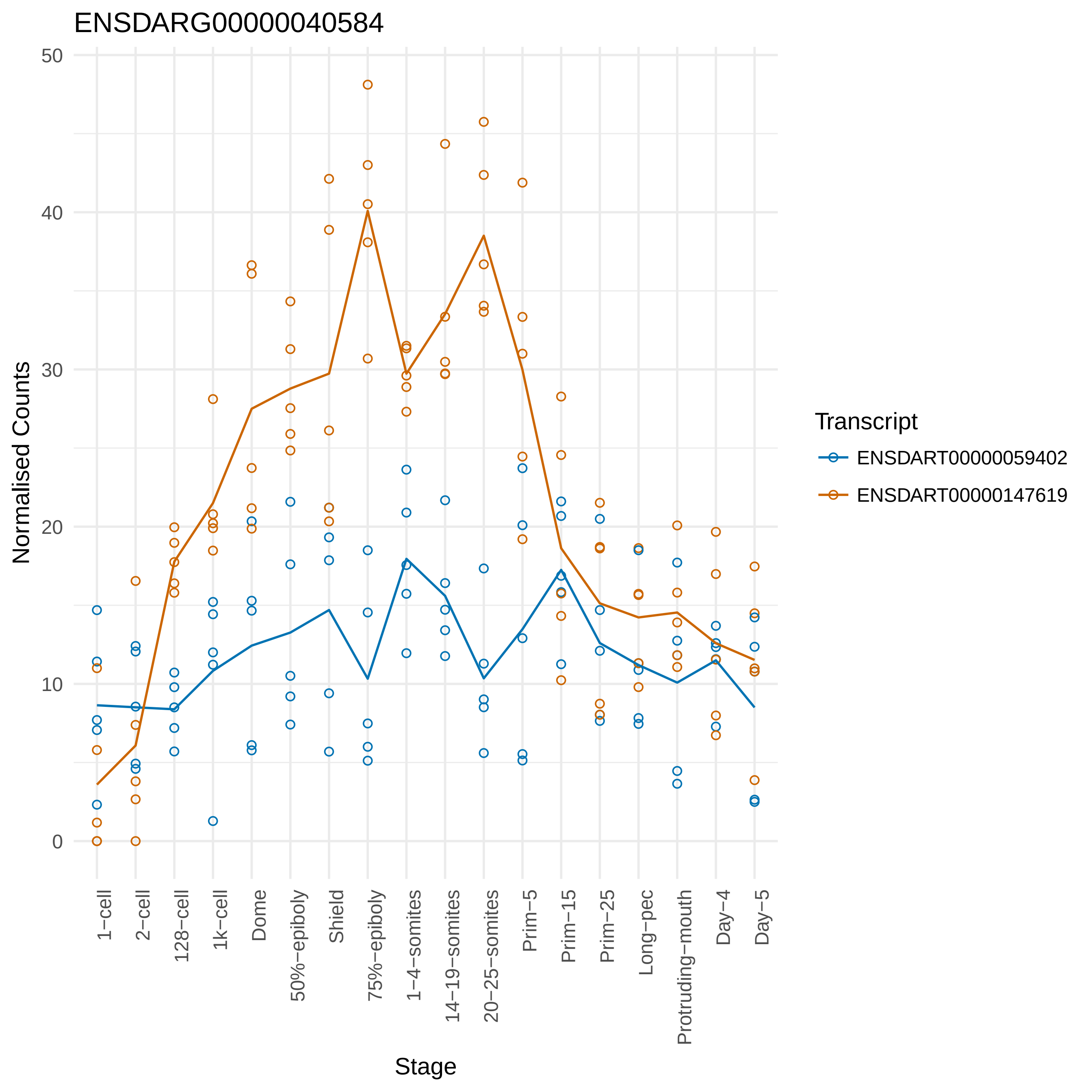

ENSDARG00000040584
Normalised Counts
0
10
20
30
40
50
Stage
1−cell
2−cell
128−cell
1k−cell
Dome
50%−epiboly
Shield
75%−epiboly
1−4−somites
14−19−somites
20−25−somites
Prim−5
Prim−15
Prim−25
Long−pec
Protruding−mouth
Day−4
Day−5
Transcript
ENSDART00000059402
ENSDART00000147619

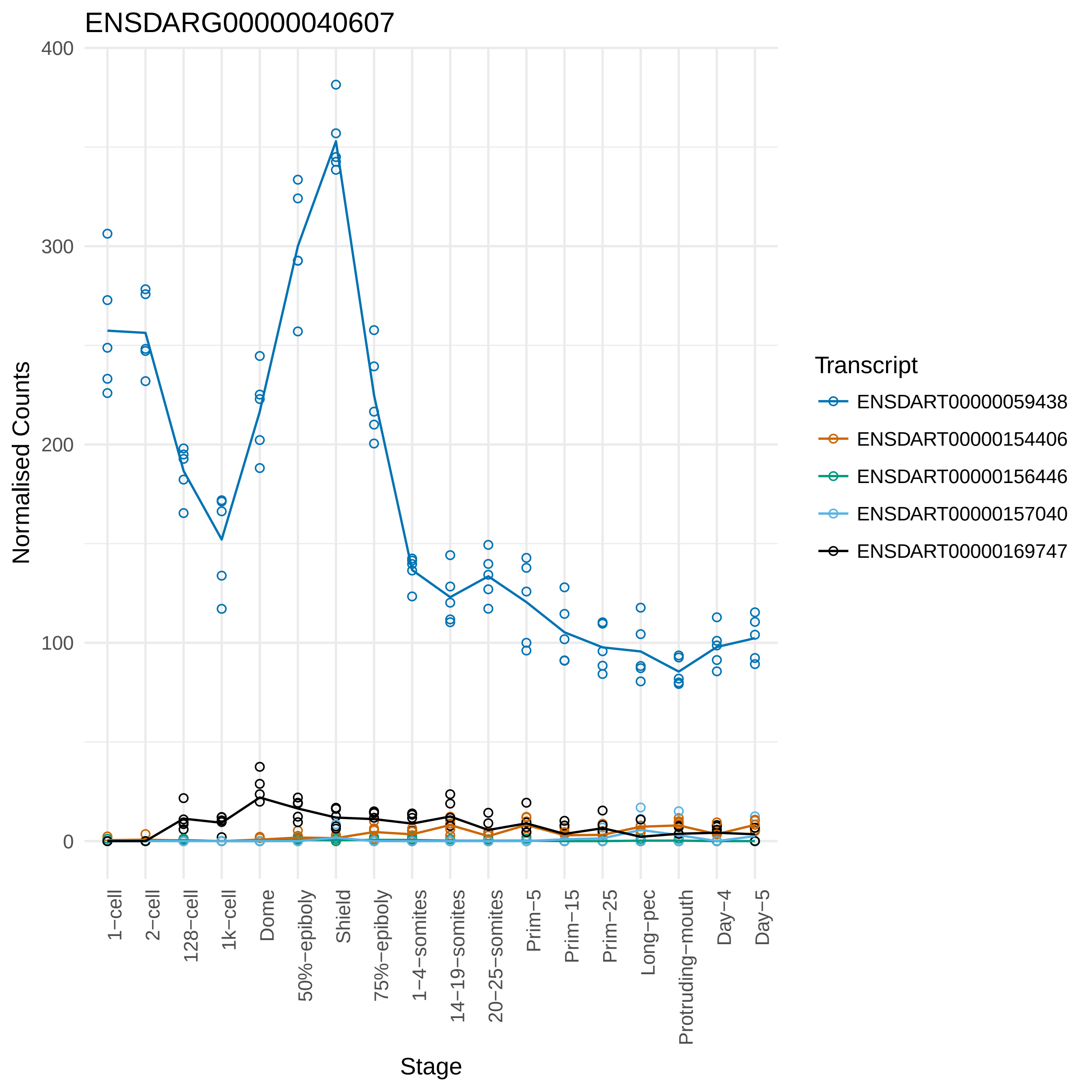

ENSDARG00000040607
Normalised Counts
400
300
200
100
0
1−cell
2−cell
128−cell
1k−cell
Dome
50%−epiboly
Shield
75%−epiboly
1−4−somites
14−19−somites
20−25−somites
Prim−5
Prim−15
Prim−25
Long−pec
Protruding−mouth
Day−4
Day−5
Stage
Transcript
ENSDART00000059438
ENSDART00000154406
ENSDART00000156446
ENSDART00000157040
ENSDART00000169747

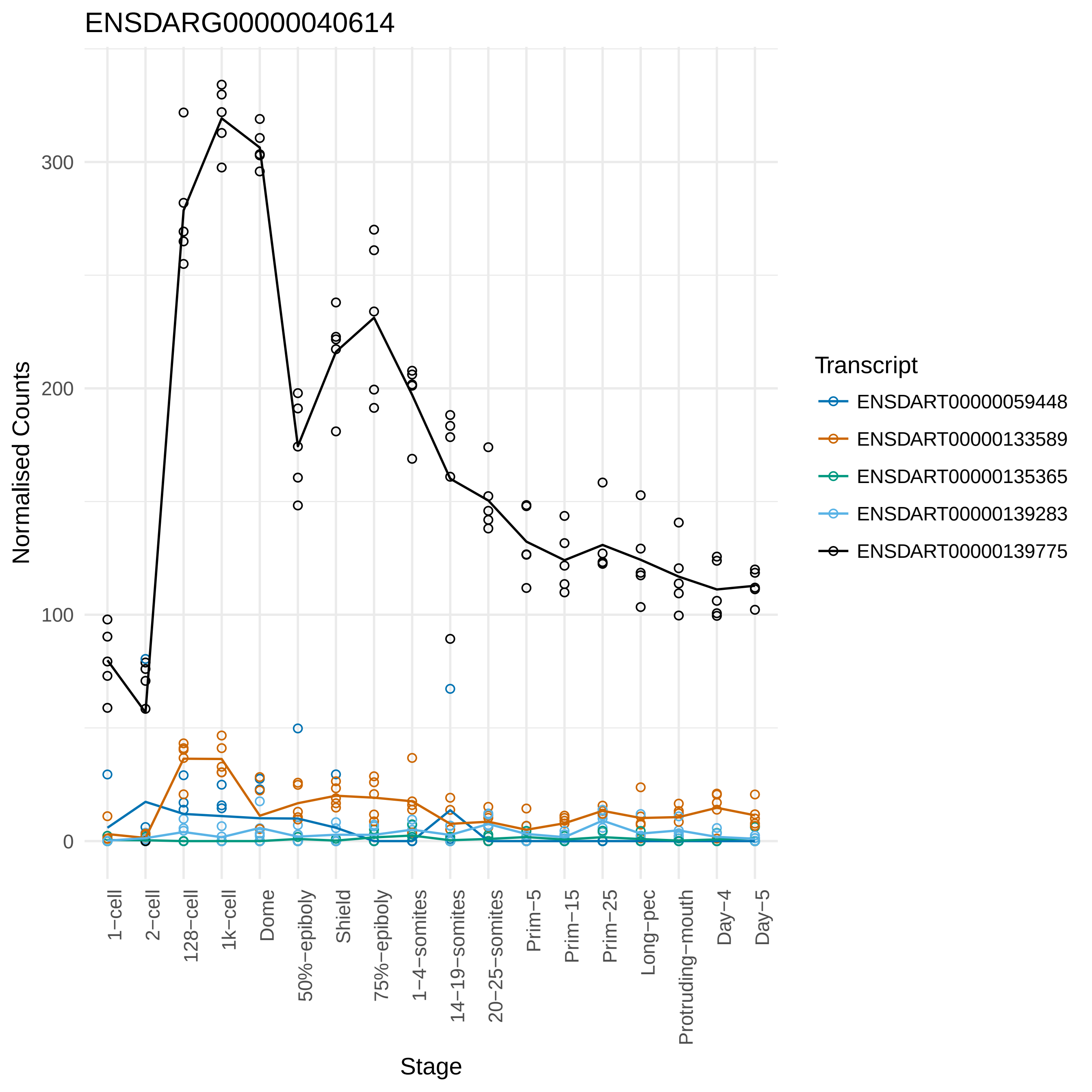
ENSDARG00000040614
Normalised Counts
300
200
100
0
1−cell
2−cell
128−cell
1k−cell
Dome
50%−epiboly
Shield
75%−epiboly
1−4−somites
14−19−somites
20−25−somites
Prim−5
Prim−15
Prim−25
Long−pec
Protruding−mouth
Day−4
Day−5
Stage
Transcript
ENSDART00000059448
ENSDART00000133589
ENSDART00000135365
ENSDART00000139283
ENSDART00000139775

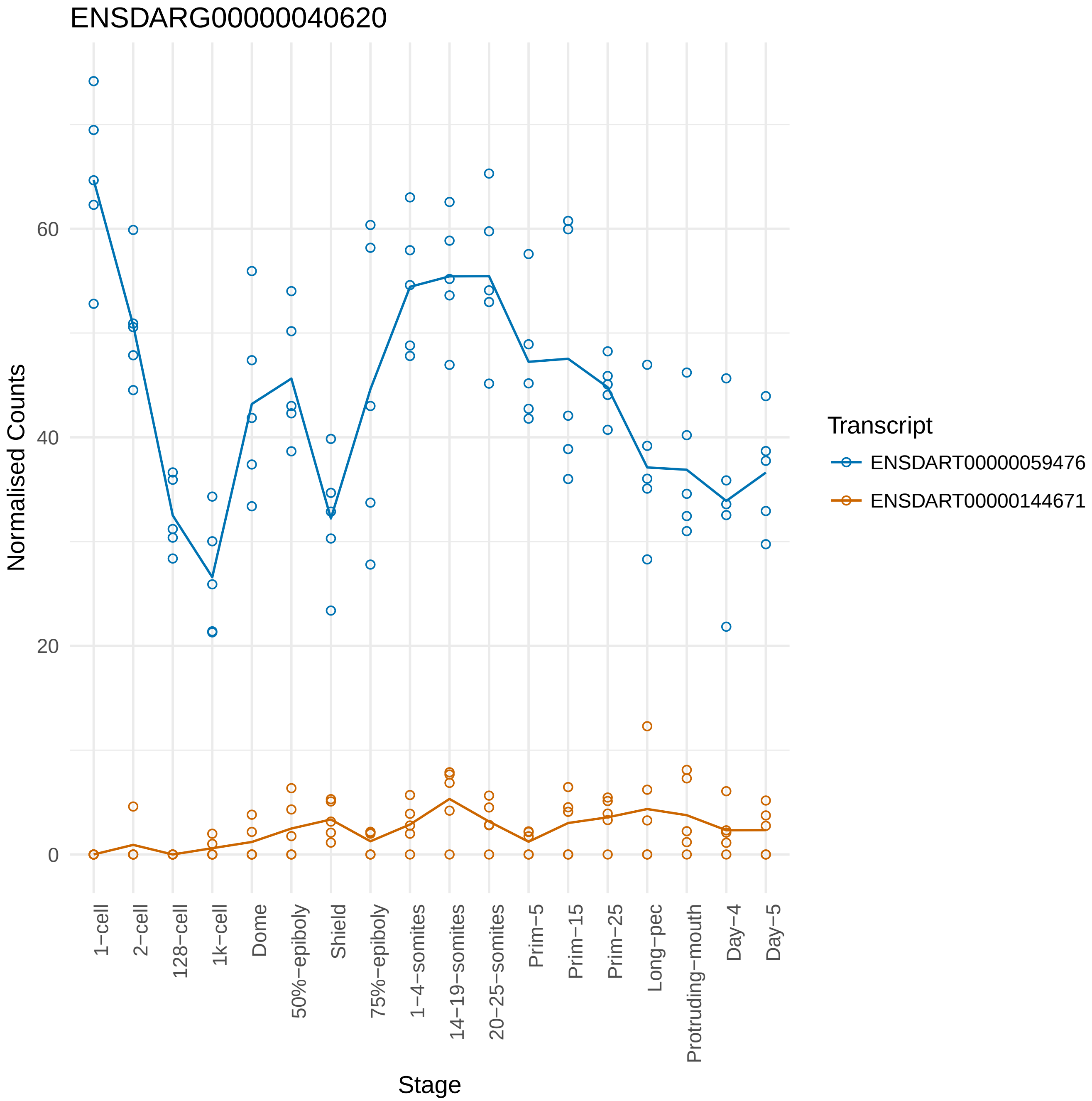
ENSDARG00000040620
Normalised Counts
60
40
20
0
Stage
1−cell
2−cell
128−cell
1k−cell
Dome
50%−epiboly
Shield
75%−epiboly
1−4−somites
14−19−somites
20−25−somites
Prim−5
Prim−15
Prim−25
Long−pec
Protruding−mouth
Day−4
Day−5
Transcript
ENSDART00000059476
ENSDART00000144671

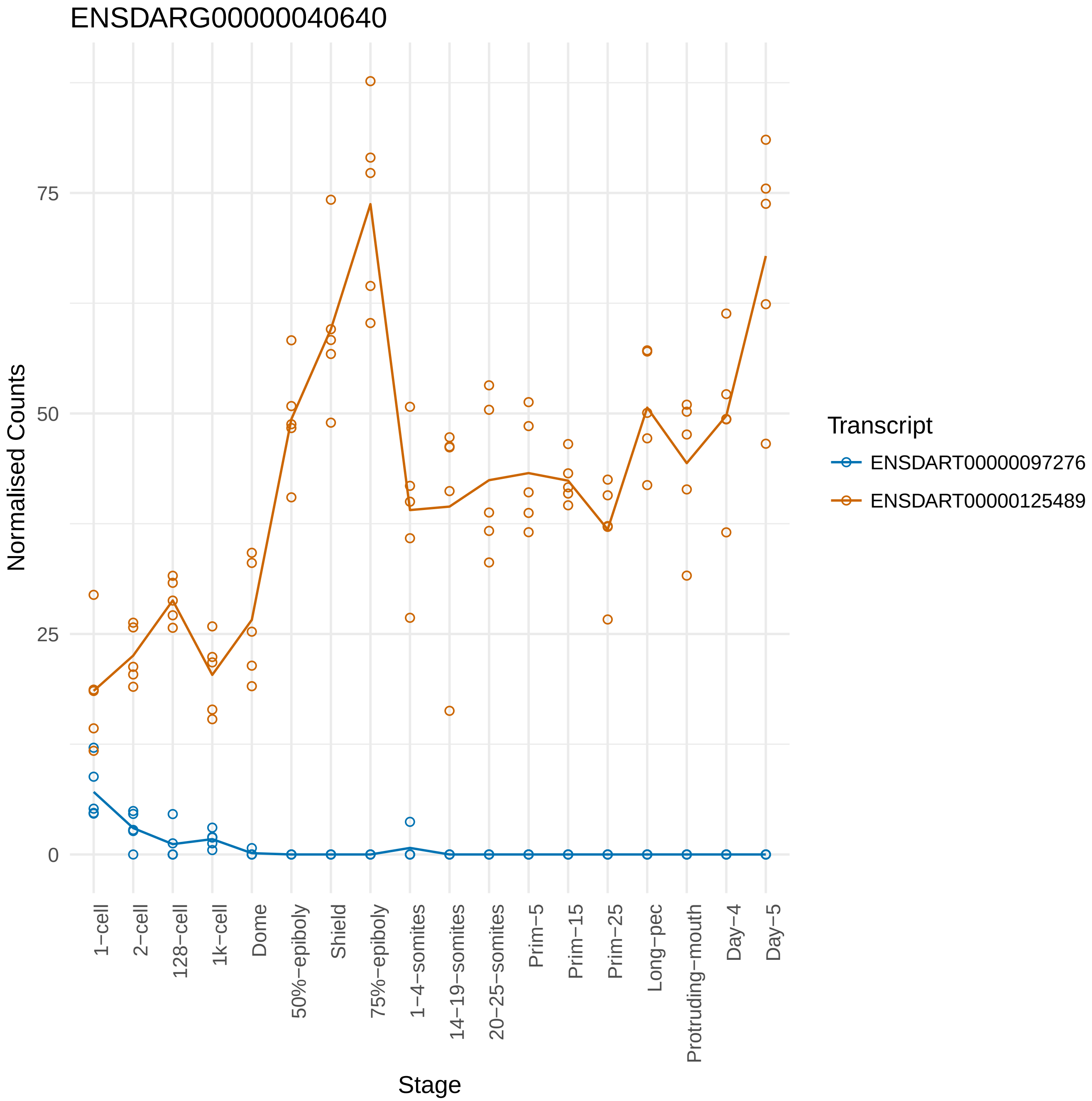
ENSDARG00000040640
Normalised Counts
0
25
50
75
1−cell
2−cell
128−cell
1k−cell
Dome
50%−epiboly
Shield
75%−epiboly
1−4−somites
14−19−somites
20−25−somites
Prim−5
Prim−15
Prim−25
Long−pec
Protruding−mouth
Day−4
Day−5
Stage
Transcript
ENSDART00000097276
ENSDART00000125489

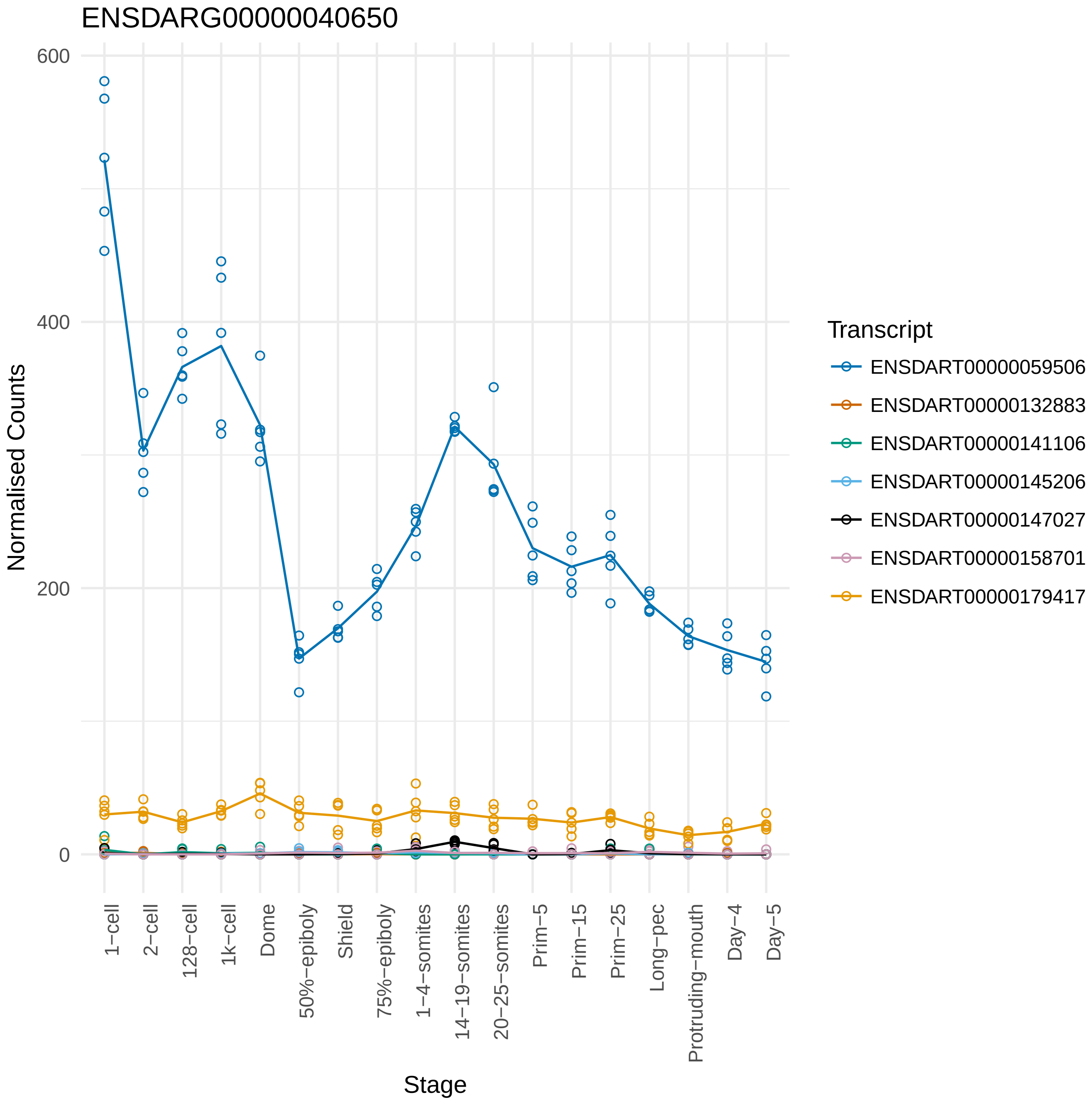
ENSDARG00000040650
Normalised Counts
600
400
200
0
1−cell
2−cell
128−cell
1k−cell
Dome
50%−epiboly
Shield
75%−epiboly
1−4−somites
14−19−somites
20−25−somites
Prim−5
Prim−15
Prim−25
Long−pec
Protruding−mouth
Day−4
Day−5
Stage
Transcript
ENSDART00000059506
ENSDART00000132883
ENSDART00000141106
ENSDART00000145206
ENSDART00000147027
ENSDART00000158701
ENSDART00000179417

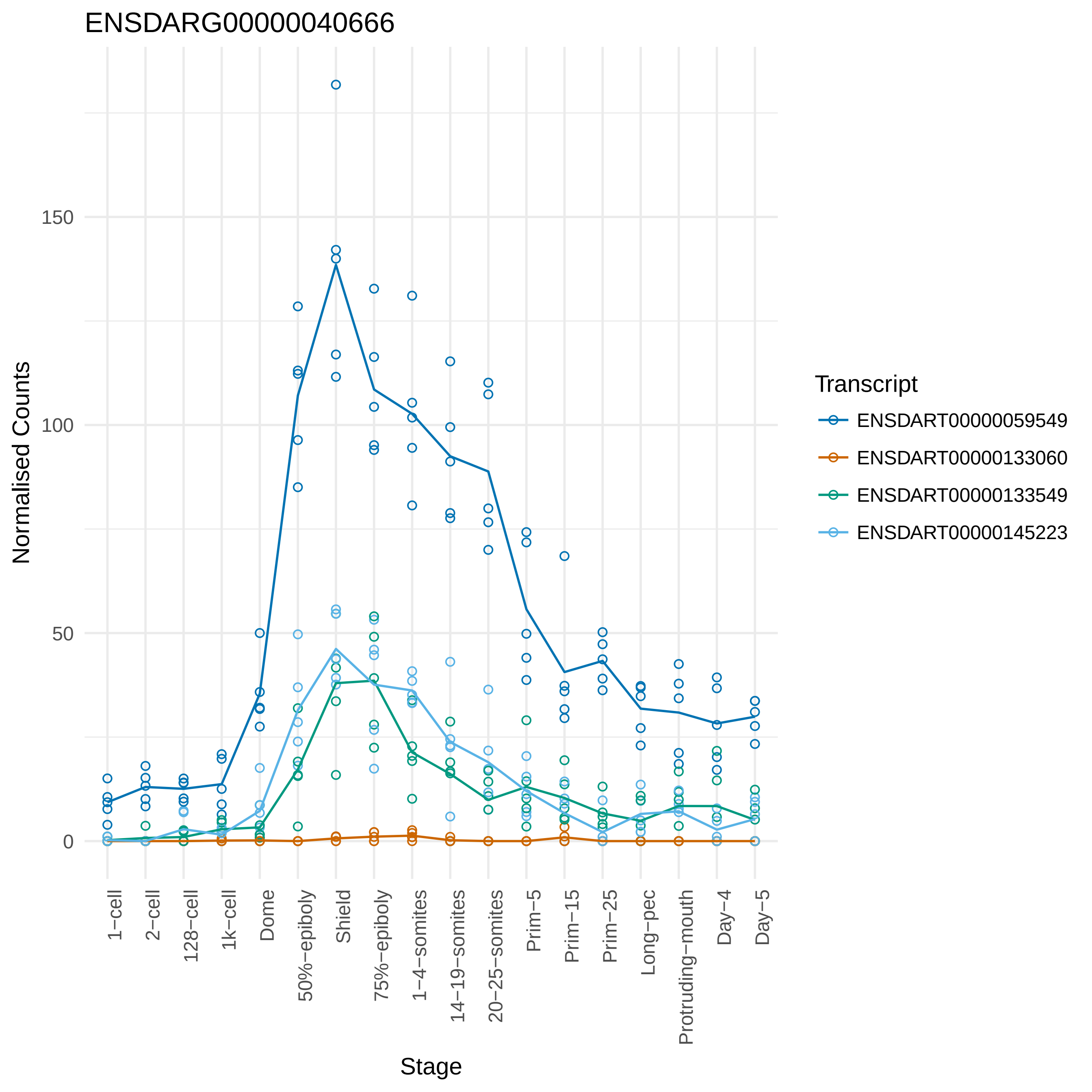
ENSDARG00000040666
Normalised Counts
0
50
100
150
Stage
1−cell
2−cell
128−cell
1k−cell
Dome
50%−epiboly
Shield
75%−epiboly
1−4−somites
14−19−somites
20−25−somites
Prim−5
Prim−15
Prim−25
Long−pec
Protruding−mouth
Day−4
Day−5
Transcript
ENSDART00000059549
ENSDART00000133060
ENSDART00000133549
ENSDART00000145223

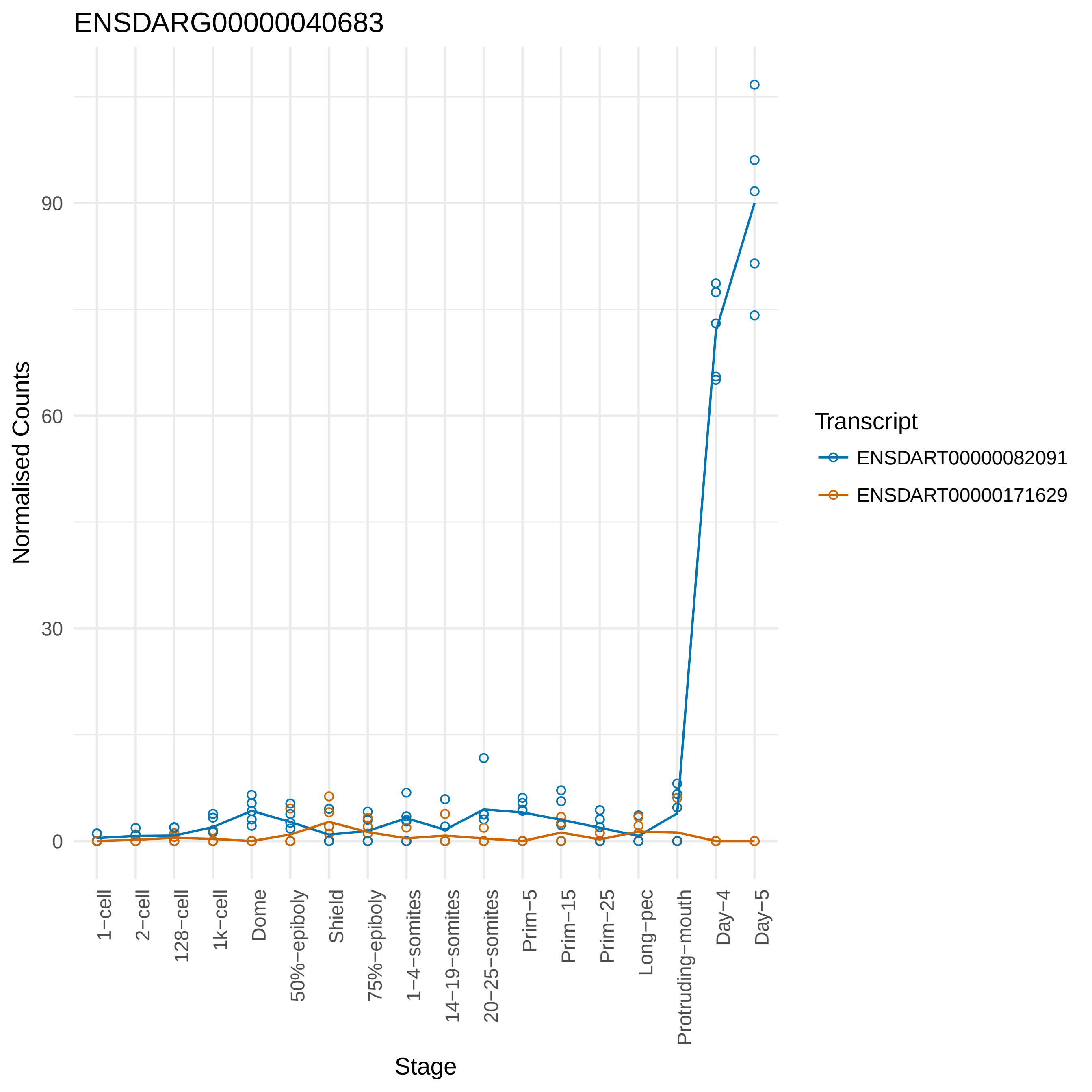

ENSDARG00000040683
Normalised Counts
0
30
60
90
Stage
1−cell
2−cell
128−cell
1k−cell
Dome
50%−epiboly
Shield
75%−epiboly
1−4−somites
14−19−somites
20−25−somites
Prim−5
Prim−15
Prim−25
Long−pec
Protruding−mouth
Day−4
Day−5
Transcript
ENSDART00000082091
ENSDART00000171629

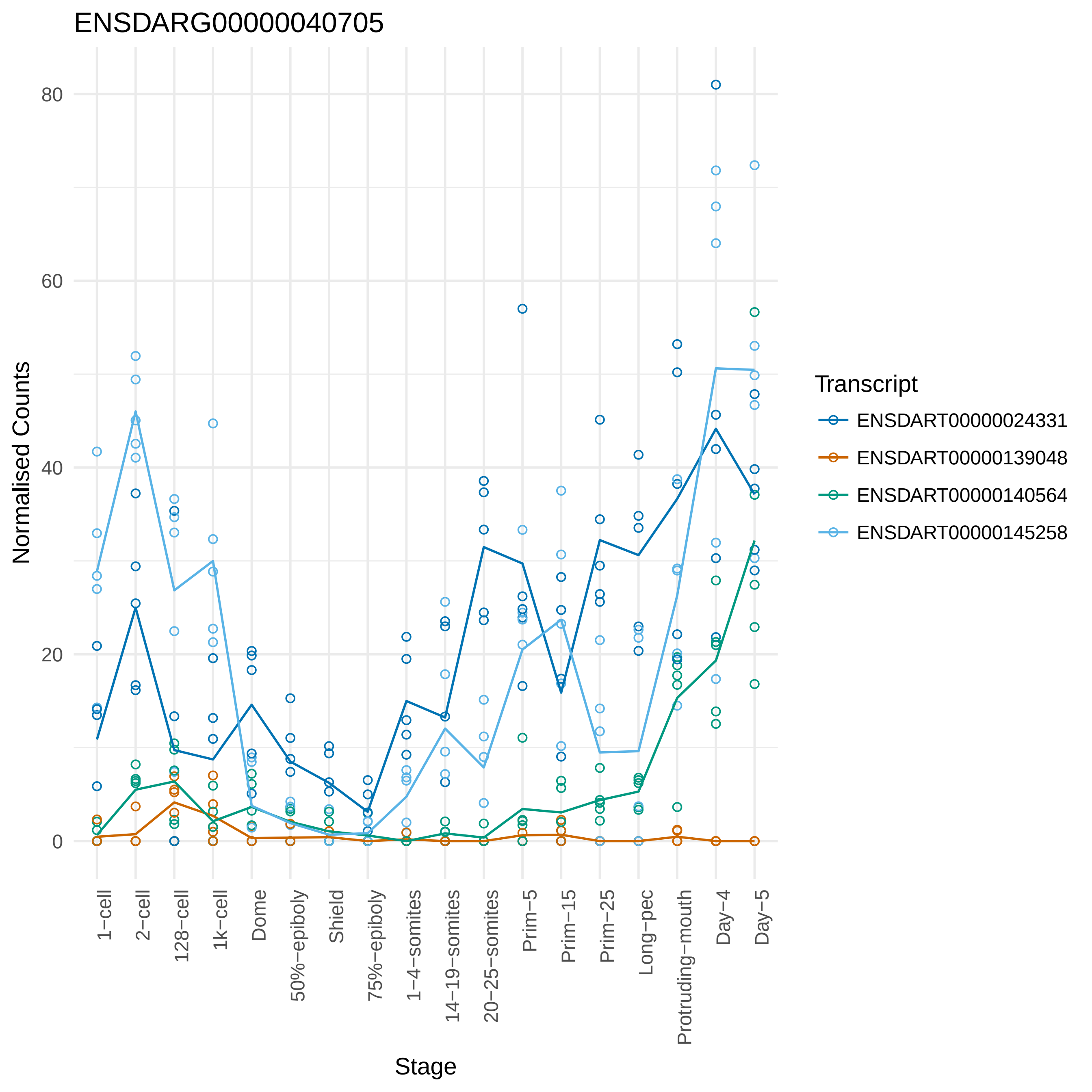

ENSDARG00000040705
Normalised Counts
0
20
40
60
80
1−cell
2−cell
128−cell
1k−cell
Dome
50%−epiboly
Shield
75%−epiboly
1−4−somites
14−19−somites
20−25−somites
Prim−5
Prim−15
Prim−25
Long−pec
Protruding−mouth
Day−4
Day−5
Stage
Transcript
ENSDART00000024331
ENSDART00000139048
ENSDART00000140564
ENSDART00000145258

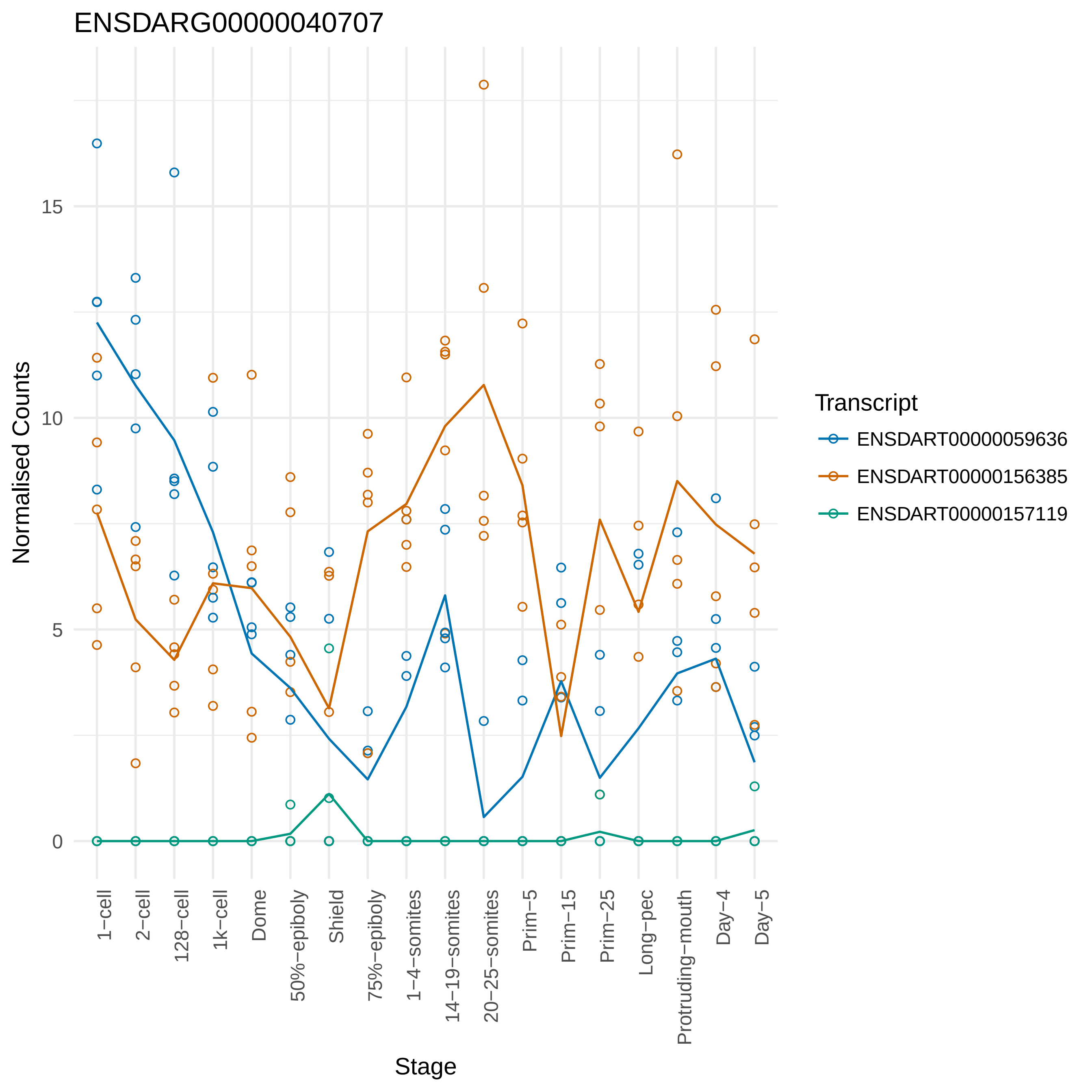

ENSDARG00000040707
Normalised Counts
15
10
5
0
1−cell
2−cell
128−cell
1k−cell
Dome
50%−epiboly
Shield
75%−epiboly
1−4−somites
14−19−somites
20−25−somites
Prim−5
Prim−15
Prim−25
Long−pec
Protruding−mouth
Day−4
Day−5
Stage
Transcript
ENSDART00000059636
ENSDART00000156385
ENSDART00000157119

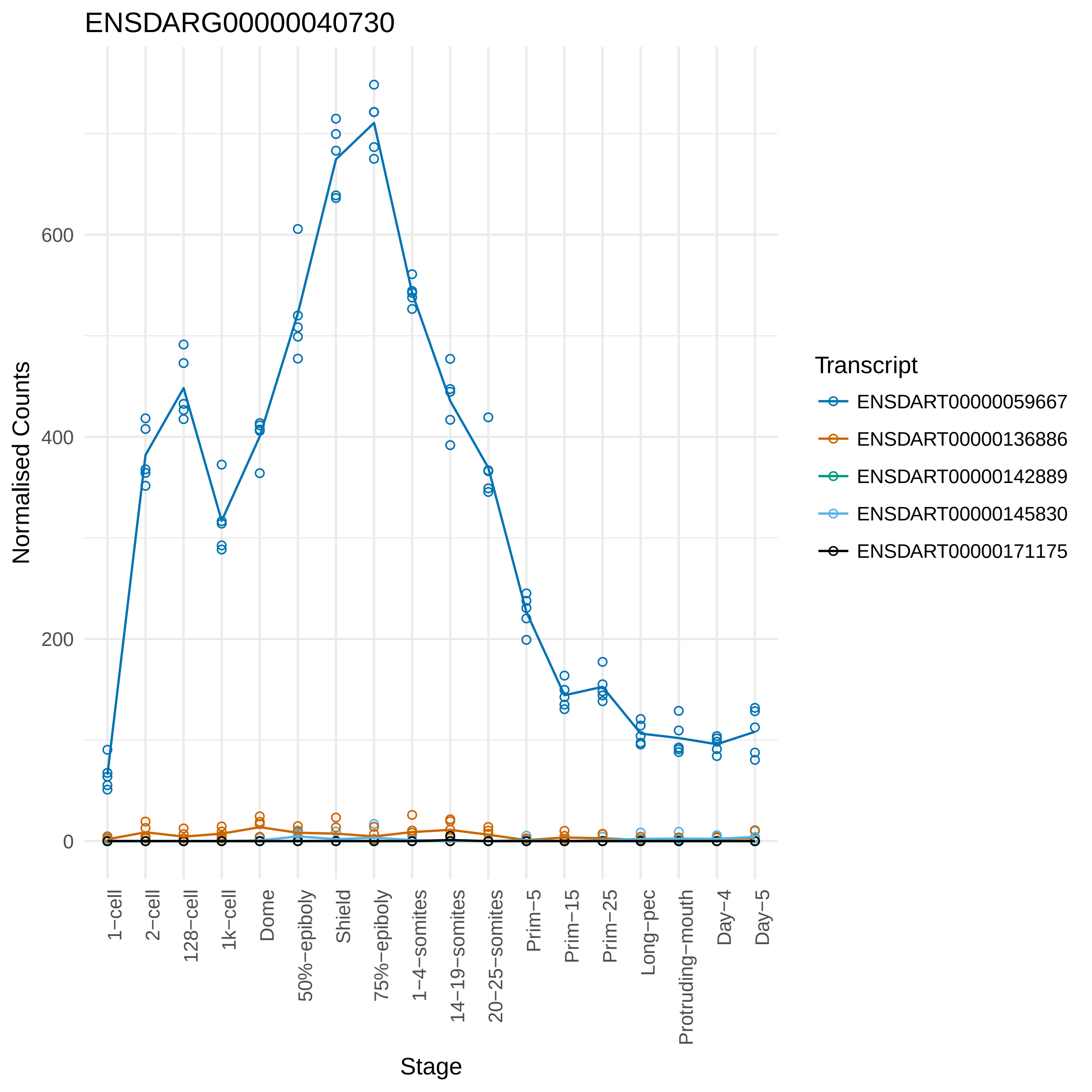

ENSDARG00000040730
Normalised Counts
600
400
200
0
Stage
1−cell
2−cell
128−cell
1k−cell
Dome
50%−epiboly
Shield
75%−epiboly
1−4−somites
14−19−somites
20−25−somites
Prim−5
Prim−15
Prim−25
Long−pec
Protruding−mouth
Day−4
Day−5
Transcript
ENSDART00000059667
ENSDART00000136886
ENSDART00000142889
ENSDART00000145830
ENSDART00000171175

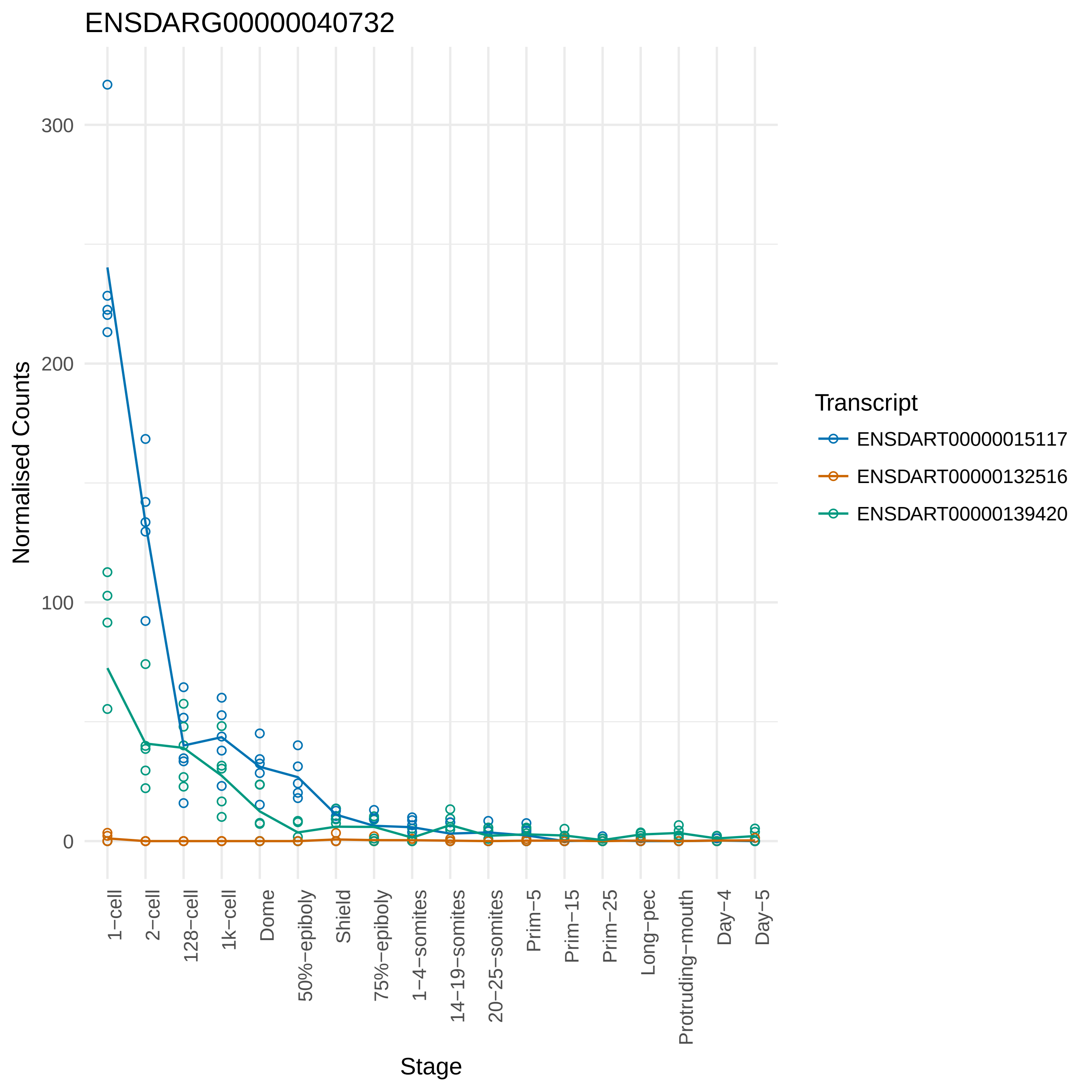

ENSDARG00000040732
Normalised Counts
300
200
100
0
1−cell
2−cell
128−cell
1k−cell
Dome
50%−epiboly
Shield
75%−epiboly
1−4−somites
14−19−somites
20−25−somites
Prim−5
Prim−15
Prim−25
Long−pec
Protruding−mouth
Day−4
Day−5
Stage
Transcript
ENSDART00000015117
ENSDART00000132516
ENSDART00000139420

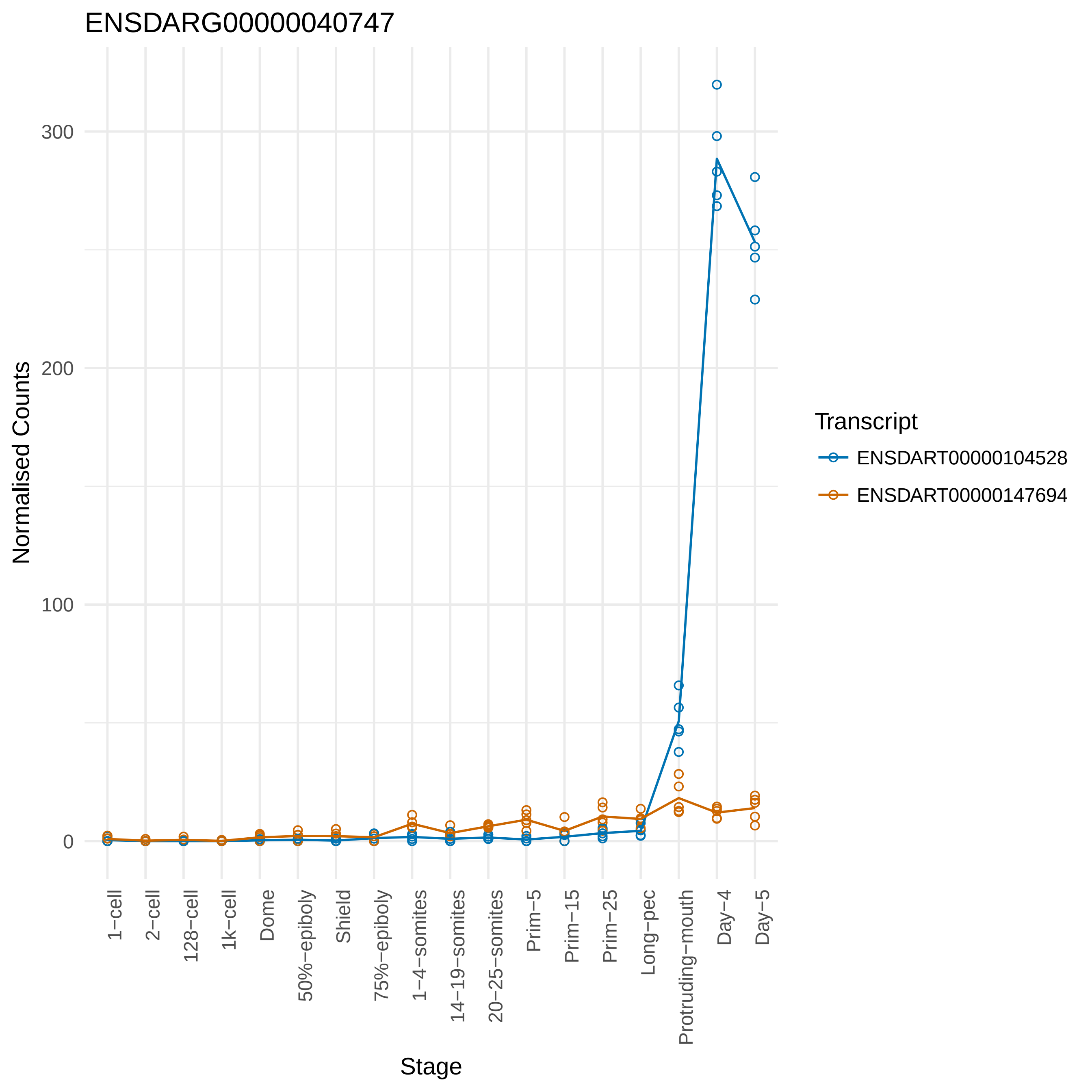
ENSDARG00000040747
Normalised Counts
300
200
100
0
1−cell
2−cell
128−cell
1k−cell
Dome
50%−epiboly
Shield
75%−epiboly
1−4−somites
14−19−somites
20−25−somites
Prim−5
Prim−15
Prim−25
Long−pec
Protruding−mouth
Day−4
Day−5
Stage
Transcript
ENSDART00000104528
ENSDART00000147694

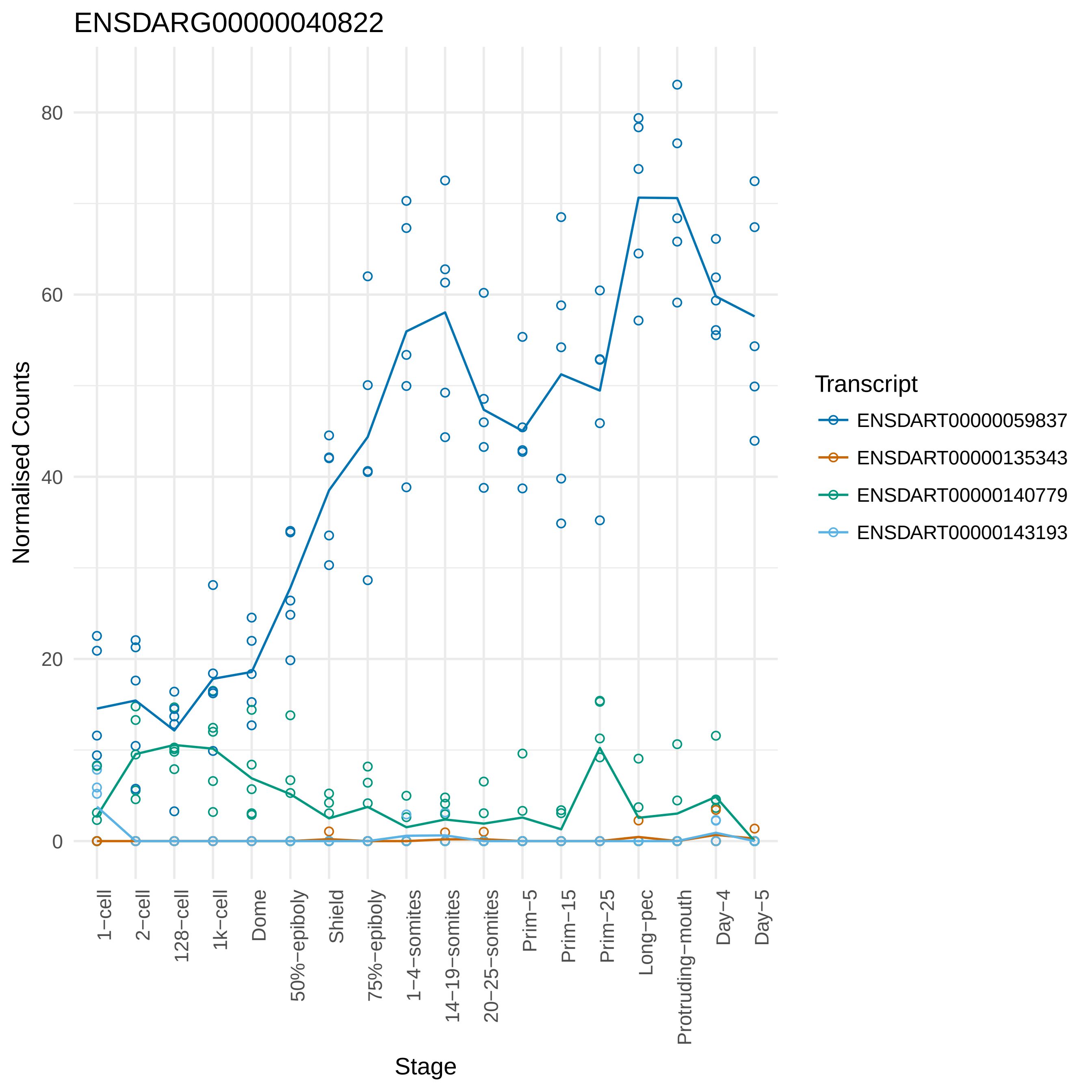

ENSDARG00000040822
Normalised Counts
0
20
40
60
80
Stage
1−cell
2−cell
128−cell
1k−cell
Dome
50%−epiboly
Shield
75%−epiboly
1−4−somites
14−19−somites
20−25−somites
Prim−5
Prim−15
Prim−25
Long−pec
Protruding−mouth
Day−4
Day−5
Transcript
ENSDART00000059837
ENSDART00000135343
ENSDART00000140779
ENSDART00000143193

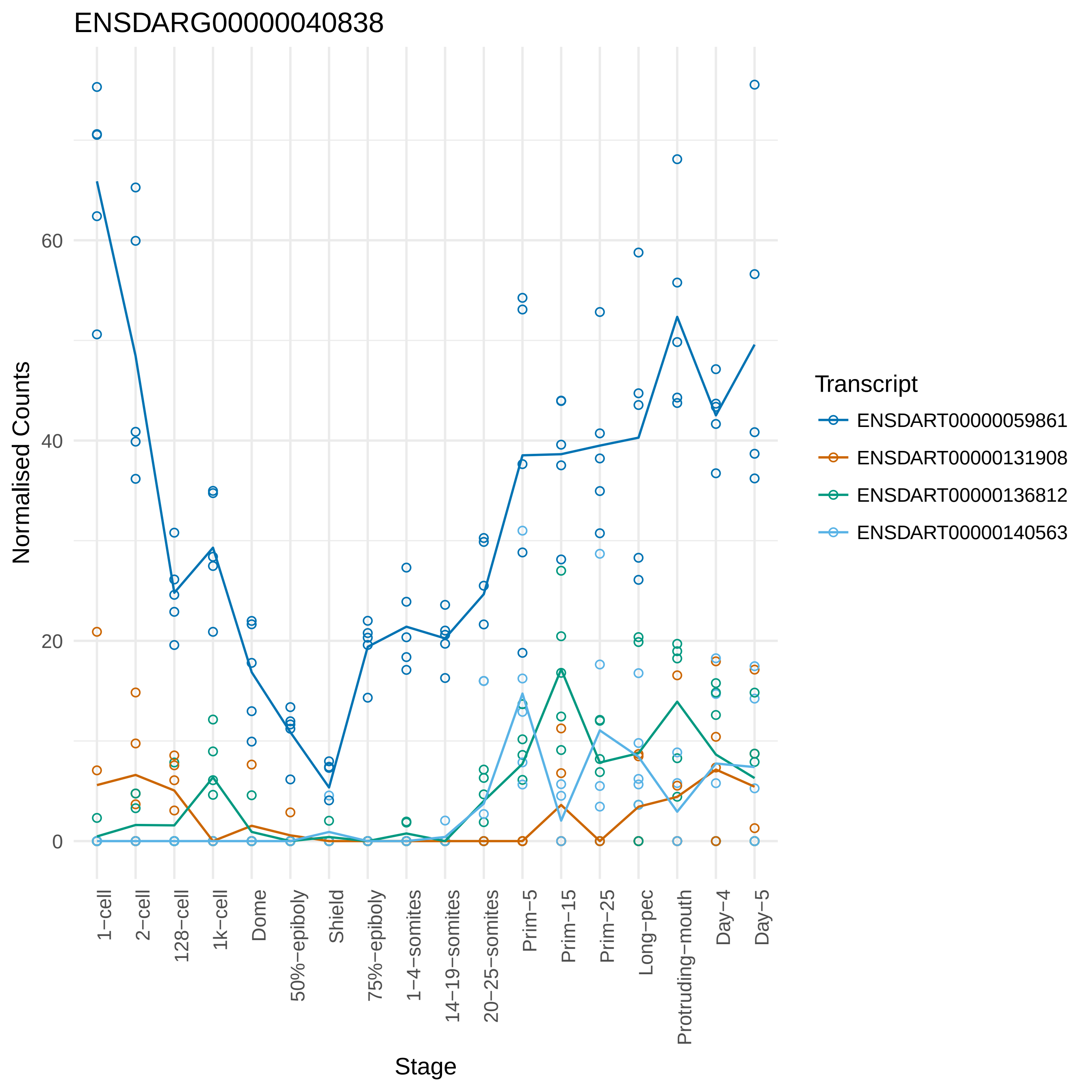
ENSDARG00000040838
Normalised Counts
60
40
20
0
1−cell
2−cell
128−cell
1k−cell
Dome
50%−epiboly
Shield
75%−epiboly
1−4−somites
14−19−somites
20−25−somites
Prim−5
Prim−15
Prim−25
Long−pec
Protruding−mouth
Day−4
Day−5
Stage
Transcript
ENSDART00000059861
ENSDART00000131908
ENSDART00000136812
ENSDART00000140563

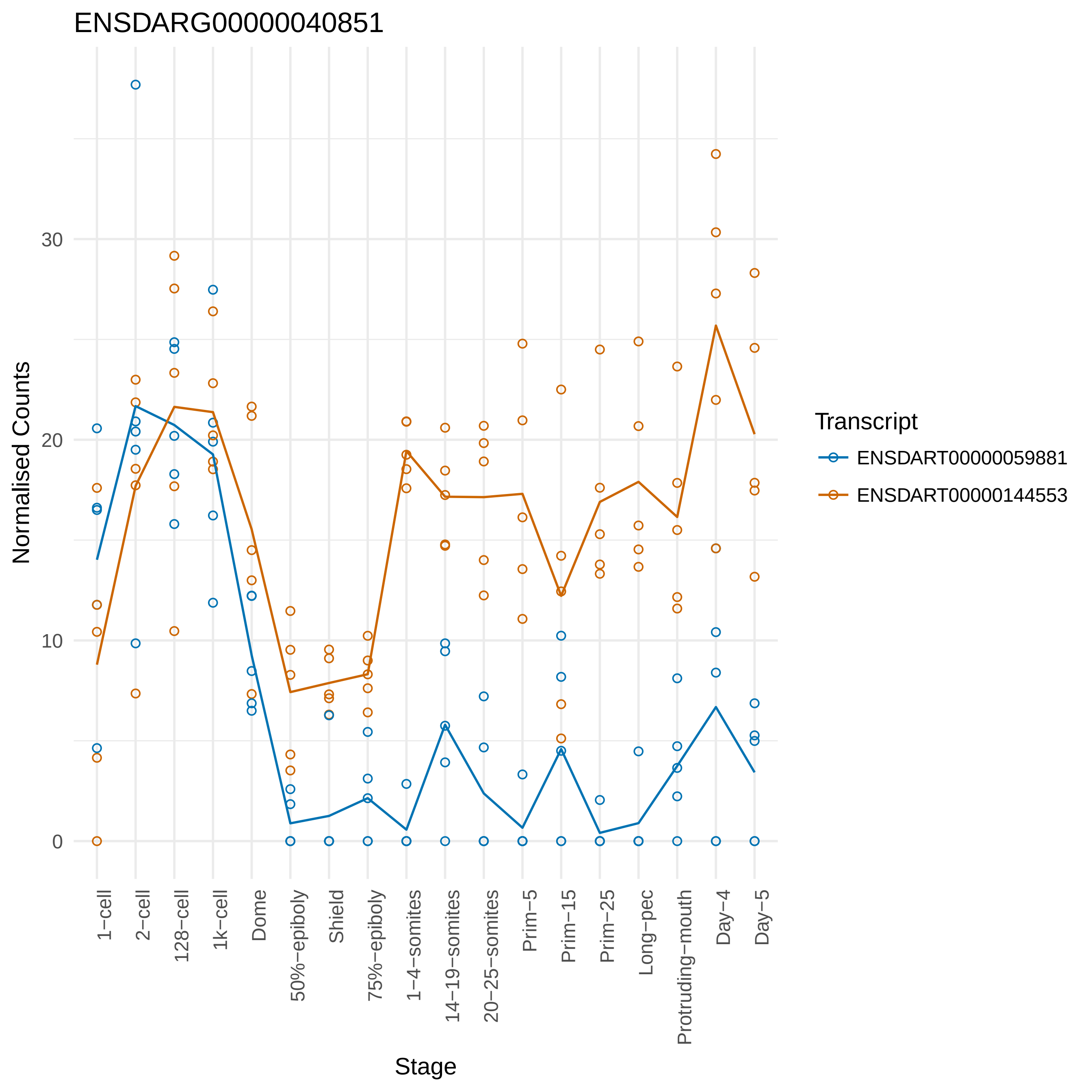
ENSDARG00000040851
Normalised Counts
0
10
20
30
1−cell
2−cell
128−cell
1k−cell
Dome
50%−epiboly
Shield
75%−epiboly
1−4−somites
14−19−somites
20−25−somites
Prim−5
Prim−15
Prim−25
Long−pec
Protruding−mouth
Day−4
Day−5
Stage
Transcript
ENSDART00000059881
ENSDART00000144553

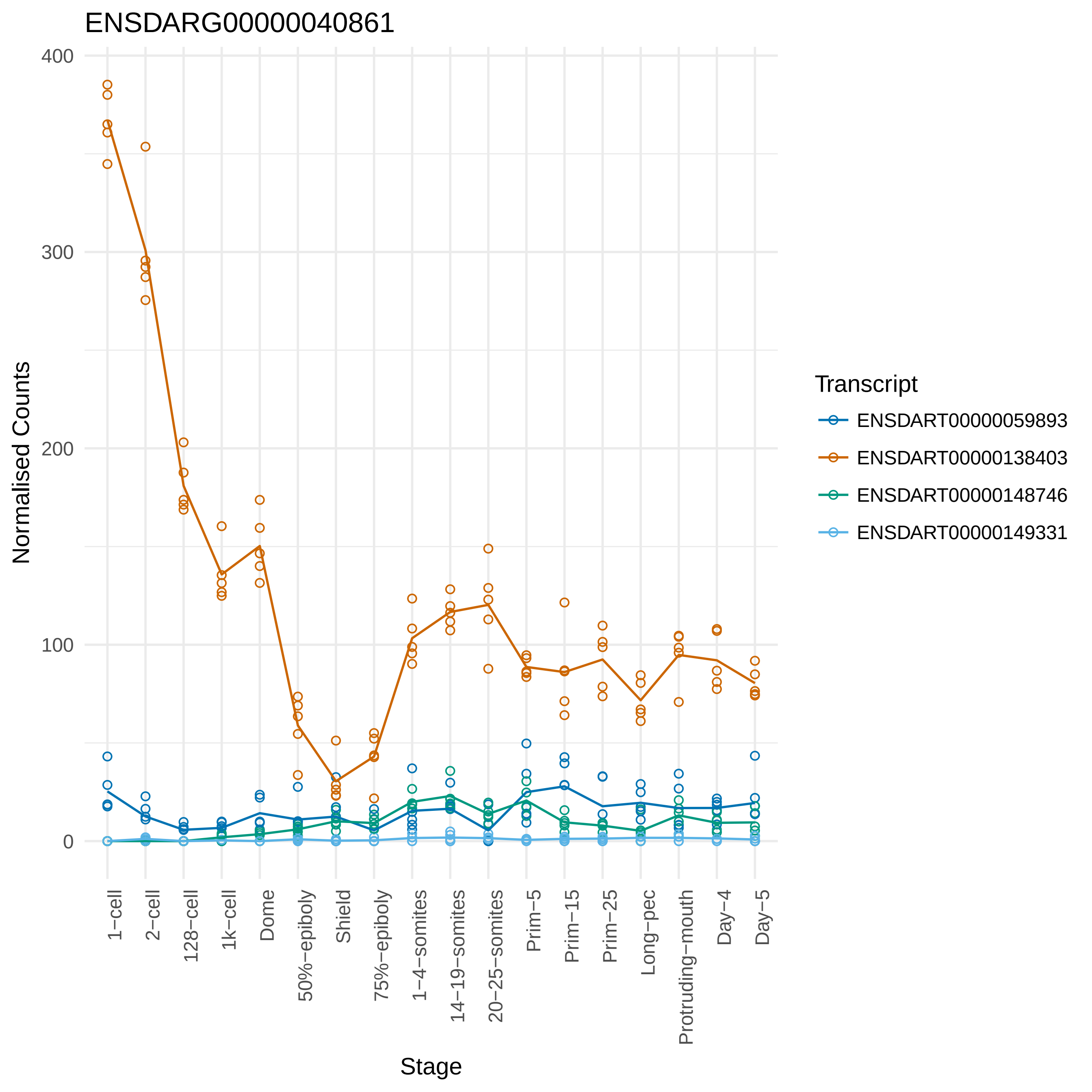

ENSDARG00000040861
Normalised Counts
400
300
200
100
0
1−cell
2−cell
128−cell
1k−cell
Dome
50%−epiboly
Shield
75%−epiboly
1−4−somites
14−19−somites
20−25−somites
Prim−5
Prim−15
Prim−25
Long−pec
Protruding−mouth
Day−4
Day−5
Stage
Transcript
ENSDART00000059893
ENSDART00000138403
ENSDART00000148746
ENSDART00000149331

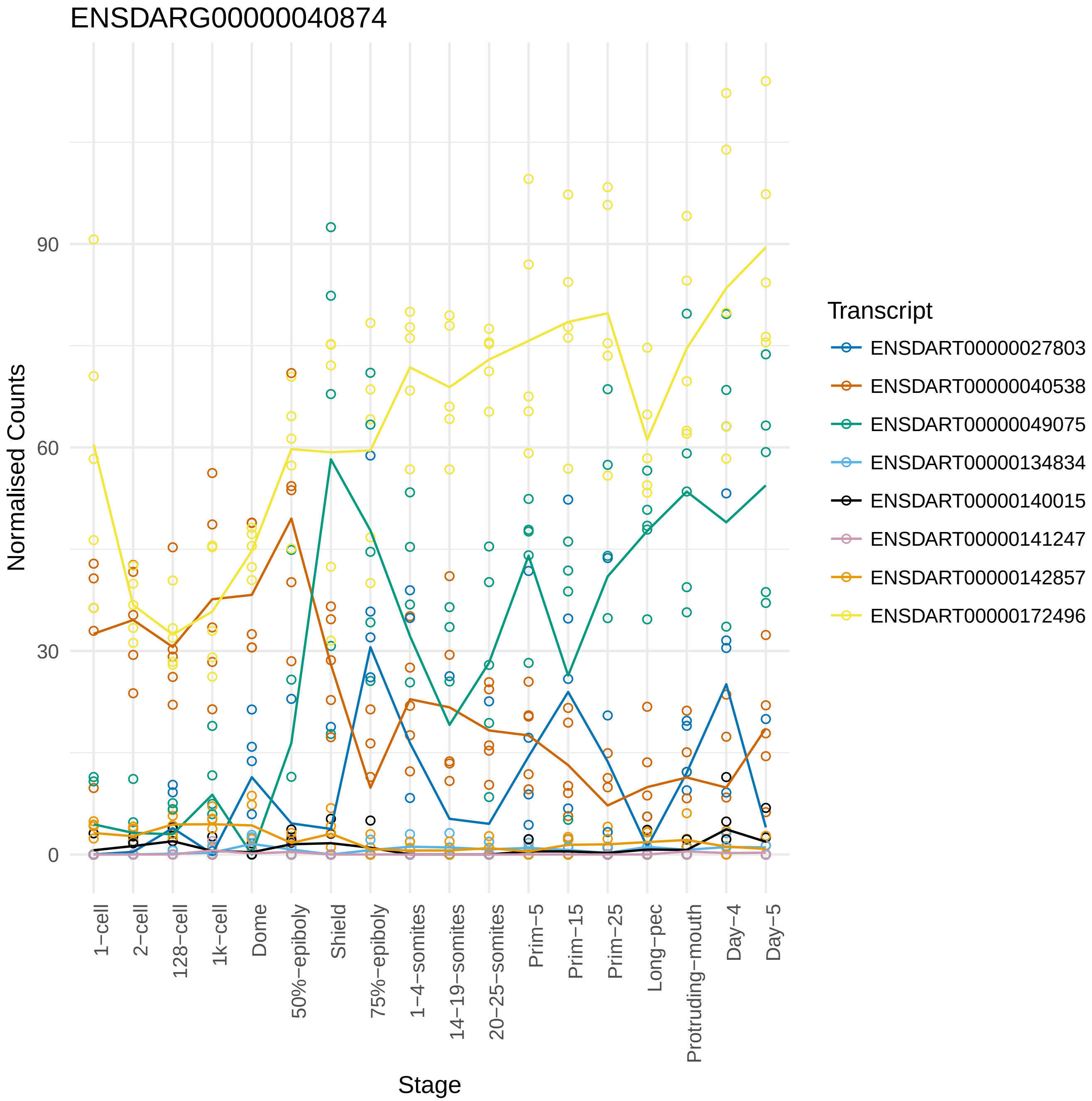

ENSDARG00000040874
Normalised Counts
90
60
30
0
1−cell
2−cell
128−cell
1k−cell
Dome
50%−epiboly
Shield
75%−epiboly
1−4−somites
14−19−somites
20−25−somites
Prim−5
Prim−15
Prim−25
Long−pec
Protruding−mouth
Day−4
Day−5
Stage
Transcript
ENSDART00000027803
ENSDART00000040538
ENSDART00000049075
ENSDART00000134834
ENSDART00000140015
ENSDART00000141247
ENSDART00000142857
ENSDART00000172496

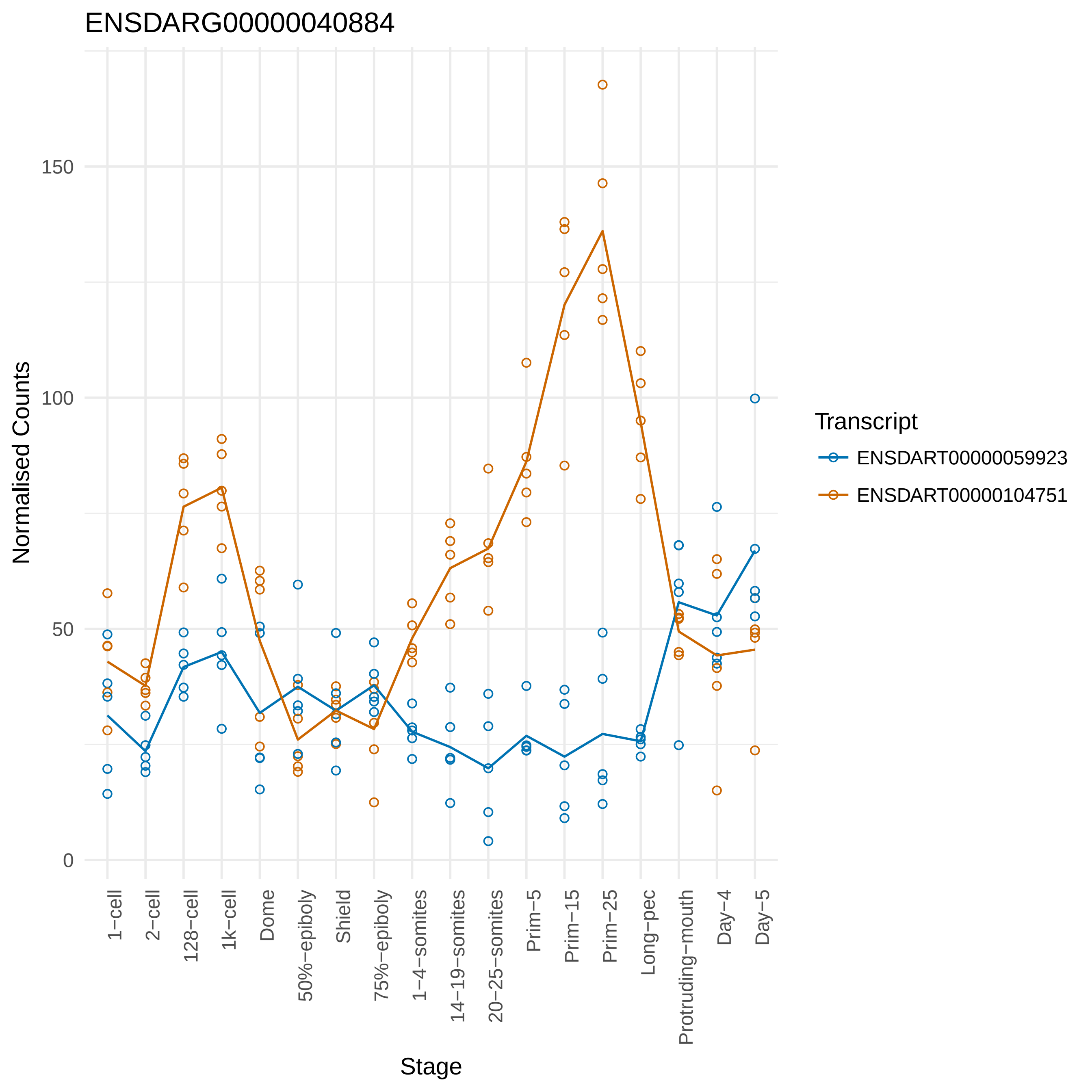

ENSDARG00000040884
Normalised Counts
150
100
50
0
1−cell
2−cell
128−cell
1k−cell
Dome
50%−epiboly
Shield
75%−epiboly
1−4−somites
14−19−somites
20−25−somites
Prim−5
Prim−15
Prim−25
Long−pec
Protruding−mouth
Day−4
Day−5
Stage
Transcript
ENSDART00000059923
ENSDART00000104751

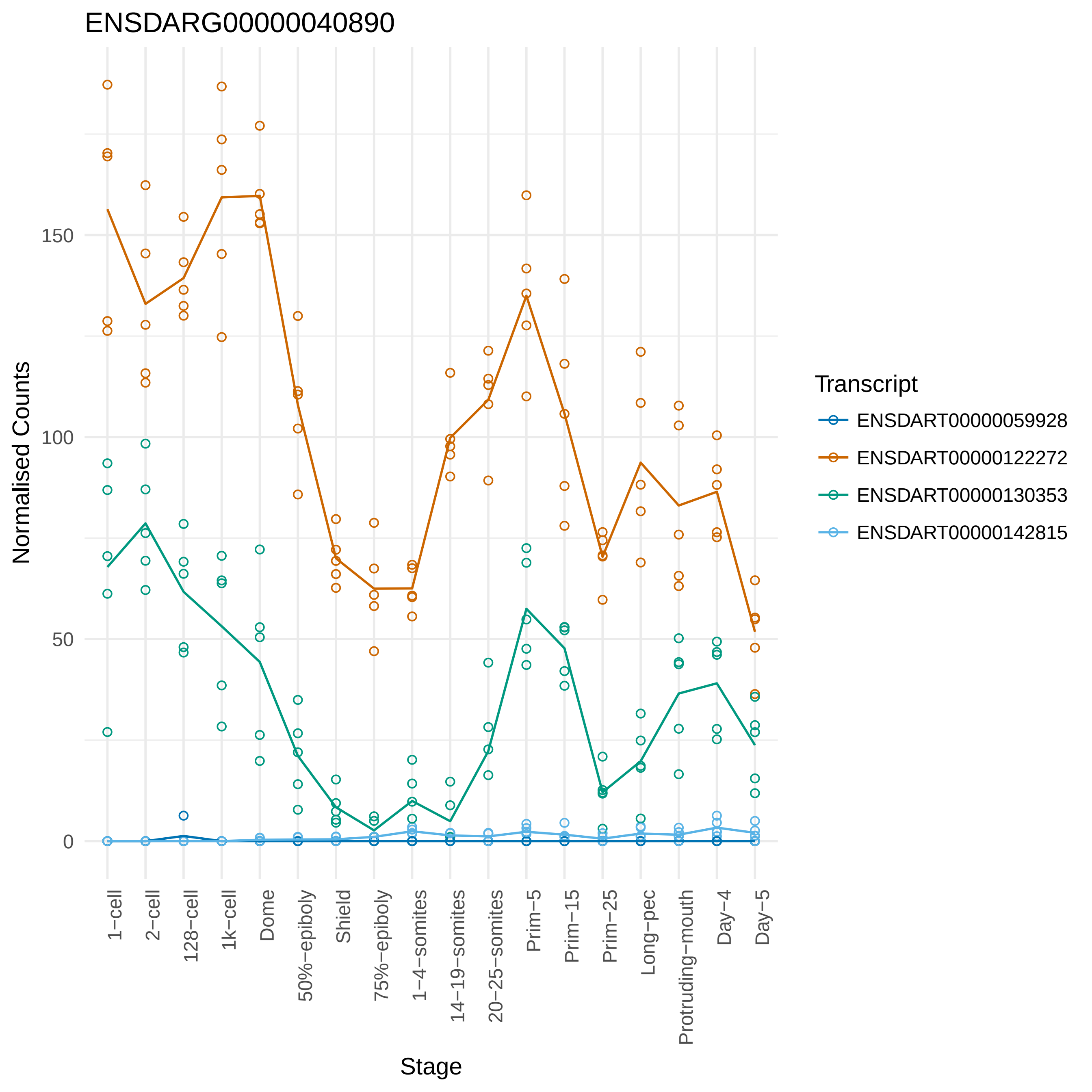

ENSDARG00000040890
Normalised Counts
150
100
50
0
1−cell
2−cell
128−cell
1k−cell
Dome
50%−epiboly
Shield
75%−epiboly
1−4−somites
14−19−somites
20−25−somites
Prim−5
Prim−15
Prim−25
Long−pec
Protruding−mouth
Day−4
Day−5
Stage
Transcript
ENSDART00000059928
ENSDART00000122272
ENSDART00000130353
ENSDART00000142815

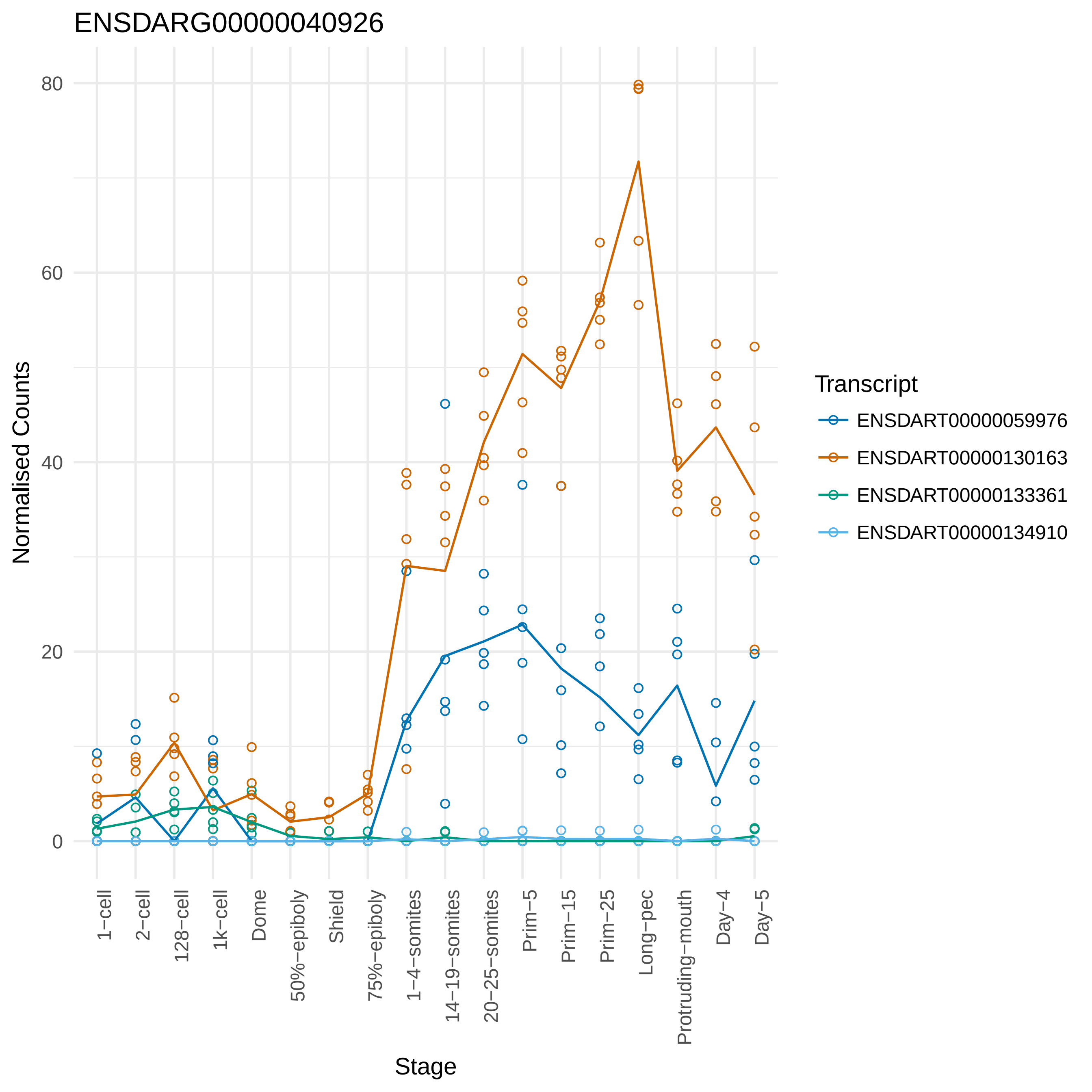

ENSDARG00000040926
Normalised Counts
80
60
40
20
0
1−cell
2−cell
128−cell
1k−cell
Dome
50%−epiboly
Shield
75%−epiboly
1−4−somites
14−19−somites
20−25−somites
Prim−5
Prim−15
Prim−25
Long−pec
Protruding−mouth
Day−4
Day−5
Stage
Transcript
ENSDART00000059976
ENSDART00000130163
ENSDART00000133361
ENSDART00000134910

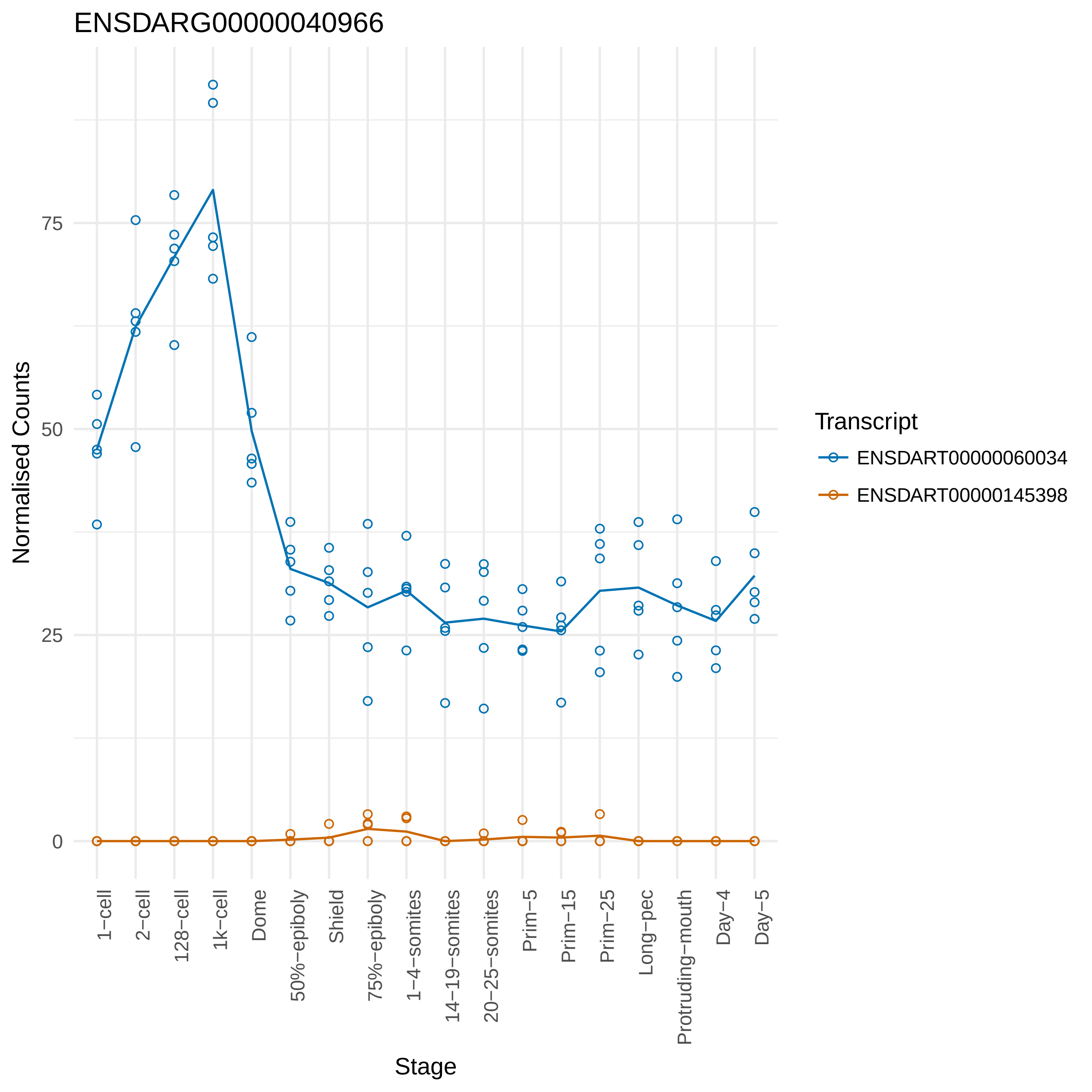

ENSDARG00000040966
Normalised Counts
75
50
25
0
1−cell
2−cell
128−cell
1k−cell
Dome
50%−epiboly
Shield
75%−epiboly
1−4−somites
14−19−somites
20−25−somites
Prim−5
Prim−15
Prim−25
Long−pec
Protruding−mouth
Day−4
Day−5
Stage
Transcript
ENSDART00000060034
ENSDART00000145398

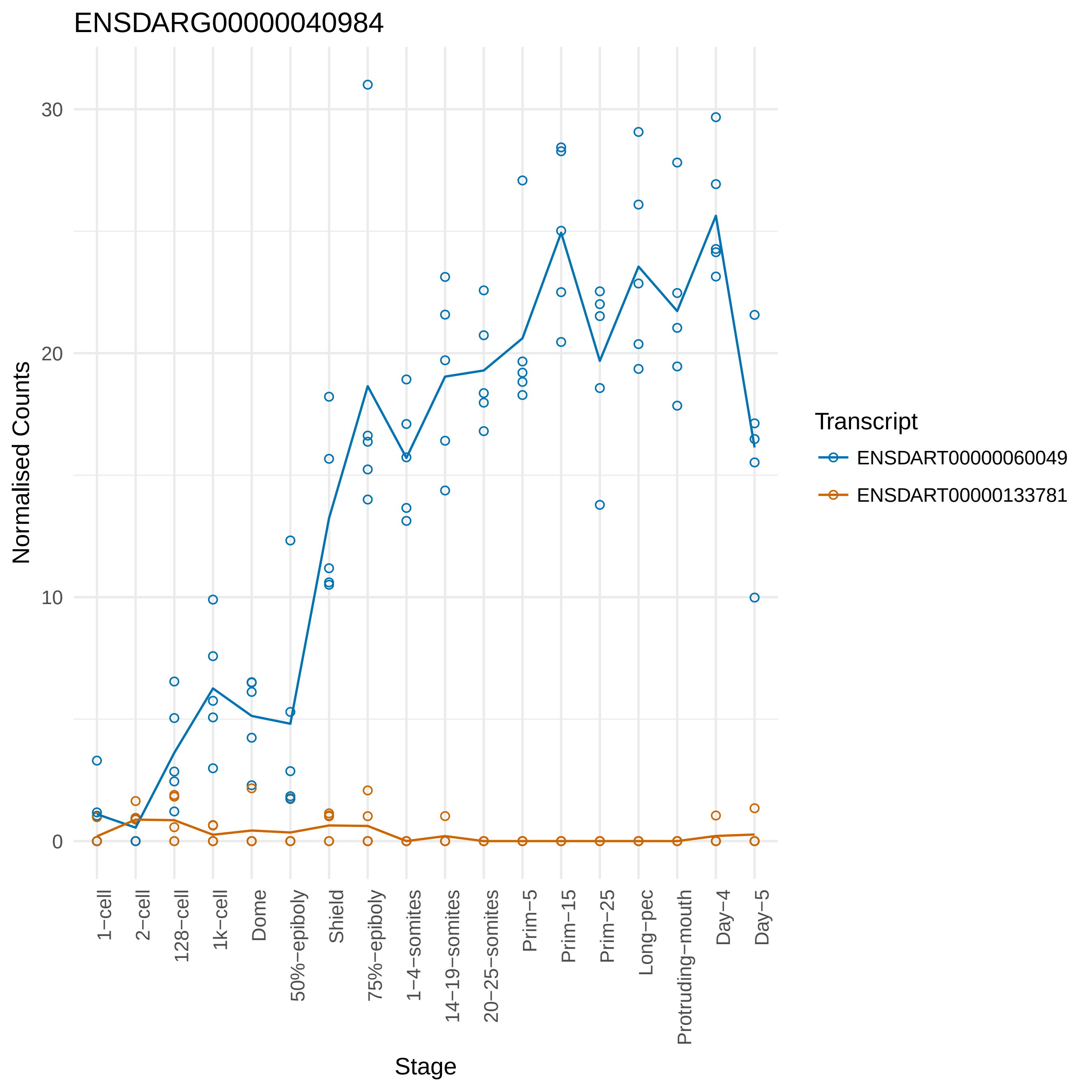
ENSDARG00000040984
Normalised Counts
30
20
10
0
1−cell
2−cell
128−cell
1k−cell
Dome
50%−epiboly
Shield
75%−epiboly
1−4−somites
14−19−somites
20−25−somites
Prim−5
Prim−15
Prim−25
Long−pec
Protruding−mouth
Day−4
Day−5
Stage
Transcript
ENSDART00000060049
ENSDART00000133781

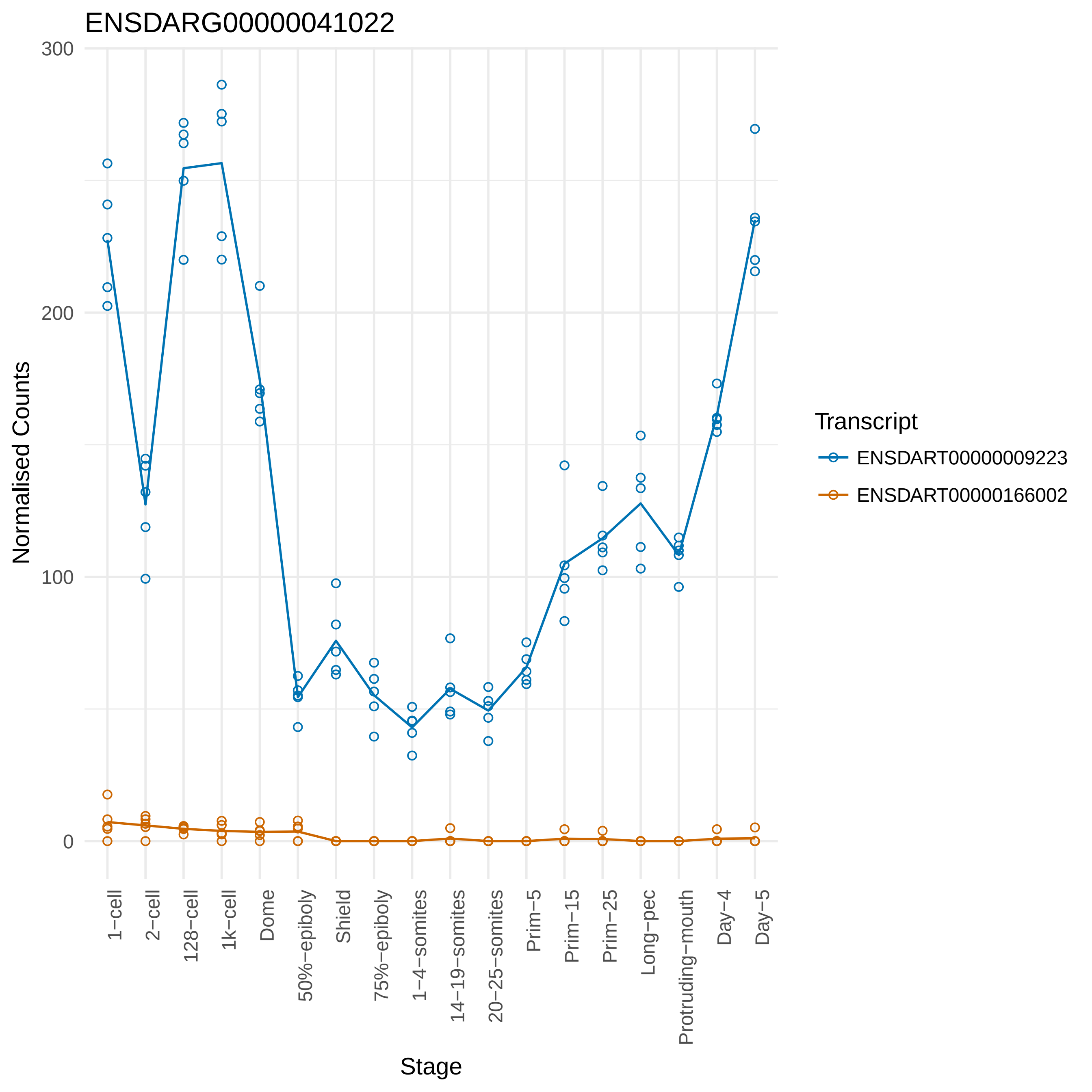
ENSDARG00000041022
Normalised Counts
300
200
100
0
1−cell
2−cell
128−cell
1k−cell
Dome
50%−epiboly
Shield
75%−epiboly
1−4−somites
14−19−somites
20−25−somites
Prim−5
Prim−15
Prim−25
Long−pec
Protruding−mouth
Day−4
Day−5
Stage
Transcript
ENSDART00000009223
ENSDART00000166002

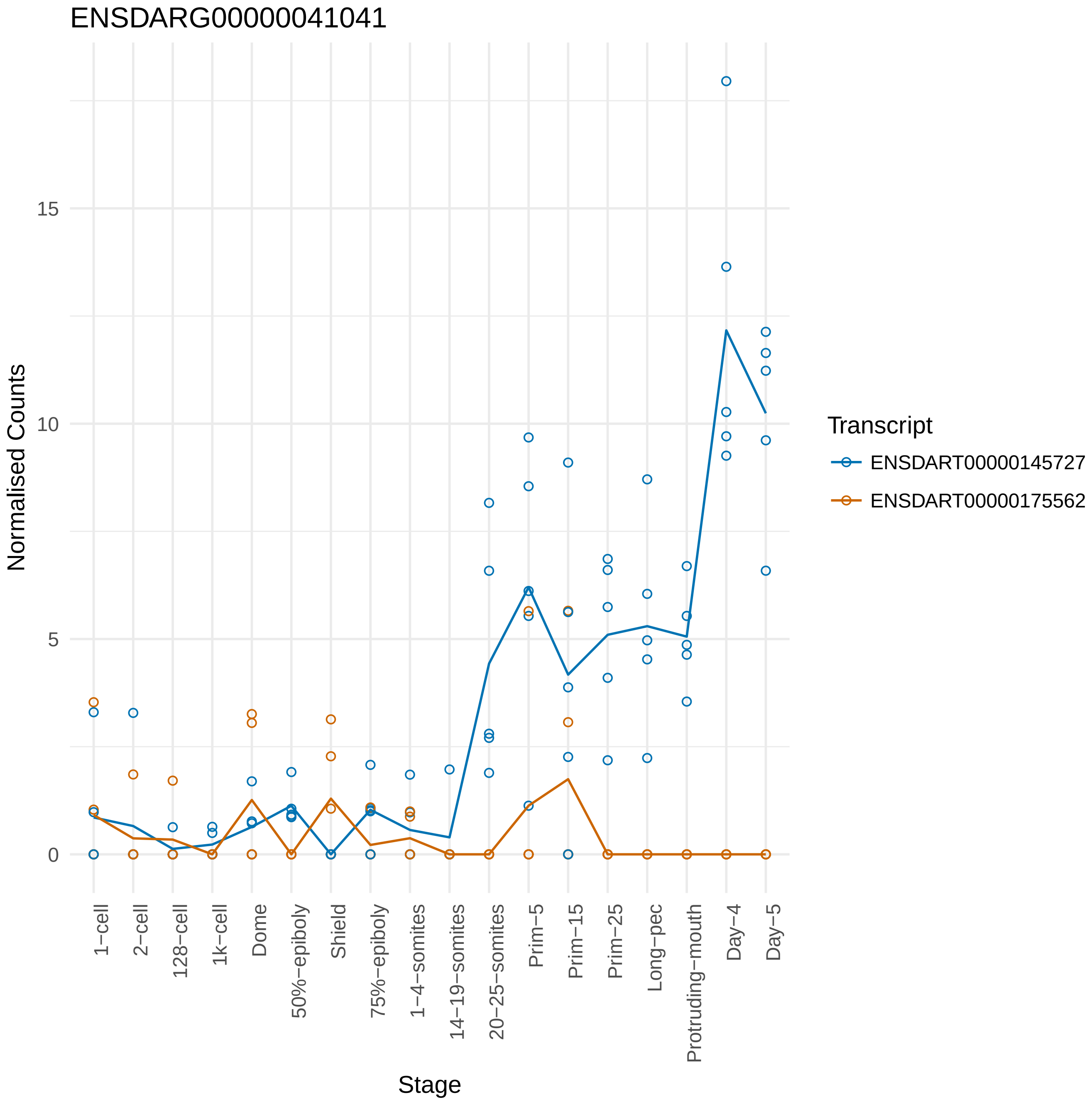

ENSDARG00000041041
Normalised Counts
0
5
10
15
1−cell
2−cell
128−cell
1k−cell
Dome
50%−epiboly
Shield
75%−epiboly
1−4−somites
14−19−somites
20−25−somites
Prim−5
Prim−15
Prim−25
Long−pec
Protruding−mouth
Day−4
Day−5
Stage
Transcript
ENSDART00000145727
ENSDART00000175562

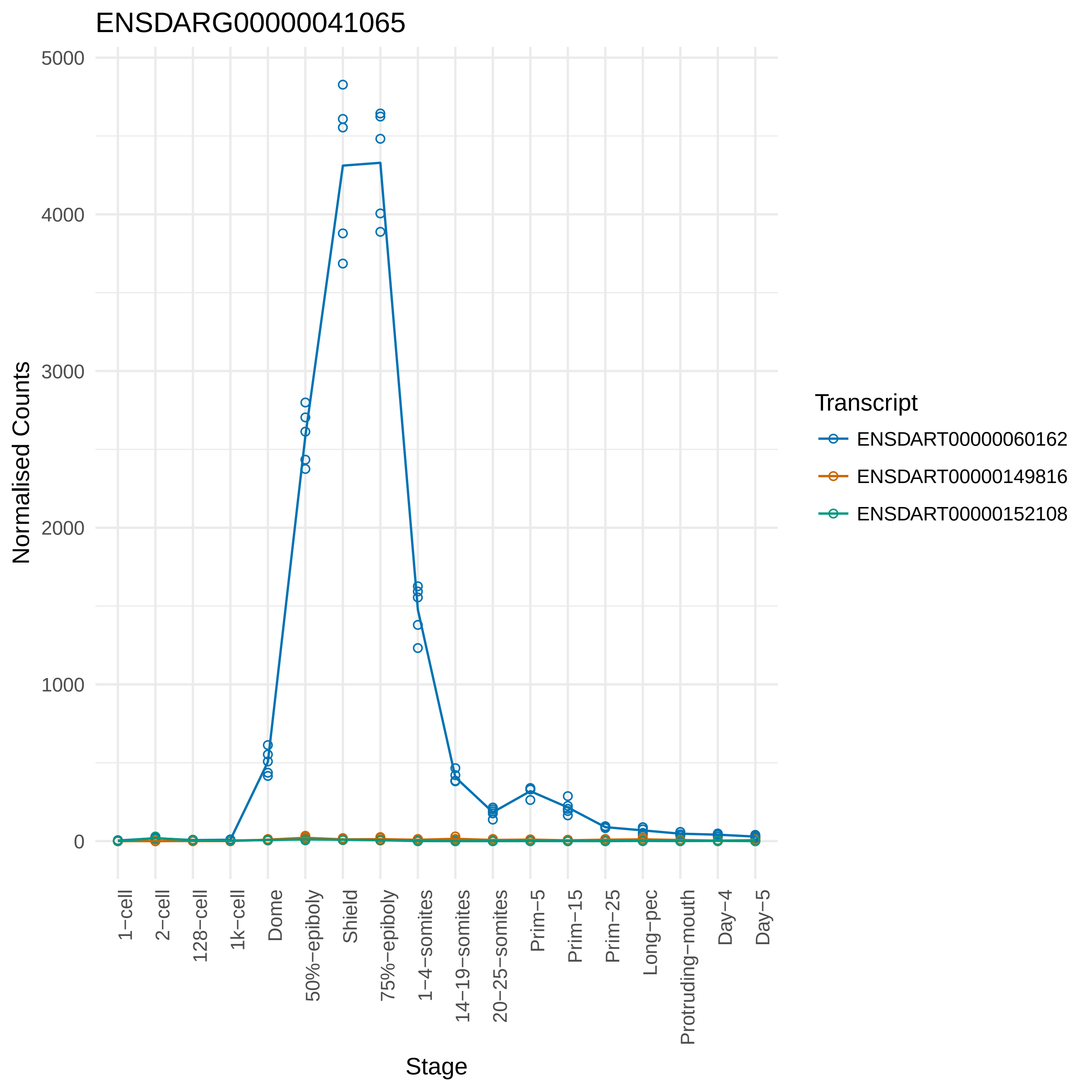

ENSDARG00000041065
Normalised Counts
0
1000
2000
3000
4000
5000
Stage
1−cell
2−cell
128−cell
1k−cell
Dome
50%−epiboly
Shield
75%−epiboly
1−4−somites
14−19−somites
20−25−somites
Prim−5
Prim−15
Prim−25
Long−pec
Protruding−mouth
Day−4
Day−5
Transcript
ENSDART00000060162
ENSDART00000149816
ENSDART00000152108

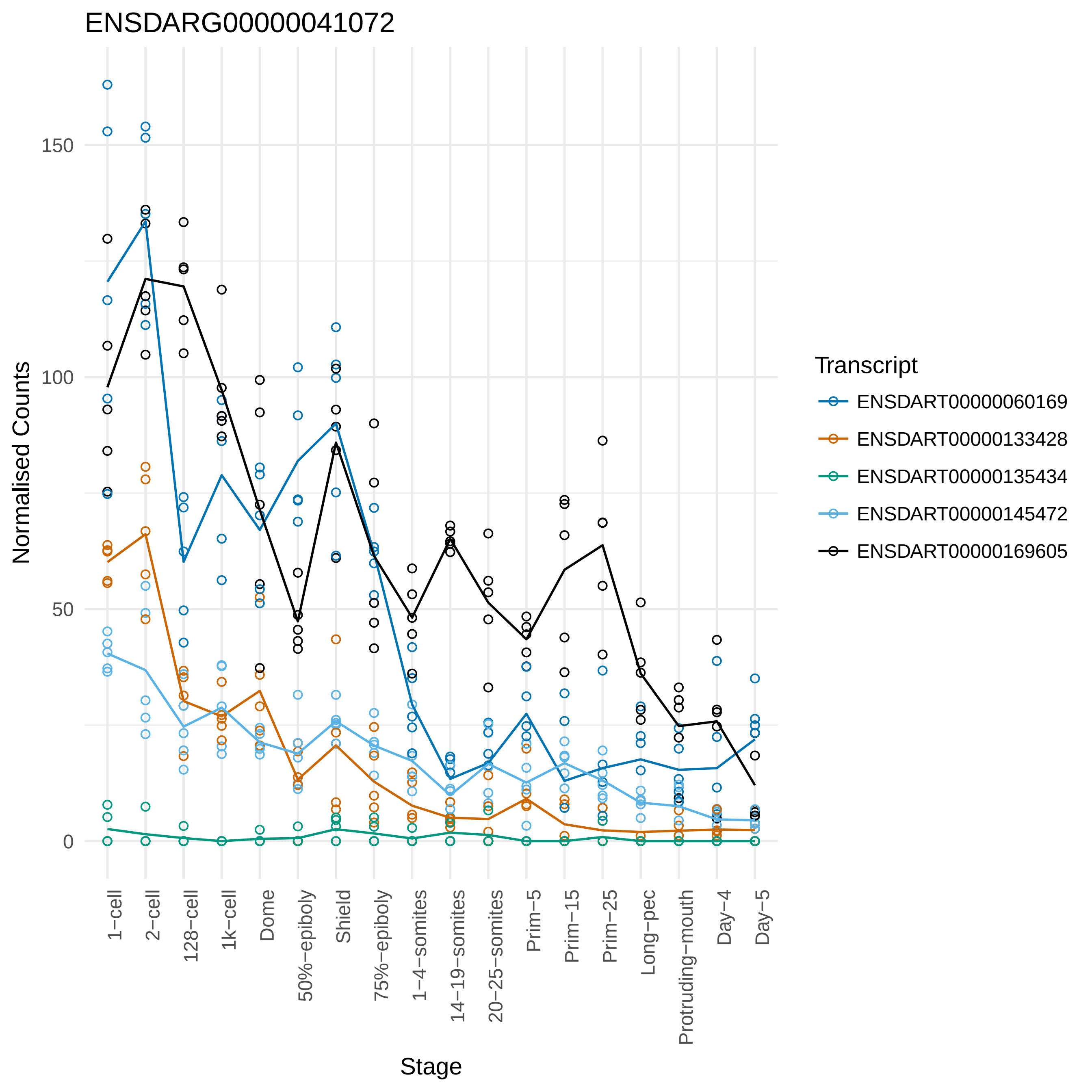

ENSDARG00000041072
Normalised Counts
150
100
50
0
1−cell
2−cell
128−cell
1k−cell
Dome
50%−epiboly
Shield
75%−epiboly
1−4−somites
14−19−somites
20−25−somites
Prim−5
Prim−15
Prim−25
Long−pec
Protruding−mouth
Day−4
Day−5
Stage
Transcript
ENSDART00000060169
ENSDART00000133428
ENSDART00000135434
ENSDART00000145472
ENSDART00000169605

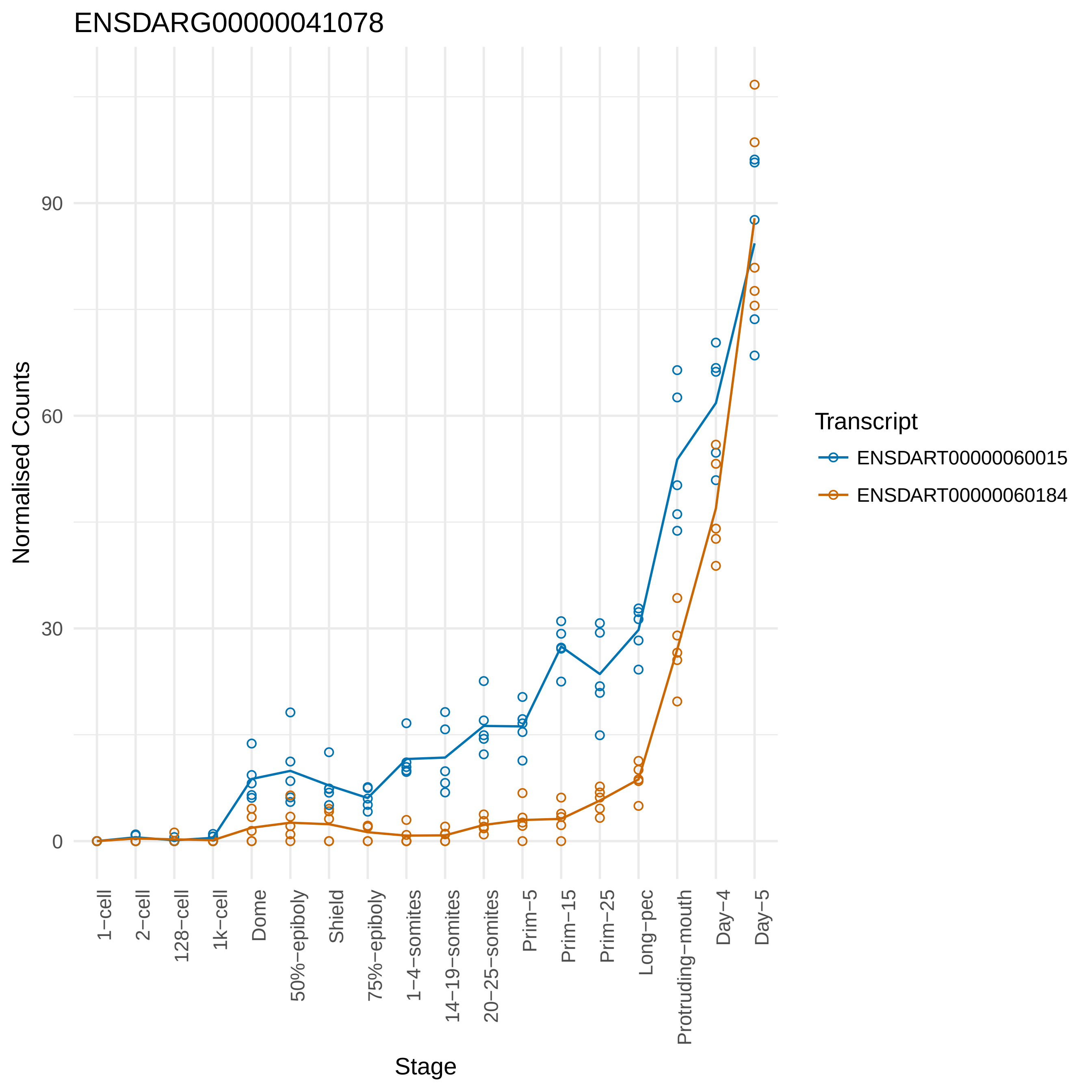

ENSDARG00000041078
Normalised Counts
0
30
60
90
Stage
1−cell
2−cell
128−cell
1k−cell
Dome
50%−epiboly
Shield
75%−epiboly
1−4−somites
14−19−somites
20−25−somites
Prim−5
Prim−15
Prim−25
Long−pec
Protruding−mouth
Day−4
Day−5
Transcript
ENSDART00000060015
ENSDART00000060184

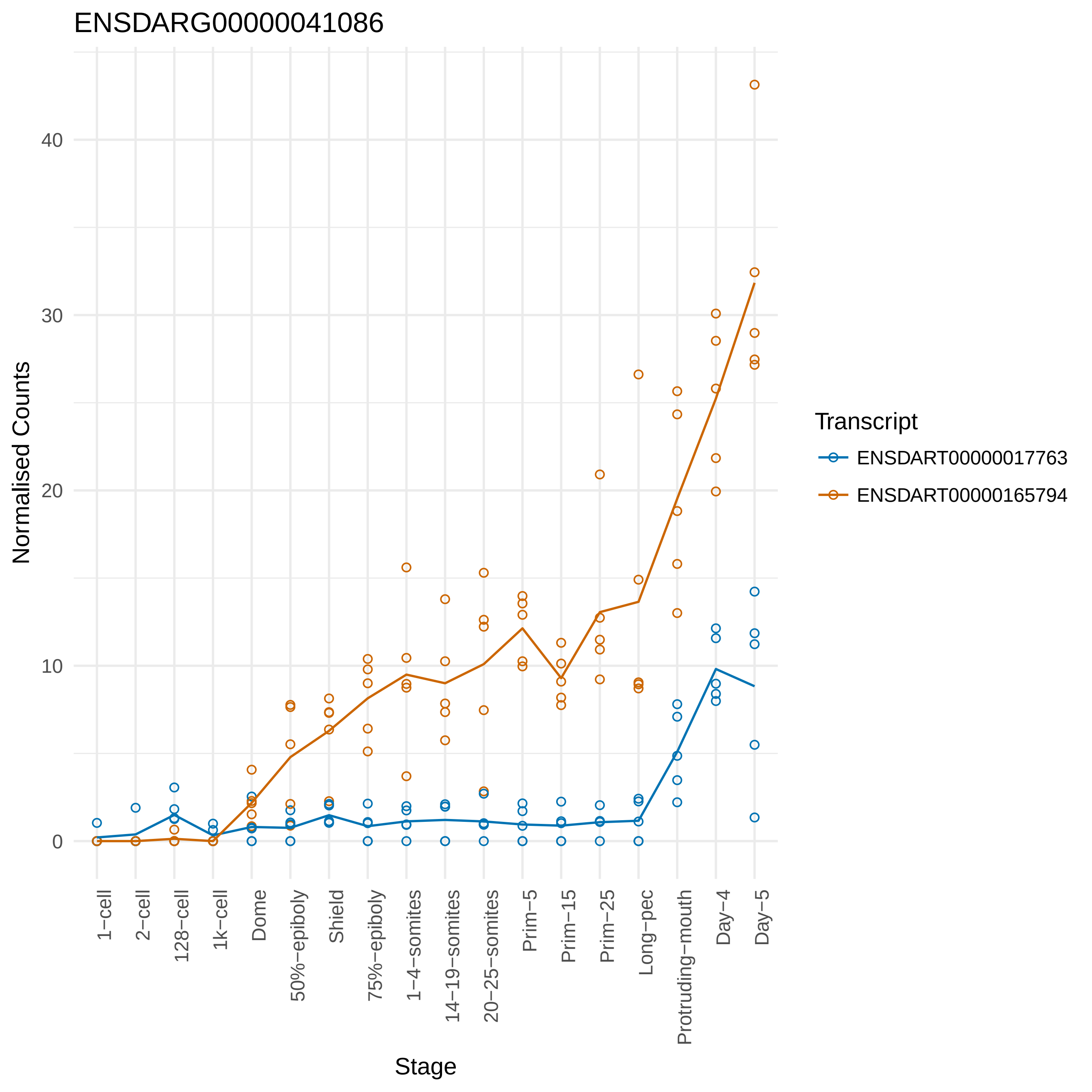

ENSDARG00000041086
Normalised Counts
0
10
20
30
40
Stage
1−cell
2−cell
128−cell
1k−cell
Dome
50%−epiboly
Shield
75%−epiboly
1−4−somites
14−19−somites
20−25−somites
Prim−5
Prim−15
Prim−25
Long−pec
Protruding−mouth
Day−4
Day−5
Transcript
ENSDART00000017763
ENSDART00000165794

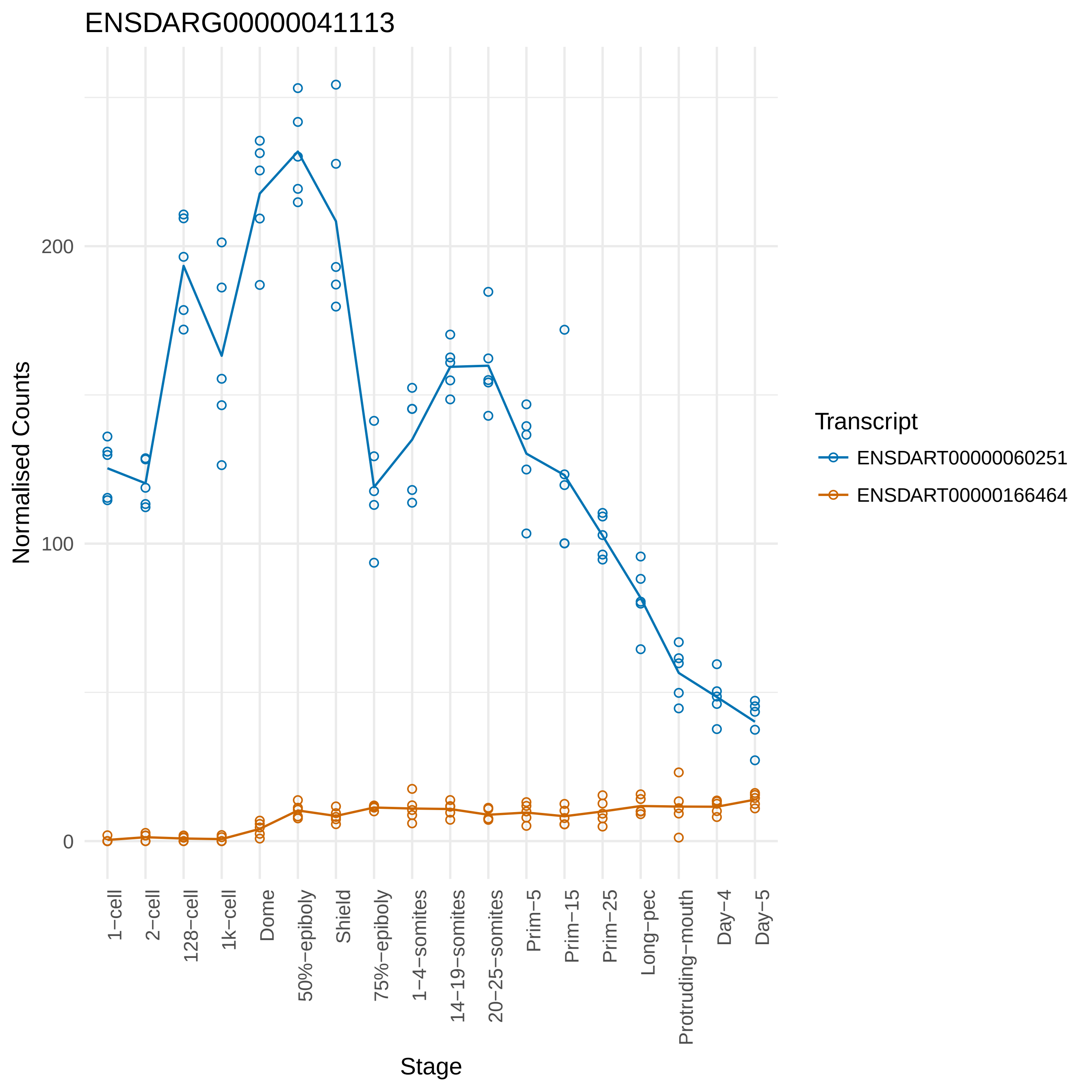

ENSDARG00000041113
Normalised Counts
200
100
0
1−cell
2−cell
128−cell
1k−cell
Dome
50%−epiboly
Shield
75%−epiboly
1−4−somites
14−19−somites
20−25−somites
Prim−5
Prim−15
Prim−25
Long−pec
Protruding−mouth
Day−4
Day−5
Stage
Transcript
ENSDART00000060251
ENSDART00000166464

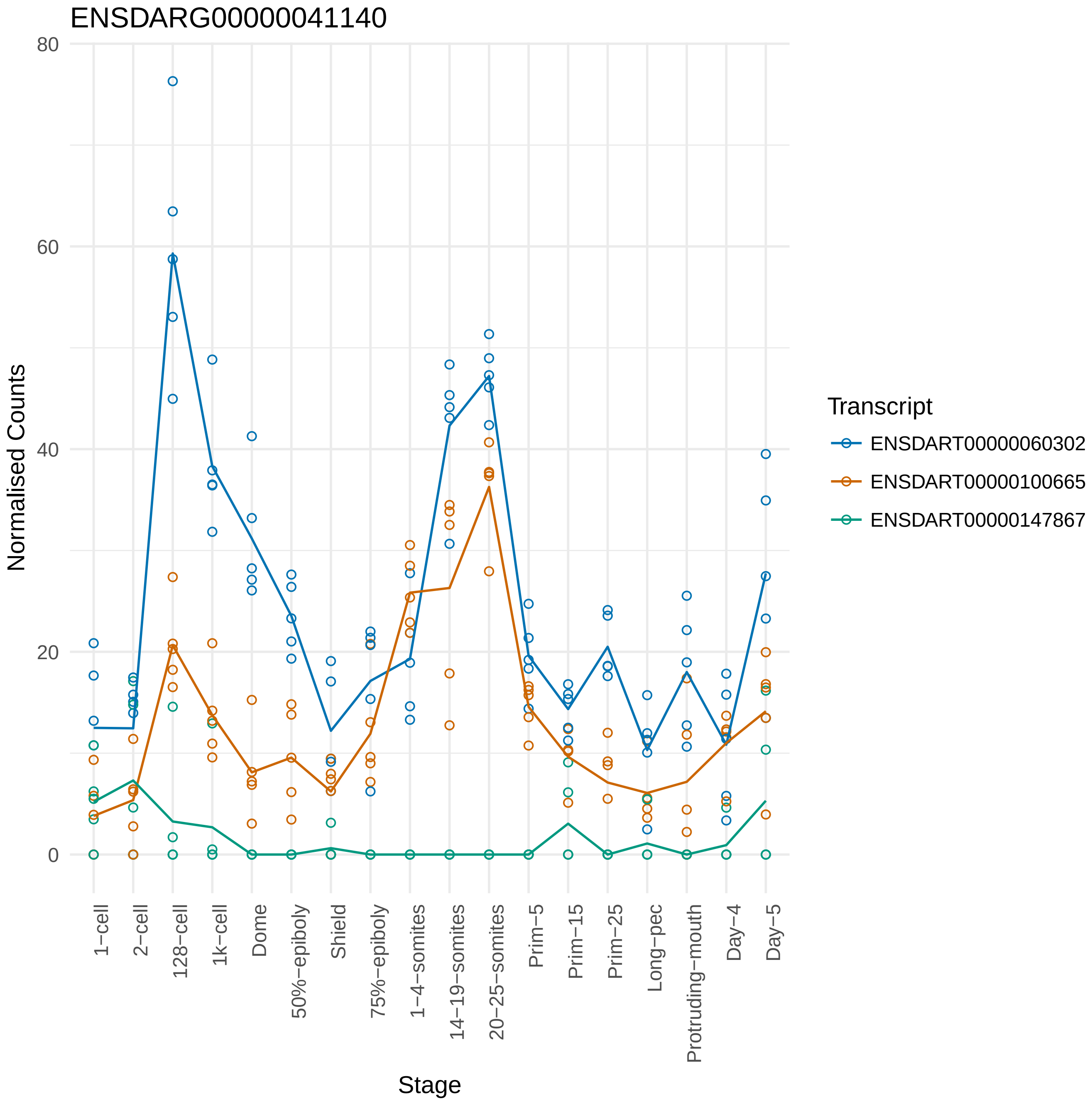

ENSDARG00000041140
Normalised Counts
0
20
40
60
80
Stage
1−cell
2−cell
128−cell
1k−cell
Dome
50%−epiboly
Shield
75%−epiboly
1−4−somites
14−19−somites
20−25−somites
Prim−5
Prim−15
Prim−25
Long−pec
Protruding−mouth
Day−4
Day−5
Transcript
ENSDART00000060302
ENSDART00000100665
ENSDART00000147867

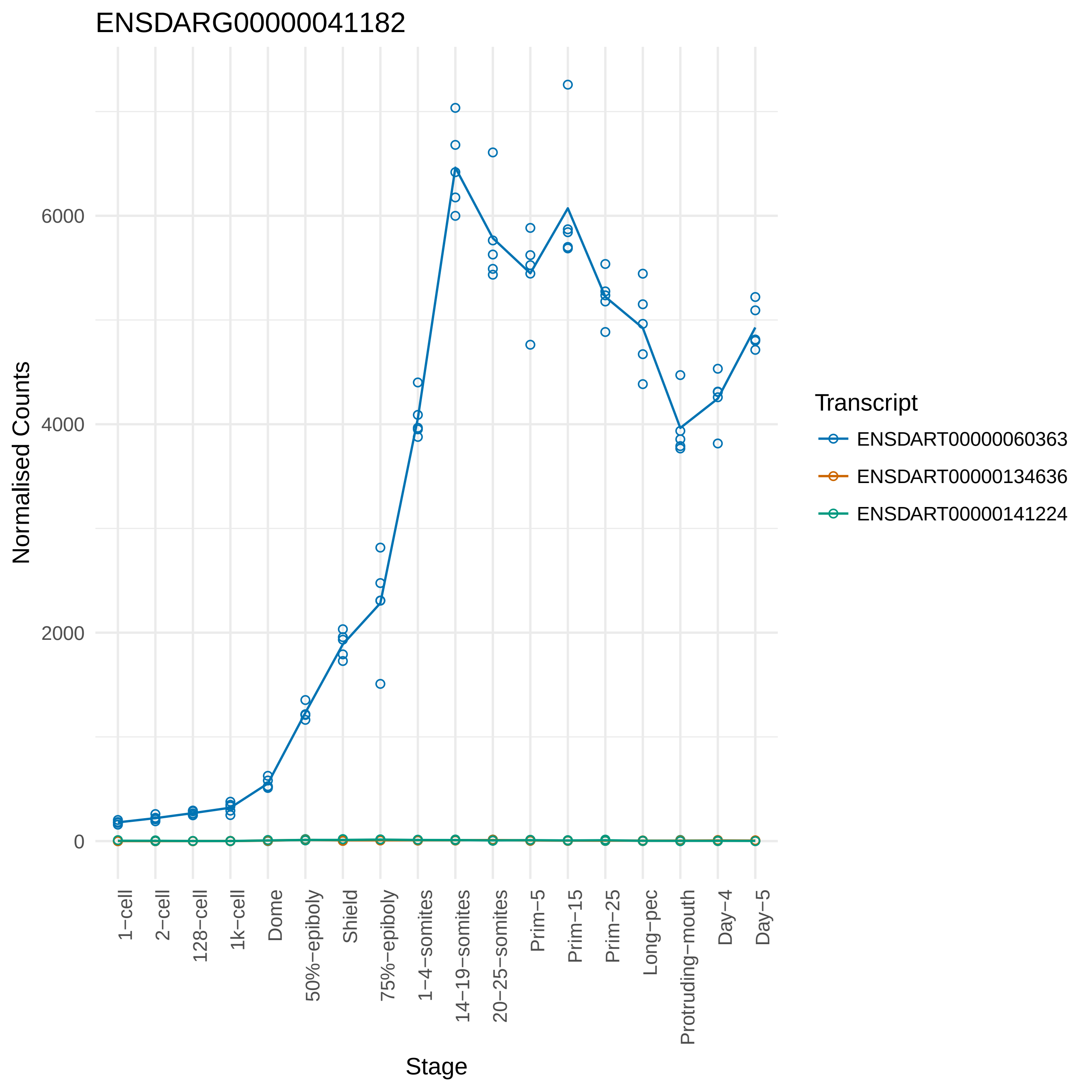
ENSDARG00000041182
Normalised Counts
6000
4000
2000
0
1−cell
2−cell
128−cell
1k−cell
Dome
50%−epiboly
Shield
75%−epiboly
1−4−somites
14−19−somites
20−25−somites
Prim−5
Prim−15
Prim−25
Long−pec
Protruding−mouth
Day−4
Day−5
Stage
Transcript
ENSDART00000060363
ENSDART00000134636
ENSDART00000141224

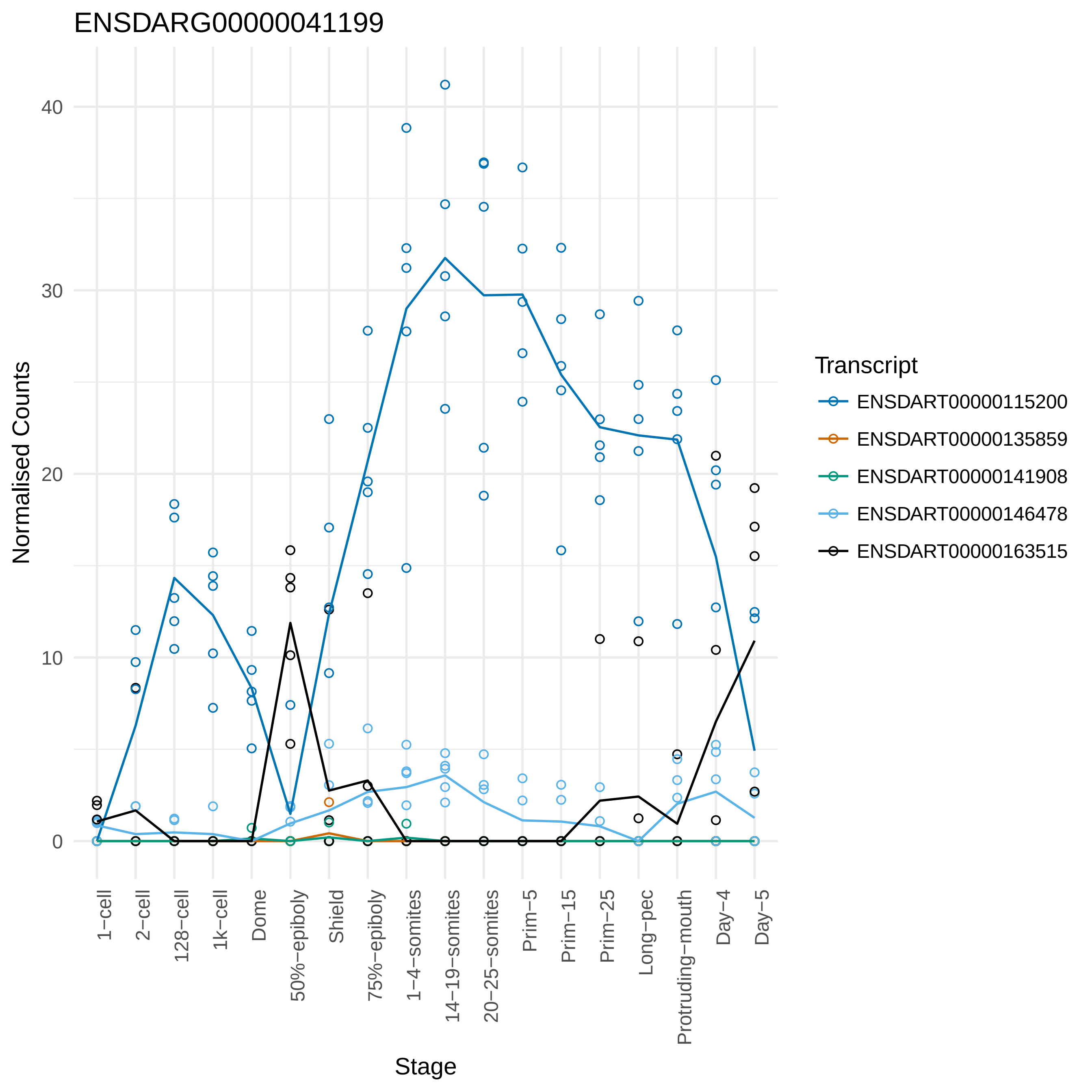
ENSDARG00000041199
Normalised Counts
40
30
20
10
0
Stage
1−cell
2−cell
128−cell
1k−cell
Dome
50%−epiboly
Shield
75%−epiboly
1−4−somites
14−19−somites
20−25−somites
Prim−5
Prim−15
Prim−25
Long−pec
Protruding−mouth
Day−4
Day−5
Transcript
ENSDART00000115200
ENSDART00000135859
ENSDART00000141908
ENSDART00000146478
ENSDART00000163515

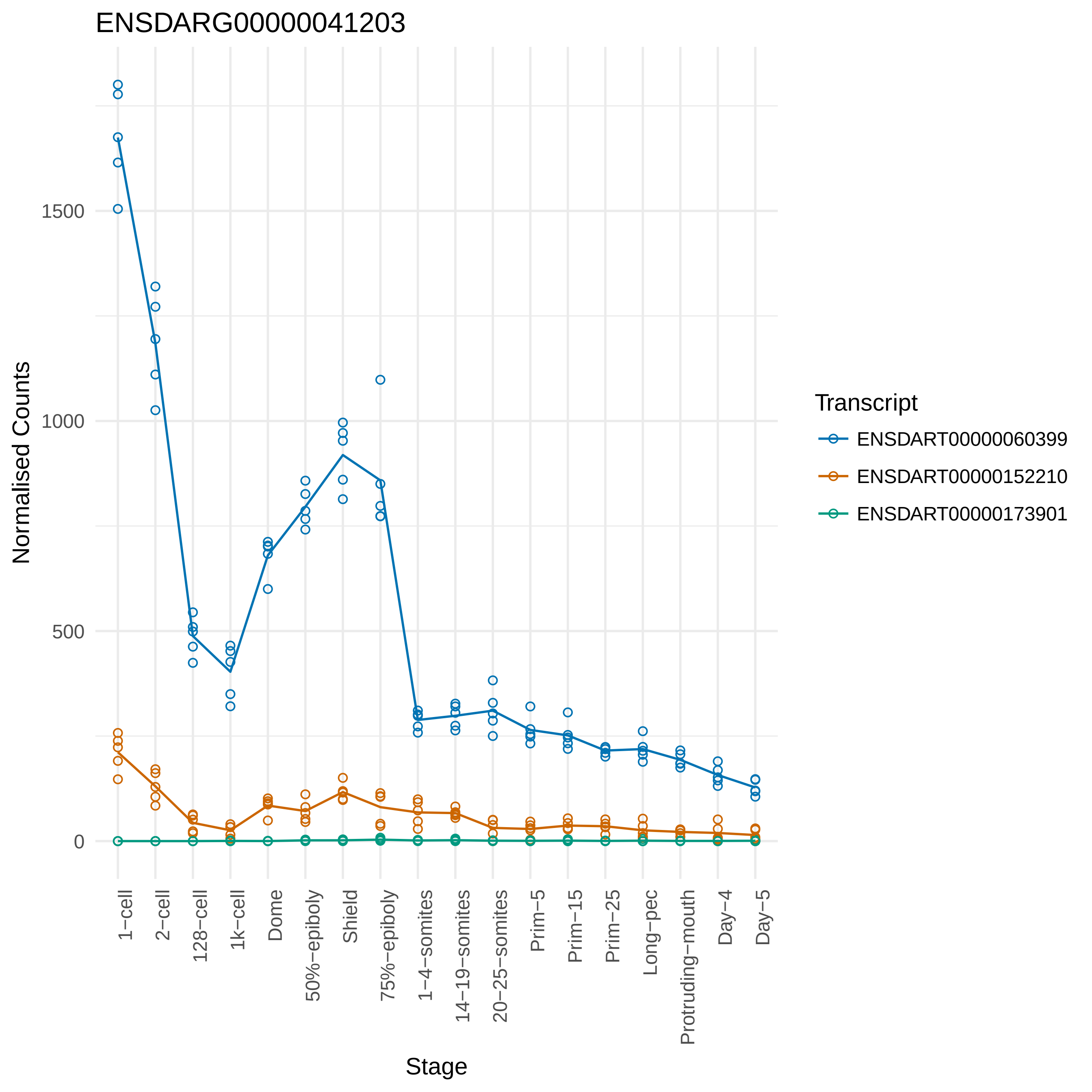

ENSDARG00000041203
Normalised Counts
1500
1000
500
0
1−cell
2−cell
128−cell
1k−cell
Dome
50%−epiboly
Shield
75%−epiboly
1−4−somites
14−19−somites
20−25−somites
Prim−5
Prim−15
Prim−25
Long−pec
Protruding−mouth
Day−4
Day−5
Stage
Transcript
ENSDART00000060399
ENSDART00000152210
ENSDART00000173901

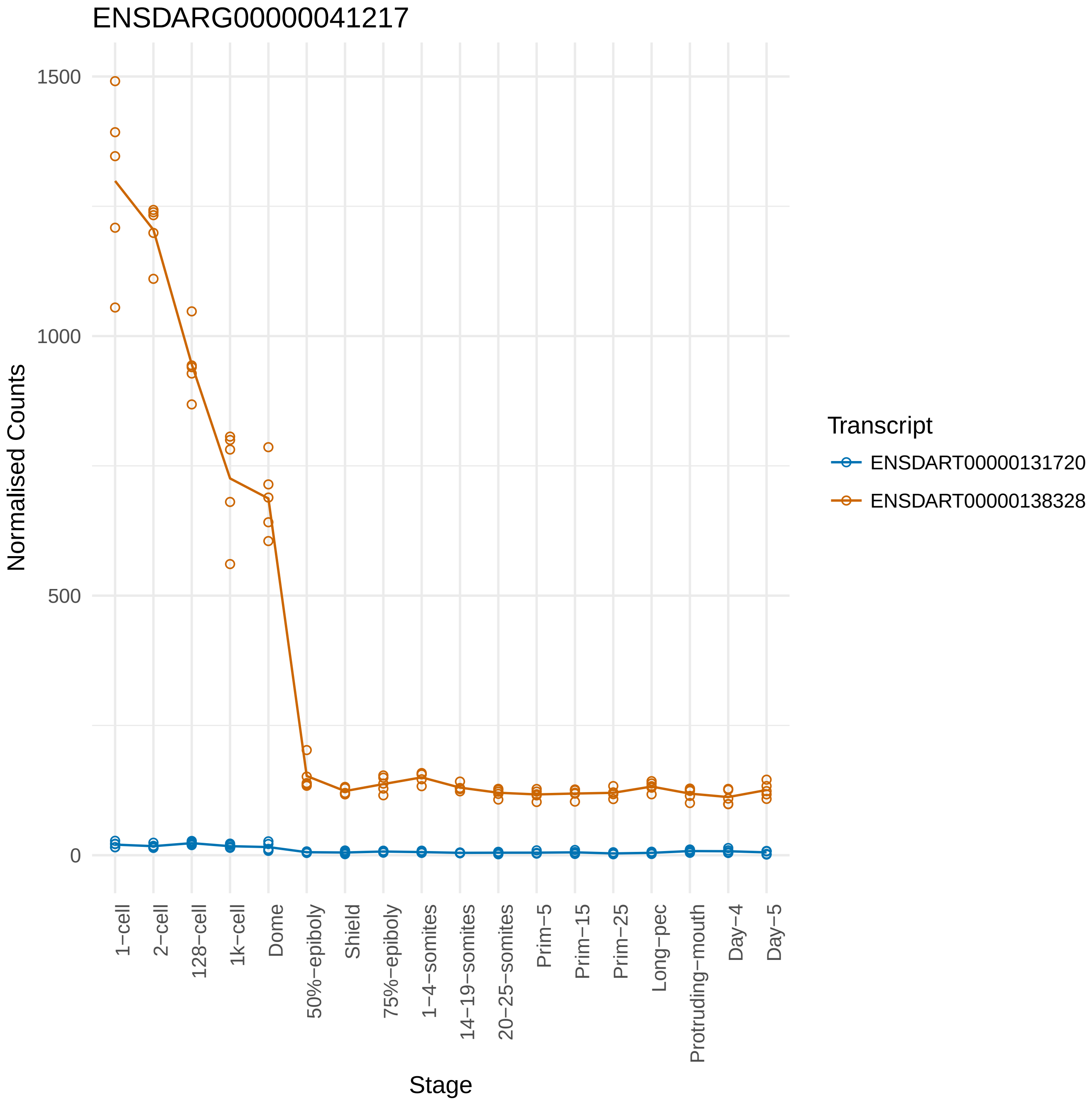
ENSDARG00000041217
Normalised Counts
1500
1000
500
0
Stage
1−cell
2−cell
128−cell
1k−cell
Dome
50%−epiboly
Shield
75%−epiboly
1−4−somites
14−19−somites
20−25−somites
Prim−5
Prim−15
Prim−25
Long−pec
Protruding−mouth
Day−4
Day−5
Transcript
ENSDART00000131720
ENSDART00000138328

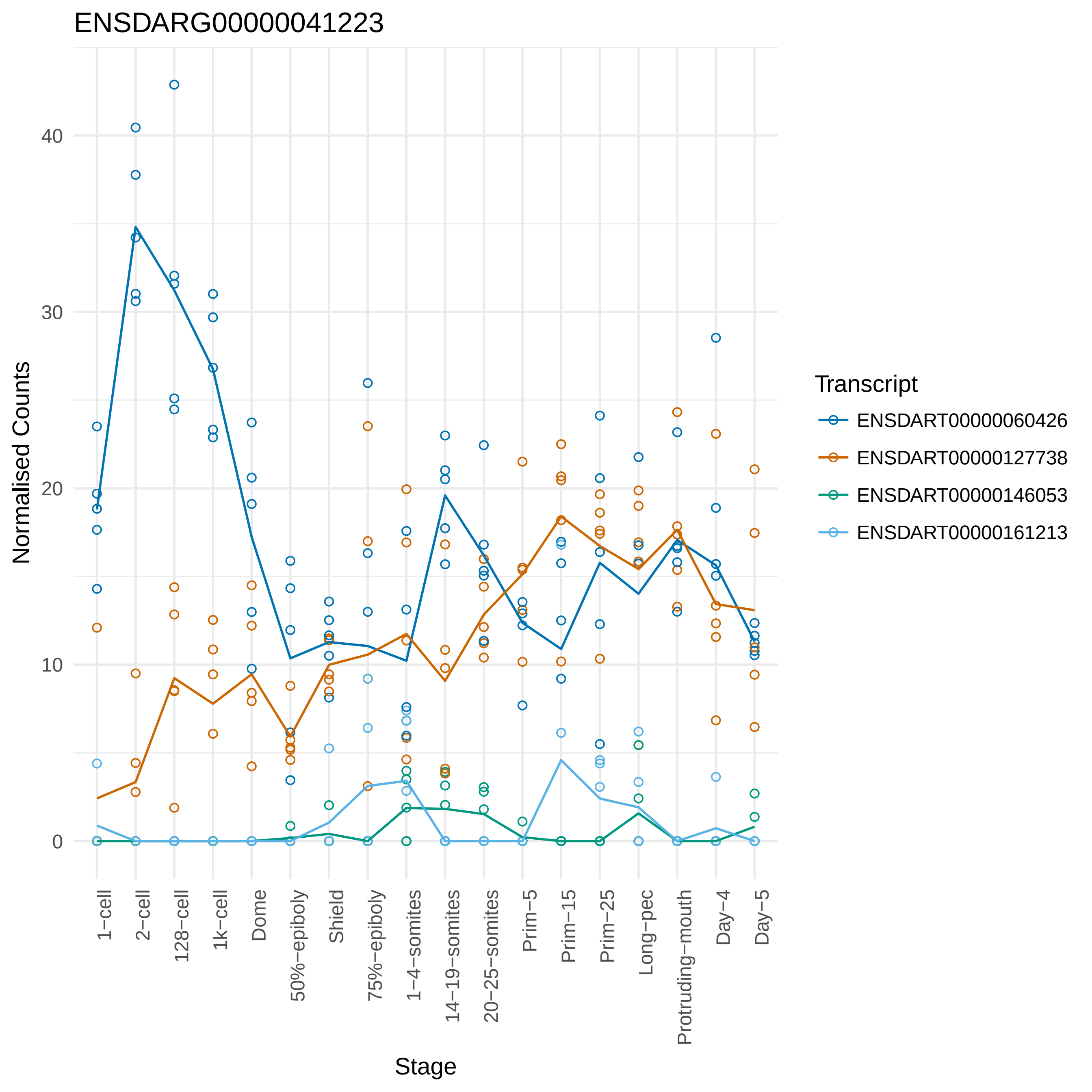
ENSDARG00000041223
Normalised Counts
0
10
20
30
40
Stage
1−cell
2−cell
128−cell
1k−cell
Dome
50%−epiboly
Shield
75%−epiboly
1−4−somites
14−19−somites
20−25−somites
Prim−5
Prim−15
Prim−25
Long−pec
Protruding−mouth
Day−4
Day−5
Transcript
ENSDART00000060426
ENSDART00000127738
ENSDART00000146053
ENSDART00000161213

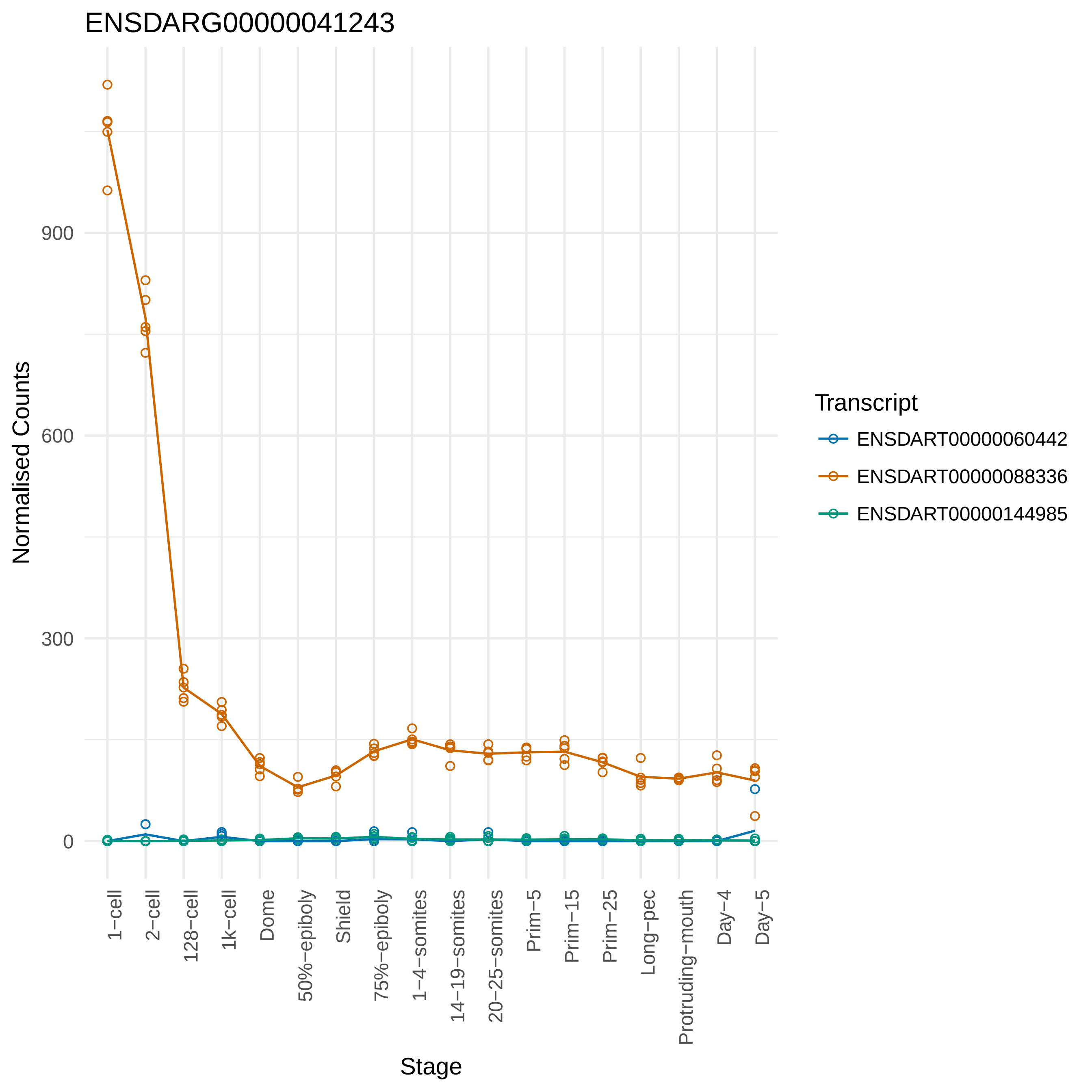

ENSDARG00000041243
Normalised Counts
900
600
300
0
1−cell
2−cell
128−cell
1k−cell
Dome
50%−epiboly
Shield
75%−epiboly
1−4−somites
14−19−somites
20−25−somites
Prim−5
Prim−15
Prim−25
Long−pec
Protruding−mouth
Day−4
Day−5
Stage
Transcript
ENSDART00000060442
ENSDART00000088336
ENSDART00000144985

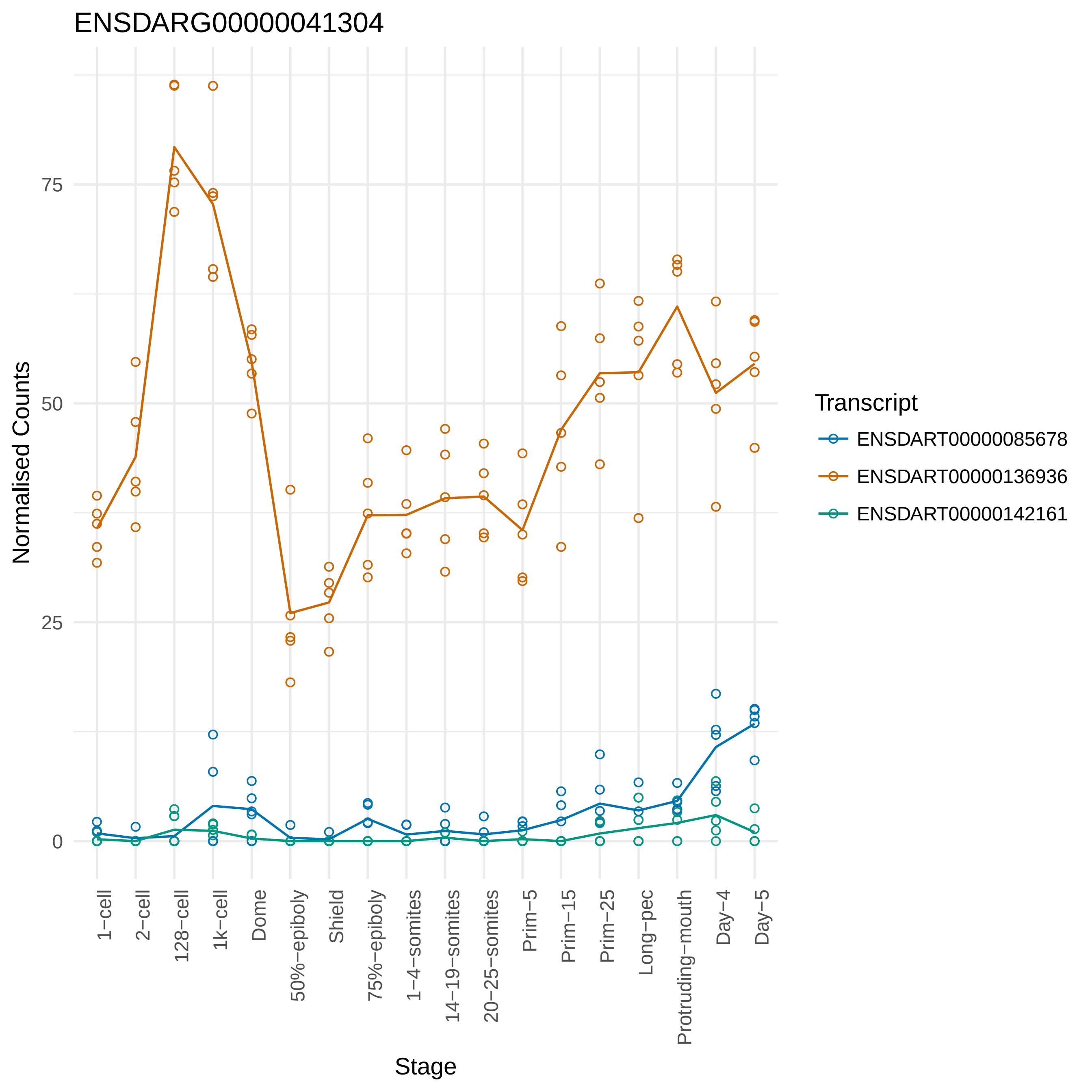

ENSDARG00000041304
Normalised Counts
75
50
25
0
1−cell
2−cell
128−cell
1k−cell
Dome
50%−epiboly
Shield
75%−epiboly
1−4−somites
14−19−somites
20−25−somites
Prim−5
Prim−15
Prim−25
Long−pec
Protruding−mouth
Day−4
Day−5
Stage
Transcript
ENSDART00000085678
ENSDART00000136936
ENSDART00000142161

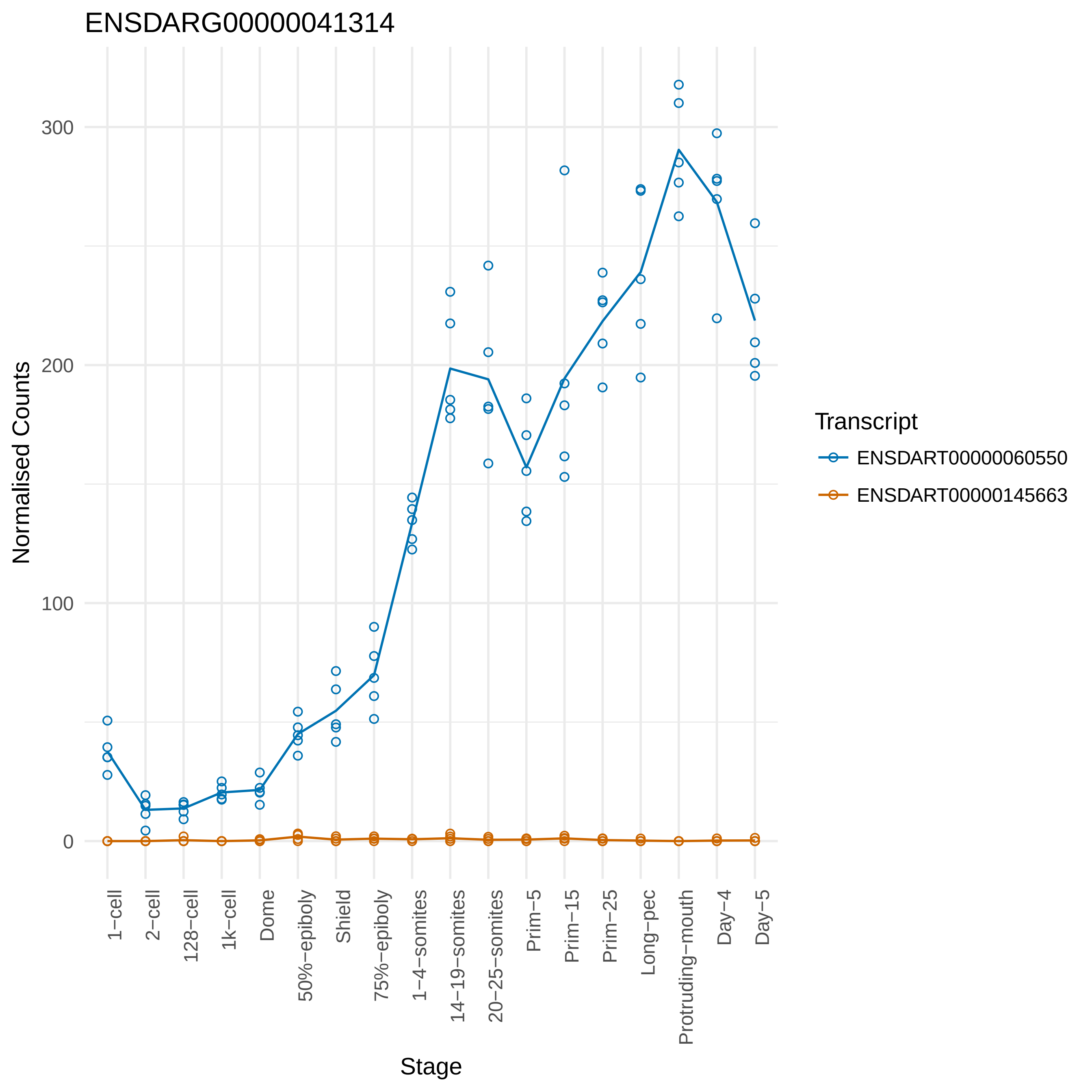

ENSDARG00000041314
Normalised Counts
300
200
100
0
1−cell
2−cell
128−cell
1k−cell
Dome
50%−epiboly
Shield
75%−epiboly
1−4−somites
14−19−somites
20−25−somites
Prim−5
Prim−15
Prim−25
Long−pec
Protruding−mouth
Day−4
Day−5
Stage
Transcript
ENSDART00000060550
ENSDART00000145663

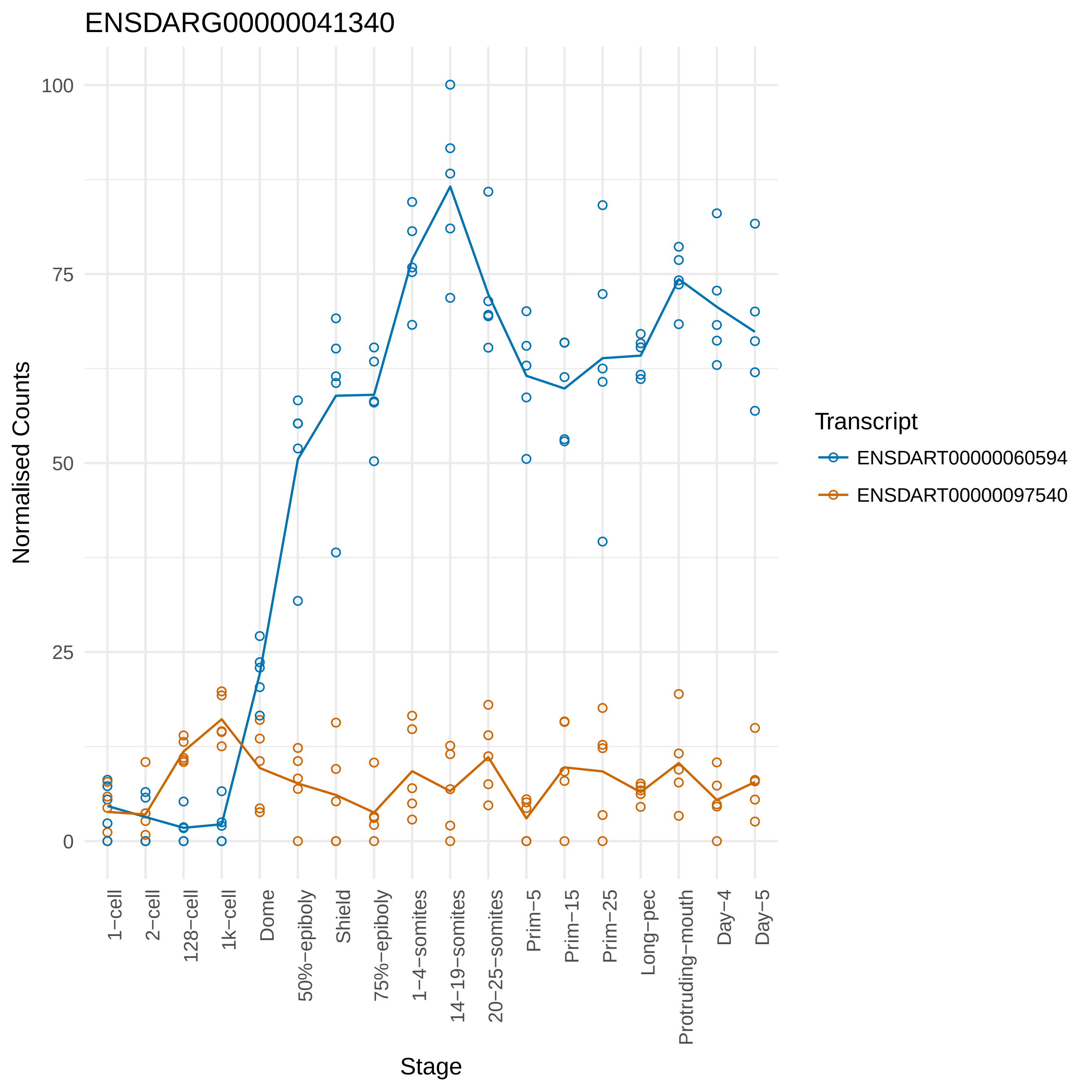
ENSDARG00000041340
Normalised Counts
100
75
50
25
0
1−cell
2−cell
128−cell
1k−cell
Dome
50%−epiboly
Shield
75%−epiboly
1−4−somites
14−19−somites
20−25−somites
Prim−5
Prim−15
Prim−25
Long−pec
Protruding−mouth
Day−4
Day−5
Stage
Transcript
ENSDART00000060594
ENSDART00000097540

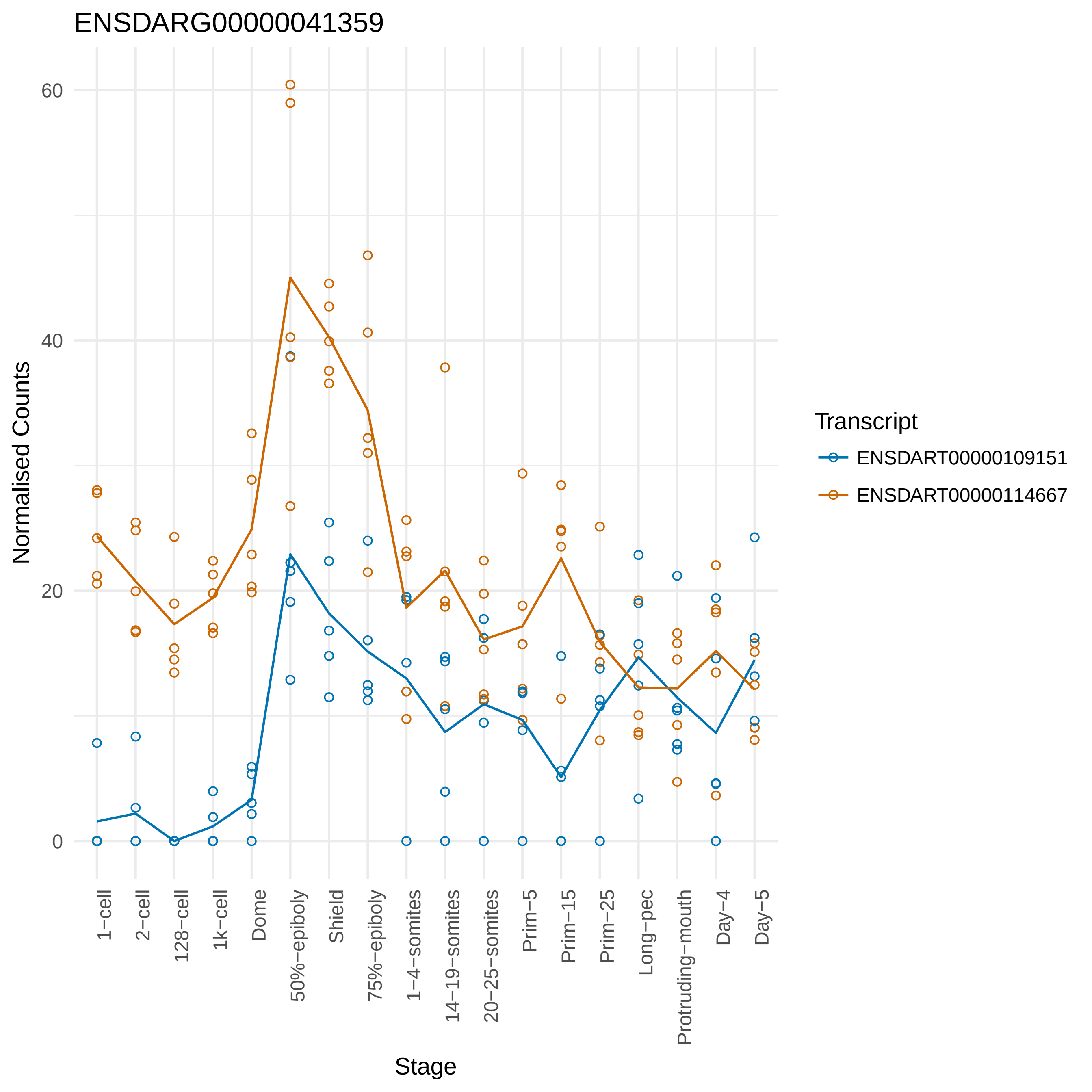
ENSDARG00000041359
Normalised Counts
0
20
40
60
Stage
1−cell
2−cell
128−cell
1k−cell
Dome
50%−epiboly
Shield
75%−epiboly
1−4−somites
14−19−somites
20−25−somites
Prim−5
Prim−15
Prim−25
Long−pec
Protruding−mouth
Day−4
Day−5
Transcript
ENSDART00000109151
ENSDART00000114667

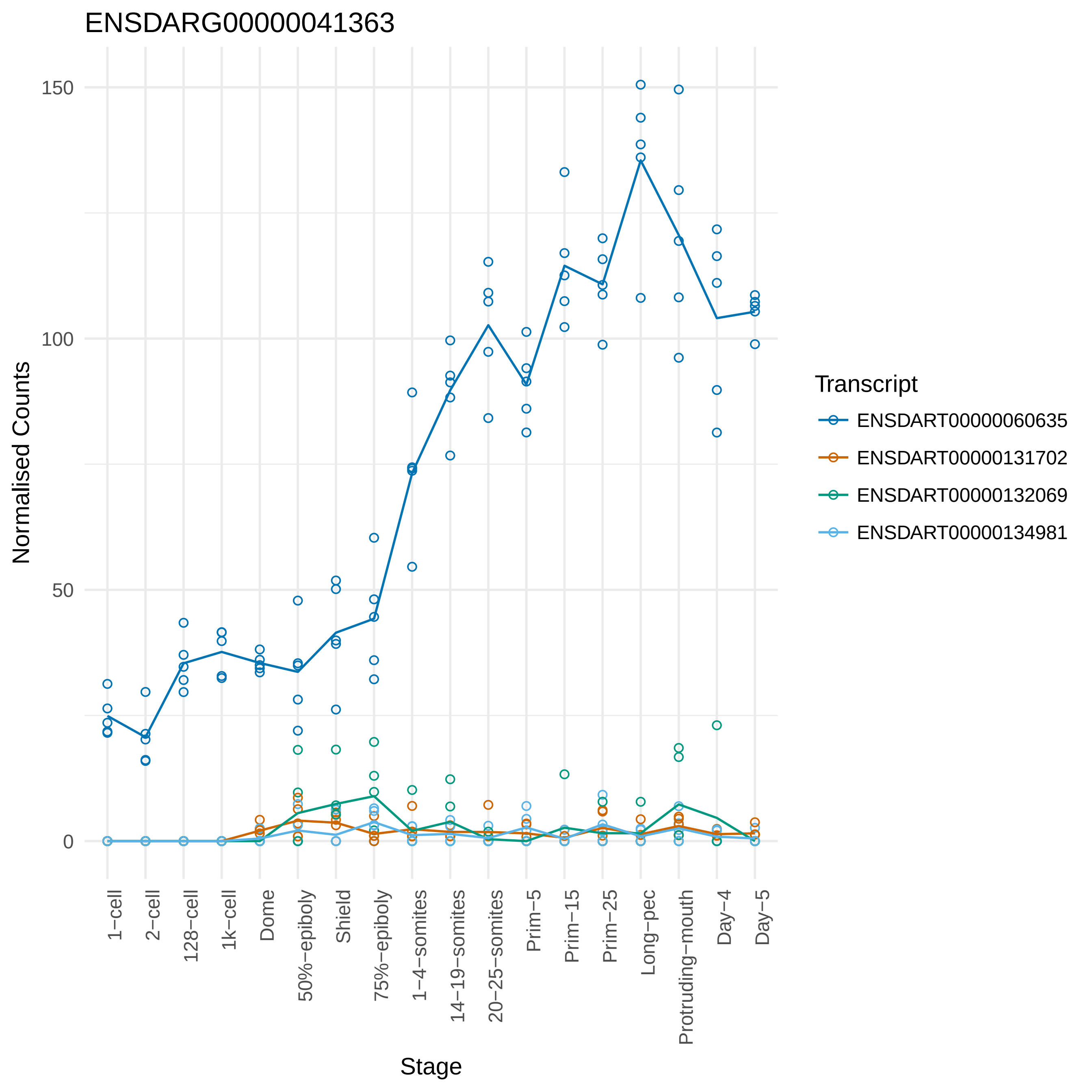

ENSDARG00000041363
Normalised Counts
150
100
50
0
1−cell
2−cell
128−cell
1k−cell
Dome
50%−epiboly
Shield
75%−epiboly
1−4−somites
14−19−somites
20−25−somites
Prim−5
Prim−15
Prim−25
Long−pec
Protruding−mouth
Day−4
Day−5
Stage
Transcript
ENSDART00000060635
ENSDART00000131702
ENSDART00000132069
ENSDART00000134981

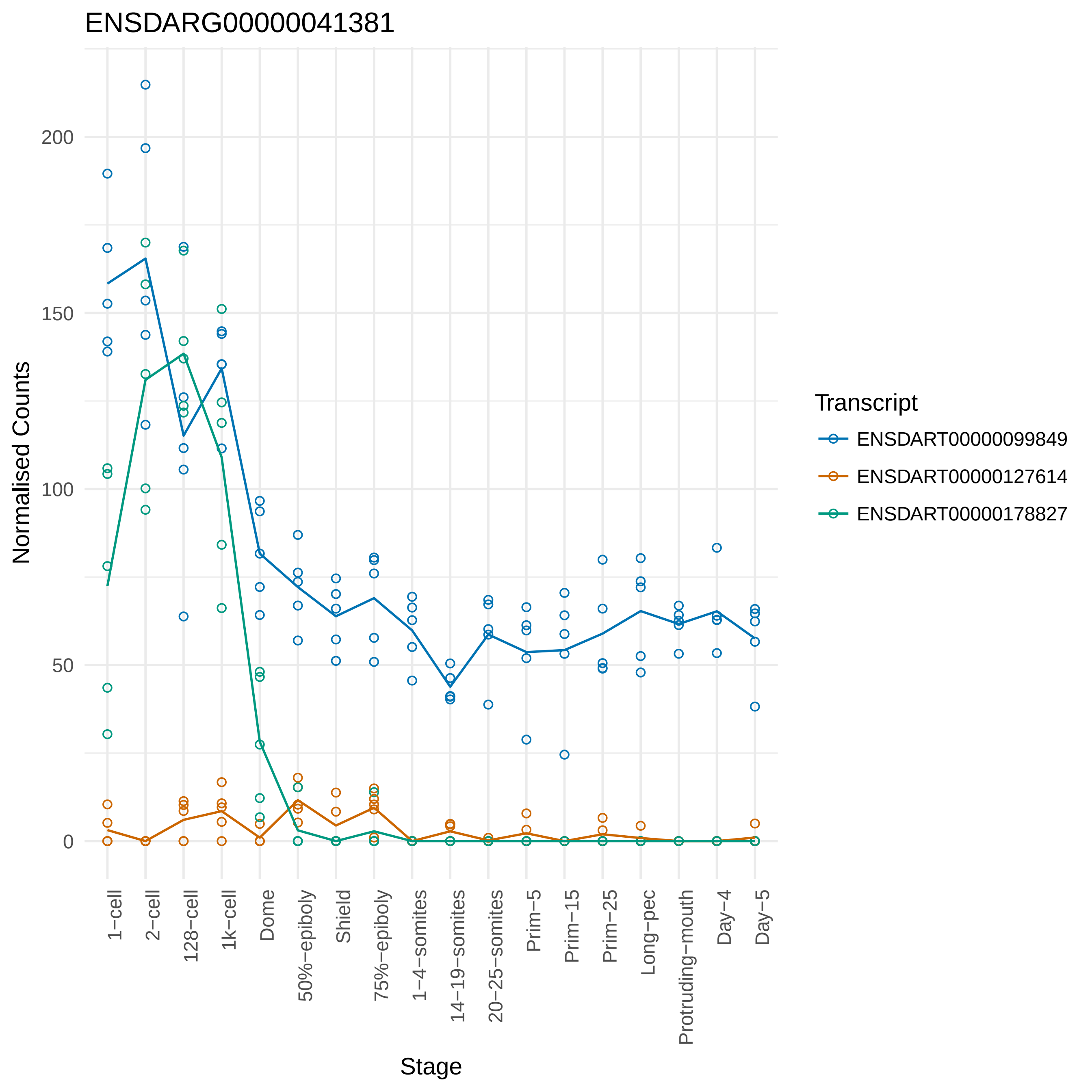

ENSDARG00000041381
Normalised Counts
200
150
100
50
0
1−cell
2−cell
128−cell
1k−cell
Dome
50%−epiboly
Shield
75%−epiboly
1−4−somites
14−19−somites
20−25−somites
Prim−5
Prim−15
Prim−25
Long−pec
Protruding−mouth
Day−4
Day−5
Stage
Transcript
ENSDART00000099849
ENSDART00000127614
ENSDART00000178827

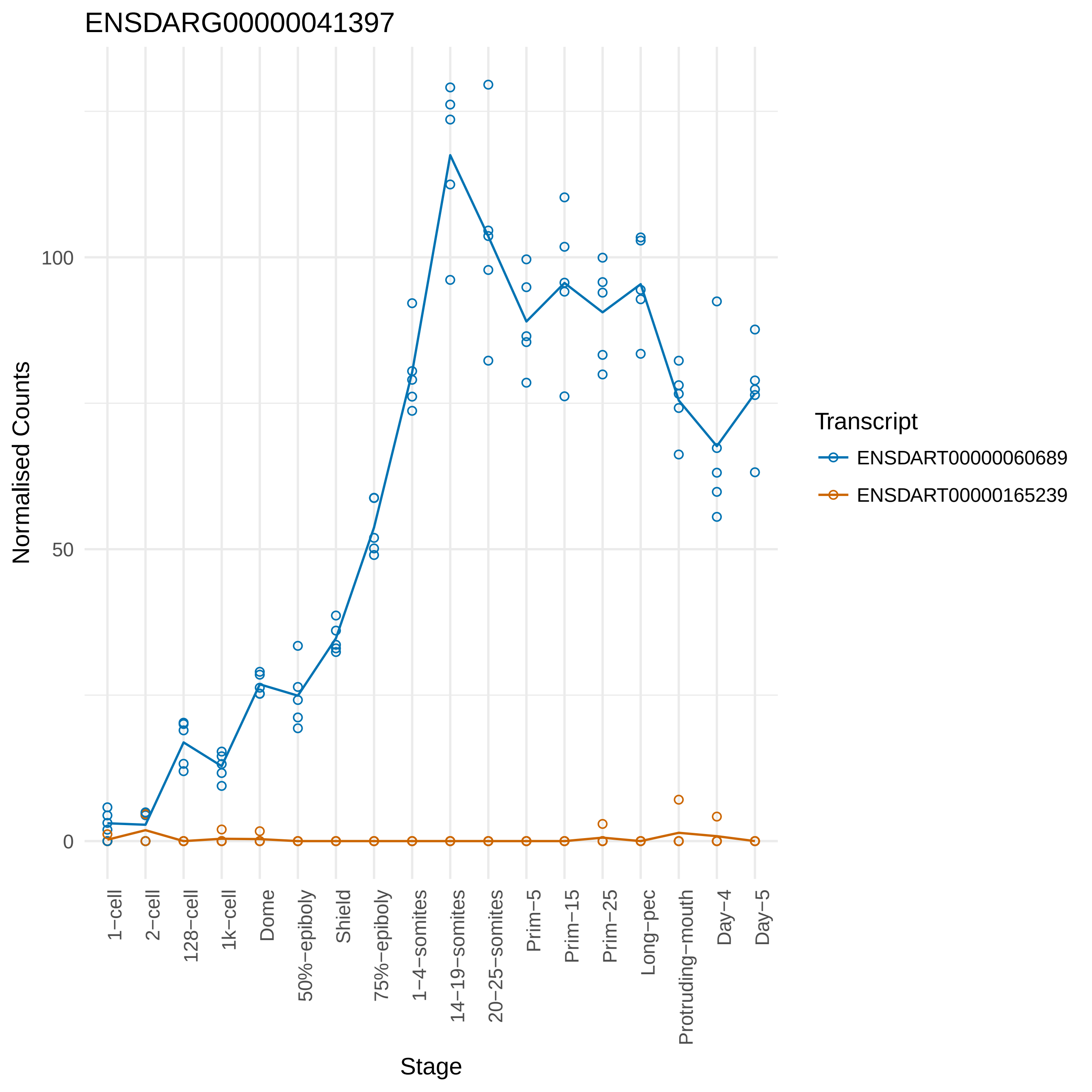

ENSDARG00000041397
Normalised Counts
100
50
0
1−cell
2−cell
128−cell
1k−cell
Dome
50%−epiboly
Shield
75%−epiboly
1−4−somites
14−19−somites
20−25−somites
Prim−5
Prim−15
Prim−25
Long−pec
Protruding−mouth
Day−4
Day−5
Stage
Transcript
ENSDART00000060689
ENSDART00000165239

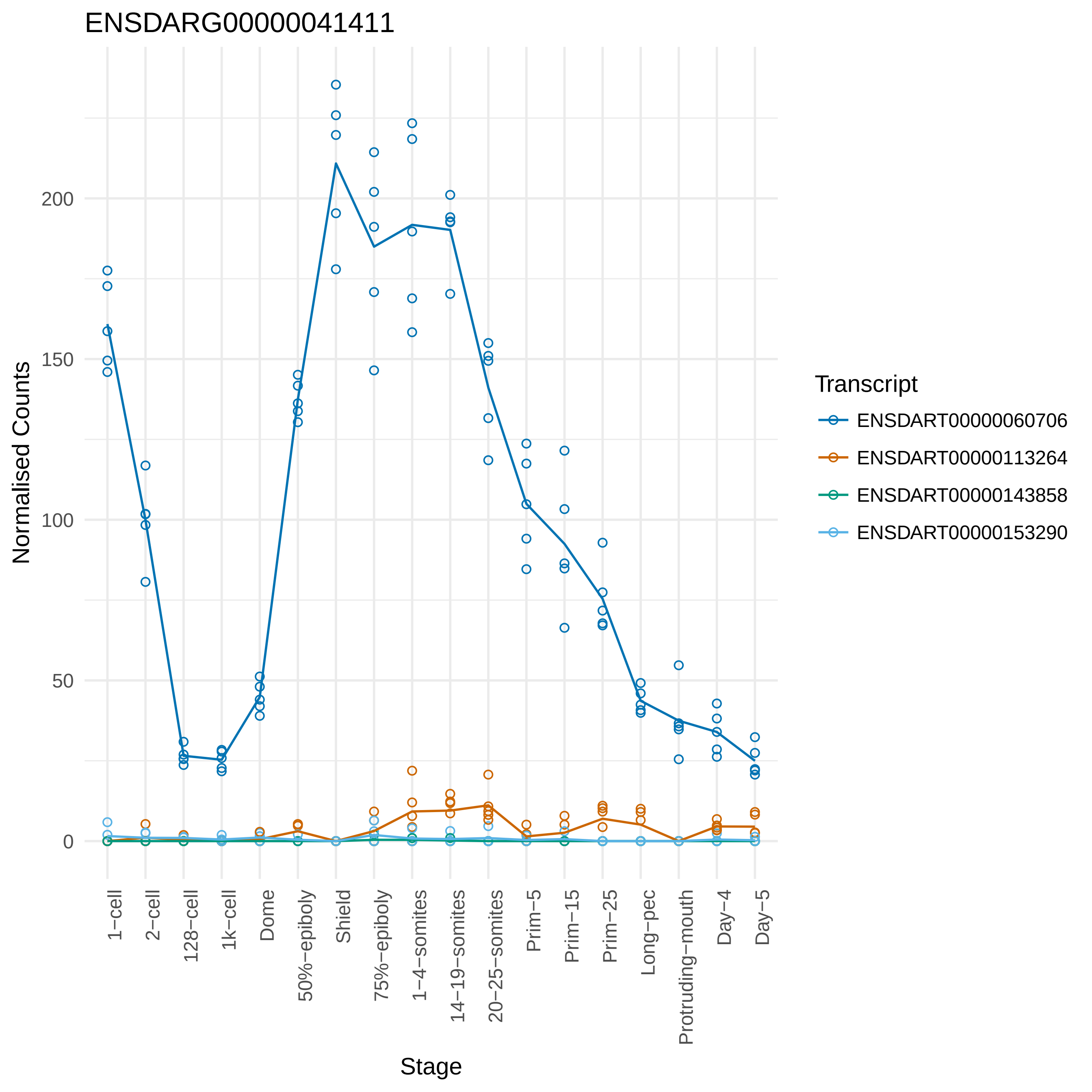

ENSDARG00000041411
Normalised Counts
0
50
100
150
200
Stage
1−cell
2−cell
128−cell
1k−cell
Dome
50%−epiboly
Shield
75%−epiboly
1−4−somites
14−19−somites
20−25−somites
Prim−5
Prim−15
Prim−25
Long−pec
Protruding−mouth
Day−4
Day−5
Transcript
ENSDART00000060706
ENSDART00000113264
ENSDART00000143858
ENSDART00000153290

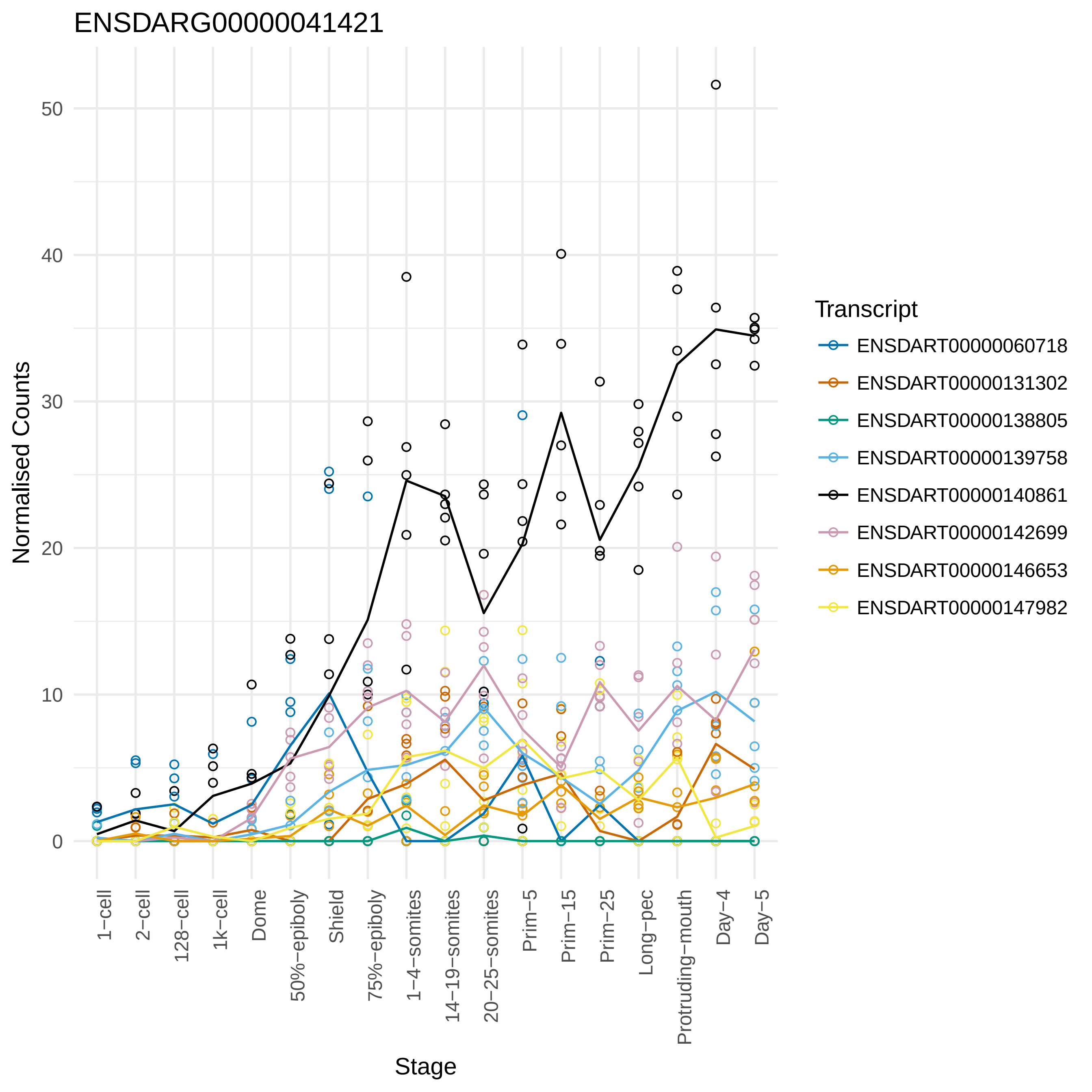

ENSDARG00000041421
Normalised Counts
0
10
20
30
40
50
Stage
1−cell
2−cell
128−cell
1k−cell
Dome
50%−epiboly
Shield
75%−epiboly
1−4−somites
14−19−somites
20−25−somites
Prim−5
Prim−15
Prim−25
Long−pec
Protruding−mouth
Day−4
Day−5
Transcript
ENSDART00000060718
ENSDART00000131302
ENSDART00000138805
ENSDART00000139758
ENSDART00000140861
ENSDART00000142699
ENSDART00000146653
ENSDART00000147982

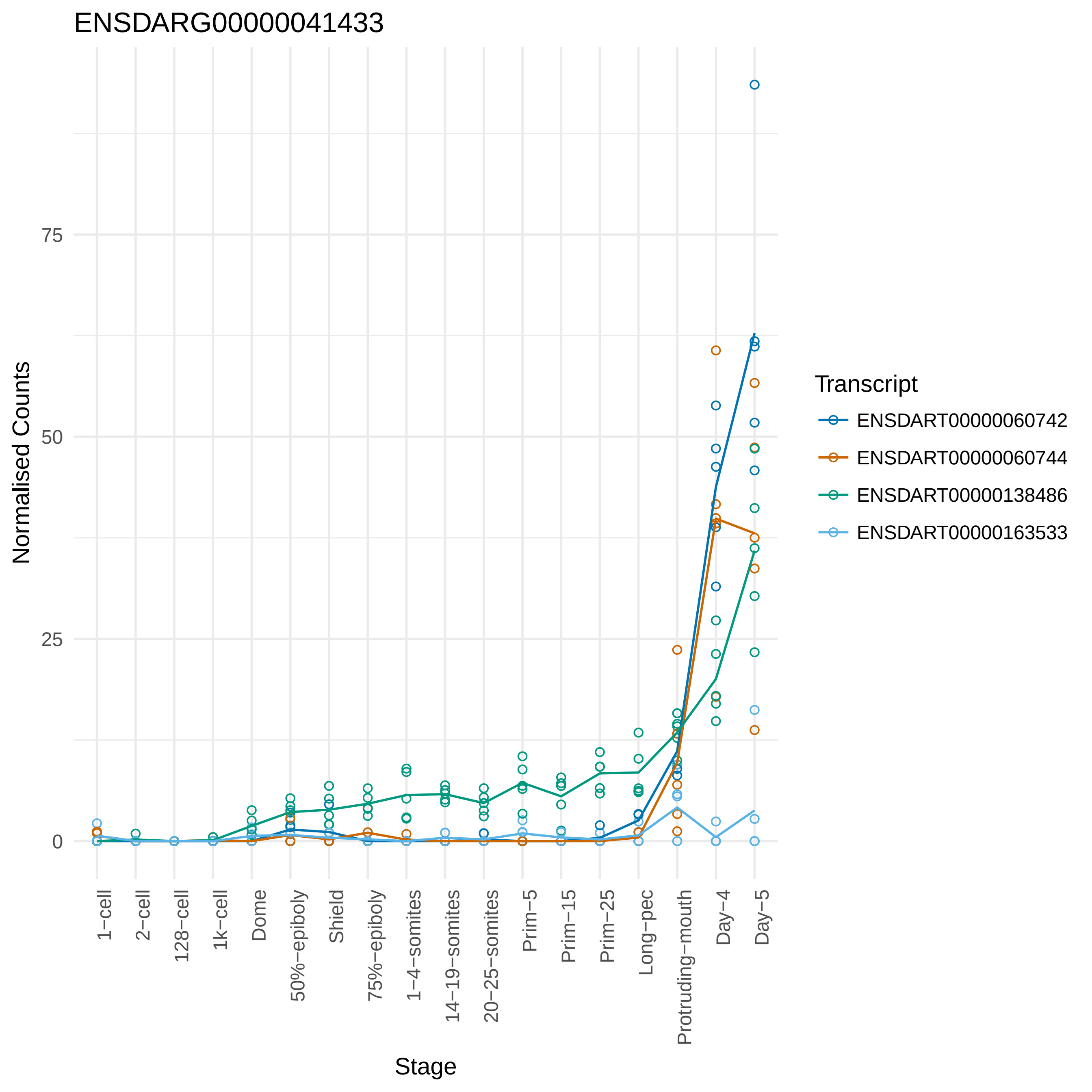

ENSDARG00000041433
Normalised Counts
75
50
25
0
1−cell
2−cell
128−cell
1k−cell
Dome
50%−epiboly
Shield
75%−epiboly
1−4−somites
14−19−somites
20−25−somites
Prim−5
Prim−15
Prim−25
Long−pec
Protruding−mouth
Day−4
Day−5
Stage
Transcript
ENSDART00000060742
ENSDART00000060744
ENSDART00000138486
ENSDART00000163533

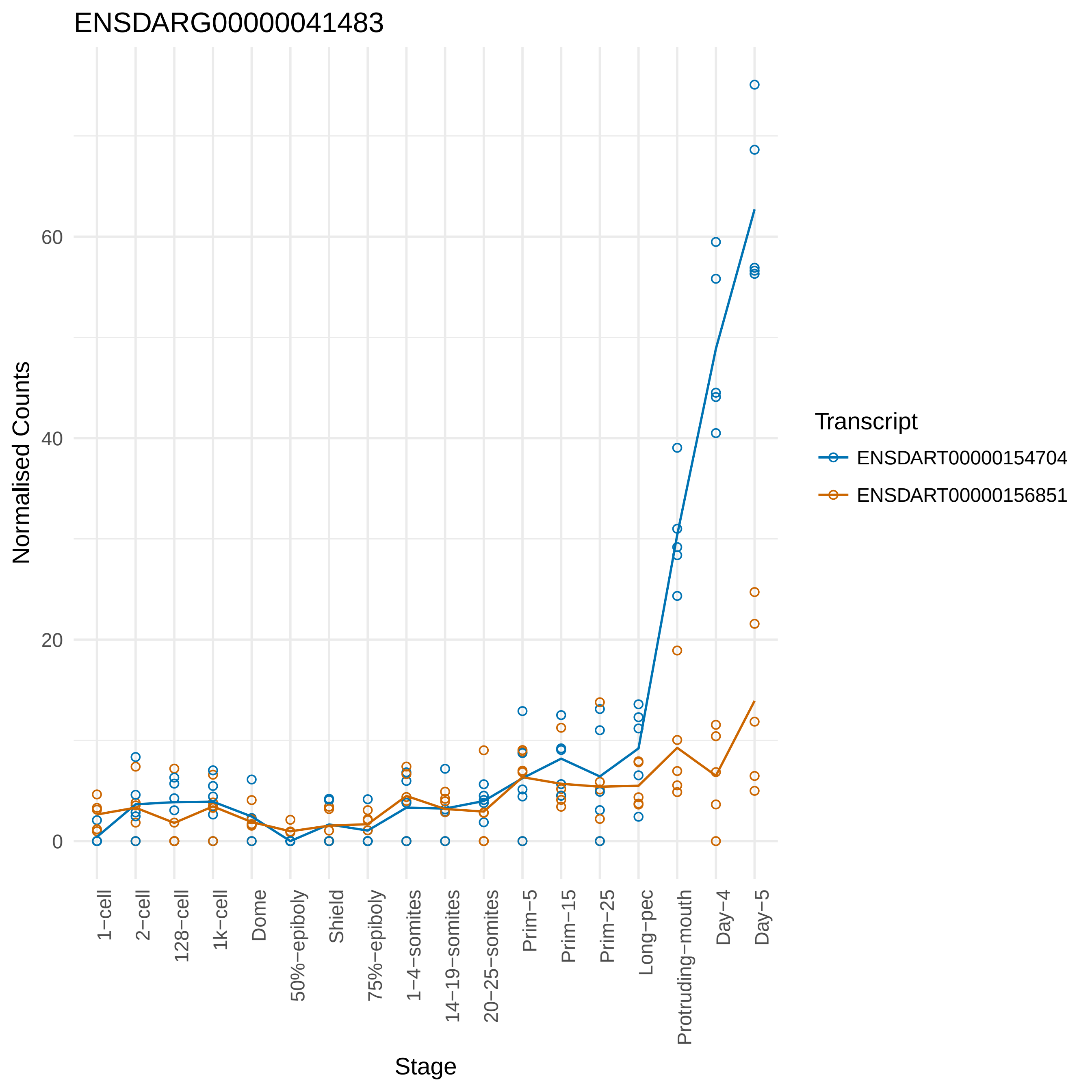

ENSDARG00000041483
Normalised Counts
0
20
40
60
1−cell
2−cell
128−cell
1k−cell
Dome
50%−epiboly
Shield
75%−epiboly
1−4−somites
14−19−somites
20−25−somites
Prim−5
Prim−15
Prim−25
Long−pec
Protruding−mouth
Day−4
Day−5
Stage
Transcript
ENSDART00000154704
ENSDART00000156851

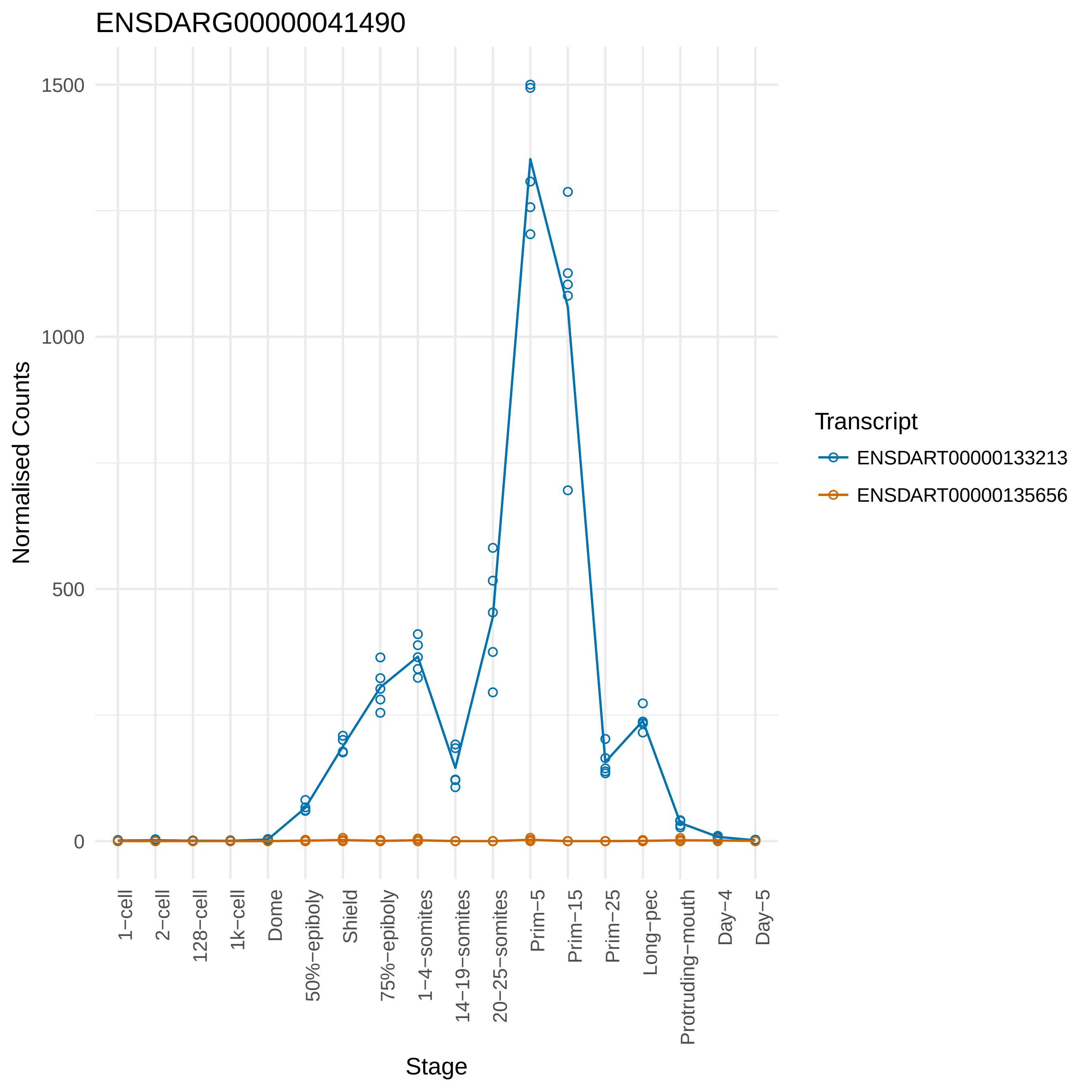
ENSDARG00000041490
Normalised Counts
1500
1000
500
0
1−cell
2−cell
128−cell
1k−cell
Dome
50%−epiboly
Shield
75%−epiboly
1−4−somites
14−19−somites
20−25−somites
Prim−5
Prim−15
Prim−25
Long−pec
Protruding−mouth
Day−4
Day−5
Stage
Transcript
ENSDART00000133213
ENSDART00000135656

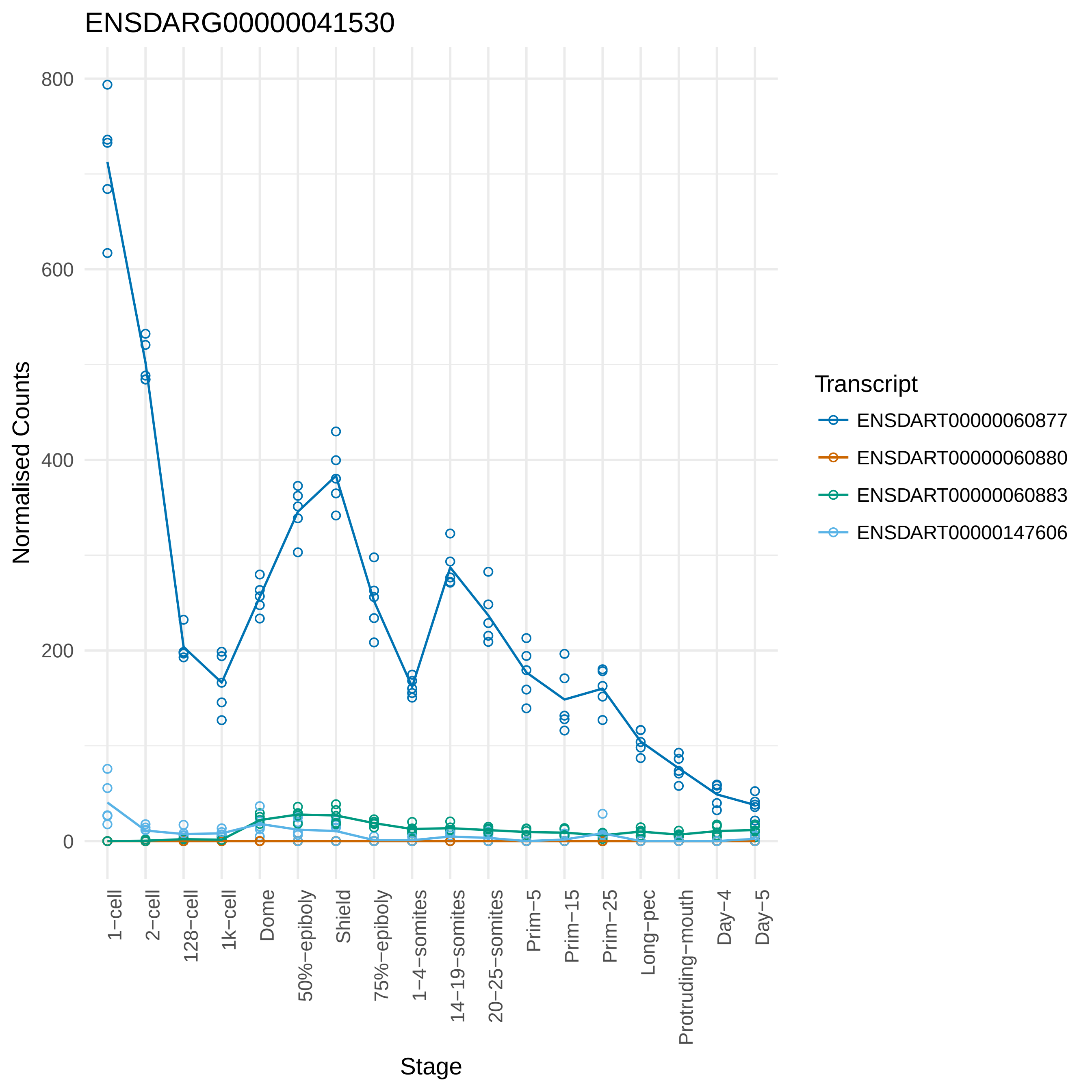
ENSDARG00000041530
Normalised Counts
800
600
400
200
0
1−cell
2−cell
128−cell
1k−cell
Dome
50%−epiboly
Shield
75%−epiboly
1−4−somites
14−19−somites
20−25−somites
Prim−5
Prim−15
Prim−25
Long−pec
Protruding−mouth
Day−4
Day−5
Stage
Transcript
ENSDART00000060877
ENSDART00000060880
ENSDART00000060883
ENSDART00000147606

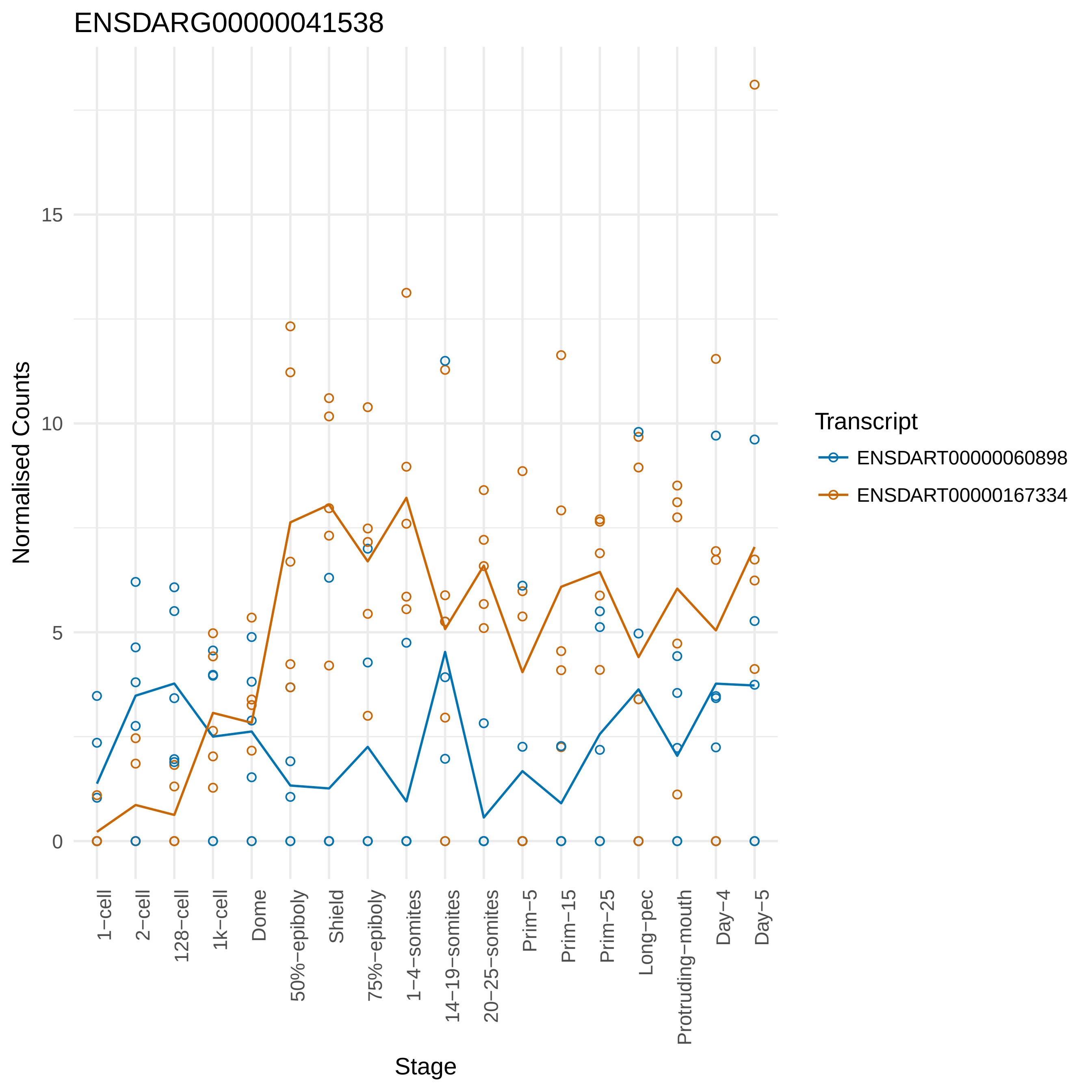
ENSDARG00000041538
Normalised Counts
0
5
10
15
1−cell
2−cell
128−cell
1k−cell
Dome
50%−epiboly
Shield
75%−epiboly
1−4−somites
14−19−somites
20−25−somites
Prim−5
Prim−15
Prim−25
Long−pec
Protruding−mouth
Day−4
Day−5
Stage
Transcript
ENSDART00000060898
ENSDART00000167334

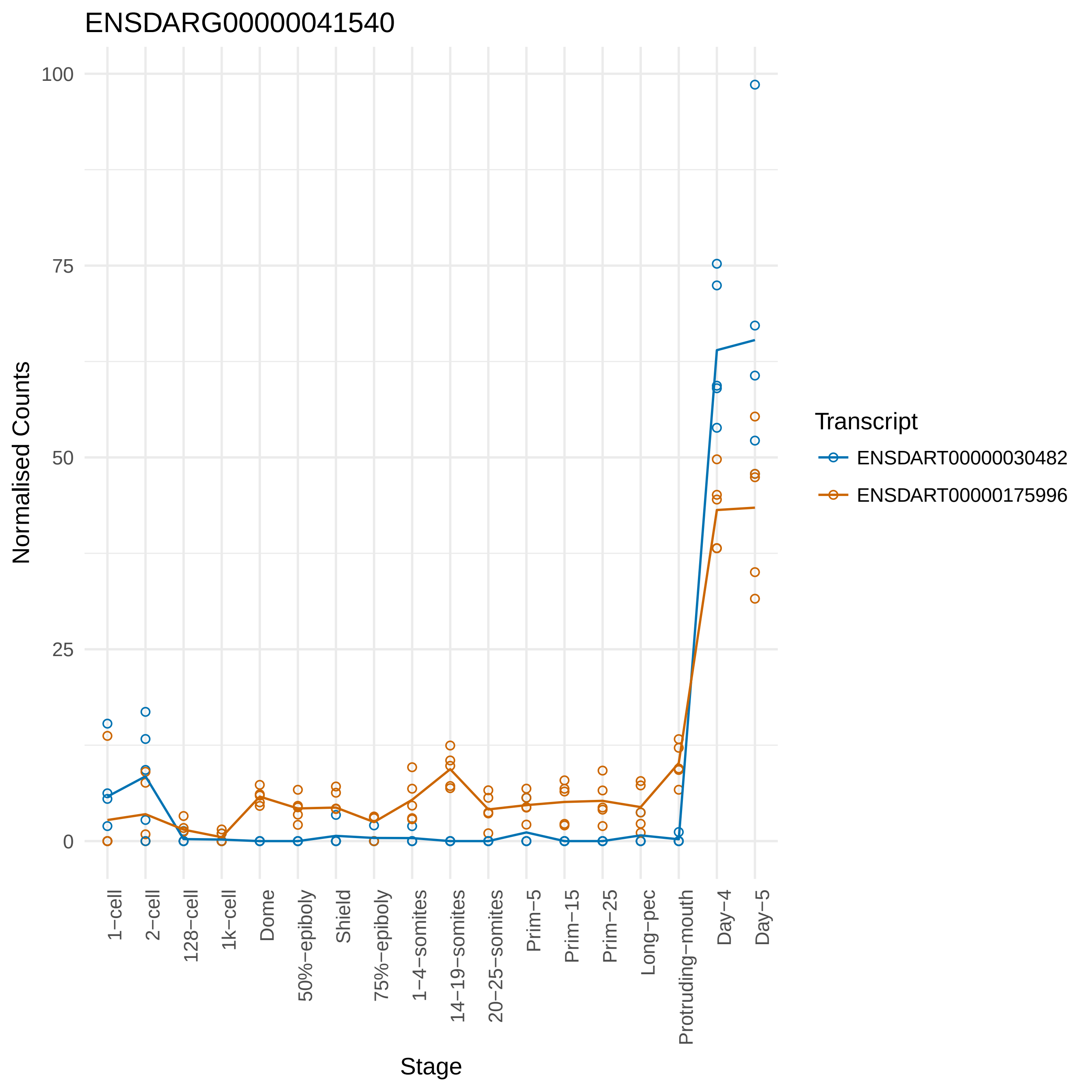
ENSDARG00000041540
Normalised Counts
100
75
50
25
0
1−cell
2−cell
128−cell
1k−cell
Dome
50%−epiboly
Shield
75%−epiboly
1−4−somites
14−19−somites
20−25−somites
Prim−5
Prim−15
Prim−25
Long−pec
Protruding−mouth
Day−4
Day−5
Stage
Transcript
ENSDART00000030482
ENSDART00000175996

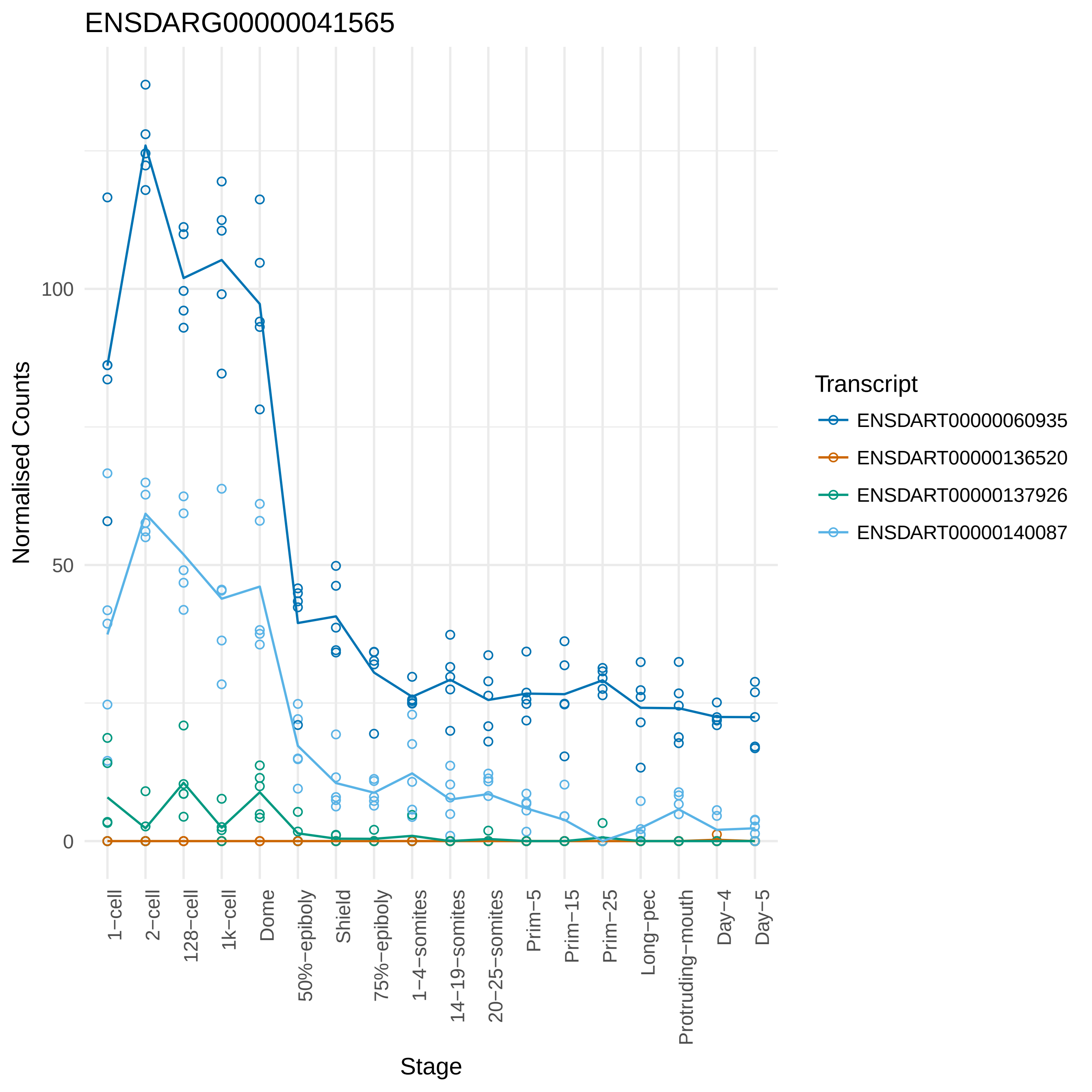

ENSDARG00000041565
Normalised Counts
100
50
0
Stage
1−cell
2−cell
128−cell
1k−cell
Dome
50%−epiboly
Shield
75%−epiboly
1−4−somites
14−19−somites
20−25−somites
Prim−5
Prim−15
Prim−25
Long−pec
Protruding−mouth
Day−4
Day−5
Transcript
ENSDART00000060935
ENSDART00000136520
ENSDART00000137926
ENSDART00000140087

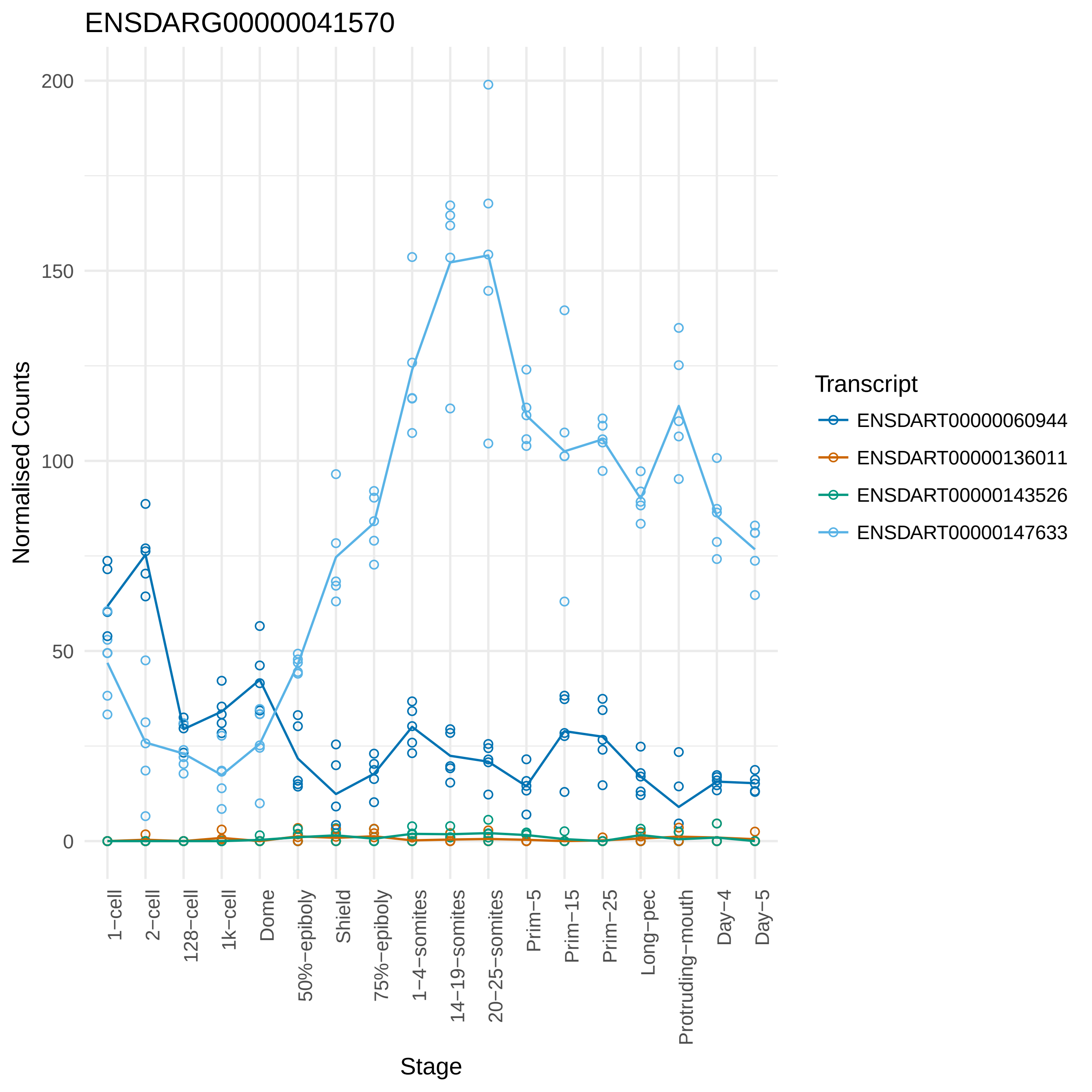

ENSDARG00000041570
Normalised Counts
200
150
100
50
0
1−cell
2−cell
128−cell
1k−cell
Dome
50%−epiboly
Shield
75%−epiboly
1−4−somites
14−19−somites
20−25−somites
Prim−5
Prim−15
Prim−25
Long−pec
Protruding−mouth
Day−4
Day−5
Stage
Transcript
ENSDART00000060944
ENSDART00000136011
ENSDART00000143526
ENSDART00000147633

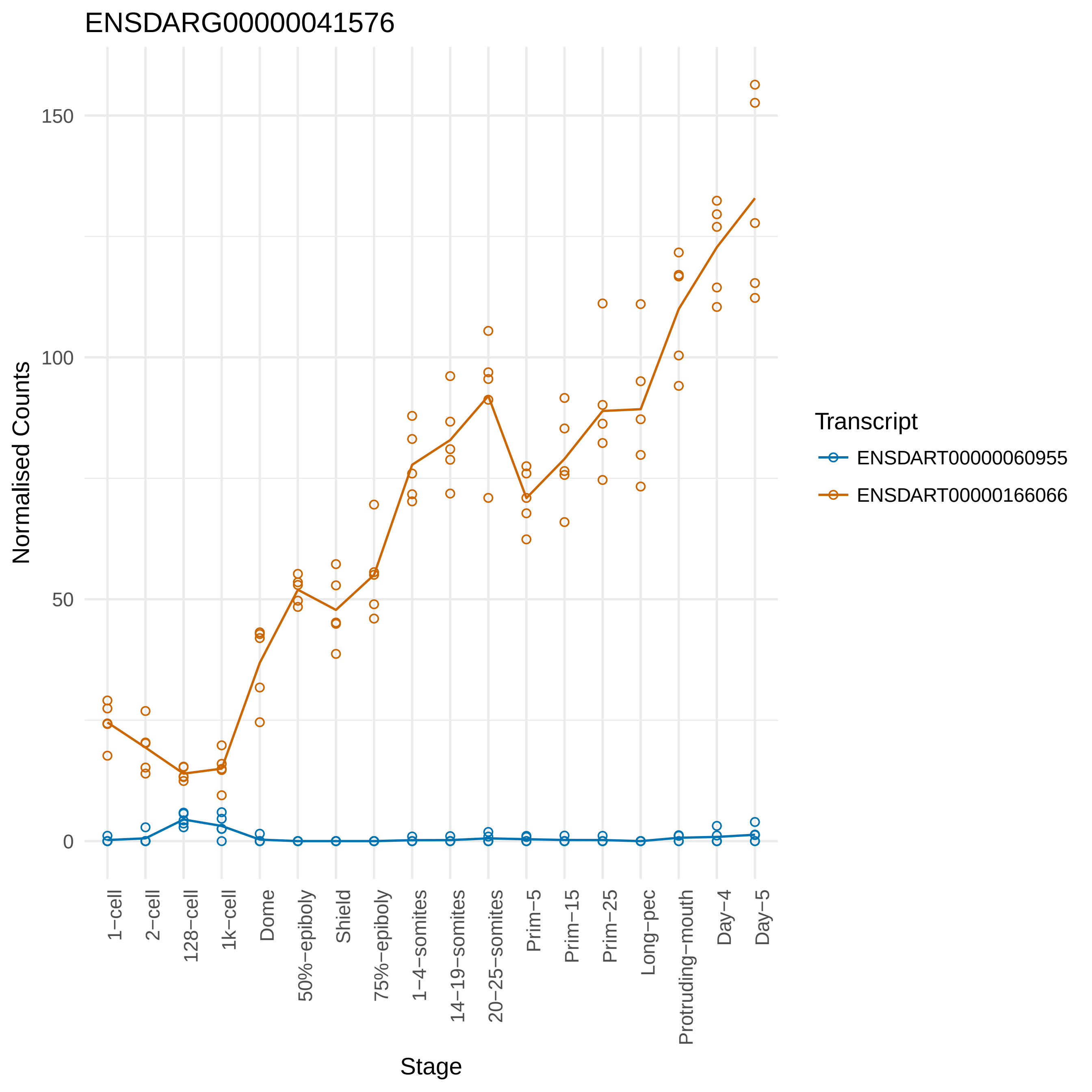

ENSDARG00000041576
Normalised Counts
150
100
50
0
1−cell
2−cell
128−cell
1k−cell
Dome
50%−epiboly
Shield
75%−epiboly
1−4−somites
14−19−somites
20−25−somites
Prim−5
Prim−15
Prim−25
Long−pec
Protruding−mouth
Day−4
Day−5
Stage
Transcript
ENSDART00000060955
ENSDART00000166066

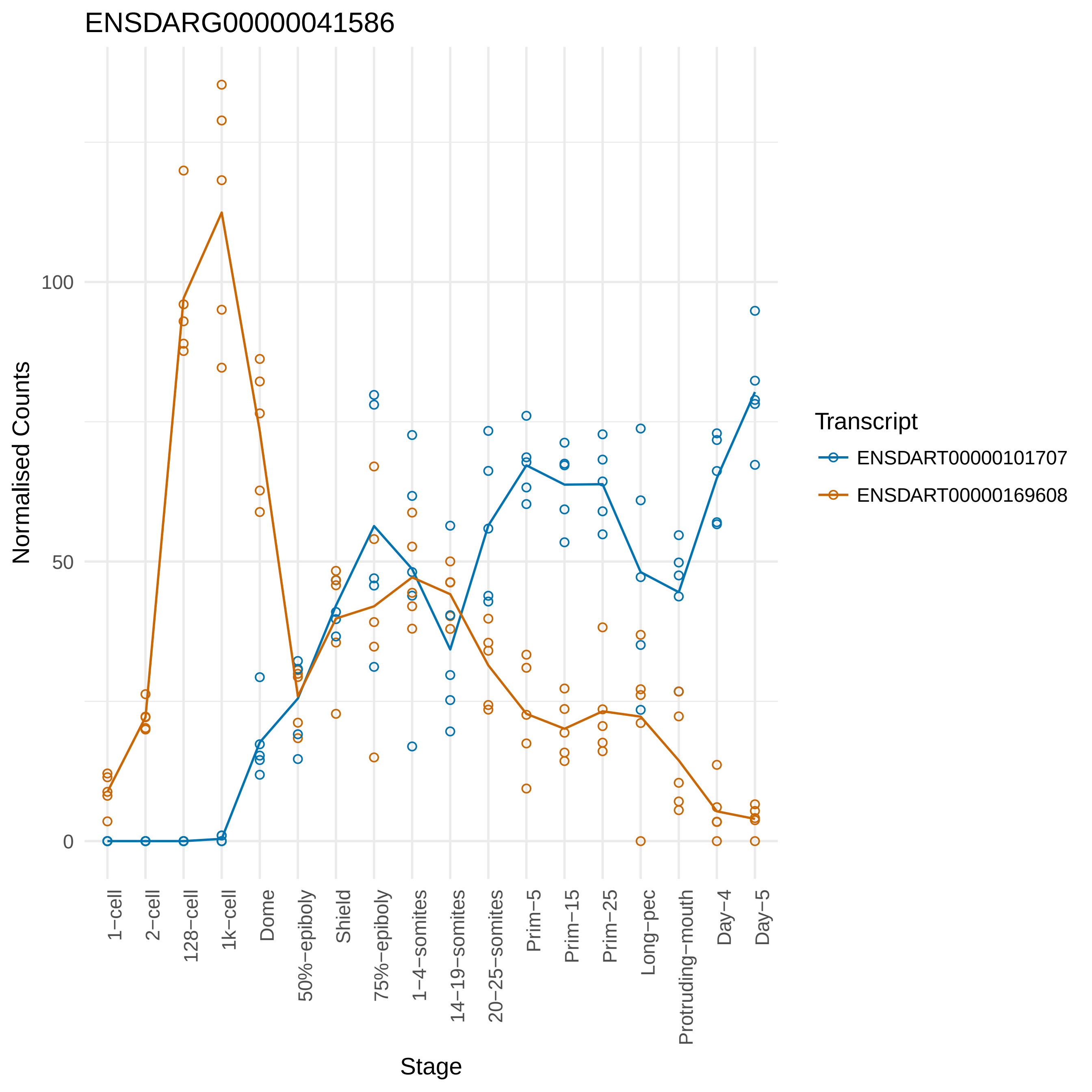

ENSDARG00000041586
Normalised Counts
100
50
0
1−cell
2−cell
128−cell
1k−cell
Dome
50%−epiboly
Shield
75%−epiboly
1−4−somites
14−19−somites
20−25−somites
Prim−5
Prim−15
Prim−25
Long−pec
Protruding−mouth
Day−4
Day−5
Stage
Transcript
ENSDART00000101707
ENSDART00000169608

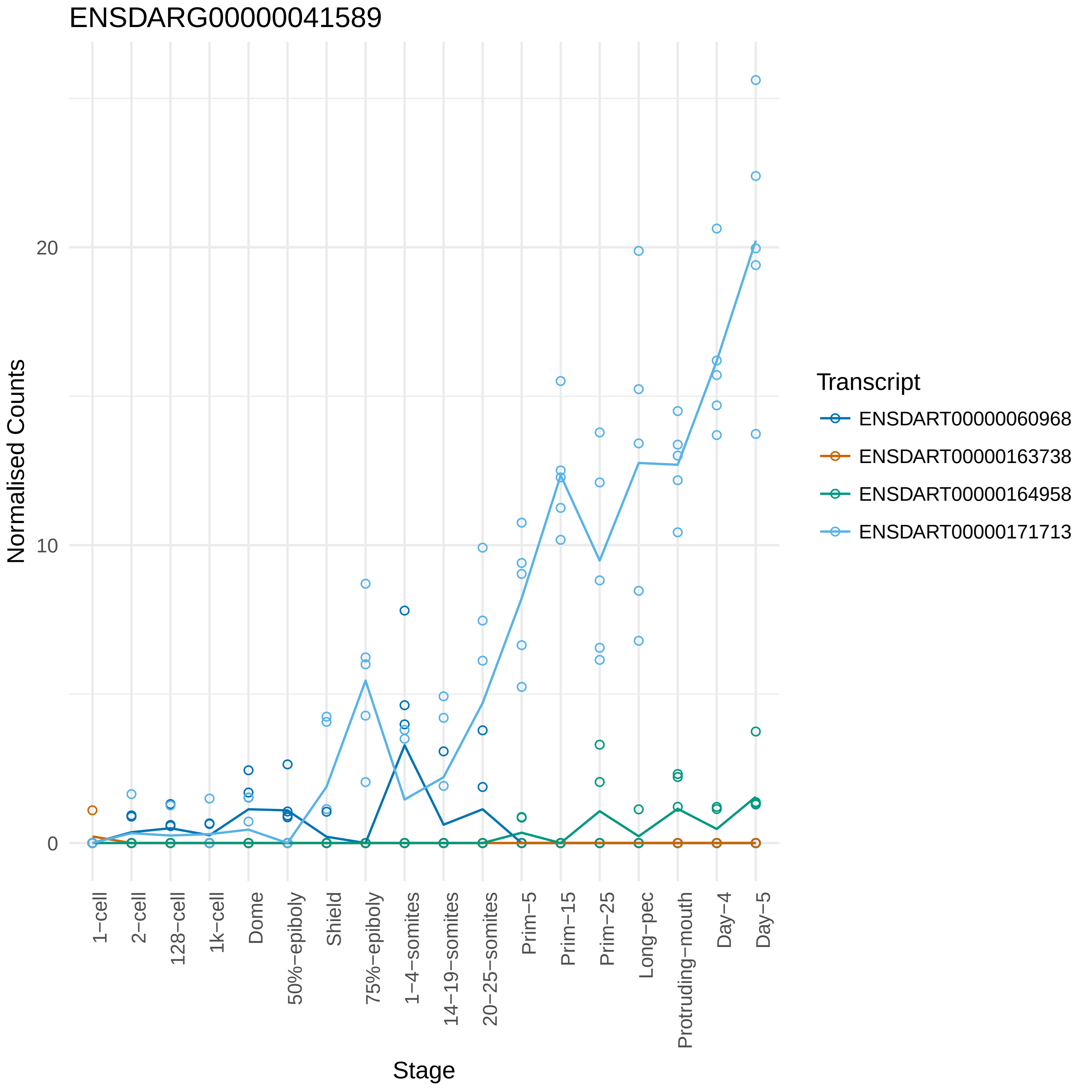

ENSDARG00000041589
Normalised Counts
20
10
0
1−cell
2−cell
128−cell
1k−cell
Dome
50%−epiboly
Shield
75%−epiboly
1−4−somites
14−19−somites
20−25−somites
Prim−5
Prim−15
Prim−25
Long−pec
Protruding−mouth
Day−4
Day−5
Stage
Transcript
ENSDART00000060968
ENSDART00000163738
ENSDART00000164958
ENSDART00000171713

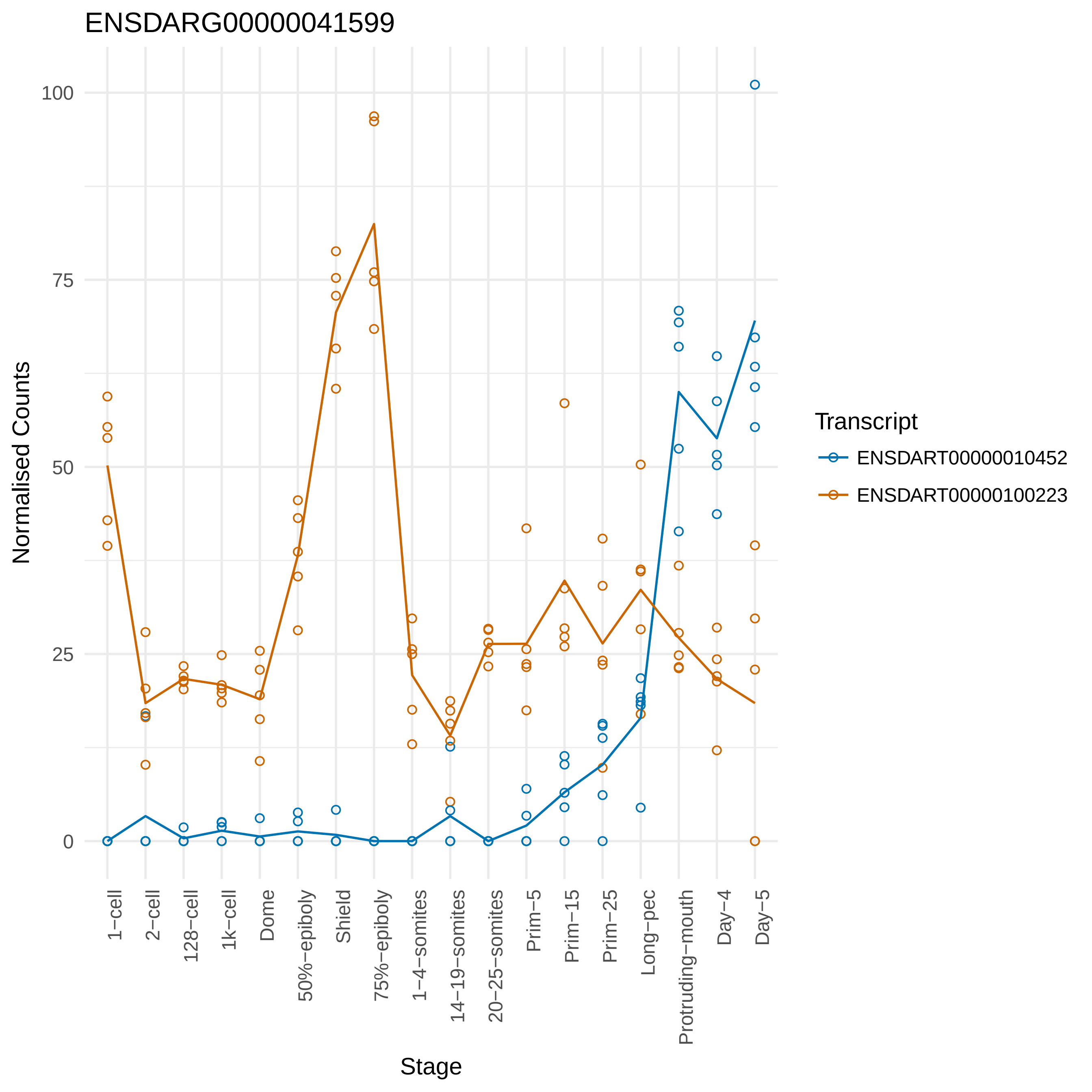
ENSDARG00000041599
Normalised Counts
100
75
50
25
0
1−cell
2−cell
128−cell
1k−cell
Dome
50%−epiboly
Shield
75%−epiboly
1−4−somites
14−19−somites
20−25−somites
Prim−5
Prim−15
Prim−25
Long−pec
Protruding−mouth
Day−4
Day−5
Stage
Transcript
ENSDART00000010452
ENSDART00000100223

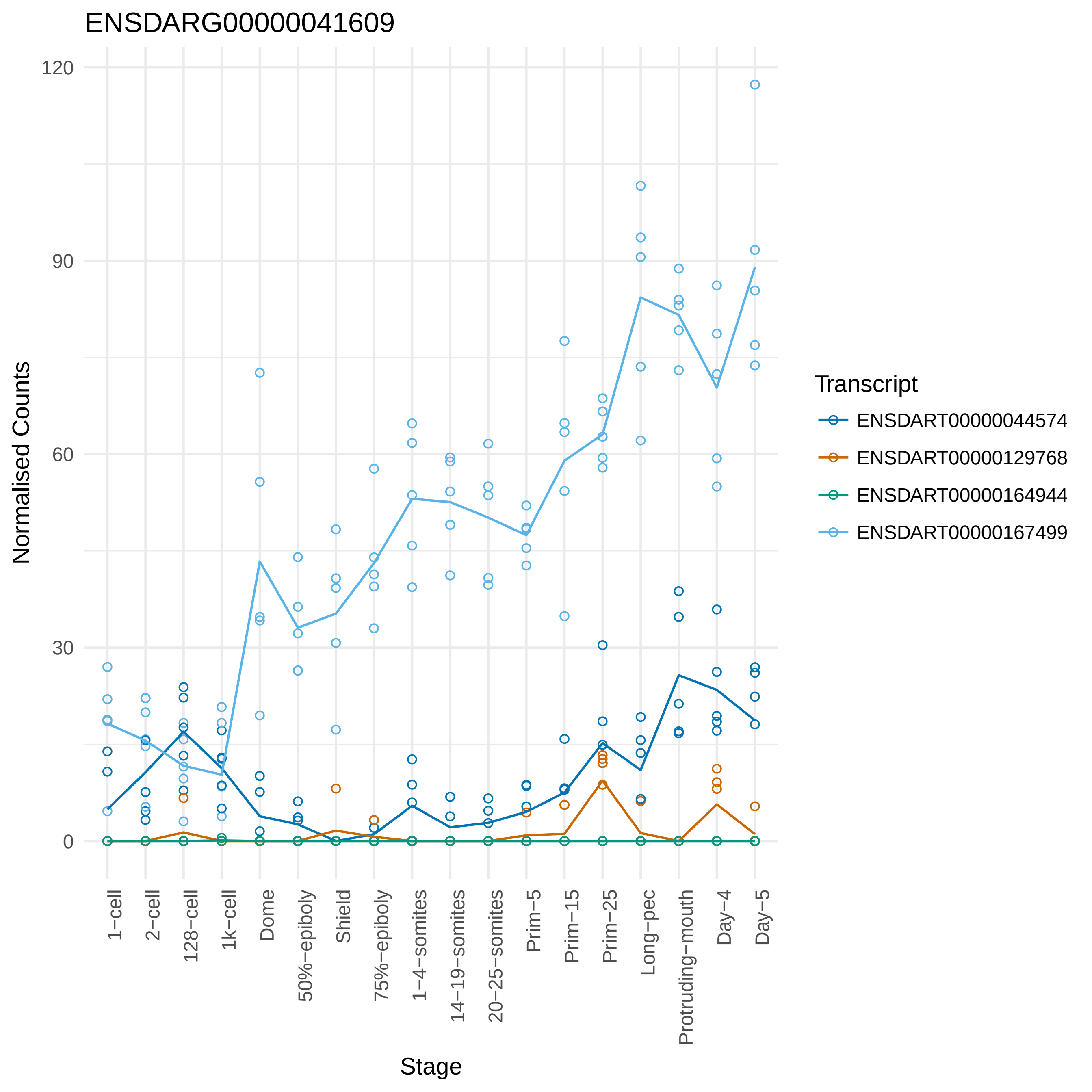

ENSDARG00000041609
Normalised Counts
120
90
60
30
0
1−cell
2−cell
128−cell
1k−cell
Dome
50%−epiboly
Shield
75%−epiboly
1−4−somites
14−19−somites
20−25−somites
Prim−5
Prim−15
Prim−25
Long−pec
Protruding−mouth
Day−4
Day−5
Stage
Transcript
ENSDART00000044574
ENSDART00000129768
ENSDART00000164944
ENSDART00000167499

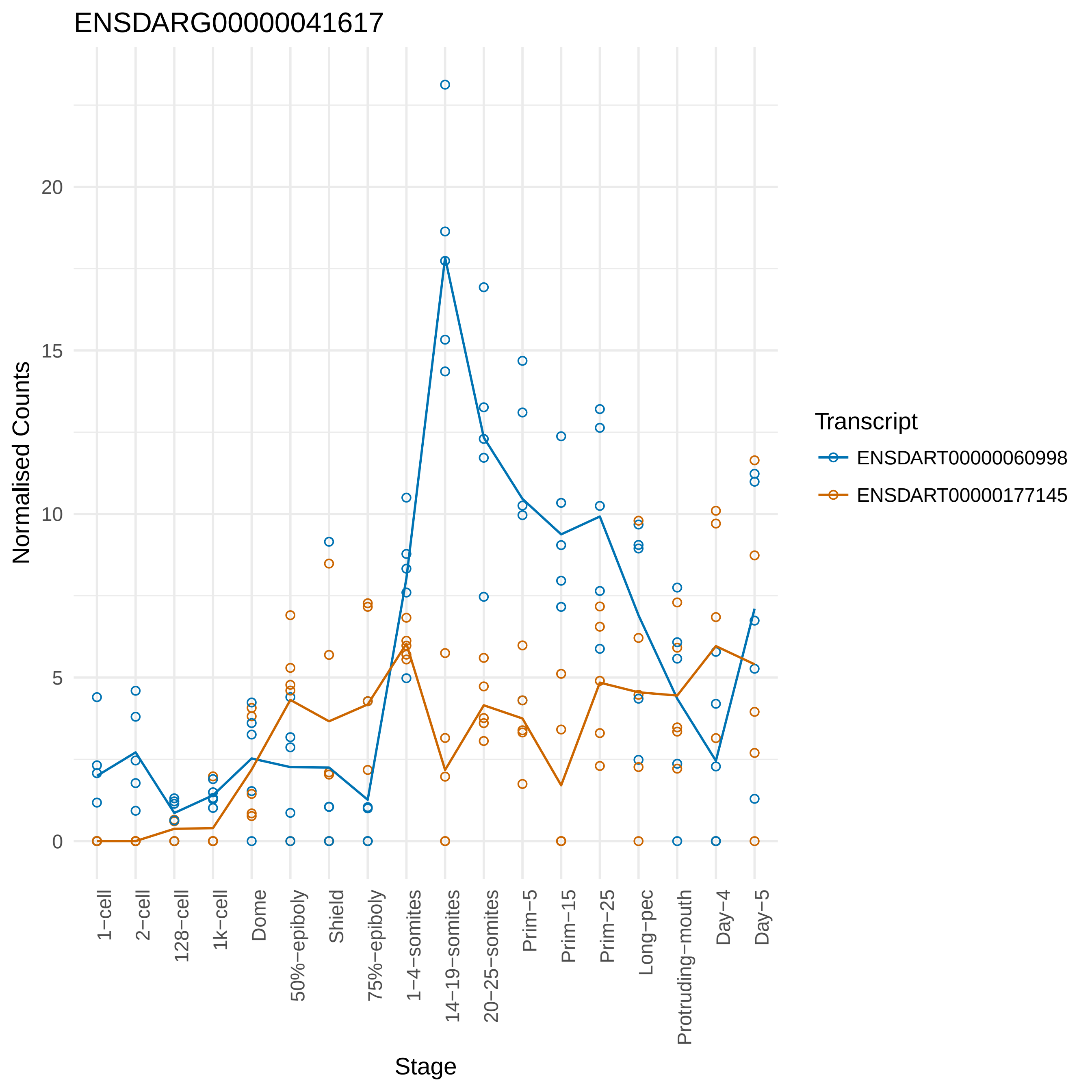

ENSDARG00000041617
Normalised Counts
0
5
10
15
20
Stage
1−cell
2−cell
128−cell
1k−cell
Dome
50%−epiboly
Shield
75%−epiboly
1−4−somites
14−19−somites
20−25−somites
Prim−5
Prim−15
Prim−25
Long−pec
Protruding−mouth
Day−4
Day−5
Transcript
ENSDART00000060998
ENSDART00000177145

ENSDARG00000041631

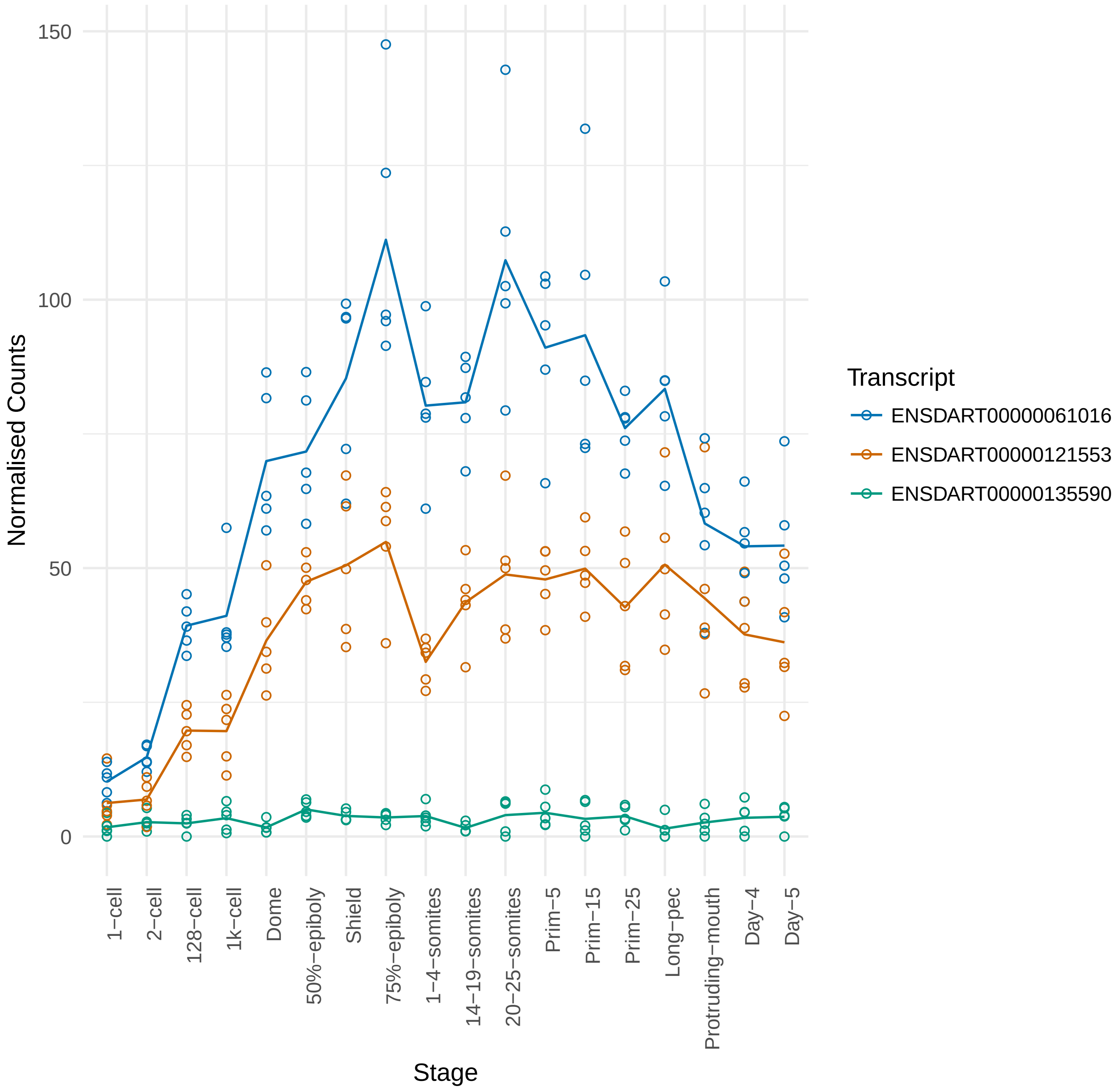

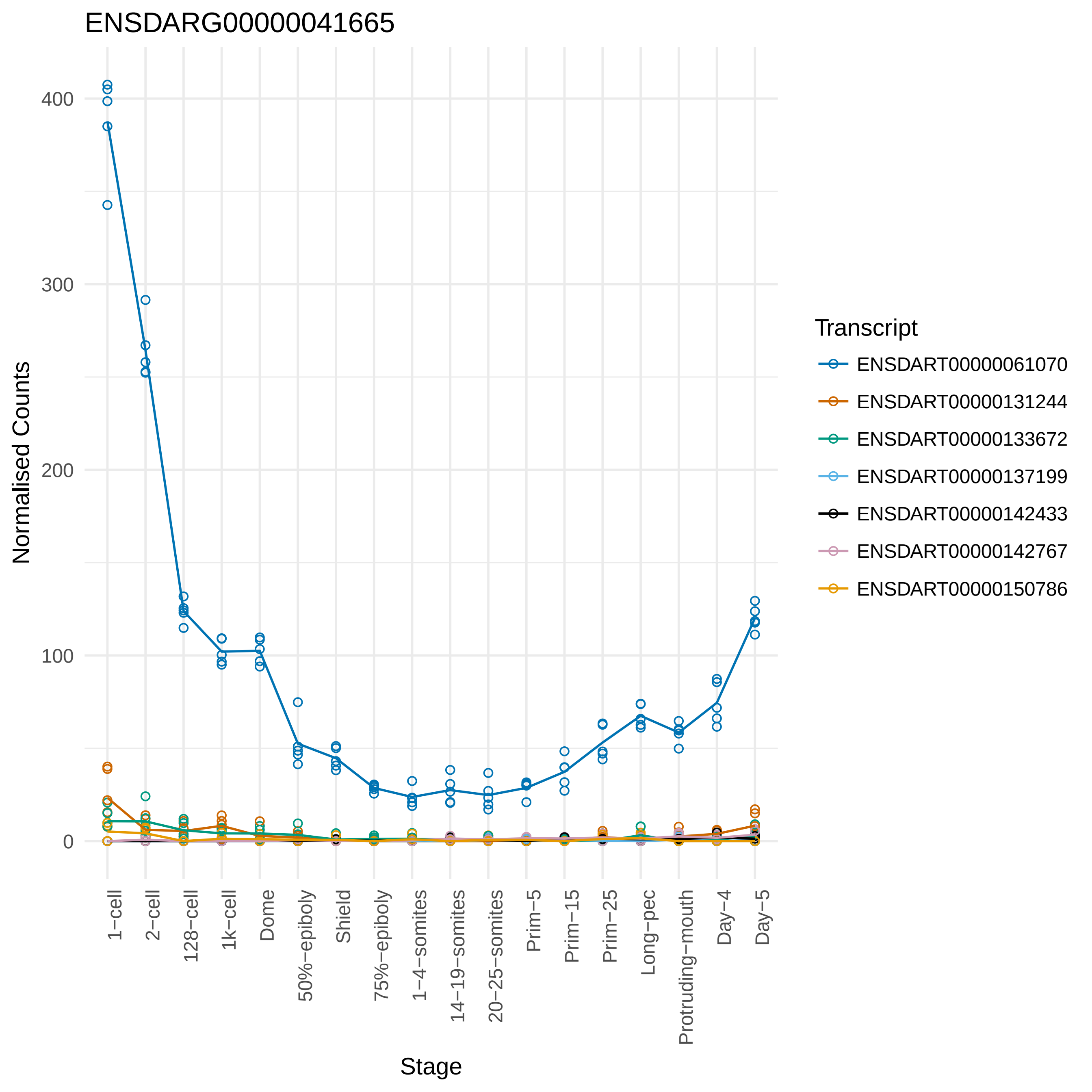

ENSDARG00000041665
Normalised Counts
400
300
200
100
0
1−cell
2−cell
128−cell
1k−cell
Dome
50%−epiboly
Shield
75%−epiboly
1−4−somites
14−19−somites
20−25−somites
Prim−5
Prim−15
Prim−25
Long−pec
Protruding−mouth
Day−4
Day−5
Stage
Transcript
ENSDART00000061070
ENSDART00000131244
ENSDART00000133672
ENSDART00000137199
ENSDART00000142433
ENSDART00000142767
ENSDART00000150786

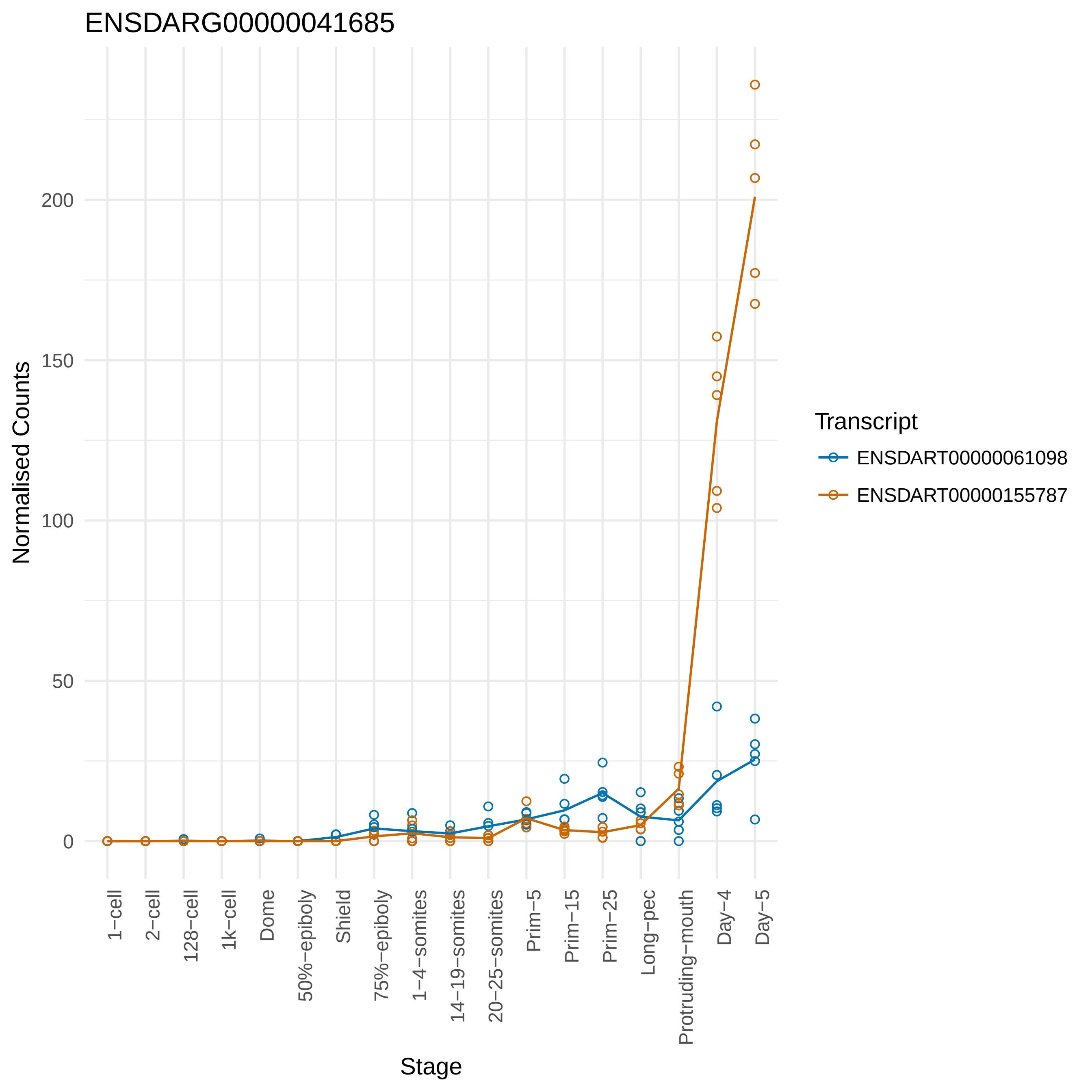

ENSDARG00000041685
Normalised Counts
200
150
100
50
0
1−cell
2−cell
128−cell
1k−cell
Dome
50%−epiboly
Shield
75%−epiboly
1−4−somites
14−19−somites
20−25−somites
Prim−5
Prim−15
Prim−25
Long−pec
Protruding−mouth
Day−4
Day−5
Stage
Transcript
ENSDART00000061098
ENSDART00000155787

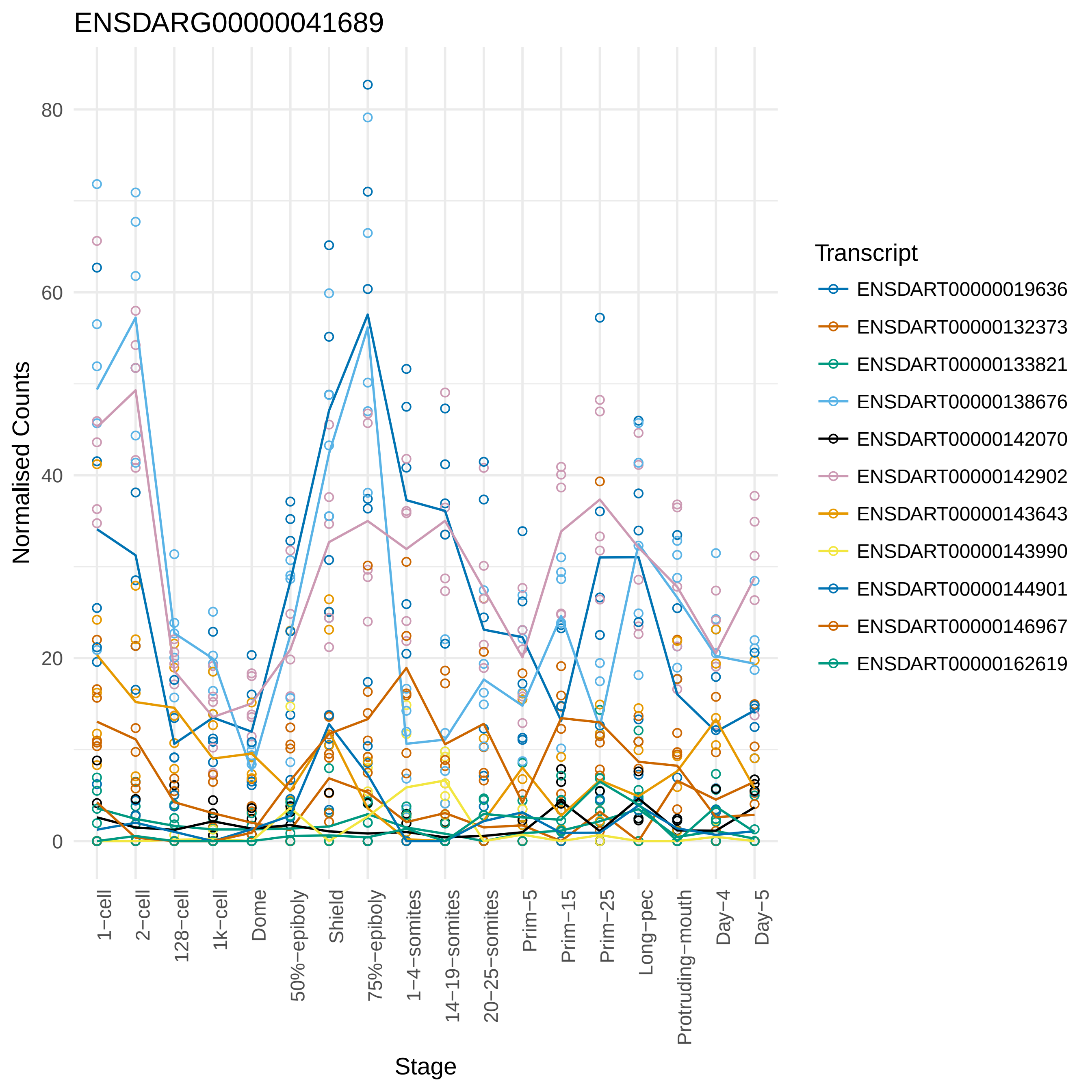

ENSDARG00000041689
Normalised Counts
0
20
40
60
80
Stage
1−cell
2−cell
128−cell
1k−cell
Dome
50%−epiboly
Shield
75%−epiboly
1−4−somites
14−19−somites
20−25−somites
Prim−5
Prim−15
Prim−25
Long−pec
Protruding−mouth
Day−4
Day−5
Transcript
ENSDART00000019636
ENSDART00000132373
ENSDART00000133821
ENSDART00000138676
ENSDART00000142070
ENSDART00000142902
ENSDART00000143643
ENSDART00000143990
ENSDART00000144901
ENSDART00000146967
ENSDART00000162619

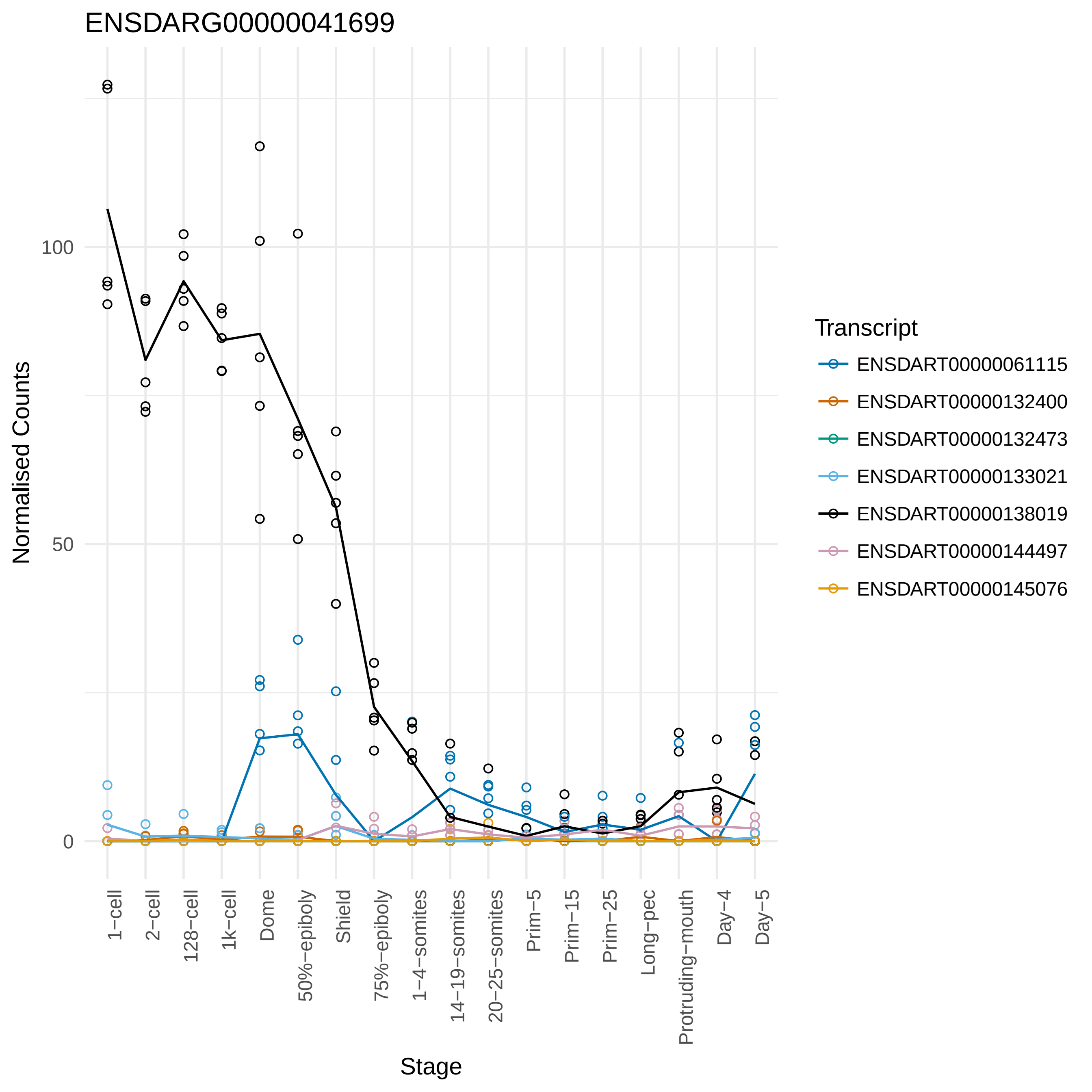

ENSDARG00000041699
Normalised Counts
100
50
0
1−cell
2−cell
128−cell
1k−cell
Dome
50%−epiboly
Shield
75%−epiboly
1−4−somites
14−19−somites
20−25−somites
Prim−5
Prim−15
Prim−25
Long−pec
Protruding−mouth
Day−4
Day−5
Stage
Transcript
ENSDART00000061115
ENSDART00000132400
ENSDART00000132473
ENSDART00000133021
ENSDART00000138019
ENSDART00000144497
ENSDART00000145076

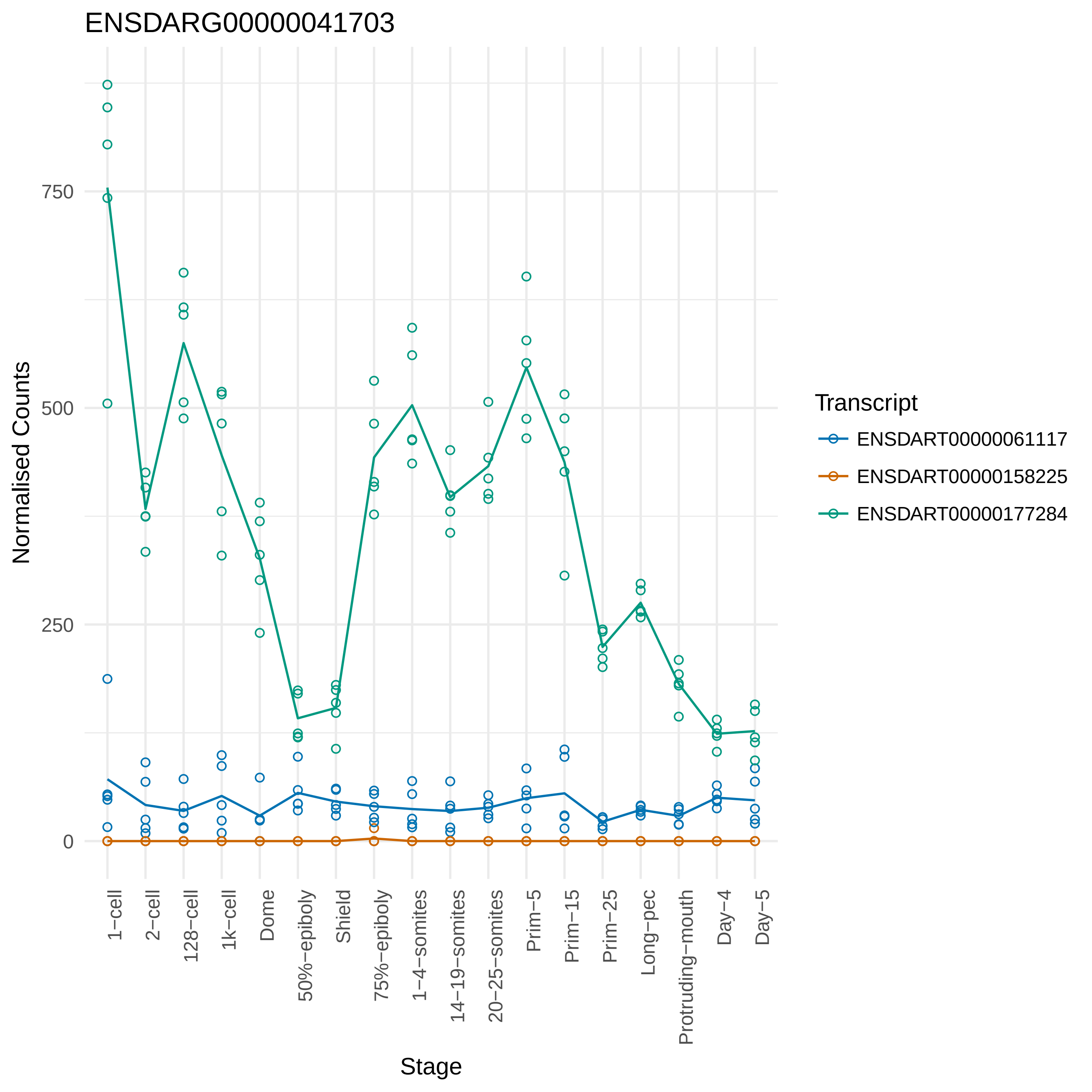

ENSDARG00000041703
Normalised Counts
750
500
250
0
1−cell
2−cell
128−cell
1k−cell
Dome
50%−epiboly
Shield
75%−epiboly
1−4−somites
14−19−somites
20−25−somites
Prim−5
Prim−15
Prim−25
Long−pec
Protruding−mouth
Day−4
Day−5
Stage
Transcript
ENSDART00000061117
ENSDART00000158225
ENSDART00000177284

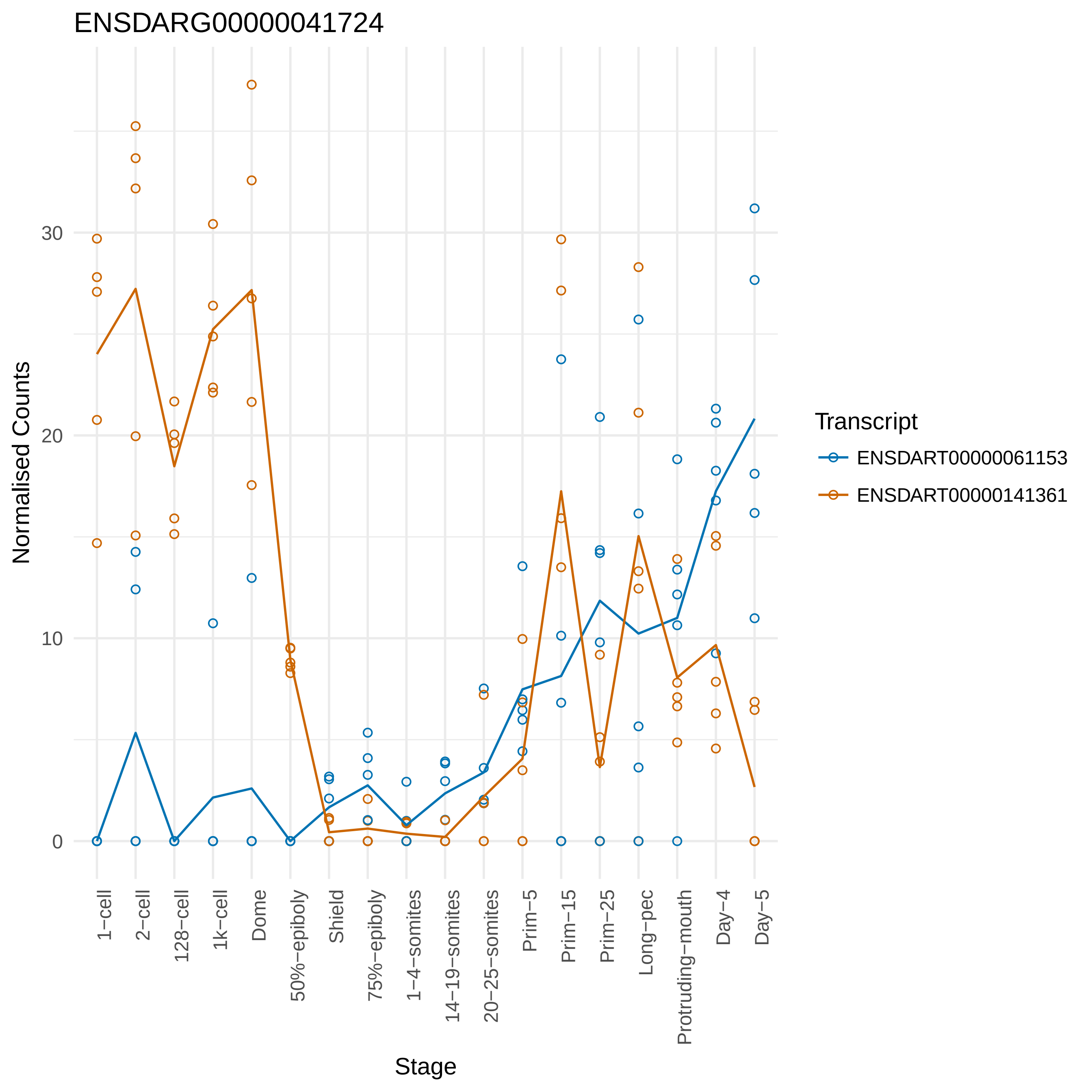

ENSDARG00000041724
Normalised Counts
0
10
20
30
Stage
1−cell
2−cell
128−cell
1k−cell
Dome
50%−epiboly
Shield
75%−epiboly
1−4−somites
14−19−somites
20−25−somites
Prim−5
Prim−15
Prim−25
Long−pec
Protruding−mouth
Day−4
Day−5
Transcript
ENSDART00000061153
ENSDART00000141361

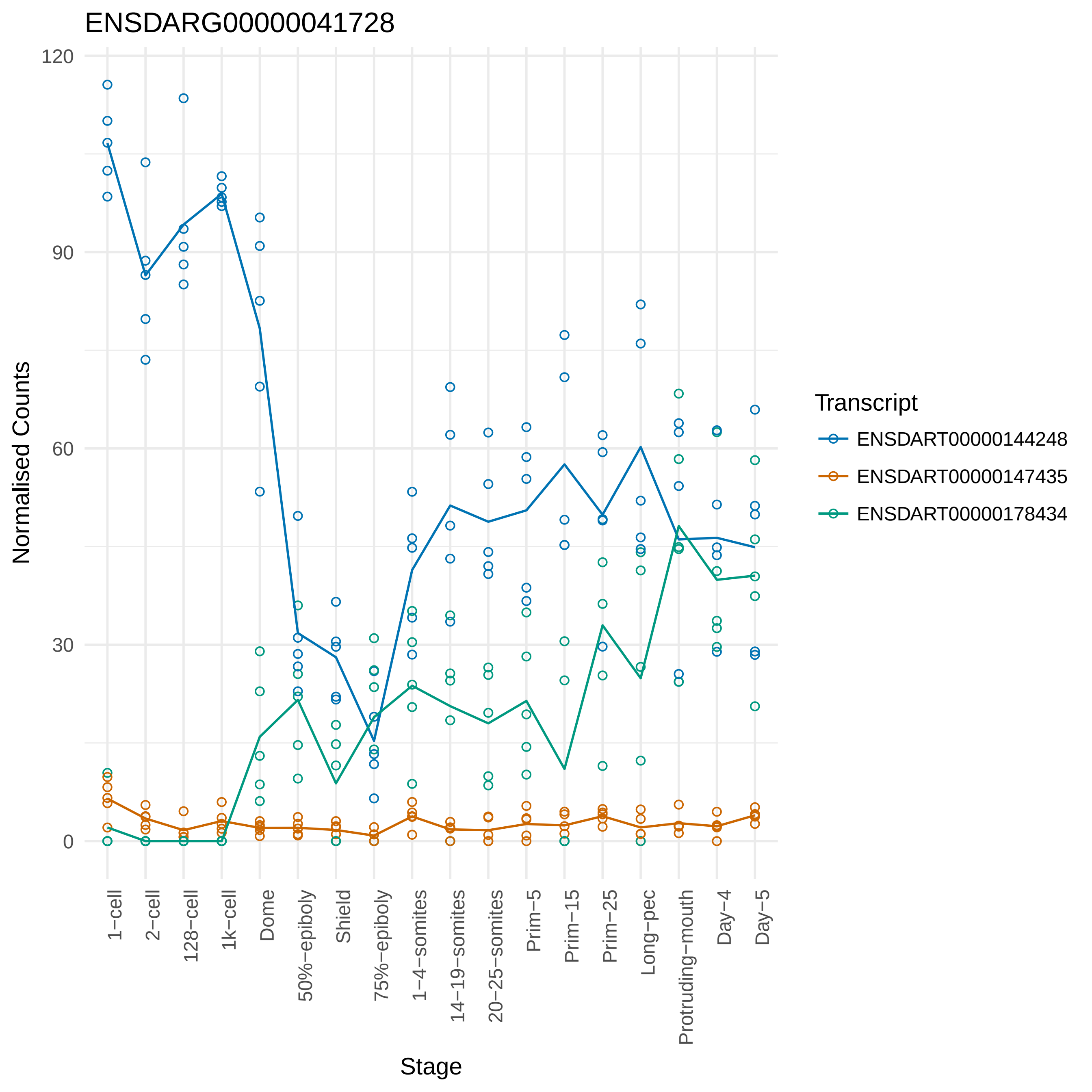

ENSDARG00000041728
Normalised Counts
120
90
60
30
0
1−cell
2−cell
128−cell
1k−cell
Dome
50%−epiboly
Shield
75%−epiboly
1−4−somites
14−19−somites
20−25−somites
Prim−5
Prim−15
Prim−25
Long−pec
Protruding−mouth
Day−4
Day−5
Stage
Transcript
ENSDART00000144248
ENSDART00000147435
ENSDART00000178434

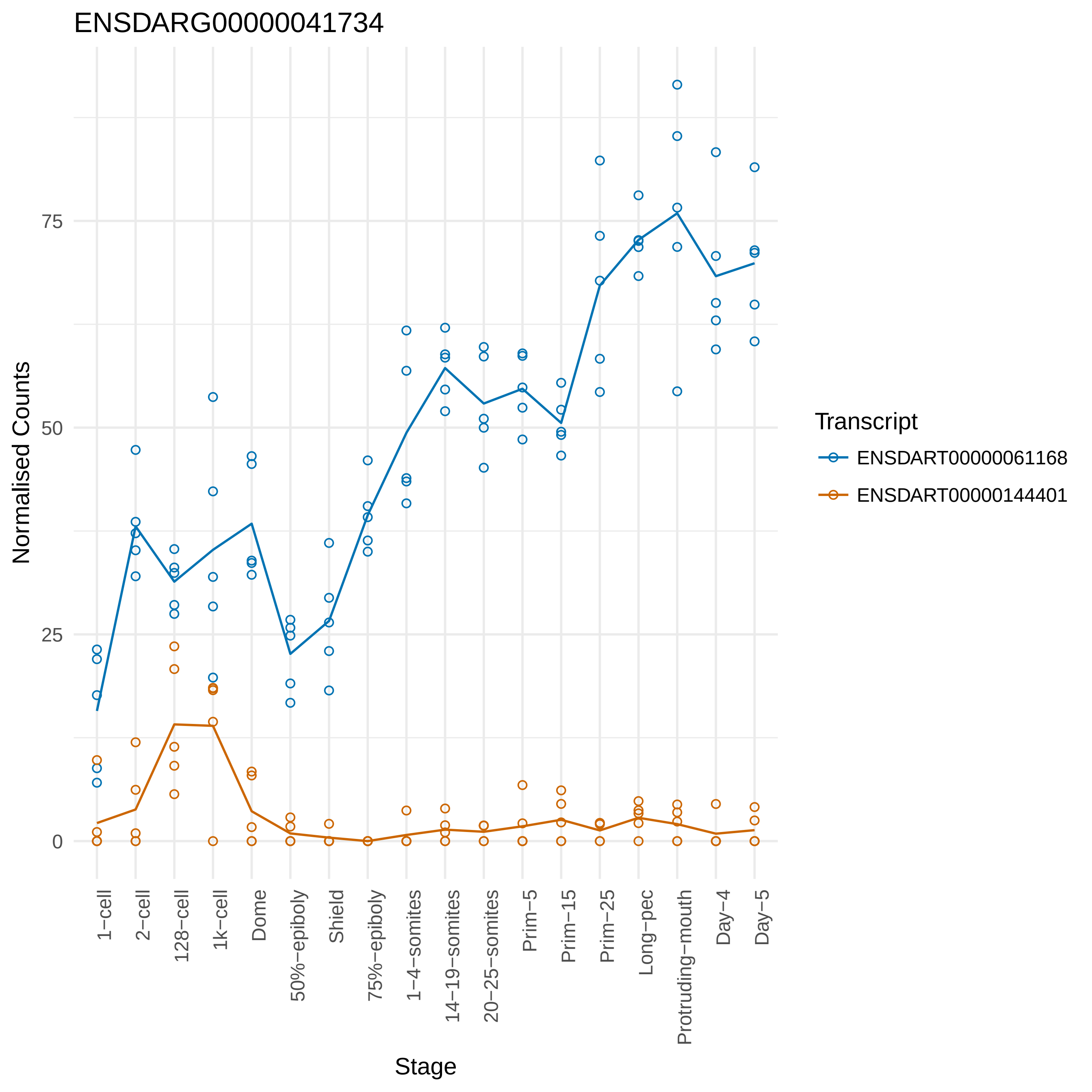
ENSDARG00000041734
Normalised Counts
0
25
50
75
Stage
1−cell
2−cell
128−cell
1k−cell
Dome
50%−epiboly
Shield
75%−epiboly
1−4−somites
14−19−somites
20−25−somites
Prim−5
Prim−15
Prim−25
Long−pec
Protruding−mouth
Day−4
Day−5
Transcript
ENSDART00000061168
ENSDART00000144401

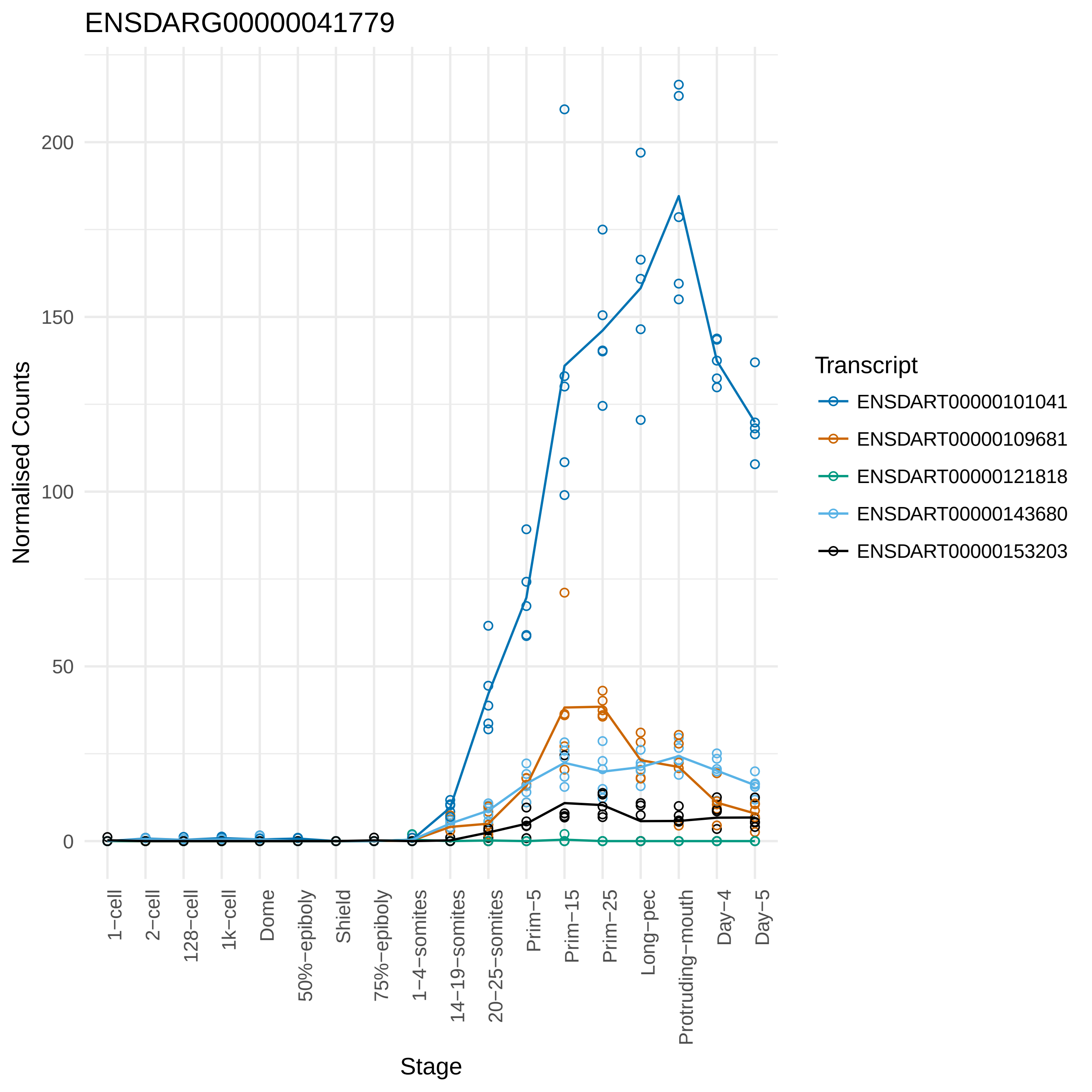

ENSDARG00000041779
Normalised Counts
200
150
100
50
0
1−cell
2−cell
128−cell
1k−cell
Dome
50%−epiboly
Shield
75%−epiboly
1−4−somites
14−19−somites
20−25−somites
Prim−5
Prim−15
Prim−25
Long−pec
Protruding−mouth
Day−4
Day−5
Stage
Transcript
ENSDART00000101041
ENSDART00000109681
ENSDART00000121818
ENSDART00000143680
ENSDART00000153203

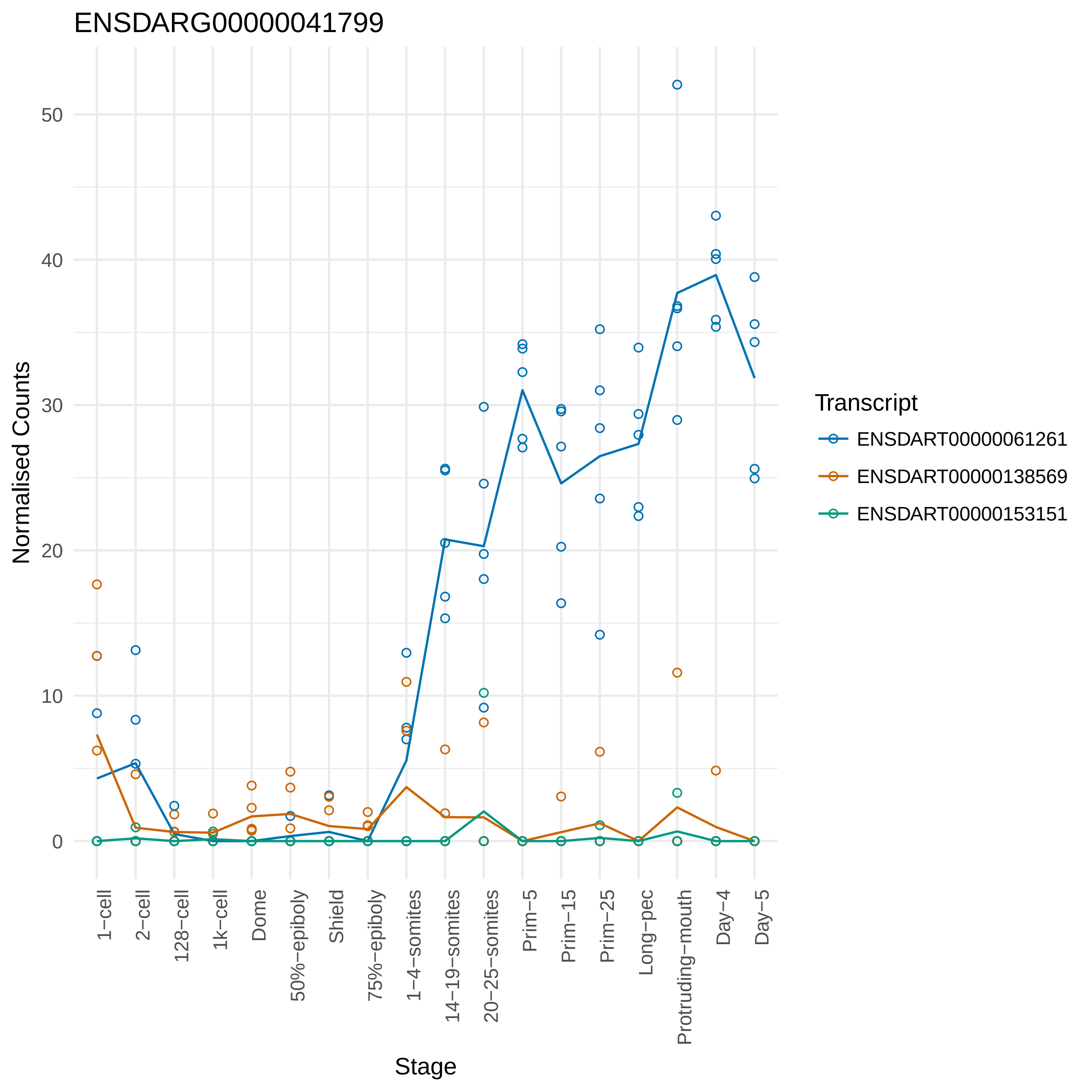

ENSDARG00000041799
Normalised Counts
50
40
30
20
10
0
1−cell
2−cell
128−cell
1k−cell
Dome
50%−epiboly
Shield
75%−epiboly
1−4−somites
14−19−somites
20−25−somites
Prim−5
Prim−15
Prim−25
Long−pec
Protruding−mouth
Day−4
Day−5
Stage
Transcript
ENSDART00000061261
ENSDART00000138569
ENSDART00000153151

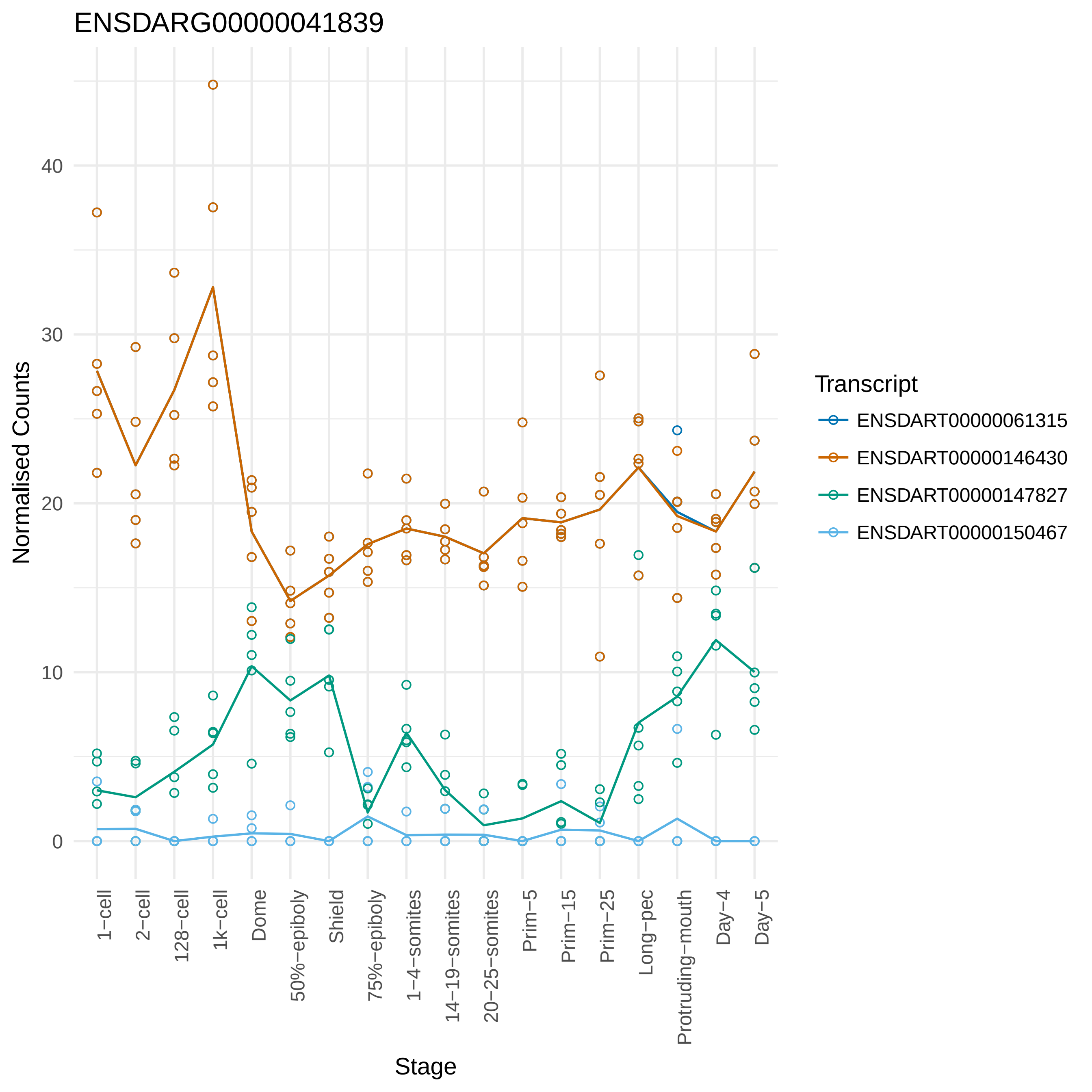

ENSDARG00000041839
Normalised Counts
0
10
20
30
40
1−cell
2−cell
128−cell
1k−cell
Dome
50%−epiboly
Shield
75%−epiboly
1−4−somites
14−19−somites
20−25−somites
Prim−5
Prim−15
Prim−25
Long−pec
Protruding−mouth
Day−4
Day−5
Stage
Transcript
ENSDART00000061315
ENSDART00000146430
ENSDART00000147827
ENSDART00000150467

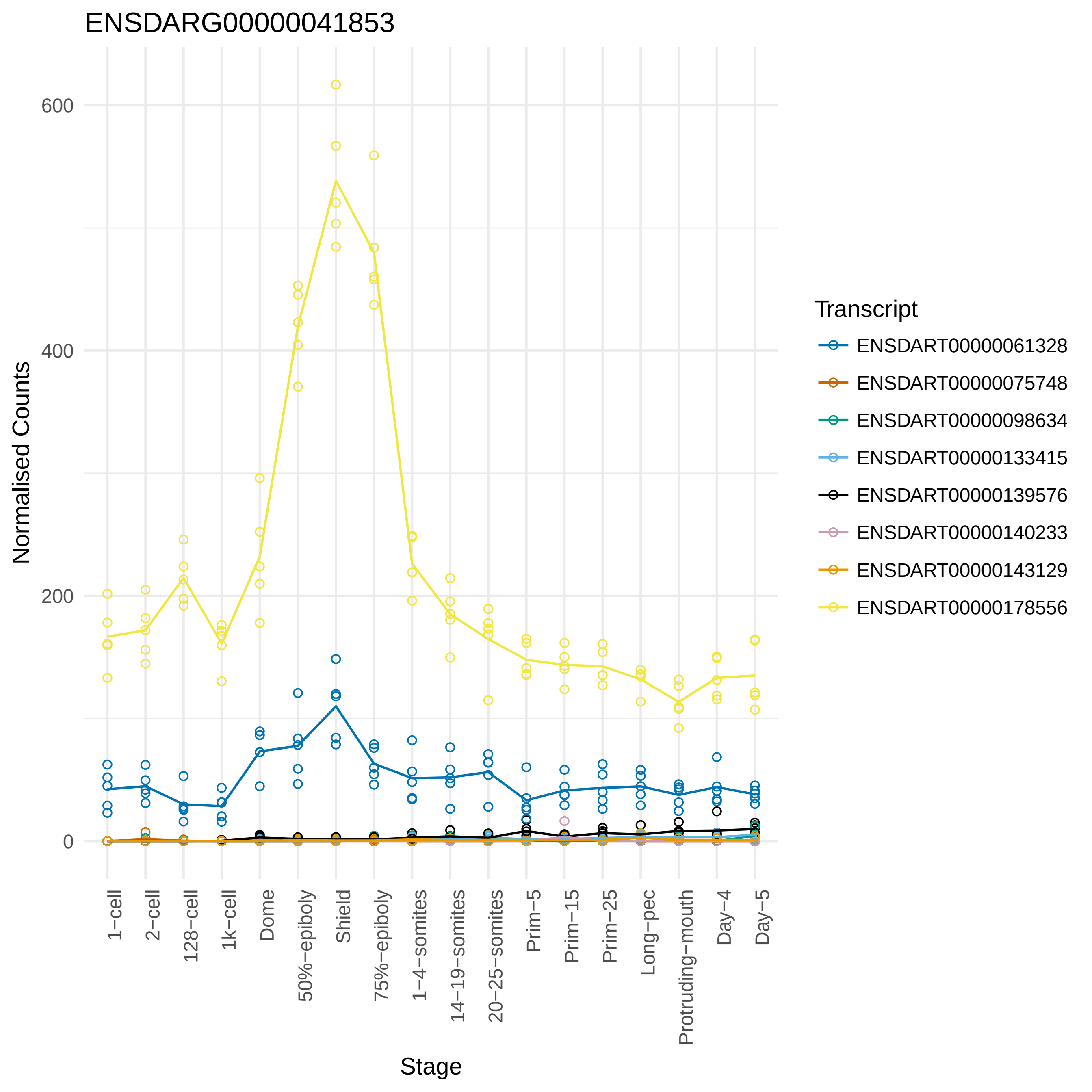

ENSDARG00000041853
Normalised Counts
600
400
200
0
1−cell
2−cell
128−cell
1k−cell
Dome
50%−epiboly
Shield
75%−epiboly
1−4−somites
14−19−somites
20−25−somites
Prim−5
Prim−15
Prim−25
Long−pec
Protruding−mouth
Day−4
Day−5
Stage
Transcript
ENSDART00000061328
ENSDART00000075748
ENSDART00000098634
ENSDART00000133415
ENSDART00000139576
ENSDART00000140233
ENSDART00000143129
ENSDART00000178556

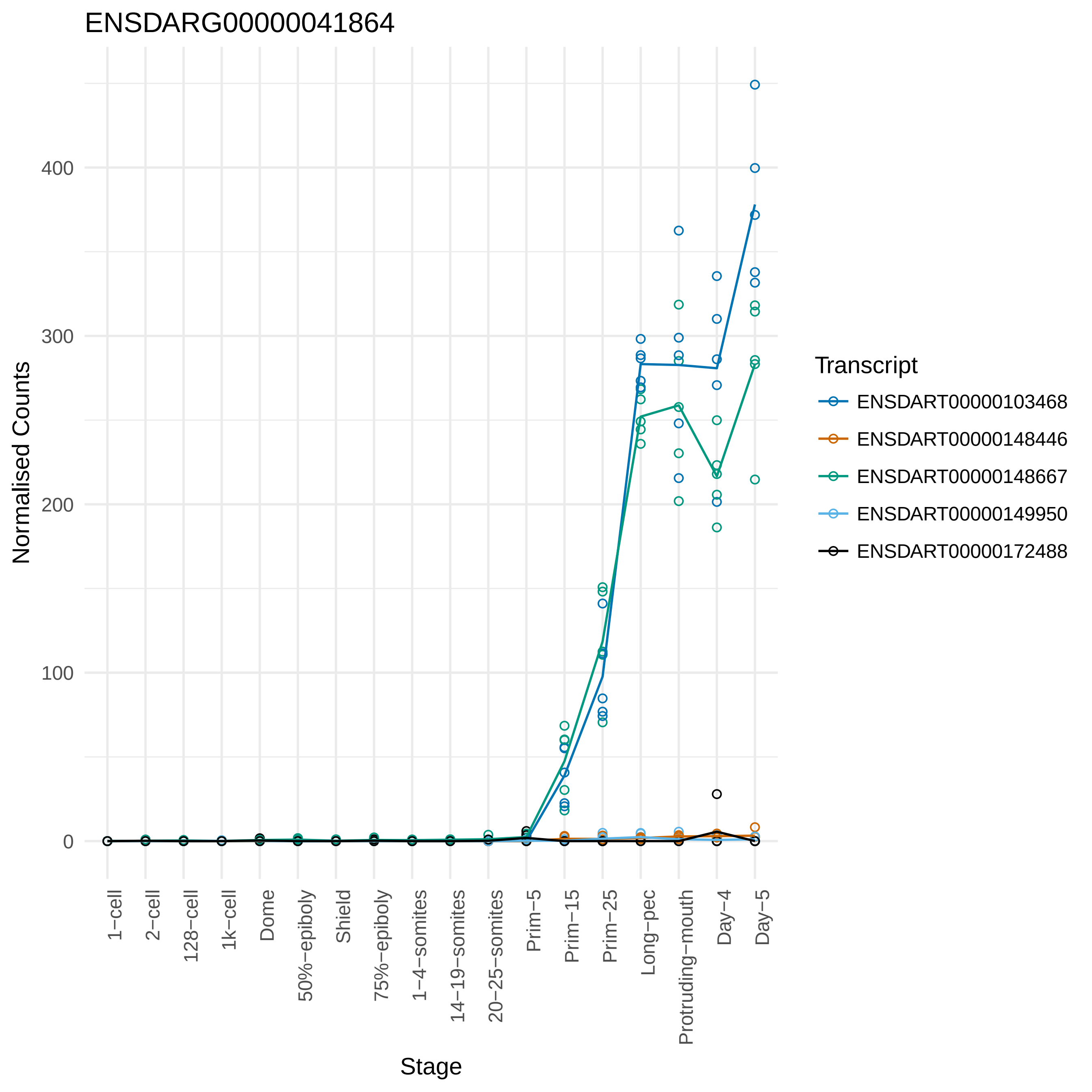
ENSDARG00000041864
Normalised Counts
400
300
200
100
0
1−cell
2−cell
128−cell
1k−cell
Dome
50%−epiboly
Shield
75%−epiboly
1−4−somites
14−19−somites
20−25−somites
Prim−5
Prim−15
Prim−25
Long−pec
Protruding−mouth
Day−4
Day−5
Stage
Transcript
ENSDART00000103468
ENSDART00000148446
ENSDART00000148667
ENSDART00000149950
ENSDART00000172488

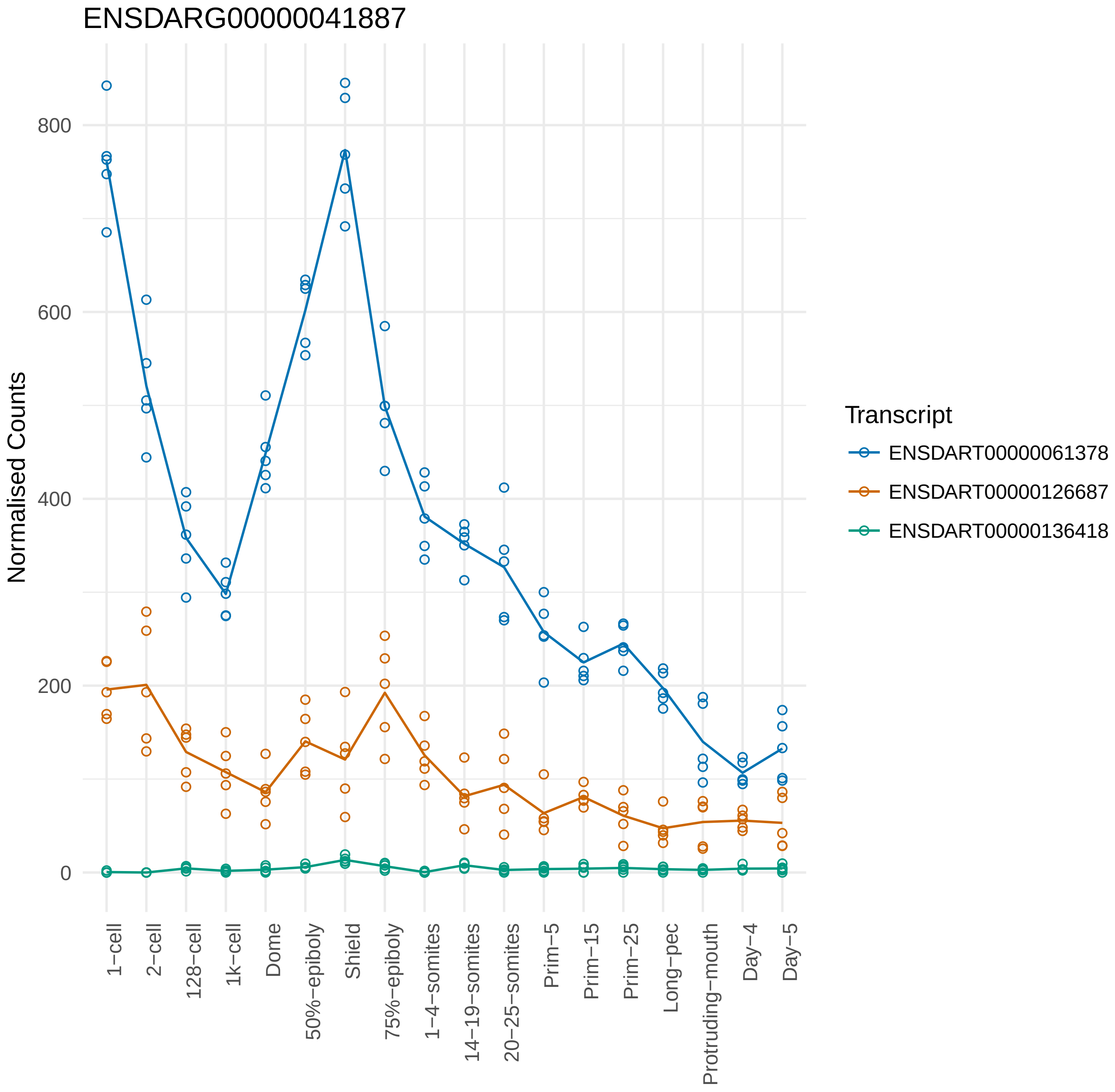

ENSDARG00000041887
Normalised Counts
800
600
400
200
0
1−cell
2−cell
128−cell
1k−cell
Dome
50%−epiboly
Shield
75%−epiboly
1−4−somites
14−19−somites
20−25−somites
Prim−5
Prim−15
Prim−25
Long−pec
Protruding−mouth
Day−4
Day−5
Stage
Transcript
ENSDART00000061378
ENSDART00000126687
ENSDART00000136418

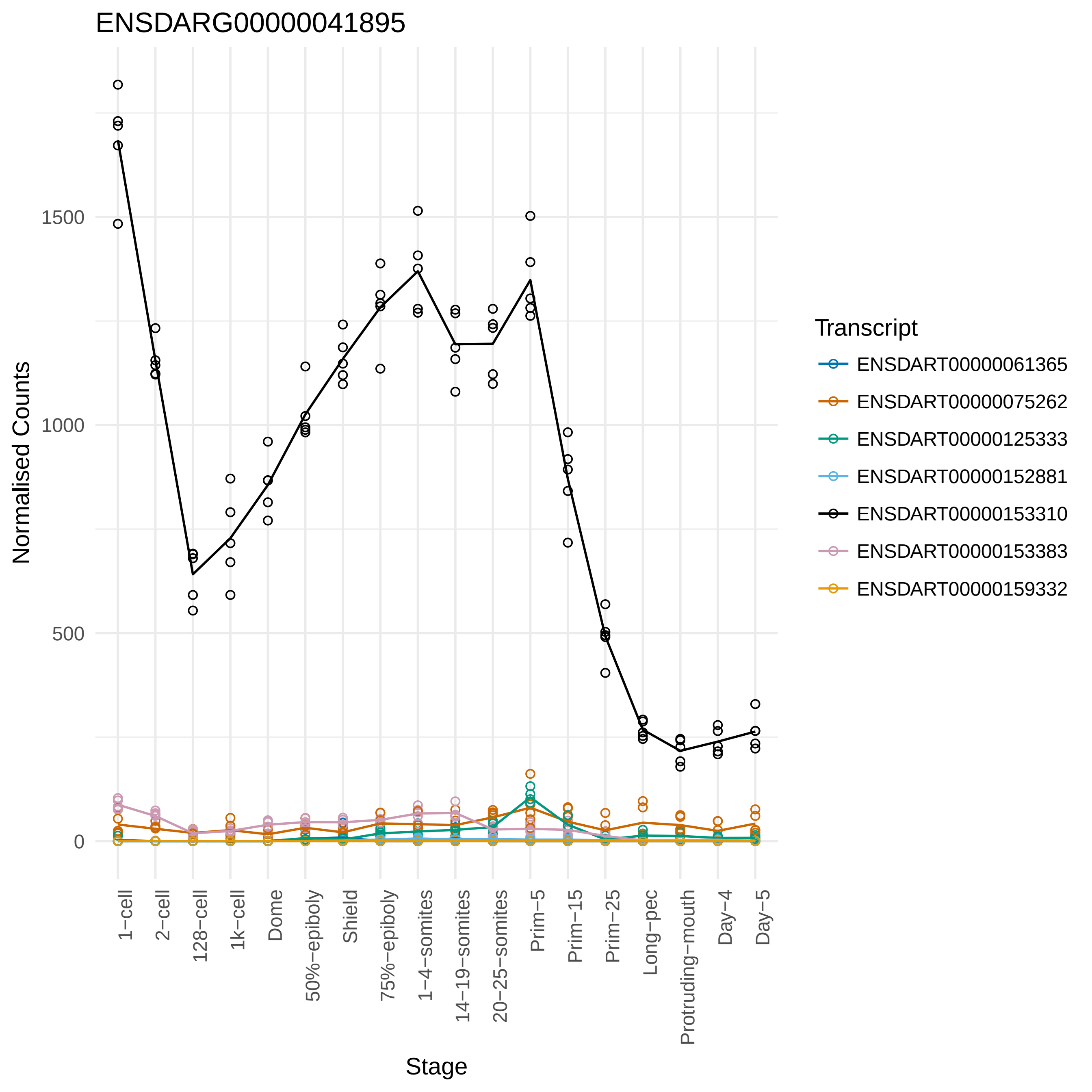

ENSDARG00000041895
Normalised Counts
1500
1000
500
0
1−cell
2−cell
128−cell
1k−cell
Dome
50%−epiboly
Shield
75%−epiboly
1−4−somites
14−19−somites
20−25−somites
Prim−5
Prim−15
Prim−25
Long−pec
Protruding−mouth
Day−4
Day−5
Stage
Transcript
ENSDART00000061365
ENSDART00000075262
ENSDART00000125333
ENSDART00000152881
ENSDART00000153310
ENSDART00000153383
ENSDART00000159332

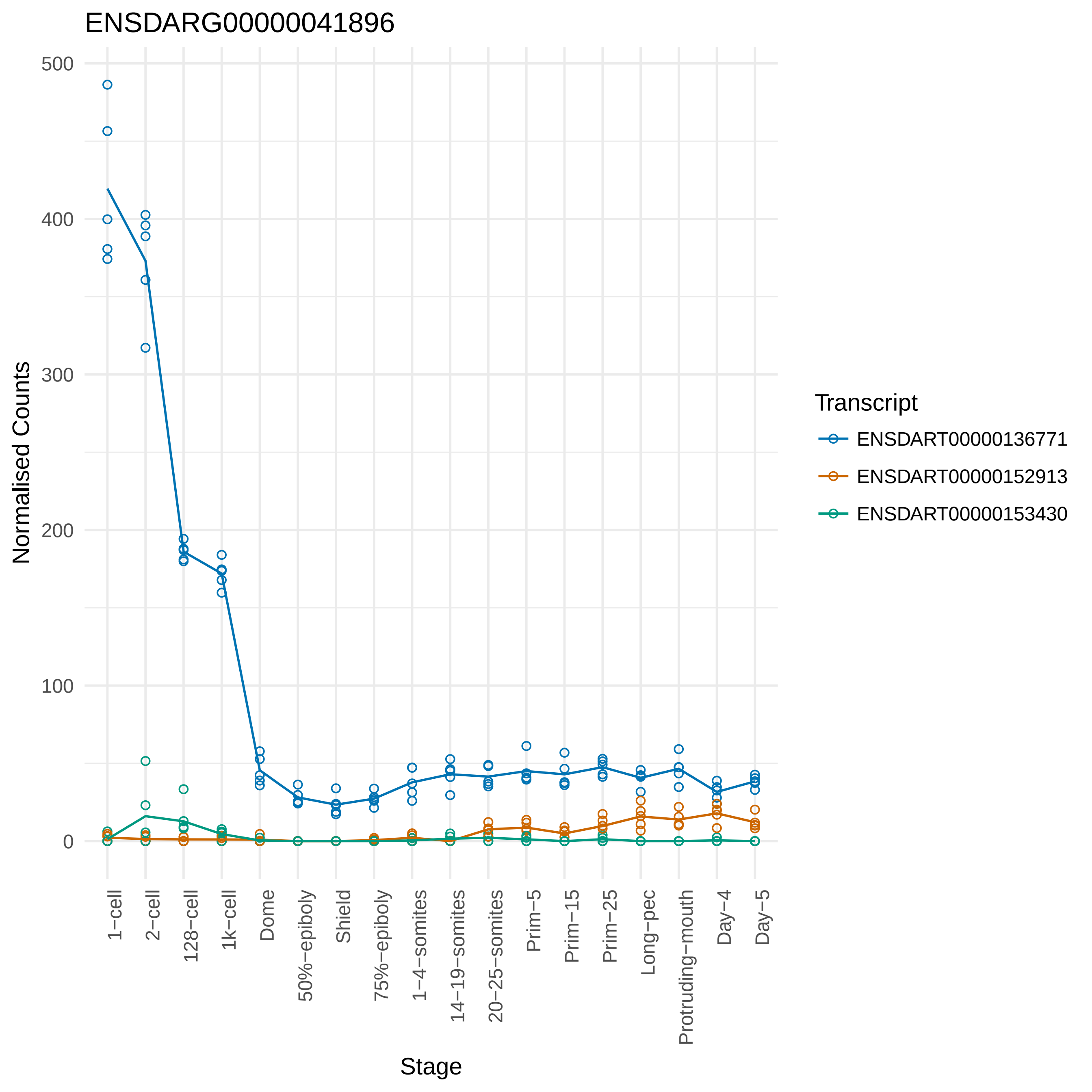

ENSDARG00000041896
Normalised Counts
500
400
300
200
100
0
1−cell
2−cell
128−cell
1k−cell
Dome
50%−epiboly
Shield
75%−epiboly
1−4−somites
14−19−somites
20−25−somites
Prim−5
Prim−15
Prim−25
Long−pec
Protruding−mouth
Day−4
Day−5
Stage
Transcript
ENSDART00000136771
ENSDART00000152913
ENSDART00000153430

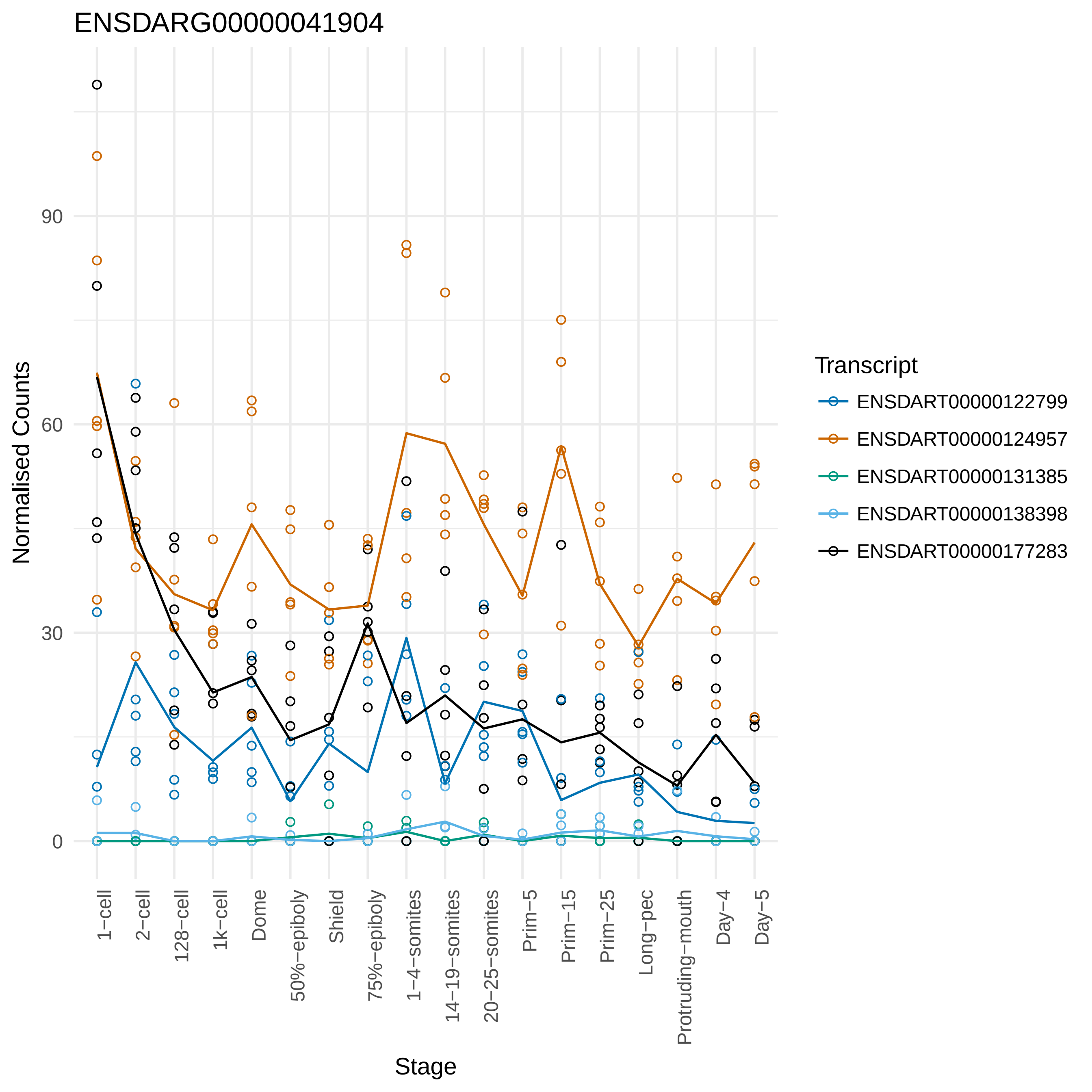

ENSDARG00000041904
Normalised Counts
0
30
60
90
Stage
1−cell
2−cell
128−cell
1k−cell
Dome
50%−epiboly
Shield
75%−epiboly
1−4−somites
14−19−somites
20−25−somites
Prim−5
Prim−15
Prim−25
Long−pec
Protruding−mouth
Day−4
Day−5
Transcript
ENSDART00000122799
ENSDART00000124957
ENSDART00000131385
ENSDART00000138398
ENSDART00000177283

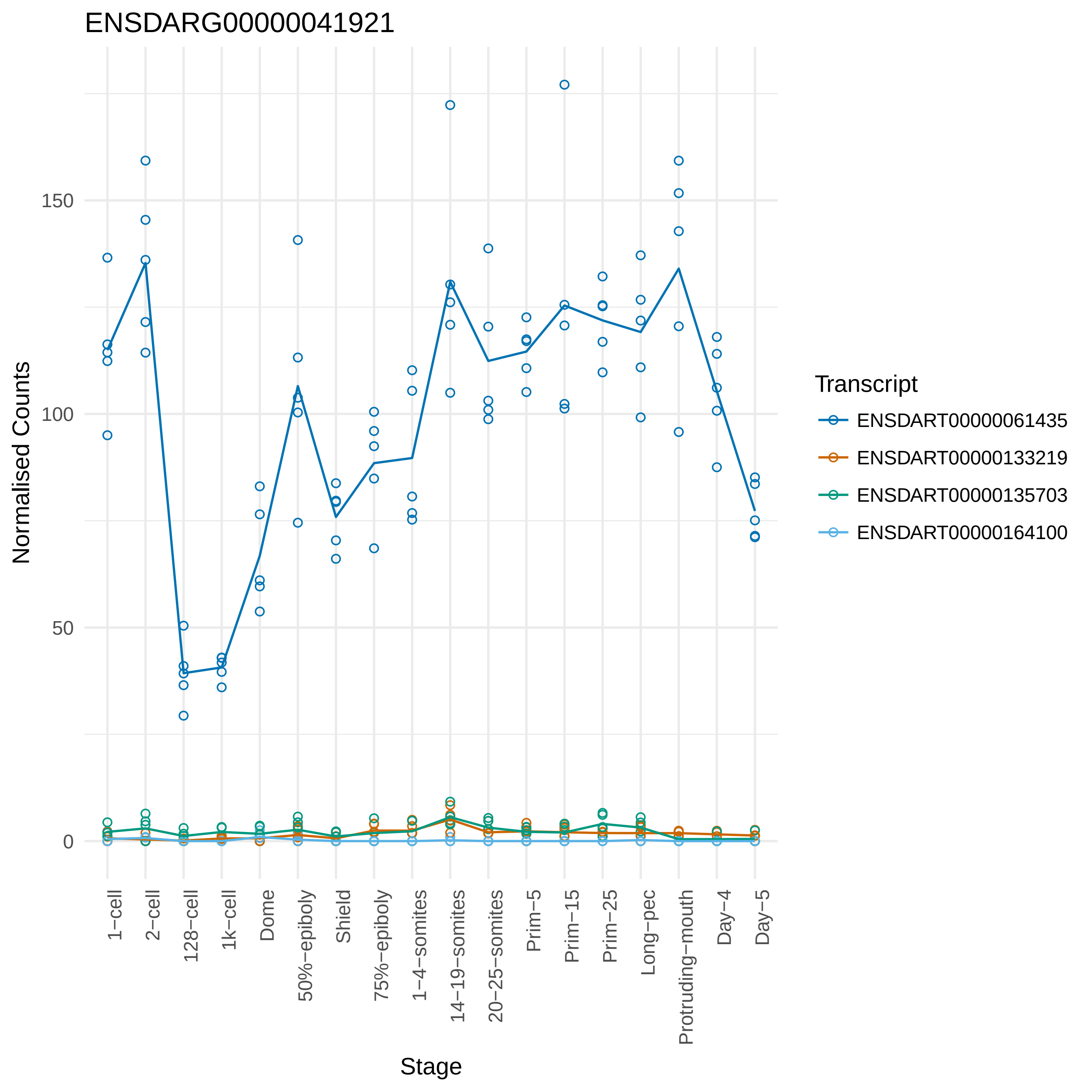
ENSDARG00000041921
Normalised Counts
150
100
50
0
1−cell
2−cell
128−cell
1k−cell
Dome
50%−epiboly
Shield
75%−epiboly
1−4−somites
14−19−somites
20−25−somites
Prim−5
Prim−15
Prim−25
Long−pec
Protruding−mouth
Day−4
Day−5
Stage
Transcript
ENSDART00000061435
ENSDART00000133219
ENSDART00000135703
ENSDART00000164100

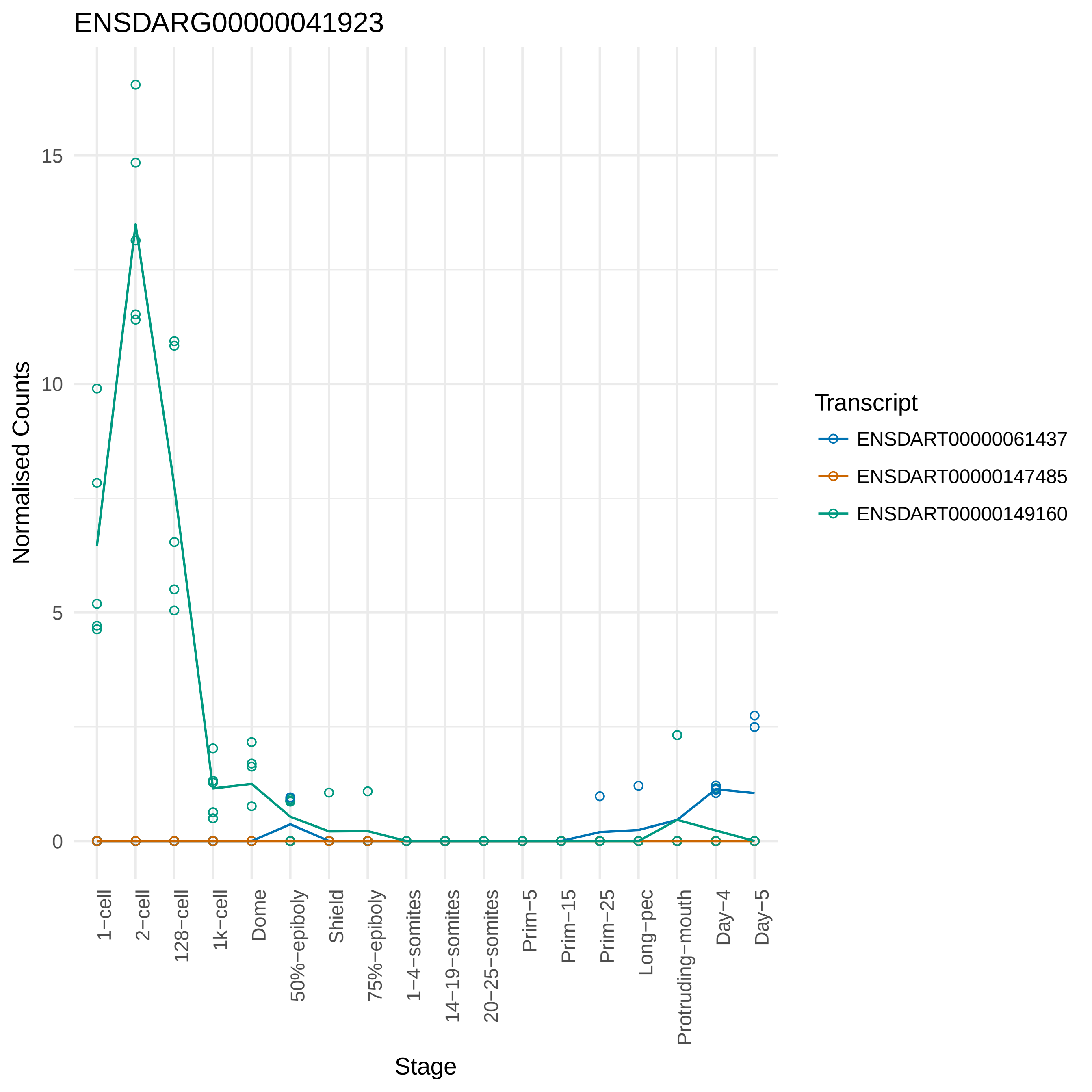

ENSDARG00000041923
Normalised Counts
15
10
5
0
1−cell
2−cell
128−cell
1k−cell
Dome
50%−epiboly
Shield
75%−epiboly
1−4−somites
14−19−somites
20−25−somites
Prim−5
Prim−15
Prim−25
Long−pec
Protruding−mouth
Day−4
Day−5
Stage
Transcript
ENSDART00000061437
ENSDART00000147485
ENSDART00000149160

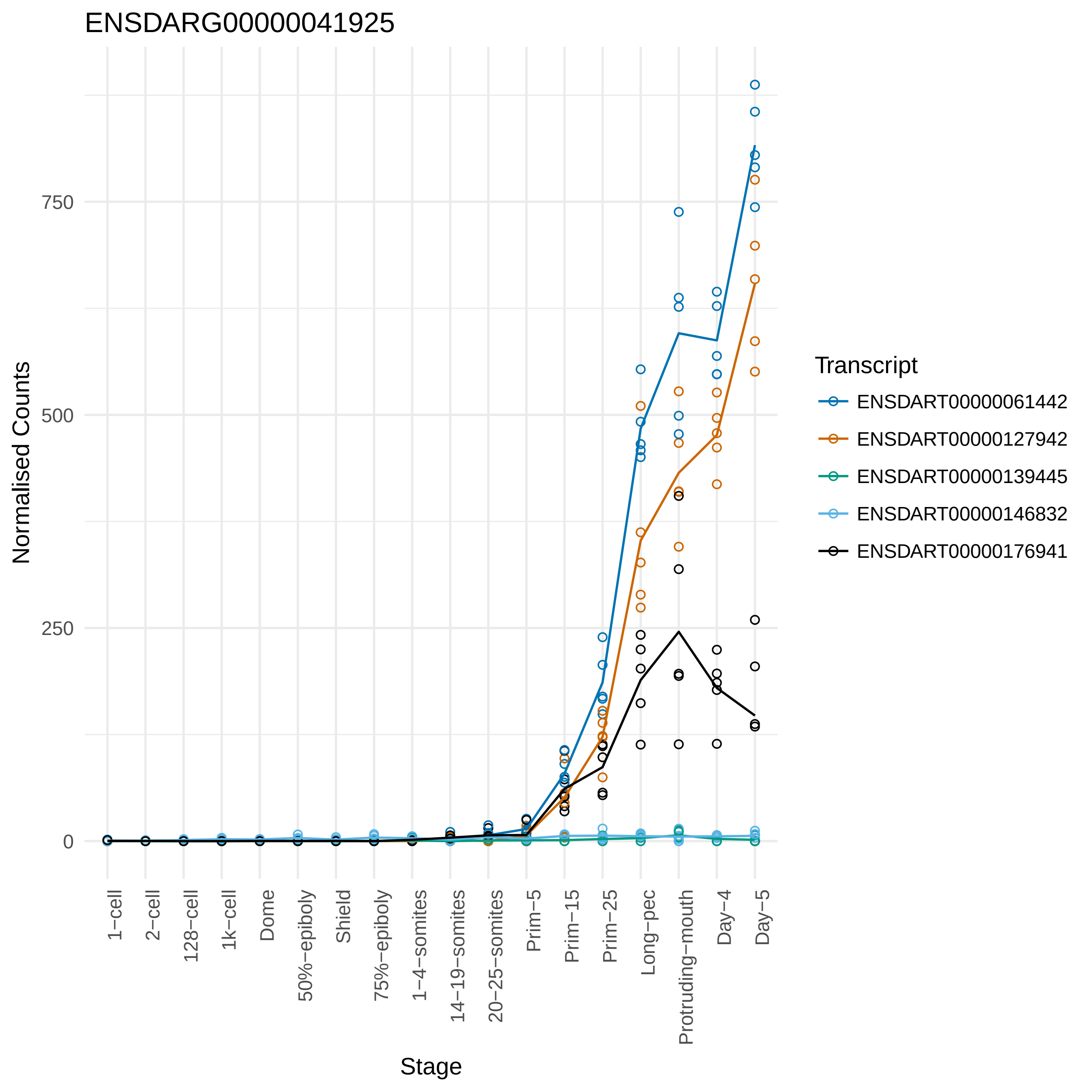

ENSDARG00000041925
Normalised Counts
750
500
250
0
1−cell
2−cell
128−cell
1k−cell
Dome
50%−epiboly
Shield
75%−epiboly
1−4−somites
14−19−somites
20−25−somites
Prim−5
Prim−15
Prim−25
Long−pec
Protruding−mouth
Day−4
Day−5
Stage
Transcript
ENSDART00000061442
ENSDART00000127942
ENSDART00000139445
ENSDART00000146832
ENSDART00000176941

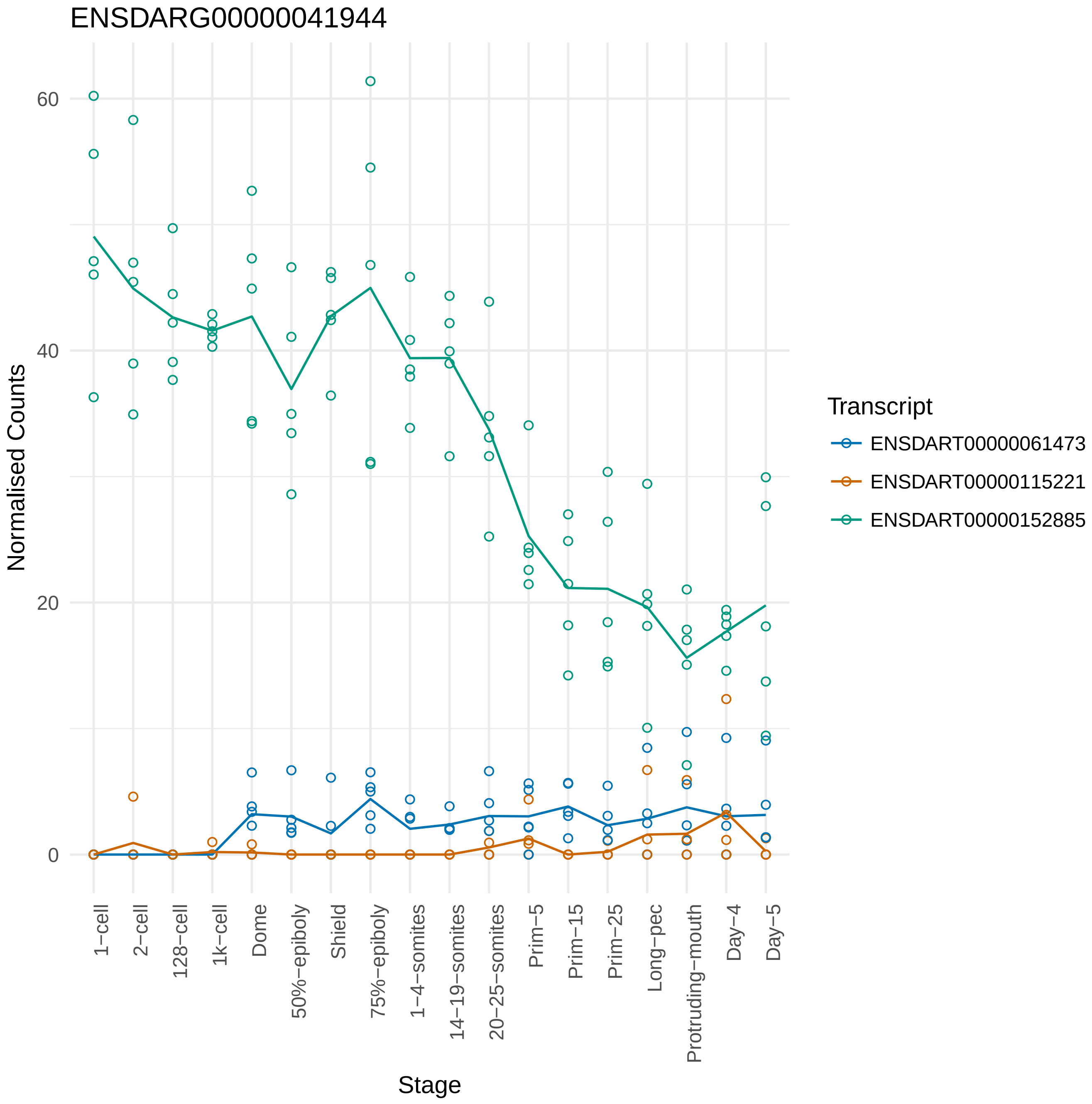
ENSDARG00000041944
Normalised Counts
0
20
40
60
Stage
1−cell
2−cell
128−cell
1k−cell
Dome
50%−epiboly
Shield
75%−epiboly
1−4−somites
14−19−somites
20−25−somites
Prim−5
Prim−15
Prim−25
Long−pec
Protruding−mouth
Day−4
Day−5
Transcript
ENSDART00000061473
ENSDART00000115221
ENSDART00000152885

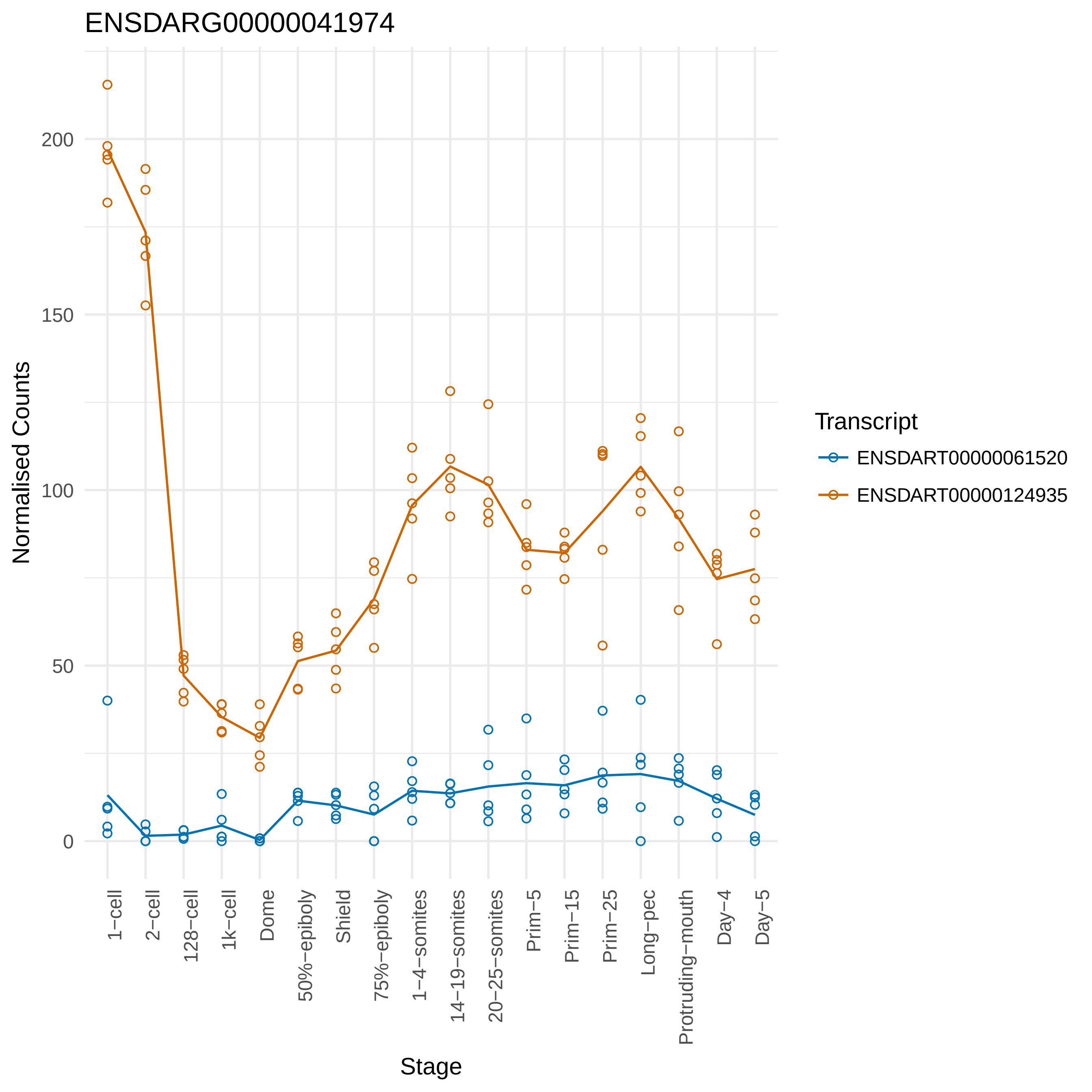

ENSDARG00000041974
Normalised Counts
0
50
100
150
200
1−cell
2−cell
128−cell
1k−cell
Dome
50%−epiboly
Shield
75%−epiboly
1−4−somites
14−19−somites
20−25−somites
Prim−5
Prim−15
Prim−25
Long−pec
Protruding−mouth
Day−4
Day−5
Stage
Transcript
ENSDART00000061520
ENSDART00000124935

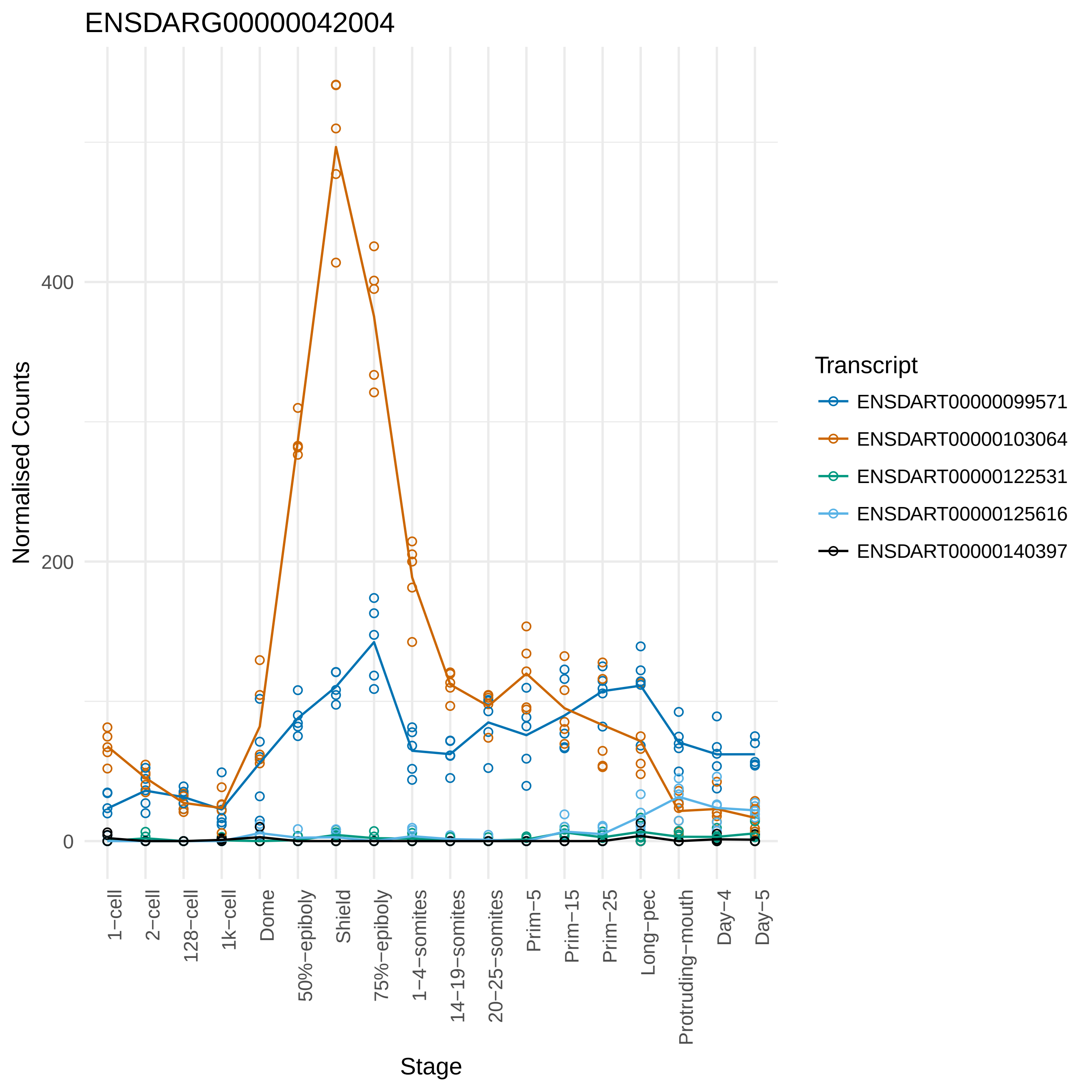
ENSDARG00000042004
Normalised Counts
400
200
0
Stage
1−cell
2−cell
128−cell
1k−cell
Dome
50%−epiboly
Shield
75%−epiboly
1−4−somites
14−19−somites
20−25−somites
Prim−5
Prim−15
Prim−25
Long−pec
Protruding−mouth
Day−4
Day−5
Transcript
ENSDART00000099571
ENSDART00000103064
ENSDART00000122531
ENSDART00000125616
ENSDART00000140397

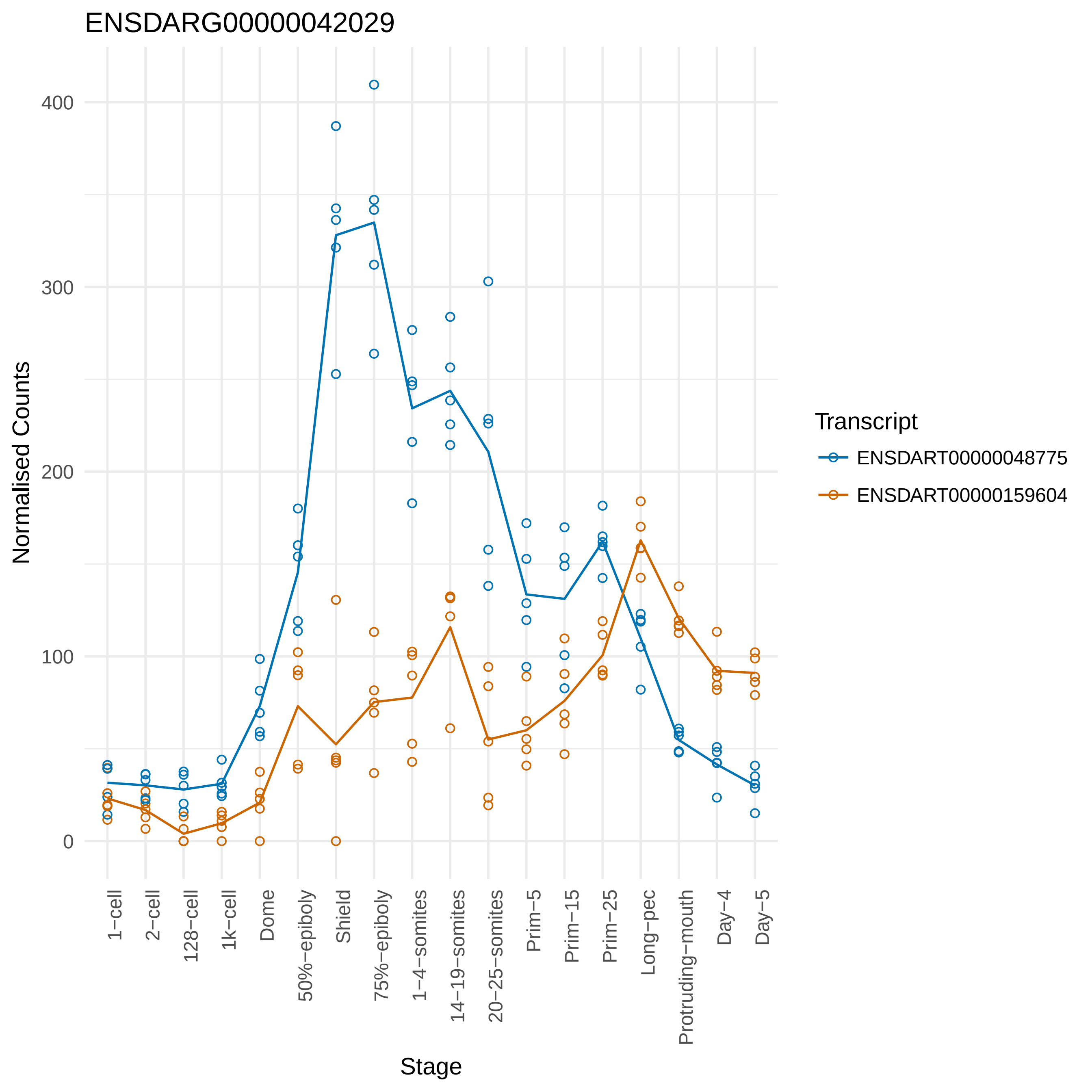
ENSDARG00000042029
Normalised Counts
0
100
200
300
400
1−cell
2−cell
128−cell
1k−cell
Dome
50%−epiboly
Shield
75%−epiboly
1−4−somites
14−19−somites
20−25−somites
Prim−5
Prim−15
Prim−25
Long−pec
Protruding−mouth
Day−4
Day−5
Stage
Transcript
ENSDART00000048775
ENSDART00000159604

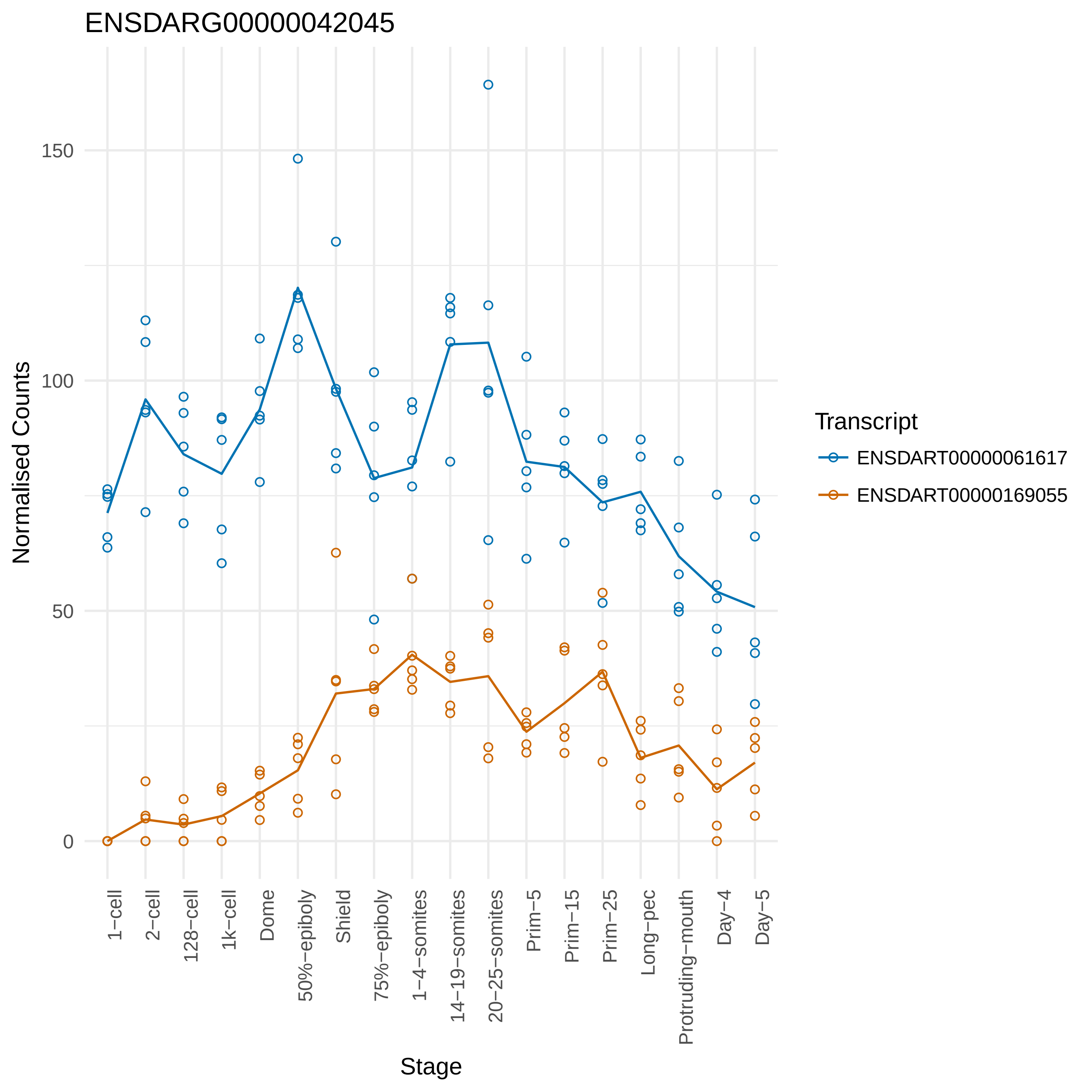
ENSDARG00000042045
Normalised Counts
150
100
50
0
1−cell
2−cell
128−cell
1k−cell
Dome
50%−epiboly
Shield
75%−epiboly
1−4−somites
14−19−somites
20−25−somites
Prim−5
Prim−15
Prim−25
Long−pec
Protruding−mouth
Day−4
Day−5
Stage
Transcript
ENSDART00000061617
ENSDART00000169055

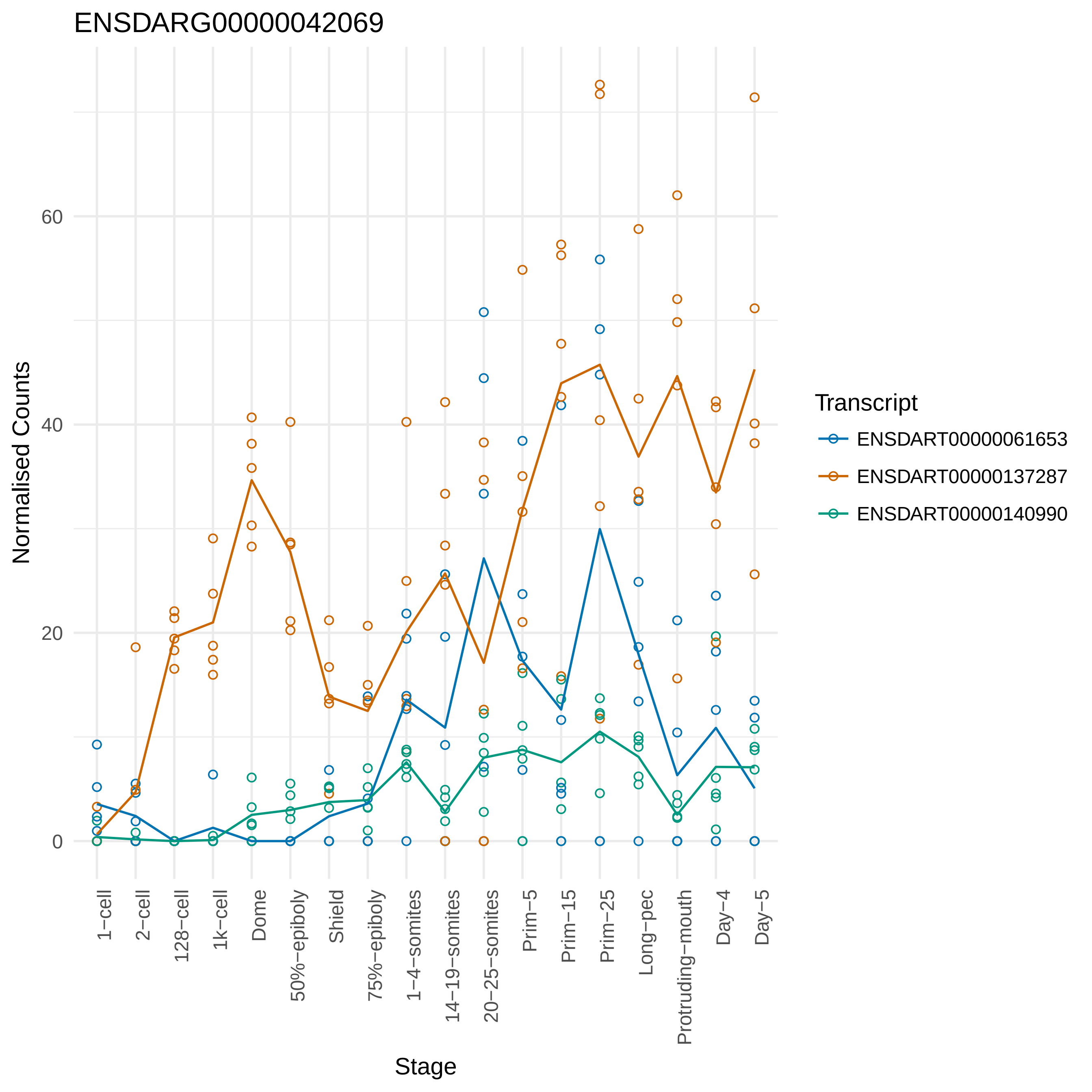
ENSDARG00000042069
Normalised Counts
0
20
40
60
1−cell
2−cell
128−cell
1k−cell
Dome
50%−epiboly
Shield
75%−epiboly
1−4−somites
14−19−somites
20−25−somites
Prim−5
Prim−15
Prim−25
Long−pec
Protruding−mouth
Day−4
Day−5
Stage
Transcript
ENSDART00000061653
ENSDART00000137287
ENSDART00000140990

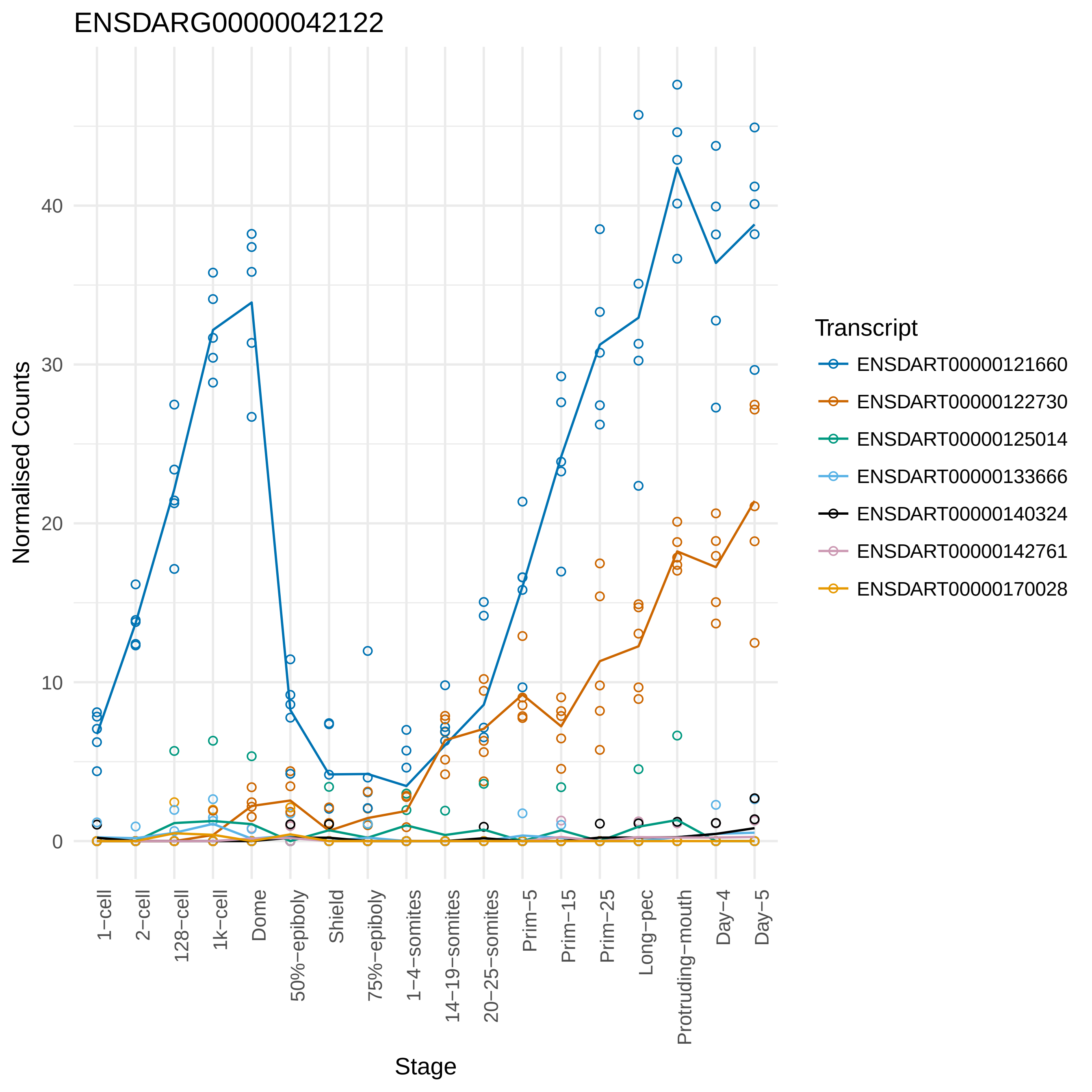
ENSDARG00000042122
Normalised Counts
0
10
20
30
40
1−cell
2−cell
128−cell
1k−cell
Dome
50%−epiboly
Shield
75%−epiboly
1−4−somites
14−19−somites
20−25−somites
Prim−5
Prim−15
Prim−25
Long−pec
Protruding−mouth
Day−4
Day−5
Stage
Transcript
ENSDART00000121660
ENSDART00000122730
ENSDART00000125014
ENSDART00000133666
ENSDART00000140324
ENSDART00000142761
ENSDART00000170028

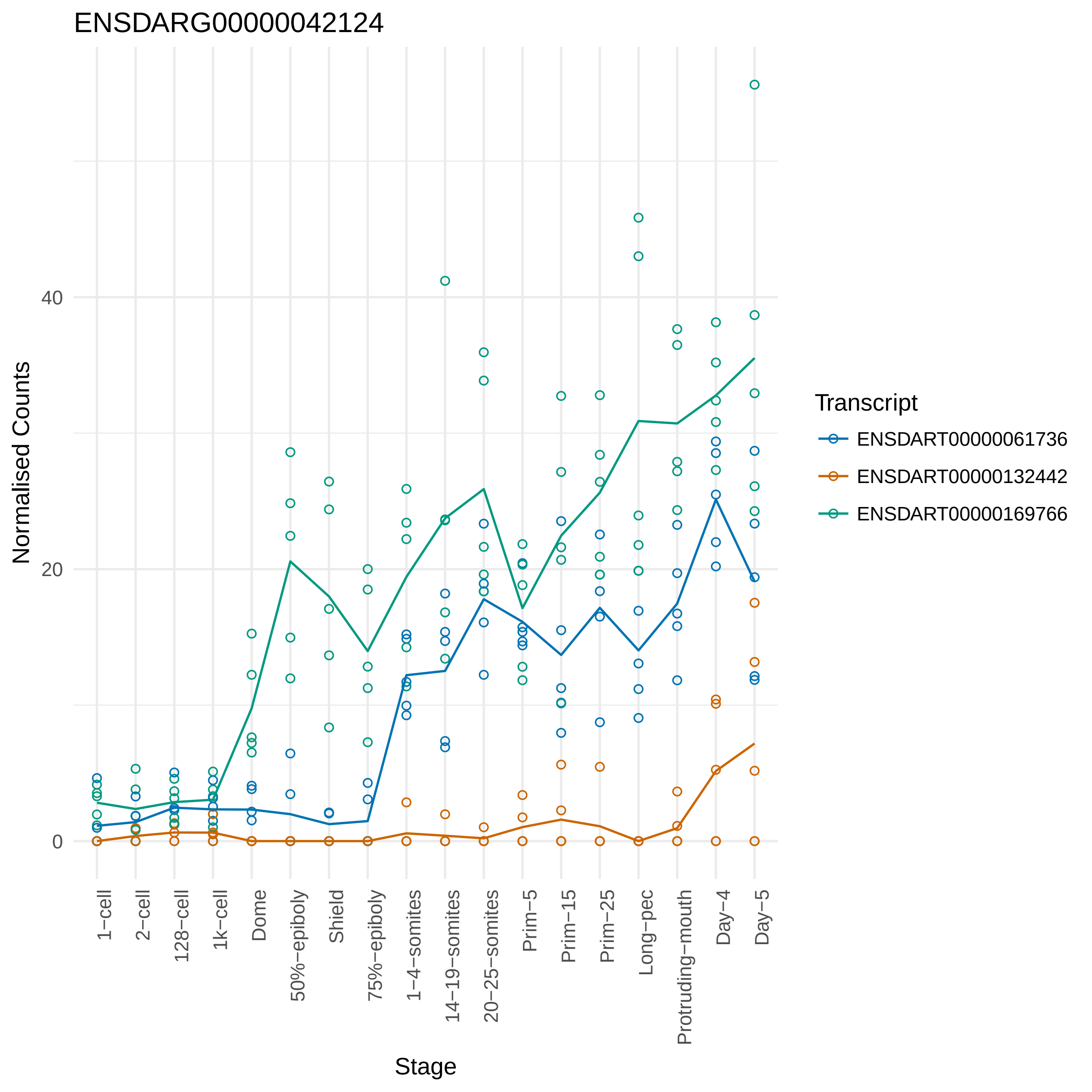

ENSDARG00000042124
Normalised Counts
0
20
40
Stage
1−cell
2−cell
128−cell
1k−cell
Dome
50%−epiboly
Shield
75%−epiboly
1−4−somites
14−19−somites
20−25−somites
Prim−5
Prim−15
Prim−25
Long−pec
Protruding−mouth
Day−4
Day−5
Transcript
ENSDART00000061736
ENSDART00000132442
ENSDART00000169766

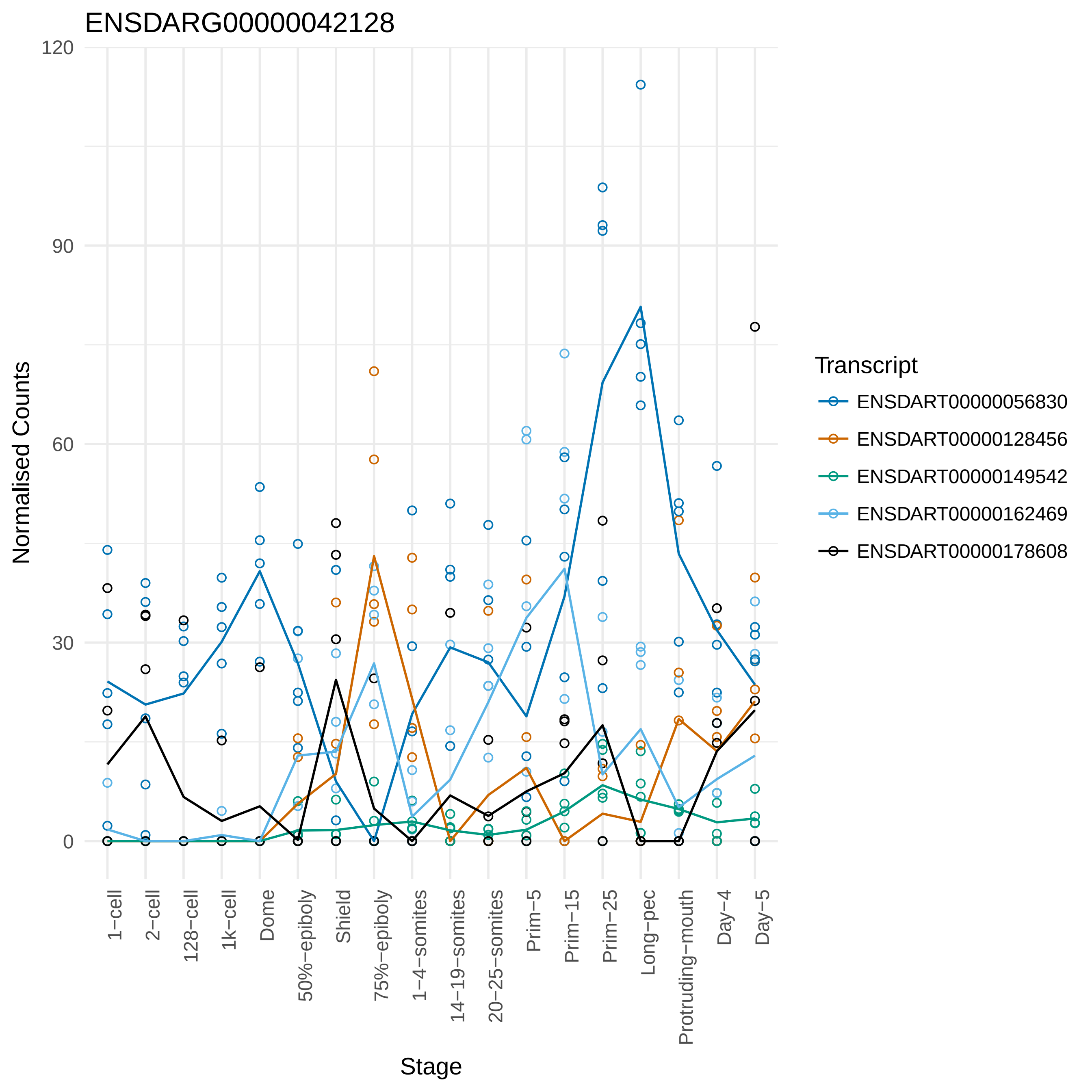

ENSDARG00000042128
Normalised Counts
120
90
60
30
0
1−cell
2−cell
128−cell
1k−cell
Dome
50%−epiboly
Shield
75%−epiboly
1−4−somites
14−19−somites
20−25−somites
Prim−5
Prim−15
Prim−25
Long−pec
Protruding−mouth
Day−4
Day−5
Stage
Transcript
ENSDART00000056830
ENSDART00000128456
ENSDART00000149542
ENSDART00000162469
ENSDART00000178608

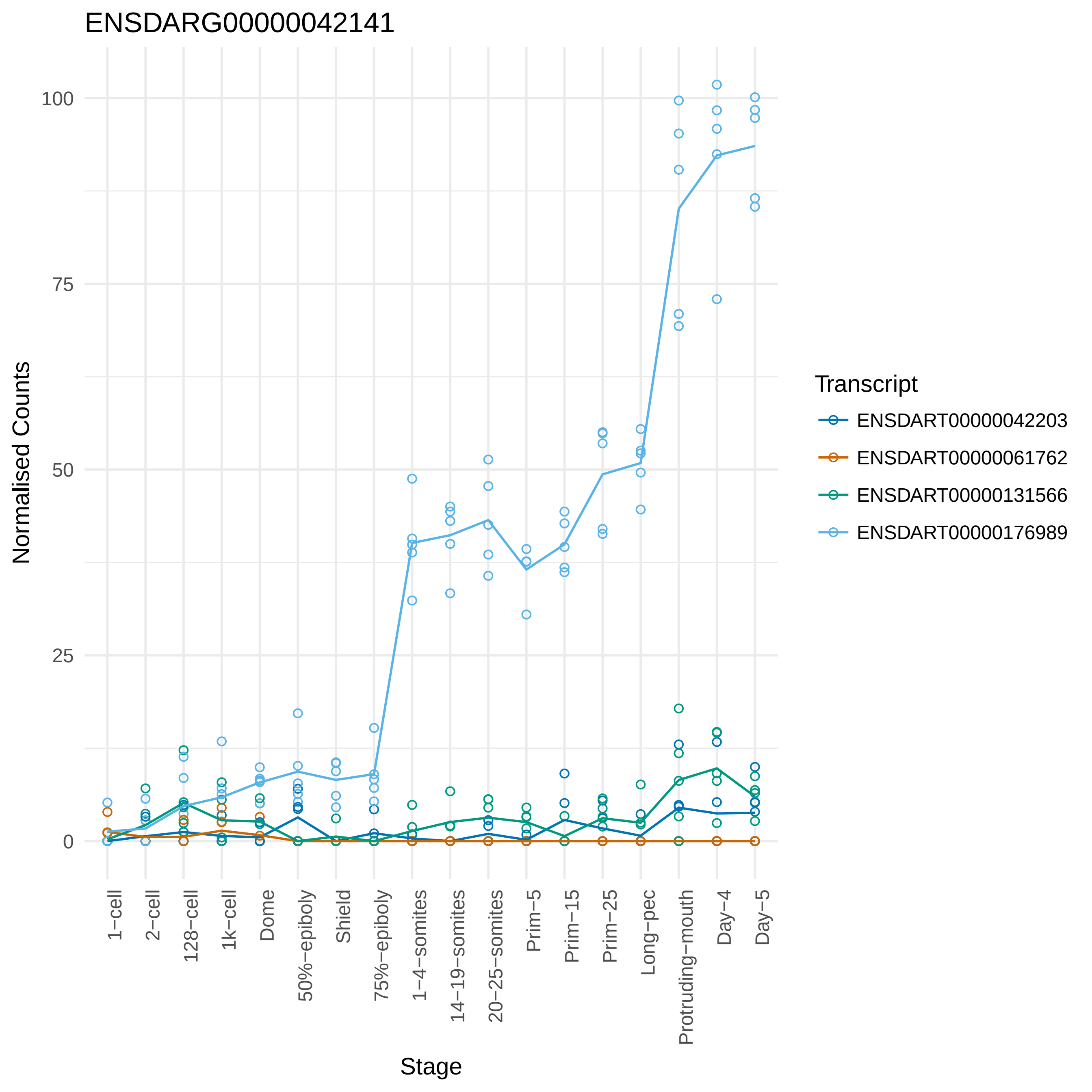

ENSDARG00000042141
Normalised Counts
100
75
50
25
0
1−cell
2−cell
128−cell
1k−cell
Dome
50%−epiboly
Shield
75%−epiboly
1−4−somites
14−19−somites
20−25−somites
Prim−5
Prim−15
Prim−25
Long−pec
Protruding−mouth
Day−4
Day−5
Stage
Transcript
ENSDART00000042203
ENSDART00000061762
ENSDART00000131566
ENSDART00000176989

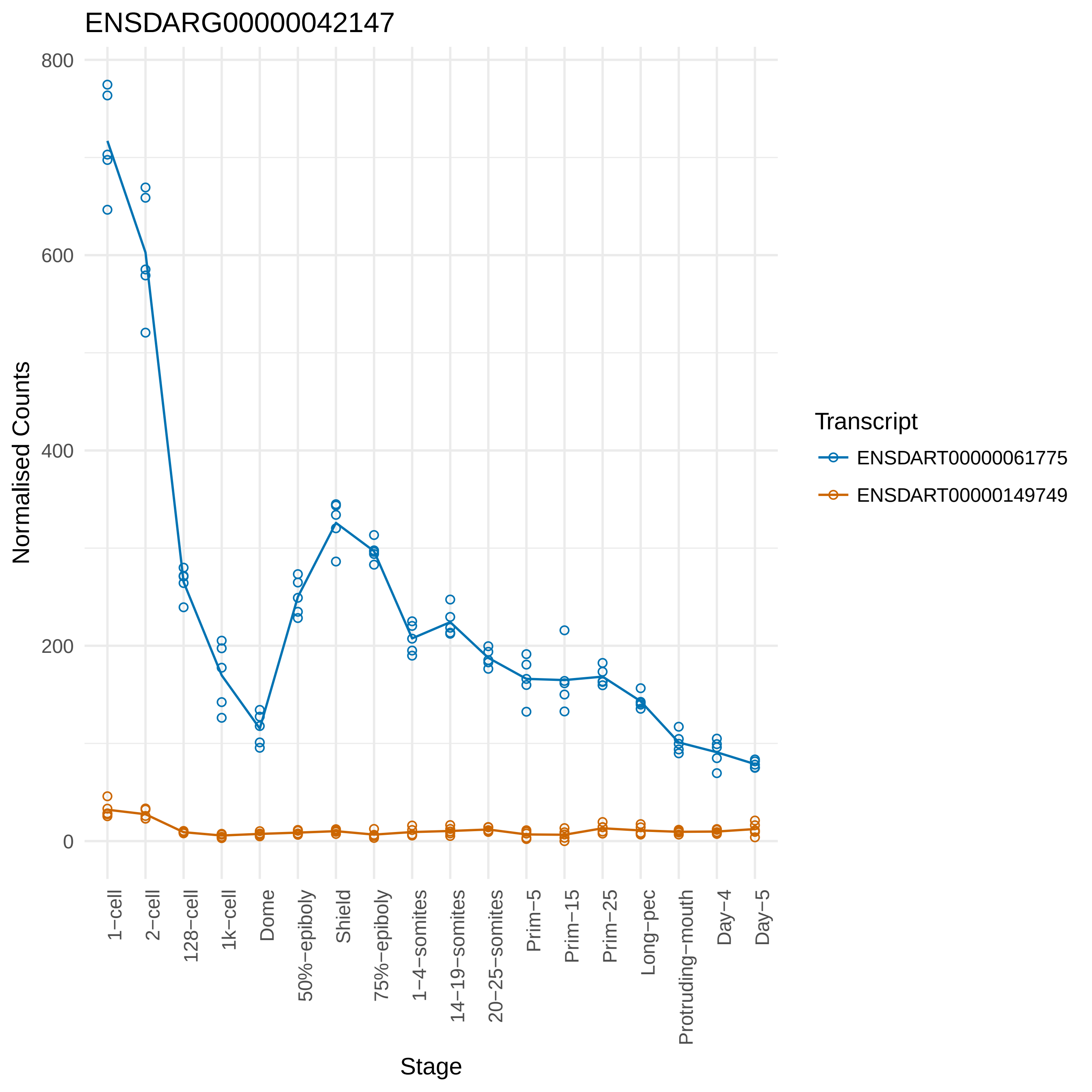
ENSDARG00000042147
Normalised Counts
800
600
400
200
0
Stage
1−cell
2−cell
128−cell
1k−cell
Dome
50%−epiboly
Shield
75%−epiboly
1−4−somites
14−19−somites
20−25−somites
Prim−5
Prim−15
Prim−25
Long−pec
Protruding−mouth
Day−4
Day−5
Transcript
ENSDART00000061775
ENSDART00000149749

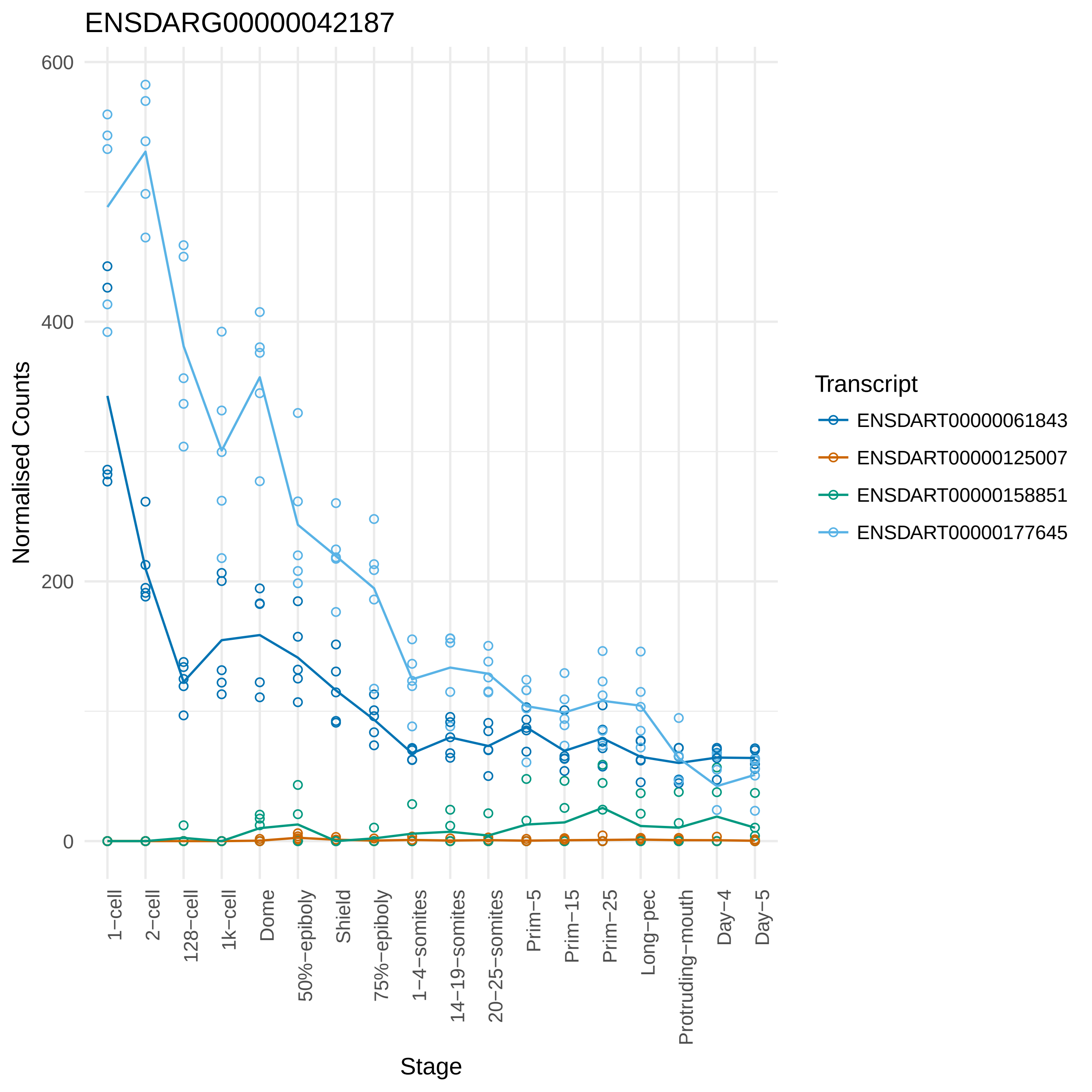

ENSDARG00000042187
Normalised Counts
600
400
200
0
1−cell
2−cell
128−cell
1k−cell
Dome
50%−epiboly
Shield
75%−epiboly
1−4−somites
14−19−somites
20−25−somites
Prim−5
Prim−15
Prim−25
Long−pec
Protruding−mouth
Day−4
Day−5
Stage
Transcript
ENSDART00000061843
ENSDART00000125007
ENSDART00000158851
ENSDART00000177645

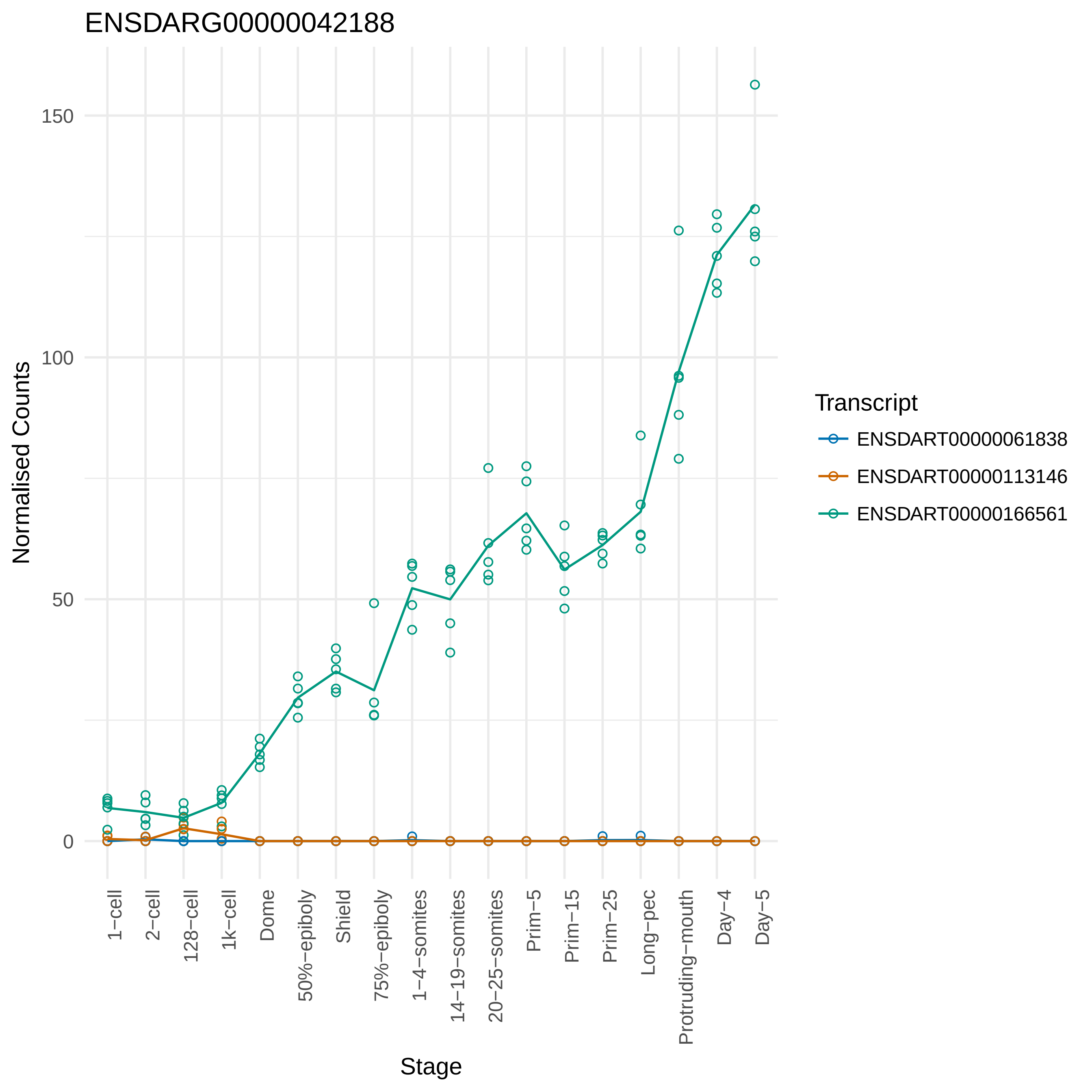

ENSDARG00000042188
Normalised Counts
150
100
50
0
1−cell
2−cell
128−cell
1k−cell
Dome
50%−epiboly
Shield
75%−epiboly
1−4−somites
14−19−somites
20−25−somites
Prim−5
Prim−15
Prim−25
Long−pec
Protruding−mouth
Day−4
Day−5
Stage
Transcript
ENSDART00000061838
ENSDART00000113146
ENSDART00000166561

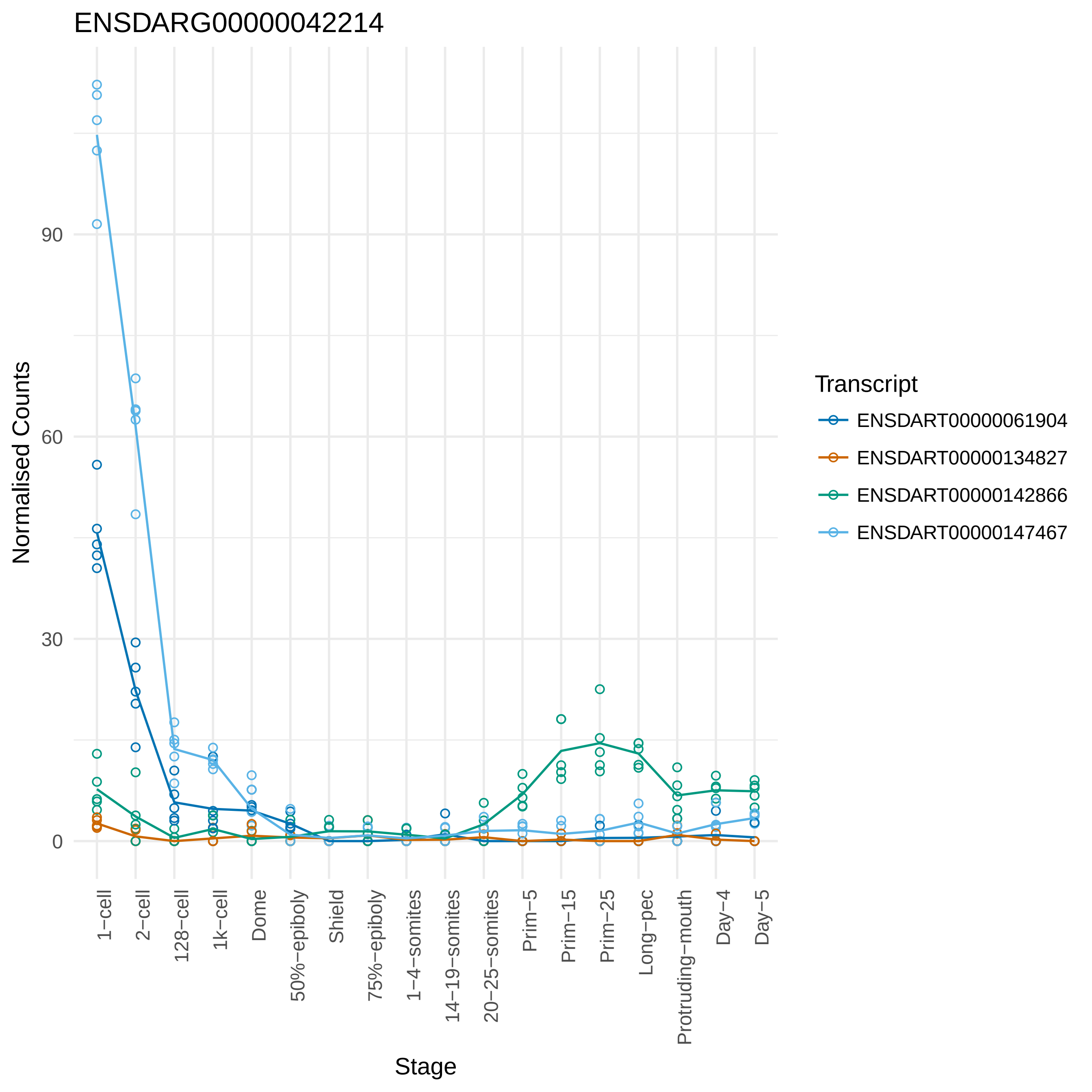
ENSDARG00000042214
Normalised Counts
0
30
60
90
Stage
1−cell
2−cell
128−cell
1k−cell
Dome
50%−epiboly
Shield
75%−epiboly
1−4−somites
14−19−somites
20−25−somites
Prim−5
Prim−15
Prim−25
Long−pec
Protruding−mouth
Day−4
Day−5
Transcript
ENSDART00000061904
ENSDART00000134827
ENSDART00000142866
ENSDART00000147467

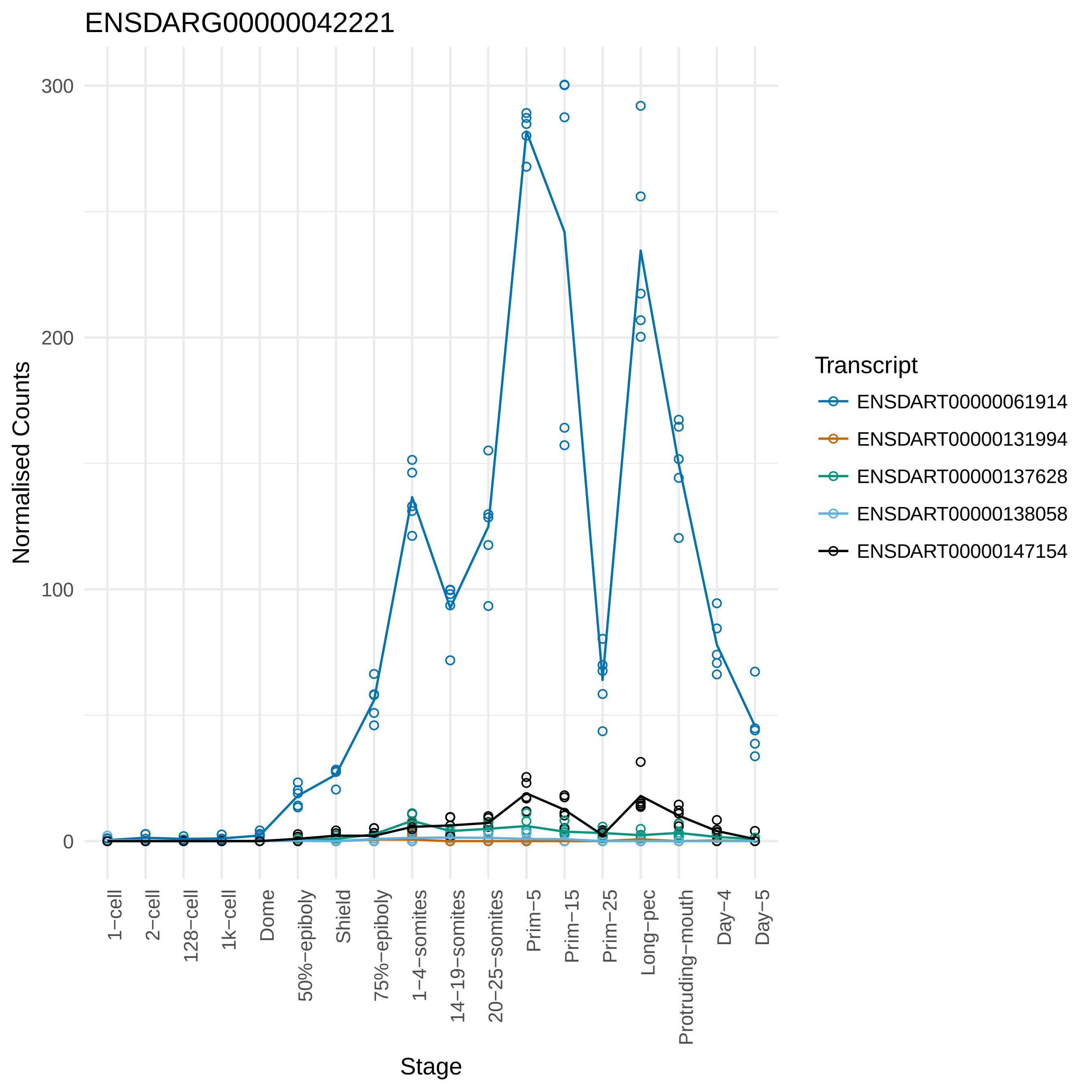
ENSDARG00000042221
Normalised Counts
300
200
100
0
1−cell
2−cell
128−cell
1k−cell
Dome
50%−epiboly
Shield
75%−epiboly
1−4−somites
14−19−somites
20−25−somites
Prim−5
Prim−15
Prim−25
Long−pec
Protruding−mouth
Day−4
Day−5
Stage
Transcript
ENSDART00000061914
ENSDART00000131994
ENSDART00000137628
ENSDART00000138058
ENSDART00000147154

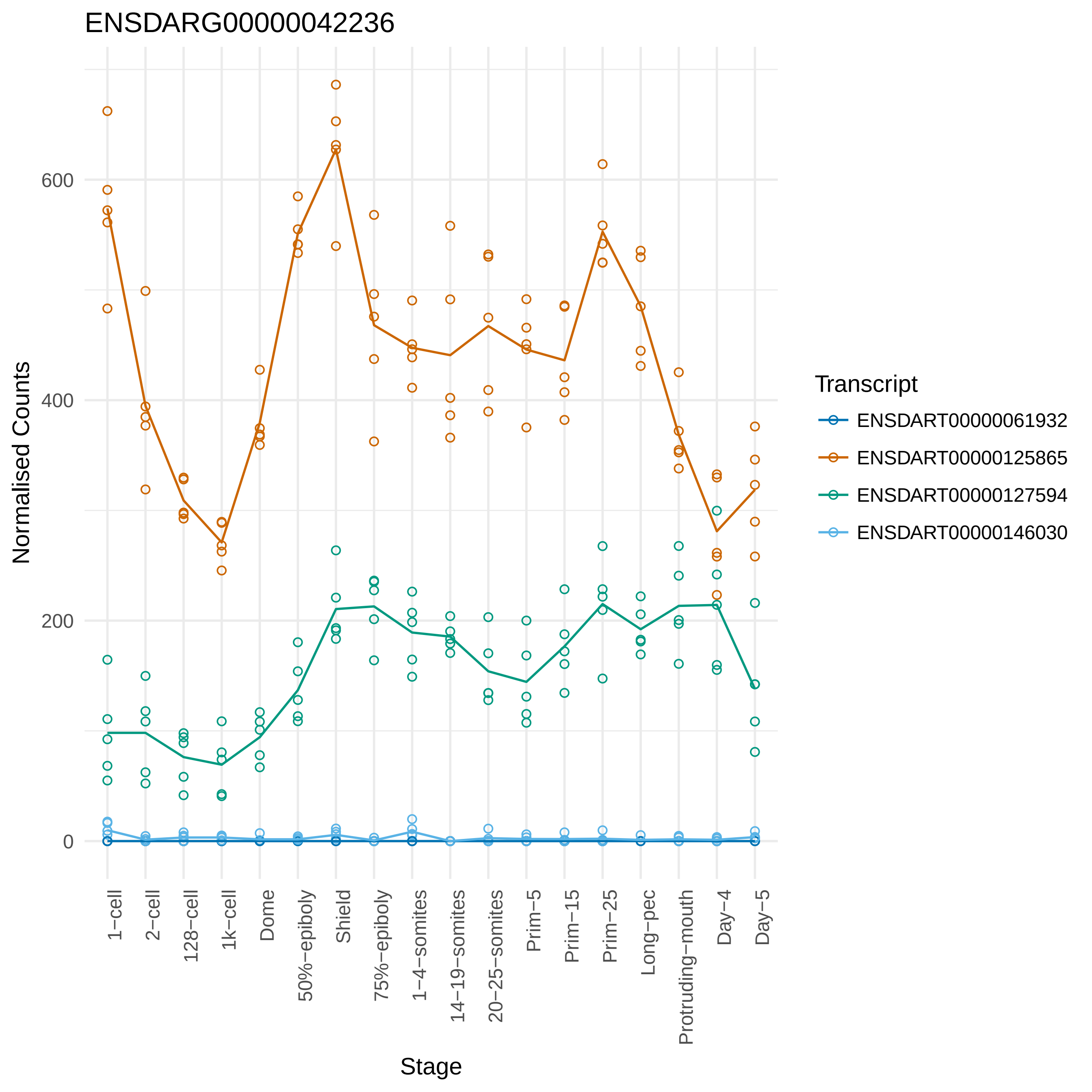

ENSDARG00000042236
Normalised Counts
0
200
400
600
Stage
1−cell
2−cell
128−cell
1k−cell
Dome
50%−epiboly
Shield
75%−epiboly
1−4−somites
14−19−somites
20−25−somites
Prim−5
Prim−15
Prim−25
Long−pec
Protruding−mouth
Day−4
Day−5
Transcript
ENSDART00000061932
ENSDART00000125865
ENSDART00000127594
ENSDART00000146030

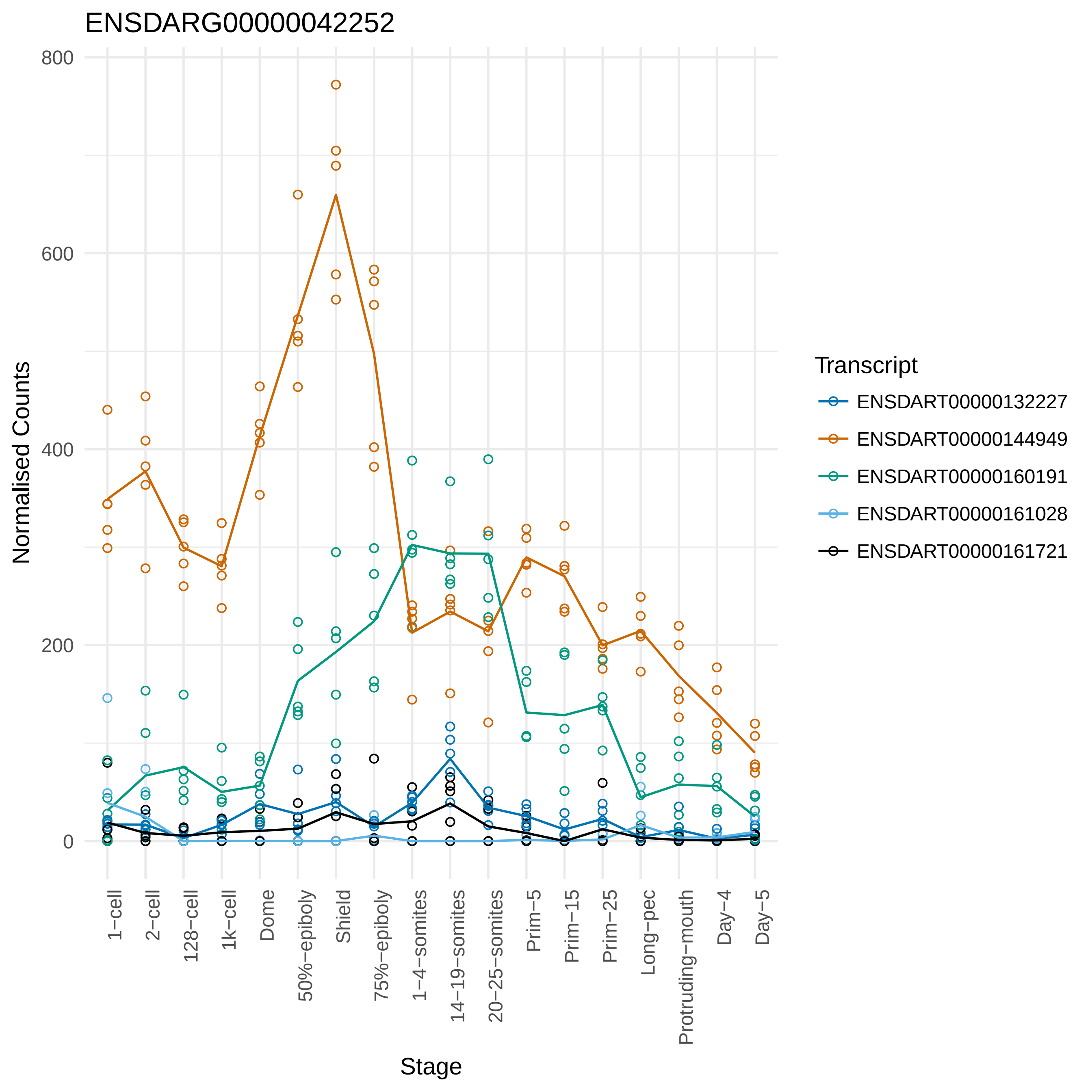
ENSDARG00000042252
Normalised Counts
800
600
400
200
0
1−cell
2−cell
128−cell
1k−cell
Dome
50%−epiboly
Shield
75%−epiboly
1−4−somites
14−19−somites
20−25−somites
Prim−5
Prim−15
Prim−25
Long−pec
Protruding−mouth
Day−4
Day−5
Stage
Transcript
ENSDART00000132227
ENSDART00000144949
ENSDART00000160191
ENSDART00000161028
ENSDART00000161721

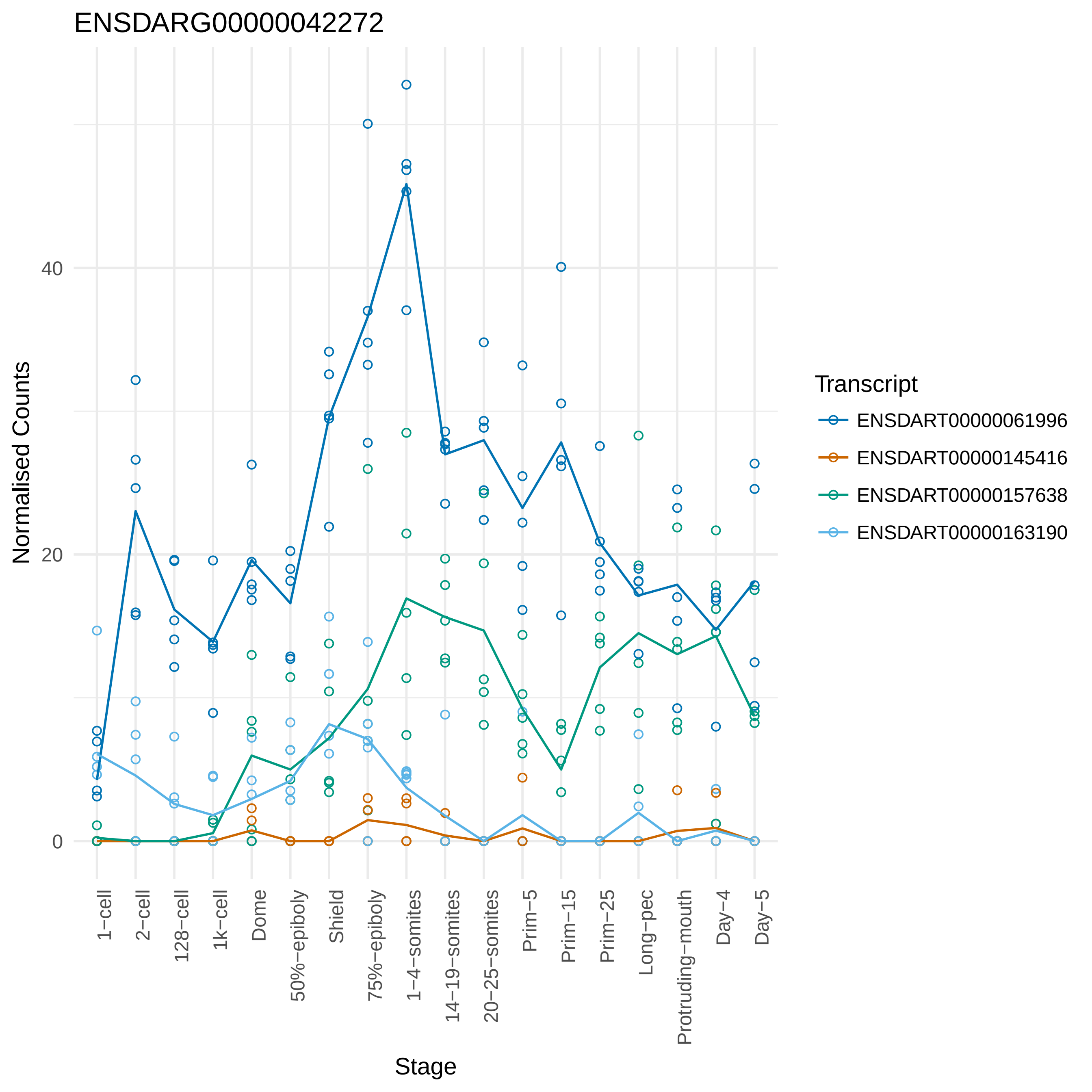
ENSDARG00000042272
Normalised Counts
0
20
40
Stage
1−cell
2−cell
128−cell
1k−cell
Dome
50%−epiboly
Shield
75%−epiboly
1−4−somites
14−19−somites
20−25−somites
Prim−5
Prim−15
Prim−25
Long−pec
Protruding−mouth
Day−4
Day−5
Transcript
ENSDART00000061996
ENSDART00000145416
ENSDART00000157638
ENSDART00000163190

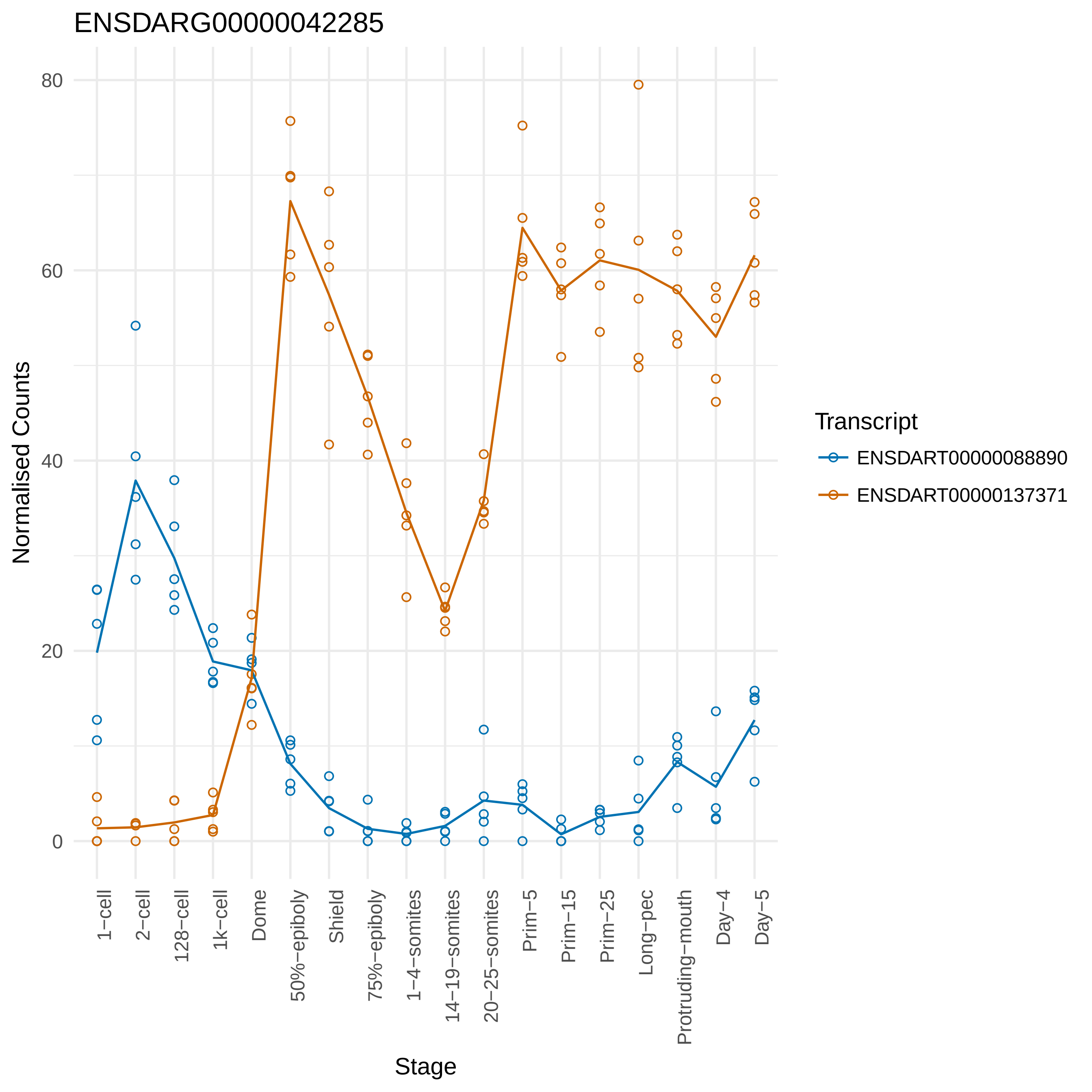
ENSDARG00000042285
Normalised Counts
0
20
40
60
80
Stage
1−cell
2−cell
128−cell
1k−cell
Dome
50%−epiboly
Shield
75%−epiboly
1−4−somites
14−19−somites
20−25−somites
Prim−5
Prim−15
Prim−25
Long−pec
Protruding−mouth
Day−4
Day−5
Transcript
ENSDART00000088890
ENSDART00000137371

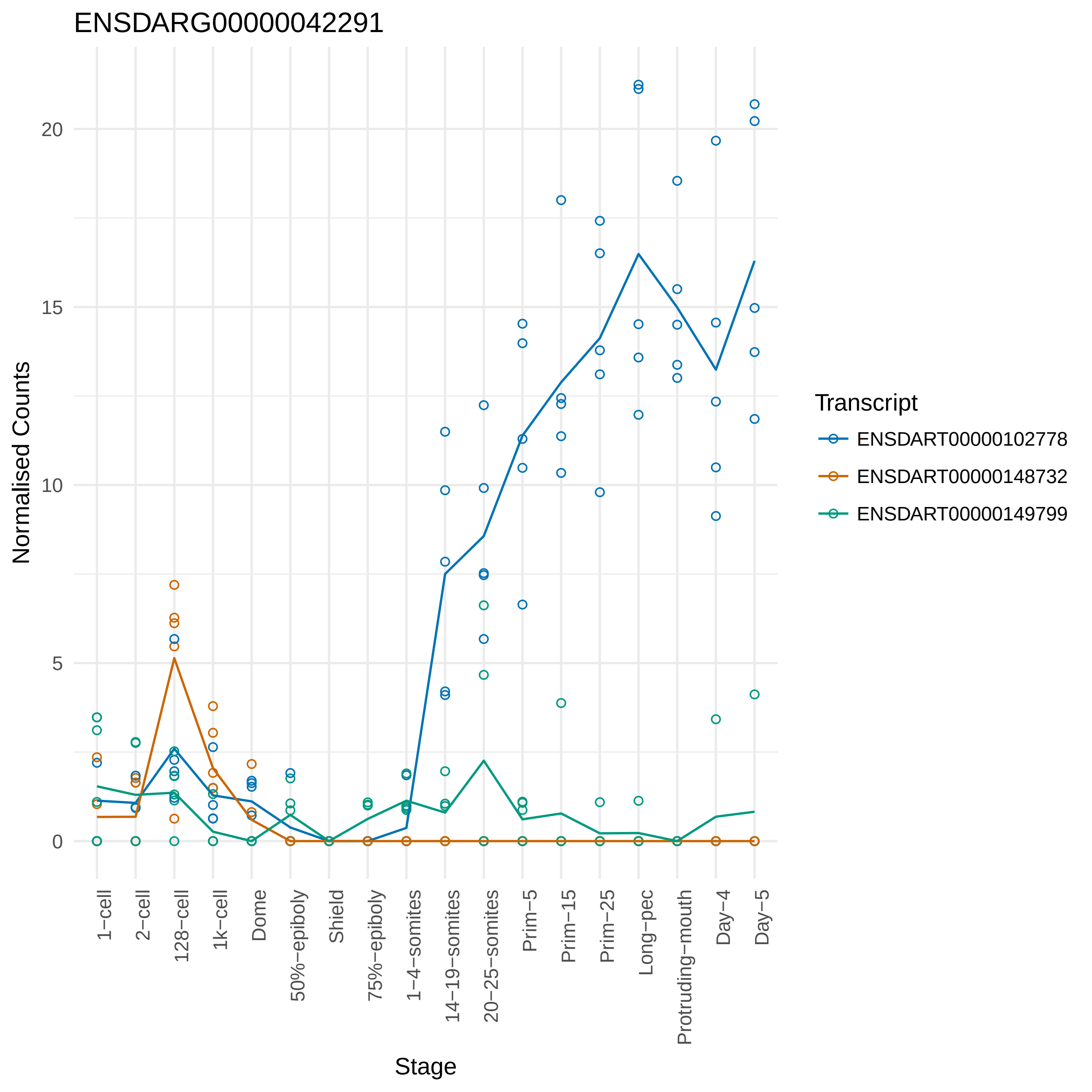

ENSDARG00000042291
Normalised Counts
20
15
10
5
0
1−cell
2−cell
128−cell
1k−cell
Dome
50%−epiboly
Shield
75%−epiboly
1−4−somites
14−19−somites
20−25−somites
Prim−5
Prim−15
Prim−25
Long−pec
Protruding−mouth
Day−4
Day−5
Stage
Transcript
ENSDART00000102778
ENSDART00000148732
ENSDART00000149799

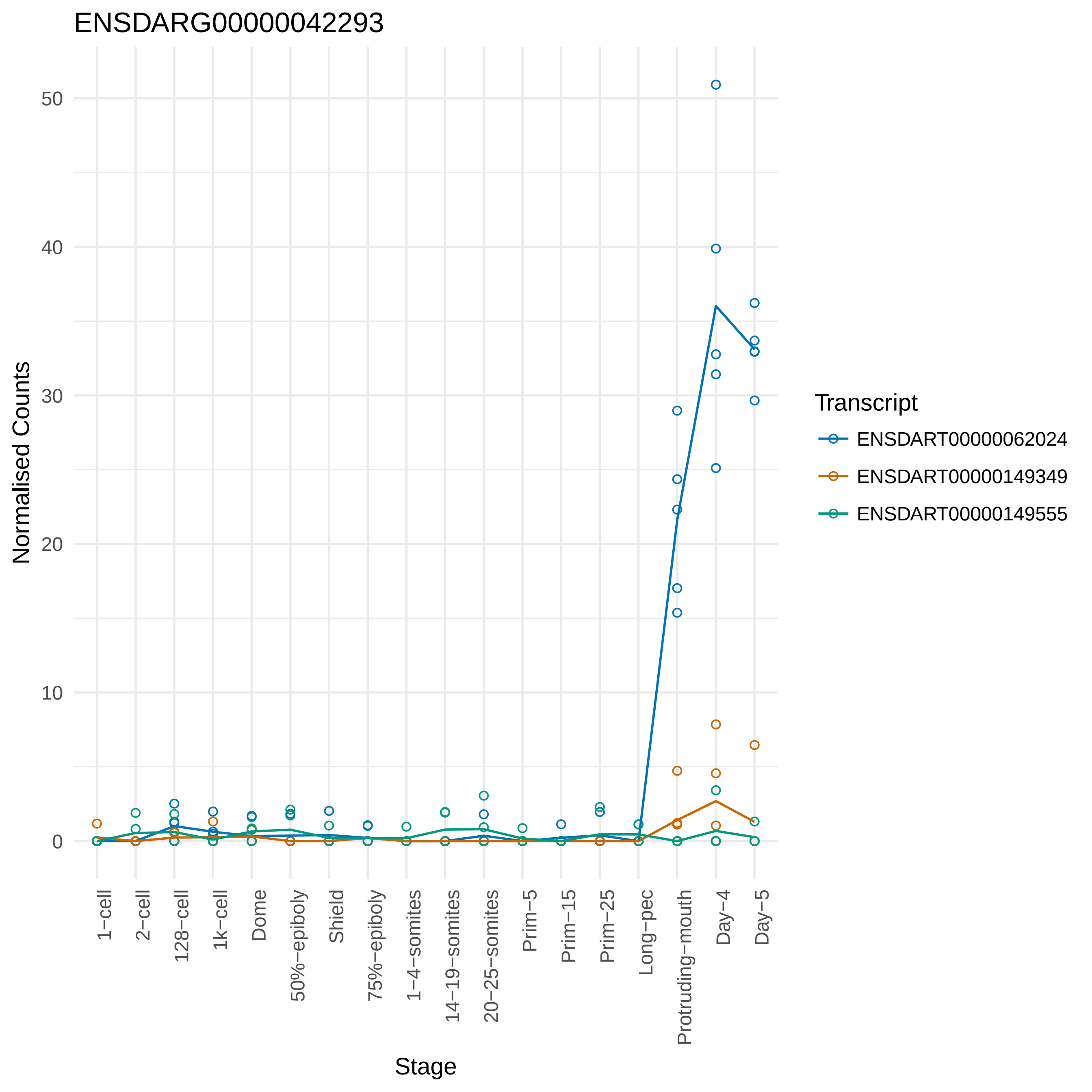
ENSDARG00000042293
Normalised Counts
0
10
20
30
40
50
1−cell
2−cell
128−cell
1k−cell
Dome
50%−epiboly
Shield
75%−epiboly
1−4−somites
14−19−somites
20−25−somites
Prim−5
Prim−15
Prim−25
Long−pec
Protruding−mouth
Day−4
Day−5
Stage
Transcript
ENSDART00000062024
ENSDART00000149349
ENSDART00000149555

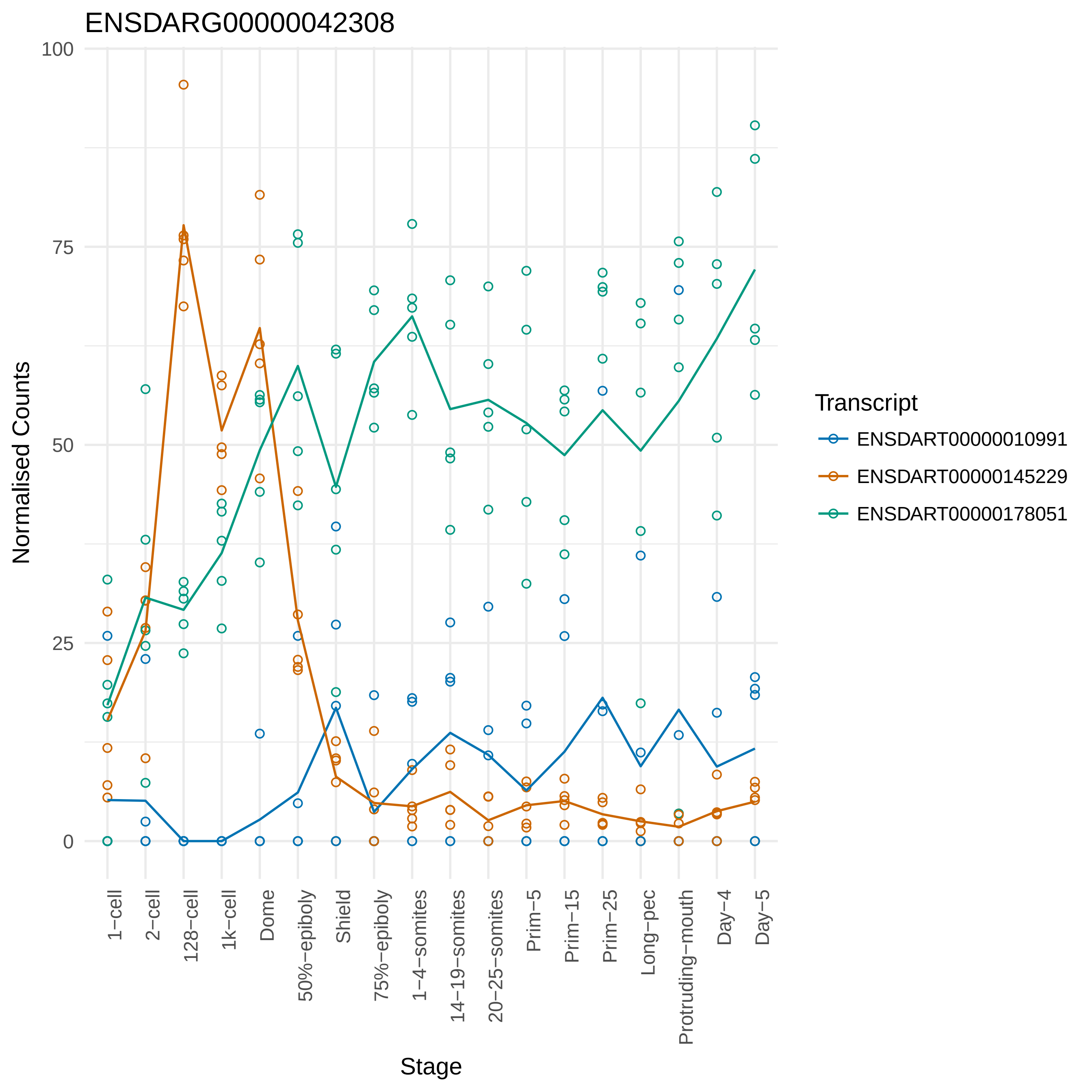
ENSDARG00000042308
Normalised Counts
100
75
50
25
0
1−cell
2−cell
128−cell
1k−cell
Dome
50%−epiboly
Shield
75%−epiboly
1−4−somites
14−19−somites
20−25−somites
Prim−5
Prim−15
Prim−25
Long−pec
Protruding−mouth
Day−4
Day−5
Stage
Transcript
ENSDART00000010991
ENSDART00000145229
ENSDART00000178051

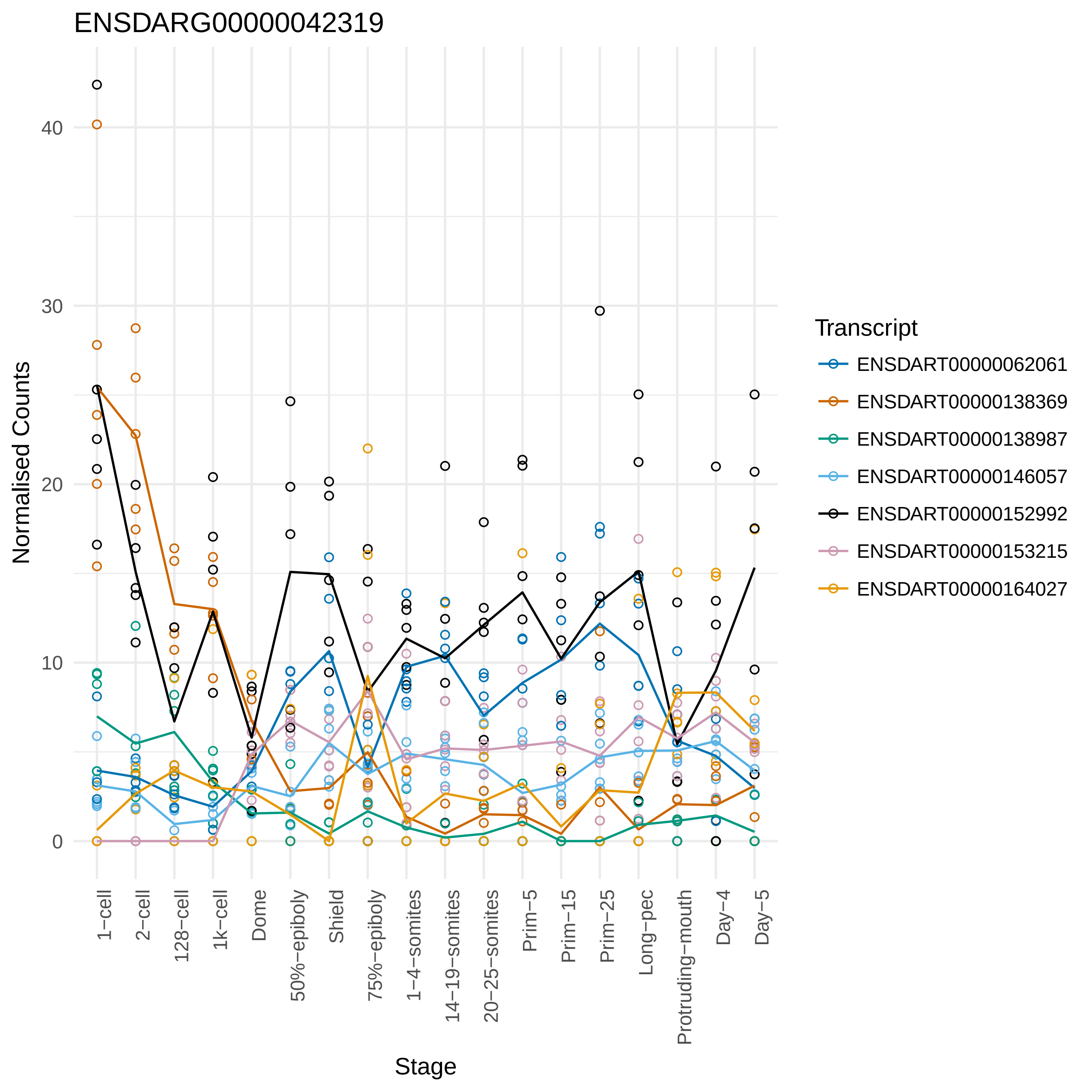

ENSDARG00000042319
Normalised Counts
40
30
20
10
0
1−cell
2−cell
128−cell
1k−cell
Dome
50%−epiboly
Shield
75%−epiboly
1−4−somites
14−19−somites
20−25−somites
Prim−5
Prim−15
Prim−25
Long−pec
Protruding−mouth
Day−4
Day−5
Stage
Transcript
ENSDART00000062061
ENSDART00000138369
ENSDART00000138987
ENSDART00000146057
ENSDART00000152992
ENSDART00000153215
ENSDART00000164027

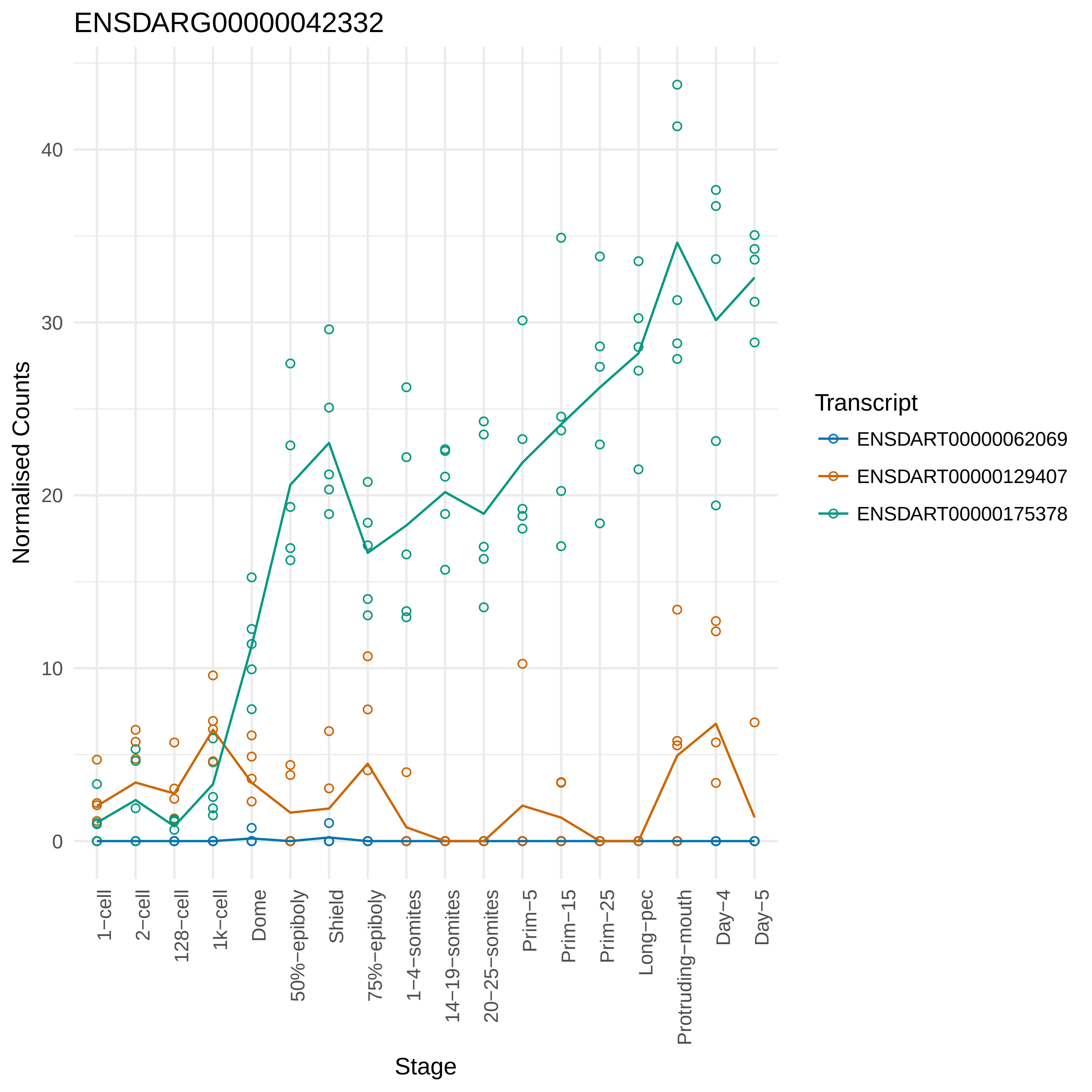

ENSDARG00000042332
Normalised Counts
0
10
20
30
40
Stage
1−cell
2−cell
128−cell
1k−cell
Dome
50%−epiboly
Shield
75%−epiboly
1−4−somites
14−19−somites
20−25−somites
Prim−5
Prim−15
Prim−25
Long−pec
Protruding−mouth
Day−4
Day−5
Transcript
ENSDART00000062069
ENSDART00000129407
ENSDART00000175378

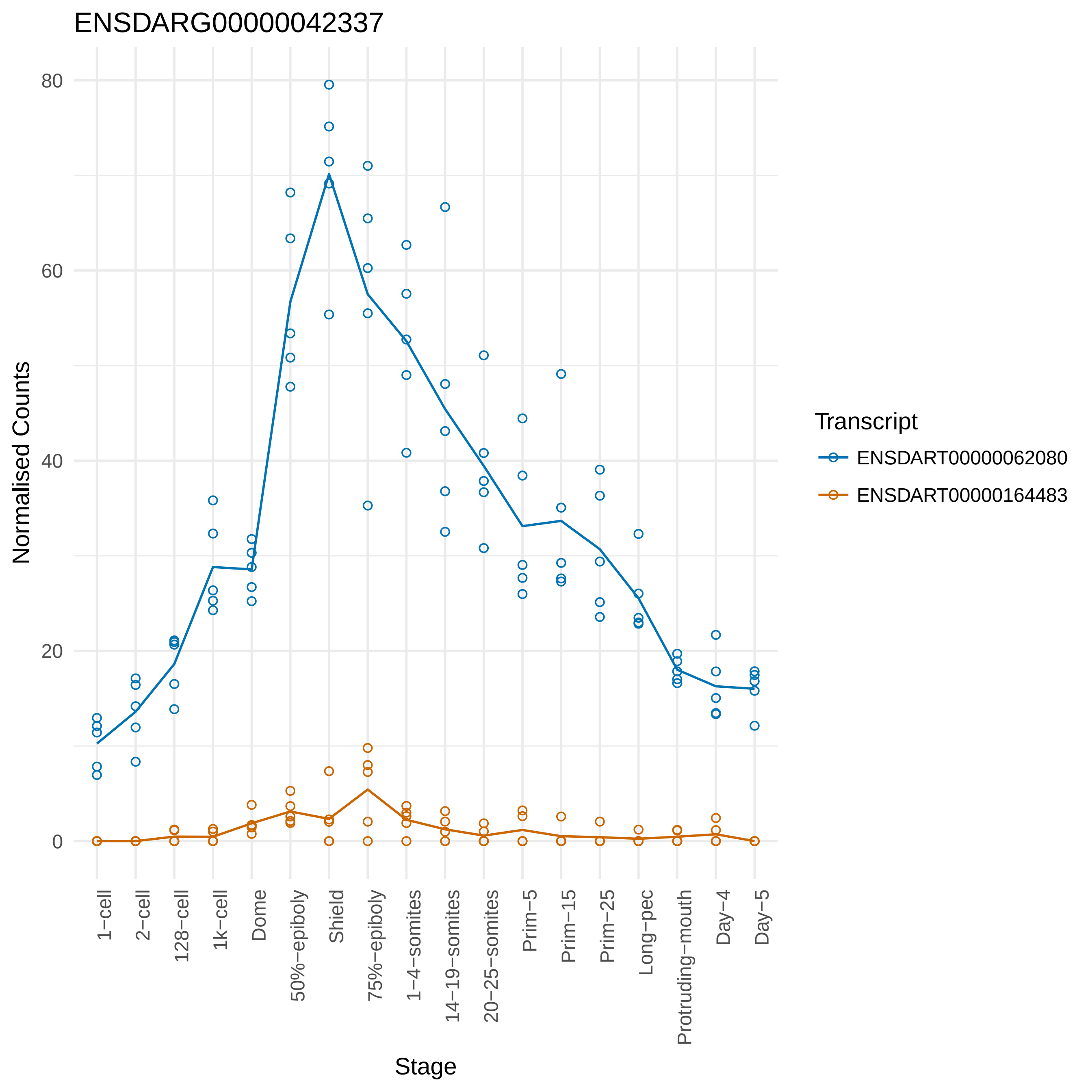

ENSDARG00000042337
Normalised Counts
80
60
40
20
0
1−cell
2−cell
128−cell
1k−cell
Dome
50%−epiboly
Shield
75%−epiboly
1−4−somites
14−19−somites
20−25−somites
Prim−5
Prim−15
Prim−25
Long−pec
Protruding−mouth
Day−4
Day−5
Stage
Transcript
ENSDART00000062080
ENSDART00000164483

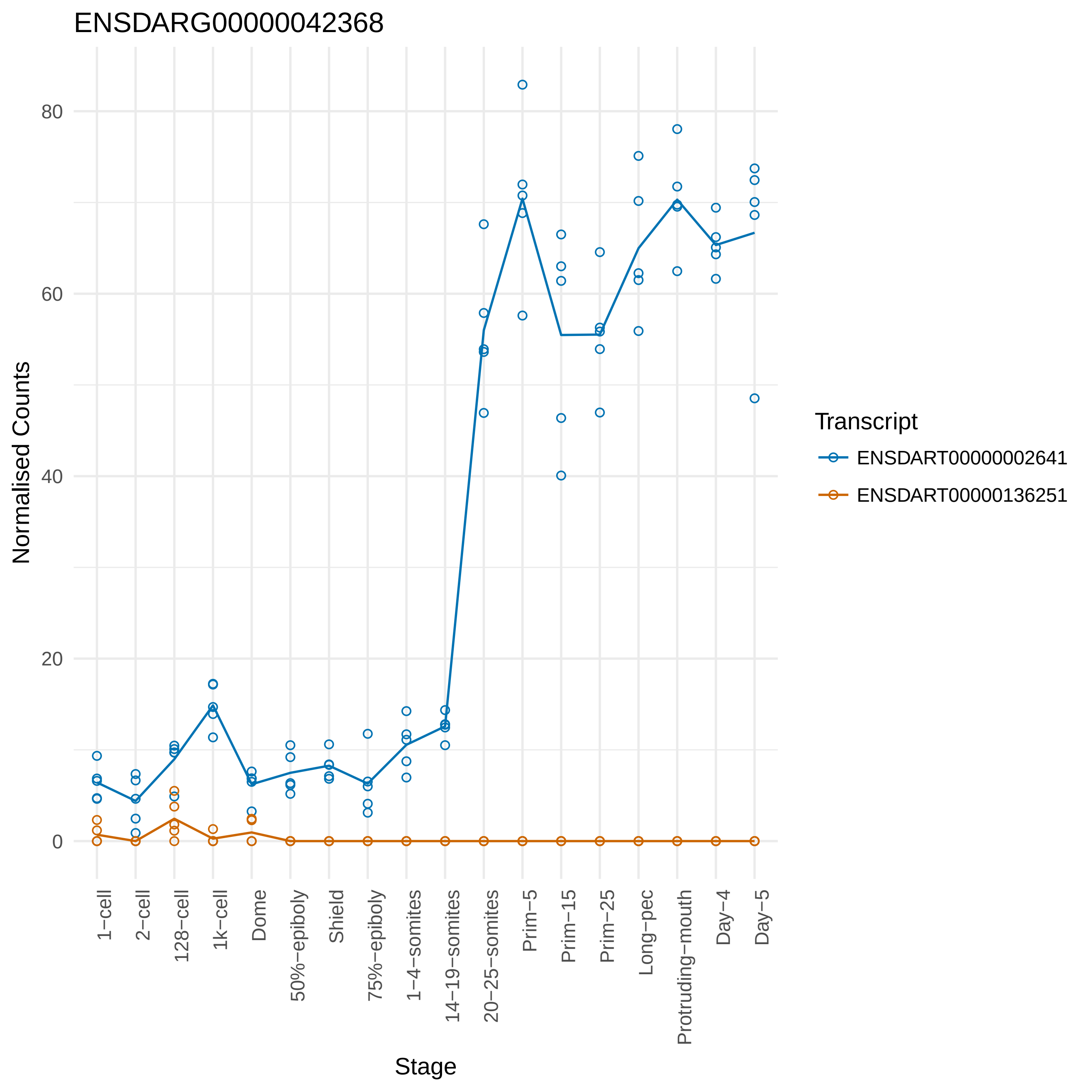

ENSDARG00000042368
Normalised Counts
80
60
40
20
0
1−cell
2−cell
128−cell
1k−cell
Dome
50%−epiboly
Shield
75%−epiboly
1−4−somites
14−19−somites
20−25−somites
Prim−5
Prim−15
Prim−25
Long−pec
Protruding−mouth
Day−4
Day−5
Stage
Transcript
ENSDART00000002641
ENSDART00000136251

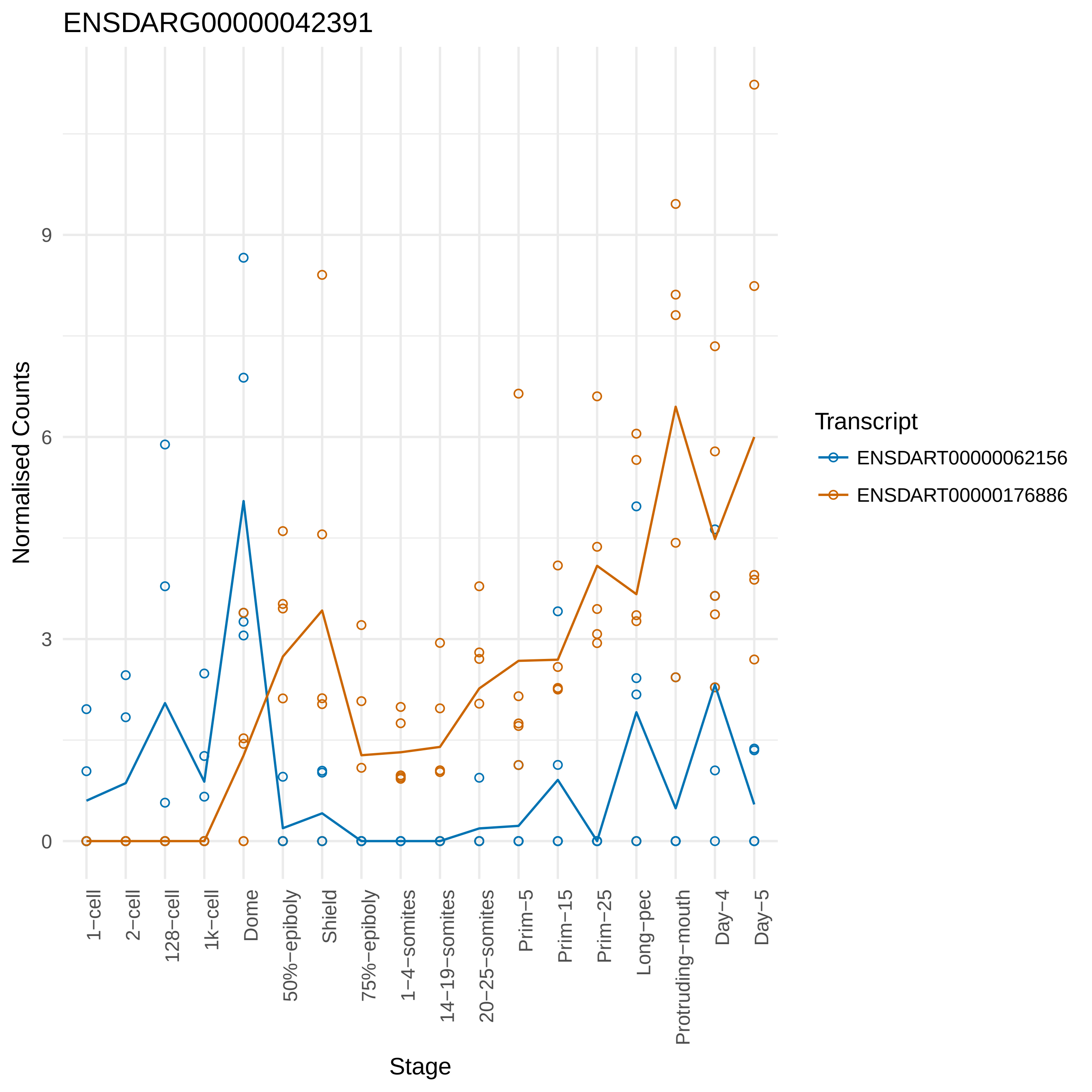

ENSDARG00000042391
Normalised Counts
9
6
3
0
1−cell
2−cell
128−cell
1k−cell
Dome
50%−epiboly
Shield
75%−epiboly
1−4−somites
14−19−somites
20−25−somites
Prim−5
Prim−15
Prim−25
Long−pec
Protruding−mouth
Day−4
Day−5
Stage
Transcript
ENSDART00000062156
ENSDART00000176886

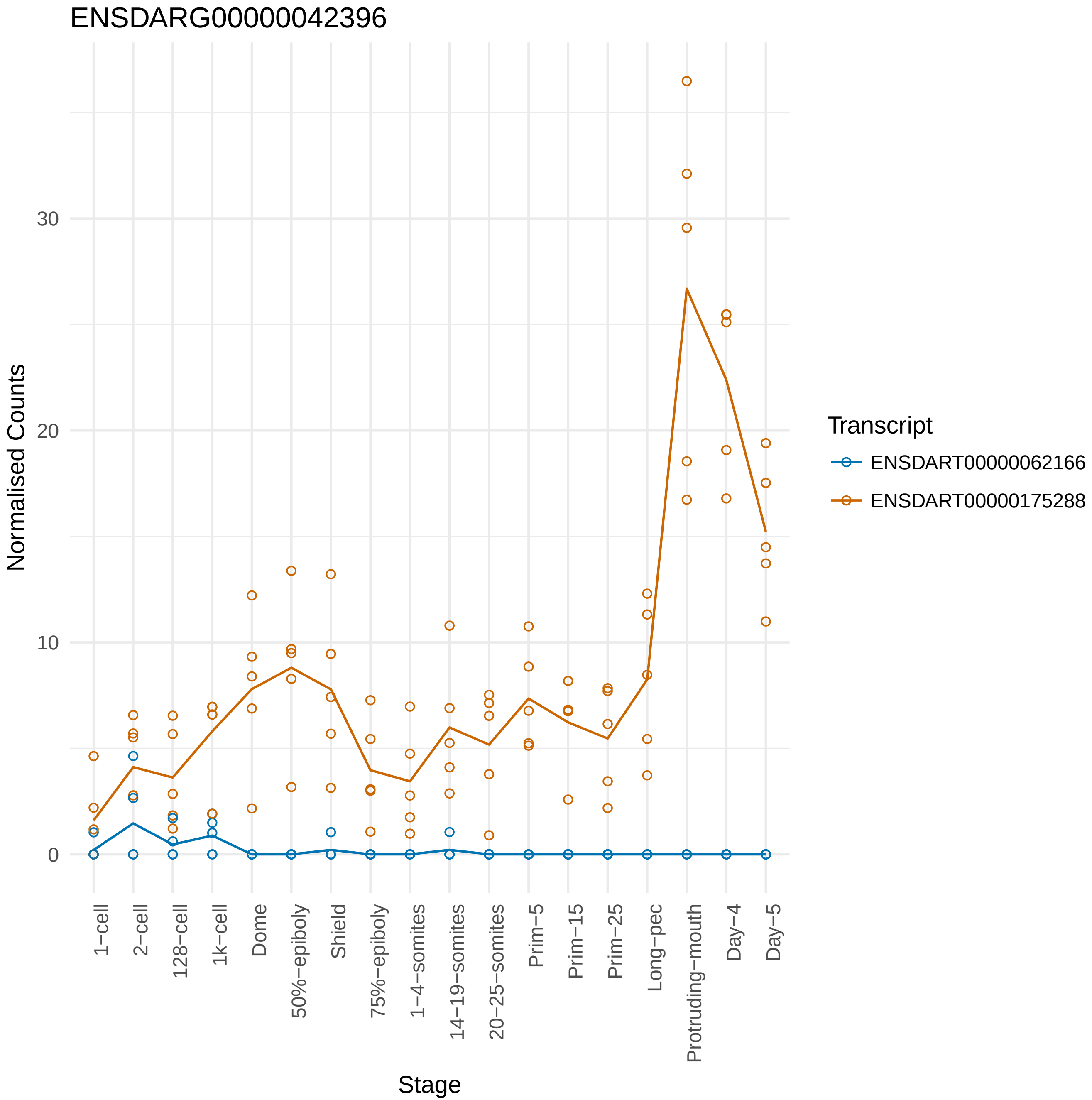

ENSDARG00000042396
Normalised Counts
0
10
20
30
1−cell
2−cell
128−cell
1k−cell
Dome
50%−epiboly
Shield
75%−epiboly
1−4−somites
14−19−somites
20−25−somites
Prim−5
Prim−15
Prim−25
Long−pec
Protruding−mouth
Day−4
Day−5
Stage
Transcript
ENSDART00000062166
ENSDART00000175288

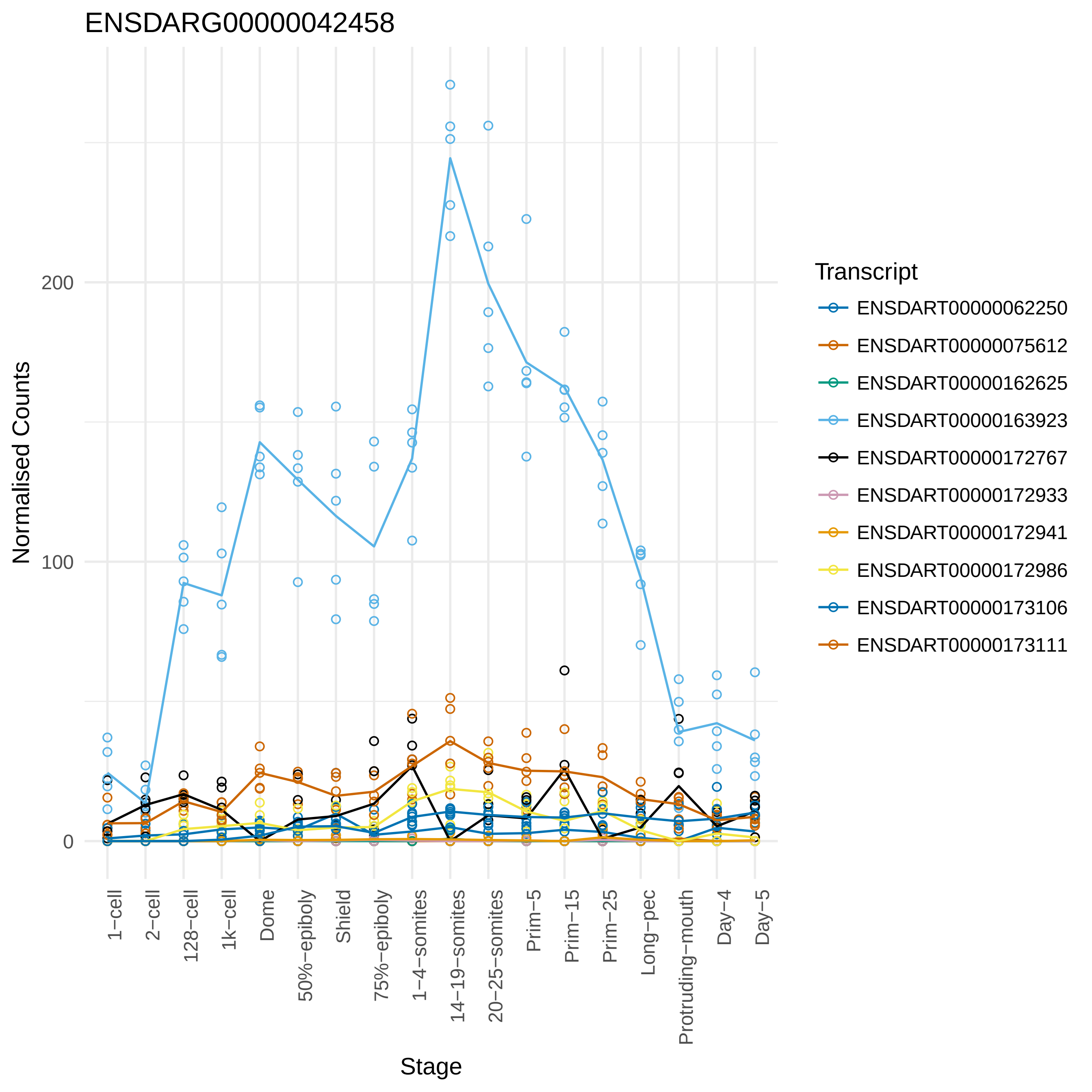

ENSDARG00000042458
Normalised Counts
0
100
200
Stage
1−cell
2−cell
128−cell
1k−cell
Dome
50%−epiboly
Shield
75%−epiboly
1−4−somites
14−19−somites
20−25−somites
Prim−5
Prim−15
Prim−25
Long−pec
Protruding−mouth
Day−4
Day−5
Transcript
ENSDART00000062250
ENSDART00000075612
ENSDART00000162625
ENSDART00000163923
ENSDART00000172767
ENSDART00000172933
ENSDART00000172941
ENSDART00000172986
ENSDART00000173106
ENSDART00000173111

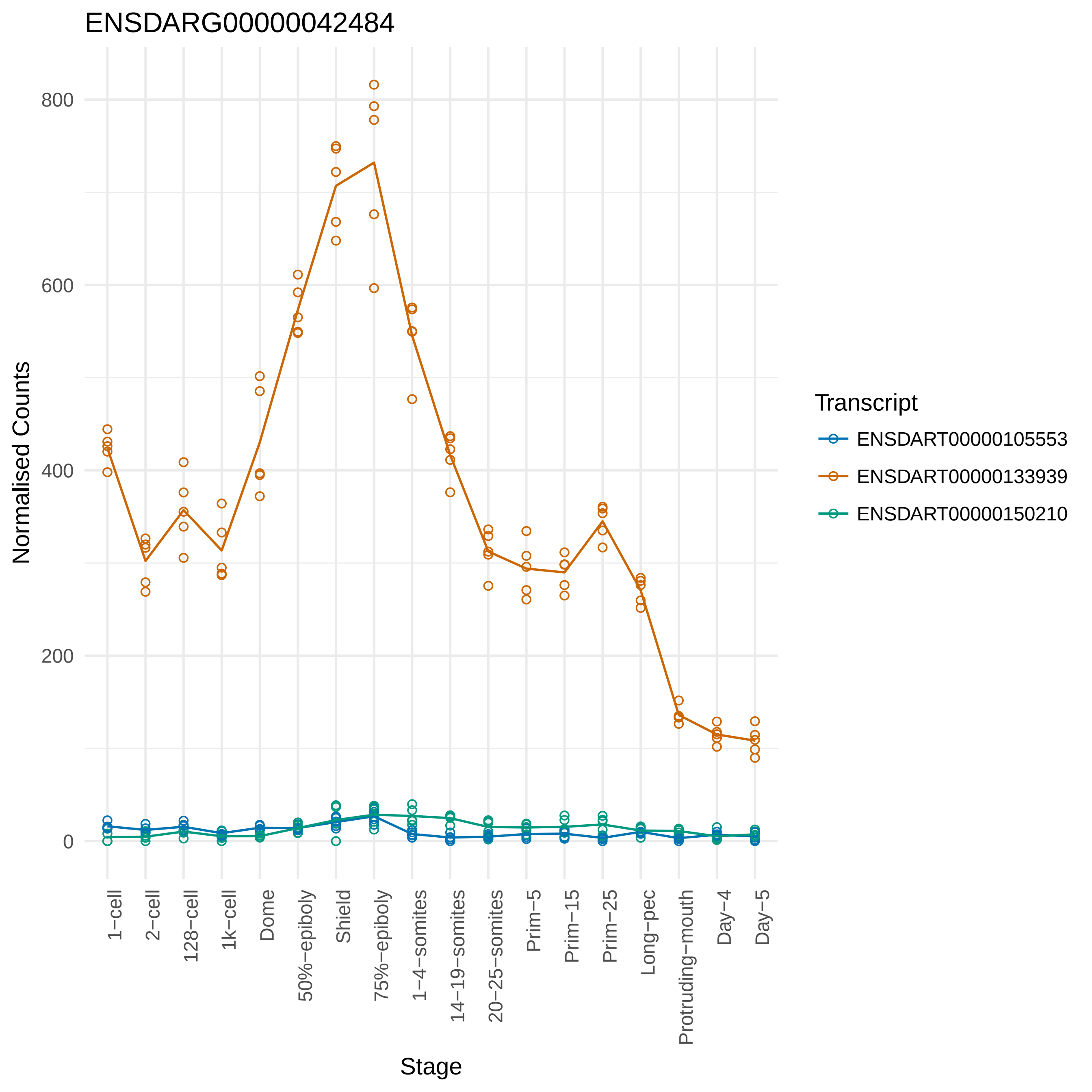
ENSDARG00000042484
Normalised Counts
800
600
400
200
0
1−cell
2−cell
128−cell
1k−cell
Dome
50%−epiboly
Shield
75%−epiboly
1−4−somites
14−19−somites
20−25−somites
Prim−5
Prim−15
Prim−25
Long−pec
Protruding−mouth
Day−4
Day−5
Stage
Transcript
ENSDART00000105553
ENSDART00000133939
ENSDART00000150210

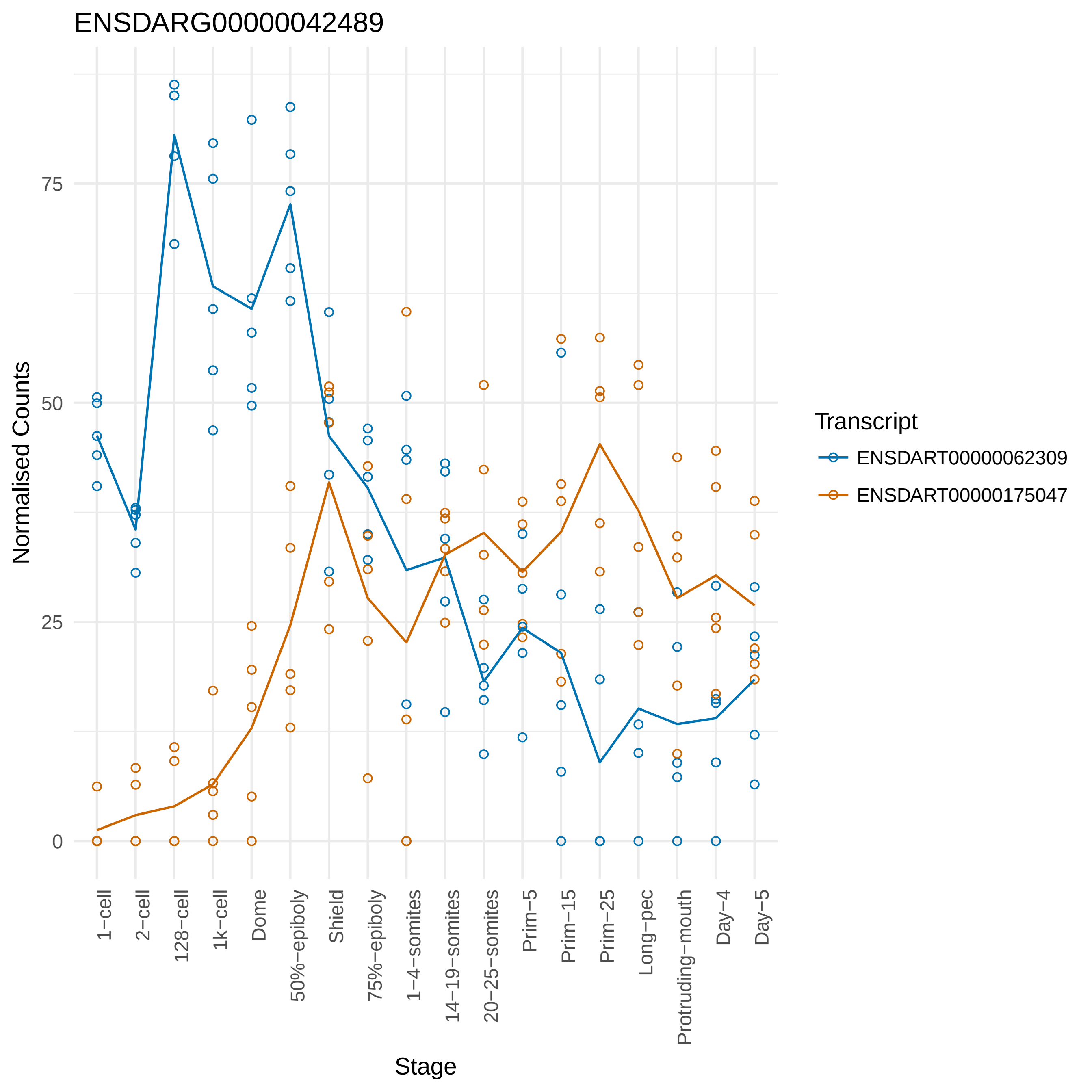

ENSDARG00000042489
Normalised Counts
0
25
50
75
1−cell
2−cell
128−cell
1k−cell
Dome
50%−epiboly
Shield
75%−epiboly
1−4−somites
14−19−somites
20−25−somites
Prim−5
Prim−15
Prim−25
Long−pec
Protruding−mouth
Day−4
Day−5
Stage
Transcript
ENSDART00000062309
ENSDART00000175047

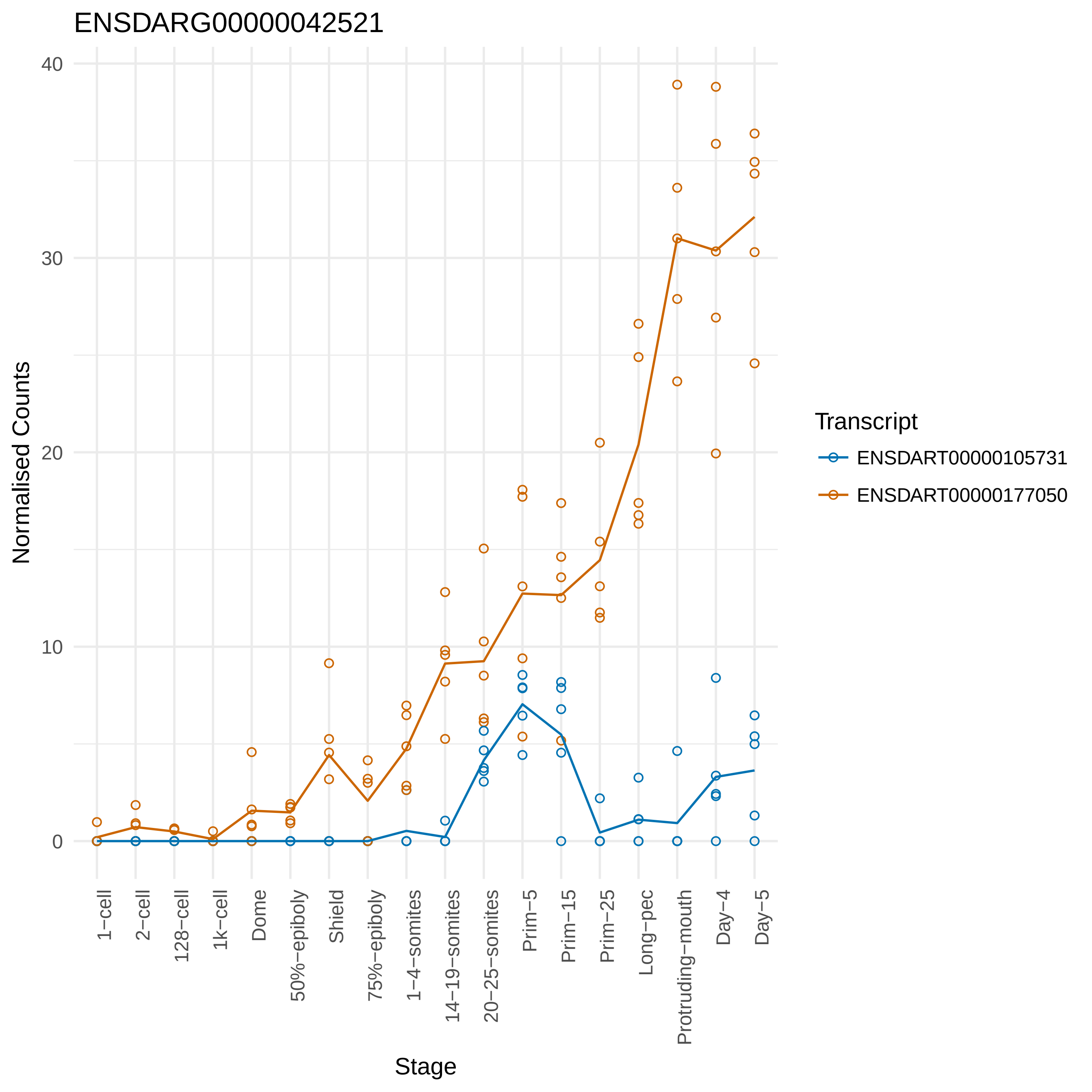

ENSDARG00000042521
Normalised Counts
40
30
20
10
0
1−cell
2−cell
128−cell
1k−cell
Dome
50%−epiboly
Shield
75%−epiboly
1−4−somites
14−19−somites
20−25−somites
Prim−5
Prim−15
Prim−25
Long−pec
Protruding−mouth
Day−4
Day−5
Stage
Transcript
ENSDART00000105731
ENSDART00000177050

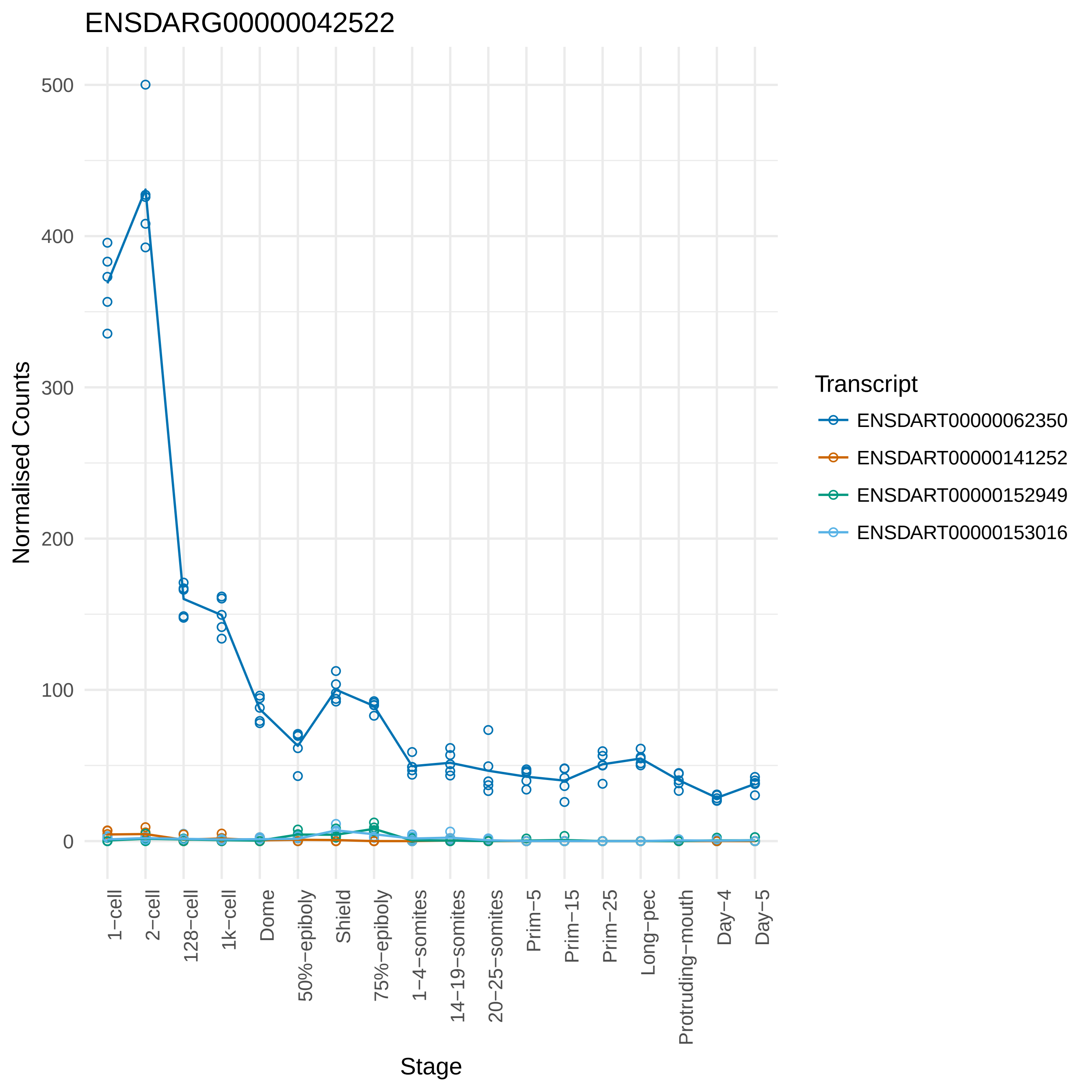

ENSDARG00000042522
Normalised Counts
500
400
300
200
100
0
1−cell
2−cell
128−cell
1k−cell
Dome
50%−epiboly
Shield
75%−epiboly
1−4−somites
14−19−somites
20−25−somites
Prim−5
Prim−15
Prim−25
Long−pec
Protruding−mouth
Day−4
Day−5
Stage
Transcript
ENSDART00000062350
ENSDART00000141252
ENSDART00000152949
ENSDART00000153016

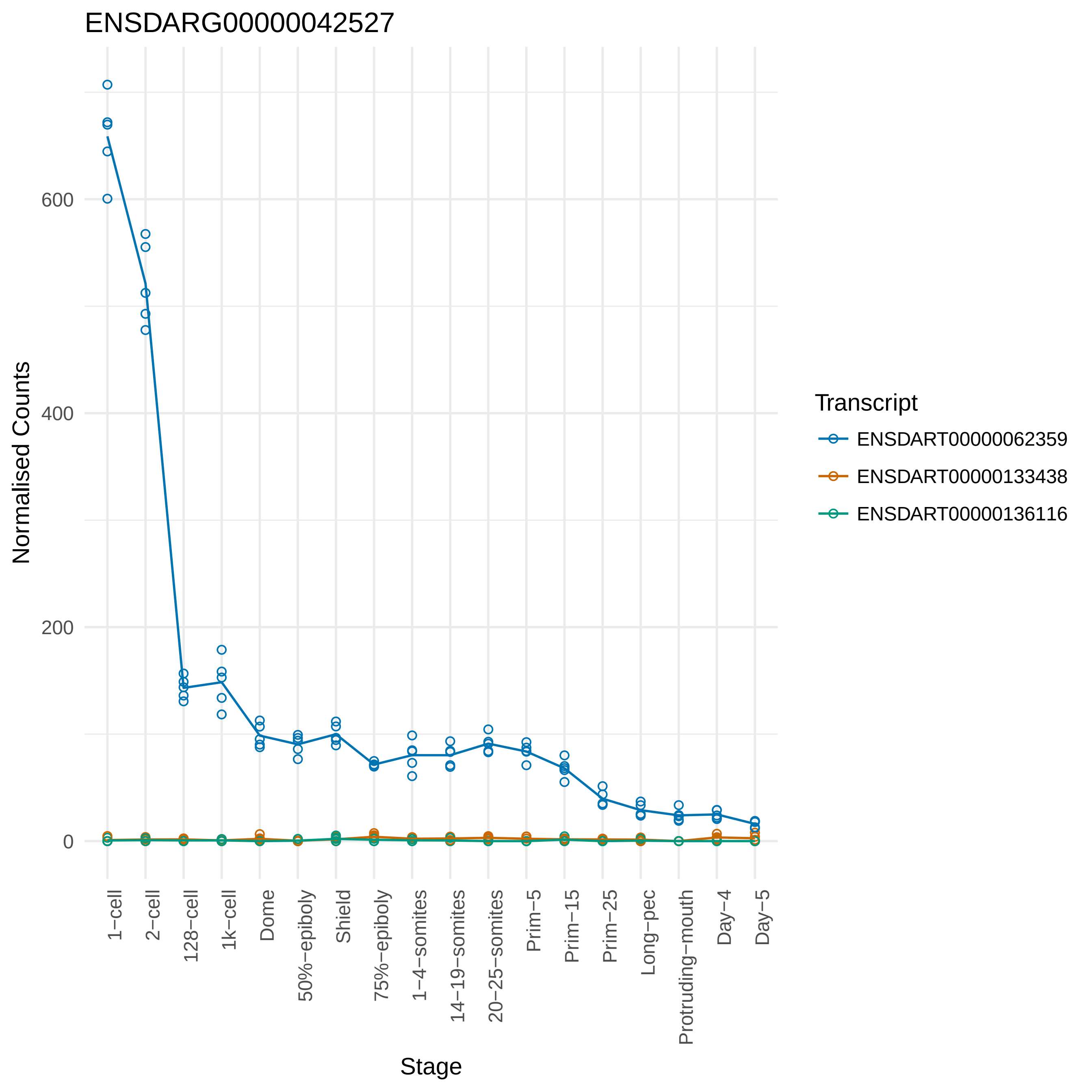
ENSDARG00000042527
Normalised Counts
600
400
200
0
1−cell
2−cell
128−cell
1k−cell
Dome
50%−epiboly
Shield
75%−epiboly
1−4−somites
14−19−somites
20−25−somites
Prim−5
Prim−15
Prim−25
Long−pec
Protruding−mouth
Day−4
Day−5
Stage
Transcript
ENSDART00000062359
ENSDART00000133438
ENSDART00000136116

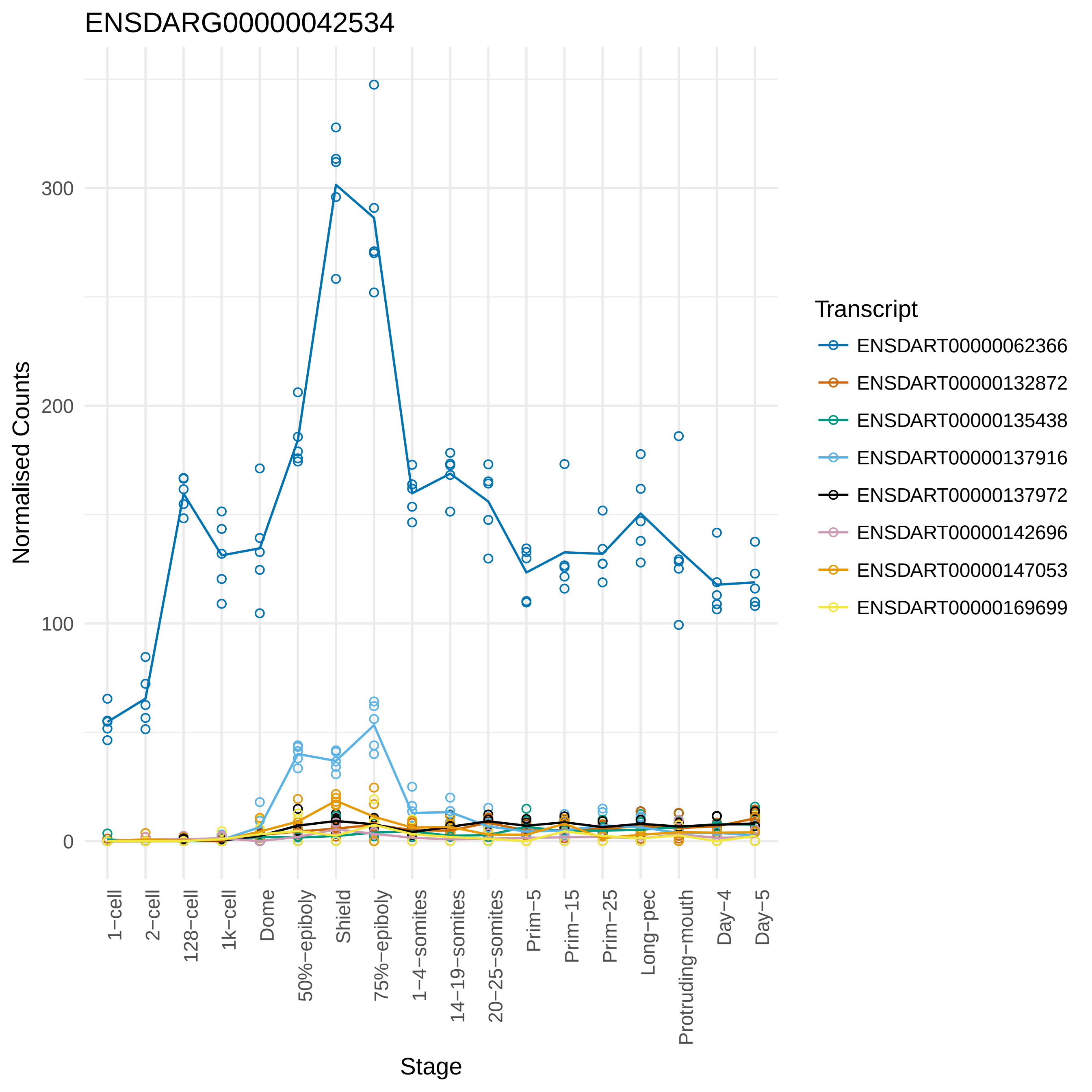

ENSDARG00000042534
Normalised Counts
300
200
100
0
1−cell
2−cell
128−cell
1k−cell
Dome
50%−epiboly
Shield
75%−epiboly
1−4−somites
14−19−somites
20−25−somites
Prim−5
Prim−15
Prim−25
Long−pec
Protruding−mouth
Day−4
Day−5
Stage
Transcript
ENSDART00000062366
ENSDART00000132872
ENSDART00000135438
ENSDART00000137916
ENSDART00000137972
ENSDART00000142696
ENSDART00000147053
ENSDART00000169699

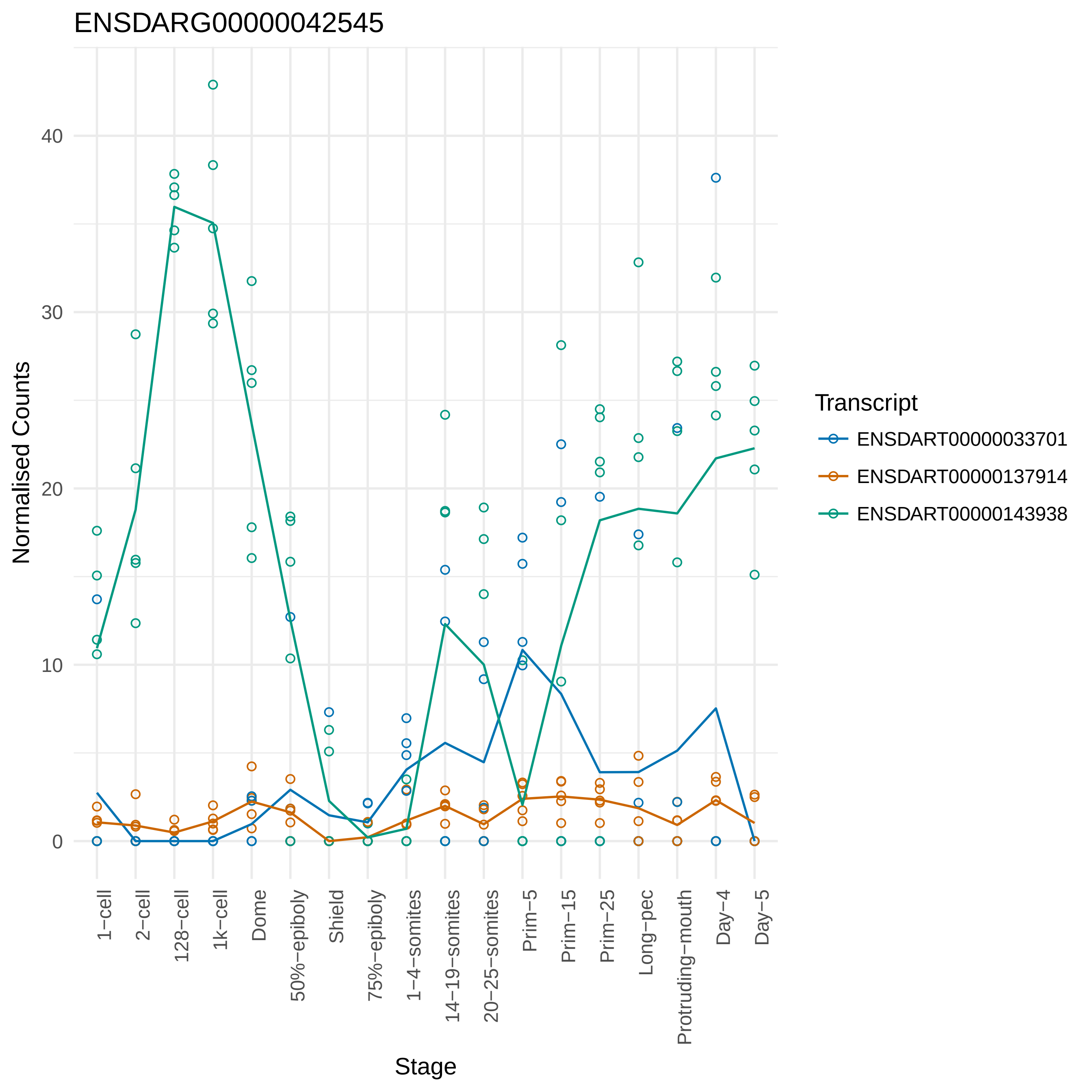

ENSDARG00000042545
Normalised Counts
40
30
20
10
0
1−cell
2−cell
128−cell
1k−cell
Dome
50%−epiboly
Shield
75%−epiboly
1−4−somites
14−19−somites
20−25−somites
Prim−5
Prim−15
Prim−25
Long−pec
Protruding−mouth
Day−4
Day−5
Stage
Transcript
ENSDART00000033701
ENSDART00000137914
ENSDART00000143938

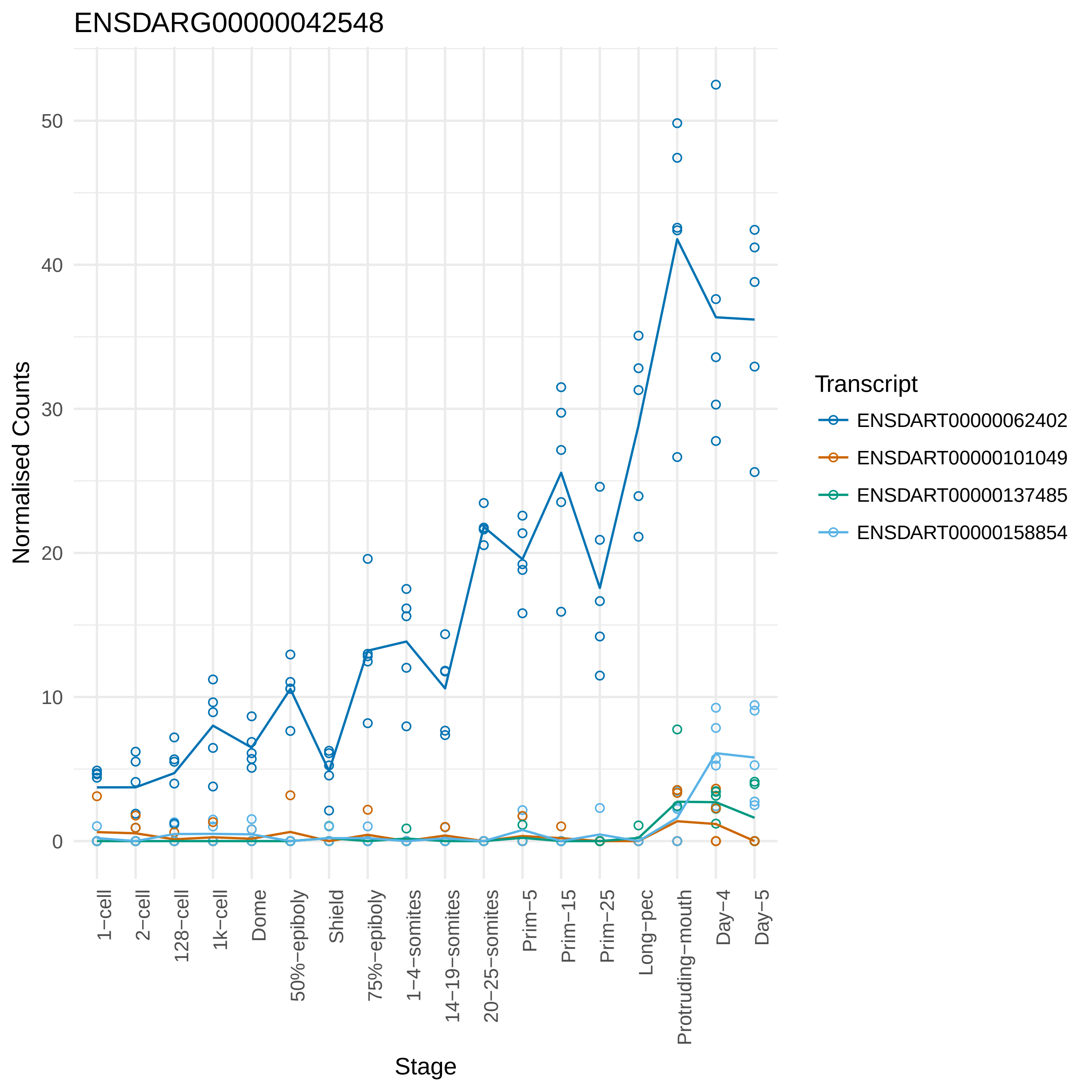

ENSDARG00000042548
Normalised Counts
0
10
20
30
40
50
1−cell
2−cell
128−cell
1k−cell
Dome
50%−epiboly
Shield
75%−epiboly
1−4−somites
14−19−somites
20−25−somites
Prim−5
Prim−15
Prim−25
Long−pec
Protruding−mouth
Day−4
Day−5
Stage
Transcript
ENSDART00000062402
ENSDART00000101049
ENSDART00000137485
ENSDART00000158854

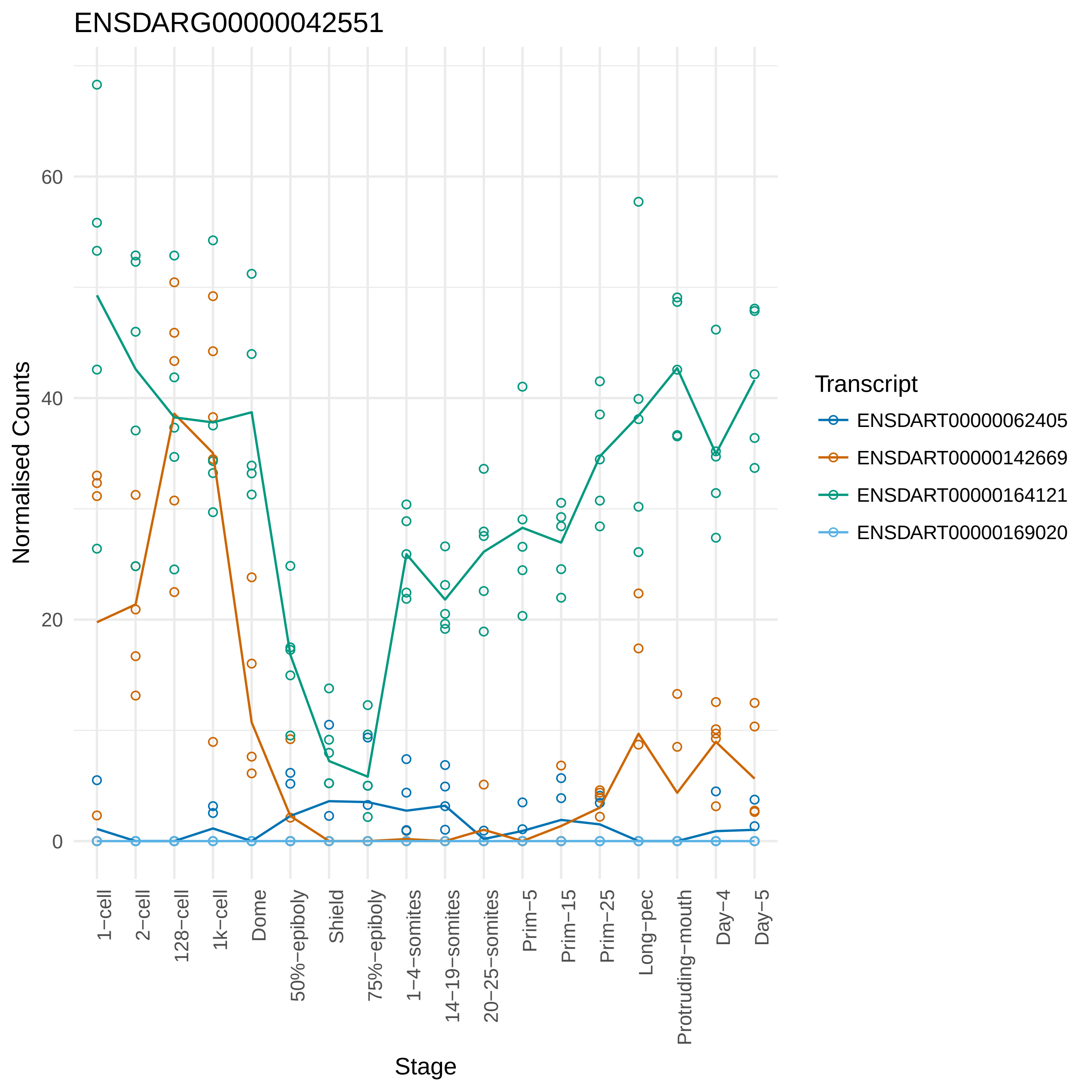

ENSDARG00000042551
Normalised Counts
0
20
40
60
Stage
1−cell
2−cell
128−cell
1k−cell
Dome
50%−epiboly
Shield
75%−epiboly
1−4−somites
14−19−somites
20−25−somites
Prim−5
Prim−15
Prim−25
Long−pec
Protruding−mouth
Day−4
Day−5
Transcript
ENSDART00000062405
ENSDART00000142669
ENSDART00000164121
ENSDART00000169020

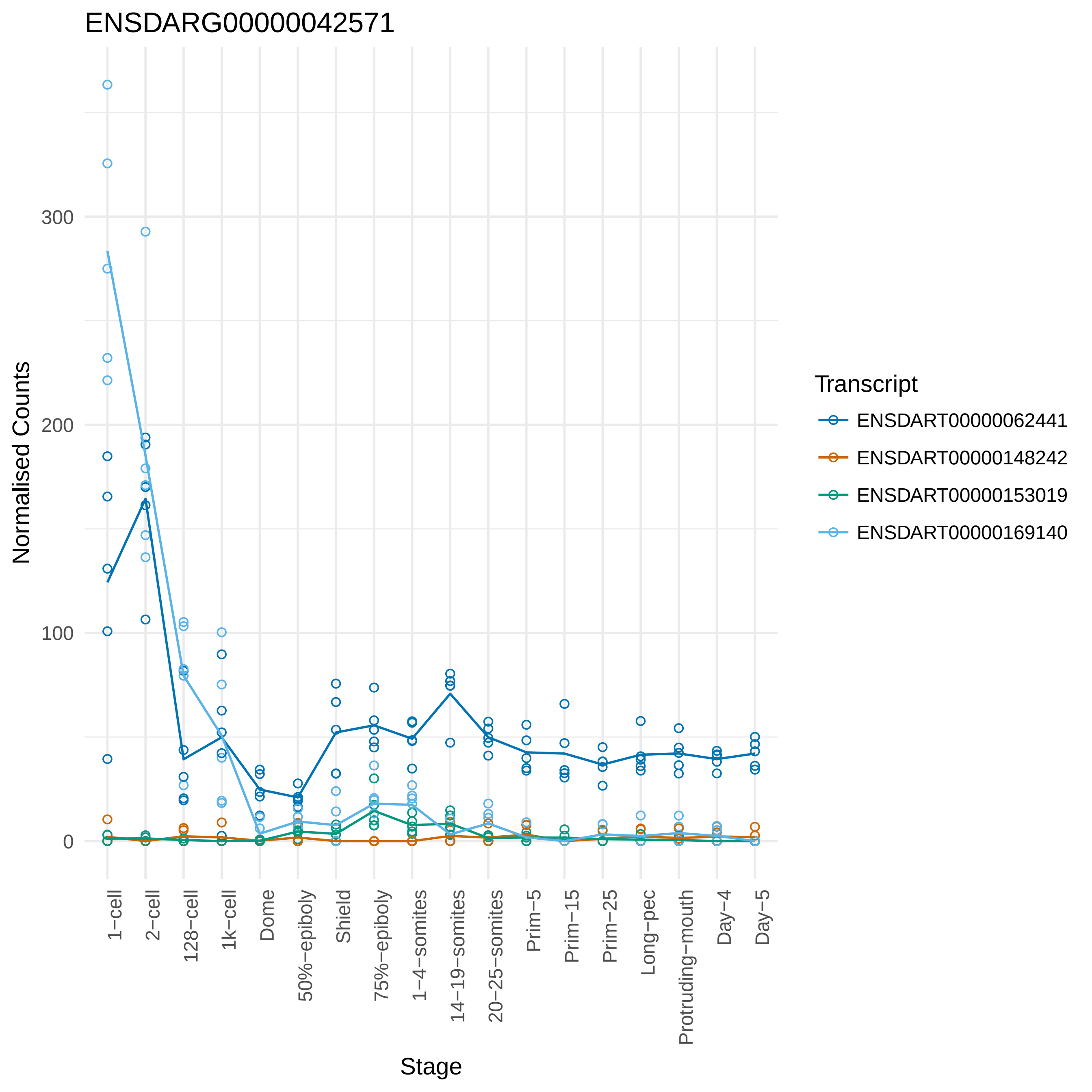

ENSDARG00000042571
Normalised Counts
300
200
100
0
1−cell
2−cell
128−cell
1k−cell
Dome
50%−epiboly
Shield
75%−epiboly
1−4−somites
14−19−somites
20−25−somites
Prim−5
Prim−15
Prim−25
Long−pec
Protruding−mouth
Day−4
Day−5
Stage
Transcript
ENSDART00000062441
ENSDART00000148242
ENSDART00000153019
ENSDART00000169140

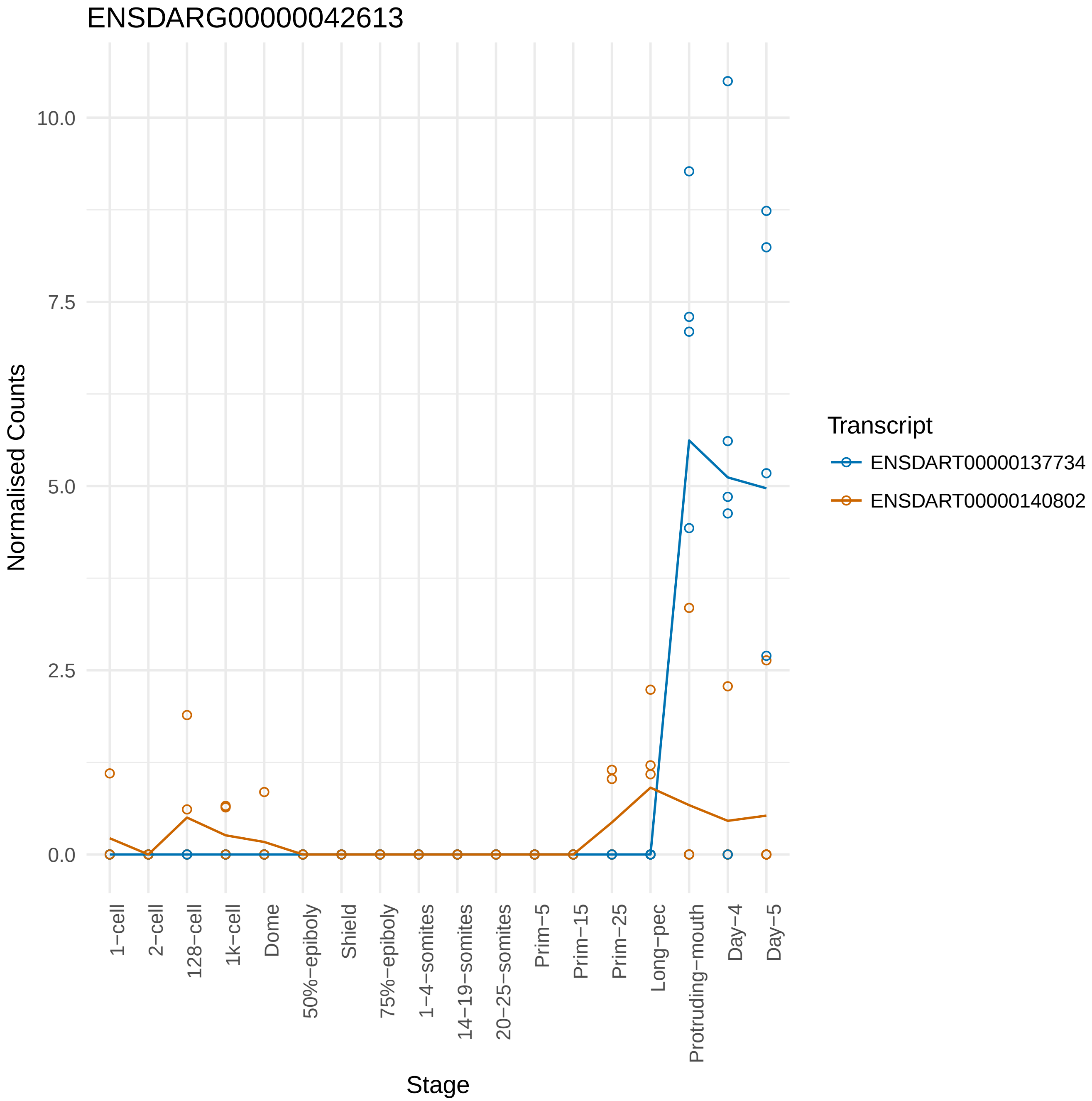

ENSDARG00000042613
Normalised Counts
10.0
7.5
5.0
2.5
0.0
1−cell
2−cell
128−cell
1k−cell
Dome
50%−epiboly
Shield
75%−epiboly
1−4−somites
14−19−somites
20−25−somites
Prim−5
Prim−15
Prim−25
Long−pec
Protruding−mouth
Day−4
Day−5
Stage
Transcript
ENSDART00000137734
ENSDART00000140802

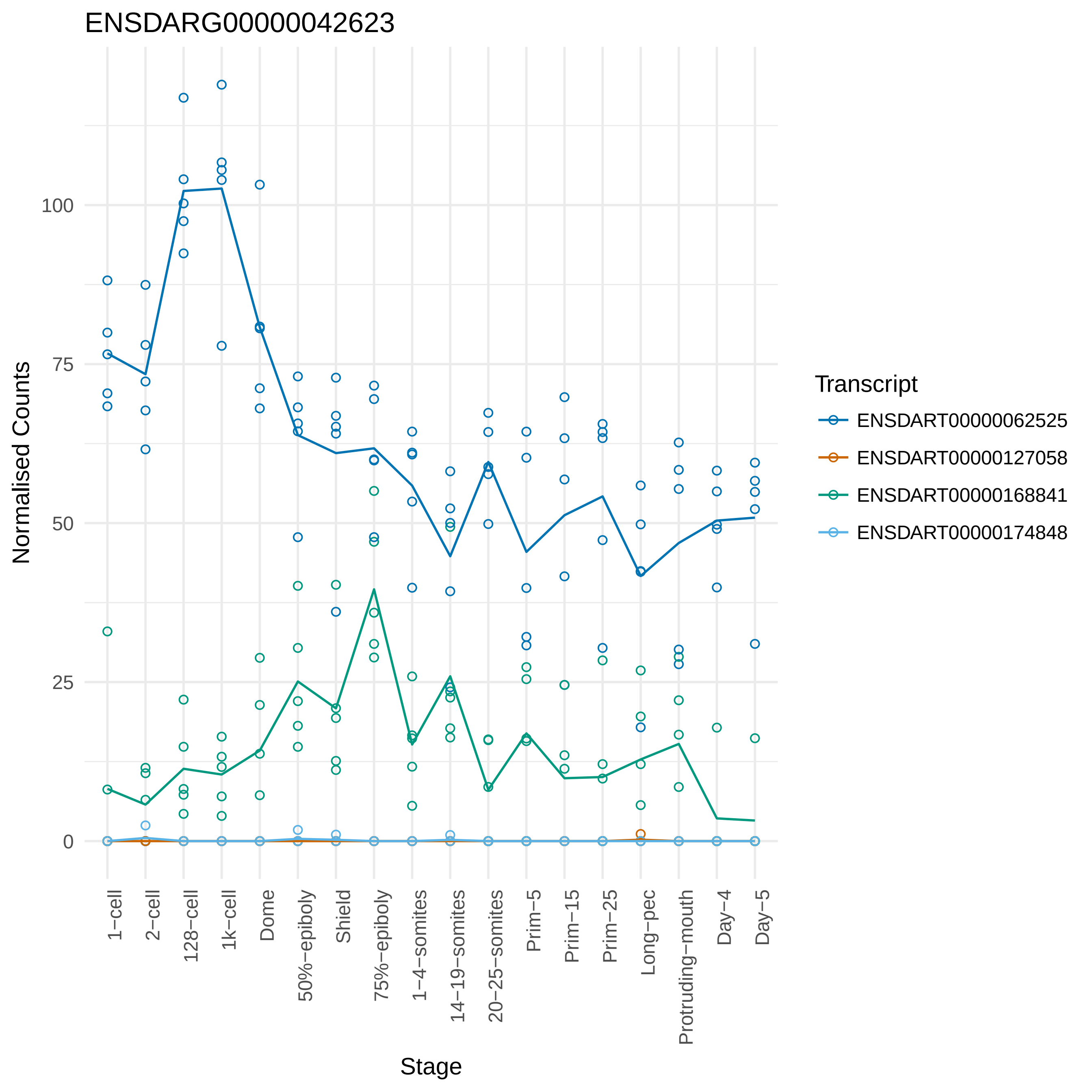
ENSDARG00000042623
Normalised Counts
100
75
50
25
0
1−cell
2−cell
128−cell
1k−cell
Dome
50%−epiboly
Shield
75%−epiboly
1−4−somites
14−19−somites
20−25−somites
Prim−5
Prim−15
Prim−25
Long−pec
Protruding−mouth
Day−4
Day−5
Stage
Transcript
ENSDART00000062525
ENSDART00000127058
ENSDART00000168841
ENSDART00000174848

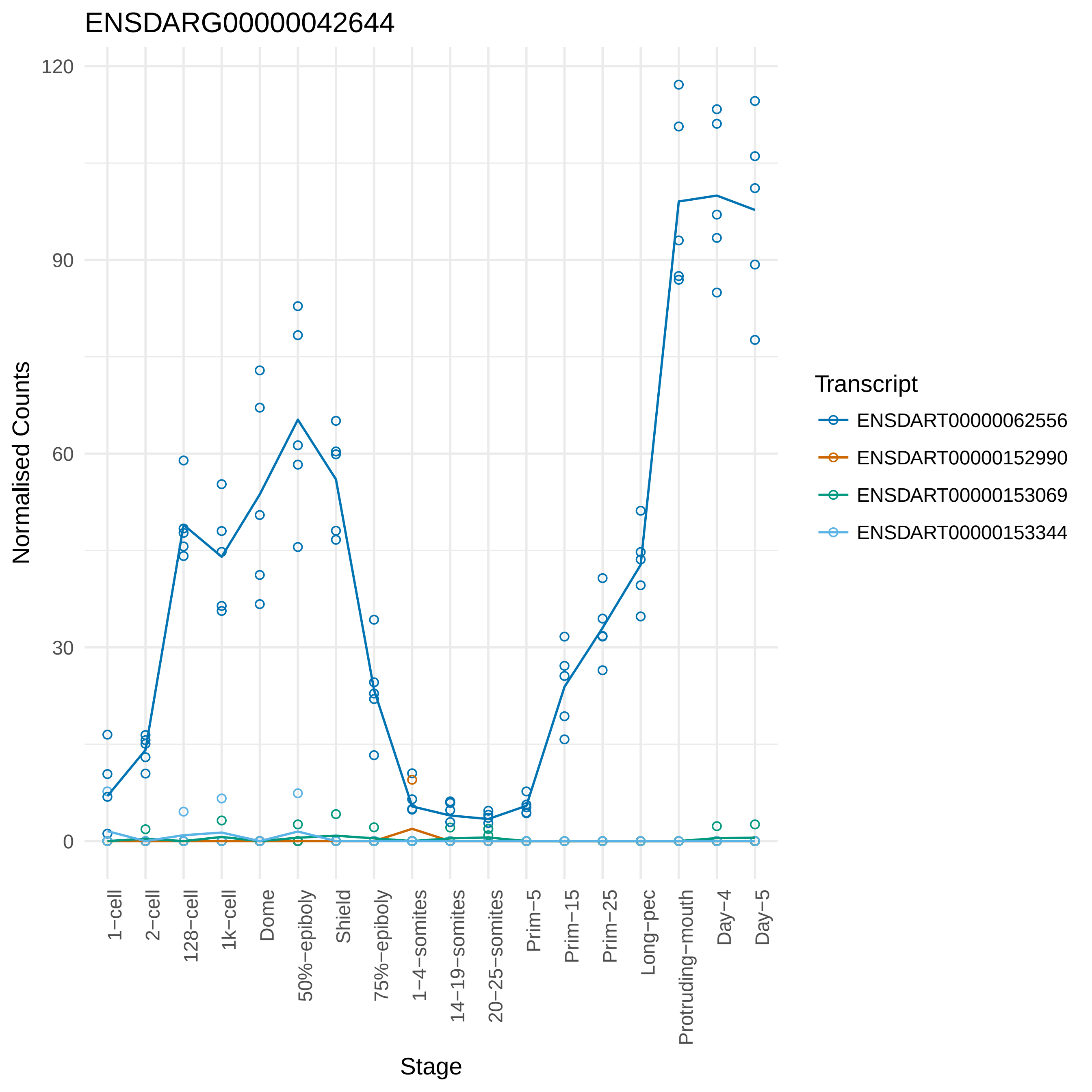

ENSDARG00000042644
Normalised Counts
120
90
60
30
0
1−cell
2−cell
128−cell
1k−cell
Dome
50%−epiboly
Shield
75%−epiboly
1−4−somites
14−19−somites
20−25−somites
Prim−5
Prim−15
Prim−25
Long−pec
Protruding−mouth
Day−4
Day−5
Stage
Transcript
ENSDART00000062556
ENSDART00000152990
ENSDART00000153069
ENSDART00000153344

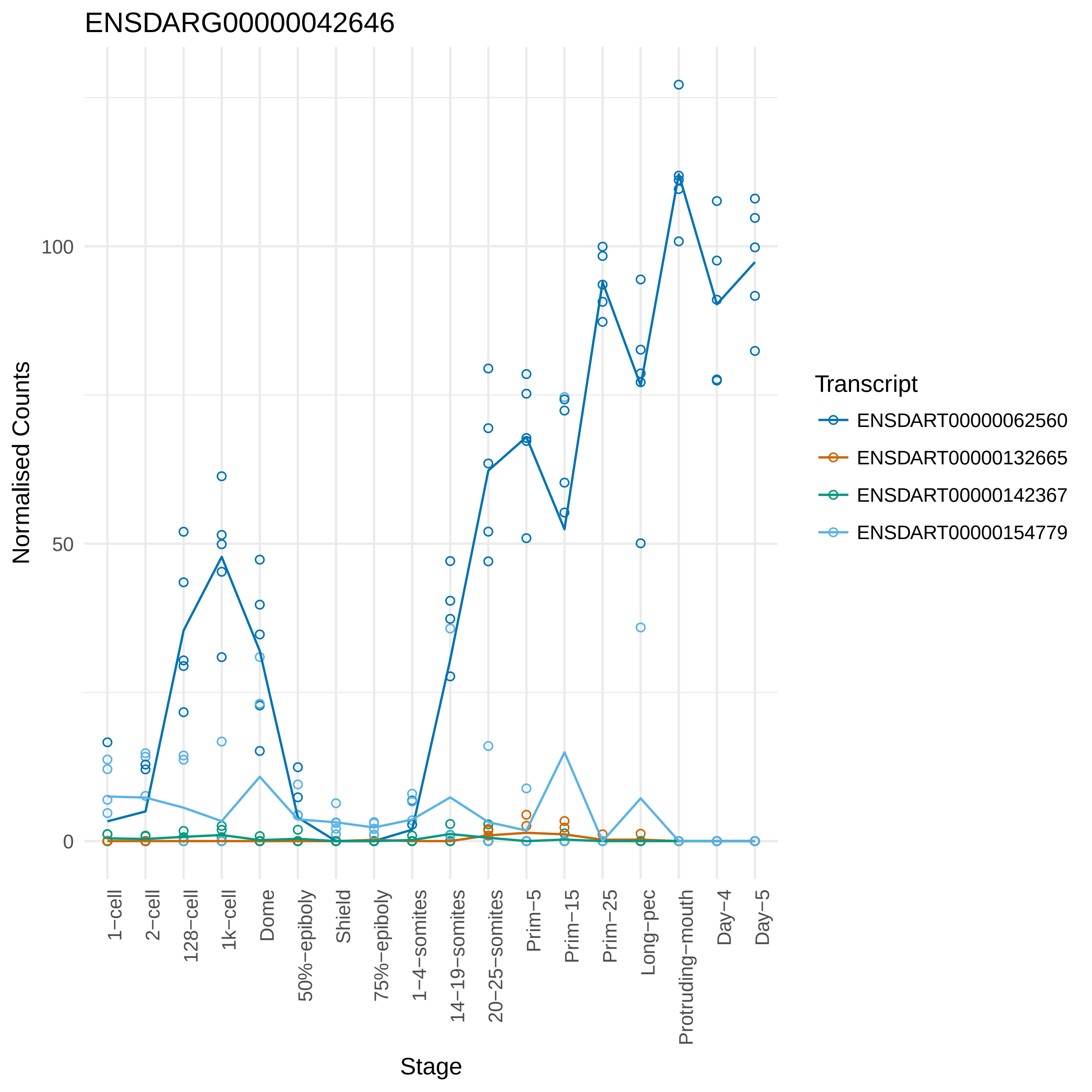
ENSDARG00000042646
Normalised Counts
100
50
0
Stage
1−cell
2−cell
128−cell
1k−cell
Dome
50%−epiboly
Shield
75%−epiboly
1−4−somites
14−19−somites
20−25−somites
Prim−5
Prim−15
Prim−25
Long−pec
Protruding−mouth
Day−4
Day−5
Transcript
ENSDART00000062560
ENSDART00000132665
ENSDART00000142367
ENSDART00000154779

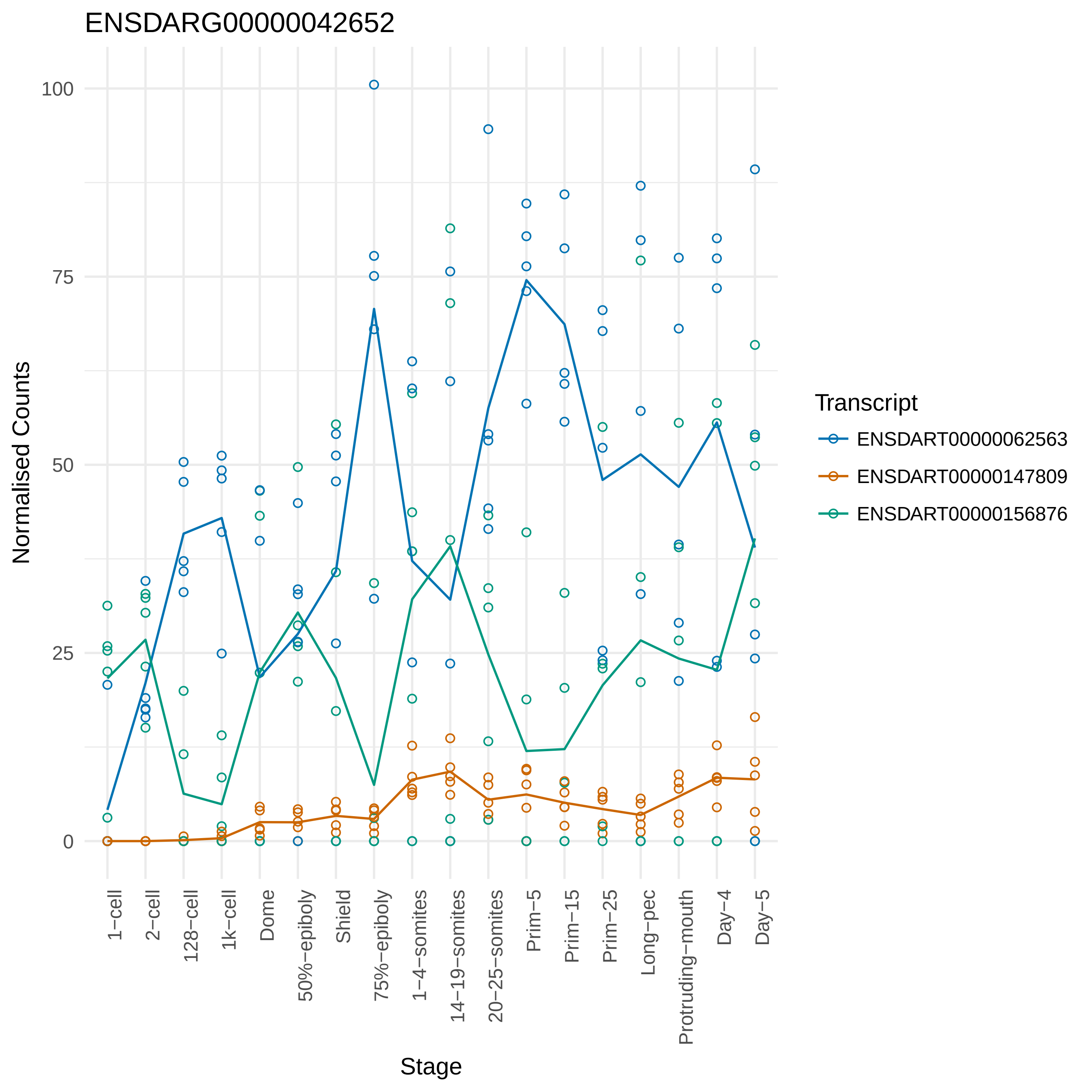

ENSDARG00000042652
Normalised Counts
100
75
50
25
0
1−cell
2−cell
128−cell
1k−cell
Dome
50%−epiboly
Shield
75%−epiboly
1−4−somites
14−19−somites
20−25−somites
Prim−5
Prim−15
Prim−25
Long−pec
Protruding−mouth
Day−4
Day−5
Stage
Transcript
ENSDART00000062563
ENSDART00000147809
ENSDART00000156876

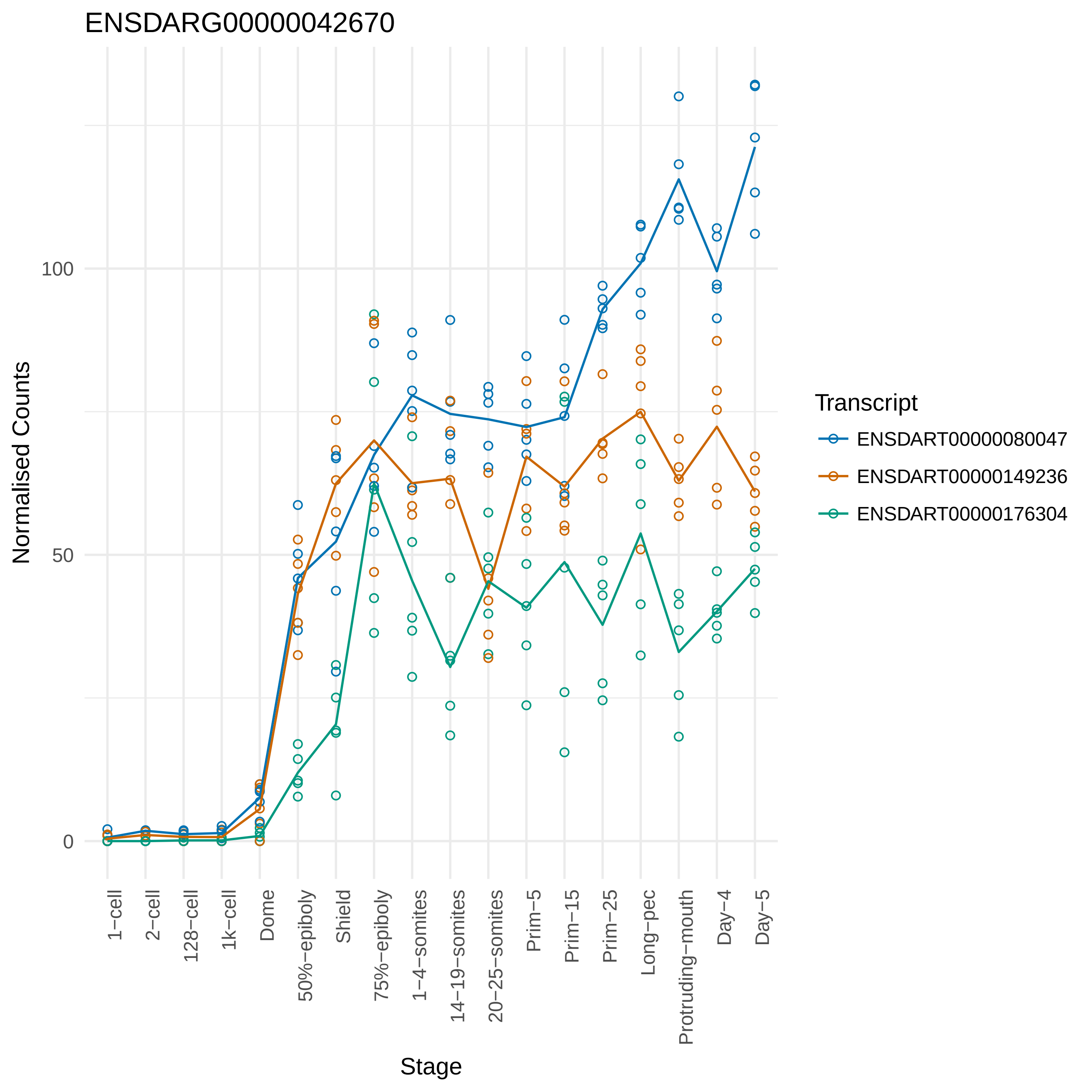

ENSDARG00000042670
Normalised Counts
100
50
0
1−cell
2−cell
128−cell
1k−cell
Dome
50%−epiboly
Shield
75%−epiboly
1−4−somites
14−19−somites
20−25−somites
Prim−5
Prim−15
Prim−25
Long−pec
Protruding−mouth
Day−4
Day−5
Stage
Transcript
ENSDART00000080047
ENSDART00000149236
ENSDART00000176304

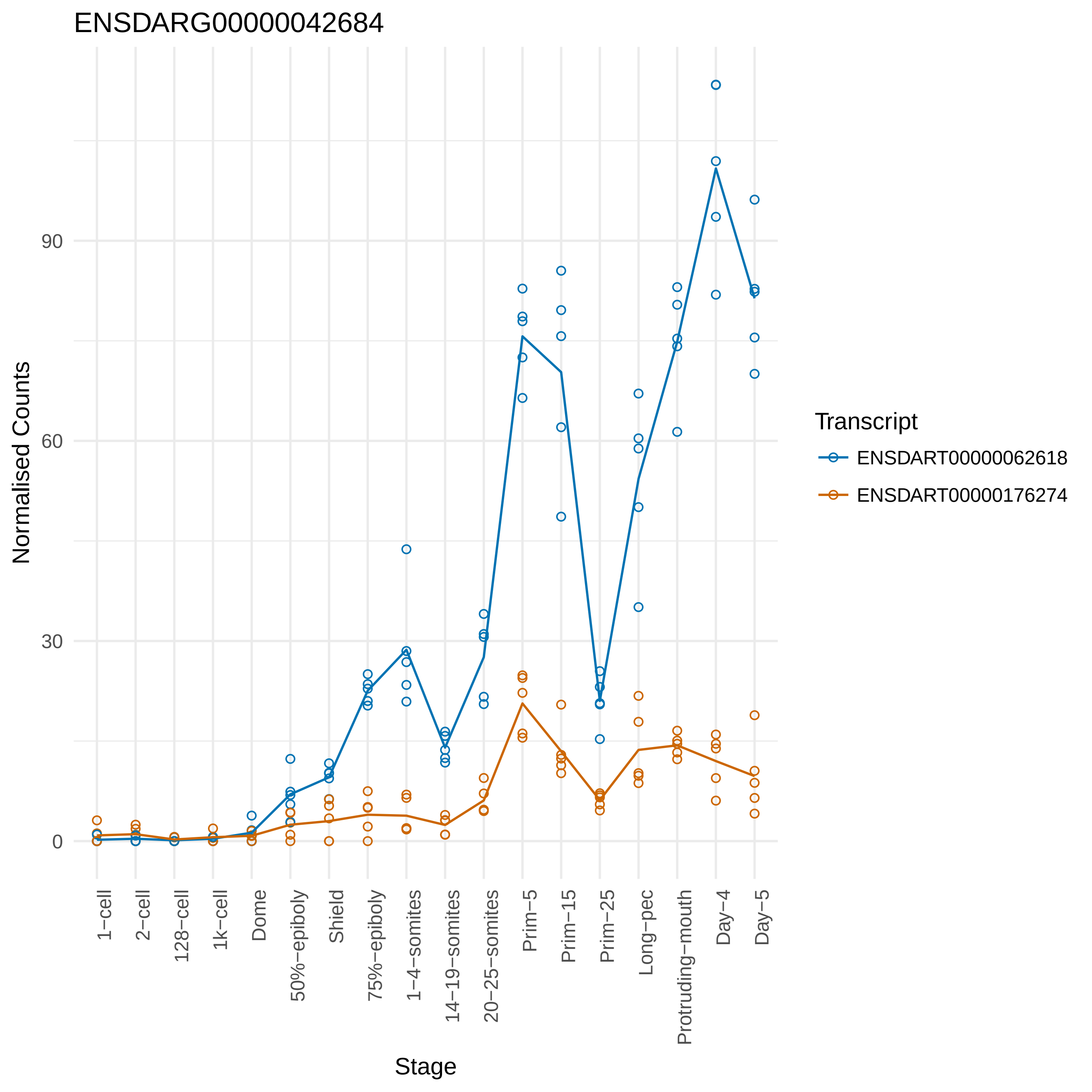

ENSDARG00000042684
Normalised Counts
90
60
30
0
1−cell
2−cell
128−cell
1k−cell
Dome
50%−epiboly
Shield
75%−epiboly
1−4−somites
14−19−somites
20−25−somites
Prim−5
Prim−15
Prim−25
Long−pec
Protruding−mouth
Day−4
Day−5
Stage
Transcript
ENSDART00000062618
ENSDART00000176274

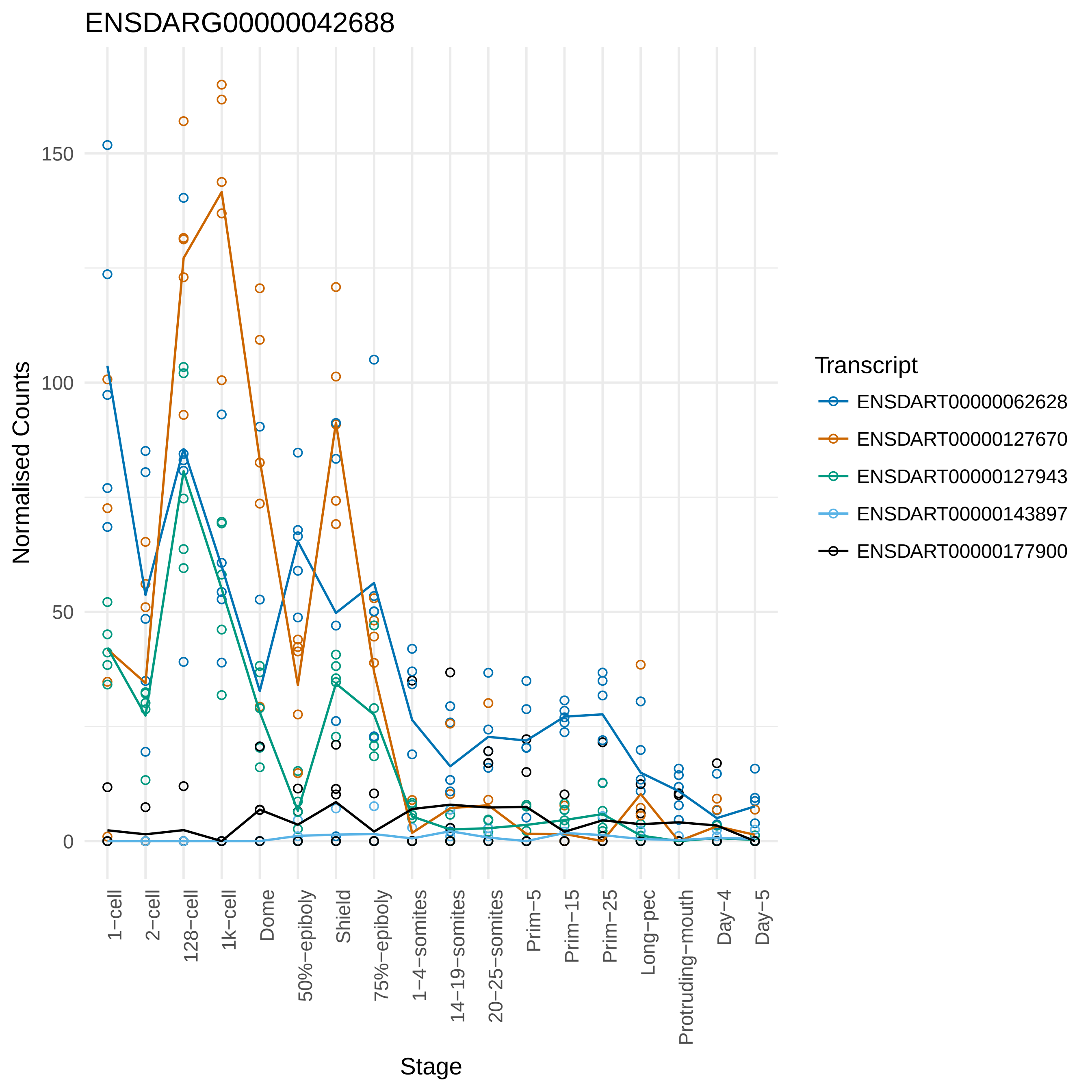
ENSDARG00000042688
Normalised Counts
150
100
50
0
1−cell
2−cell
128−cell
1k−cell
Dome
50%−epiboly
Shield
75%−epiboly
1−4−somites
14−19−somites
20−25−somites
Prim−5
Prim−15
Prim−25
Long−pec
Protruding−mouth
Day−4
Day−5
Stage
Transcript
ENSDART00000062628
ENSDART00000127670
ENSDART00000127943
ENSDART00000143897
ENSDART00000177900

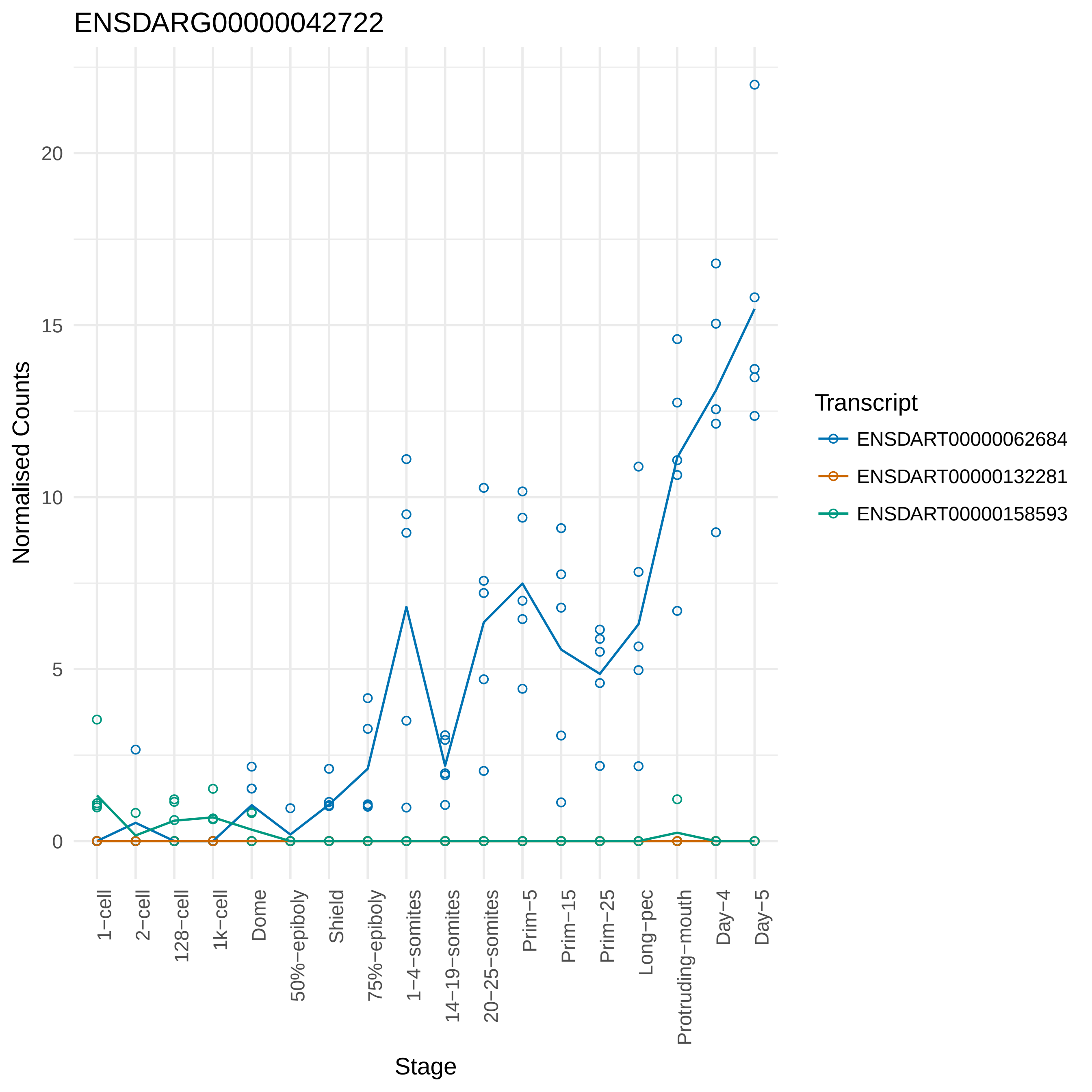
ENSDARG00000042722
Normalised Counts
0
5
10
15
20
Stage
1−cell
2−cell
128−cell
1k−cell
Dome
50%−epiboly
Shield
75%−epiboly
1−4−somites
14−19−somites
20−25−somites
Prim−5
Prim−15
Prim−25
Long−pec
Protruding−mouth
Day−4
Day−5
Transcript
ENSDART00000062684
ENSDART00000132281
ENSDART00000158593

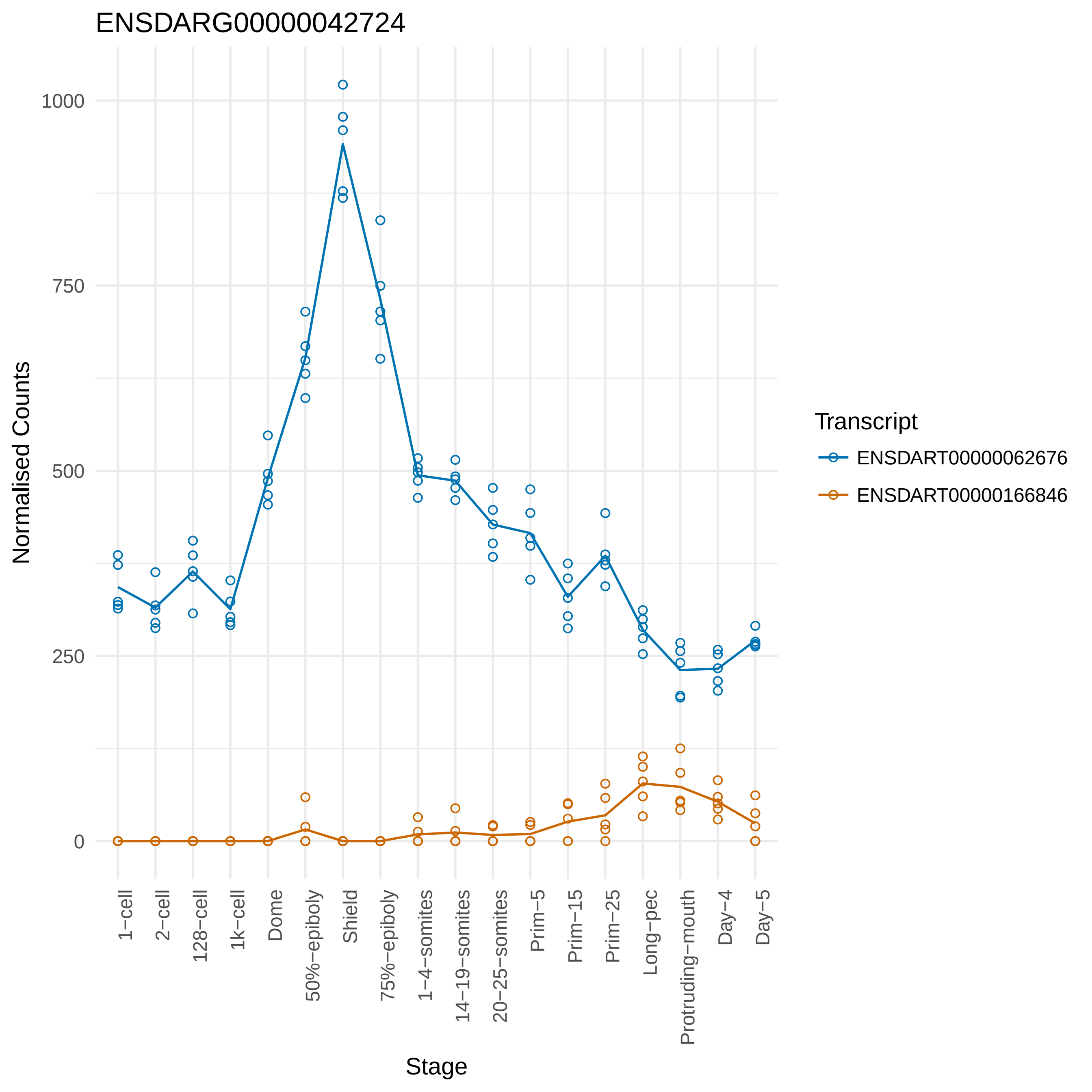

ENSDARG00000042724
Normalised Counts
1000
750
500
250
0
1−cell
2−cell
128−cell
1k−cell
Dome
50%−epiboly
Shield
75%−epiboly
1−4−somites
14−19−somites
20−25−somites
Prim−5
Prim−15
Prim−25
Long−pec
Protruding−mouth
Day−4
Day−5
Stage
Transcript
ENSDART00000062676
ENSDART00000166846

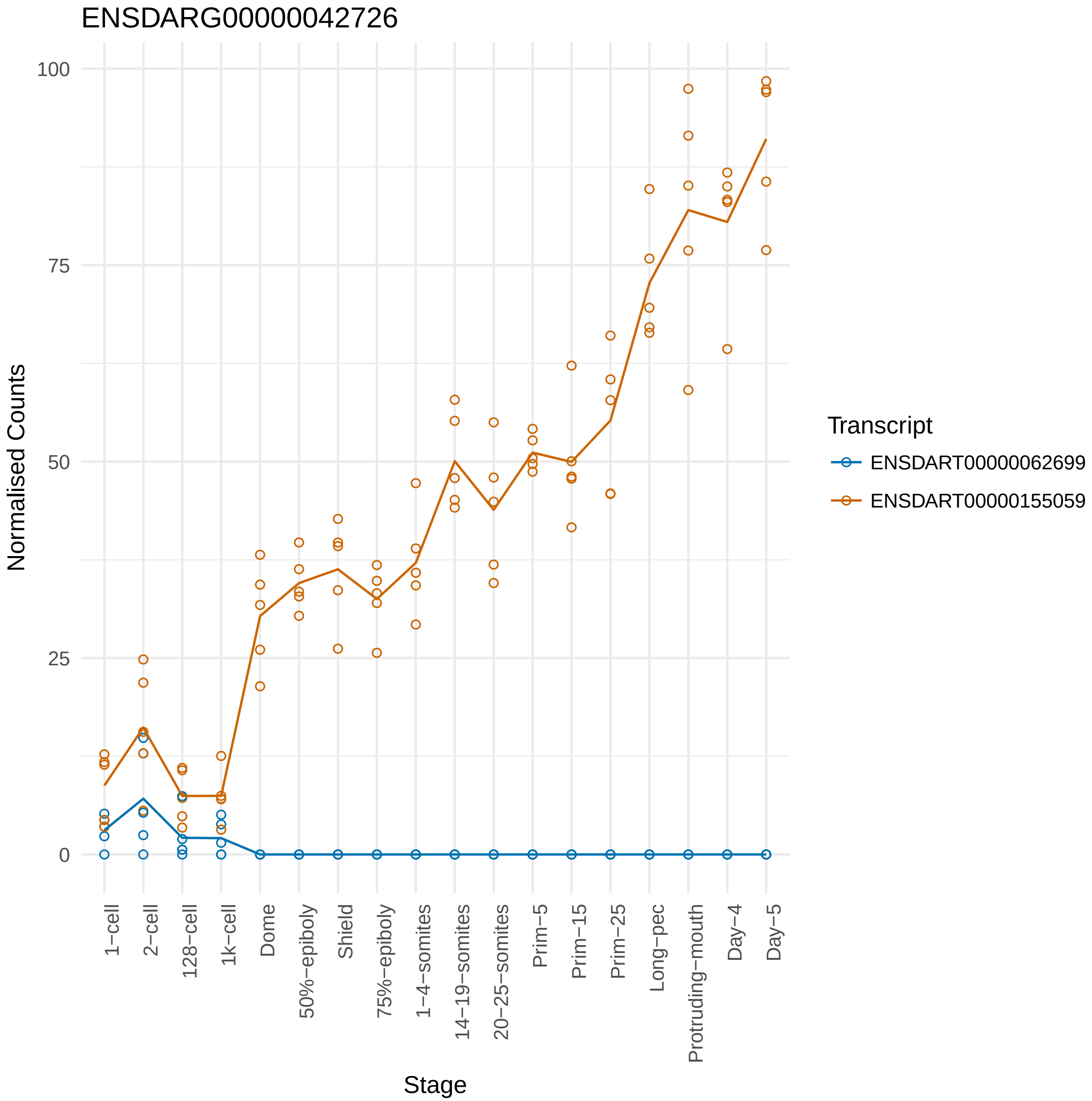
ENSDARG00000042726
Normalised Counts
100
75
50
25
0
Stage
1−cell
2−cell
128−cell
1k−cell
Dome
50%−epiboly
Shield
75%−epiboly
1−4−somites
14−19−somites
20−25−somites
Prim−5
Prim−15
Prim−25
Long−pec
Protruding−mouth
Day−4
Day−5
Transcript
ENSDART00000062699
ENSDART00000155059

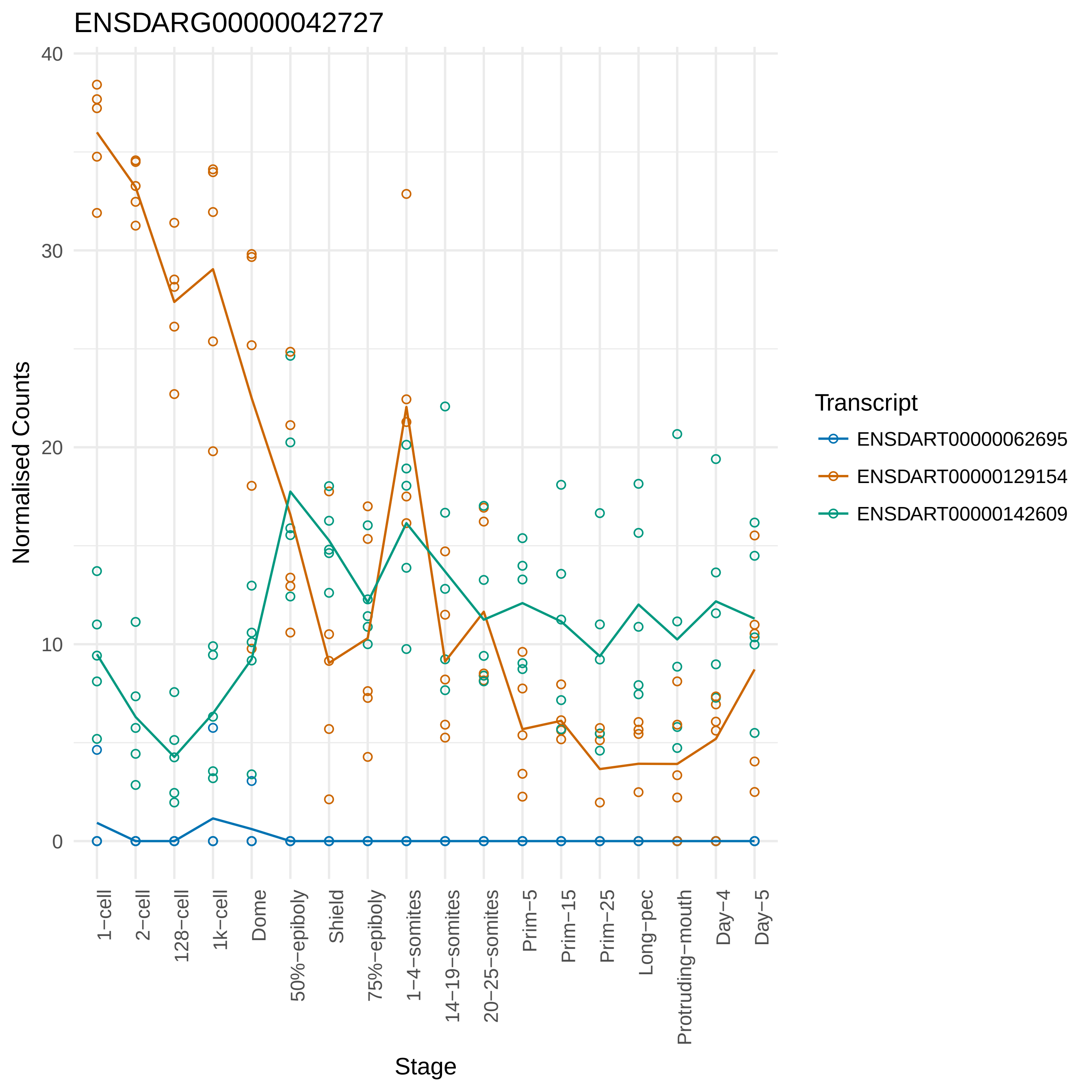

ENSDARG00000042727
Normalised Counts
40
30
20
10
0
1−cell
2−cell
128−cell
1k−cell
Dome
50%−epiboly
Shield
75%−epiboly
1−4−somites
14−19−somites
20−25−somites
Prim−5
Prim−15
Prim−25
Long−pec
Protruding−mouth
Day−4
Day−5
Stage
Transcript
ENSDART00000062695
ENSDART00000129154
ENSDART00000142609

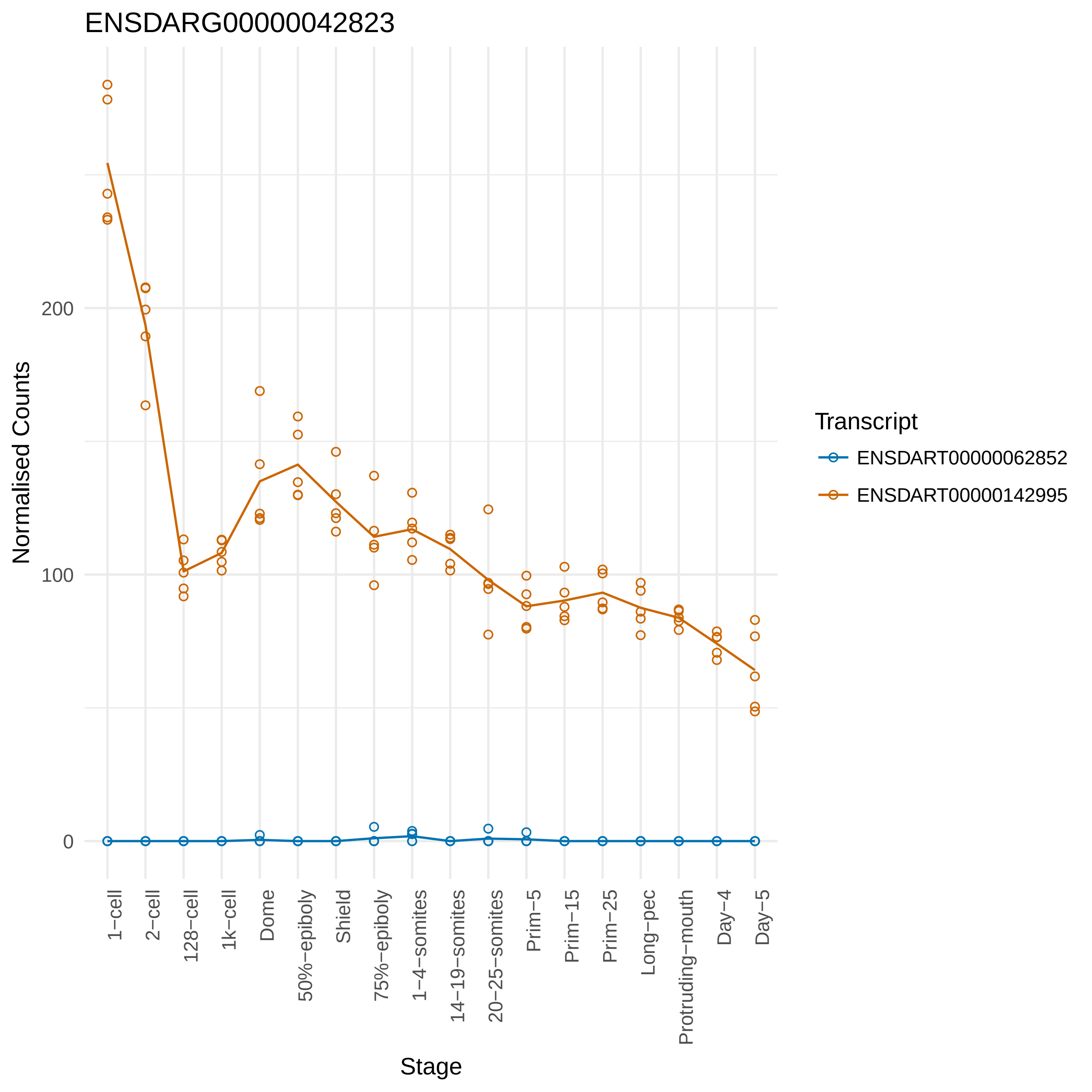

ENSDARG00000042823
Normalised Counts
200
100
0
1−cell
2−cell
128−cell
1k−cell
Dome
50%−epiboly
Shield
75%−epiboly
1−4−somites
14−19−somites
20−25−somites
Prim−5
Prim−15
Prim−25
Long−pec
Protruding−mouth
Day−4
Day−5
Stage
Transcript
ENSDART00000062852
ENSDART00000142995

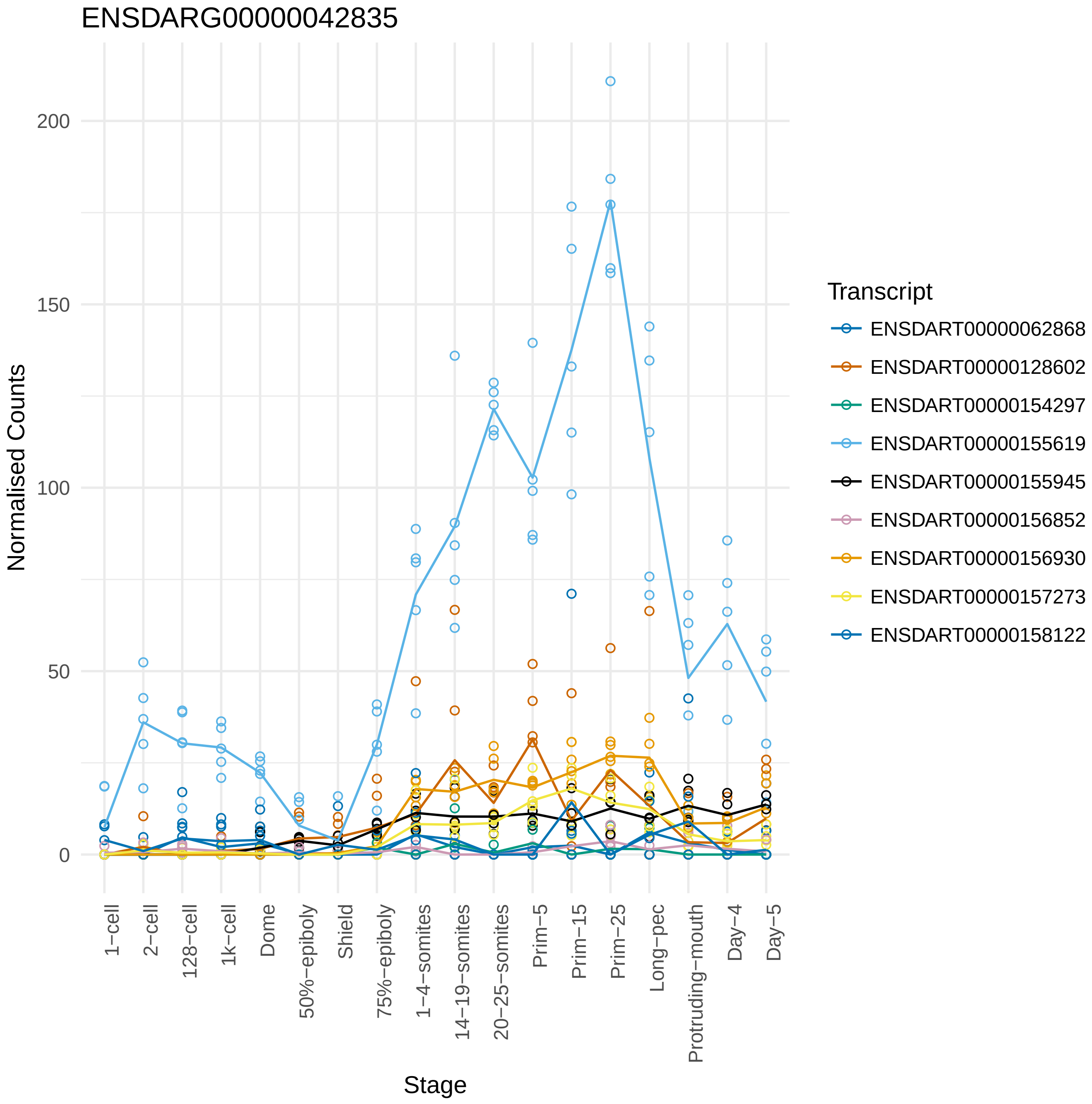

ENSDARG00000042835
Normalised Counts
200
150
100
50
0
1−cell
2−cell
128−cell
1k−cell
Dome
50%−epiboly
Shield
75%−epiboly
1−4−somites
14−19−somites
20−25−somites
Prim−5
Prim−15
Prim−25
Long−pec
Protruding−mouth
Day−4
Day−5
Stage
Transcript
ENSDART00000062868
ENSDART00000128602
ENSDART00000154297
ENSDART00000155619
ENSDART00000155945
ENSDART00000156852
ENSDART00000156930
ENSDART00000157273
ENSDART00000158122

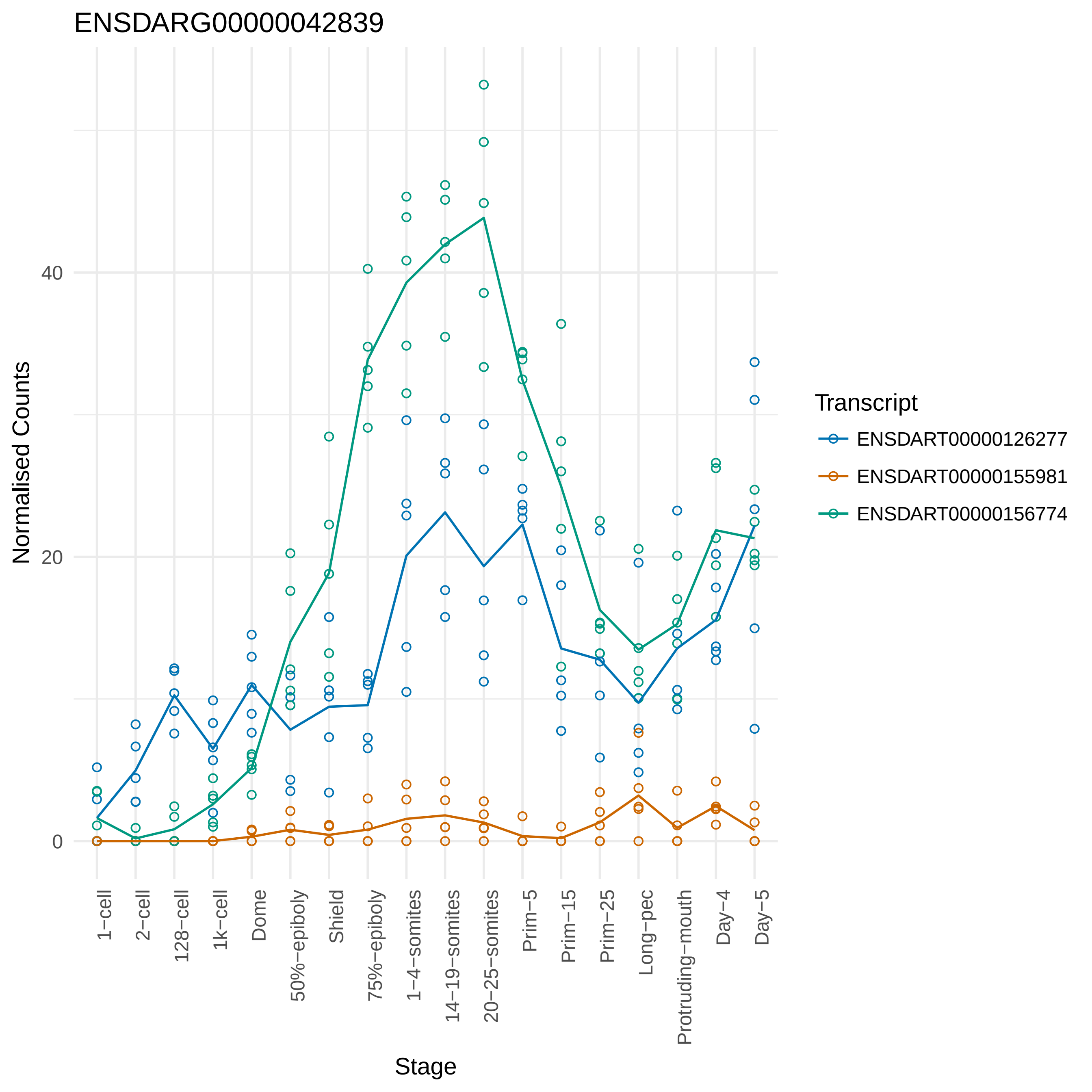
ENSDARG00000042839
Normalised Counts
0
20
40
Stage
1−cell
2−cell
128−cell
1k−cell
Dome
50%−epiboly
Shield
75%−epiboly
1−4−somites
14−19−somites
20−25−somites
Prim−5
Prim−15
Prim−25
Long−pec
Protruding−mouth
Day−4
Day−5
Transcript
ENSDART00000126277
ENSDART00000155981
ENSDART00000156774

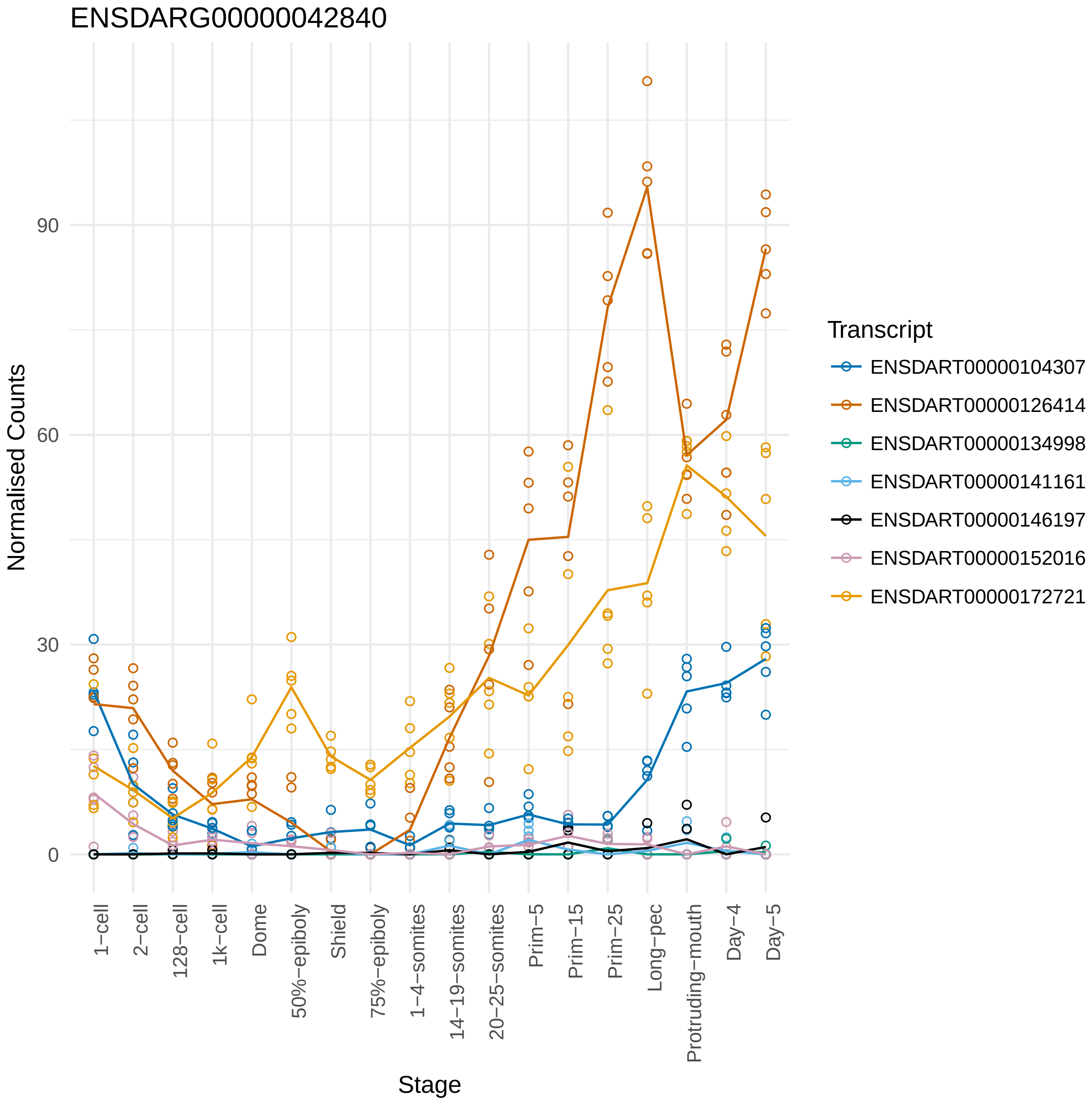

ENSDARG00000042840
Normalised Counts
0
30
60
90
1−cell
2−cell
128−cell
1k−cell
Dome
50%−epiboly
Shield
75%−epiboly
1−4−somites
14−19−somites
20−25−somites
Prim−5
Prim−15
Prim−25
Long−pec
Protruding−mouth
Day−4
Day−5
Stage
Transcript
ENSDART00000104307
ENSDART00000126414
ENSDART00000134998
ENSDART00000141161
ENSDART00000146197
ENSDART00000152016
ENSDART00000172721

ENSDARG00000042861

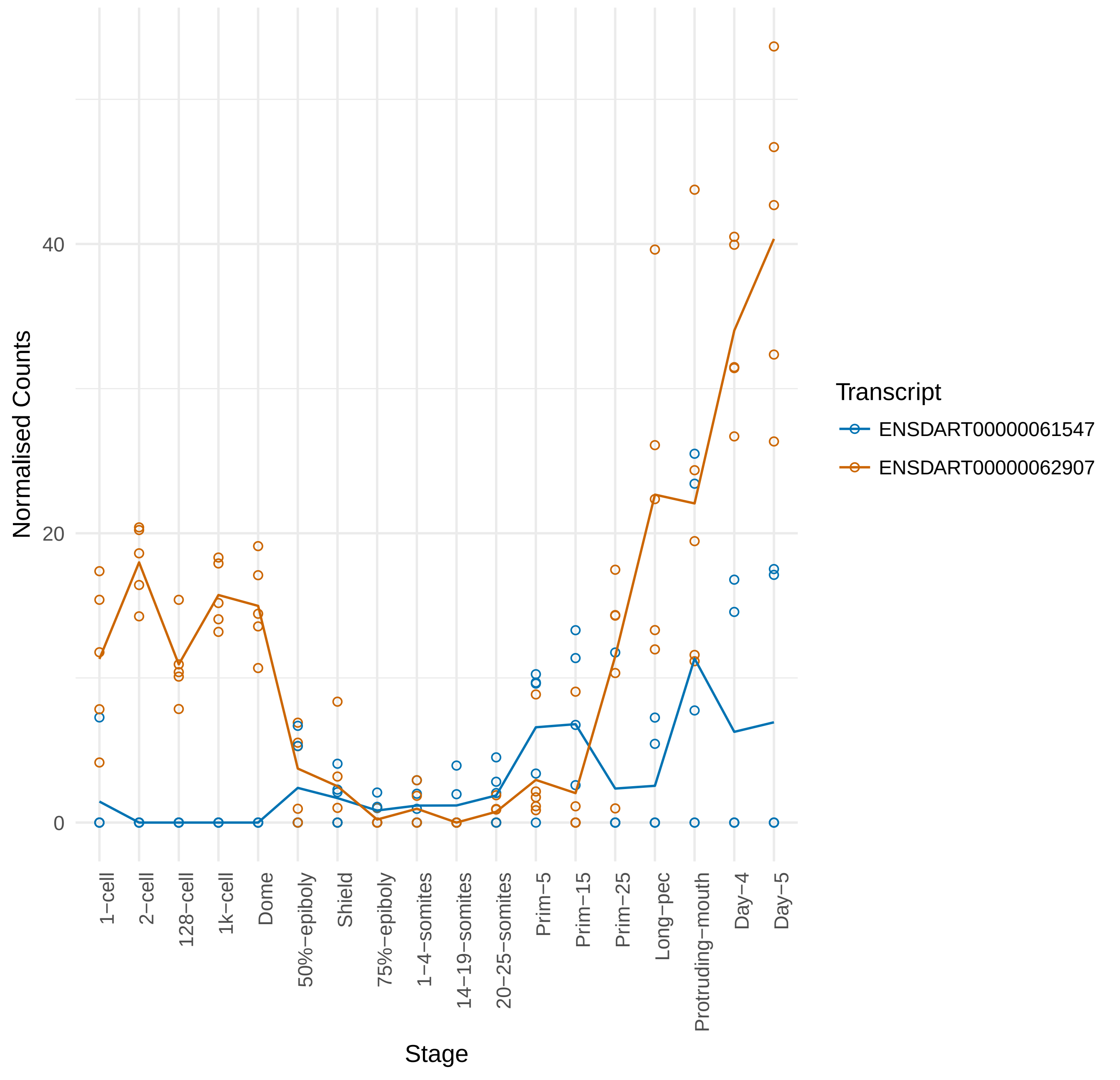

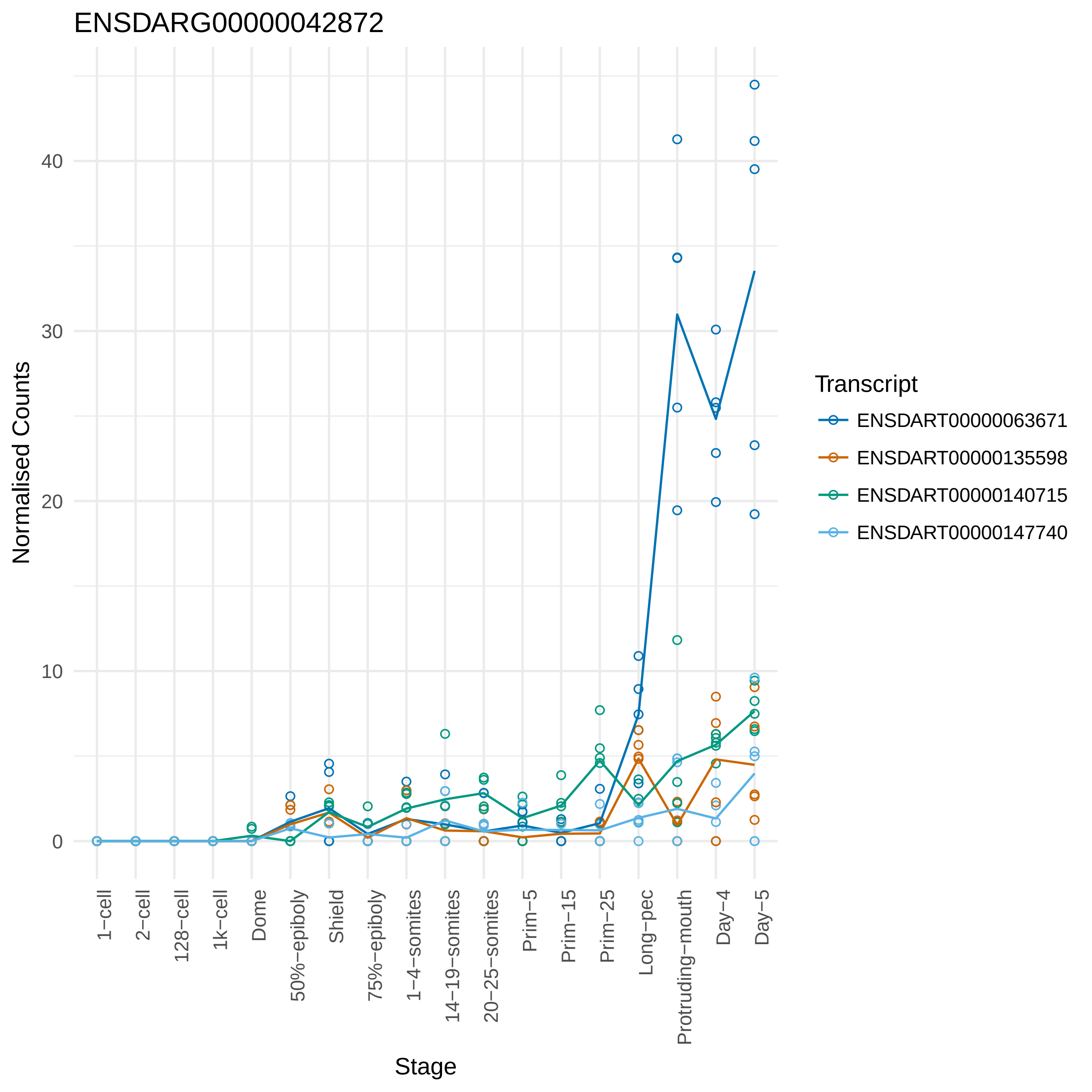
ENSDARG00000042872
Normalised Counts
0
10
20
30
40
1−cell
2−cell
128−cell
1k−cell
Dome
50%−epiboly
Shield
75%−epiboly
1−4−somites
14−19−somites
20−25−somites
Prim−5
Prim−15
Prim−25
Long−pec
Protruding−mouth
Day−4
Day−5
Stage
Transcript
ENSDART00000063671
ENSDART00000135598
ENSDART00000140715
ENSDART00000147740

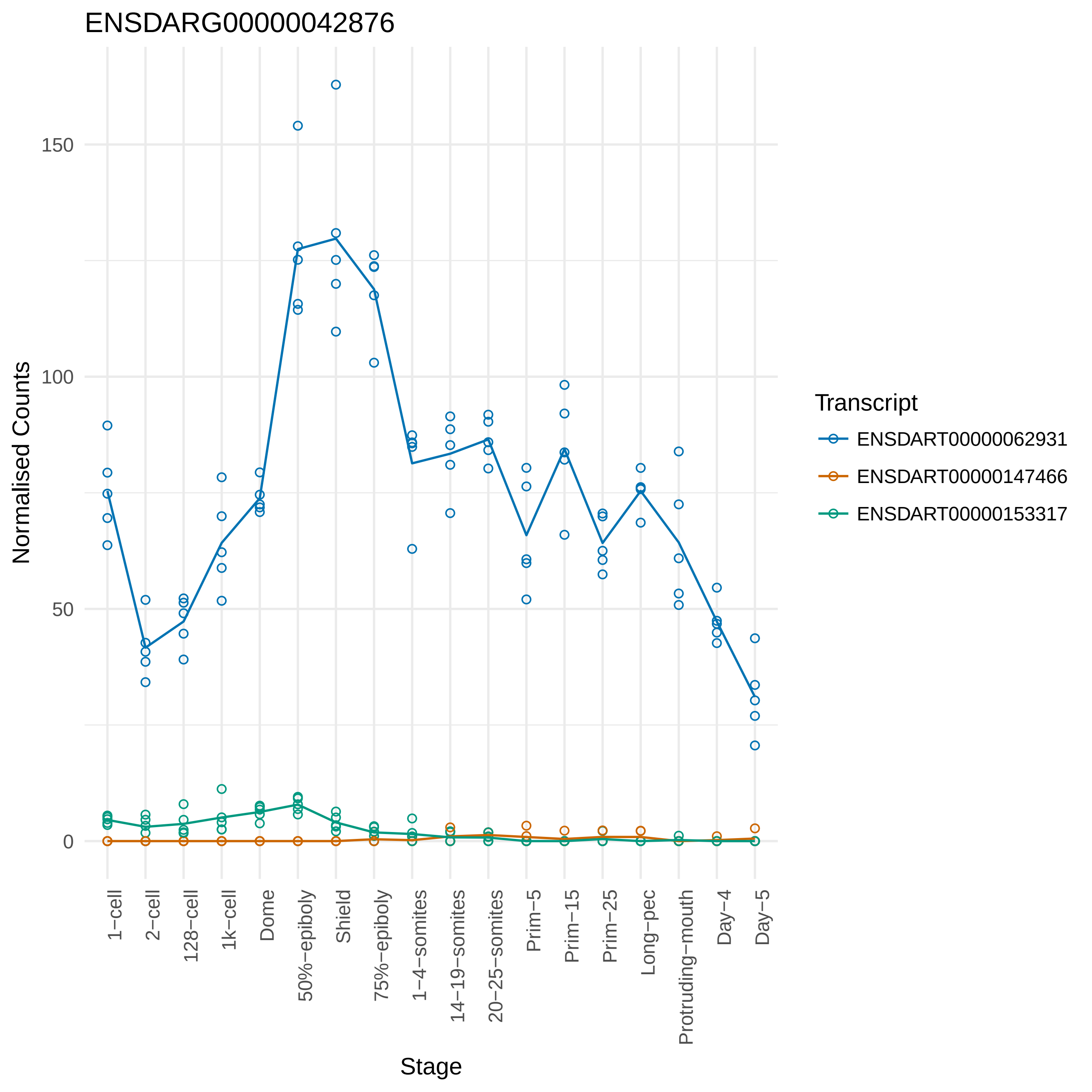

ENSDARG00000042876
Normalised Counts
150
100
50
0
1−cell
2−cell
128−cell
1k−cell
Dome
50%−epiboly
Shield
75%−epiboly
1−4−somites
14−19−somites
20−25−somites
Prim−5
Prim−15
Prim−25
Long−pec
Protruding−mouth
Day−4
Day−5
Stage
Transcript
ENSDART00000062931
ENSDART00000147466
ENSDART00000153317

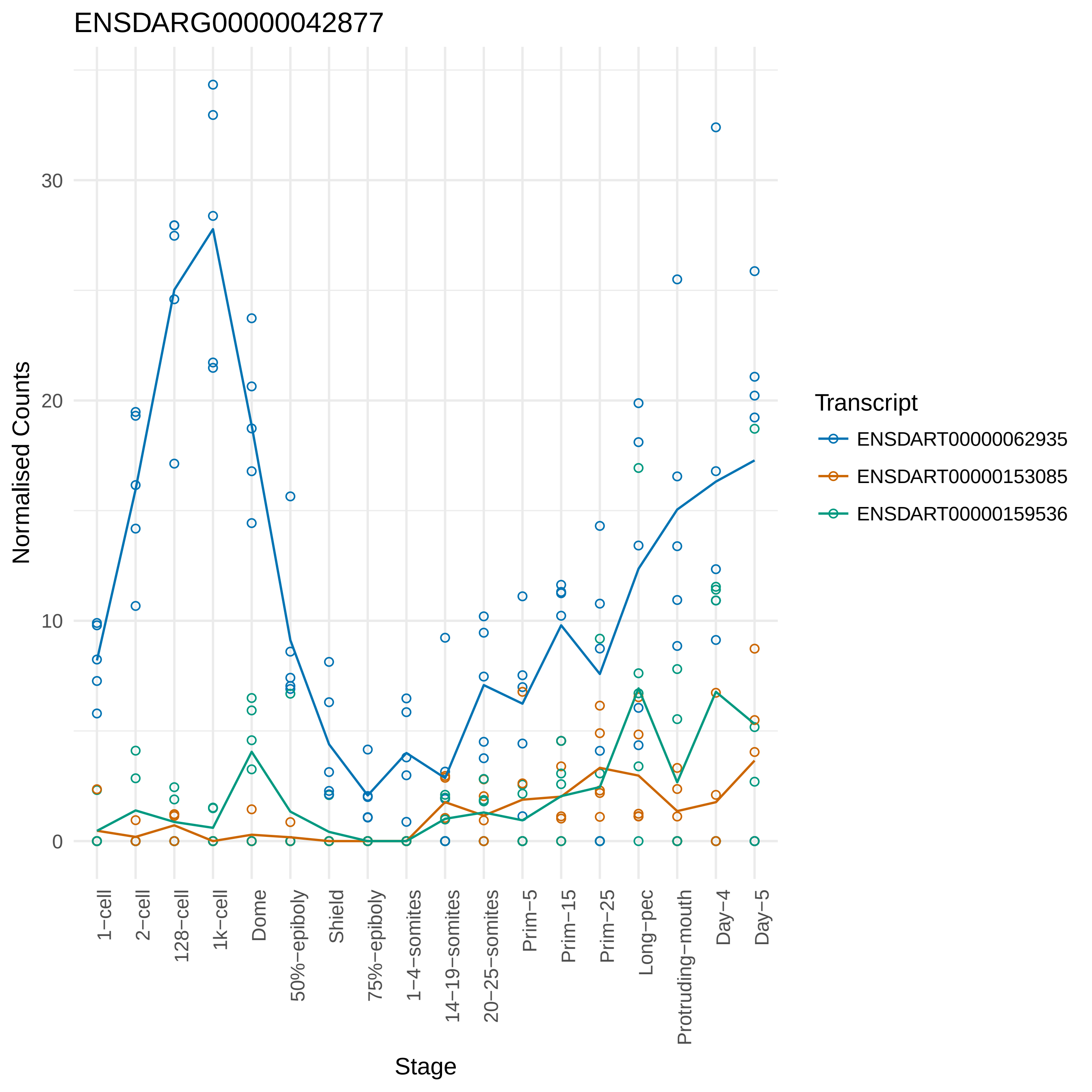

ENSDARG00000042877
Normalised Counts
30
20
10
0
1−cell
2−cell
128−cell
1k−cell
Dome
50%−epiboly
Shield
75%−epiboly
1−4−somites
14−19−somites
20−25−somites
Prim−5
Prim−15
Prim−25
Long−pec
Protruding−mouth
Day−4
Day−5
Stage
Transcript
ENSDART00000062935
ENSDART00000153085
ENSDART00000159536

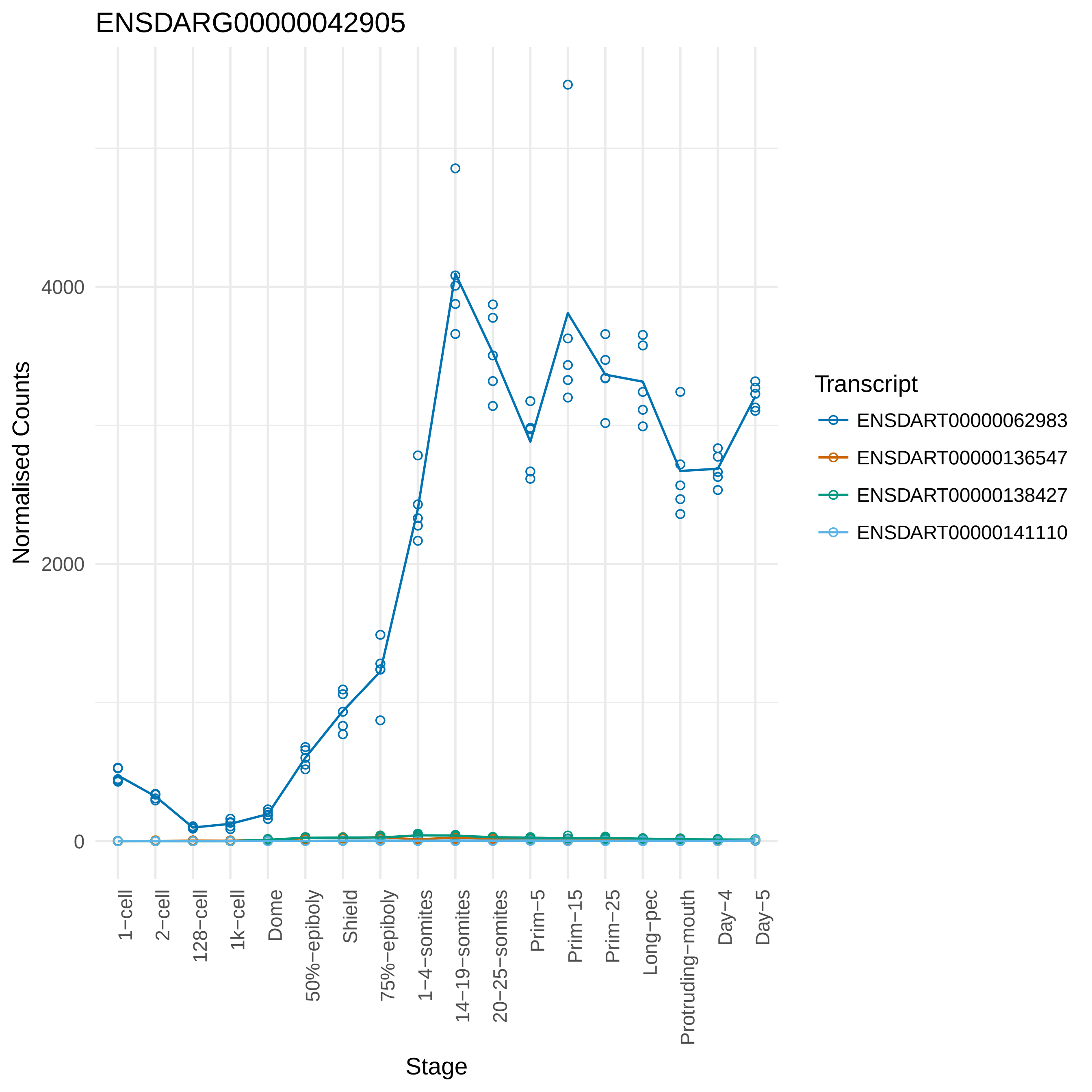
ENSDARG00000042905
Normalised Counts
4000
2000
0
Stage
1−cell
2−cell
128−cell
1k−cell
Dome
50%−epiboly
Shield
75%−epiboly
1−4−somites
14−19−somites
20−25−somites
Prim−5
Prim−15
Prim−25
Long−pec
Protruding−mouth
Day−4
Day−5
Transcript
ENSDART00000062983
ENSDART00000136547
ENSDART00000138427
ENSDART00000141110

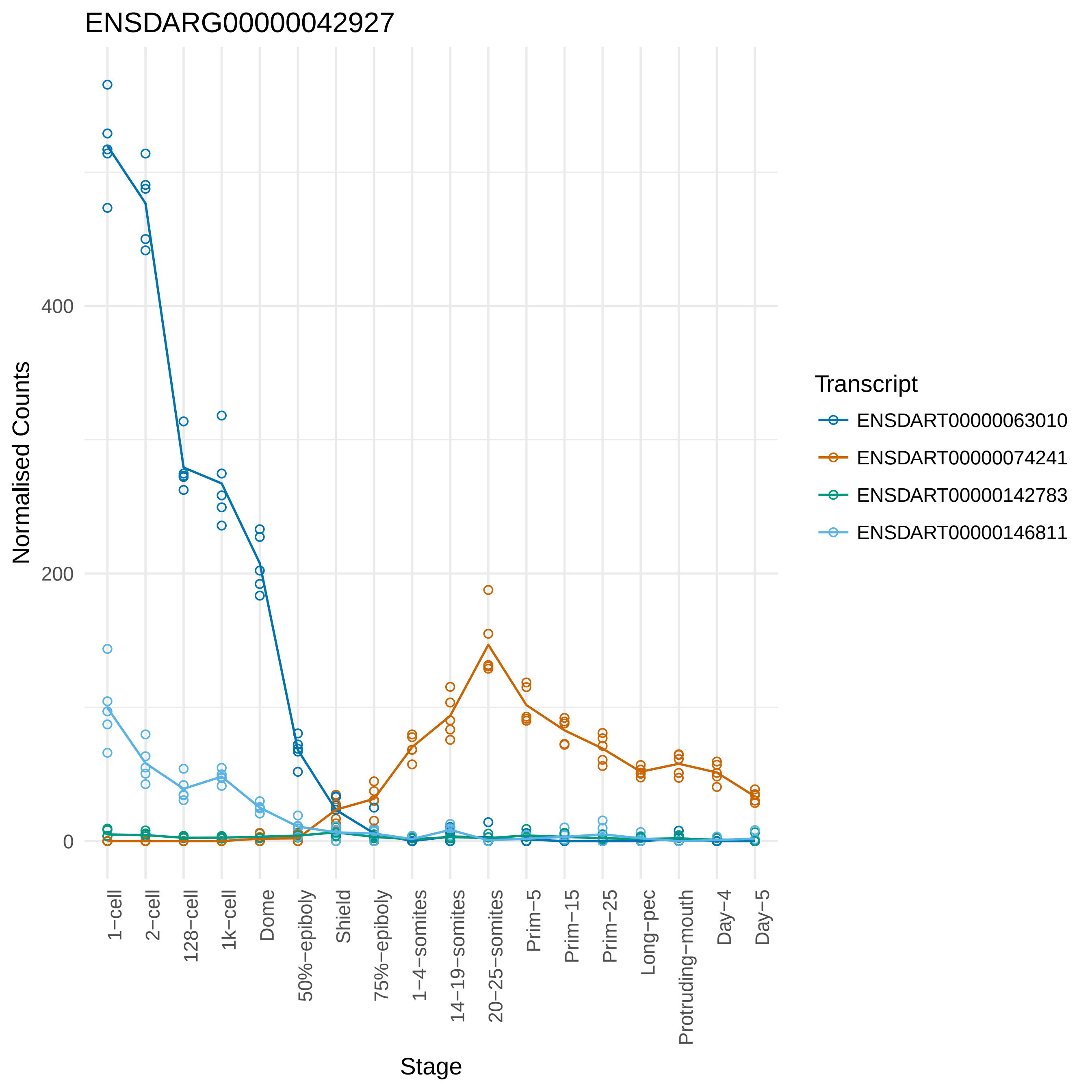

ENSDARG00000042927
Normalised Counts
400
200
0
1−cell
2−cell
128−cell
1k−cell
Dome
50%−epiboly
Shield
75%−epiboly
1−4−somites
14−19−somites
20−25−somites
Prim−5
Prim−15
Prim−25
Long−pec
Protruding−mouth
Day−4
Day−5
Stage
Transcript
ENSDART00000063010
ENSDART00000074241
ENSDART00000142783
ENSDART00000146811

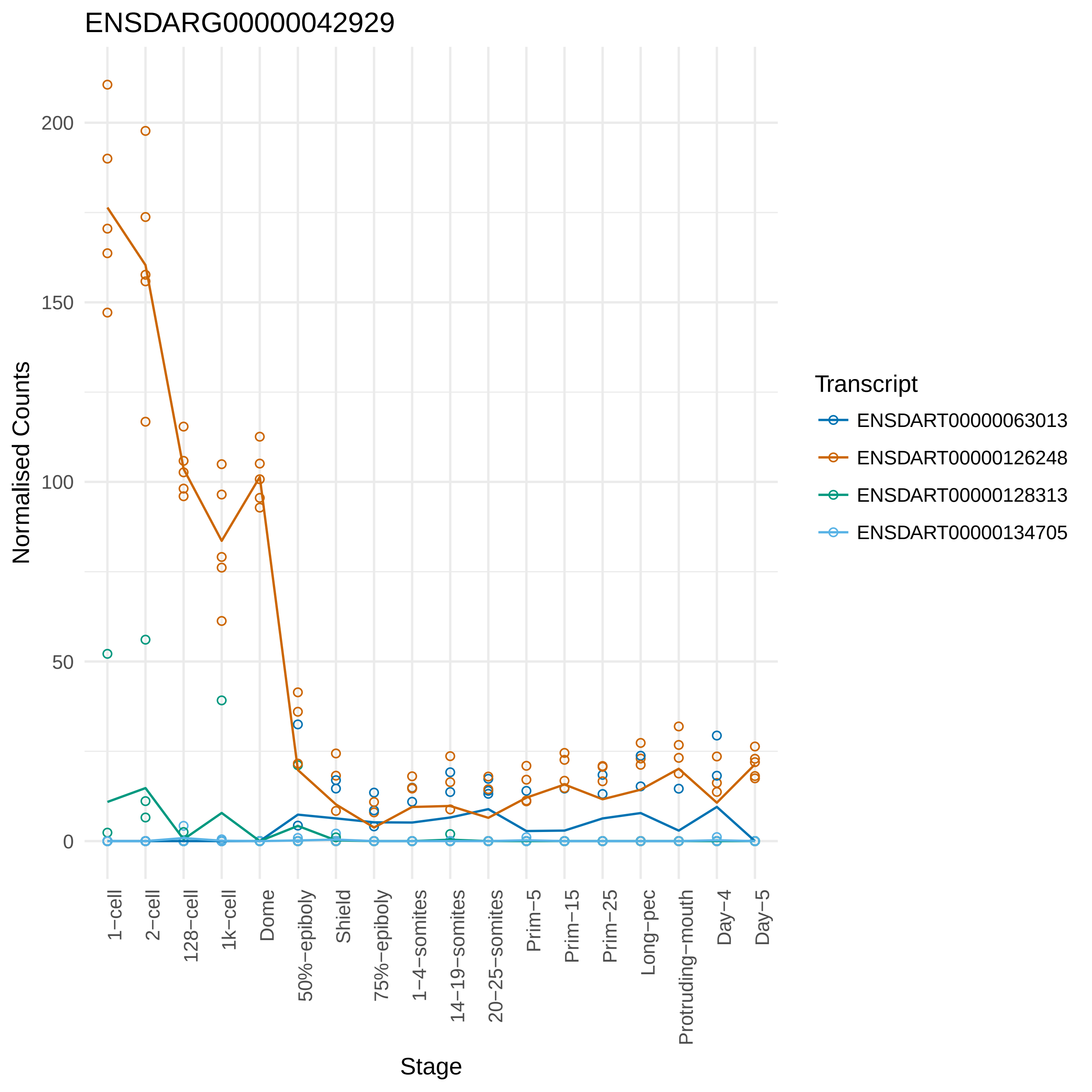
ENSDARG00000042929
Normalised Counts
200
150
100
50
0
1−cell
2−cell
128−cell
1k−cell
Dome
50%−epiboly
Shield
75%−epiboly
1−4−somites
14−19−somites
20−25−somites
Prim−5
Prim−15
Prim−25
Long−pec
Protruding−mouth
Day−4
Day−5
Stage
Transcript
ENSDART00000063013
ENSDART00000126248
ENSDART00000128313
ENSDART00000134705

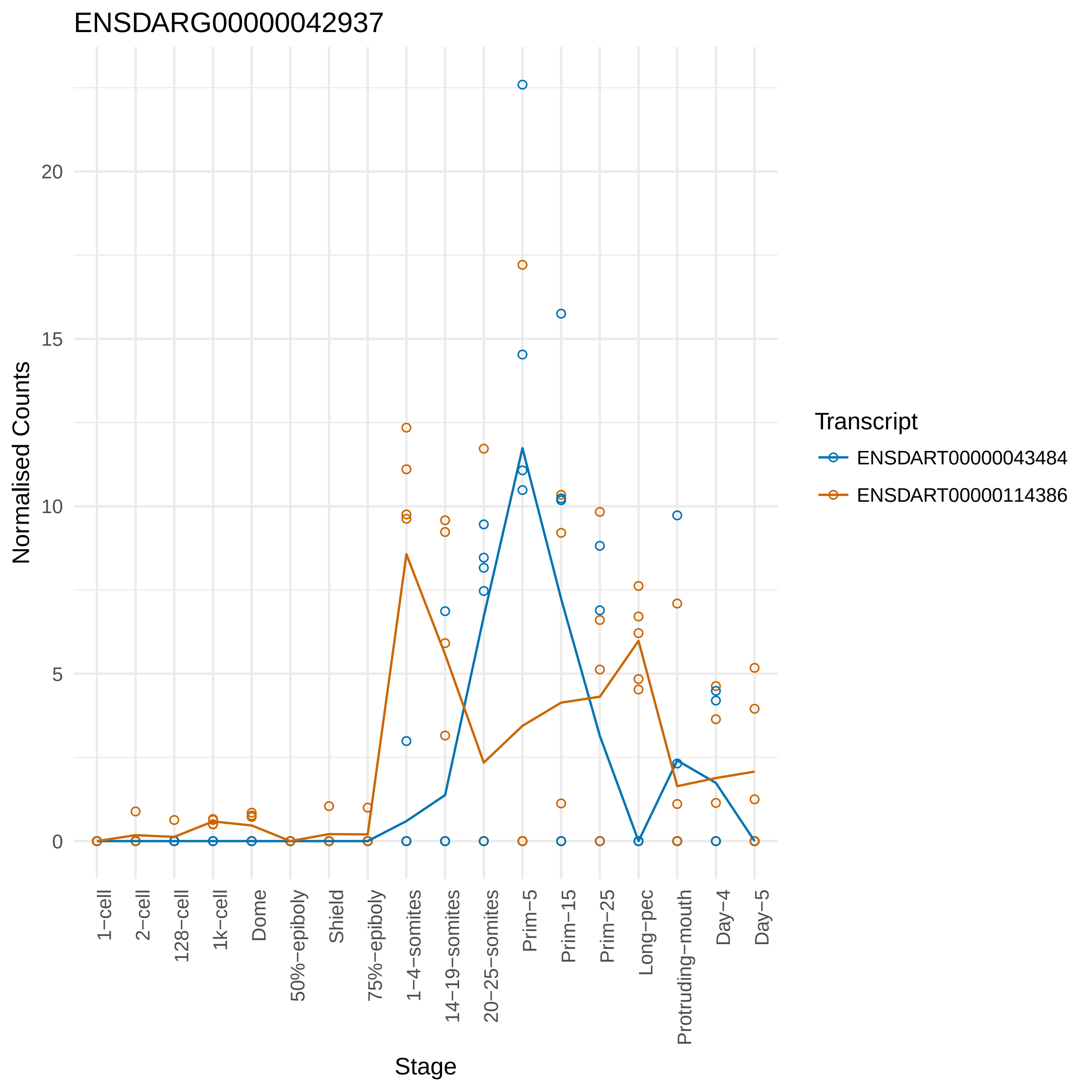

ENSDARG00000042937
Normalised Counts
0
5
10
15
20
Stage
1−cell
2−cell
128−cell
1k−cell
Dome
50%−epiboly
Shield
75%−epiboly
1−4−somites
14−19−somites
20−25−somites
Prim−5
Prim−15
Prim−25
Long−pec
Protruding−mouth
Day−4
Day−5
Transcript
ENSDART00000043484
ENSDART00000114386

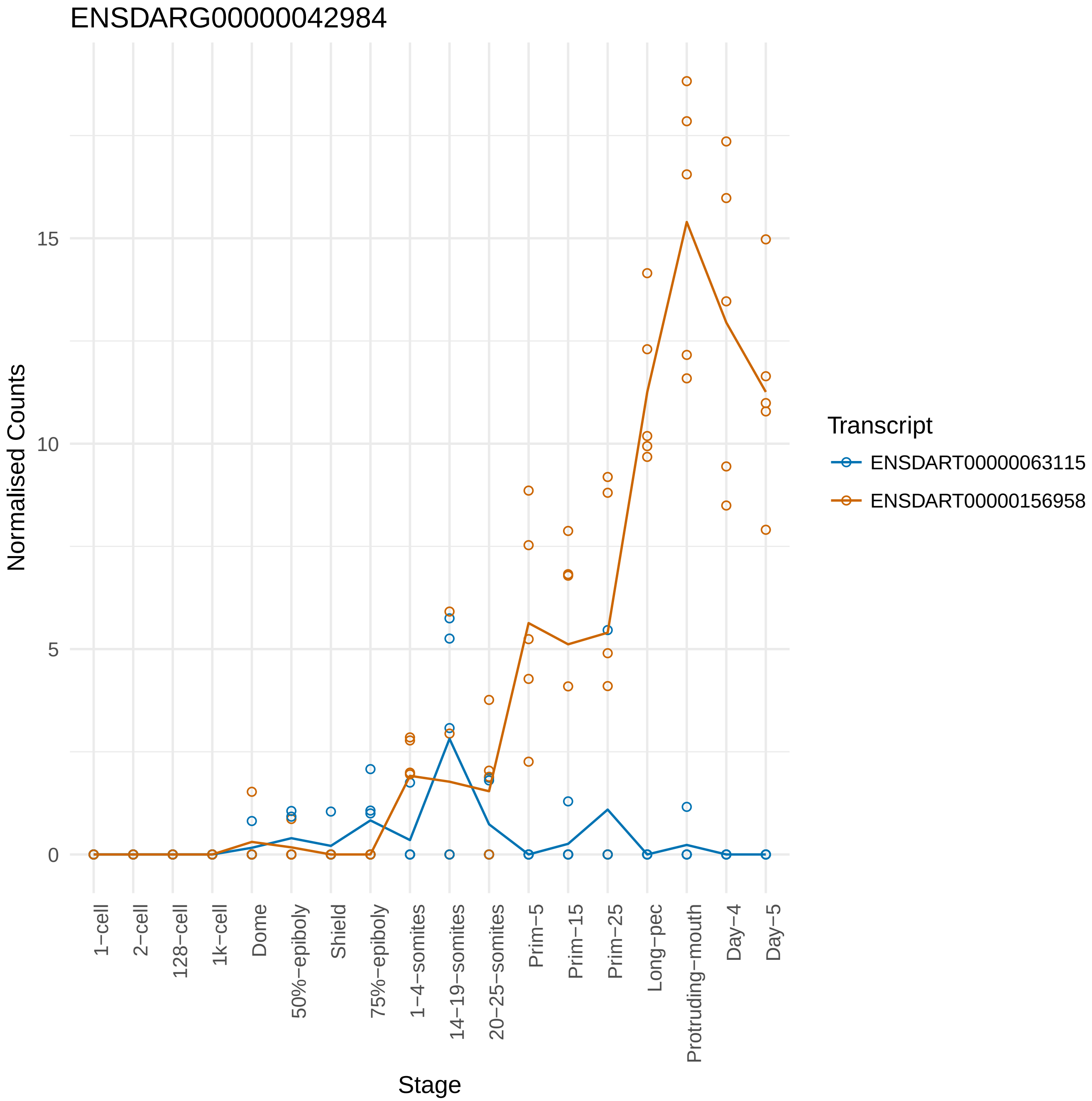
ENSDARG00000042984
Normalised Counts
0
5
10
15
Stage
1−cell
2−cell
128−cell
1k−cell
Dome
50%−epiboly
Shield
75%−epiboly
1−4−somites
14−19−somites
20−25−somites
Prim−5
Prim−15
Prim−25
Long−pec
Protruding−mouth
Day−4
Day−5
Transcript
ENSDART00000063115
ENSDART00000156958

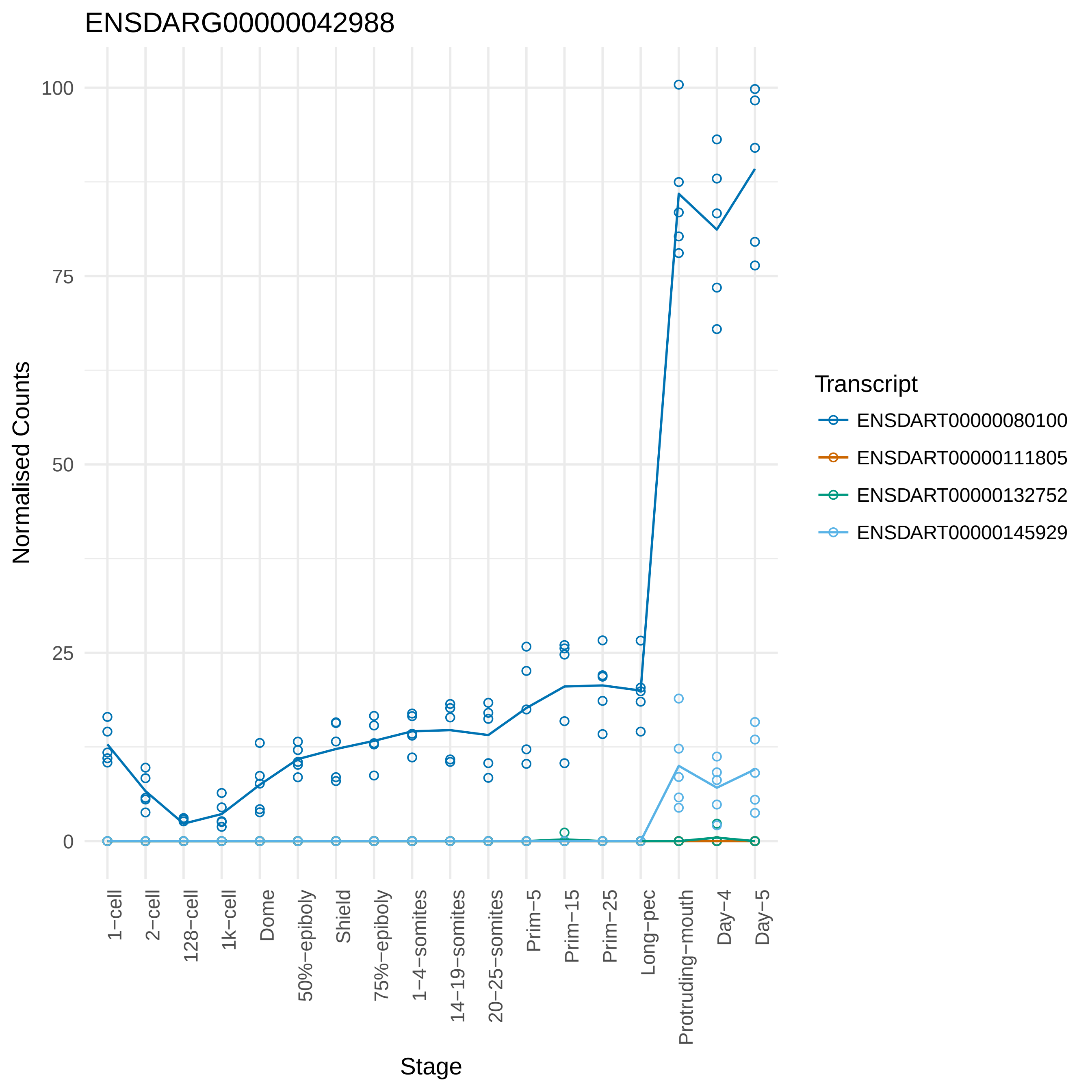

ENSDARG00000042988
Normalised Counts
100
75
50
25
0
Stage
1−cell
2−cell
128−cell
1k−cell
Dome
50%−epiboly
Shield
75%−epiboly
1−4−somites
14−19−somites
20−25−somites
Prim−5
Prim−15
Prim−25
Long−pec
Protruding−mouth
Day−4
Day−5
Transcript
ENSDART00000080100
ENSDART00000111805
ENSDART00000132752
ENSDART00000145929

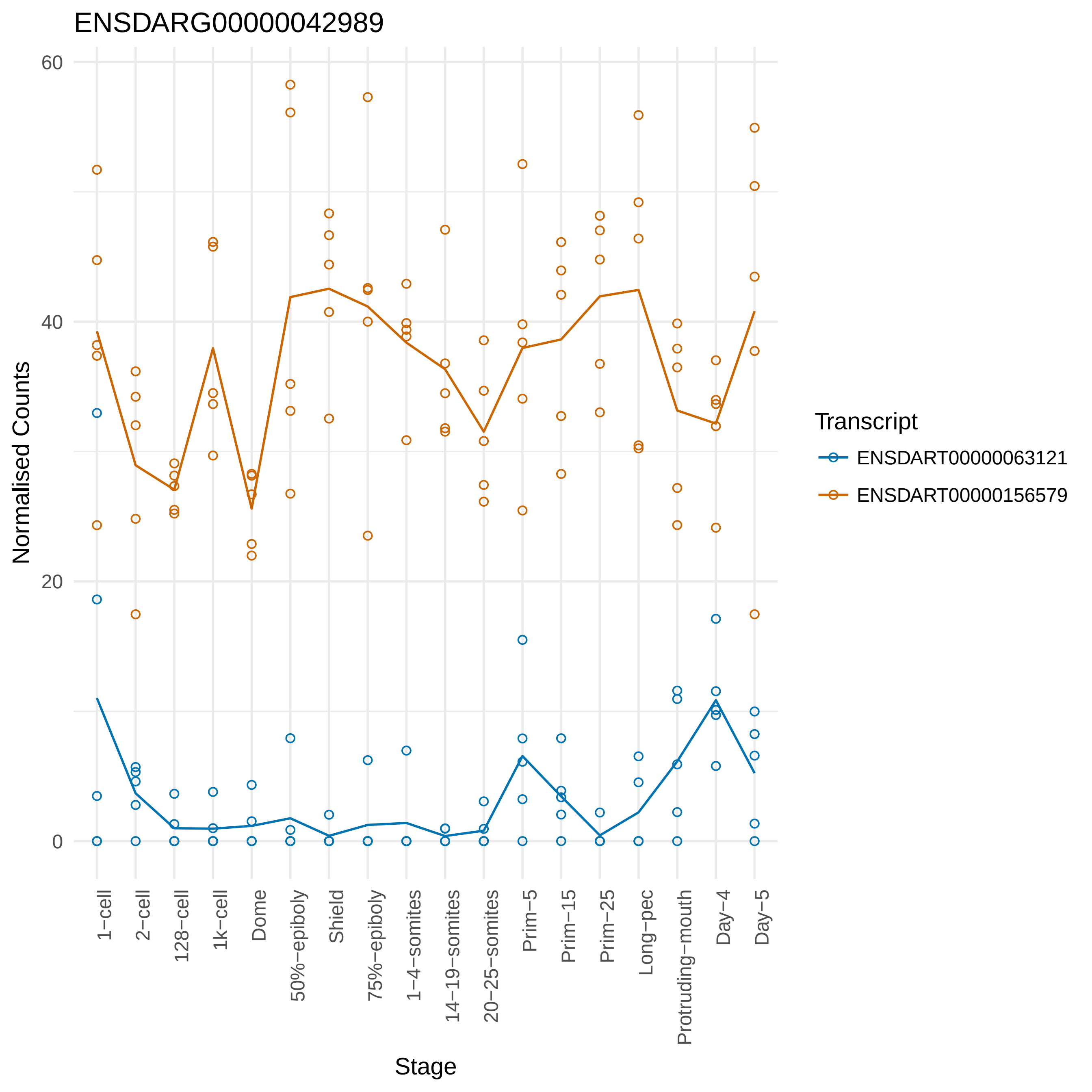

ENSDARG00000042989
Normalised Counts
0
20
40
60
Stage
1−cell
2−cell
128−cell
1k−cell
Dome
50%−epiboly
Shield
75%−epiboly
1−4−somites
14−19−somites
20−25−somites
Prim−5
Prim−15
Prim−25
Long−pec
Protruding−mouth
Day−4
Day−5
Transcript
ENSDART00000063121
ENSDART00000156579

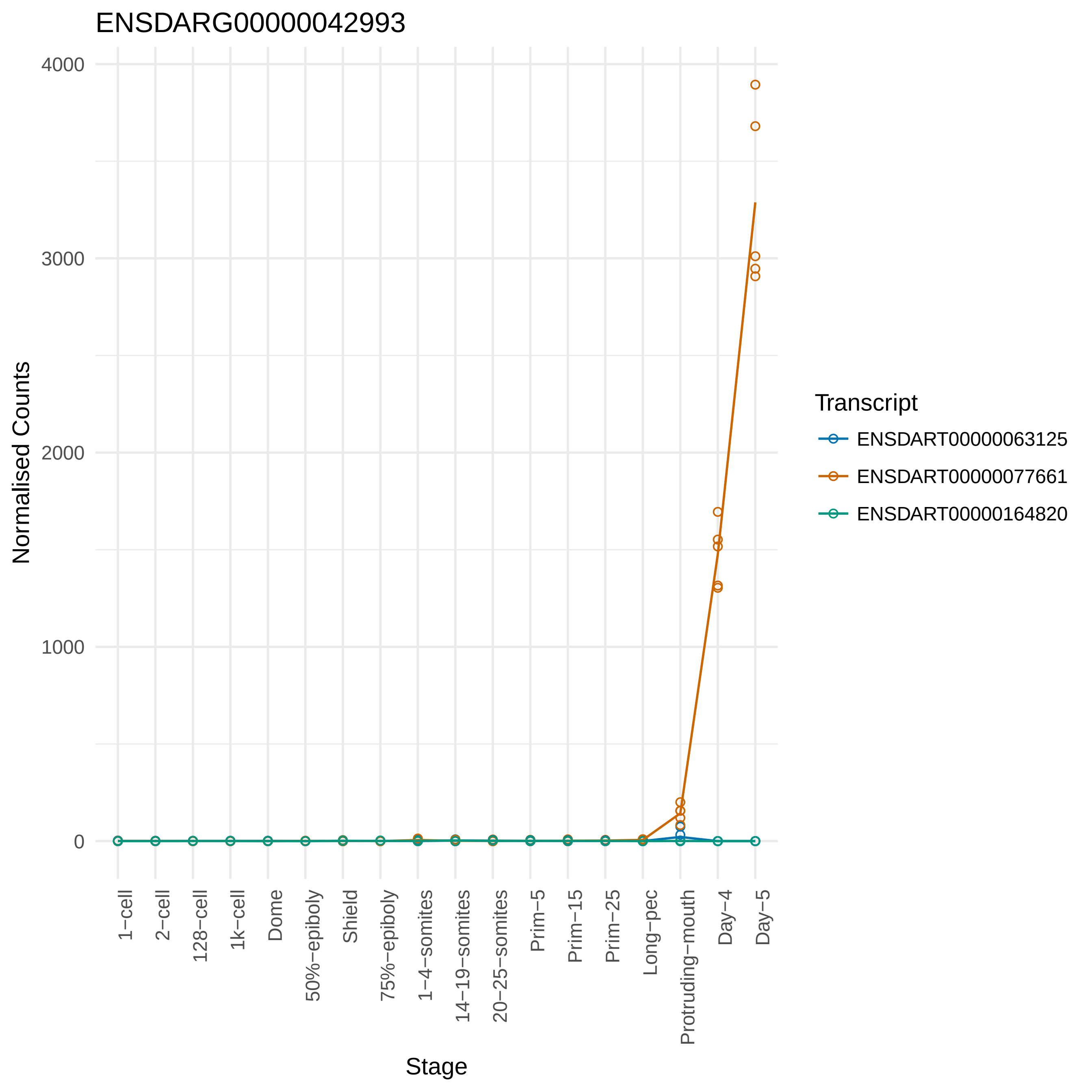
ENSDARG00000042993
Normalised Counts
4000
3000
2000
1000
0
Stage
1−cell
2−cell
128−cell
1k−cell
Dome
50%−epiboly
Shield
75%−epiboly
1−4−somites
14−19−somites
20−25−somites
Prim−5
Prim−15
Prim−25
Long−pec
Protruding−mouth
Day−4
Day−5
Transcript
ENSDART00000063125
ENSDART00000077661
ENSDART00000164820

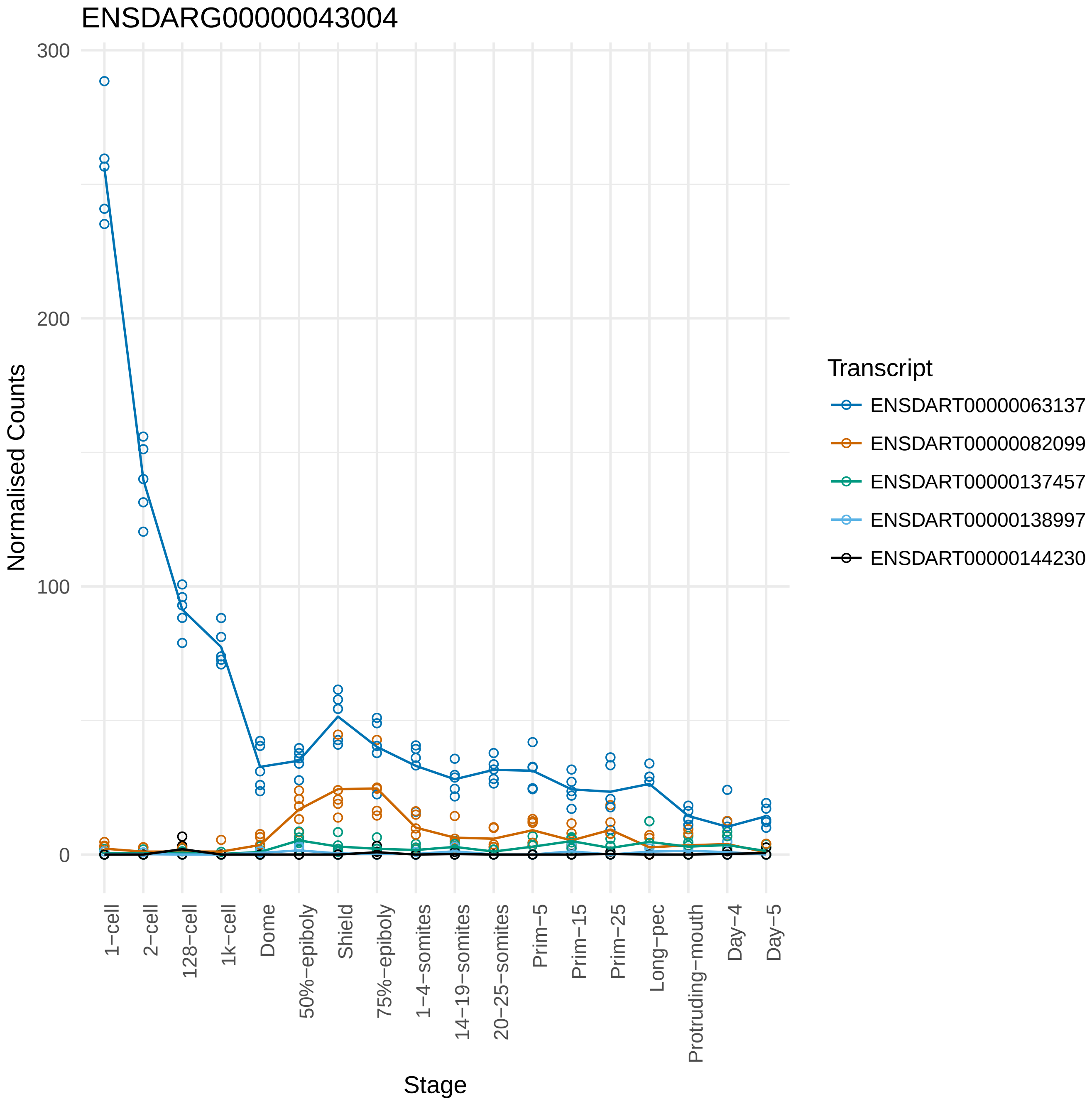
ENSDARG00000043004
Normalised Counts
300
200
100
0
1−cell
2−cell
128−cell
1k−cell
Dome
50%−epiboly
Shield
75%−epiboly
1−4−somites
14−19−somites
20−25−somites
Prim−5
Prim−15
Prim−25
Long−pec
Protruding−mouth
Day−4
Day−5
Stage
Transcript
ENSDART00000063137
ENSDART00000082099
ENSDART00000137457
ENSDART00000138997
ENSDART00000144230

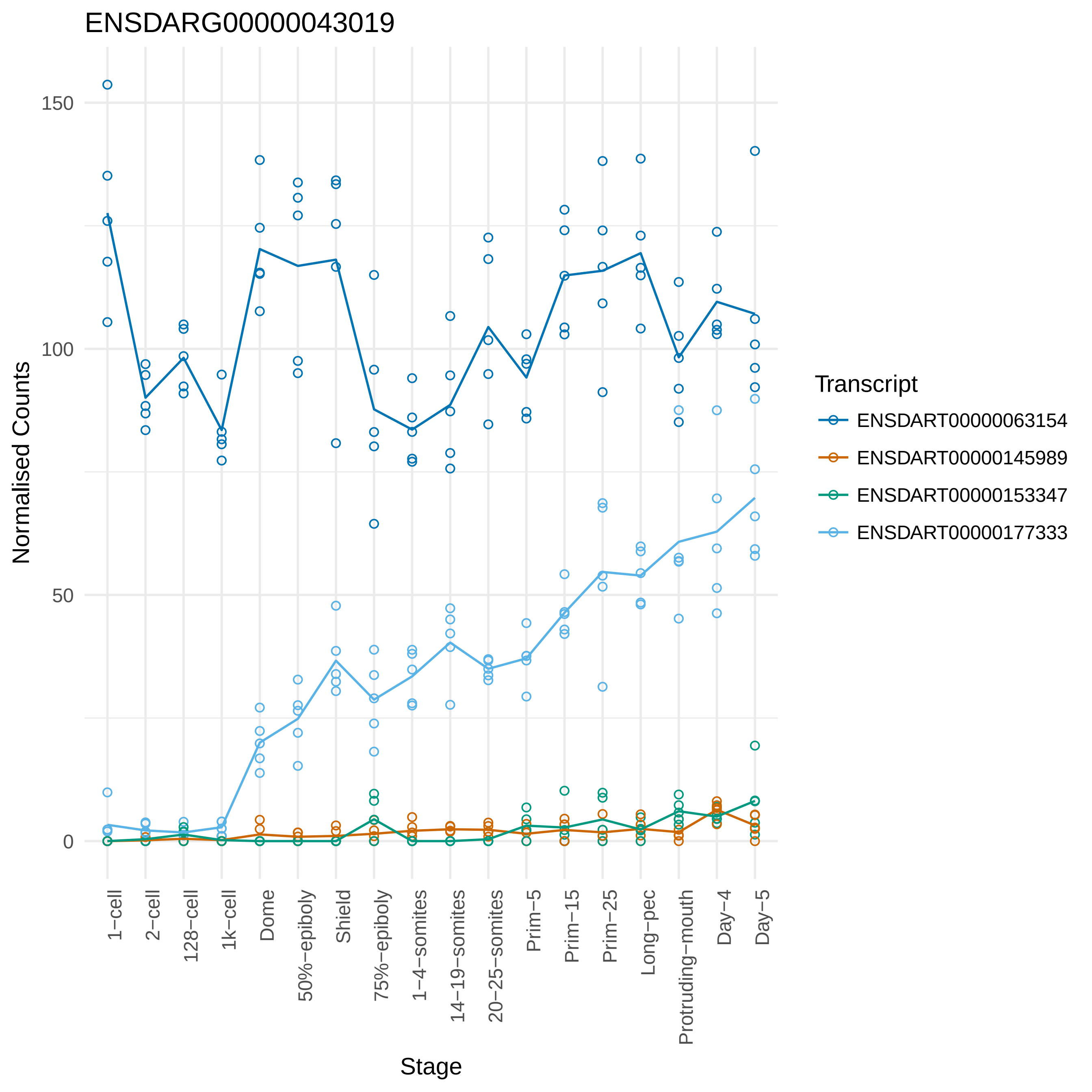

ENSDARG00000043019
Normalised Counts
150
100
50
0
1−cell
2−cell
128−cell
1k−cell
Dome
50%−epiboly
Shield
75%−epiboly
1−4−somites
14−19−somites
20−25−somites
Prim−5
Prim−15
Prim−25
Long−pec
Protruding−mouth
Day−4
Day−5
Stage
Transcript
ENSDART00000063154
ENSDART00000145989
ENSDART00000153347
ENSDART00000177333

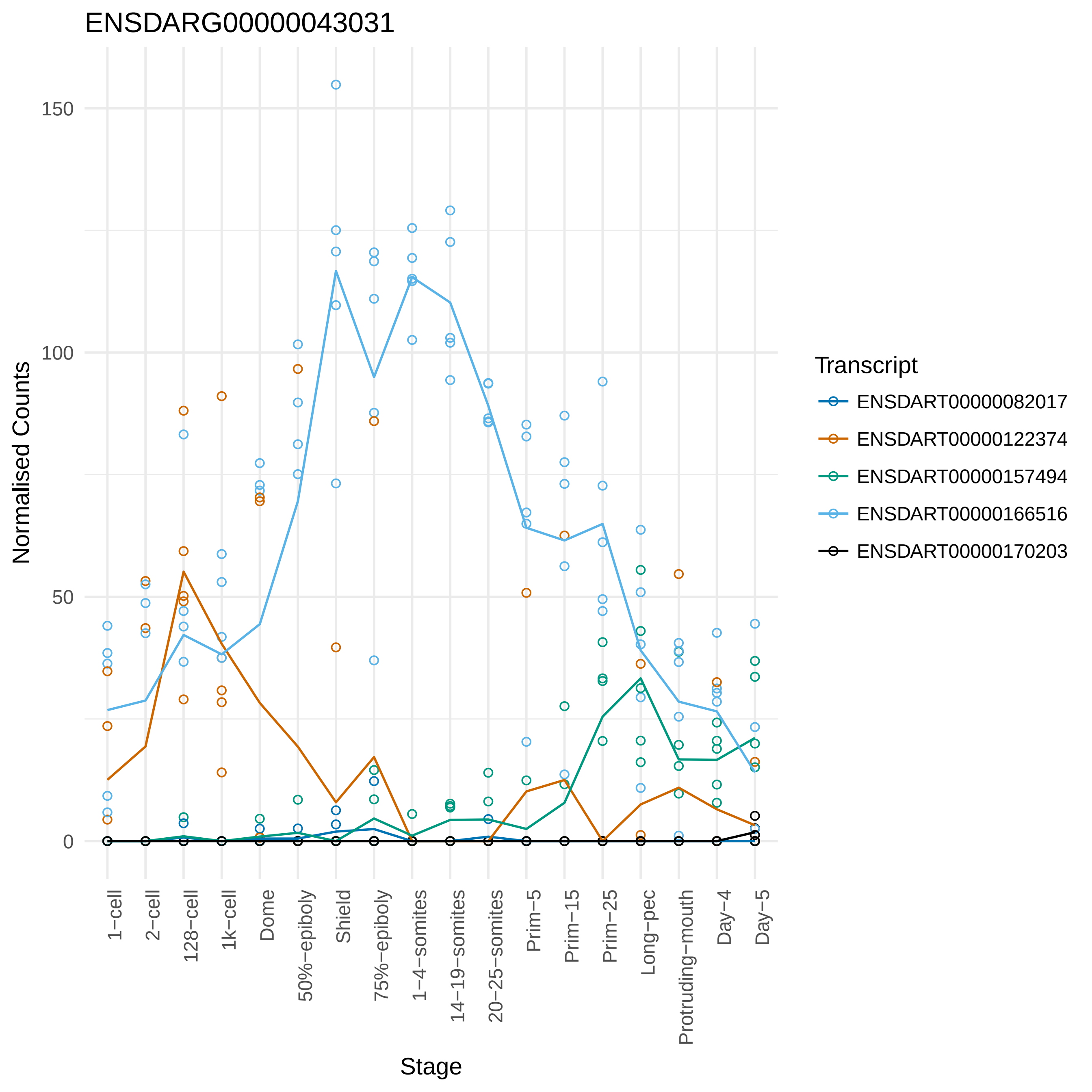
ENSDARG00000043031
Normalised Counts
150
100
50
0
1−cell
2−cell
128−cell
1k−cell
Dome
50%−epiboly
Shield
75%−epiboly
1−4−somites
14−19−somites
20−25−somites
Prim−5
Prim−15
Prim−25
Long−pec
Protruding−mouth
Day−4
Day−5
Stage
Transcript
ENSDART00000082017
ENSDART00000122374
ENSDART00000157494
ENSDART00000166516
ENSDART00000170203

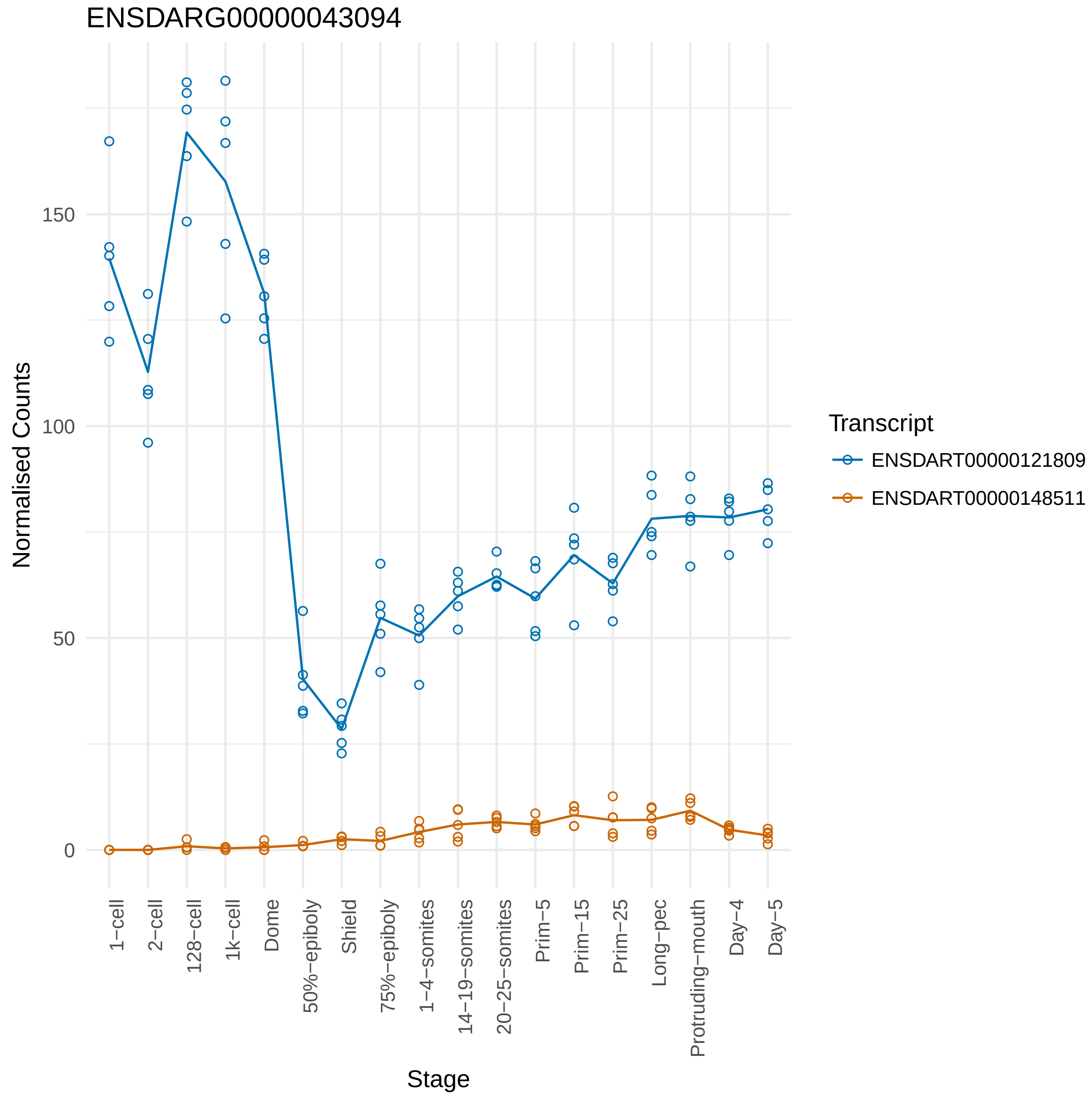
ENSDARG00000043094
Normalised Counts
150
100
50
0
1−cell
2−cell
128−cell
1k−cell
Dome
50%−epiboly
Shield
75%−epiboly
1−4−somites
14−19−somites
20−25−somites
Prim−5
Prim−15
Prim−25
Long−pec
Protruding−mouth
Day−4
Day−5
Stage
Transcript
ENSDART00000121809
ENSDART00000148511

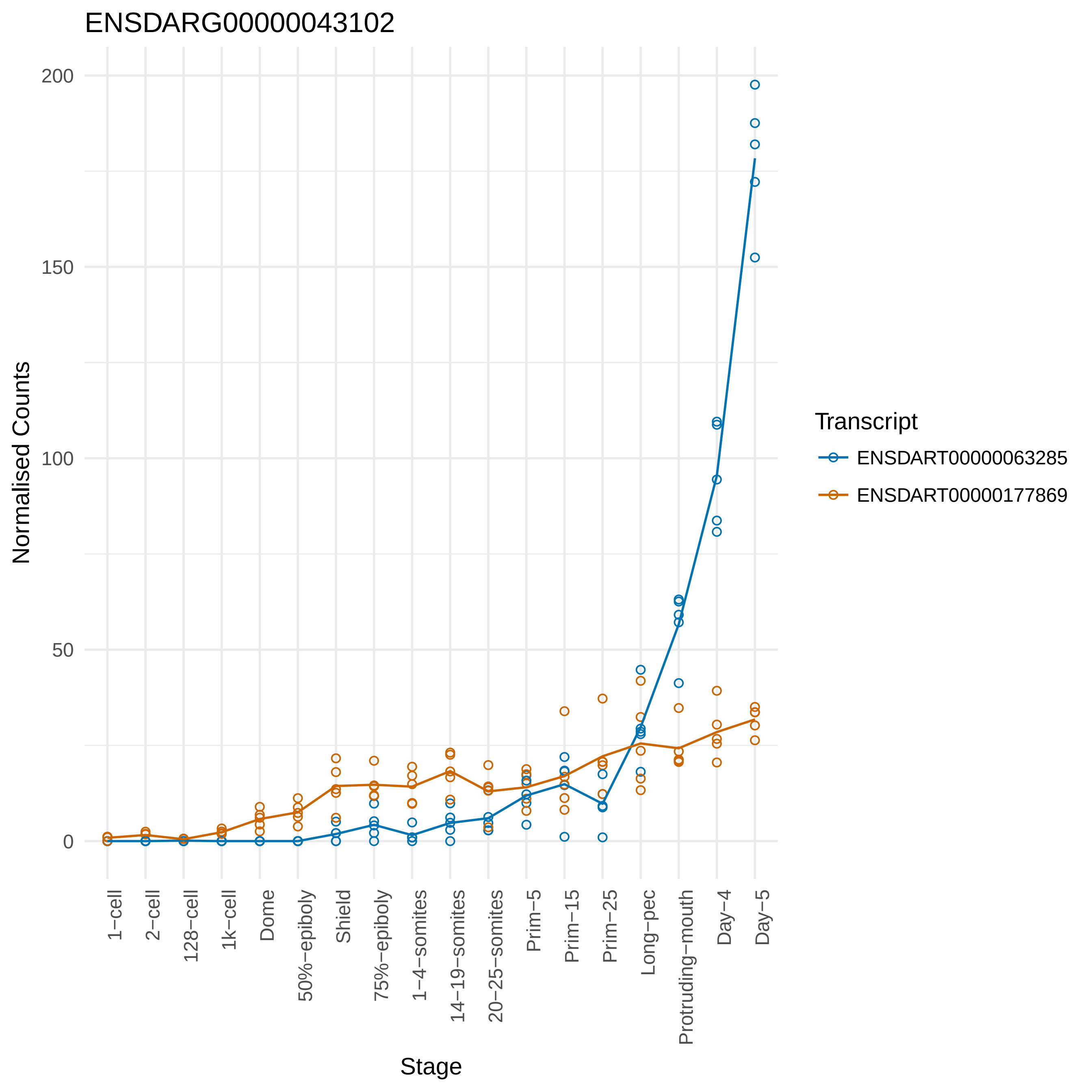

ENSDARG00000043102
Normalised Counts
200
150
100
50
0
1−cell
2−cell
128−cell
1k−cell
Dome
50%−epiboly
Shield
75%−epiboly
1−4−somites
14−19−somites
20−25−somites
Prim−5
Prim−15
Prim−25
Long−pec
Protruding−mouth
Day−4
Day−5
Stage
Transcript
ENSDART00000063285
ENSDART00000177869

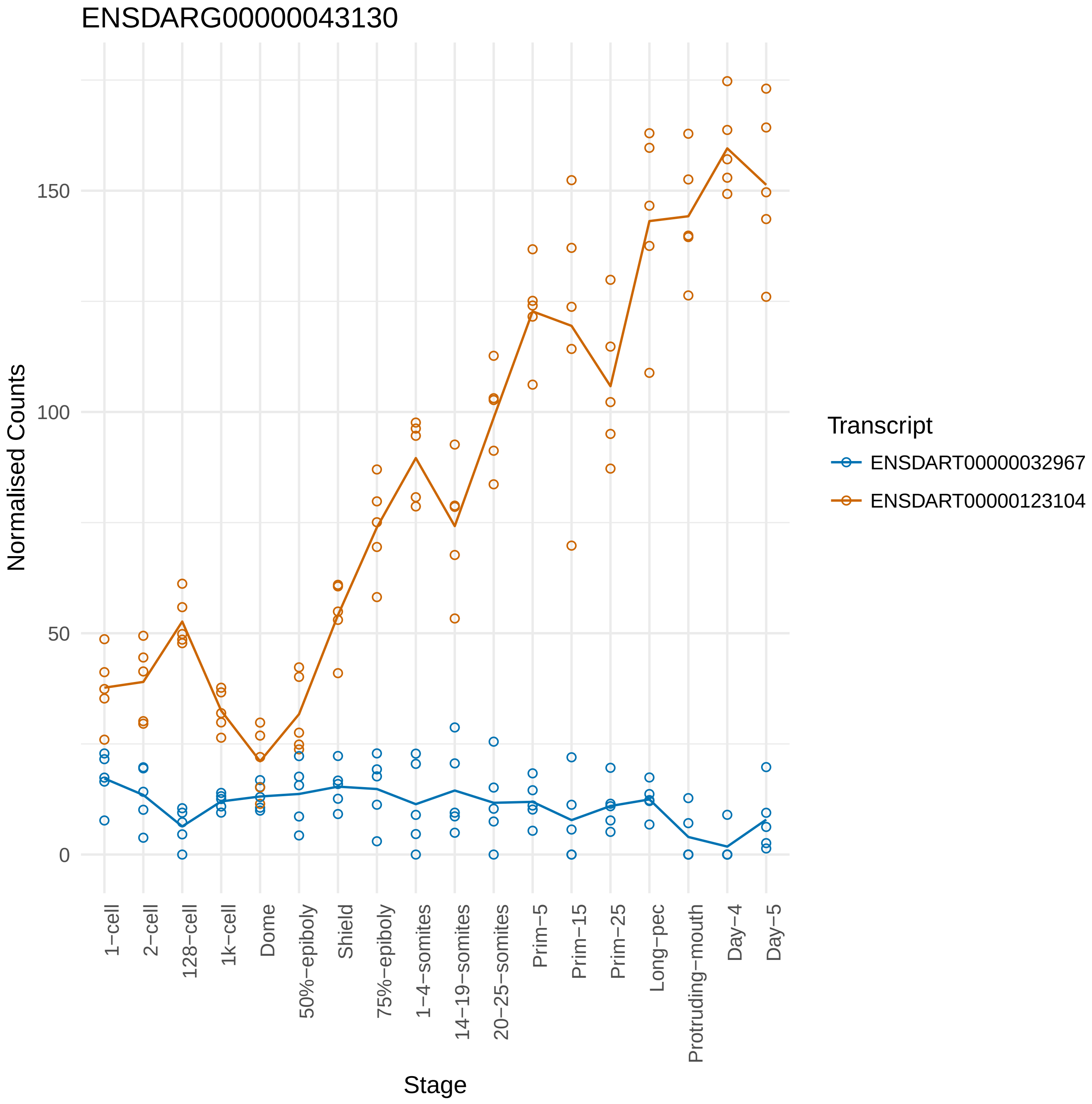
ENSDARG00000043130
Normalised Counts
150
100
50
0
1−cell
2−cell
128−cell
1k−cell
Dome
50%−epiboly
Shield
75%−epiboly
1−4−somites
14−19−somites
20−25−somites
Prim−5
Prim−15
Prim−25
Long−pec
Protruding−mouth
Day−4
Day−5
Stage
Transcript
ENSDART00000032967
ENSDART00000123104

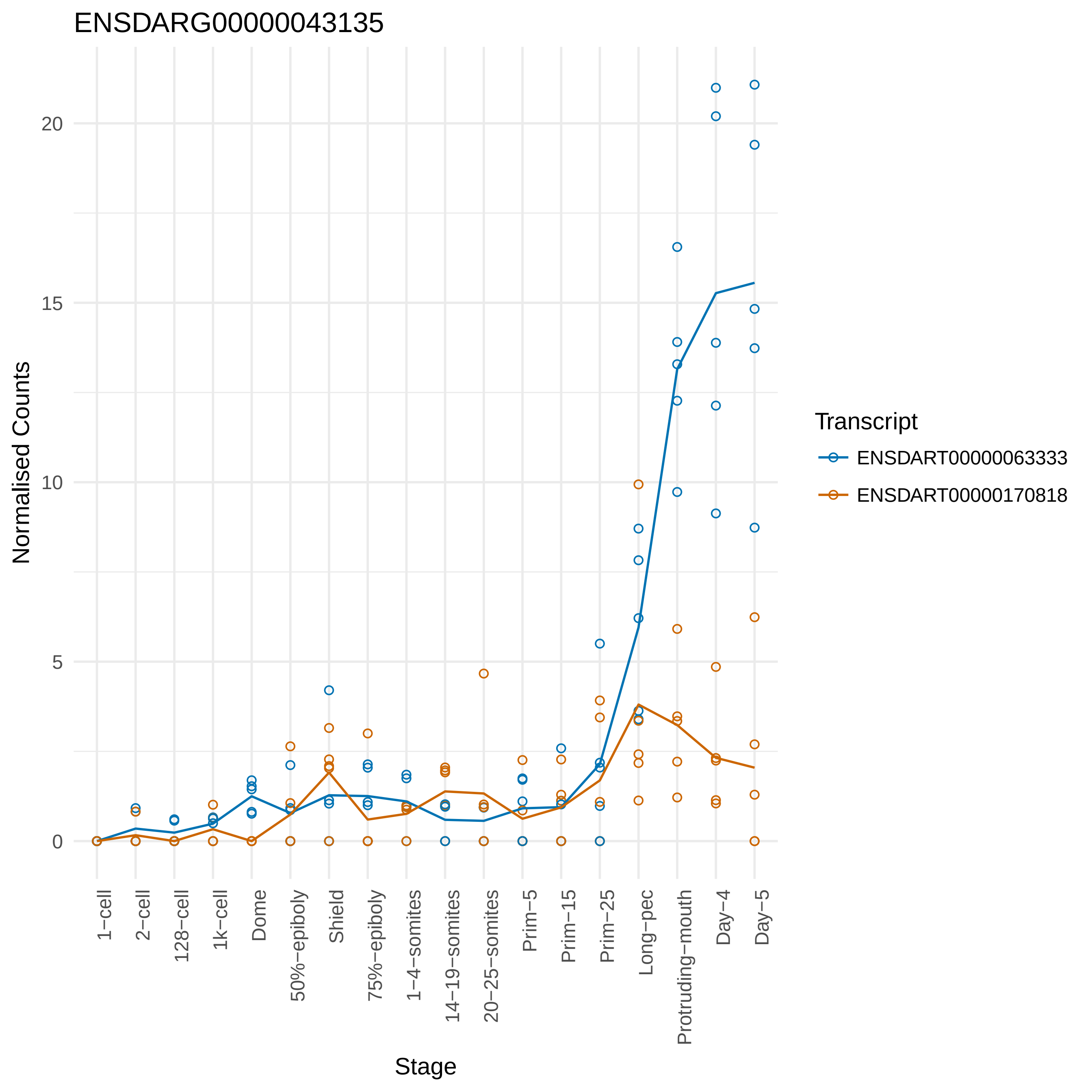

ENSDARG00000043135
Normalised Counts
20
15
10
5
0
1−cell
2−cell
128−cell
1k−cell
Dome
50%−epiboly
Shield
75%−epiboly
1−4−somites
14−19−somites
20−25−somites
Prim−5
Prim−15
Prim−25
Long−pec
Protruding−mouth
Day−4
Day−5
Stage
Transcript
ENSDART00000063333
ENSDART00000170818

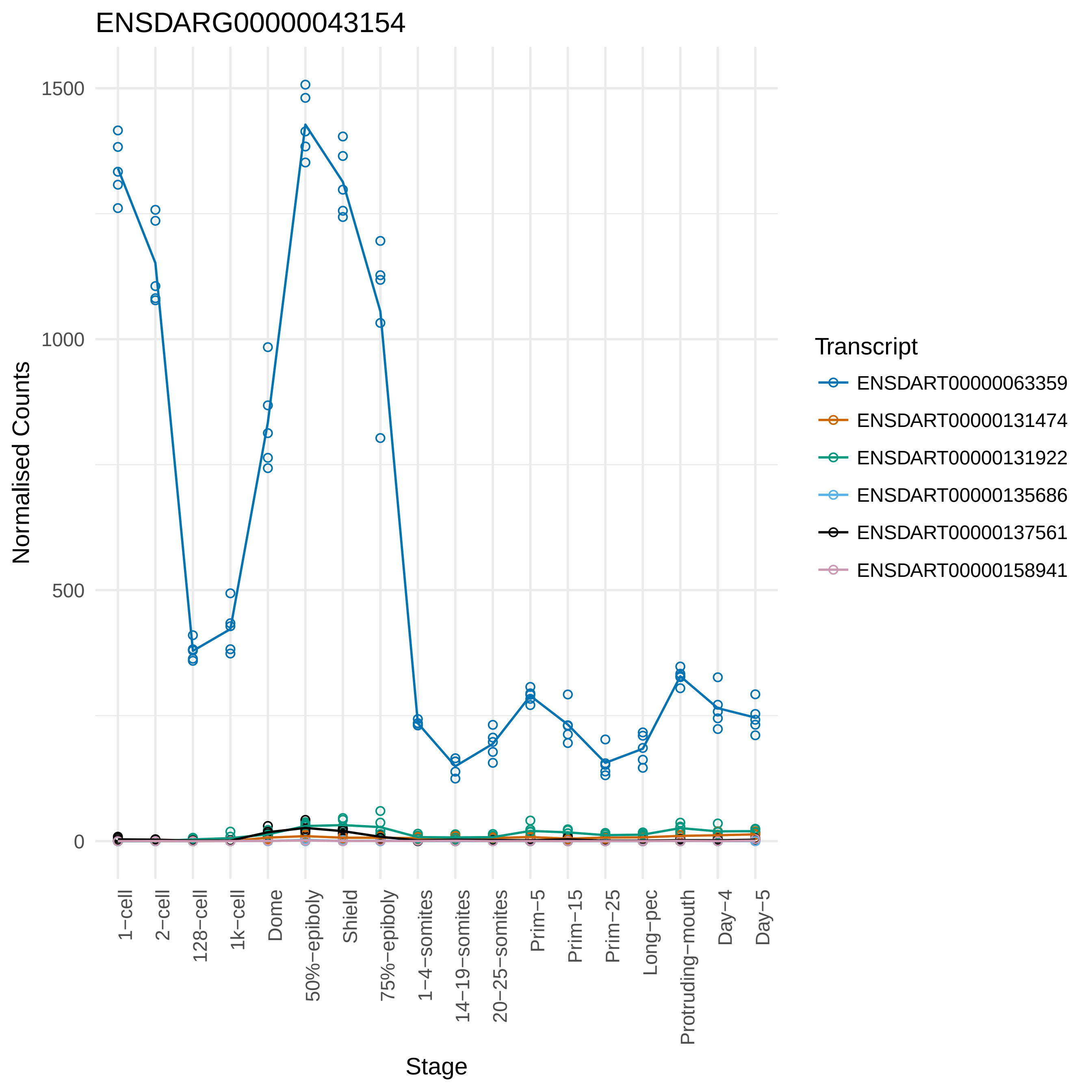

ENSDARG00000043154
Normalised Counts
1500
1000
500
0
1−cell
2−cell
128−cell
1k−cell
Dome
50%−epiboly
Shield
75%−epiboly
1−4−somites
14−19−somites
20−25−somites
Prim−5
Prim−15
Prim−25
Long−pec
Protruding−mouth
Day−4
Day−5
Stage
Transcript
ENSDART00000063359
ENSDART00000131474
ENSDART00000131922
ENSDART00000135686
ENSDART00000137561
ENSDART00000158941

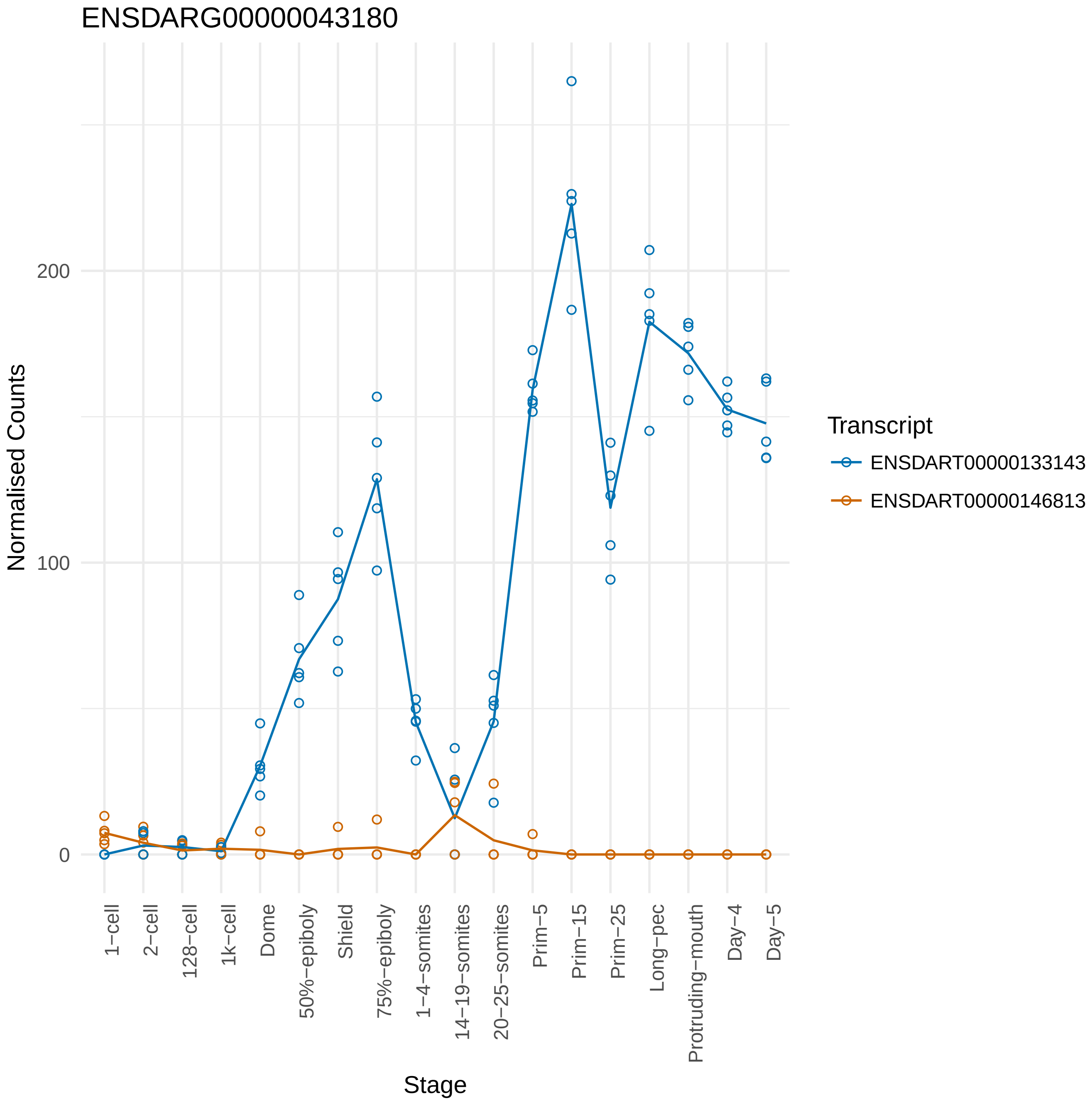
ENSDARG00000043180
Normalised Counts
200
100
0
Stage
1−cell
2−cell
128−cell
1k−cell
Dome
50%−epiboly
Shield
75%−epiboly
1−4−somites
14−19−somites
20−25−somites
Prim−5
Prim−15
Prim−25
Long−pec
Protruding−mouth
Day−4
Day−5
Transcript
ENSDART00000133143
ENSDART00000146813

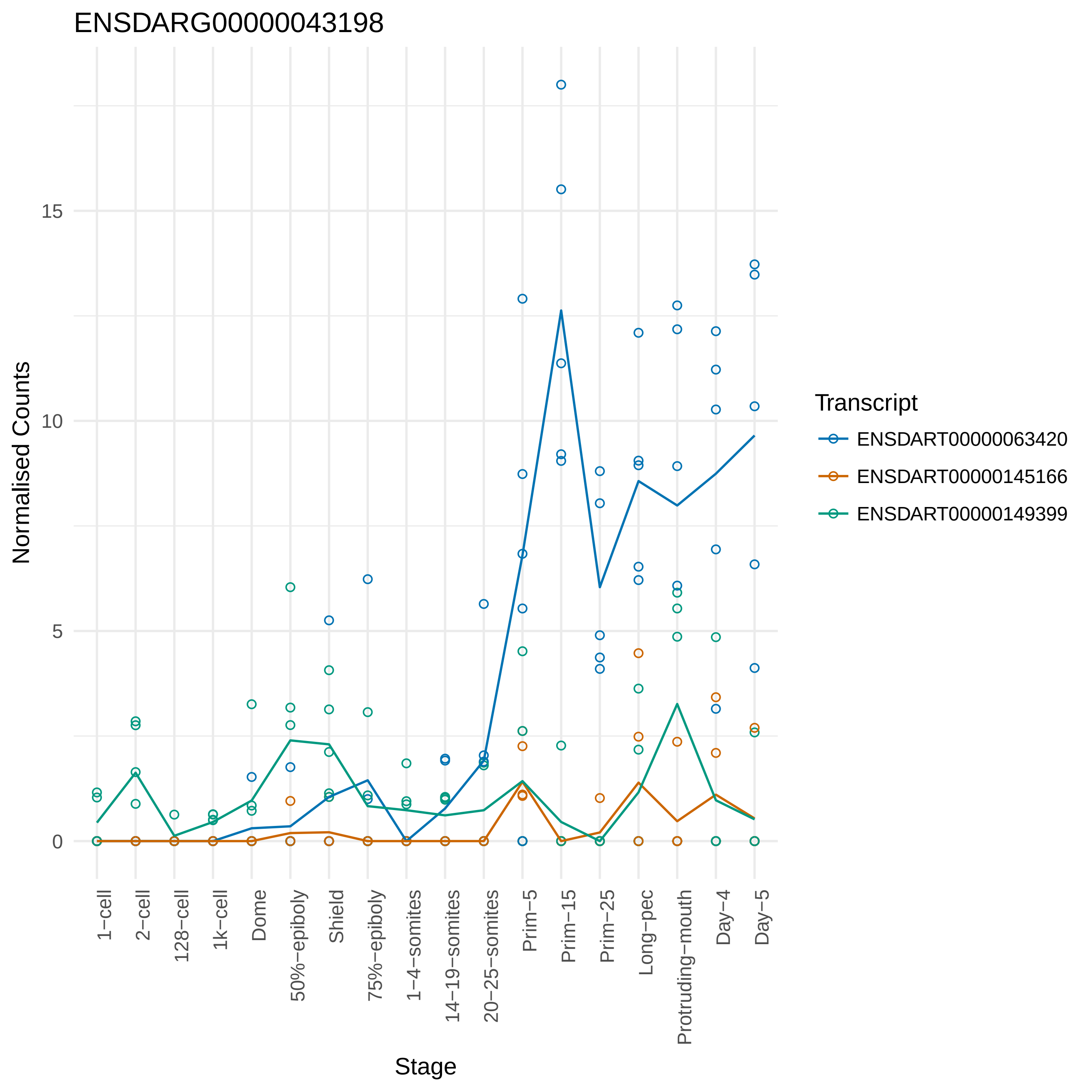
ENSDARG00000043198
Normalised Counts
15
10
5
0
1−cell
2−cell
128−cell
1k−cell
Dome
50%−epiboly
Shield
75%−epiboly
1−4−somites
14−19−somites
20−25−somites
Prim−5
Prim−15
Prim−25
Long−pec
Protruding−mouth
Day−4
Day−5
Stage
Transcript
ENSDART00000063420
ENSDART00000145166
ENSDART00000149399

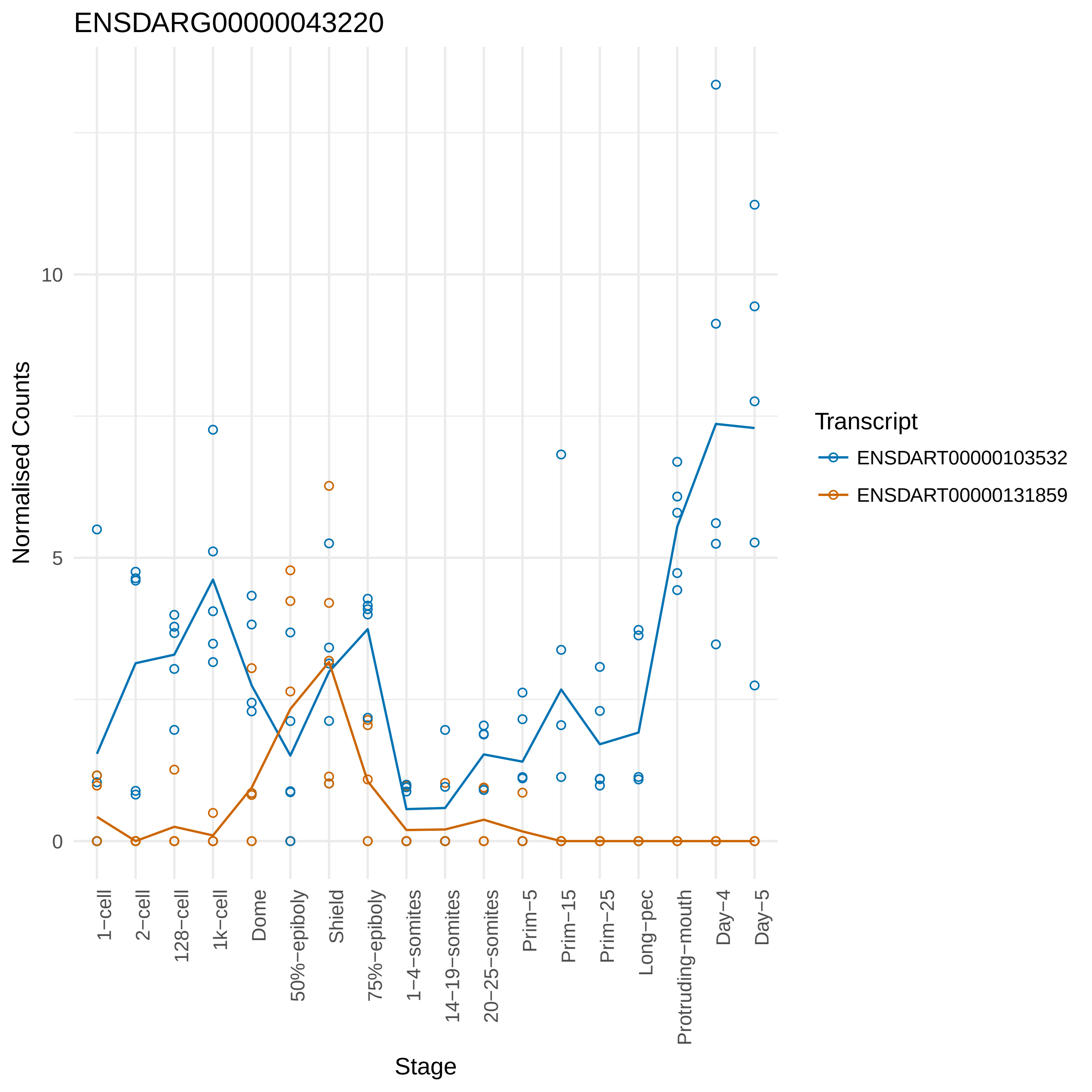
ENSDARG00000043220
Normalised Counts
0
5
10
1−cell
2−cell
128−cell
1k−cell
Dome
50%−epiboly
Shield
75%−epiboly
1−4−somites
14−19−somites
20−25−somites
Prim−5
Prim−15
Prim−25
Long−pec
Protruding−mouth
Day−4
Day−5
Stage
Transcript
ENSDART00000103532
ENSDART00000131859

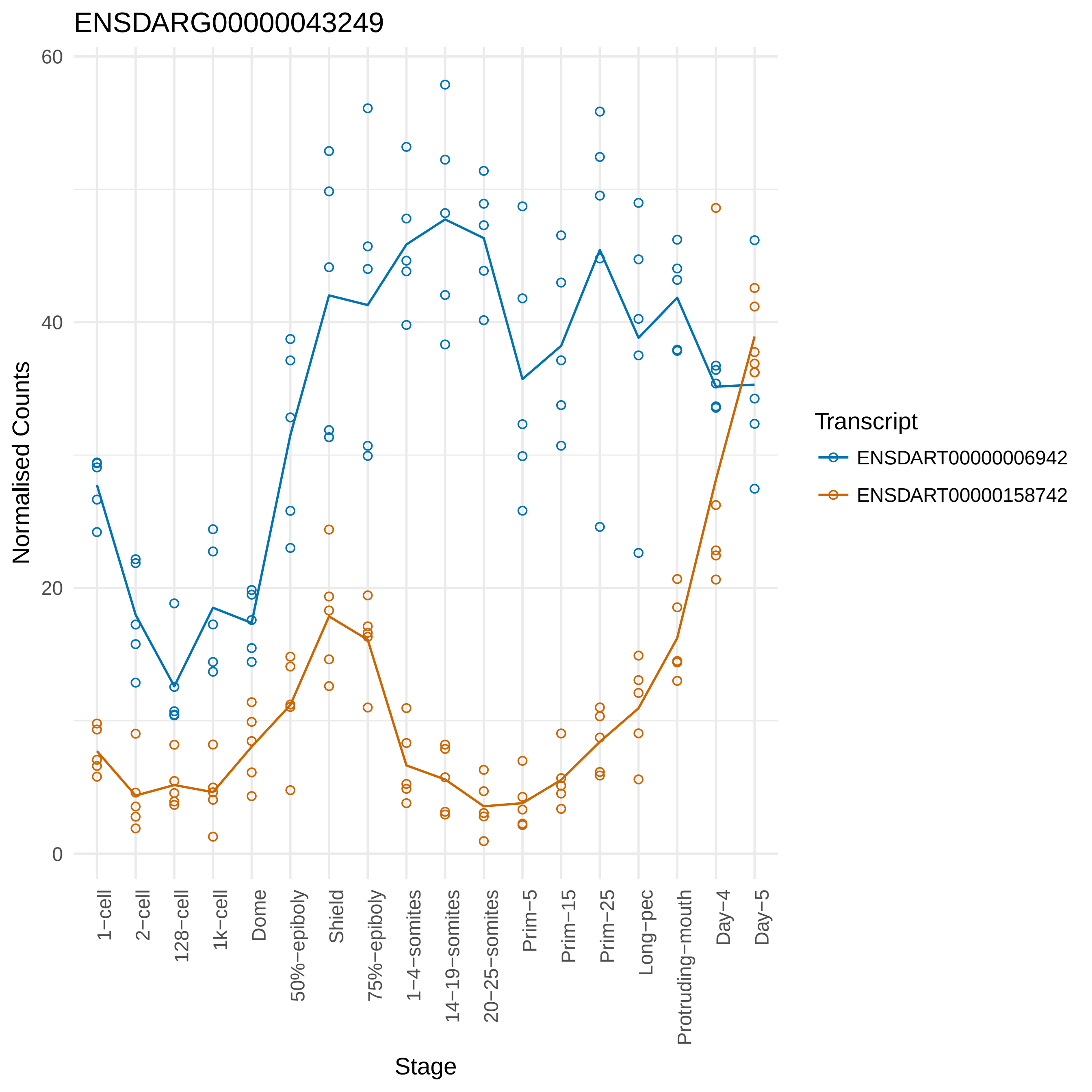
ENSDARG00000043249
Normalised Counts
60
40
20
0
1−cell
2−cell
128−cell
1k−cell
Dome
50%−epiboly
Shield
75%−epiboly
1−4−somites
14−19−somites
20−25−somites
Prim−5
Prim−15
Prim−25
Long−pec
Protruding−mouth
Day−4
Day−5
Stage
Transcript
ENSDART00000006942
ENSDART00000158742

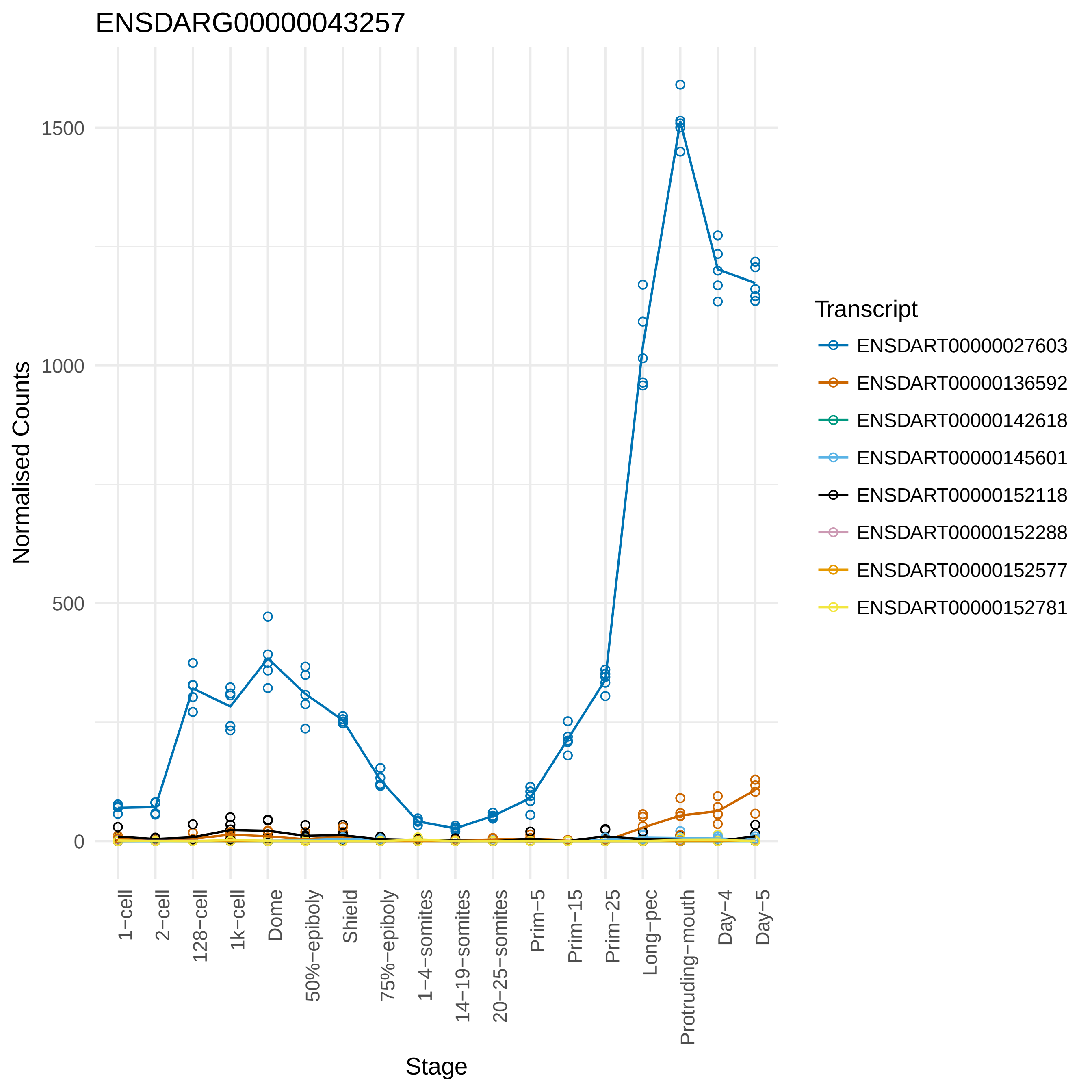

ENSDARG00000043257
Normalised Counts
1500
1000
500
0
Stage
1−cell
2−cell
128−cell
1k−cell
Dome
50%−epiboly
Shield
75%−epiboly
1−4−somites
14−19−somites
20−25−somites
Prim−5
Prim−15
Prim−25
Long−pec
Protruding−mouth
Day−4
Day−5
Transcript
ENSDART00000027603
ENSDART00000136592
ENSDART00000142618
ENSDART00000145601
ENSDART00000152118
ENSDART00000152288
ENSDART00000152577
ENSDART00000152781

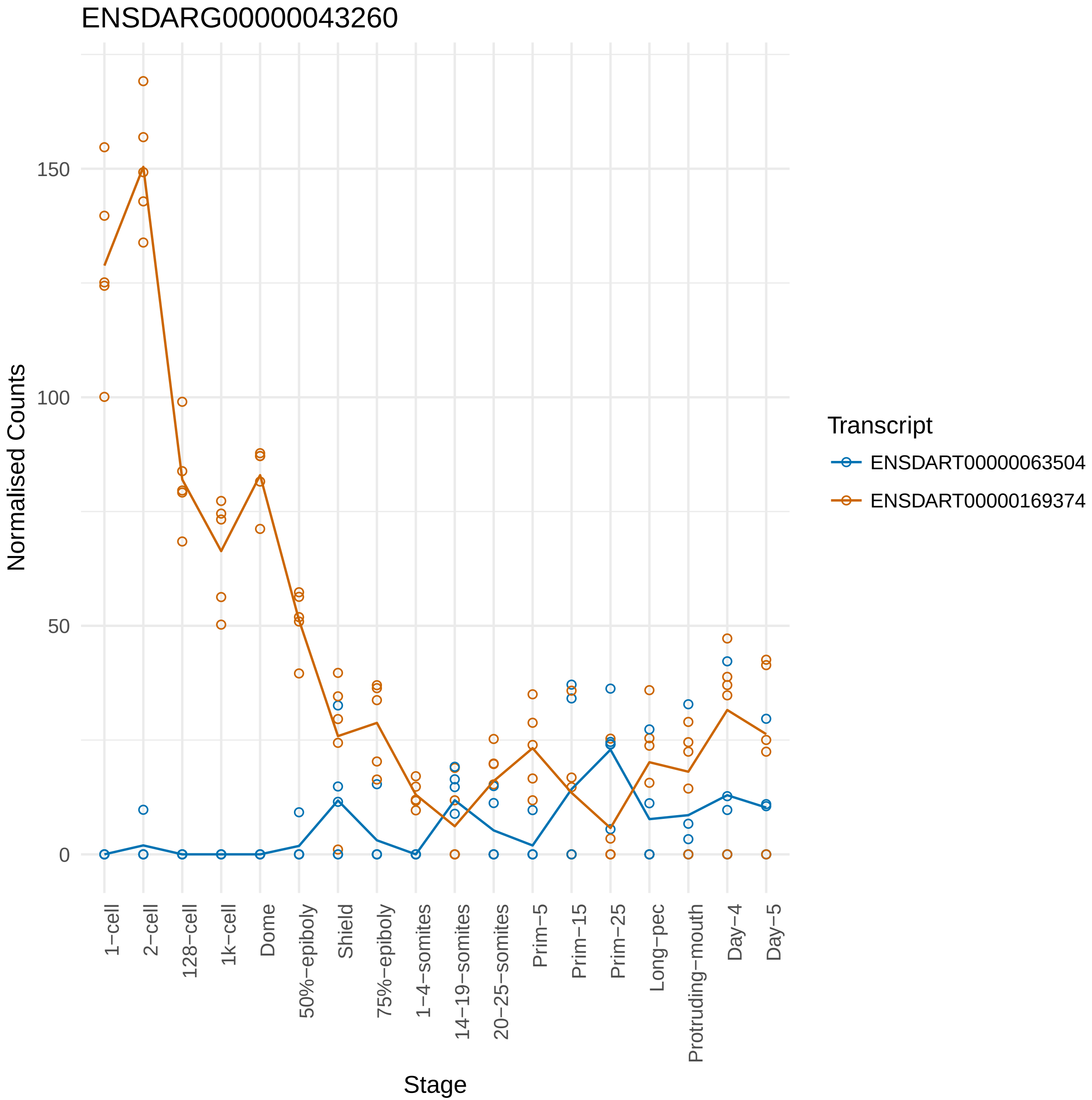

ENSDARG00000043260
Normalised Counts
150
100
50
0
1−cell
2−cell
128−cell
1k−cell
Dome
50%−epiboly
Shield
75%−epiboly
1−4−somites
14−19−somites
20−25−somites
Prim−5
Prim−15
Prim−25
Long−pec
Protruding−mouth
Day−4
Day−5
Stage
Transcript
ENSDART00000063504
ENSDART00000169374

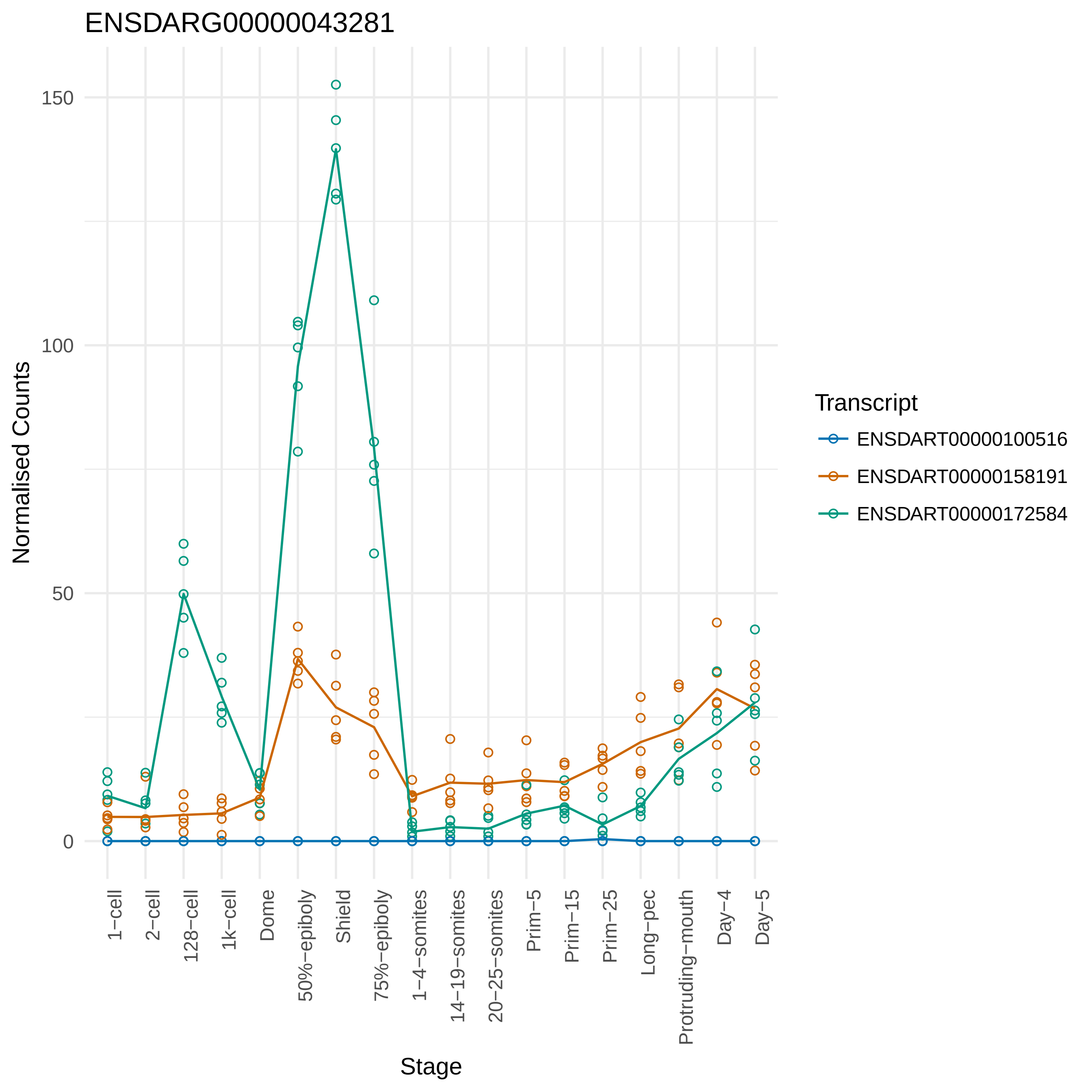

ENSDARG00000043281
Normalised Counts
0
50
100
150
Stage
1−cell
2−cell
128−cell
1k−cell
Dome
50%−epiboly
Shield
75%−epiboly
1−4−somites
14−19−somites
20−25−somites
Prim−5
Prim−15
Prim−25
Long−pec
Protruding−mouth
Day−4
Day−5
Transcript
ENSDART00000100516
ENSDART00000158191
ENSDART00000172584

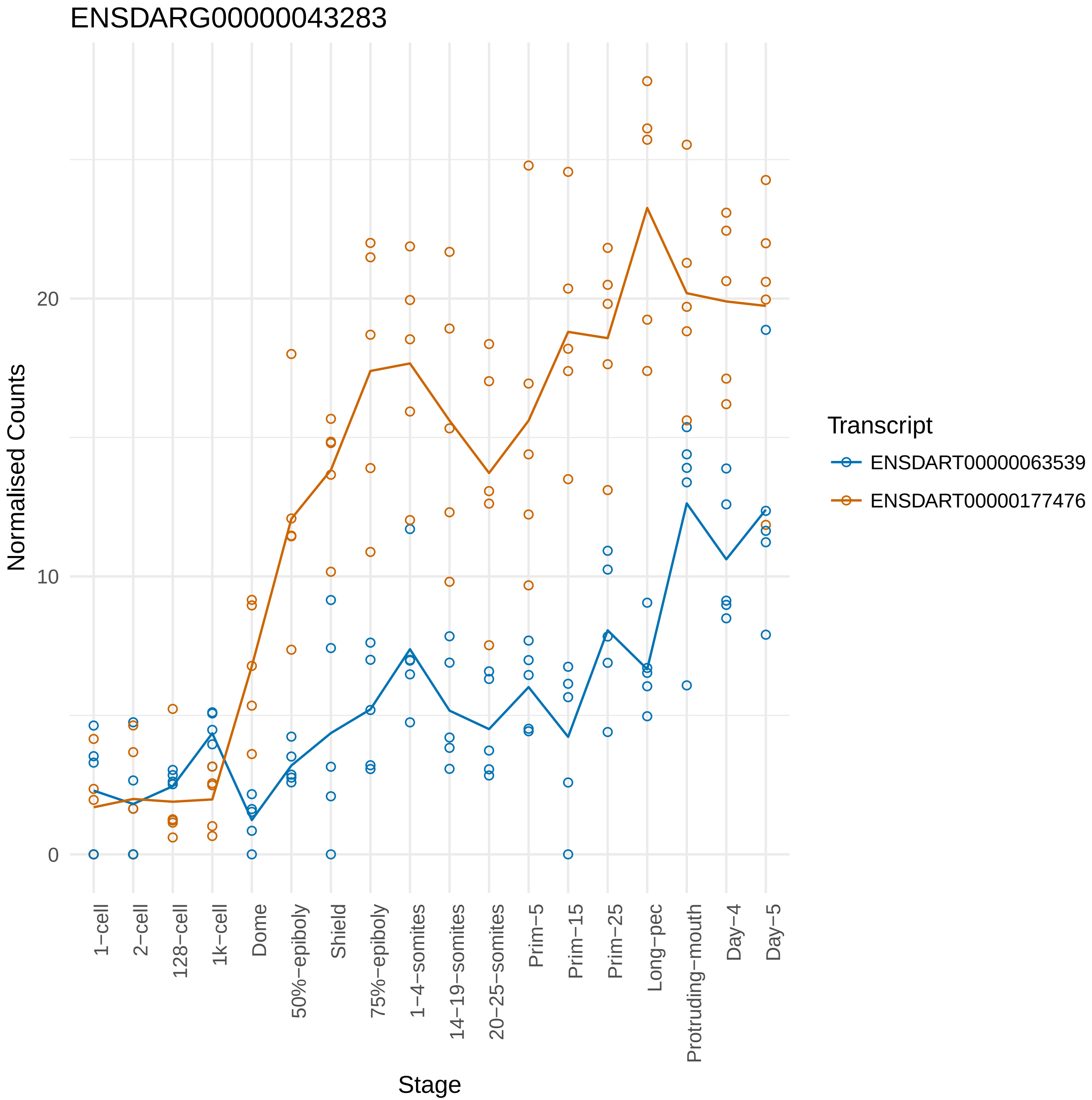

ENSDARG00000043283
Normalised Counts
0
10
20
Stage
1−cell
2−cell
128−cell
1k−cell
Dome
50%−epiboly
Shield
75%−epiboly
1−4−somites
14−19−somites
20−25−somites
Prim−5
Prim−15
Prim−25
Long−pec
Protruding−mouth
Day−4
Day−5
Transcript
ENSDART00000063539
ENSDART00000177476

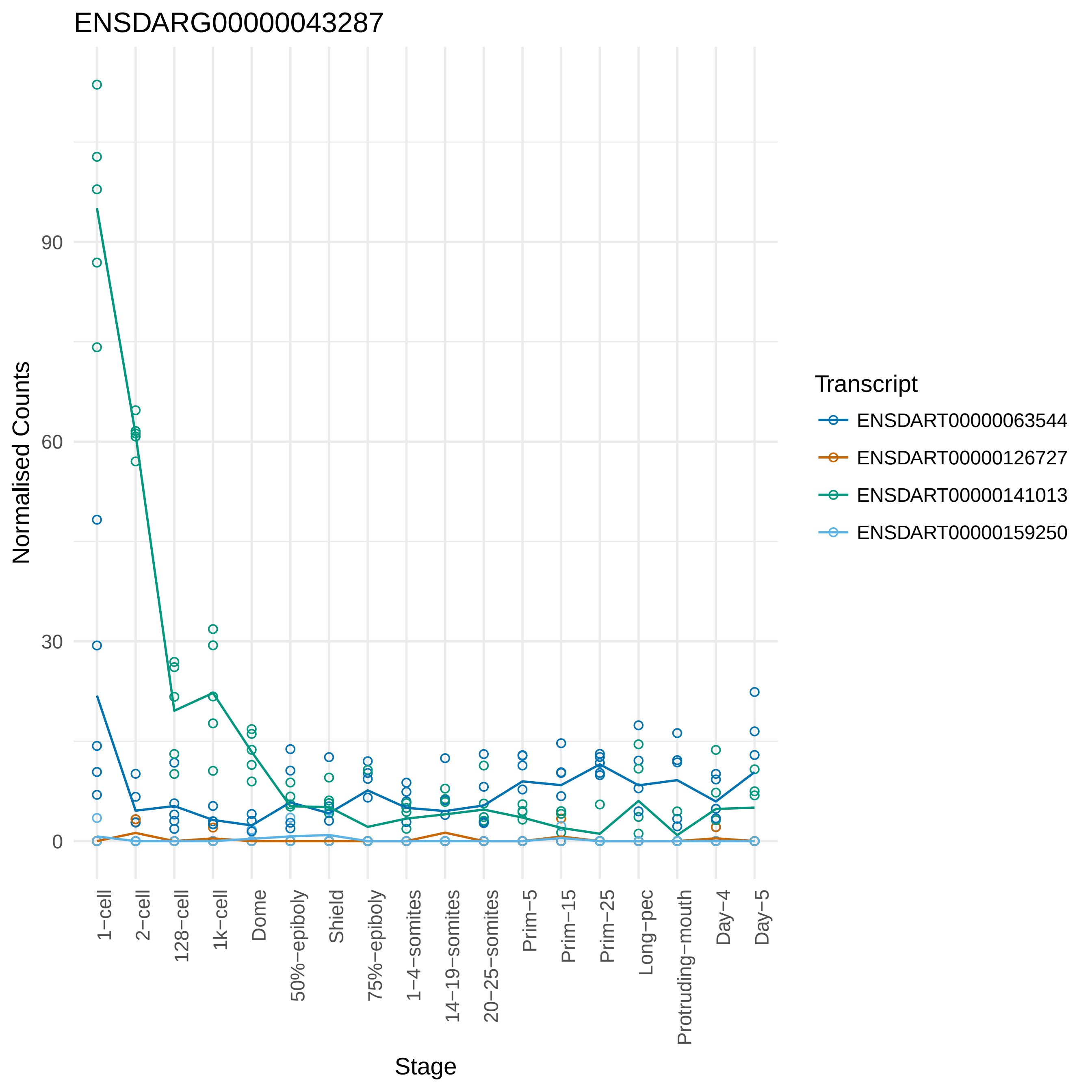
ENSDARG00000043287
Normalised Counts
0
30
60
90
1−cell
2−cell
128−cell
1k−cell
Dome
50%−epiboly
Shield
75%−epiboly
1−4−somites
14−19−somites
20−25−somites
Prim−5
Prim−15
Prim−25
Long−pec
Protruding−mouth
Day−4
Day−5
Stage
Transcript
ENSDART00000063544
ENSDART00000126727
ENSDART00000141013
ENSDART00000159250

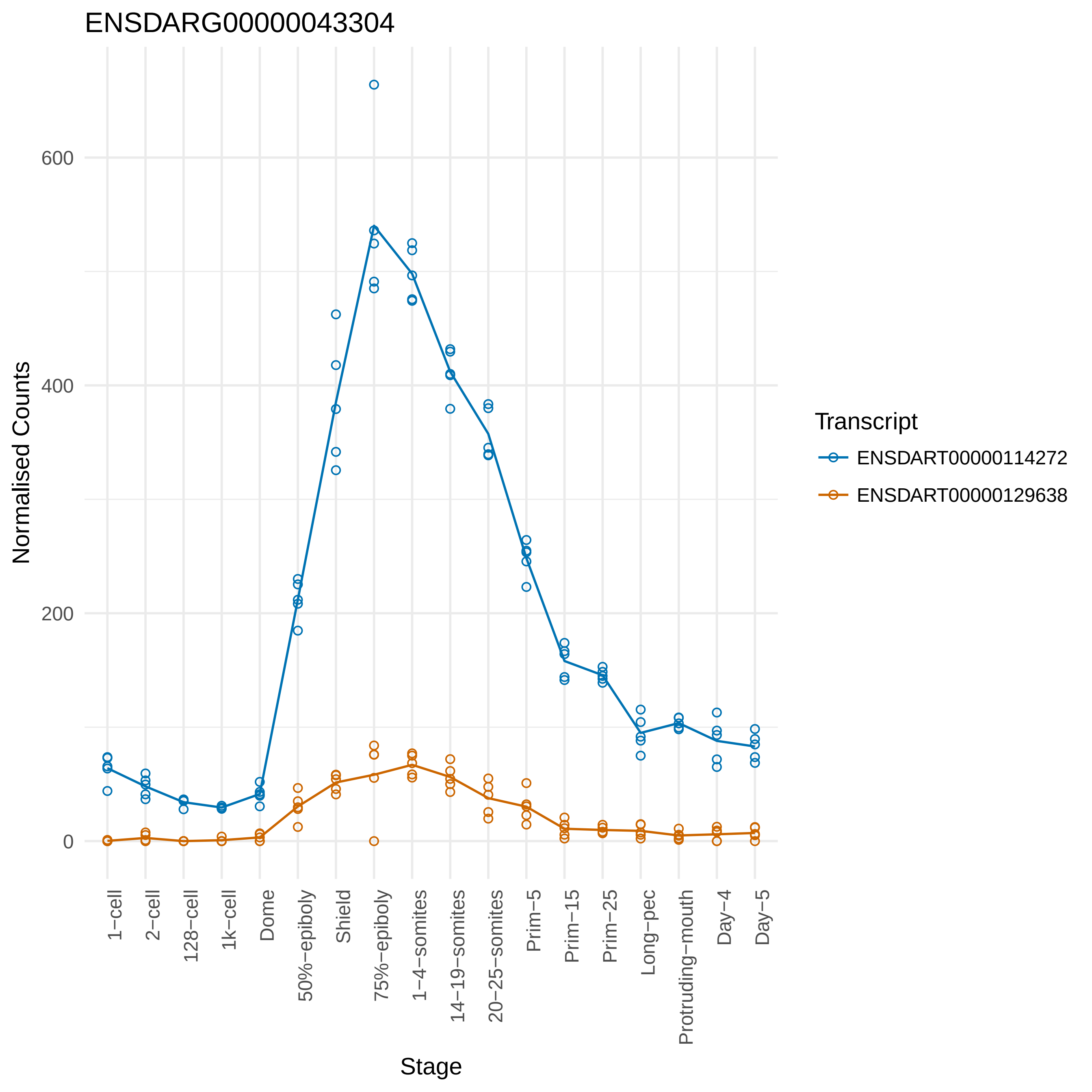

ENSDARG00000043304
Normalised Counts
600
400
200
0
1−cell
2−cell
128−cell
1k−cell
Dome
50%−epiboly
Shield
75%−epiboly
1−4−somites
14−19−somites
20−25−somites
Prim−5
Prim−15
Prim−25
Long−pec
Protruding−mouth
Day−4
Day−5
Stage
Transcript
ENSDART00000114272
ENSDART00000129638

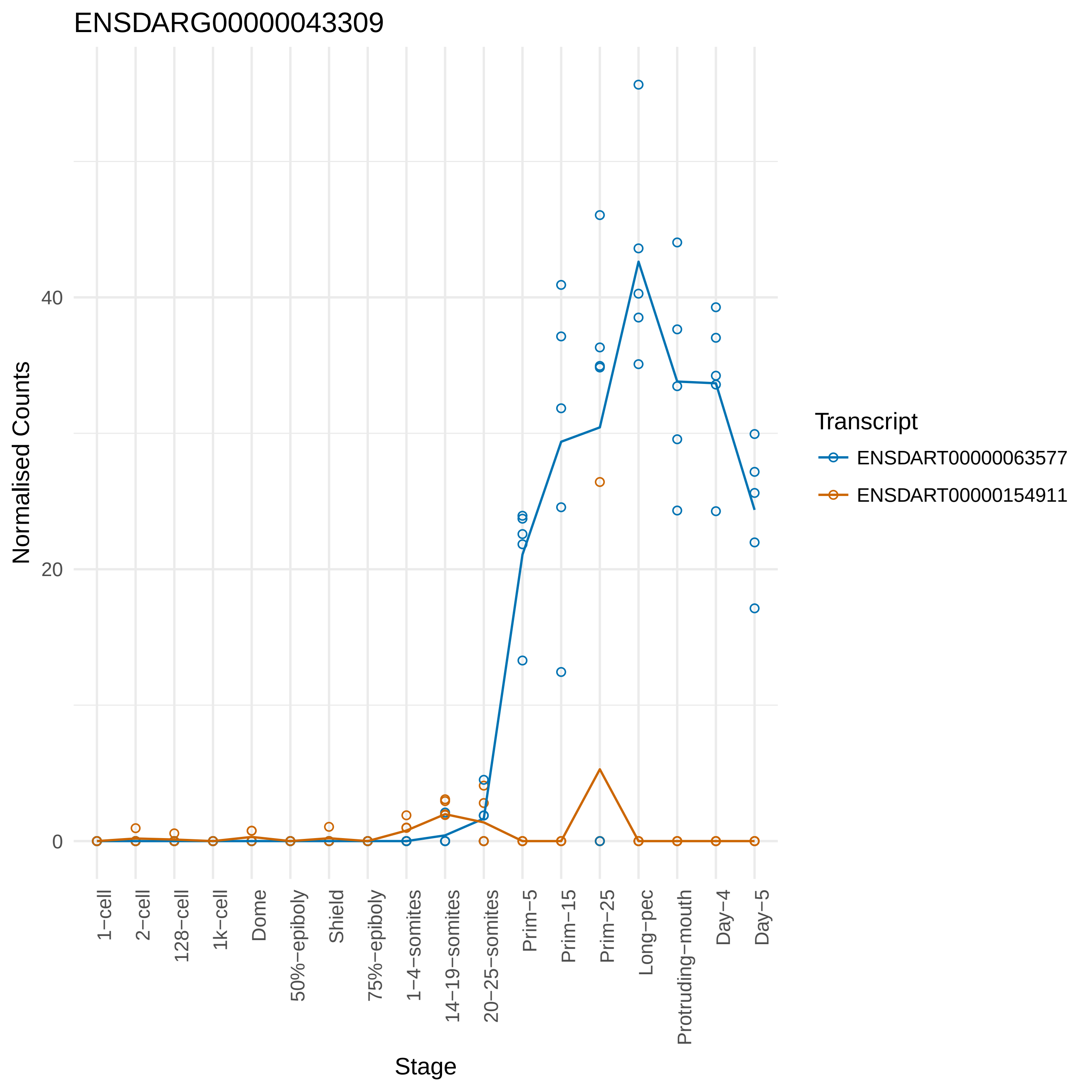
ENSDARG00000043309
Normalised Counts
0
20
40
Stage
1−cell
2−cell
128−cell
1k−cell
Dome
50%−epiboly
Shield
75%−epiboly
1−4−somites
14−19−somites
20−25−somites
Prim−5
Prim−15
Prim−25
Long−pec
Protruding−mouth
Day−4
Day−5
Transcript
ENSDART00000063577
ENSDART00000154911

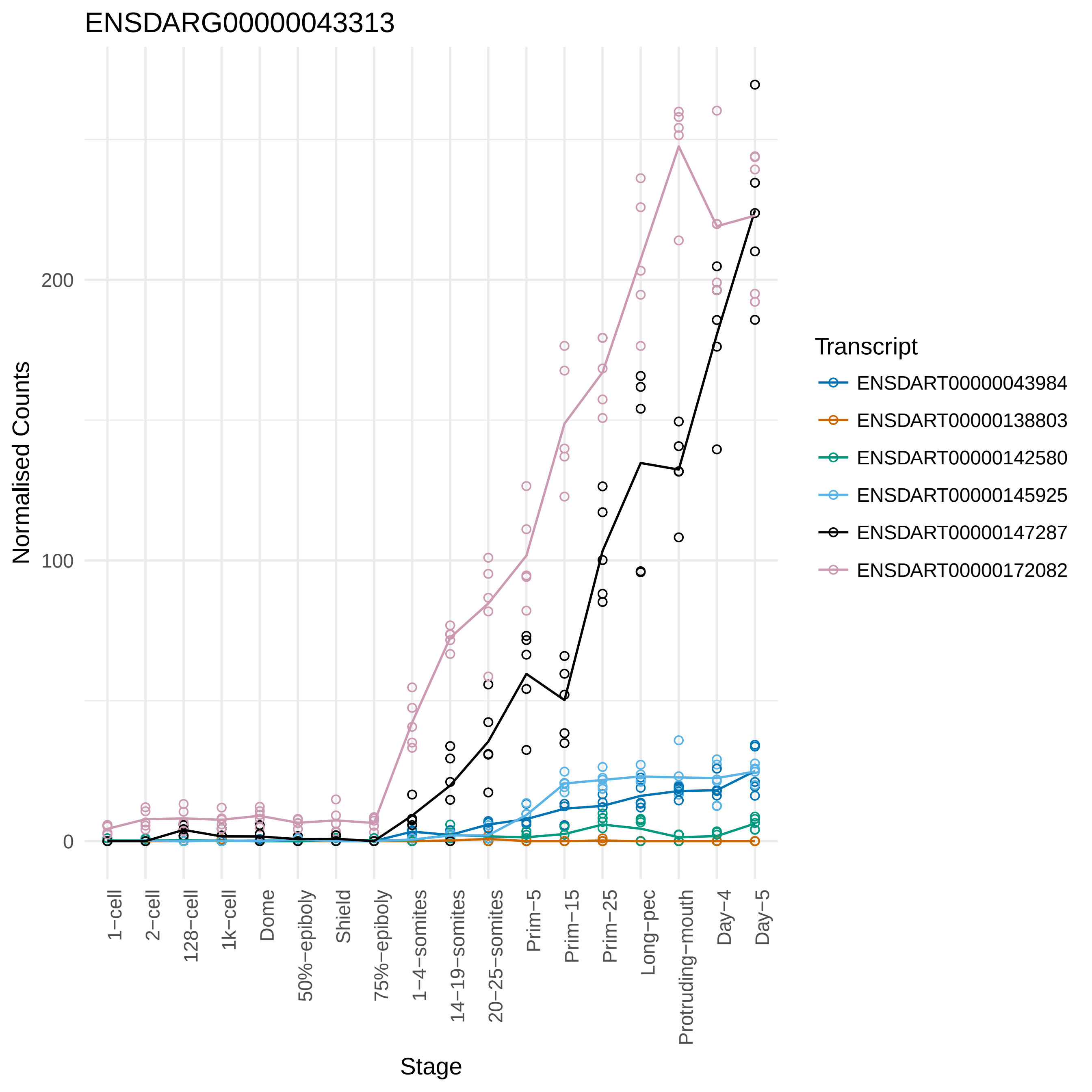

ENSDARG00000043313
Normalised Counts
200
100
0
1−cell
2−cell
128−cell
1k−cell
Dome
50%−epiboly
Shield
75%−epiboly
1−4−somites
14−19−somites
20−25−somites
Prim−5
Prim−15
Prim−25
Long−pec
Protruding−mouth
Day−4
Day−5
Stage
Transcript
ENSDART00000043984
ENSDART00000138803
ENSDART00000142580
ENSDART00000145925
ENSDART00000147287
ENSDART00000172082

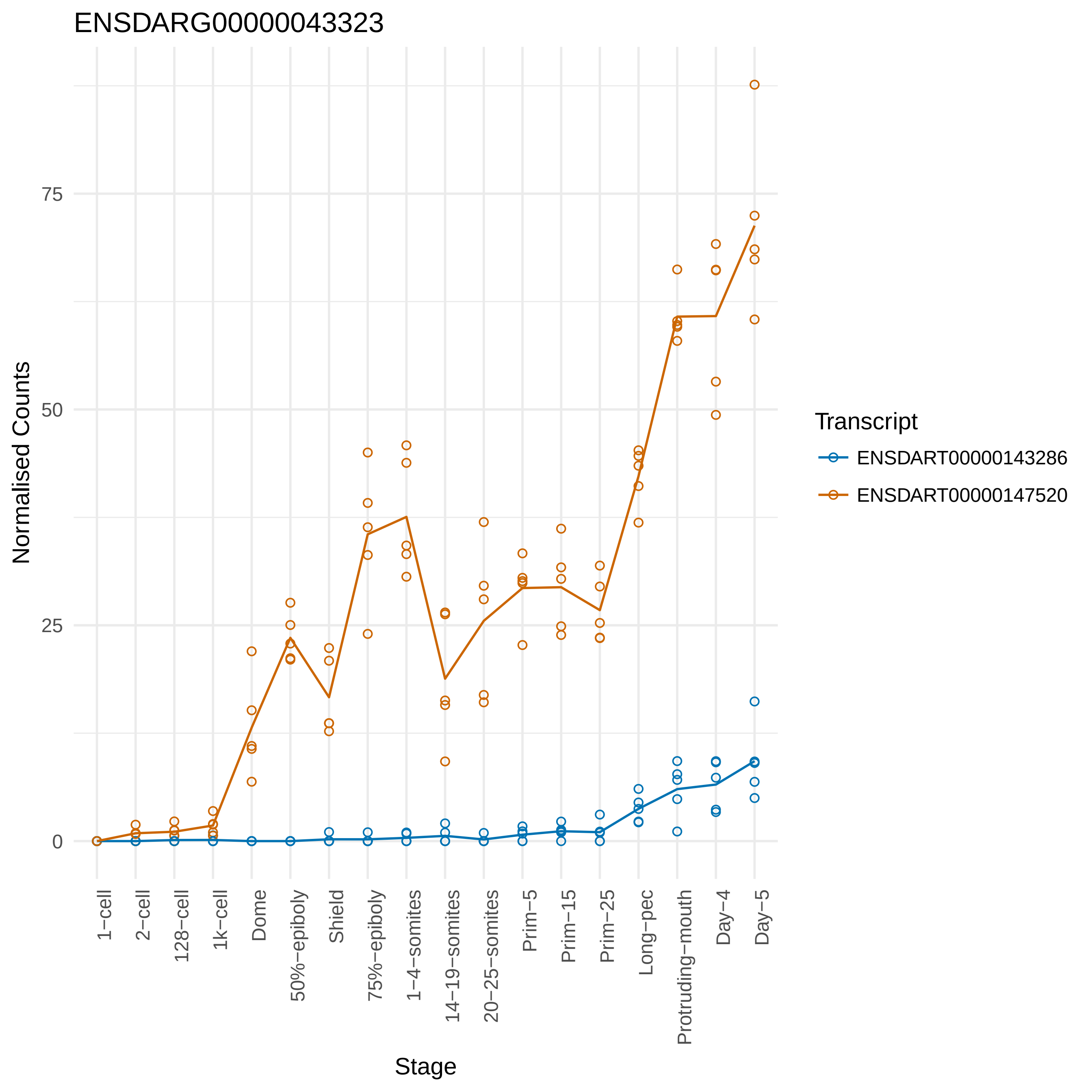

ENSDARG00000043323
Normalised Counts
75
50
25
0
1−cell
2−cell
128−cell
1k−cell
Dome
50%−epiboly
Shield
75%−epiboly
1−4−somites
14−19−somites
20−25−somites
Prim−5
Prim−15
Prim−25
Long−pec
Protruding−mouth
Day−4
Day−5
Stage
Transcript
ENSDART00000143286
ENSDART00000147520

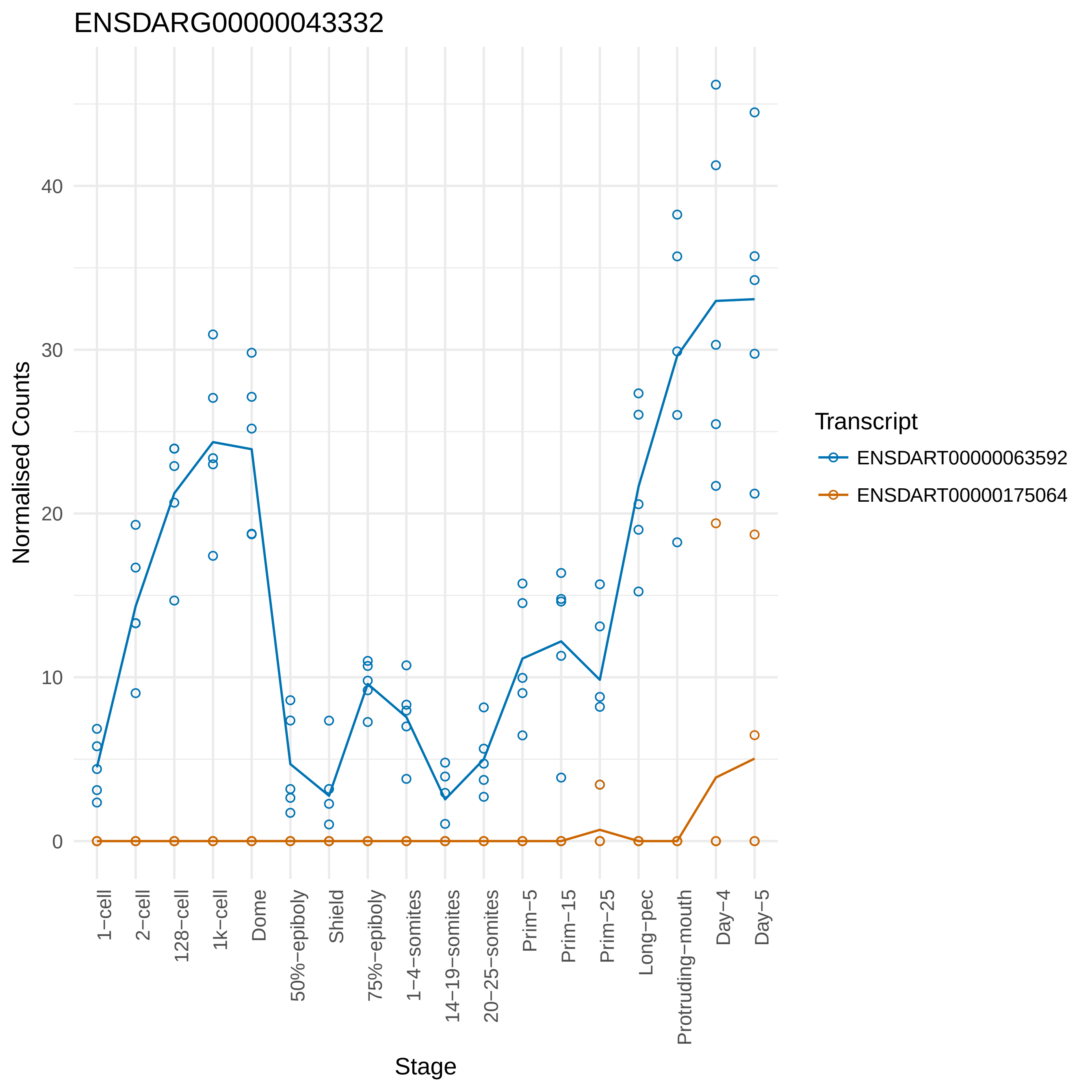

ENSDARG00000043332
Normalised Counts
0
10
20
30
40
Stage
1−cell
2−cell
128−cell
1k−cell
Dome
50%−epiboly
Shield
75%−epiboly
1−4−somites
14−19−somites
20−25−somites
Prim−5
Prim−15
Prim−25
Long−pec
Protruding−mouth
Day−4
Day−5
Transcript
ENSDART00000063592
ENSDART00000175064

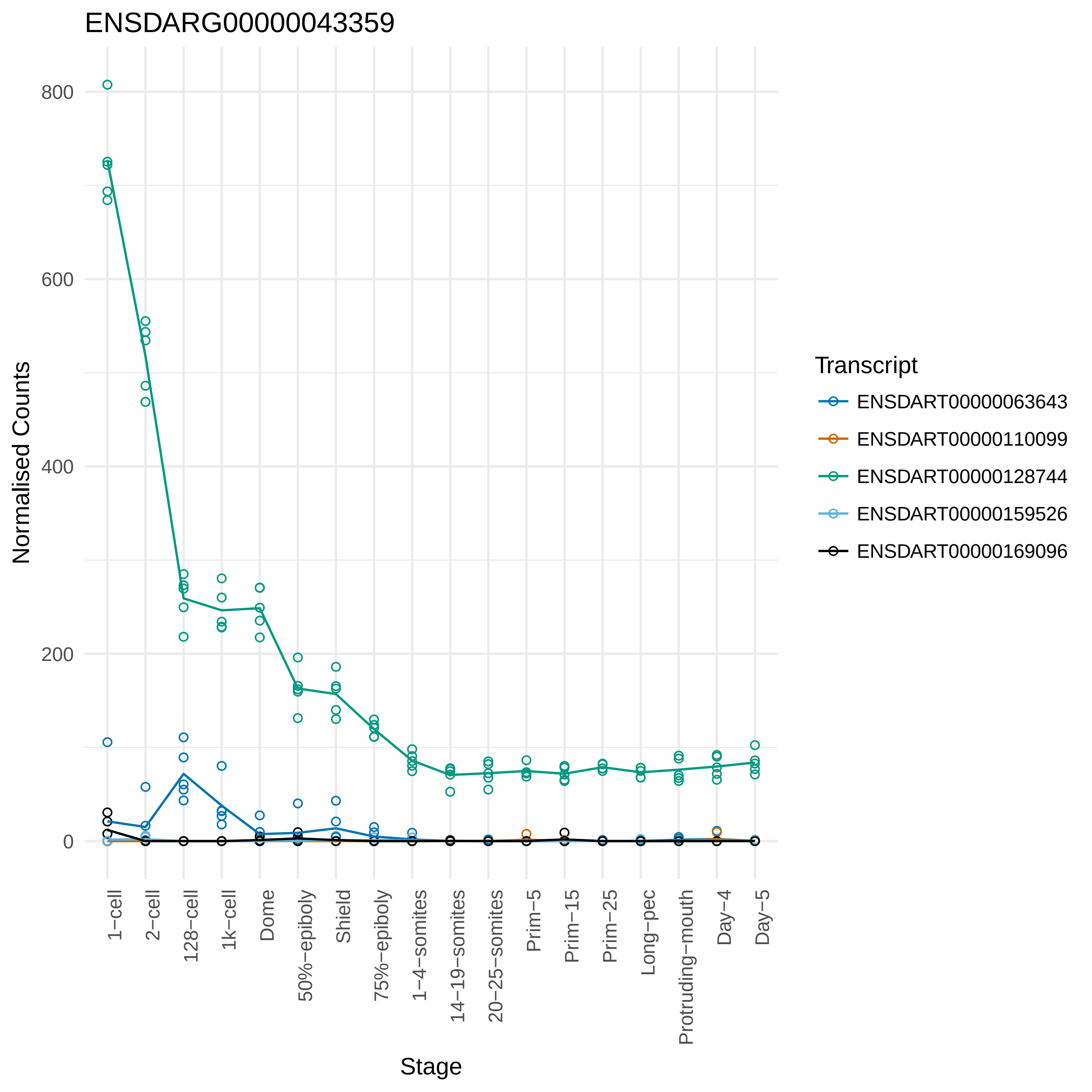
ENSDARG00000043359
Normalised Counts
800
600
400
200
0
1−cell
2−cell
128−cell
1k−cell
Dome
50%−epiboly
Shield
75%−epiboly
1−4−somites
14−19−somites
20−25−somites
Prim−5
Prim−15
Prim−25
Long−pec
Protruding−mouth
Day−4
Day−5
Stage
Transcript
ENSDART00000063643
ENSDART00000110099
ENSDART00000128744
ENSDART00000159526
ENSDART00000169096

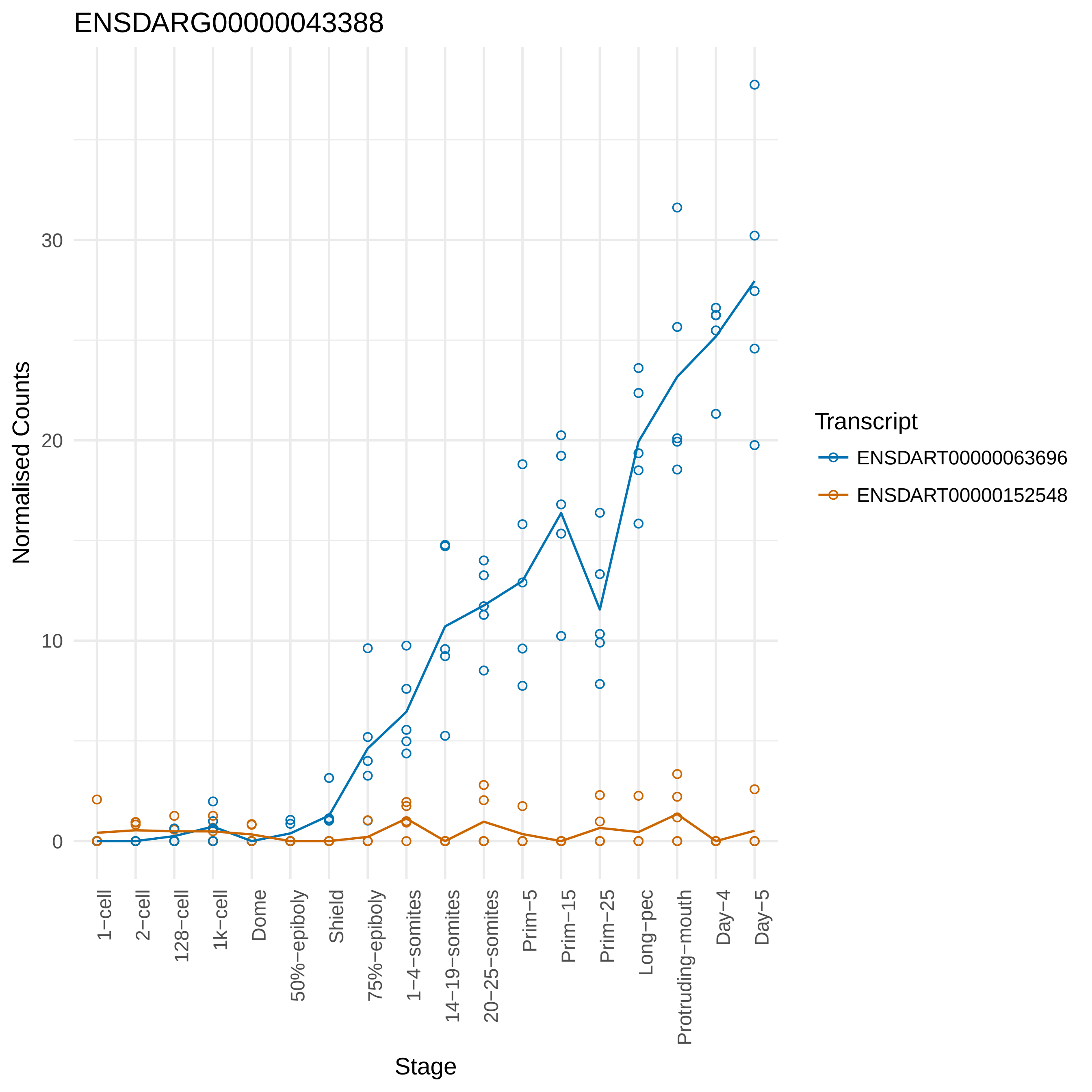
ENSDARG00000043388
Normalised Counts
0
10
20
30
Stage
1−cell
2−cell
128−cell
1k−cell
Dome
50%−epiboly
Shield
75%−epiboly
1−4−somites
14−19−somites
20−25−somites
Prim−5
Prim−15
Prim−25
Long−pec
Protruding−mouth
Day−4
Day−5
Transcript
ENSDART00000063696
ENSDART00000152548

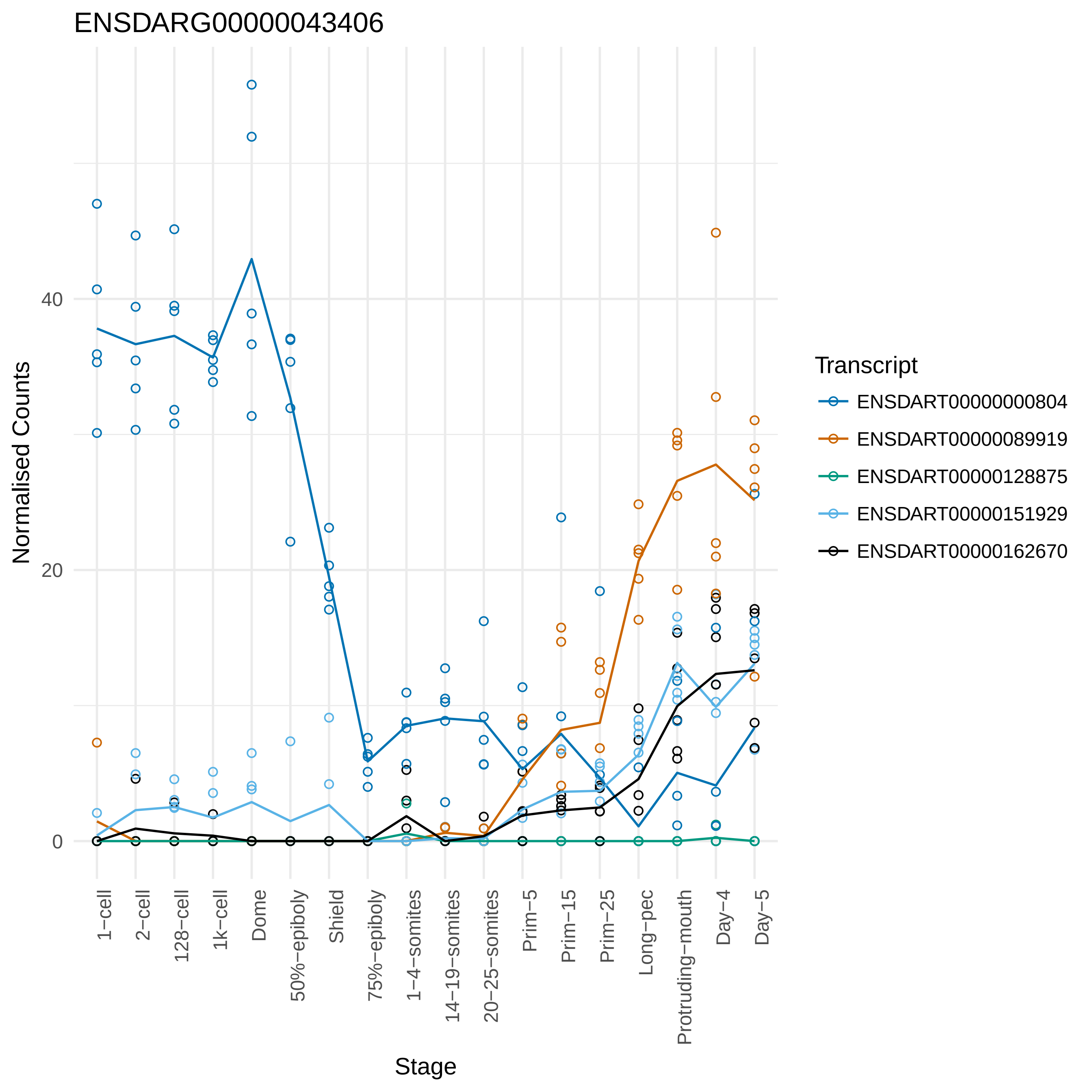

ENSDARG00000043406
Normalised Counts
0
20
40
Stage
1−cell
2−cell
128−cell
1k−cell
Dome
50%−epiboly
Shield
75%−epiboly
1−4−somites
14−19−somites
20−25−somites
Prim−5
Prim−15
Prim−25
Long−pec
Protruding−mouth
Day−4
Day−5
Transcript
ENSDART00000000804
ENSDART00000089919
ENSDART00000128875
ENSDART00000151929
ENSDART00000162670

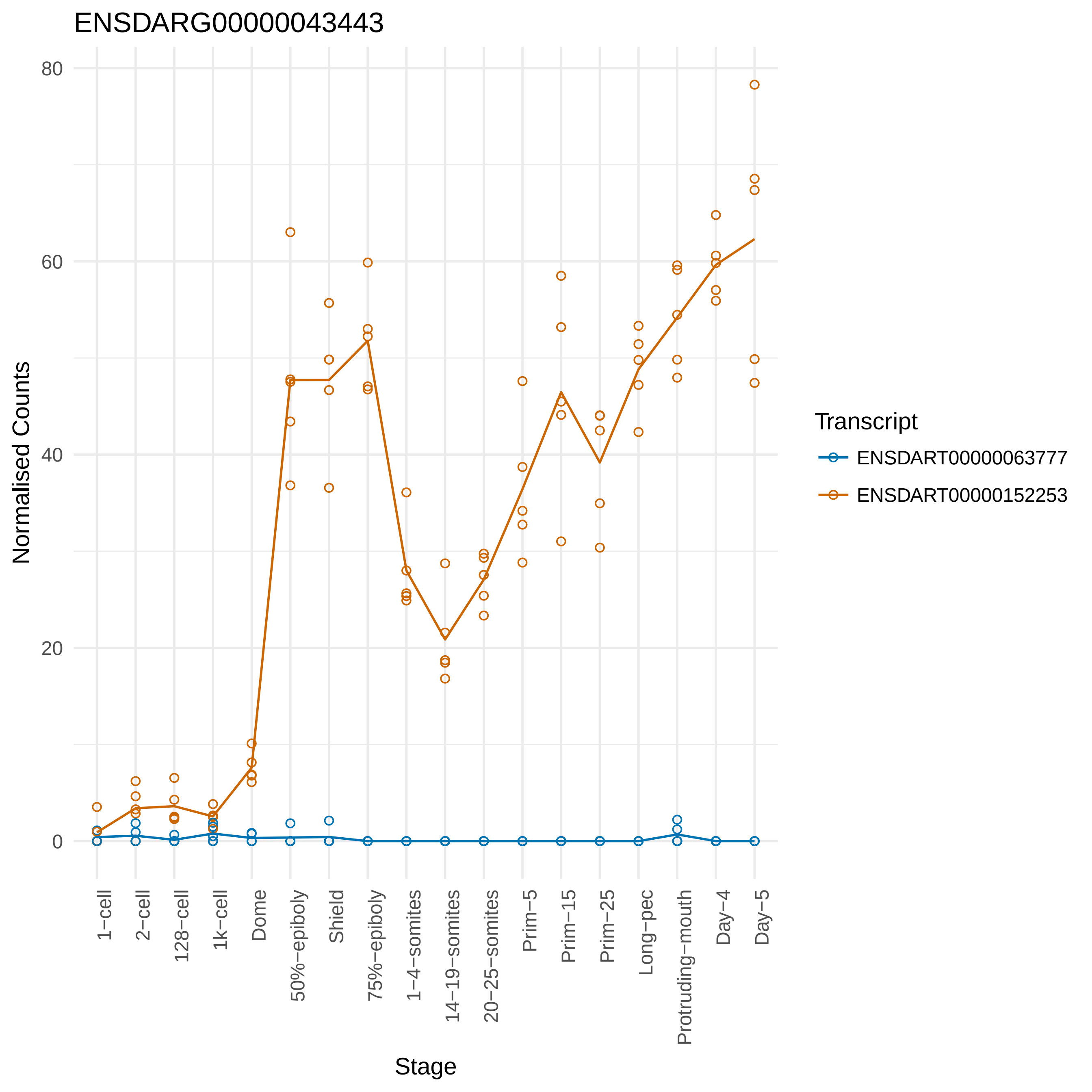

ENSDARG00000043443
Normalised Counts
80
60
40
20
0
1−cell
2−cell
128−cell
1k−cell
Dome
50%−epiboly
Shield
75%−epiboly
1−4−somites
14−19−somites
20−25−somites
Prim−5
Prim−15
Prim−25
Long−pec
Protruding−mouth
Day−4
Day−5
Stage
Transcript
ENSDART00000063777
ENSDART00000152253

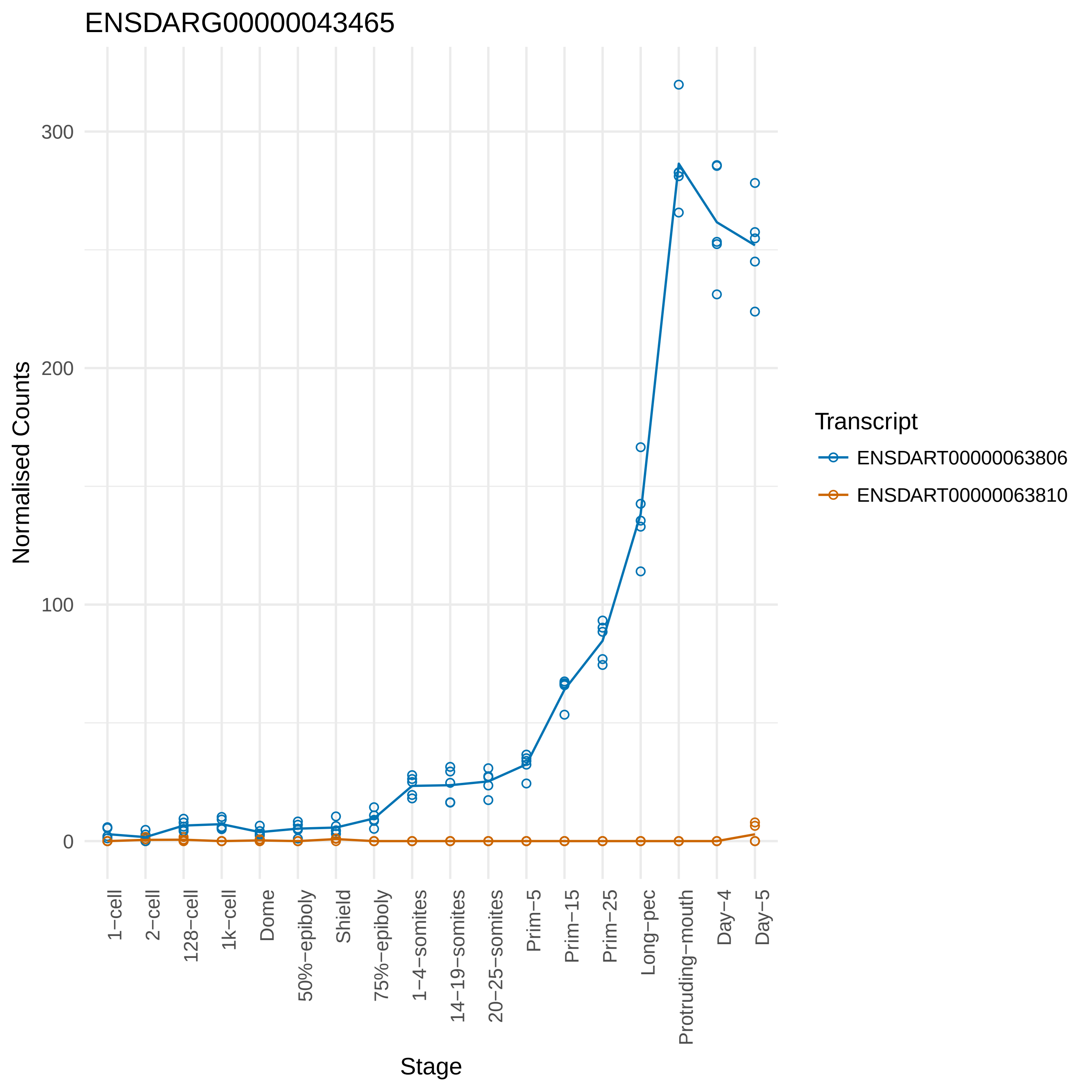
ENSDARG00000043465
Normalised Counts
300
200
100
0
1−cell
2−cell
128−cell
1k−cell
Dome
50%−epiboly
Shield
75%−epiboly
1−4−somites
14−19−somites
20−25−somites
Prim−5
Prim−15
Prim−25
Long−pec
Protruding−mouth
Day−4
Day−5
Stage
Transcript
ENSDART00000063806
ENSDART00000063810

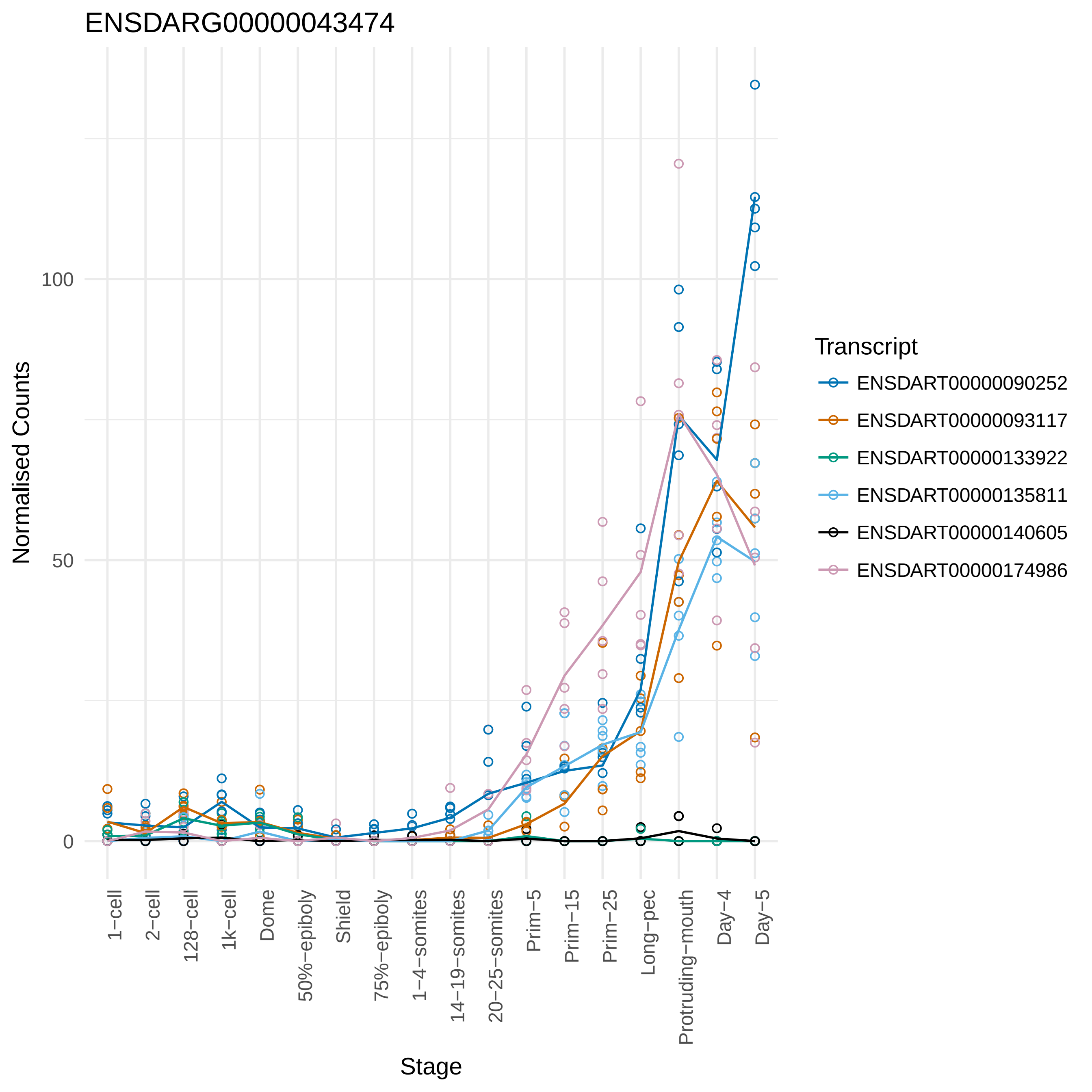
ENSDARG00000043474
Normalised Counts
100
50
0
1−cell
2−cell
128−cell
1k−cell
Dome
50%−epiboly
Shield
75%−epiboly
1−4−somites
14−19−somites
20−25−somites
Prim−5
Prim−15
Prim−25
Long−pec
Protruding−mouth
Day−4
Day−5
Stage
Transcript
ENSDART00000090252
ENSDART00000093117
ENSDART00000133922
ENSDART00000135811
ENSDART00000140605
ENSDART00000174986

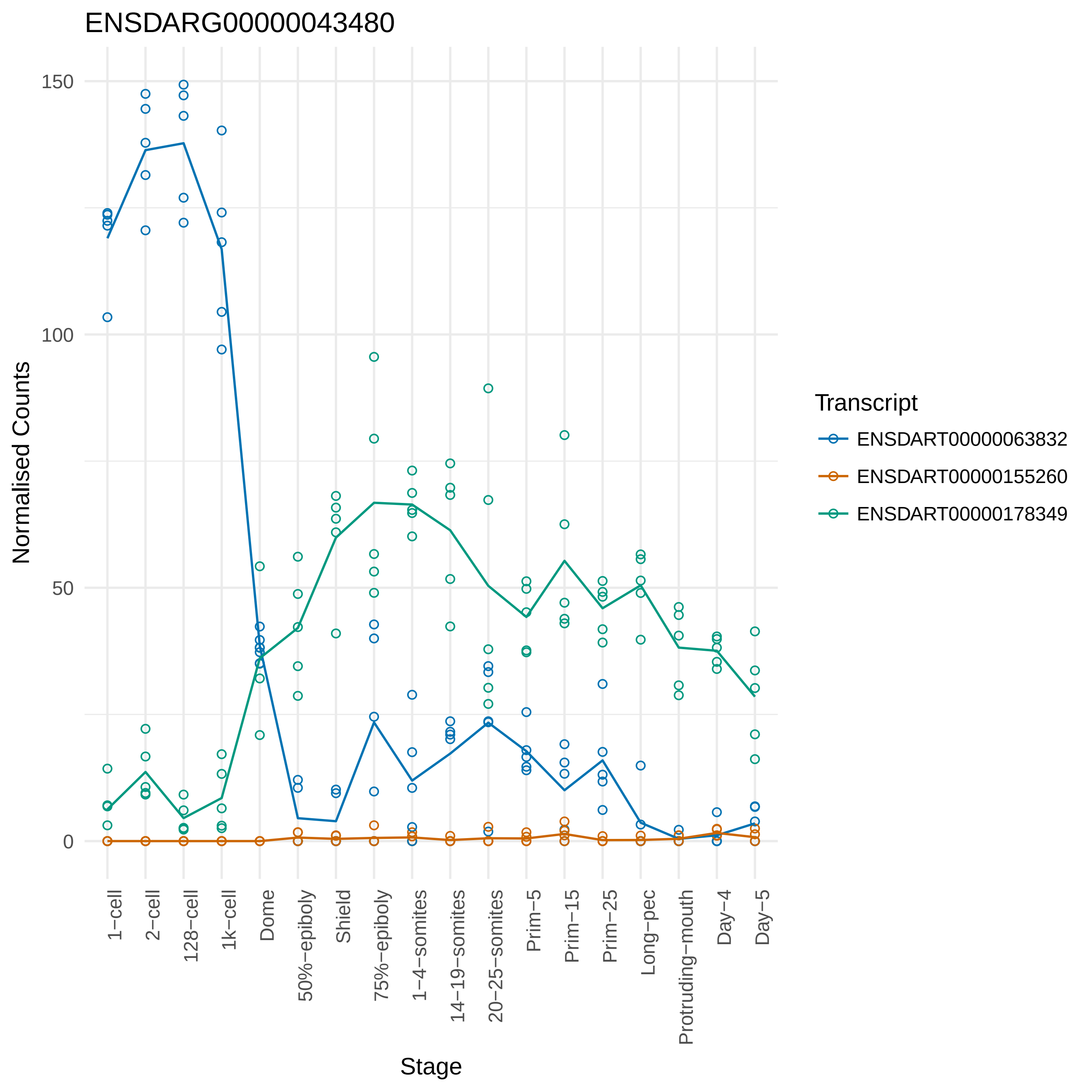

ENSDARG00000043480
Normalised Counts
150
100
50
0
1−cell
2−cell
128−cell
1k−cell
Dome
50%−epiboly
Shield
75%−epiboly
1−4−somites
14−19−somites
20−25−somites
Prim−5
Prim−15
Prim−25
Long−pec
Protruding−mouth
Day−4
Day−5
Stage
Transcript
ENSDART00000063832
ENSDART00000155260
ENSDART00000178349

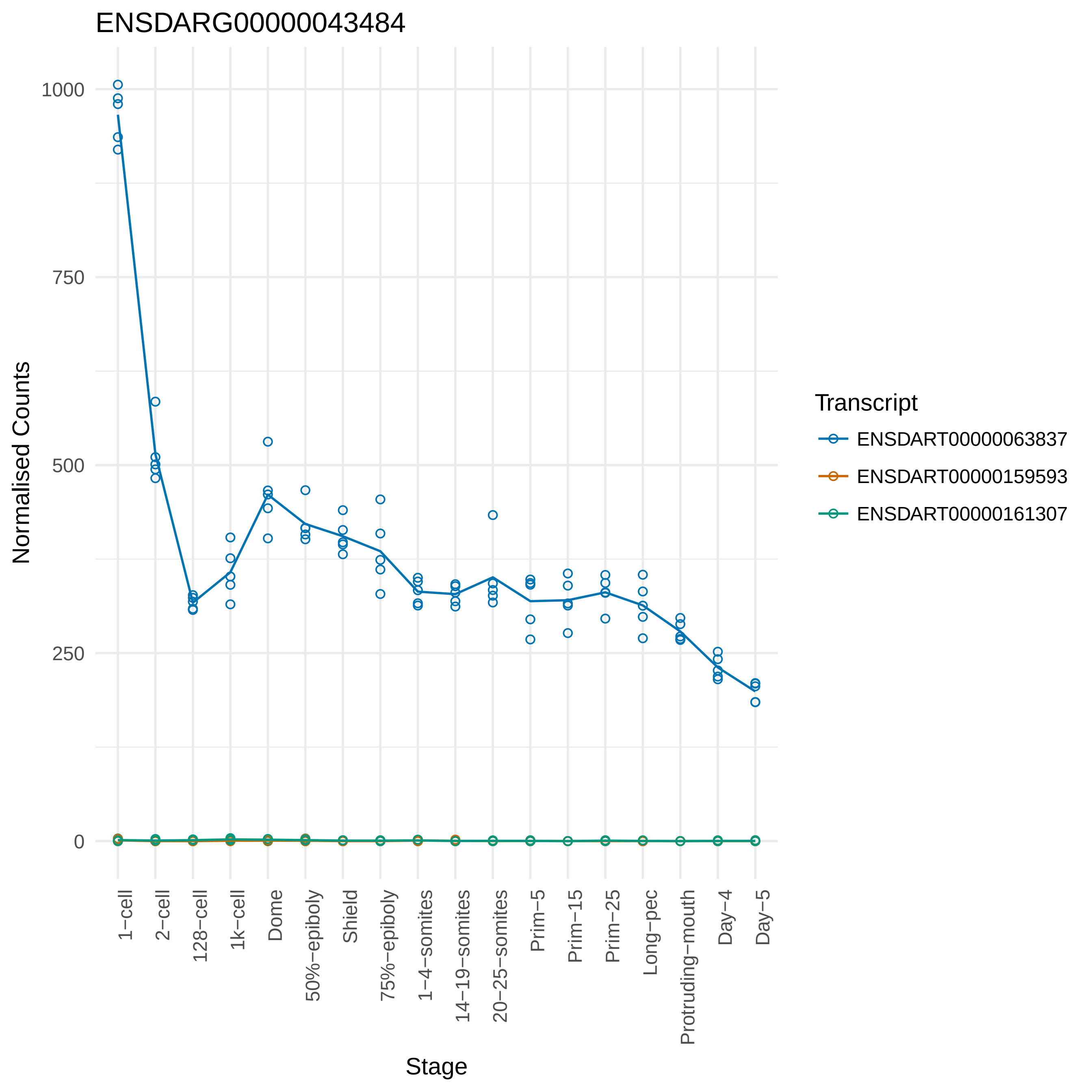

ENSDARG00000043484
Normalised Counts
1000
750
500
250
0
1−cell
2−cell
128−cell
1k−cell
Dome
50%−epiboly
Shield
75%−epiboly
1−4−somites
14−19−somites
20−25−somites
Prim−5
Prim−15
Prim−25
Long−pec
Protruding−mouth
Day−4
Day−5
Stage
Transcript
ENSDART00000063837
ENSDART00000159593
ENSDART00000161307

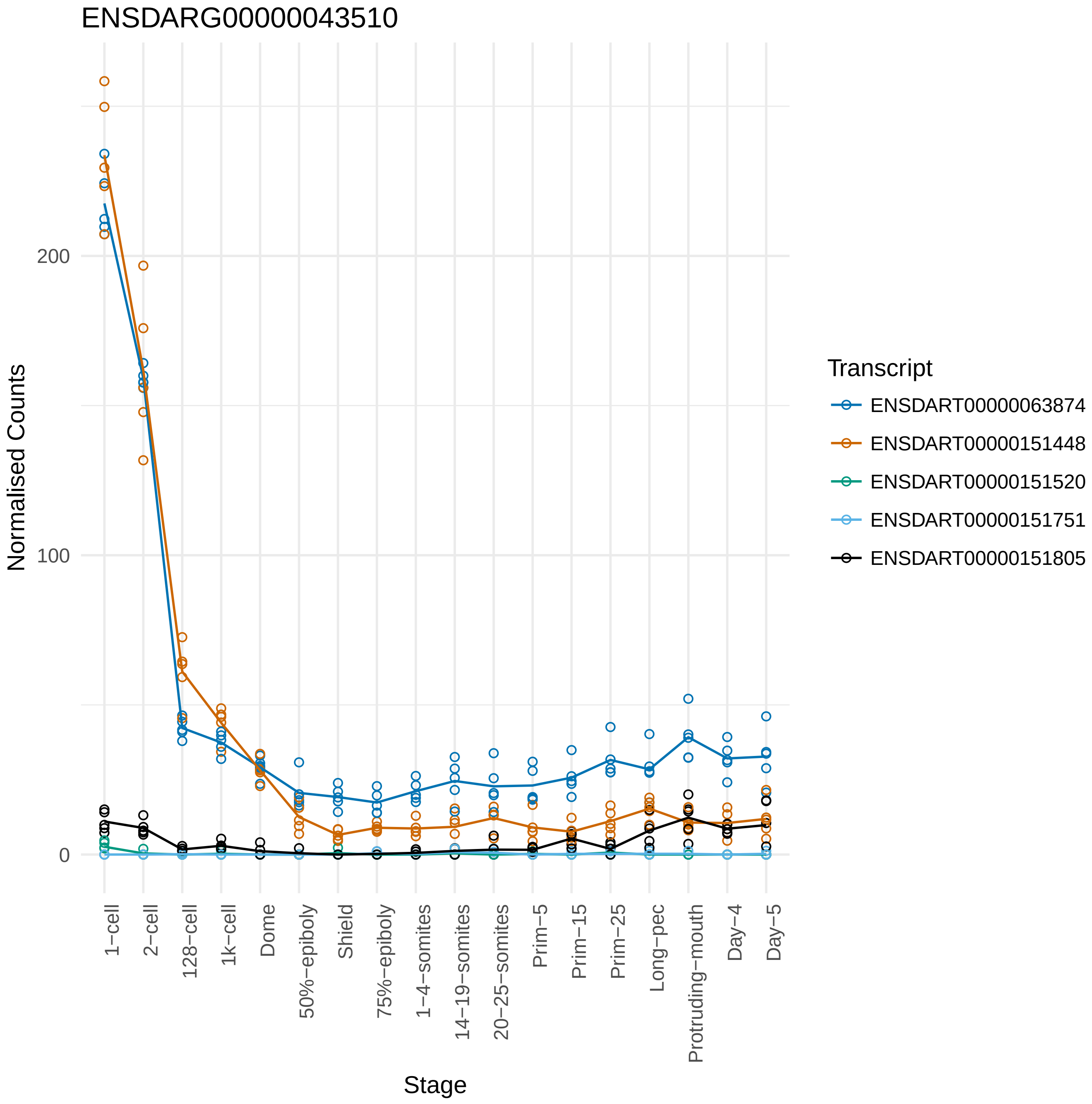
ENSDARG00000043510
Normalised Counts
200
100
0
1−cell
2−cell
128−cell
1k−cell
Dome
50%−epiboly
Shield
75%−epiboly
1−4−somites
14−19−somites
20−25−somites
Prim−5
Prim−15
Prim−25
Long−pec
Protruding−mouth
Day−4
Day−5
Stage
Transcript
ENSDART00000063874
ENSDART00000151448
ENSDART00000151520
ENSDART00000151751
ENSDART00000151805

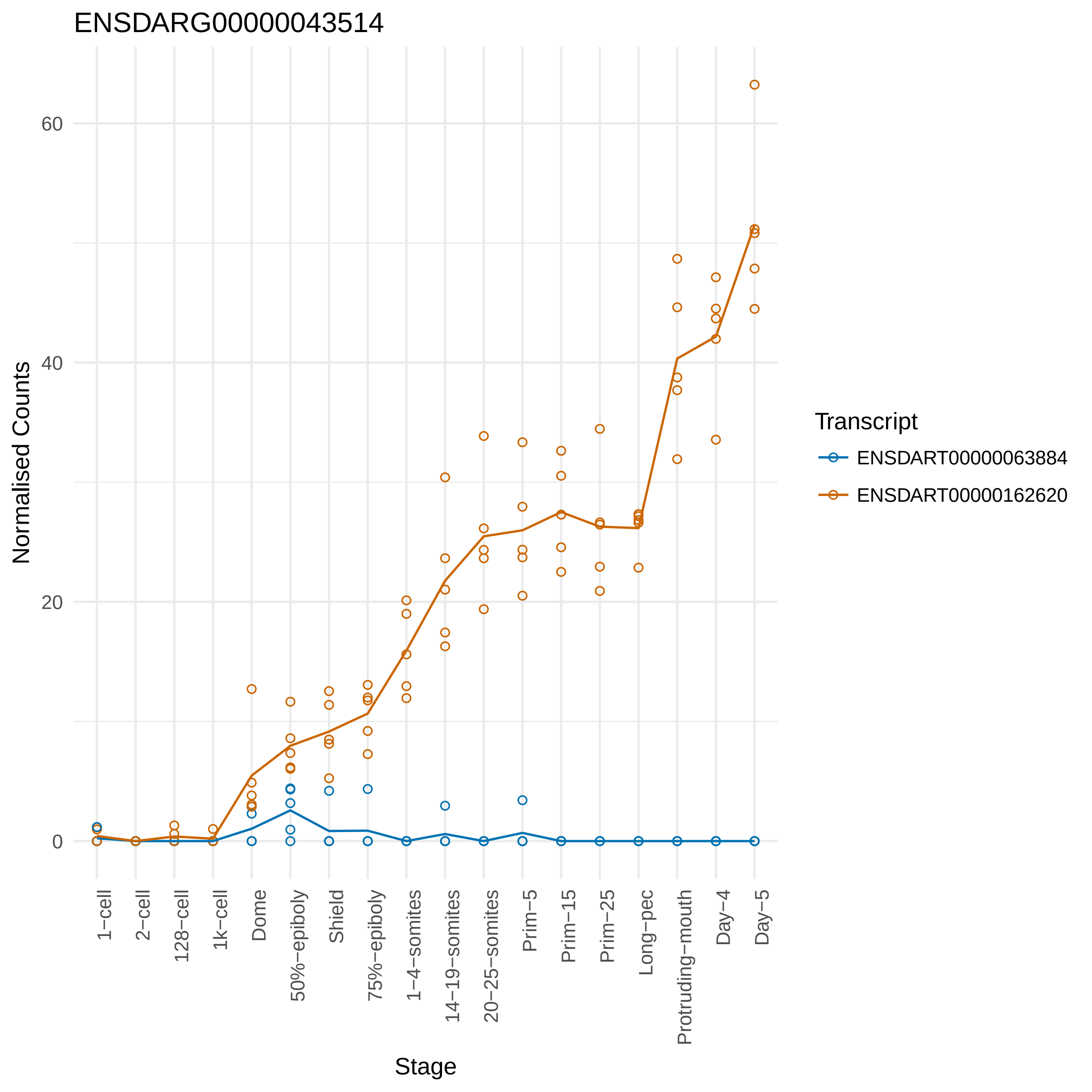

ENSDARG00000043514
Normalised Counts
0
20
40
60
Stage
1−cell
2−cell
128−cell
1k−cell
Dome
50%−epiboly
Shield
75%−epiboly
1−4−somites
14−19−somites
20−25−somites
Prim−5
Prim−15
Prim−25
Long−pec
Protruding−mouth
Day−4
Day−5
Transcript
ENSDART00000063884
ENSDART00000162620

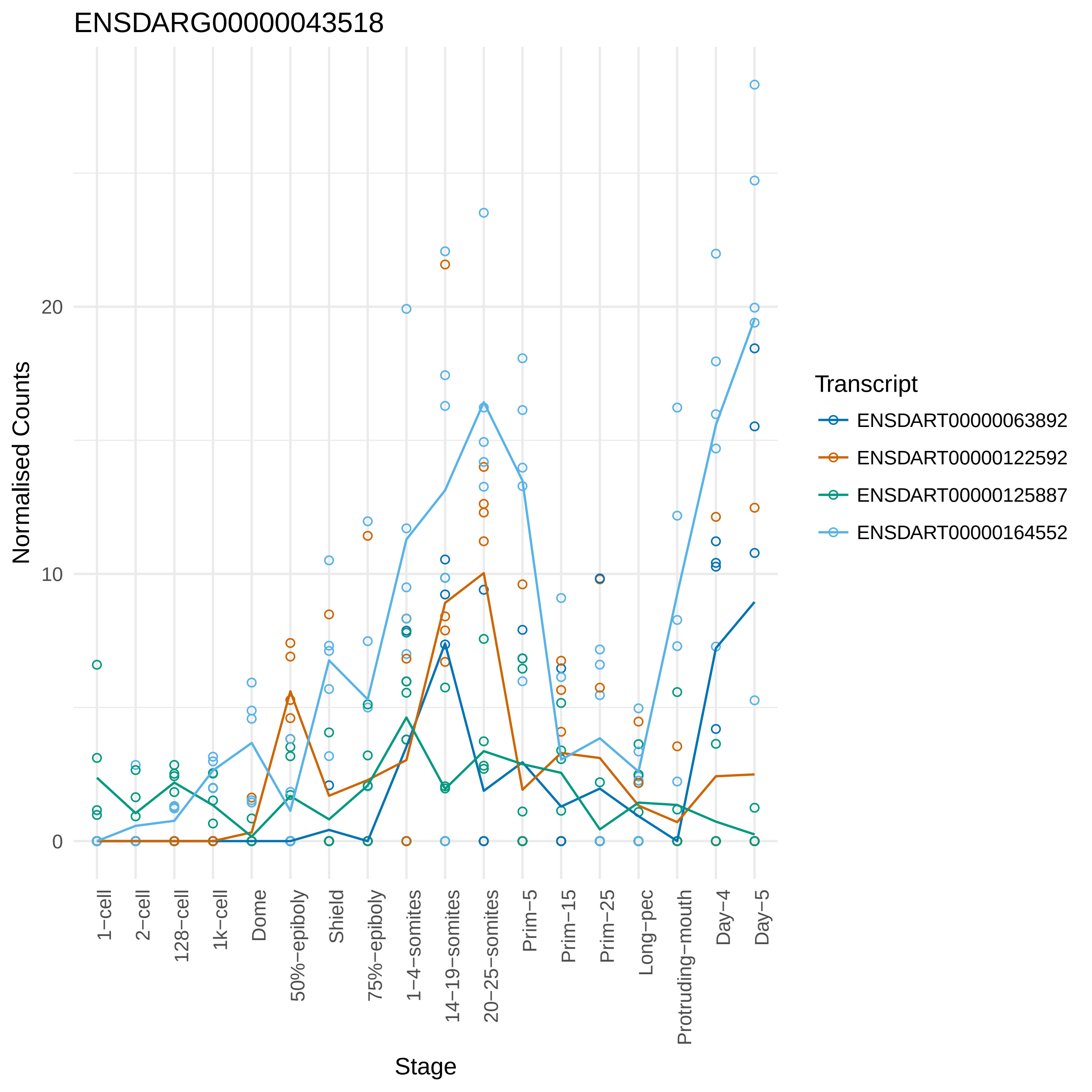

ENSDARG00000043518
Normalised Counts
20
10
0
1−cell
2−cell
128−cell
1k−cell
Dome
50%−epiboly
Shield
75%−epiboly
1−4−somites
14−19−somites
20−25−somites
Prim−5
Prim−15
Prim−25
Long−pec
Protruding−mouth
Day−4
Day−5
Stage
Transcript
ENSDART00000063892
ENSDART00000122592
ENSDART00000125887
ENSDART00000164552

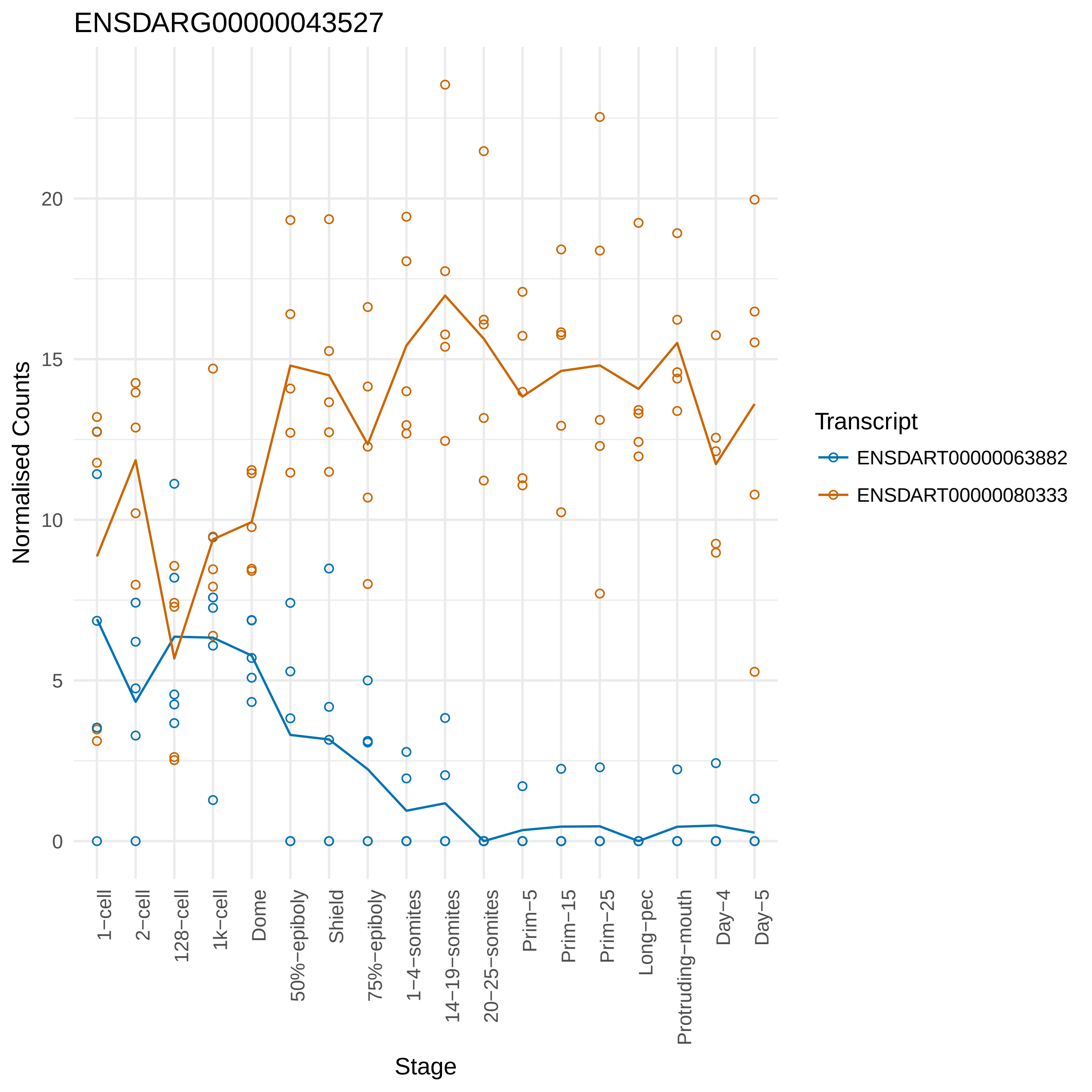

ENSDARG00000043527
Normalised Counts
0
5
10
15
20
1−cell
2−cell
128−cell
1k−cell
Dome
50%−epiboly
Shield
75%−epiboly
1−4−somites
14−19−somites
20−25−somites
Prim−5
Prim−15
Prim−25
Long−pec
Protruding−mouth
Day−4
Day−5
Stage
Transcript
ENSDART00000063882
ENSDART00000080333

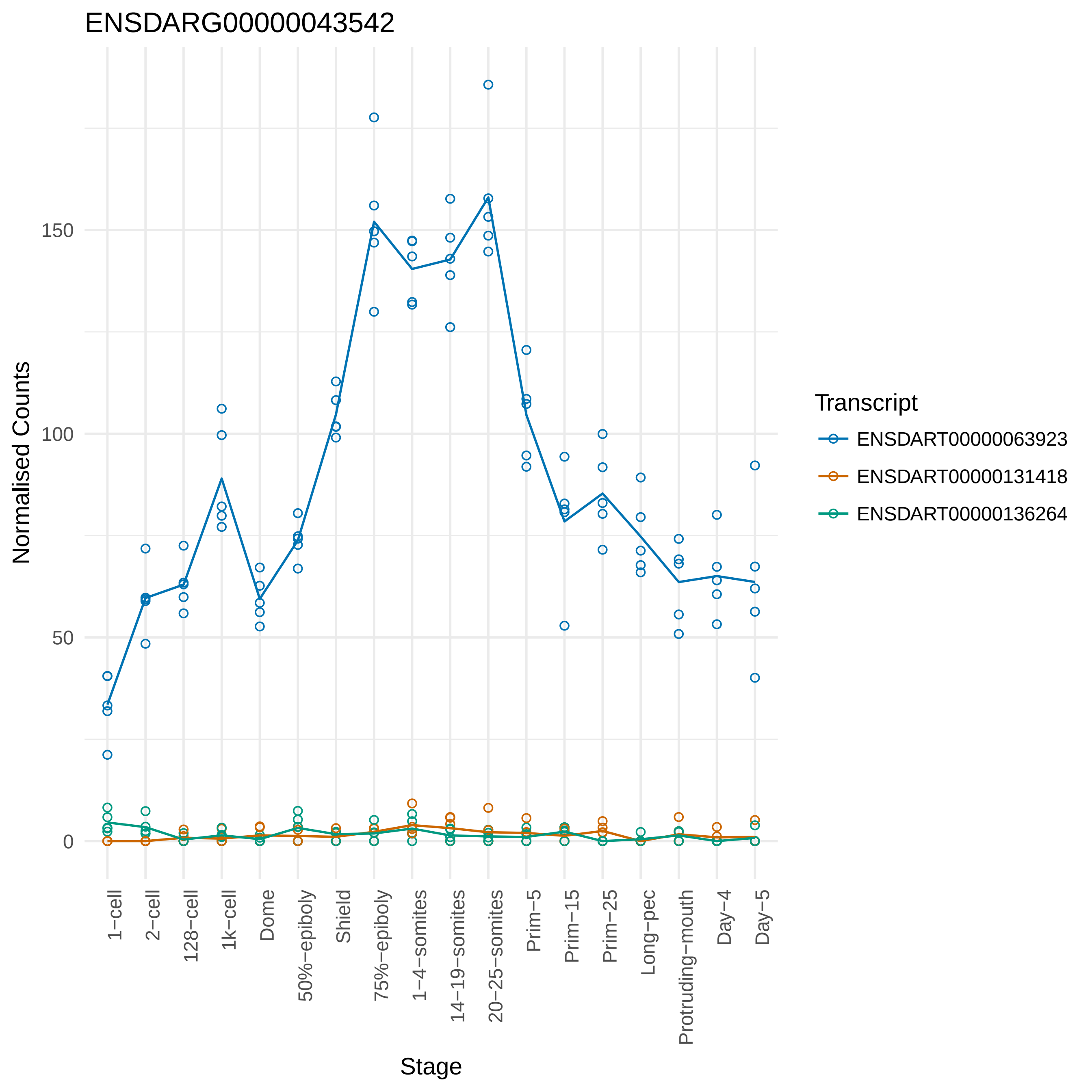

ENSDARG00000043542
Normalised Counts
150
100
50
0
1−cell
2−cell
128−cell
1k−cell
Dome
50%−epiboly
Shield
75%−epiboly
1−4−somites
14−19−somites
20−25−somites
Prim−5
Prim−15
Prim−25
Long−pec
Protruding−mouth
Day−4
Day−5
Stage
Transcript
ENSDART00000063923
ENSDART00000131418
ENSDART00000136264

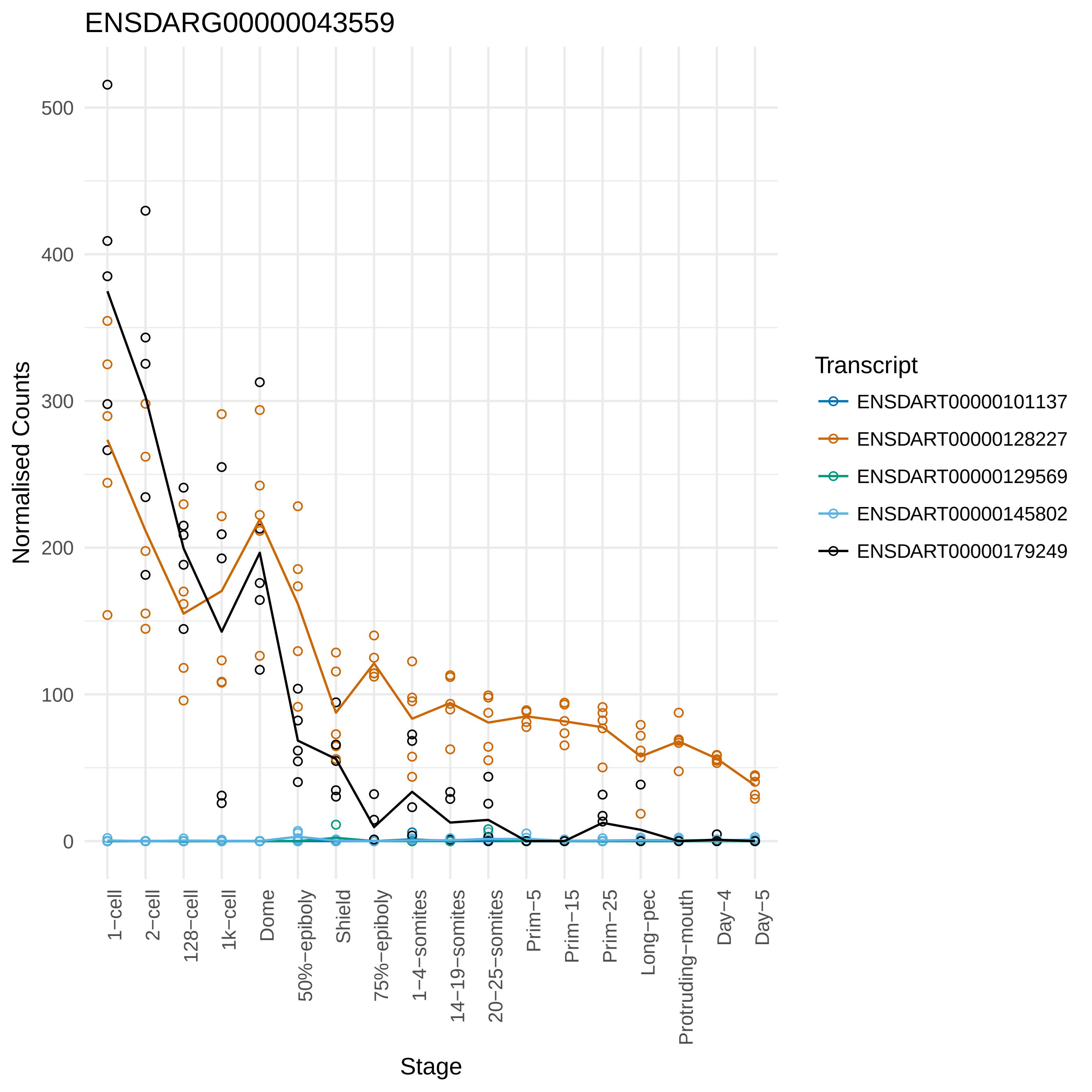

ENSDARG00000043559
Normalised Counts
500
400
300
200
100
0
1−cell
2−cell
128−cell
1k−cell
Dome
50%−epiboly
Shield
75%−epiboly
1−4−somites
14−19−somites
20−25−somites
Prim−5
Prim−15
Prim−25
Long−pec
Protruding−mouth
Day−4
Day−5
Stage
Transcript
ENSDART00000101137
ENSDART00000128227
ENSDART00000129569
ENSDART00000145802
ENSDART00000179249

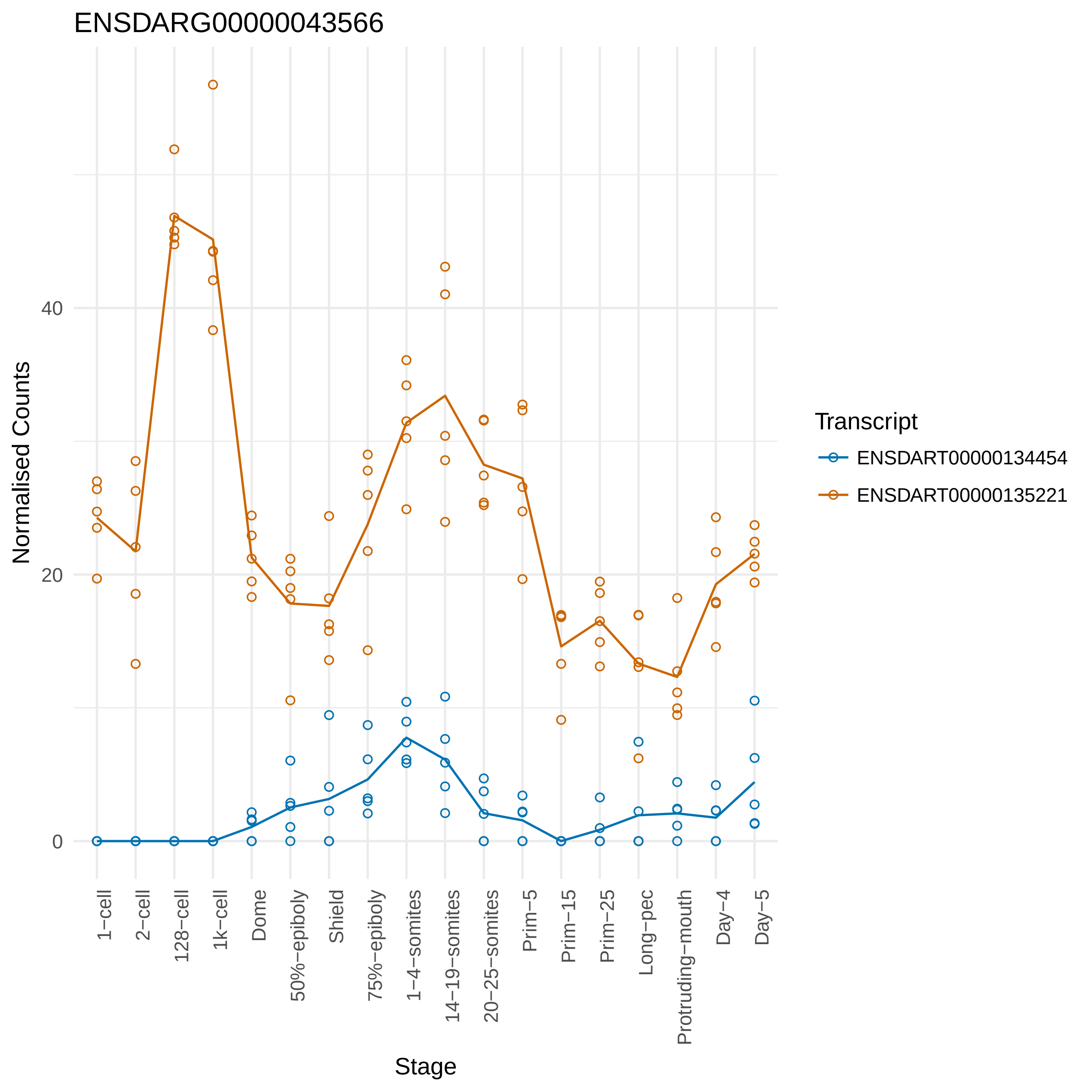

ENSDARG00000043566
Normalised Counts
40
20
0
1−cell
2−cell
128−cell
1k−cell
Dome
50%−epiboly
Shield
75%−epiboly
1−4−somites
14−19−somites
20−25−somites
Prim−5
Prim−15
Prim−25
Long−pec
Protruding−mouth
Day−4
Day−5
Stage
Transcript
ENSDART00000134454
ENSDART00000135221

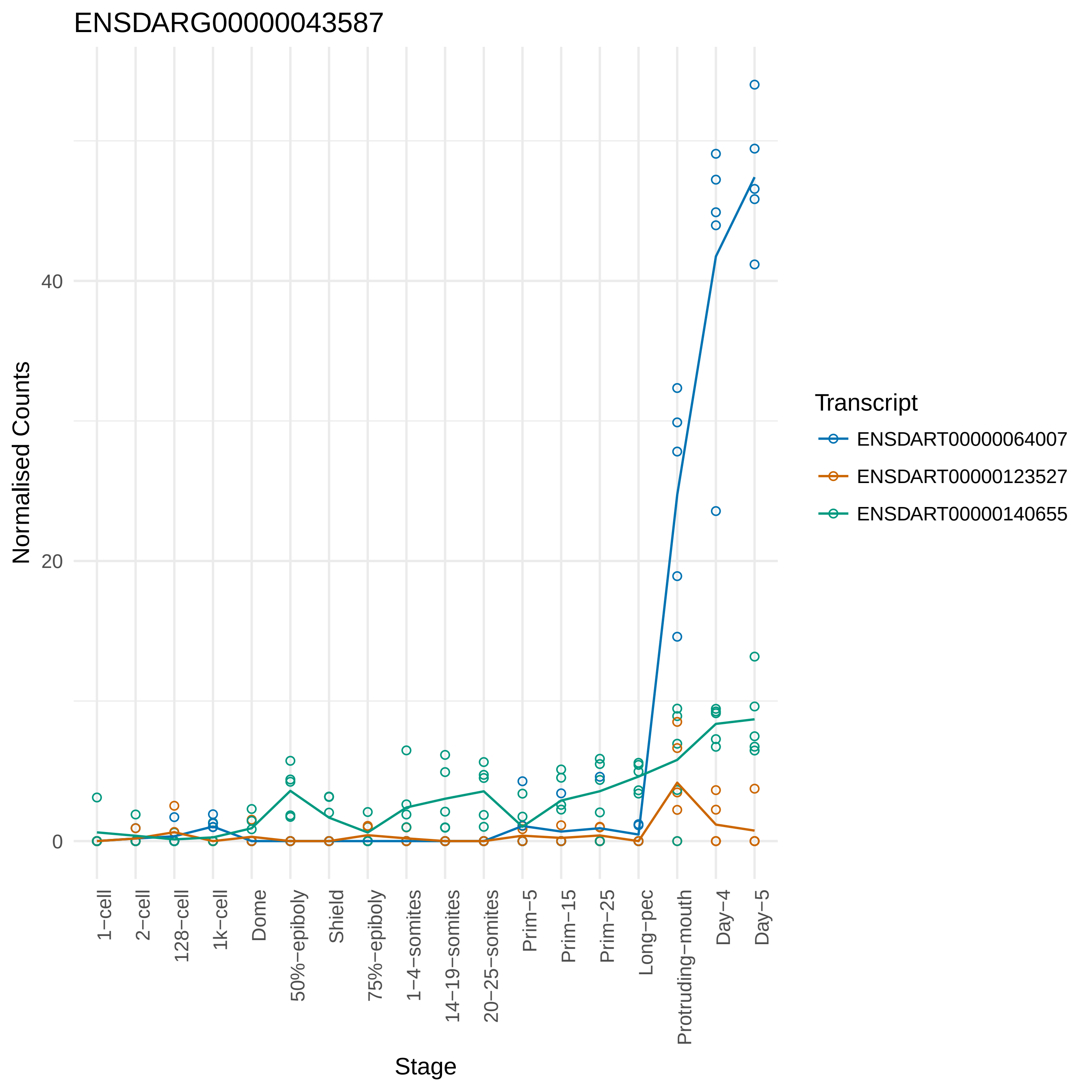

ENSDARG00000043587
Normalised Counts
0
20
40
Stage
1−cell
2−cell
128−cell
1k−cell
Dome
50%−epiboly
Shield
75%−epiboly
1−4−somites
14−19−somites
20−25−somites
Prim−5
Prim−15
Prim−25
Long−pec
Protruding−mouth
Day−4
Day−5
Transcript
ENSDART00000064007
ENSDART00000123527
ENSDART00000140655

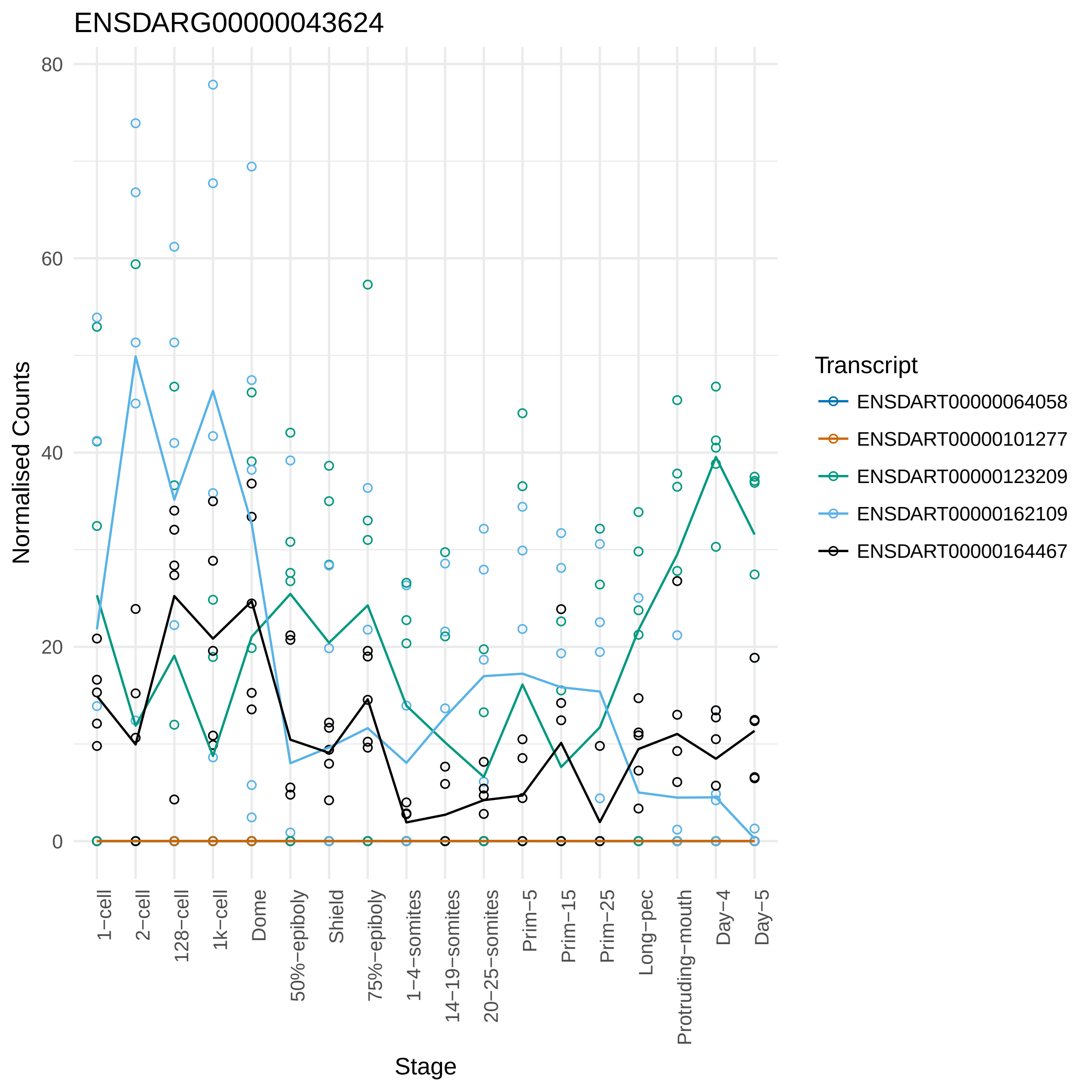

ENSDARG00000043624
Normalised Counts
80
60
40
20
0
Stage
1−cell
2−cell
128−cell
1k−cell
Dome
50%−epiboly
Shield
75%−epiboly
1−4−somites
14−19−somites
20−25−somites
Prim−5
Prim−15
Prim−25
Long−pec
Protruding−mouth
Day−4
Day−5
Transcript
ENSDART00000064058
ENSDART00000101277
ENSDART00000123209
ENSDART00000162109
ENSDART00000164467

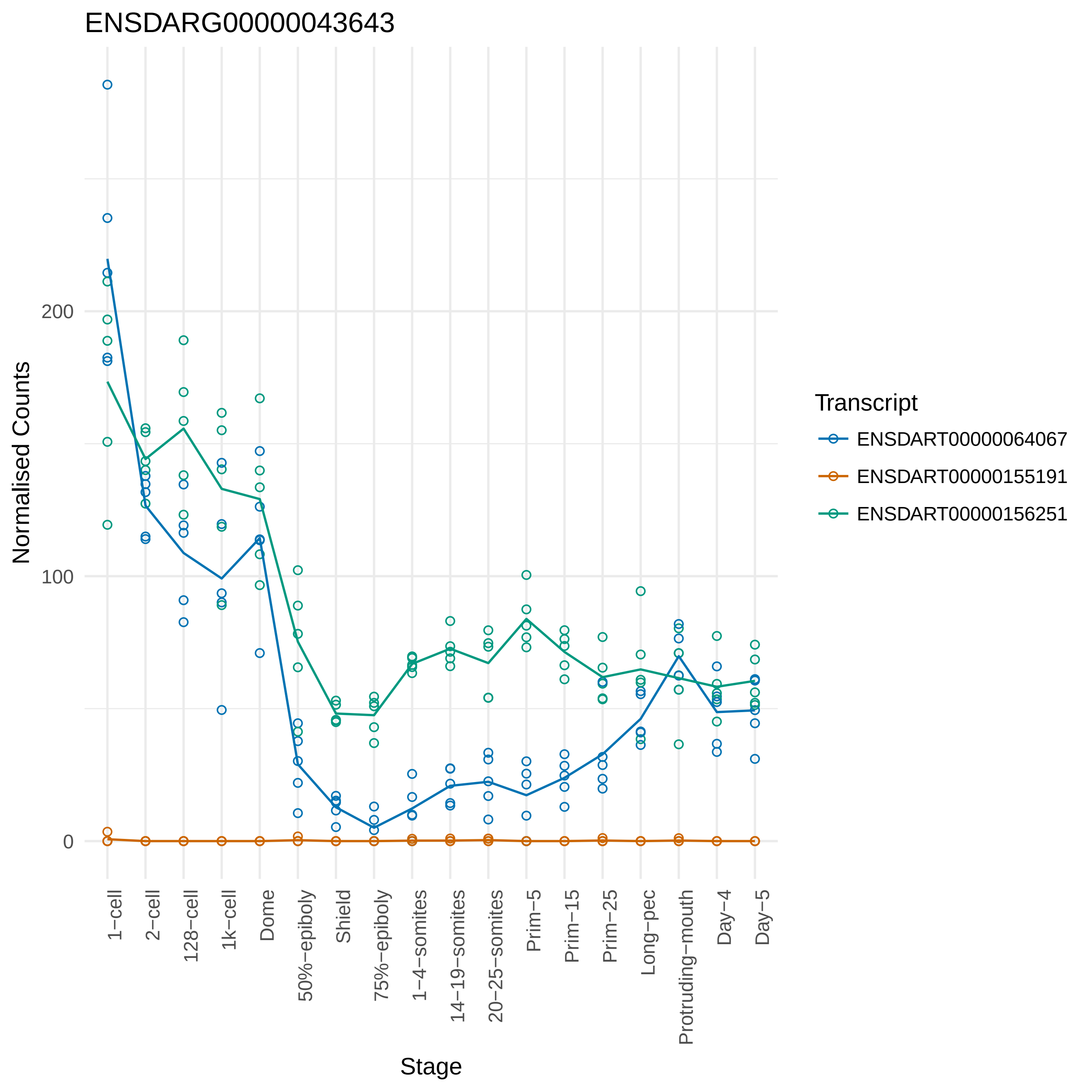

ENSDARG00000043643
Normalised Counts
200
100
0
1−cell
2−cell
128−cell
1k−cell
Dome
50%−epiboly
Shield
75%−epiboly
1−4−somites
14−19−somites
20−25−somites
Prim−5
Prim−15
Prim−25
Long−pec
Protruding−mouth
Day−4
Day−5
Stage
Transcript
ENSDART00000064067
ENSDART00000155191
ENSDART00000156251

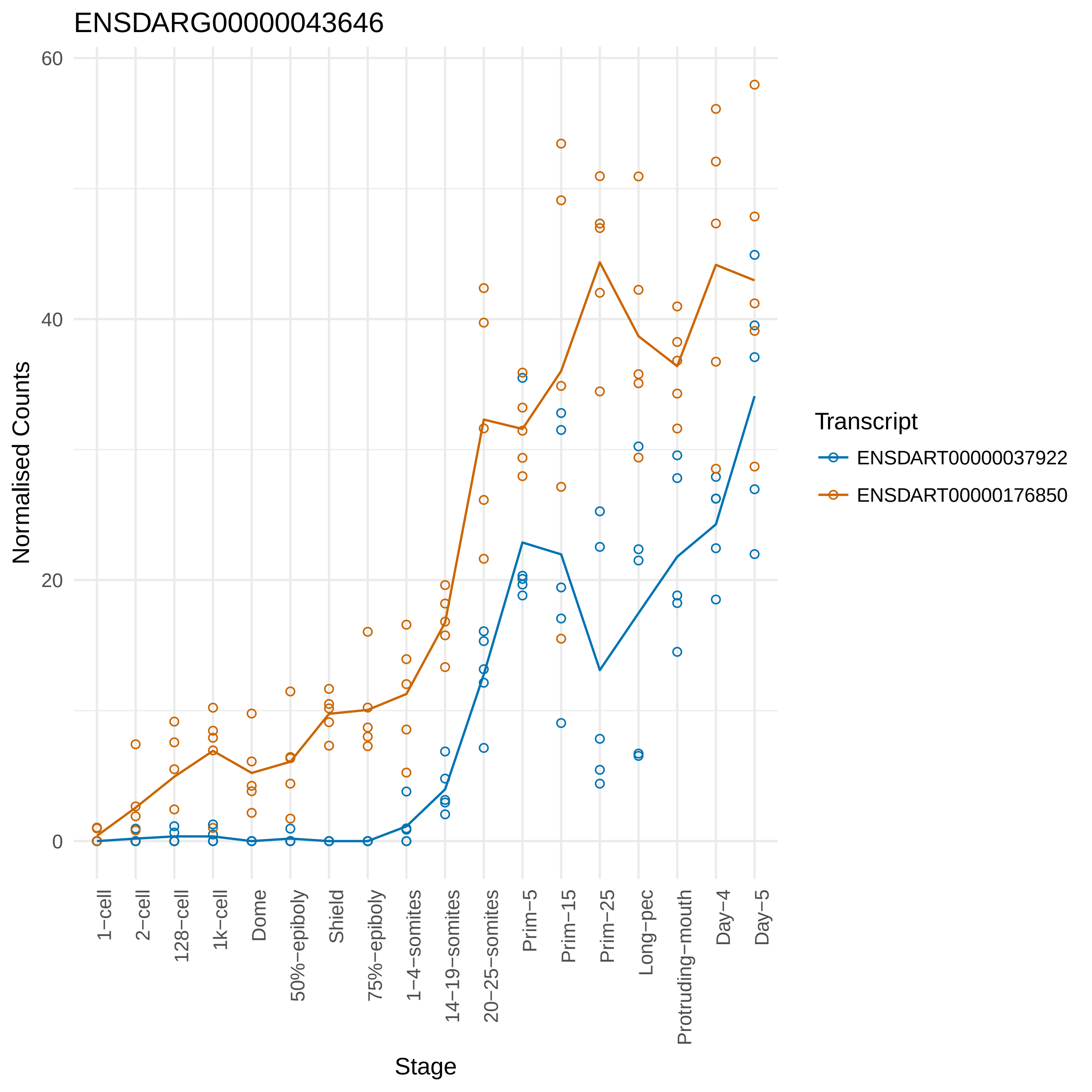
ENSDARG00000043646
Normalised Counts
60
40
20
0
Stage
1−cell
2−cell
128−cell
1k−cell
Dome
50%−epiboly
Shield
75%−epiboly
1−4−somites
14−19−somites
20−25−somites
Prim−5
Prim−15
Prim−25
Long−pec
Protruding−mouth
Day−4
Day−5
Transcript
ENSDART00000037922
ENSDART00000176850

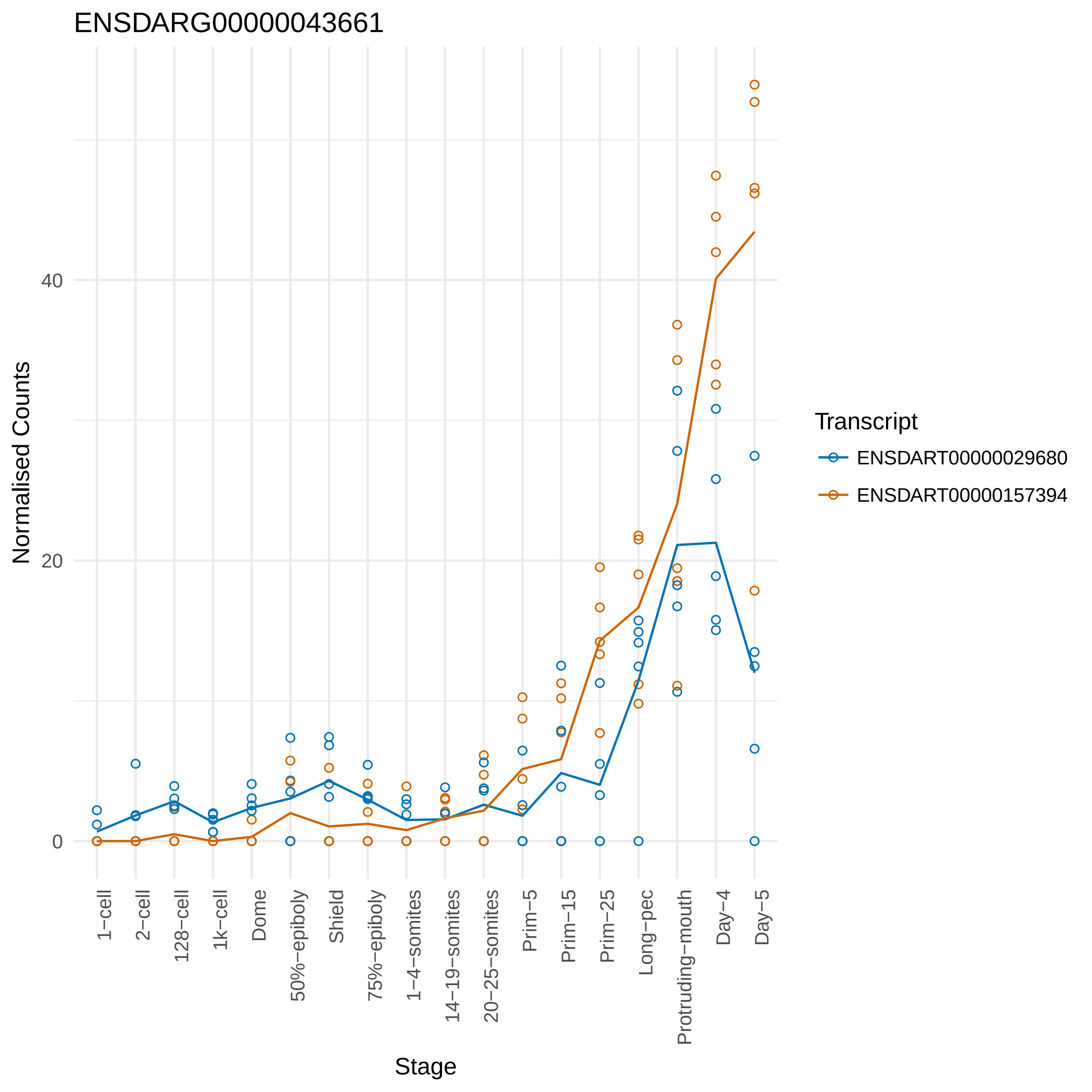

ENSDARG00000043661
Normalised Counts
0
20
40
1−cell
2−cell
128−cell
1k−cell
Dome
50%−epiboly
Shield
75%−epiboly
1−4−somites
14−19−somites
20−25−somites
Prim−5
Prim−15
Prim−25
Long−pec
Protruding−mouth
Day−4
Day−5
Stage
Transcript
ENSDART00000029680
ENSDART00000157394

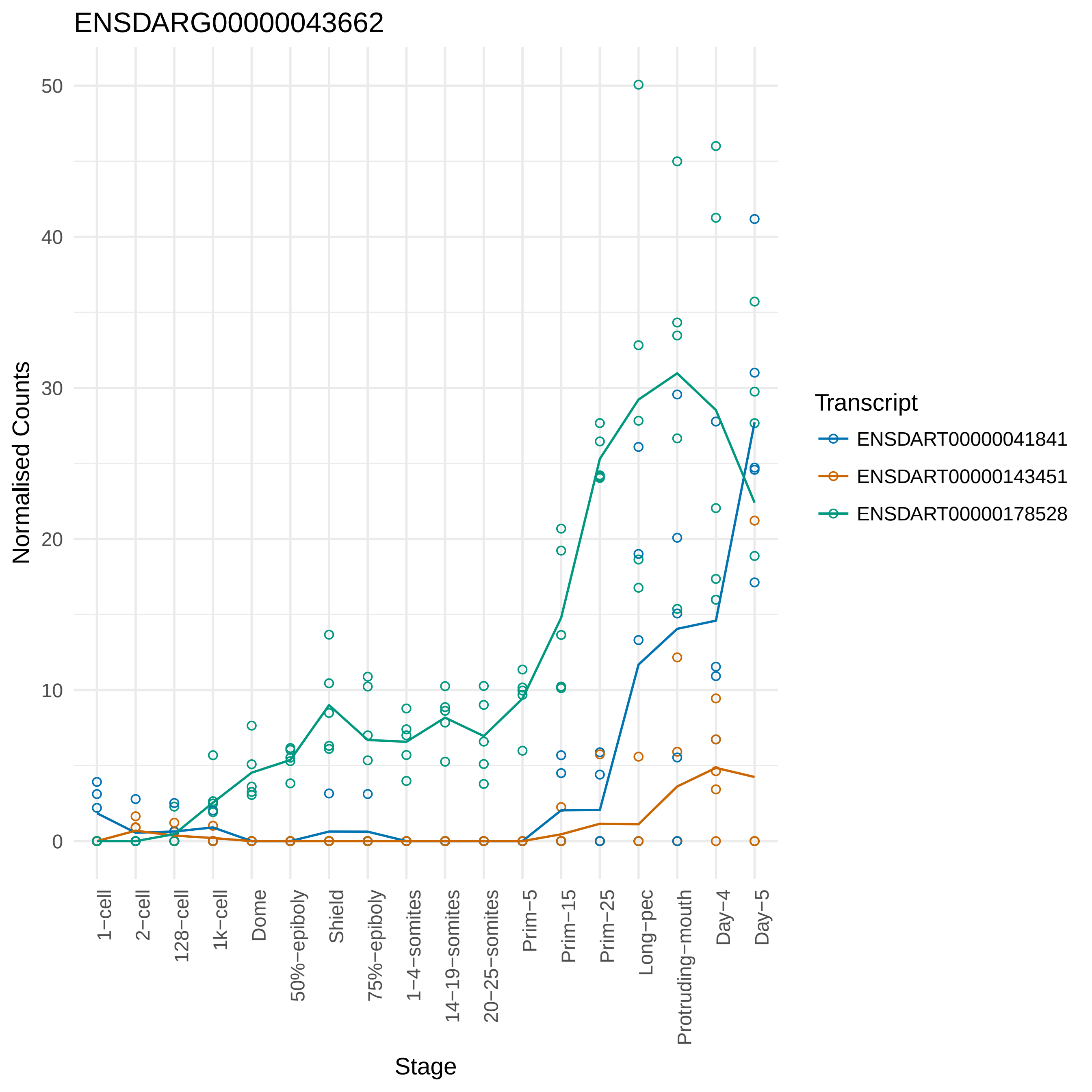

ENSDARG00000043662
Normalised Counts
0
10
20
30
40
50
1−cell
2−cell
128−cell
1k−cell
Dome
50%−epiboly
Shield
75%−epiboly
1−4−somites
14−19−somites
20−25−somites
Prim−5
Prim−15
Prim−25
Long−pec
Protruding−mouth
Day−4
Day−5
Stage
Transcript
ENSDART00000041841
ENSDART00000143451
ENSDART00000178528

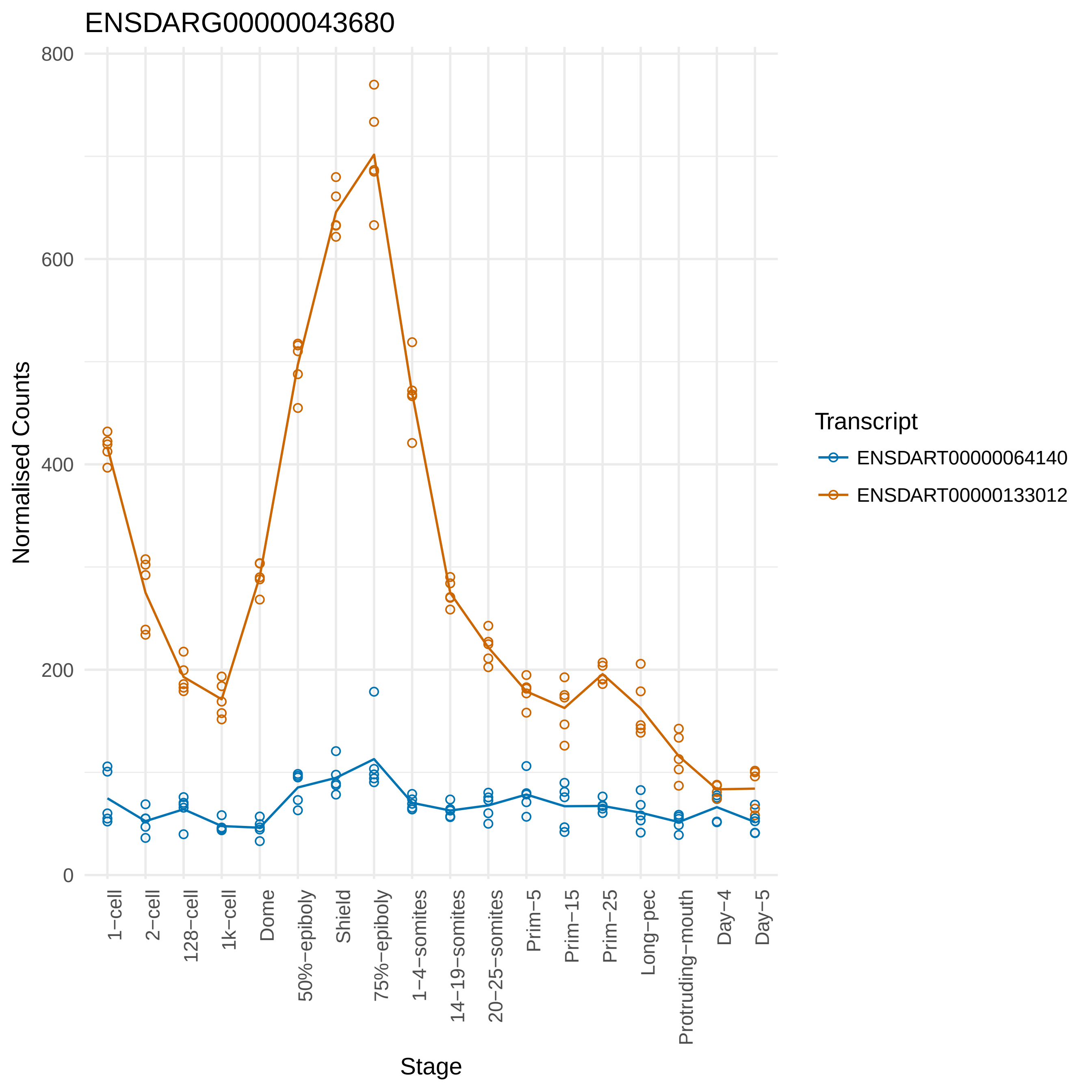

ENSDARG00000043680
Normalised Counts
800
600
400
200
0
1−cell
2−cell
128−cell
1k−cell
Dome
50%−epiboly
Shield
75%−epiboly
1−4−somites
14−19−somites
20−25−somites
Prim−5
Prim−15
Prim−25
Long−pec
Protruding−mouth
Day−4
Day−5
Stage
Transcript
ENSDART00000064140
ENSDART00000133012

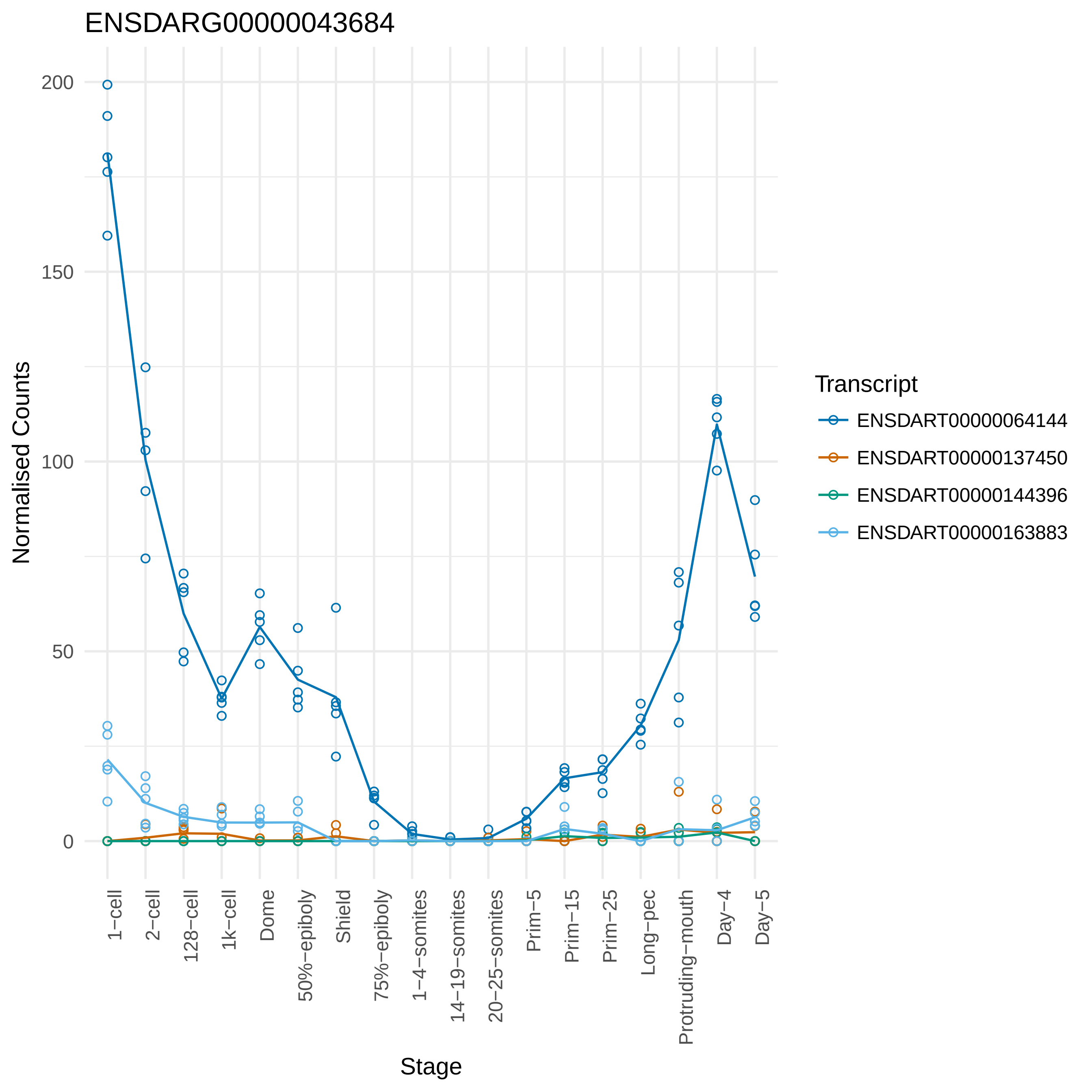

ENSDARG00000043684
Normalised Counts
200
150
100
50
0
1−cell
2−cell
128−cell
1k−cell
Dome
50%−epiboly
Shield
75%−epiboly
1−4−somites
14−19−somites
20−25−somites
Prim−5
Prim−15
Prim−25
Long−pec
Protruding−mouth
Day−4
Day−5
Stage
Transcript
ENSDART00000064144
ENSDART00000137450
ENSDART00000144396
ENSDART00000163883

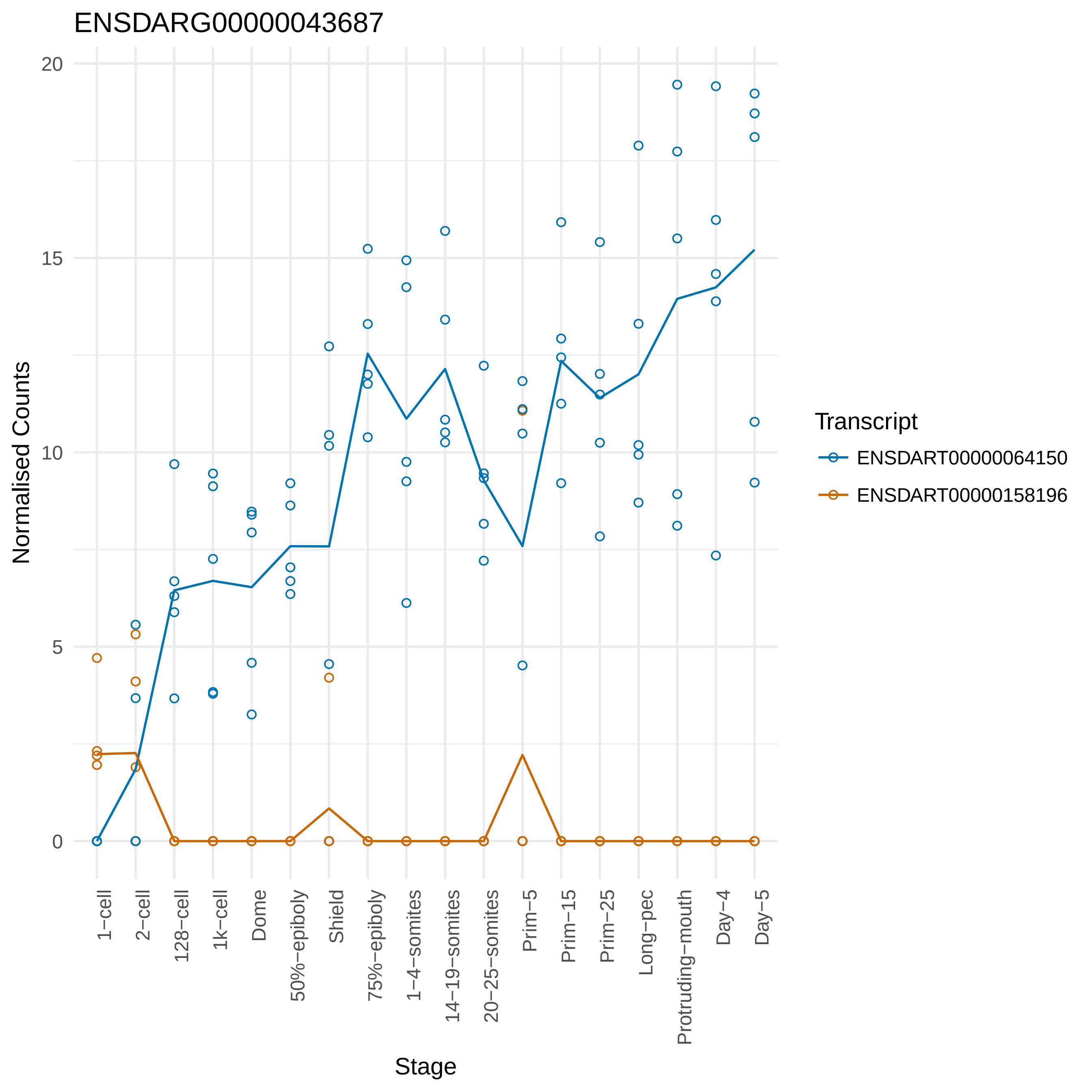

ENSDARG00000043687
Normalised Counts
0
5
10
15
20
1−cell
2−cell
128−cell
1k−cell
Dome
50%−epiboly
Shield
75%−epiboly
1−4−somites
14−19−somites
20−25−somites
Prim−5
Prim−15
Prim−25
Long−pec
Protruding−mouth
Day−4
Day−5
Stage
Transcript
ENSDART00000064150
ENSDART00000158196

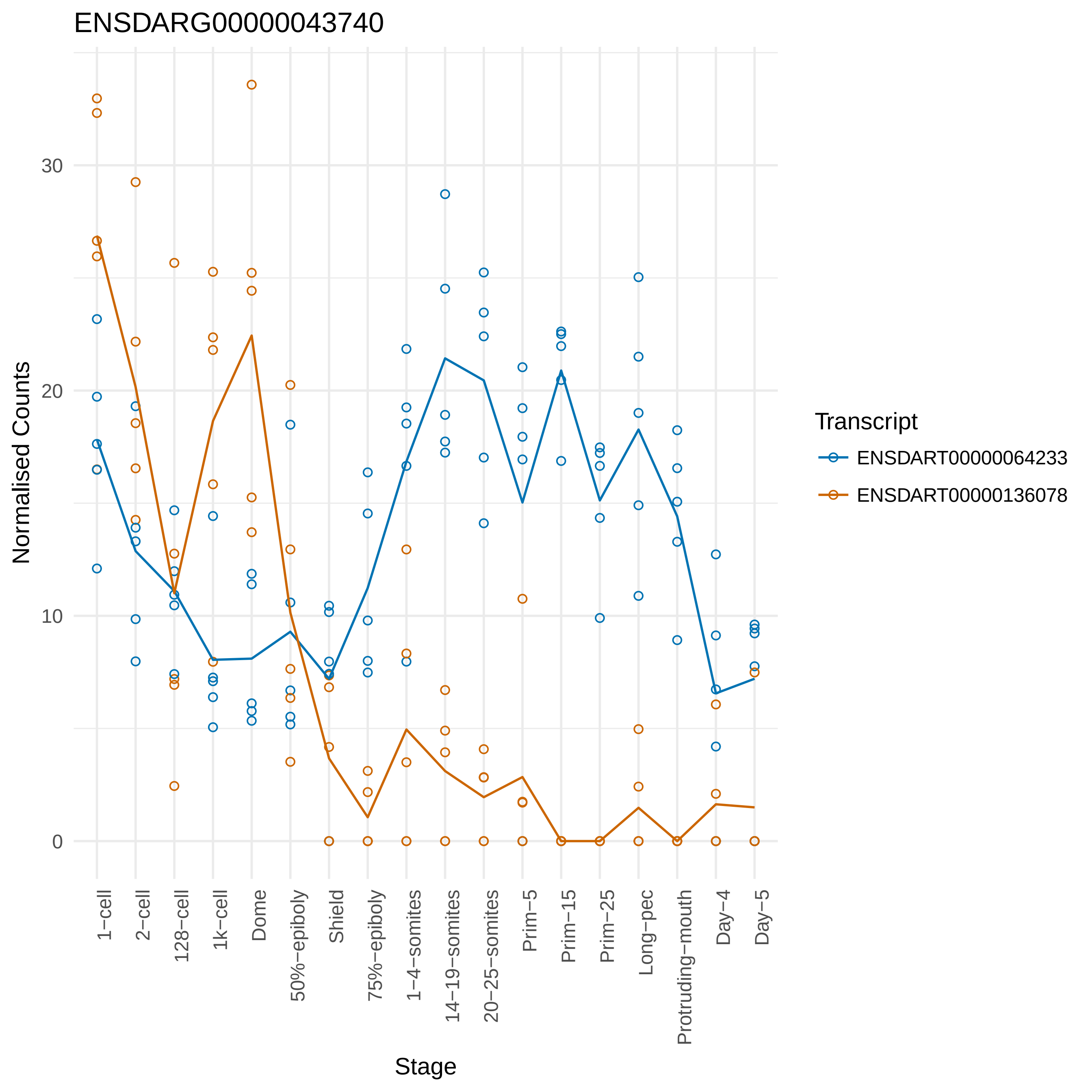

ENSDARG00000043740
Normalised Counts
30
20
10
0
1−cell
2−cell
128−cell
1k−cell
Dome
50%−epiboly
Shield
75%−epiboly
1−4−somites
14−19−somites
20−25−somites
Prim−5
Prim−15
Prim−25
Long−pec
Protruding−mouth
Day−4
Day−5
Stage
Transcript
ENSDART00000064233
ENSDART00000136078

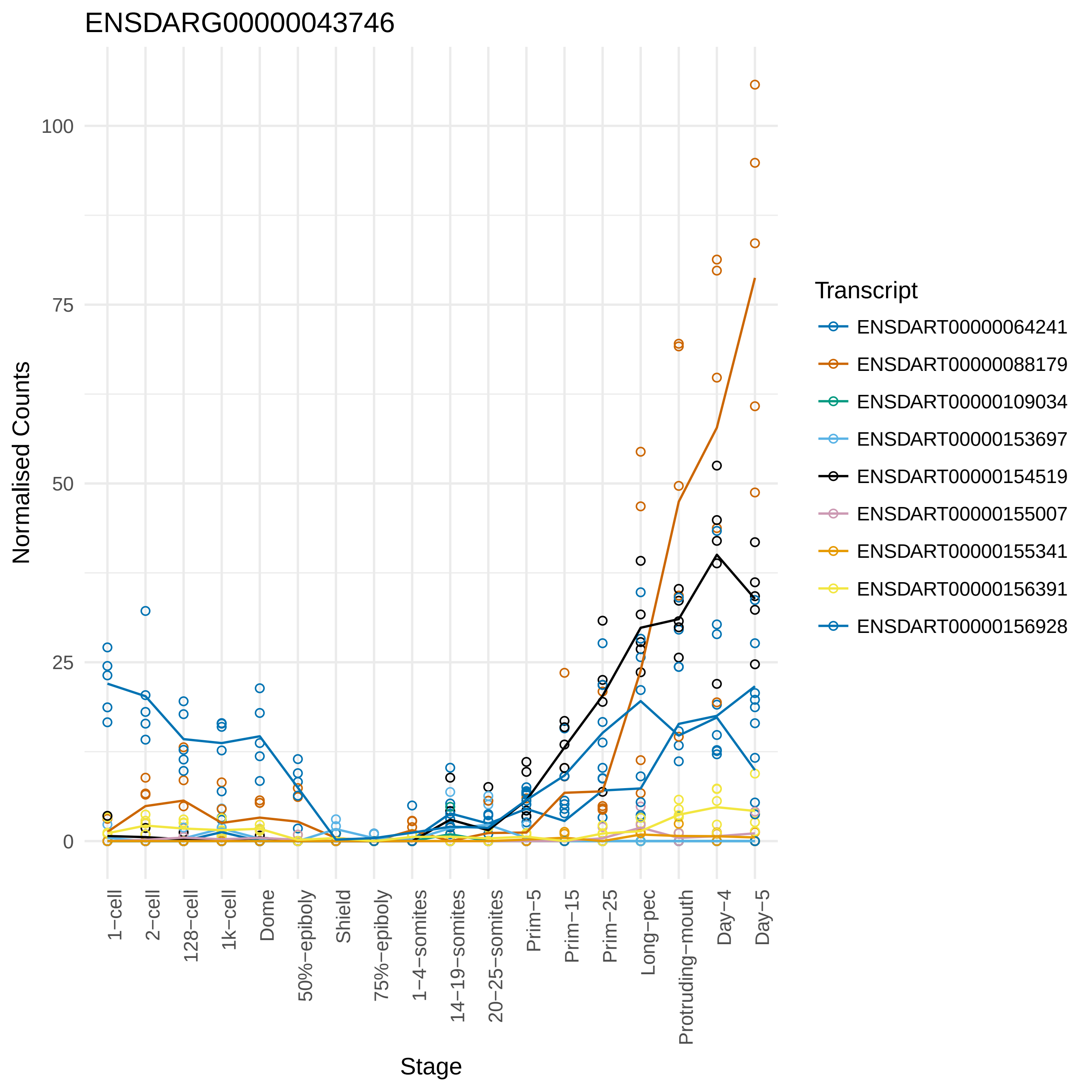

ENSDARG00000043746
Normalised Counts
0
25
50
75
100
1−cell
2−cell
128−cell
1k−cell
Dome
50%−epiboly
Shield
75%−epiboly
1−4−somites
14−19−somites
20−25−somites
Prim−5
Prim−15
Prim−25
Long−pec
Protruding−mouth
Day−4
Day−5
Stage
Transcript
ENSDART00000064241
ENSDART00000088179
ENSDART00000109034
ENSDART00000153697
ENSDART00000154519
ENSDART00000155007
ENSDART00000155341
ENSDART00000156391
ENSDART00000156928

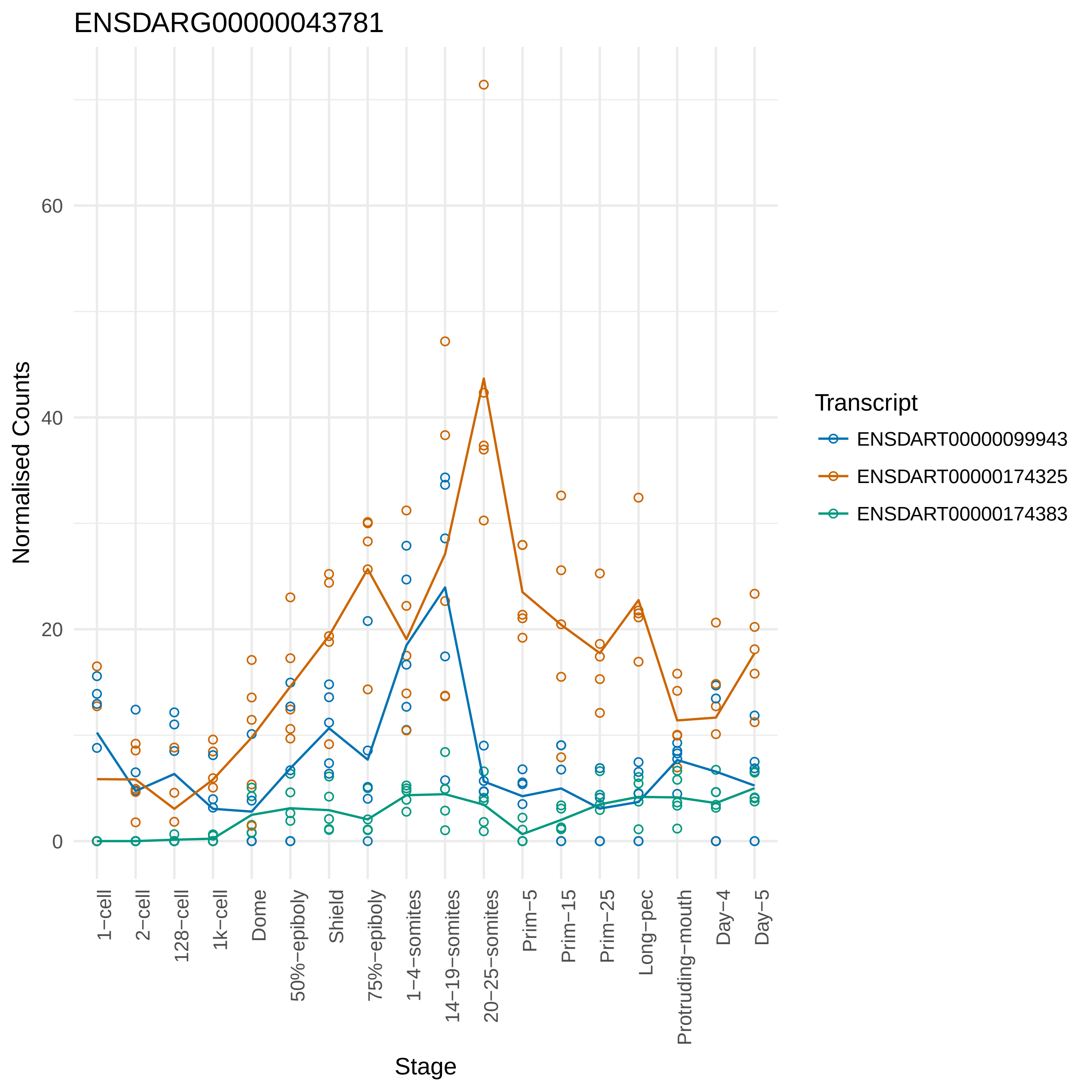
ENSDARG00000043781
Normalised Counts
0
20
40
60
1−cell
2−cell
128−cell
1k−cell
Dome
50%−epiboly
Shield
75%−epiboly
1−4−somites
14−19−somites
20−25−somites
Prim−5
Prim−15
Prim−25
Long−pec
Protruding−mouth
Day−4
Day−5
Stage
Transcript
ENSDART00000099943
ENSDART00000174325
ENSDART00000174383

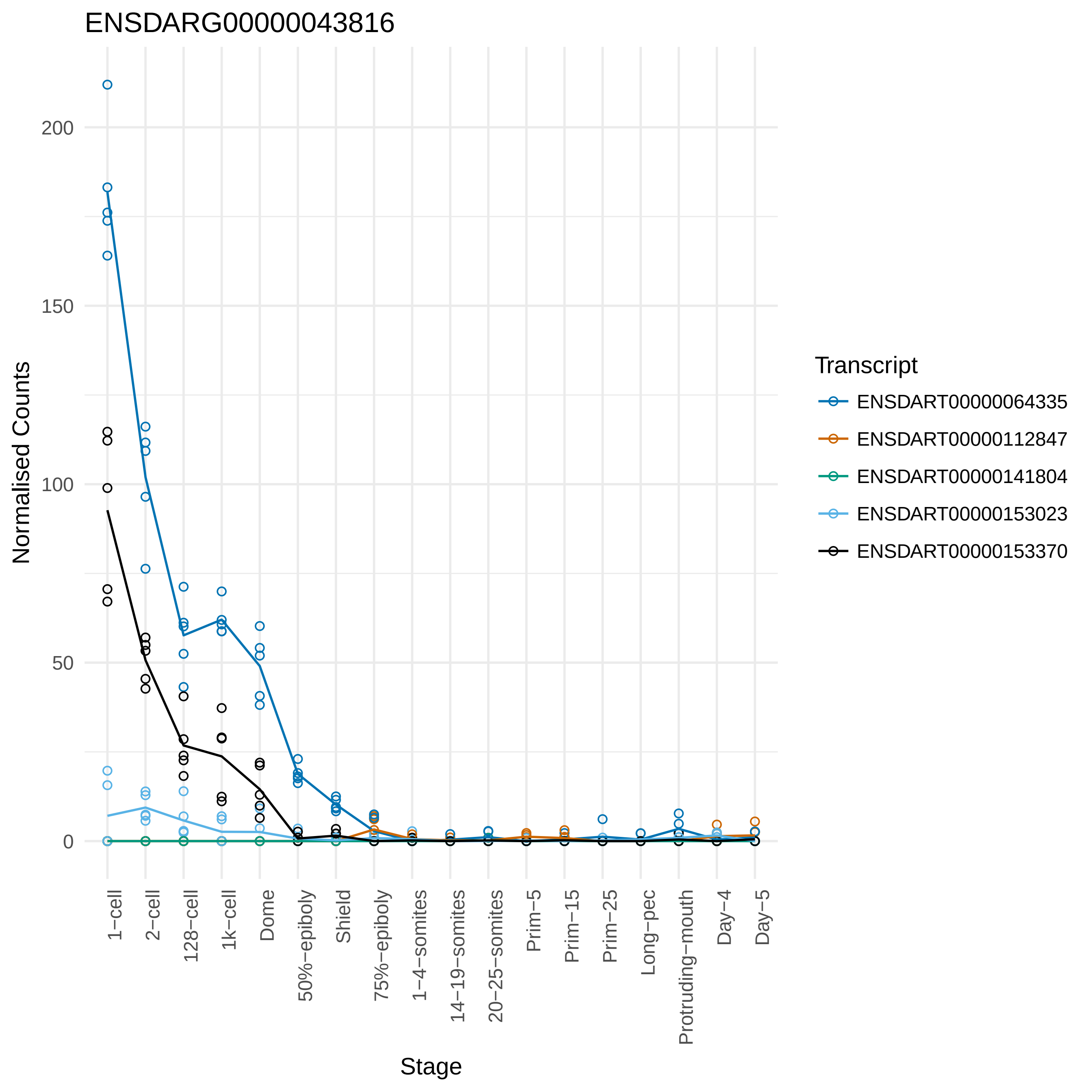

ENSDARG00000043816
Normalised Counts
200
150
100
50
0
1−cell
2−cell
128−cell
1k−cell
Dome
50%−epiboly
Shield
75%−epiboly
1−4−somites
14−19−somites
20−25−somites
Prim−5
Prim−15
Prim−25
Long−pec
Protruding−mouth
Day−4
Day−5
Stage
Transcript
ENSDART00000064335
ENSDART00000112847
ENSDART00000141804
ENSDART00000153023
ENSDART00000153370

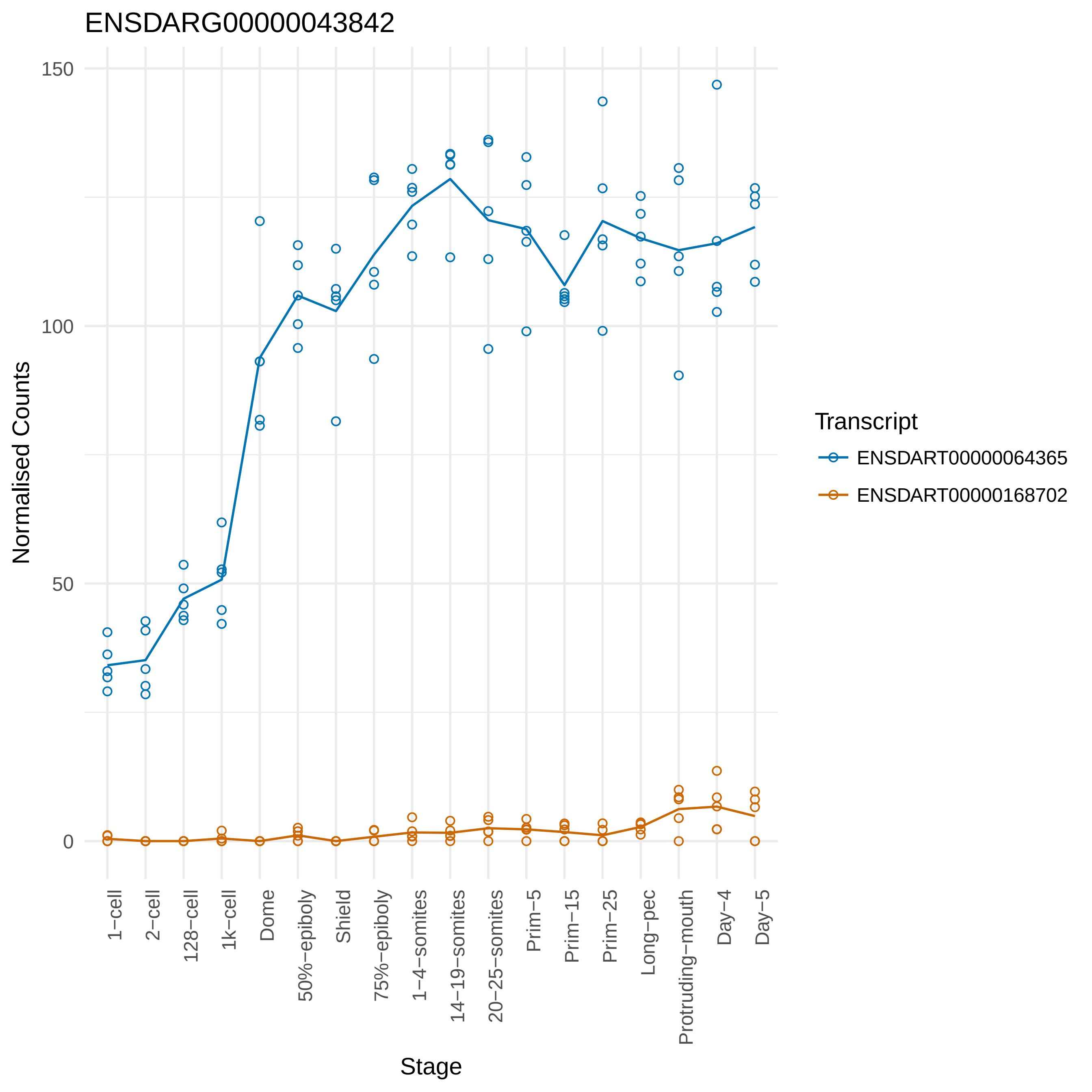

ENSDARG00000043842
Normalised Counts
150
100
50
0
1−cell
2−cell
128−cell
1k−cell
Dome
50%−epiboly
Shield
75%−epiboly
1−4−somites
14−19−somites
20−25−somites
Prim−5
Prim−15
Prim−25
Long−pec
Protruding−mouth
Day−4
Day−5
Stage
Transcript
ENSDART00000064365
ENSDART00000168702

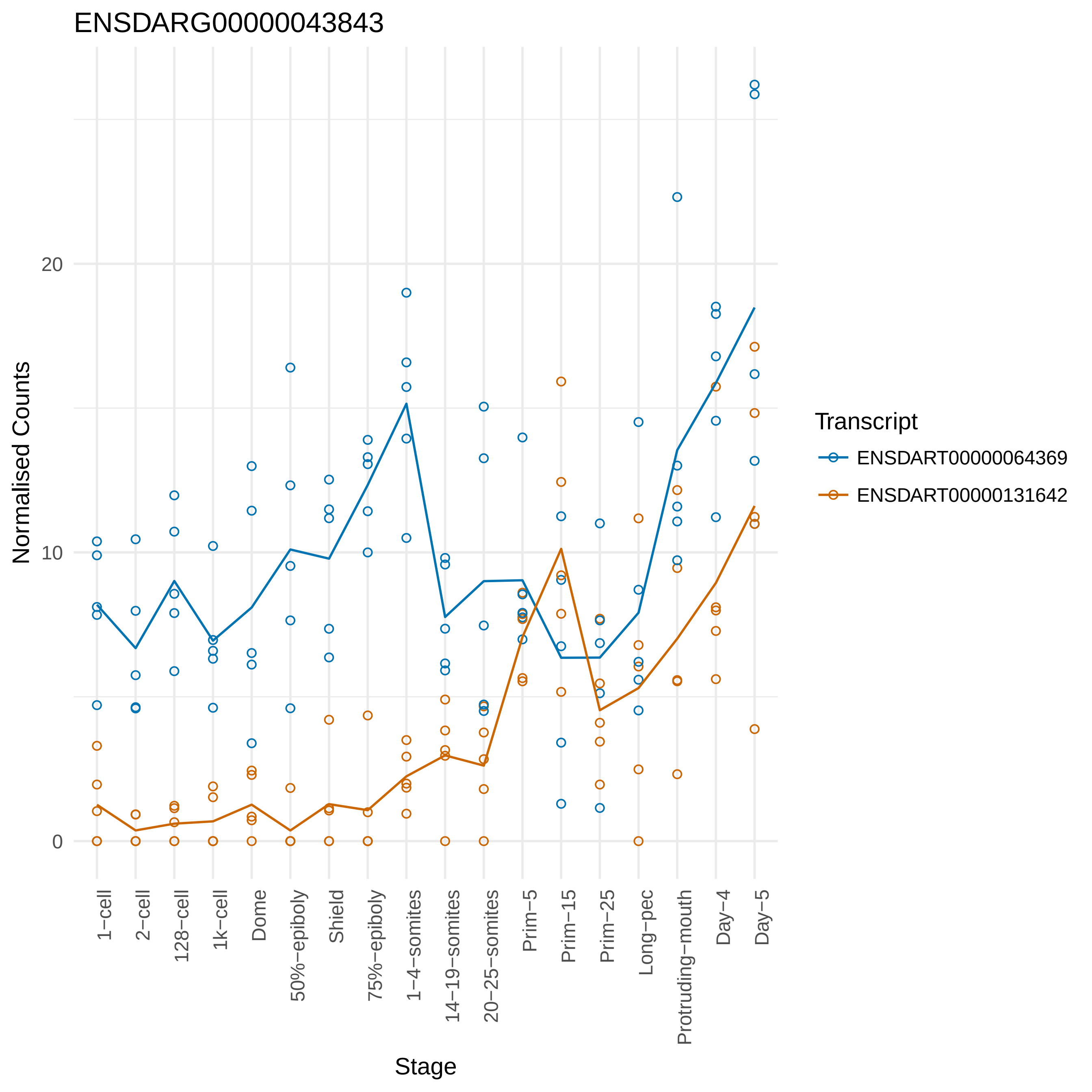

ENSDARG00000043843
Normalised Counts
20
10
0
1−cell
2−cell
128−cell
1k−cell
Dome
50%−epiboly
Shield
75%−epiboly
1−4−somites
14−19−somites
20−25−somites
Prim−5
Prim−15
Prim−25
Long−pec
Protruding−mouth
Day−4
Day−5
Stage
Transcript
ENSDART00000064369
ENSDART00000131642

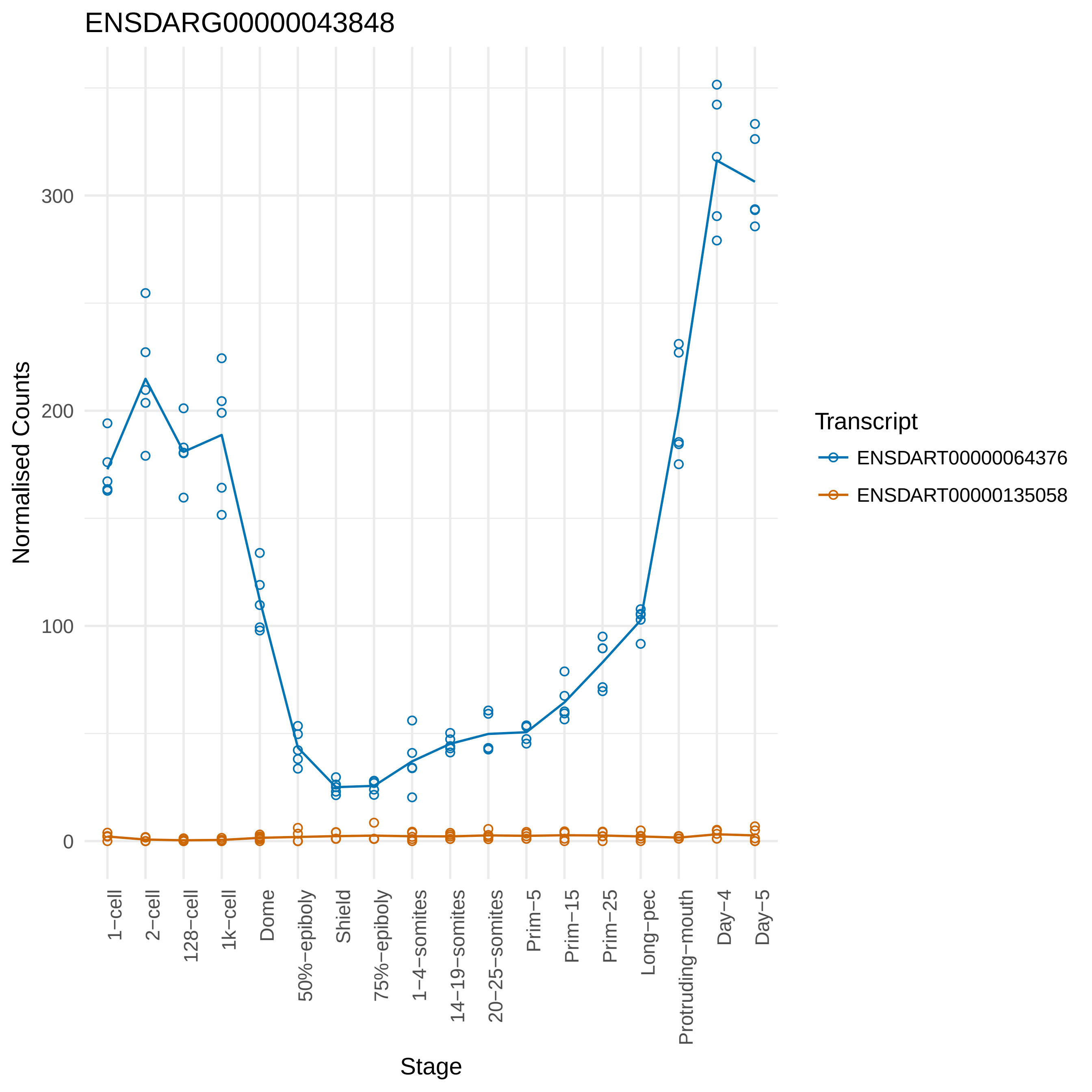

ENSDARG00000043848
Normalised Counts
300
200
100
0
1−cell
2−cell
128−cell
1k−cell
Dome
50%−epiboly
Shield
75%−epiboly
1−4−somites
14−19−somites
20−25−somites
Prim−5
Prim−15
Prim−25
Long−pec
Protruding−mouth
Day−4
Day−5
Stage
Transcript
ENSDART00000064376
ENSDART00000135058

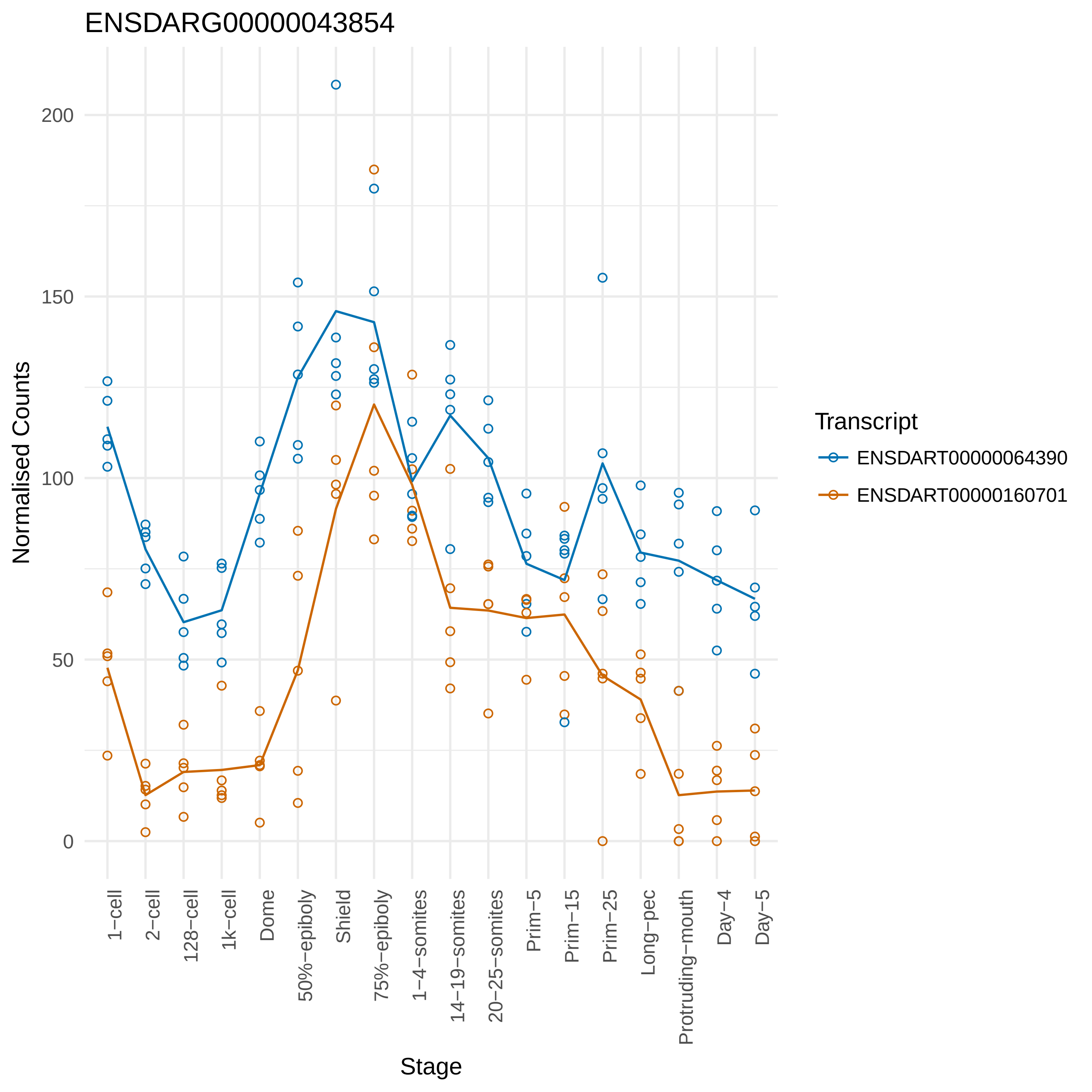

ENSDARG00000043854
Normalised Counts
200
150
100
50
0
1−cell
2−cell
128−cell
1k−cell
Dome
50%−epiboly
Shield
75%−epiboly
1−4−somites
14−19−somites
20−25−somites
Prim−5
Prim−15
Prim−25
Long−pec
Protruding−mouth
Day−4
Day−5
Stage
Transcript
ENSDART00000064390
ENSDART00000160701

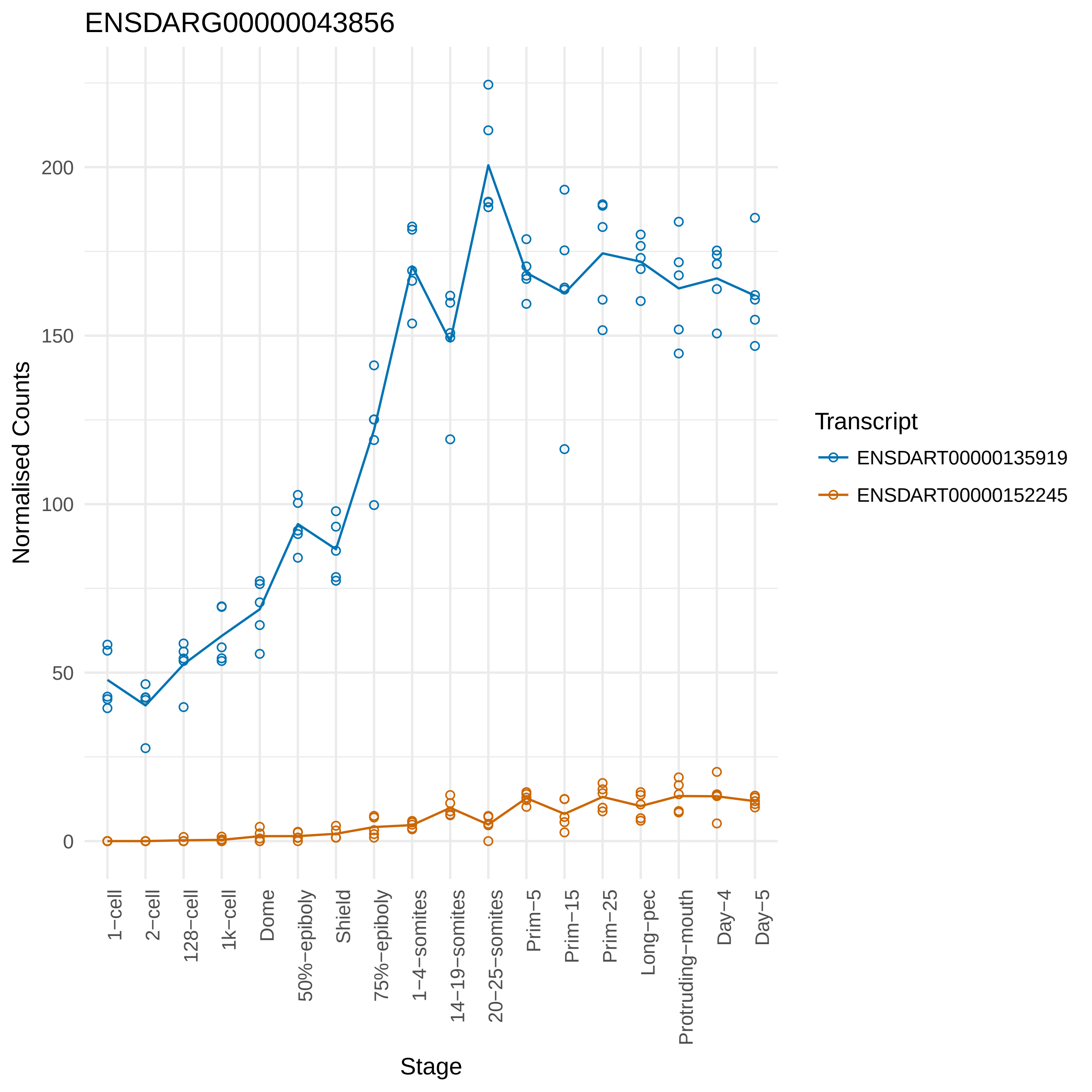
ENSDARG00000043856
Normalised Counts
200
150
100
50
0
1−cell
2−cell
128−cell
1k−cell
Dome
50%−epiboly
Shield
75%−epiboly
1−4−somites
14−19−somites
20−25−somites
Prim−5
Prim−15
Prim−25
Long−pec
Protruding−mouth
Day−4
Day−5
Stage
Transcript
ENSDART00000135919
ENSDART00000152245

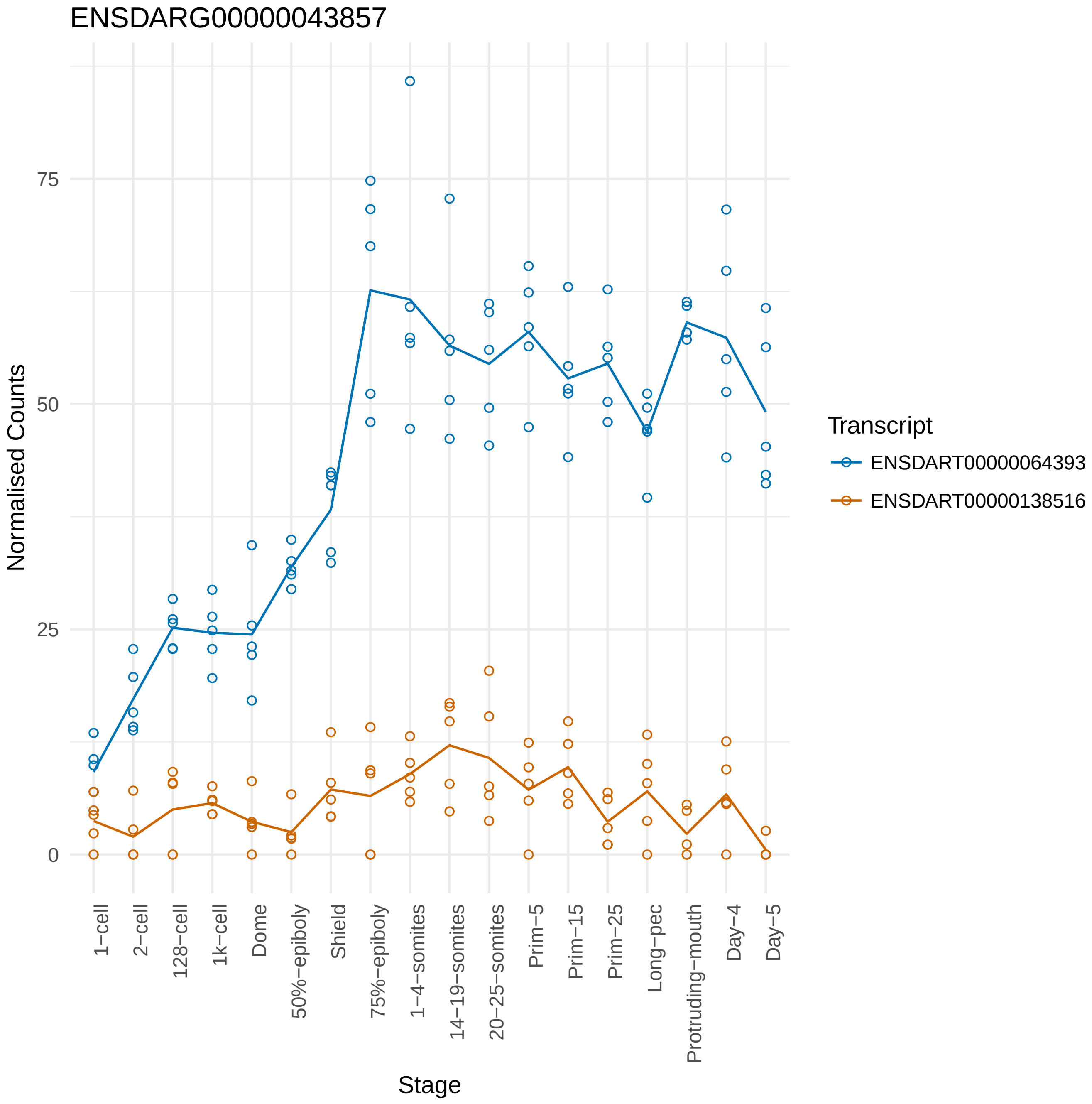

ENSDARG00000043857
Normalised Counts
0
25
50
75
1−cell
2−cell
128−cell
1k−cell
Dome
50%−epiboly
Shield
75%−epiboly
1−4−somites
14−19−somites
20−25−somites
Prim−5
Prim−15
Prim−25
Long−pec
Protruding−mouth
Day−4
Day−5
Stage
Transcript
ENSDART00000064393
ENSDART00000138516

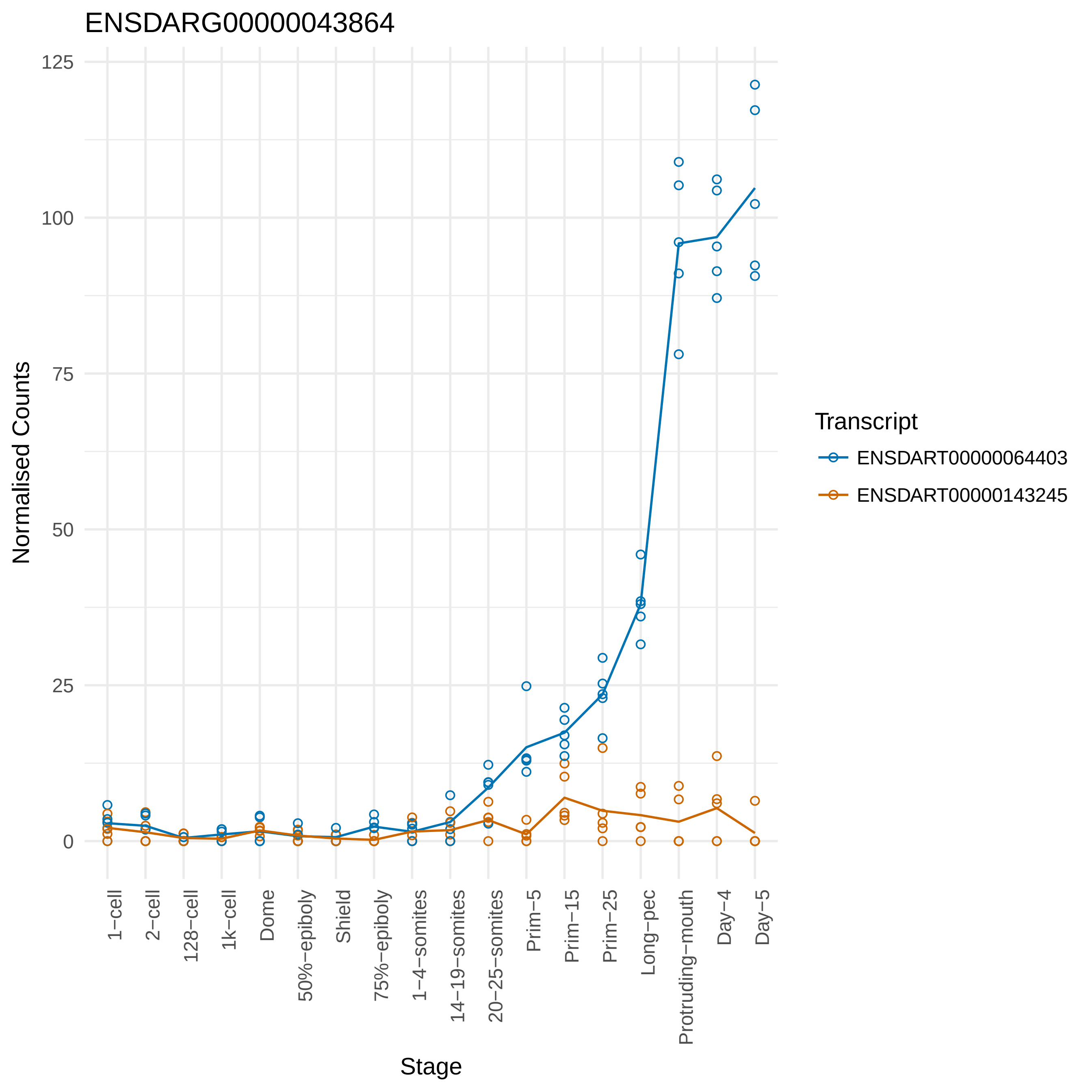
ENSDARG00000043864
Normalised Counts
125
100
75
50
25
0
1−cell
2−cell
128−cell
1k−cell
Dome
50%−epiboly
Shield
75%−epiboly
1−4−somites
14−19−somites
20−25−somites
Prim−5
Prim−15
Prim−25
Long−pec
Protruding−mouth
Day−4
Day−5
Stage
Transcript
ENSDART00000064403
ENSDART00000143245

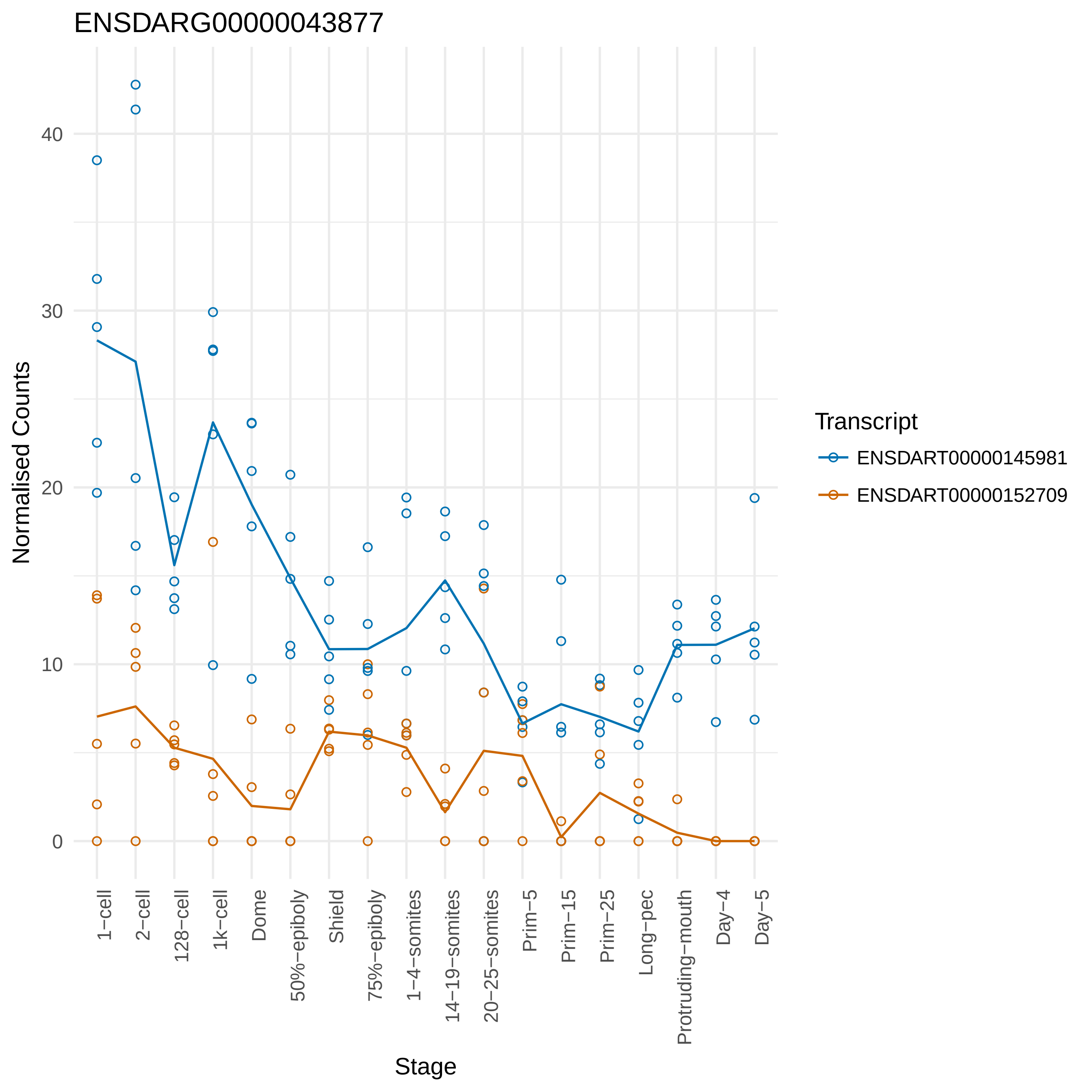

ENSDARG00000043877
Normalised Counts
40
30
20
10
0
1−cell
2−cell
128−cell
1k−cell
Dome
50%−epiboly
Shield
75%−epiboly
1−4−somites
14−19−somites
20−25−somites
Prim−5
Prim−15
Prim−25
Long−pec
Protruding−mouth
Day−4
Day−5
Stage
Transcript
ENSDART00000145981
ENSDART00000152709

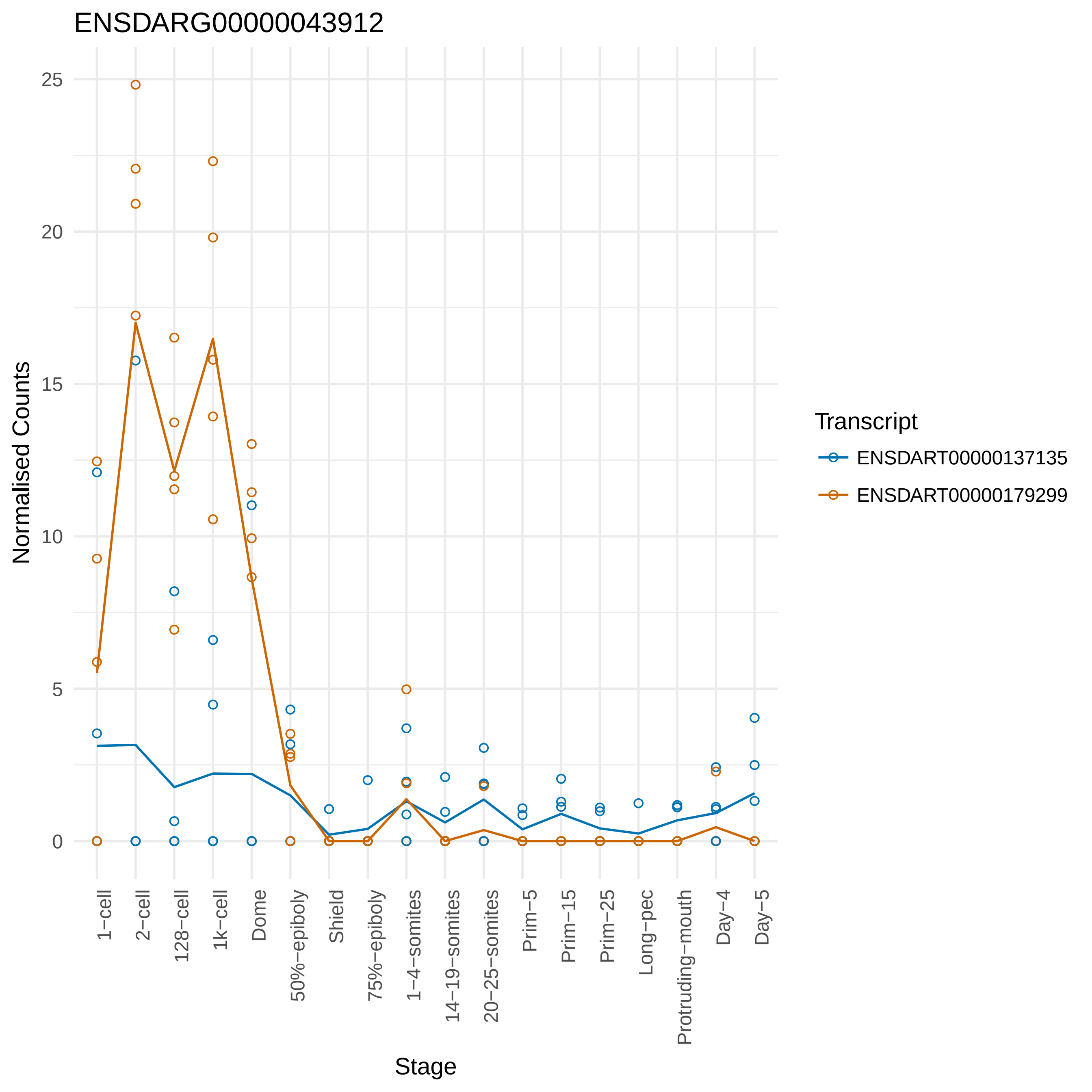

ENSDARG00000043912
Normalised Counts
0
5
10
15
20
25
1−cell
2−cell
128−cell
1k−cell
Dome
50%−epiboly
Shield
75%−epiboly
1−4−somites
14−19−somites
20−25−somites
Prim−5
Prim−15
Prim−25
Long−pec
Protruding−mouth
Day−4
Day−5
Stage
Transcript
ENSDART00000137135
ENSDART00000179299

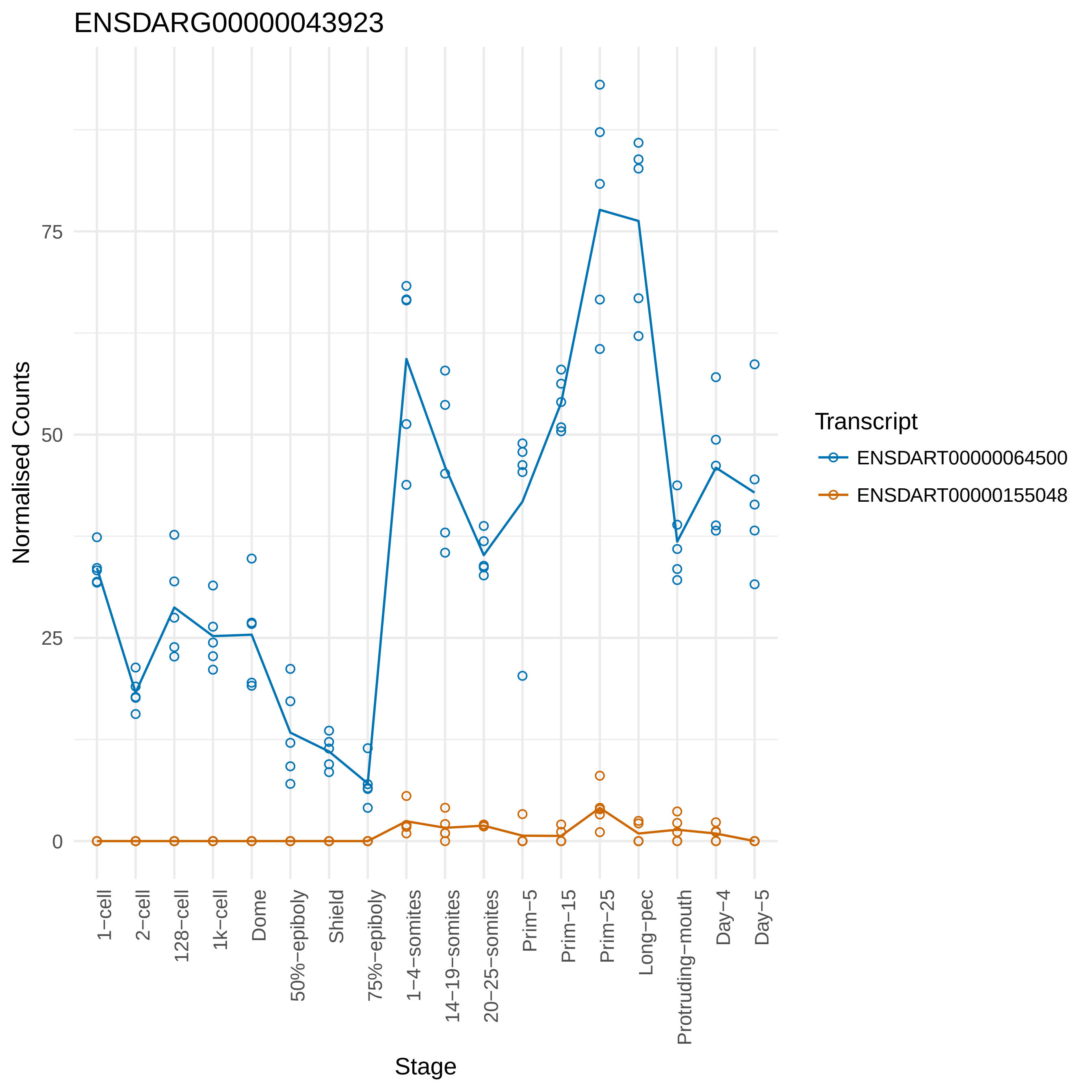

ENSDARG00000043923
Normalised Counts
75
50
25
0
1−cell
2−cell
128−cell
1k−cell
Dome
50%−epiboly
Shield
75%−epiboly
1−4−somites
14−19−somites
20−25−somites
Prim−5
Prim−15
Prim−25
Long−pec
Protruding−mouth
Day−4
Day−5
Stage
Transcript
ENSDART00000064500
ENSDART00000155048

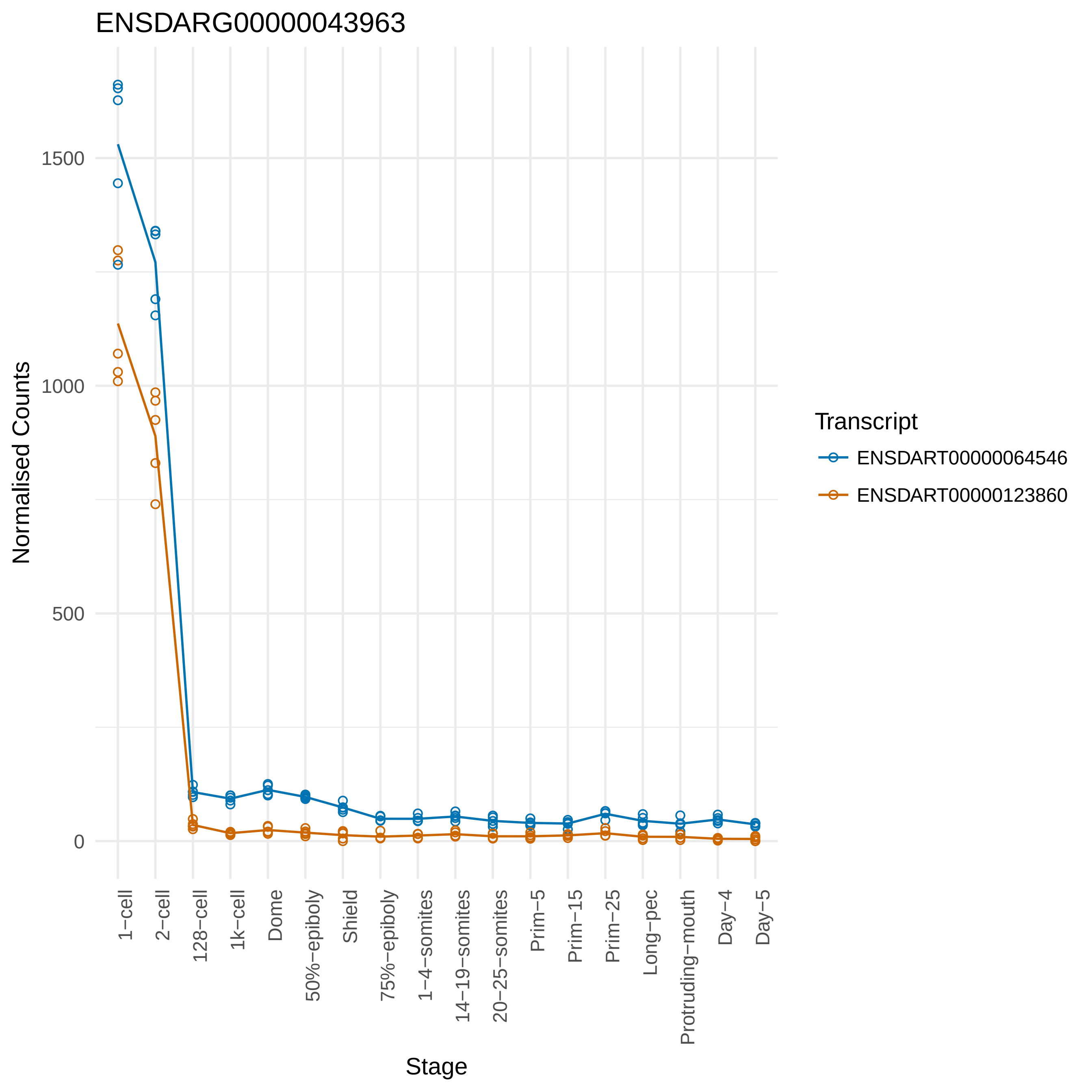
ENSDARG00000043963
Normalised Counts
1500
1000
500
0
1−cell
2−cell
128−cell
1k−cell
Dome
50%−epiboly
Shield
75%−epiboly
1−4−somites
14−19−somites
20−25−somites
Prim−5
Prim−15
Prim−25
Long−pec
Protruding−mouth
Day−4
Day−5
Stage
Transcript
ENSDART00000064546
ENSDART00000123860

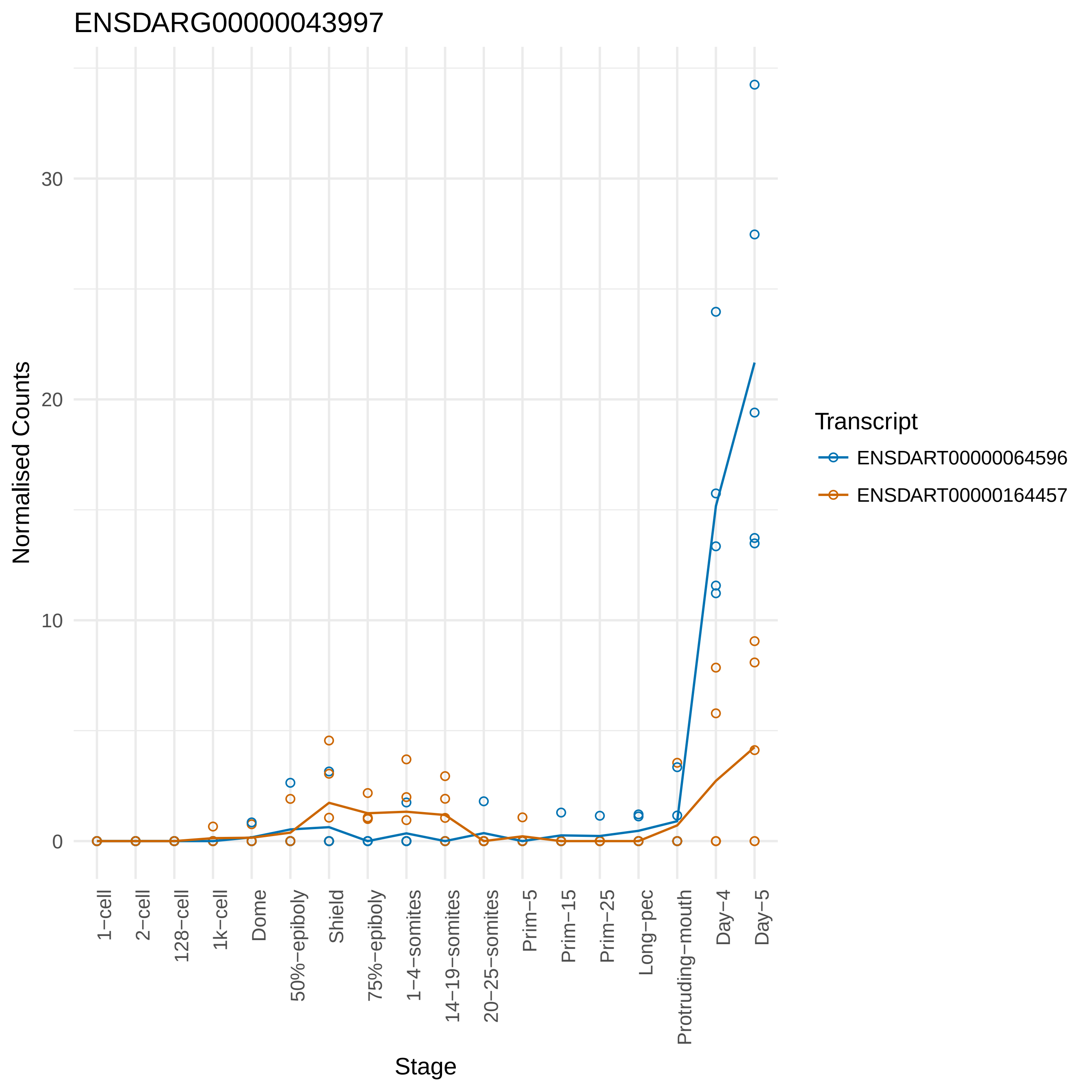
ENSDARG00000043997
Normalised Counts
0
10
20
30
Stage
1−cell
2−cell
128−cell
1k−cell
Dome
50%−epiboly
Shield
75%−epiboly
1−4−somites
14−19−somites
20−25−somites
Prim−5
Prim−15
Prim−25
Long−pec
Protruding−mouth
Day−4
Day−5
Transcript
ENSDART00000064596
ENSDART00000164457

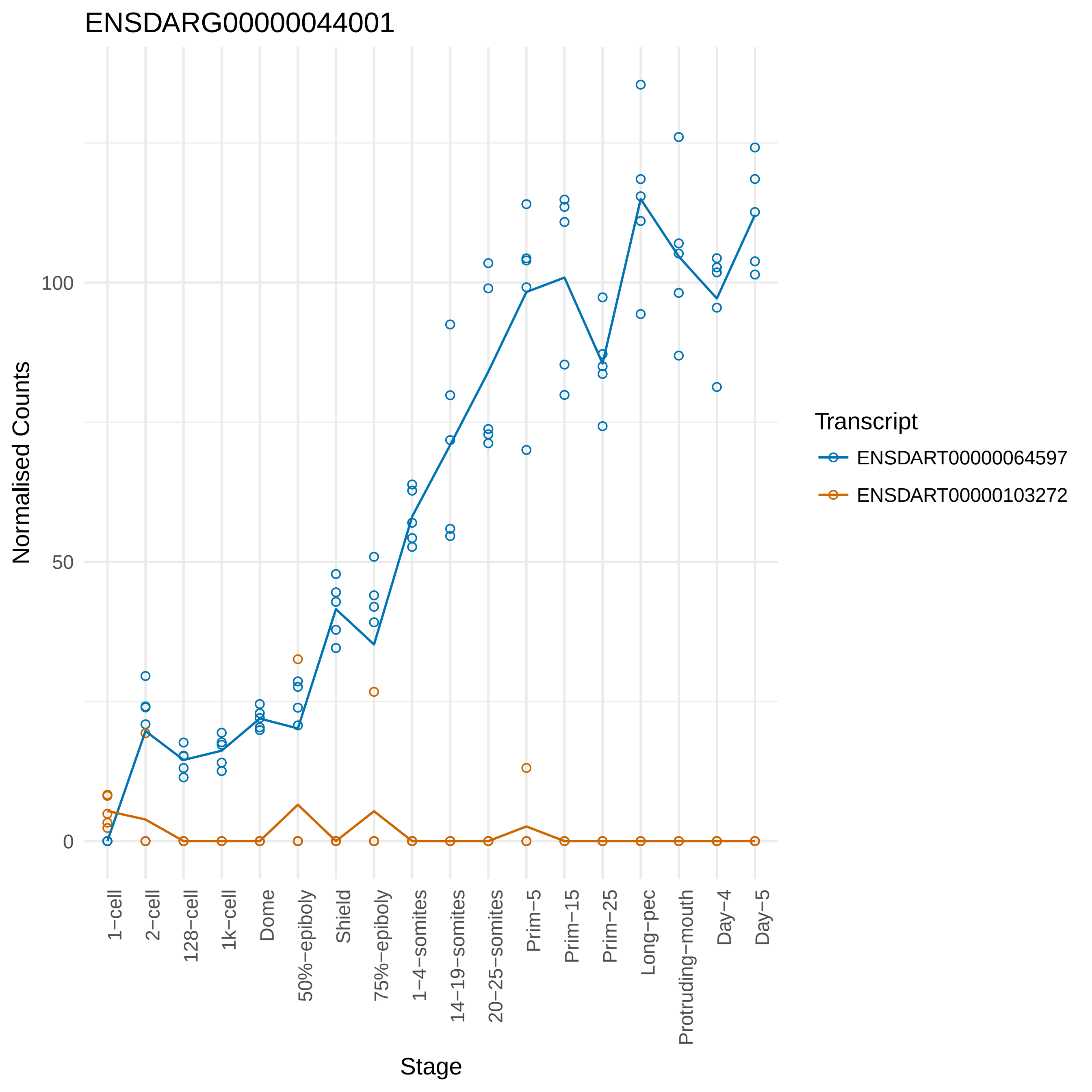

ENSDARG00000044001
Normalised Counts
100
50
0
1−cell
2−cell
128−cell
1k−cell
Dome
50%−epiboly
Shield
75%−epiboly
1−4−somites
14−19−somites
20−25−somites
Prim−5
Prim−15
Prim−25
Long−pec
Protruding−mouth
Day−4
Day−5
Stage
Transcript
ENSDART00000064597
ENSDART00000103272

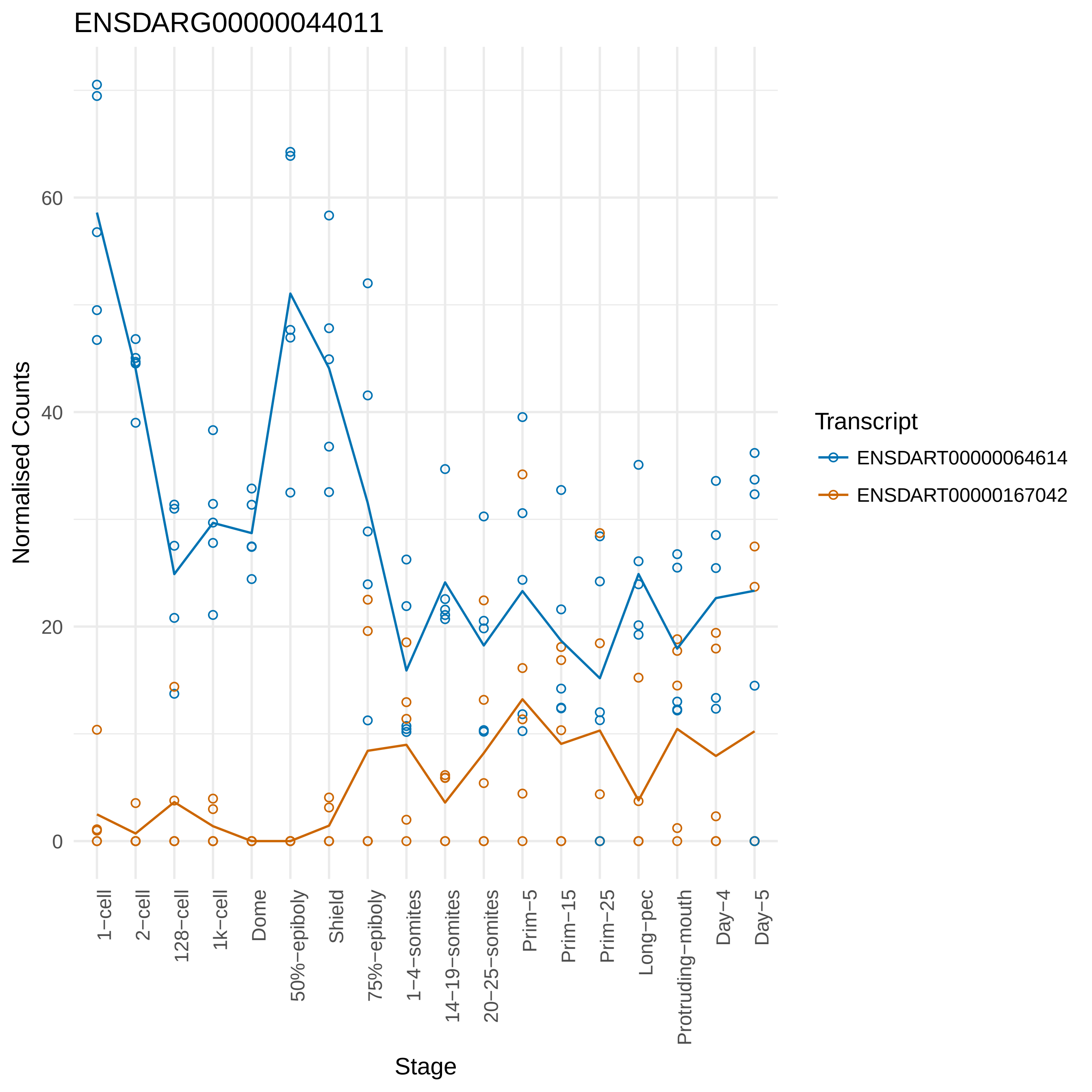

ENSDARG00000044011
Normalised Counts
0
20
40
60
1−cell
2−cell
128−cell
1k−cell
Dome
50%−epiboly
Shield
75%−epiboly
1−4−somites
14−19−somites
20−25−somites
Prim−5
Prim−15
Prim−25
Long−pec
Protruding−mouth
Day−4
Day−5
Stage
Transcript
ENSDART00000064614
ENSDART00000167042

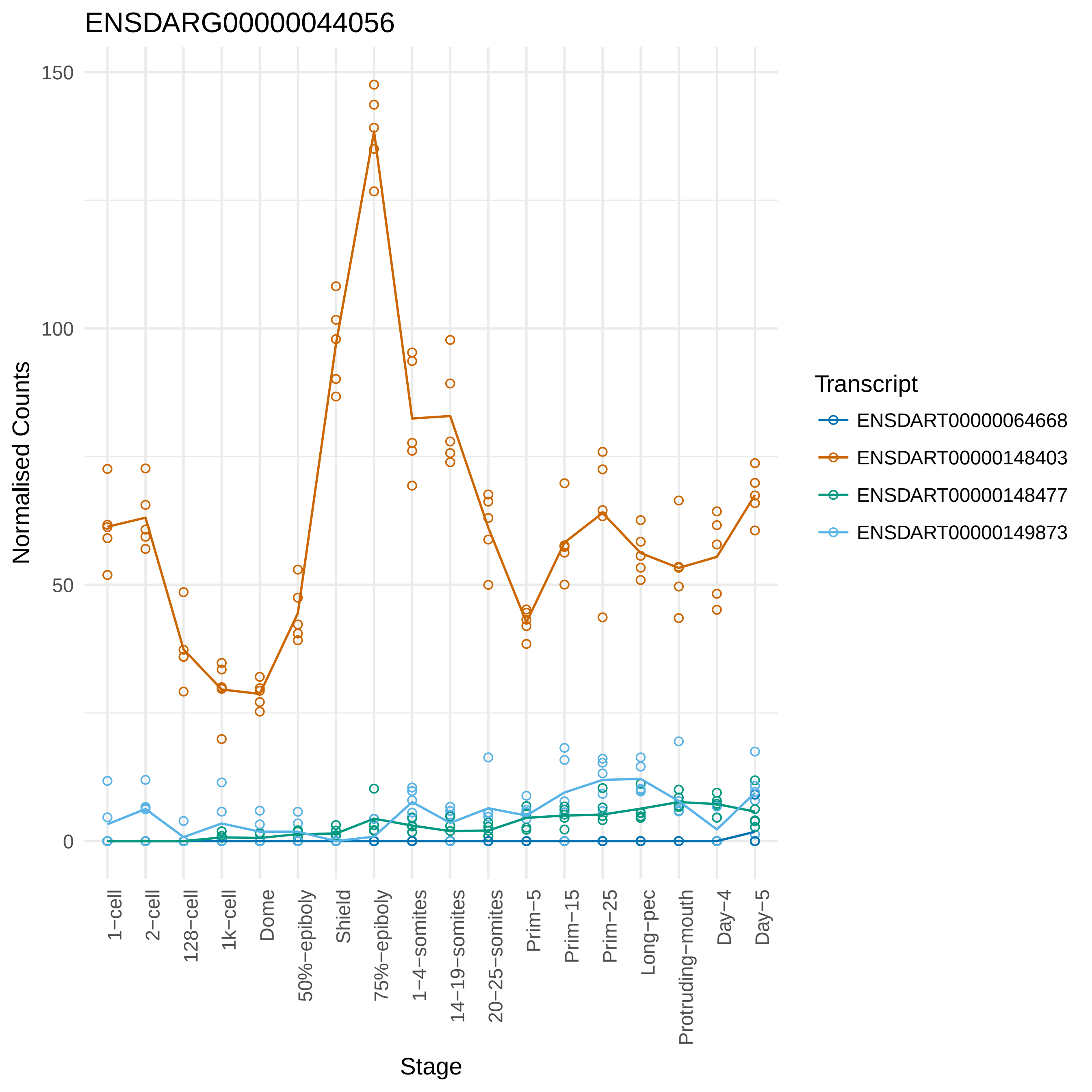

ENSDARG00000044056
Normalised Counts
150
100
50
0
1−cell
2−cell
128−cell
1k−cell
Dome
50%−epiboly
Shield
75%−epiboly
1−4−somites
14−19−somites
20−25−somites
Prim−5
Prim−15
Prim−25
Long−pec
Protruding−mouth
Day−4
Day−5
Stage
Transcript
ENSDART00000064668
ENSDART00000148403
ENSDART00000148477
ENSDART00000149873

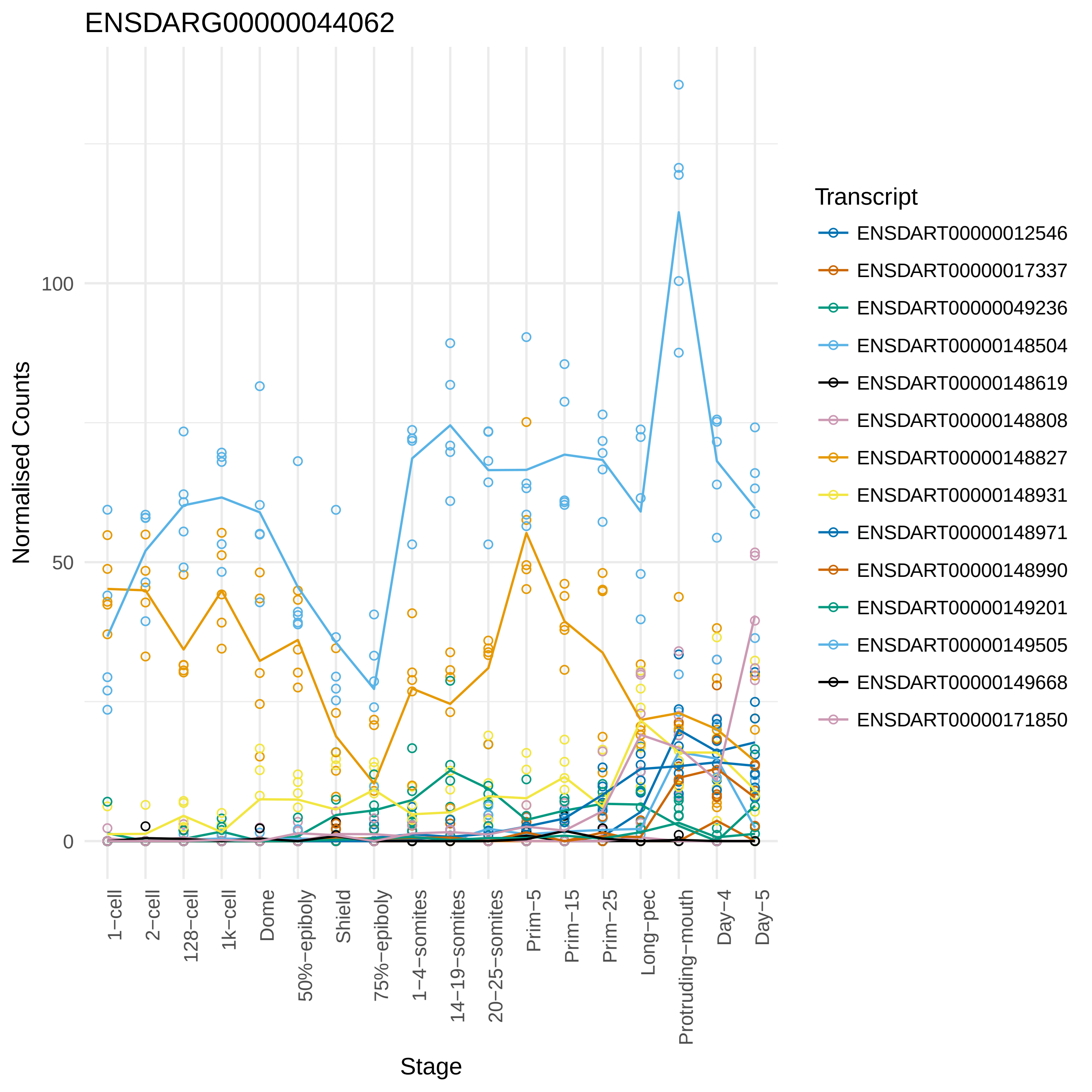

ENSDARG00000044062
Normalised Counts
0
50
100
Stage
1−cell
2−cell
128−cell
1k−cell
Dome
50%−epiboly
Shield
75%−epiboly
1−4−somites
14−19−somites
20−25−somites
Prim−5
Prim−15
Prim−25
Long−pec
Protruding−mouth
Day−4
Day−5
Transcript
ENSDART00000012546
ENSDART00000017337
ENSDART00000049236
ENSDART00000148504
ENSDART00000148619
ENSDART00000148808
ENSDART00000148827
ENSDART00000148931
ENSDART00000148971
ENSDART00000148990
ENSDART00000149201
ENSDART00000149505
ENSDART00000149668
ENSDART00000171850

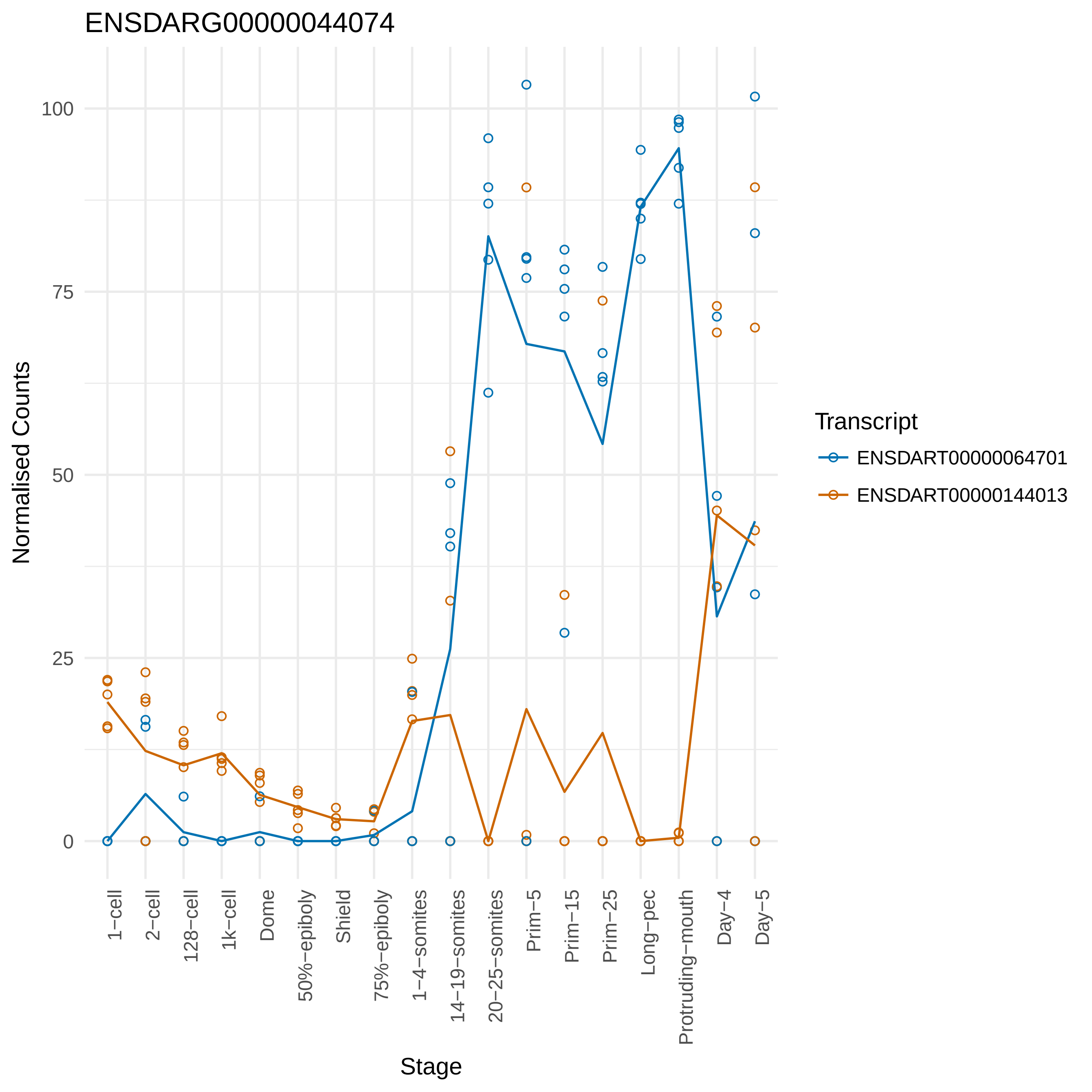

ENSDARG00000044074
Normalised Counts
100
75
50
25
0
1−cell
2−cell
128−cell
1k−cell
Dome
50%−epiboly
Shield
75%−epiboly
1−4−somites
14−19−somites
20−25−somites
Prim−5
Prim−15
Prim−25
Long−pec
Protruding−mouth
Day−4
Day−5
Stage
Transcript
ENSDART00000064701
ENSDART00000144013

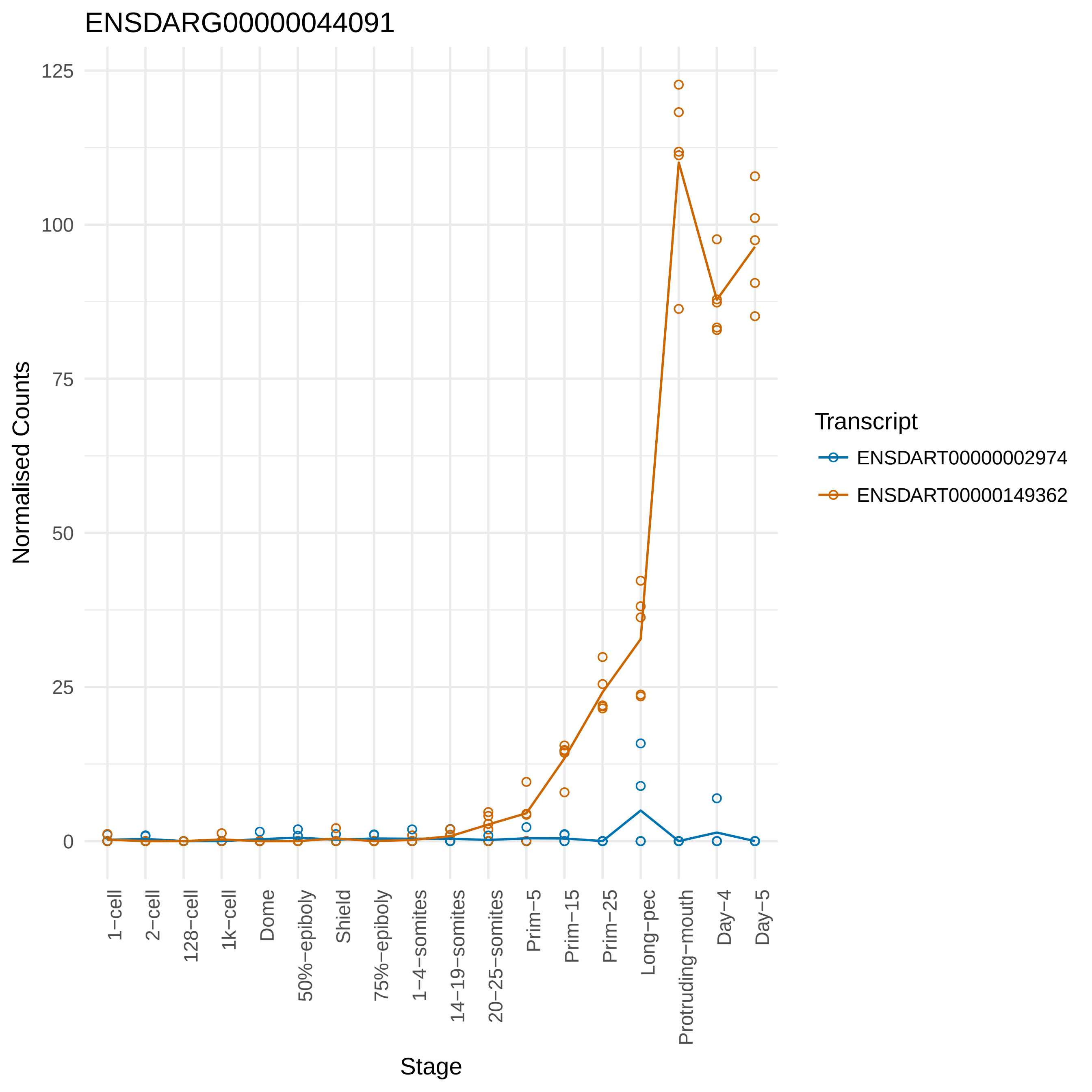

ENSDARG00000044091
Normalised Counts
125
100
75
50
25
0
1−cell
2−cell
128−cell
1k−cell
Dome
50%−epiboly
Shield
75%−epiboly
1−4−somites
14−19−somites
20−25−somites
Prim−5
Prim−15
Prim−25
Long−pec
Protruding−mouth
Day−4
Day−5
Stage
Transcript
ENSDART00000002974
ENSDART00000149362

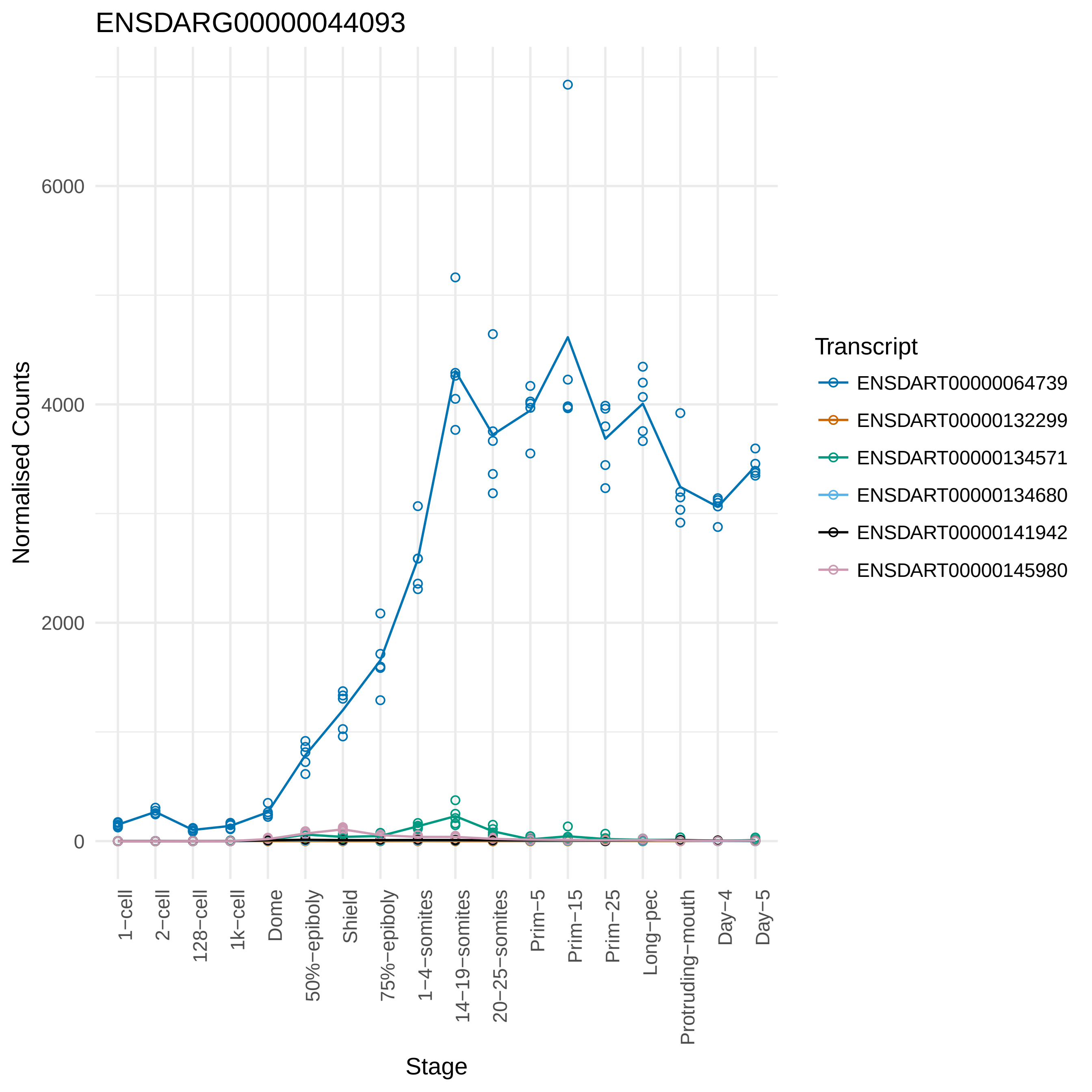
ENSDARG00000044093
Normalised Counts
0
2000
4000
6000
Stage
1−cell
2−cell
128−cell
1k−cell
Dome
50%−epiboly
Shield
75%−epiboly
1−4−somites
14−19−somites
20−25−somites
Prim−5
Prim−15
Prim−25
Long−pec
Protruding−mouth
Day−4
Day−5
Transcript
ENSDART00000064739
ENSDART00000132299
ENSDART00000134571
ENSDART00000134680
ENSDART00000141942
ENSDART00000145980

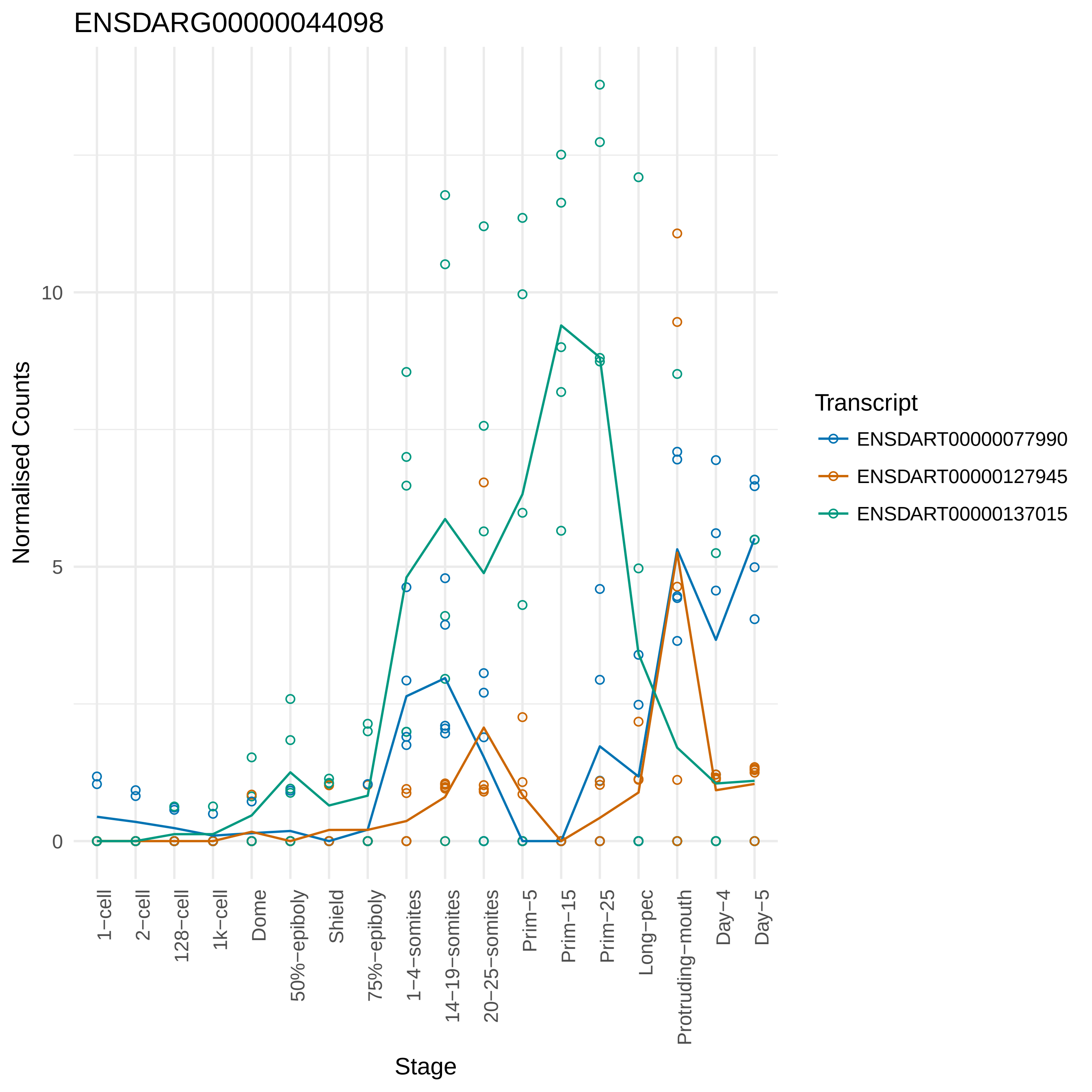
ENSDARG00000044098
Normalised Counts
0
5
10
Stage
1−cell
2−cell
128−cell
1k−cell
Dome
50%−epiboly
Shield
75%−epiboly
1−4−somites
14−19−somites
20−25−somites
Prim−5
Prim−15
Prim−25
Long−pec
Protruding−mouth
Day−4
Day−5
Transcript
ENSDART00000077990
ENSDART00000127945
ENSDART00000137015

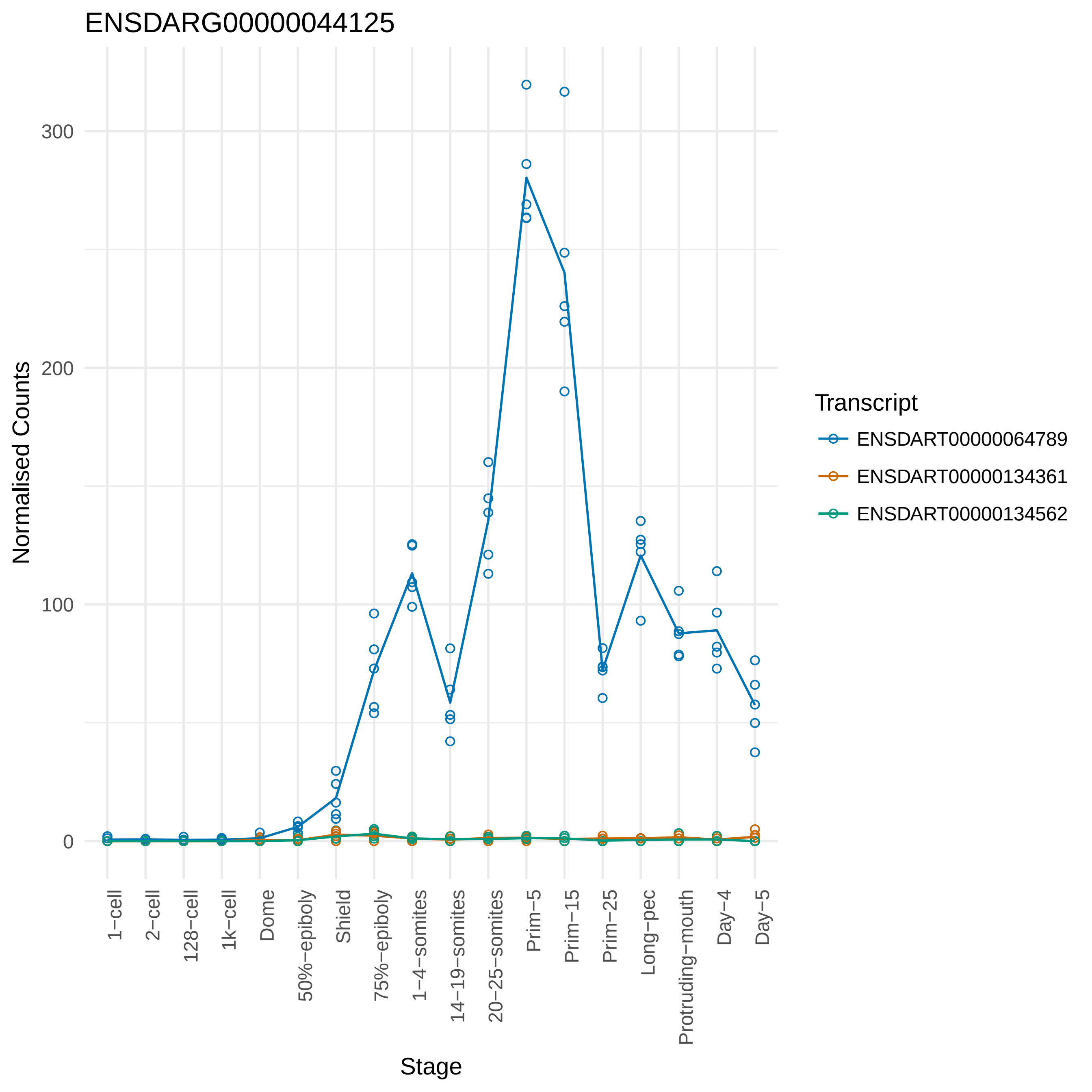
ENSDARG00000044125
Normalised Counts
0
100
200
300
1−cell
2−cell
128−cell
1k−cell
Dome
50%−epiboly
Shield
75%−epiboly
1−4−somites
14−19−somites
20−25−somites
Prim−5
Prim−15
Prim−25
Long−pec
Protruding−mouth
Day−4
Day−5
Stage
Transcript
ENSDART00000064789
ENSDART00000134361
ENSDART00000134562

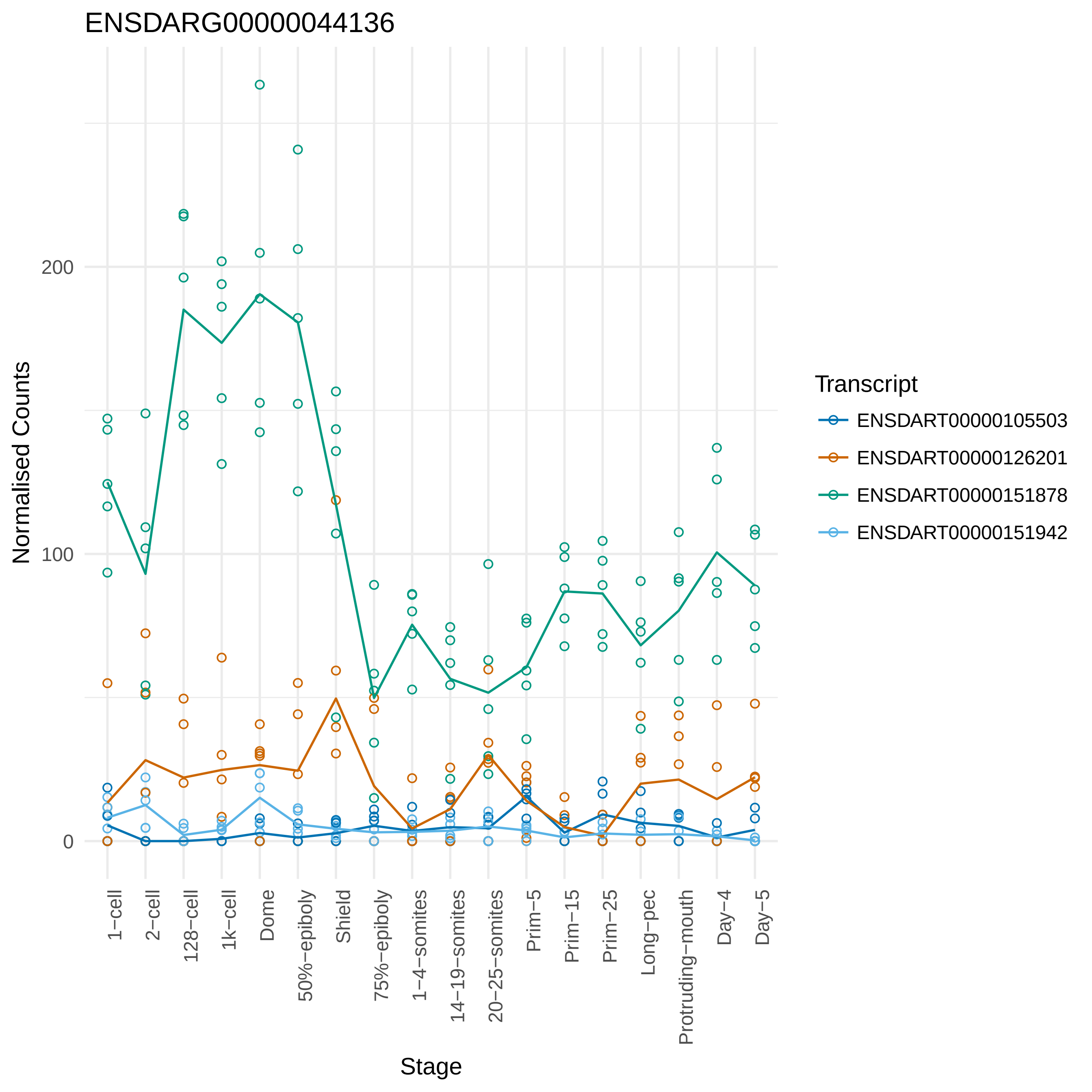
ENSDARG00000044136
Normalised Counts
200
100
0
1−cell
2−cell
128−cell
1k−cell
Dome
50%−epiboly
Shield
75%−epiboly
1−4−somites
14−19−somites
20−25−somites
Prim−5
Prim−15
Prim−25
Long−pec
Protruding−mouth
Day−4
Day−5
Stage
Transcript
ENSDART00000105503
ENSDART00000126201
ENSDART00000151878
ENSDART00000151942

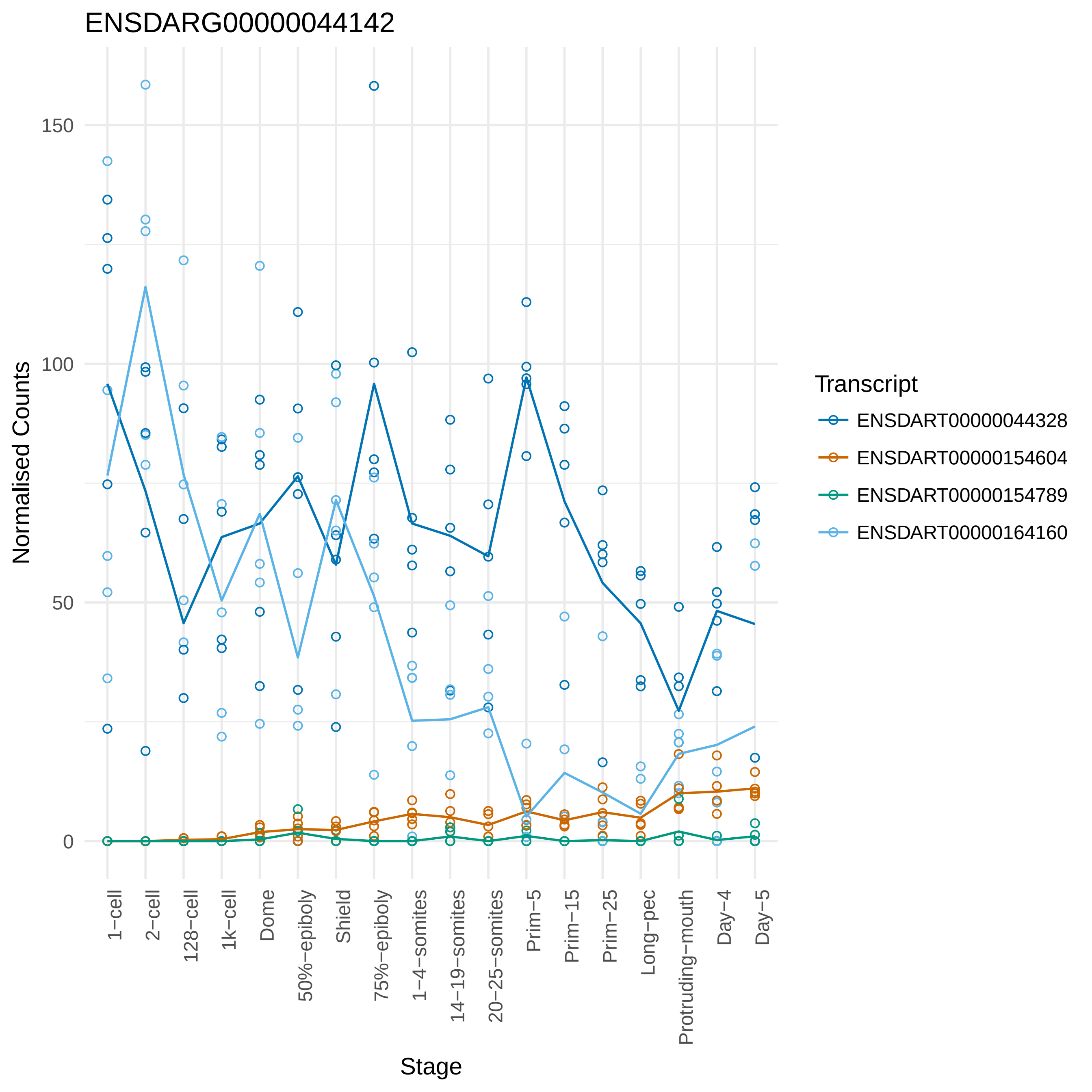

ENSDARG00000044142
Normalised Counts
0
50
100
150
Stage
1−cell
2−cell
128−cell
1k−cell
Dome
50%−epiboly
Shield
75%−epiboly
1−4−somites
14−19−somites
20−25−somites
Prim−5
Prim−15
Prim−25
Long−pec
Protruding−mouth
Day−4
Day−5
Transcript
ENSDART00000044328
ENSDART00000154604
ENSDART00000154789
ENSDART00000164160

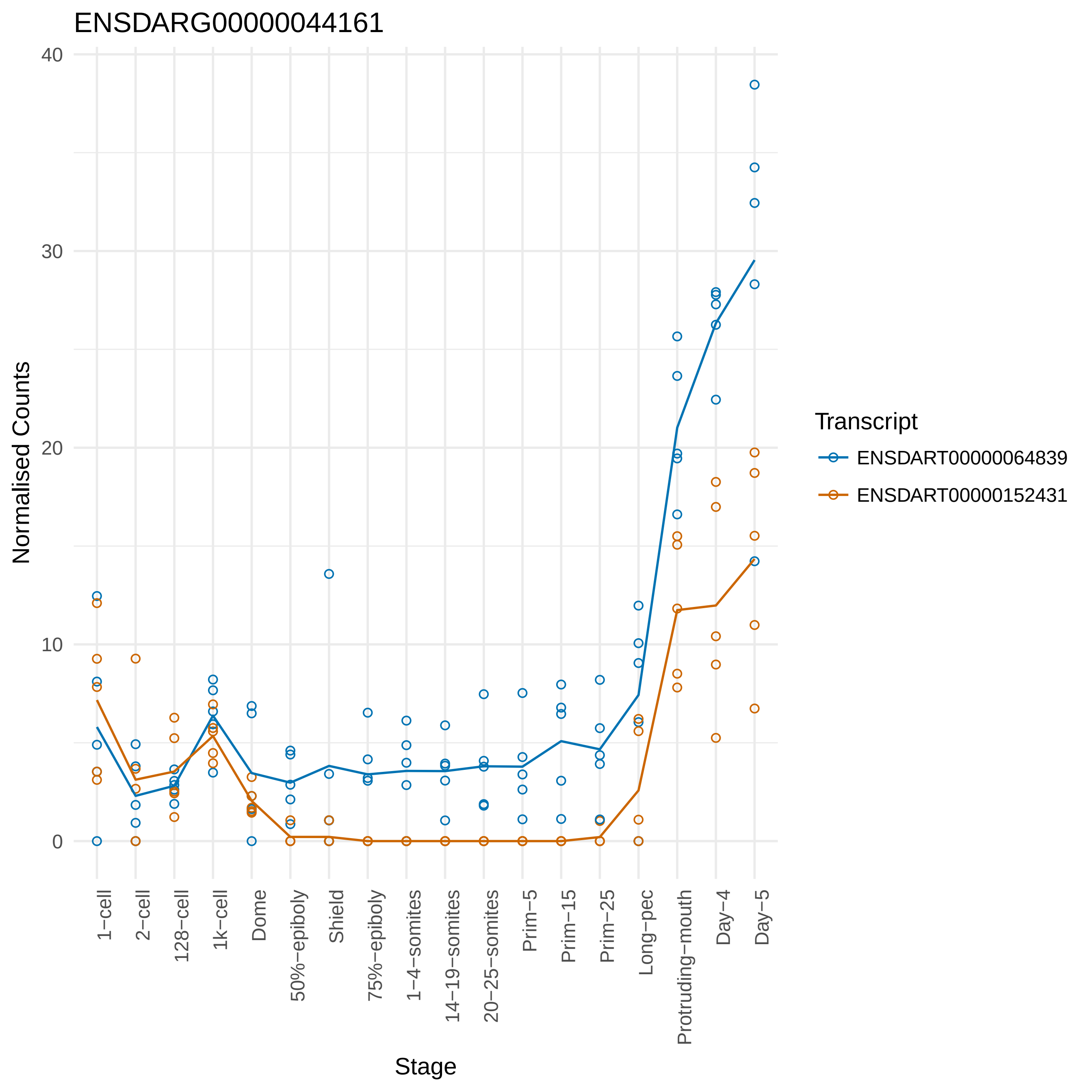
ENSDARG00000044161
Normalised Counts
40
30
20
10
0
1−cell
2−cell
128−cell
1k−cell
Dome
50%−epiboly
Shield
75%−epiboly
1−4−somites
14−19−somites
20−25−somites
Prim−5
Prim−15
Prim−25
Long−pec
Protruding−mouth
Day−4
Day−5
Stage
Transcript
ENSDART00000064839
ENSDART00000152431

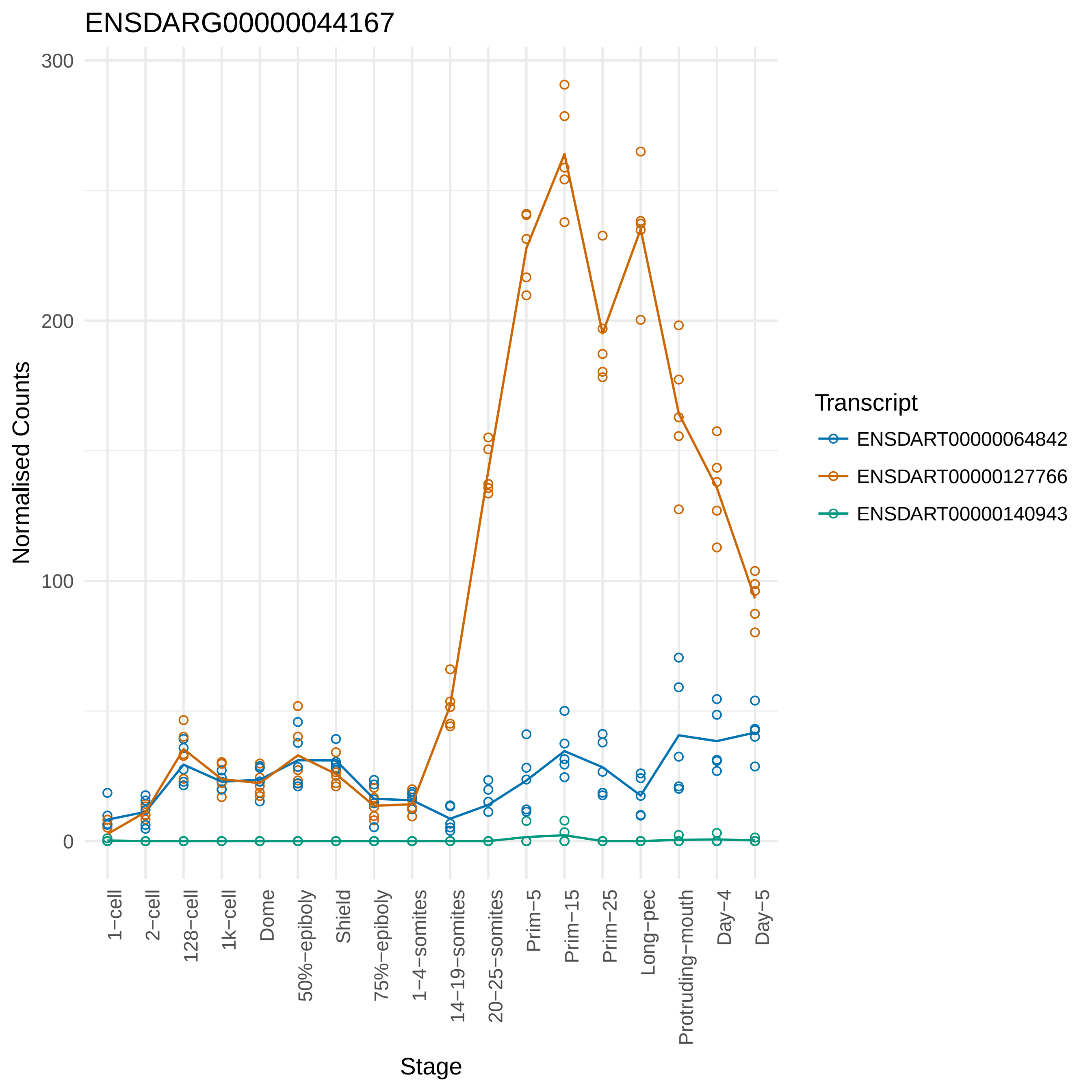
ENSDARG00000044167
300
200
100
0
Normalised Counts
1−cell
2−cell
128−cell
1k−cell
Dome
50%−epiboly
Shield
75%−epiboly
1−4−somites
14−19−somites
20−25−somites
Prim−5
Prim−15
Prim−25
Long−pec
Protruding−mouth
Day−4
Day−5
Stage
Transcript
ENSDART00000064842
ENSDART00000127766
ENSDART00000140943

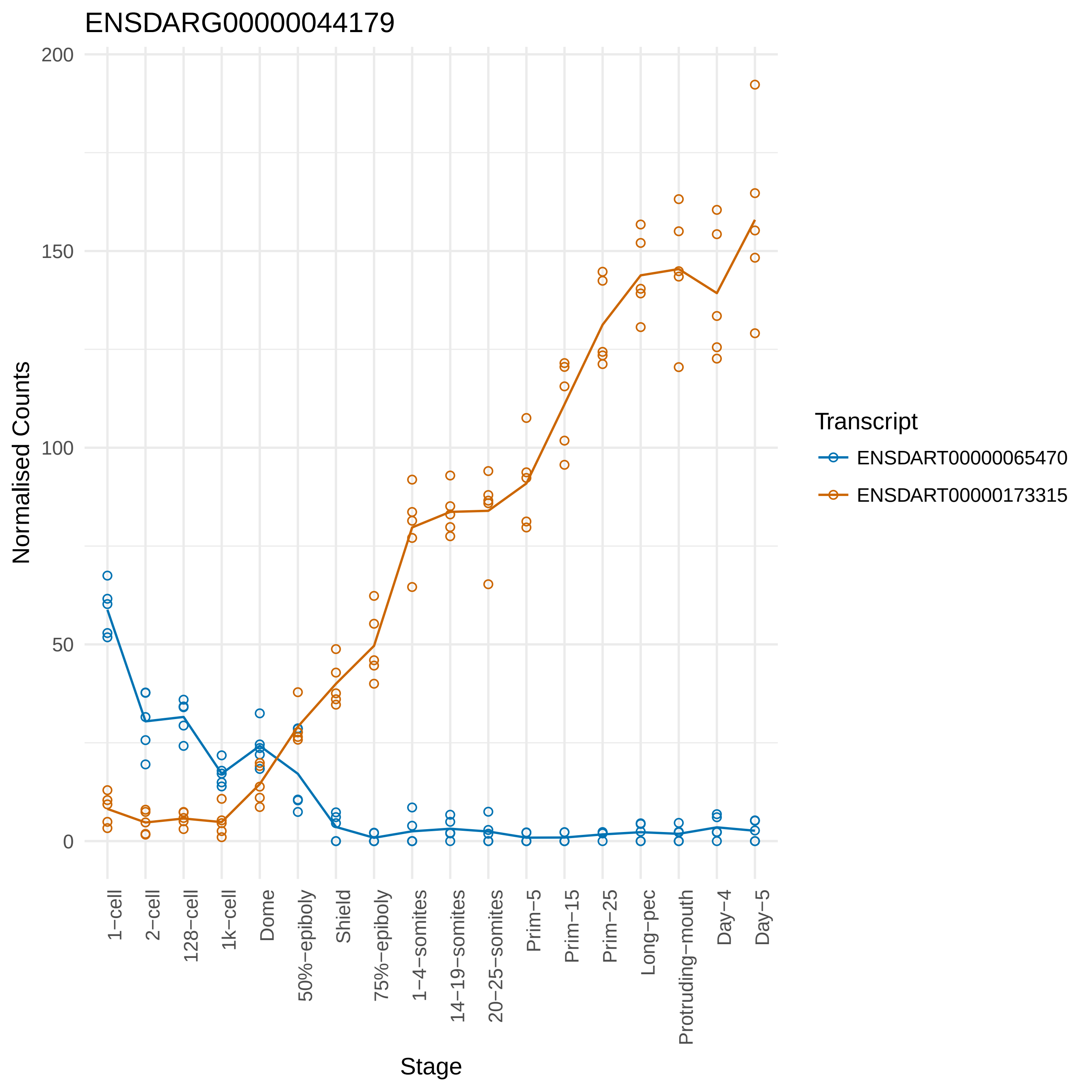
ENSDARG00000044179
Normalised Counts
200
150
100
50
0
1−cell
2−cell
128−cell
1k−cell
Dome
50%−epiboly
Shield
75%−epiboly
1−4−somites
14−19−somites
20−25−somites
Prim−5
Prim−15
Prim−25
Long−pec
Protruding−mouth
Day−4
Day−5
Stage
Transcript
ENSDART00000065470
ENSDART00000173315

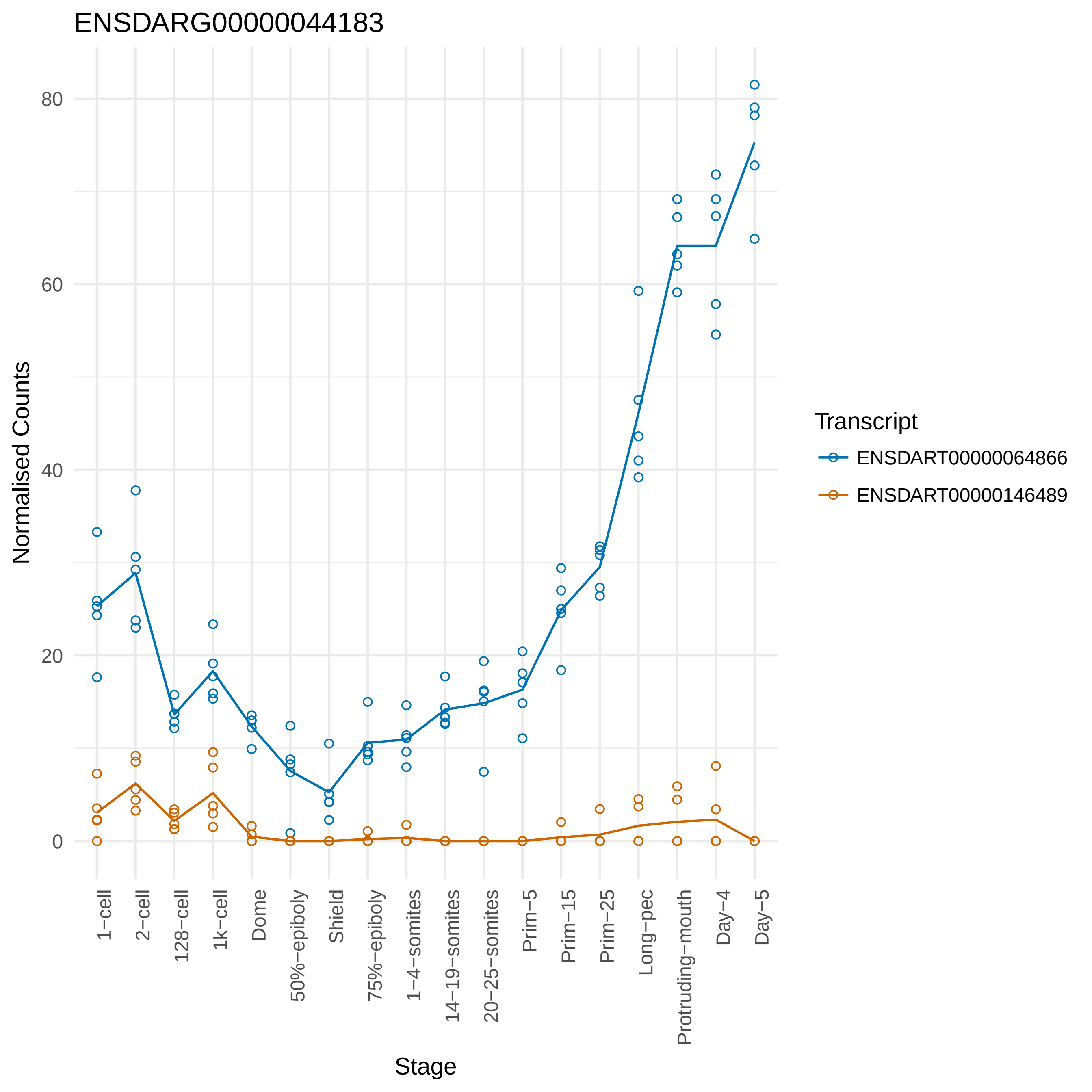

ENSDARG00000044183
Normalised Counts
0
20
40
60
80
1−cell
2−cell
128−cell
1k−cell
Dome
50%−epiboly
Shield
75%−epiboly
1−4−somites
14−19−somites
20−25−somites
Prim−5
Prim−15
Prim−25
Long−pec
Protruding−mouth
Day−4
Day−5
Stage
Transcript
ENSDART00000064866
ENSDART00000146489

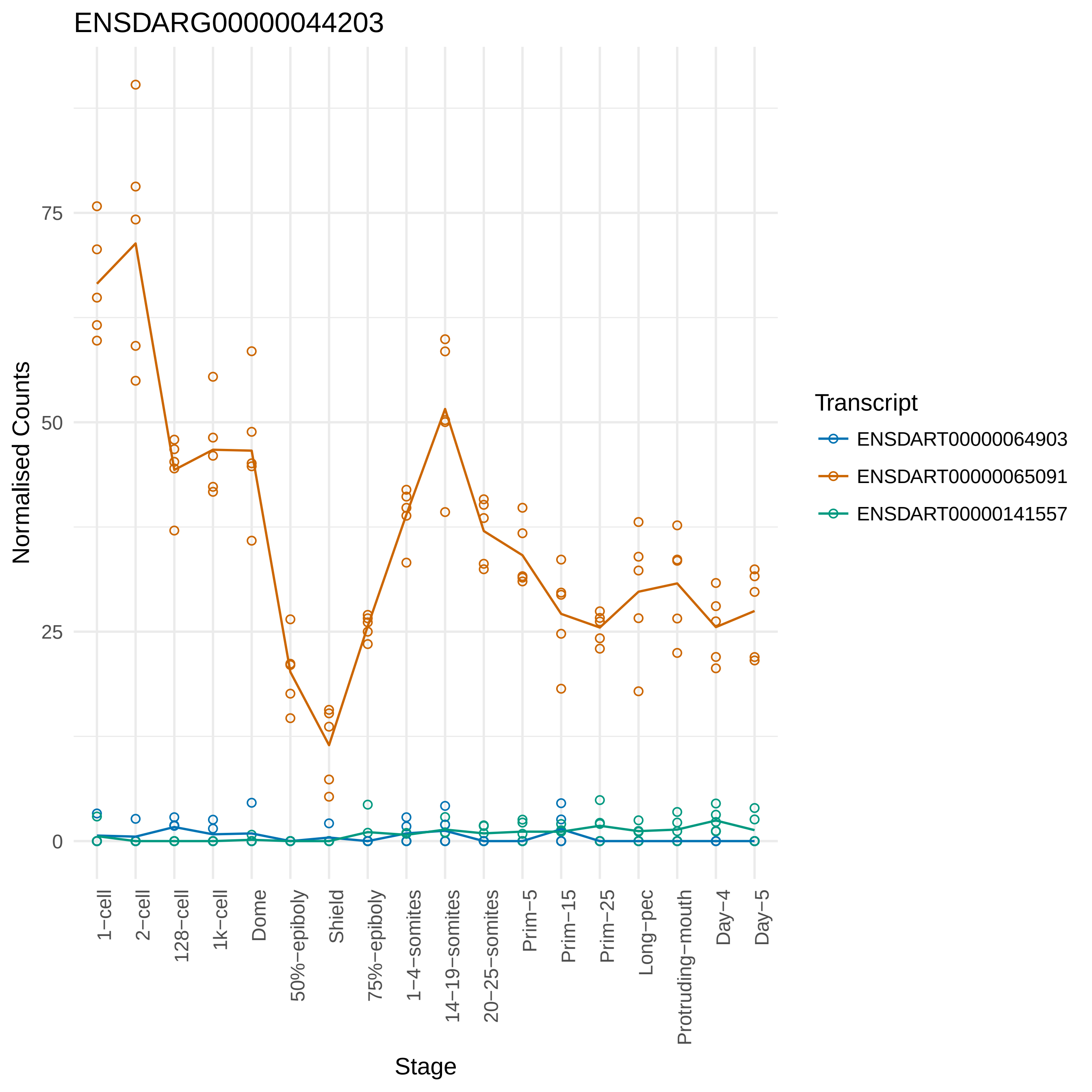
ENSDARG00000044203
Normalised Counts
75
50
25
0
1−cell
2−cell
128−cell
1k−cell
Dome
50%−epiboly
Shield
75%−epiboly
1−4−somites
14−19−somites
20−25−somites
Prim−5
Prim−15
Prim−25
Long−pec
Protruding−mouth
Day−4
Day−5
Stage
Transcript
ENSDART00000064903
ENSDART00000065091
ENSDART00000141557

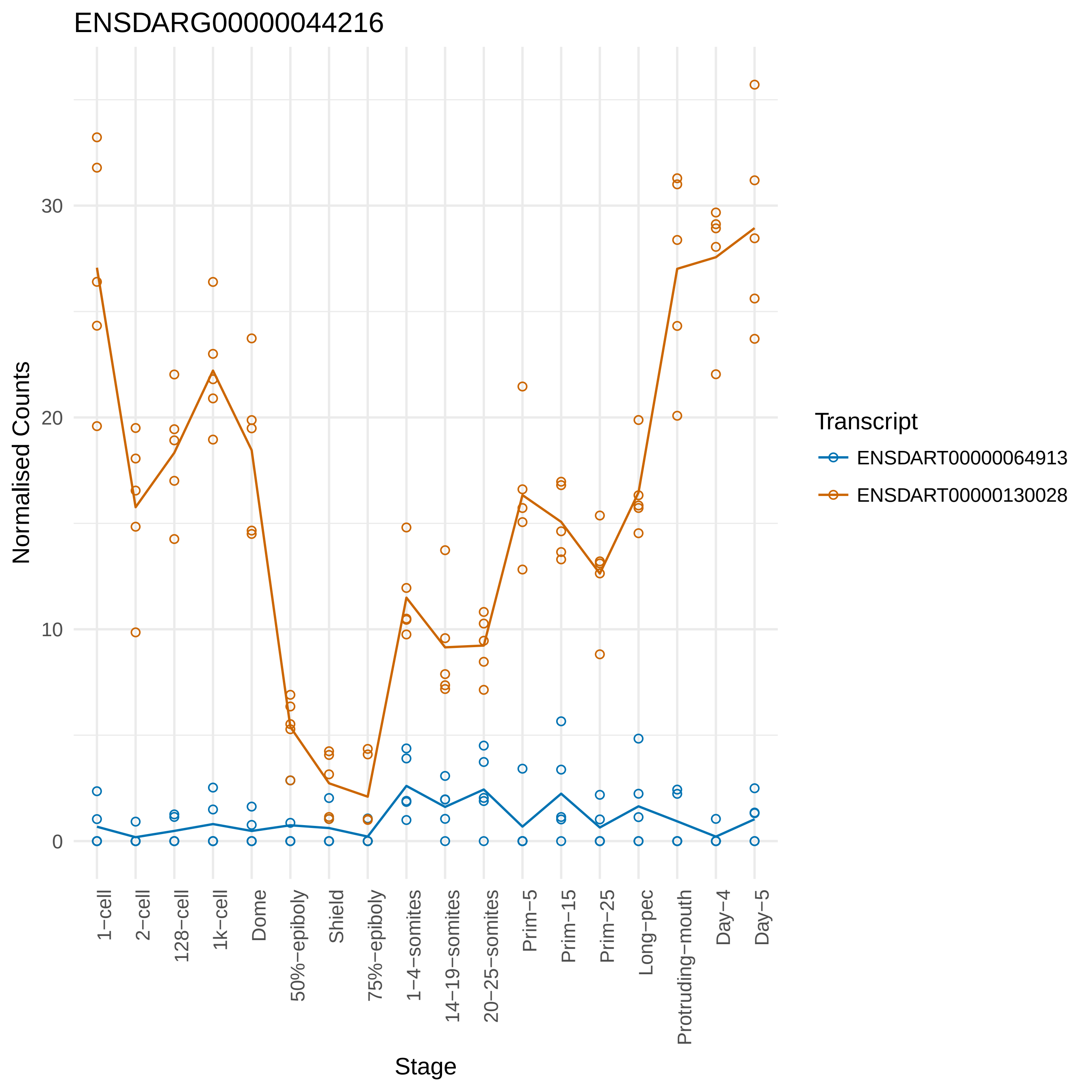
ENSDARG00000044216
Normalised Counts
0
10
20
30
1−cell
2−cell
128−cell
1k−cell
Dome
50%−epiboly
Shield
75%−epiboly
1−4−somites
14−19−somites
20−25−somites
Prim−5
Prim−15
Prim−25
Long−pec
Protruding−mouth
Day−4
Day−5
Stage
Transcript
ENSDART00000064913
ENSDART00000130028

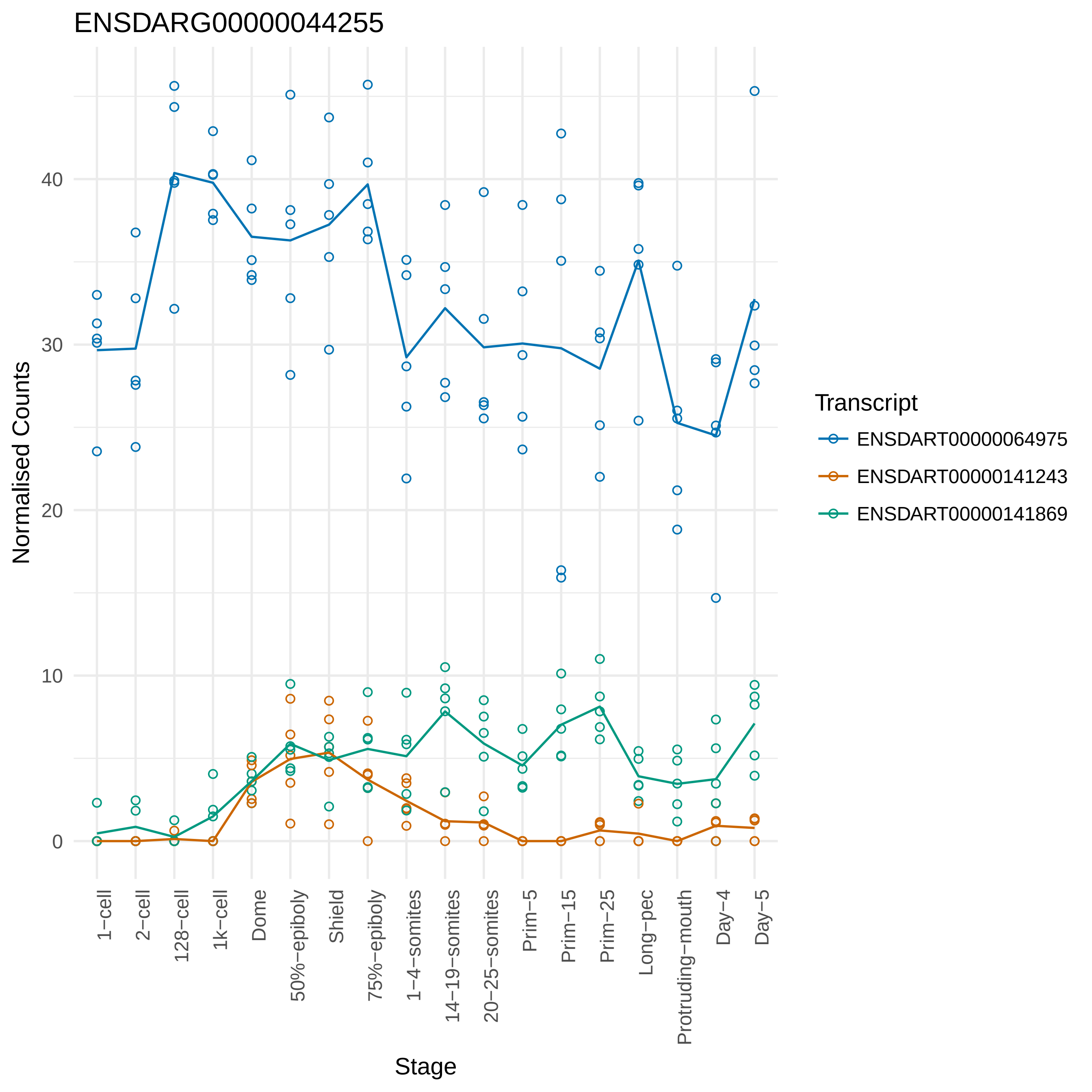

ENSDARG00000044255
Normalised Counts
0
10
20
30
40
1−cell
2−cell
128−cell
1k−cell
Dome
50%−epiboly
Shield
75%−epiboly
1−4−somites
14−19−somites
20−25−somites
Prim−5
Prim−15
Prim−25
Long−pec
Protruding−mouth
Day−4
Day−5
Stage
Transcript
ENSDART00000064975
ENSDART00000141243
ENSDART00000141869

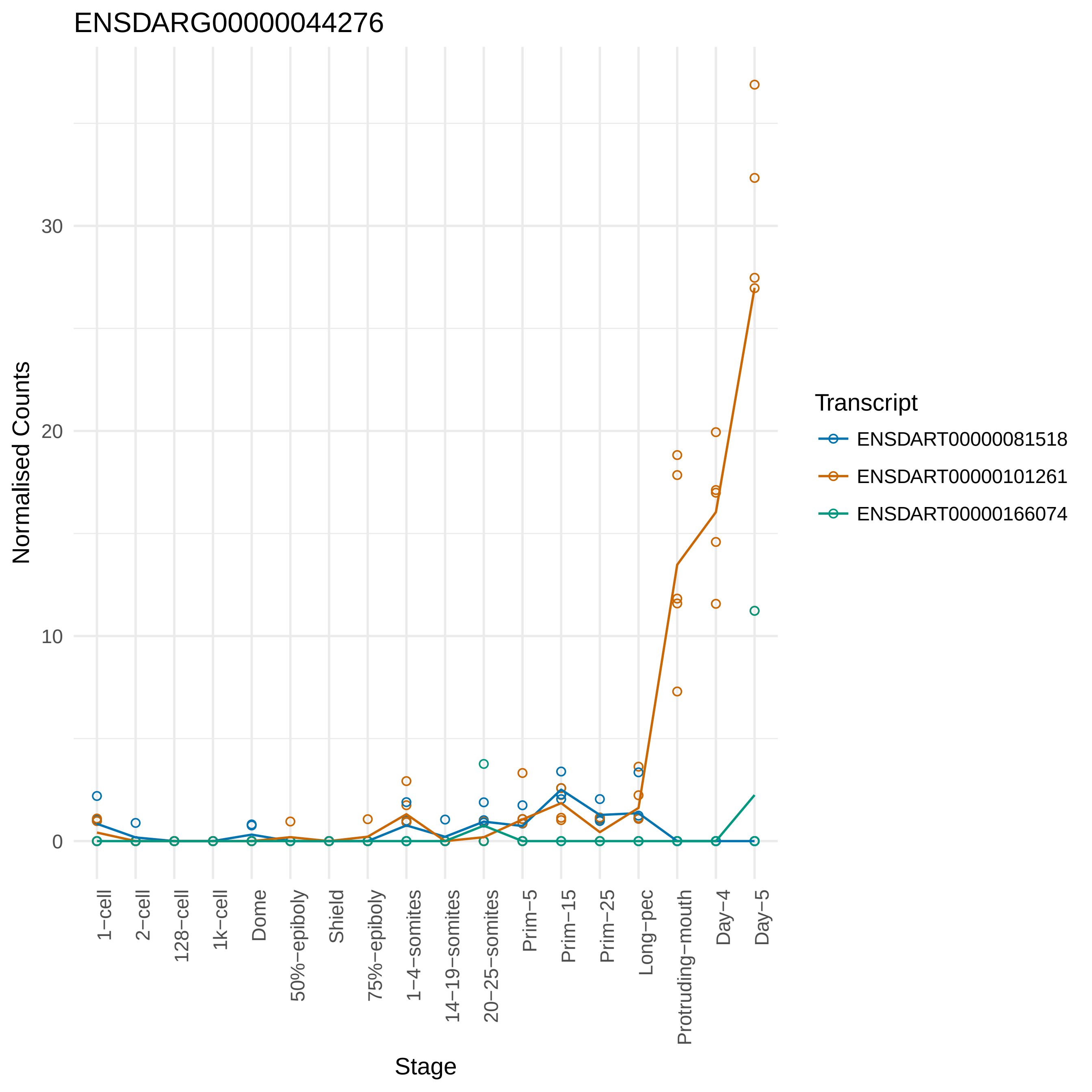
ENSDARG00000044276
Normalised Counts
0
10
20
30
Stage
1−cell
2−cell
128−cell
1k−cell
Dome
50%−epiboly
Shield
75%−epiboly
1−4−somites
14−19−somites
20−25−somites
Prim−5
Prim−15
Prim−25
Long−pec
Protruding−mouth
Day−4
Day−5
Transcript
ENSDART00000081518
ENSDART00000101261
ENSDART00000166074

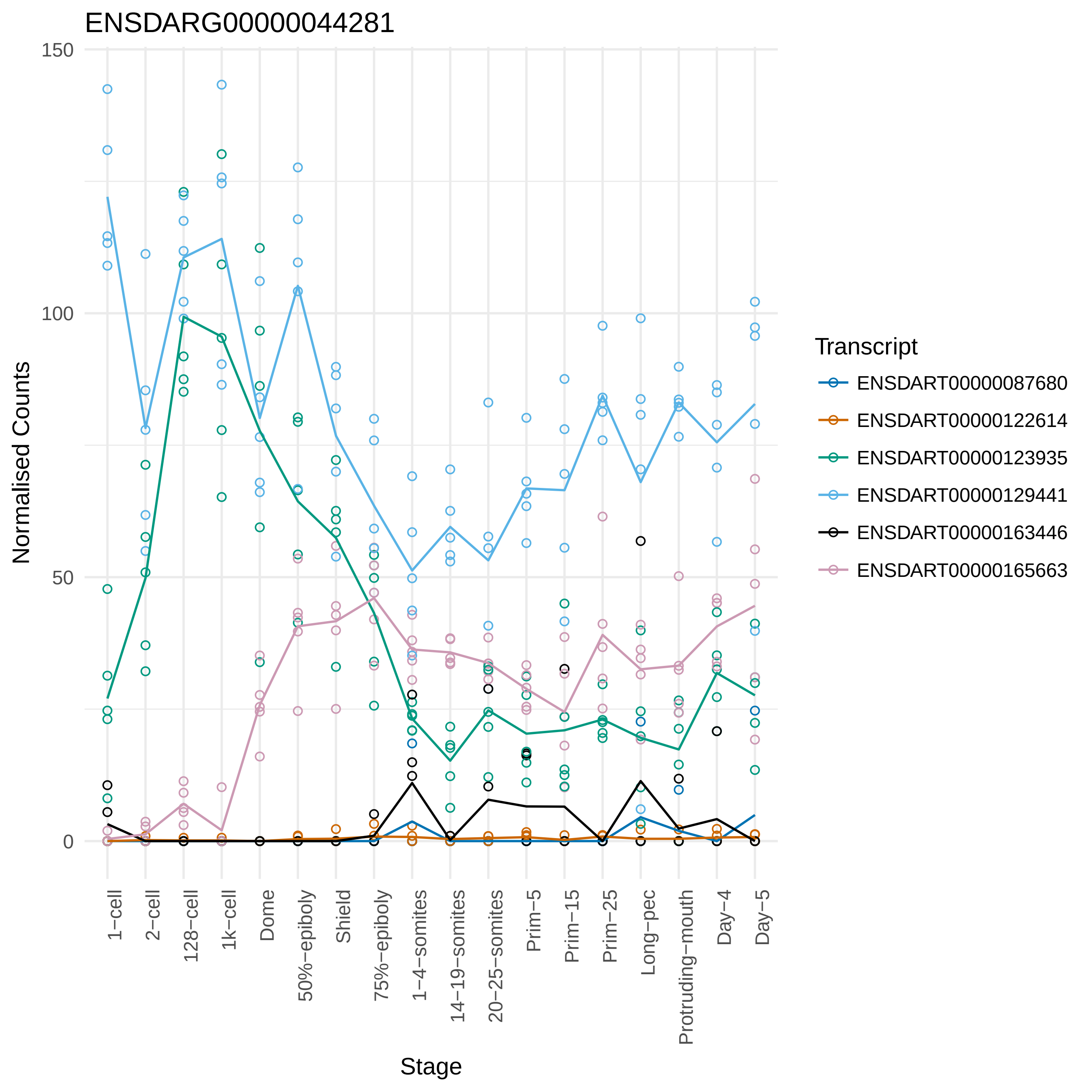
ENSDARG00000044281
Normalised Counts
150
100
50
0
1−cell
2−cell
128−cell
1k−cell
Dome
50%−epiboly
Shield
75%−epiboly
1−4−somites
14−19−somites
20−25−somites
Prim−5
Prim−15
Prim−25
Long−pec
Protruding−mouth
Day−4
Day−5
Stage
Transcript
ENSDART00000087680
ENSDART00000122614
ENSDART00000123935
ENSDART00000129441
ENSDART00000163446
ENSDART00000165663

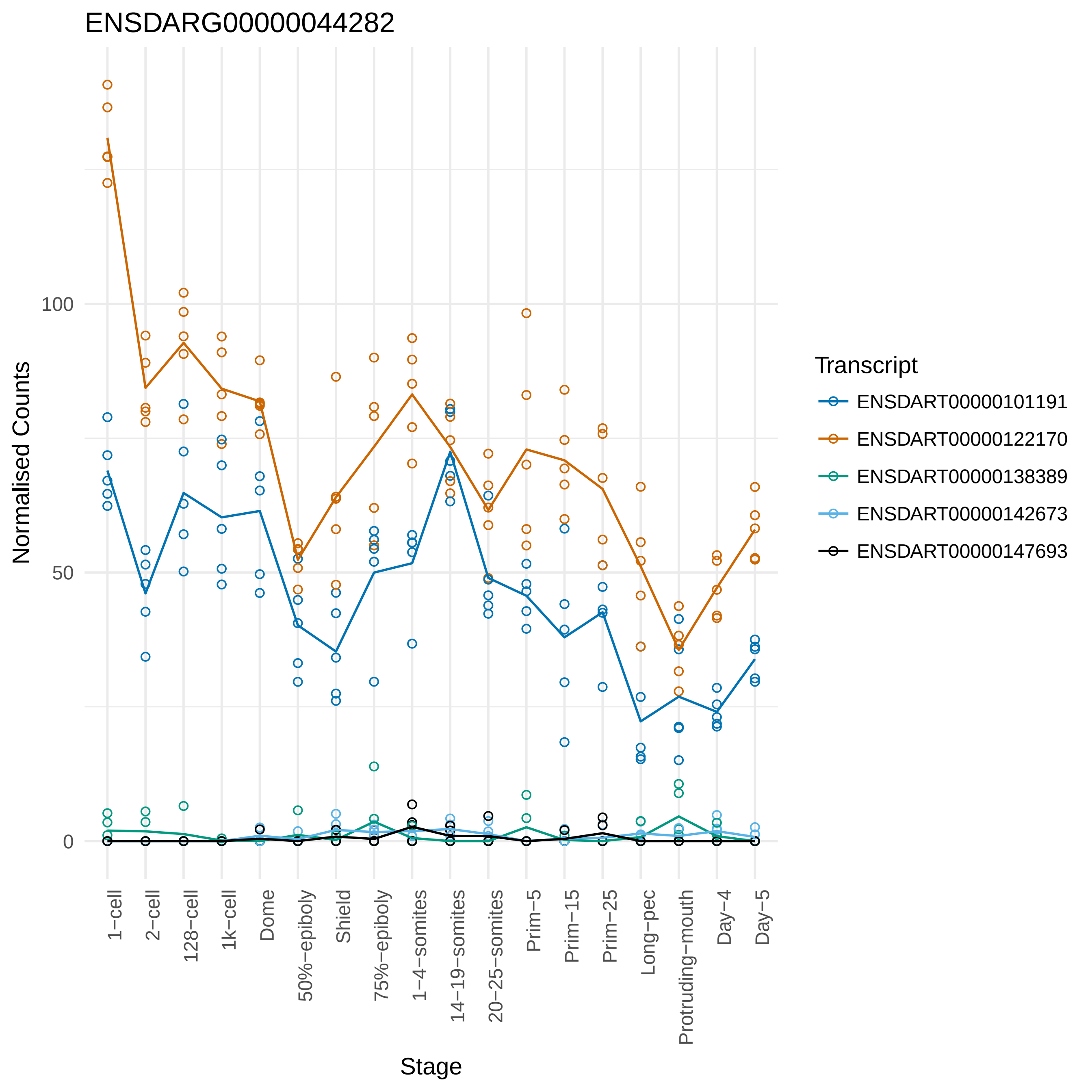

ENSDARG00000044282
Normalised Counts
100
50
0
1−cell
2−cell
128−cell
1k−cell
Dome
50%−epiboly
Shield
75%−epiboly
1−4−somites
14−19−somites
20−25−somites
Prim−5
Prim−15
Prim−25
Long−pec
Protruding−mouth
Day−4
Day−5
Stage
Transcript
ENSDART00000101191
ENSDART00000122170
ENSDART00000138389
ENSDART00000142673
ENSDART00000147693

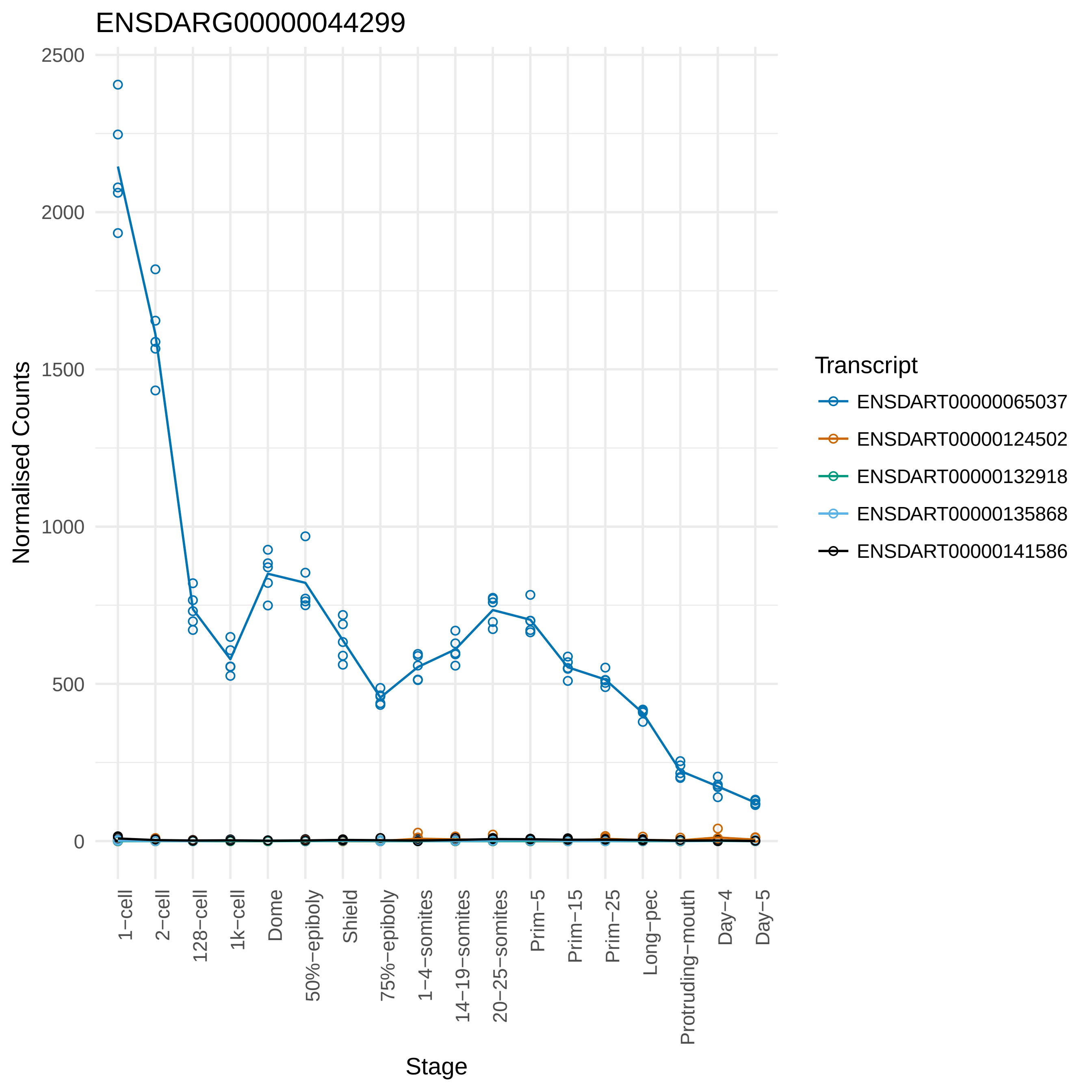

ENSDARG00000044299
Normalised Counts
2500
2000
1500
1000
500
0
Stage
1−cell
2−cell
128−cell
1k−cell
Dome
50%−epiboly
Shield
75%−epiboly
1−4−somites
14−19−somites
20−25−somites
Prim−5
Prim−15
Prim−25
Long−pec
Protruding−mouth
Day−4
Day−5
Transcript
ENSDART00000065037
ENSDART00000124502
ENSDART00000132918
ENSDART00000135868
ENSDART00000141586

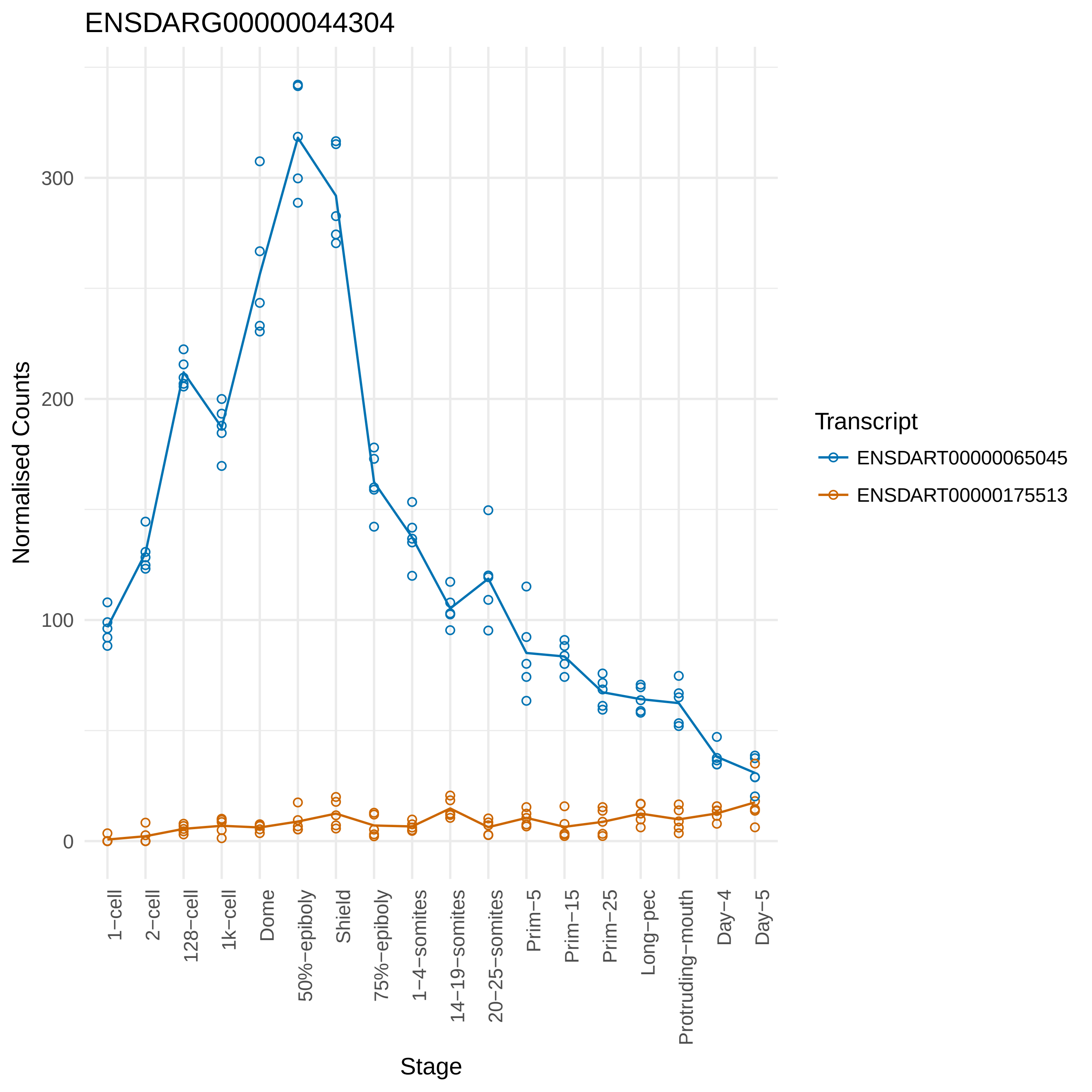

ENSDARG00000044304
Normalised Counts
0
100
200
300
Stage
1−cell
2−cell
128−cell
1k−cell
Dome
50%−epiboly
Shield
75%−epiboly
1−4−somites
14−19−somites
20−25−somites
Prim−5
Prim−15
Prim−25
Long−pec
Protruding−mouth
Day−4
Day−5
Transcript
ENSDART00000065045
ENSDART00000175513

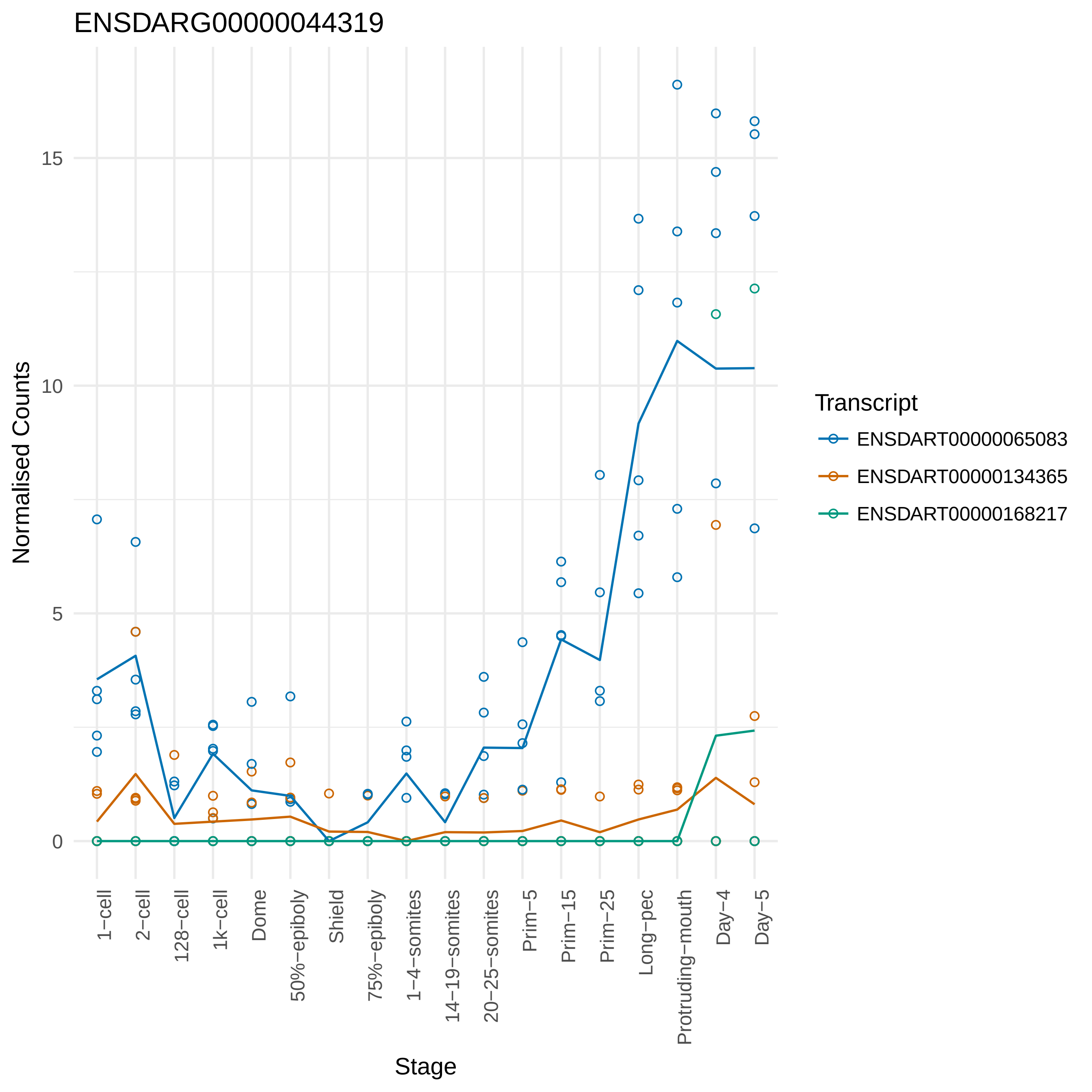

ENSDARG00000044319
Normalised Counts
15
10
5
0
1−cell
2−cell
128−cell
1k−cell
Dome
50%−epiboly
Shield
75%−epiboly
1−4−somites
14−19−somites
20−25−somites
Prim−5
Prim−15
Prim−25
Long−pec
Protruding−mouth
Day−4
Day−5
Stage
Transcript
ENSDART00000065083
ENSDART00000134365
ENSDART00000168217

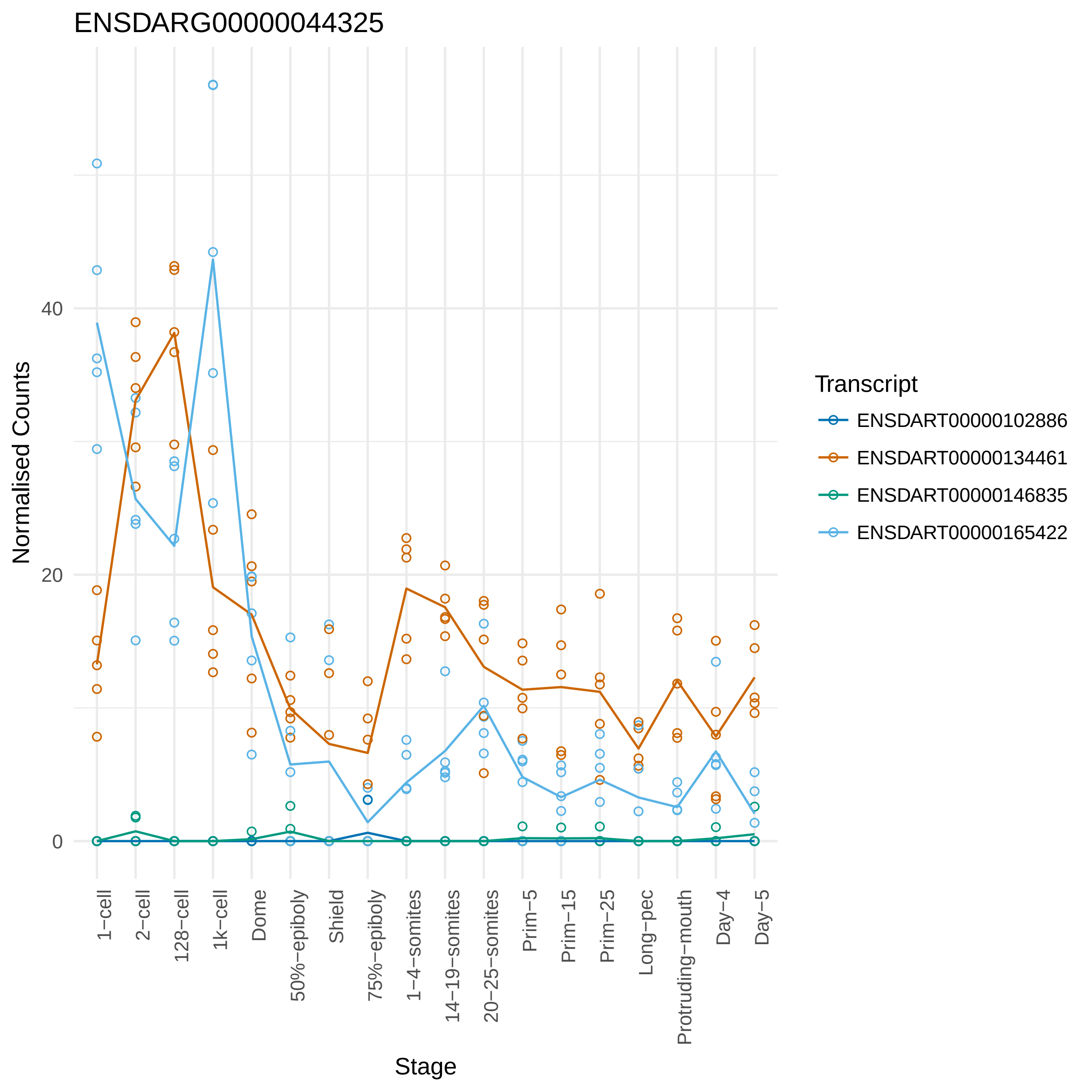
ENSDARG00000044325
Normalised Counts
0
20
40
Stage
1−cell
2−cell
128−cell
1k−cell
Dome
50%−epiboly
Shield
75%−epiboly
1−4−somites
14−19−somites
20−25−somites
Prim−5
Prim−15
Prim−25
Long−pec
Protruding−mouth
Day−4
Day−5
Transcript
ENSDART00000102886
ENSDART00000134461
ENSDART00000146835
ENSDART00000165422

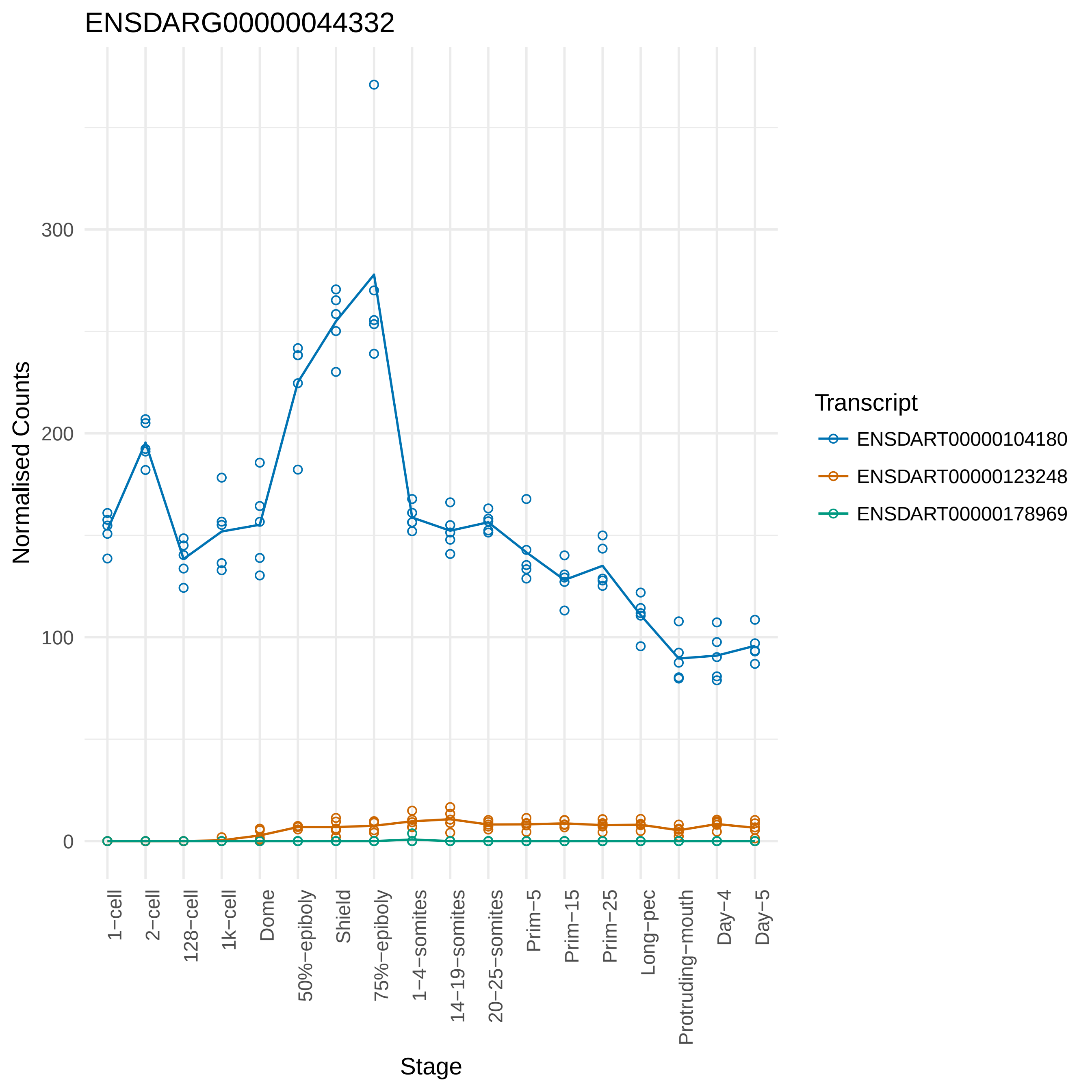

ENSDARG00000044332
Normalised Counts
300
200
100
0
1−cell
2−cell
128−cell
1k−cell
Dome
50%−epiboly
Shield
75%−epiboly
1−4−somites
14−19−somites
20−25−somites
Prim−5
Prim−15
Prim−25
Long−pec
Protruding−mouth
Day−4
Day−5
Stage
Transcript
ENSDART00000104180
ENSDART00000123248
ENSDART00000178969

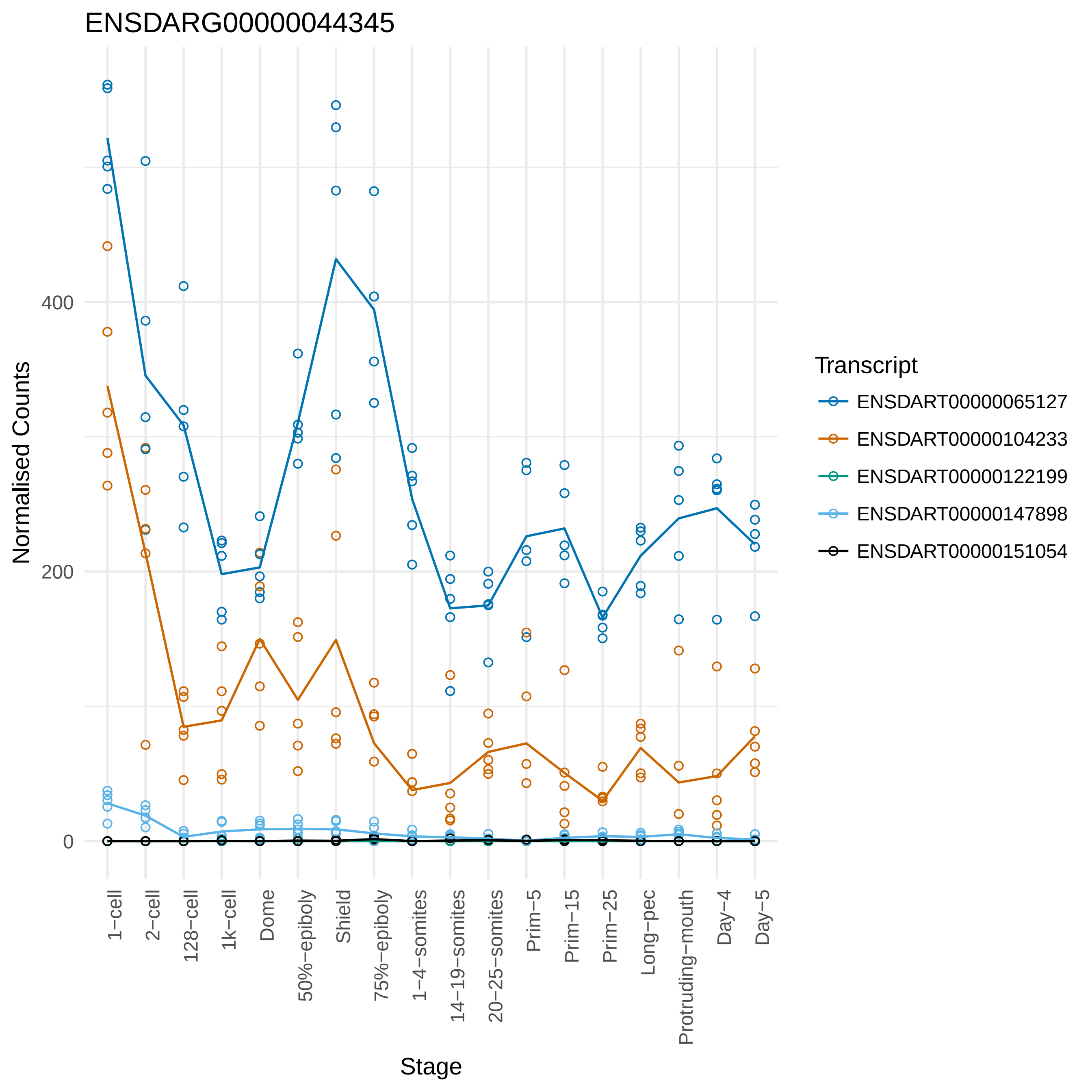
ENSDARG00000044345
Normalised Counts
0
200
400
Stage
1−cell
2−cell
128−cell
1k−cell
Dome
50%−epiboly
Shield
75%−epiboly
1−4−somites
14−19−somites
20−25−somites
Prim−5
Prim−15
Prim−25
Long−pec
Protruding−mouth
Day−4
Day−5
Transcript
ENSDART00000065127
ENSDART00000104233
ENSDART00000122199
ENSDART00000147898
ENSDART00000151054

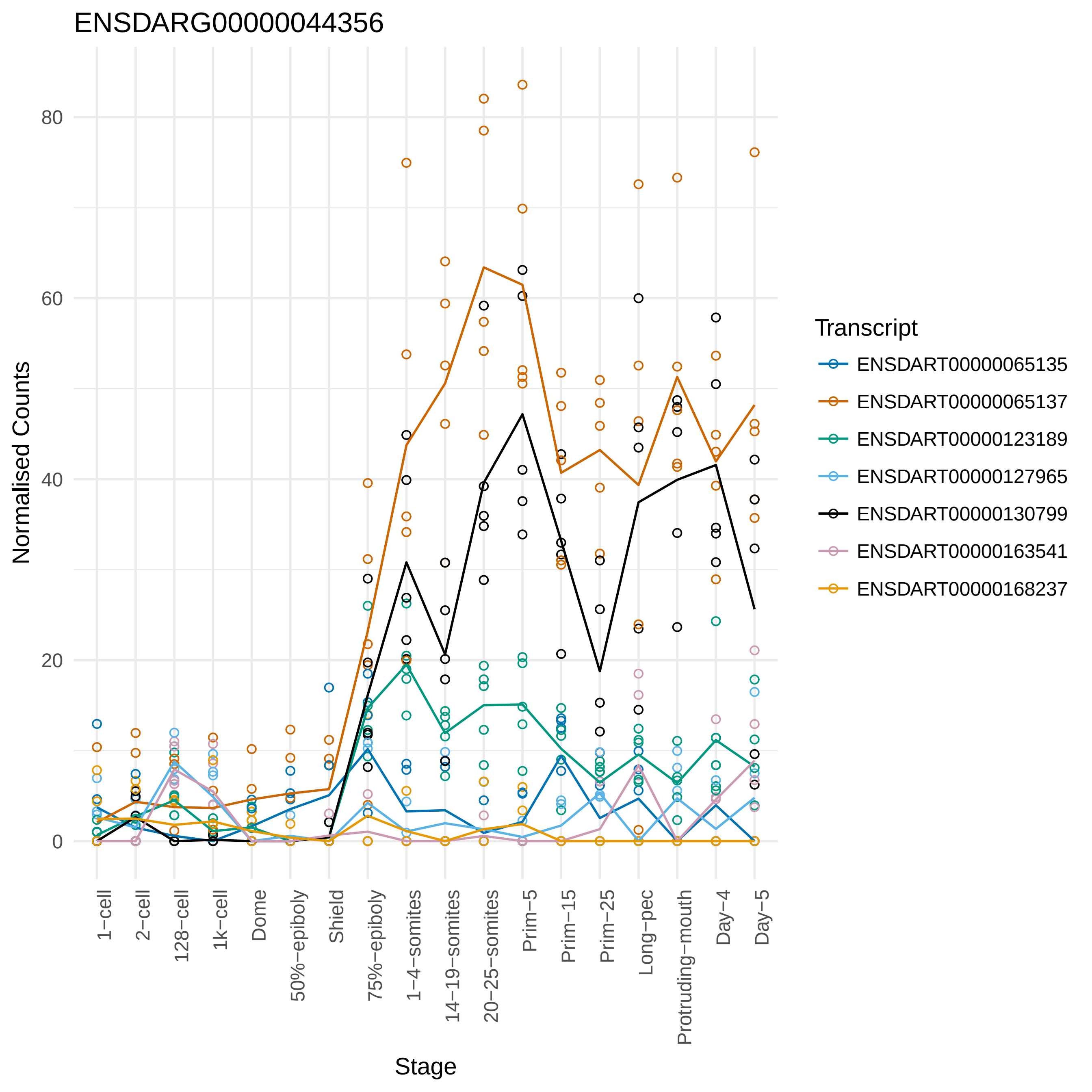

ENSDARG00000044356
Normalised Counts
0
20
40
60
80
Stage
1−cell
2−cell
128−cell
1k−cell
Dome
50%−epiboly
Shield
75%−epiboly
1−4−somites
14−19−somites
20−25−somites
Prim−5
Prim−15
Prim−25
Long−pec
Protruding−mouth
Day−4
Day−5
Transcript
ENSDART00000065135
ENSDART00000065137
ENSDART00000123189
ENSDART00000127965
ENSDART00000130799
ENSDART00000163541
ENSDART00000168237

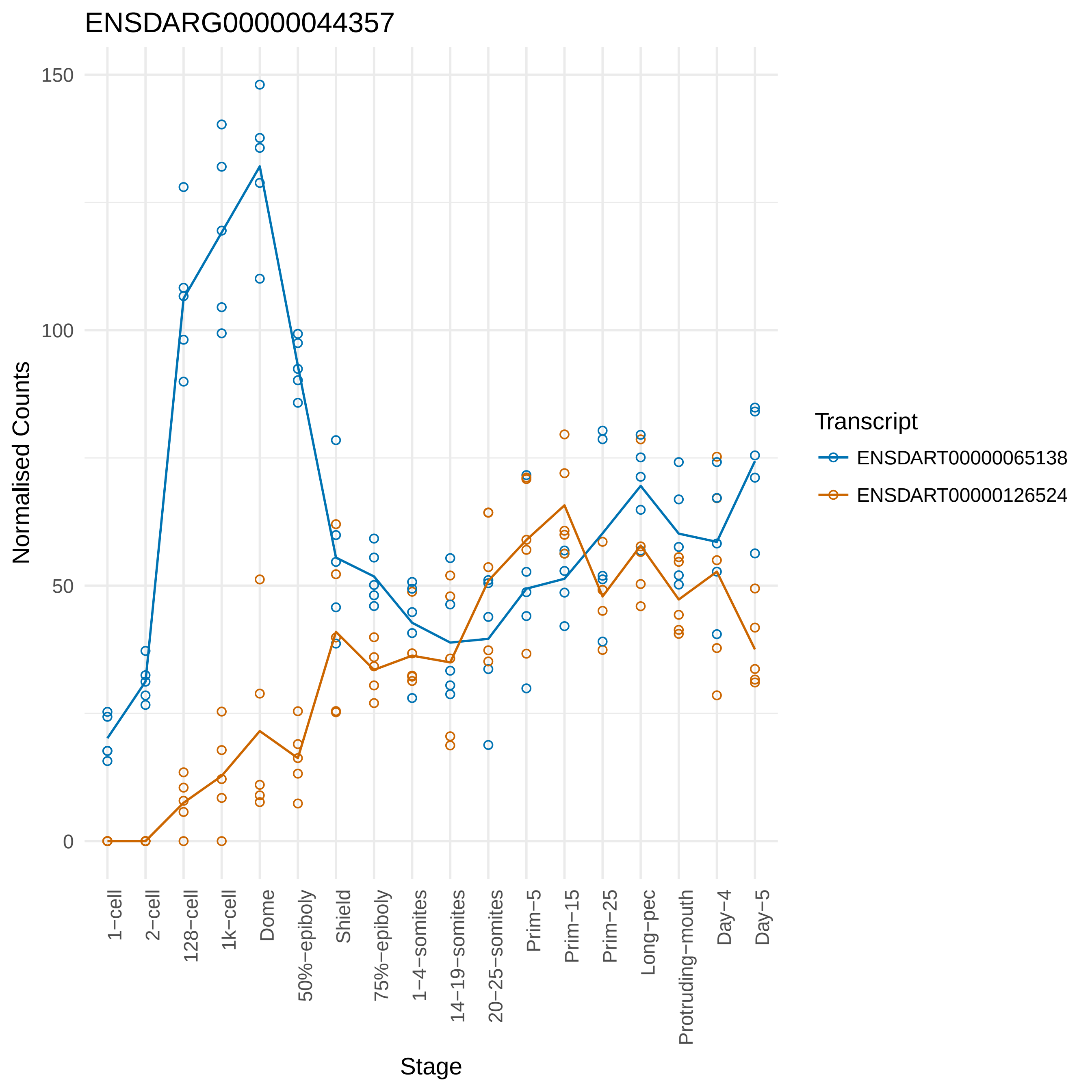

ENSDARG00000044357
150
100
50
0
Normalised Counts
1−cell
2−cell
128−cell
1k−cell
Dome
50%−epiboly
Shield
75%−epiboly
1−4−somites
14−19−somites
20−25−somites
Prim−5
Prim−15
Prim−25
Long−pec
Protruding−mouth
Day−4
Day−5
Stage
Transcript
ENSDART00000065138
ENSDART00000126524

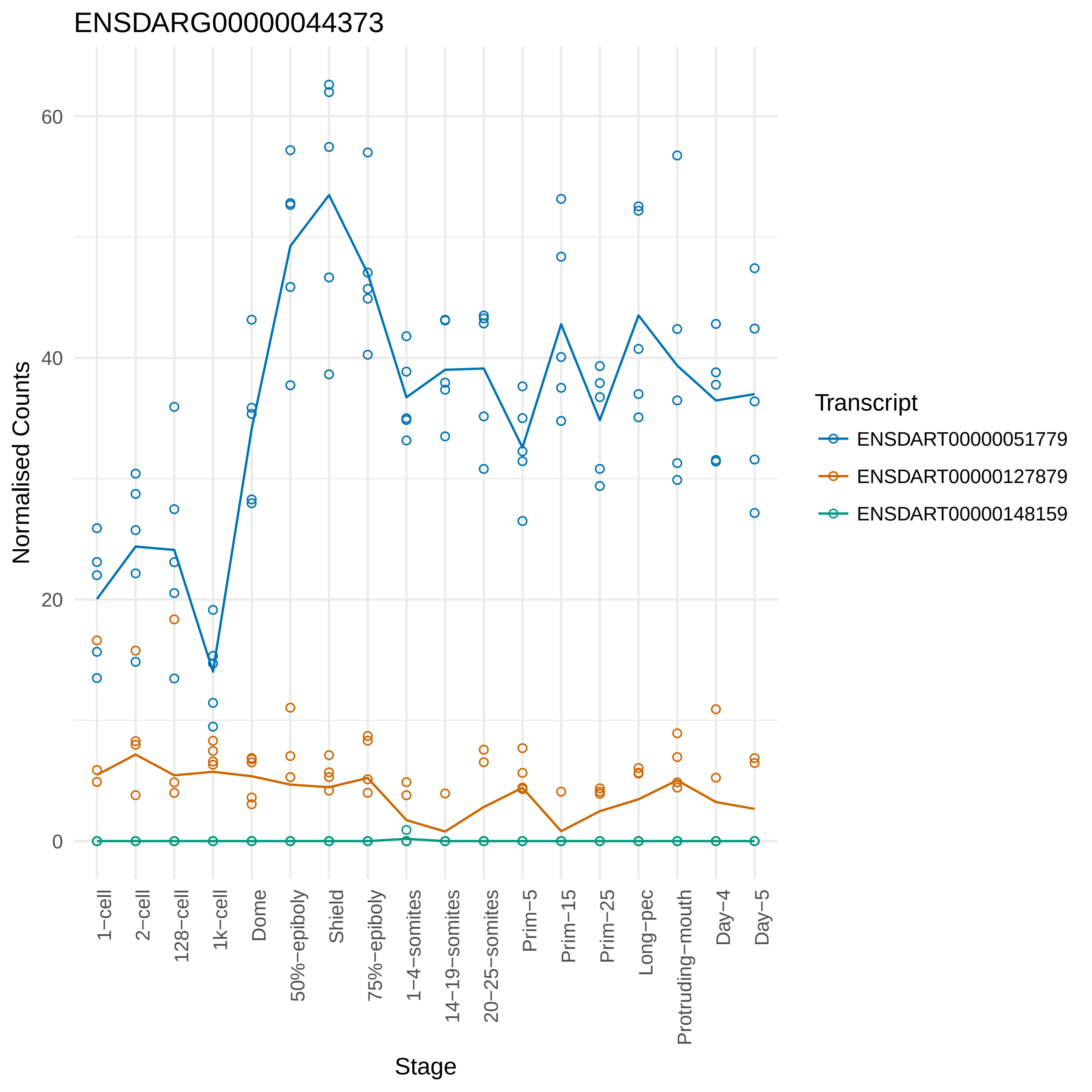
ENSDARG00000044373
Normalised Counts
60
40
20
0
Stage
1−cell
2−cell
128−cell
1k−cell
Dome
50%−epiboly
Shield
75%−epiboly
1−4−somites
14−19−somites
20−25−somites
Prim−5
Prim−15
Prim−25
Long−pec
Protruding−mouth
Day−4
Day−5
Transcript
ENSDART00000051779
ENSDART00000127879
ENSDART00000148159

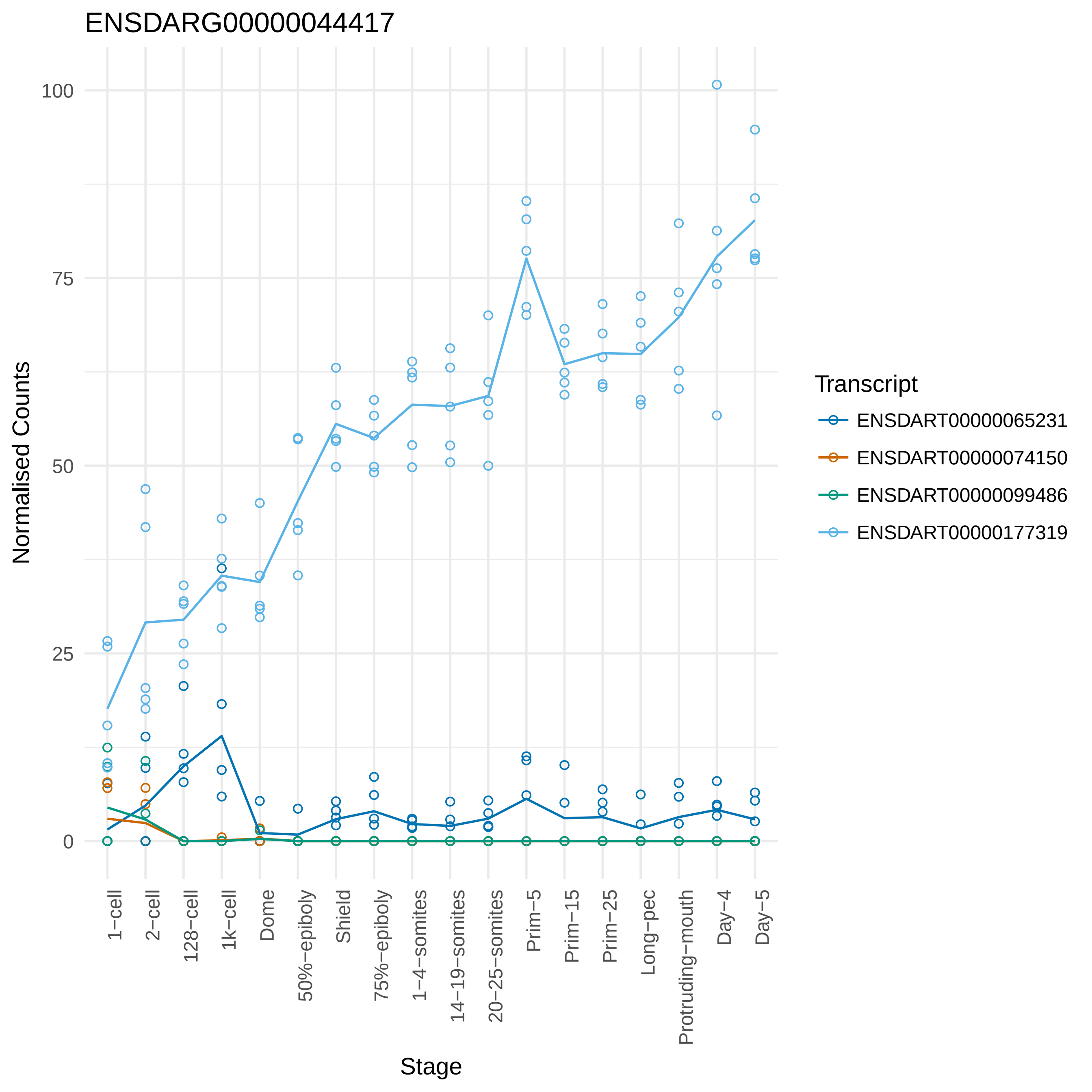

ENSDARG00000044417
Normalised Counts
100
75
50
25
0
1−cell
2−cell
128−cell
1k−cell
Dome
50%−epiboly
Shield
75%−epiboly
1−4−somites
14−19−somites
20−25−somites
Prim−5
Prim−15
Prim−25
Long−pec
Protruding−mouth
Day−4
Day−5
Stage
Transcript
ENSDART00000065231
ENSDART00000074150
ENSDART00000099486
ENSDART00000177319

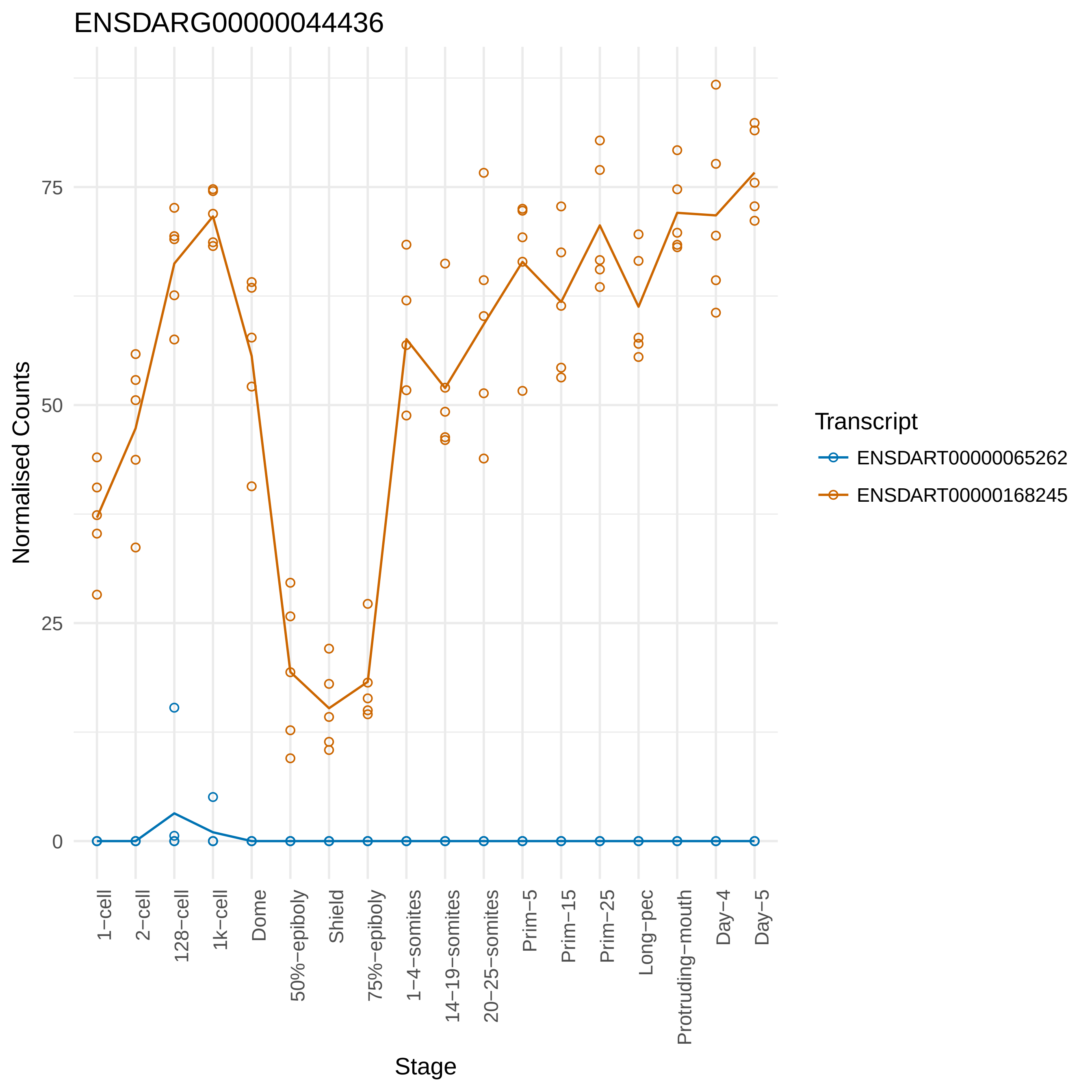

ENSDARG00000044436
Normalised Counts
0
25
50
75
Stage
1−cell
2−cell
128−cell
1k−cell
Dome
50%−epiboly
Shield
75%−epiboly
1−4−somites
14−19−somites
20−25−somites
Prim−5
Prim−15
Prim−25
Long−pec
Protruding−mouth
Day−4
Day−5
Transcript
ENSDART00000065262
ENSDART00000168245

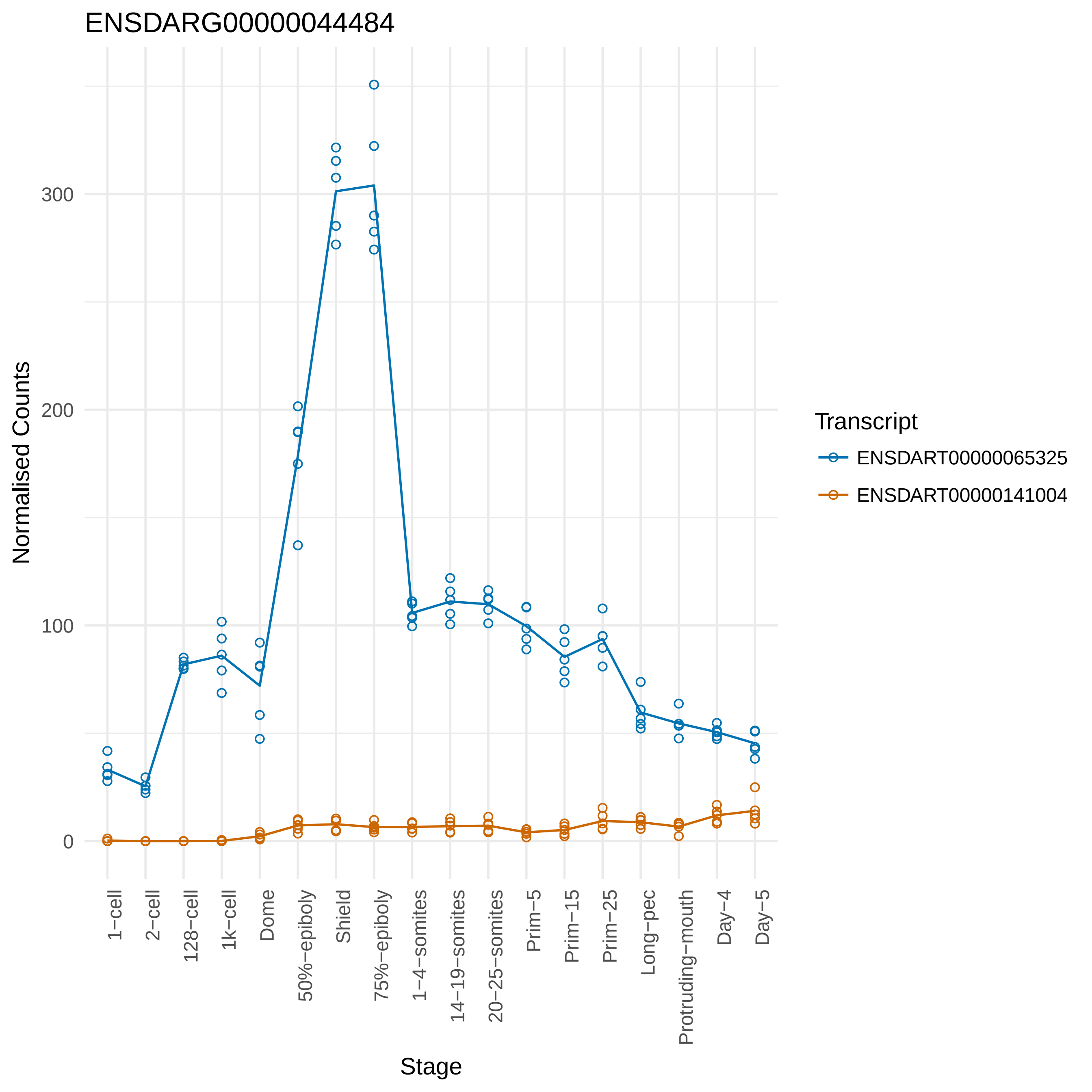

ENSDARG00000044484
Normalised Counts
300
200
100
0
1−cell
2−cell
128−cell
1k−cell
Dome
50%−epiboly
Shield
75%−epiboly
1−4−somites
14−19−somites
20−25−somites
Prim−5
Prim−15
Prim−25
Long−pec
Protruding−mouth
Day−4
Day−5
Stage
Transcript
ENSDART00000065325
ENSDART00000141004

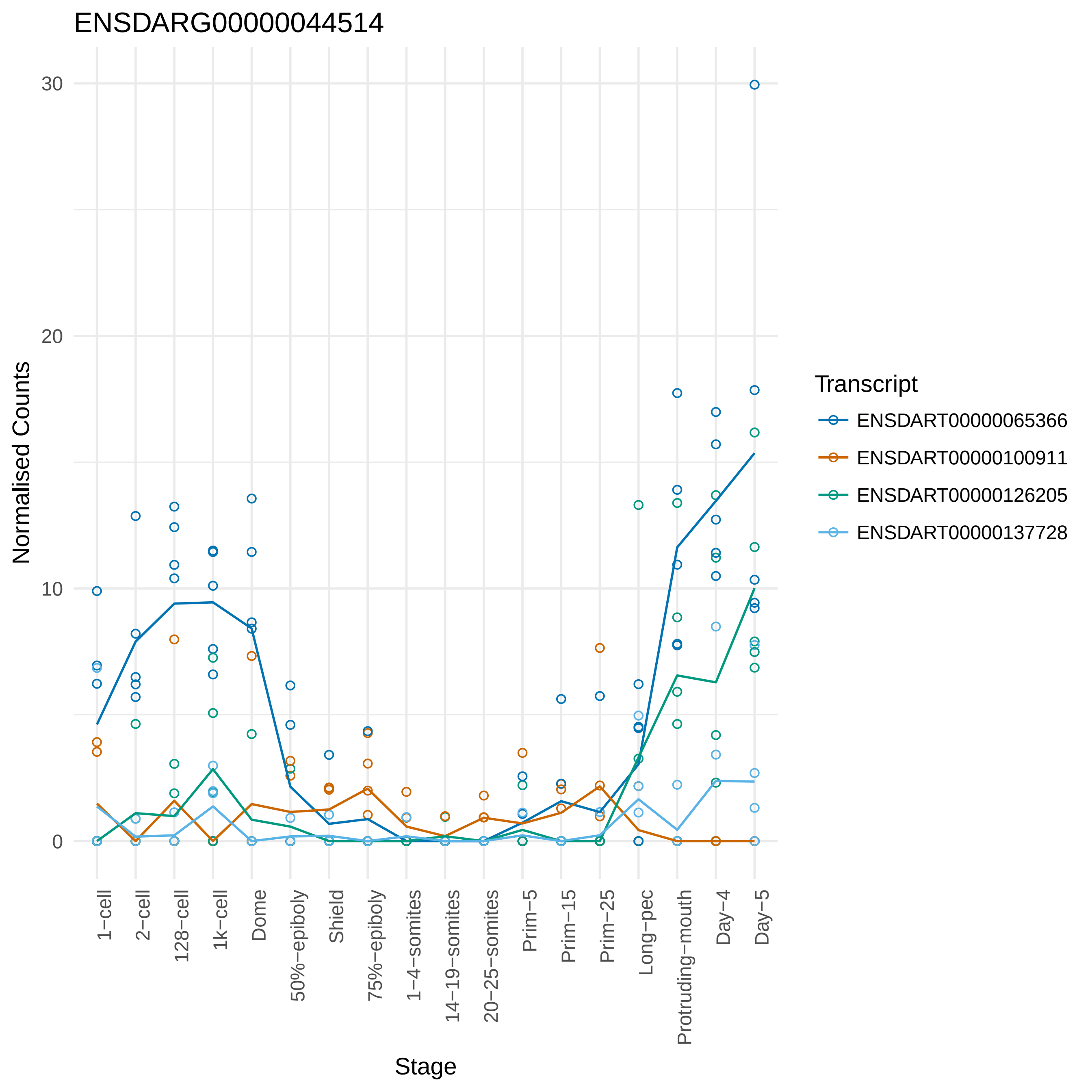

ENSDARG00000044514
Normalised Counts
30
20
10
0
1−cell
2−cell
128−cell
1k−cell
Dome
50%−epiboly
Shield
75%−epiboly
1−4−somites
14−19−somites
20−25−somites
Prim−5
Prim−15
Prim−25
Long−pec
Protruding−mouth
Day−4
Day−5
Stage
Transcript
ENSDART00000065366
ENSDART00000100911
ENSDART00000126205
ENSDART00000137728

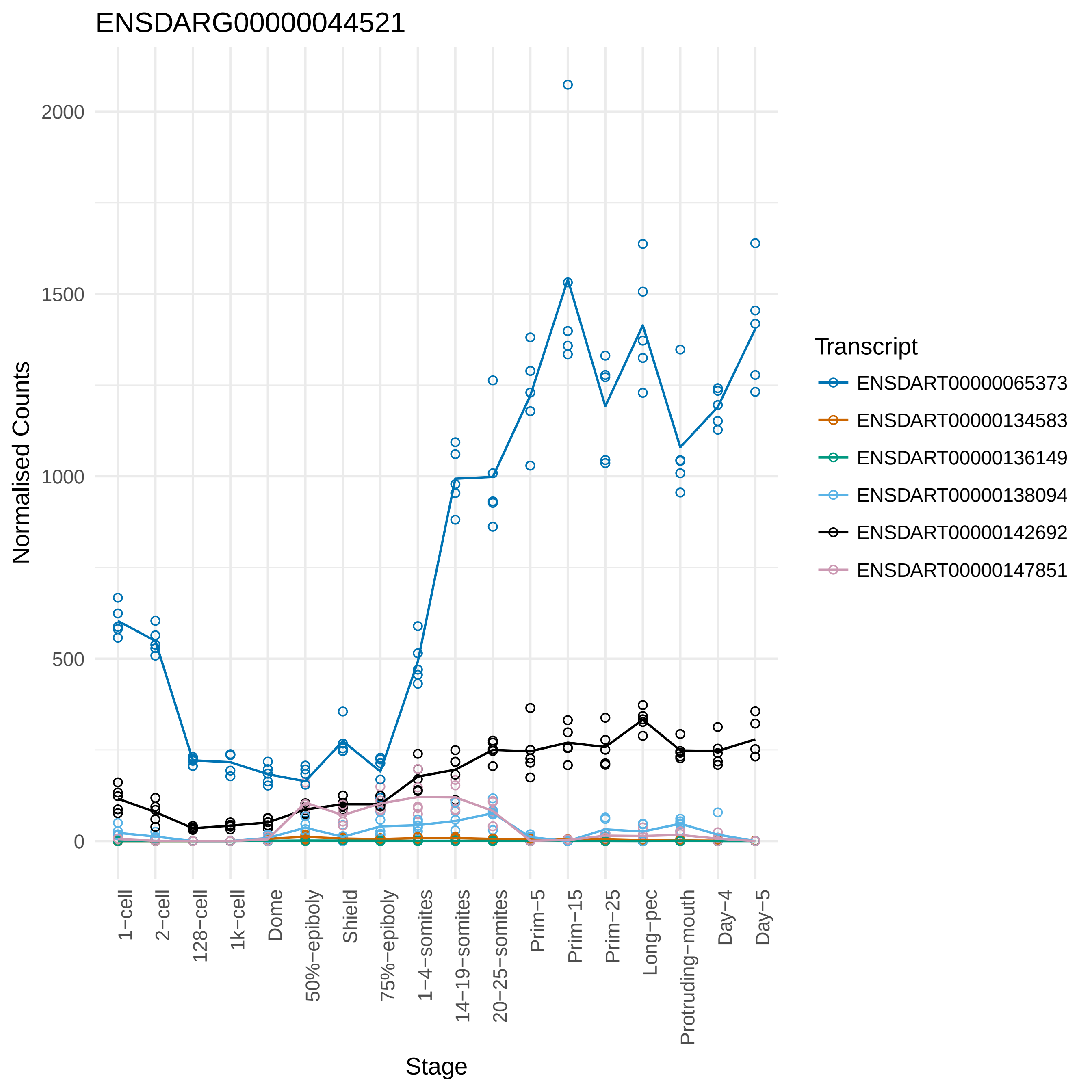

ENSDARG00000044521
Normalised Counts
2000
1500
1000
500
0
1−cell
2−cell
128−cell
1k−cell
Dome
50%−epiboly
Shield
75%−epiboly
1−4−somites
14−19−somites
20−25−somites
Prim−5
Prim−15
Prim−25
Long−pec
Protruding−mouth
Day−4
Day−5
Stage
Transcript
ENSDART00000065373
ENSDART00000134583
ENSDART00000136149
ENSDART00000138094
ENSDART00000142692
ENSDART00000147851

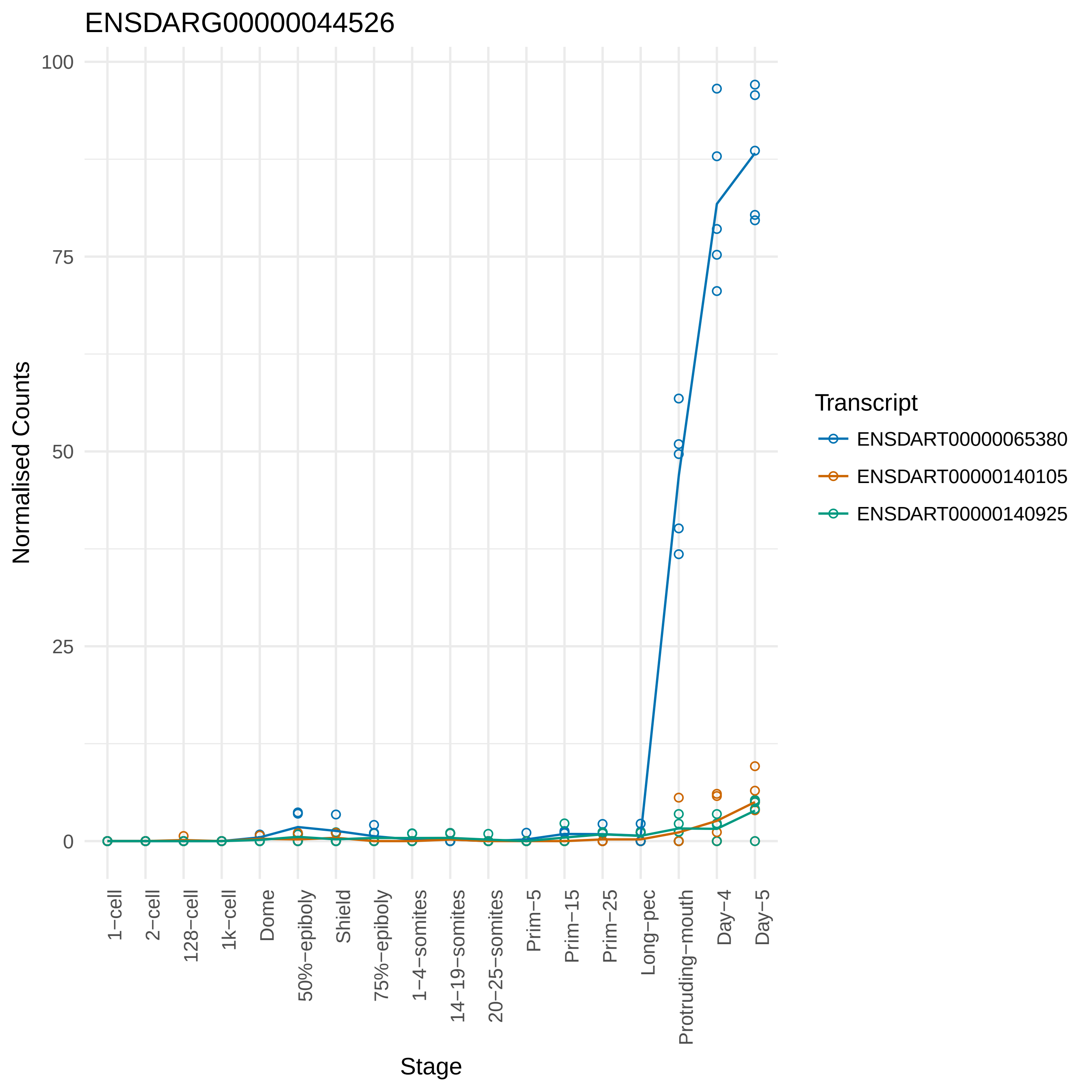
ENSDARG00000044526
Normalised Counts
100
75
50
25
0
1−cell
2−cell
128−cell
1k−cell
Dome
50%−epiboly
Shield
75%−epiboly
1−4−somites
14−19−somites
20−25−somites
Prim−5
Prim−15
Prim−25
Long−pec
Protruding−mouth
Day−4
Day−5
Stage
Transcript
ENSDART00000065380
ENSDART00000140105
ENSDART00000140925

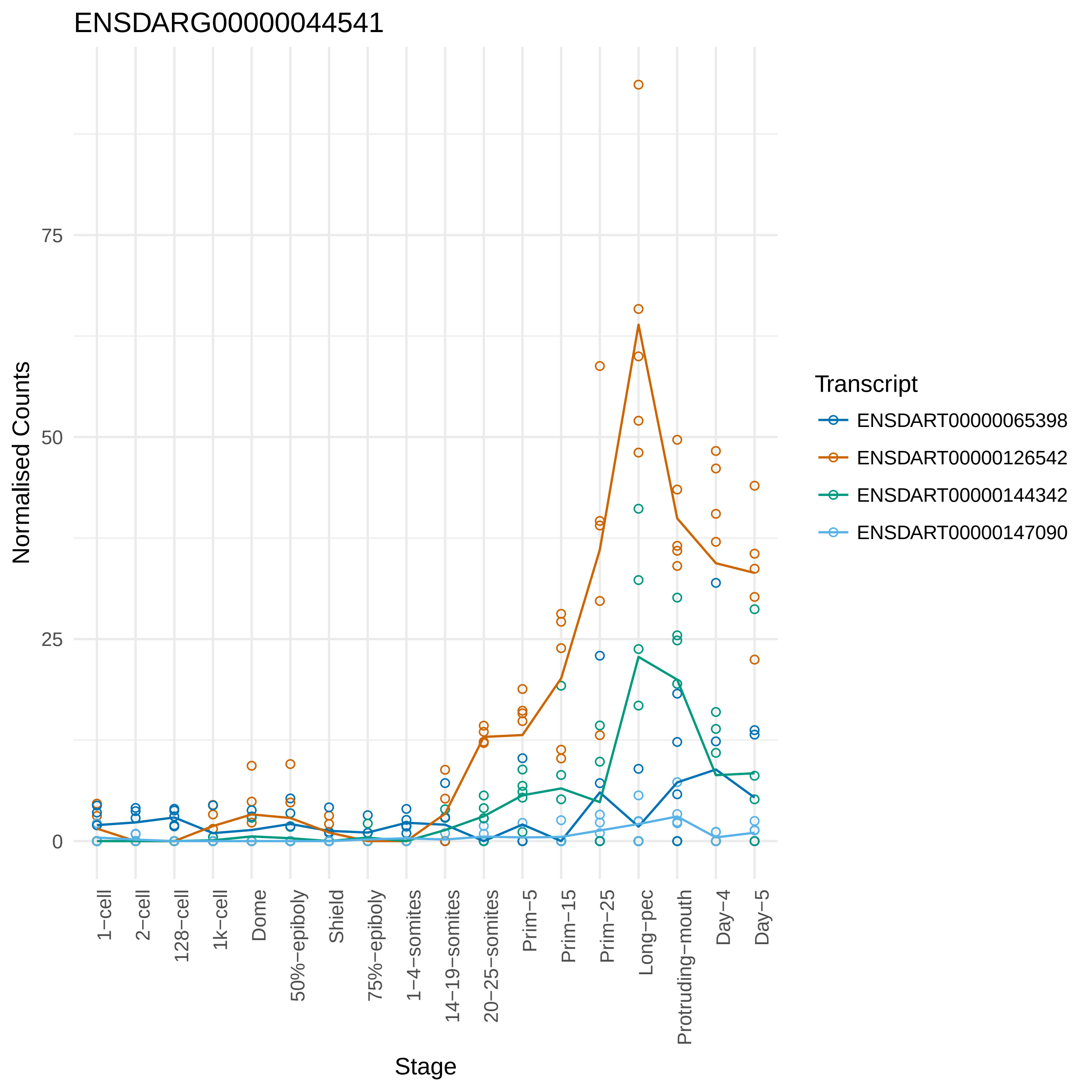

ENSDARG00000044541
Normalised Counts
75
50
25
0
1−cell
2−cell
128−cell
1k−cell
Dome
50%−epiboly
Shield
75%−epiboly
1−4−somites
14−19−somites
20−25−somites
Prim−5
Prim−15
Prim−25
Long−pec
Protruding−mouth
Day−4
Day−5
Stage
Transcript
ENSDART00000065398
ENSDART00000126542
ENSDART00000144342
ENSDART00000147090

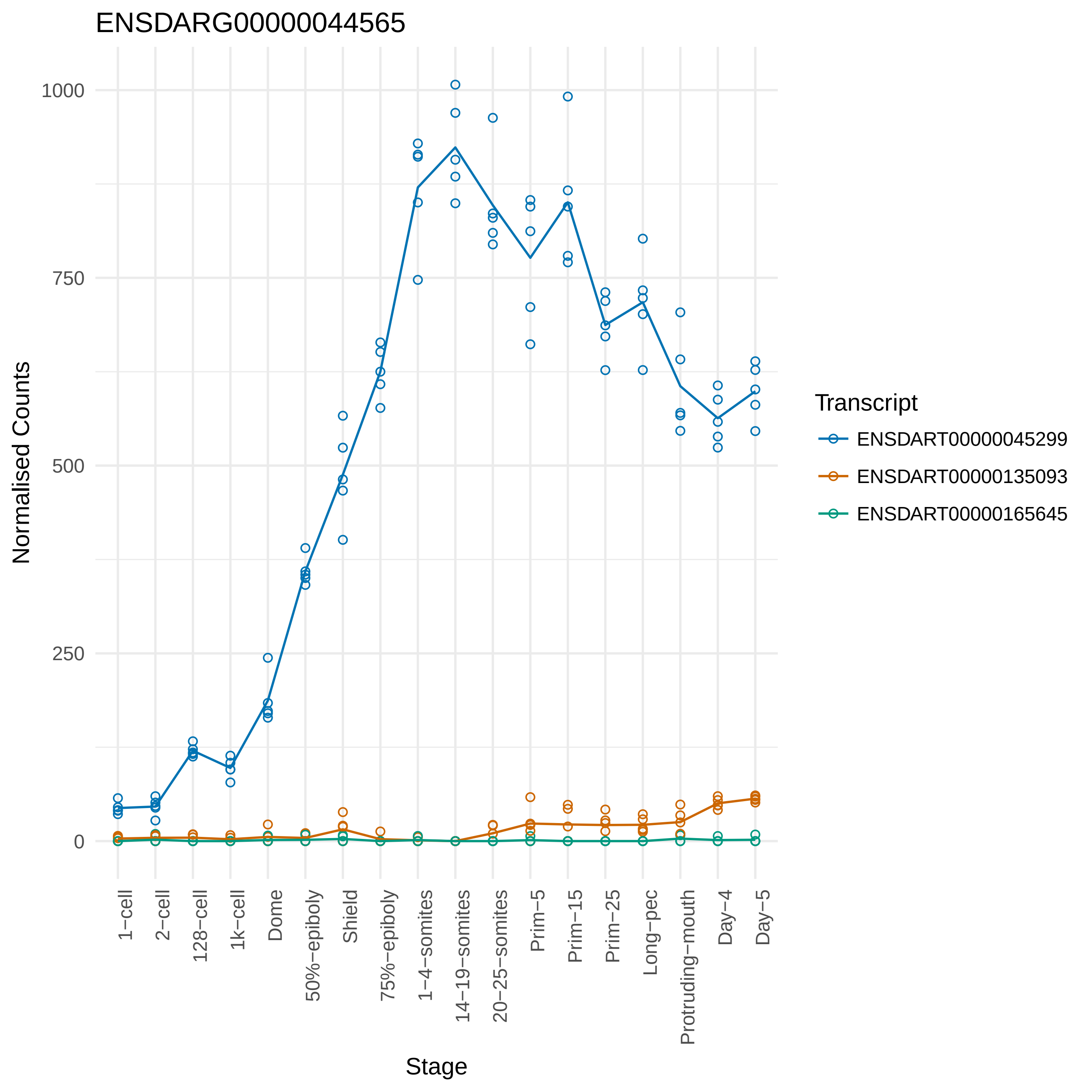
ENSDARG00000044565
Normalised Counts
1000
750
500
250
0
Stage
1−cell
2−cell
128−cell
1k−cell
Dome
50%−epiboly
Shield
75%−epiboly
1−4−somites
14−19−somites
20−25−somites
Prim−5
Prim−15
Prim−25
Long−pec
Protruding−mouth
Day−4
Day−5
Transcript
ENSDART00000045299
ENSDART00000135093
ENSDART00000165645

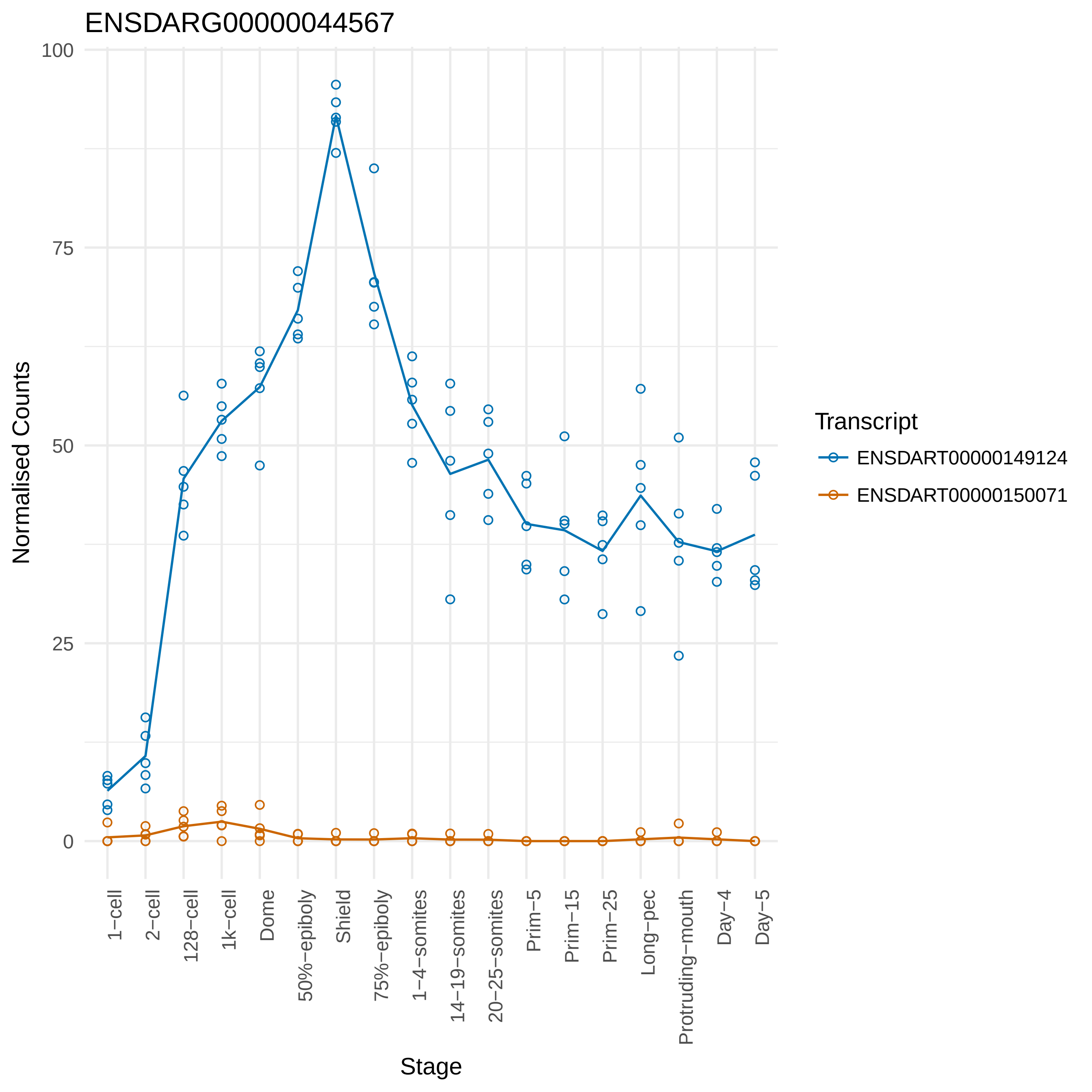

ENSDARG00000044567
100
75
50
25
0
Normalised Counts
1−cell
2−cell
128−cell
1k−cell
Dome
50%−epiboly
Shield
75%−epiboly
1−4−somites
14−19−somites
20−25−somites
Prim−5
Prim−15
Prim−25
Long−pec
Protruding−mouth
Day−4
Day−5
Stage
Transcript
ENSDART00000149124
ENSDART00000150071

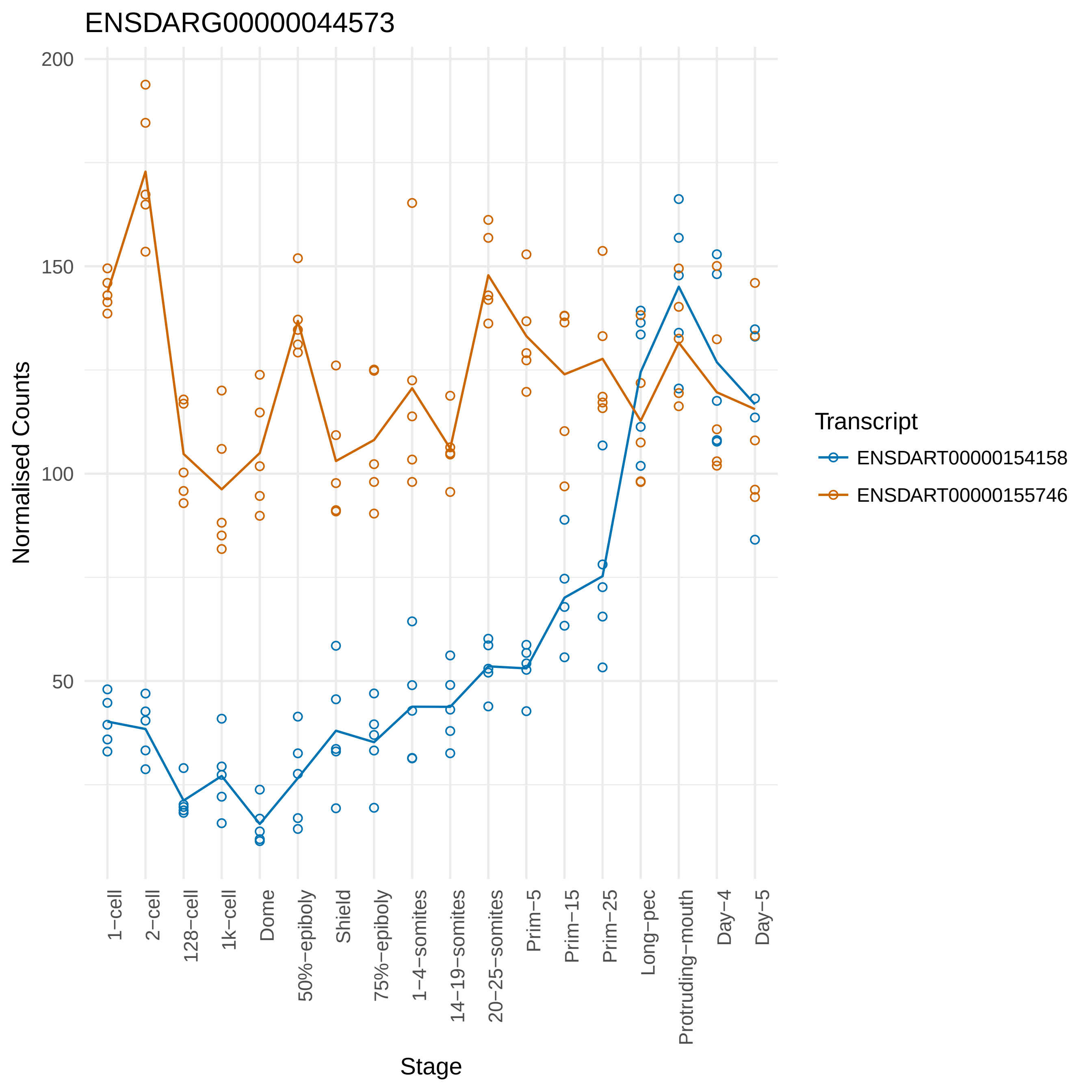
ENSDARG00000044573
Normalised Counts
200
150
100
50
Stage
1−cell
2−cell
128−cell
1k−cell
Dome
50%−epiboly
Shield
75%−epiboly
1−4−somites
14−19−somites
20−25−somites
Prim−5
Prim−15
Prim−25
Long−pec
Protruding−mouth
Day−4
Day−5
Transcript
ENSDART00000154158
ENSDART00000155746

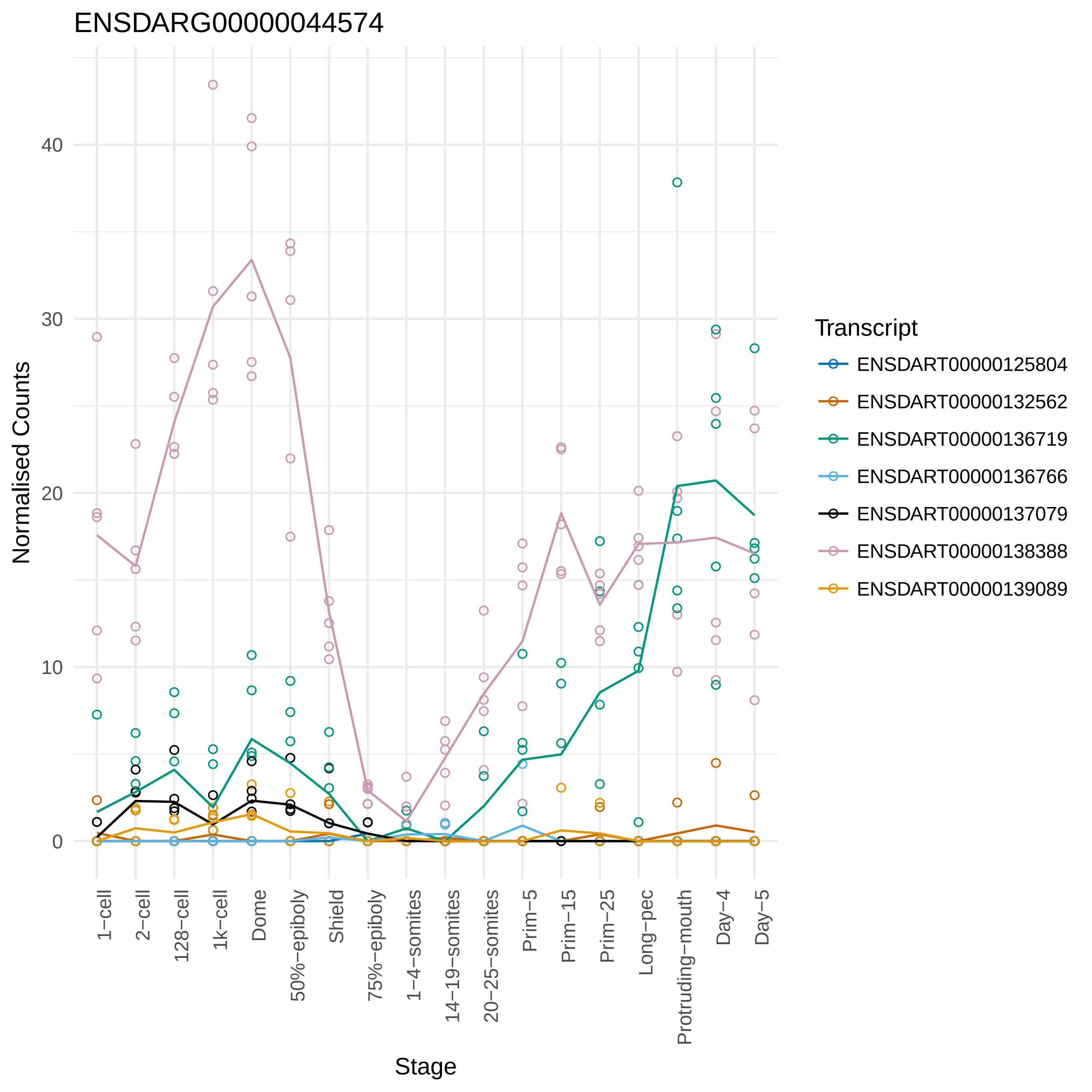

ENSDARG00000044574
Normalised Counts
0
10
20
30
40
Stage
1−cell
2−cell
128−cell
1k−cell
Dome
50%−epiboly
Shield
75%−epiboly
1−4−somites
14−19−somites
20−25−somites
Prim−5
Prim−15
Prim−25
Long−pec
Protruding−mouth
Day−4
Day−5
Transcript
ENSDART00000125804
ENSDART00000132562
ENSDART00000136719
ENSDART00000136766
ENSDART00000137079
ENSDART00000138388
ENSDART00000139089

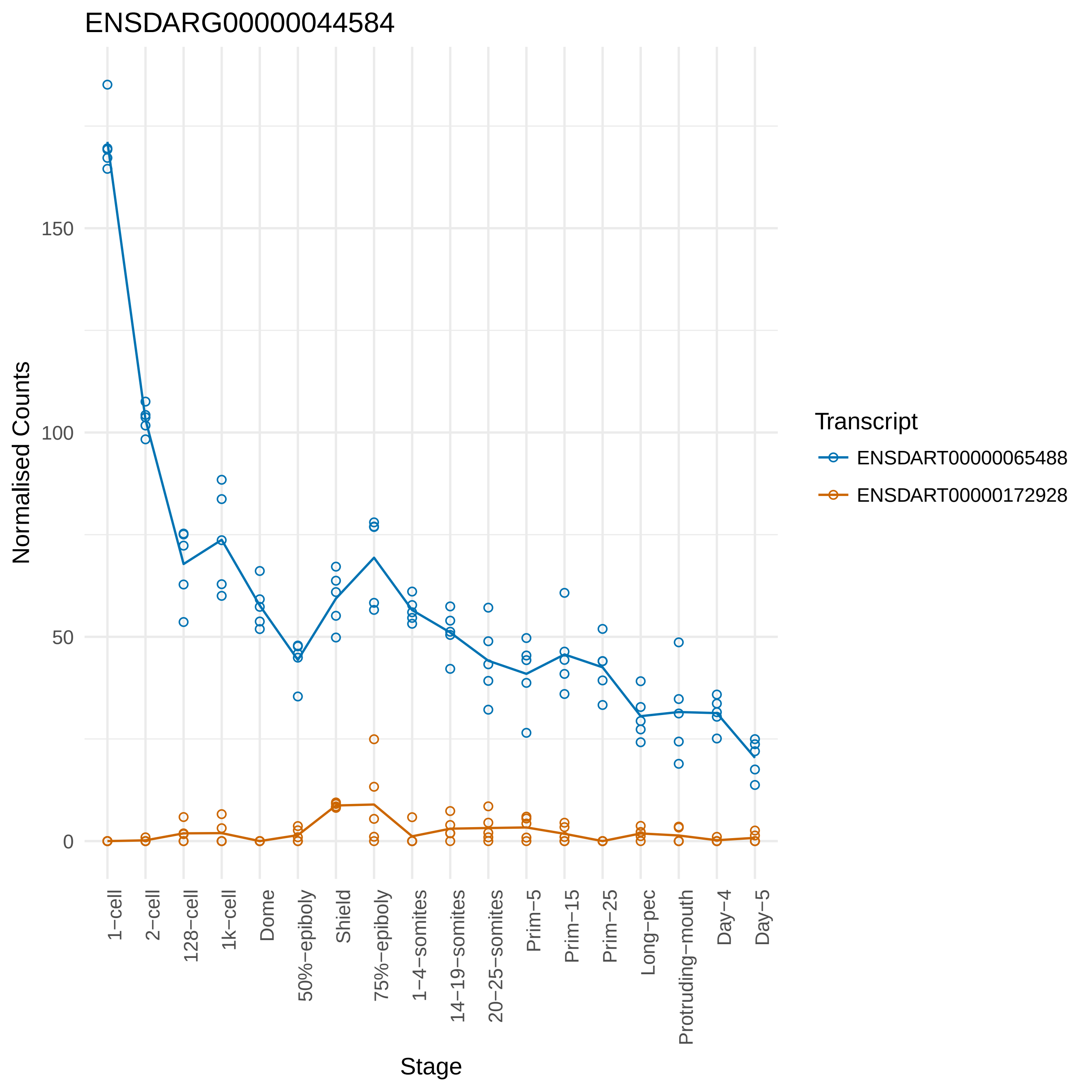

ENSDARG00000044584
Normalised Counts
150
100
50
0
1−cell
2−cell
128−cell
1k−cell
Dome
50%−epiboly
Shield
75%−epiboly
1−4−somites
14−19−somites
20−25−somites
Prim−5
Prim−15
Prim−25
Long−pec
Protruding−mouth
Day−4
Day−5
Stage
Transcript
ENSDART00000065488
ENSDART00000172928

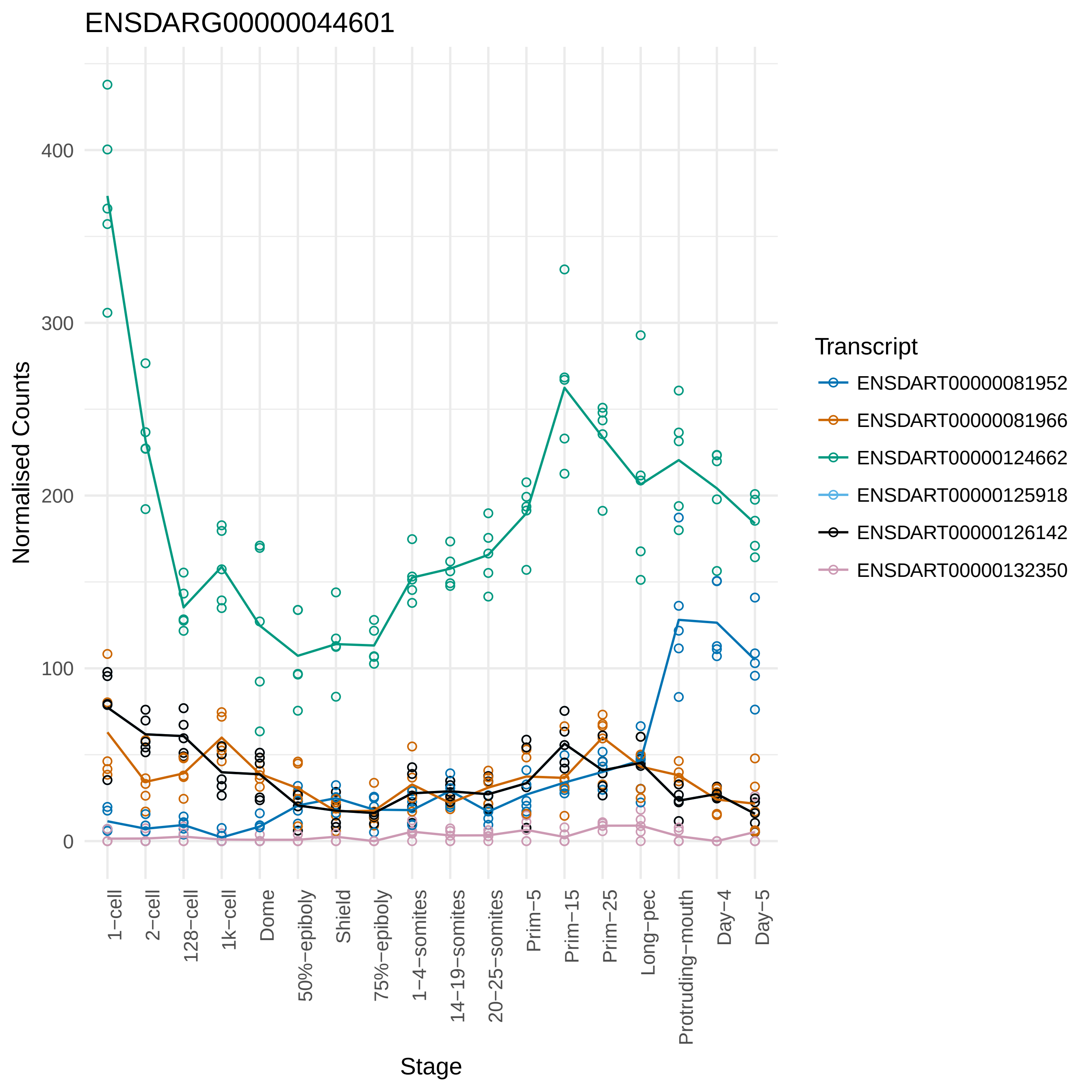

ENSDARG00000044601
Normalised Counts
400
300
200
100
0
1−cell
2−cell
128−cell
1k−cell
Dome
50%−epiboly
Shield
75%−epiboly
1−4−somites
14−19−somites
20−25−somites
Prim−5
Prim−15
Prim−25
Long−pec
Protruding−mouth
Day−4
Day−5
Stage
Transcript
ENSDART00000081952
ENSDART00000081966
ENSDART00000124662
ENSDART00000125918
ENSDART00000126142
ENSDART00000132350

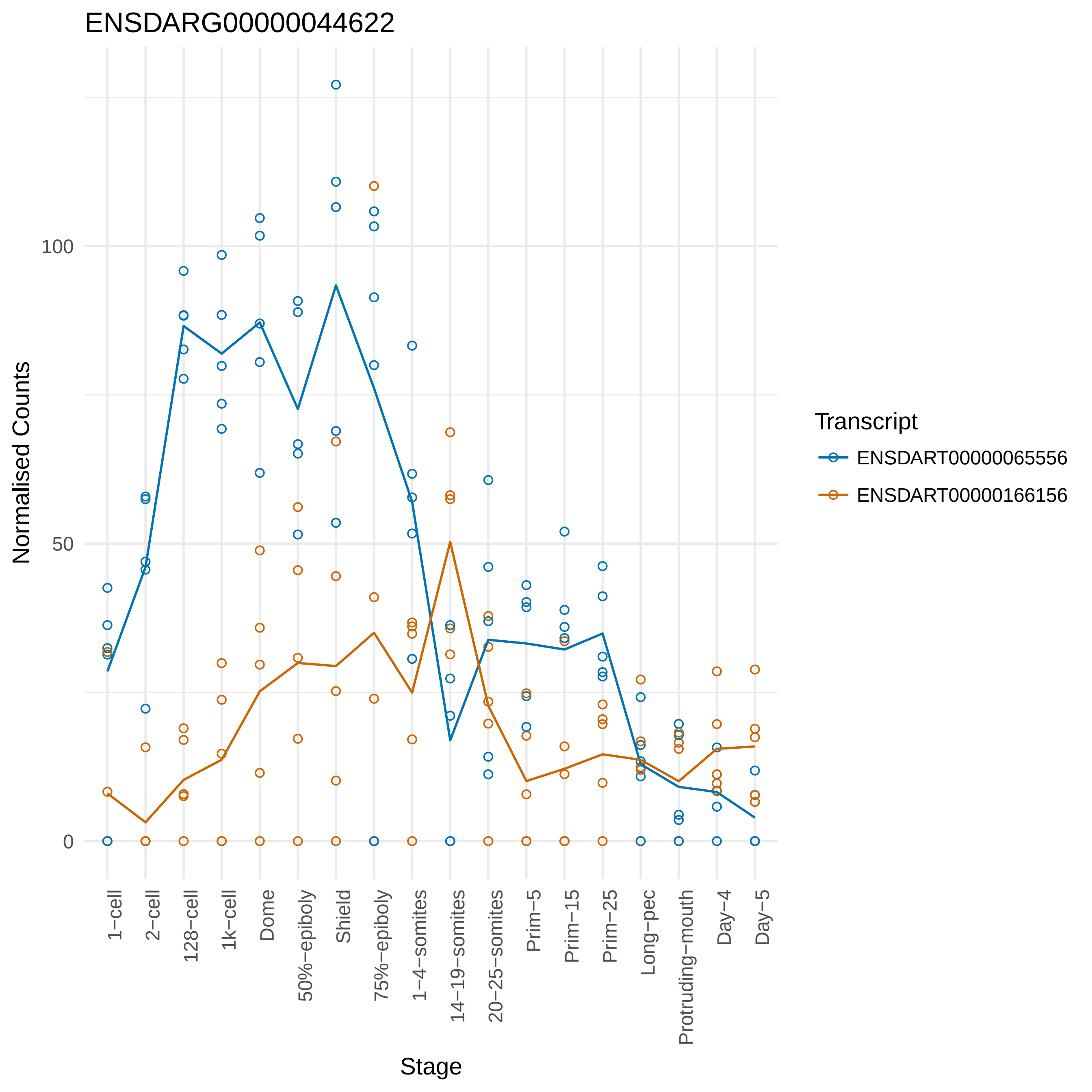

ENSDARG00000044622
Normalised Counts
0
50
100
Stage
1−cell
2−cell
128−cell
1k−cell
Dome
50%−epiboly
Shield
75%−epiboly
1−4−somites
14−19−somites
20−25−somites
Prim−5
Prim−15
Prim−25
Long−pec
Protruding−mouth
Day−4
Day−5
Transcript
ENSDART00000065556
ENSDART00000166156

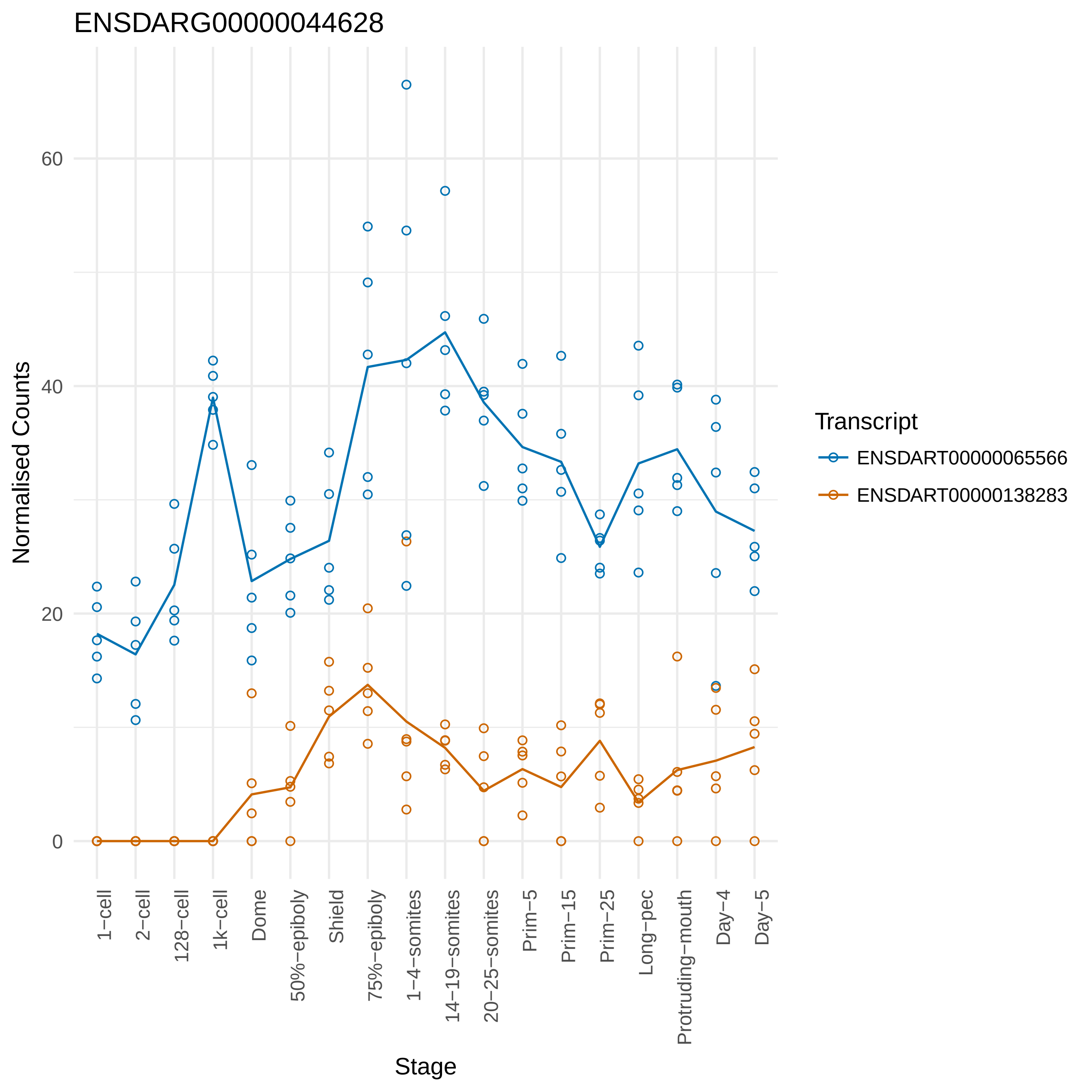

ENSDARG00000044628
Normalised Counts
60
40
20
0
1−cell
2−cell
128−cell
1k−cell
Dome
50%−epiboly
Shield
75%−epiboly
1−4−somites
14−19−somites
20−25−somites
Prim−5
Prim−15
Prim−25
Long−pec
Protruding−mouth
Day−4
Day−5
Stage
Transcript
ENSDART00000065566
ENSDART00000138283

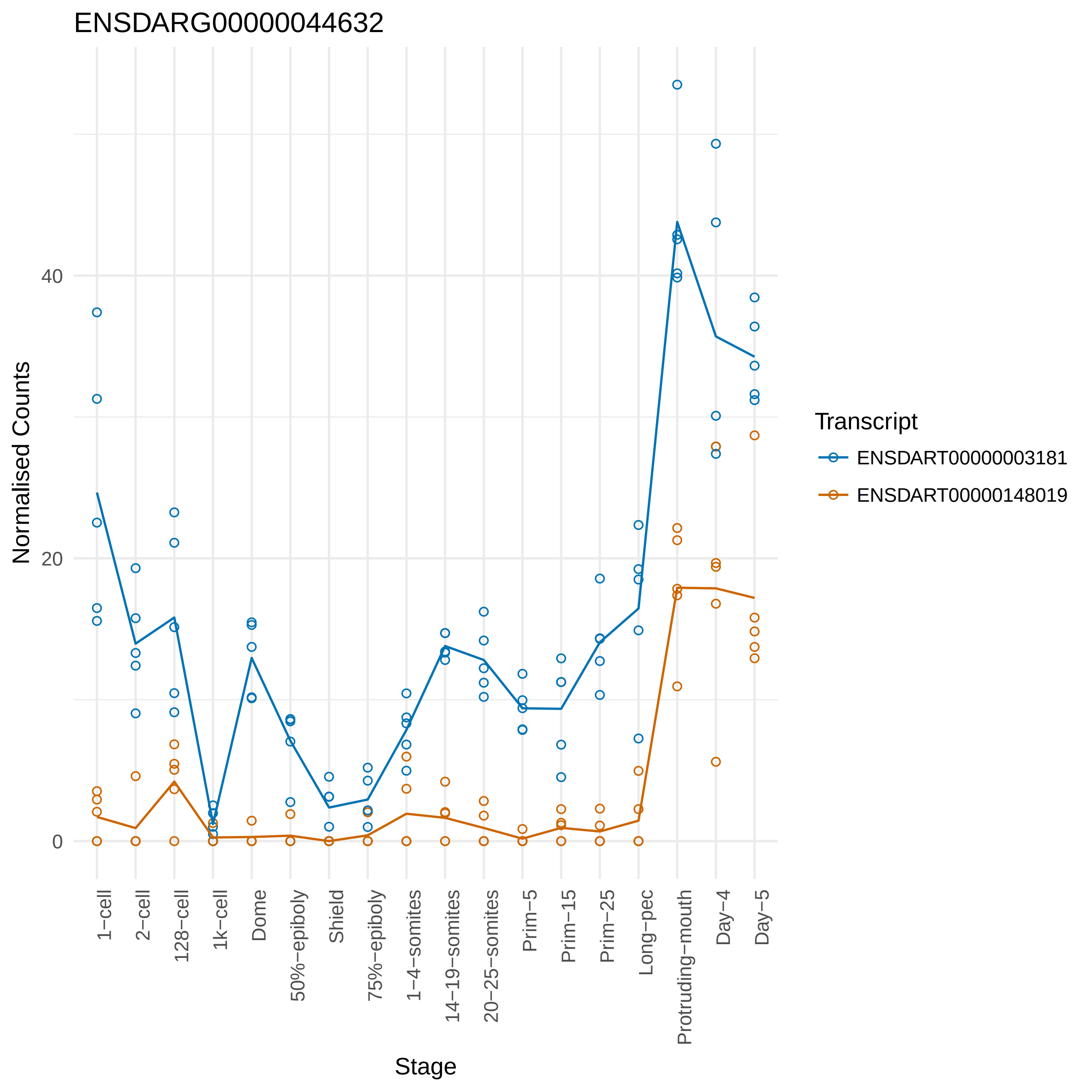
ENSDARG00000044632
Normalised Counts
0
20
40
1−cell
2−cell
128−cell
1k−cell
Dome
50%−epiboly
Shield
75%−epiboly
1−4−somites
14−19−somites
20−25−somites
Prim−5
Prim−15
Prim−25
Long−pec
Protruding−mouth
Day−4
Day−5
Stage
Transcript
ENSDART00000003181
ENSDART00000148019

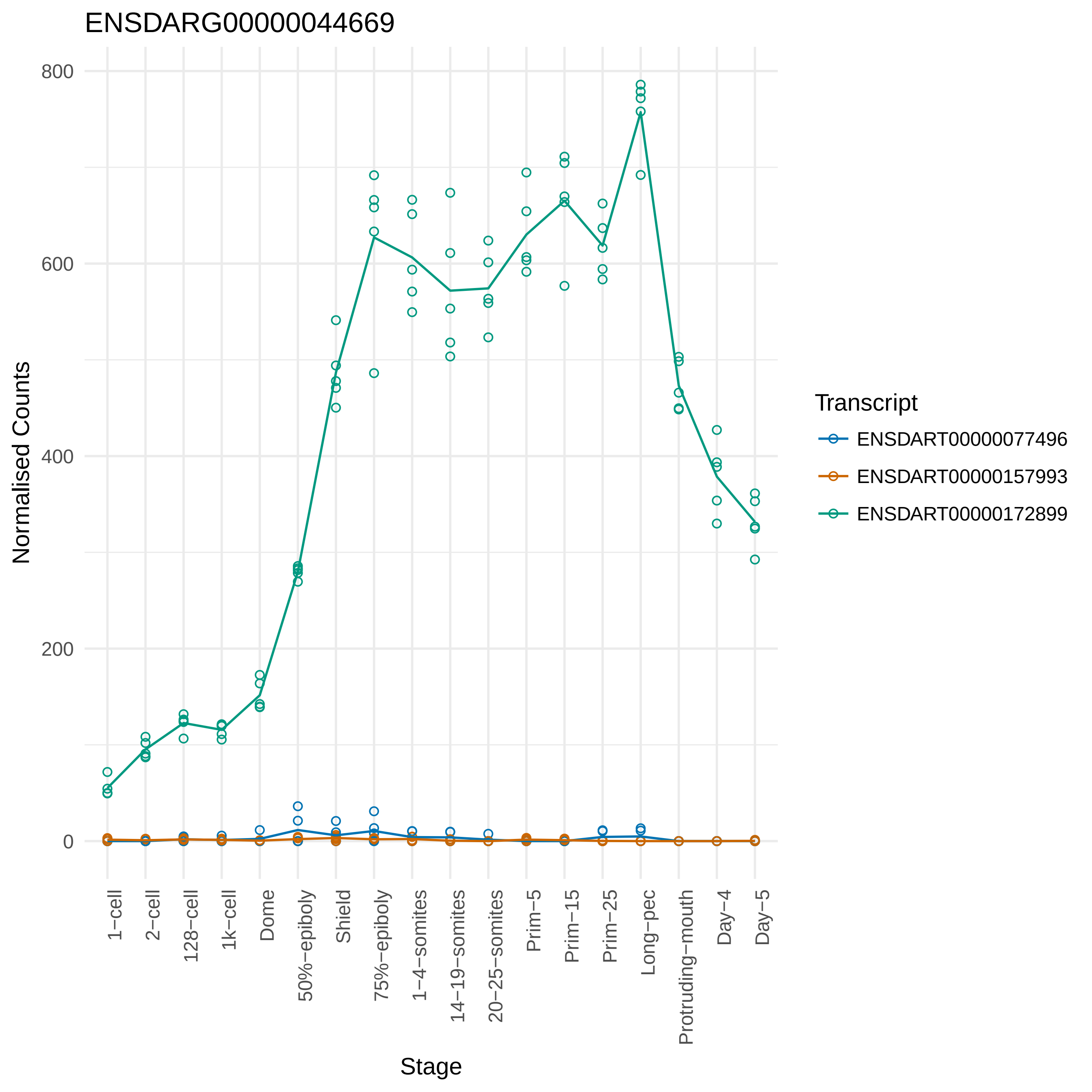
ENSDARG00000044669
Normalised Counts
800
600
400
200
0
1−cell
2−cell
128−cell
1k−cell
Dome
50%−epiboly
Shield
75%−epiboly
1−4−somites
14−19−somites
20−25−somites
Prim−5
Prim−15
Prim−25
Long−pec
Protruding−mouth
Day−4
Day−5
Stage
Transcript
ENSDART00000077496
ENSDART00000157993
ENSDART00000172899

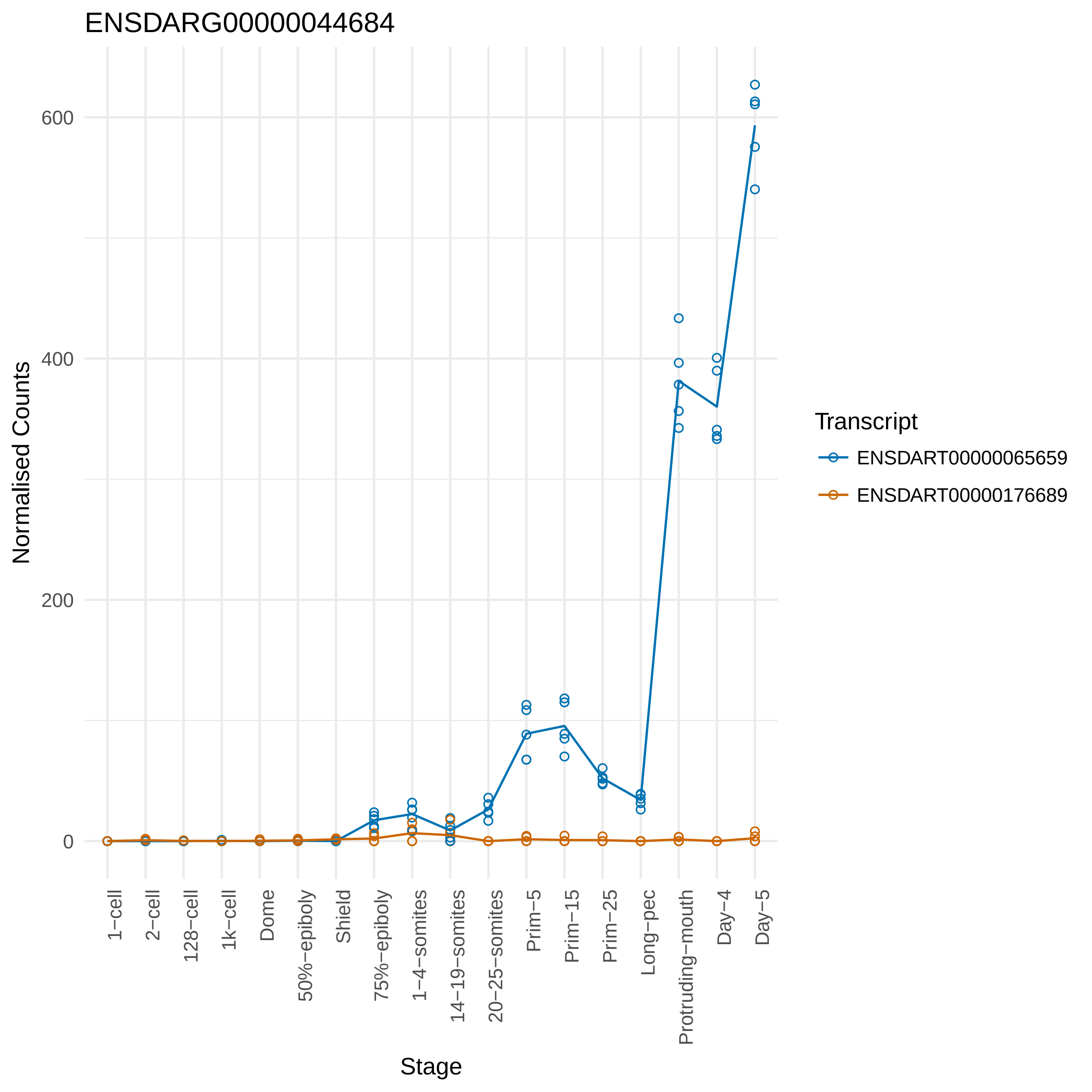

ENSDARG00000044684
Normalised Counts
600
400
200
0
1−cell
2−cell
128−cell
1k−cell
Dome
50%−epiboly
Shield
75%−epiboly
1−4−somites
14−19−somites
20−25−somites
Prim−5
Prim−15
Prim−25
Long−pec
Protruding−mouth
Day−4
Day−5
Stage
Transcript
ENSDART00000065659
ENSDART00000176689

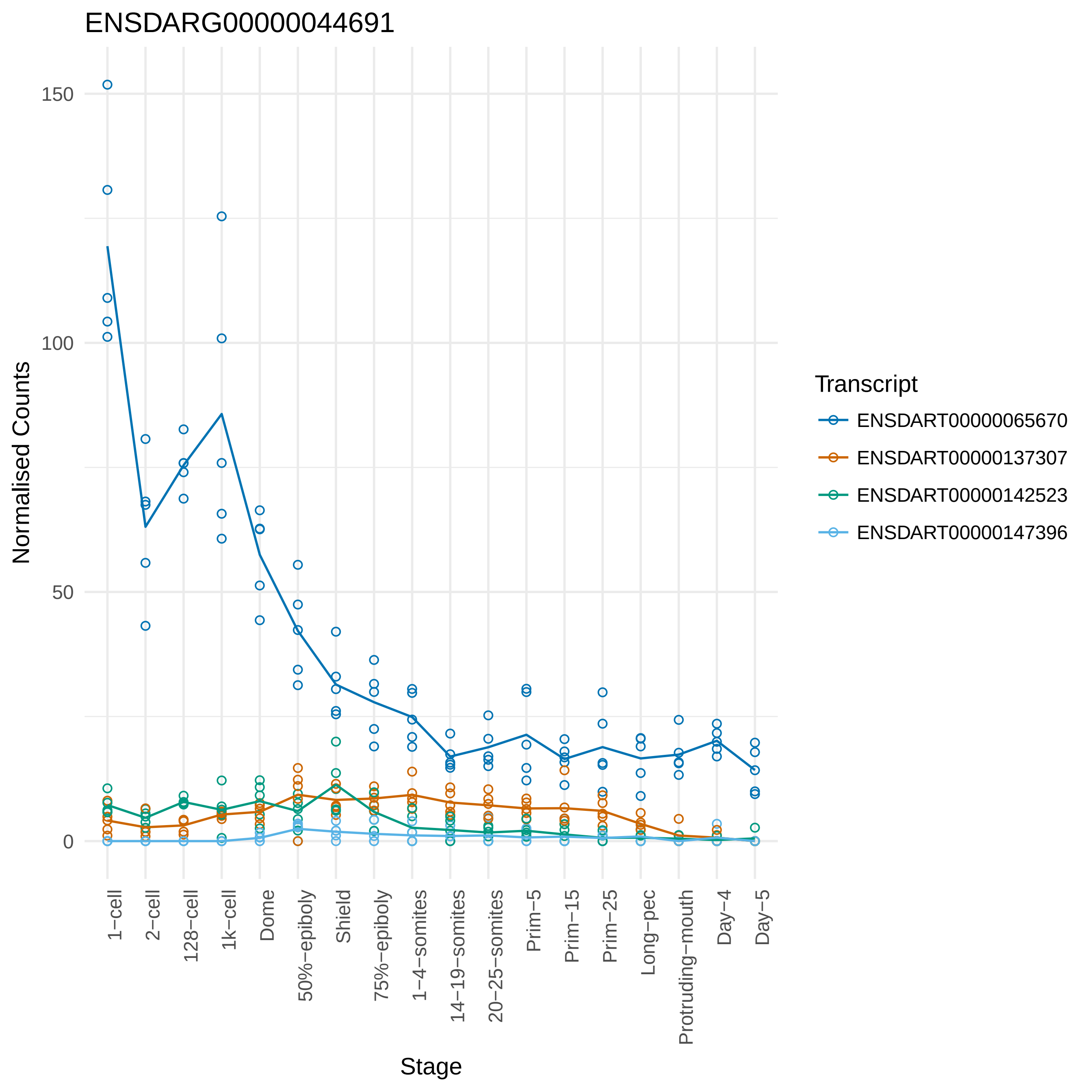
ENSDARG00000044691
Normalised Counts
150
100
50
0
1−cell
2−cell
128−cell
1k−cell
Dome
50%−epiboly
Shield
75%−epiboly
1−4−somites
14−19−somites
20−25−somites
Prim−5
Prim−15
Prim−25
Long−pec
Protruding−mouth
Day−4
Day−5
Stage
Transcript
ENSDART00000065670
ENSDART00000137307
ENSDART00000142523
ENSDART00000147396

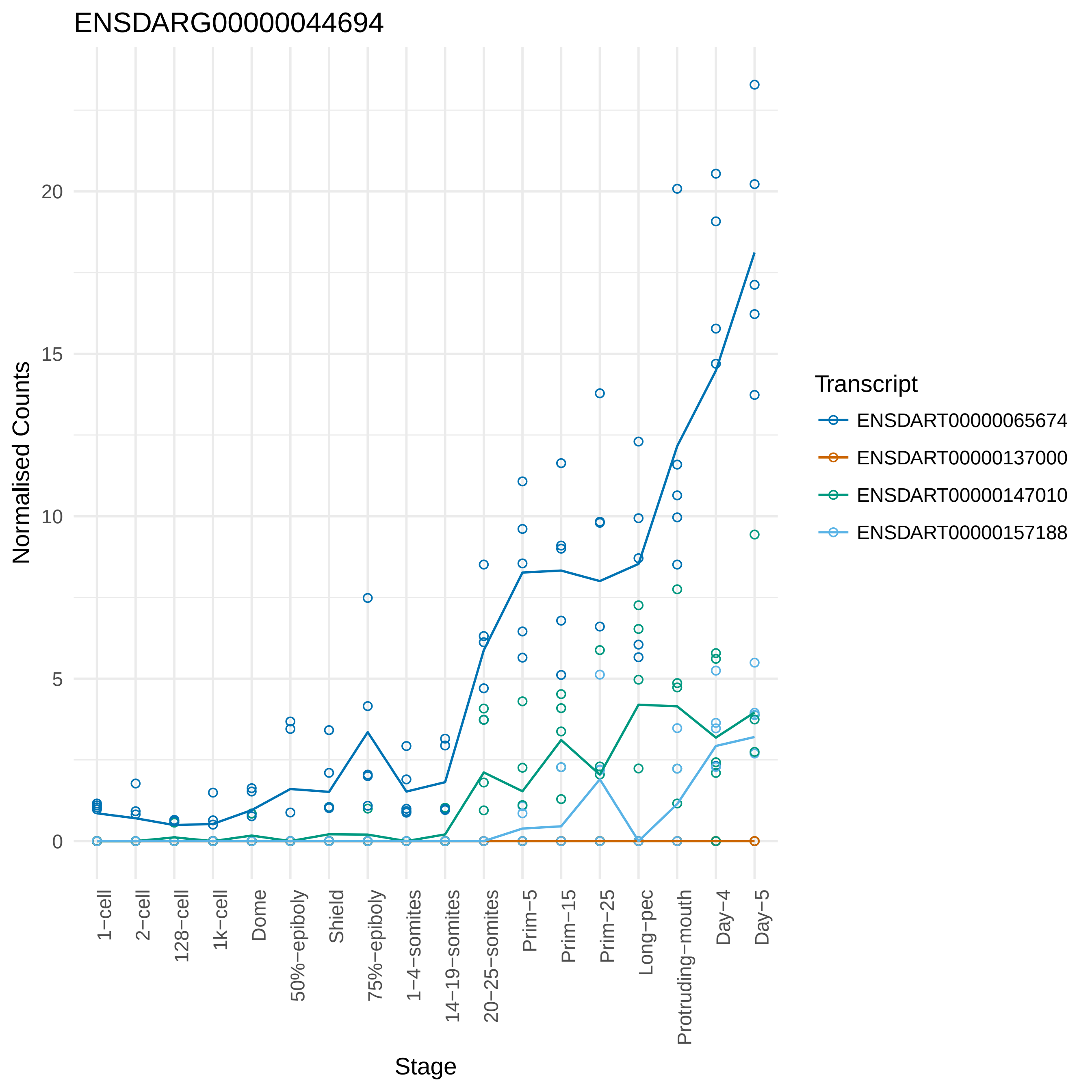

ENSDARG00000044694
Normalised Counts
0
5
10
15
20
1−cell
2−cell
128−cell
1k−cell
Dome
50%−epiboly
Shield
75%−epiboly
1−4−somites
14−19−somites
20−25−somites
Prim−5
Prim−15
Prim−25
Long−pec
Protruding−mouth
Day−4
Day−5
Stage
Transcript
ENSDART00000065674
ENSDART00000137000
ENSDART00000147010
ENSDART00000157188

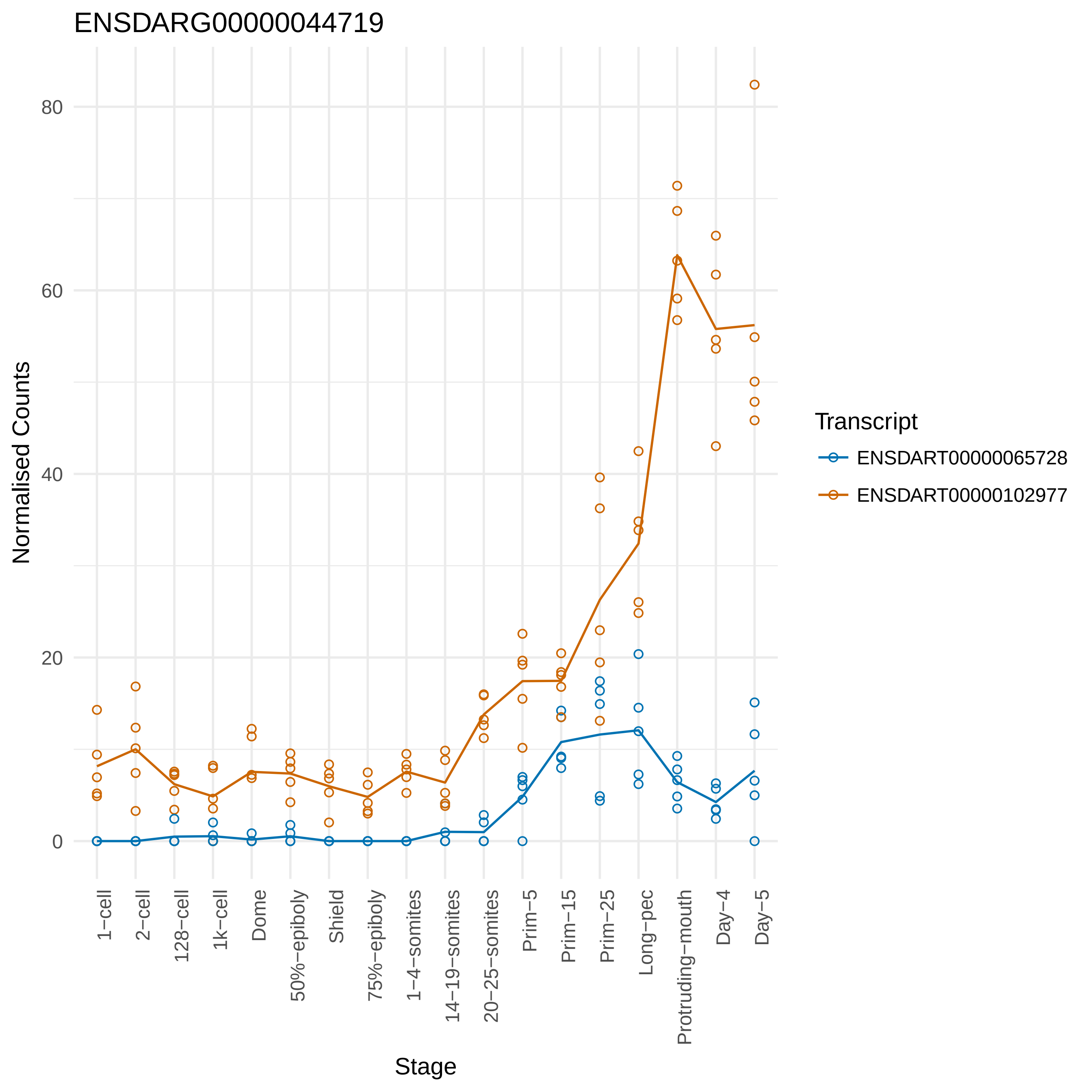
ENSDARG00000044719
Normalised Counts
80
60
40
20
0
1−cell
2−cell
128−cell
1k−cell
Dome
50%−epiboly
Shield
75%−epiboly
1−4−somites
14−19−somites
20−25−somites
Prim−5
Prim−15
Prim−25
Long−pec
Protruding−mouth
Day−4
Day−5
Stage
Transcript
ENSDART00000065728
ENSDART00000102977

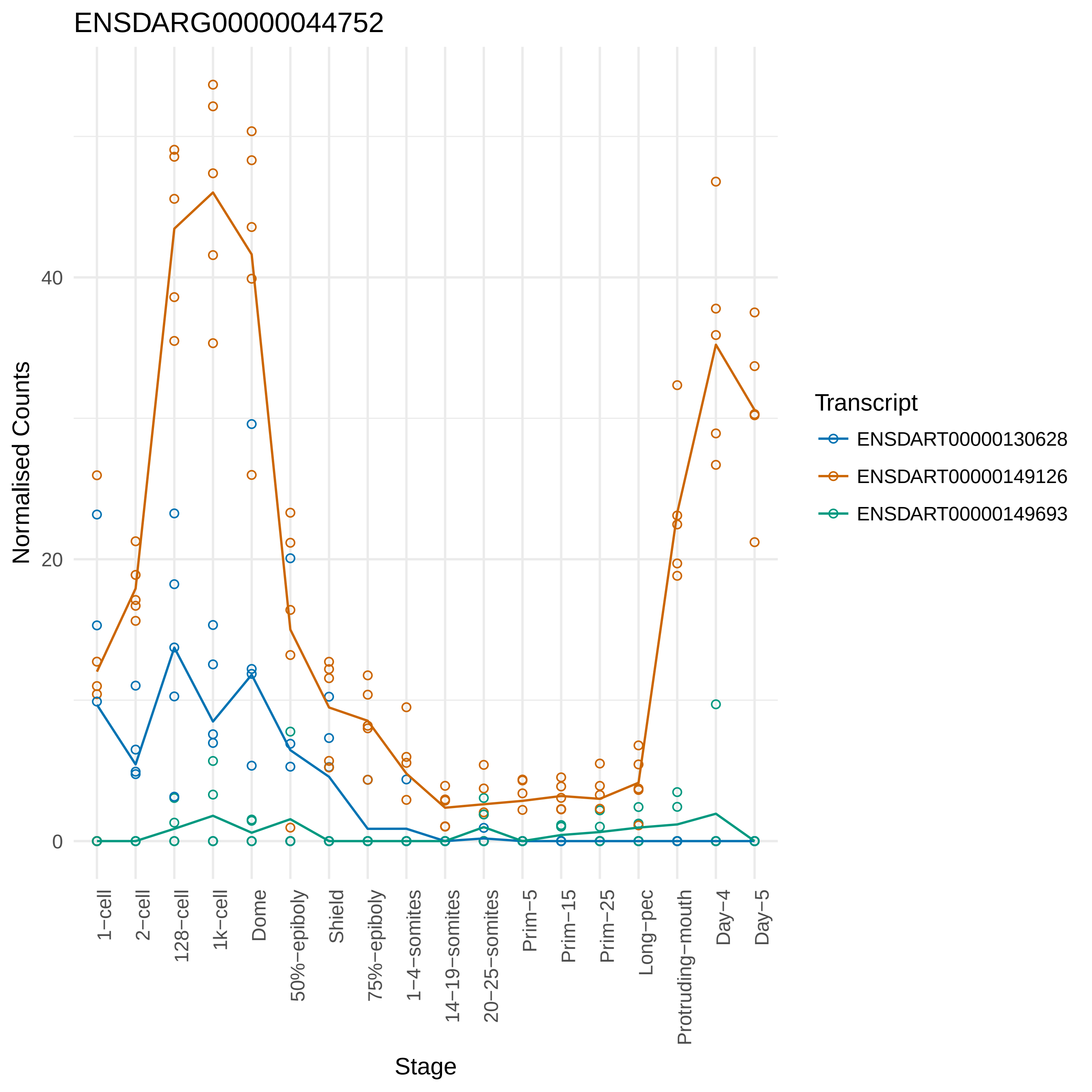

ENSDARG00000044752
Normalised Counts
0
20
40
Stage
1−cell
2−cell
128−cell
1k−cell
Dome
50%−epiboly
Shield
75%−epiboly
1−4−somites
14−19−somites
20−25−somites
Prim−5
Prim−15
Prim−25
Long−pec
Protruding−mouth
Day−4
Day−5
Transcript
ENSDART00000130628
ENSDART00000149126
ENSDART00000149693

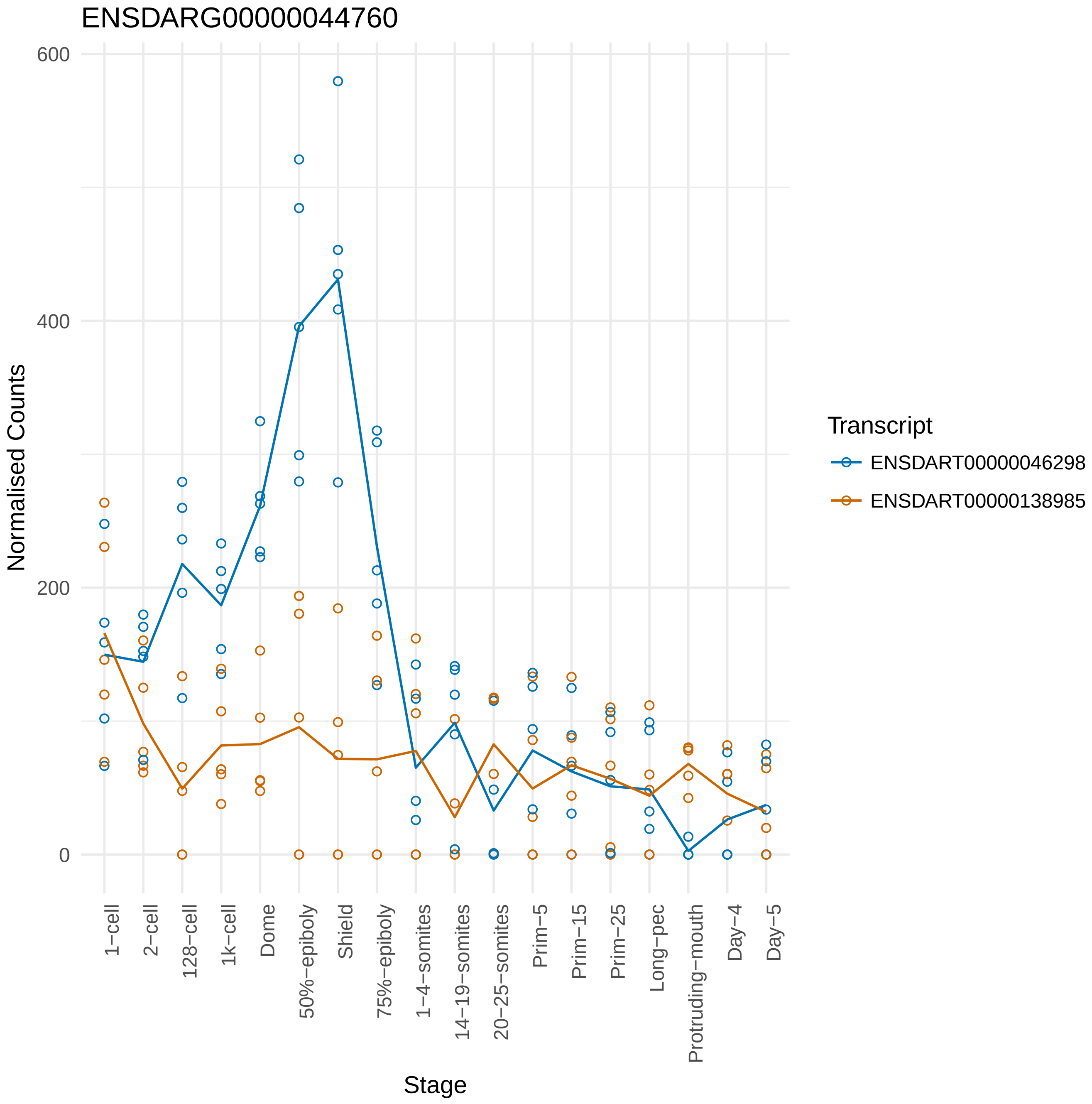

ENSDARG00000044760
Normalised Counts
600
400
200
0
1−cell
2−cell
128−cell
1k−cell
Dome
50%−epiboly
Shield
75%−epiboly
1−4−somites
14−19−somites
20−25−somites
Prim−5
Prim−15
Prim−25
Long−pec
Protruding−mouth
Day−4
Day−5
Stage
Transcript
ENSDART00000046298
ENSDART00000138985

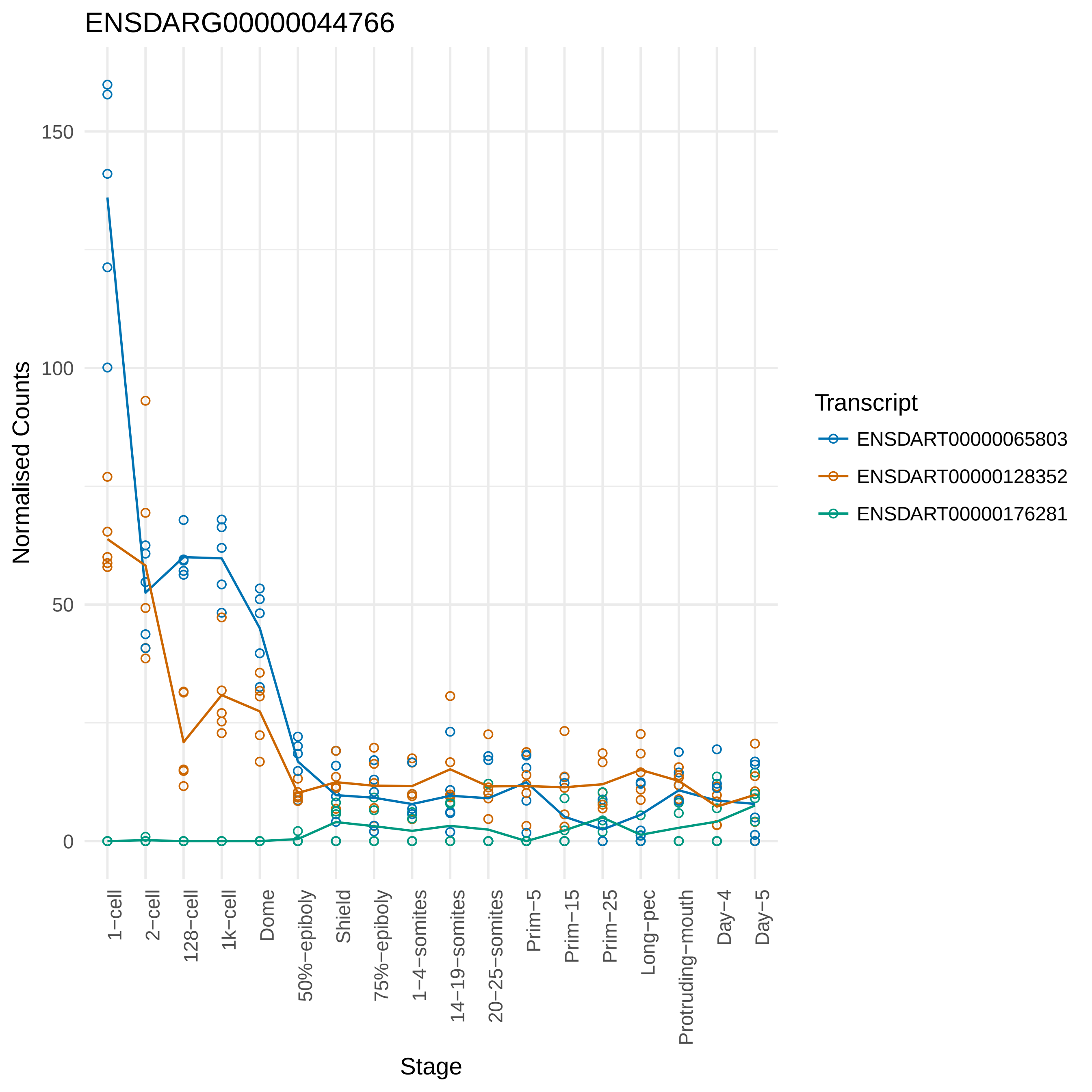

ENSDARG00000044766
Normalised Counts
150
100
50
0
1−cell
2−cell
128−cell
1k−cell
Dome
50%−epiboly
Shield
75%−epiboly
1−4−somites
14−19−somites
20−25−somites
Prim−5
Prim−15
Prim−25
Long−pec
Protruding−mouth
Day−4
Day−5
Stage
Transcript
ENSDART00000065803
ENSDART00000128352
ENSDART00000176281

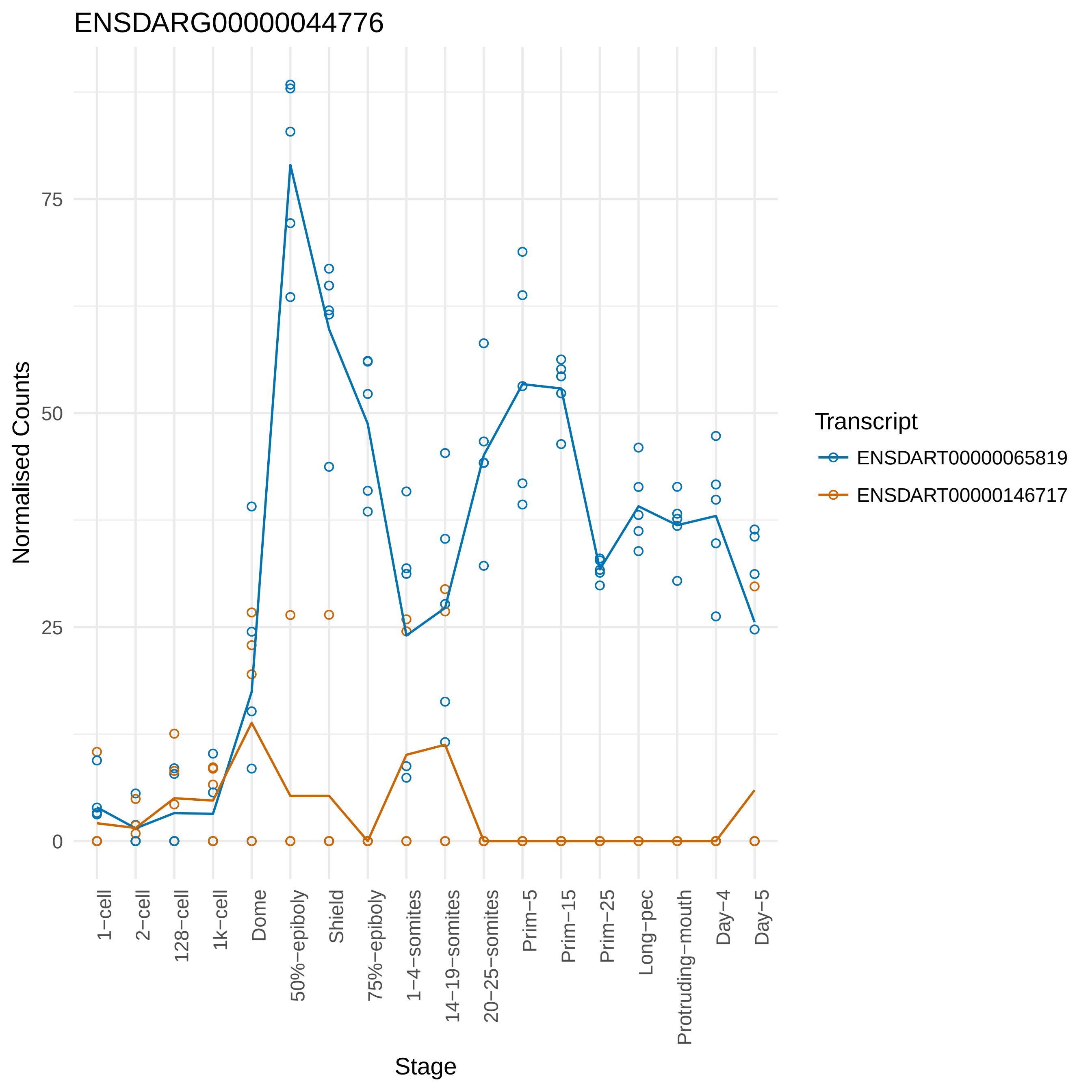

ENSDARG00000044776
Normalised Counts
0
25
50
75
Stage
1−cell
2−cell
128−cell
1k−cell
Dome
50%−epiboly
Shield
75%−epiboly
1−4−somites
14−19−somites
20−25−somites
Prim−5
Prim−15
Prim−25
Long−pec
Protruding−mouth
Day−4
Day−5
Transcript
ENSDART00000065819
ENSDART00000146717

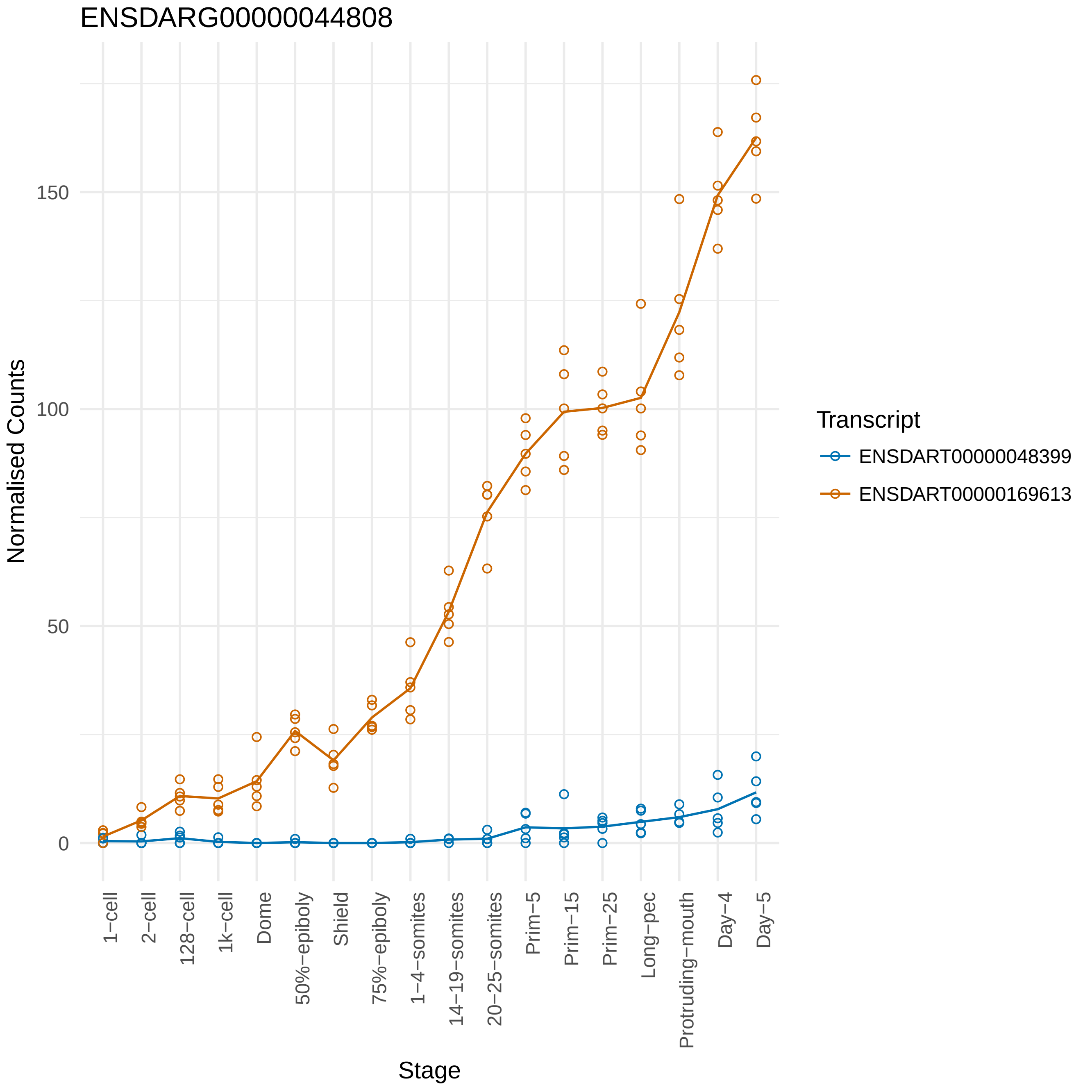

ENSDARG00000044808
Normalised Counts
0
50
100
150
Stage
1−cell
2−cell
128−cell
1k−cell
Dome
50%−epiboly
Shield
75%−epiboly
1−4−somites
14−19−somites
20−25−somites
Prim−5
Prim−15
Prim−25
Long−pec
Protruding−mouth
Day−4
Day−5
Transcript
ENSDART00000048399
ENSDART00000169613

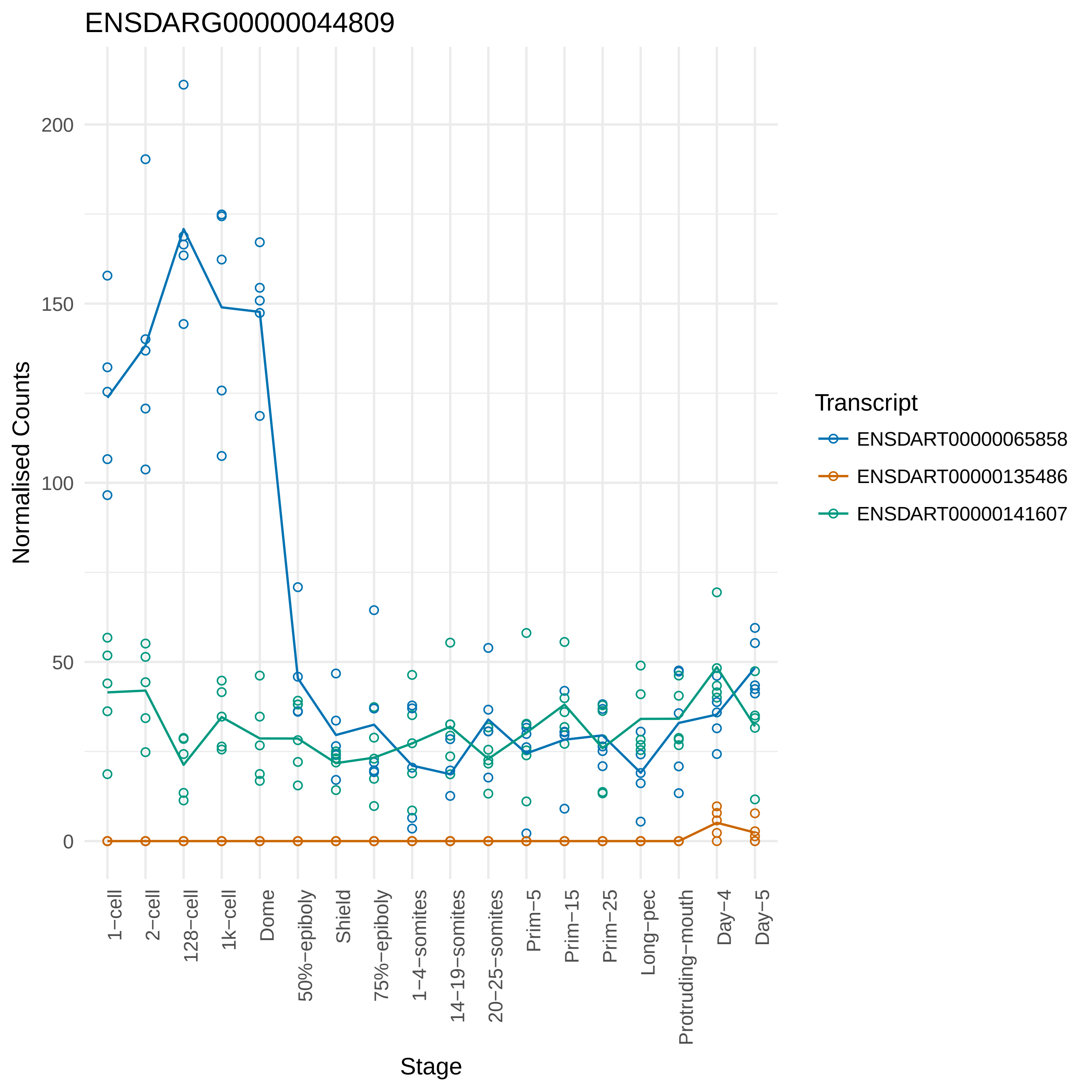

ENSDARG00000044809
Normalised Counts
200
150
100
50
0
1−cell
2−cell
128−cell
1k−cell
Dome
50%−epiboly
Shield
75%−epiboly
1−4−somites
14−19−somites
20−25−somites
Prim−5
Prim−15
Prim−25
Long−pec
Protruding−mouth
Day−4
Day−5
Stage
Transcript
ENSDART00000065858
ENSDART00000135486
ENSDART00000141607

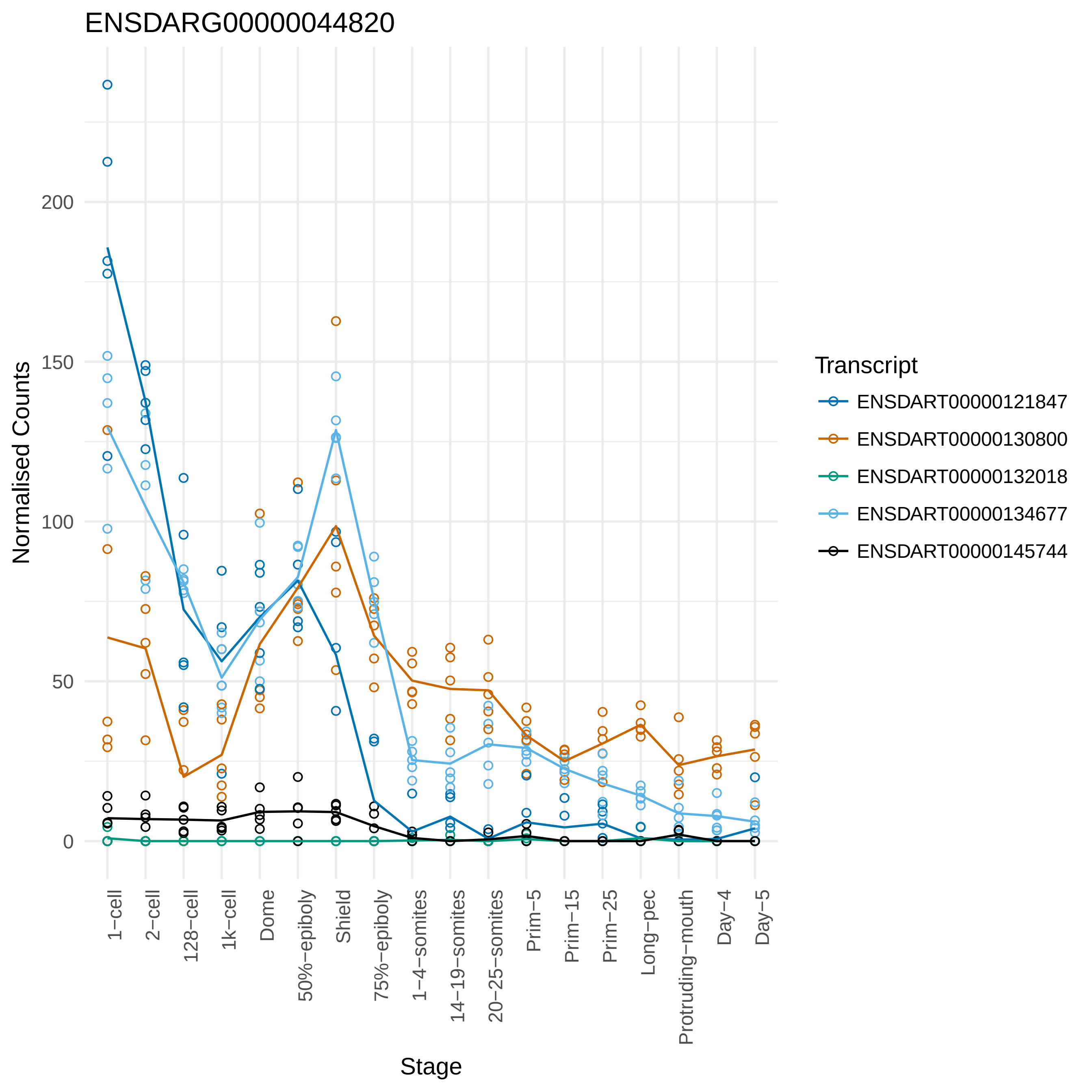
ENSDARG00000044820
Normalised Counts
0
50
100
150
200
1−cell
2−cell
128−cell
1k−cell
Dome
50%−epiboly
Shield
75%−epiboly
1−4−somites
14−19−somites
20−25−somites
Prim−5
Prim−15
Prim−25
Long−pec
Protruding−mouth
Day−4
Day−5
Stage
Transcript
ENSDART00000121847
ENSDART00000130800
ENSDART00000132018
ENSDART00000134677
ENSDART00000145744

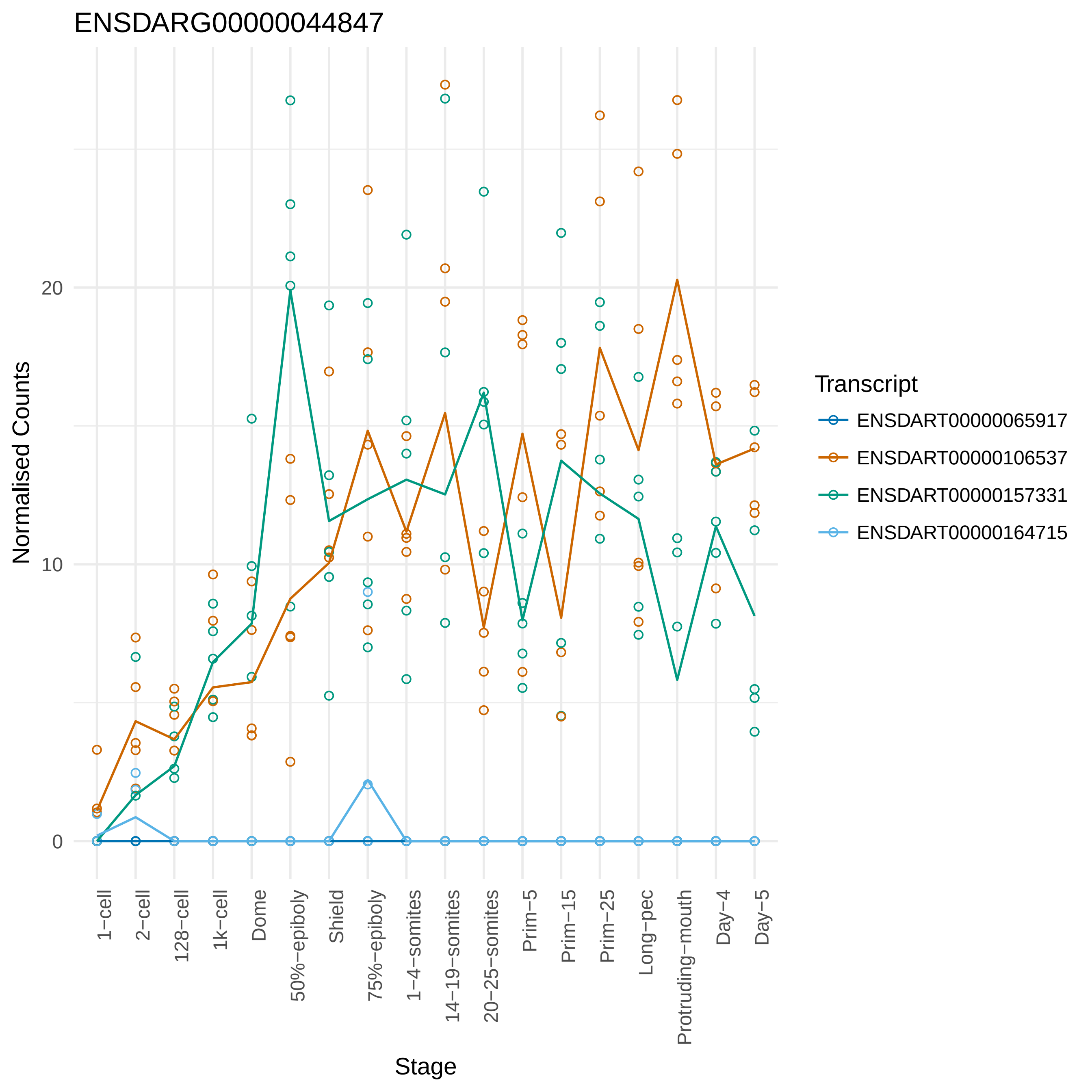

ENSDARG00000044847
Normalised Counts
20
10
0
1−cell
2−cell
128−cell
1k−cell
Dome
50%−epiboly
Shield
75%−epiboly
1−4−somites
14−19−somites
20−25−somites
Prim−5
Prim−15
Prim−25
Long−pec
Protruding−mouth
Day−4
Day−5
Stage
Transcript
ENSDART00000065917
ENSDART00000106537
ENSDART00000157331
ENSDART00000164715

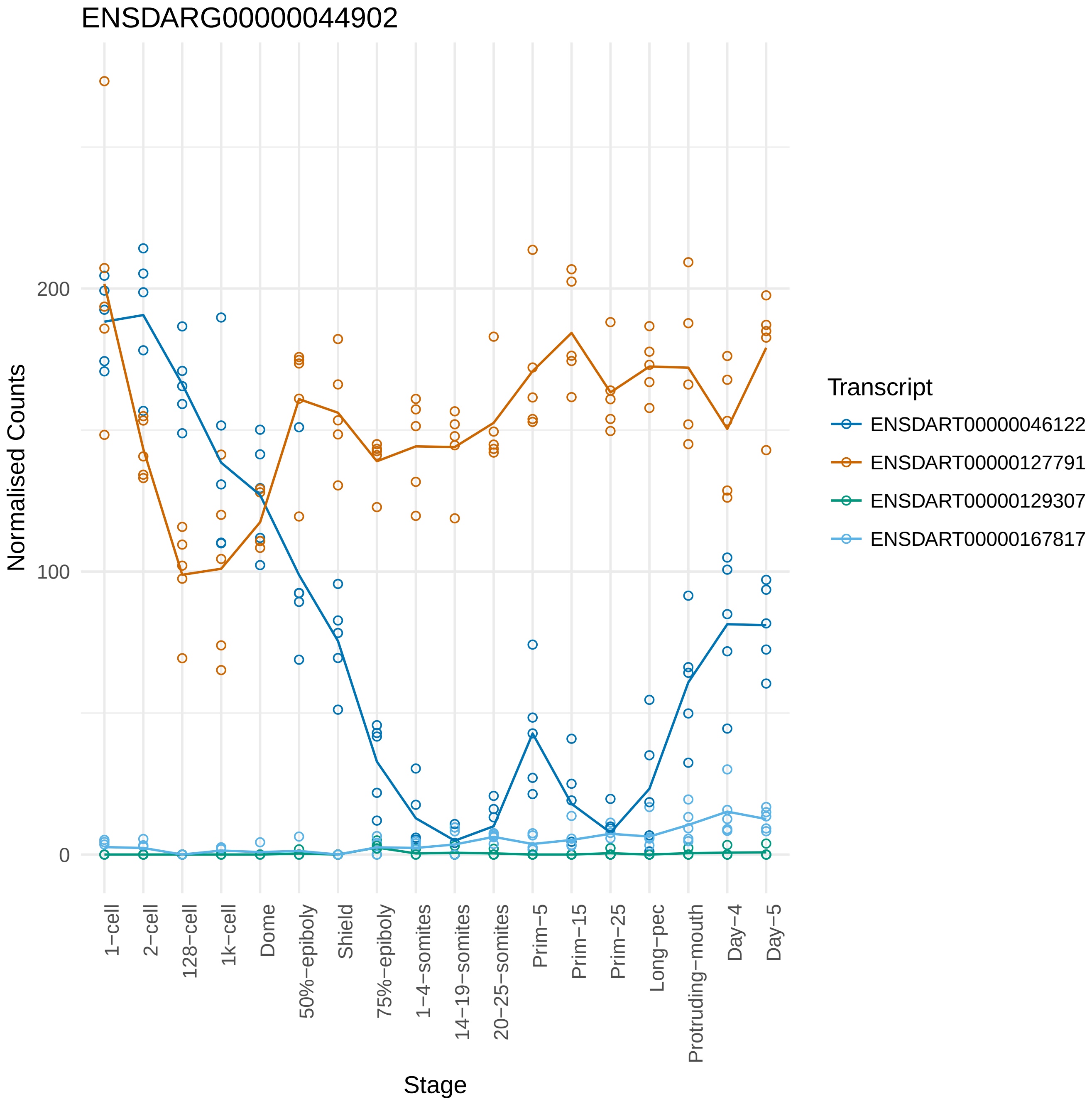

ENSDARG00000044902
Normalised Counts
200
100
0
1−cell
2−cell
128−cell
1k−cell
Dome
50%−epiboly
Shield
75%−epiboly
1−4−somites
14−19−somites
20−25−somites
Prim−5
Prim−15
Prim−25
Long−pec
Protruding−mouth
Day−4
Day−5
Stage
Transcript
ENSDART00000046122
ENSDART00000127791
ENSDART00000129307
ENSDART00000167817

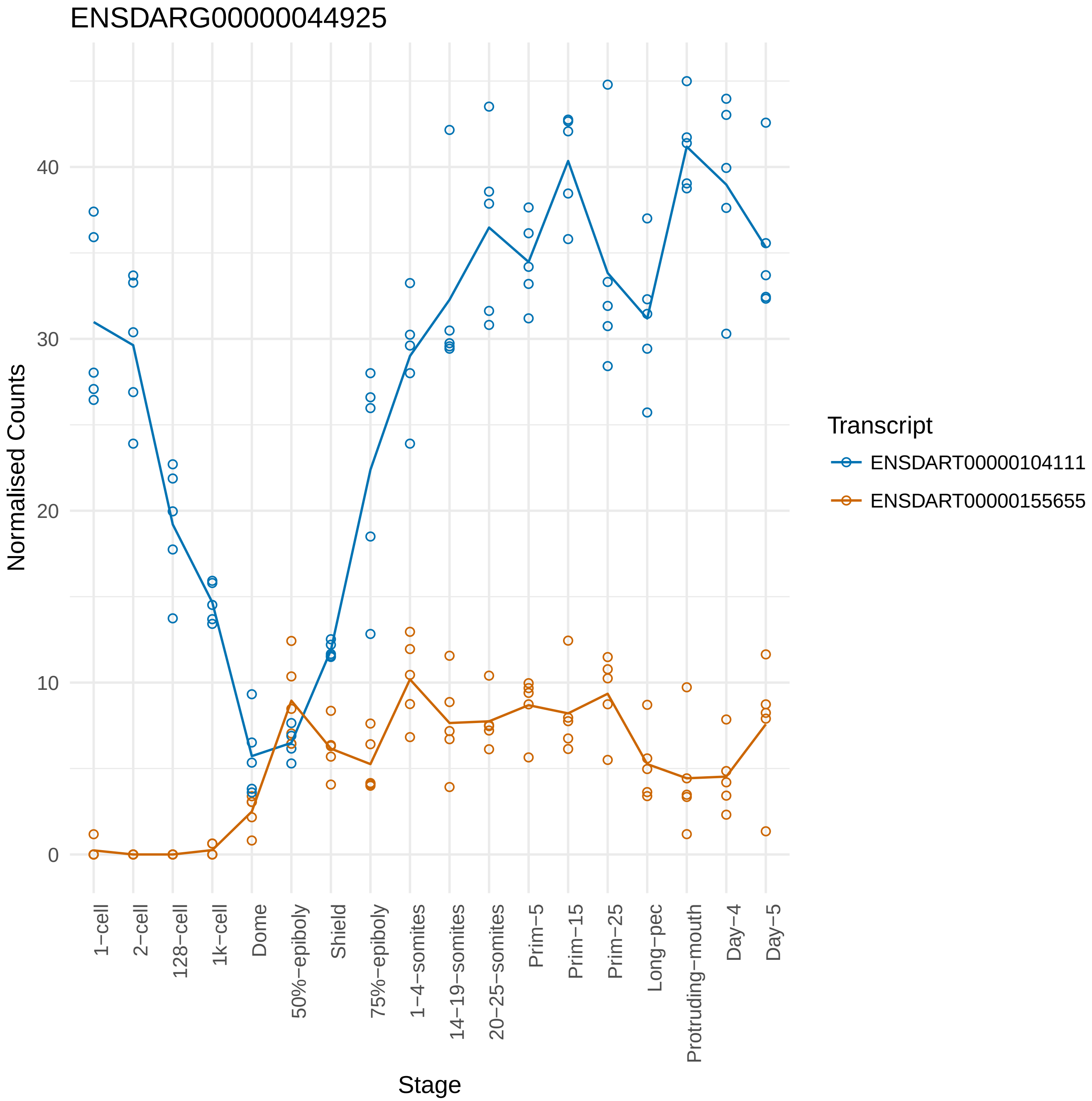

ENSDARG00000044925
Normalised Counts
0
10
20
30
40
1−cell
2−cell
128−cell
1k−cell
Dome
50%−epiboly
Shield
75%−epiboly
1−4−somites
14−19−somites
20−25−somites
Prim−5
Prim−15
Prim−25
Long−pec
Protruding−mouth
Day−4
Day−5
Stage
Transcript
ENSDART00000104111
ENSDART00000155655

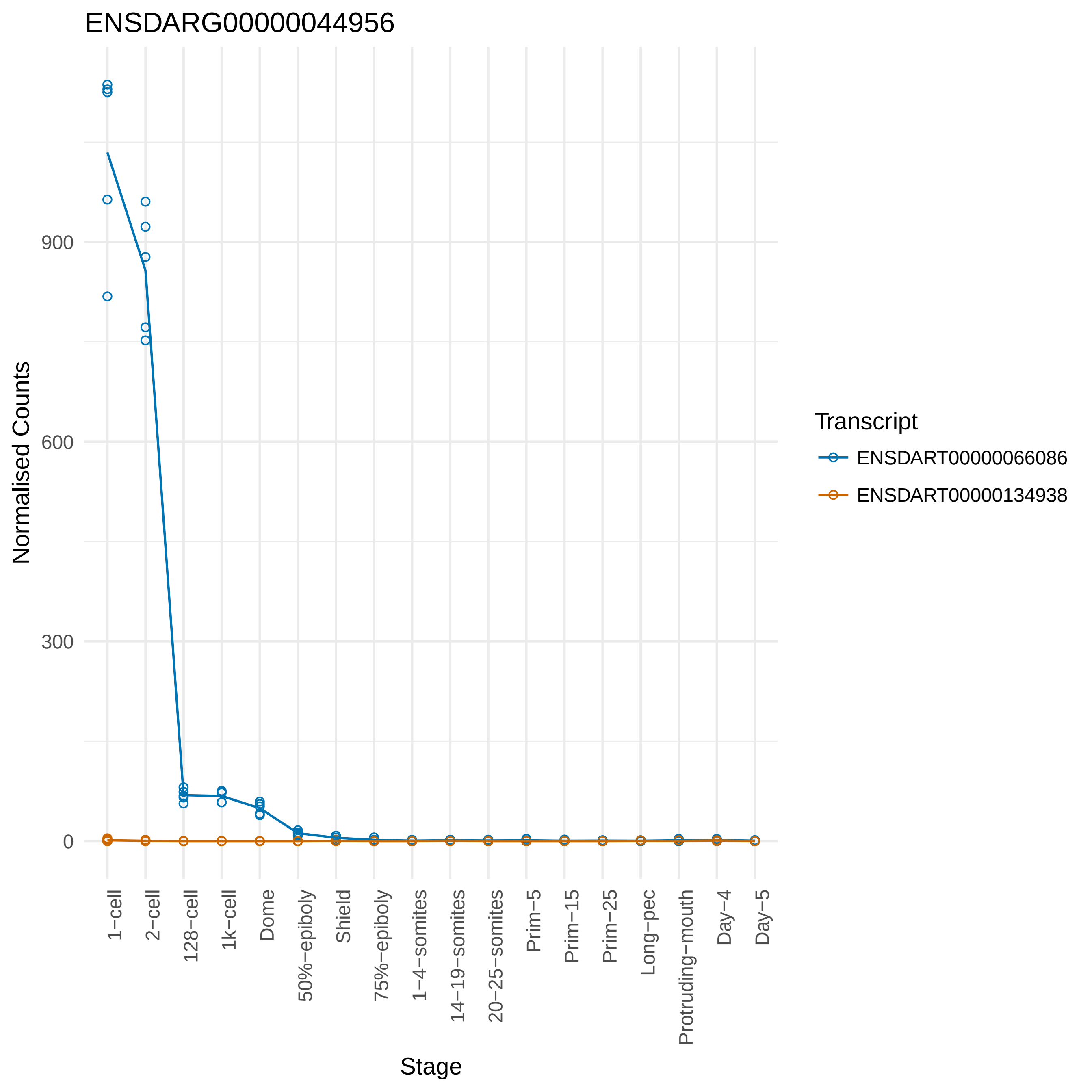
ENSDARG00000044956
Normalised Counts
900
600
300
0
1−cell
2−cell
128−cell
1k−cell
Dome
50%−epiboly
Shield
75%−epiboly
1−4−somites
14−19−somites
20−25−somites
Prim−5
Prim−15
Prim−25
Long−pec
Protruding−mouth
Day−4
Day−5
Stage
Transcript
ENSDART00000066086
ENSDART00000134938

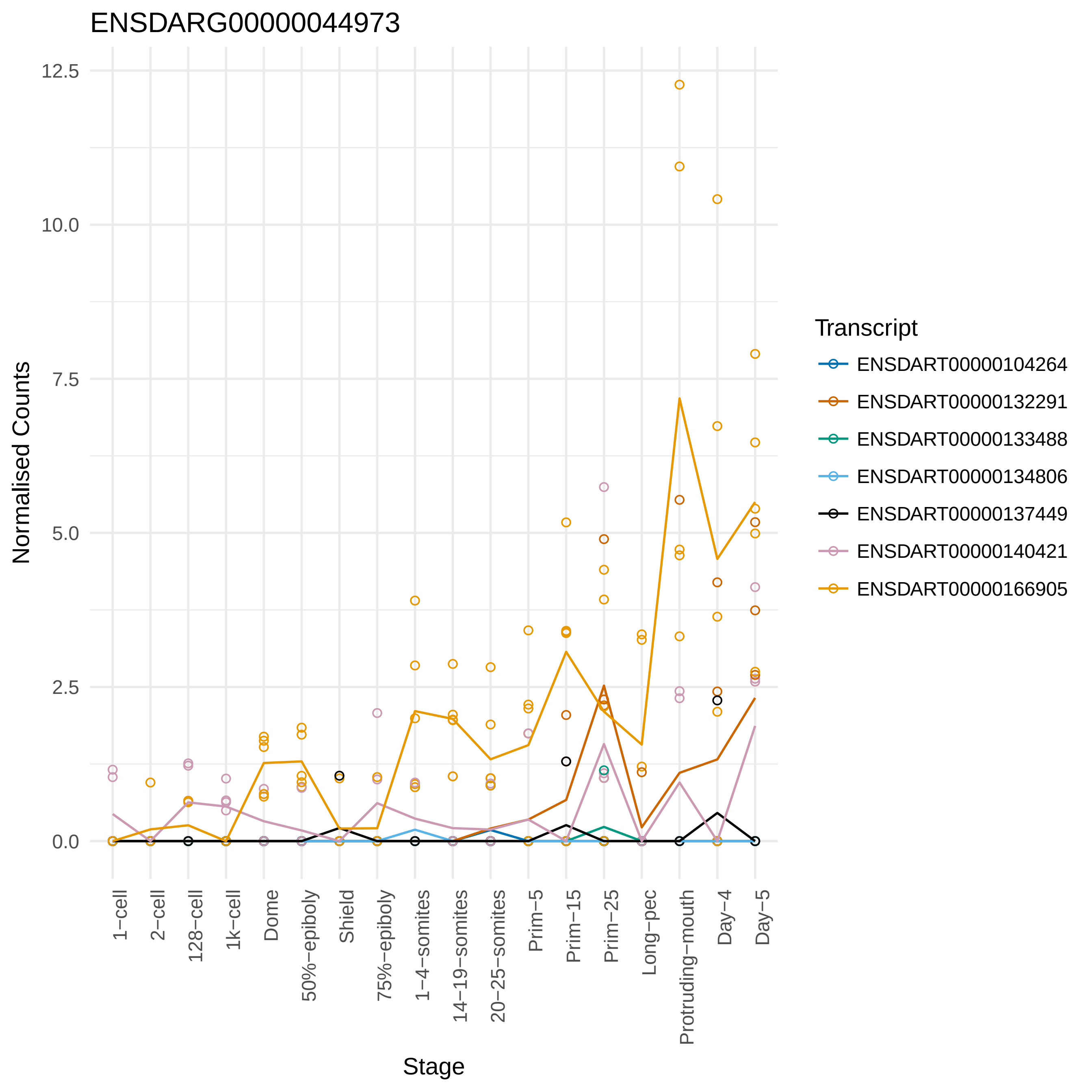

ENSDARG00000044973
Normalised Counts
12.5
10.0
7.5
5.0
2.5
0.0
1−cell
2−cell
128−cell
1k−cell
Dome
50%−epiboly
Shield
75%−epiboly
1−4−somites
14−19−somites
20−25−somites
Prim−5
Prim−15
Prim−25
Long−pec
Protruding−mouth
Day−4
Day−5
Stage
Transcript
ENSDART00000104264
ENSDART00000132291
ENSDART00000133488
ENSDART00000134806
ENSDART00000137449
ENSDART00000140421
ENSDART00000166905

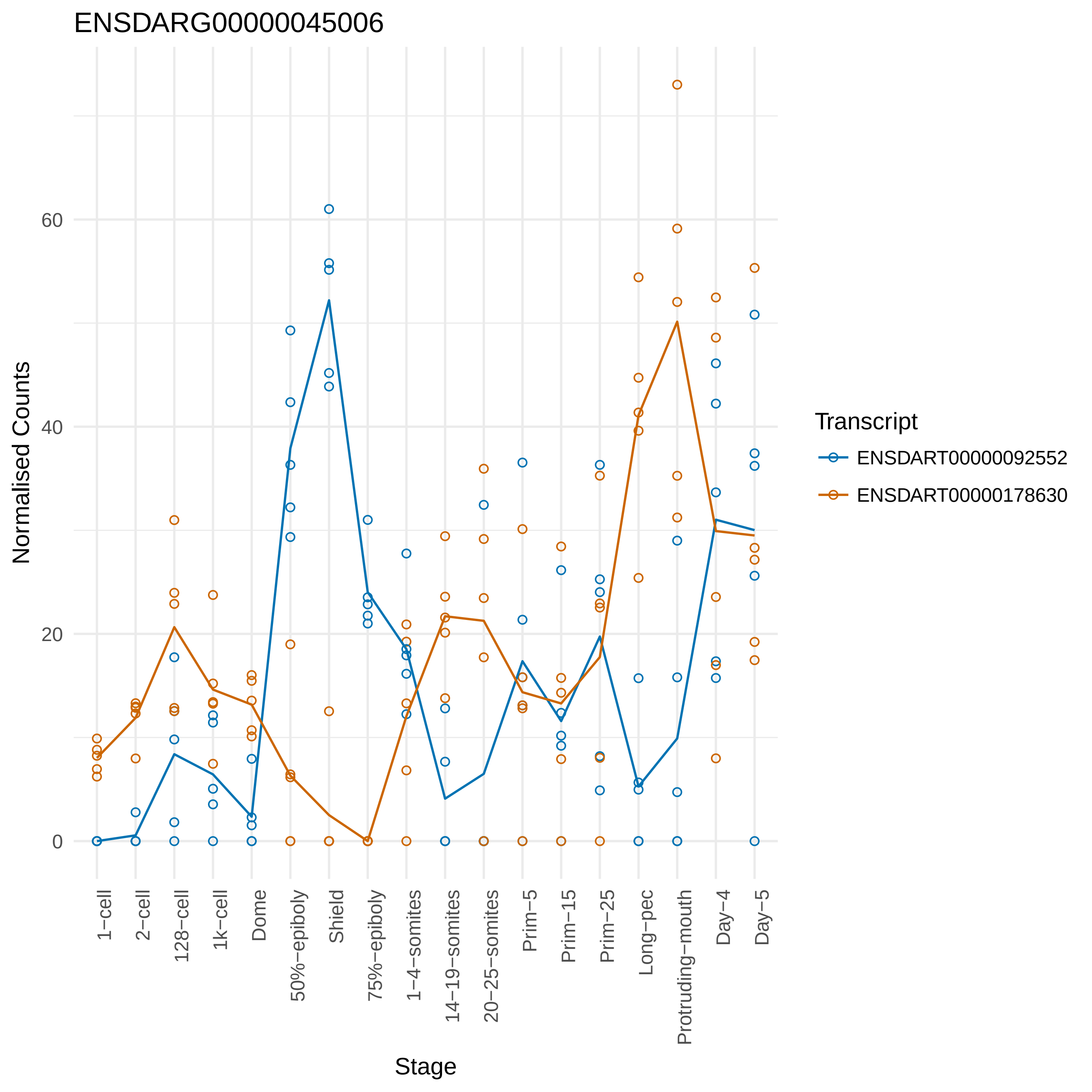

ENSDARG00000045006
Normalised Counts
0
20
40
60
Stage
1−cell
2−cell
128−cell
1k−cell
Dome
50%−epiboly
Shield
75%−epiboly
1−4−somites
14−19−somites
20−25−somites
Prim−5
Prim−15
Prim−25
Long−pec
Protruding−mouth
Day−4
Day−5
Transcript
ENSDART00000092552
ENSDART00000178630

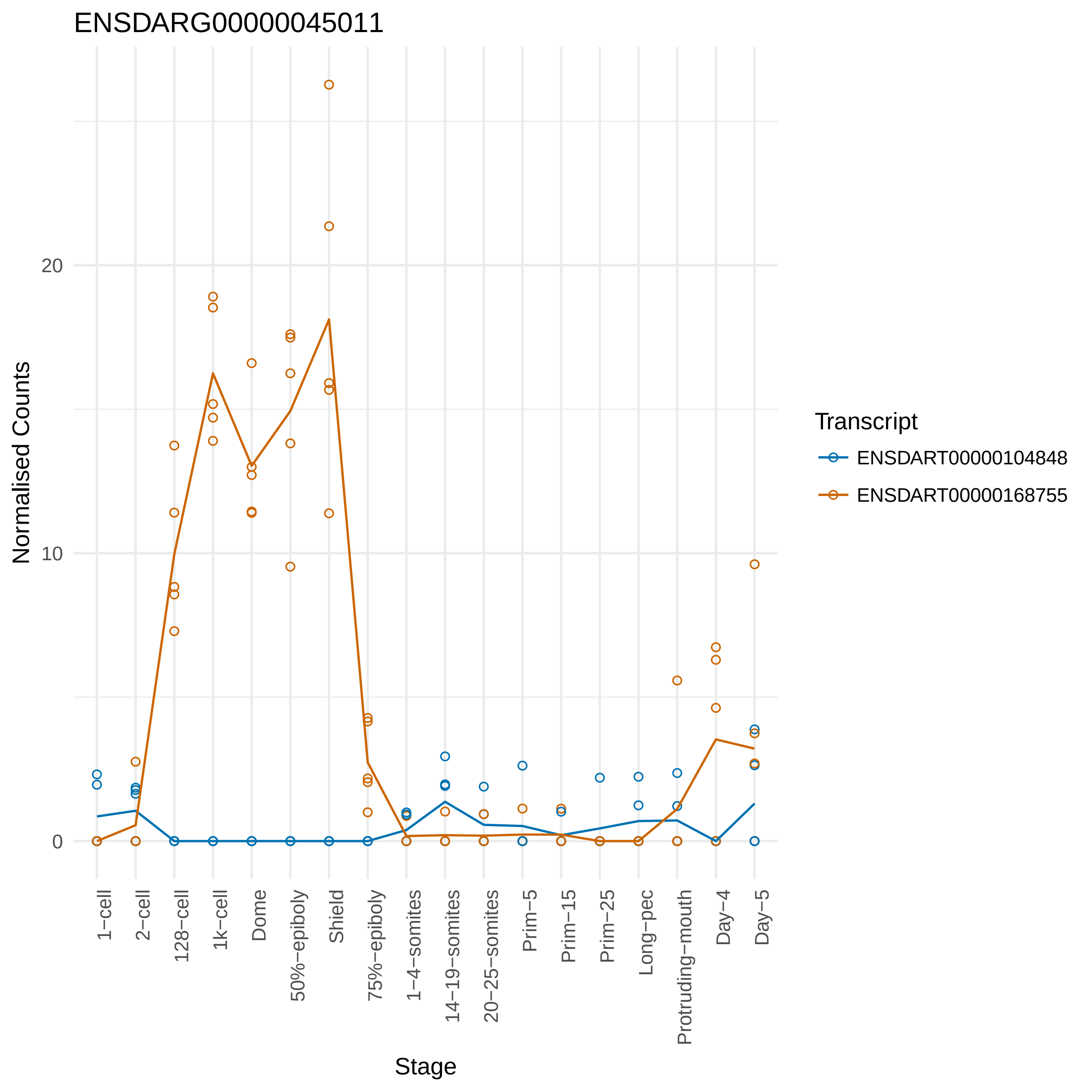

ENSDARG00000045011
Normalised Counts
0
10
20
Stage
1−cell
2−cell
128−cell
1k−cell
Dome
50%−epiboly
Shield
75%−epiboly
1−4−somites
14−19−somites
20−25−somites
Prim−5
Prim−15
Prim−25
Long−pec
Protruding−mouth
Day−4
Day−5
Transcript
ENSDART00000104848
ENSDART00000168755

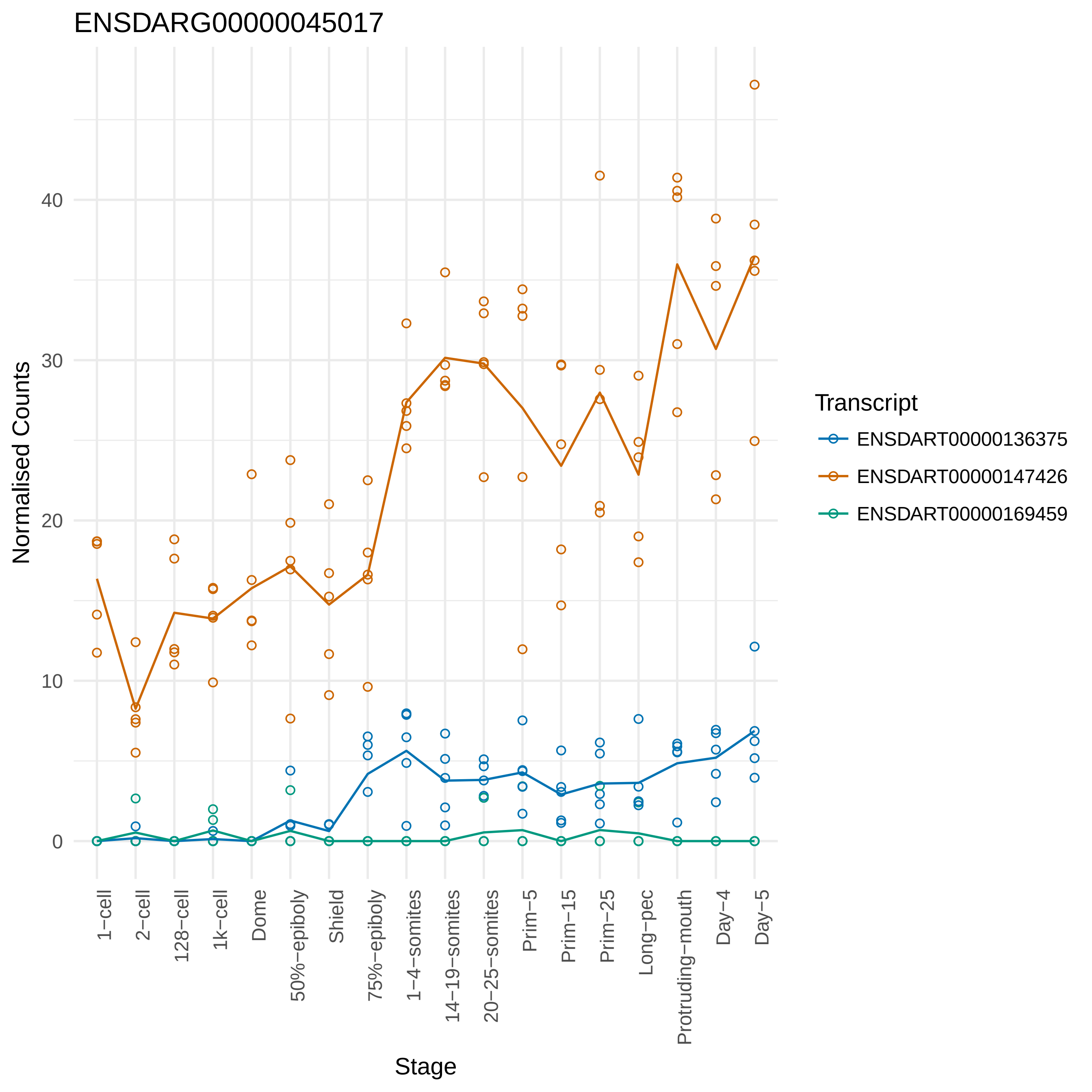
ENSDARG00000045017
Normalised Counts
0
10
20
30
40
Stage
1−cell
2−cell
128−cell
1k−cell
Dome
50%−epiboly
Shield
75%−epiboly
1−4−somites
14−19−somites
20−25−somites
Prim−5
Prim−15
Prim−25
Long−pec
Protruding−mouth
Day−4
Day−5
Transcript
ENSDART00000136375
ENSDART00000147426
ENSDART00000169459

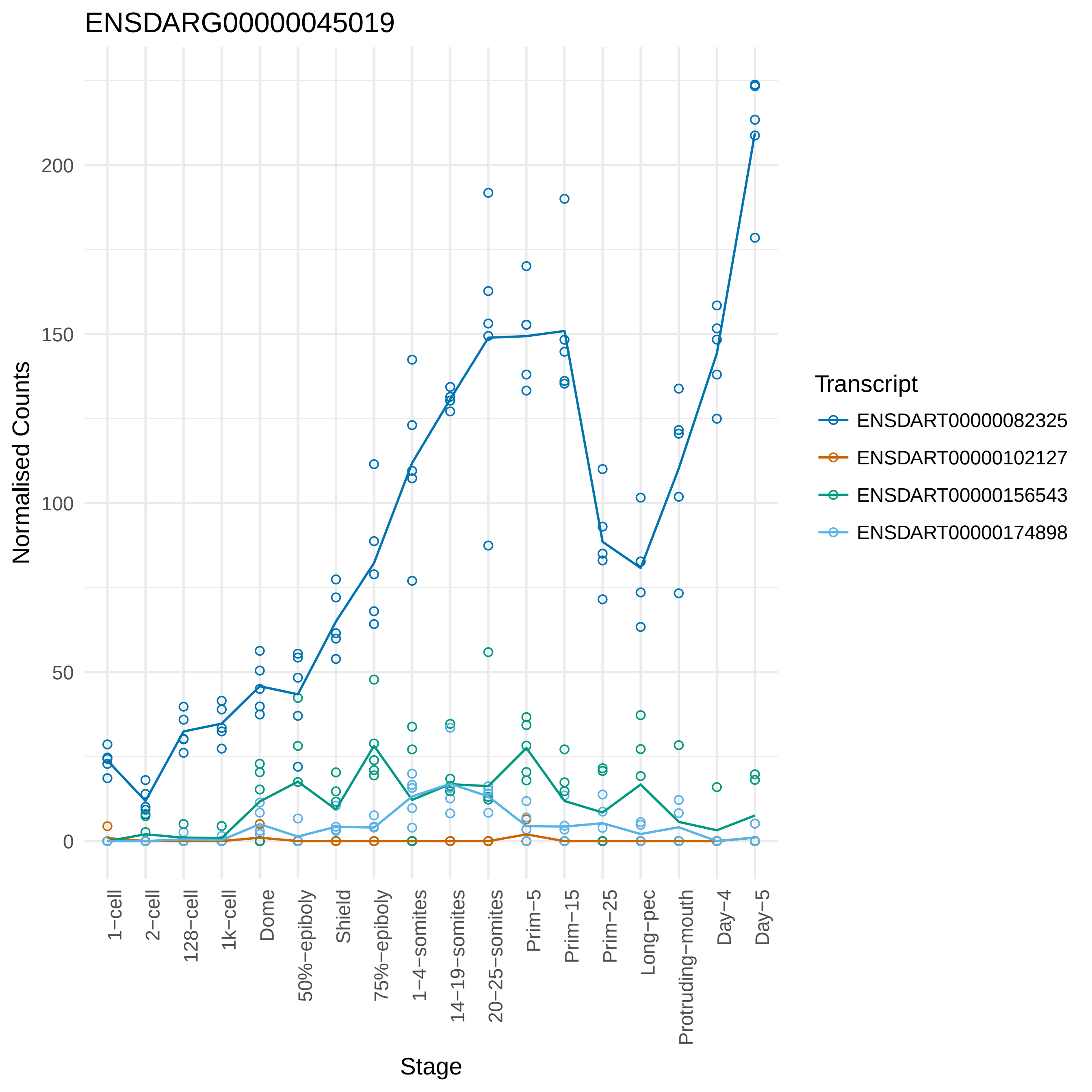
ENSDARG00000045019
Normalised Counts
0
50
100
150
200
Stage
1−cell
2−cell
128−cell
1k−cell
Dome
50%−epiboly
Shield
75%−epiboly
1−4−somites
14−19−somites
20−25−somites
Prim−5
Prim−15
Prim−25
Long−pec
Protruding−mouth
Day−4
Day−5
Transcript
ENSDART00000082325
ENSDART00000102127
ENSDART00000156543
ENSDART00000174898

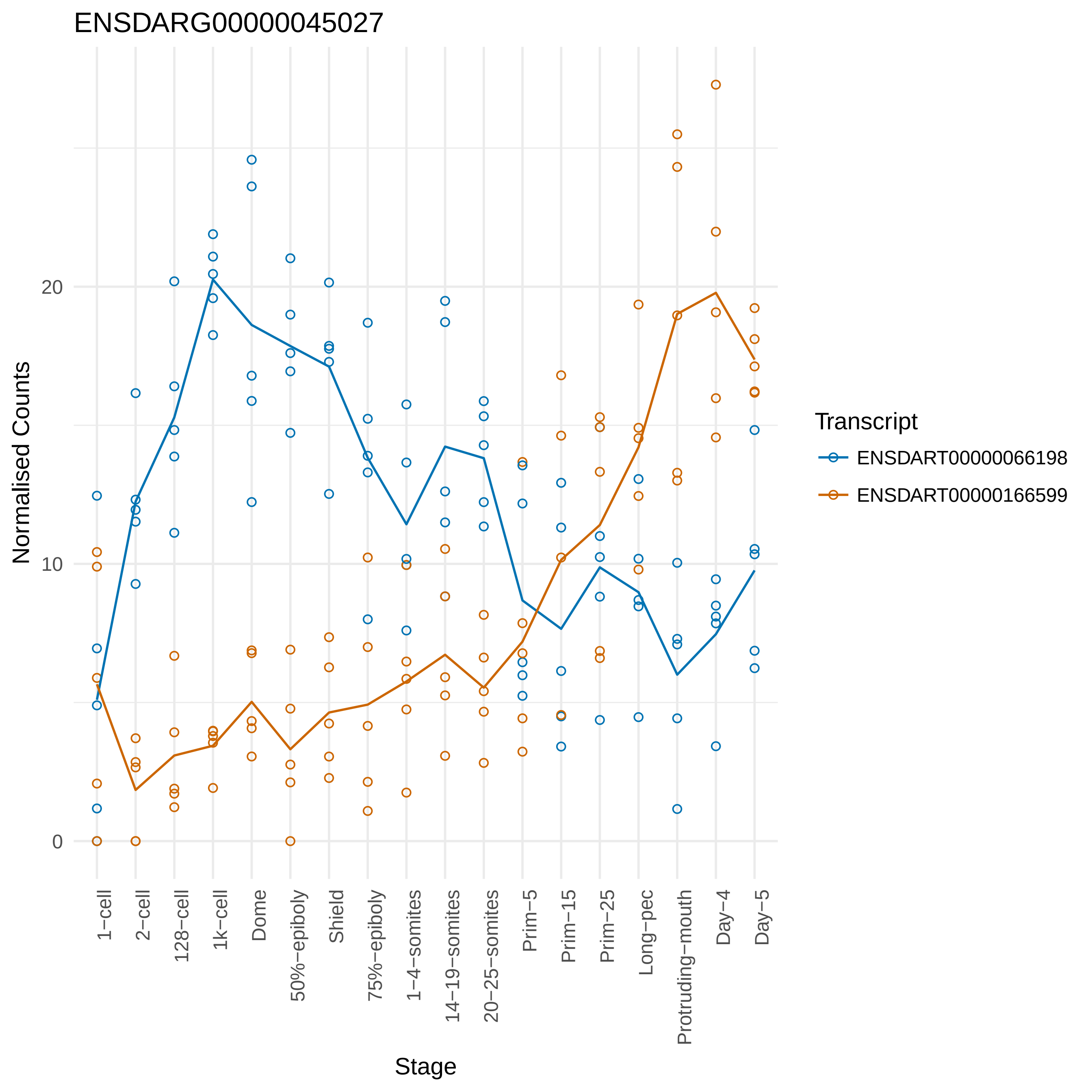

ENSDARG00000045027
Normalised Counts
0
10
20
1−cell
2−cell
128−cell
1k−cell
Dome
50%−epiboly
Shield
75%−epiboly
1−4−somites
14−19−somites
20−25−somites
Prim−5
Prim−15
Prim−25
Long−pec
Protruding−mouth
Day−4
Day−5
Stage
Transcript
ENSDART00000066198
ENSDART00000166599

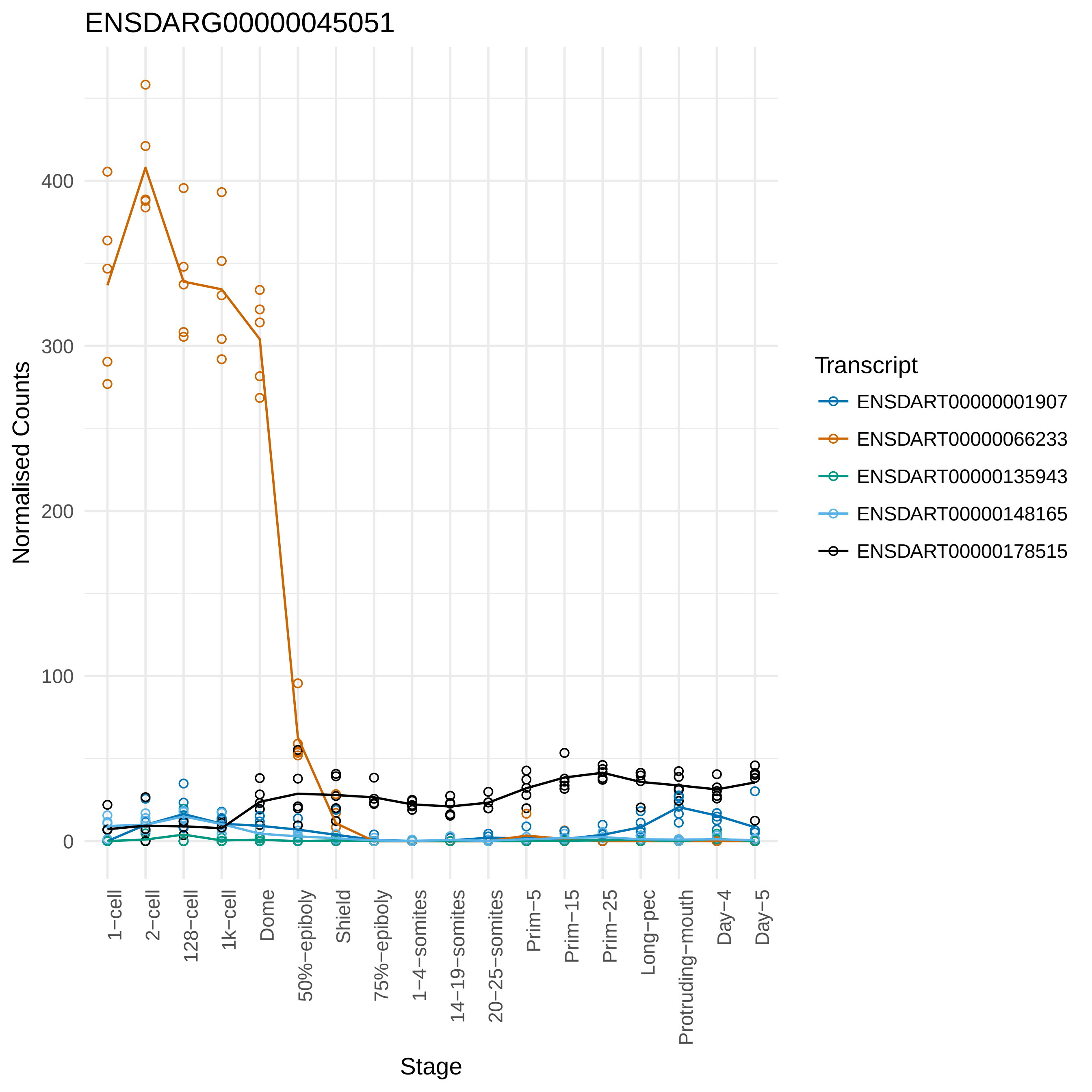

ENSDARG00000045051
Normalised Counts
0
100
200
300
400
Stage
1−cell
2−cell
128−cell
1k−cell
Dome
50%−epiboly
Shield
75%−epiboly
1−4−somites
14−19−somites
20−25−somites
Prim−5
Prim−15
Prim−25
Long−pec
Protruding−mouth
Day−4
Day−5
Transcript
ENSDART00000001907
ENSDART00000066233
ENSDART00000135943
ENSDART00000148165
ENSDART00000178515

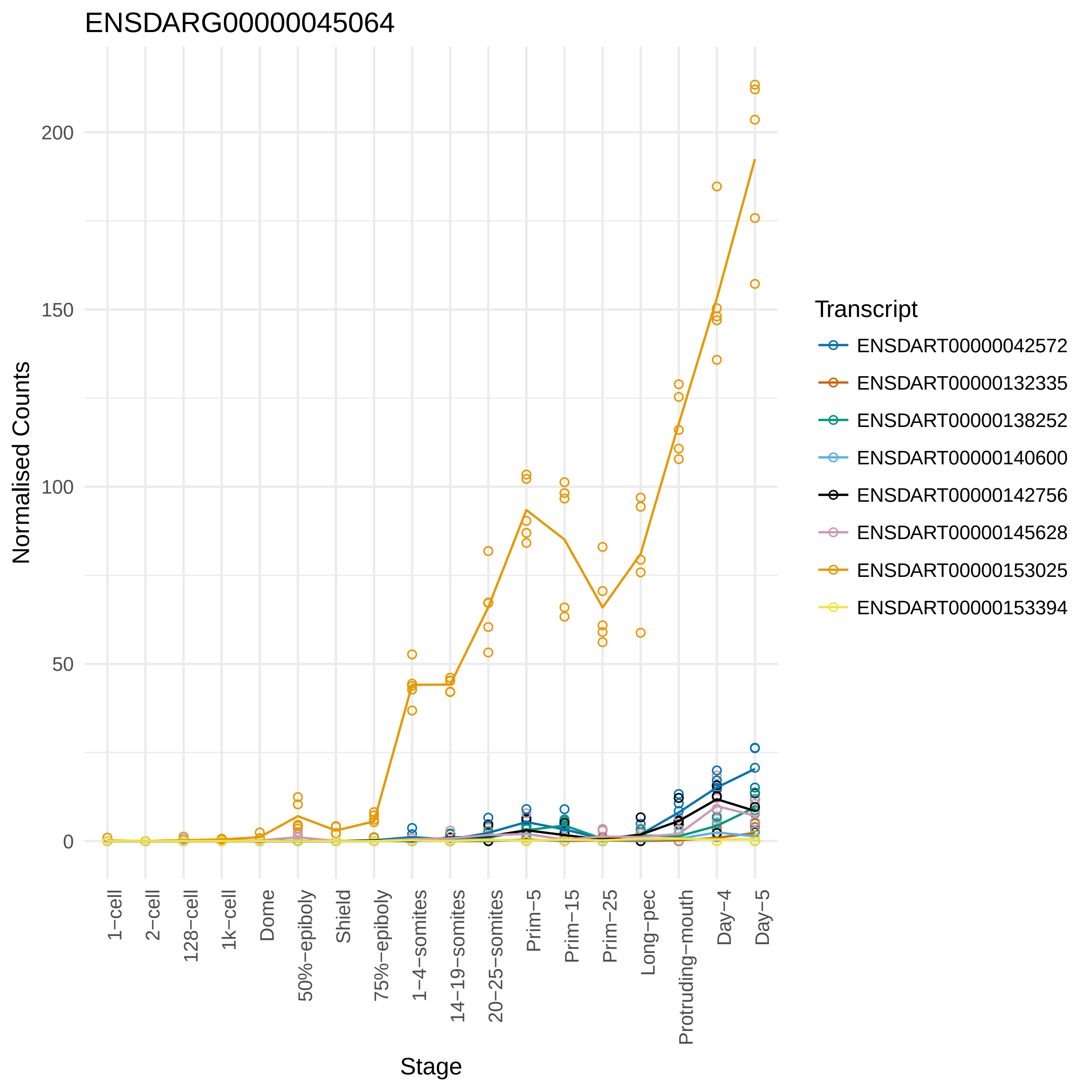

ENSDARG00000045064
Normalised Counts
0
50
100
150
200
1−cell
2−cell
128−cell
1k−cell
Dome
50%−epiboly
Shield
75%−epiboly
1−4−somites
14−19−somites
20−25−somites
Prim−5
Prim−15
Prim−25
Long−pec
Protruding−mouth
Day−4
Day−5
Stage
Transcript
ENSDART00000042572
ENSDART00000132335
ENSDART00000138252
ENSDART00000140600
ENSDART00000142756
ENSDART00000145628
ENSDART00000153025
ENSDART00000153394

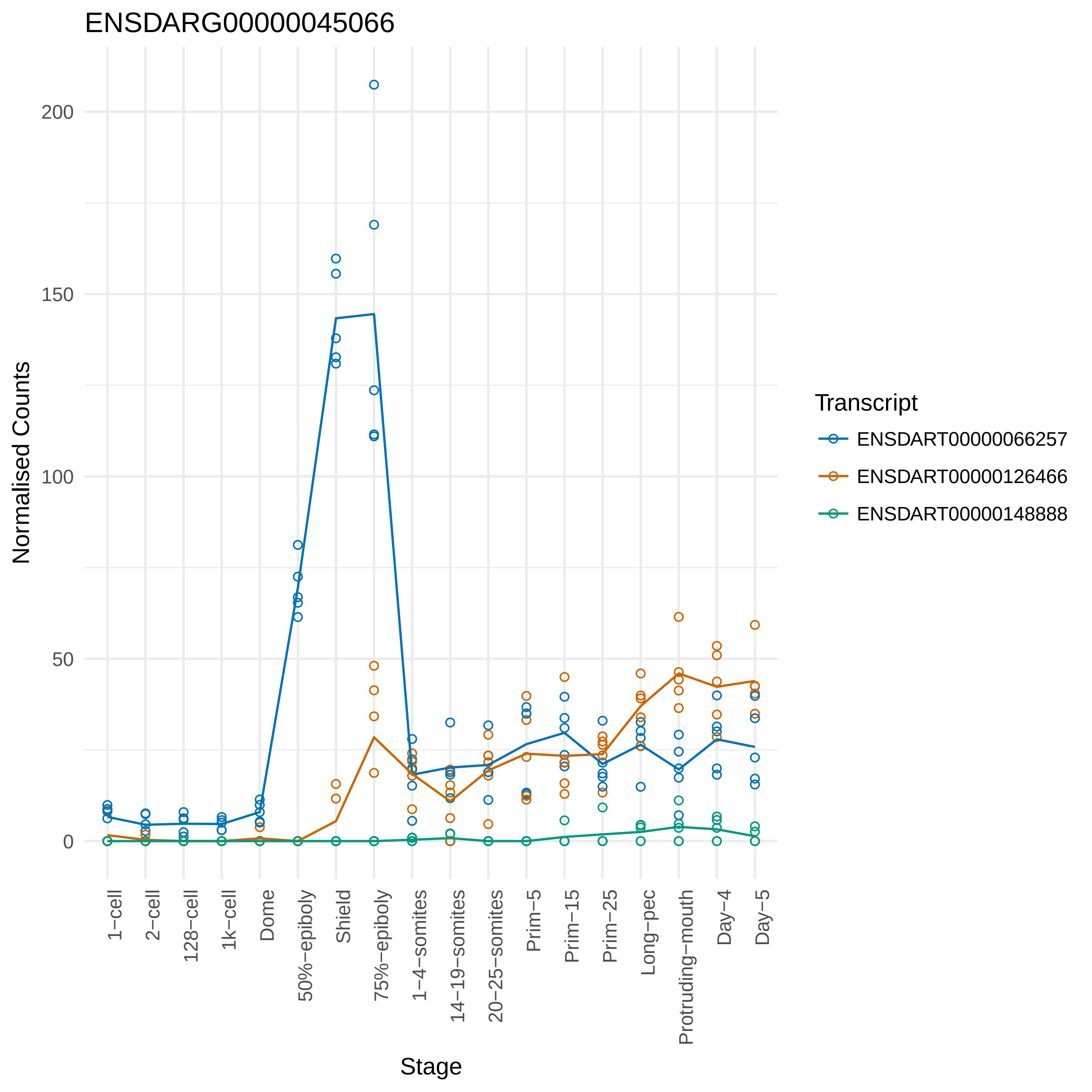
ENSDARG00000045066
Normalised Counts
0
50
100
150
200
Stage
1−cell
2−cell
128−cell
1k−cell
Dome
50%−epiboly
Shield
75%−epiboly
1−4−somites
14−19−somites
20−25−somites
Prim−5
Prim−15
Prim−25
Long−pec
Protruding−mouth
Day−4
Day−5
Transcript
ENSDART00000066257
ENSDART00000126466
ENSDART00000148888

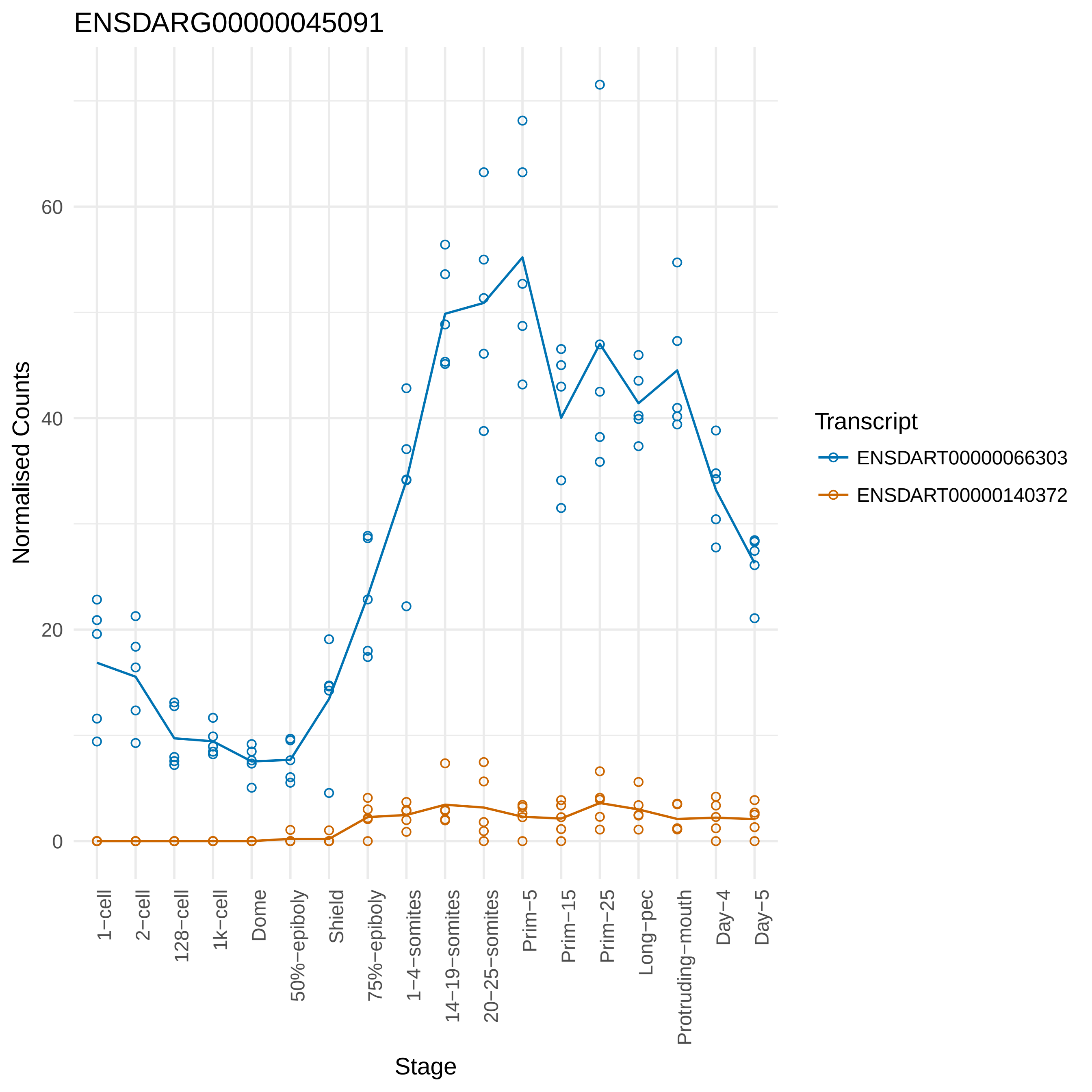
ENSDARG00000045091
Normalised Counts
0
20
40
60
Stage
1−cell
2−cell
128−cell
1k−cell
Dome
50%−epiboly
Shield
75%−epiboly
1−4−somites
14−19−somites
20−25−somites
Prim−5
Prim−15
Prim−25
Long−pec
Protruding−mouth
Day−4
Day−5
Transcript
ENSDART00000066303
ENSDART00000140372

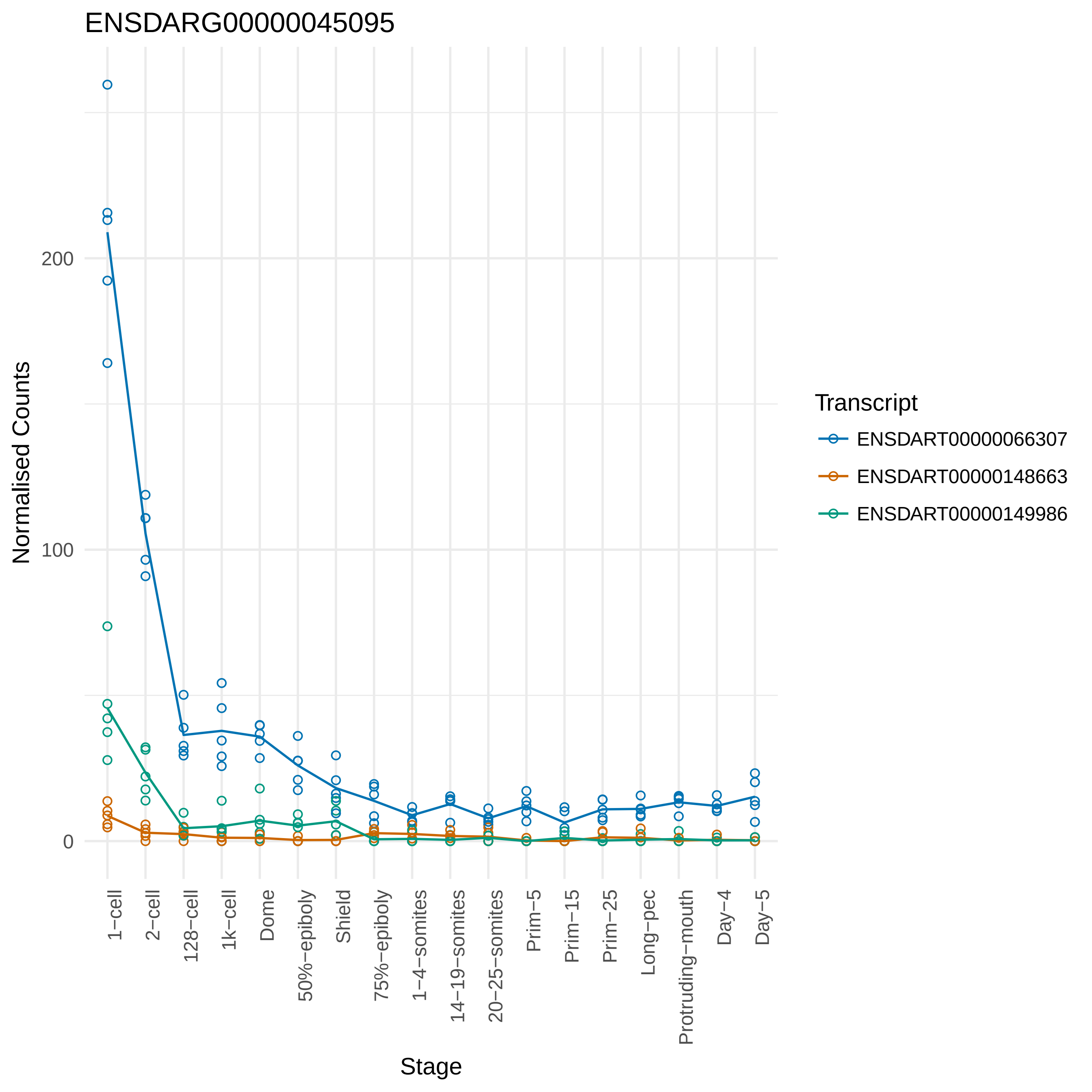

ENSDARG00000045095
Normalised Counts
200
100
0
1−cell
2−cell
128−cell
1k−cell
Dome
50%−epiboly
Shield
75%−epiboly
1−4−somites
14−19−somites
20−25−somites
Prim−5
Prim−15
Prim−25
Long−pec
Protruding−mouth
Day−4
Day−5
Stage
Transcript
ENSDART00000066307
ENSDART00000148663
ENSDART00000149986

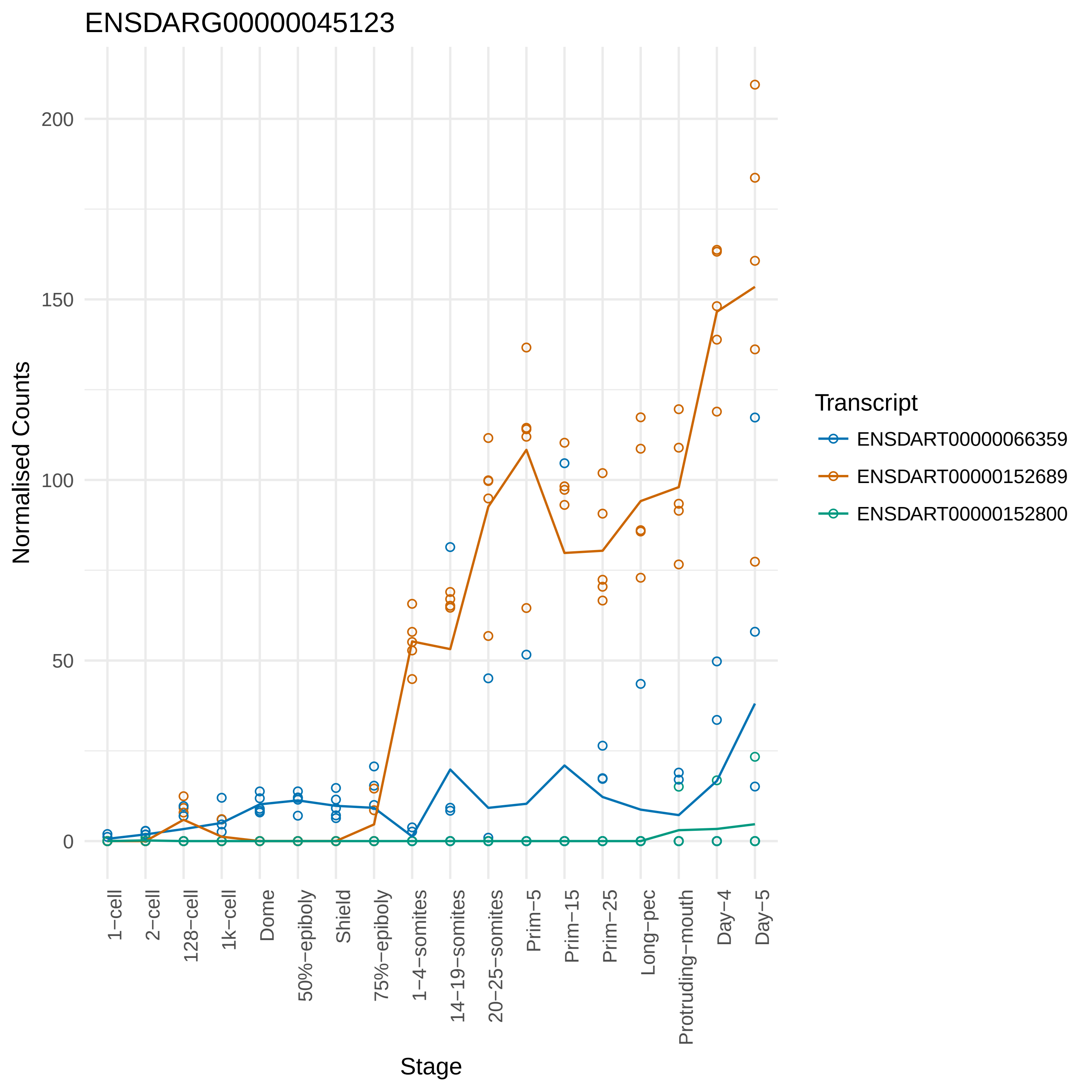

ENSDARG00000045123
Normalised Counts
200
150
100
50
0
1−cell
2−cell
128−cell
1k−cell
Dome
50%−epiboly
Shield
75%−epiboly
1−4−somites
14−19−somites
20−25−somites
Prim−5
Prim−15
Prim−25
Long−pec
Protruding−mouth
Day−4
Day−5
Stage
Transcript
ENSDART00000066359
ENSDART00000152689
ENSDART00000152800

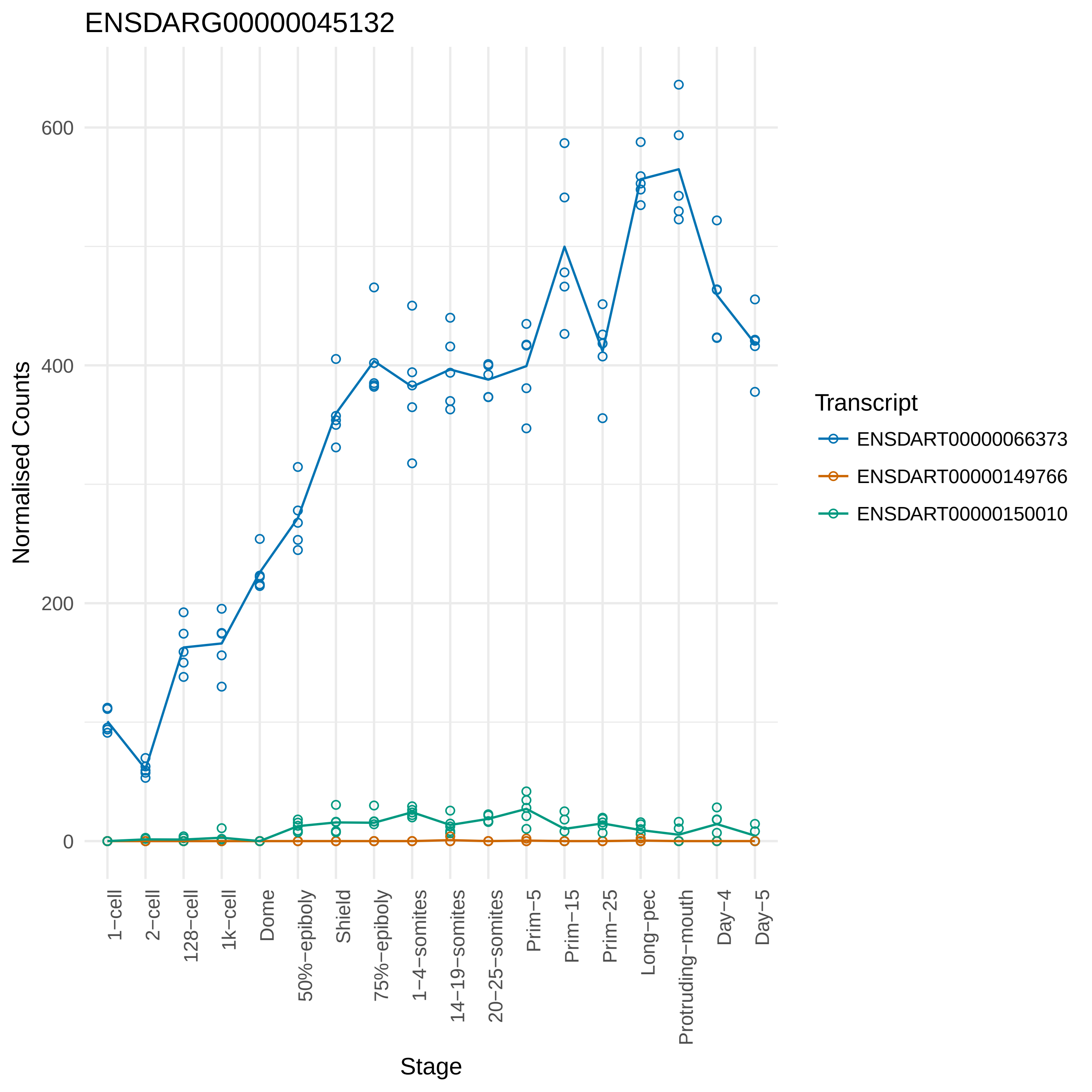

ENSDARG00000045132
Normalised Counts
600
400
200
0
1−cell
2−cell
128−cell
1k−cell
Dome
50%−epiboly
Shield
75%−epiboly
1−4−somites
14−19−somites
20−25−somites
Prim−5
Prim−15
Prim−25
Long−pec
Protruding−mouth
Day−4
Day−5
Stage
Transcript
ENSDART00000066373
ENSDART00000149766
ENSDART00000150010

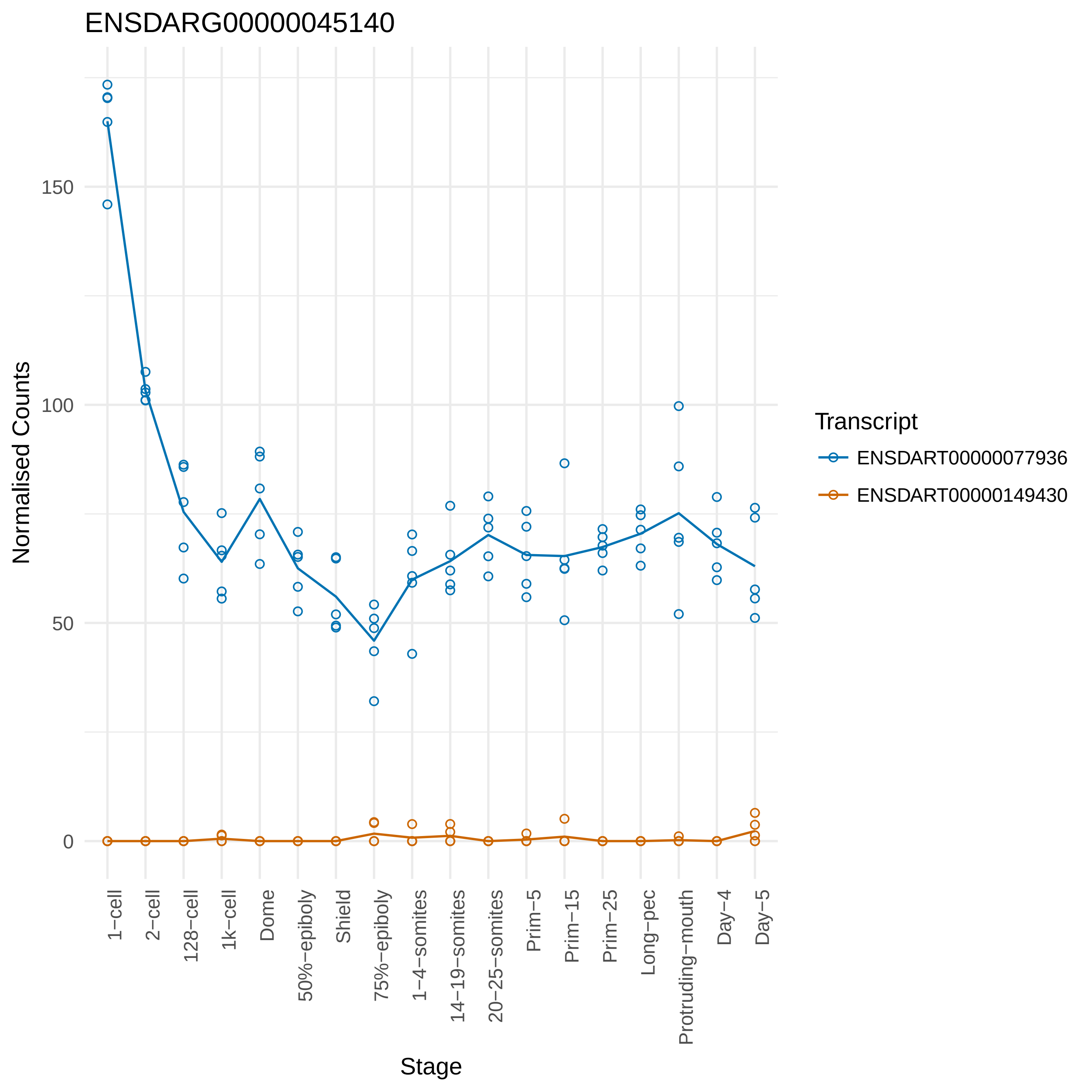
ENSDARG00000045140
Normalised Counts
150
100
50
0
1−cell
2−cell
128−cell
1k−cell
Dome
50%−epiboly
Shield
75%−epiboly
1−4−somites
14−19−somites
20−25−somites
Prim−5
Prim−15
Prim−25
Long−pec
Protruding−mouth
Day−4
Day−5
Stage
Transcript
ENSDART00000077936
ENSDART00000149430

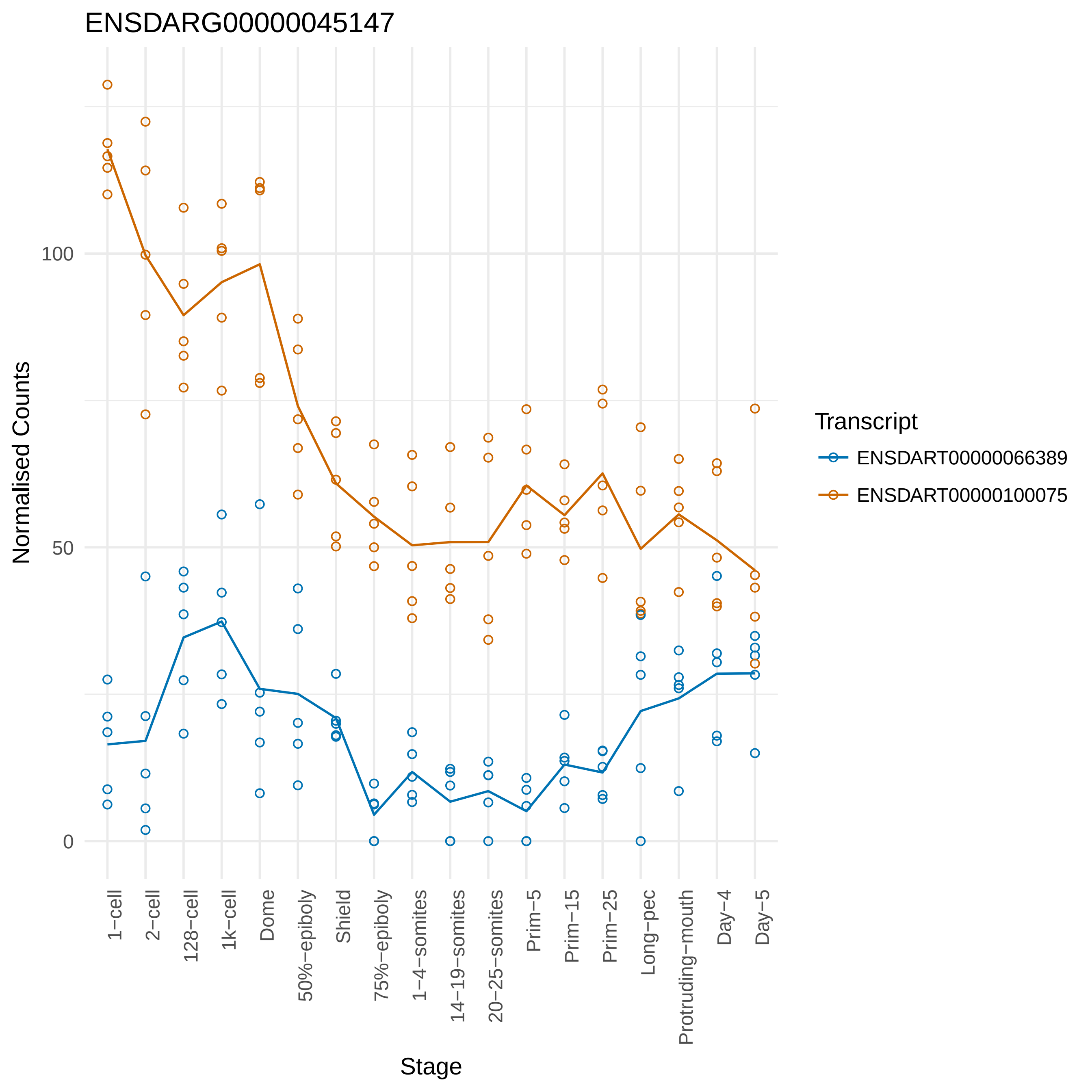

ENSDARG00000045147
Normalised Counts
100
50
0
1−cell
2−cell
128−cell
1k−cell
Dome
50%−epiboly
Shield
75%−epiboly
1−4−somites
14−19−somites
20−25−somites
Prim−5
Prim−15
Prim−25
Long−pec
Protruding−mouth
Day−4
Day−5
Stage
Transcript
ENSDART00000066389
ENSDART00000100075

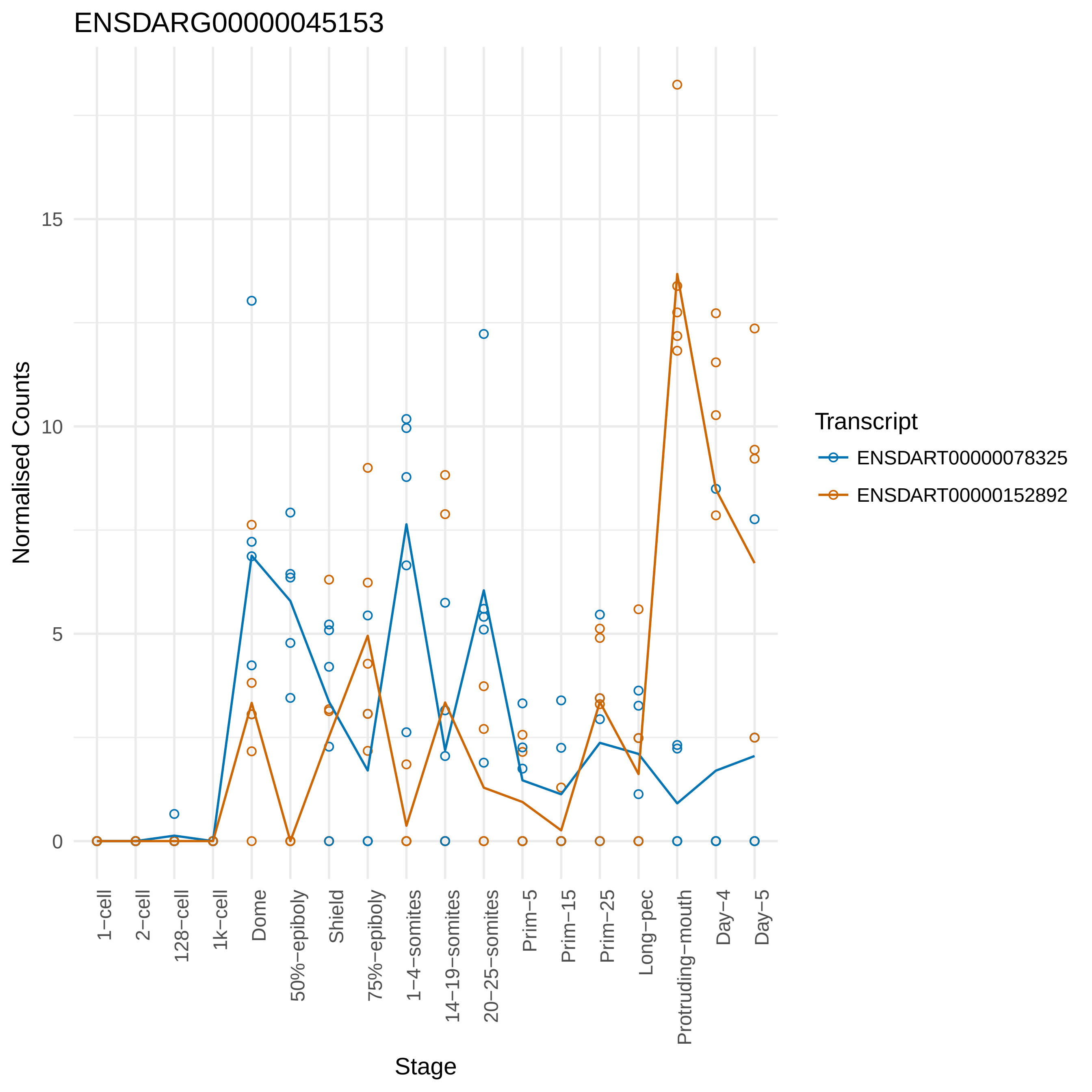

ENSDARG00000045153
Normalised Counts
15
10
5
0
1−cell
2−cell
128−cell
1k−cell
Dome
50%−epiboly
Shield
75%−epiboly
1−4−somites
14−19−somites
20−25−somites
Prim−5
Prim−15
Prim−25
Long−pec
Protruding−mouth
Day−4
Day−5
Stage
Transcript
ENSDART00000078325
ENSDART00000152892

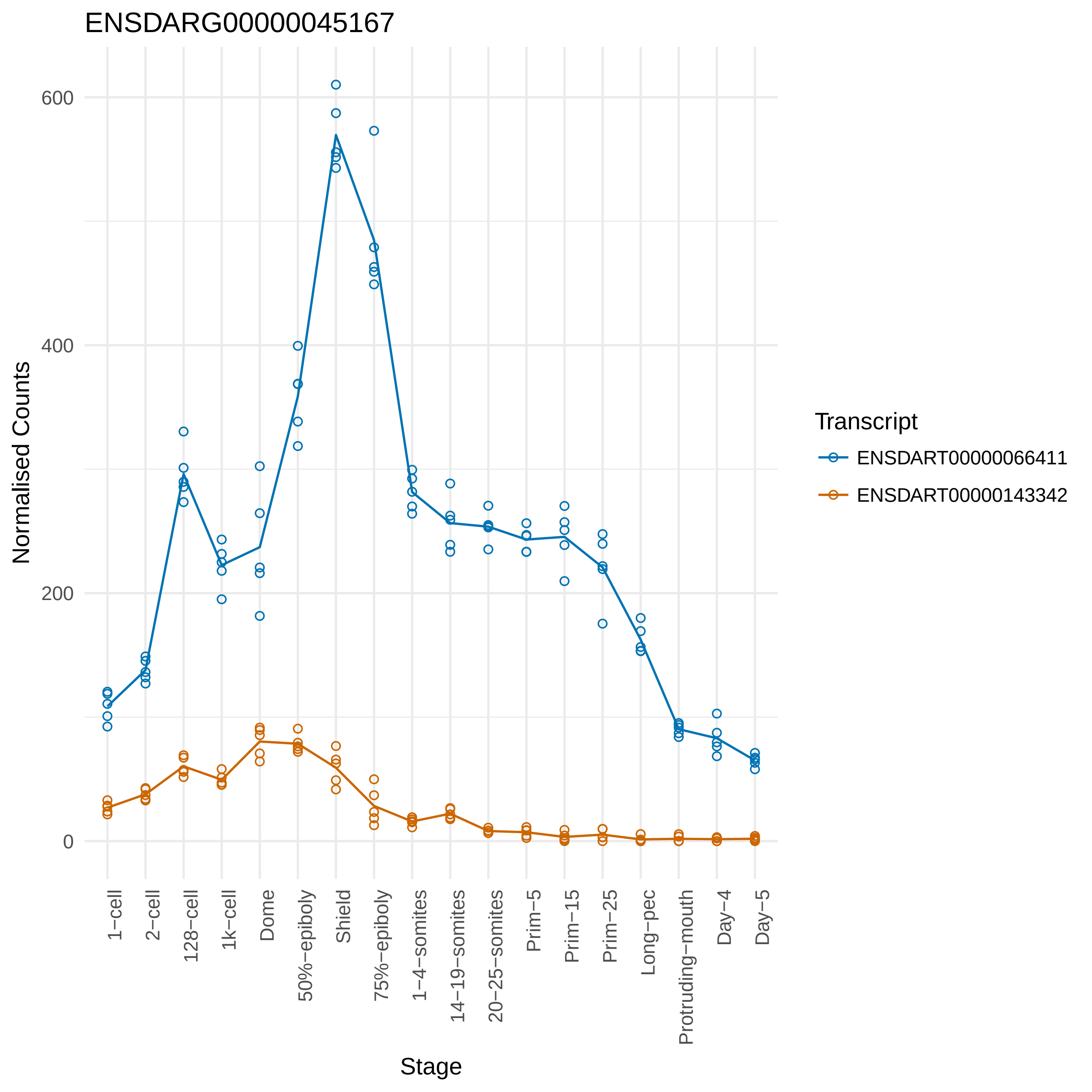

ENSDARG00000045167
Normalised Counts
600
400
200
0
1−cell
2−cell
128−cell
1k−cell
Dome
50%−epiboly
Shield
75%−epiboly
1−4−somites
14−19−somites
20−25−somites
Prim−5
Prim−15
Prim−25
Long−pec
Protruding−mouth
Day−4
Day−5
Stage
Transcript
ENSDART00000066411
ENSDART00000143342

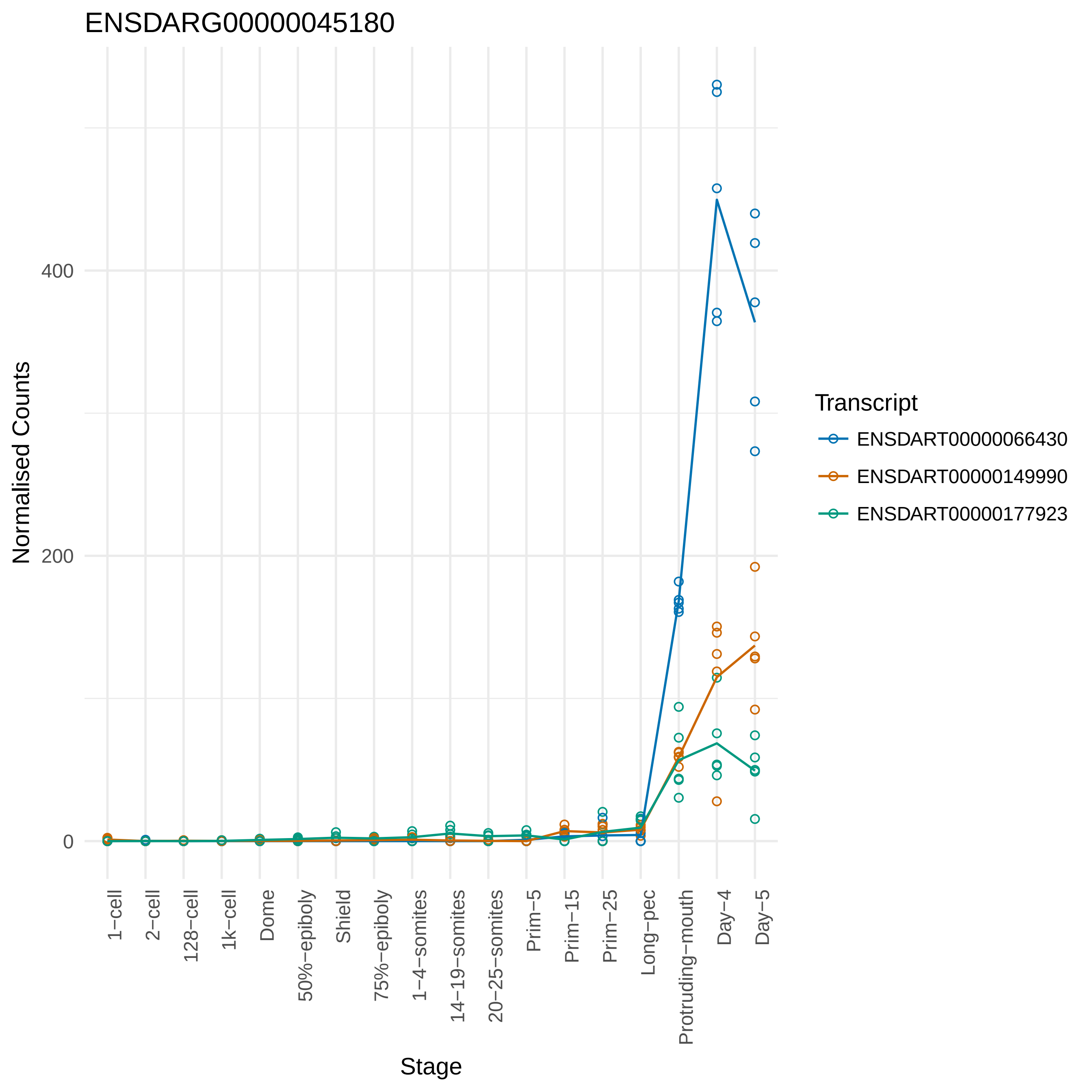

ENSDARG00000045180
Normalised Counts
400
200
0
1−cell
2−cell
128−cell
1k−cell
Dome
50%−epiboly
Shield
75%−epiboly
1−4−somites
14−19−somites
20−25−somites
Prim−5
Prim−15
Prim−25
Long−pec
Protruding−mouth
Day−4
Day−5
Stage
Transcript
ENSDART00000066430
ENSDART00000149990
ENSDART00000177923

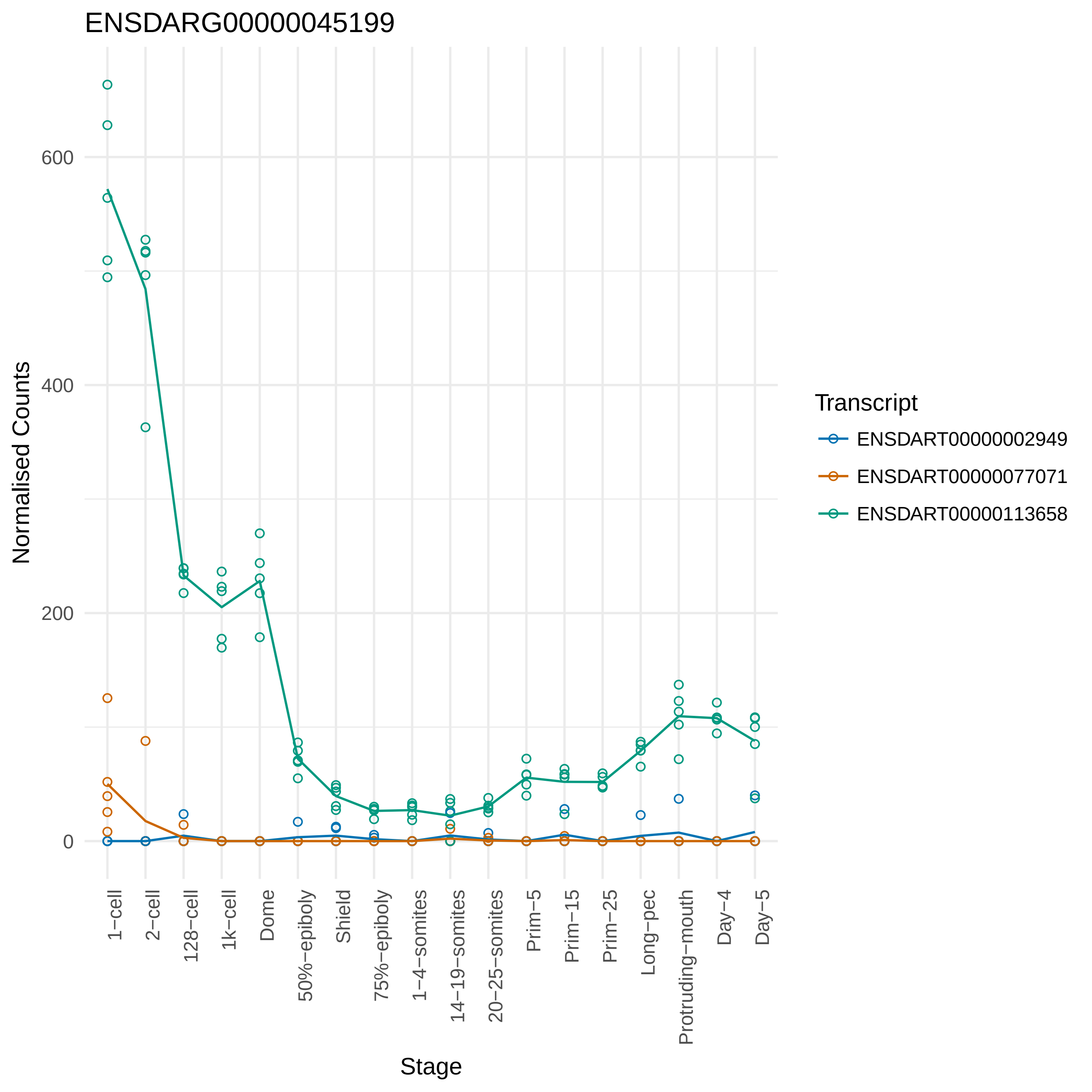

ENSDARG00000045199
Normalised Counts
600
400
200
0
1−cell
2−cell
128−cell
1k−cell
Dome
50%−epiboly
Shield
75%−epiboly
1−4−somites
14−19−somites
20−25−somites
Prim−5
Prim−15
Prim−25
Long−pec
Protruding−mouth
Day−4
Day−5
Stage
Transcript
ENSDART00000002949
ENSDART00000077071
ENSDART00000113658

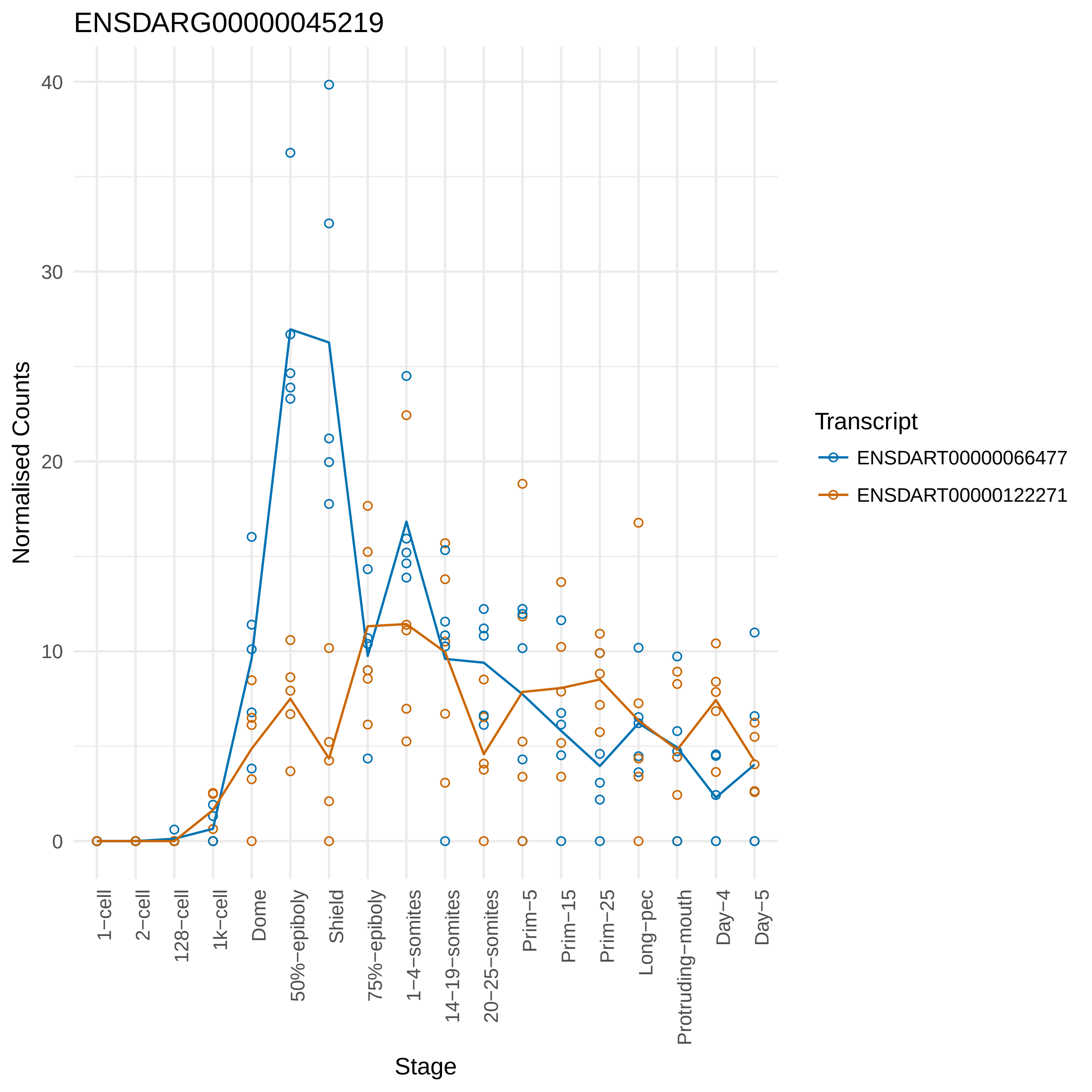

ENSDARG00000045219
Normalised Counts
40
30
20
10
0
1−cell
2−cell
128−cell
1k−cell
Dome
50%−epiboly
Shield
75%−epiboly
1−4−somites
14−19−somites
20−25−somites
Prim−5
Prim−15
Prim−25
Long−pec
Protruding−mouth
Day−4
Day−5
Stage
Transcript
ENSDART00000066477
ENSDART00000122271

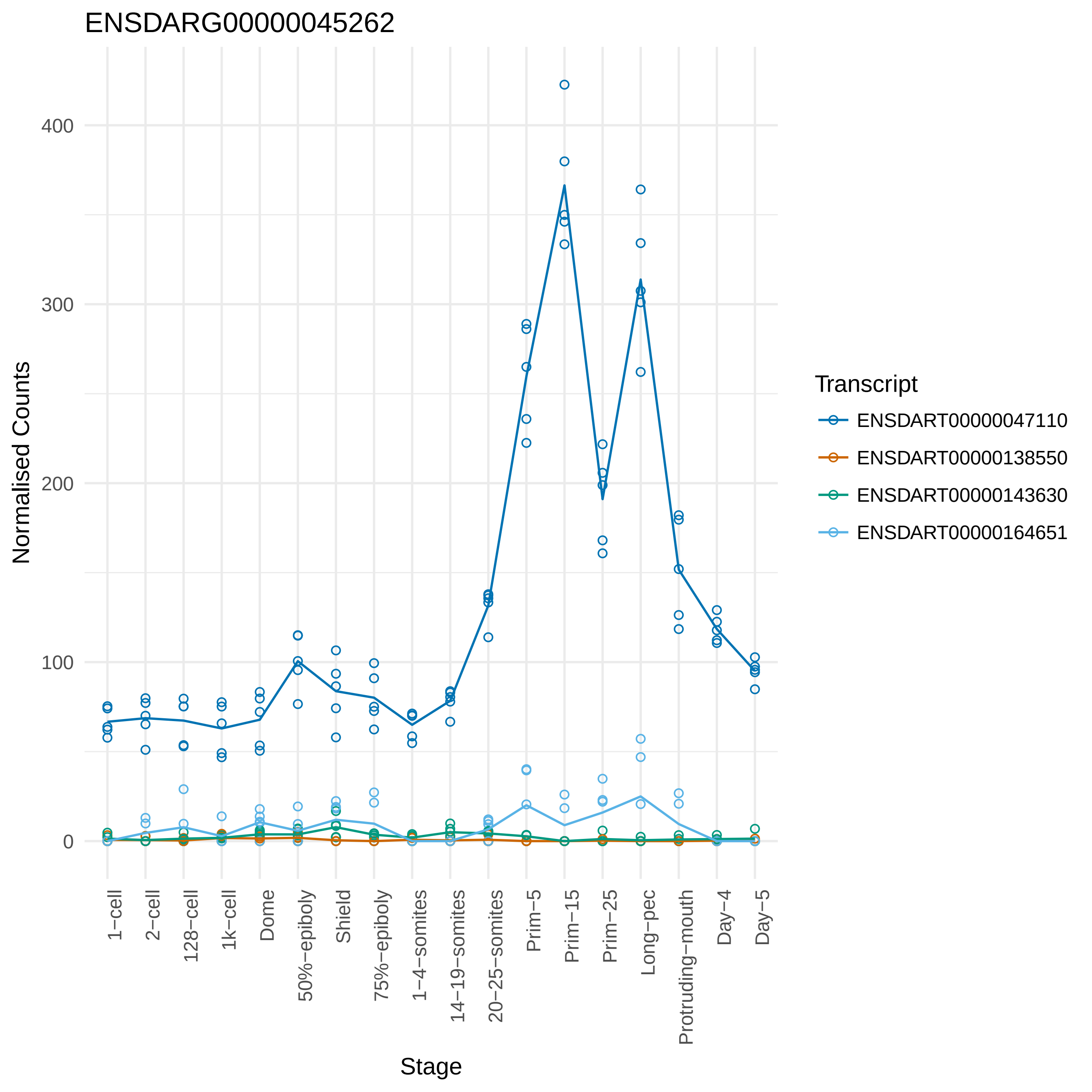
ENSDARG00000045262
Normalised Counts
400
300
200
100
0
1−cell
2−cell
128−cell
1k−cell
Dome
50%−epiboly
Shield
75%−epiboly
1−4−somites
14−19−somites
20−25−somites
Prim−5
Prim−15
Prim−25
Long−pec
Protruding−mouth
Day−4
Day−5
Stage
Transcript
ENSDART00000047110
ENSDART00000138550
ENSDART00000143630
ENSDART00000164651

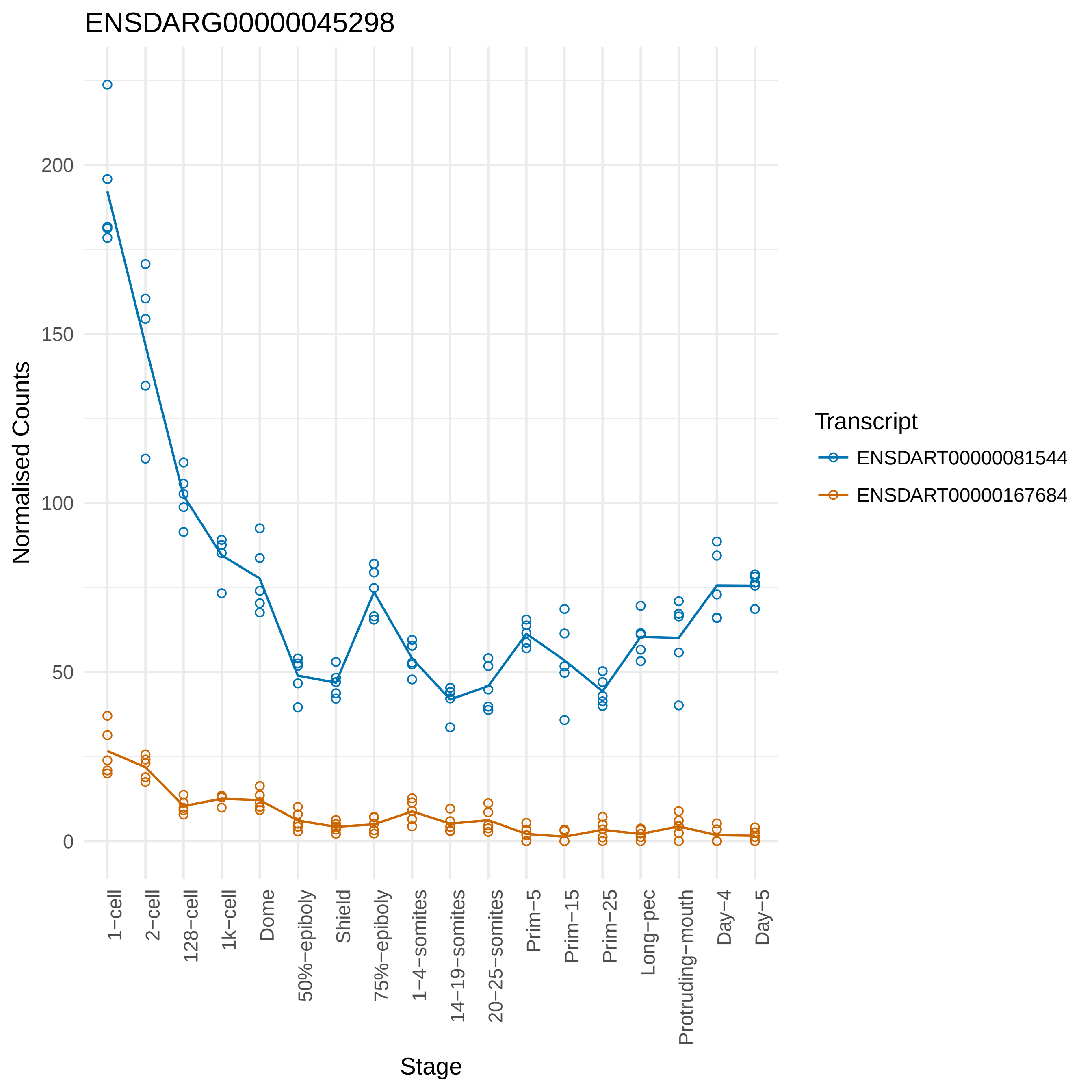
ENSDARG00000045298
Normalised Counts
200
150
100
50
0
1−cell
2−cell
128−cell
1k−cell
Dome
50%−epiboly
Shield
75%−epiboly
1−4−somites
14−19−somites
20−25−somites
Prim−5
Prim−15
Prim−25
Long−pec
Protruding−mouth
Day−4
Day−5
Stage
Transcript
ENSDART00000081544
ENSDART00000167684

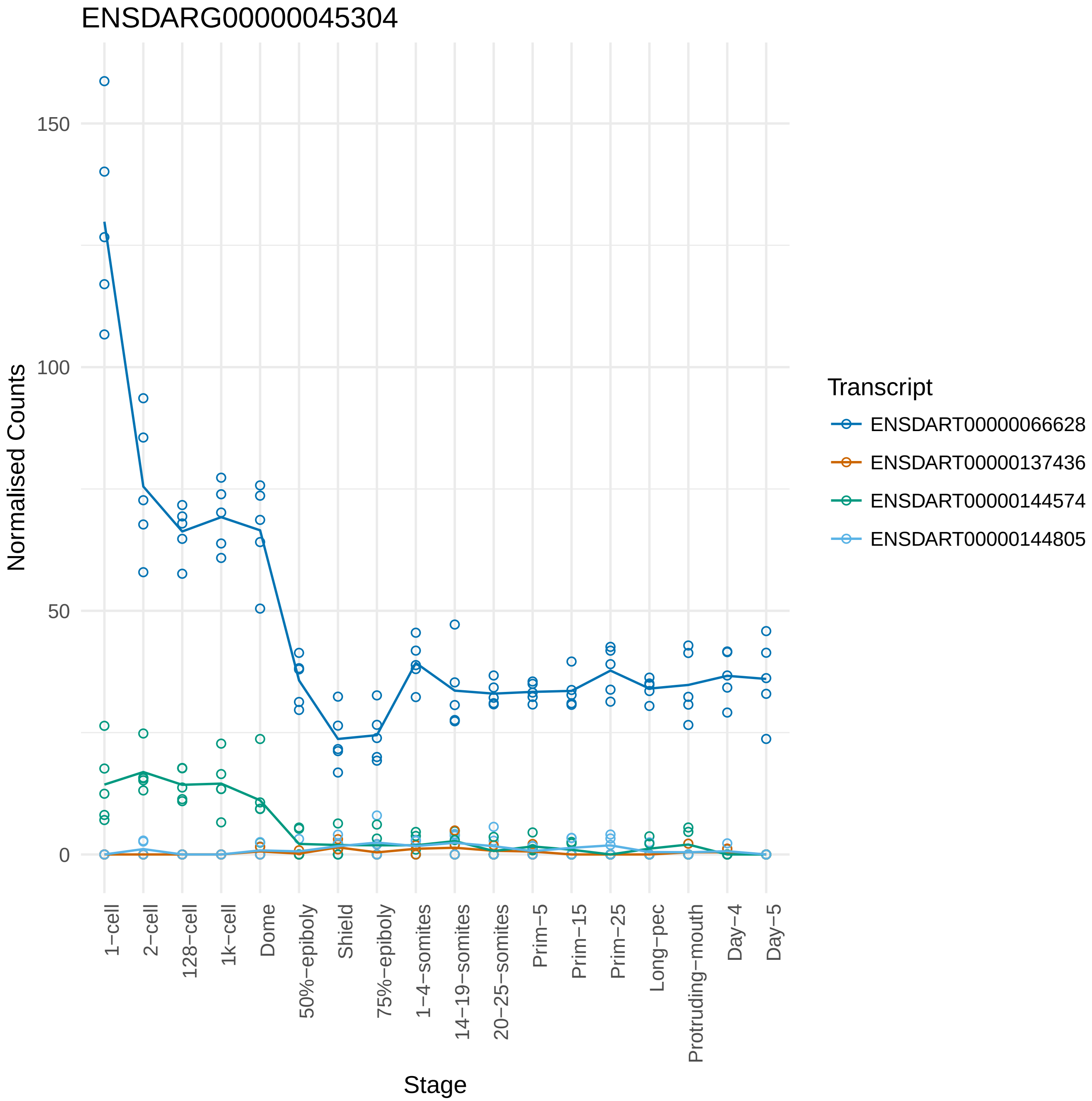

ENSDARG00000045304
Normalised Counts
150
100
50
0
1−cell
2−cell
128−cell
1k−cell
Dome
50%−epiboly
Shield
75%−epiboly
1−4−somites
14−19−somites
20−25−somites
Prim−5
Prim−15
Prim−25
Long−pec
Protruding−mouth
Day−4
Day−5
Stage
Transcript
ENSDART00000066628
ENSDART00000137436
ENSDART00000144574
ENSDART00000144805

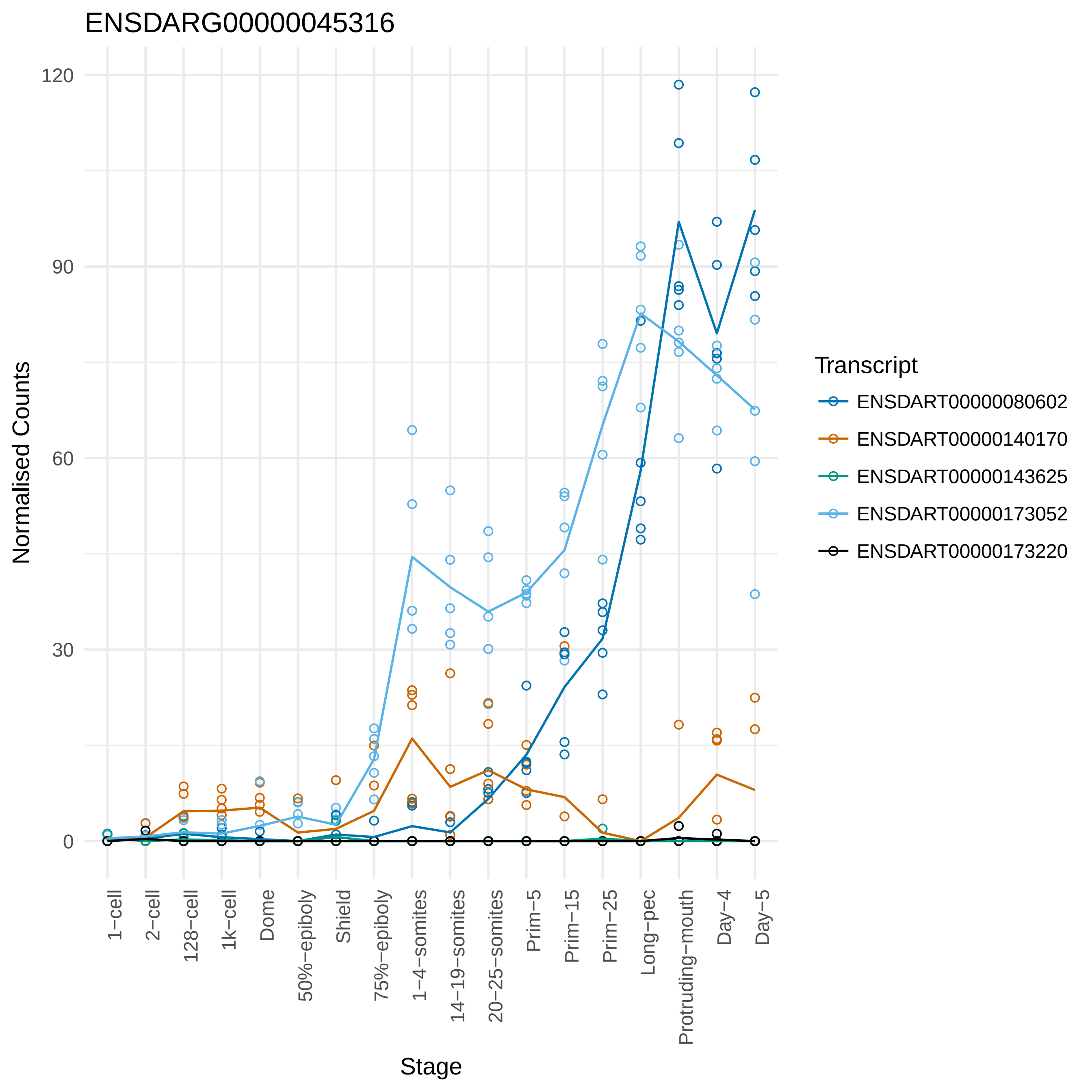
ENSDARG00000045316
Normalised Counts
120
90
60
30
0
1−cell
2−cell
128−cell
1k−cell
Dome
50%−epiboly
Shield
75%−epiboly
1−4−somites
14−19−somites
20−25−somites
Prim−5
Prim−15
Prim−25
Long−pec
Protruding−mouth
Day−4
Day−5
Stage
Transcript
ENSDART00000080602
ENSDART00000140170
ENSDART00000143625
ENSDART00000173052
ENSDART00000173220

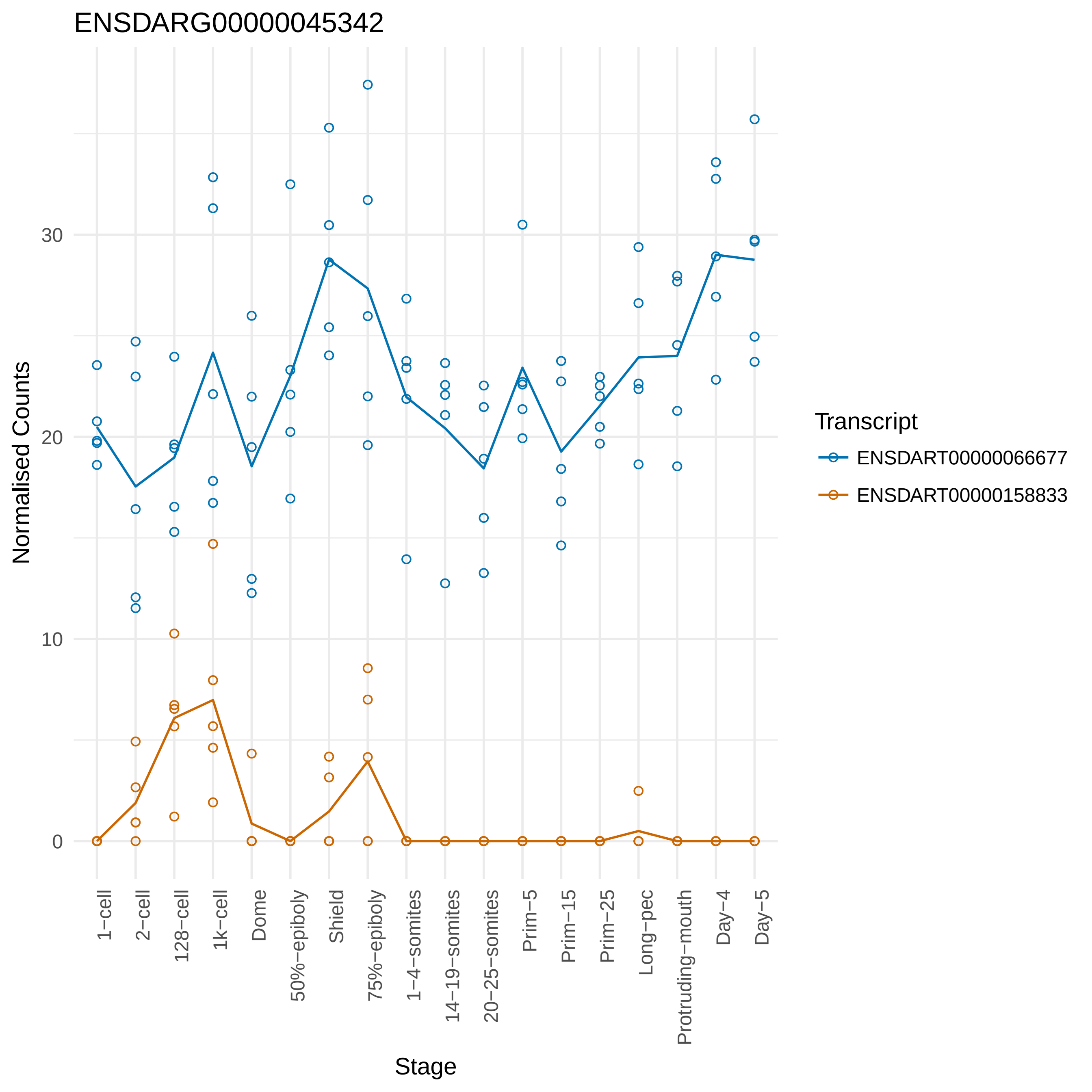

ENSDARG00000045342
Normalised Counts
30
20
10
0
1−cell
2−cell
128−cell
1k−cell
Dome
50%−epiboly
Shield
75%−epiboly
1−4−somites
14−19−somites
20−25−somites
Prim−5
Prim−15
Prim−25
Long−pec
Protruding−mouth
Day−4
Day−5
Stage
Transcript
ENSDART00000066677
ENSDART00000158833

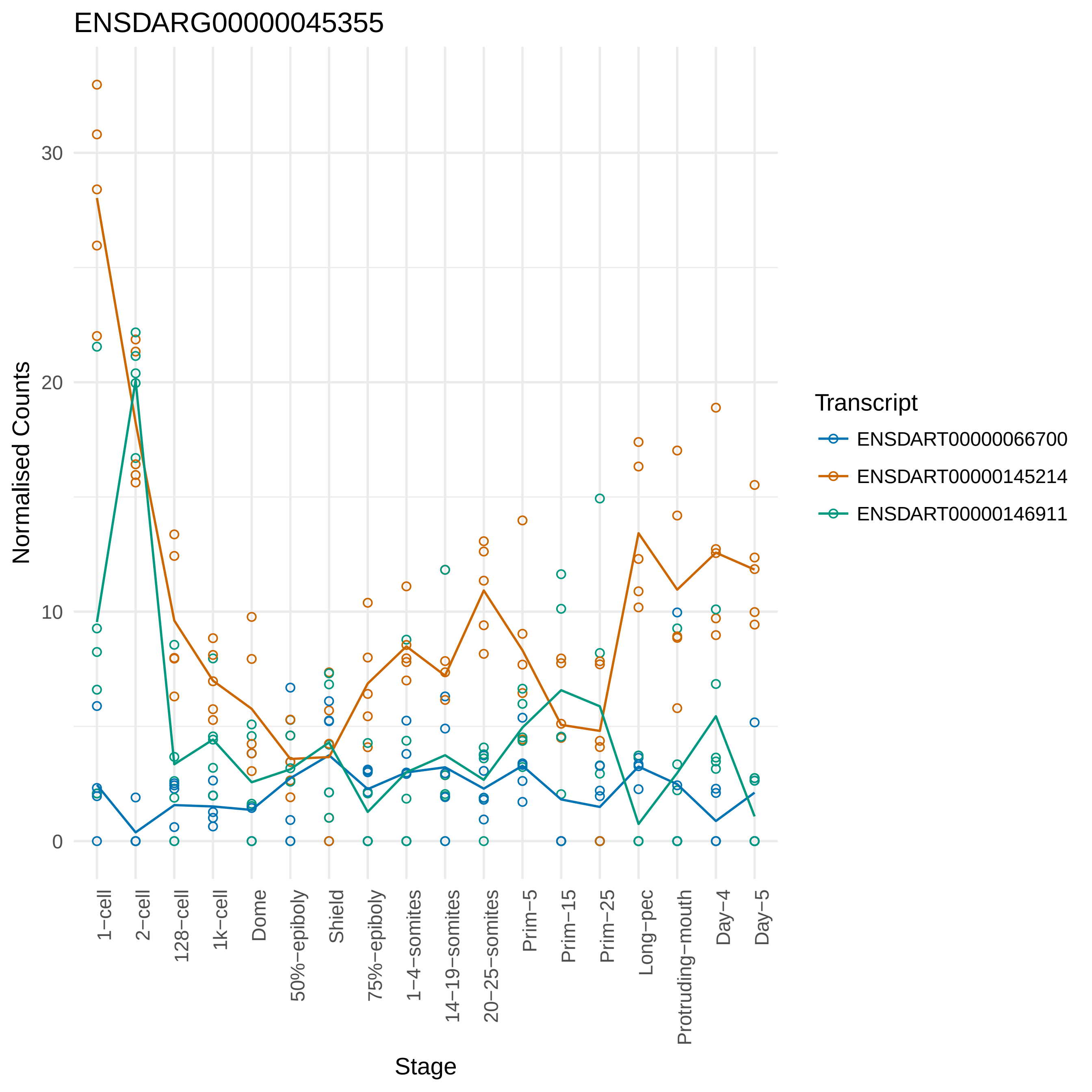

ENSDARG00000045355
Normalised Counts
30
20
10
0
1−cell
2−cell
128−cell
1k−cell
Dome
50%−epiboly
Shield
75%−epiboly
1−4−somites
14−19−somites
20−25−somites
Prim−5
Prim−15
Prim−25
Long−pec
Protruding−mouth
Day−4
Day−5
Stage
Transcript
ENSDART00000066700
ENSDART00000145214
ENSDART00000146911

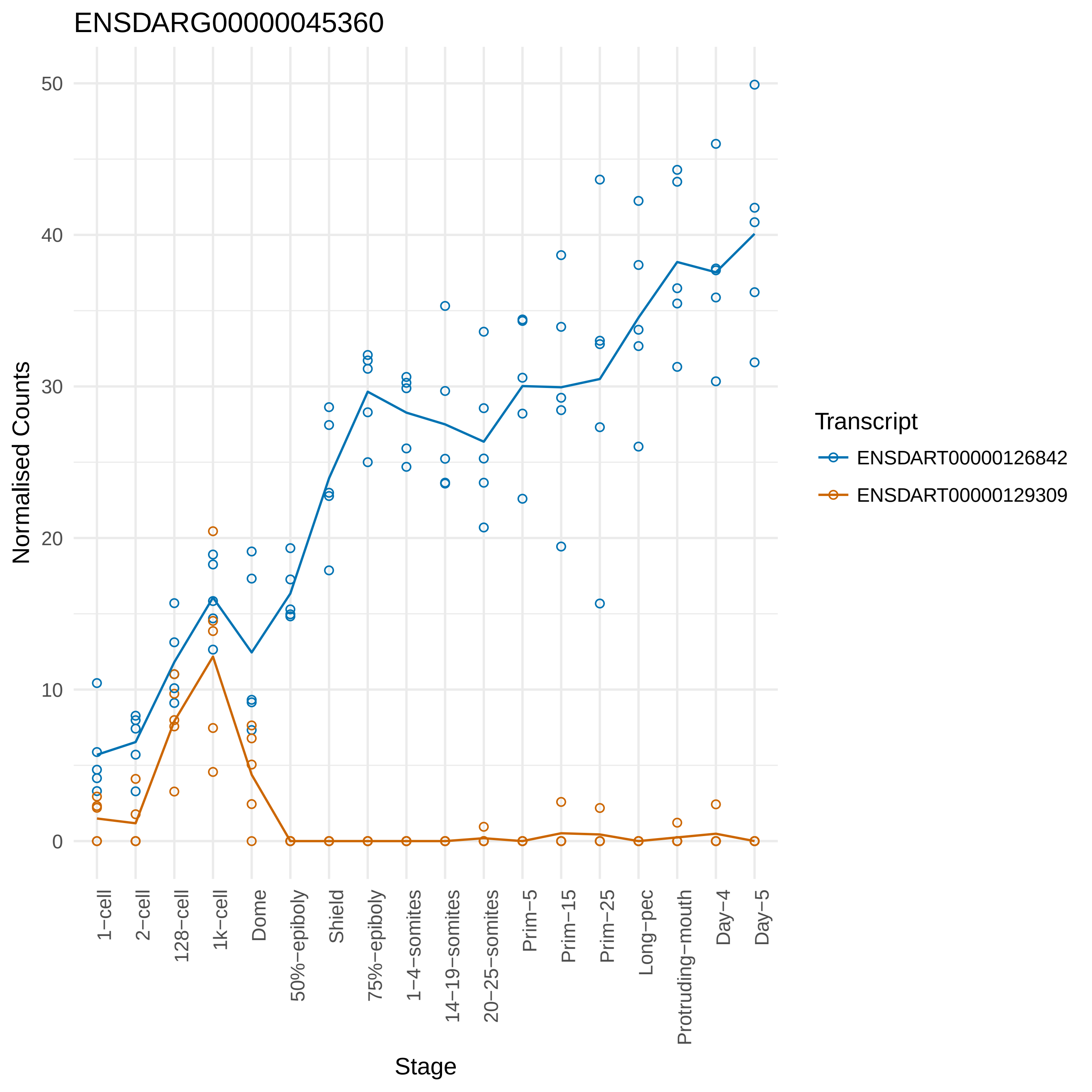

ENSDARG00000045360
Normalised Counts
0
10
20
30
40
50
1−cell
2−cell
128−cell
1k−cell
Dome
50%−epiboly
Shield
75%−epiboly
1−4−somites
14−19−somites
20−25−somites
Prim−5
Prim−15
Prim−25
Long−pec
Protruding−mouth
Day−4
Day−5
Stage
Transcript
ENSDART00000126842
ENSDART00000129309

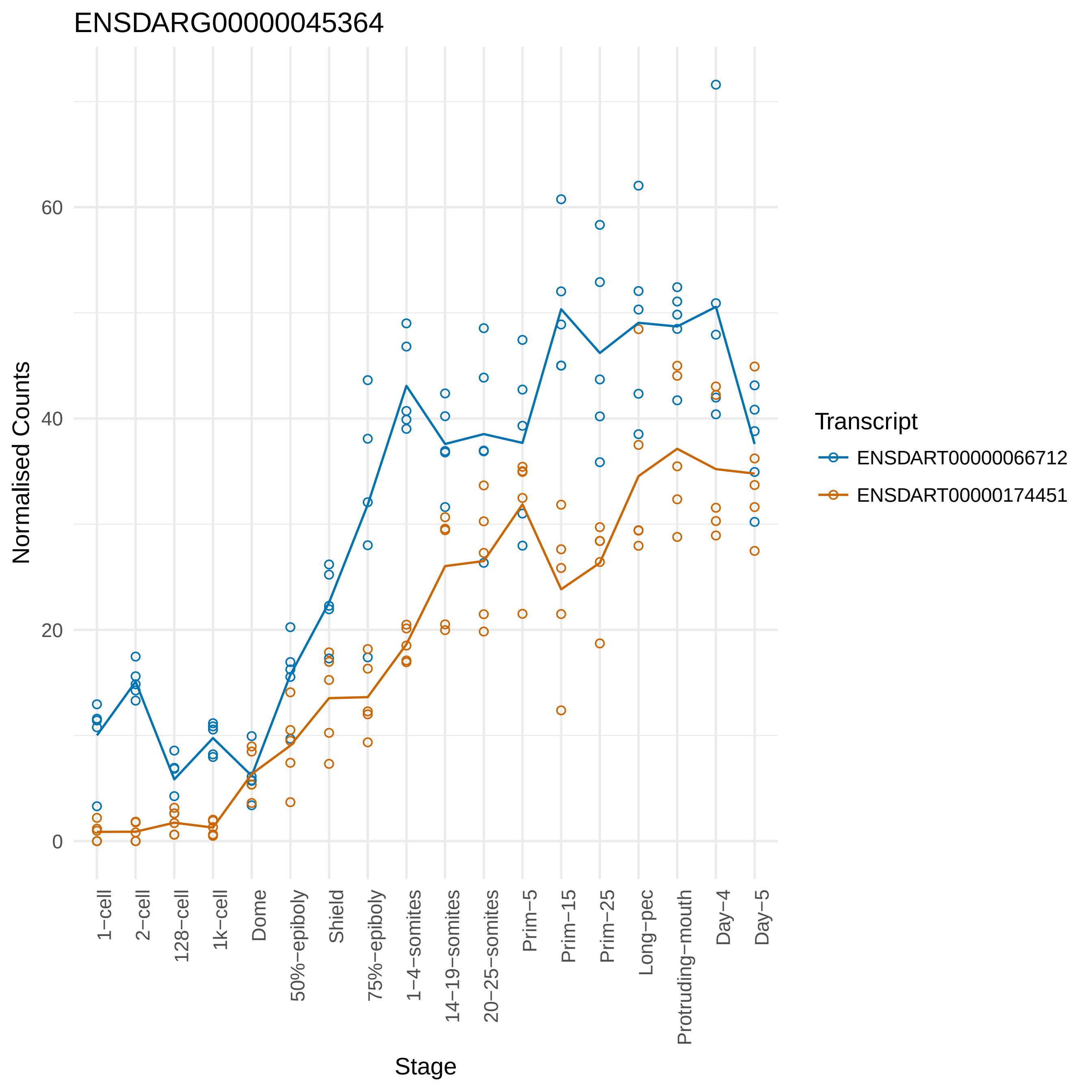
ENSDARG00000045364
Normalised Counts
0
20
40
60
Stage
1−cell
2−cell
128−cell
1k−cell
Dome
50%−epiboly
Shield
75%−epiboly
1−4−somites
14−19−somites
20−25−somites
Prim−5
Prim−15
Prim−25
Long−pec
Protruding−mouth
Day−4
Day−5
Transcript
ENSDART00000066712
ENSDART00000174451

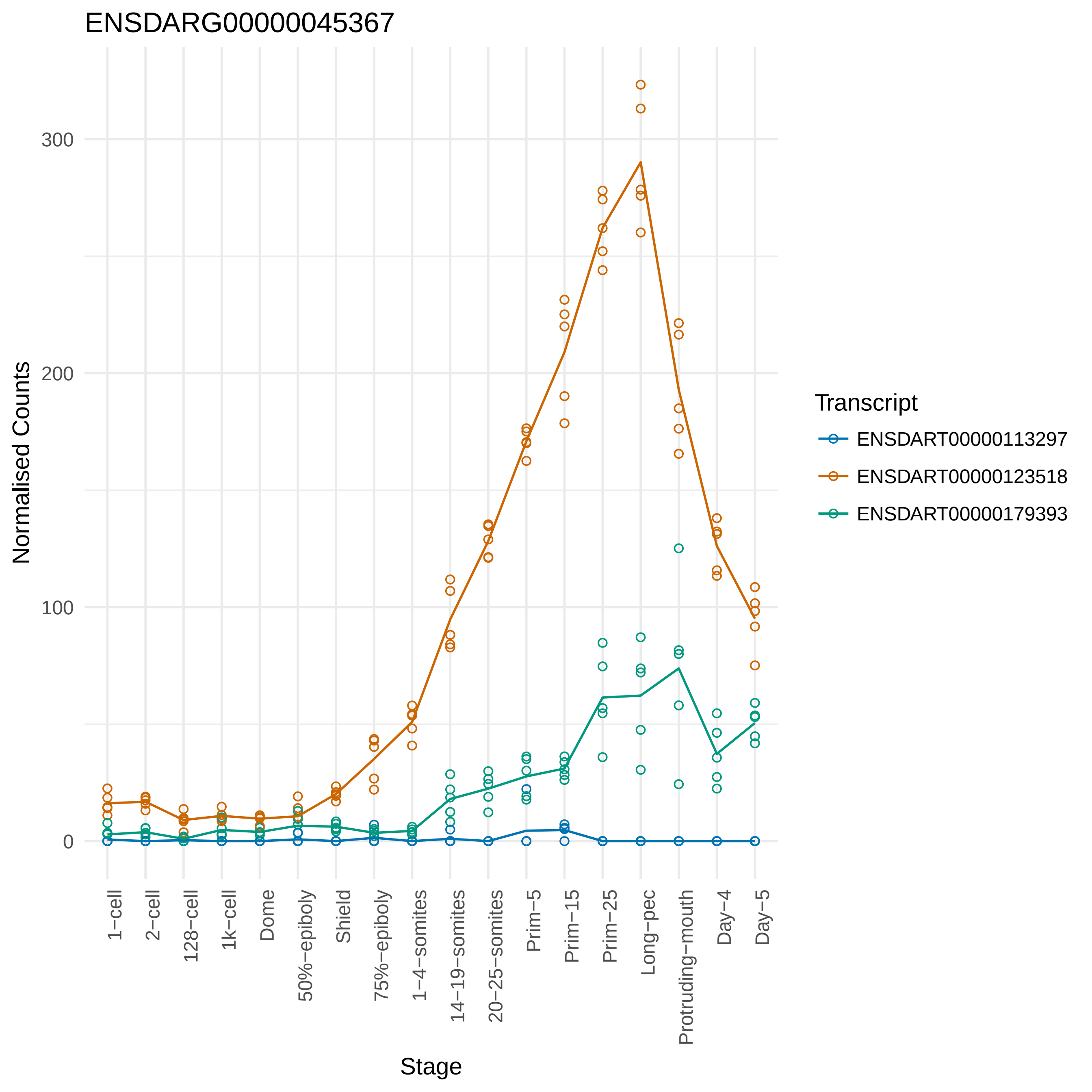

ENSDARG00000045367
Normalised Counts
300
200
100
0
1−cell
2−cell
128−cell
1k−cell
Dome
50%−epiboly
Shield
75%−epiboly
1−4−somites
14−19−somites
20−25−somites
Prim−5
Prim−15
Prim−25
Long−pec
Protruding−mouth
Day−4
Day−5
Stage
Transcript
ENSDART00000113297
ENSDART00000123518
ENSDART00000179393

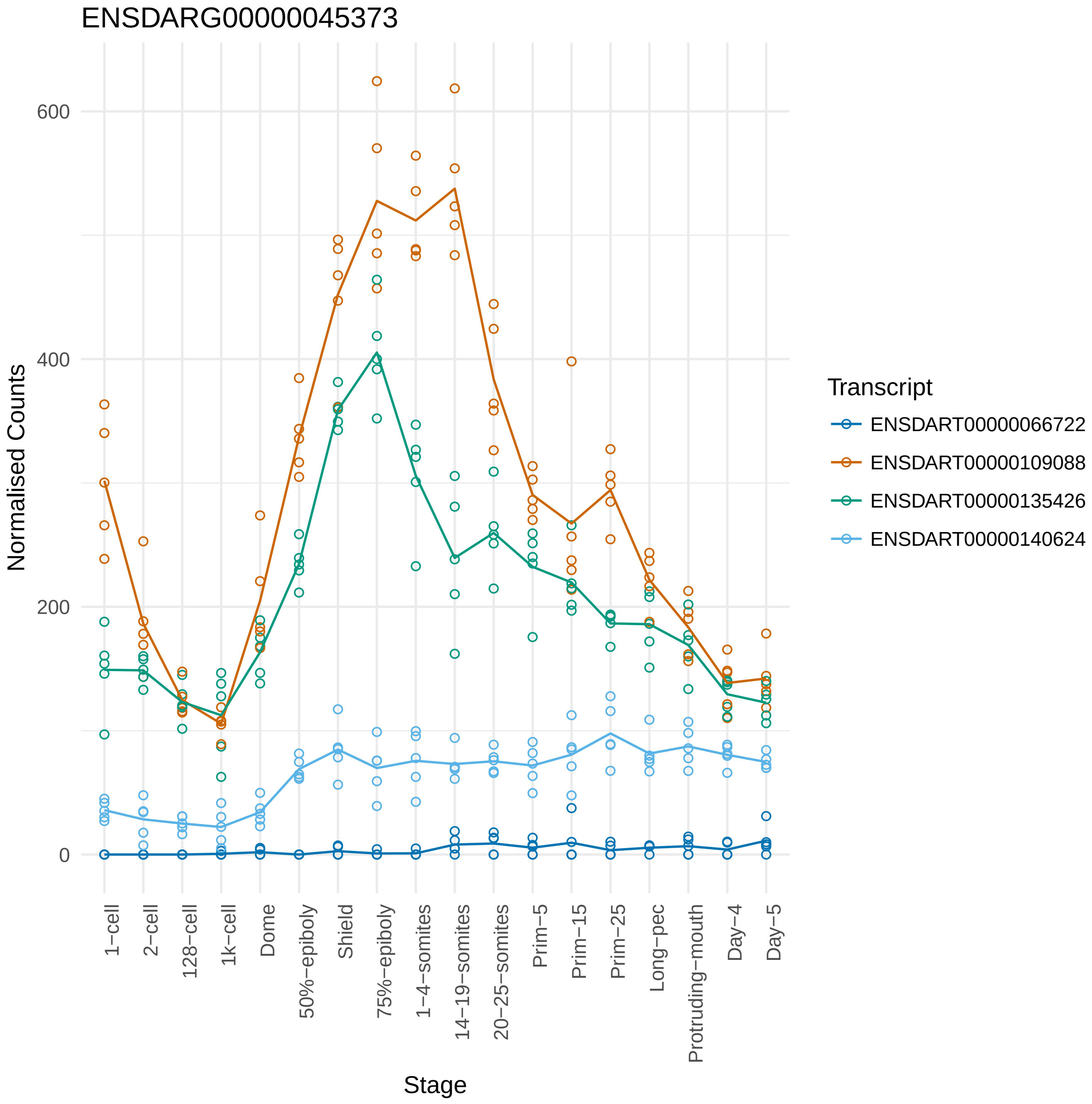

ENSDARG00000045373
Normalised Counts
600
400
200
0
1−cell
2−cell
128−cell
1k−cell
Dome
50%−epiboly
Shield
75%−epiboly
1−4−somites
14−19−somites
20−25−somites
Prim−5
Prim−15
Prim−25
Long−pec
Protruding−mouth
Day−4
Day−5
Stage
Transcript
ENSDART00000066722
ENSDART00000109088
ENSDART00000135426
ENSDART00000140624

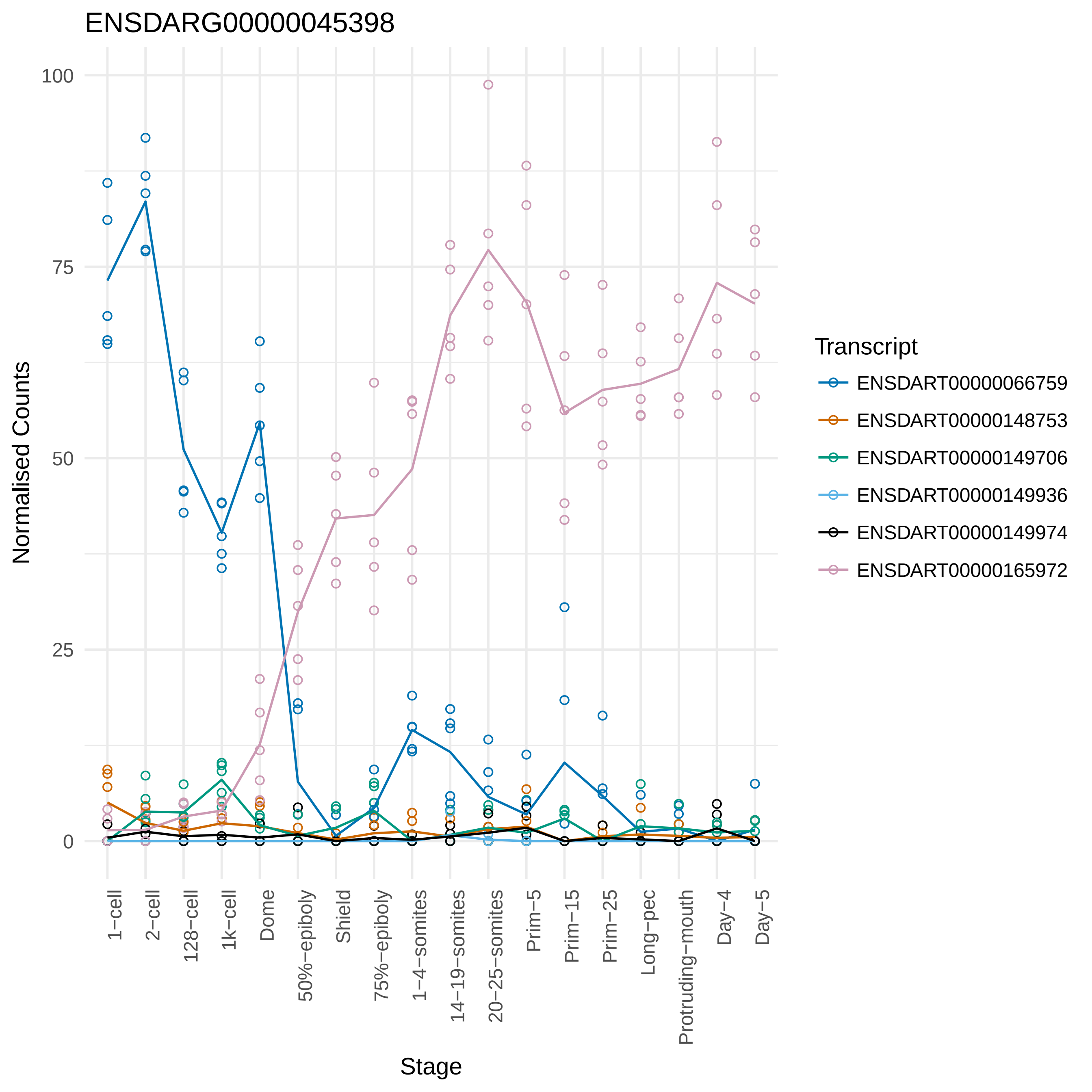
ENSDARG00000045398
Normalised Counts
100
75
50
25
0
1−cell
2−cell
128−cell
1k−cell
Dome
50%−epiboly
Shield
75%−epiboly
1−4−somites
14−19−somites
20−25−somites
Prim−5
Prim−15
Prim−25
Long−pec
Protruding−mouth
Day−4
Day−5
Stage
Transcript
ENSDART00000066759
ENSDART00000148753
ENSDART00000149706
ENSDART00000149936
ENSDART00000149974
ENSDART00000165972

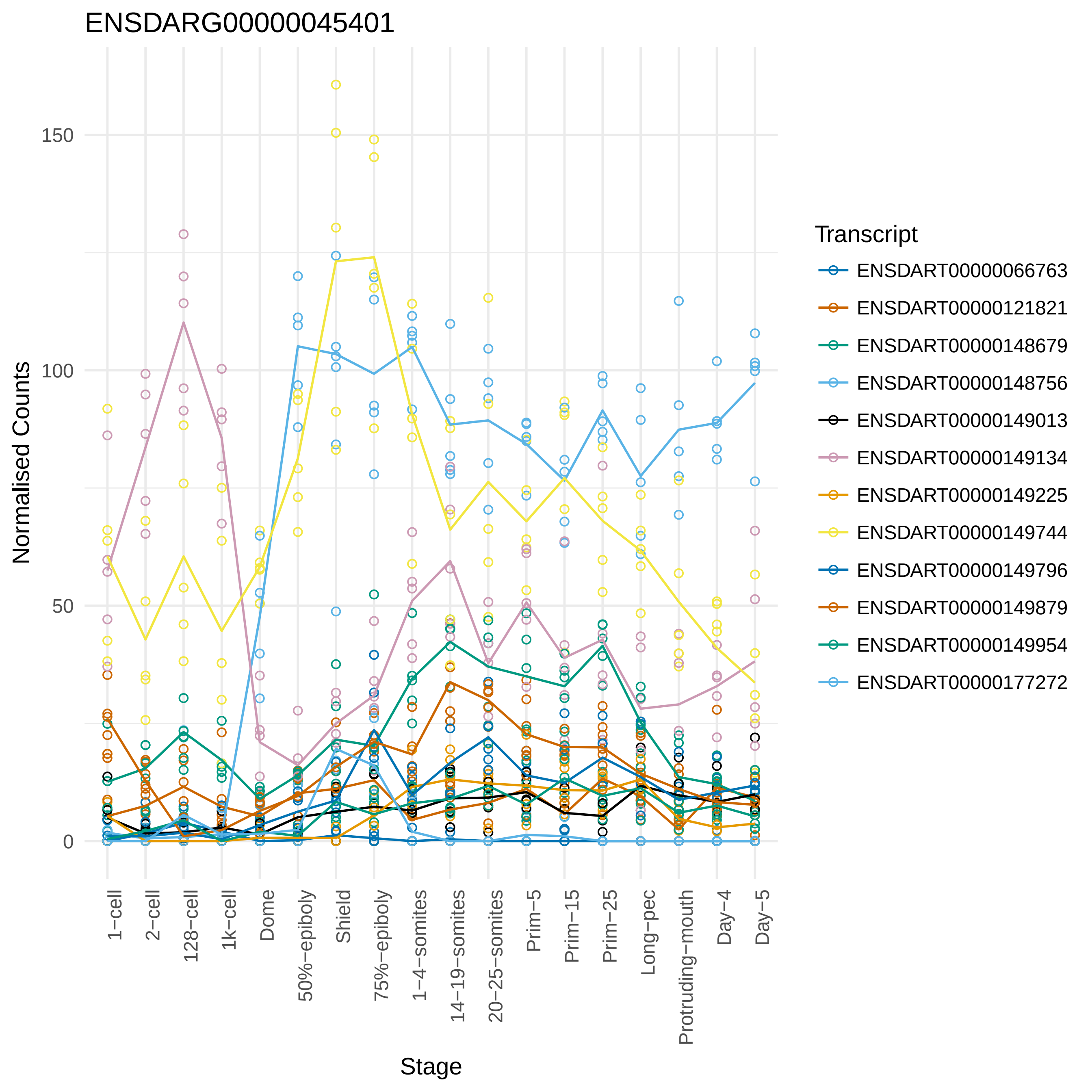

ENSDARG00000045401
Normalised Counts
150
100
50
0
1−cell
2−cell
128−cell
1k−cell
Dome
50%−epiboly
Shield
75%−epiboly
1−4−somites
14−19−somites
20−25−somites
Prim−5
Prim−15
Prim−25
Long−pec
Protruding−mouth
Day−4
Day−5
Stage
Transcript
ENSDART00000066763
ENSDART00000121821
ENSDART00000148679
ENSDART00000148756
ENSDART00000149013
ENSDART00000149134
ENSDART00000149225
ENSDART00000149744
ENSDART00000149796
ENSDART00000149879
ENSDART00000149954
ENSDART00000177272

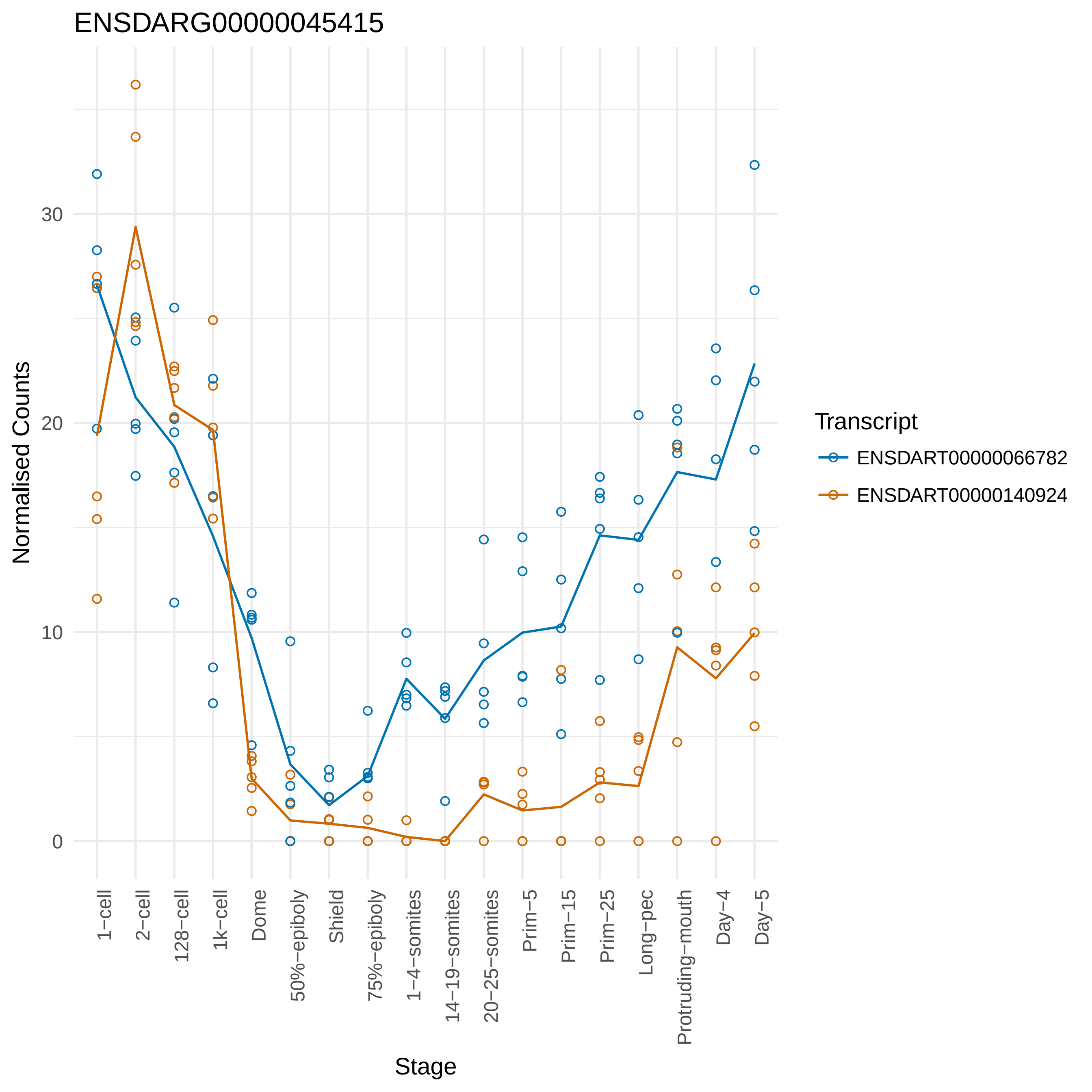

ENSDARG00000045415
Normalised Counts
0
10
20
30
Stage
1−cell
2−cell
128−cell
1k−cell
Dome
50%−epiboly
Shield
75%−epiboly
1−4−somites
14−19−somites
20−25−somites
Prim−5
Prim−15
Prim−25
Long−pec
Protruding−mouth
Day−4
Day−5
Transcript
ENSDART00000066782
ENSDART00000140924

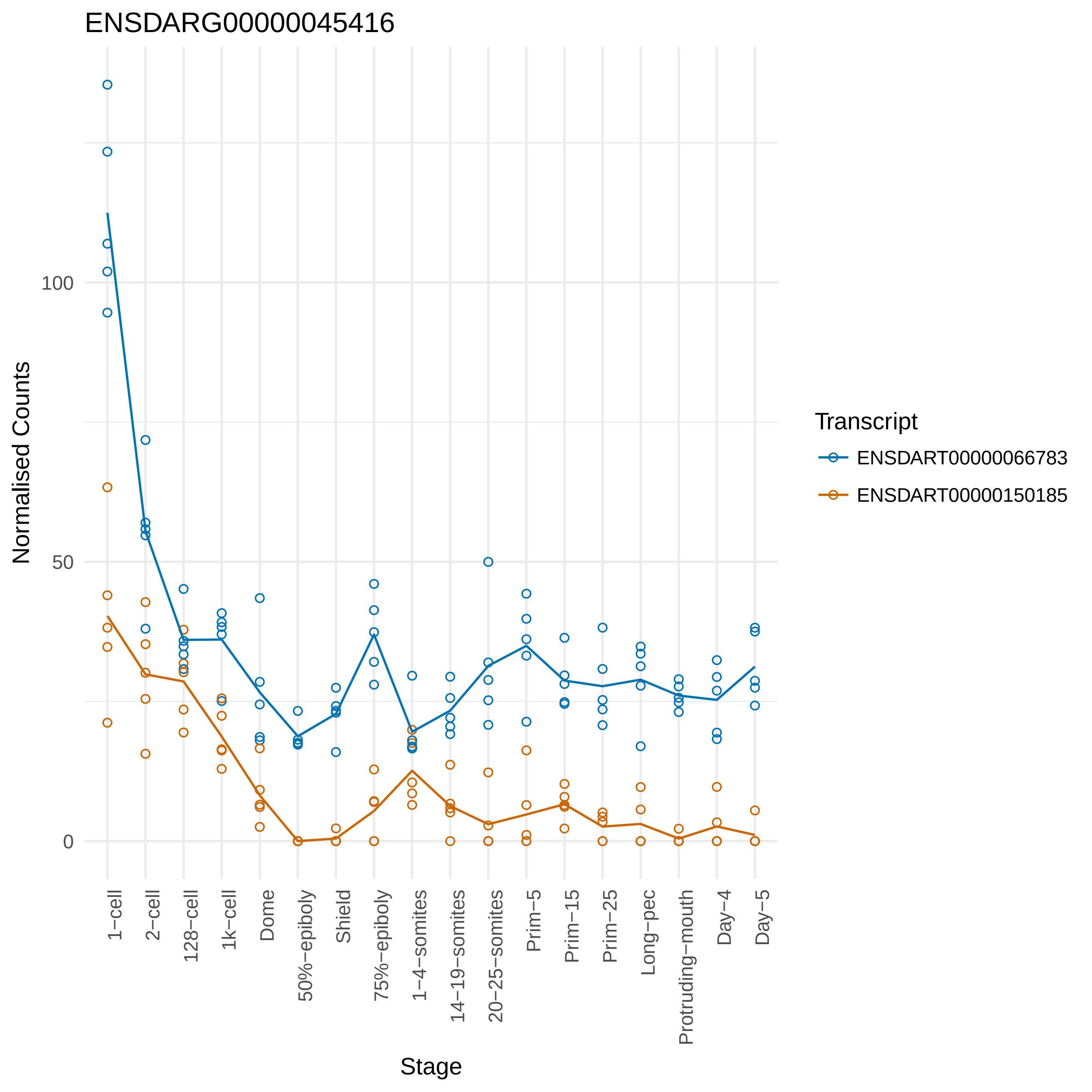
ENSDARG00000045416
Normalised Counts
100
50
0
1−cell
2−cell
128−cell
1k−cell
Dome
50%−epiboly
Shield
75%−epiboly
1−4−somites
14−19−somites
20−25−somites
Prim−5
Prim−15
Prim−25
Long−pec
Protruding−mouth
Day−4
Day−5
Stage
Transcript
ENSDART00000066783
ENSDART00000150185

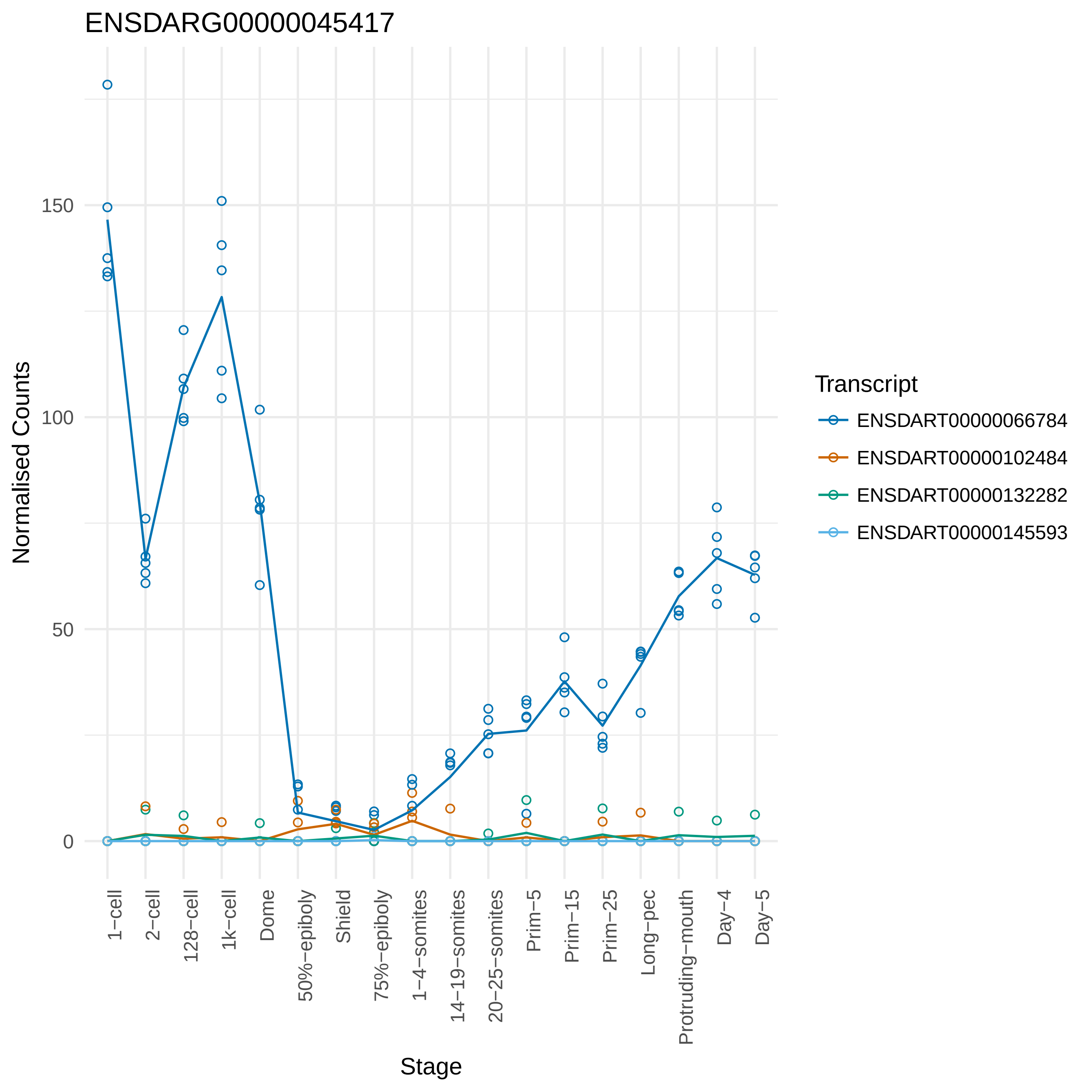
ENSDARG00000045417
Normalised Counts
150
100
50
0
1−cell
2−cell
128−cell
1k−cell
Dome
50%−epiboly
Shield
75%−epiboly
1−4−somites
14−19−somites
20−25−somites
Prim−5
Prim−15
Prim−25
Long−pec
Protruding−mouth
Day−4
Day−5
Stage
Transcript
ENSDART00000066784
ENSDART00000102484
ENSDART00000132282
ENSDART00000145593

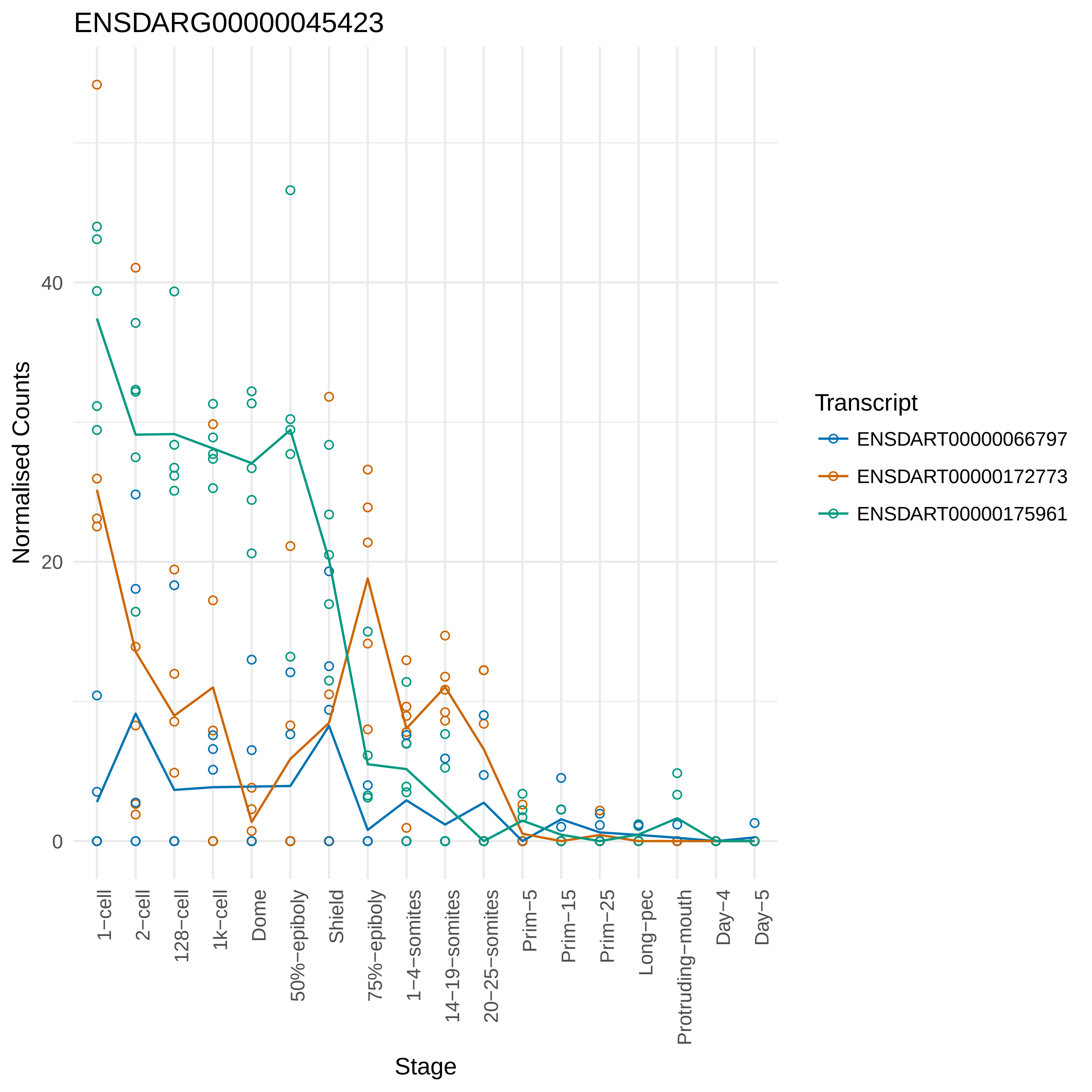
ENSDARG00000045423
Normalised Counts
0
20
40
Stage
1−cell
2−cell
128−cell
1k−cell
Dome
50%−epiboly
Shield
75%−epiboly
1−4−somites
14−19−somites
20−25−somites
Prim−5
Prim−15
Prim−25
Long−pec
Protruding−mouth
Day−4
Day−5
Transcript
ENSDART00000066797
ENSDART00000172773
ENSDART00000175961

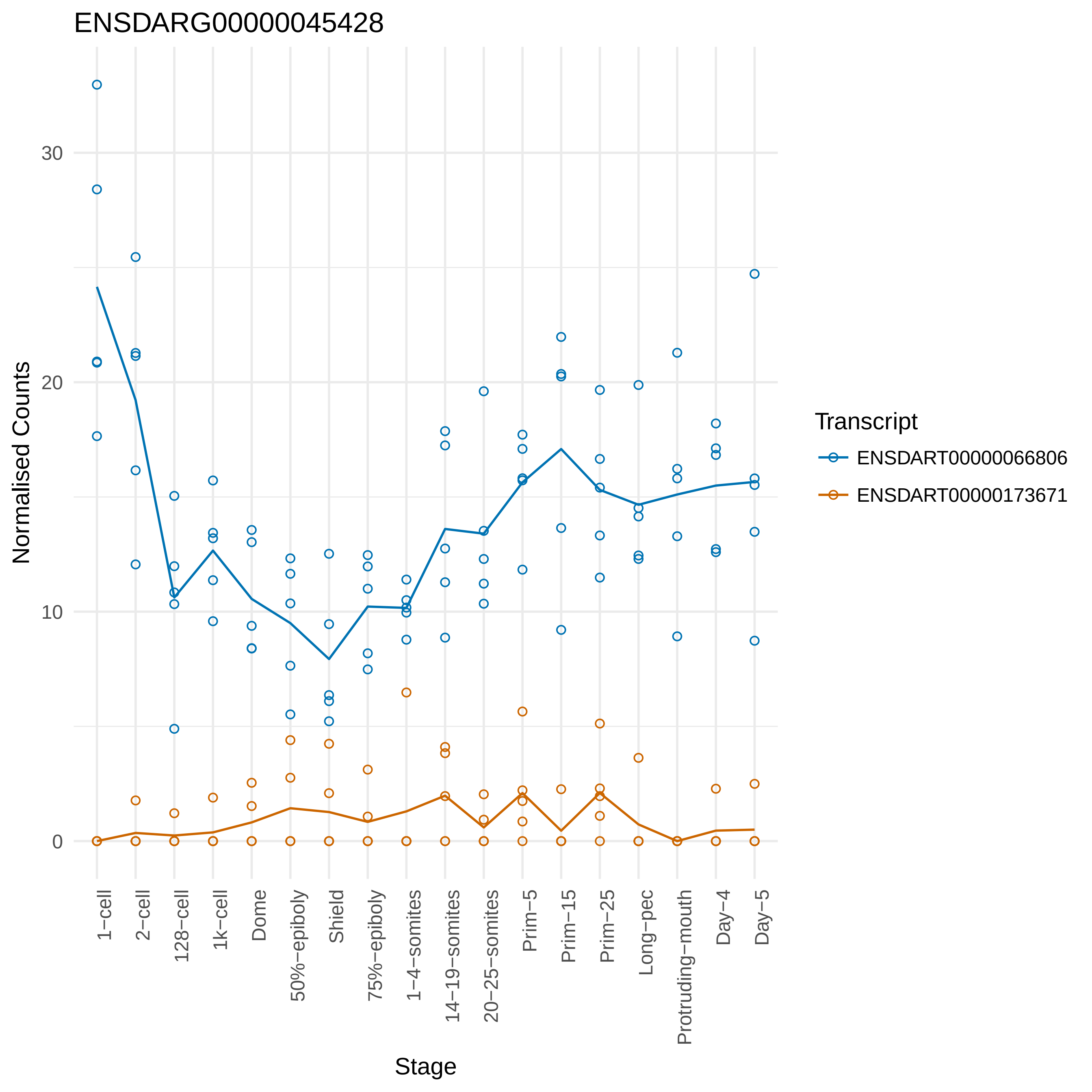

ENSDARG00000045428
Normalised Counts
30
20
10
0
1−cell
2−cell
128−cell
1k−cell
Dome
50%−epiboly
Shield
75%−epiboly
1−4−somites
14−19−somites
20−25−somites
Prim−5
Prim−15
Prim−25
Long−pec
Protruding−mouth
Day−4
Day−5
Stage
Transcript
ENSDART00000066806
ENSDART00000173671

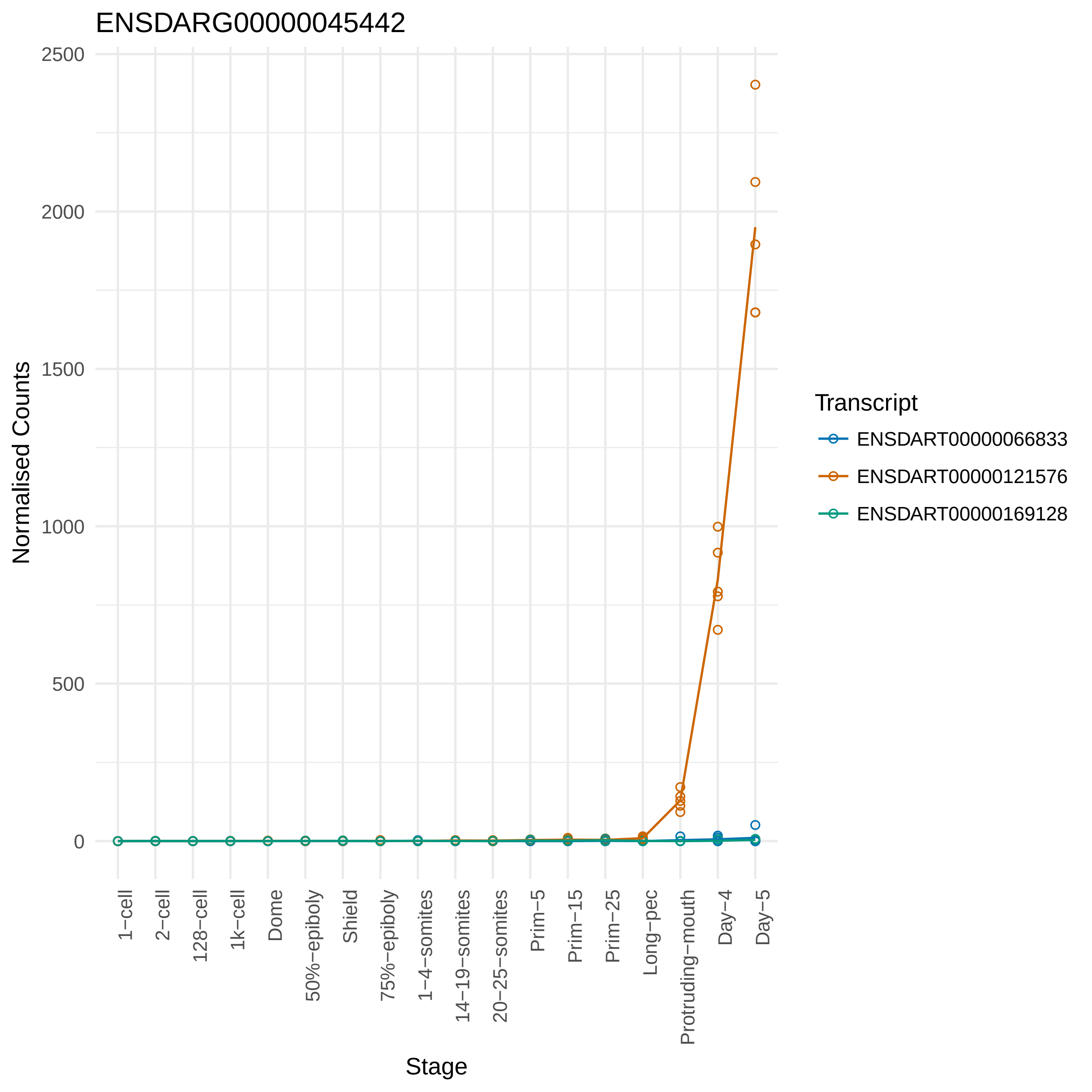
ENSDARG00000045442
Normalised Counts
2500
2000
1500
1000
500
0
1−cell
2−cell
128−cell
1k−cell
Dome
50%−epiboly
Shield
75%−epiboly
1−4−somites
14−19−somites
20−25−somites
Prim−5
Prim−15
Prim−25
Long−pec
Protruding−mouth
Day−4
Day−5
Stage
Transcript
ENSDART00000066833
ENSDART00000121576
ENSDART00000169128

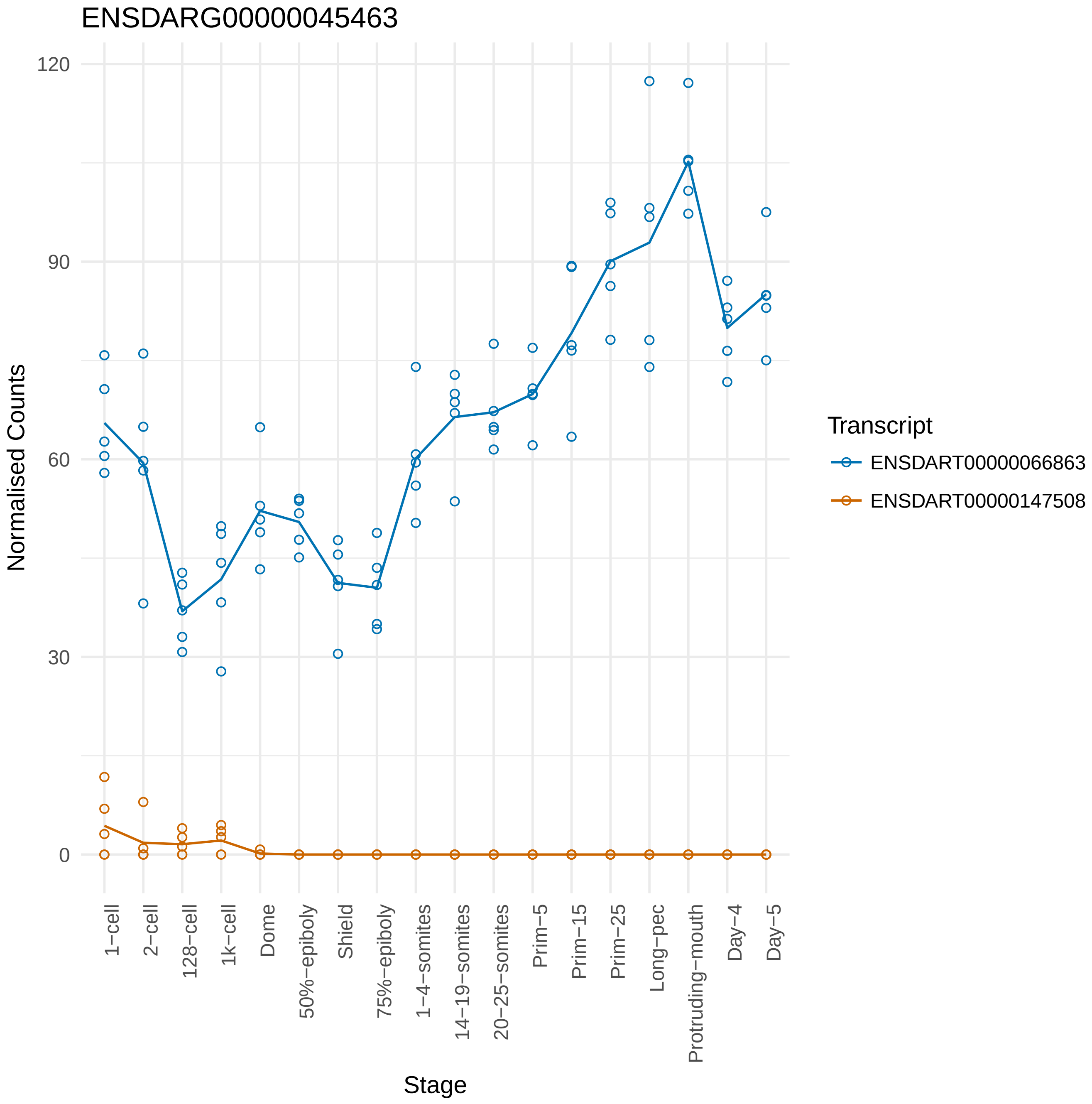

ENSDARG00000045463
Normalised Counts
120
90
60
30
0
Stage
1−cell
2−cell
128−cell
1k−cell
Dome
50%−epiboly
Shield
75%−epiboly
1−4−somites
14−19−somites
20−25−somites
Prim−5
Prim−15
Prim−25
Long−pec
Protruding−mouth
Day−4
Day−5
Transcript
ENSDART00000066863
ENSDART00000147508

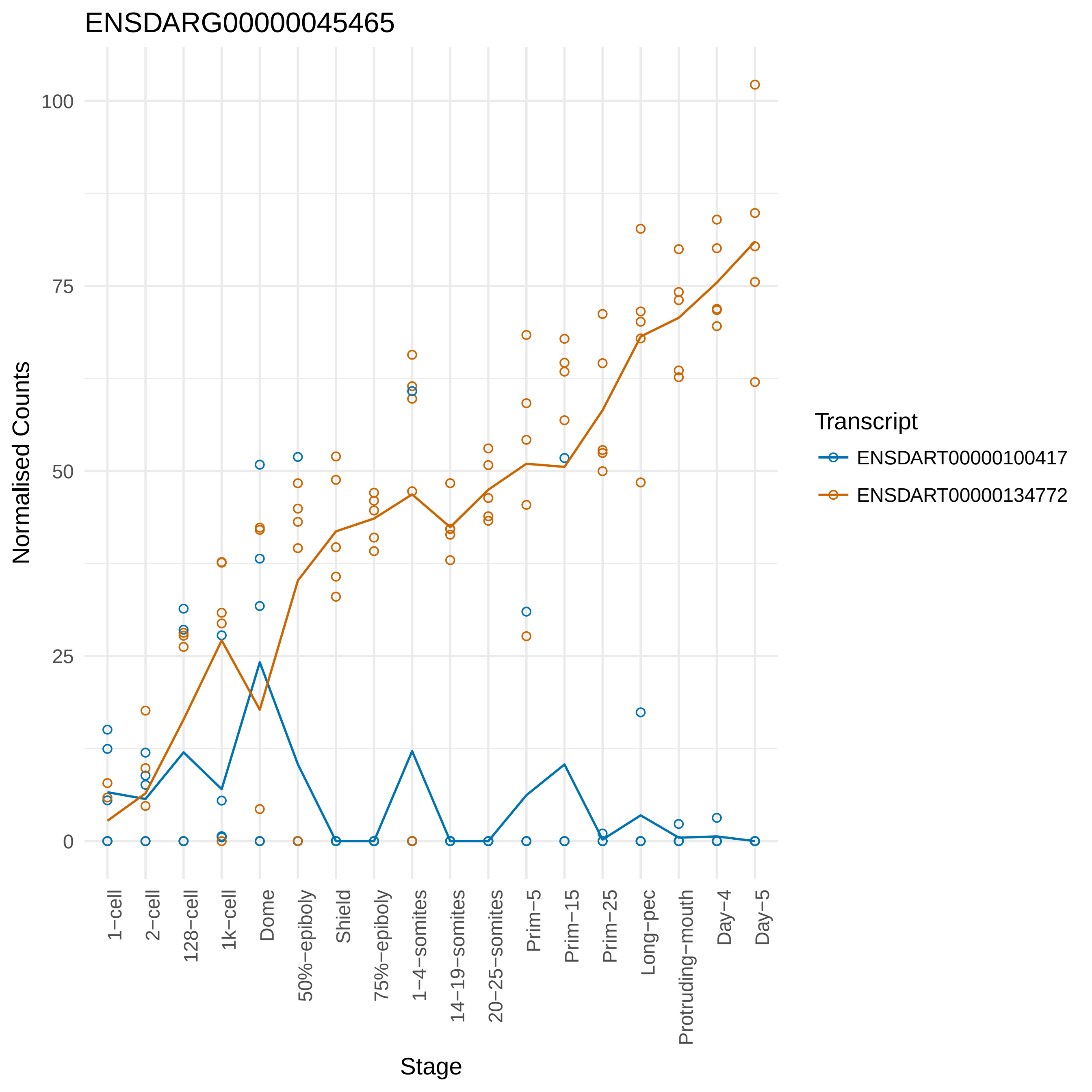

ENSDARG00000045465
Normalised Counts
100
75
50
25
0
1−cell
2−cell
128−cell
1k−cell
Dome
50%−epiboly
Shield
75%−epiboly
1−4−somites
14−19−somites
20−25−somites
Prim−5
Prim−15
Prim−25
Long−pec
Protruding−mouth
Day−4
Day−5
Stage
Transcript
ENSDART00000100417
ENSDART00000134772

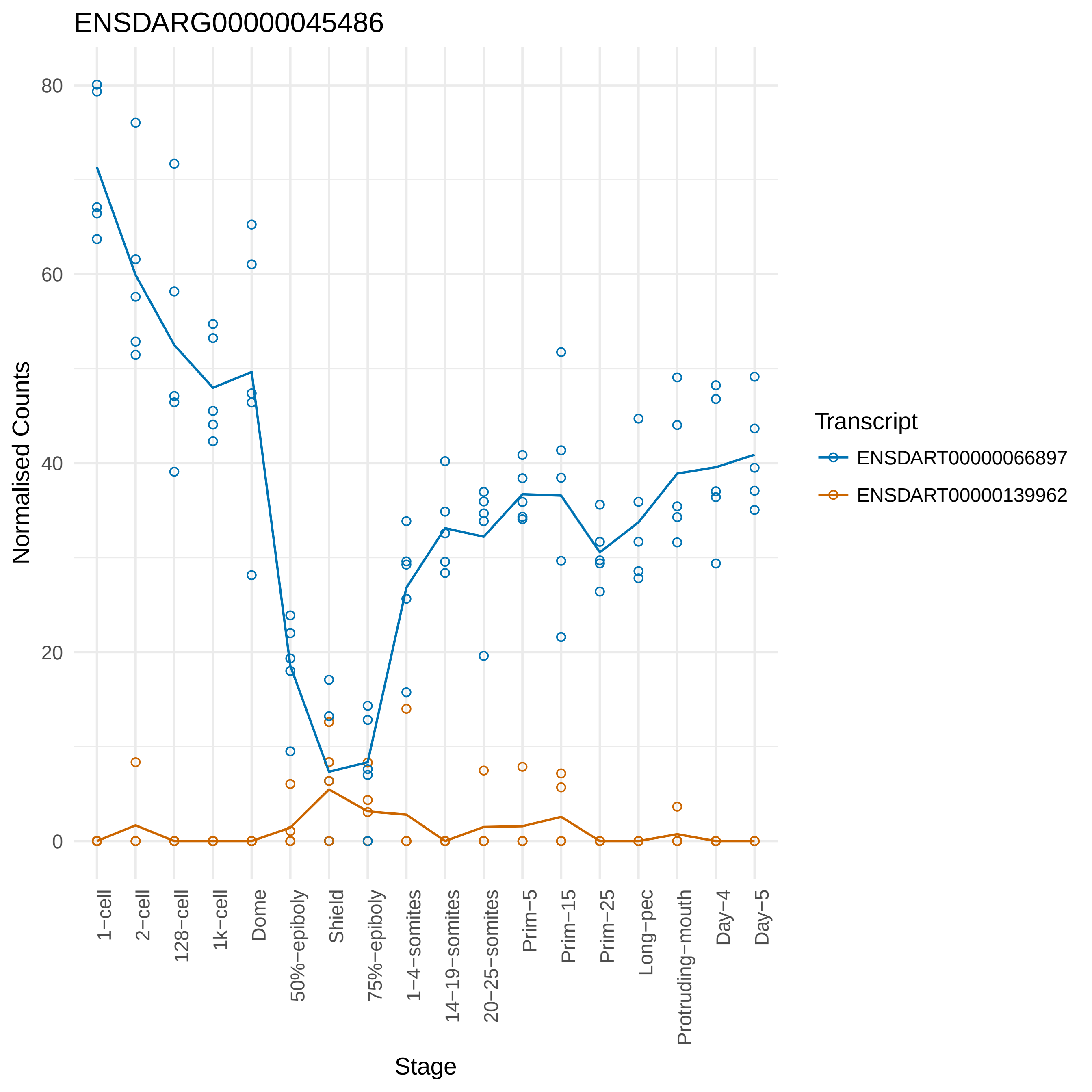
ENSDARG00000045486
Normalised Counts
80
60
40
20
0
Stage
1−cell
2−cell
128−cell
1k−cell
Dome
50%−epiboly
Shield
75%−epiboly
1−4−somites
14−19−somites
20−25−somites
Prim−5
Prim−15
Prim−25
Long−pec
Protruding−mouth
Day−4
Day−5
Transcript
ENSDART00000066897
ENSDART00000139962

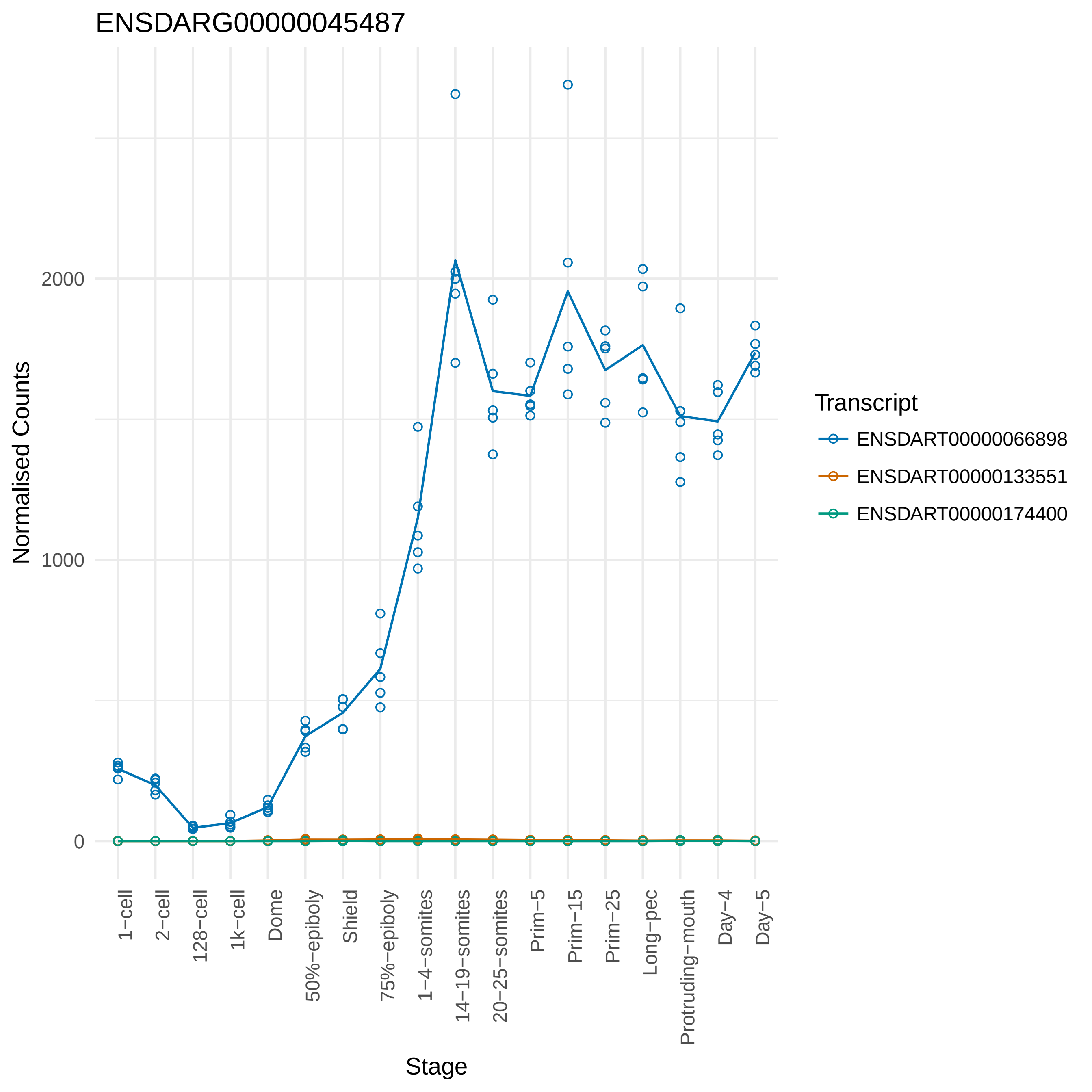
ENSDARG00000045487
Normalised Counts
2000
1000
0
1−cell
2−cell
128−cell
1k−cell
Dome
50%−epiboly
Shield
75%−epiboly
1−4−somites
14−19−somites
20−25−somites
Prim−5
Prim−15
Prim−25
Long−pec
Protruding−mouth
Day−4
Day−5
Stage
Transcript
ENSDART00000066898
ENSDART00000133551
ENSDART00000174400

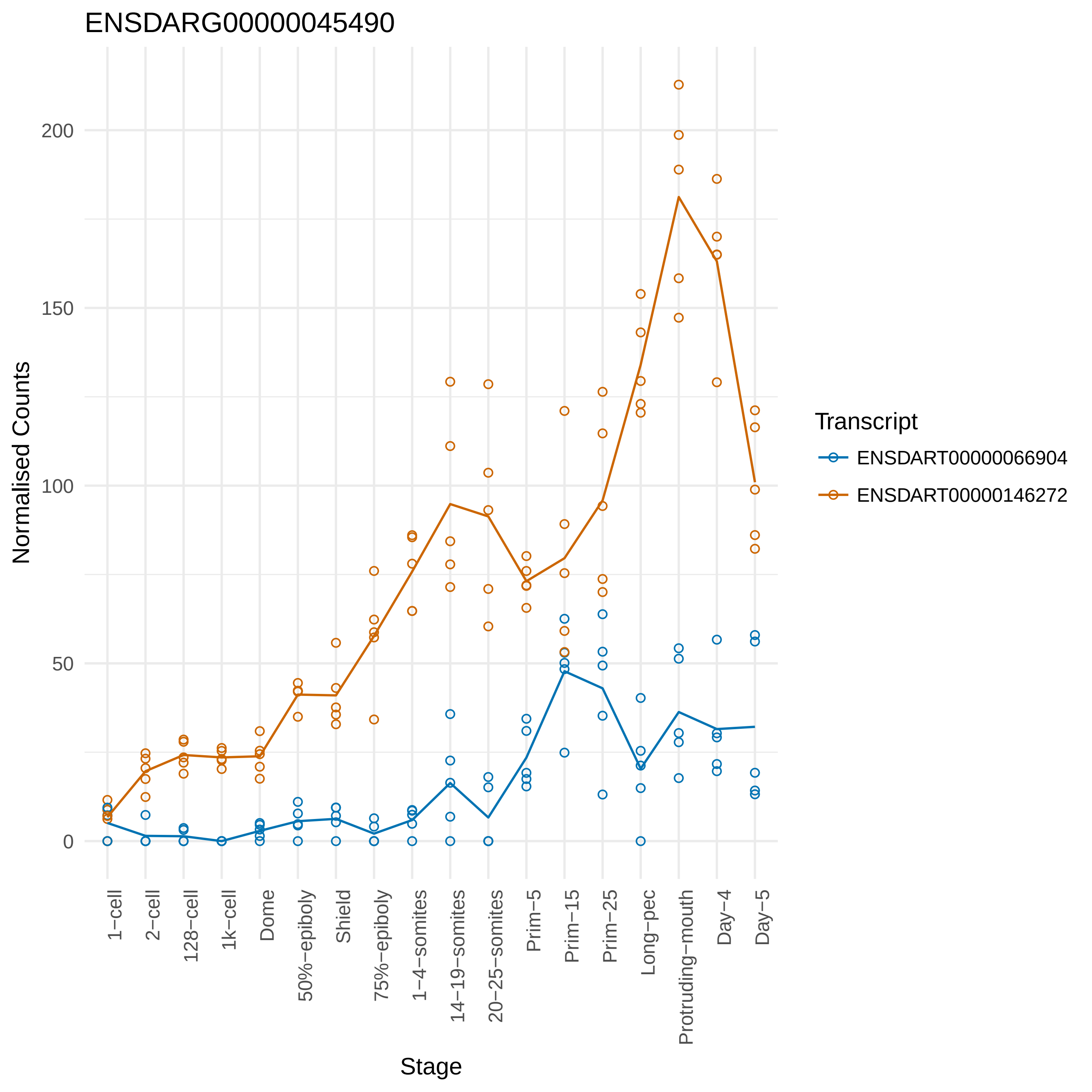
ENSDARG00000045490
Normalised Counts
0
50
100
150
200
Stage
1−cell
2−cell
128−cell
1k−cell
Dome
50%−epiboly
Shield
75%−epiboly
1−4−somites
14−19−somites
20−25−somites
Prim−5
Prim−15
Prim−25
Long−pec
Protruding−mouth
Day−4
Day−5
Transcript
ENSDART00000066904
ENSDART00000146272

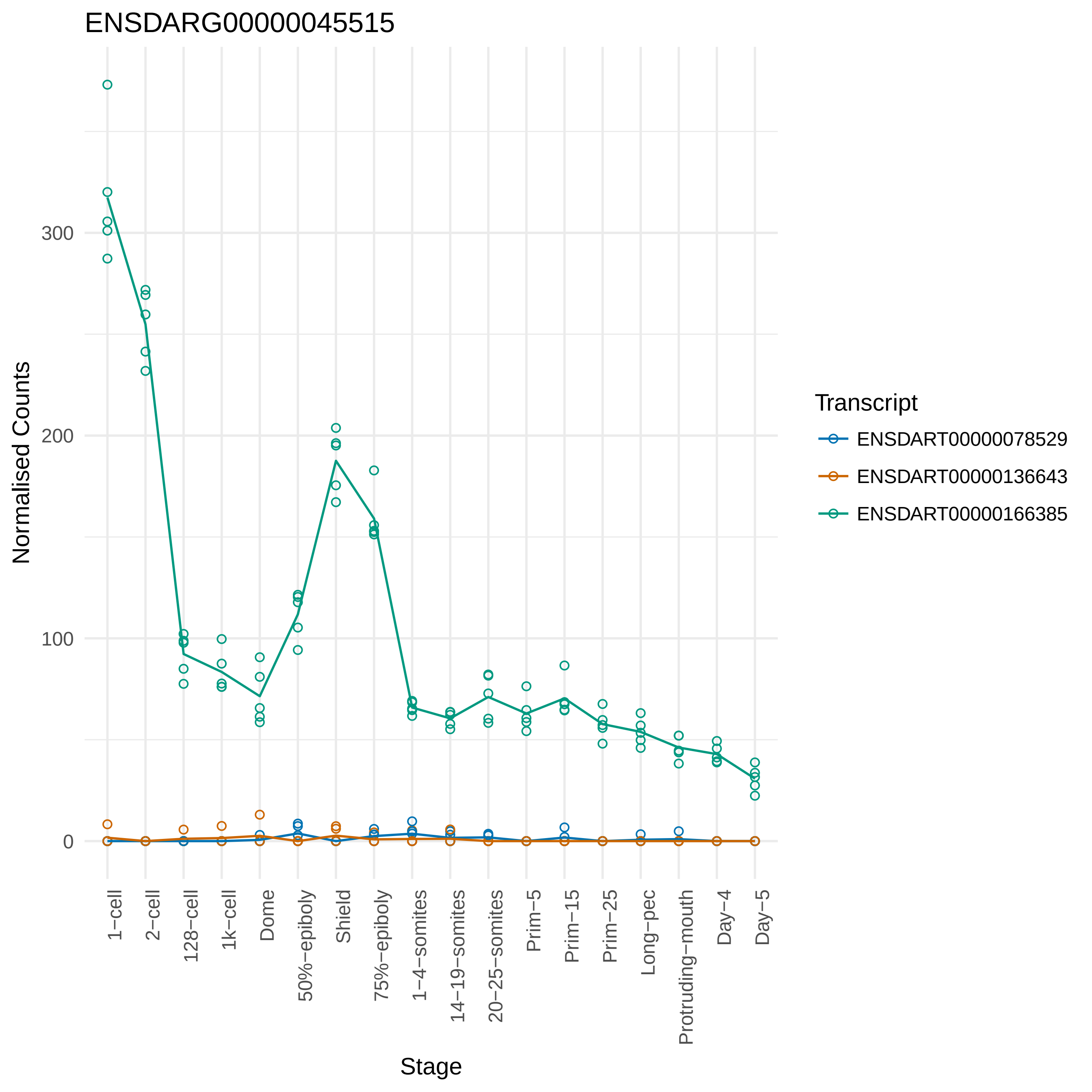

ENSDARG00000045515
Normalised Counts
300
200
100
0
1−cell
2−cell
128−cell
1k−cell
Dome
50%−epiboly
Shield
75%−epiboly
1−4−somites
14−19−somites
20−25−somites
Prim−5
Prim−15
Prim−25
Long−pec
Protruding−mouth
Day−4
Day−5
Stage
Transcript
ENSDART00000078529
ENSDART00000136643
ENSDART00000166385

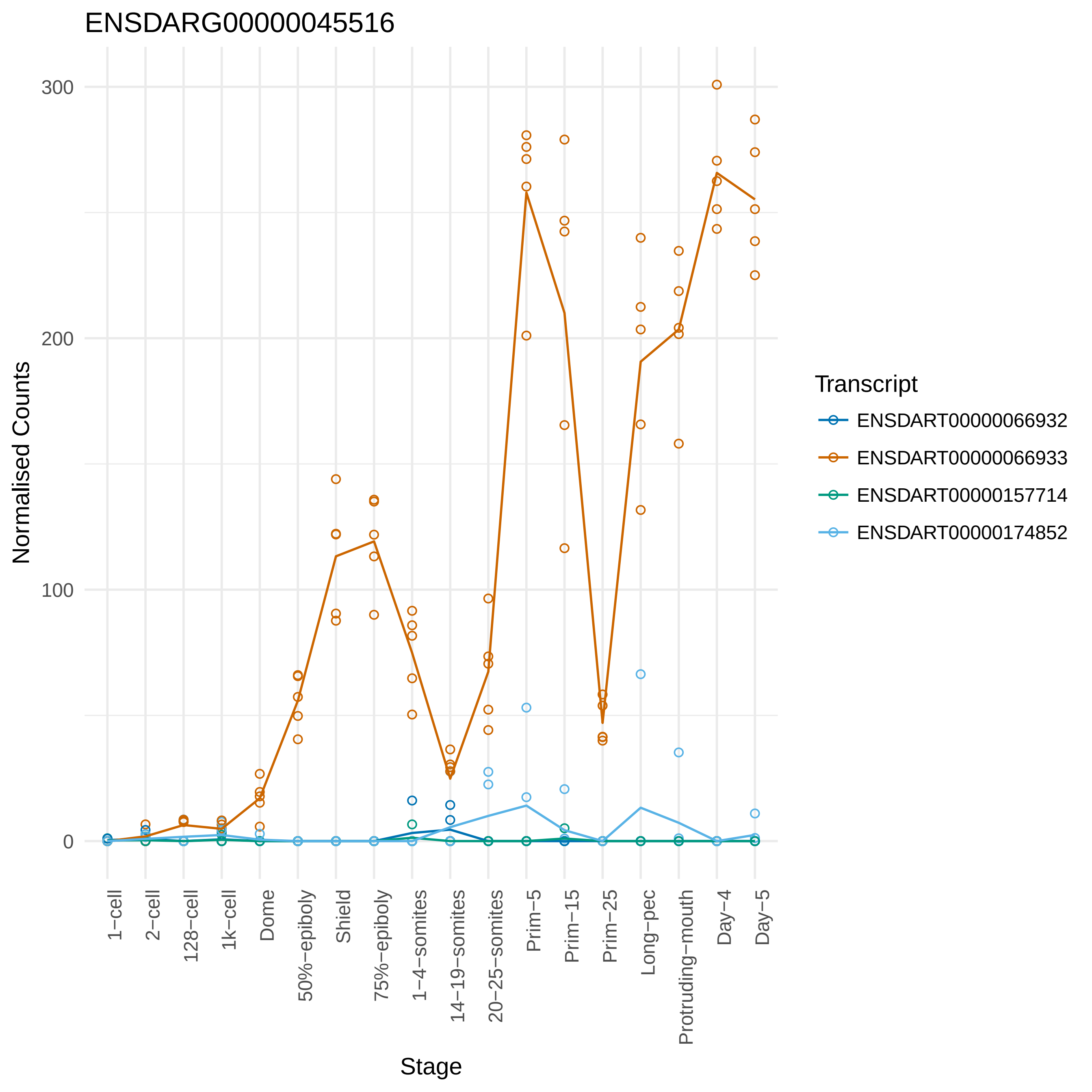

ENSDARG00000045516
Normalised Counts
300
200
100
0
1−cell
2−cell
128−cell
1k−cell
Dome
50%−epiboly
Shield
75%−epiboly
1−4−somites
14−19−somites
20−25−somites
Prim−5
Prim−15
Prim−25
Long−pec
Protruding−mouth
Day−4
Day−5
Stage
Transcript
ENSDART00000066932
ENSDART00000066933
ENSDART00000157714
ENSDART00000174852

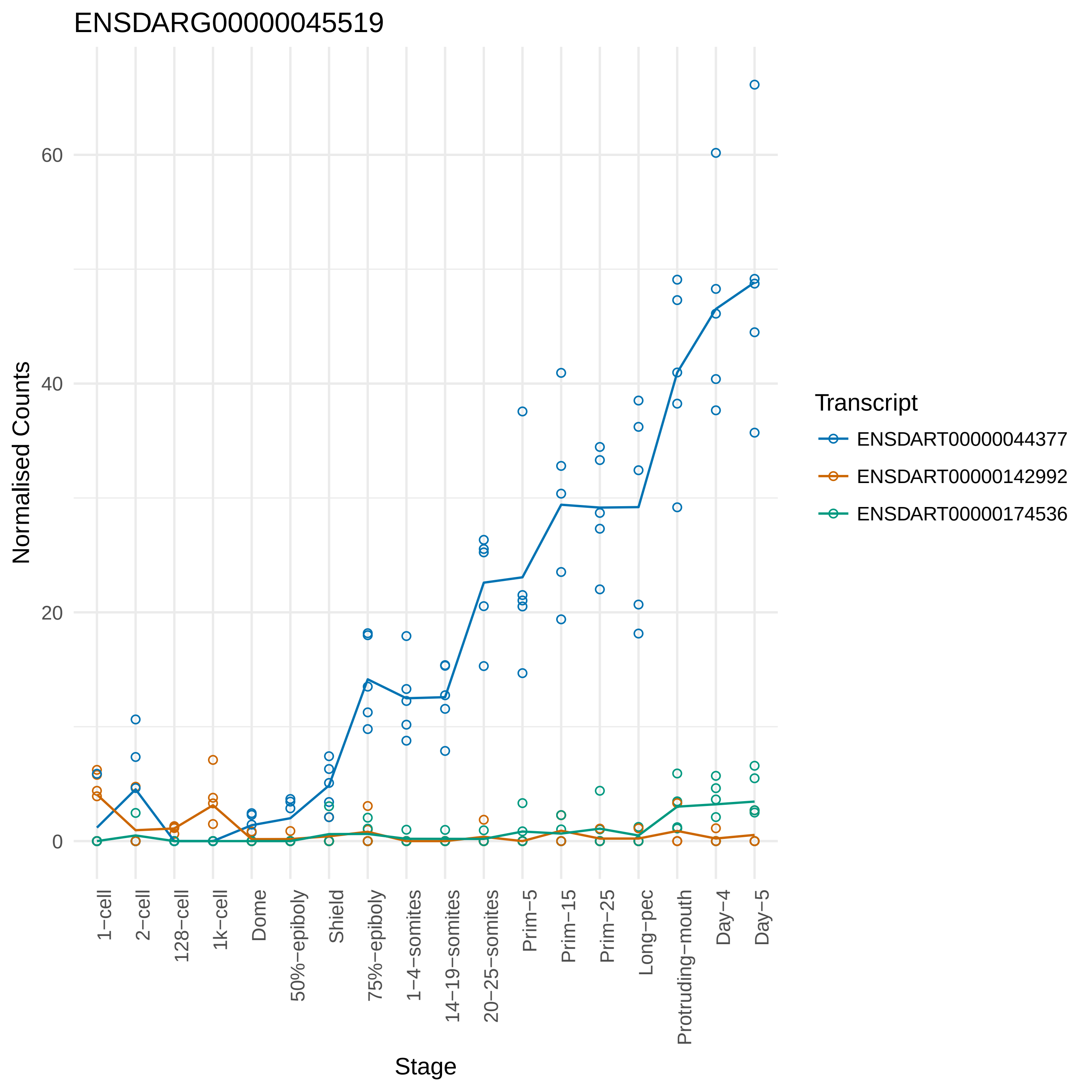

ENSDARG00000045519
Normalised Counts
0
20
40
60
Stage
1−cell
2−cell
128−cell
1k−cell
Dome
50%−epiboly
Shield
75%−epiboly
1−4−somites
14−19−somites
20−25−somites
Prim−5
Prim−15
Prim−25
Long−pec
Protruding−mouth
Day−4
Day−5
Transcript
ENSDART00000044377
ENSDART00000142992
ENSDART00000174536

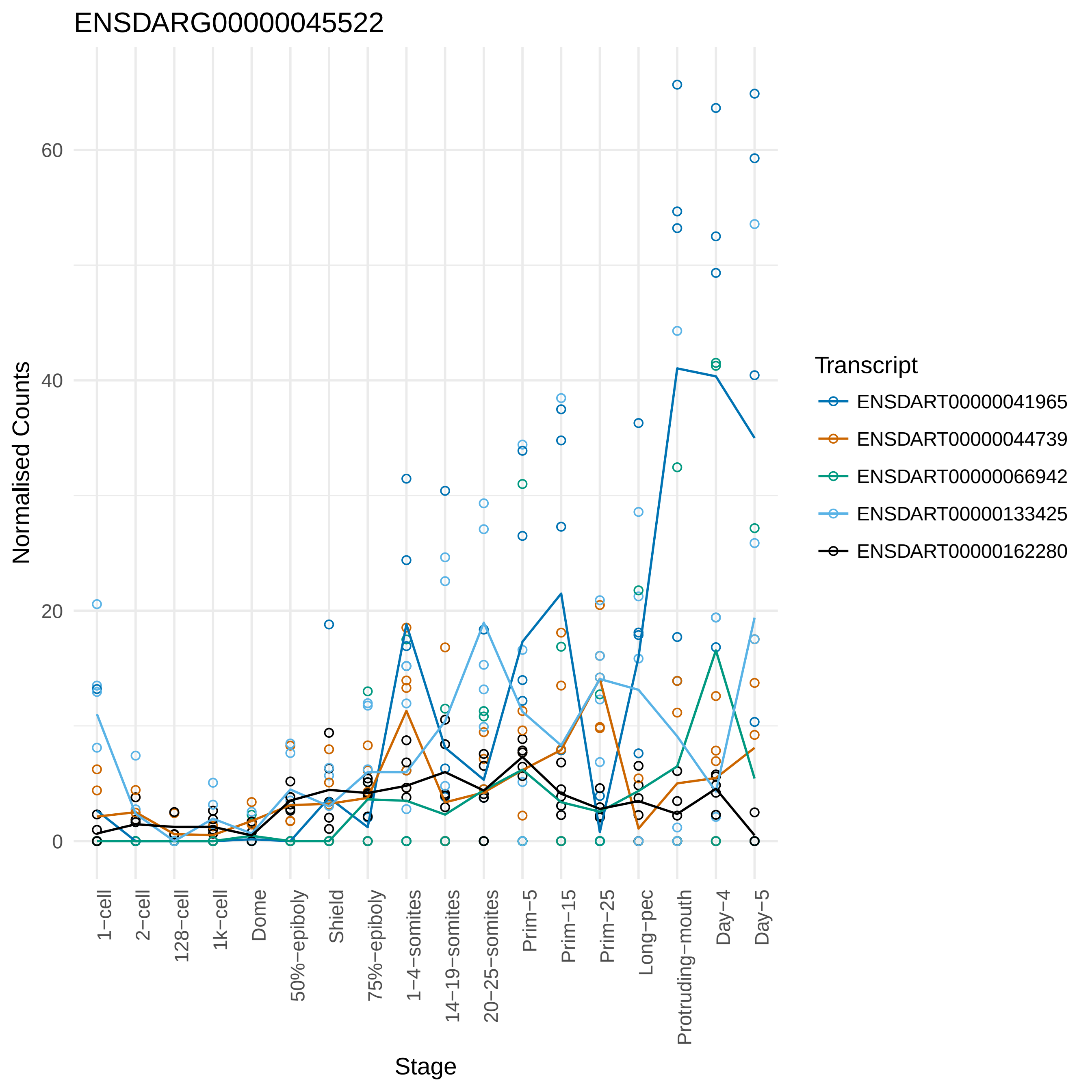

ENSDARG00000045522
Normalised Counts
0
20
40
60
1−cell
2−cell
128−cell
1k−cell
Dome
50%−epiboly
Shield
75%−epiboly
1−4−somites
14−19−somites
20−25−somites
Prim−5
Prim−15
Prim−25
Long−pec
Protruding−mouth
Day−4
Day−5
Stage
Transcript
ENSDART00000041965
ENSDART00000044739
ENSDART00000066942
ENSDART00000133425
ENSDART00000162280

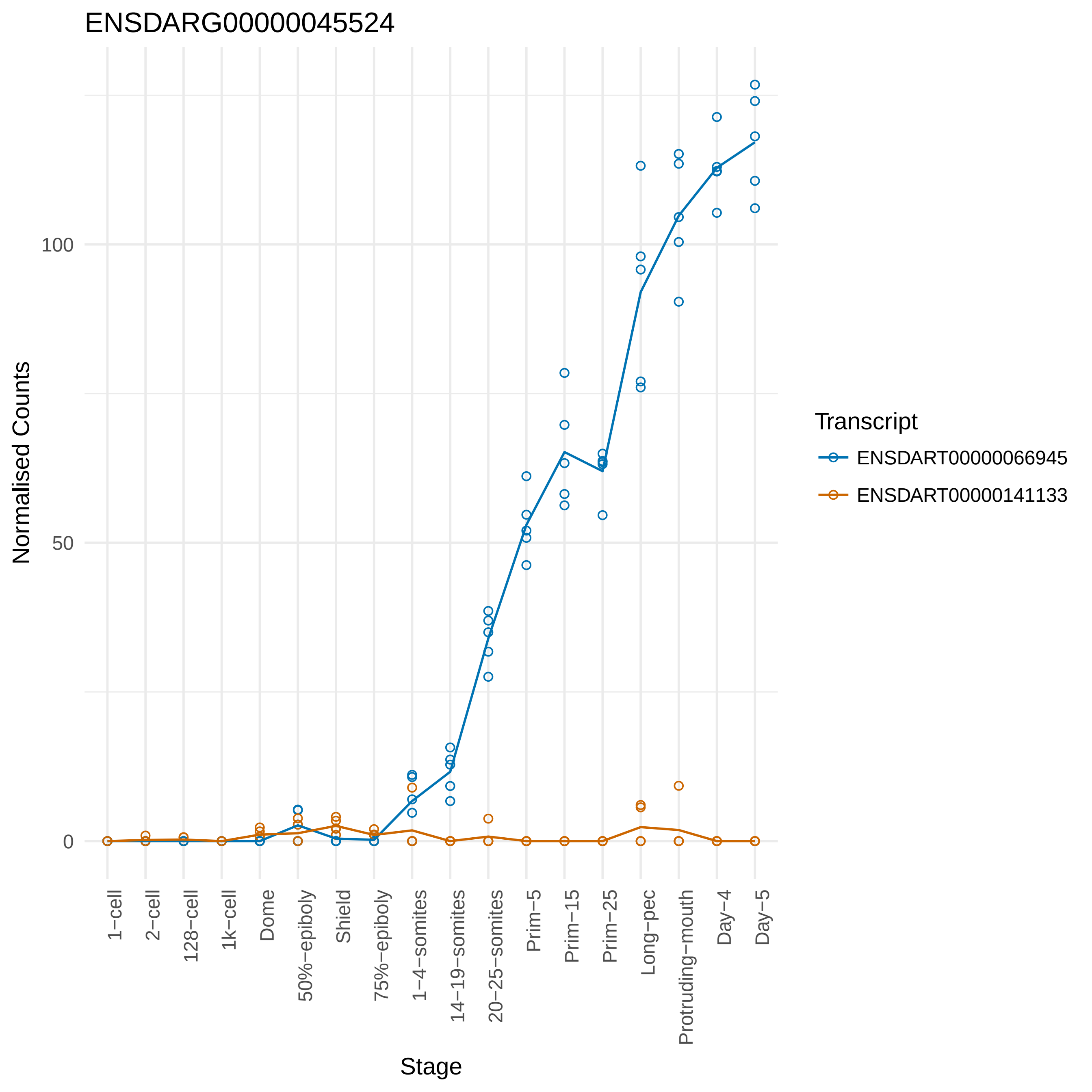
ENSDARG00000045524
Normalised Counts
100
50
0
1−cell
2−cell
128−cell
1k−cell
Dome
50%−epiboly
Shield
75%−epiboly
1−4−somites
14−19−somites
20−25−somites
Prim−5
Prim−15
Prim−25
Long−pec
Protruding−mouth
Day−4
Day−5
Stage
Transcript
ENSDART00000066945
ENSDART00000141133

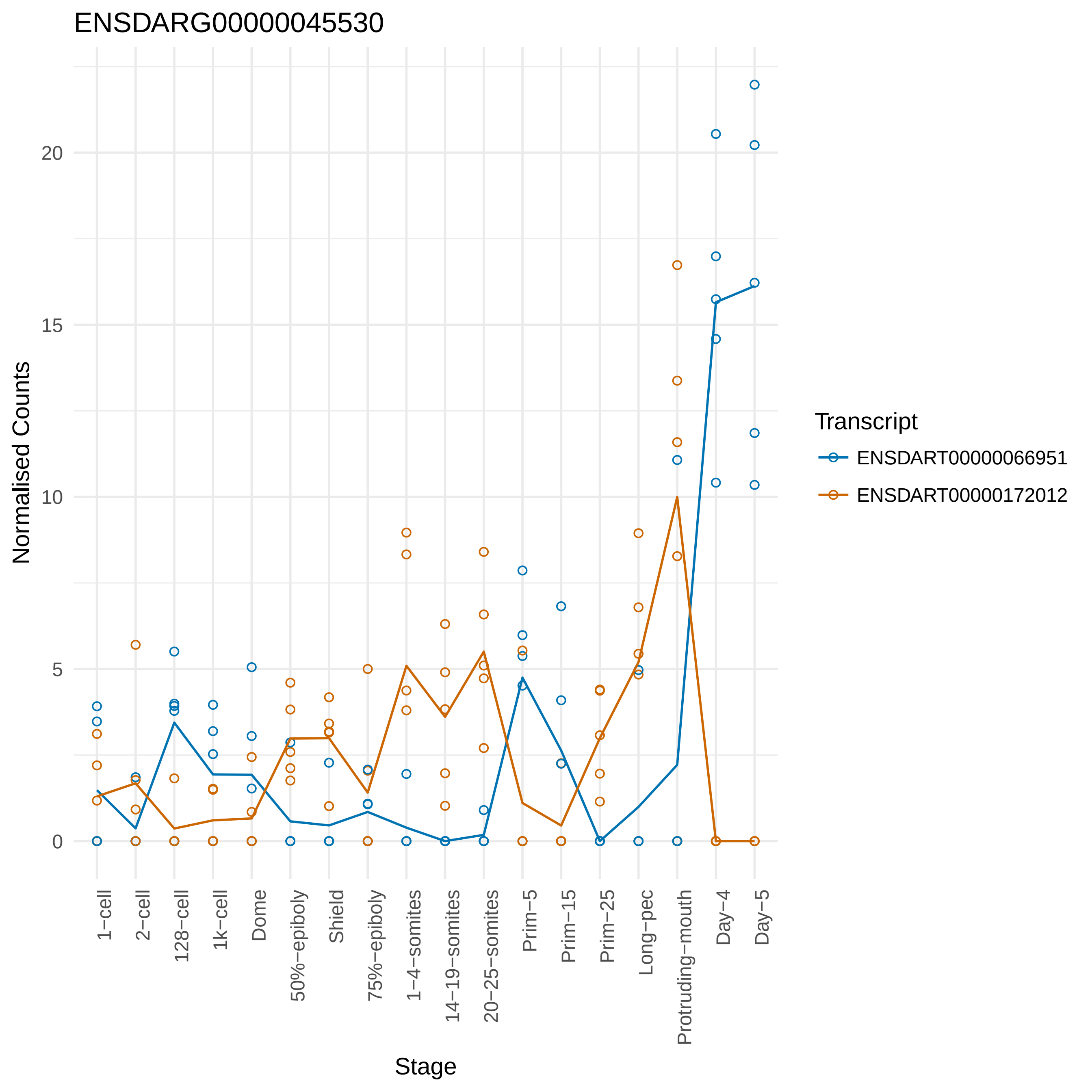

ENSDARG00000045530
Normalised Counts
0
5
10
15
20
Stage
1−cell
2−cell
128−cell
1k−cell
Dome
50%−epiboly
Shield
75%−epiboly
1−4−somites
14−19−somites
20−25−somites
Prim−5
Prim−15
Prim−25
Long−pec
Protruding−mouth
Day−4
Day−5
Transcript
ENSDART00000066951
ENSDART00000172012

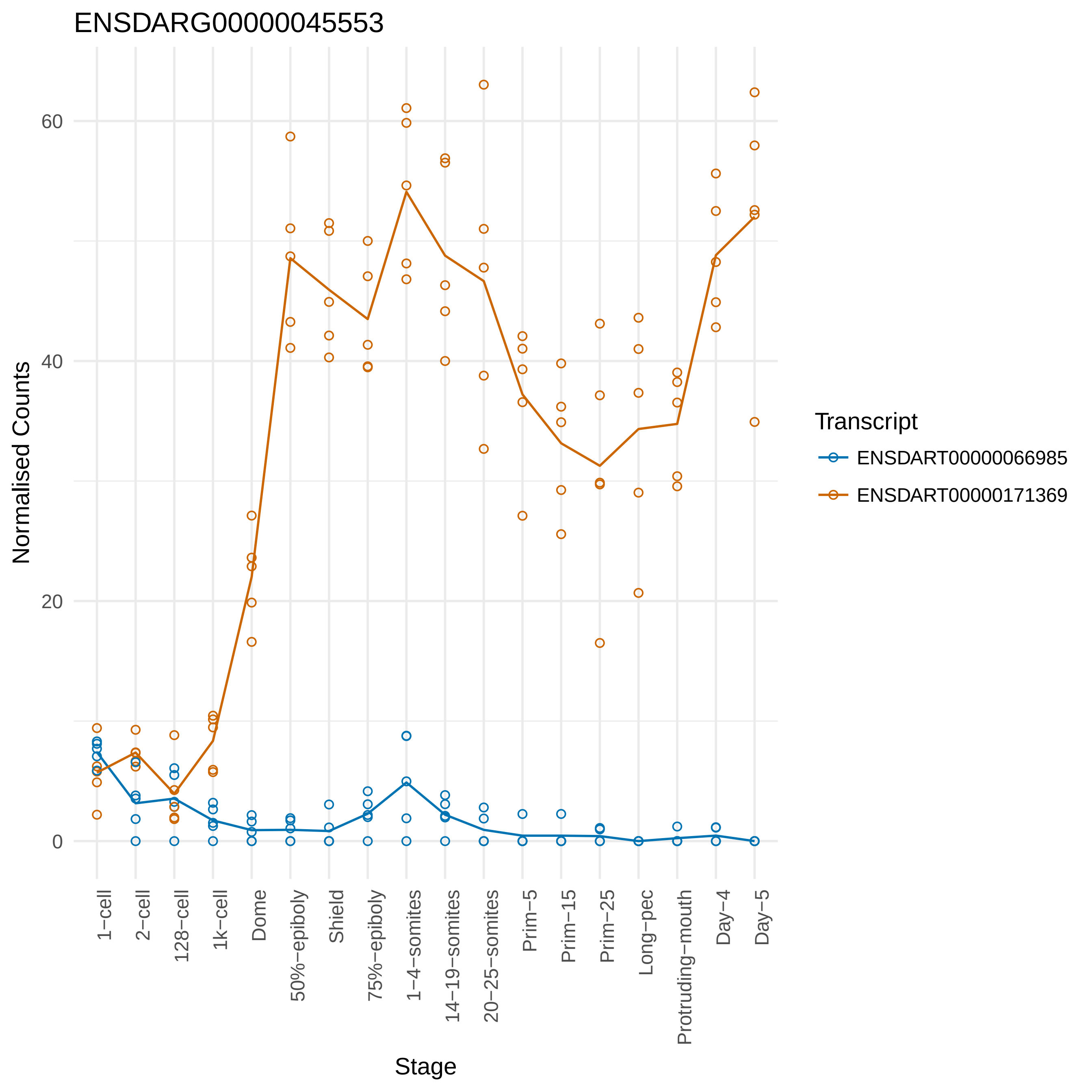

ENSDARG00000045553
Normalised Counts
60
40
20
0
1−cell
2−cell
128−cell
1k−cell
Dome
50%−epiboly
Shield
75%−epiboly
1−4−somites
14−19−somites
20−25−somites
Prim−5
Prim−15
Prim−25
Long−pec
Protruding−mouth
Day−4
Day−5
Stage
Transcript
ENSDART00000066985
ENSDART00000171369

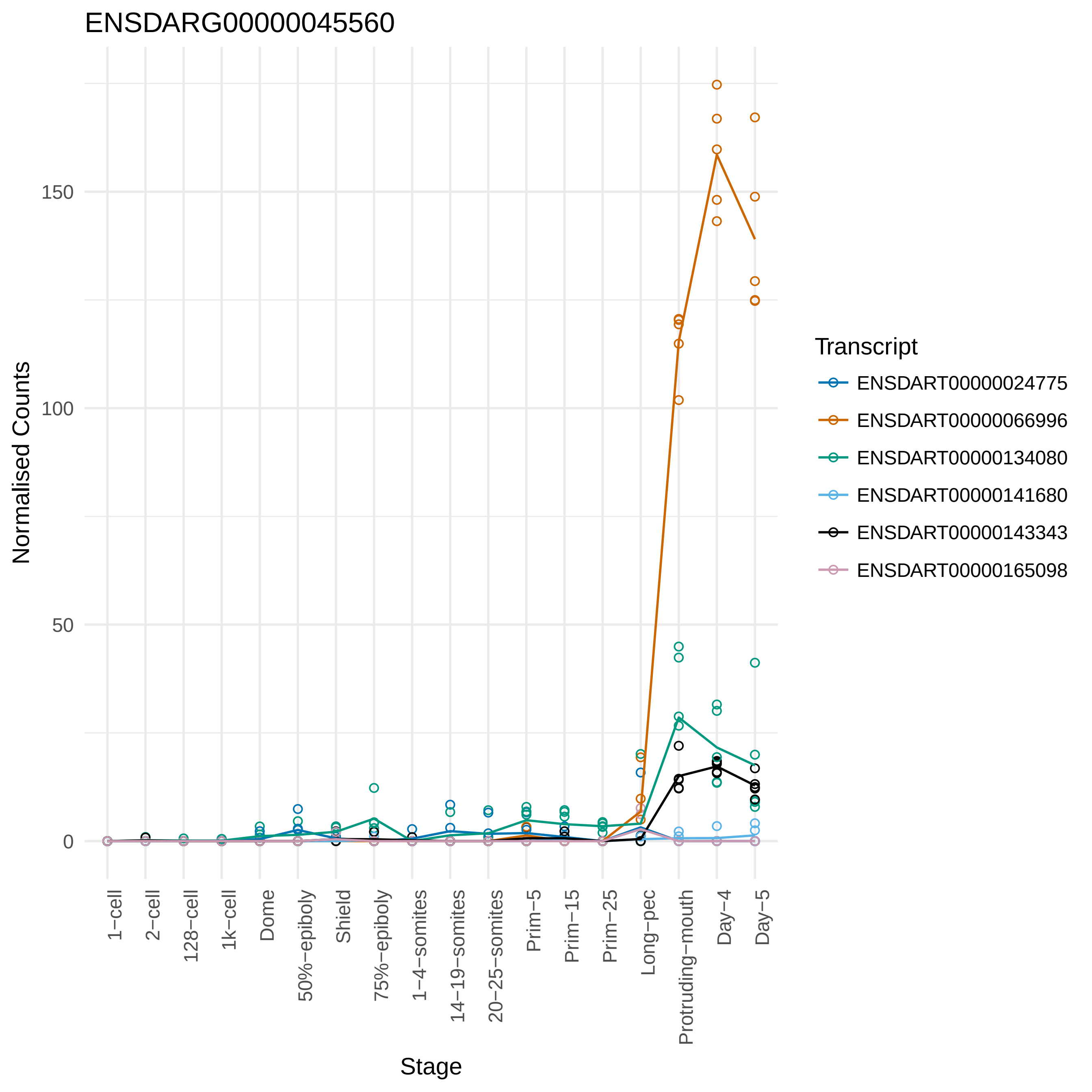

ENSDARG00000045560
Normalised Counts
150
100
50
0
1−cell
2−cell
128−cell
1k−cell
Dome
50%−epiboly
Shield
75%−epiboly
1−4−somites
14−19−somites
20−25−somites
Prim−5
Prim−15
Prim−25
Long−pec
Protruding−mouth
Day−4
Day−5
Stage
Transcript
ENSDART00000024775
ENSDART00000066996
ENSDART00000134080
ENSDART00000141680
ENSDART00000143343
ENSDART00000165098

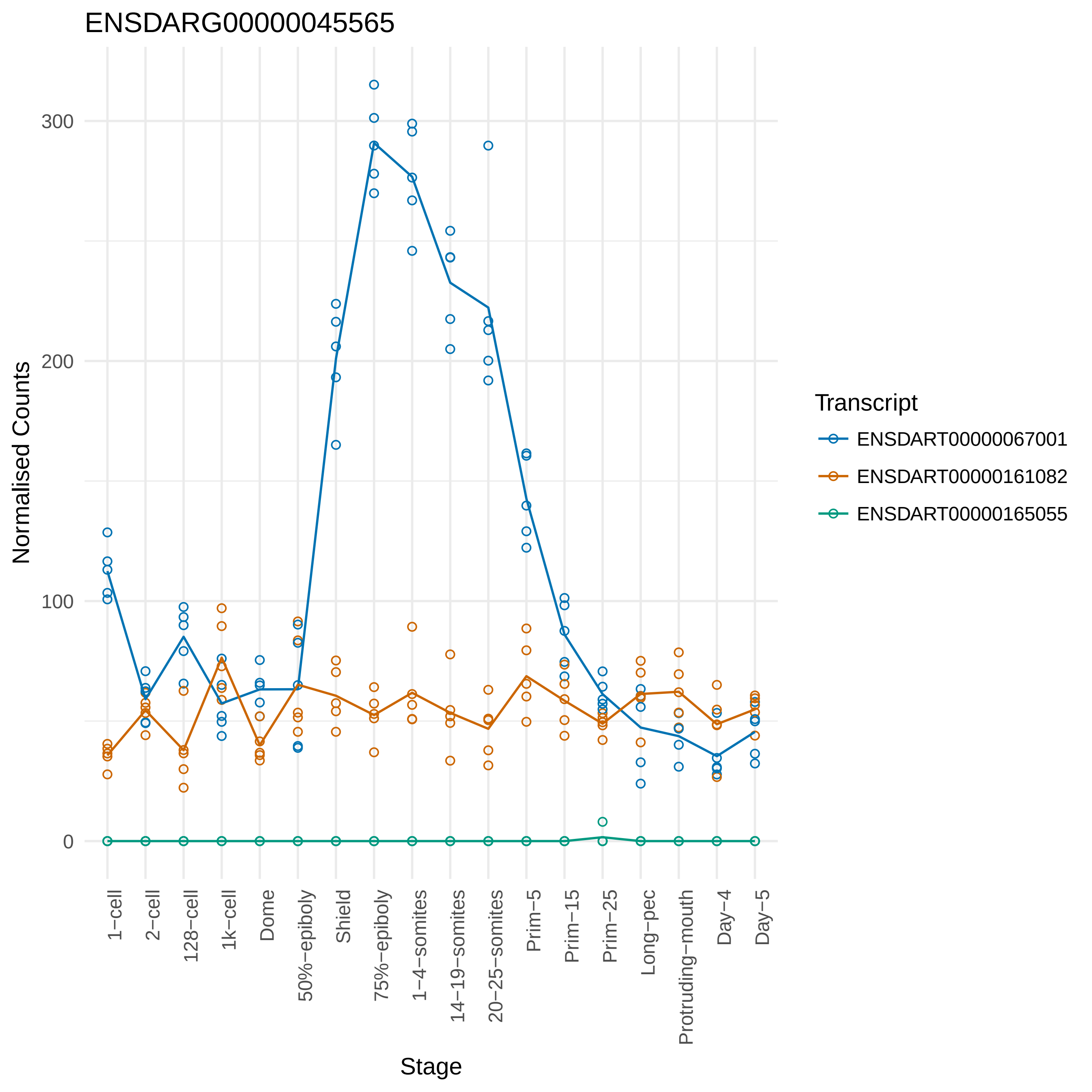

ENSDARG00000045565
Normalised Counts
300
200
100
0
1−cell
2−cell
128−cell
1k−cell
Dome
50%−epiboly
Shield
75%−epiboly
1−4−somites
14−19−somites
20−25−somites
Prim−5
Prim−15
Prim−25
Long−pec
Protruding−mouth
Day−4
Day−5
Stage
Transcript
ENSDART00000067001
ENSDART00000161082
ENSDART00000165055

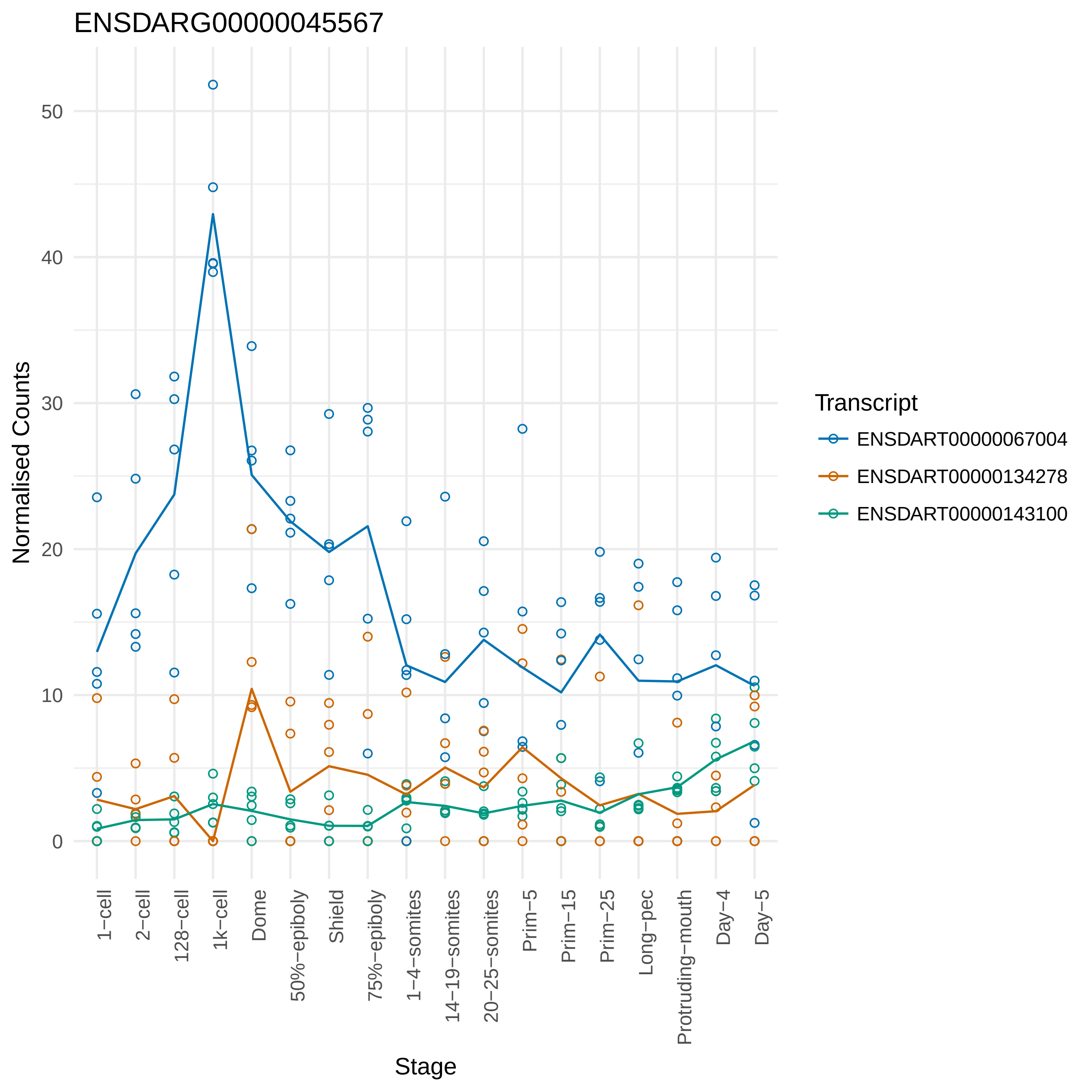
ENSDARG00000045567
Normalised Counts
0
10
20
30
40
50
1−cell
2−cell
128−cell
1k−cell
Dome
50%−epiboly
Shield
75%−epiboly
1−4−somites
14−19−somites
20−25−somites
Prim−5
Prim−15
Prim−25
Long−pec
Protruding−mouth
Day−4
Day−5
Stage
Transcript
ENSDART00000067004
ENSDART00000134278
ENSDART00000143100

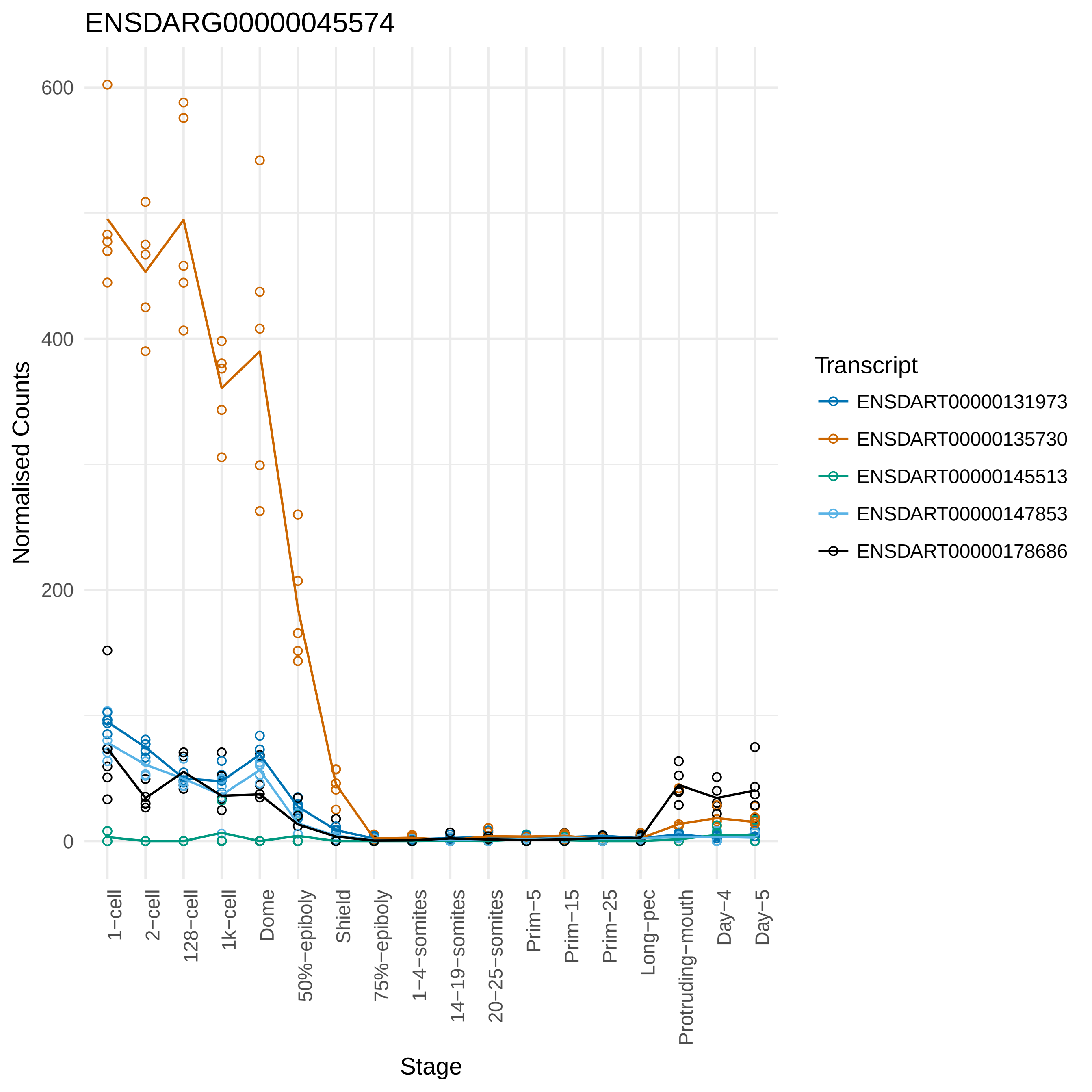

ENSDARG00000045574
Normalised Counts
0
200
400
600
Stage
1−cell
2−cell
128−cell
1k−cell
Dome
50%−epiboly
Shield
75%−epiboly
1−4−somites
14−19−somites
20−25−somites
Prim−5
Prim−15
Prim−25
Long−pec
Protruding−mouth
Day−4
Day−5
Transcript
ENSDART00000131973
ENSDART00000135730
ENSDART00000145513
ENSDART00000147853
ENSDART00000178686

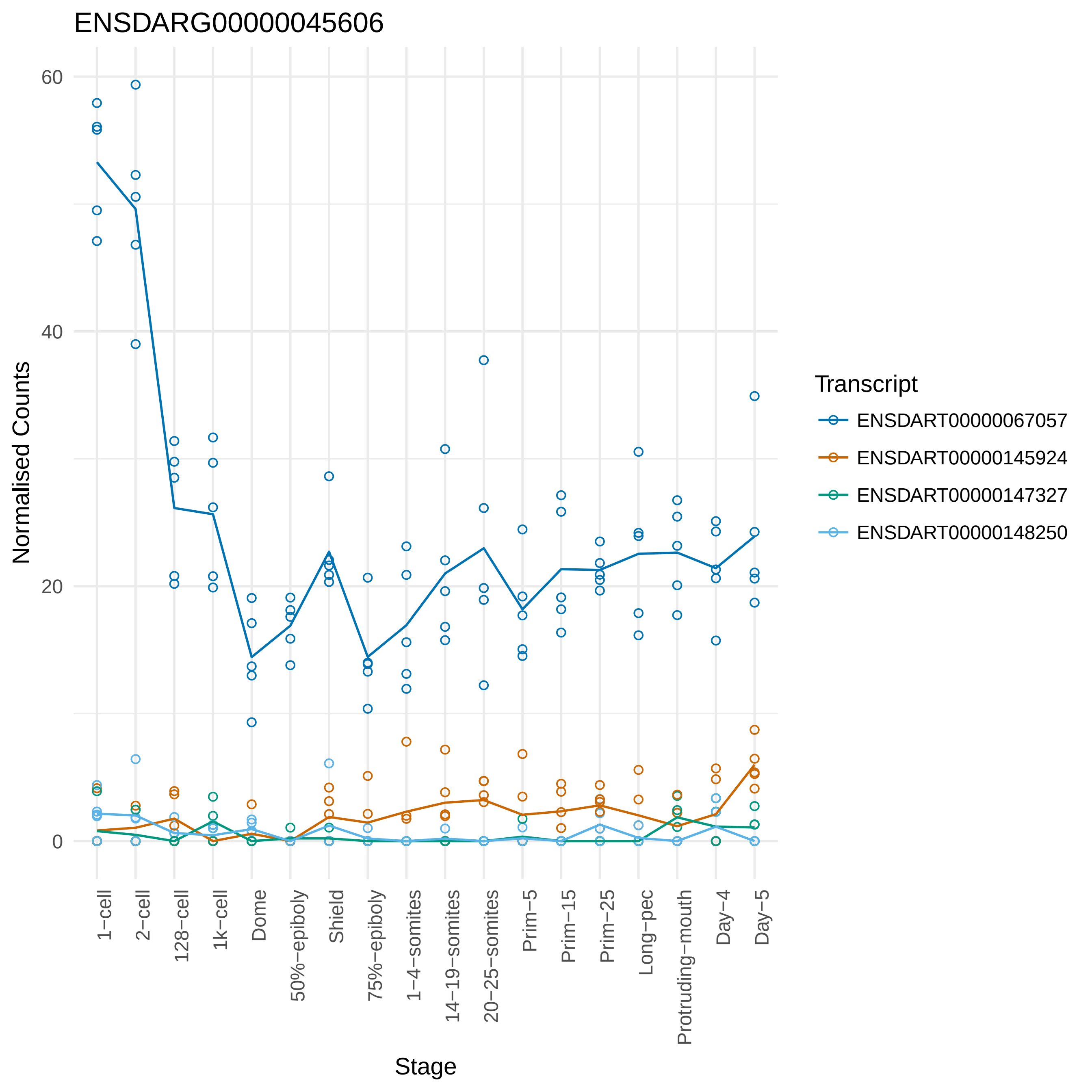

ENSDARG00000045606
Normalised Counts
60
40
20
0
1−cell
2−cell
128−cell
1k−cell
Dome
50%−epiboly
Shield
75%−epiboly
1−4−somites
14−19−somites
20−25−somites
Prim−5
Prim−15
Prim−25
Long−pec
Protruding−mouth
Day−4
Day−5
Stage
Transcript
ENSDART00000067057
ENSDART00000145924
ENSDART00000147327
ENSDART00000148250

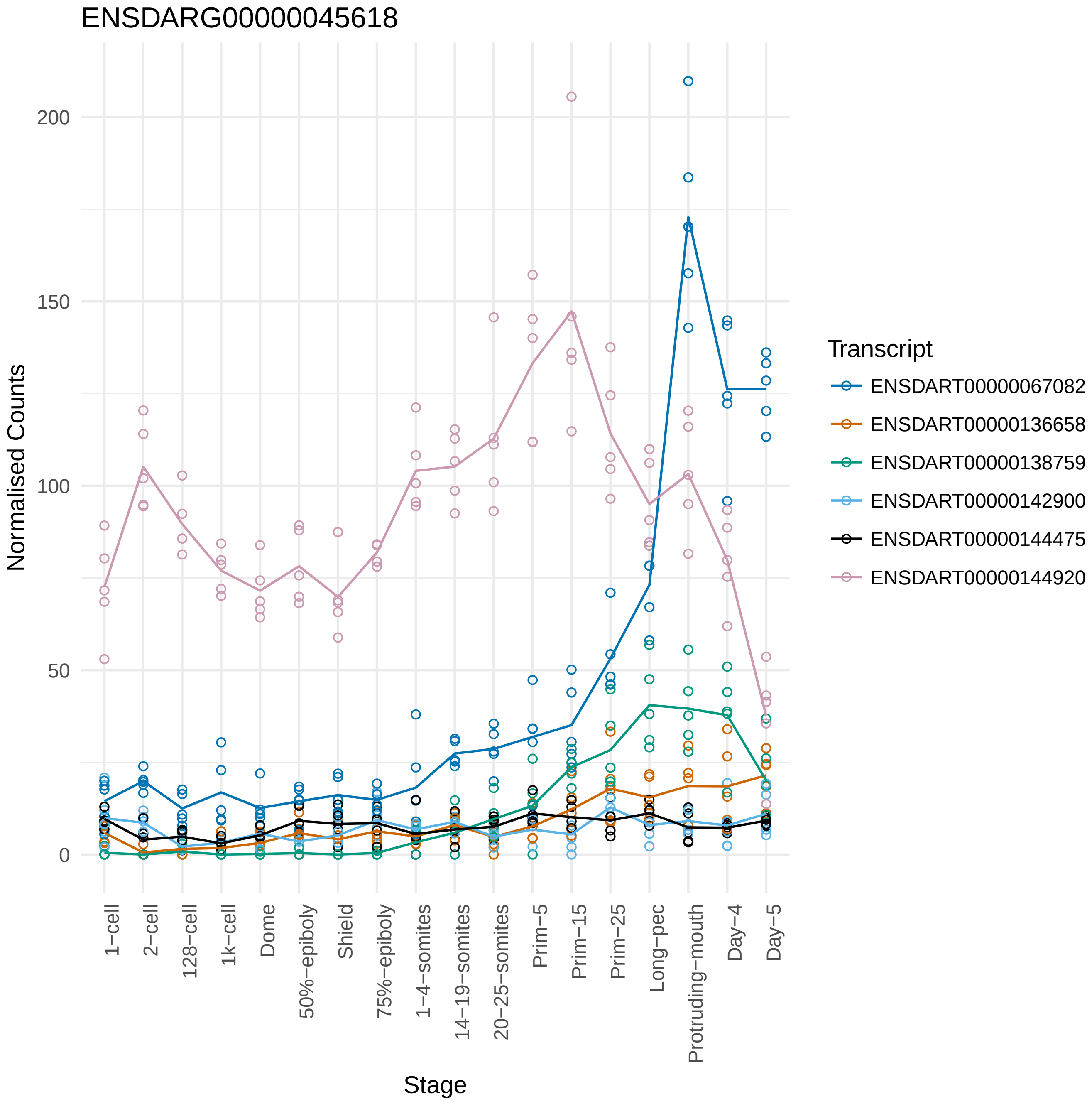
ENSDARG00000045618
Normalised Counts
200
150
100
50
0
1−cell
2−cell
128−cell
1k−cell
Dome
50%−epiboly
Shield
75%−epiboly
1−4−somites
14−19−somites
20−25−somites
Prim−5
Prim−15
Prim−25
Long−pec
Protruding−mouth
Day−4
Day−5
Stage
Transcript
ENSDART00000067082
ENSDART00000136658
ENSDART00000138759
ENSDART00000142900
ENSDART00000144475
ENSDART00000144920

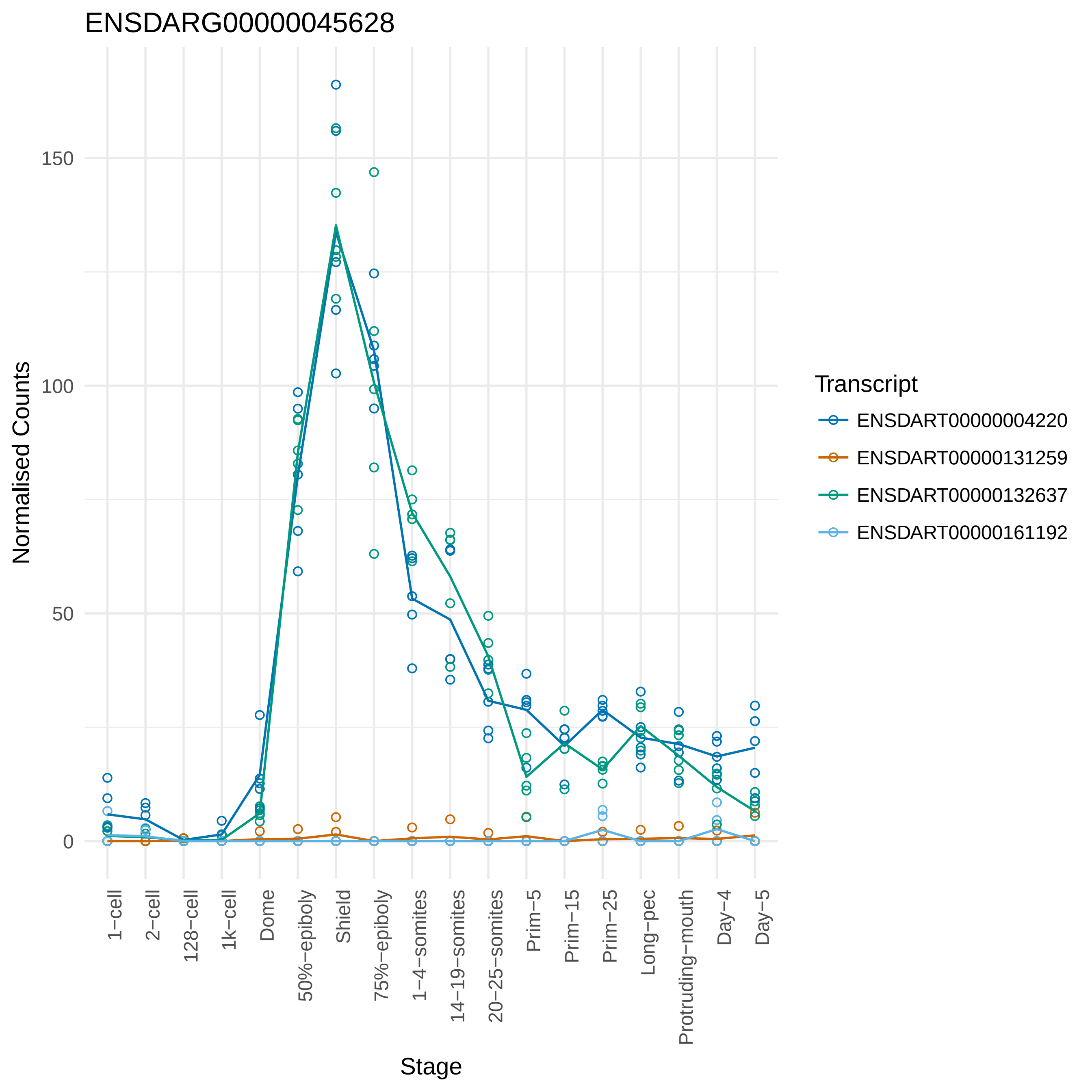

ENSDARG00000045628
Normalised Counts
150
100
50
0
1−cell
2−cell
128−cell
1k−cell
Dome
50%−epiboly
Shield
75%−epiboly
1−4−somites
14−19−somites
20−25−somites
Prim−5
Prim−15
Prim−25
Long−pec
Protruding−mouth
Day−4
Day−5
Stage
Transcript
ENSDART00000004220
ENSDART00000131259
ENSDART00000132637
ENSDART00000161192

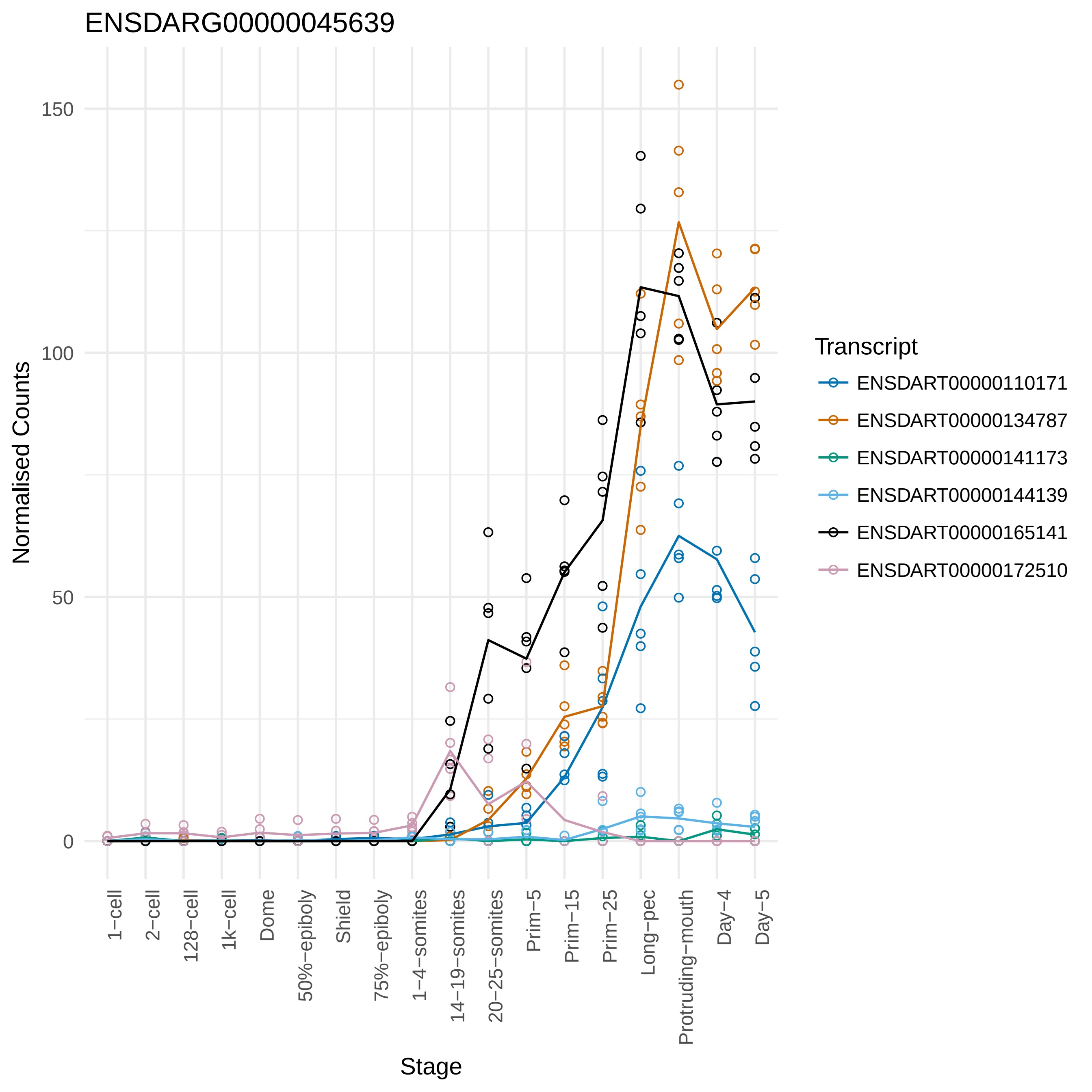

ENSDARG00000045639
Normalised Counts
150
100
50
0
1−cell
2−cell
128−cell
1k−cell
Dome
50%−epiboly
Shield
75%−epiboly
1−4−somites
14−19−somites
20−25−somites
Prim−5
Prim−15
Prim−25
Long−pec
Protruding−mouth
Day−4
Day−5
Stage
Transcript
ENSDART00000110171
ENSDART00000134787
ENSDART00000141173
ENSDART00000144139
ENSDART00000165141
ENSDART00000172510

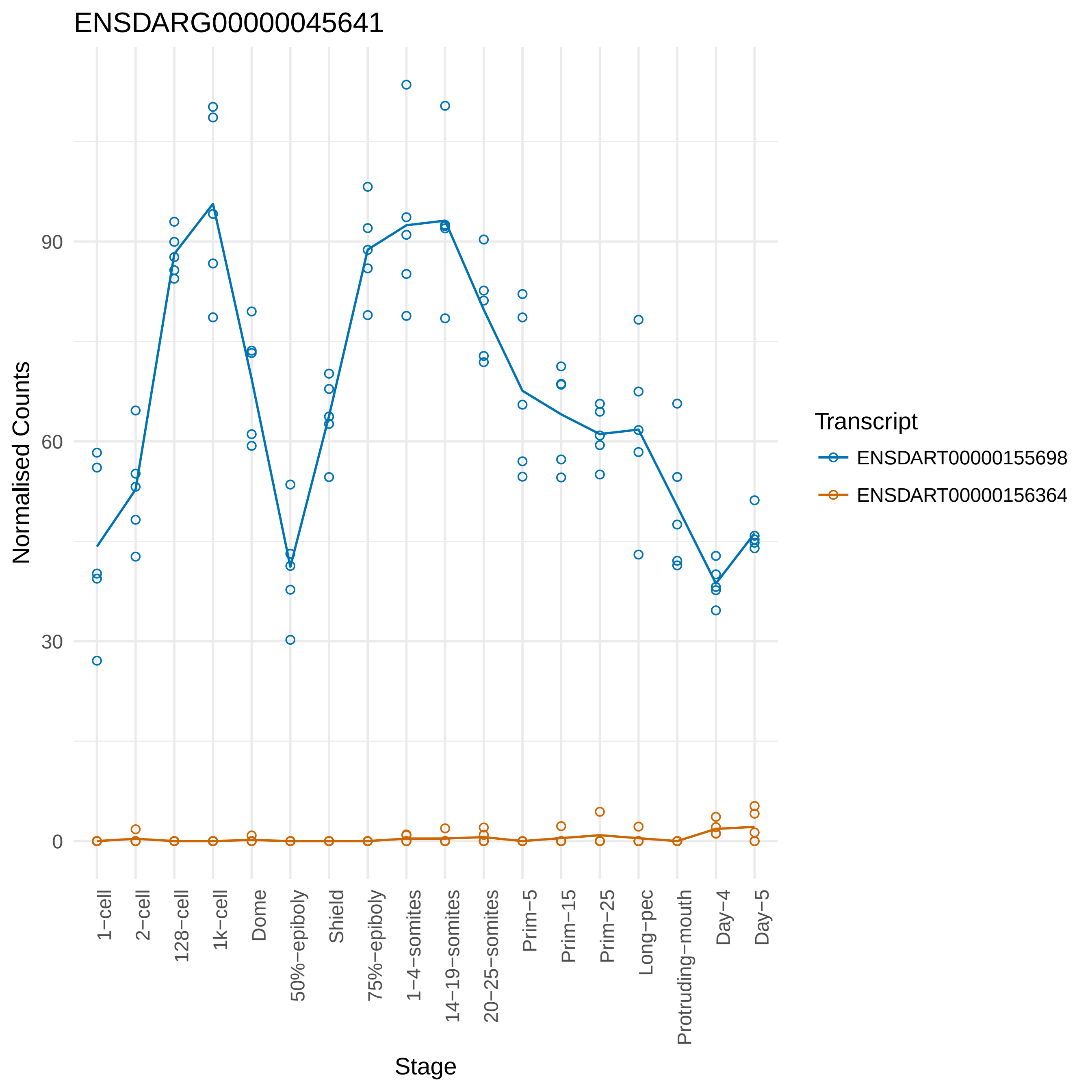

ENSDARG00000045641
Normalised Counts
0
30
60
90
Stage
1−cell
2−cell
128−cell
1k−cell
Dome
50%−epiboly
Shield
75%−epiboly
1−4−somites
14−19−somites
20−25−somites
Prim−5
Prim−15
Prim−25
Long−pec
Protruding−mouth
Day−4
Day−5
Transcript
ENSDART00000155698
ENSDART00000156364

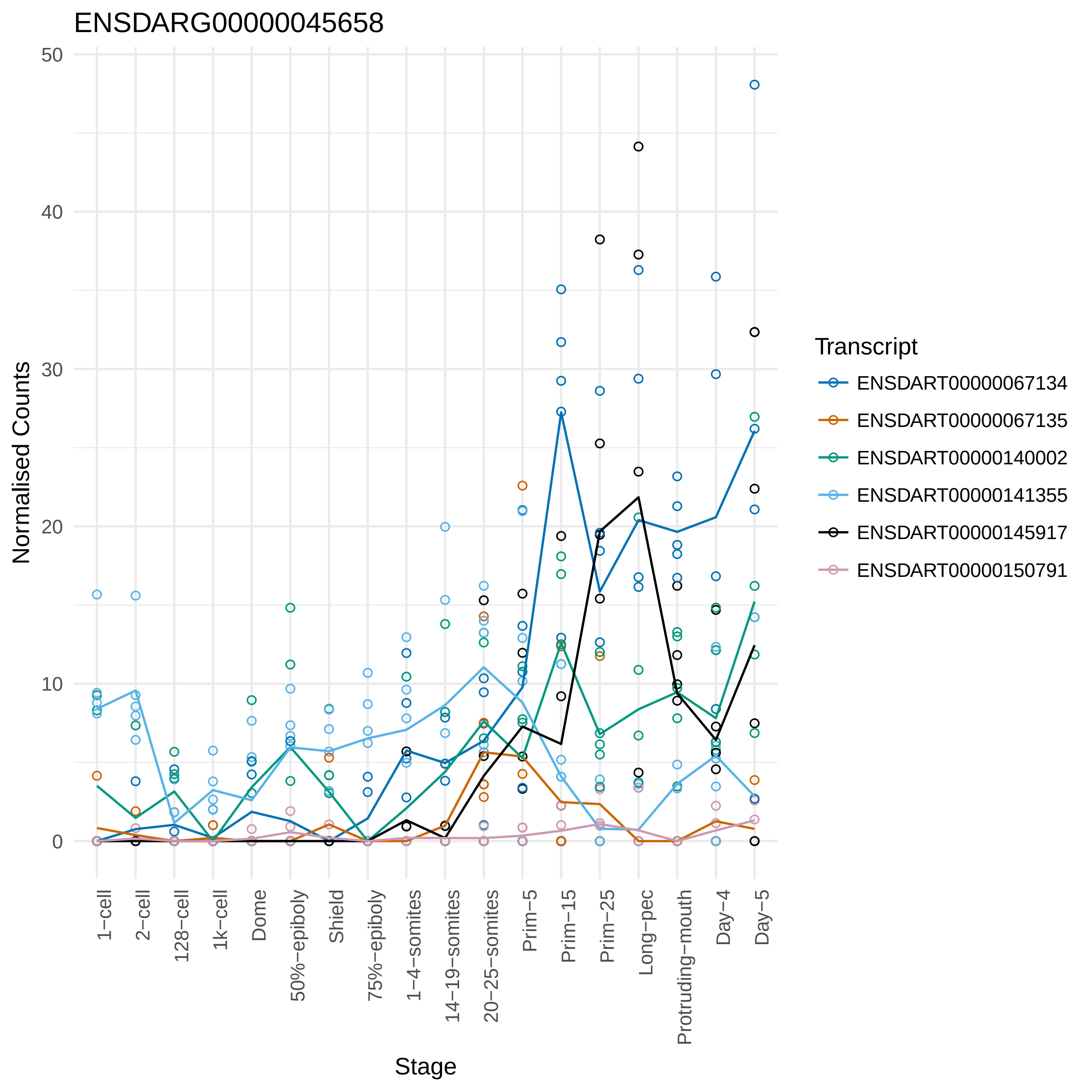

ENSDARG00000045658
Normalised Counts
0
10
20
30
40
50
1−cell
2−cell
128−cell
1k−cell
Dome
50%−epiboly
Shield
75%−epiboly
1−4−somites
14−19−somites
20−25−somites
Prim−5
Prim−15
Prim−25
Long−pec
Protruding−mouth
Day−4
Day−5
Stage
Transcript
ENSDART00000067134
ENSDART00000067135
ENSDART00000140002
ENSDART00000141355
ENSDART00000145917
ENSDART00000150791

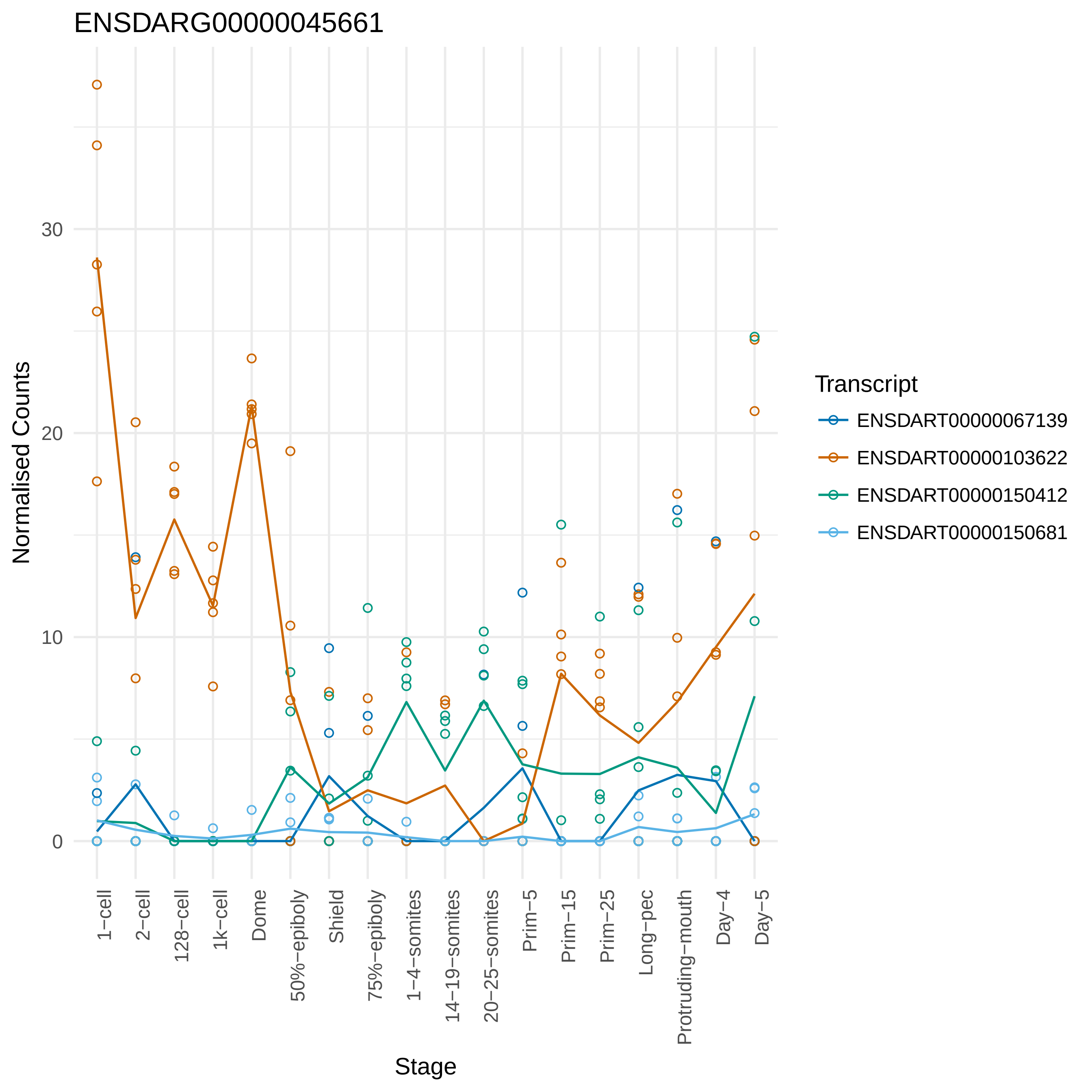

ENSDARG00000045661
Normalised Counts
30
20
10
0
1−cell
2−cell
128−cell
1k−cell
Dome
50%−epiboly
Shield
75%−epiboly
1−4−somites
14−19−somites
20−25−somites
Prim−5
Prim−15
Prim−25
Long−pec
Protruding−mouth
Day−4
Day−5
Stage
Transcript
ENSDART00000067139
ENSDART00000103622
ENSDART00000150412
ENSDART00000150681

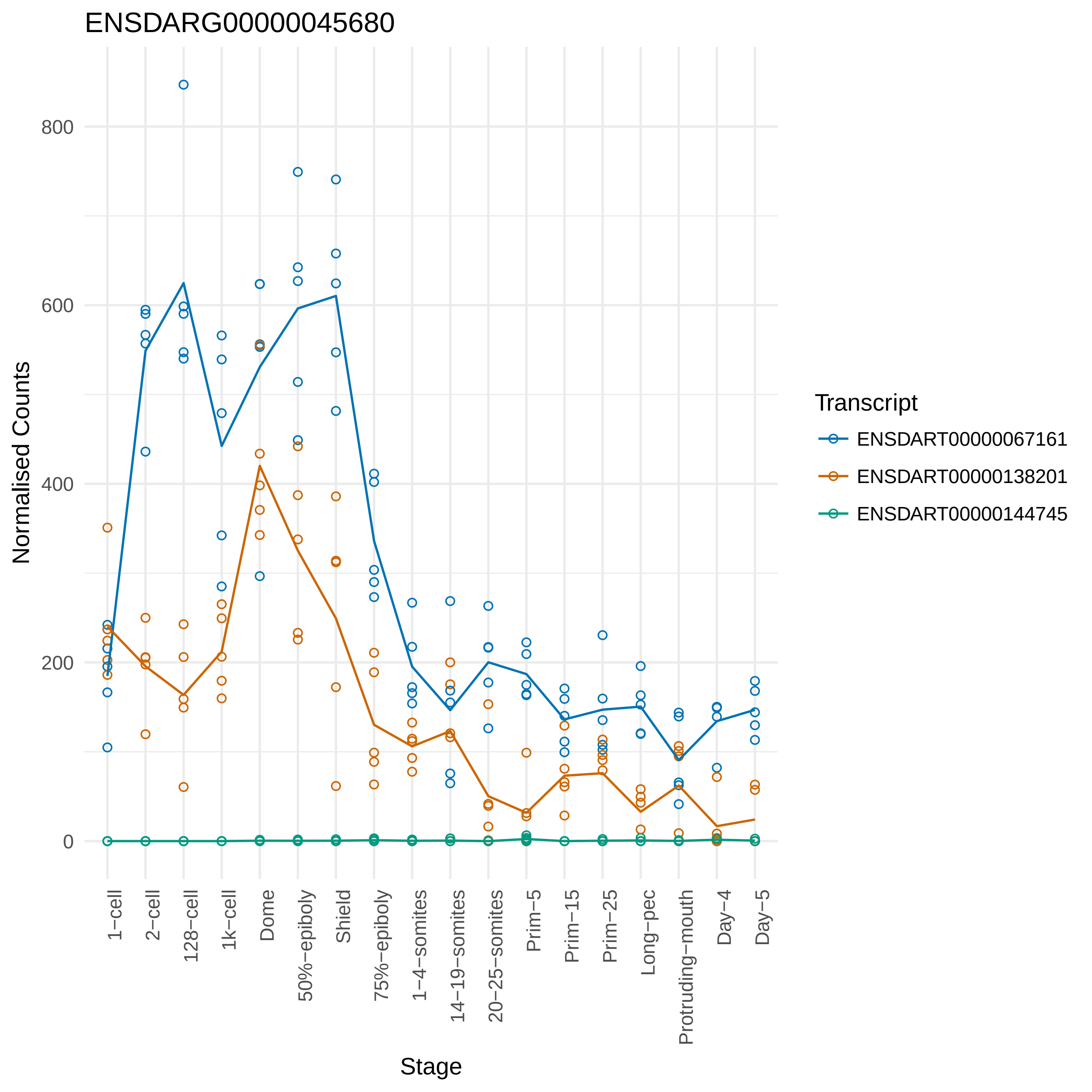

ENSDARG00000045680
Normalised Counts
800
600
400
200
0
1−cell
2−cell
128−cell
1k−cell
Dome
50%−epiboly
Shield
75%−epiboly
1−4−somites
14−19−somites
20−25−somites
Prim−5
Prim−15
Prim−25
Long−pec
Protruding−mouth
Day−4
Day−5
Stage
Transcript
ENSDART00000067161
ENSDART00000138201
ENSDART00000144745

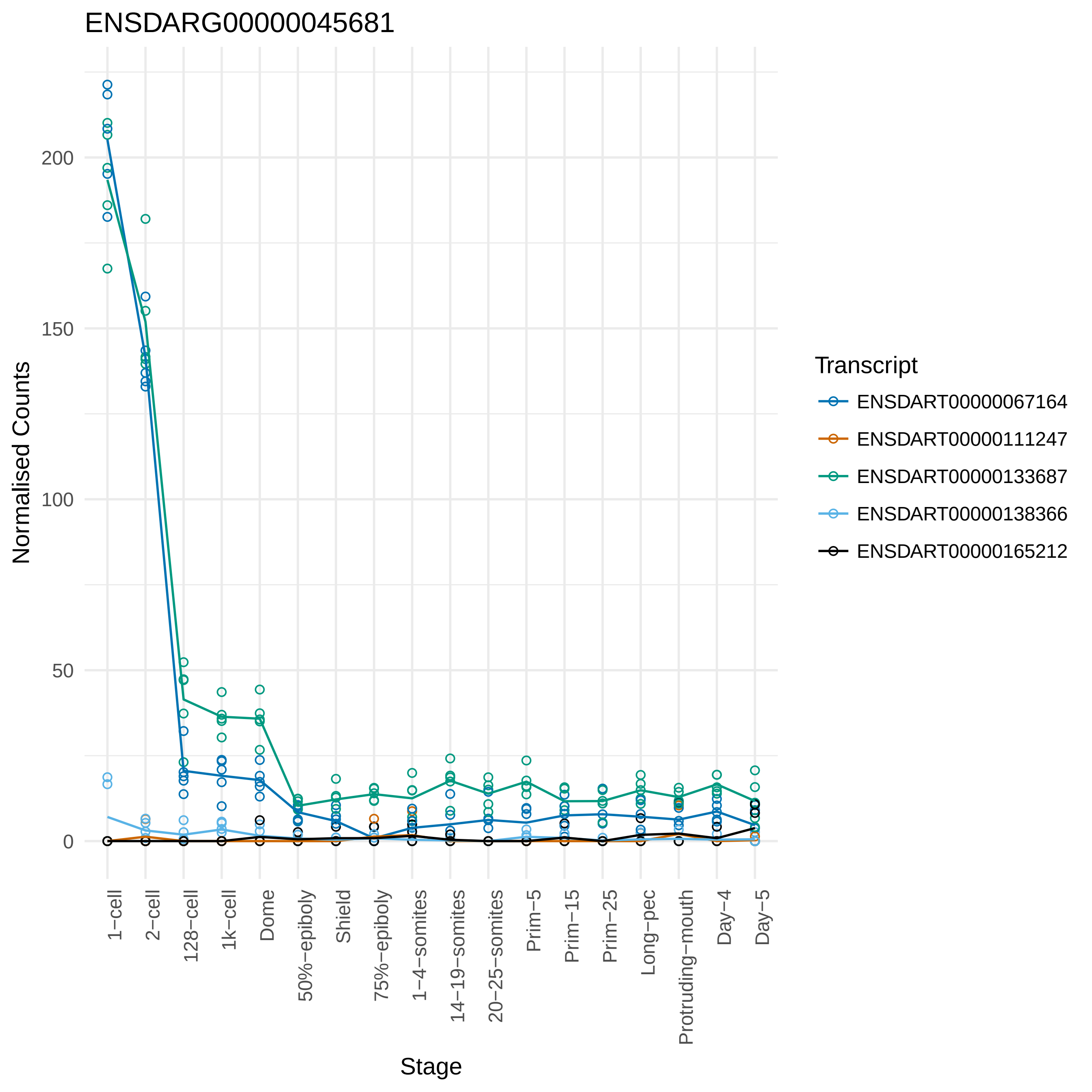

ENSDARG00000045681
Normalised Counts
0
50
100
150
200
1−cell
2−cell
128−cell
1k−cell
Dome
50%−epiboly
Shield
75%−epiboly
1−4−somites
14−19−somites
20−25−somites
Prim−5
Prim−15
Prim−25
Long−pec
Protruding−mouth
Day−4
Day−5
Stage
Transcript
ENSDART00000067164
ENSDART00000111247
ENSDART00000133687
ENSDART00000138366
ENSDART00000165212

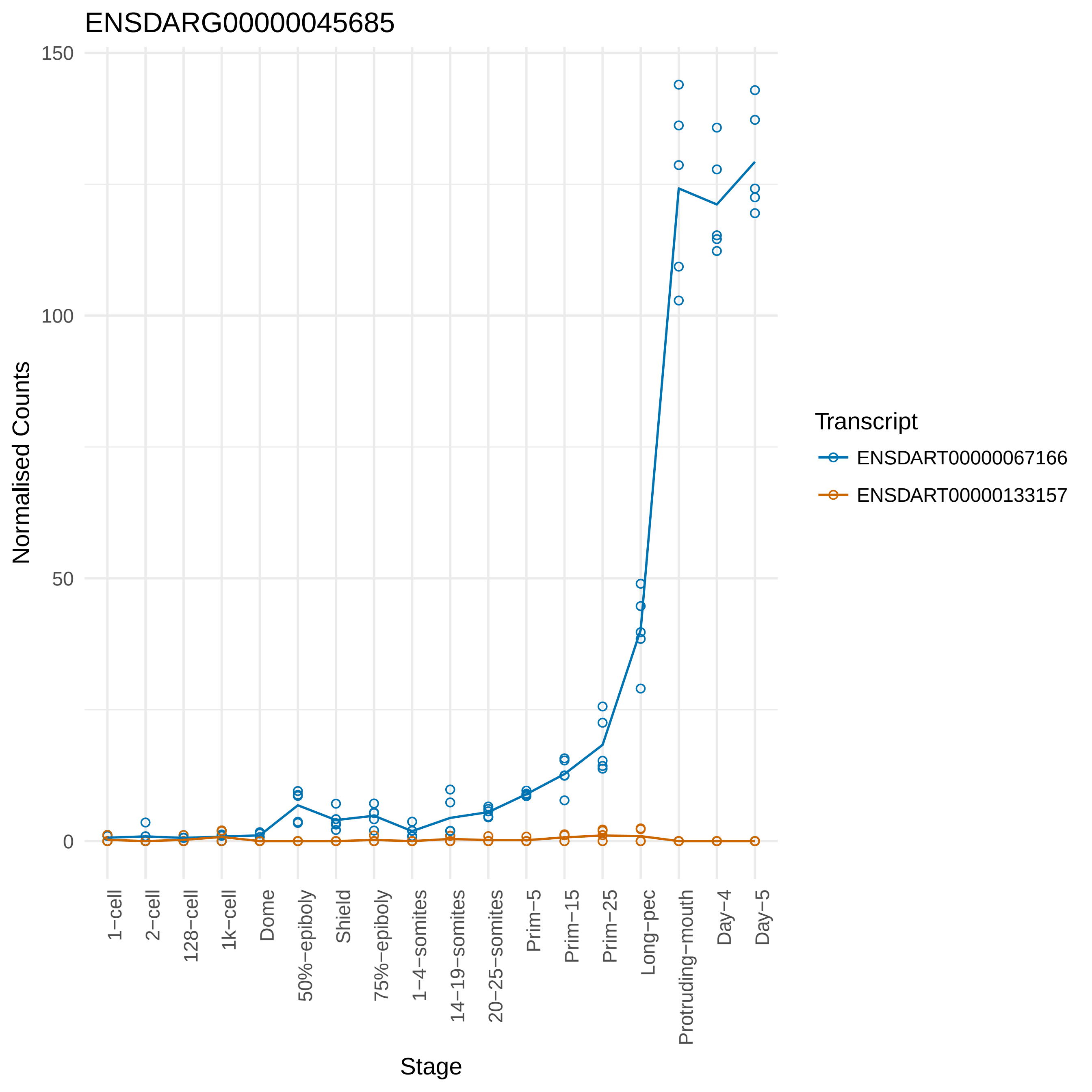

ENSDARG00000045685
Normalised Counts
150
100
50
0
1−cell
2−cell
128−cell
1k−cell
Dome
50%−epiboly
Shield
75%−epiboly
1−4−somites
14−19−somites
20−25−somites
Prim−5
Prim−15
Prim−25
Long−pec
Protruding−mouth
Day−4
Day−5
Stage
Transcript
ENSDART00000067166
ENSDART00000133157

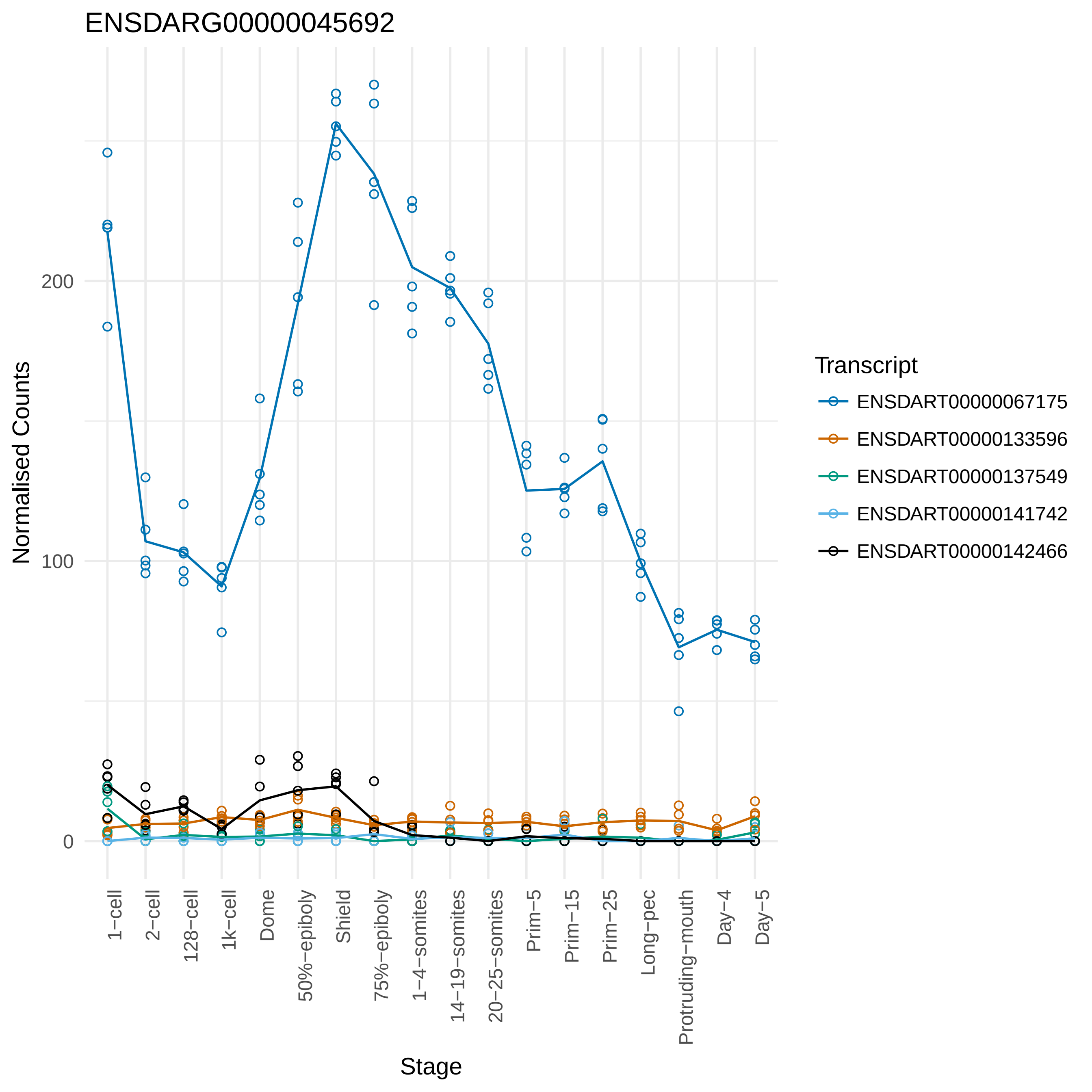
ENSDARG00000045692
Normalised Counts
200
100
0
1−cell
2−cell
128−cell
1k−cell
Dome
50%−epiboly
Shield
75%−epiboly
1−4−somites
14−19−somites
20−25−somites
Prim−5
Prim−15
Prim−25
Long−pec
Protruding−mouth
Day−4
Day−5
Stage
Transcript
ENSDART00000067175
ENSDART00000133596
ENSDART00000137549
ENSDART00000141742
ENSDART00000142466

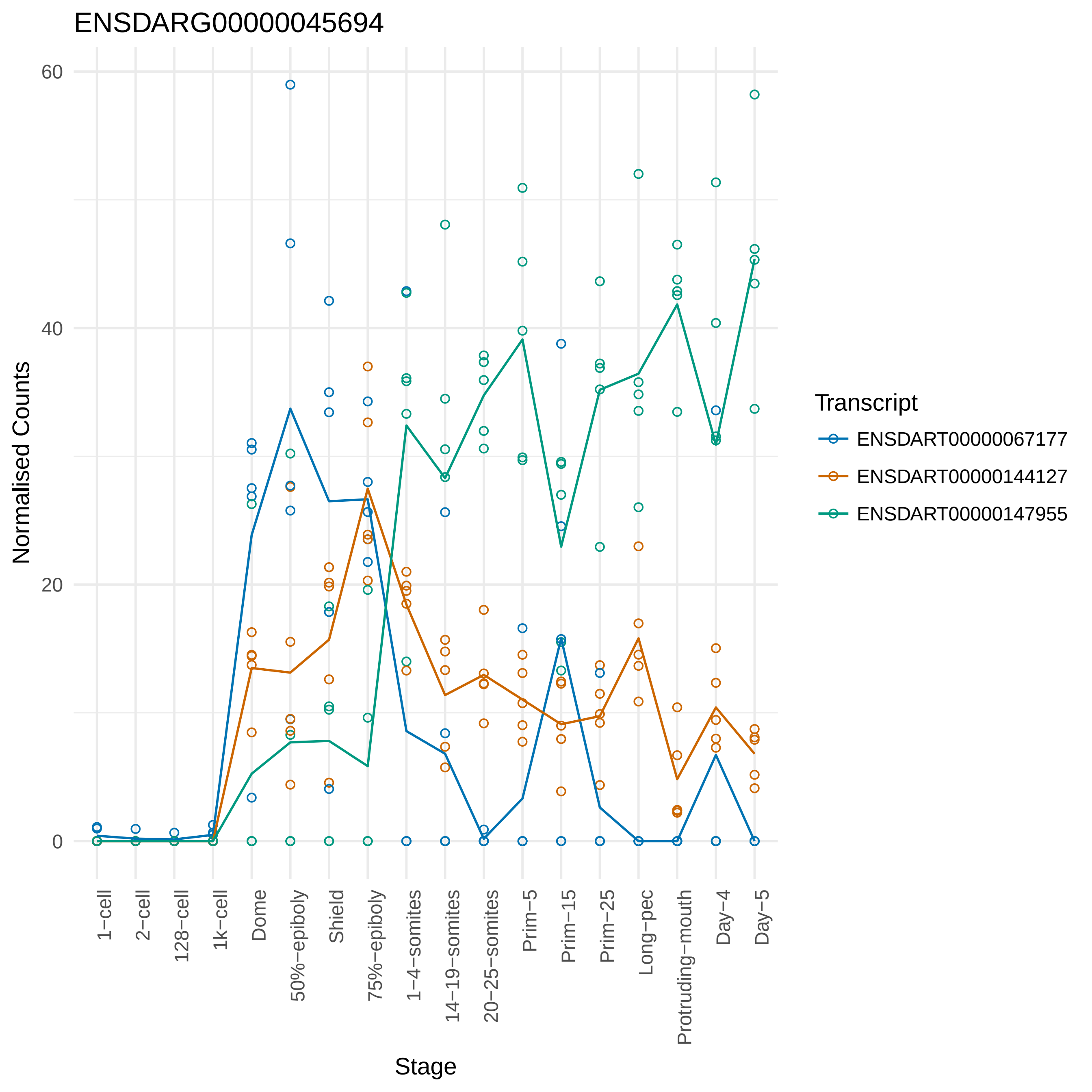

ENSDARG00000045694
Normalised Counts
60
40
20
0
1−cell
2−cell
128−cell
1k−cell
Dome
50%−epiboly
Shield
75%−epiboly
1−4−somites
14−19−somites
20−25−somites
Prim−5
Prim−15
Prim−25
Long−pec
Protruding−mouth
Day−4
Day−5
Stage
Transcript
ENSDART00000067177
ENSDART00000144127
ENSDART00000147955

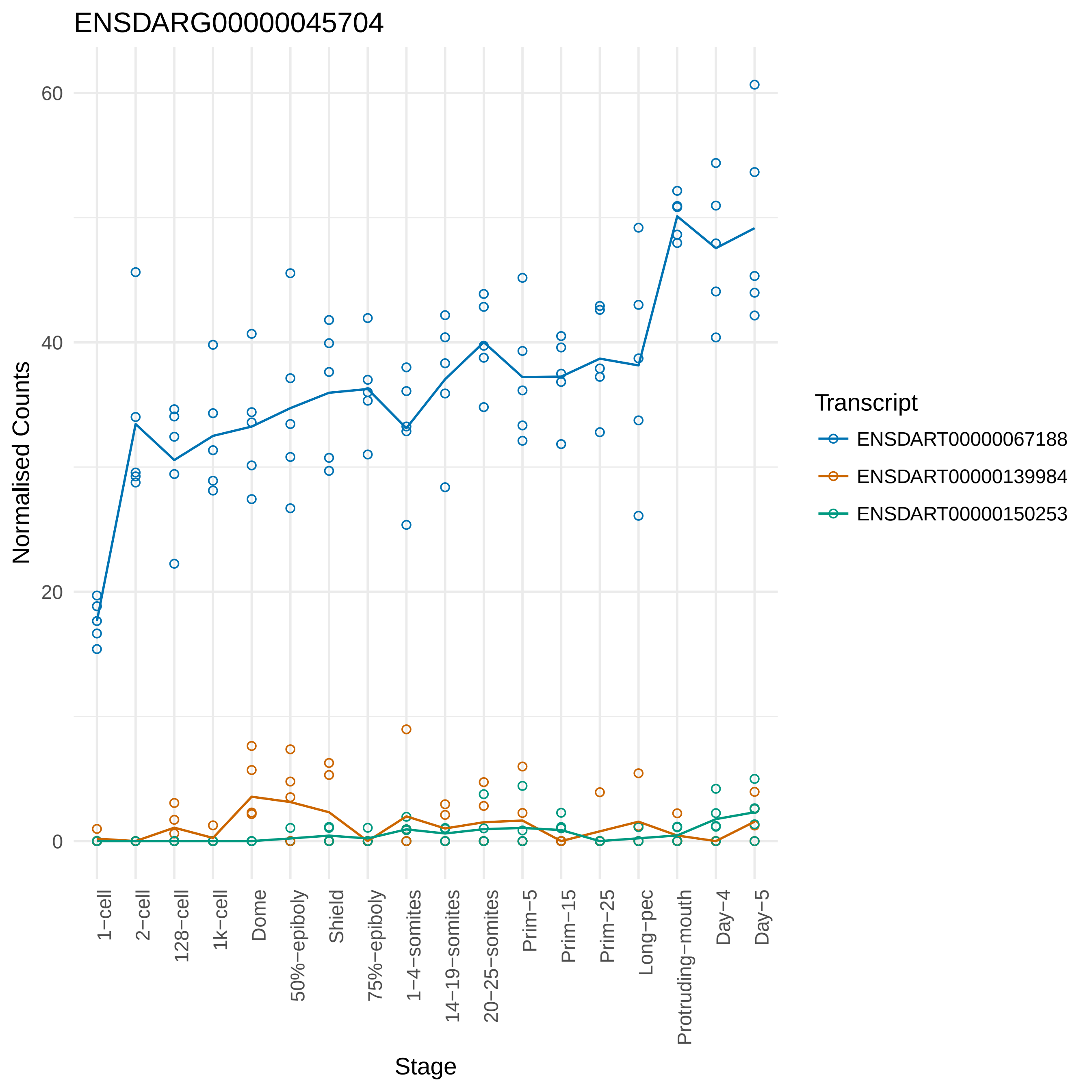

ENSDARG00000045704
Normalised Counts
60
40
20
0
1−cell
2−cell
128−cell
1k−cell
Dome
50%−epiboly
Shield
75%−epiboly
1−4−somites
14−19−somites
20−25−somites
Prim−5
Prim−15
Prim−25
Long−pec
Protruding−mouth
Day−4
Day−5
Stage
Transcript
ENSDART00000067188
ENSDART00000139984
ENSDART00000150253

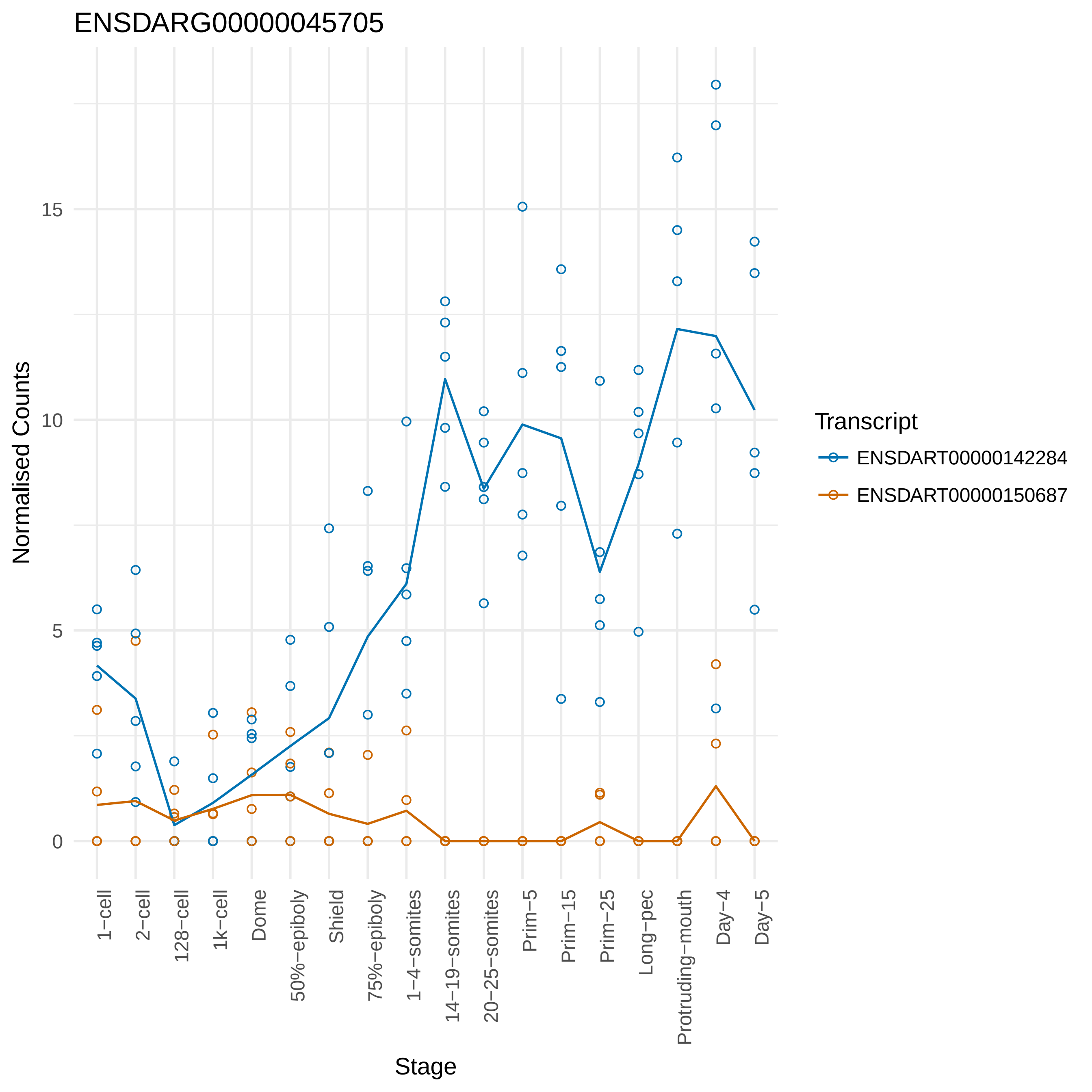
ENSDARG00000045705
Normalised Counts
0
5
10
15
Stage
1−cell
2−cell
128−cell
1k−cell
Dome
50%−epiboly
Shield
75%−epiboly
1−4−somites
14−19−somites
20−25−somites
Prim−5
Prim−15
Prim−25
Long−pec
Protruding−mouth
Day−4
Day−5
Transcript
ENSDART00000142284
ENSDART00000150687

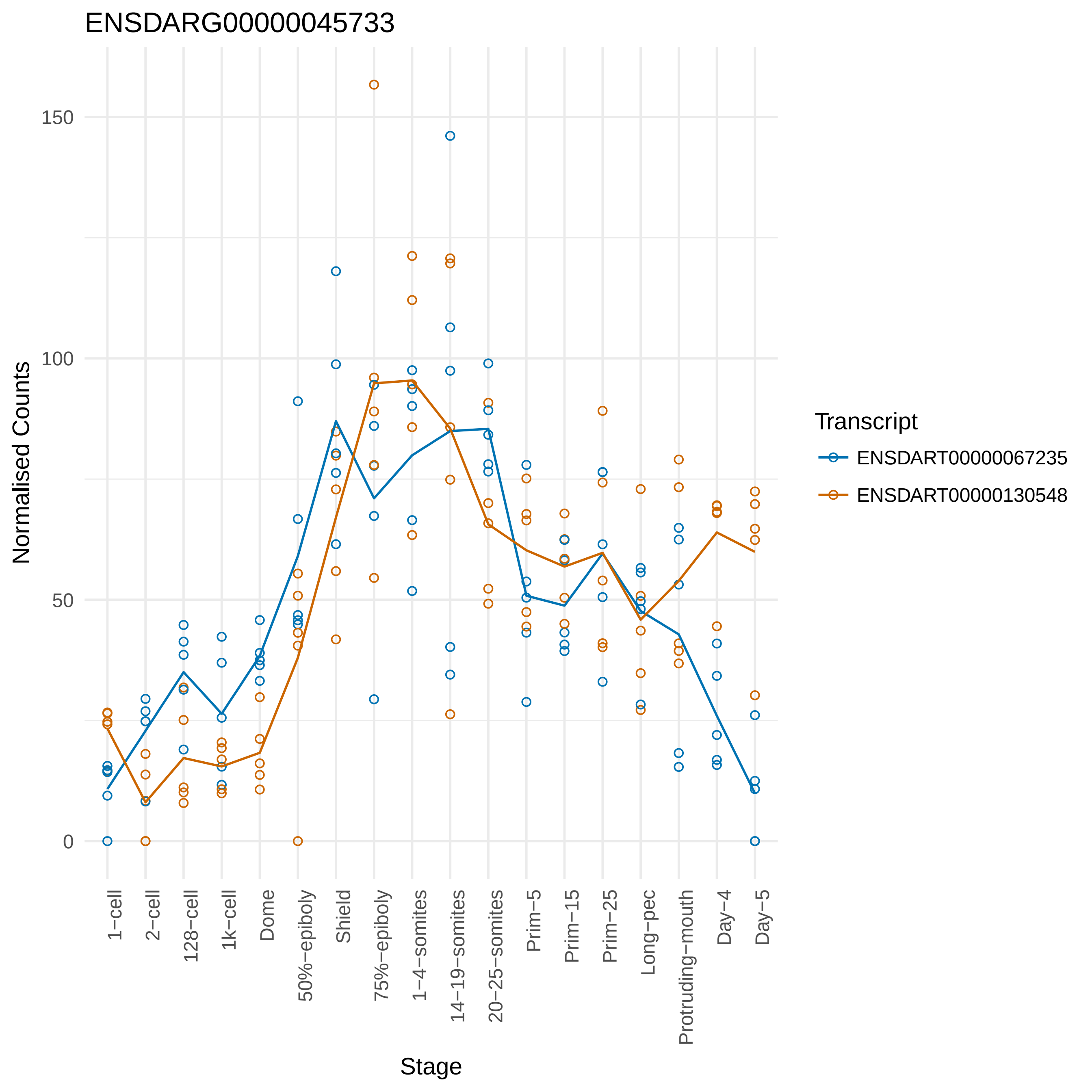

ENSDARG00000045733
Normalised Counts
150
100
50
0
1−cell
2−cell
128−cell
1k−cell
Dome
50%−epiboly
Shield
75%−epiboly
1−4−somites
14−19−somites
20−25−somites
Prim−5
Prim−15
Prim−25
Long−pec
Protruding−mouth
Day−4
Day−5
Stage
Transcript
ENSDART00000067235
ENSDART00000130548

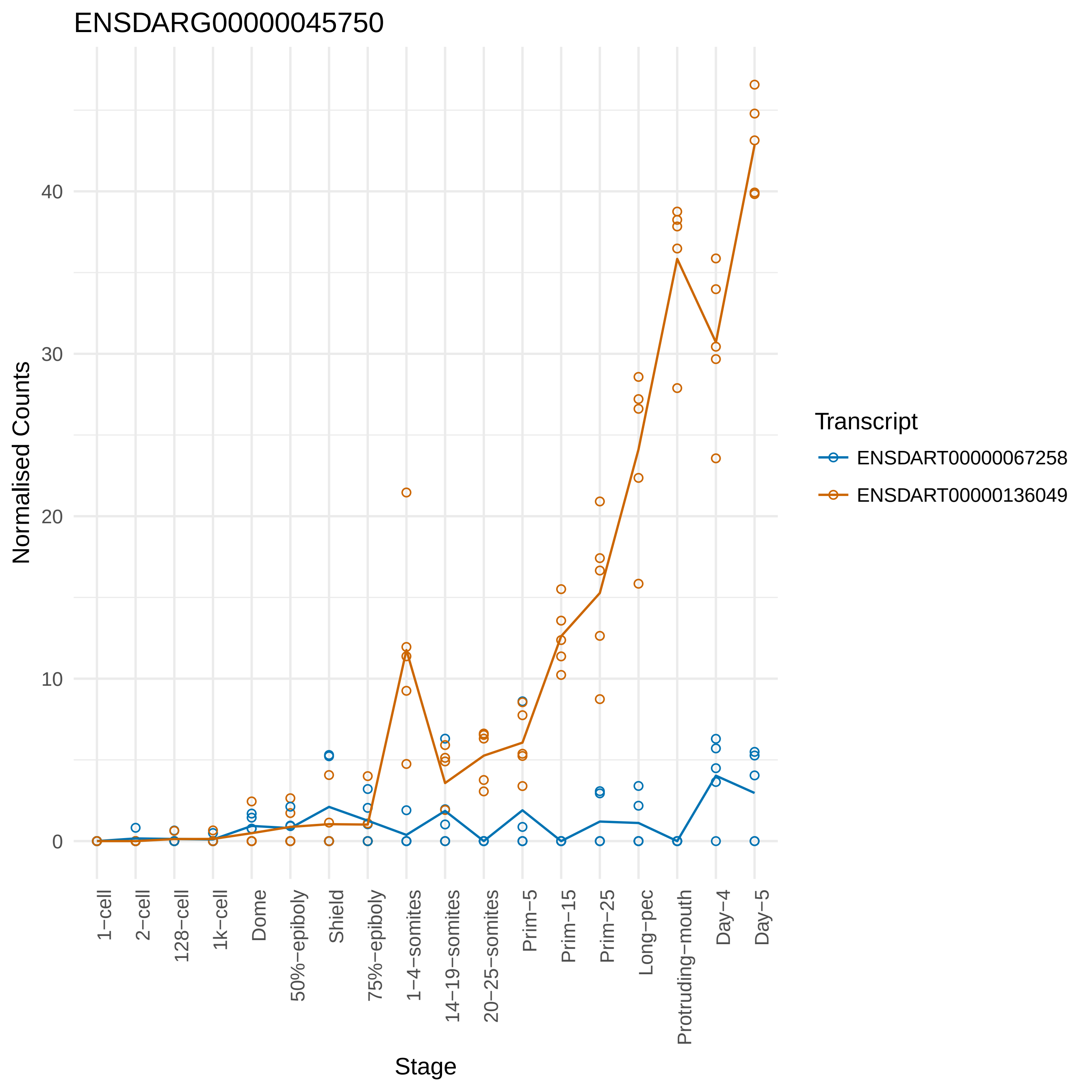
ENSDARG00000045750
Normalised Counts
0
10
20
30
40
Stage
1−cell
2−cell
128−cell
1k−cell
Dome
50%−epiboly
Shield
75%−epiboly
1−4−somites
14−19−somites
20−25−somites
Prim−5
Prim−15
Prim−25
Long−pec
Protruding−mouth
Day−4
Day−5
Transcript
ENSDART00000067258
ENSDART00000136049

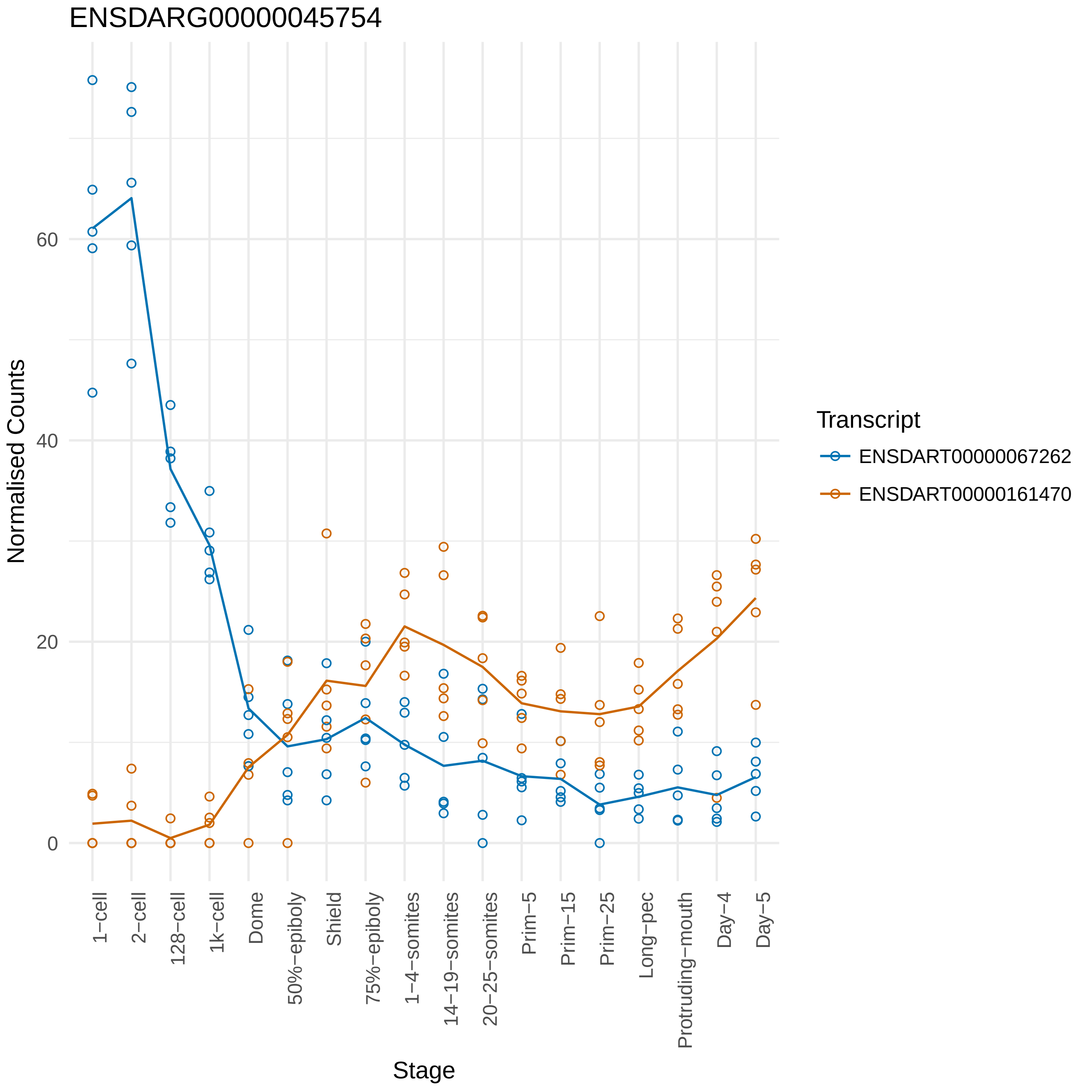

ENSDARG00000045754
Normalised Counts
0
20
40
60
1−cell
2−cell
128−cell
1k−cell
Dome
50%−epiboly
Shield
75%−epiboly
1−4−somites
14−19−somites
20−25−somites
Prim−5
Prim−15
Prim−25
Long−pec
Protruding−mouth
Day−4
Day−5
Stage
Transcript
ENSDART00000067262
ENSDART00000161470

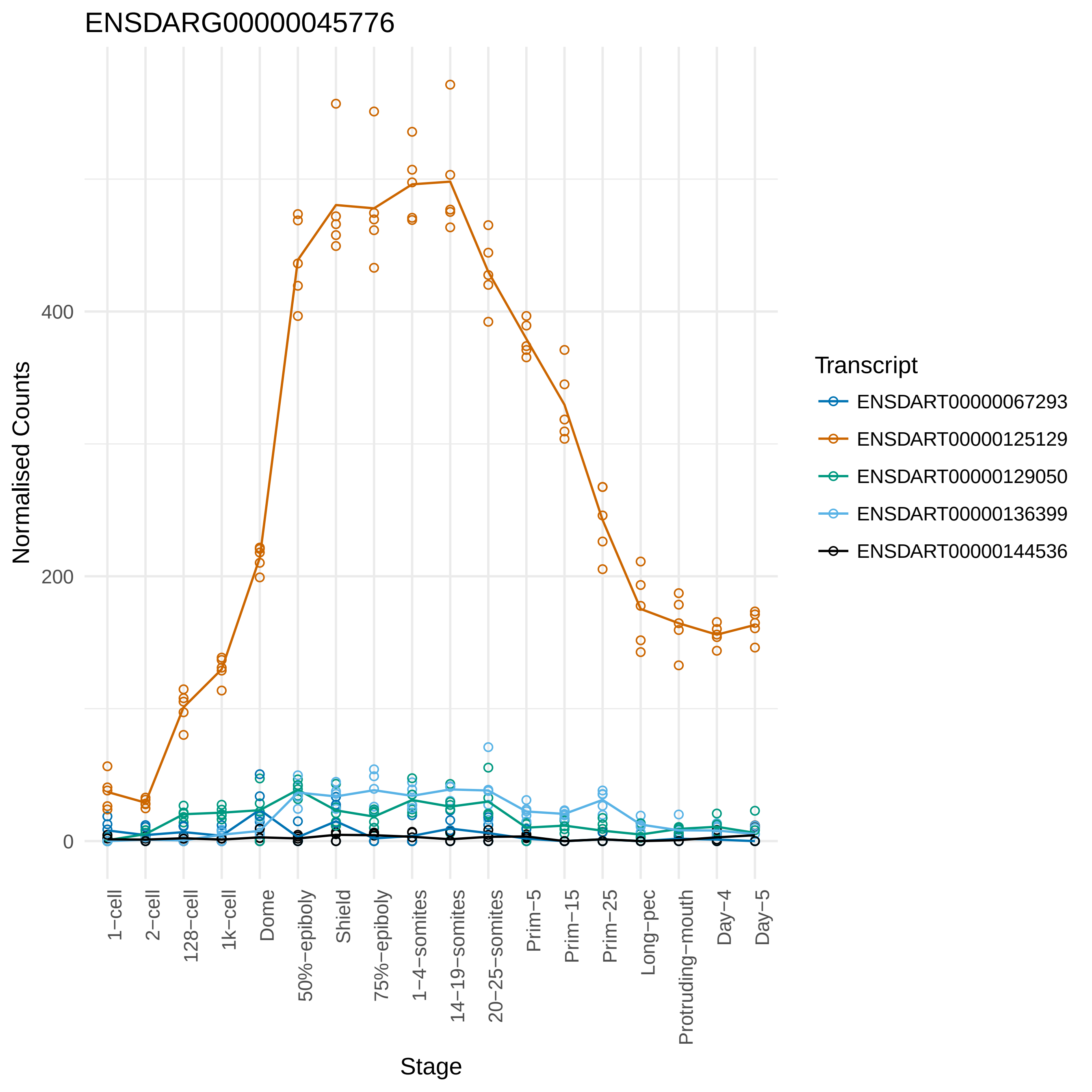

ENSDARG00000045776
Normalised Counts
400
200
0
1−cell
2−cell
128−cell
1k−cell
Dome
50%−epiboly
Shield
75%−epiboly
1−4−somites
14−19−somites
20−25−somites
Prim−5
Prim−15
Prim−25
Long−pec
Protruding−mouth
Day−4
Day−5
Stage
Transcript
ENSDART00000067293
ENSDART00000125129
ENSDART00000129050
ENSDART00000136399
ENSDART00000144536

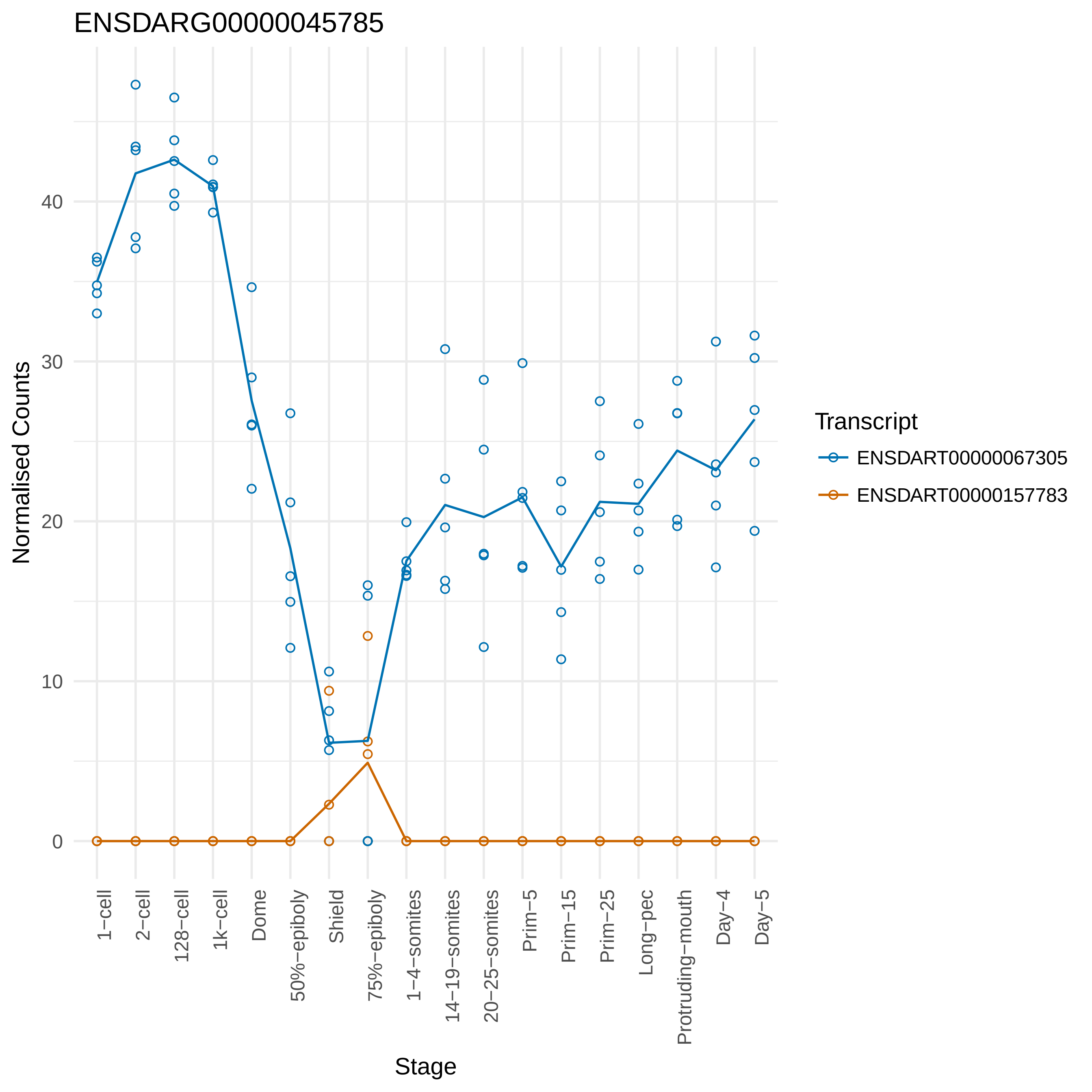

ENSDARG00000045785
Normalised Counts
0
10
20
30
40
1−cell
2−cell
128−cell
1k−cell
Dome
50%−epiboly
Shield
75%−epiboly
1−4−somites
14−19−somites
20−25−somites
Prim−5
Prim−15
Prim−25
Long−pec
Protruding−mouth
Day−4
Day−5
Stage
Transcript
ENSDART00000067305
ENSDART00000157783

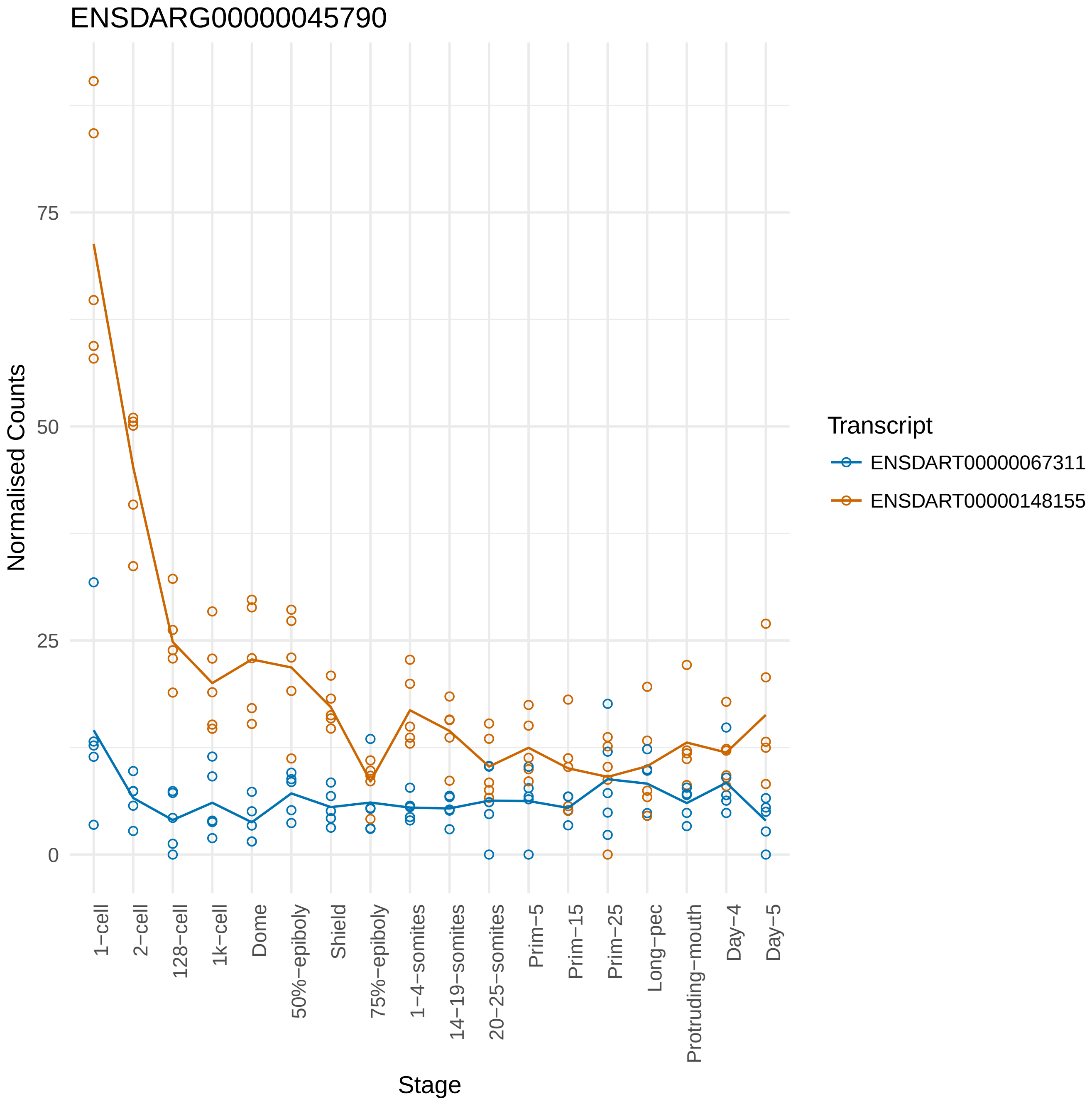
ENSDARG00000045790
Normalised Counts
75
50
25
0
1−cell
2−cell
128−cell
1k−cell
Dome
50%−epiboly
Shield
75%−epiboly
1−4−somites
14−19−somites
20−25−somites
Prim−5
Prim−15
Prim−25
Long−pec
Protruding−mouth
Day−4
Day−5
Stage
Transcript
ENSDART00000067311
ENSDART00000148155

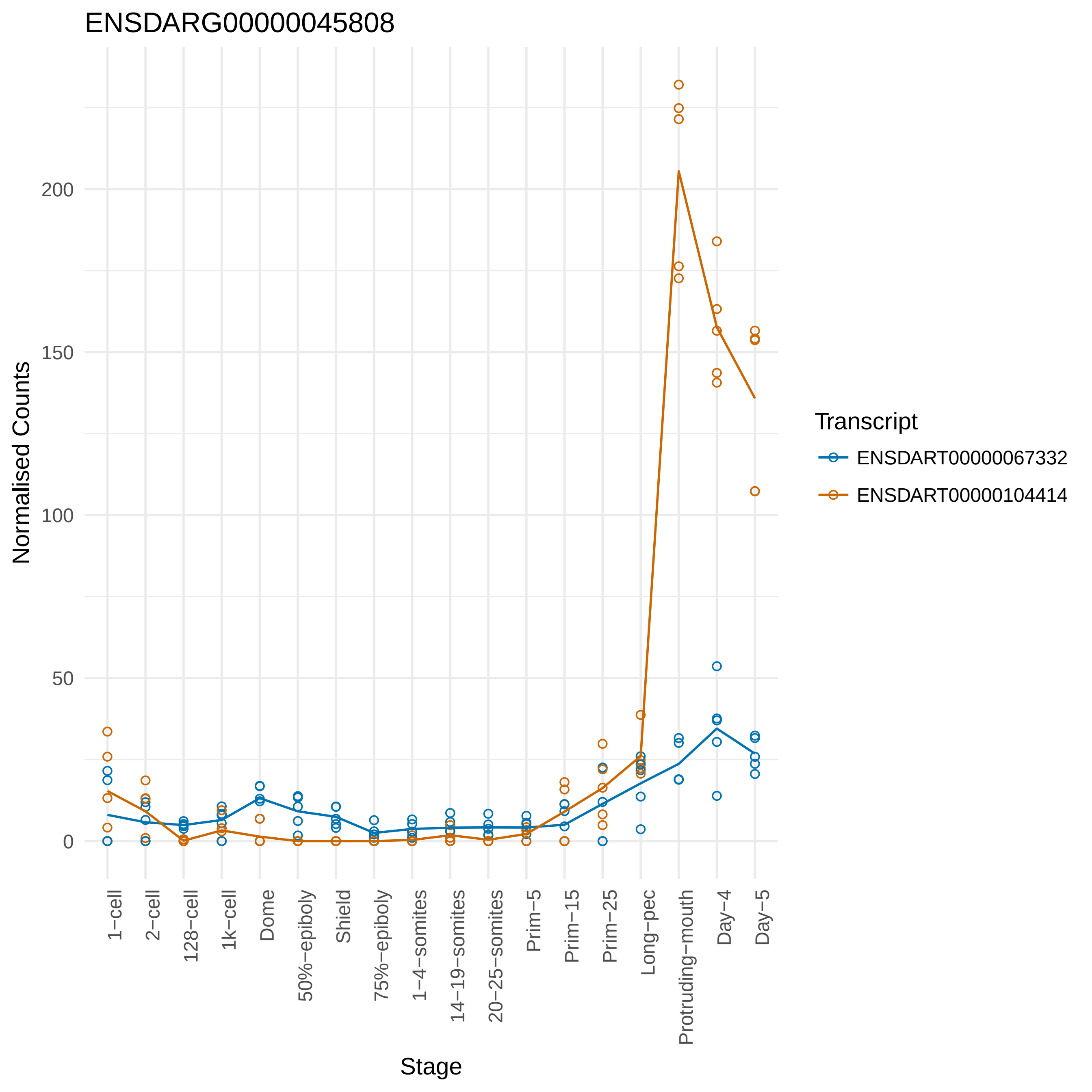

ENSDARG00000045808
Normalised Counts
0
50
100
150
200
Stage
1−cell
2−cell
128−cell
1k−cell
Dome
50%−epiboly
Shield
75%−epiboly
1−4−somites
14−19−somites
20−25−somites
Prim−5
Prim−15
Prim−25
Long−pec
Protruding−mouth
Day−4
Day−5
Transcript
ENSDART00000067332
ENSDART00000104414

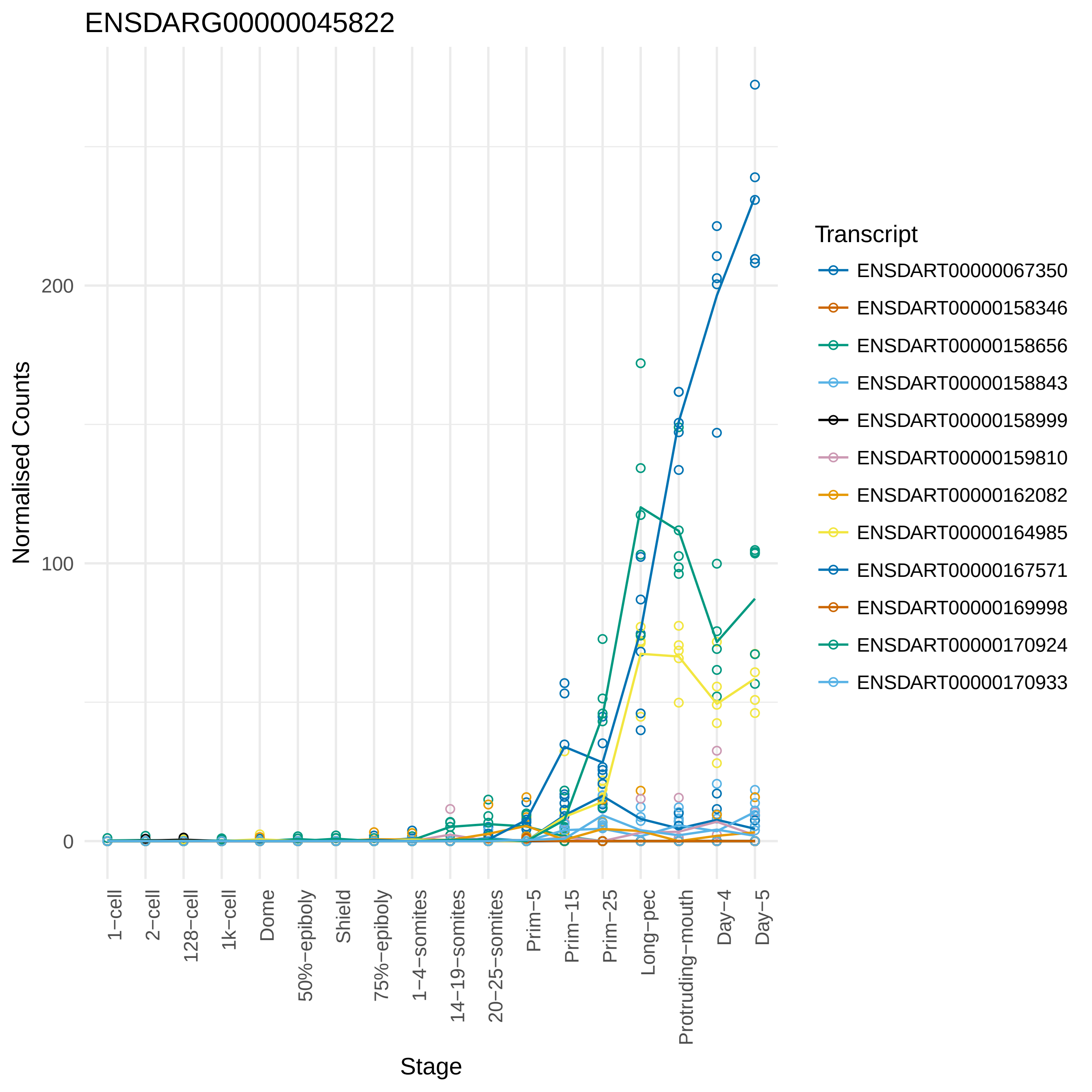

ENSDARG00000045822
Normalised Counts
200
100
0
1−cell
2−cell
128−cell
1k−cell
Dome
50%−epiboly
Shield
75%−epiboly
1−4−somites
14−19−somites
20−25−somites
Prim−5
Prim−15
Prim−25
Long−pec
Protruding−mouth
Day−4
Day−5
Stage
Transcript
ENSDART00000067350
ENSDART00000158346
ENSDART00000158656
ENSDART00000158843
ENSDART00000158999
ENSDART00000159810
ENSDART00000162082
ENSDART00000164985
ENSDART00000167571
ENSDART00000169998
ENSDART00000170924
ENSDART00000170933

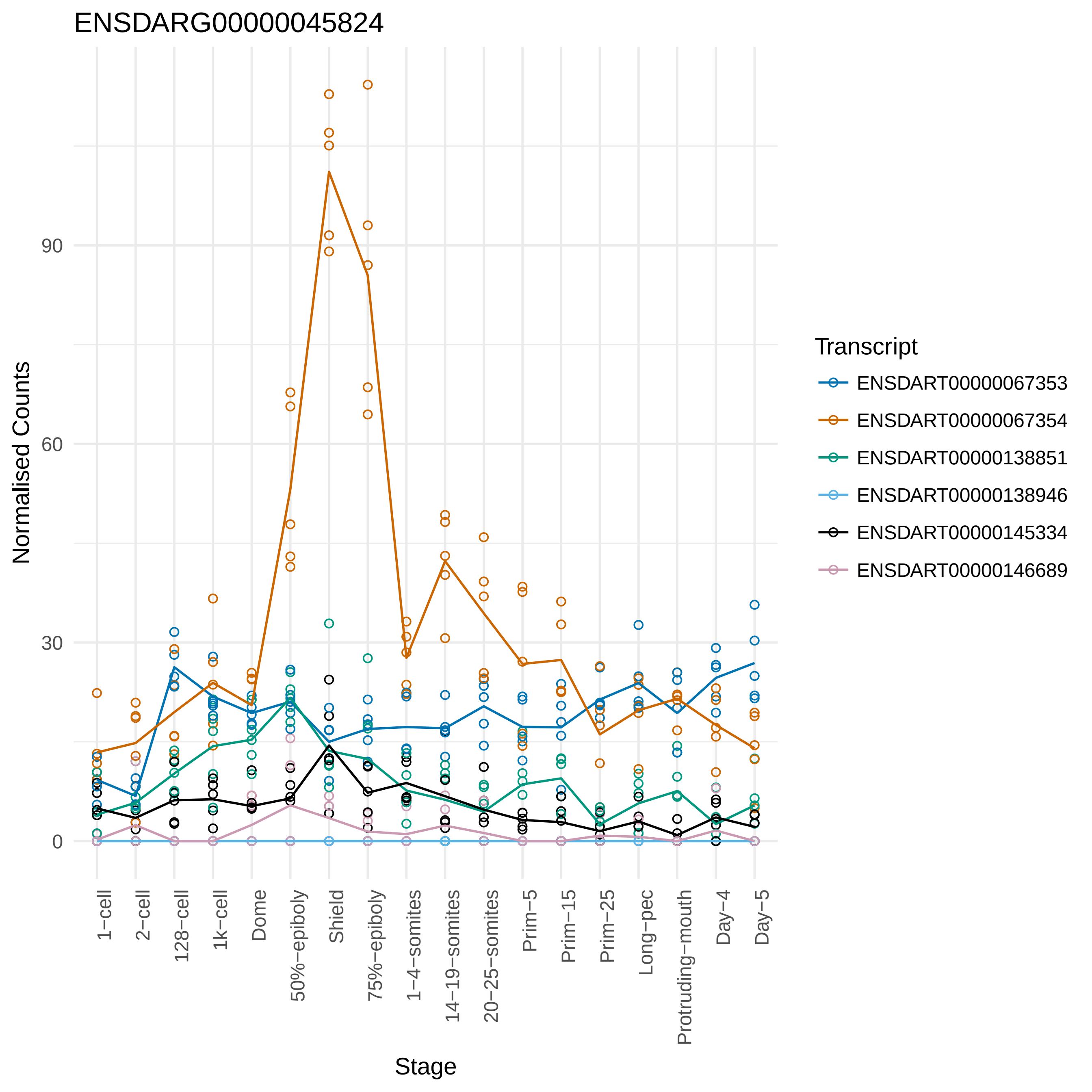

ENSDARG00000045824
Normalised Counts
90
60
30
0
1−cell
2−cell
128−cell
1k−cell
Dome
50%−epiboly
Shield
75%−epiboly
1−4−somites
14−19−somites
20−25−somites
Prim−5
Prim−15
Prim−25
Long−pec
Protruding−mouth
Day−4
Day−5
Stage
Transcript
ENSDART00000067353
ENSDART00000067354
ENSDART00000138851
ENSDART00000138946
ENSDART00000145334
ENSDART00000146689

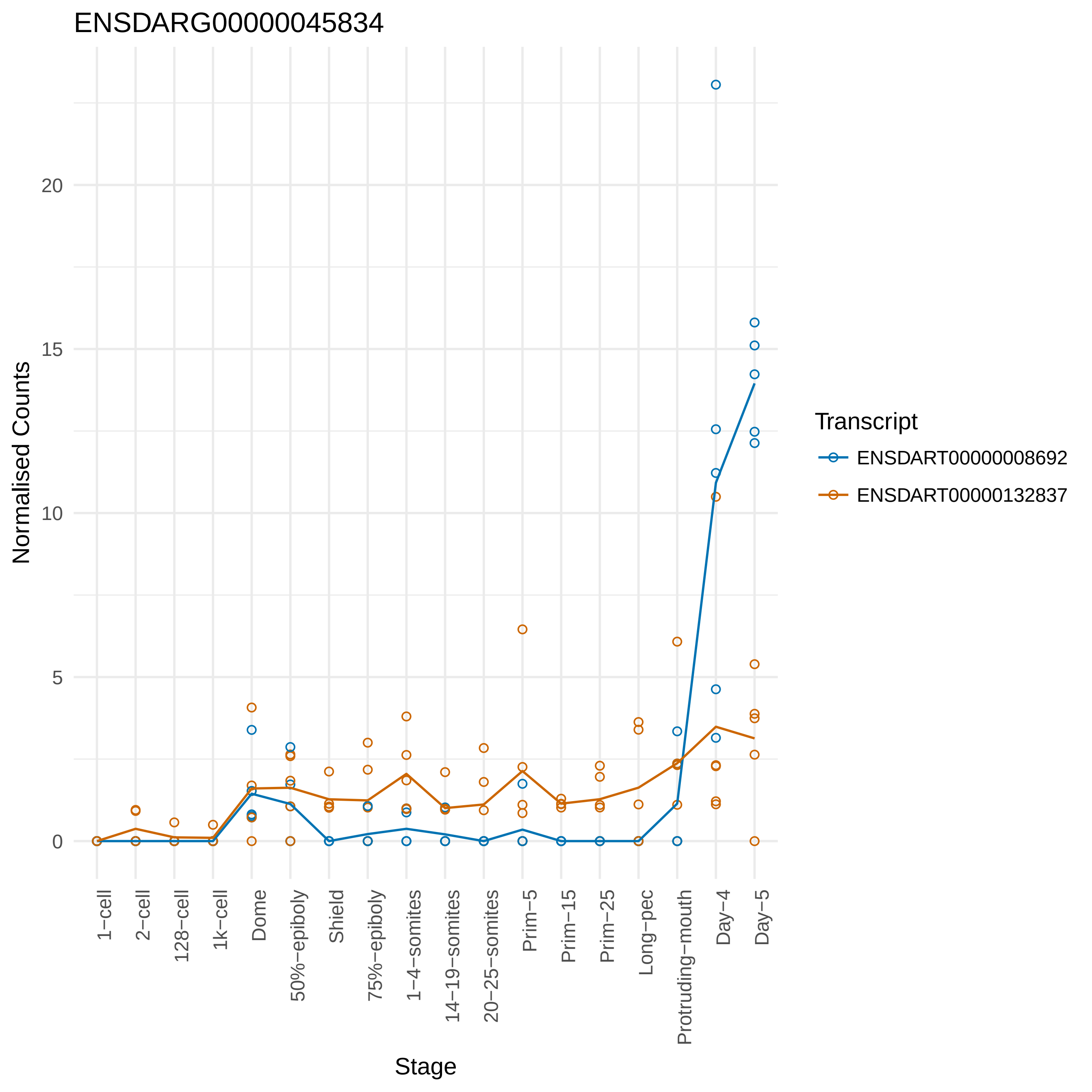
ENSDARG00000045834
Normalised Counts
20
15
10
5
0
1−cell
2−cell
128−cell
1k−cell
Dome
50%−epiboly
Shield
75%−epiboly
1−4−somites
14−19−somites
20−25−somites
Prim−5
Prim−15
Prim−25
Long−pec
Protruding−mouth
Day−4
Day−5
Stage
Transcript
ENSDART00000008692
ENSDART00000132837

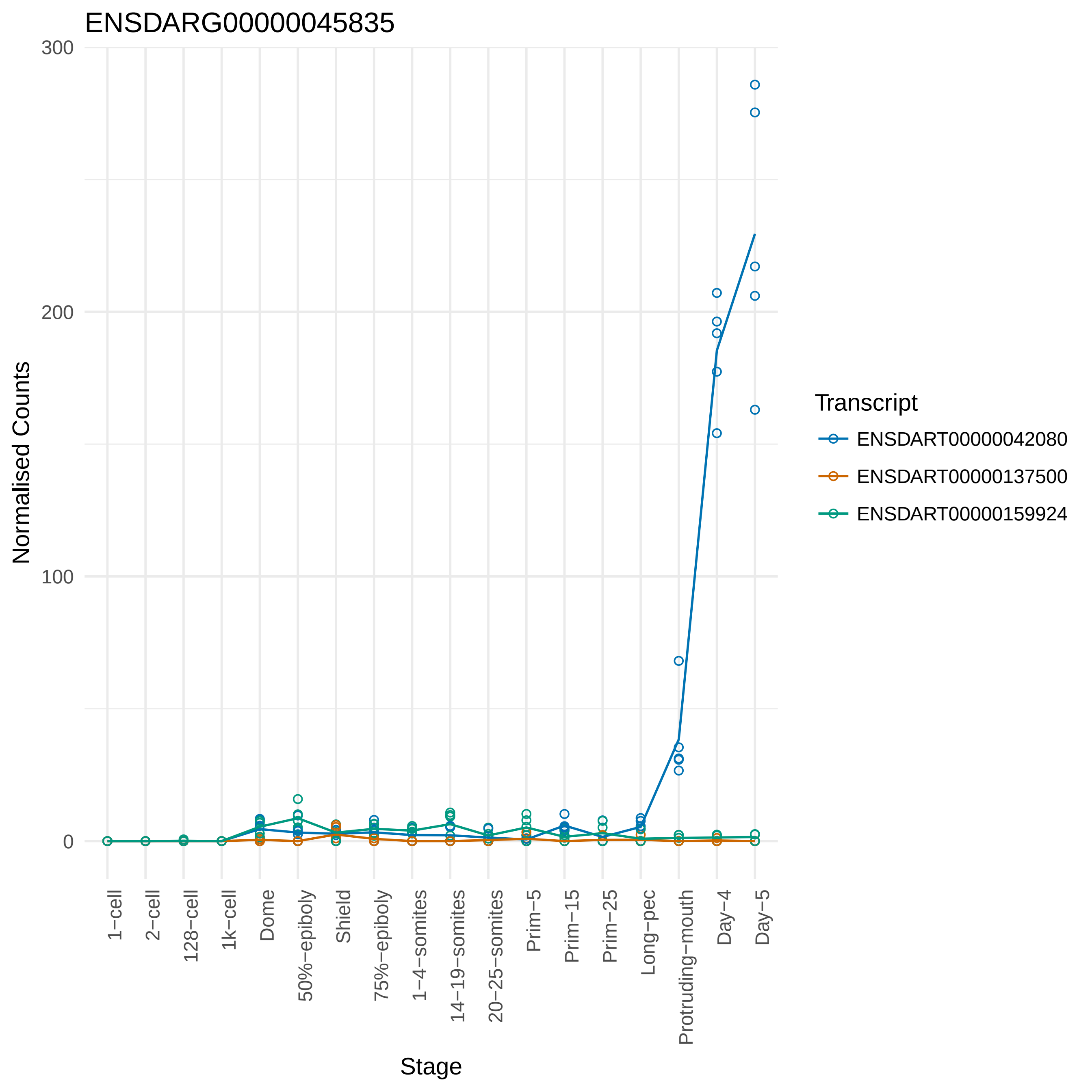
ENSDARG00000045835
300
200
100
0
Normalised Counts
1−cell
2−cell
128−cell
1k−cell
Dome
50%−epiboly
Shield
75%−epiboly
1−4−somites
14−19−somites
20−25−somites
Prim−5
Prim−15
Prim−25
Long−pec
Protruding−mouth
Day−4
Day−5
Stage
Transcript
ENSDART00000042080
ENSDART00000137500
ENSDART00000159924

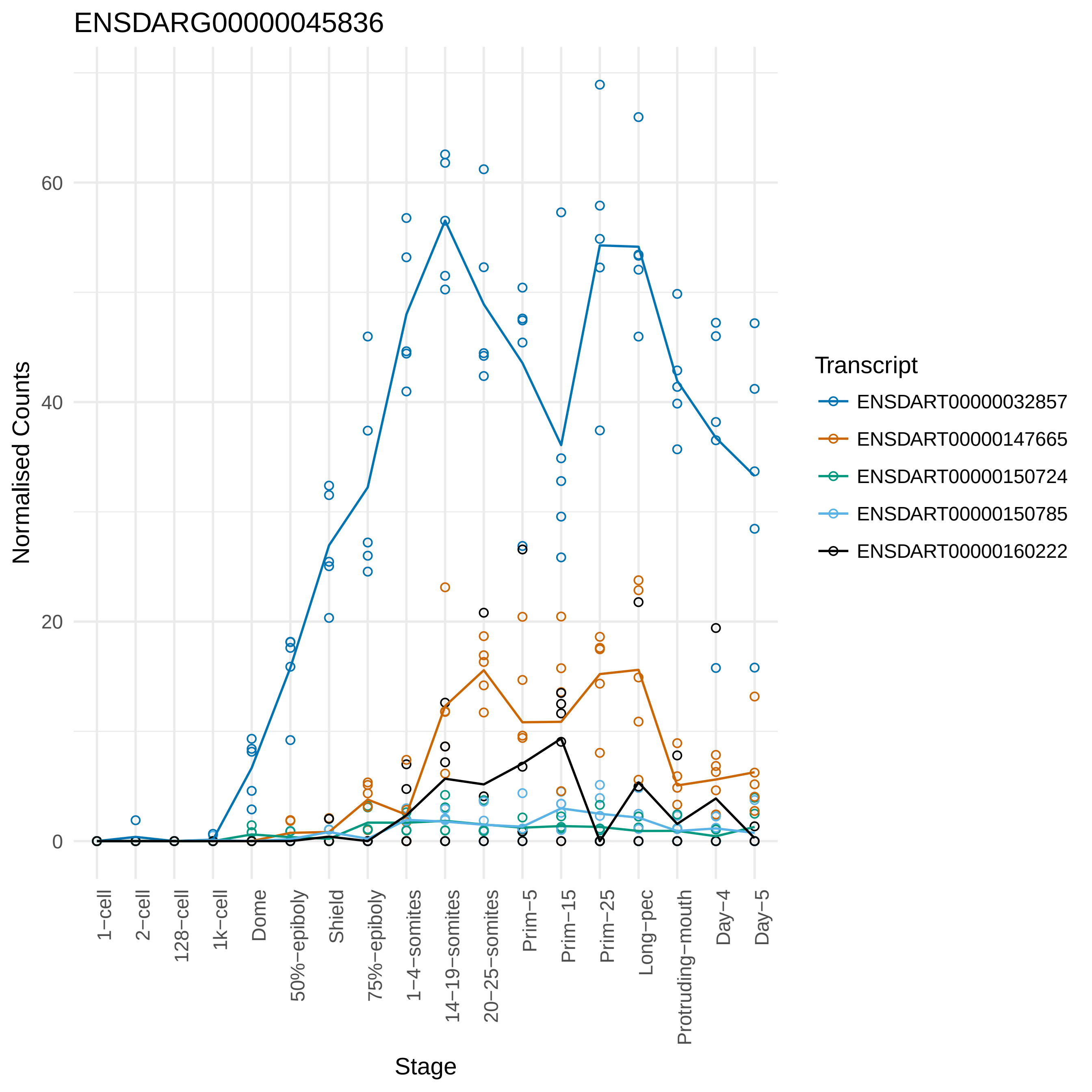

ENSDARG00000045836
Normalised Counts
60
40
20
0
1−cell
2−cell
128−cell
1k−cell
Dome
50%−epiboly
Shield
75%−epiboly
1−4−somites
14−19−somites
20−25−somites
Prim−5
Prim−15
Prim−25
Long−pec
Protruding−mouth
Day−4
Day−5
Stage
Transcript
ENSDART00000032857
ENSDART00000147665
ENSDART00000150724
ENSDART00000150785
ENSDART00000160222

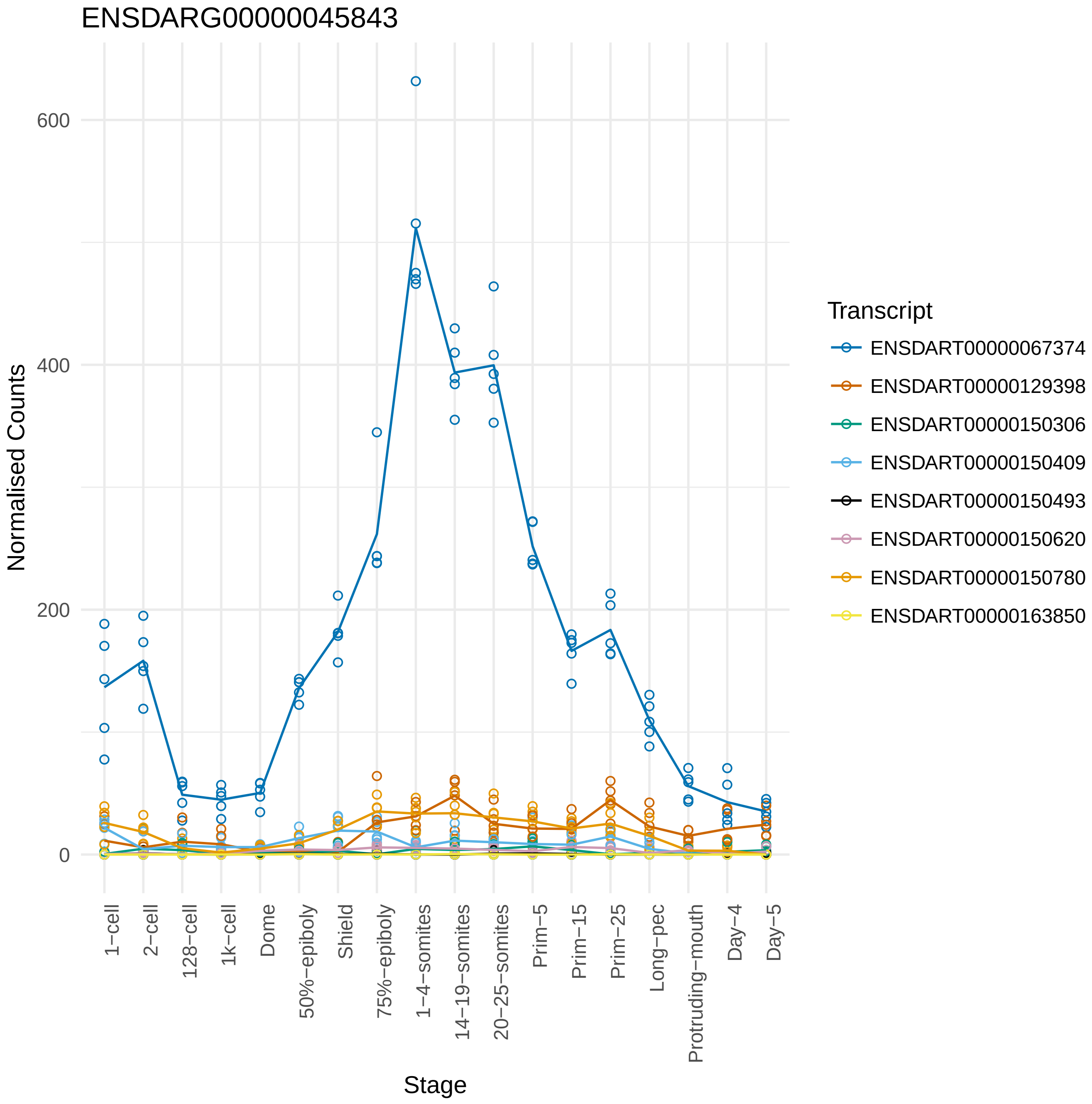
ENSDARG00000045843
Normalised Counts
600
400
200
0
1−cell
2−cell
128−cell
1k−cell
Dome
50%−epiboly
Shield
75%−epiboly
1−4−somites
14−19−somites
20−25−somites
Prim−5
Prim−15
Prim−25
Long−pec
Protruding−mouth
Day−4
Day−5
Stage
Transcript
ENSDART00000067374
ENSDART00000129398
ENSDART00000150306
ENSDART00000150409
ENSDART00000150493
ENSDART00000150620
ENSDART00000150780
ENSDART00000163850

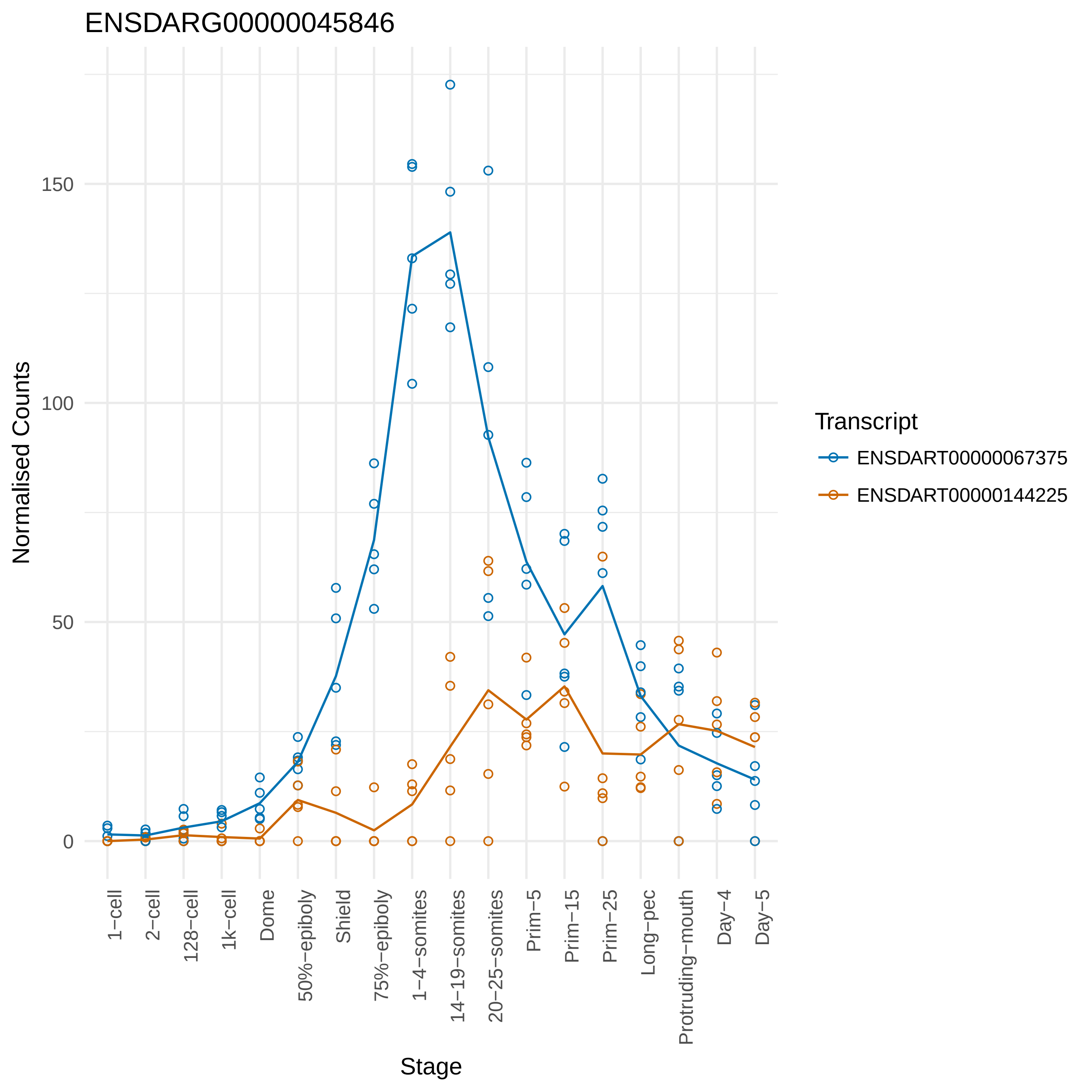
ENSDARG00000045846
Normalised Counts
150
100
50
0
1−cell
2−cell
128−cell
1k−cell
Dome
50%−epiboly
Shield
75%−epiboly
1−4−somites
14−19−somites
20−25−somites
Prim−5
Prim−15
Prim−25
Long−pec
Protruding−mouth
Day−4
Day−5
Stage
Transcript
ENSDART00000067375
ENSDART00000144225

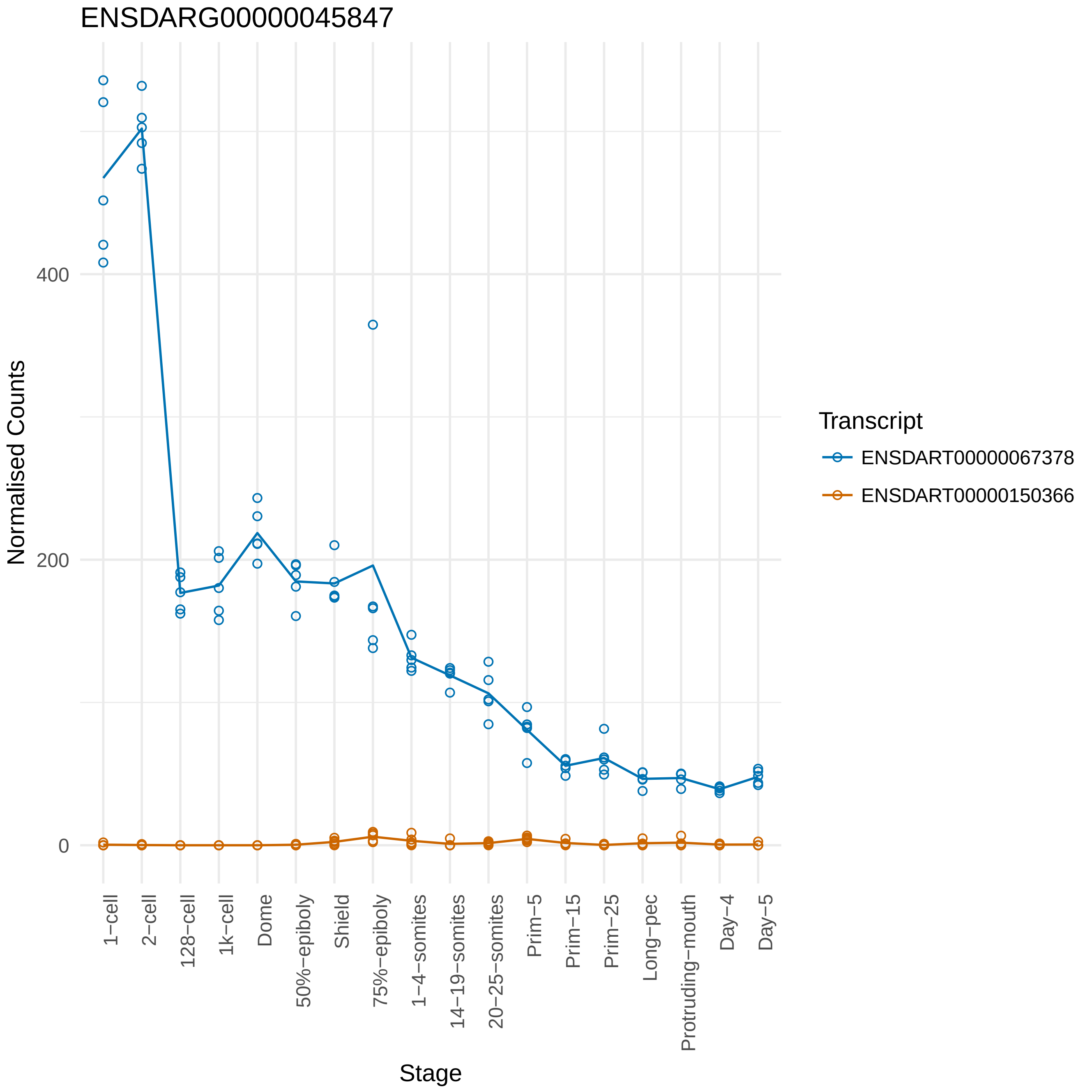

ENSDARG00000045847
Normalised Counts
400
200
0
1−cell
2−cell
128−cell
1k−cell
Dome
50%−epiboly
Shield
75%−epiboly
1−4−somites
14−19−somites
20−25−somites
Prim−5
Prim−15
Prim−25
Long−pec
Protruding−mouth
Day−4
Day−5
Stage
Transcript
ENSDART00000067378
ENSDART00000150366

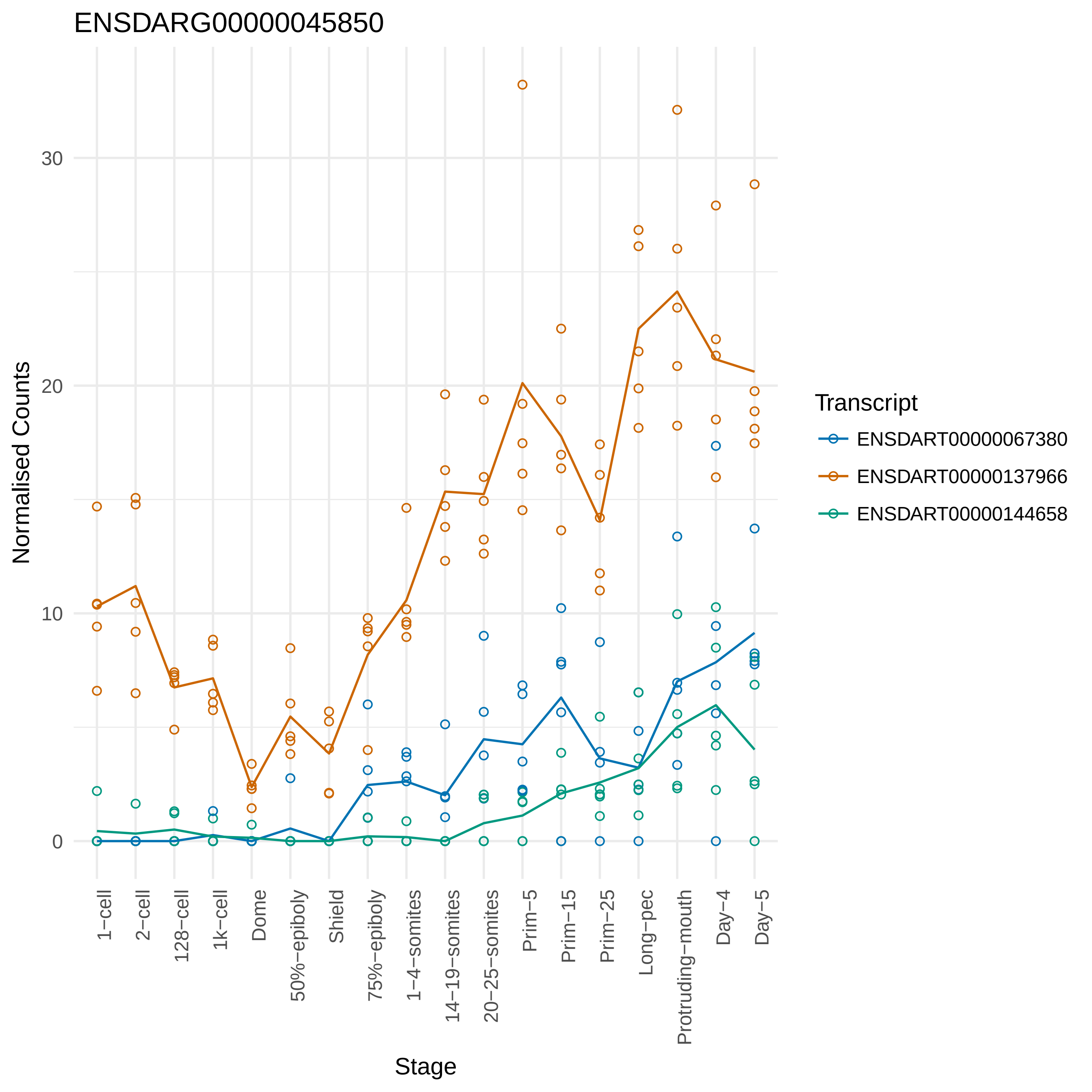

ENSDARG00000045850
Normalised Counts
0
10
20
30
1−cell
2−cell
128−cell
1k−cell
Dome
50%−epiboly
Shield
75%−epiboly
1−4−somites
14−19−somites
20−25−somites
Prim−5
Prim−15
Prim−25
Long−pec
Protruding−mouth
Day−4
Day−5
Stage
Transcript
ENSDART00000067380
ENSDART00000137966
ENSDART00000144658

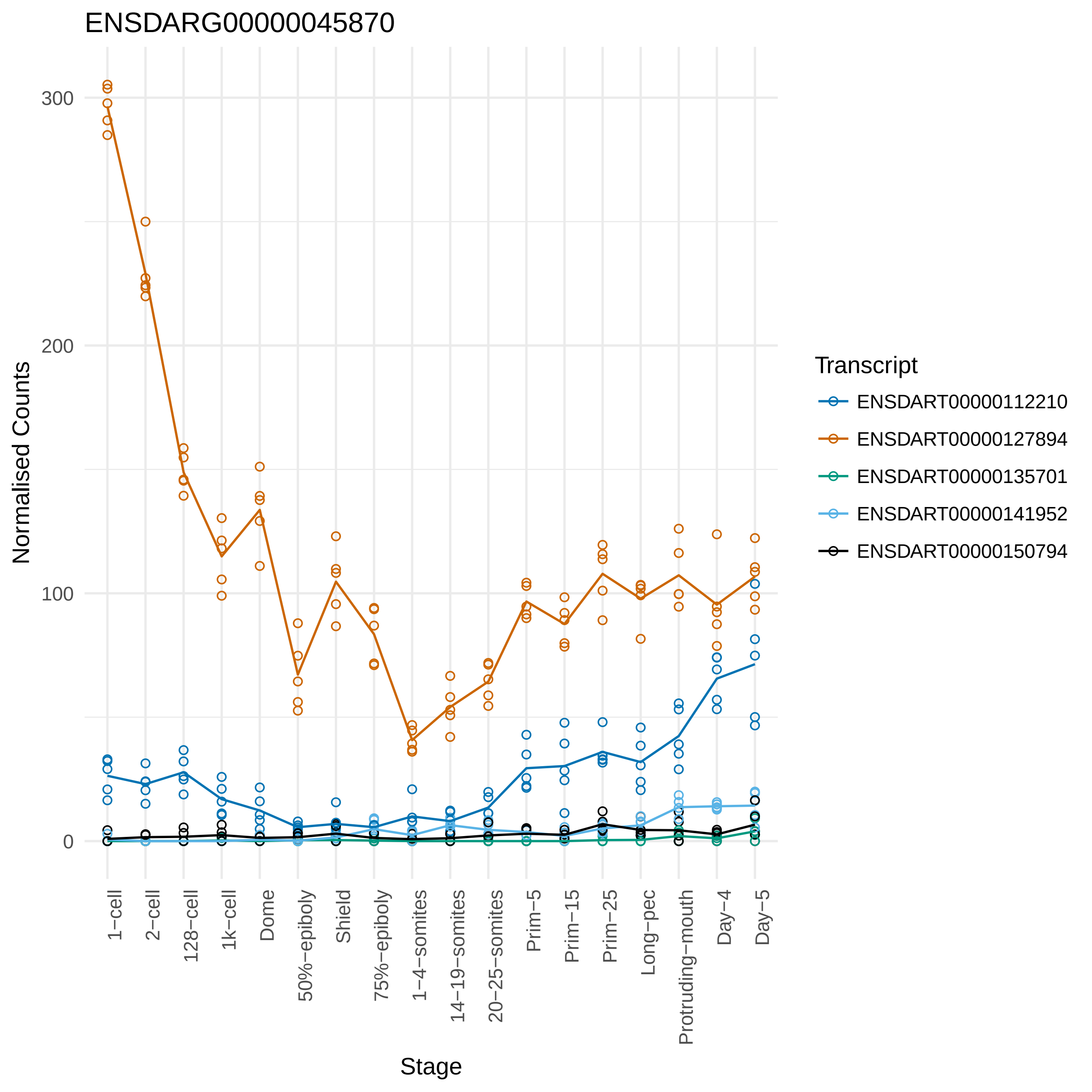

ENSDARG00000045870
Normalised Counts
300
200
100
0
1−cell
2−cell
128−cell
1k−cell
Dome
50%−epiboly
Shield
75%−epiboly
1−4−somites
14−19−somites
20−25−somites
Prim−5
Prim−15
Prim−25
Long−pec
Protruding−mouth
Day−4
Day−5
Stage
Transcript
ENSDART00000112210
ENSDART00000127894
ENSDART00000135701
ENSDART00000141952
ENSDART00000150794

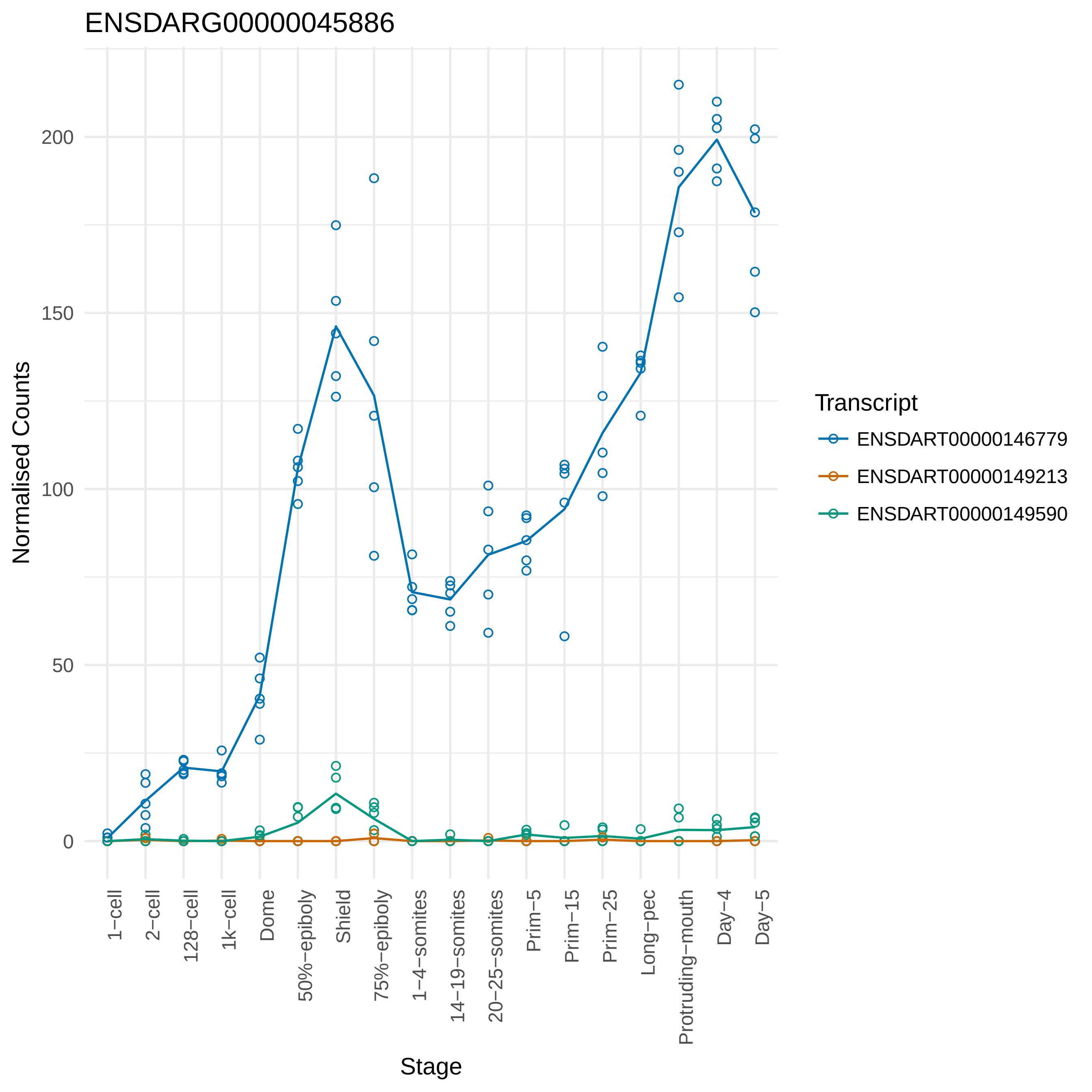

ENSDARG00000045886
Normalised Counts
0
50
100
150
200
Stage
1−cell
2−cell
128−cell
1k−cell
Dome
50%−epiboly
Shield
75%−epiboly
1−4−somites
14−19−somites
20−25−somites
Prim−5
Prim−15
Prim−25
Long−pec
Protruding−mouth
Day−4
Day−5
Transcript
ENSDART00000146779
ENSDART00000149213
ENSDART00000149590

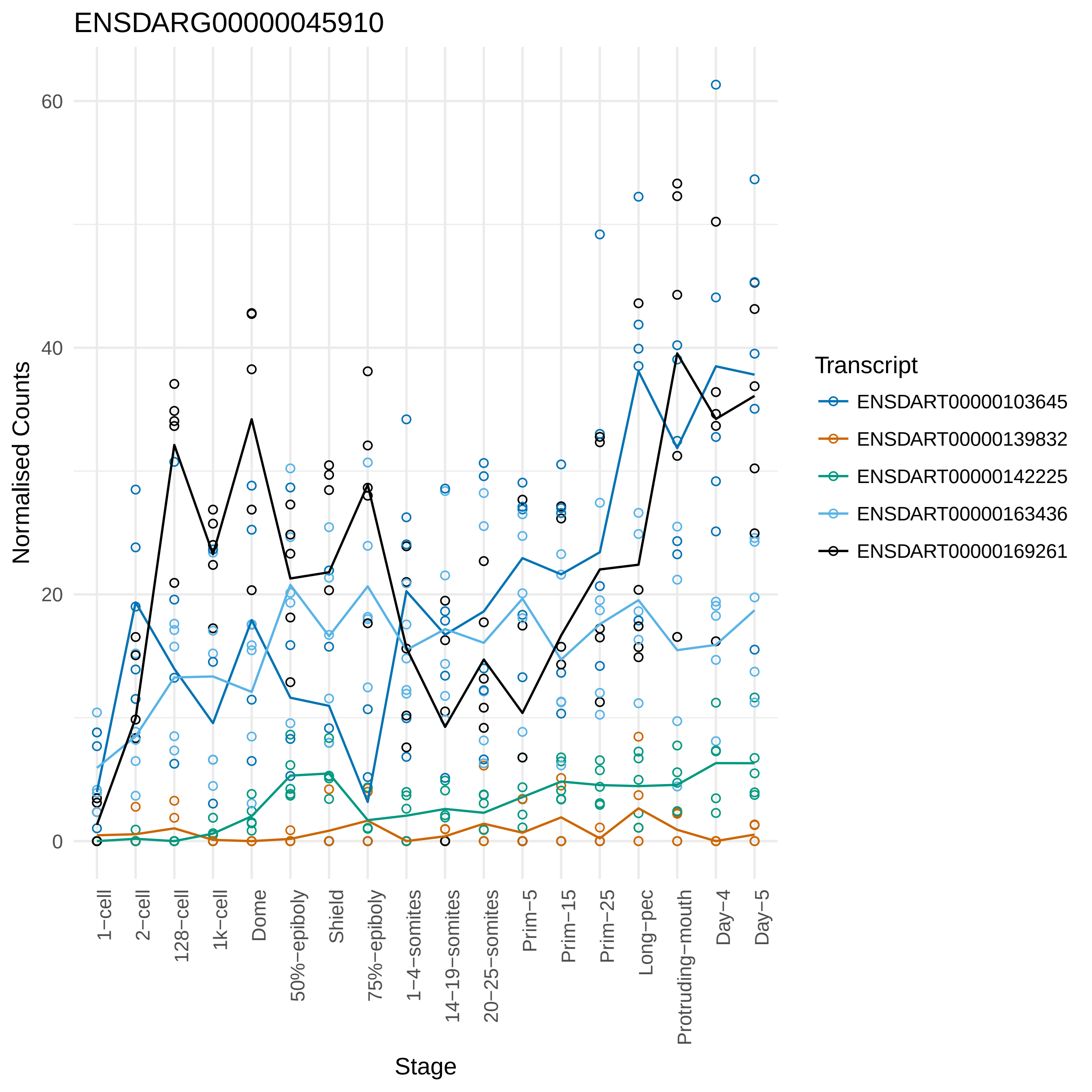
ENSDARG00000045910
Normalised Counts
60
40
20
0
1−cell
2−cell
128−cell
1k−cell
Dome
50%−epiboly
Shield
75%−epiboly
1−4−somites
14−19−somites
20−25−somites
Prim−5
Prim−15
Prim−25
Long−pec
Protruding−mouth
Day−4
Day−5
Stage
Transcript
ENSDART00000103645
ENSDART00000139832
ENSDART00000142225
ENSDART00000163436
ENSDART00000169261

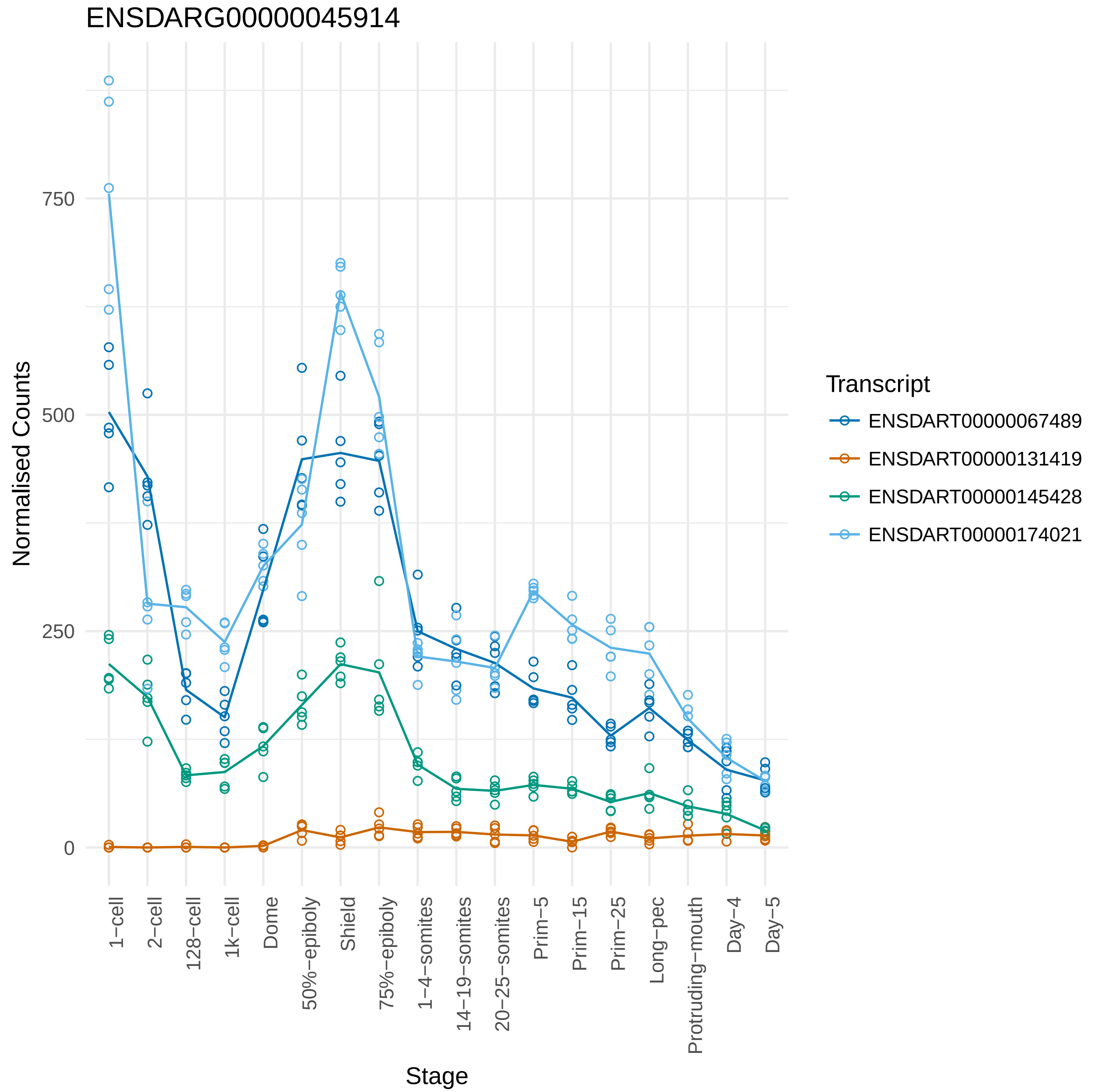

ENSDARG00000045914
Normalised Counts
750
500
250
0
1−cell
2−cell
128−cell
1k−cell
Dome
50%−epiboly
Shield
75%−epiboly
1−4−somites
14−19−somites
20−25−somites
Prim−5
Prim−15
Prim−25
Long−pec
Protruding−mouth
Day−4
Day−5
Stage
Transcript
ENSDART00000067489
ENSDART00000131419
ENSDART00000145428
ENSDART00000174021

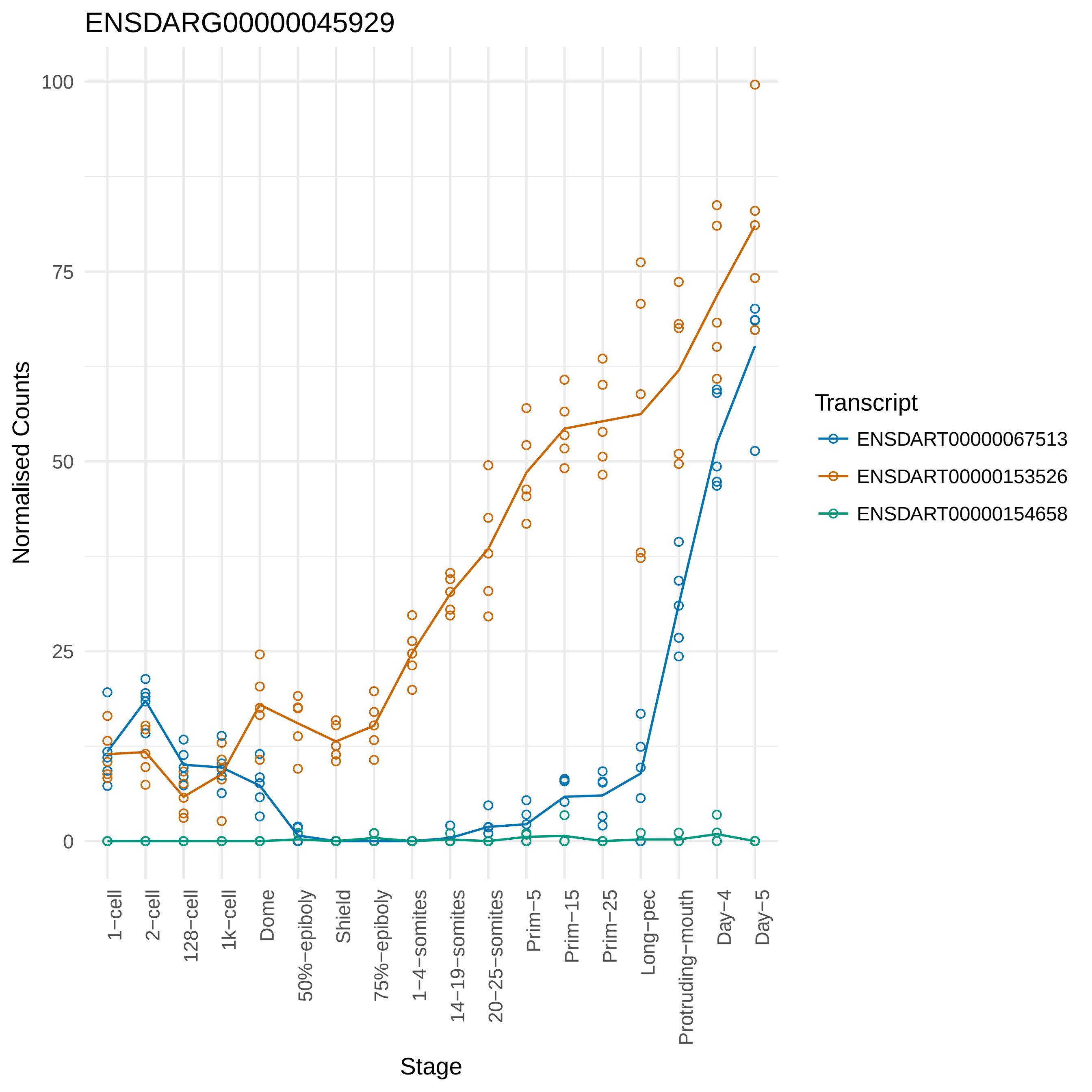

ENSDARG00000045929
Normalised Counts
100
75
50
25
0
1−cell
2−cell
128−cell
1k−cell
Dome
50%−epiboly
Shield
75%−epiboly
1−4−somites
14−19−somites
20−25−somites
Prim−5
Prim−15
Prim−25
Long−pec
Protruding−mouth
Day−4
Day−5
Stage
Transcript
ENSDART00000067513
ENSDART00000153526
ENSDART00000154658

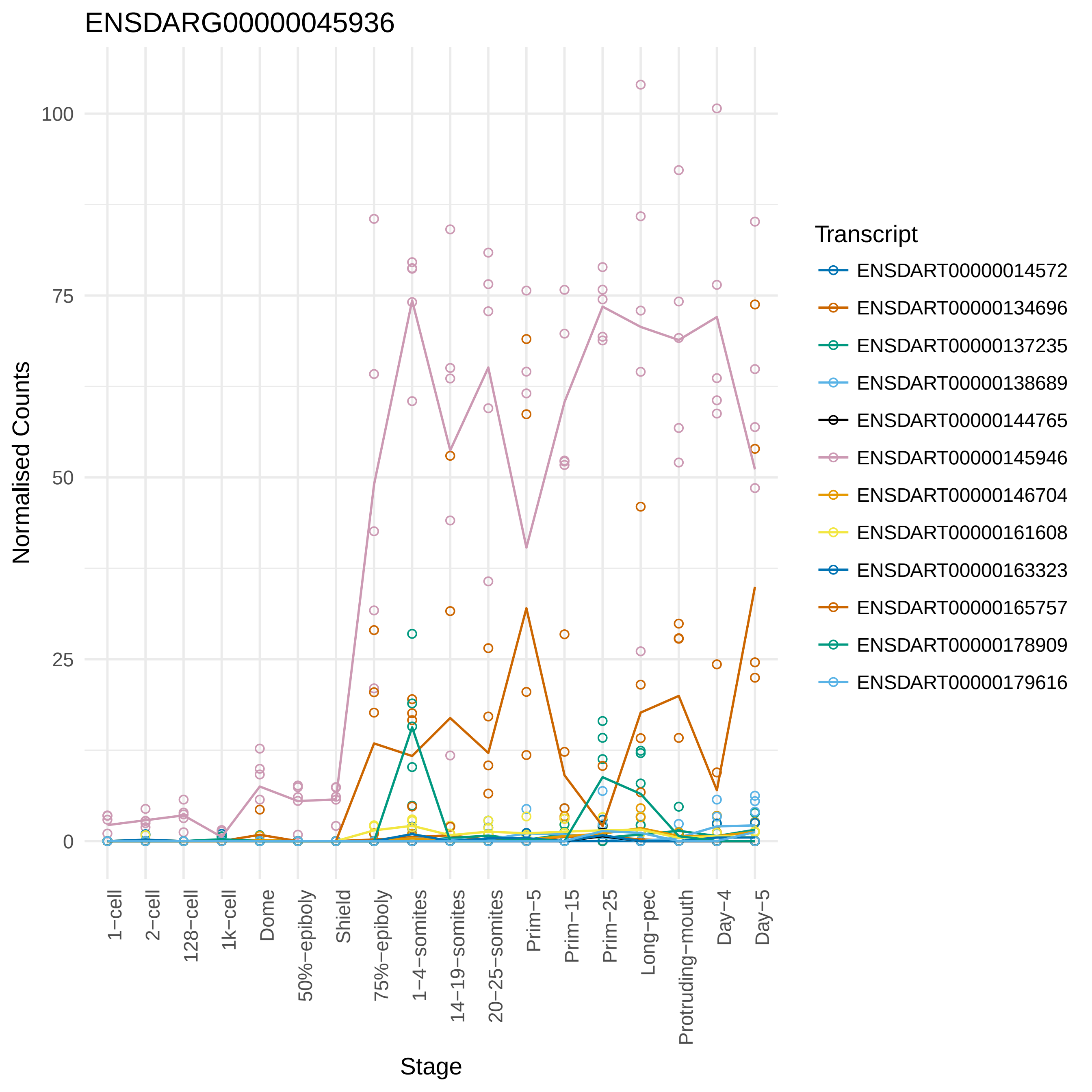

ENSDARG00000045936
Normalised Counts
0
25
50
75
100
Stage
1−cell
2−cell
128−cell
1k−cell
Dome
50%−epiboly
Shield
75%−epiboly
1−4−somites
14−19−somites
20−25−somites
Prim−5
Prim−15
Prim−25
Long−pec
Protruding−mouth
Day−4
Day−5
Transcript
ENSDART00000014572
ENSDART00000134696
ENSDART00000137235
ENSDART00000138689
ENSDART00000144765
ENSDART00000145946
ENSDART00000146704
ENSDART00000161608
ENSDART00000163323
ENSDART00000165757
ENSDART00000178909
ENSDART00000179616

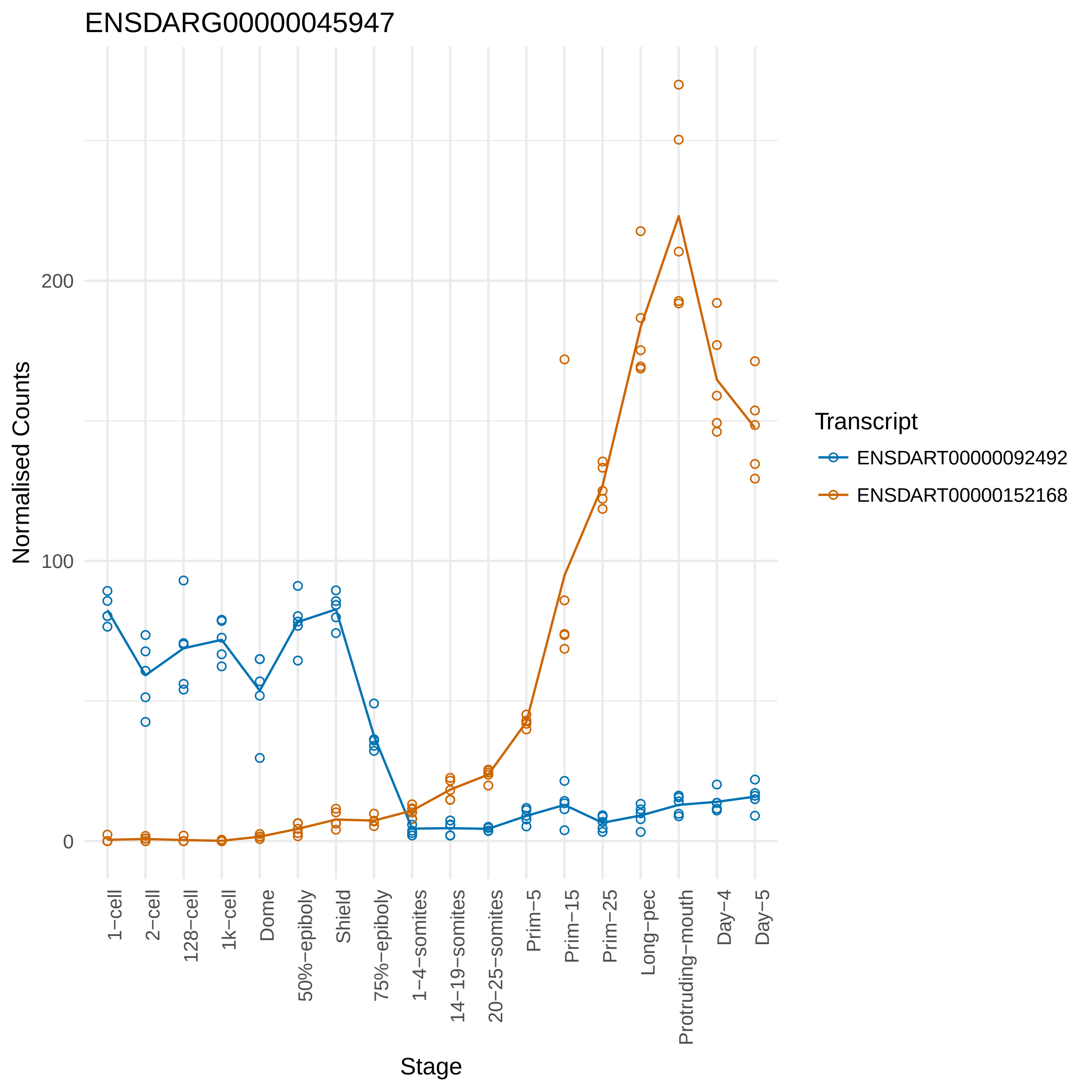
ENSDARG00000045947
Normalised Counts
200
100
0
1−cell
2−cell
128−cell
1k−cell
Dome
50%−epiboly
Shield
75%−epiboly
1−4−somites
14−19−somites
20−25−somites
Prim−5
Prim−15
Prim−25
Long−pec
Protruding−mouth
Day−4
Day−5
Stage
Transcript
ENSDART00000092492
ENSDART00000152168

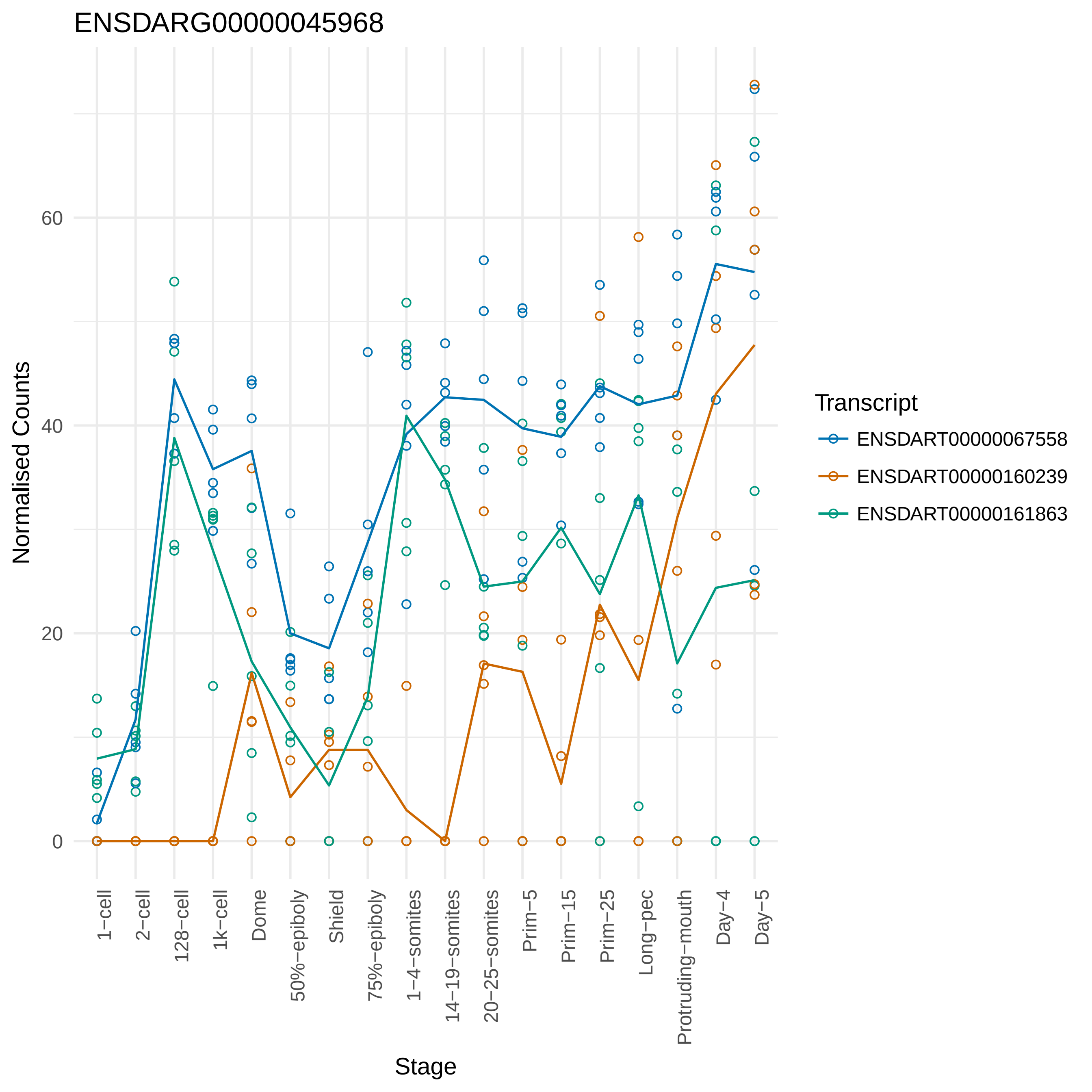

ENSDARG00000045968
Normalised Counts
60
40
20
0
1−cell
2−cell
128−cell
1k−cell
Dome
50%−epiboly
Shield
75%−epiboly
1−4−somites
14−19−somites
20−25−somites
Prim−5
Prim−15
Prim−25
Long−pec
Protruding−mouth
Day−4
Day−5
Stage
Transcript
ENSDART00000067558
ENSDART00000160239
ENSDART00000161863

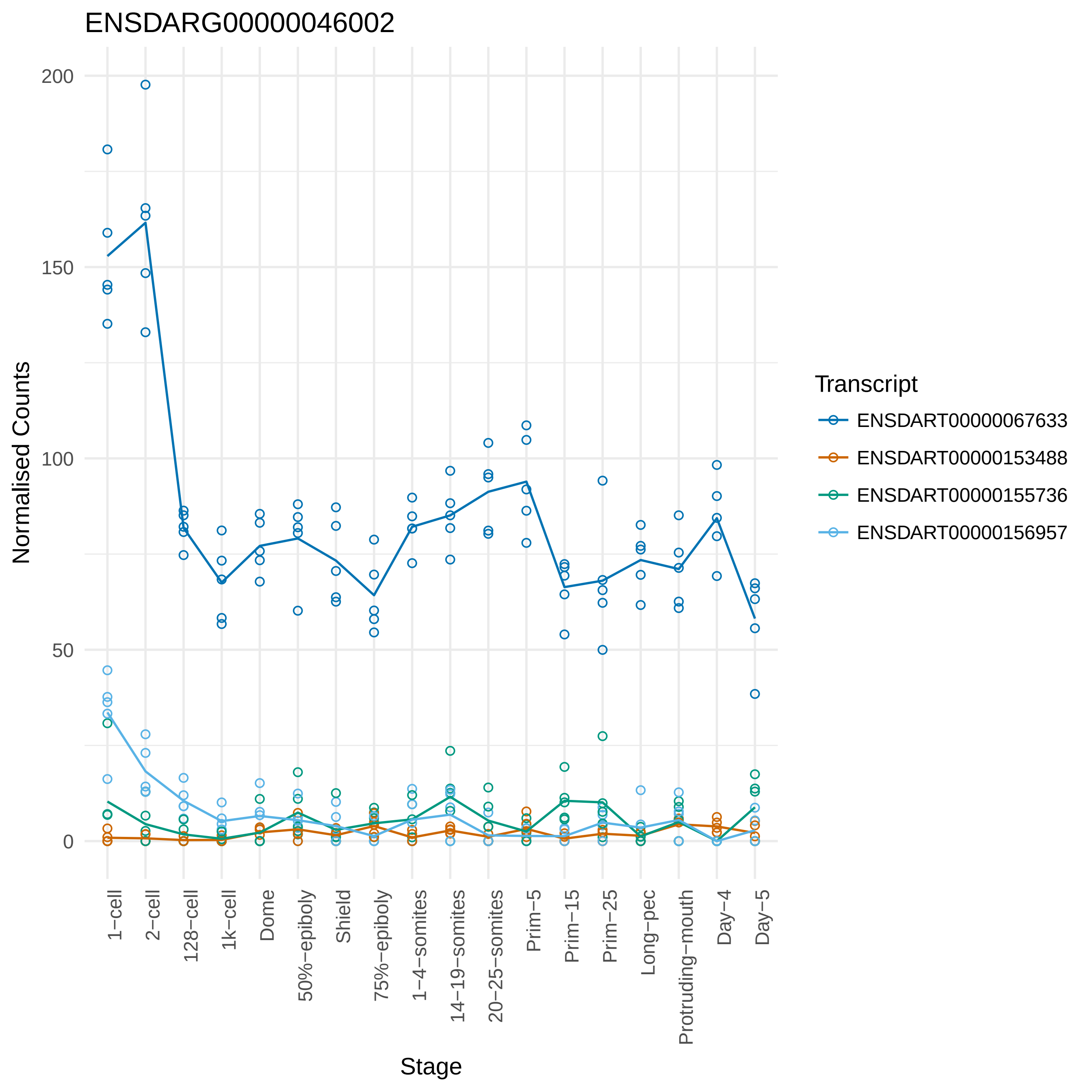

ENSDARG00000046002
Normalised Counts
200
150
100
50
0
1−cell
2−cell
128−cell
1k−cell
Dome
50%−epiboly
Shield
75%−epiboly
1−4−somites
14−19−somites
20−25−somites
Prim−5
Prim−15
Prim−25
Long−pec
Protruding−mouth
Day−4
Day−5
Stage
Transcript
ENSDART00000067633
ENSDART00000153488
ENSDART00000155736
ENSDART00000156957

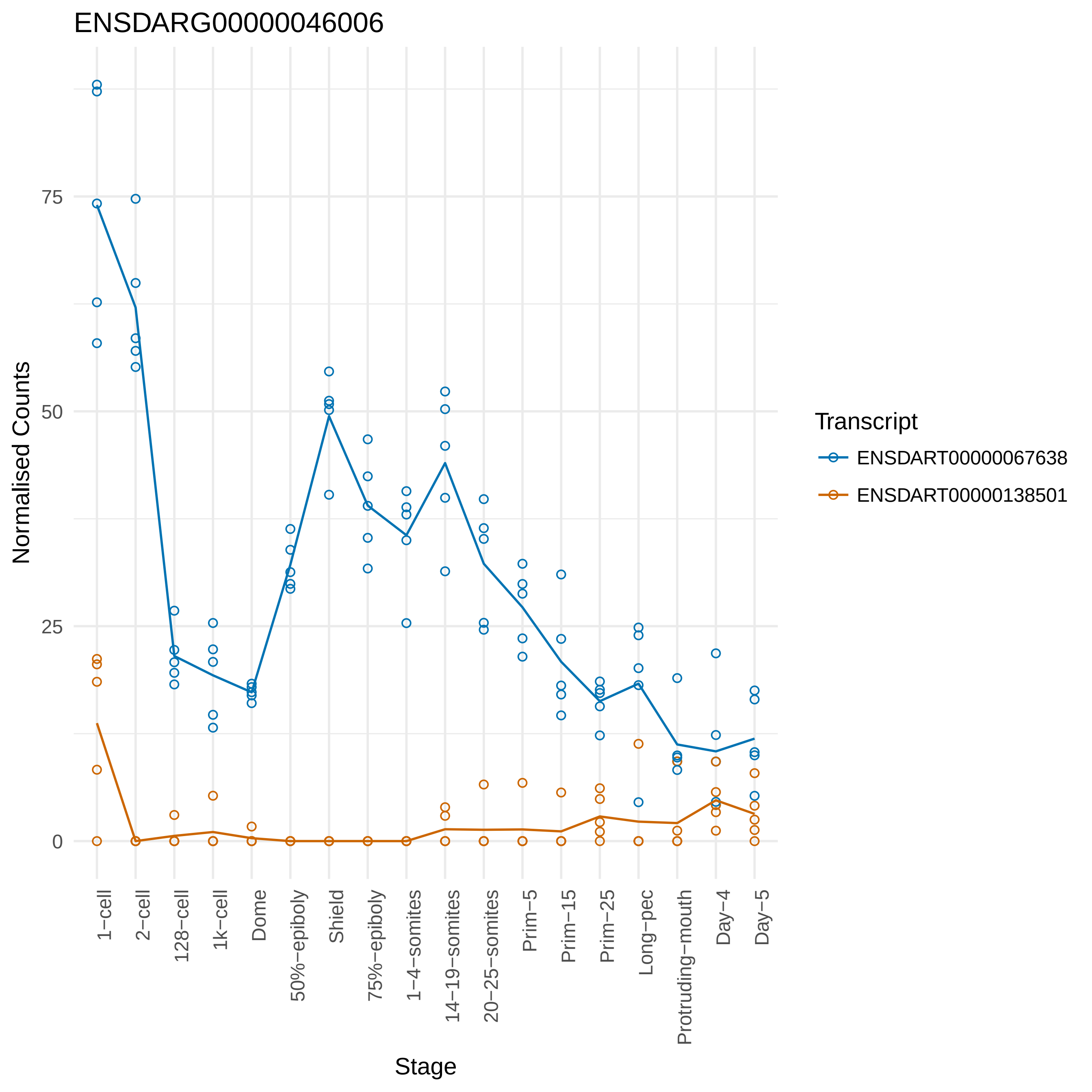
ENSDARG00000046006
Normalised Counts
75
50
25
0
1−cell
2−cell
128−cell
1k−cell
Dome
50%−epiboly
Shield
75%−epiboly
1−4−somites
14−19−somites
20−25−somites
Prim−5
Prim−15
Prim−25
Long−pec
Protruding−mouth
Day−4
Day−5
Stage
Transcript
ENSDART00000067638
ENSDART00000138501

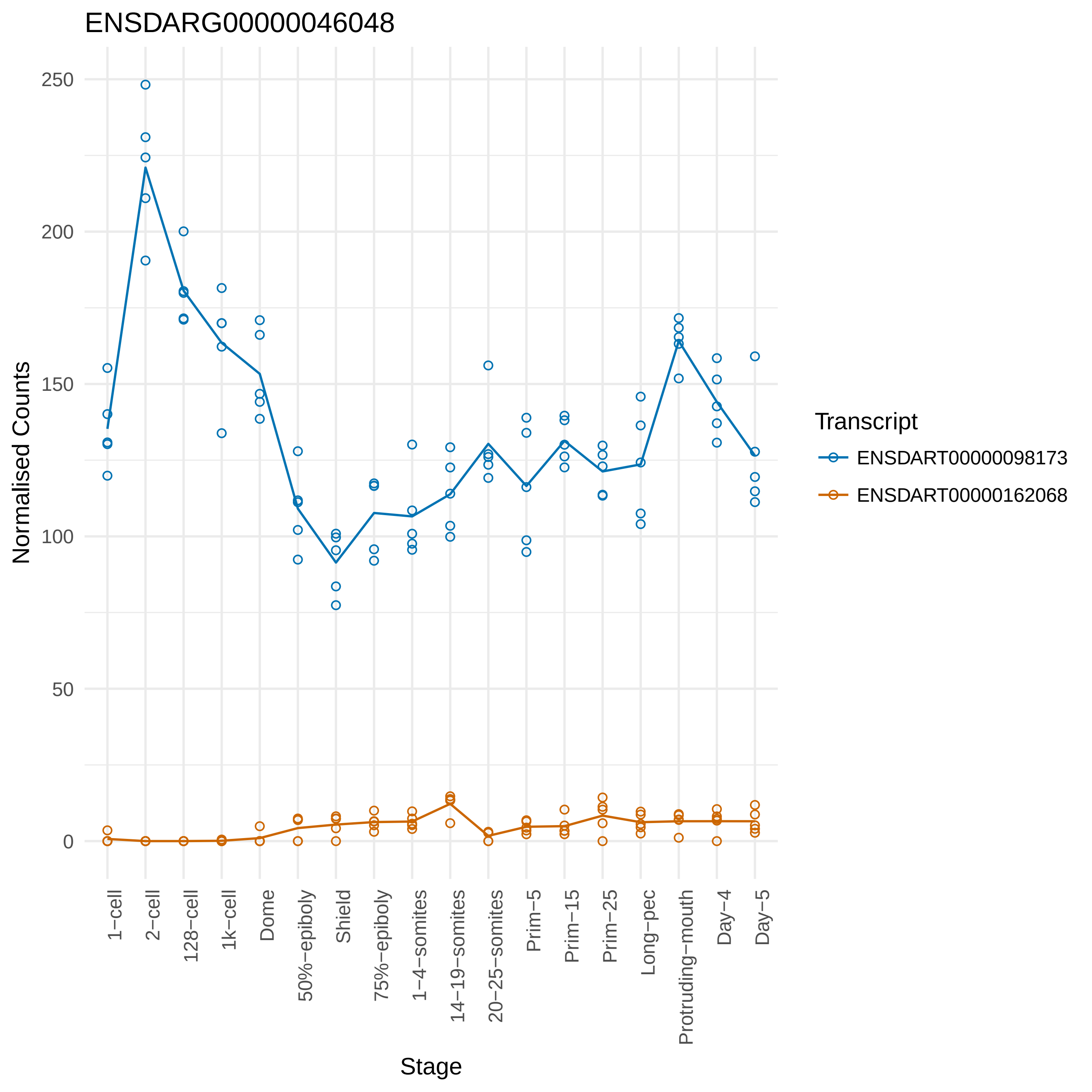

ENSDARG00000046048
Normalised Counts
250
200
150
100
50
0
1−cell
2−cell
128−cell
1k−cell
Dome
50%−epiboly
Shield
75%−epiboly
1−4−somites
14−19−somites
20−25−somites
Prim−5
Prim−15
Prim−25
Long−pec
Protruding−mouth
Day−4
Day−5
Stage
Transcript
ENSDART00000098173
ENSDART00000162068

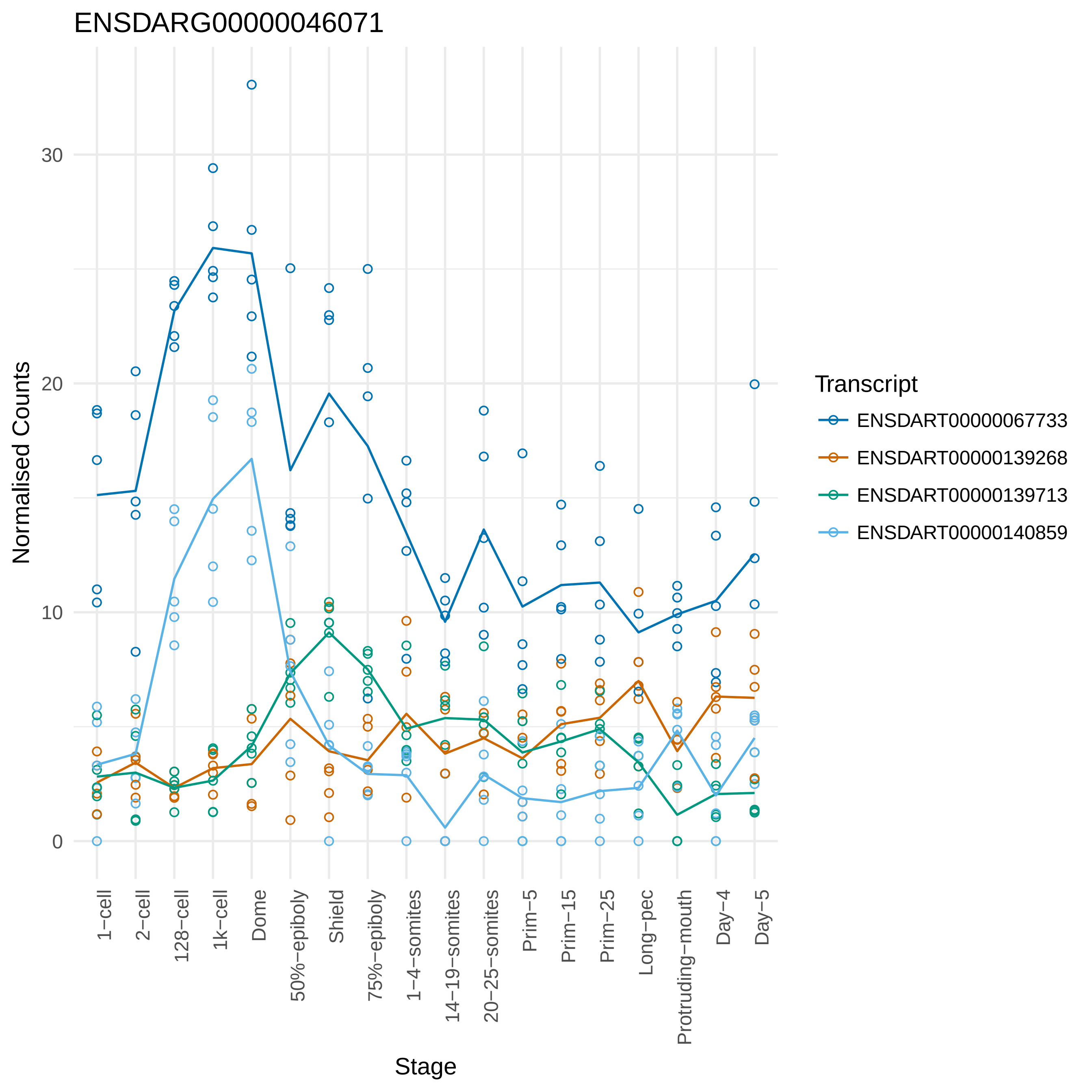

ENSDARG00000046071
Normalised Counts
30
20
10
0
1−cell
2−cell
128−cell
1k−cell
Dome
50%−epiboly
Shield
75%−epiboly
1−4−somites
14−19−somites
20−25−somites
Prim−5
Prim−15
Prim−25
Long−pec
Protruding−mouth
Day−4
Day−5
Stage
Transcript
ENSDART00000067733
ENSDART00000139268
ENSDART00000139713
ENSDART00000140859

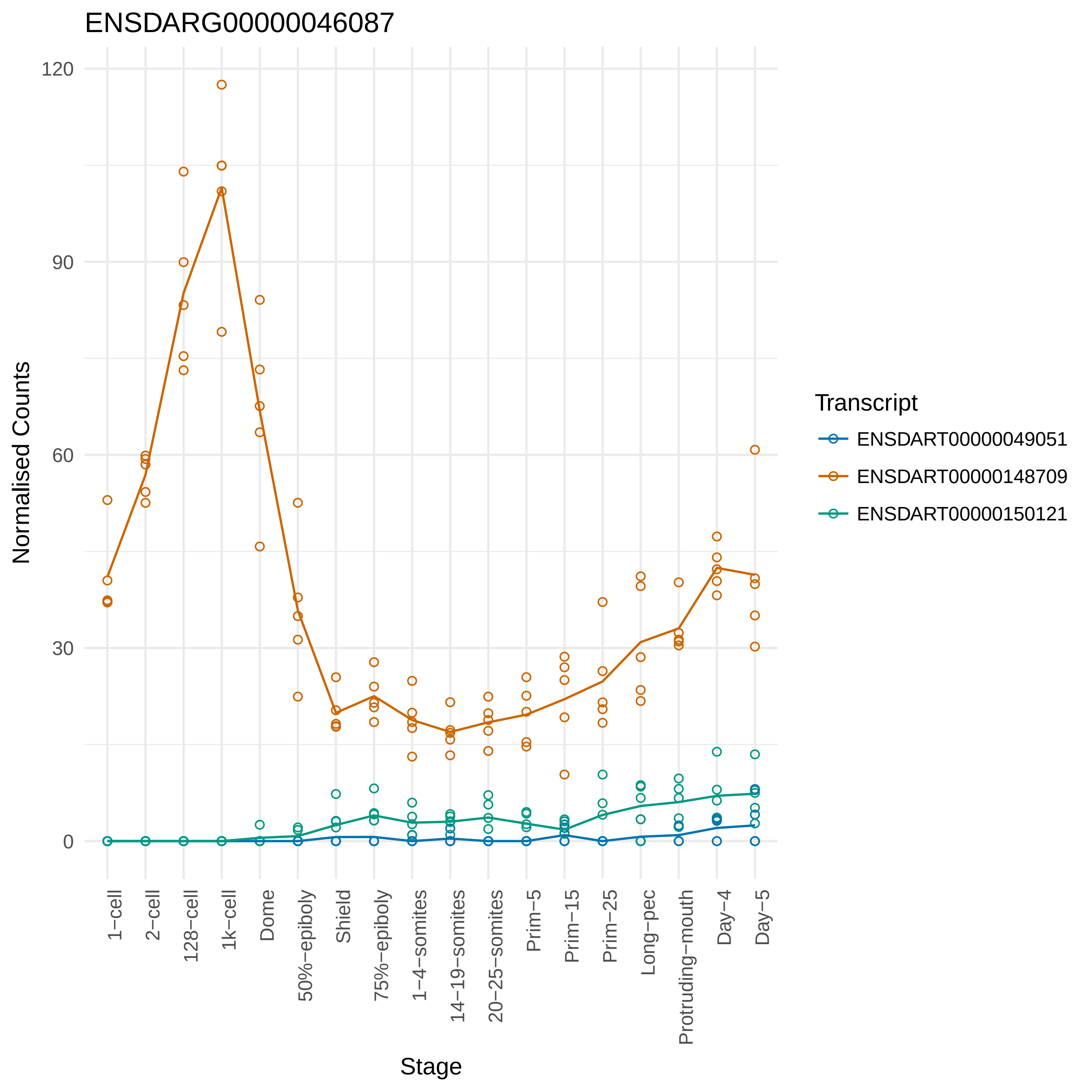

ENSDARG00000046087
Normalised Counts
120
90
60
30
0
1−cell
2−cell
128−cell
1k−cell
Dome
50%−epiboly
Shield
75%−epiboly
1−4−somites
14−19−somites
20−25−somites
Prim−5
Prim−15
Prim−25
Long−pec
Protruding−mouth
Day−4
Day−5
Stage
Transcript
ENSDART00000049051
ENSDART00000148709
ENSDART00000150121

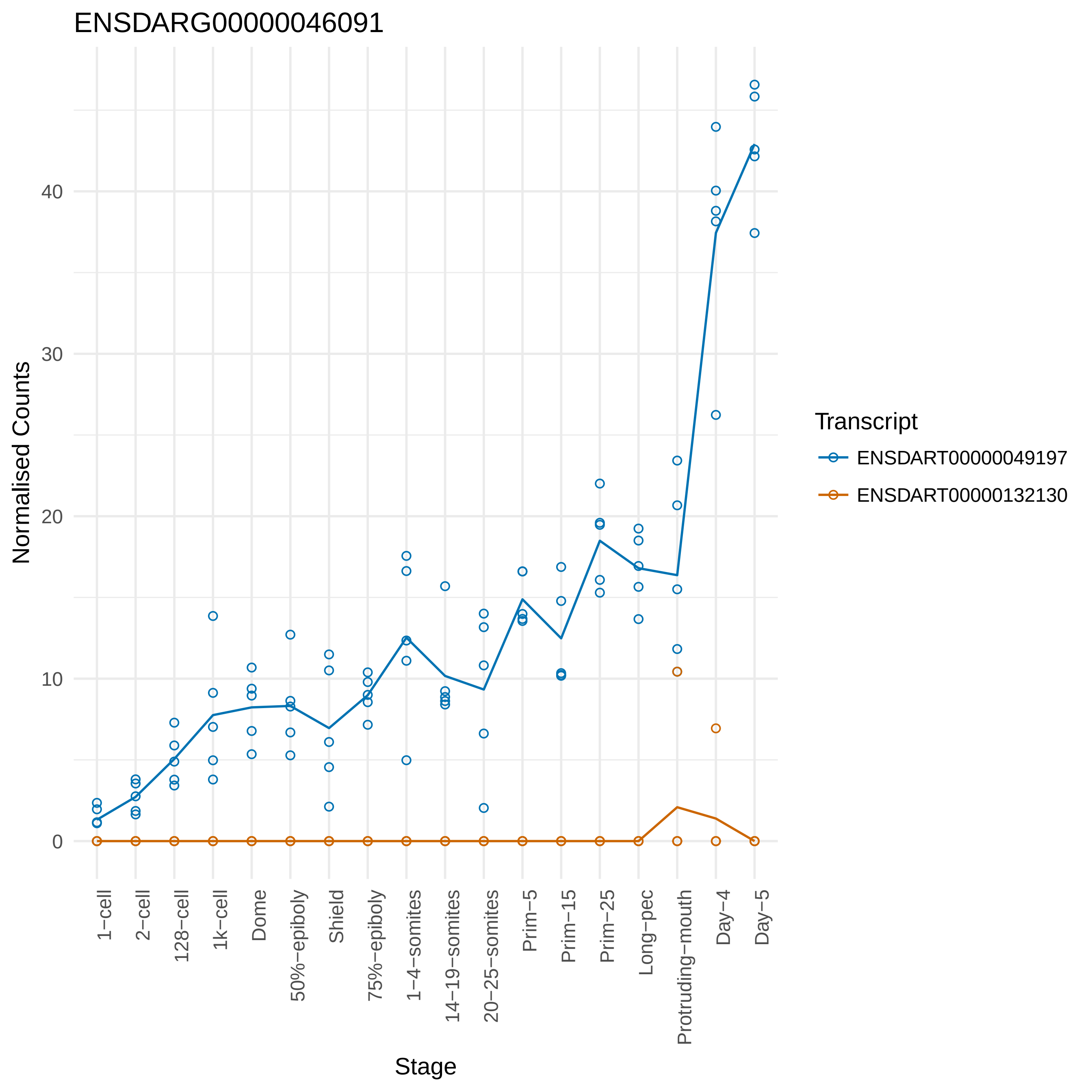
ENSDARG00000046091
Normalised Counts
0
10
20
30
40
Stage
1−cell
2−cell
128−cell
1k−cell
Dome
50%−epiboly
Shield
75%−epiboly
1−4−somites
14−19−somites
20−25−somites
Prim−5
Prim−15
Prim−25
Long−pec
Protruding−mouth
Day−4
Day−5
Transcript
ENSDART00000049197
ENSDART00000132130

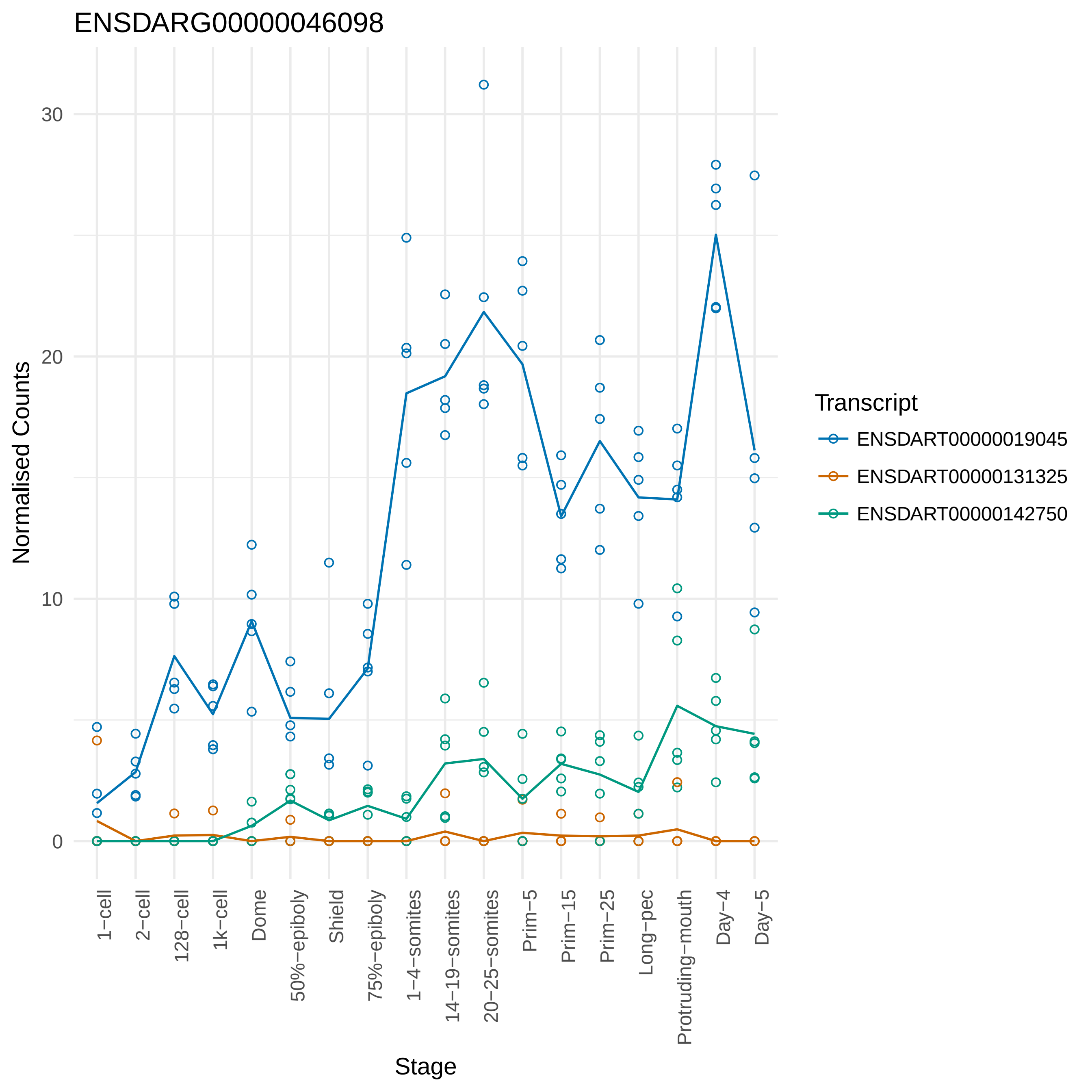

ENSDARG00000046098
Normalised Counts
0
10
20
30
1−cell
2−cell
128−cell
1k−cell
Dome
50%−epiboly
Shield
75%−epiboly
1−4−somites
14−19−somites
20−25−somites
Prim−5
Prim−15
Prim−25
Long−pec
Protruding−mouth
Day−4
Day−5
Stage
Transcript
ENSDART00000019045
ENSDART00000131325
ENSDART00000142750

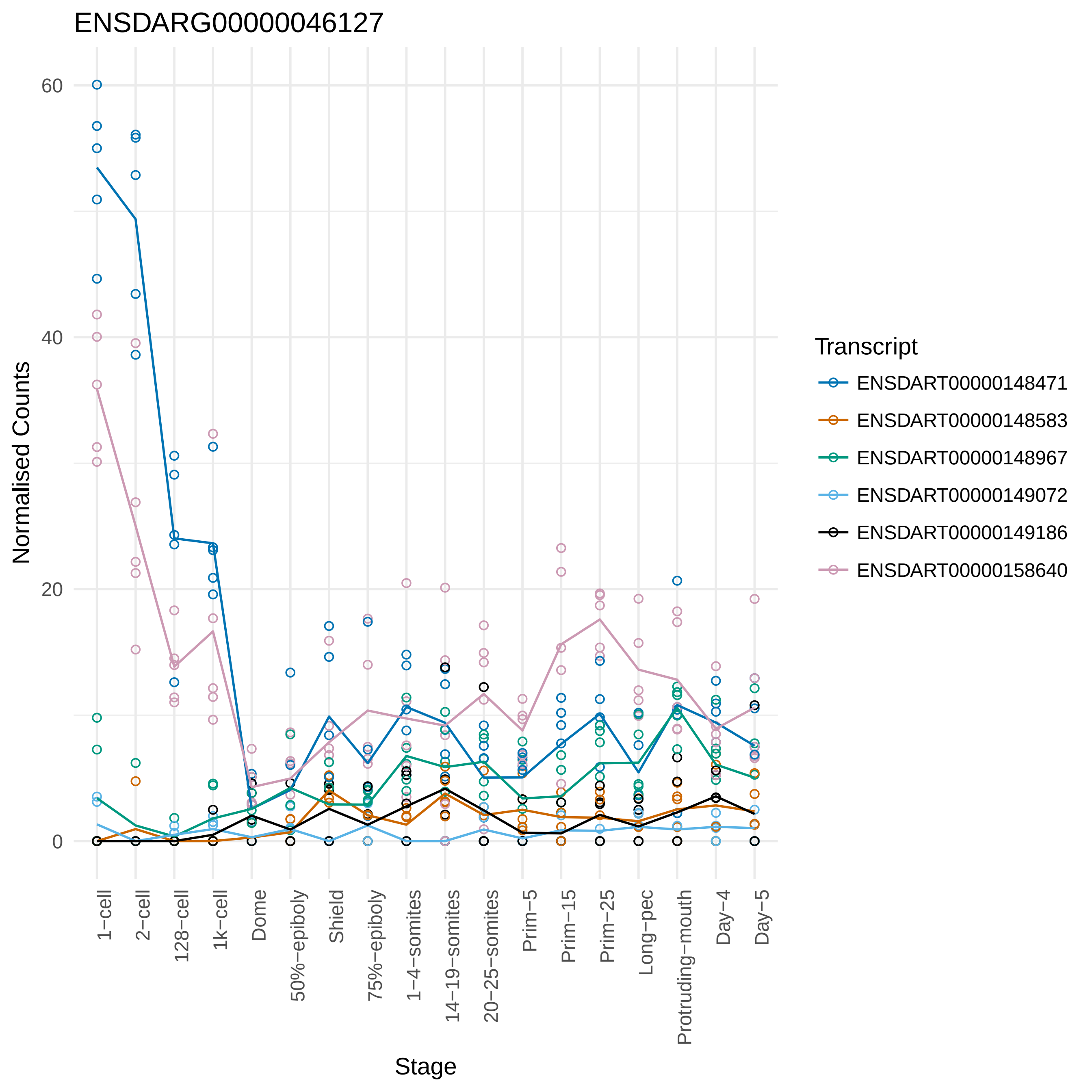
ENSDARG00000046127
Normalised Counts
60
40
20
0
Stage
1−cell
2−cell
128−cell
1k−cell
Dome
50%−epiboly
Shield
75%−epiboly
1−4−somites
14−19−somites
20−25−somites
Prim−5
Prim−15
Prim−25
Long−pec
Protruding−mouth
Day−4
Day−5
Transcript
ENSDART00000148471
ENSDART00000148583
ENSDART00000148967
ENSDART00000149072
ENSDART00000149186
ENSDART00000158640

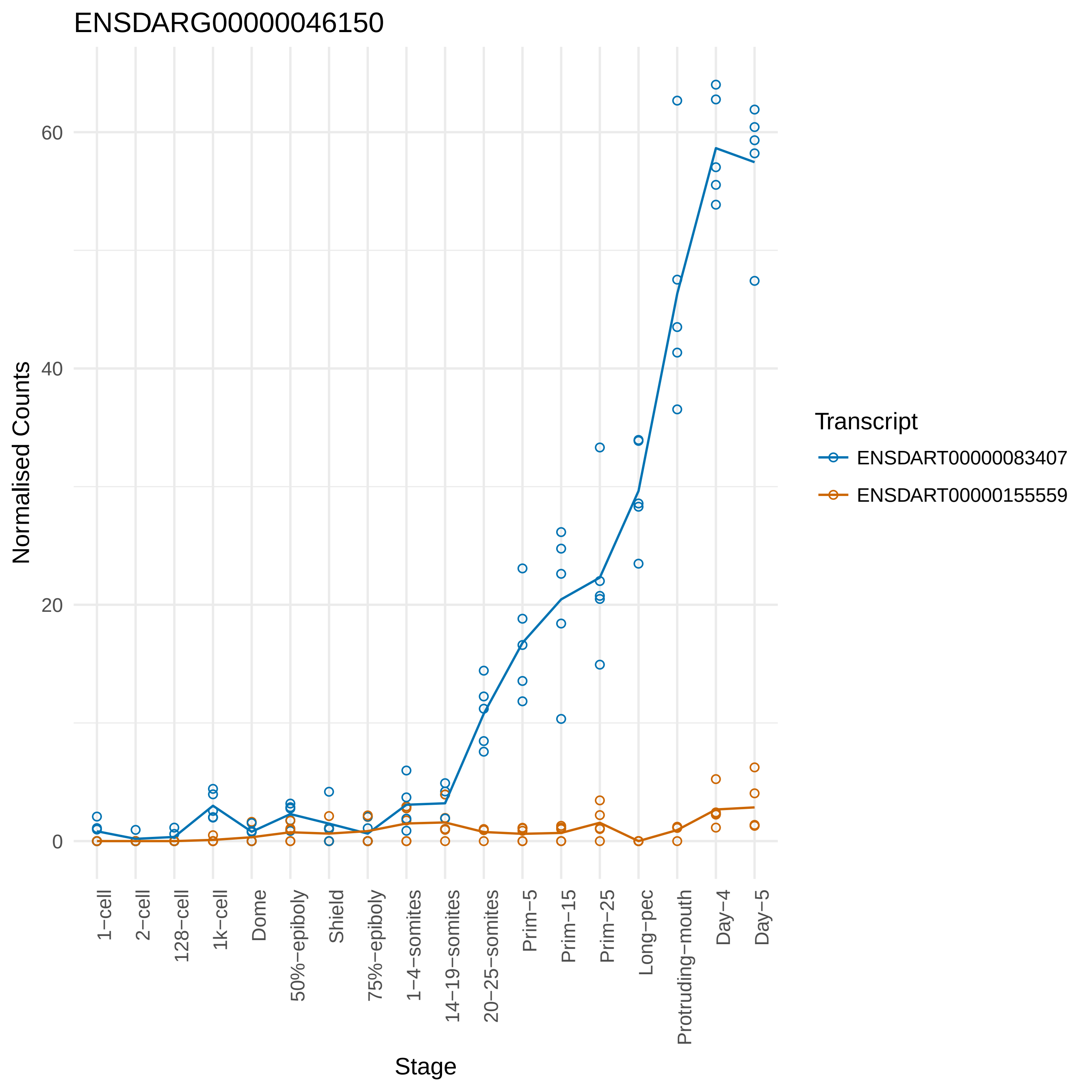

ENSDARG00000046150
Normalised Counts
0
20
40
60
Stage
1−cell
2−cell
128−cell
1k−cell
Dome
50%−epiboly
Shield
75%−epiboly
1−4−somites
14−19−somites
20−25−somites
Prim−5
Prim−15
Prim−25
Long−pec
Protruding−mouth
Day−4
Day−5
Transcript
ENSDART00000083407
ENSDART00000155559

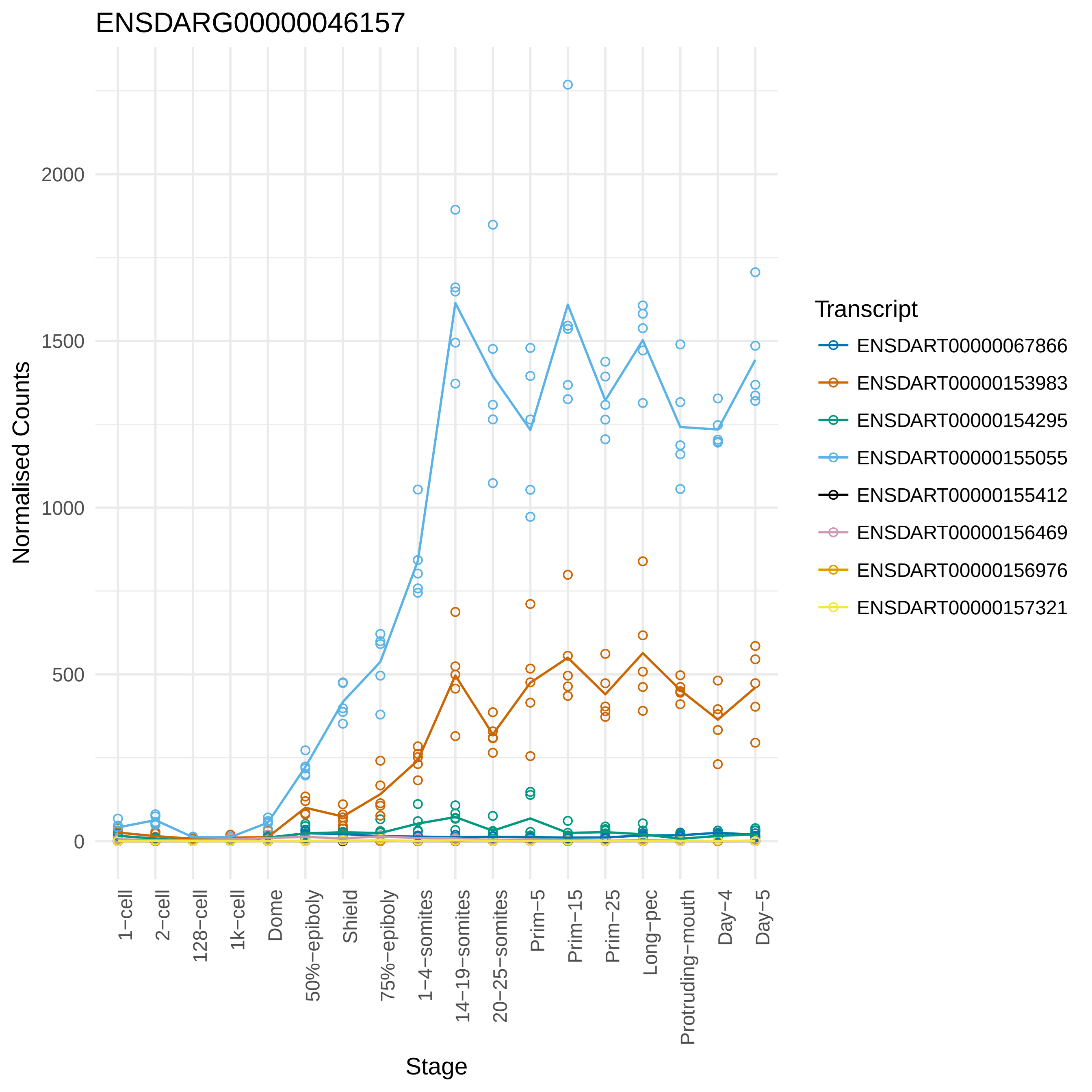

ENSDARG00000046157
Normalised Counts
0
500
1000
1500
2000
Stage
1−cell
2−cell
128−cell
1k−cell
Dome
50%−epiboly
Shield
75%−epiboly
1−4−somites
14−19−somites
20−25−somites
Prim−5
Prim−15
Prim−25
Long−pec
Protruding−mouth
Day−4
Day−5
Transcript
ENSDART00000067866
ENSDART00000153983
ENSDART00000154295
ENSDART00000155055
ENSDART00000155412
ENSDART00000156469
ENSDART00000156976
ENSDART00000157321

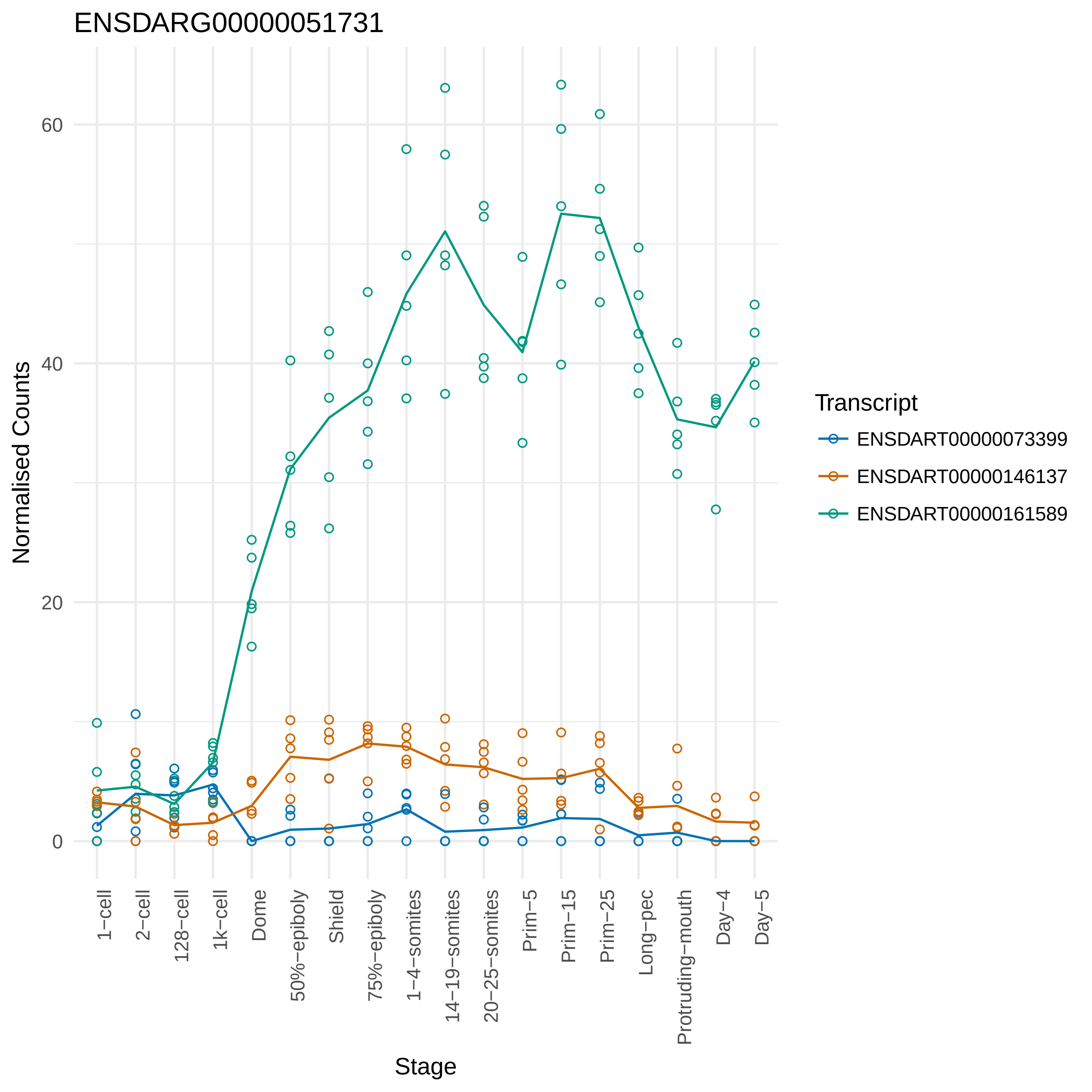
ENSDARG00000051731
Normalised Counts
0
20
40
60
Stage
1−cell
2−cell
128−cell
1k−cell
Dome
50%−epiboly
Shield
75%−epiboly
1−4−somites
14−19−somites
20−25−somites
Prim−5
Prim−15
Prim−25
Long−pec
Protruding−mouth
Day−4
Day−5
Transcript
ENSDART00000073399
ENSDART00000146137
ENSDART00000161589

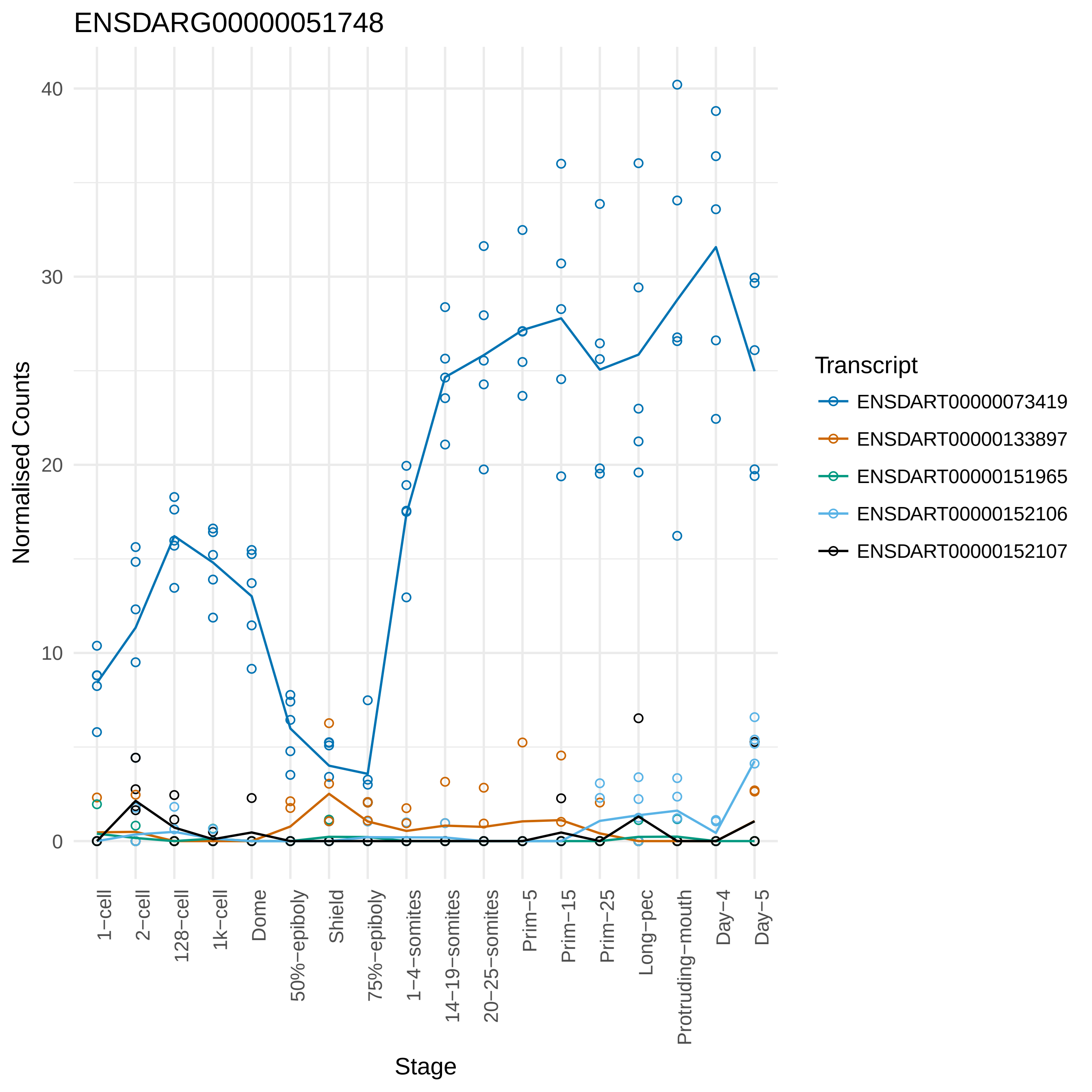

ENSDARG00000051748
Normalised Counts
0
10
20
30
40
1−cell
2−cell
128−cell
1k−cell
Dome
50%−epiboly
Shield
75%−epiboly
1−4−somites
14−19−somites
20−25−somites
Prim−5
Prim−15
Prim−25
Long−pec
Protruding−mouth
Day−4
Day−5
Stage
Transcript
ENSDART00000073419
ENSDART00000133897
ENSDART00000151965
ENSDART00000152106
ENSDART00000152107

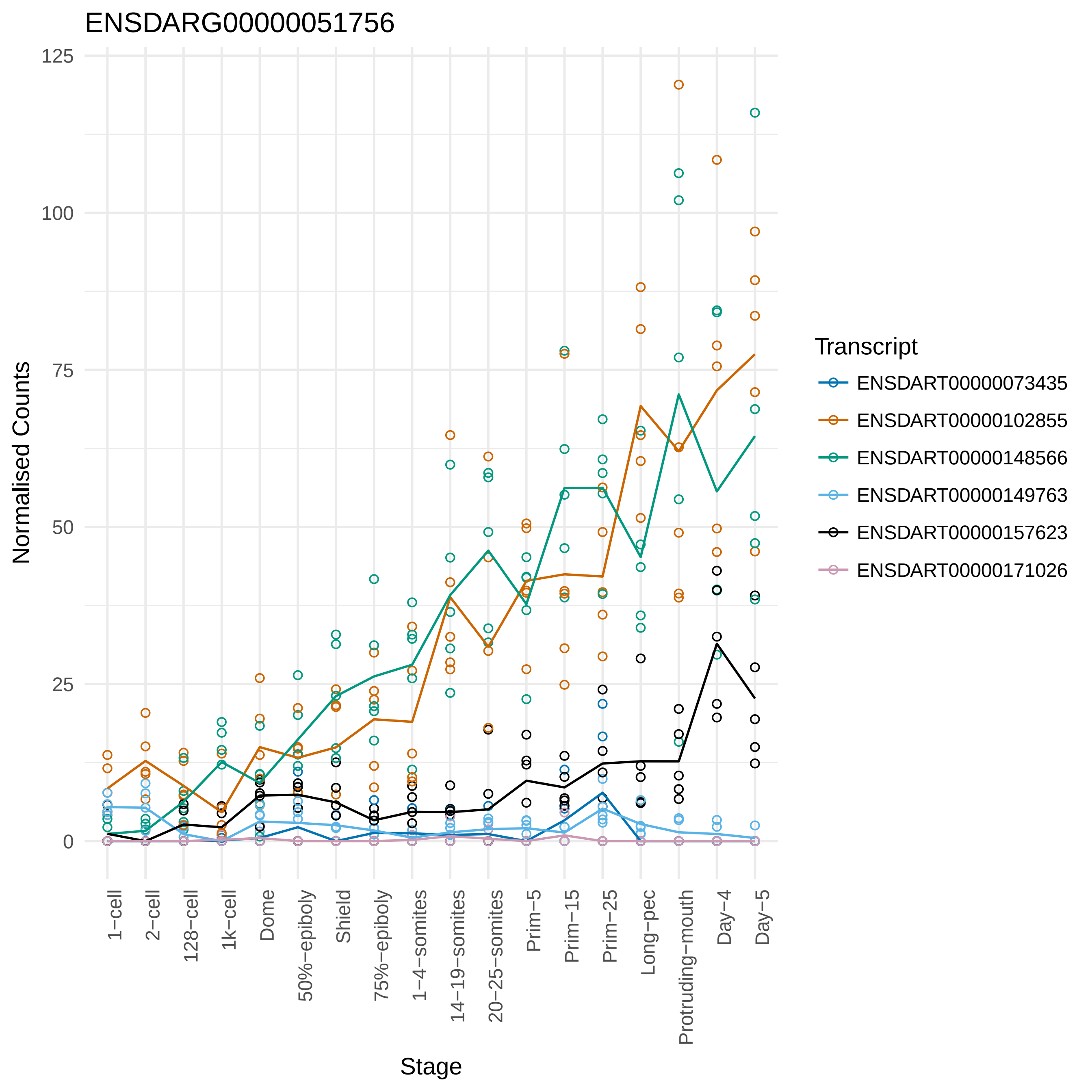
ENSDARG00000051756
Normalised Counts
125
100
75
50
25
0
1−cell
2−cell
128−cell
1k−cell
Dome
50%−epiboly
Shield
75%−epiboly
1−4−somites
14−19−somites
20−25−somites
Prim−5
Prim−15
Prim−25
Long−pec
Protruding−mouth
Day−4
Day−5
Stage
Transcript
ENSDART00000073435
ENSDART00000102855
ENSDART00000148566
ENSDART00000149763
ENSDART00000157623
ENSDART00000171026

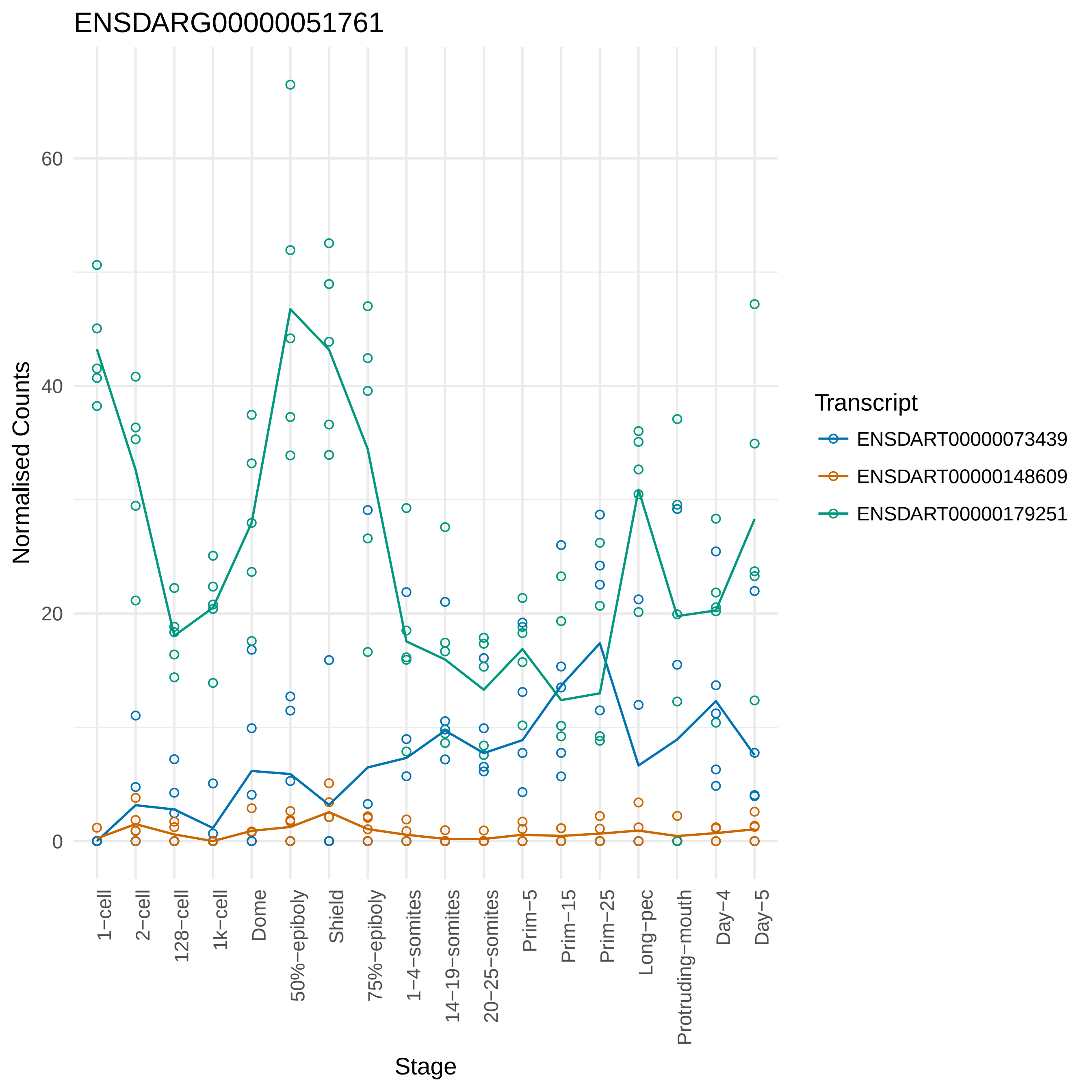

ENSDARG00000051761
Normalised Counts
60
40
20
0
1−cell
2−cell
128−cell
1k−cell
Dome
50%−epiboly
Shield
75%−epiboly
1−4−somites
14−19−somites
20−25−somites
Prim−5
Prim−15
Prim−25
Long−pec
Protruding−mouth
Day−4
Day−5
Stage
Transcript
ENSDART00000073439
ENSDART00000148609
ENSDART00000179251

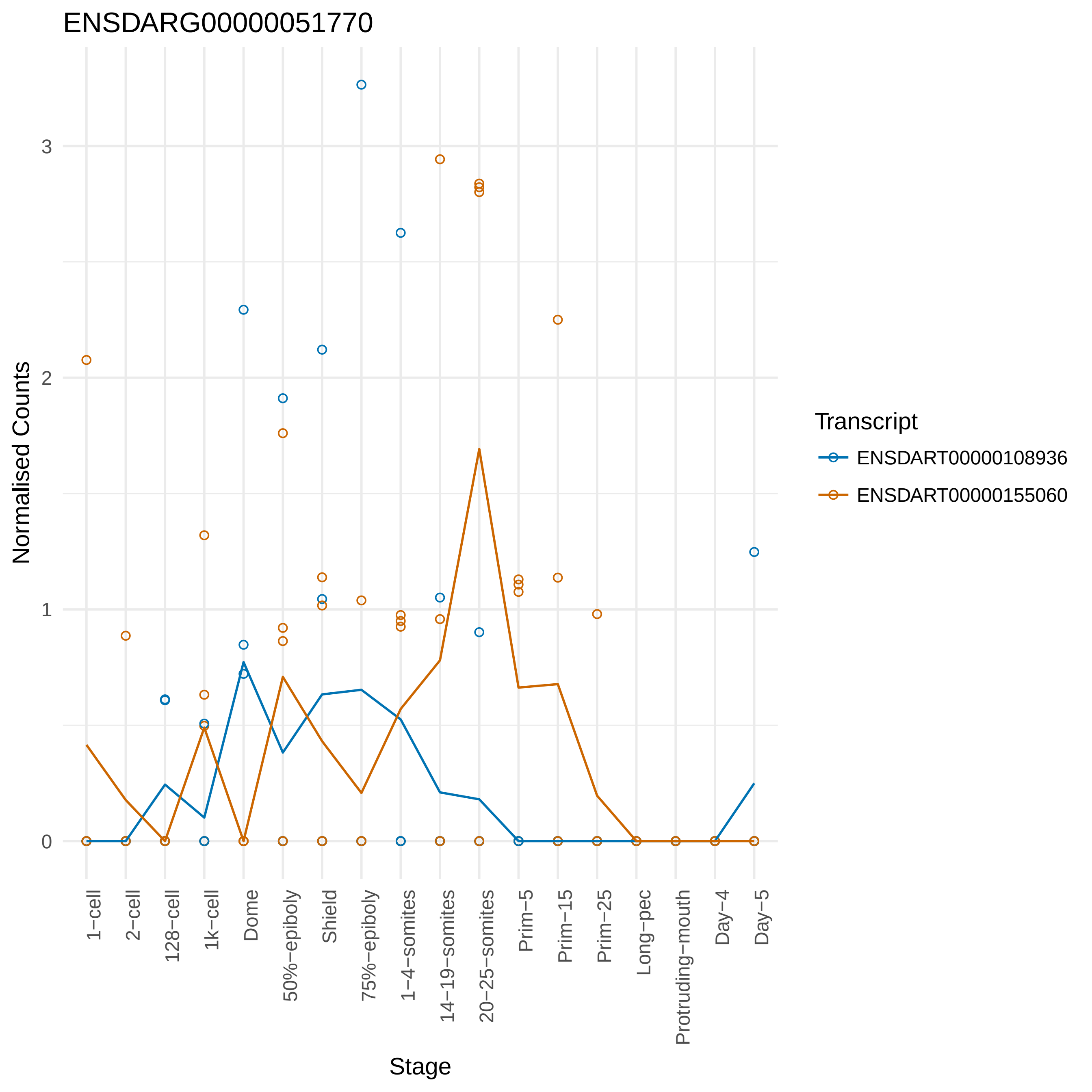

ENSDARG00000051770
Normalised Counts
3
2
1
0
1−cell
2−cell
128−cell
1k−cell
Dome
50%−epiboly
Shield
75%−epiboly
1−4−somites
14−19−somites
20−25−somites
Prim−5
Prim−15
Prim−25
Long−pec
Protruding−mouth
Day−4
Day−5
Stage
Transcript
ENSDART00000108936
ENSDART00000155060

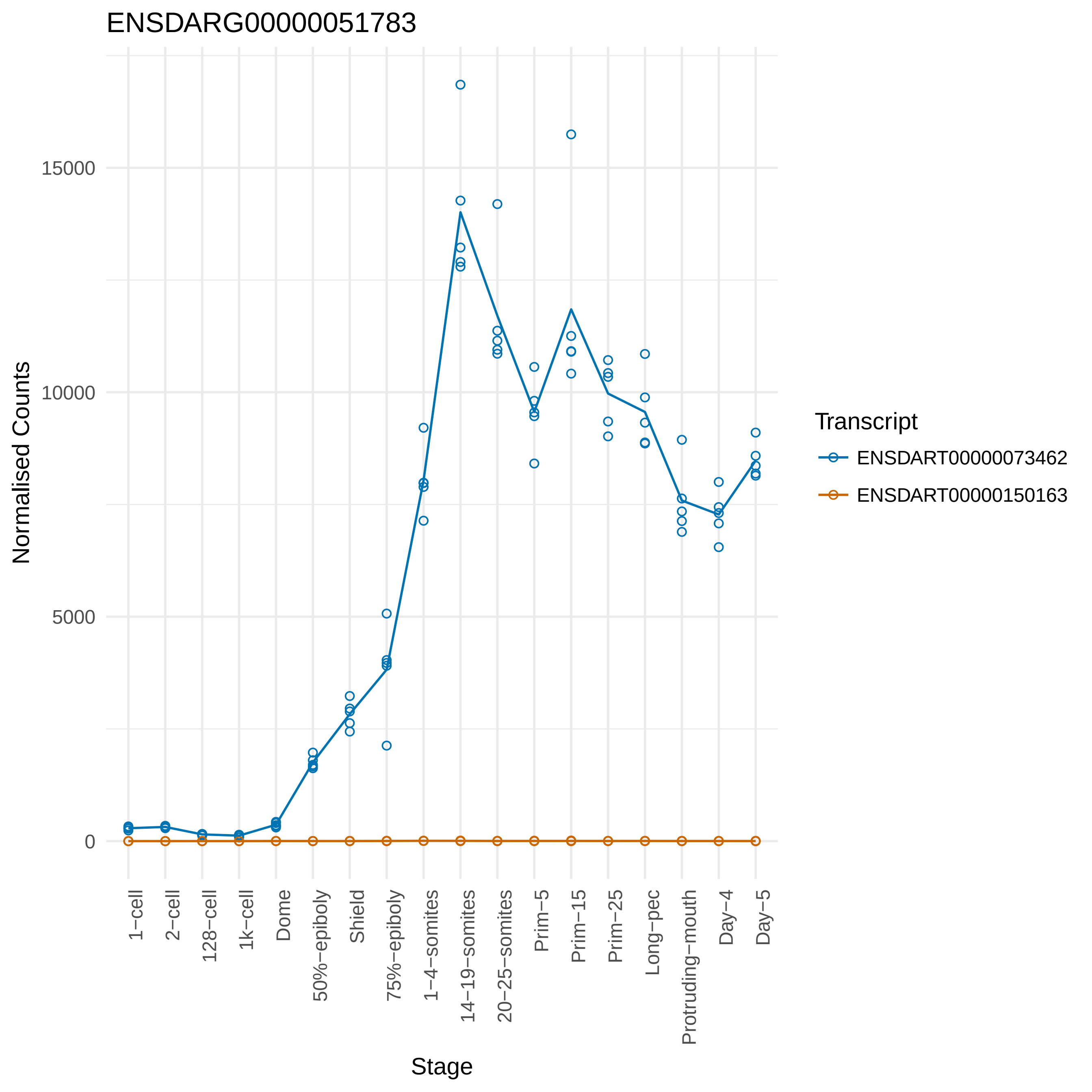

ENSDARG00000051783
Normalised Counts
15000
10000
5000
0
1−cell
2−cell
128−cell
1k−cell
Dome
50%−epiboly
Shield
75%−epiboly
1−4−somites
14−19−somites
20−25−somites
Prim−5
Prim−15
Prim−25
Long−pec
Protruding−mouth
Day−4
Day−5
Stage
Transcript
ENSDART00000073462
ENSDART00000150163

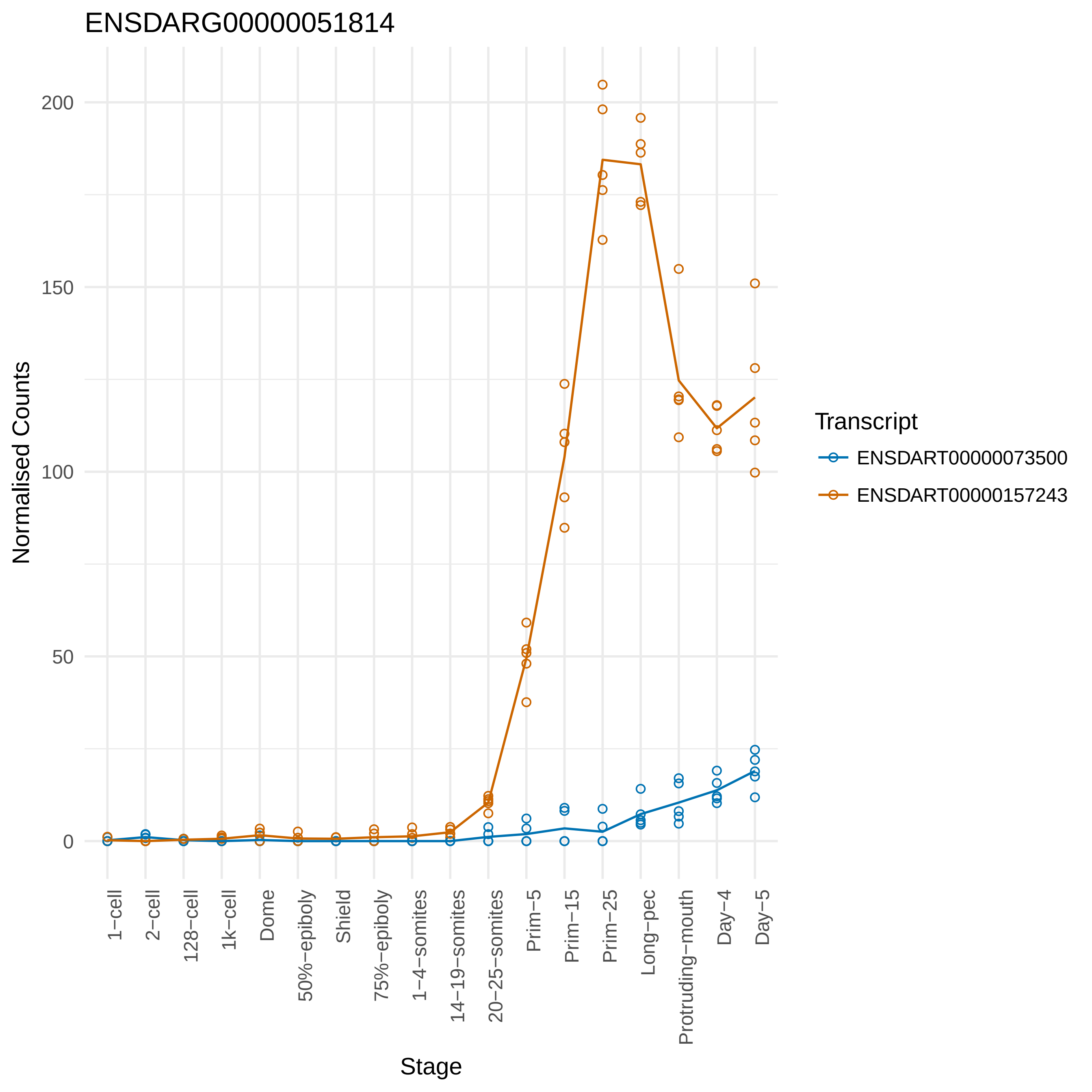

ENSDARG00000051814
Normalised Counts
200
150
100
50
0
1−cell
2−cell
128−cell
1k−cell
Dome
50%−epiboly
Shield
75%−epiboly
1−4−somites
14−19−somites
20−25−somites
Prim−5
Prim−15
Prim−25
Long−pec
Protruding−mouth
Day−4
Day−5
Stage
Transcript
ENSDART00000073500
ENSDART00000157243

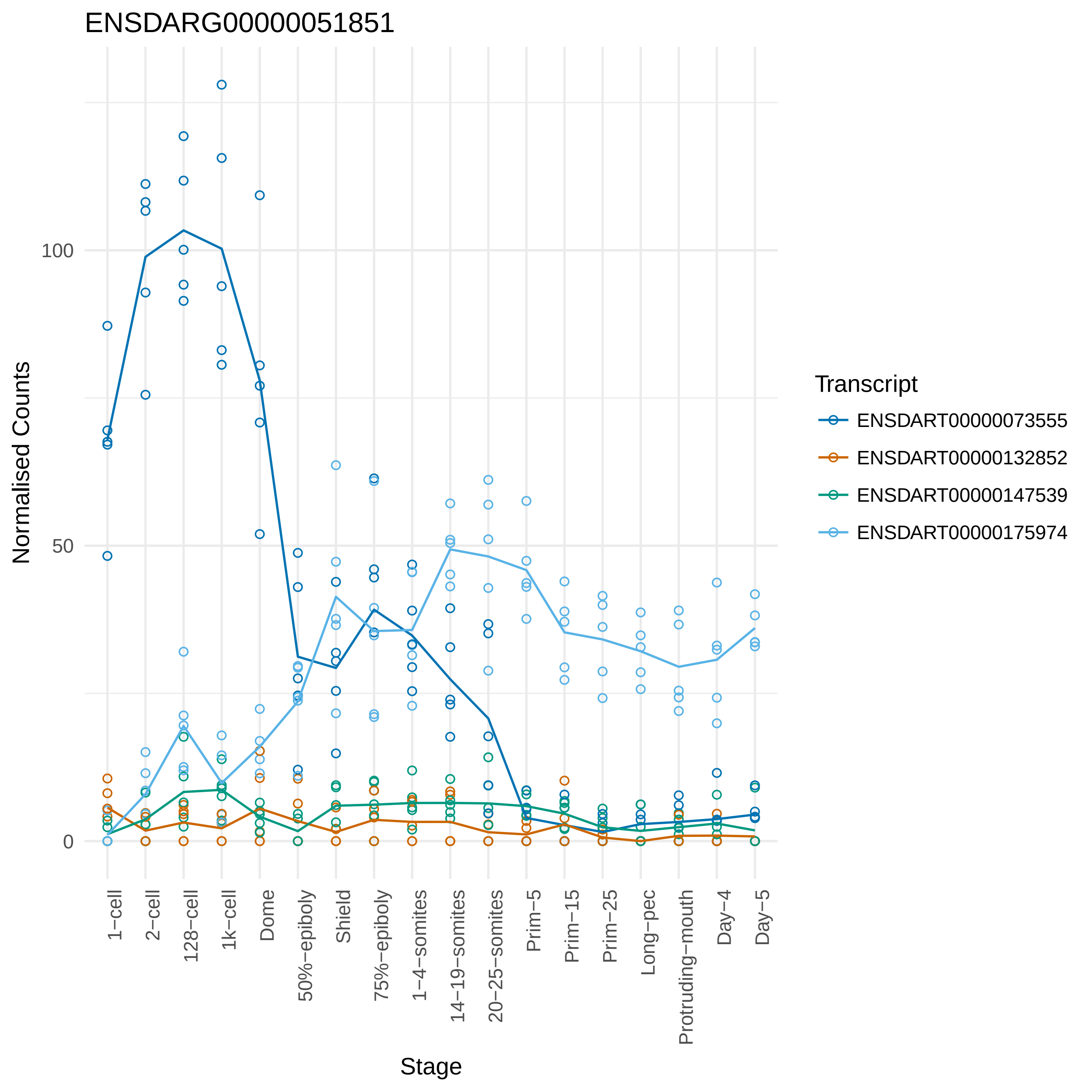
ENSDARG00000051851
Normalised Counts
100
50
0
1−cell
2−cell
128−cell
1k−cell
Dome
50%−epiboly
Shield
75%−epiboly
1−4−somites
14−19−somites
20−25−somites
Prim−5
Prim−15
Prim−25
Long−pec
Protruding−mouth
Day−4
Day−5
Stage
Transcript
ENSDART00000073555
ENSDART00000132852
ENSDART00000147539
ENSDART00000175974

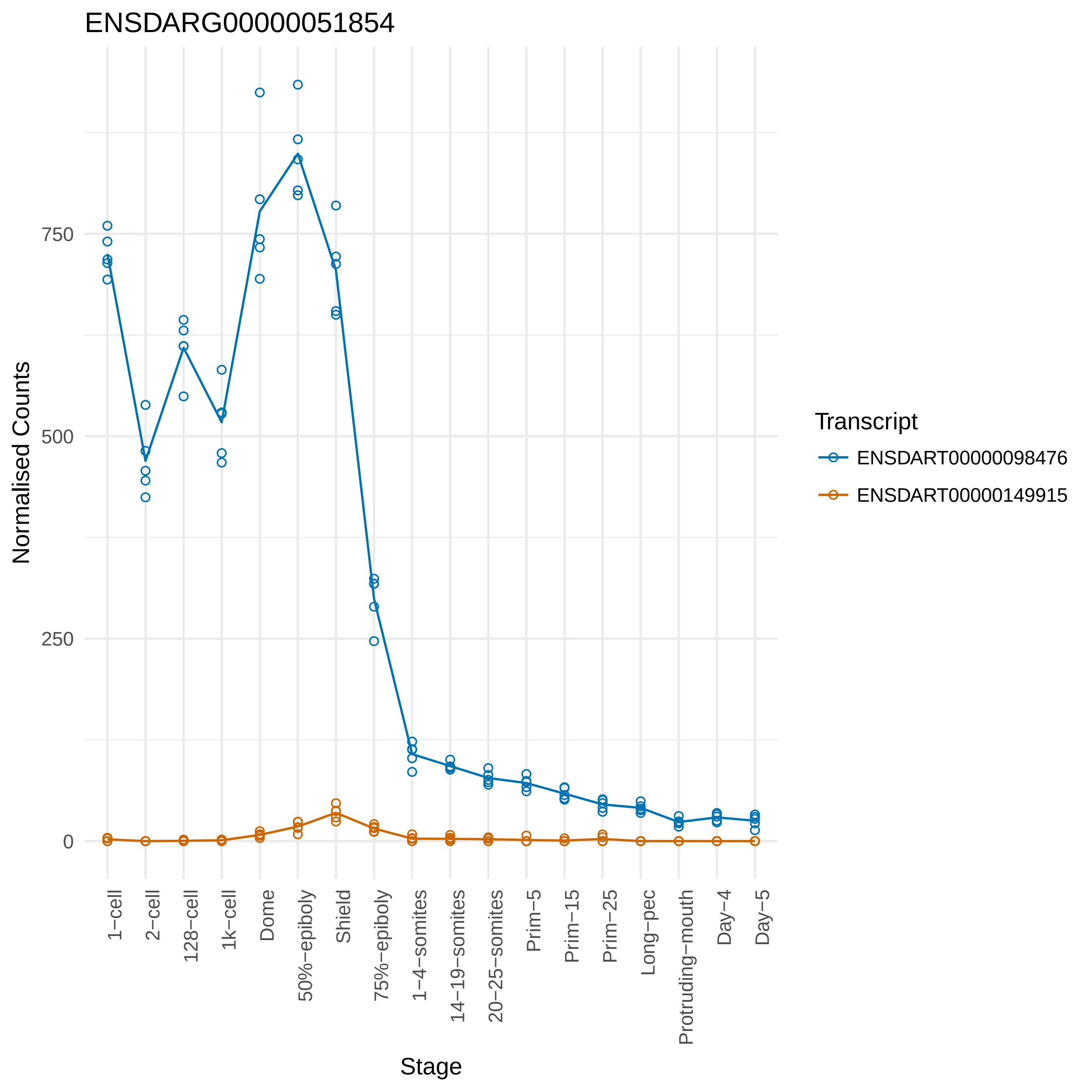
ENSDARG00000051854
Normalised Counts
0
250
500
750
1−cell
2−cell
128−cell
1k−cell
Dome
50%−epiboly
Shield
75%−epiboly
1−4−somites
14−19−somites
20−25−somites
Prim−5
Prim−15
Prim−25
Long−pec
Protruding−mouth
Day−4
Day−5
Stage
Transcript
ENSDART00000098476
ENSDART00000149915

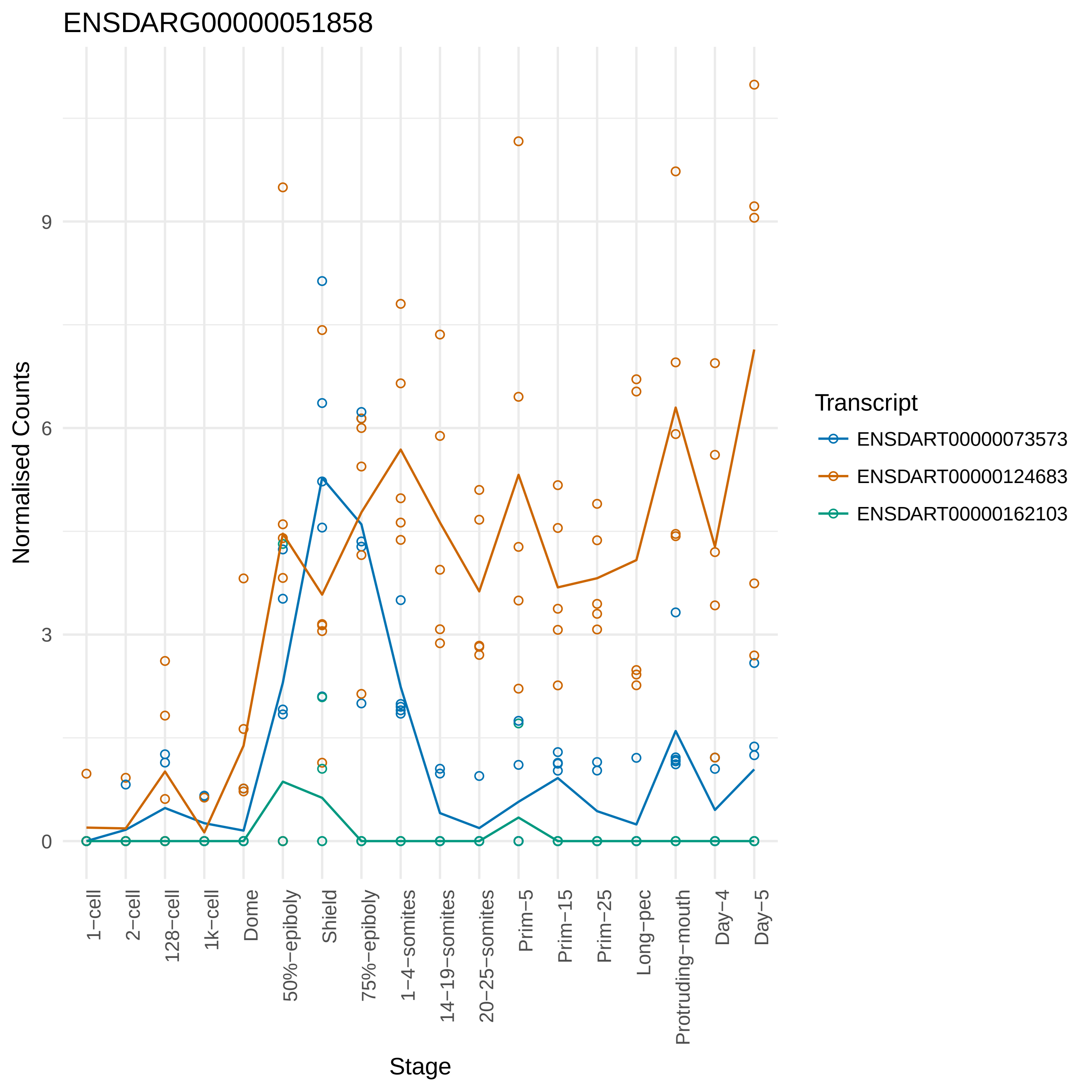

ENSDARG00000051858
Normalised Counts
0
3
6
9
1−cell
2−cell
128−cell
1k−cell
Dome
50%−epiboly
Shield
75%−epiboly
1−4−somites
14−19−somites
20−25−somites
Prim−5
Prim−15
Prim−25
Long−pec
Protruding−mouth
Day−4
Day−5
Stage
Transcript
ENSDART00000073573
ENSDART00000124683
ENSDART00000162103

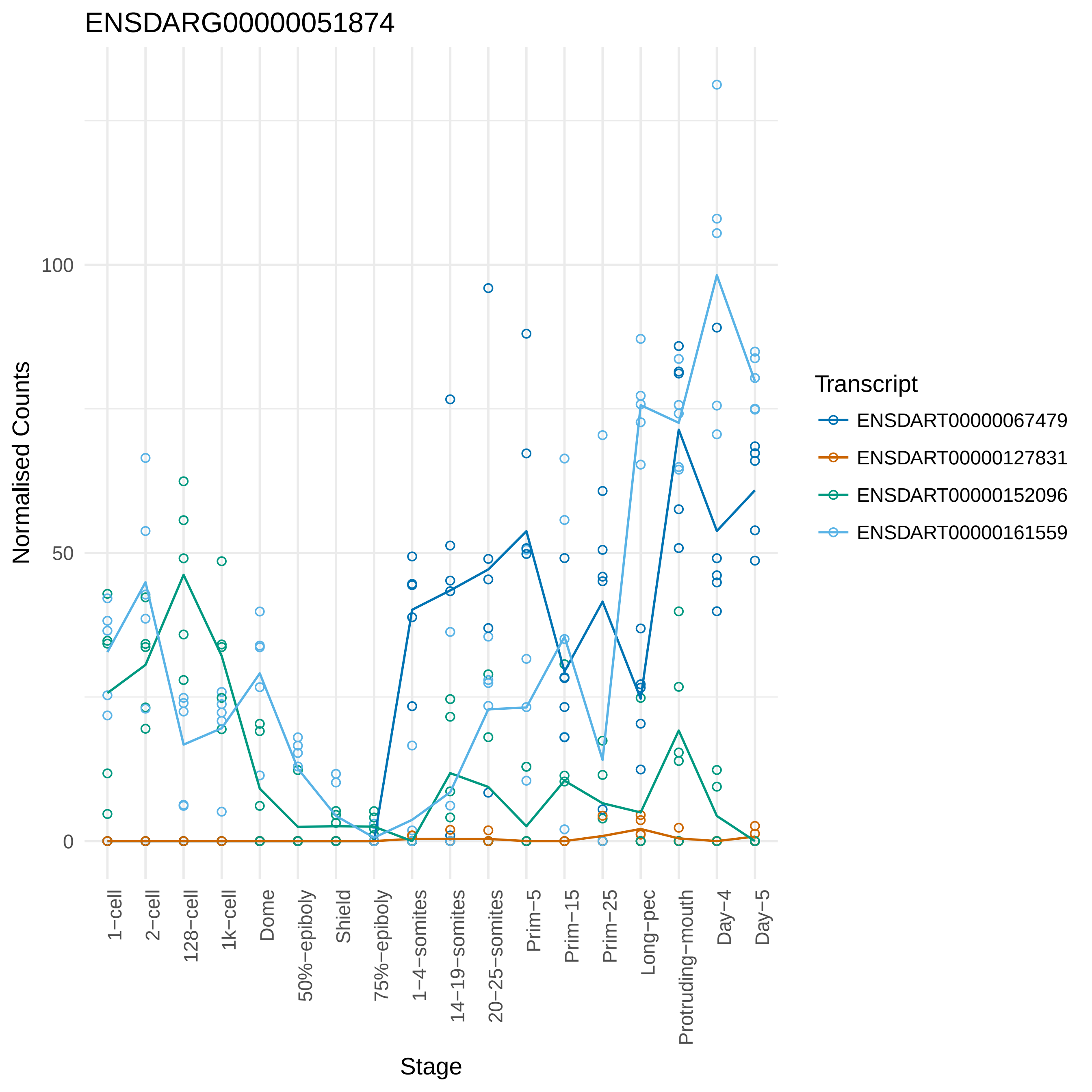

ENSDARG00000051874
Normalised Counts
0
50
100
1−cell
2−cell
128−cell
1k−cell
Dome
50%−epiboly
Shield
75%−epiboly
1−4−somites
14−19−somites
20−25−somites
Prim−5
Prim−15
Prim−25
Long−pec
Protruding−mouth
Day−4
Day−5
Stage
Transcript
ENSDART00000067479
ENSDART00000127831
ENSDART00000152096
ENSDART00000161559

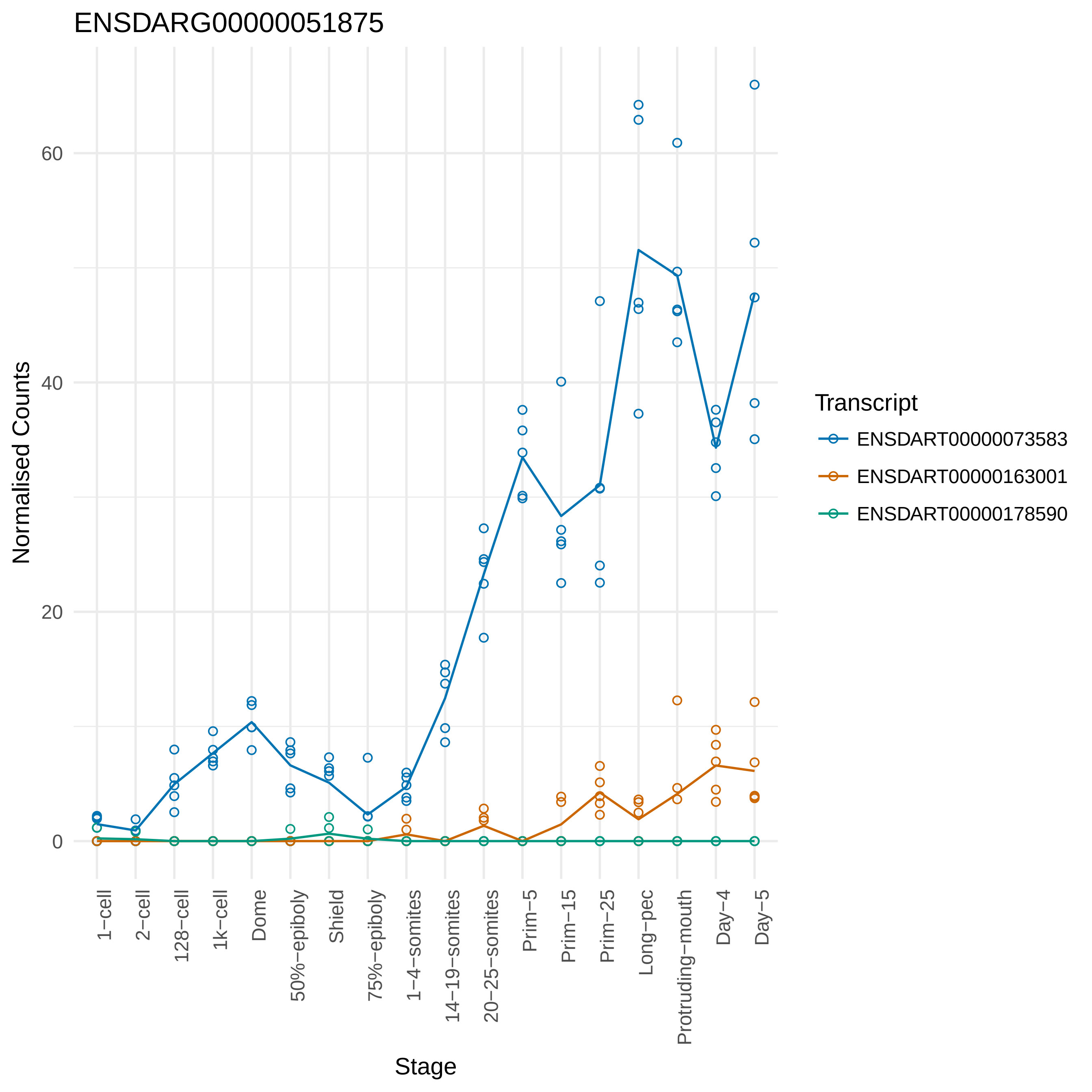
ENSDARG00000051875
Normalised Counts
0
20
40
60
1−cell
2−cell
128−cell
1k−cell
Dome
50%−epiboly
Shield
75%−epiboly
1−4−somites
14−19−somites
20−25−somites
Prim−5
Prim−15
Prim−25
Long−pec
Protruding−mouth
Day−4
Day−5
Stage
Transcript
ENSDART00000073583
ENSDART00000163001
ENSDART00000178590

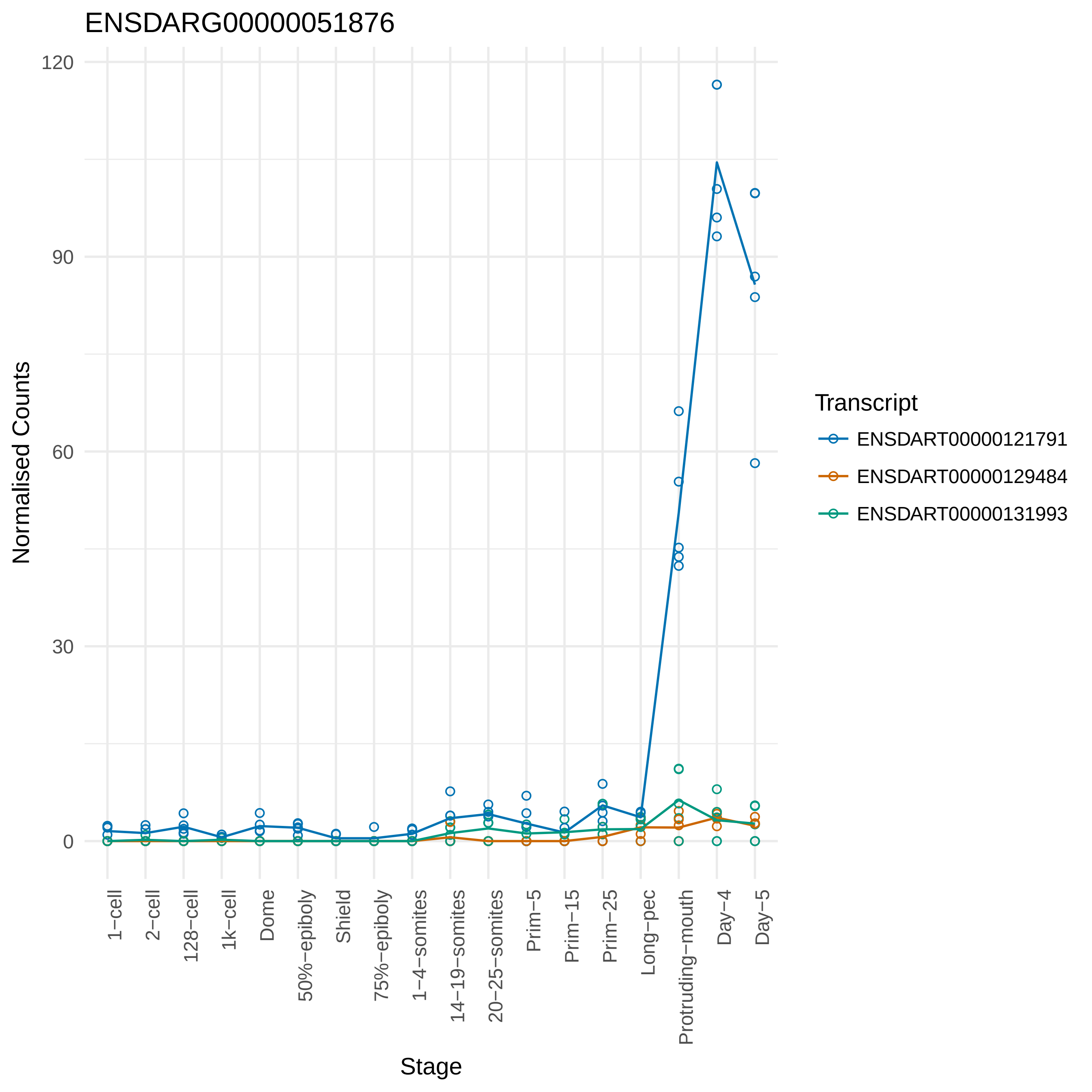

ENSDARG00000051876
Normalised Counts
120
90
60
30
0
1−cell
2−cell
128−cell
1k−cell
Dome
50%−epiboly
Shield
75%−epiboly
1−4−somites
14−19−somites
20−25−somites
Prim−5
Prim−15
Prim−25
Long−pec
Protruding−mouth
Day−4
Day−5
Stage
Transcript
ENSDART00000121791
ENSDART00000129484
ENSDART00000131993

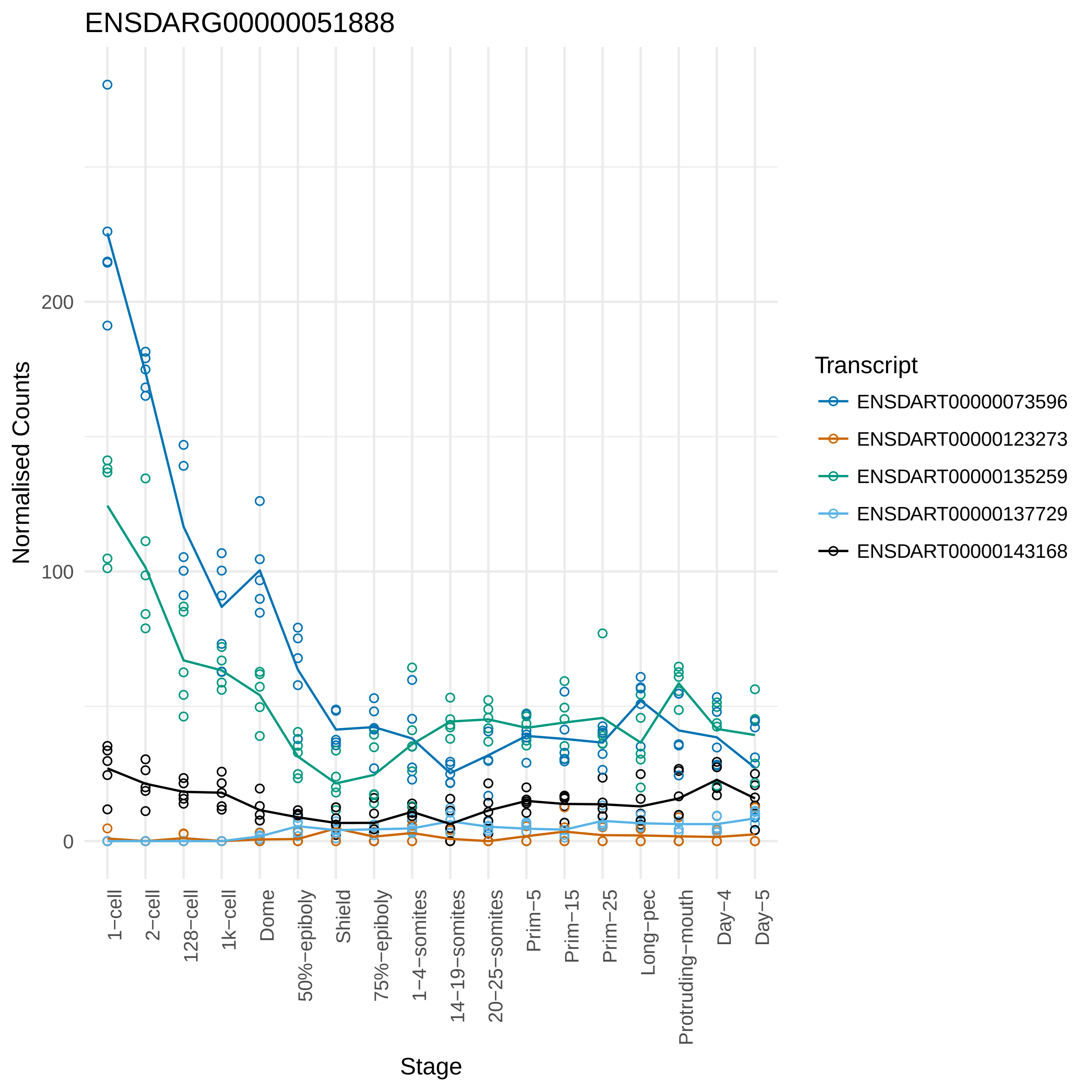

ENSDARG00000051888
Normalised Counts
200
100
0
1−cell
2−cell
128−cell
1k−cell
Dome
50%−epiboly
Shield
75%−epiboly
1−4−somites
14−19−somites
20−25−somites
Prim−5
Prim−15
Prim−25
Long−pec
Protruding−mouth
Day−4
Day−5
Stage
Transcript
ENSDART00000073596
ENSDART00000123273
ENSDART00000135259
ENSDART00000137729
ENSDART00000143168

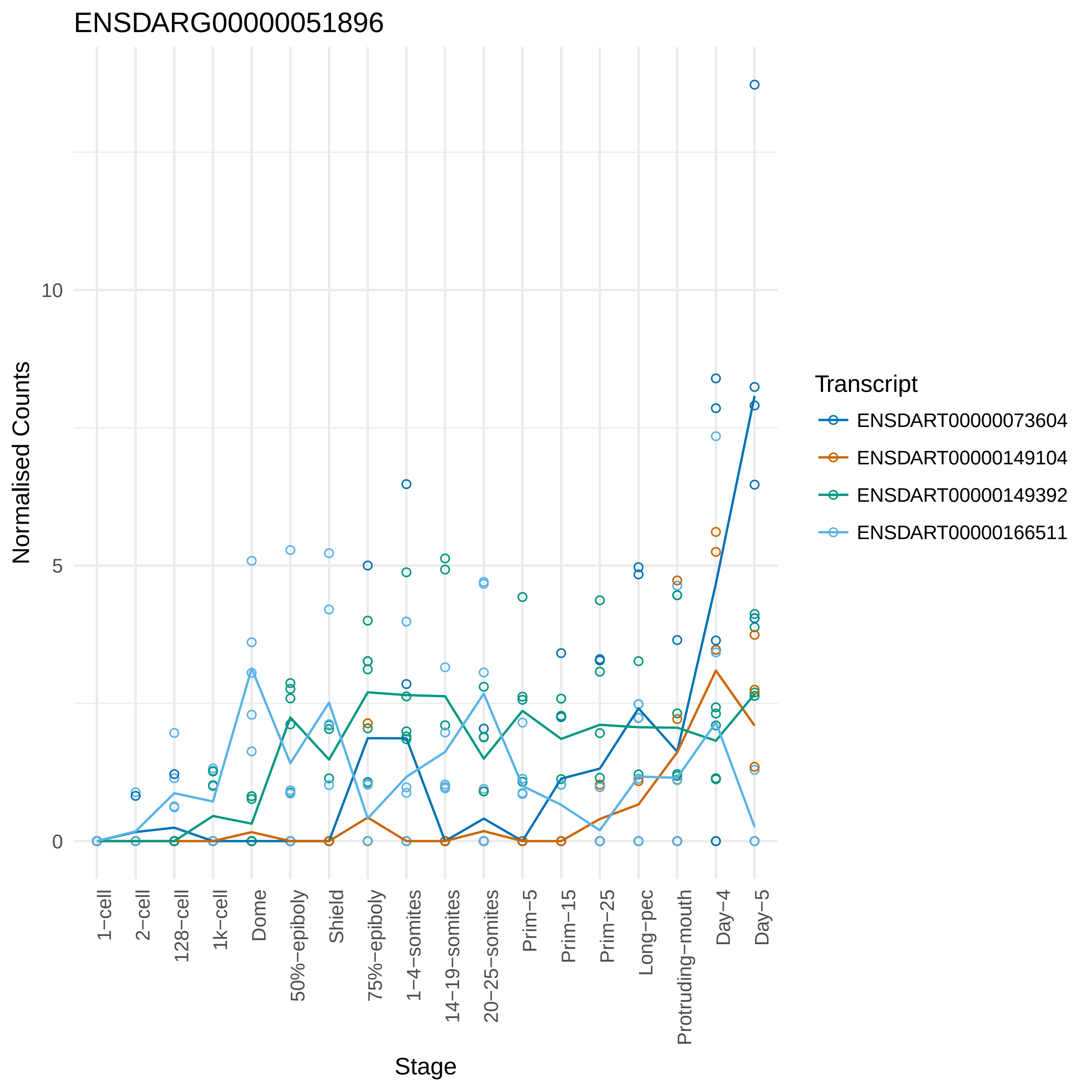
ENSDARG00000051896
Normalised Counts
0
5
10
1−cell
2−cell
128−cell
1k−cell
Dome
50%−epiboly
Shield
75%−epiboly
1−4−somites
14−19−somites
20−25−somites
Prim−5
Prim−15
Prim−25
Long−pec
Protruding−mouth
Day−4
Day−5
Stage
Transcript
ENSDART00000073604
ENSDART00000149104
ENSDART00000149392
ENSDART00000166511

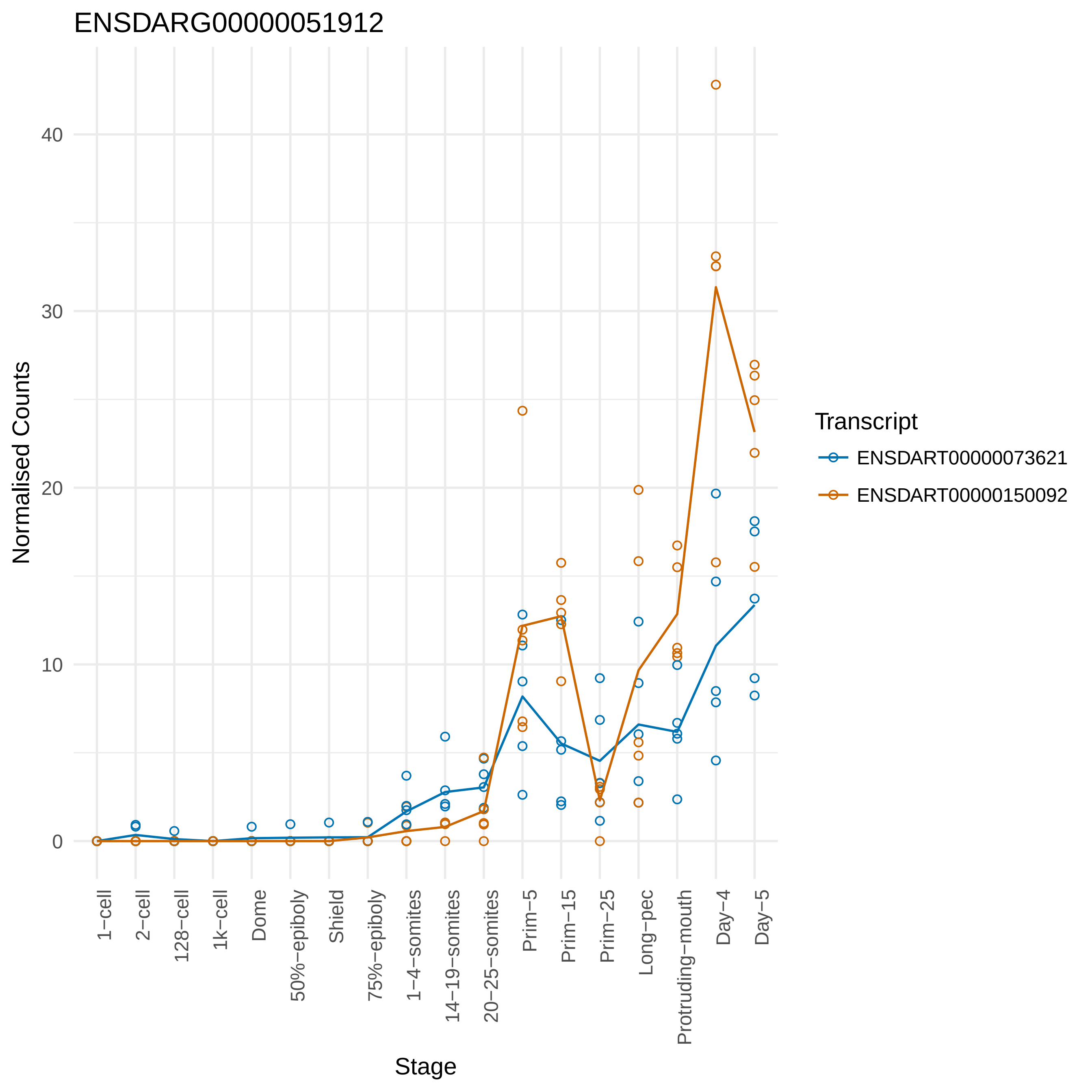

ENSDARG00000051912
Normalised Counts
0
10
20
30
40
1−cell
2−cell
128−cell
1k−cell
Dome
50%−epiboly
Shield
75%−epiboly
1−4−somites
14−19−somites
20−25−somites
Prim−5
Prim−15
Prim−25
Long−pec
Protruding−mouth
Day−4
Day−5
Stage
Transcript
ENSDART00000073621
ENSDART00000150092

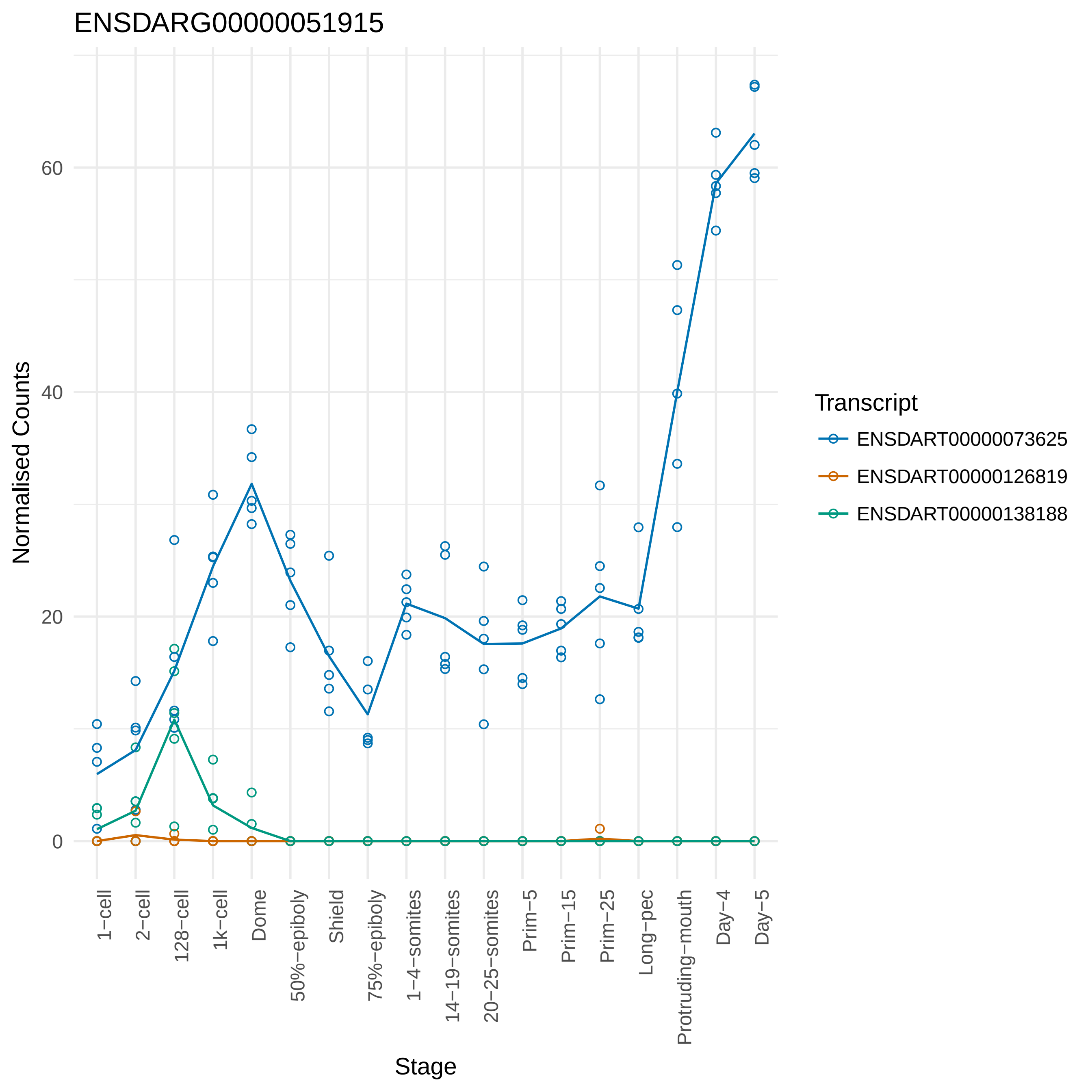

ENSDARG00000051915
Normalised Counts
0
20
40
60
Stage
1−cell
2−cell
128−cell
1k−cell
Dome
50%−epiboly
Shield
75%−epiboly
1−4−somites
14−19−somites
20−25−somites
Prim−5
Prim−15
Prim−25
Long−pec
Protruding−mouth
Day−4
Day−5
Transcript
ENSDART00000073625
ENSDART00000126819
ENSDART00000138188

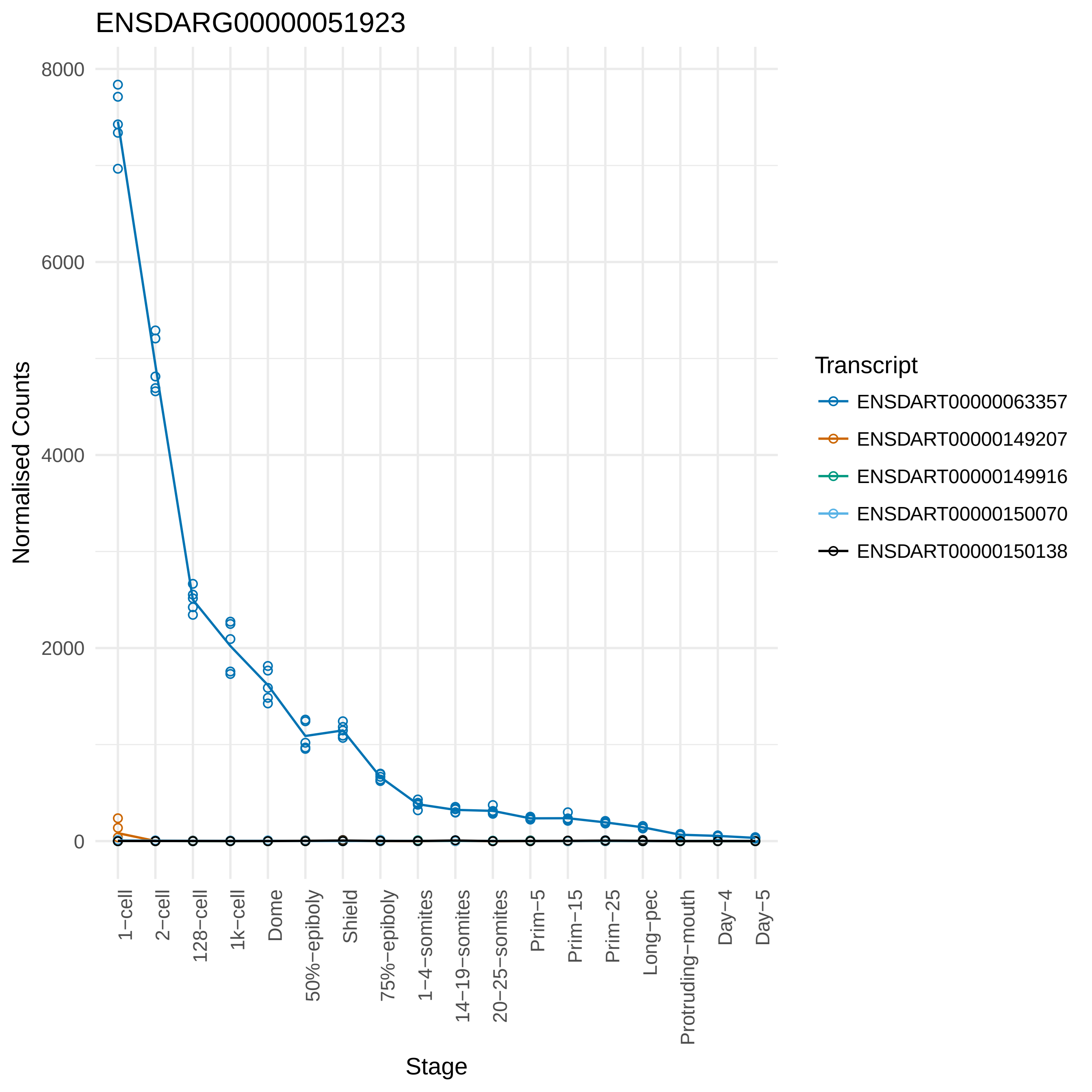

ENSDARG00000051923
Normalised Counts
8000
6000
4000
2000
0
1−cell
2−cell
128−cell
1k−cell
Dome
50%−epiboly
Shield
75%−epiboly
1−4−somites
14−19−somites
20−25−somites
Prim−5
Prim−15
Prim−25
Long−pec
Protruding−mouth
Day−4
Day−5
Stage
Transcript
ENSDART00000063357
ENSDART00000149207
ENSDART00000149916
ENSDART00000150070
ENSDART00000150138

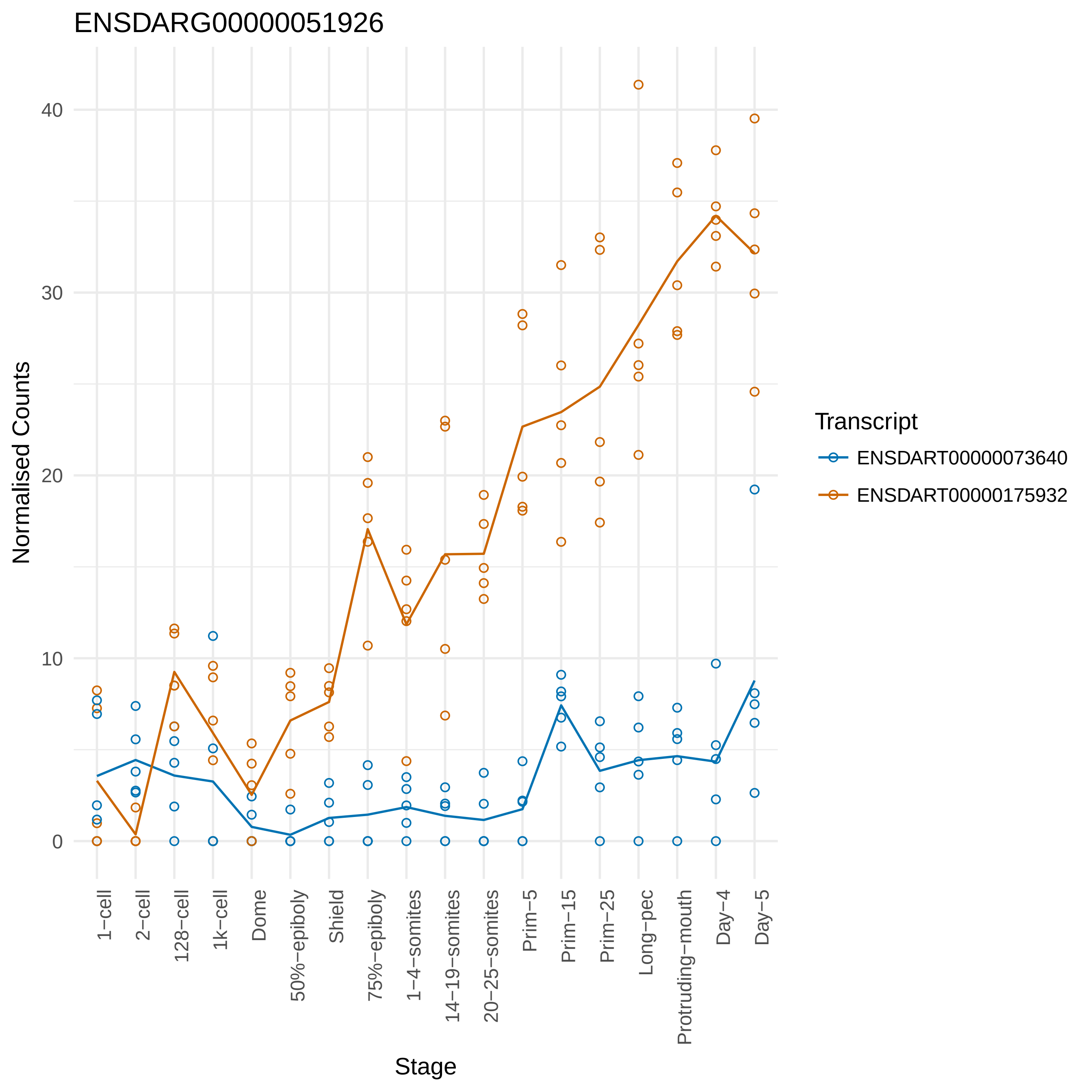

ENSDARG00000051926
Normalised Counts
40
30
20
10
0
1−cell
2−cell
128−cell
1k−cell
Dome
50%−epiboly
Shield
75%−epiboly
1−4−somites
14−19−somites
20−25−somites
Prim−5
Prim−15
Prim−25
Long−pec
Protruding−mouth
Day−4
Day−5
Stage
Transcript
ENSDART00000073640
ENSDART00000175932

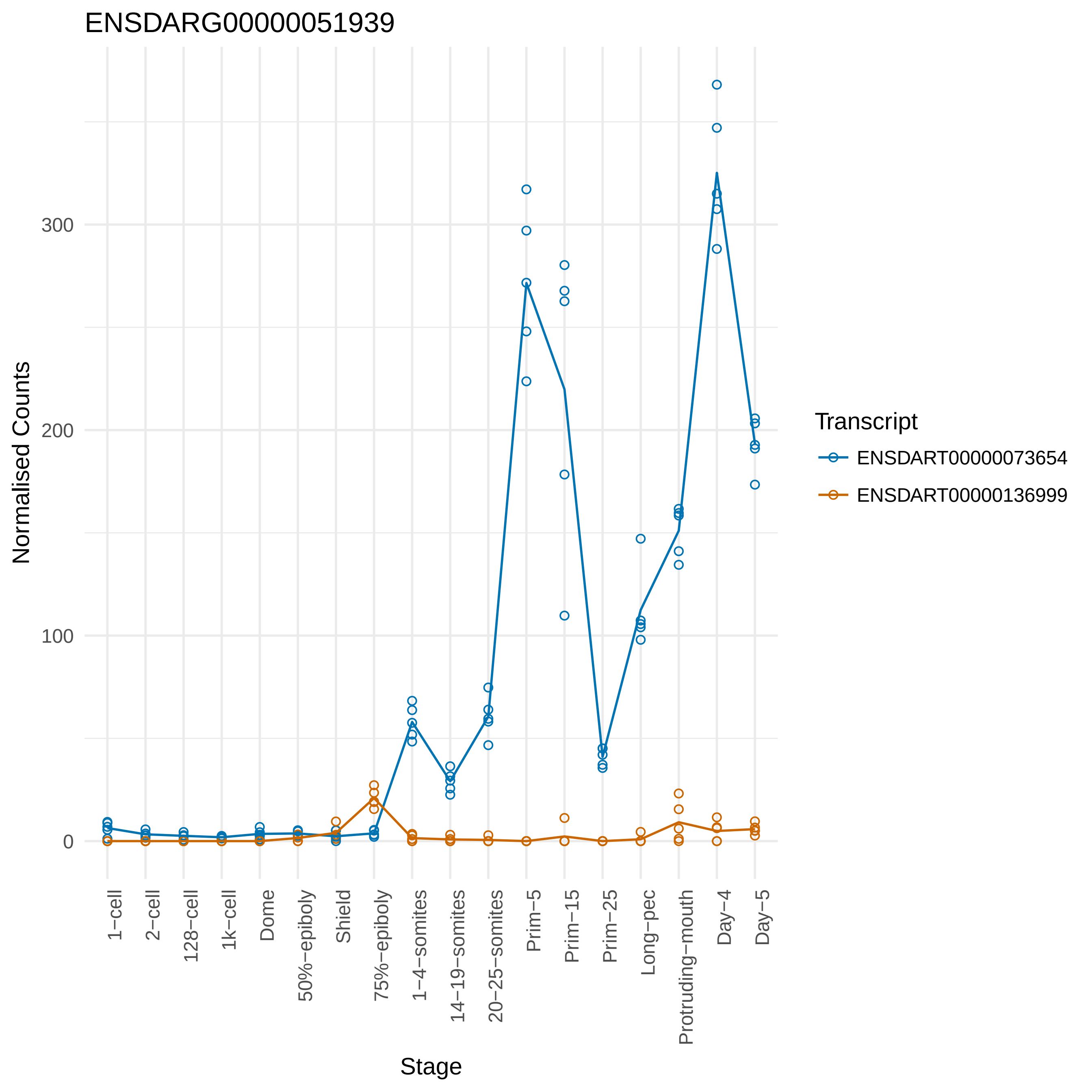

ENSDARG00000051939
Normalised Counts
0
100
200
300
Stage
1−cell
2−cell
128−cell
1k−cell
Dome
50%−epiboly
Shield
75%−epiboly
1−4−somites
14−19−somites
20−25−somites
Prim−5
Prim−15
Prim−25
Long−pec
Protruding−mouth
Day−4
Day−5
Transcript
ENSDART00000073654
ENSDART00000136999

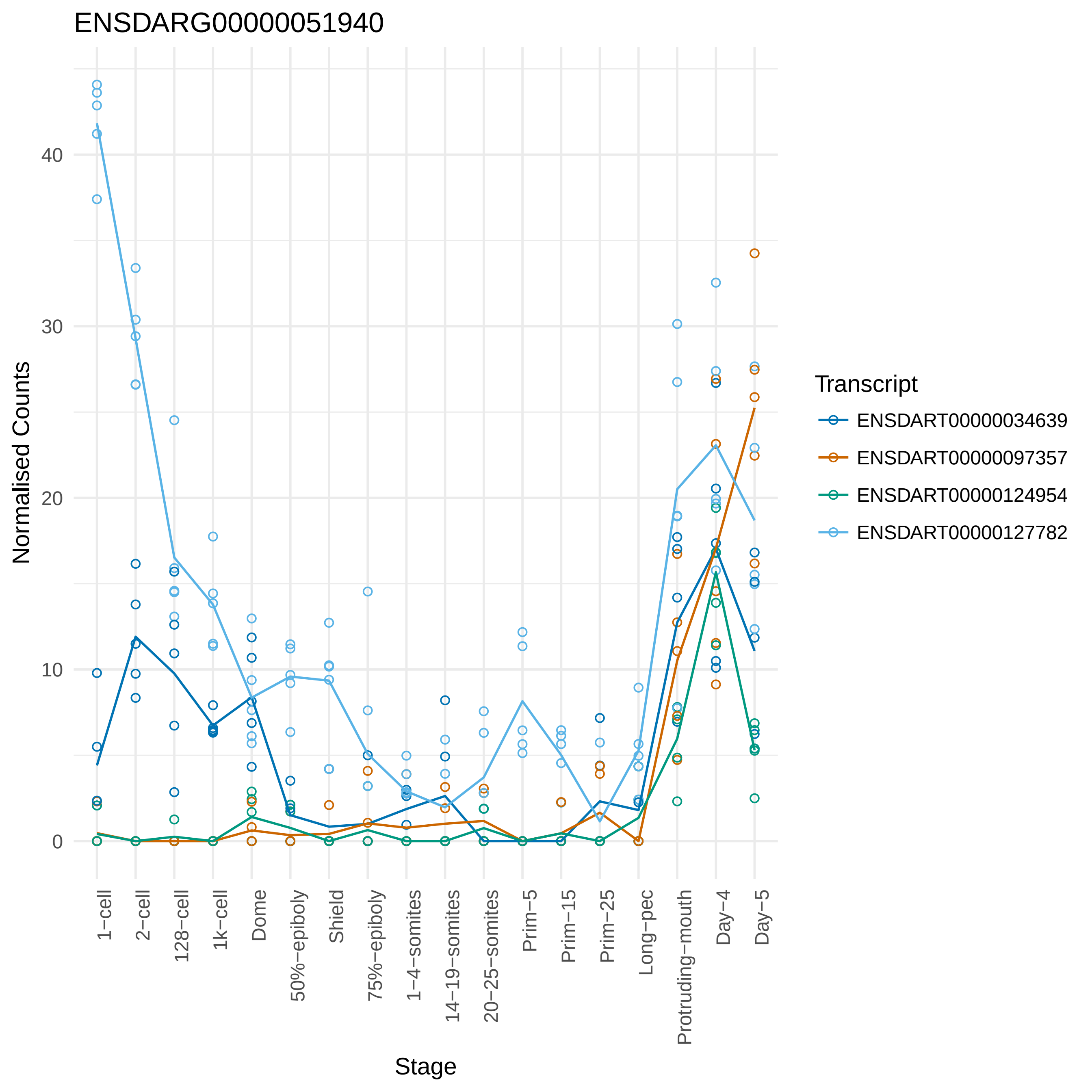

ENSDARG00000051940
Normalised Counts
0
10
20
30
40
1−cell
2−cell
128−cell
1k−cell
Dome
50%−epiboly
Shield
75%−epiboly
1−4−somites
14−19−somites
20−25−somites
Prim−5
Prim−15
Prim−25
Long−pec
Protruding−mouth
Day−4
Day−5
Stage
Transcript
ENSDART00000034639
ENSDART00000097357
ENSDART00000124954
ENSDART00000127782

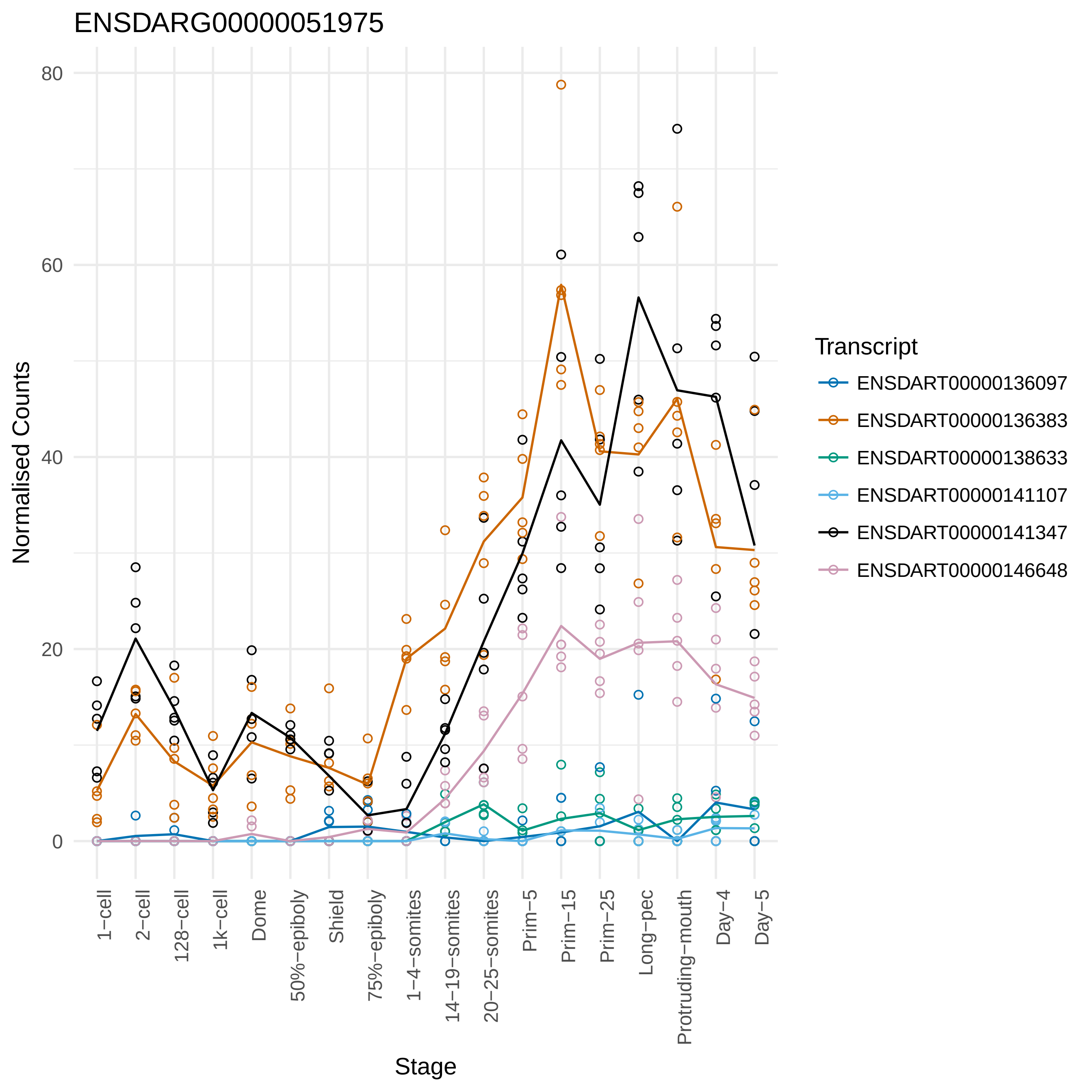

ENSDARG00000051975
Normalised Counts
80
60
40
20
0
1−cell
2−cell
128−cell
1k−cell
Dome
50%−epiboly
Shield
75%−epiboly
1−4−somites
14−19−somites
20−25−somites
Prim−5
Prim−15
Prim−25
Long−pec
Protruding−mouth
Day−4
Day−5
Stage
Transcript
ENSDART00000136097
ENSDART00000136383
ENSDART00000138633
ENSDART00000141107
ENSDART00000141347
ENSDART00000146648

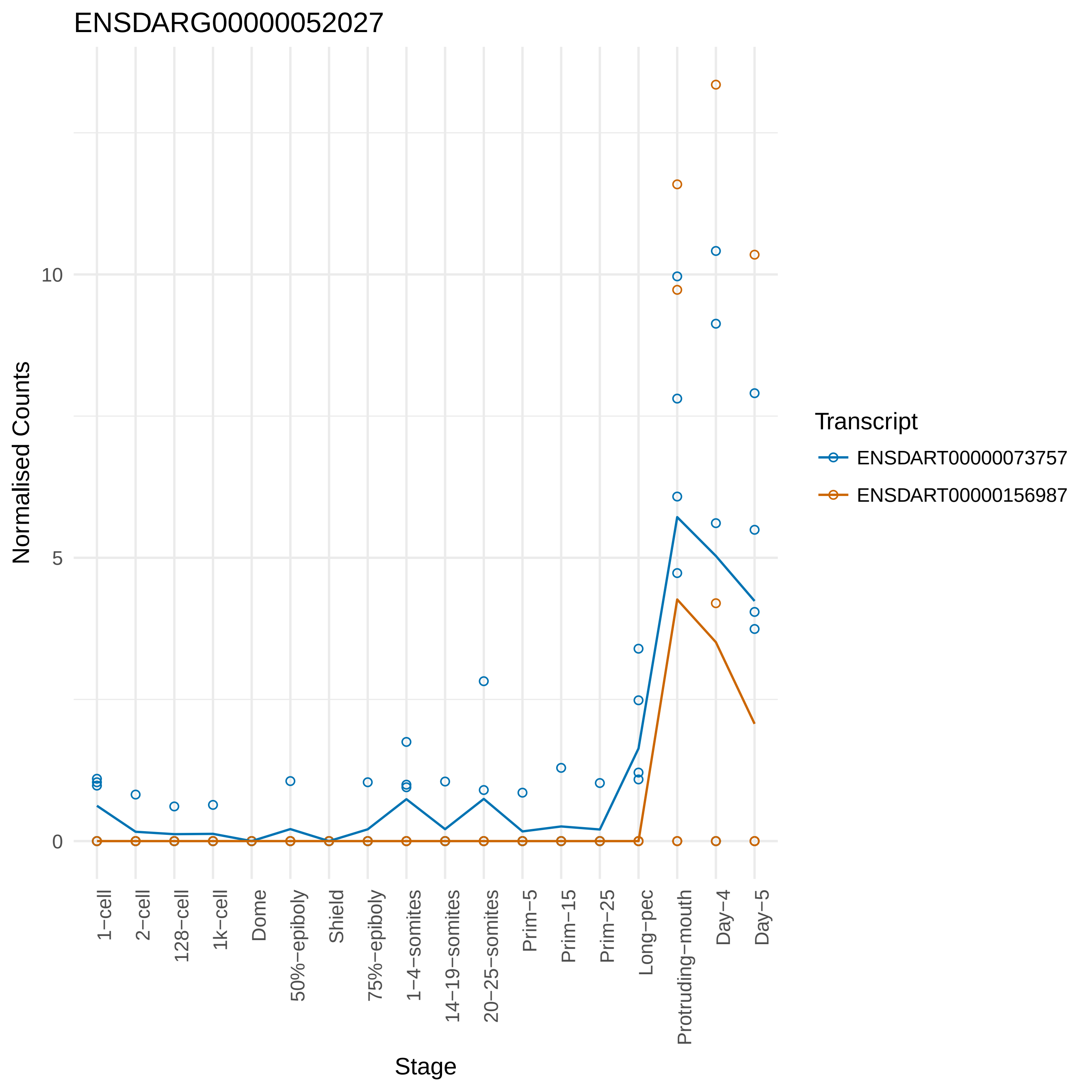
ENSDARG00000052027
Normalised Counts
0
5
10
Stage
1−cell
2−cell
128−cell
1k−cell
Dome
50%−epiboly
Shield
75%−epiboly
1−4−somites
14−19−somites
20−25−somites
Prim−5
Prim−15
Prim−25
Long−pec
Protruding−mouth
Day−4
Day−5
Transcript
ENSDART00000073757
ENSDART00000156987

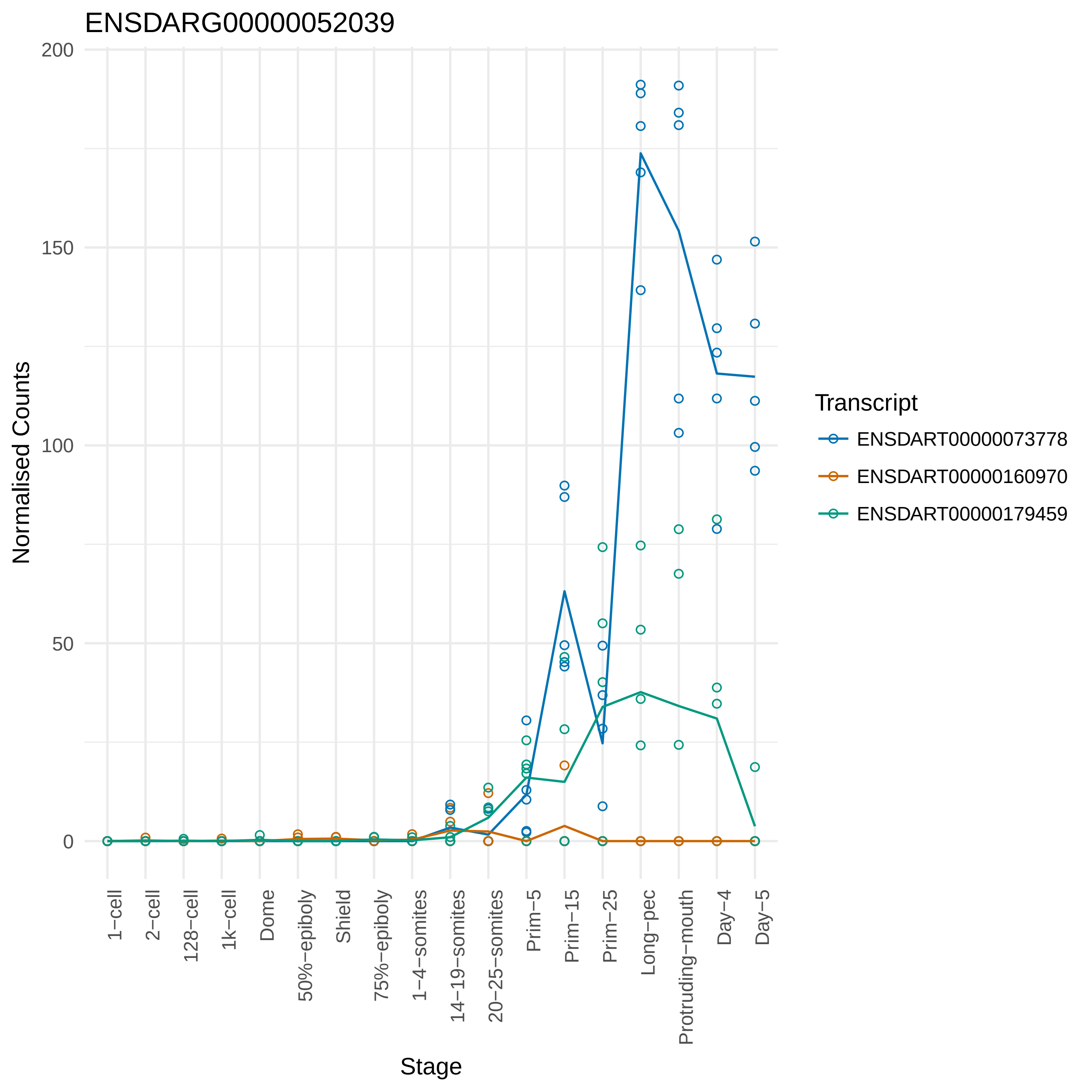

ENSDARG00000052039
Normalised Counts
200
150
100
50
0
1−cell
2−cell
128−cell
1k−cell
Dome
50%−epiboly
Shield
75%−epiboly
1−4−somites
14−19−somites
20−25−somites
Prim−5
Prim−15
Prim−25
Long−pec
Protruding−mouth
Day−4
Day−5
Stage
Transcript
ENSDART00000073778
ENSDART00000160970
ENSDART00000179459

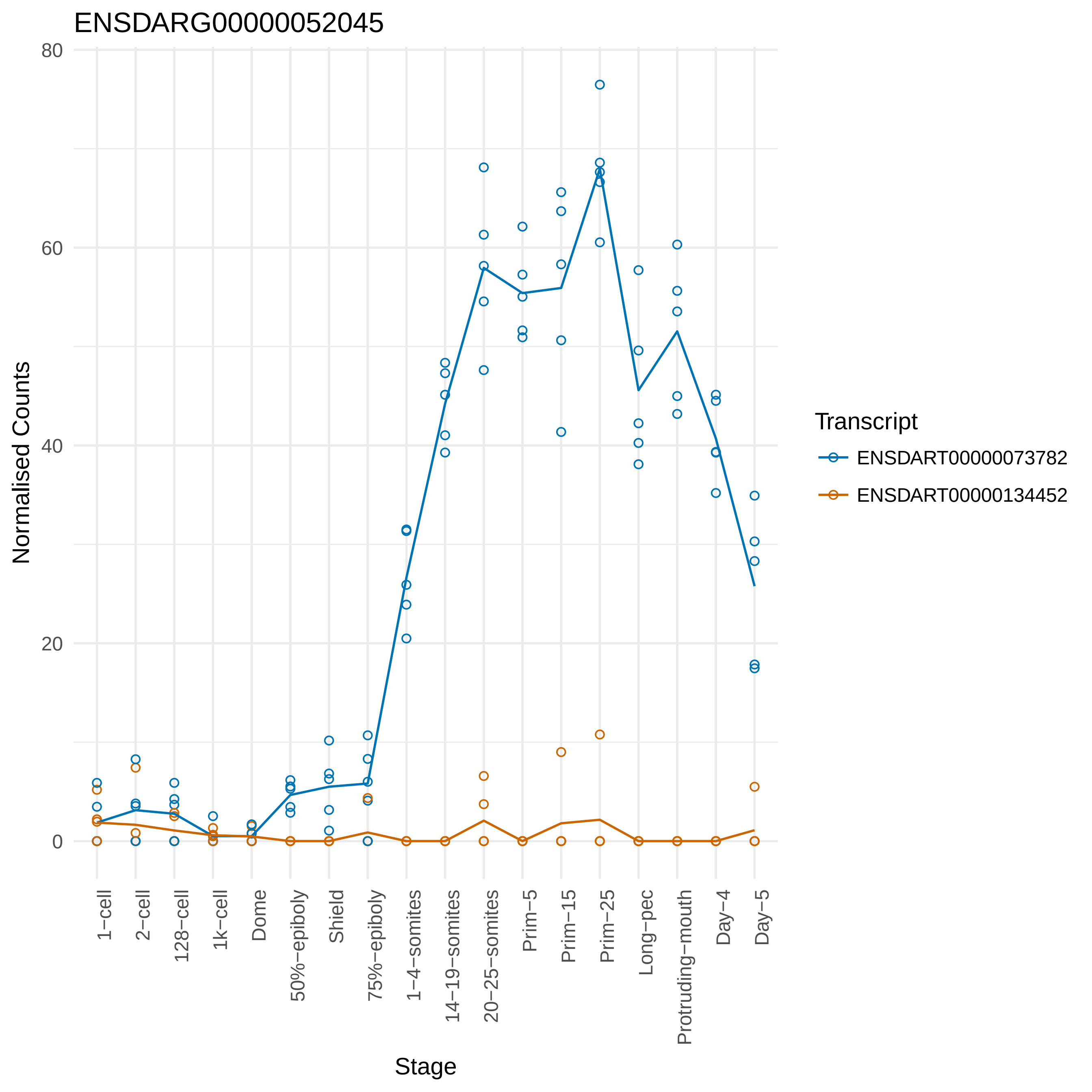
ENSDARG00000052045
Normalised Counts
80
60
40
20
0
1−cell
2−cell
128−cell
1k−cell
Dome
50%−epiboly
Shield
75%−epiboly
1−4−somites
14−19−somites
20−25−somites
Prim−5
Prim−15
Prim−25
Long−pec
Protruding−mouth
Day−4
Day−5
Stage
Transcript
ENSDART00000073782
ENSDART00000134452

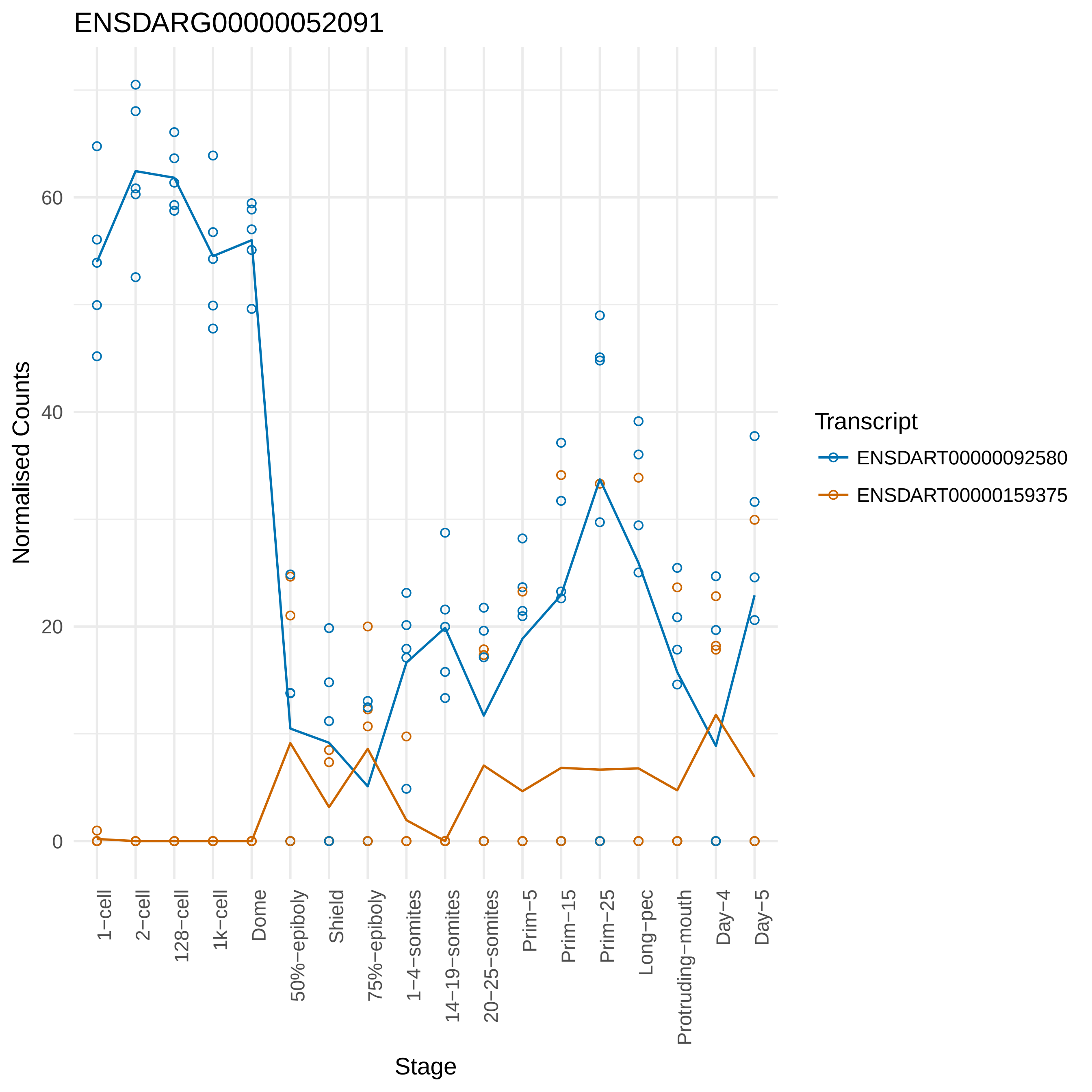

ENSDARG00000052091
Normalised Counts
0
20
40
60
1−cell
2−cell
128−cell
1k−cell
Dome
50%−epiboly
Shield
75%−epiboly
1−4−somites
14−19−somites
20−25−somites
Prim−5
Prim−15
Prim−25
Long−pec
Protruding−mouth
Day−4
Day−5
Stage
Transcript
ENSDART00000092580
ENSDART00000159375

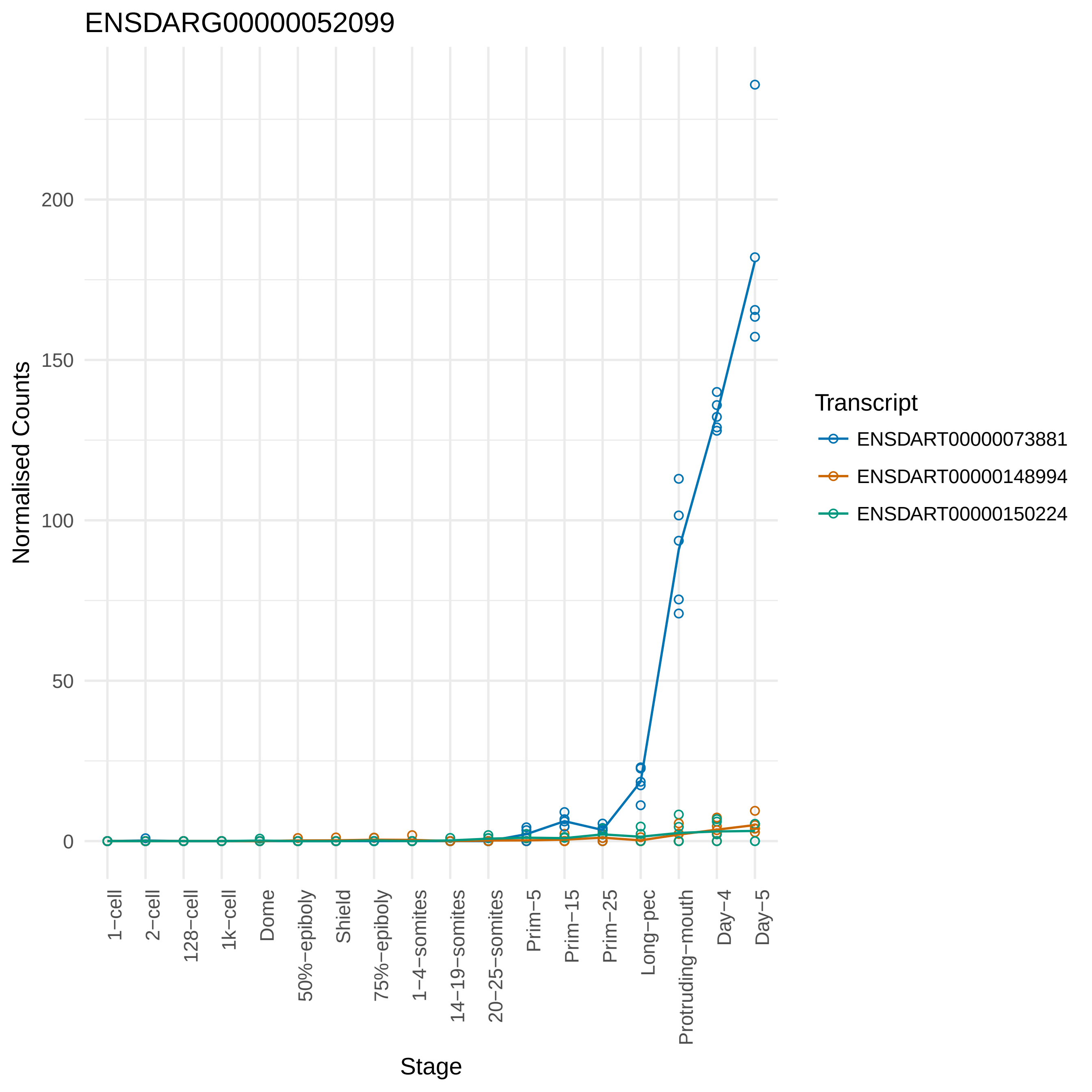
ENSDARG00000052099
Normalised Counts
0
50
100
150
200
Stage
1−cell
2−cell
128−cell
1k−cell
Dome
50%−epiboly
Shield
75%−epiboly
1−4−somites
14−19−somites
20−25−somites
Prim−5
Prim−15
Prim−25
Long−pec
Protruding−mouth
Day−4
Day−5
Transcript
ENSDART00000073881
ENSDART00000148994
ENSDART00000150224

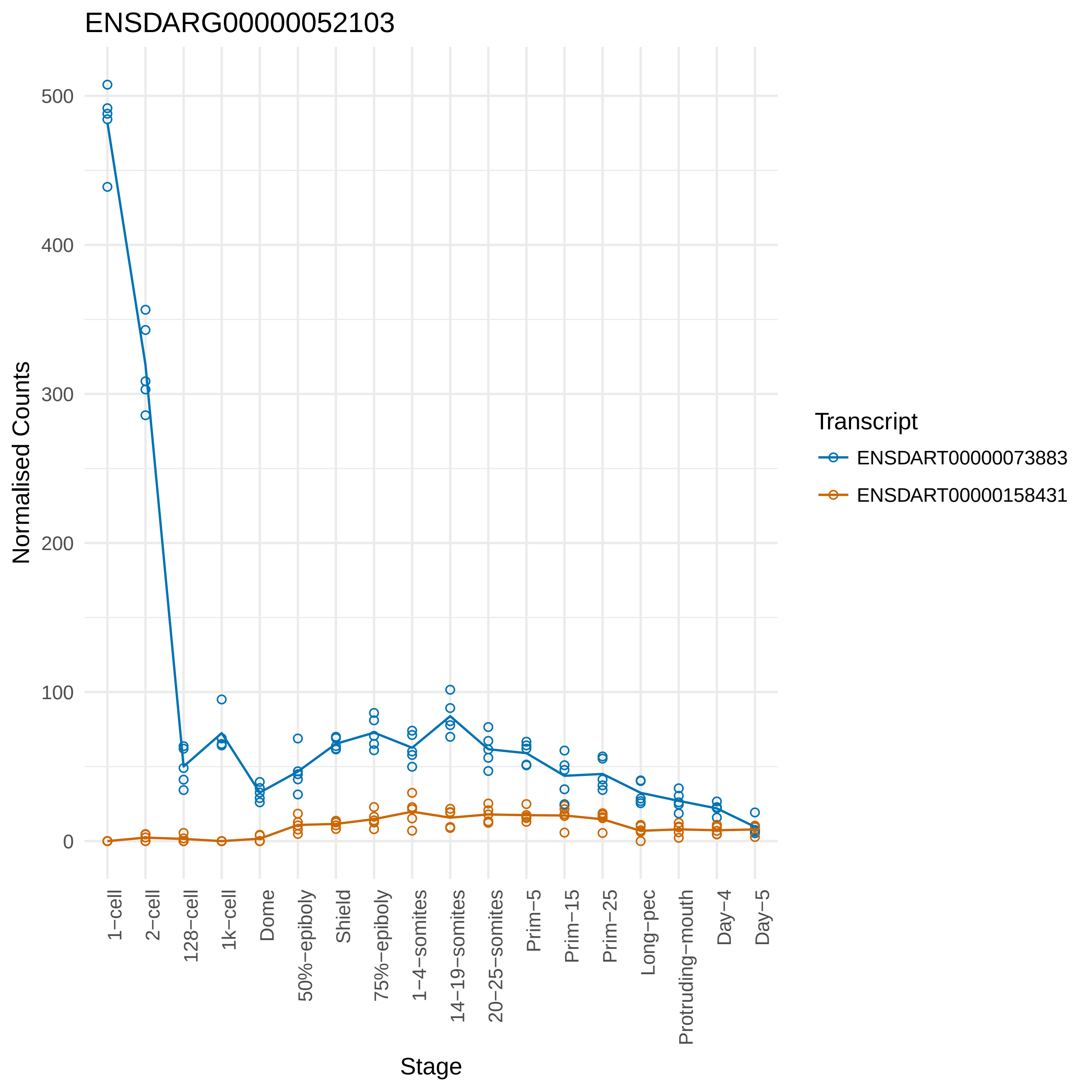

ENSDARG00000052103
Normalised Counts
0
100
200
300
400
500
Stage
1−cell
2−cell
128−cell
1k−cell
Dome
50%−epiboly
Shield
75%−epiboly
1−4−somites
14−19−somites
20−25−somites
Prim−5
Prim−15
Prim−25
Long−pec
Protruding−mouth
Day−4
Day−5
Transcript
ENSDART00000073883
ENSDART00000158431

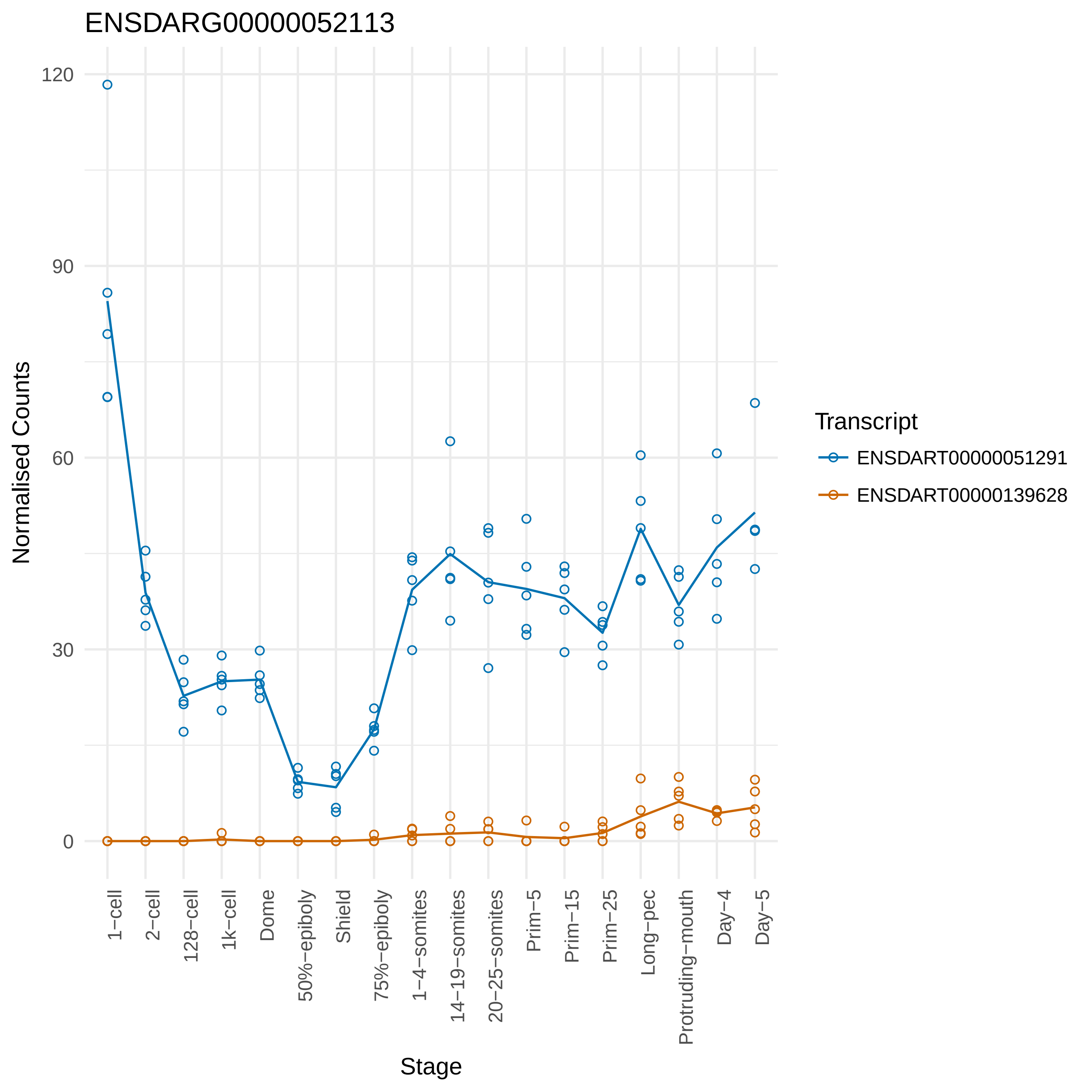

ENSDARG00000052113
Normalised Counts
120
90
60
30
0
1−cell
2−cell
128−cell
1k−cell
Dome
50%−epiboly
Shield
75%−epiboly
1−4−somites
14−19−somites
20−25−somites
Prim−5
Prim−15
Prim−25
Long−pec
Protruding−mouth
Day−4
Day−5
Stage
Transcript
ENSDART00000051291
ENSDART00000139628

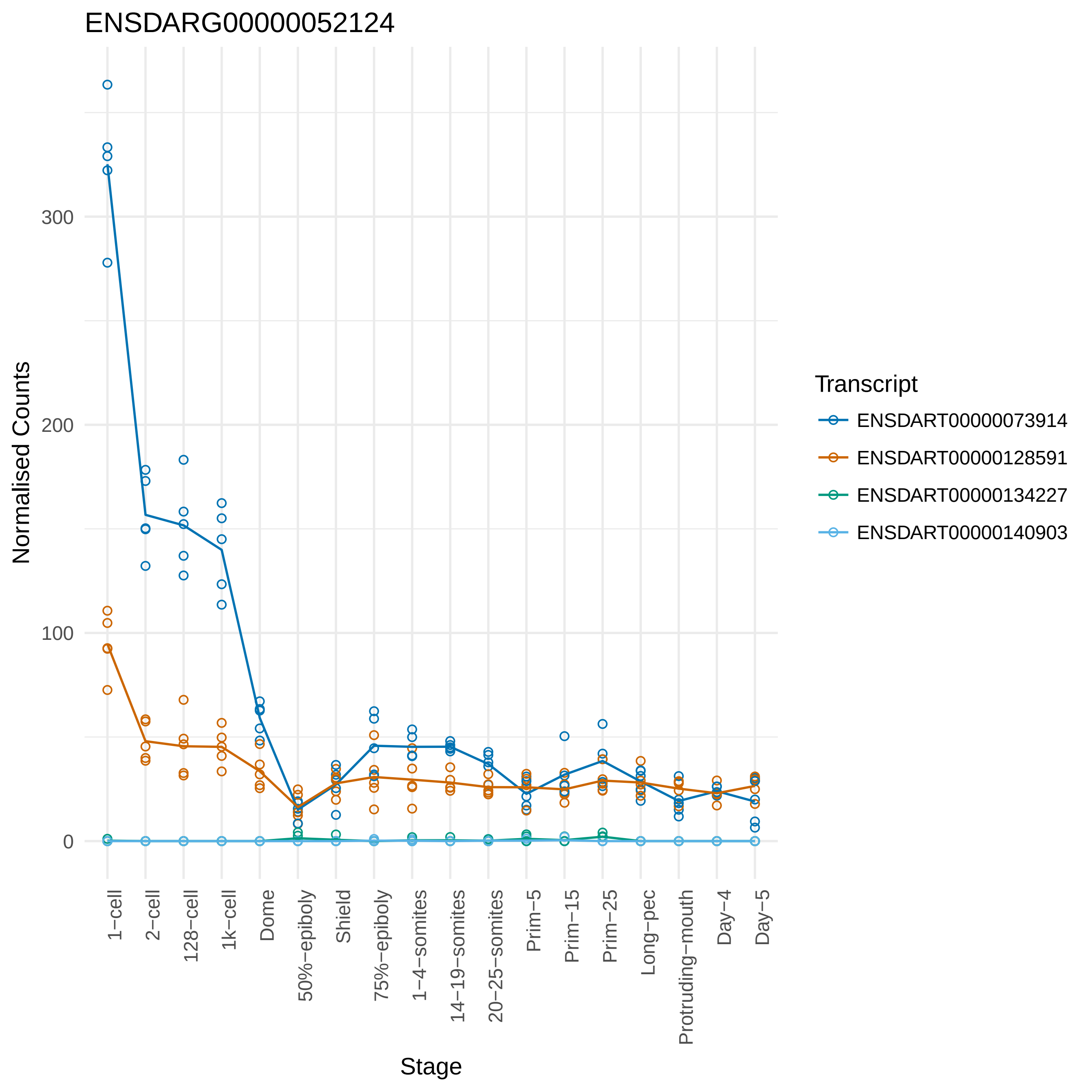

ENSDARG00000052124
Normalised Counts
300
200
100
0
1−cell
2−cell
128−cell
1k−cell
Dome
50%−epiboly
Shield
75%−epiboly
1−4−somites
14−19−somites
20−25−somites
Prim−5
Prim−15
Prim−25
Long−pec
Protruding−mouth
Day−4
Day−5
Stage
Transcript
ENSDART00000073914
ENSDART00000128591
ENSDART00000134227
ENSDART00000140903

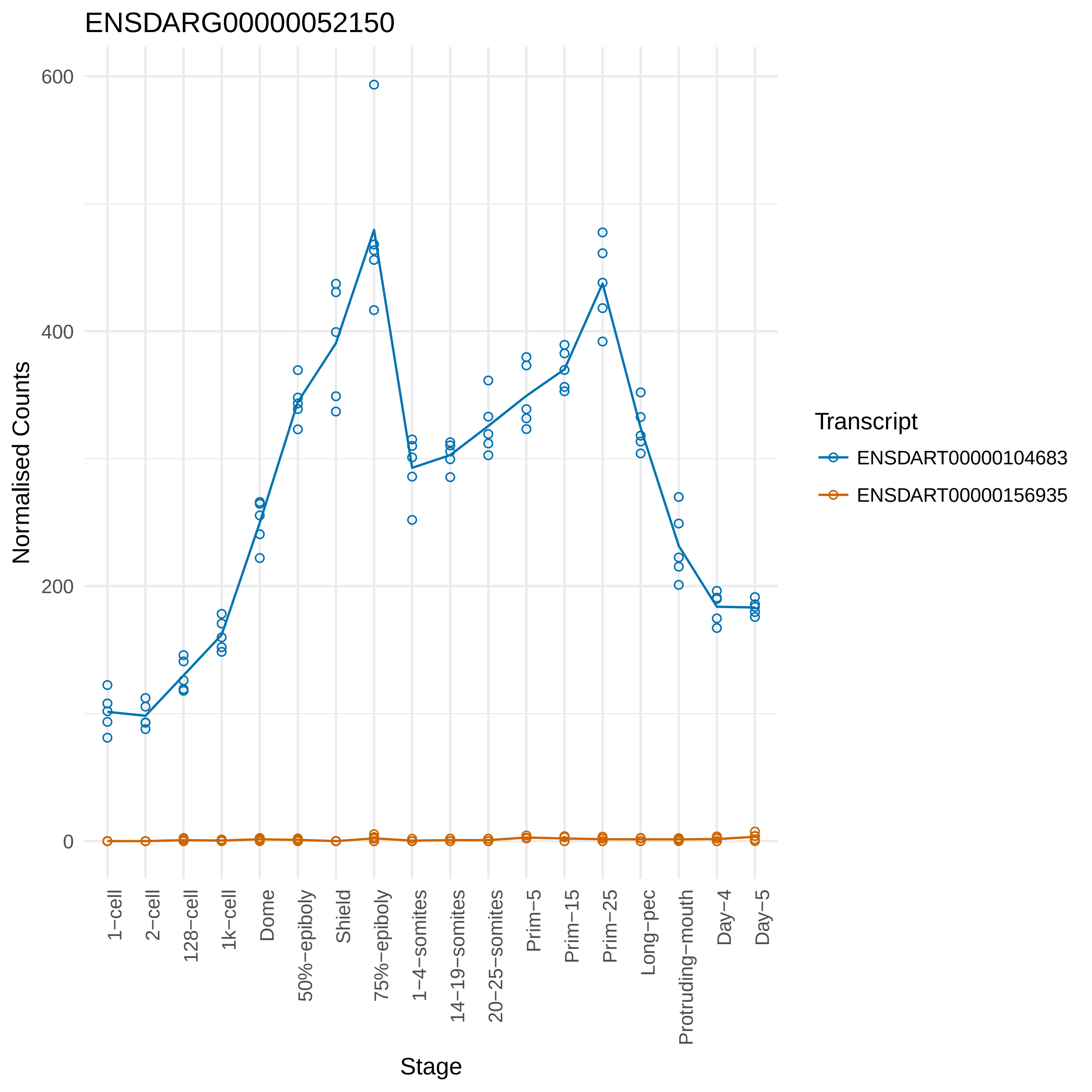

ENSDARG00000052150
Normalised Counts
600
400
200
0
1−cell
2−cell
128−cell
1k−cell
Dome
50%−epiboly
Shield
75%−epiboly
1−4−somites
14−19−somites
20−25−somites
Prim−5
Prim−15
Prim−25
Long−pec
Protruding−mouth
Day−4
Day−5
Stage
Transcript
ENSDART00000104683
ENSDART00000156935

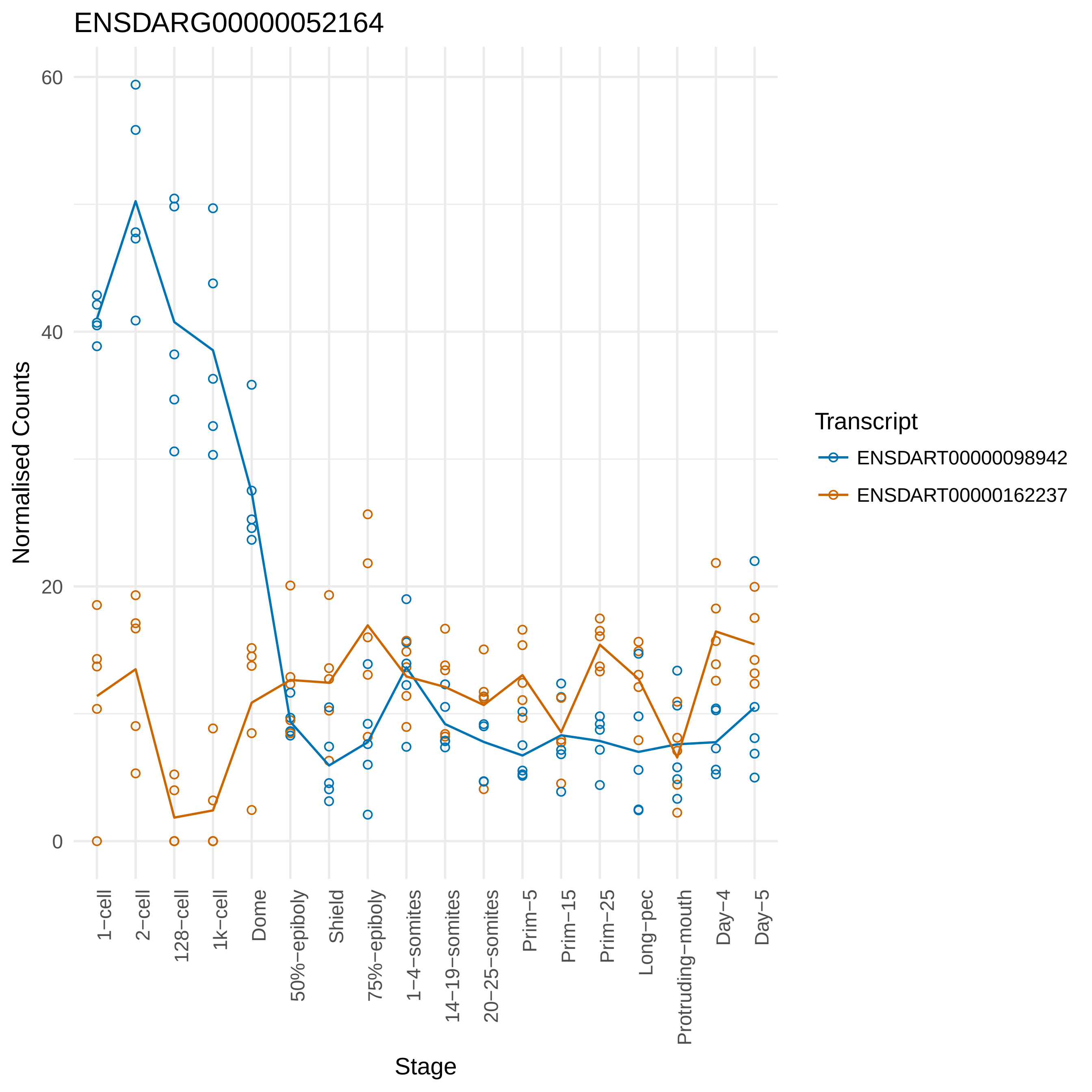
ENSDARG00000052164
Normalised Counts
60
40
20
0
Stage
1−cell
2−cell
128−cell
1k−cell
Dome
50%−epiboly
Shield
75%−epiboly
1−4−somites
14−19−somites
20−25−somites
Prim−5
Prim−15
Prim−25
Long−pec
Protruding−mouth
Day−4
Day−5
Transcript
ENSDART00000098942
ENSDART00000162237

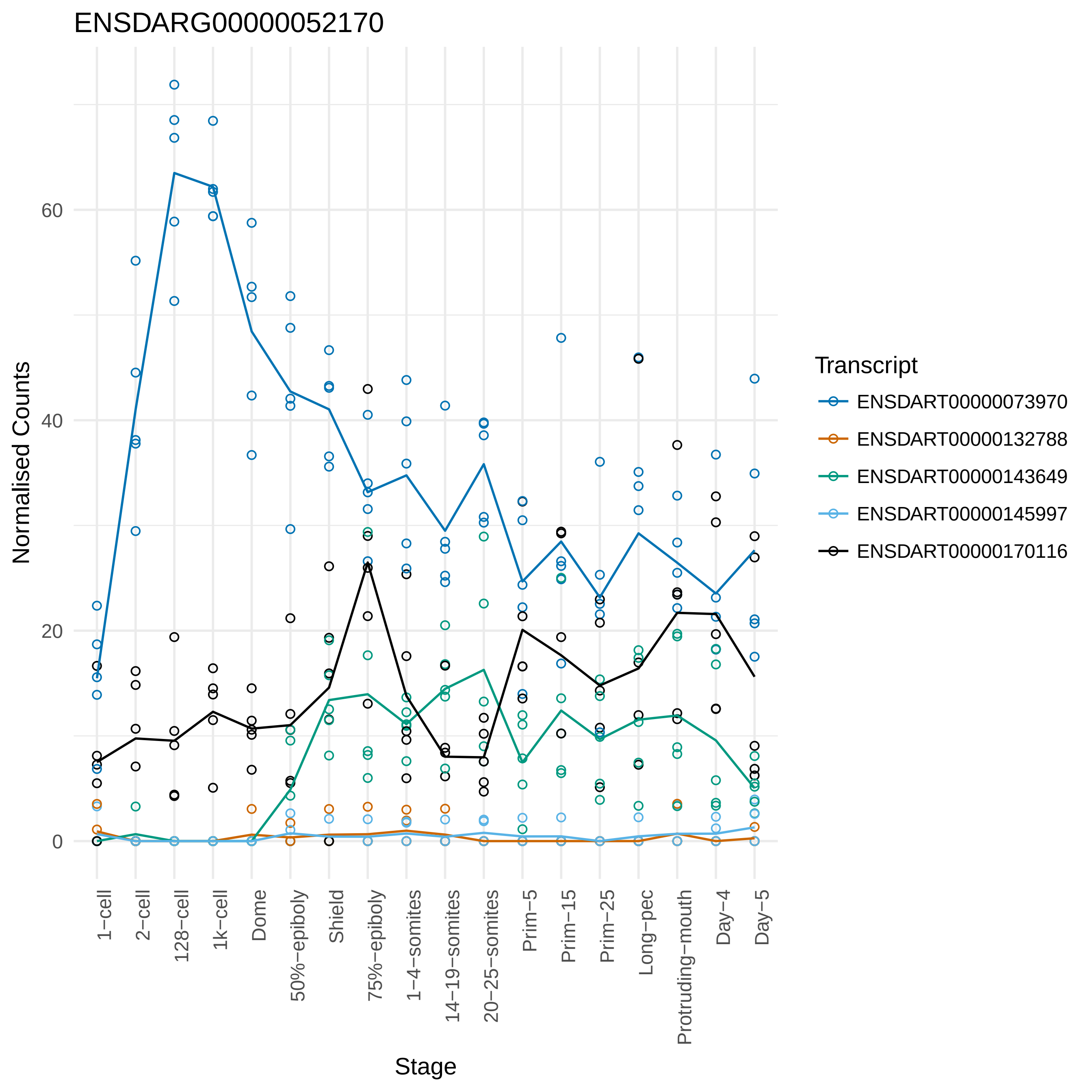

ENSDARG00000052170
Normalised Counts
60
40
20
0
1−cell
2−cell
128−cell
1k−cell
Dome
50%−epiboly
Shield
75%−epiboly
1−4−somites
14−19−somites
20−25−somites
Prim−5
Prim−15
Prim−25
Long−pec
Protruding−mouth
Day−4
Day−5
Stage
Transcript
ENSDART00000073970
ENSDART00000132788
ENSDART00000143649
ENSDART00000145997
ENSDART00000170116

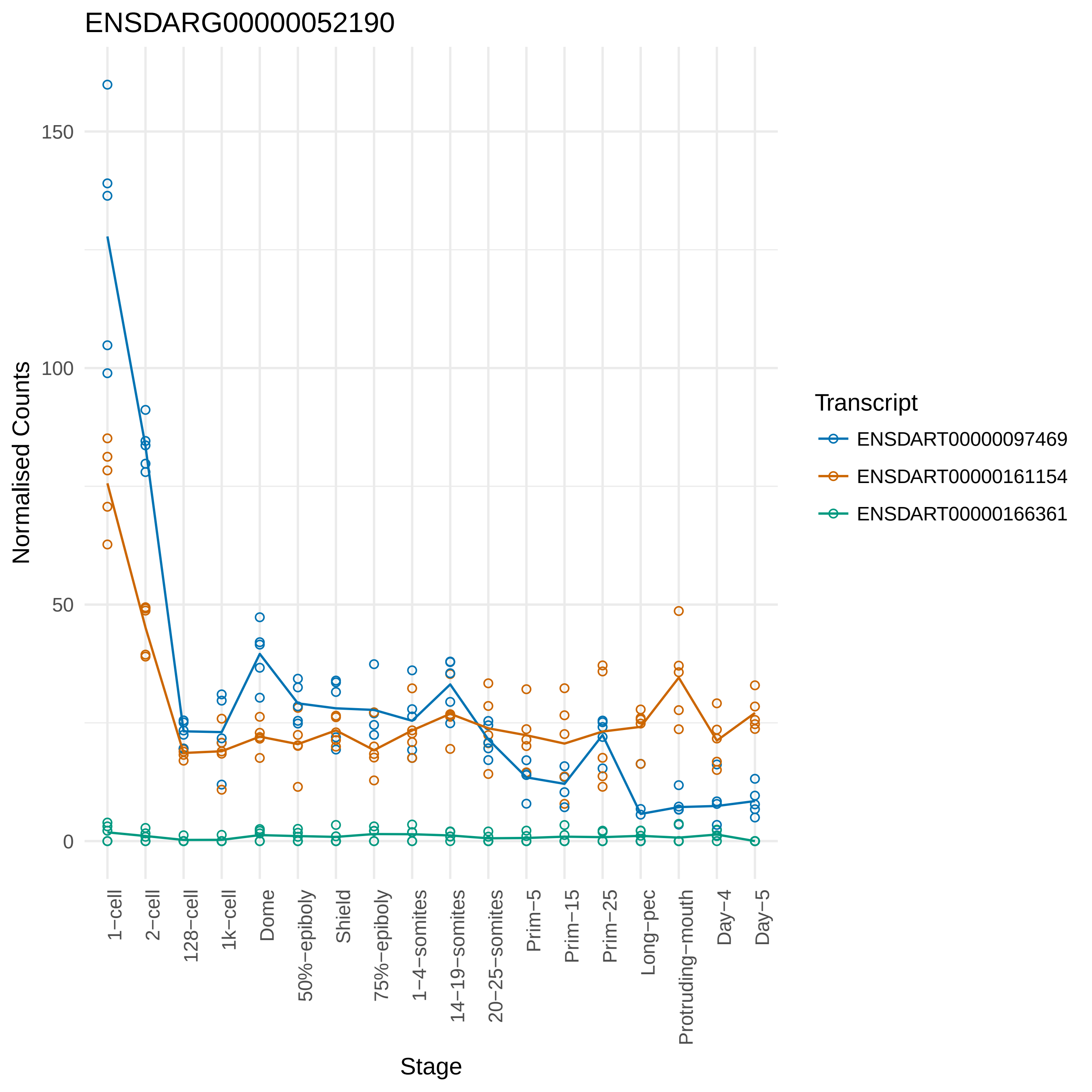

ENSDARG00000052190
Normalised Counts
150
100
50
0
1−cell
2−cell
128−cell
1k−cell
Dome
50%−epiboly
Shield
75%−epiboly
1−4−somites
14−19−somites
20−25−somites
Prim−5
Prim−15
Prim−25
Long−pec
Protruding−mouth
Day−4
Day−5
Stage
Transcript
ENSDART00000097469
ENSDART00000161154
ENSDART00000166361

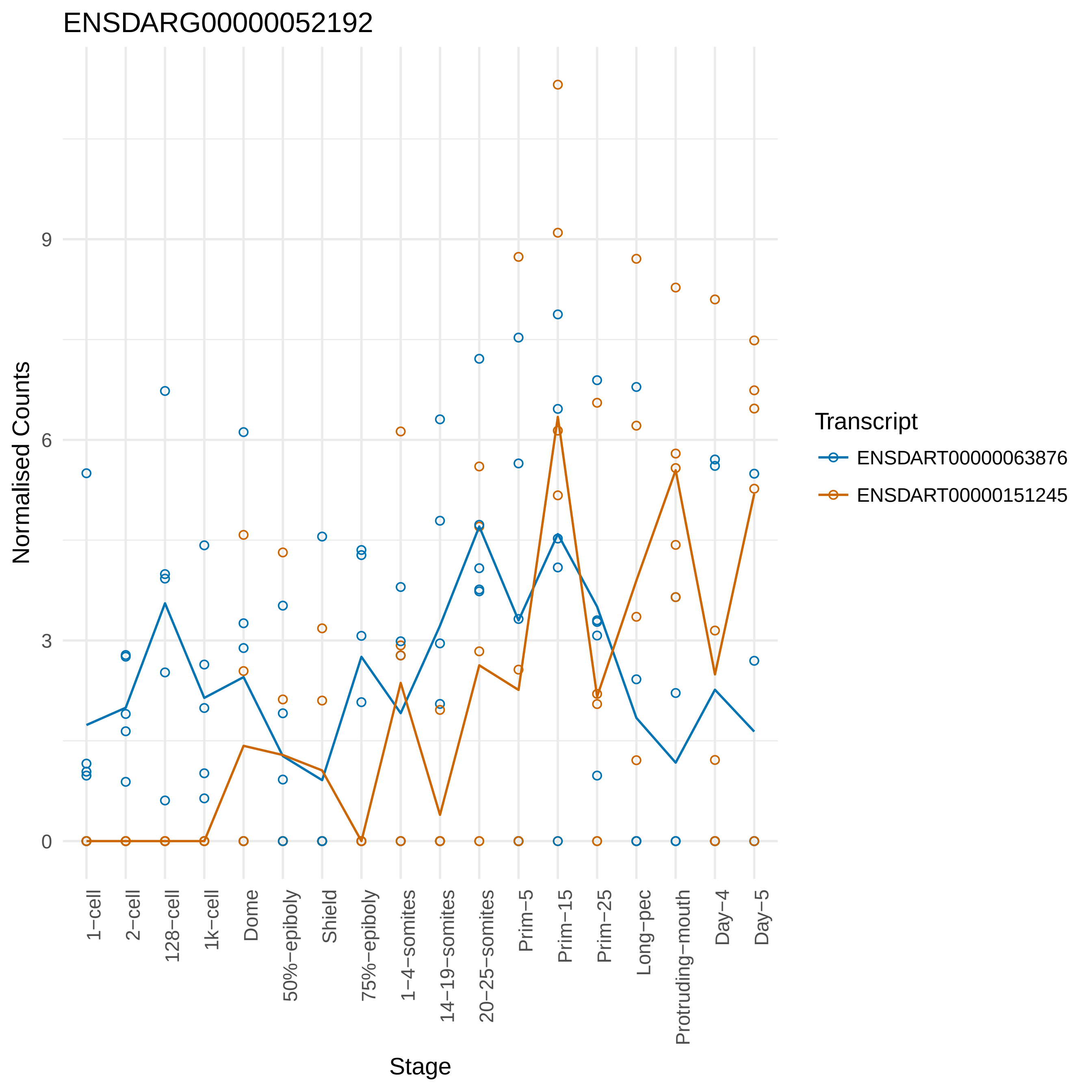
ENSDARG00000052192
Normalised Counts
0
3
6
9
Stage
1−cell
2−cell
128−cell
1k−cell
Dome
50%−epiboly
Shield
75%−epiboly
1−4−somites
14−19−somites
20−25−somites
Prim−5
Prim−15
Prim−25
Long−pec
Protruding−mouth
Day−4
Day−5
Transcript
ENSDART00000063876
ENSDART00000151245

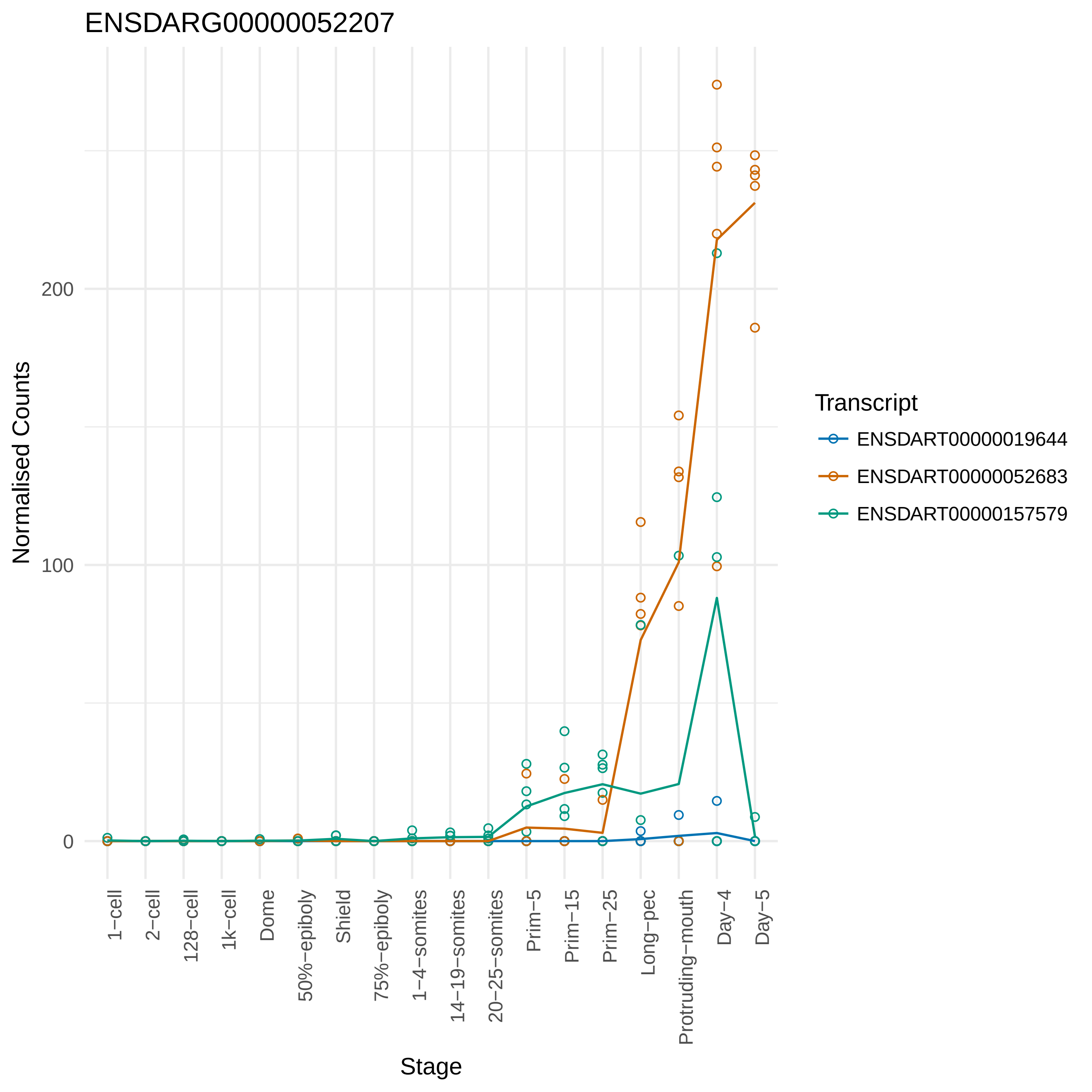
ENSDARG00000052207
Normalised Counts
0
100
200
Stage
1−cell
2−cell
128−cell
1k−cell
Dome
50%−epiboly
Shield
75%−epiboly
1−4−somites
14−19−somites
20−25−somites
Prim−5
Prim−15
Prim−25
Long−pec
Protruding−mouth
Day−4
Day−5
Transcript
ENSDART00000019644
ENSDART00000052683
ENSDART00000157579

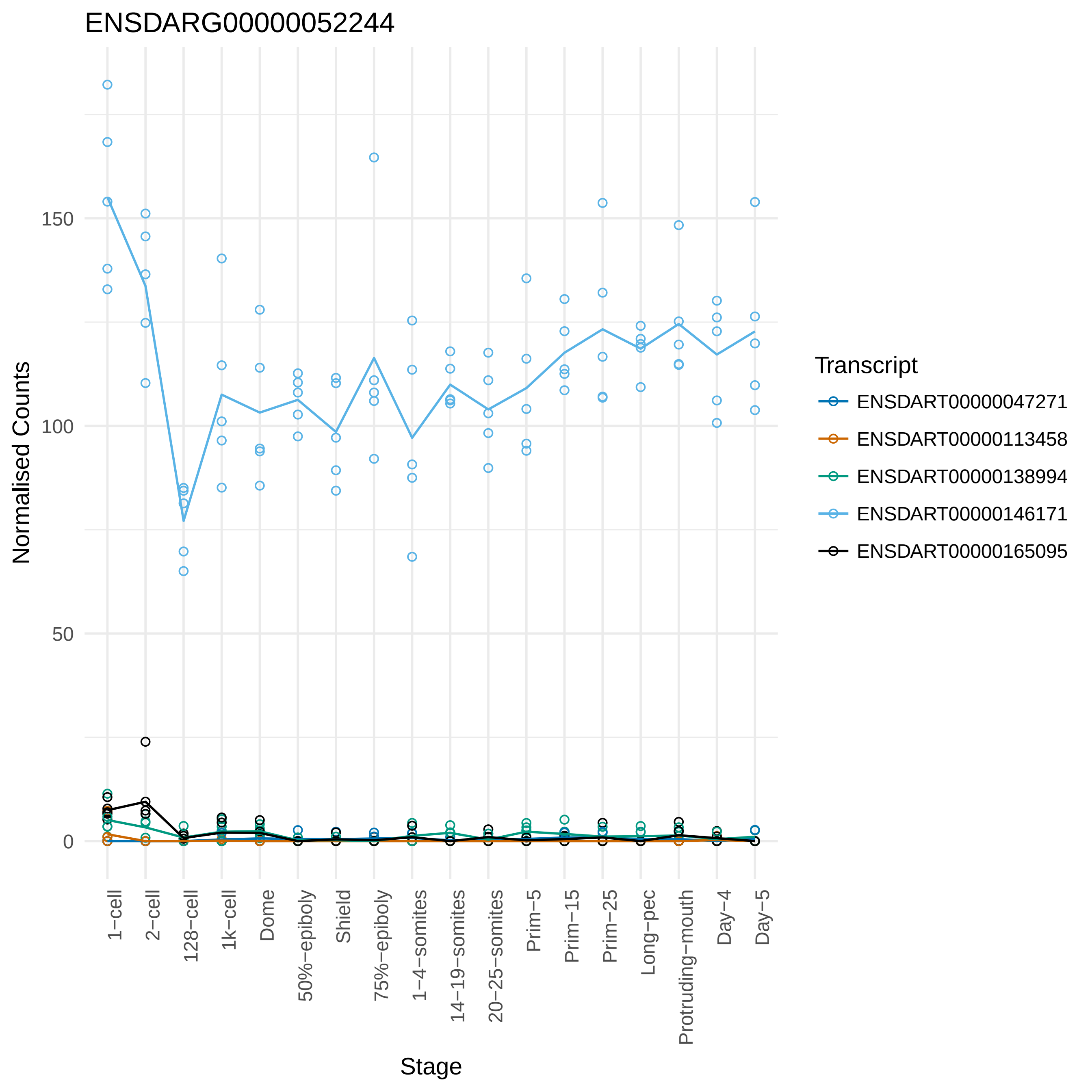
ENSDARG00000052244
Normalised Counts
150
100
50
0
1−cell
2−cell
128−cell
1k−cell
Dome
50%−epiboly
Shield
75%−epiboly
1−4−somites
14−19−somites
20−25−somites
Prim−5
Prim−15
Prim−25
Long−pec
Protruding−mouth
Day−4
Day−5
Stage
Transcript
ENSDART00000047271
ENSDART00000113458
ENSDART00000138994
ENSDART00000146171
ENSDART00000165095

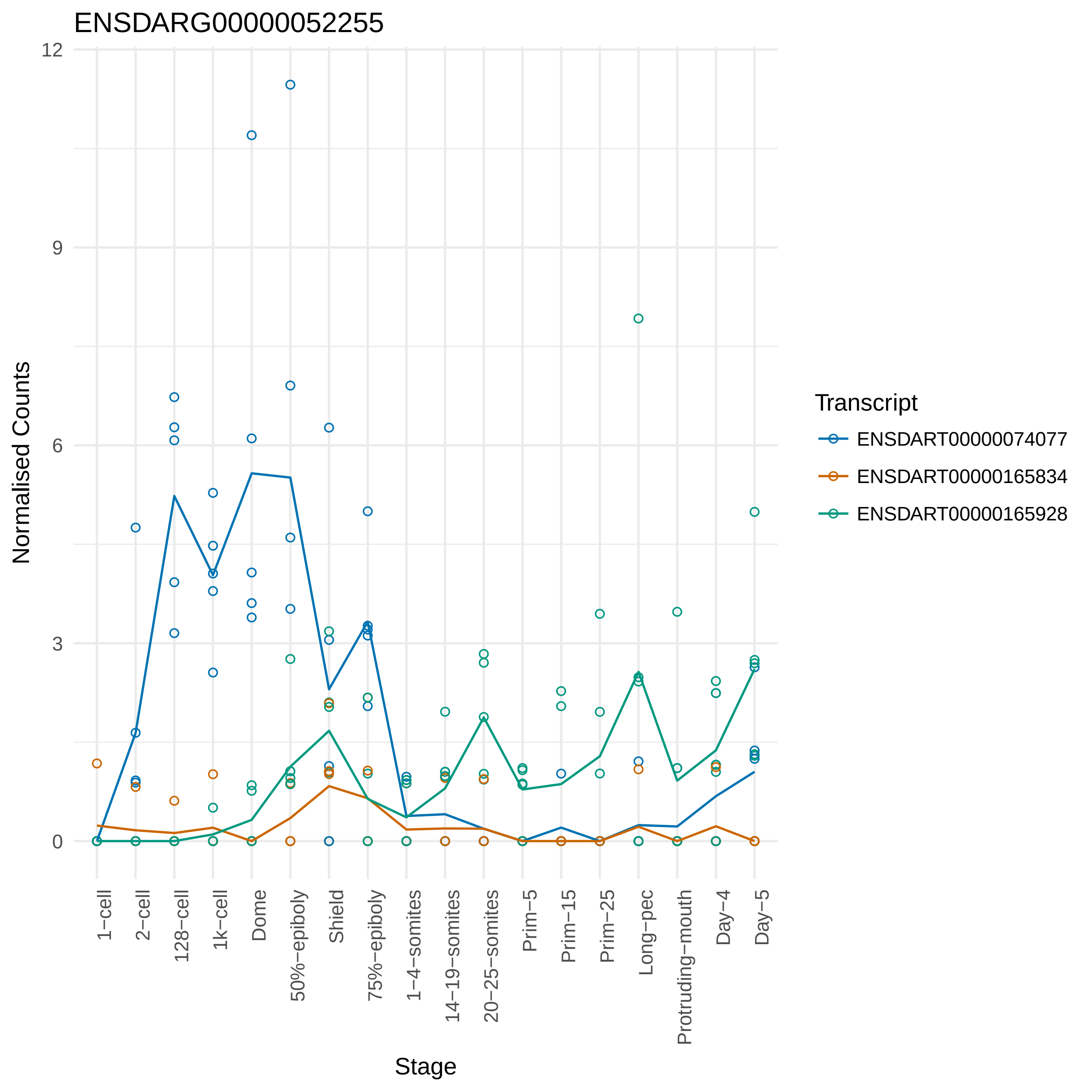
ENSDARG00000052255
Normalised Counts
12
9
6
3
0
1−cell
2−cell
128−cell
1k−cell
Dome
50%−epiboly
Shield
75%−epiboly
1−4−somites
14−19−somites
20−25−somites
Prim−5
Prim−15
Prim−25
Long−pec
Protruding−mouth
Day−4
Day−5
Stage
Transcript
ENSDART00000074077
ENSDART00000165834
ENSDART00000165928

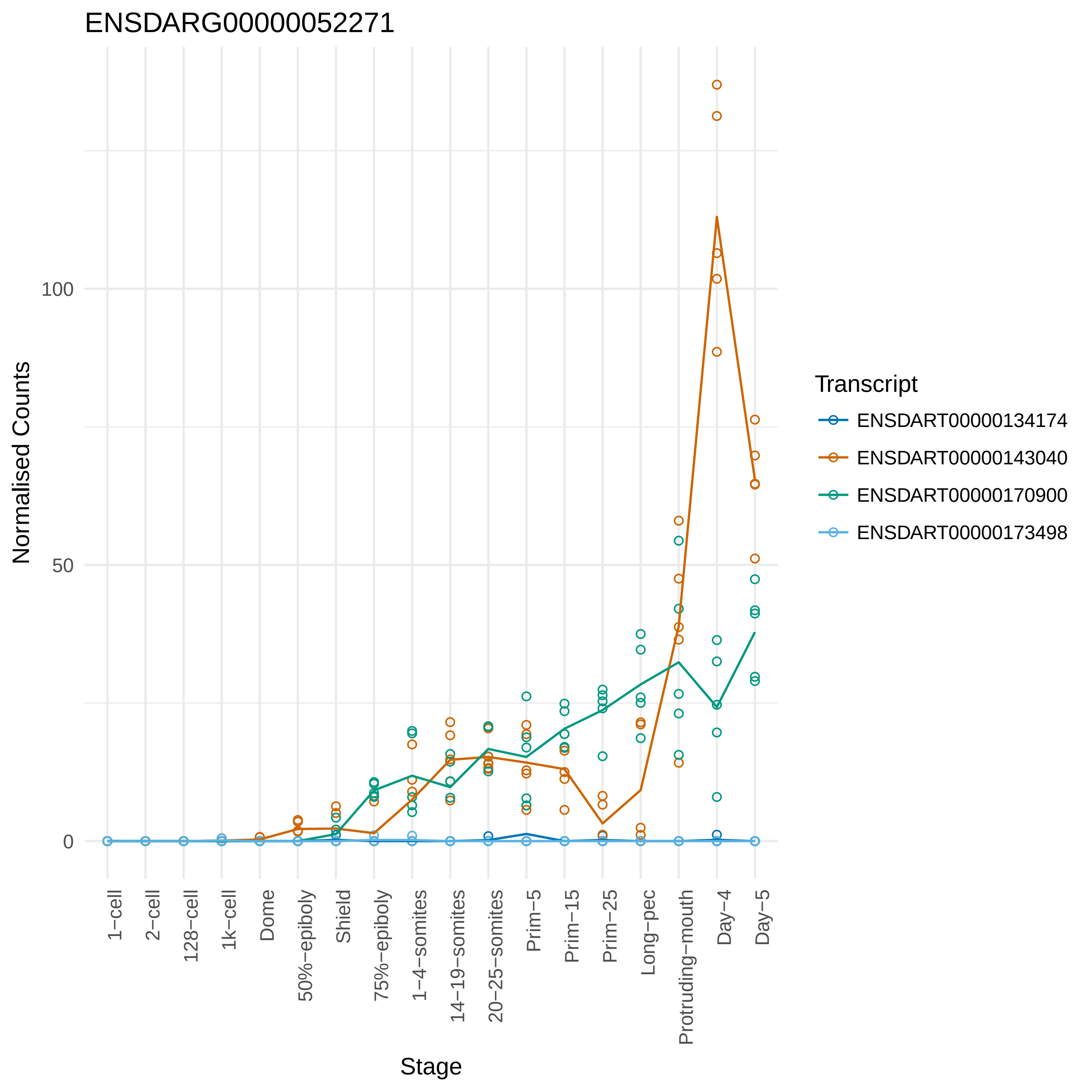
ENSDARG00000052271
Normalised Counts
0
50
100
Stage
1−cell
2−cell
128−cell
1k−cell
Dome
50%−epiboly
Shield
75%−epiboly
1−4−somites
14−19−somites
20−25−somites
Prim−5
Prim−15
Prim−25
Long−pec
Protruding−mouth
Day−4
Day−5
Transcript
ENSDART00000134174
ENSDART00000143040
ENSDART00000170900
ENSDART00000173498

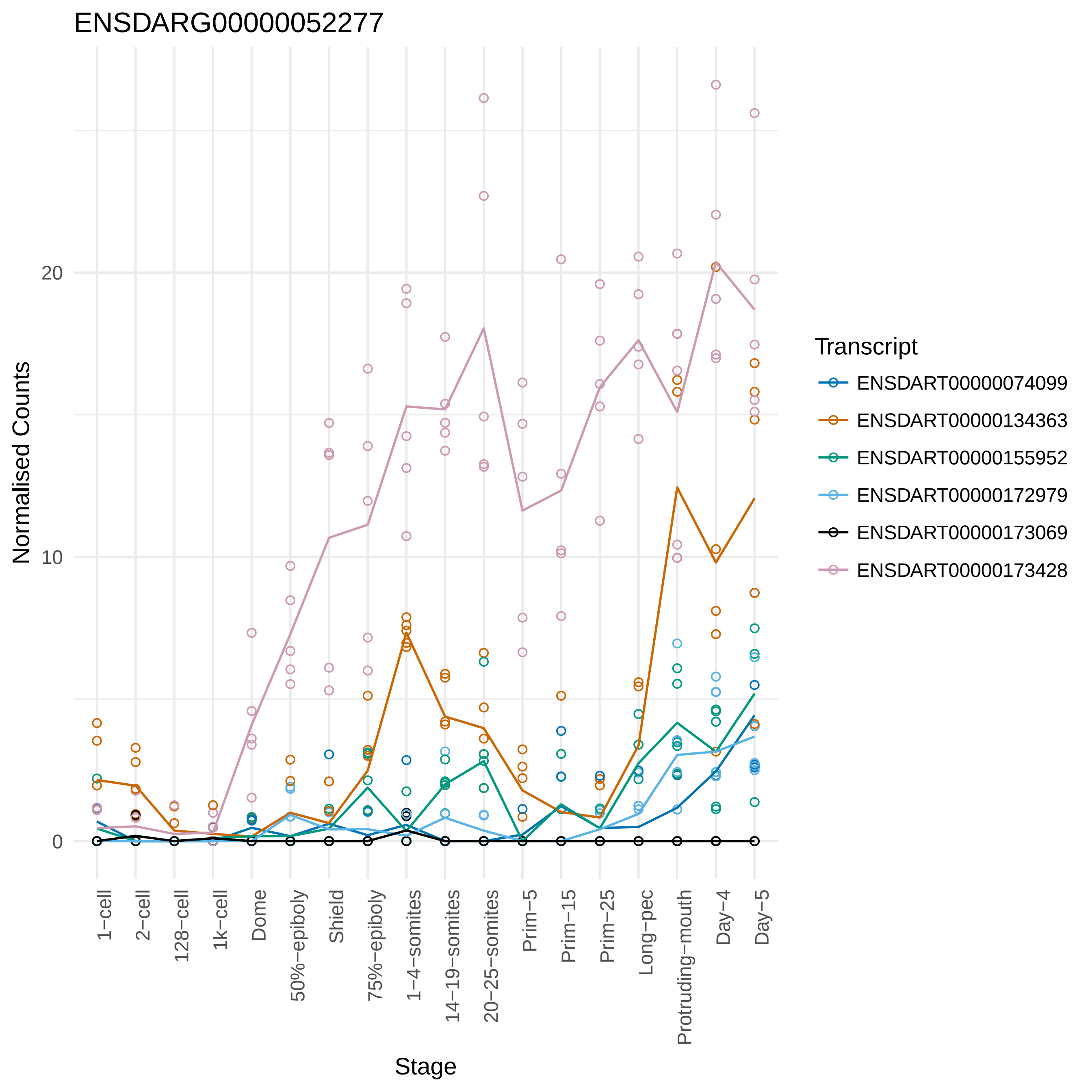

ENSDARG00000052277
Normalised Counts
20
10
0
1−cell
2−cell
128−cell
1k−cell
Dome
50%−epiboly
Shield
75%−epiboly
1−4−somites
14−19−somites
20−25−somites
Prim−5
Prim−15
Prim−25
Long−pec
Protruding−mouth
Day−4
Day−5
Stage
Transcript
ENSDART00000074099
ENSDART00000134363
ENSDART00000155952
ENSDART00000172979
ENSDART00000173069
ENSDART00000173428

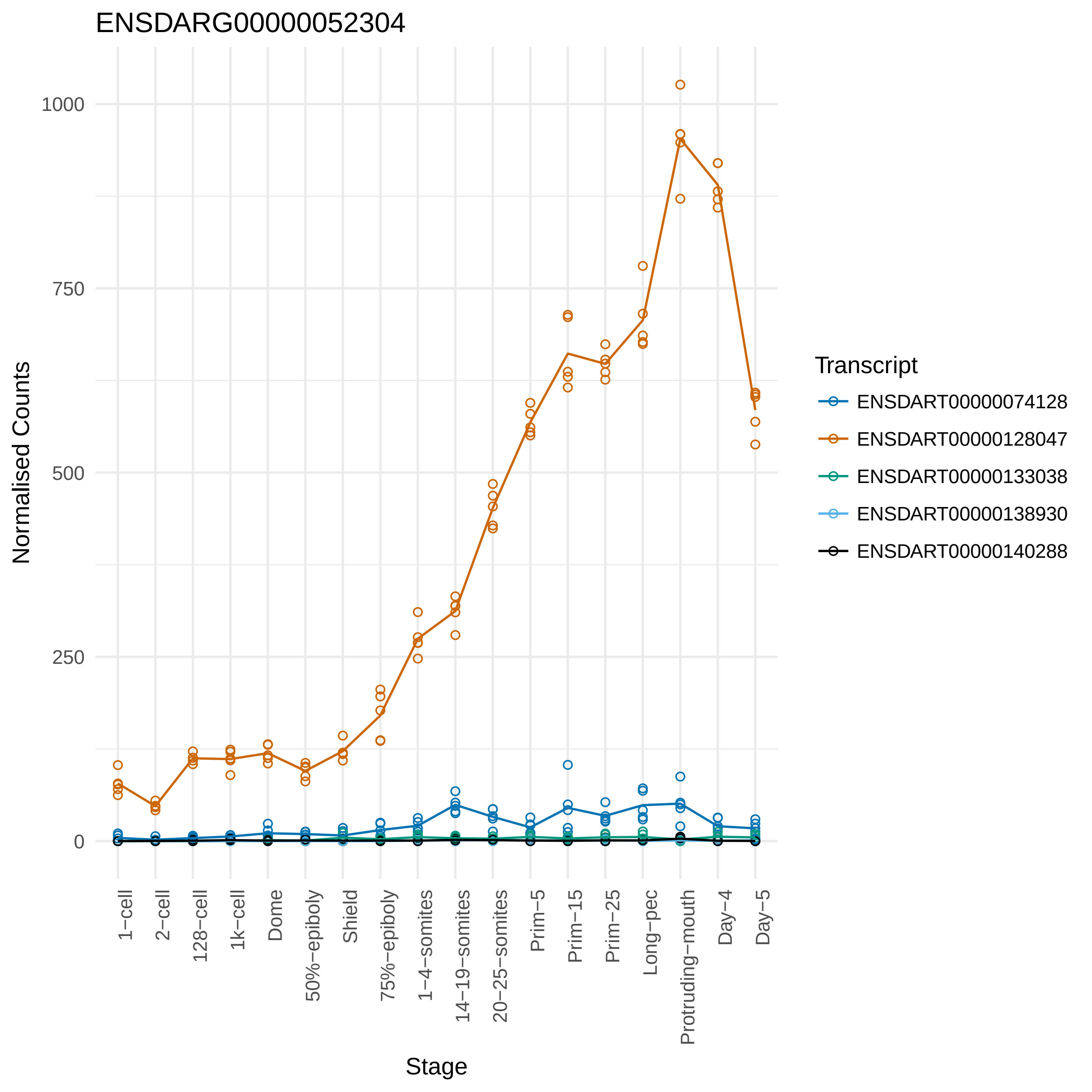

ENSDARG00000052304
Normalised Counts
1000
750
500
250
0
1−cell
2−cell
128−cell
1k−cell
Dome
50%−epiboly
Shield
75%−epiboly
1−4−somites
14−19−somites
20−25−somites
Prim−5
Prim−15
Prim−25
Long−pec
Protruding−mouth
Day−4
Day−5
Stage
Transcript
ENSDART00000074128
ENSDART00000128047
ENSDART00000133038
ENSDART00000138930
ENSDART00000140288

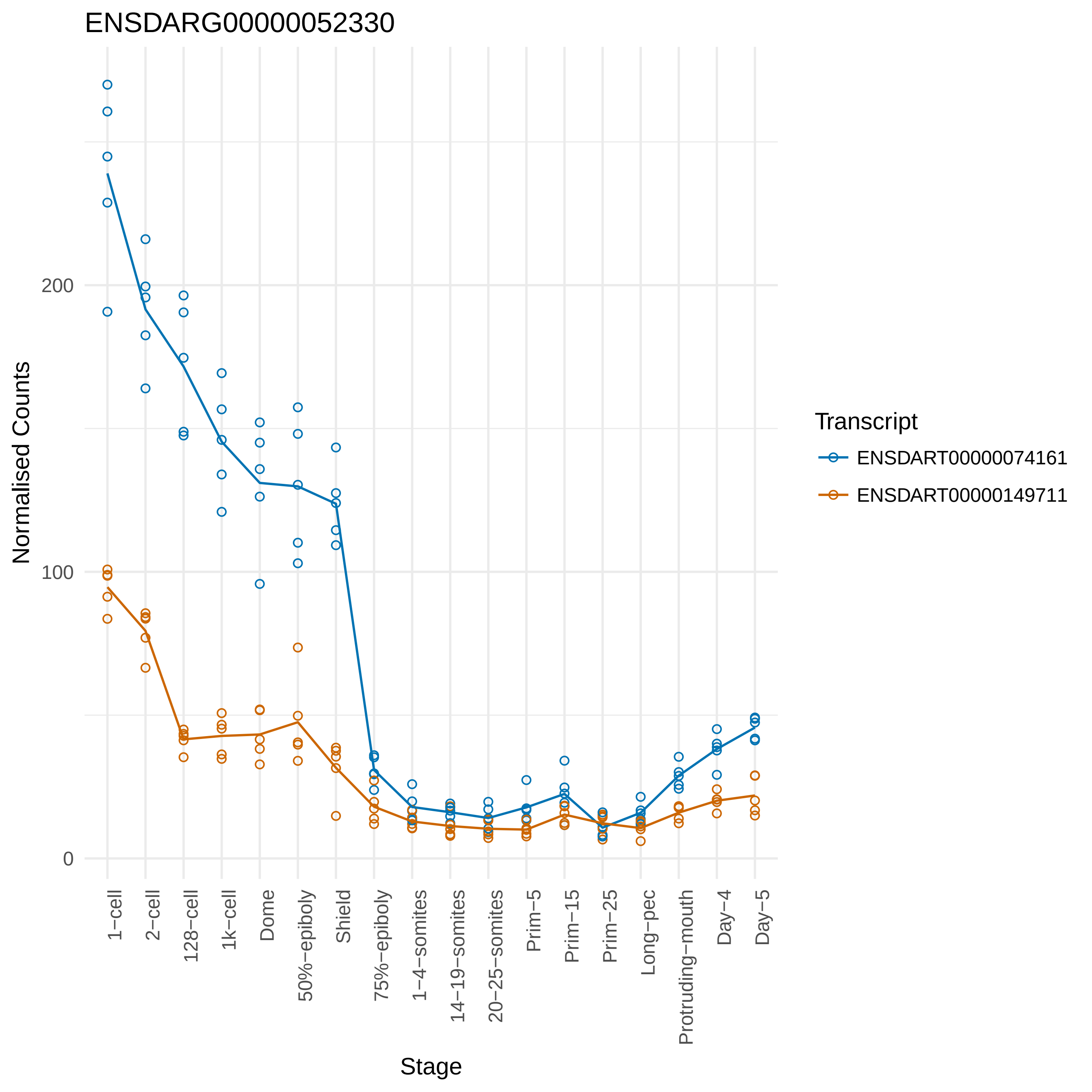

ENSDARG00000052330
Normalised Counts
200
100
0
1−cell
2−cell
128−cell
1k−cell
Dome
50%−epiboly
Shield
75%−epiboly
1−4−somites
14−19−somites
20−25−somites
Prim−5
Prim−15
Prim−25
Long−pec
Protruding−mouth
Day−4
Day−5
Stage
Transcript
ENSDART00000074161
ENSDART00000149711

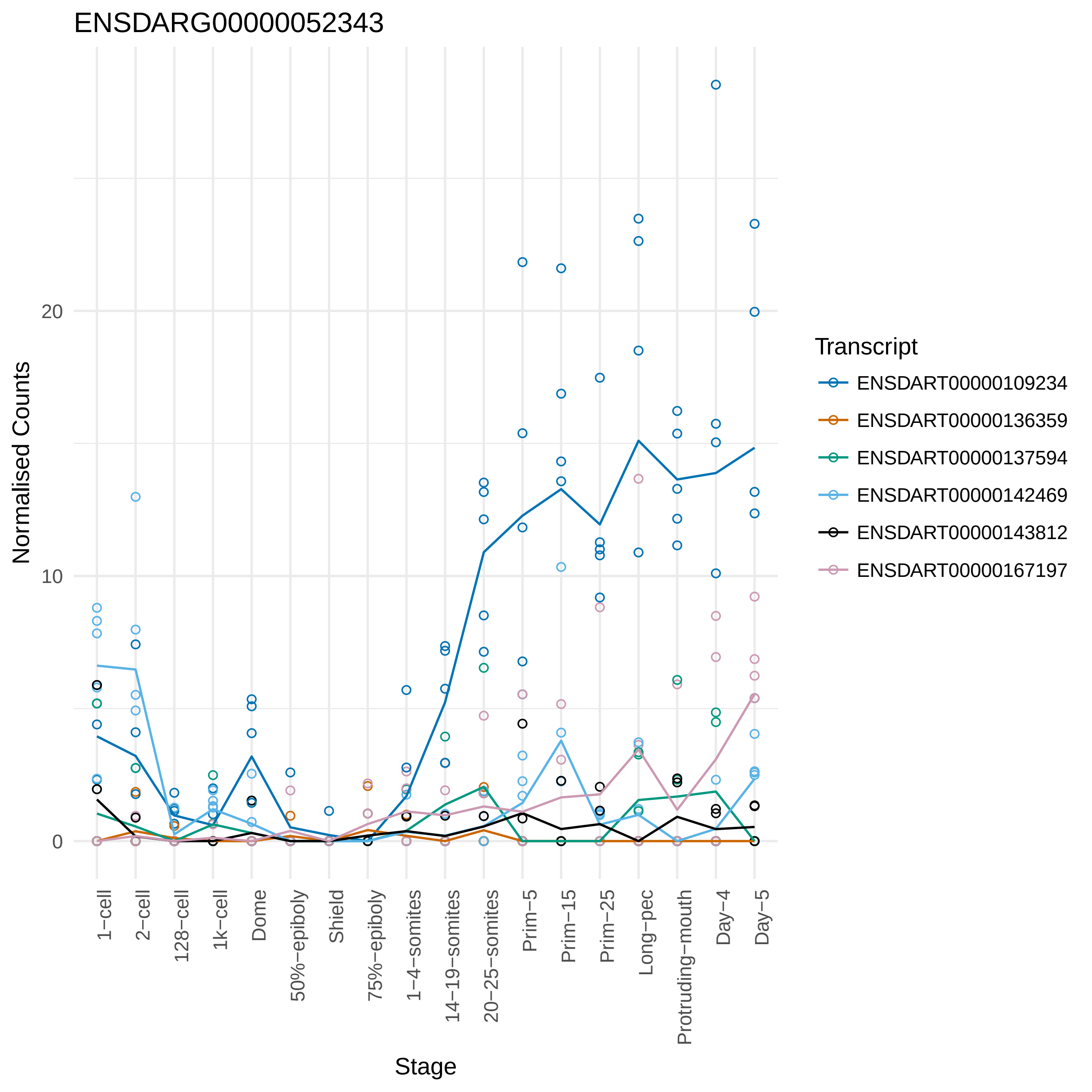

ENSDARG00000052343
Normalised Counts
0
10
20
1−cell
2−cell
128−cell
1k−cell
Dome
50%−epiboly
Shield
75%−epiboly
1−4−somites
14−19−somites
20−25−somites
Prim−5
Prim−15
Prim−25
Long−pec
Protruding−mouth
Day−4
Day−5
Stage
Transcript
ENSDART00000109234
ENSDART00000136359
ENSDART00000137594
ENSDART00000142469
ENSDART00000143812
ENSDART00000167197

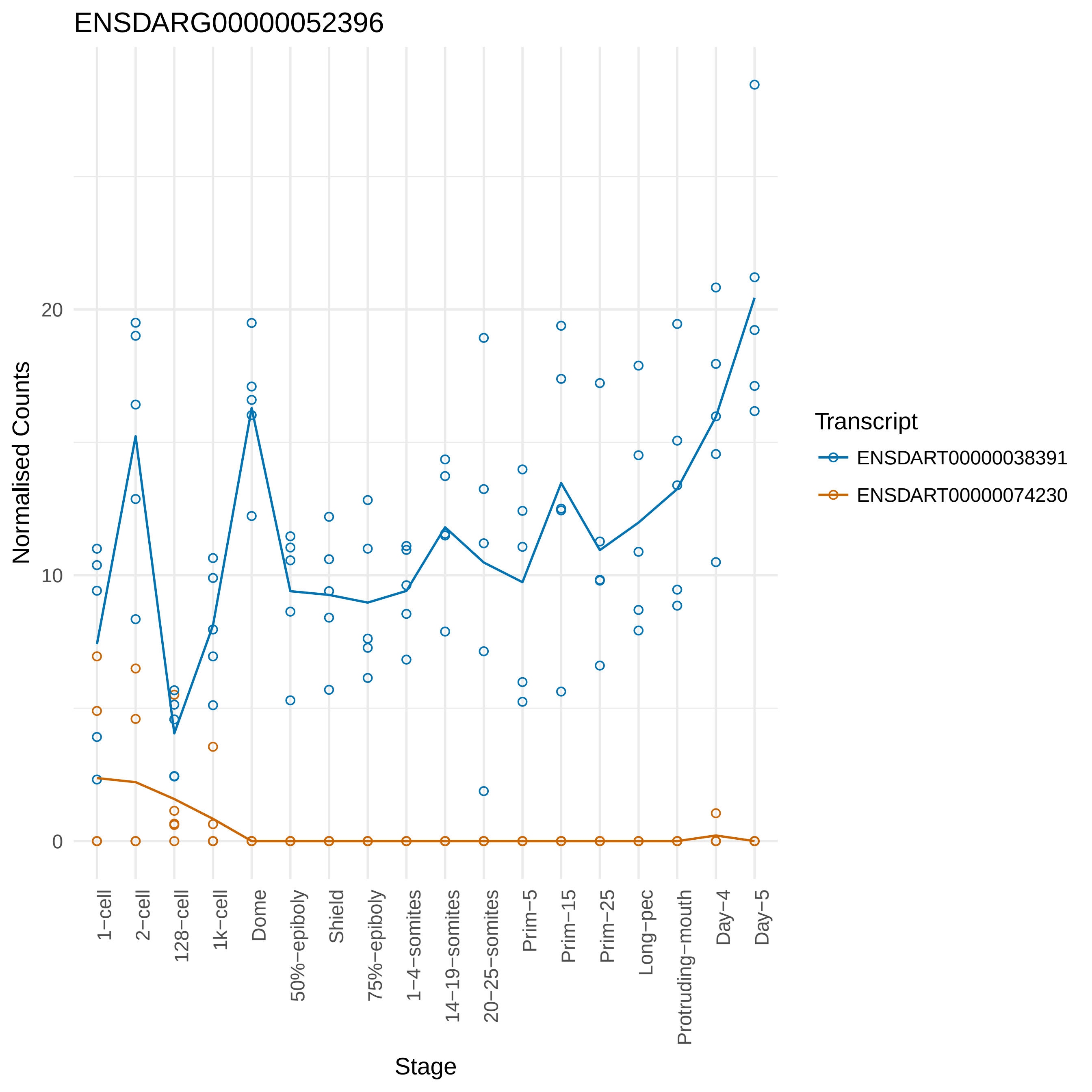

ENSDARG00000052396
Normalised Counts
0
10
20
Stage
1−cell
2−cell
128−cell
1k−cell
Dome
50%−epiboly
Shield
75%−epiboly
1−4−somites
14−19−somites
20−25−somites
Prim−5
Prim−15
Prim−25
Long−pec
Protruding−mouth
Day−4
Day−5
Transcript
ENSDART00000038391
ENSDART00000074230

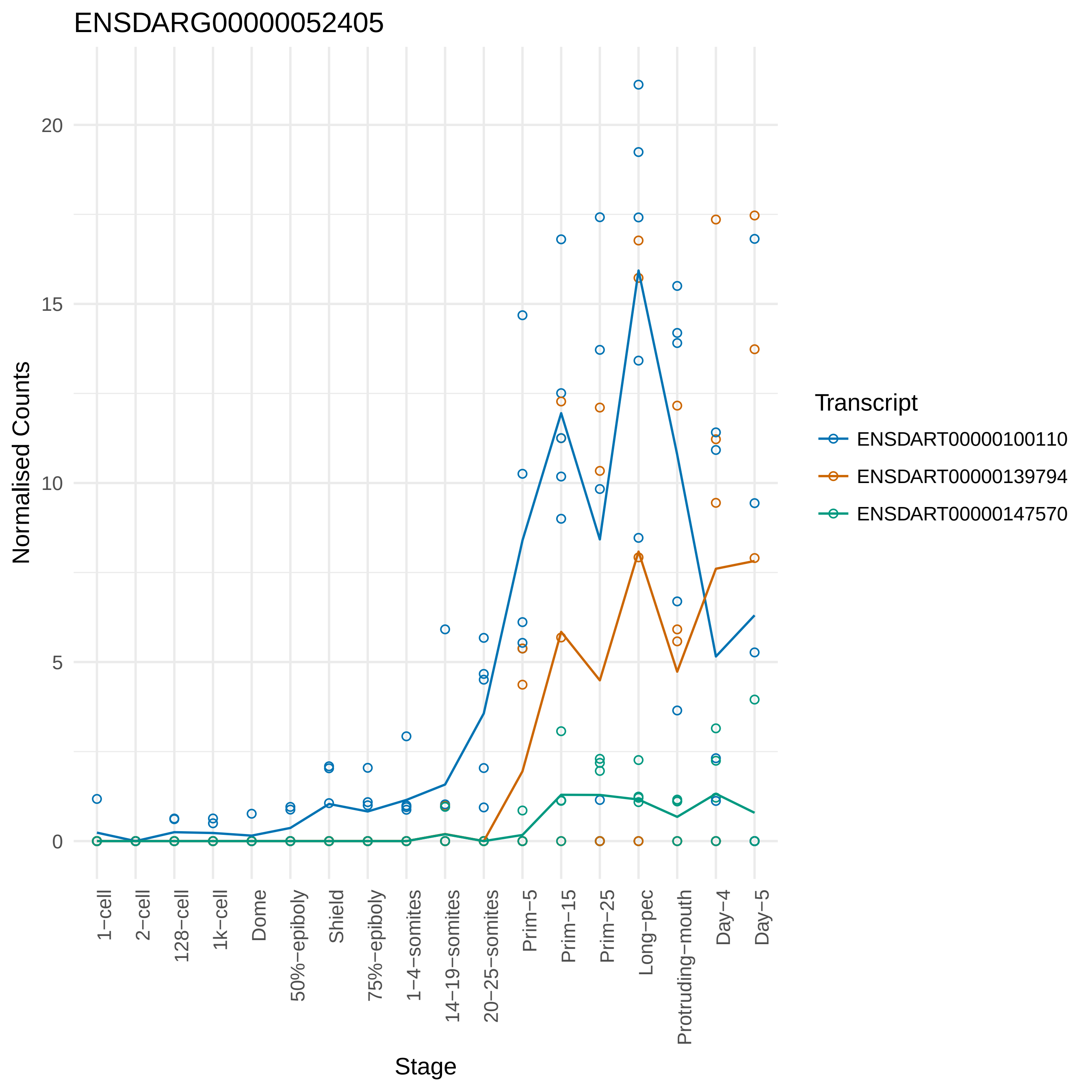
ENSDARG00000052405
Normalised Counts
20
15
10
5
0
1−cell
2−cell
128−cell
1k−cell
Dome
50%−epiboly
Shield
75%−epiboly
1−4−somites
14−19−somites
20−25−somites
Prim−5
Prim−15
Prim−25
Long−pec
Protruding−mouth
Day−4
Day−5
Stage
Transcript
ENSDART00000100110
ENSDART00000139794
ENSDART00000147570

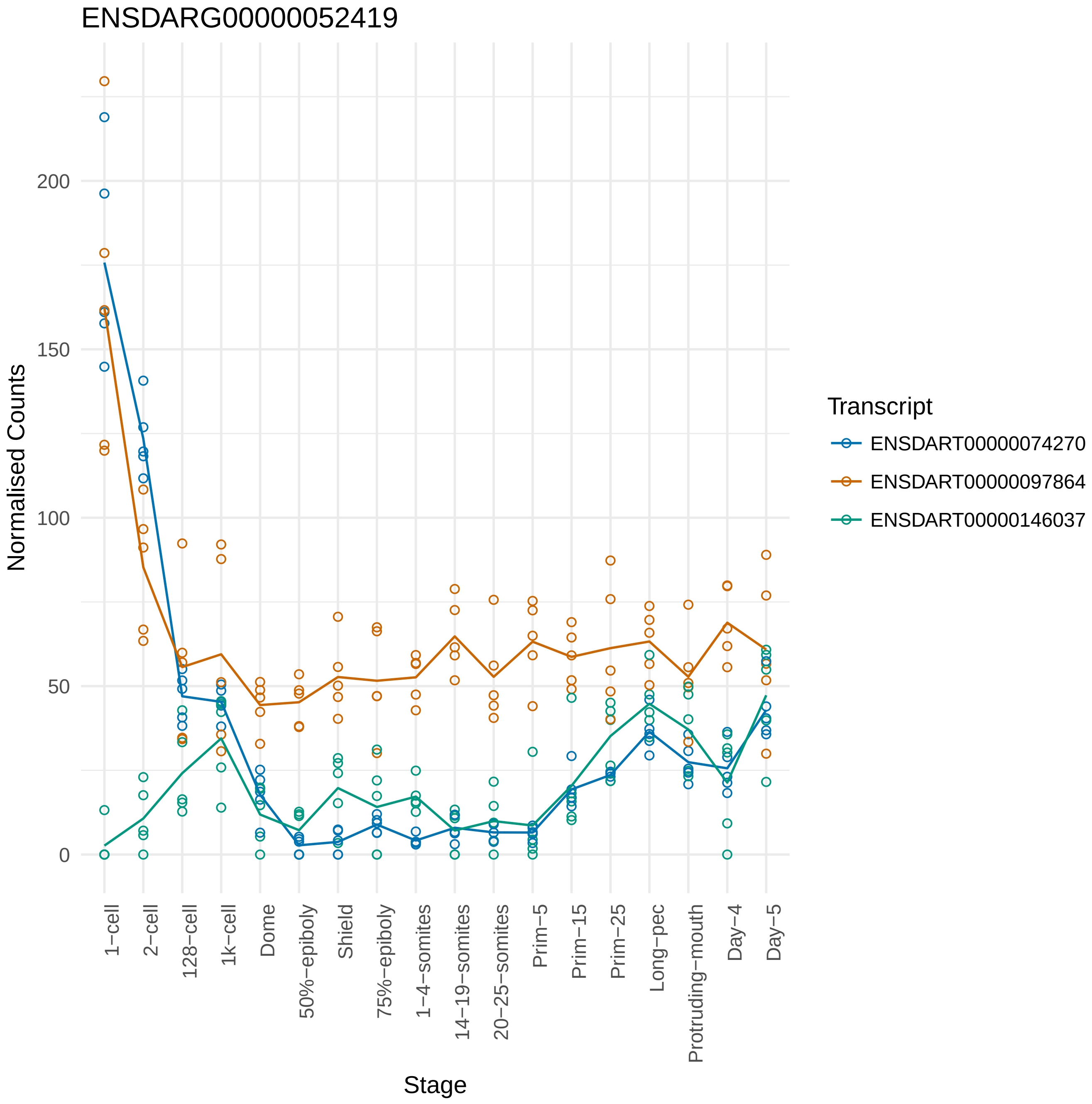

ENSDARG00000052419
Normalised Counts
0
50
100
150
200
1−cell
2−cell
128−cell
1k−cell
Dome
50%−epiboly
Shield
75%−epiboly
1−4−somites
14−19−somites
20−25−somites
Prim−5
Prim−15
Prim−25
Long−pec
Protruding−mouth
Day−4
Day−5
Stage
Transcript
ENSDART00000074270
ENSDART00000097864
ENSDART00000146037

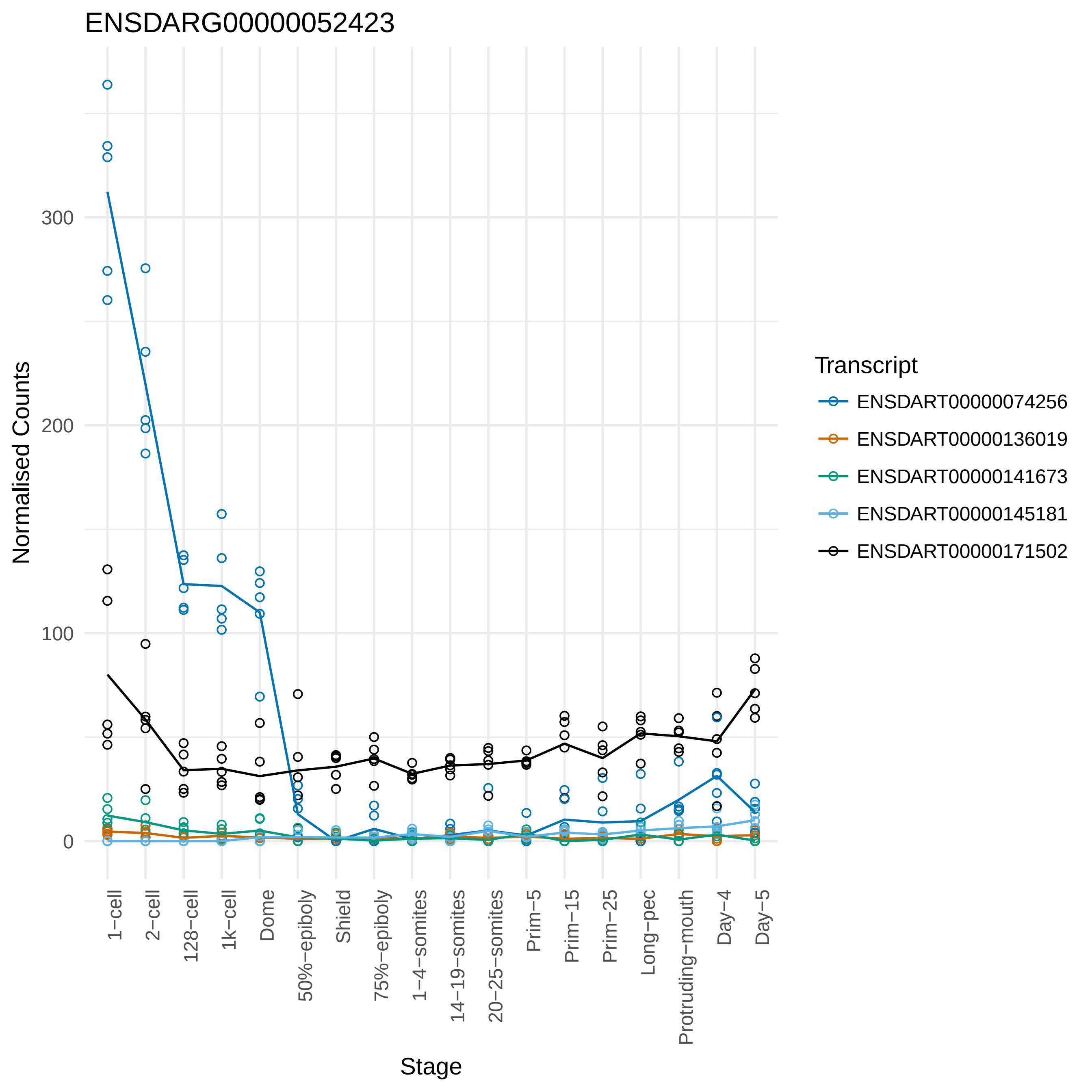
ENSDARG00000052423
Normalised Counts
300
200
100
0
1−cell
2−cell
128−cell
1k−cell
Dome
50%−epiboly
Shield
75%−epiboly
1−4−somites
14−19−somites
20−25−somites
Prim−5
Prim−15
Prim−25
Long−pec
Protruding−mouth
Day−4
Day−5
Stage
Transcript
ENSDART00000074256
ENSDART00000136019
ENSDART00000141673
ENSDART00000145181
ENSDART00000171502

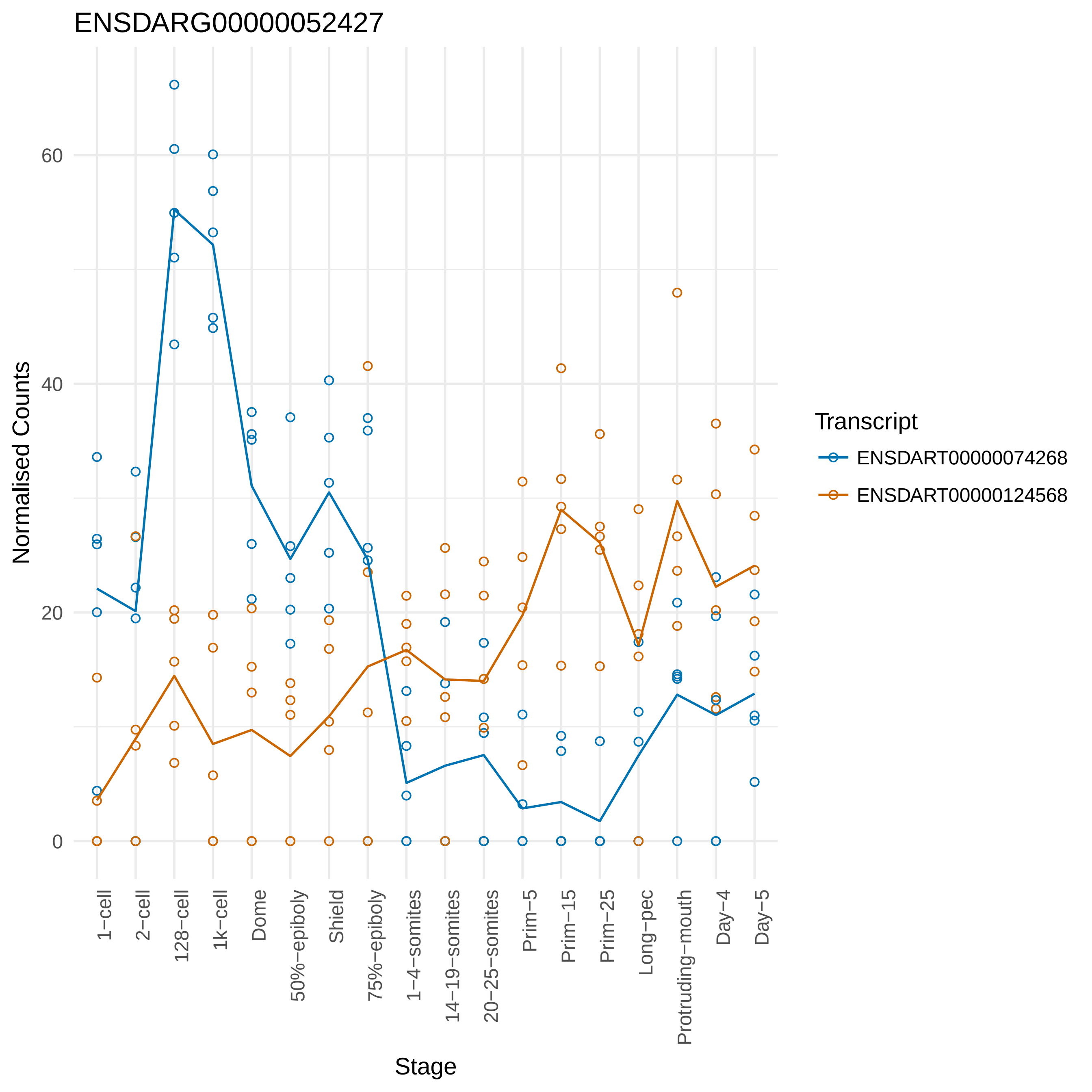
ENSDARG00000052427
Normalised Counts
0
20
40
60
1−cell
2−cell
128−cell
1k−cell
Dome
50%−epiboly
Shield
75%−epiboly
1−4−somites
14−19−somites
20−25−somites
Prim−5
Prim−15
Prim−25
Long−pec
Protruding−mouth
Day−4
Day−5
Stage
Transcript
ENSDART00000074268
ENSDART00000124568

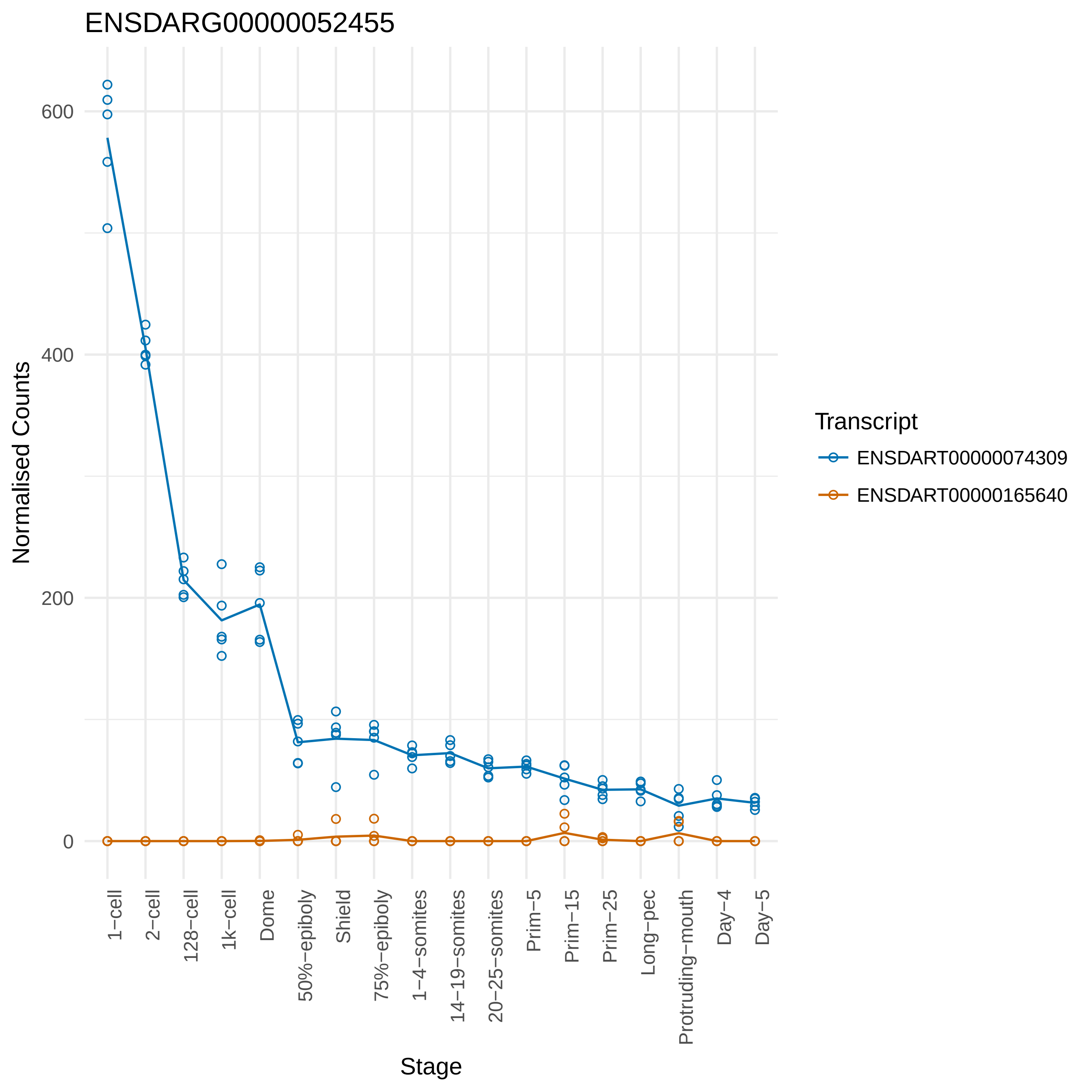
ENSDARG00000052455
Normalised Counts
600
400
200
0
1−cell
2−cell
128−cell
1k−cell
Dome
50%−epiboly
Shield
75%−epiboly
1−4−somites
14−19−somites
20−25−somites
Prim−5
Prim−15
Prim−25
Long−pec
Protruding−mouth
Day−4
Day−5
Stage
Transcript
ENSDART00000074309
ENSDART00000165640

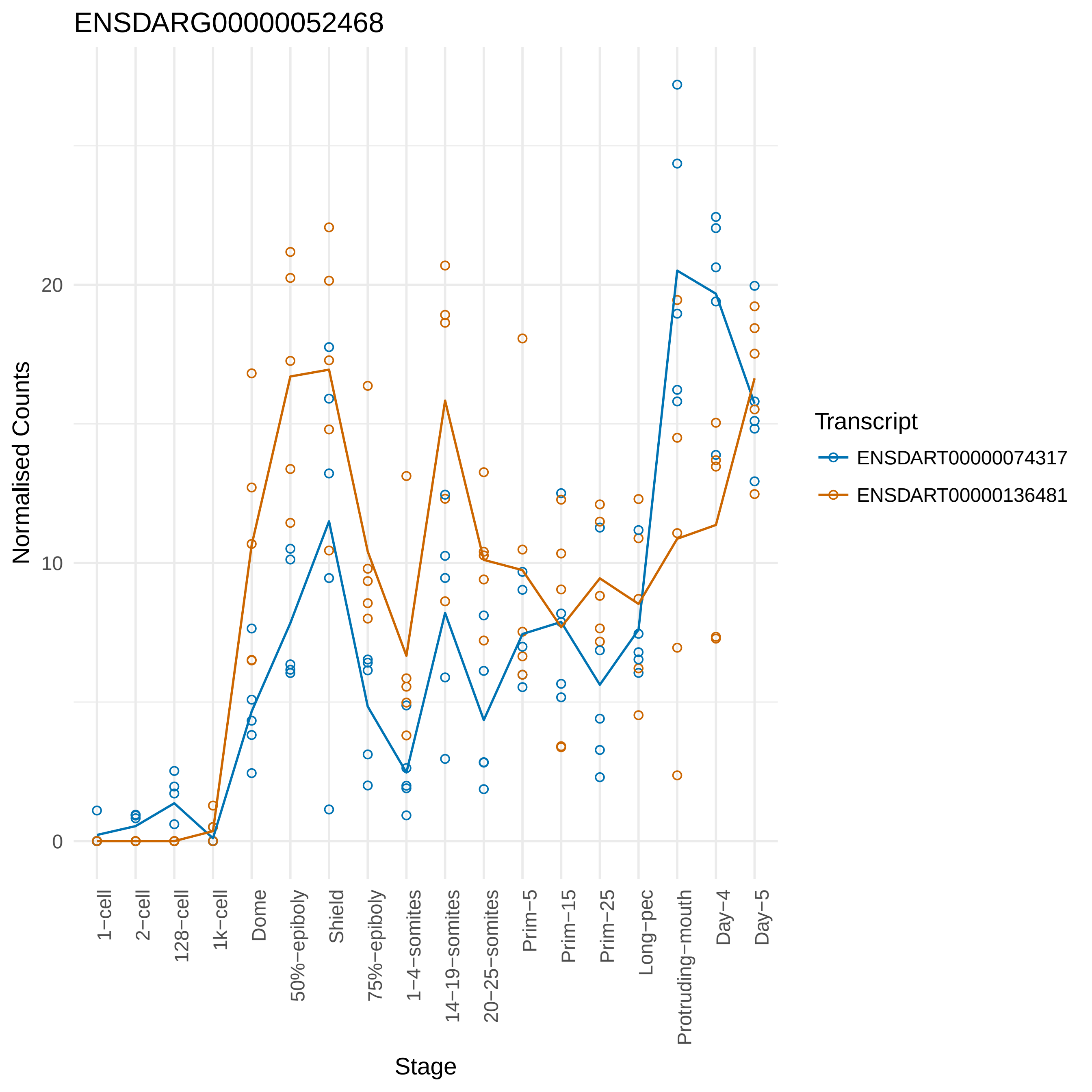

ENSDARG00000052468
Normalised Counts
20
10
0
1−cell
2−cell
128−cell
1k−cell
Dome
50%−epiboly
Shield
75%−epiboly
1−4−somites
14−19−somites
20−25−somites
Prim−5
Prim−15
Prim−25
Long−pec
Protruding−mouth
Day−4
Day−5
Stage
Transcript
ENSDART00000074317
ENSDART00000136481

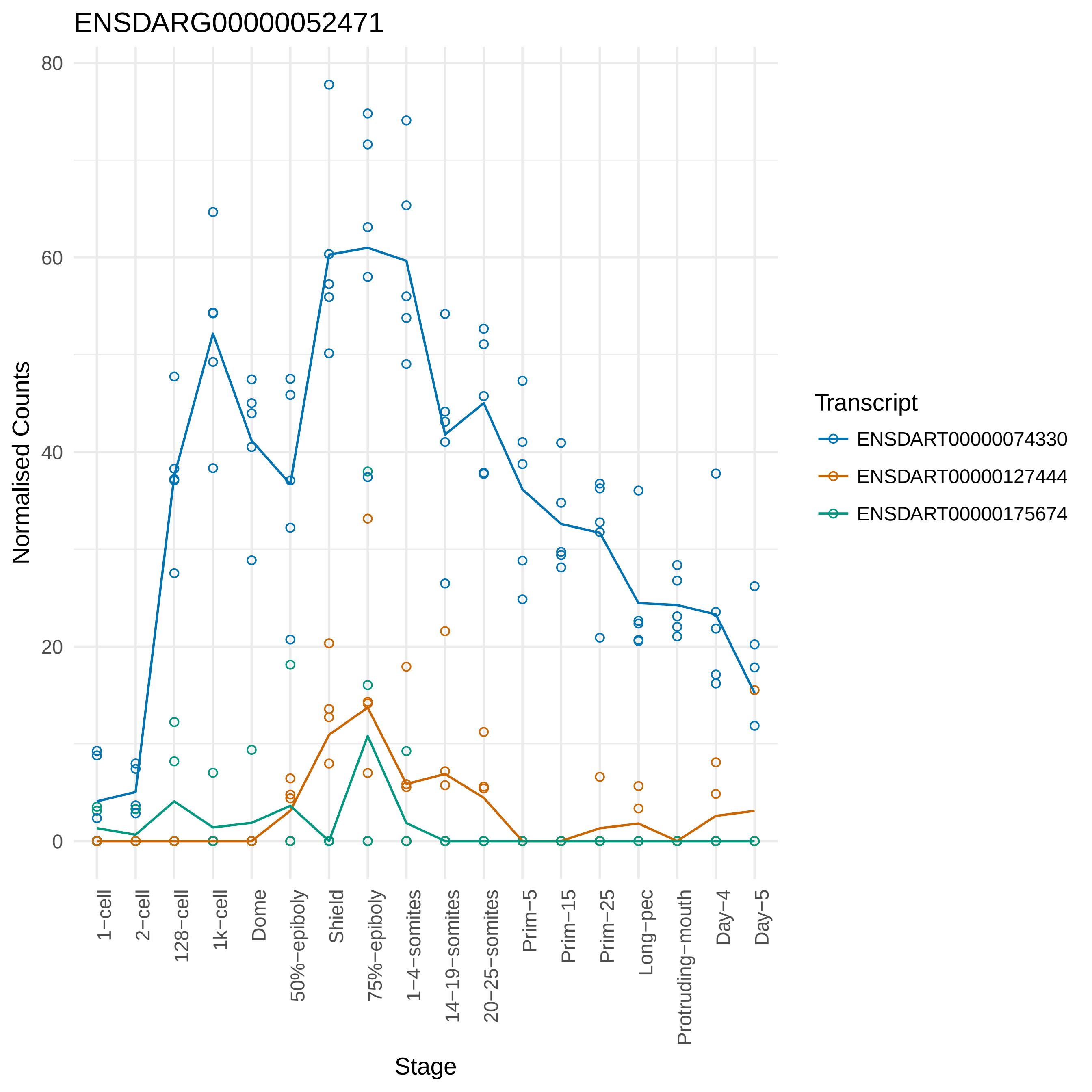

ENSDARG00000052471
Normalised Counts
80
60
40
20
0
1−cell
2−cell
128−cell
1k−cell
Dome
50%−epiboly
Shield
75%−epiboly
1−4−somites
14−19−somites
20−25−somites
Prim−5
Prim−15
Prim−25
Long−pec
Protruding−mouth
Day−4
Day−5
Stage
Transcript
ENSDART00000074330
ENSDART00000127444
ENSDART00000175674

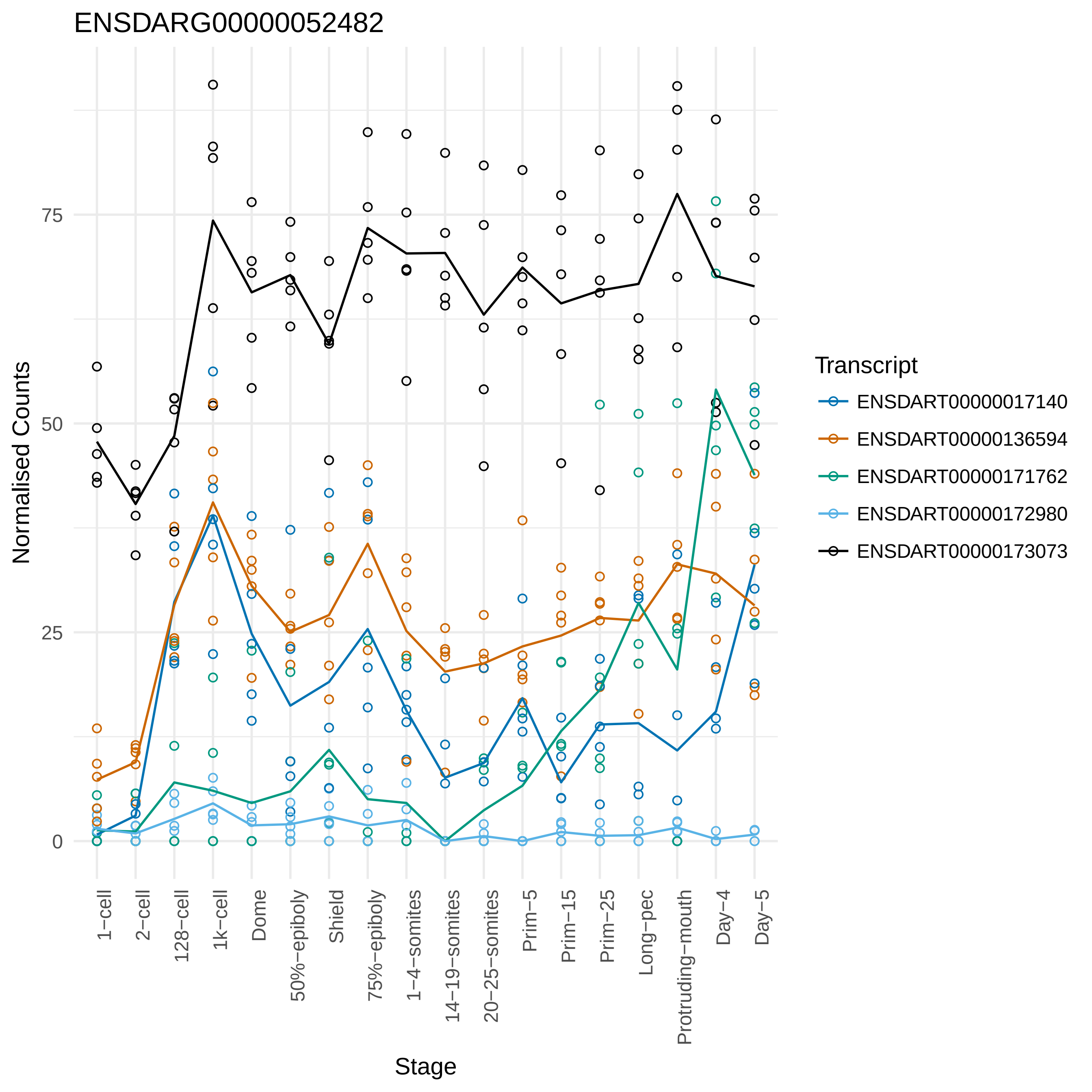

ENSDARG00000052482
Normalised Counts
0
25
50
75
Stage
1−cell
2−cell
128−cell
1k−cell
Dome
50%−epiboly
Shield
75%−epiboly
1−4−somites
14−19−somites
20−25−somites
Prim−5
Prim−15
Prim−25
Long−pec
Protruding−mouth
Day−4
Day−5
Transcript
ENSDART00000017140
ENSDART00000136594
ENSDART00000171762
ENSDART00000172980
ENSDART00000173073

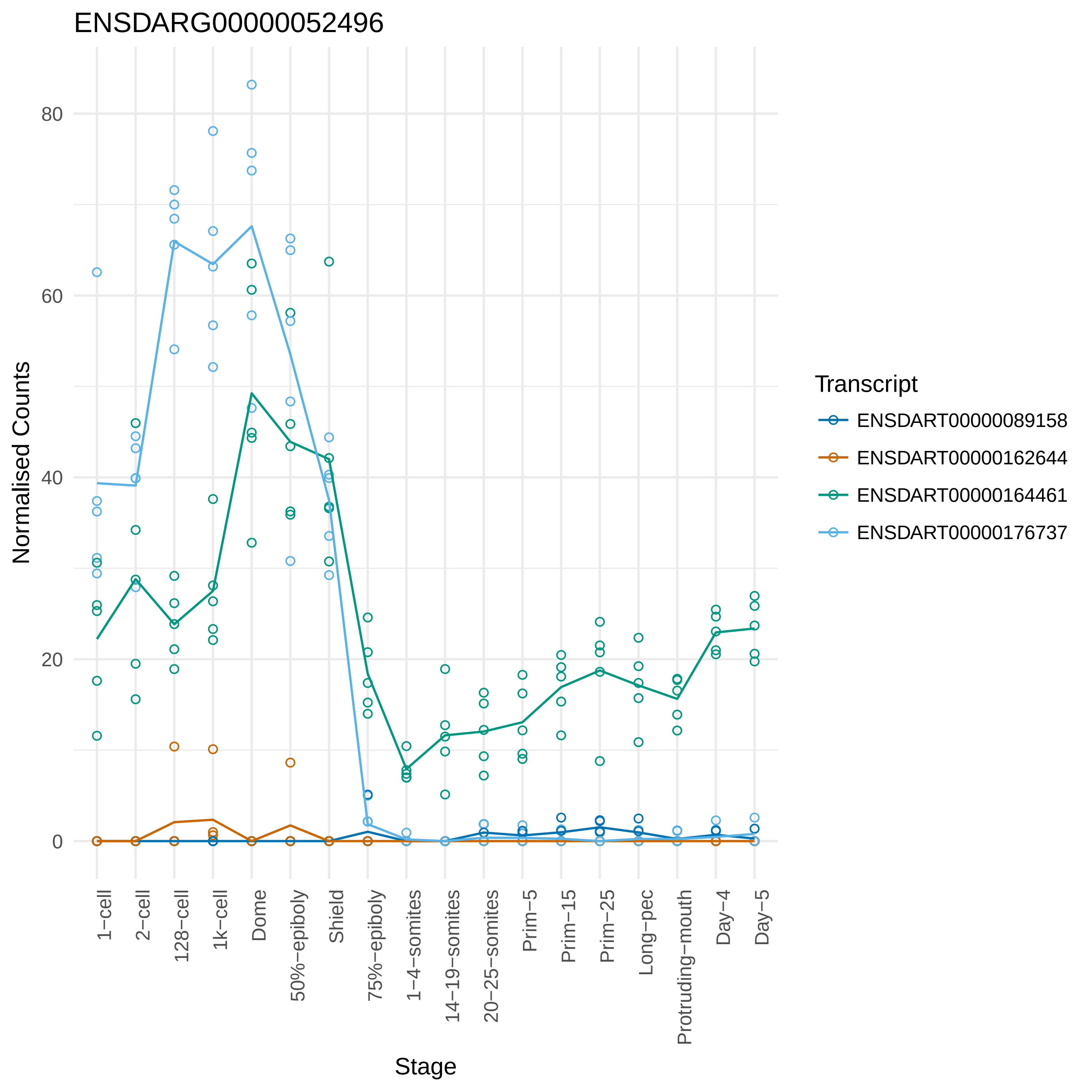

ENSDARG00000052496
Normalised Counts
0
20
40
60
80
1−cell
2−cell
128−cell
1k−cell
Dome
50%−epiboly
Shield
75%−epiboly
1−4−somites
14−19−somites
20−25−somites
Prim−5
Prim−15
Prim−25
Long−pec
Protruding−mouth
Day−4
Day−5
Stage
Transcript
ENSDART00000089158
ENSDART00000162644
ENSDART00000164461
ENSDART00000176737

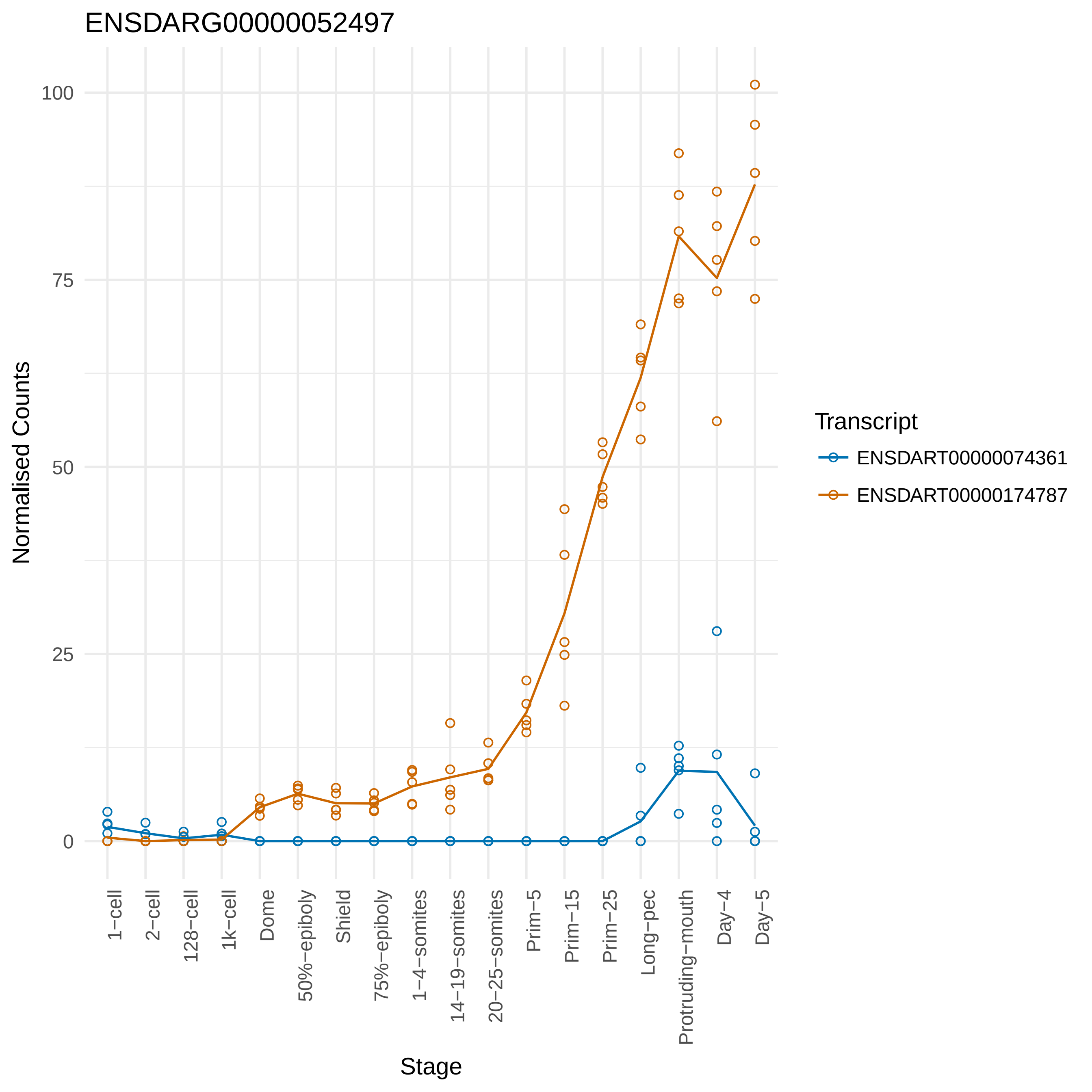
ENSDARG00000052497
Normalised Counts
100
75
50
25
0
1−cell
2−cell
128−cell
1k−cell
Dome
50%−epiboly
Shield
75%−epiboly
1−4−somites
14−19−somites
20−25−somites
Prim−5
Prim−15
Prim−25
Long−pec
Protruding−mouth
Day−4
Day−5
Stage
Transcript
ENSDART00000074361
ENSDART00000174787

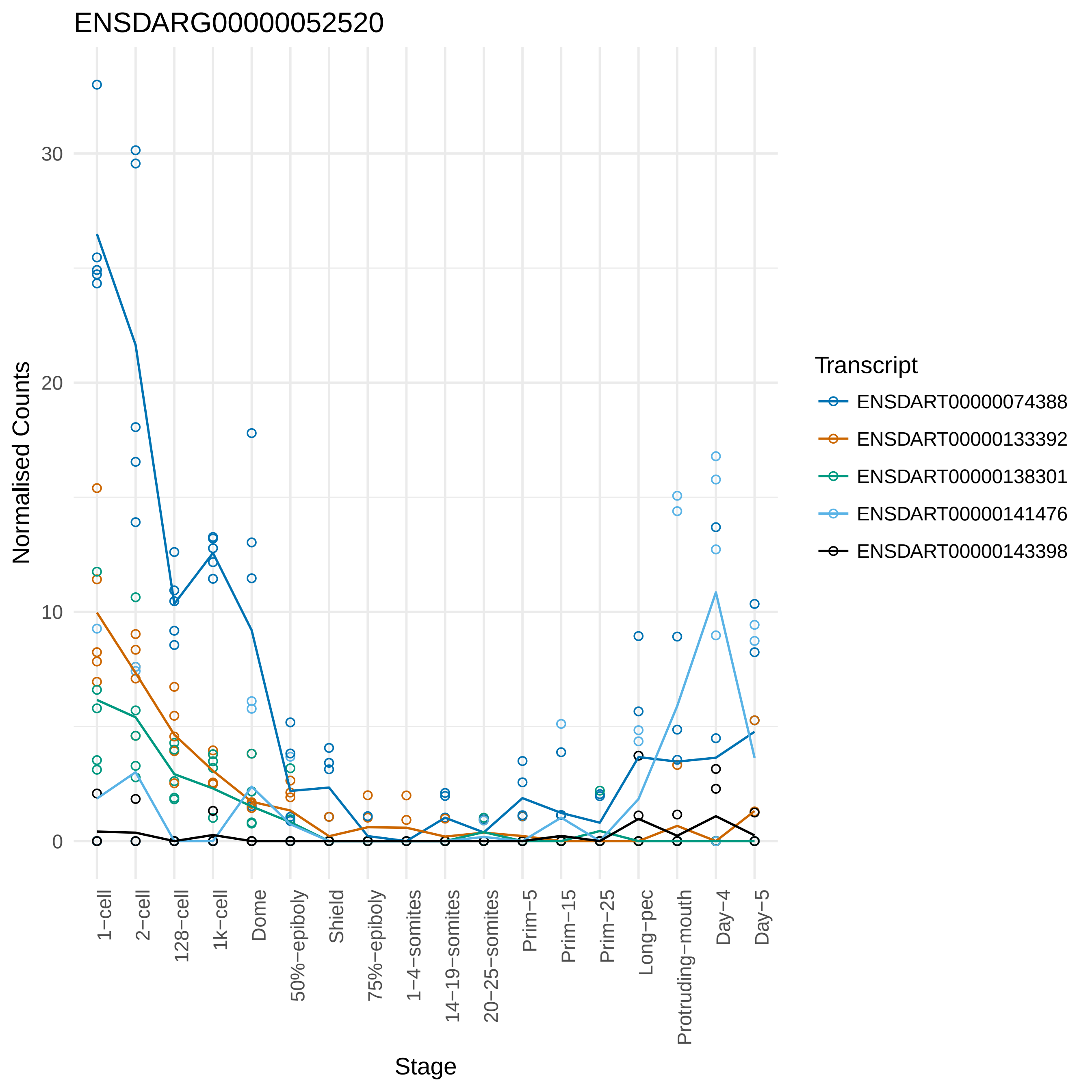

ENSDARG00000052520
Normalised Counts
30
20
10
0
1−cell
2−cell
128−cell
1k−cell
Dome
50%−epiboly
Shield
75%−epiboly
1−4−somites
14−19−somites
20−25−somites
Prim−5
Prim−15
Prim−25
Long−pec
Protruding−mouth
Day−4
Day−5
Stage
Transcript
ENSDART00000074388
ENSDART00000133392
ENSDART00000138301
ENSDART00000141476
ENSDART00000143398

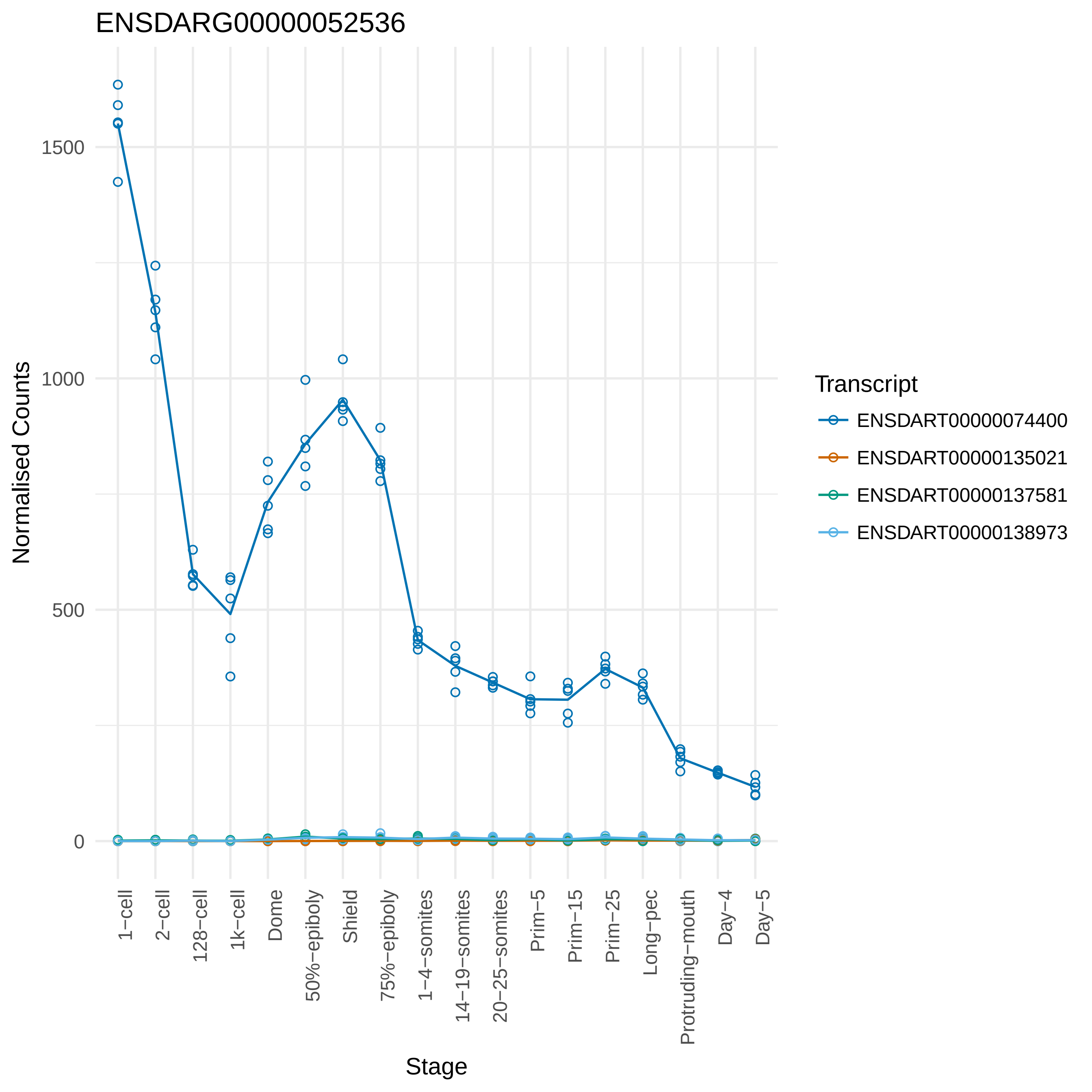

ENSDARG00000052536
Normalised Counts
1500
1000
500
0
1−cell
2−cell
128−cell
1k−cell
Dome
50%−epiboly
Shield
75%−epiboly
1−4−somites
14−19−somites
20−25−somites
Prim−5
Prim−15
Prim−25
Long−pec
Protruding−mouth
Day−4
Day−5
Stage
Transcript
ENSDART00000074400
ENSDART00000135021
ENSDART00000137581
ENSDART00000138973

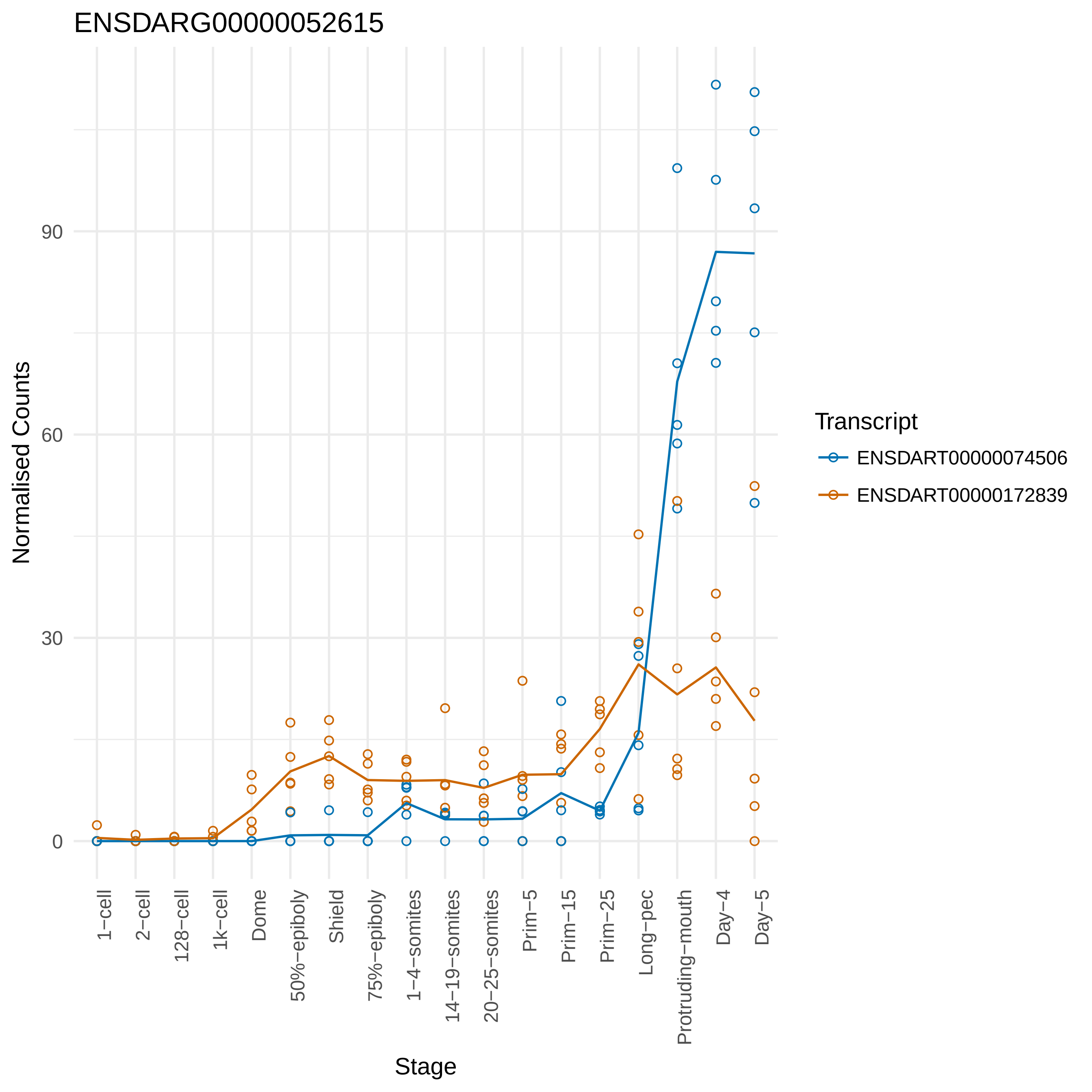

ENSDARG00000052615
Normalised Counts
90
60
30
0
1−cell
2−cell
128−cell
1k−cell
Dome
50%−epiboly
Shield
75%−epiboly
1−4−somites
14−19−somites
20−25−somites
Prim−5
Prim−15
Prim−25
Long−pec
Protruding−mouth
Day−4
Day−5
Stage
Transcript
ENSDART00000074506
ENSDART00000172839

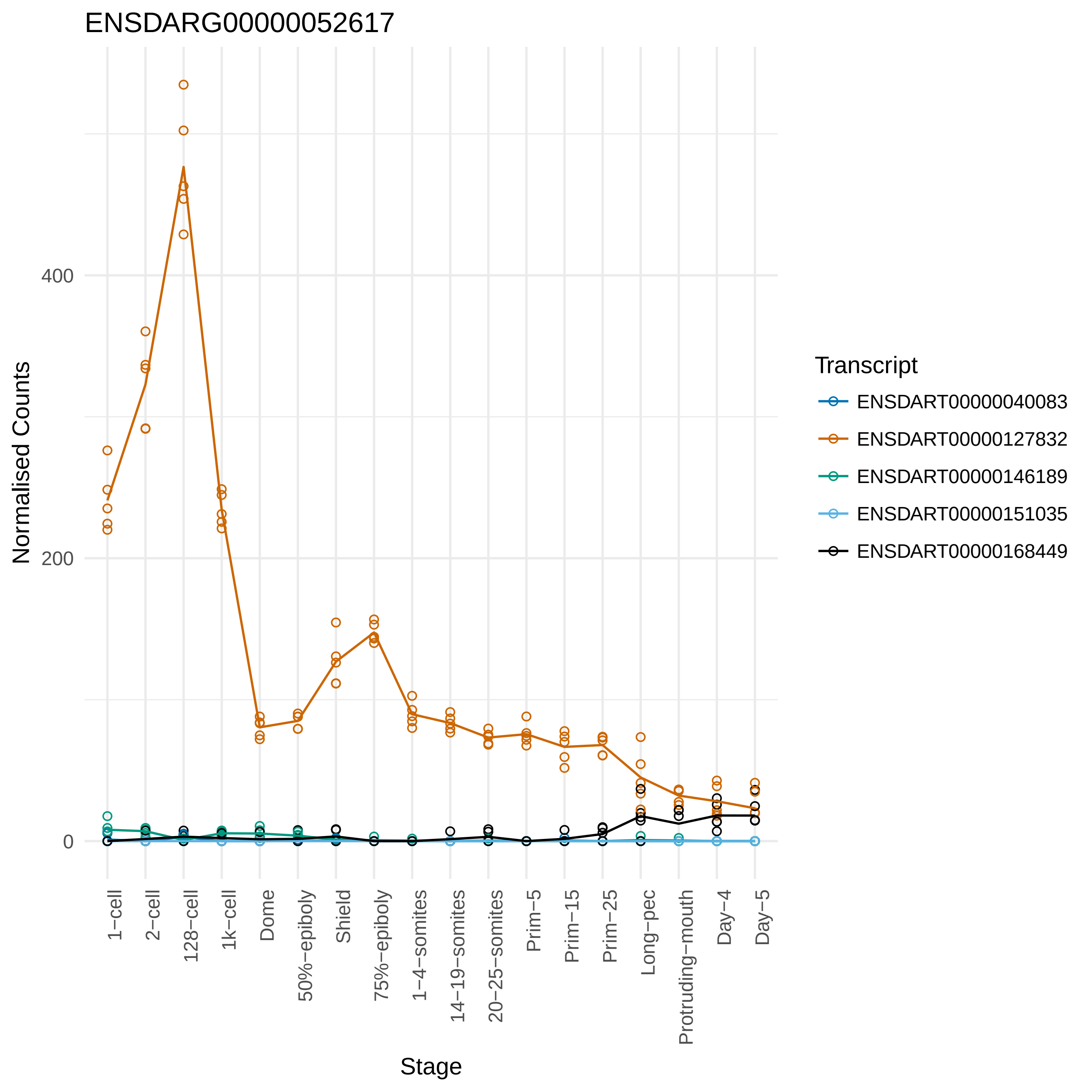

ENSDARG00000052617
Normalised Counts
400
200
0
Stage
1−cell
2−cell
128−cell
1k−cell
Dome
50%−epiboly
Shield
75%−epiboly
1−4−somites
14−19−somites
20−25−somites
Prim−5
Prim−15
Prim−25
Long−pec
Protruding−mouth
Day−4
Day−5
Transcript
ENSDART00000040083
ENSDART00000127832
ENSDART00000146189
ENSDART00000151035
ENSDART00000168449

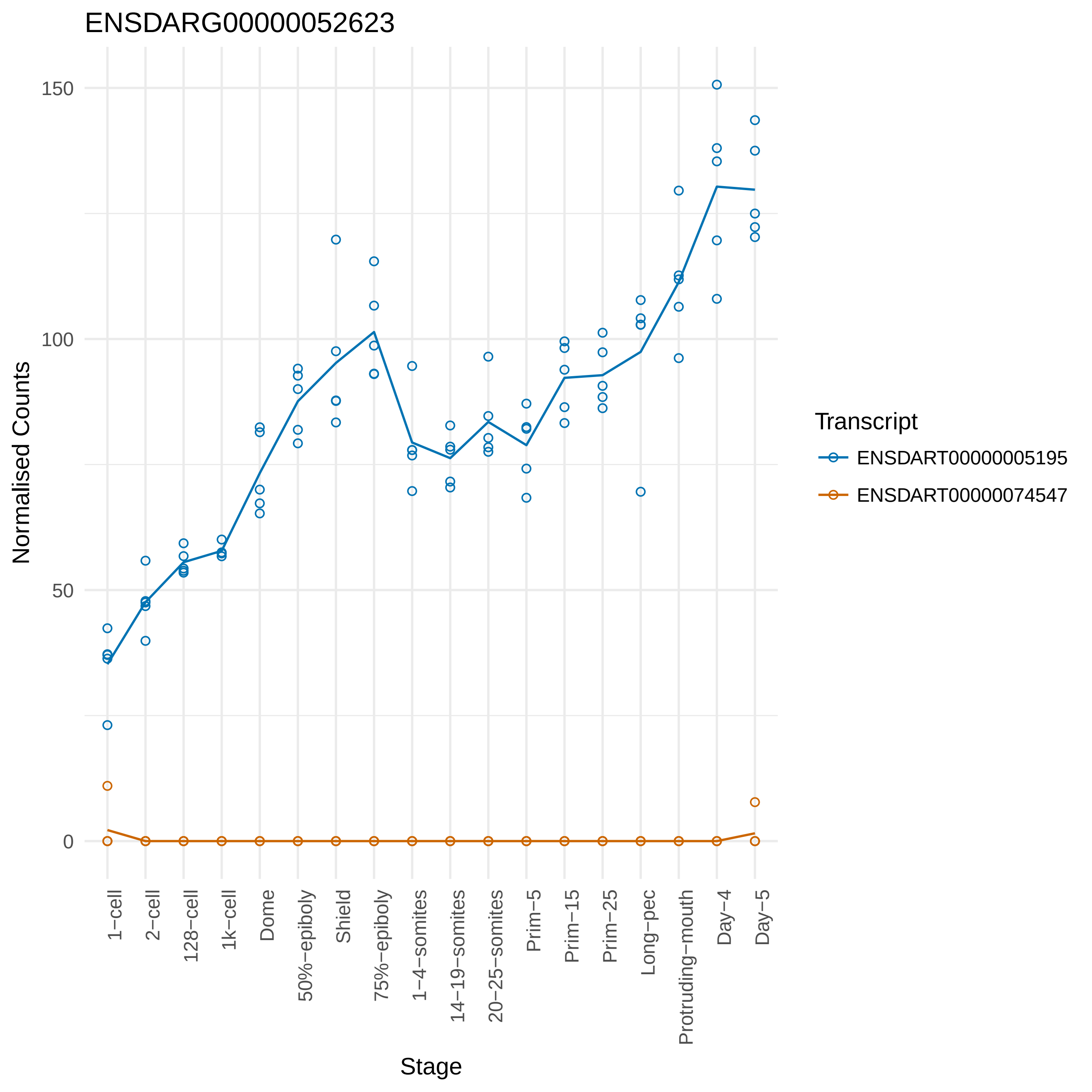

ENSDARG00000052623
150
100
50
0
Normalised Counts
1−cell
2−cell
128−cell
1k−cell
Dome
50%−epiboly
Shield
75%−epiboly
1−4−somites
14−19−somites
20−25−somites
Prim−5
Prim−15
Prim−25
Long−pec
Protruding−mouth
Day−4
Day−5
Stage
Transcript
ENSDART00000005195
ENSDART00000074547

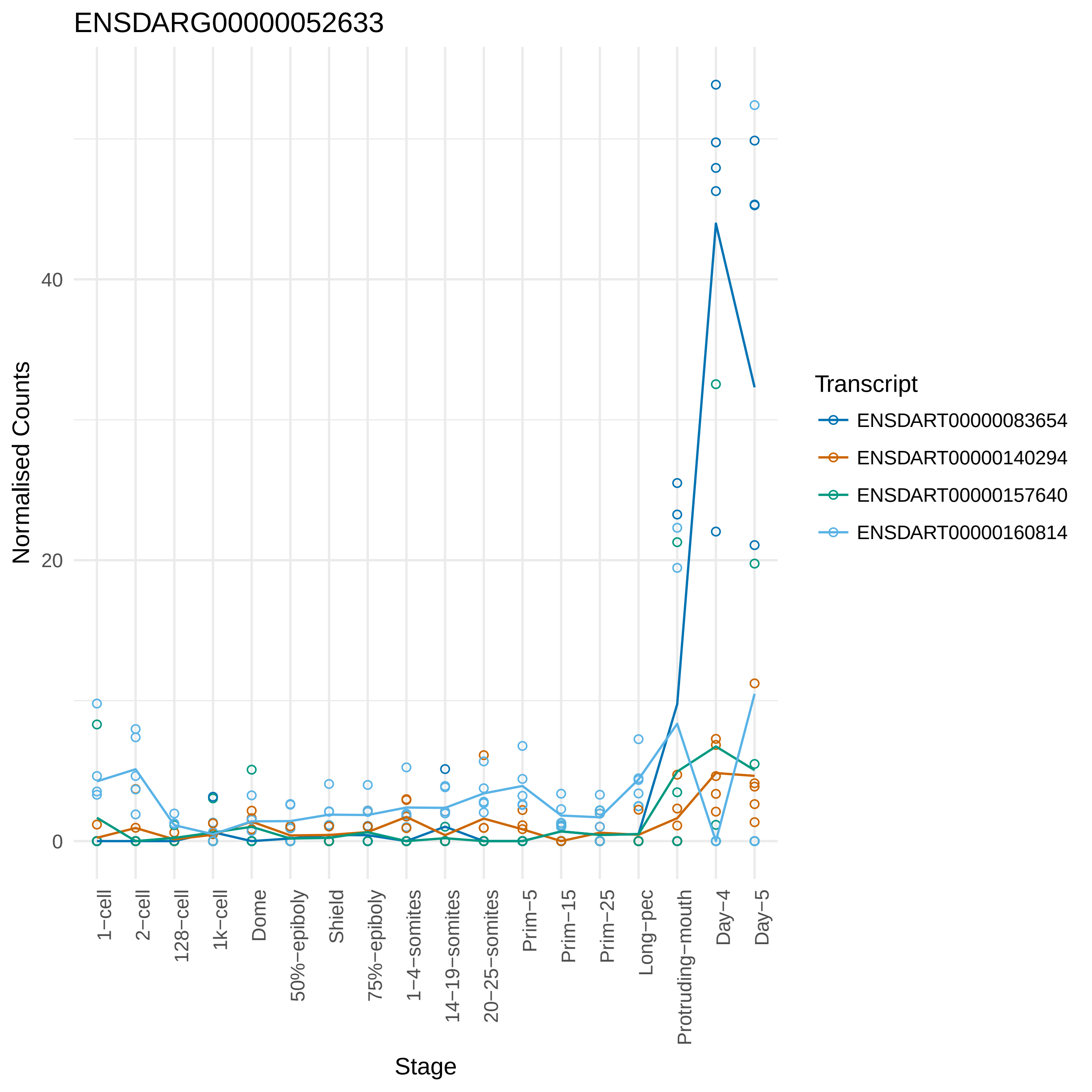

ENSDARG00000052633
Normalised Counts
0
20
40
Stage
1−cell
2−cell
128−cell
1k−cell
Dome
50%−epiboly
Shield
75%−epiboly
1−4−somites
14−19−somites
20−25−somites
Prim−5
Prim−15
Prim−25
Long−pec
Protruding−mouth
Day−4
Day−5
Transcript
ENSDART00000083654
ENSDART00000140294
ENSDART00000157640
ENSDART00000160814

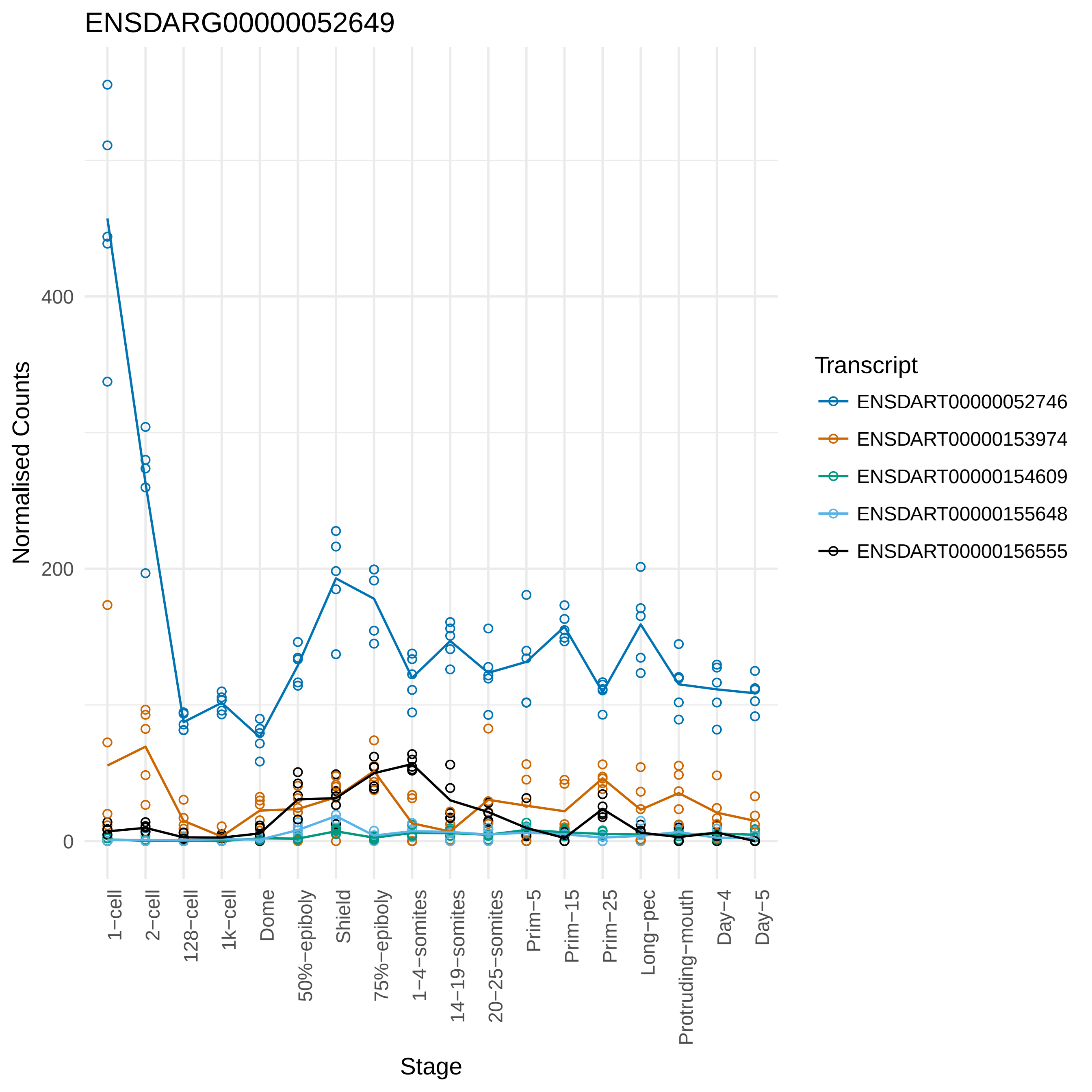
ENSDARG00000052649
Normalised Counts
0
200
400
1−cell
2−cell
128−cell
1k−cell
Dome
50%−epiboly
Shield
75%−epiboly
1−4−somites
14−19−somites
20−25−somites
Prim−5
Prim−15
Prim−25
Long−pec
Protruding−mouth
Day−4
Day−5
Stage
Transcript
ENSDART00000052746
ENSDART00000153974
ENSDART00000154609
ENSDART00000155648
ENSDART00000156555

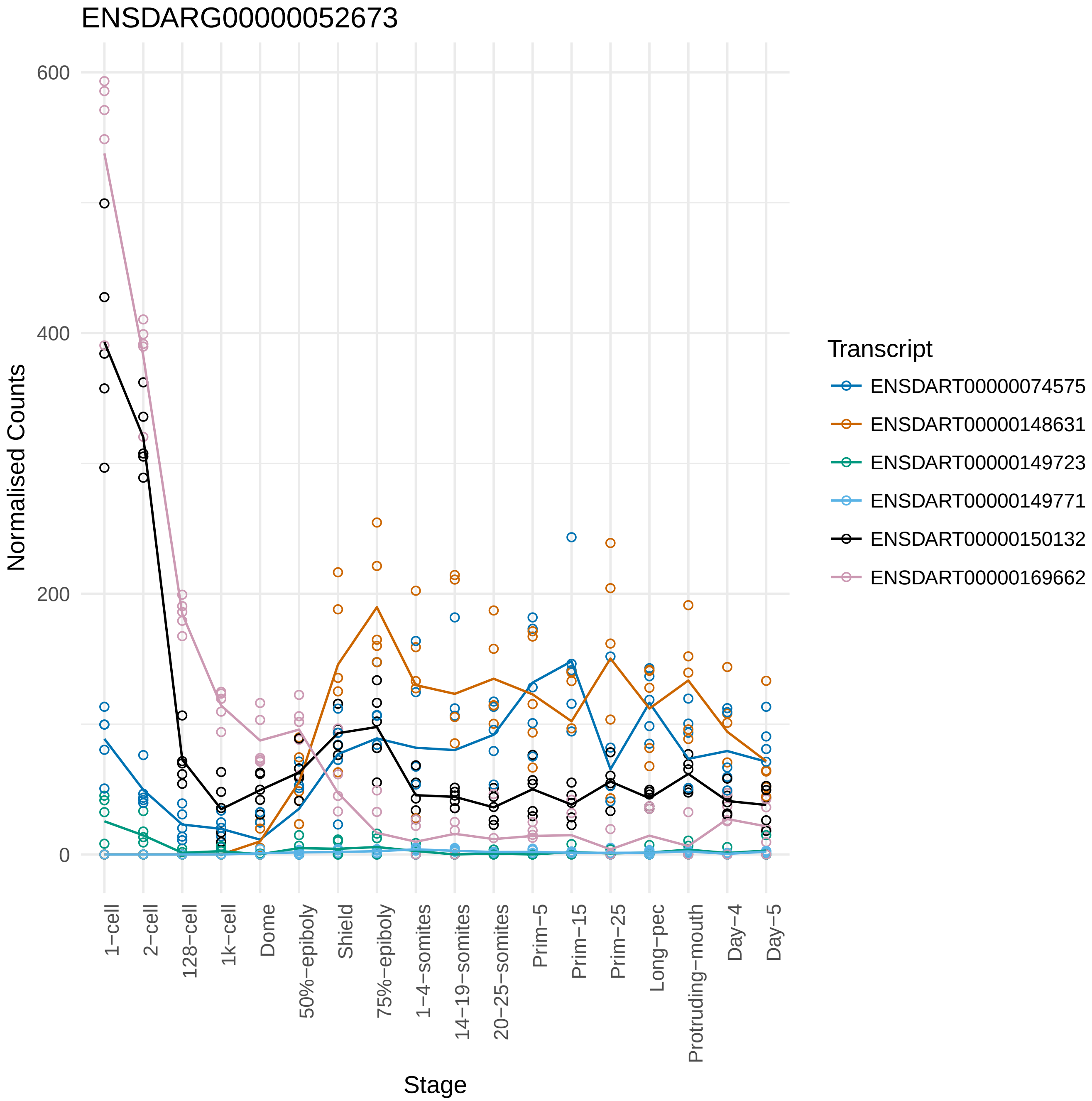

ENSDARG00000052673
Normalised Counts
600
400
200
0
Stage
1−cell
2−cell
128−cell
1k−cell
Dome
50%−epiboly
Shield
75%−epiboly
1−4−somites
14−19−somites
20−25−somites
Prim−5
Prim−15
Prim−25
Long−pec
Protruding−mouth
Day−4
Day−5
Transcript
ENSDART00000074575
ENSDART00000148631
ENSDART00000149723
ENSDART00000149771
ENSDART00000150132
ENSDART00000169662

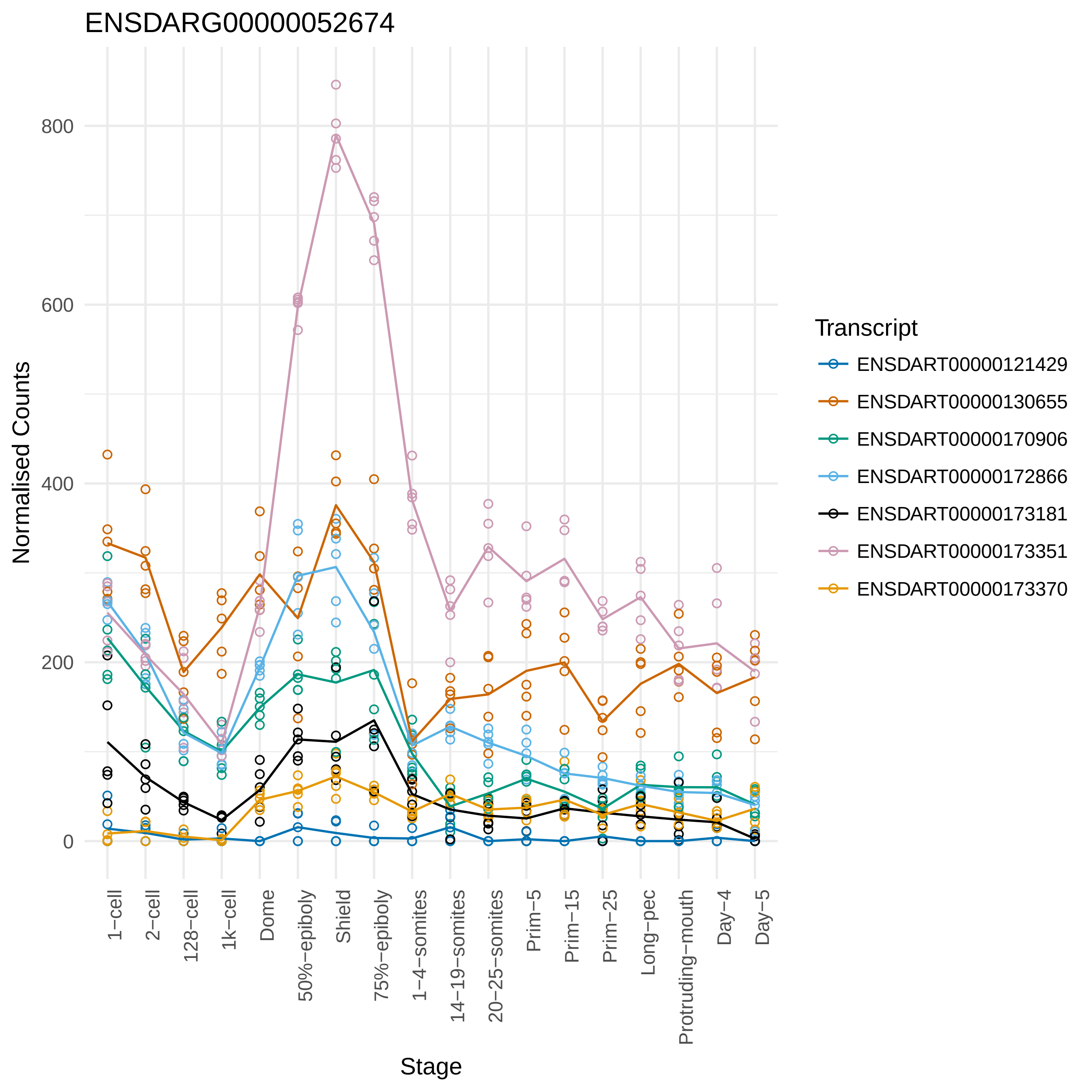
ENSDARG00000052674
Normalised Counts
800
600
400
200
0
1−cell
2−cell
128−cell
1k−cell
Dome
50%−epiboly
Shield
75%−epiboly
1−4−somites
14−19−somites
20−25−somites
Prim−5
Prim−15
Prim−25
Long−pec
Protruding−mouth
Day−4
Day−5
Stage
Transcript
ENSDART00000121429
ENSDART00000130655
ENSDART00000170906
ENSDART00000172866
ENSDART00000173181
ENSDART00000173351
ENSDART00000173370

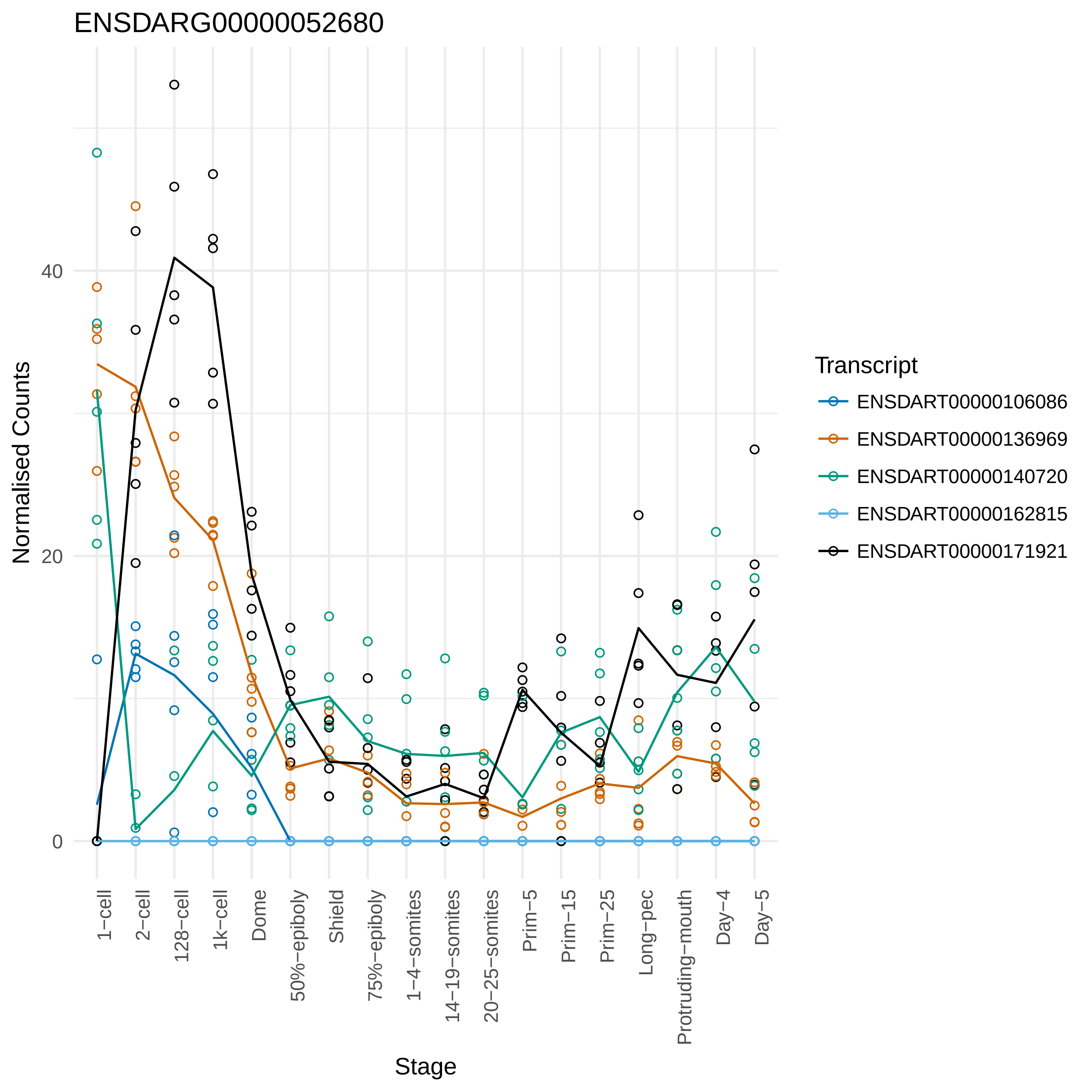

ENSDARG00000052680
Normalised Counts
0
20
40
1−cell
2−cell
128−cell
1k−cell
Dome
50%−epiboly
Shield
75%−epiboly
1−4−somites
14−19−somites
20−25−somites
Prim−5
Prim−15
Prim−25
Long−pec
Protruding−mouth
Day−4
Day−5
Stage
Transcript
ENSDART00000106086
ENSDART00000136969
ENSDART00000140720
ENSDART00000162815
ENSDART00000171921

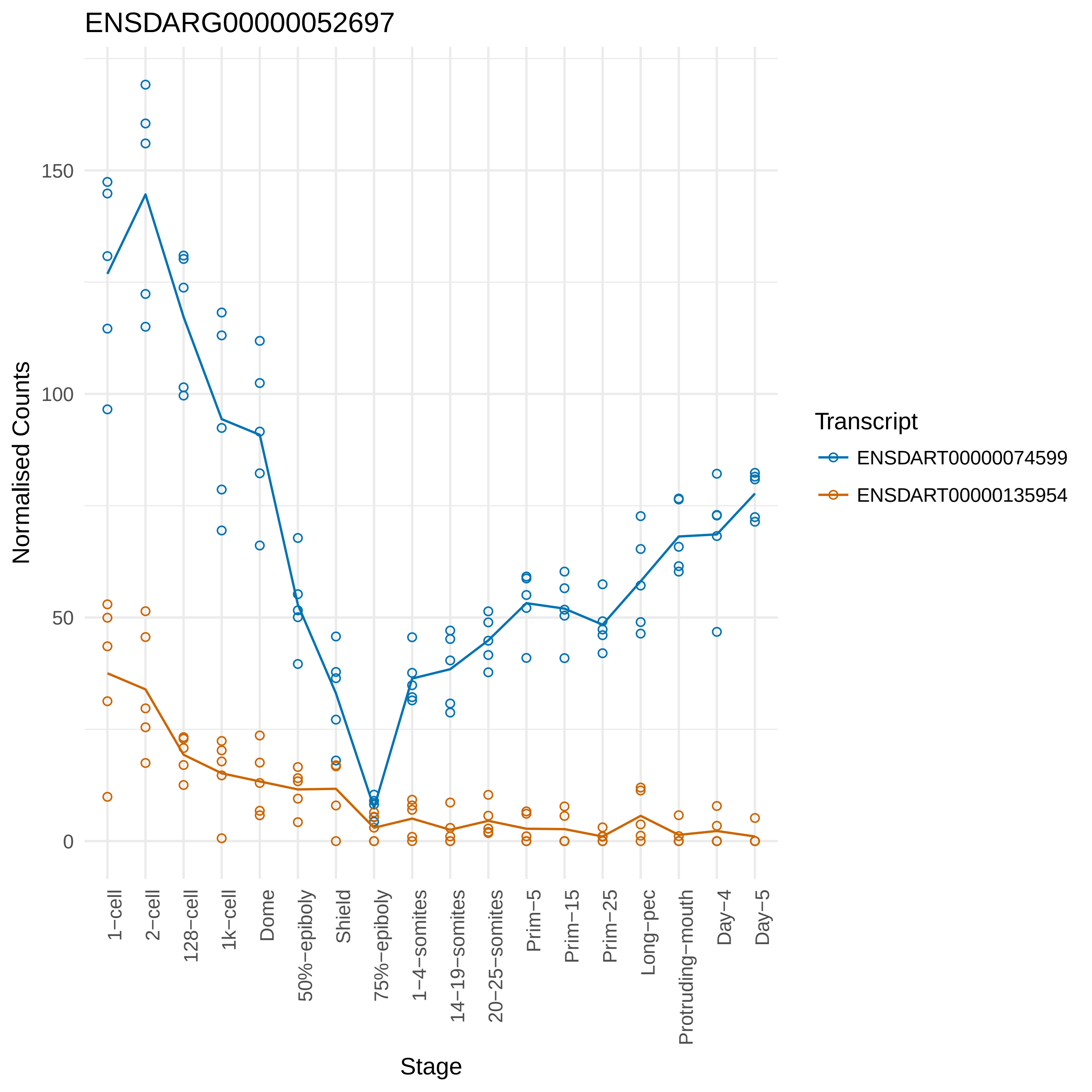
ENSDARG00000052697
Normalised Counts
150
100
50
0
1−cell
2−cell
128−cell
1k−cell
Dome
50%−epiboly
Shield
75%−epiboly
1−4−somites
14−19−somites
20−25−somites
Prim−5
Prim−15
Prim−25
Long−pec
Protruding−mouth
Day−4
Day−5
Stage
Transcript
ENSDART00000074599
ENSDART00000135954

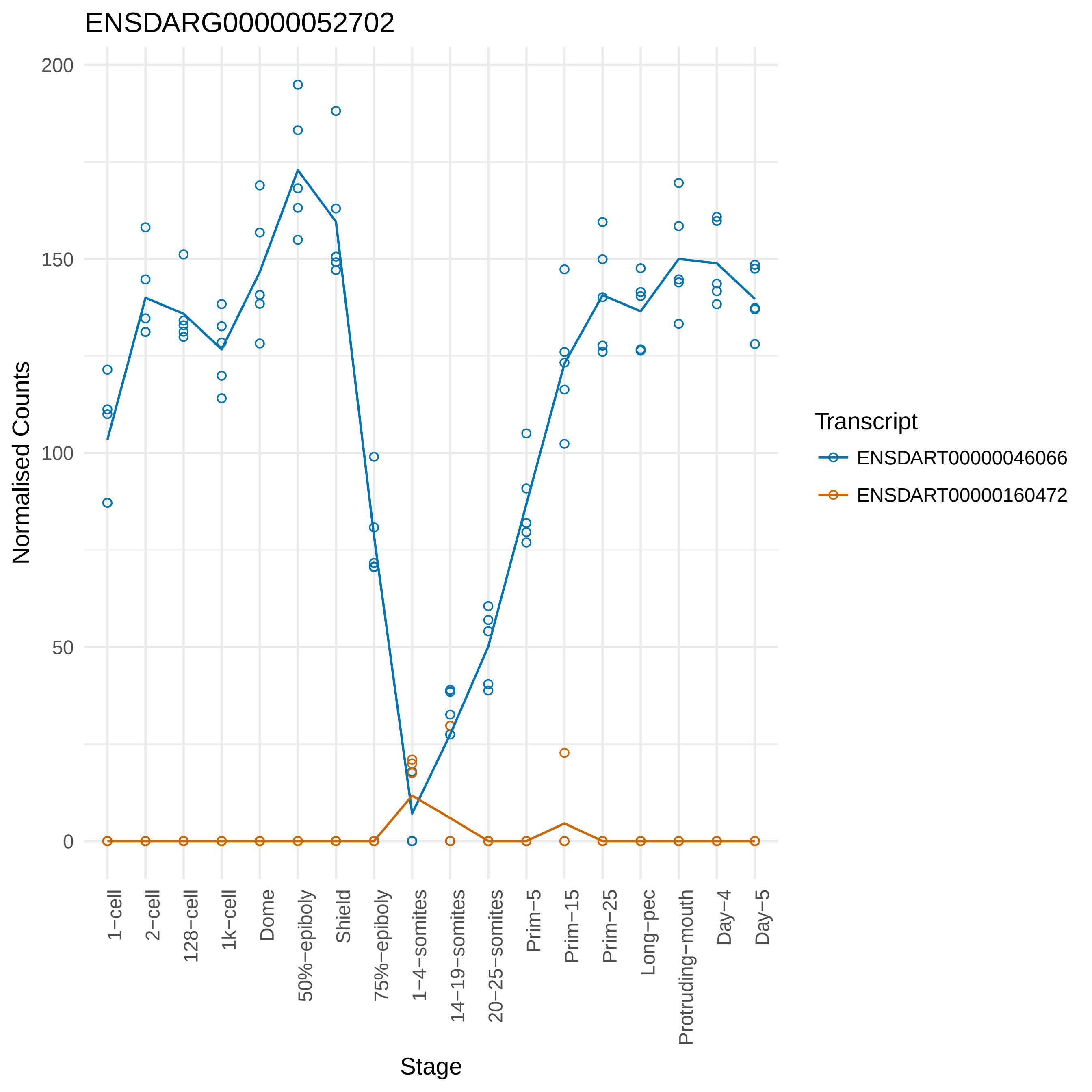
ENSDARG00000052702
Normalised Counts
200
150
100
50
0
1−cell
2−cell
128−cell
1k−cell
Dome
50%−epiboly
Shield
75%−epiboly
1−4−somites
14−19−somites
20−25−somites
Prim−5
Prim−15
Prim−25
Long−pec
Protruding−mouth
Day−4
Day−5
Stage
Transcript
ENSDART00000046066
ENSDART00000160472

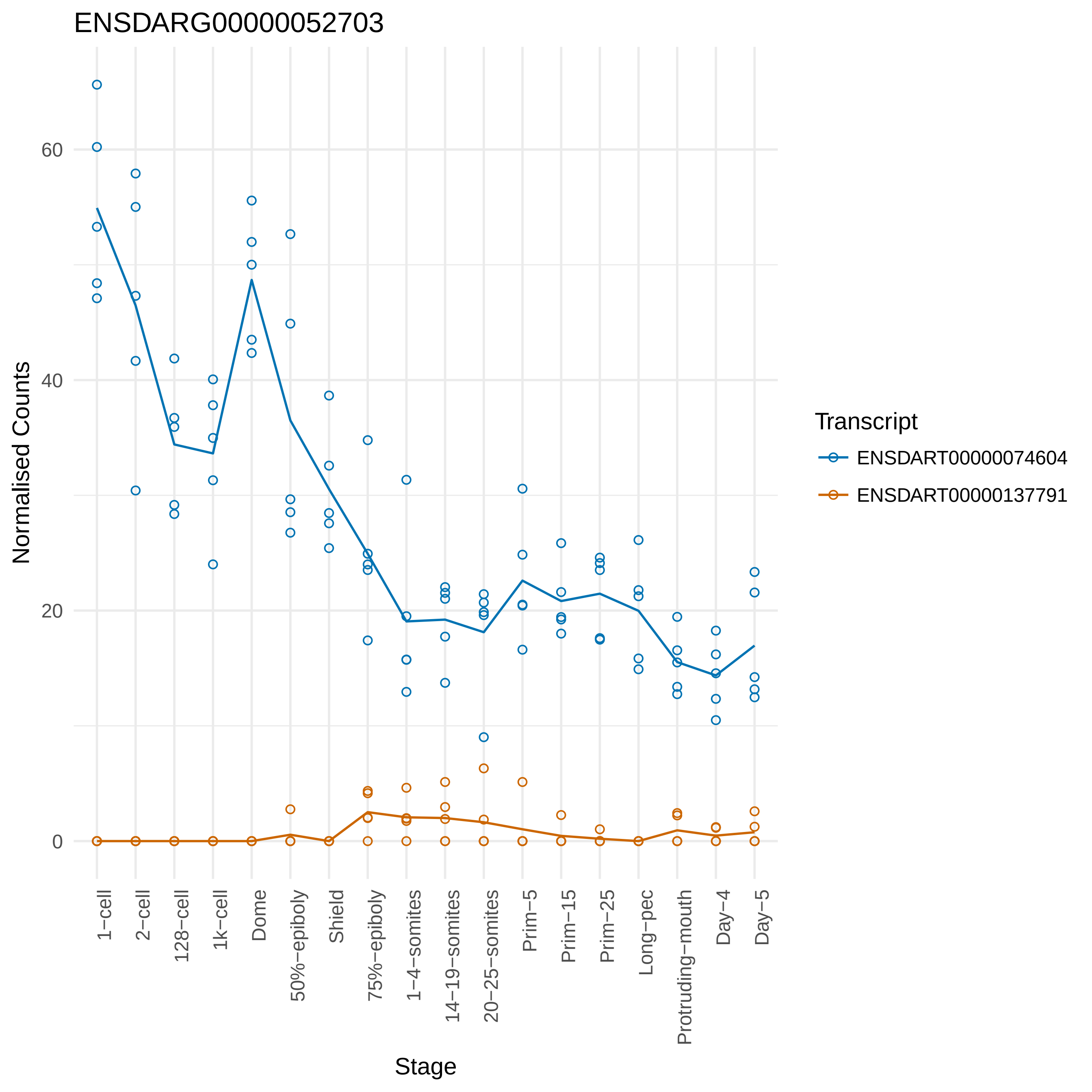
ENSDARG00000052703
Normalised Counts
60
40
20
0
Stage
1−cell
2−cell
128−cell
1k−cell
Dome
50%−epiboly
Shield
75%−epiboly
1−4−somites
14−19−somites
20−25−somites
Prim−5
Prim−15
Prim−25
Long−pec
Protruding−mouth
Day−4
Day−5
Transcript
ENSDART00000074604
ENSDART00000137791

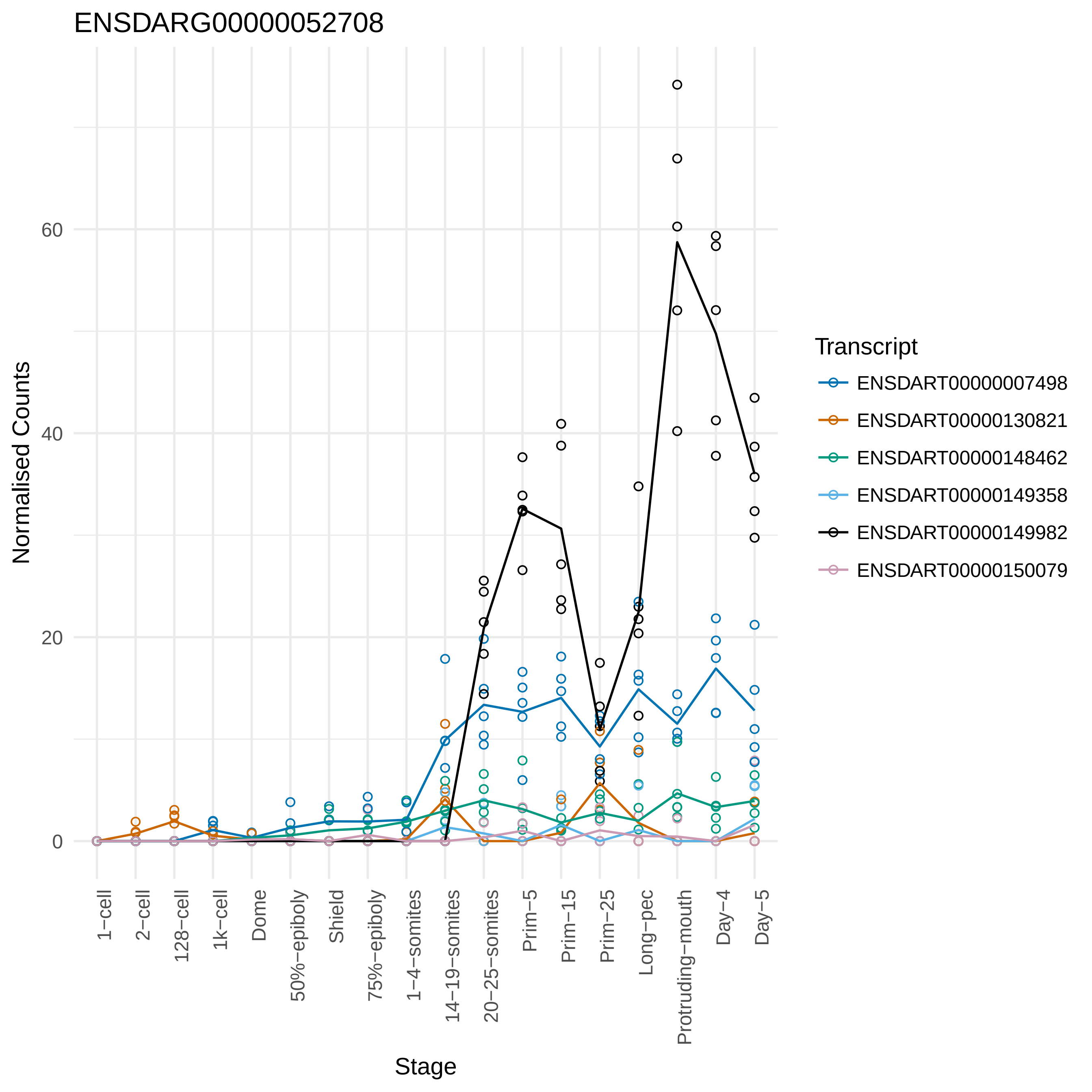
ENSDARG00000052708
Normalised Counts
0
20
40
60
Stage
1−cell
2−cell
128−cell
1k−cell
Dome
50%−epiboly
Shield
75%−epiboly
1−4−somites
14−19−somites
20−25−somites
Prim−5
Prim−15
Prim−25
Long−pec
Protruding−mouth
Day−4
Day−5
Transcript
ENSDART00000007498
ENSDART00000130821
ENSDART00000148462
ENSDART00000149358
ENSDART00000149982
ENSDART00000150079

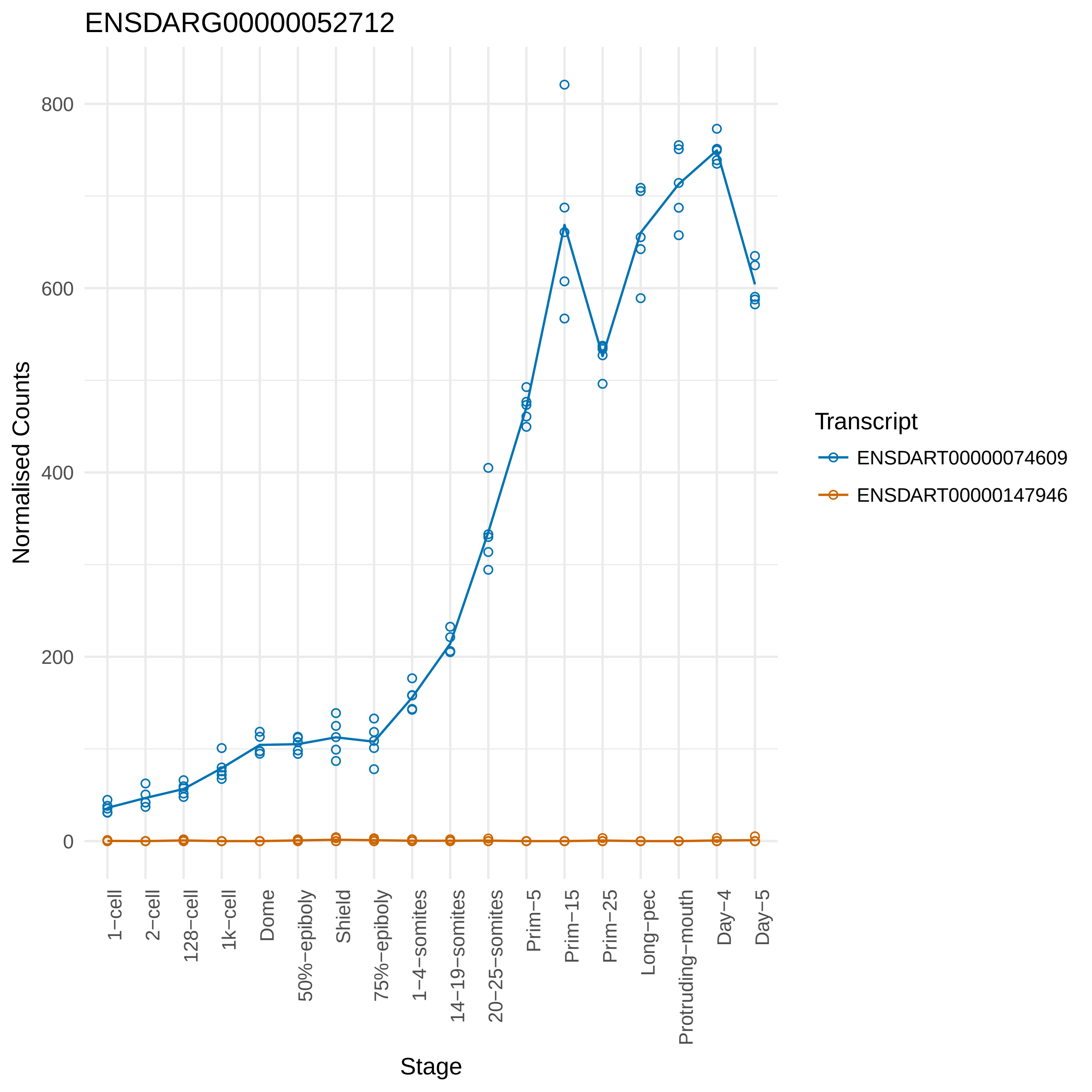
ENSDARG00000052712
Normalised Counts
800
600
400
200
0
1−cell
2−cell
128−cell
1k−cell
Dome
50%−epiboly
Shield
75%−epiboly
1−4−somites
14−19−somites
20−25−somites
Prim−5
Prim−15
Prim−25
Long−pec
Protruding−mouth
Day−4
Day−5
Stage
Transcript
ENSDART00000074609
ENSDART00000147946

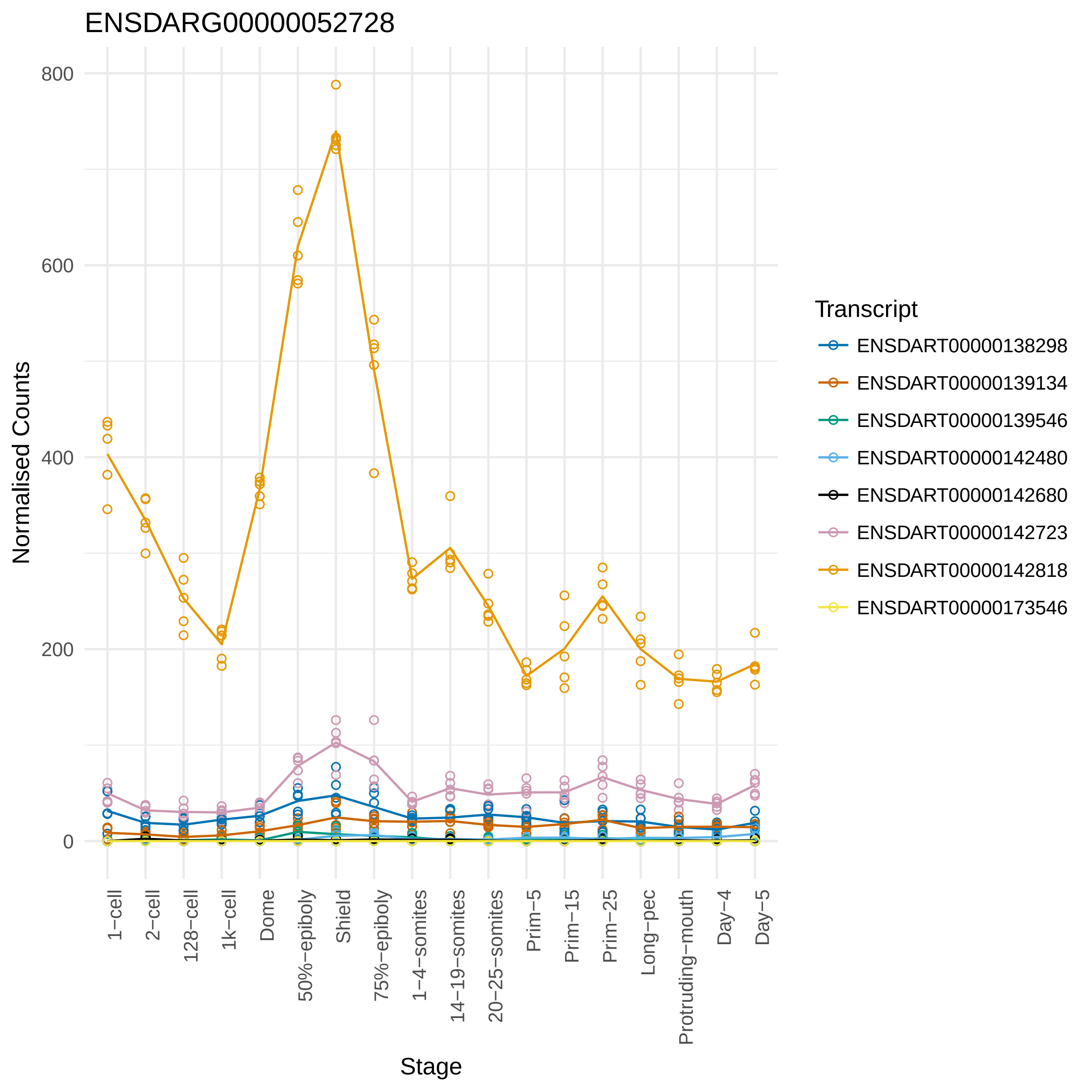

ENSDARG00000052728
Normalised Counts
800
600
400
200
0
1−cell
2−cell
128−cell
1k−cell
Dome
50%−epiboly
Shield
75%−epiboly
1−4−somites
14−19−somites
20−25−somites
Prim−5
Prim−15
Prim−25
Long−pec
Protruding−mouth
Day−4
Day−5
Stage
Transcript
ENSDART00000138298
ENSDART00000139134
ENSDART00000139546
ENSDART00000142480
ENSDART00000142680
ENSDART00000142723
ENSDART00000142818
ENSDART00000173546

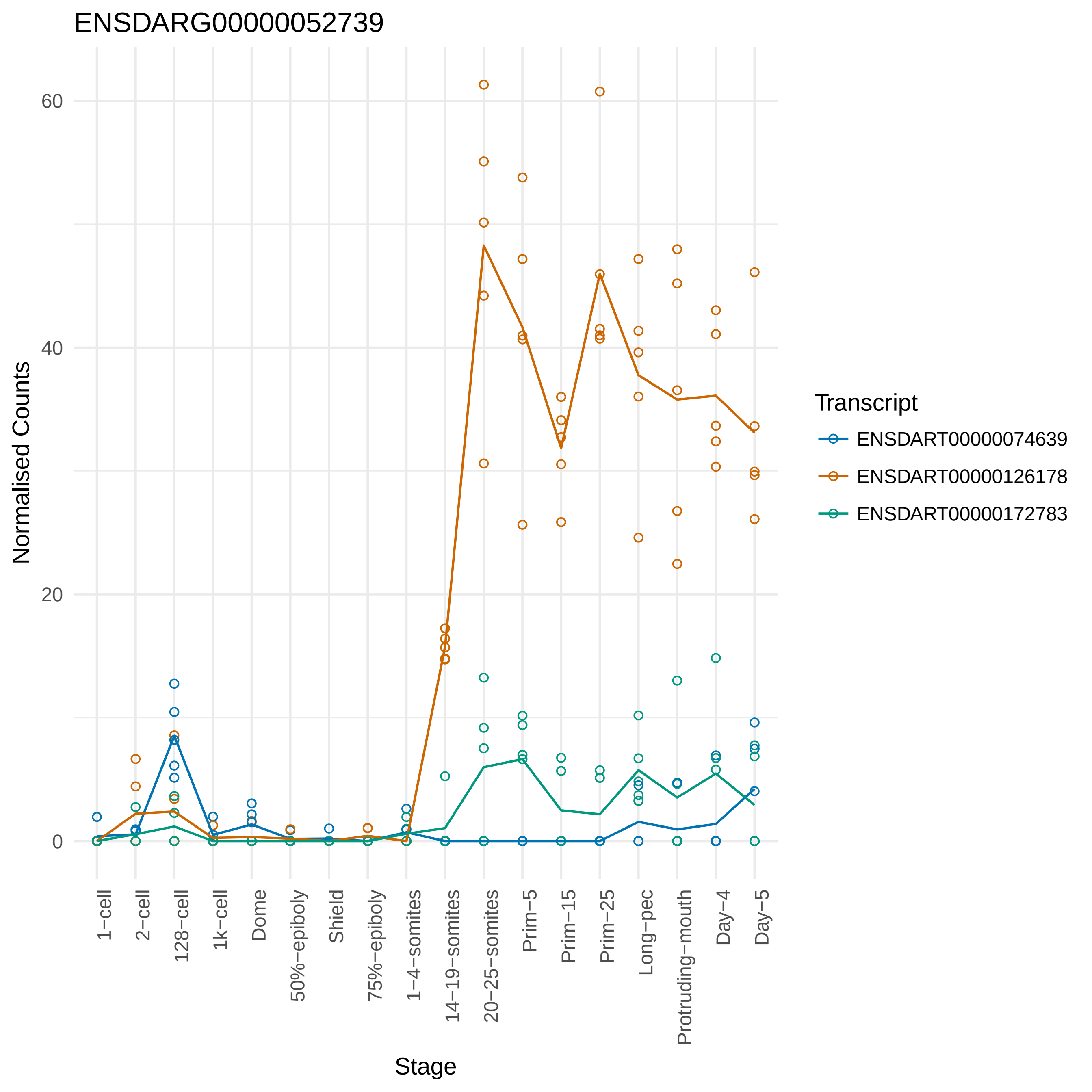

ENSDARG00000052739
Normalised Counts
60
40
20
0
1−cell
2−cell
128−cell
1k−cell
Dome
50%−epiboly
Shield
75%−epiboly
1−4−somites
14−19−somites
20−25−somites
Prim−5
Prim−15
Prim−25
Long−pec
Protruding−mouth
Day−4
Day−5
Stage
Transcript
ENSDART00000074639
ENSDART00000126178
ENSDART00000172783

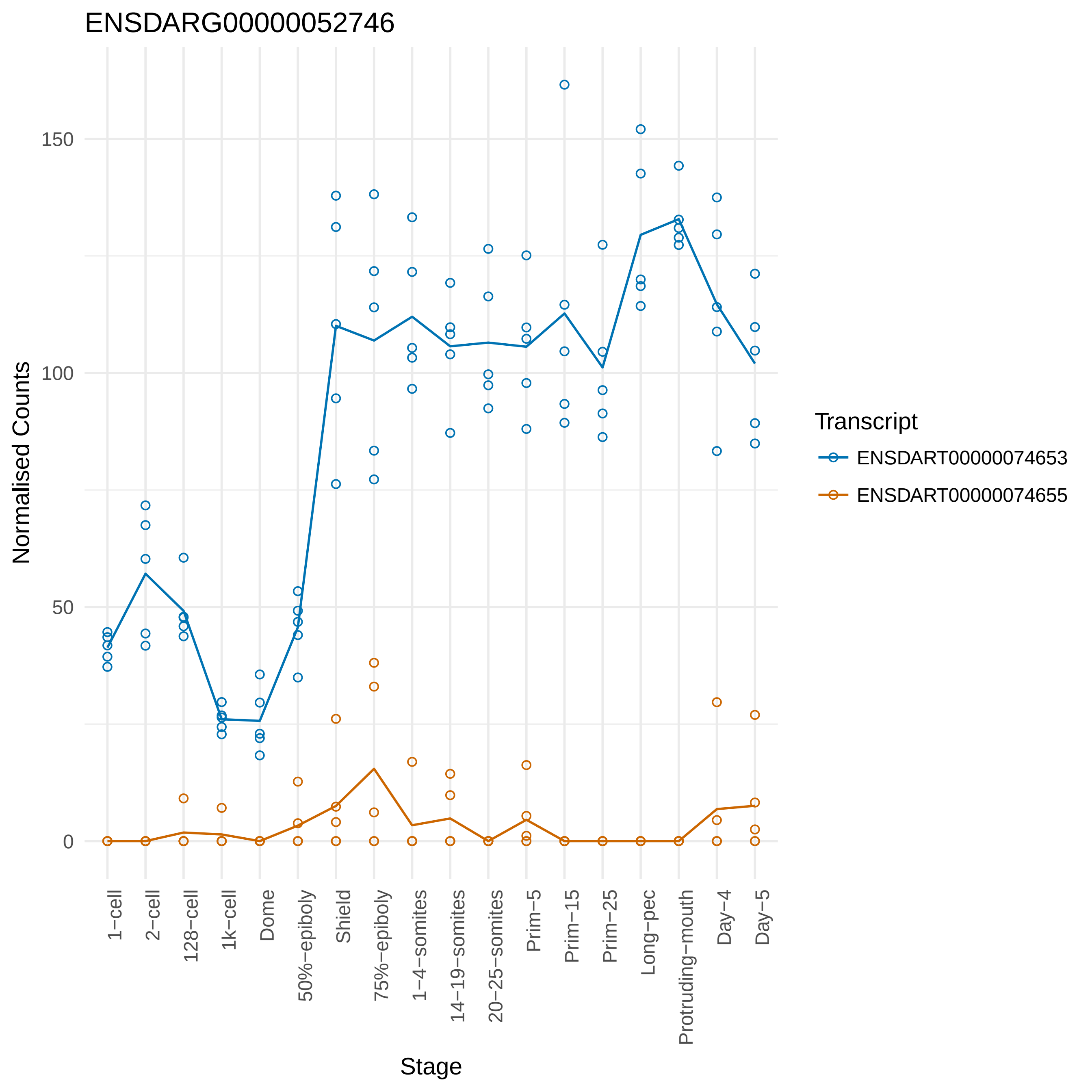

ENSDARG00000052746
Normalised Counts
150
100
50
0
1−cell
2−cell
128−cell
1k−cell
Dome
50%−epiboly
Shield
75%−epiboly
1−4−somites
14−19−somites
20−25−somites
Prim−5
Prim−15
Prim−25
Long−pec
Protruding−mouth
Day−4
Day−5
Stage
Transcript
ENSDART00000074653
ENSDART00000074655

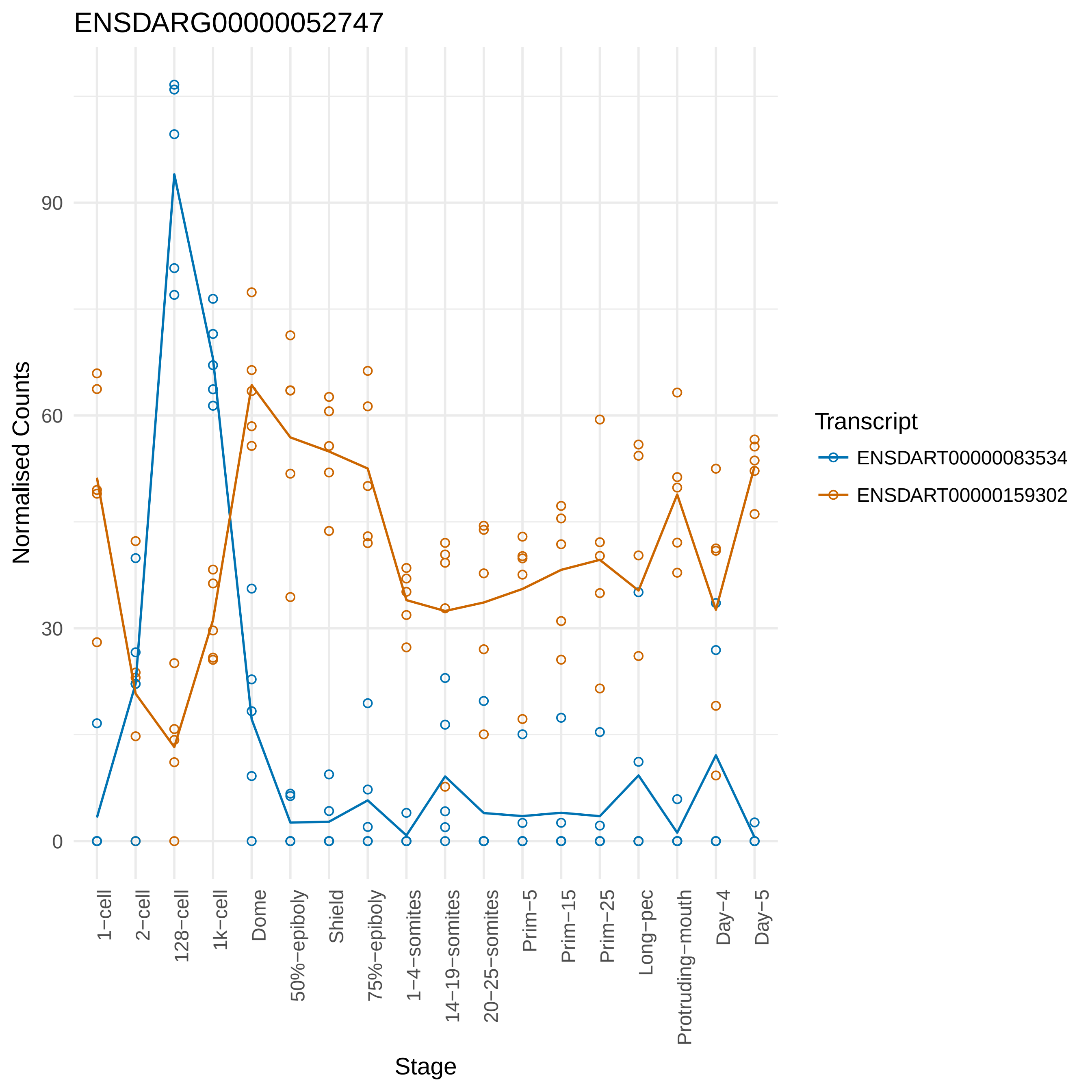
ENSDARG00000052747
Normalised Counts
90
60
30
0
1−cell
2−cell
128−cell
1k−cell
Dome
50%−epiboly
Shield
75%−epiboly
1−4−somites
14−19−somites
20−25−somites
Prim−5
Prim−15
Prim−25
Long−pec
Protruding−mouth
Day−4
Day−5
Stage
Transcript
ENSDART00000083534
ENSDART00000159302

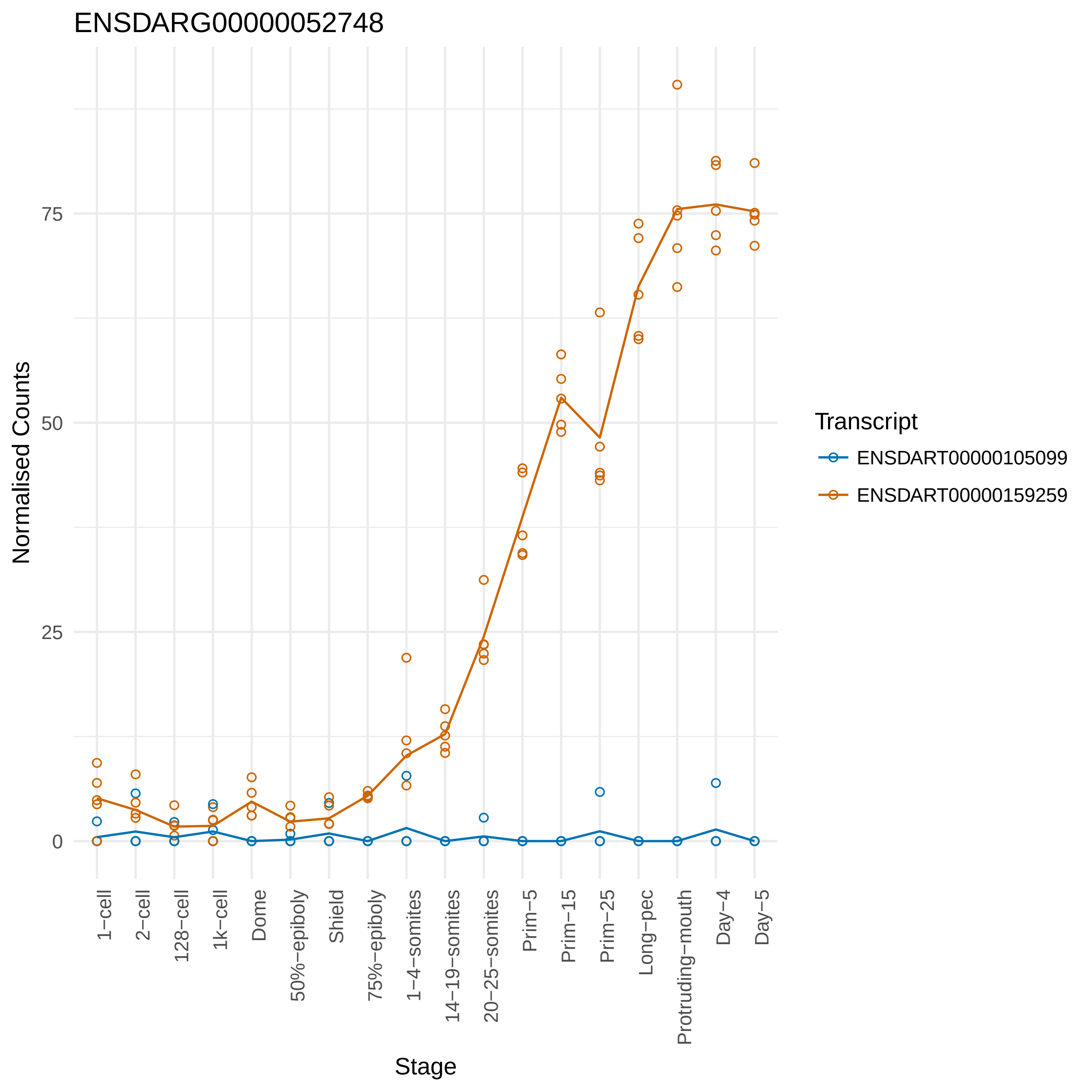

ENSDARG00000052748
Normalised Counts
0
25
50
75
1−cell
2−cell
128−cell
1k−cell
Dome
50%−epiboly
Shield
75%−epiboly
1−4−somites
14−19−somites
20−25−somites
Prim−5
Prim−15
Prim−25
Long−pec
Protruding−mouth
Day−4
Day−5
Stage
Transcript
ENSDART00000105099
ENSDART00000159259

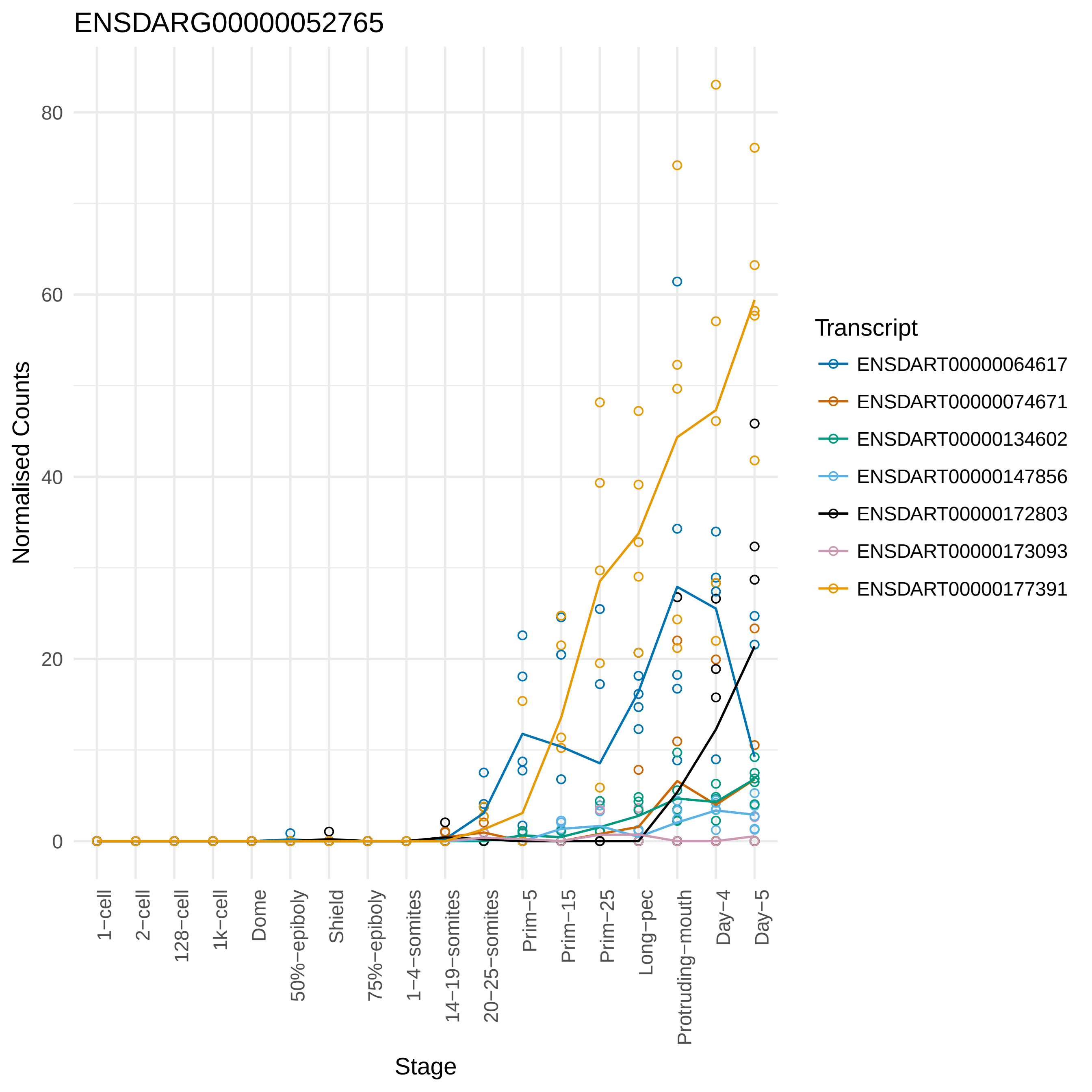

ENSDARG00000052765
Normalised Counts
0
20
40
60
80
Stage
1−cell
2−cell
128−cell
1k−cell
Dome
50%−epiboly
Shield
75%−epiboly
1−4−somites
14−19−somites
20−25−somites
Prim−5
Prim−15
Prim−25
Long−pec
Protruding−mouth
Day−4
Day−5
Transcript
ENSDART00000064617
ENSDART00000074671
ENSDART00000134602
ENSDART00000147856
ENSDART00000172803
ENSDART00000173093
ENSDART00000177391

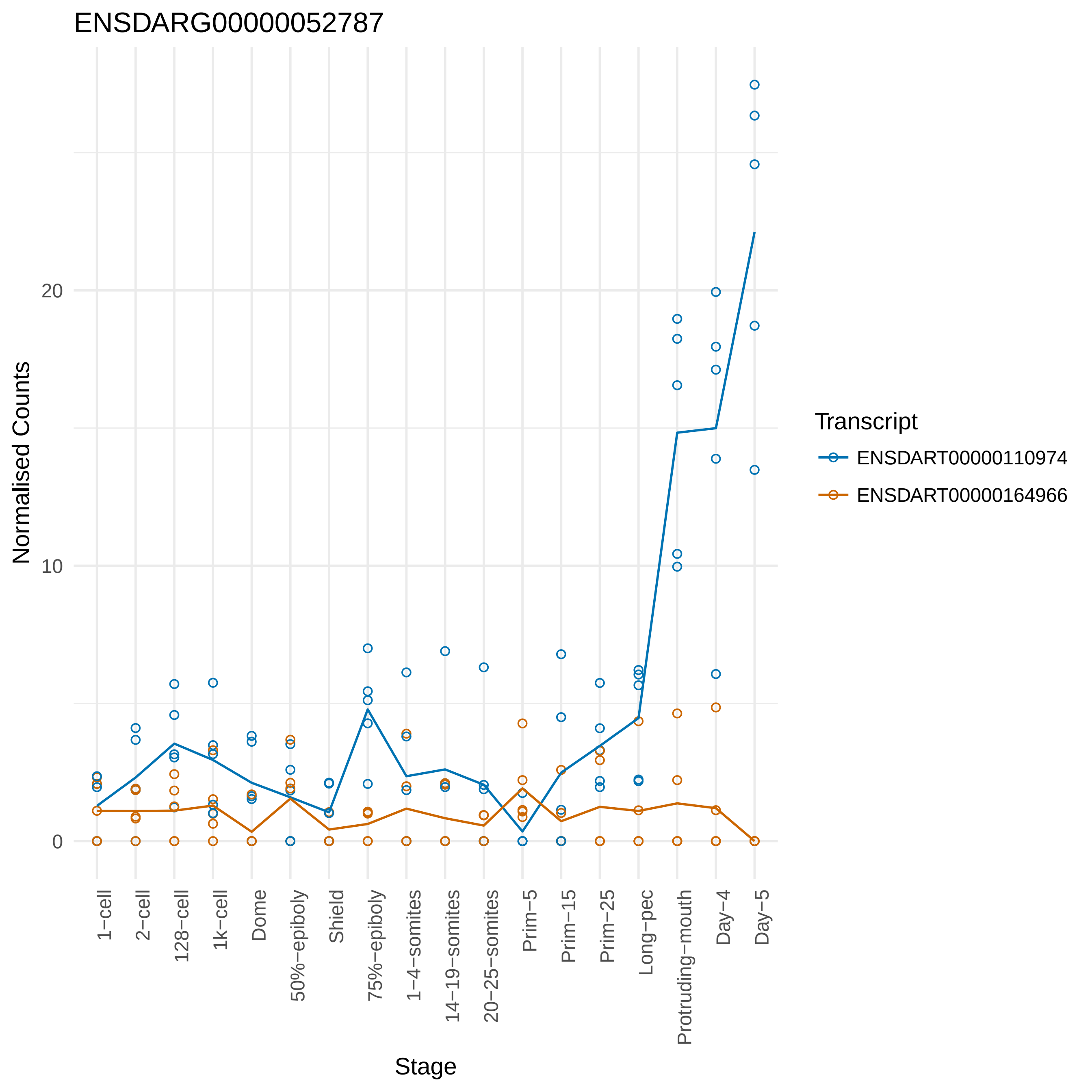
ENSDARG00000052787
Normalised Counts
20
10
0
1−cell
2−cell
128−cell
1k−cell
Dome
50%−epiboly
Shield
75%−epiboly
1−4−somites
14−19−somites
20−25−somites
Prim−5
Prim−15
Prim−25
Long−pec
Protruding−mouth
Day−4
Day−5
Stage
Transcript
ENSDART00000110974
ENSDART00000164966

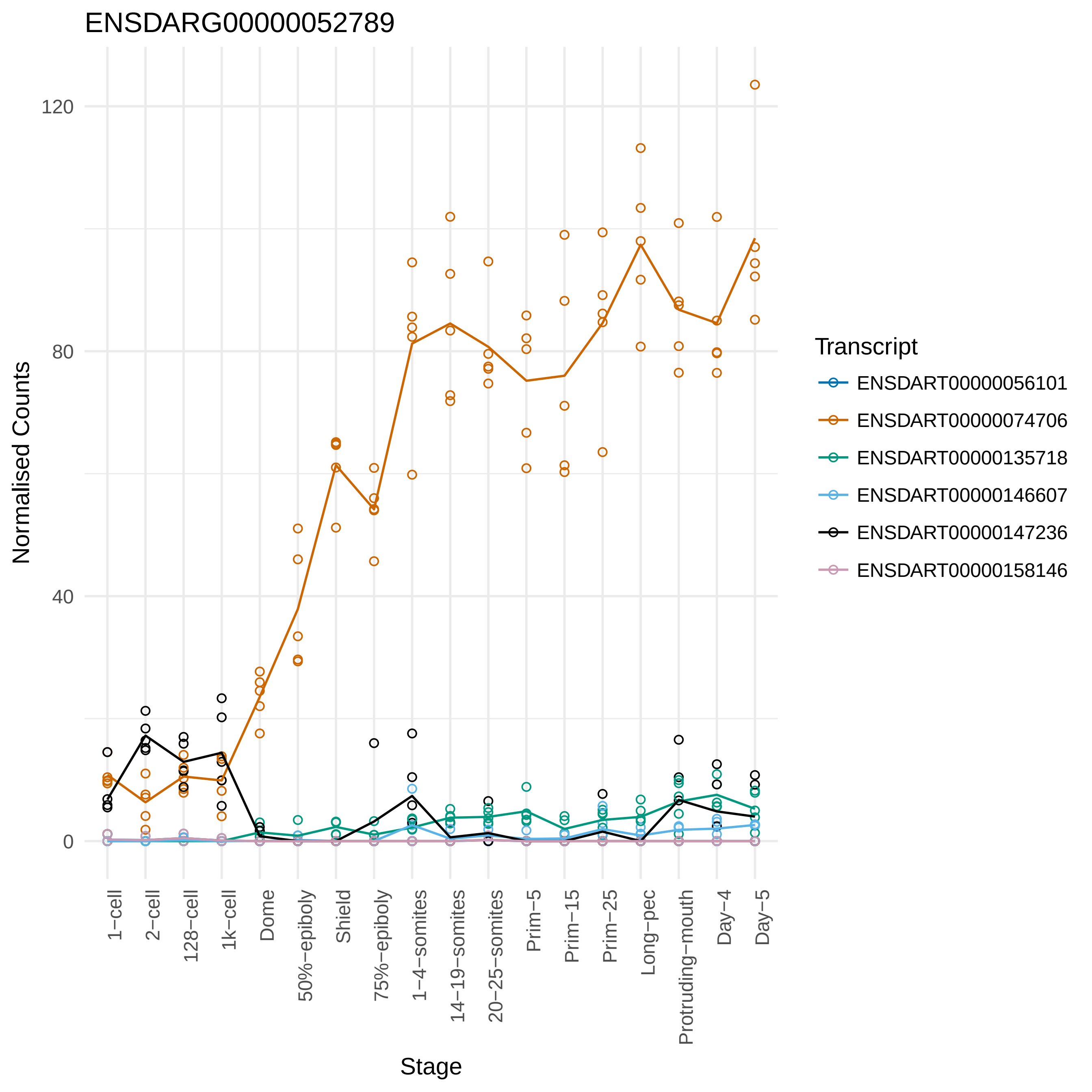
ENSDARG00000052789
Normalised Counts
120
80
40
0
1−cell
2−cell
128−cell
1k−cell
Dome
50%−epiboly
Shield
75%−epiboly
1−4−somites
14−19−somites
20−25−somites
Prim−5
Prim−15
Prim−25
Long−pec
Protruding−mouth
Day−4
Day−5
Stage
Transcript
ENSDART00000056101
ENSDART00000074706
ENSDART00000135718
ENSDART00000146607
ENSDART00000147236
ENSDART00000158146

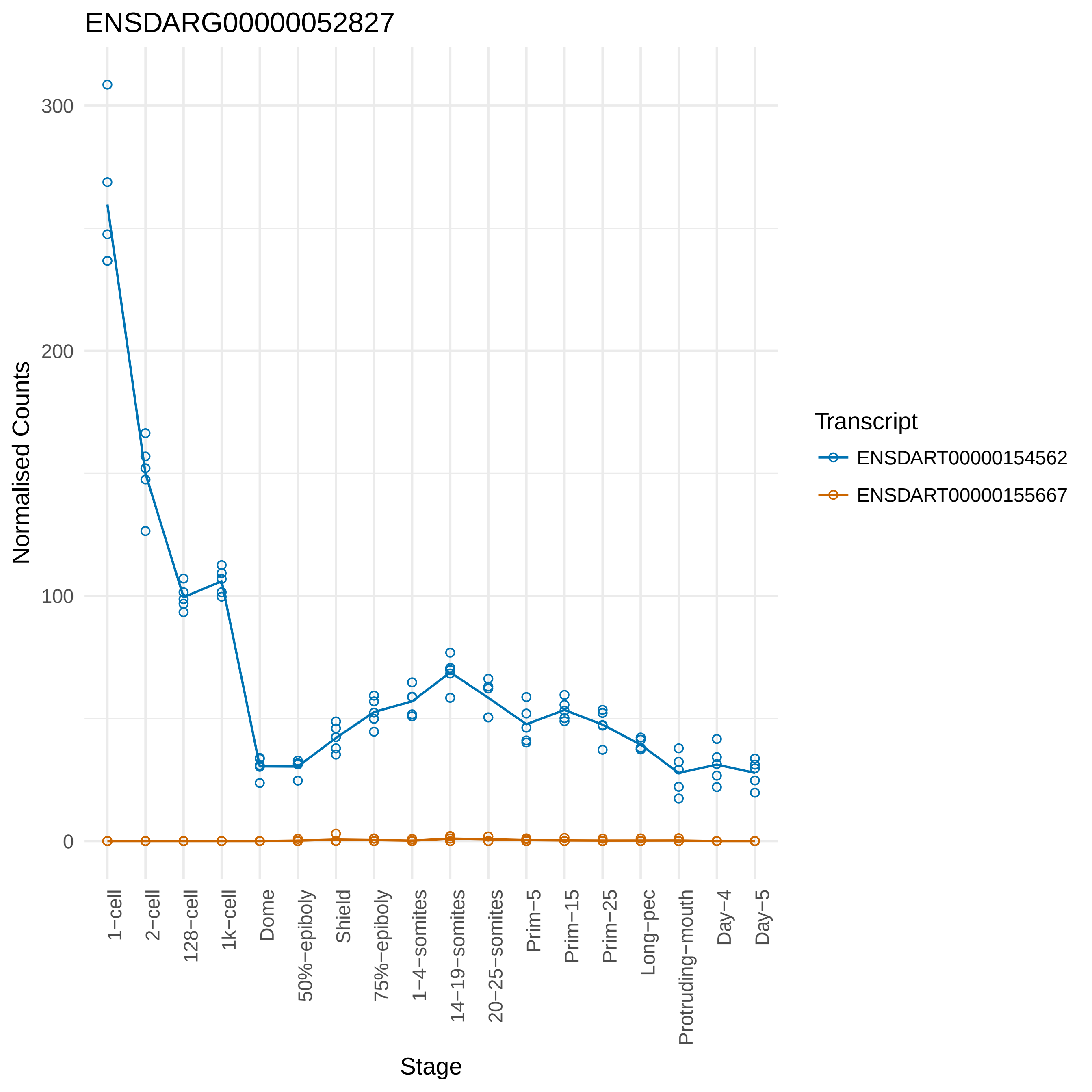
ENSDARG00000052827
Normalised Counts
300
200
100
0
1−cell
2−cell
128−cell
1k−cell
Dome
50%−epiboly
Shield
75%−epiboly
1−4−somites
14−19−somites
20−25−somites
Prim−5
Prim−15
Prim−25
Long−pec
Protruding−mouth
Day−4
Day−5
Stage
Transcript
ENSDART00000154562
ENSDART00000155667

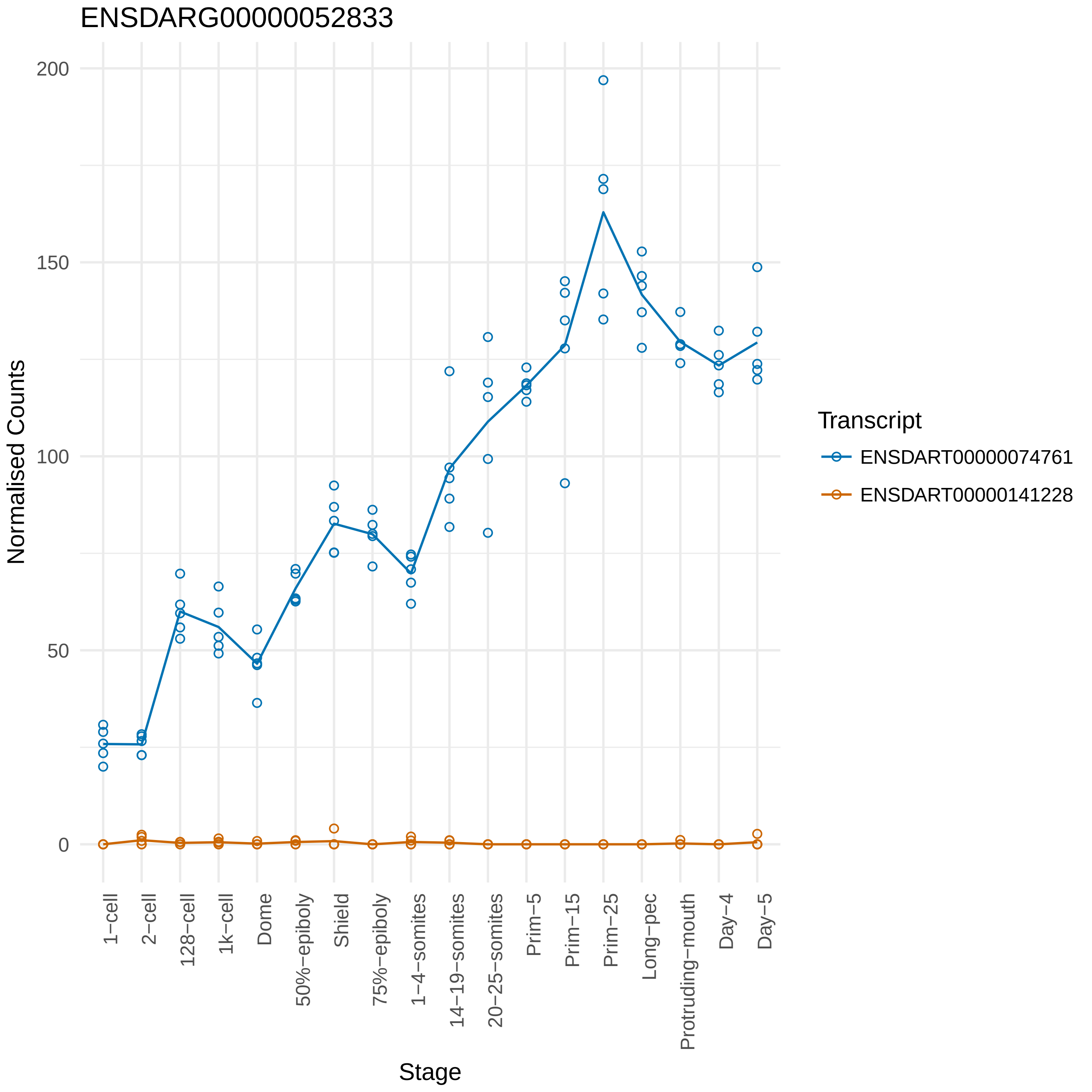

ENSDARG00000052833
Normalised Counts
Stage
Transcript
ENSDART00000074761
ENSDART00000141228
0
50
100
150
200
1−cell
2−cell
128−cell
1k−cell
Dome
50%−epiboly
Shield
75%−epiboly
1−4−somites
14−19−somites
20−25−somites
Prim−5
Prim−15
Prim−25
Long−pec
Protruding−mouth
Day−4
Day−5

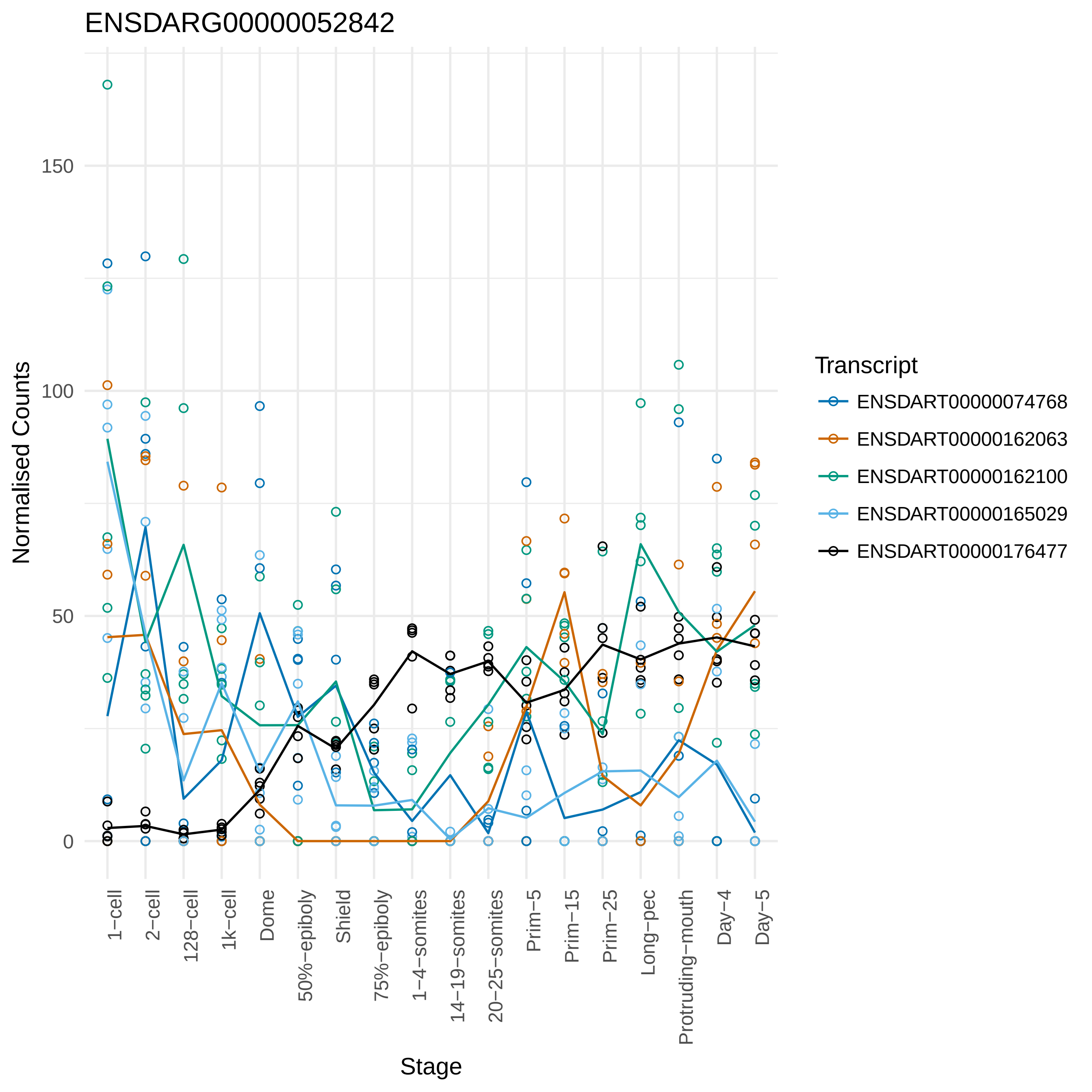
ENSDARG00000052842
Normalised Counts
150
100
50
0
1−cell
2−cell
128−cell
1k−cell
Dome
50%−epiboly
Shield
75%−epiboly
1−4−somites
14−19−somites
20−25−somites
Prim−5
Prim−15
Prim−25
Long−pec
Protruding−mouth
Day−4
Day−5
Stage
Transcript
ENSDART00000074768
ENSDART00000162063
ENSDART00000162100
ENSDART00000165029
ENSDART00000176477

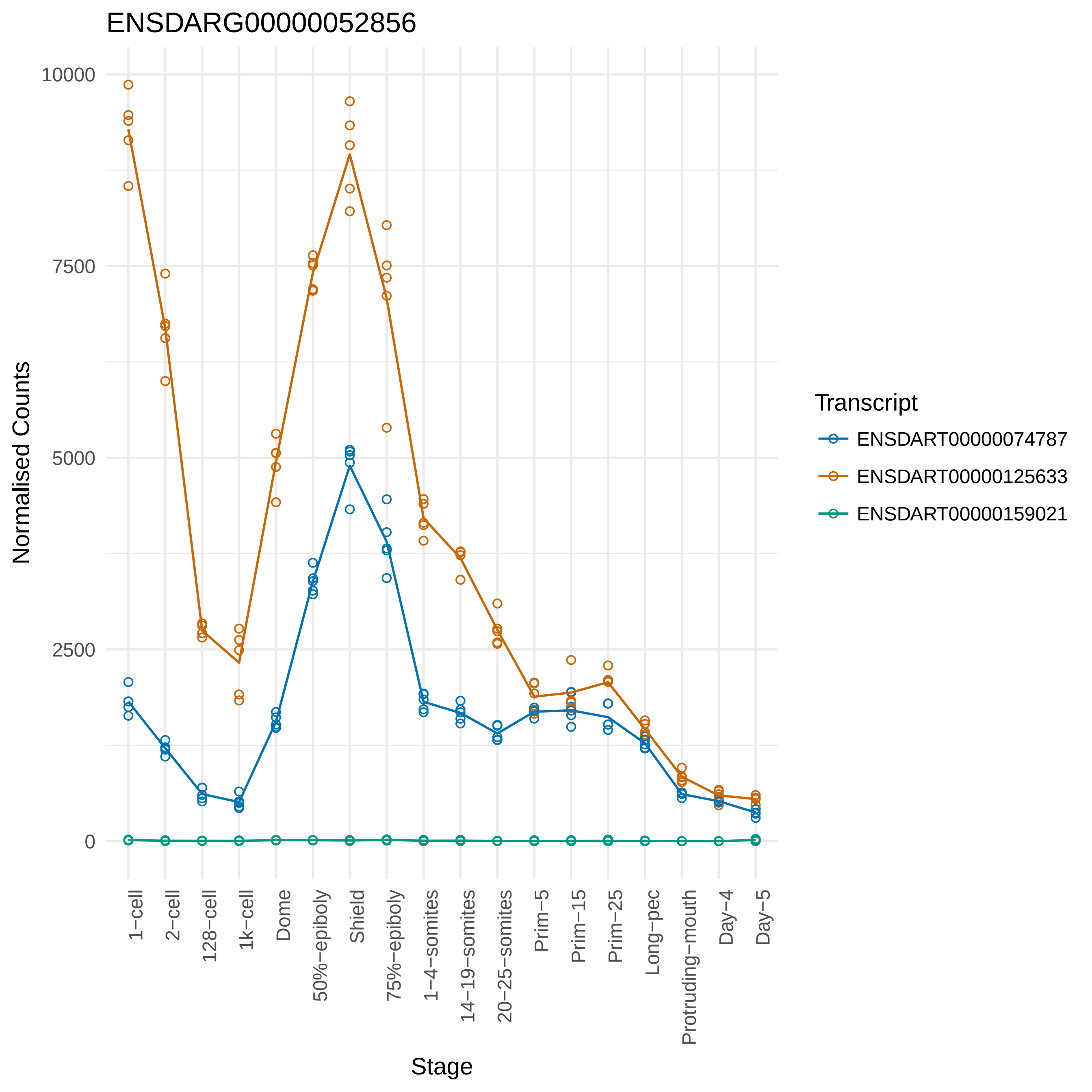

ENSDARG00000052856
Normalised Counts
10000
7500
5000
2500
0
1−cell
2−cell
128−cell
1k−cell
Dome
50%−epiboly
Shield
75%−epiboly
1−4−somites
14−19−somites
20−25−somites
Prim−5
Prim−15
Prim−25
Long−pec
Protruding−mouth
Day−4
Day−5
Stage
Transcript
ENSDART00000074787
ENSDART00000125633
ENSDART00000159021

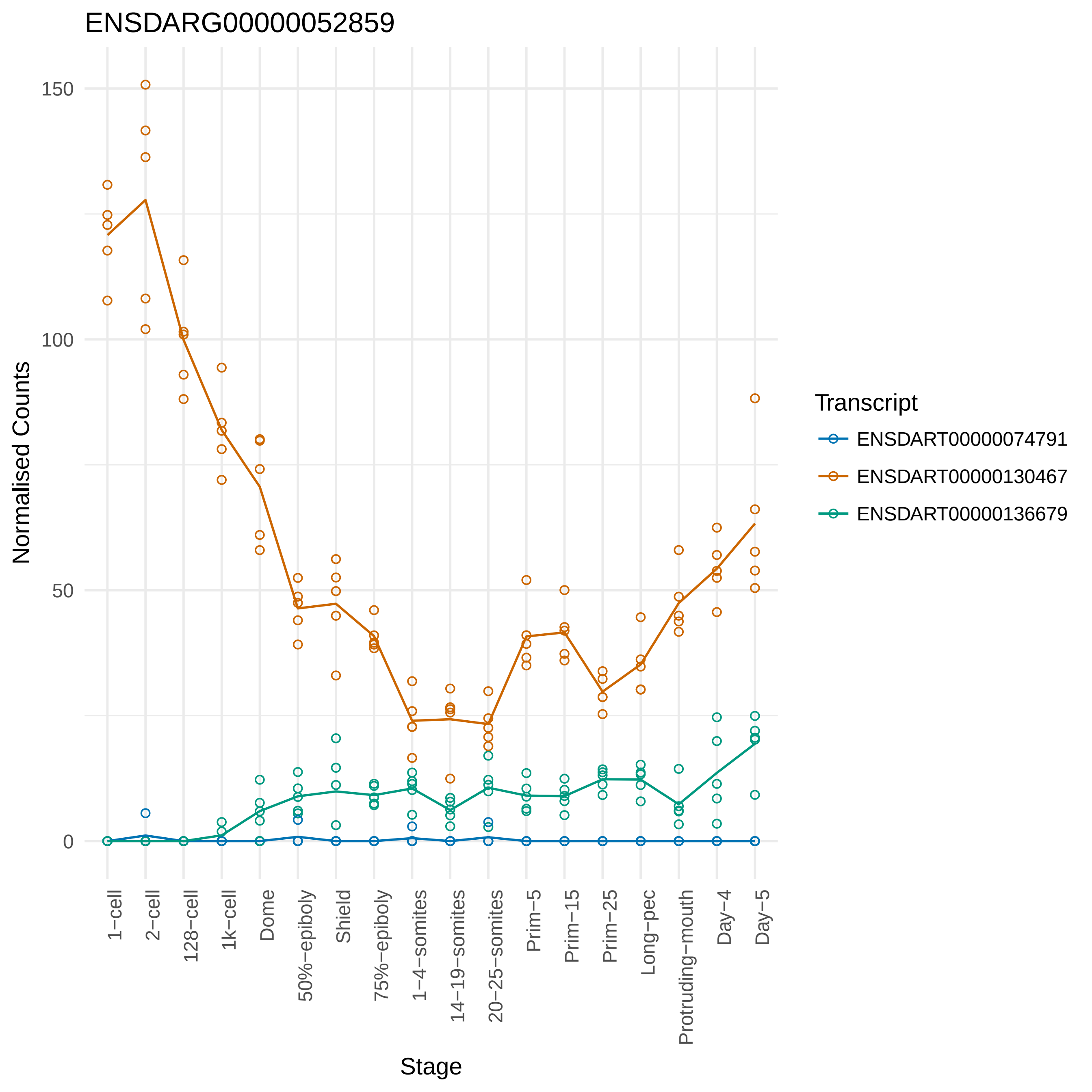
ENSDARG00000052859
Normalised Counts
150
100
50
0
Stage
1−cell
2−cell
128−cell
1k−cell
Dome
50%−epiboly
Shield
75%−epiboly
1−4−somites
14−19−somites
20−25−somites
Prim−5
Prim−15
Prim−25
Long−pec
Protruding−mouth
Day−4
Day−5
Transcript
ENSDART00000074791
ENSDART00000130467
ENSDART00000136679

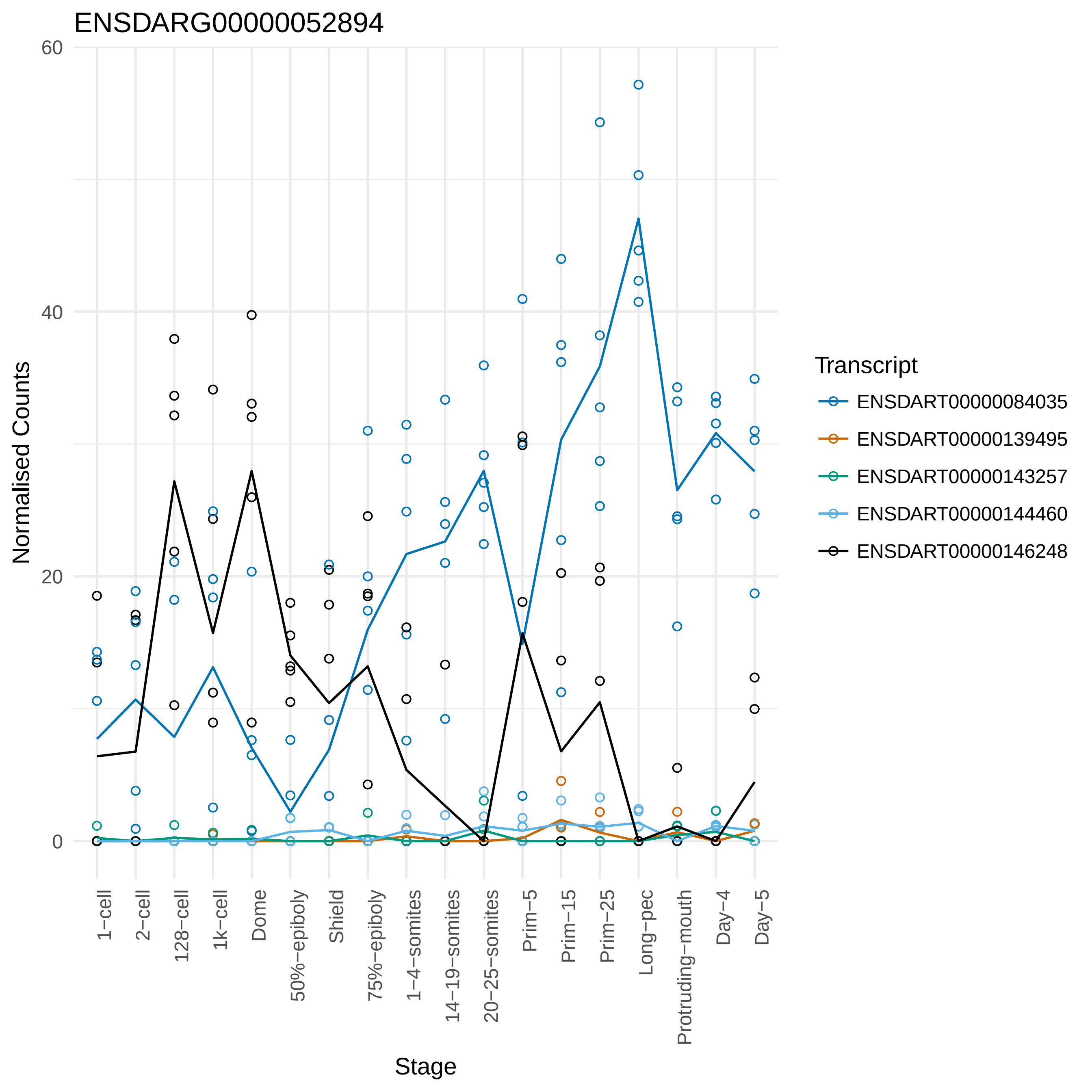
ENSDARG00000052894
Normalised Counts
60
40
20
0
1−cell
2−cell
128−cell
1k−cell
Dome
50%−epiboly
Shield
75%−epiboly
1−4−somites
14−19−somites
20−25−somites
Prim−5
Prim−15
Prim−25
Long−pec
Protruding−mouth
Day−4
Day−5
Stage
Transcript
ENSDART00000084035
ENSDART00000139495
ENSDART00000143257
ENSDART00000144460
ENSDART00000146248

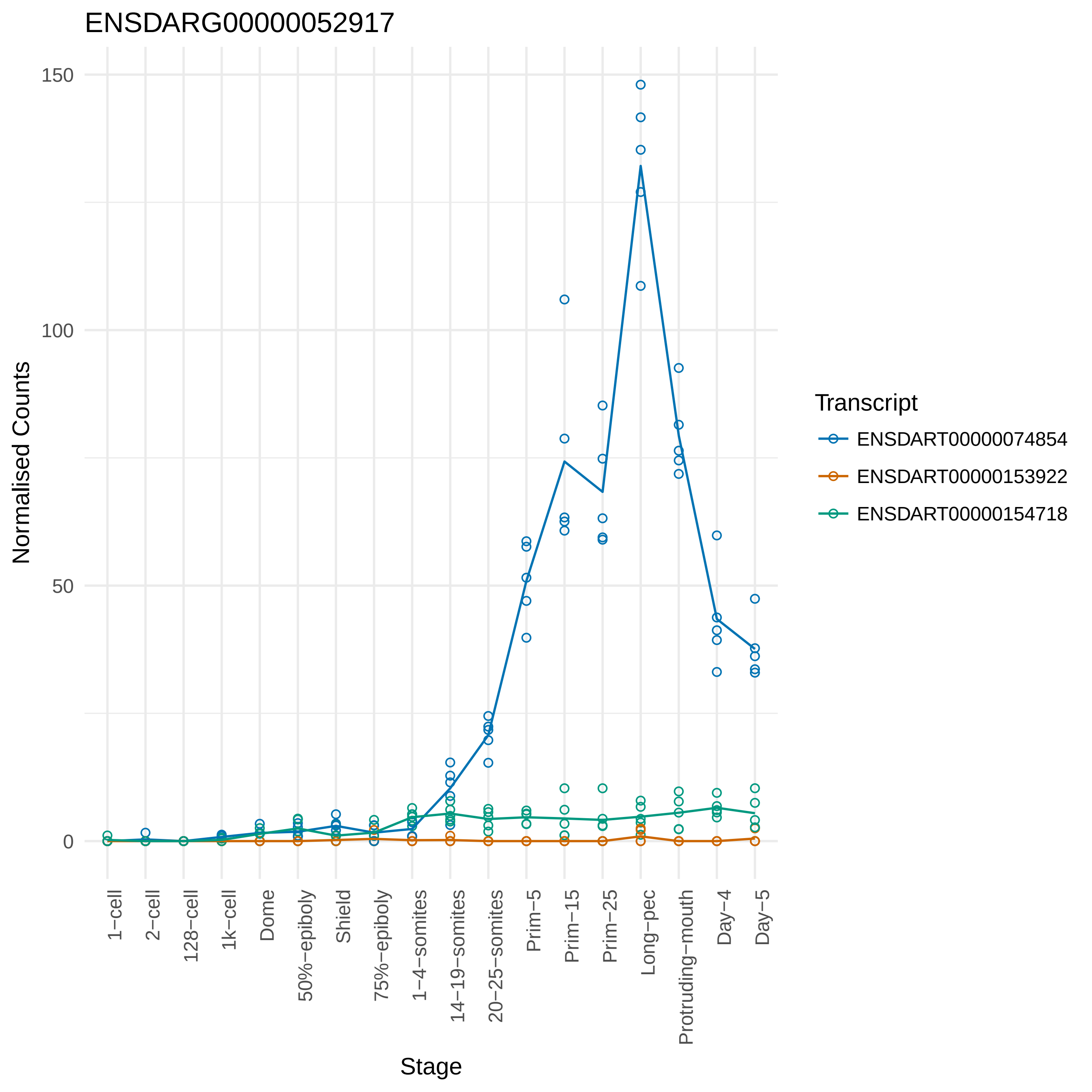
ENSDARG00000052917
Normalised Counts
150
100
50
0
1−cell
2−cell
128−cell
1k−cell
Dome
50%−epiboly
Shield
75%−epiboly
1−4−somites
14−19−somites
20−25−somites
Prim−5
Prim−15
Prim−25
Long−pec
Protruding−mouth
Day−4
Day−5
Stage
Transcript
ENSDART00000074854
ENSDART00000153922
ENSDART00000154718

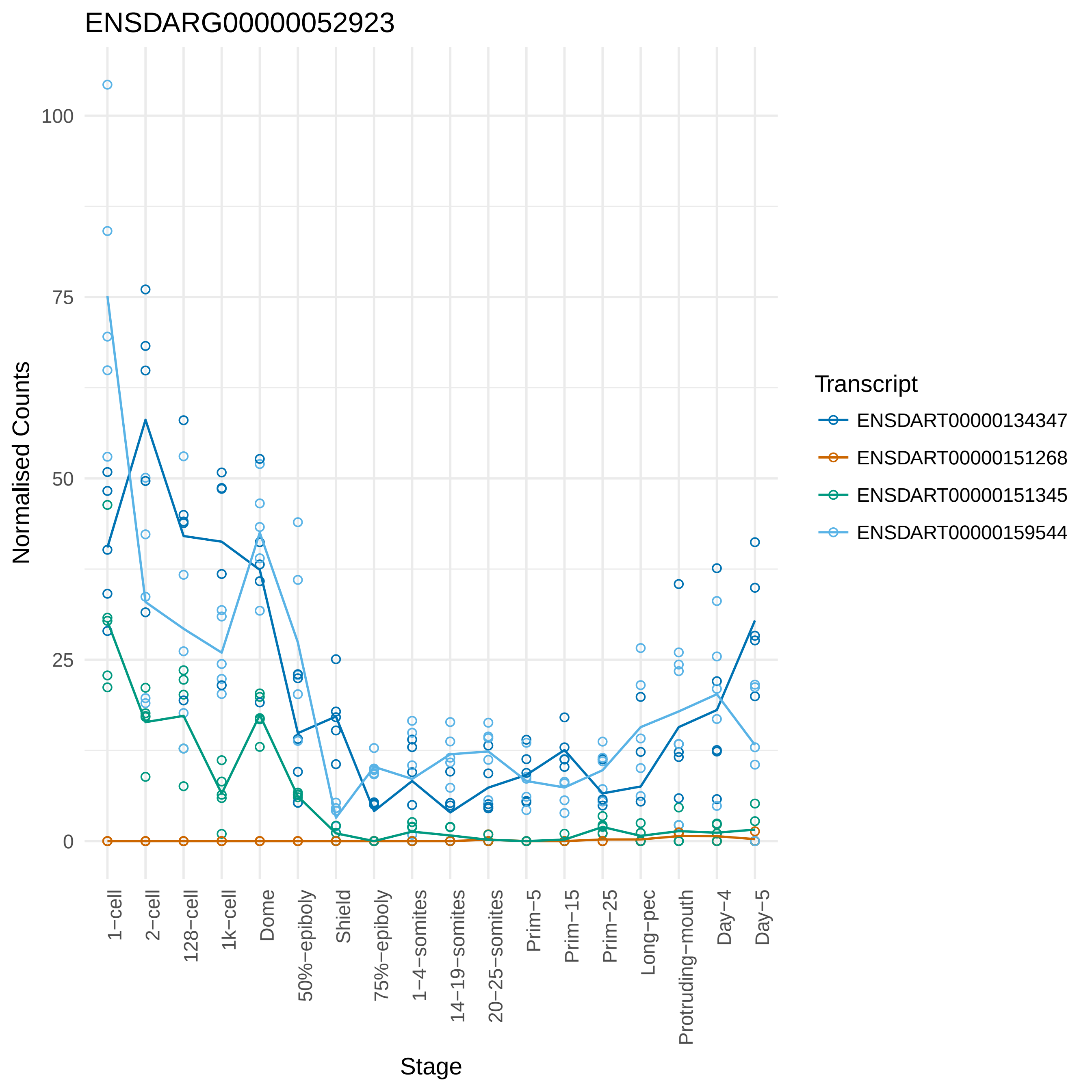

ENSDARG00000052923
Normalised Counts
100
75
50
25
0
1−cell
2−cell
128−cell
1k−cell
Dome
50%−epiboly
Shield
75%−epiboly
1−4−somites
14−19−somites
20−25−somites
Prim−5
Prim−15
Prim−25
Long−pec
Protruding−mouth
Day−4
Day−5
Stage
Transcript
ENSDART00000134347
ENSDART00000151268
ENSDART00000151345
ENSDART00000159544

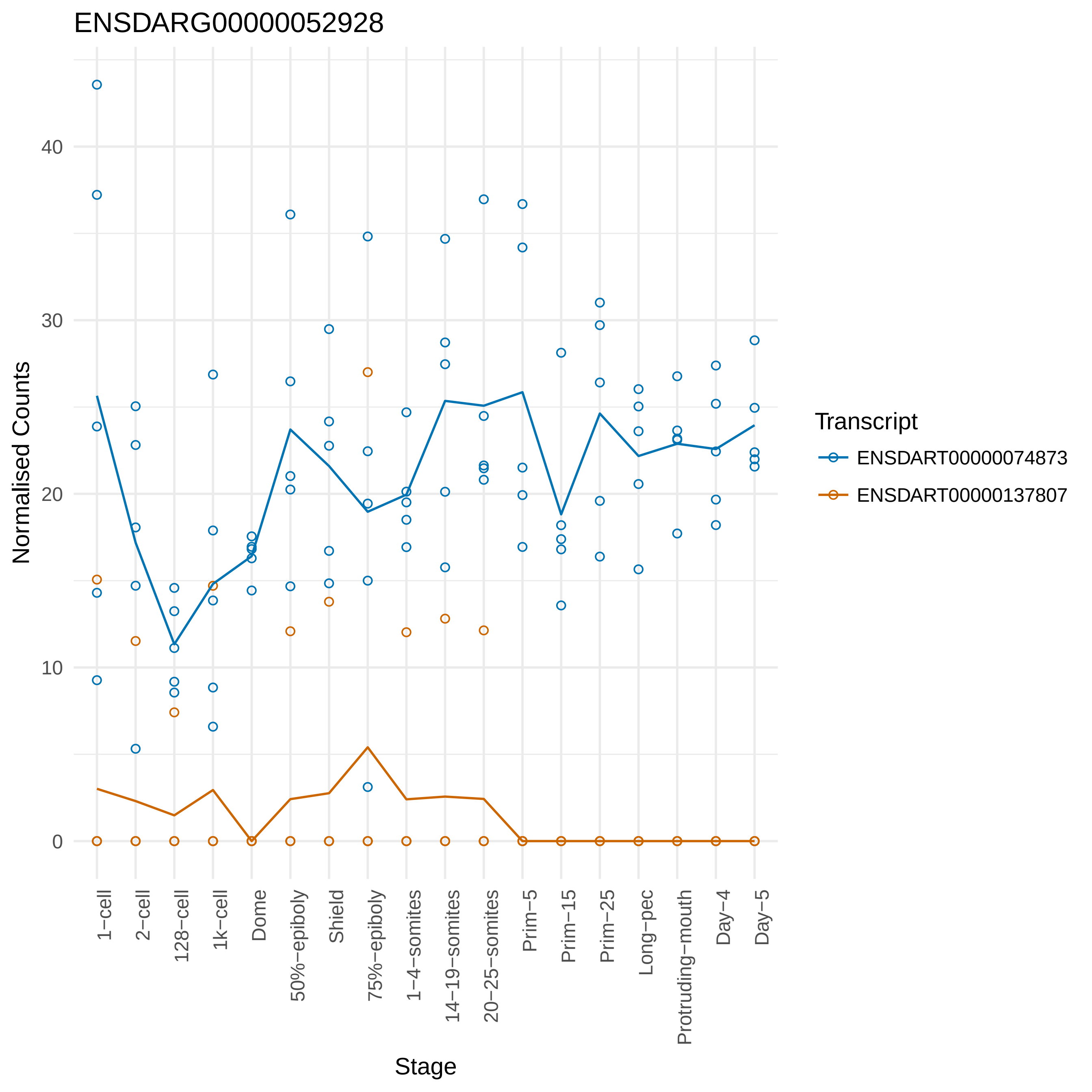

ENSDARG00000052928
Normalised Counts
0
10
20
30
40
1−cell
2−cell
128−cell
1k−cell
Dome
50%−epiboly
Shield
75%−epiboly
1−4−somites
14−19−somites
20−25−somites
Prim−5
Prim−15
Prim−25
Long−pec
Protruding−mouth
Day−4
Day−5
Stage
Transcript
ENSDART00000074873
ENSDART00000137807

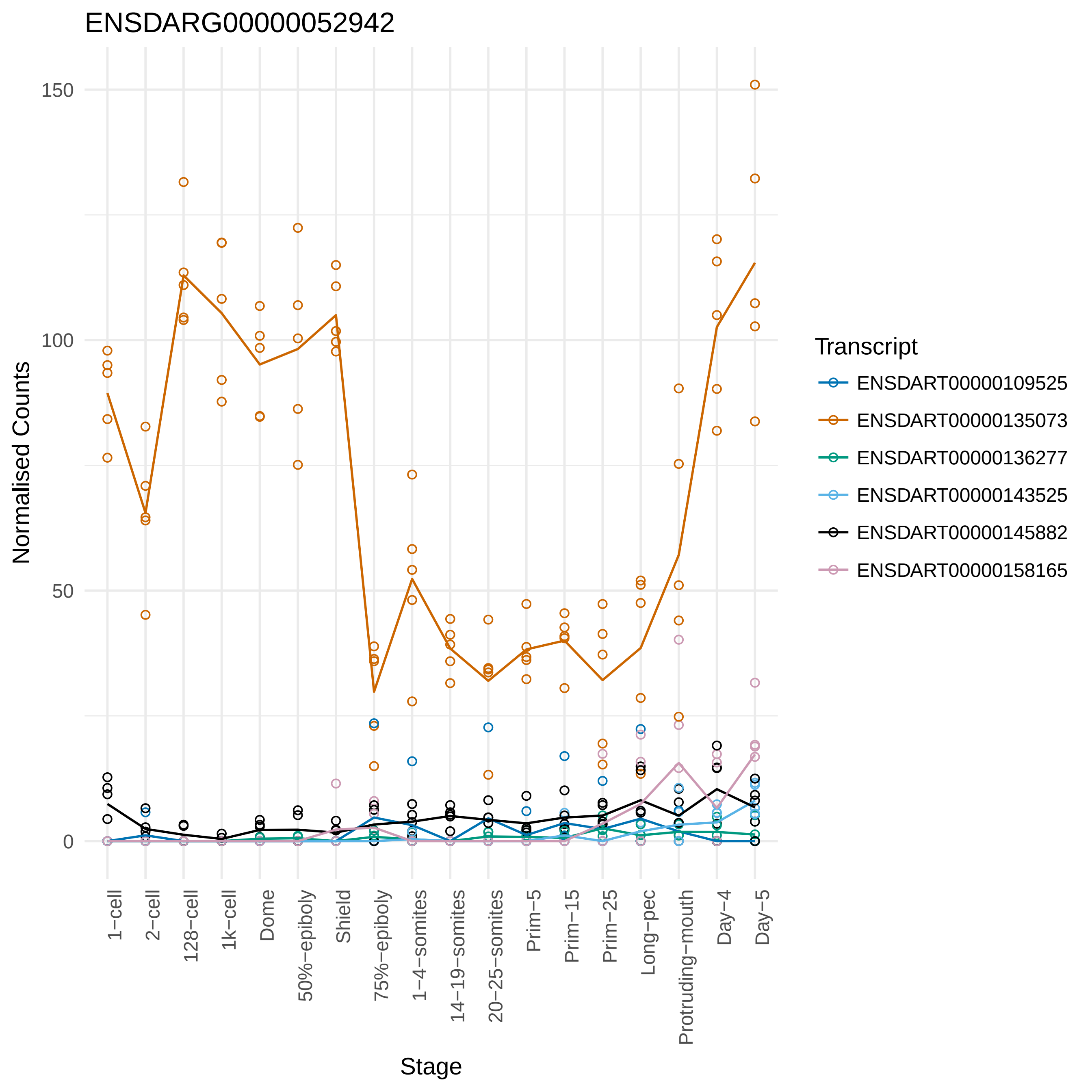

ENSDARG00000052942
Normalised Counts
150
100
50
0
Stage
1−cell
2−cell
128−cell
1k−cell
Dome
50%−epiboly
Shield
75%−epiboly
1−4−somites
14−19−somites
20−25−somites
Prim−5
Prim−15
Prim−25
Long−pec
Protruding−mouth
Day−4
Day−5
Transcript
ENSDART00000109525
ENSDART00000135073
ENSDART00000136277
ENSDART00000143525
ENSDART00000145882
ENSDART00000158165

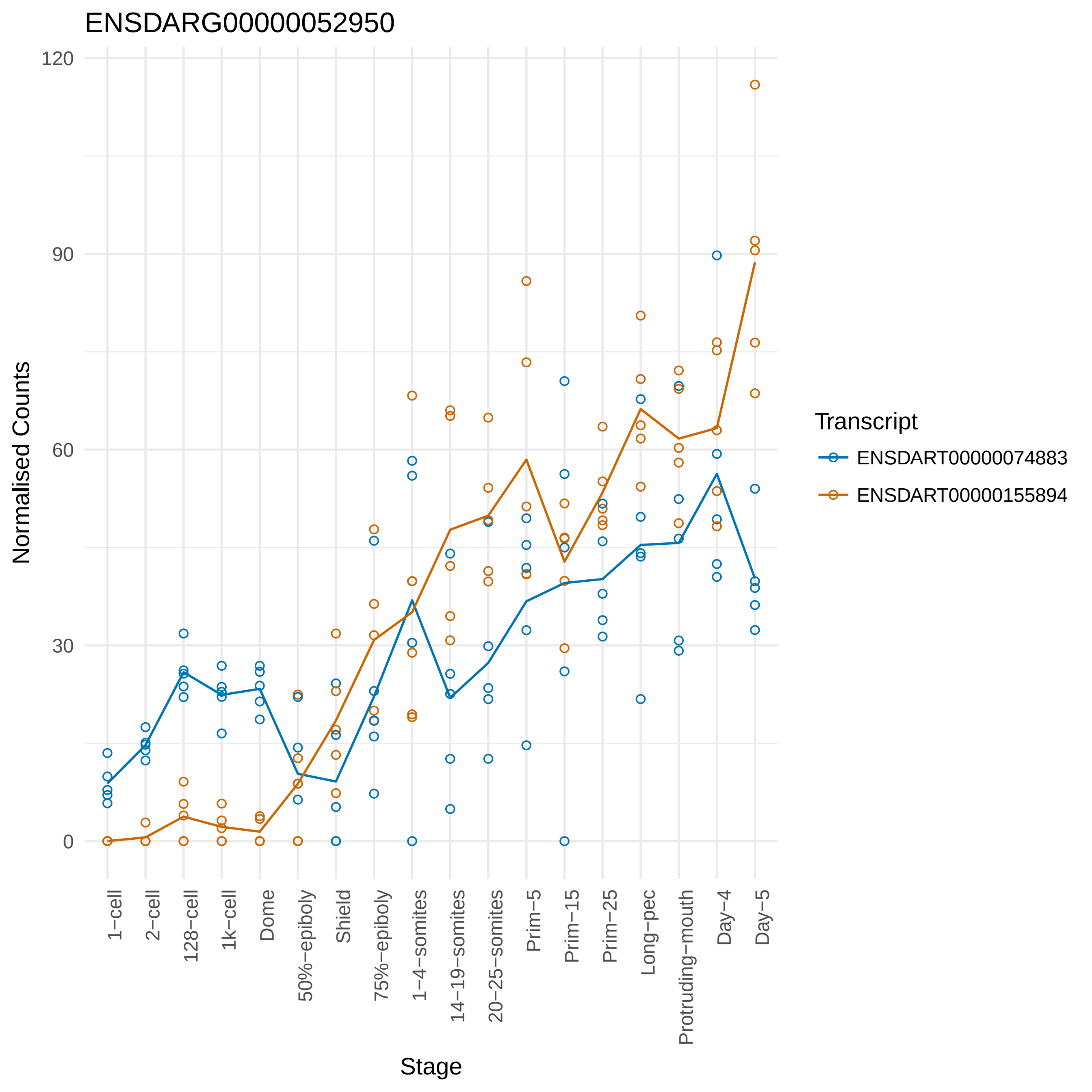

ENSDARG00000052950
Normalised Counts
120
90
60
30
0
1−cell
2−cell
128−cell
1k−cell
Dome
50%−epiboly
Shield
75%−epiboly
1−4−somites
14−19−somites
20−25−somites
Prim−5
Prim−15
Prim−25
Long−pec
Protruding−mouth
Day−4
Day−5
Stage
Transcript
ENSDART00000074883
ENSDART00000155894

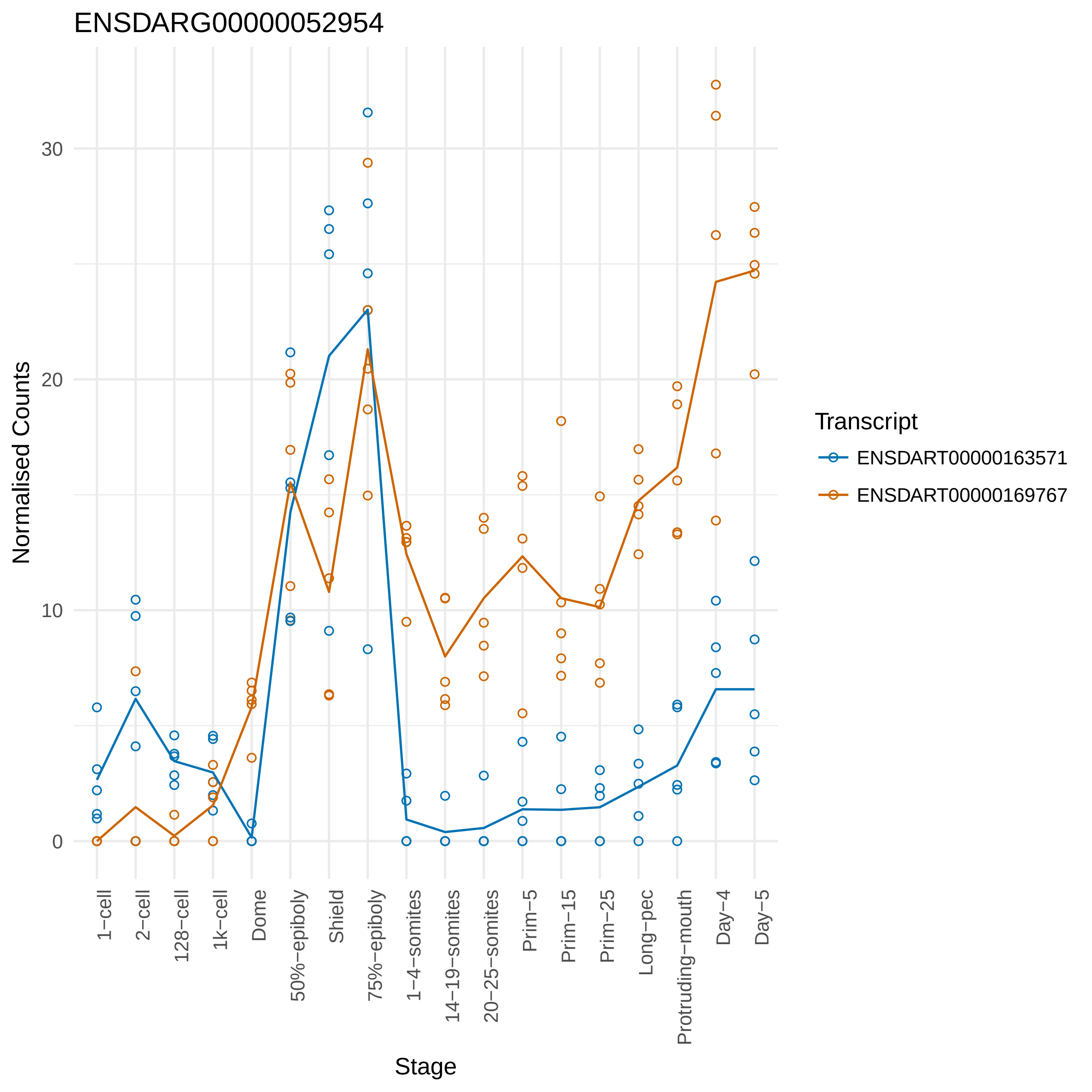

ENSDARG00000052954
Normalised Counts
0
10
20
30
1−cell
2−cell
128−cell
1k−cell
Dome
50%−epiboly
Shield
75%−epiboly
1−4−somites
14−19−somites
20−25−somites
Prim−5
Prim−15
Prim−25
Long−pec
Protruding−mouth
Day−4
Day−5
Stage
Transcript
ENSDART00000163571
ENSDART00000169767

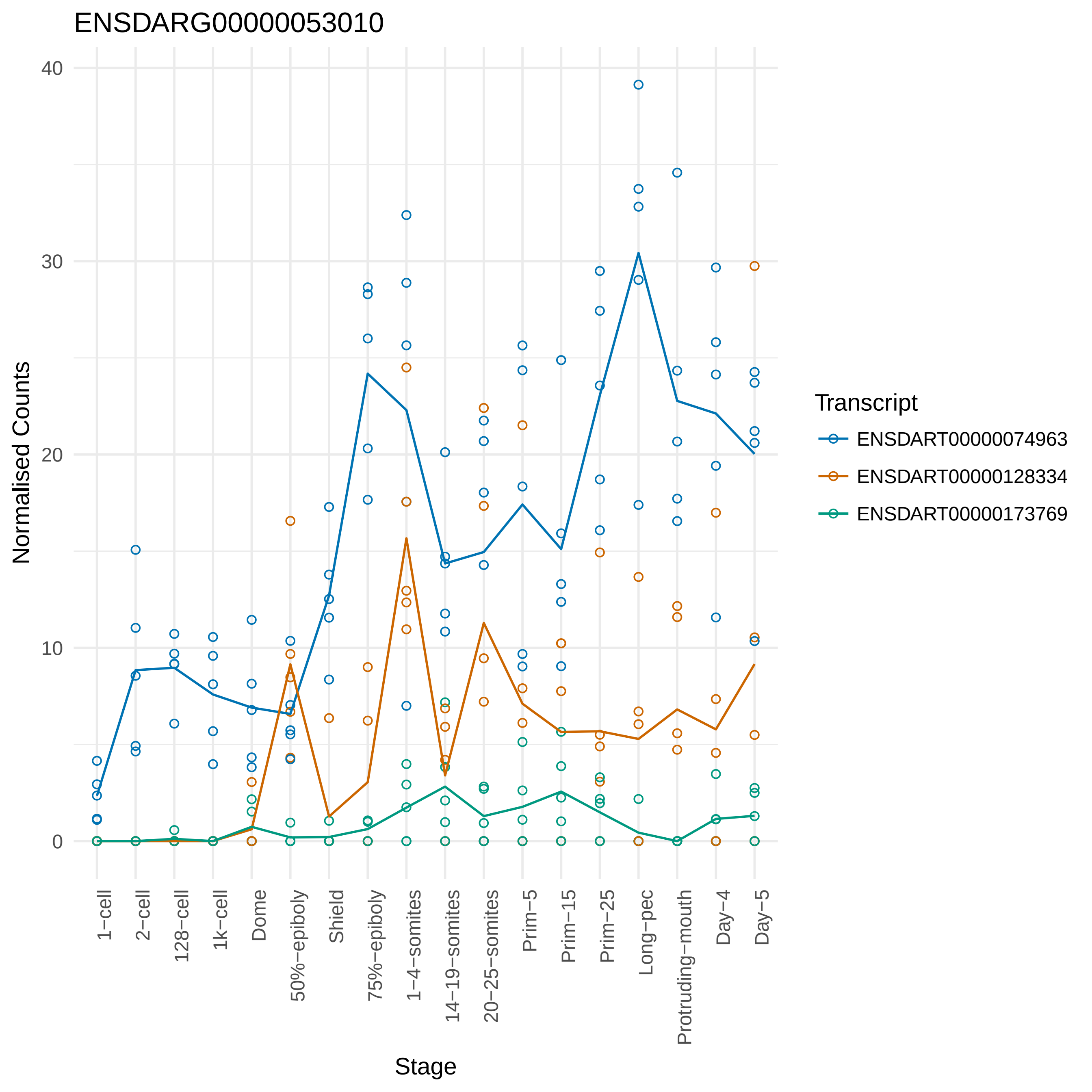
ENSDARG00000053010
Normalised Counts
40
30
20
10
0
1−cell
2−cell
128−cell
1k−cell
Dome
50%−epiboly
Shield
75%−epiboly
1−4−somites
14−19−somites
20−25−somites
Prim−5
Prim−15
Prim−25
Long−pec
Protruding−mouth
Day−4
Day−5
Stage
Transcript
ENSDART00000074963
ENSDART00000128334
ENSDART00000173769

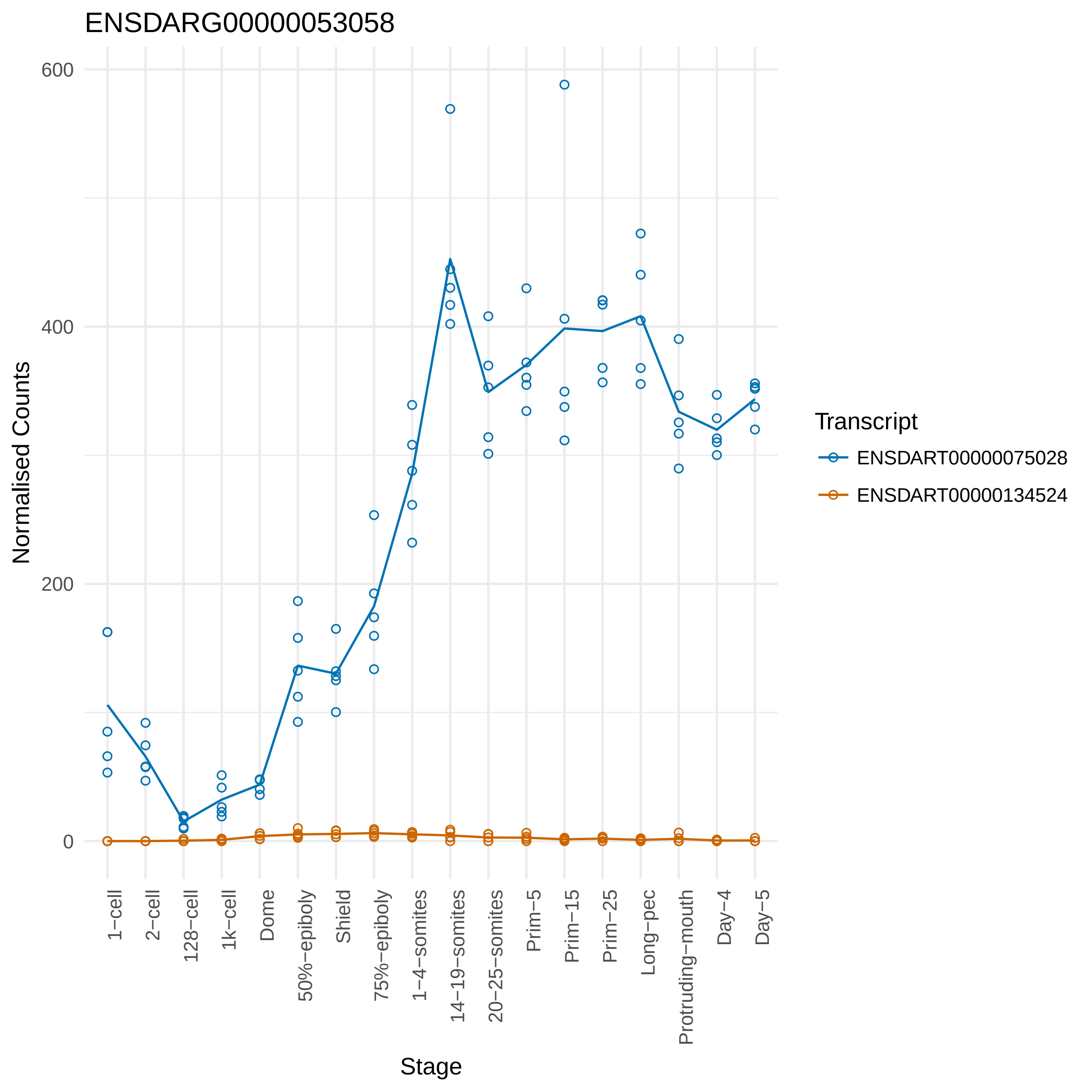

ENSDARG00000053058
Normalised Counts
600
400
200
0
1−cell
2−cell
128−cell
1k−cell
Dome
50%−epiboly
Shield
75%−epiboly
1−4−somites
14−19−somites
20−25−somites
Prim−5
Prim−15
Prim−25
Long−pec
Protruding−mouth
Day−4
Day−5
Stage
Transcript
ENSDART00000075028
ENSDART00000134524

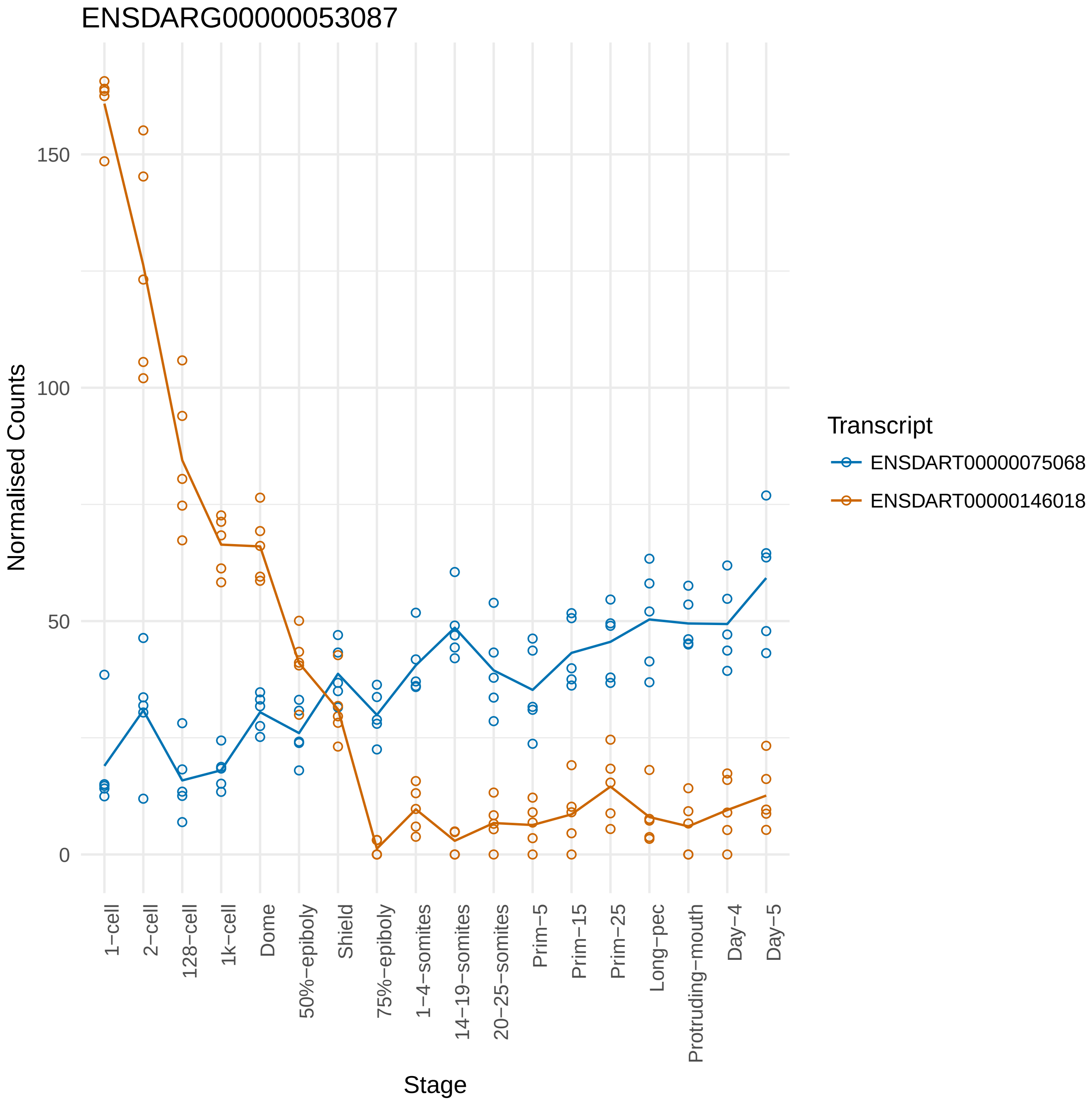

ENSDARG00000053087
Normalised Counts
150
100
50
0
1−cell
2−cell
128−cell
1k−cell
Dome
50%−epiboly
Shield
75%−epiboly
1−4−somites
14−19−somites
20−25−somites
Prim−5
Prim−15
Prim−25
Long−pec
Protruding−mouth
Day−4
Day−5
Stage
Transcript
ENSDART00000075068
ENSDART00000146018

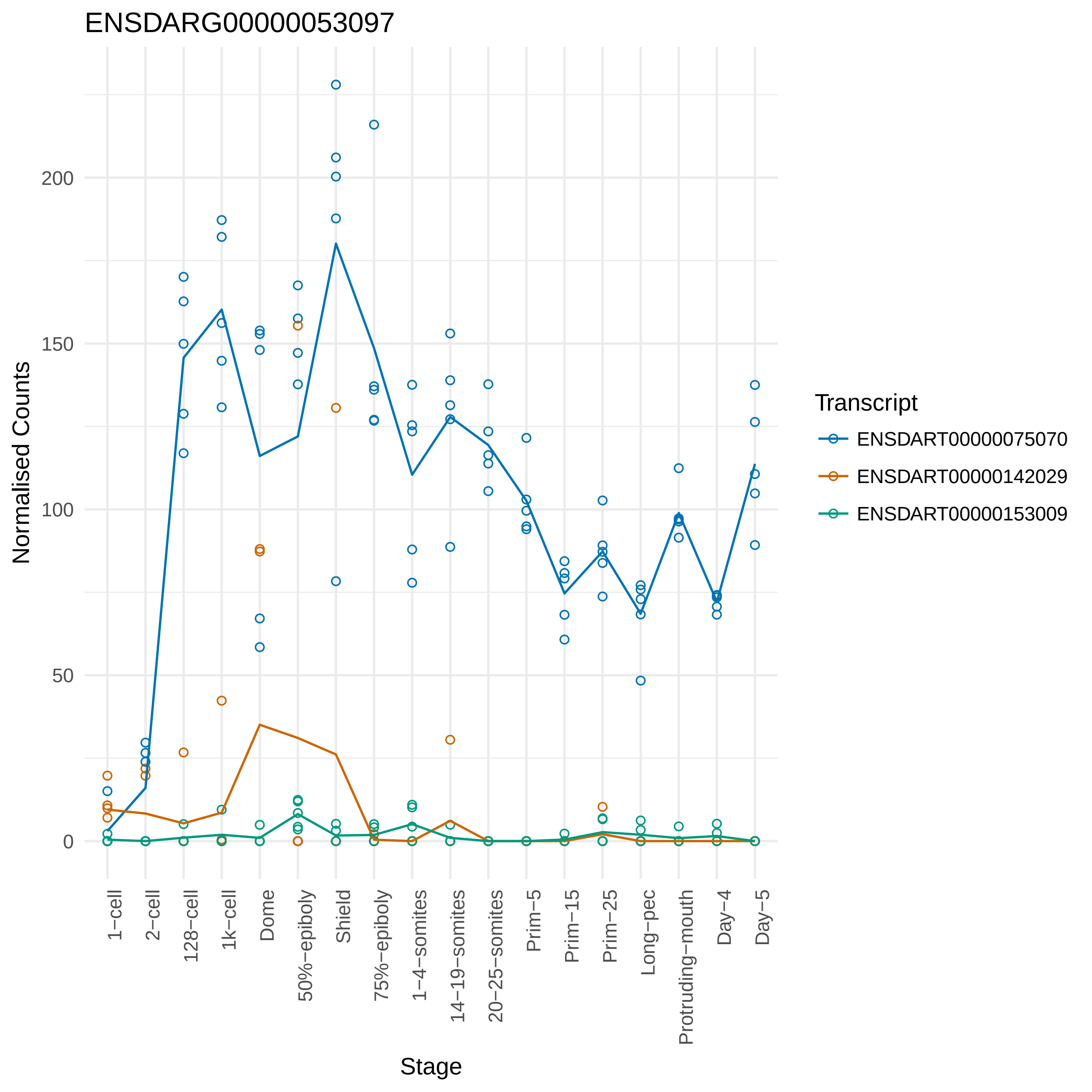
ENSDARG00000053097
Normalised Counts
200
150
100
50
0
1−cell
2−cell
128−cell
1k−cell
Dome
50%−epiboly
Shield
75%−epiboly
1−4−somites
14−19−somites
20−25−somites
Prim−5
Prim−15
Prim−25
Long−pec
Protruding−mouth
Day−4
Day−5
Stage
Transcript
ENSDART00000075070
ENSDART00000142029
ENSDART00000153009

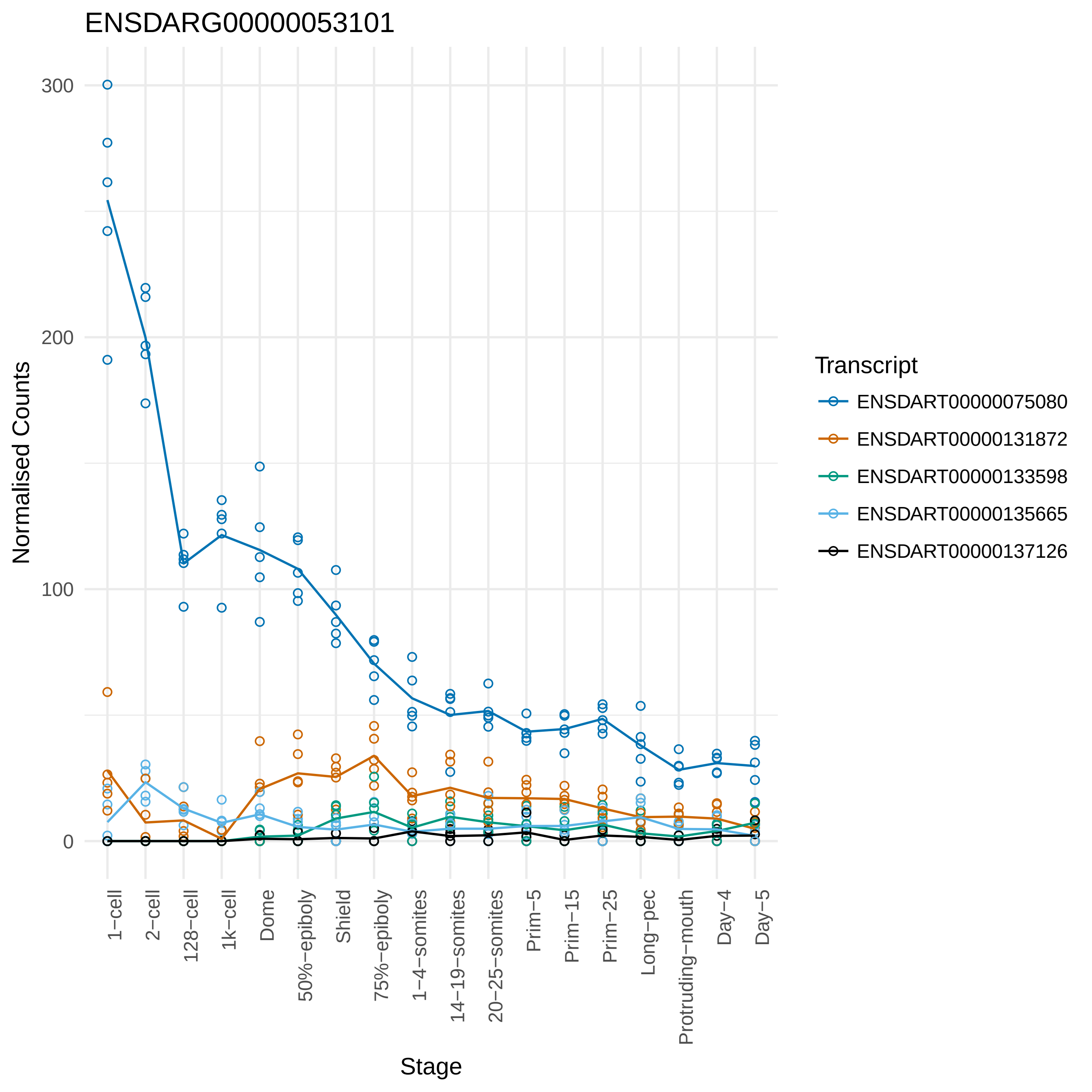

ENSDARG00000053101
Normalised Counts
300
200
100
0
1−cell
2−cell
128−cell
1k−cell
Dome
50%−epiboly
Shield
75%−epiboly
1−4−somites
14−19−somites
20−25−somites
Prim−5
Prim−15
Prim−25
Long−pec
Protruding−mouth
Day−4
Day−5
Stage
Transcript
ENSDART00000075080
ENSDART00000131872
ENSDART00000133598
ENSDART00000135665
ENSDART00000137126

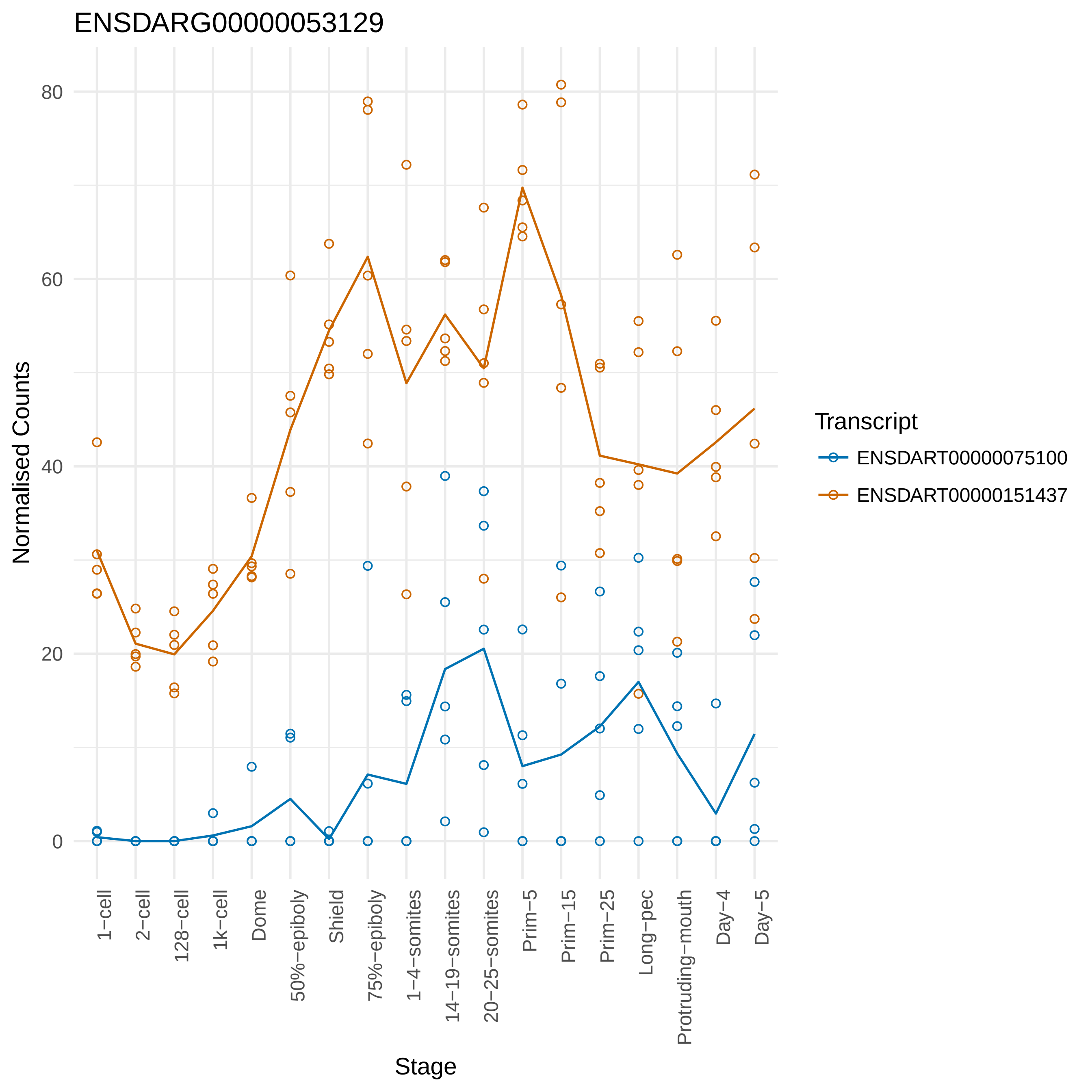
ENSDARG00000053129
Normalised Counts
80
60
40
20
0
1−cell
2−cell
128−cell
1k−cell
Dome
50%−epiboly
Shield
75%−epiboly
1−4−somites
14−19−somites
20−25−somites
Prim−5
Prim−15
Prim−25
Long−pec
Protruding−mouth
Day−4
Day−5
Stage
Transcript
ENSDART00000075100
ENSDART00000151437

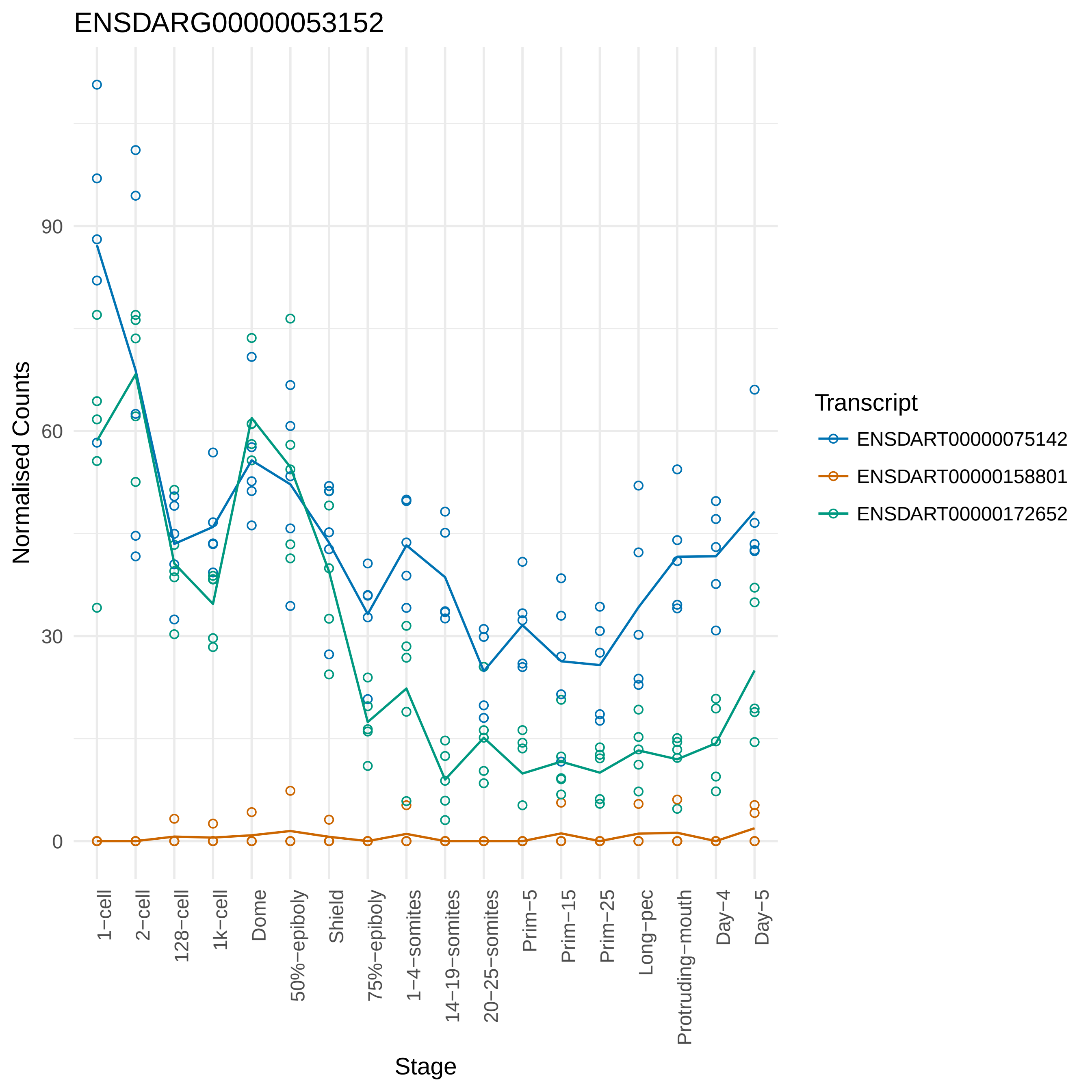
ENSDARG00000053152
Normalised Counts
90
60
30
0
1−cell
2−cell
128−cell
1k−cell
Dome
50%−epiboly
Shield
75%−epiboly
1−4−somites
14−19−somites
20−25−somites
Prim−5
Prim−15
Prim−25
Long−pec
Protruding−mouth
Day−4
Day−5
Stage
Transcript
ENSDART00000075142
ENSDART00000158801
ENSDART00000172652

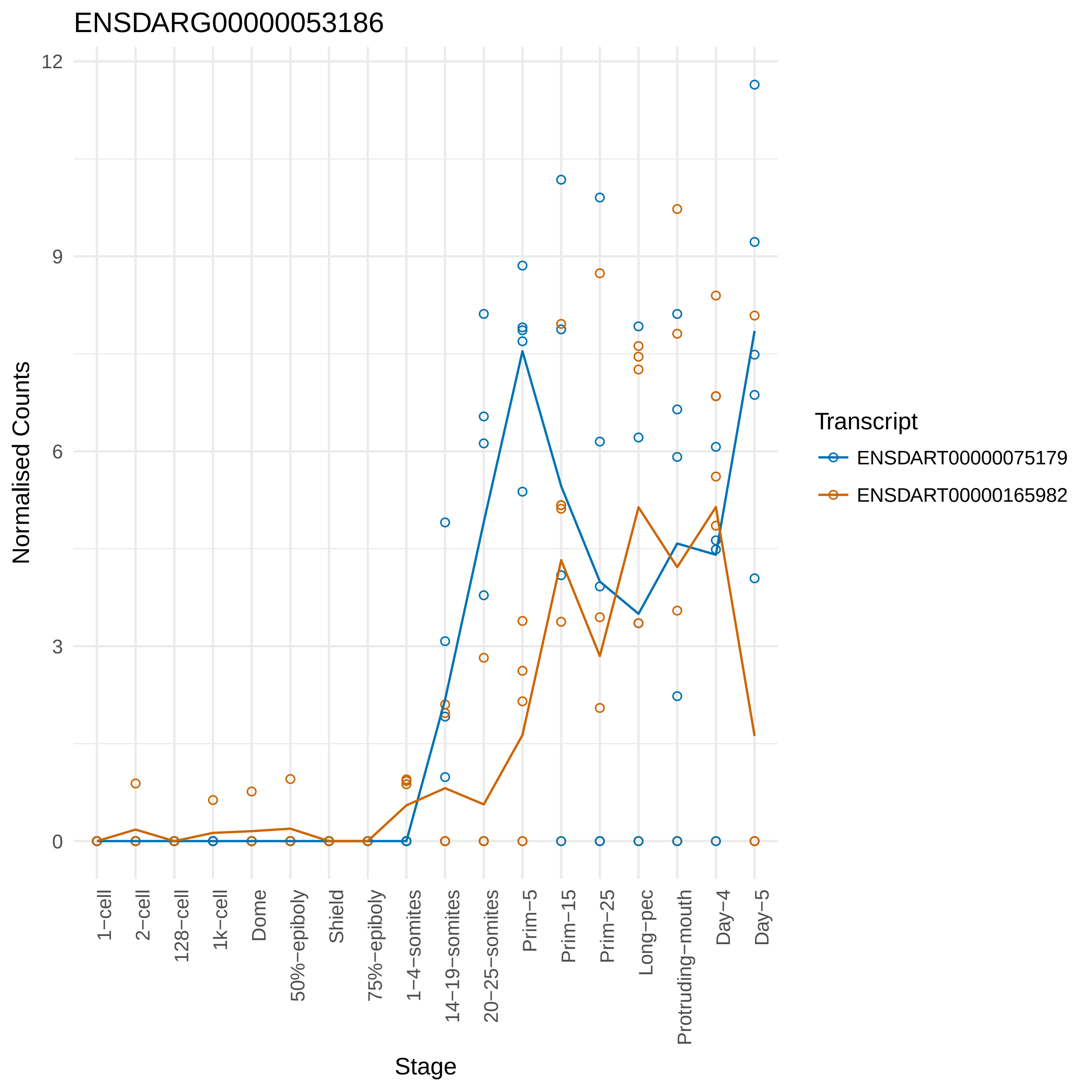

ENSDARG00000053186
Normalised Counts
12
9
6
3
0
1−cell
2−cell
128−cell
1k−cell
Dome
50%−epiboly
Shield
75%−epiboly
1−4−somites
14−19−somites
20−25−somites
Prim−5
Prim−15
Prim−25
Long−pec
Protruding−mouth
Day−4
Day−5
Stage
Transcript
ENSDART00000075179
ENSDART00000165982

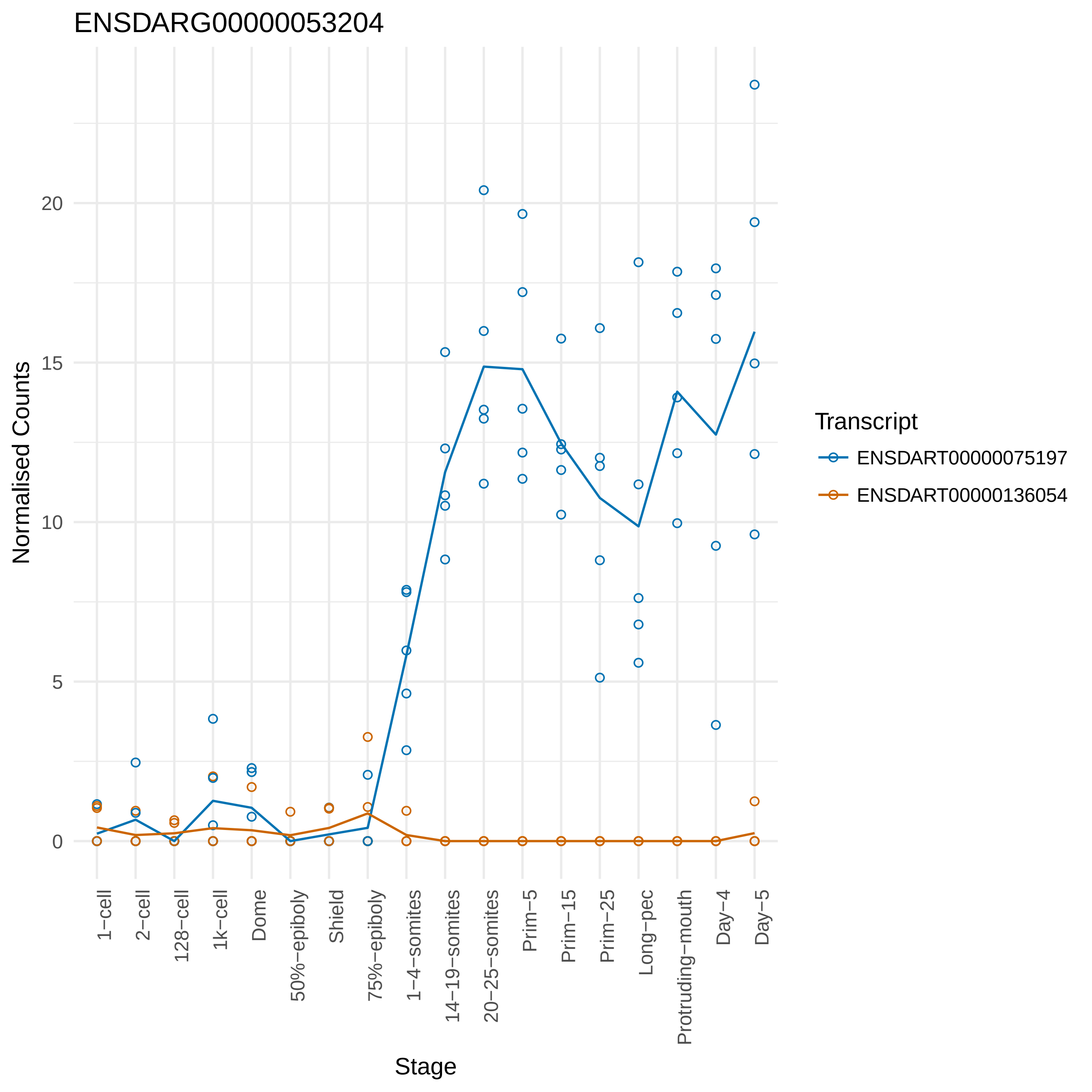

ENSDARG00000053204
Normalised Counts
0
5
10
15
20
1−cell
2−cell
128−cell
1k−cell
Dome
50%−epiboly
Shield
75%−epiboly
1−4−somites
14−19−somites
20−25−somites
Prim−5
Prim−15
Prim−25
Long−pec
Protruding−mouth
Day−4
Day−5
Stage
Transcript
ENSDART00000075197
ENSDART00000136054

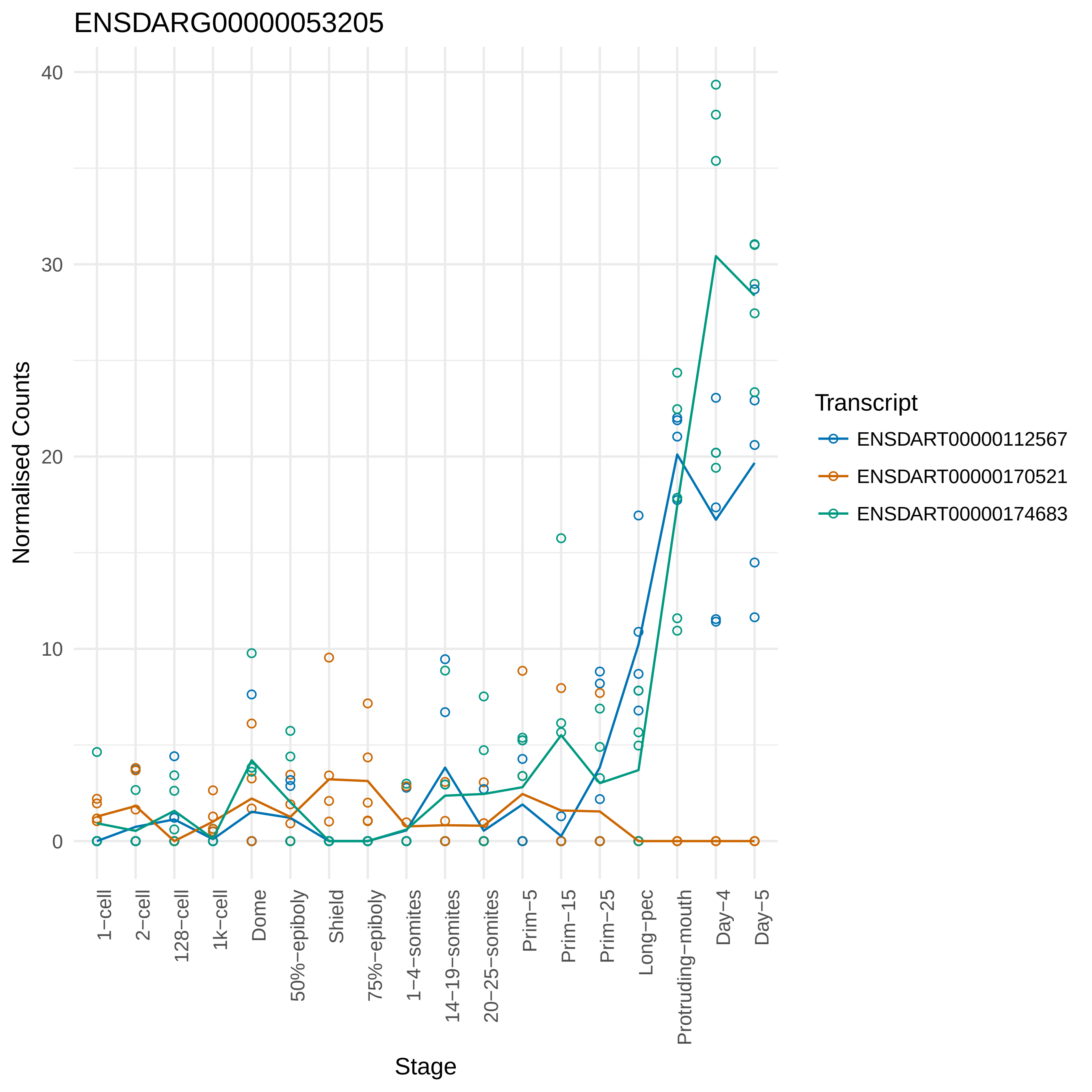
ENSDARG00000053205
Normalised Counts
40
30
20
10
0
1−cell
2−cell
128−cell
1k−cell
Dome
50%−epiboly
Shield
75%−epiboly
1−4−somites
14−19−somites
20−25−somites
Prim−5
Prim−15
Prim−25
Long−pec
Protruding−mouth
Day−4
Day−5
Stage
Transcript
ENSDART00000112567
ENSDART00000170521
ENSDART00000174683

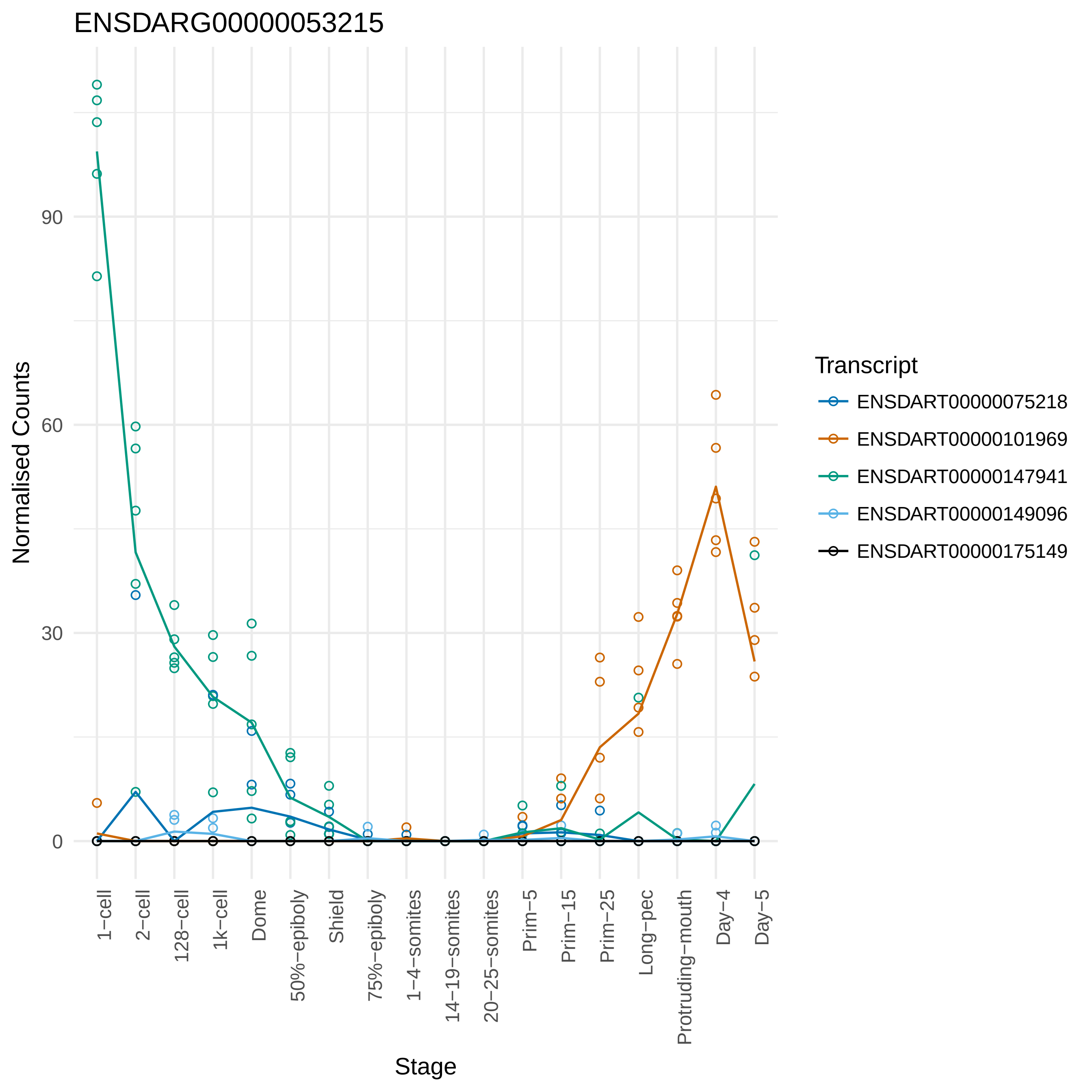

ENSDARG00000053215
Normalised Counts
90
60
30
0
1−cell
2−cell
128−cell
1k−cell
Dome
50%−epiboly
Shield
75%−epiboly
1−4−somites
14−19−somites
20−25−somites
Prim−5
Prim−15
Prim−25
Long−pec
Protruding−mouth
Day−4
Day−5
Stage
Transcript
ENSDART00000075218
ENSDART00000101969
ENSDART00000147941
ENSDART00000149096
ENSDART00000175149

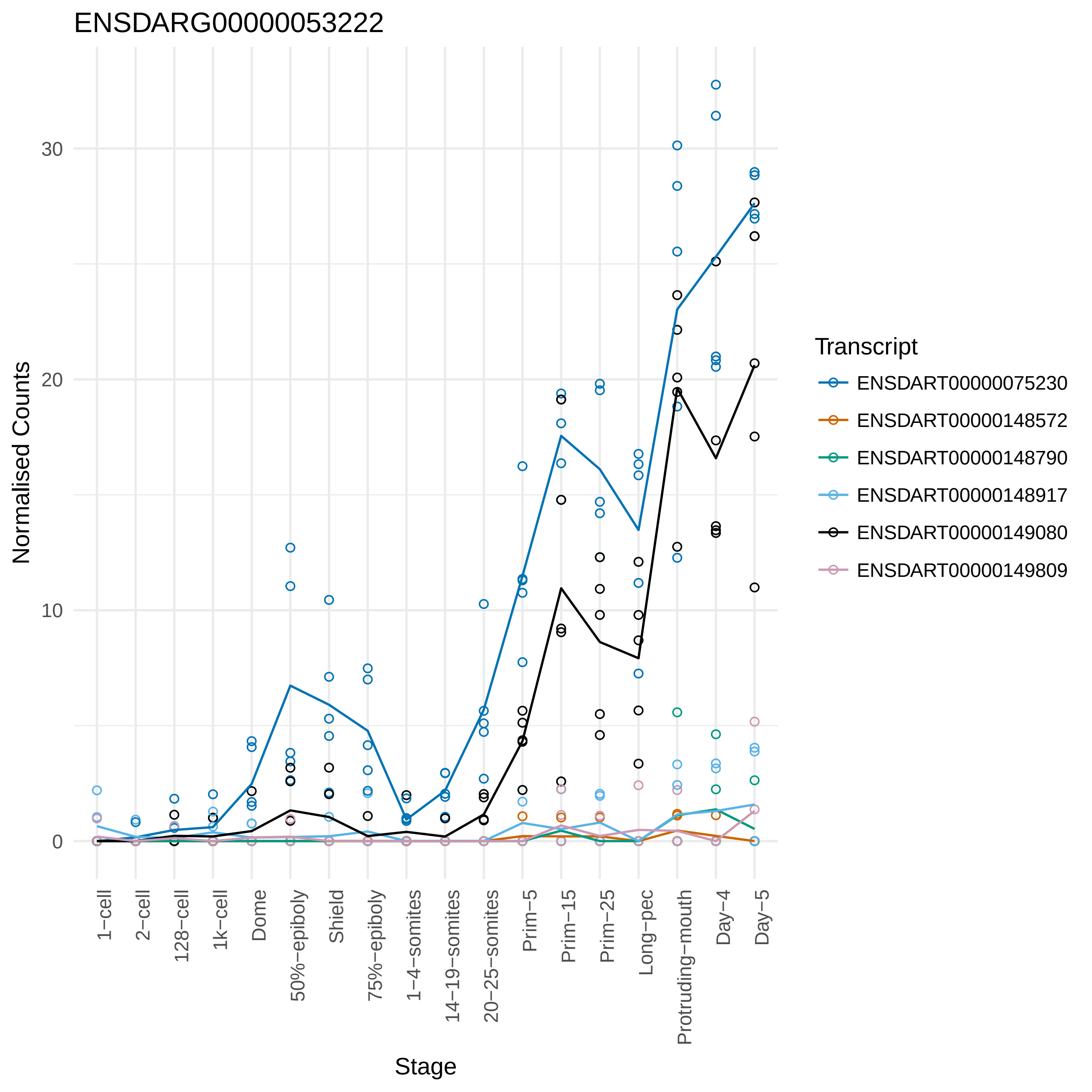

ENSDARG00000053222
Normalised Counts
0
10
20
30
1−cell
2−cell
128−cell
1k−cell
Dome
50%−epiboly
Shield
75%−epiboly
1−4−somites
14−19−somites
20−25−somites
Prim−5
Prim−15
Prim−25
Long−pec
Protruding−mouth
Day−4
Day−5
Stage
Transcript
ENSDART00000075230
ENSDART00000148572
ENSDART00000148790
ENSDART00000148917
ENSDART00000149080
ENSDART00000149809

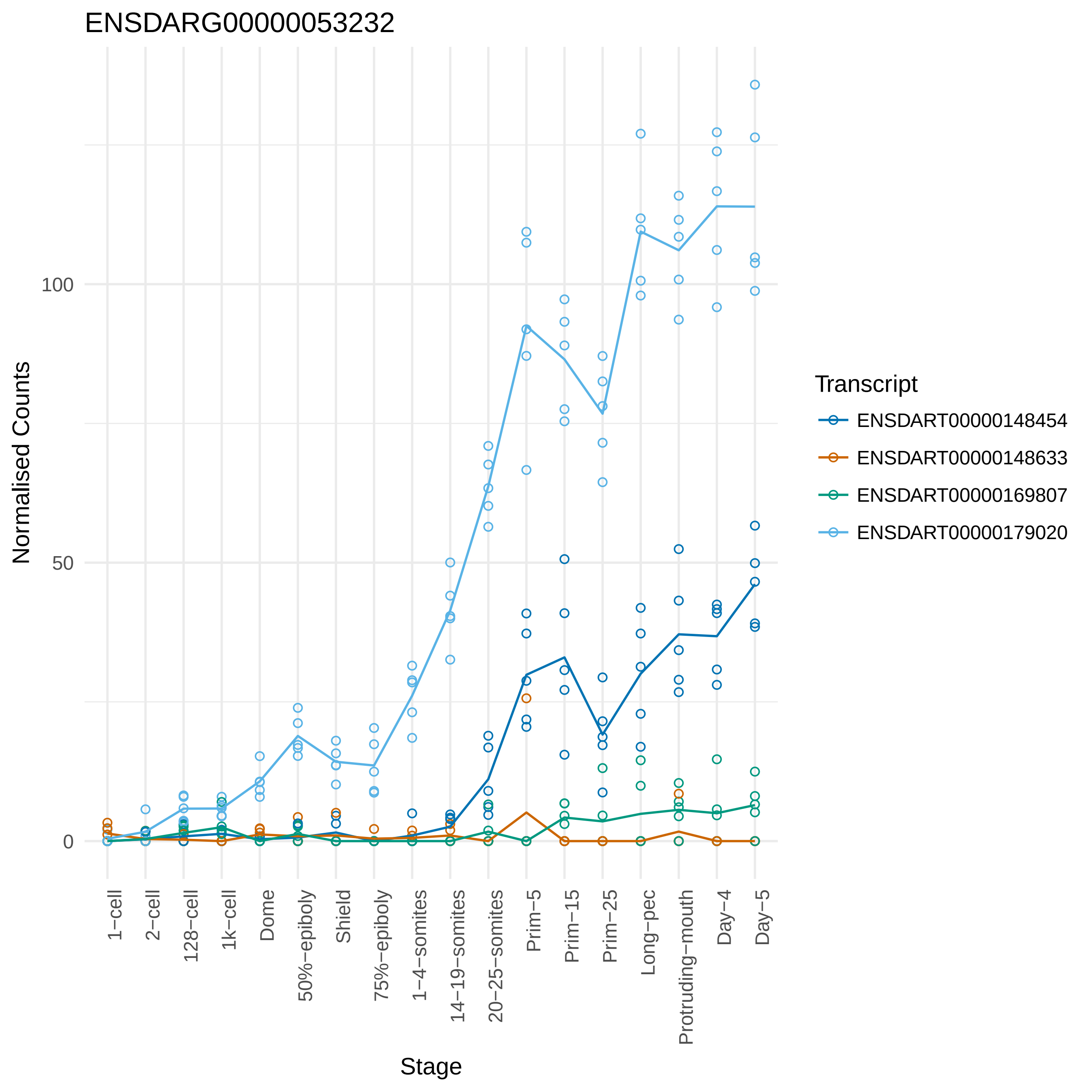
ENSDARG00000053232
Normalised Counts
0
50
100
Stage
1−cell
2−cell
128−cell
1k−cell
Dome
50%−epiboly
Shield
75%−epiboly
1−4−somites
14−19−somites
20−25−somites
Prim−5
Prim−15
Prim−25
Long−pec
Protruding−mouth
Day−4
Day−5
Transcript
ENSDART00000148454
ENSDART00000148633
ENSDART00000169807
ENSDART00000179020

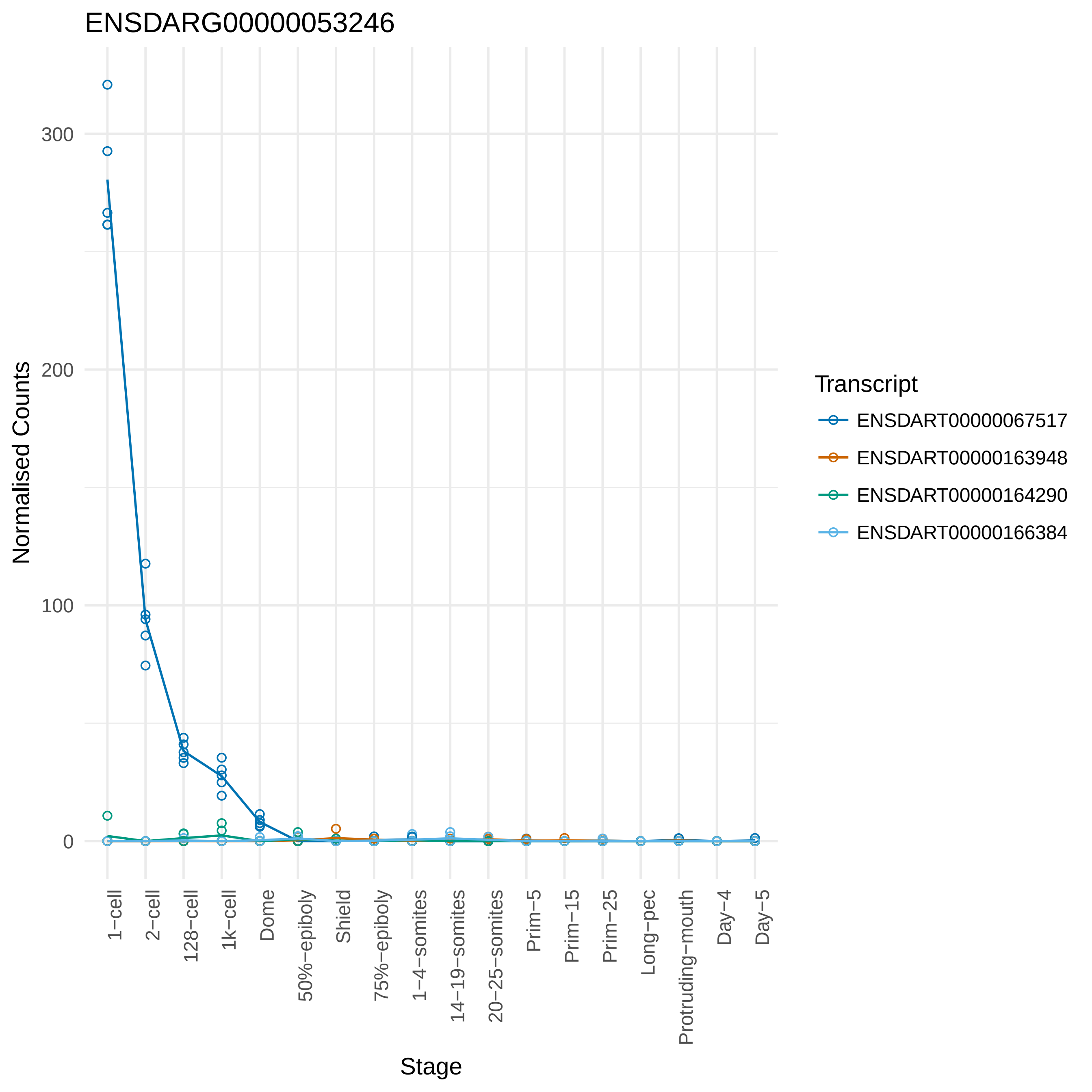

ENSDARG00000053246
Normalised Counts
300
200
100
0
1−cell
2−cell
128−cell
1k−cell
Dome
50%−epiboly
Shield
75%−epiboly
1−4−somites
14−19−somites
20−25−somites
Prim−5
Prim−15
Prim−25
Long−pec
Protruding−mouth
Day−4
Day−5
Stage
Transcript
ENSDART00000067517
ENSDART00000163948
ENSDART00000164290
ENSDART00000166384

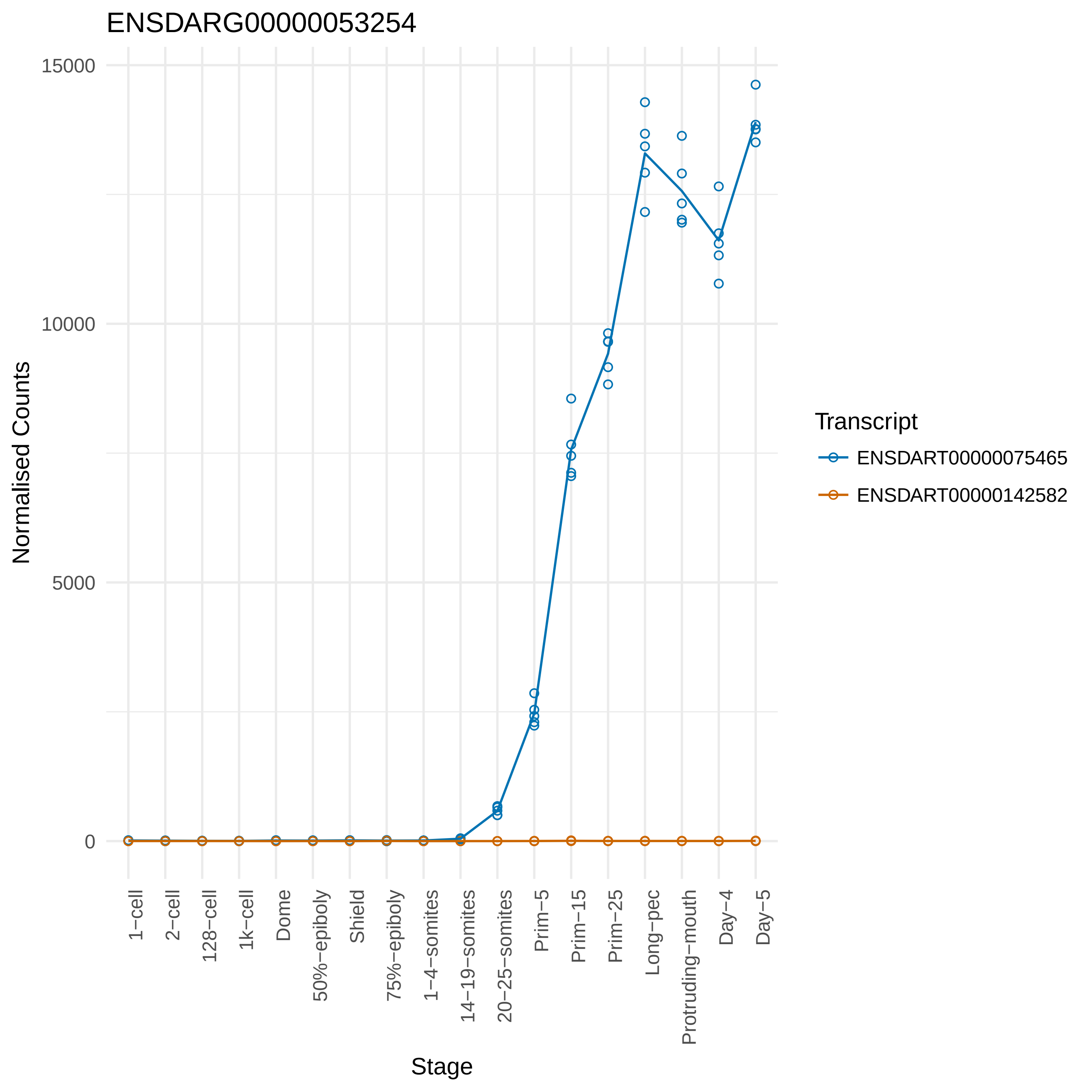
ENSDARG00000053254
Normalised Counts
15000
10000
5000
0
Stage
1−cell
2−cell
128−cell
1k−cell
Dome
50%−epiboly
Shield
75%−epiboly
1−4−somites
14−19−somites
20−25−somites
Prim−5
Prim−15
Prim−25
Long−pec
Protruding−mouth
Day−4
Day−5
Transcript
ENSDART00000075465
ENSDART00000142582

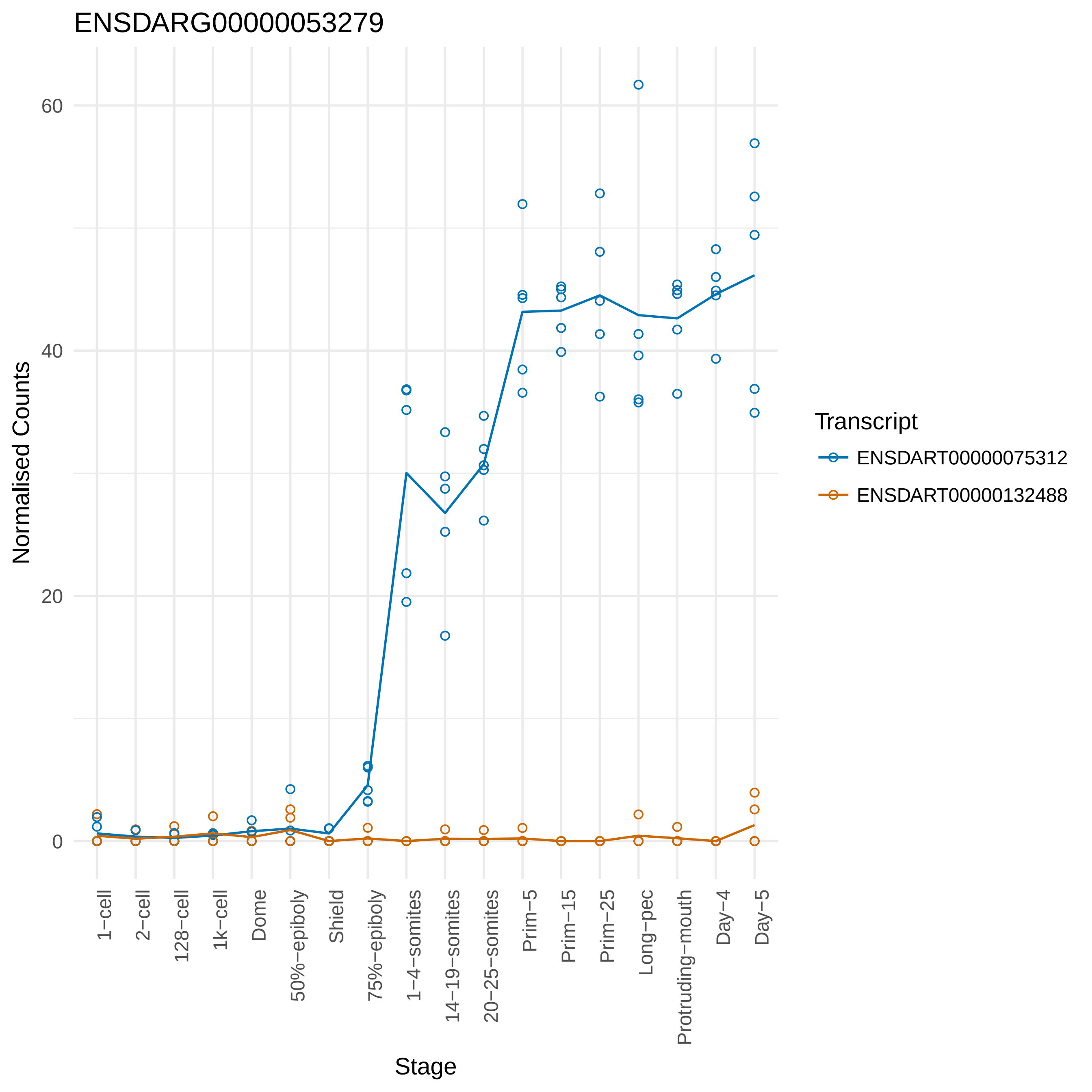
ENSDARG00000053279
Normalised Counts
0
20
40
60
Stage
1−cell
2−cell
128−cell
1k−cell
Dome
50%−epiboly
Shield
75%−epiboly
1−4−somites
14−19−somites
20−25−somites
Prim−5
Prim−15
Prim−25
Long−pec
Protruding−mouth
Day−4
Day−5
Transcript
ENSDART00000075312
ENSDART00000132488

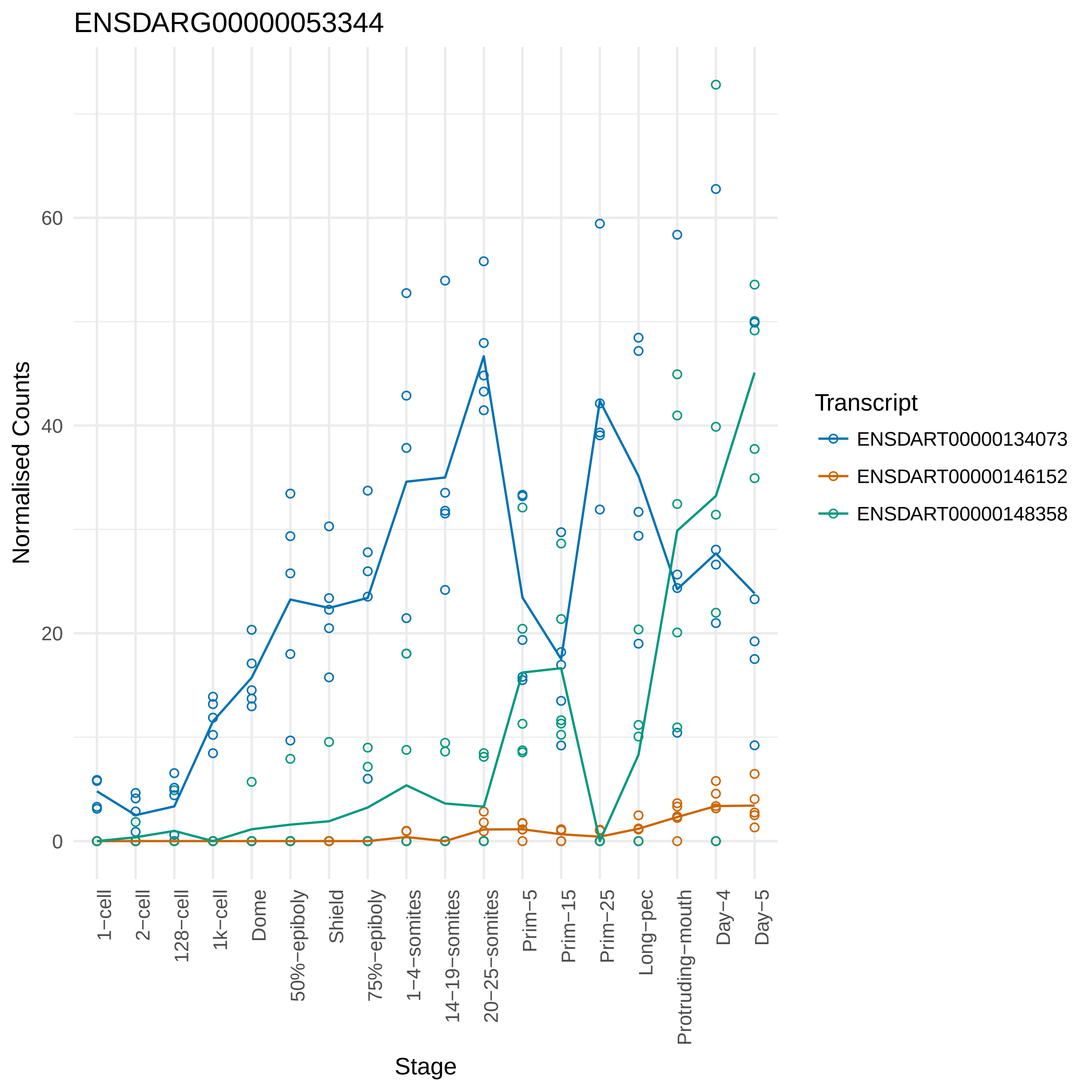

ENSDARG00000053344
Normalised Counts
0
20
40
60
1−cell
2−cell
128−cell
1k−cell
Dome
50%−epiboly
Shield
75%−epiboly
1−4−somites
14−19−somites
20−25−somites
Prim−5
Prim−15
Prim−25
Long−pec
Protruding−mouth
Day−4
Day−5
Stage
Transcript
ENSDART00000134073
ENSDART00000146152
ENSDART00000148358

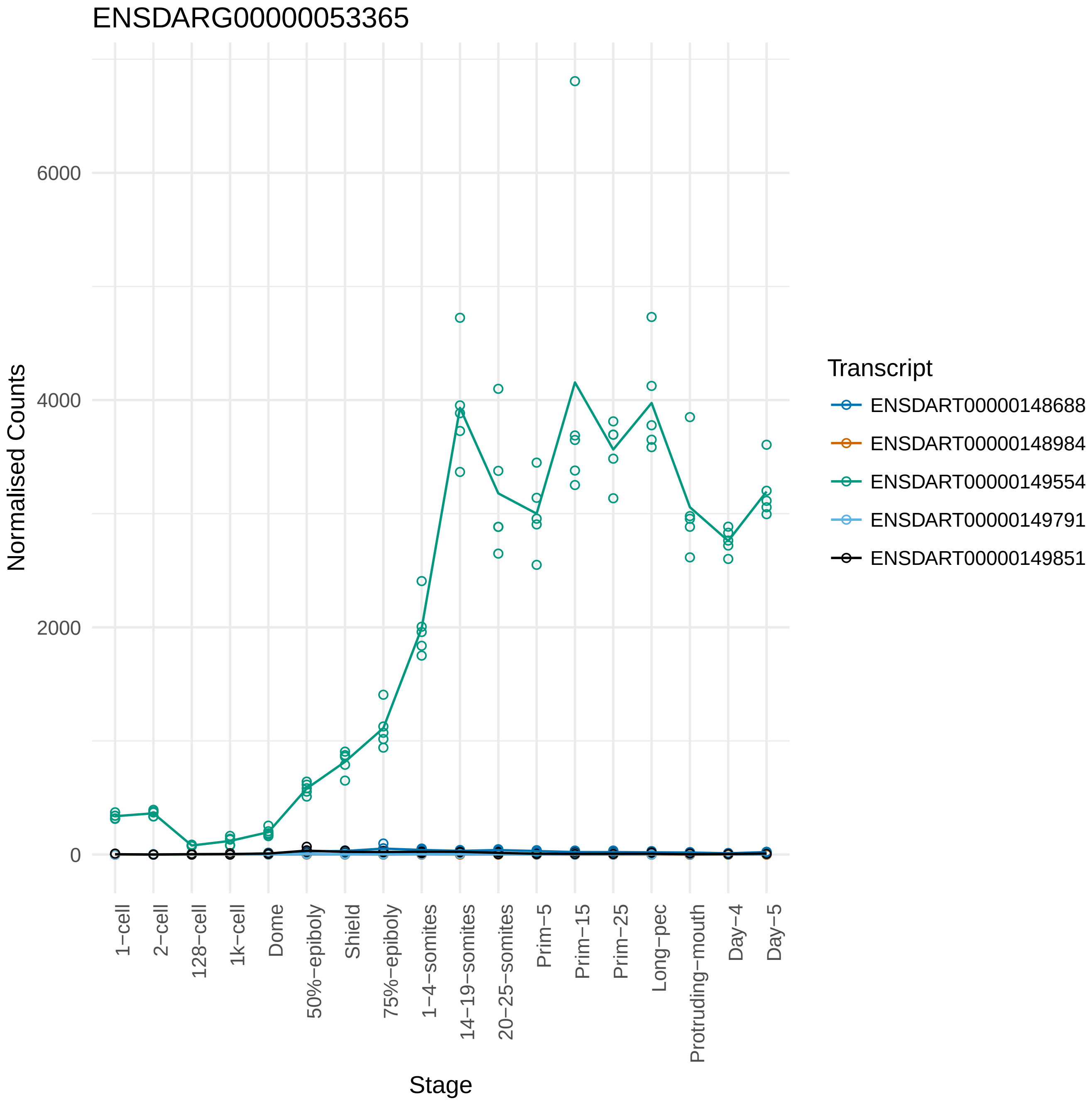

ENSDARG00000053365
Normalised Counts
6000
4000
2000
0
1−cell
2−cell
128−cell
1k−cell
Dome
50%−epiboly
Shield
75%−epiboly
1−4−somites
14−19−somites
20−25−somites
Prim−5
Prim−15
Prim−25
Long−pec
Protruding−mouth
Day−4
Day−5
Stage
Transcript
ENSDART00000148688
ENSDART00000148984
ENSDART00000149554
ENSDART00000149791
ENSDART00000149851

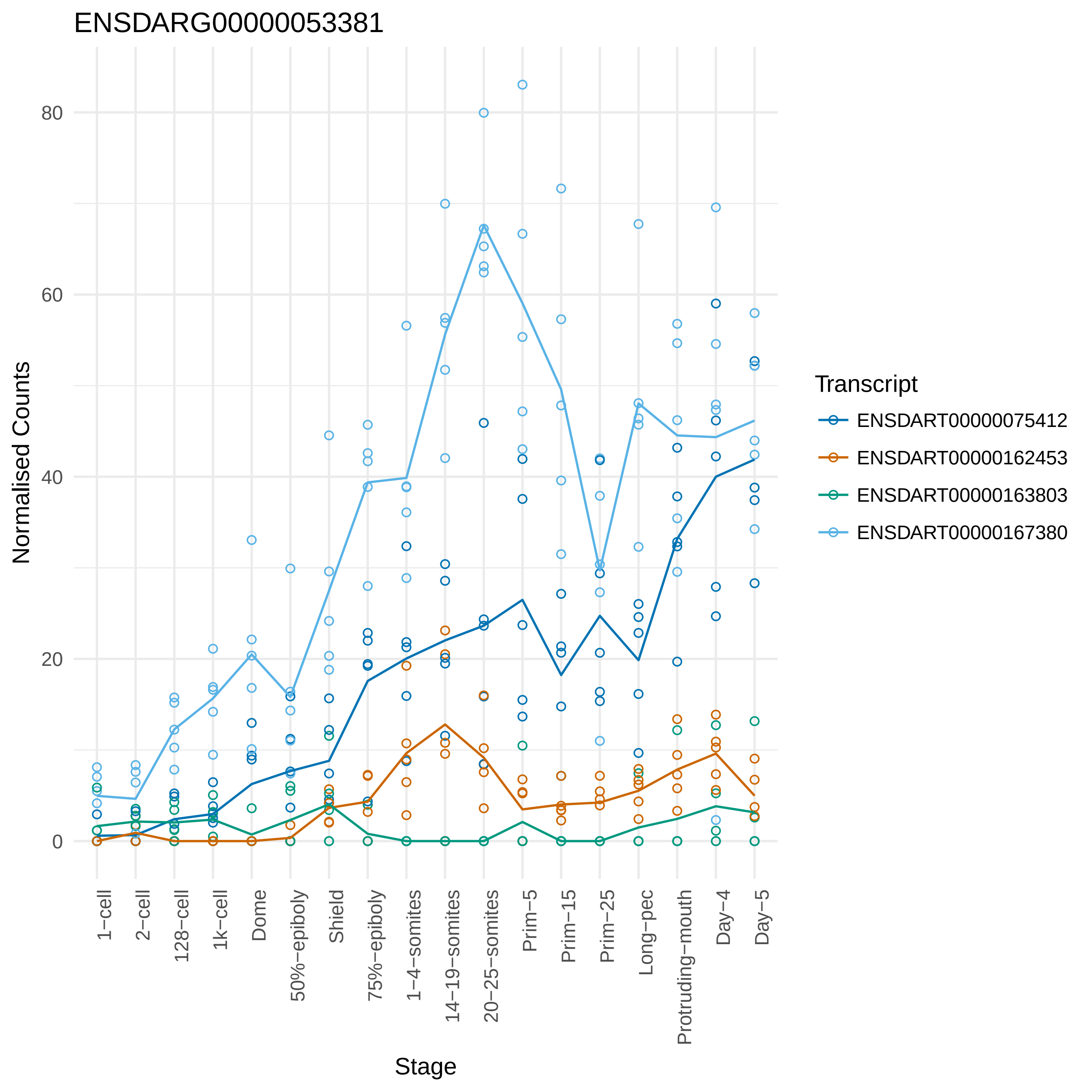
ENSDARG00000053381
Normalised Counts
0
20
40
60
80
Stage
1−cell
2−cell
128−cell
1k−cell
Dome
50%−epiboly
Shield
75%−epiboly
1−4−somites
14−19−somites
20−25−somites
Prim−5
Prim−15
Prim−25
Long−pec
Protruding−mouth
Day−4
Day−5
Transcript
ENSDART00000075412
ENSDART00000162453
ENSDART00000163803
ENSDART00000167380

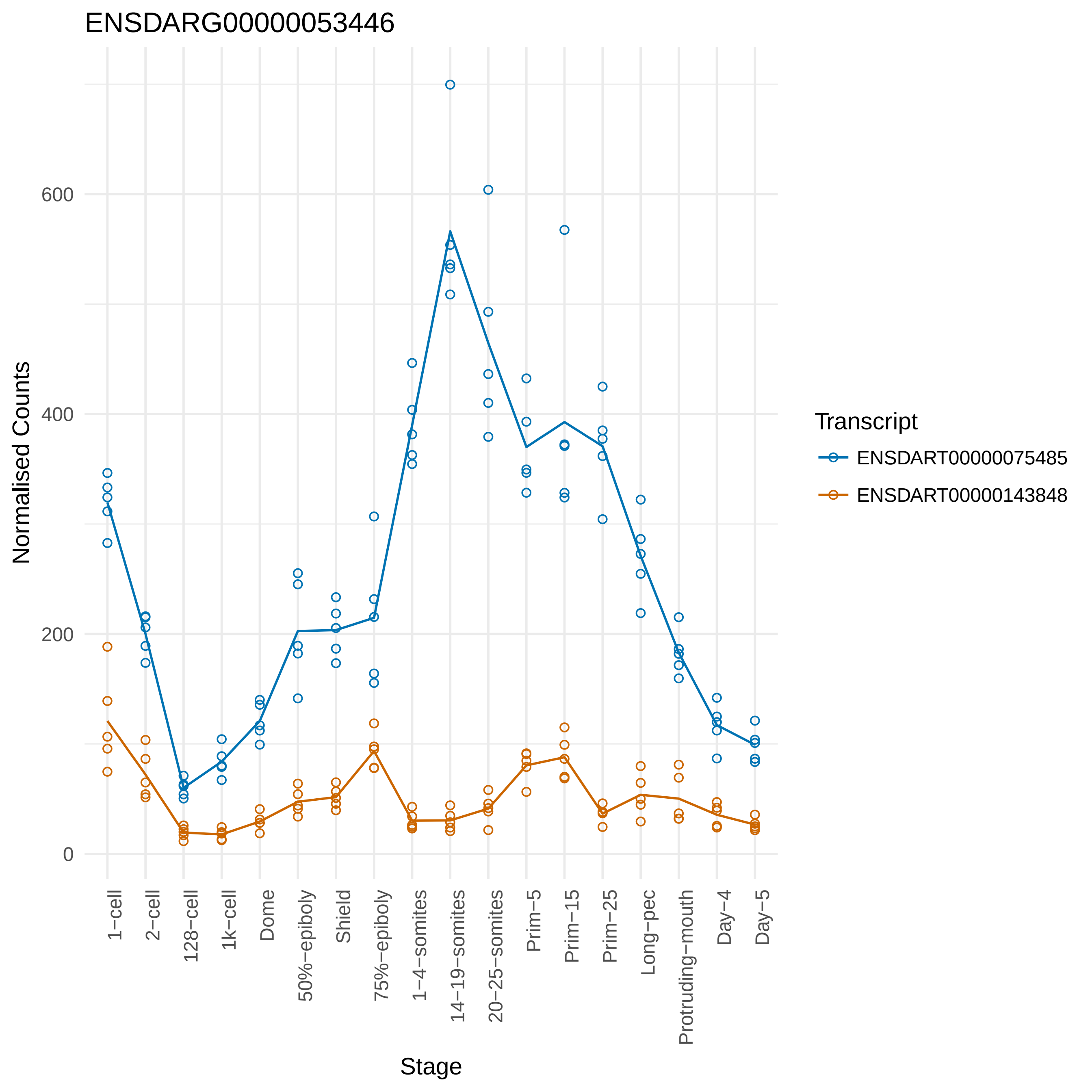
ENSDARG00000053446
Normalised Counts
600
400
200
0
Stage
1−cell
2−cell
128−cell
1k−cell
Dome
50%−epiboly
Shield
75%−epiboly
1−4−somites
14−19−somites
20−25−somites
Prim−5
Prim−15
Prim−25
Long−pec
Protruding−mouth
Day−4
Day−5
Transcript
ENSDART00000075485
ENSDART00000143848

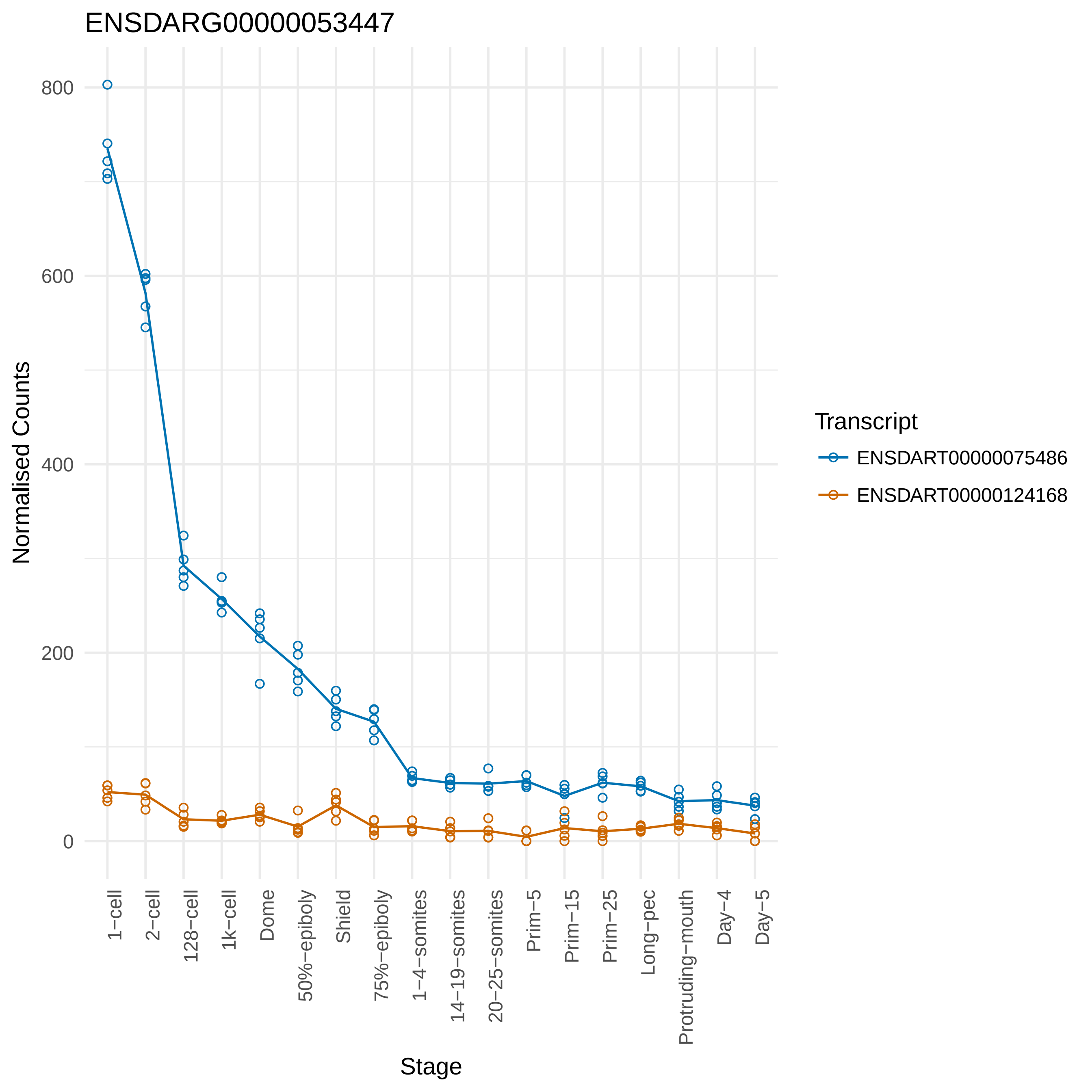
ENSDARG00000053447
Normalised Counts
800
600
400
200
0
1−cell
2−cell
128−cell
1k−cell
Dome
50%−epiboly
Shield
75%−epiboly
1−4−somites
14−19−somites
20−25−somites
Prim−5
Prim−15
Prim−25
Long−pec
Protruding−mouth
Day−4
Day−5
Stage
Transcript
ENSDART00000075486
ENSDART00000124168

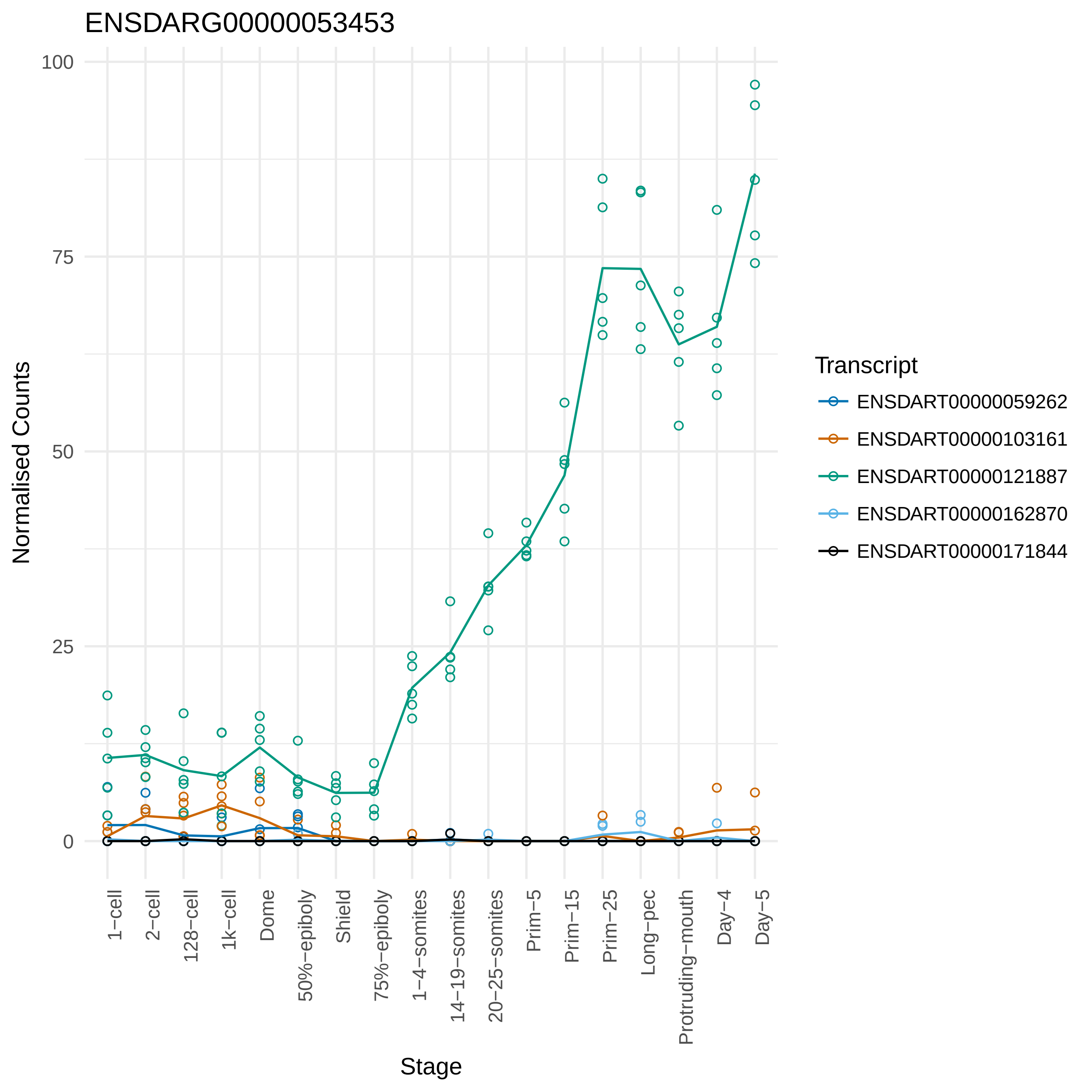
ENSDARG00000053453
Normalised Counts
100
75
50
25
0
1−cell
2−cell
128−cell
1k−cell
Dome
50%−epiboly
Shield
75%−epiboly
1−4−somites
14−19−somites
20−25−somites
Prim−5
Prim−15
Prim−25
Long−pec
Protruding−mouth
Day−4
Day−5
Stage
Transcript
ENSDART00000059262
ENSDART00000103161
ENSDART00000121887
ENSDART00000162870
ENSDART00000171844

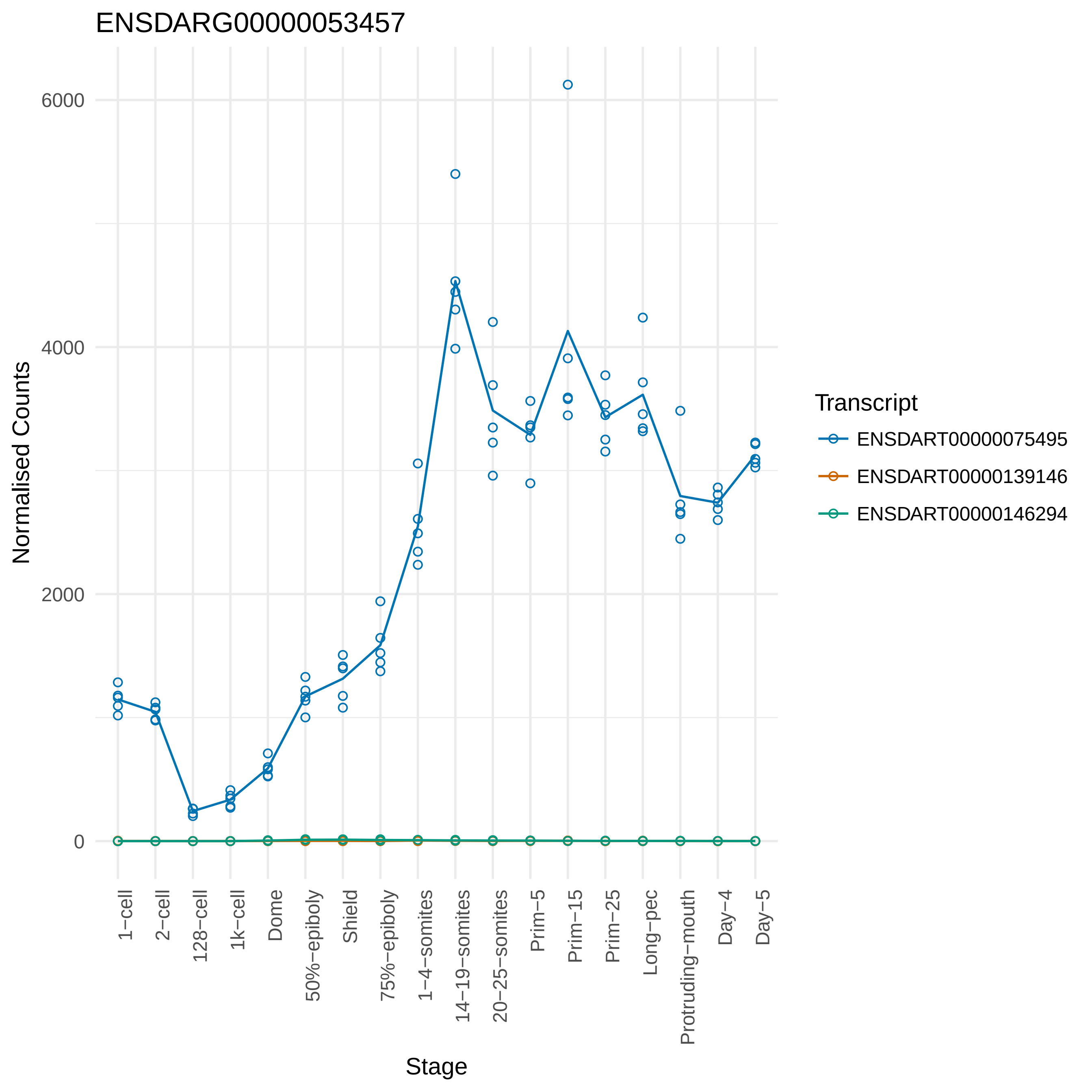
ENSDARG00000053457
Normalised Counts
6000
4000
2000
0
1−cell
2−cell
128−cell
1k−cell
Dome
50%−epiboly
Shield
75%−epiboly
1−4−somites
14−19−somites
20−25−somites
Prim−5
Prim−15
Prim−25
Long−pec
Protruding−mouth
Day−4
Day−5
Stage
Transcript
ENSDART00000075495
ENSDART00000139146
ENSDART00000146294

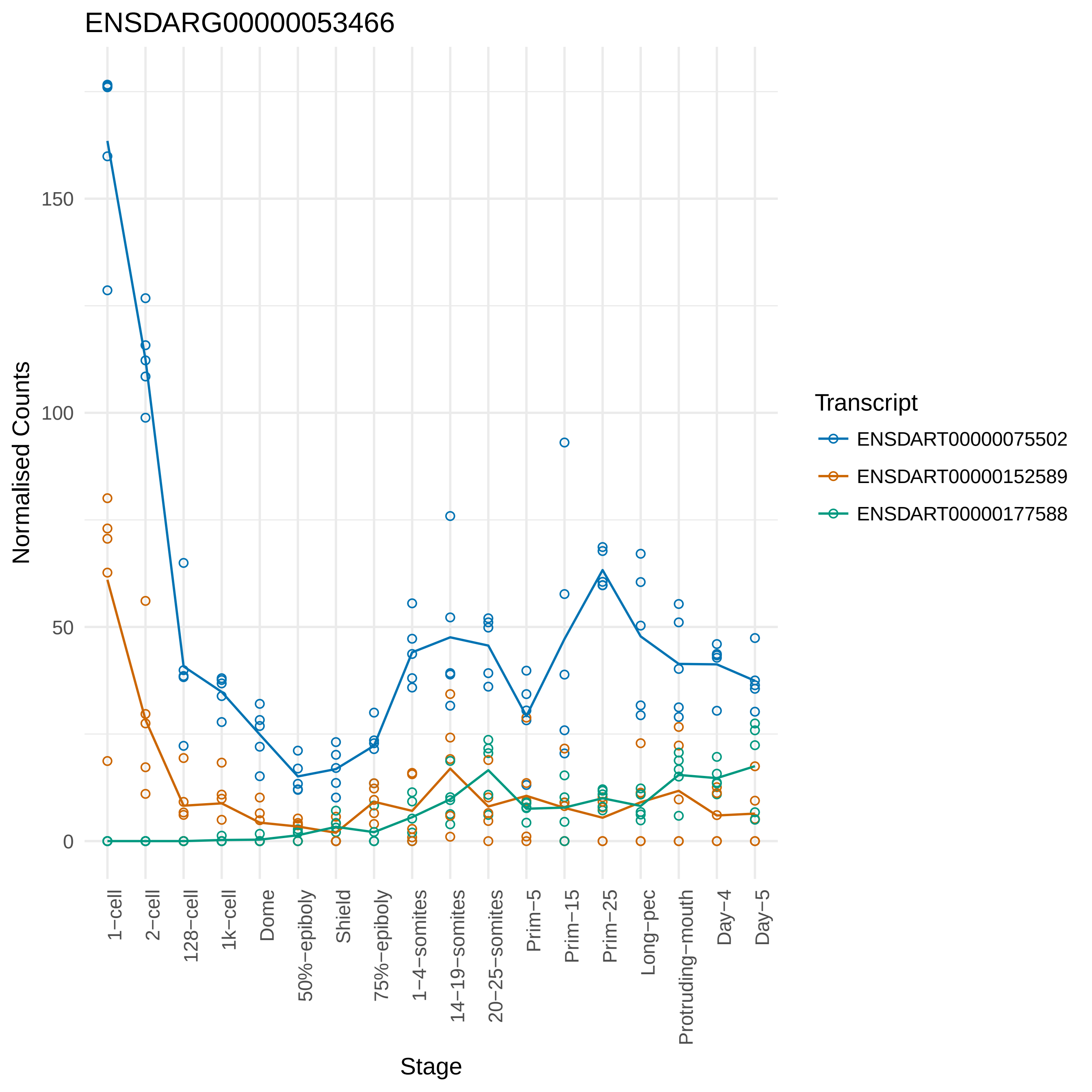
ENSDARG00000053466
Normalised Counts
150
100
50
0
1−cell
2−cell
128−cell
1k−cell
Dome
50%−epiboly
Shield
75%−epiboly
1−4−somites
14−19−somites
20−25−somites
Prim−5
Prim−15
Prim−25
Long−pec
Protruding−mouth
Day−4
Day−5
Stage
Transcript
ENSDART00000075502
ENSDART00000152589
ENSDART00000177588

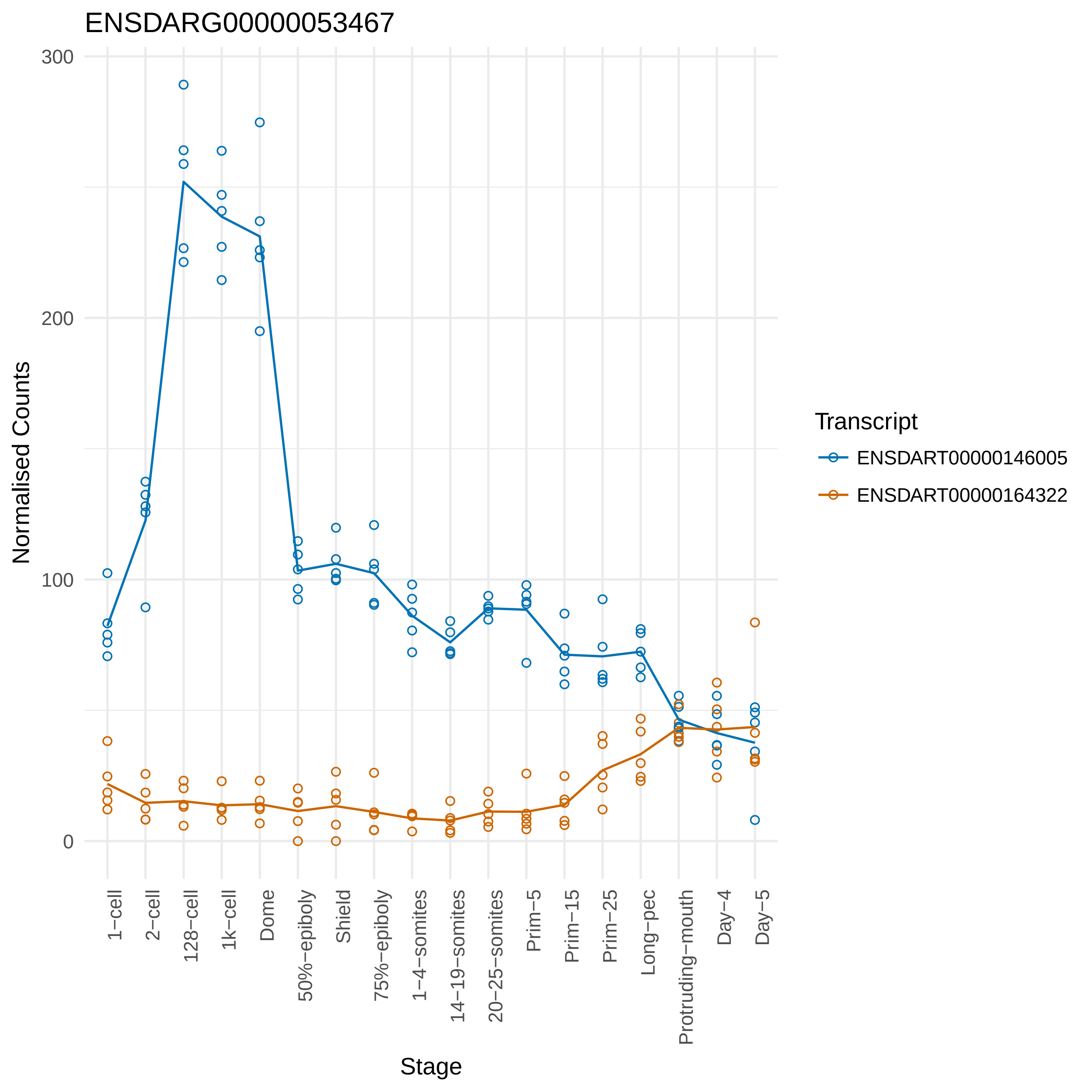

ENSDARG00000053467
Normalised Counts
300
200
100
0
1−cell
2−cell
128−cell
1k−cell
Dome
50%−epiboly
Shield
75%−epiboly
1−4−somites
14−19−somites
20−25−somites
Prim−5
Prim−15
Prim−25
Long−pec
Protruding−mouth
Day−4
Day−5
Stage
Transcript
ENSDART00000146005
ENSDART00000164322

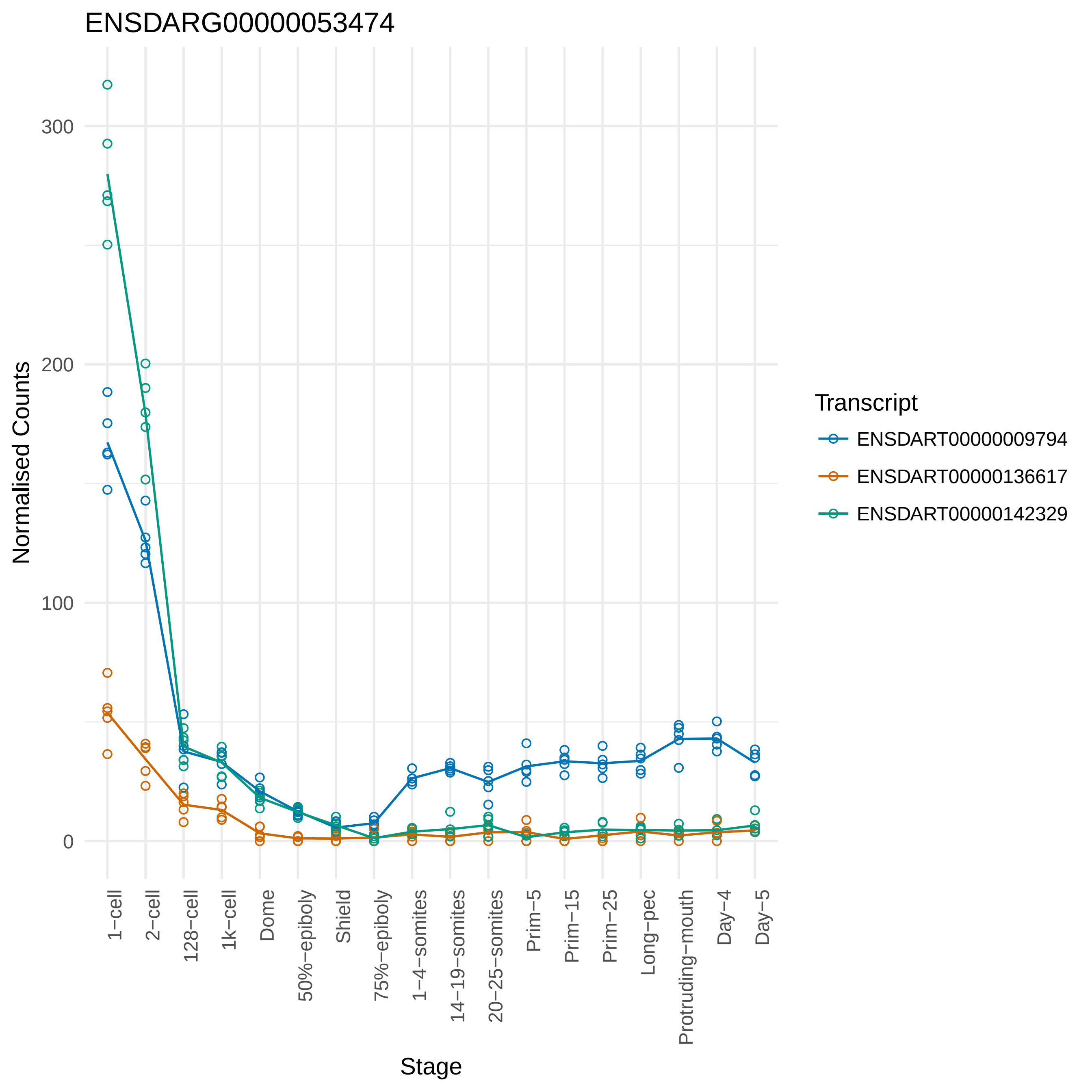
ENSDARG00000053474
Normalised Counts
0
100
200
300
Stage
1−cell
2−cell
128−cell
1k−cell
Dome
50%−epiboly
Shield
75%−epiboly
1−4−somites
14−19−somites
20−25−somites
Prim−5
Prim−15
Prim−25
Long−pec
Protruding−mouth
Day−4
Day−5
Transcript
ENSDART00000009794
ENSDART00000136617
ENSDART00000142329

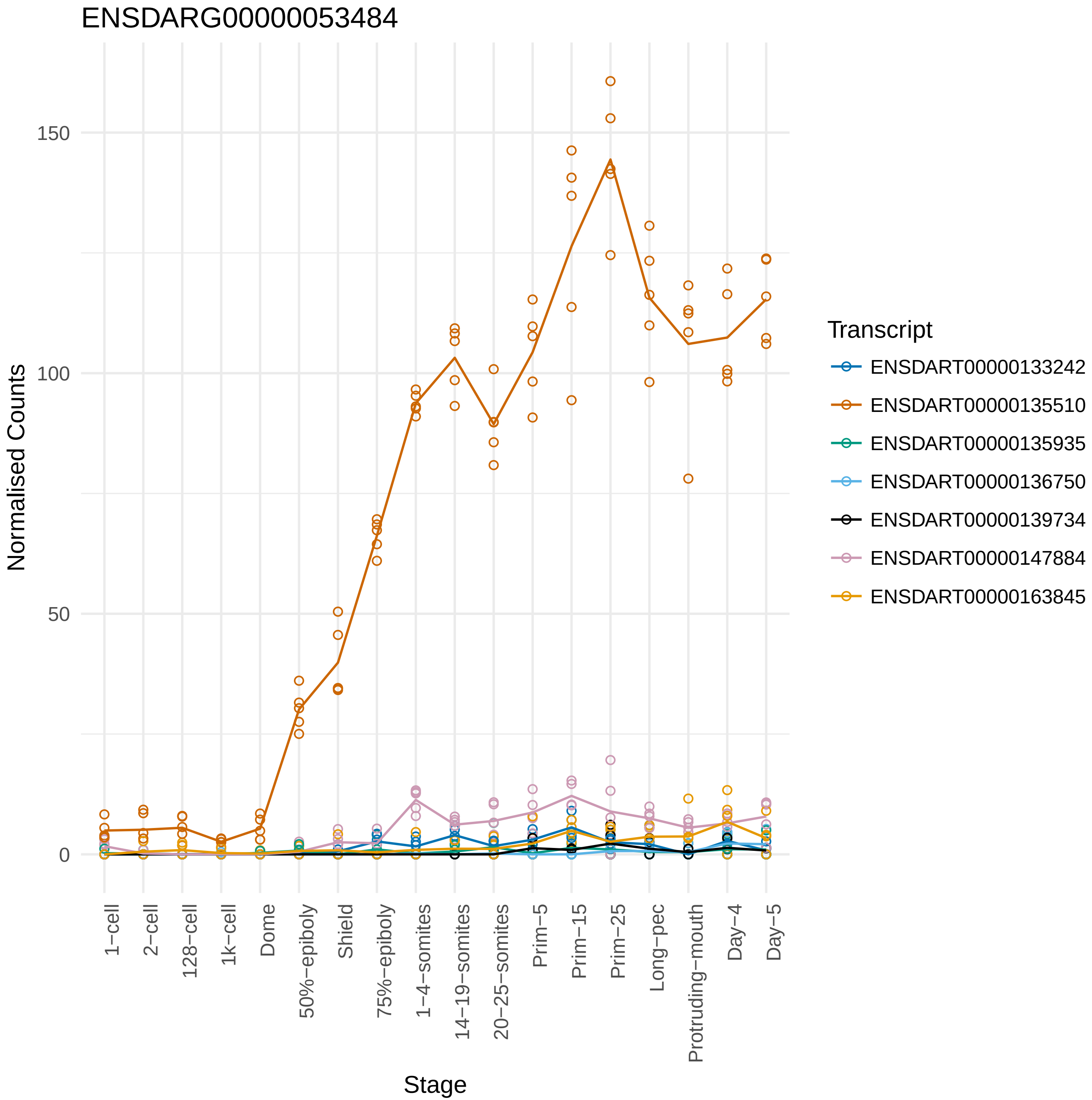

ENSDARG00000053484
Normalised Counts
150
100
50
0
1−cell
2−cell
128−cell
1k−cell
Dome
50%−epiboly
Shield
75%−epiboly
1−4−somites
14−19−somites
20−25−somites
Prim−5
Prim−15
Prim−25
Long−pec
Protruding−mouth
Day−4
Day−5
Stage
Transcript
ENSDART00000133242
ENSDART00000135510
ENSDART00000135935
ENSDART00000136750
ENSDART00000139734
ENSDART00000147884
ENSDART00000163845

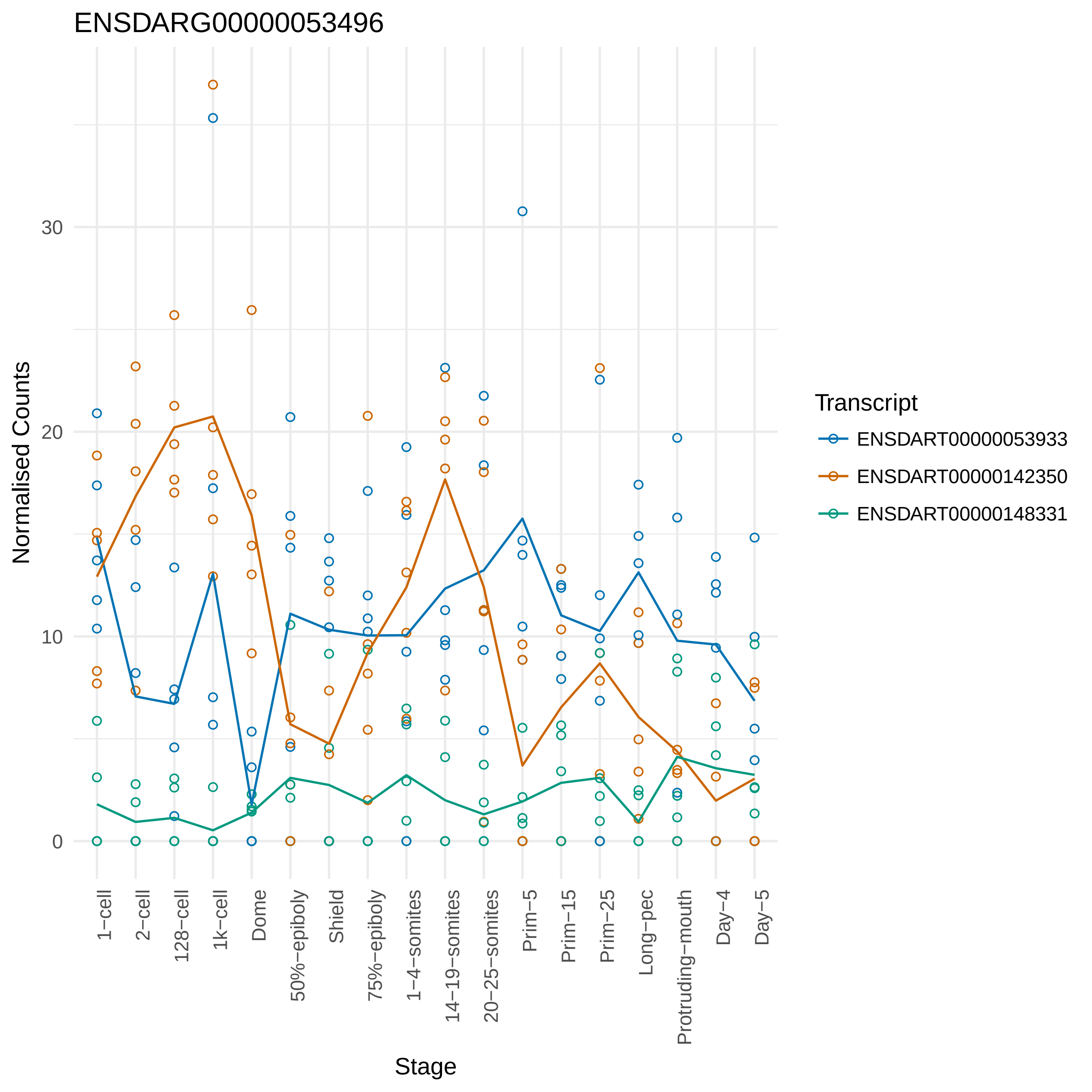

ENSDARG00000053496
Normalised Counts
0
10
20
30
Stage
1−cell
2−cell
128−cell
1k−cell
Dome
50%−epiboly
Shield
75%−epiboly
1−4−somites
14−19−somites
20−25−somites
Prim−5
Prim−15
Prim−25
Long−pec
Protruding−mouth
Day−4
Day−5
Transcript
ENSDART00000053933
ENSDART00000142350
ENSDART00000148331

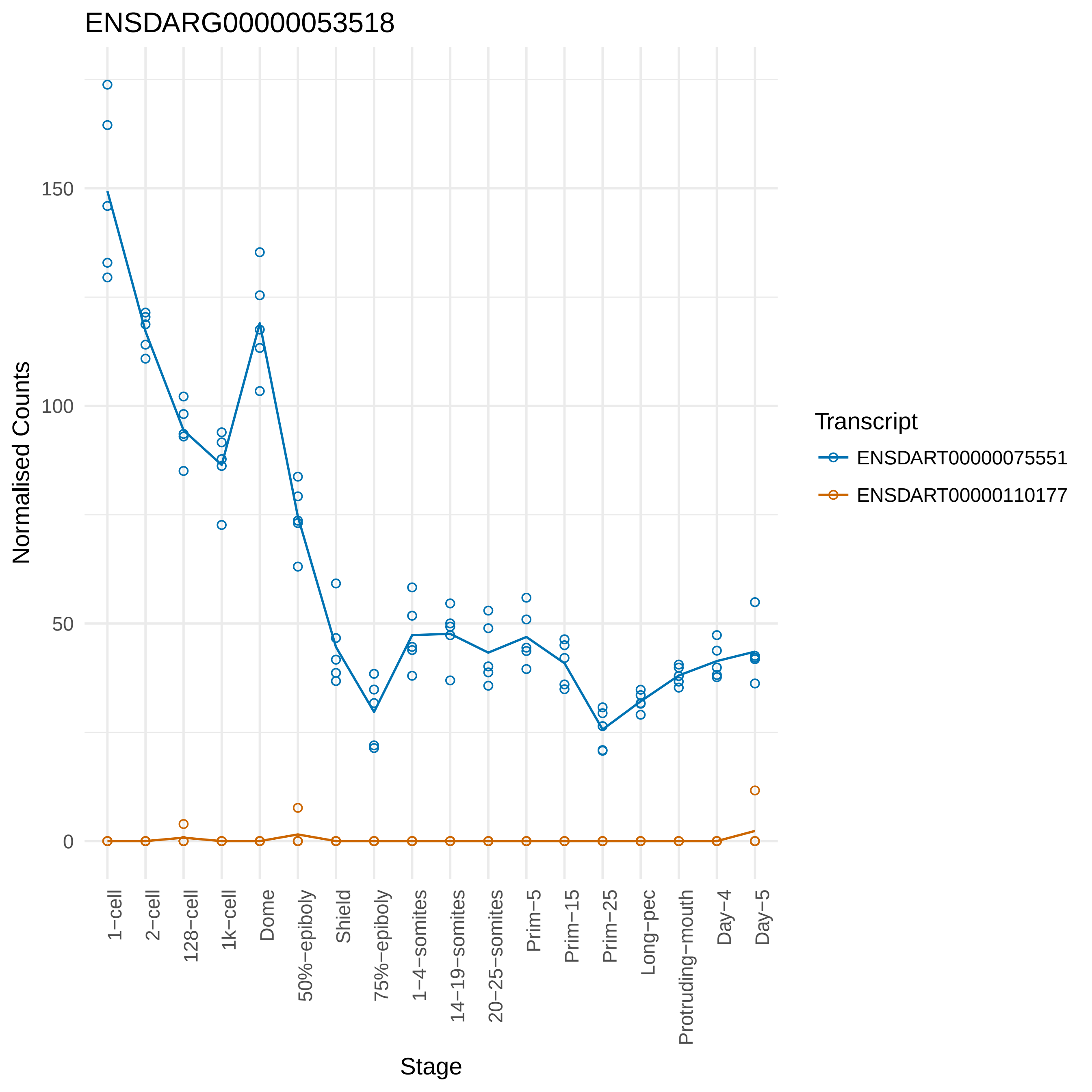

ENSDARG00000053518
Normalised Counts
150
100
50
0
1−cell
2−cell
128−cell
1k−cell
Dome
50%−epiboly
Shield
75%−epiboly
1−4−somites
14−19−somites
20−25−somites
Prim−5
Prim−15
Prim−25
Long−pec
Protruding−mouth
Day−4
Day−5
Stage
Transcript
ENSDART00000075551
ENSDART00000110177

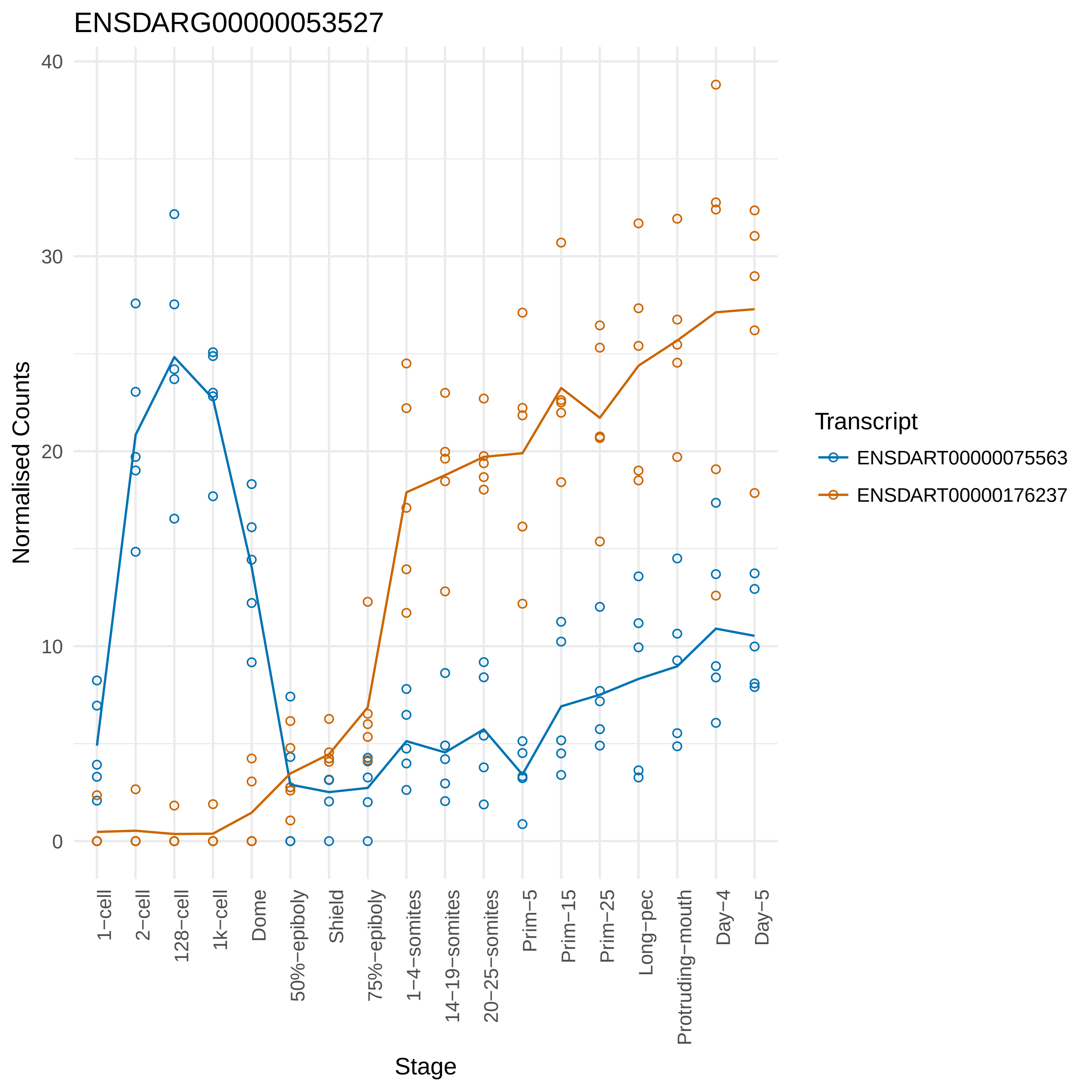
ENSDARG00000053527
Normalised Counts
0
10
20
30
40
Stage
1−cell
2−cell
128−cell
1k−cell
Dome
50%−epiboly
Shield
75%−epiboly
1−4−somites
14−19−somites
20−25−somites
Prim−5
Prim−15
Prim−25
Long−pec
Protruding−mouth
Day−4
Day−5
Transcript
ENSDART00000075563
ENSDART00000176237

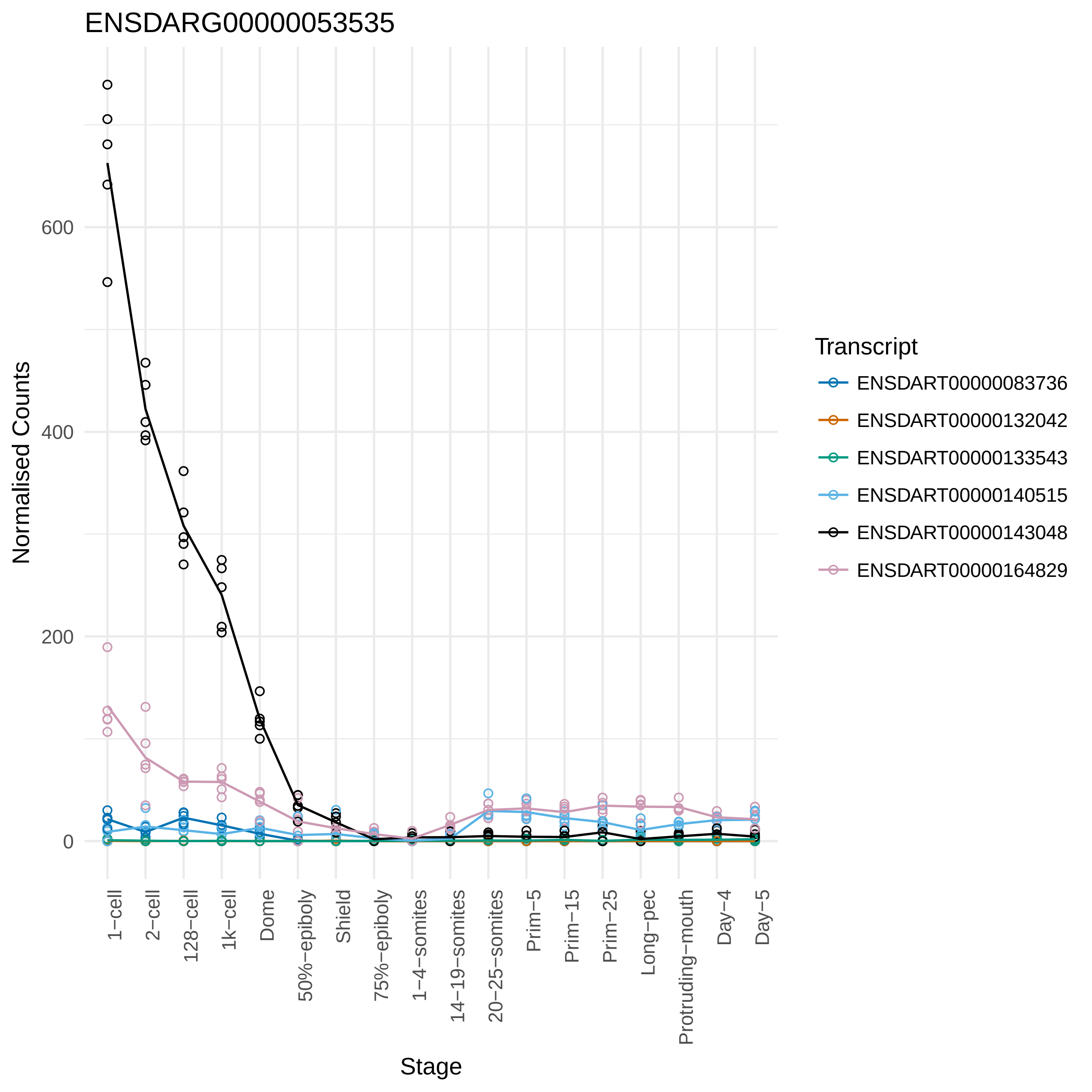

ENSDARG00000053535
Normalised Counts
600
400
200
0
1−cell
2−cell
128−cell
1k−cell
Dome
50%−epiboly
Shield
75%−epiboly
1−4−somites
14−19−somites
20−25−somites
Prim−5
Prim−15
Prim−25
Long−pec
Protruding−mouth
Day−4
Day−5
Stage
Transcript
ENSDART00000083736
ENSDART00000132042
ENSDART00000133543
ENSDART00000140515
ENSDART00000143048
ENSDART00000164829

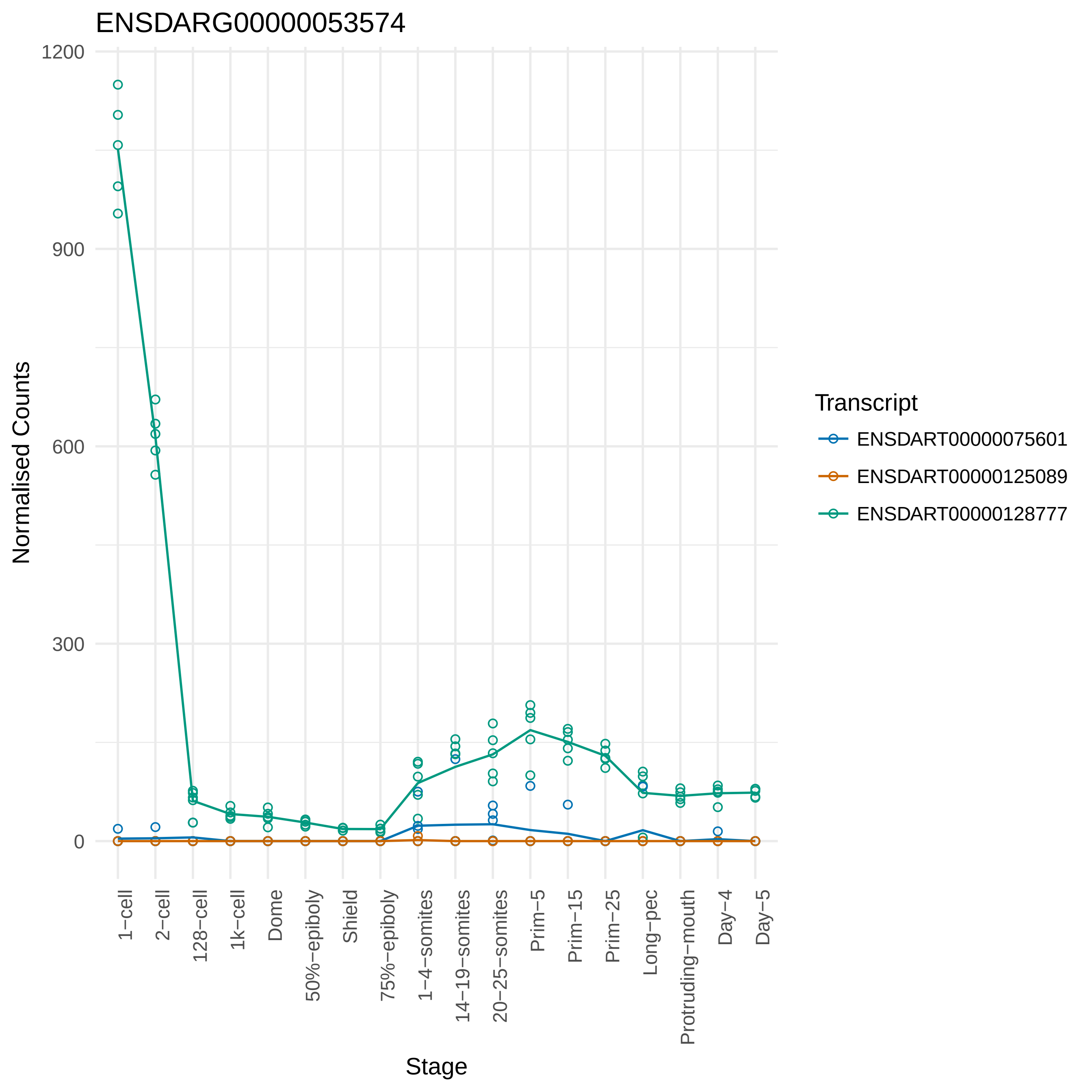

ENSDARG00000053574
Normalised Counts
1200
900
600
300
0
1−cell
2−cell
128−cell
1k−cell
Dome
50%−epiboly
Shield
75%−epiboly
1−4−somites
14−19−somites
20−25−somites
Prim−5
Prim−15
Prim−25
Long−pec
Protruding−mouth
Day−4
Day−5
Stage
Transcript
ENSDART00000075601
ENSDART00000125089
ENSDART00000128777

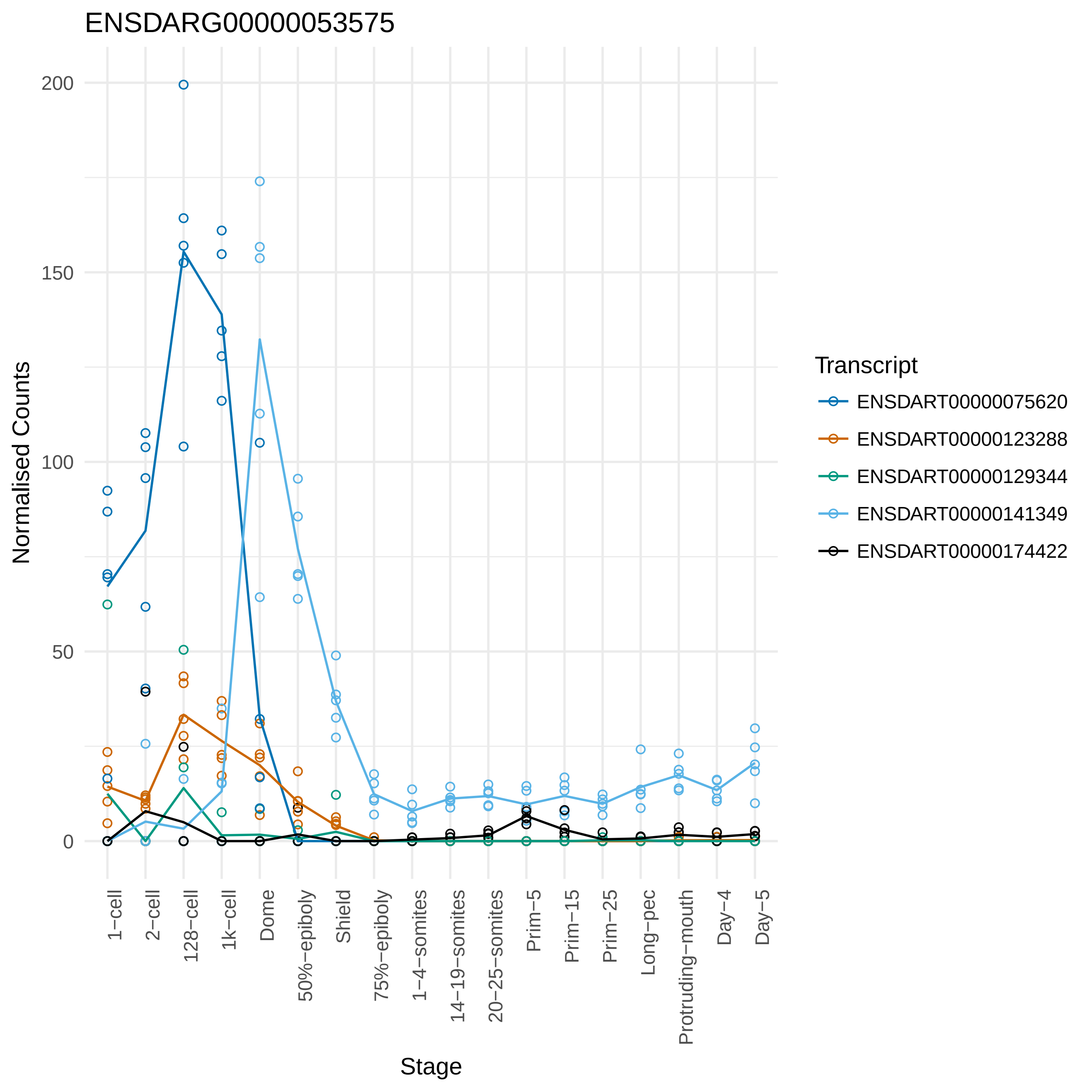

ENSDARG00000053575
Normalised Counts
200
150
100
50
0
1−cell
2−cell
128−cell
1k−cell
Dome
50%−epiboly
Shield
75%−epiboly
1−4−somites
14−19−somites
20−25−somites
Prim−5
Prim−15
Prim−25
Long−pec
Protruding−mouth
Day−4
Day−5
Stage
Transcript
ENSDART00000075620
ENSDART00000123288
ENSDART00000129344
ENSDART00000141349
ENSDART00000174422

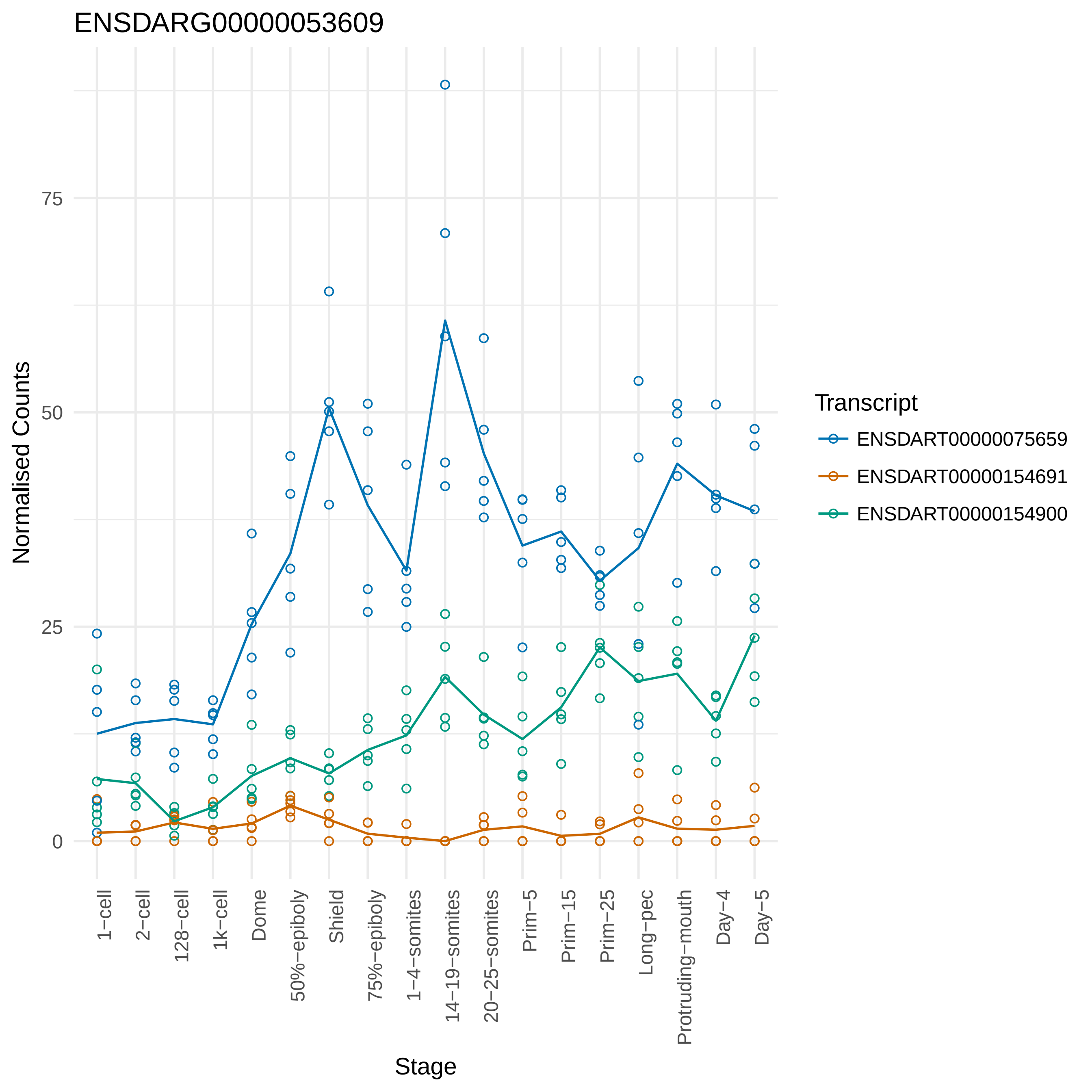

ENSDARG00000053609
Normalised Counts
0
25
50
75
1−cell
2−cell
128−cell
1k−cell
Dome
50%−epiboly
Shield
75%−epiboly
1−4−somites
14−19−somites
20−25−somites
Prim−5
Prim−15
Prim−25
Long−pec
Protruding−mouth
Day−4
Day−5
Stage
Transcript
ENSDART00000075659
ENSDART00000154691
ENSDART00000154900

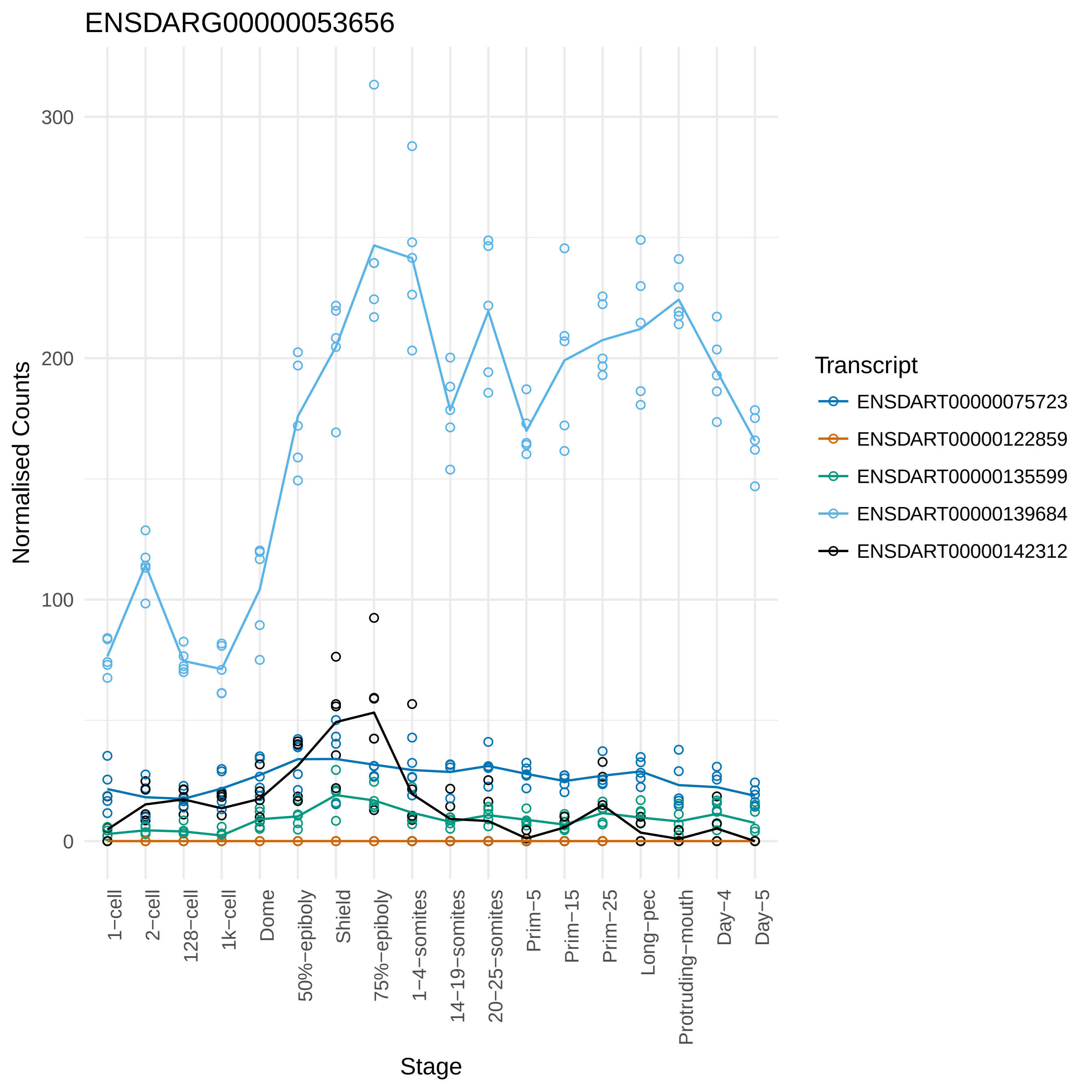
ENSDARG00000053656
Normalised Counts
300
200
100
0
Stage
1−cell
2−cell
128−cell
1k−cell
Dome
50%−epiboly
Shield
75%−epiboly
1−4−somites
14−19−somites
20−25−somites
Prim−5
Prim−15
Prim−25
Long−pec
Protruding−mouth
Day−4
Day−5
Transcript
ENSDART00000075723
ENSDART00000122859
ENSDART00000135599
ENSDART00000139684
ENSDART00000142312

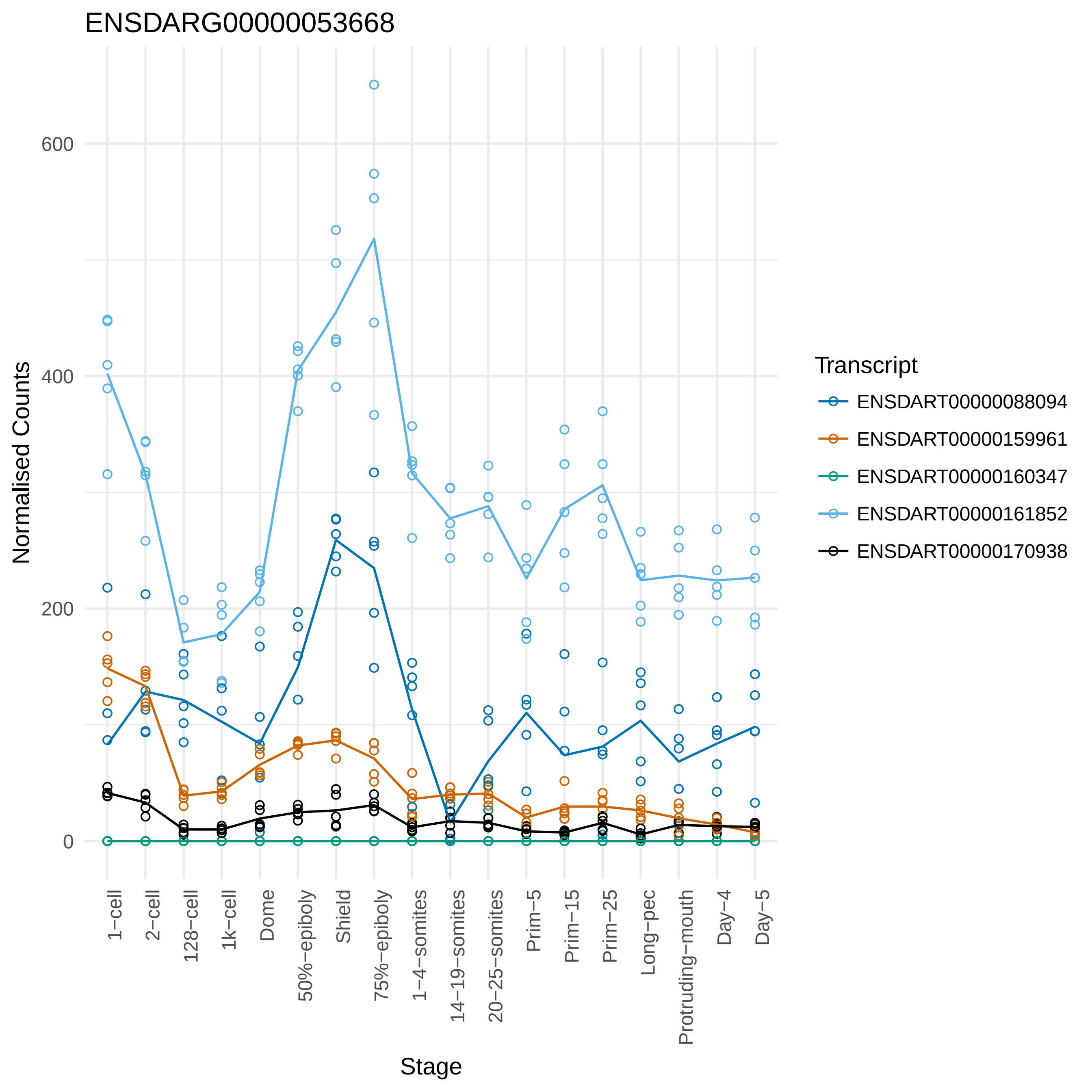
ENSDARG00000053668
Normalised Counts
0
200
400
600
Stage
1−cell
2−cell
128−cell
1k−cell
Dome
50%−epiboly
Shield
75%−epiboly
1−4−somites
14−19−somites
20−25−somites
Prim−5
Prim−15
Prim−25
Long−pec
Protruding−mouth
Day−4
Day−5
Transcript
ENSDART00000088094
ENSDART00000159961
ENSDART00000160347
ENSDART00000161852
ENSDART00000170938

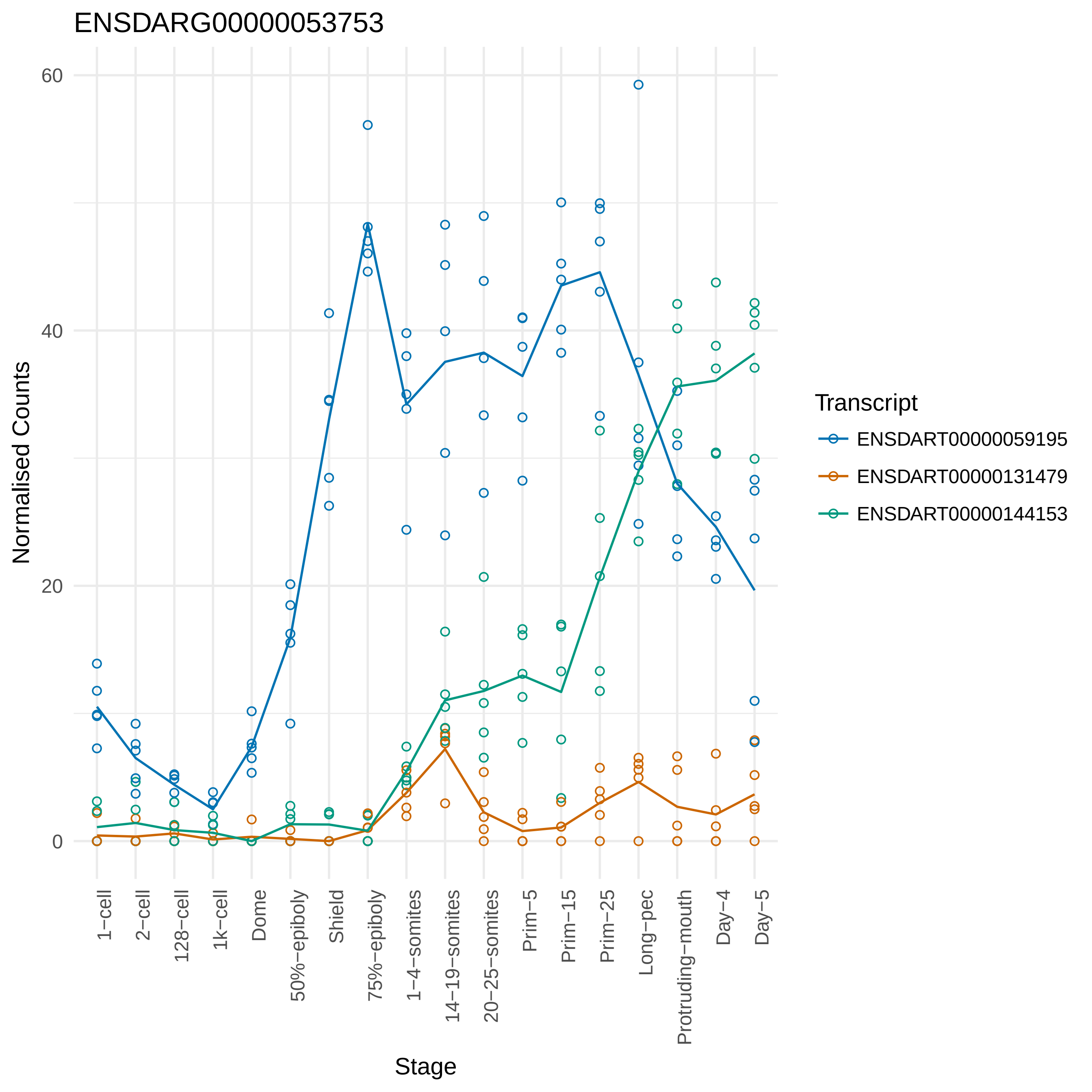

ENSDARG00000053753
Normalised Counts
60
40
20
0
1−cell
2−cell
128−cell
1k−cell
Dome
50%−epiboly
Shield
75%−epiboly
1−4−somites
14−19−somites
20−25−somites
Prim−5
Prim−15
Prim−25
Long−pec
Protruding−mouth
Day−4
Day−5
Stage
Transcript
ENSDART00000059195
ENSDART00000131479
ENSDART00000144153

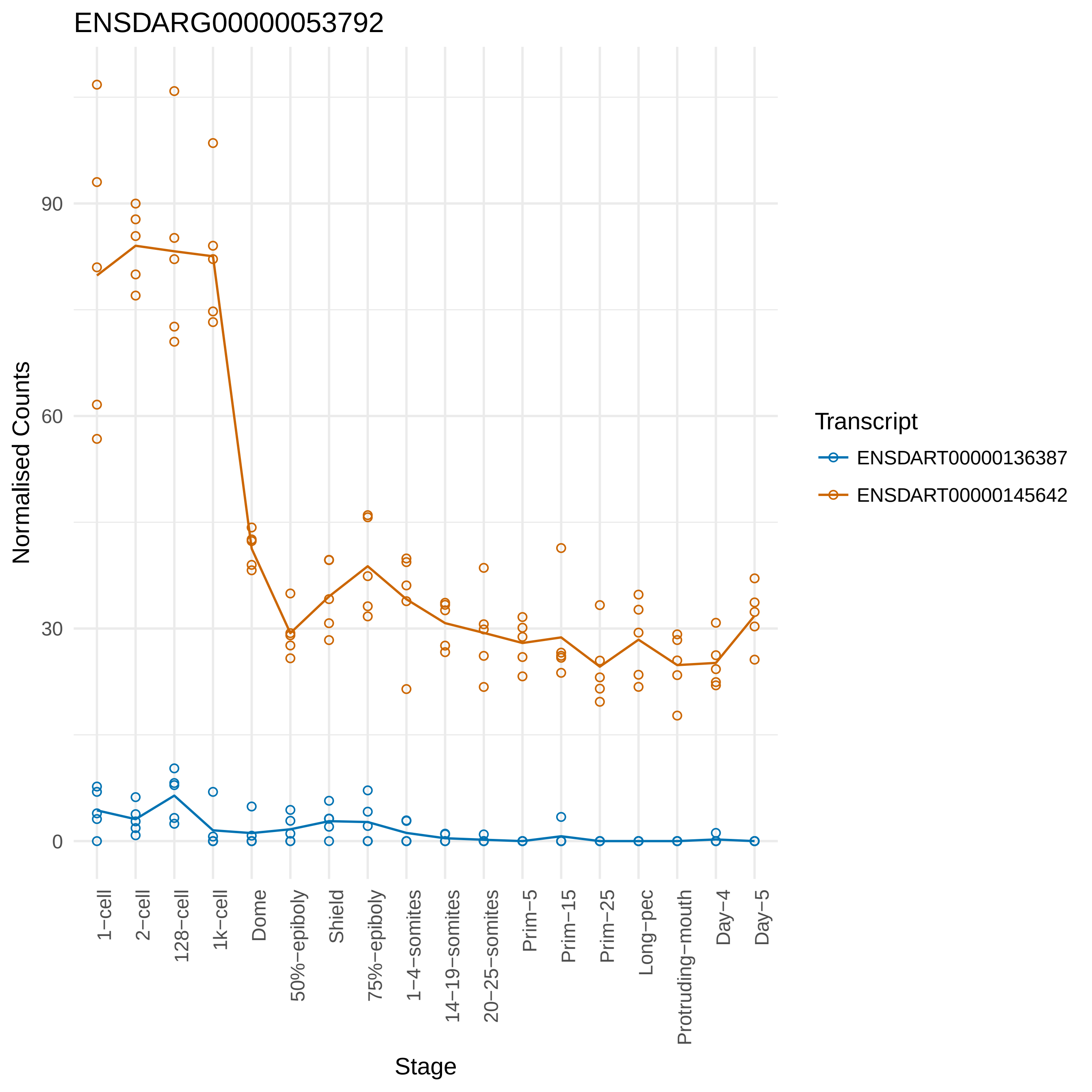

ENSDARG00000053792
Normalised Counts
90
60
30
0
1−cell
2−cell
128−cell
1k−cell
Dome
50%−epiboly
Shield
75%−epiboly
1−4−somites
14−19−somites
20−25−somites
Prim−5
Prim−15
Prim−25
Long−pec
Protruding−mouth
Day−4
Day−5
Stage
Transcript
ENSDART00000136387
ENSDART00000145642

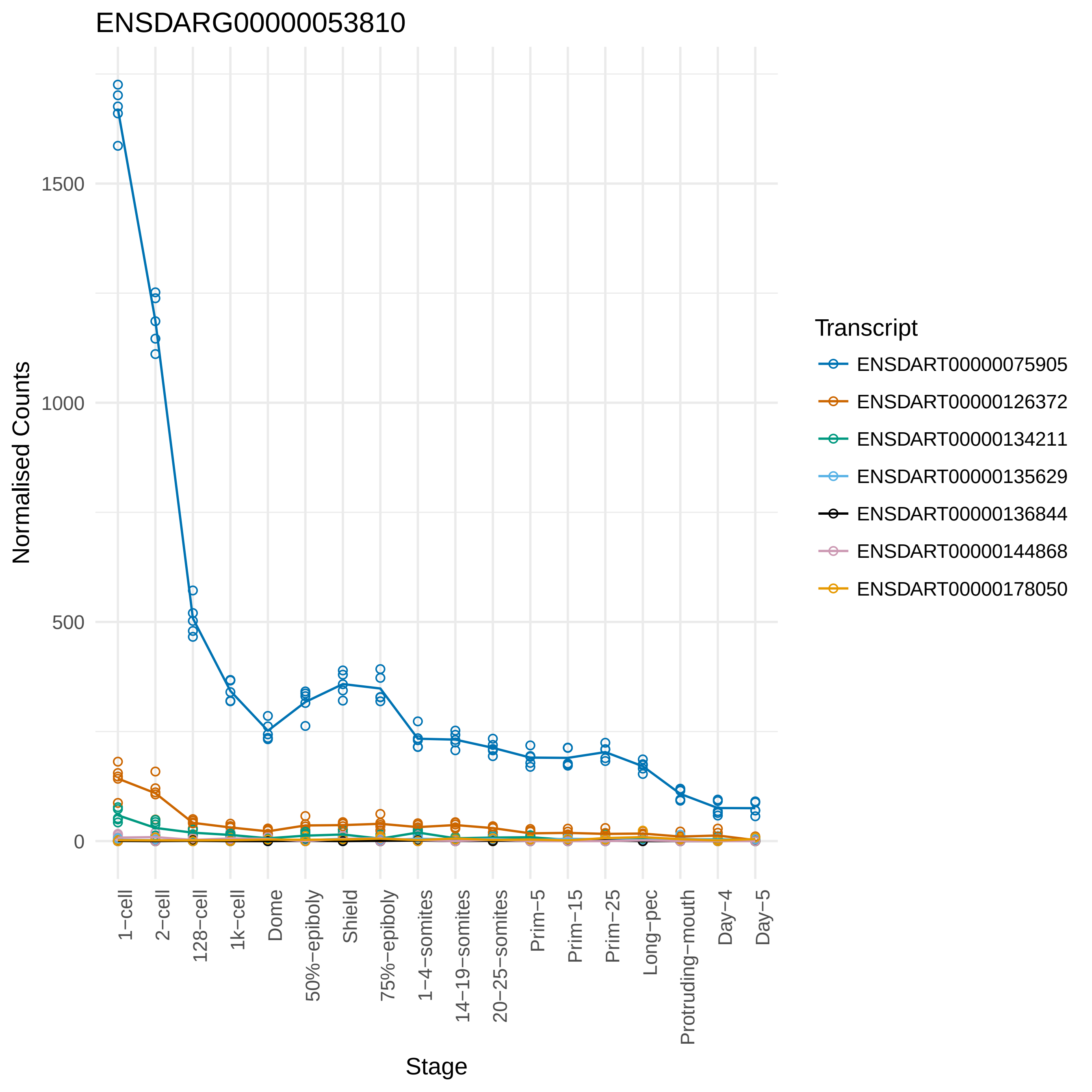

ENSDARG00000053810
Normalised Counts
1500
1000
500
0
1−cell
2−cell
128−cell
1k−cell
Dome
50%−epiboly
Shield
75%−epiboly
1−4−somites
14−19−somites
20−25−somites
Prim−5
Prim−15
Prim−25
Long−pec
Protruding−mouth
Day−4
Day−5
Stage
Transcript
ENSDART00000075905
ENSDART00000126372
ENSDART00000134211
ENSDART00000135629
ENSDART00000136844
ENSDART00000144868
ENSDART00000178050

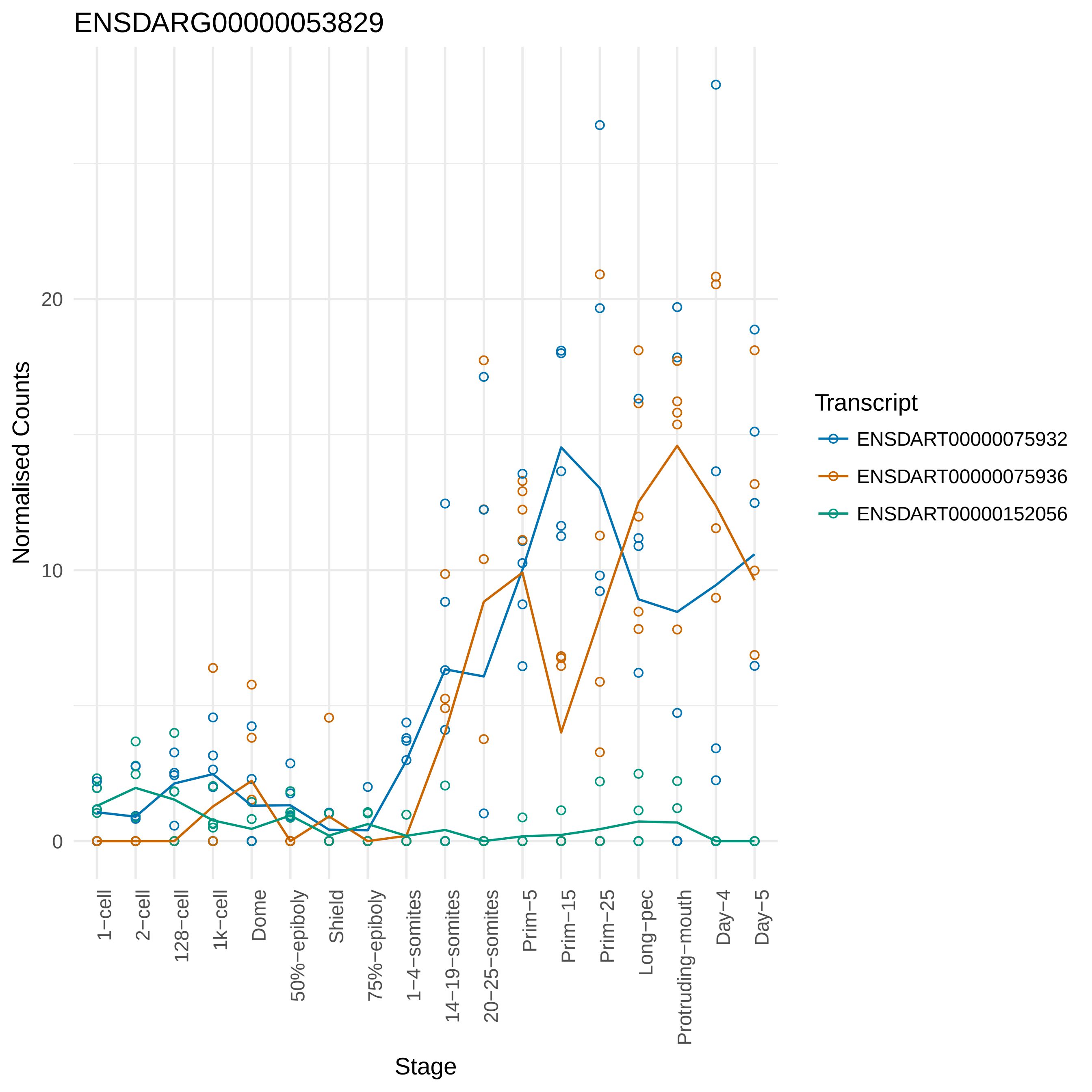

ENSDARG00000053829
Normalised Counts
0
10
20
1−cell
2−cell
128−cell
1k−cell
Dome
50%−epiboly
Shield
75%−epiboly
1−4−somites
14−19−somites
20−25−somites
Prim−5
Prim−15
Prim−25
Long−pec
Protruding−mouth
Day−4
Day−5
Stage
Transcript
ENSDART00000075932
ENSDART00000075936
ENSDART00000152056

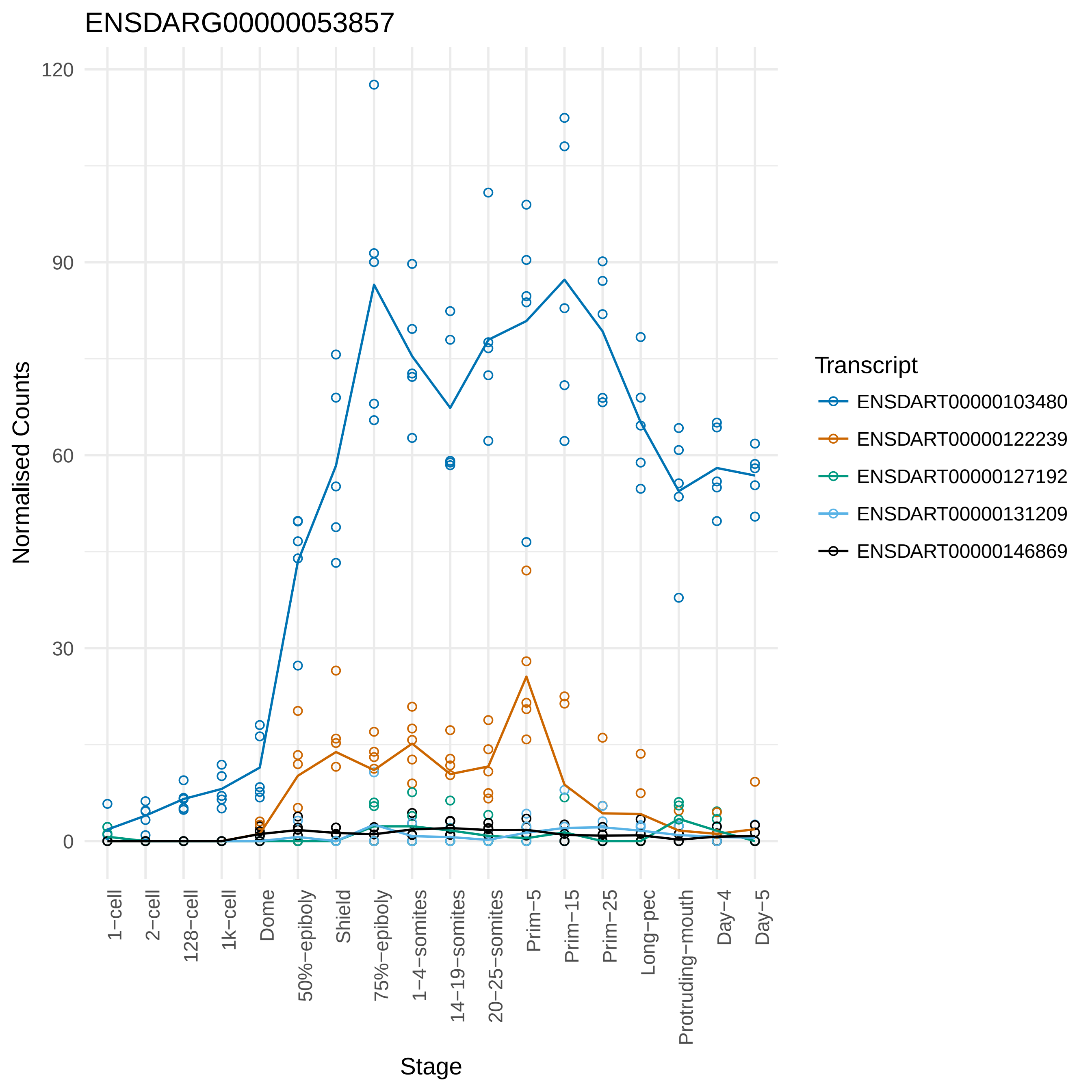
ENSDARG00000053857
Normalised Counts
120
90
60
30
0
Stage
1−cell
2−cell
128−cell
1k−cell
Dome
50%−epiboly
Shield
75%−epiboly
1−4−somites
14−19−somites
20−25−somites
Prim−5
Prim−15
Prim−25
Long−pec
Protruding−mouth
Day−4
Day−5
Transcript
ENSDART00000103480
ENSDART00000122239
ENSDART00000127192
ENSDART00000131209
ENSDART00000146869

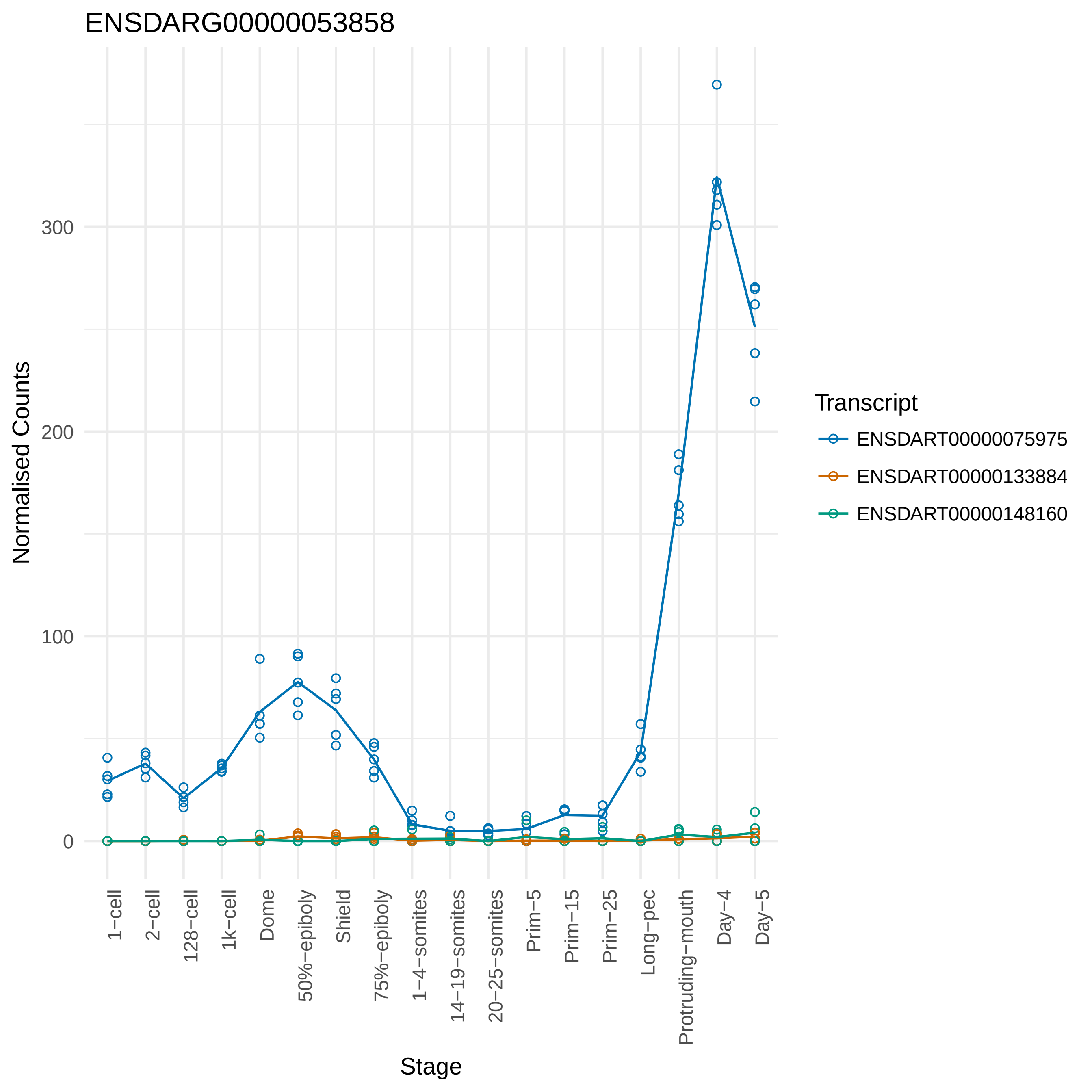

ENSDARG00000053858
Normalised Counts
300
200
100
0
1−cell
2−cell
128−cell
1k−cell
Dome
50%−epiboly
Shield
75%−epiboly
1−4−somites
14−19−somites
20−25−somites
Prim−5
Prim−15
Prim−25
Long−pec
Protruding−mouth
Day−4
Day−5
Stage
Transcript
ENSDART00000075975
ENSDART00000133884
ENSDART00000148160

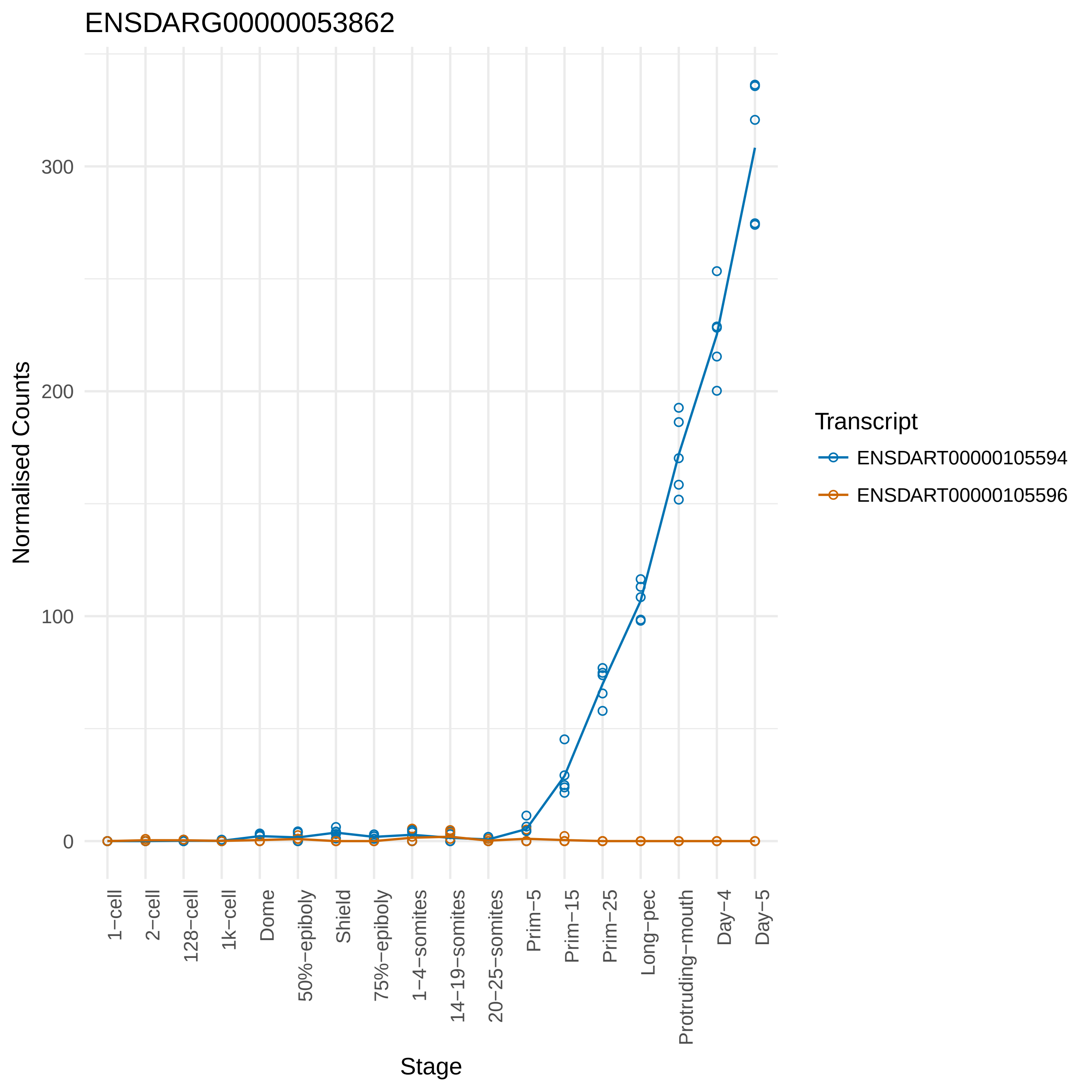
ENSDARG00000053862
Normalised Counts
300
200
100
0
1−cell
2−cell
128−cell
1k−cell
Dome
50%−epiboly
Shield
75%−epiboly
1−4−somites
14−19−somites
20−25−somites
Prim−5
Prim−15
Prim−25
Long−pec
Protruding−mouth
Day−4
Day−5
Stage
Transcript
ENSDART00000105594
ENSDART00000105596

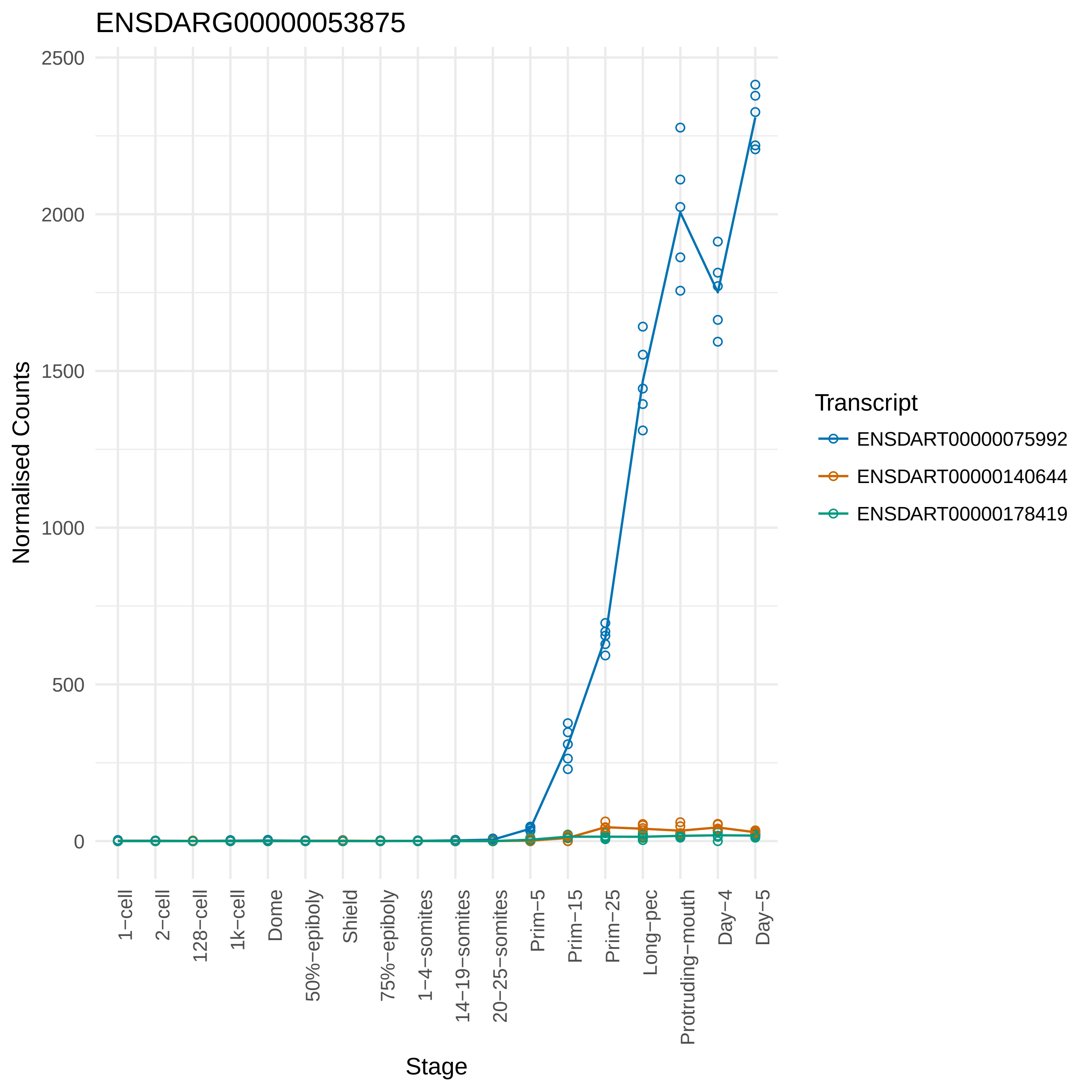
ENSDARG00000053875
Normalised Counts
2500
2000
1500
1000
500
0
1−cell
2−cell
128−cell
1k−cell
Dome
50%−epiboly
Shield
75%−epiboly
1−4−somites
14−19−somites
20−25−somites
Prim−5
Prim−15
Prim−25
Long−pec
Protruding−mouth
Day−4
Day−5
Stage
Transcript
ENSDART00000075992
ENSDART00000140644
ENSDART00000178419

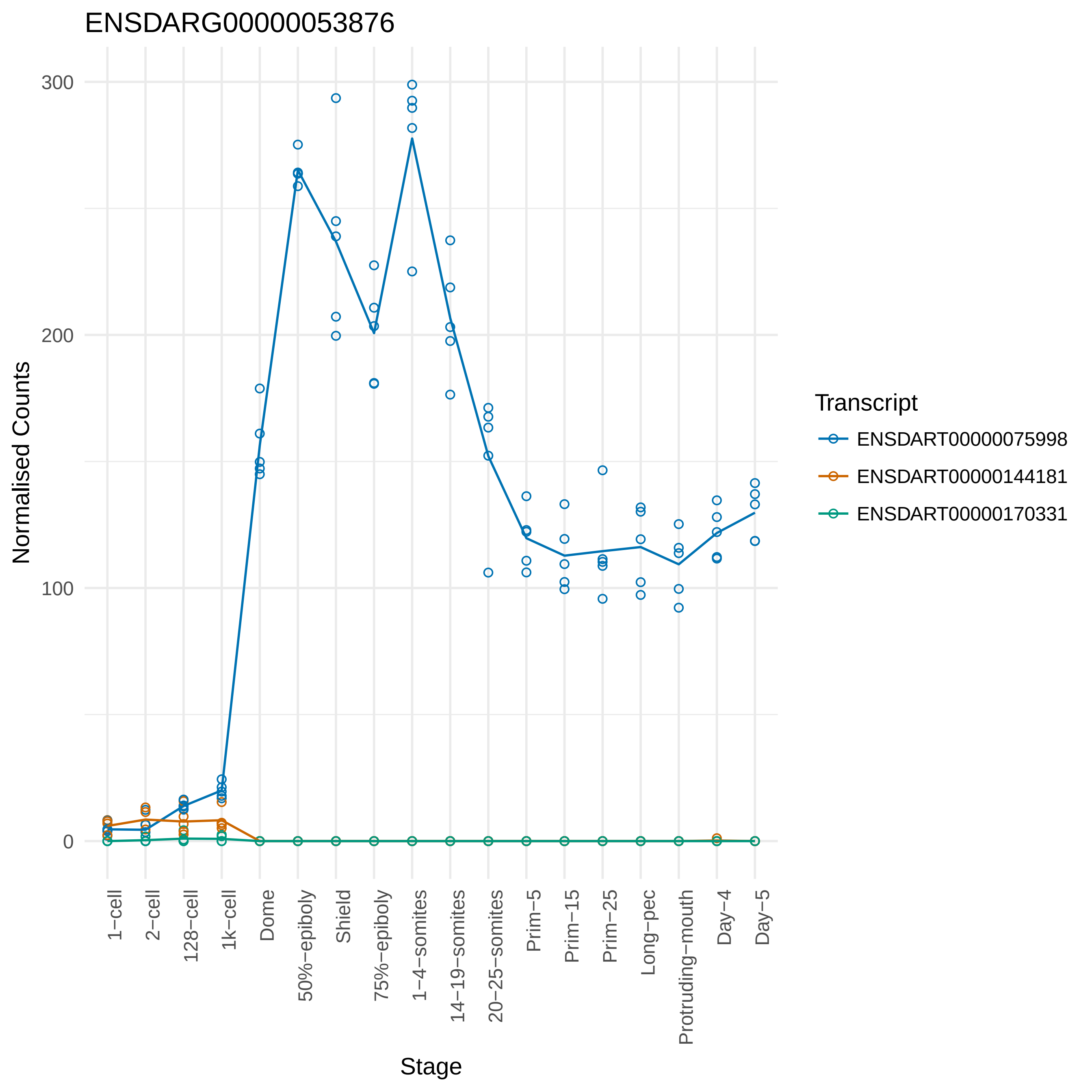
ENSDARG00000053876
Normalised Counts
300
200
100
0
1−cell
2−cell
128−cell
1k−cell
Dome
50%−epiboly
Shield
75%−epiboly
1−4−somites
14−19−somites
20−25−somites
Prim−5
Prim−15
Prim−25
Long−pec
Protruding−mouth
Day−4
Day−5
Stage
Transcript
ENSDART00000075998
ENSDART00000144181
ENSDART00000170331

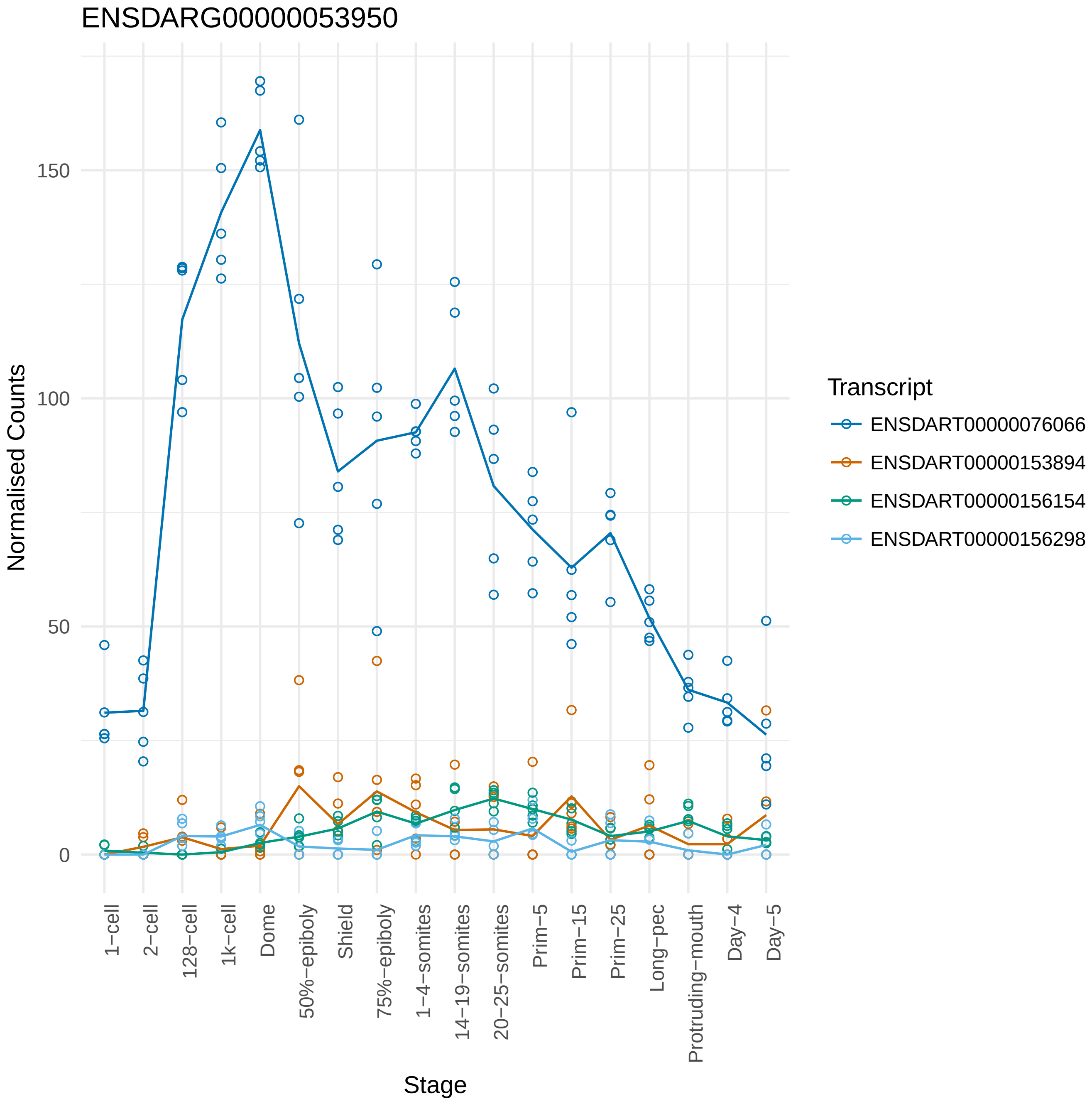
ENSDARG00000053950
Normalised Counts
150
100
50
0
1−cell
2−cell
128−cell
1k−cell
Dome
50%−epiboly
Shield
75%−epiboly
1−4−somites
14−19−somites
20−25−somites
Prim−5
Prim−15
Prim−25
Long−pec
Protruding−mouth
Day−4
Day−5
Stage
Transcript
ENSDART00000076066
ENSDART00000153894
ENSDART00000156154
ENSDART00000156298

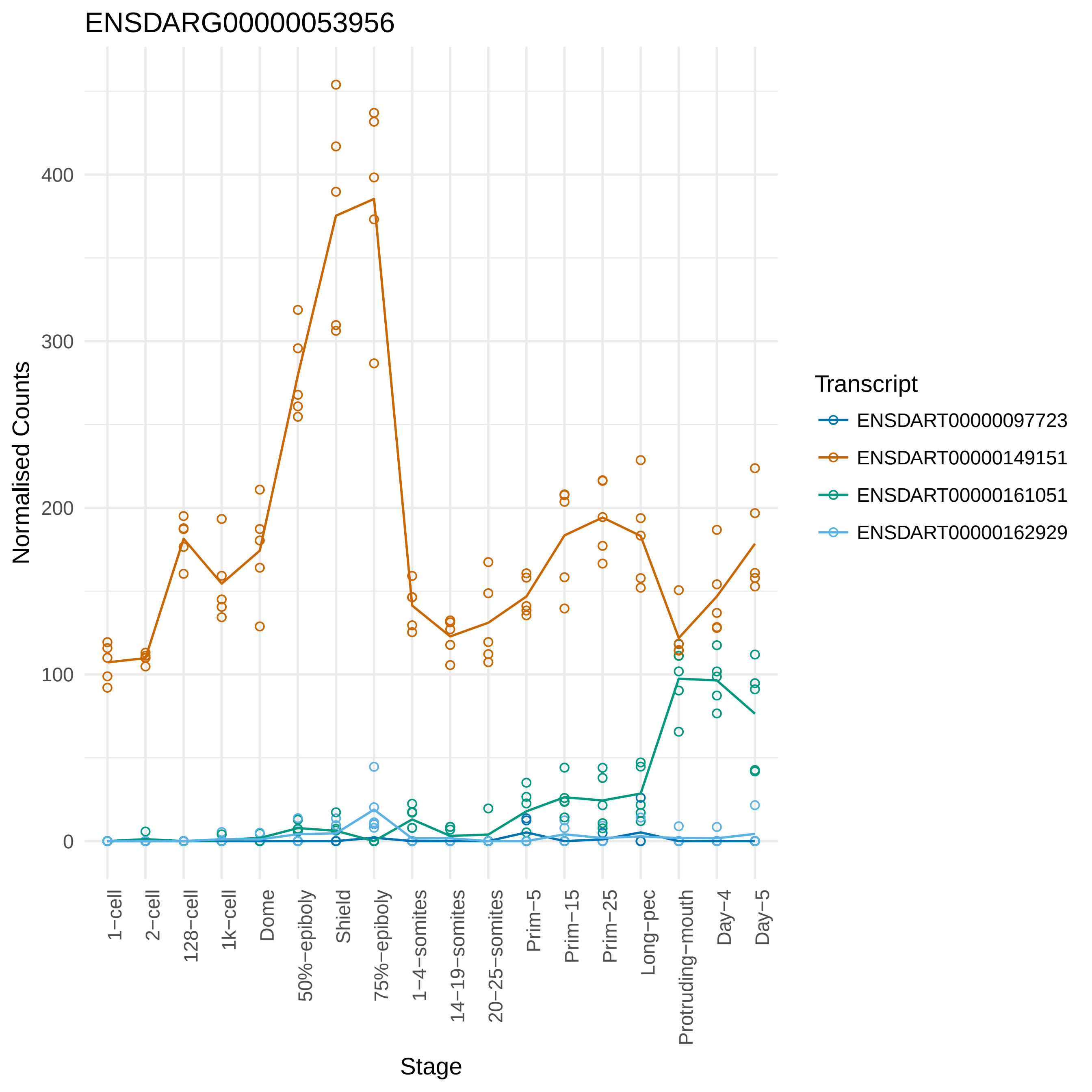
ENSDARG00000053956
Normalised Counts
0
100
200
300
400
Stage
1−cell
2−cell
128−cell
1k−cell
Dome
50%−epiboly
Shield
75%−epiboly
1−4−somites
14−19−somites
20−25−somites
Prim−5
Prim−15
Prim−25
Long−pec
Protruding−mouth
Day−4
Day−5
Transcript
ENSDART00000097723
ENSDART00000149151
ENSDART00000161051
ENSDART00000162929

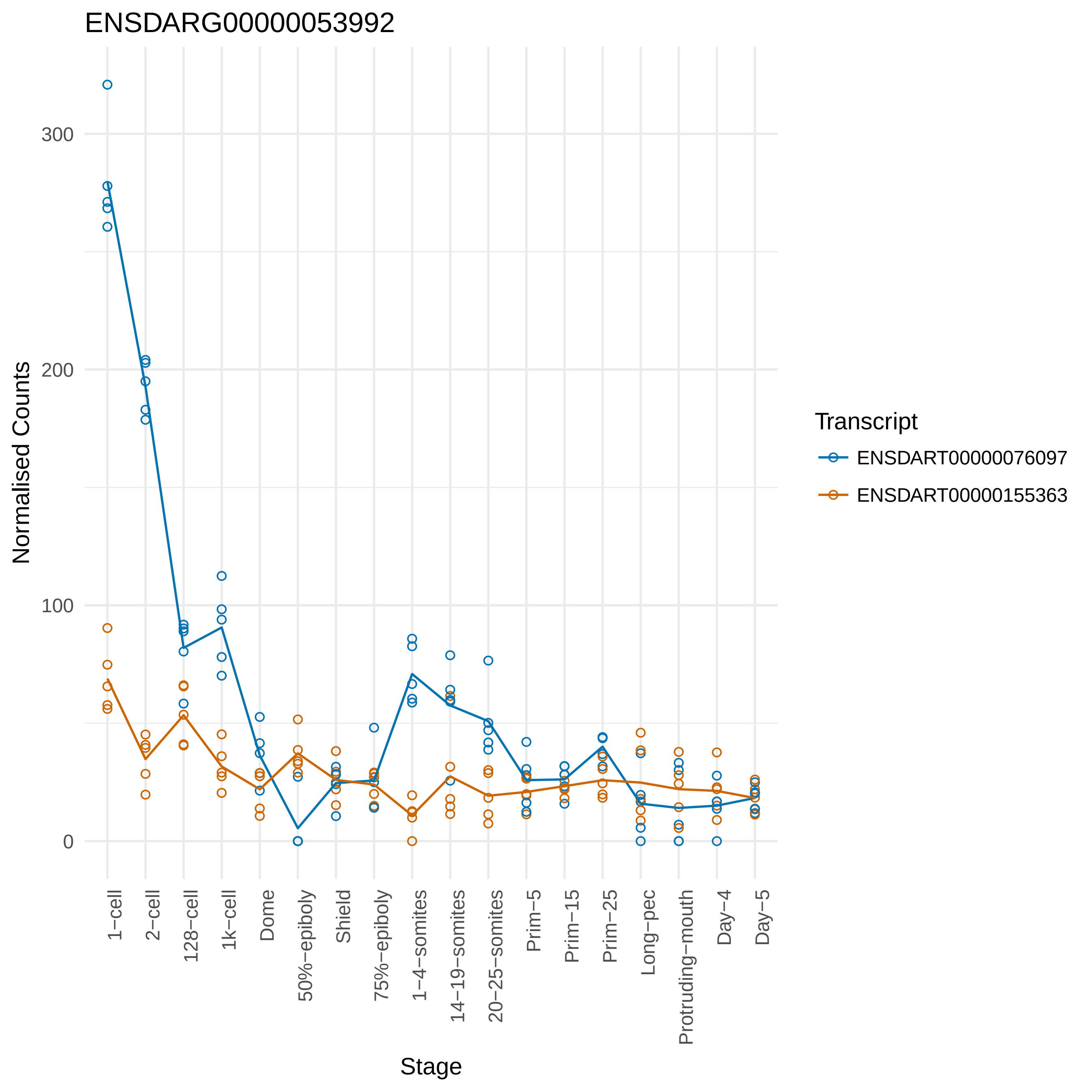
ENSDARG00000053992
Normalised Counts
300
200
100
0
1−cell
2−cell
128−cell
1k−cell
Dome
50%−epiboly
Shield
75%−epiboly
1−4−somites
14−19−somites
20−25−somites
Prim−5
Prim−15
Prim−25
Long−pec
Protruding−mouth
Day−4
Day−5
Stage
Transcript
ENSDART00000076097
ENSDART00000155363

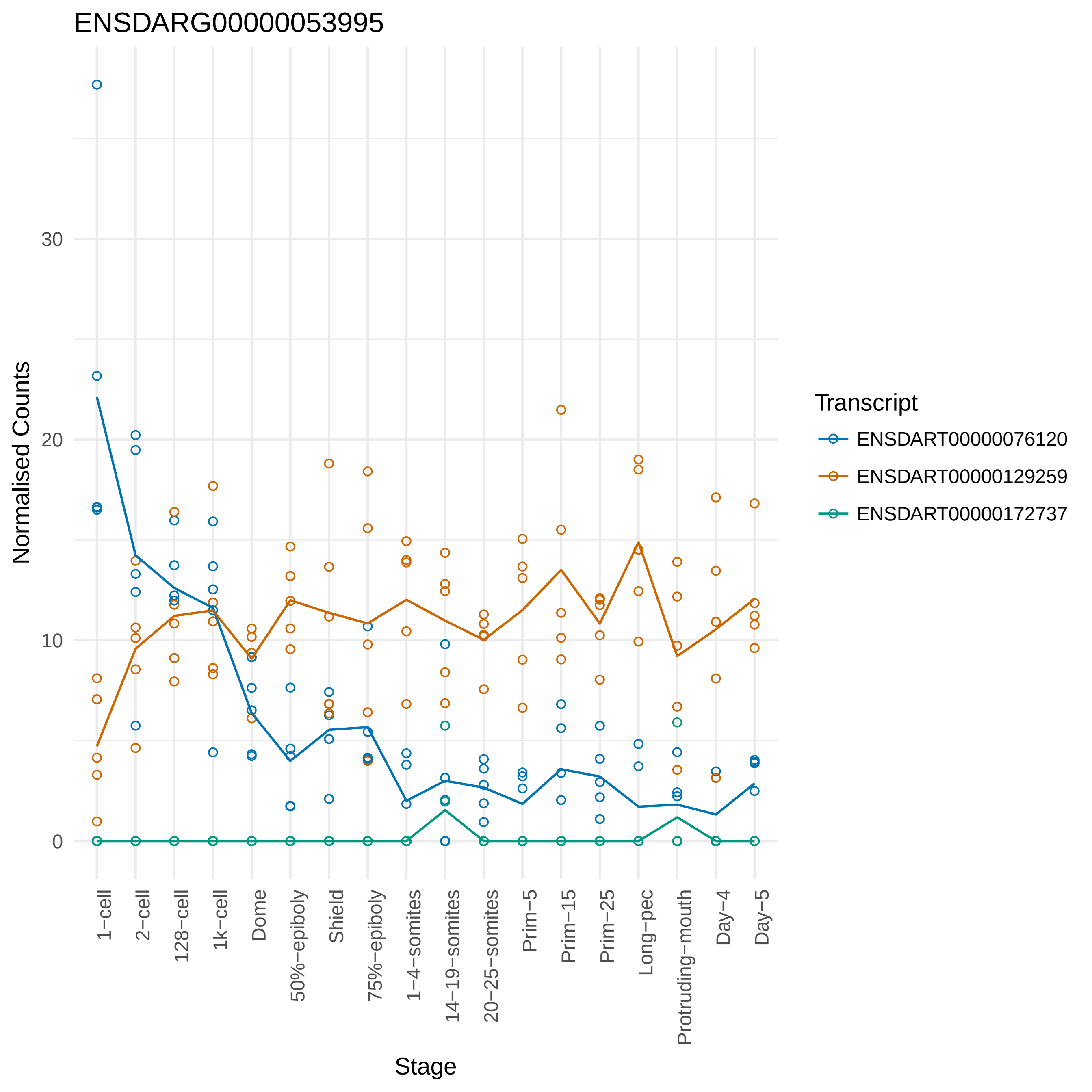
ENSDARG00000053995
Normalised Counts
0
10
20
30
Stage
1−cell
2−cell
128−cell
1k−cell
Dome
50%−epiboly
Shield
75%−epiboly
1−4−somites
14−19−somites
20−25−somites
Prim−5
Prim−15
Prim−25
Long−pec
Protruding−mouth
Day−4
Day−5
Transcript
ENSDART00000076120
ENSDART00000129259
ENSDART00000172737

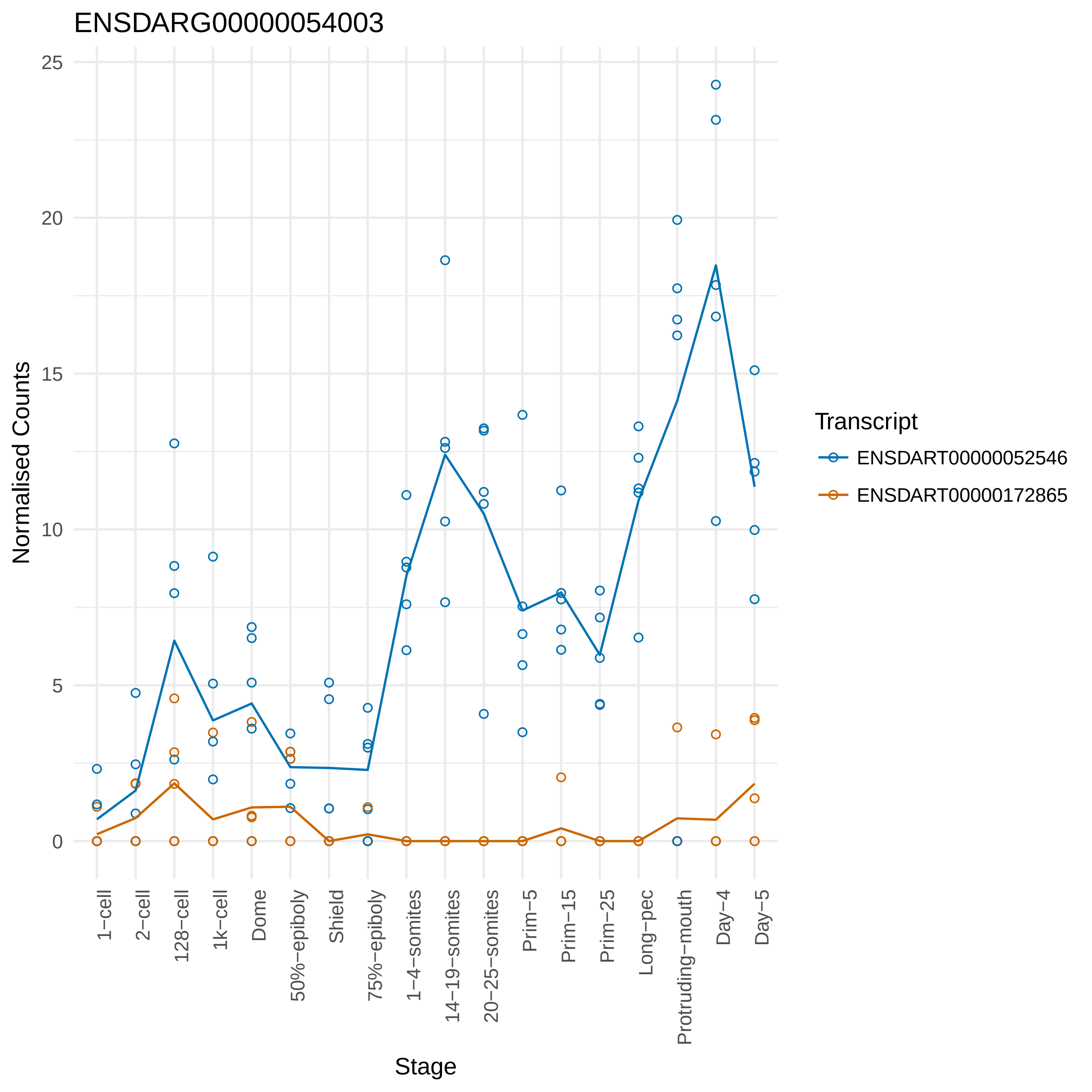

ENSDARG00000054003
Normalised Counts
25
20
15
10
5
0
1−cell
2−cell
128−cell
1k−cell
Dome
50%−epiboly
Shield
75%−epiboly
1−4−somites
14−19−somites
20−25−somites
Prim−5
Prim−15
Prim−25
Long−pec
Protruding−mouth
Day−4
Day−5
Stage
Transcript
ENSDART00000052546
ENSDART00000172865

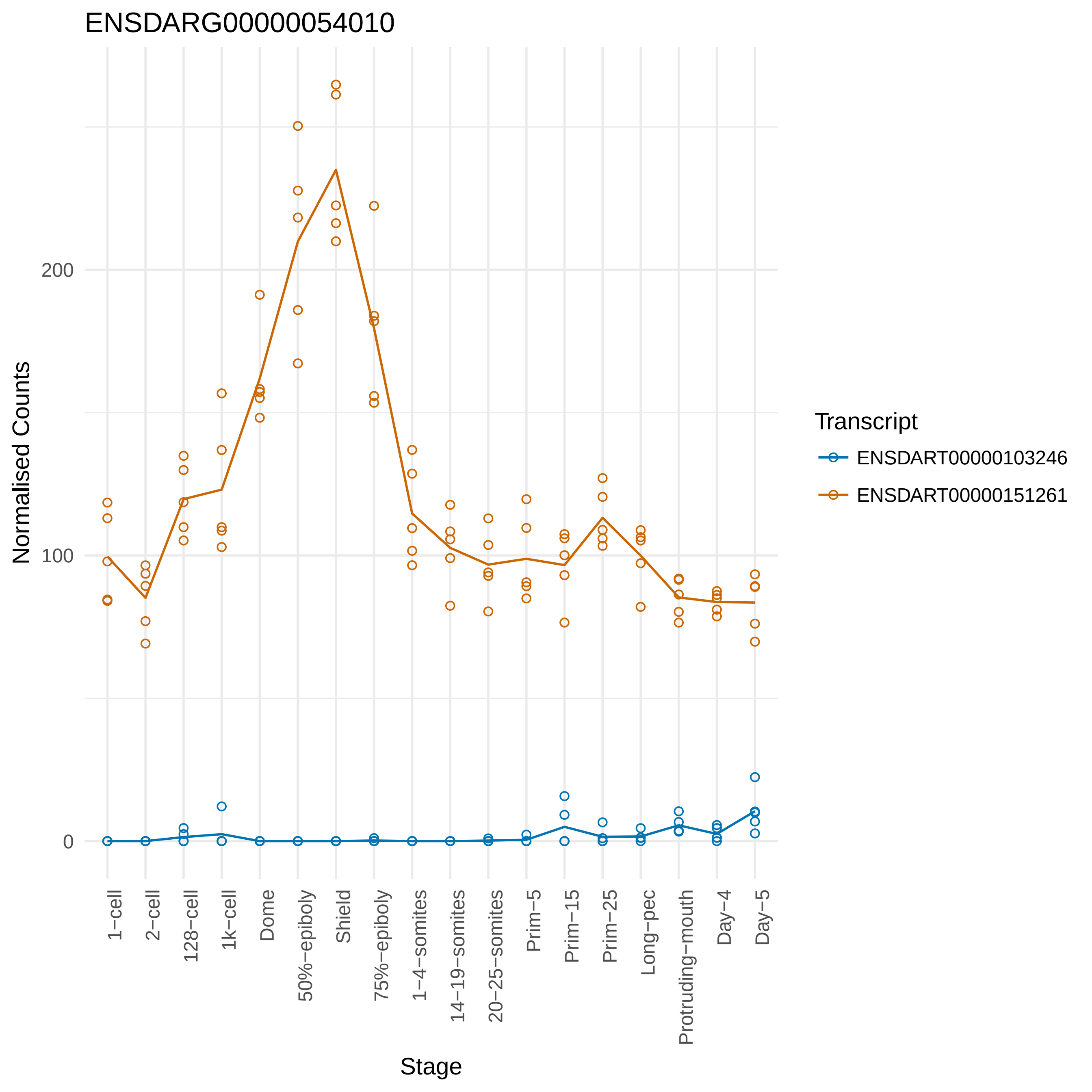

ENSDARG00000054010
Normalised Counts
200
100
0
1−cell
2−cell
128−cell
1k−cell
Dome
50%−epiboly
Shield
75%−epiboly
1−4−somites
14−19−somites
20−25−somites
Prim−5
Prim−15
Prim−25
Long−pec
Protruding−mouth
Day−4
Day−5
Stage
Transcript
ENSDART00000103246
ENSDART00000151261

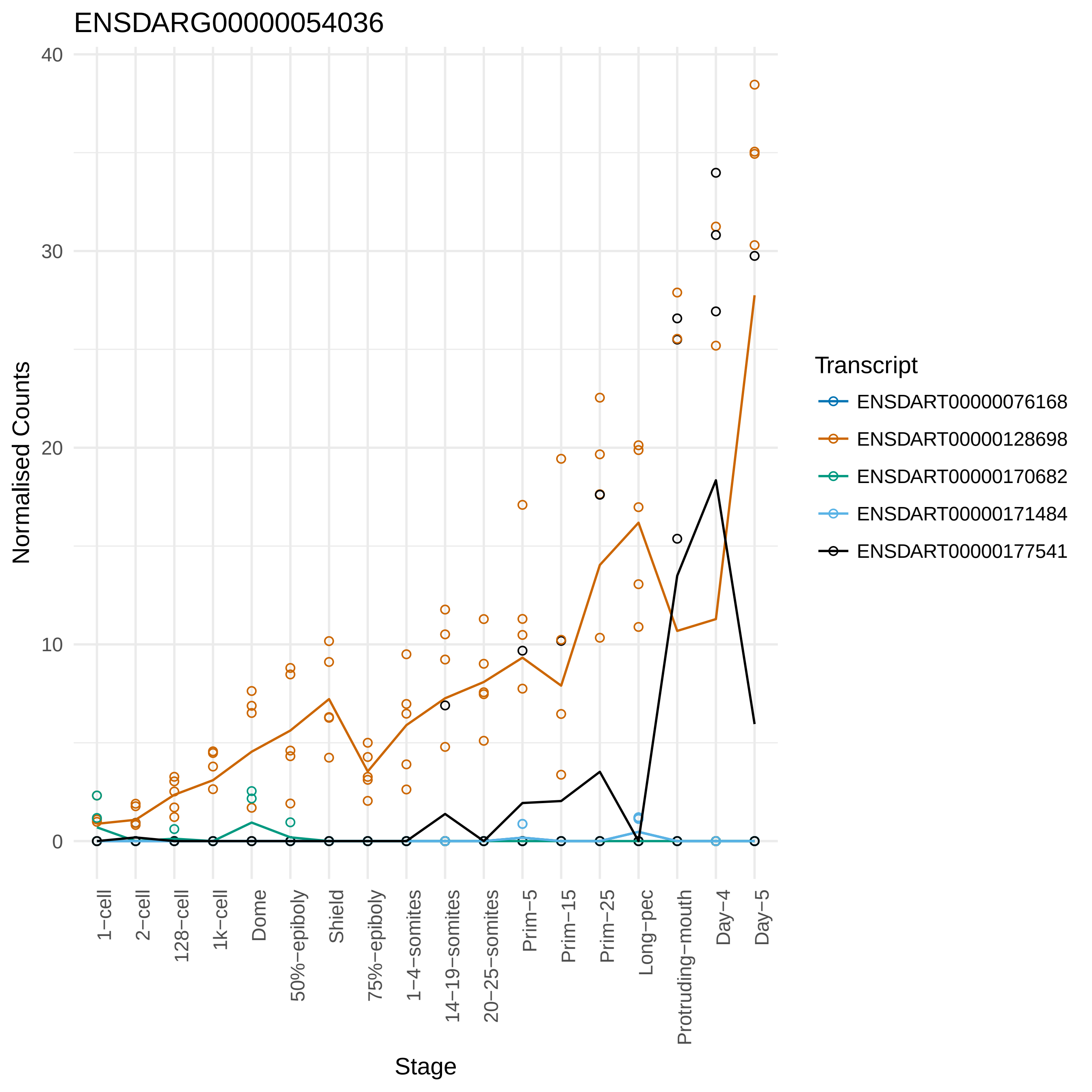

ENSDARG00000054036
Normalised Counts
40
30
20
10
0
1−cell
2−cell
128−cell
1k−cell
Dome
50%−epiboly
Shield
75%−epiboly
1−4−somites
14−19−somites
20−25−somites
Prim−5
Prim−15
Prim−25
Long−pec
Protruding−mouth
Day−4
Day−5
Stage
Transcript
ENSDART00000076168
ENSDART00000128698
ENSDART00000170682
ENSDART00000171484
ENSDART00000177541

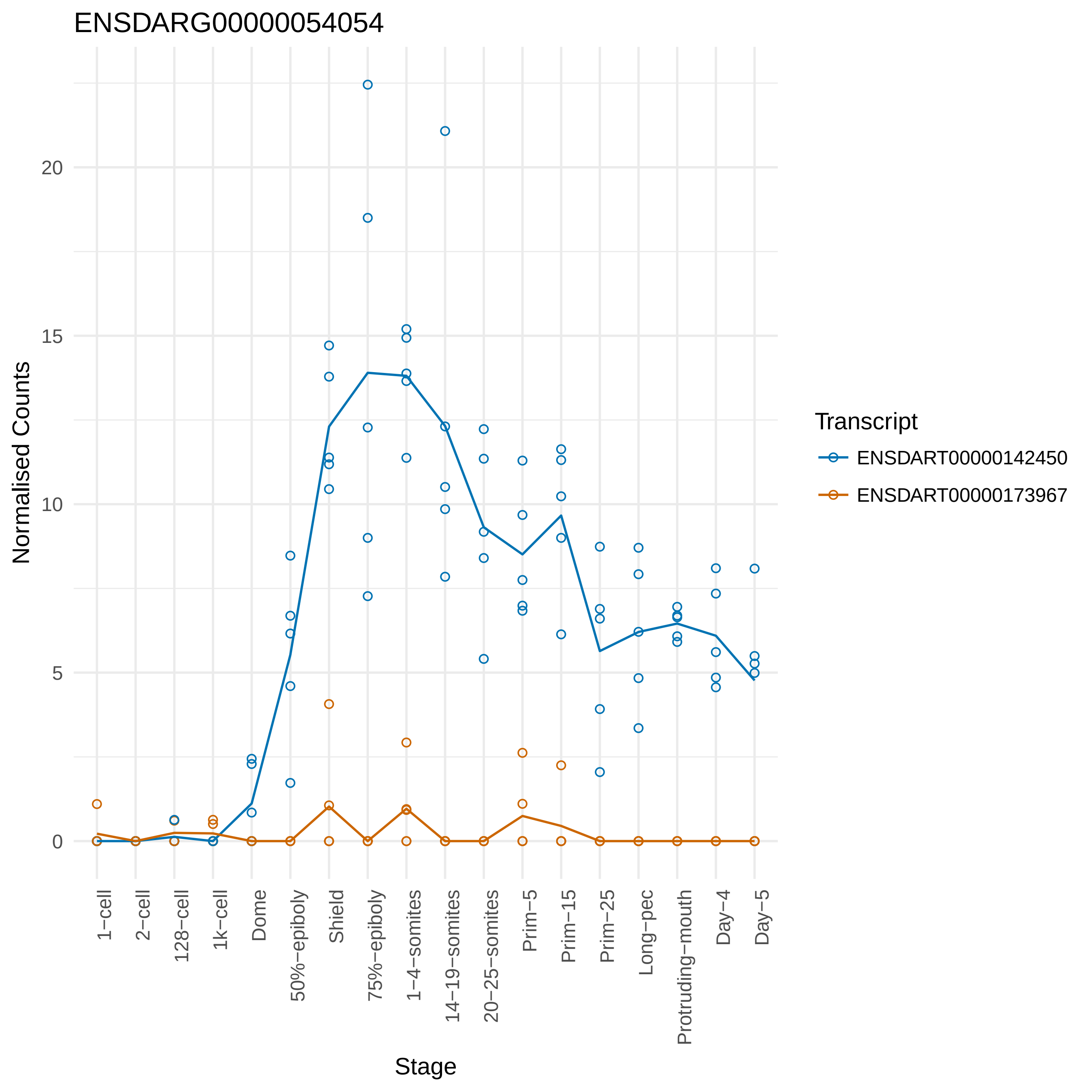

ENSDARG00000054054
Normalised Counts
0
5
10
15
20
Stage
1−cell
2−cell
128−cell
1k−cell
Dome
50%−epiboly
Shield
75%−epiboly
1−4−somites
14−19−somites
20−25−somites
Prim−5
Prim−15
Prim−25
Long−pec
Protruding−mouth
Day−4
Day−5
Transcript
ENSDART00000142450
ENSDART00000173967

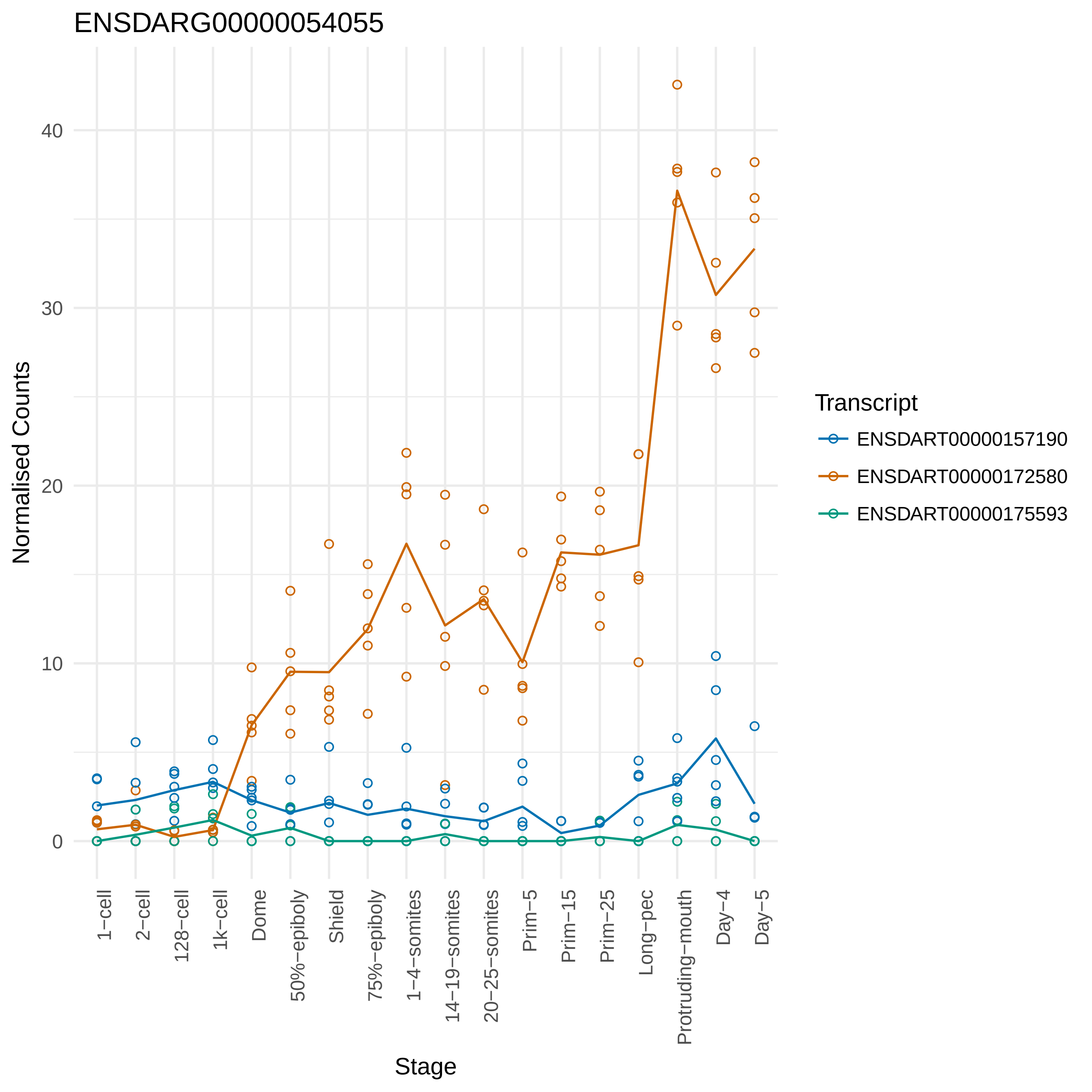
ENSDARG00000054055
Normalised Counts
0
10
20
30
40
Stage
1−cell
2−cell
128−cell
1k−cell
Dome
50%−epiboly
Shield
75%−epiboly
1−4−somites
14−19−somites
20−25−somites
Prim−5
Prim−15
Prim−25
Long−pec
Protruding−mouth
Day−4
Day−5
Transcript
ENSDART00000157190
ENSDART00000172580
ENSDART00000175593

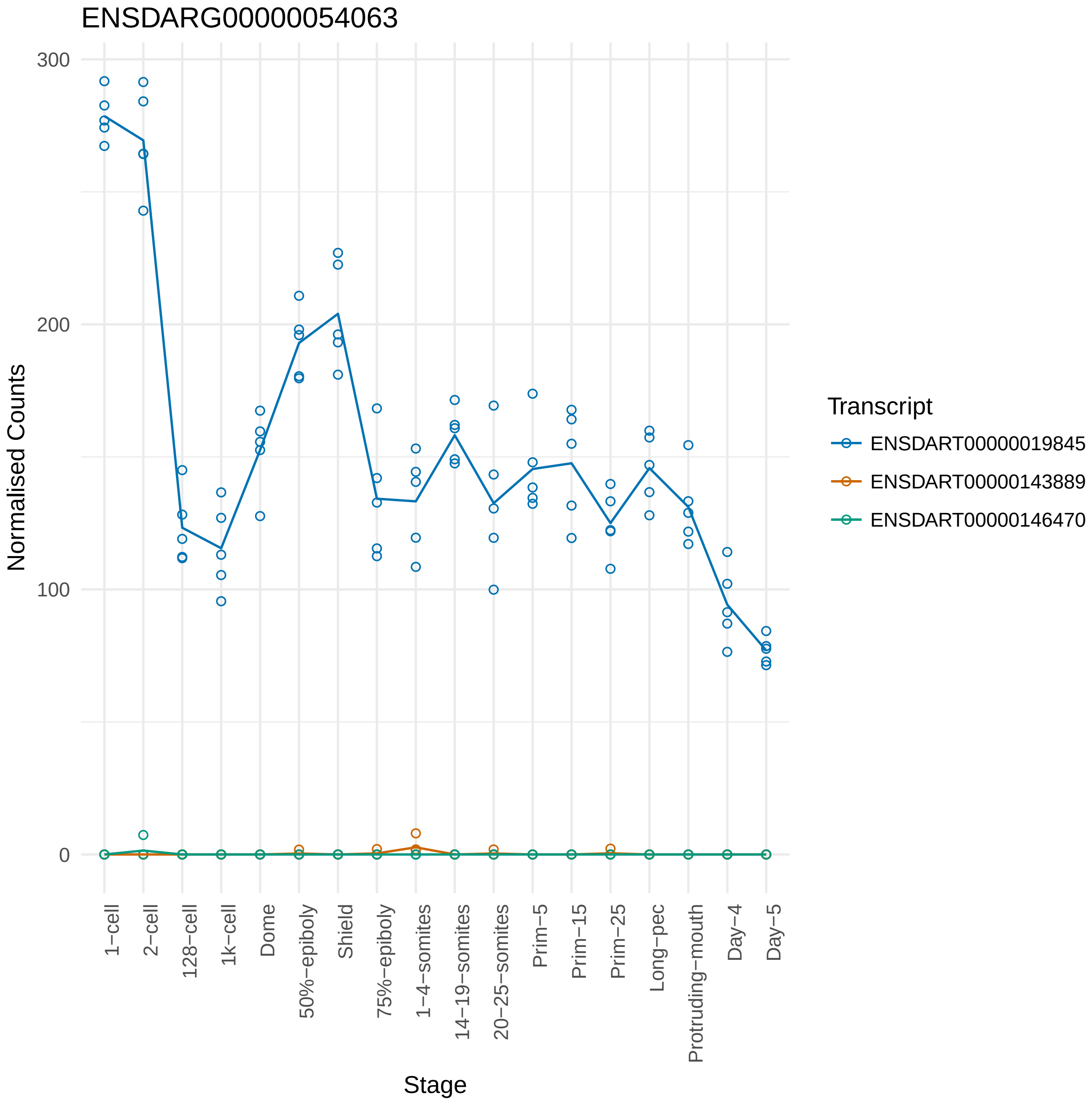
ENSDARG00000054063
Normalised Counts
300
200
100
0
1−cell
2−cell
128−cell
1k−cell
Dome
50%−epiboly
Shield
75%−epiboly
1−4−somites
14−19−somites
20−25−somites
Prim−5
Prim−15
Prim−25
Long−pec
Protruding−mouth
Day−4
Day−5
Stage
Transcript
ENSDART00000019845
ENSDART00000143889
ENSDART00000146470

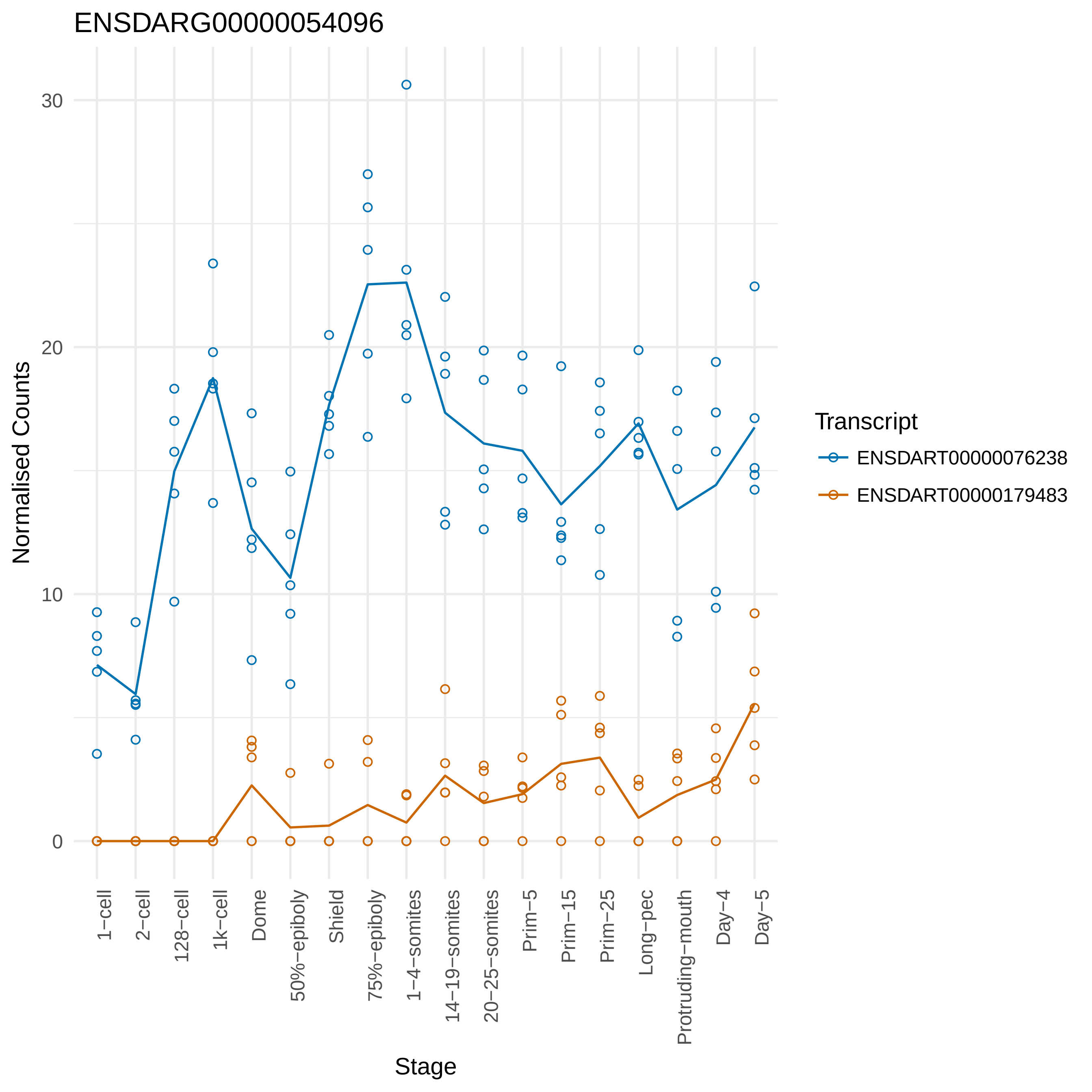
ENSDARG00000054096
Normalised Counts
30
20
10
0
1−cell
2−cell
128−cell
1k−cell
Dome
50%−epiboly
Shield
75%−epiboly
1−4−somites
14−19−somites
20−25−somites
Prim−5
Prim−15
Prim−25
Long−pec
Protruding−mouth
Day−4
Day−5
Stage
Transcript
ENSDART00000076238
ENSDART00000179483

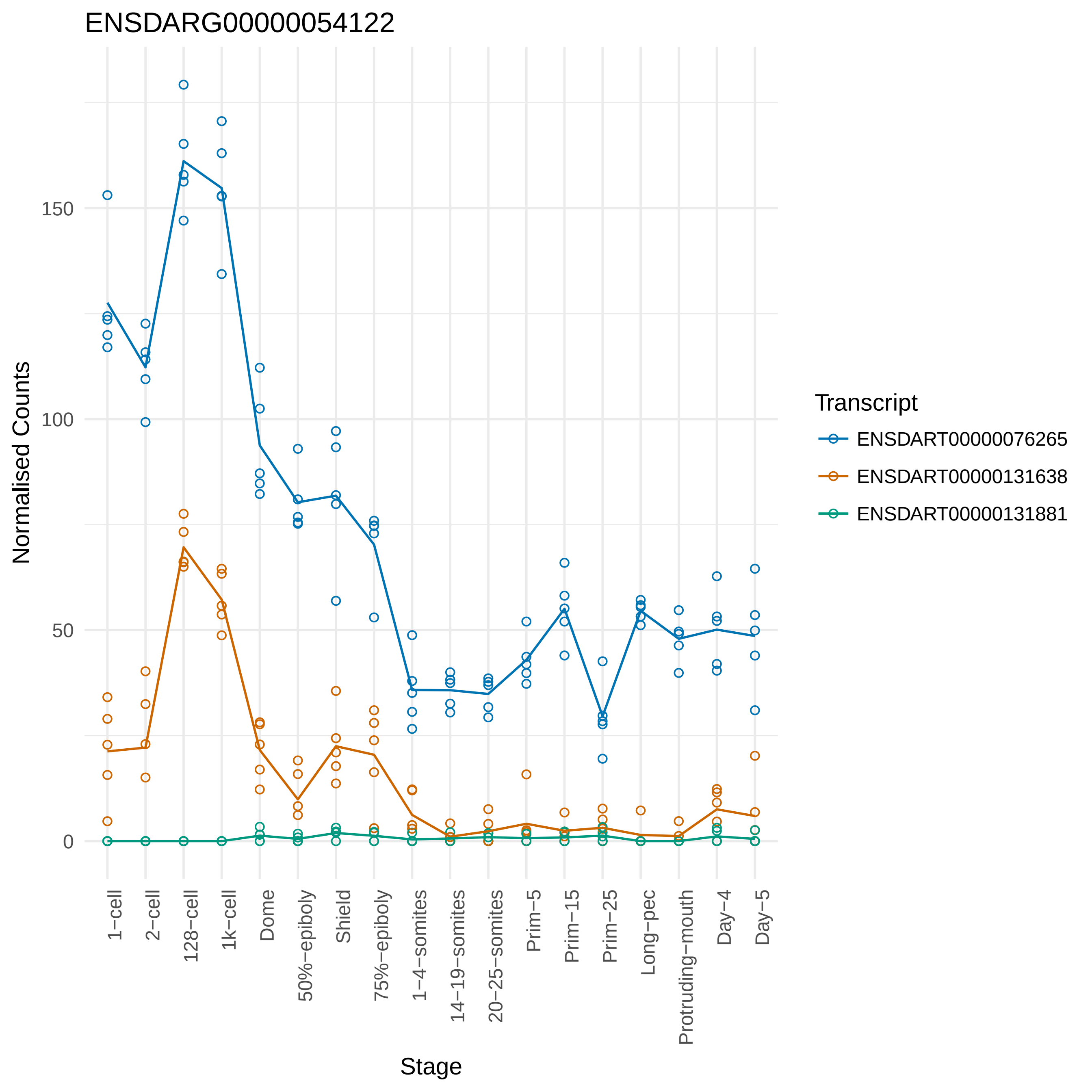
ENSDARG00000054122
Normalised Counts
150
100
50
0
1−cell
2−cell
128−cell
1k−cell
Dome
50%−epiboly
Shield
75%−epiboly
1−4−somites
14−19−somites
20−25−somites
Prim−5
Prim−15
Prim−25
Long−pec
Protruding−mouth
Day−4
Day−5
Stage
Transcript
ENSDART00000076265
ENSDART00000131638
ENSDART00000131881

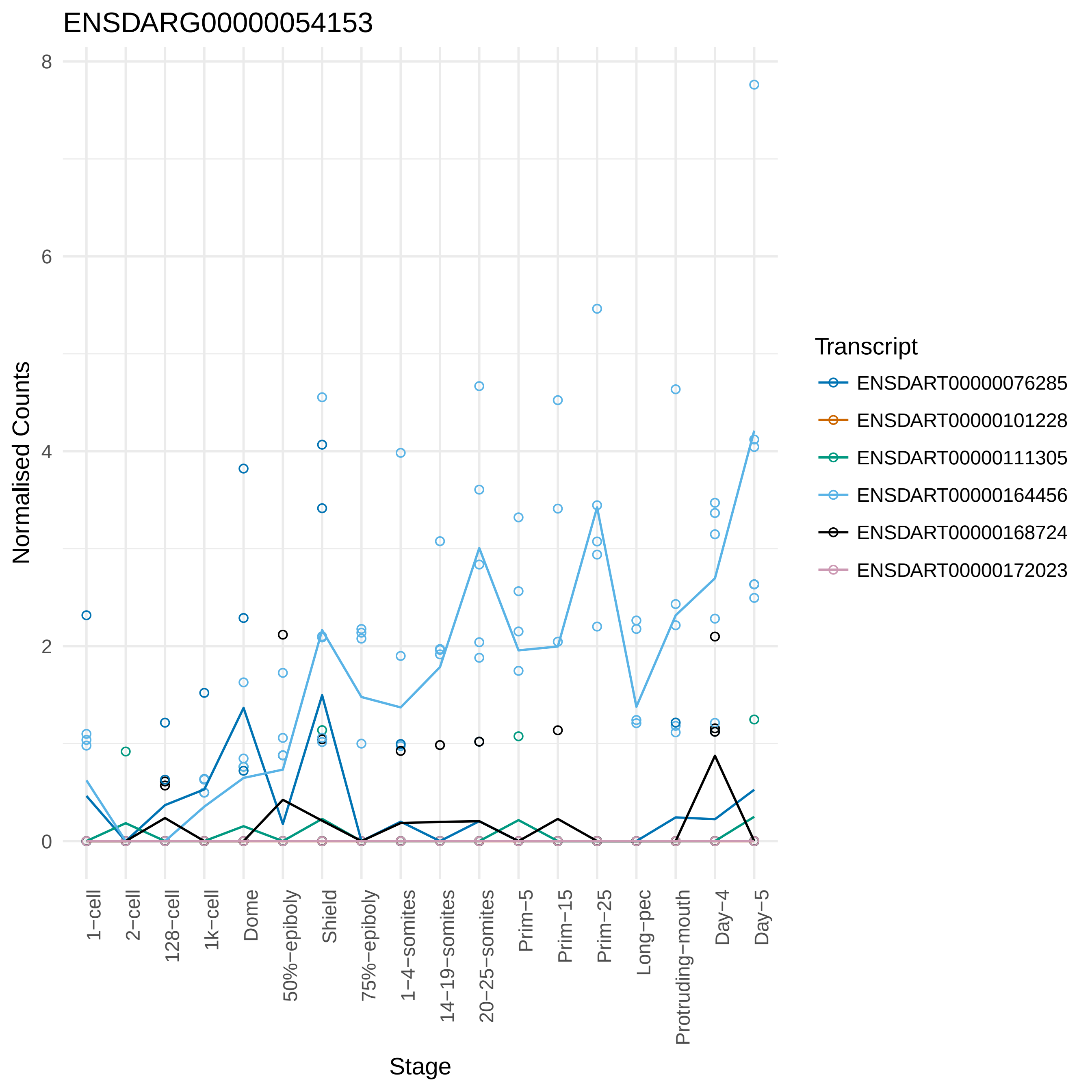
ENSDARG00000054153
Normalised Counts
0
2
4
6
8
Stage
1−cell
2−cell
128−cell
1k−cell
Dome
50%−epiboly
Shield
75%−epiboly
1−4−somites
14−19−somites
20−25−somites
Prim−5
Prim−15
Prim−25
Long−pec
Protruding−mouth
Day−4
Day−5
Transcript
ENSDART00000076285
ENSDART00000101228
ENSDART00000111305
ENSDART00000164456
ENSDART00000168724
ENSDART00000172023

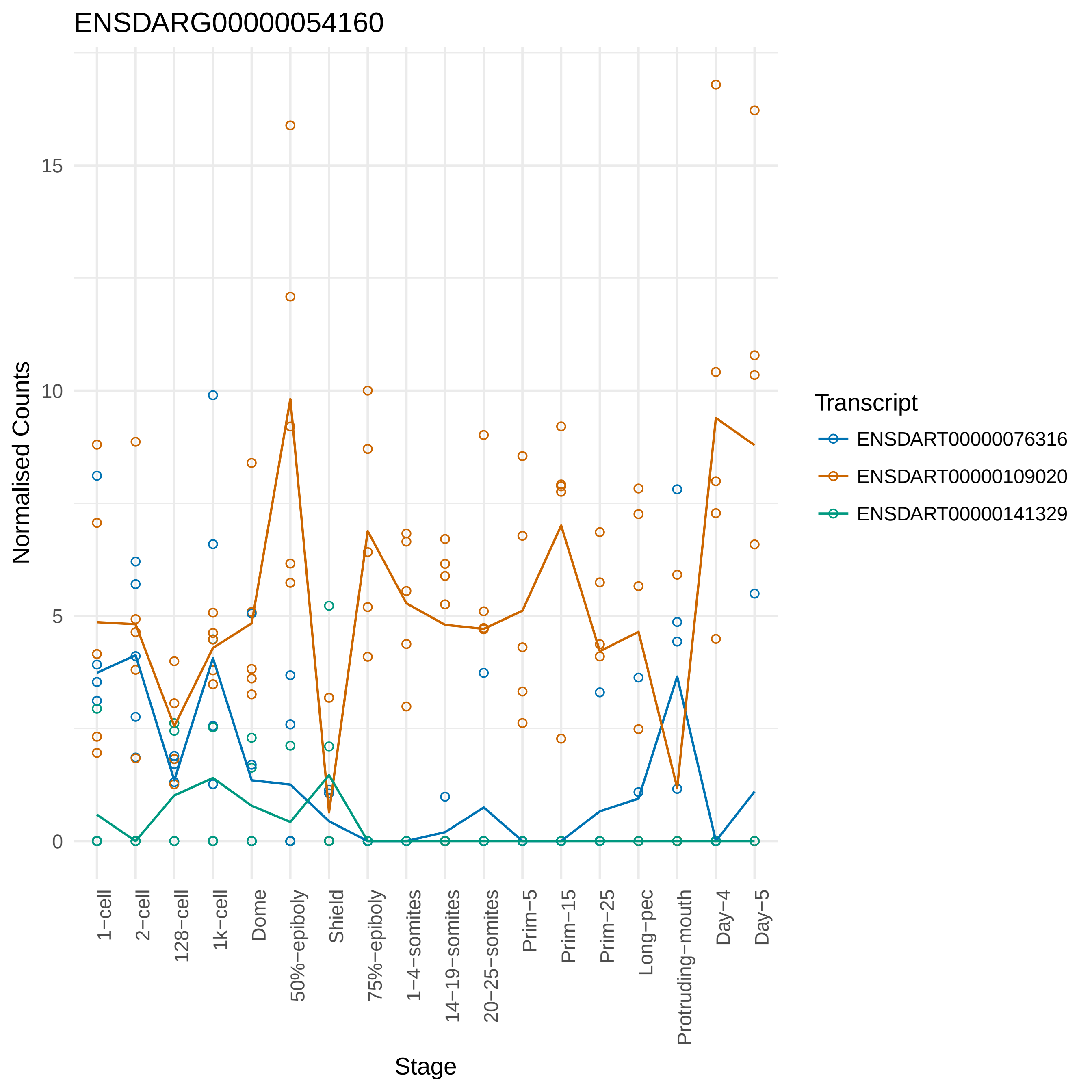

ENSDARG00000054160
Normalised Counts
0
5
10
15
Stage
1−cell
2−cell
128−cell
1k−cell
Dome
50%−epiboly
Shield
75%−epiboly
1−4−somites
14−19−somites
20−25−somites
Prim−5
Prim−15
Prim−25
Long−pec
Protruding−mouth
Day−4
Day−5
Transcript
ENSDART00000076316
ENSDART00000109020
ENSDART00000141329

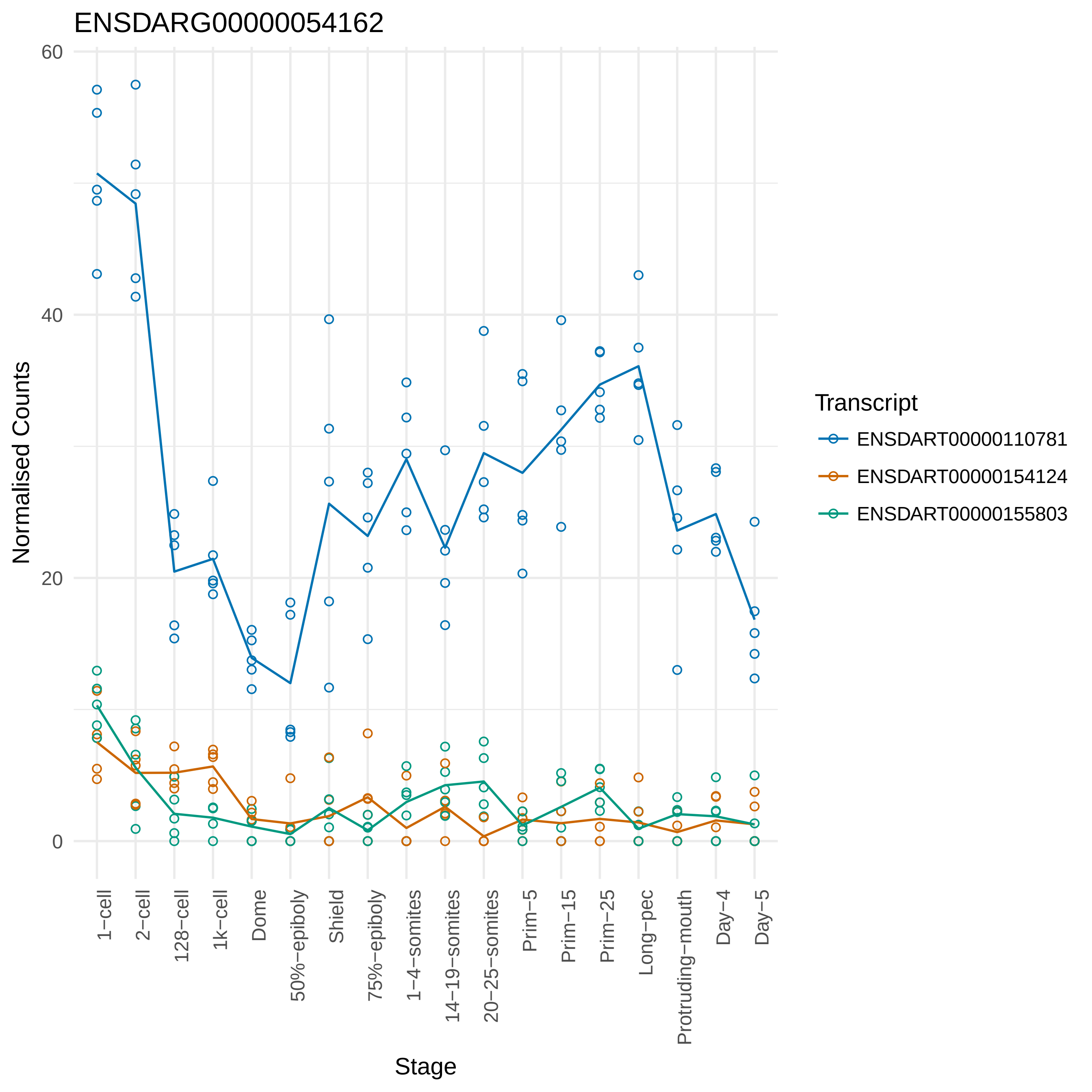
ENSDARG00000054162
Normalised Counts
60
40
20
0
1−cell
2−cell
128−cell
1k−cell
Dome
50%−epiboly
Shield
75%−epiboly
1−4−somites
14−19−somites
20−25−somites
Prim−5
Prim−15
Prim−25
Long−pec
Protruding−mouth
Day−4
Day−5
Stage
Transcript
ENSDART00000110781
ENSDART00000154124
ENSDART00000155803

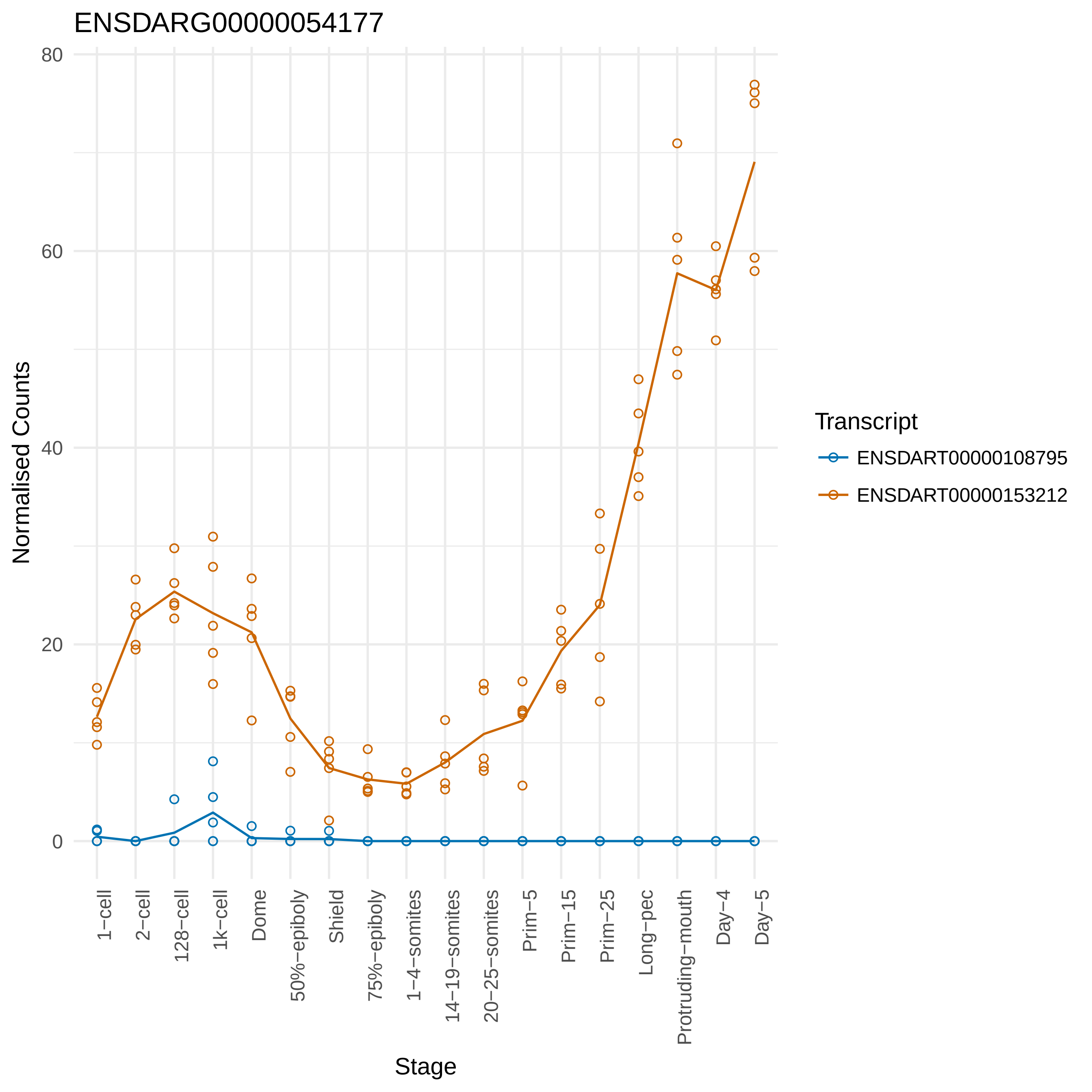

ENSDARG00000054177
Normalised Counts
80
60
40
20
0
1−cell
2−cell
128−cell
1k−cell
Dome
50%−epiboly
Shield
75%−epiboly
1−4−somites
14−19−somites
20−25−somites
Prim−5
Prim−15
Prim−25
Long−pec
Protruding−mouth
Day−4
Day−5
Stage
Transcript
ENSDART00000108795
ENSDART00000153212

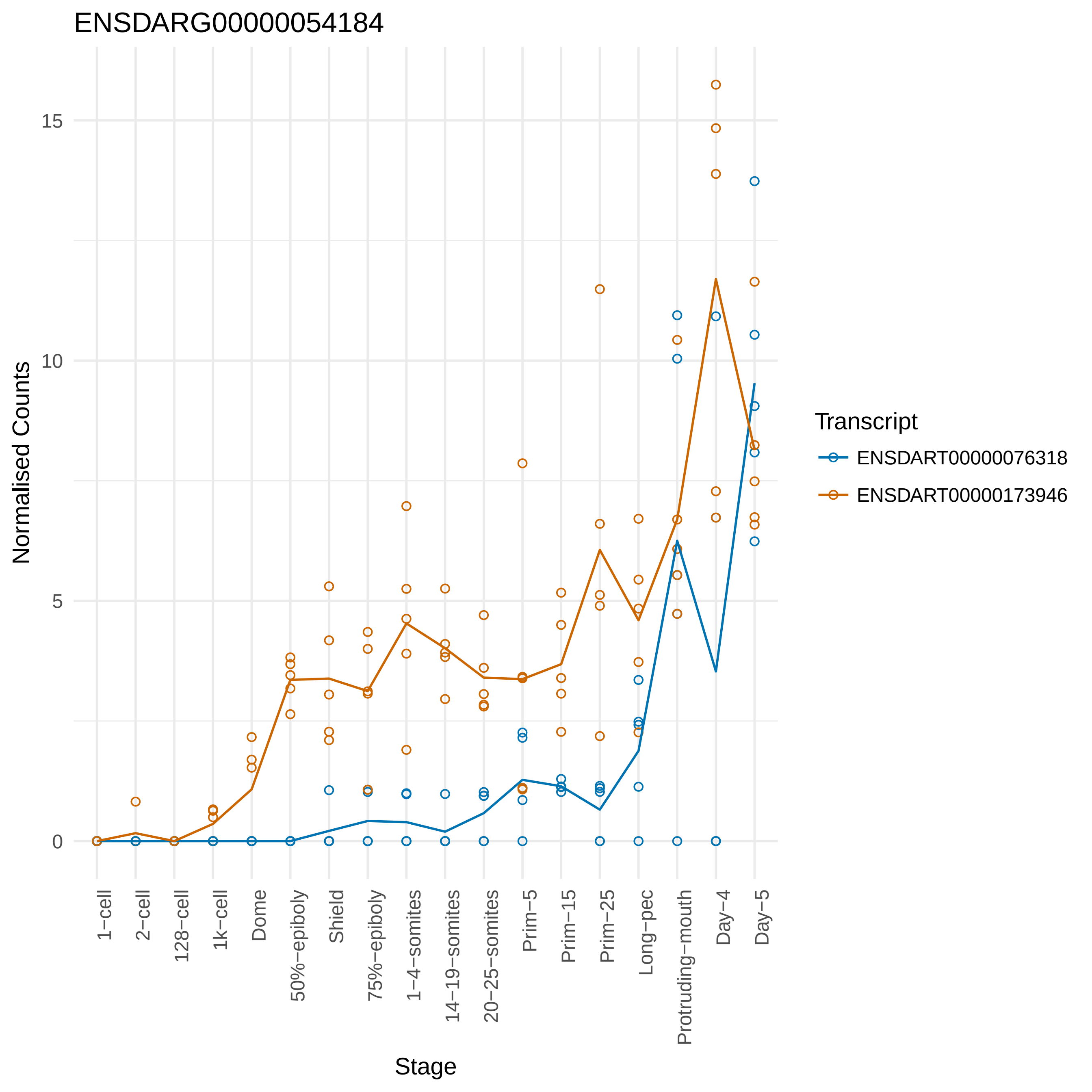

ENSDARG00000054184
Normalised Counts
15
10
5
0
1−cell
2−cell
128−cell
1k−cell
Dome
50%−epiboly
Shield
75%−epiboly
1−4−somites
14−19−somites
20−25−somites
Prim−5
Prim−15
Prim−25
Long−pec
Protruding−mouth
Day−4
Day−5
Stage
Transcript
ENSDART00000076318
ENSDART00000173946

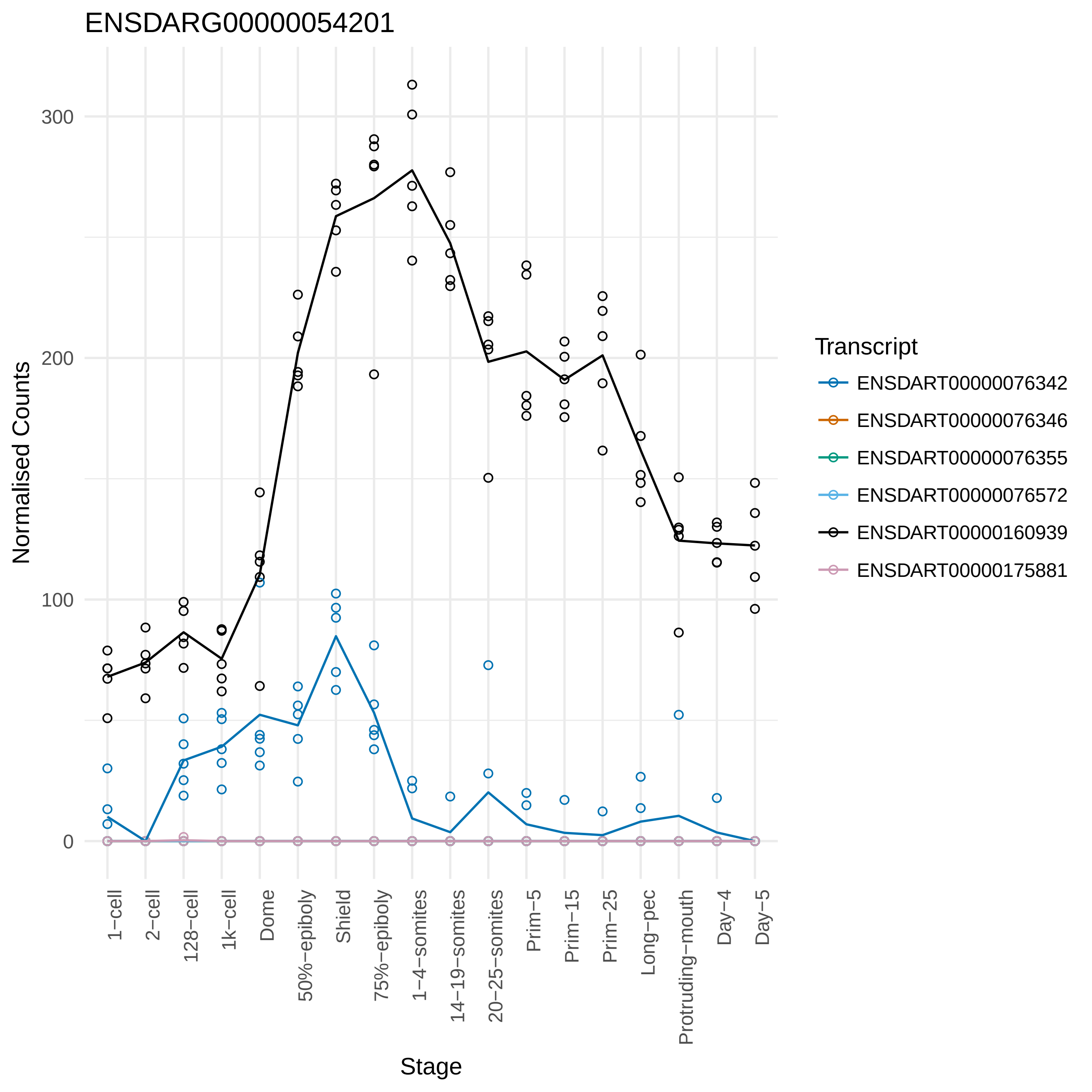

ENSDARG00000054201
Normalised Counts
300
200
100
0
Stage
1−cell
2−cell
128−cell
1k−cell
Dome
50%−epiboly
Shield
75%−epiboly
1−4−somites
14−19−somites
20−25−somites
Prim−5
Prim−15
Prim−25
Long−pec
Protruding−mouth
Day−4
Day−5
Transcript
ENSDART00000076342
ENSDART00000076346
ENSDART00000076355
ENSDART00000076572
ENSDART00000160939
ENSDART00000175881

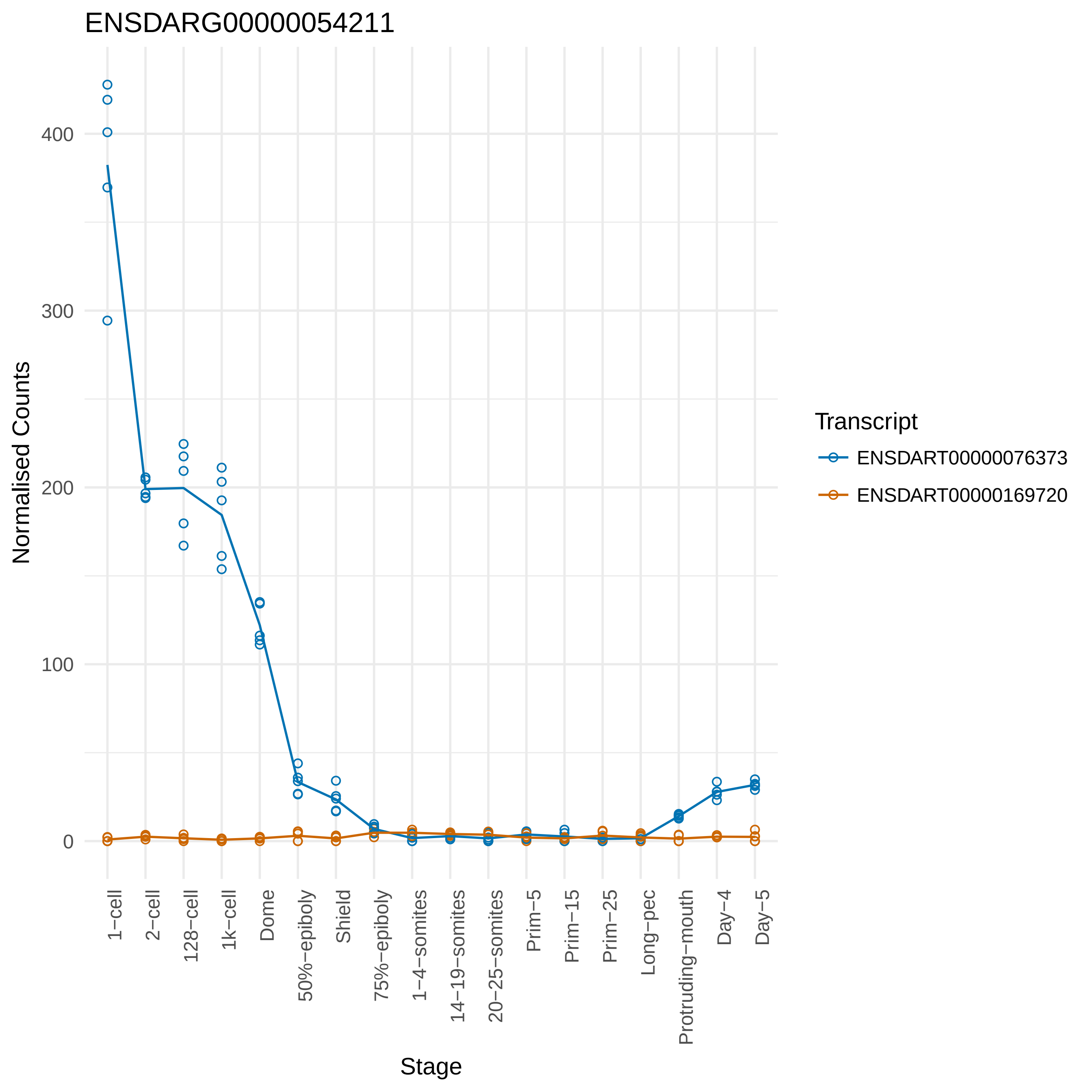
ENSDARG00000054211
Normalised Counts
400
300
200
100
0
1−cell
2−cell
128−cell
1k−cell
Dome
50%−epiboly
Shield
75%−epiboly
1−4−somites
14−19−somites
20−25−somites
Prim−5
Prim−15
Prim−25
Long−pec
Protruding−mouth
Day−4
Day−5
Stage
Transcript
ENSDART00000076373
ENSDART00000169720

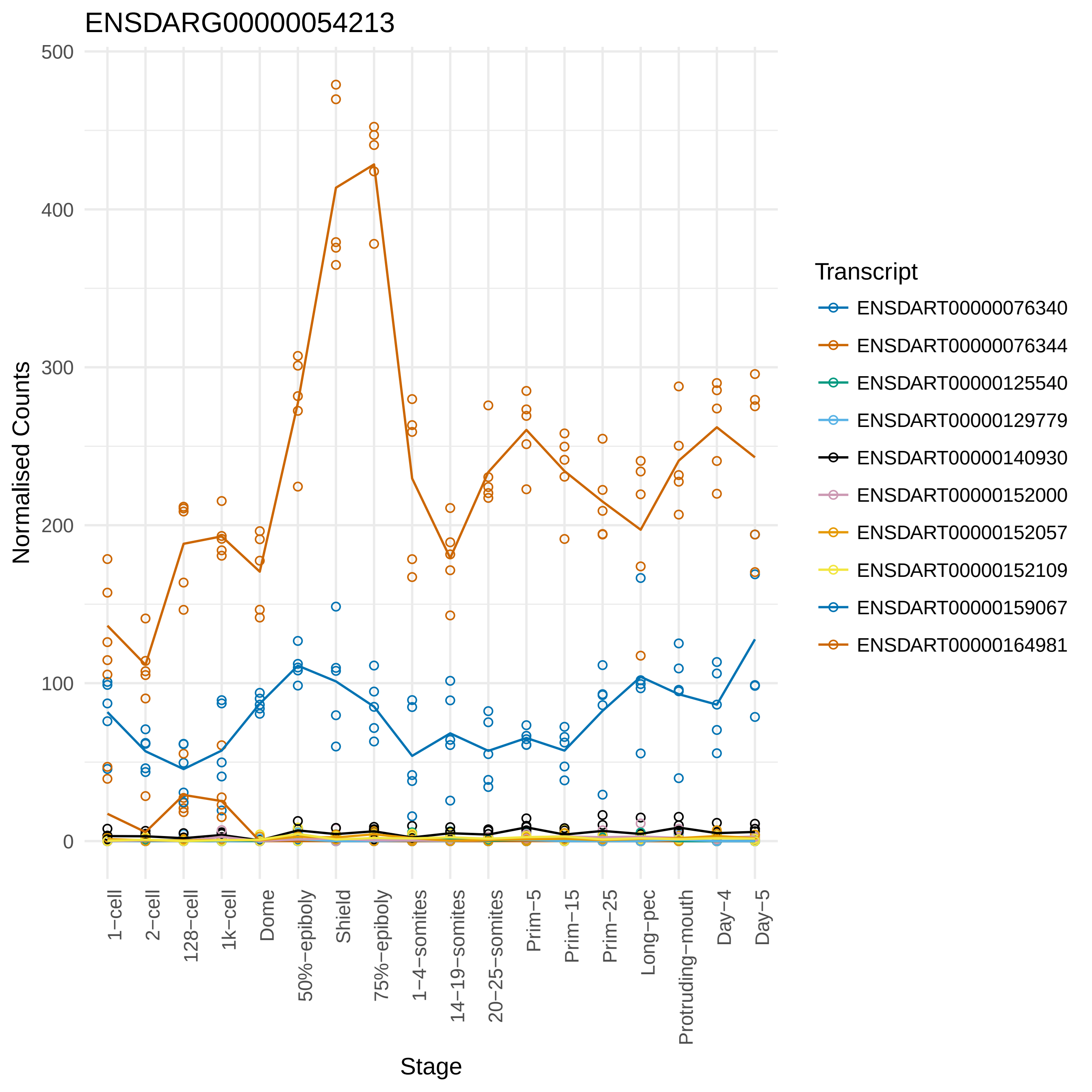
ENSDARG00000054213
Normalised Counts
0
100
200
300
400
500
1−cell
2−cell
128−cell
1k−cell
Dome
50%−epiboly
Shield
75%−epiboly
1−4−somites
14−19−somites
20−25−somites
Prim−5
Prim−15
Prim−25
Long−pec
Protruding−mouth
Day−4
Day−5
Stage
Transcript
ENSDART00000076340
ENSDART00000076344
ENSDART00000125540
ENSDART00000129779
ENSDART00000140930
ENSDART00000152000
ENSDART00000152057
ENSDART00000152109
ENSDART00000159067
ENSDART00000164981

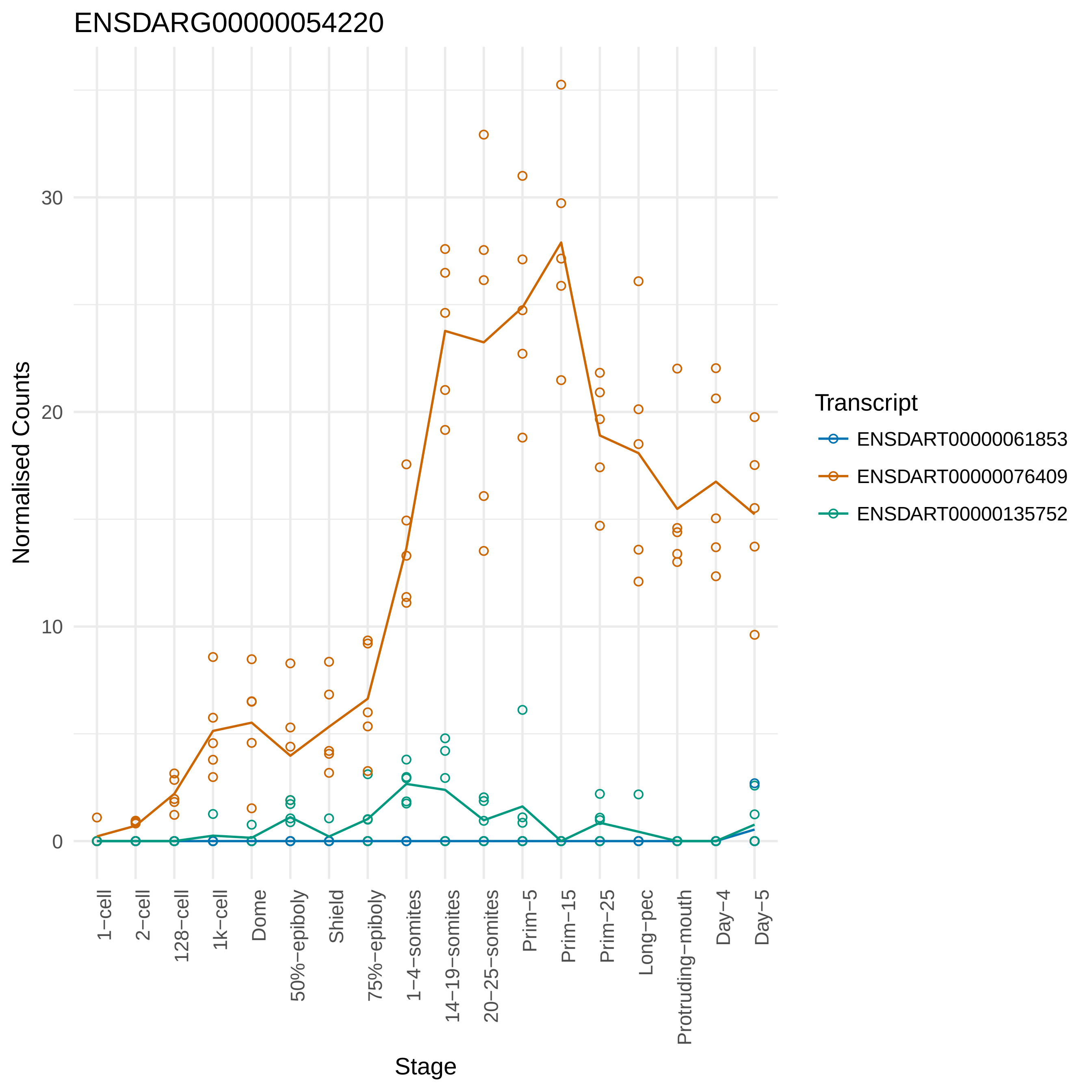

ENSDARG00000054220
Normalised Counts
30
20
10
0
1−cell
2−cell
128−cell
1k−cell
Dome
50%−epiboly
Shield
75%−epiboly
1−4−somites
14−19−somites
20−25−somites
Prim−5
Prim−15
Prim−25
Long−pec
Protruding−mouth
Day−4
Day−5
Stage
Transcript
ENSDART00000061853
ENSDART00000076409
ENSDART00000135752

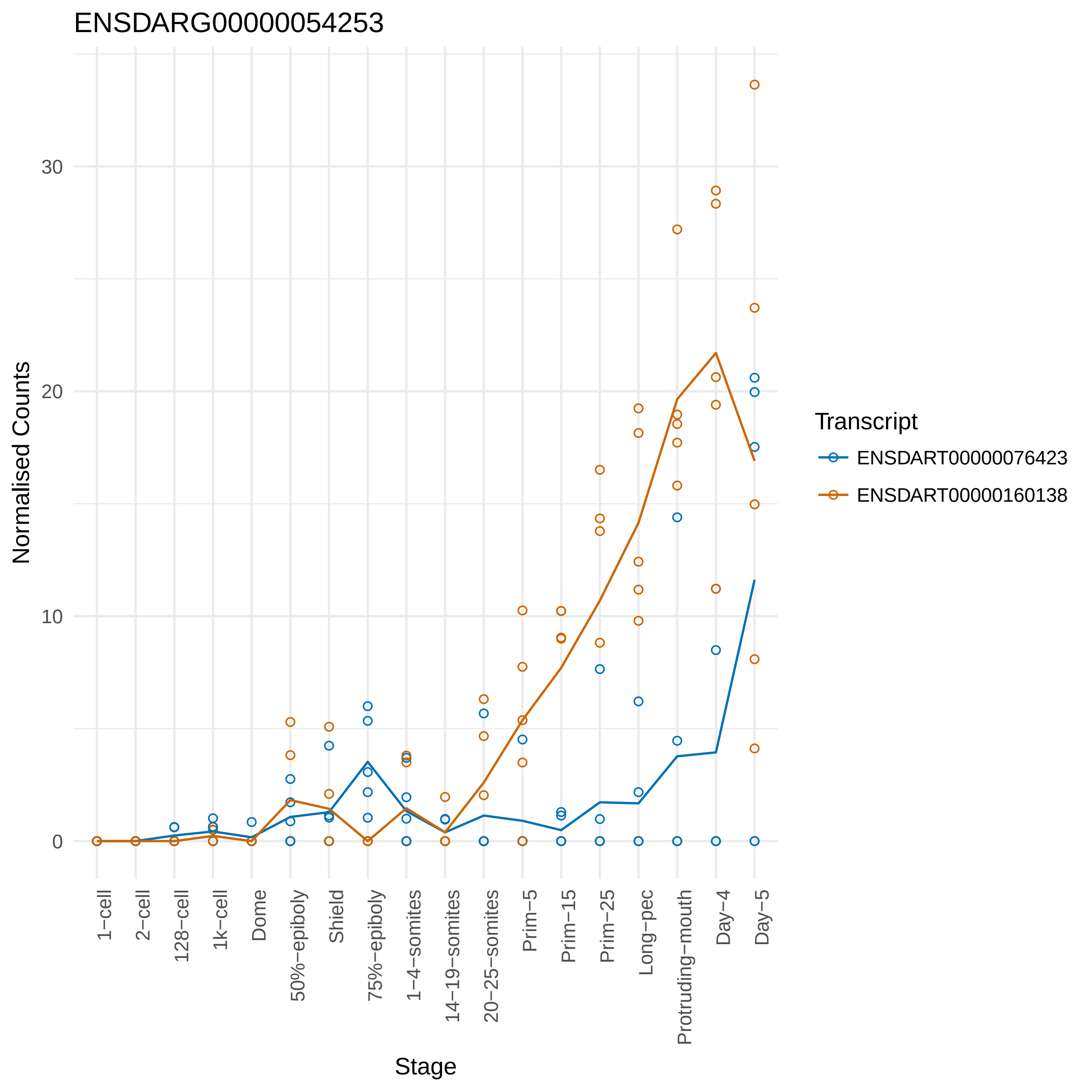
ENSDARG00000054253
Normalised Counts
0
10
20
30
1−cell
2−cell
128−cell
1k−cell
Dome
50%−epiboly
Shield
75%−epiboly
1−4−somites
14−19−somites
20−25−somites
Prim−5
Prim−15
Prim−25
Long−pec
Protruding−mouth
Day−4
Day−5
Stage
Transcript
ENSDART00000076423
ENSDART00000160138

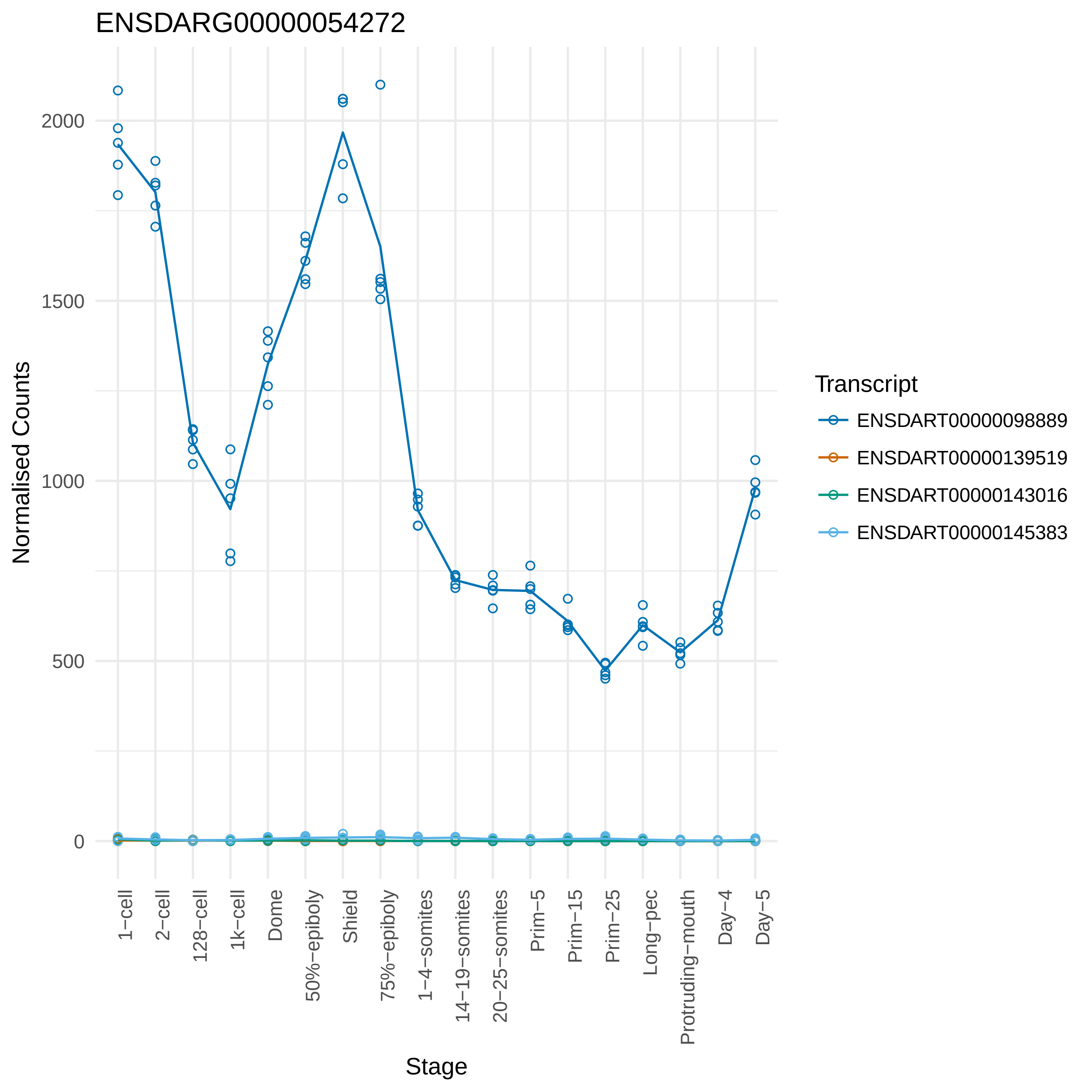

ENSDARG00000054272
Normalised Counts
2000
1500
1000
500
0
Stage
1−cell
2−cell
128−cell
1k−cell
Dome
50%−epiboly
Shield
75%−epiboly
1−4−somites
14−19−somites
20−25−somites
Prim−5
Prim−15
Prim−25
Long−pec
Protruding−mouth
Day−4
Day−5
Transcript
ENSDART00000098889
ENSDART00000139519
ENSDART00000143016
ENSDART00000145383

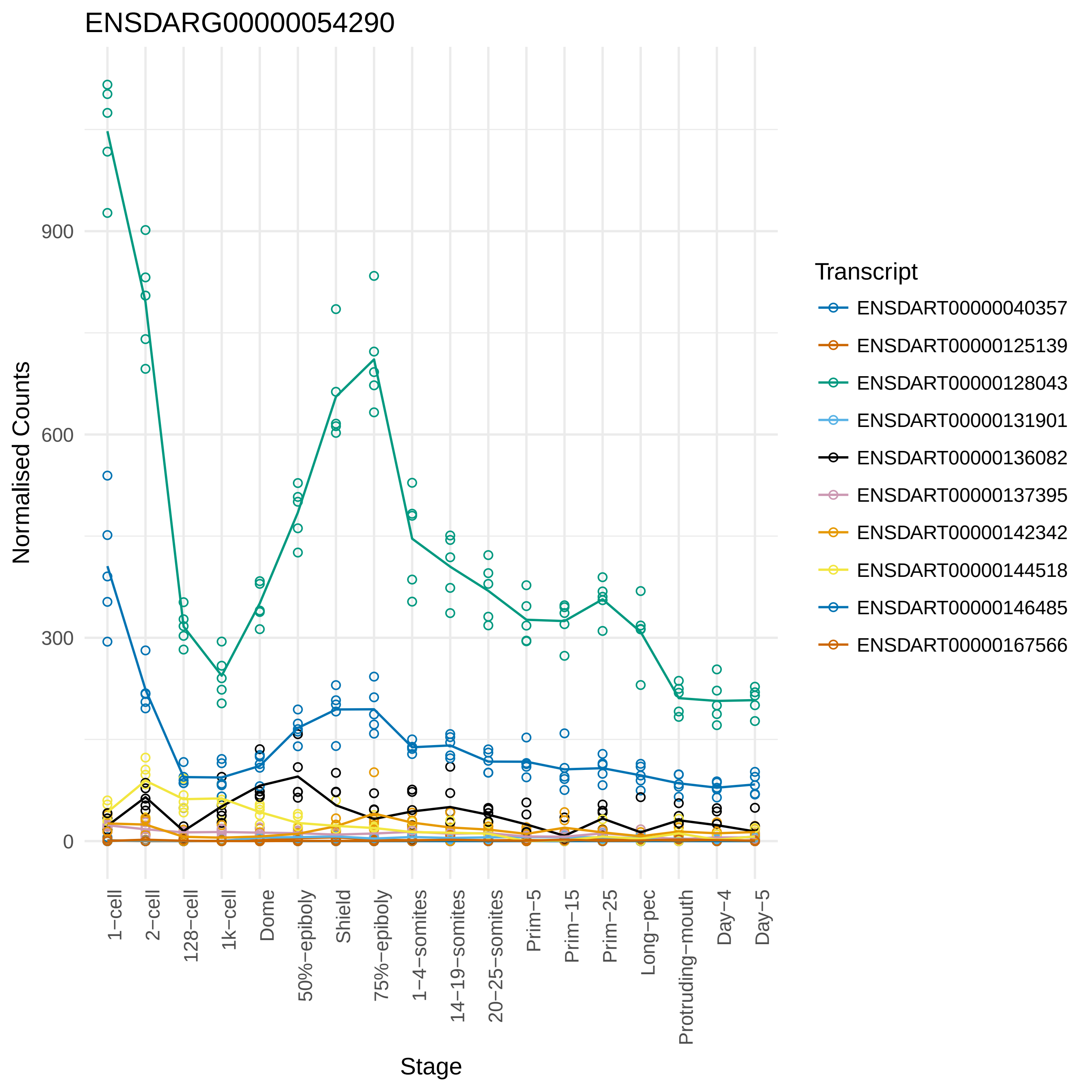

ENSDARG00000054290
Normalised Counts
900
600
300
0
1−cell
2−cell
128−cell
1k−cell
Dome
50%−epiboly
Shield
75%−epiboly
1−4−somites
14−19−somites
20−25−somites
Prim−5
Prim−15
Prim−25
Long−pec
Protruding−mouth
Day−4
Day−5
Stage
Transcript
ENSDART00000040357
ENSDART00000125139
ENSDART00000128043
ENSDART00000131901
ENSDART00000136082
ENSDART00000137395
ENSDART00000142342
ENSDART00000144518
ENSDART00000146485
ENSDART00000167566

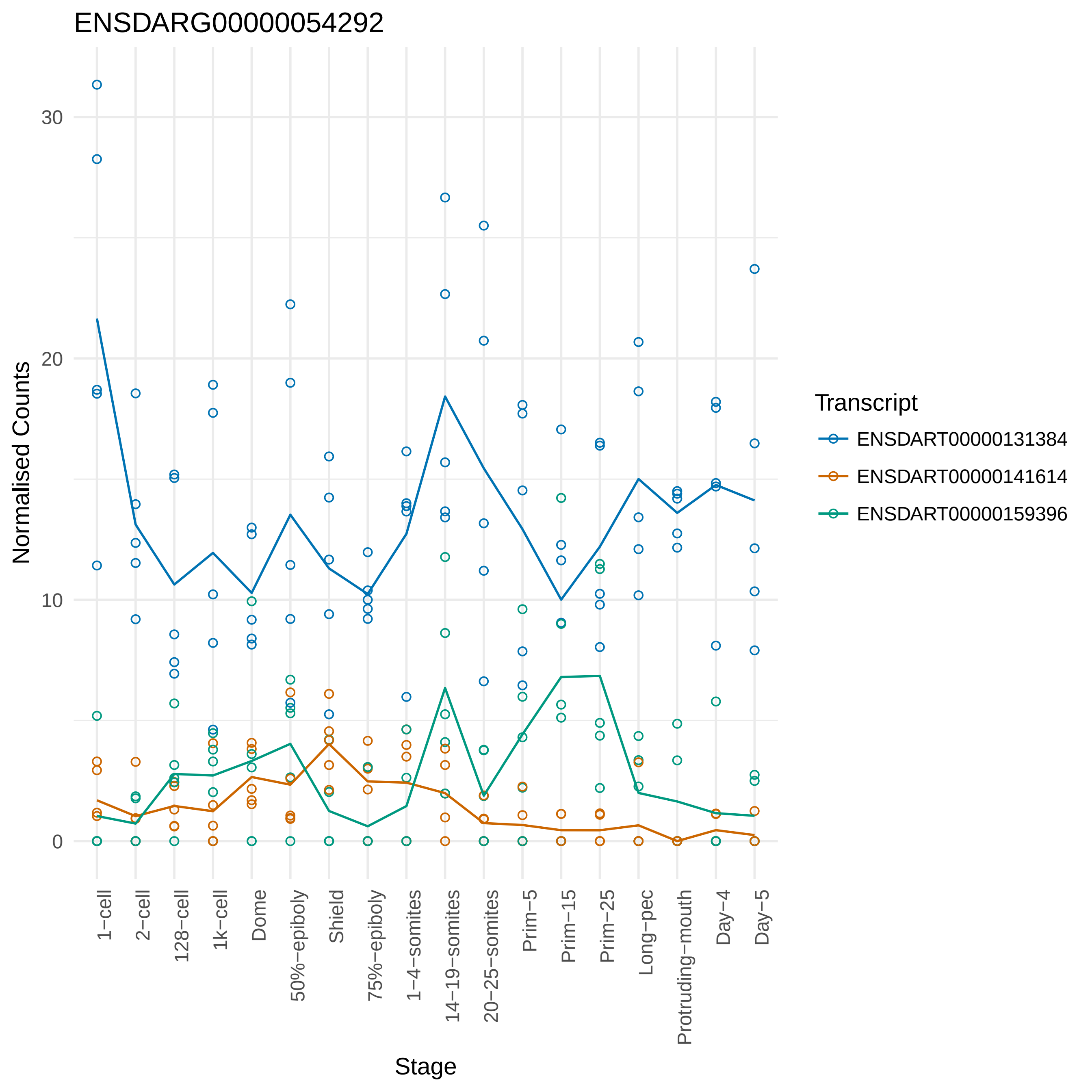

ENSDARG00000054292
Normalised Counts
30
20
10
0
Stage
1−cell
2−cell
128−cell
1k−cell
Dome
50%−epiboly
Shield
75%−epiboly
1−4−somites
14−19−somites
20−25−somites
Prim−5
Prim−15
Prim−25
Long−pec
Protruding−mouth
Day−4
Day−5
Transcript
ENSDART00000131384
ENSDART00000141614
ENSDART00000159396

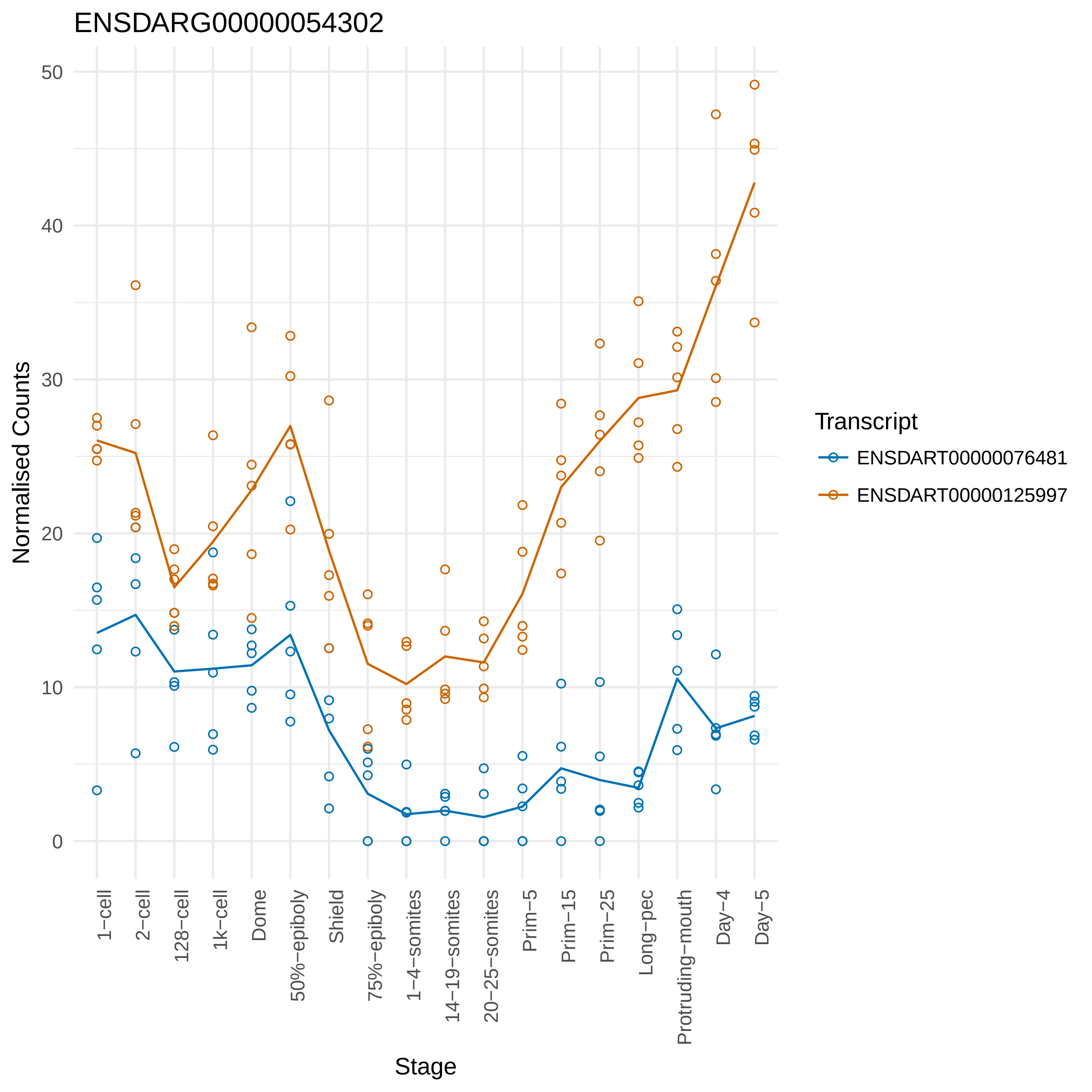

ENSDARG00000054302
Normalised Counts
0
10
20
30
40
50
1−cell
2−cell
128−cell
1k−cell
Dome
50%−epiboly
Shield
75%−epiboly
1−4−somites
14−19−somites
20−25−somites
Prim−5
Prim−15
Prim−25
Long−pec
Protruding−mouth
Day−4
Day−5
Stage
Transcript
ENSDART00000076481
ENSDART00000125997

ENSDARG00000054312

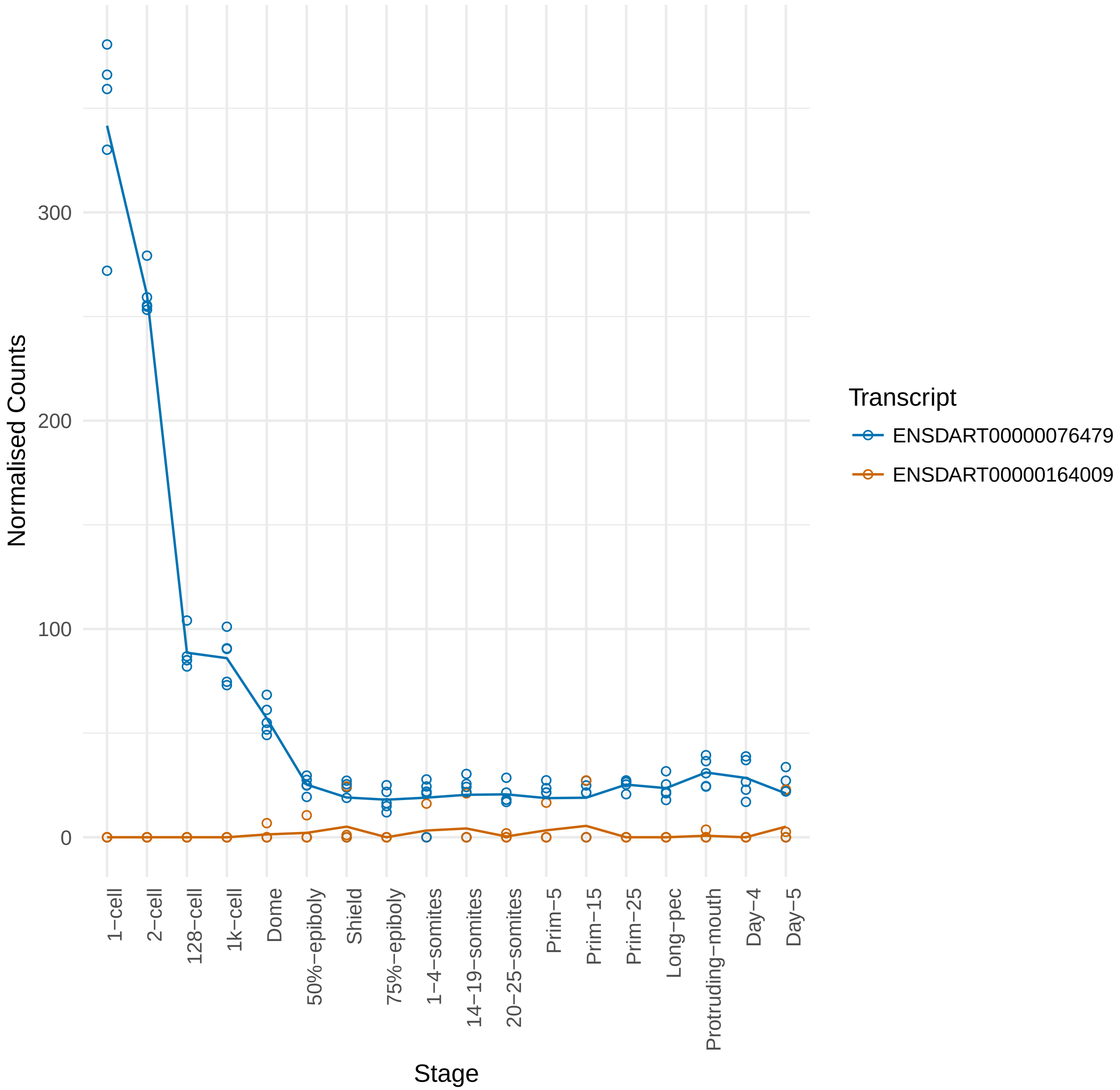

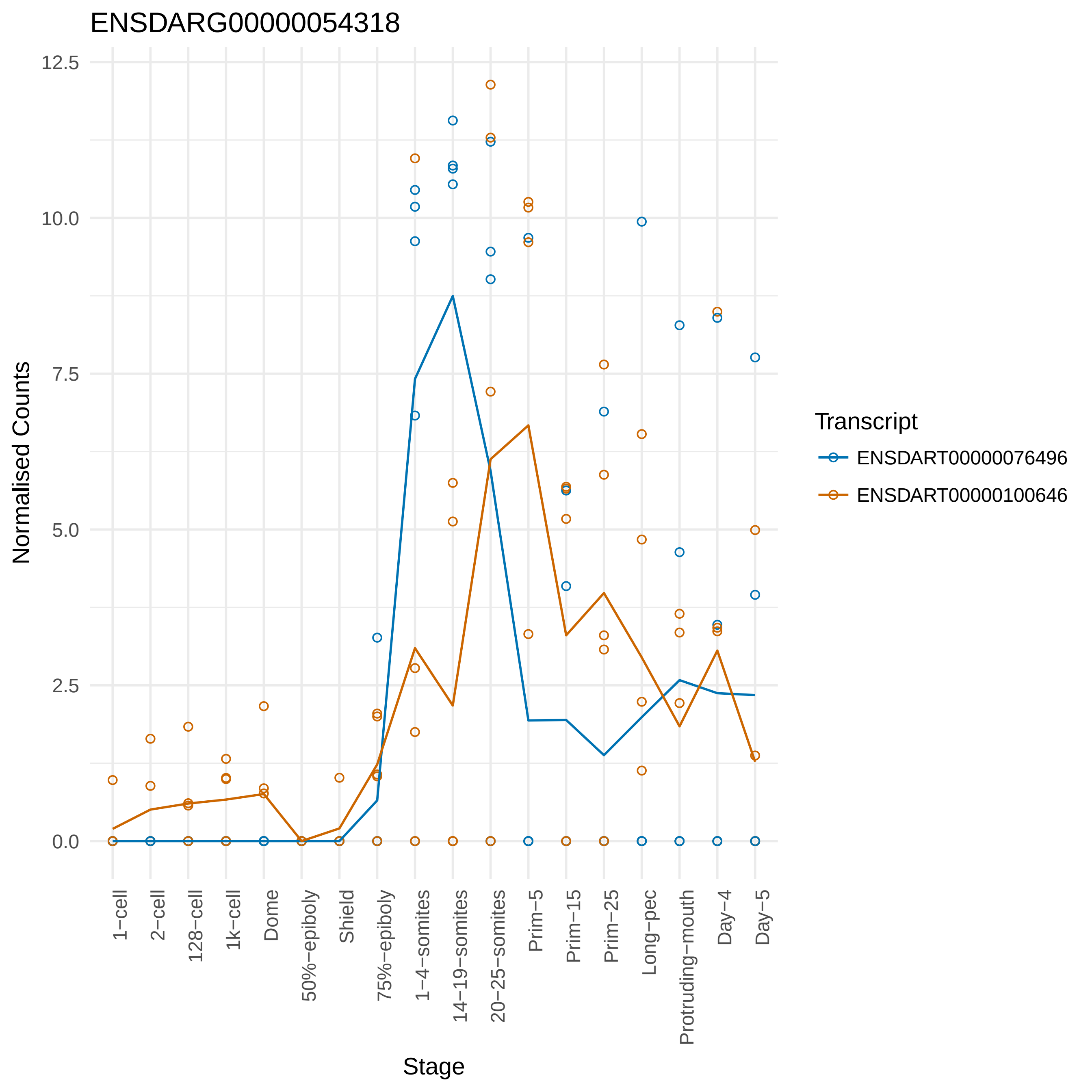

ENSDARG00000054318
Normalised Counts
12.5
10.0
7.5
5.0
2.5
0.0
1−cell
2−cell
128−cell
1k−cell
Dome
50%−epiboly
Shield
75%−epiboly
1−4−somites
14−19−somites
20−25−somites
Prim−5
Prim−15
Prim−25
Long−pec
Protruding−mouth
Day−4
Day−5
Stage
Transcript
ENSDART00000076496
ENSDART00000100646

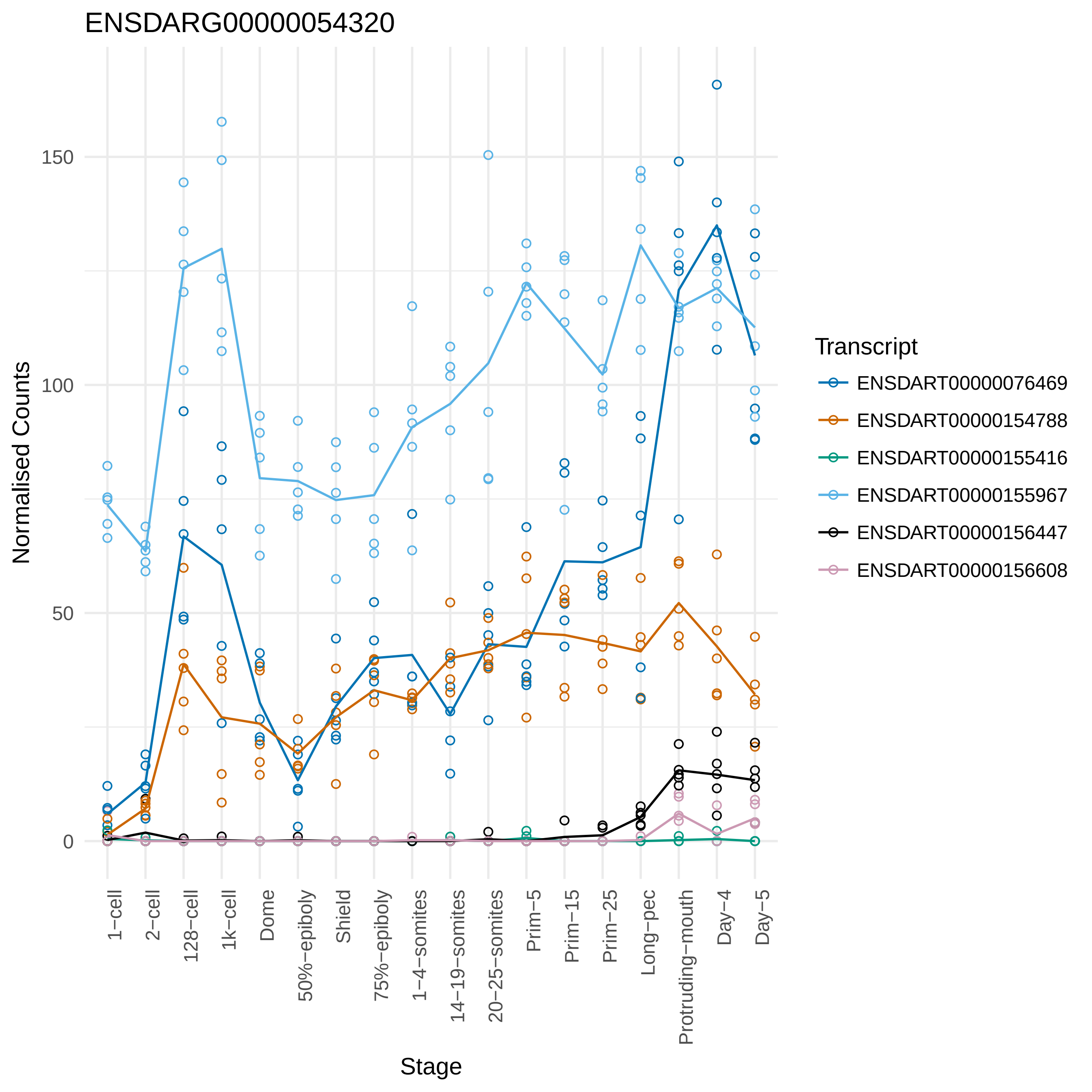
ENSDARG00000054320
Normalised Counts
0
50
100
150
Stage
1−cell
2−cell
128−cell
1k−cell
Dome
50%−epiboly
Shield
75%−epiboly
1−4−somites
14−19−somites
20−25−somites
Prim−5
Prim−15
Prim−25
Long−pec
Protruding−mouth
Day−4
Day−5
Transcript
ENSDART00000076469
ENSDART00000154788
ENSDART00000155416
ENSDART00000155967
ENSDART00000156447
ENSDART00000156608

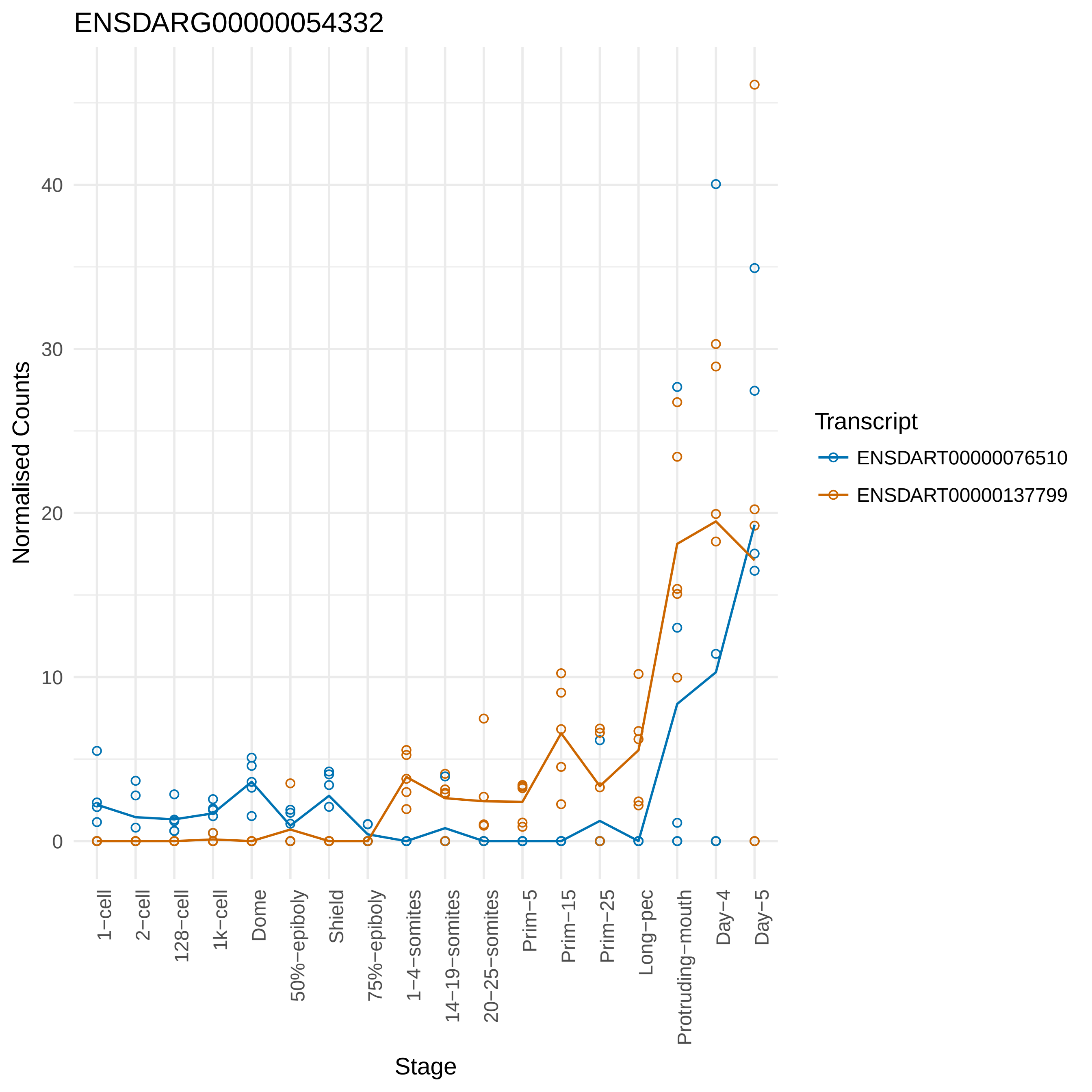

ENSDARG00000054332
Normalised Counts
0
10
20
30
40
1−cell
2−cell
128−cell
1k−cell
Dome
50%−epiboly
Shield
75%−epiboly
1−4−somites
14−19−somites
20−25−somites
Prim−5
Prim−15
Prim−25
Long−pec
Protruding−mouth
Day−4
Day−5
Stage
Transcript
ENSDART00000076510
ENSDART00000137799

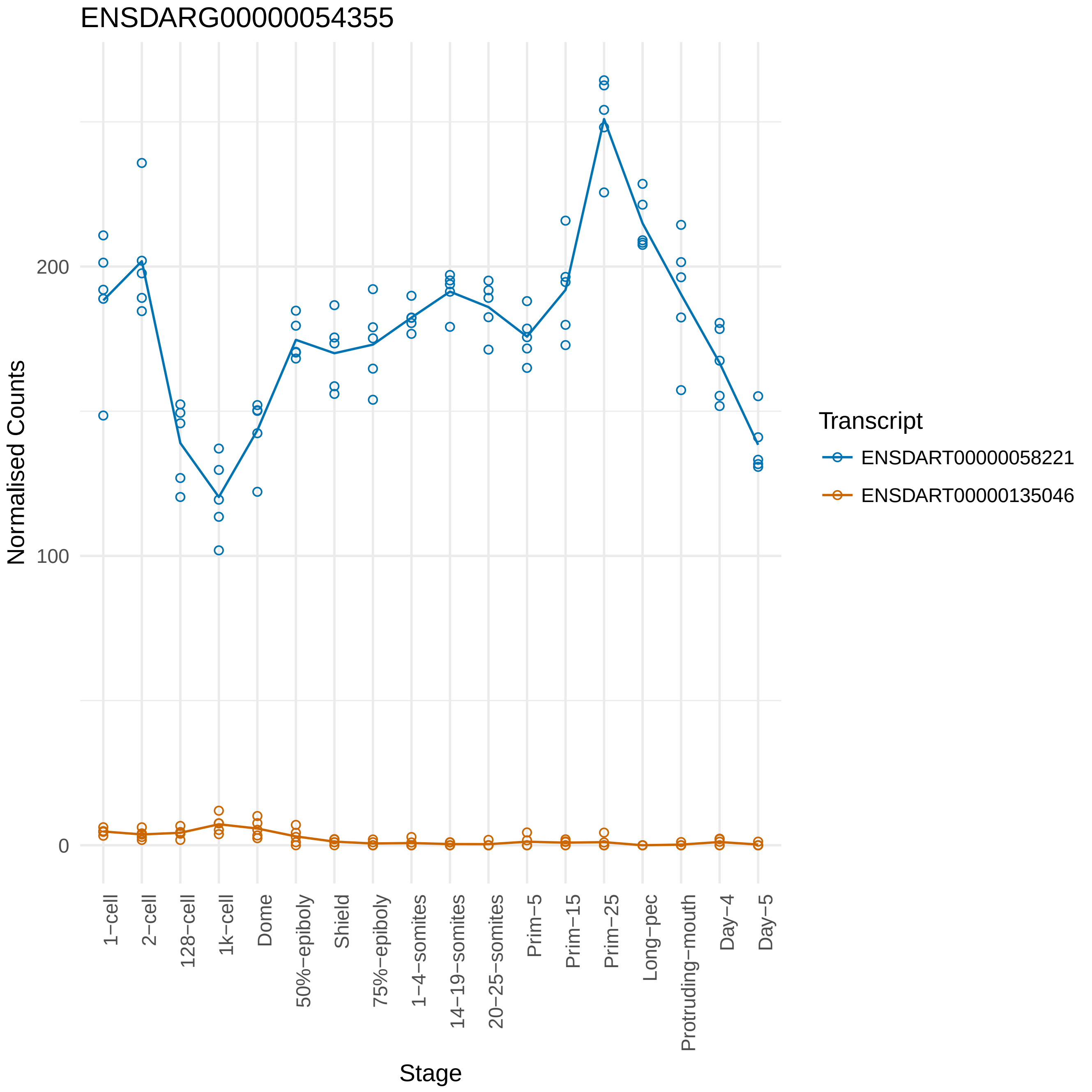

ENSDARG00000054355
Normalised Counts
200
100
0
Stage
1−cell
2−cell
128−cell
1k−cell
Dome
50%−epiboly
Shield
75%−epiboly
1−4−somites
14−19−somites
20−25−somites
Prim−5
Prim−15
Prim−25
Long−pec
Protruding−mouth
Day−4
Day−5
Transcript
ENSDART00000058221
ENSDART00000135046

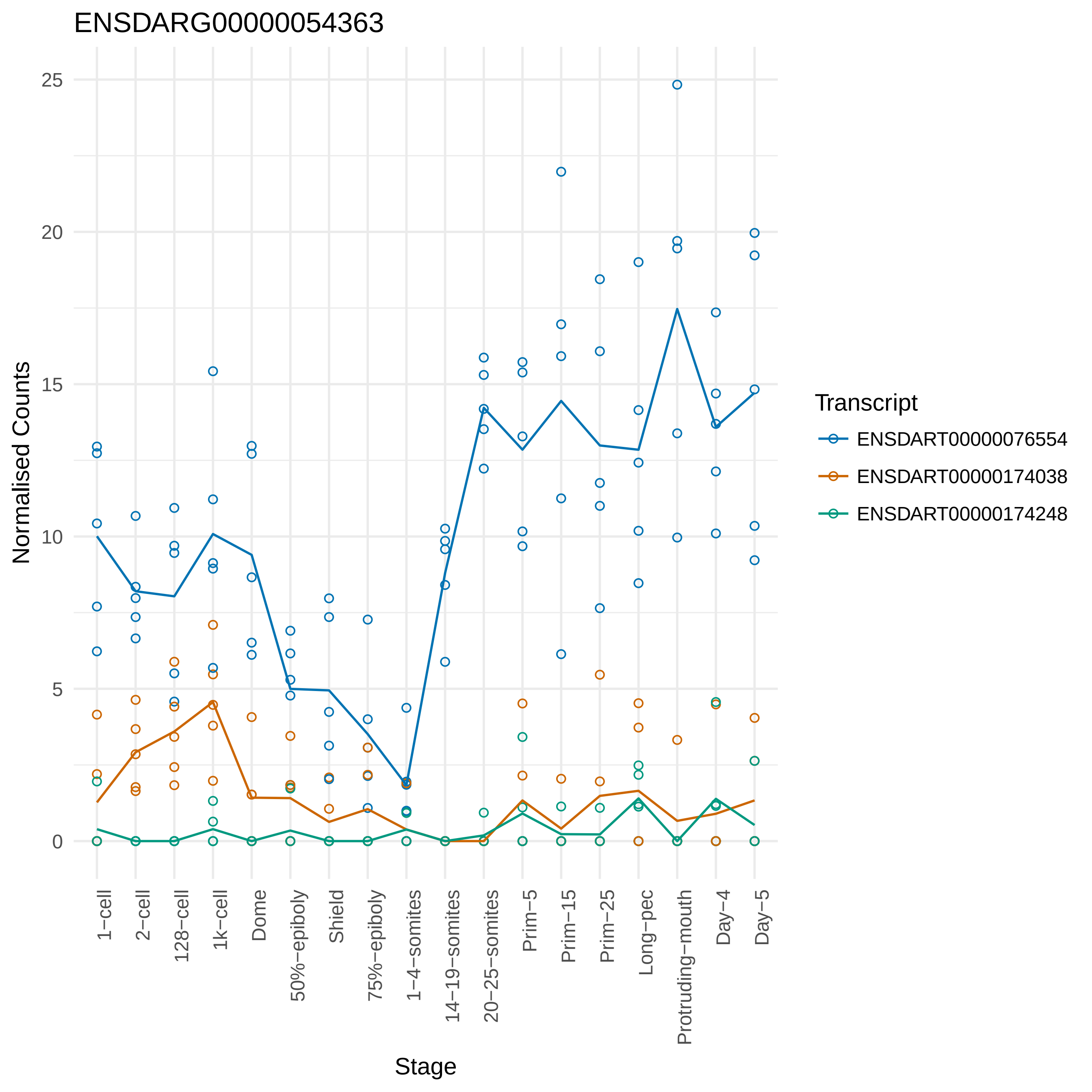

ENSDARG00000054363
Normalised Counts
0
5
10
15
20
25
Stage
1−cell
2−cell
128−cell
1k−cell
Dome
50%−epiboly
Shield
75%−epiboly
1−4−somites
14−19−somites
20−25−somites
Prim−5
Prim−15
Prim−25
Long−pec
Protruding−mouth
Day−4
Day−5
Transcript
ENSDART00000076554
ENSDART00000174038
ENSDART00000174248

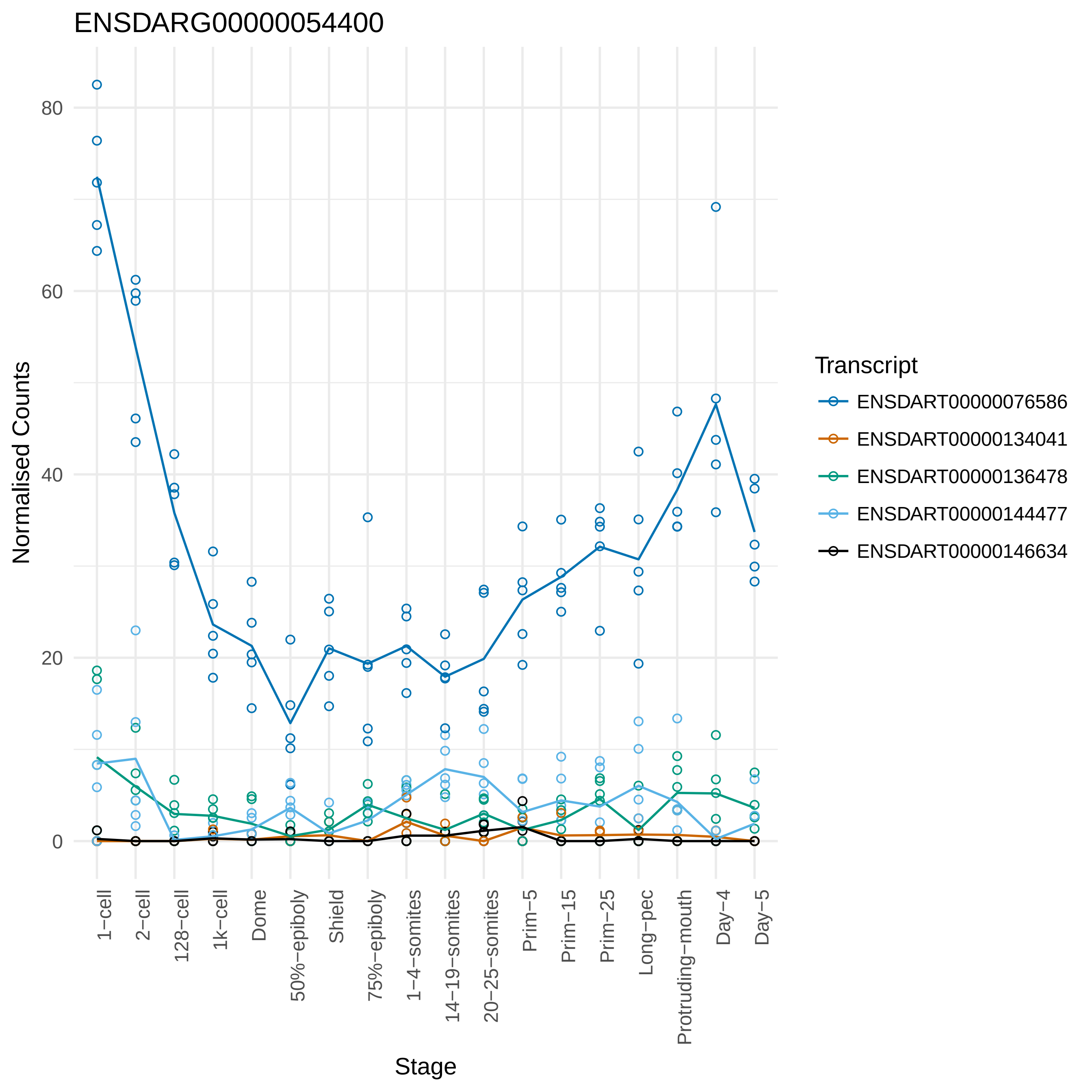

ENSDARG00000054400
Normalised Counts
0
20
40
60
80
Stage
1−cell
2−cell
128−cell
1k−cell
Dome
50%−epiboly
Shield
75%−epiboly
1−4−somites
14−19−somites
20−25−somites
Prim−5
Prim−15
Prim−25
Long−pec
Protruding−mouth
Day−4
Day−5
Transcript
ENSDART00000076586
ENSDART00000134041
ENSDART00000136478
ENSDART00000144477
ENSDART00000146634

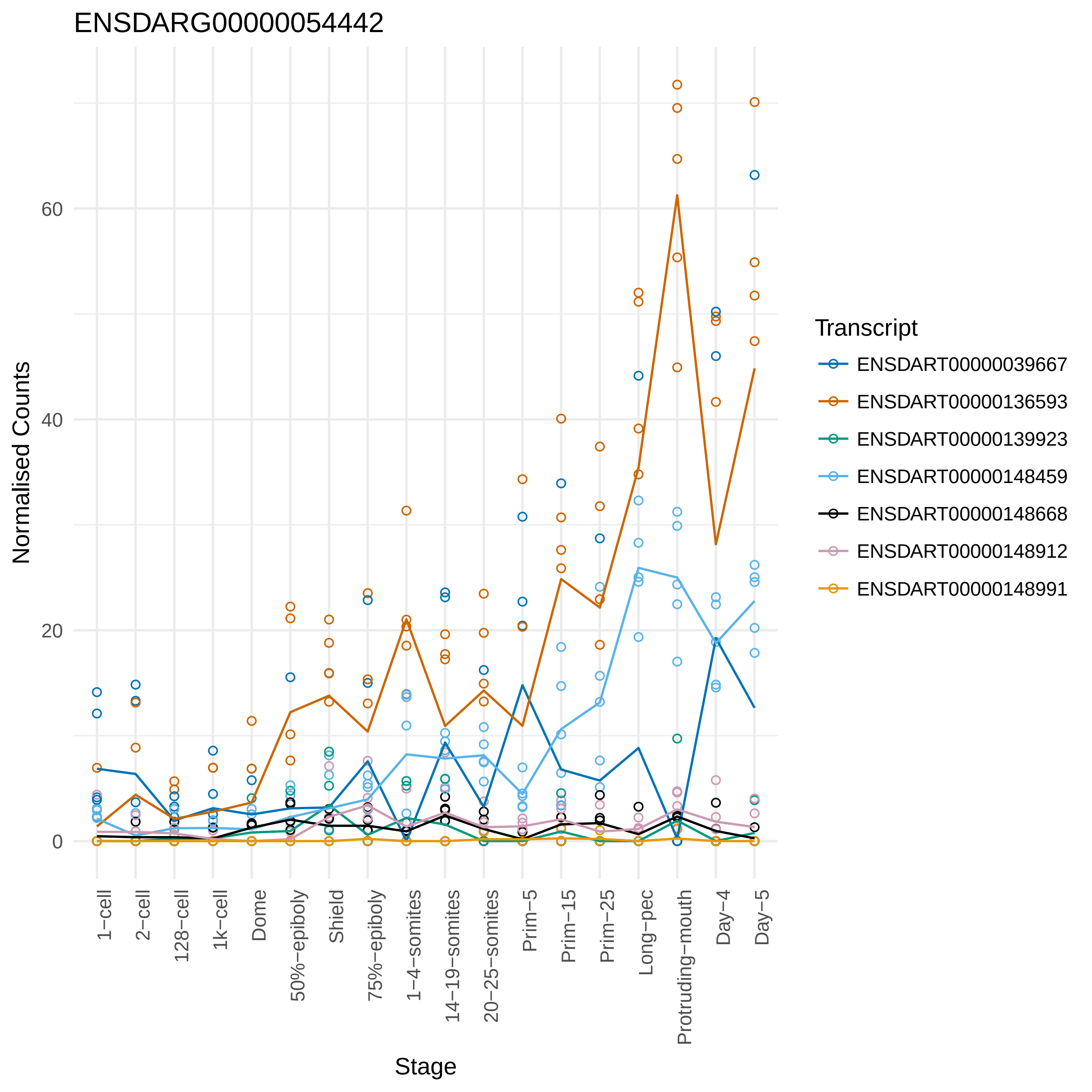

ENSDARG00000054442
Normalised Counts
0
20
40
60
1−cell
2−cell
128−cell
1k−cell
Dome
50%−epiboly
Shield
75%−epiboly
1−4−somites
14−19−somites
20−25−somites
Prim−5
Prim−15
Prim−25
Long−pec
Protruding−mouth
Day−4
Day−5
Stage
Transcript
ENSDART00000039667
ENSDART00000136593
ENSDART00000139923
ENSDART00000148459
ENSDART00000148668
ENSDART00000148912
ENSDART00000148991

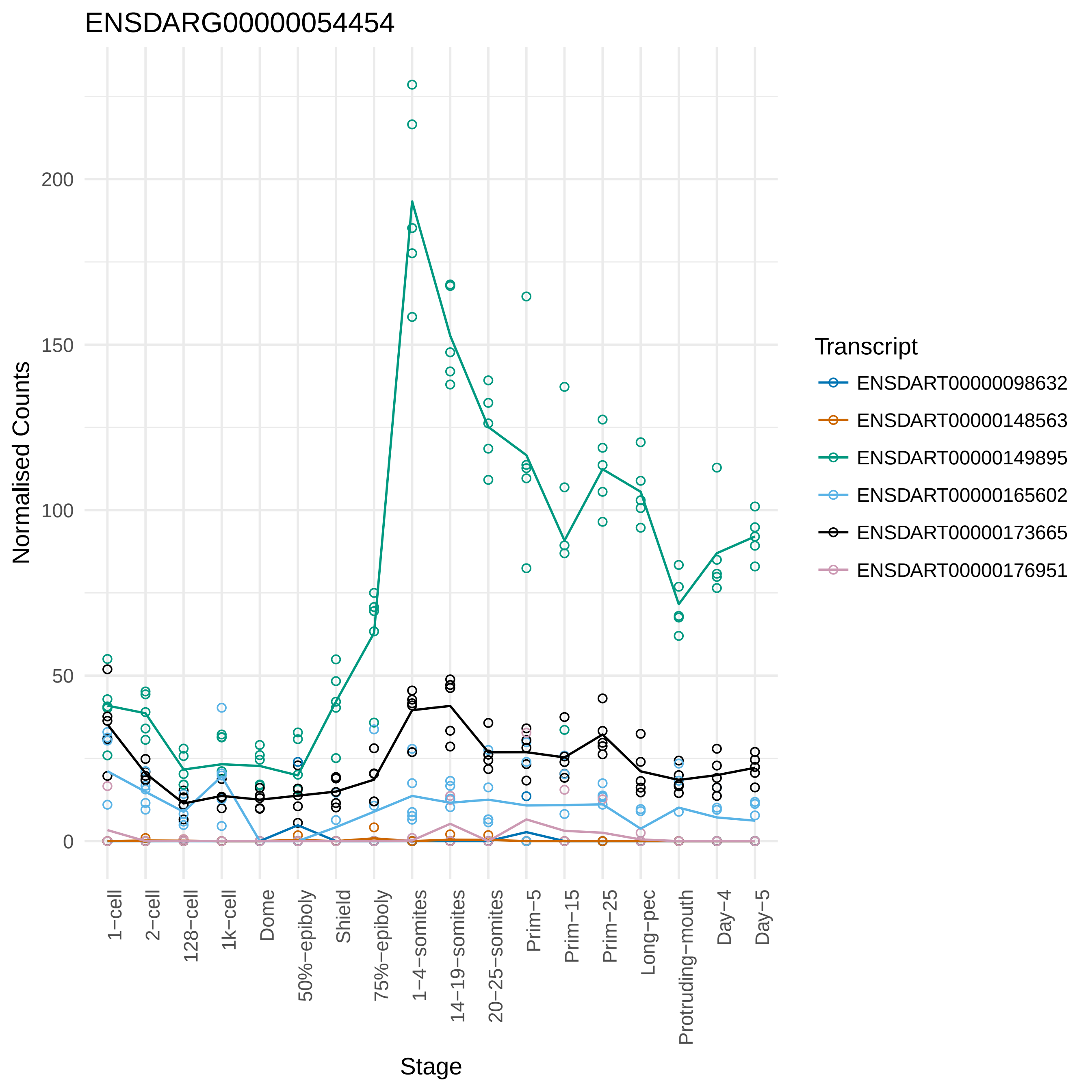
ENSDARG00000054454
Normalised Counts
0
50
100
150
200
Stage
1−cell
2−cell
128−cell
1k−cell
Dome
50%−epiboly
Shield
75%−epiboly
1−4−somites
14−19−somites
20−25−somites
Prim−5
Prim−15
Prim−25
Long−pec
Protruding−mouth
Day−4
Day−5
Transcript
ENSDART00000098632
ENSDART00000148563
ENSDART00000149895
ENSDART00000165602
ENSDART00000173665
ENSDART00000176951

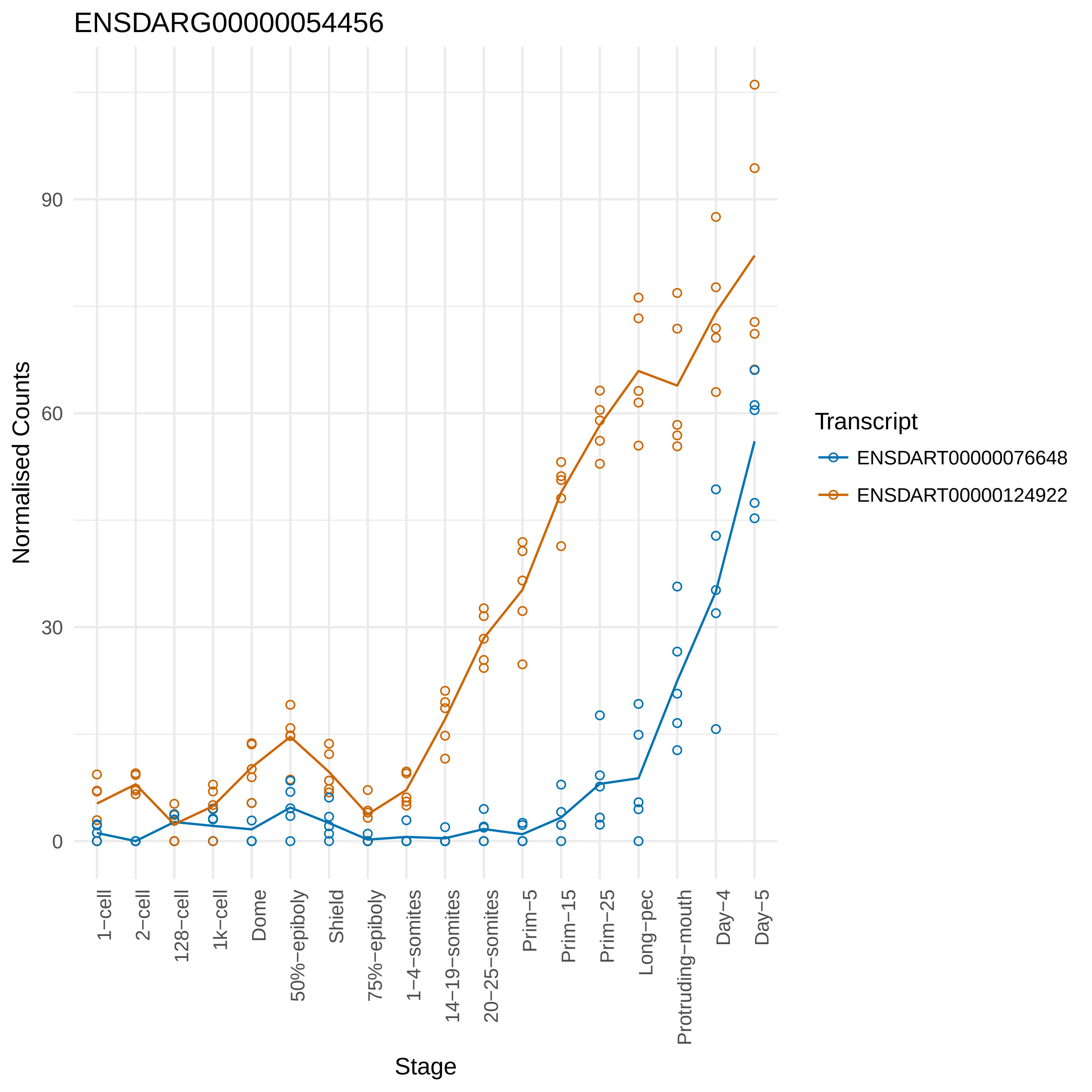

ENSDARG00000054456
Normalised Counts
0
30
60
90
Stage
1−cell
2−cell
128−cell
1k−cell
Dome
50%−epiboly
Shield
75%−epiboly
1−4−somites
14−19−somites
20−25−somites
Prim−5
Prim−15
Prim−25
Long−pec
Protruding−mouth
Day−4
Day−5
Transcript
ENSDART00000076648
ENSDART00000124922

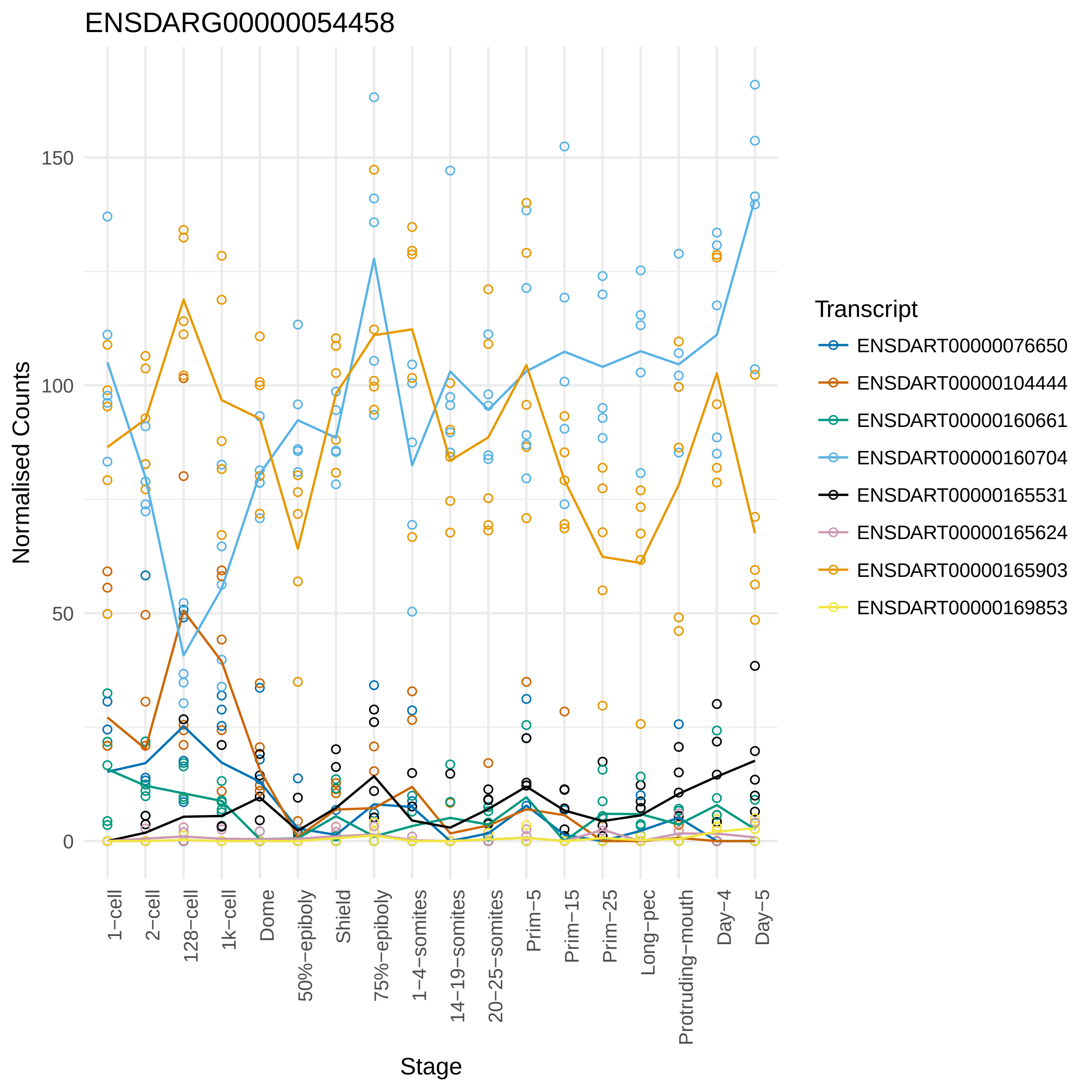

ENSDARG00000054458
Normalised Counts
150
100
50
0
1−cell
2−cell
128−cell
1k−cell
Dome
50%−epiboly
Shield
75%−epiboly
1−4−somites
14−19−somites
20−25−somites
Prim−5
Prim−15
Prim−25
Long−pec
Protruding−mouth
Day−4
Day−5
Stage
Transcript
ENSDART00000076650
ENSDART00000104444
ENSDART00000160661
ENSDART00000160704
ENSDART00000165531
ENSDART00000165624
ENSDART00000165903
ENSDART00000169853

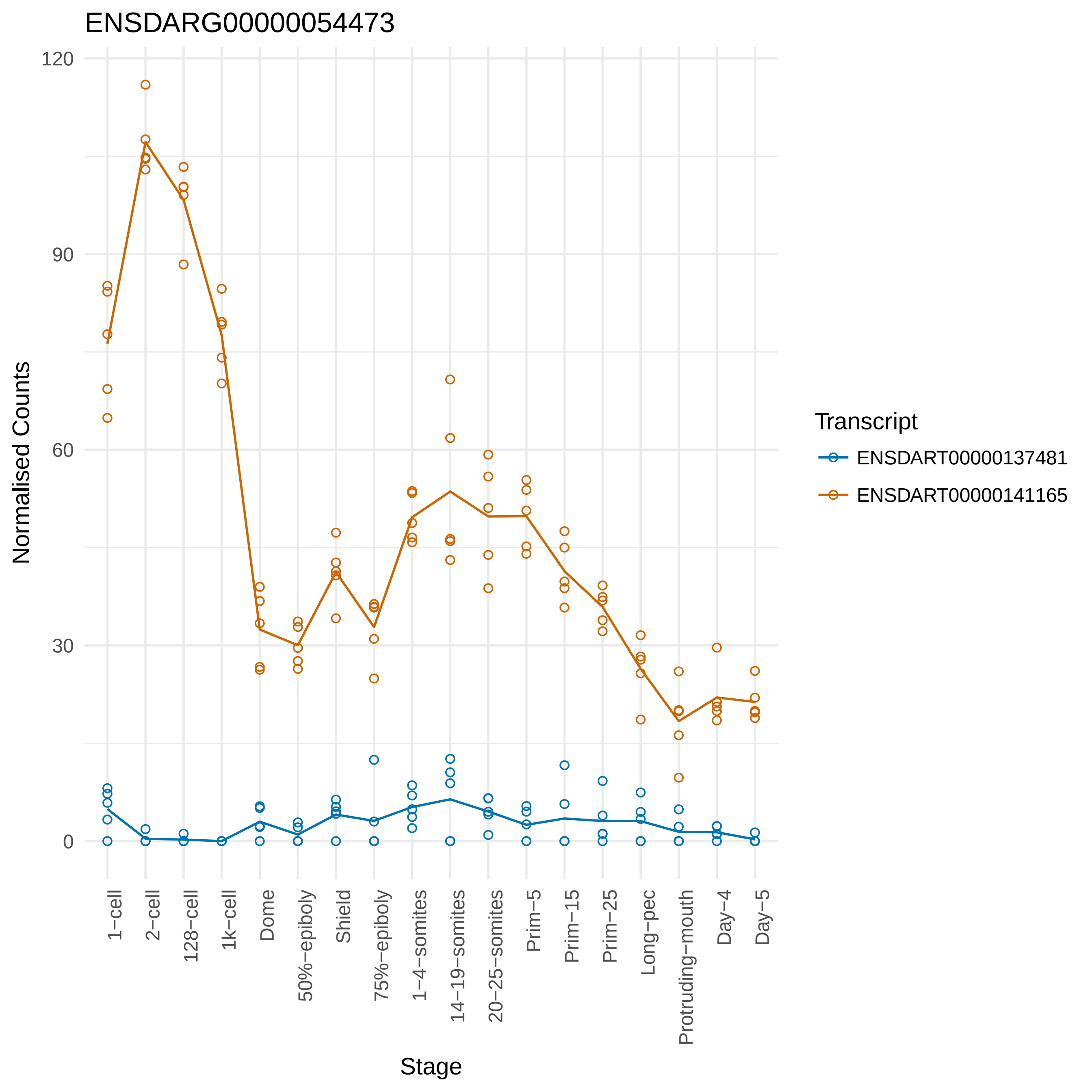

ENSDARG00000054473
120
90
60
30
0
Normalised Counts
1−cell
2−cell
128−cell
1k−cell
Dome
50%−epiboly
Shield
75%−epiboly
1−4−somites
14−19−somites
20−25−somites
Prim−5
Prim−15
Prim−25
Long−pec
Protruding−mouth
Day−4
Day−5
Stage
Transcript
ENSDART00000137481
ENSDART00000141165

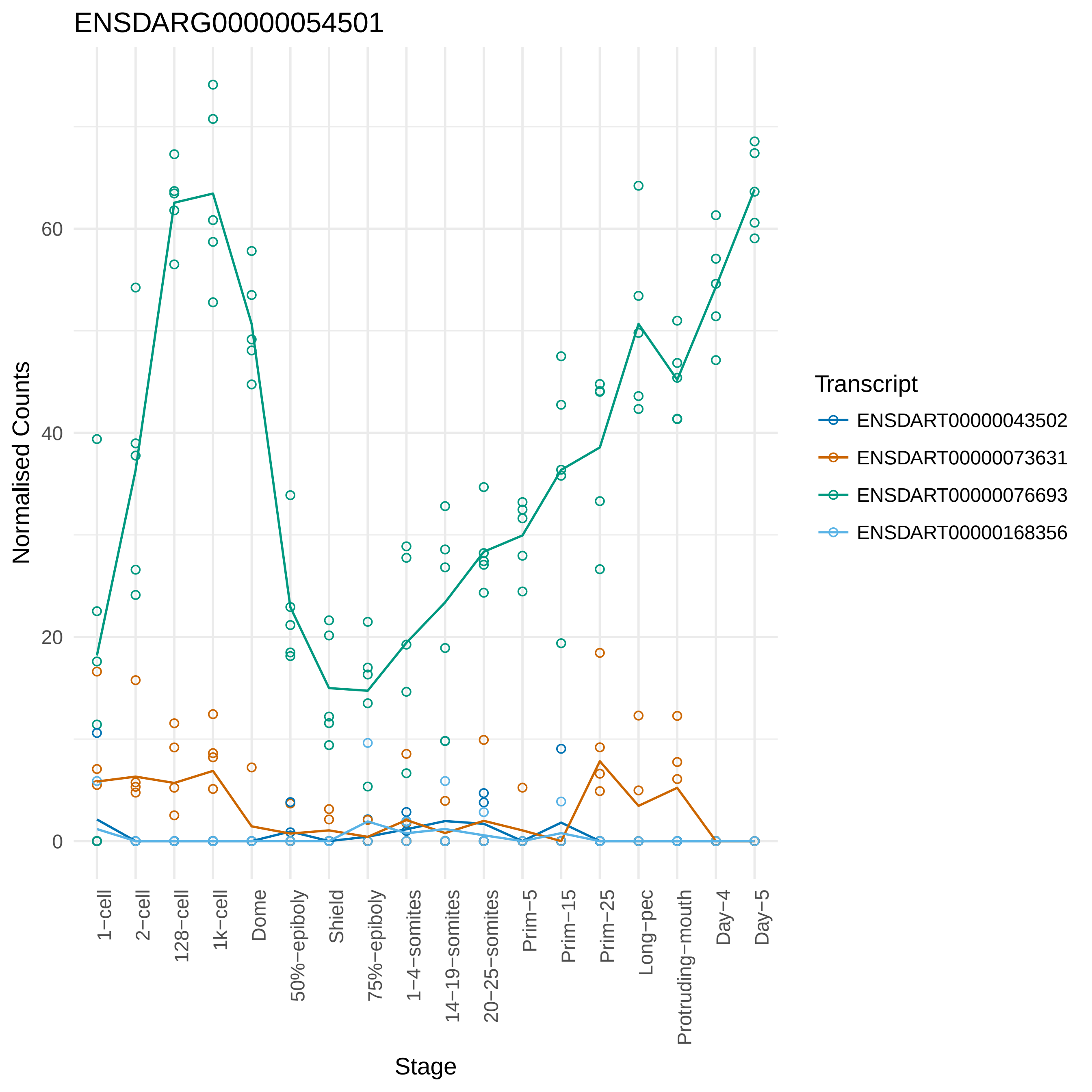

ENSDARG00000054501
Normalised Counts
0
20
40
60
Stage
1−cell
2−cell
128−cell
1k−cell
Dome
50%−epiboly
Shield
75%−epiboly
1−4−somites
14−19−somites
20−25−somites
Prim−5
Prim−15
Prim−25
Long−pec
Protruding−mouth
Day−4
Day−5
Transcript
ENSDART00000043502
ENSDART00000073631
ENSDART00000076693
ENSDART00000168356

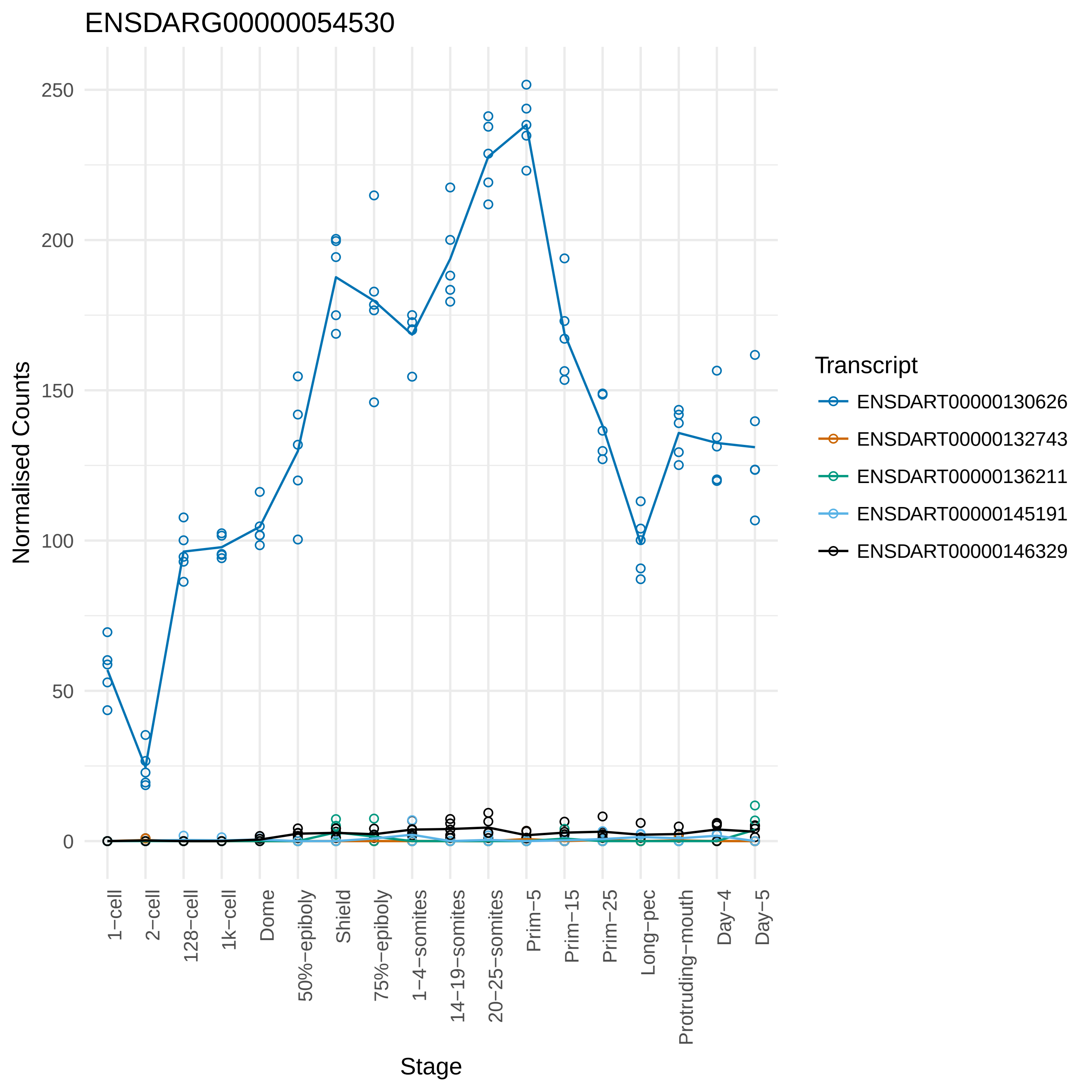

ENSDARG00000054530
Normalised Counts
250
200
150
100
50
0
1−cell
2−cell
128−cell
1k−cell
Dome
50%−epiboly
Shield
75%−epiboly
1−4−somites
14−19−somites
20−25−somites
Prim−5
Prim−15
Prim−25
Long−pec
Protruding−mouth
Day−4
Day−5
Stage
Transcript
ENSDART00000130626
ENSDART00000132743
ENSDART00000136211
ENSDART00000145191
ENSDART00000146329

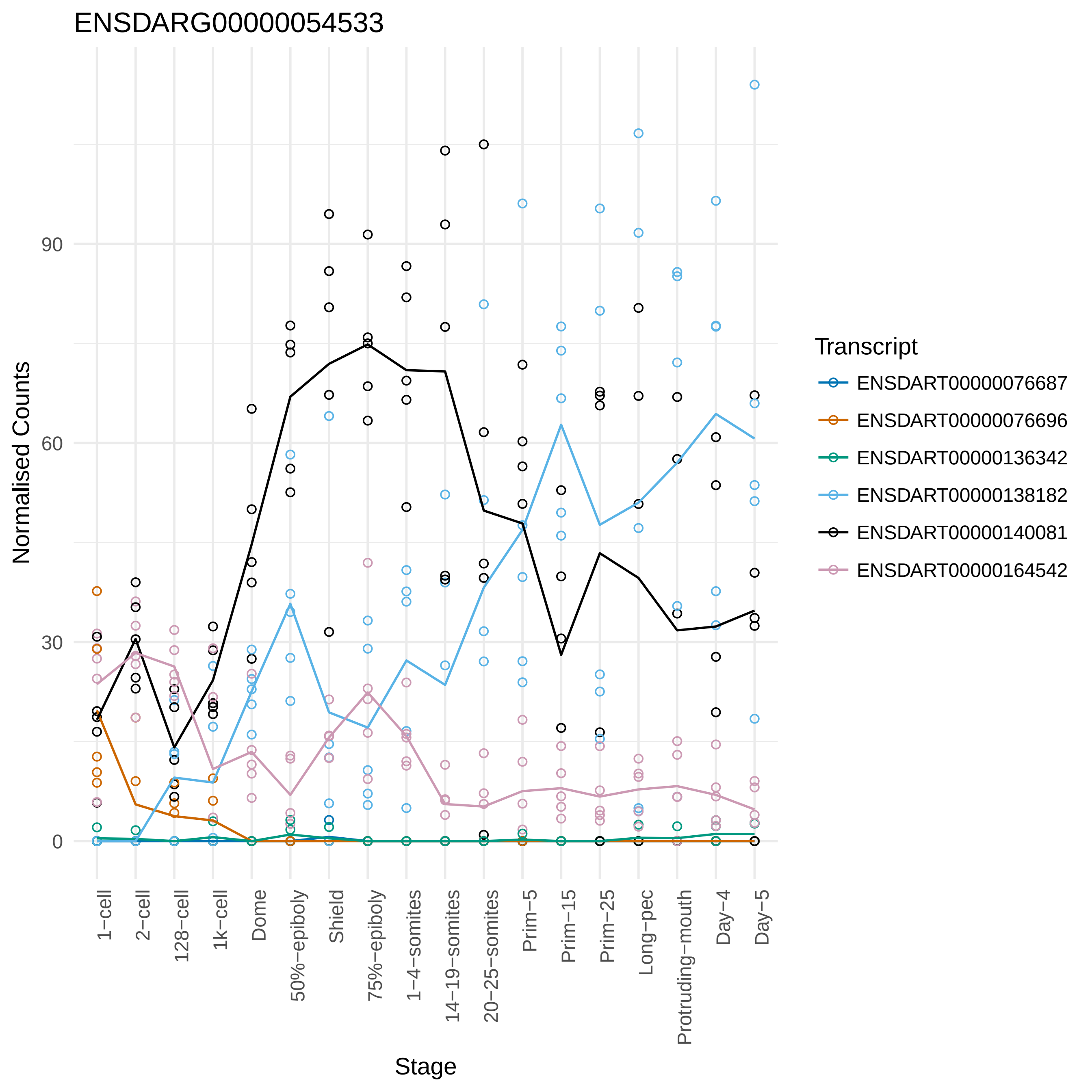

ENSDARG00000054533
Normalised Counts
0
30
60
90
1−cell
2−cell
128−cell
1k−cell
Dome
50%−epiboly
Shield
75%−epiboly
1−4−somites
14−19−somites
20−25−somites
Prim−5
Prim−15
Prim−25
Long−pec
Protruding−mouth
Day−4
Day−5
Stage
Transcript
ENSDART00000076687
ENSDART00000076696
ENSDART00000136342
ENSDART00000138182
ENSDART00000140081
ENSDART00000164542

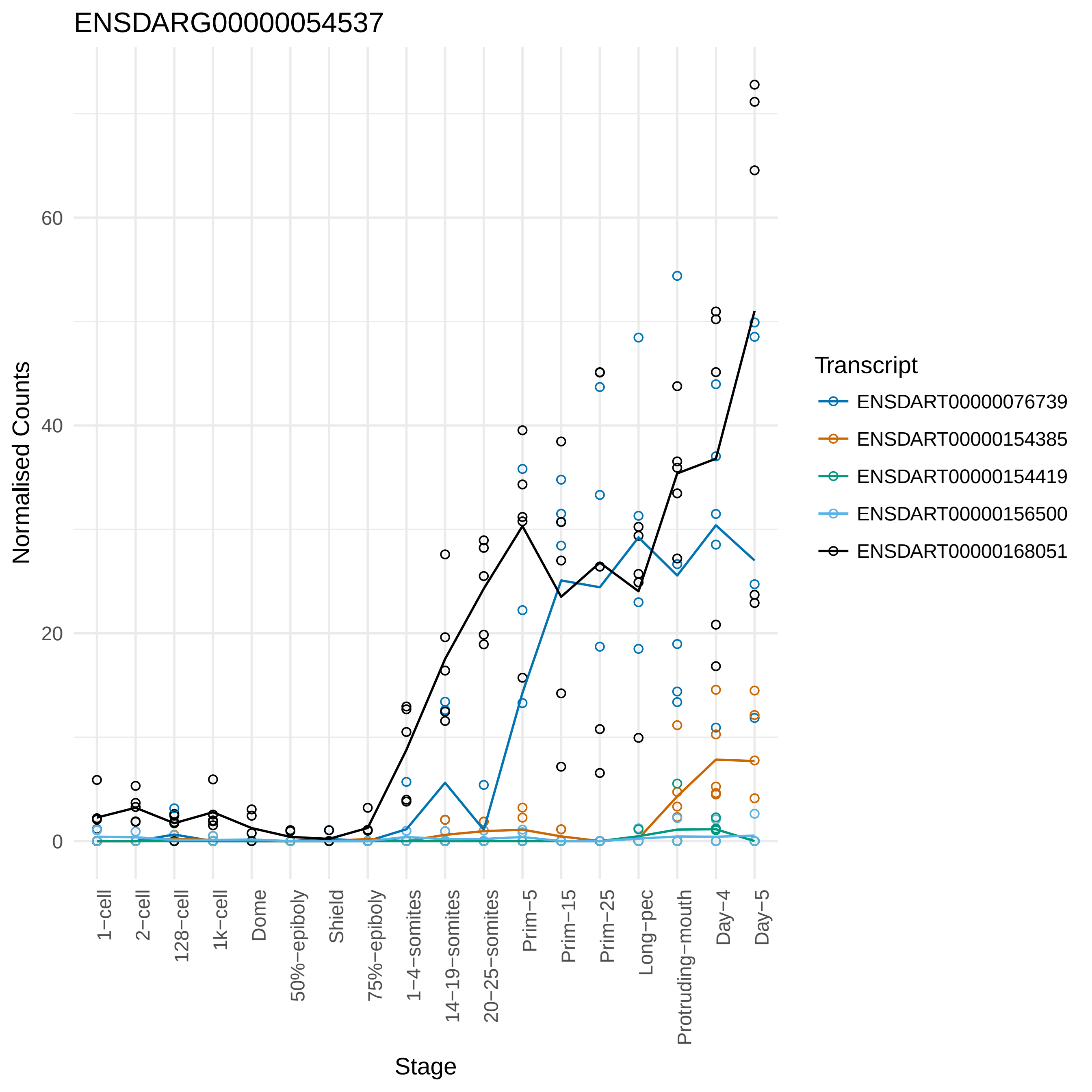

ENSDARG00000054537
Normalised Counts
0
20
40
60
Stage
1−cell
2−cell
128−cell
1k−cell
Dome
50%−epiboly
Shield
75%−epiboly
1−4−somites
14−19−somites
20−25−somites
Prim−5
Prim−15
Prim−25
Long−pec
Protruding−mouth
Day−4
Day−5
Transcript
ENSDART00000076739
ENSDART00000154385
ENSDART00000154419
ENSDART00000156500
ENSDART00000168051

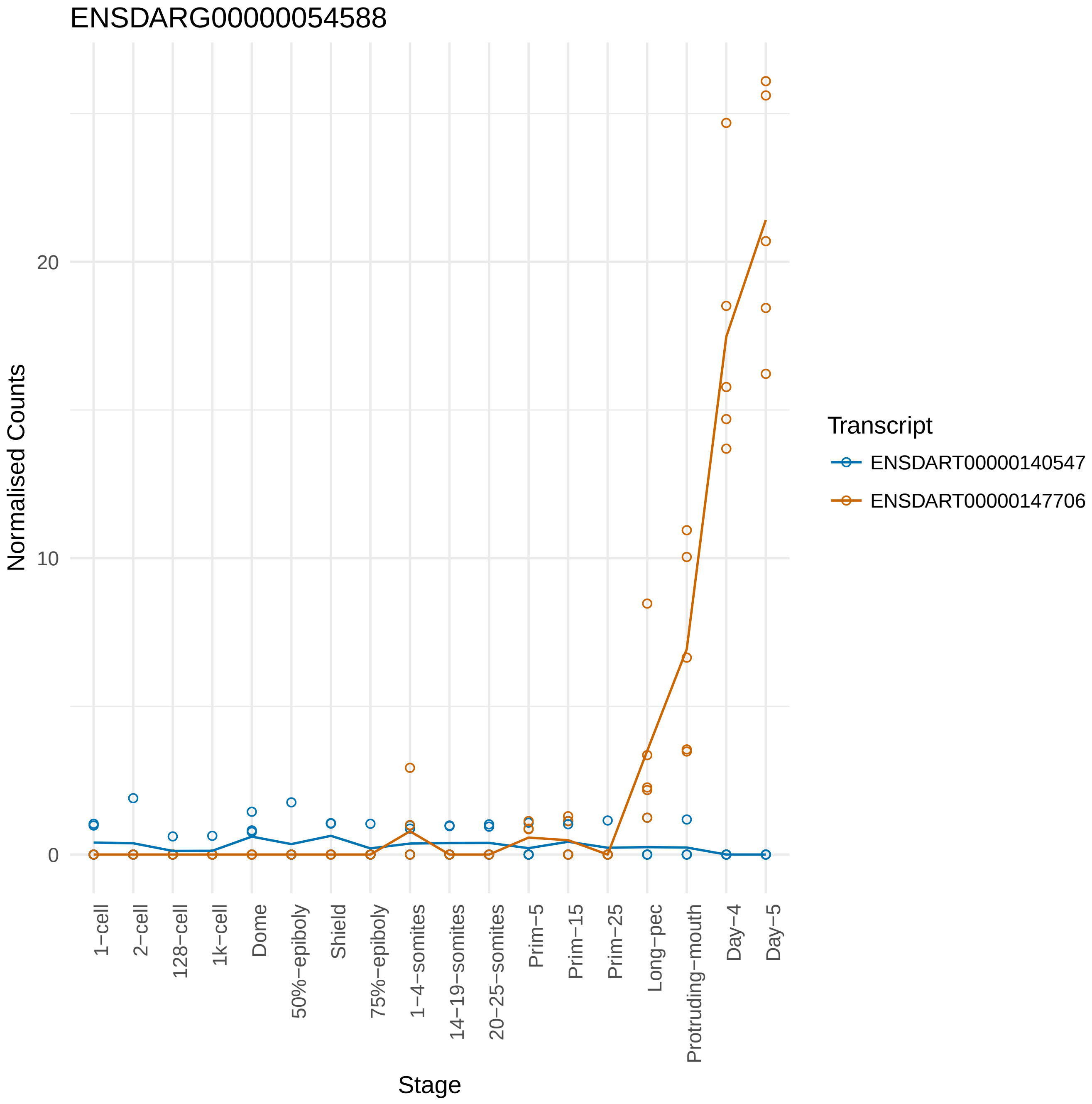

ENSDARG00000054588
Normalised Counts
Stage
0
10
20
1−cell
2−cell
128−cell
1k−cell
Dome
50%−epiboly
Shield
75%−epiboly
1−4−somites
14−19−somites
20−25−somites
Prim−5
Prim−15
Prim−25
Long−pec
Protruding−mouth
Day−4
Day−5
Transcript
ENSDART00000140547
ENSDART00000147706

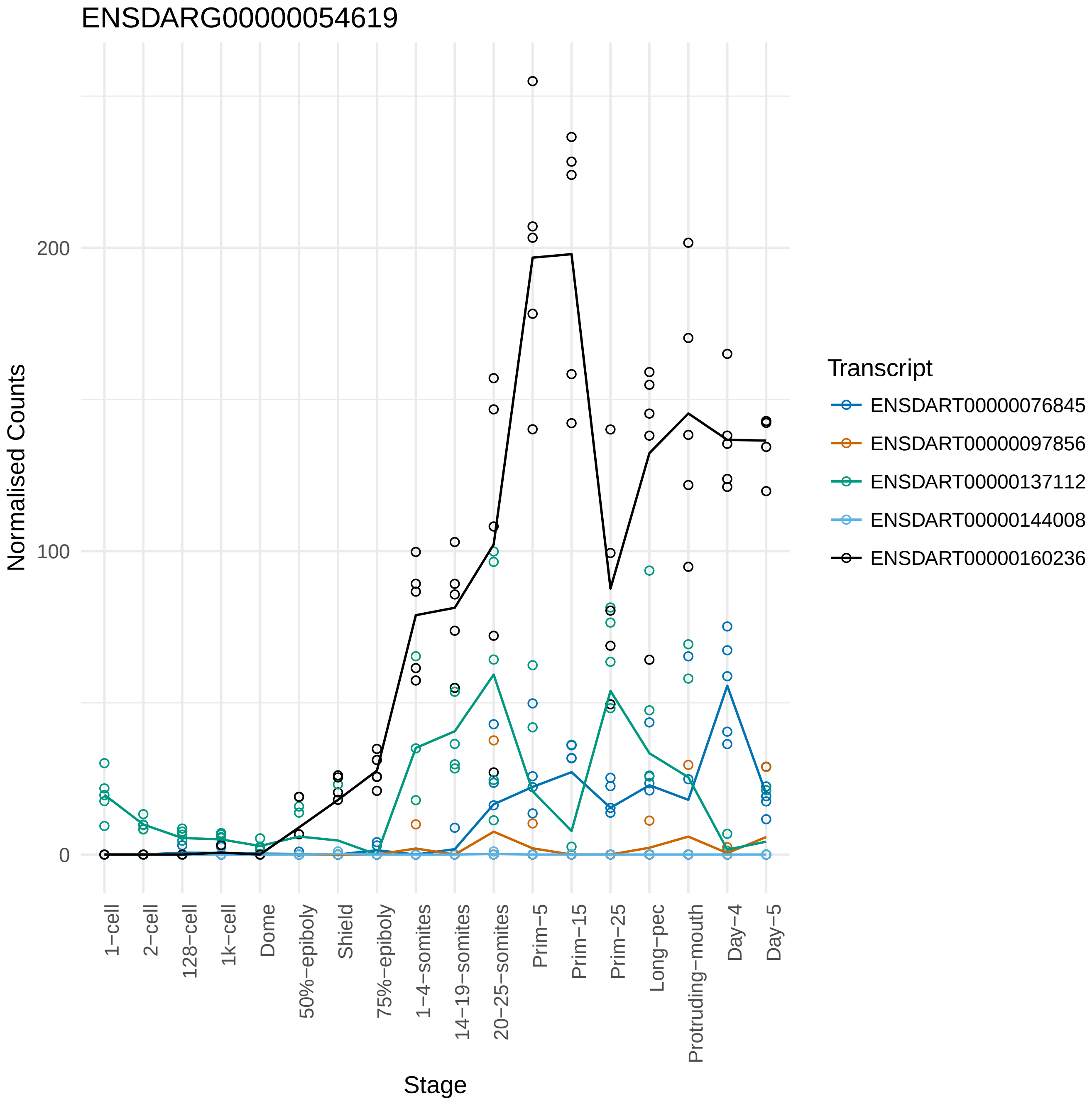
ENSDARG00000054619
Normalised Counts
200
100
0
1−cell
2−cell
128−cell
1k−cell
Dome
50%−epiboly
Shield
75%−epiboly
1−4−somites
14−19−somites
20−25−somites
Prim−5
Prim−15
Prim−25
Long−pec
Protruding−mouth
Day−4
Day−5
Stage
Transcript
ENSDART00000076845
ENSDART00000097856
ENSDART00000137112
ENSDART00000144008
ENSDART00000160236

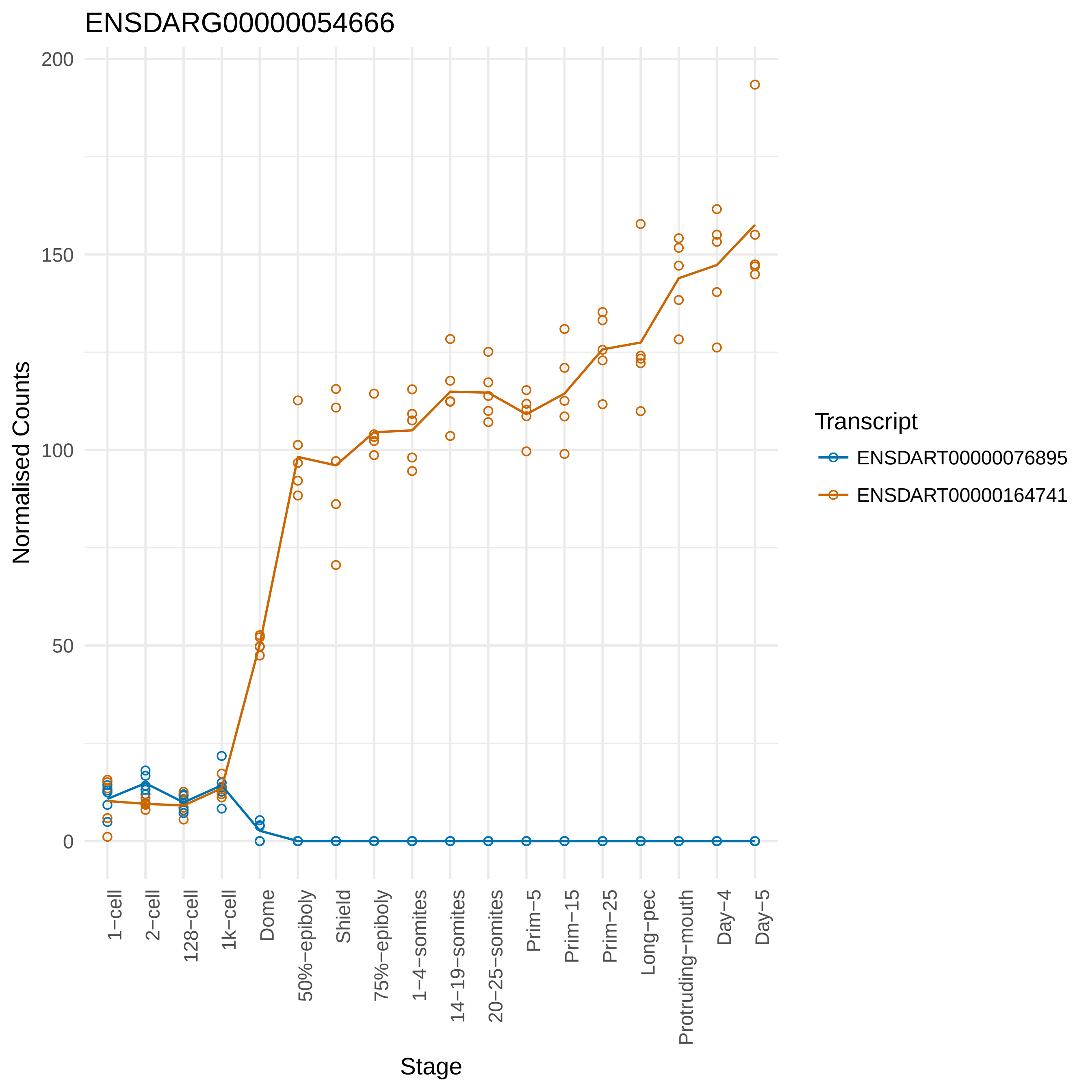
ENSDARG00000054666
Normalised Counts
200
150
100
50
0
1−cell
2−cell
128−cell
1k−cell
Dome
50%−epiboly
Shield
75%−epiboly
1−4−somites
14−19−somites
20−25−somites
Prim−5
Prim−15
Prim−25
Long−pec
Protruding−mouth
Day−4
Day−5
Stage
Transcript
ENSDART00000076895
ENSDART00000164741

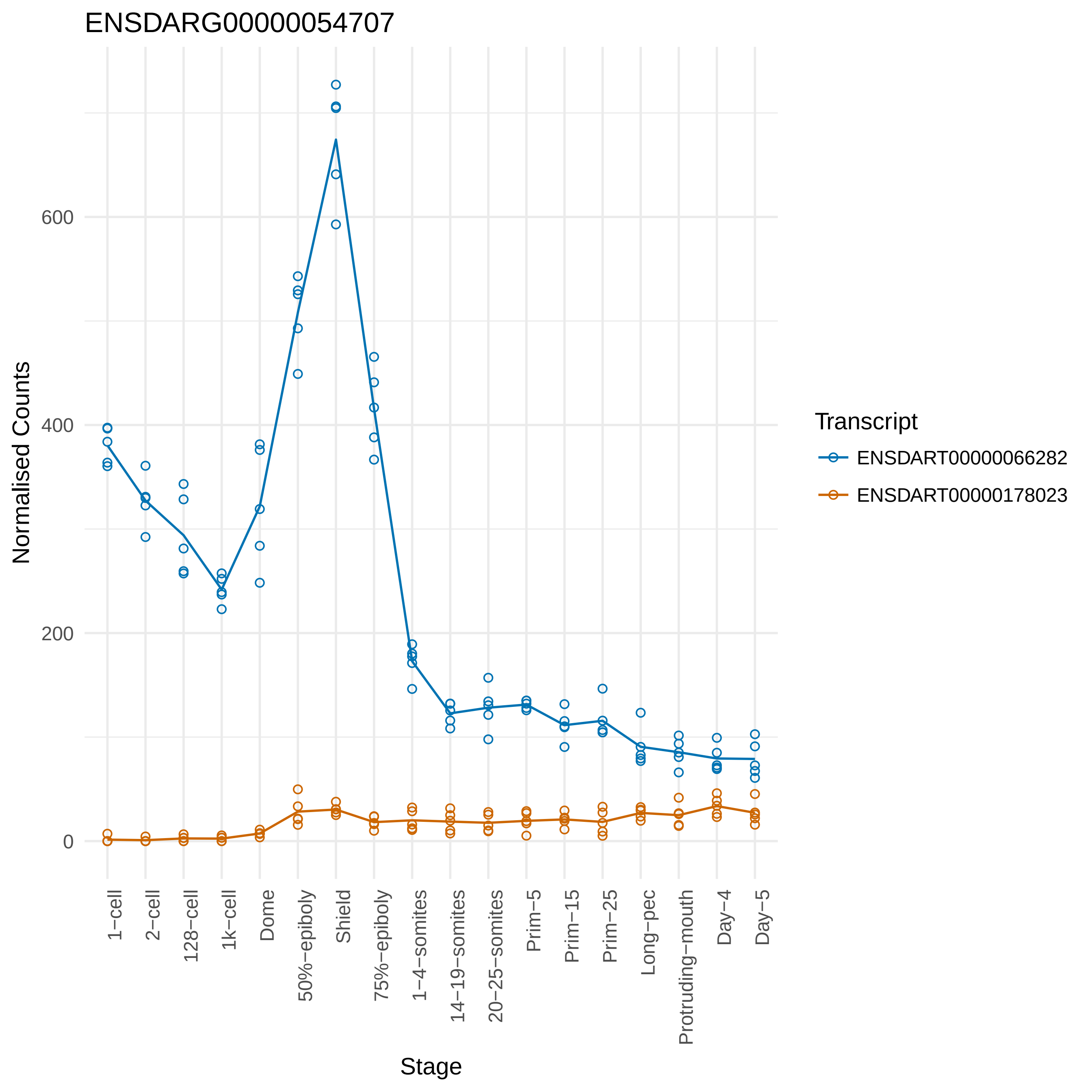
ENSDARG00000054707
Normalised Counts
0
200
400
600
1−cell
2−cell
128−cell
1k−cell
Dome
50%−epiboly
Shield
75%−epiboly
1−4−somites
14−19−somites
20−25−somites
Prim−5
Prim−15
Prim−25
Long−pec
Protruding−mouth
Day−4
Day−5
Stage
Transcript
ENSDART00000066282
ENSDART00000178023

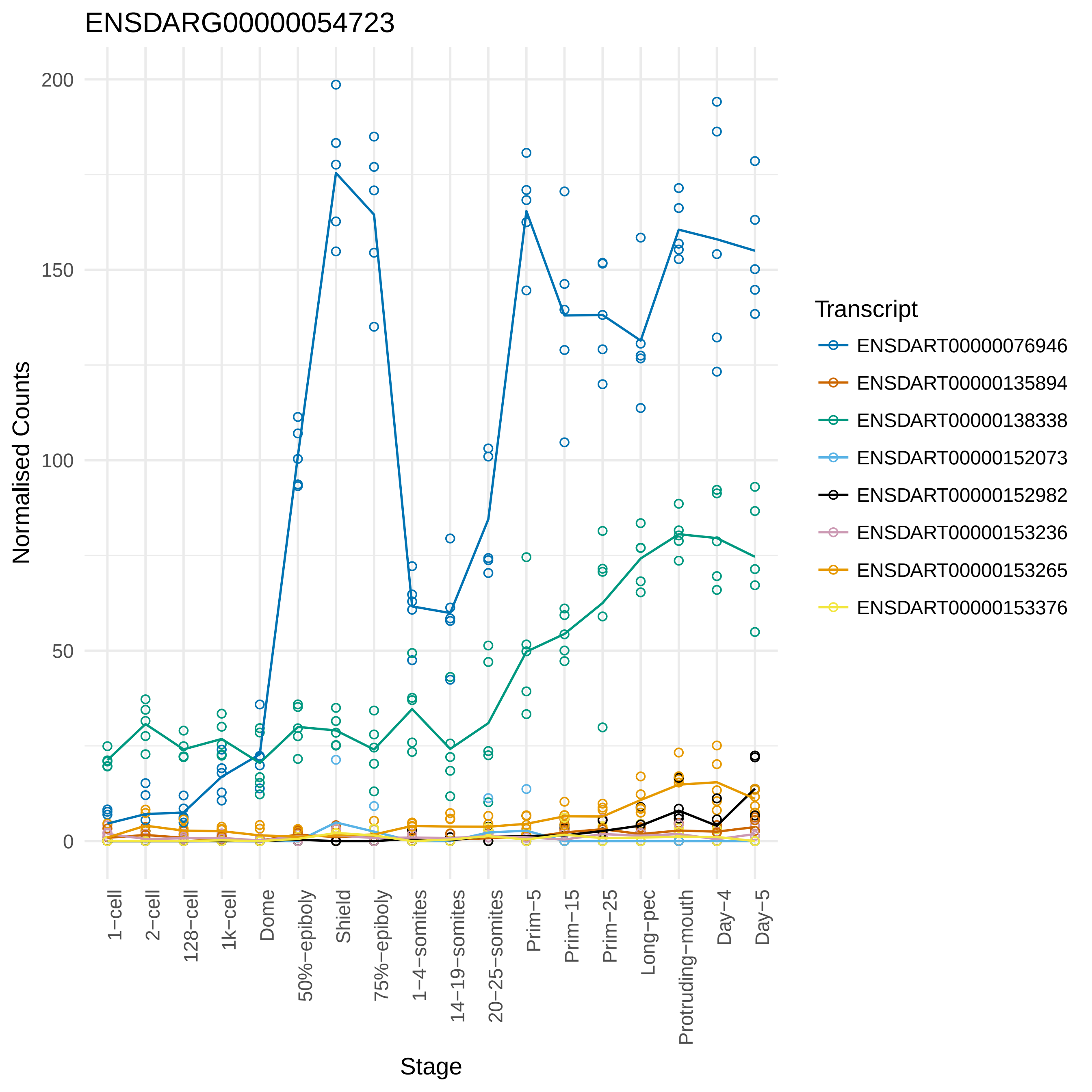
ENSDARG00000054723
Normalised Counts
200
150
100
50
0
1−cell
2−cell
128−cell
1k−cell
Dome
50%−epiboly
Shield
75%−epiboly
1−4−somites
14−19−somites
20−25−somites
Prim−5
Prim−15
Prim−25
Long−pec
Protruding−mouth
Day−4
Day−5
Stage
Transcript
ENSDART00000076946
ENSDART00000135894
ENSDART00000138338
ENSDART00000152073
ENSDART00000152982
ENSDART00000153236
ENSDART00000153265
ENSDART00000153376

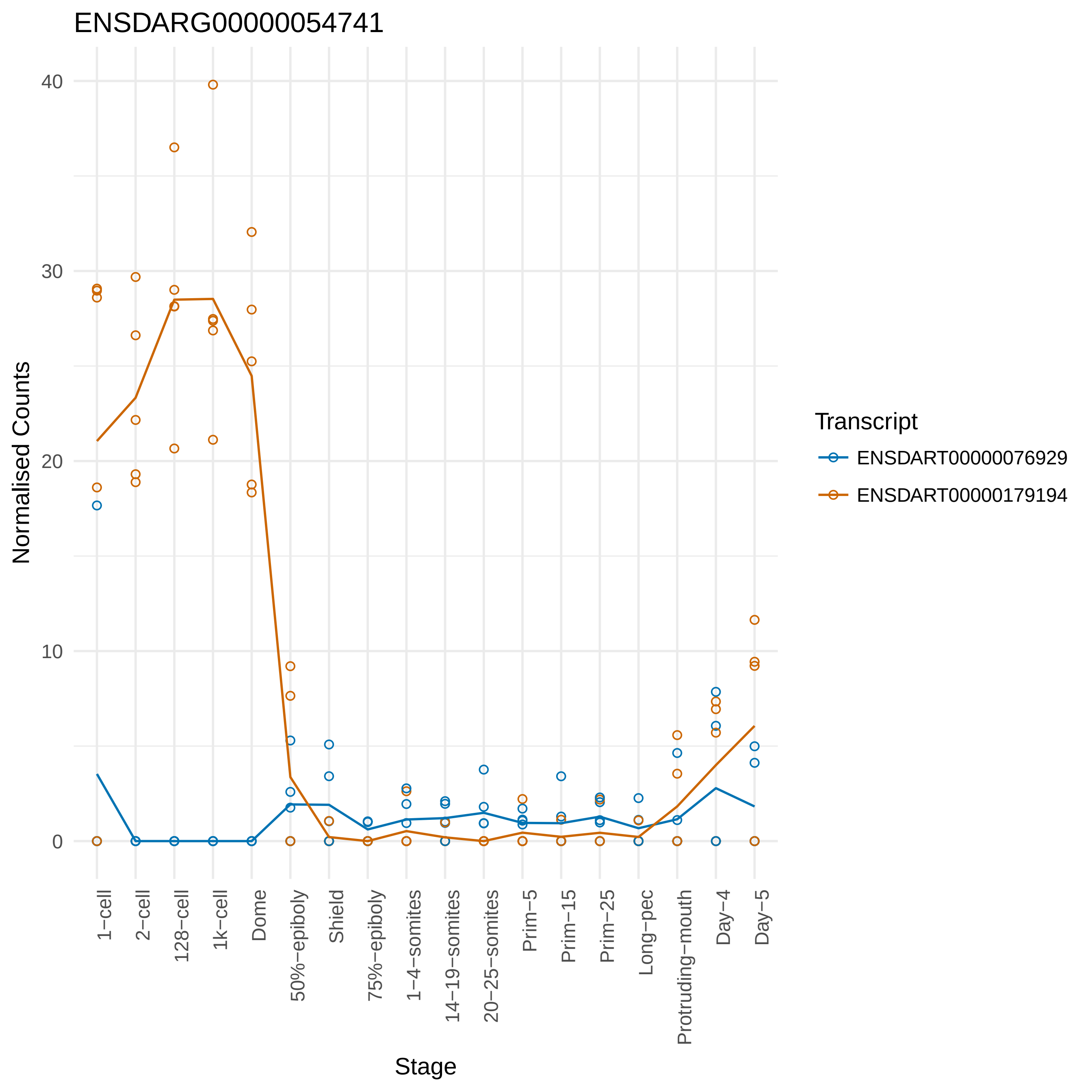

ENSDARG00000054741
Normalised Counts
40
30
20
10
0
1−cell
2−cell
128−cell
1k−cell
Dome
50%−epiboly
Shield
75%−epiboly
1−4−somites
14−19−somites
20−25−somites
Prim−5
Prim−15
Prim−25
Long−pec
Protruding−mouth
Day−4
Day−5
Stage
Transcript
ENSDART00000076929
ENSDART00000179194

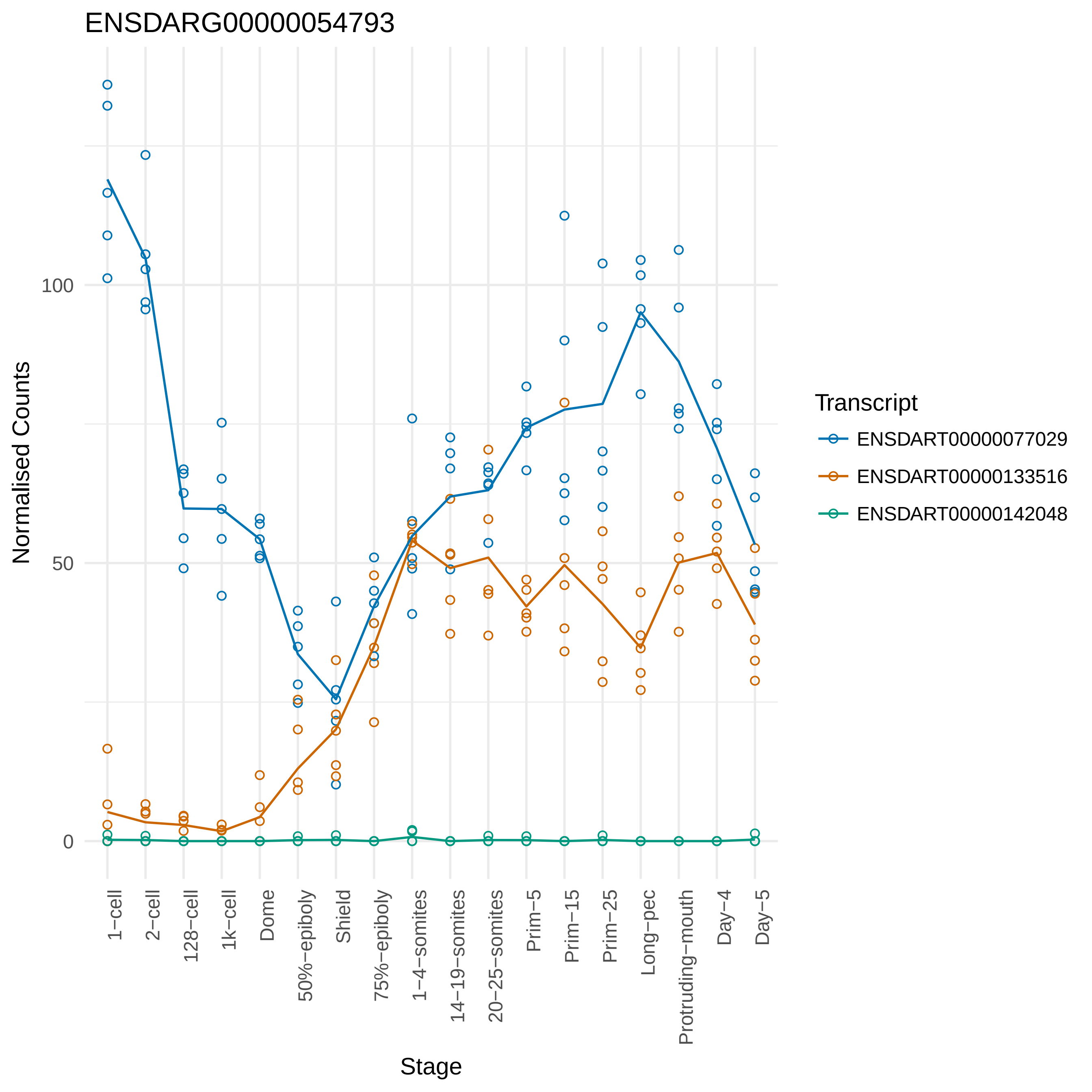

ENSDARG00000054793
Normalised Counts
100
50
0
Stage
1−cell
2−cell
128−cell
1k−cell
Dome
50%−epiboly
Shield
75%−epiboly
1−4−somites
14−19−somites
20−25−somites
Prim−5
Prim−15
Prim−25
Long−pec
Protruding−mouth
Day−4
Day−5
Transcript
ENSDART00000077029
ENSDART00000133516
ENSDART00000142048

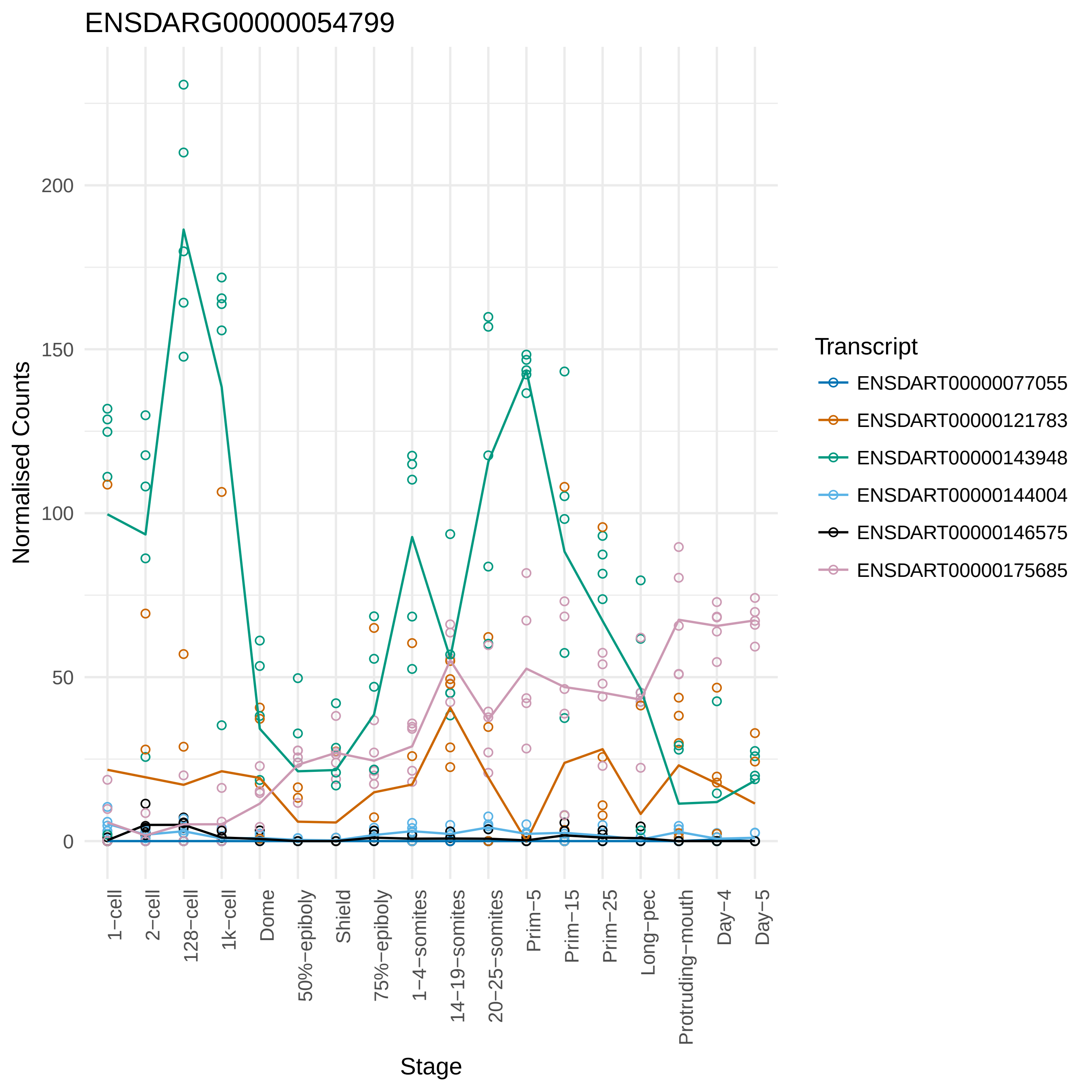

ENSDARG00000054799
Normalised Counts
0
50
100
150
200
1−cell
2−cell
128−cell
1k−cell
Dome
50%−epiboly
Shield
75%−epiboly
1−4−somites
14−19−somites
20−25−somites
Prim−5
Prim−15
Prim−25
Long−pec
Protruding−mouth
Day−4
Day−5
Stage
Transcript
ENSDART00000077055
ENSDART00000121783
ENSDART00000143948
ENSDART00000144004
ENSDART00000146575
ENSDART00000175685

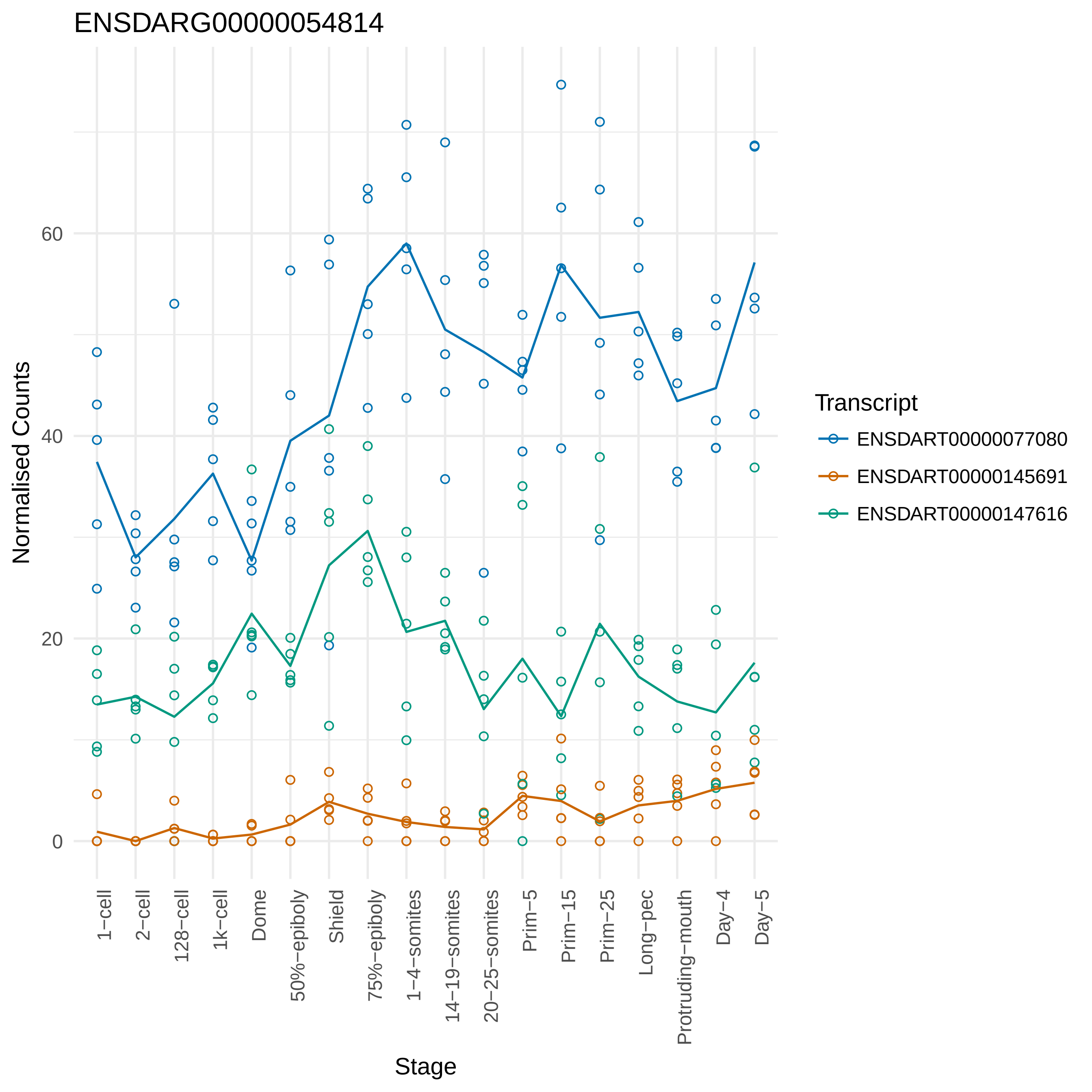

ENSDARG00000054814
Normalised Counts
0
20
40
60
1−cell
2−cell
128−cell
1k−cell
Dome
50%−epiboly
Shield
75%−epiboly
1−4−somites
14−19−somites
20−25−somites
Prim−5
Prim−15
Prim−25
Long−pec
Protruding−mouth
Day−4
Day−5
Stage
Transcript
ENSDART00000077080
ENSDART00000145691
ENSDART00000147616

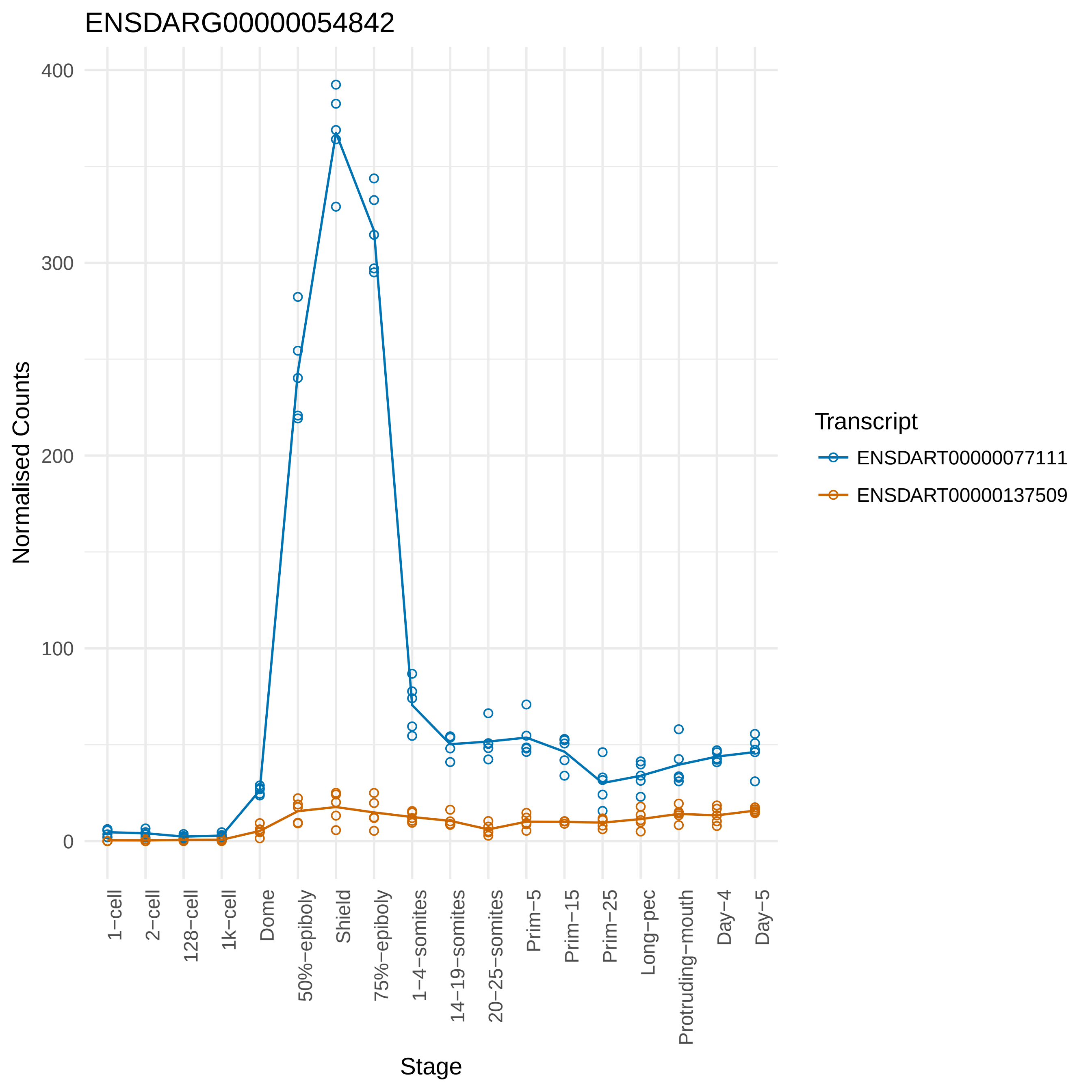

ENSDARG00000054842
Normalised Counts
400
300
200
100
0
1−cell
2−cell
128−cell
1k−cell
Dome
50%−epiboly
Shield
75%−epiboly
1−4−somites
14−19−somites
20−25−somites
Prim−5
Prim−15
Prim−25
Long−pec
Protruding−mouth
Day−4
Day−5
Stage
Transcript
ENSDART00000077111
ENSDART00000137509

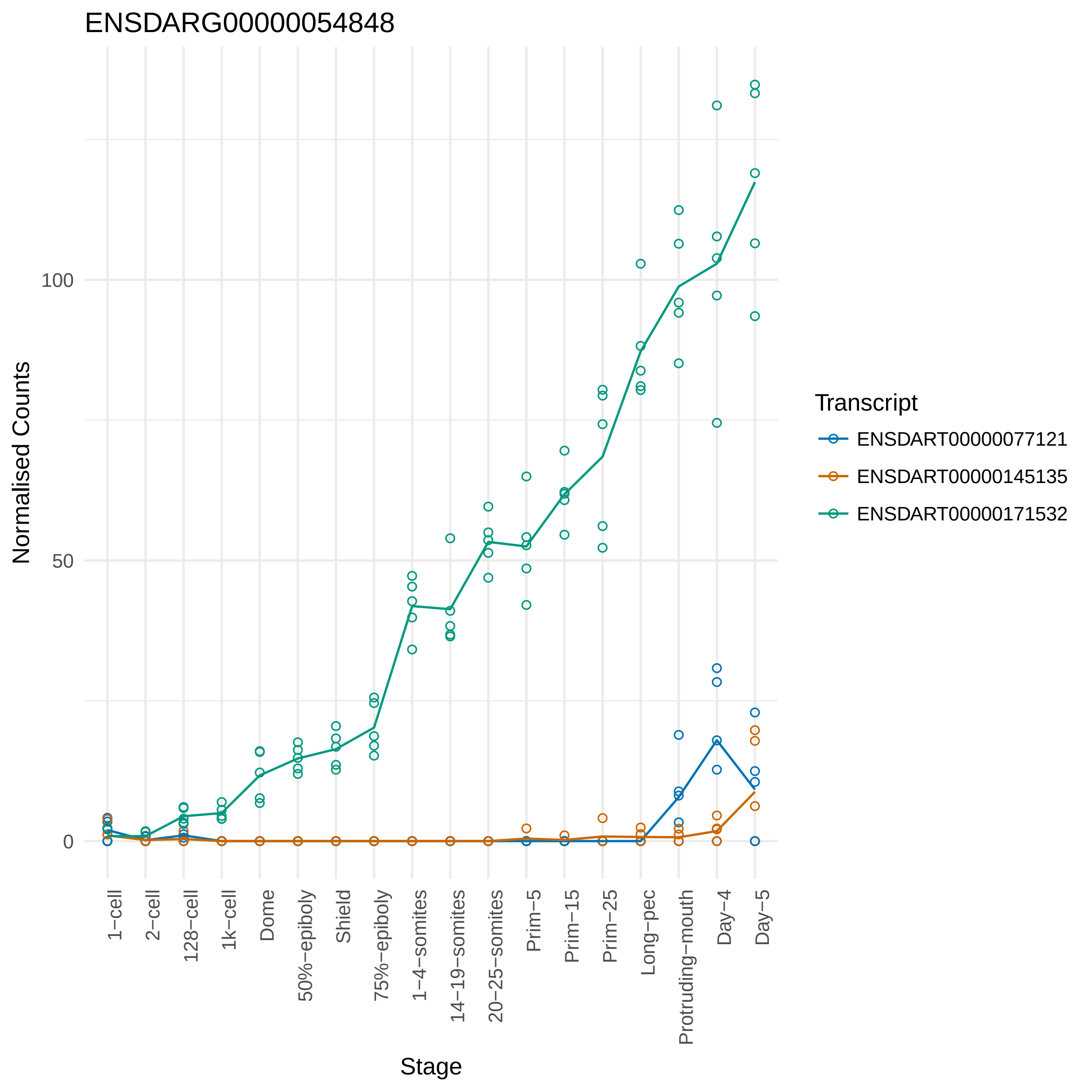

ENSDARG00000054848
Normalised Counts
100
50
0
1−cell
2−cell
128−cell
1k−cell
Dome
50%−epiboly
Shield
75%−epiboly
1−4−somites
14−19−somites
20−25−somites
Prim−5
Prim−15
Prim−25
Long−pec
Protruding−mouth
Day−4
Day−5
Stage
Transcript
ENSDART00000077121
ENSDART00000145135
ENSDART00000171532

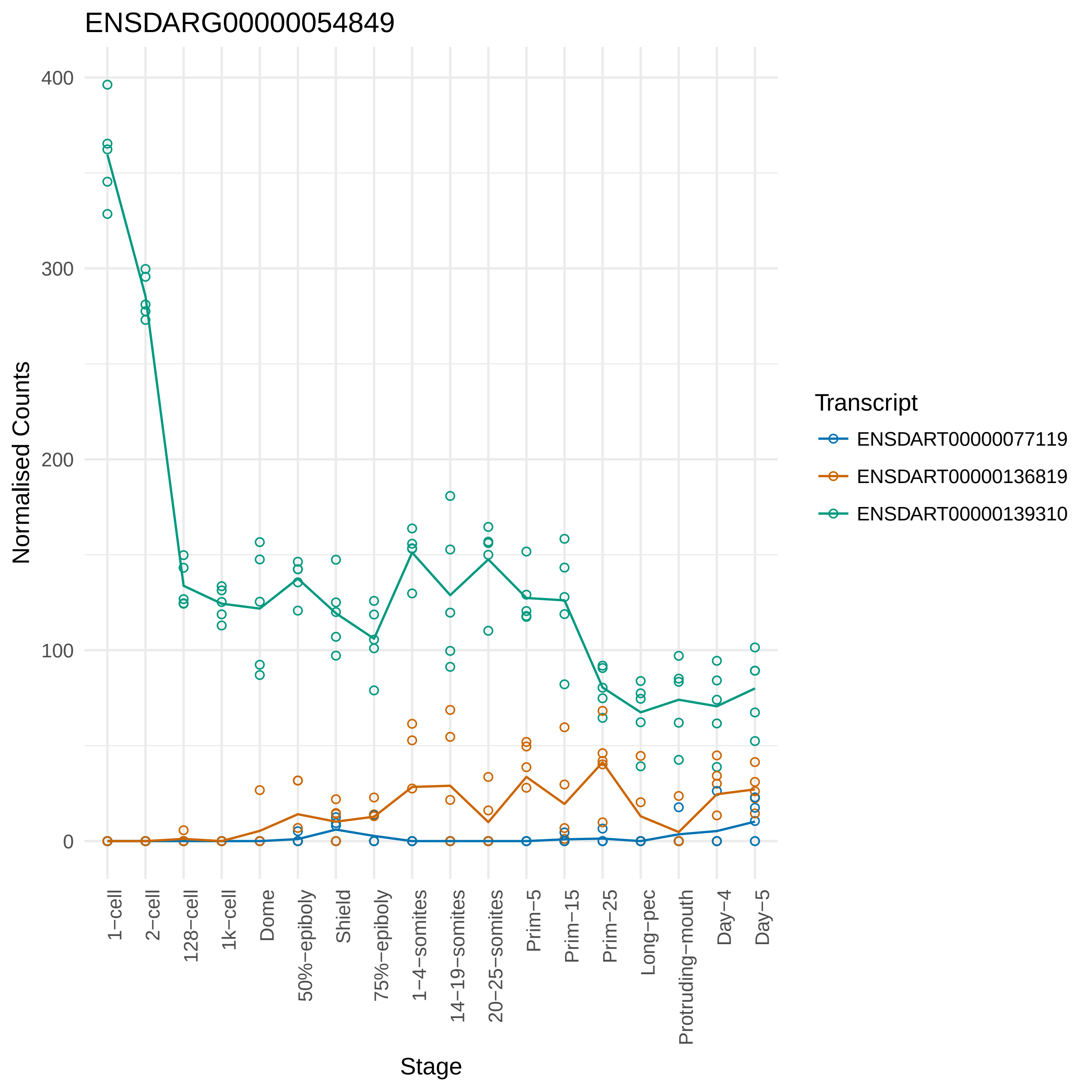
ENSDARG00000054849
Normalised Counts
400
300
200
100
0
1−cell
2−cell
128−cell
1k−cell
Dome
50%−epiboly
Shield
75%−epiboly
1−4−somites
14−19−somites
20−25−somites
Prim−5
Prim−15
Prim−25
Long−pec
Protruding−mouth
Day−4
Day−5
Stage
Transcript
ENSDART00000077119
ENSDART00000136819
ENSDART00000139310

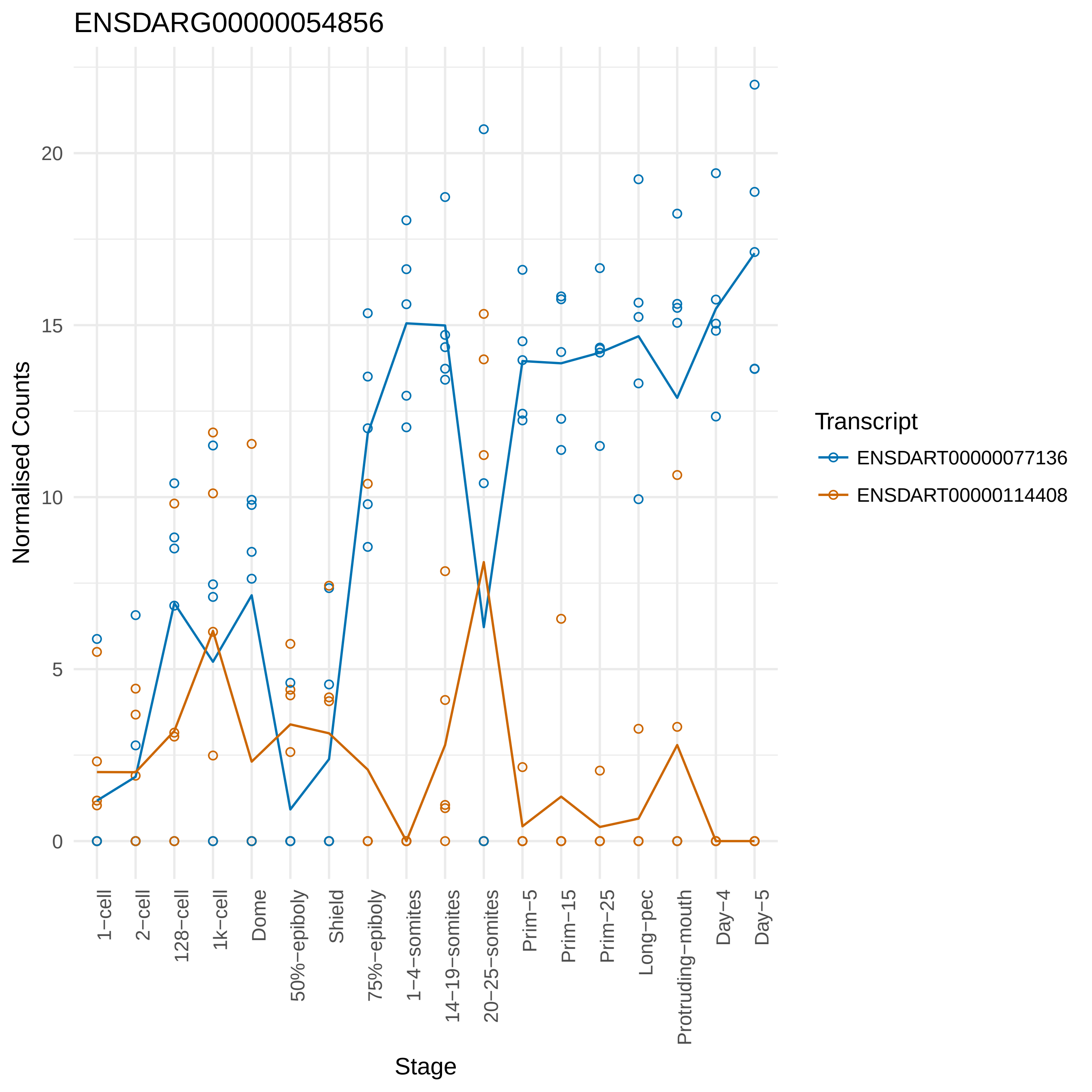
ENSDARG00000054856
Normalised Counts
0
5
10
15
20
Stage
1−cell
2−cell
128−cell
1k−cell
Dome
50%−epiboly
Shield
75%−epiboly
1−4−somites
14−19−somites
20−25−somites
Prim−5
Prim−15
Prim−25
Long−pec
Protruding−mouth
Day−4
Day−5
Transcript
ENSDART00000077136
ENSDART00000114408

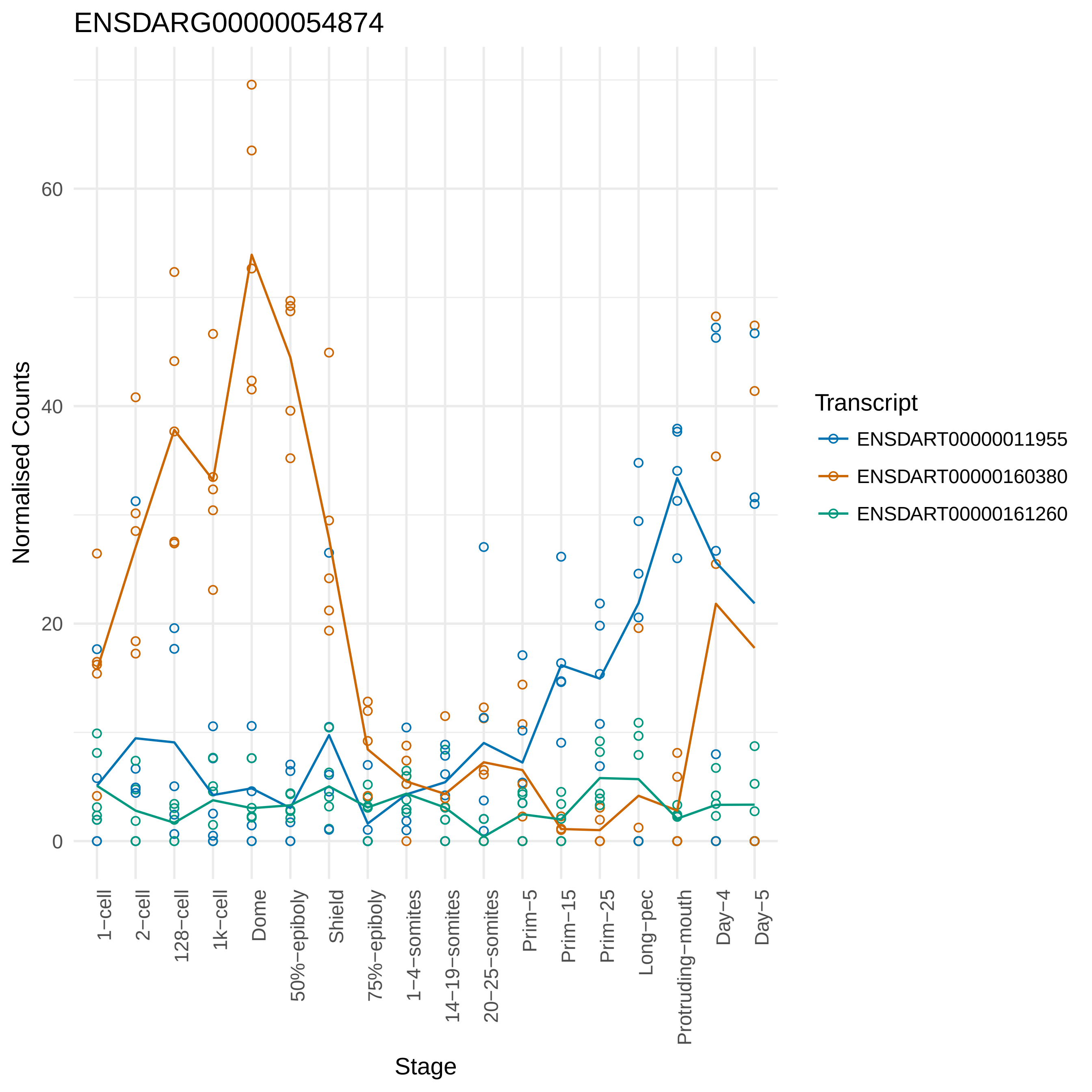
ENSDARG00000054874
Normalised Counts
60
40
20
0
1−cell
2−cell
128−cell
1k−cell
Dome
50%−epiboly
Shield
75%−epiboly
1−4−somites
14−19−somites
20−25−somites
Prim−5
Prim−15
Prim−25
Long−pec
Protruding−mouth
Day−4
Day−5
Stage
Transcript
ENSDART00000011955
ENSDART00000160380
ENSDART00000161260

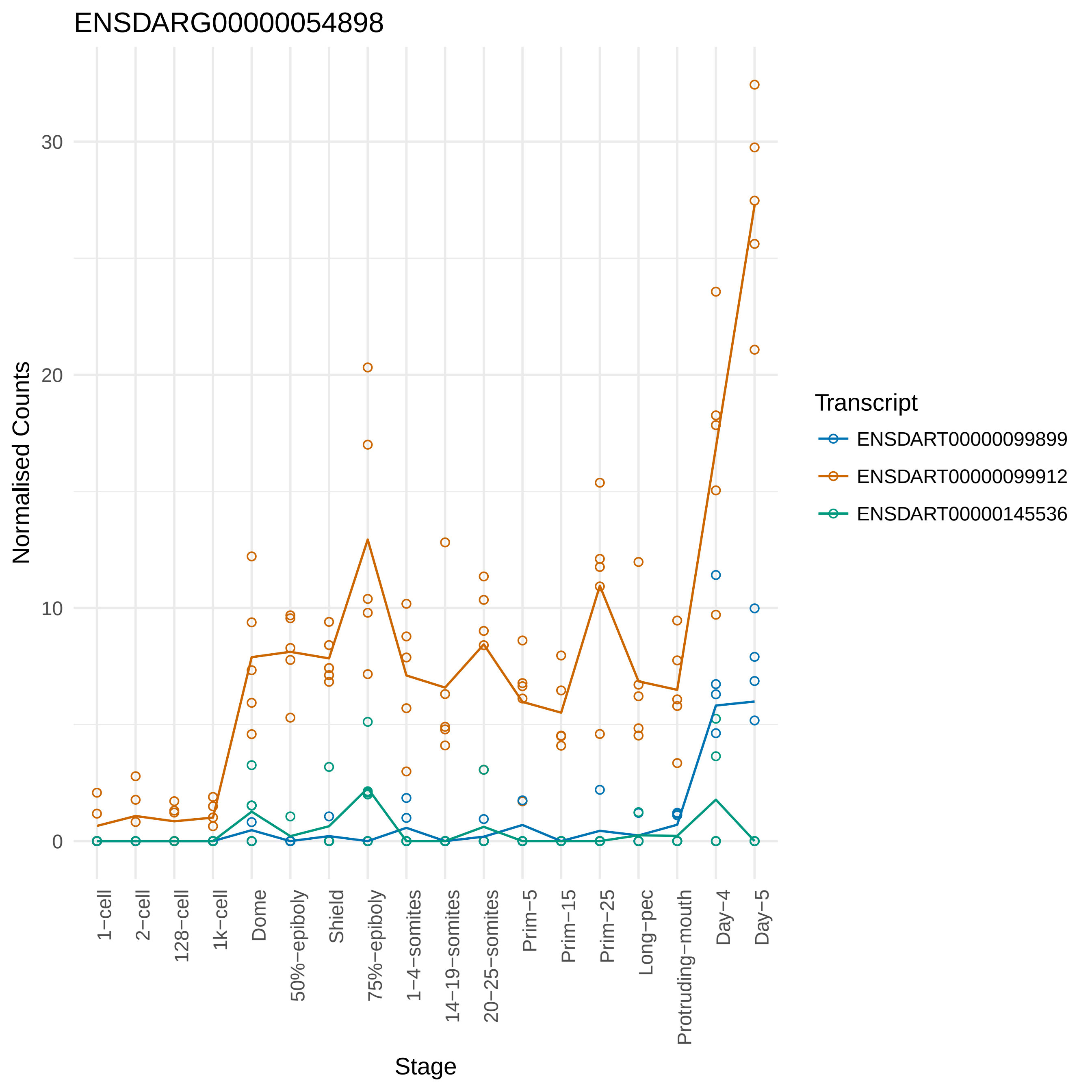
ENSDARG00000054898
Normalised Counts
30
20
10
0
1−cell
2−cell
128−cell
1k−cell
Dome
50%−epiboly
Shield
75%−epiboly
1−4−somites
14−19−somites
20−25−somites
Prim−5
Prim−15
Prim−25
Long−pec
Protruding−mouth
Day−4
Day−5
Stage
Transcript
ENSDART00000099899
ENSDART00000099912
ENSDART00000145536

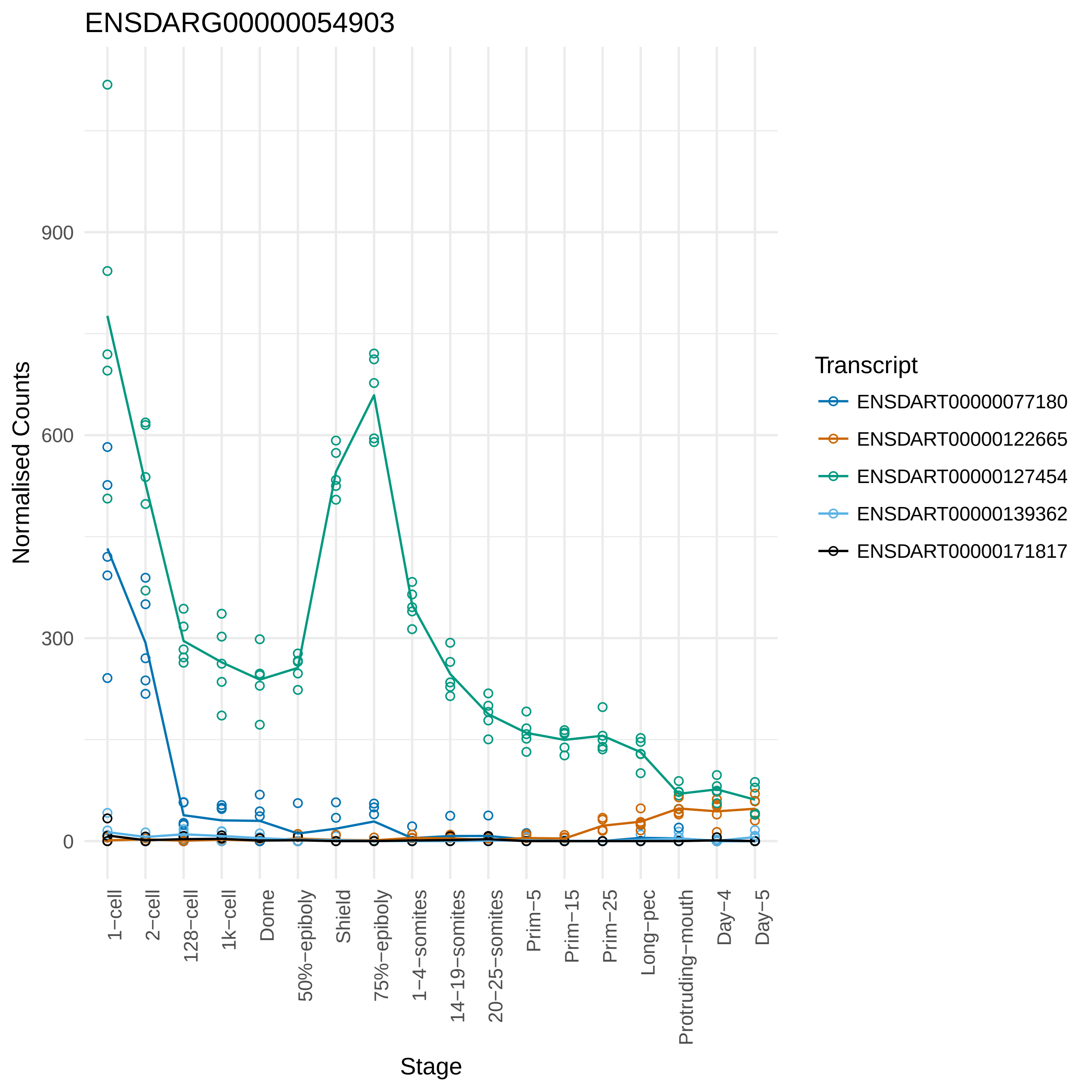
ENSDARG00000054903
Normalised Counts
900
600
300
0
1−cell
2−cell
128−cell
1k−cell
Dome
50%−epiboly
Shield
75%−epiboly
1−4−somites
14−19−somites
20−25−somites
Prim−5
Prim−15
Prim−25
Long−pec
Protruding−mouth
Day−4
Day−5
Stage
Transcript
ENSDART00000077180
ENSDART00000122665
ENSDART00000127454
ENSDART00000139362
ENSDART00000171817

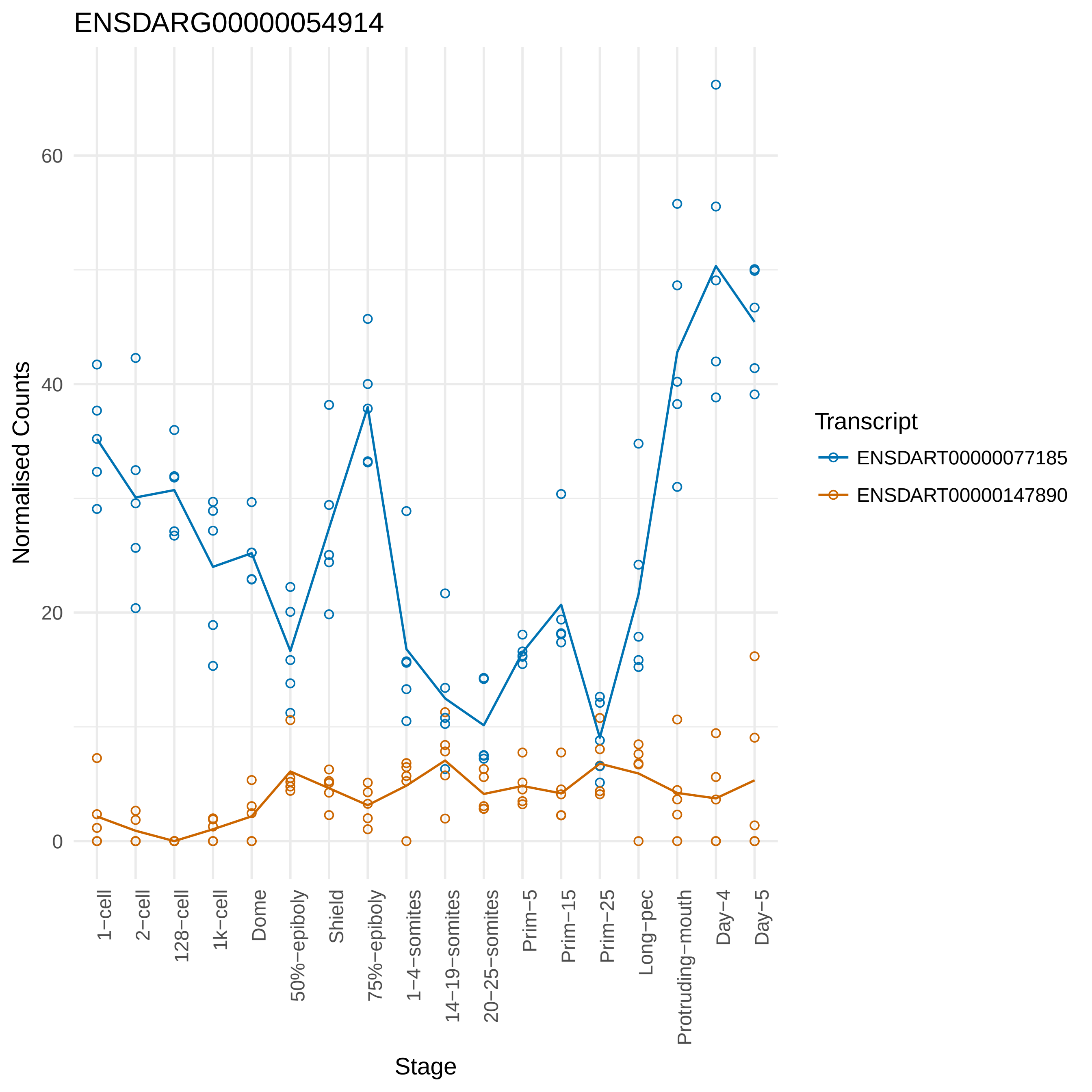

ENSDARG00000054914
Normalised Counts
60
40
20
0
1−cell
2−cell
128−cell
1k−cell
Dome
50%−epiboly
Shield
75%−epiboly
1−4−somites
14−19−somites
20−25−somites
Prim−5
Prim−15
Prim−25
Long−pec
Protruding−mouth
Day−4
Day−5
Stage
Transcript
ENSDART00000077185
ENSDART00000147890

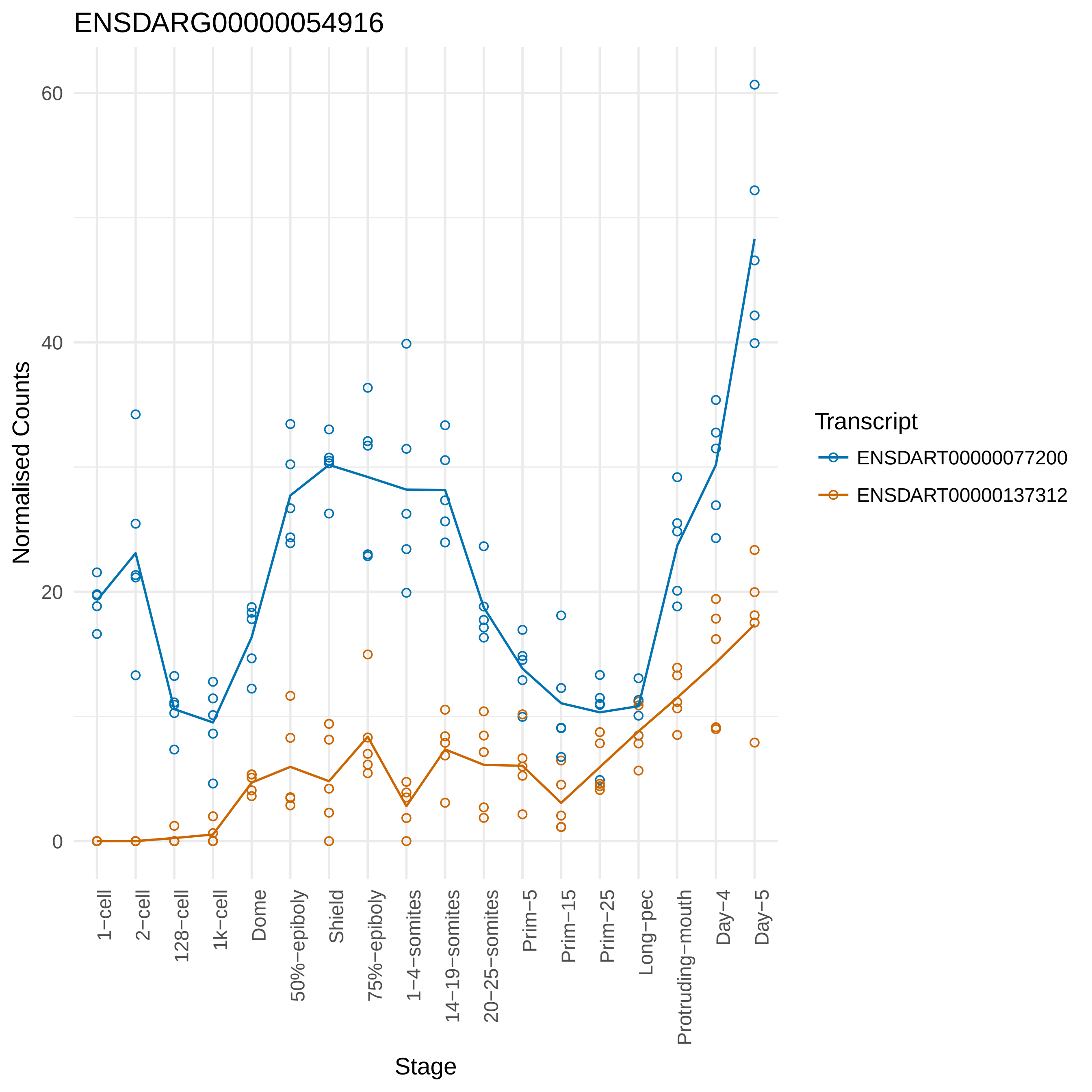

ENSDARG00000054916
Normalised Counts
0
20
40
60
1−cell
2−cell
128−cell
1k−cell
Dome
50%−epiboly
Shield
75%−epiboly
1−4−somites
14−19−somites
20−25−somites
Prim−5
Prim−15
Prim−25
Long−pec
Protruding−mouth
Day−4
Day−5
Stage
Transcript
ENSDART00000077200
ENSDART00000137312

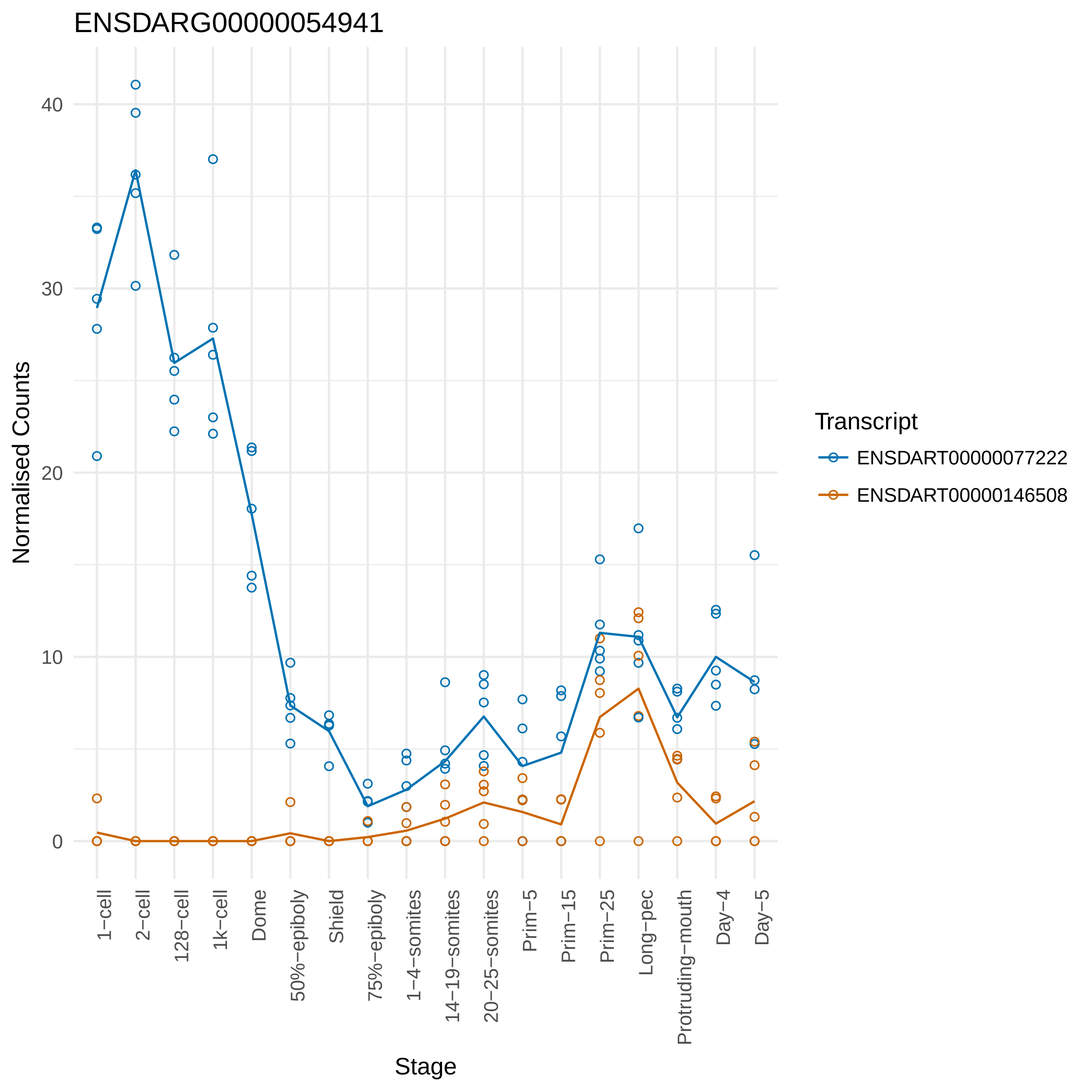
ENSDARG00000054941
Normalised Counts
0
10
20
30
40
Stage
1−cell
2−cell
128−cell
1k−cell
Dome
50%−epiboly
Shield
75%−epiboly
1−4−somites
14−19−somites
20−25−somites
Prim−5
Prim−15
Prim−25
Long−pec
Protruding−mouth
Day−4
Day−5
Transcript
ENSDART00000077222
ENSDART00000146508

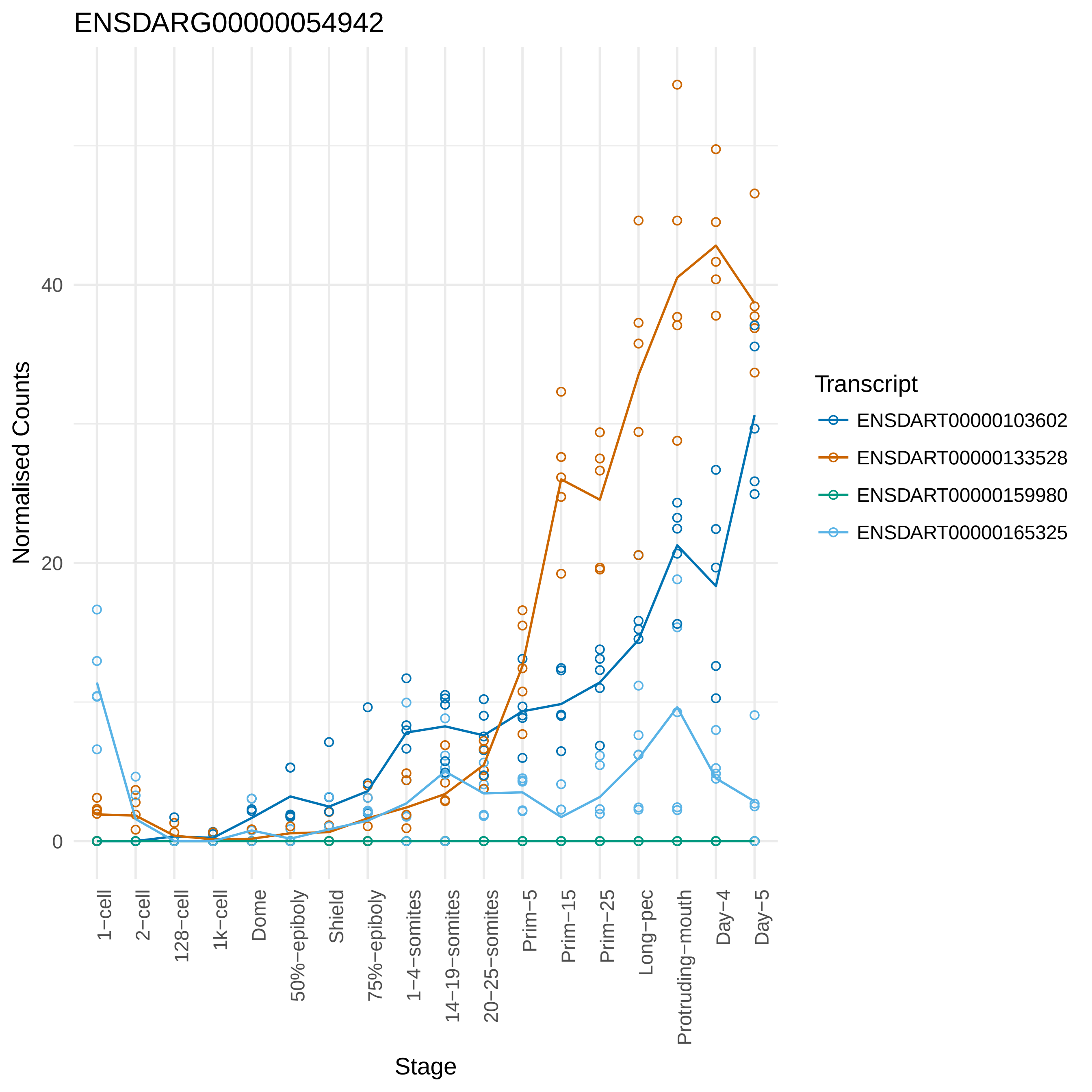

ENSDARG00000054942
Normalised Counts
0
20
40
1−cell
2−cell
128−cell
1k−cell
Dome
50%−epiboly
Shield
75%−epiboly
1−4−somites
14−19−somites
20−25−somites
Prim−5
Prim−15
Prim−25
Long−pec
Protruding−mouth
Day−4
Day−5
Stage
Transcript
ENSDART00000103602
ENSDART00000133528
ENSDART00000159980
ENSDART00000165325

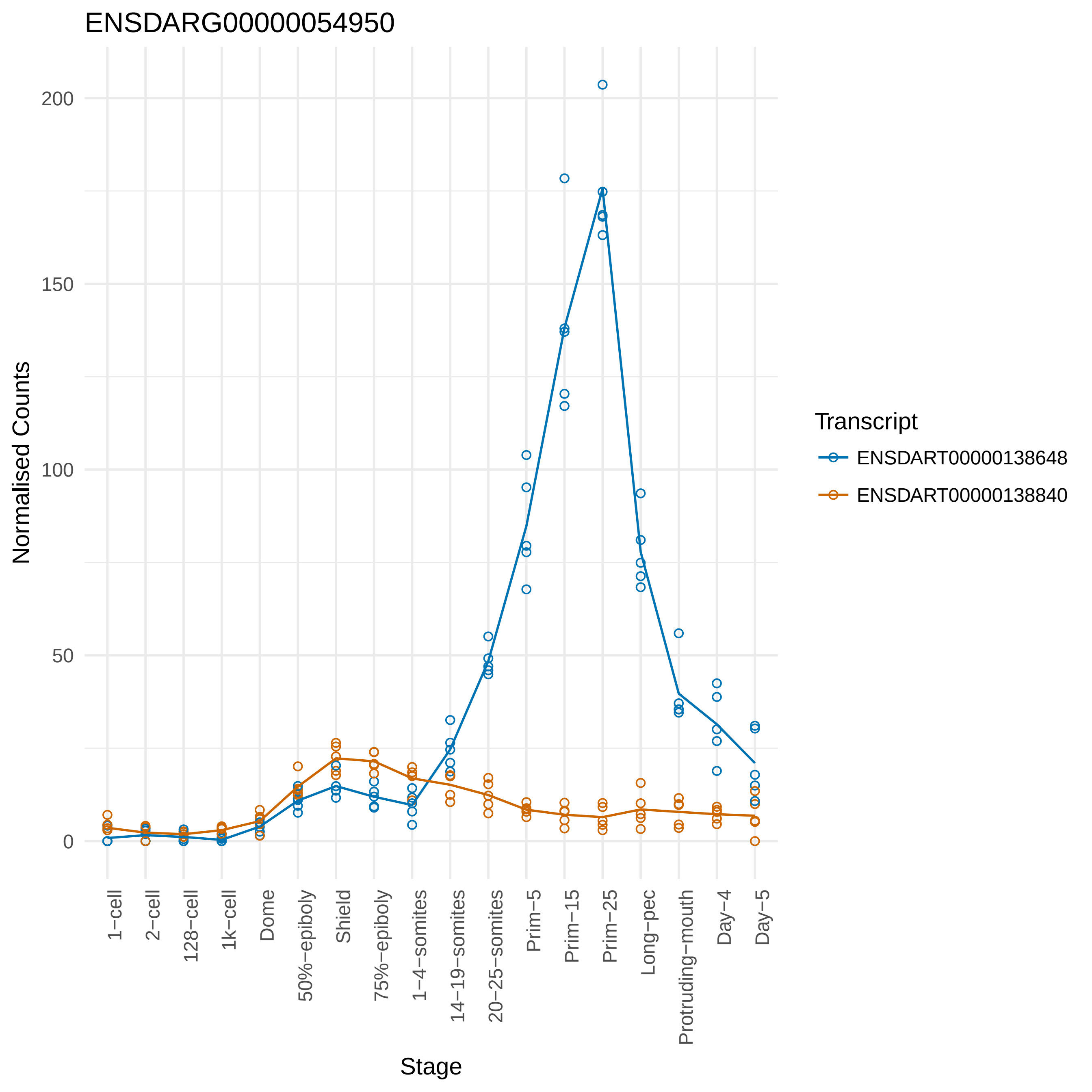

ENSDARG00000054950
Normalised Counts
200
150
100
50
0
1−cell
2−cell
128−cell
1k−cell
Dome
50%−epiboly
Shield
75%−epiboly
1−4−somites
14−19−somites
20−25−somites
Prim−5
Prim−15
Prim−25
Long−pec
Protruding−mouth
Day−4
Day−5
Stage
Transcript
ENSDART00000138648
ENSDART00000138840

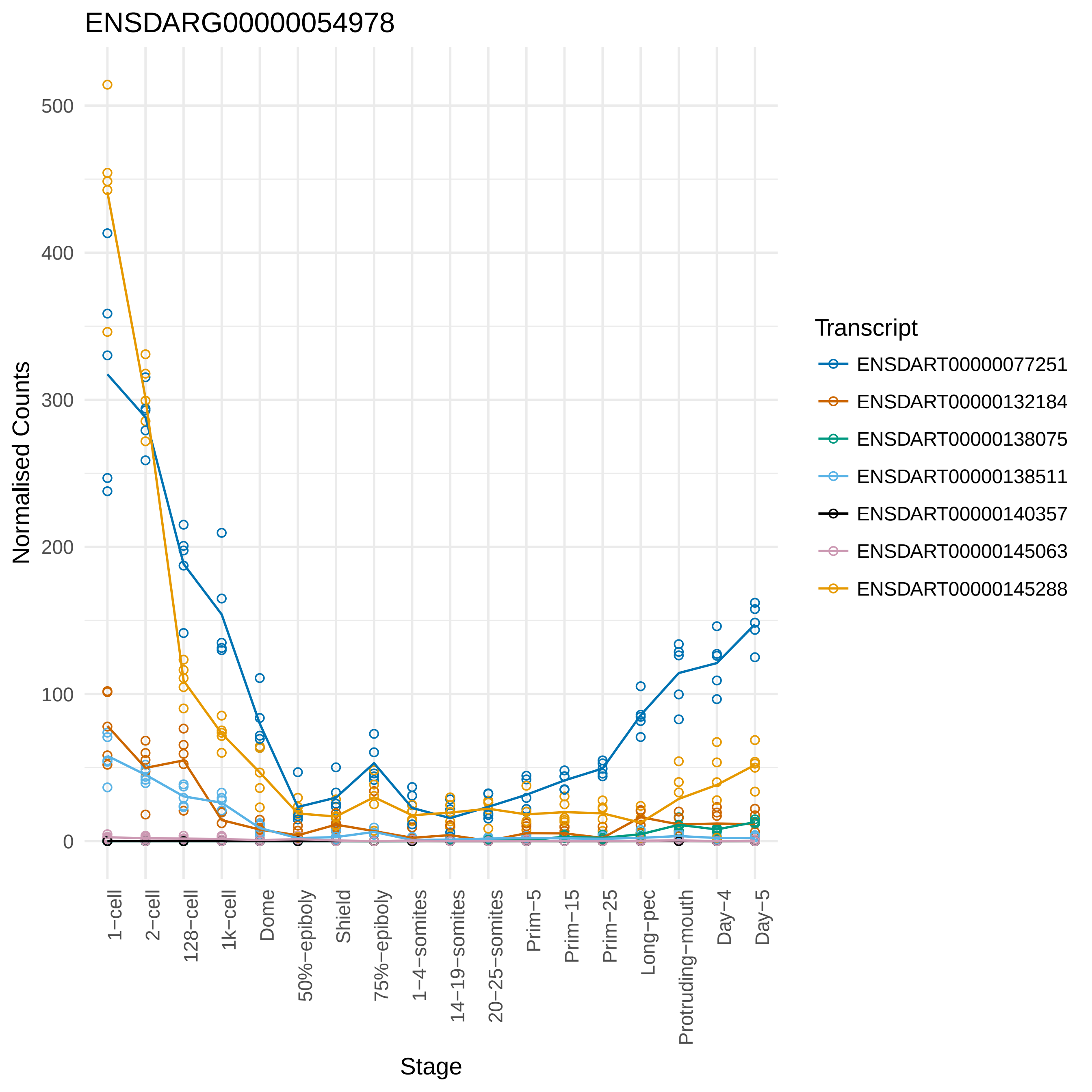

ENSDARG00000054978
Normalised Counts
500
400
300
200
100
0
1−cell
2−cell
128−cell
1k−cell
Dome
50%−epiboly
Shield
75%−epiboly
1−4−somites
14−19−somites
20−25−somites
Prim−5
Prim−15
Prim−25
Long−pec
Protruding−mouth
Day−4
Day−5
Stage
Transcript
ENSDART00000077251
ENSDART00000132184
ENSDART00000138075
ENSDART00000138511
ENSDART00000140357
ENSDART00000145063
ENSDART00000145288

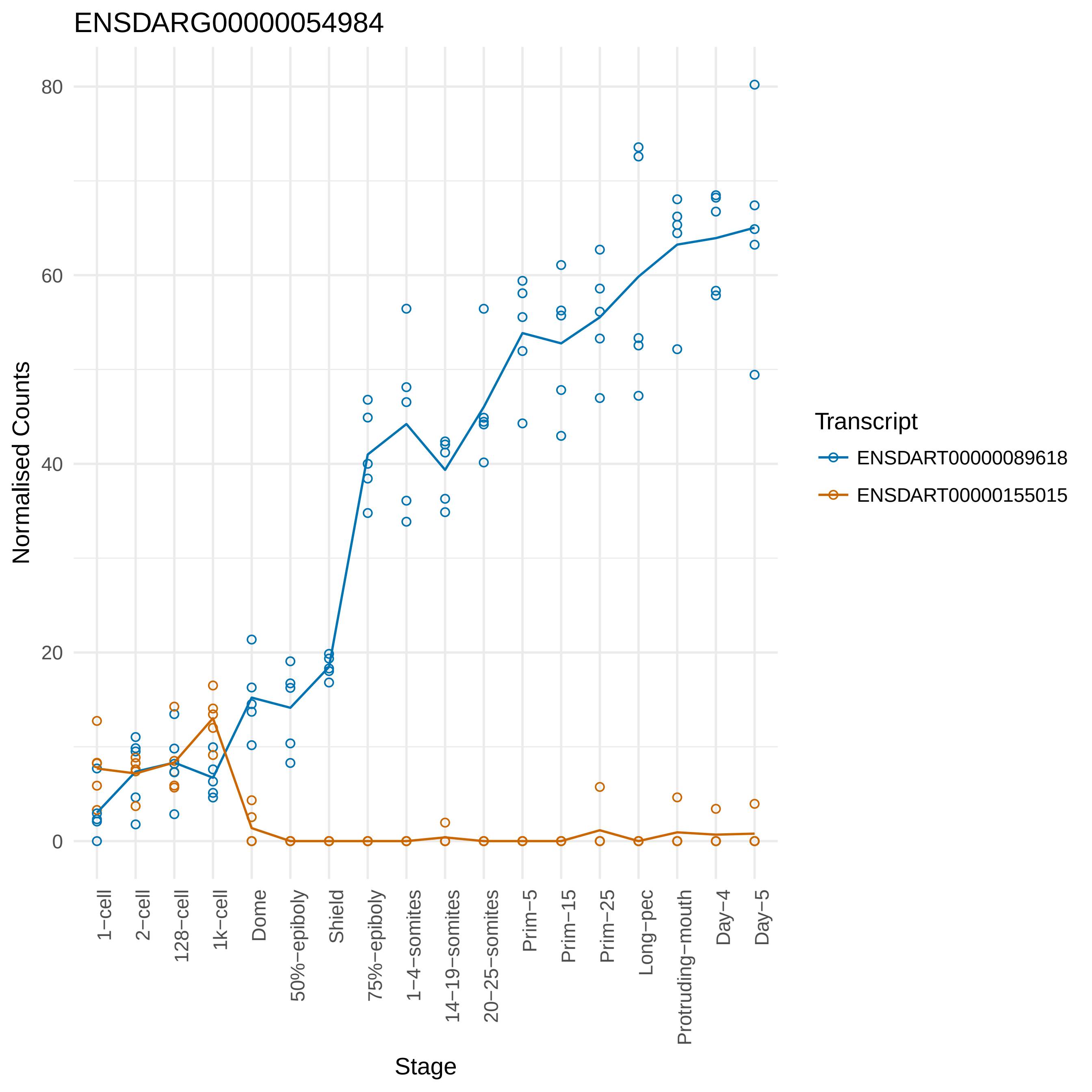

ENSDARG00000054984
Normalised Counts
0
20
40
60
80
Stage
1−cell
2−cell
128−cell
1k−cell
Dome
50%−epiboly
Shield
75%−epiboly
1−4−somites
14−19−somites
20−25−somites
Prim−5
Prim−15
Prim−25
Long−pec
Protruding−mouth
Day−4
Day−5
Transcript
ENSDART00000089618
ENSDART00000155015

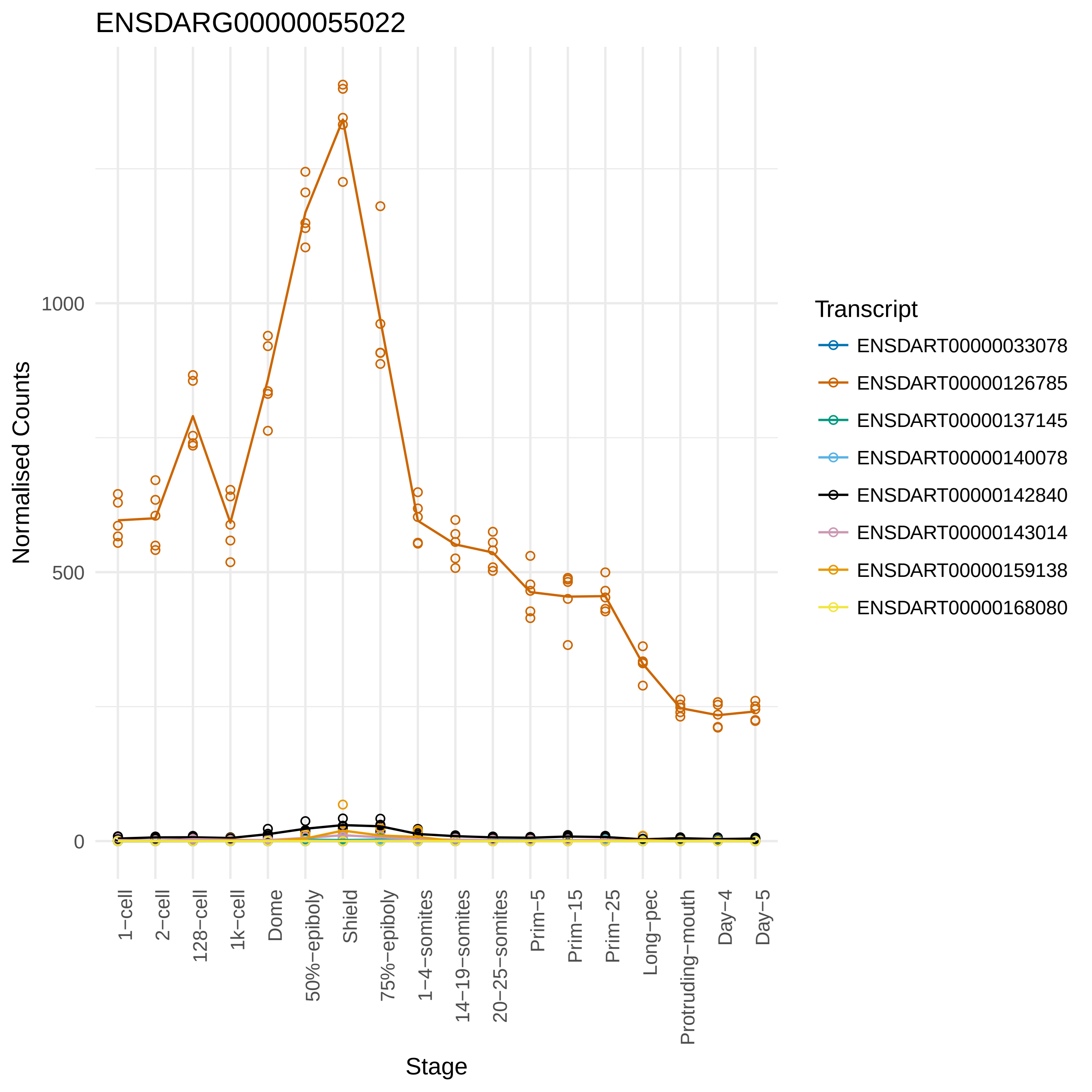

ENSDARG00000055022
Normalised Counts
1000
500
0
1−cell
2−cell
128−cell
1k−cell
Dome
50%−epiboly
Shield
75%−epiboly
1−4−somites
14−19−somites
20−25−somites
Prim−5
Prim−15
Prim−25
Long−pec
Protruding−mouth
Day−4
Day−5
Stage
Transcript
ENSDART00000033078
ENSDART00000126785
ENSDART00000137145
ENSDART00000140078
ENSDART00000142840
ENSDART00000143014
ENSDART00000159138
ENSDART00000168080

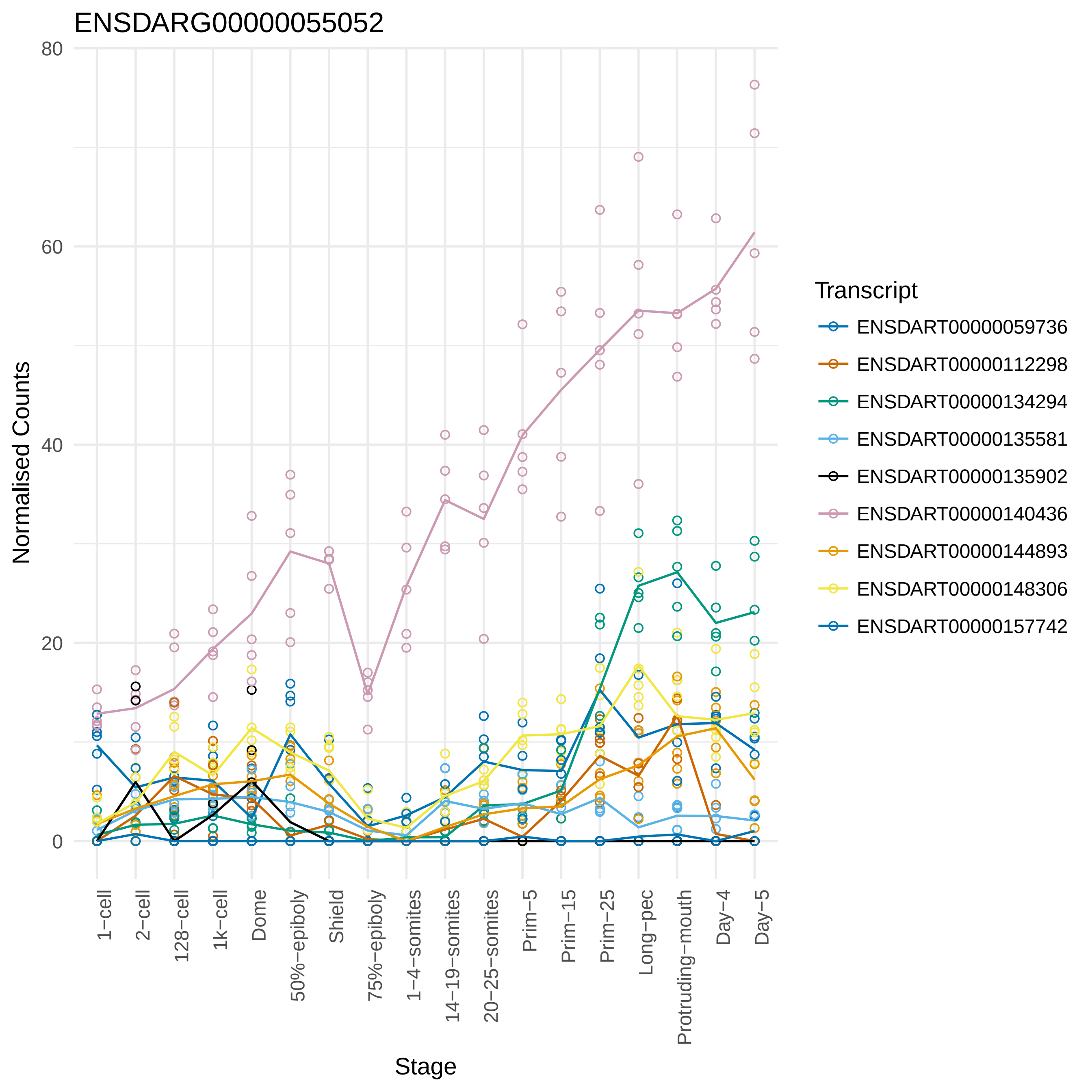
ENSDARG00000055052
Normalised Counts
0
20
40
60
80
1−cell
2−cell
128−cell
1k−cell
Dome
50%−epiboly
Shield
75%−epiboly
1−4−somites
14−19−somites
20−25−somites
Prim−5
Prim−15
Prim−25
Long−pec
Protruding−mouth
Day−4
Day−5
Stage
Transcript
ENSDART00000059736
ENSDART00000112298
ENSDART00000134294
ENSDART00000135581
ENSDART00000135902
ENSDART00000140436
ENSDART00000144893
ENSDART00000148306
ENSDART00000157742

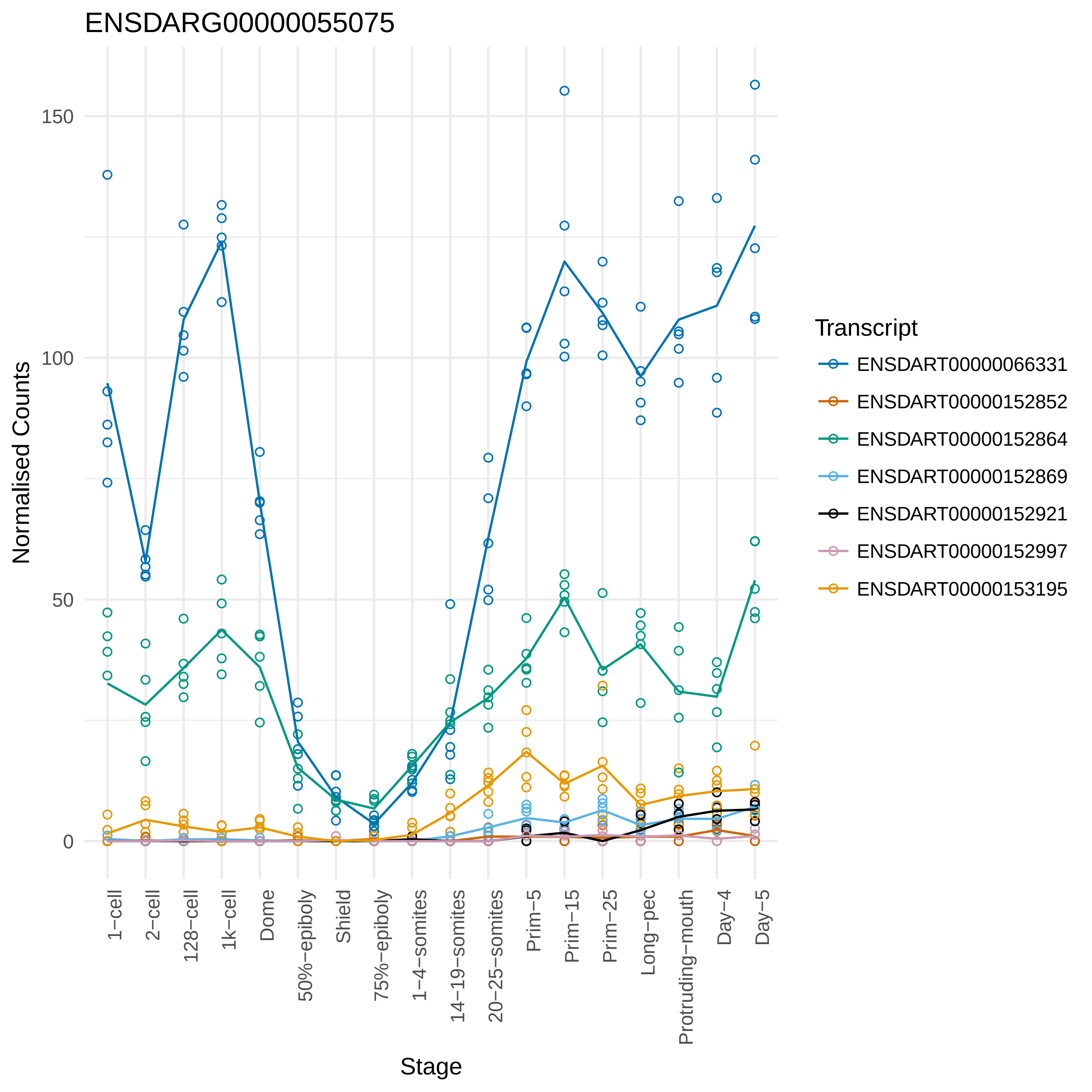

ENSDARG00000055075
Normalised Counts
150
100
50
0
1−cell
2−cell
128−cell
1k−cell
Dome
50%−epiboly
Shield
75%−epiboly
1−4−somites
14−19−somites
20−25−somites
Prim−5
Prim−15
Prim−25
Long−pec
Protruding−mouth
Day−4
Day−5
Stage
Transcript
ENSDART00000066331
ENSDART00000152852
ENSDART00000152864
ENSDART00000152869
ENSDART00000152921
ENSDART00000152997
ENSDART00000153195

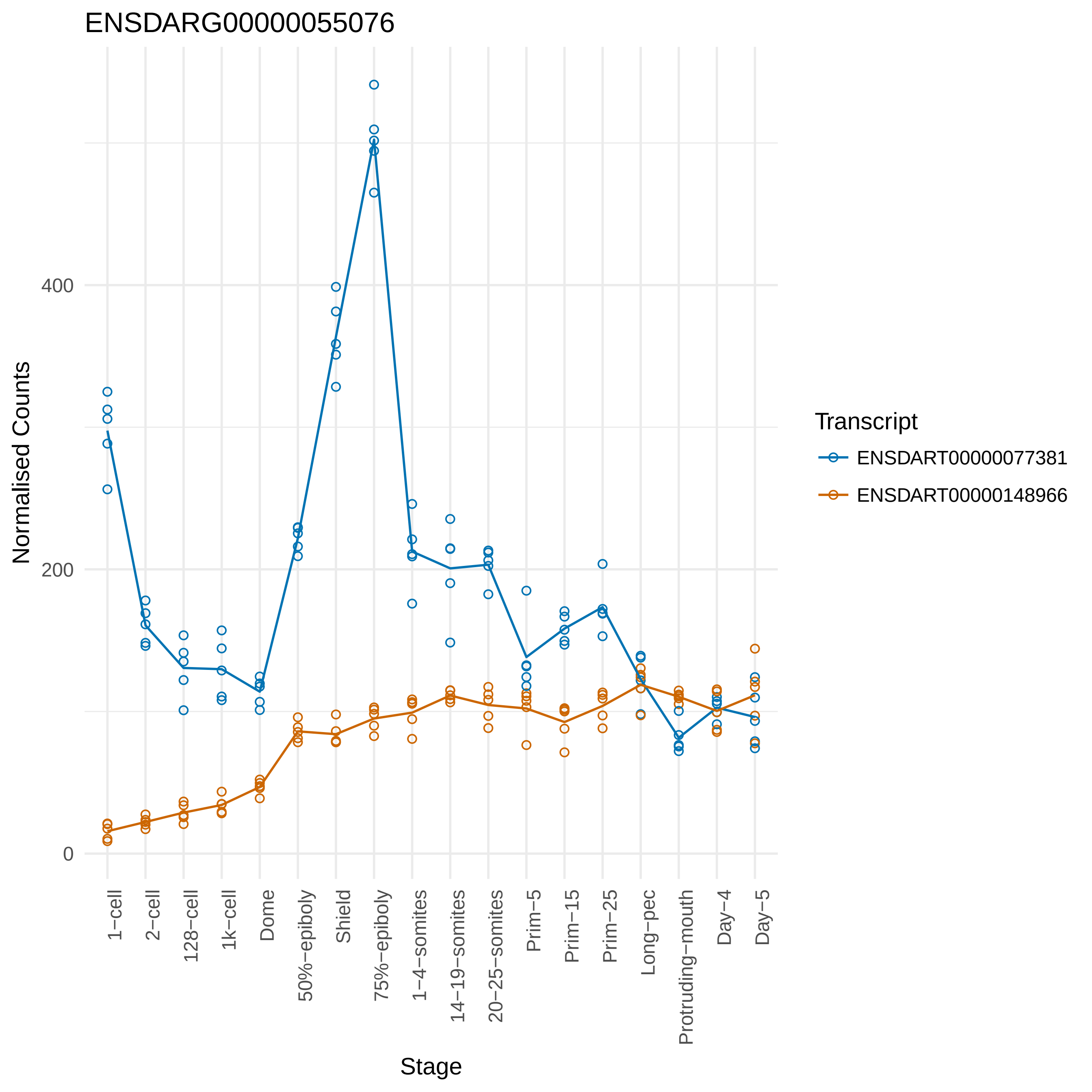
ENSDARG00000055076
Normalised Counts
0
200
400
Stage
1−cell
2−cell
128−cell
1k−cell
Dome
50%−epiboly
Shield
75%−epiboly
1−4−somites
14−19−somites
20−25−somites
Prim−5
Prim−15
Prim−25
Long−pec
Protruding−mouth
Day−4
Day−5
Transcript
ENSDART00000077381
ENSDART00000148966

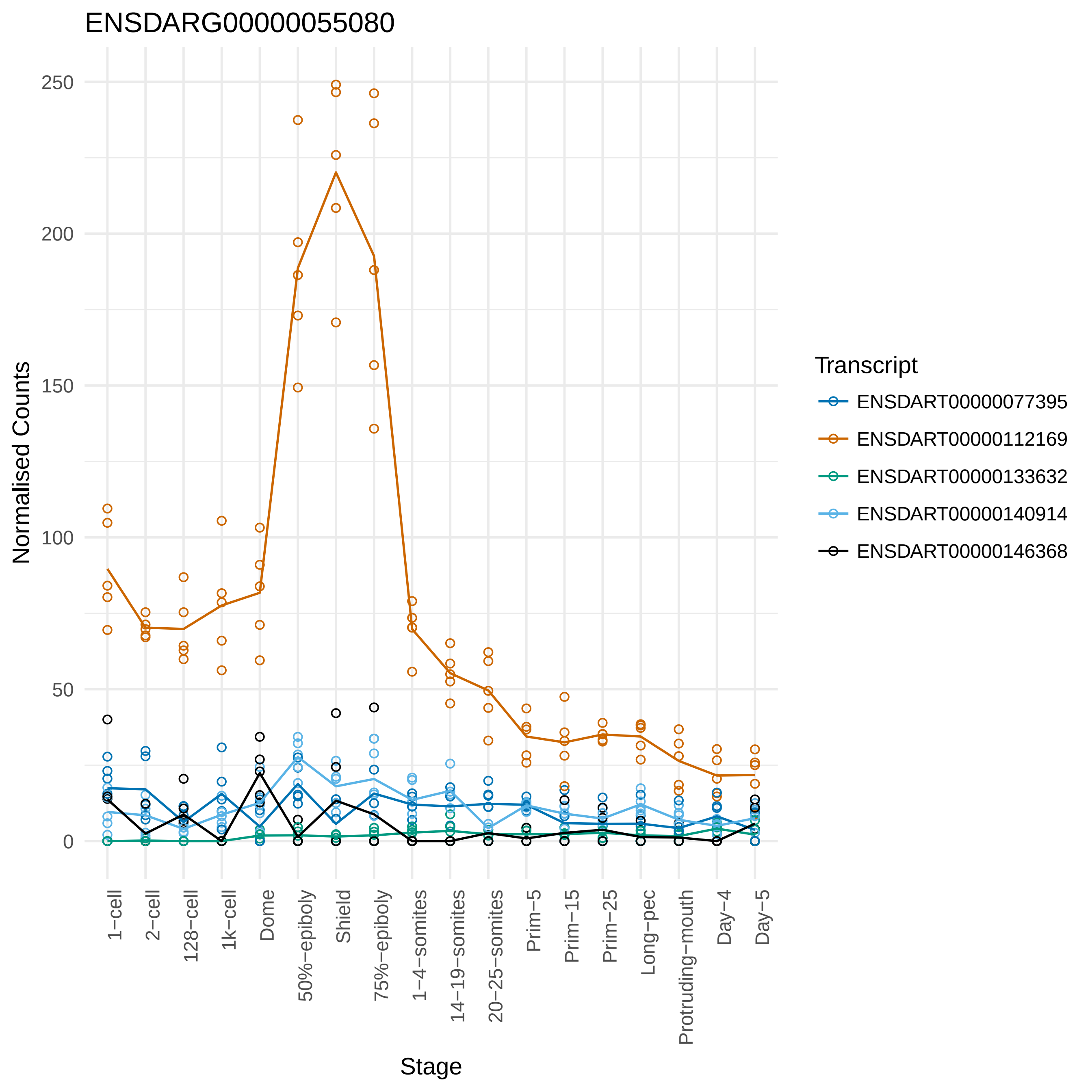
ENSDARG00000055080
Normalised Counts
250
200
150
100
50
0
1−cell
2−cell
128−cell
1k−cell
Dome
50%−epiboly
Shield
75%−epiboly
1−4−somites
14−19−somites
20−25−somites
Prim−5
Prim−15
Prim−25
Long−pec
Protruding−mouth
Day−4
Day−5
Stage
Transcript
ENSDART00000077395
ENSDART00000112169
ENSDART00000133632
ENSDART00000140914
ENSDART00000146368

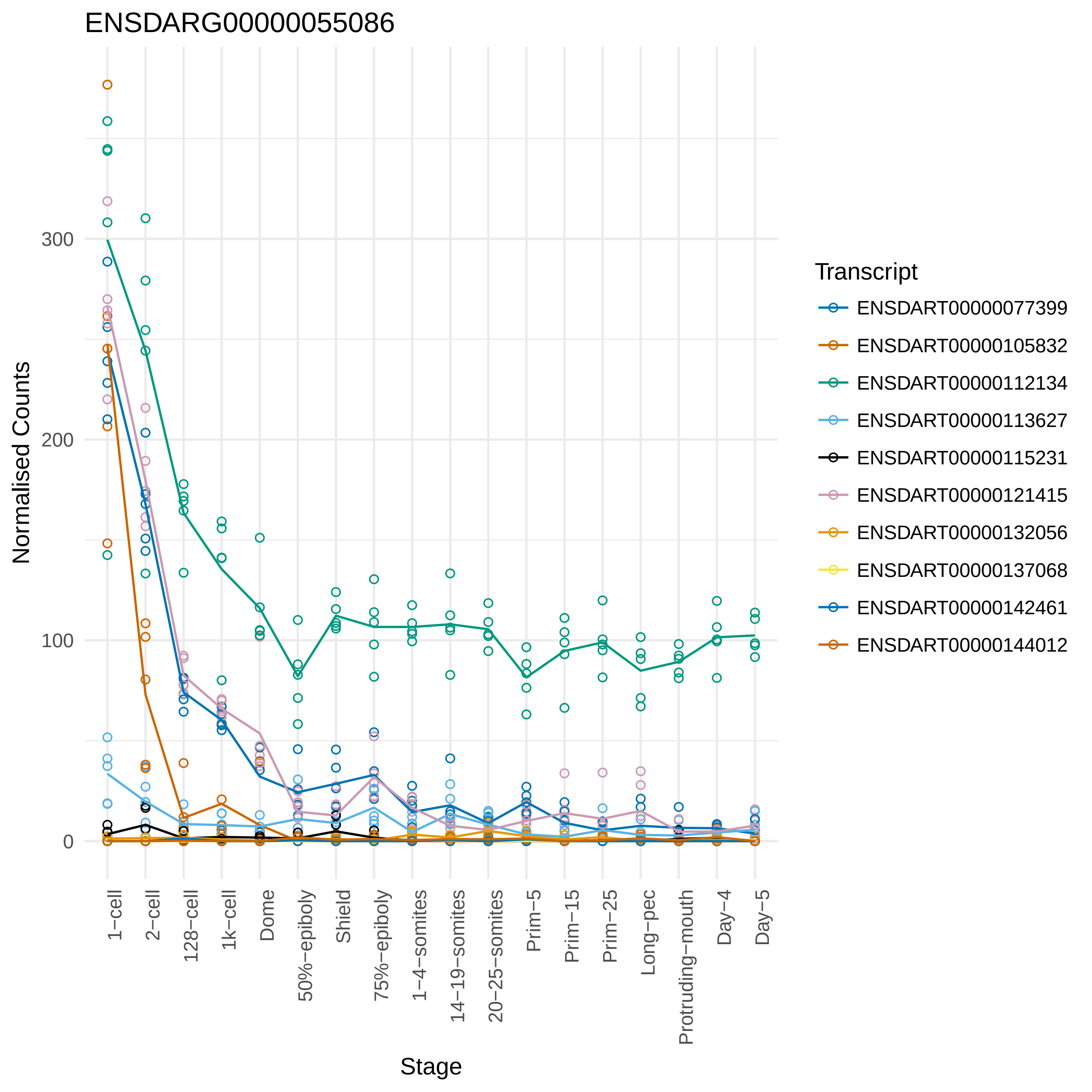

ENSDARG00000055086
Normalised Counts
300
200
100
0
1−cell
2−cell
128−cell
1k−cell
Dome
50%−epiboly
Shield
75%−epiboly
1−4−somites
14−19−somites
20−25−somites
Prim−5
Prim−15
Prim−25
Long−pec
Protruding−mouth
Day−4
Day−5
Stage
Transcript
ENSDART00000077399
ENSDART00000105832
ENSDART00000112134
ENSDART00000113627
ENSDART00000115231
ENSDART00000121415
ENSDART00000132056
ENSDART00000137068
ENSDART00000142461
ENSDART00000144012

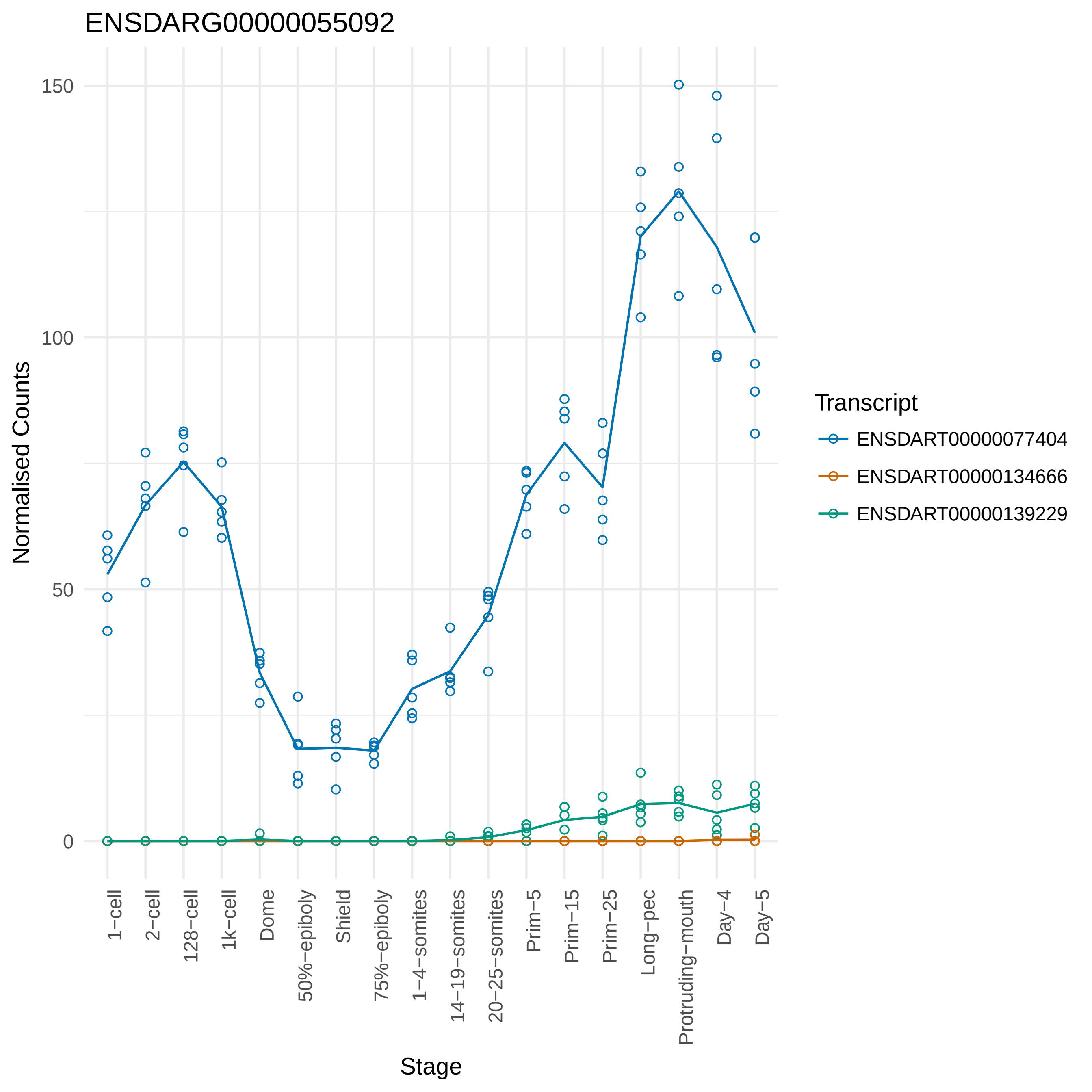
ENSDARG00000055092
Normalised Counts
150
100
50
0
Stage
1−cell
2−cell
128−cell
1k−cell
Dome
50%−epiboly
Shield
75%−epiboly
1−4−somites
14−19−somites
20−25−somites
Prim−5
Prim−15
Prim−25
Long−pec
Protruding−mouth
Day−4
Day−5
Transcript
ENSDART00000077404
ENSDART00000134666
ENSDART00000139229

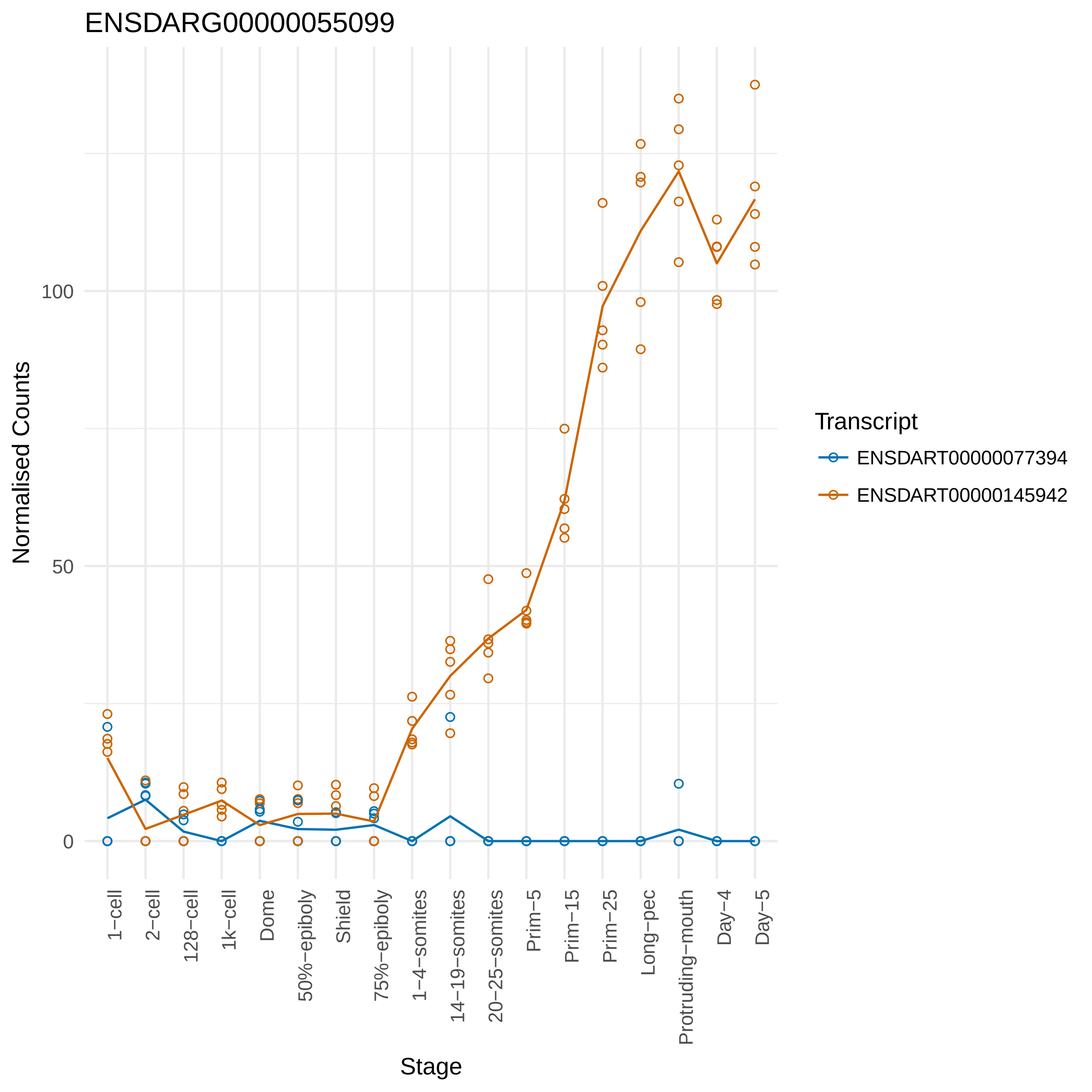

ENSDARG00000055099
Normalised Counts
100
50
0
1−cell
2−cell
128−cell
1k−cell
Dome
50%−epiboly
Shield
75%−epiboly
1−4−somites
14−19−somites
20−25−somites
Prim−5
Prim−15
Prim−25
Long−pec
Protruding−mouth
Day−4
Day−5
Stage
Transcript
ENSDART00000077394
ENSDART00000145942

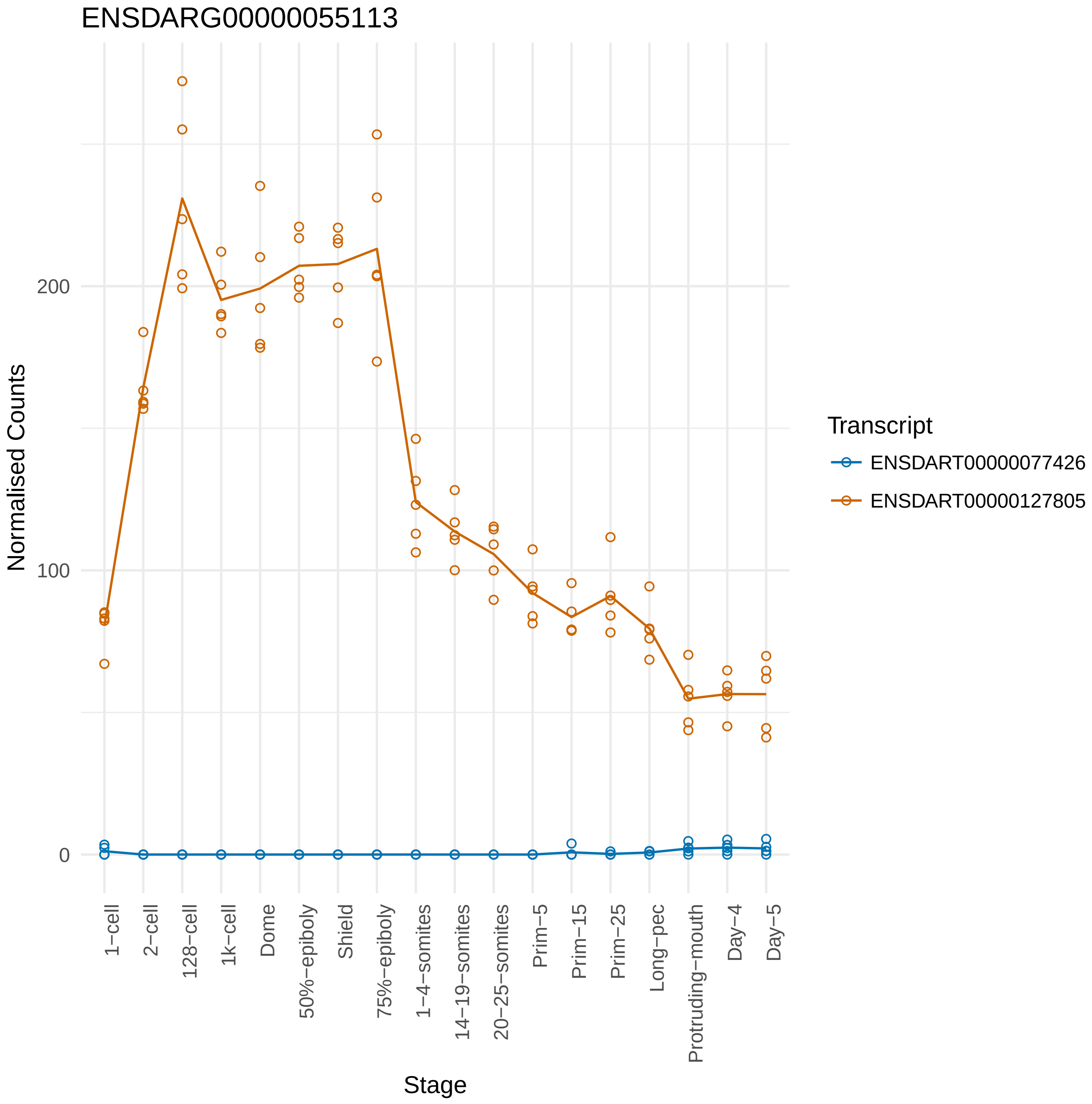

ENSDARG00000055113
Normalised Counts
200
100
0
1−cell
2−cell
128−cell
1k−cell
Dome
50%−epiboly
Shield
75%−epiboly
1−4−somites
14−19−somites
20−25−somites
Prim−5
Prim−15
Prim−25
Long−pec
Protruding−mouth
Day−4
Day−5
Stage
Transcript
ENSDART00000077426
ENSDART00000127805

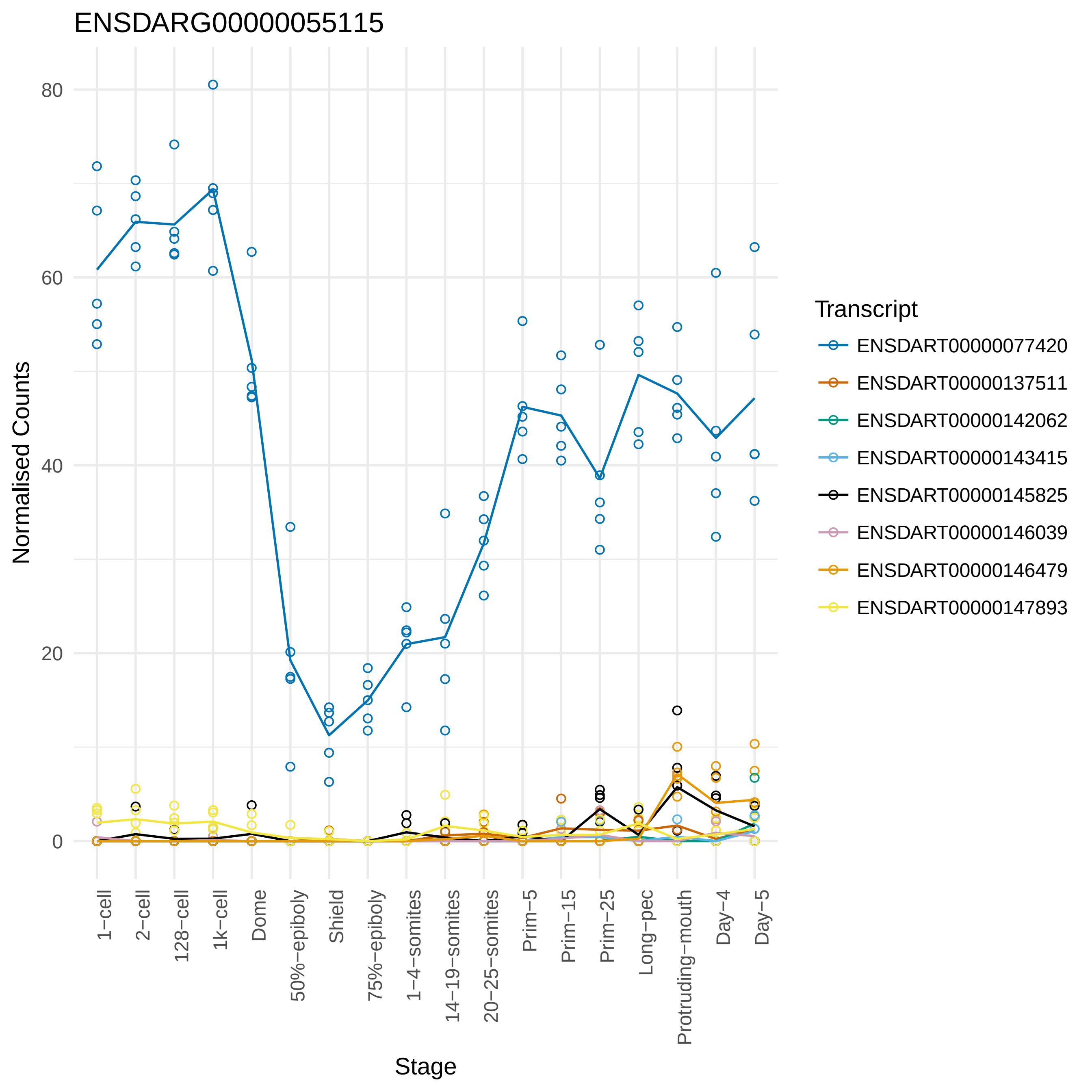

ENSDARG00000055115
Normalised Counts
0
20
40
60
80
Stage
1−cell
2−cell
128−cell
1k−cell
Dome
50%−epiboly
Shield
75%−epiboly
1−4−somites
14−19−somites
20−25−somites
Prim−5
Prim−15
Prim−25
Long−pec
Protruding−mouth
Day−4
Day−5
Transcript
ENSDART00000077420
ENSDART00000137511
ENSDART00000142062
ENSDART00000143415
ENSDART00000145825
ENSDART00000146039
ENSDART00000146479
ENSDART00000147893

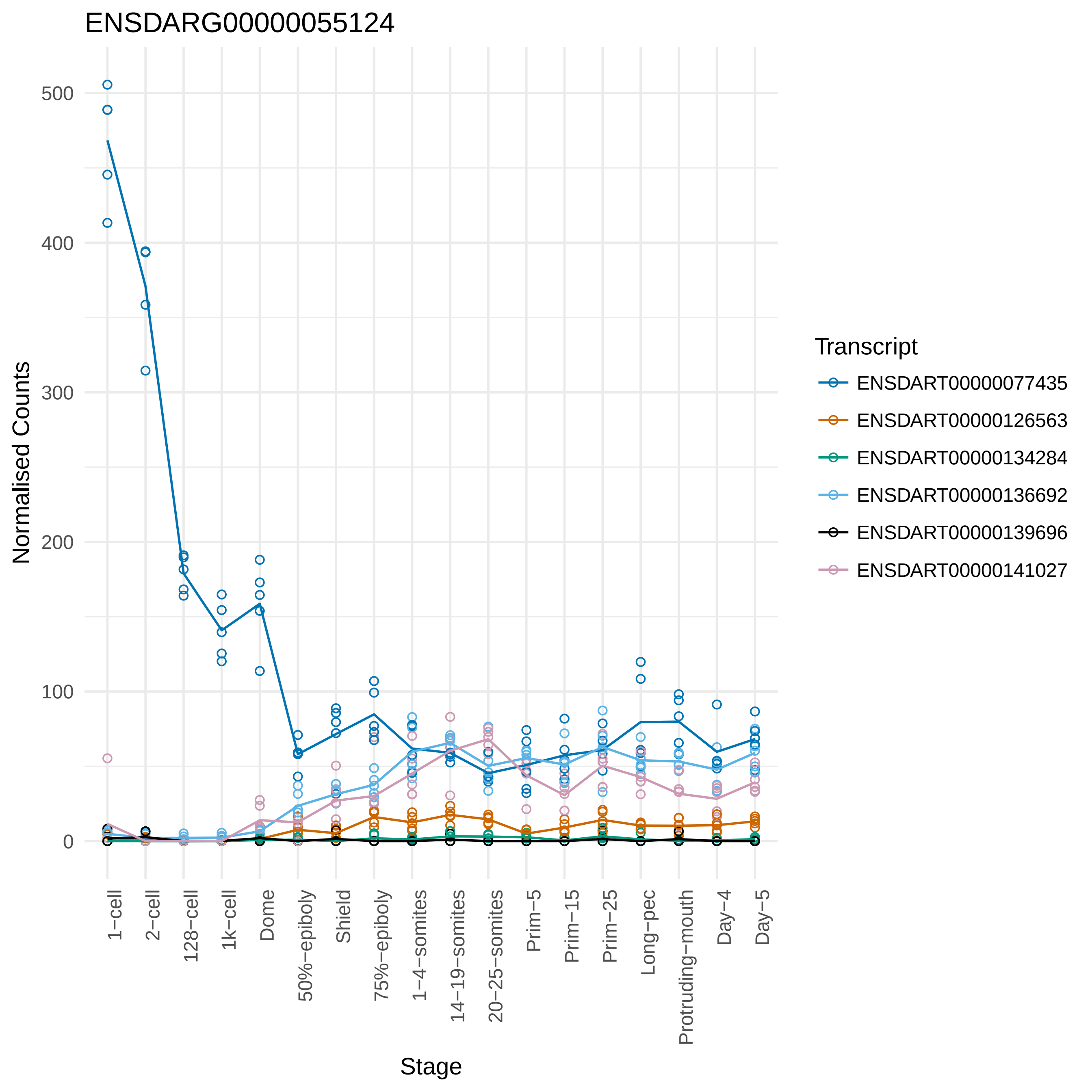

ENSDARG00000055124
Normalised Counts
500
400
300
200
100
0
1−cell
2−cell
128−cell
1k−cell
Dome
50%−epiboly
Shield
75%−epiboly
1−4−somites
14−19−somites
20−25−somites
Prim−5
Prim−15
Prim−25
Long−pec
Protruding−mouth
Day−4
Day−5
Stage
Transcript
ENSDART00000077435
ENSDART00000126563
ENSDART00000134284
ENSDART00000136692
ENSDART00000139696
ENSDART00000141027

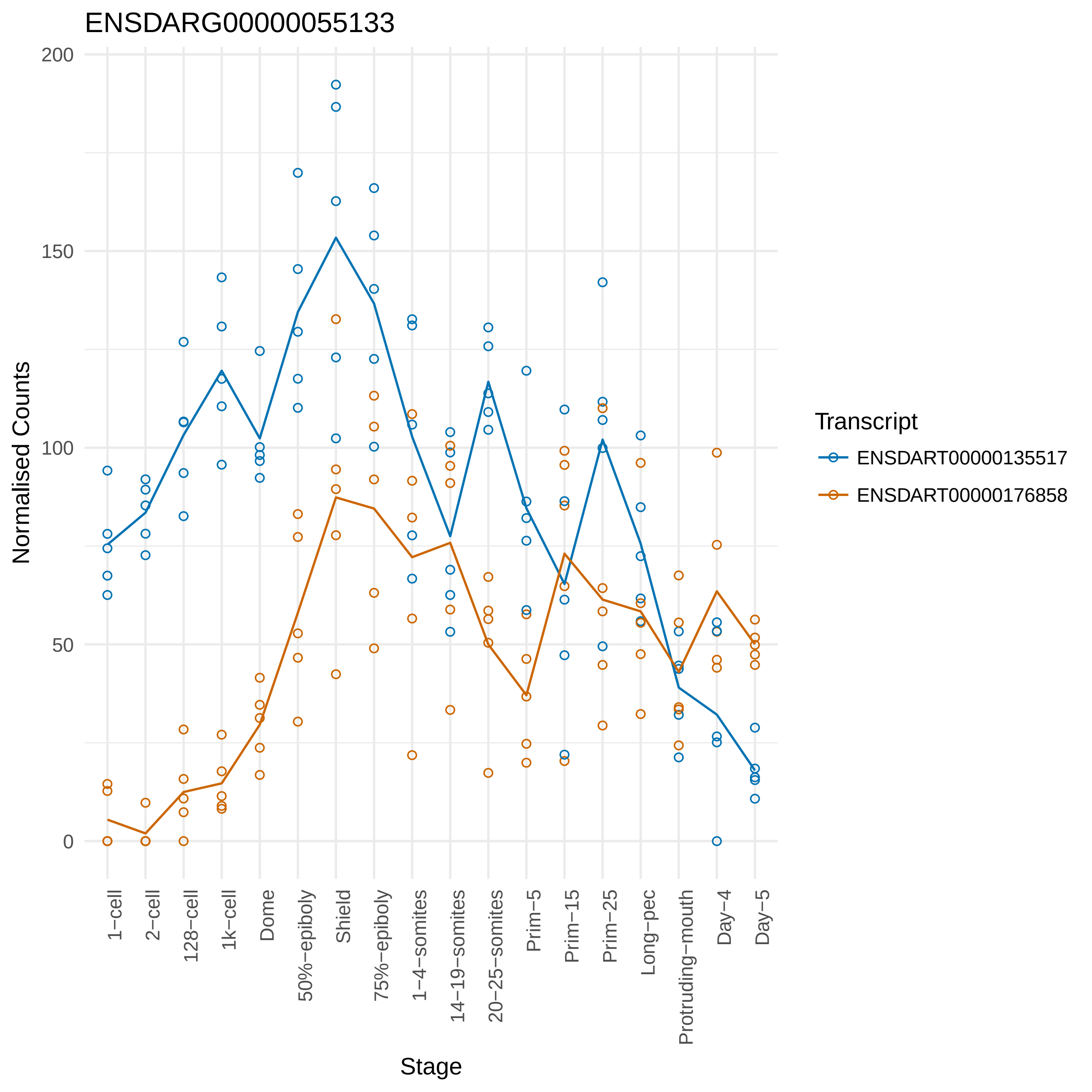

ENSDARG00000055133
Normalised Counts
200
150
100
50
0
1−cell
2−cell
128−cell
1k−cell
Dome
50%−epiboly
Shield
75%−epiboly
1−4−somites
14−19−somites
20−25−somites
Prim−5
Prim−15
Prim−25
Long−pec
Protruding−mouth
Day−4
Day−5
Stage
Transcript
ENSDART00000135517
ENSDART00000176858

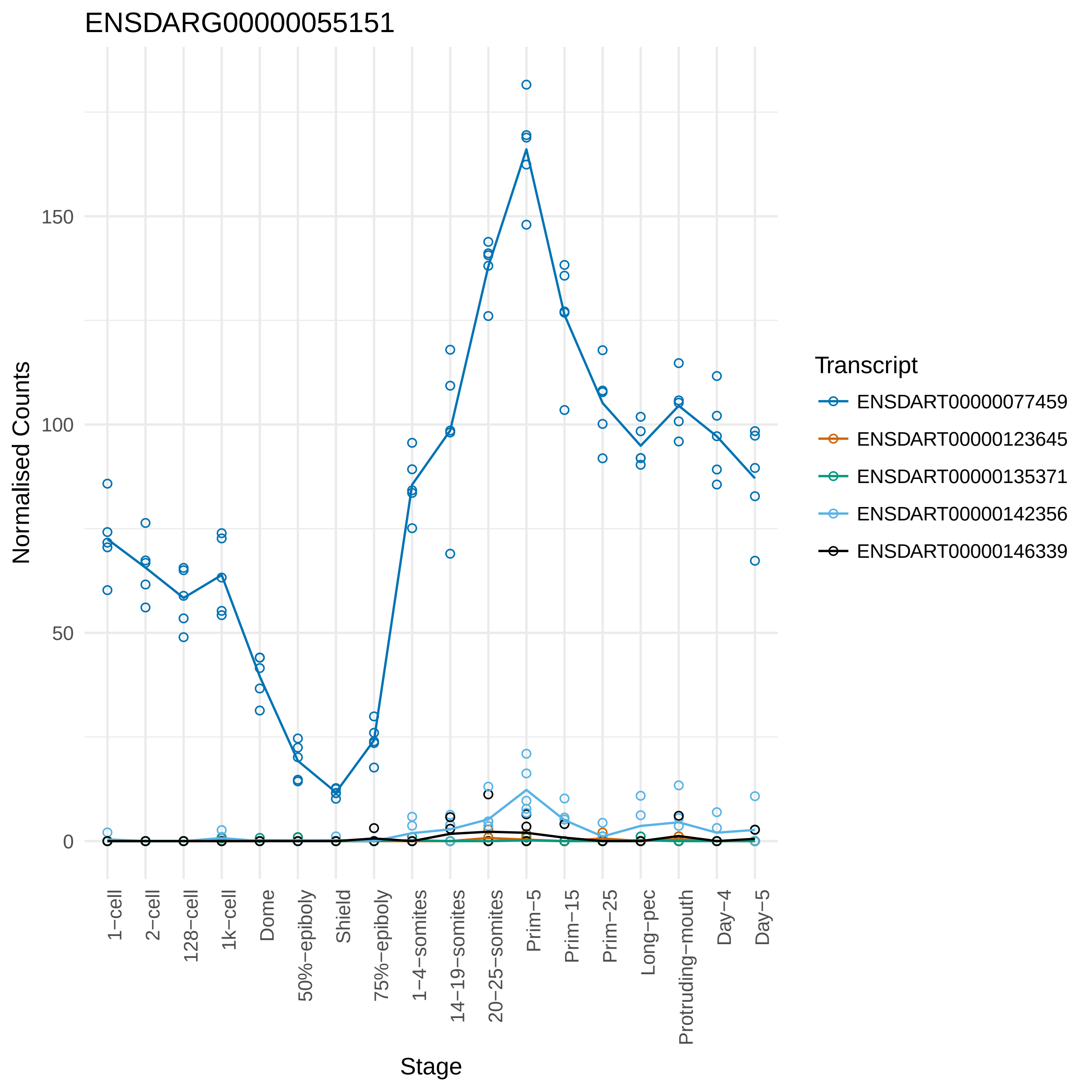

ENSDARG00000055151
Normalised Counts
150
100
50
0
1−cell
2−cell
128−cell
1k−cell
Dome
50%−epiboly
Shield
75%−epiboly
1−4−somites
14−19−somites
20−25−somites
Prim−5
Prim−15
Prim−25
Long−pec
Protruding−mouth
Day−4
Day−5
Stage
Transcript
ENSDART00000077459
ENSDART00000123645
ENSDART00000135371
ENSDART00000142356
ENSDART00000146339

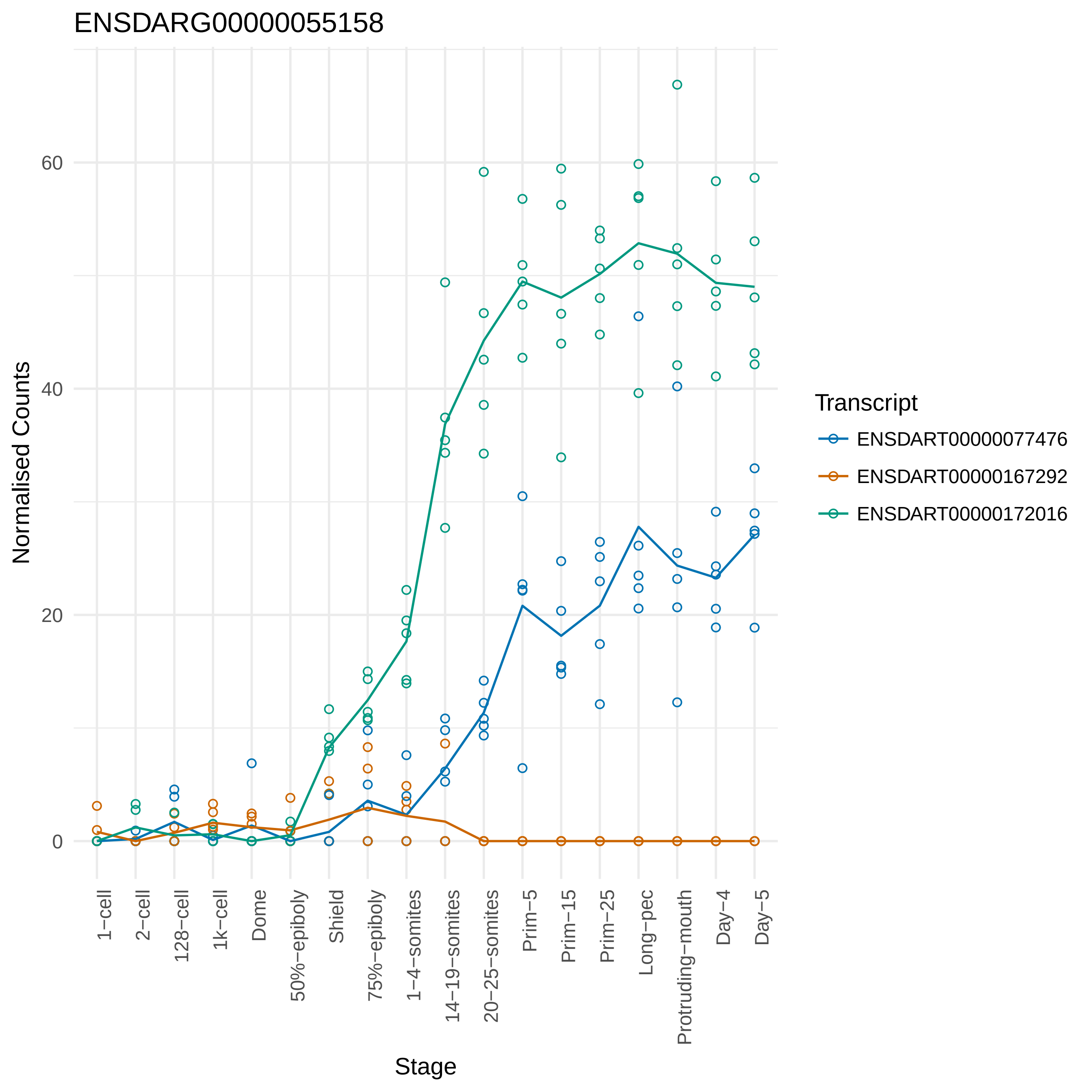
ENSDARG00000055158
Normalised Counts
0
20
40
60
Stage
1−cell
2−cell
128−cell
1k−cell
Dome
50%−epiboly
Shield
75%−epiboly
1−4−somites
14−19−somites
20−25−somites
Prim−5
Prim−15
Prim−25
Long−pec
Protruding−mouth
Day−4
Day−5
Transcript
ENSDART00000077476
ENSDART00000167292
ENSDART00000172016

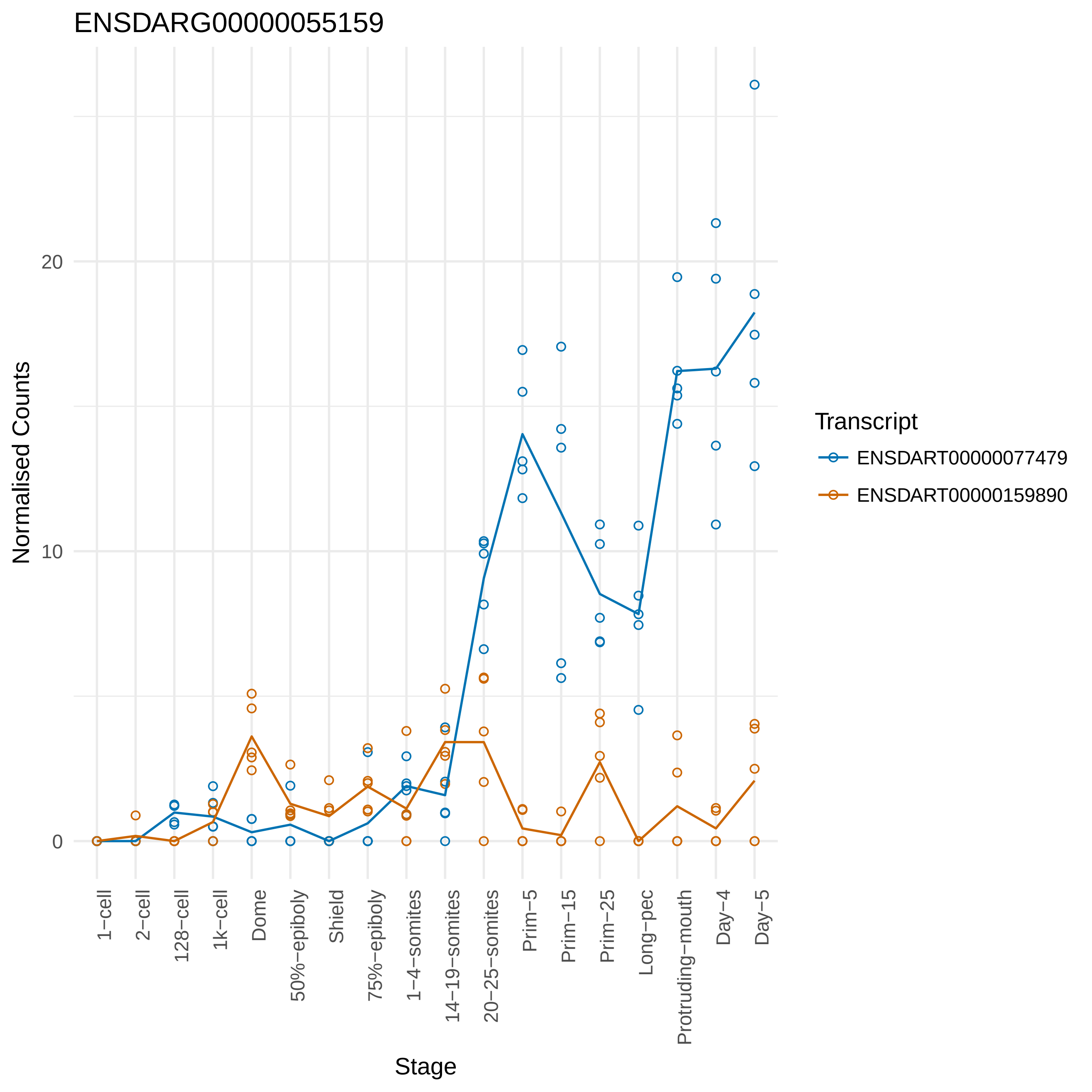
ENSDARG00000055159
Normalised Counts
20
10
0
1−cell
2−cell
128−cell
1k−cell
Dome
50%−epiboly
Shield
75%−epiboly
1−4−somites
14−19−somites
20−25−somites
Prim−5
Prim−15
Prim−25
Long−pec
Protruding−mouth
Day−4
Day−5
Stage
Transcript
ENSDART00000077479
ENSDART00000159890

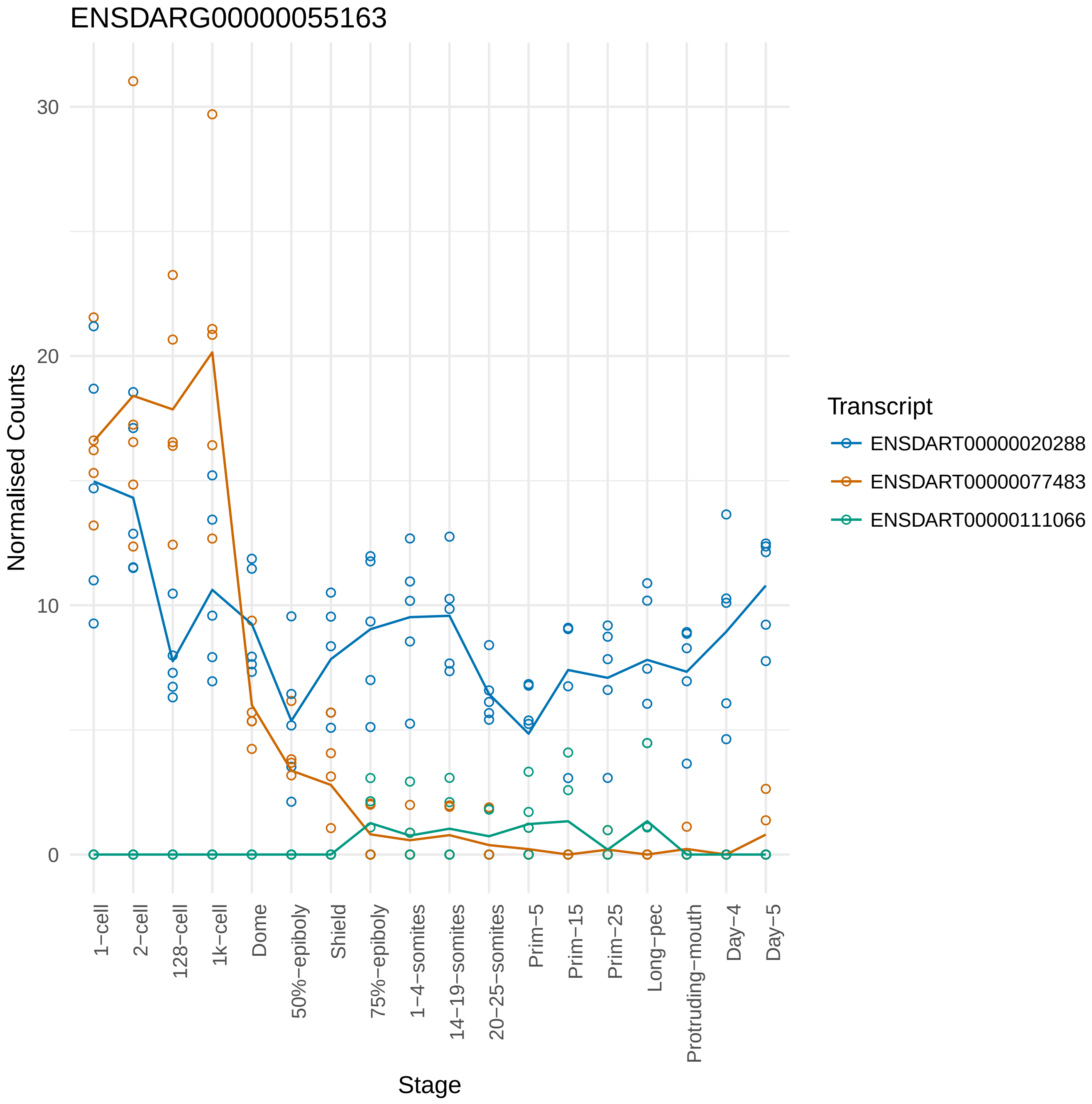
ENSDARG00000055163
Normalised Counts
30
20
10
0
1−cell
2−cell
128−cell
1k−cell
Dome
50%−epiboly
Shield
75%−epiboly
1−4−somites
14−19−somites
20−25−somites
Prim−5
Prim−15
Prim−25
Long−pec
Protruding−mouth
Day−4
Day−5
Stage
Transcript
ENSDART00000020288
ENSDART00000077483
ENSDART00000111066

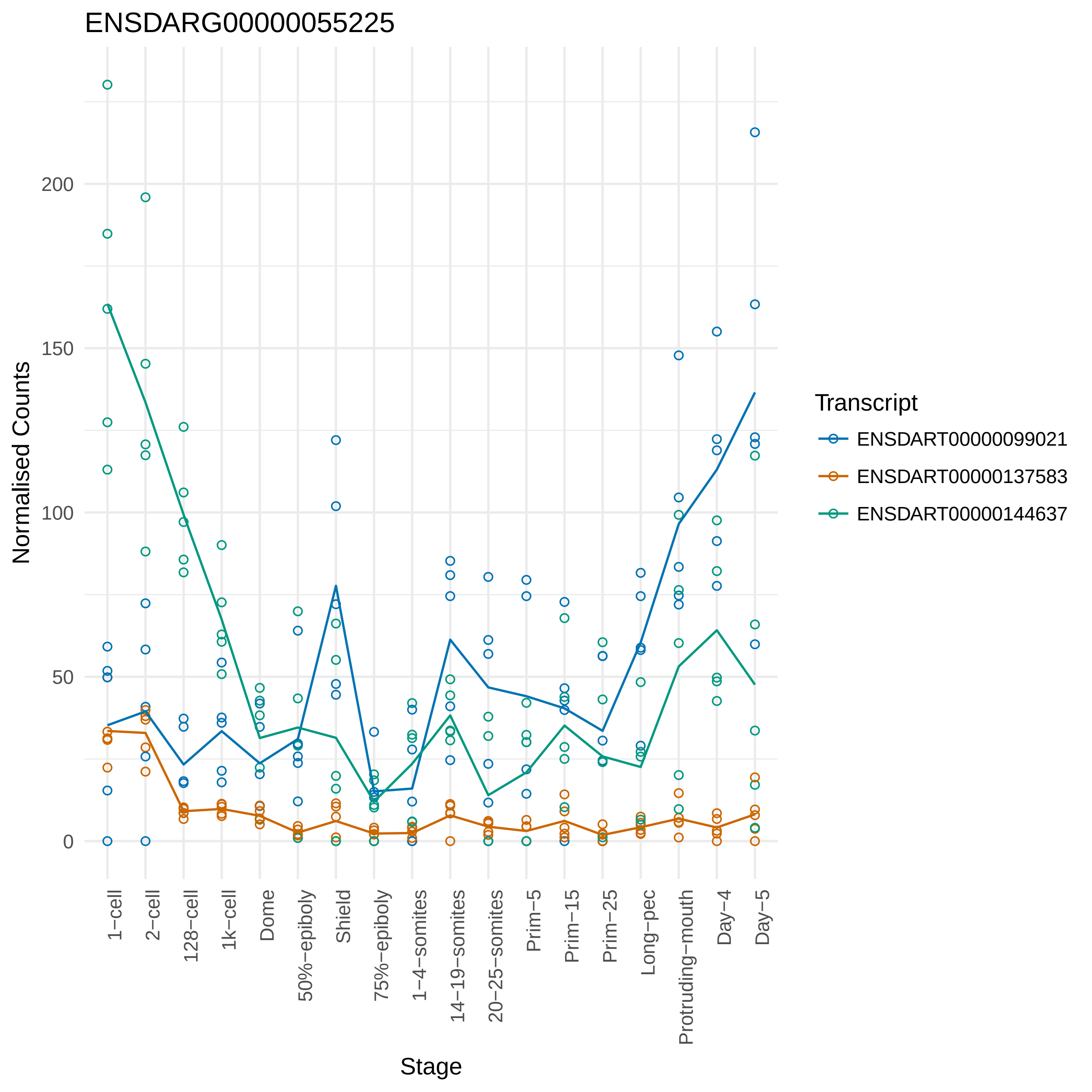
ENSDARG00000055225
Normalised Counts
0
50
100
150
200
Stage
1−cell
2−cell
128−cell
1k−cell
Dome
50%−epiboly
Shield
75%−epiboly
1−4−somites
14−19−somites
20−25−somites
Prim−5
Prim−15
Prim−25
Long−pec
Protruding−mouth
Day−4
Day−5
Transcript
ENSDART00000099021
ENSDART00000137583
ENSDART00000144637

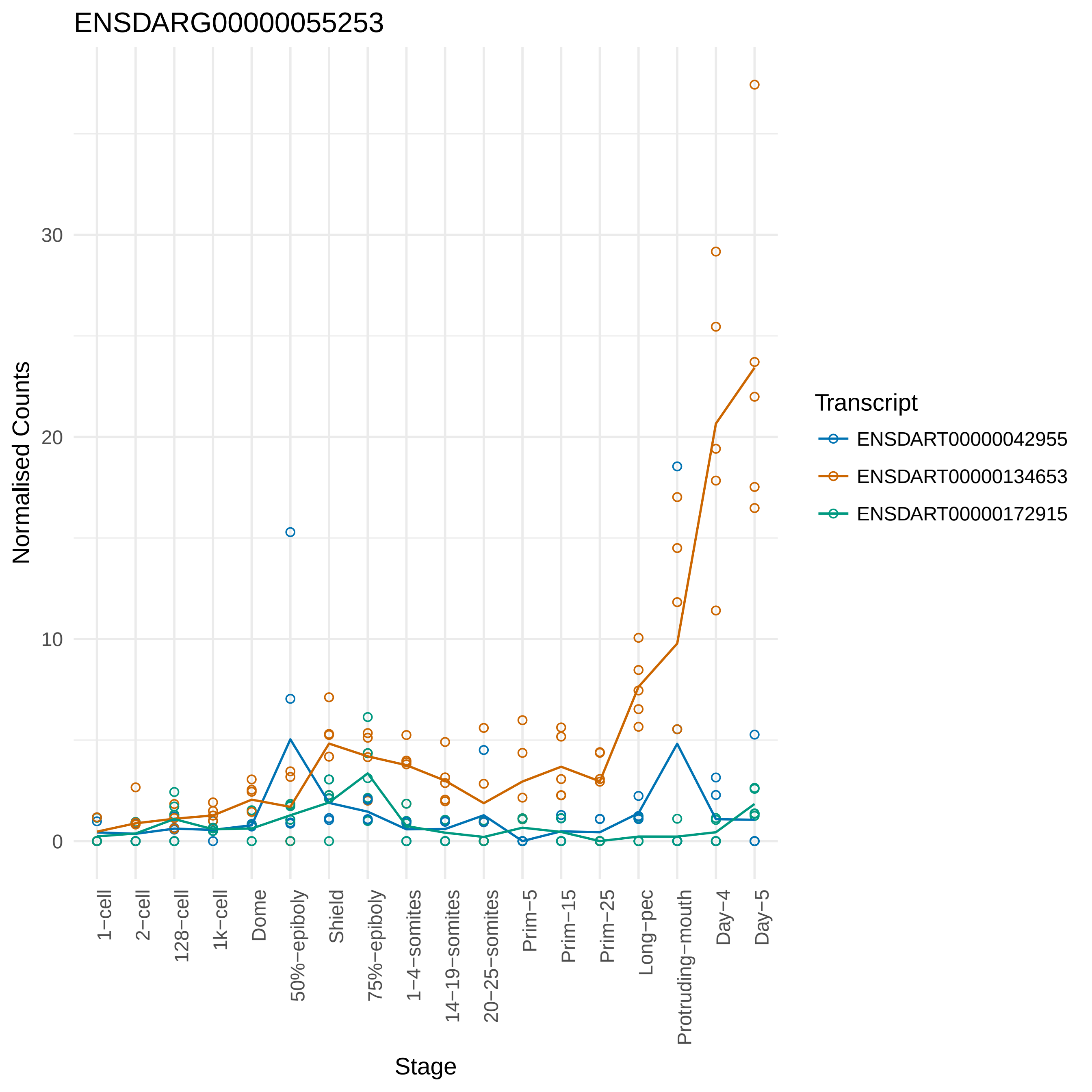

ENSDARG00000055253
Normalised Counts
0
10
20
30
Stage
1−cell
2−cell
128−cell
1k−cell
Dome
50%−epiboly
Shield
75%−epiboly
1−4−somites
14−19−somites
20−25−somites
Prim−5
Prim−15
Prim−25
Long−pec
Protruding−mouth
Day−4
Day−5
Transcript
ENSDART00000042955
ENSDART00000134653
ENSDART00000172915

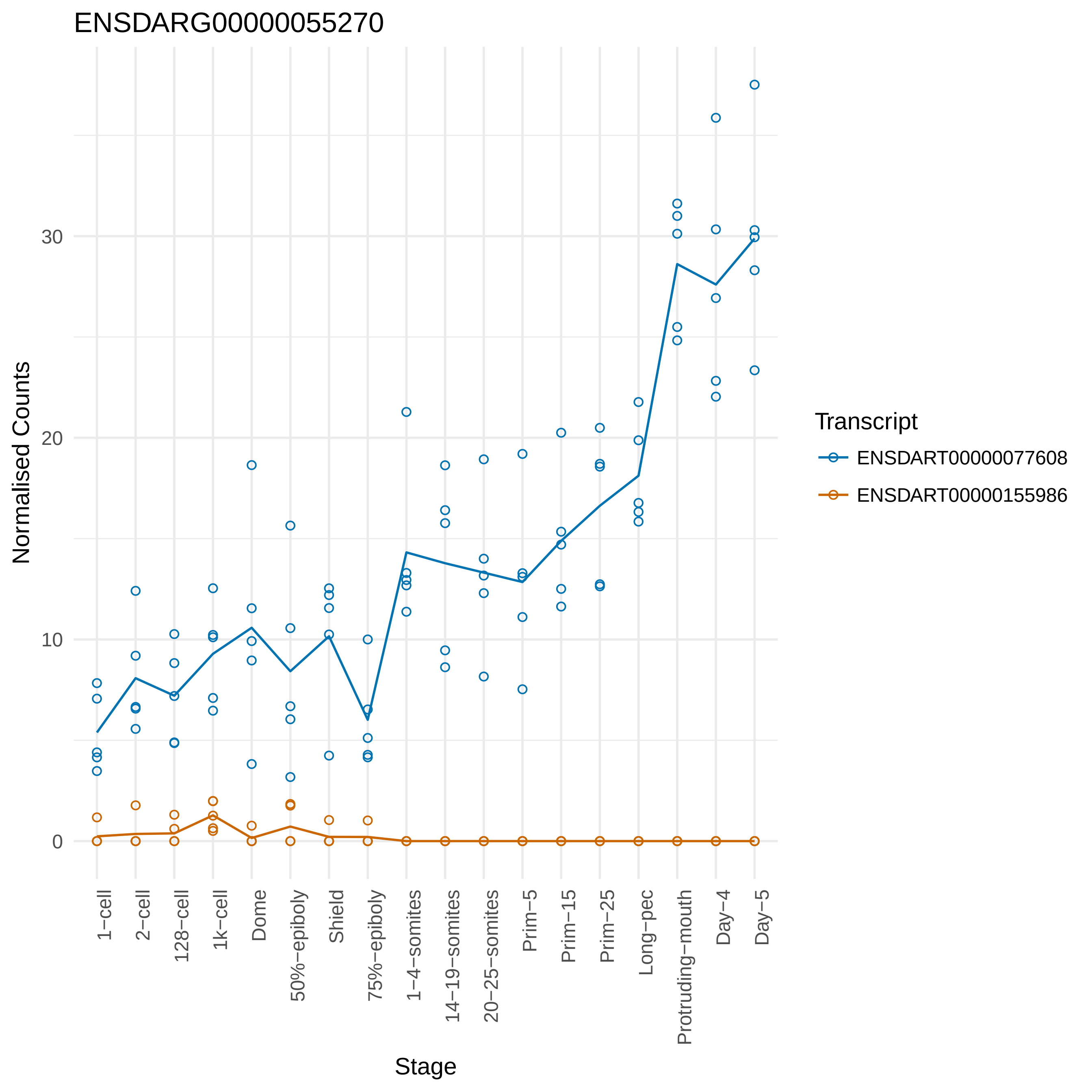
ENSDARG00000055270
Normalised Counts
0
10
20
30
1−cell
2−cell
128−cell
1k−cell
Dome
50%−epiboly
Shield
75%−epiboly
1−4−somites
14−19−somites
20−25−somites
Prim−5
Prim−15
Prim−25
Long−pec
Protruding−mouth
Day−4
Day−5
Stage
Transcript
ENSDART00000077608
ENSDART00000155986

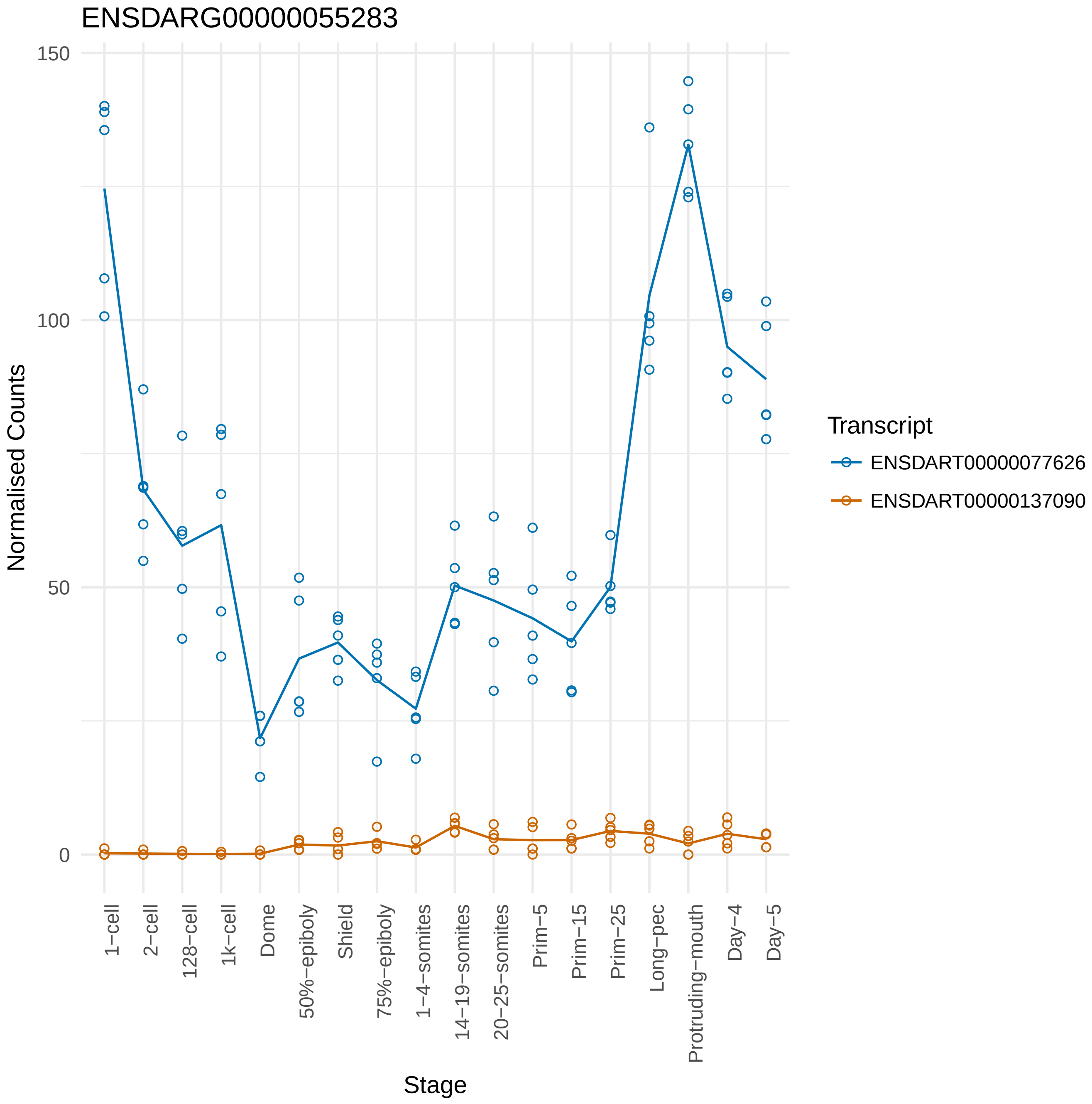

ENSDARG00000055283
Normalised Counts
150
100
50
0
1−cell
2−cell
128−cell
1k−cell
Dome
50%−epiboly
Shield
75%−epiboly
1−4−somites
14−19−somites
20−25−somites
Prim−5
Prim−15
Prim−25
Long−pec
Protruding−mouth
Day−4
Day−5
Stage
Transcript
ENSDART00000077626
ENSDART00000137090

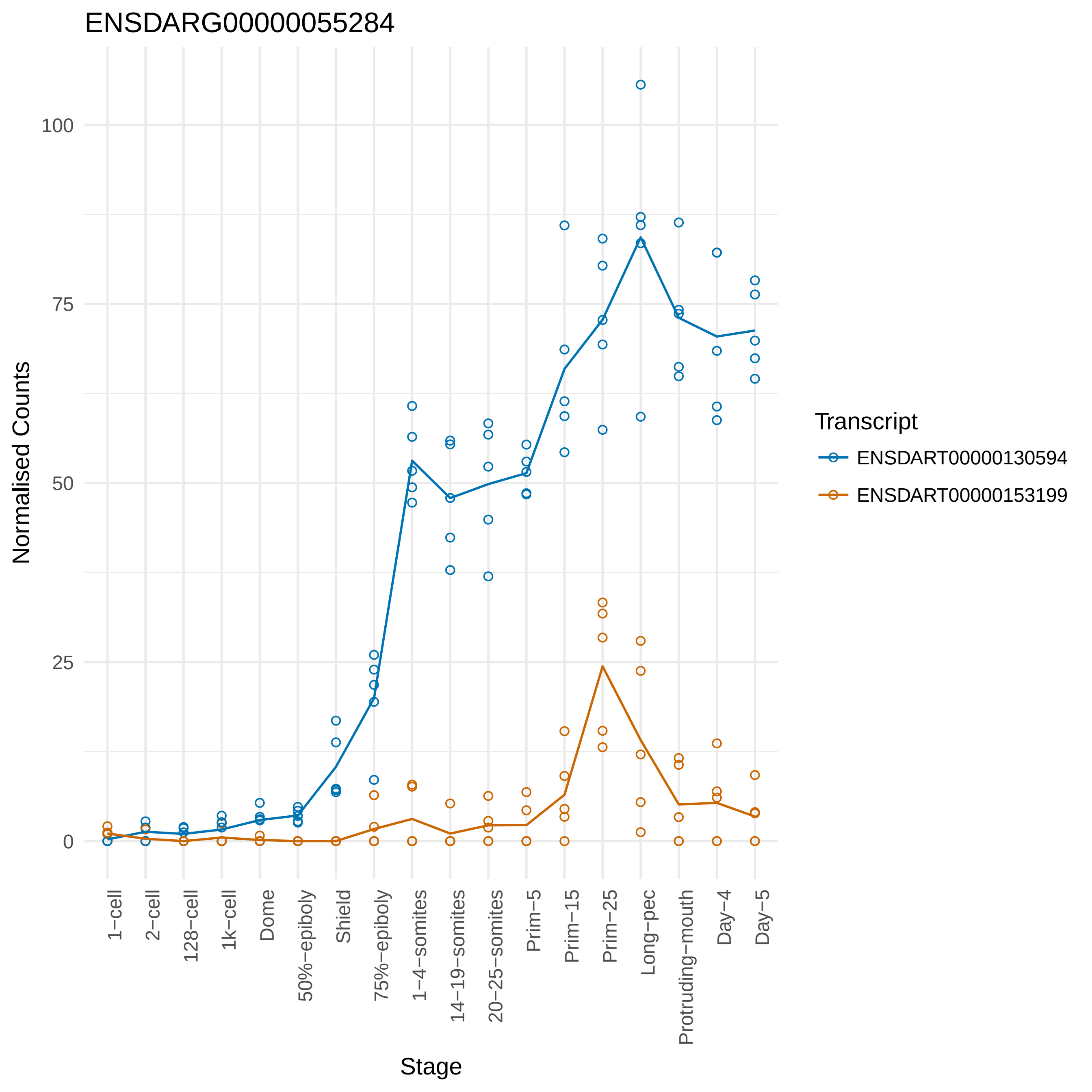

ENSDARG00000055284
Normalised Counts
100
75
50
25
0
1−cell
2−cell
128−cell
1k−cell
Dome
50%−epiboly
Shield
75%−epiboly
1−4−somites
14−19−somites
20−25−somites
Prim−5
Prim−15
Prim−25
Long−pec
Protruding−mouth
Day−4
Day−5
Stage
Transcript
ENSDART00000130594
ENSDART00000153199

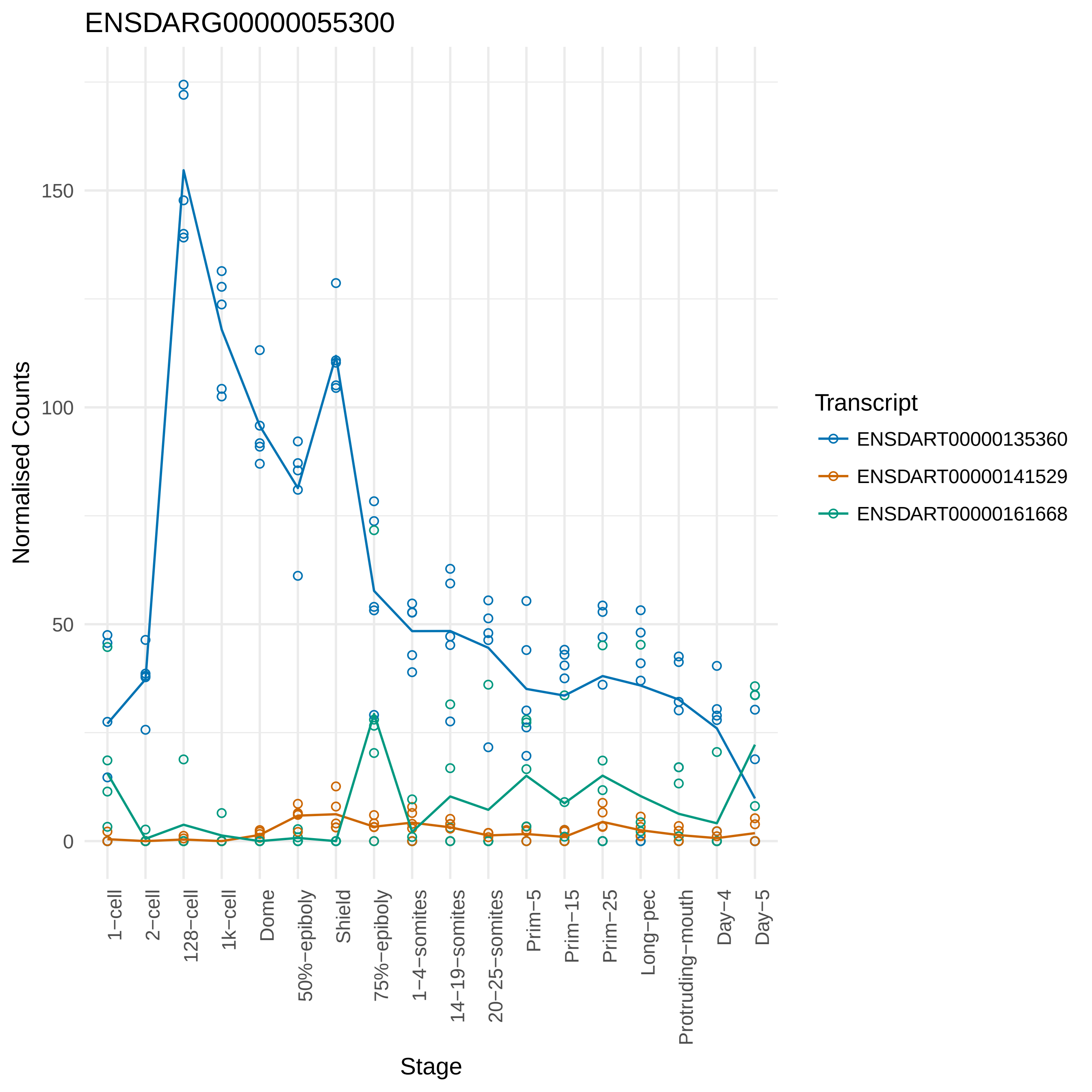

ENSDARG00000055300
Normalised Counts
150
100
50
0
1−cell
2−cell
128−cell
1k−cell
Dome
50%−epiboly
Shield
75%−epiboly
1−4−somites
14−19−somites
20−25−somites
Prim−5
Prim−15
Prim−25
Long−pec
Protruding−mouth
Day−4
Day−5
Stage
Transcript
ENSDART00000135360
ENSDART00000141529
ENSDART00000161668

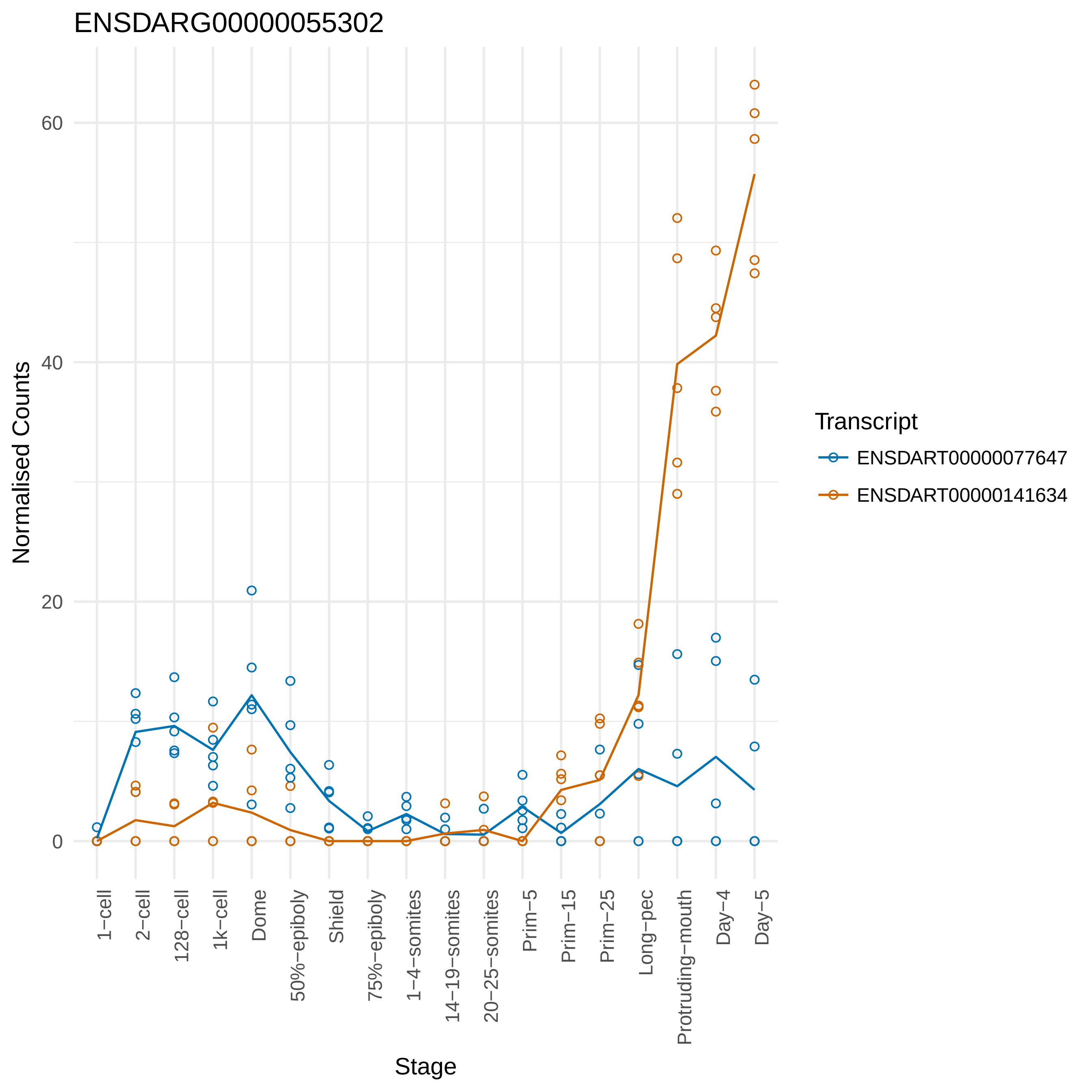

ENSDARG00000055302
Normalised Counts
0
20
40
60
Stage
1−cell
2−cell
128−cell
1k−cell
Dome
50%−epiboly
Shield
75%−epiboly
1−4−somites
14−19−somites
20−25−somites
Prim−5
Prim−15
Prim−25
Long−pec
Protruding−mouth
Day−4
Day−5
Transcript
ENSDART00000077647
ENSDART00000141634

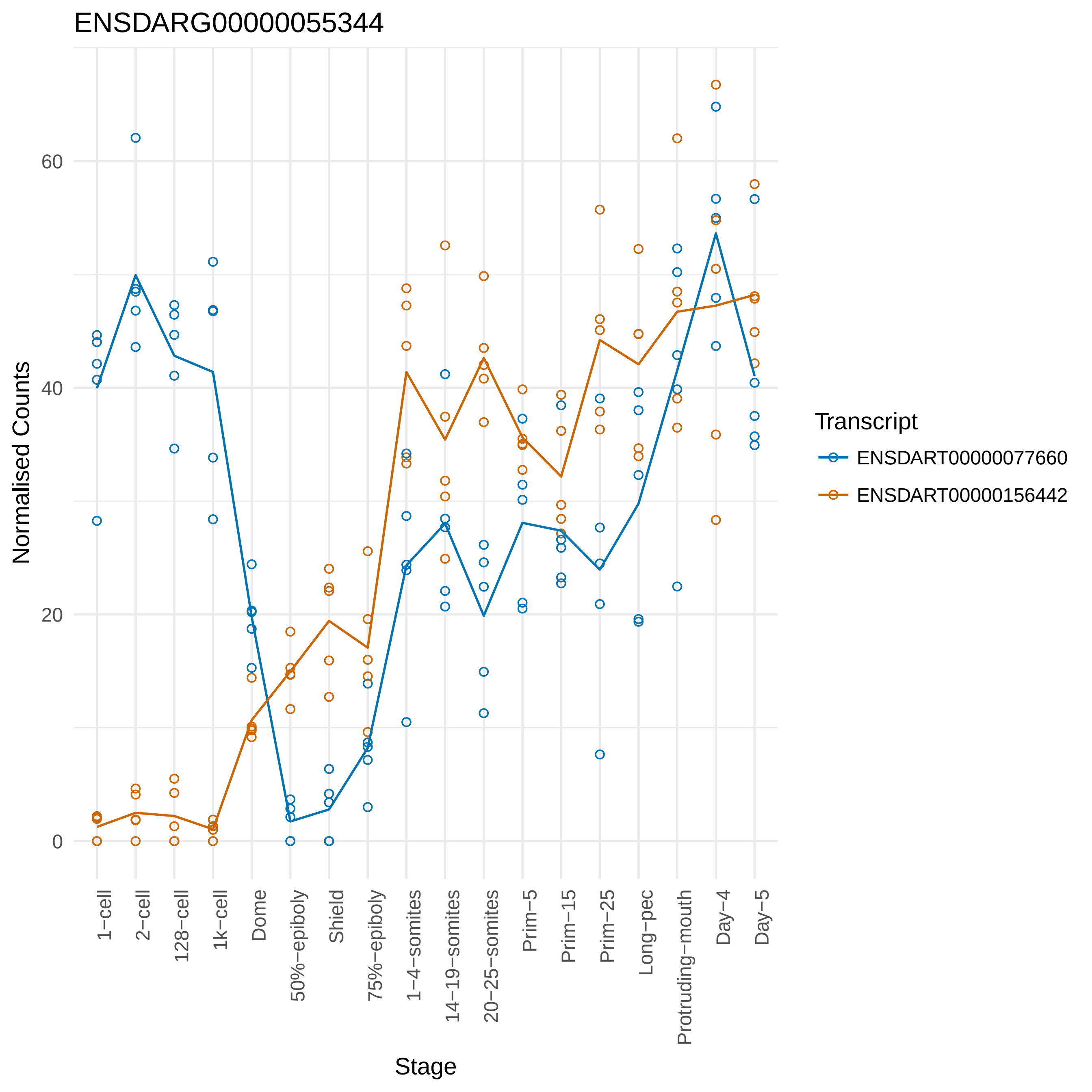
ENSDARG00000055344
Normalised Counts
0
20
40
60
Stage
1−cell
2−cell
128−cell
1k−cell
Dome
50%−epiboly
Shield
75%−epiboly
1−4−somites
14−19−somites
20−25−somites
Prim−5
Prim−15
Prim−25
Long−pec
Protruding−mouth
Day−4
Day−5
Transcript
ENSDART00000077660
ENSDART00000156442

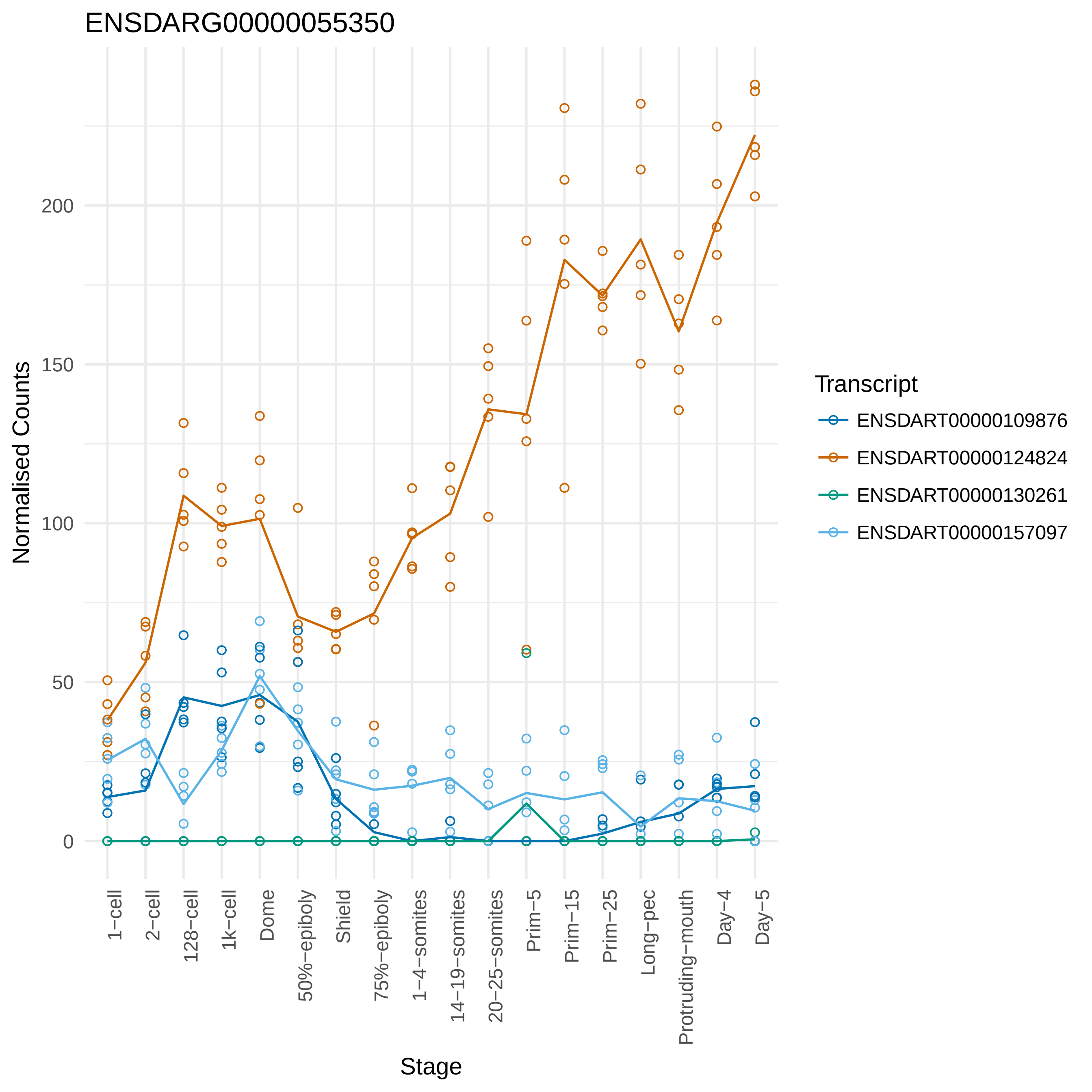

ENSDARG00000055350
Normalised Counts
0
50
100
150
200
Stage
1−cell
2−cell
128−cell
1k−cell
Dome
50%−epiboly
Shield
75%−epiboly
1−4−somites
14−19−somites
20−25−somites
Prim−5
Prim−15
Prim−25
Long−pec
Protruding−mouth
Day−4
Day−5
Transcript
ENSDART00000109876
ENSDART00000124824
ENSDART00000130261
ENSDART00000157097

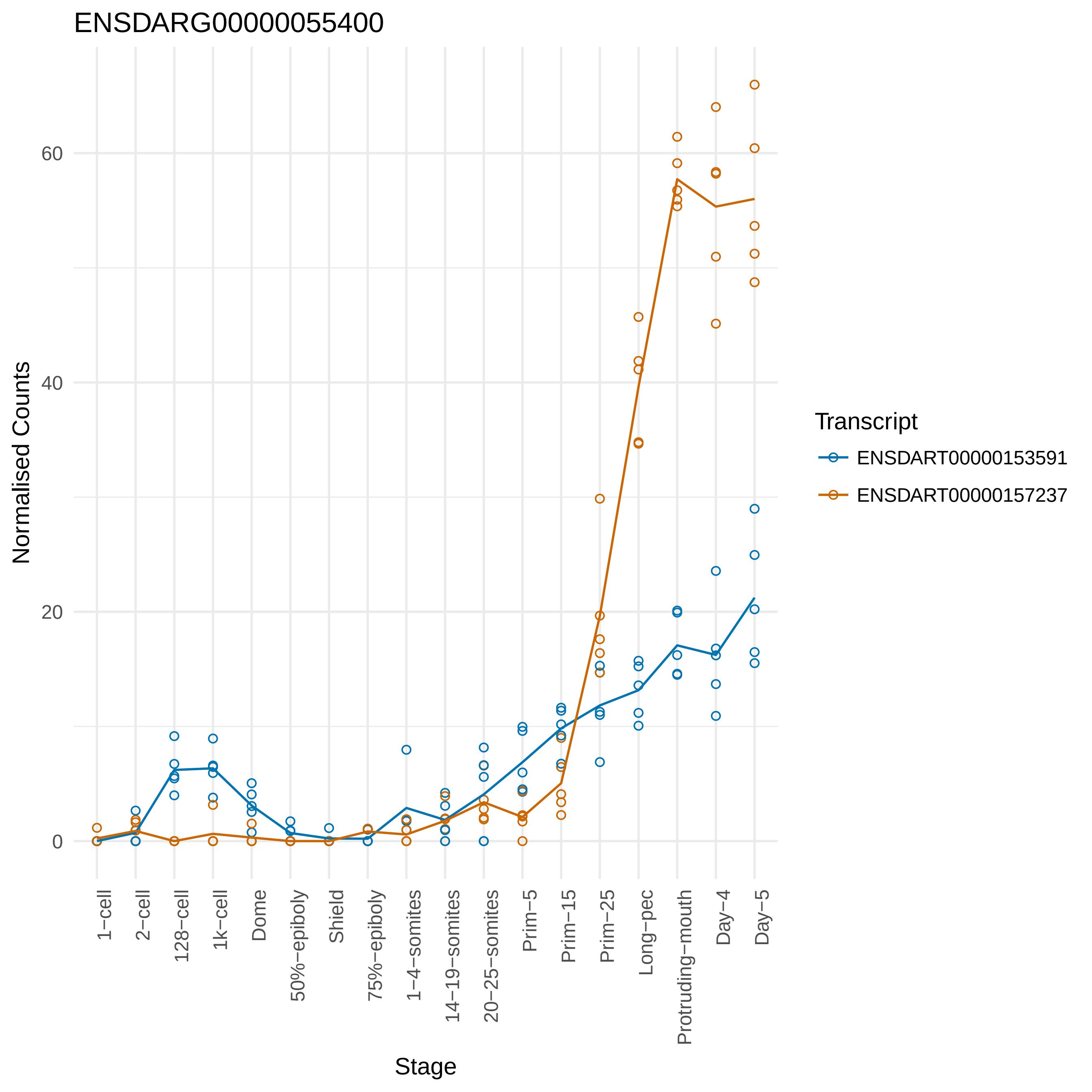
ENSDARG00000055400
Normalised Counts
0
20
40
60
1−cell
2−cell
128−cell
1k−cell
Dome
50%−epiboly
Shield
75%−epiboly
1−4−somites
14−19−somites
20−25−somites
Prim−5
Prim−15
Prim−25
Long−pec
Protruding−mouth
Day−4
Day−5
Stage
Transcript
ENSDART00000153591
ENSDART00000157237

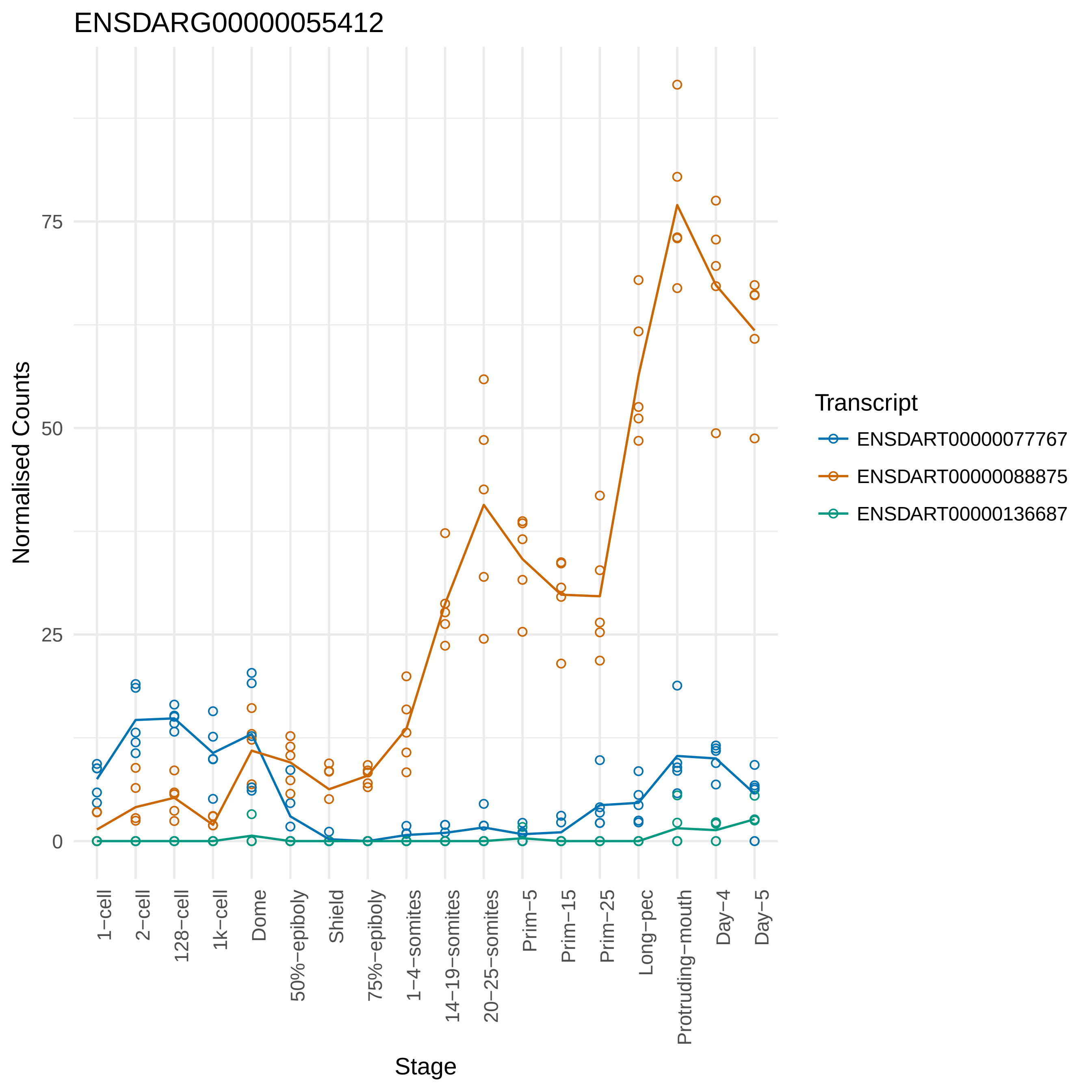
ENSDARG00000055412
Normalised Counts
0
25
50
75
Stage
1−cell
2−cell
128−cell
1k−cell
Dome
50%−epiboly
Shield
75%−epiboly
1−4−somites
14−19−somites
20−25−somites
Prim−5
Prim−15
Prim−25
Long−pec
Protruding−mouth
Day−4
Day−5
Transcript
ENSDART00000077767
ENSDART00000088875
ENSDART00000136687

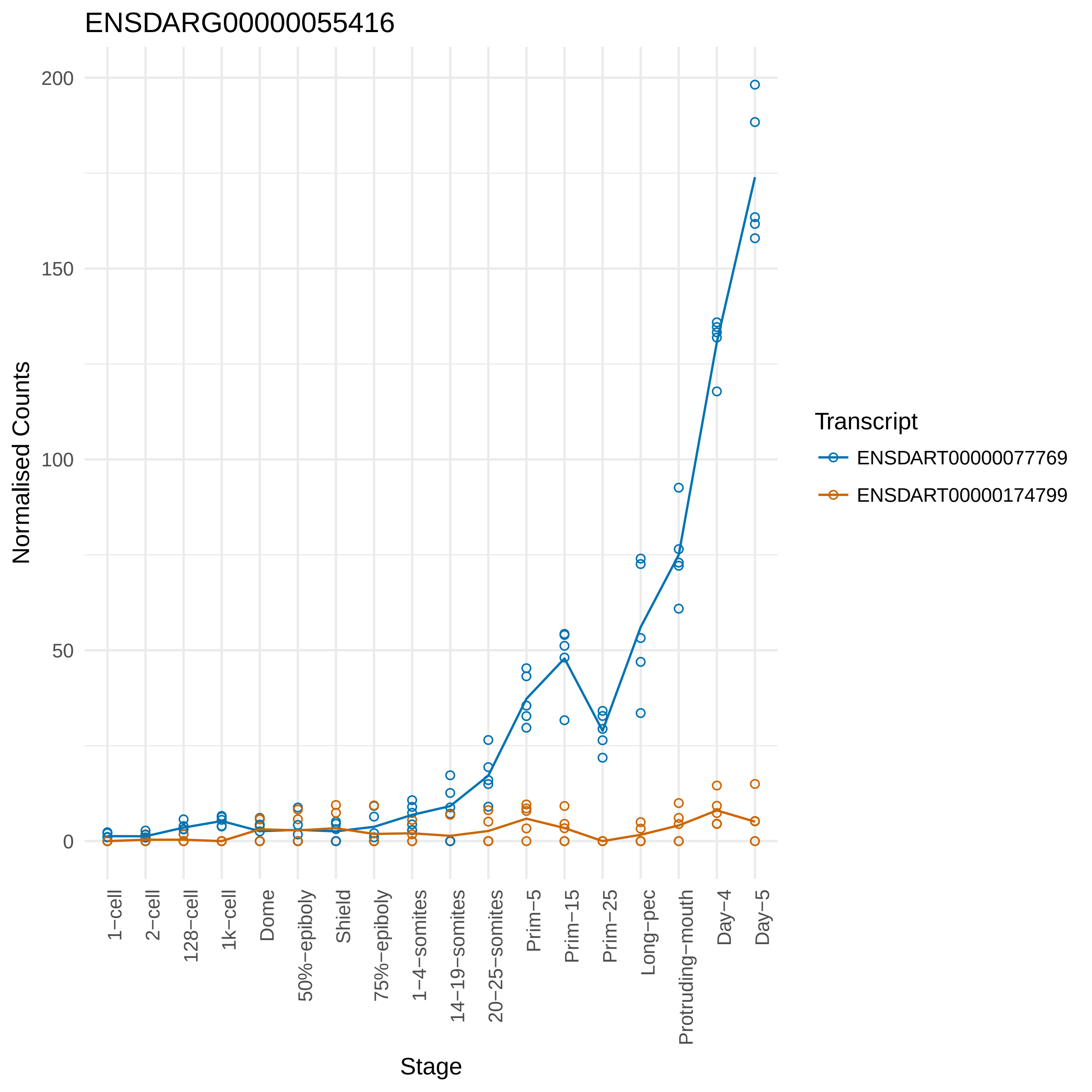
ENSDARG00000055416
Normalised Counts
200
150
100
50
0
1−cell
2−cell
128−cell
1k−cell
Dome
50%−epiboly
Shield
75%−epiboly
1−4−somites
14−19−somites
20−25−somites
Prim−5
Prim−15
Prim−25
Long−pec
Protruding−mouth
Day−4
Day−5
Stage
Transcript
ENSDART00000077769
ENSDART00000174799

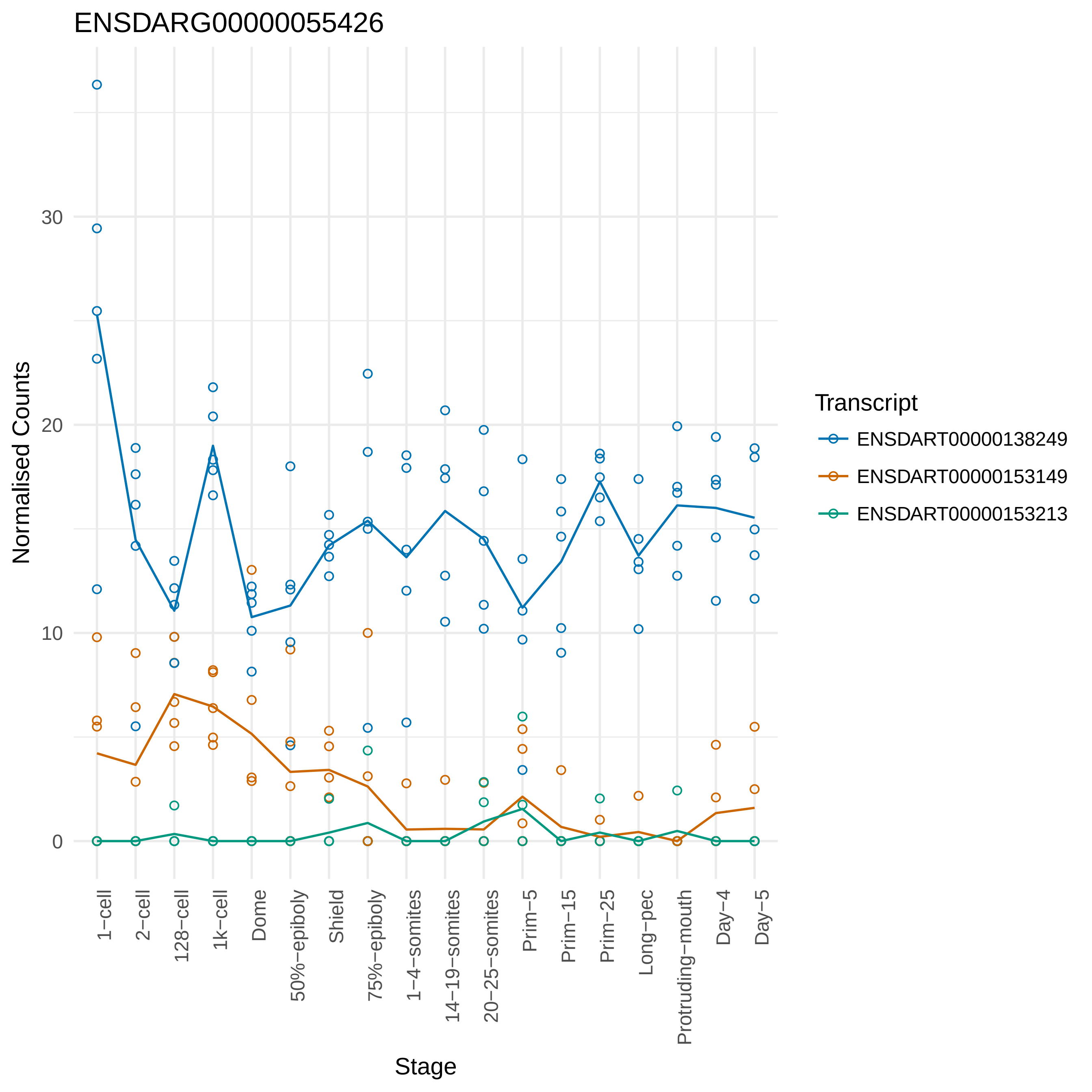

ENSDARG00000055426
Normalised Counts
30
20
10
0
1−cell
2−cell
128−cell
1k−cell
Dome
50%−epiboly
Shield
75%−epiboly
1−4−somites
14−19−somites
20−25−somites
Prim−5
Prim−15
Prim−25
Long−pec
Protruding−mouth
Day−4
Day−5
Stage
Transcript
ENSDART00000138249
ENSDART00000153149
ENSDART00000153213

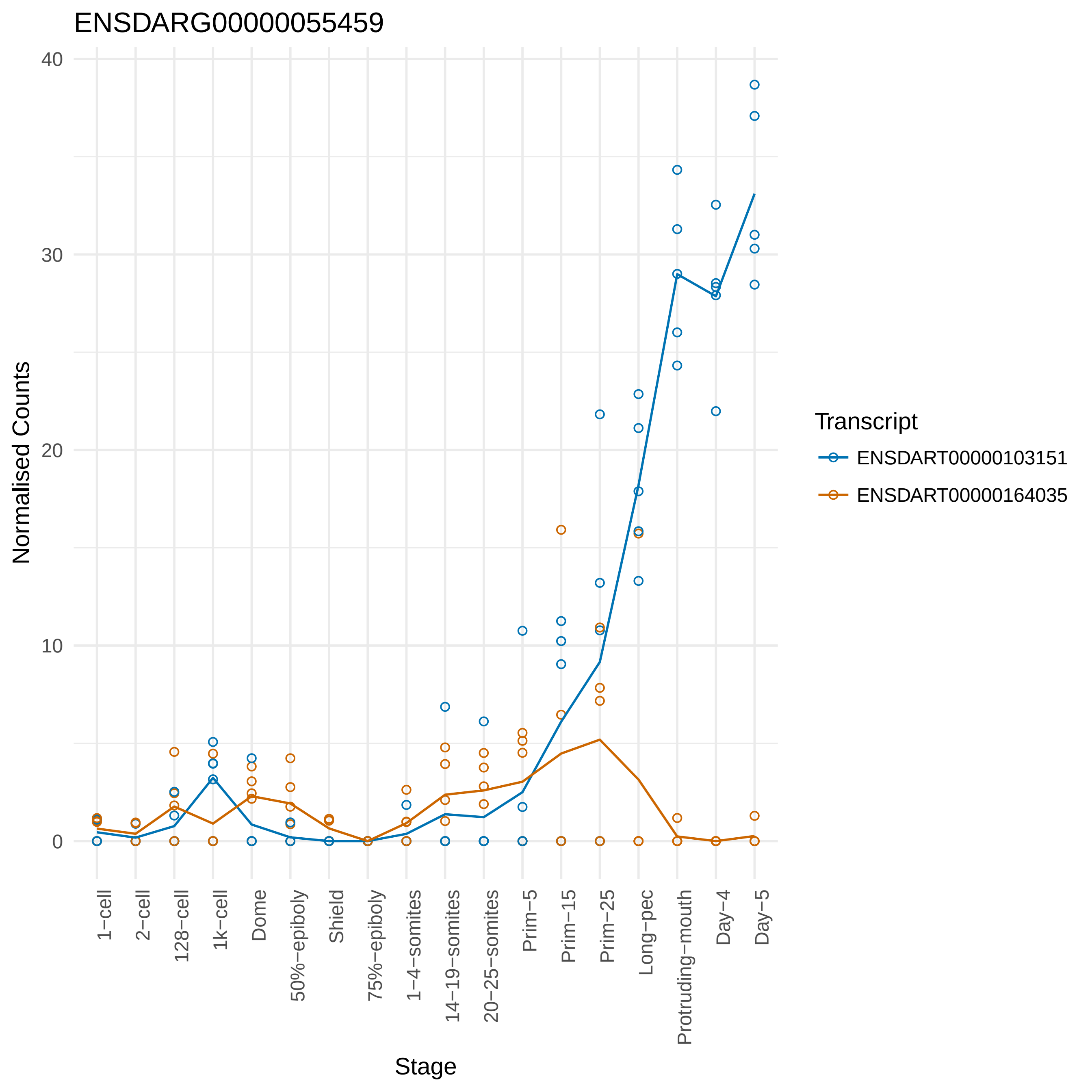
ENSDARG00000055459
Normalised Counts
40
30
20
10
0
1−cell
2−cell
128−cell
1k−cell
Dome
50%−epiboly
Shield
75%−epiboly
1−4−somites
14−19−somites
20−25−somites
Prim−5
Prim−15
Prim−25
Long−pec
Protruding−mouth
Day−4
Day−5
Stage
Transcript
ENSDART00000103151
ENSDART00000164035

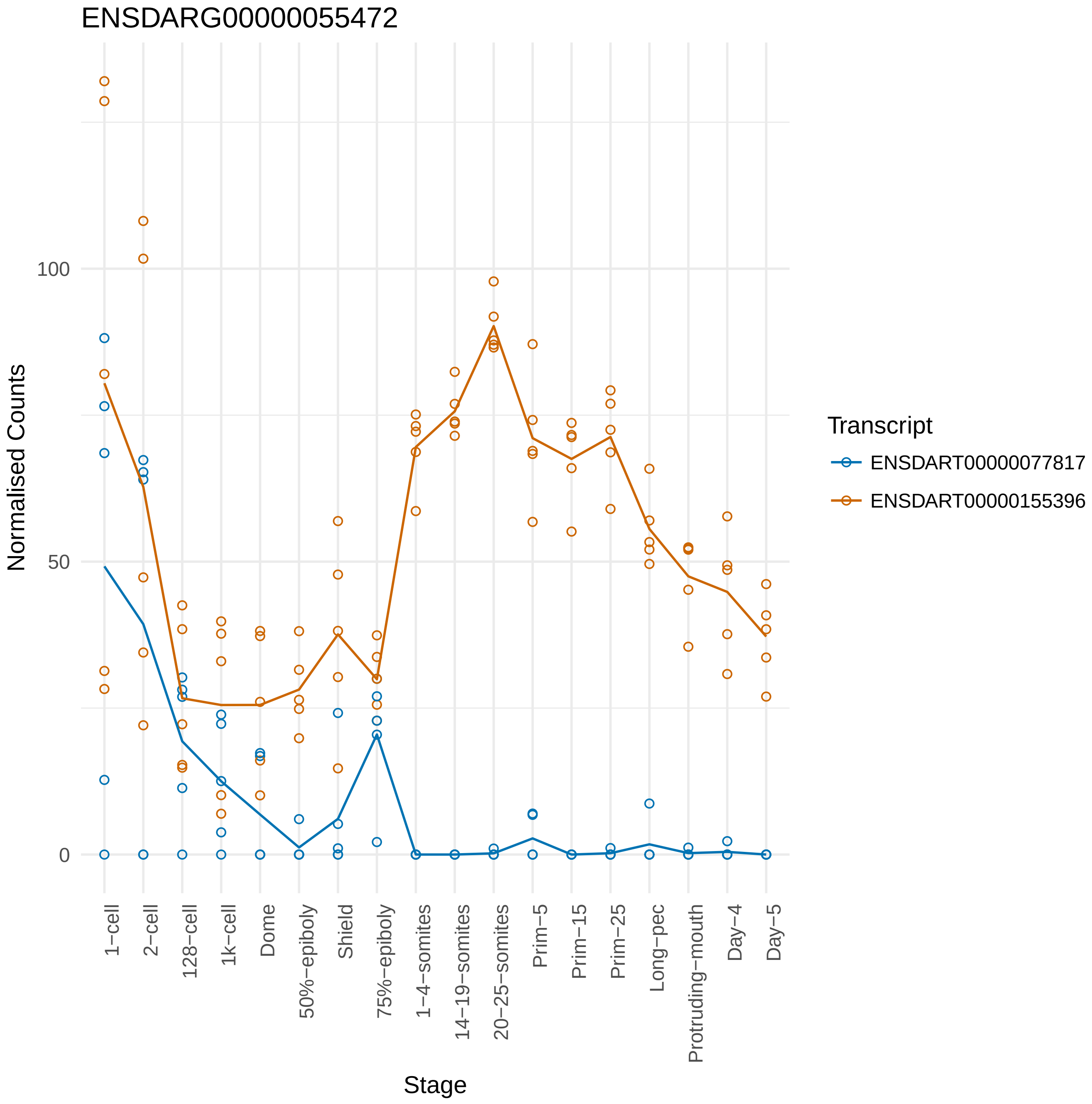

ENSDARG00000055472
Normalised Counts
100
50
0
1−cell
2−cell
128−cell
1k−cell
Dome
50%−epiboly
Shield
75%−epiboly
1−4−somites
14−19−somites
20−25−somites
Prim−5
Prim−15
Prim−25
Long−pec
Protruding−mouth
Day−4
Day−5
Stage
Transcript
ENSDART00000077817
ENSDART00000155396

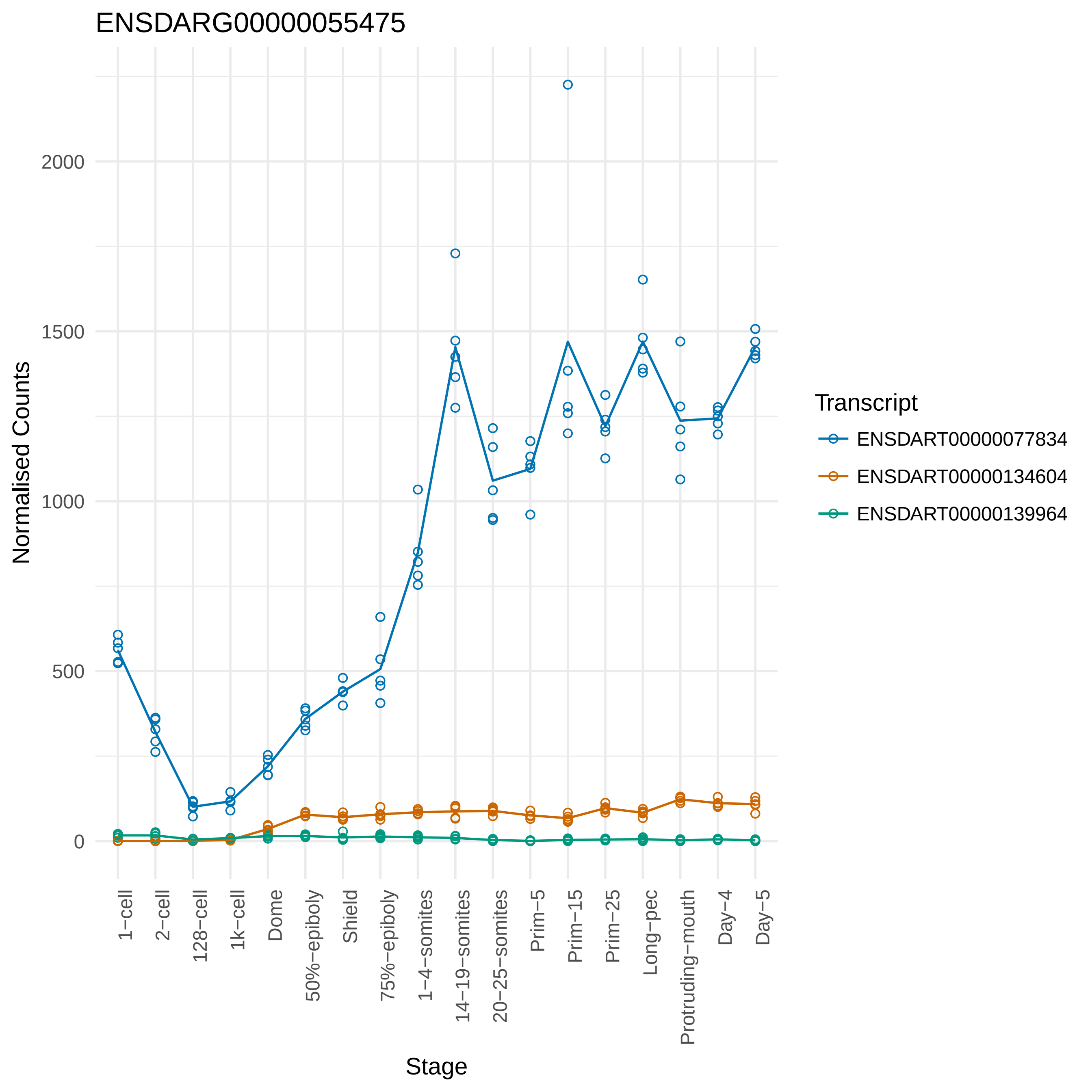
ENSDARG00000055475
Normalised Counts
2000
1500
1000
500
0
1−cell
2−cell
128−cell
1k−cell
Dome
50%−epiboly
Shield
75%−epiboly
1−4−somites
14−19−somites
20−25−somites
Prim−5
Prim−15
Prim−25
Long−pec
Protruding−mouth
Day−4
Day−5
Stage
Transcript
ENSDART00000077834
ENSDART00000134604
ENSDART00000139964

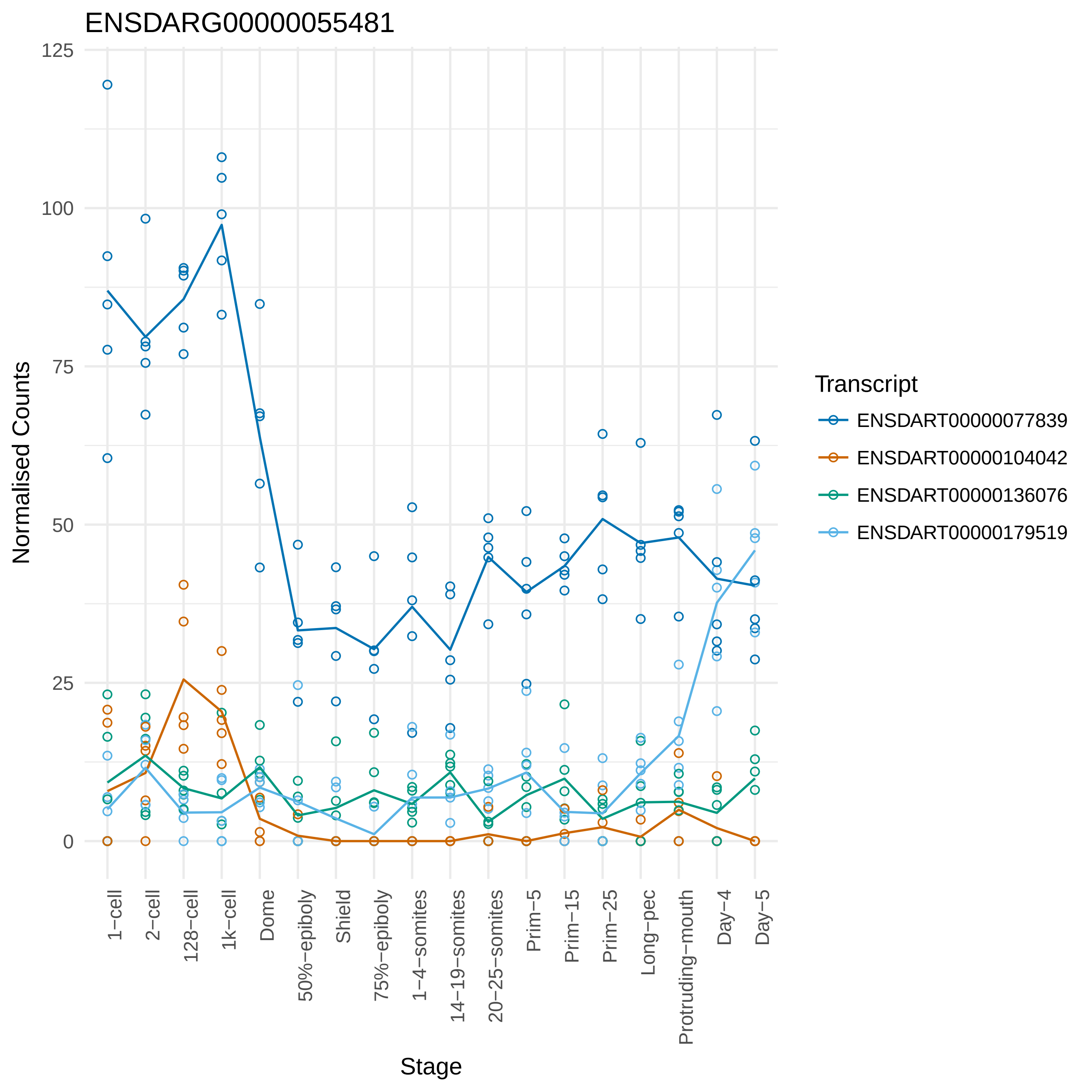
ENSDARG00000055481
Normalised Counts
125
100
75
50
25
0
Stage
1−cell
2−cell
128−cell
1k−cell
Dome
50%−epiboly
Shield
75%−epiboly
1−4−somites
14−19−somites
20−25−somites
Prim−5
Prim−15
Prim−25
Long−pec
Protruding−mouth
Day−4
Day−5
Transcript
ENSDART00000077839
ENSDART00000104042
ENSDART00000136076
ENSDART00000179519

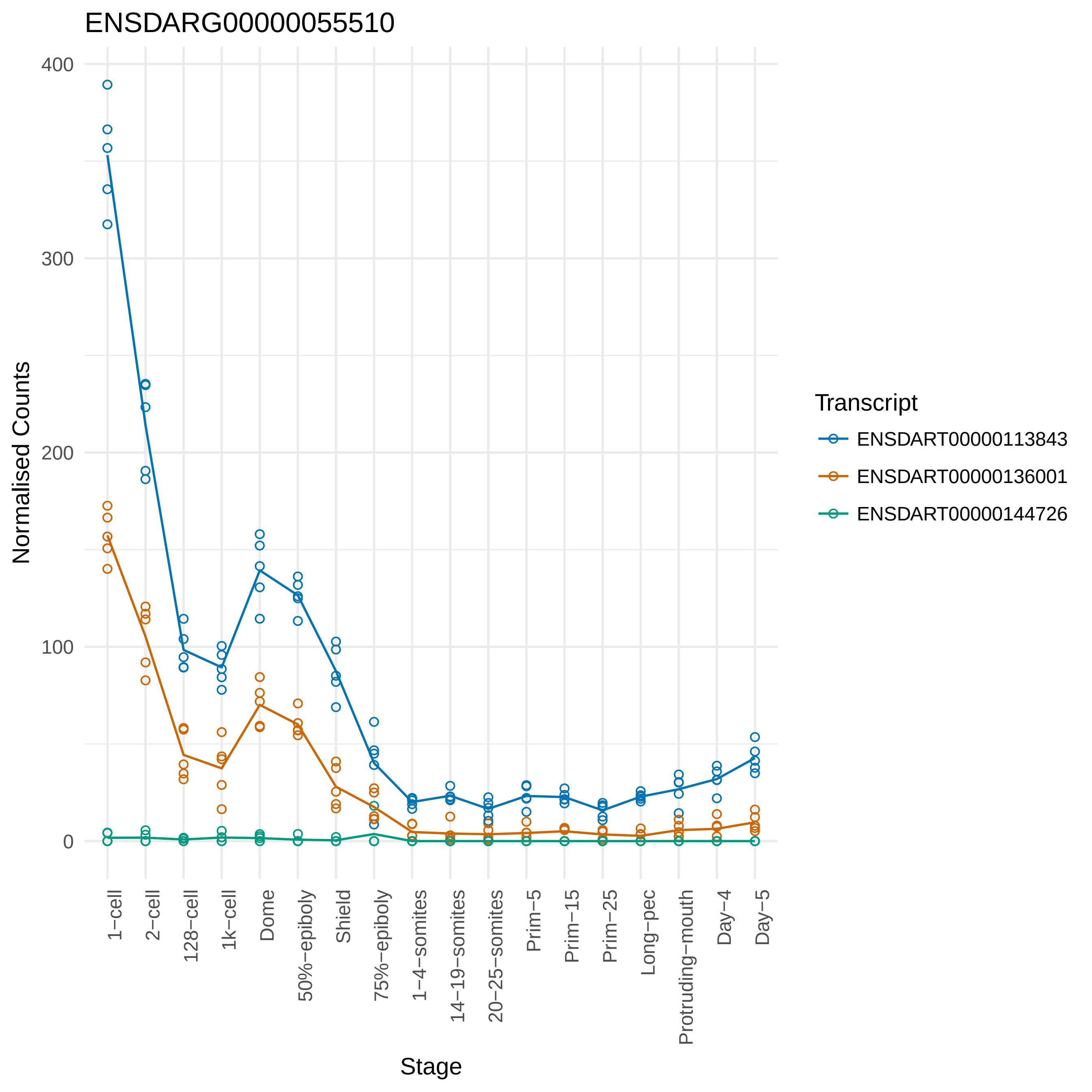
ENSDARG00000055510
Normalised Counts
400
300
200
100
0
1−cell
2−cell
128−cell
1k−cell
Dome
50%−epiboly
Shield
75%−epiboly
1−4−somites
14−19−somites
20−25−somites
Prim−5
Prim−15
Prim−25
Long−pec
Protruding−mouth
Day−4
Day−5
Stage
Transcript
ENSDART00000113843
ENSDART00000136001
ENSDART00000144726

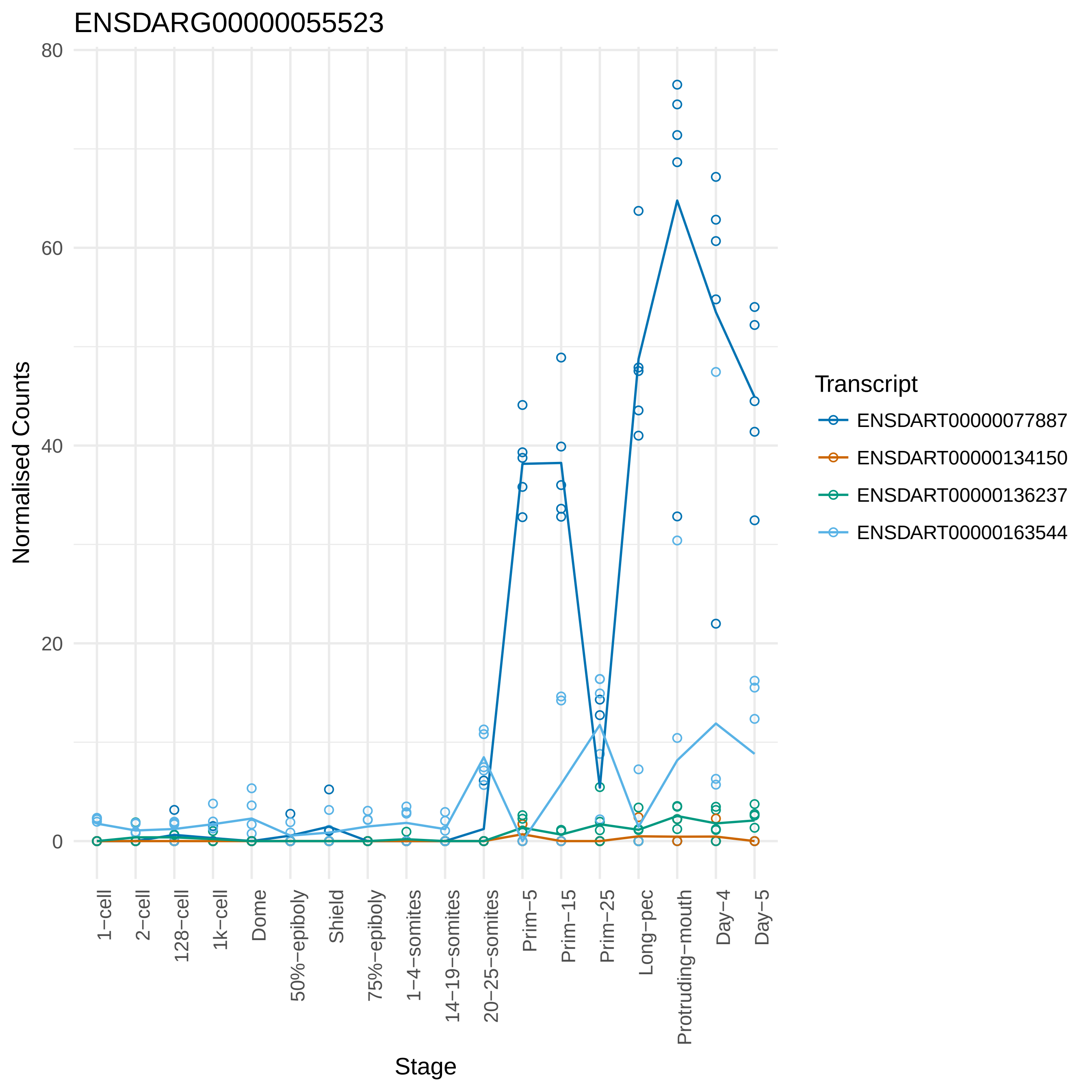

ENSDARG00000055523
80
60
40
20
0
Normalised Counts
1−cell
2−cell
128−cell
1k−cell
Dome
50%−epiboly
Shield
75%−epiboly
1−4−somites
14−19−somites
20−25−somites
Prim−5
Prim−15
Prim−25
Long−pec
Protruding−mouth
Day−4
Day−5
Stage
Transcript
ENSDART00000077887
ENSDART00000134150
ENSDART00000136237
ENSDART00000163544

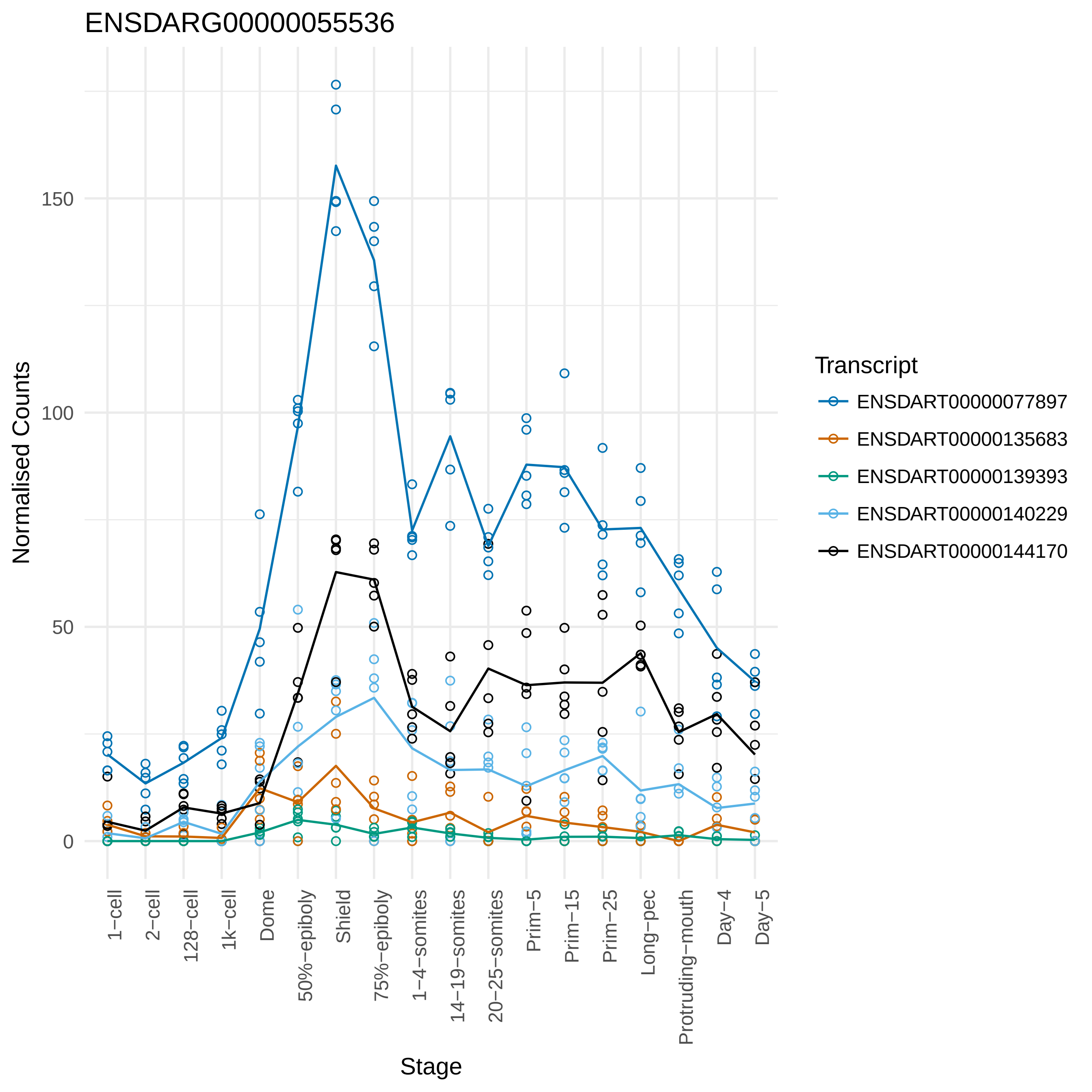

ENSDARG00000055536
Normalised Counts
150
100
50
0
1−cell
2−cell
128−cell
1k−cell
Dome
50%−epiboly
Shield
75%−epiboly
1−4−somites
14−19−somites
20−25−somites
Prim−5
Prim−15
Prim−25
Long−pec
Protruding−mouth
Day−4
Day−5
Stage
Transcript
ENSDART00000077897
ENSDART00000135683
ENSDART00000139393
ENSDART00000140229
ENSDART00000144170

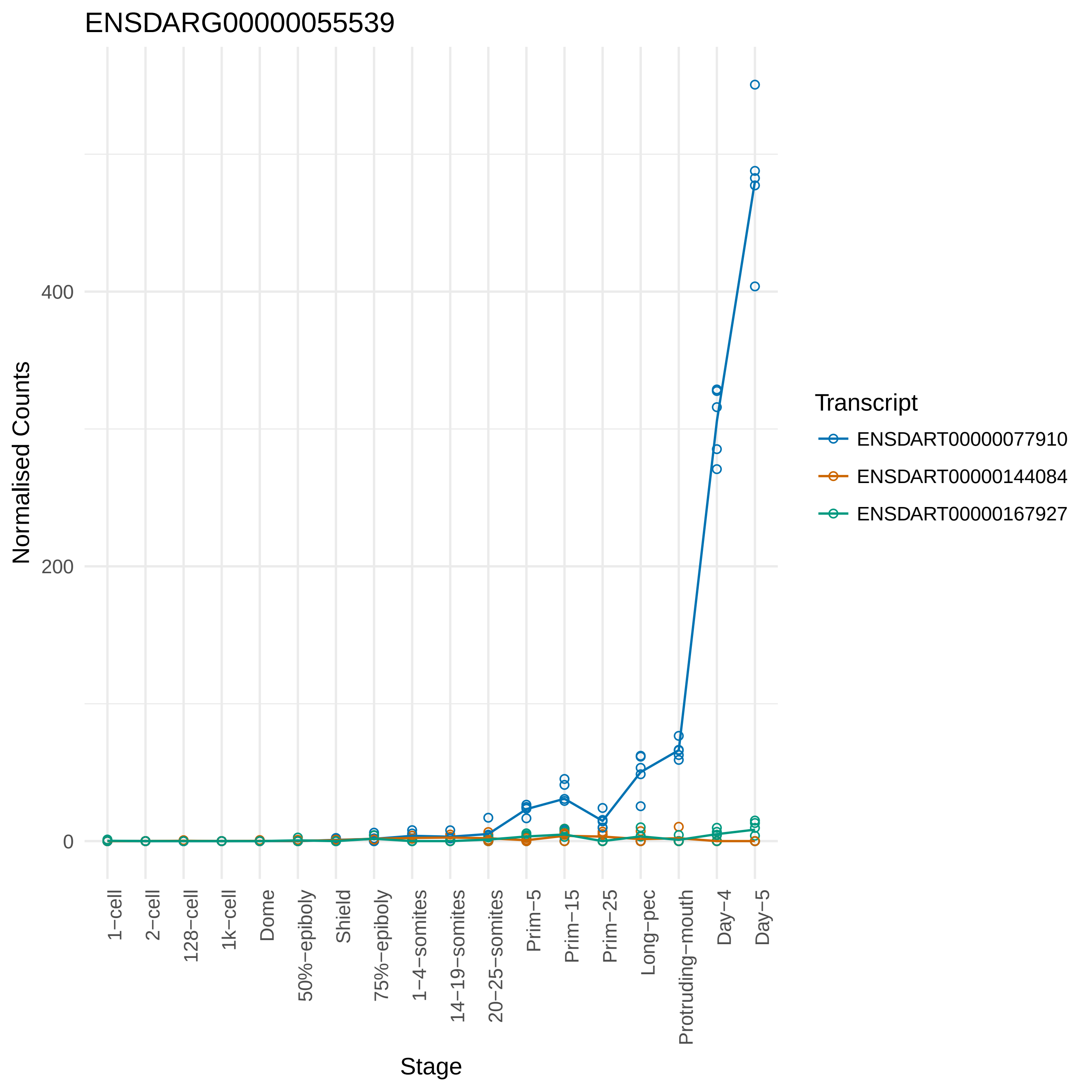

ENSDARG00000055539
Normalised Counts
400
200
0
1−cell
2−cell
128−cell
1k−cell
Dome
50%−epiboly
Shield
75%−epiboly
1−4−somites
14−19−somites
20−25−somites
Prim−5
Prim−15
Prim−25
Long−pec
Protruding−mouth
Day−4
Day−5
Stage
Transcript
ENSDART00000077910
ENSDART00000144084
ENSDART00000167927

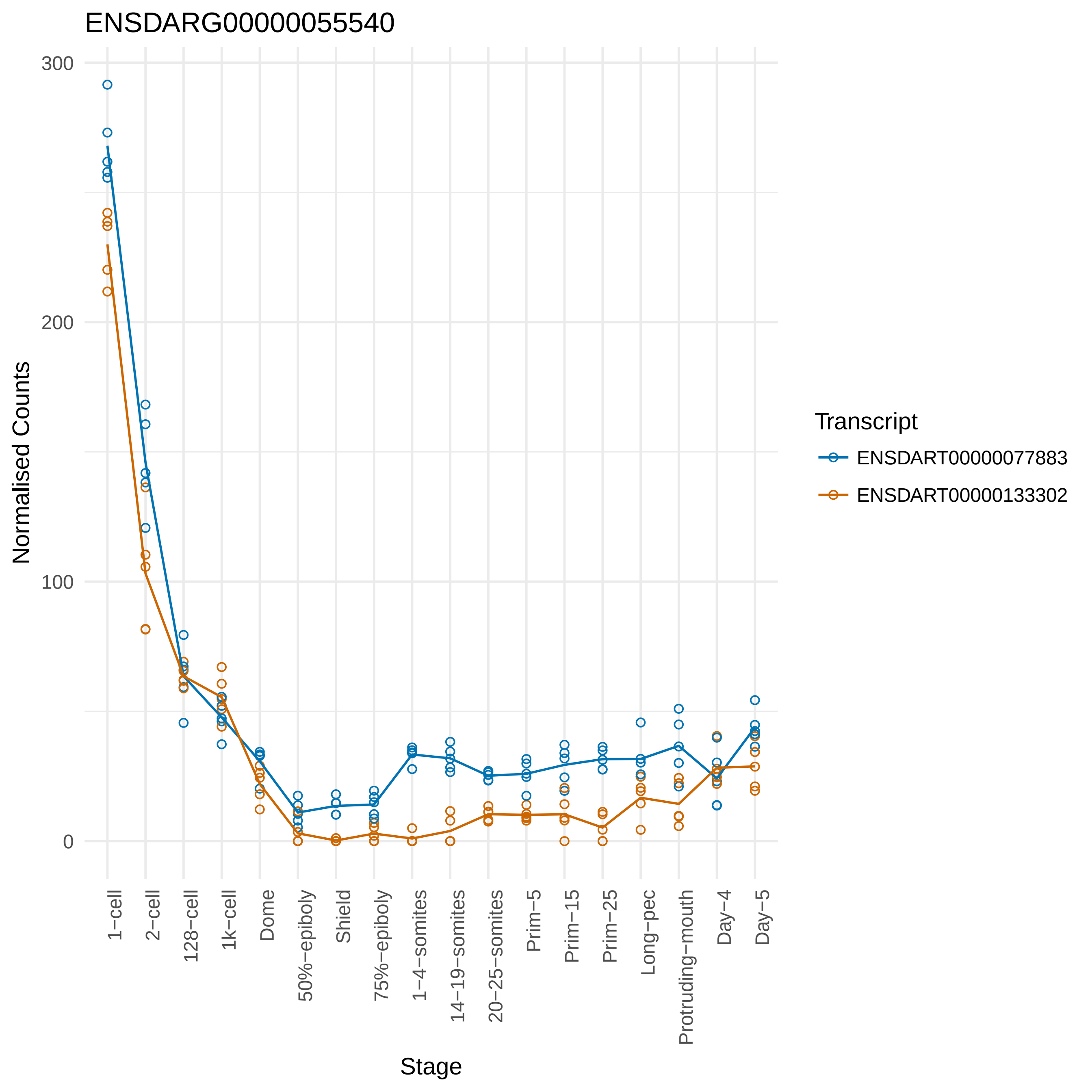
ENSDARG00000055540
Normalised Counts
300
200
100
0
1−cell
2−cell
128−cell
1k−cell
Dome
50%−epiboly
Shield
75%−epiboly
1−4−somites
14−19−somites
20−25−somites
Prim−5
Prim−15
Prim−25
Long−pec
Protruding−mouth
Day−4
Day−5
Stage
Transcript
ENSDART00000077883
ENSDART00000133302

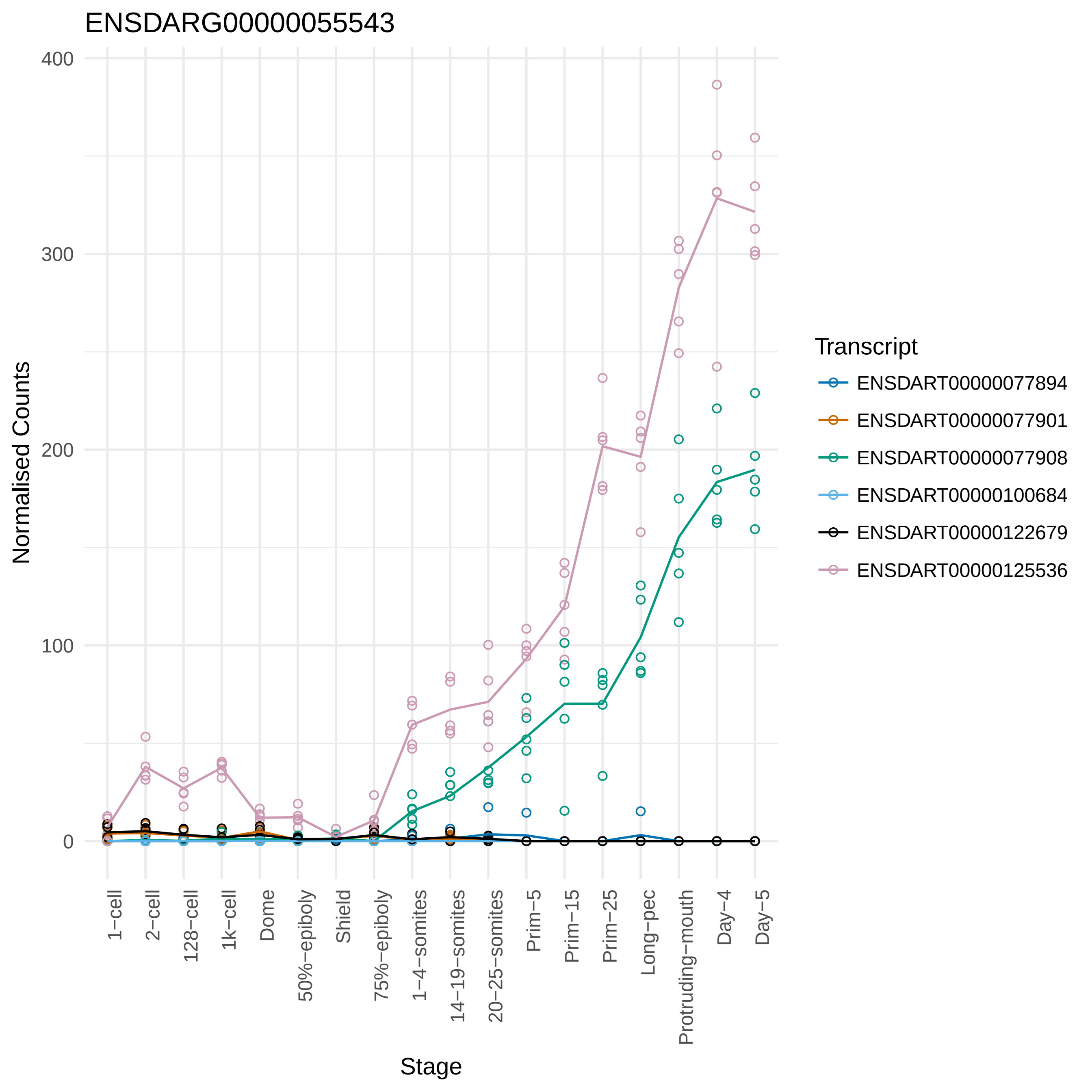

ENSDARG00000055543
Normalised Counts
400
300
200
100
0
1−cell
2−cell
128−cell
1k−cell
Dome
50%−epiboly
Shield
75%−epiboly
1−4−somites
14−19−somites
20−25−somites
Prim−5
Prim−15
Prim−25
Long−pec
Protruding−mouth
Day−4
Day−5
Stage
Transcript
ENSDART00000077894
ENSDART00000077901
ENSDART00000077908
ENSDART00000100684
ENSDART00000122679
ENSDART00000125536

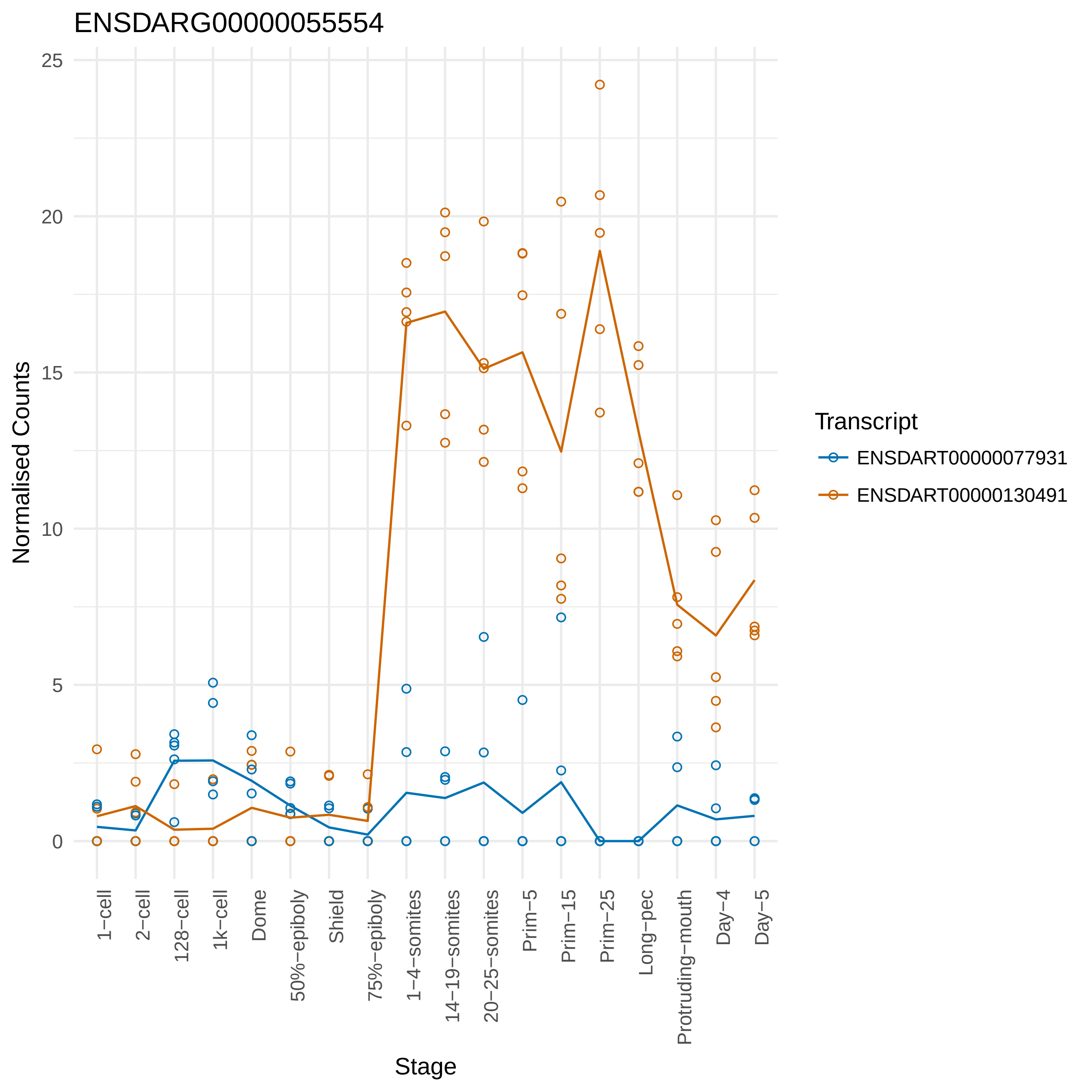

ENSDARG00000055554
Normalised Counts
25
20
15
10
5
0
1−cell
2−cell
128−cell
1k−cell
Dome
50%−epiboly
Shield
75%−epiboly
1−4−somites
14−19−somites
20−25−somites
Prim−5
Prim−15
Prim−25
Long−pec
Protruding−mouth
Day−4
Day−5
Stage
Transcript
ENSDART00000077931
ENSDART00000130491

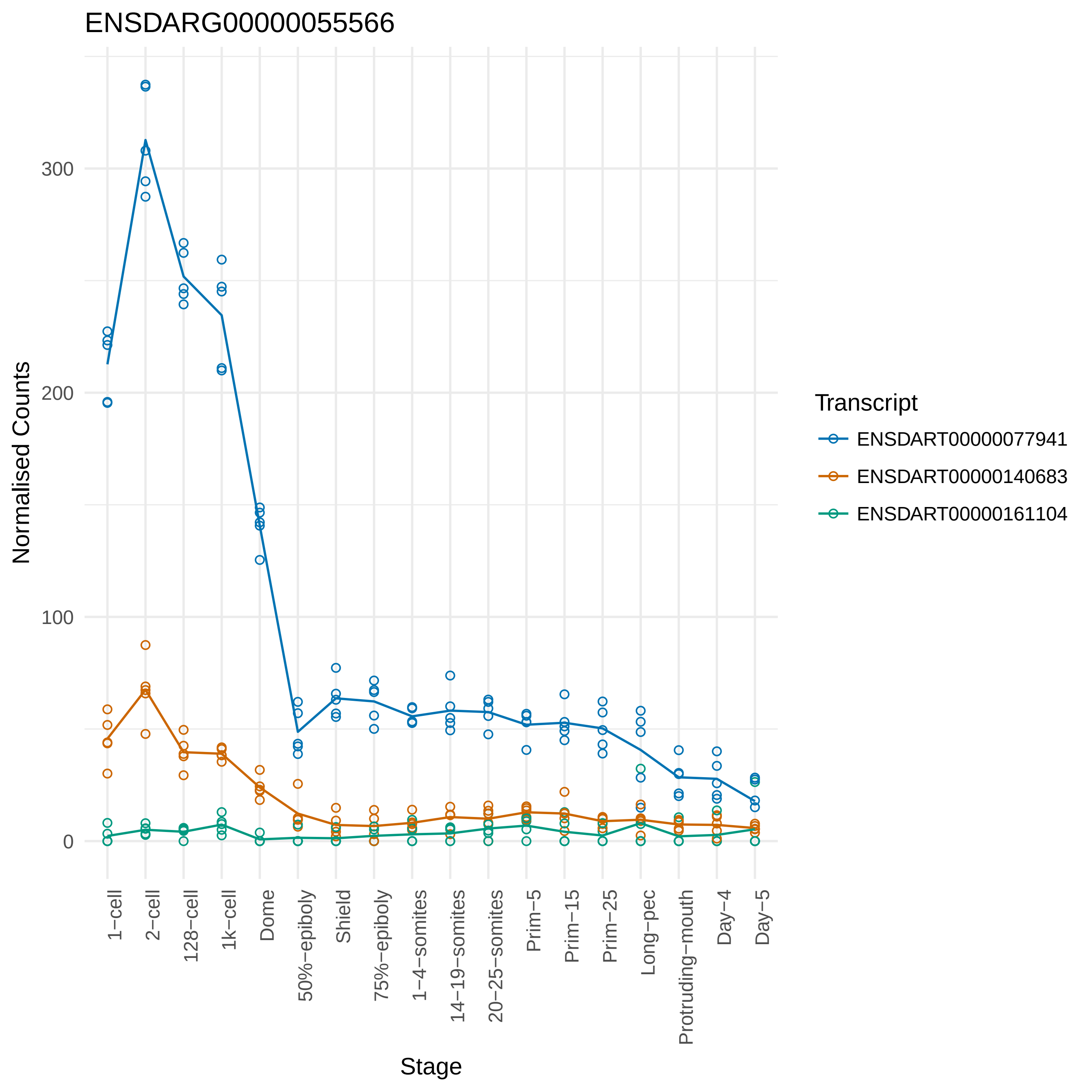
ENSDARG00000055566
Normalised Counts
300
200
100
0
1−cell
2−cell
128−cell
1k−cell
Dome
50%−epiboly
Shield
75%−epiboly
1−4−somites
14−19−somites
20−25−somites
Prim−5
Prim−15
Prim−25
Long−pec
Protruding−mouth
Day−4
Day−5
Stage
Transcript
ENSDART00000077941
ENSDART00000140683
ENSDART00000161104

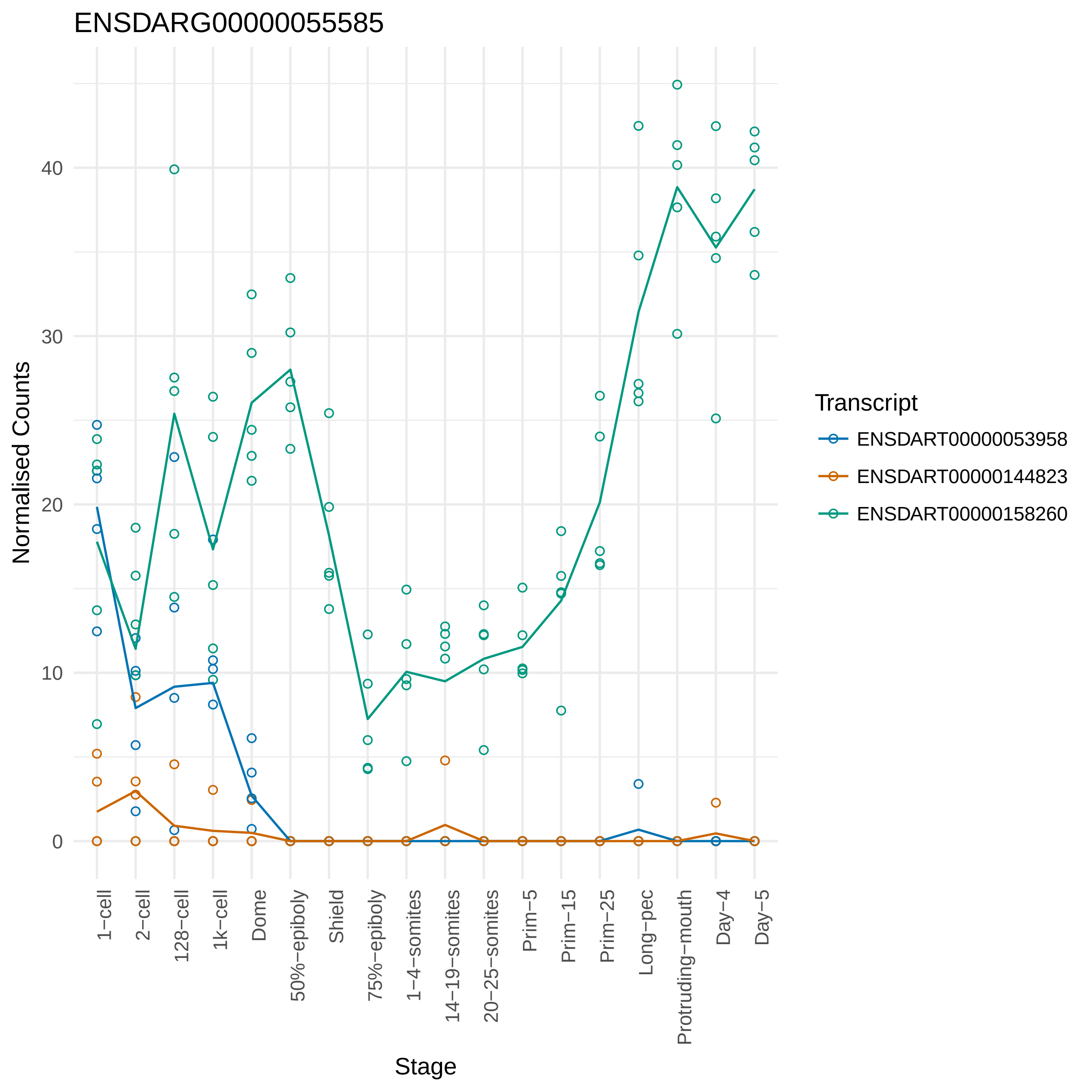

ENSDARG00000055585
Normalised Counts
0
10
20
30
40
Stage
1−cell
2−cell
128−cell
1k−cell
Dome
50%−epiboly
Shield
75%−epiboly
1−4−somites
14−19−somites
20−25−somites
Prim−5
Prim−15
Prim−25
Long−pec
Protruding−mouth
Day−4
Day−5
Transcript
ENSDART00000053958
ENSDART00000144823
ENSDART00000158260

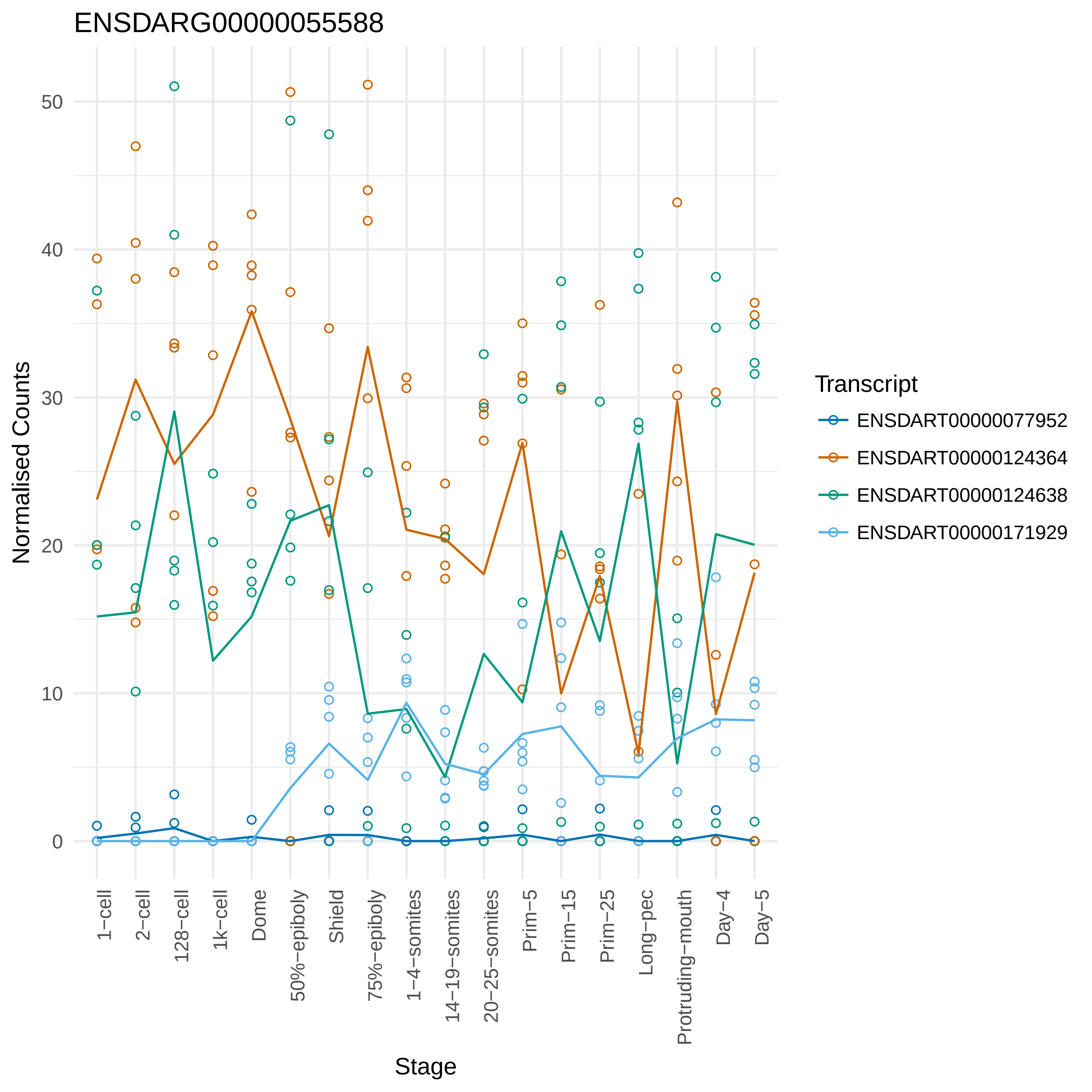

ENSDARG00000055588
Normalised Counts
0
10
20
30
40
50
1−cell
2−cell
128−cell
1k−cell
Dome
50%−epiboly
Shield
75%−epiboly
1−4−somites
14−19−somites
20−25−somites
Prim−5
Prim−15
Prim−25
Long−pec
Protruding−mouth
Day−4
Day−5
Stage
Transcript
ENSDART00000077952
ENSDART00000124364
ENSDART00000124638
ENSDART00000171929

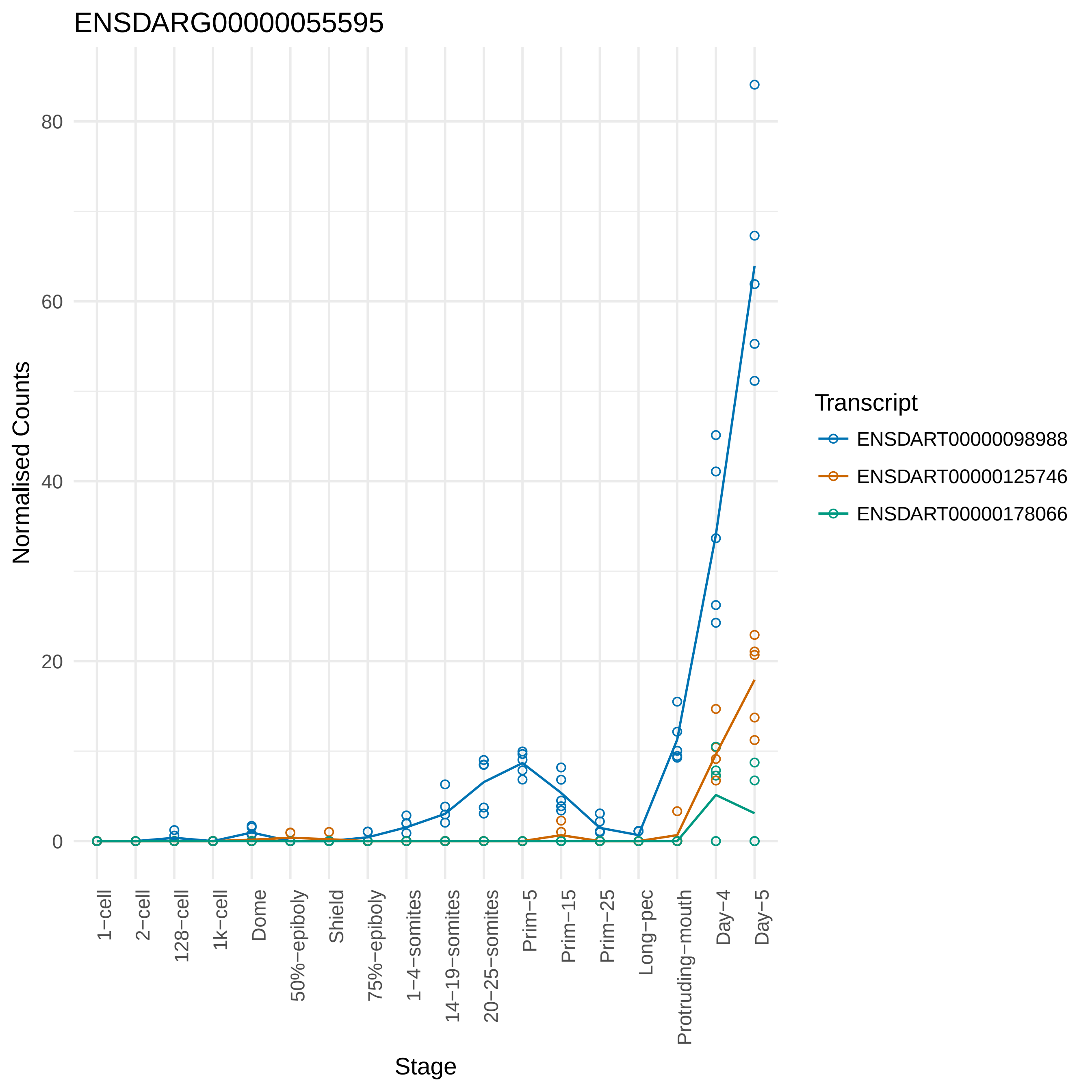
ENSDARG00000055595
Normalised Counts
0
20
40
60
80
1−cell
2−cell
128−cell
1k−cell
Dome
50%−epiboly
Shield
75%−epiboly
1−4−somites
14−19−somites
20−25−somites
Prim−5
Prim−15
Prim−25
Long−pec
Protruding−mouth
Day−4
Day−5
Stage
Transcript
ENSDART00000098988
ENSDART00000125746
ENSDART00000178066

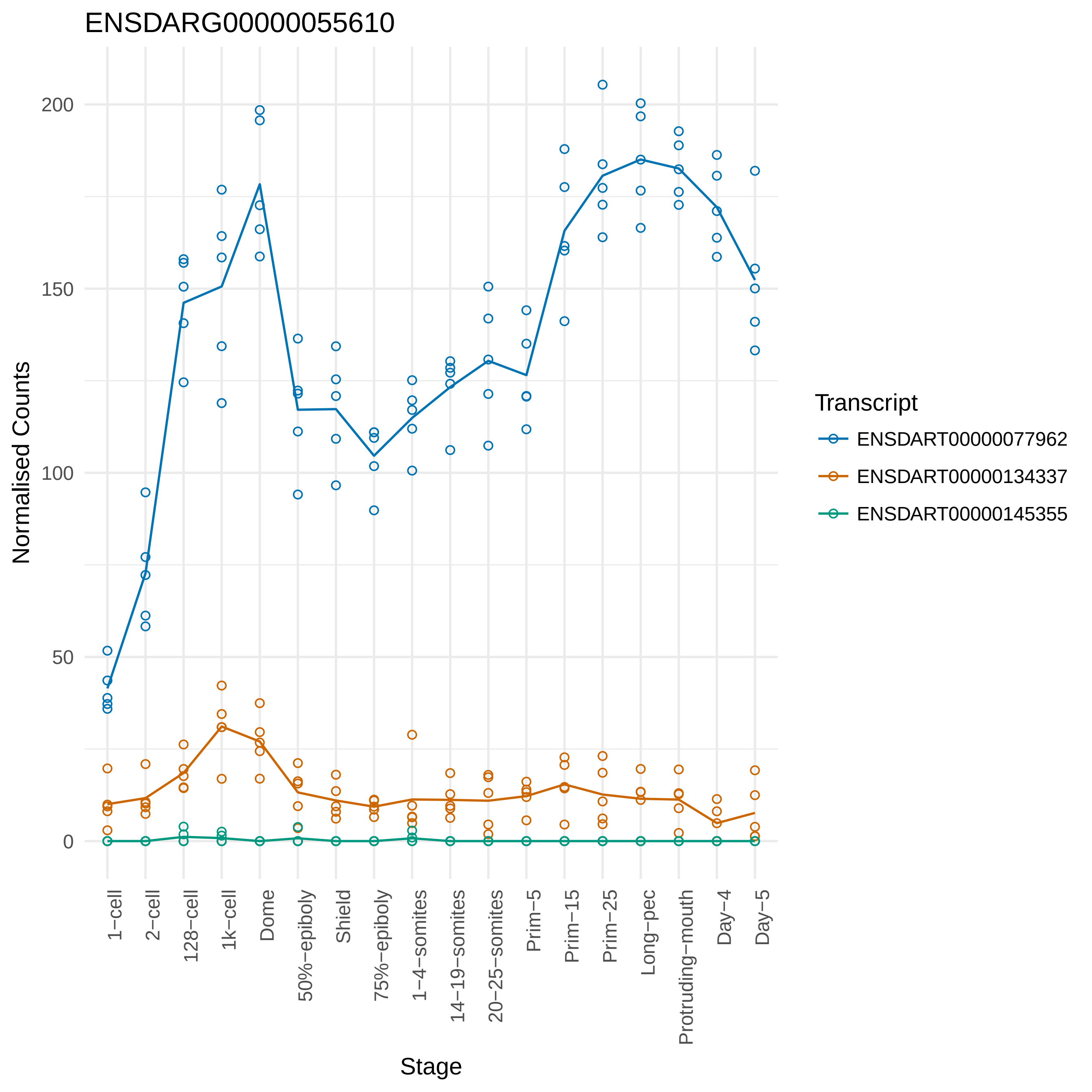
ENSDARG00000055610
Normalised Counts
200
150
100
50
0
1−cell
2−cell
128−cell
1k−cell
Dome
50%−epiboly
Shield
75%−epiboly
1−4−somites
14−19−somites
20−25−somites
Prim−5
Prim−15
Prim−25
Long−pec
Protruding−mouth
Day−4
Day−5
Stage
Transcript
ENSDART00000077962
ENSDART00000134337
ENSDART00000145355

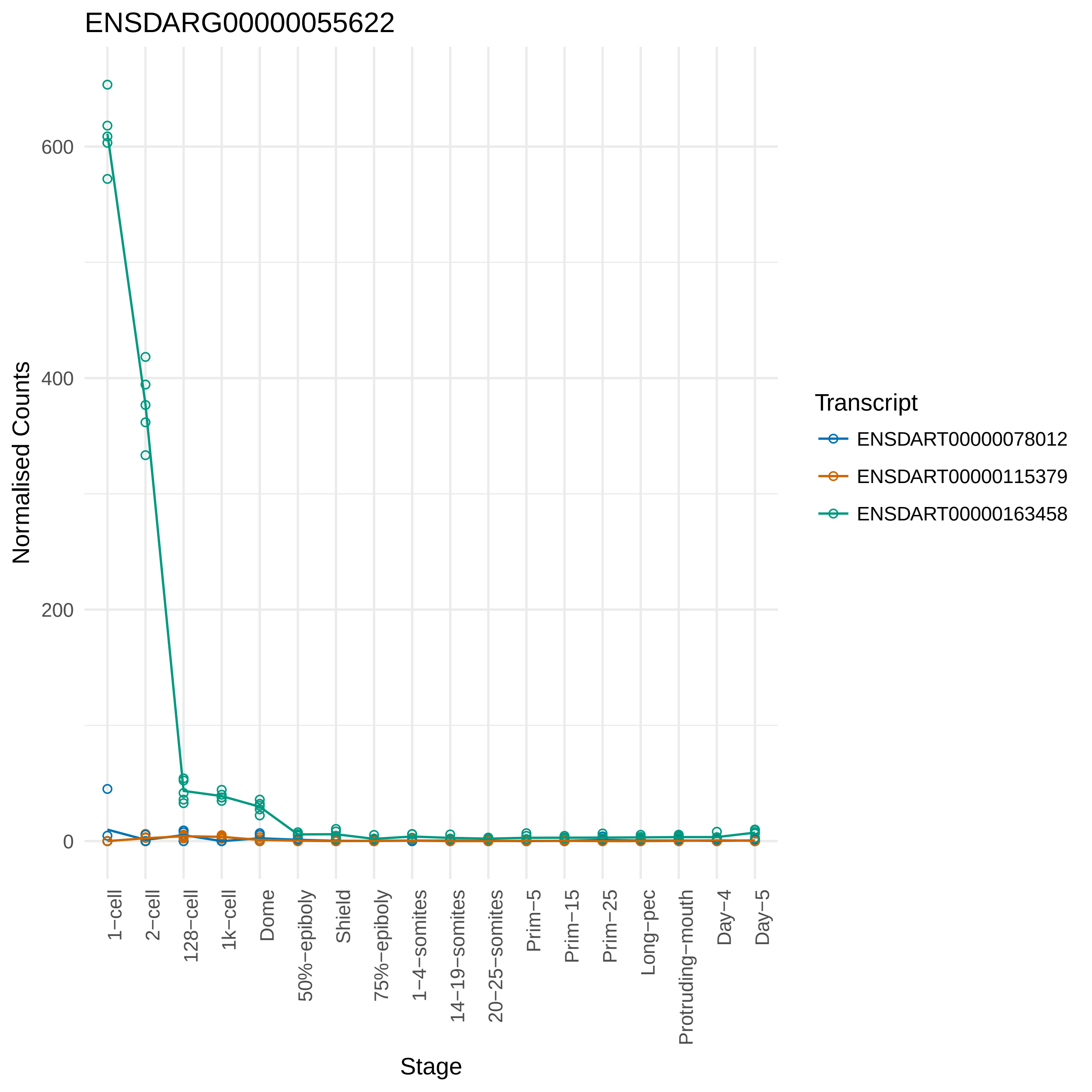
ENSDARG00000055622
Normalised Counts
0
200
400
600
Stage
1−cell
2−cell
128−cell
1k−cell
Dome
50%−epiboly
Shield
75%−epiboly
1−4−somites
14−19−somites
20−25−somites
Prim−5
Prim−15
Prim−25
Long−pec
Protruding−mouth
Day−4
Day−5
Transcript
ENSDART00000078012
ENSDART00000115379
ENSDART00000163458

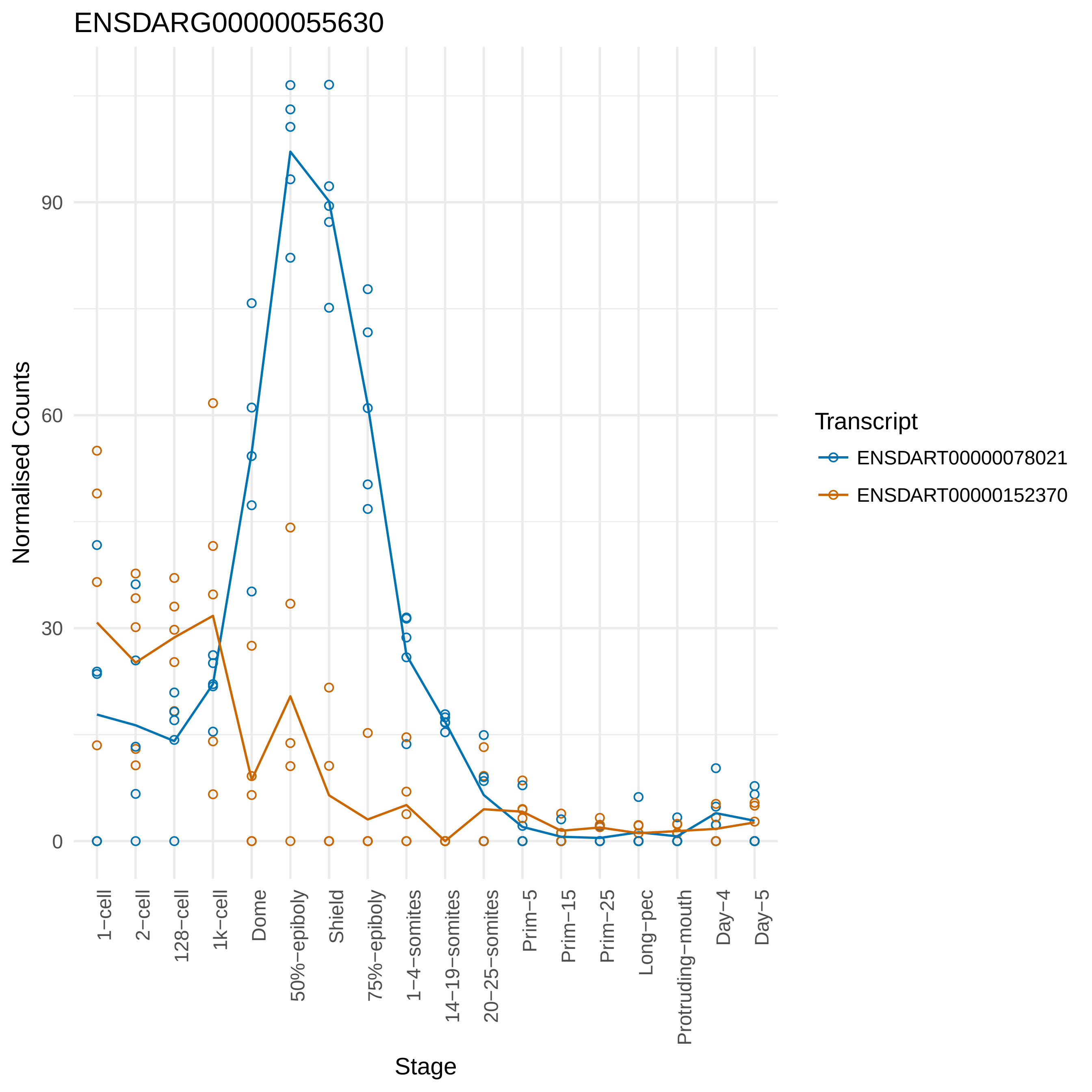

ENSDARG00000055630
Normalised Counts
0
30
60
90
1−cell
2−cell
128−cell
1k−cell
Dome
50%−epiboly
Shield
75%−epiboly
1−4−somites
14−19−somites
20−25−somites
Prim−5
Prim−15
Prim−25
Long−pec
Protruding−mouth
Day−4
Day−5
Stage
Transcript
ENSDART00000078021
ENSDART00000152370

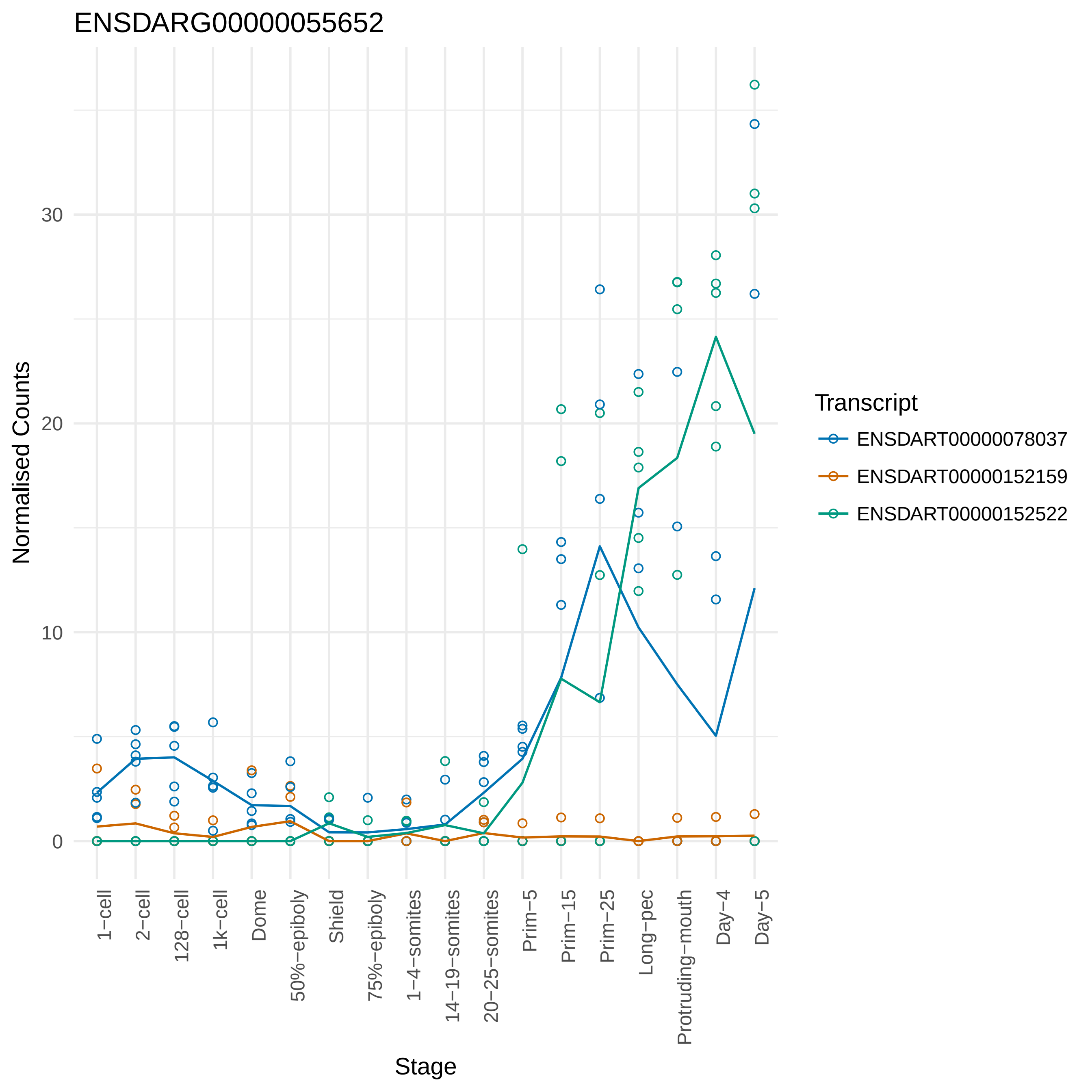
ENSDARG00000055652
Normalised Counts
30
20
10
0
1−cell
2−cell
128−cell
1k−cell
Dome
50%−epiboly
Shield
75%−epiboly
1−4−somites
14−19−somites
20−25−somites
Prim−5
Prim−15
Prim−25
Long−pec
Protruding−mouth
Day−4
Day−5
Stage
Transcript
ENSDART00000078037
ENSDART00000152159
ENSDART00000152522

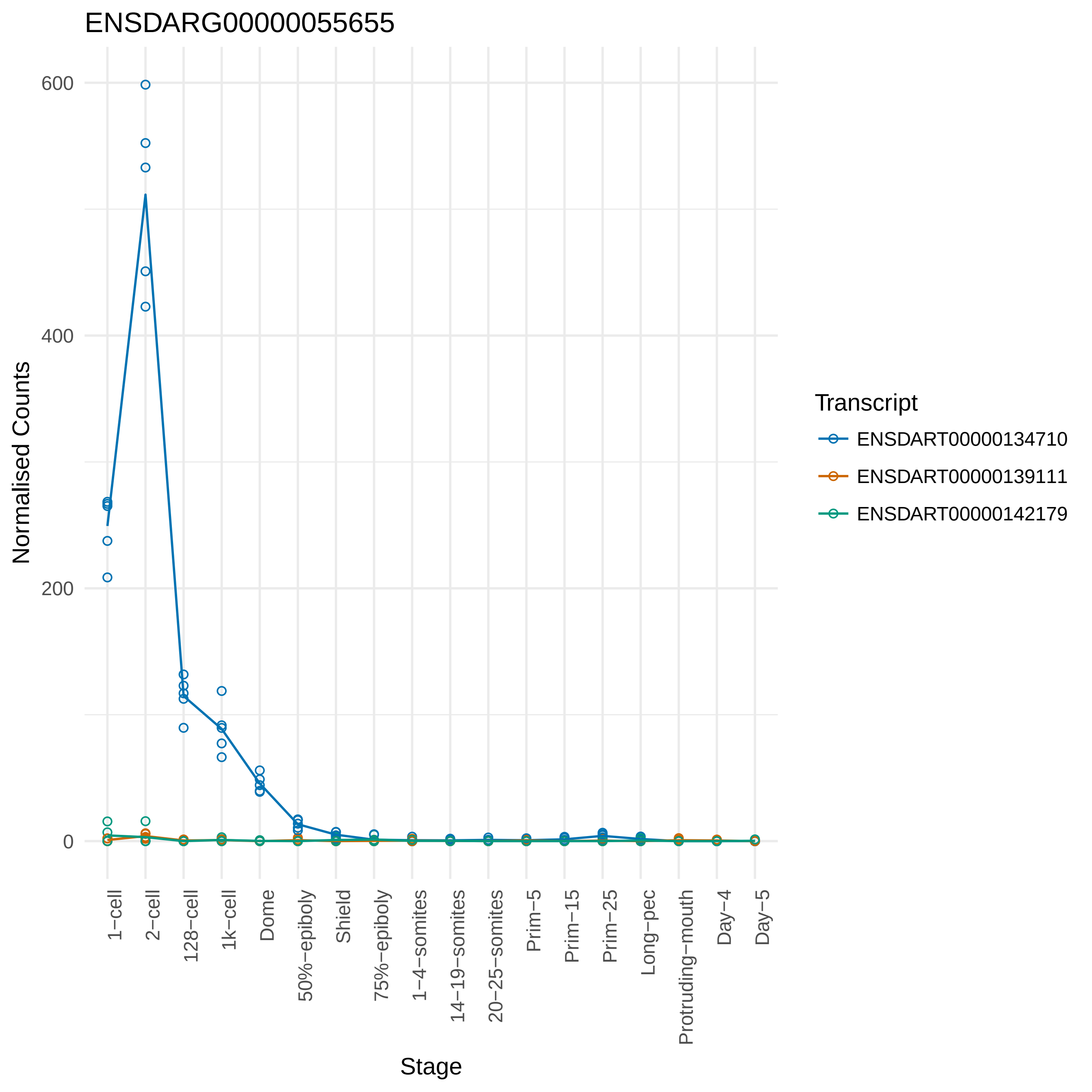

ENSDARG00000055655
600
400
200
0
Normalised Counts
1−cell
2−cell
128−cell
1k−cell
Dome
50%−epiboly
Shield
75%−epiboly
1−4−somites
14−19−somites
20−25−somites
Prim−5
Prim−15
Prim−25
Long−pec
Protruding−mouth
Day−4
Day−5
Stage
Transcript
ENSDART00000134710
ENSDART00000139111
ENSDART00000142179

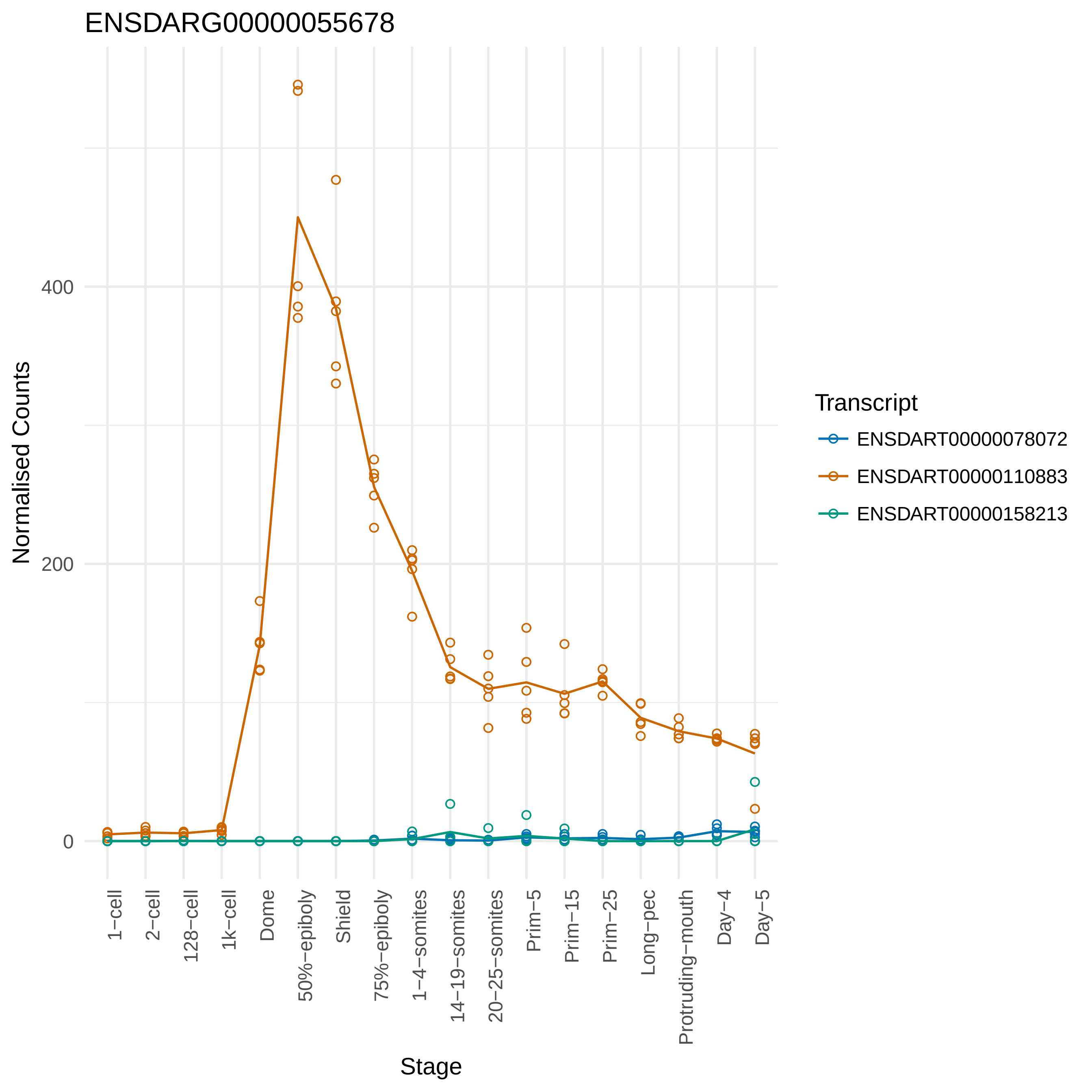

ENSDARG00000055678
Normalised Counts
400
200
0
1−cell
2−cell
128−cell
1k−cell
Dome
50%−epiboly
Shield
75%−epiboly
1−4−somites
14−19−somites
20−25−somites
Prim−5
Prim−15
Prim−25
Long−pec
Protruding−mouth
Day−4
Day−5
Stage
Transcript
ENSDART00000078072
ENSDART00000110883
ENSDART00000158213

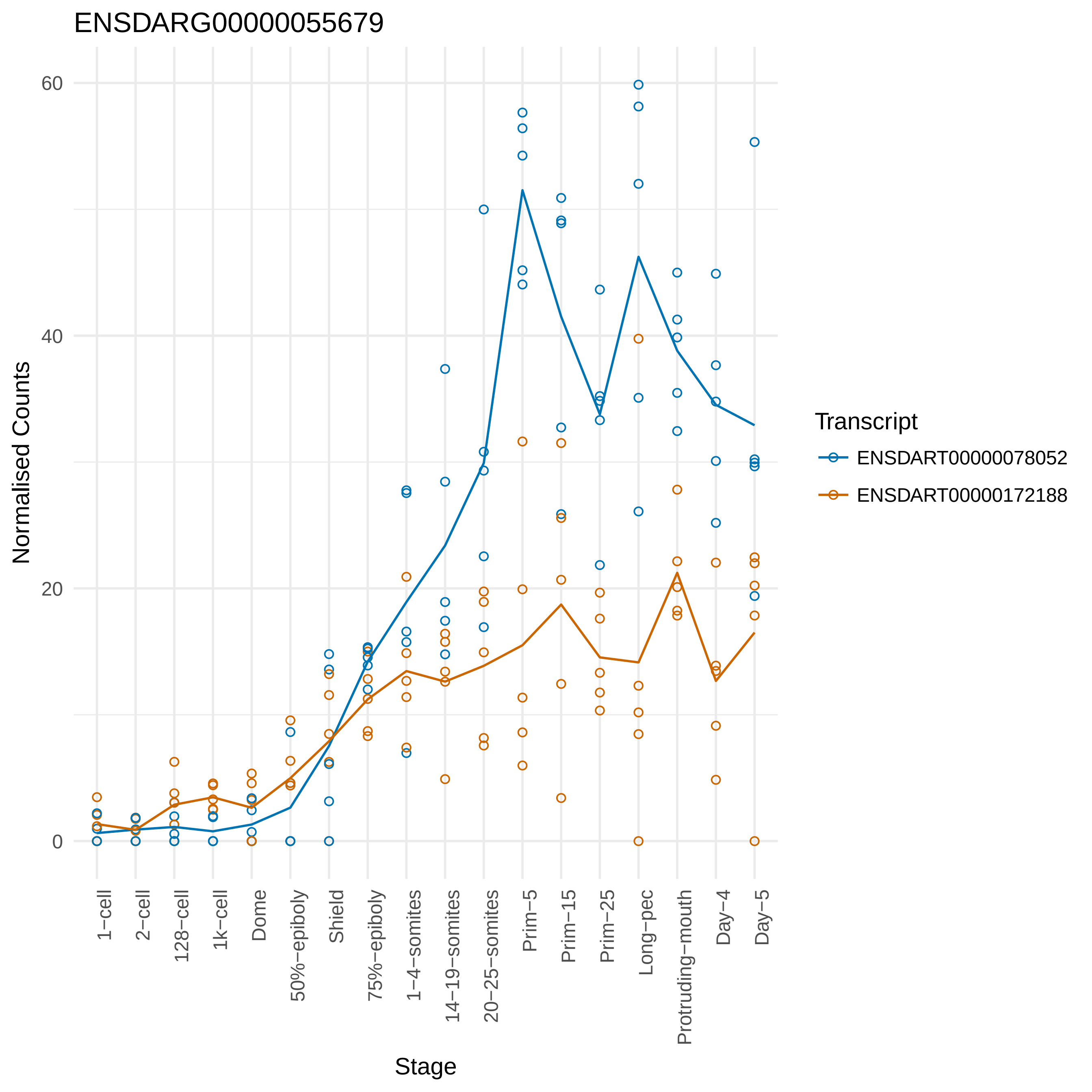
ENSDARG00000055679
Normalised Counts
60
40
20
0
1−cell
2−cell
128−cell
1k−cell
Dome
50%−epiboly
Shield
75%−epiboly
1−4−somites
14−19−somites
20−25−somites
Prim−5
Prim−15
Prim−25
Long−pec
Protruding−mouth
Day−4
Day−5
Stage
Transcript
ENSDART00000078052
ENSDART00000172188

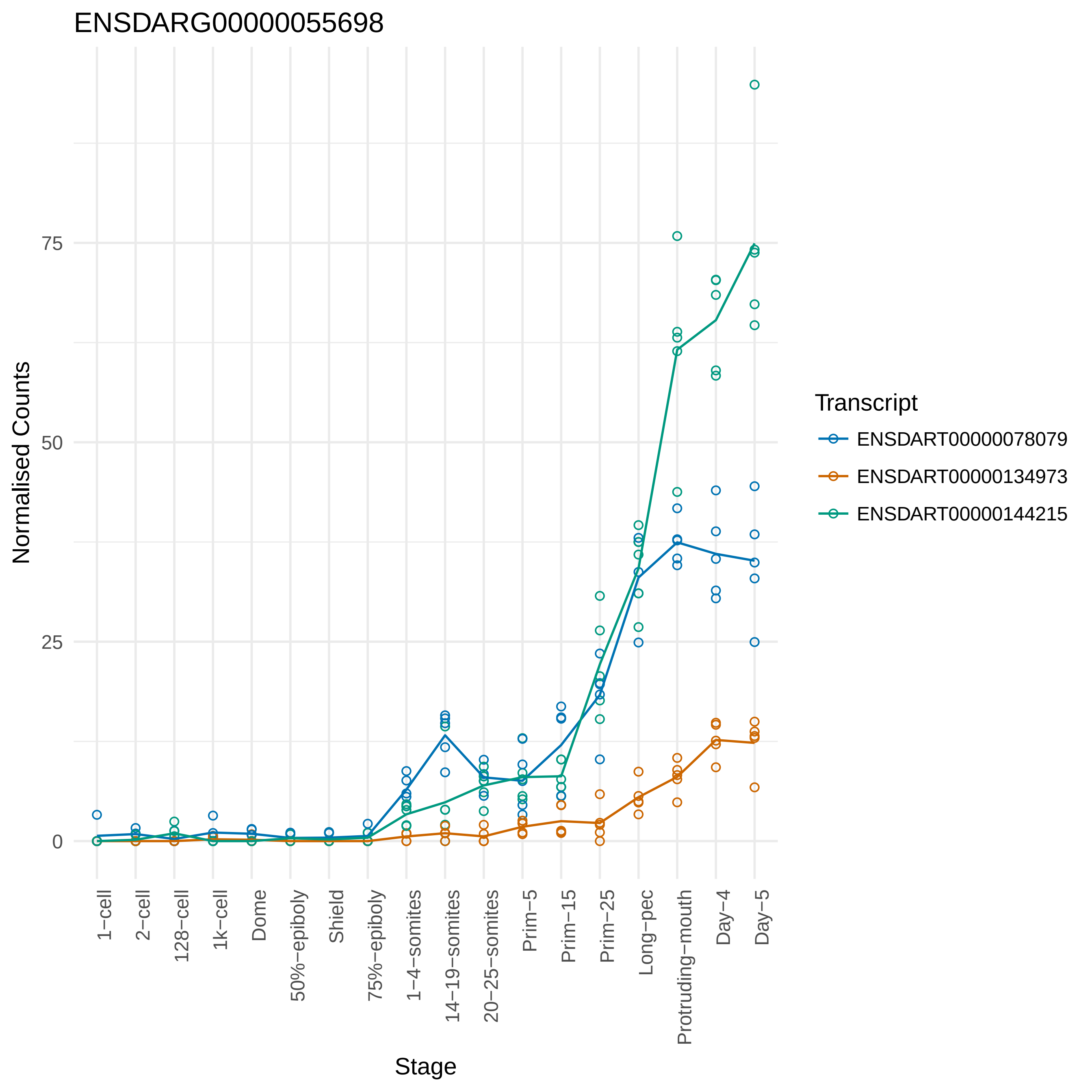

ENSDARG00000055698
Normalised Counts
0
25
50
75
1−cell
2−cell
128−cell
1k−cell
Dome
50%−epiboly
Shield
75%−epiboly
1−4−somites
14−19−somites
20−25−somites
Prim−5
Prim−15
Prim−25
Long−pec
Protruding−mouth
Day−4
Day−5
Stage
Transcript
ENSDART00000078079
ENSDART00000134973
ENSDART00000144215

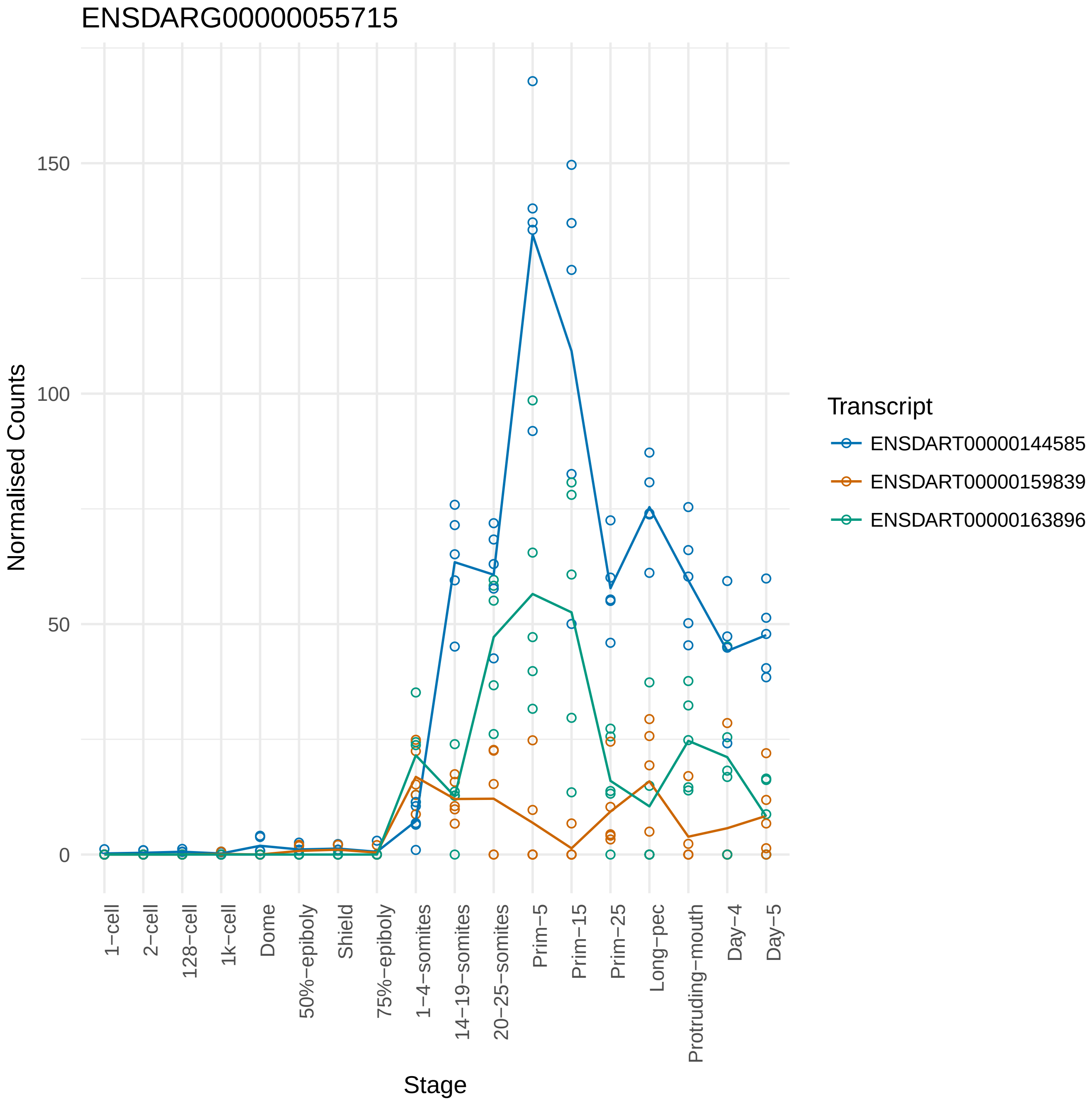

ENSDARG00000055715
Normalised Counts
150
100
50
0
1−cell
2−cell
128−cell
1k−cell
Dome
50%−epiboly
Shield
75%−epiboly
1−4−somites
14−19−somites
20−25−somites
Prim−5
Prim−15
Prim−25
Long−pec
Protruding−mouth
Day−4
Day−5
Stage
Transcript
ENSDART00000144585
ENSDART00000159839
ENSDART00000163896

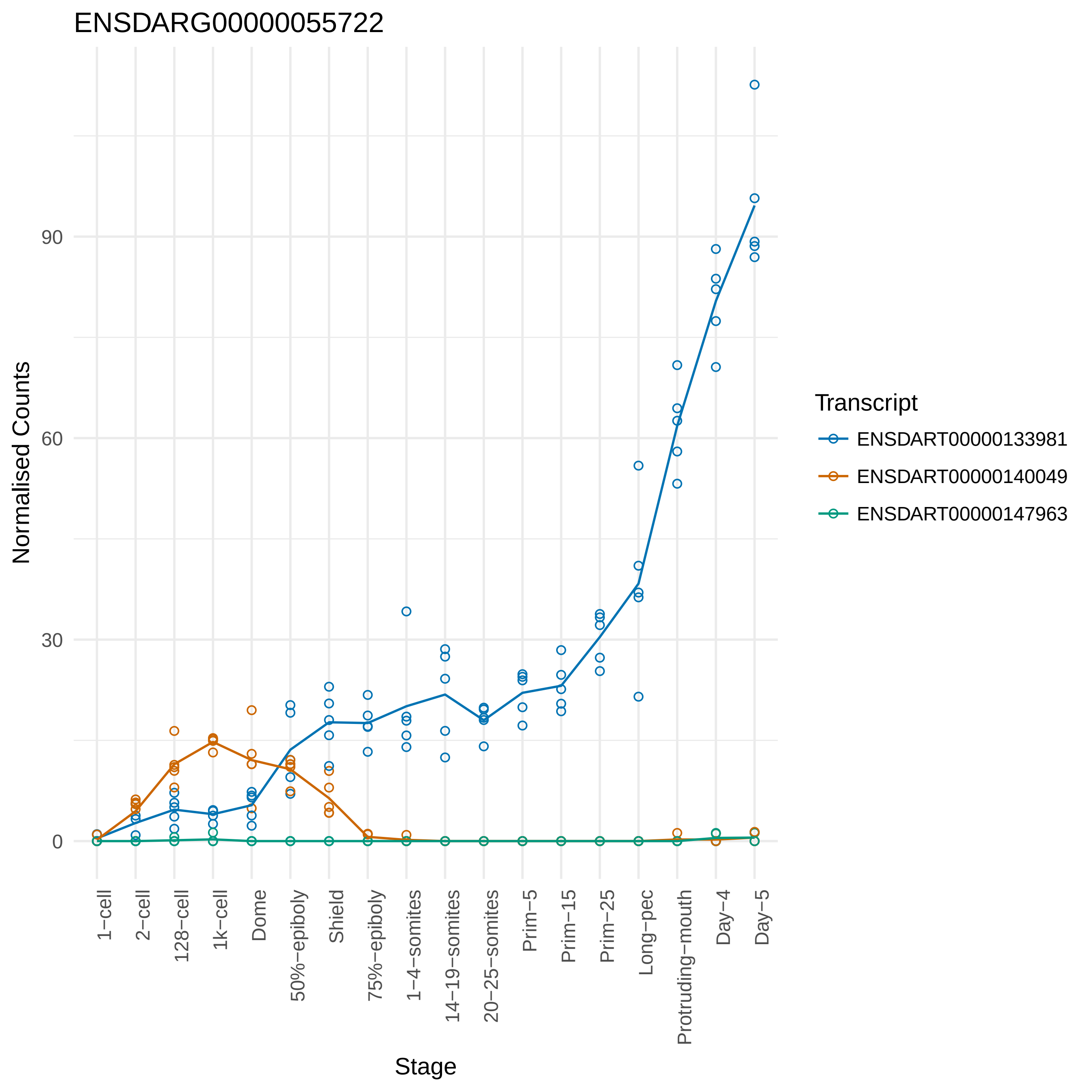

ENSDARG00000055722
Normalised Counts
0
30
60
90
Stage
1−cell
2−cell
128−cell
1k−cell
Dome
50%−epiboly
Shield
75%−epiboly
1−4−somites
14−19−somites
20−25−somites
Prim−5
Prim−15
Prim−25
Long−pec
Protruding−mouth
Day−4
Day−5
Transcript
ENSDART00000133981
ENSDART00000140049
ENSDART00000147963

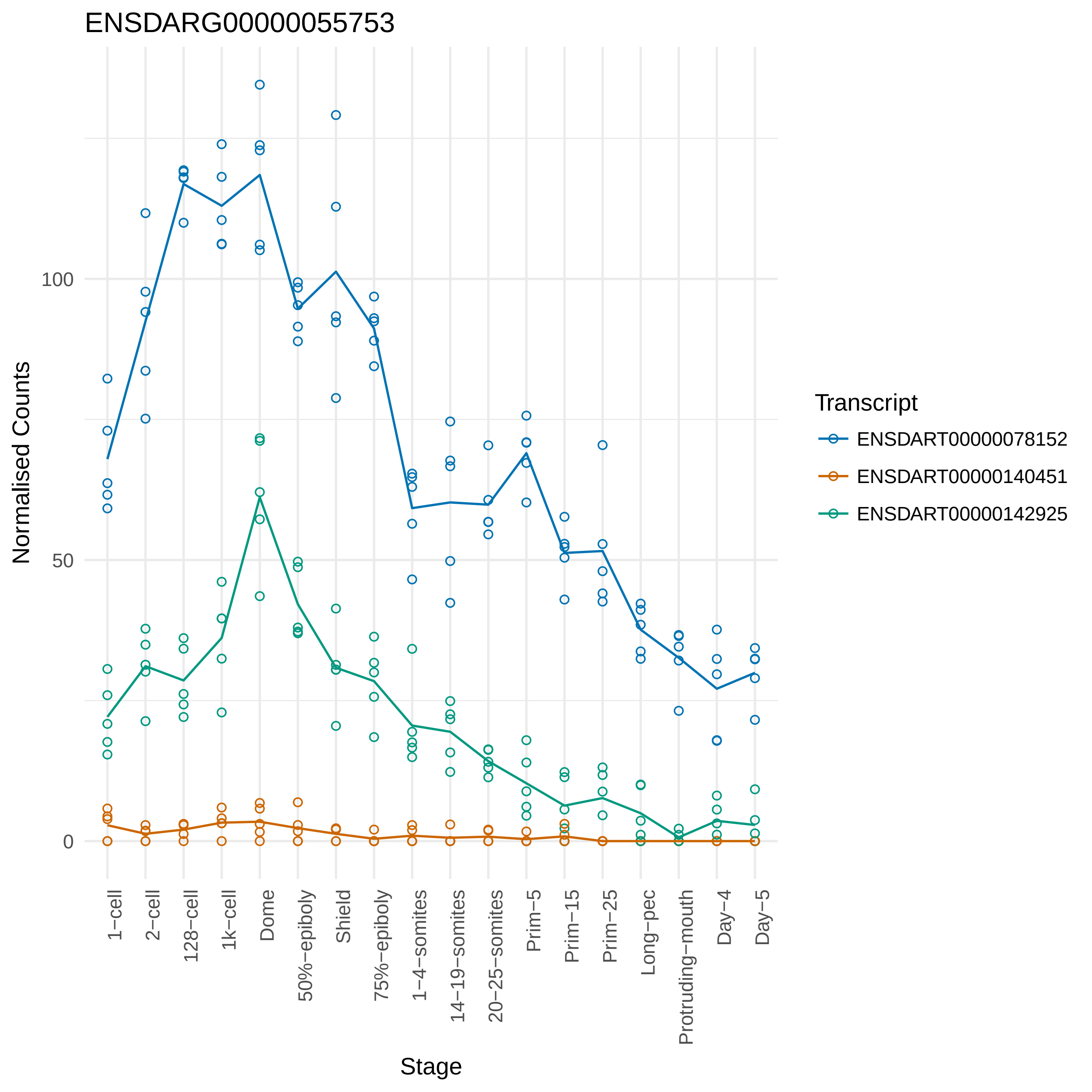

ENSDARG00000055753
Normalised Counts
0
50
100
Stage
1−cell
2−cell
128−cell
1k−cell
Dome
50%−epiboly
Shield
75%−epiboly
1−4−somites
14−19−somites
20−25−somites
Prim−5
Prim−15
Prim−25
Long−pec
Protruding−mouth
Day−4
Day−5
Transcript
ENSDART00000078152
ENSDART00000140451
ENSDART00000142925

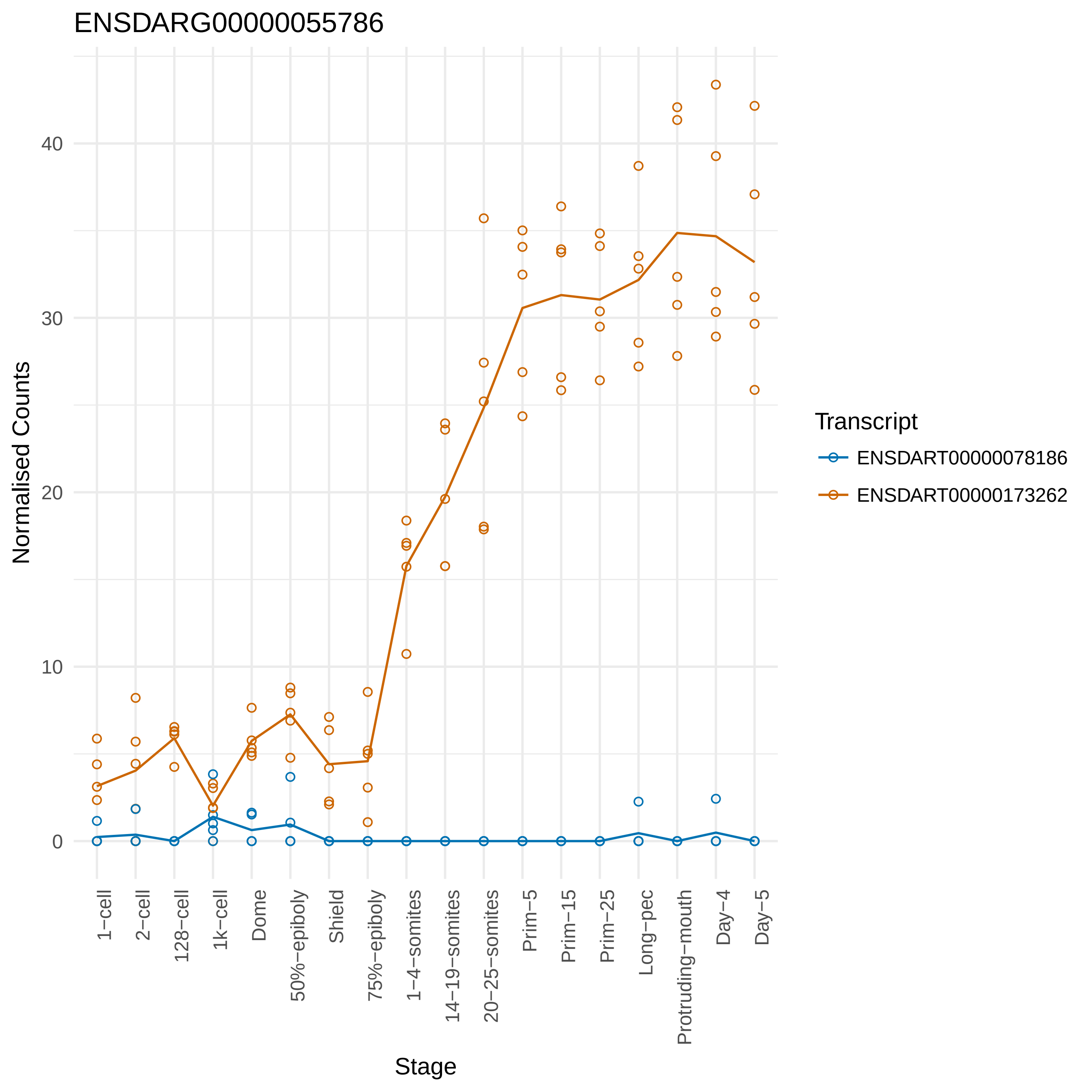
ENSDARG00000055786
Normalised Counts
Stage
Transcript
ENSDART00000078186
ENSDART00000173262
0
10
20
30
40
1−cell
2−cell
128−cell
1k−cell
Dome
50%−epiboly
Shield
75%−epiboly
1−4−somites
14−19−somites
20−25−somites
Prim−5
Prim−15
Prim−25
Long−pec
Protruding−mouth
Day−4
Day−5

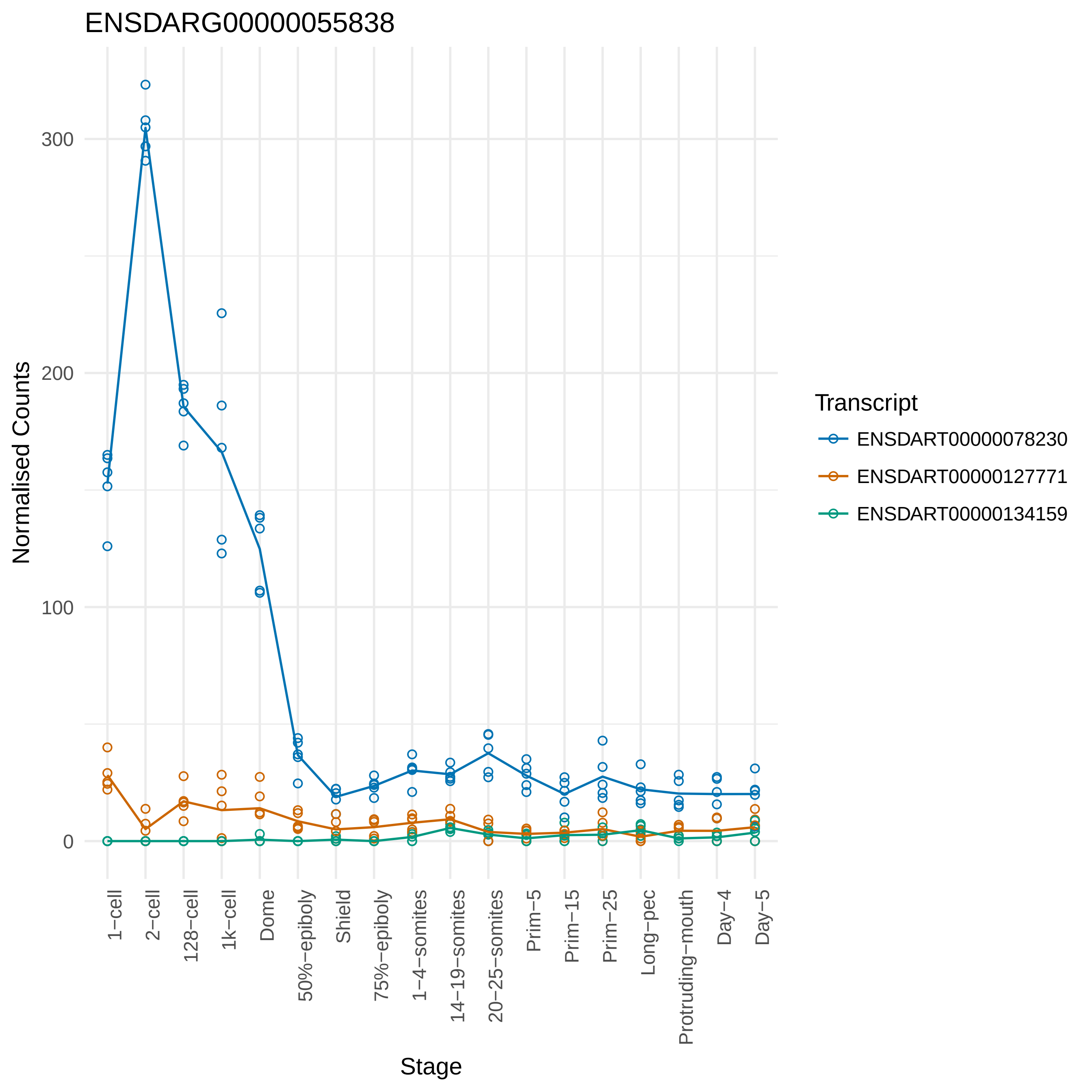
ENSDARG00000055838
Normalised Counts
300
200
100
0
1−cell
2−cell
128−cell
1k−cell
Dome
50%−epiboly
Shield
75%−epiboly
1−4−somites
14−19−somites
20−25−somites
Prim−5
Prim−15
Prim−25
Long−pec
Protruding−mouth
Day−4
Day−5
Stage
Transcript
ENSDART00000078230
ENSDART00000127771
ENSDART00000134159

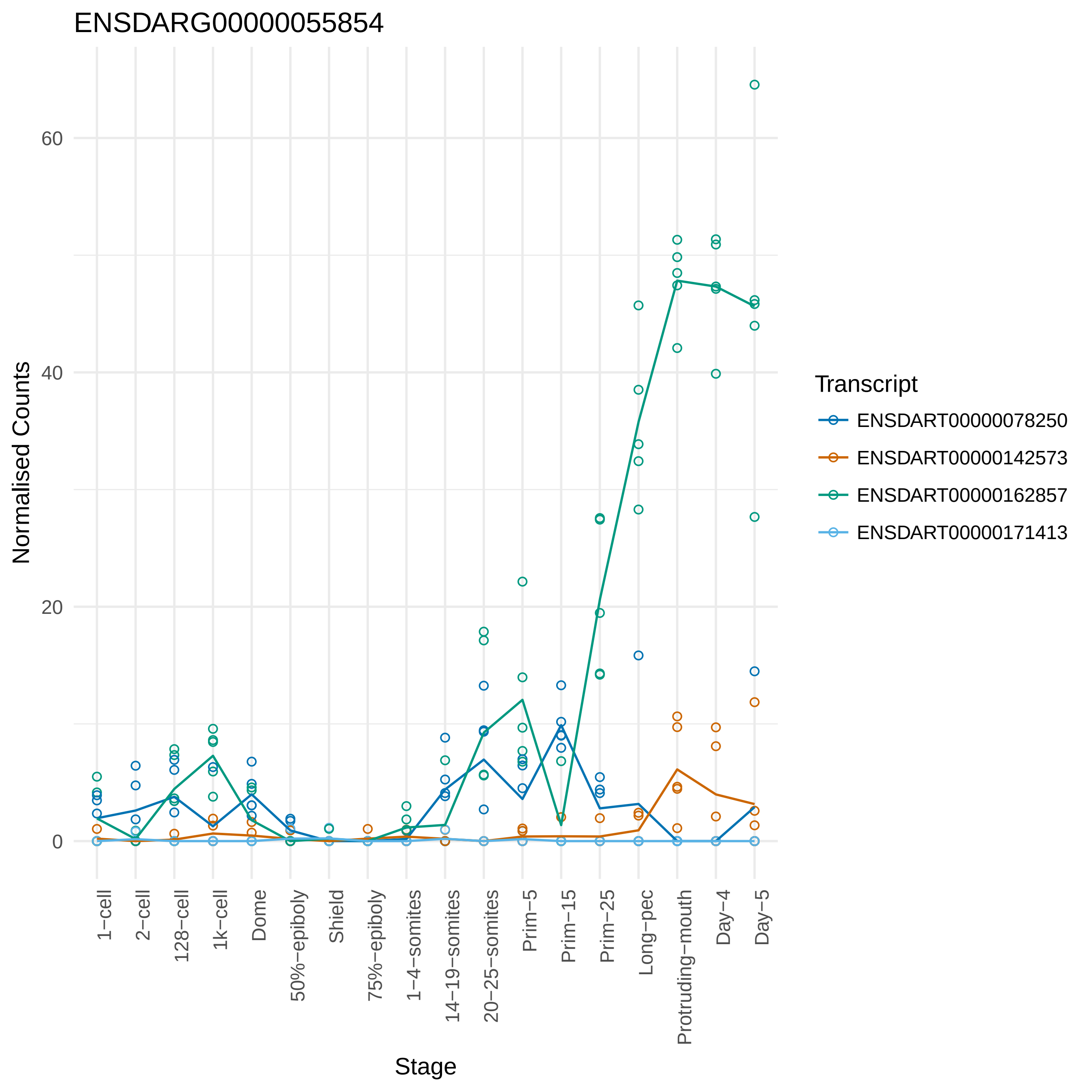
ENSDARG00000055854
Normalised Counts
0
20
40
60
Stage
1−cell
2−cell
128−cell
1k−cell
Dome
50%−epiboly
Shield
75%−epiboly
1−4−somites
14−19−somites
20−25−somites
Prim−5
Prim−15
Prim−25
Long−pec
Protruding−mouth
Day−4
Day−5
Transcript
ENSDART00000078250
ENSDART00000142573
ENSDART00000162857
ENSDART00000171413

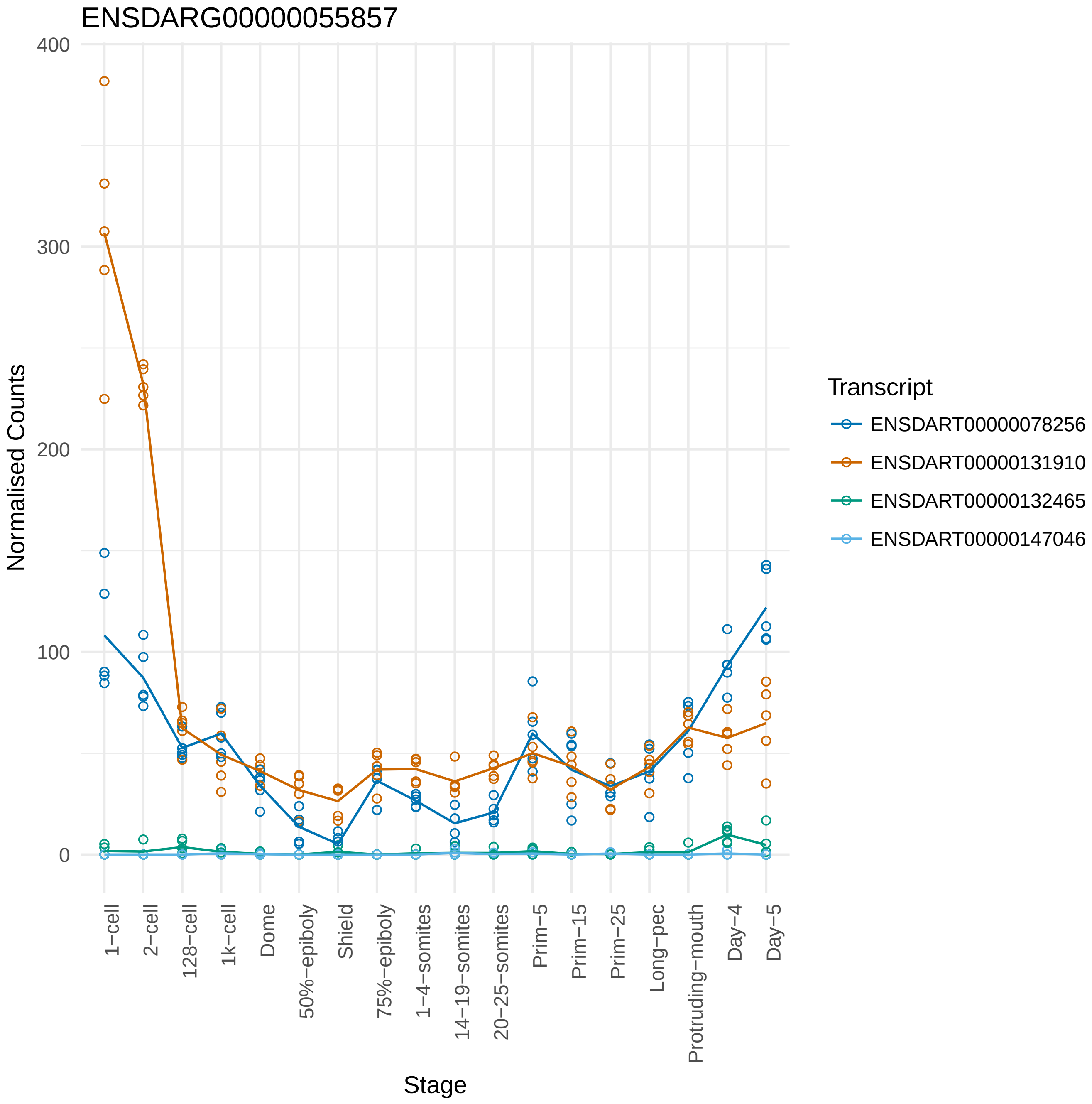
ENSDARG00000055857
Normalised Counts
400
300
200
100
0
1−cell
2−cell
128−cell
1k−cell
Dome
50%−epiboly
Shield
75%−epiboly
1−4−somites
14−19−somites
20−25−somites
Prim−5
Prim−15
Prim−25
Long−pec
Protruding−mouth
Day−4
Day−5
Stage
Transcript
ENSDART00000078256
ENSDART00000131910
ENSDART00000132465
ENSDART00000147046

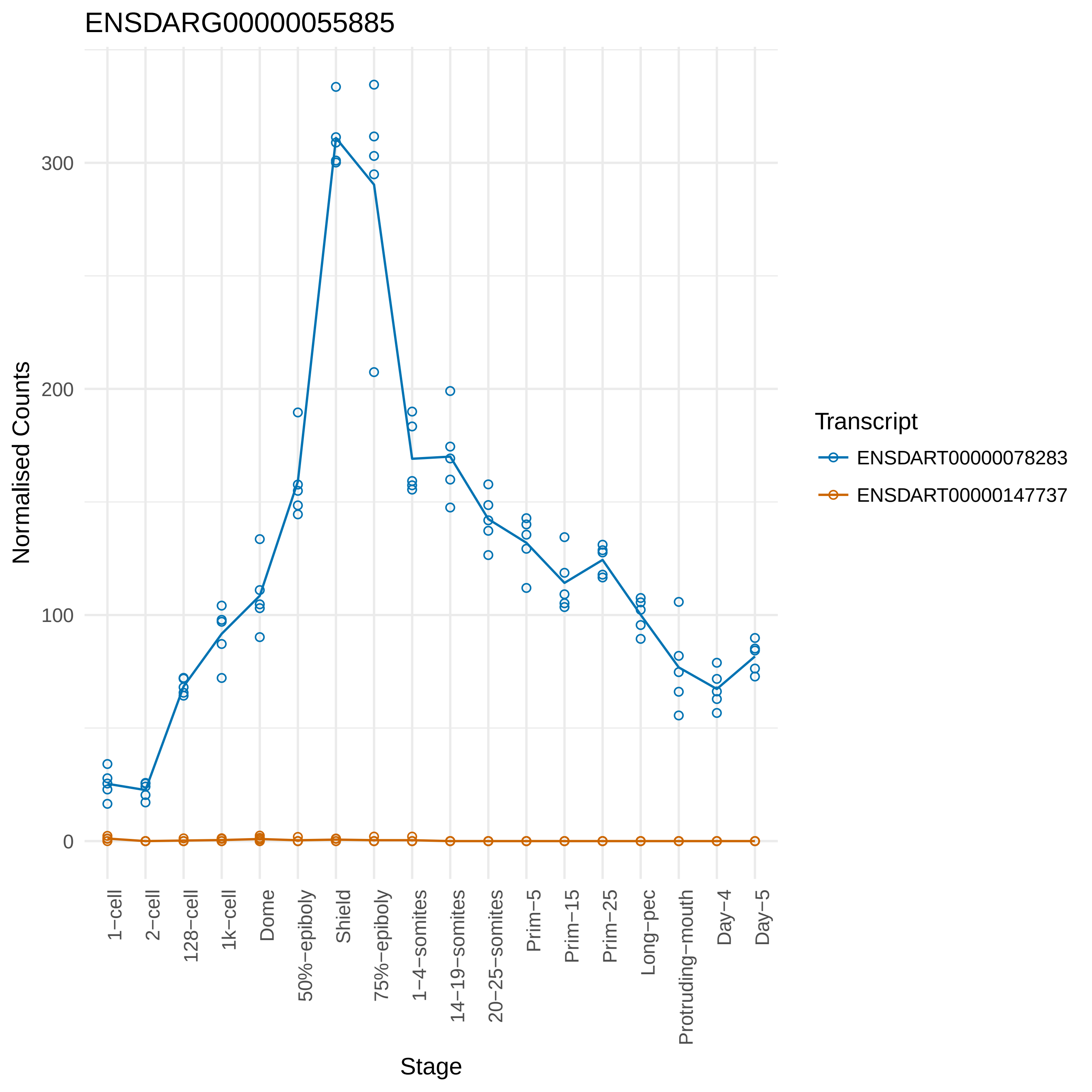

ENSDARG00000055885
Normalised Counts
300
200
100
0
1−cell
2−cell
128−cell
1k−cell
Dome
50%−epiboly
Shield
75%−epiboly
1−4−somites
14−19−somites
20−25−somites
Prim−5
Prim−15
Prim−25
Long−pec
Protruding−mouth
Day−4
Day−5
Stage
Transcript
ENSDART00000078283
ENSDART00000147737

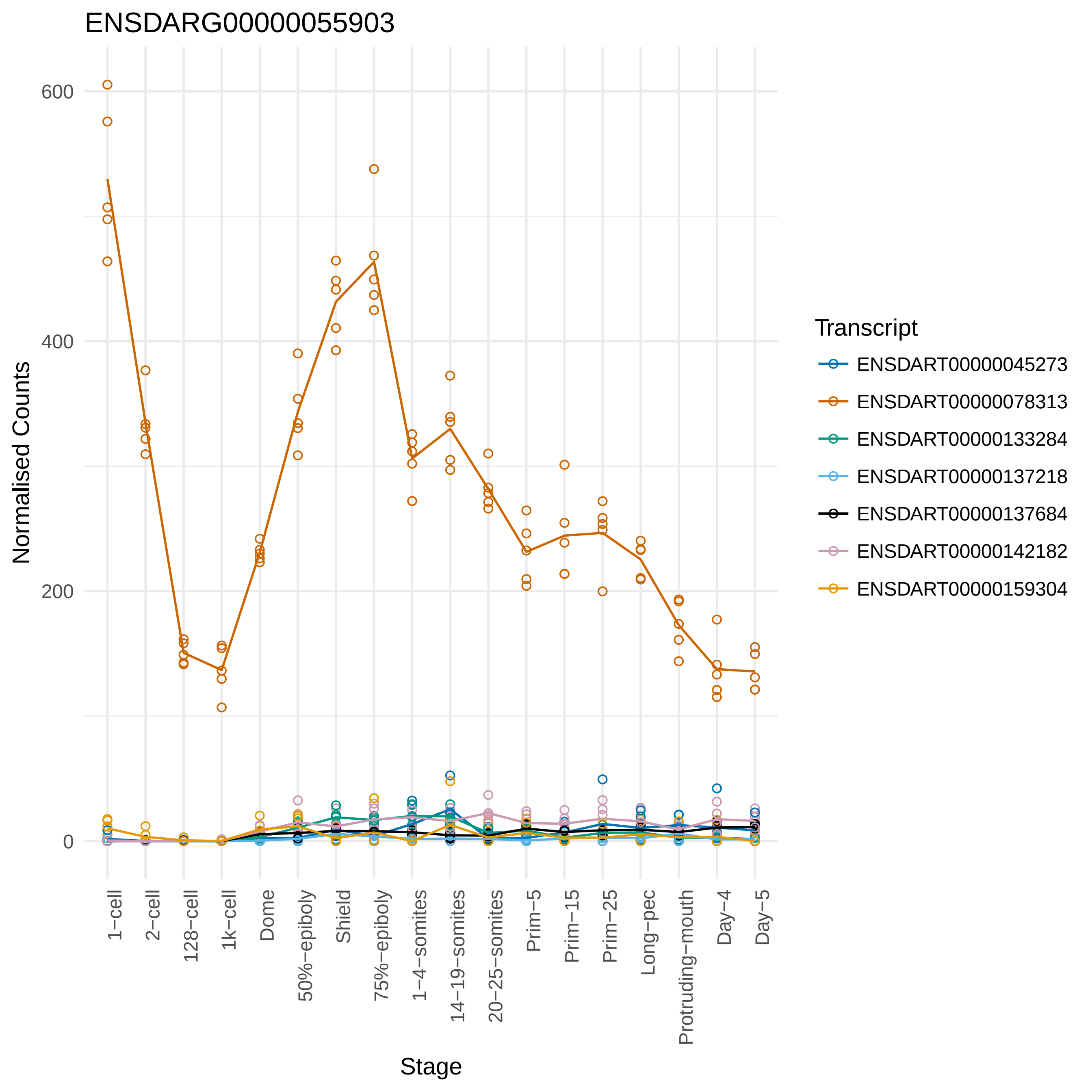

ENSDARG00000055903
Normalised Counts
0
200
400
600
Stage
1−cell
2−cell
128−cell
1k−cell
Dome
50%−epiboly
Shield
75%−epiboly
1−4−somites
14−19−somites
20−25−somites
Prim−5
Prim−15
Prim−25
Long−pec
Protruding−mouth
Day−4
Day−5
Transcript
ENSDART00000045273
ENSDART00000078313
ENSDART00000133284
ENSDART00000137218
ENSDART00000137684
ENSDART00000142182
ENSDART00000159304

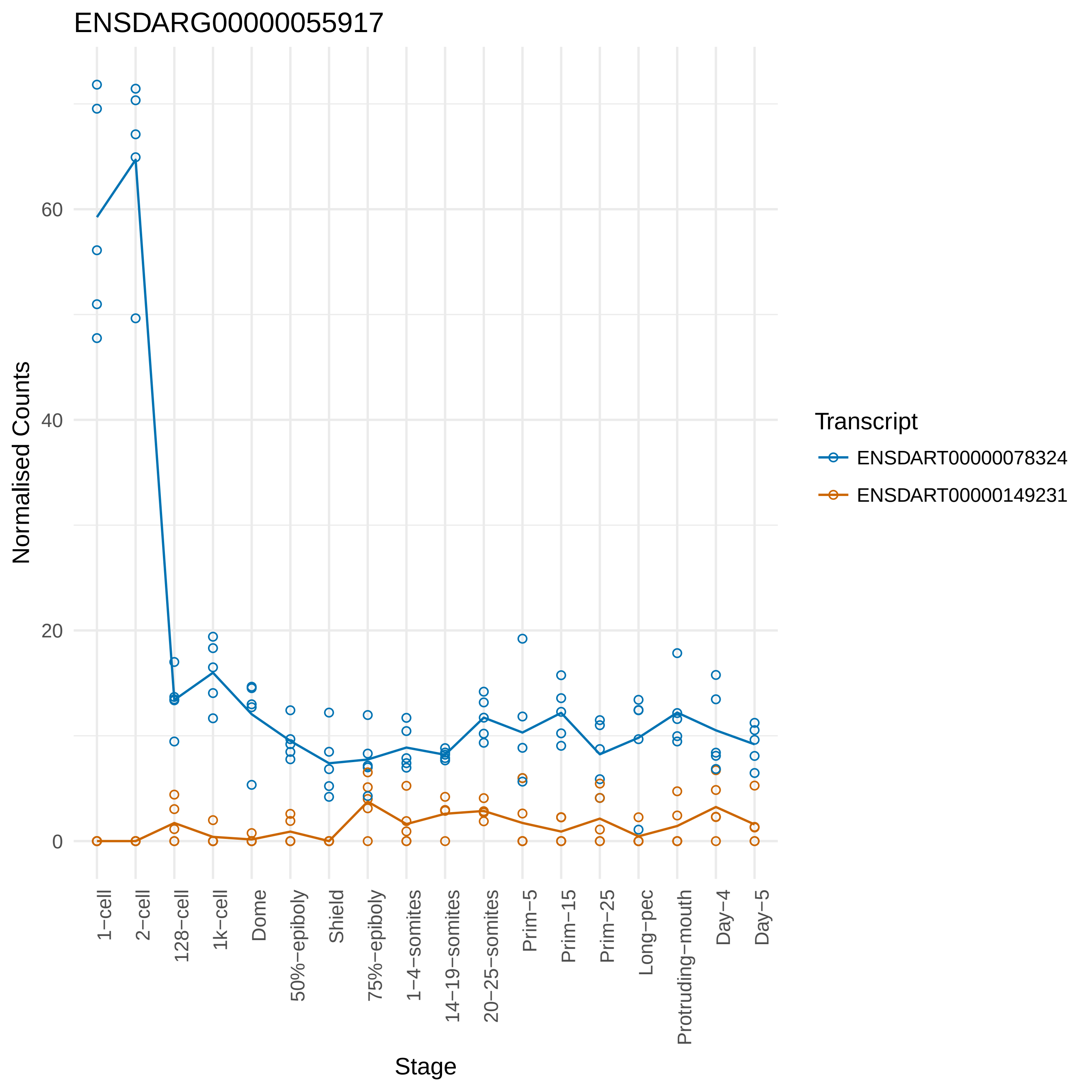
ENSDARG00000055917
Normalised Counts
0
20
40
60
Stage
1−cell
2−cell
128−cell
1k−cell
Dome
50%−epiboly
Shield
75%−epiboly
1−4−somites
14−19−somites
20−25−somites
Prim−5
Prim−15
Prim−25
Long−pec
Protruding−mouth
Day−4
Day−5
Transcript
ENSDART00000078324
ENSDART00000149231

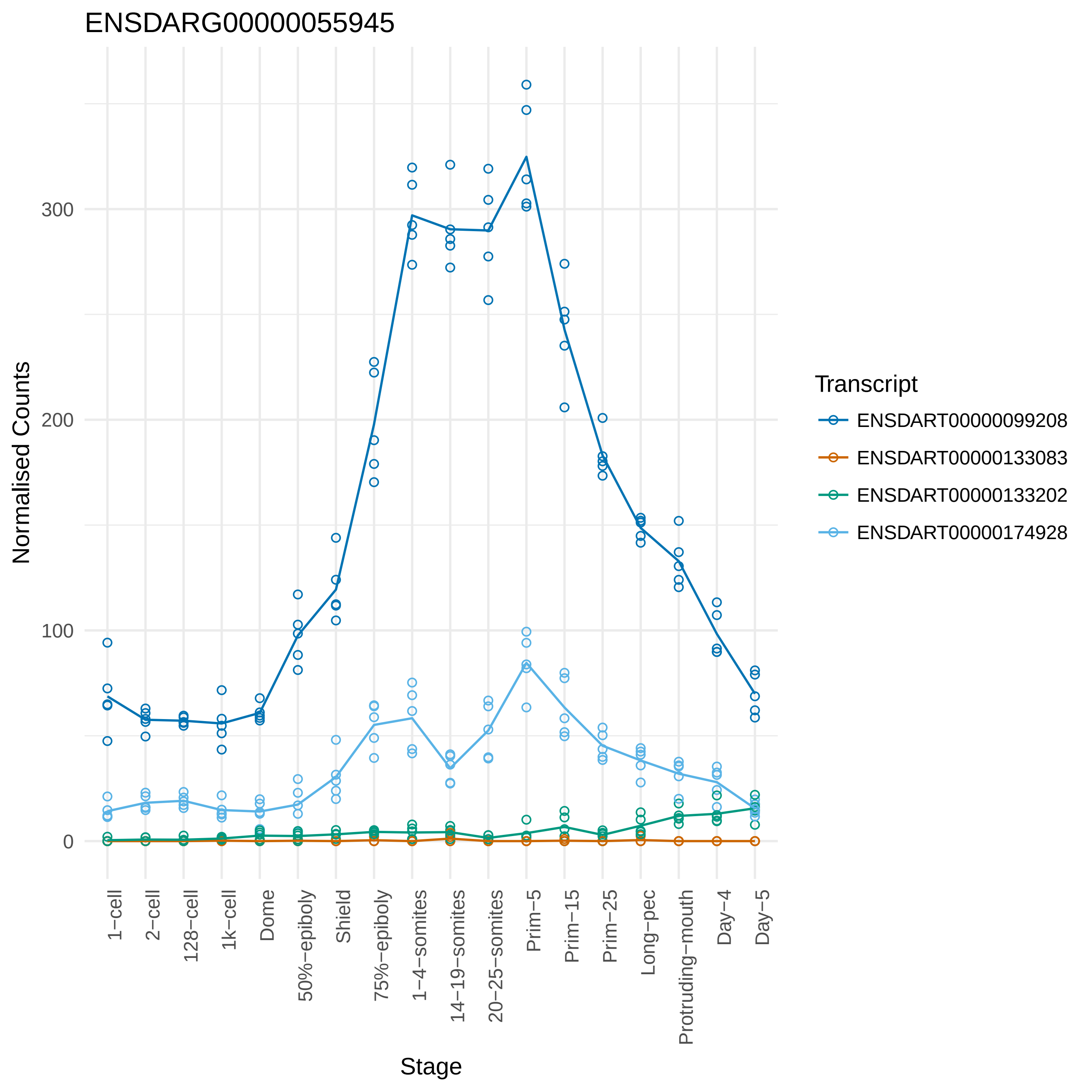
ENSDARG00000055945
Normalised Counts
300
200
100
0
Stage
1−cell
2−cell
128−cell
1k−cell
Dome
50%−epiboly
Shield
75%−epiboly
1−4−somites
14−19−somites
20−25−somites
Prim−5
Prim−15
Prim−25
Long−pec
Protruding−mouth
Day−4
Day−5
Transcript
ENSDART00000099208
ENSDART00000133083
ENSDART00000133202
ENSDART00000174928

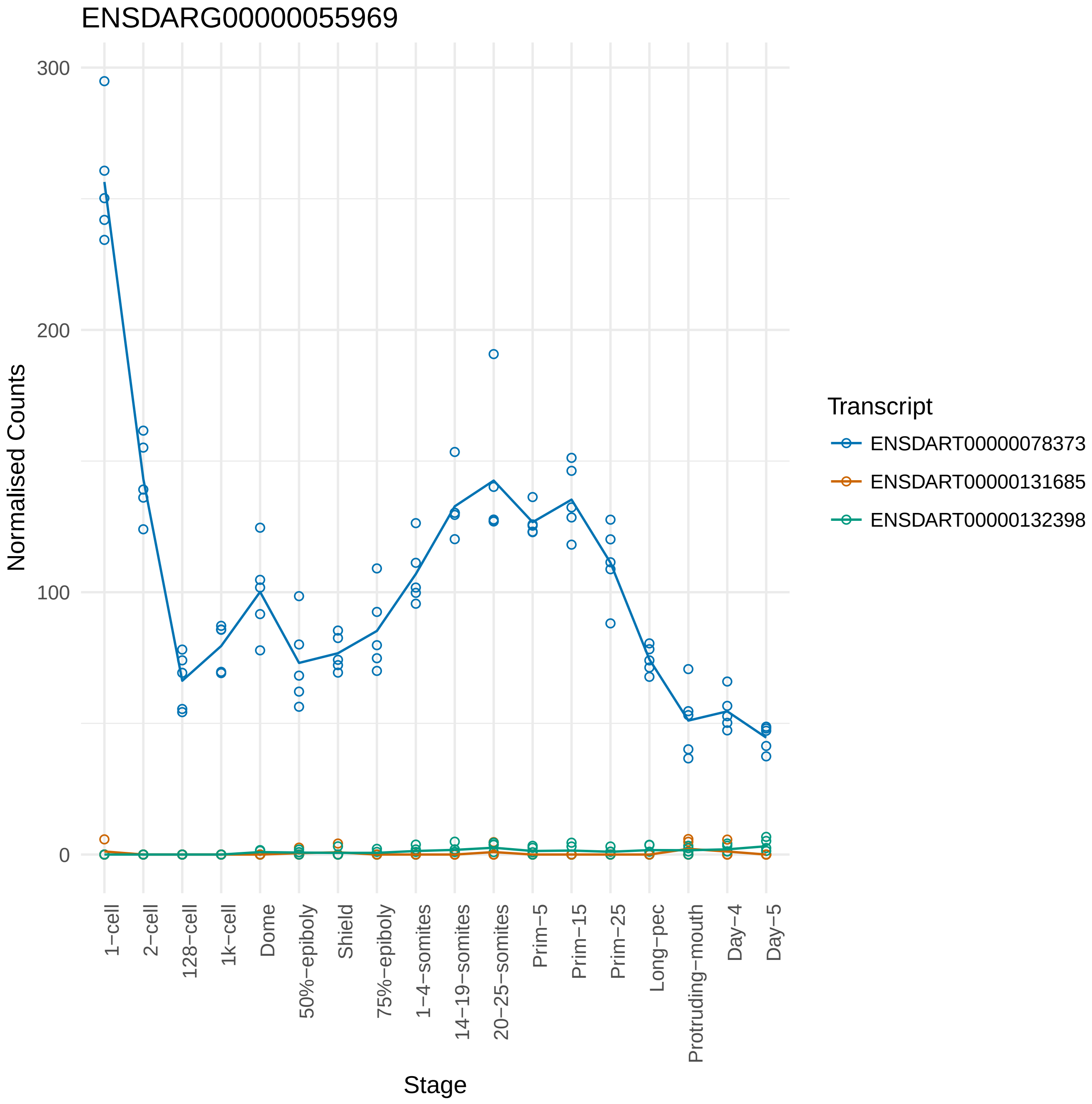

ENSDARG00000055969
Normalised Counts
300
200
100
0
1−cell
2−cell
128−cell
1k−cell
Dome
50%−epiboly
Shield
75%−epiboly
1−4−somites
14−19−somites
20−25−somites
Prim−5
Prim−15
Prim−25
Long−pec
Protruding−mouth
Day−4
Day−5
Stage
Transcript
ENSDART00000078373
ENSDART00000131685
ENSDART00000132398

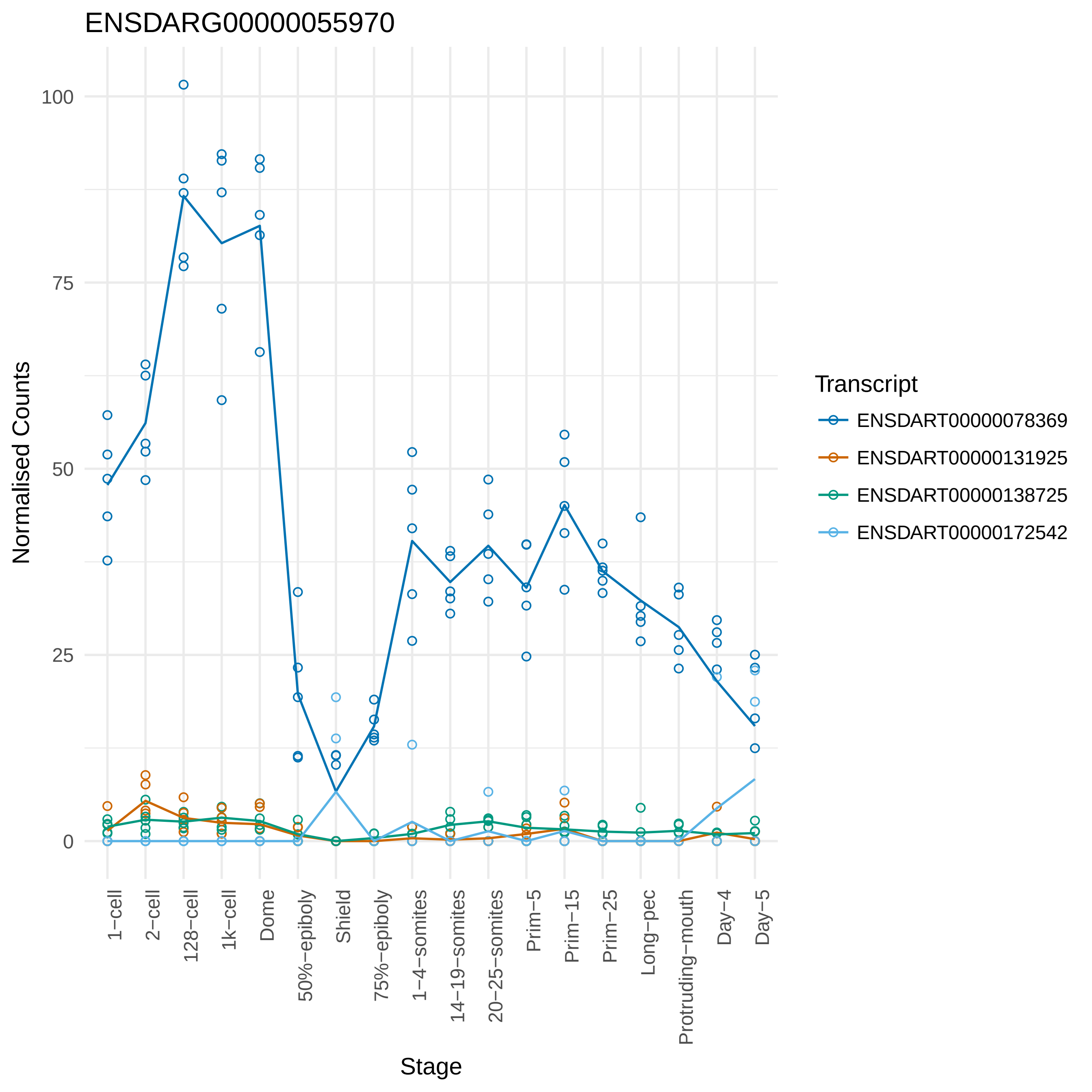
ENSDARG00000055970
Normalised Counts
100
75
50
25
0
1−cell
2−cell
128−cell
1k−cell
Dome
50%−epiboly
Shield
75%−epiboly
1−4−somites
14−19−somites
20−25−somites
Prim−5
Prim−15
Prim−25
Long−pec
Protruding−mouth
Day−4
Day−5
Stage
Transcript
ENSDART00000078369
ENSDART00000131925
ENSDART00000138725
ENSDART00000172542

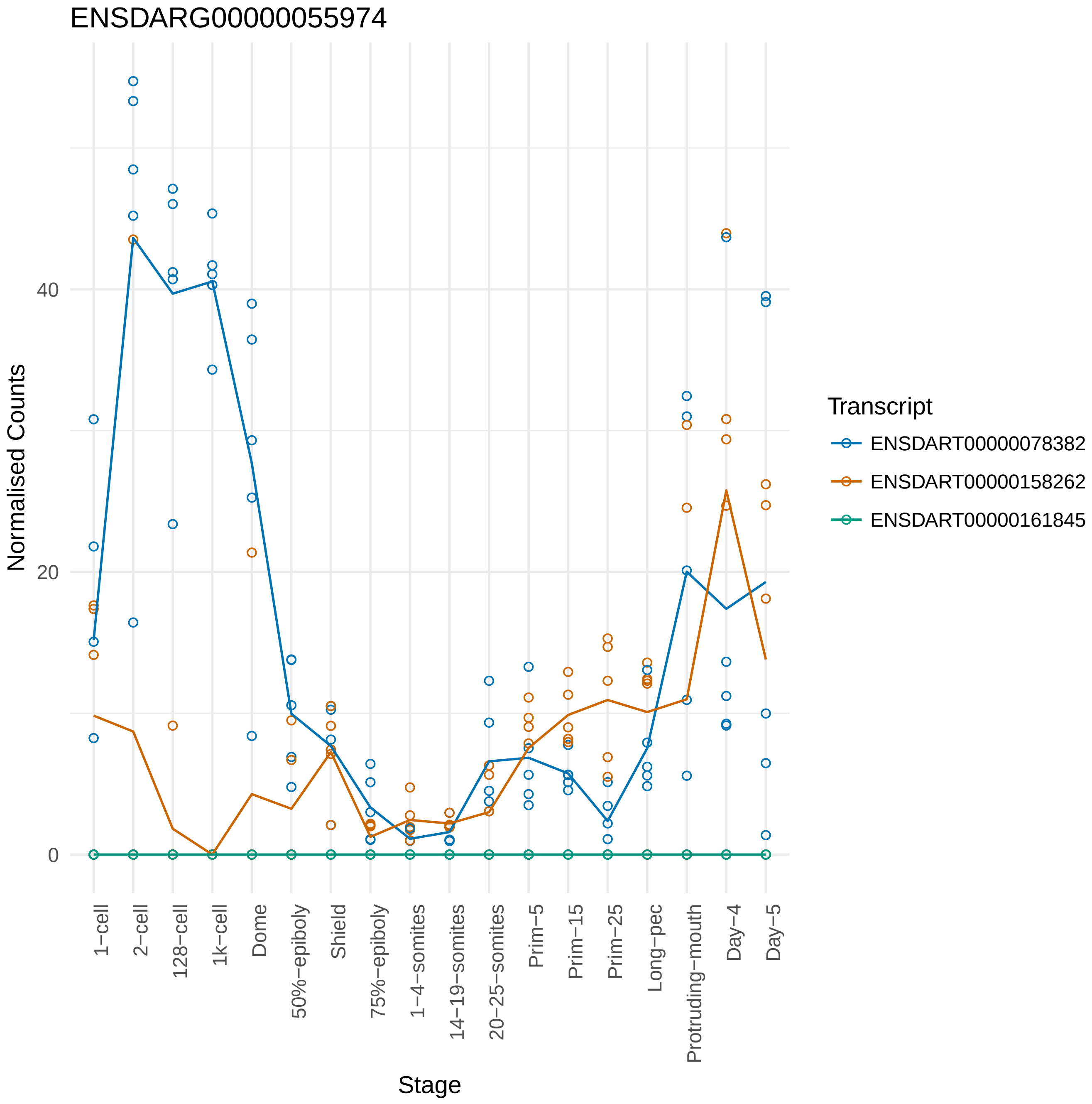

ENSDARG00000055974
Normalised Counts
0
20
40
1−cell
2−cell
128−cell
1k−cell
Dome
50%−epiboly
Shield
75%−epiboly
1−4−somites
14−19−somites
20−25−somites
Prim−5
Prim−15
Prim−25
Long−pec
Protruding−mouth
Day−4
Day−5
Stage
Transcript
ENSDART00000078382
ENSDART00000158262
ENSDART00000161845

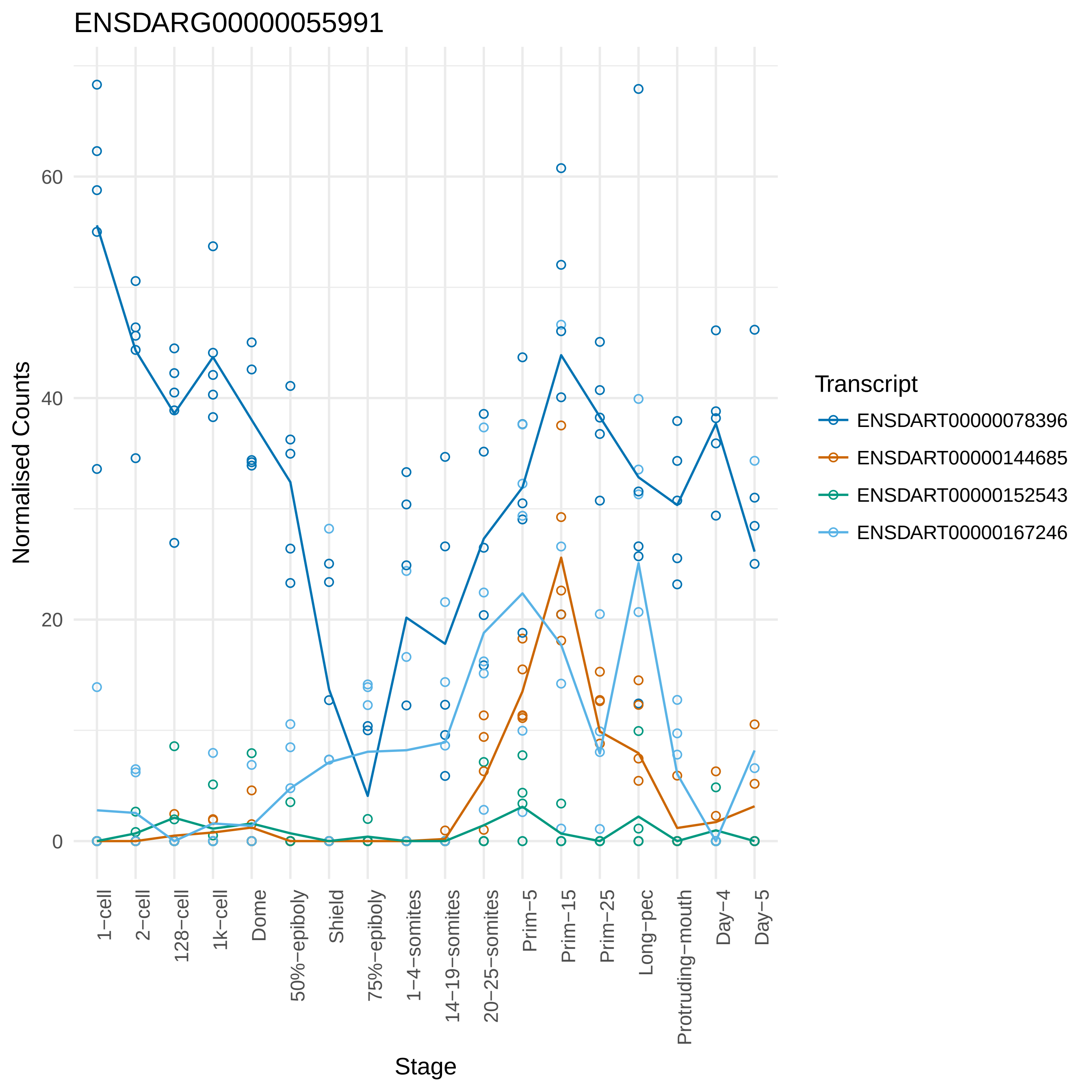

ENSDARG00000055991
Normalised Counts
60
40
20
0
1−cell
2−cell
128−cell
1k−cell
Dome
50%−epiboly
Shield
75%−epiboly
1−4−somites
14−19−somites
20−25−somites
Prim−5
Prim−15
Prim−25
Long−pec
Protruding−mouth
Day−4
Day−5
Stage
Transcript
ENSDART00000078396
ENSDART00000144685
ENSDART00000152543
ENSDART00000167246

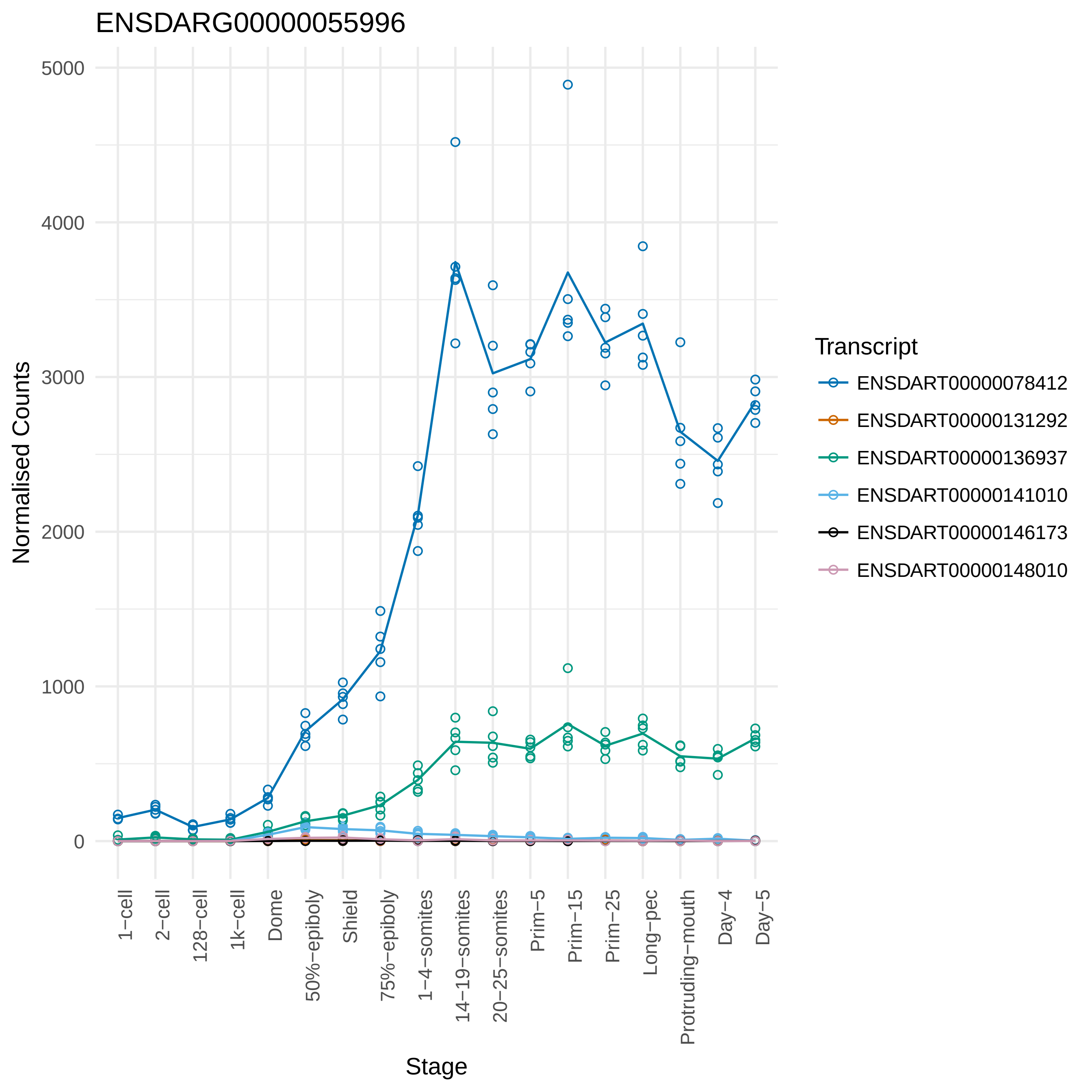

ENSDARG00000055996
Normalised Counts
5000
4000
3000
2000
1000
0
Stage
1−cell
2−cell
128−cell
1k−cell
Dome
50%−epiboly
Shield
75%−epiboly
1−4−somites
14−19−somites
20−25−somites
Prim−5
Prim−15
Prim−25
Long−pec
Protruding−mouth
Day−4
Day−5
Transcript
ENSDART00000078412
ENSDART00000131292
ENSDART00000136937
ENSDART00000141010
ENSDART00000146173
ENSDART00000148010

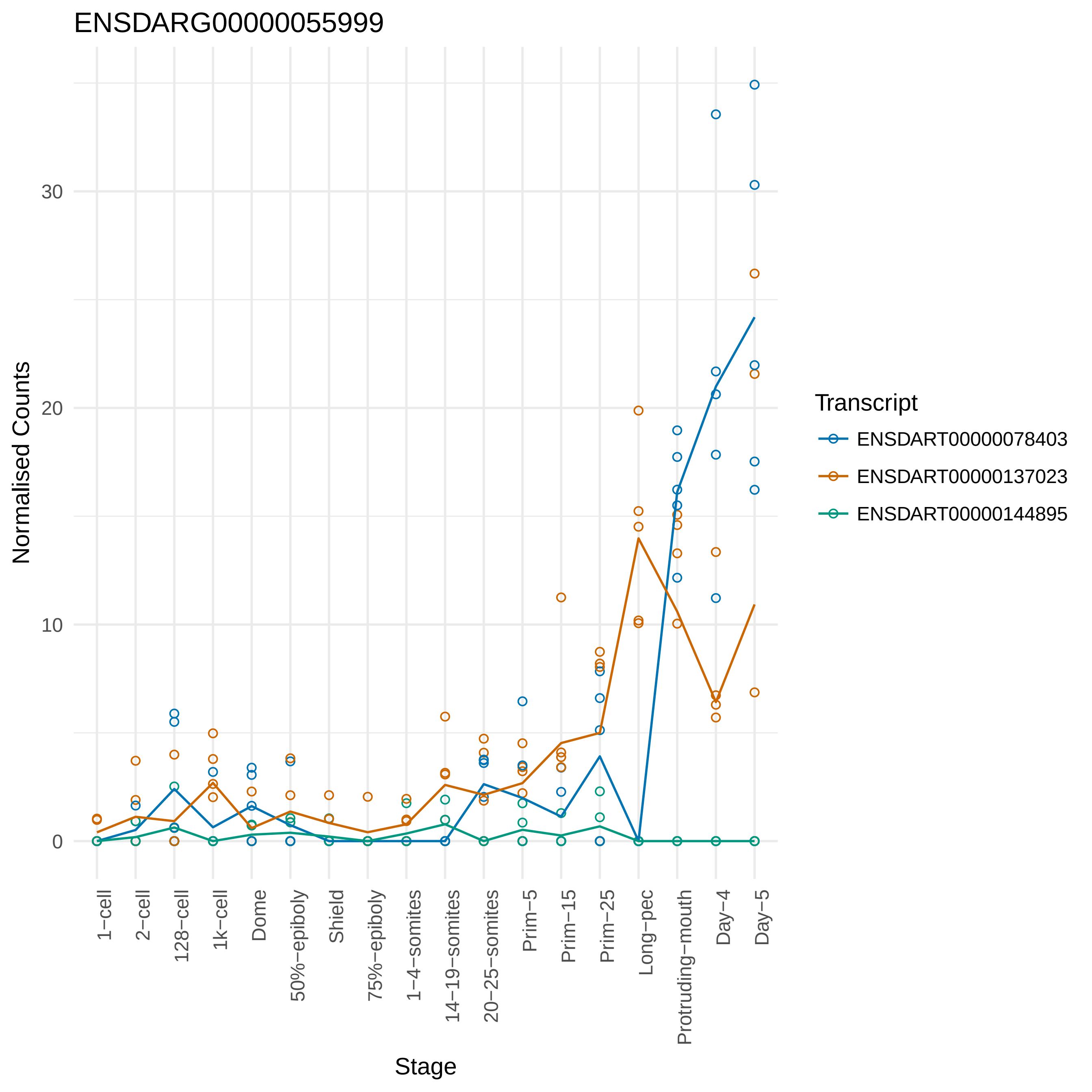
ENSDARG00000055999
Normalised Counts
0
10
20
30
Stage
1−cell
2−cell
128−cell
1k−cell
Dome
50%−epiboly
Shield
75%−epiboly
1−4−somites
14−19−somites
20−25−somites
Prim−5
Prim−15
Prim−25
Long−pec
Protruding−mouth
Day−4
Day−5
Transcript
ENSDART00000078403
ENSDART00000137023
ENSDART00000144895

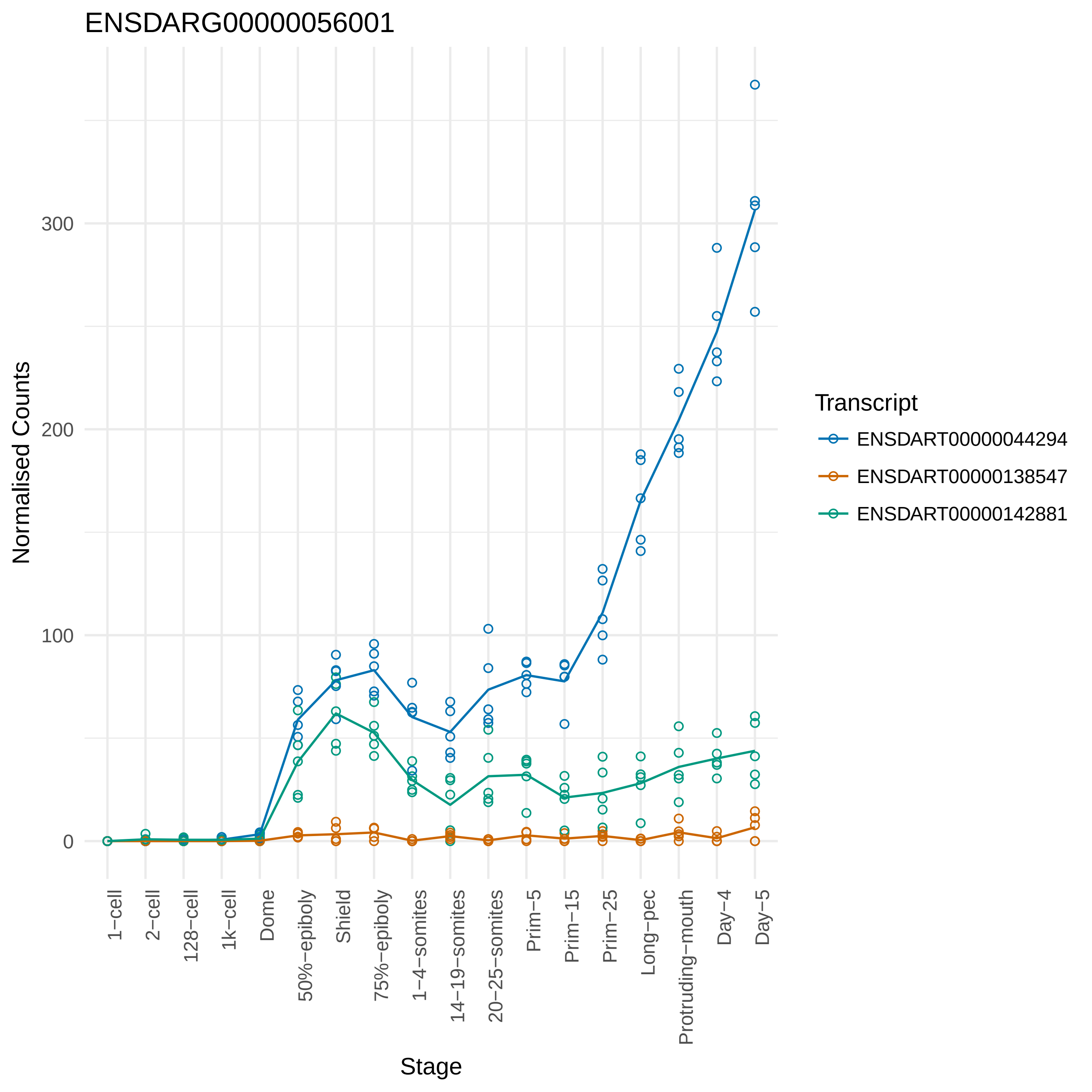
ENSDARG00000056001
Normalised Counts
300
200
100
0
1−cell
2−cell
128−cell
1k−cell
Dome
50%−epiboly
Shield
75%−epiboly
1−4−somites
14−19−somites
20−25−somites
Prim−5
Prim−15
Prim−25
Long−pec
Protruding−mouth
Day−4
Day−5
Stage
Transcript
ENSDART00000044294
ENSDART00000138547
ENSDART00000142881

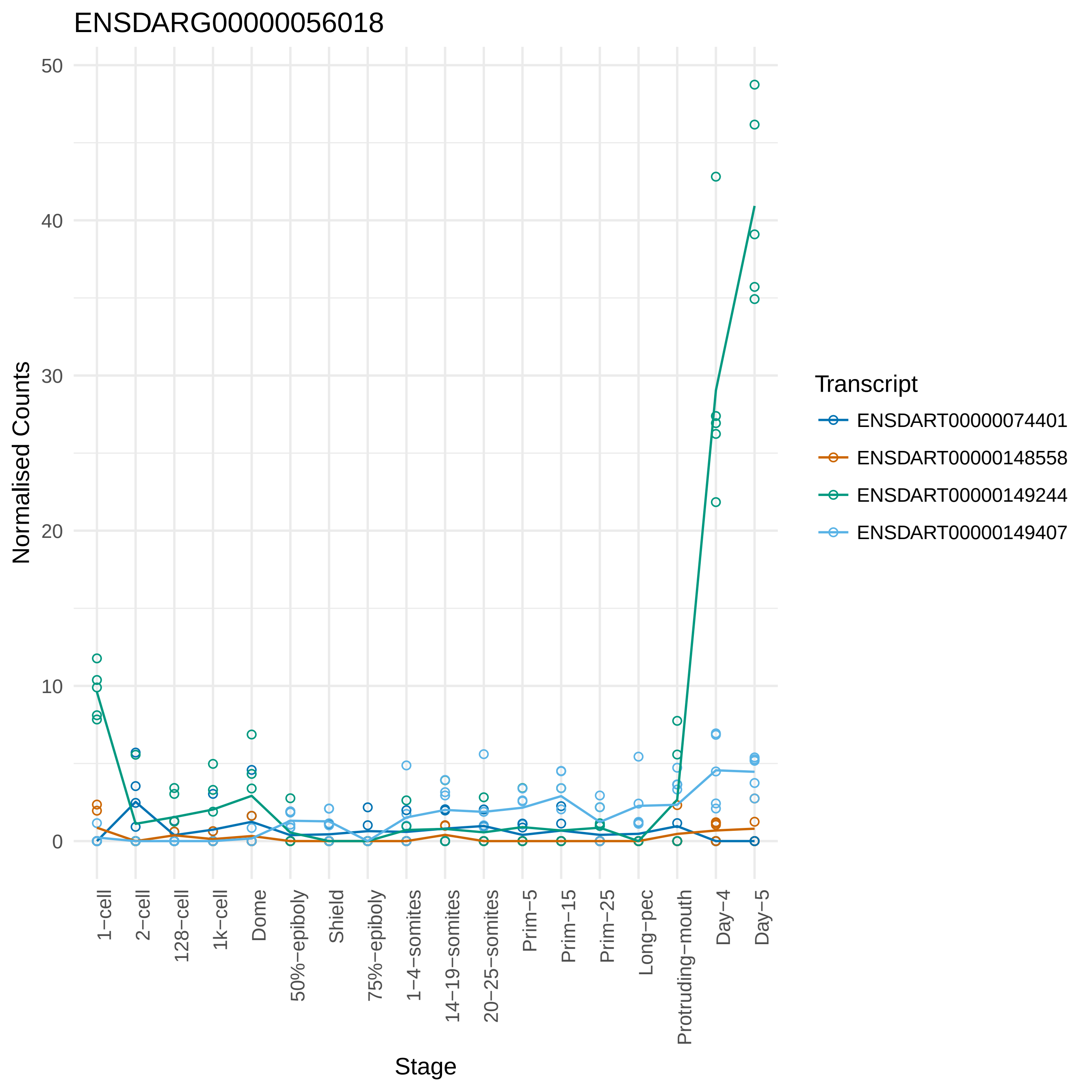
ENSDARG00000056018
Normalised Counts
0
10
20
30
40
50
1−cell
2−cell
128−cell
1k−cell
Dome
50%−epiboly
Shield
75%−epiboly
1−4−somites
14−19−somites
20−25−somites
Prim−5
Prim−15
Prim−25
Long−pec
Protruding−mouth
Day−4
Day−5
Stage
Transcript
ENSDART00000074401
ENSDART00000148558
ENSDART00000149244
ENSDART00000149407

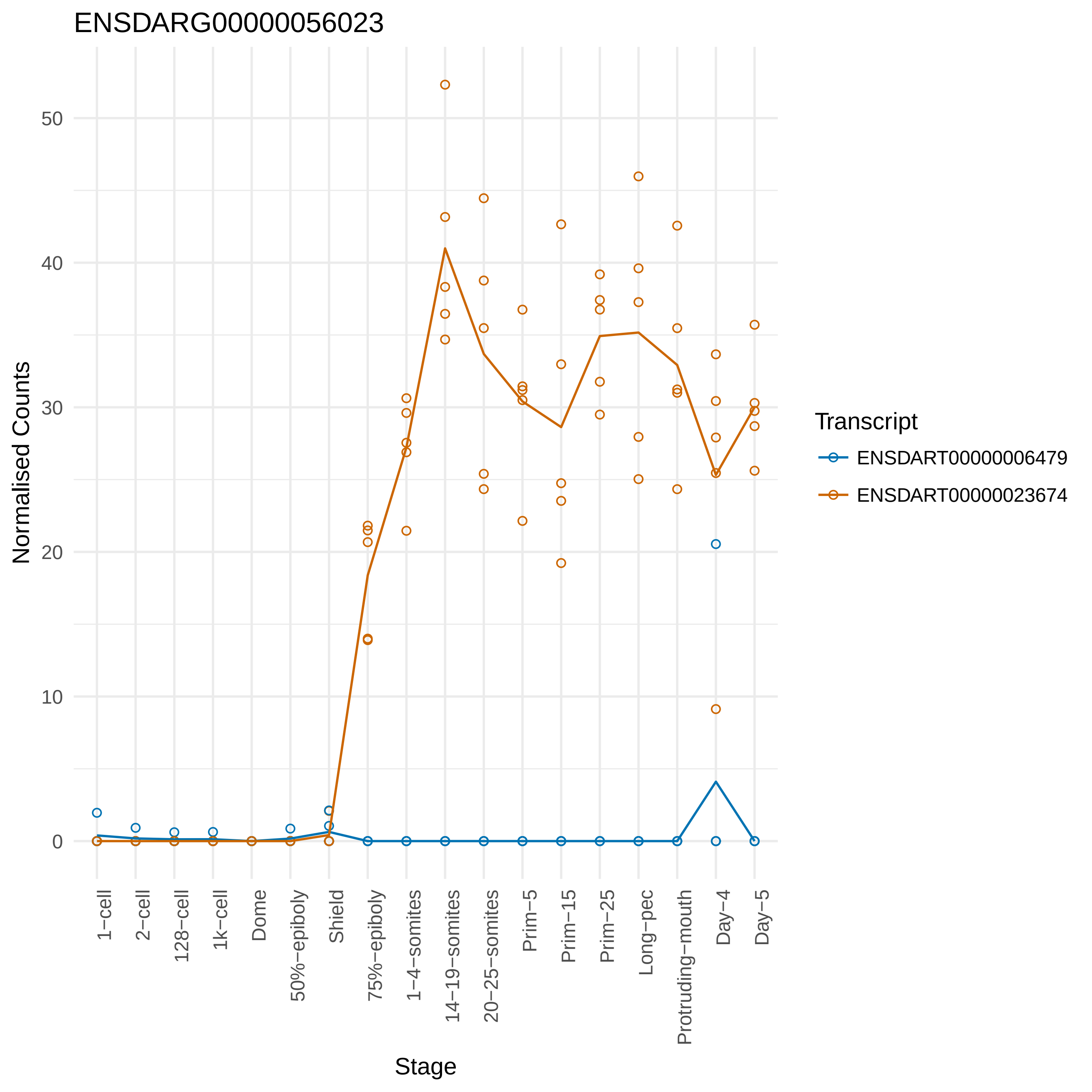

ENSDARG00000056023
Normalised Counts
0
10
20
30
40
50
1−cell
2−cell
128−cell
1k−cell
Dome
50%−epiboly
Shield
75%−epiboly
1−4−somites
14−19−somites
20−25−somites
Prim−5
Prim−15
Prim−25
Long−pec
Protruding−mouth
Day−4
Day−5
Stage
Transcript
ENSDART00000006479
ENSDART00000023674

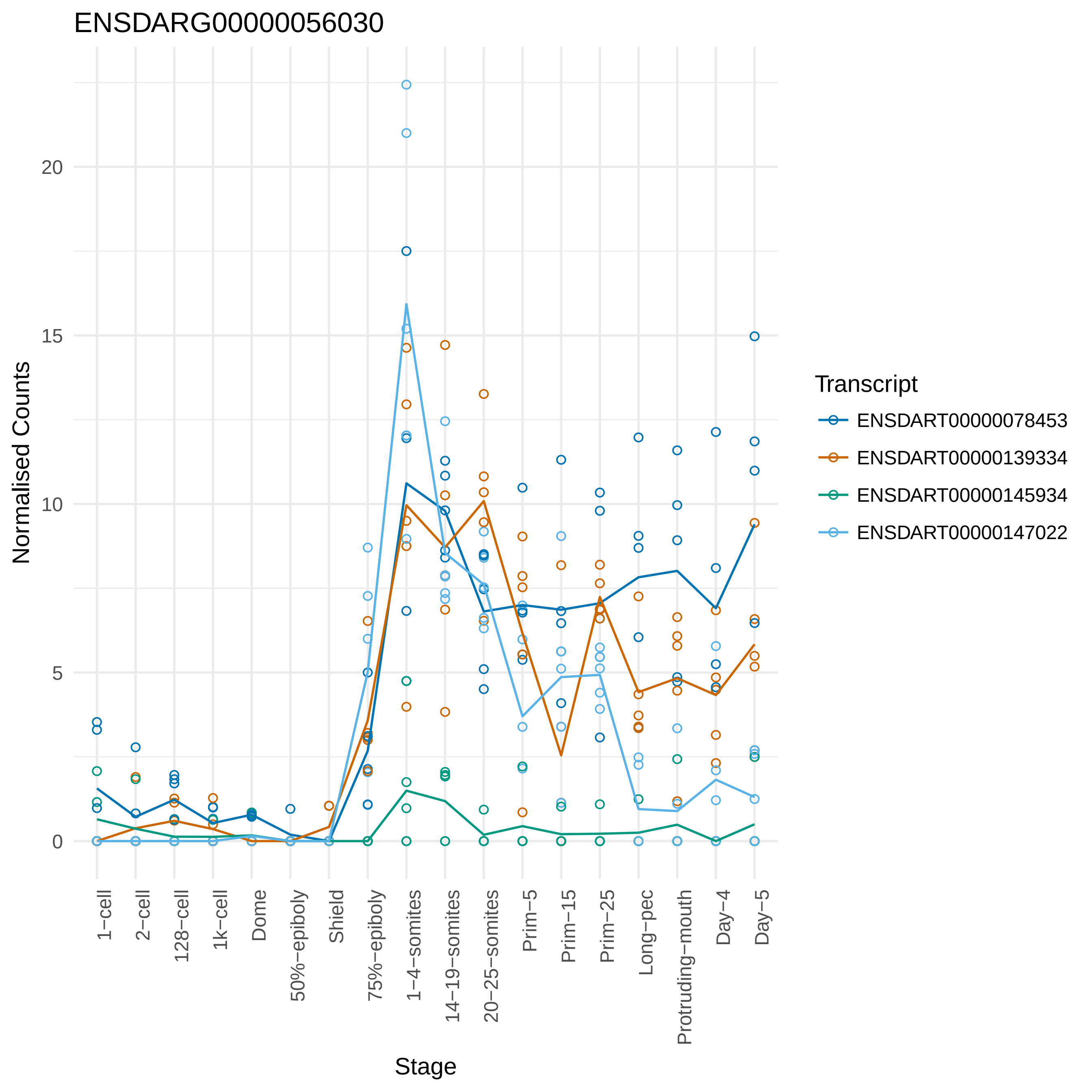
ENSDARG00000056030
Normalised Counts
0
5
10
15
20
Stage
1−cell
2−cell
128−cell
1k−cell
Dome
50%−epiboly
Shield
75%−epiboly
1−4−somites
14−19−somites
20−25−somites
Prim−5
Prim−15
Prim−25
Long−pec
Protruding−mouth
Day−4
Day−5
Transcript
ENSDART00000078453
ENSDART00000139334
ENSDART00000145934
ENSDART00000147022

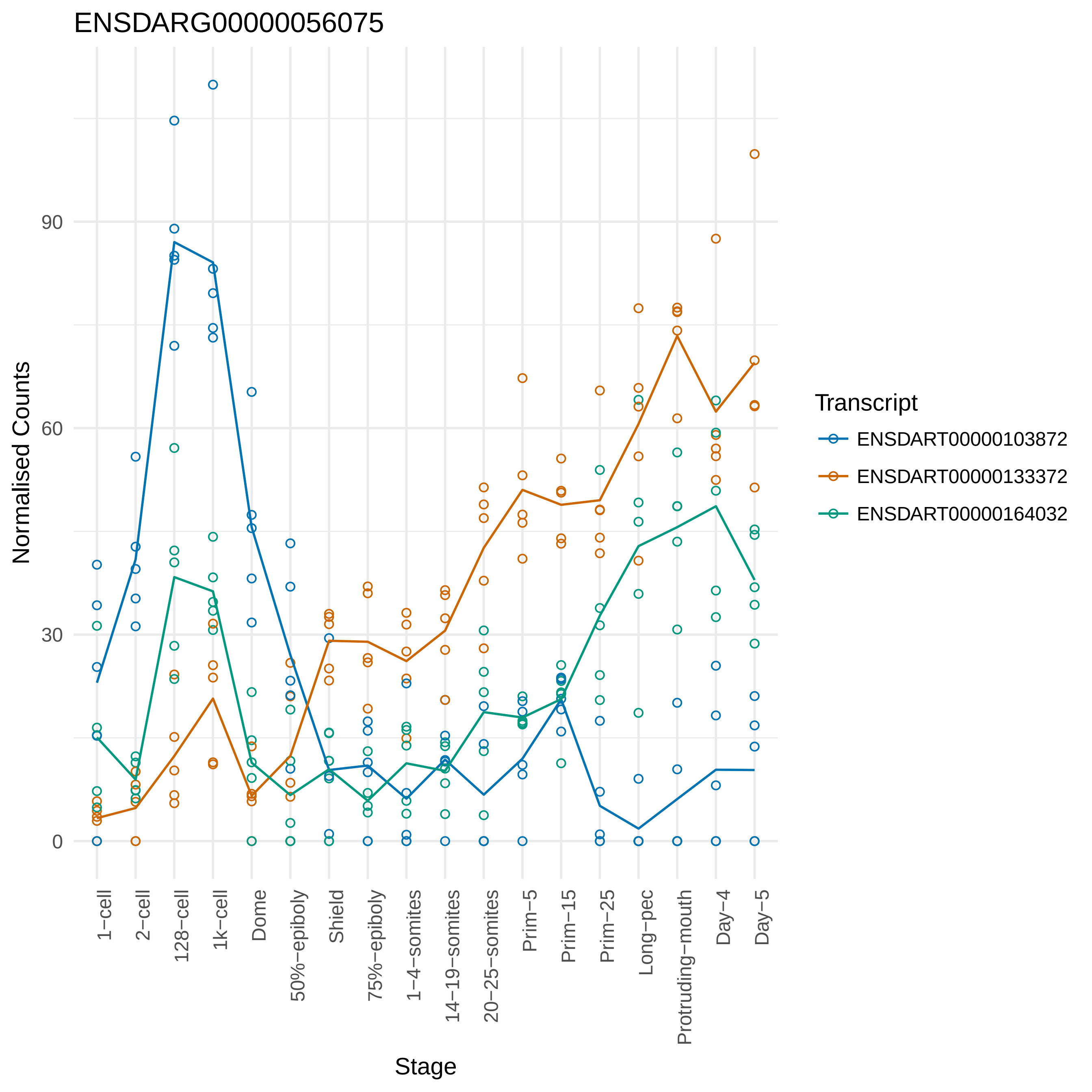
ENSDARG00000056075
Normalised Counts
0
30
60
90
Stage
1−cell
2−cell
128−cell
1k−cell
Dome
50%−epiboly
Shield
75%−epiboly
1−4−somites
14−19−somites
20−25−somites
Prim−5
Prim−15
Prim−25
Long−pec
Protruding−mouth
Day−4
Day−5
Transcript
ENSDART00000103872
ENSDART00000133372
ENSDART00000164032

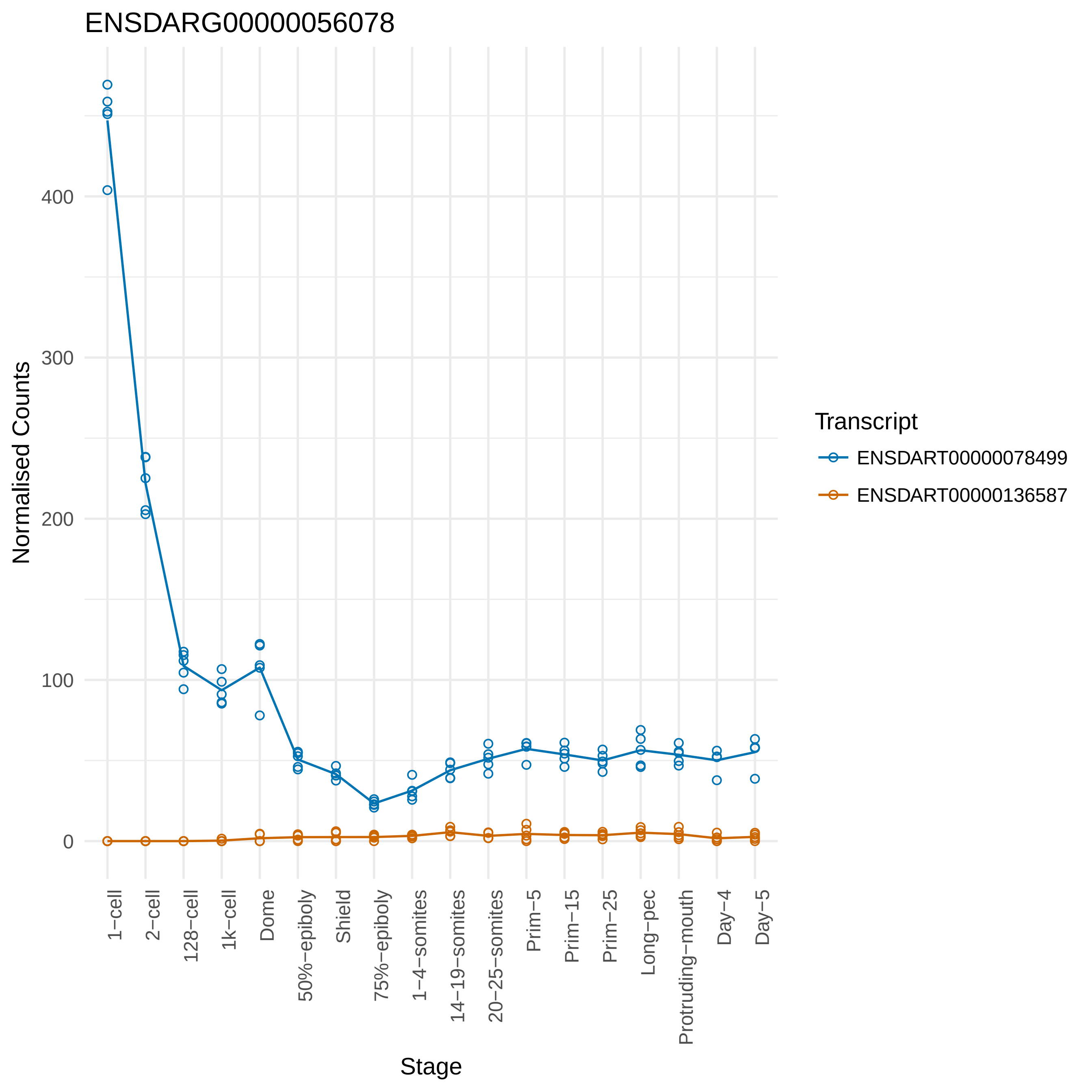

ENSDARG00000056078
Normalised Counts
0
100
200
300
400
Stage
1−cell
2−cell
128−cell
1k−cell
Dome
50%−epiboly
Shield
75%−epiboly
1−4−somites
14−19−somites
20−25−somites
Prim−5
Prim−15
Prim−25
Long−pec
Protruding−mouth
Day−4
Day−5
Transcript
ENSDART00000078499
ENSDART00000136587

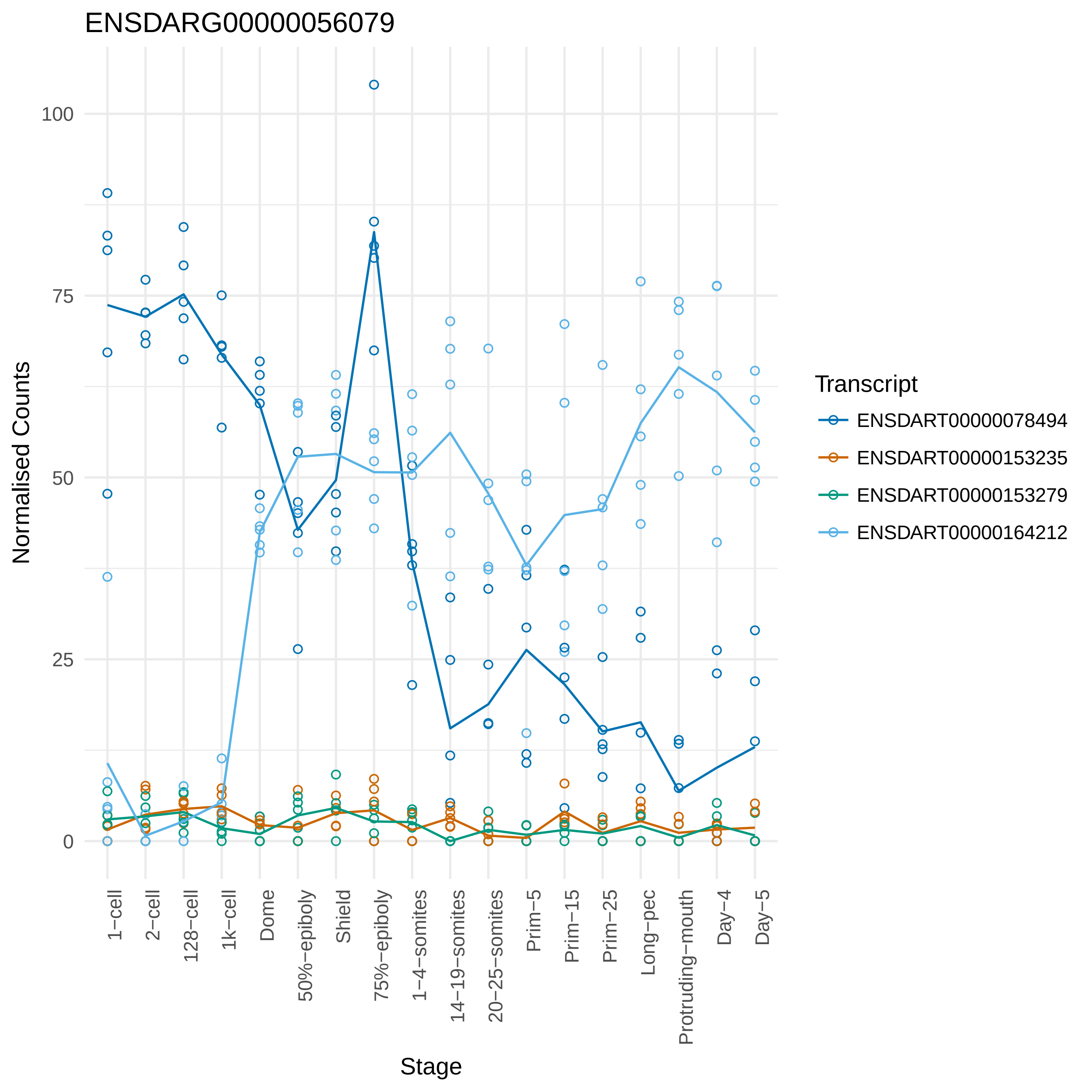

ENSDARG00000056079
Normalised Counts
100
75
50
25
0
1−cell
2−cell
128−cell
1k−cell
Dome
50%−epiboly
Shield
75%−epiboly
1−4−somites
14−19−somites
20−25−somites
Prim−5
Prim−15
Prim−25
Long−pec
Protruding−mouth
Day−4
Day−5
Stage
Transcript
ENSDART00000078494
ENSDART00000153235
ENSDART00000153279
ENSDART00000164212

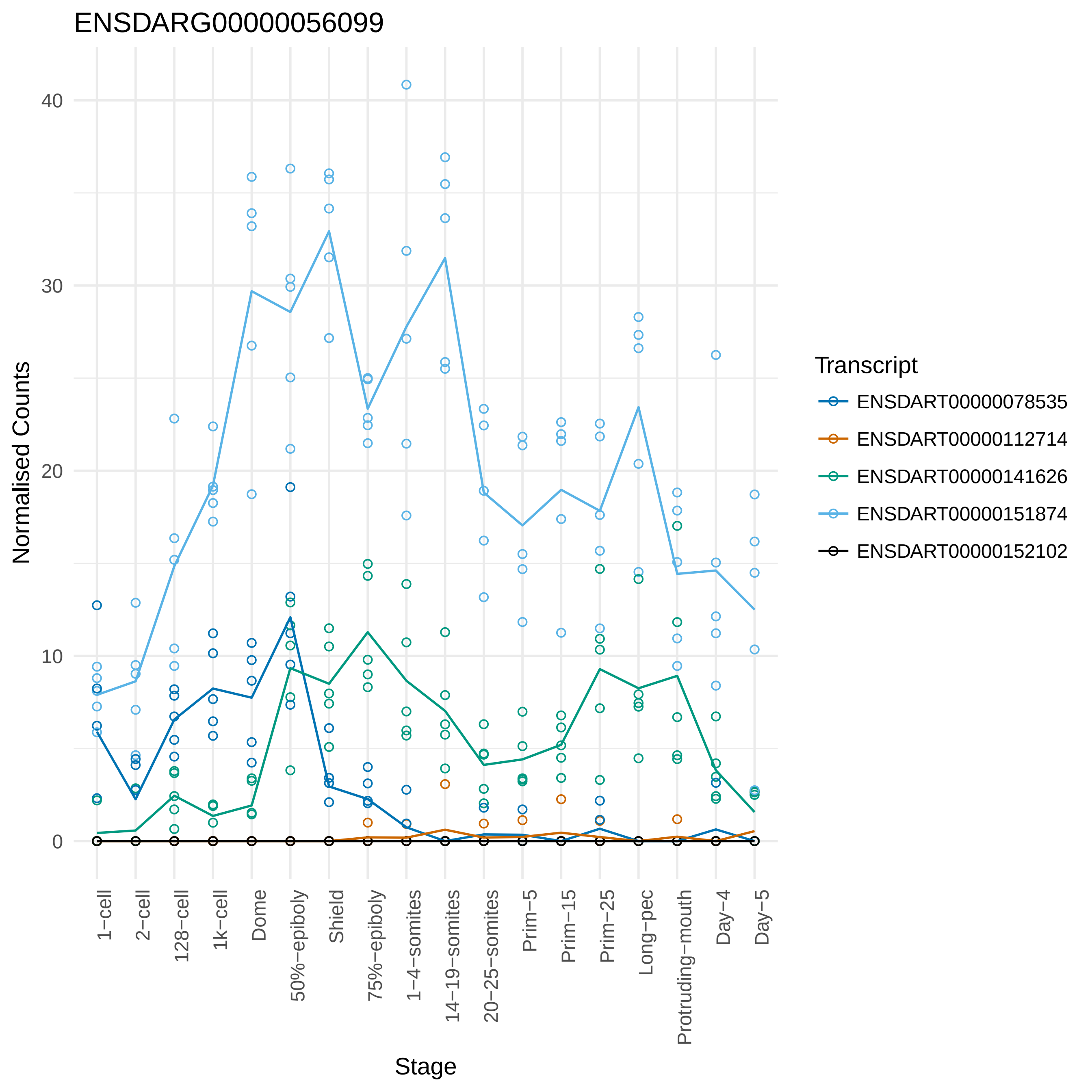

ENSDARG00000056099
Normalised Counts
0
10
20
30
40
Stage
1−cell
2−cell
128−cell
1k−cell
Dome
50%−epiboly
Shield
75%−epiboly
1−4−somites
14−19−somites
20−25−somites
Prim−5
Prim−15
Prim−25
Long−pec
Protruding−mouth
Day−4
Day−5
Transcript
ENSDART00000078535
ENSDART00000112714
ENSDART00000141626
ENSDART00000151874
ENSDART00000152102

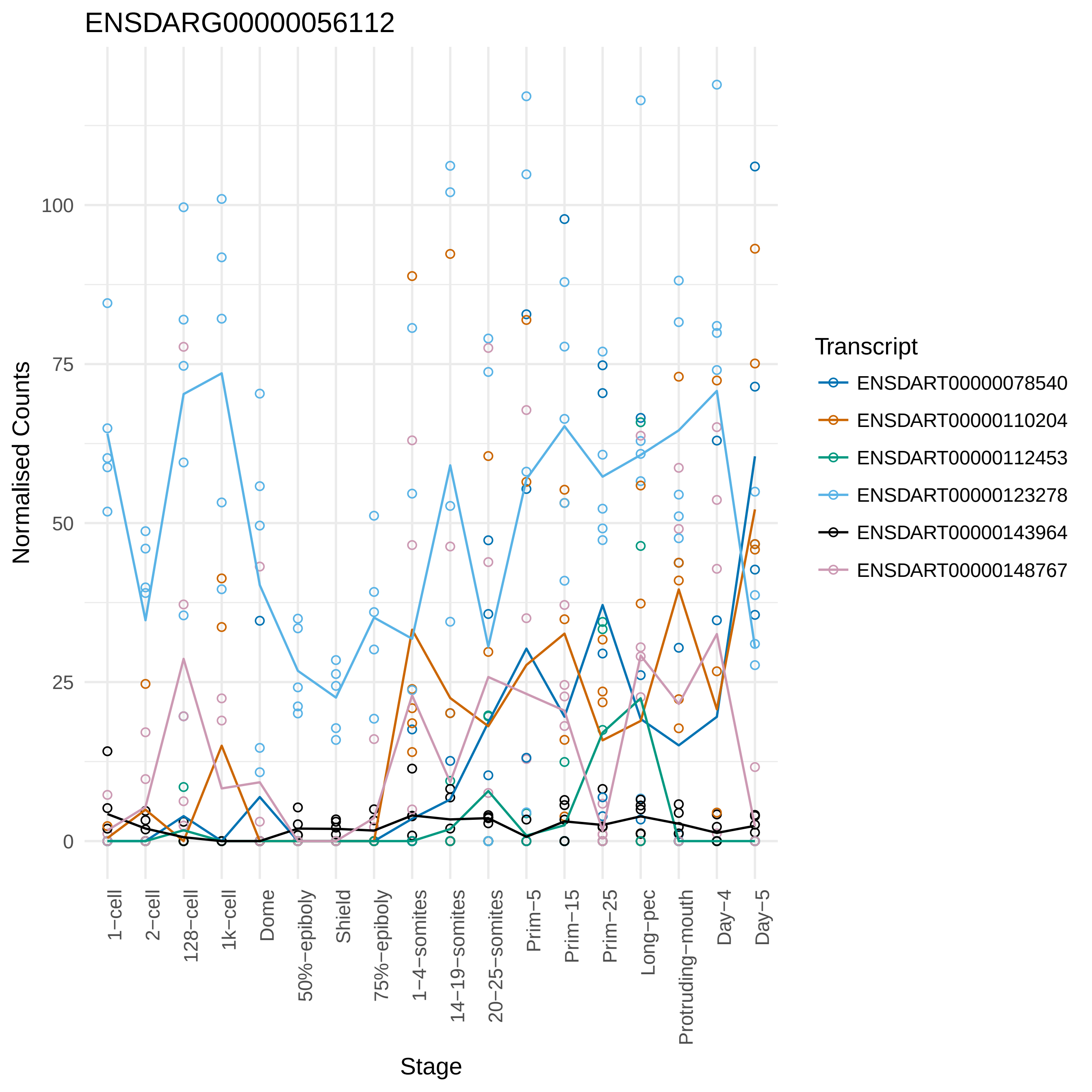

ENSDARG00000056112
Normalised Counts
100
75
50
25
0
1−cell
2−cell
128−cell
1k−cell
Dome
50%−epiboly
Shield
75%−epiboly
1−4−somites
14−19−somites
20−25−somites
Prim−5
Prim−15
Prim−25
Long−pec
Protruding−mouth
Day−4
Day−5
Stage
Transcript
ENSDART00000078540
ENSDART00000110204
ENSDART00000112453
ENSDART00000123278
ENSDART00000143964
ENSDART00000148767

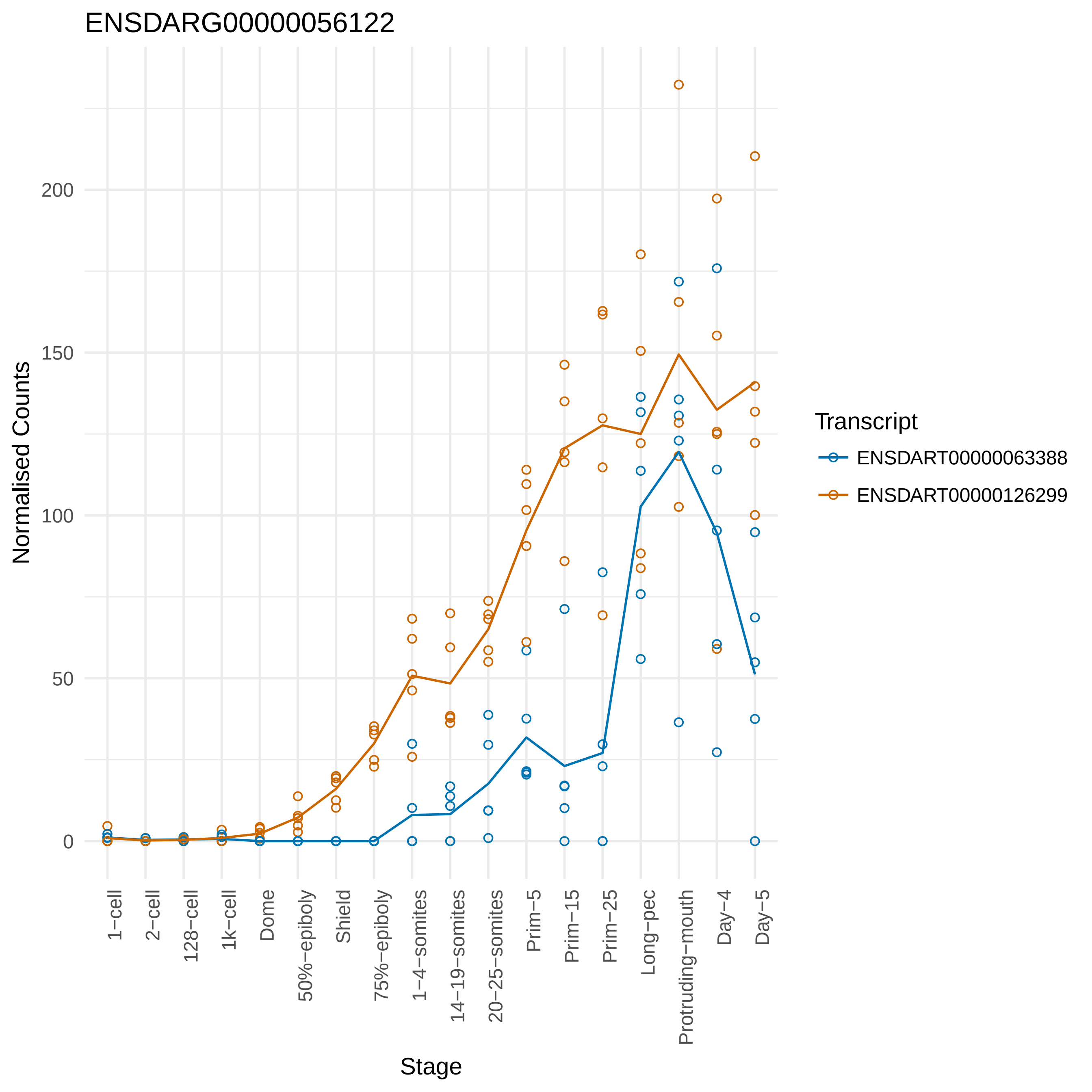
ENSDARG00000056122
Normalised Counts
0
50
100
150
200
Stage
1−cell
2−cell
128−cell
1k−cell
Dome
50%−epiboly
Shield
75%−epiboly
1−4−somites
14−19−somites
20−25−somites
Prim−5
Prim−15
Prim−25
Long−pec
Protruding−mouth
Day−4
Day−5
Transcript
ENSDART00000063388
ENSDART00000126299

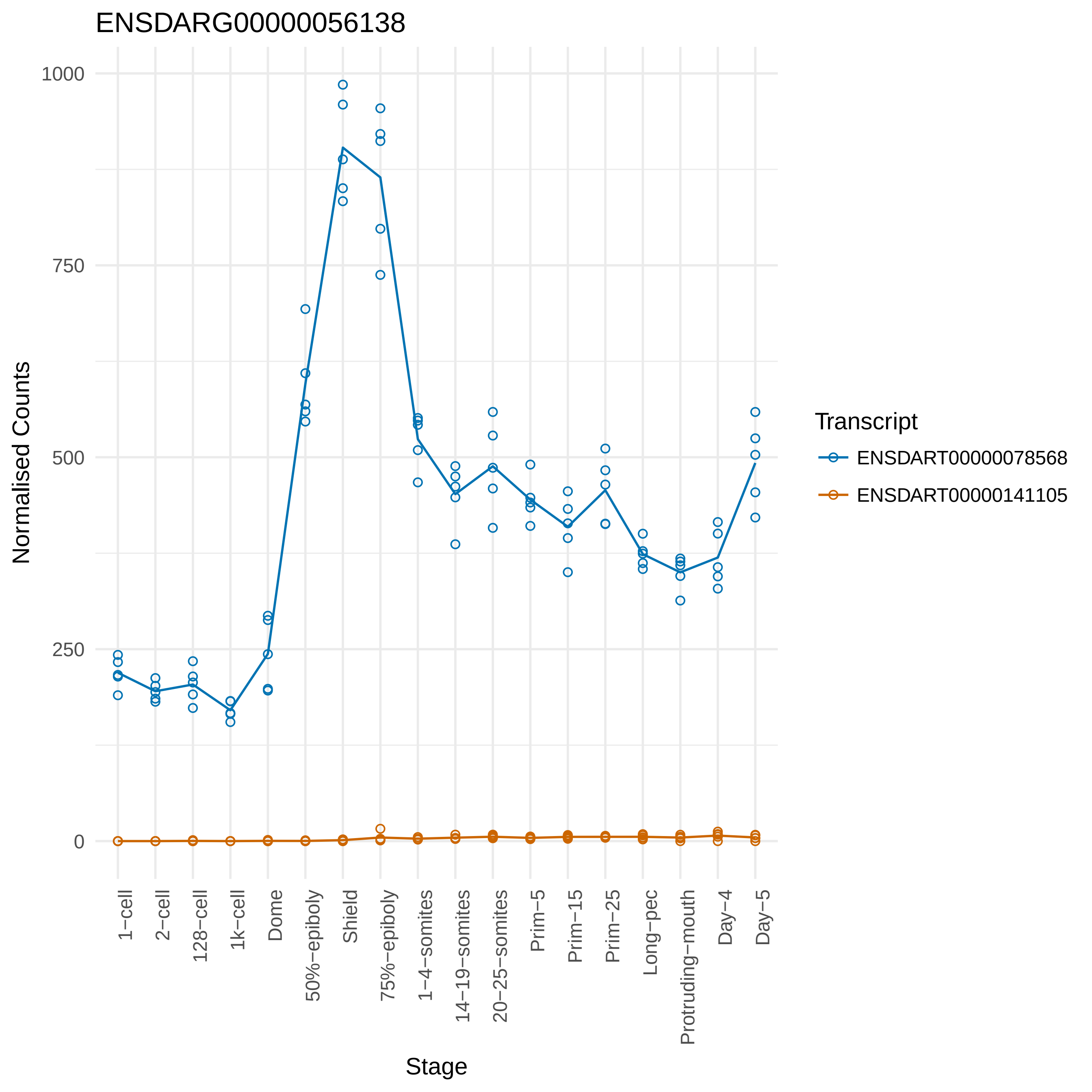

ENSDARG00000056138
Normalised Counts
1000
750
500
250
0
1−cell
2−cell
128−cell
1k−cell
Dome
50%−epiboly
Shield
75%−epiboly
1−4−somites
14−19−somites
20−25−somites
Prim−5
Prim−15
Prim−25
Long−pec
Protruding−mouth
Day−4
Day−5
Stage
Transcript
ENSDART00000078568
ENSDART00000141105

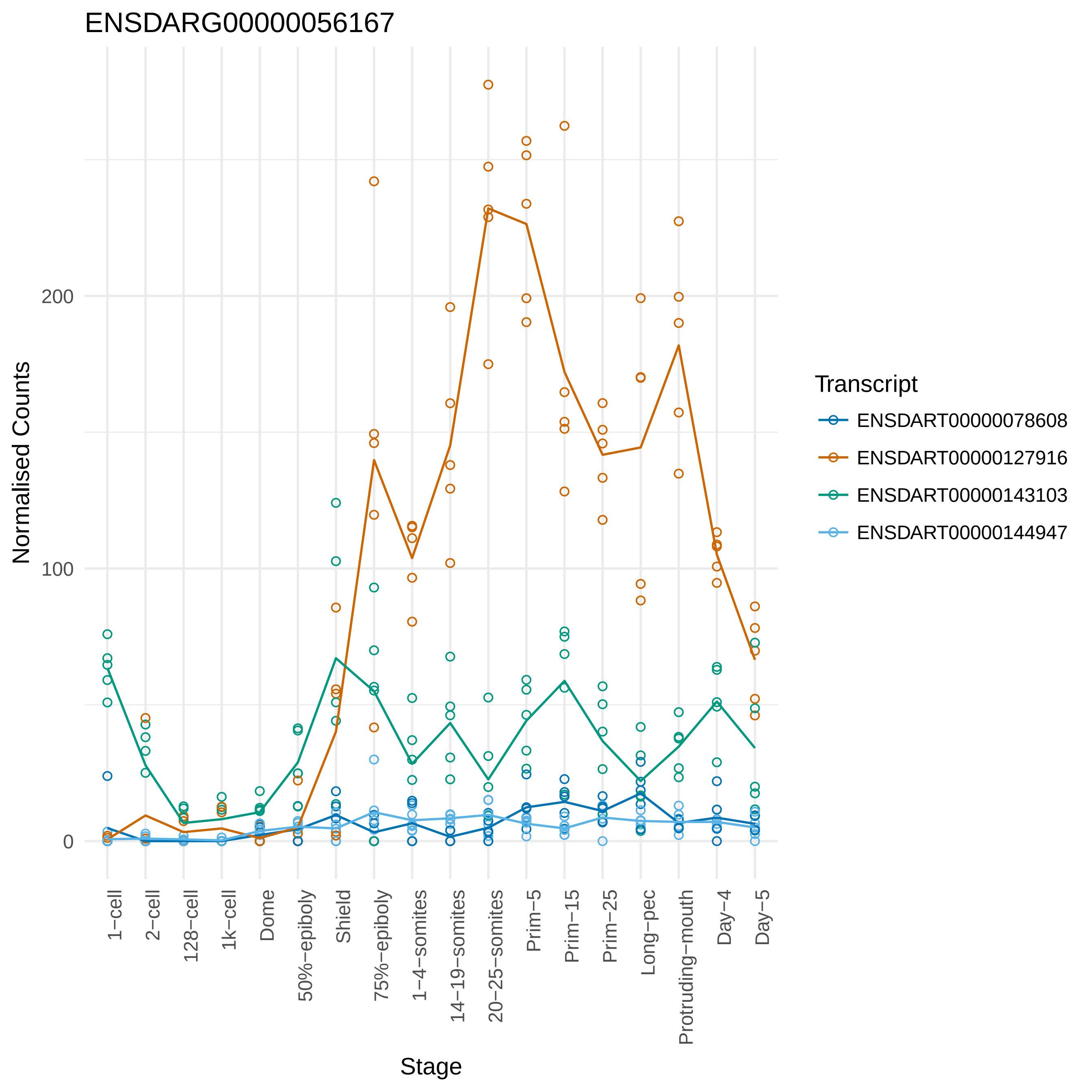
ENSDARG00000056167
Normalised Counts
0
100
200
1−cell
2−cell
128−cell
1k−cell
Dome
50%−epiboly
Shield
75%−epiboly
1−4−somites
14−19−somites
20−25−somites
Prim−5
Prim−15
Prim−25
Long−pec
Protruding−mouth
Day−4
Day−5
Stage
Transcript
ENSDART00000078608
ENSDART00000127916
ENSDART00000143103
ENSDART00000144947

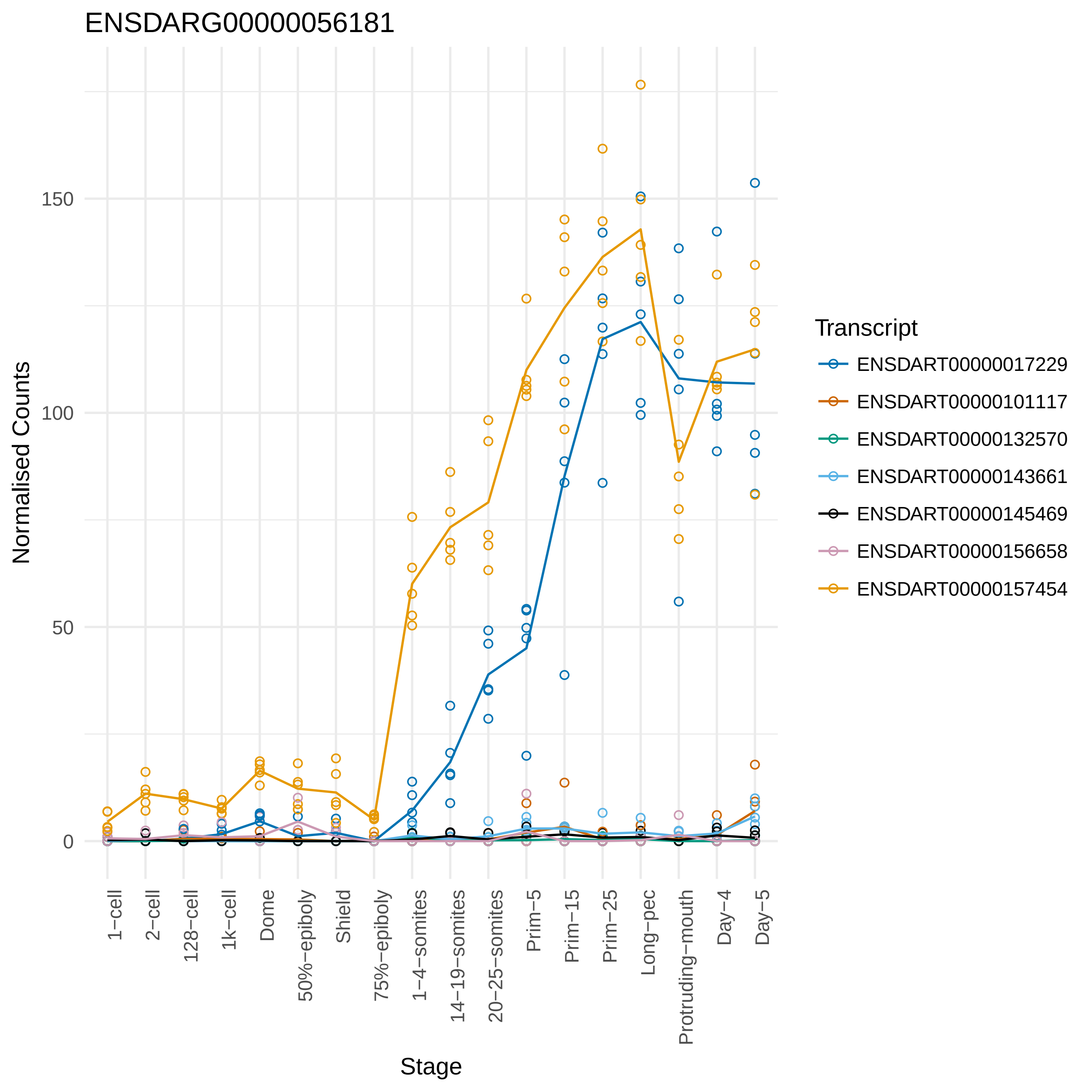

ENSDARG00000056181
Normalised Counts
150
100
50
0
1−cell
2−cell
128−cell
1k−cell
Dome
50%−epiboly
Shield
75%−epiboly
1−4−somites
14−19−somites
20−25−somites
Prim−5
Prim−15
Prim−25
Long−pec
Protruding−mouth
Day−4
Day−5
Stage
Transcript
ENSDART00000017229
ENSDART00000101117
ENSDART00000132570
ENSDART00000143661
ENSDART00000145469
ENSDART00000156658
ENSDART00000157454

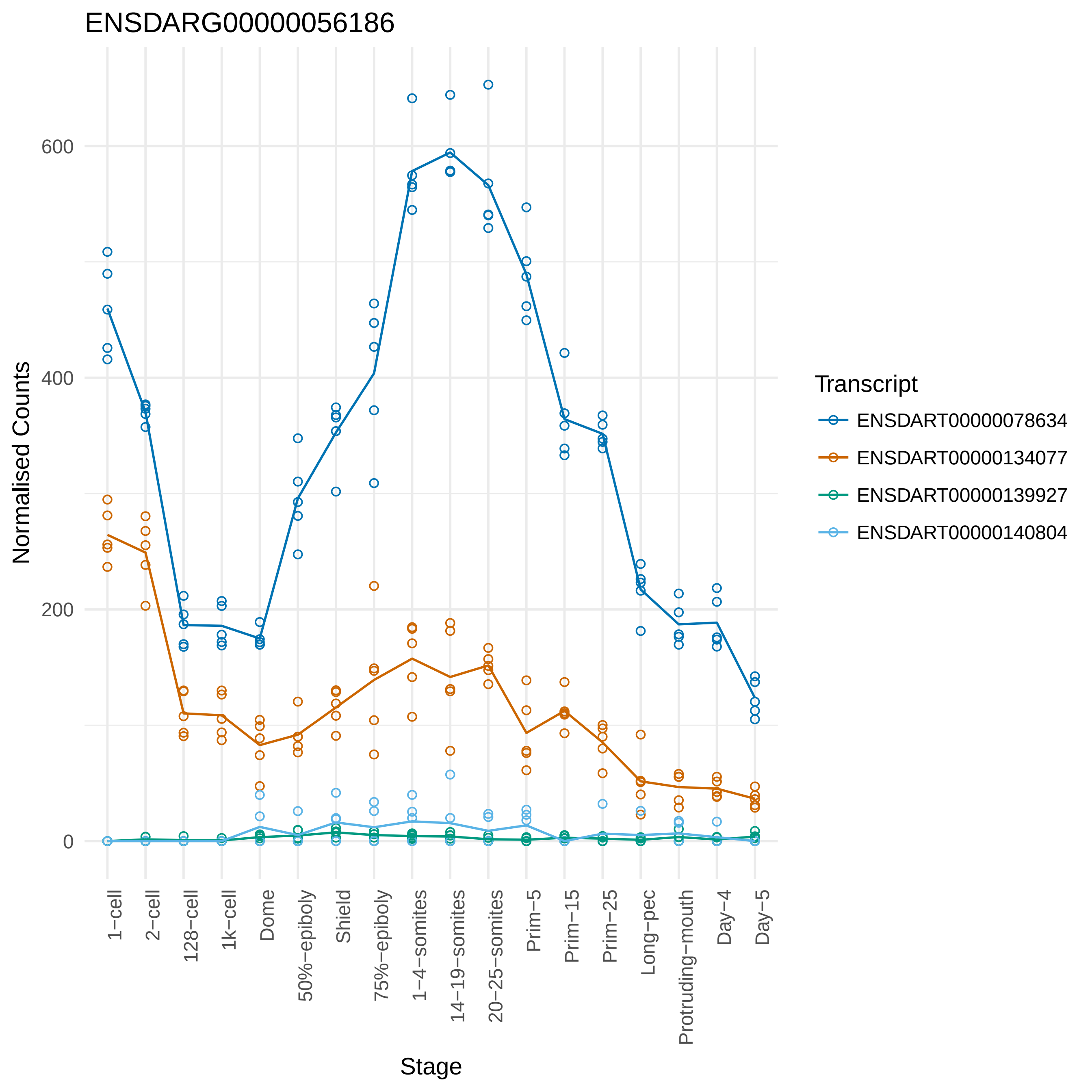
ENSDARG00000056186
Normalised Counts
600
400
200
0
1−cell
2−cell
128−cell
1k−cell
Dome
50%−epiboly
Shield
75%−epiboly
1−4−somites
14−19−somites
20−25−somites
Prim−5
Prim−15
Prim−25
Long−pec
Protruding−mouth
Day−4
Day−5
Stage
Transcript
ENSDART00000078634
ENSDART00000134077
ENSDART00000139927
ENSDART00000140804

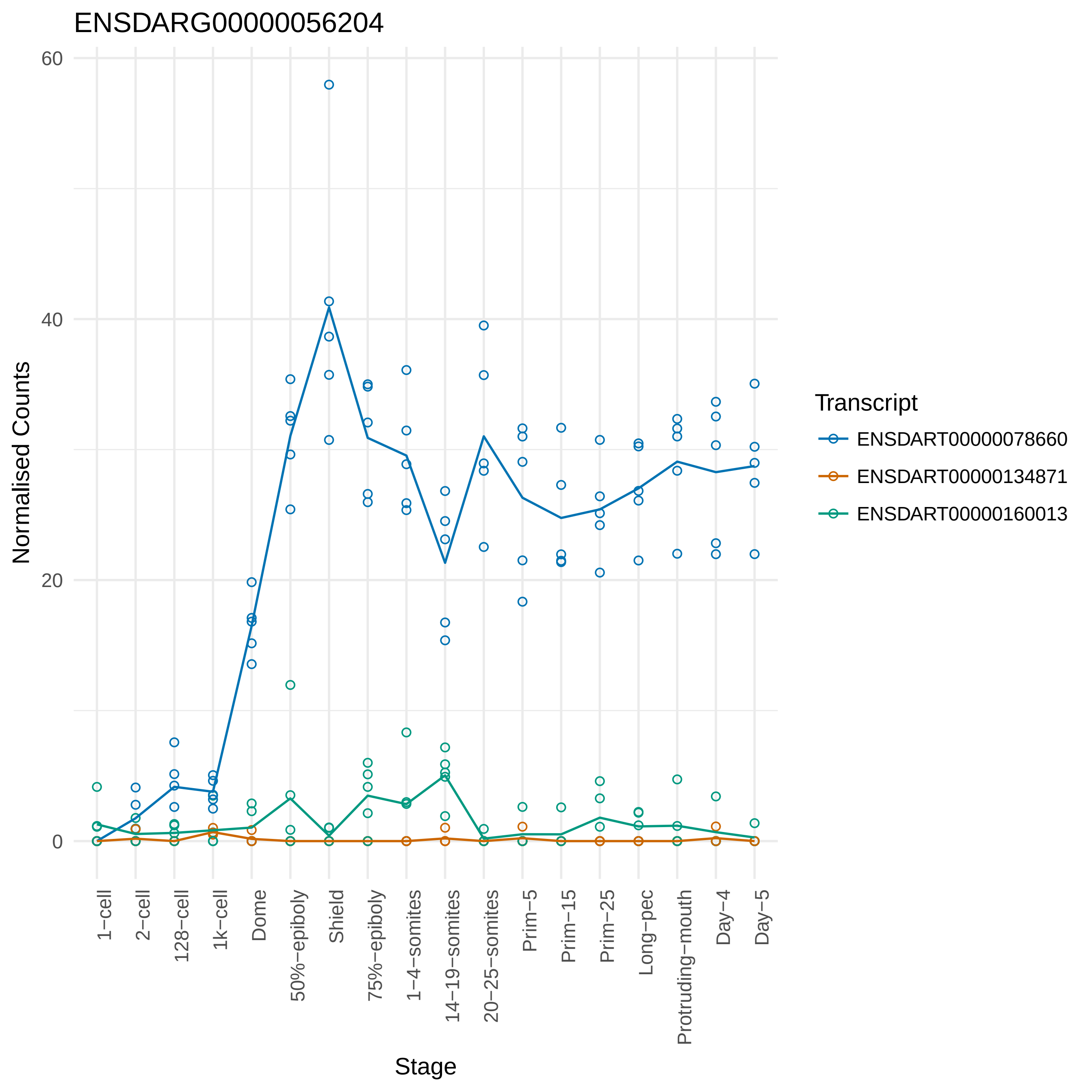
ENSDARG00000056204
Normalised Counts
0
20
40
60
Stage
1−cell
2−cell
128−cell
1k−cell
Dome
50%−epiboly
Shield
75%−epiboly
1−4−somites
14−19−somites
20−25−somites
Prim−5
Prim−15
Prim−25
Long−pec
Protruding−mouth
Day−4
Day−5
Transcript
ENSDART00000078660
ENSDART00000134871
ENSDART00000160013

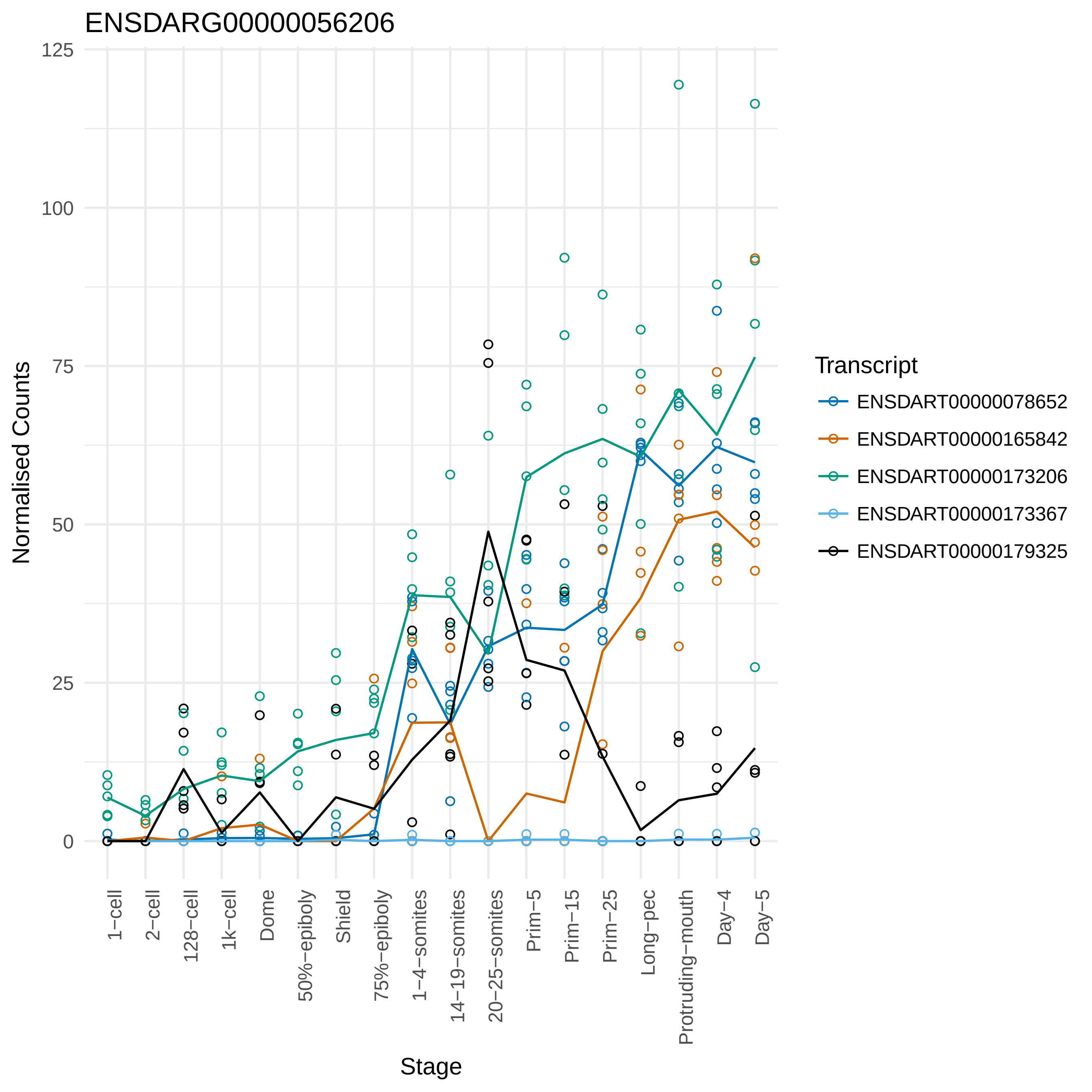

ENSDARG00000056206
Normalised Counts
125
100
75
50
25
0
1−cell
2−cell
128−cell
1k−cell
Dome
50%−epiboly
Shield
75%−epiboly
1−4−somites
14−19−somites
20−25−somites
Prim−5
Prim−15
Prim−25
Long−pec
Protruding−mouth
Day−4
Day−5
Stage
Transcript
ENSDART00000078652
ENSDART00000165842
ENSDART00000173206
ENSDART00000173367
ENSDART00000179325

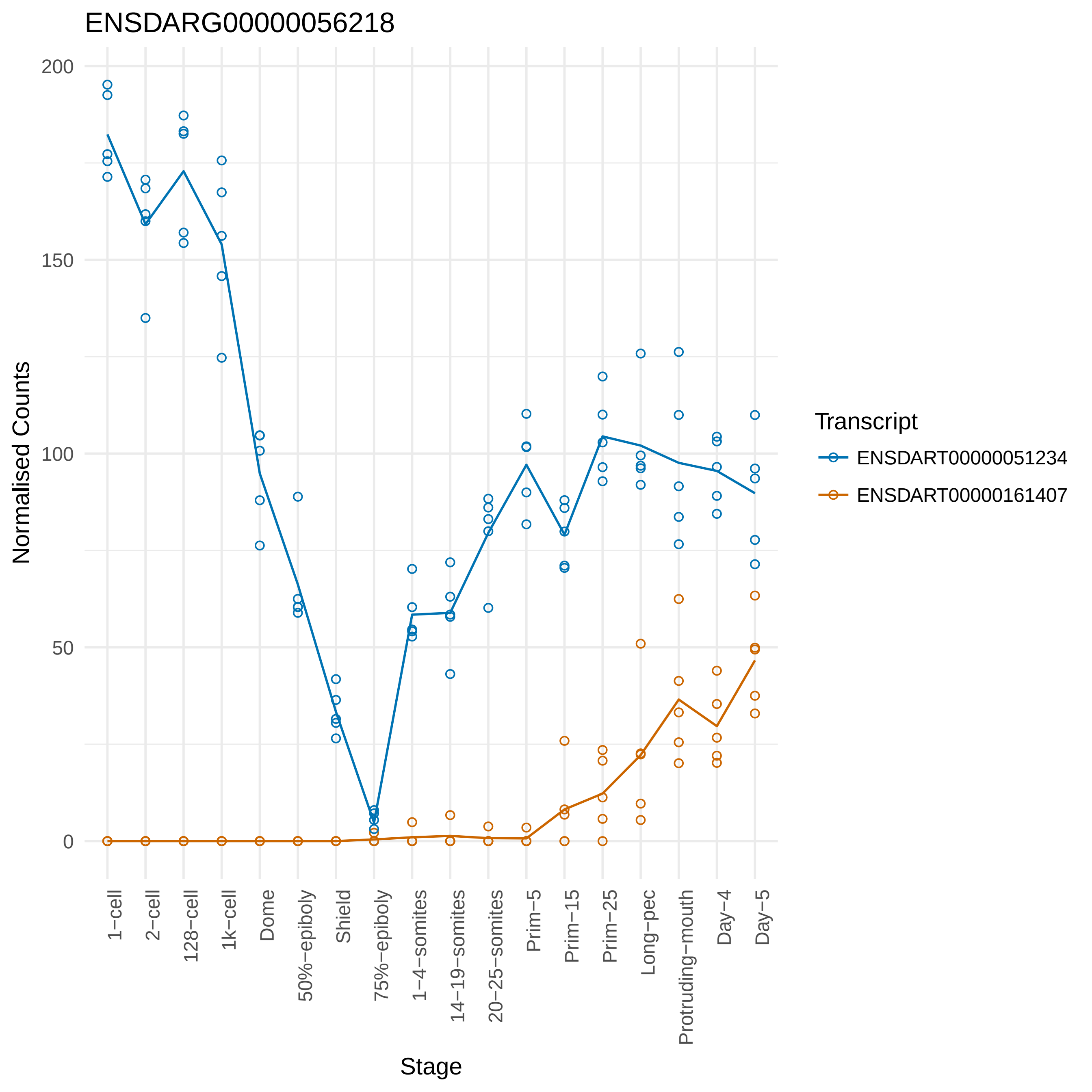
ENSDARG00000056218
Normalised Counts
200
150
100
50
0
1−cell
2−cell
128−cell
1k−cell
Dome
50%−epiboly
Shield
75%−epiboly
1−4−somites
14−19−somites
20−25−somites
Prim−5
Prim−15
Prim−25
Long−pec
Protruding−mouth
Day−4
Day−5
Stage
Transcript
ENSDART00000051234
ENSDART00000161407

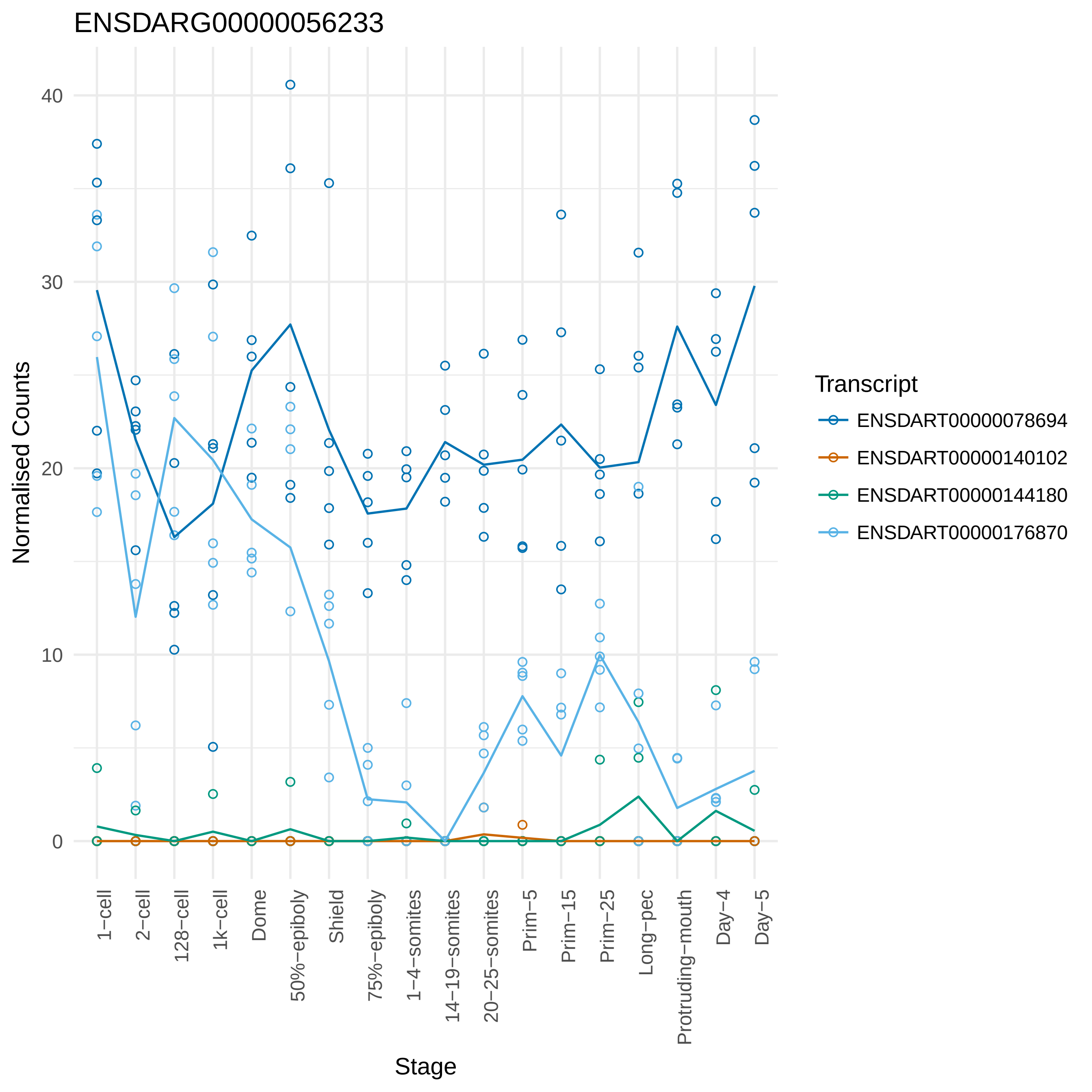

ENSDARG00000056233
Normalised Counts
0
10
20
30
40
Stage
1−cell
2−cell
128−cell
1k−cell
Dome
50%−epiboly
Shield
75%−epiboly
1−4−somites
14−19−somites
20−25−somites
Prim−5
Prim−15
Prim−25
Long−pec
Protruding−mouth
Day−4
Day−5
Transcript
ENSDART00000078694
ENSDART00000140102
ENSDART00000144180
ENSDART00000176870

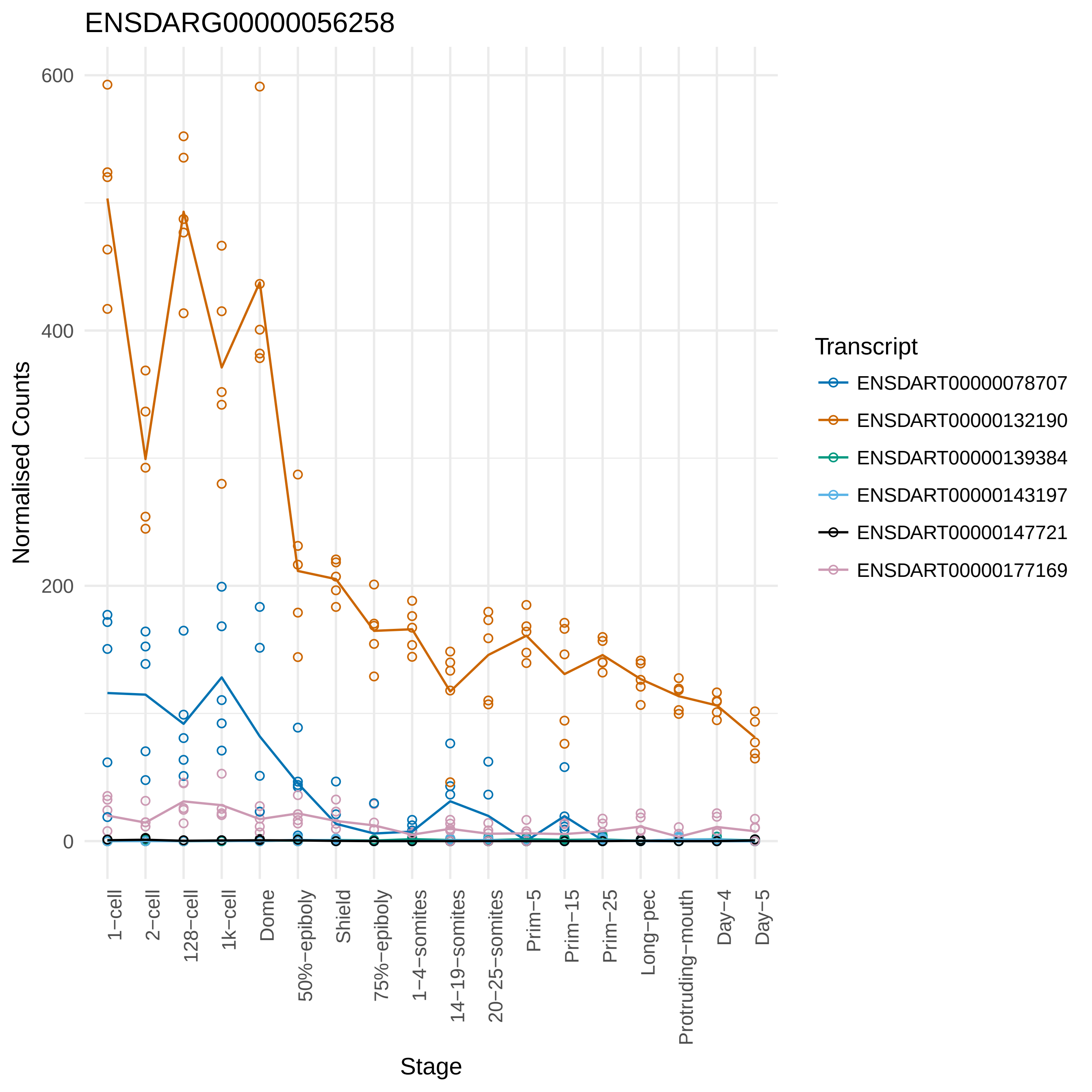
ENSDARG00000056258
Normalised Counts
600
400
200
0
1−cell
2−cell
128−cell
1k−cell
Dome
50%−epiboly
Shield
75%−epiboly
1−4−somites
14−19−somites
20−25−somites
Prim−5
Prim−15
Prim−25
Long−pec
Protruding−mouth
Day−4
Day−5
Stage
Transcript
ENSDART00000078707
ENSDART00000132190
ENSDART00000139384
ENSDART00000143197
ENSDART00000147721
ENSDART00000177169

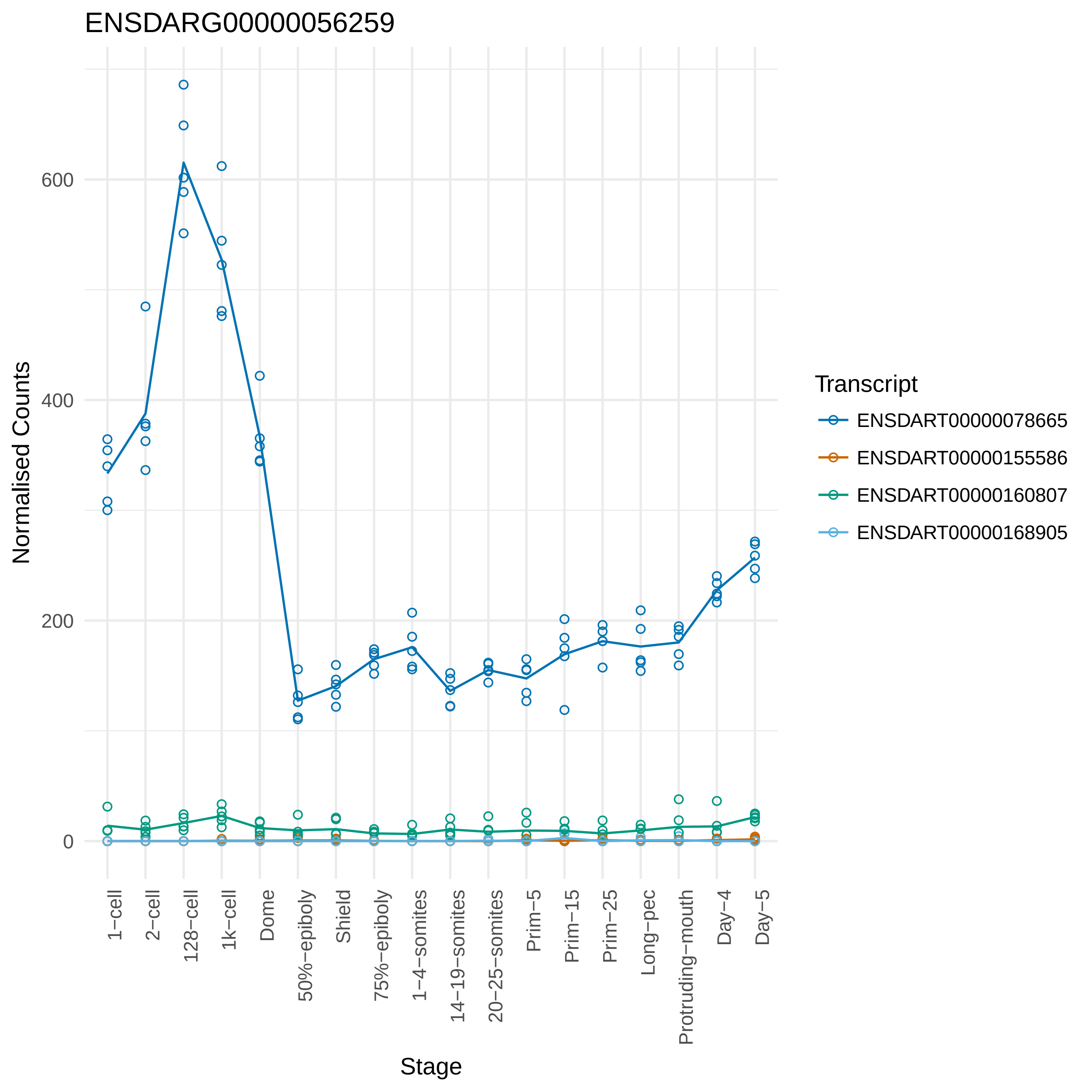

ENSDARG00000056259
Normalised Counts
600
400
200
0
1−cell
2−cell
128−cell
1k−cell
Dome
50%−epiboly
Shield
75%−epiboly
1−4−somites
14−19−somites
20−25−somites
Prim−5
Prim−15
Prim−25
Long−pec
Protruding−mouth
Day−4
Day−5
Stage
Transcript
ENSDART00000078665
ENSDART00000155586
ENSDART00000160807
ENSDART00000168905

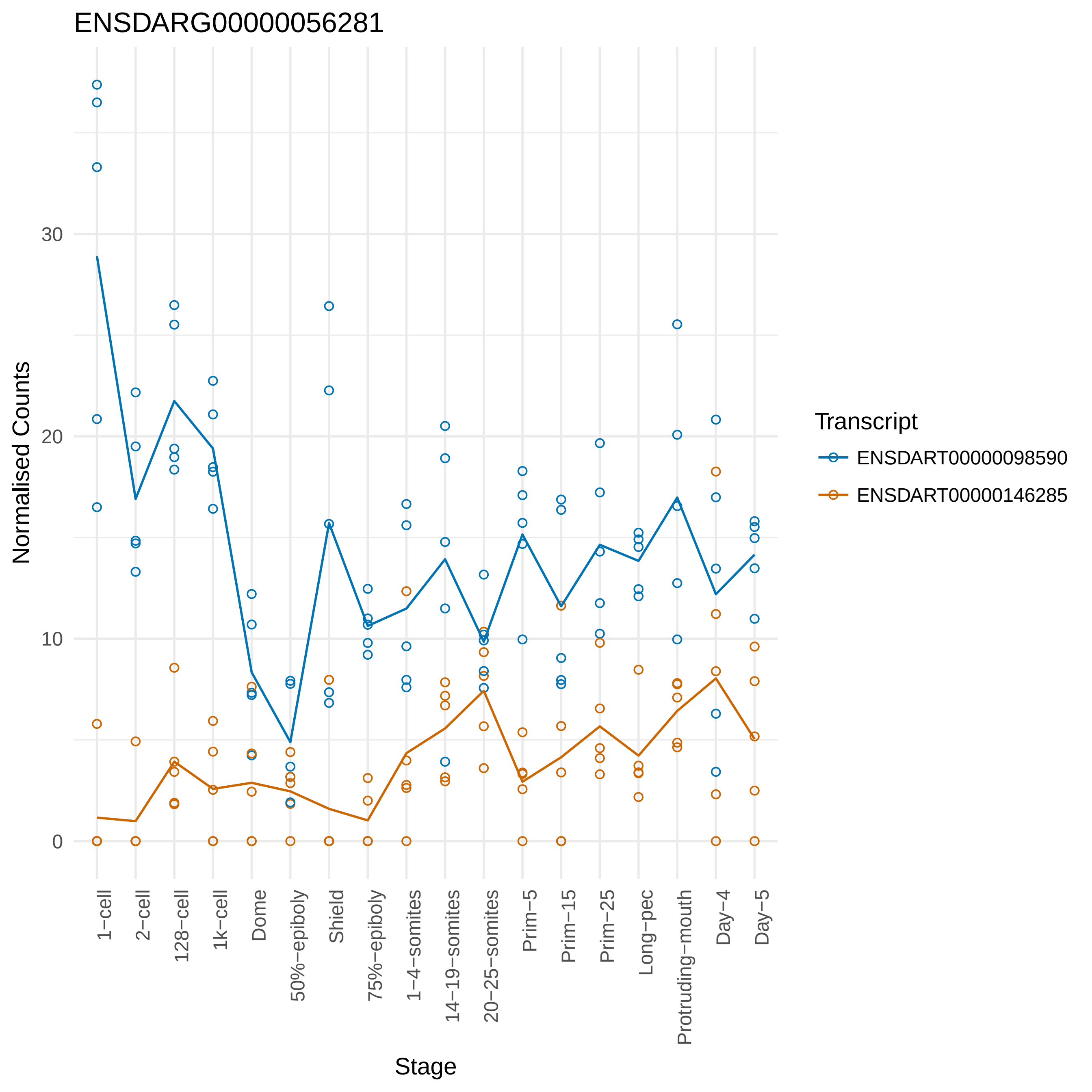
ENSDARG00000056281
Normalised Counts
0
10
20
30
Stage
1−cell
2−cell
128−cell
1k−cell
Dome
50%−epiboly
Shield
75%−epiboly
1−4−somites
14−19−somites
20−25−somites
Prim−5
Prim−15
Prim−25
Long−pec
Protruding−mouth
Day−4
Day−5
Transcript
ENSDART00000098590
ENSDART00000146285

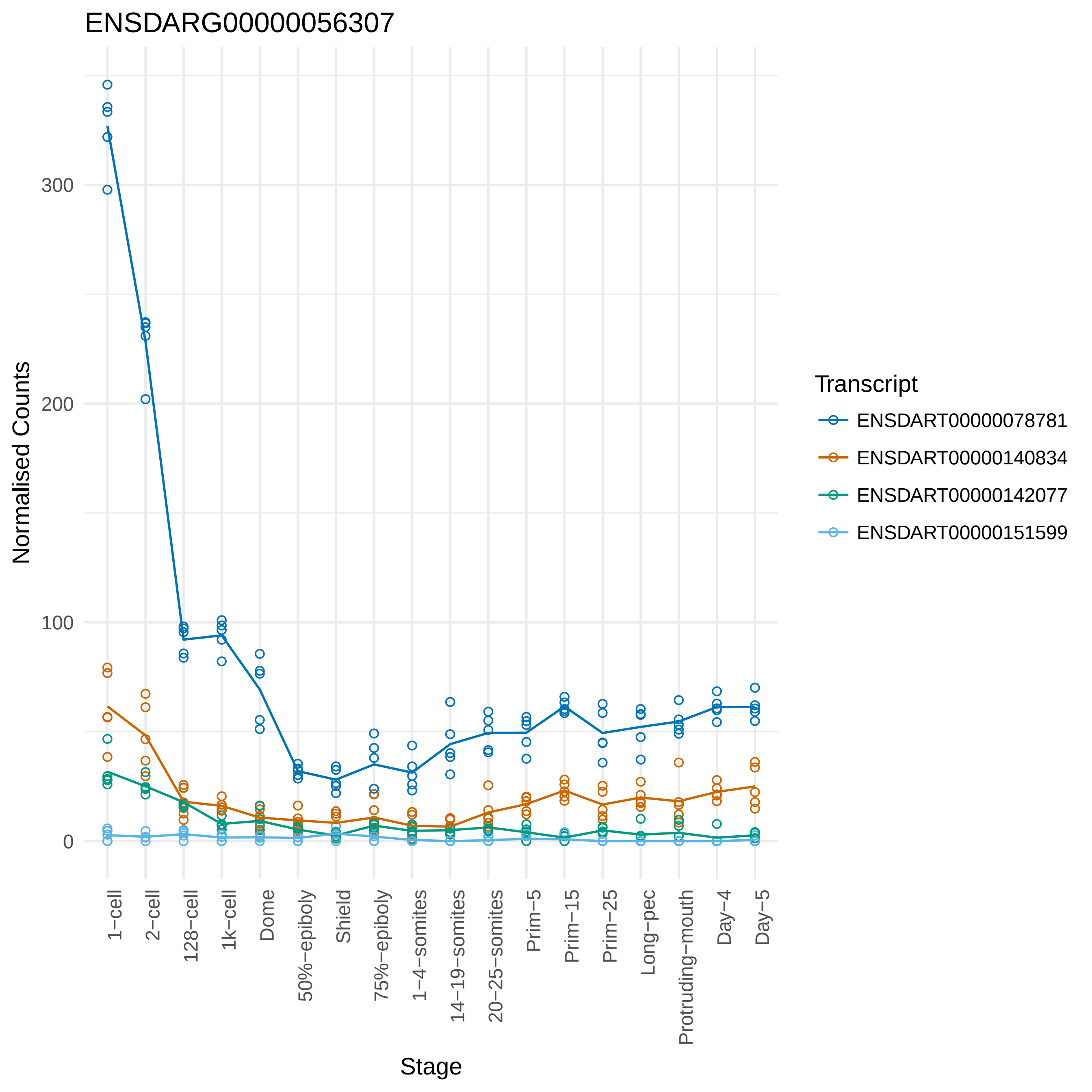
ENSDARG00000056307
Normalised Counts
300
200
100
0
1−cell
2−cell
128−cell
1k−cell
Dome
50%−epiboly
Shield
75%−epiboly
1−4−somites
14−19−somites
20−25−somites
Prim−5
Prim−15
Prim−25
Long−pec
Protruding−mouth
Day−4
Day−5
Stage
Transcript
ENSDART00000078781
ENSDART00000140834
ENSDART00000142077
ENSDART00000151599

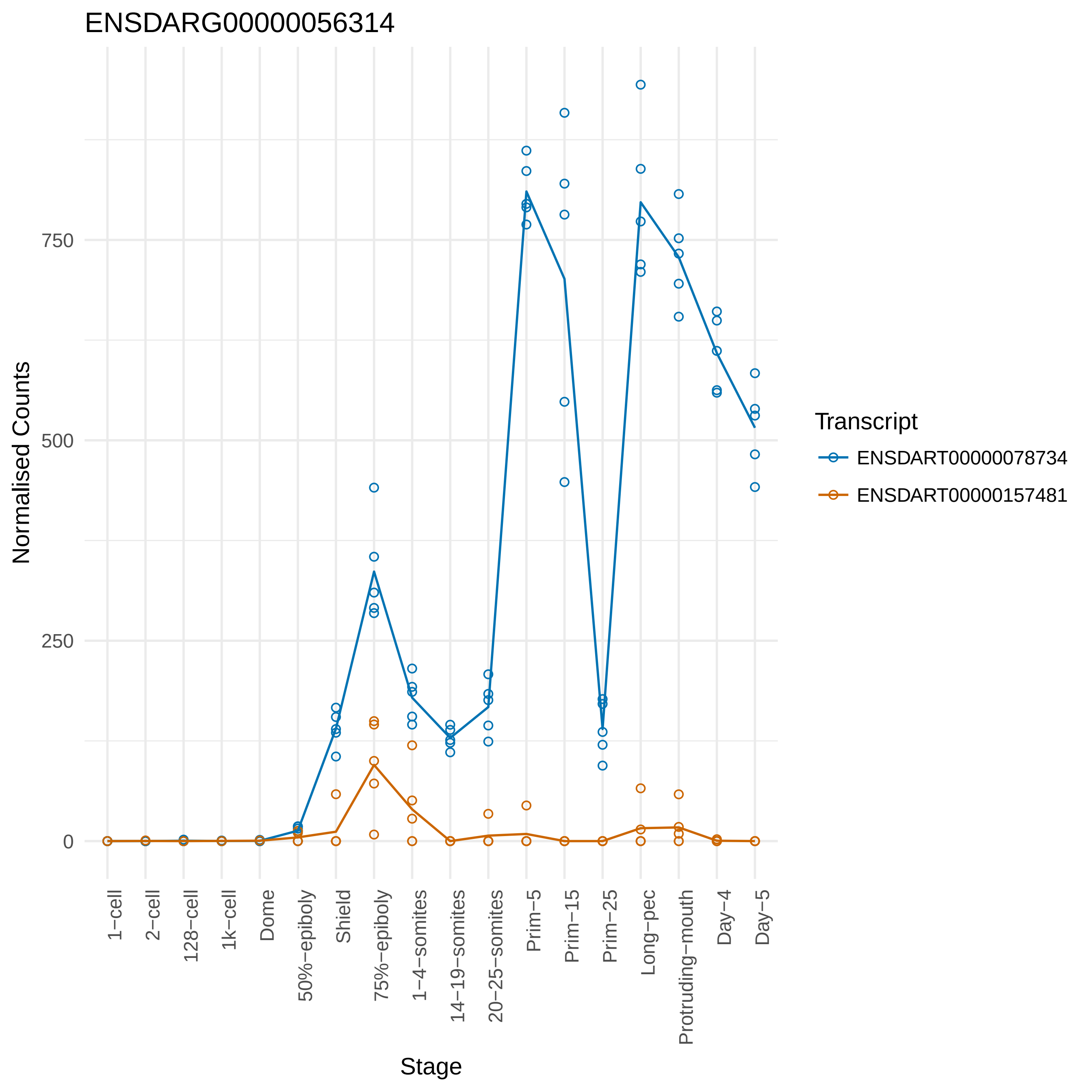

ENSDARG00000056314
Normalised Counts
0
250
500
750
Stage
1−cell
2−cell
128−cell
1k−cell
Dome
50%−epiboly
Shield
75%−epiboly
1−4−somites
14−19−somites
20−25−somites
Prim−5
Prim−15
Prim−25
Long−pec
Protruding−mouth
Day−4
Day−5
Transcript
ENSDART00000078734
ENSDART00000157481

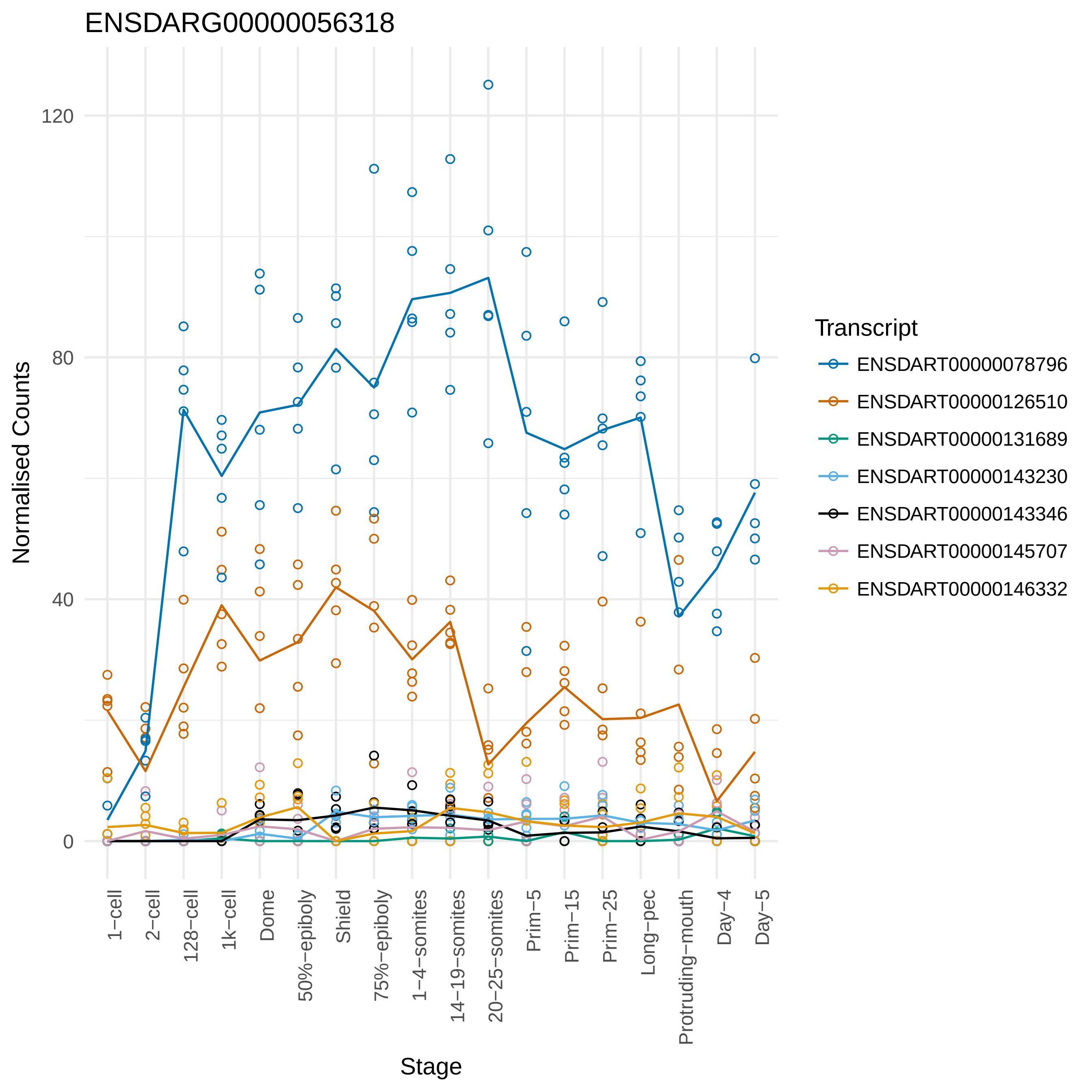
ENSDARG00000056318
Normalised Counts
120
80
40
0
1−cell
2−cell
128−cell
1k−cell
Dome
50%−epiboly
Shield
75%−epiboly
1−4−somites
14−19−somites
20−25−somites
Prim−5
Prim−15
Prim−25
Long−pec
Protruding−mouth
Day−4
Day−5
Stage
Transcript
ENSDART00000078796
ENSDART00000126510
ENSDART00000131689
ENSDART00000143230
ENSDART00000143346
ENSDART00000145707
ENSDART00000146332

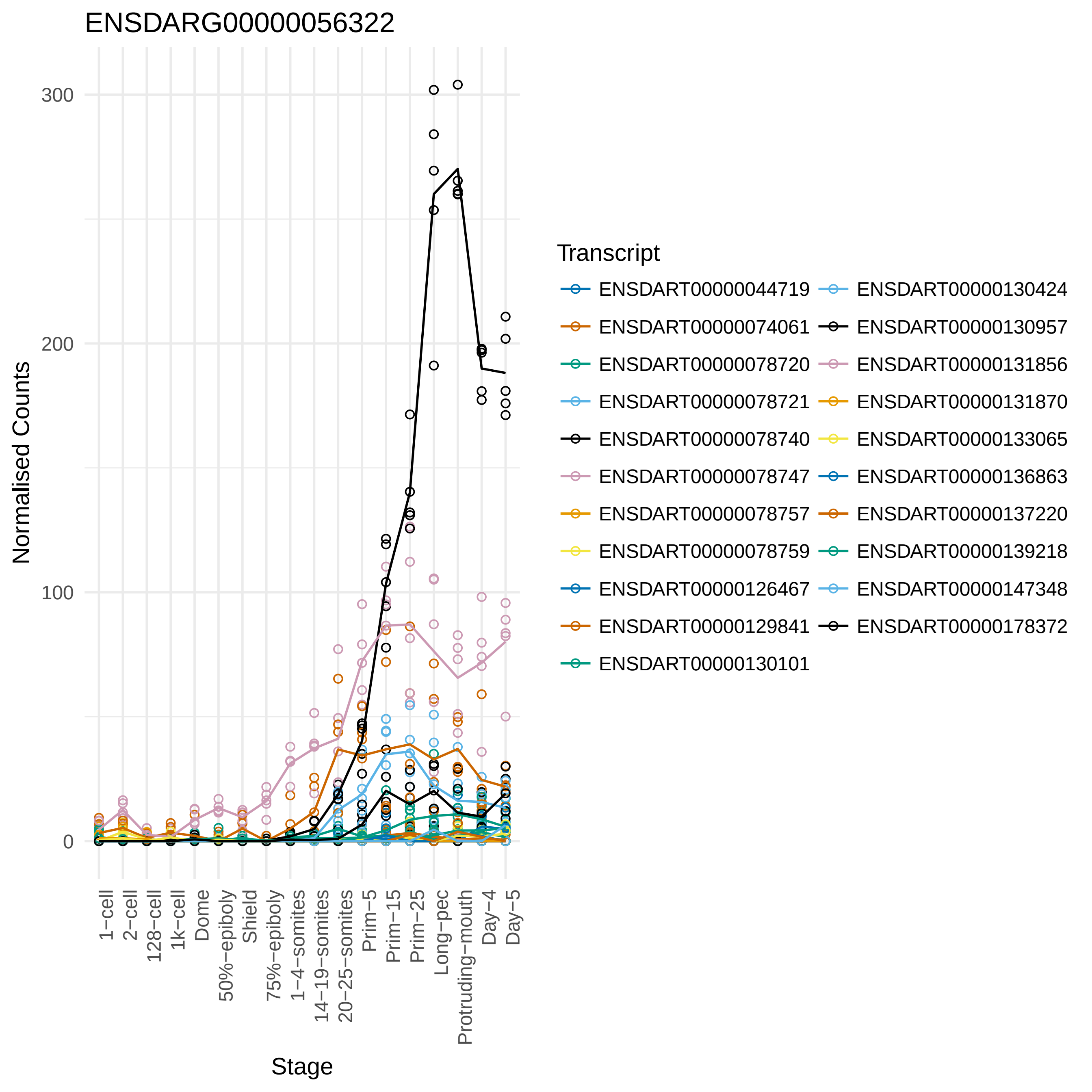

ENSDARG00000056322
Normalised Counts
300
200
100
0
1−cell
2−cell
128−cell
1k−cell
Dome
50%−epiboly
Shield
75%−epiboly
1−4−somites
14−19−somites
20−25−somites
Prim−5
Prim−15
Prim−25
Long−pec
Protruding−mouth
Day−4
Day−5
Stage
Transcript
ENSDART00000044719
ENSDART00000074061
ENSDART00000078720
ENSDART00000078721
ENSDART00000078740
ENSDART00000078747
ENSDART00000078757
ENSDART00000078759
ENSDART00000126467
ENSDART00000129841
ENSDART00000130101
ENSDART00000130424
ENSDART00000130957
ENSDART00000131856
ENSDART00000131870
ENSDART00000133065
ENSDART00000136863
ENSDART00000137220
ENSDART00000139218
ENSDART00000147348
ENSDART00000178372

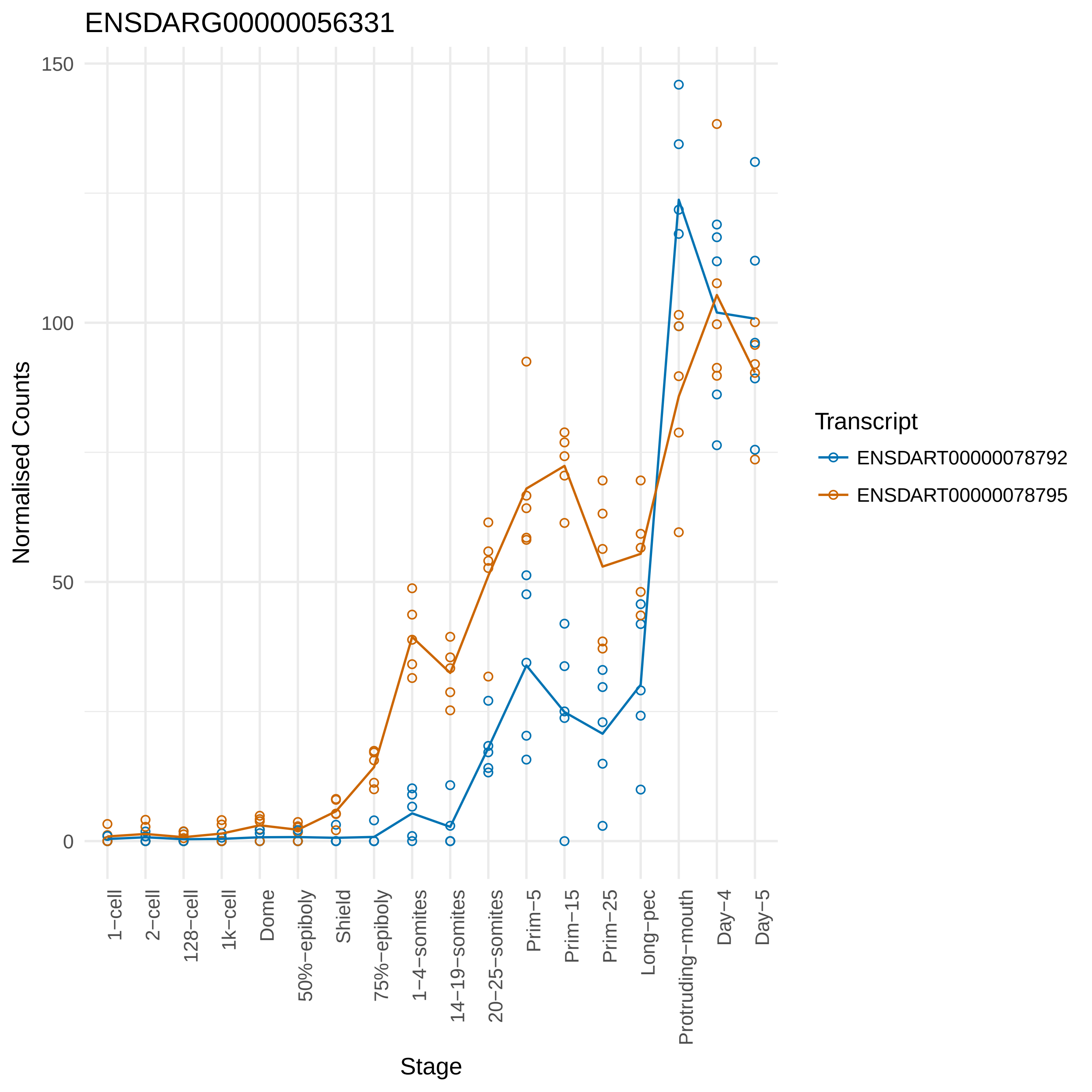
ENSDARG00000056331
Normalised Counts
150
100
50
0
1−cell
2−cell
128−cell
1k−cell
Dome
50%−epiboly
Shield
75%−epiboly
1−4−somites
14−19−somites
20−25−somites
Prim−5
Prim−15
Prim−25
Long−pec
Protruding−mouth
Day−4
Day−5
Stage
Transcript
ENSDART00000078792
ENSDART00000078795

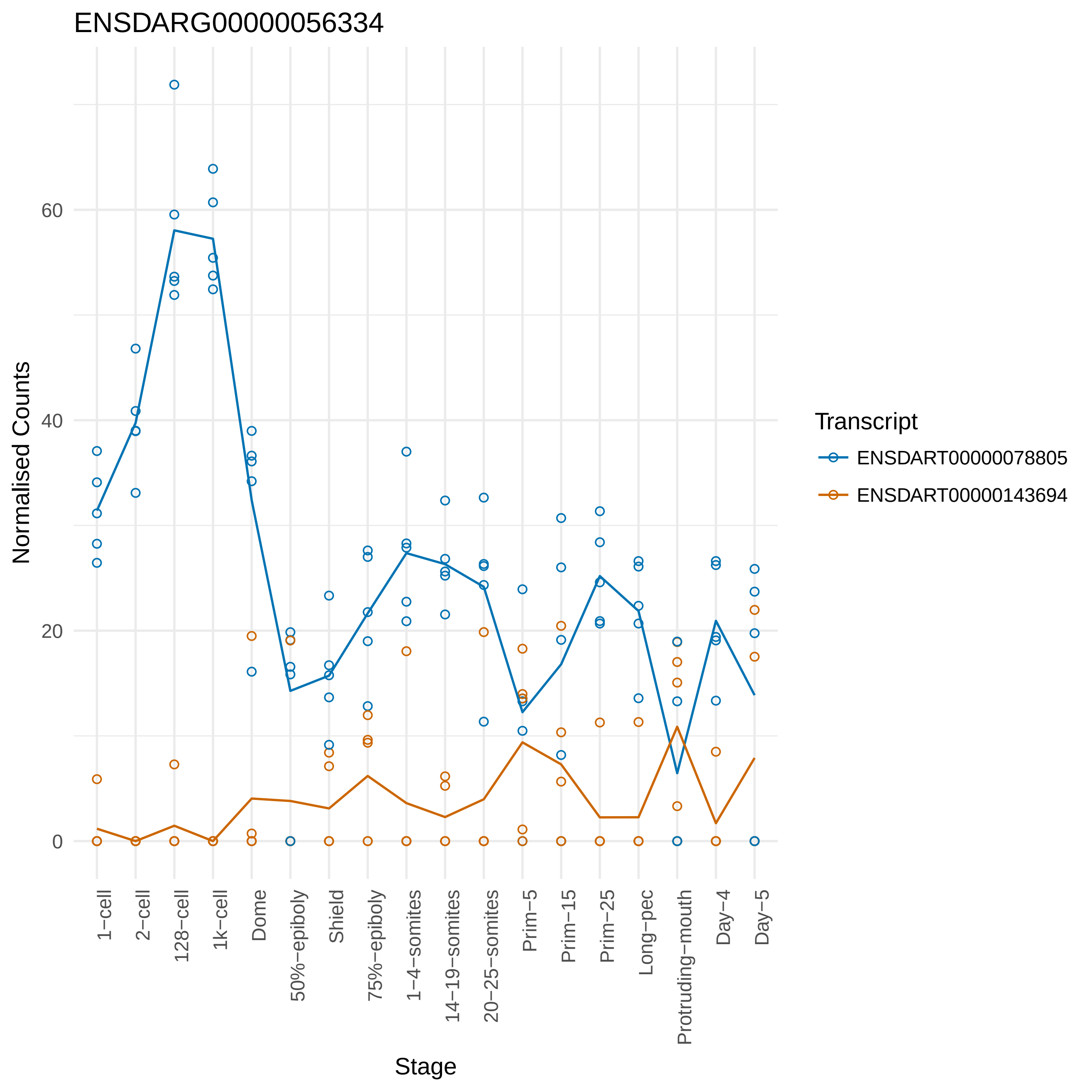

ENSDARG00000056334
Normalised Counts
0
20
40
60
Stage
1−cell
2−cell
128−cell
1k−cell
Dome
50%−epiboly
Shield
75%−epiboly
1−4−somites
14−19−somites
20−25−somites
Prim−5
Prim−15
Prim−25
Long−pec
Protruding−mouth
Day−4
Day−5
Transcript
ENSDART00000078805
ENSDART00000143694

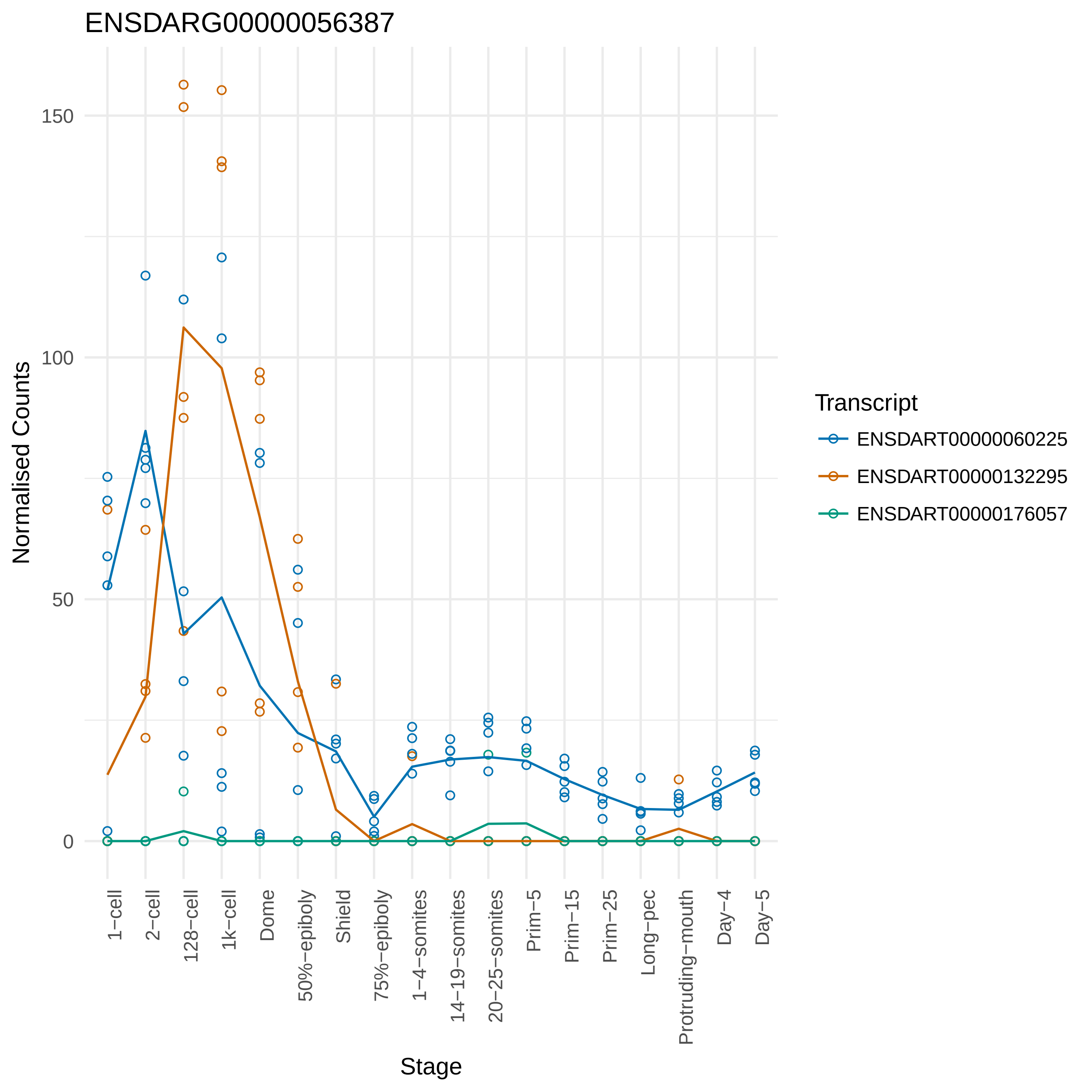

ENSDARG00000056387
Normalised Counts
150
100
50
0
1−cell
2−cell
128−cell
1k−cell
Dome
50%−epiboly
Shield
75%−epiboly
1−4−somites
14−19−somites
20−25−somites
Prim−5
Prim−15
Prim−25
Long−pec
Protruding−mouth
Day−4
Day−5
Stage
Transcript
ENSDART00000060225
ENSDART00000132295
ENSDART00000176057

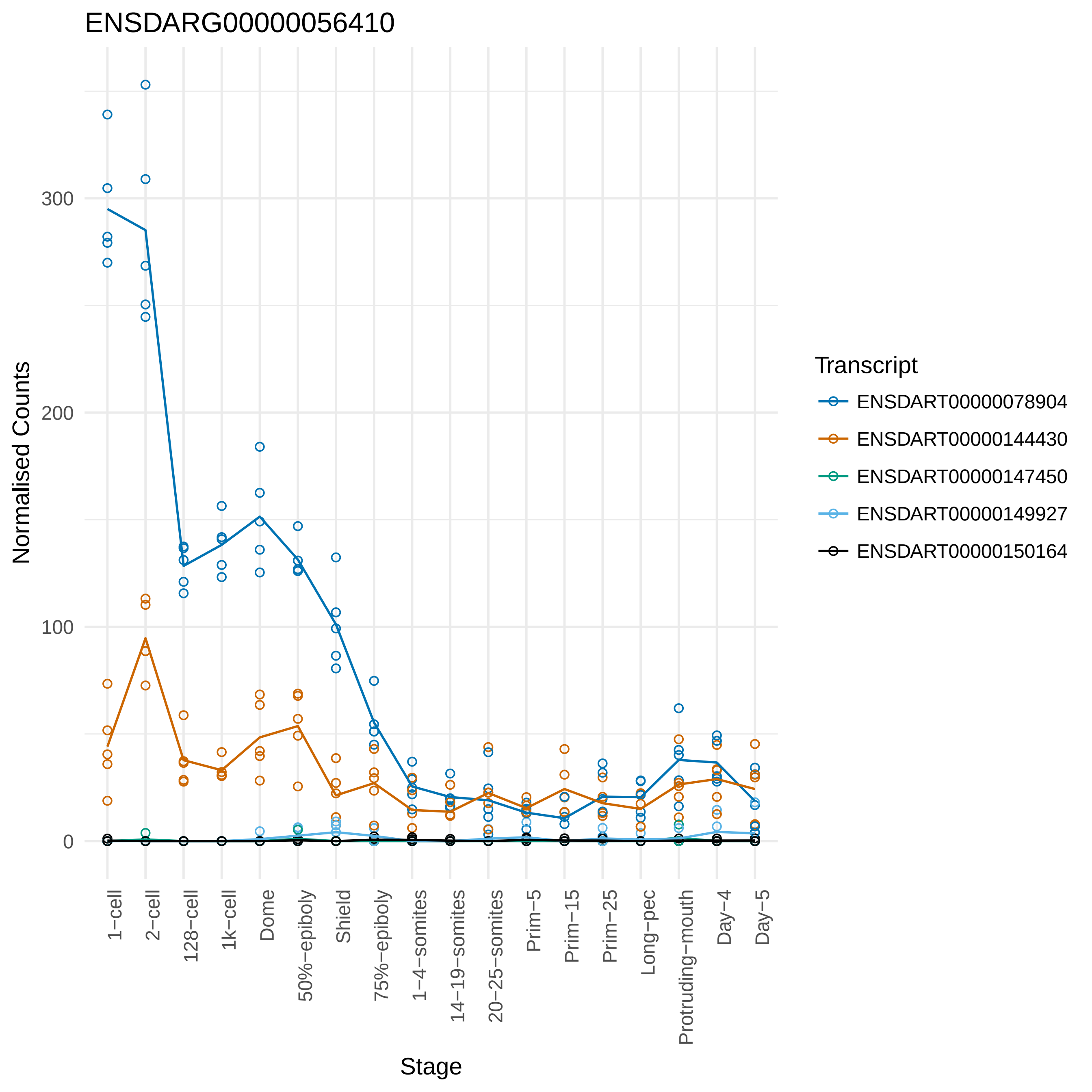
ENSDARG00000056410
Normalised Counts
300
200
100
0
1−cell
2−cell
128−cell
1k−cell
Dome
50%−epiboly
Shield
75%−epiboly
1−4−somites
14−19−somites
20−25−somites
Prim−5
Prim−15
Prim−25
Long−pec
Protruding−mouth
Day−4
Day−5
Stage
Transcript
ENSDART00000078904
ENSDART00000144430
ENSDART00000147450
ENSDART00000149927
ENSDART00000150164

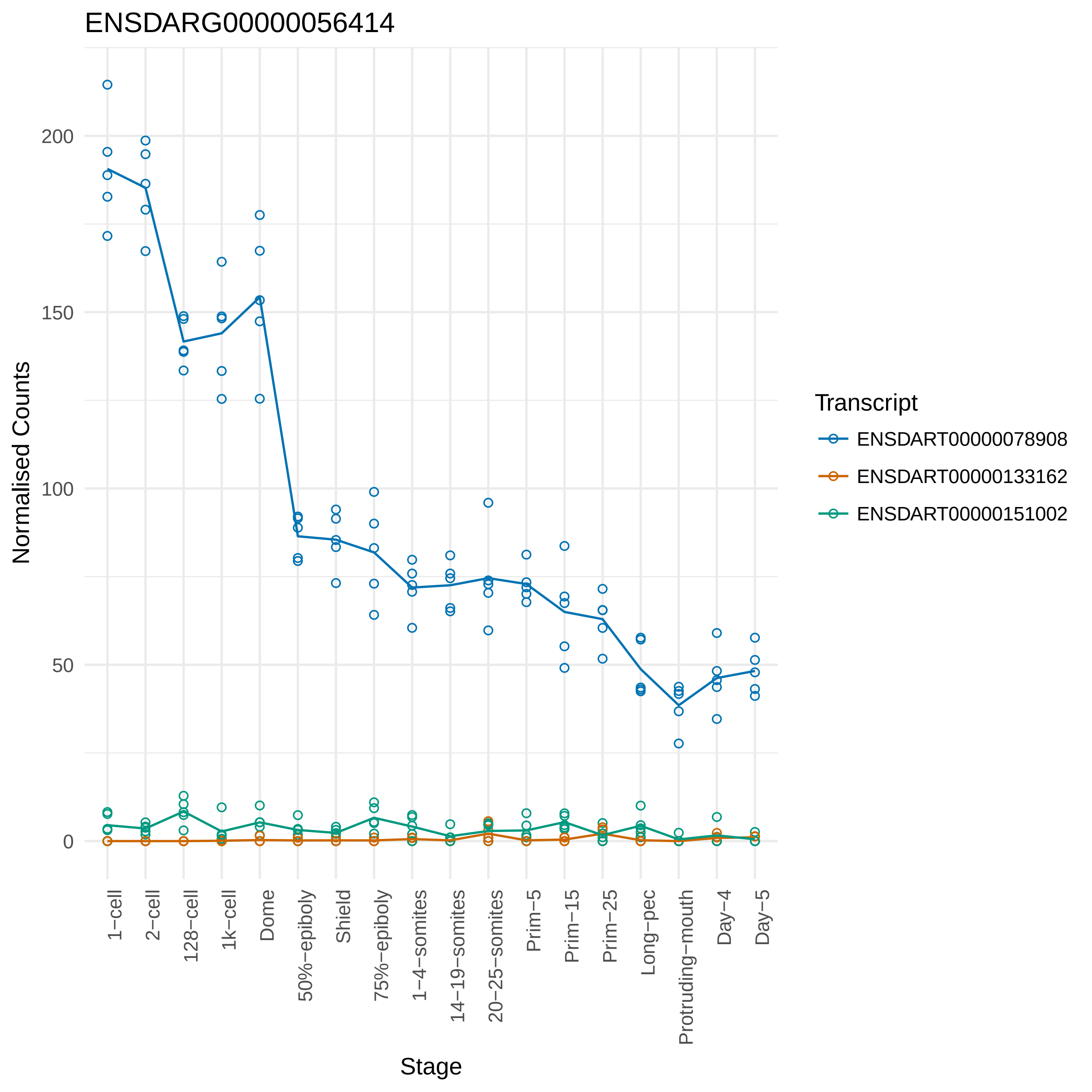

ENSDARG00000056414
Normalised Counts
200
150
100
50
0
1−cell
2−cell
128−cell
1k−cell
Dome
50%−epiboly
Shield
75%−epiboly
1−4−somites
14−19−somites
20−25−somites
Prim−5
Prim−15
Prim−25
Long−pec
Protruding−mouth
Day−4
Day−5
Stage
Transcript
ENSDART00000078908
ENSDART00000133162
ENSDART00000151002

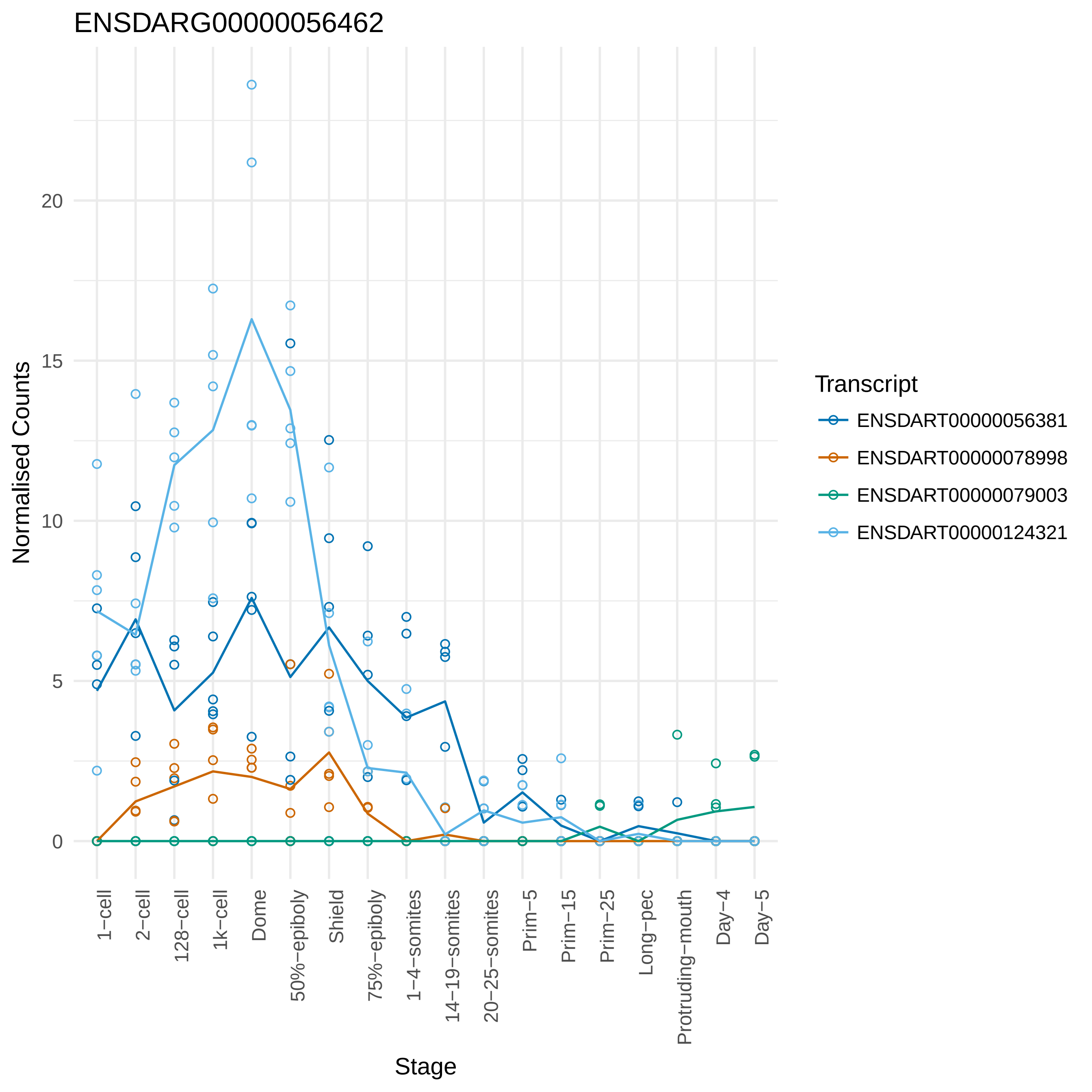

ENSDARG00000056462
Normalised Counts
0
5
10
15
20
Stage
1−cell
2−cell
128−cell
1k−cell
Dome
50%−epiboly
Shield
75%−epiboly
1−4−somites
14−19−somites
20−25−somites
Prim−5
Prim−15
Prim−25
Long−pec
Protruding−mouth
Day−4
Day−5
Transcript
ENSDART00000056381
ENSDART00000078998
ENSDART00000079003
ENSDART00000124321

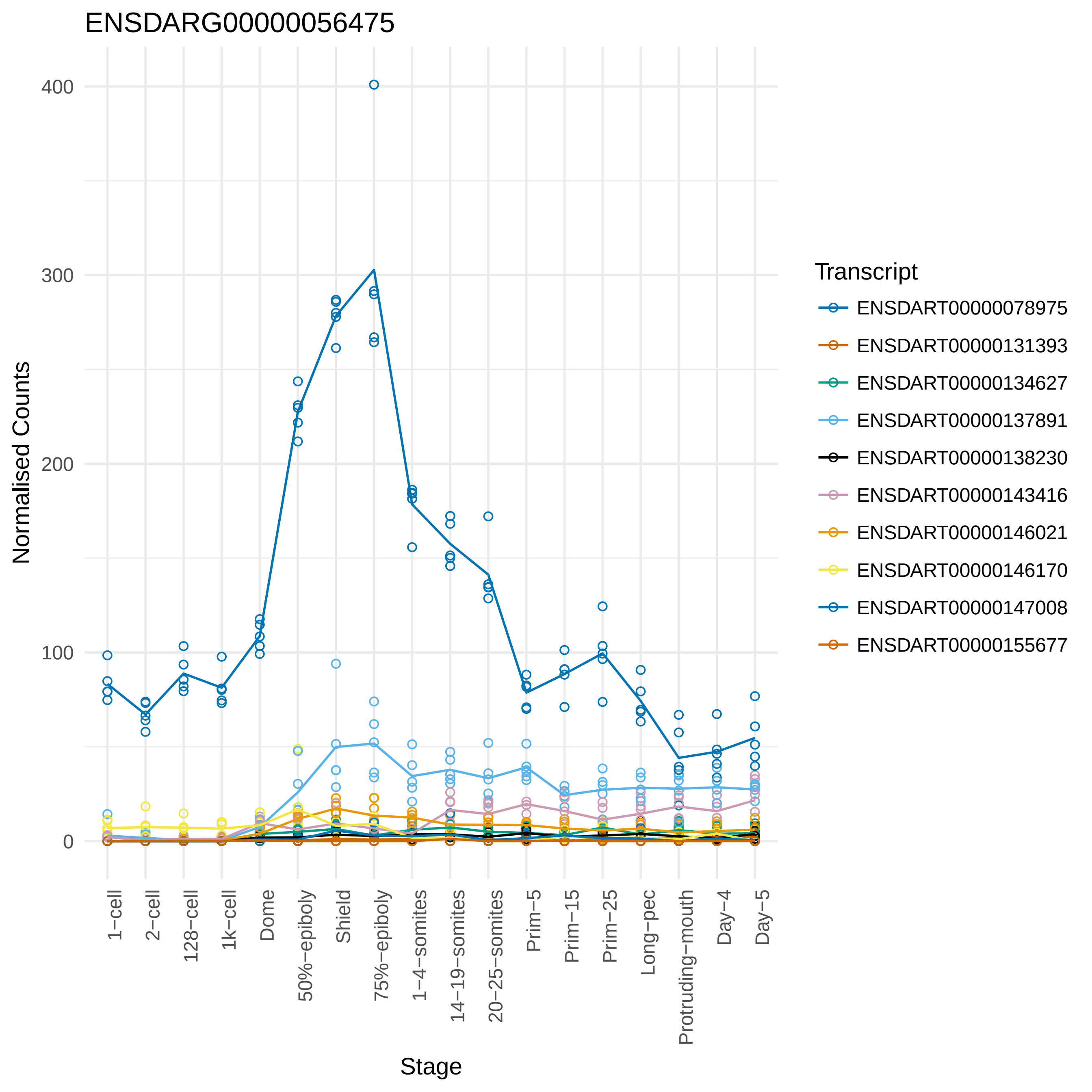

ENSDARG00000056475
Normalised Counts
400
300
200
100
0
1−cell
2−cell
128−cell
1k−cell
Dome
50%−epiboly
Shield
75%−epiboly
1−4−somites
14−19−somites
20−25−somites
Prim−5
Prim−15
Prim−25
Long−pec
Protruding−mouth
Day−4
Day−5
Stage
Transcript
ENSDART00000078975
ENSDART00000131393
ENSDART00000134627
ENSDART00000137891
ENSDART00000138230
ENSDART00000143416
ENSDART00000146021
ENSDART00000146170
ENSDART00000147008
ENSDART00000155677

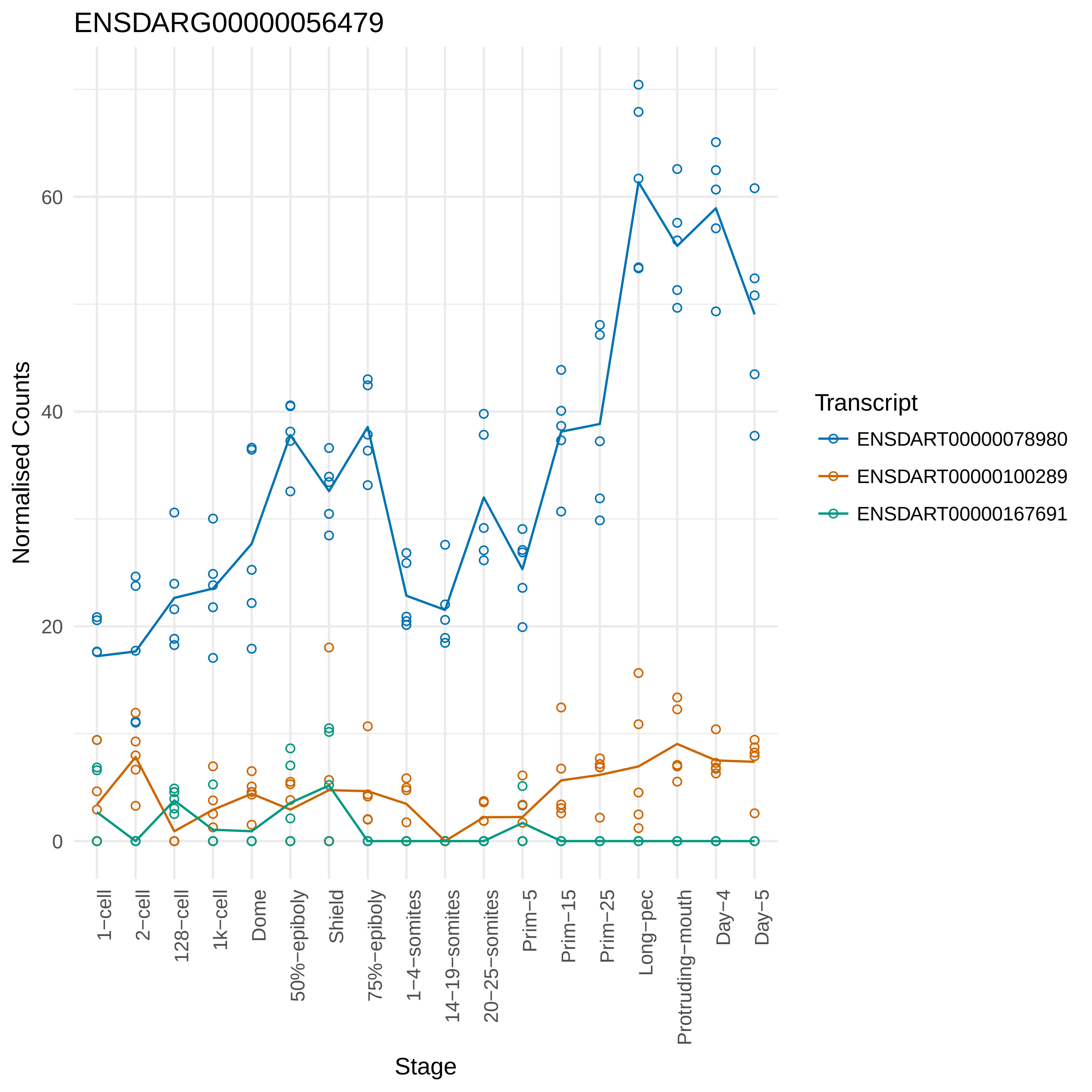

ENSDARG00000056479
Normalised Counts
0
20
40
60
Stage
1−cell
2−cell
128−cell
1k−cell
Dome
50%−epiboly
Shield
75%−epiboly
1−4−somites
14−19−somites
20−25−somites
Prim−5
Prim−15
Prim−25
Long−pec
Protruding−mouth
Day−4
Day−5
Transcript
ENSDART00000078980
ENSDART00000100289
ENSDART00000167691

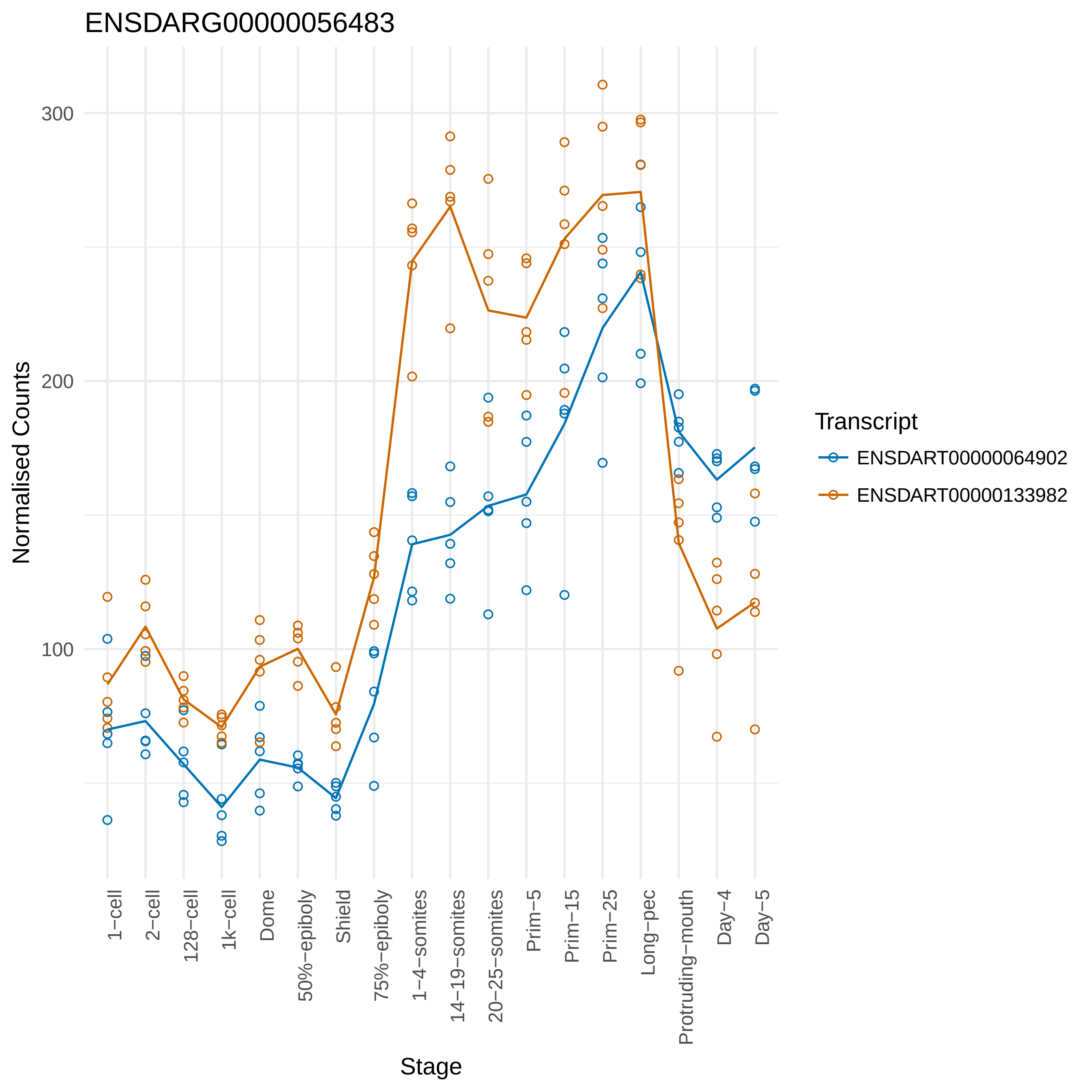
ENSDARG00000056483
Normalised Counts
300
200
100
1−cell
2−cell
128−cell
1k−cell
Dome
50%−epiboly
Shield
75%−epiboly
1−4−somites
14−19−somites
20−25−somites
Prim−5
Prim−15
Prim−25
Long−pec
Protruding−mouth
Day−4
Day−5
Stage
Transcript
ENSDART00000064902
ENSDART00000133982

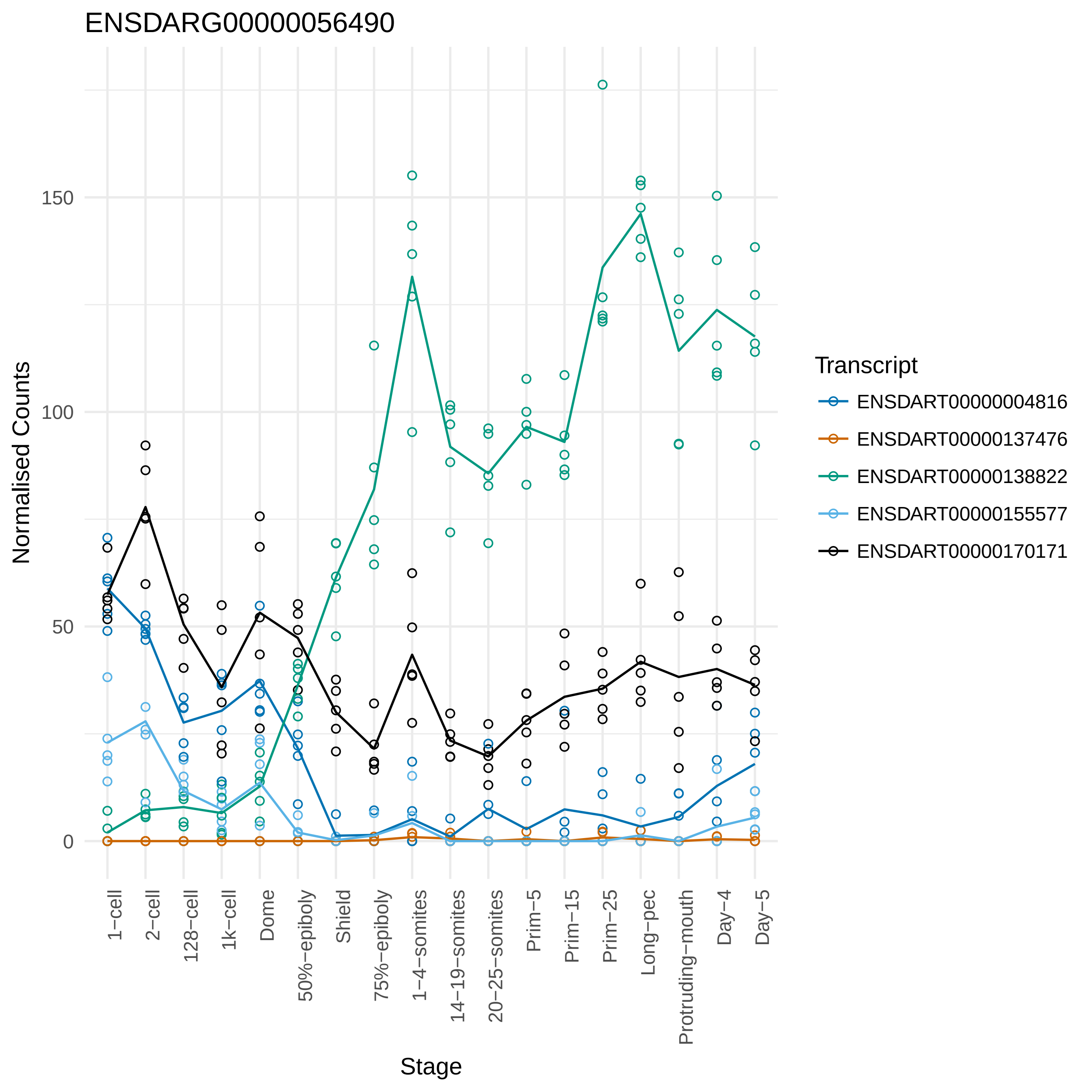

ENSDARG00000056490
Normalised Counts
0
50
100
150
Stage
1−cell
2−cell
128−cell
1k−cell
Dome
50%−epiboly
Shield
75%−epiboly
1−4−somites
14−19−somites
20−25−somites
Prim−5
Prim−15
Prim−25
Long−pec
Protruding−mouth
Day−4
Day−5
Transcript
ENSDART00000004816
ENSDART00000137476
ENSDART00000138822
ENSDART00000155577
ENSDART00000170171

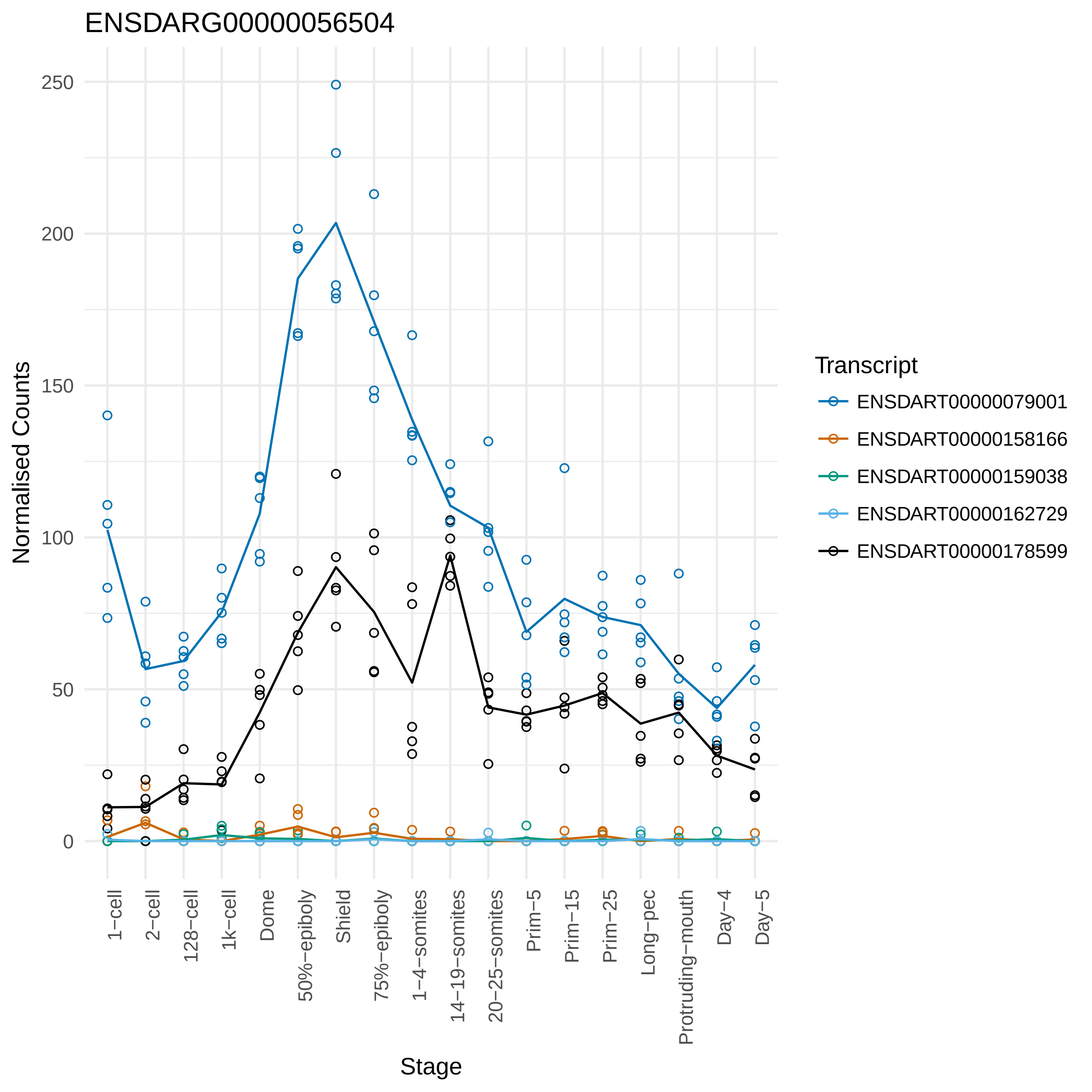
ENSDARG00000056504
Normalised Counts
250
200
150
100
50
0
1−cell
2−cell
128−cell
1k−cell
Dome
50%−epiboly
Shield
75%−epiboly
1−4−somites
14−19−somites
20−25−somites
Prim−5
Prim−15
Prim−25
Long−pec
Protruding−mouth
Day−4
Day−5
Stage
Transcript
ENSDART00000079001
ENSDART00000158166
ENSDART00000159038
ENSDART00000162729
ENSDART00000178599

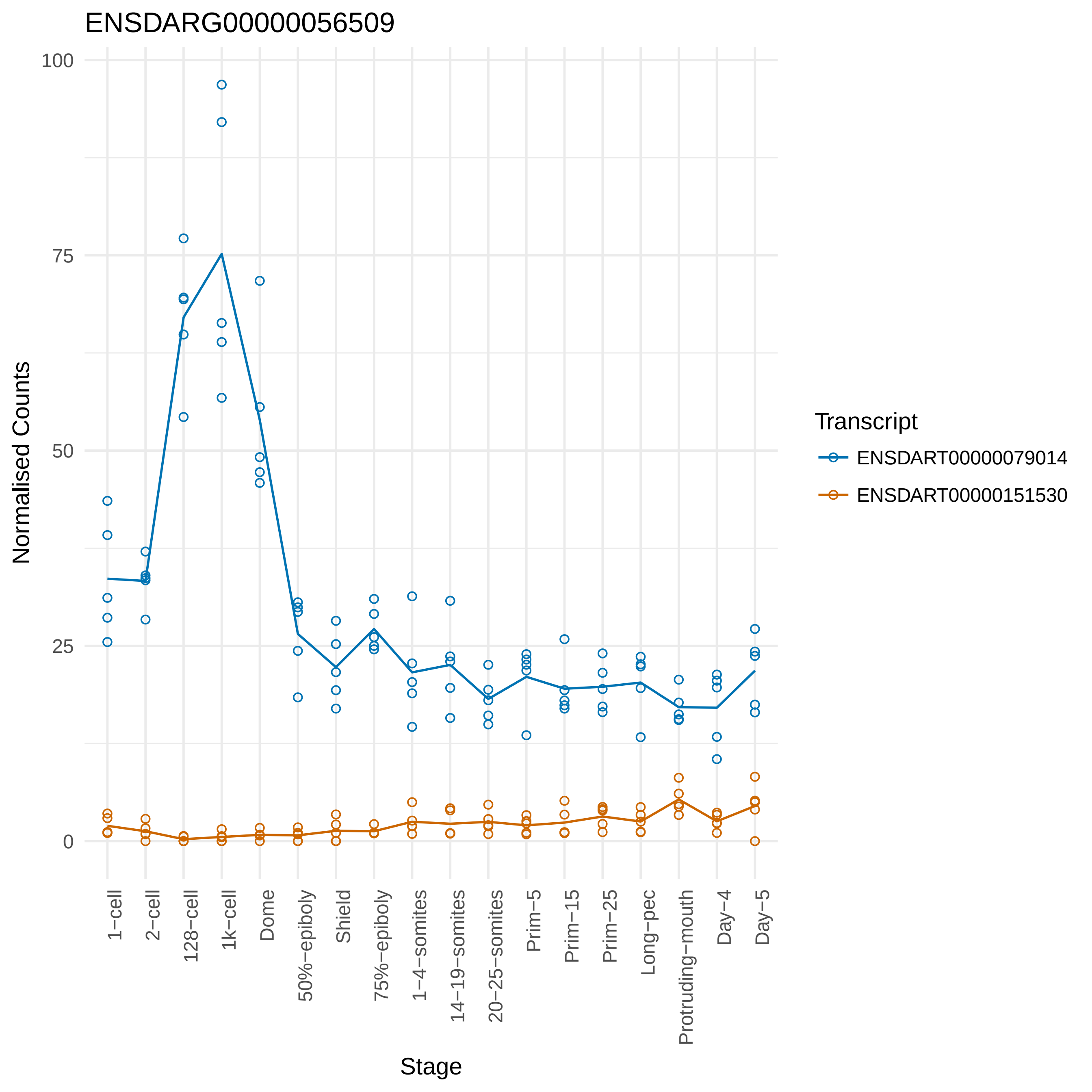
ENSDARG00000056509
Normalised Counts
100
75
50
25
0
1−cell
2−cell
128−cell
1k−cell
Dome
50%−epiboly
Shield
75%−epiboly
1−4−somites
14−19−somites
20−25−somites
Prim−5
Prim−15
Prim−25
Long−pec
Protruding−mouth
Day−4
Day−5
Stage
Transcript
ENSDART00000079014
ENSDART00000151530

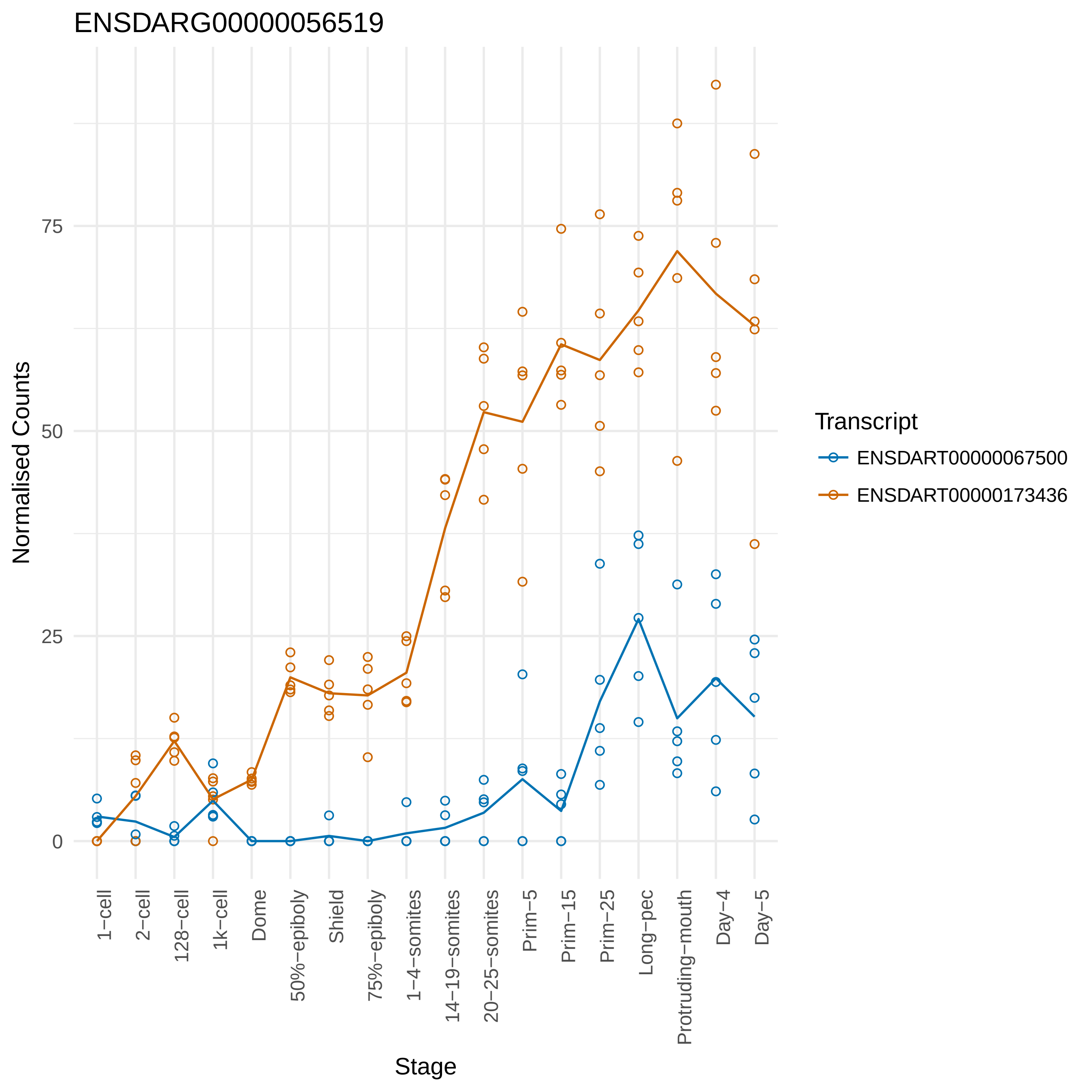

ENSDARG00000056519
Normalised Counts
75
50
25
0
1−cell
2−cell
128−cell
1k−cell
Dome
50%−epiboly
Shield
75%−epiboly
1−4−somites
14−19−somites
20−25−somites
Prim−5
Prim−15
Prim−25
Long−pec
Protruding−mouth
Day−4
Day−5
Stage
Transcript
ENSDART00000067500
ENSDART00000173436

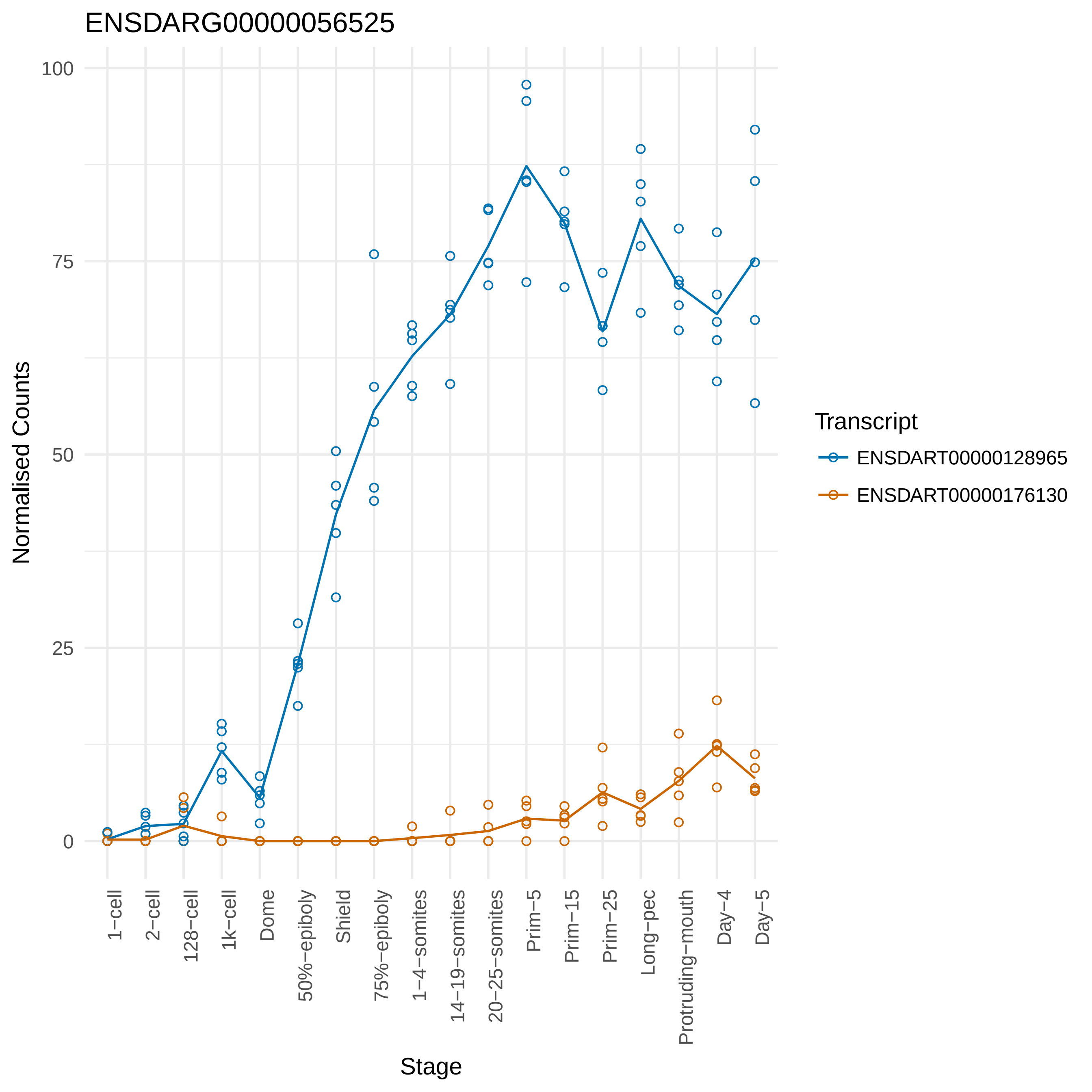

ENSDARG00000056525
Normalised Counts
100
75
50
25
0
1−cell
2−cell
128−cell
1k−cell
Dome
50%−epiboly
Shield
75%−epiboly
1−4−somites
14−19−somites
20−25−somites
Prim−5
Prim−15
Prim−25
Long−pec
Protruding−mouth
Day−4
Day−5
Stage
Transcript
ENSDART00000128965
ENSDART00000176130

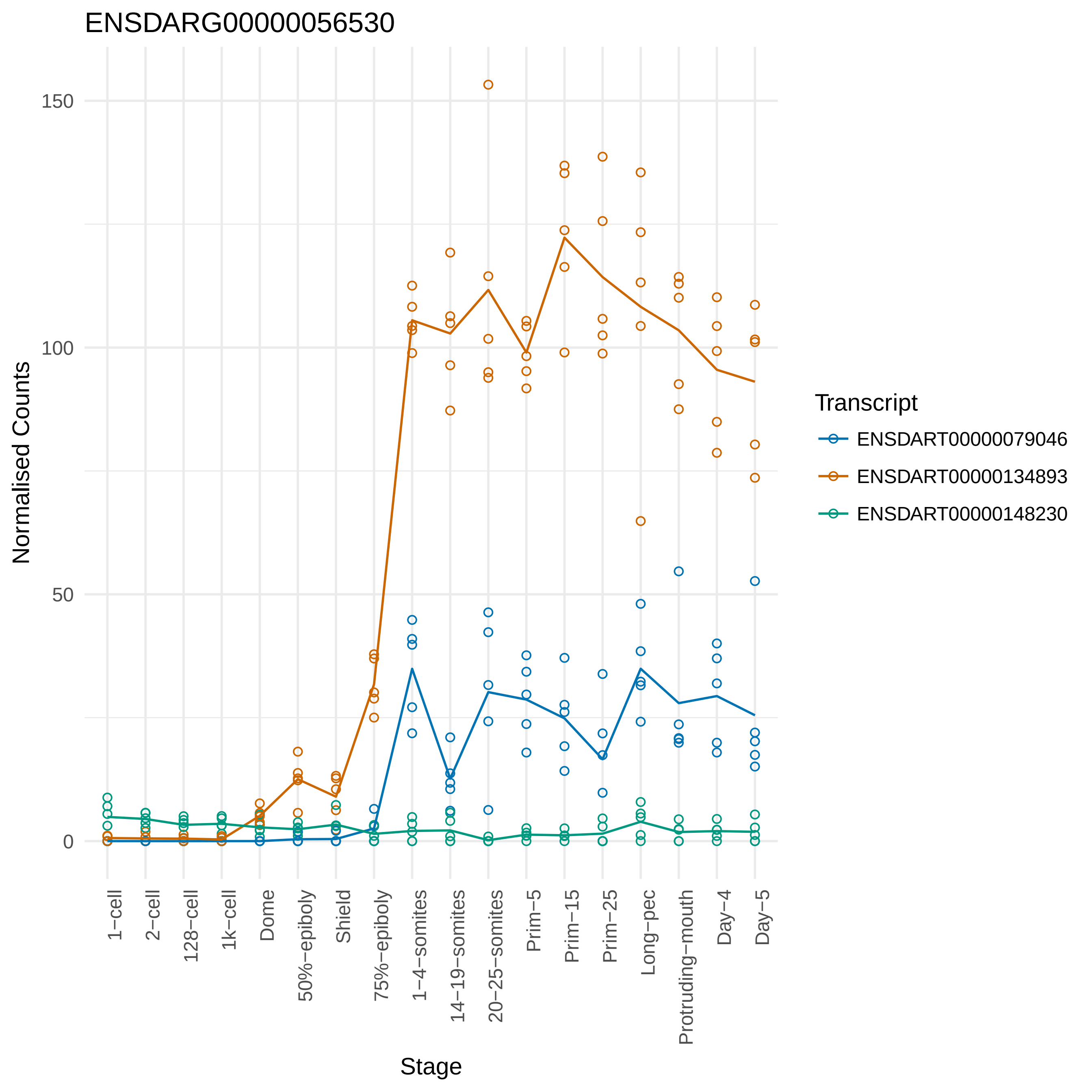

ENSDARG00000056530
Normalised Counts
150
100
50
0
1−cell
2−cell
128−cell
1k−cell
Dome
50%−epiboly
Shield
75%−epiboly
1−4−somites
14−19−somites
20−25−somites
Prim−5
Prim−15
Prim−25
Long−pec
Protruding−mouth
Day−4
Day−5
Stage
Transcript
ENSDART00000079046
ENSDART00000134893
ENSDART00000148230

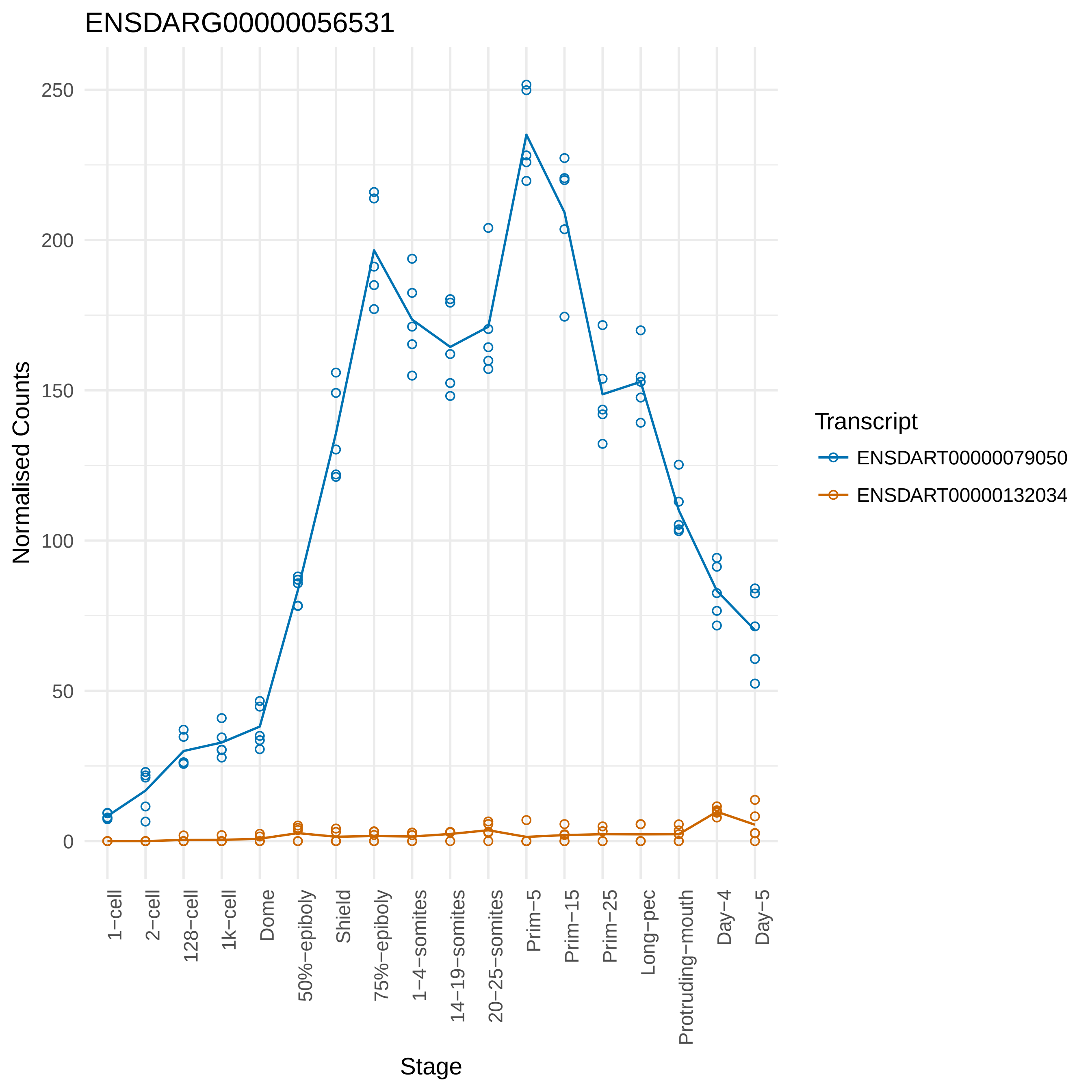

ENSDARG00000056531
Normalised Counts
250
200
150
100
50
0
1−cell
2−cell
128−cell
1k−cell
Dome
50%−epiboly
Shield
75%−epiboly
1−4−somites
14−19−somites
20−25−somites
Prim−5
Prim−15
Prim−25
Long−pec
Protruding−mouth
Day−4
Day−5
Stage
Transcript
ENSDART00000079050
ENSDART00000132034

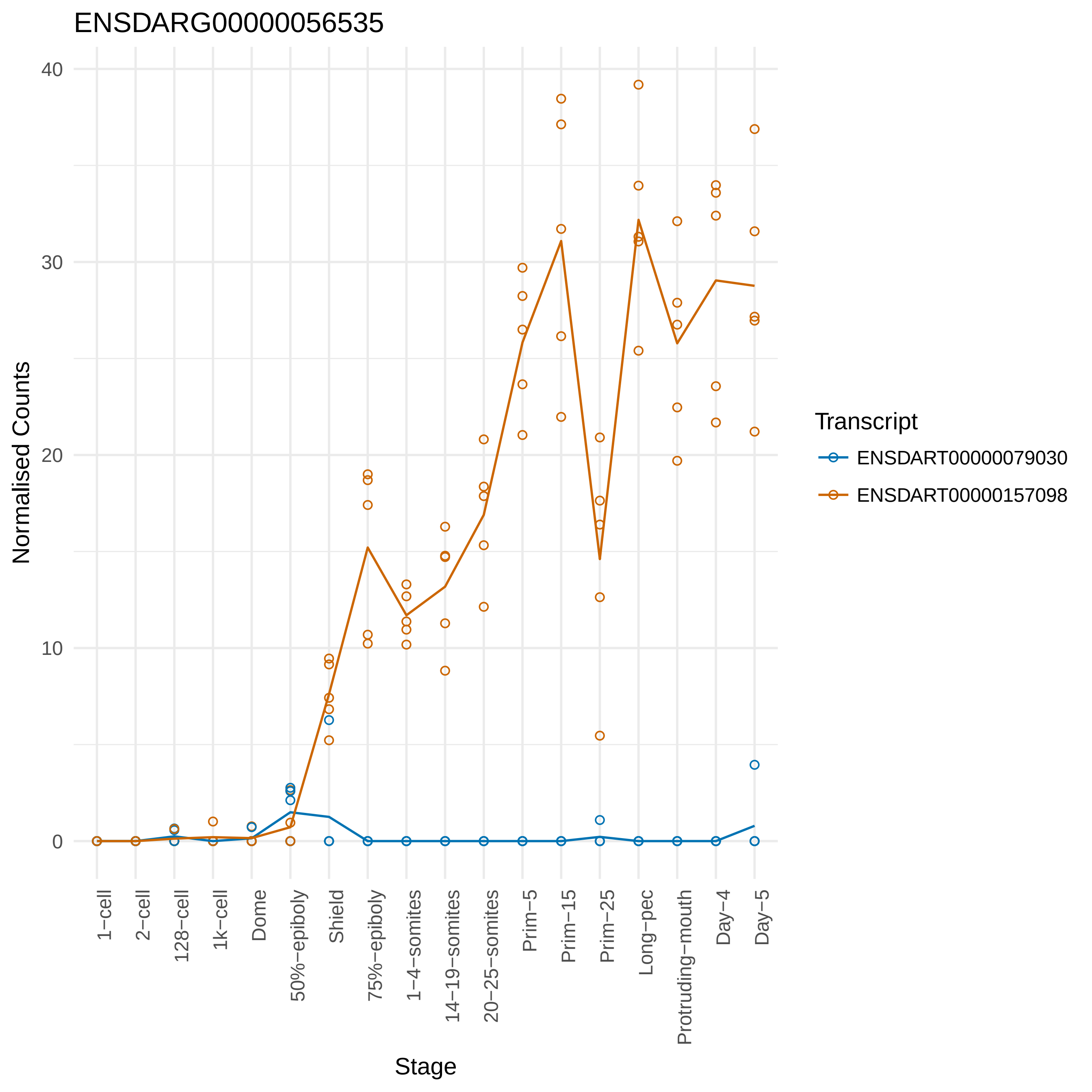

ENSDARG00000056535
Normalised Counts
0
10
20
30
40
Stage
1−cell
2−cell
128−cell
1k−cell
Dome
50%−epiboly
Shield
75%−epiboly
1−4−somites
14−19−somites
20−25−somites
Prim−5
Prim−15
Prim−25
Long−pec
Protruding−mouth
Day−4
Day−5
Transcript
ENSDART00000079030
ENSDART00000157098

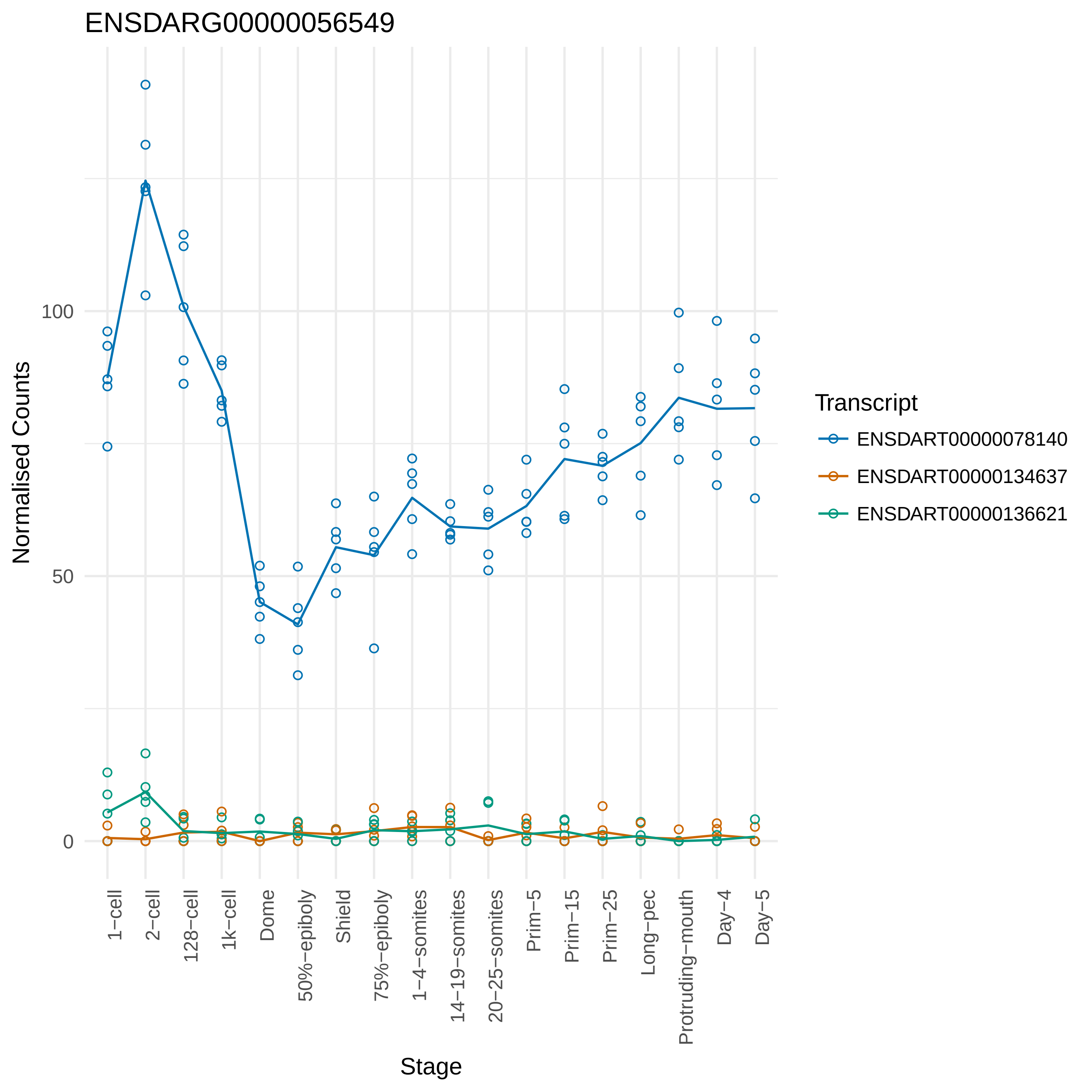

ENSDARG00000056549
Normalised Counts
100
50
0
1−cell
2−cell
128−cell
1k−cell
Dome
50%−epiboly
Shield
75%−epiboly
1−4−somites
14−19−somites
20−25−somites
Prim−5
Prim−15
Prim−25
Long−pec
Protruding−mouth
Day−4
Day−5
Stage
Transcript
ENSDART00000078140
ENSDART00000134637
ENSDART00000136621

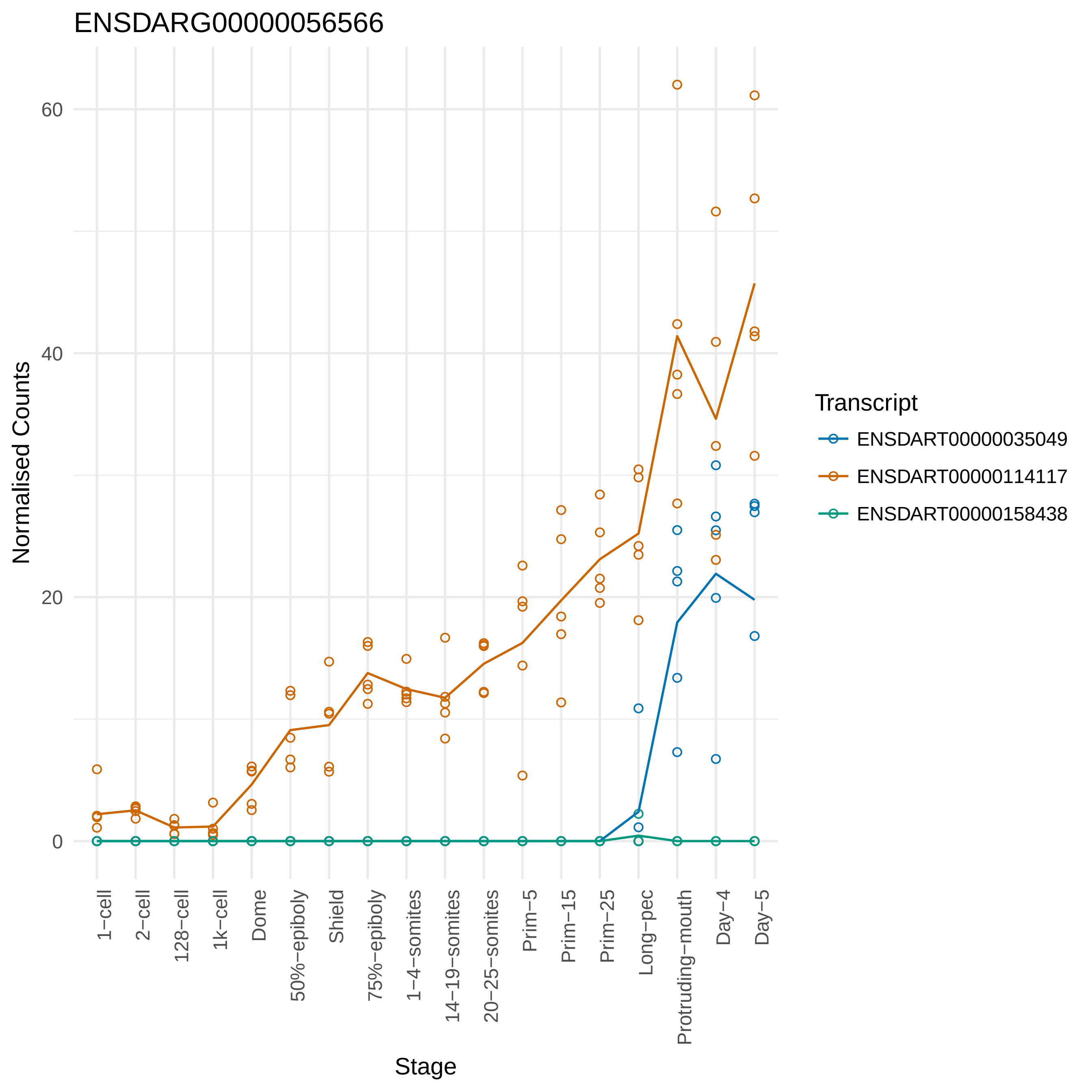

ENSDARG00000056566
Normalised Counts
0
20
40
60
1−cell
2−cell
128−cell
1k−cell
Dome
50%−epiboly
Shield
75%−epiboly
1−4−somites
14−19−somites
20−25−somites
Prim−5
Prim−15
Prim−25
Long−pec
Protruding−mouth
Day−4
Day−5
Stage
Transcript
ENSDART00000035049
ENSDART00000114117
ENSDART00000158438

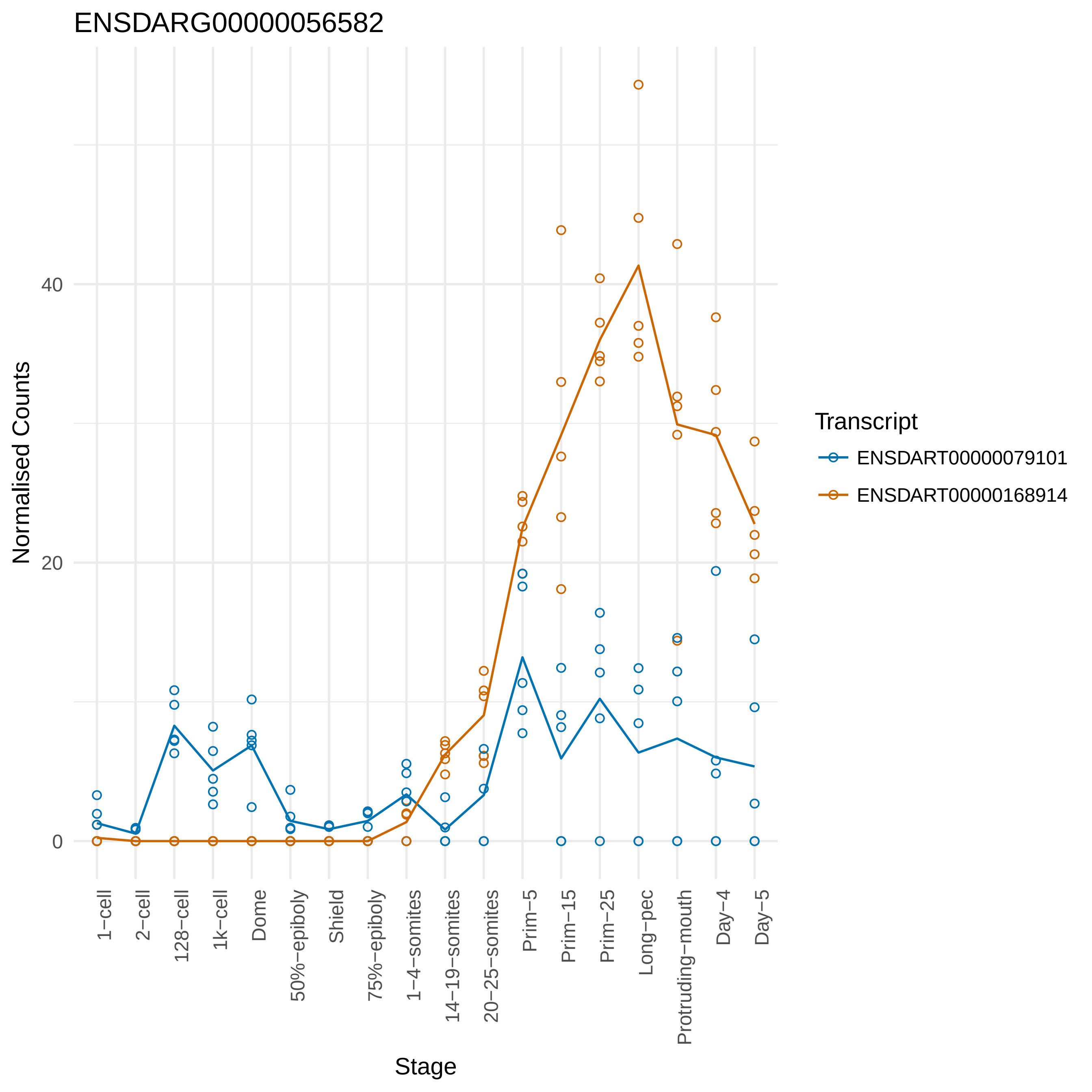
ENSDARG00000056582
Normalised Counts
0
20
40
Stage
1−cell
2−cell
128−cell
1k−cell
Dome
50%−epiboly
Shield
75%−epiboly
1−4−somites
14−19−somites
20−25−somites
Prim−5
Prim−15
Prim−25
Long−pec
Protruding−mouth
Day−4
Day−5
Transcript
ENSDART00000079101
ENSDART00000168914

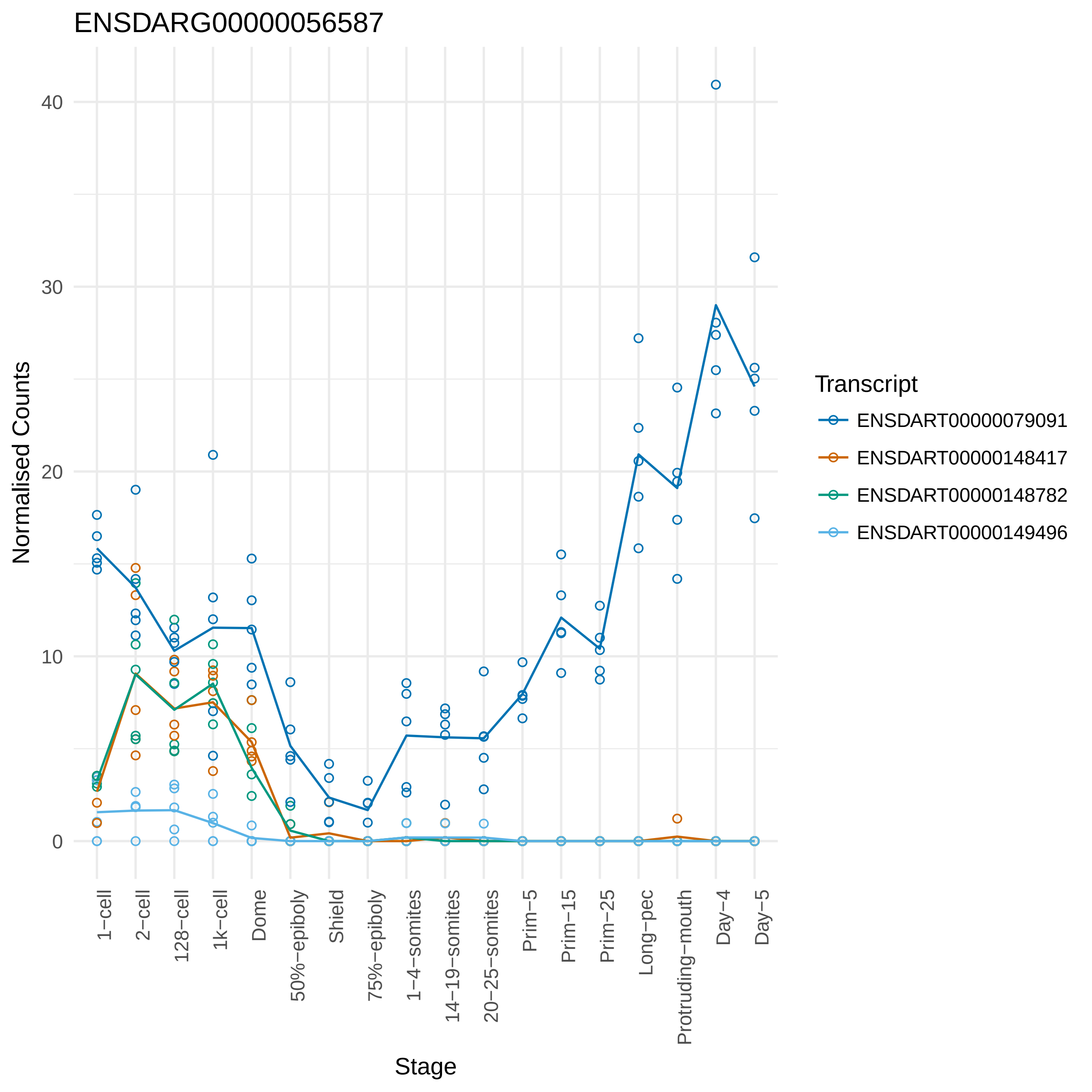
ENSDARG00000056587
Normalised Counts
0
10
20
30
40
1−cell
2−cell
128−cell
1k−cell
Dome
50%−epiboly
Shield
75%−epiboly
1−4−somites
14−19−somites
20−25−somites
Prim−5
Prim−15
Prim−25
Long−pec
Protruding−mouth
Day−4
Day−5
Stage
Transcript
ENSDART00000079091
ENSDART00000148417
ENSDART00000148782
ENSDART00000149496

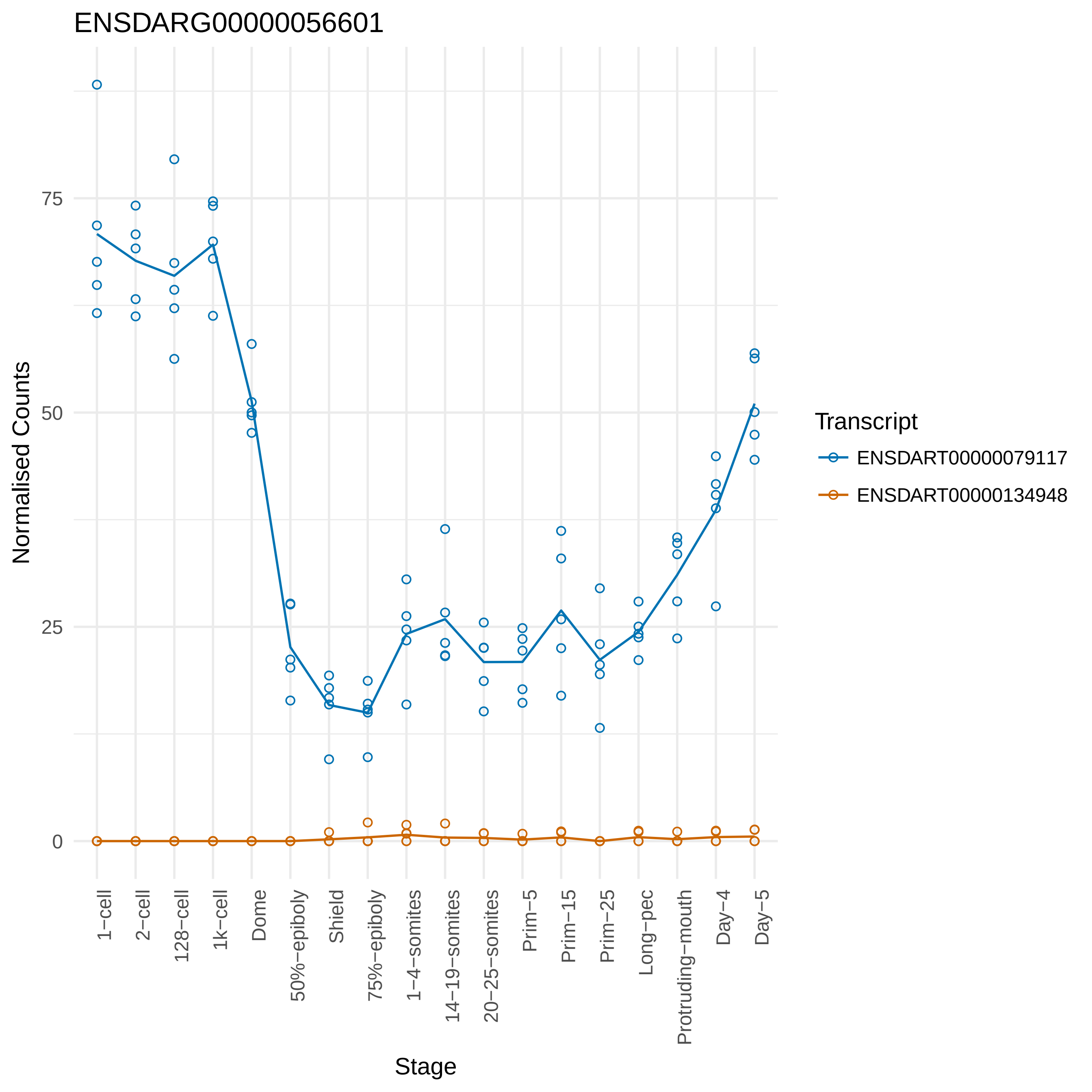
ENSDARG00000056601
Normalised Counts
0
25
50
75
Stage
1−cell
2−cell
128−cell
1k−cell
Dome
50%−epiboly
Shield
75%−epiboly
1−4−somites
14−19−somites
20−25−somites
Prim−5
Prim−15
Prim−25
Long−pec
Protruding−mouth
Day−4
Day−5
Transcript
ENSDART00000079117
ENSDART00000134948

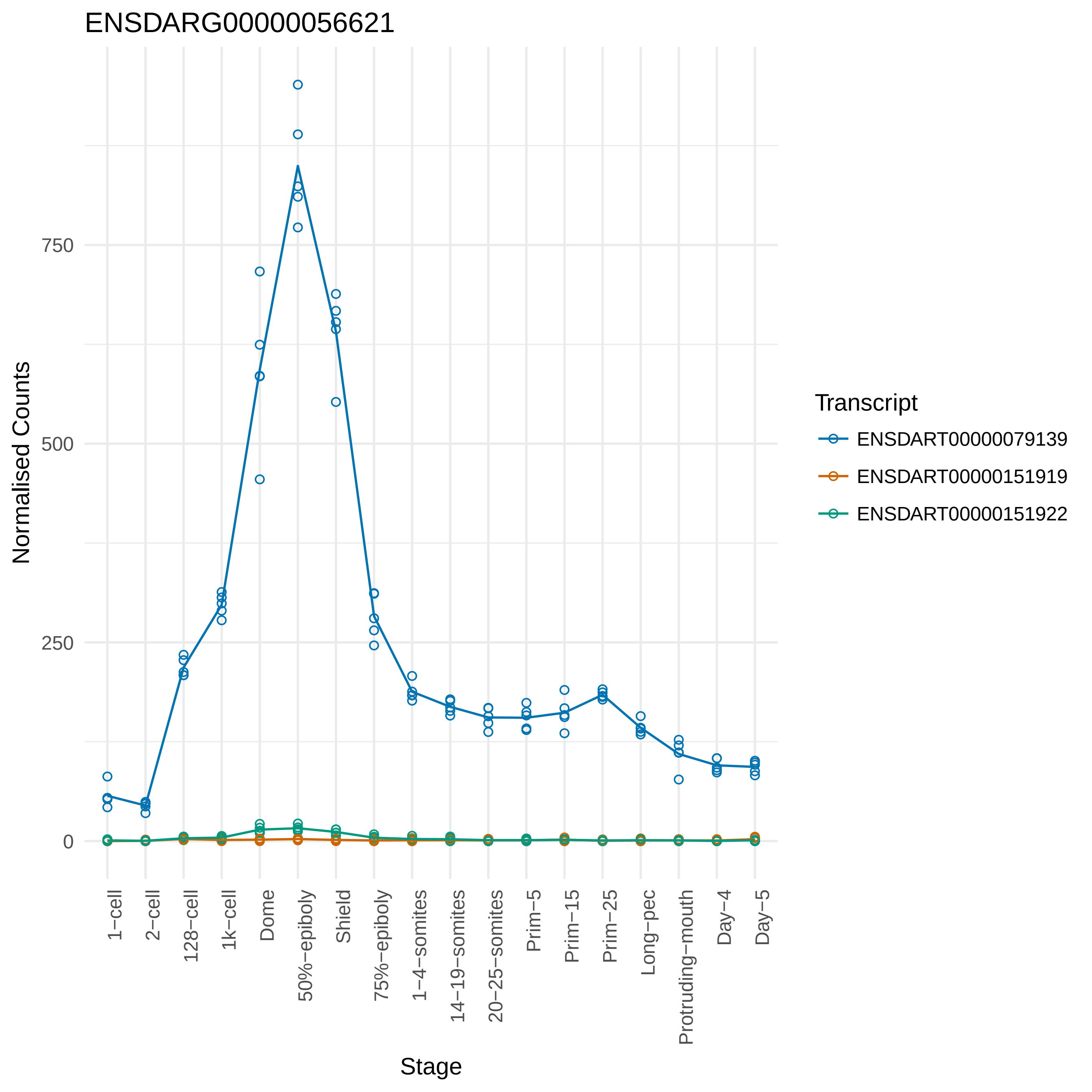

ENSDARG00000056621
Normalised Counts
750
500
250
0
1−cell
2−cell
128−cell
1k−cell
Dome
50%−epiboly
Shield
75%−epiboly
1−4−somites
14−19−somites
20−25−somites
Prim−5
Prim−15
Prim−25
Long−pec
Protruding−mouth
Day−4
Day−5
Stage
Transcript
ENSDART00000079139
ENSDART00000151919
ENSDART00000151922

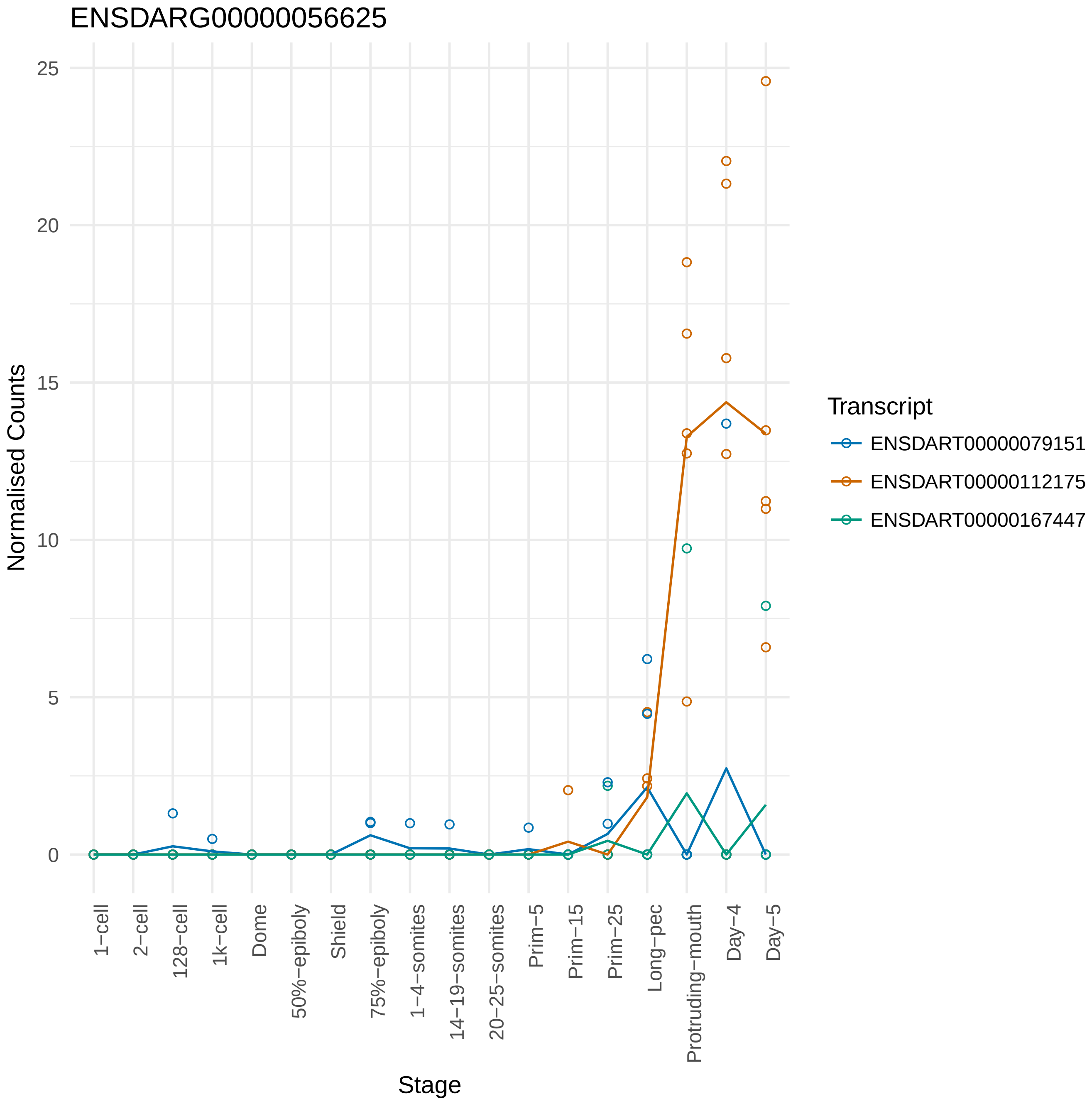

ENSDARG00000056625
Normalised Counts
25
20
15
10
5
0
1−cell
2−cell
128−cell
1k−cell
Dome
50%−epiboly
Shield
75%−epiboly
1−4−somites
14−19−somites
20−25−somites
Prim−5
Prim−15
Prim−25
Long−pec
Protruding−mouth
Day−4
Day−5
Stage
Transcript
ENSDART00000079151
ENSDART00000112175
ENSDART00000167447

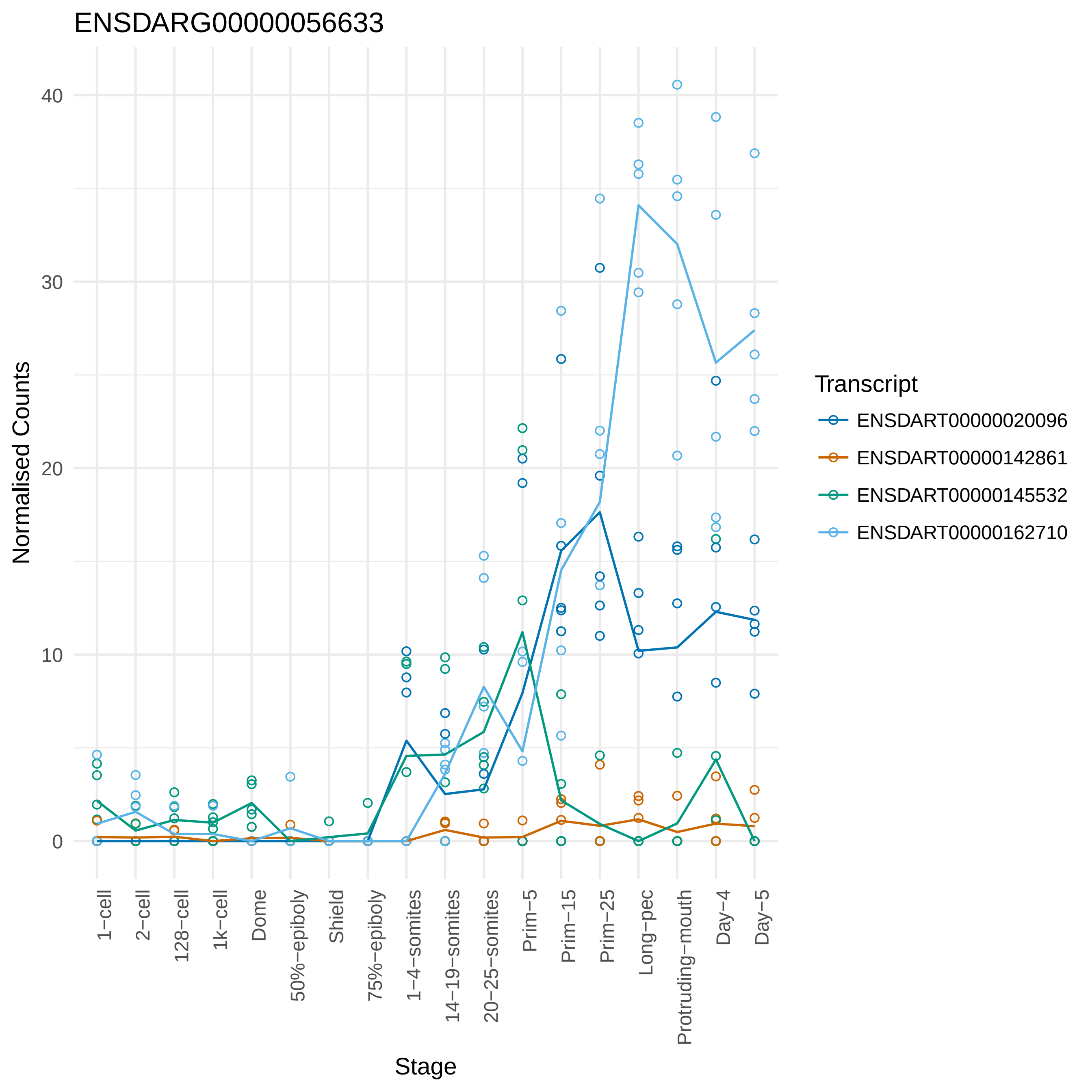

ENSDARG00000056633
Normalised Counts
40
30
20
10
0
1−cell
2−cell
128−cell
1k−cell
Dome
50%−epiboly
Shield
75%−epiboly
1−4−somites
14−19−somites
20−25−somites
Prim−5
Prim−15
Prim−25
Long−pec
Protruding−mouth
Day−4
Day−5
Stage
Transcript
ENSDART00000020096
ENSDART00000142861
ENSDART00000145532
ENSDART00000162710

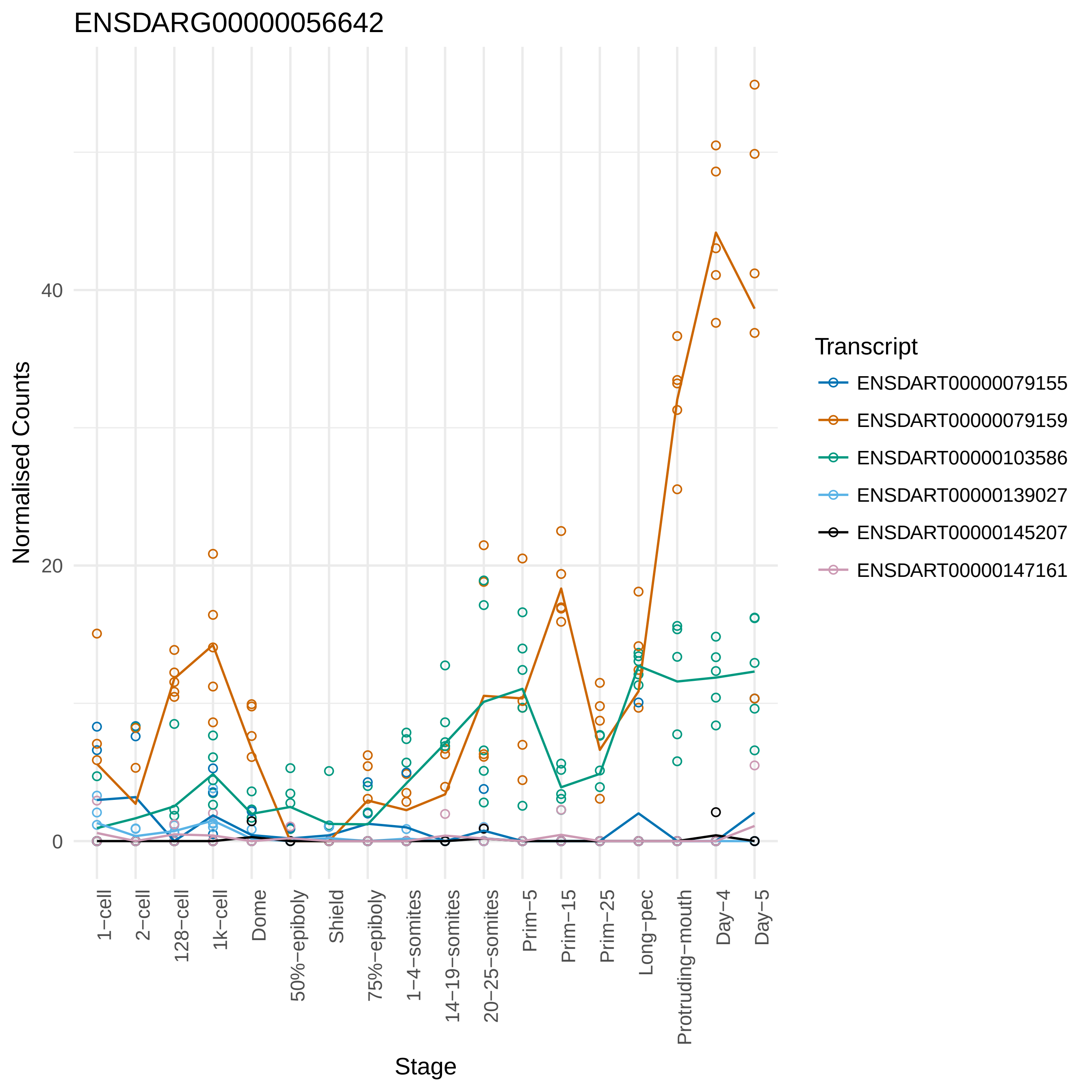
ENSDARG00000056642
Normalised Counts
0
20
40
Stage
1−cell
2−cell
128−cell
1k−cell
Dome
50%−epiboly
Shield
75%−epiboly
1−4−somites
14−19−somites
20−25−somites
Prim−5
Prim−15
Prim−25
Long−pec
Protruding−mouth
Day−4
Day−5
Transcript
ENSDART00000079155
ENSDART00000079159
ENSDART00000103586
ENSDART00000139027
ENSDART00000145207
ENSDART00000147161

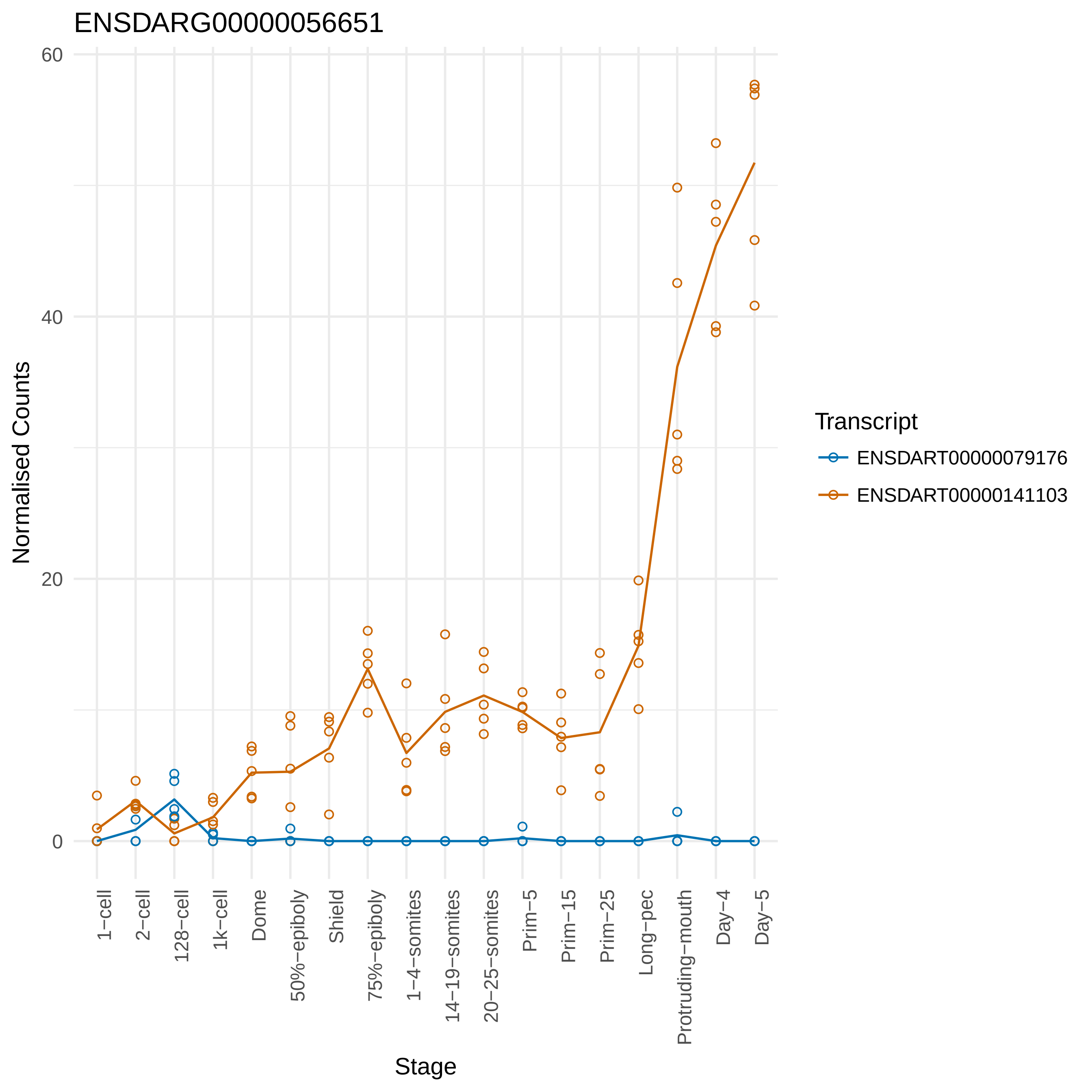
ENSDARG00000056651
Normalised Counts
60
40
20
0
1−cell
2−cell
128−cell
1k−cell
Dome
50%−epiboly
Shield
75%−epiboly
1−4−somites
14−19−somites
20−25−somites
Prim−5
Prim−15
Prim−25
Long−pec
Protruding−mouth
Day−4
Day−5
Stage
Transcript
ENSDART00000079176
ENSDART00000141103

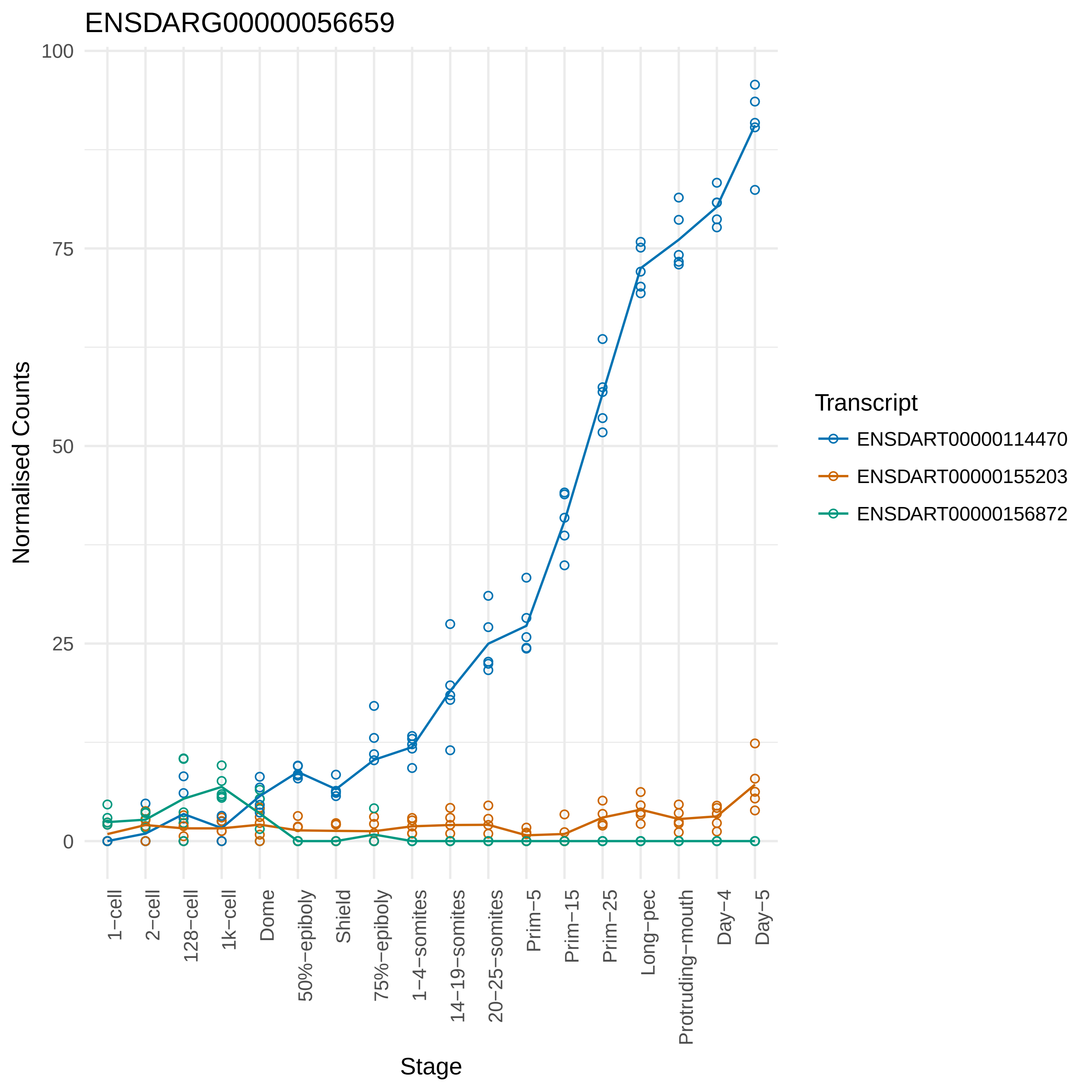

ENSDARG00000056659
Normalised Counts
100
75
50
25
0
1−cell
2−cell
128−cell
1k−cell
Dome
50%−epiboly
Shield
75%−epiboly
1−4−somites
14−19−somites
20−25−somites
Prim−5
Prim−15
Prim−25
Long−pec
Protruding−mouth
Day−4
Day−5
Stage
Transcript
ENSDART00000114470
ENSDART00000155203
ENSDART00000156872

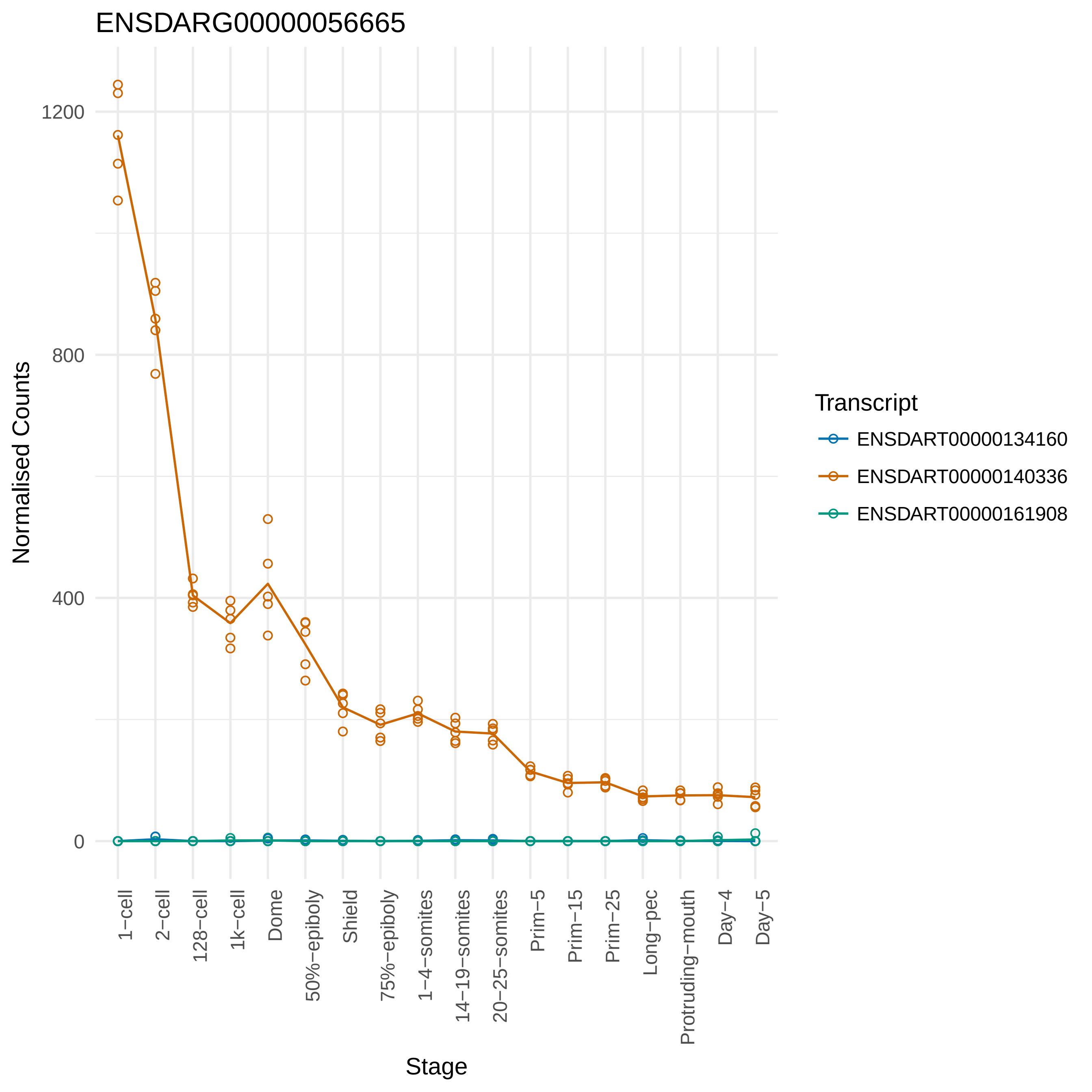
ENSDARG00000056665
Normalised Counts
1200
800
400
0
1−cell
2−cell
128−cell
1k−cell
Dome
50%−epiboly
Shield
75%−epiboly
1−4−somites
14−19−somites
20−25−somites
Prim−5
Prim−15
Prim−25
Long−pec
Protruding−mouth
Day−4
Day−5
Stage
Transcript
ENSDART00000134160
ENSDART00000140336
ENSDART00000161908

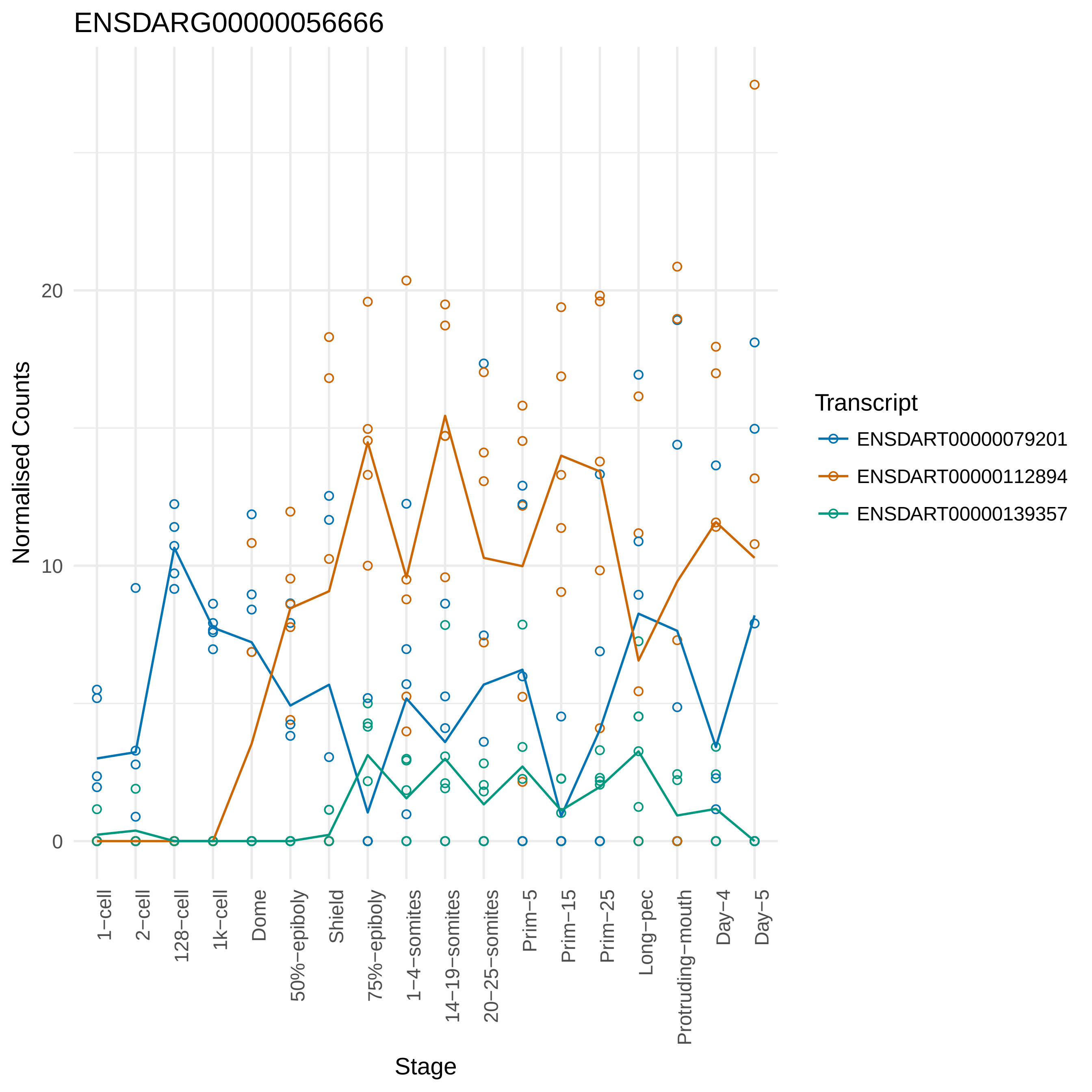

ENSDARG00000056666
Normalised Counts
20
10
0
1−cell
2−cell
128−cell
1k−cell
Dome
50%−epiboly
Shield
75%−epiboly
1−4−somites
14−19−somites
20−25−somites
Prim−5
Prim−15
Prim−25
Long−pec
Protruding−mouth
Day−4
Day−5
Stage
Transcript
ENSDART00000079201
ENSDART00000112894
ENSDART00000139357

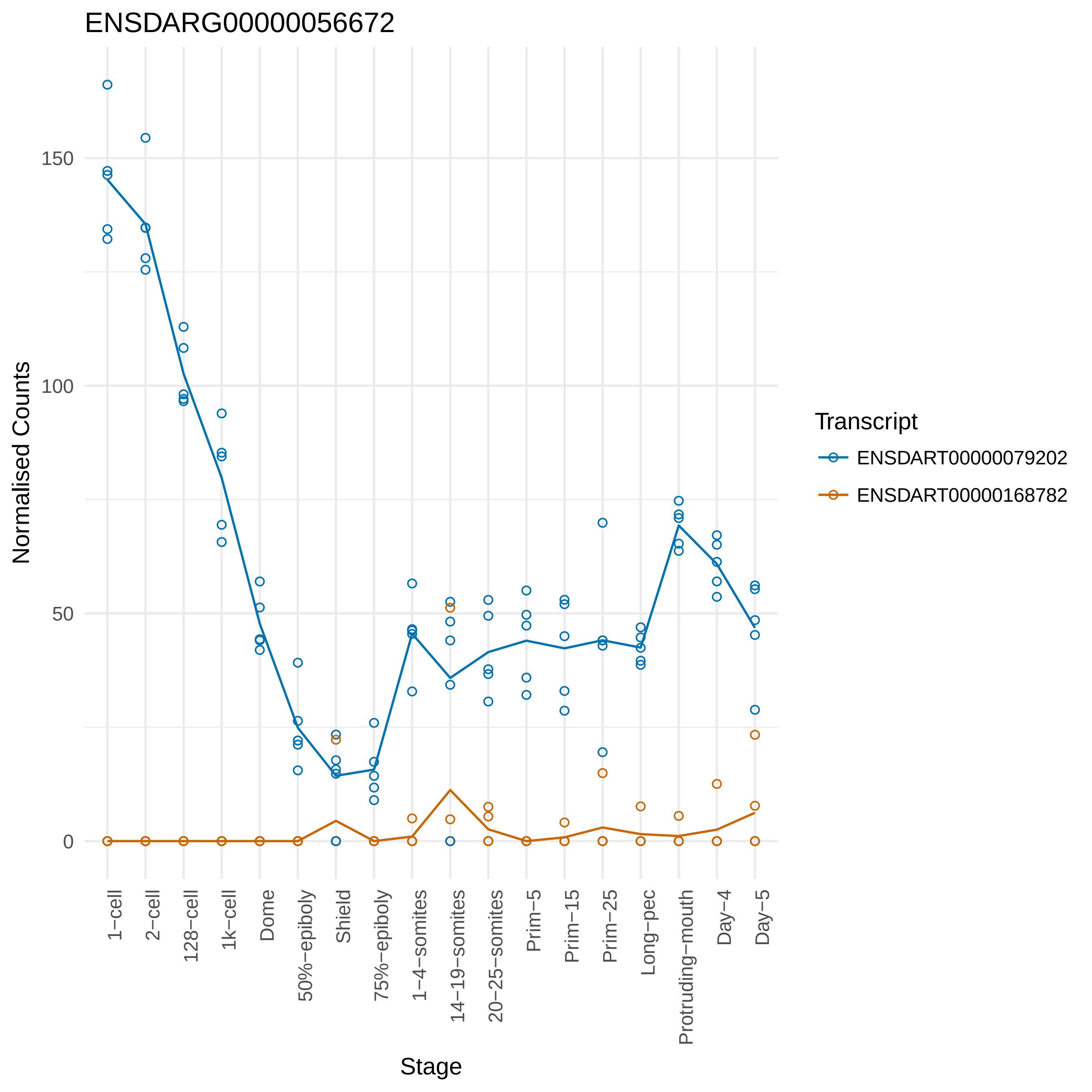

ENSDARG00000056672
Normalised Counts
150
100
50
0
1−cell
2−cell
128−cell
1k−cell
Dome
50%−epiboly
Shield
75%−epiboly
1−4−somites
14−19−somites
20−25−somites
Prim−5
Prim−15
Prim−25
Long−pec
Protruding−mouth
Day−4
Day−5
Stage
Transcript
ENSDART00000079202
ENSDART00000168782

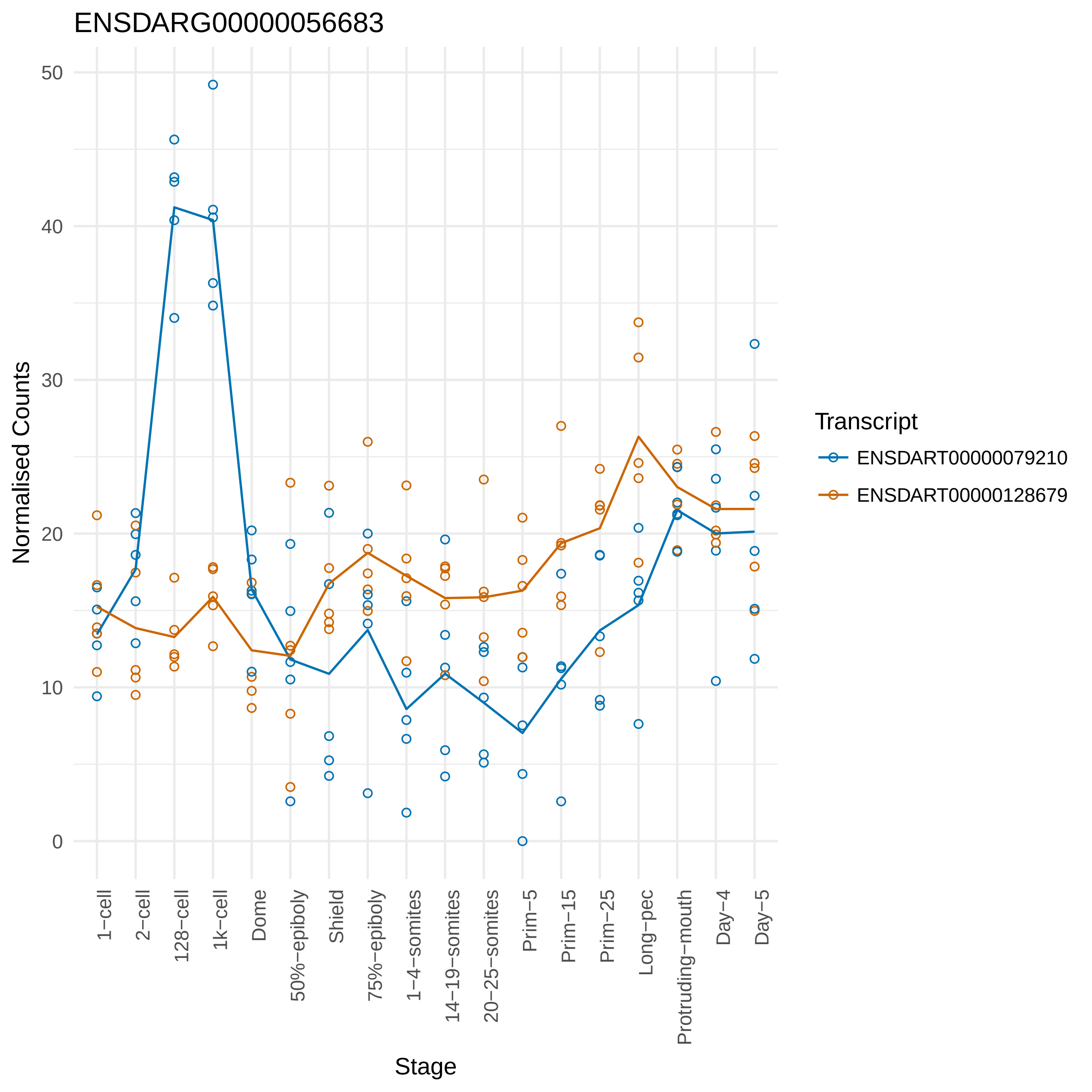
ENSDARG00000056683
Normalised Counts
0
10
20
30
40
50
1−cell
2−cell
128−cell
1k−cell
Dome
50%−epiboly
Shield
75%−epiboly
1−4−somites
14−19−somites
20−25−somites
Prim−5
Prim−15
Prim−25
Long−pec
Protruding−mouth
Day−4
Day−5
Stage
Transcript
ENSDART00000079210
ENSDART00000128679

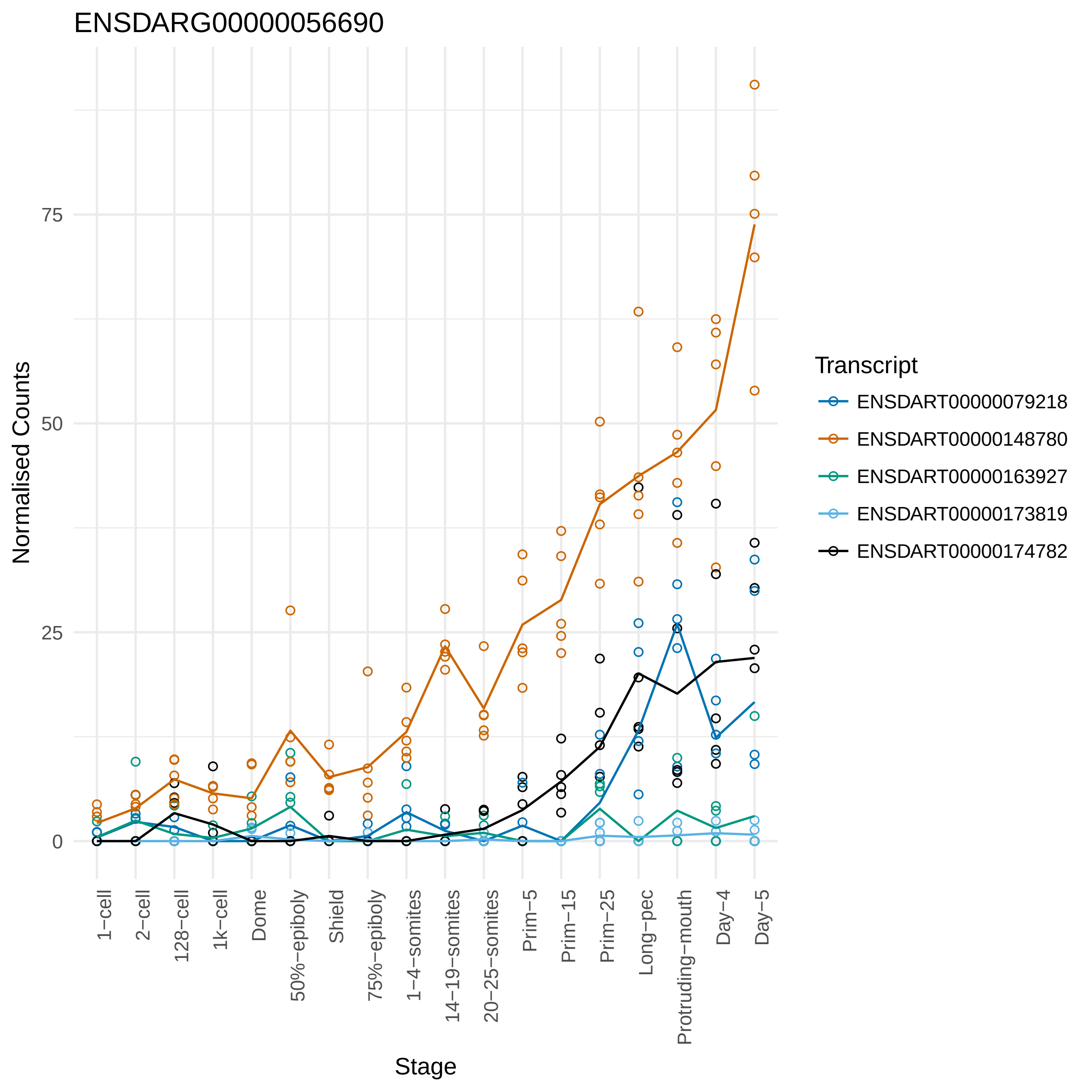
ENSDARG00000056690
Normalised Counts
0
25
50
75
1−cell
2−cell
128−cell
1k−cell
Dome
50%−epiboly
Shield
75%−epiboly
1−4−somites
14−19−somites
20−25−somites
Prim−5
Prim−15
Prim−25
Long−pec
Protruding−mouth
Day−4
Day−5
Stage
Transcript
ENSDART00000079218
ENSDART00000148780
ENSDART00000163927
ENSDART00000173819
ENSDART00000174782

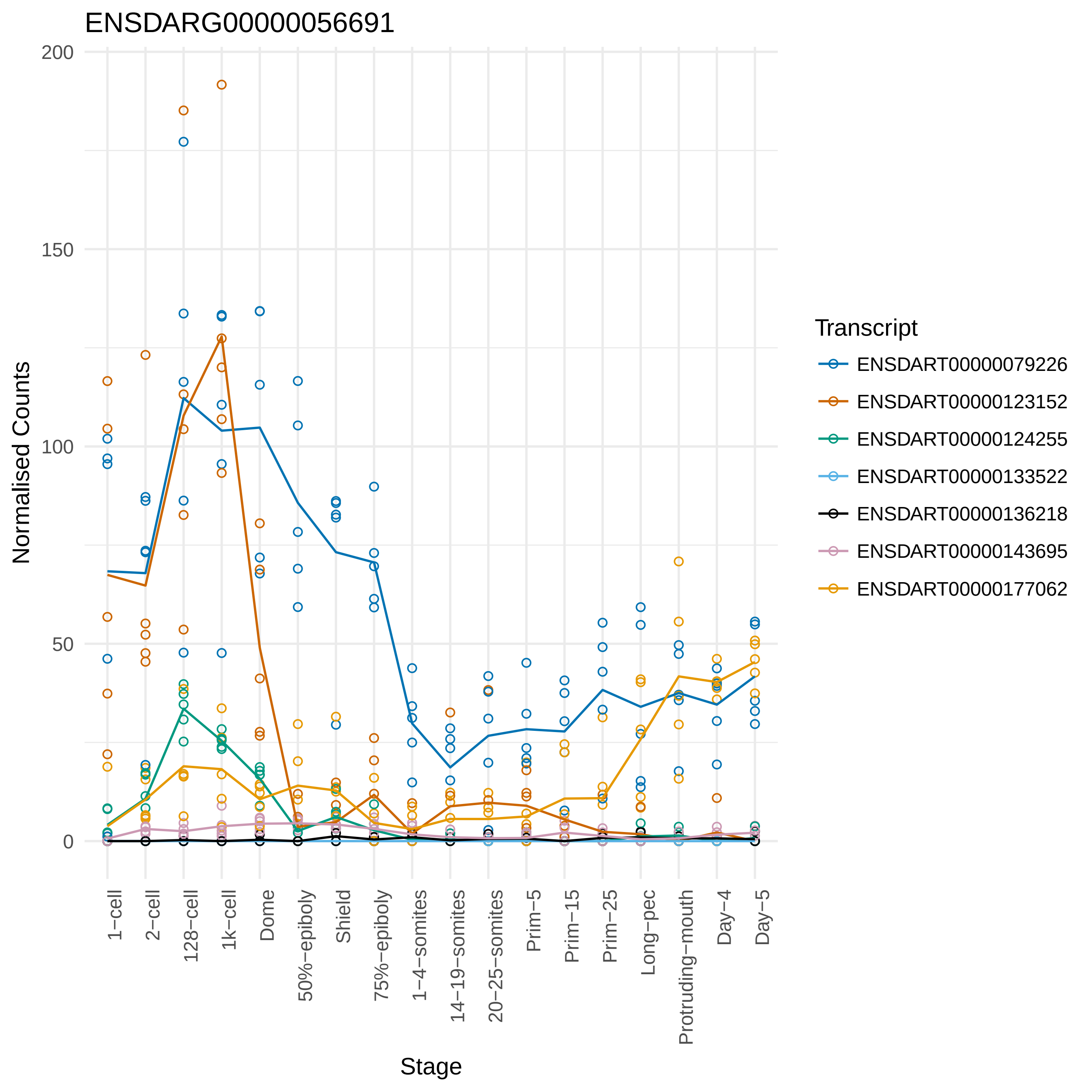

ENSDARG00000056691
Normalised Counts
200
150
100
50
0
1−cell
2−cell
128−cell
1k−cell
Dome
50%−epiboly
Shield
75%−epiboly
1−4−somites
14−19−somites
20−25−somites
Prim−5
Prim−15
Prim−25
Long−pec
Protruding−mouth
Day−4
Day−5
Stage
Transcript
ENSDART00000079226
ENSDART00000123152
ENSDART00000124255
ENSDART00000133522
ENSDART00000136218
ENSDART00000143695
ENSDART00000177062

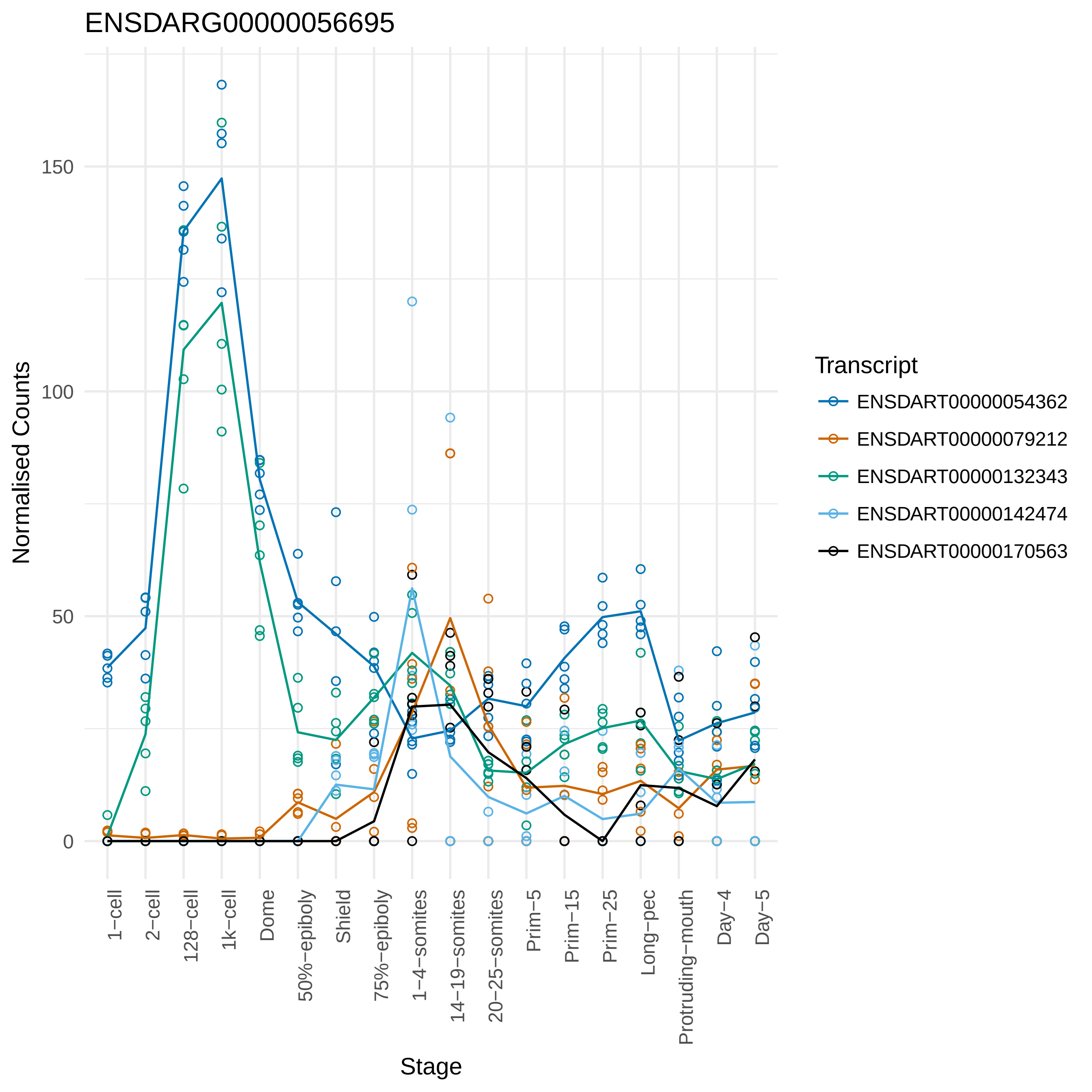

ENSDARG00000056695
Normalised Counts
150
100
50
0
1−cell
2−cell
128−cell
1k−cell
Dome
50%−epiboly
Shield
75%−epiboly
1−4−somites
14−19−somites
20−25−somites
Prim−5
Prim−15
Prim−25
Long−pec
Protruding−mouth
Day−4
Day−5
Stage
Transcript
ENSDART00000054362
ENSDART00000079212
ENSDART00000132343
ENSDART00000142474
ENSDART00000170563

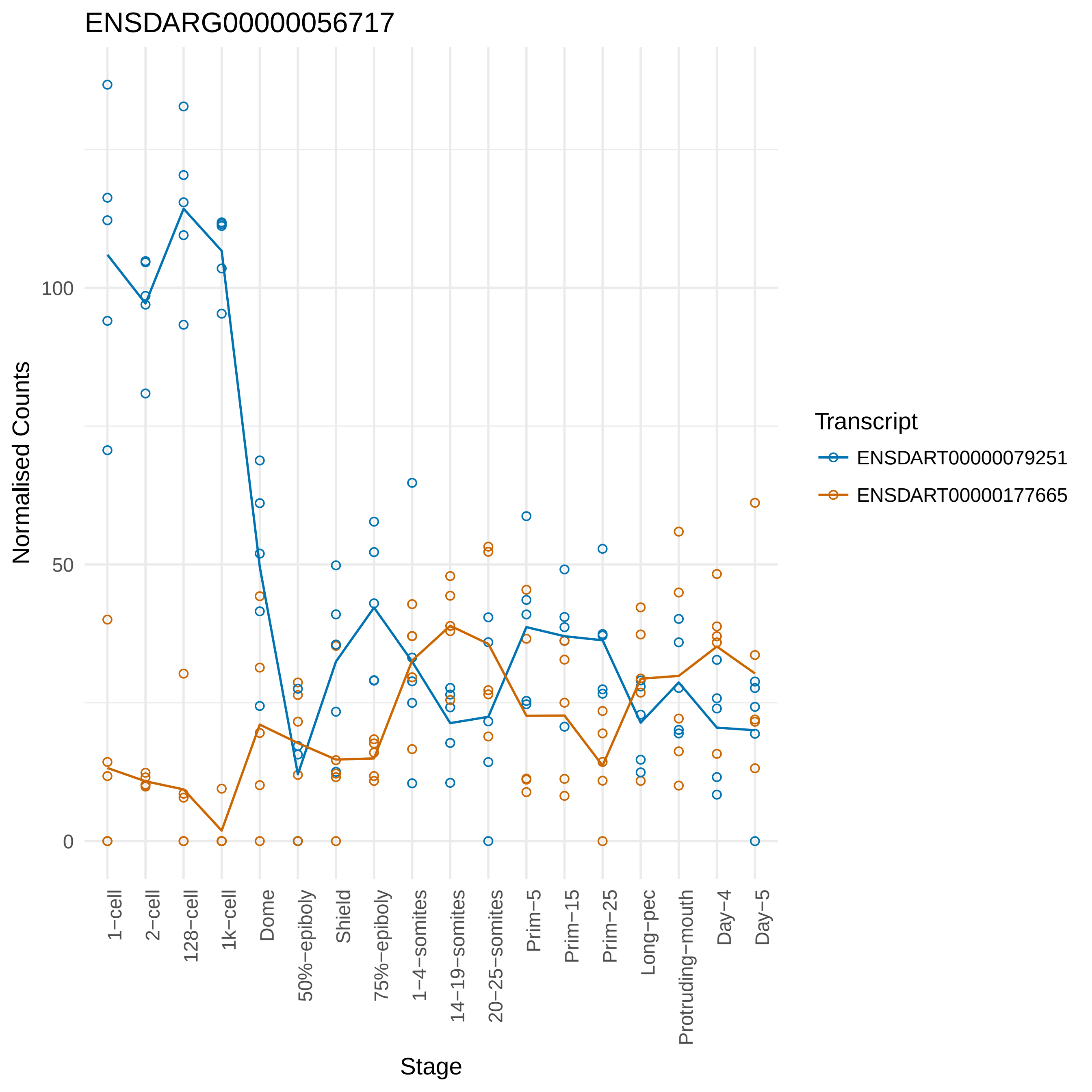
ENSDARG00000056717
Normalised Counts
100
50
0
Stage
1−cell
2−cell
128−cell
1k−cell
Dome
50%−epiboly
Shield
75%−epiboly
1−4−somites
14−19−somites
20−25−somites
Prim−5
Prim−15
Prim−25
Long−pec
Protruding−mouth
Day−4
Day−5
Transcript
ENSDART00000079251
ENSDART00000177665

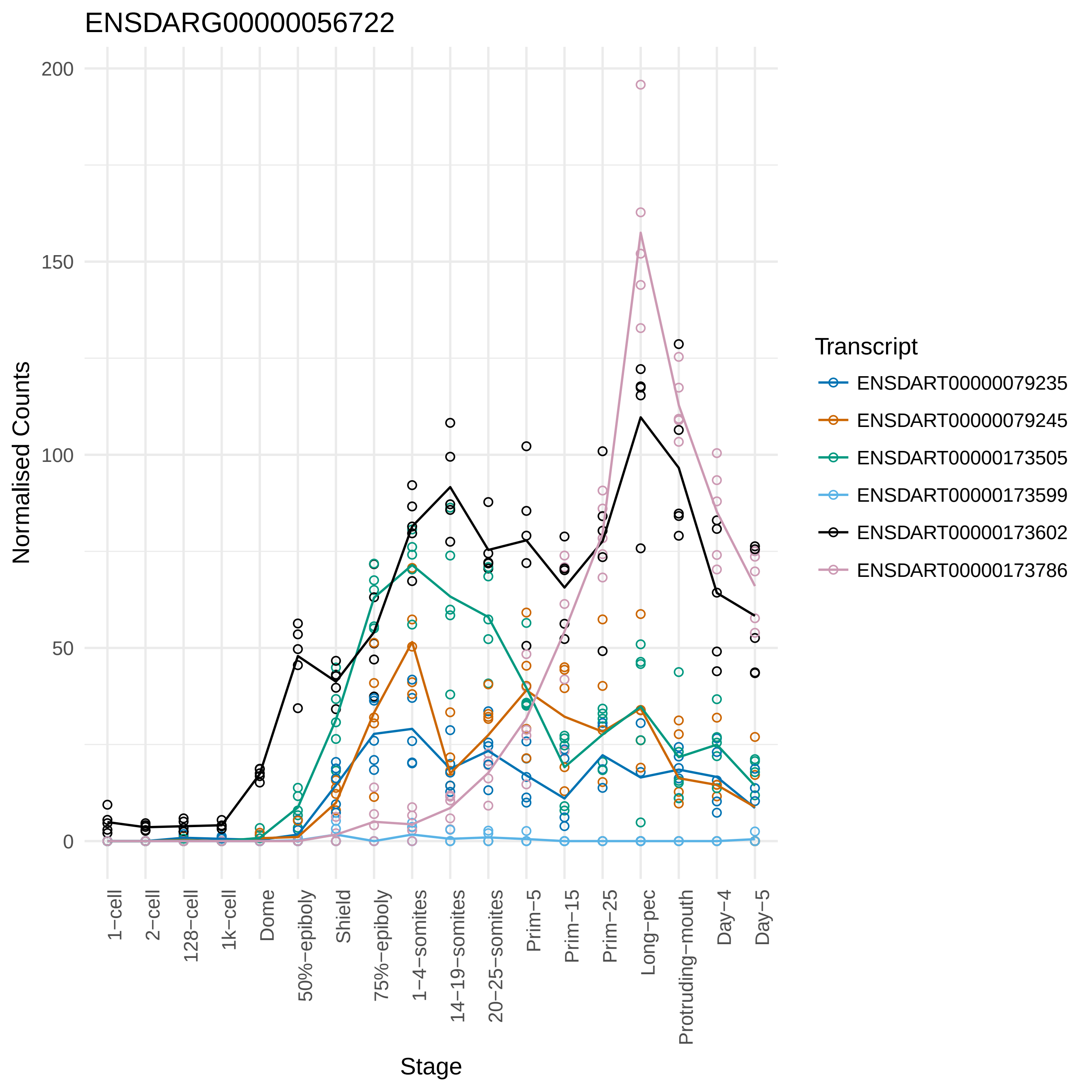

ENSDARG00000056722
Normalised Counts
200
150
100
50
0
1−cell
2−cell
128−cell
1k−cell
Dome
50%−epiboly
Shield
75%−epiboly
1−4−somites
14−19−somites
20−25−somites
Prim−5
Prim−15
Prim−25
Long−pec
Protruding−mouth
Day−4
Day−5
Stage
Transcript
ENSDART00000079235
ENSDART00000079245
ENSDART00000173505
ENSDART00000173599
ENSDART00000173602
ENSDART00000173786

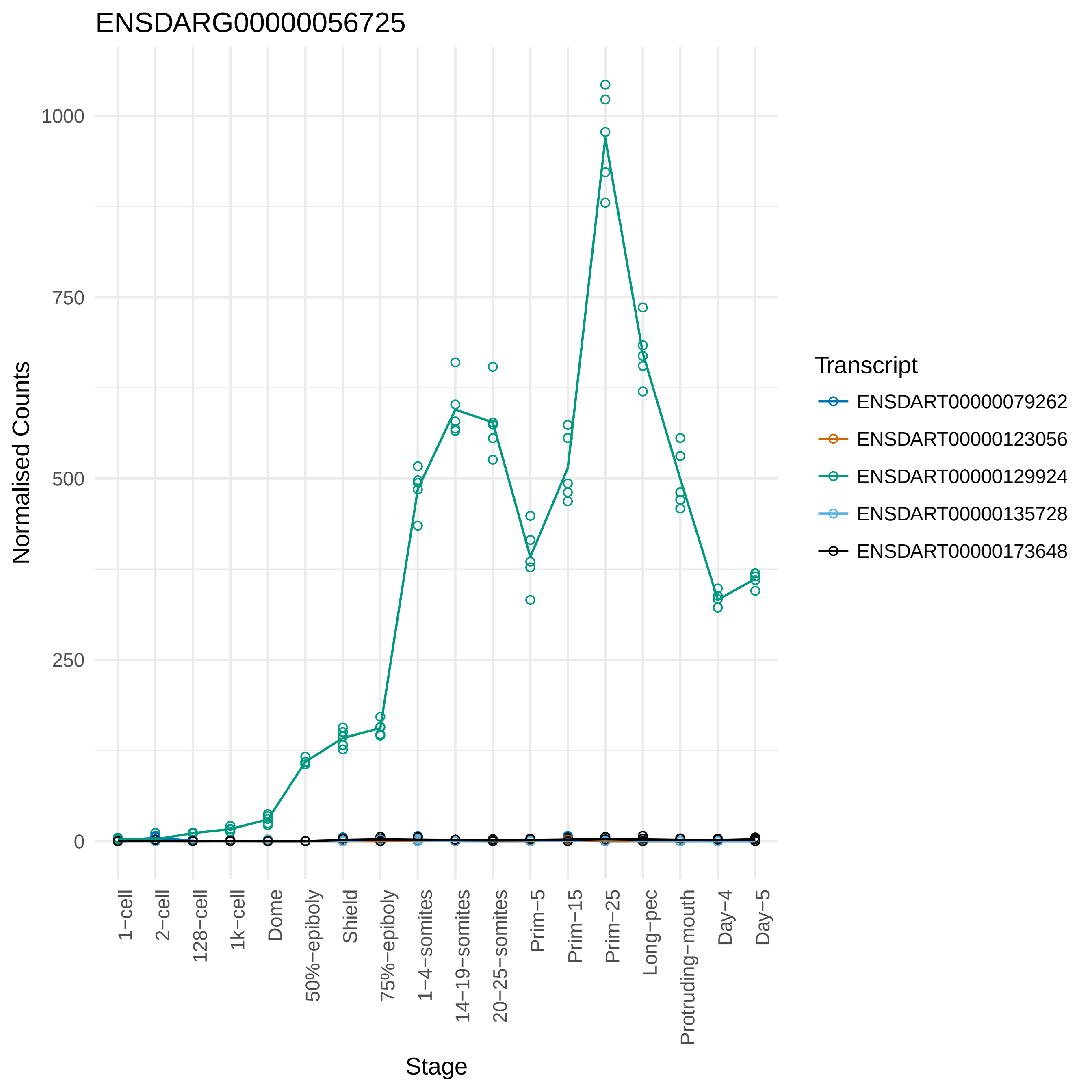

ENSDARG00000056725
Normalised Counts
1000
750
500
250
0
1−cell
2−cell
128−cell
1k−cell
Dome
50%−epiboly
Shield
75%−epiboly
1−4−somites
14−19−somites
20−25−somites
Prim−5
Prim−15
Prim−25
Long−pec
Protruding−mouth
Day−4
Day−5
Stage
Transcript
ENSDART00000079262
ENSDART00000123056
ENSDART00000129924
ENSDART00000135728
ENSDART00000173648

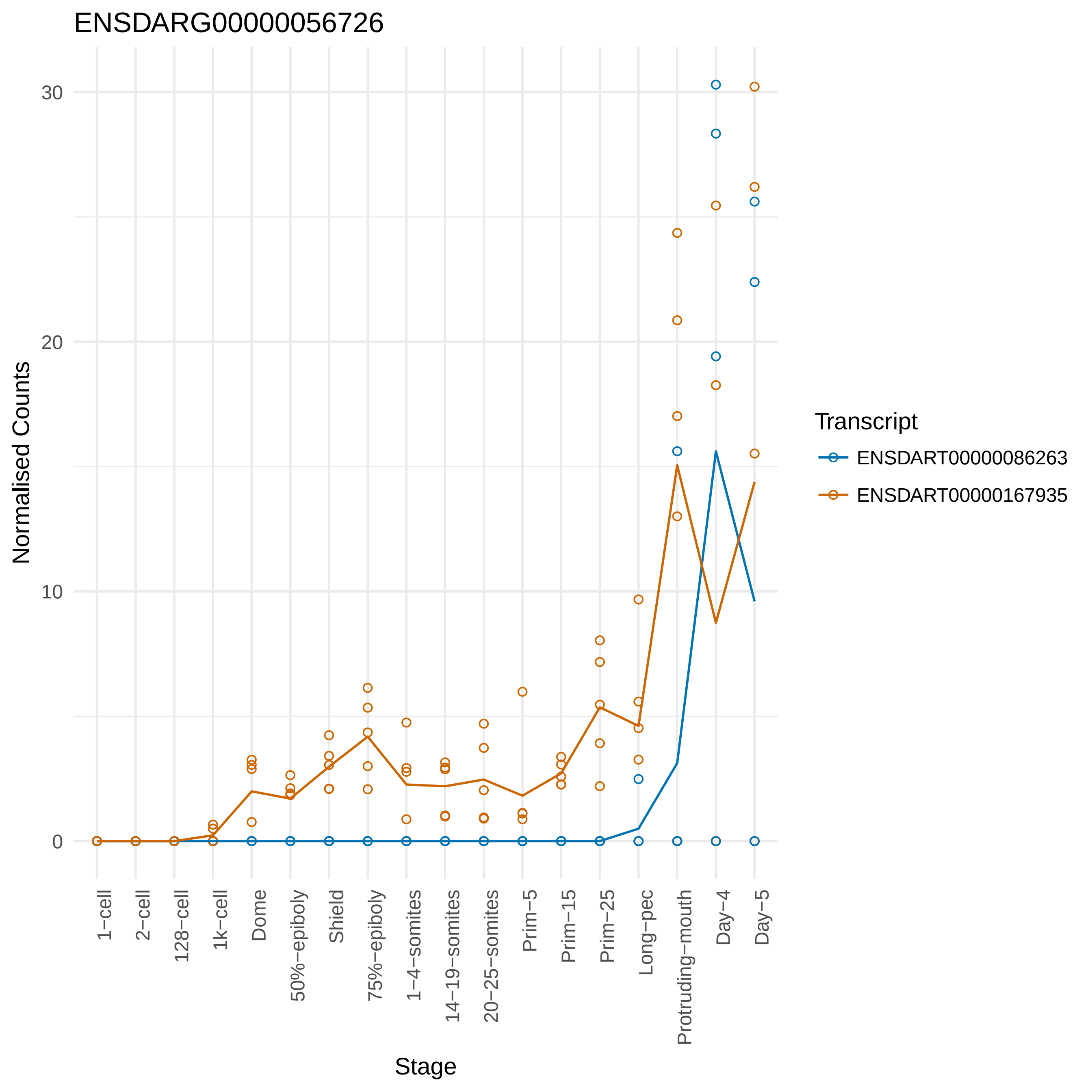

ENSDARG00000056726
Normalised Counts
30
20
10
0
1−cell
2−cell
128−cell
1k−cell
Dome
50%−epiboly
Shield
75%−epiboly
1−4−somites
14−19−somites
20−25−somites
Prim−5
Prim−15
Prim−25
Long−pec
Protruding−mouth
Day−4
Day−5
Stage
Transcript
ENSDART00000086263
ENSDART00000167935

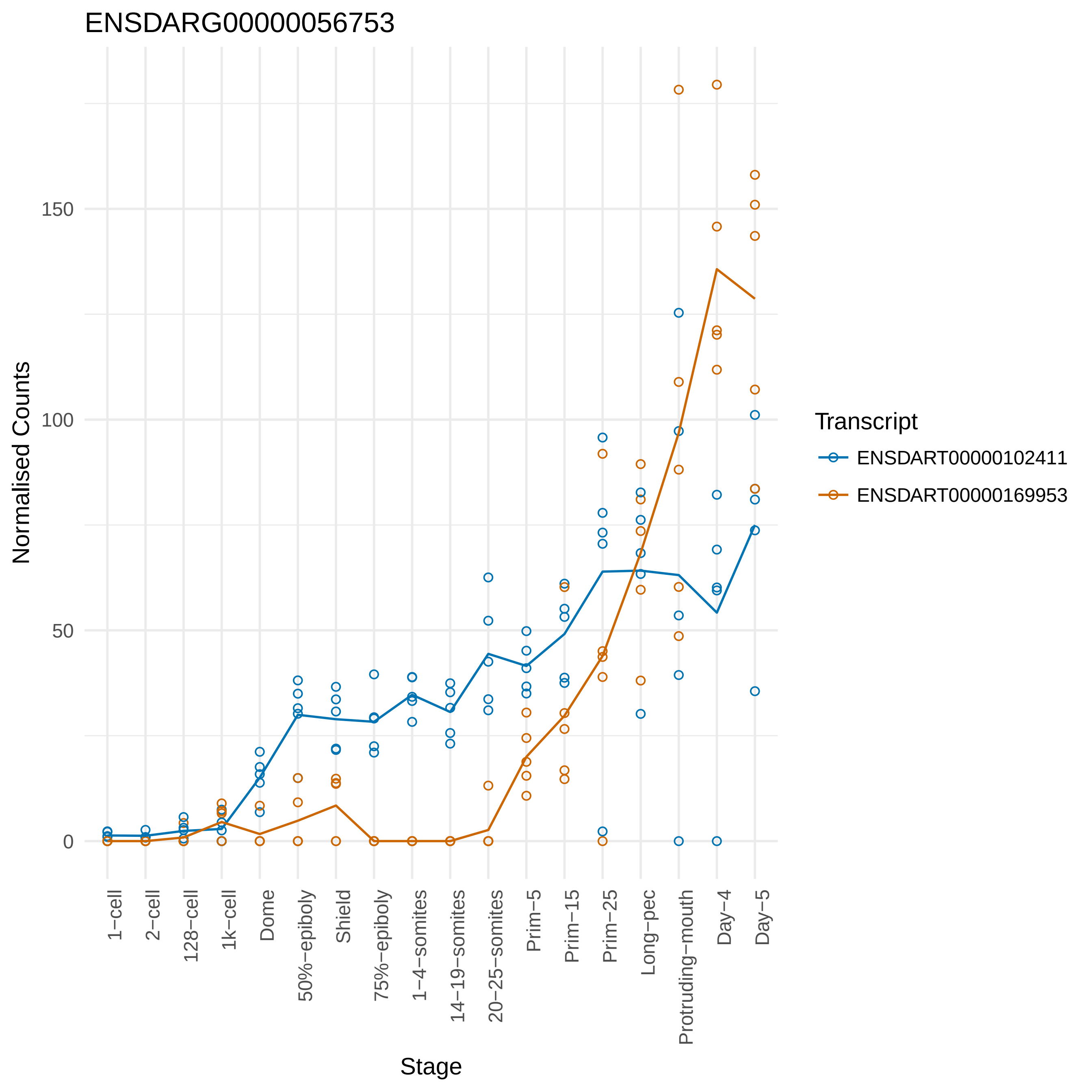
ENSDARG00000056753
Normalised Counts
0
50
100
150
Stage
1−cell
2−cell
128−cell
1k−cell
Dome
50%−epiboly
Shield
75%−epiboly
1−4−somites
14−19−somites
20−25−somites
Prim−5
Prim−15
Prim−25
Long−pec
Protruding−mouth
Day−4
Day−5
Transcript
ENSDART00000102411
ENSDART00000169953

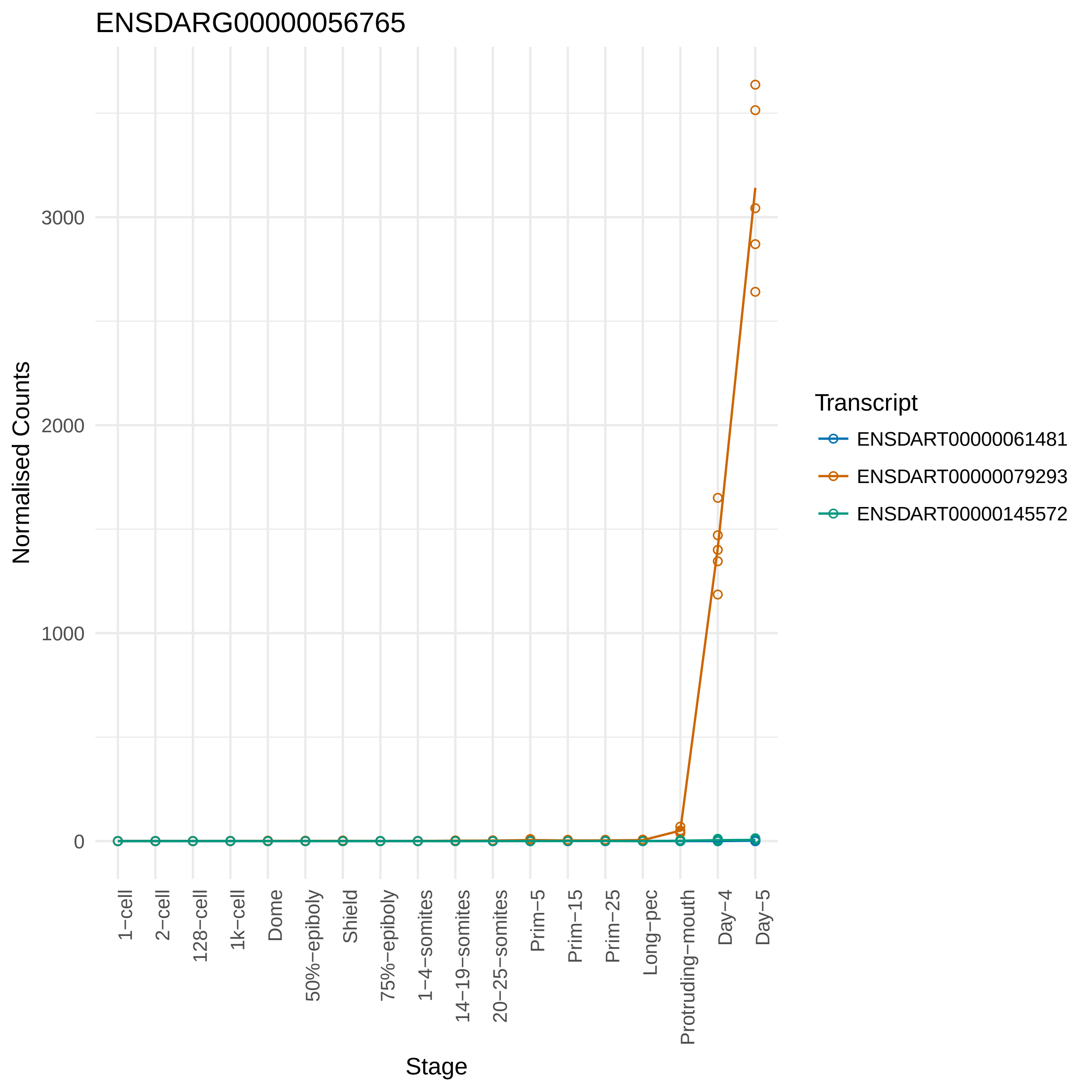
ENSDARG00000056765
Normalised Counts
3000
2000
1000
0
1−cell
2−cell
128−cell
1k−cell
Dome
50%−epiboly
Shield
75%−epiboly
1−4−somites
14−19−somites
20−25−somites
Prim−5
Prim−15
Prim−25
Long−pec
Protruding−mouth
Day−4
Day−5
Stage
Transcript
ENSDART00000061481
ENSDART00000079293
ENSDART00000145572

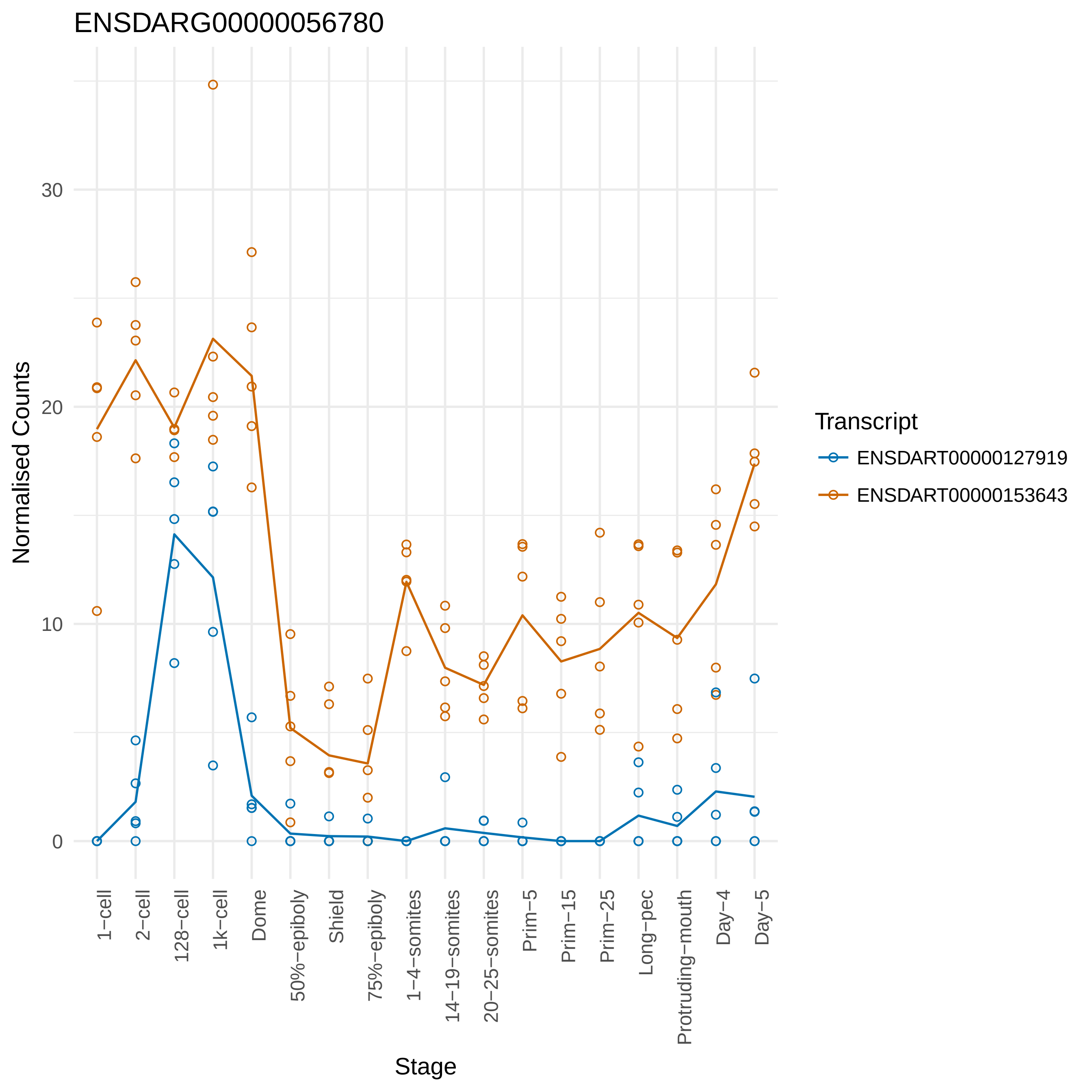

ENSDARG00000056780
Normalised Counts
0
10
20
30
1−cell
2−cell
128−cell
1k−cell
Dome
50%−epiboly
Shield
75%−epiboly
1−4−somites
14−19−somites
20−25−somites
Prim−5
Prim−15
Prim−25
Long−pec
Protruding−mouth
Day−4
Day−5
Stage
Transcript
ENSDART00000127919
ENSDART00000153643

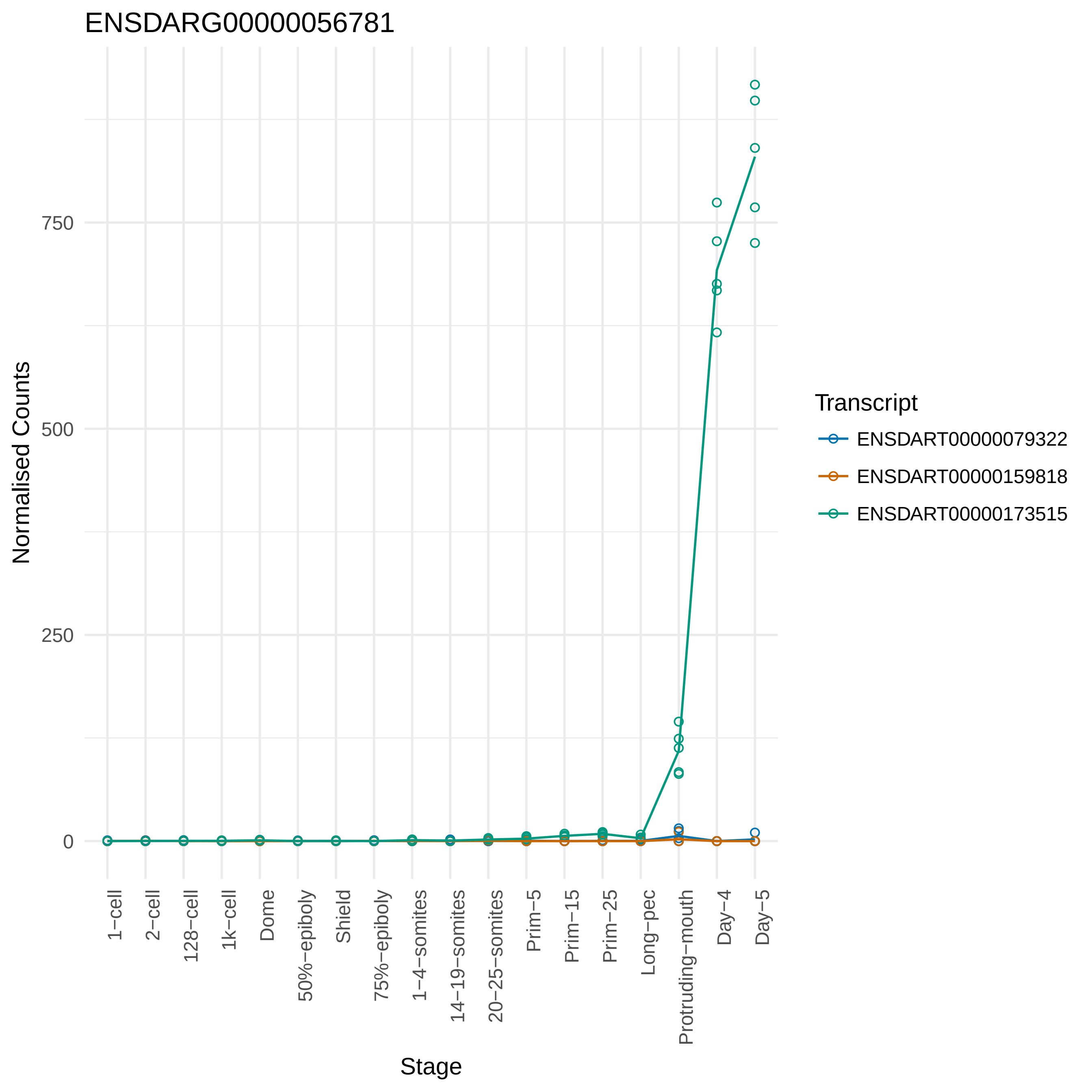
ENSDARG00000056781
Normalised Counts
0
250
500
750
Stage
1−cell
2−cell
128−cell
1k−cell
Dome
50%−epiboly
Shield
75%−epiboly
1−4−somites
14−19−somites
20−25−somites
Prim−5
Prim−15
Prim−25
Long−pec
Protruding−mouth
Day−4
Day−5
Transcript
ENSDART00000079322
ENSDART00000159818
ENSDART00000173515

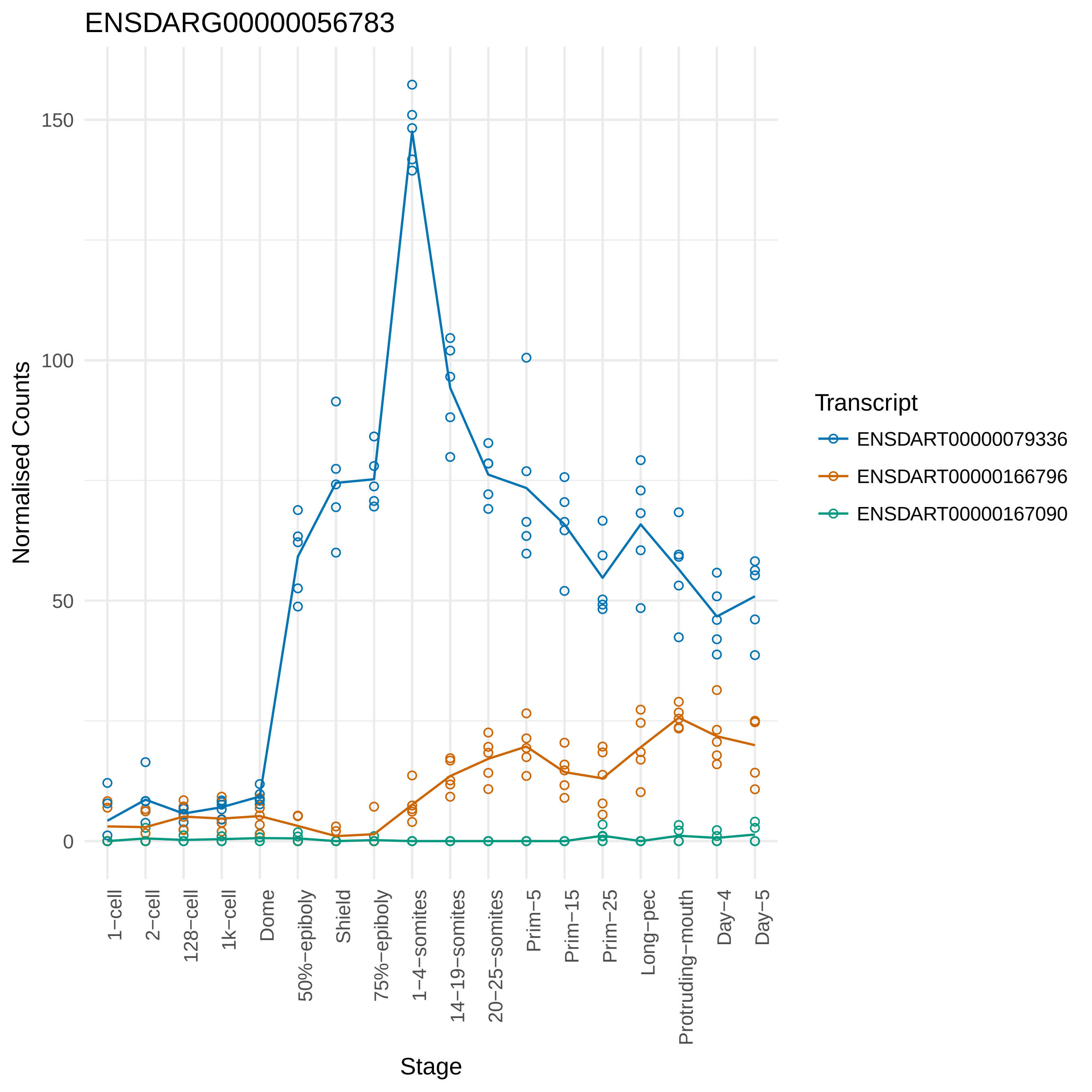

ENSDARG00000056783
Normalised Counts
150
100
50
0
1−cell
2−cell
128−cell
1k−cell
Dome
50%−epiboly
Shield
75%−epiboly
1−4−somites
14−19−somites
20−25−somites
Prim−5
Prim−15
Prim−25
Long−pec
Protruding−mouth
Day−4
Day−5
Stage
Transcript
ENSDART00000079336
ENSDART00000166796
ENSDART00000167090

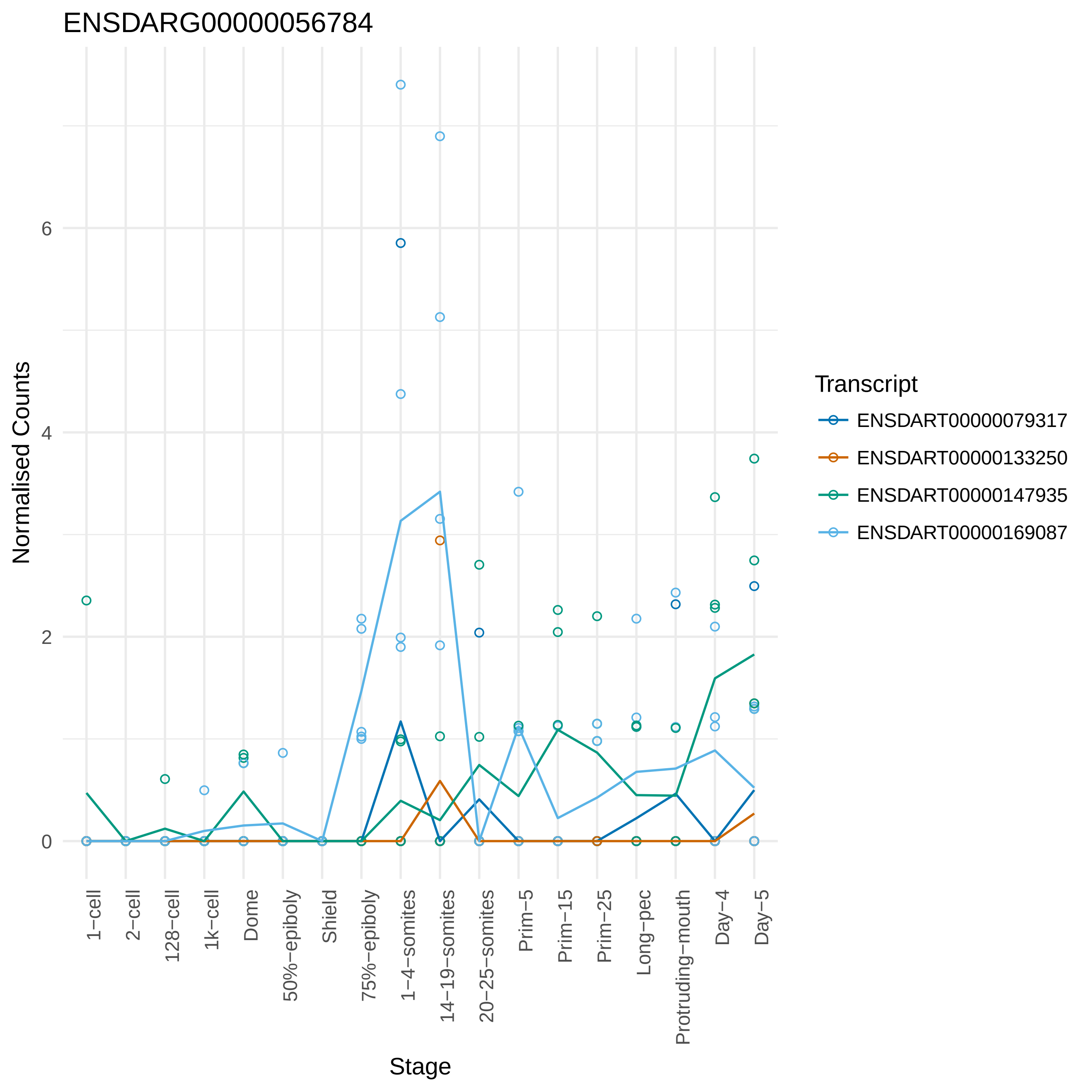

ENSDARG00000056784
Normalised Counts
0
2
4
6
1−cell
2−cell
128−cell
1k−cell
Dome
50%−epiboly
Shield
75%−epiboly
1−4−somites
14−19−somites
20−25−somites
Prim−5
Prim−15
Prim−25
Long−pec
Protruding−mouth
Day−4
Day−5
Stage
Transcript
ENSDART00000079317
ENSDART00000133250
ENSDART00000147935
ENSDART00000169087

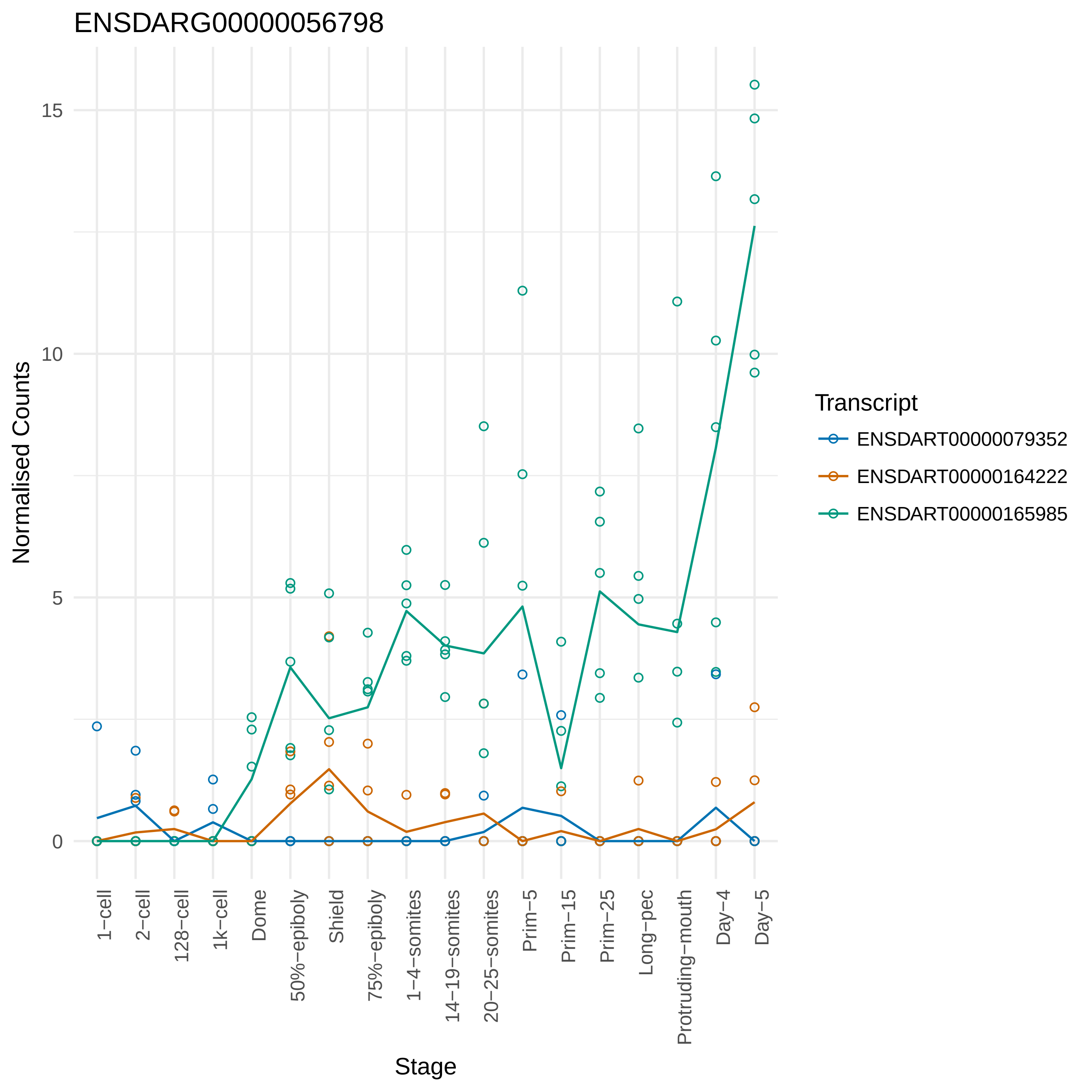
ENSDARG00000056798
Normalised Counts
15
10
5
0
Transcript
ENSDART00000079352
ENSDART00000164222
ENSDART00000165985
1−cell
2−cell
128−cell
1k−cell
Dome
50%−epiboly
Shield
75%−epiboly
1−4−somites
14−19−somites
20−25−somites
Prim−5
Prim−15
Prim−25
Long−pec
Protruding−mouth
Day−4
Day−5
Stage

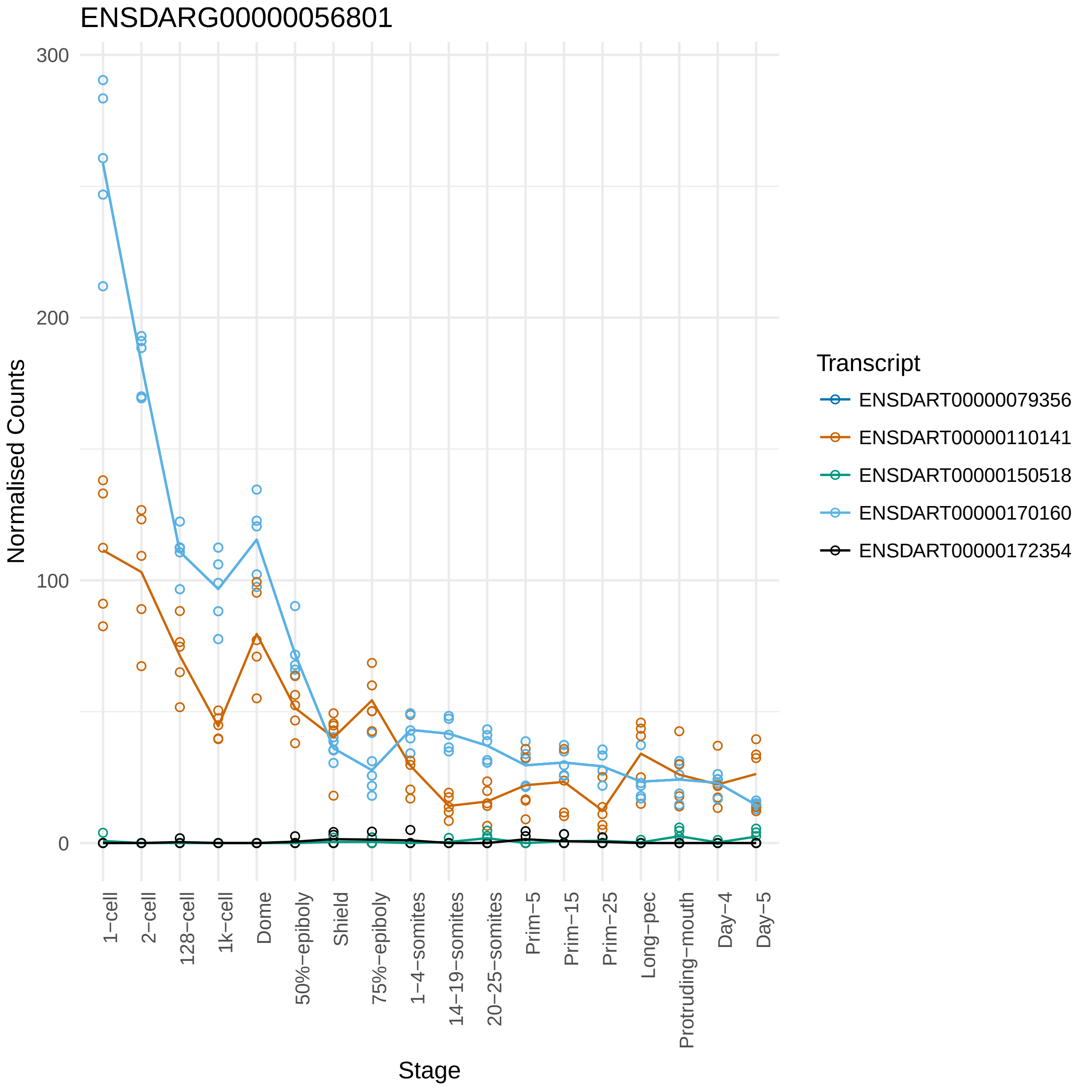
ENSDARG00000056801
Normalised Counts
300
200
100
0
1−cell
2−cell
128−cell
1k−cell
Dome
50%−epiboly
Shield
75%−epiboly
1−4−somites
14−19−somites
20−25−somites
Prim−5
Prim−15
Prim−25
Long−pec
Protruding−mouth
Day−4
Day−5
Stage
Transcript
ENSDART00000079356
ENSDART00000110141
ENSDART00000150518
ENSDART00000170160
ENSDART00000172354

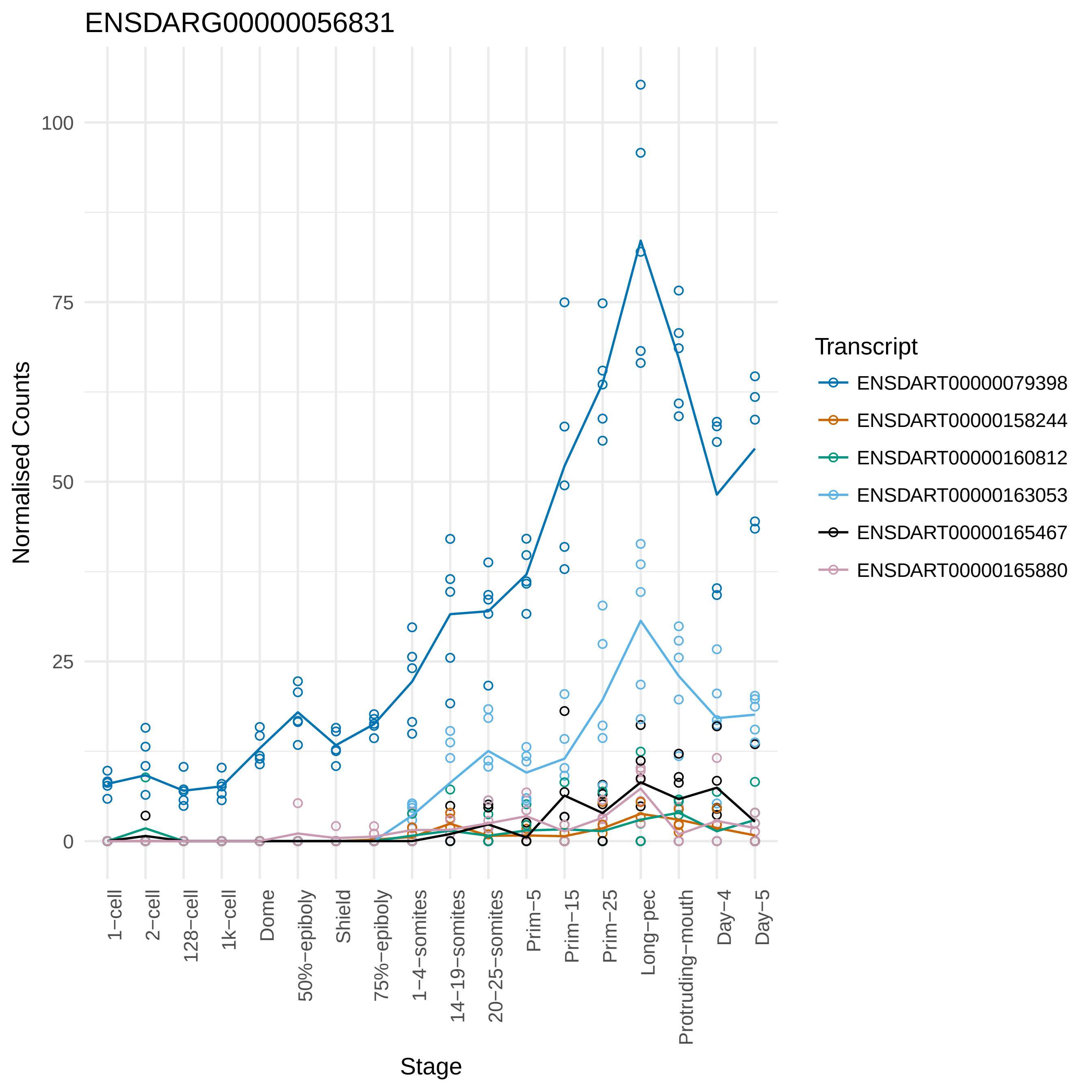
ENSDARG00000056831
Normalised Counts
100
75
50
25
0
Stage
1−cell
2−cell
128−cell
1k−cell
Dome
50%−epiboly
Shield
75%−epiboly
1−4−somites
14−19−somites
20−25−somites
Prim−5
Prim−15
Prim−25
Long−pec
Protruding−mouth
Day−4
Day−5
Transcript
ENSDART00000079398
ENSDART00000158244
ENSDART00000160812
ENSDART00000163053
ENSDART00000165467
ENSDART00000165880

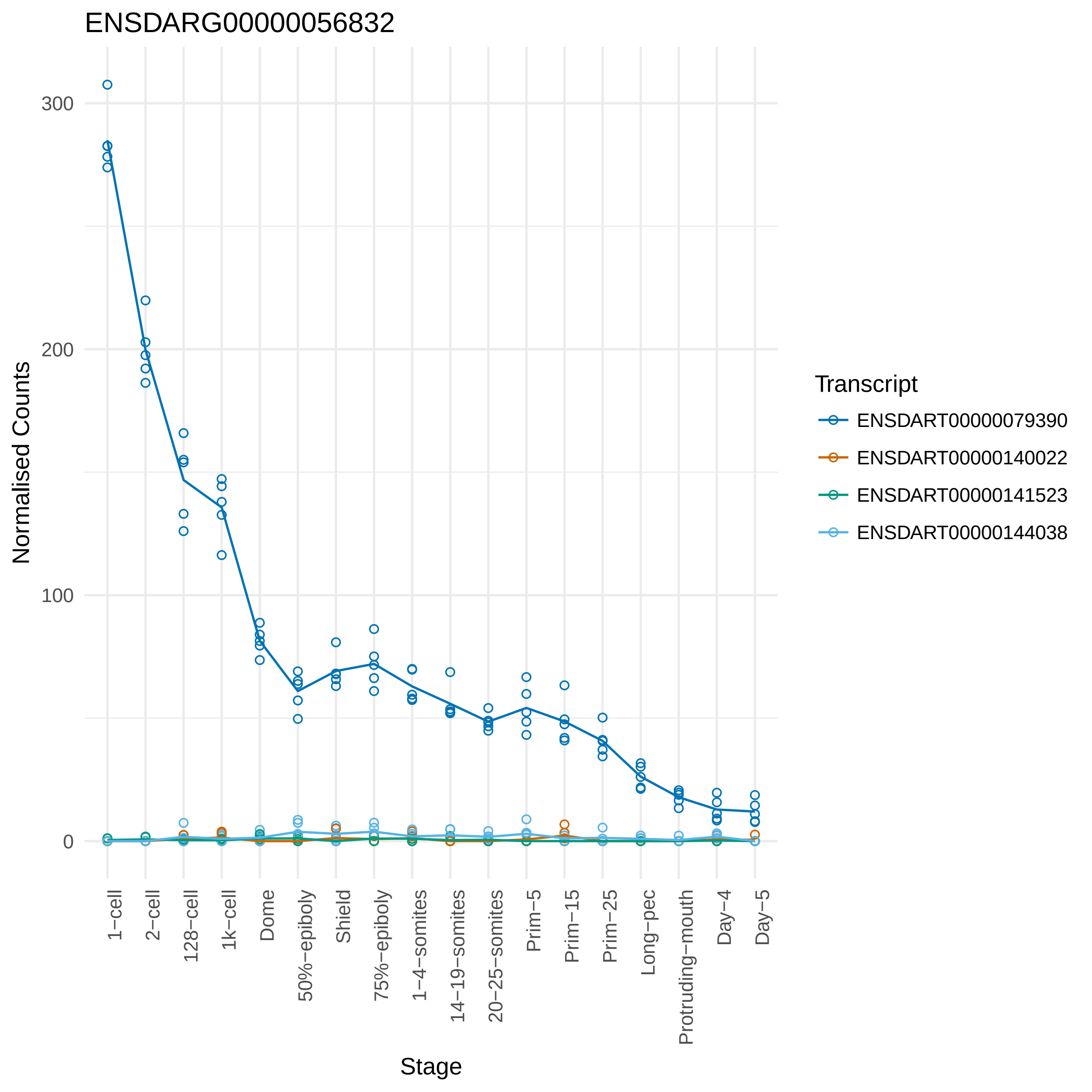
ENSDARG00000056832
Normalised Counts
300
200
100
0
1−cell
2−cell
128−cell
1k−cell
Dome
50%−epiboly
Shield
75%−epiboly
1−4−somites
14−19−somites
20−25−somites
Prim−5
Prim−15
Prim−25
Long−pec
Protruding−mouth
Day−4
Day−5
Stage
Transcript
ENSDART00000079390
ENSDART00000140022
ENSDART00000141523
ENSDART00000144038

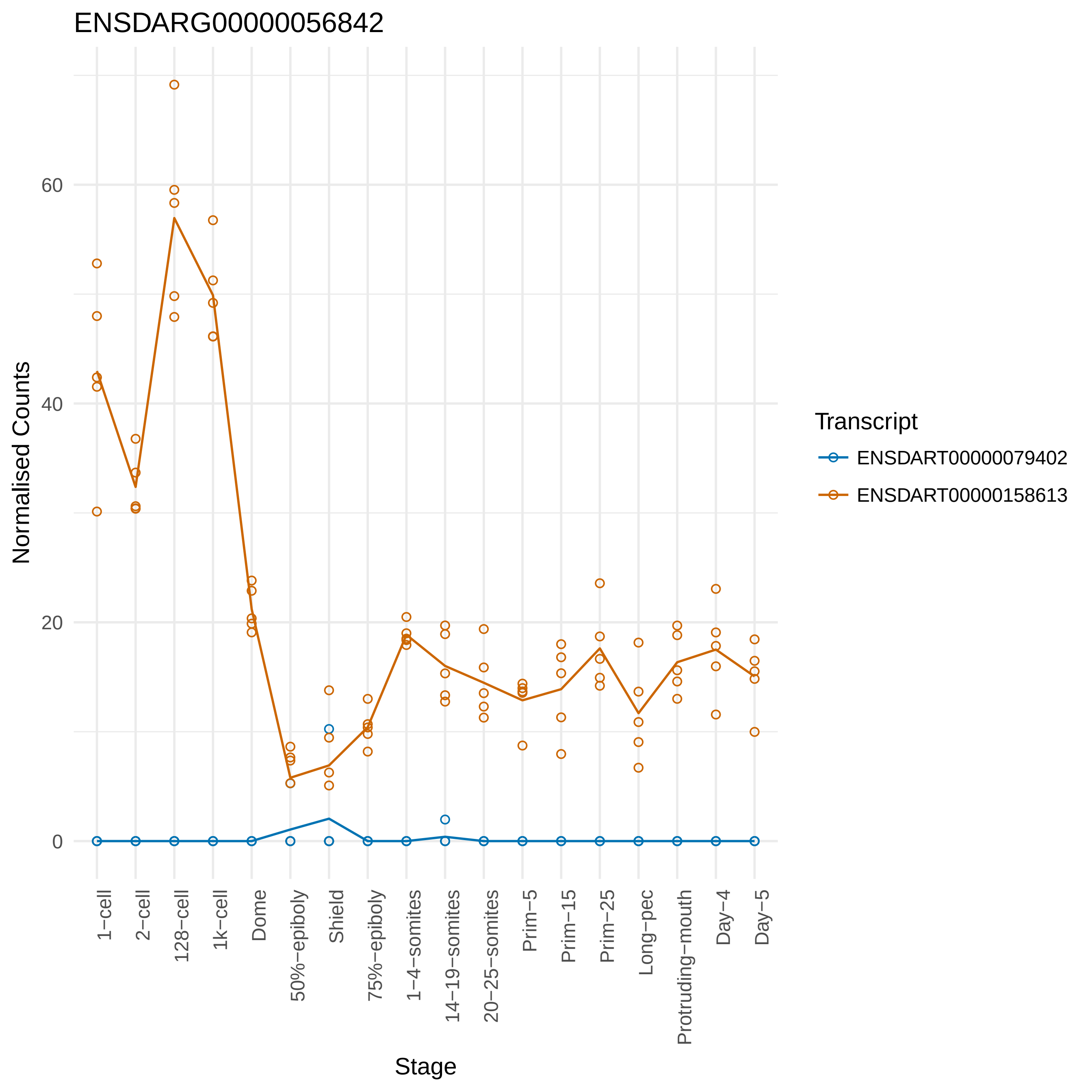

ENSDARG00000056842
Normalised Counts
60
40
20
0
1−cell
2−cell
128−cell
1k−cell
Dome
50%−epiboly
Shield
75%−epiboly
1−4−somites
14−19−somites
20−25−somites
Prim−5
Prim−15
Prim−25
Long−pec
Protruding−mouth
Day−4
Day−5
Stage
Transcript
ENSDART00000079402
ENSDART00000158613

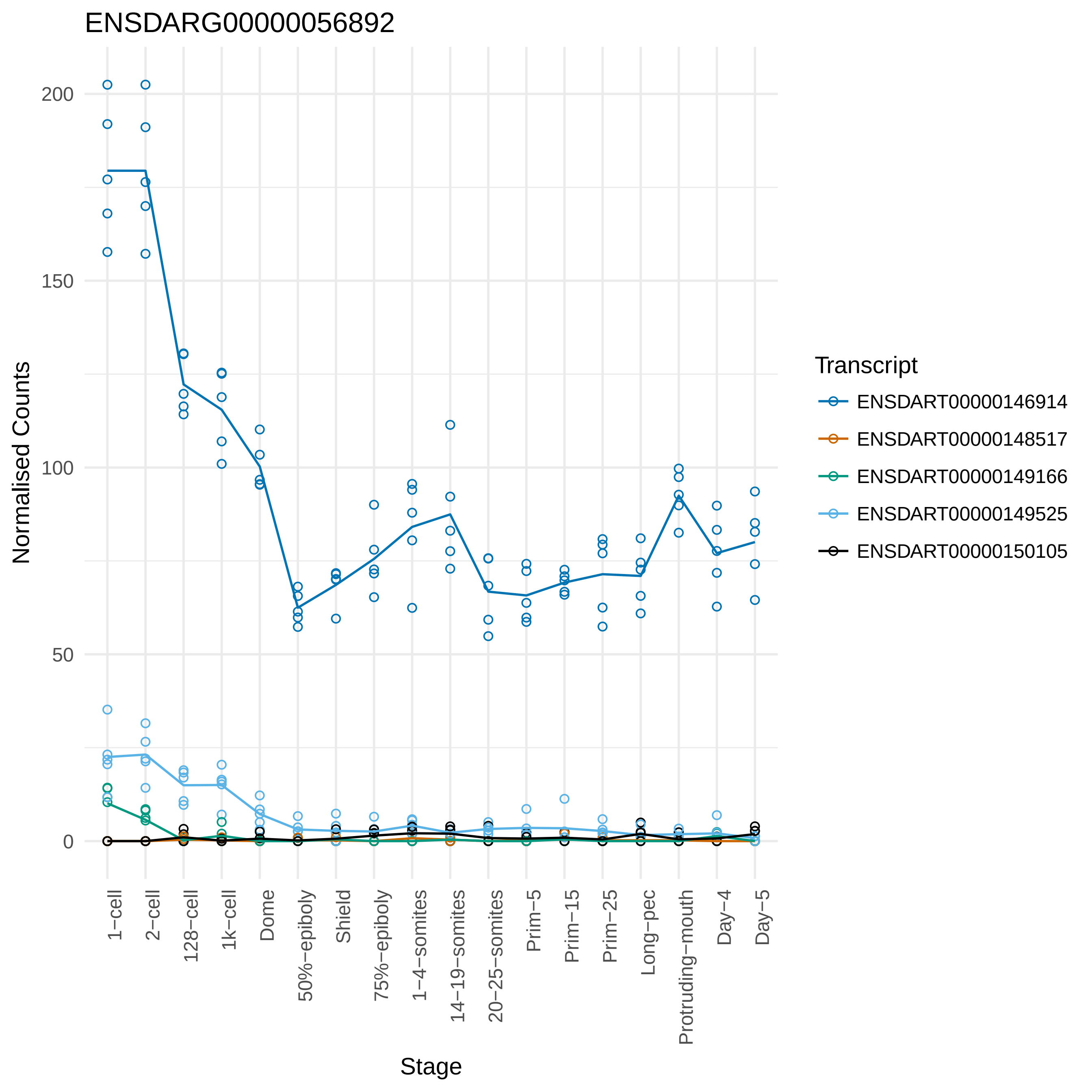

ENSDARG00000056892
Normalised Counts
200
150
100
50
0
1−cell
2−cell
128−cell
1k−cell
Dome
50%−epiboly
Shield
75%−epiboly
1−4−somites
14−19−somites
20−25−somites
Prim−5
Prim−15
Prim−25
Long−pec
Protruding−mouth
Day−4
Day−5
Stage
Transcript
ENSDART00000146914
ENSDART00000148517
ENSDART00000149166
ENSDART00000149525
ENSDART00000150105

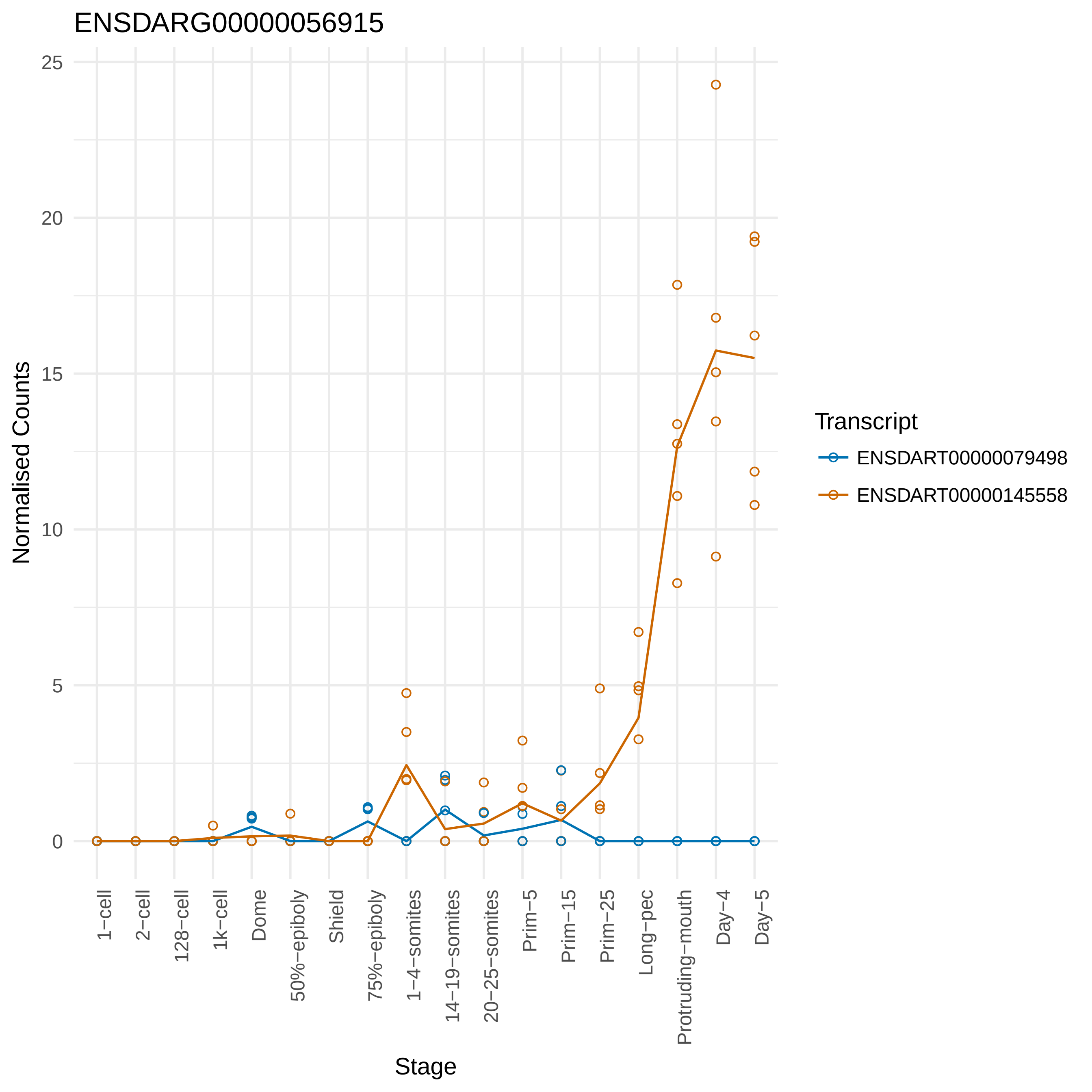
ENSDARG00000056915
Normalised Counts
0
5
10
15
20
25
Stage
1−cell
2−cell
128−cell
1k−cell
Dome
50%−epiboly
Shield
75%−epiboly
1−4−somites
14−19−somites
20−25−somites
Prim−5
Prim−15
Prim−25
Long−pec
Protruding−mouth
Day−4
Day−5
Transcript
ENSDART00000079498
ENSDART00000145558

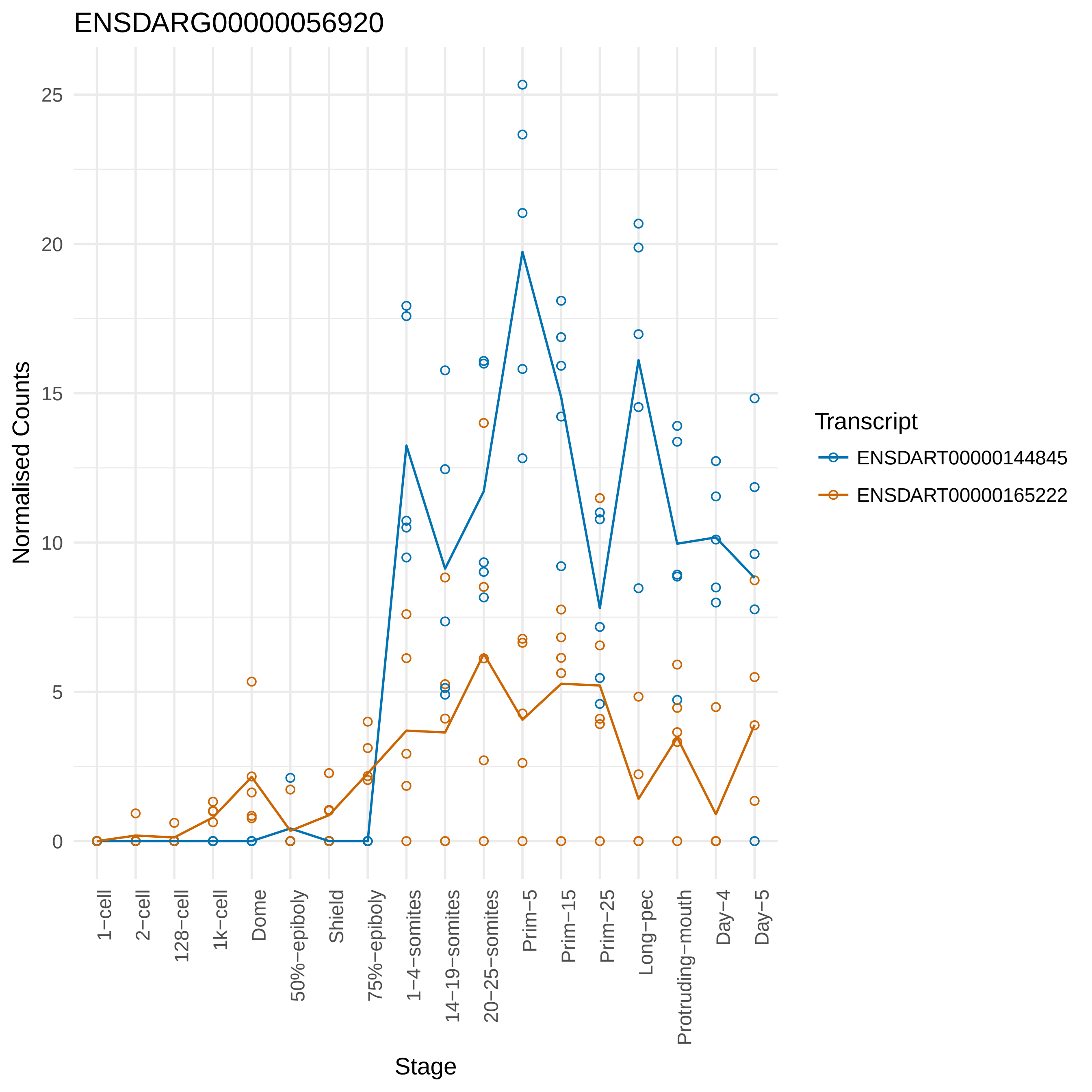

ENSDARG00000056920
Normalised Counts
0
5
10
15
20
25
1−cell
2−cell
128−cell
1k−cell
Dome
50%−epiboly
Shield
75%−epiboly
1−4−somites
14−19−somites
20−25−somites
Prim−5
Prim−15
Prim−25
Long−pec
Protruding−mouth
Day−4
Day−5
Stage
Transcript
ENSDART00000144845
ENSDART00000165222

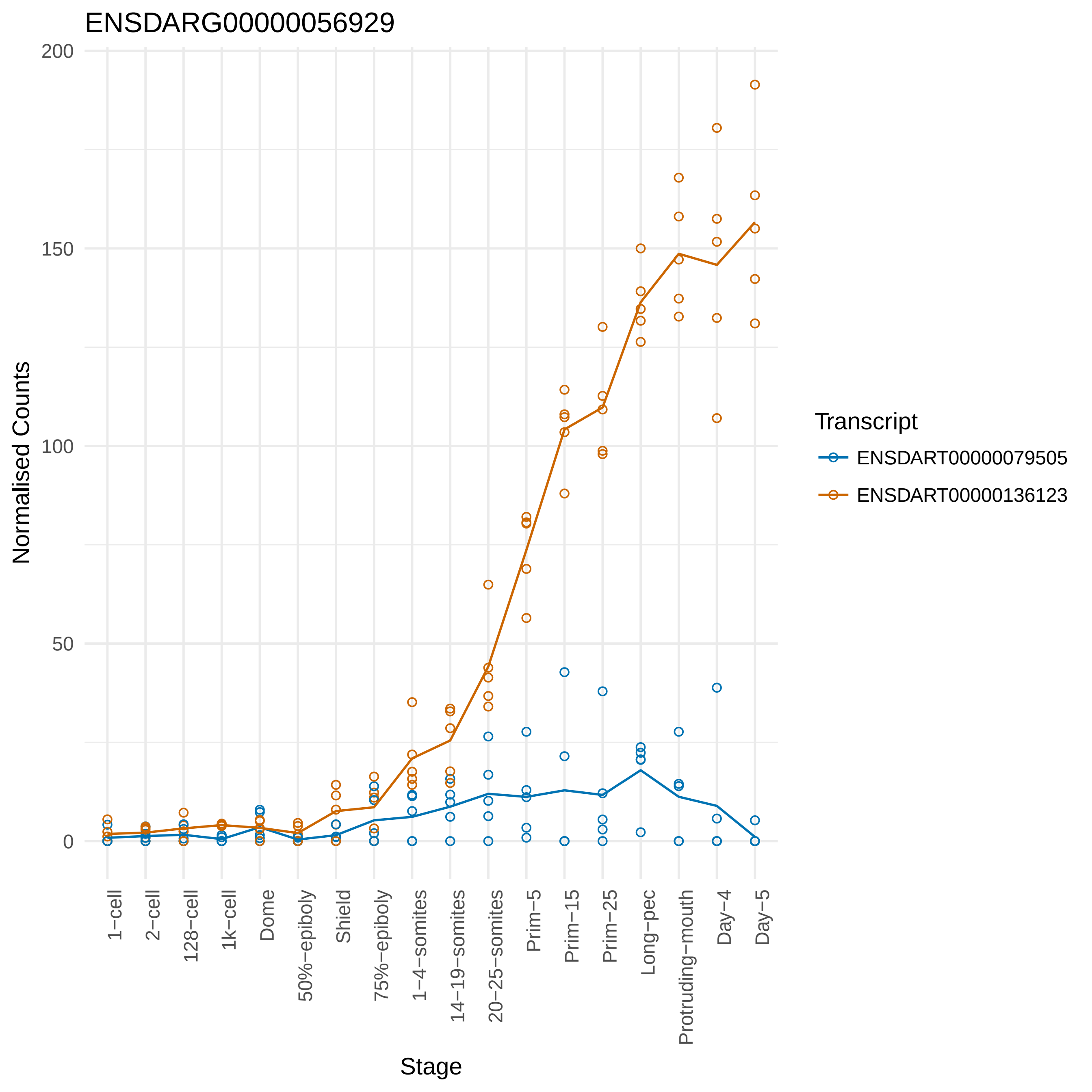

ENSDARG00000056929
200
150
100
50
0
Normalised Counts
1−cell
2−cell
128−cell
1k−cell
Dome
50%−epiboly
Shield
75%−epiboly
1−4−somites
14−19−somites
20−25−somites
Prim−5
Prim−15
Prim−25
Long−pec
Protruding−mouth
Day−4
Day−5
Stage
Transcript
ENSDART00000079505
ENSDART00000136123

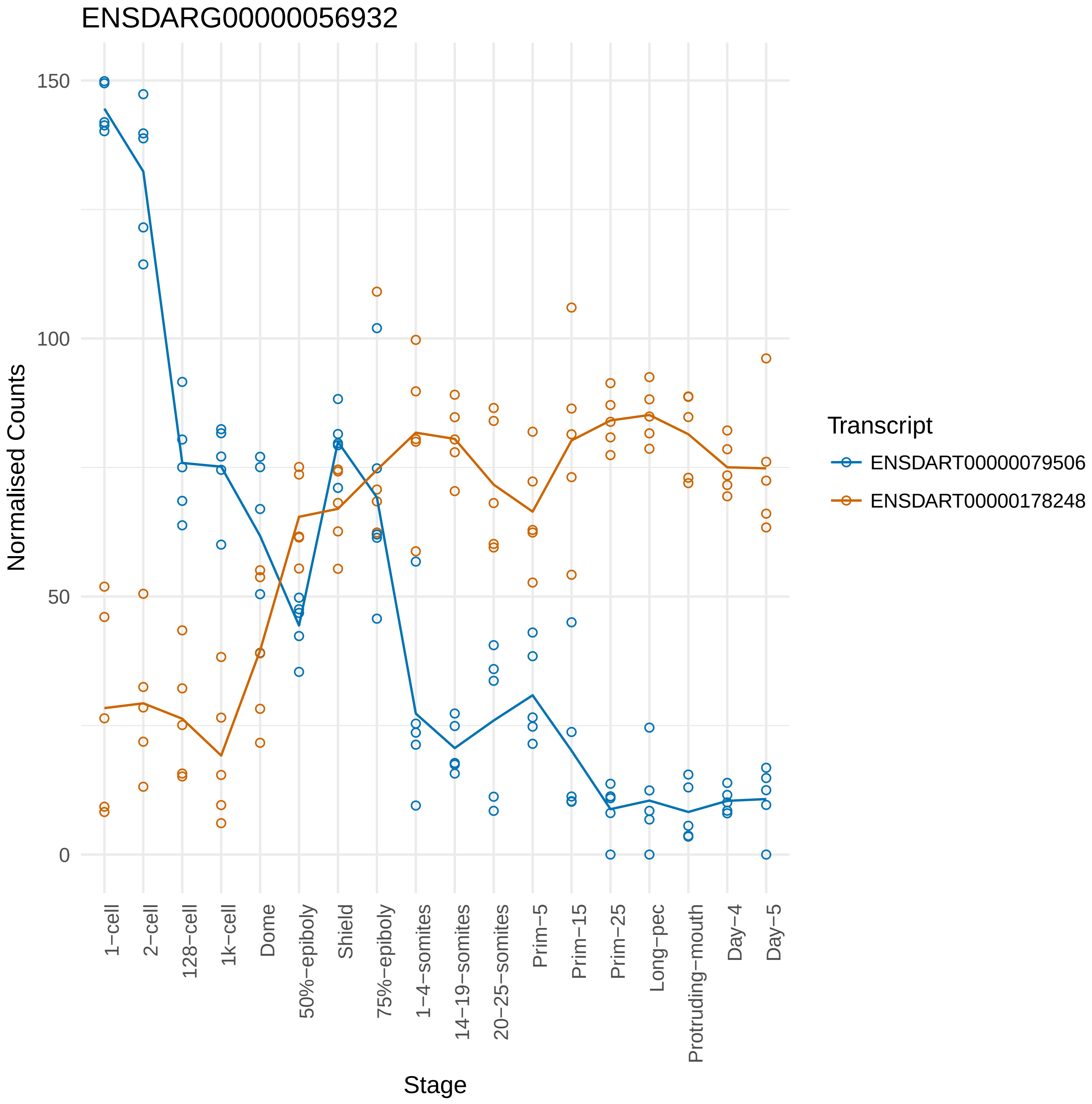

ENSDARG00000056932
Normalised Counts
150
100
50
0
1−cell
2−cell
128−cell
1k−cell
Dome
50%−epiboly
Shield
75%−epiboly
1−4−somites
14−19−somites
20−25−somites
Prim−5
Prim−15
Prim−25
Long−pec
Protruding−mouth
Day−4
Day−5
Stage
Transcript
ENSDART00000079506
ENSDART00000178248

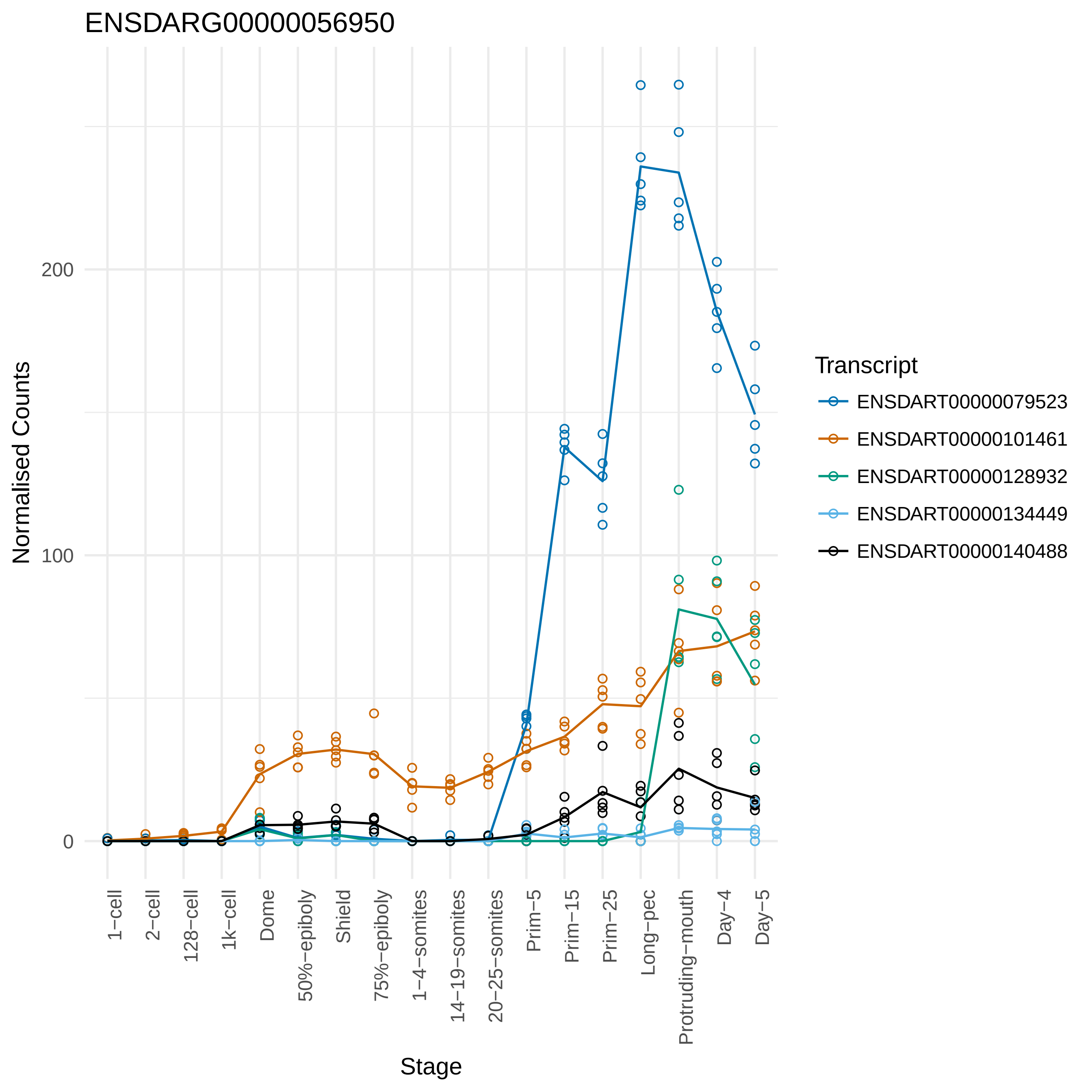
ENSDARG00000056950
Normalised Counts
200
100
0
1−cell
2−cell
128−cell
1k−cell
Dome
50%−epiboly
Shield
75%−epiboly
1−4−somites
14−19−somites
20−25−somites
Prim−5
Prim−15
Prim−25
Long−pec
Protruding−mouth
Day−4
Day−5
Stage
Transcript
ENSDART00000079523
ENSDART00000101461
ENSDART00000128932
ENSDART00000134449
ENSDART00000140488

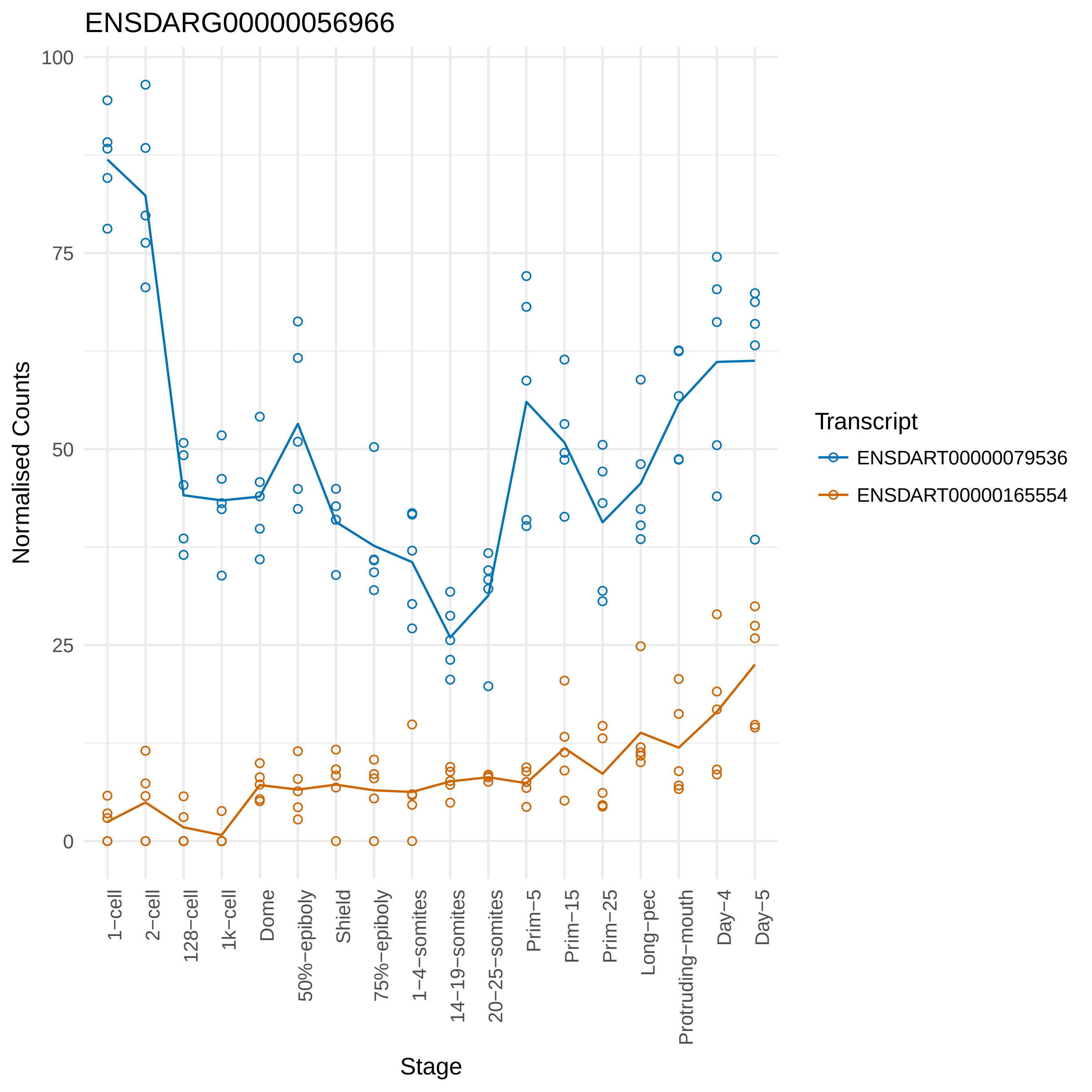

ENSDARG00000056966
Normalised Counts
100
75
50
25
0
1−cell
2−cell
128−cell
1k−cell
Dome
50%−epiboly
Shield
75%−epiboly
1−4−somites
14−19−somites
20−25−somites
Prim−5
Prim−15
Prim−25
Long−pec
Protruding−mouth
Day−4
Day−5
Stage
Transcript
ENSDART00000079536
ENSDART00000165554

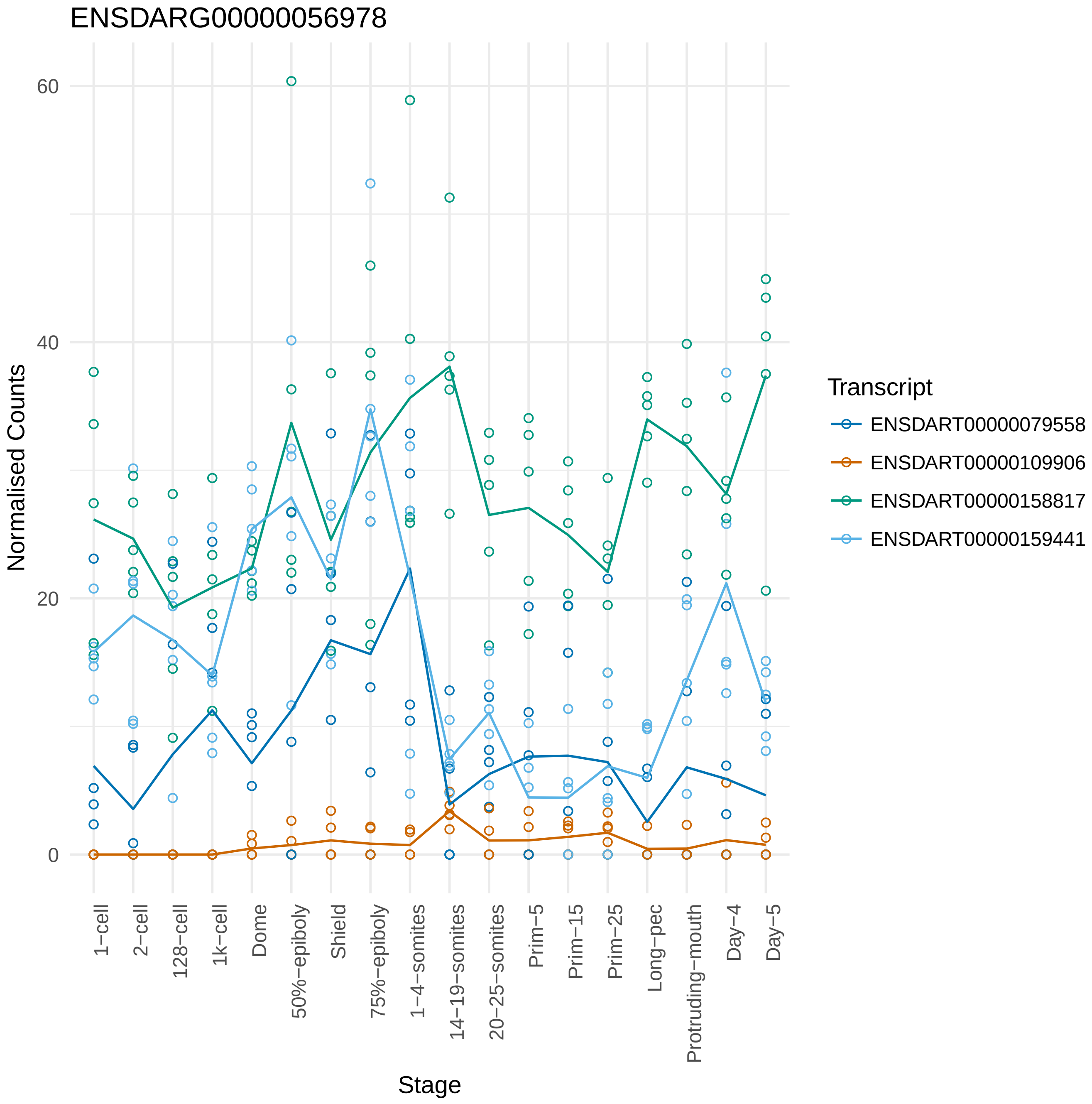

ENSDARG00000056978
Normalised Counts
60
40
20
0
1−cell
2−cell
128−cell
1k−cell
Dome
50%−epiboly
Shield
75%−epiboly
1−4−somites
14−19−somites
20−25−somites
Prim−5
Prim−15
Prim−25
Long−pec
Protruding−mouth
Day−4
Day−5
Stage
Transcript
ENSDART00000079558
ENSDART00000109906
ENSDART00000158817
ENSDART00000159441

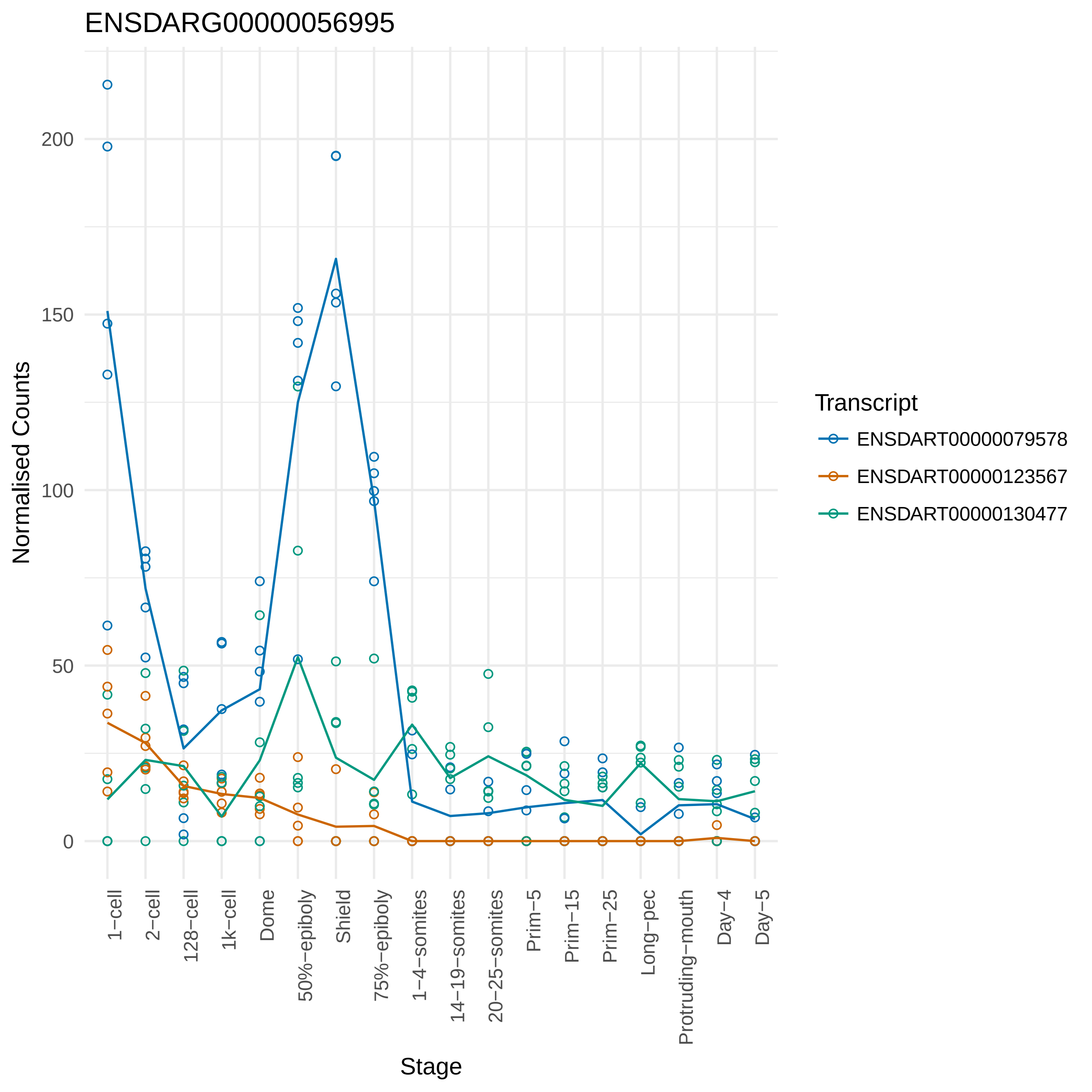

ENSDARG00000056995
Normalised Counts
0
50
100
150
200
Stage
1−cell
2−cell
128−cell
1k−cell
Dome
50%−epiboly
Shield
75%−epiboly
1−4−somites
14−19−somites
20−25−somites
Prim−5
Prim−15
Prim−25
Long−pec
Protruding−mouth
Day−4
Day−5
Transcript
ENSDART00000079578
ENSDART00000123567
ENSDART00000130477

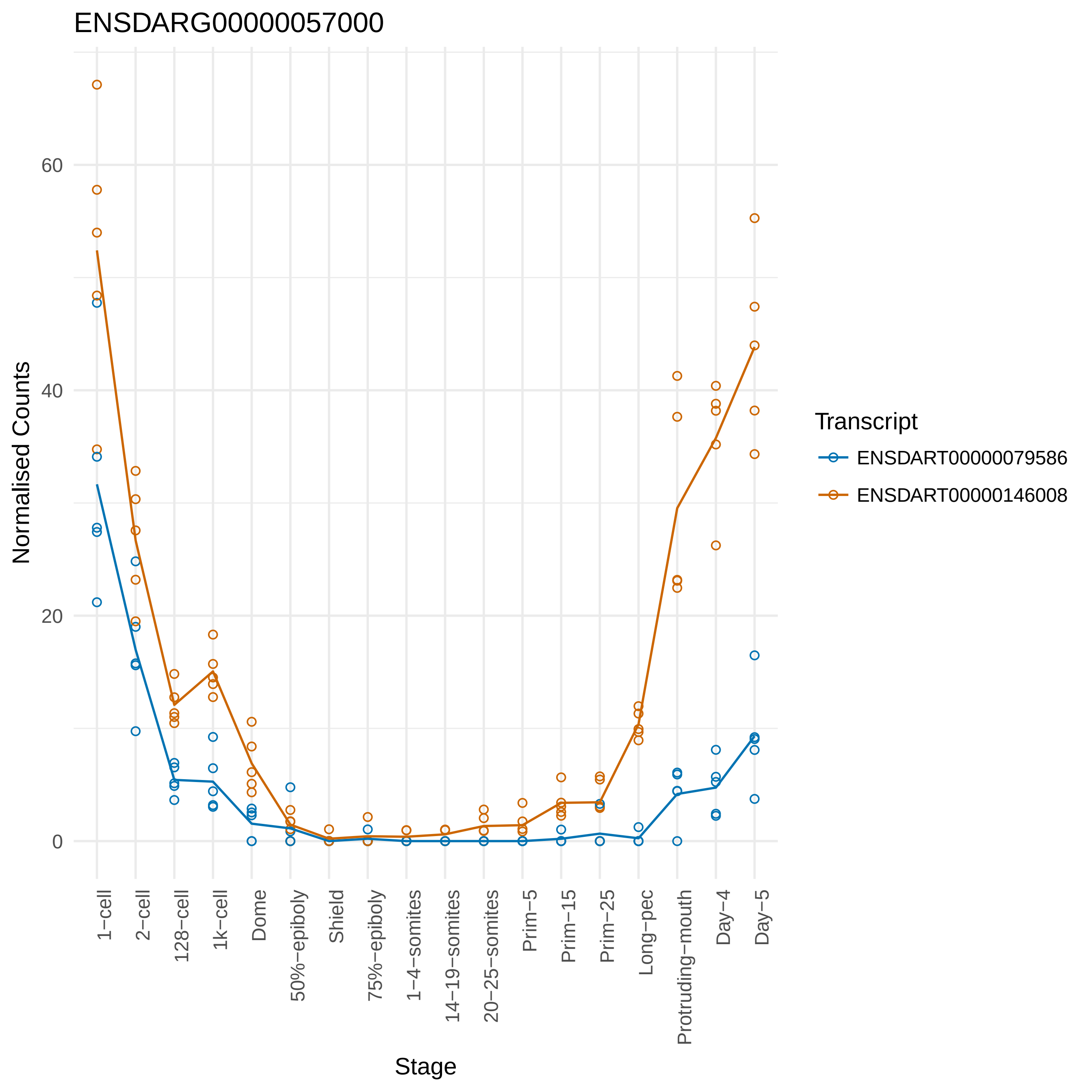

ENSDARG00000057000
Normalised Counts
0
20
40
60
1−cell
2−cell
128−cell
1k−cell
Dome
50%−epiboly
Shield
75%−epiboly
1−4−somites
14−19−somites
20−25−somites
Prim−5
Prim−15
Prim−25
Long−pec
Protruding−mouth
Day−4
Day−5
Stage
Transcript
ENSDART00000079586
ENSDART00000146008

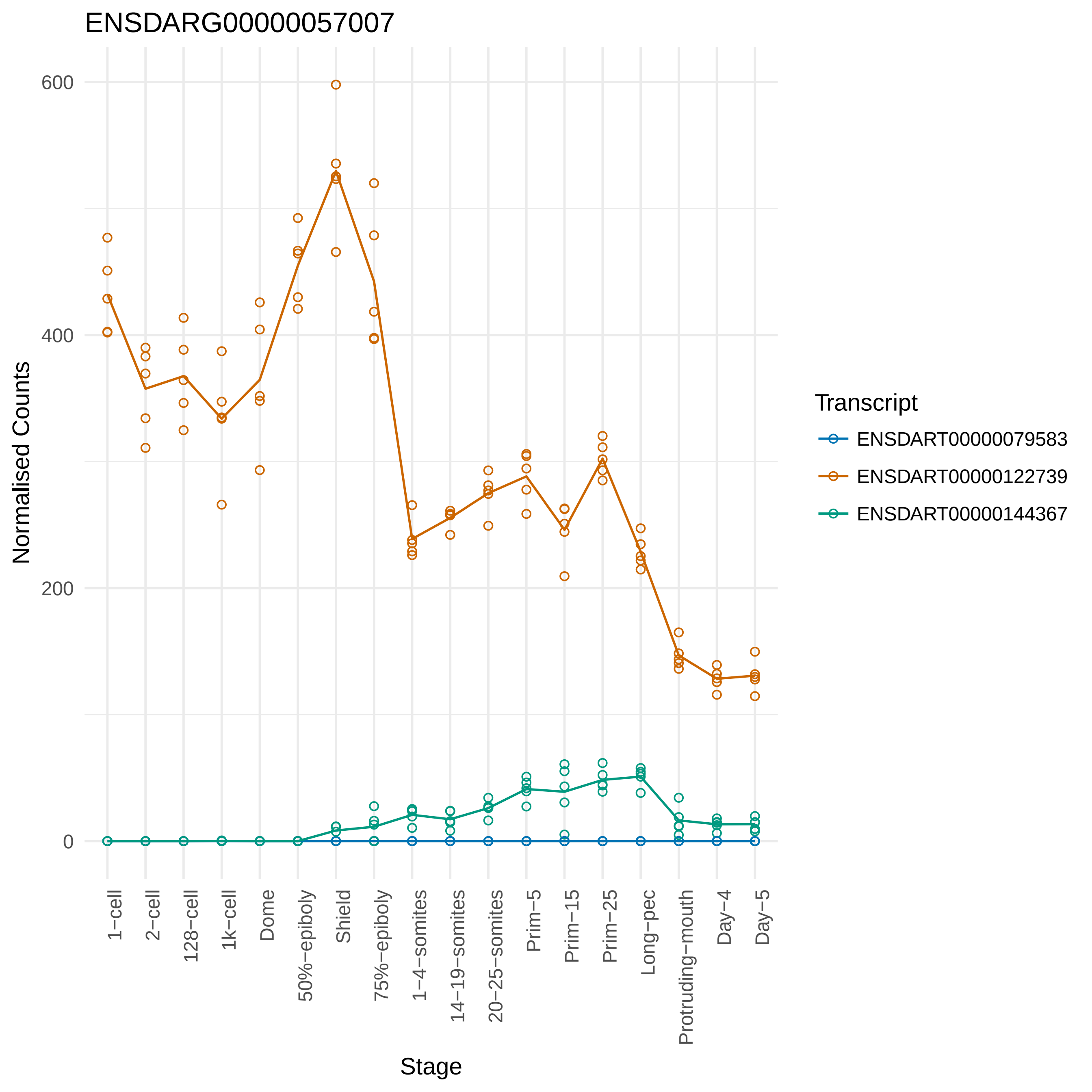

ENSDARG00000057007
Normalised Counts
600
400
200
0
1−cell
2−cell
128−cell
1k−cell
Dome
50%−epiboly
Shield
75%−epiboly
1−4−somites
14−19−somites
20−25−somites
Prim−5
Prim−15
Prim−25
Long−pec
Protruding−mouth
Day−4
Day−5
Stage
Transcript
ENSDART00000079583
ENSDART00000122739
ENSDART00000144367

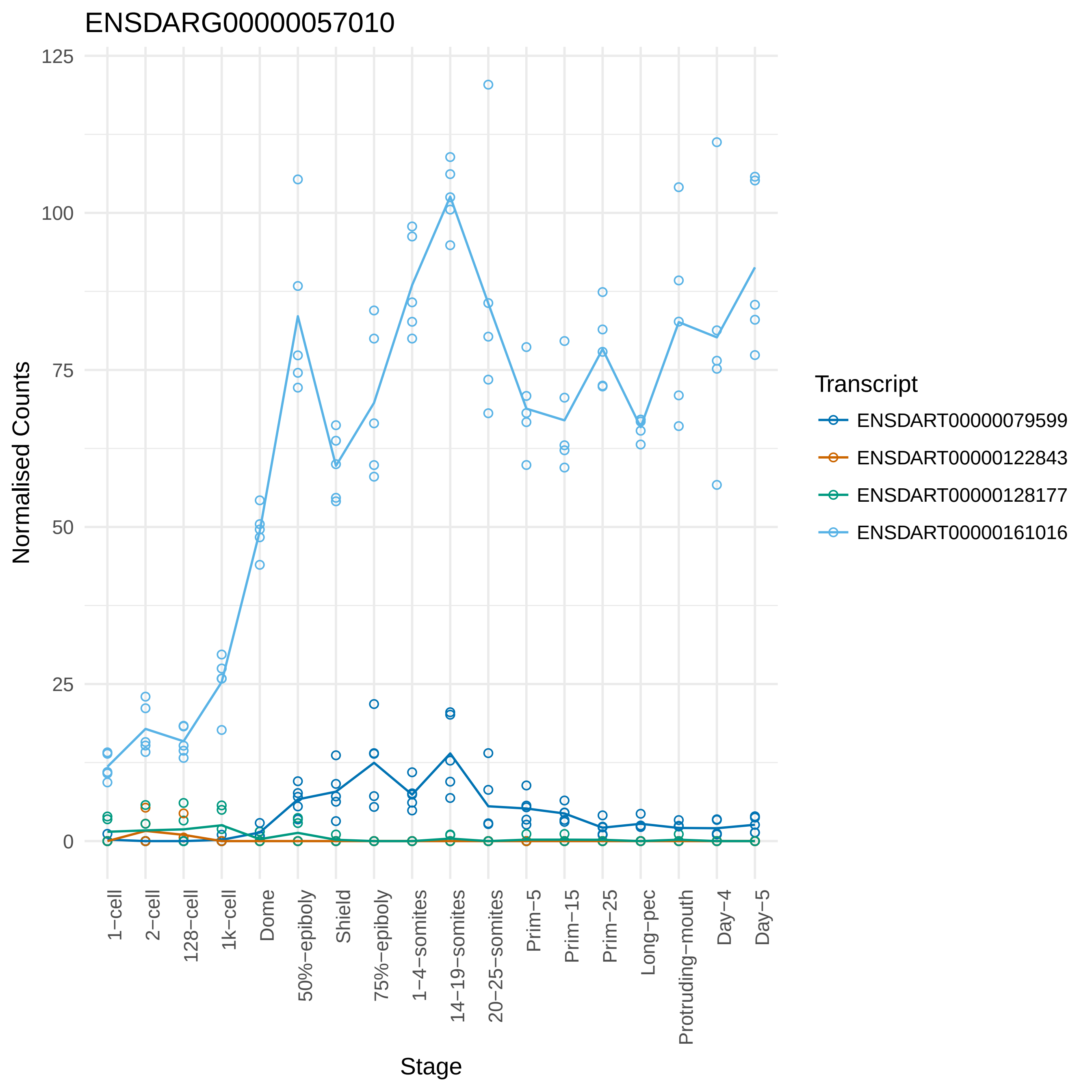

ENSDARG00000057010
Normalised Counts
125
100
75
50
25
0
1−cell
2−cell
128−cell
1k−cell
Dome
50%−epiboly
Shield
75%−epiboly
1−4−somites
14−19−somites
20−25−somites
Prim−5
Prim−15
Prim−25
Long−pec
Protruding−mouth
Day−4
Day−5
Stage
Transcript
ENSDART00000079599
ENSDART00000122843
ENSDART00000128177
ENSDART00000161016

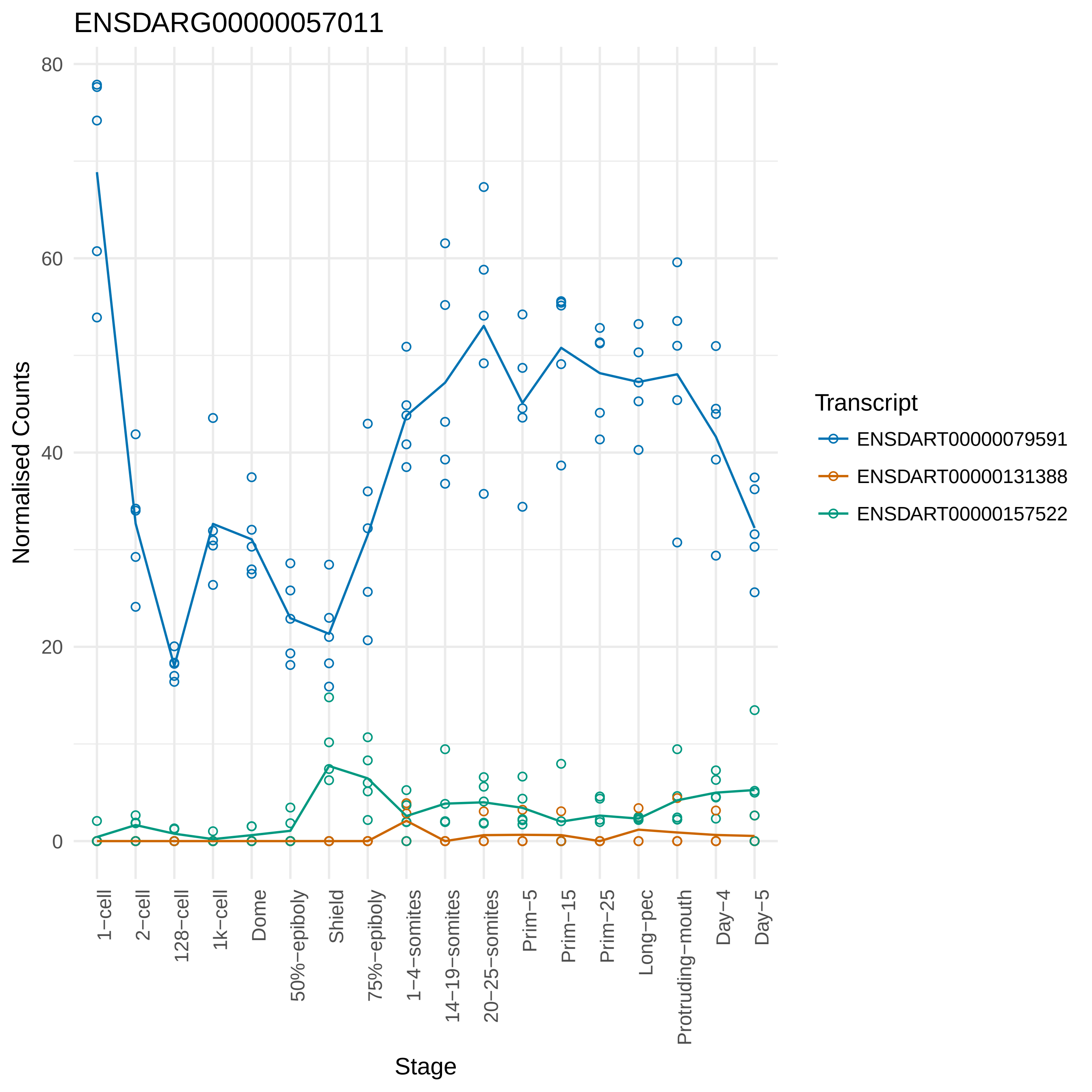
ENSDARG00000057011
Normalised Counts
0
20
40
60
80
1−cell
2−cell
128−cell
1k−cell
Dome
50%−epiboly
Shield
75%−epiboly
1−4−somites
14−19−somites
20−25−somites
Prim−5
Prim−15
Prim−25
Long−pec
Protruding−mouth
Day−4
Day−5
Stage
Transcript
ENSDART00000079591
ENSDART00000131388
ENSDART00000157522

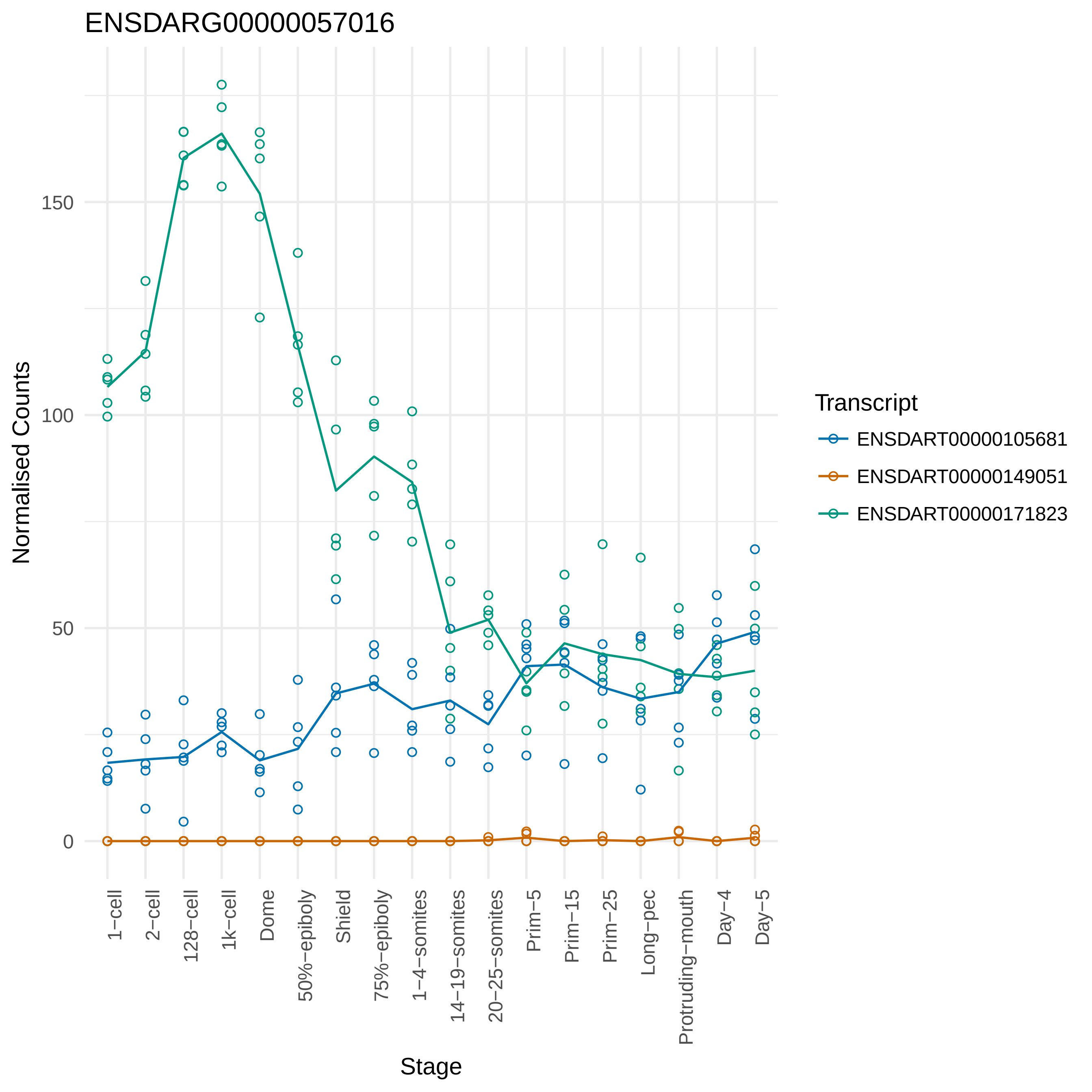

ENSDARG00000057016
Normalised Counts
150
100
50
0
1−cell
2−cell
128−cell
1k−cell
Dome
50%−epiboly
Shield
75%−epiboly
1−4−somites
14−19−somites
20−25−somites
Prim−5
Prim−15
Prim−25
Long−pec
Protruding−mouth
Day−4
Day−5
Stage
Transcript
ENSDART00000105681
ENSDART00000149051
ENSDART00000171823

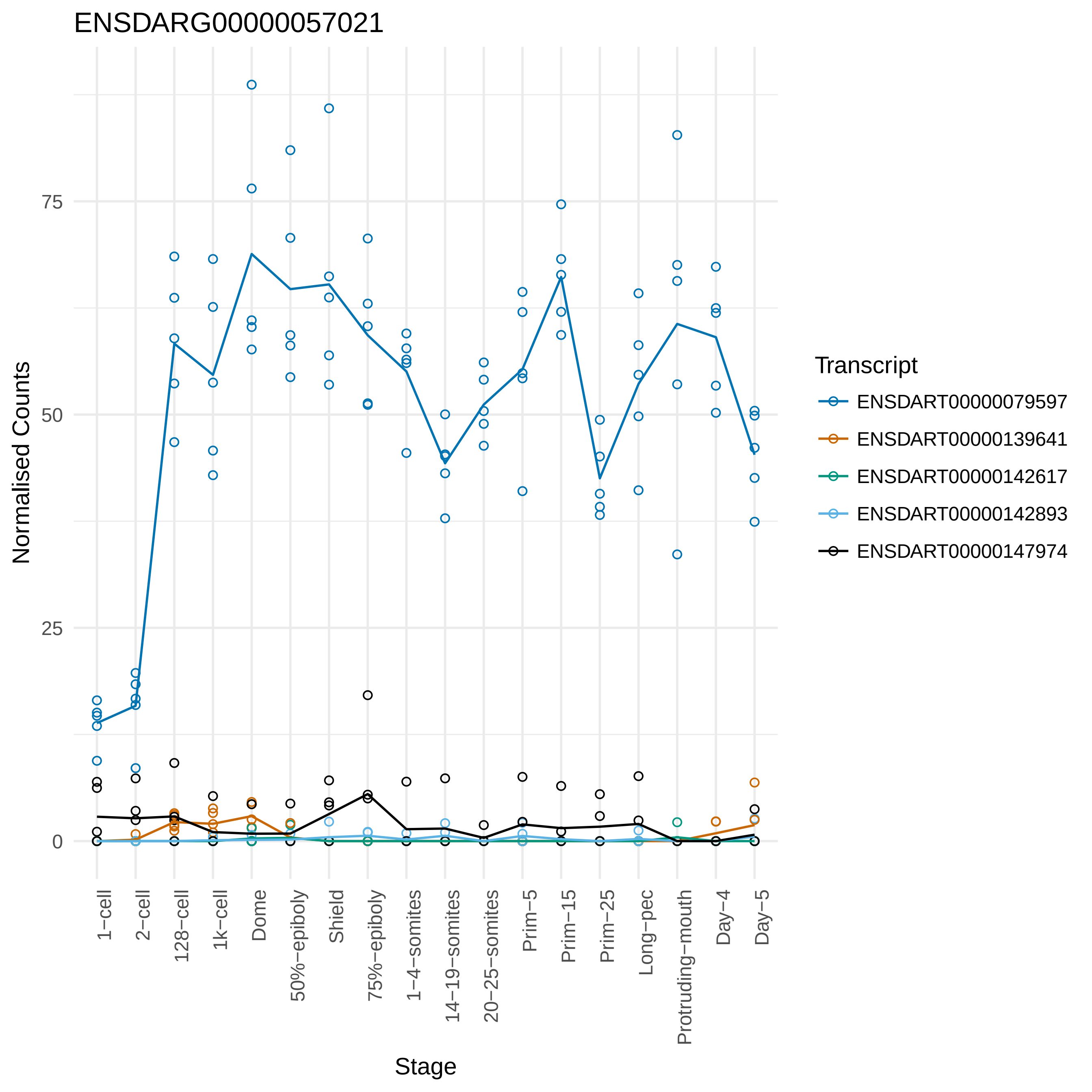
ENSDARG00000057021
Normalised Counts
75
50
25
0
Stage
1−cell
2−cell
128−cell
1k−cell
Dome
50%−epiboly
Shield
75%−epiboly
1−4−somites
14−19−somites
20−25−somites
Prim−5
Prim−15
Prim−25
Long−pec
Protruding−mouth
Day−4
Day−5
Transcript
ENSDART00000079597
ENSDART00000139641
ENSDART00000142617
ENSDART00000142893
ENSDART00000147974

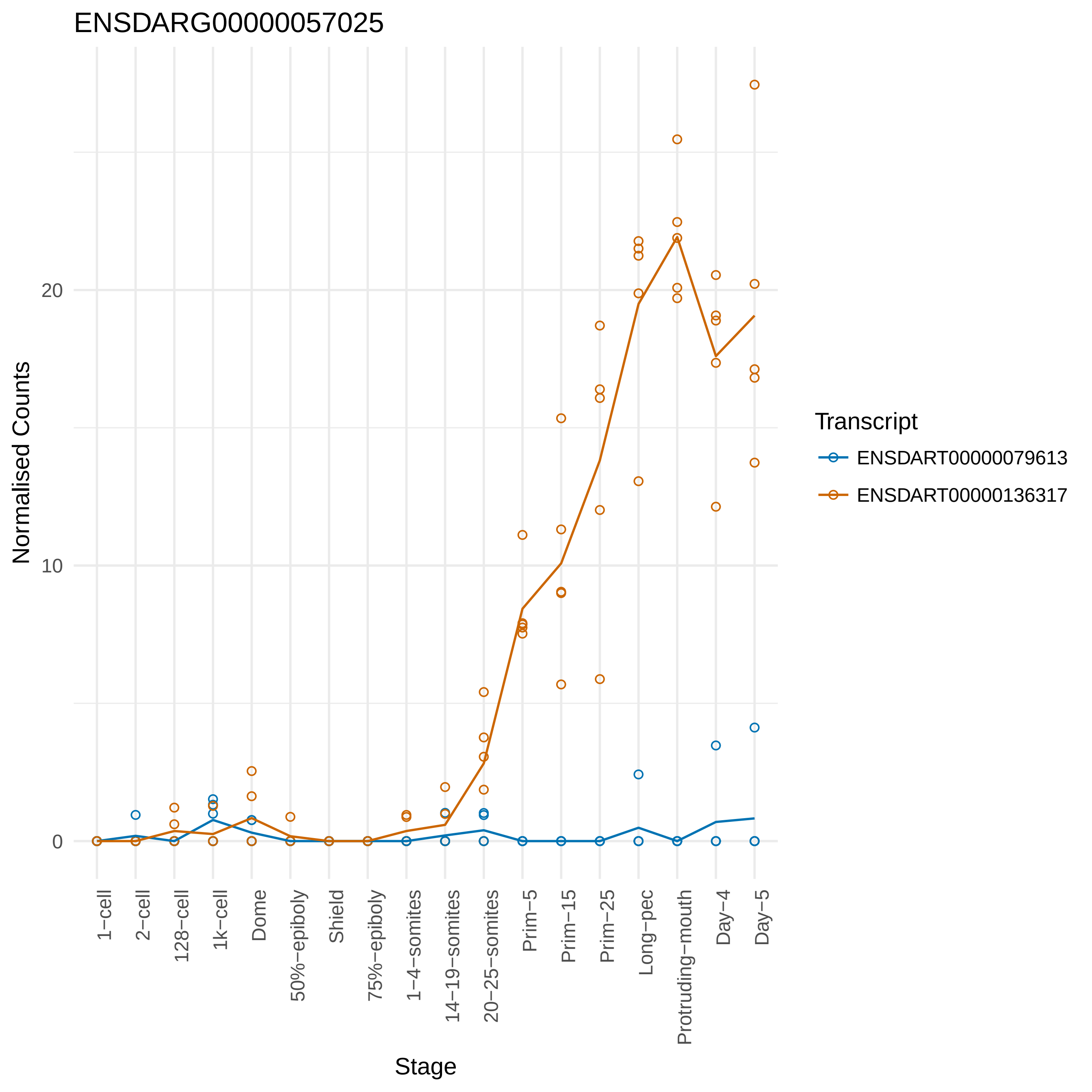
ENSDARG00000057025
Normalised Counts
0
10
20
1−cell
2−cell
128−cell
1k−cell
Dome
50%−epiboly
Shield
75%−epiboly
1−4−somites
14−19−somites
20−25−somites
Prim−5
Prim−15
Prim−25
Long−pec
Protruding−mouth
Day−4
Day−5
Stage
Transcript
ENSDART00000079613
ENSDART00000136317

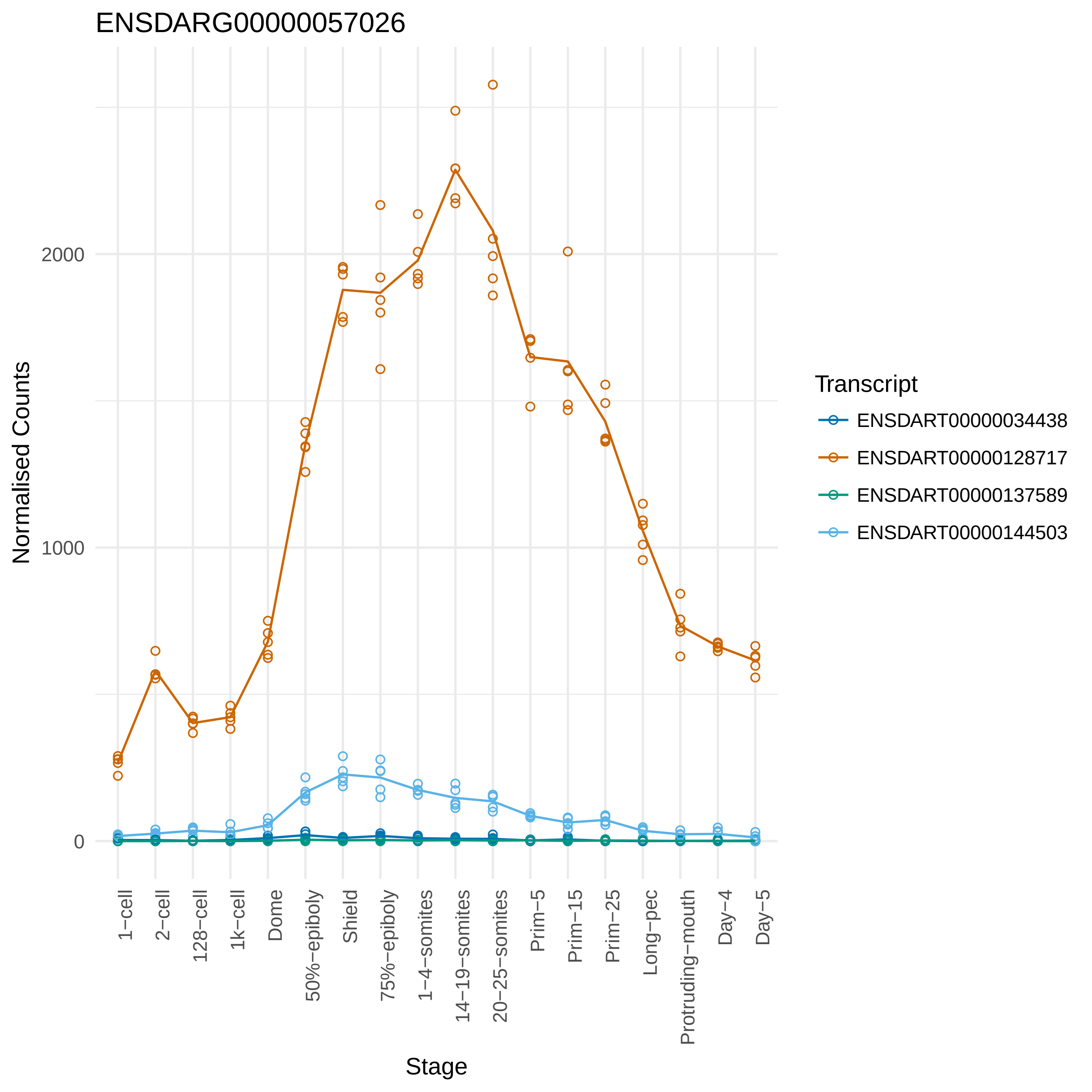
ENSDARG00000057026
Normalised Counts
2000
1000
0
Stage
1−cell
2−cell
128−cell
1k−cell
Dome
50%−epiboly
Shield
75%−epiboly
1−4−somites
14−19−somites
20−25−somites
Prim−5
Prim−15
Prim−25
Long−pec
Protruding−mouth
Day−4
Day−5
Transcript
ENSDART00000034438
ENSDART00000128717
ENSDART00000137589
ENSDART00000144503

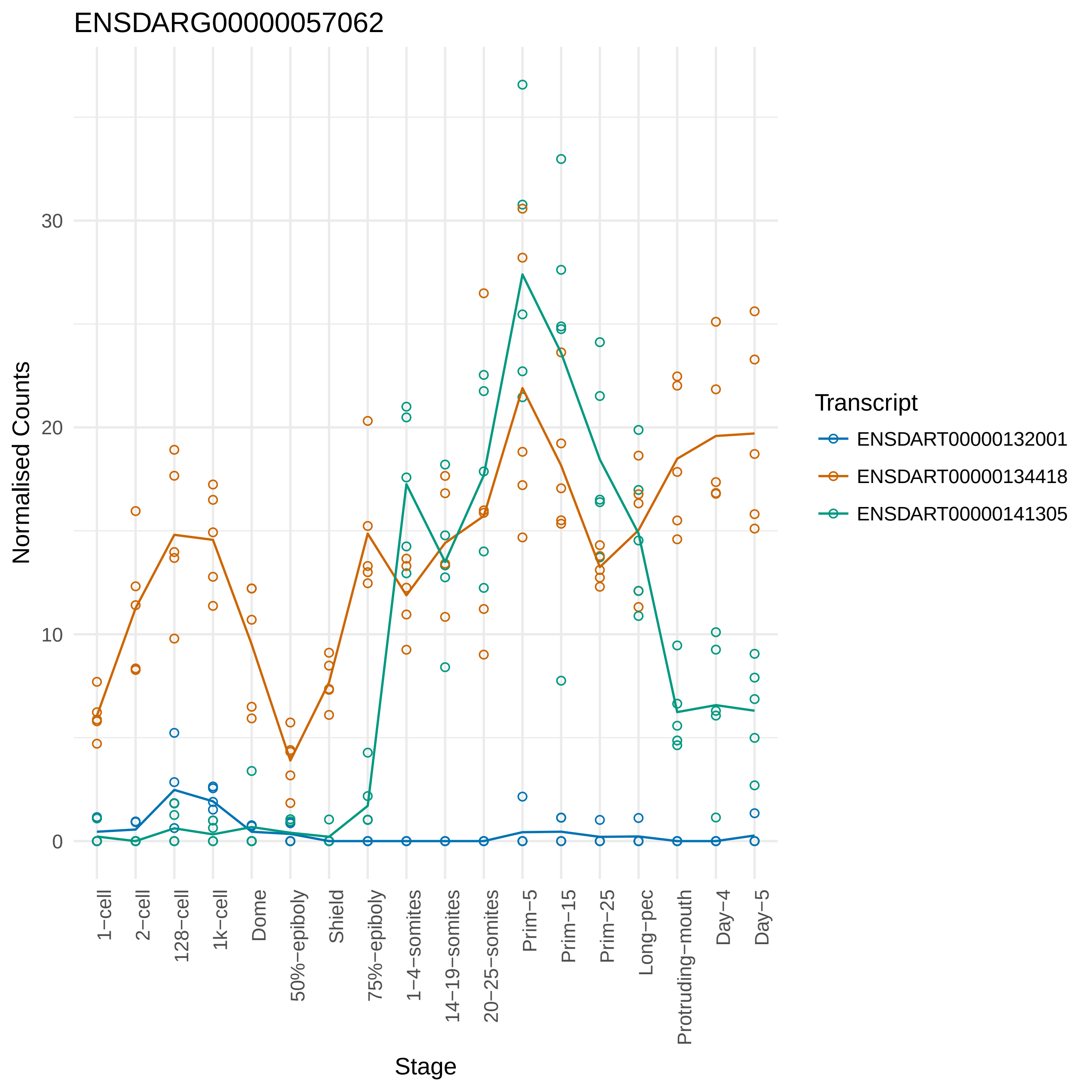

ENSDARG00000057062
Normalised Counts
0
10
20
30
1−cell
2−cell
128−cell
1k−cell
Dome
50%−epiboly
Shield
75%−epiboly
1−4−somites
14−19−somites
20−25−somites
Prim−5
Prim−15
Prim−25
Long−pec
Protruding−mouth
Day−4
Day−5
Stage
Transcript
ENSDART00000132001
ENSDART00000134418
ENSDART00000141305

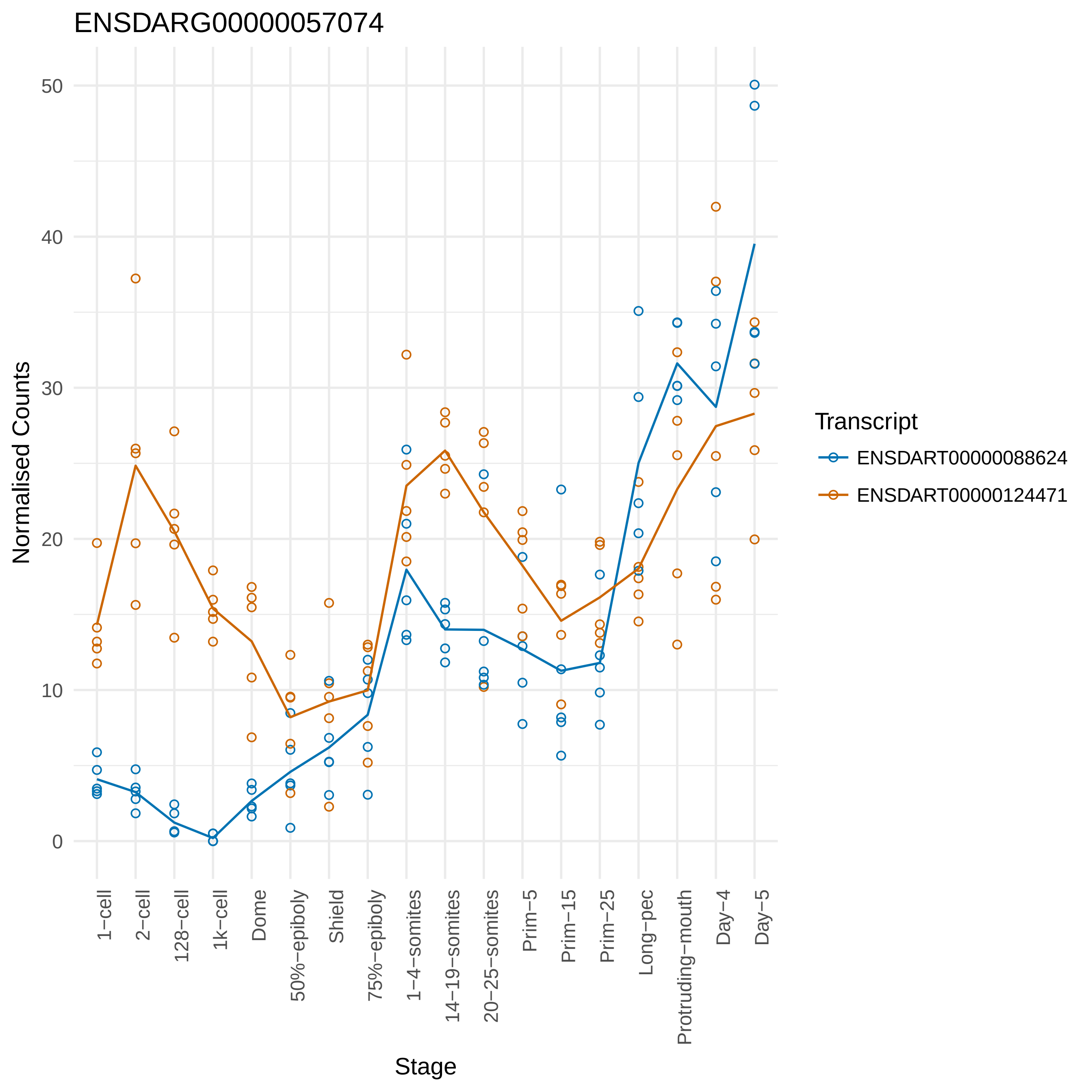

ENSDARG00000057074
Normalised Counts
0
10
20
30
40
50
1−cell
2−cell
128−cell
1k−cell
Dome
50%−epiboly
Shield
75%−epiboly
1−4−somites
14−19−somites
20−25−somites
Prim−5
Prim−15
Prim−25
Long−pec
Protruding−mouth
Day−4
Day−5
Stage
Transcript
ENSDART00000088624
ENSDART00000124471

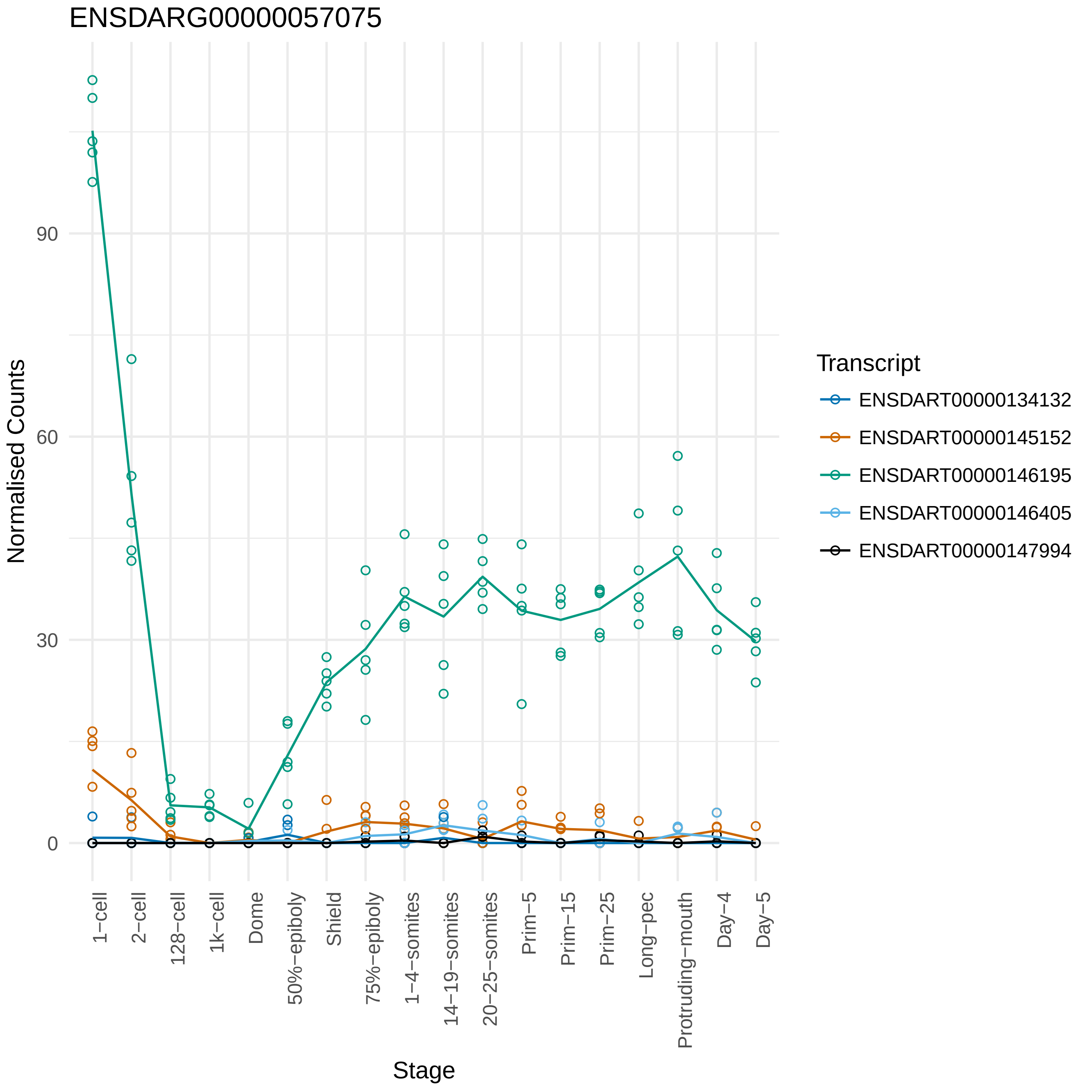

ENSDARG00000057075
Normalised Counts
90
60
30
0
1−cell
2−cell
128−cell
1k−cell
Dome
50%−epiboly
Shield
75%−epiboly
1−4−somites
14−19−somites
20−25−somites
Prim−5
Prim−15
Prim−25
Long−pec
Protruding−mouth
Day−4
Day−5
Stage
Transcript
ENSDART00000134132
ENSDART00000145152
ENSDART00000146195
ENSDART00000146405
ENSDART00000147994

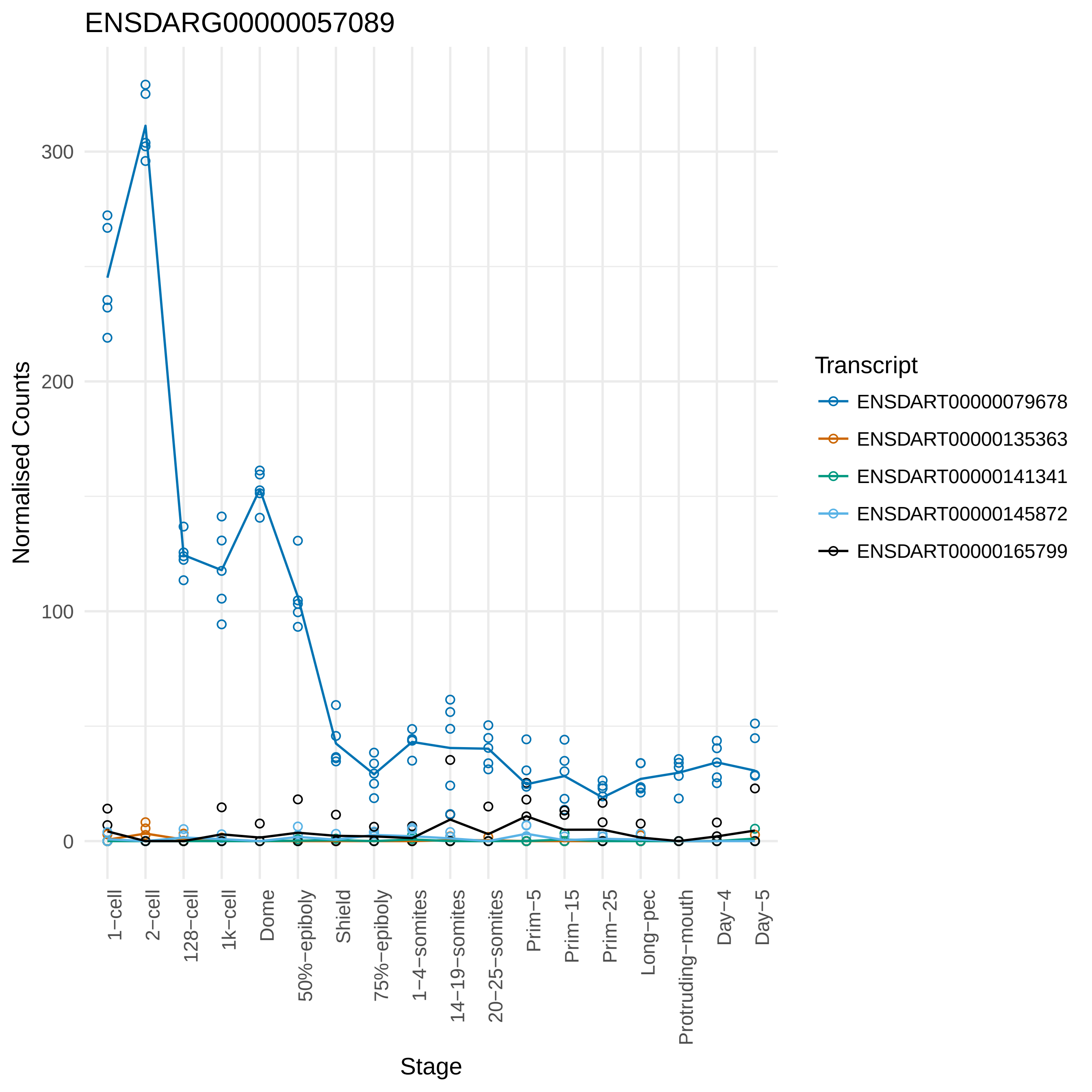

ENSDARG00000057089
Normalised Counts
300
200
100
0
1−cell
2−cell
128−cell
1k−cell
Dome
50%−epiboly
Shield
75%−epiboly
1−4−somites
14−19−somites
20−25−somites
Prim−5
Prim−15
Prim−25
Long−pec
Protruding−mouth
Day−4
Day−5
Stage
Transcript
ENSDART00000079678
ENSDART00000135363
ENSDART00000141341
ENSDART00000145872
ENSDART00000165799

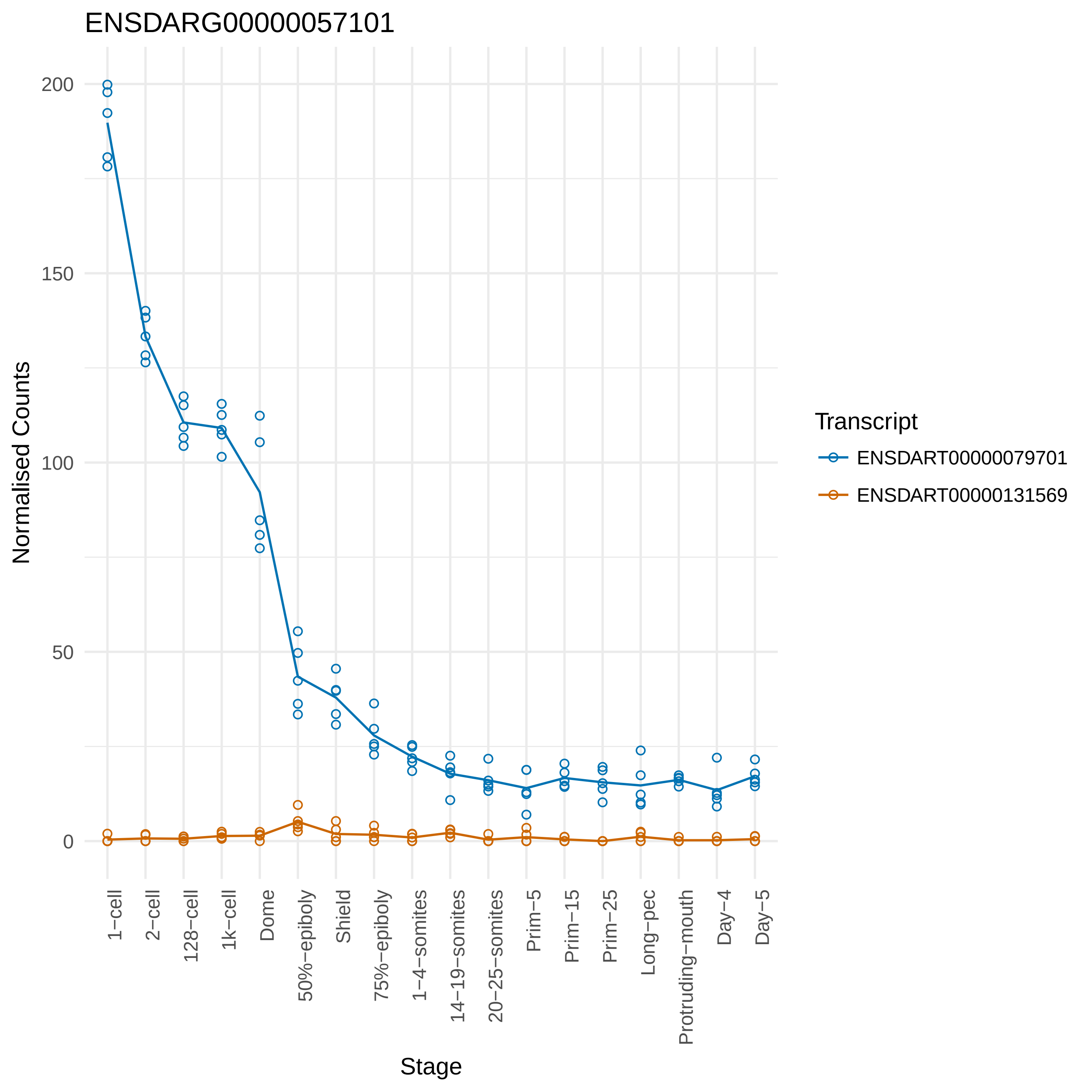
ENSDARG00000057101
Normalised Counts
200
150
100
50
0
1−cell
2−cell
128−cell
1k−cell
Dome
50%−epiboly
Shield
75%−epiboly
1−4−somites
14−19−somites
20−25−somites
Prim−5
Prim−15
Prim−25
Long−pec
Protruding−mouth
Day−4
Day−5
Stage
Transcript
ENSDART00000079701
ENSDART00000131569

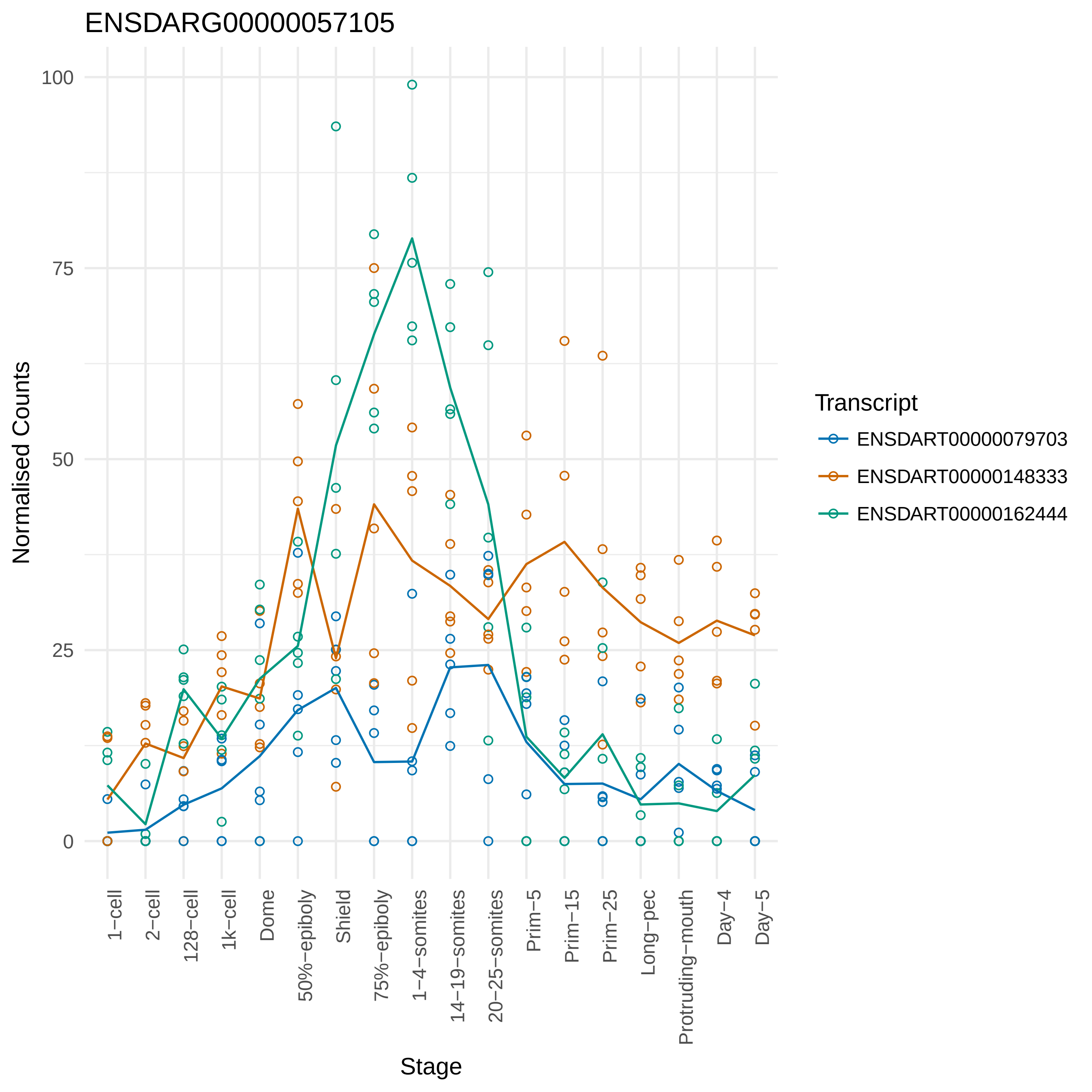

ENSDARG00000057105
Normalised Counts
100
75
50
25
0
1−cell
2−cell
128−cell
1k−cell
Dome
50%−epiboly
Shield
75%−epiboly
1−4−somites
14−19−somites
20−25−somites
Prim−5
Prim−15
Prim−25
Long−pec
Protruding−mouth
Day−4
Day−5
Stage
Transcript
ENSDART00000079703
ENSDART00000148333
ENSDART00000162444

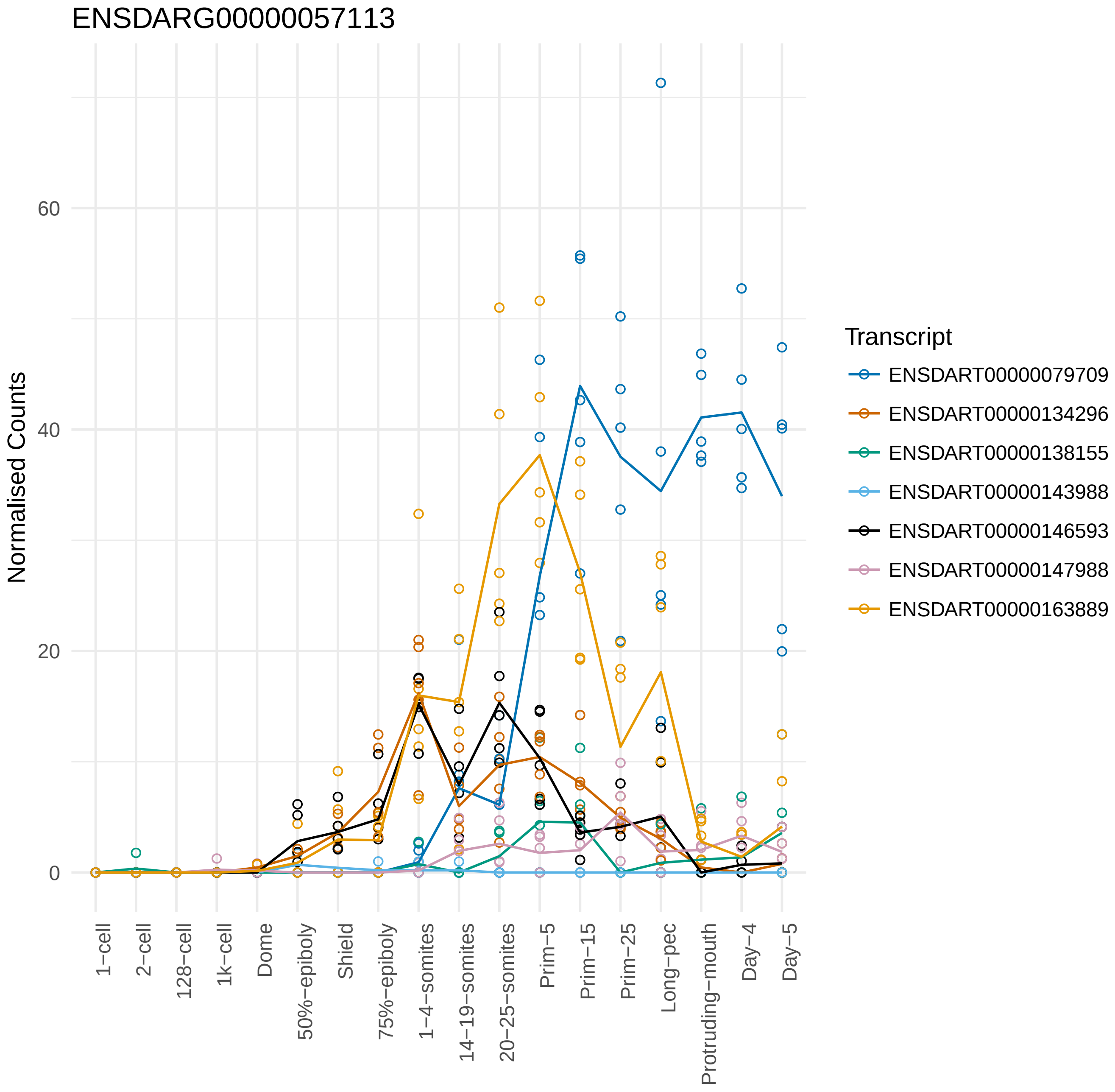

ENSDARG00000057113
Normalised Counts
Stage
0
20
40
60
1−cell
2−cell
128−cell
1k−cell
Dome
50%−epiboly
Shield
75%−epiboly
1−4−somites
14−19−somites
20−25−somites
Prim−5
Prim−15
Prim−25
Long−pec
Protruding−mouth
Day−4
Day−5
Transcript
ENSDART00000079709
ENSDART00000134296
ENSDART00000138155
ENSDART00000143988
ENSDART00000146593
ENSDART00000147988
ENSDART00000163889

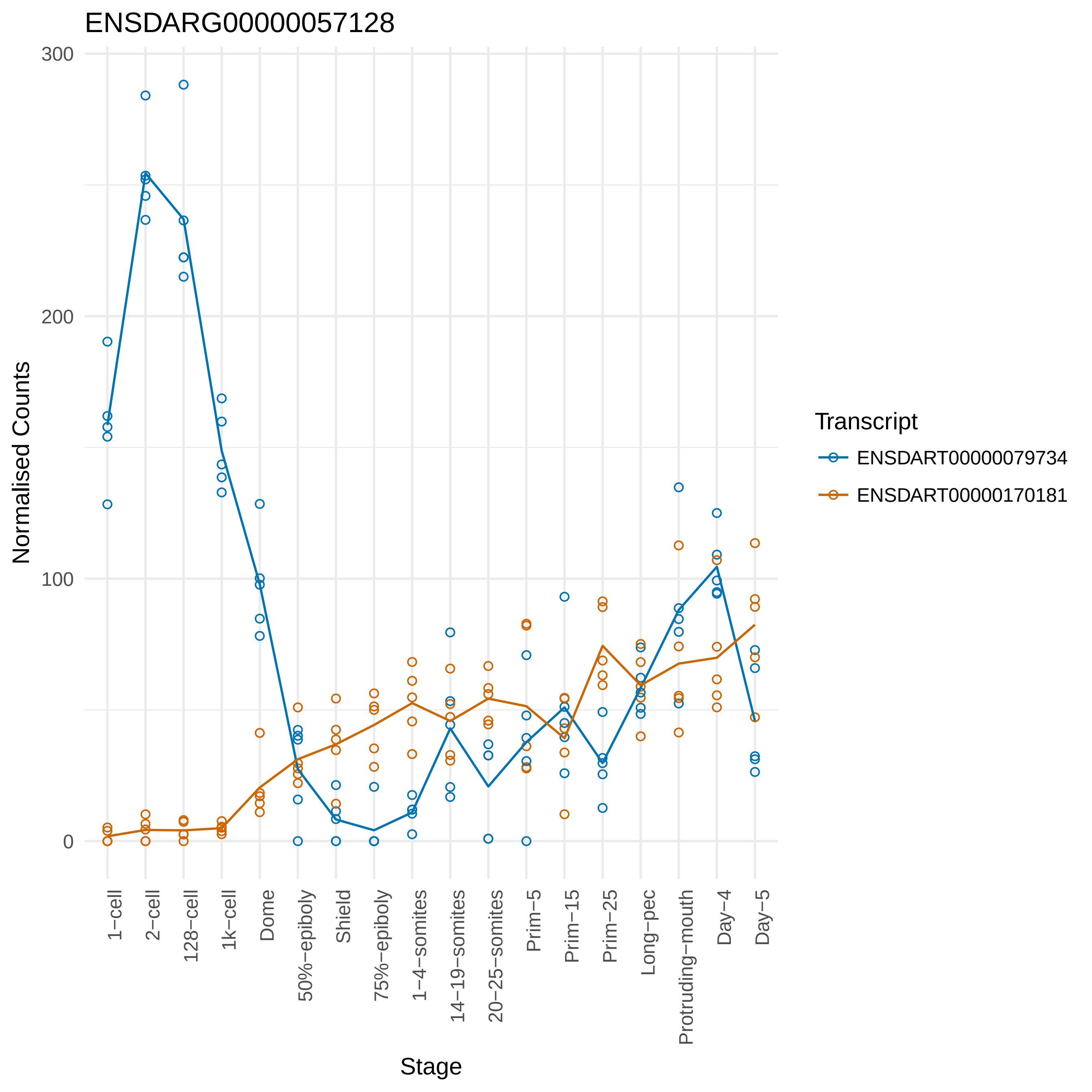

ENSDARG00000057128
Normalised Counts
300
200
100
0
Stage
1−cell
2−cell
128−cell
1k−cell
Dome
50%−epiboly
Shield
75%−epiboly
1−4−somites
14−19−somites
20−25−somites
Prim−5
Prim−15
Prim−25
Long−pec
Protruding−mouth
Day−4
Day−5
Transcript
ENSDART00000079734
ENSDART00000170181

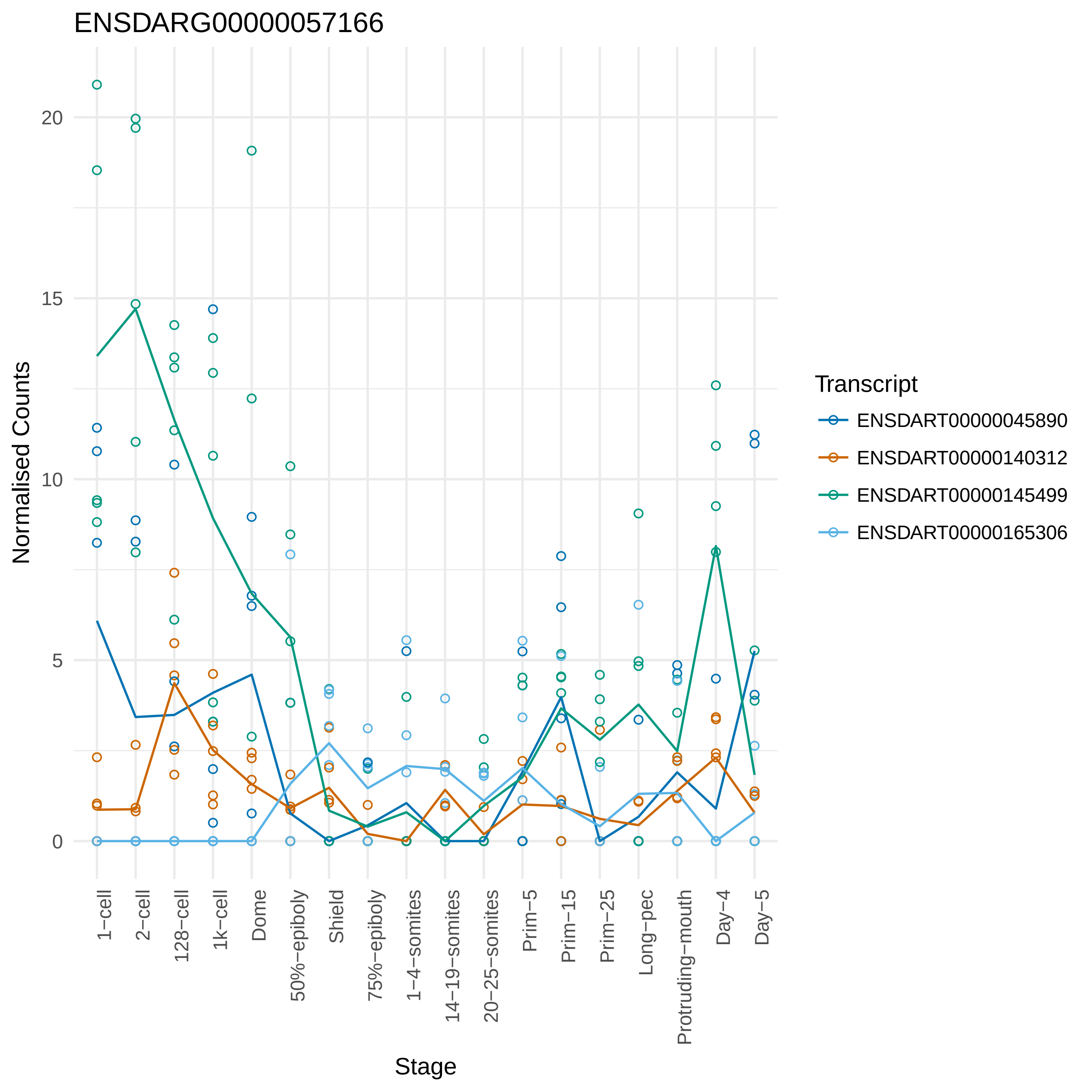
ENSDARG00000057166
Normalised Counts
20
15
10
5
0
1−cell
2−cell
128−cell
1k−cell
Dome
50%−epiboly
Shield
75%−epiboly
1−4−somites
14−19−somites
20−25−somites
Prim−5
Prim−15
Prim−25
Long−pec
Protruding−mouth
Day−4
Day−5
Stage
Transcript
ENSDART00000045890
ENSDART00000140312
ENSDART00000145499
ENSDART00000165306

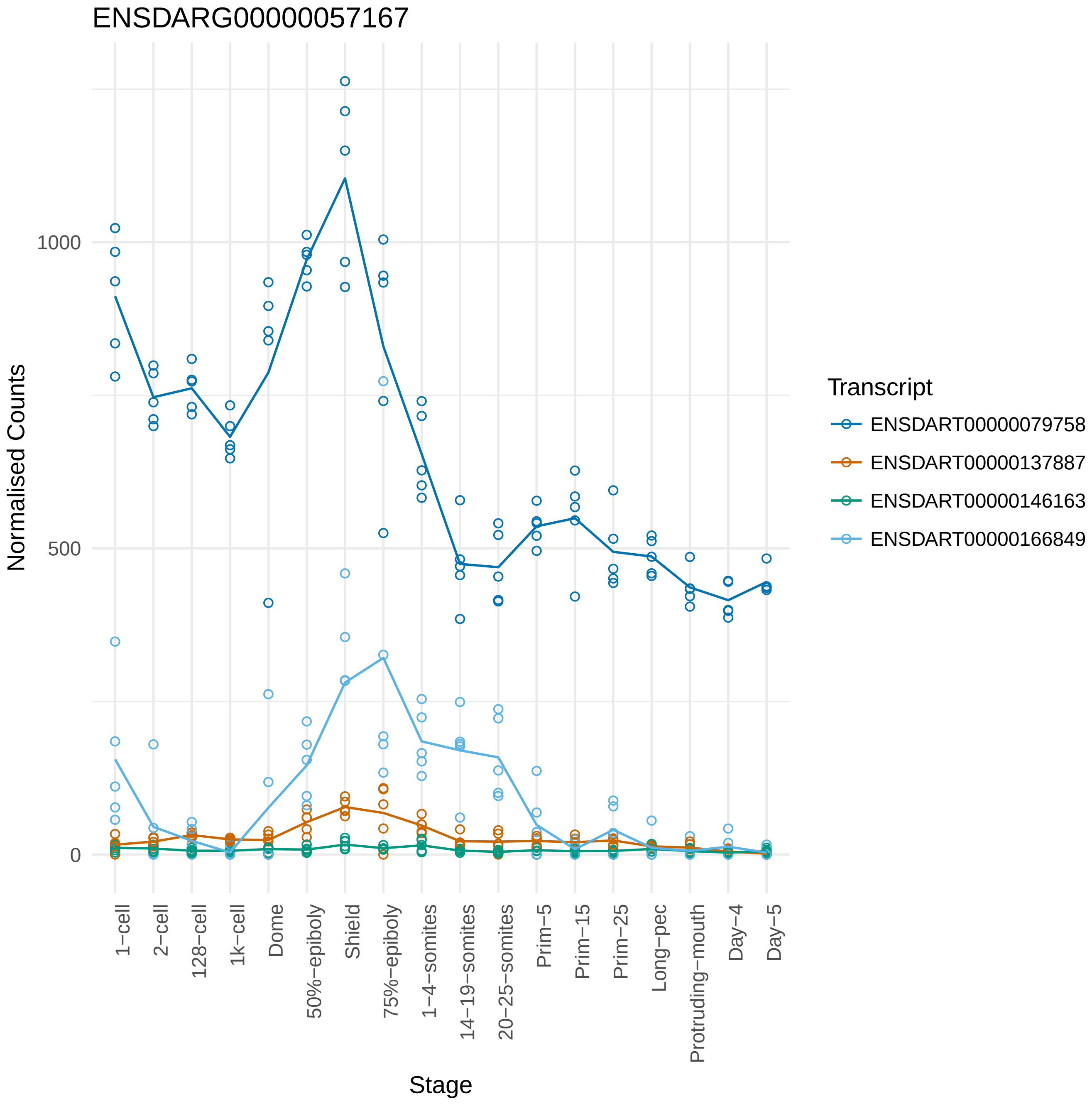

ENSDARG00000057167
Normalised Counts
1000
500
0
1−cell
2−cell
128−cell
1k−cell
Dome
50%−epiboly
Shield
75%−epiboly
1−4−somites
14−19−somites
20−25−somites
Prim−5
Prim−15
Prim−25
Long−pec
Protruding−mouth
Day−4
Day−5
Stage
Transcript
ENSDART00000079758
ENSDART00000137887
ENSDART00000146163
ENSDART00000166849

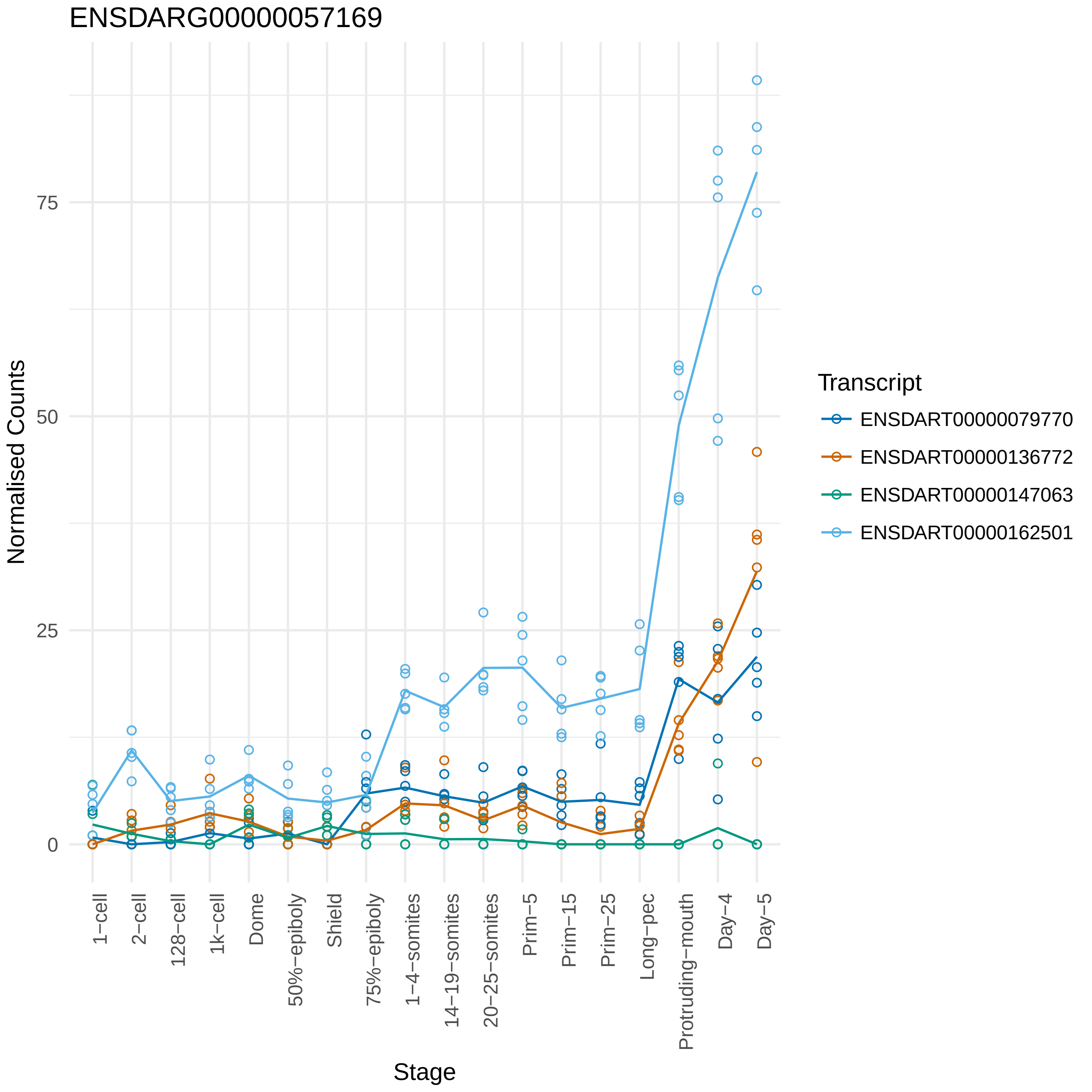

ENSDARG00000057169
Normalised Counts
0
25
50
75
Stage
1−cell
2−cell
128−cell
1k−cell
Dome
50%−epiboly
Shield
75%−epiboly
1−4−somites
14−19−somites
20−25−somites
Prim−5
Prim−15
Prim−25
Long−pec
Protruding−mouth
Day−4
Day−5
Transcript
ENSDART00000079770
ENSDART00000136772
ENSDART00000147063
ENSDART00000162501

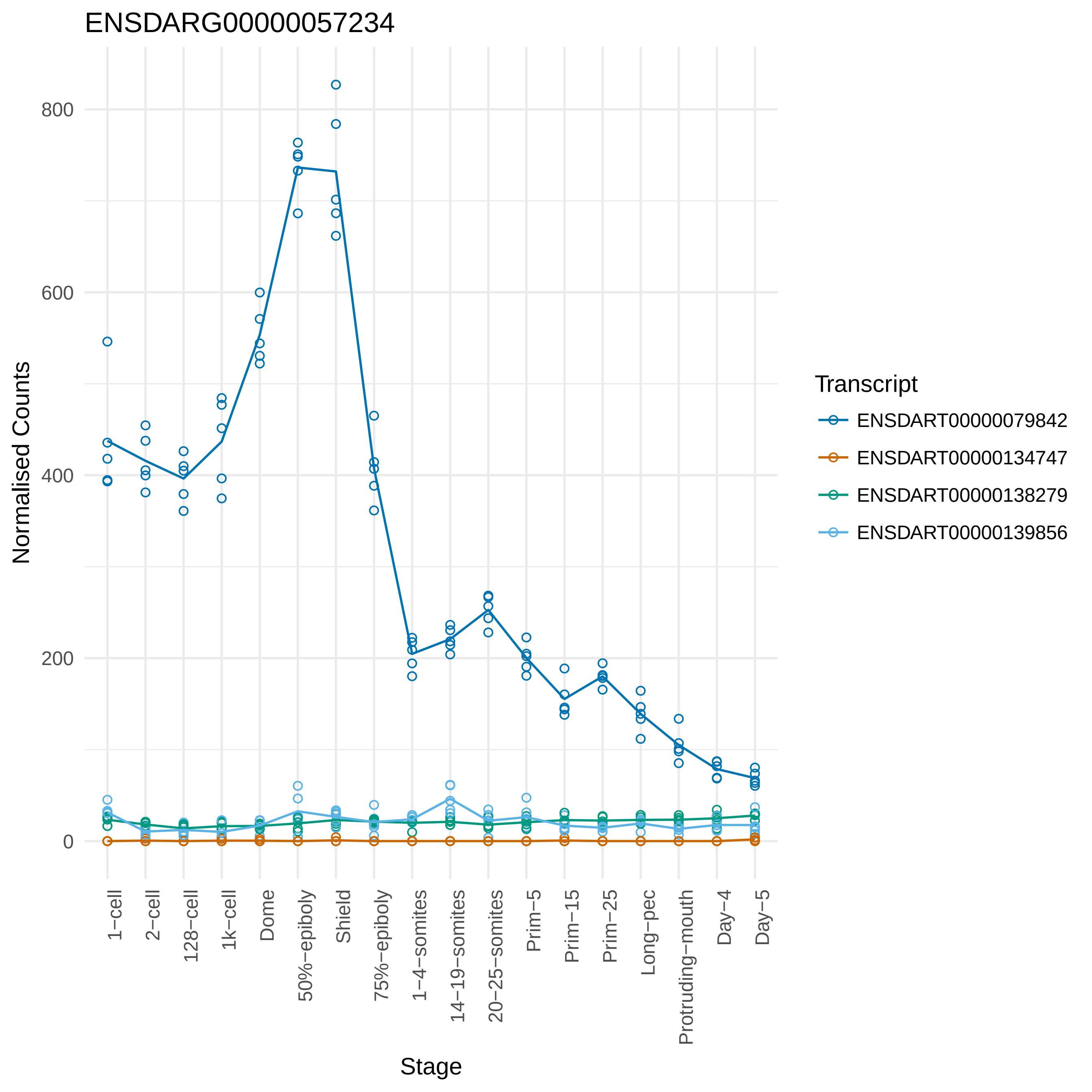

ENSDARG00000057234
Normalised Counts
0
200
400
600
800
Stage
1−cell
2−cell
128−cell
1k−cell
Dome
50%−epiboly
Shield
75%−epiboly
1−4−somites
14−19−somites
20−25−somites
Prim−5
Prim−15
Prim−25
Long−pec
Protruding−mouth
Day−4
Day−5
Transcript
ENSDART00000079842
ENSDART00000134747
ENSDART00000138279
ENSDART00000139856

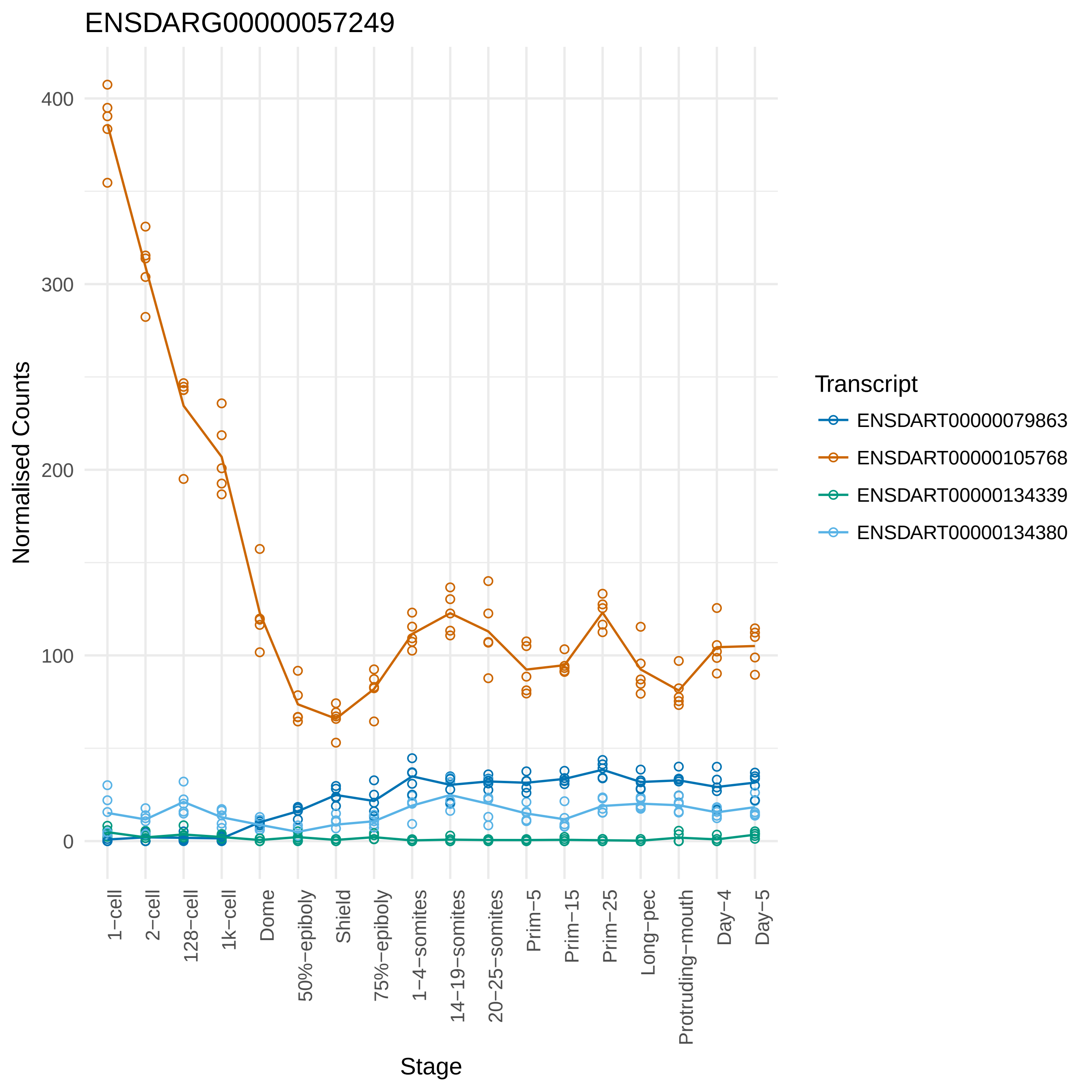

ENSDARG00000057249
Normalised Counts
400
300
200
100
0
1−cell
2−cell
128−cell
1k−cell
Dome
50%−epiboly
Shield
75%−epiboly
1−4−somites
14−19−somites
20−25−somites
Prim−5
Prim−15
Prim−25
Long−pec
Protruding−mouth
Day−4
Day−5
Stage
Transcript
ENSDART00000079863
ENSDART00000105768
ENSDART00000134339
ENSDART00000134380

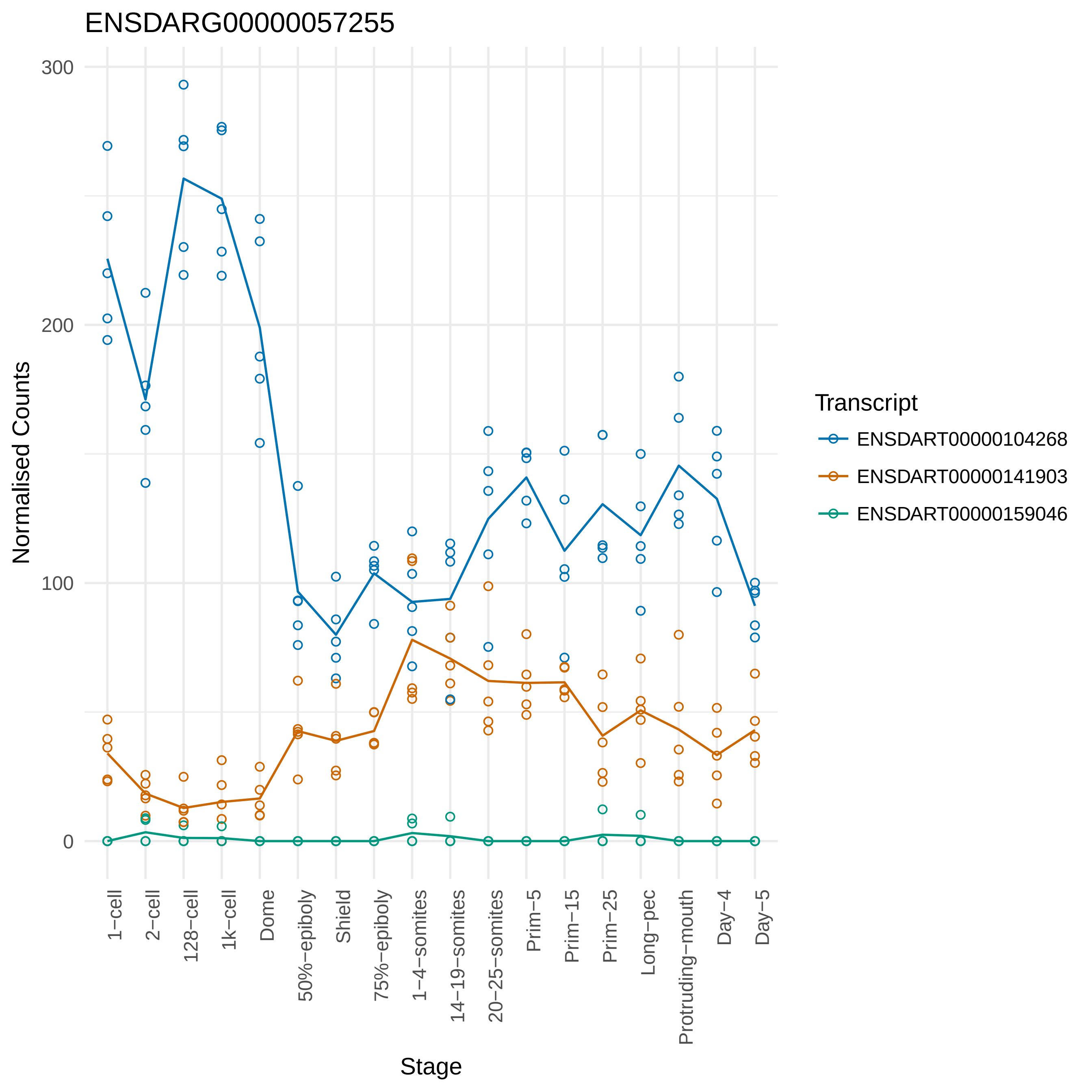
ENSDARG00000057255
Normalised Counts
300
200
100
0
1−cell
2−cell
128−cell
1k−cell
Dome
50%−epiboly
Shield
75%−epiboly
1−4−somites
14−19−somites
20−25−somites
Prim−5
Prim−15
Prim−25
Long−pec
Protruding−mouth
Day−4
Day−5
Stage
Transcript
ENSDART00000104268
ENSDART00000141903
ENSDART00000159046

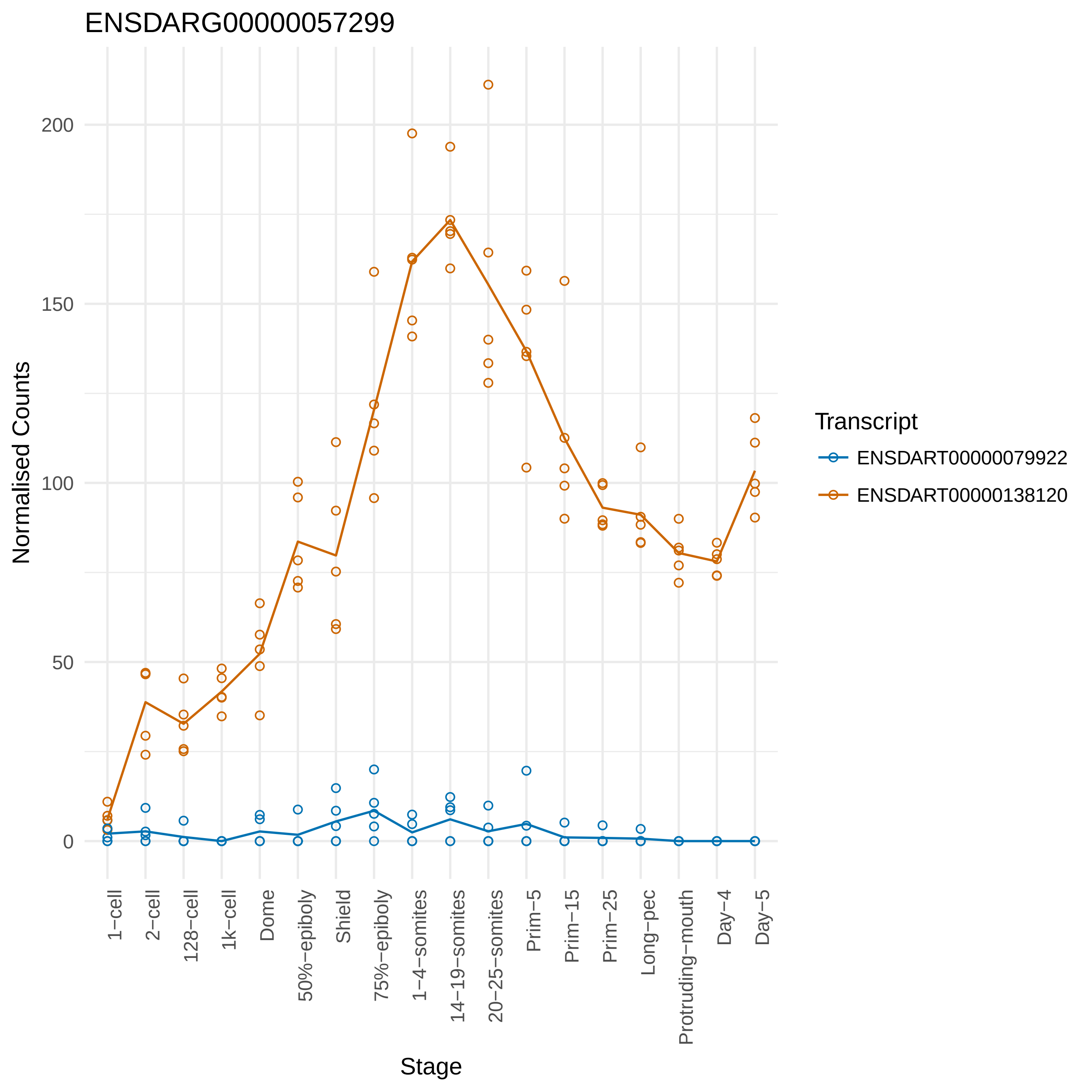

ENSDARG00000057299
Normalised Counts
200
150
100
50
0
Stage
1−cell
2−cell
128−cell
1k−cell
Dome
50%−epiboly
Shield
75%−epiboly
1−4−somites
14−19−somites
20−25−somites
Prim−5
Prim−15
Prim−25
Long−pec
Protruding−mouth
Day−4
Day−5
Transcript
ENSDART00000079922
ENSDART00000138120

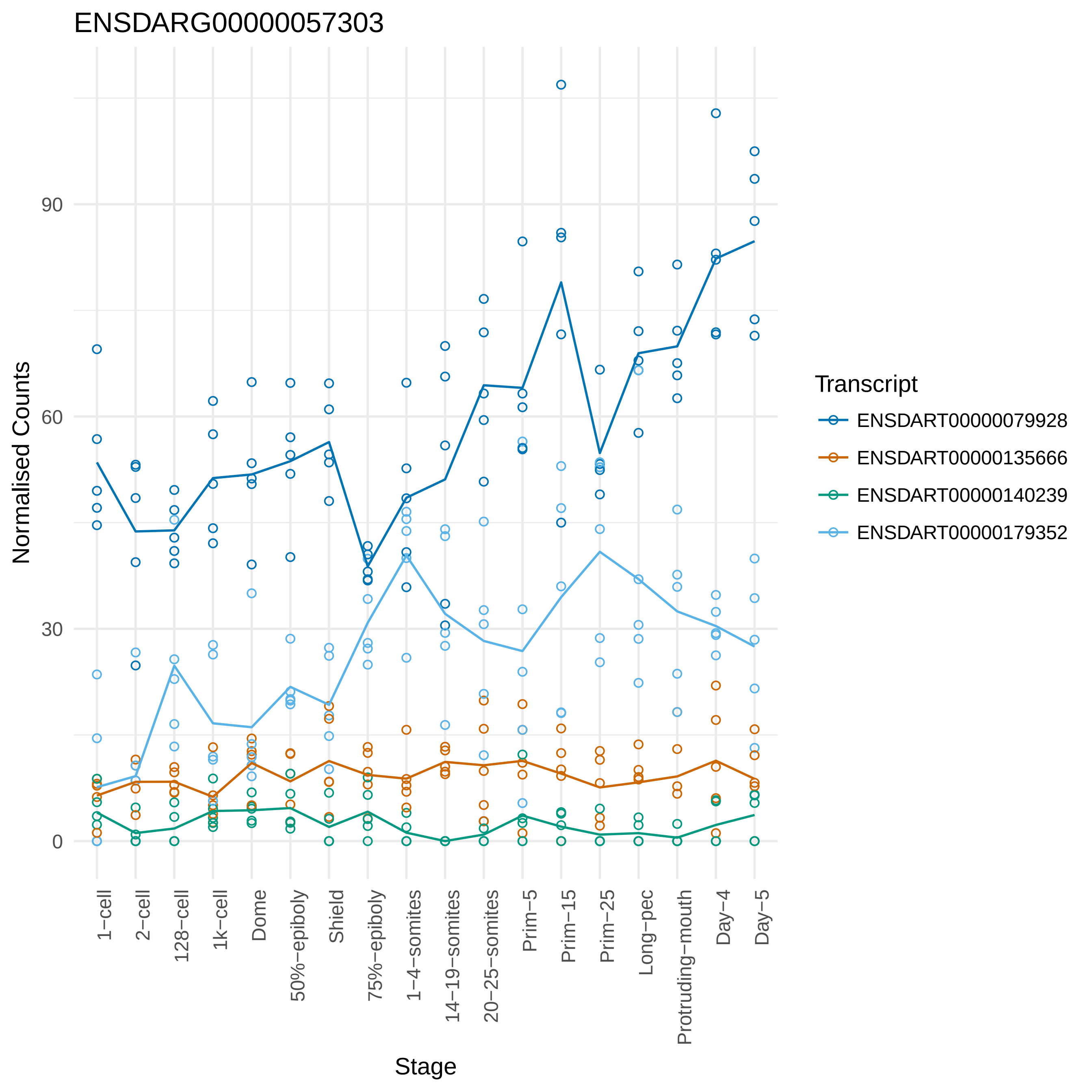

ENSDARG00000057303
Normalised Counts
0
30
60
90
Stage
1−cell
2−cell
128−cell
1k−cell
Dome
50%−epiboly
Shield
75%−epiboly
1−4−somites
14−19−somites
20−25−somites
Prim−5
Prim−15
Prim−25
Long−pec
Protruding−mouth
Day−4
Day−5
Transcript
ENSDART00000079928
ENSDART00000135666
ENSDART00000140239
ENSDART00000179352

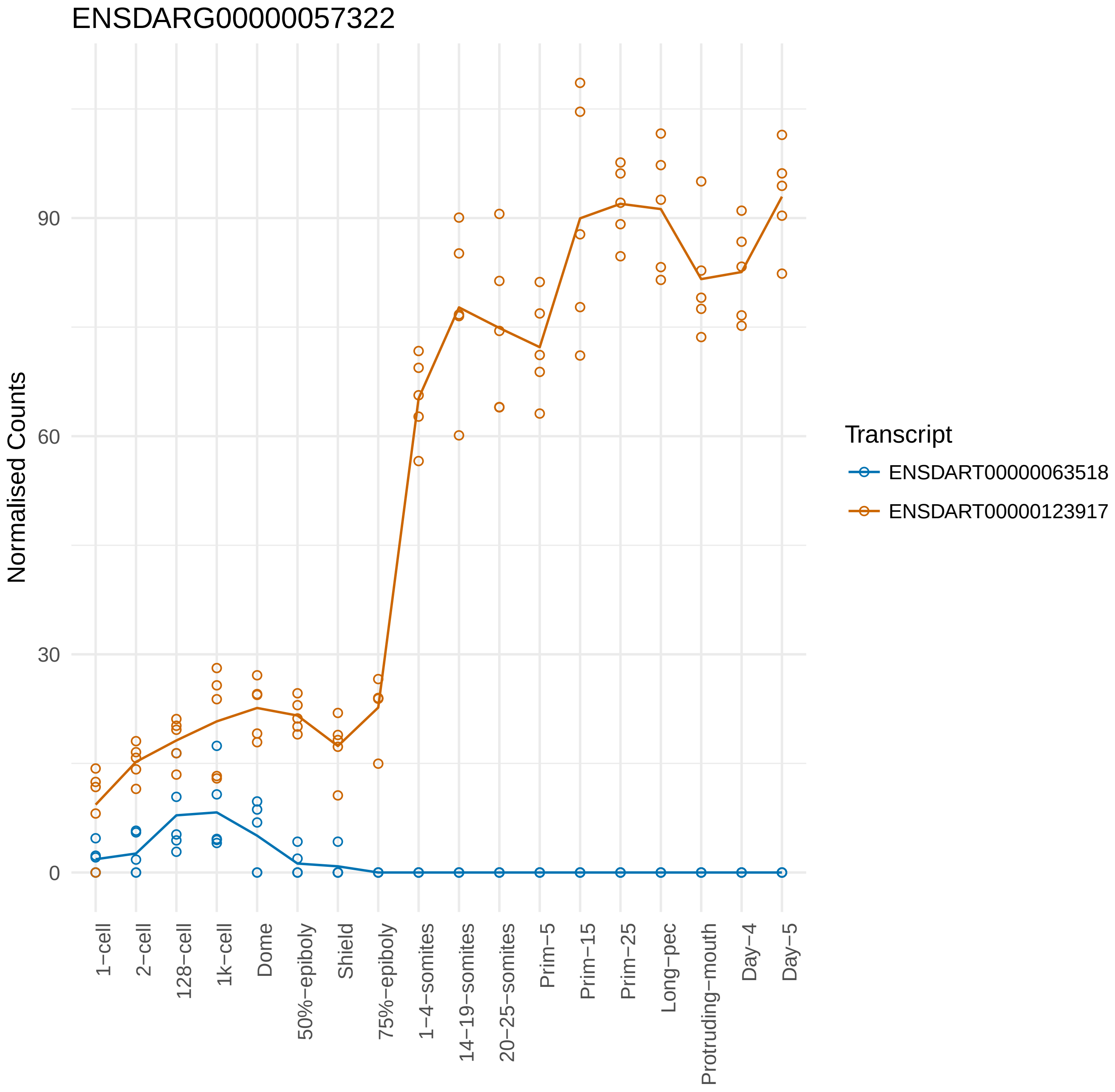
ENSDARG00000057322
Normalised Counts
0
30
60
90
Stage
1−cell
2−cell
128−cell
1k−cell
Dome
50%−epiboly
Shield
75%−epiboly
1−4−somites
14−19−somites
20−25−somites
Prim−5
Prim−15
Prim−25
Long−pec
Protruding−mouth
Day−4
Day−5
Transcript
ENSDART00000063518
ENSDART00000123917

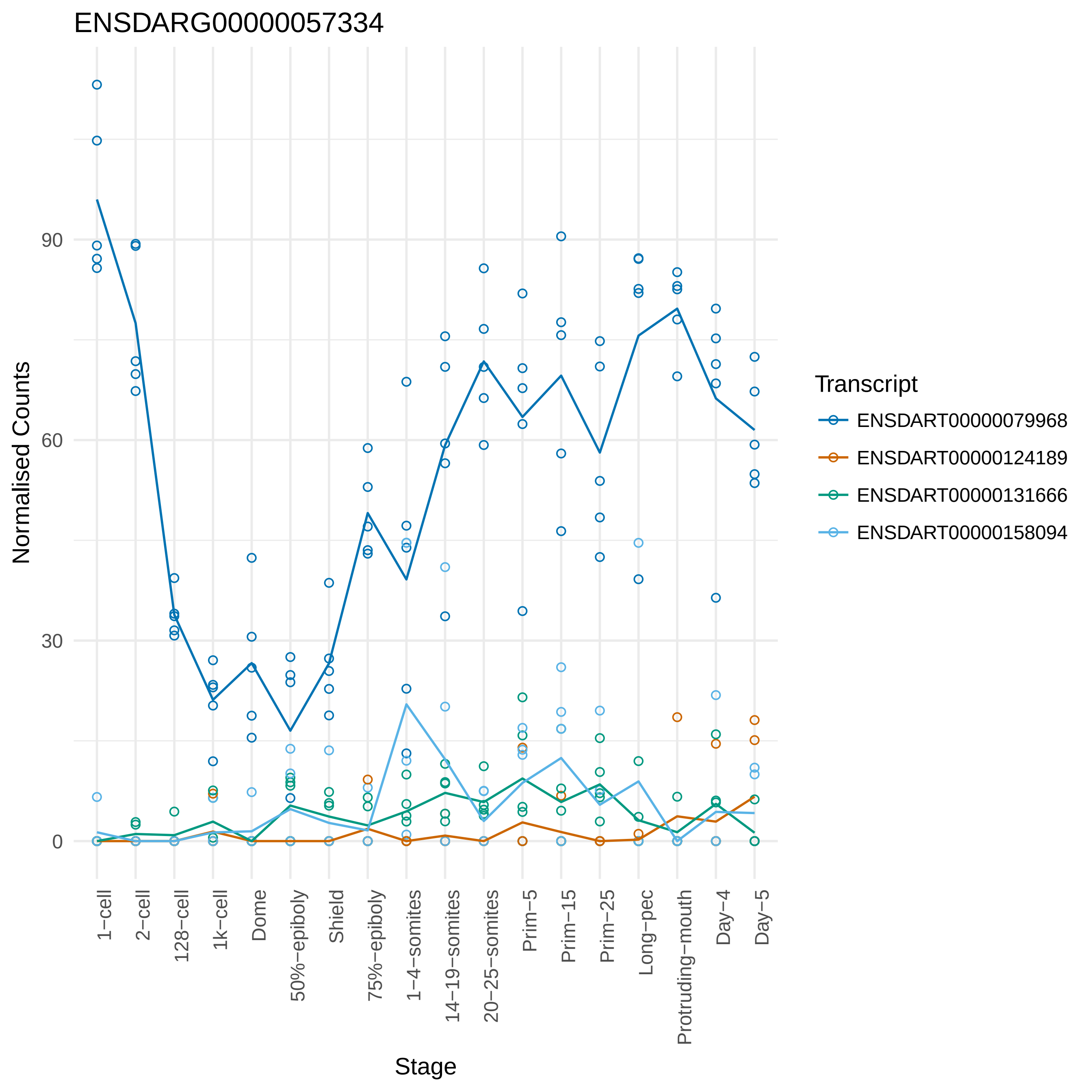
ENSDARG00000057334
Normalised Counts
0
30
60
90
Stage
1−cell
2−cell
128−cell
1k−cell
Dome
50%−epiboly
Shield
75%−epiboly
1−4−somites
14−19−somites
20−25−somites
Prim−5
Prim−15
Prim−25
Long−pec
Protruding−mouth
Day−4
Day−5
Transcript
ENSDART00000079968
ENSDART00000124189
ENSDART00000131666
ENSDART00000158094

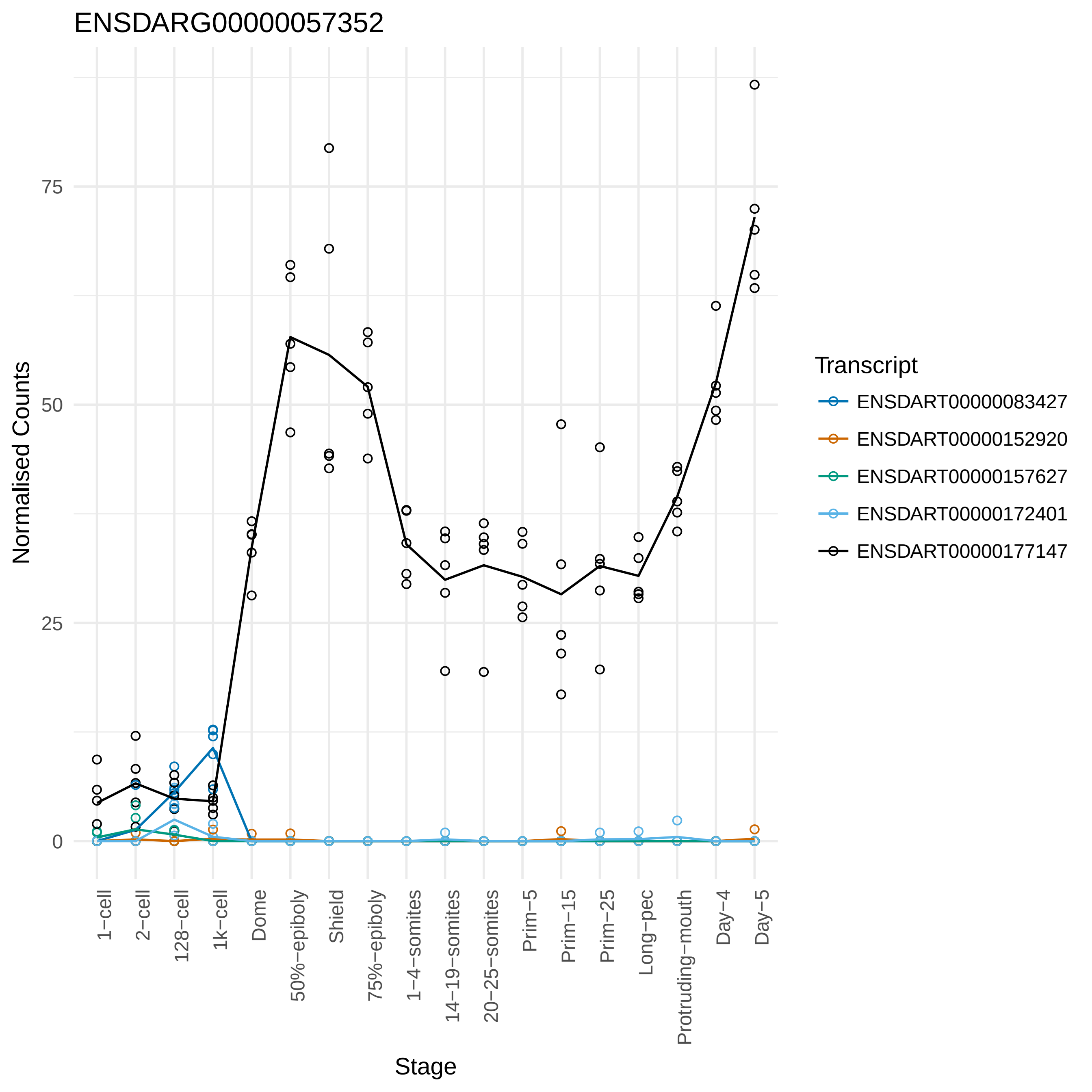
ENSDARG00000057352
Normalised Counts
0
25
50
75
Stage
1−cell
2−cell
128−cell
1k−cell
Dome
50%−epiboly
Shield
75%−epiboly
1−4−somites
14−19−somites
20−25−somites
Prim−5
Prim−15
Prim−25
Long−pec
Protruding−mouth
Day−4
Day−5
Transcript
ENSDART00000083427
ENSDART00000152920
ENSDART00000157627
ENSDART00000172401
ENSDART00000177147

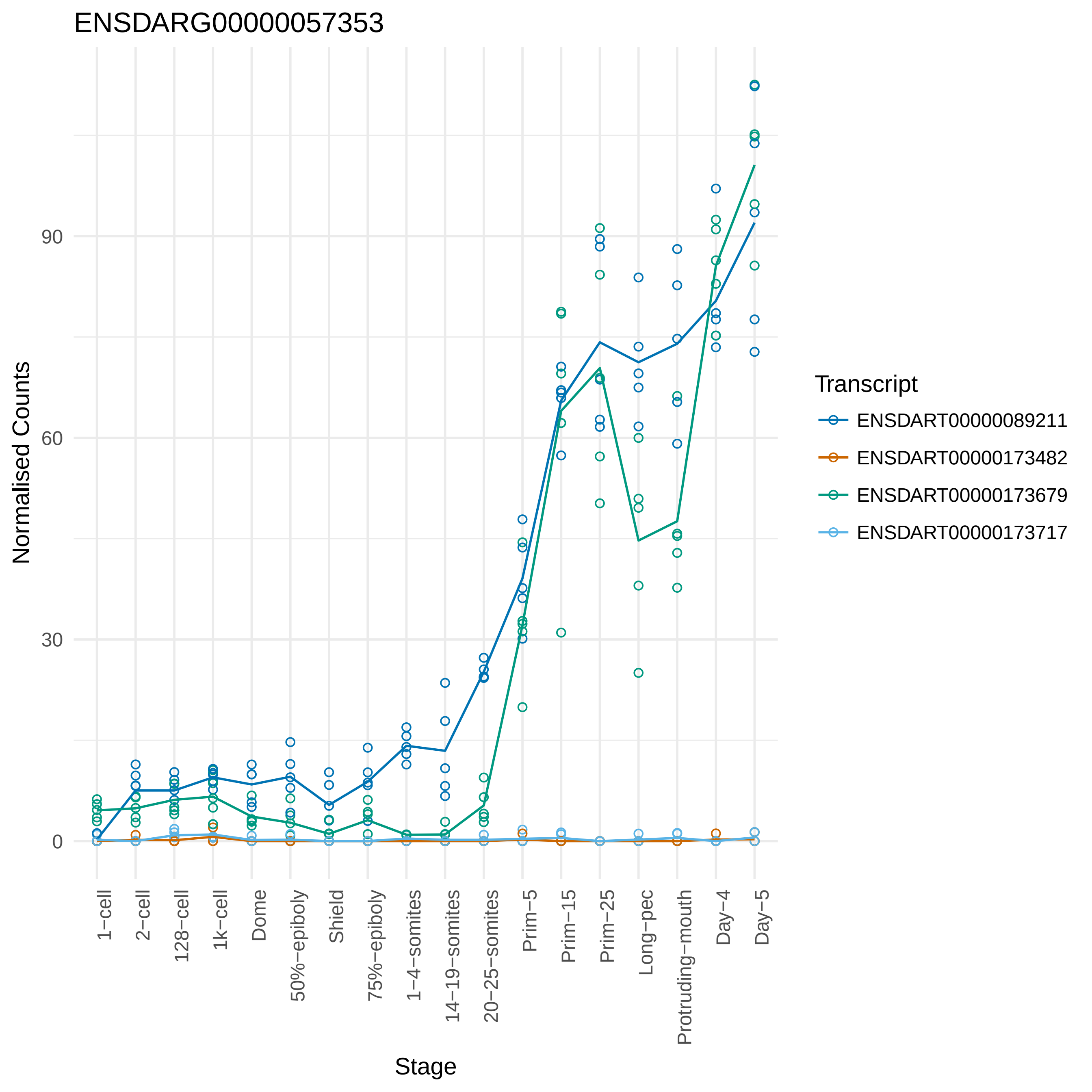
ENSDARG00000057353
Normalised Counts
0
30
60
90
Stage
1−cell
2−cell
128−cell
1k−cell
Dome
50%−epiboly
Shield
75%−epiboly
1−4−somites
14−19−somites
20−25−somites
Prim−5
Prim−15
Prim−25
Long−pec
Protruding−mouth
Day−4
Day−5
Transcript
ENSDART00000089211
ENSDART00000173482
ENSDART00000173679
ENSDART00000173717

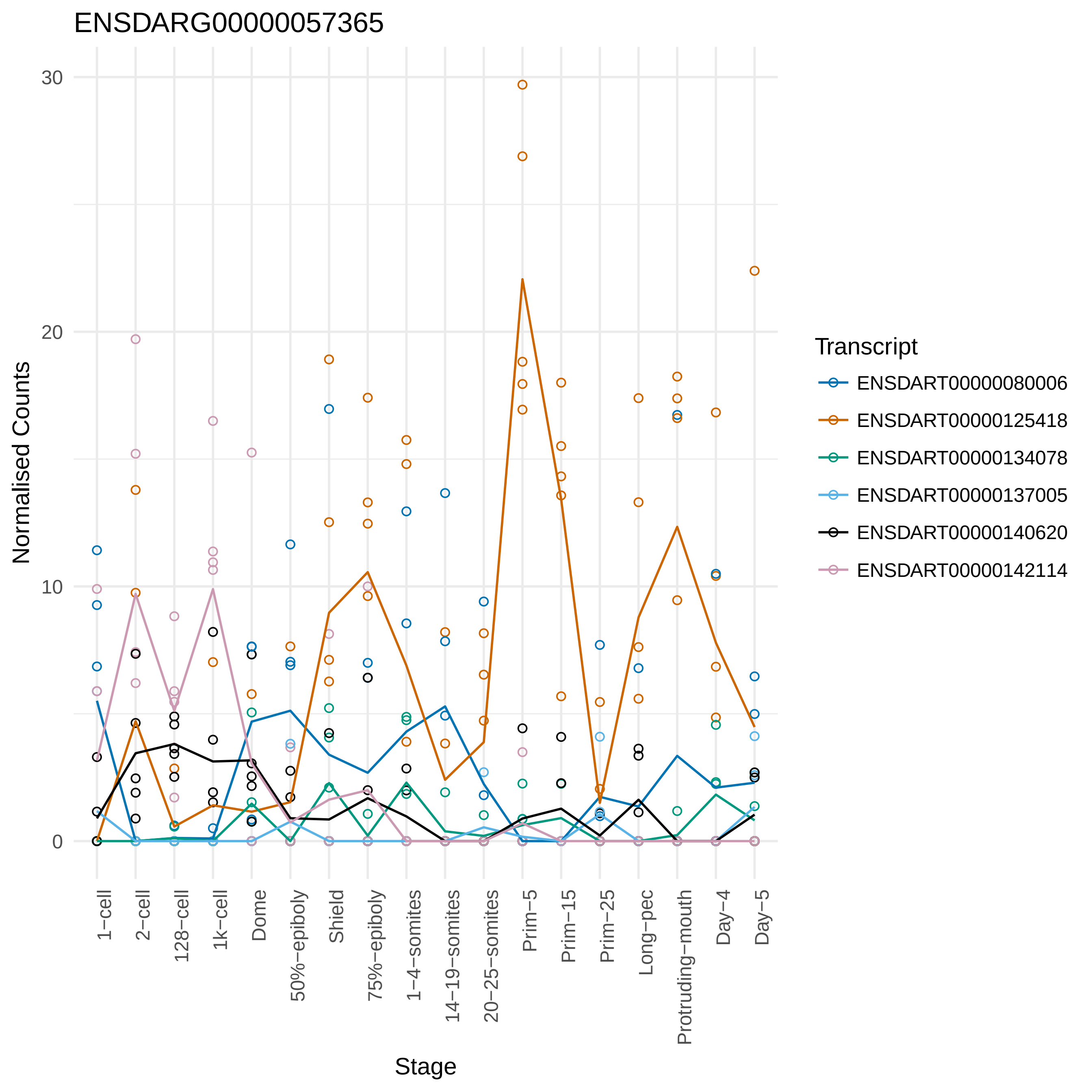

ENSDARG00000057365
Normalised Counts
30
20
10
0
1−cell
2−cell
128−cell
1k−cell
Dome
50%−epiboly
Shield
75%−epiboly
1−4−somites
14−19−somites
20−25−somites
Prim−5
Prim−15
Prim−25
Long−pec
Protruding−mouth
Day−4
Day−5
Stage
Transcript
ENSDART00000080006
ENSDART00000125418
ENSDART00000134078
ENSDART00000137005
ENSDART00000140620
ENSDART00000142114

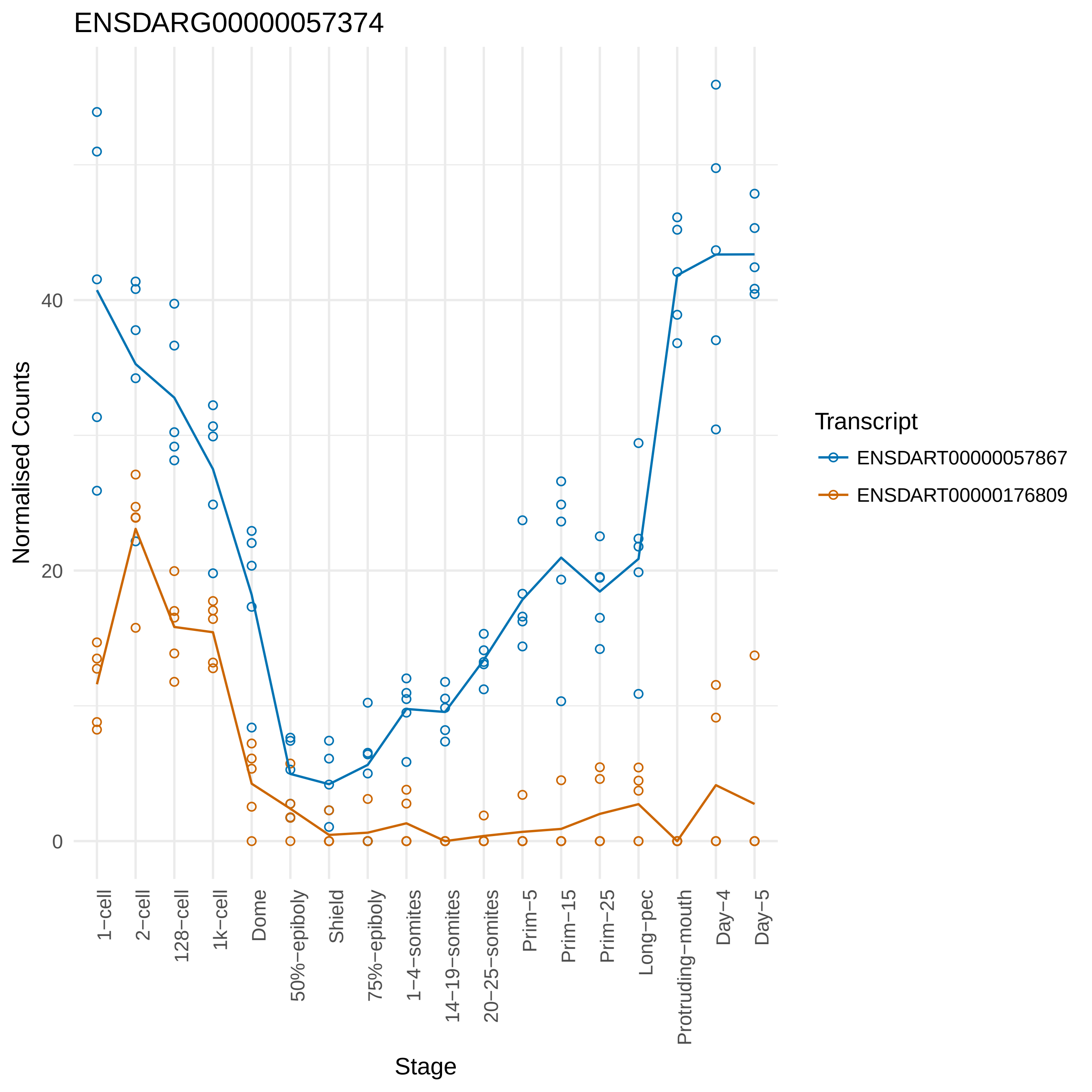

ENSDARG00000057374
Normalised Counts
0
20
40
Stage
1−cell
2−cell
128−cell
1k−cell
Dome
50%−epiboly
Shield
75%−epiboly
1−4−somites
14−19−somites
20−25−somites
Prim−5
Prim−15
Prim−25
Long−pec
Protruding−mouth
Day−4
Day−5
Transcript
ENSDART00000057867
ENSDART00000176809

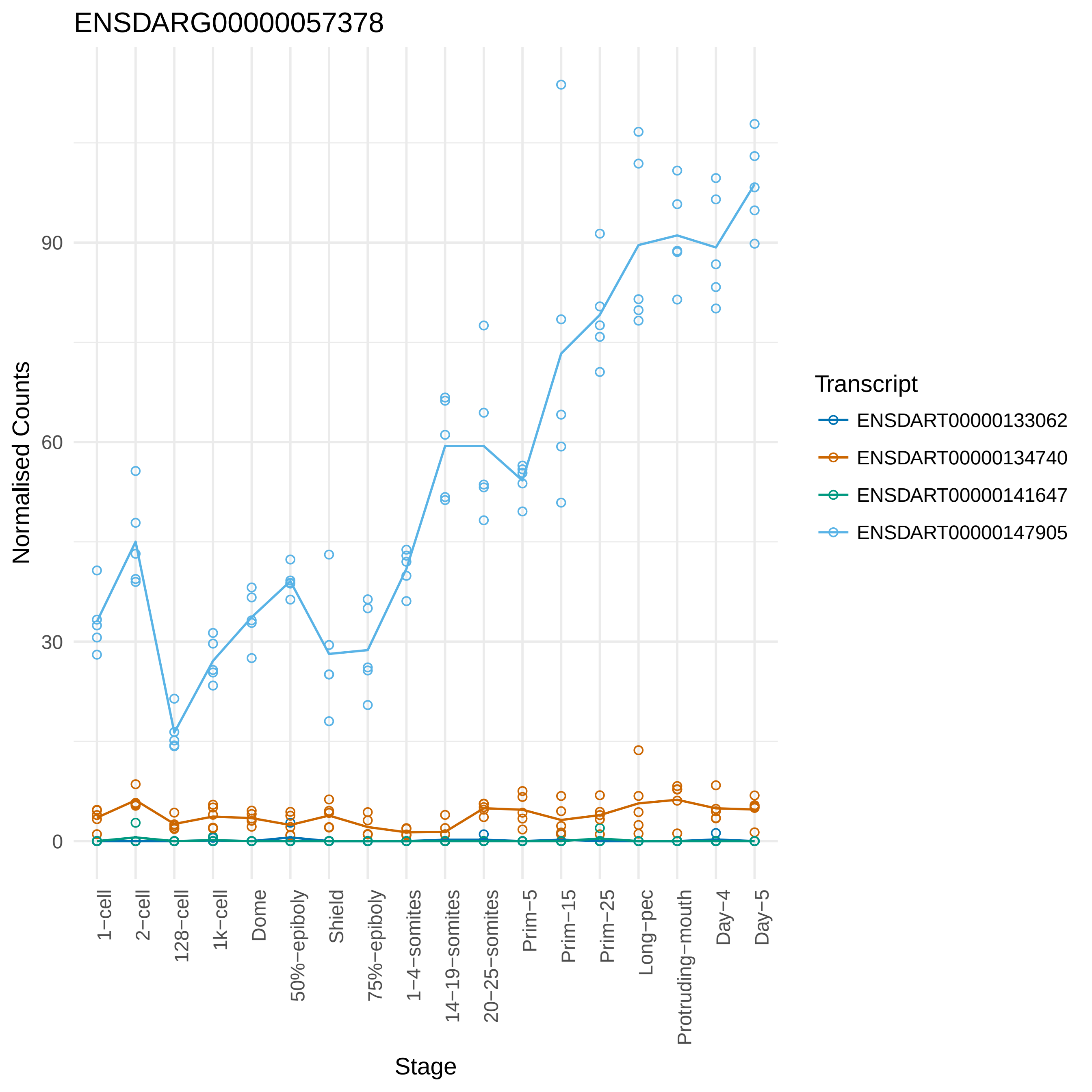
ENSDARG00000057378
Normalised Counts
90
60
30
0
1−cell
2−cell
128−cell
1k−cell
Dome
50%−epiboly
Shield
75%−epiboly
1−4−somites
14−19−somites
20−25−somites
Prim−5
Prim−15
Prim−25
Long−pec
Protruding−mouth
Day−4
Day−5
Stage
Transcript
ENSDART00000133062
ENSDART00000134740
ENSDART00000141647
ENSDART00000147905

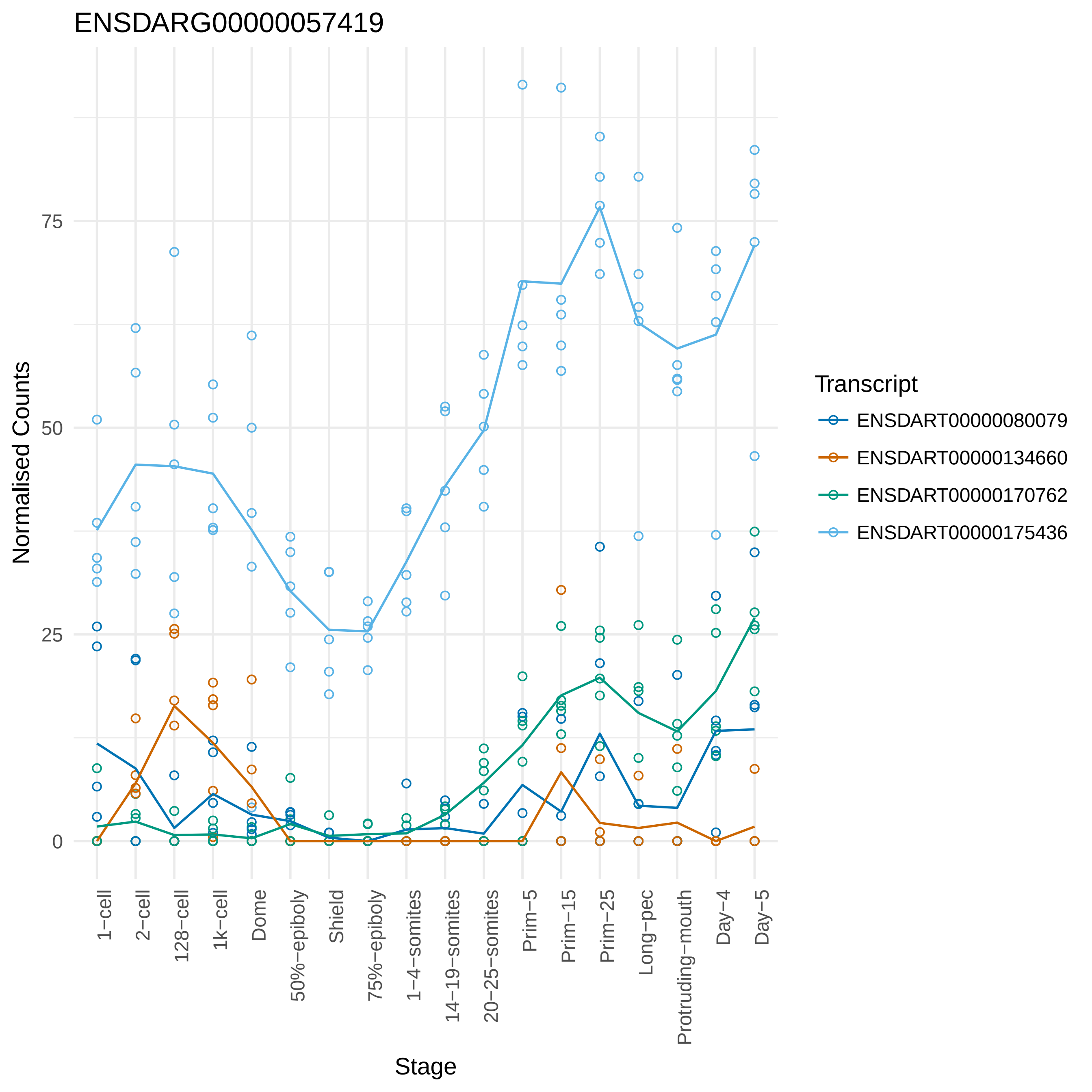

ENSDARG00000057419
Normalised Counts
75
50
25
0
1−cell
2−cell
128−cell
1k−cell
Dome
50%−epiboly
Shield
75%−epiboly
1−4−somites
14−19−somites
20−25−somites
Prim−5
Prim−15
Prim−25
Long−pec
Protruding−mouth
Day−4
Day−5
Stage
Transcript
ENSDART00000080079
ENSDART00000134660
ENSDART00000170762
ENSDART00000175436

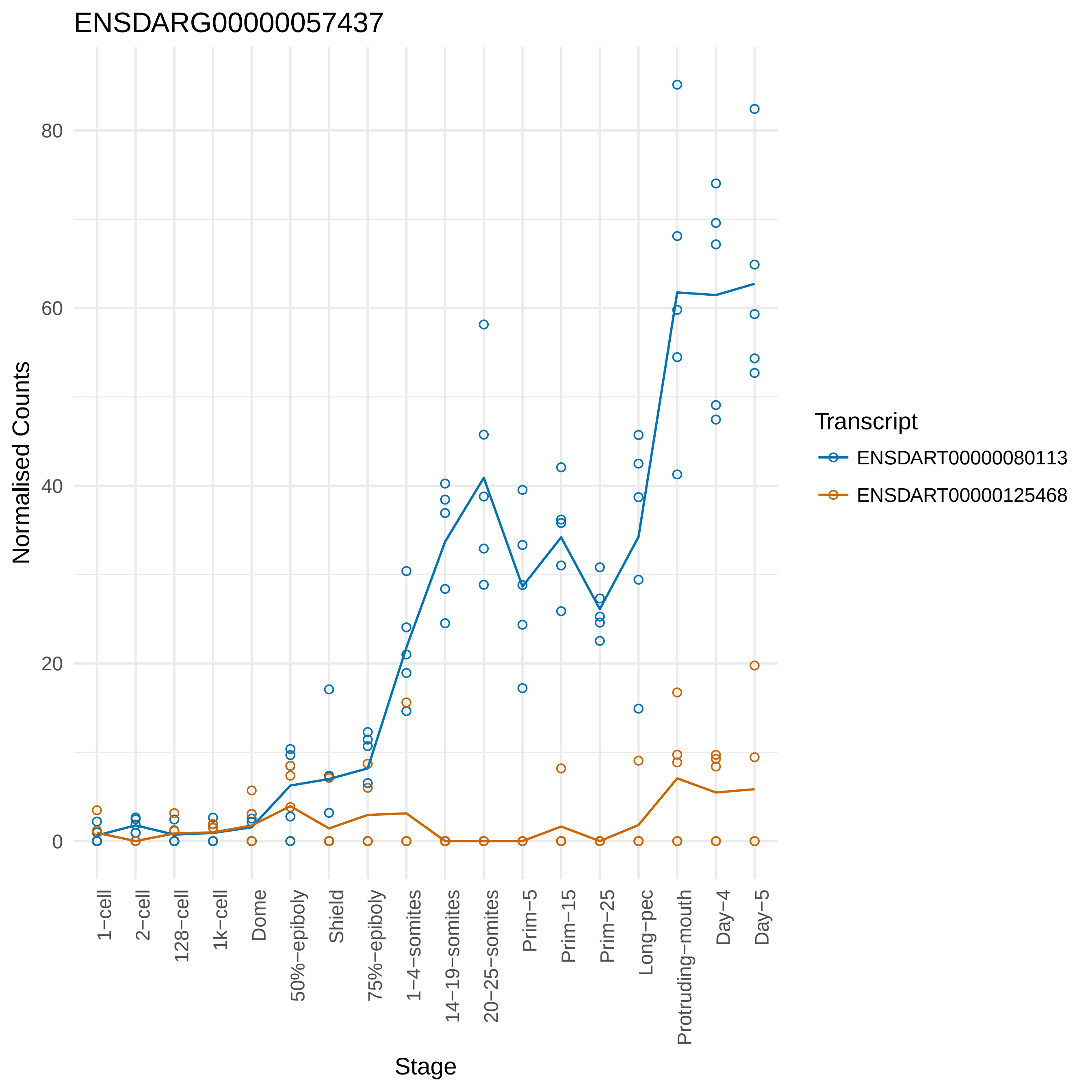
ENSDARG00000057437
Normalised Counts
0
20
40
60
80
Stage
1−cell
2−cell
128−cell
1k−cell
Dome
50%−epiboly
Shield
75%−epiboly
1−4−somites
14−19−somites
20−25−somites
Prim−5
Prim−15
Prim−25
Long−pec
Protruding−mouth
Day−4
Day−5
Transcript
ENSDART00000080113
ENSDART00000125468

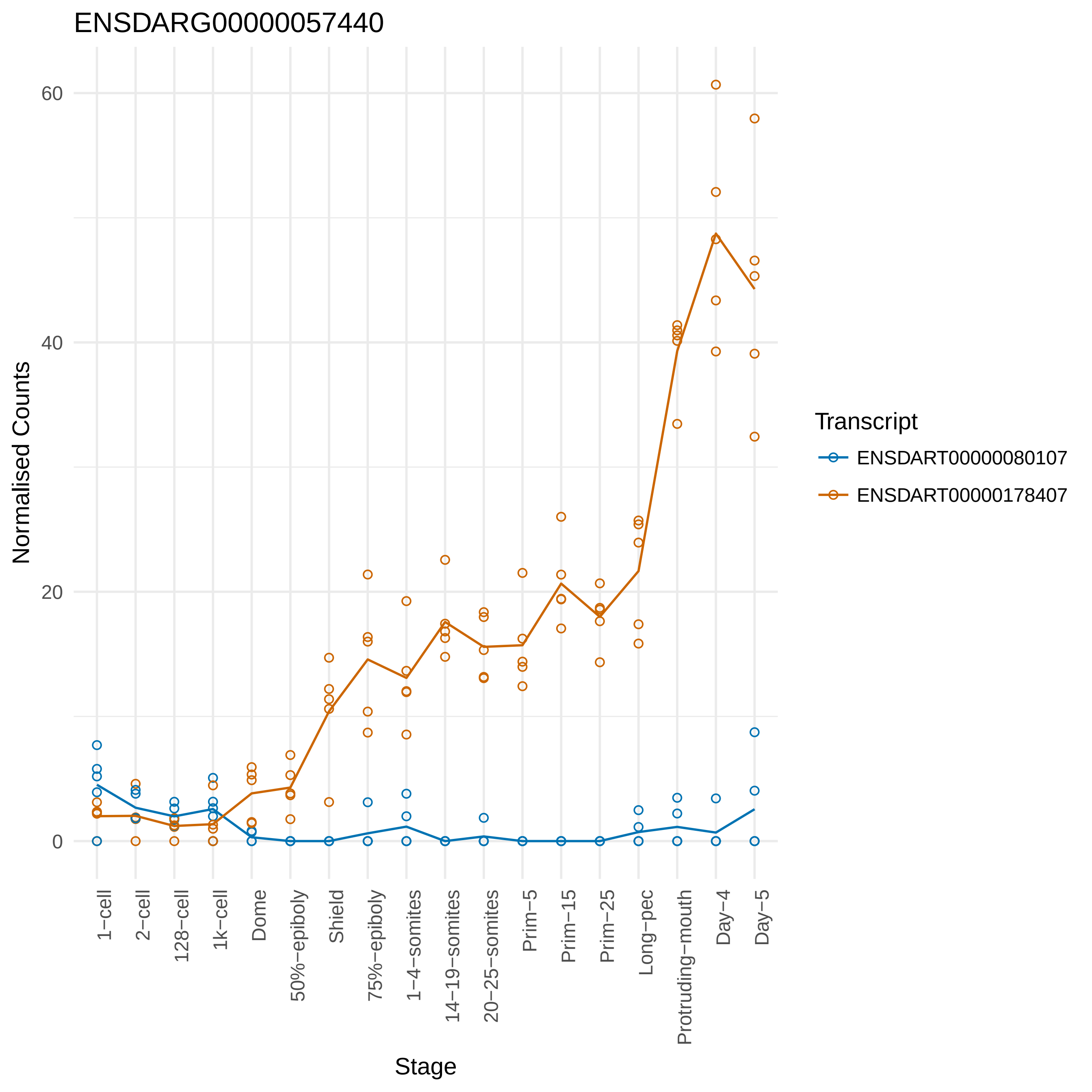

ENSDARG00000057440
Normalised Counts
60
40
20
0
1−cell
2−cell
128−cell
1k−cell
Dome
50%−epiboly
Shield
75%−epiboly
1−4−somites
14−19−somites
20−25−somites
Prim−5
Prim−15
Prim−25
Long−pec
Protruding−mouth
Day−4
Day−5
Stage
Transcript
ENSDART00000080107
ENSDART00000178407

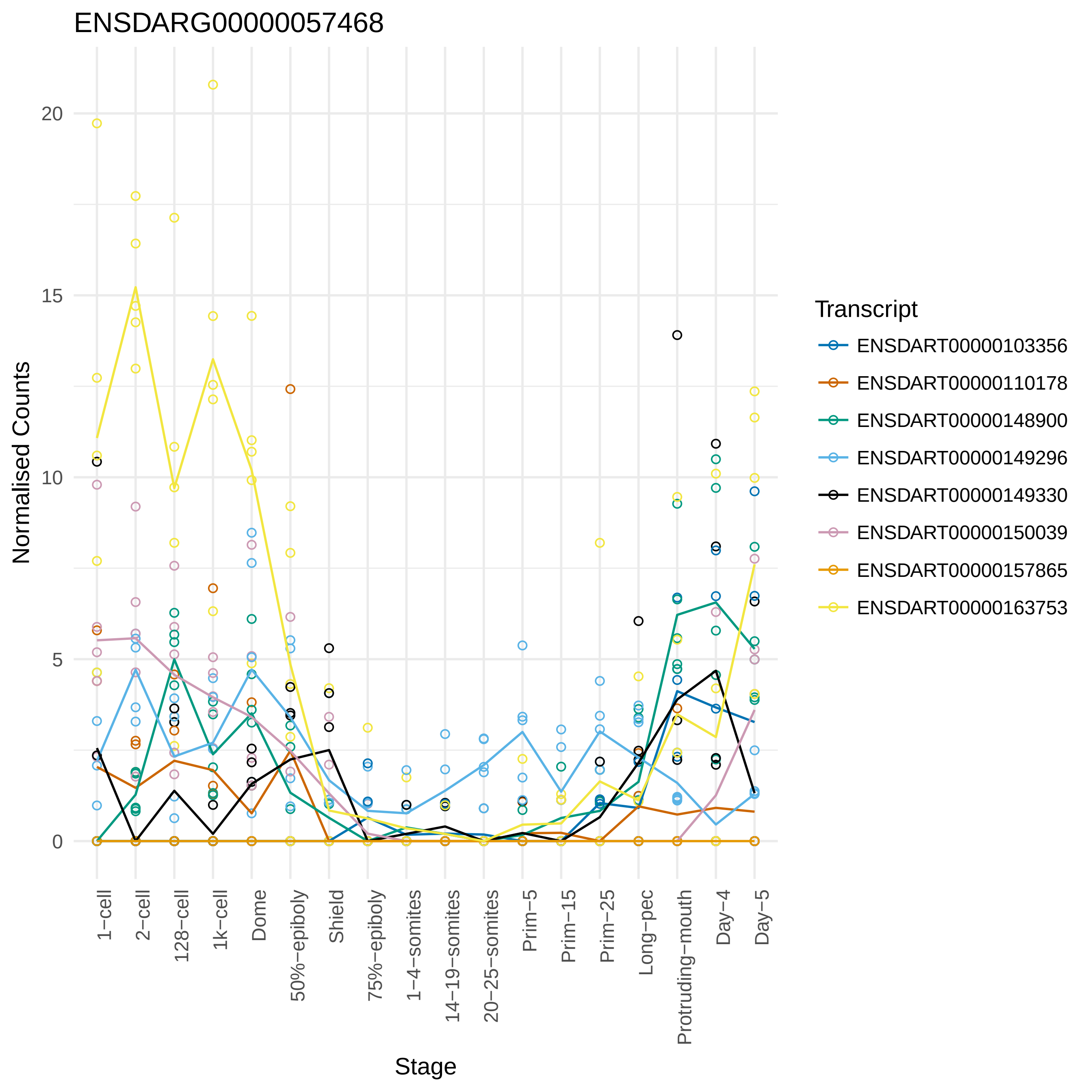

ENSDARG00000057468
Normalised Counts
0
5
10
15
20
Stage
1−cell
2−cell
128−cell
1k−cell
Dome
50%−epiboly
Shield
75%−epiboly
1−4−somites
14−19−somites
20−25−somites
Prim−5
Prim−15
Prim−25
Long−pec
Protruding−mouth
Day−4
Day−5
Transcript
ENSDART00000103356
ENSDART00000110178
ENSDART00000148900
ENSDART00000149296
ENSDART00000149330
ENSDART00000150039
ENSDART00000157865
ENSDART00000163753

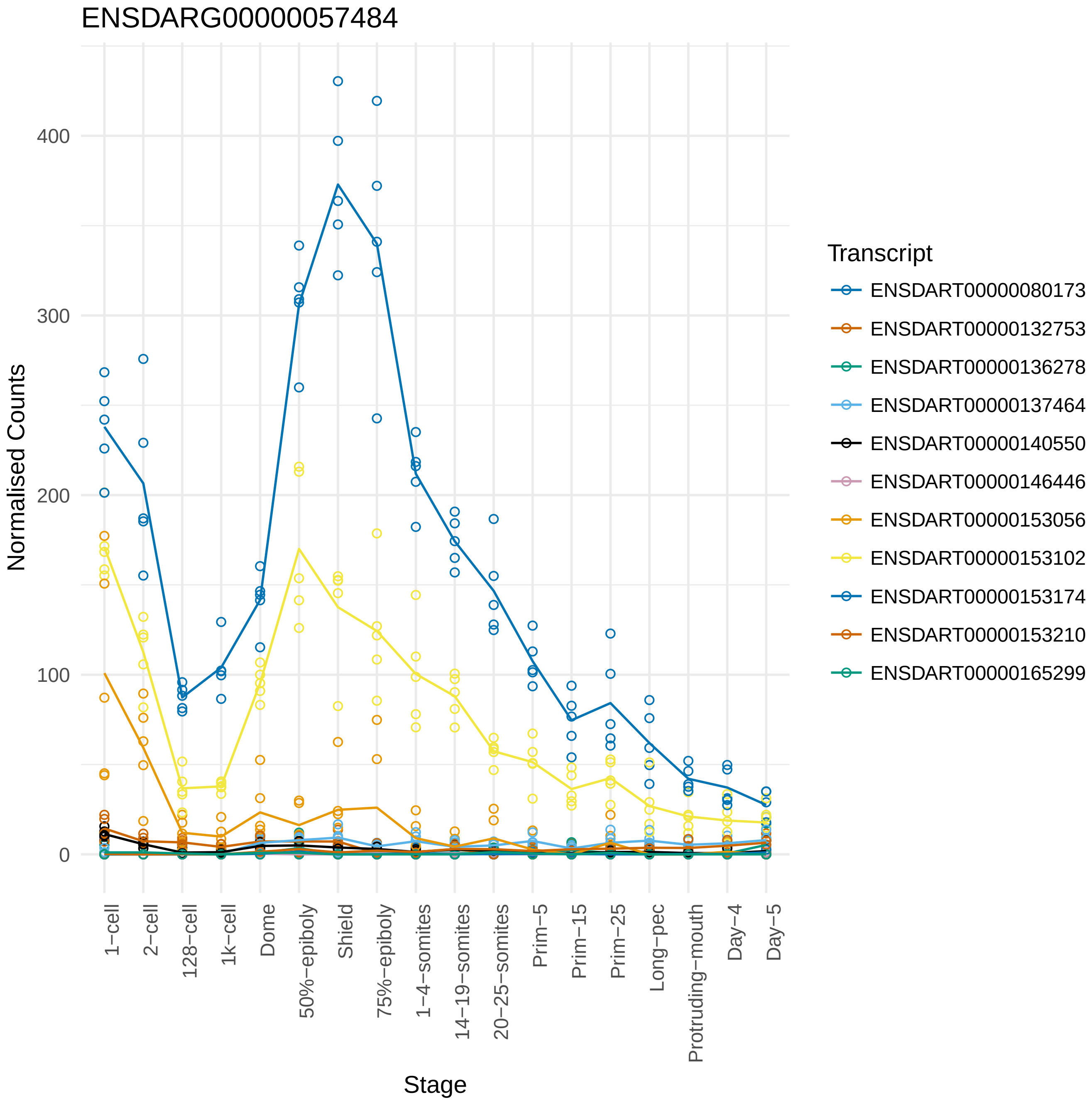

ENSDARG00000057484
Normalised Counts
0
100
200
300
400
1−cell
2−cell
128−cell
1k−cell
Dome
50%−epiboly
Shield
75%−epiboly
1−4−somites
14−19−somites
20−25−somites
Prim−5
Prim−15
Prim−25
Long−pec
Protruding−mouth
Day−4
Day−5
Stage
Transcript
ENSDART00000080173
ENSDART00000132753
ENSDART00000136278
ENSDART00000137464
ENSDART00000140550
ENSDART00000146446
ENSDART00000153056
ENSDART00000153102
ENSDART00000153174
ENSDART00000153210
ENSDART00000165299

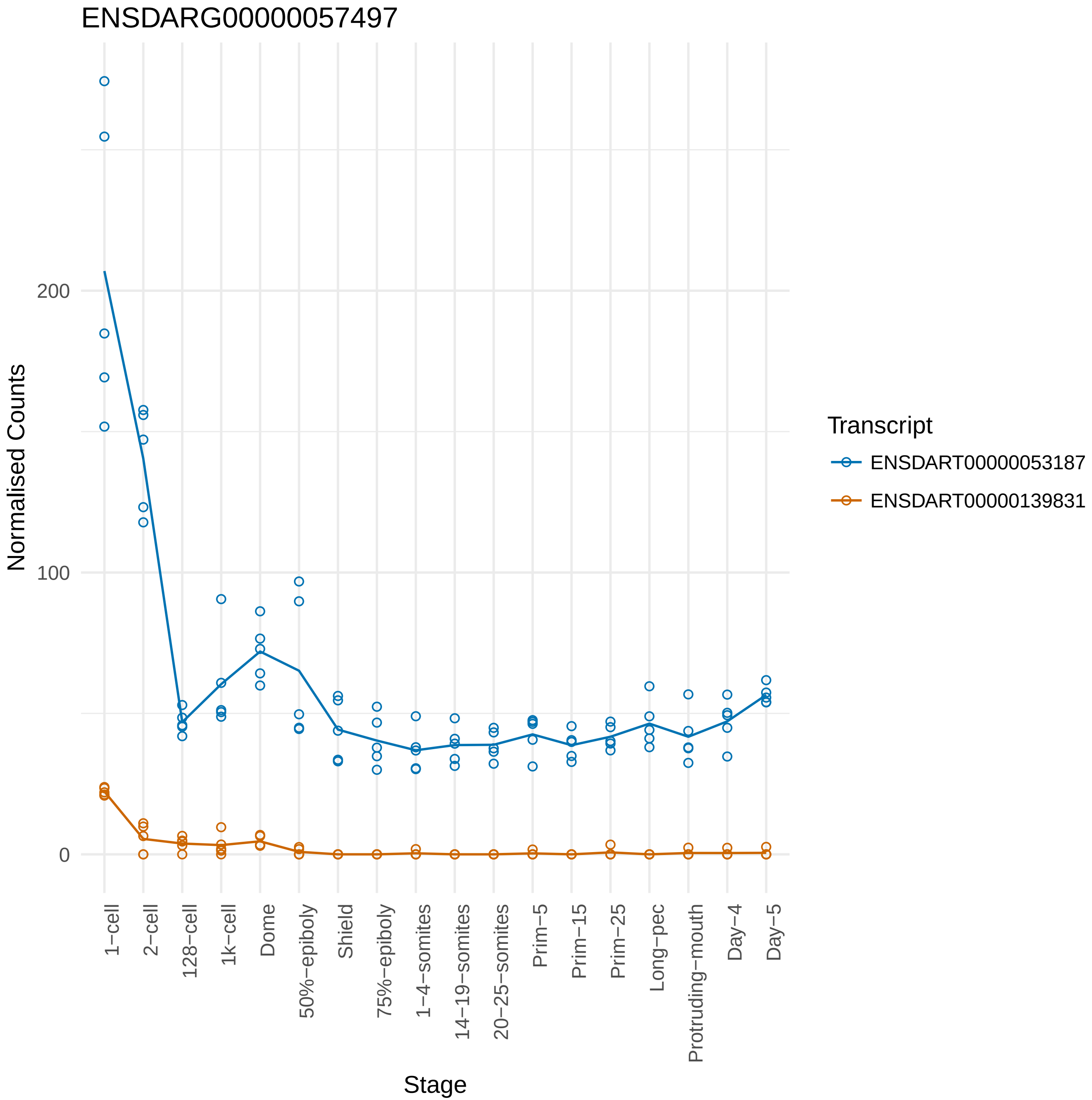

ENSDARG00000057497
Normalised Counts
0
100
200
Stage
1−cell
2−cell
128−cell
1k−cell
Dome
50%−epiboly
Shield
75%−epiboly
1−4−somites
14−19−somites
20−25−somites
Prim−5
Prim−15
Prim−25
Long−pec
Protruding−mouth
Day−4
Day−5
Transcript
ENSDART00000053187
ENSDART00000139831

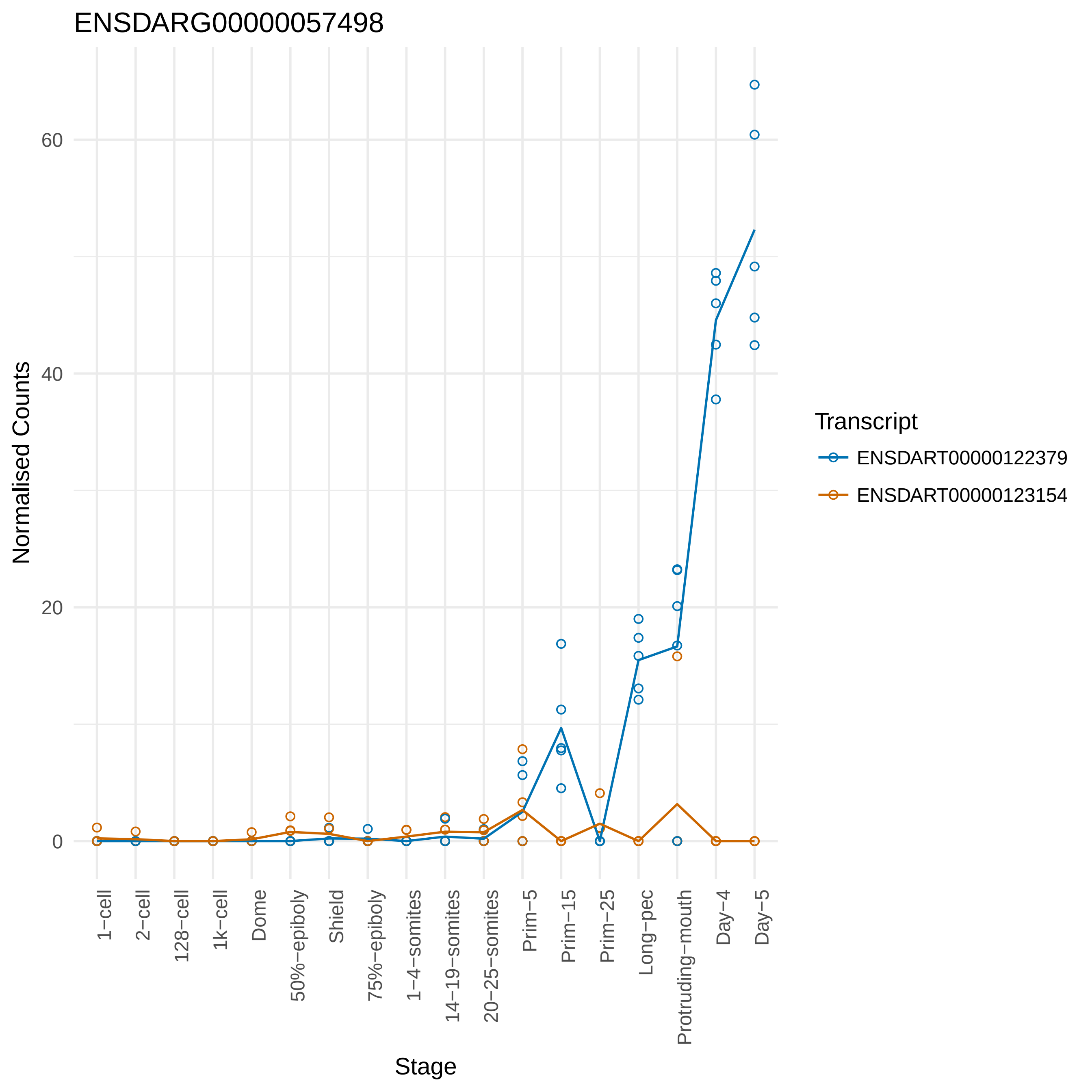

ENSDARG00000057498
Normalised Counts
60
40
20
0
1−cell
2−cell
128−cell
1k−cell
Dome
50%−epiboly
Shield
75%−epiboly
1−4−somites
14−19−somites
20−25−somites
Prim−5
Prim−15
Prim−25
Long−pec
Protruding−mouth
Day−4
Day−5
Stage
Transcript
ENSDART00000122379
ENSDART00000123154

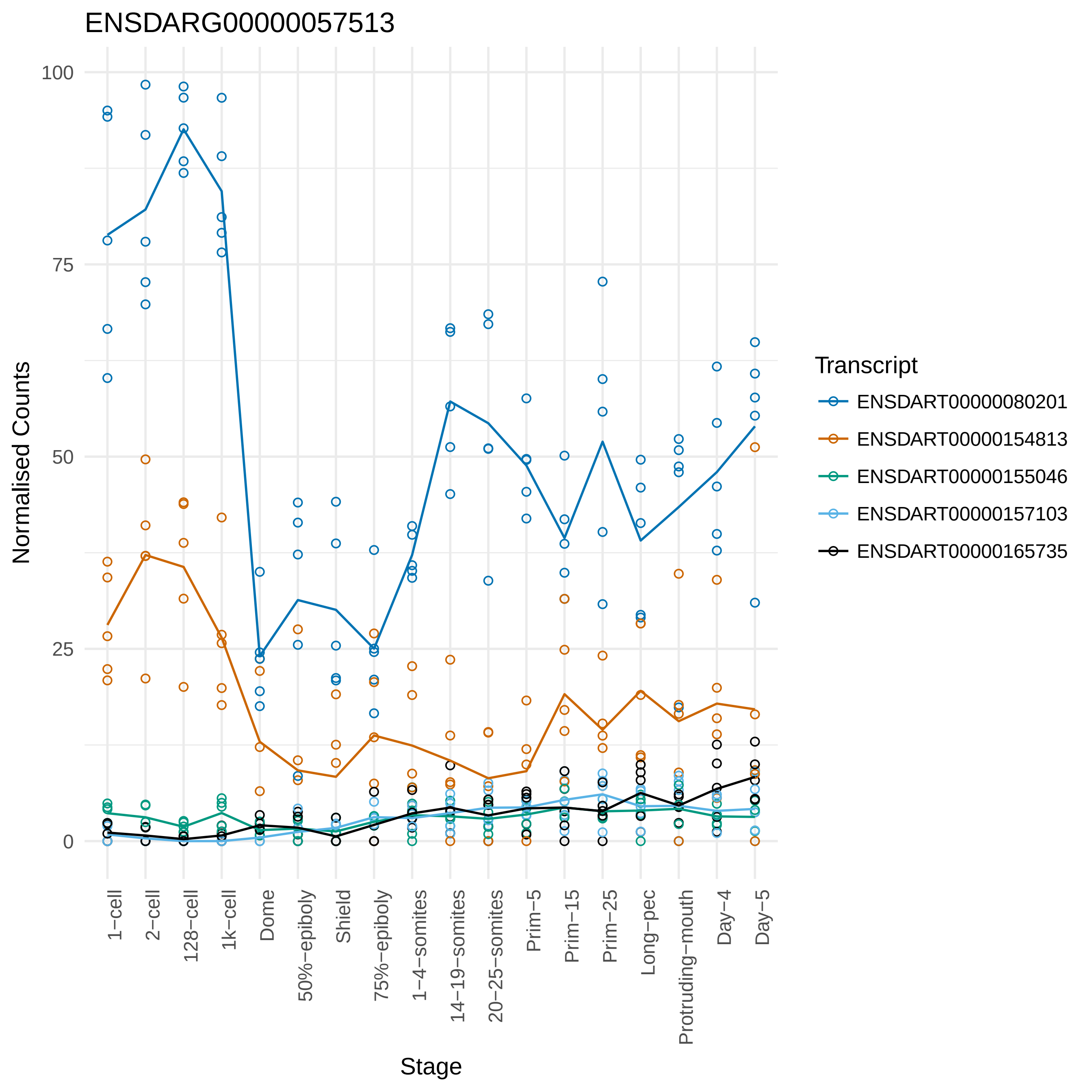

ENSDARG00000057513
Normalised Counts
100
75
50
25
0
1−cell
2−cell
128−cell
1k−cell
Dome
50%−epiboly
Shield
75%−epiboly
1−4−somites
14−19−somites
20−25−somites
Prim−5
Prim−15
Prim−25
Long−pec
Protruding−mouth
Day−4
Day−5
Stage
Transcript
ENSDART00000080201
ENSDART00000154813
ENSDART00000155046
ENSDART00000157103
ENSDART00000165735

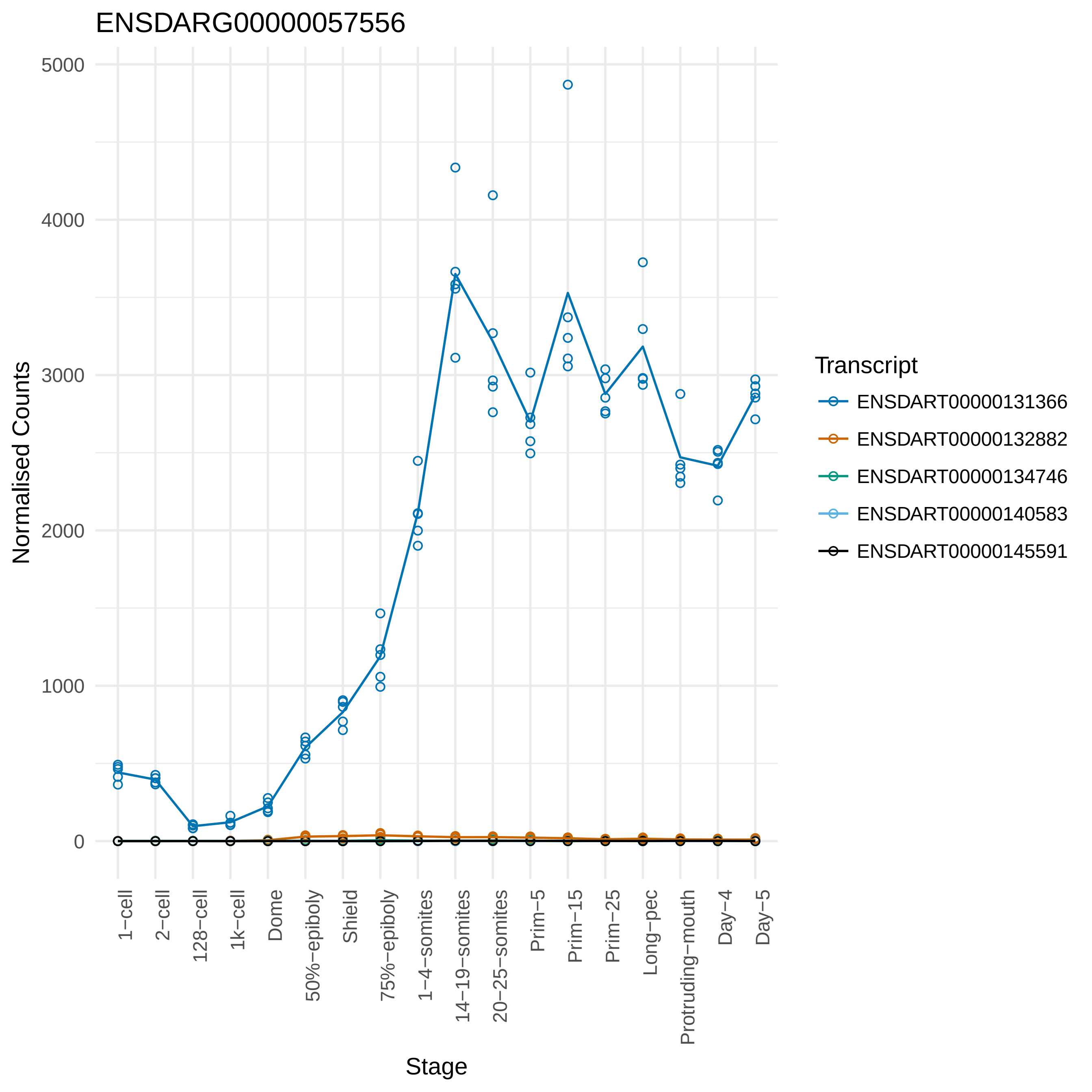

ENSDARG00000057556
Normalised Counts
5000
4000
3000
2000
1000
0
1−cell
2−cell
128−cell
1k−cell
Dome
50%−epiboly
Shield
75%−epiboly
1−4−somites
14−19−somites
20−25−somites
Prim−5
Prim−15
Prim−25
Long−pec
Protruding−mouth
Day−4
Day−5
Stage
Transcript
ENSDART00000131366
ENSDART00000132882
ENSDART00000134746
ENSDART00000140583
ENSDART00000145591

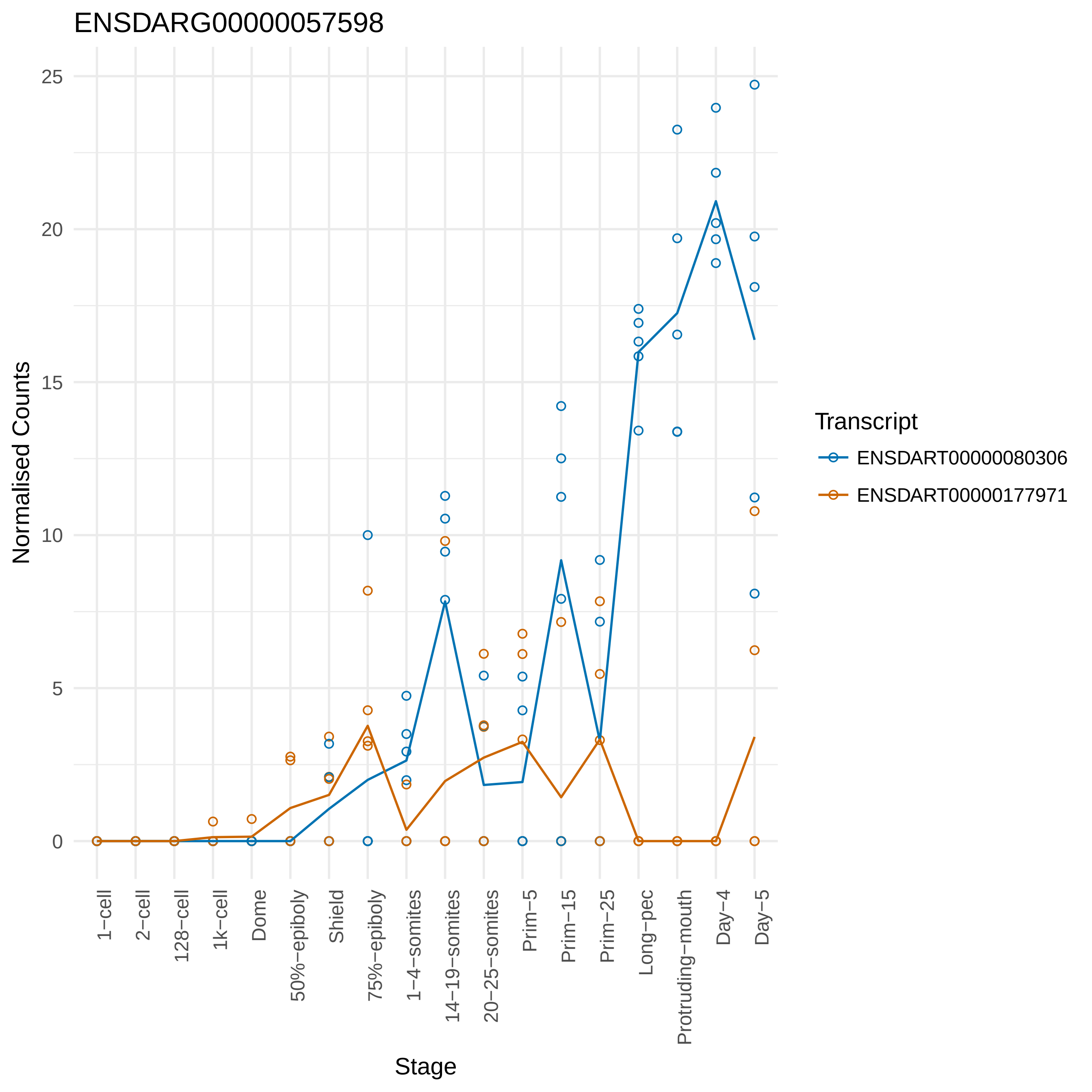
ENSDARG00000057598
Normalised Counts
0
5
10
15
20
25
1−cell
2−cell
128−cell
1k−cell
Dome
50%−epiboly
Shield
75%−epiboly
1−4−somites
14−19−somites
20−25−somites
Prim−5
Prim−15
Prim−25
Long−pec
Protruding−mouth
Day−4
Day−5
Stage
Transcript
ENSDART00000080306
ENSDART00000177971

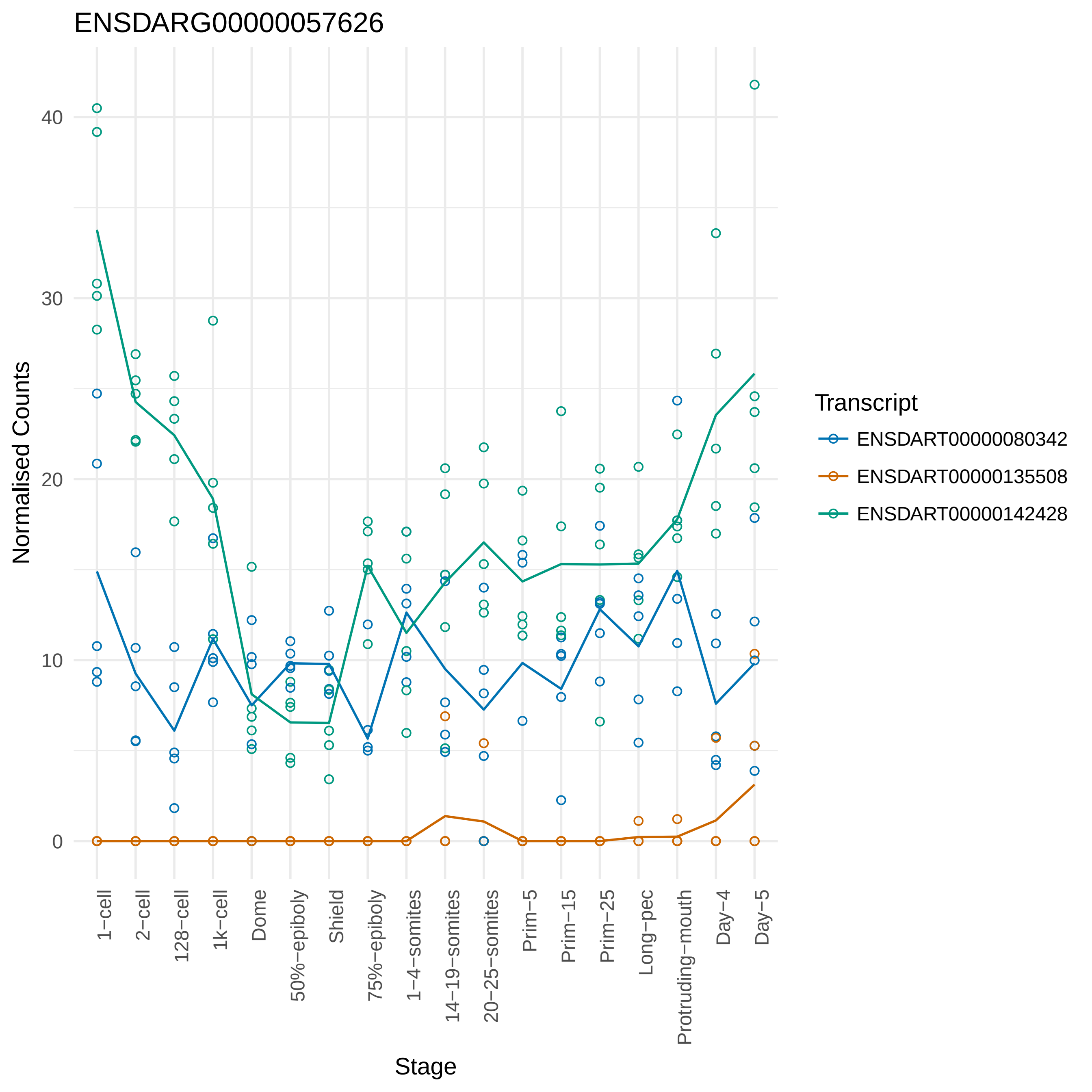
ENSDARG00000057626
Normalised Counts
0
10
20
30
40
Stage
1−cell
2−cell
128−cell
1k−cell
Dome
50%−epiboly
Shield
75%−epiboly
1−4−somites
14−19−somites
20−25−somites
Prim−5
Prim−15
Prim−25
Long−pec
Protruding−mouth
Day−4
Day−5
Transcript
ENSDART00000080342
ENSDART00000135508
ENSDART00000142428

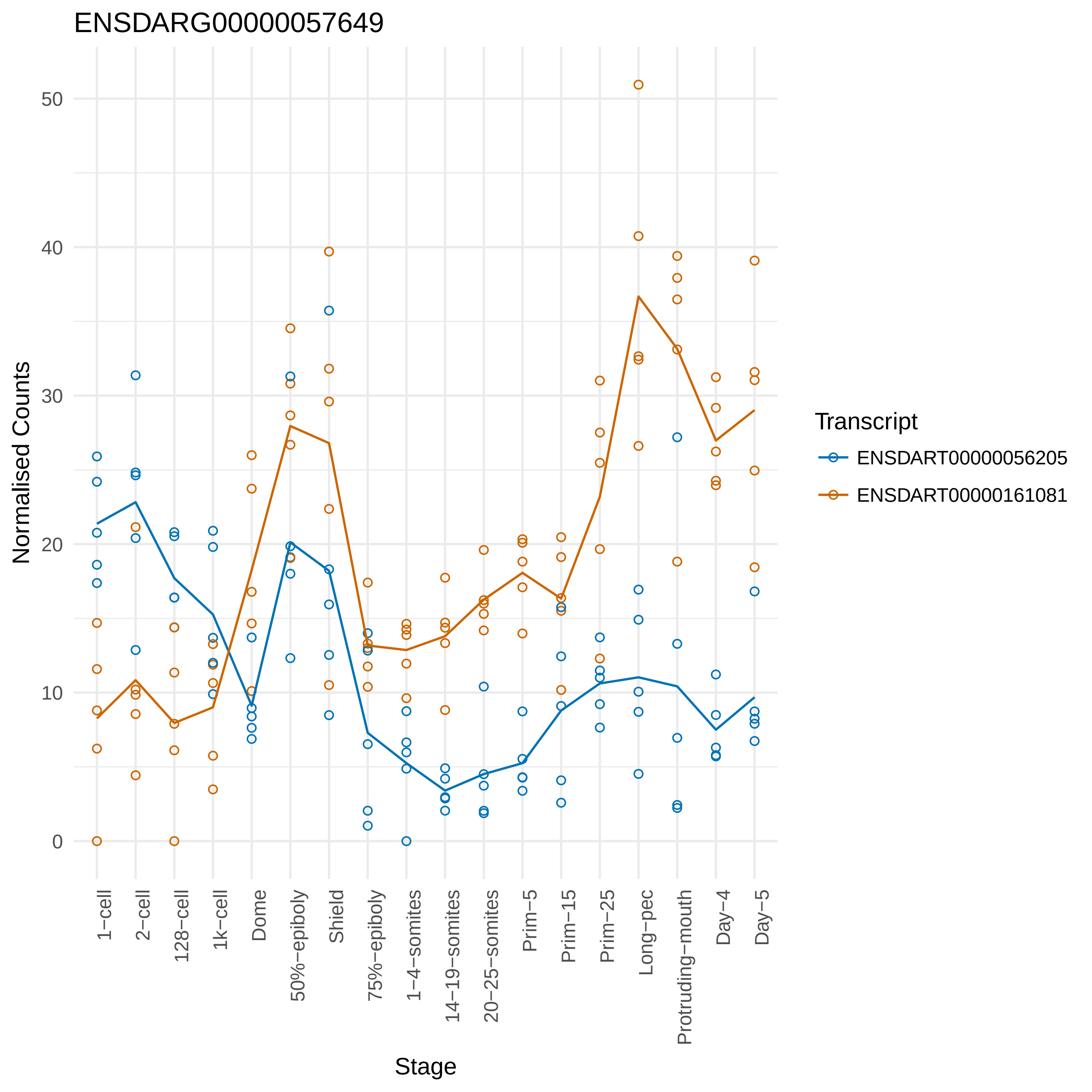

ENSDARG00000057649
Normalised Counts
0
10
20
30
40
50
1−cell
2−cell
128−cell
1k−cell
Dome
50%−epiboly
Shield
75%−epiboly
1−4−somites
14−19−somites
20−25−somites
Prim−5
Prim−15
Prim−25
Long−pec
Protruding−mouth
Day−4
Day−5
Stage
Transcript
ENSDART00000056205
ENSDART00000161081

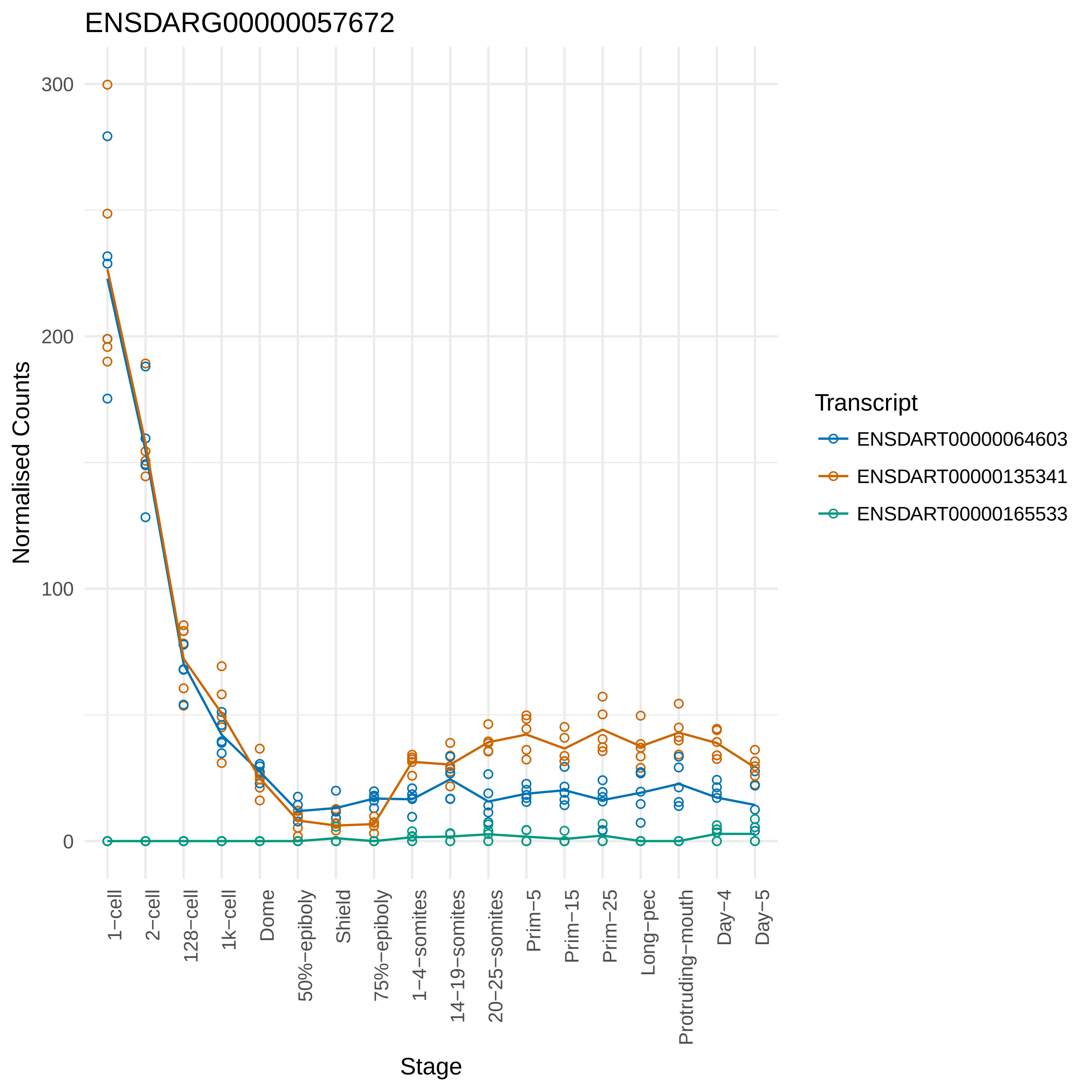
ENSDARG00000057672
Normalised Counts
300
200
100
0
1−cell
2−cell
128−cell
1k−cell
Dome
50%−epiboly
Shield
75%−epiboly
1−4−somites
14−19−somites
20−25−somites
Prim−5
Prim−15
Prim−25
Long−pec
Protruding−mouth
Day−4
Day−5
Stage
Transcript
ENSDART00000064603
ENSDART00000135341
ENSDART00000165533

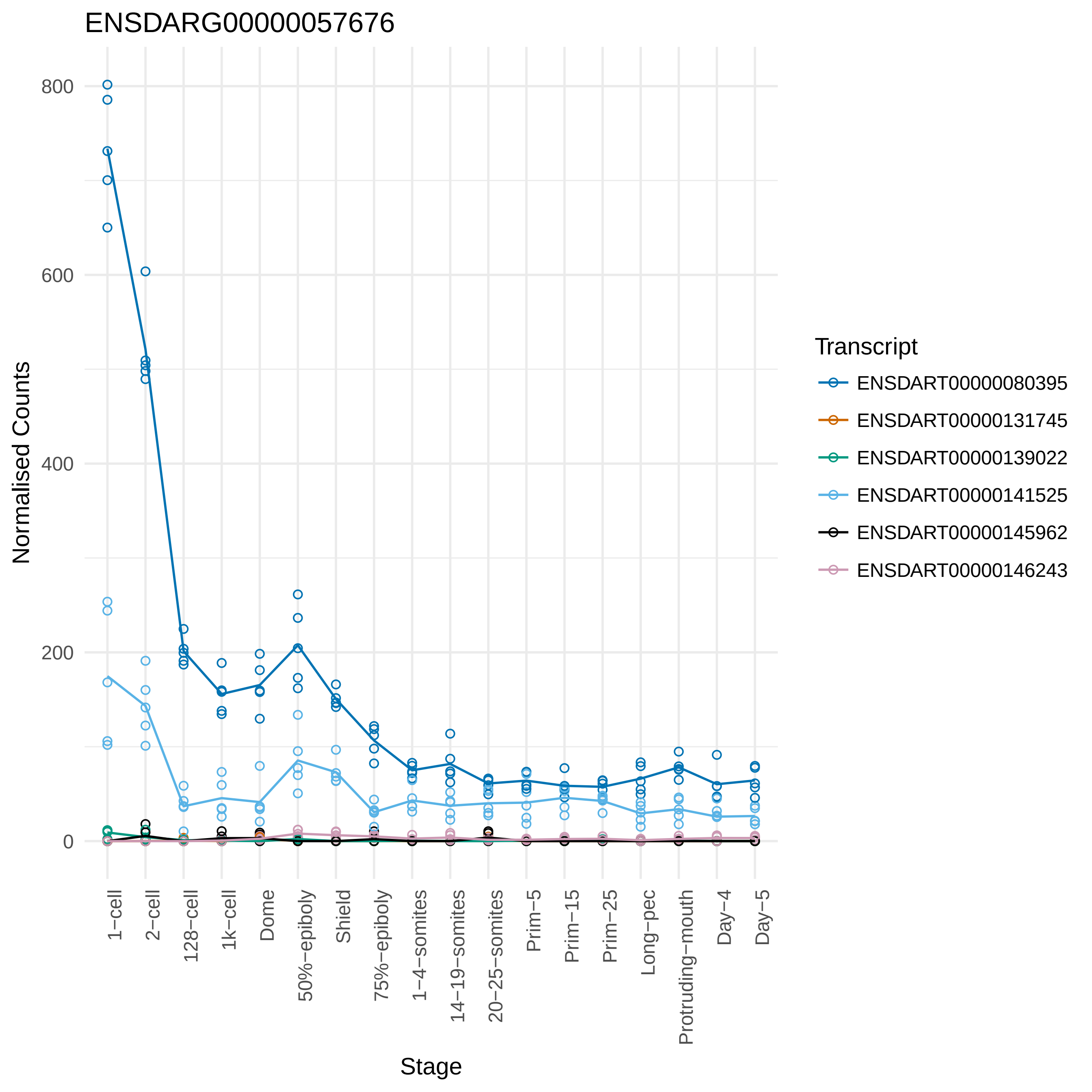
ENSDARG00000057676
Normalised Counts
800
600
400
200
0
1−cell
2−cell
128−cell
1k−cell
Dome
50%−epiboly
Shield
75%−epiboly
1−4−somites
14−19−somites
20−25−somites
Prim−5
Prim−15
Prim−25
Long−pec
Protruding−mouth
Day−4
Day−5
Stage
Transcript
ENSDART00000080395
ENSDART00000131745
ENSDART00000139022
ENSDART00000141525
ENSDART00000145962
ENSDART00000146243

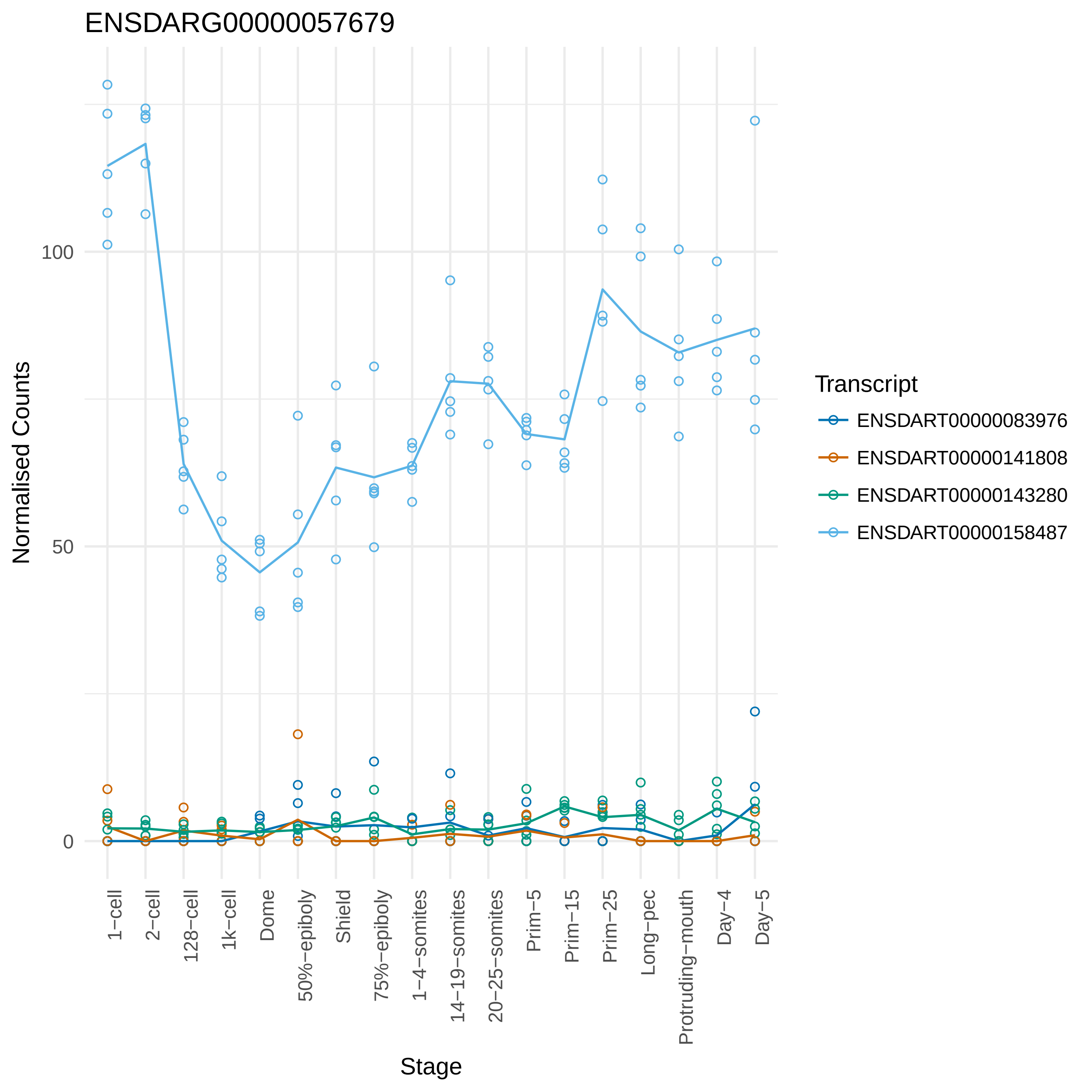

ENSDARG00000057679
Normalised Counts
100
50
0
Stage
1−cell
2−cell
128−cell
1k−cell
Dome
50%−epiboly
Shield
75%−epiboly
1−4−somites
14−19−somites
20−25−somites
Prim−5
Prim−15
Prim−25
Long−pec
Protruding−mouth
Day−4
Day−5
Transcript
ENSDART00000083976
ENSDART00000141808
ENSDART00000143280
ENSDART00000158487

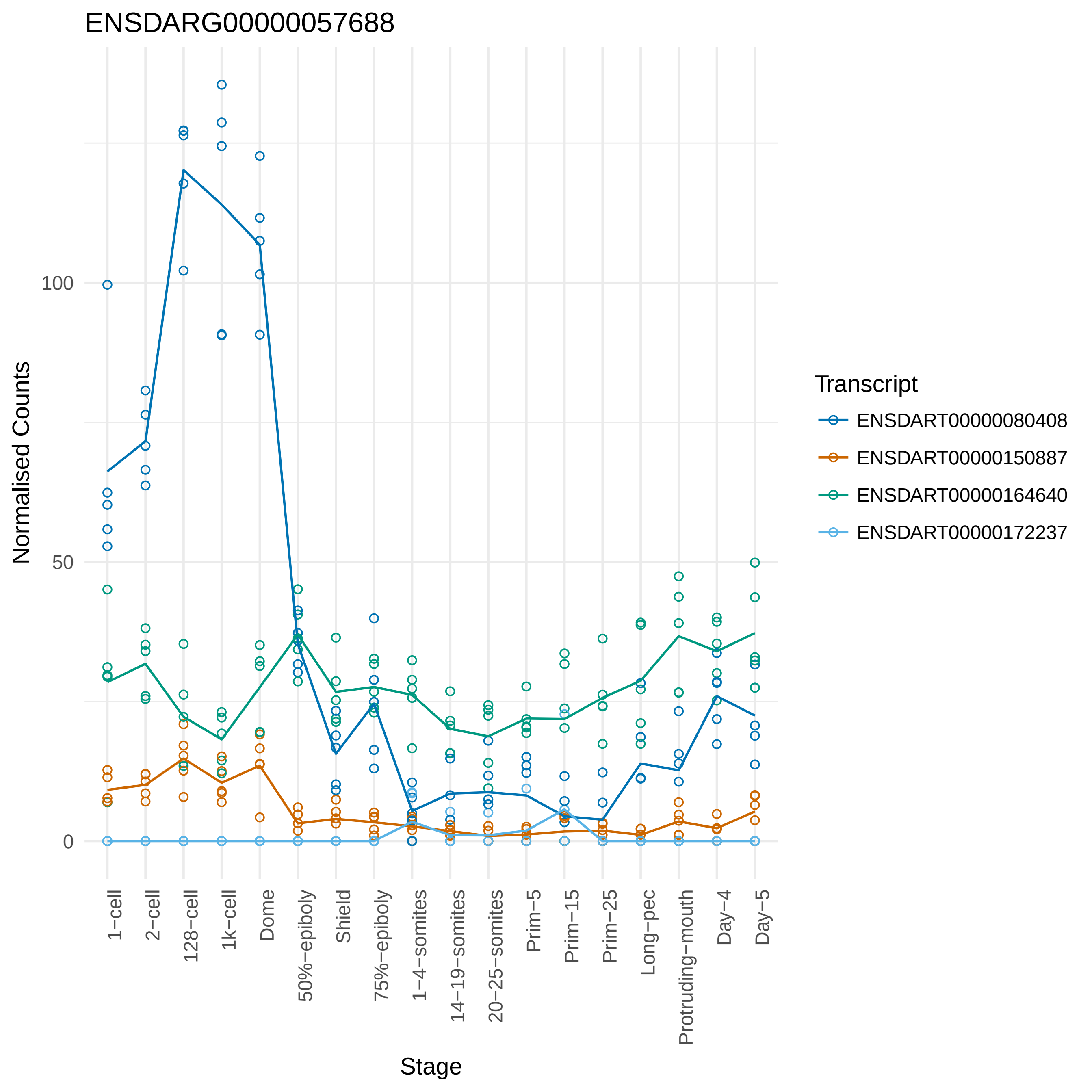

ENSDARG00000057688
Normalised Counts
100
50
0
1−cell
2−cell
128−cell
1k−cell
Dome
50%−epiboly
Shield
75%−epiboly
1−4−somites
14−19−somites
20−25−somites
Prim−5
Prim−15
Prim−25
Long−pec
Protruding−mouth
Day−4
Day−5
Stage
Transcript
ENSDART00000080408
ENSDART00000150887
ENSDART00000164640
ENSDART00000172237

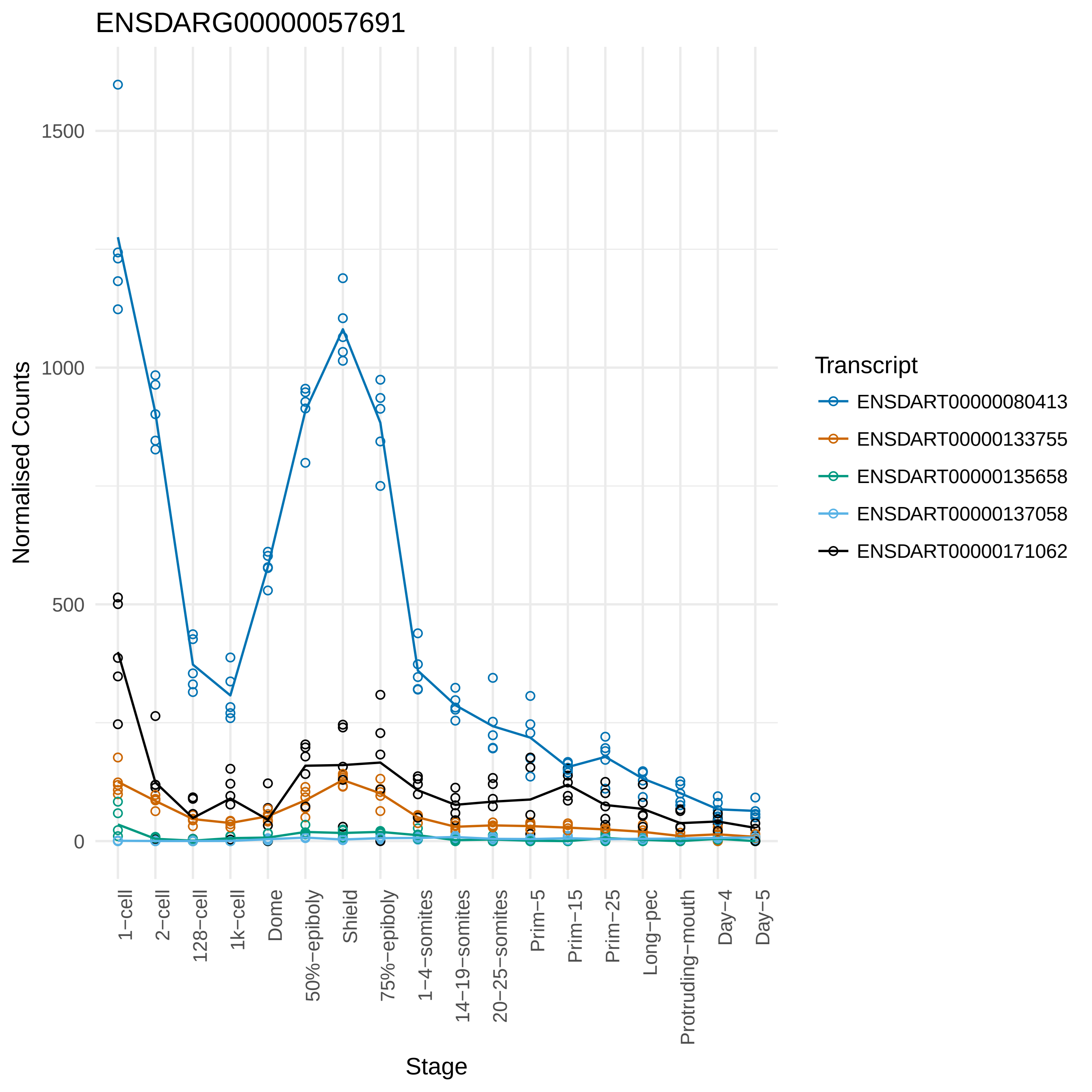

ENSDARG00000057691
Normalised Counts
1500
1000
500
0
1−cell
2−cell
128−cell
1k−cell
Dome
50%−epiboly
Shield
75%−epiboly
1−4−somites
14−19−somites
20−25−somites
Prim−5
Prim−15
Prim−25
Long−pec
Protruding−mouth
Day−4
Day−5
Stage
Transcript
ENSDART00000080413
ENSDART00000133755
ENSDART00000135658
ENSDART00000137058
ENSDART00000171062

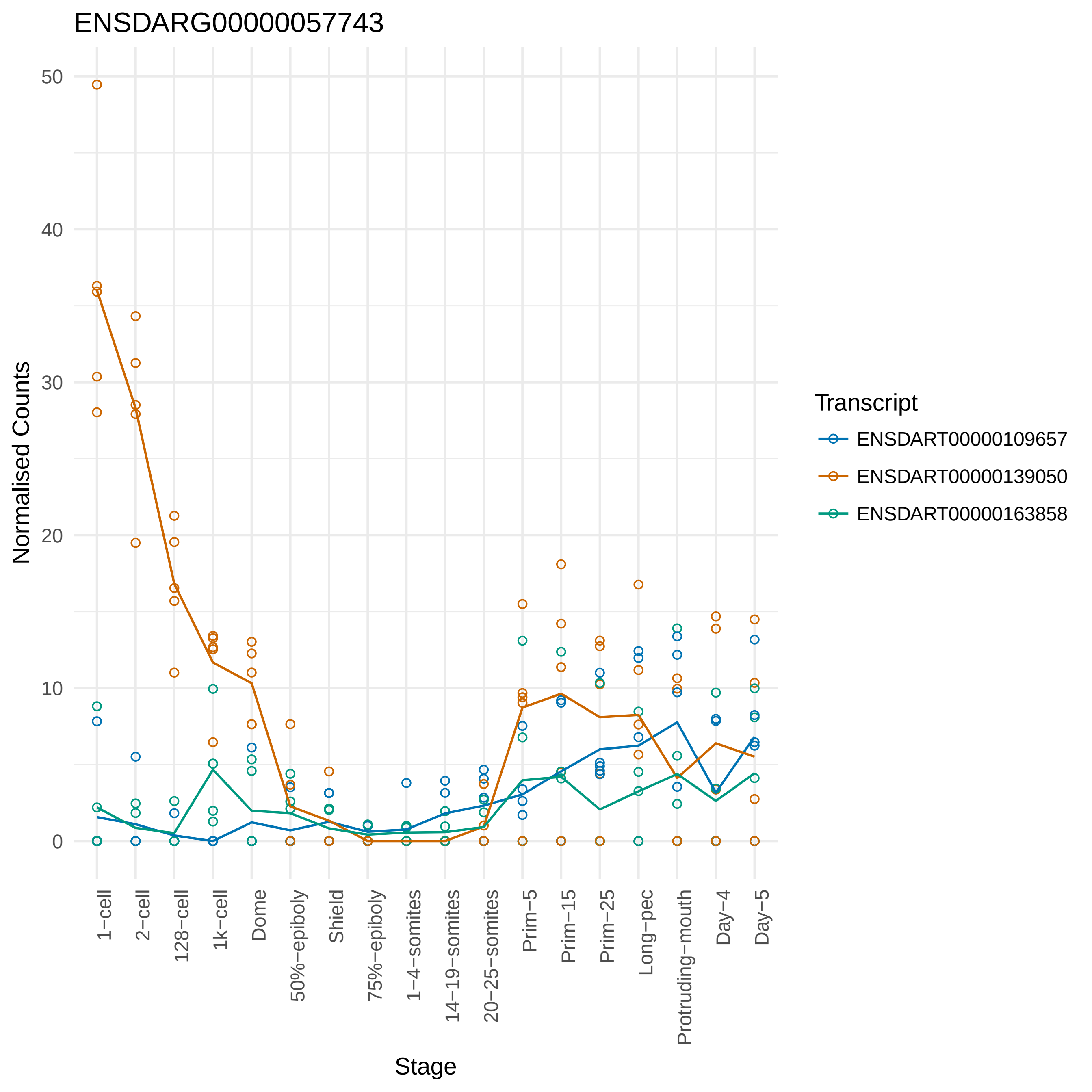

ENSDARG00000057743
Normalised Counts
50
40
30
20
10
0
1−cell
2−cell
128−cell
1k−cell
Dome
50%−epiboly
Shield
75%−epiboly
1−4−somites
14−19−somites
20−25−somites
Prim−5
Prim−15
Prim−25
Long−pec
Protruding−mouth
Day−4
Day−5
Stage
Transcript
ENSDART00000109657
ENSDART00000139050
ENSDART00000163858

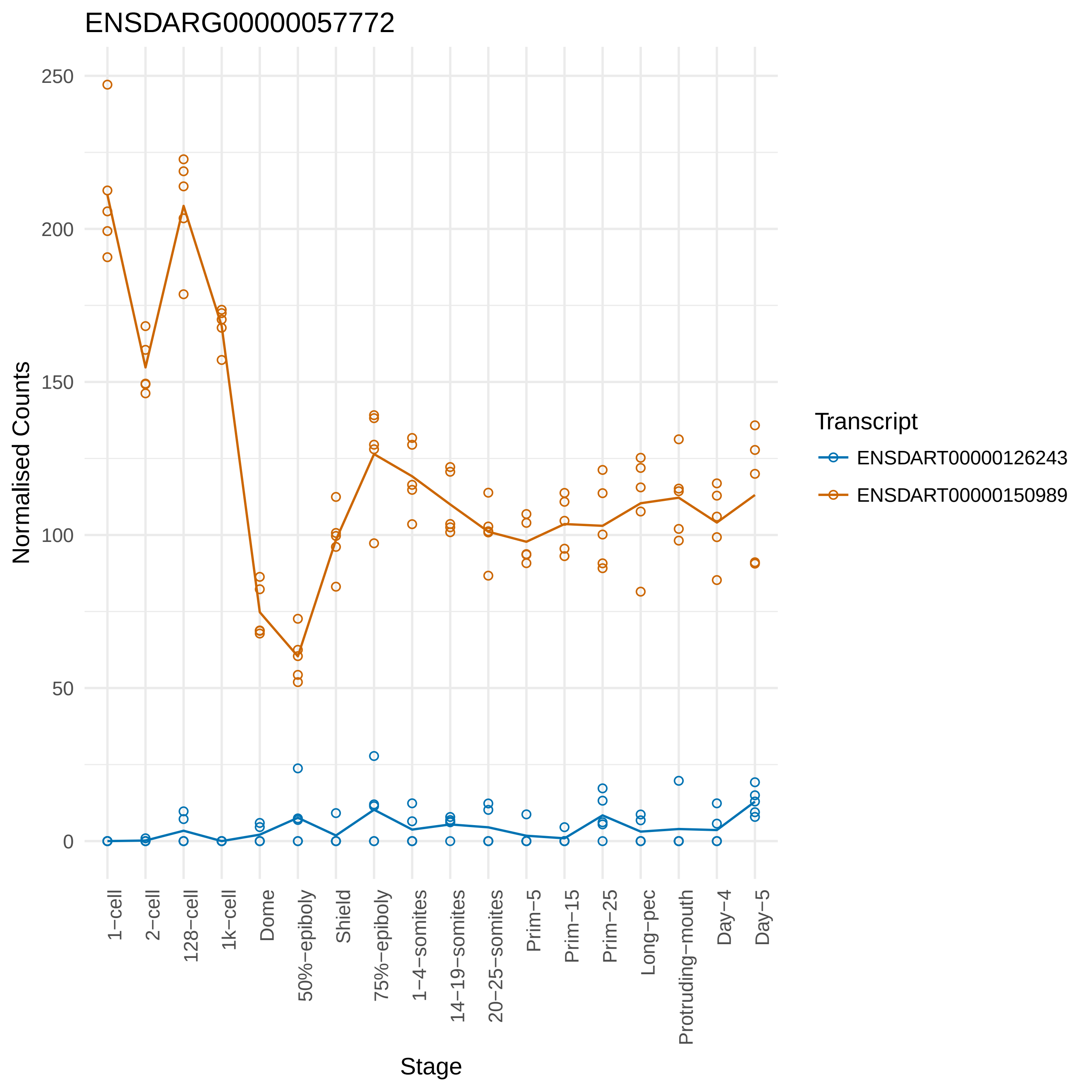

ENSDARG00000057772
Normalised Counts
250
200
150
100
50
0
1−cell
2−cell
128−cell
1k−cell
Dome
50%−epiboly
Shield
75%−epiboly
1−4−somites
14−19−somites
20−25−somites
Prim−5
Prim−15
Prim−25
Long−pec
Protruding−mouth
Day−4
Day−5
Stage
Transcript
ENSDART00000126243
ENSDART00000150989

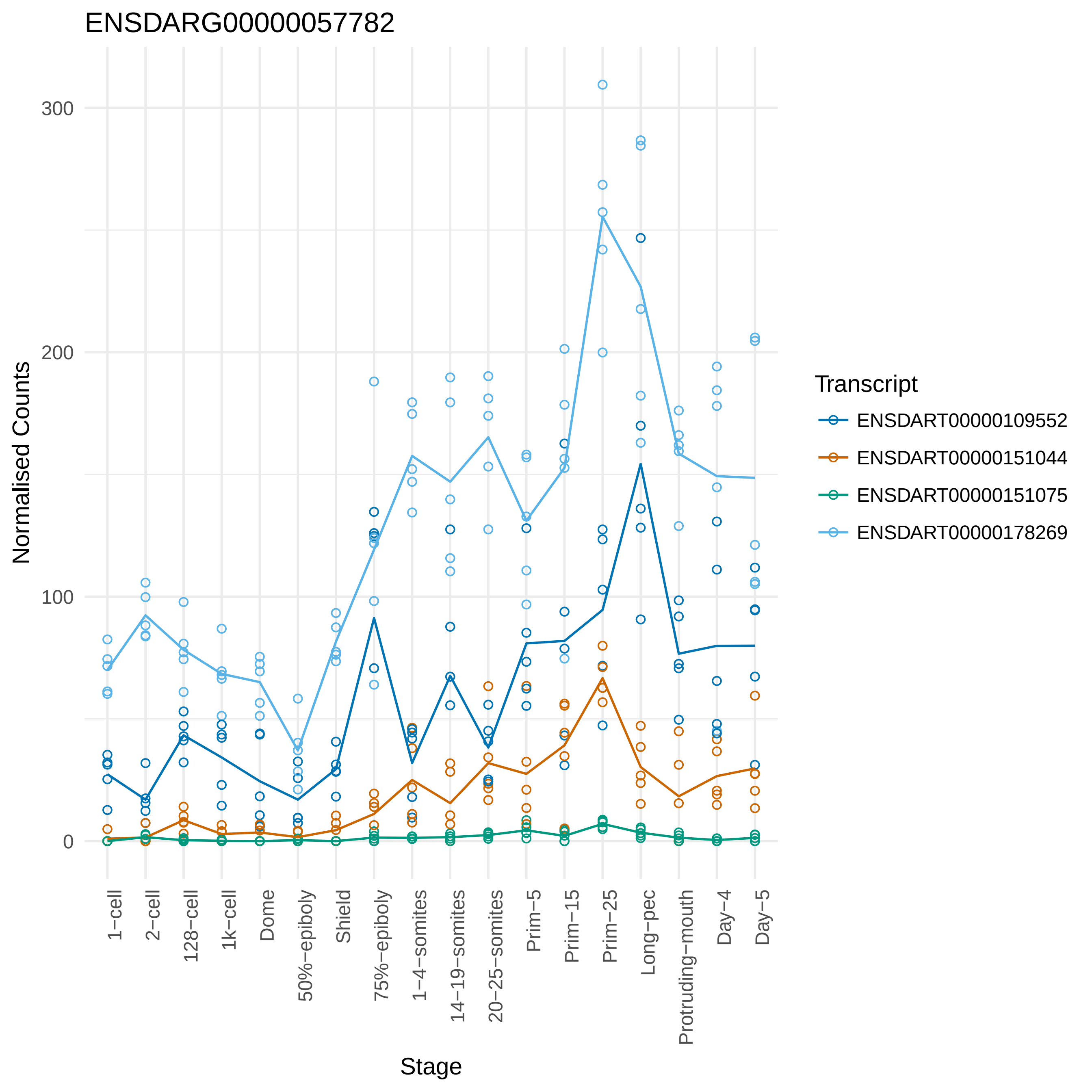
ENSDARG00000057782
Normalised Counts
300
200
100
0
1−cell
2−cell
128−cell
1k−cell
Dome
50%−epiboly
Shield
75%−epiboly
1−4−somites
14−19−somites
20−25−somites
Prim−5
Prim−15
Prim−25
Long−pec
Protruding−mouth
Day−4
Day−5
Stage
Transcript
ENSDART00000109552
ENSDART00000151044
ENSDART00000151075
ENSDART00000178269

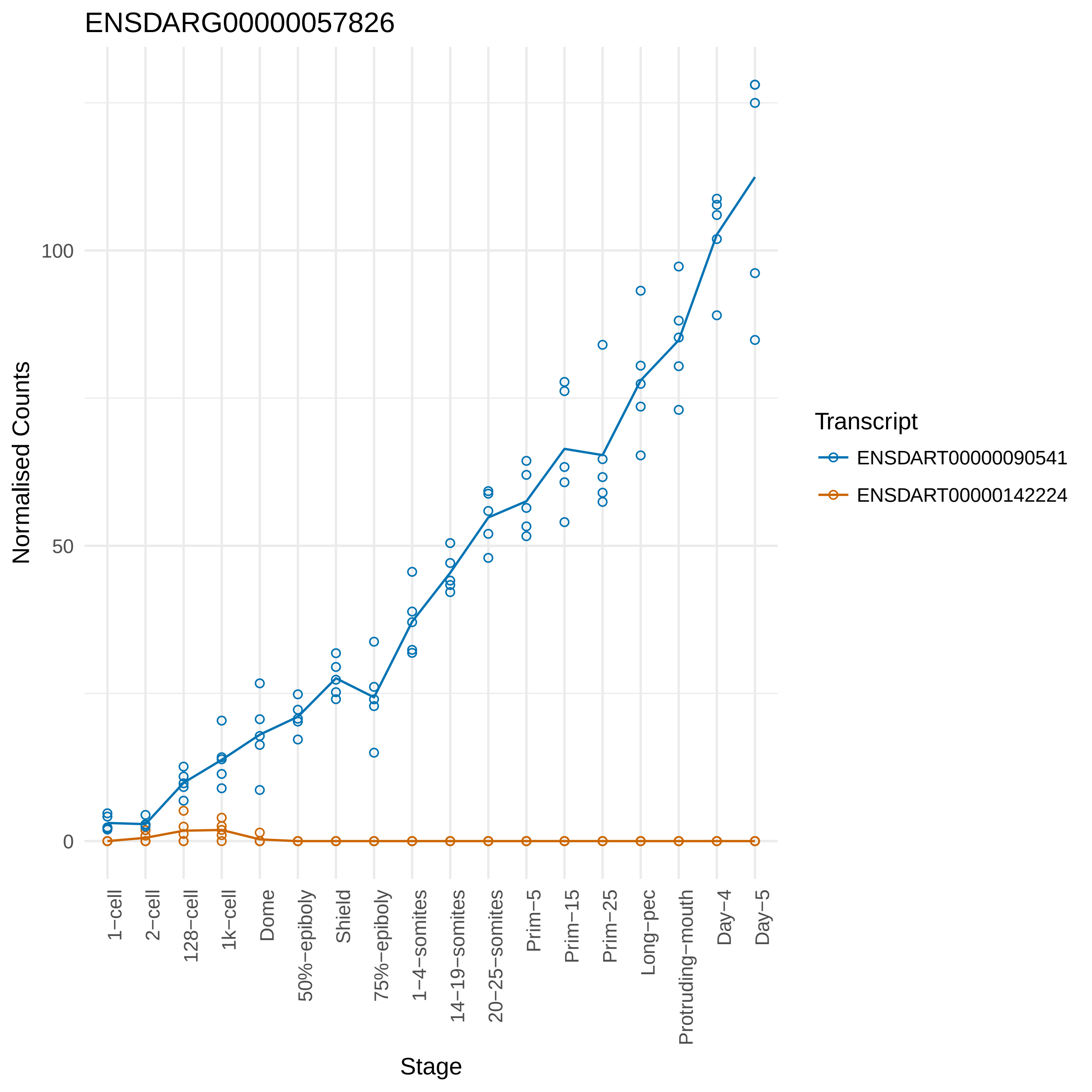
ENSDARG00000057826
Normalised Counts
100
50
0
1−cell
2−cell
128−cell
1k−cell
Dome
50%−epiboly
Shield
75%−epiboly
1−4−somites
14−19−somites
20−25−somites
Prim−5
Prim−15
Prim−25
Long−pec
Protruding−mouth
Day−4
Day−5
Stage
Transcript
ENSDART00000090541
ENSDART00000142224

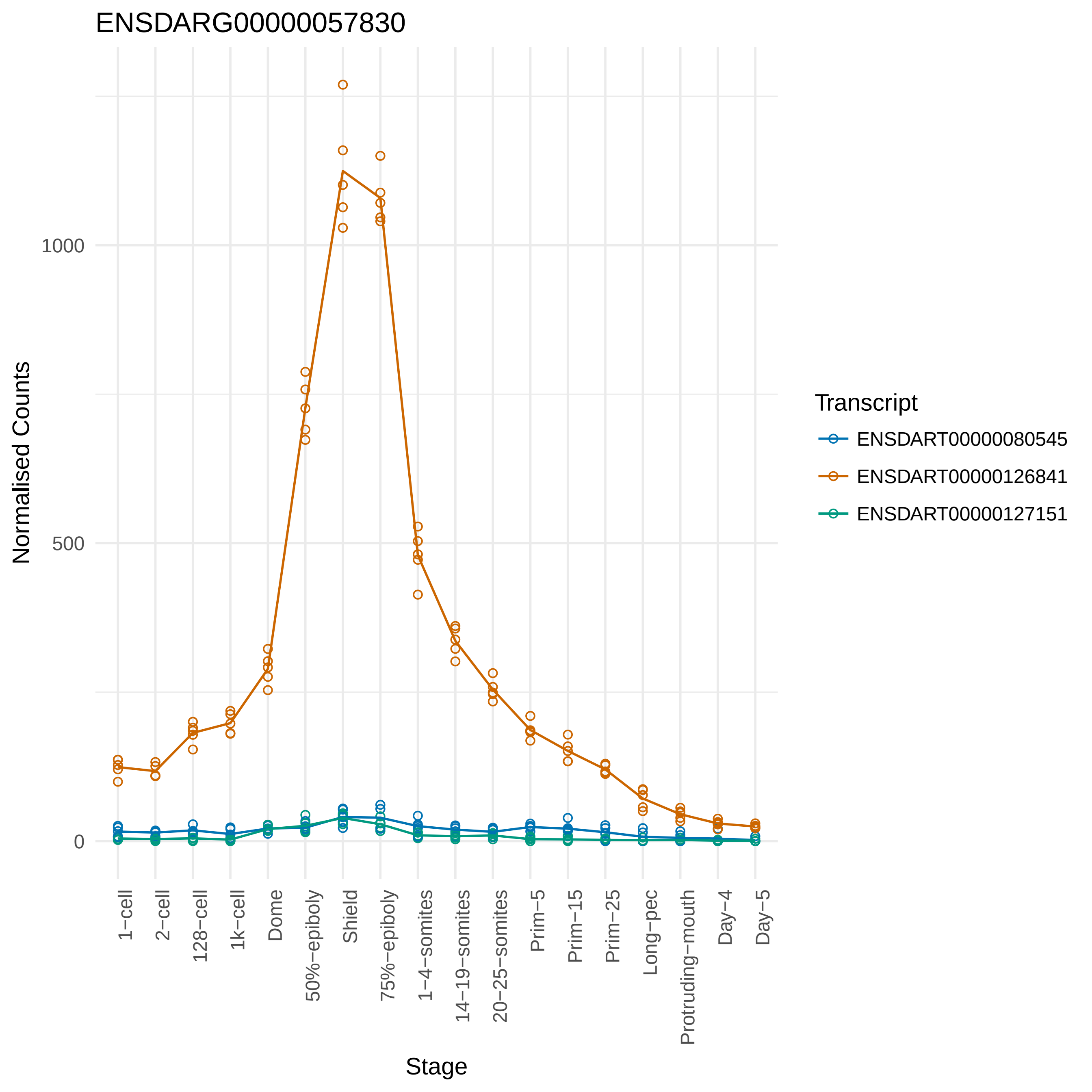
ENSDARG00000057830
Normalised Counts
1000
500
0
1−cell
2−cell
128−cell
1k−cell
Dome
50%−epiboly
Shield
75%−epiboly
1−4−somites
14−19−somites
20−25−somites
Prim−5
Prim−15
Prim−25
Long−pec
Protruding−mouth
Day−4
Day−5
Stage
Transcript
ENSDART00000080545
ENSDART00000126841
ENSDART00000127151

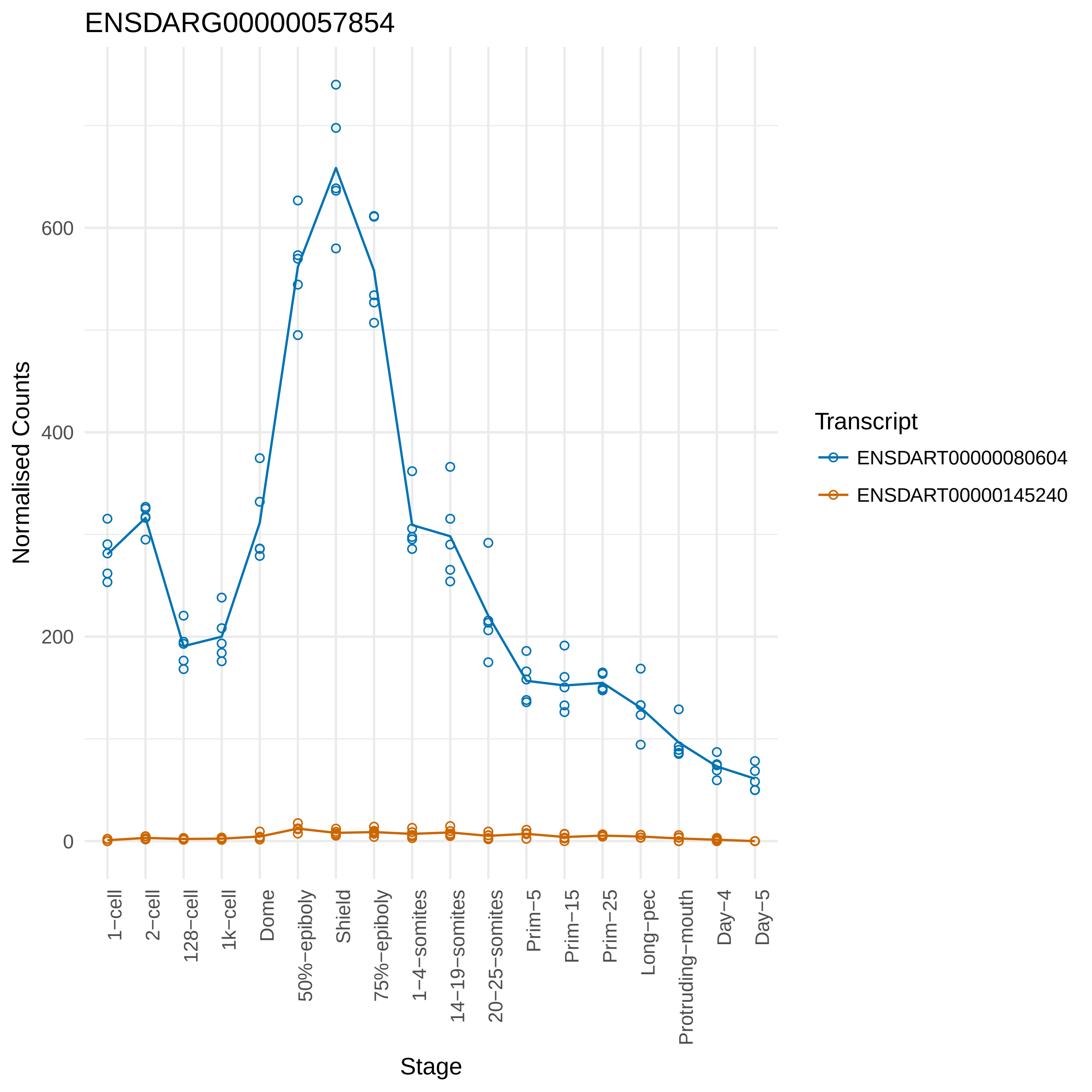
ENSDARG00000057854
Normalised Counts
600
400
200
0
1−cell
2−cell
128−cell
1k−cell
Dome
50%−epiboly
Shield
75%−epiboly
1−4−somites
14−19−somites
20−25−somites
Prim−5
Prim−15
Prim−25
Long−pec
Protruding−mouth
Day−4
Day−5
Stage
Transcript
ENSDART00000080604
ENSDART00000145240

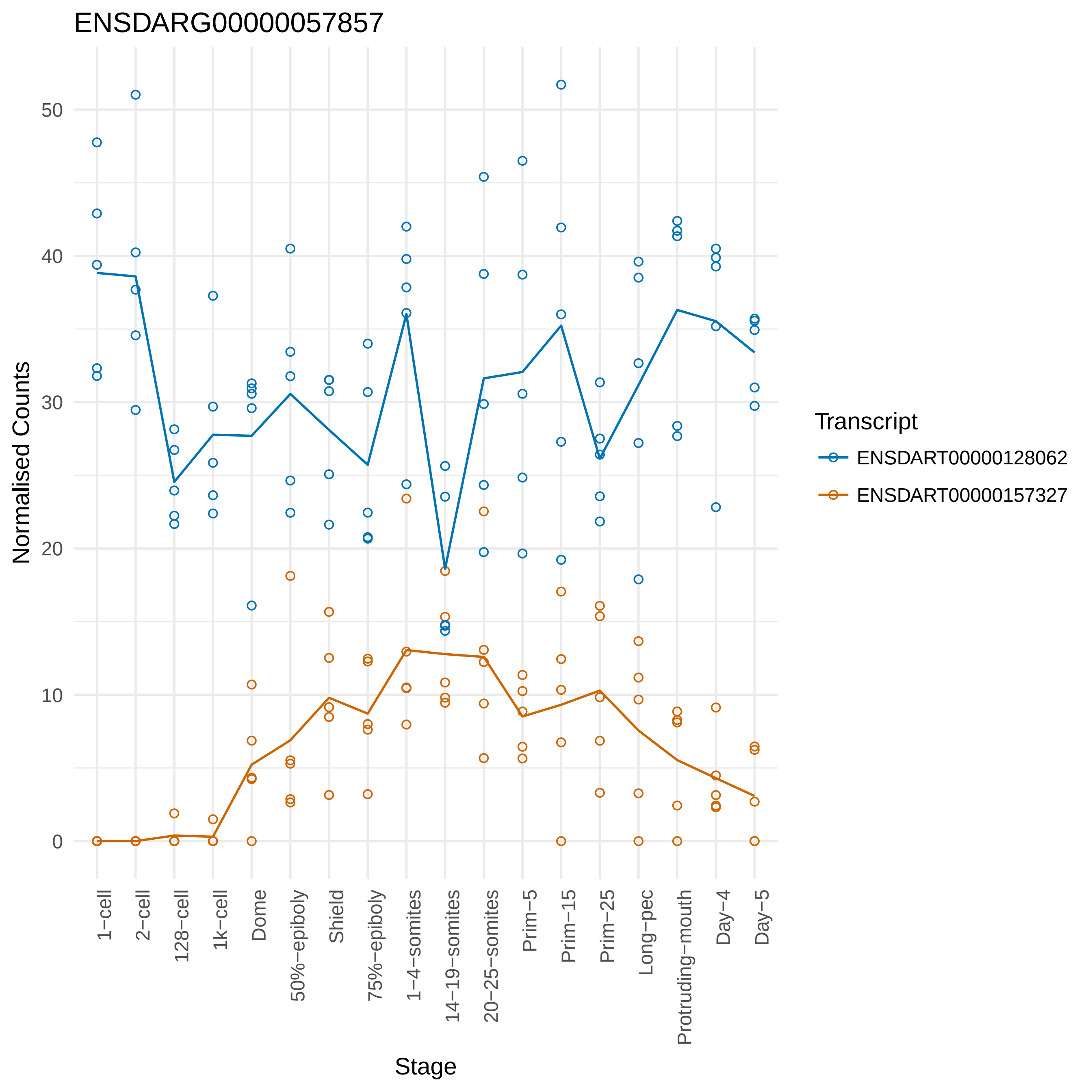

ENSDARG00000057857
Normalised Counts
0
10
20
30
40
50
Stage
1−cell
2−cell
128−cell
1k−cell
Dome
50%−epiboly
Shield
75%−epiboly
1−4−somites
14−19−somites
20−25−somites
Prim−5
Prim−15
Prim−25
Long−pec
Protruding−mouth
Day−4
Day−5
Transcript
ENSDART00000128062
ENSDART00000157327

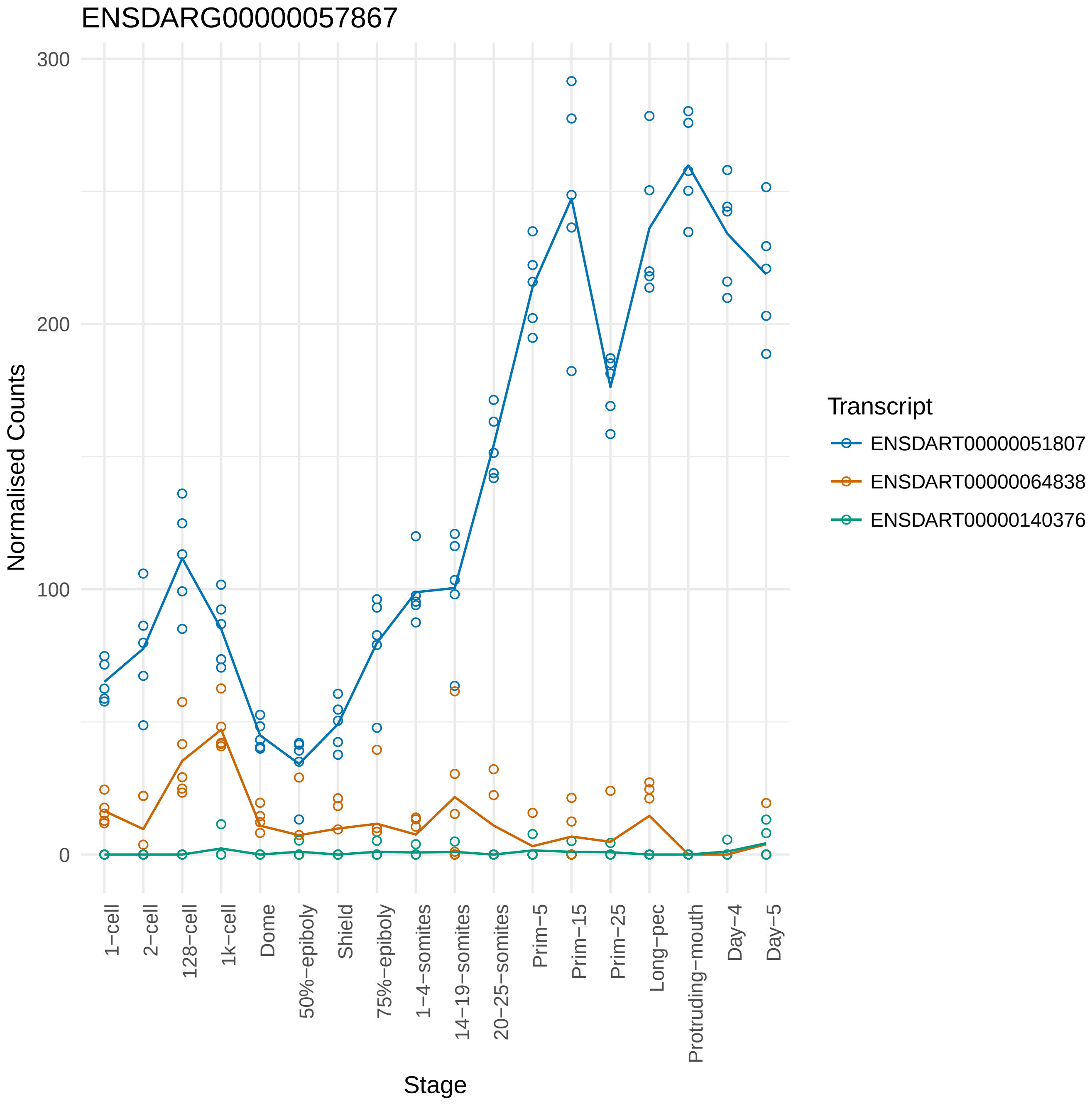

ENSDARG00000057867
Normalised Counts
300
200
100
0
1−cell
2−cell
128−cell
1k−cell
Dome
50%−epiboly
Shield
75%−epiboly
1−4−somites
14−19−somites
20−25−somites
Prim−5
Prim−15
Prim−25
Long−pec
Protruding−mouth
Day−4
Day−5
Stage
Transcript
ENSDART00000051807
ENSDART00000064838
ENSDART00000140376

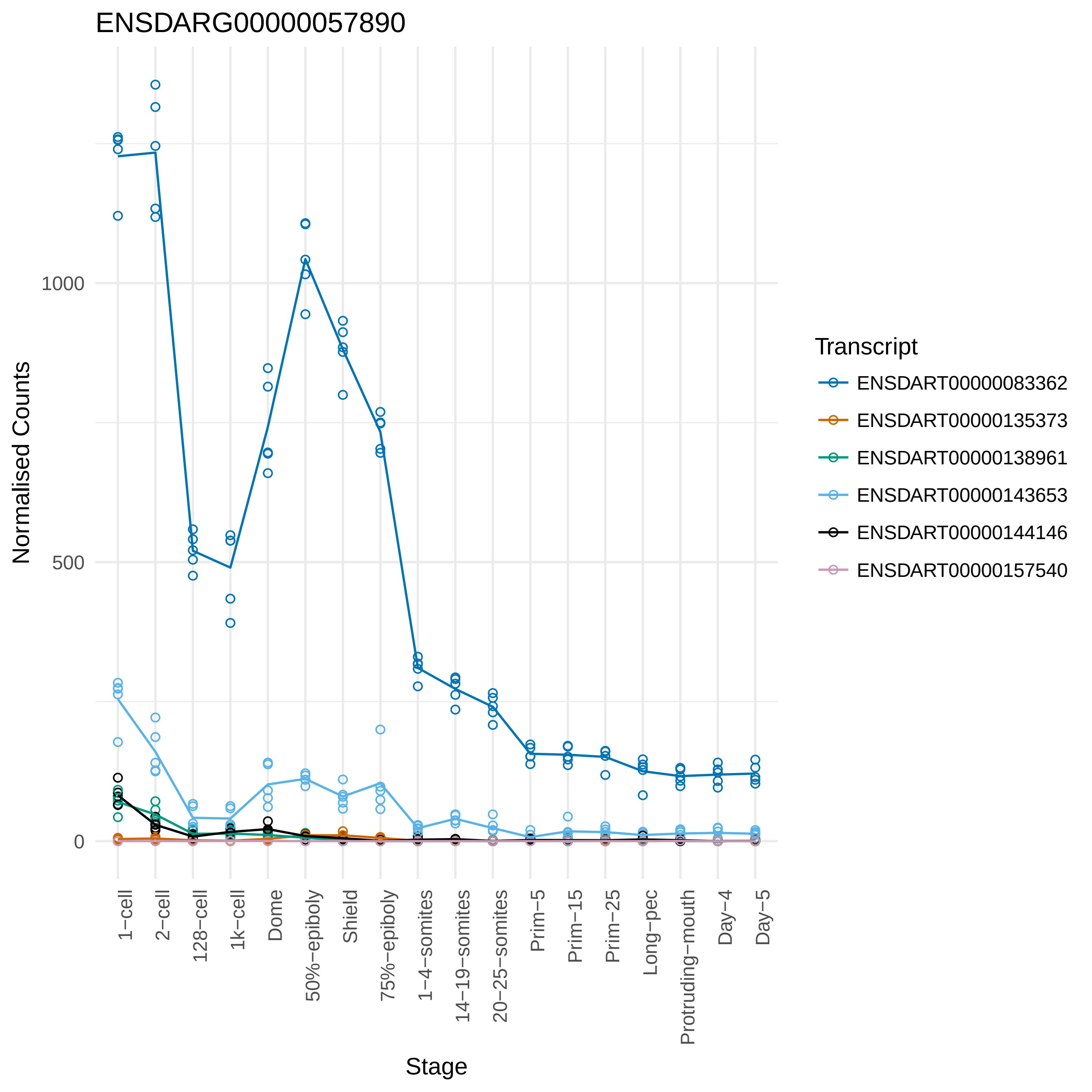
ENSDARG00000057890
Normalised Counts
1000
500
0
1−cell
2−cell
128−cell
1k−cell
Dome
50%−epiboly
Shield
75%−epiboly
1−4−somites
14−19−somites
20−25−somites
Prim−5
Prim−15
Prim−25
Long−pec
Protruding−mouth
Day−4
Day−5
Stage
Transcript
ENSDART00000083362
ENSDART00000135373
ENSDART00000138961
ENSDART00000143653
ENSDART00000144146
ENSDART00000157540

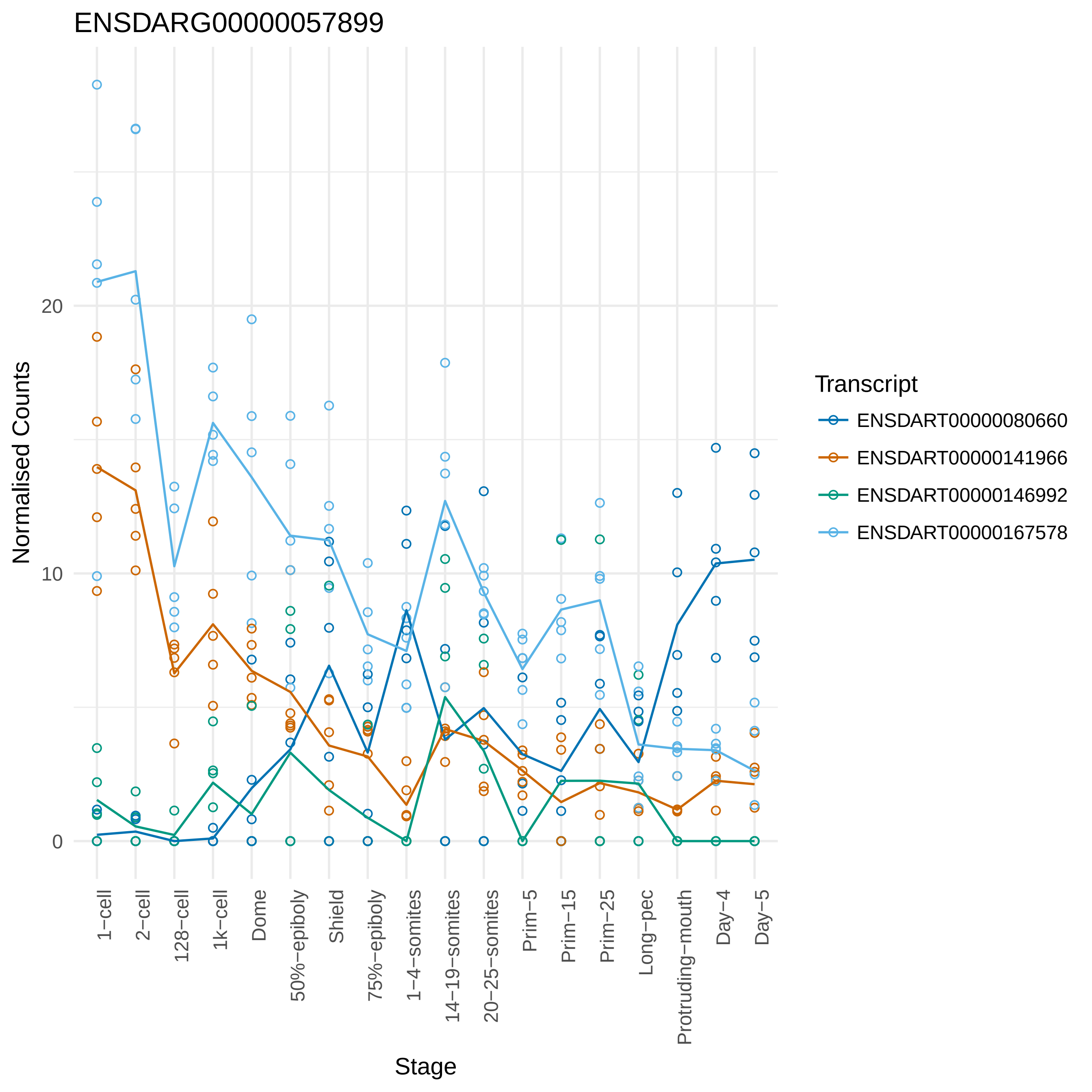

ENSDARG00000057899
Normalised Counts
20
10
0
1−cell
2−cell
128−cell
1k−cell
Dome
50%−epiboly
Shield
75%−epiboly
1−4−somites
14−19−somites
20−25−somites
Prim−5
Prim−15
Prim−25
Long−pec
Protruding−mouth
Day−4
Day−5
Stage
Transcript
ENSDART00000080660
ENSDART00000141966
ENSDART00000146992
ENSDART00000167578

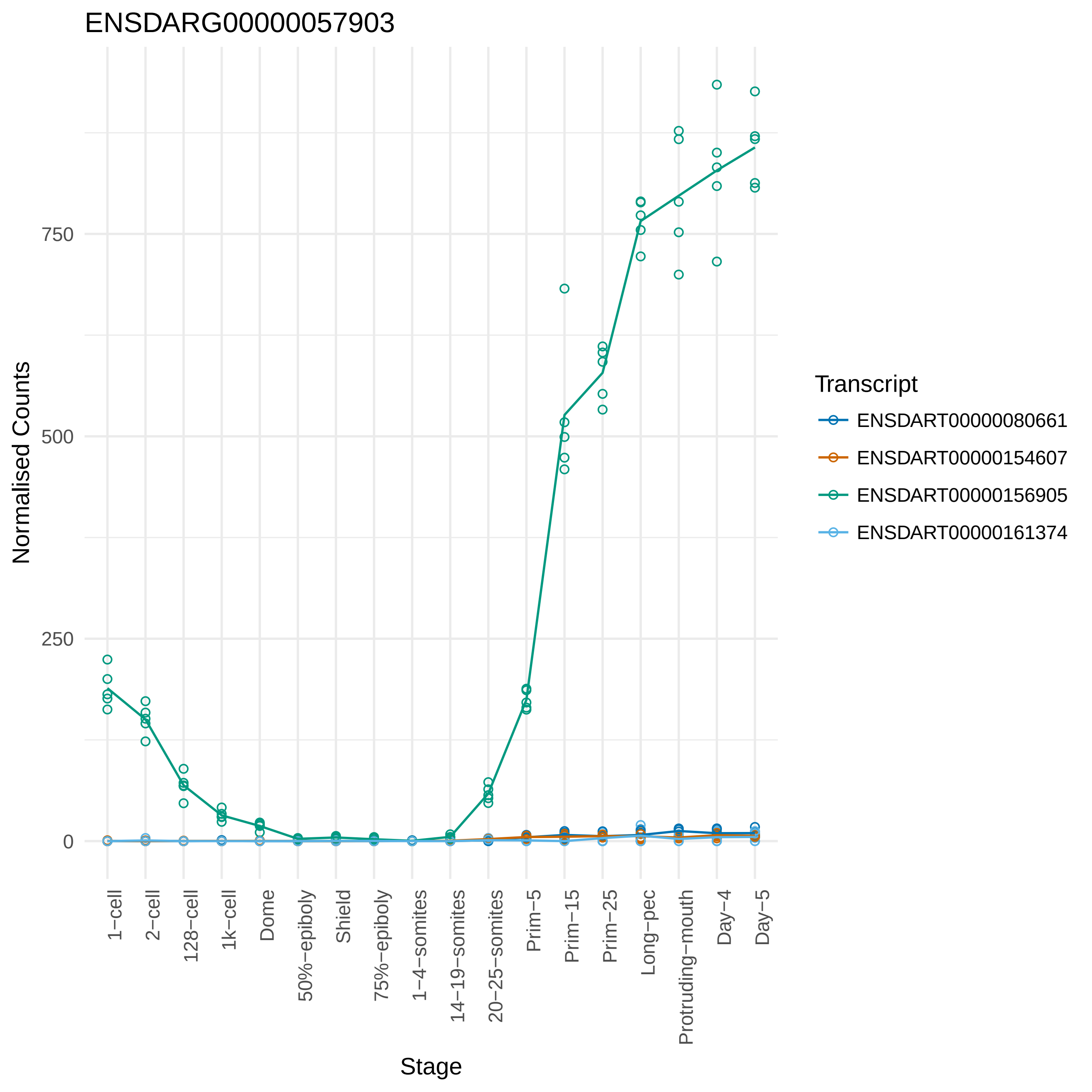

ENSDARG00000057903
Normalised Counts
750
500
250
0
1−cell
2−cell
128−cell
1k−cell
Dome
50%−epiboly
Shield
75%−epiboly
1−4−somites
14−19−somites
20−25−somites
Prim−5
Prim−15
Prim−25
Long−pec
Protruding−mouth
Day−4
Day−5
Stage
Transcript
ENSDART00000080661
ENSDART00000154607
ENSDART00000156905
ENSDART00000161374

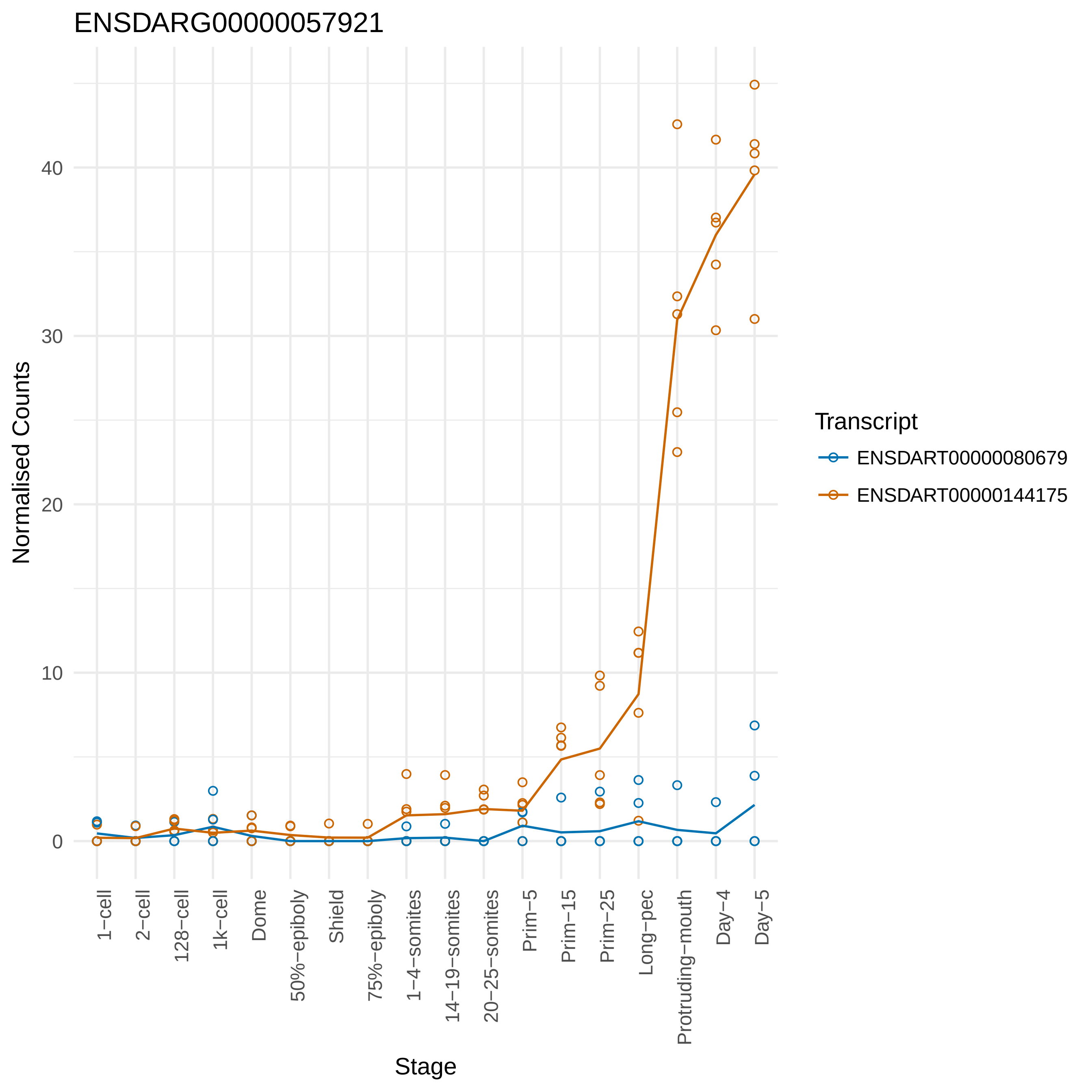
ENSDARG00000057921
Normalised Counts
0
10
20
30
40
Stage
1−cell
2−cell
128−cell
1k−cell
Dome
50%−epiboly
Shield
75%−epiboly
1−4−somites
14−19−somites
20−25−somites
Prim−5
Prim−15
Prim−25
Long−pec
Protruding−mouth
Day−4
Day−5
Transcript
ENSDART00000080679
ENSDART00000144175

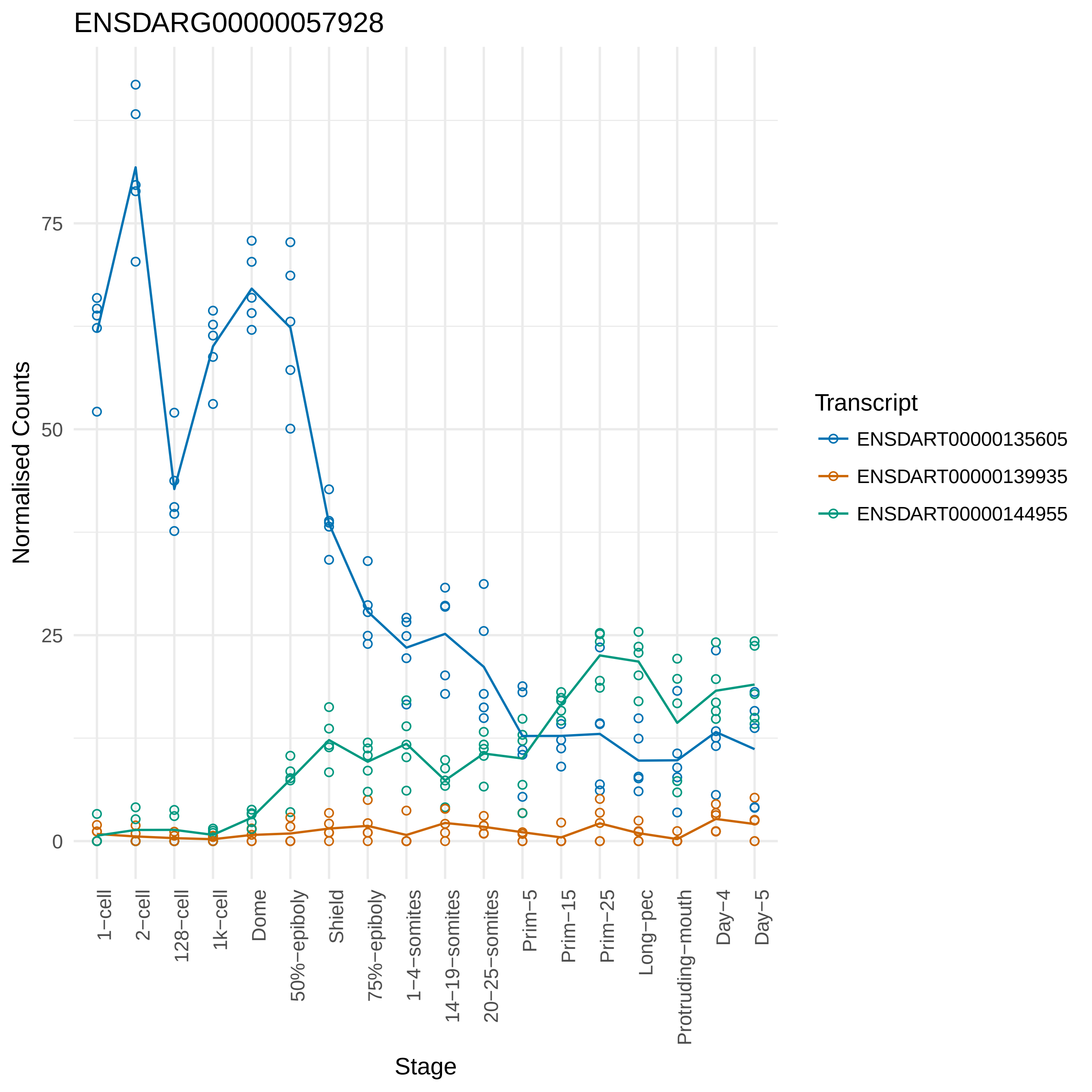

ENSDARG00000057928
Normalised Counts
0
25
50
75
1−cell
2−cell
128−cell
1k−cell
Dome
50%−epiboly
Shield
75%−epiboly
1−4−somites
14−19−somites
20−25−somites
Prim−5
Prim−15
Prim−25
Long−pec
Protruding−mouth
Day−4
Day−5
Stage
Transcript
ENSDART00000135605
ENSDART00000139935
ENSDART00000144955

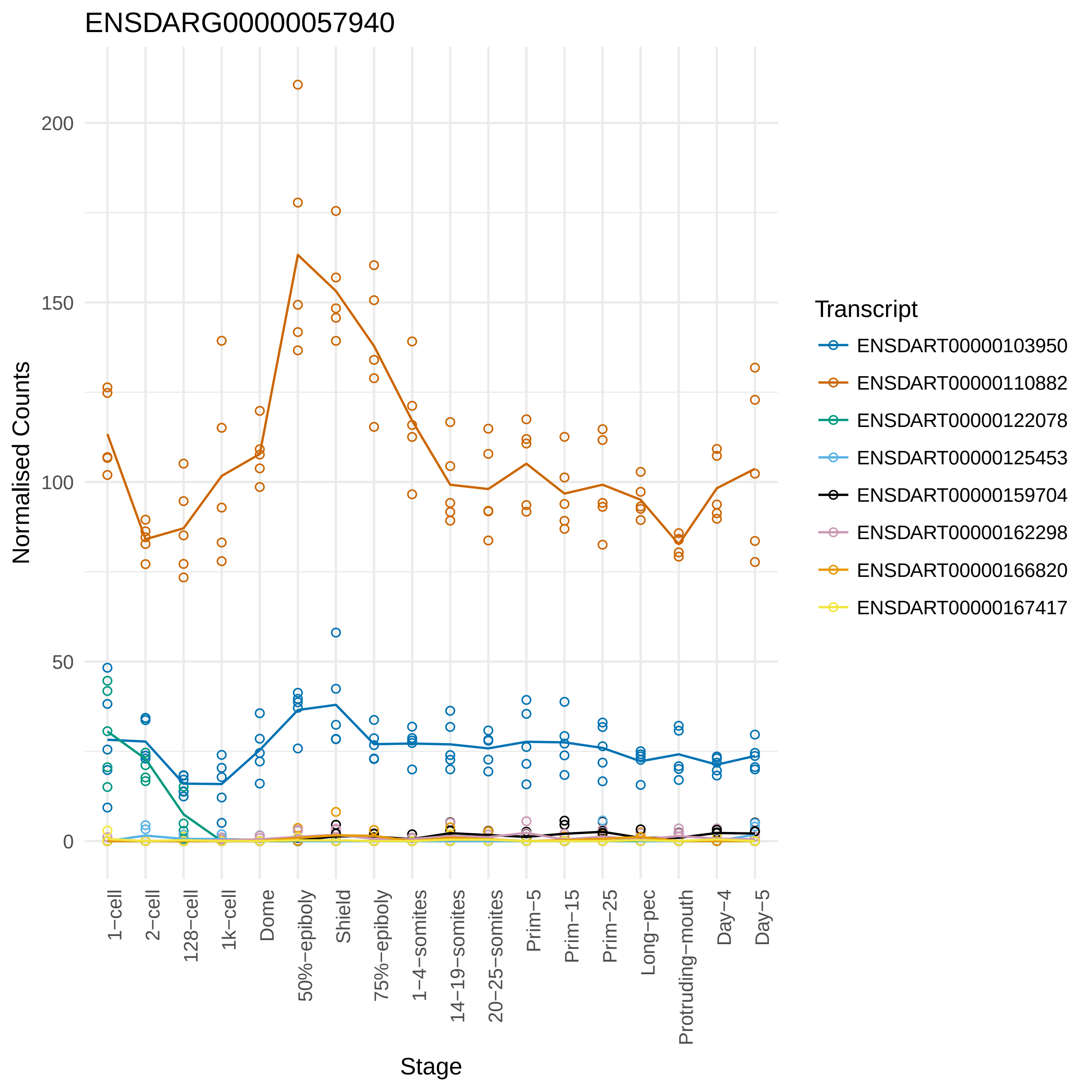
ENSDARG00000057940
Normalised Counts
200
150
100
50
0
1−cell
2−cell
128−cell
1k−cell
Dome
50%−epiboly
Shield
75%−epiboly
1−4−somites
14−19−somites
20−25−somites
Prim−5
Prim−15
Prim−25
Long−pec
Protruding−mouth
Day−4
Day−5
Stage
Transcript
ENSDART00000103950
ENSDART00000110882
ENSDART00000122078
ENSDART00000125453
ENSDART00000159704
ENSDART00000162298
ENSDART00000166820
ENSDART00000167417

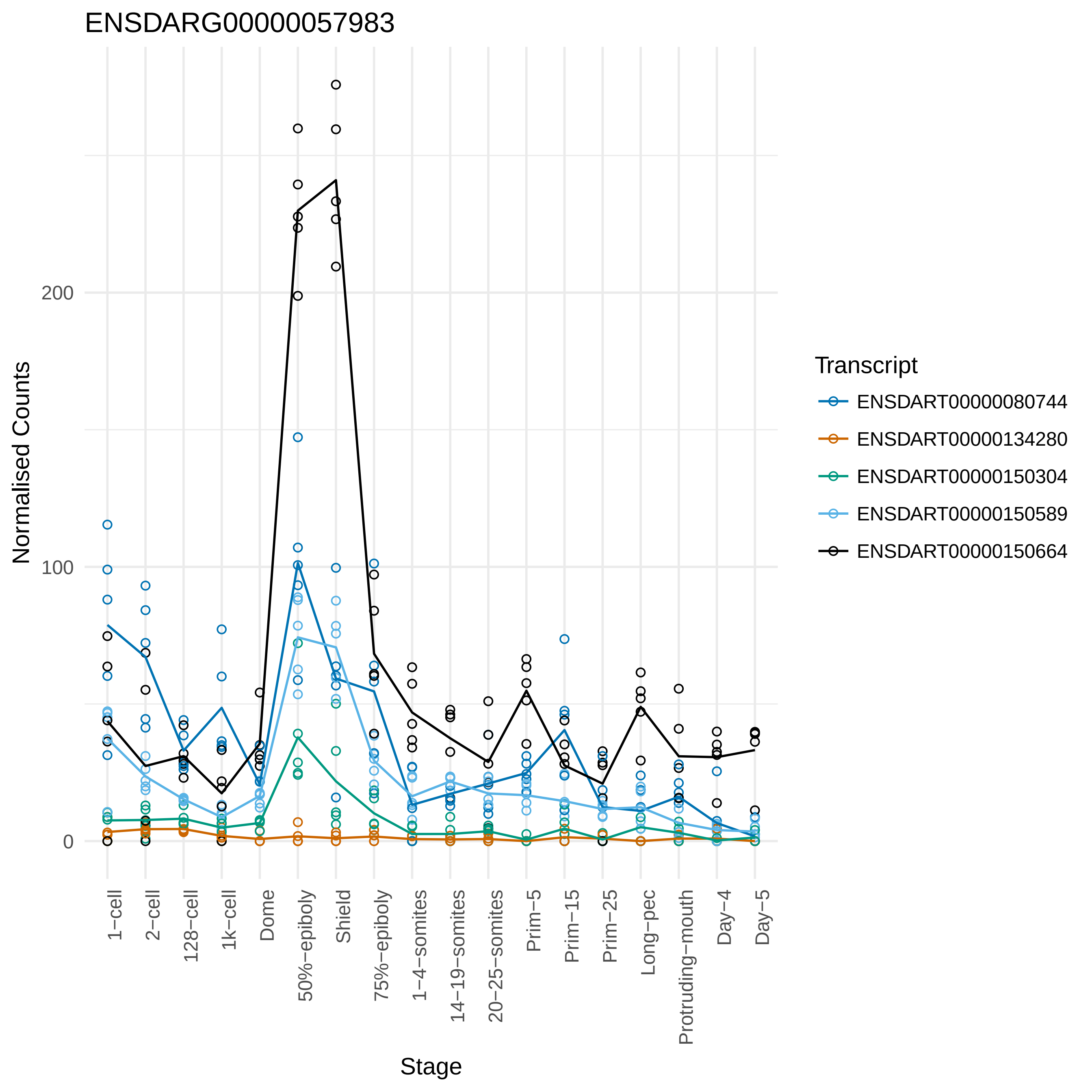

ENSDARG00000057983
Normalised Counts
200
100
0
1−cell
2−cell
128−cell
1k−cell
Dome
50%−epiboly
Shield
75%−epiboly
1−4−somites
14−19−somites
20−25−somites
Prim−5
Prim−15
Prim−25
Long−pec
Protruding−mouth
Day−4
Day−5
Stage
Transcript
ENSDART00000080744
ENSDART00000134280
ENSDART00000150304
ENSDART00000150589
ENSDART00000150664

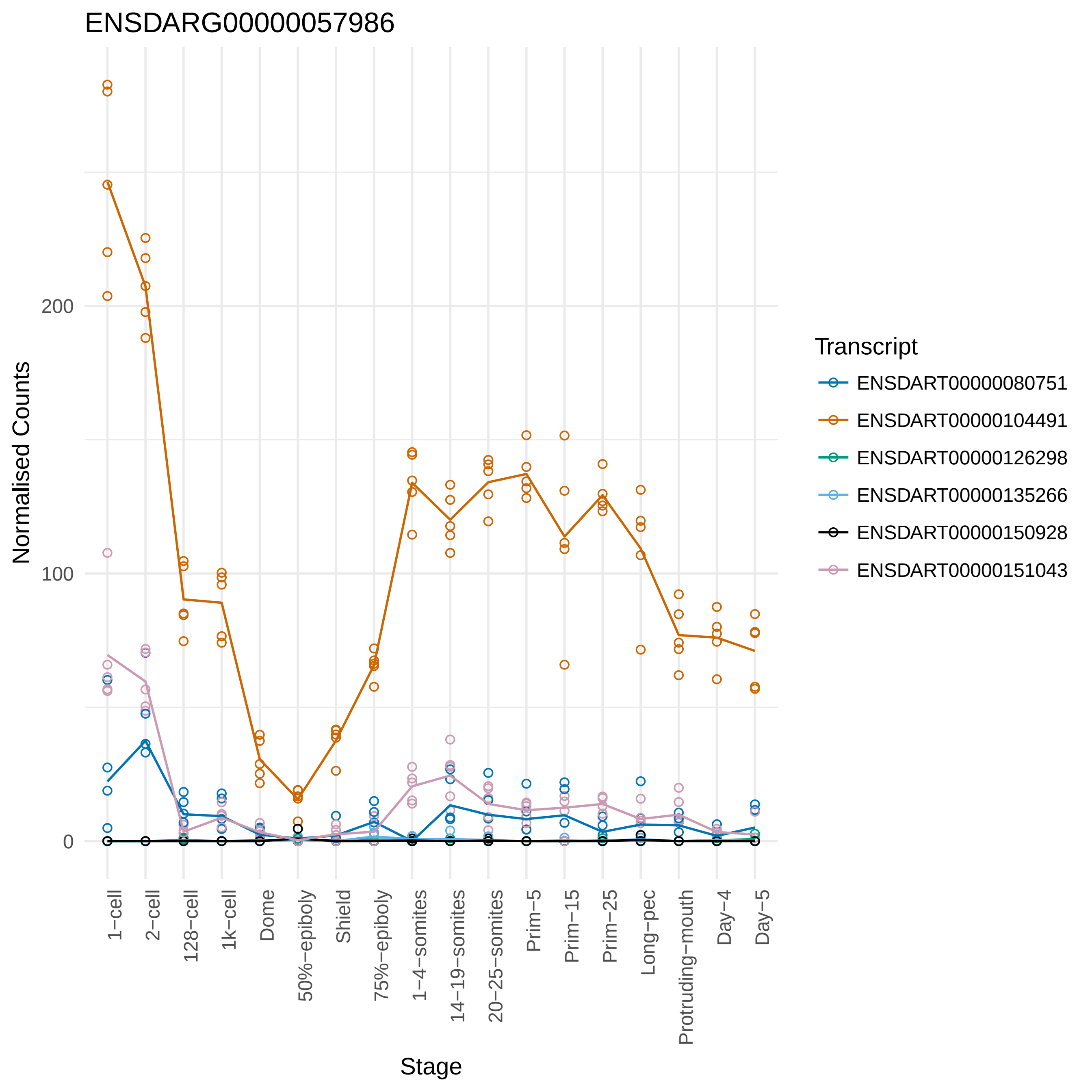

ENSDARG00000057986
Normalised Counts
200
100
0
Stage
1−cell
2−cell
128−cell
1k−cell
Dome
50%−epiboly
Shield
75%−epiboly
1−4−somites
14−19−somites
20−25−somites
Prim−5
Prim−15
Prim−25
Long−pec
Protruding−mouth
Day−4
Day−5
Transcript
ENSDART00000080751
ENSDART00000104491
ENSDART00000126298
ENSDART00000135266
ENSDART00000150928
ENSDART00000151043

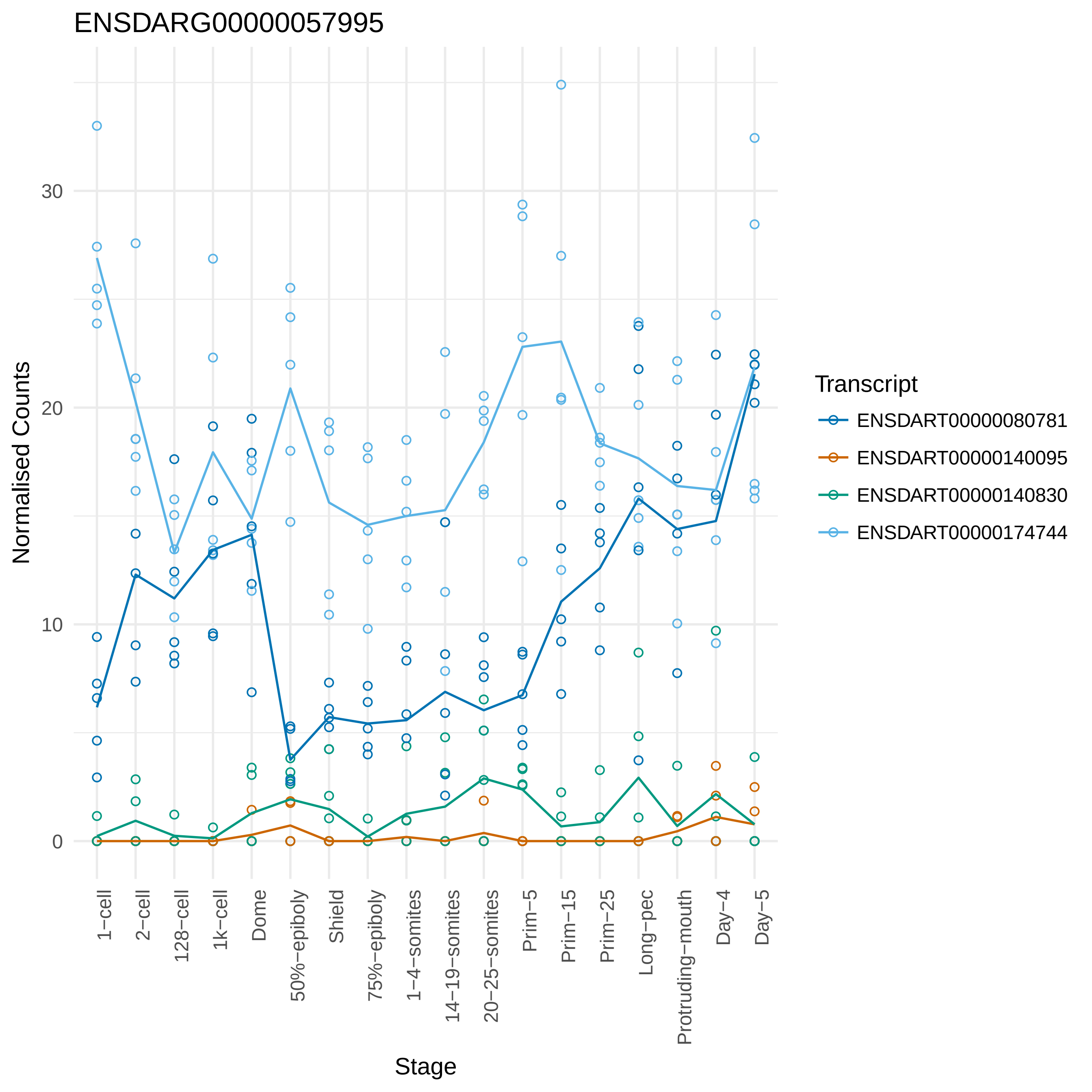
ENSDARG00000057995
Normalised Counts
0
10
20
30
1−cell
2−cell
128−cell
1k−cell
Dome
50%−epiboly
Shield
75%−epiboly
1−4−somites
14−19−somites
20−25−somites
Prim−5
Prim−15
Prim−25
Long−pec
Protruding−mouth
Day−4
Day−5
Stage
Transcript
ENSDART00000080781
ENSDART00000140095
ENSDART00000140830
ENSDART00000174744

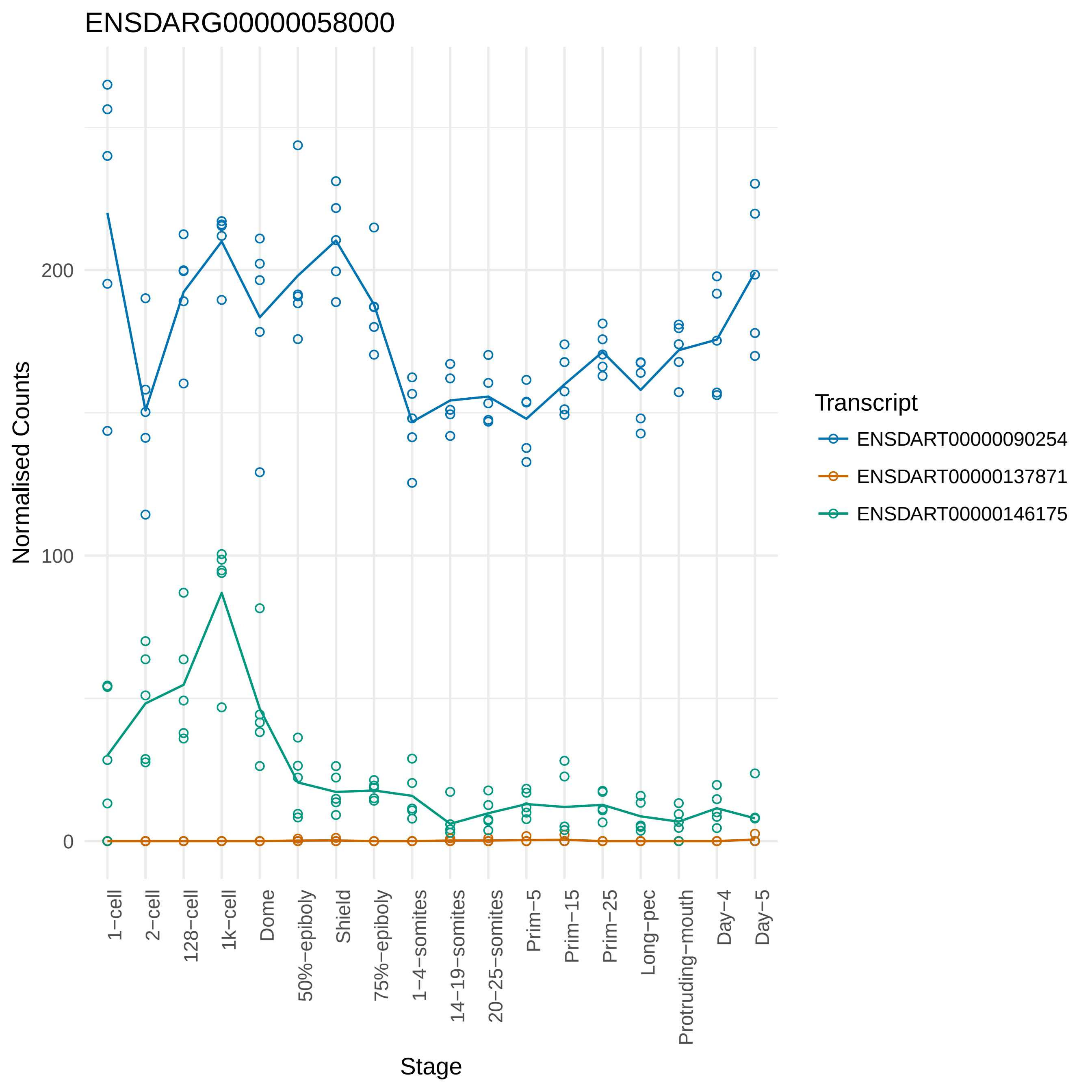
ENSDARG00000058000
Normalised Counts
0
100
200
1−cell
2−cell
128−cell
1k−cell
Dome
50%−epiboly
Shield
75%−epiboly
1−4−somites
14−19−somites
20−25−somites
Prim−5
Prim−15
Prim−25
Long−pec
Protruding−mouth
Day−4
Day−5
Stage
Transcript
ENSDART00000090254
ENSDART00000137871
ENSDART00000146175

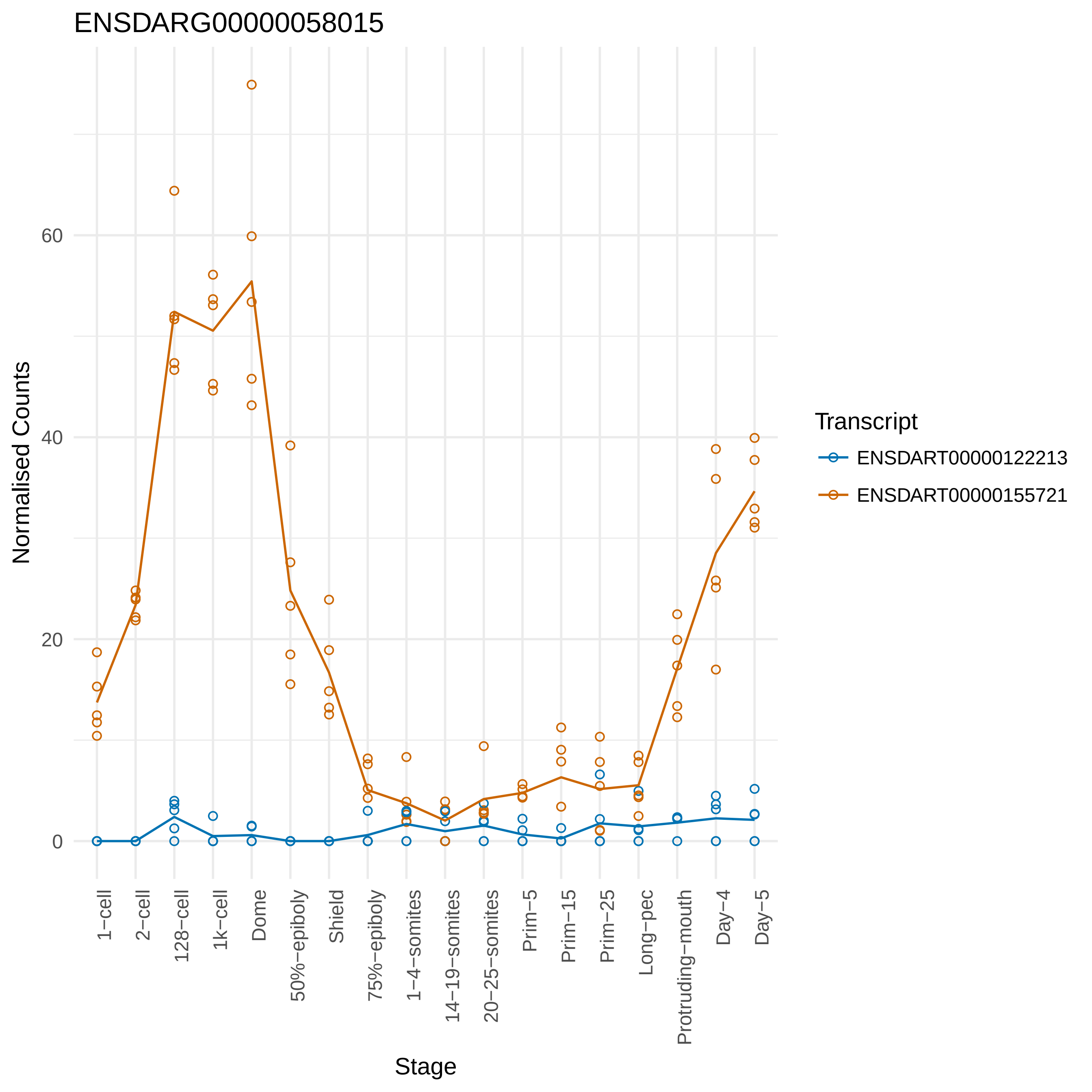
ENSDARG00000058015
Normalised Counts
0
20
40
60
1−cell
2−cell
128−cell
1k−cell
Dome
50%−epiboly
Shield
75%−epiboly
1−4−somites
14−19−somites
20−25−somites
Prim−5
Prim−15
Prim−25
Long−pec
Protruding−mouth
Day−4
Day−5
Stage
Transcript
ENSDART00000122213
ENSDART00000155721

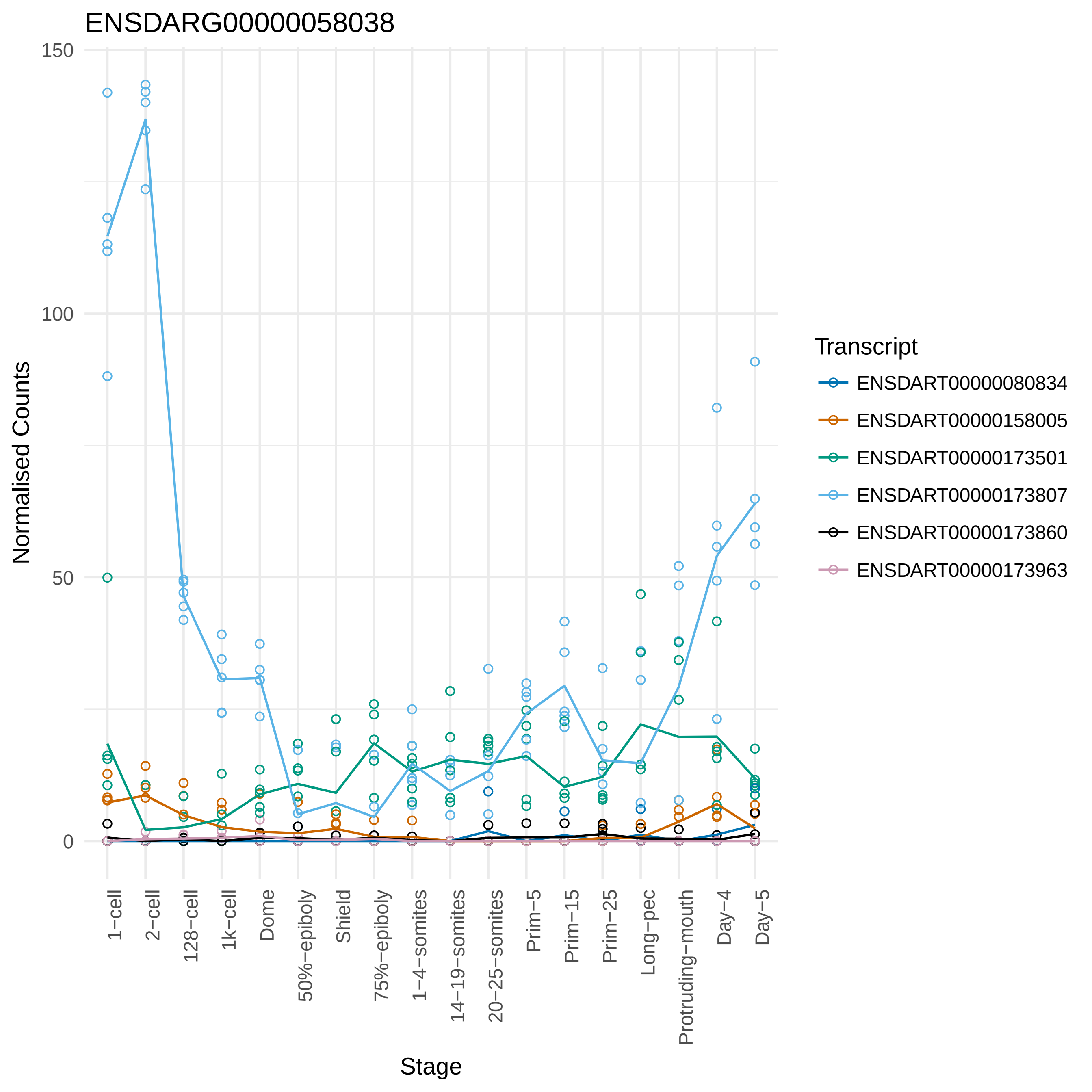
ENSDARG00000058038
Normalised Counts
150
100
50
0
1−cell
2−cell
128−cell
1k−cell
Dome
50%−epiboly
Shield
75%−epiboly
1−4−somites
14−19−somites
20−25−somites
Prim−5
Prim−15
Prim−25
Long−pec
Protruding−mouth
Day−4
Day−5
Stage
Transcript
ENSDART00000080834
ENSDART00000158005
ENSDART00000173501
ENSDART00000173807
ENSDART00000173860
ENSDART00000173963

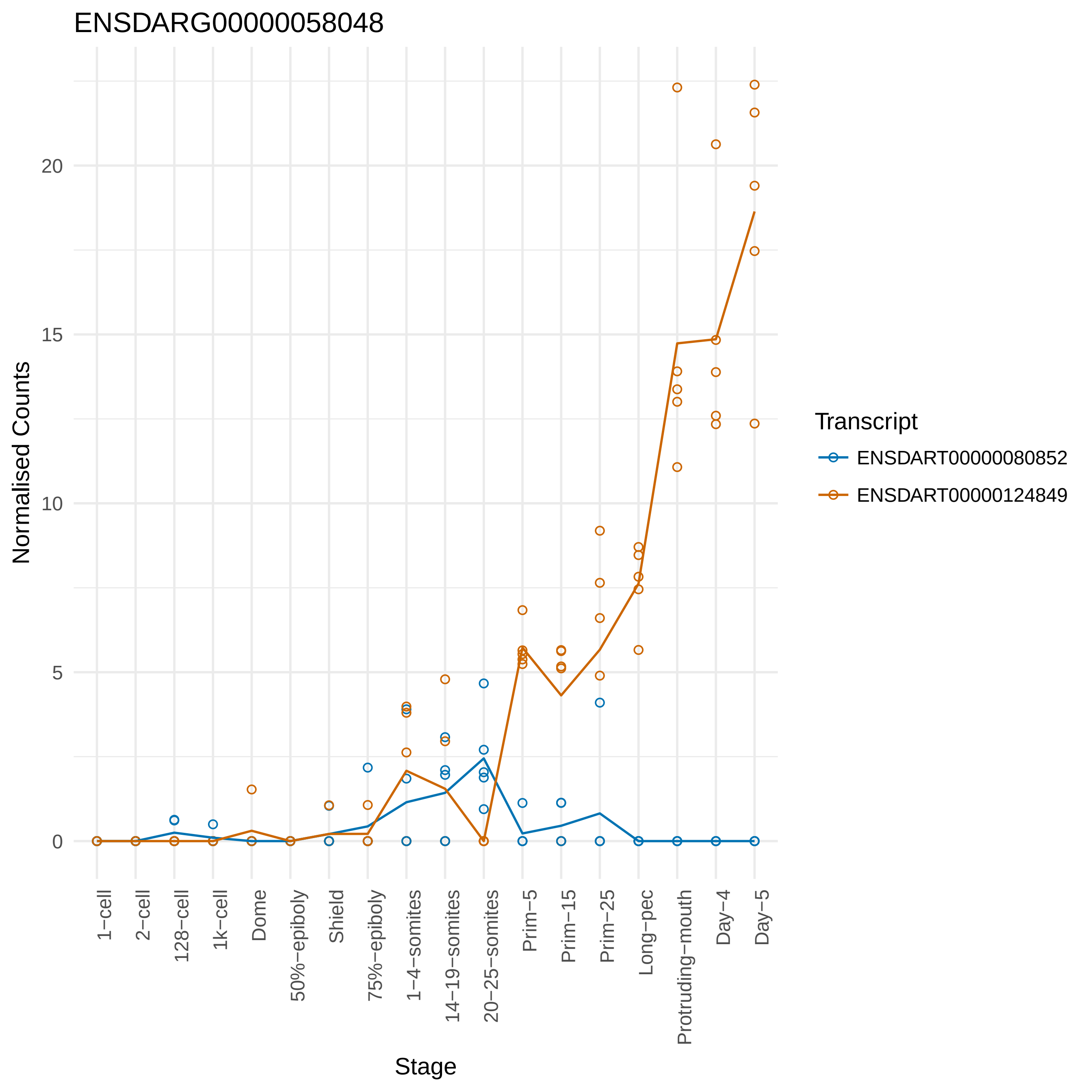

ENSDARG00000058048
Normalised Counts
Stage
Transcript
ENSDART00000080852
ENSDART00000124849
0
5
10
15
20
1−cell
2−cell
128−cell
1k−cell
Dome
50%−epiboly
Shield
75%−epiboly
1−4−somites
14−19−somites
20−25−somites
Prim−5
Prim−15
Prim−25
Long−pec
Protruding−mouth
Day−4
Day−5

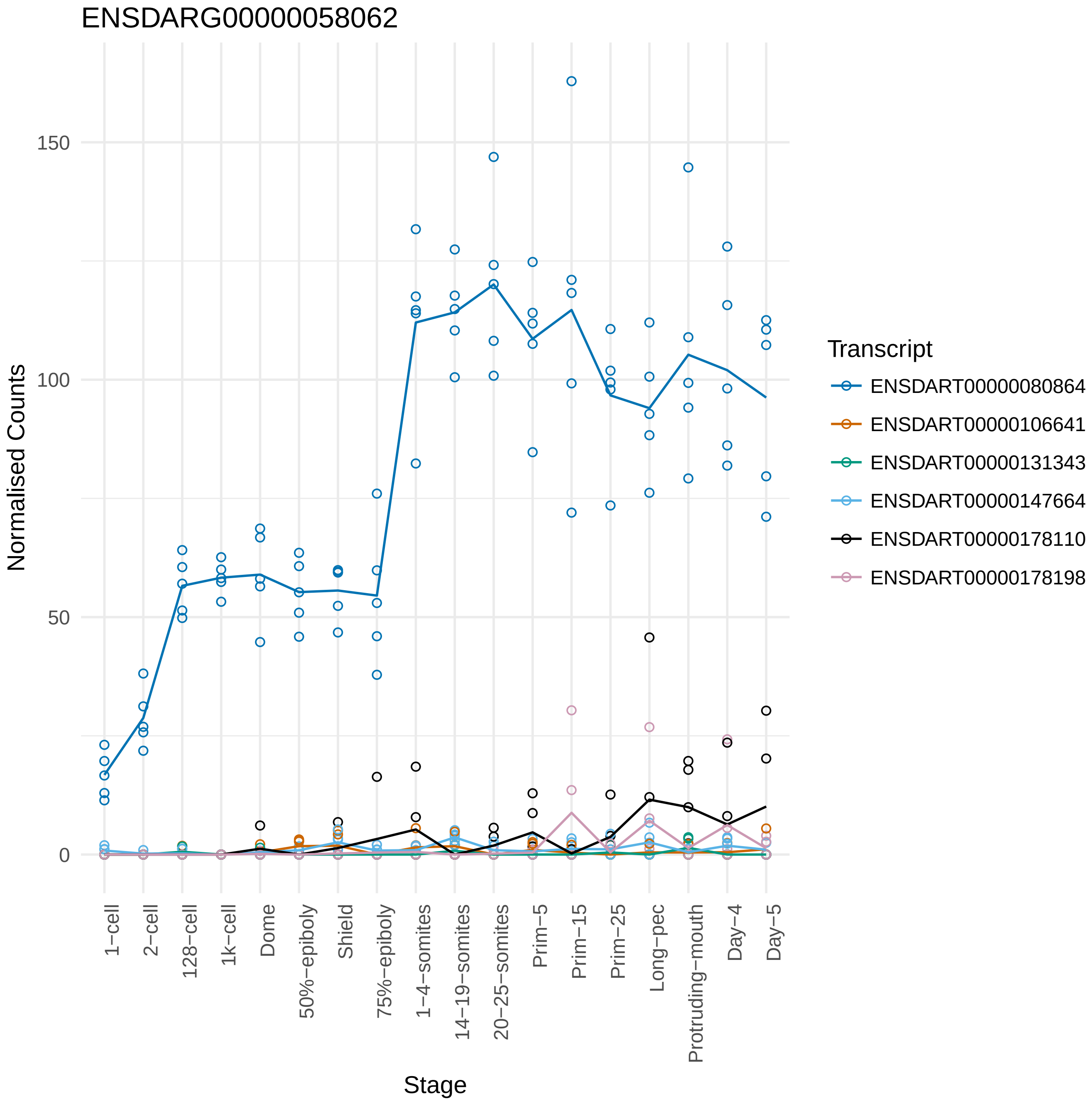
ENSDARG00000058062
Normalised Counts
150
100
50
0
1−cell
2−cell
128−cell
1k−cell
Dome
50%−epiboly
Shield
75%−epiboly
1−4−somites
14−19−somites
20−25−somites
Prim−5
Prim−15
Prim−25
Long−pec
Protruding−mouth
Day−4
Day−5
Stage
Transcript
ENSDART00000080864
ENSDART00000106641
ENSDART00000131343
ENSDART00000147664
ENSDART00000178110
ENSDART00000178198

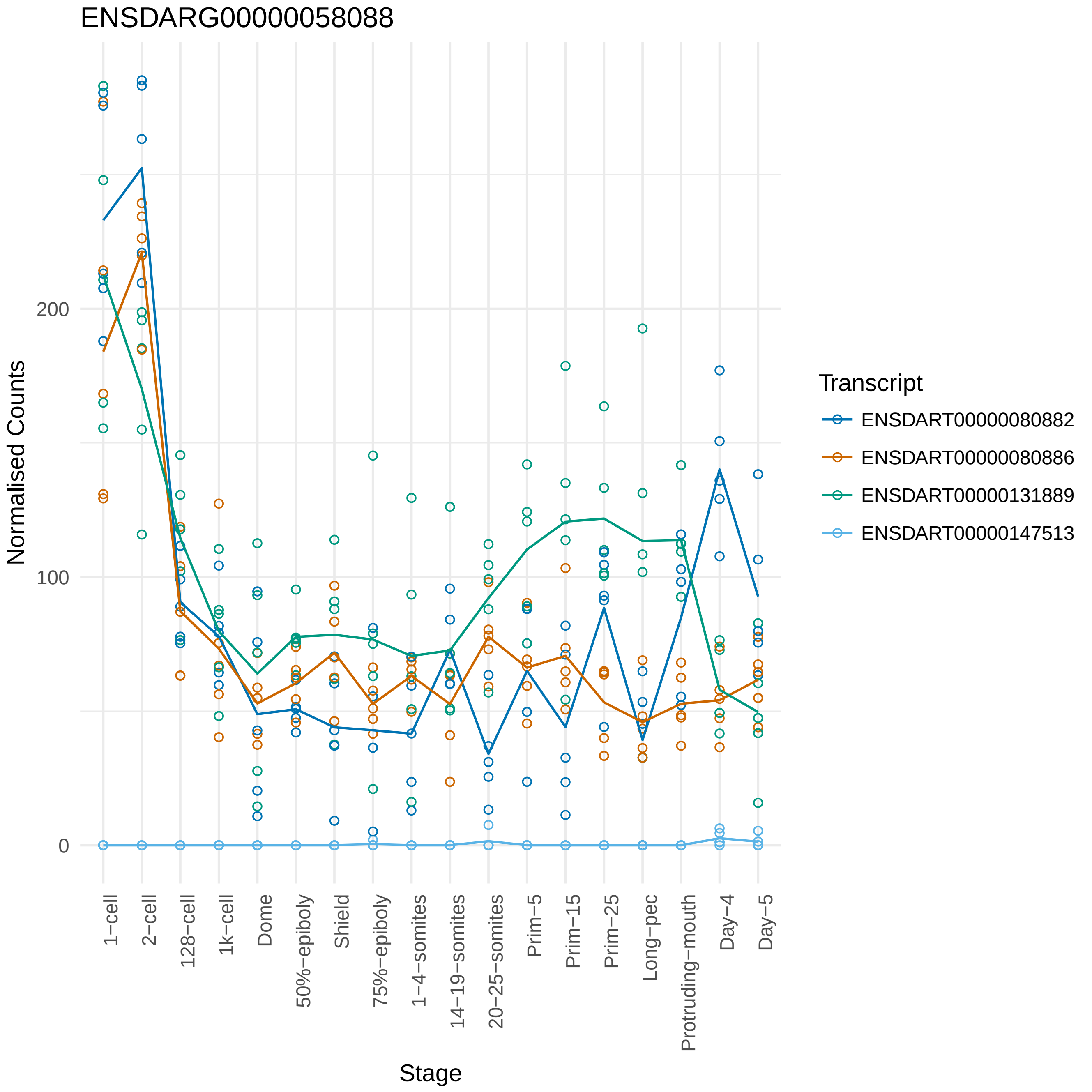
ENSDARG00000058088
Normalised Counts
0
100
200
Stage
1−cell
2−cell
128−cell
1k−cell
Dome
50%−epiboly
Shield
75%−epiboly
1−4−somites
14−19−somites
20−25−somites
Prim−5
Prim−15
Prim−25
Long−pec
Protruding−mouth
Day−4
Day−5
Transcript
ENSDART00000080882
ENSDART00000080886
ENSDART00000131889
ENSDART00000147513

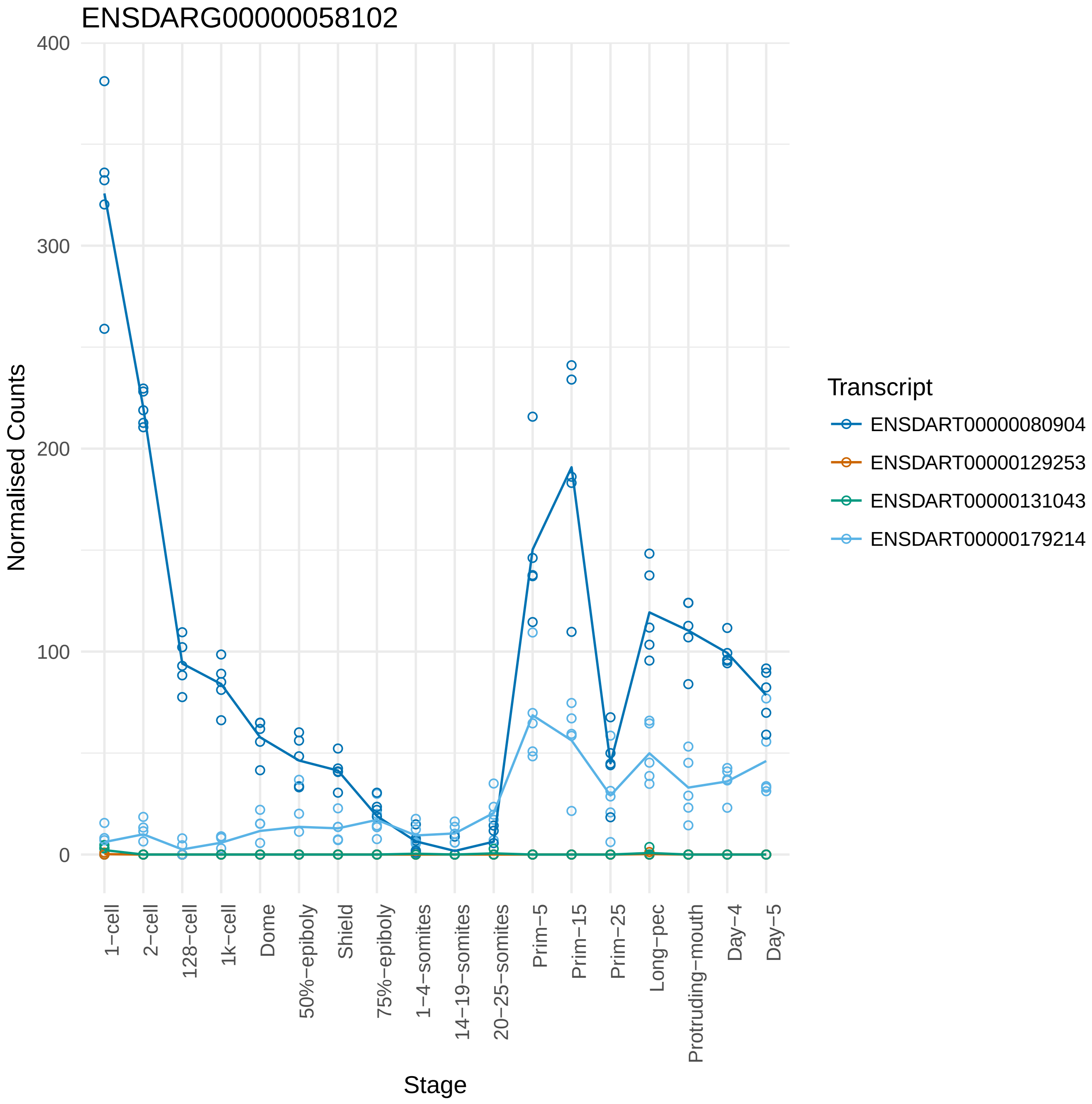
ENSDARG00000058102
Normalised Counts
400
300
200
100
0
Stage
1−cell
2−cell
128−cell
1k−cell
Dome
50%−epiboly
Shield
75%−epiboly
1−4−somites
14−19−somites
20−25−somites
Prim−5
Prim−15
Prim−25
Long−pec
Protruding−mouth
Day−4
Day−5
Transcript
ENSDART00000080904
ENSDART00000129253
ENSDART00000131043
ENSDART00000179214

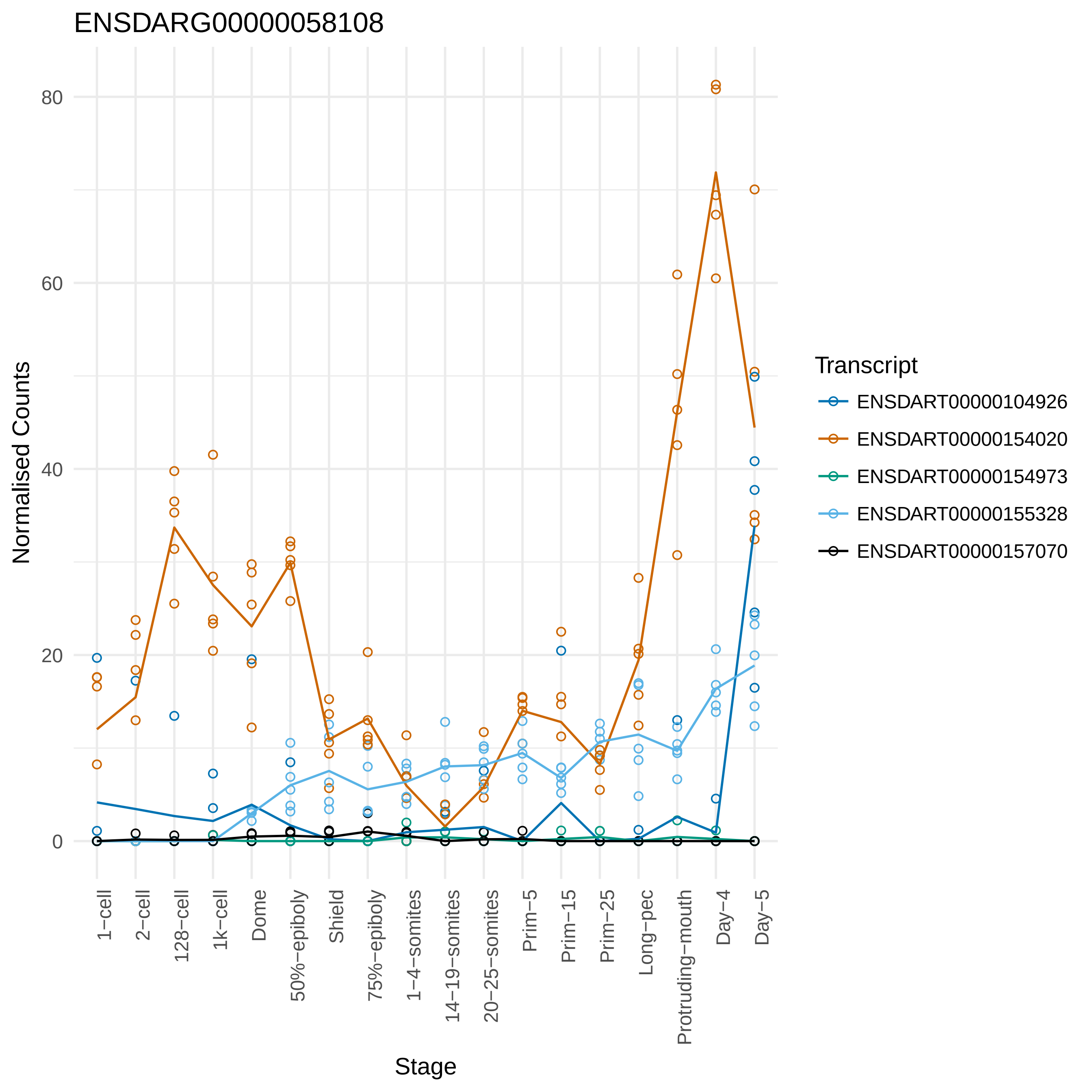
ENSDARG00000058108
Normalised Counts
0
20
40
60
80
1−cell
2−cell
128−cell
1k−cell
Dome
50%−epiboly
Shield
75%−epiboly
1−4−somites
14−19−somites
20−25−somites
Prim−5
Prim−15
Prim−25
Long−pec
Protruding−mouth
Day−4
Day−5
Stage
Transcript
ENSDART00000104926
ENSDART00000154020
ENSDART00000154973
ENSDART00000155328
ENSDART00000157070

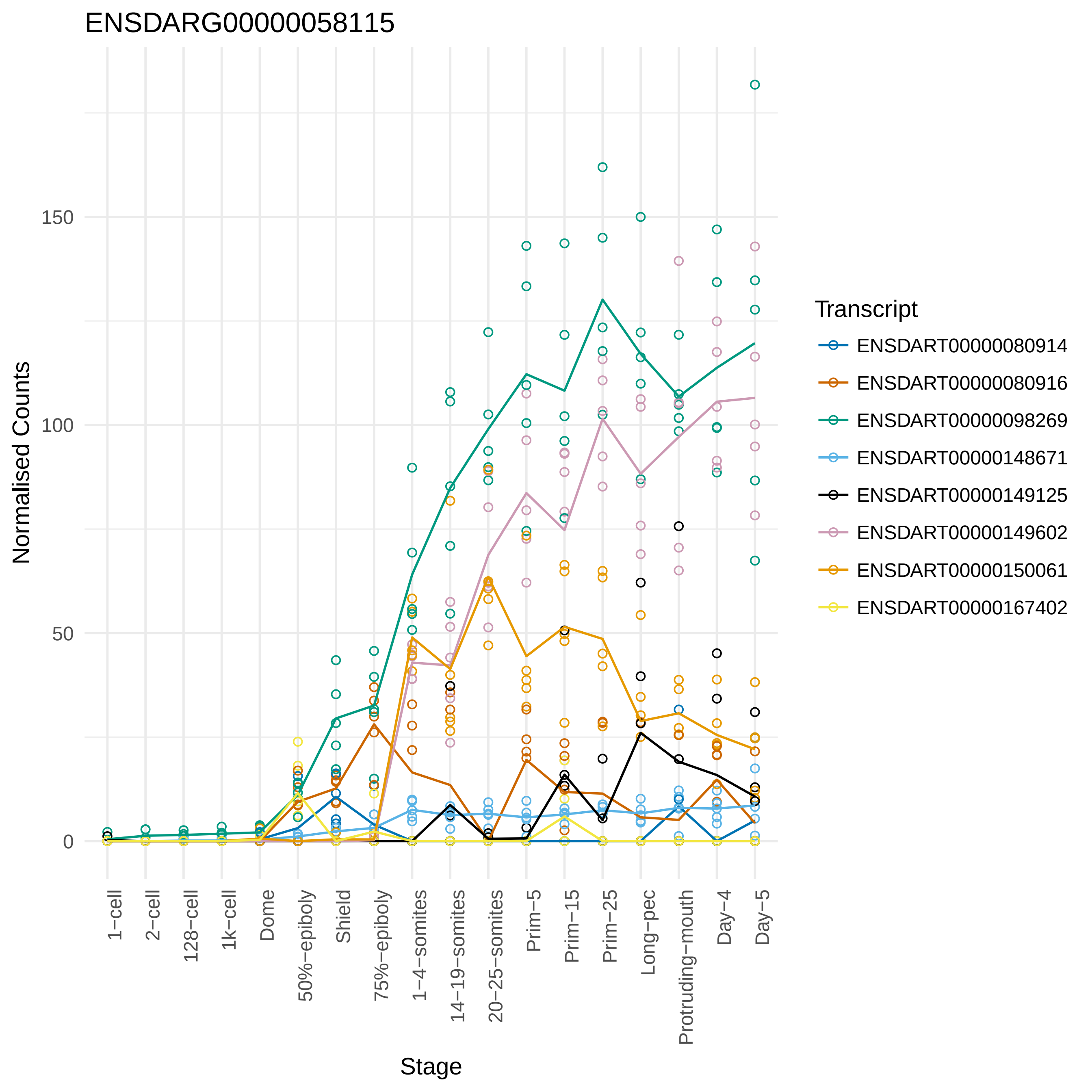

ENSDARG00000058115
Normalised Counts
0
50
100
150
1−cell
2−cell
128−cell
1k−cell
Dome
50%−epiboly
Shield
75%−epiboly
1−4−somites
14−19−somites
20−25−somites
Prim−5
Prim−15
Prim−25
Long−pec
Protruding−mouth
Day−4
Day−5
Stage
Transcript
ENSDART00000080914
ENSDART00000080916
ENSDART00000098269
ENSDART00000148671
ENSDART00000149125
ENSDART00000149602
ENSDART00000150061
ENSDART00000167402

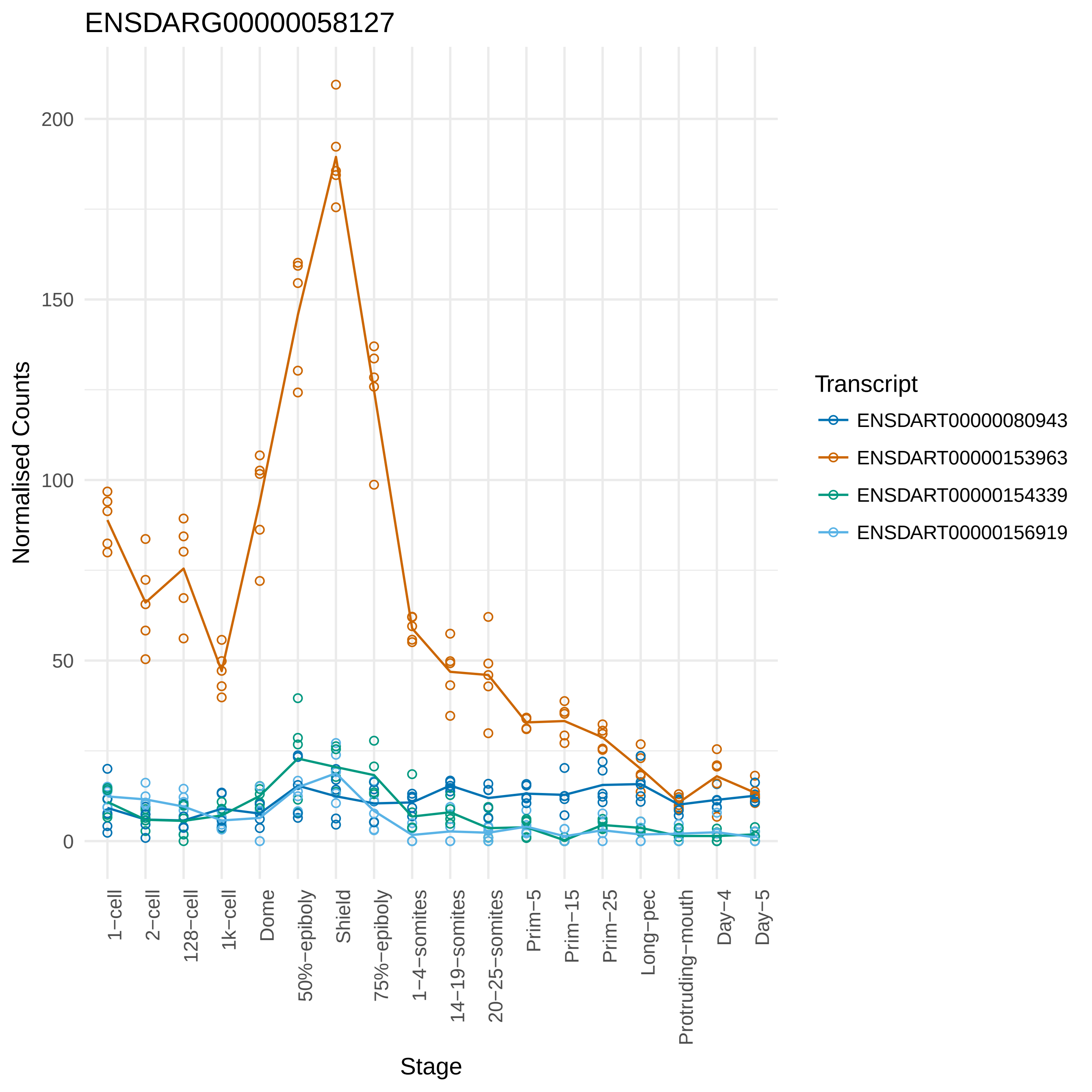

ENSDARG00000058127
Normalised Counts
0
50
100
150
200
1−cell
2−cell
128−cell
1k−cell
Dome
50%−epiboly
Shield
75%−epiboly
1−4−somites
14−19−somites
20−25−somites
Prim−5
Prim−15
Prim−25
Long−pec
Protruding−mouth
Day−4
Day−5
Stage
Transcript
ENSDART00000080943
ENSDART00000153963
ENSDART00000154339
ENSDART00000156919

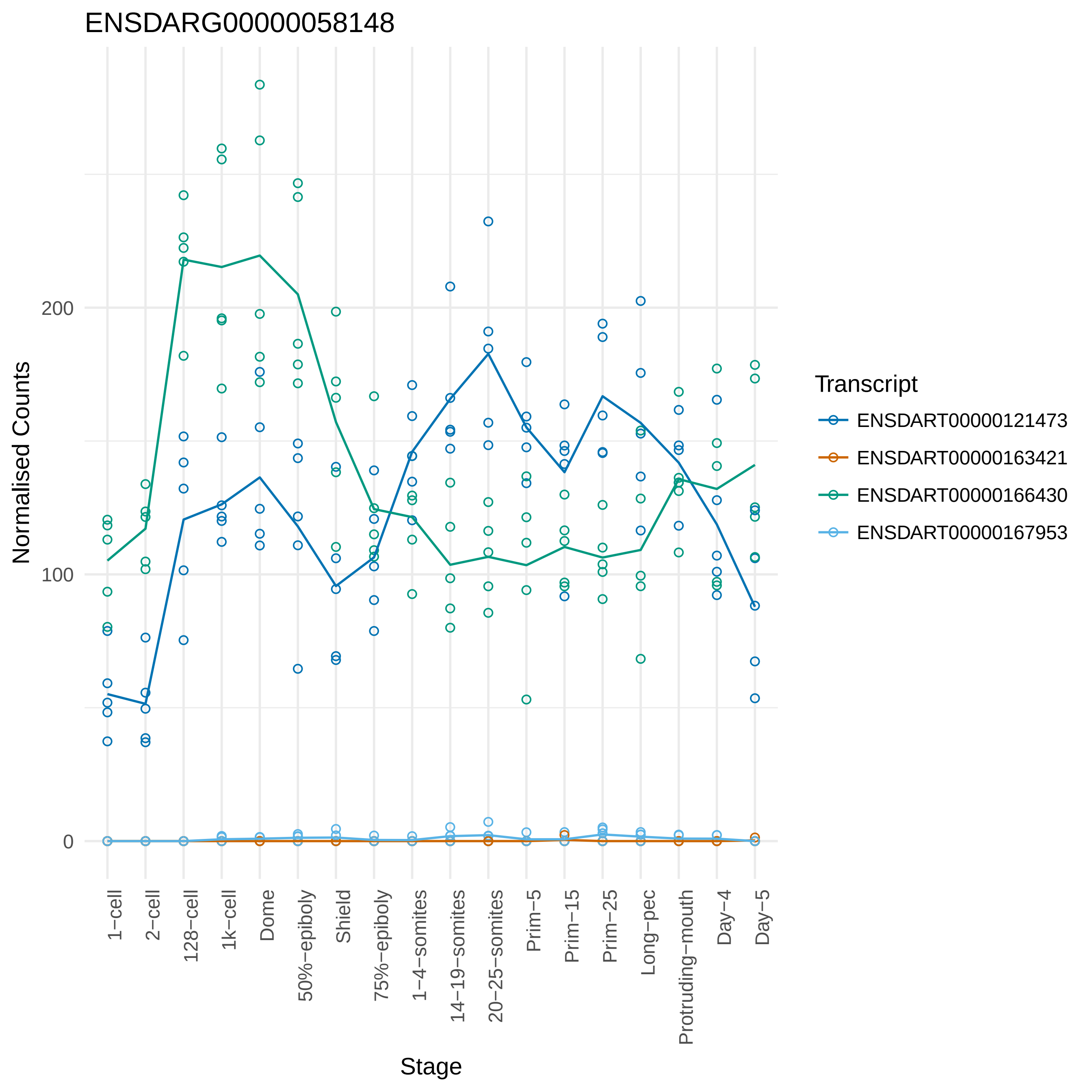

ENSDARG00000058148
Normalised Counts
200
100
0
1−cell
2−cell
128−cell
1k−cell
Dome
50%−epiboly
Shield
75%−epiboly
1−4−somites
14−19−somites
20−25−somites
Prim−5
Prim−15
Prim−25
Long−pec
Protruding−mouth
Day−4
Day−5
Stage
Transcript
ENSDART00000121473
ENSDART00000163421
ENSDART00000166430
ENSDART00000167953

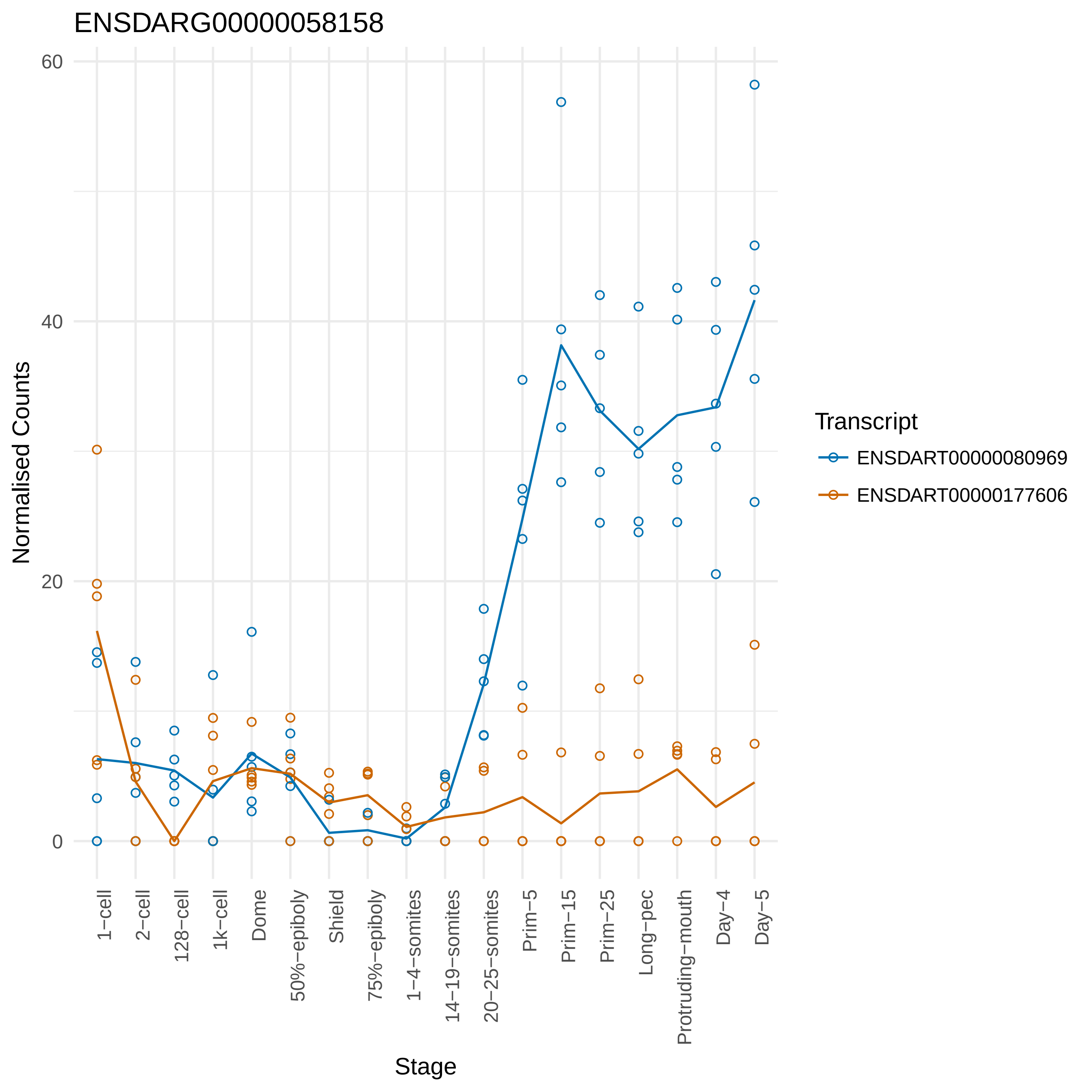
ENSDARG00000058158
Normalised Counts
60
40
20
0
1−cell
2−cell
128−cell
1k−cell
Dome
50%−epiboly
Shield
75%−epiboly
1−4−somites
14−19−somites
20−25−somites
Prim−5
Prim−15
Prim−25
Long−pec
Protruding−mouth
Day−4
Day−5
Stage
Transcript
ENSDART00000080969
ENSDART00000177606

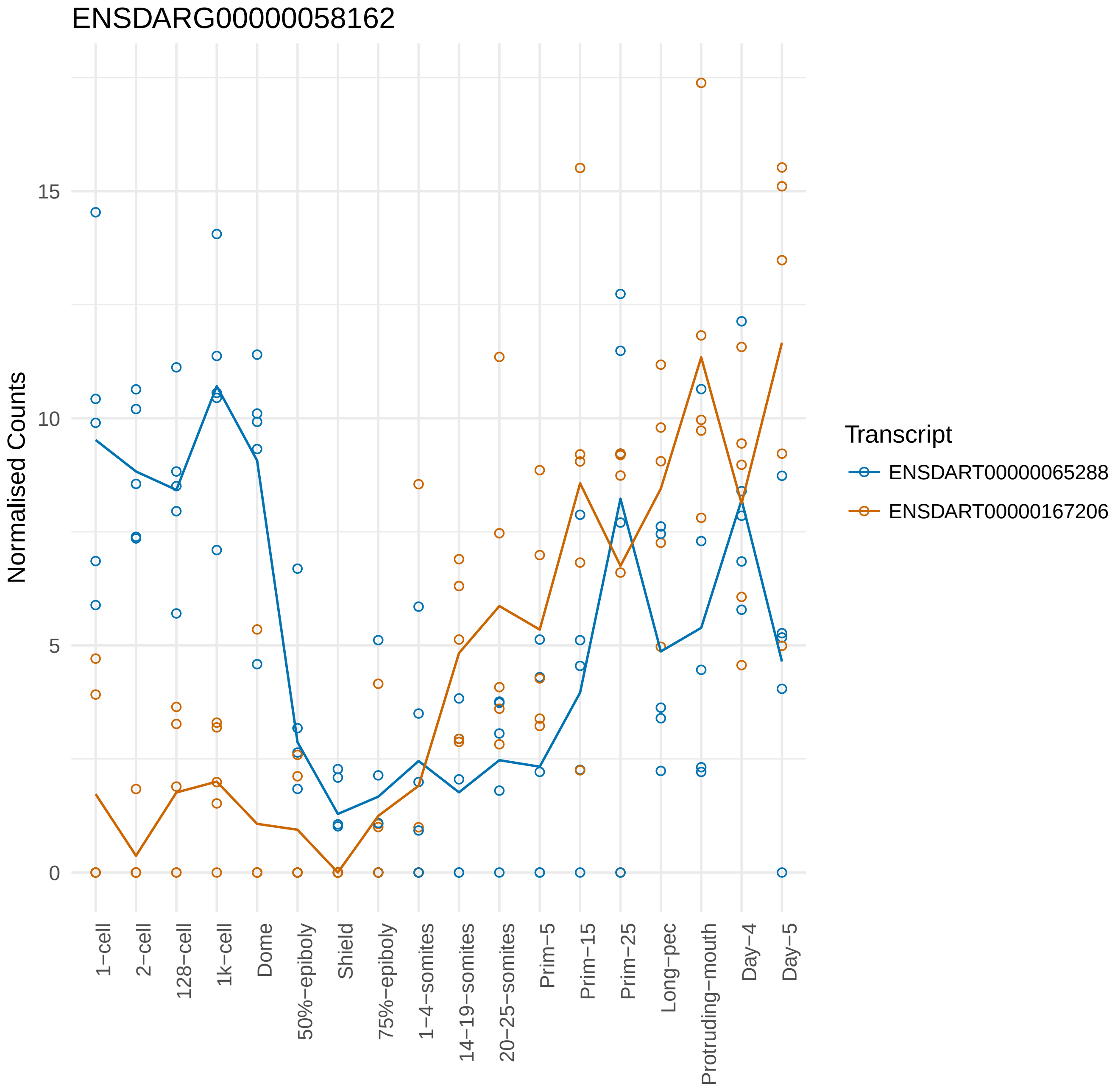

ENSDARG00000058162
Normalised Counts
15
10
5
0
1−cell
2−cell
128−cell
1k−cell
Dome
50%−epiboly
Shield
75%−epiboly
1−4−somites
14−19−somites
20−25−somites
Prim−5
Prim−15
Prim−25
Long−pec
Protruding−mouth
Day−4
Day−5
Stage
Transcript
ENSDART00000065288
ENSDART00000167206

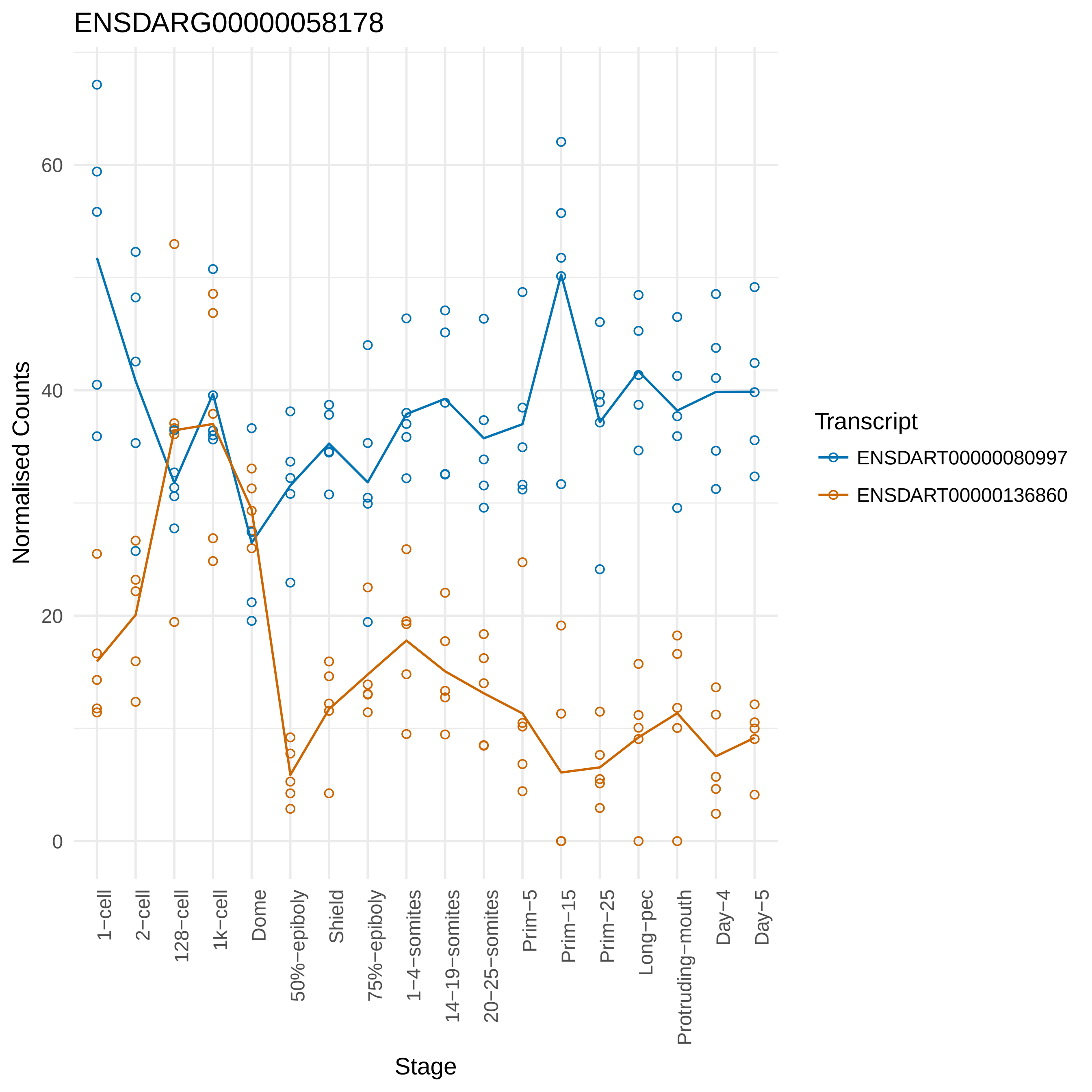
ENSDARG00000058178
Normalised Counts
0
20
40
60
Stage
1−cell
2−cell
128−cell
1k−cell
Dome
50%−epiboly
Shield
75%−epiboly
1−4−somites
14−19−somites
20−25−somites
Prim−5
Prim−15
Prim−25
Long−pec
Protruding−mouth
Day−4
Day−5
Transcript
ENSDART00000080997
ENSDART00000136860

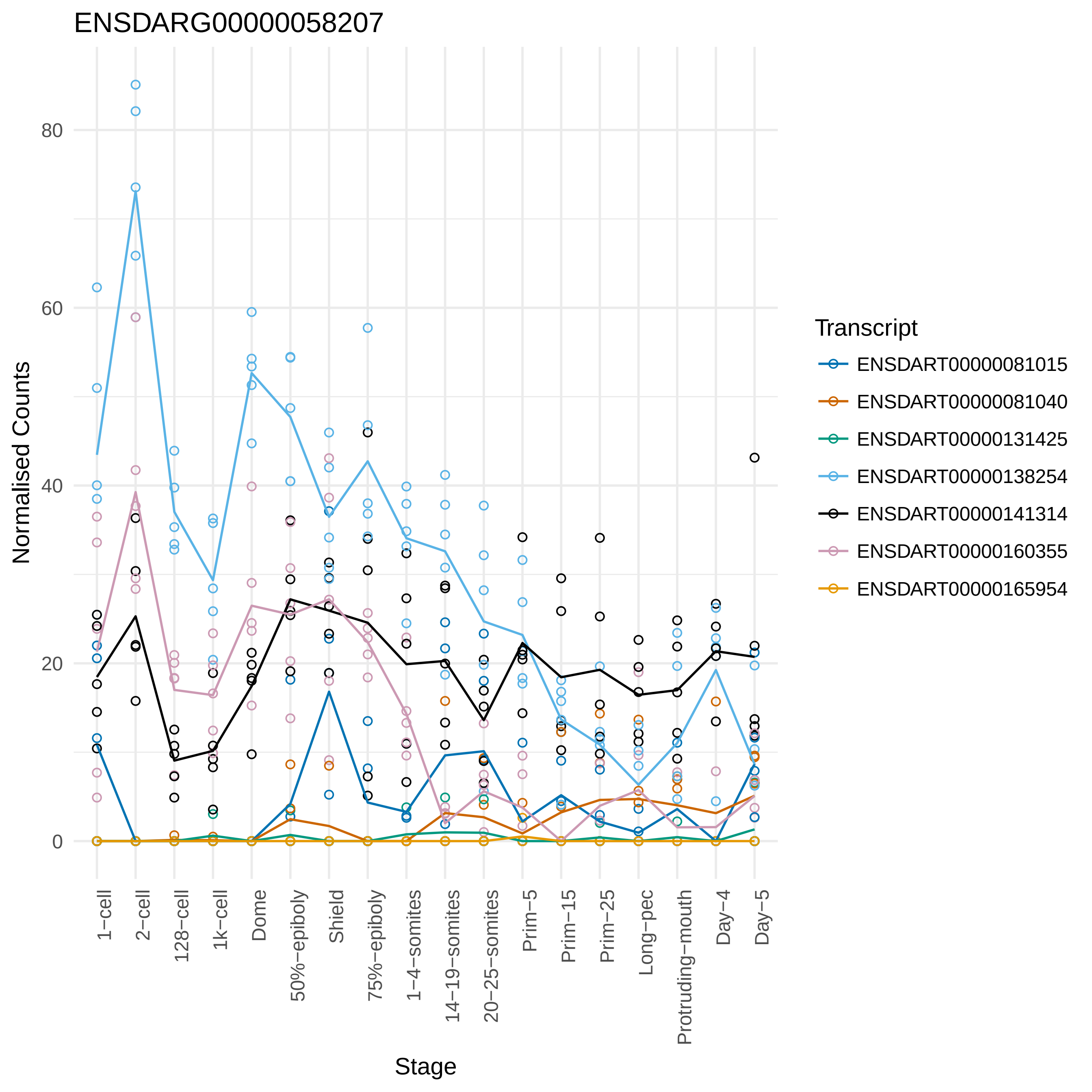

ENSDARG00000058207
Normalised Counts
0
20
40
60
80
1−cell
2−cell
128−cell
1k−cell
Dome
50%−epiboly
Shield
75%−epiboly
1−4−somites
14−19−somites
20−25−somites
Prim−5
Prim−15
Prim−25
Long−pec
Protruding−mouth
Day−4
Day−5
Stage
Transcript
ENSDART00000081015
ENSDART00000081040
ENSDART00000131425
ENSDART00000138254
ENSDART00000141314
ENSDART00000160355
ENSDART00000165954

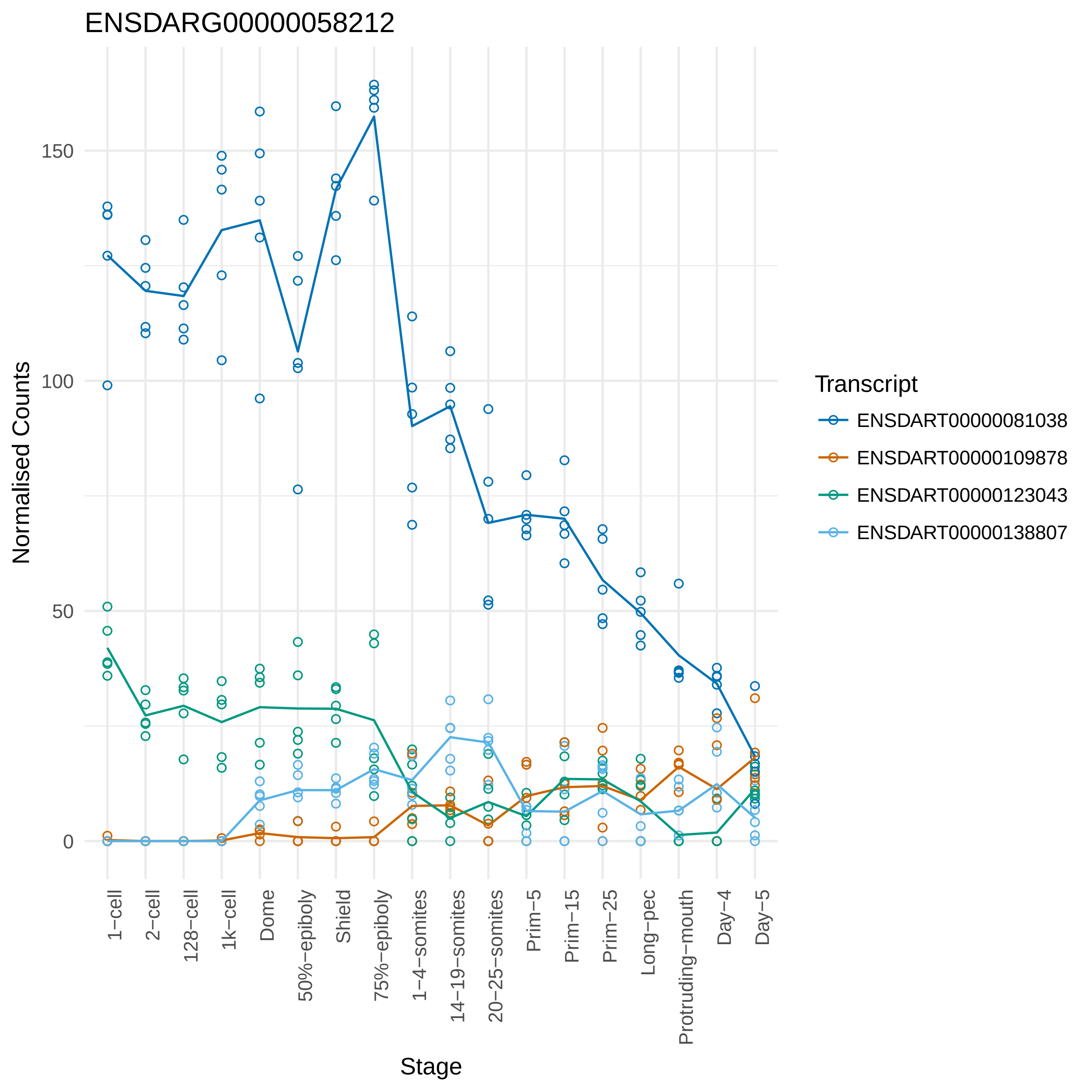

ENSDARG00000058212
Normalised Counts
150
100
50
0
1−cell
2−cell
128−cell
1k−cell
Dome
50%−epiboly
Shield
75%−epiboly
1−4−somites
14−19−somites
20−25−somites
Prim−5
Prim−15
Prim−25
Long−pec
Protruding−mouth
Day−4
Day−5
Stage
Transcript
ENSDART00000081038
ENSDART00000109878
ENSDART00000123043
ENSDART00000138807

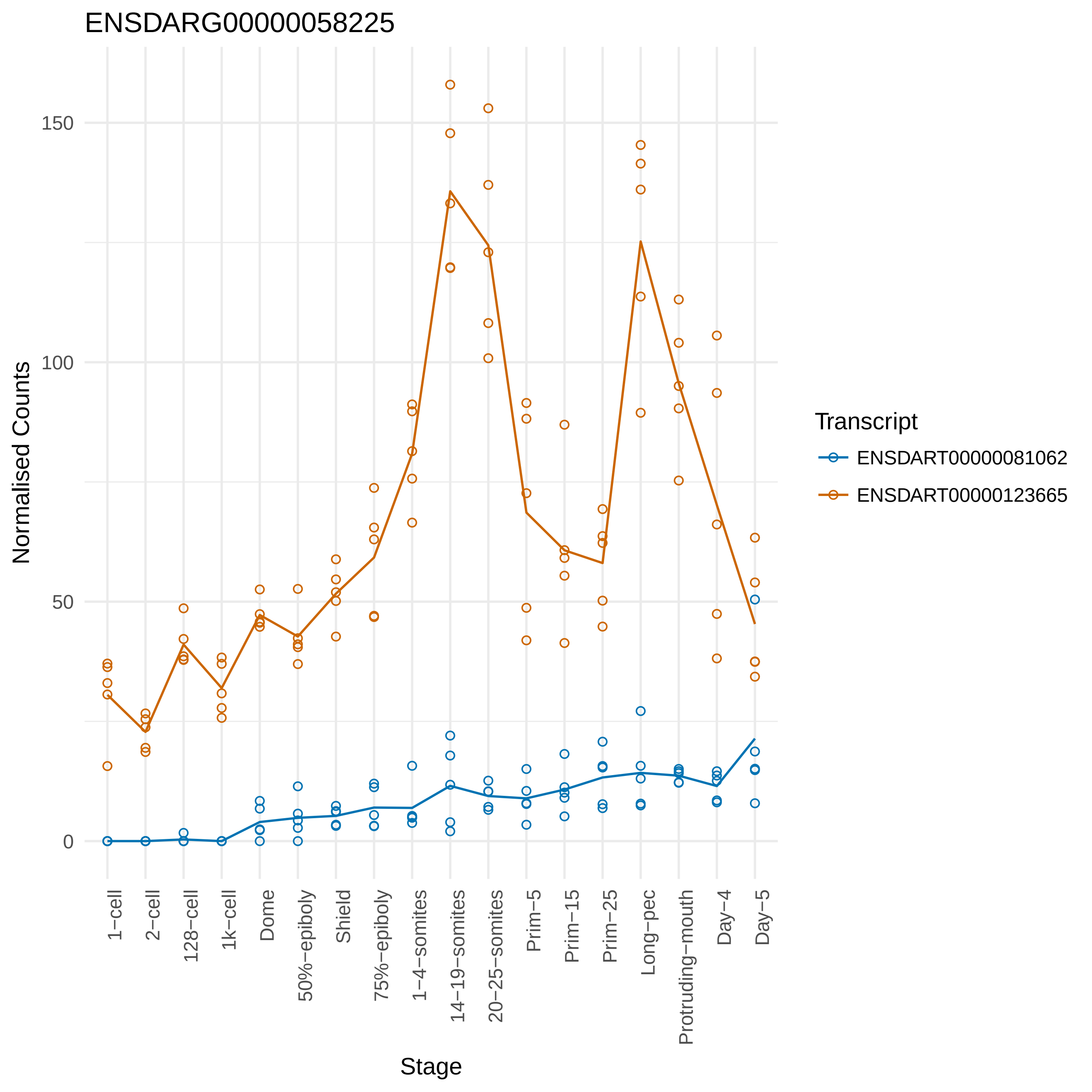

ENSDARG00000058225
Normalised Counts
150
100
50
0
1−cell
2−cell
128−cell
1k−cell
Dome
50%−epiboly
Shield
75%−epiboly
1−4−somites
14−19−somites
20−25−somites
Prim−5
Prim−15
Prim−25
Long−pec
Protruding−mouth
Day−4
Day−5
Stage
Transcript
ENSDART00000081062
ENSDART00000123665

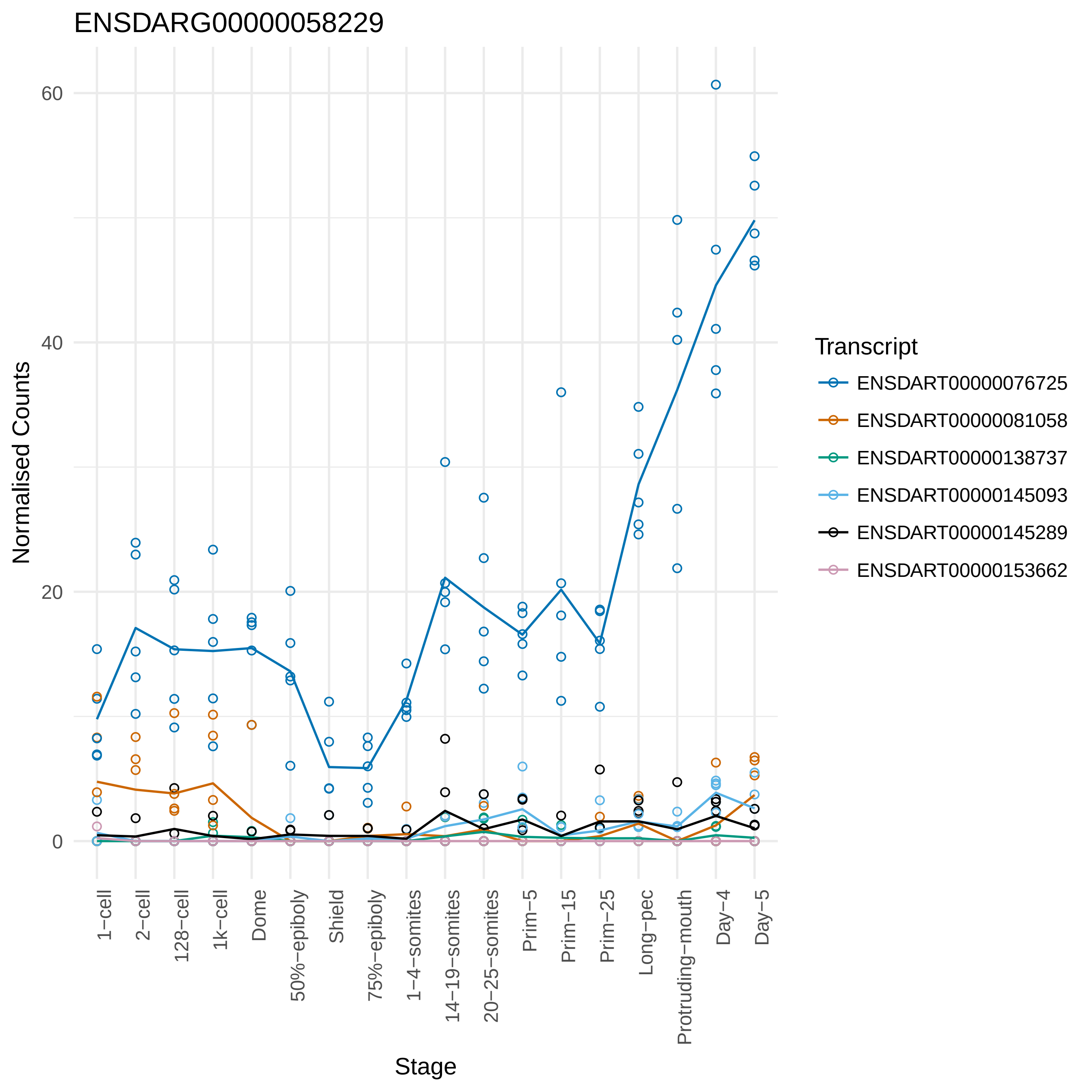
ENSDARG00000058229
Normalised Counts
0
20
40
60
Stage
1−cell
2−cell
128−cell
1k−cell
Dome
50%−epiboly
Shield
75%−epiboly
1−4−somites
14−19−somites
20−25−somites
Prim−5
Prim−15
Prim−25
Long−pec
Protruding−mouth
Day−4
Day−5
Transcript
ENSDART00000076725
ENSDART00000081058
ENSDART00000138737
ENSDART00000145093
ENSDART00000145289
ENSDART00000153662

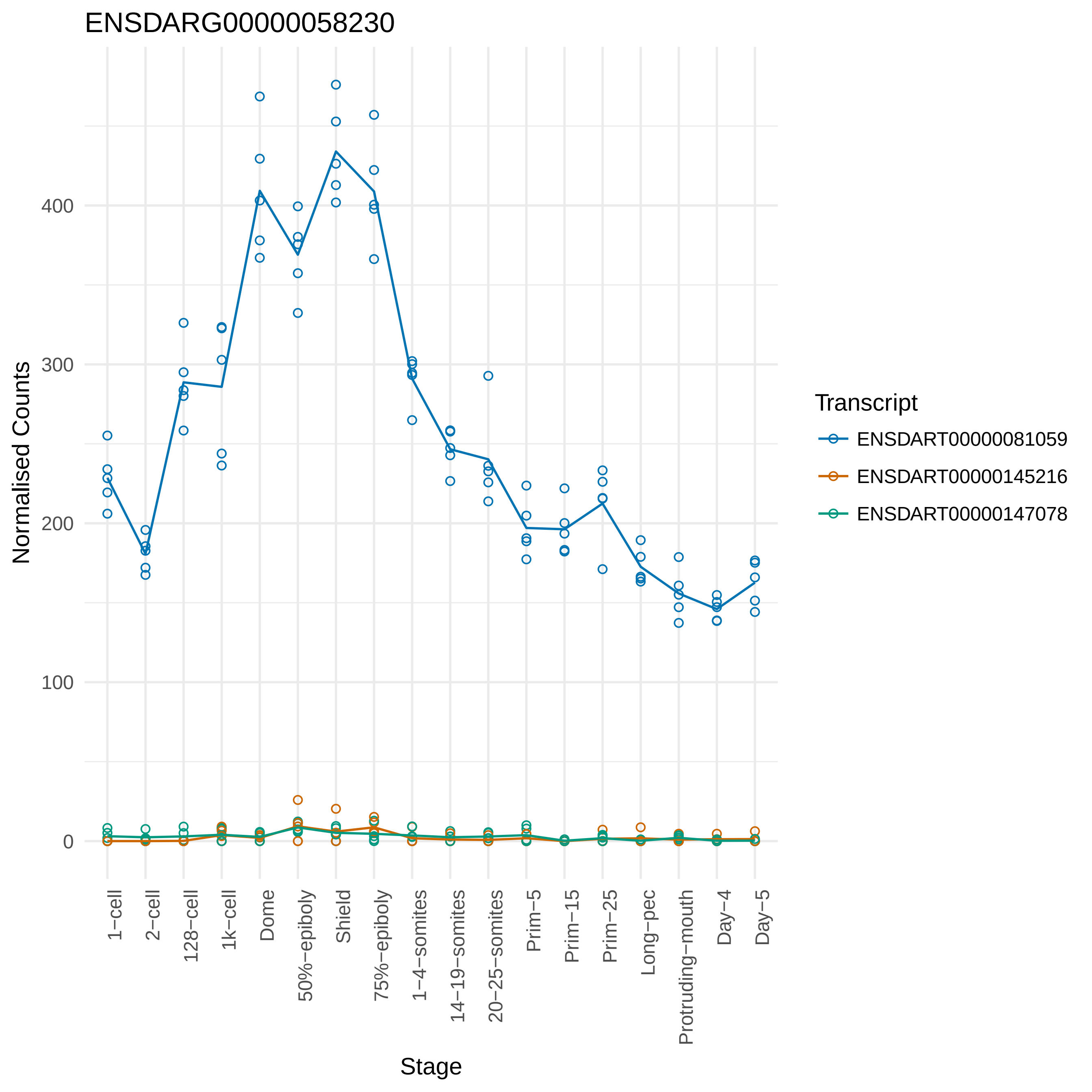

ENSDARG00000058230
Normalised Counts
400
300
200
100
0
1−cell
2−cell
128−cell
1k−cell
Dome
50%−epiboly
Shield
75%−epiboly
1−4−somites
14−19−somites
20−25−somites
Prim−5
Prim−15
Prim−25
Long−pec
Protruding−mouth
Day−4
Day−5
Stage
Transcript
ENSDART00000081059
ENSDART00000145216
ENSDART00000147078

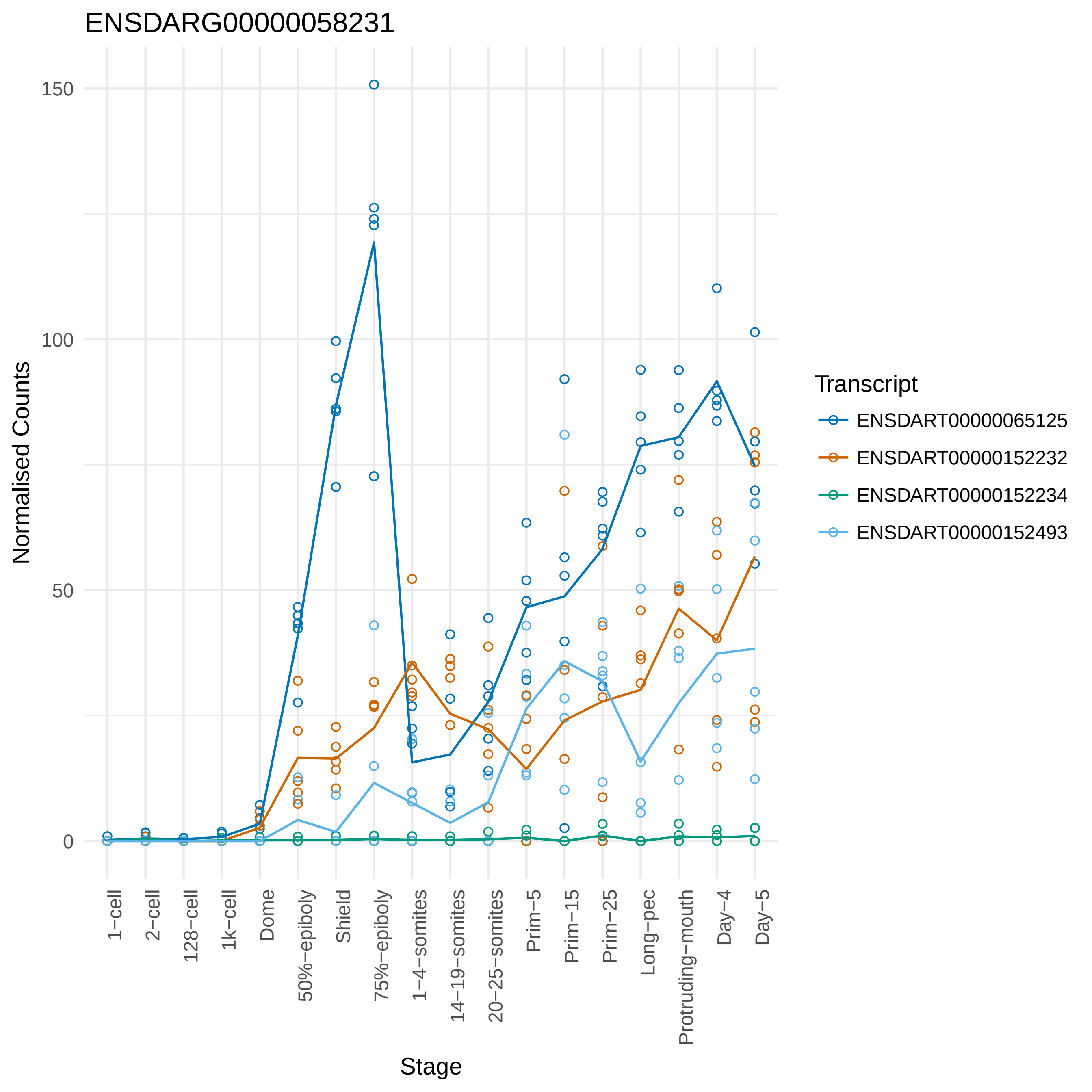

ENSDARG00000058231
Normalised Counts
150
100
50
0
1−cell
2−cell
128−cell
1k−cell
Dome
50%−epiboly
Shield
75%−epiboly
1−4−somites
14−19−somites
20−25−somites
Prim−5
Prim−15
Prim−25
Long−pec
Protruding−mouth
Day−4
Day−5
Stage
Transcript
ENSDART00000065125
ENSDART00000152232
ENSDART00000152234
ENSDART00000152493

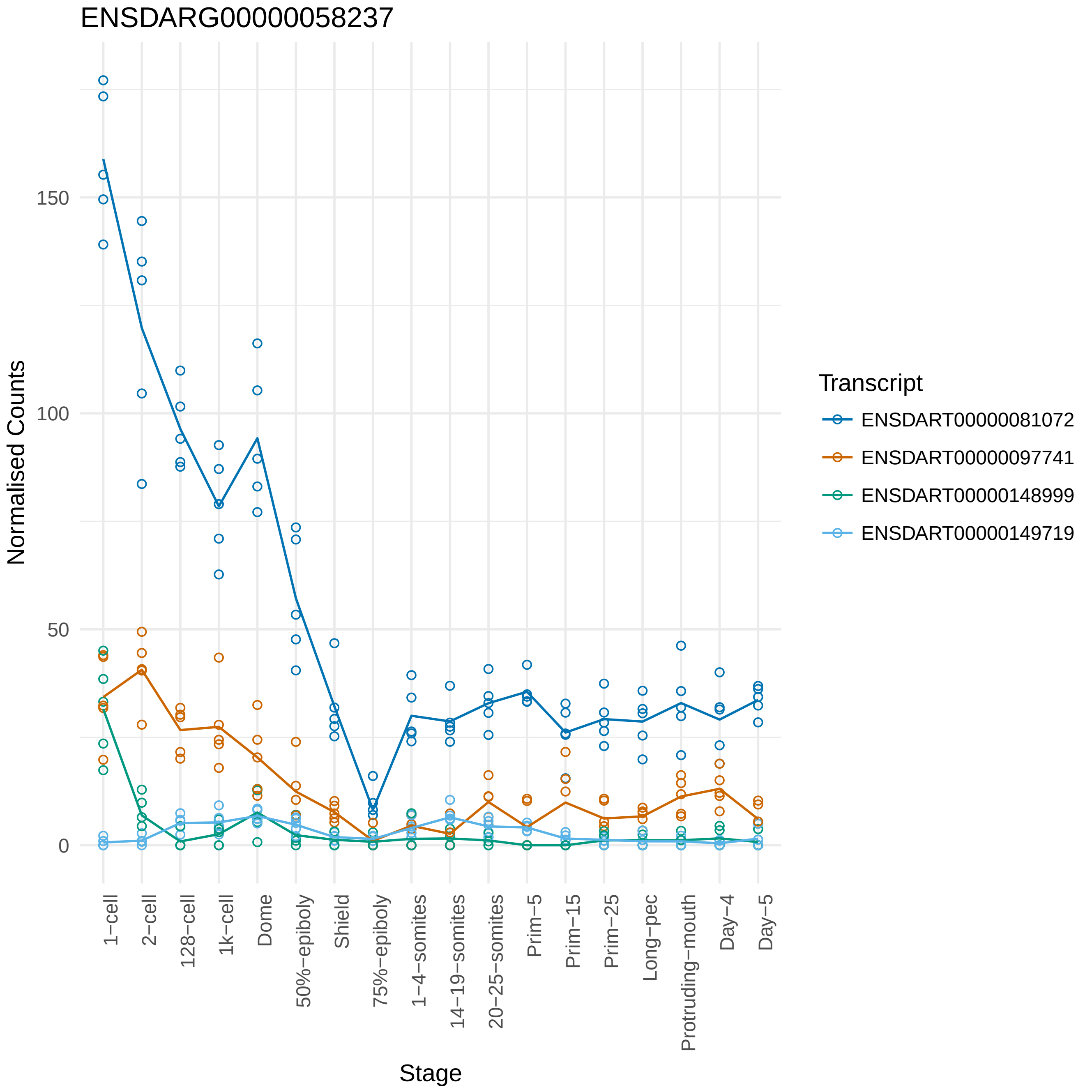
ENSDARG00000058237
Normalised Counts
150
100
50
0
1−cell
2−cell
128−cell
1k−cell
Dome
50%−epiboly
Shield
75%−epiboly
1−4−somites
14−19−somites
20−25−somites
Prim−5
Prim−15
Prim−25
Long−pec
Protruding−mouth
Day−4
Day−5
Stage
Transcript
ENSDART00000081072
ENSDART00000097741
ENSDART00000148999
ENSDART00000149719

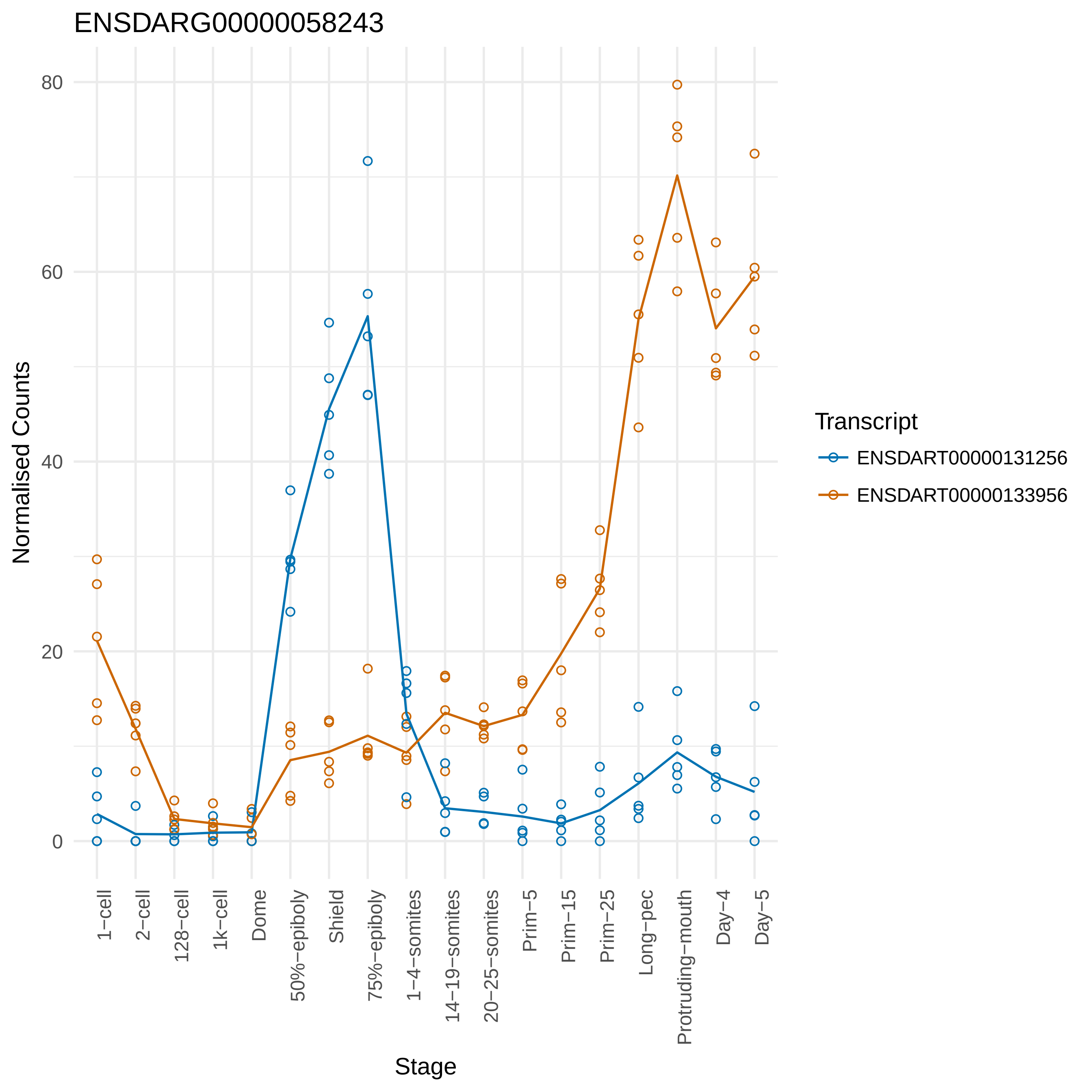

ENSDARG00000058243
Normalised Counts
80
60
40
20
0
1−cell
2−cell
128−cell
1k−cell
Dome
50%−epiboly
Shield
75%−epiboly
1−4−somites
14−19−somites
20−25−somites
Prim−5
Prim−15
Prim−25
Long−pec
Protruding−mouth
Day−4
Day−5
Stage
Transcript
ENSDART00000131256
ENSDART00000133956

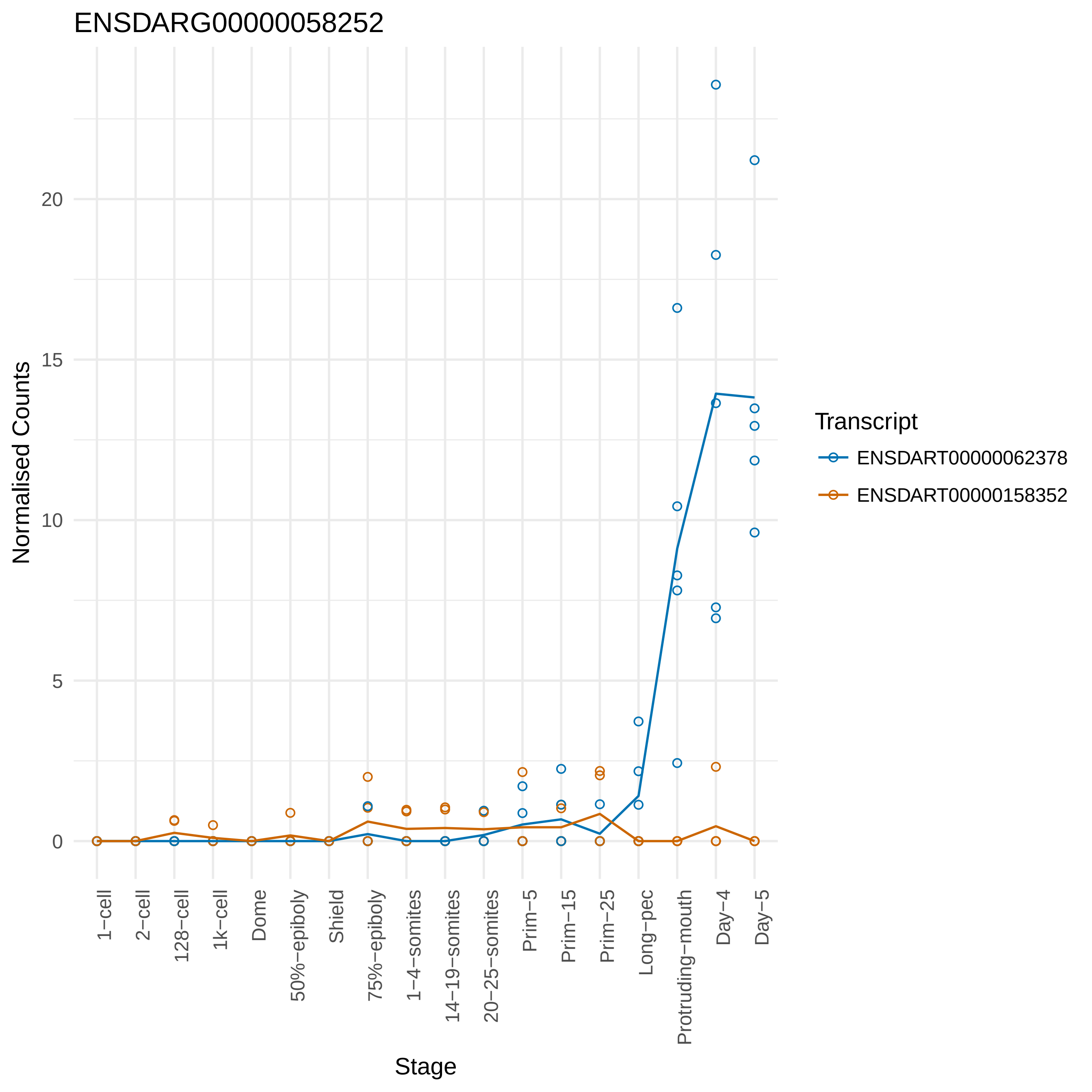

ENSDARG00000058252
Normalised Counts
0
5
10
15
20
Stage
1−cell
2−cell
128−cell
1k−cell
Dome
50%−epiboly
Shield
75%−epiboly
1−4−somites
14−19−somites
20−25−somites
Prim−5
Prim−15
Prim−25
Long−pec
Protruding−mouth
Day−4
Day−5
Transcript
ENSDART00000062378
ENSDART00000158352

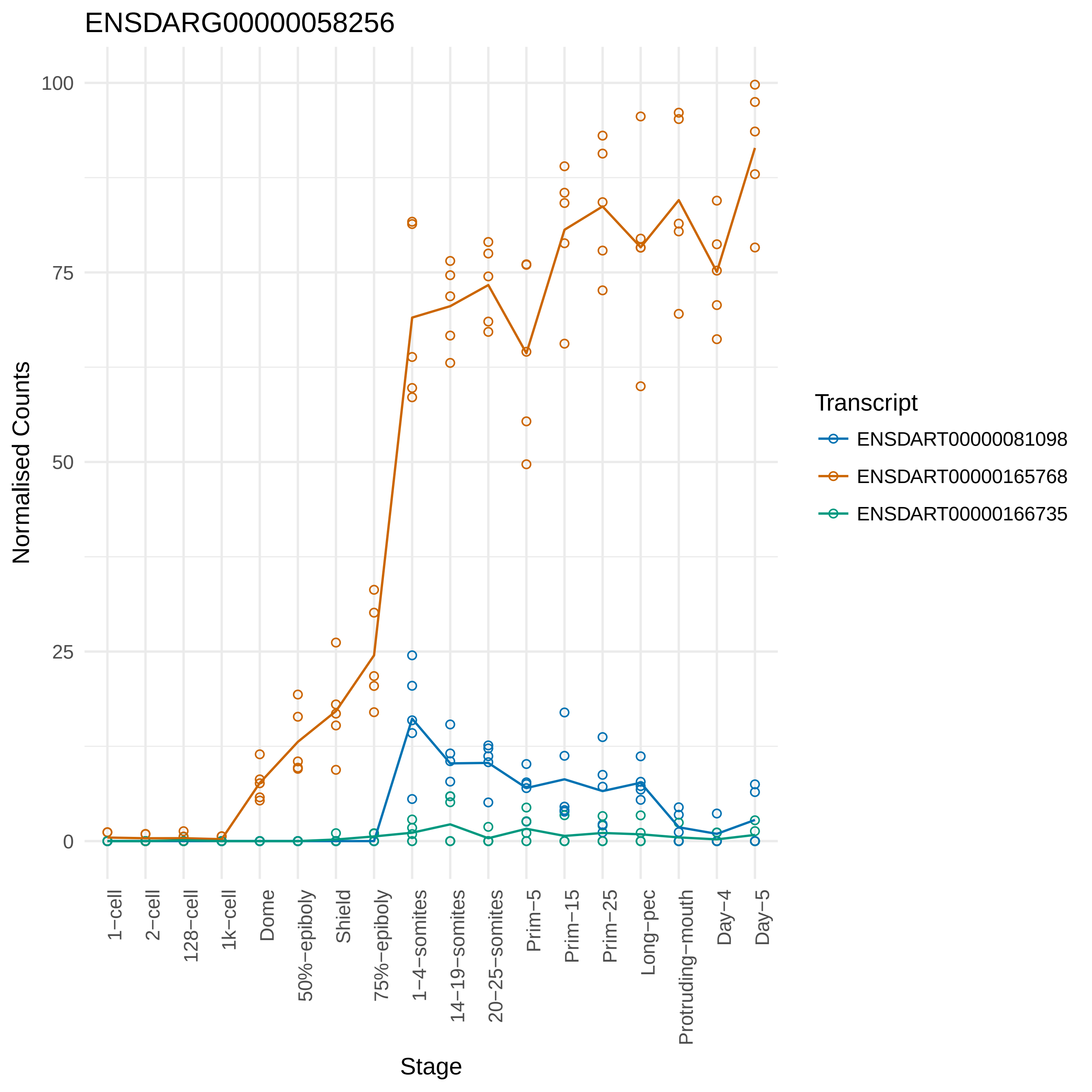

ENSDARG00000058256
Normalised Counts
100
75
50
25
0
1−cell
2−cell
128−cell
1k−cell
Dome
50%−epiboly
Shield
75%−epiboly
1−4−somites
14−19−somites
20−25−somites
Prim−5
Prim−15
Prim−25
Long−pec
Protruding−mouth
Day−4
Day−5
Stage
Transcript
ENSDART00000081098
ENSDART00000165768
ENSDART00000166735

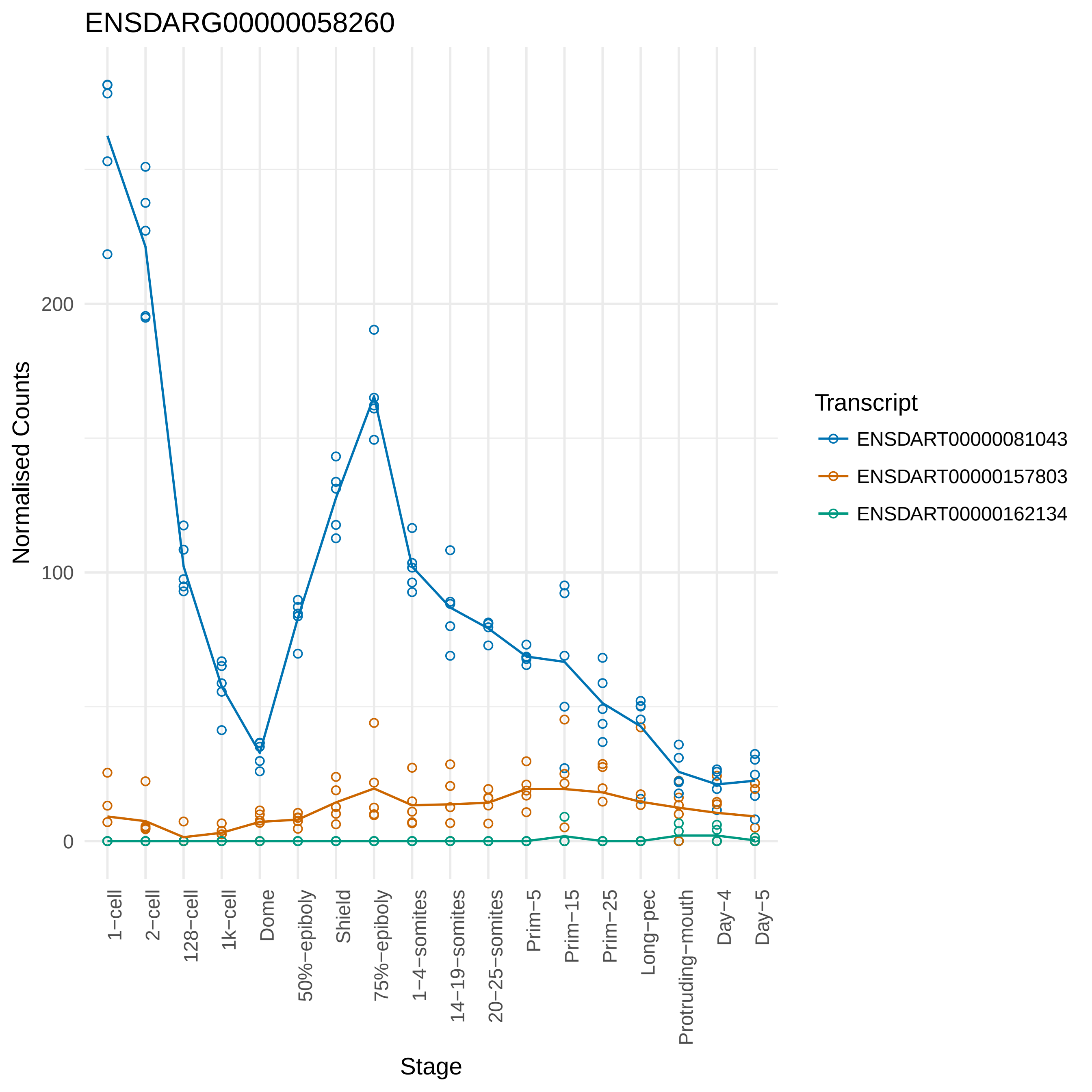

ENSDARG00000058260
Normalised Counts
200
100
0
1−cell
2−cell
128−cell
1k−cell
Dome
50%−epiboly
Shield
75%−epiboly
1−4−somites
14−19−somites
20−25−somites
Prim−5
Prim−15
Prim−25
Long−pec
Protruding−mouth
Day−4
Day−5
Stage
Transcript
ENSDART00000081043
ENSDART00000157803
ENSDART00000162134

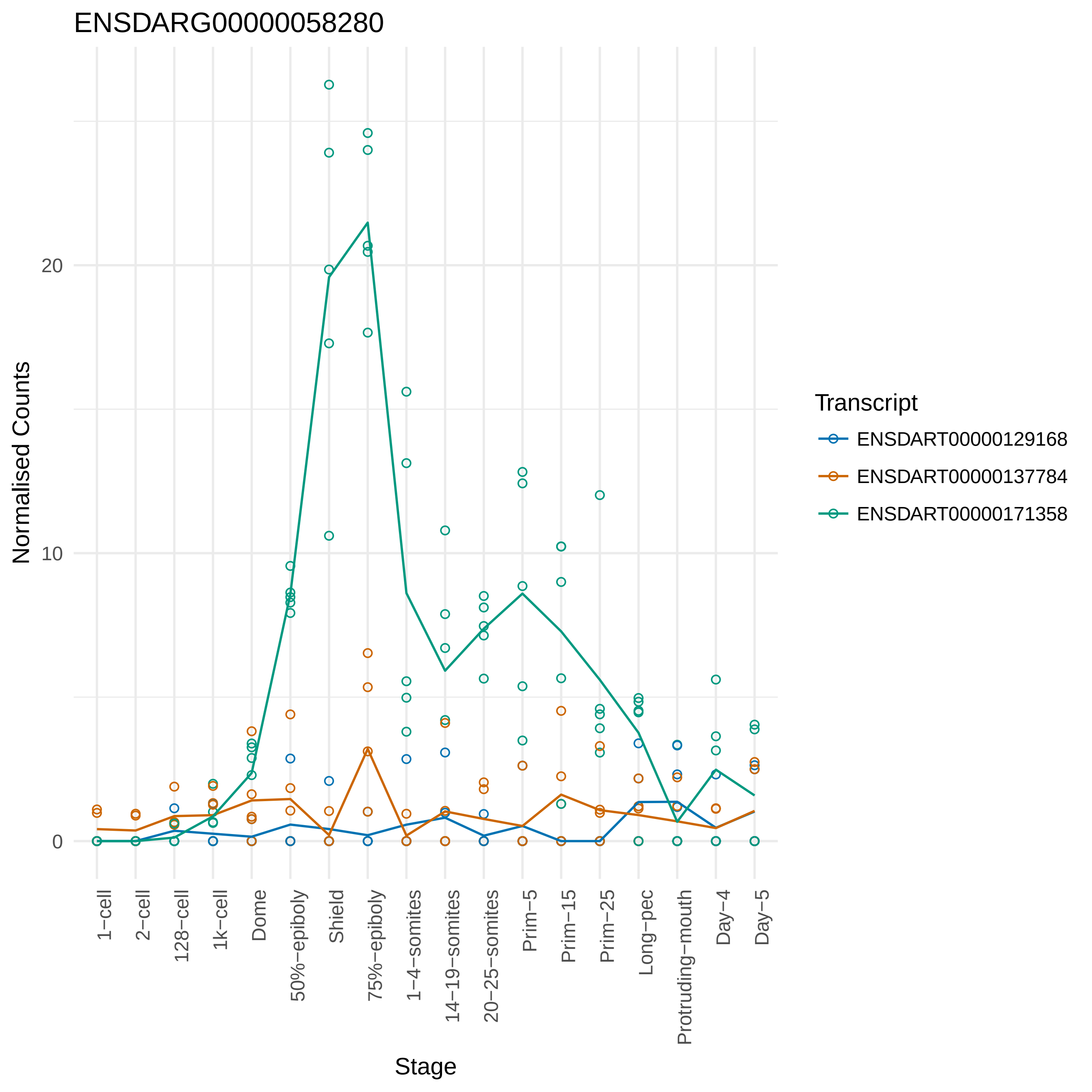
ENSDARG00000058280
Normalised Counts
0
10
20
Stage
1−cell
2−cell
128−cell
1k−cell
Dome
50%−epiboly
Shield
75%−epiboly
1−4−somites
14−19−somites
20−25−somites
Prim−5
Prim−15
Prim−25
Long−pec
Protruding−mouth
Day−4
Day−5
Transcript
ENSDART00000129168
ENSDART00000137784
ENSDART00000171358

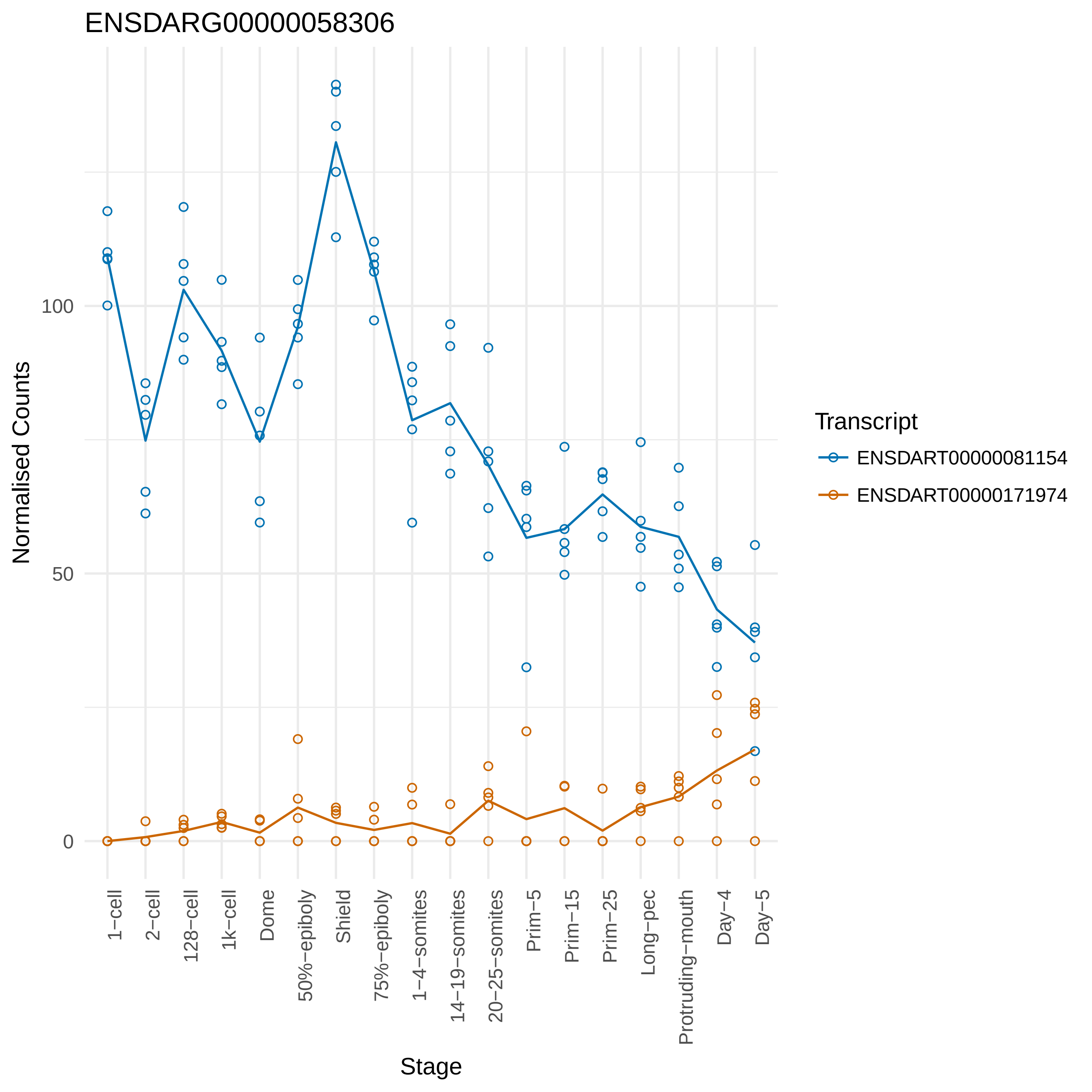
ENSDARG00000058306
Normalised Counts
100
50
0
1−cell
2−cell
128−cell
1k−cell
Dome
50%−epiboly
Shield
75%−epiboly
1−4−somites
14−19−somites
20−25−somites
Prim−5
Prim−15
Prim−25
Long−pec
Protruding−mouth
Day−4
Day−5
Stage
Transcript
ENSDART00000081154
ENSDART00000171974

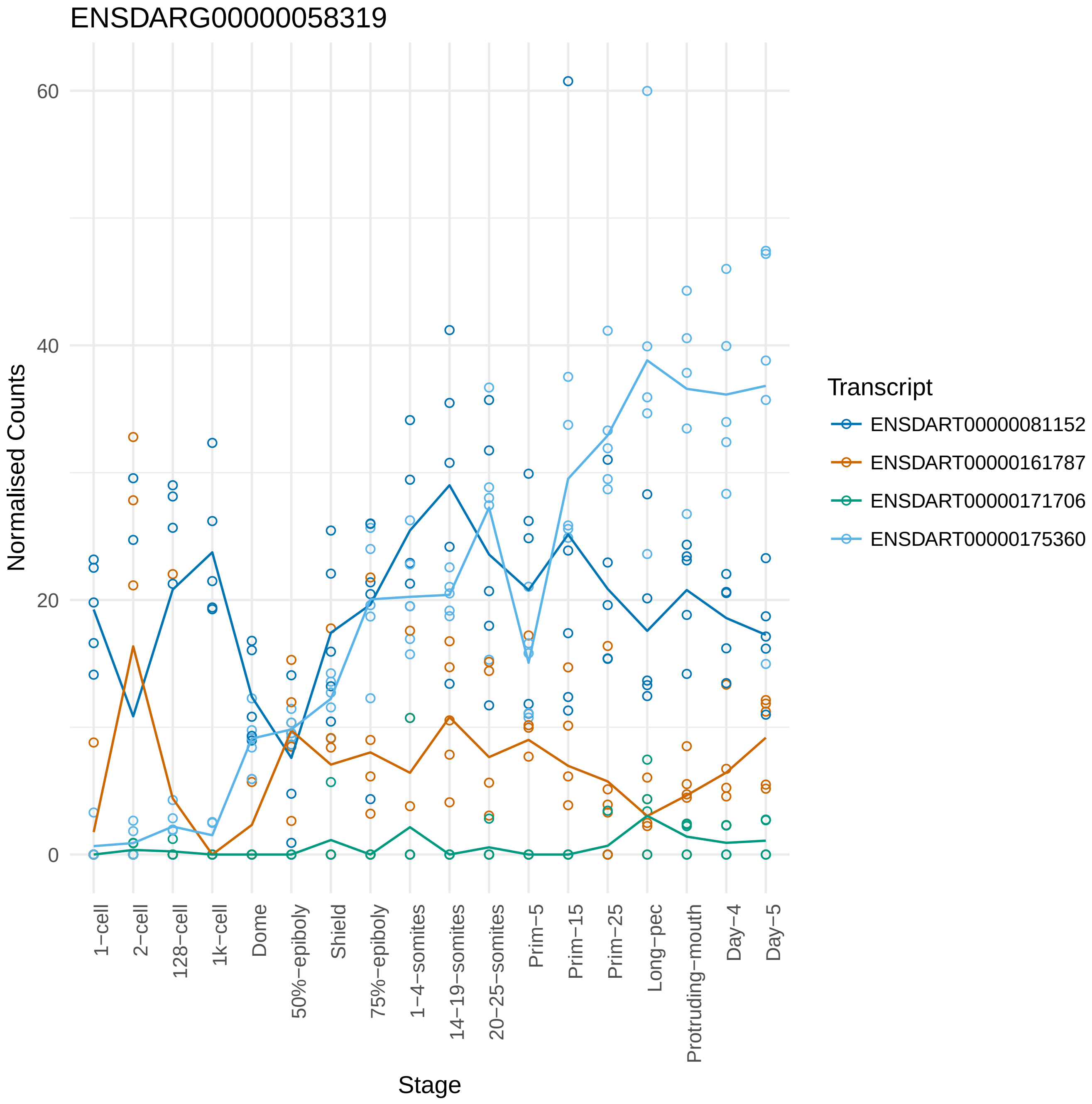

ENSDARG00000058319
Normalised Counts
60
40
20
0
1−cell
2−cell
128−cell
1k−cell
Dome
50%−epiboly
Shield
75%−epiboly
1−4−somites
14−19−somites
20−25−somites
Prim−5
Prim−15
Prim−25
Long−pec
Protruding−mouth
Day−4
Day−5
Stage
Transcript
ENSDART00000081152
ENSDART00000161787
ENSDART00000171706
ENSDART00000175360

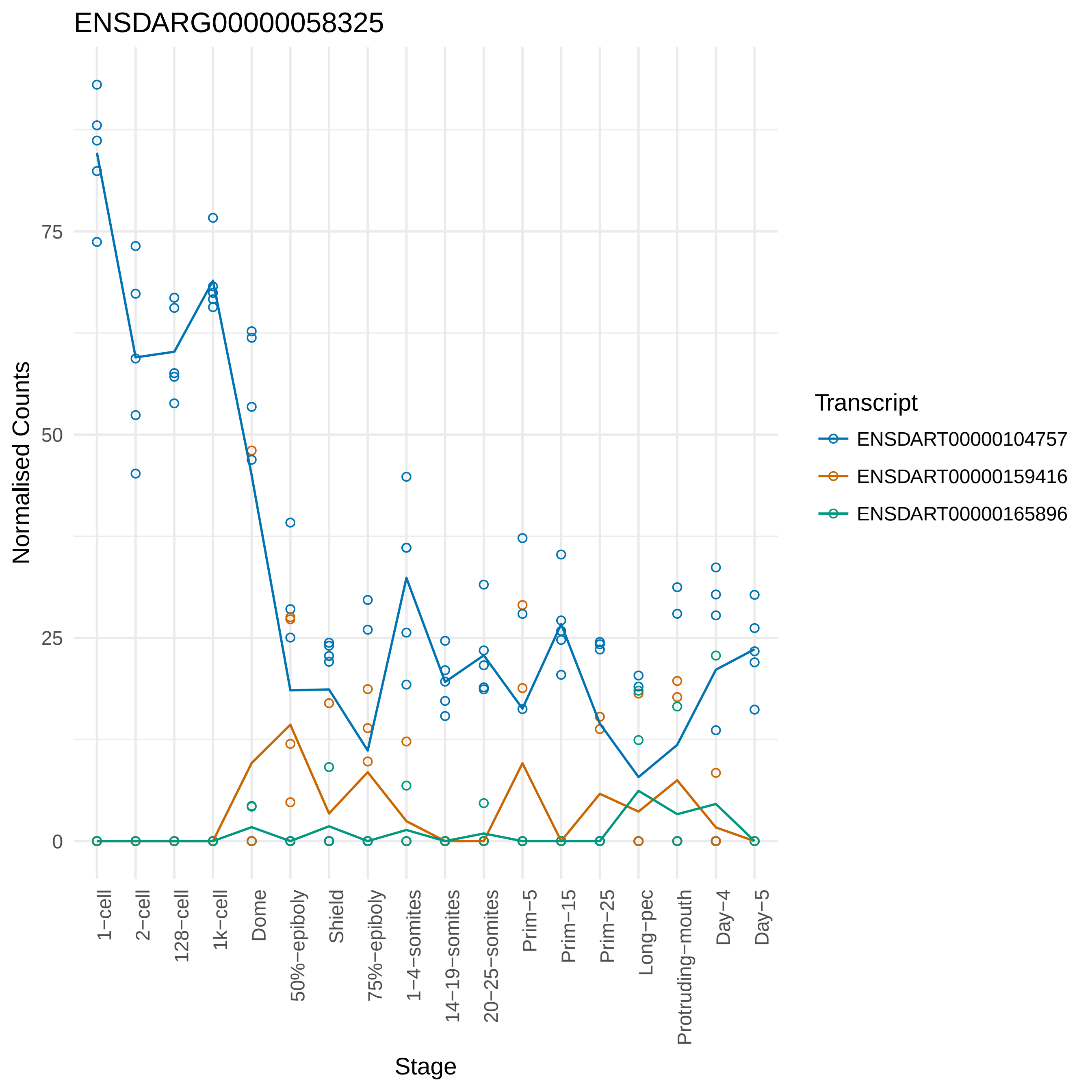

ENSDARG00000058325
Normalised Counts
75
50
25
0
1−cell
2−cell
128−cell
1k−cell
Dome
50%−epiboly
Shield
75%−epiboly
1−4−somites
14−19−somites
20−25−somites
Prim−5
Prim−15
Prim−25
Long−pec
Protruding−mouth
Day−4
Day−5
Stage
Transcript
ENSDART00000104757
ENSDART00000159416
ENSDART00000165896

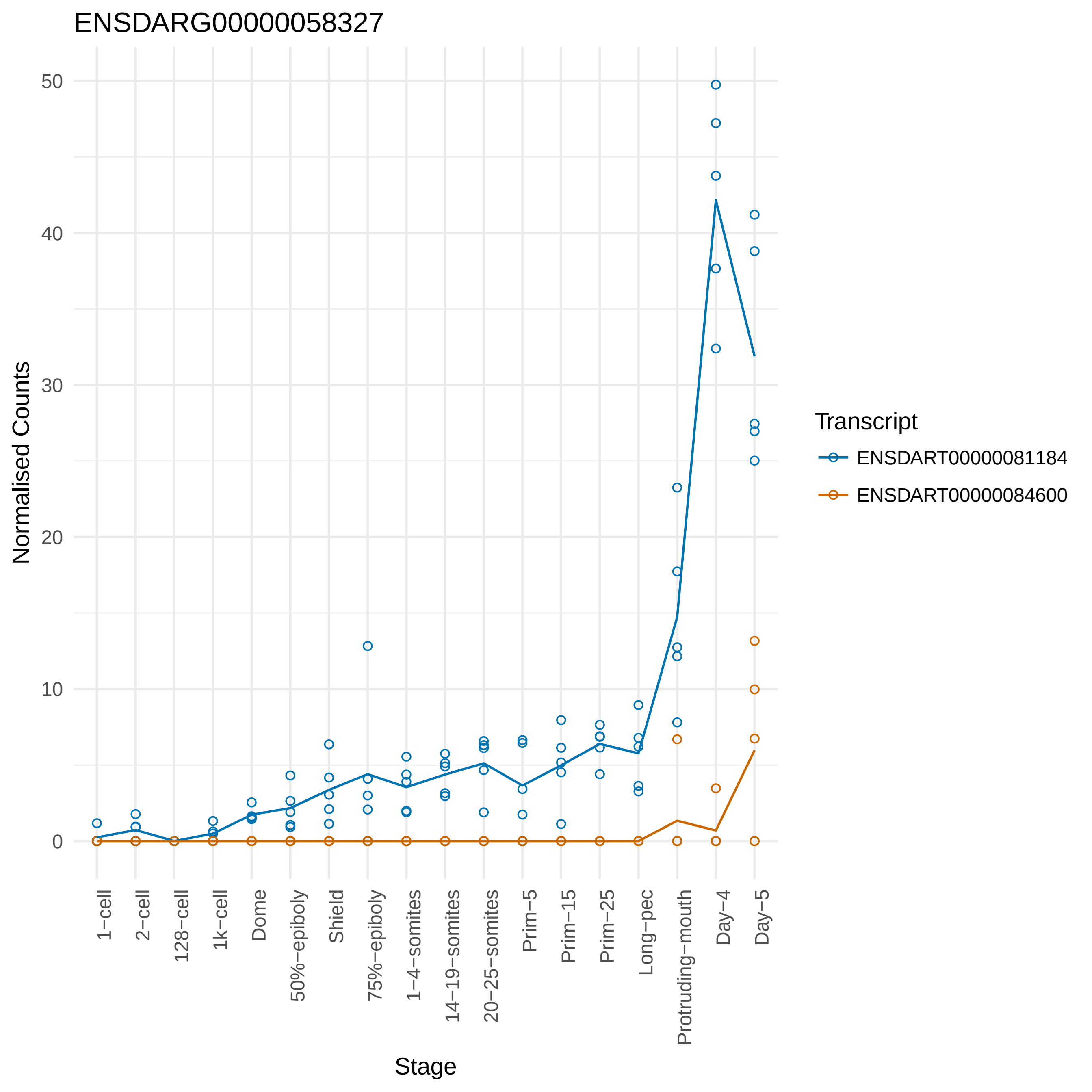

ENSDARG00000058327
Normalised Counts
50
40
30
20
10
0
1−cell
2−cell
128−cell
1k−cell
Dome
50%−epiboly
Shield
75%−epiboly
1−4−somites
14−19−somites
20−25−somites
Prim−5
Prim−15
Prim−25
Long−pec
Protruding−mouth
Day−4
Day−5
Stage
Transcript
ENSDART00000081184
ENSDART00000084600

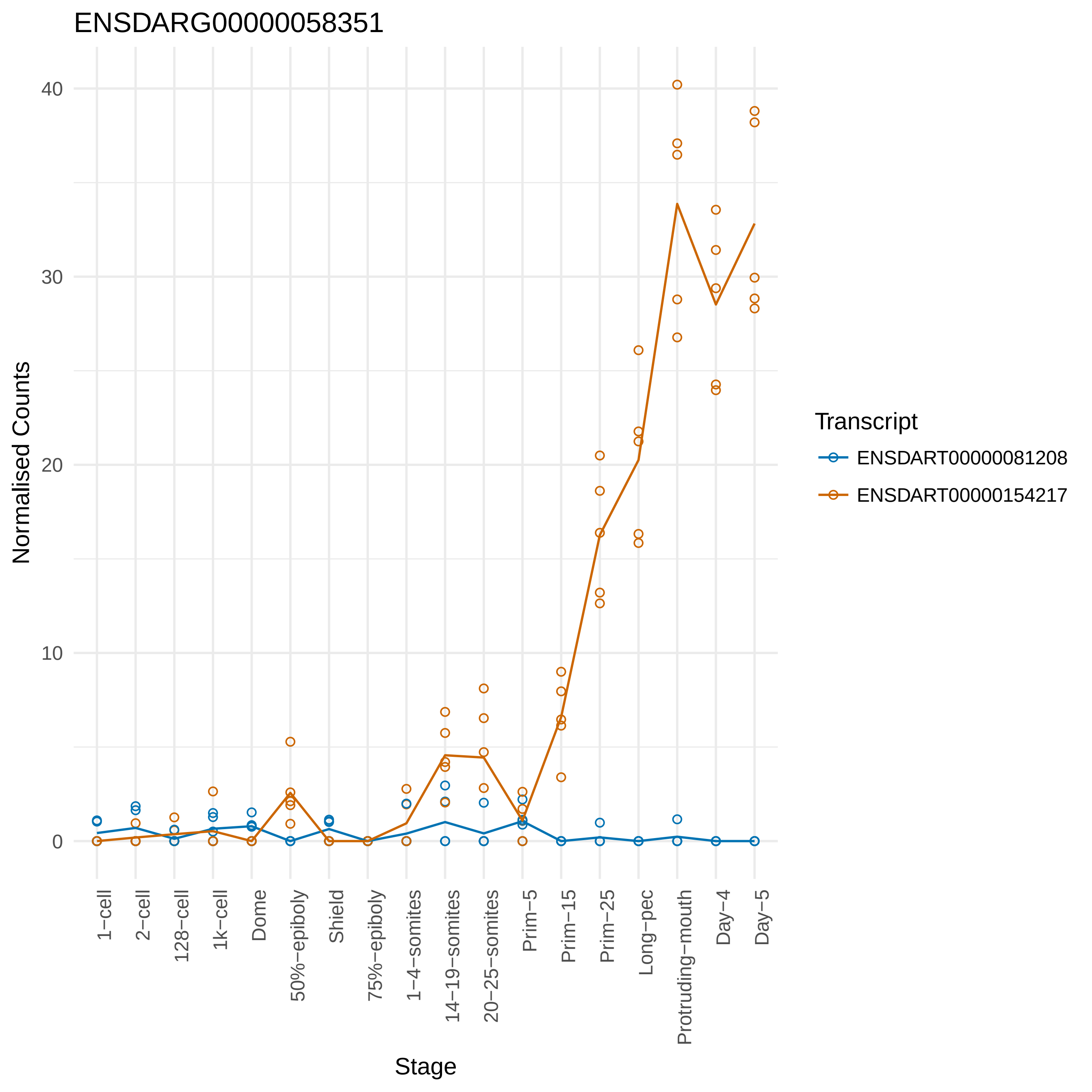

ENSDARG00000058351
Normalised Counts
40
30
20
10
0
1−cell
2−cell
128−cell
1k−cell
Dome
50%−epiboly
Shield
75%−epiboly
1−4−somites
14−19−somites
20−25−somites
Prim−5
Prim−15
Prim−25
Long−pec
Protruding−mouth
Day−4
Day−5
Stage
Transcript
ENSDART00000081208
ENSDART00000154217

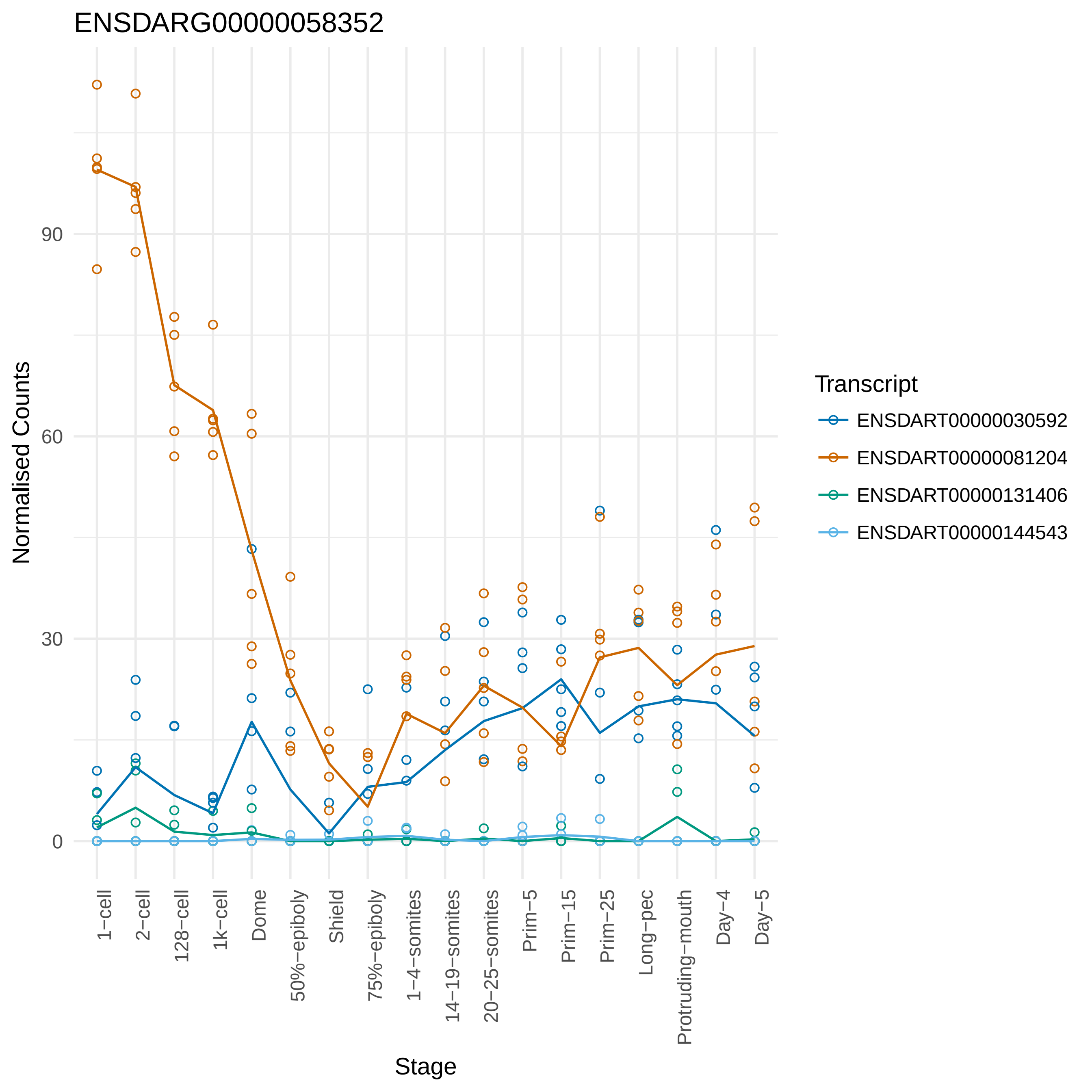
ENSDARG00000058352
Normalised Counts
90
60
30
0
1−cell
2−cell
128−cell
1k−cell
Dome
50%−epiboly
Shield
75%−epiboly
1−4−somites
14−19−somites
20−25−somites
Prim−5
Prim−15
Prim−25
Long−pec
Protruding−mouth
Day−4
Day−5
Stage
Transcript
ENSDART00000030592
ENSDART00000081204
ENSDART00000131406
ENSDART00000144543

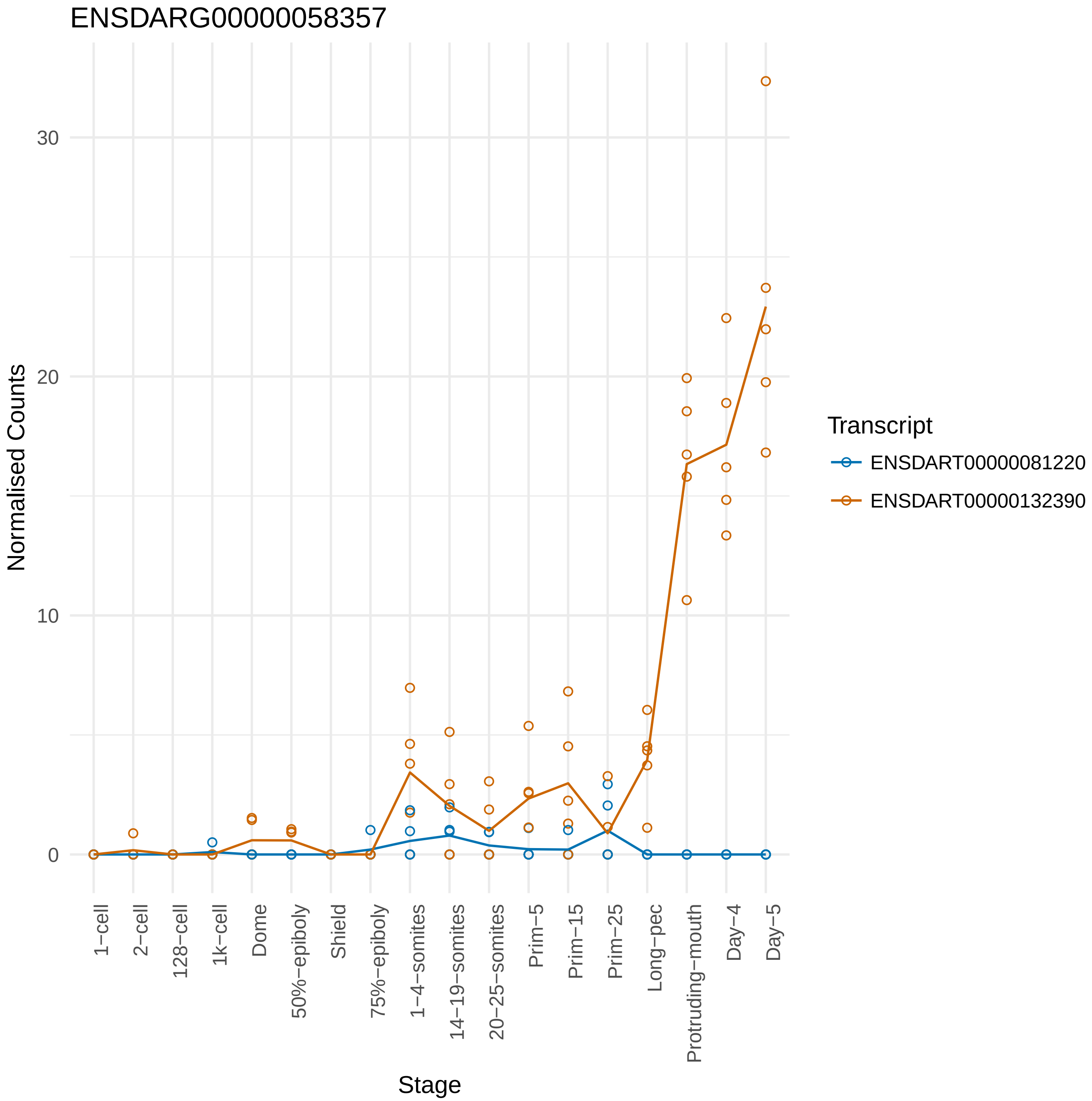

ENSDARG00000058357
Normalised Counts
0
10
20
30
1−cell
2−cell
128−cell
1k−cell
Dome
50%−epiboly
Shield
75%−epiboly
1−4−somites
14−19−somites
20−25−somites
Prim−5
Prim−15
Prim−25
Long−pec
Protruding−mouth
Day−4
Day−5
Stage
Transcript
ENSDART00000081220
ENSDART00000132390

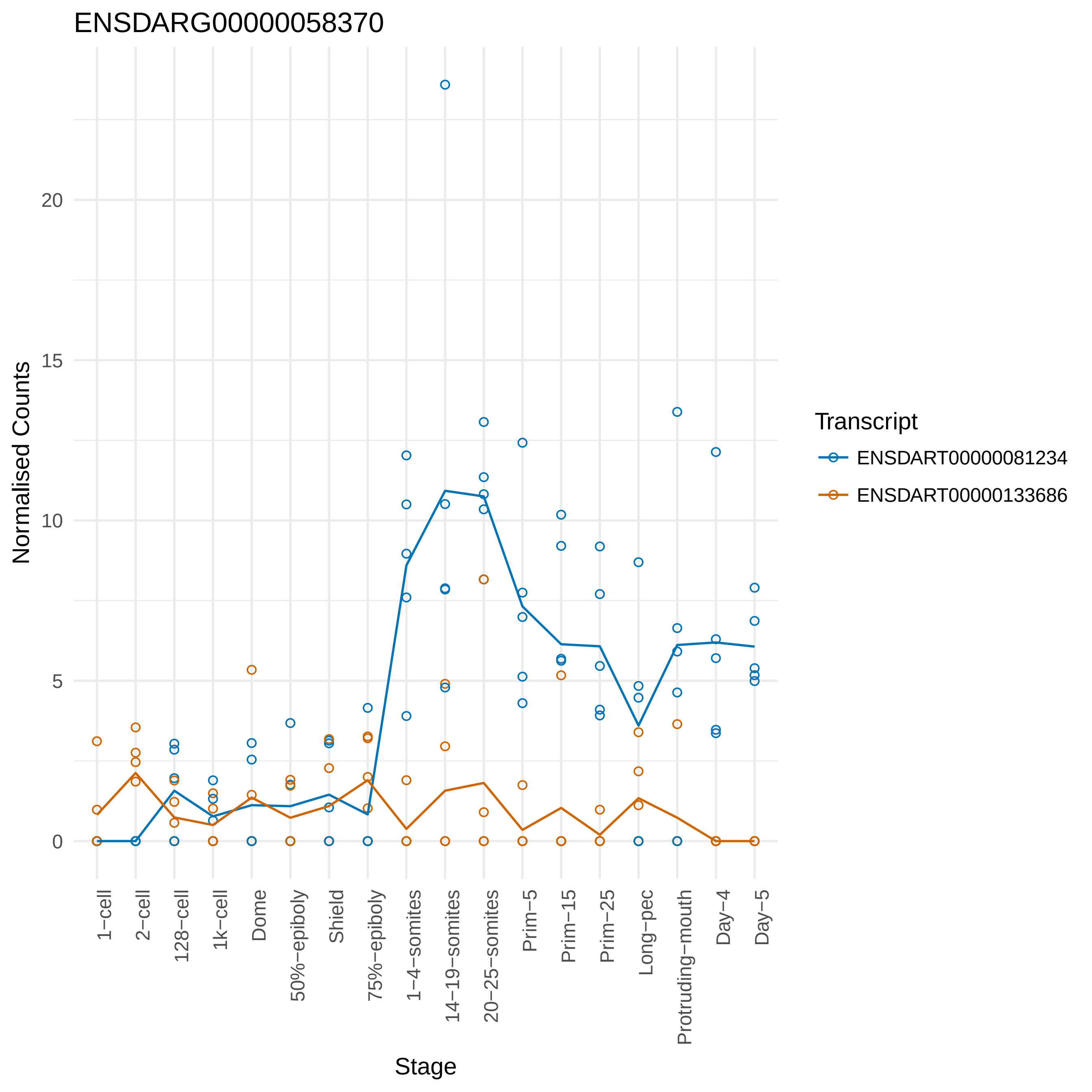

ENSDARG00000058370
Normalised Counts
0
5
10
15
20
Stage
1−cell
2−cell
128−cell
1k−cell
Dome
50%−epiboly
Shield
75%−epiboly
1−4−somites
14−19−somites
20−25−somites
Prim−5
Prim−15
Prim−25
Long−pec
Protruding−mouth
Day−4
Day−5
Transcript
ENSDART00000081234
ENSDART00000133686

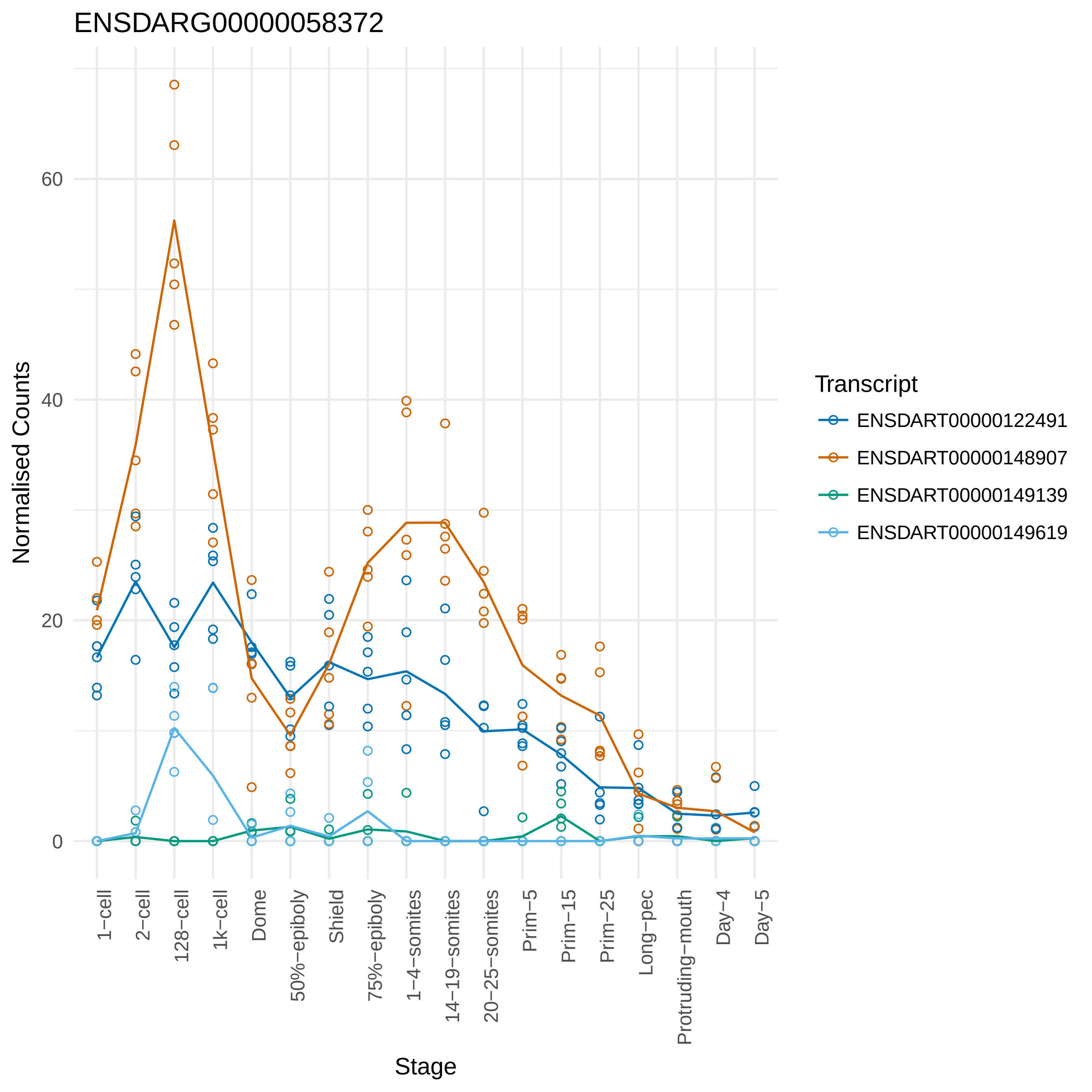
ENSDARG00000058372
Normalised Counts
0
20
40
60
1−cell
2−cell
128−cell
1k−cell
Dome
50%−epiboly
Shield
75%−epiboly
1−4−somites
14−19−somites
20−25−somites
Prim−5
Prim−15
Prim−25
Long−pec
Protruding−mouth
Day−4
Day−5
Stage
Transcript
ENSDART00000122491
ENSDART00000148907
ENSDART00000149139
ENSDART00000149619

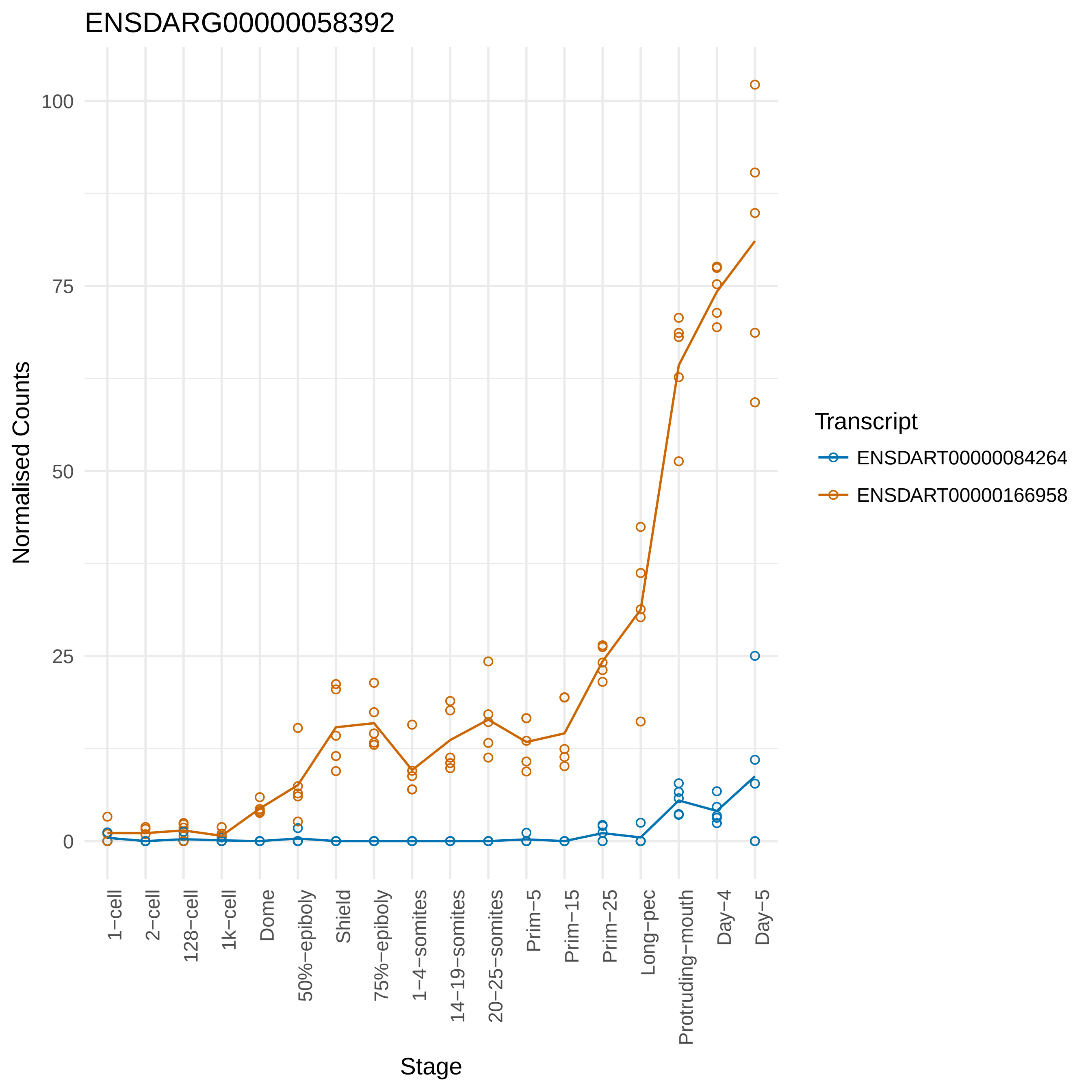

ENSDARG00000058392
Normalised Counts
100
75
50
25
0
1−cell
2−cell
128−cell
1k−cell
Dome
50%−epiboly
Shield
75%−epiboly
1−4−somites
14−19−somites
20−25−somites
Prim−5
Prim−15
Prim−25
Long−pec
Protruding−mouth
Day−4
Day−5
Stage
Transcript
ENSDART00000084264
ENSDART00000166958

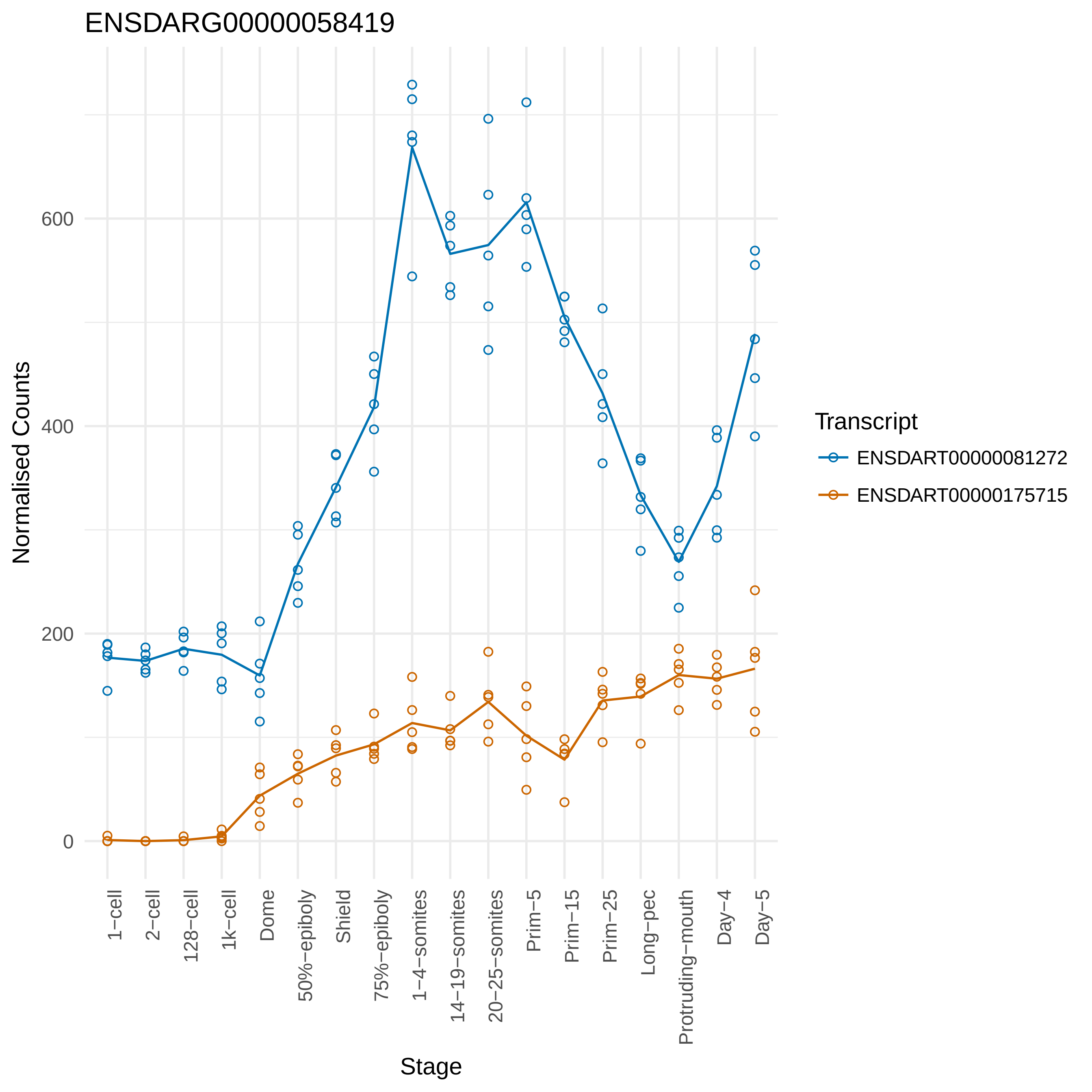

ENSDARG00000058419
Normalised Counts
600
400
200
0
1−cell
2−cell
128−cell
1k−cell
Dome
50%−epiboly
Shield
75%−epiboly
1−4−somites
14−19−somites
20−25−somites
Prim−5
Prim−15
Prim−25
Long−pec
Protruding−mouth
Day−4
Day−5
Stage
Transcript
ENSDART00000081272
ENSDART00000175715

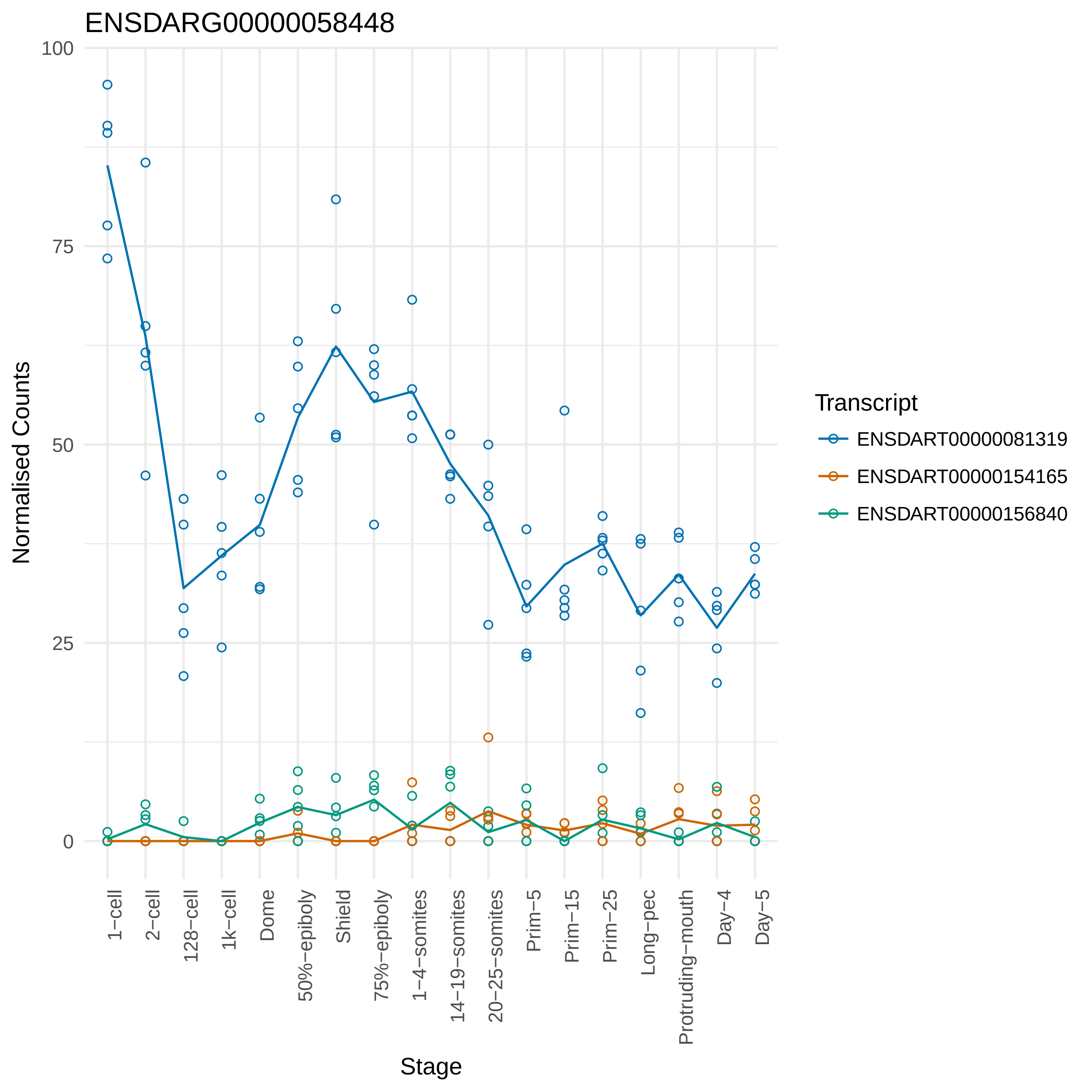

ENSDARG00000058448
100
75
50
25
0
Normalised Counts
1−cell
2−cell
128−cell
1k−cell
Dome
50%−epiboly
Shield
75%−epiboly
1−4−somites
14−19−somites
20−25−somites
Prim−5
Prim−15
Prim−25
Long−pec
Protruding−mouth
Day−4
Day−5
Stage
Transcript
ENSDART00000081319
ENSDART00000154165
ENSDART00000156840

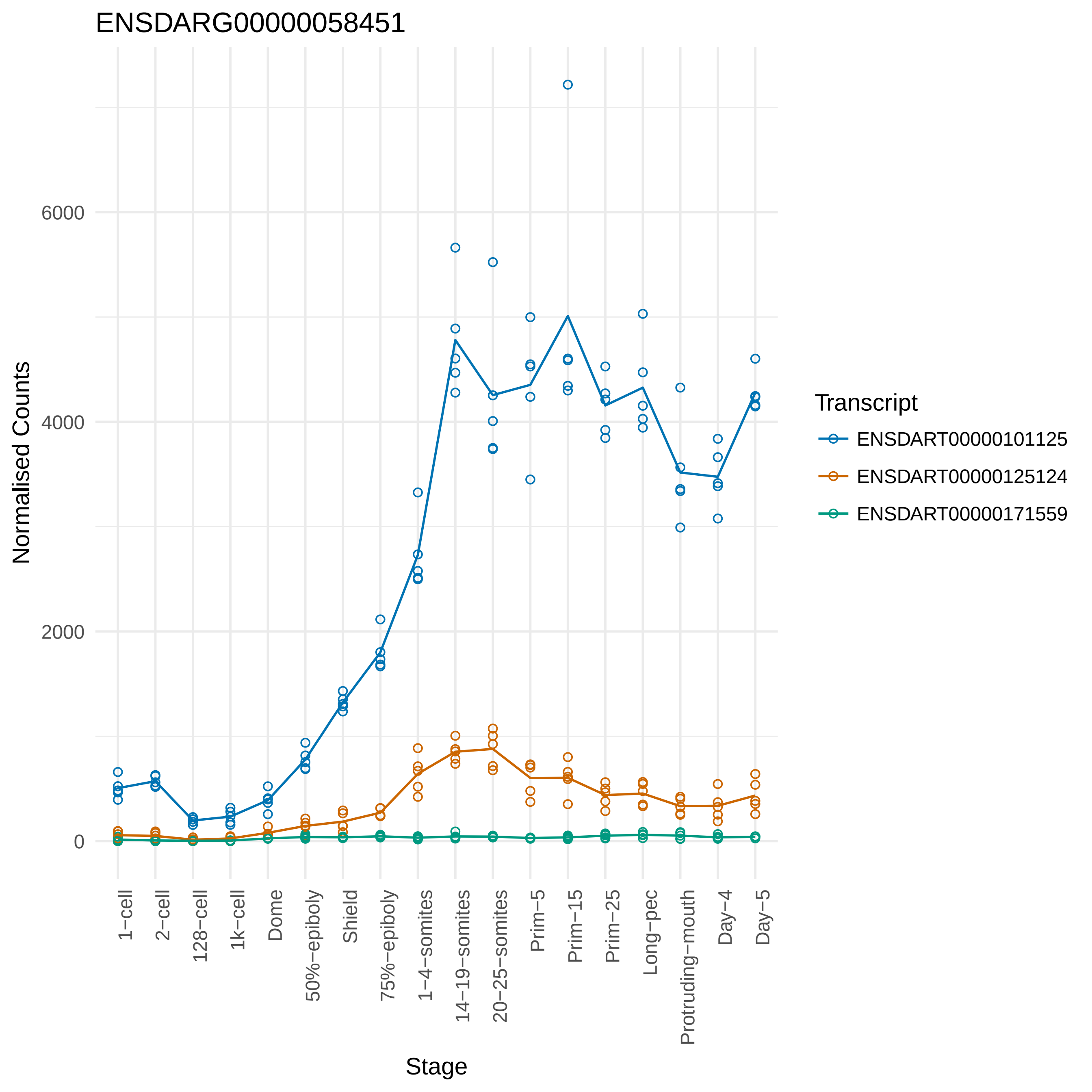

ENSDARG00000058451
Normalised Counts
0
2000
4000
6000
Stage
1−cell
2−cell
128−cell
1k−cell
Dome
50%−epiboly
Shield
75%−epiboly
1−4−somites
14−19−somites
20−25−somites
Prim−5
Prim−15
Prim−25
Long−pec
Protruding−mouth
Day−4
Day−5
Transcript
ENSDART00000101125
ENSDART00000125124
ENSDART00000171559

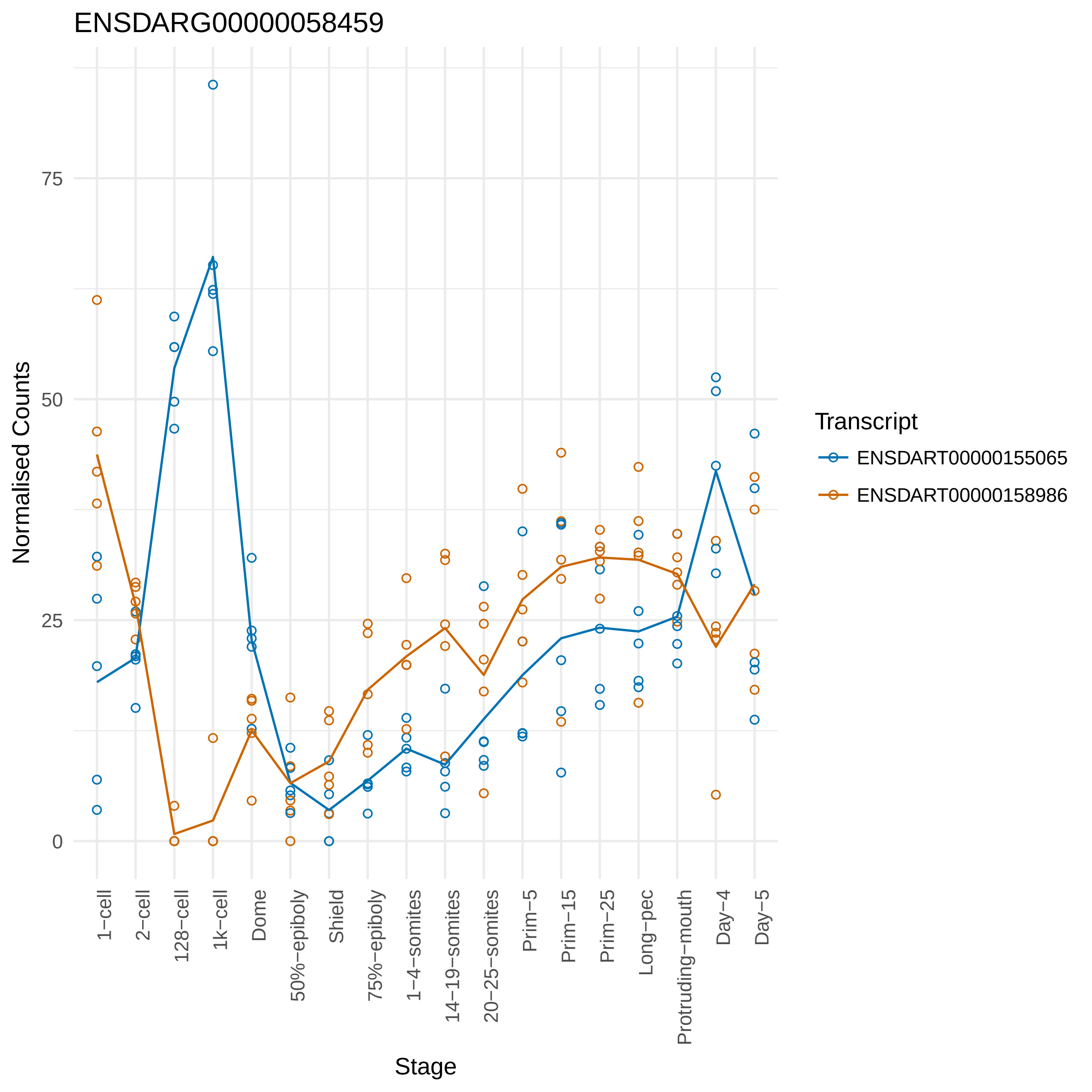
ENSDARG00000058459
Normalised Counts
75
50
25
0
1−cell
2−cell
128−cell
1k−cell
Dome
50%−epiboly
Shield
75%−epiboly
1−4−somites
14−19−somites
20−25−somites
Prim−5
Prim−15
Prim−25
Long−pec
Protruding−mouth
Day−4
Day−5
Stage
Transcript
ENSDART00000155065
ENSDART00000158986

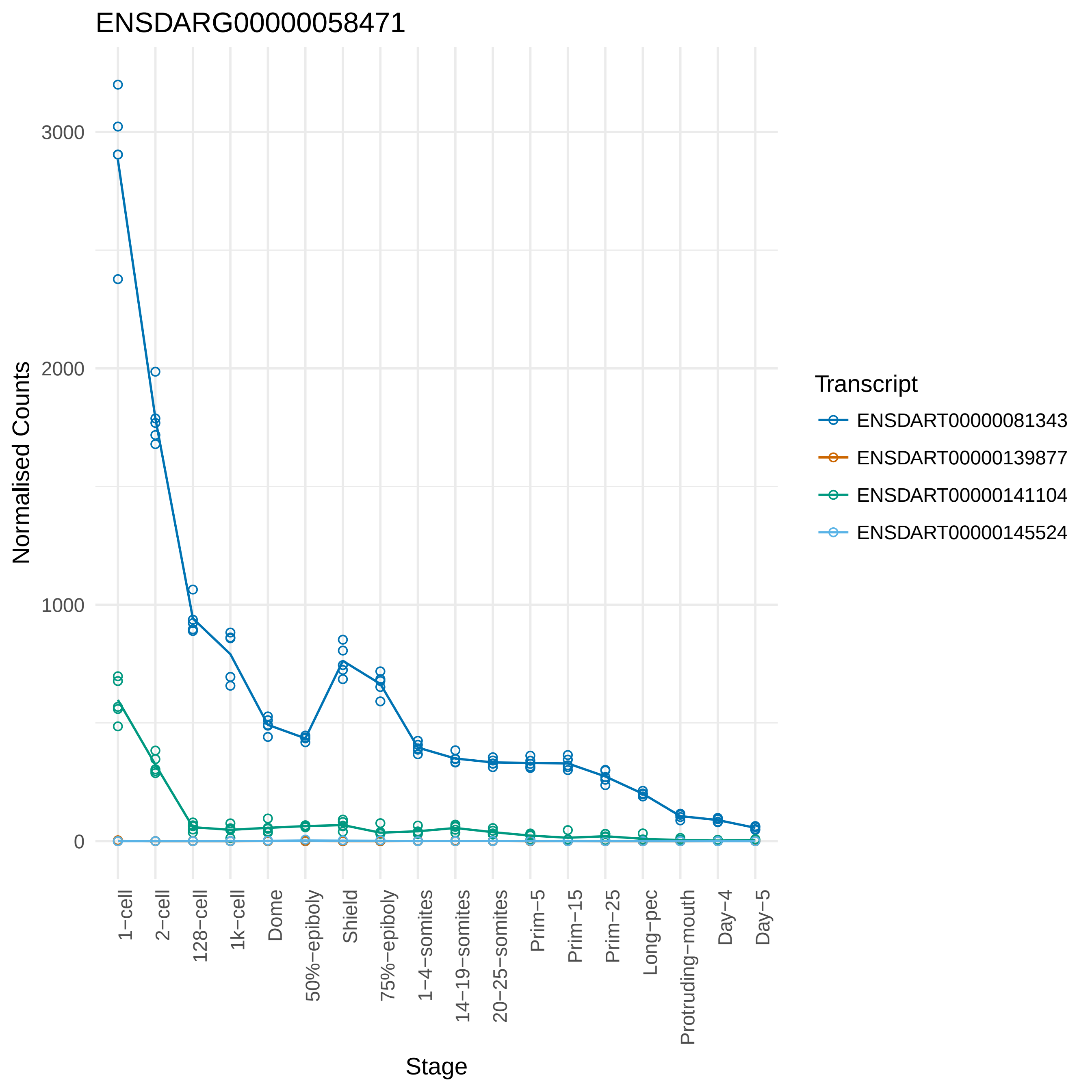

ENSDARG00000058471
Normalised Counts
3000
2000
1000
0
1−cell
2−cell
128−cell
1k−cell
Dome
50%−epiboly
Shield
75%−epiboly
1−4−somites
14−19−somites
20−25−somites
Prim−5
Prim−15
Prim−25
Long−pec
Protruding−mouth
Day−4
Day−5
Stage
Transcript
ENSDART00000081343
ENSDART00000139877
ENSDART00000141104
ENSDART00000145524

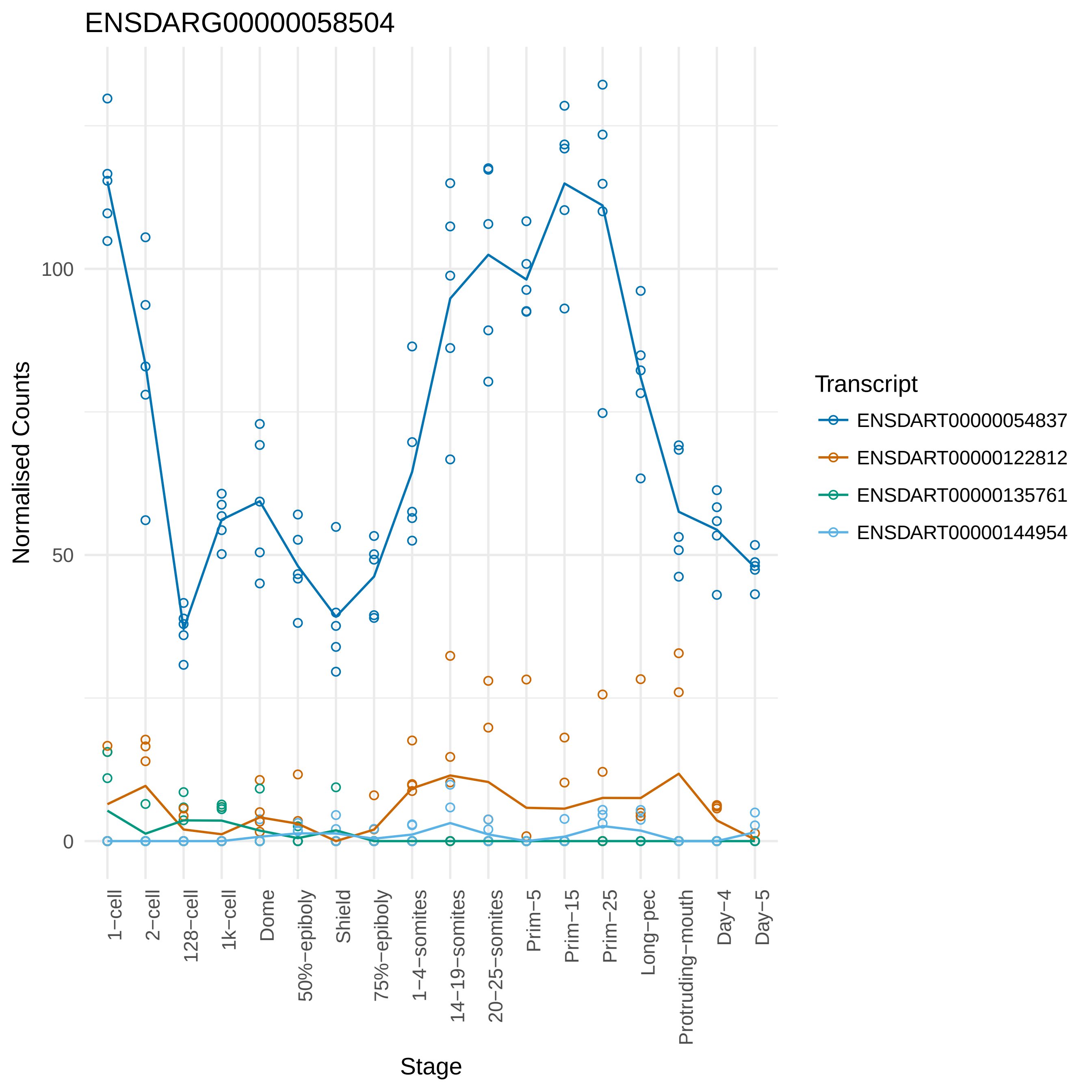

ENSDARG00000058504
Normalised Counts
100
50
0
1−cell
2−cell
128−cell
1k−cell
Dome
50%−epiboly
Shield
75%−epiboly
1−4−somites
14−19−somites
20−25−somites
Prim−5
Prim−15
Prim−25
Long−pec
Protruding−mouth
Day−4
Day−5
Stage
Transcript
ENSDART00000054837
ENSDART00000122812
ENSDART00000135761
ENSDART00000144954

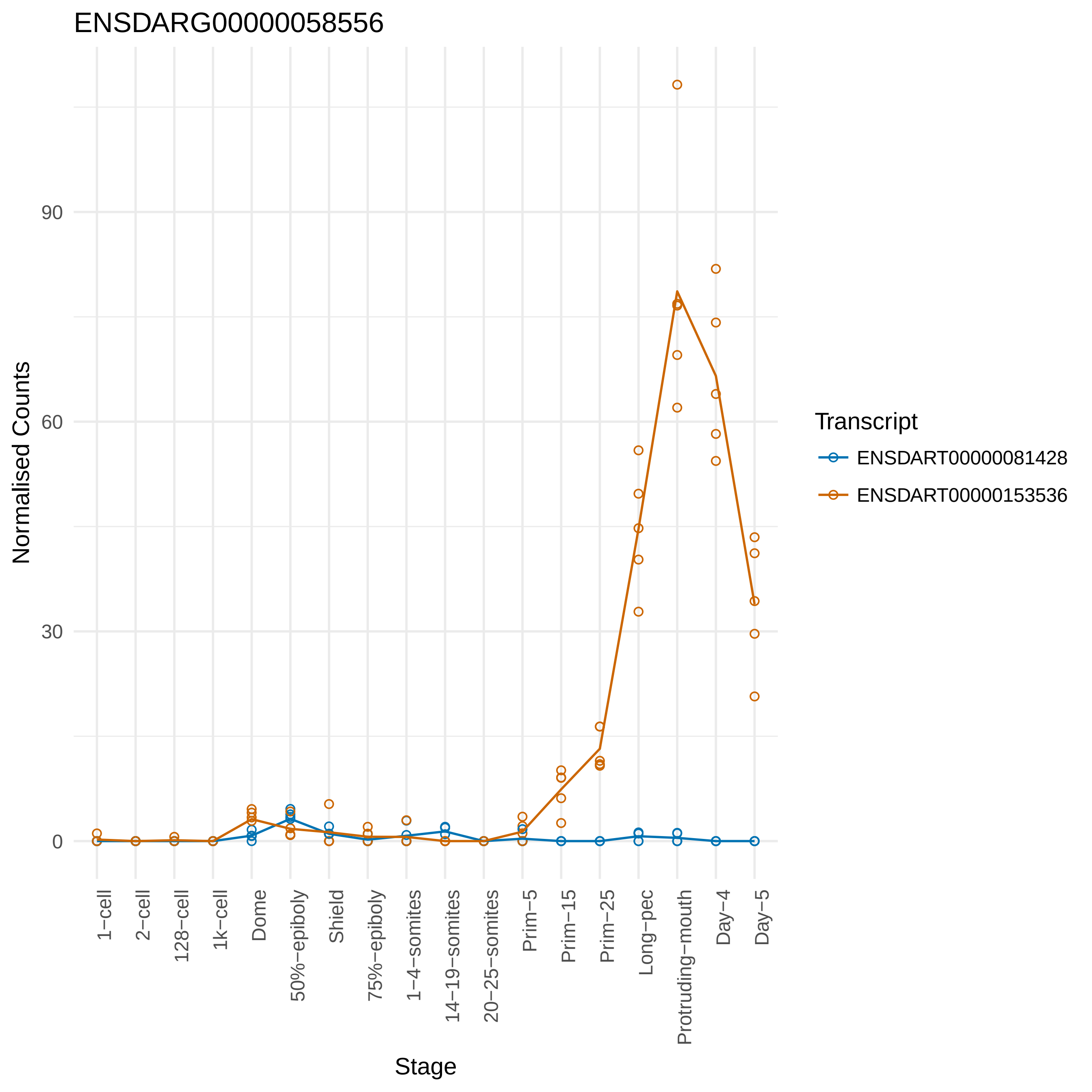
ENSDARG00000058556
Normalised Counts
0
30
60
90
1−cell
2−cell
128−cell
1k−cell
Dome
50%−epiboly
Shield
75%−epiboly
1−4−somites
14−19−somites
20−25−somites
Prim−5
Prim−15
Prim−25
Long−pec
Protruding−mouth
Day−4
Day−5
Stage
Transcript
ENSDART00000081428
ENSDART00000153536

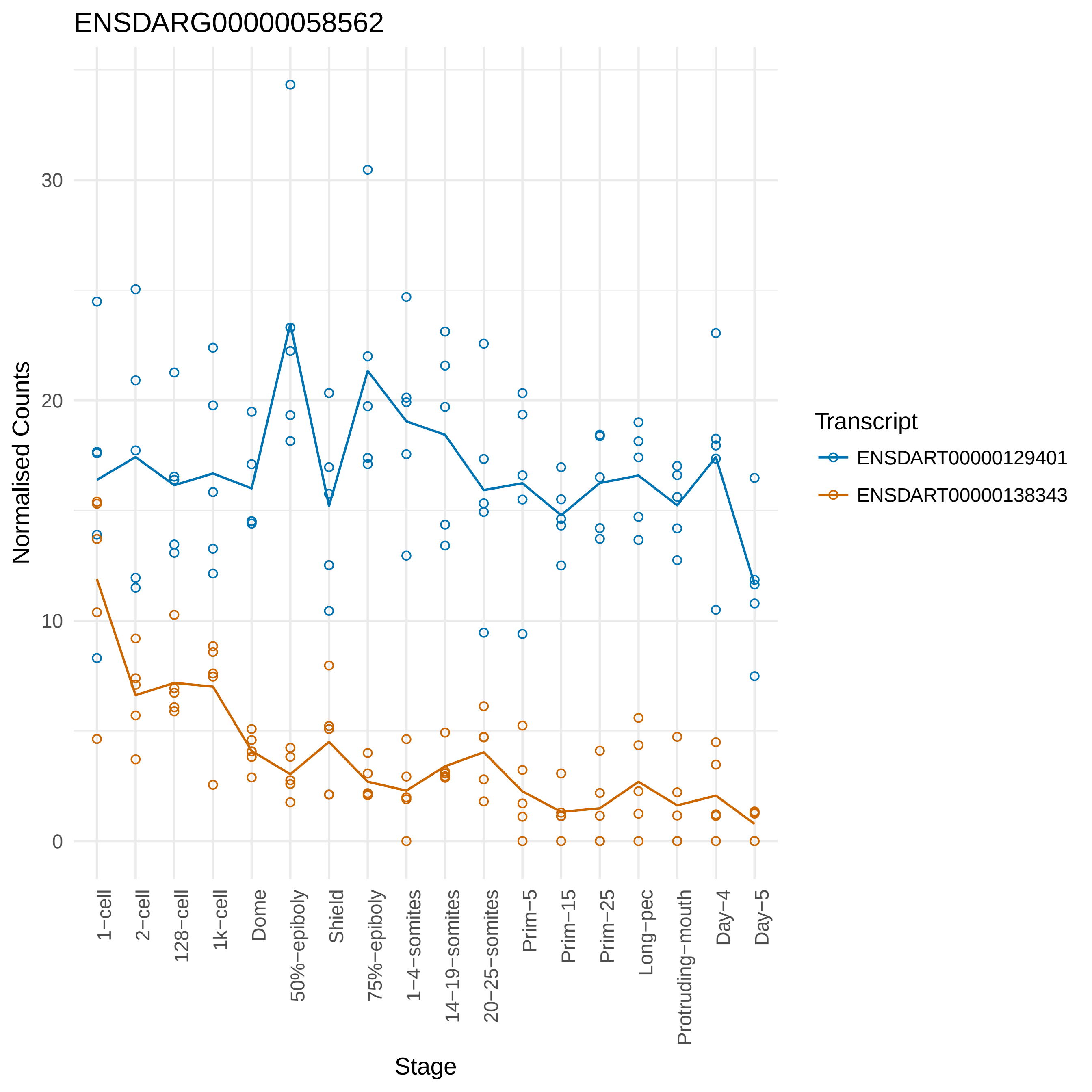

ENSDARG00000058562
Normalised Counts
0
10
20
30
Stage
1−cell
2−cell
128−cell
1k−cell
Dome
50%−epiboly
Shield
75%−epiboly
1−4−somites
14−19−somites
20−25−somites
Prim−5
Prim−15
Prim−25
Long−pec
Protruding−mouth
Day−4
Day−5
Transcript
ENSDART00000129401
ENSDART00000138343

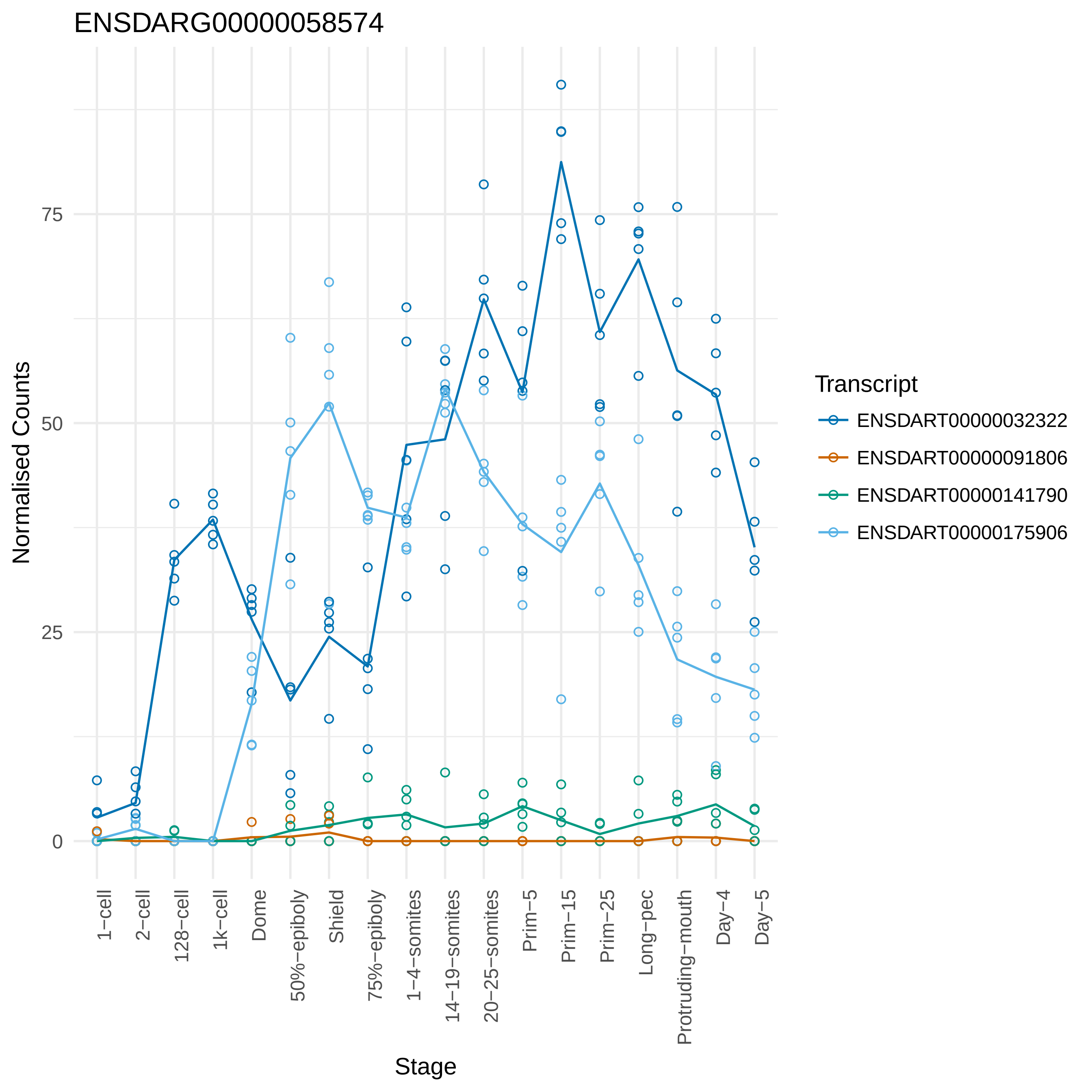

ENSDARG00000058574
Normalised Counts
0
25
50
75
Stage
1−cell
2−cell
128−cell
1k−cell
Dome
50%−epiboly
Shield
75%−epiboly
1−4−somites
14−19−somites
20−25−somites
Prim−5
Prim−15
Prim−25
Long−pec
Protruding−mouth
Day−4
Day−5
Transcript
ENSDART00000032322
ENSDART00000091806
ENSDART00000141790
ENSDART00000175906

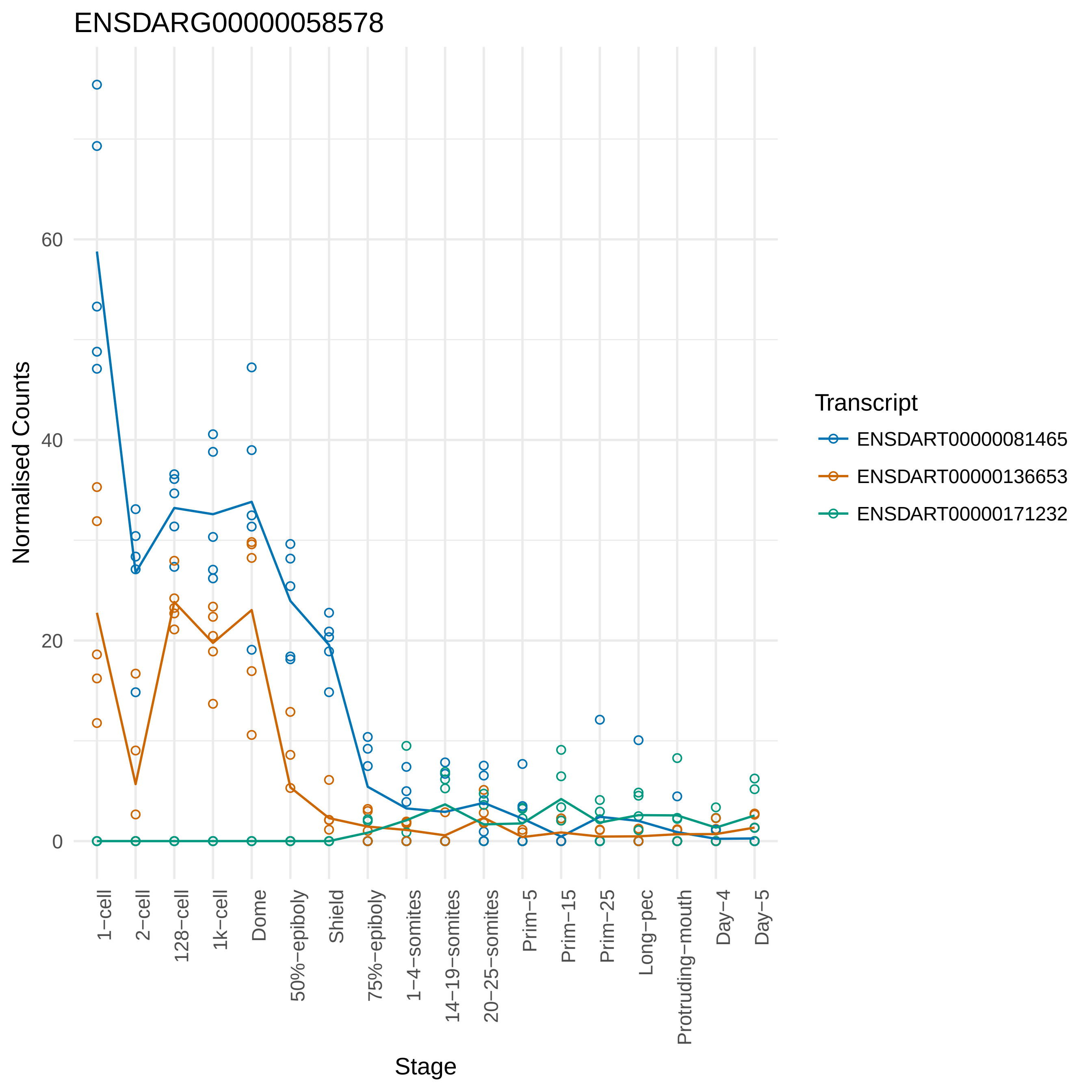

ENSDARG00000058578
Normalised Counts
0
20
40
60
Stage
1−cell
2−cell
128−cell
1k−cell
Dome
50%−epiboly
Shield
75%−epiboly
1−4−somites
14−19−somites
20−25−somites
Prim−5
Prim−15
Prim−25
Long−pec
Protruding−mouth
Day−4
Day−5
Transcript
ENSDART00000081465
ENSDART00000136653
ENSDART00000171232

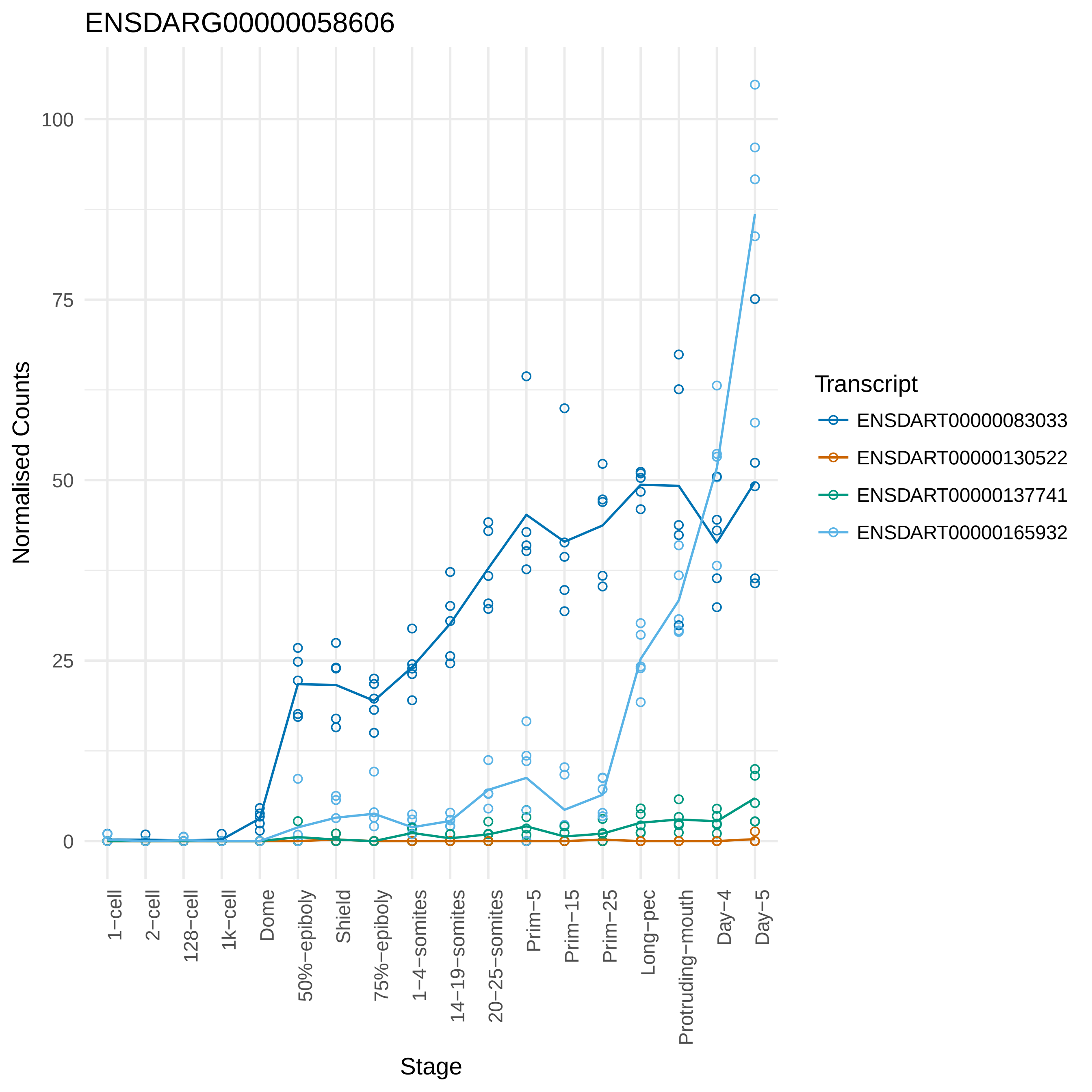
ENSDARG00000058606
Normalised Counts
0
25
50
75
100
1−cell
2−cell
128−cell
1k−cell
Dome
50%−epiboly
Shield
75%−epiboly
1−4−somites
14−19−somites
20−25−somites
Prim−5
Prim−15
Prim−25
Long−pec
Protruding−mouth
Day−4
Day−5
Stage
Transcript
ENSDART00000083033
ENSDART00000130522
ENSDART00000137741
ENSDART00000165932

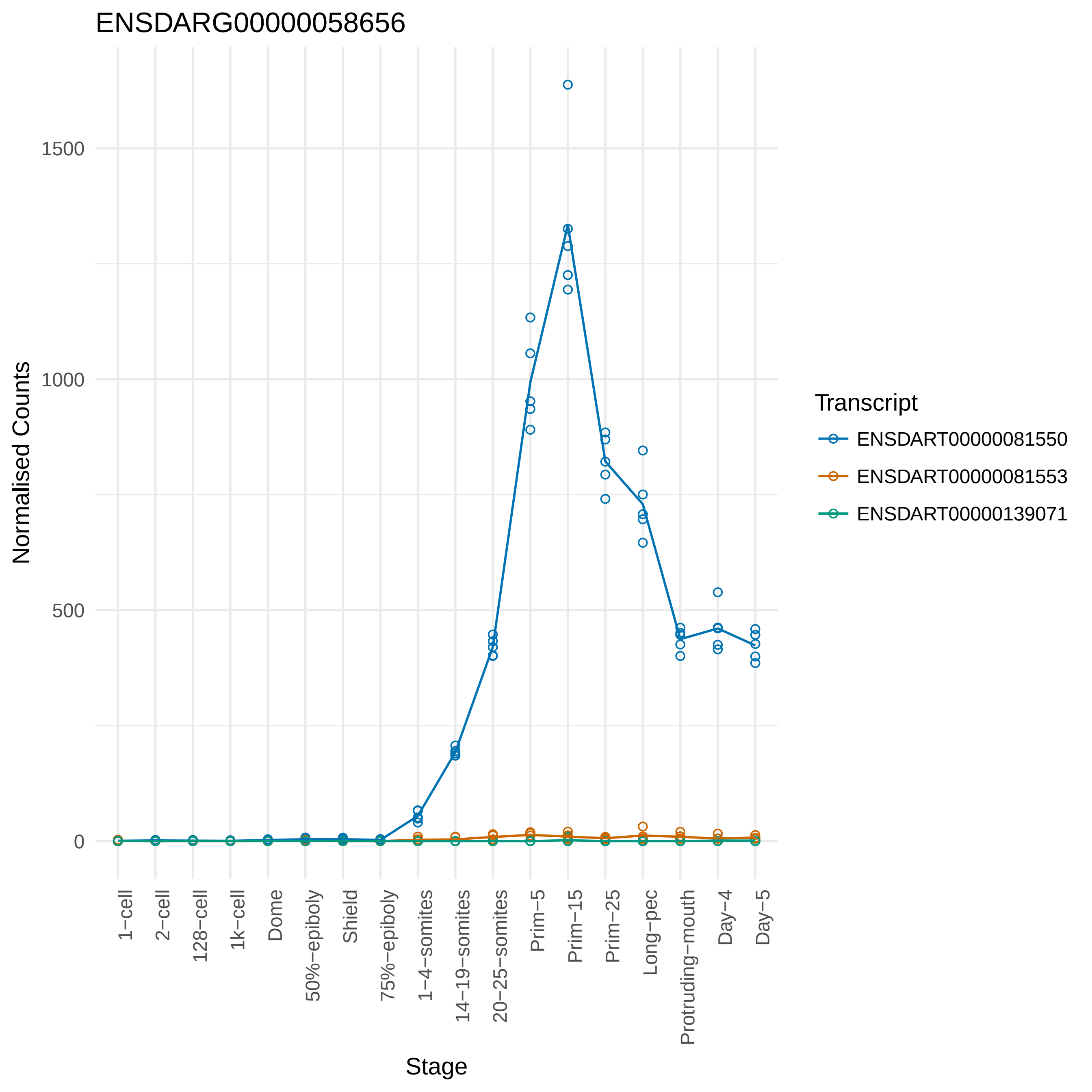
ENSDARG00000058656
Normalised Counts
1500
1000
500
0
1−cell
2−cell
128−cell
1k−cell
Dome
50%−epiboly
Shield
75%−epiboly
1−4−somites
14−19−somites
20−25−somites
Prim−5
Prim−15
Prim−25
Long−pec
Protruding−mouth
Day−4
Day−5
Stage
Transcript
ENSDART00000081550
ENSDART00000081553
ENSDART00000139071

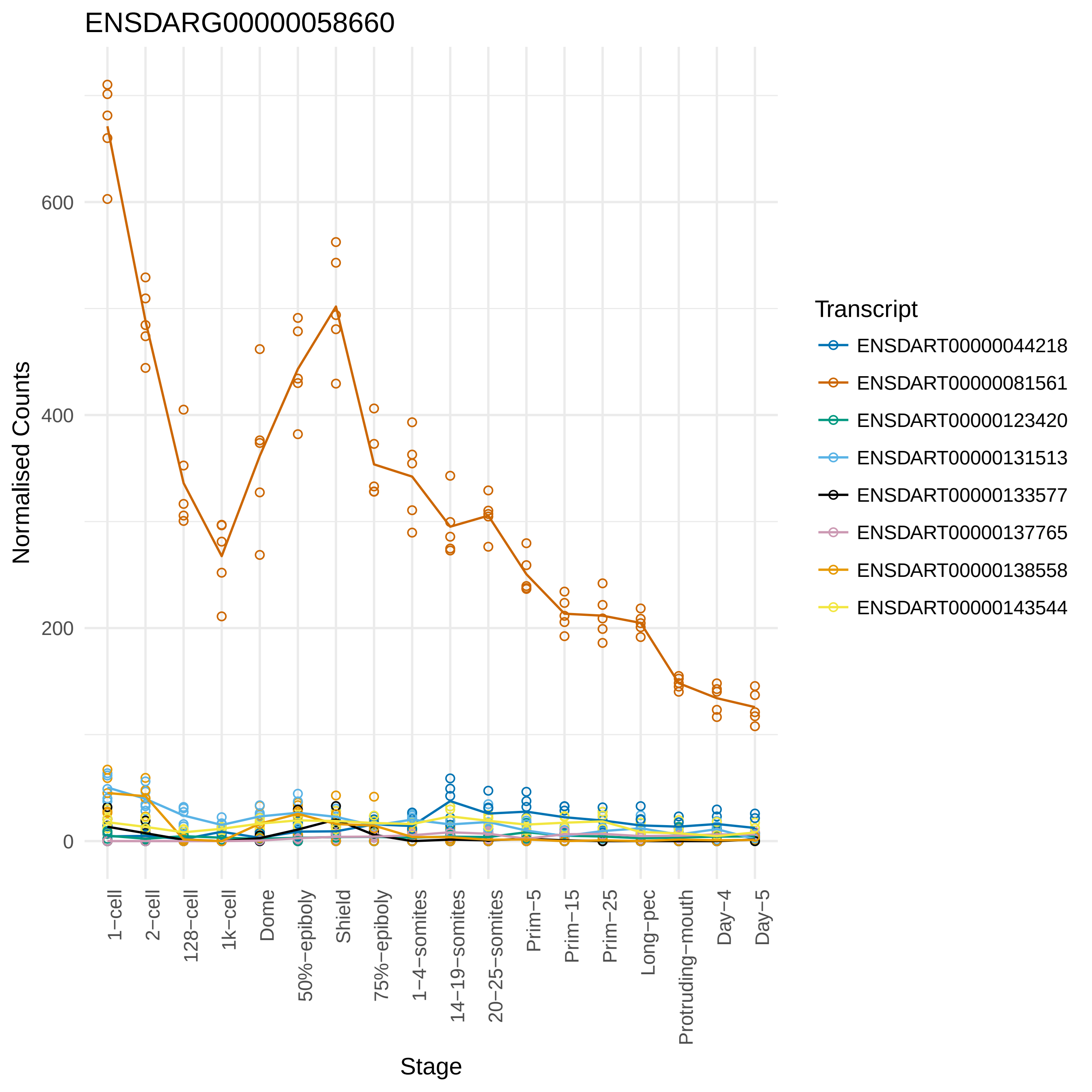
ENSDARG00000058660
Normalised Counts
600
400
200
0
1−cell
2−cell
128−cell
1k−cell
Dome
50%−epiboly
Shield
75%−epiboly
1−4−somites
14−19−somites
20−25−somites
Prim−5
Prim−15
Prim−25
Long−pec
Protruding−mouth
Day−4
Day−5
Stage
Transcript
ENSDART00000044218
ENSDART00000081561
ENSDART00000123420
ENSDART00000131513
ENSDART00000133577
ENSDART00000137765
ENSDART00000138558
ENSDART00000143544

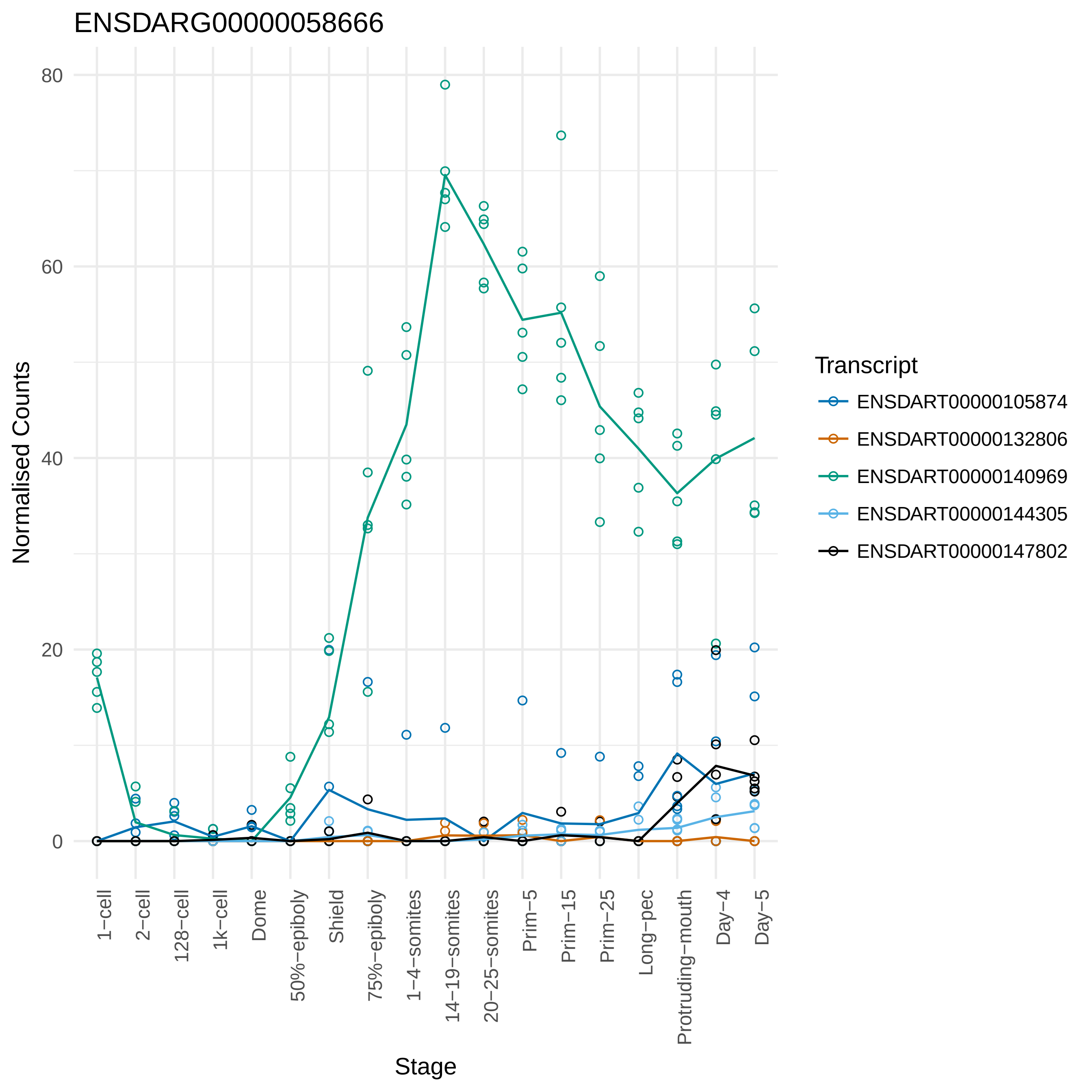
ENSDARG00000058666
Normalised Counts
80
60
40
20
0
1−cell
2−cell
128−cell
1k−cell
Dome
50%−epiboly
Shield
75%−epiboly
1−4−somites
14−19−somites
20−25−somites
Prim−5
Prim−15
Prim−25
Long−pec
Protruding−mouth
Day−4
Day−5
Stage
Transcript
ENSDART00000105874
ENSDART00000132806
ENSDART00000140969
ENSDART00000144305
ENSDART00000147802

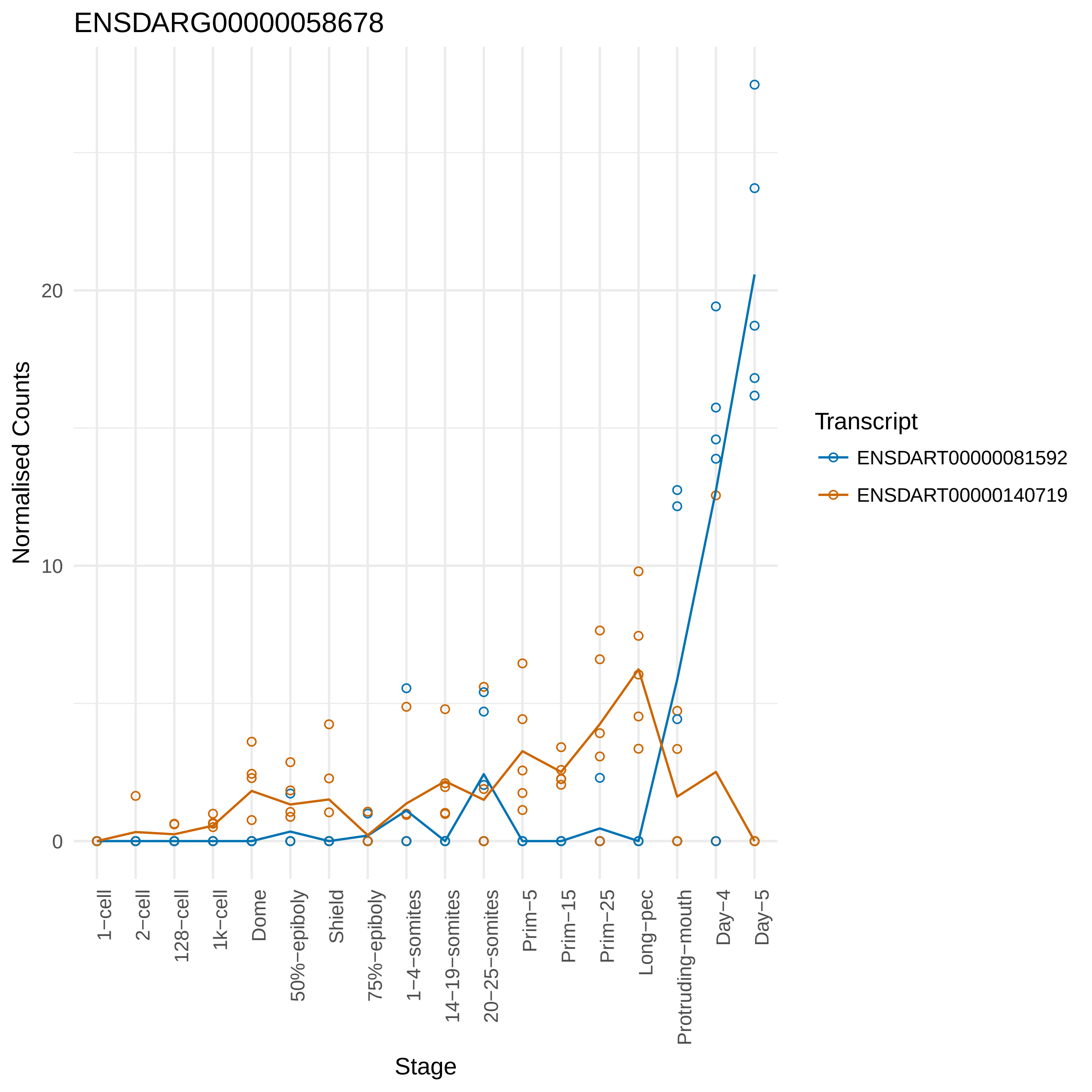

ENSDARG00000058678
Normalised Counts
20
10
0
1−cell
2−cell
128−cell
1k−cell
Dome
50%−epiboly
Shield
75%−epiboly
1−4−somites
14−19−somites
20−25−somites
Prim−5
Prim−15
Prim−25
Long−pec
Protruding−mouth
Day−4
Day−5
Stage
Transcript
ENSDART00000081592
ENSDART00000140719

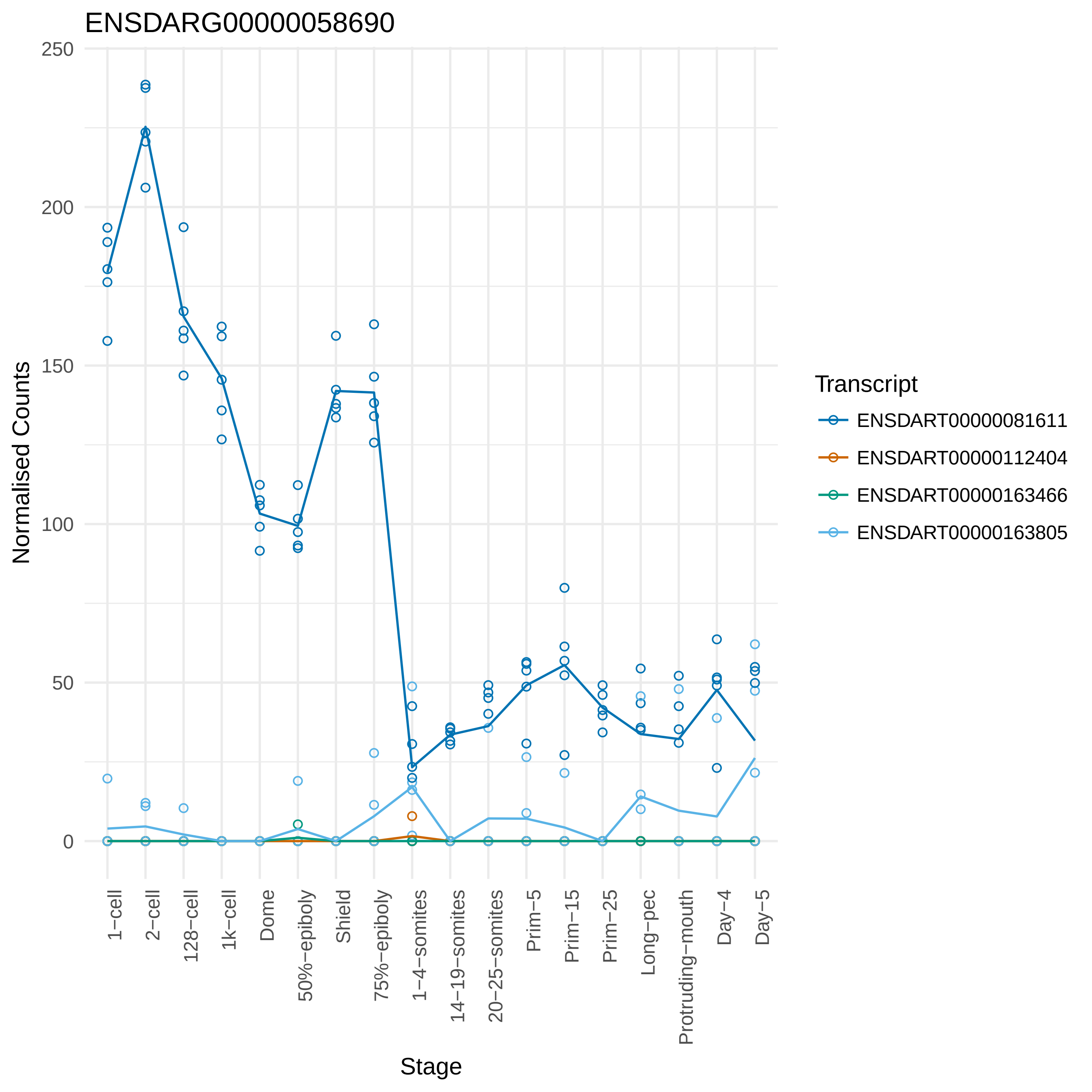
ENSDARG00000058690
Normalised Counts
250
200
150
100
50
0
Stage
1−cell
2−cell
128−cell
1k−cell
Dome
50%−epiboly
Shield
75%−epiboly
1−4−somites
14−19−somites
20−25−somites
Prim−5
Prim−15
Prim−25
Long−pec
Protruding−mouth
Day−4
Day−5
Transcript
ENSDART00000081611
ENSDART00000112404
ENSDART00000163466
ENSDART00000163805

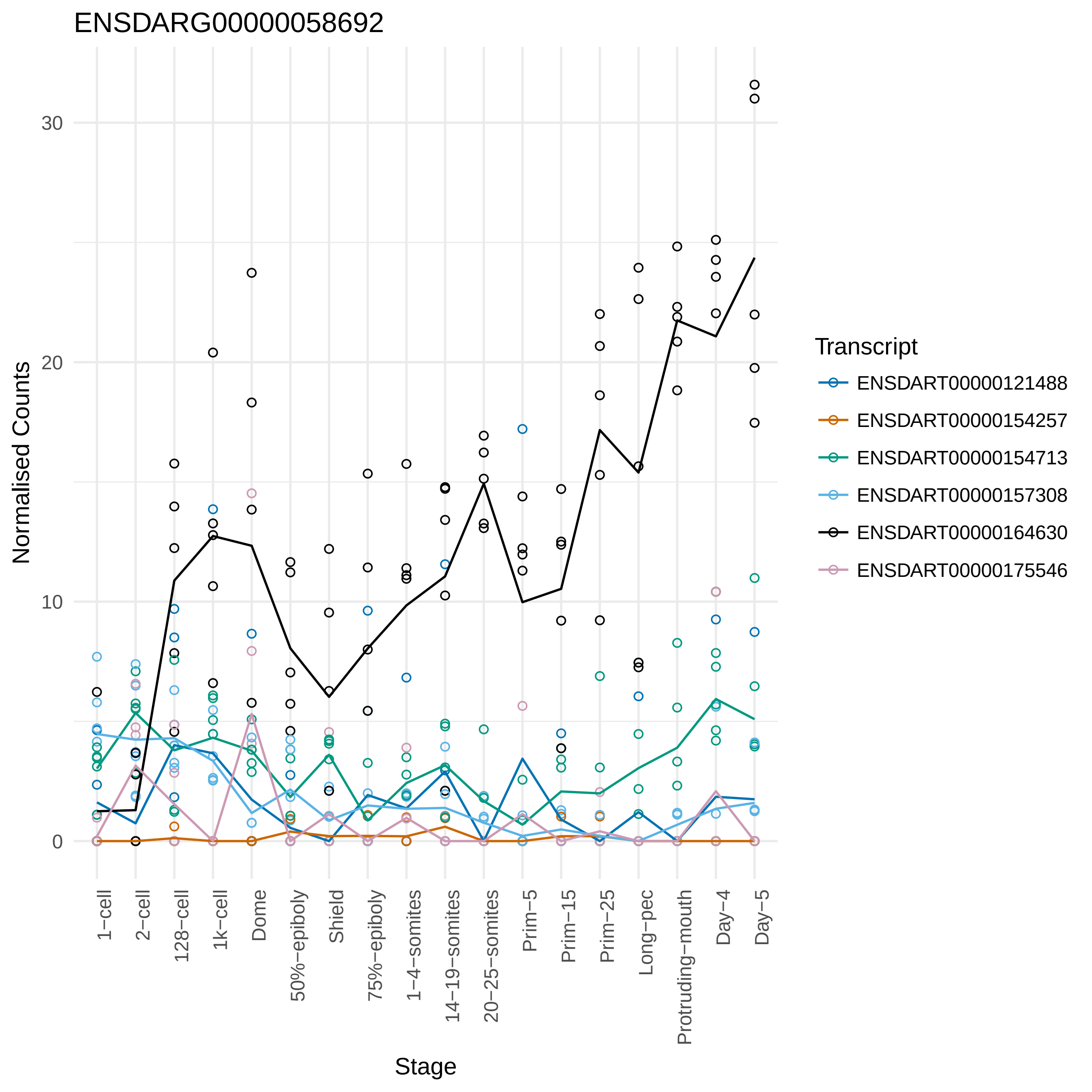

ENSDARG00000058692
Normalised Counts
0
10
20
30
1−cell
2−cell
128−cell
1k−cell
Dome
50%−epiboly
Shield
75%−epiboly
1−4−somites
14−19−somites
20−25−somites
Prim−5
Prim−15
Prim−25
Long−pec
Protruding−mouth
Day−4
Day−5
Stage
Transcript
ENSDART00000121488
ENSDART00000154257
ENSDART00000154713
ENSDART00000157308
ENSDART00000164630
ENSDART00000175546

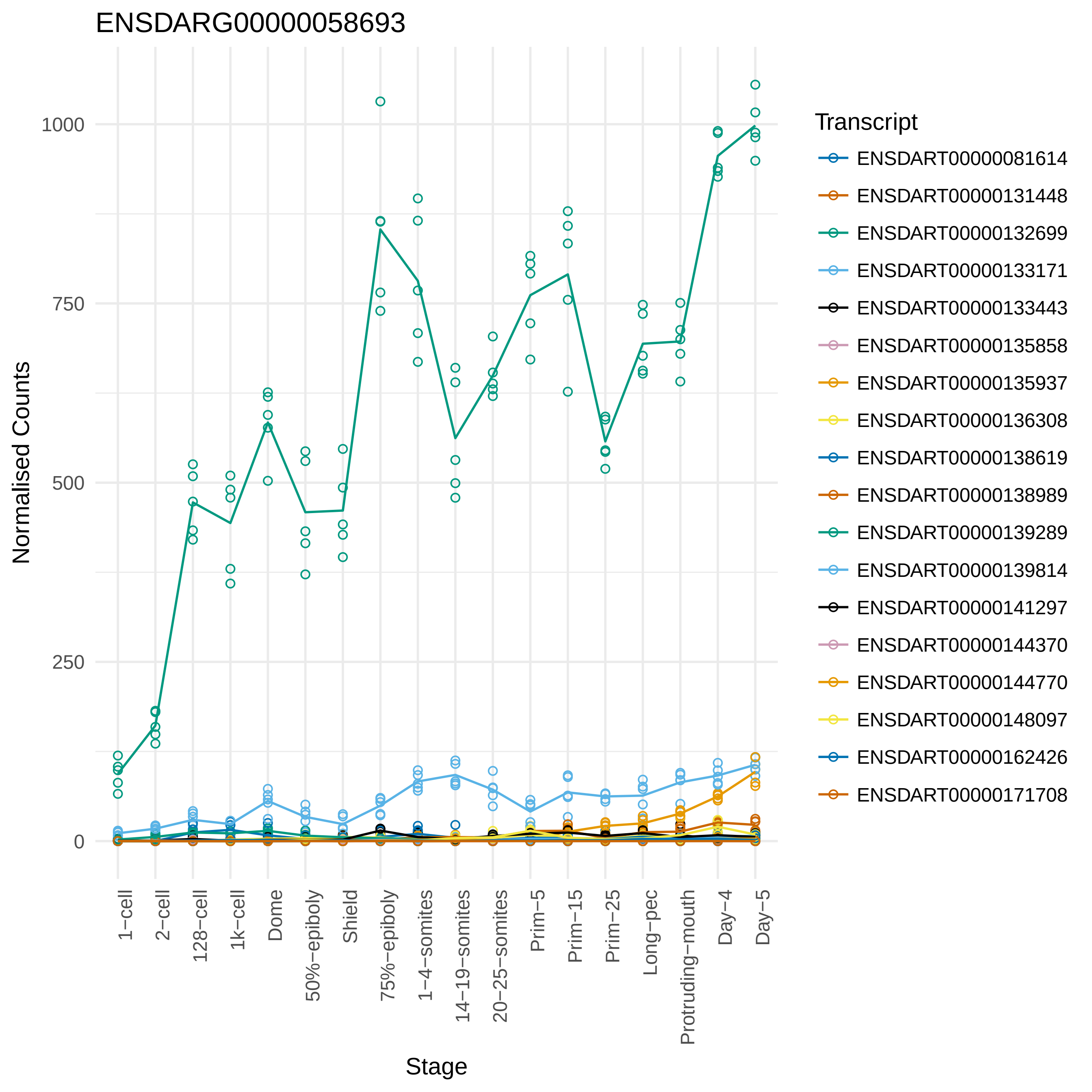
ENSDARG00000058693
Normalised Counts
1000
750
500
250
0
1−cell
2−cell
128−cell
1k−cell
Dome
50%−epiboly
Shield
75%−epiboly
1−4−somites
14−19−somites
20−25−somites
Prim−5
Prim−15
Prim−25
Long−pec
Protruding−mouth
Day−4
Day−5
Stage
Transcript
ENSDART00000081614
ENSDART00000131448
ENSDART00000132699
ENSDART00000133171
ENSDART00000133443
ENSDART00000135858
ENSDART00000135937
ENSDART00000136308
ENSDART00000138619
ENSDART00000138989
ENSDART00000139289
ENSDART00000139814
ENSDART00000141297
ENSDART00000144370
ENSDART00000144770
ENSDART00000148097
ENSDART00000162426
ENSDART00000171708

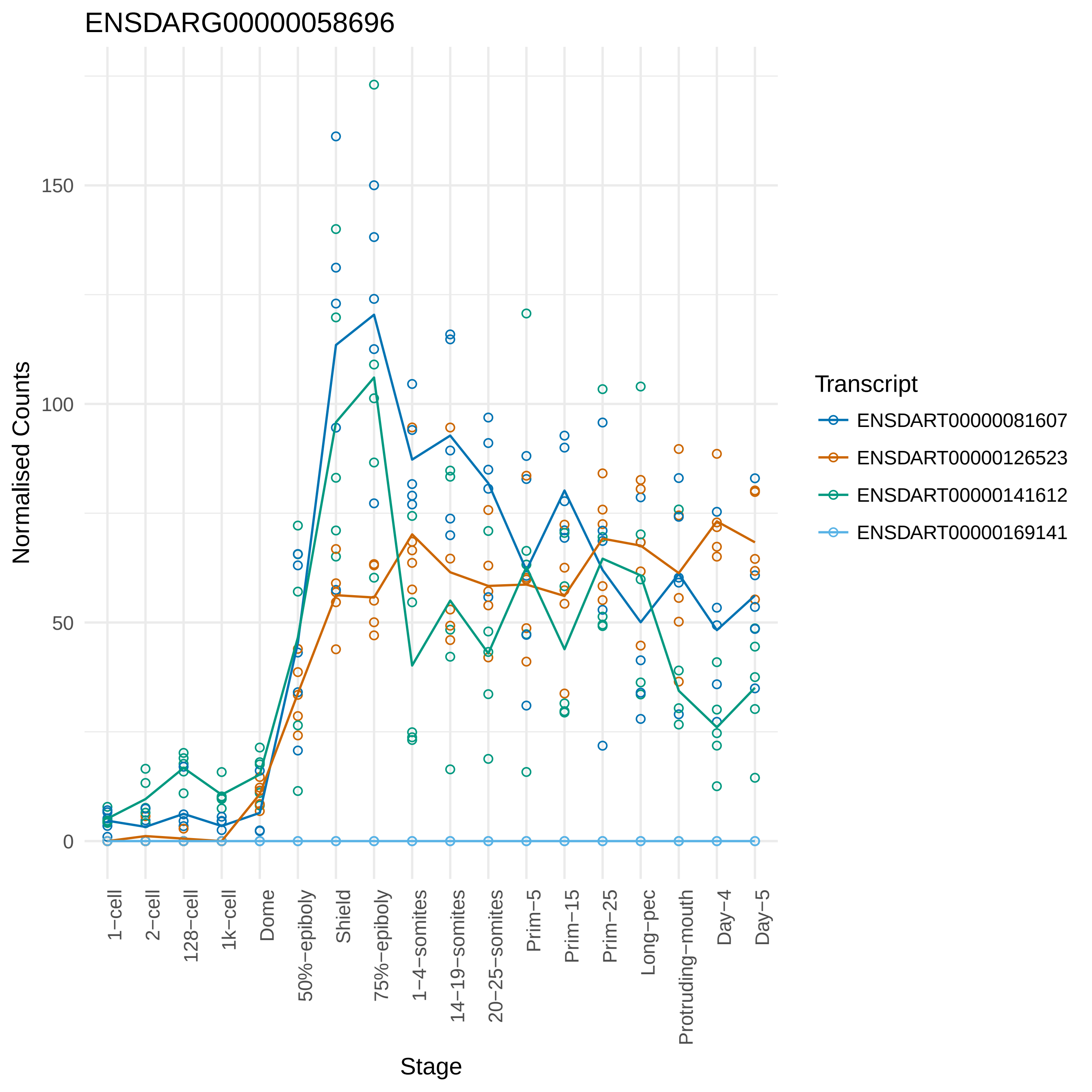
ENSDARG00000058696
Normalised Counts
150
100
50
0
1−cell
2−cell
128−cell
1k−cell
Dome
50%−epiboly
Shield
75%−epiboly
1−4−somites
14−19−somites
20−25−somites
Prim−5
Prim−15
Prim−25
Long−pec
Protruding−mouth
Day−4
Day−5
Stage
Transcript
ENSDART00000081607
ENSDART00000126523
ENSDART00000141612
ENSDART00000169141

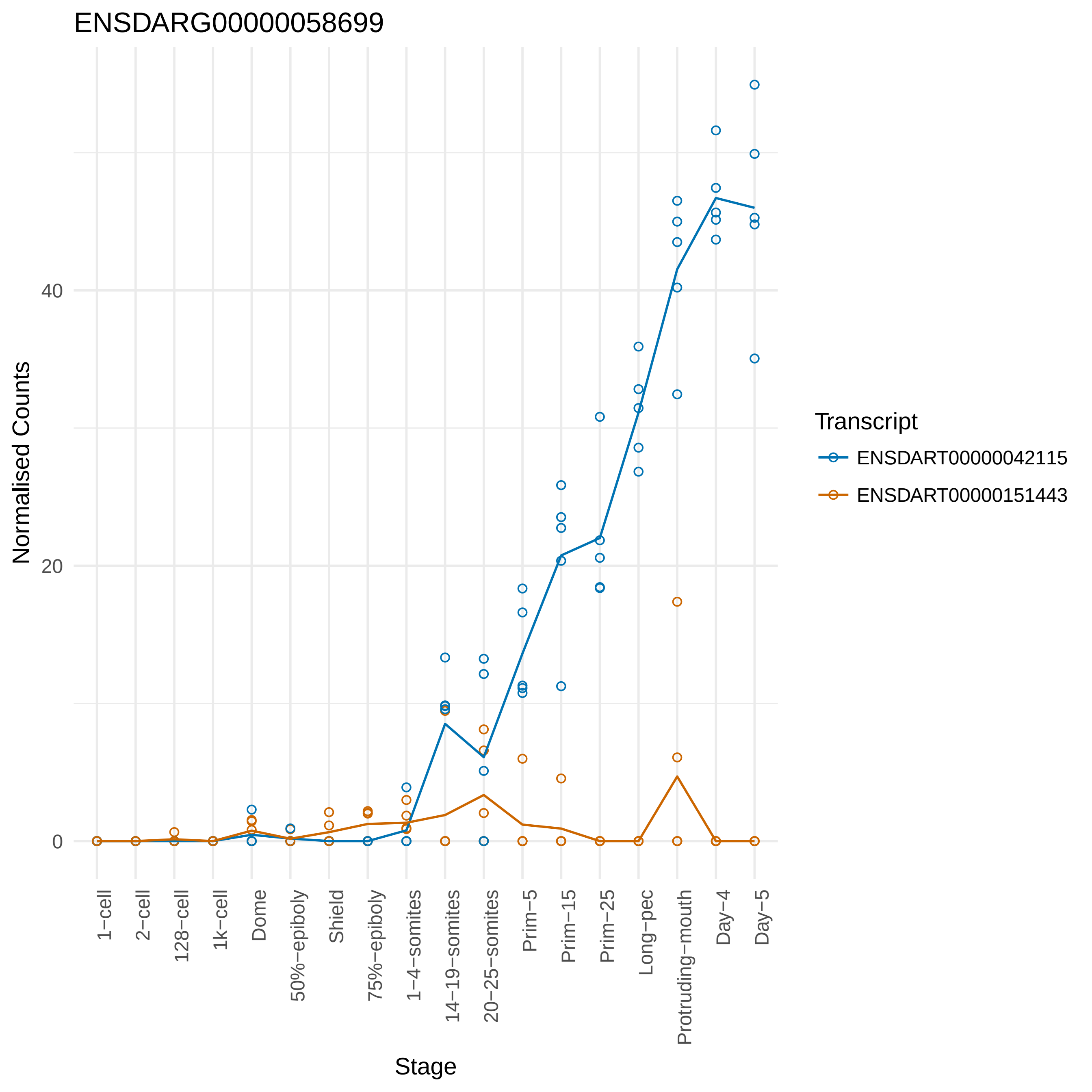
ENSDARG00000058699
Normalised Counts
0
20
40
Stage
1−cell
2−cell
128−cell
1k−cell
Dome
50%−epiboly
Shield
75%−epiboly
1−4−somites
14−19−somites
20−25−somites
Prim−5
Prim−15
Prim−25
Long−pec
Protruding−mouth
Day−4
Day−5
Transcript
ENSDART00000042115
ENSDART00000151443

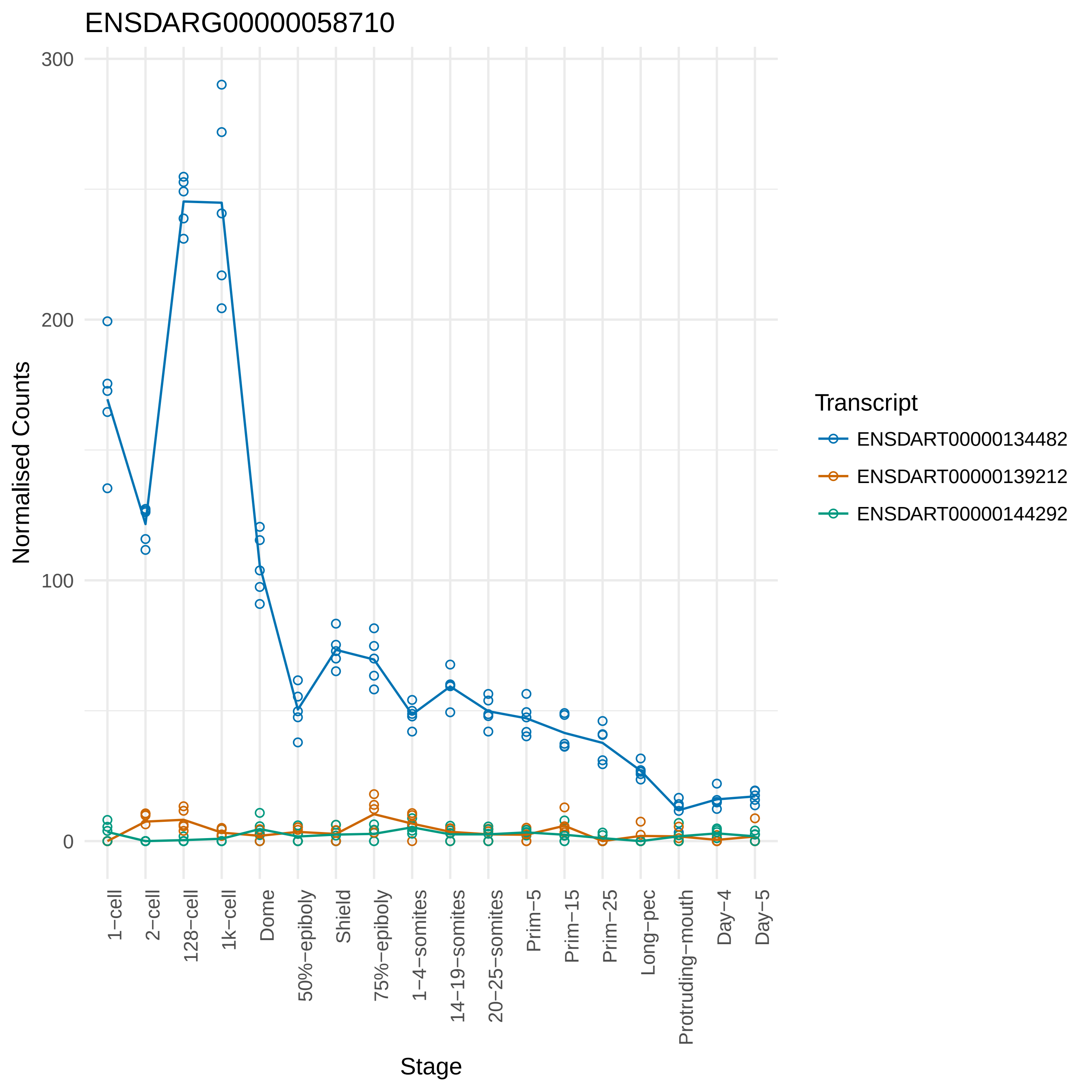

ENSDARG00000058710
Normalised Counts
300
200
100
0
1−cell
2−cell
128−cell
1k−cell
Dome
50%−epiboly
Shield
75%−epiboly
1−4−somites
14−19−somites
20−25−somites
Prim−5
Prim−15
Prim−25
Long−pec
Protruding−mouth
Day−4
Day−5
Stage
Transcript
ENSDART00000134482
ENSDART00000139212
ENSDART00000144292

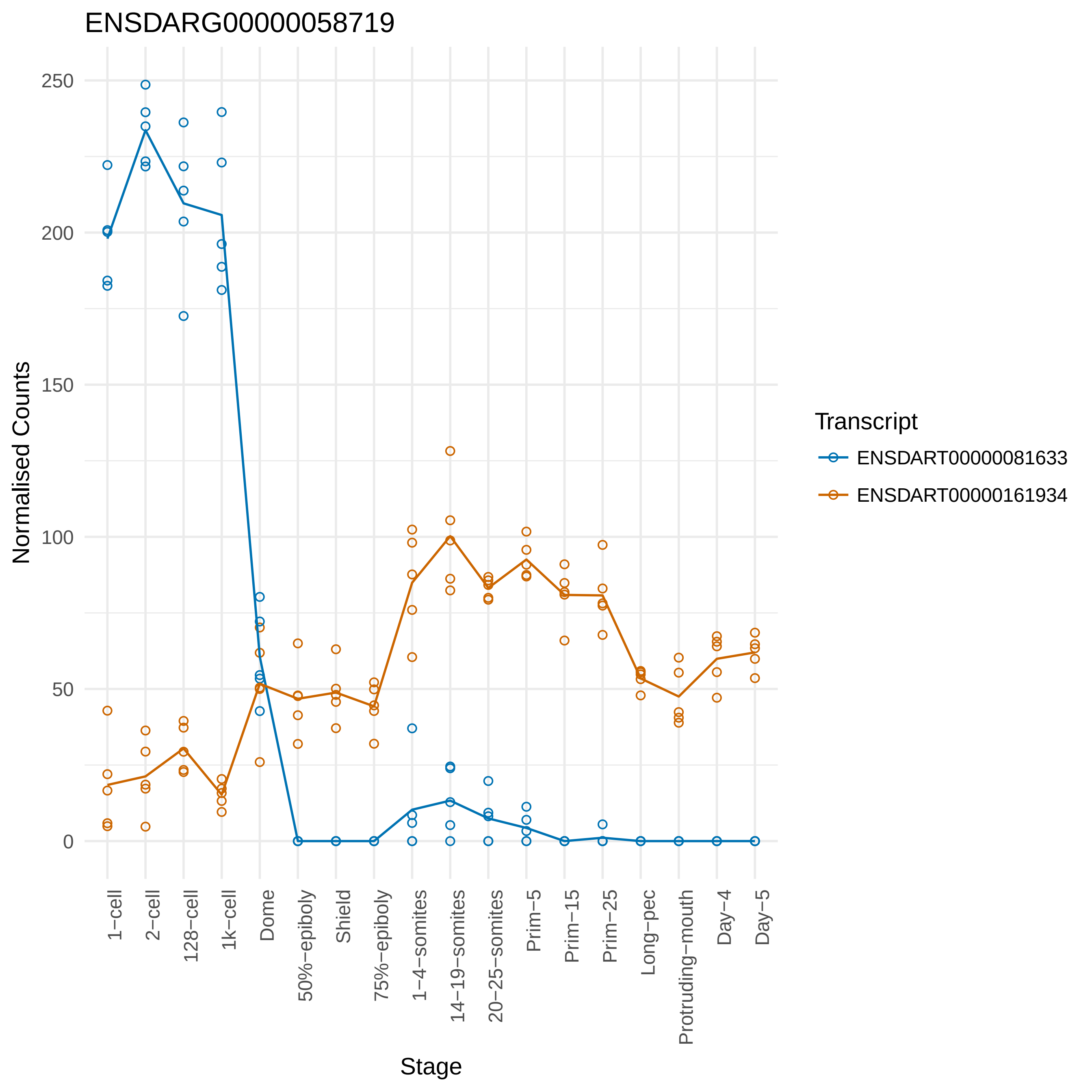

ENSDARG00000058719
Normalised Counts
250
200
150
100
50
0
1−cell
2−cell
128−cell
1k−cell
Dome
50%−epiboly
Shield
75%−epiboly
1−4−somites
14−19−somites
20−25−somites
Prim−5
Prim−15
Prim−25
Long−pec
Protruding−mouth
Day−4
Day−5
Stage
Transcript
ENSDART00000081633
ENSDART00000161934

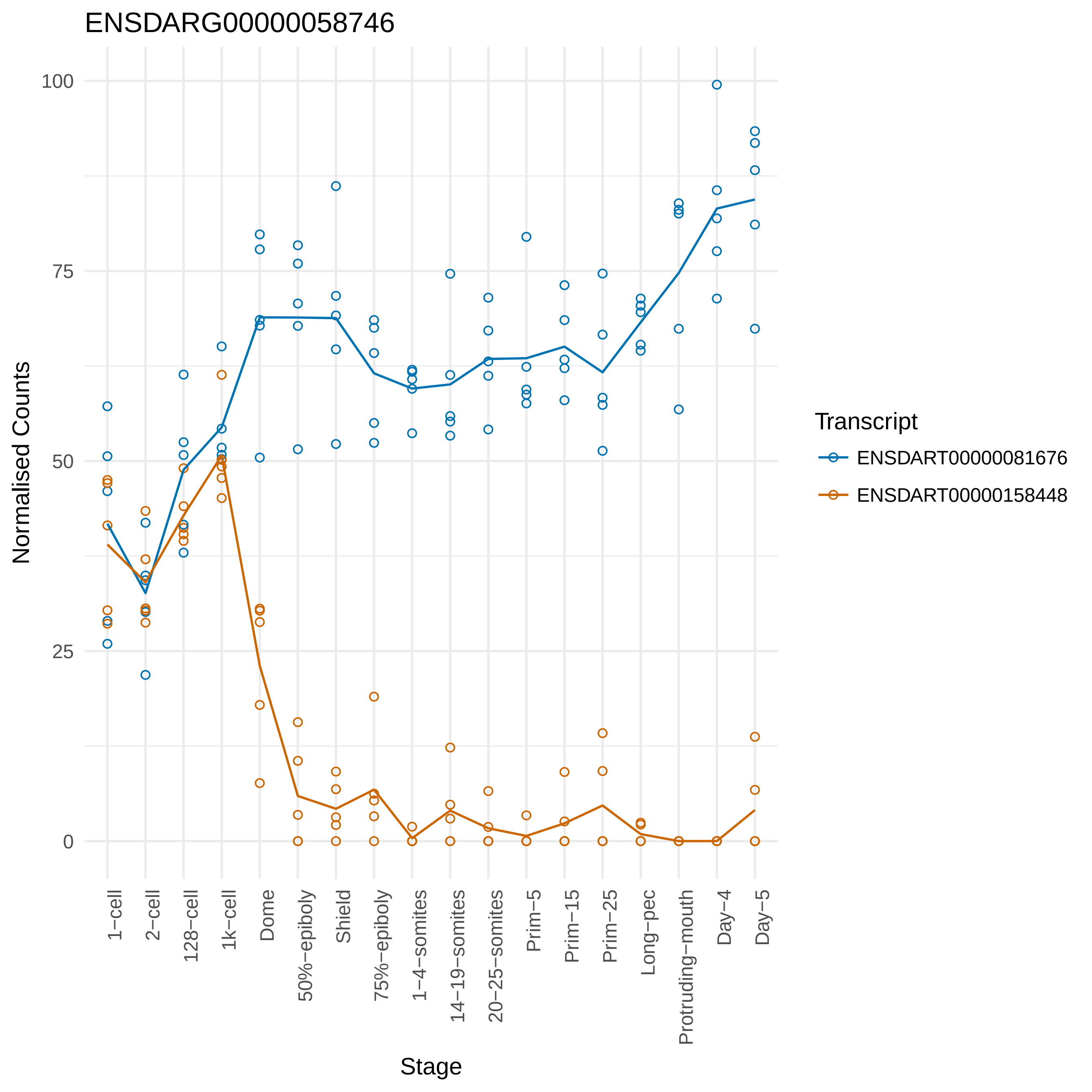

ENSDARG00000058746
Normalised Counts
100
75
50
25
0
1−cell
2−cell
128−cell
1k−cell
Dome
50%−epiboly
Shield
75%−epiboly
1−4−somites
14−19−somites
20−25−somites
Prim−5
Prim−15
Prim−25
Long−pec
Protruding−mouth
Day−4
Day−5
Stage
Transcript
ENSDART00000081676
ENSDART00000158448

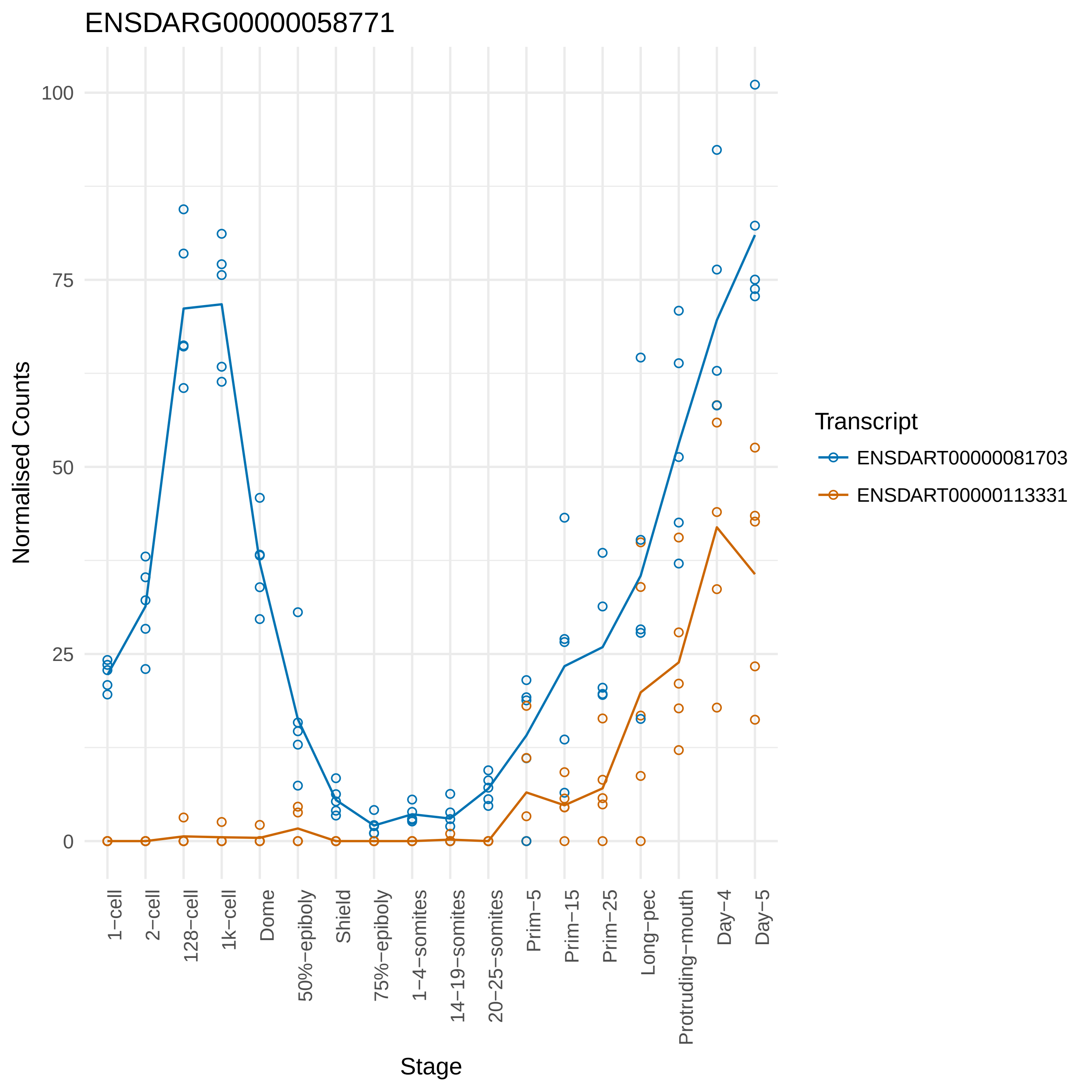
ENSDARG00000058771
Normalised Counts
100
75
50
25
0
1−cell
2−cell
128−cell
1k−cell
Dome
50%−epiboly
Shield
75%−epiboly
1−4−somites
14−19−somites
20−25−somites
Prim−5
Prim−15
Prim−25
Long−pec
Protruding−mouth
Day−4
Day−5
Stage
Transcript
ENSDART00000081703
ENSDART00000113331

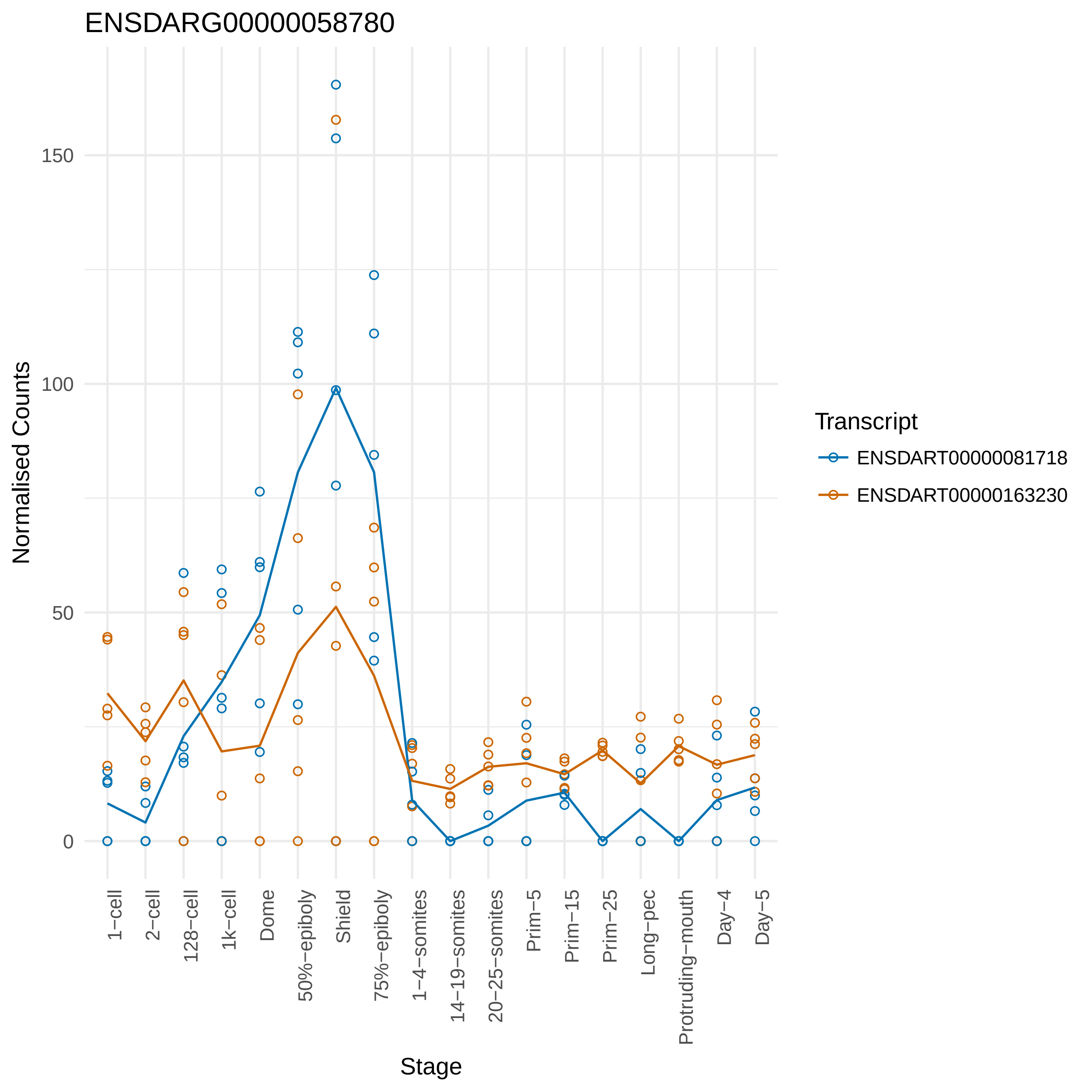

ENSDARG00000058780
Normalised Counts
150
100
50
0
1−cell
2−cell
128−cell
1k−cell
Dome
50%−epiboly
Shield
75%−epiboly
1−4−somites
14−19−somites
20−25−somites
Prim−5
Prim−15
Prim−25
Long−pec
Protruding−mouth
Day−4
Day−5
Stage
Transcript
ENSDART00000081718
ENSDART00000163230

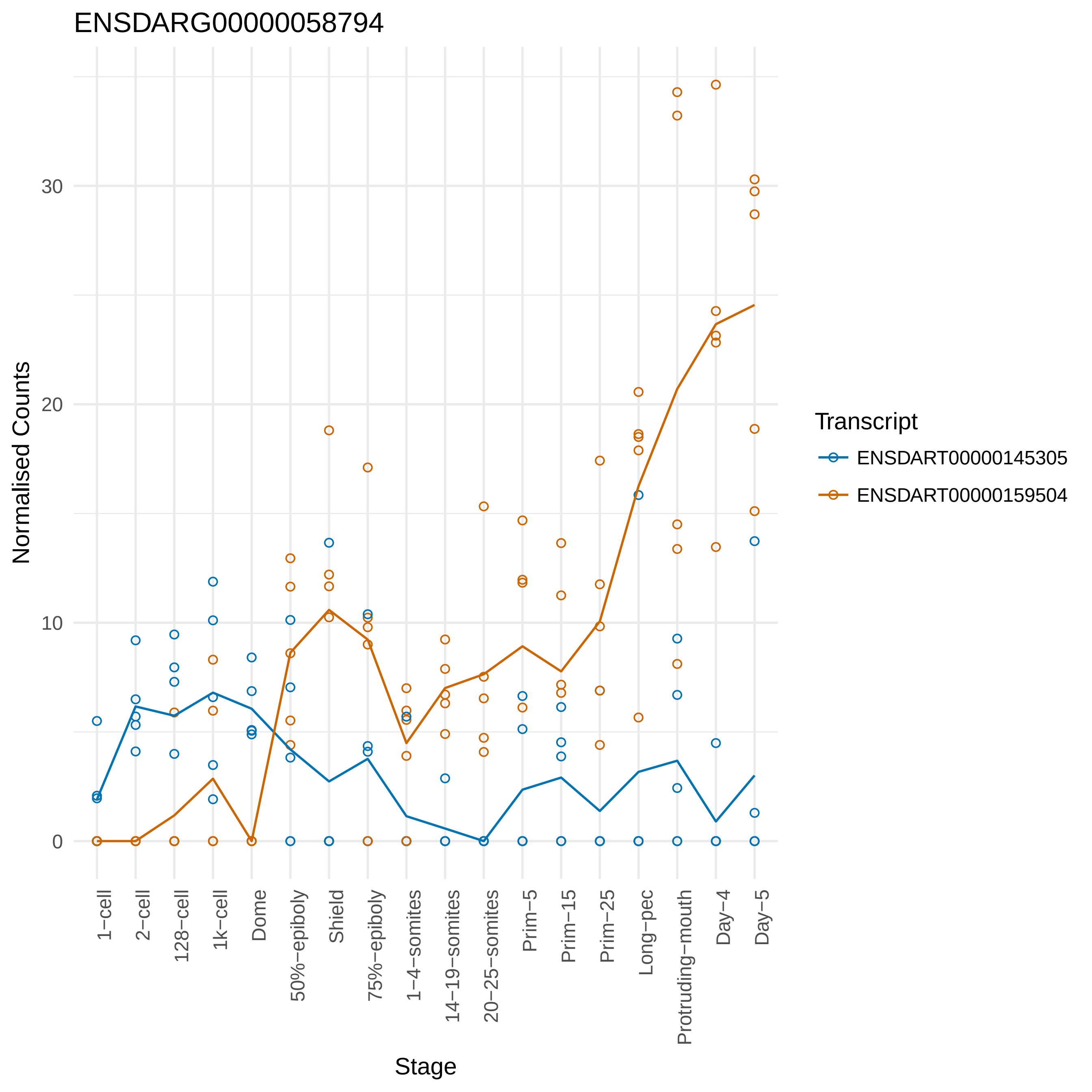

ENSDARG00000058794
Normalised Counts
0
10
20
30
1−cell
2−cell
128−cell
1k−cell
Dome
50%−epiboly
Shield
75%−epiboly
1−4−somites
14−19−somites
20−25−somites
Prim−5
Prim−15
Prim−25
Long−pec
Protruding−mouth
Day−4
Day−5
Stage
Transcript
ENSDART00000145305
ENSDART00000159504

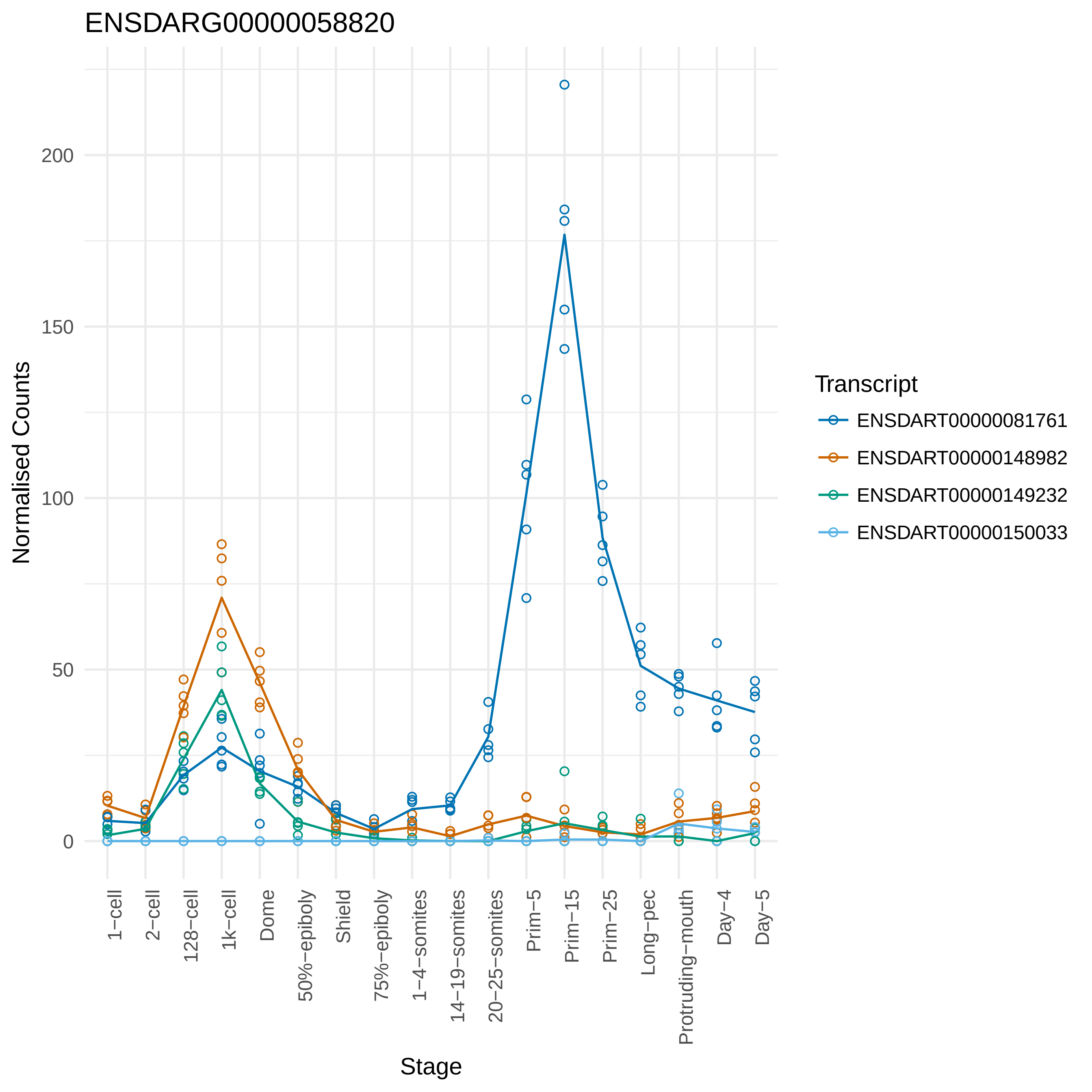
ENSDARG00000058820
Normalised Counts
0
50
100
150
200
Stage
1−cell
2−cell
128−cell
1k−cell
Dome
50%−epiboly
Shield
75%−epiboly
1−4−somites
14−19−somites
20−25−somites
Prim−5
Prim−15
Prim−25
Long−pec
Protruding−mouth
Day−4
Day−5
Transcript
ENSDART00000081761
ENSDART00000148982
ENSDART00000149232
ENSDART00000150033

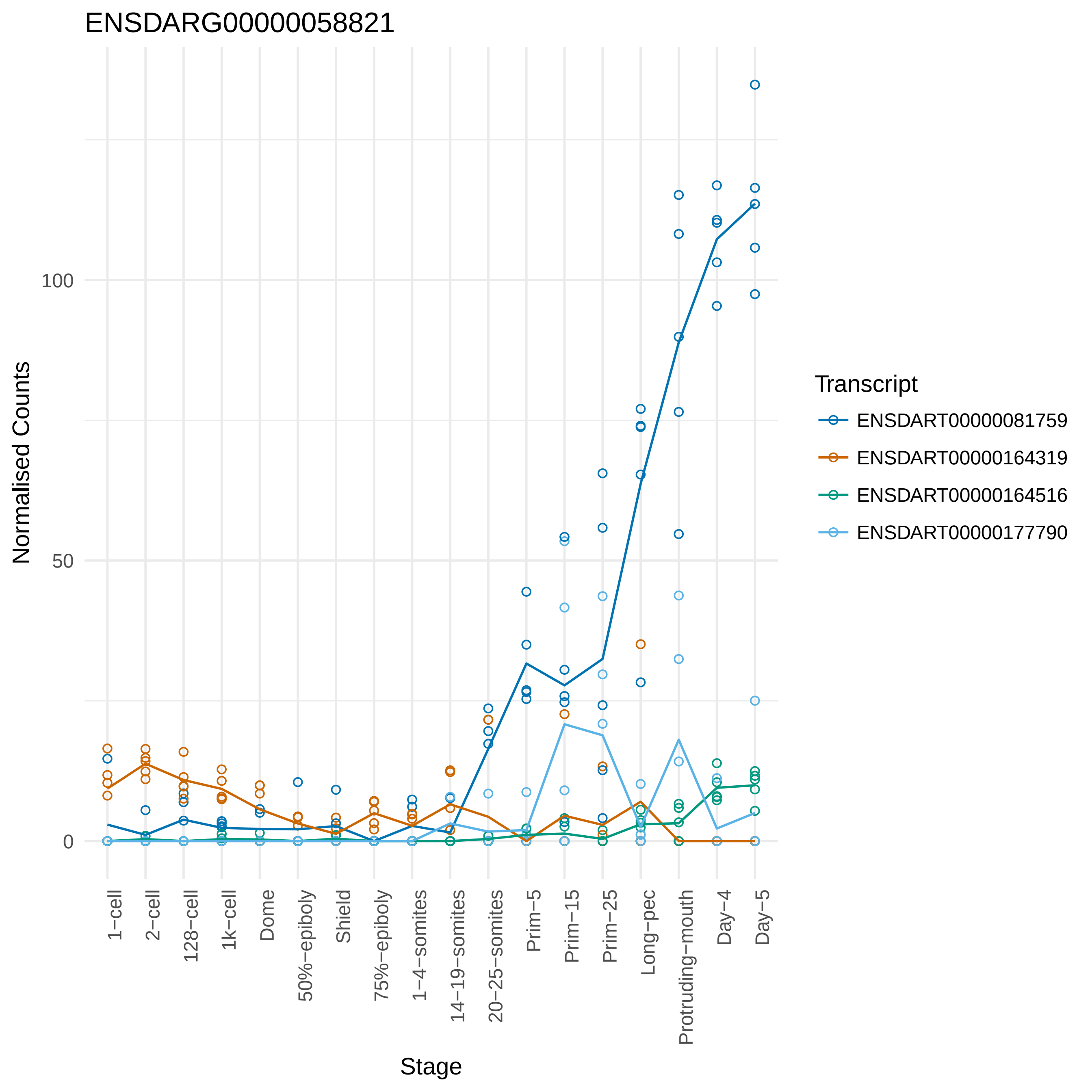

ENSDARG00000058821
Normalised Counts
100
50
0
1−cell
2−cell
128−cell
1k−cell
Dome
50%−epiboly
Shield
75%−epiboly
1−4−somites
14−19−somites
20−25−somites
Prim−5
Prim−15
Prim−25
Long−pec
Protruding−mouth
Day−4
Day−5
Stage
Transcript
ENSDART00000081759
ENSDART00000164319
ENSDART00000164516
ENSDART00000177790

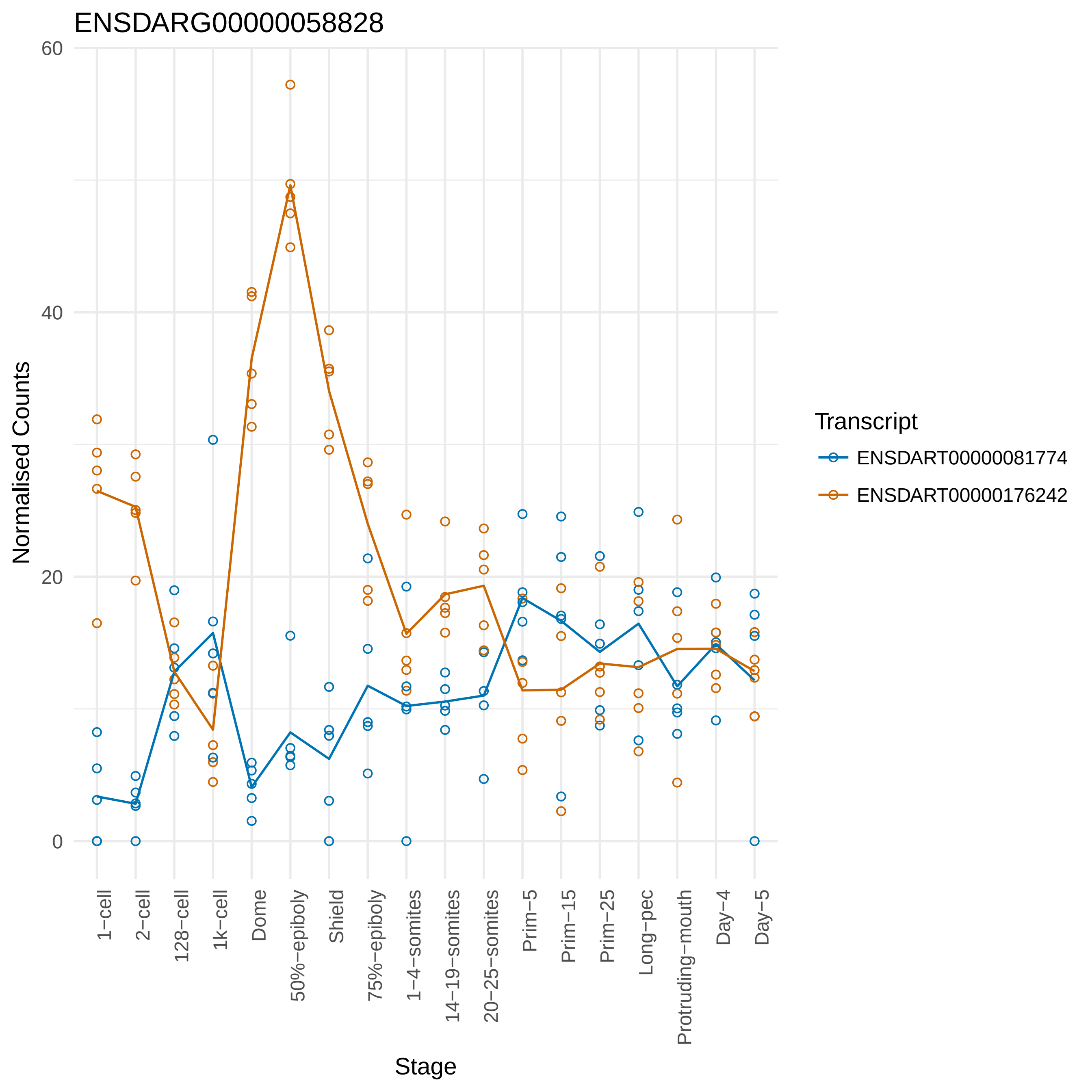
ENSDARG00000058828
Normalised Counts
60
40
20
0
1−cell
2−cell
128−cell
1k−cell
Dome
50%−epiboly
Shield
75%−epiboly
1−4−somites
14−19−somites
20−25−somites
Prim−5
Prim−15
Prim−25
Long−pec
Protruding−mouth
Day−4
Day−5
Stage
Transcript
ENSDART00000081774
ENSDART00000176242

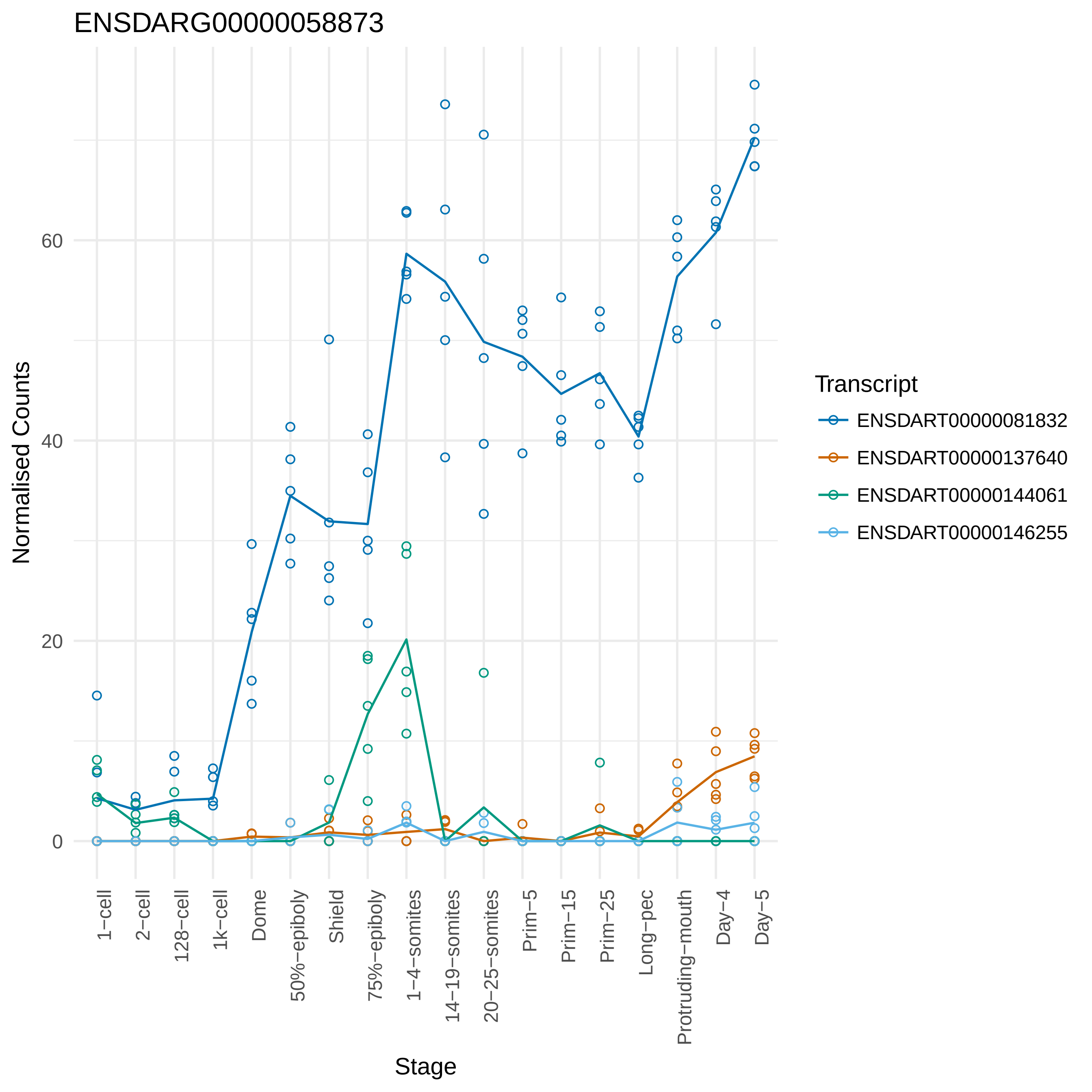
ENSDARG00000058873
Normalised Counts
0
20
40
60
Stage
1−cell
2−cell
128−cell
1k−cell
Dome
50%−epiboly
Shield
75%−epiboly
1−4−somites
14−19−somites
20−25−somites
Prim−5
Prim−15
Prim−25
Long−pec
Protruding−mouth
Day−4
Day−5
Transcript
ENSDART00000081832
ENSDART00000137640
ENSDART00000144061
ENSDART00000146255

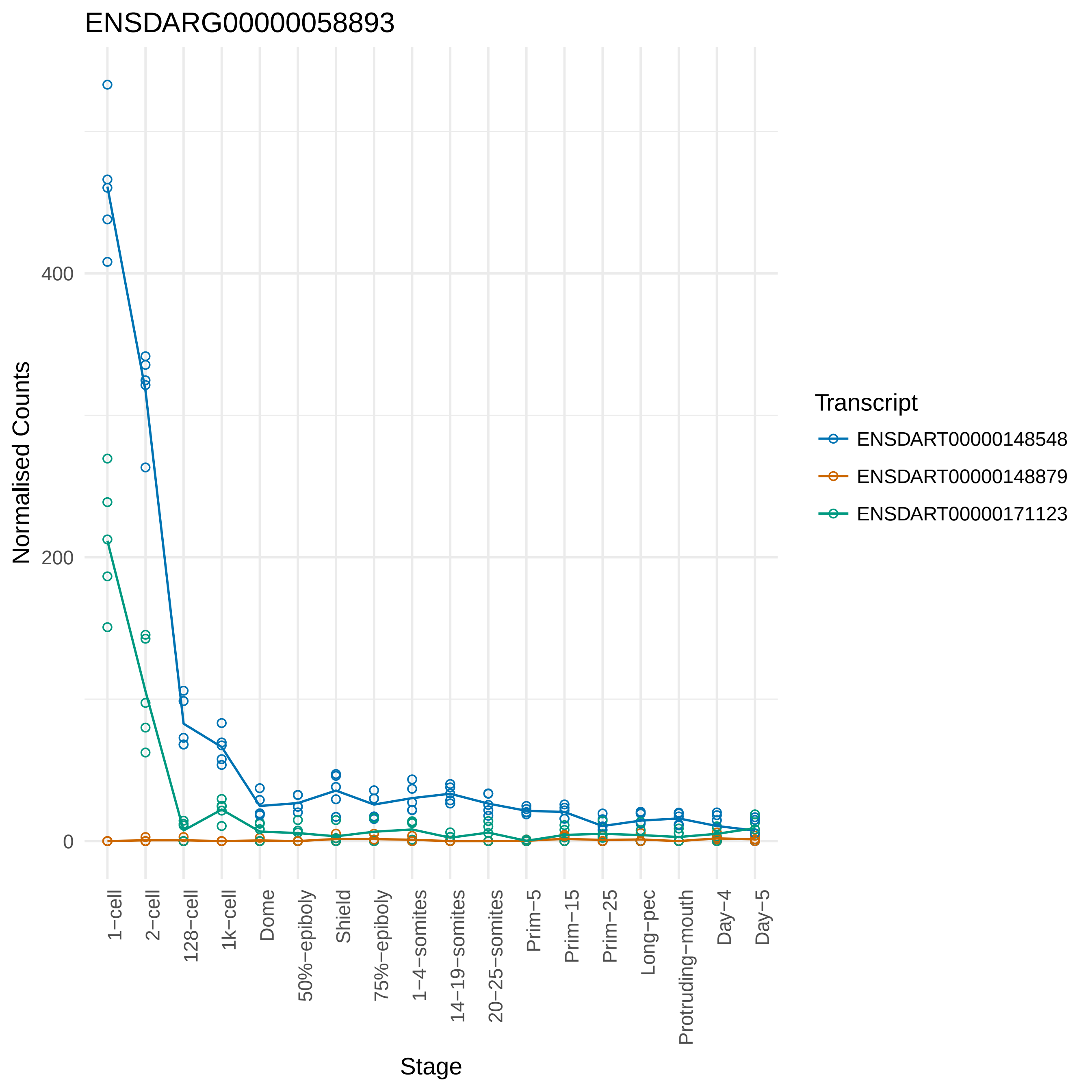
ENSDARG00000058893
Normalised Counts
400
200
0
Stage
1−cell
2−cell
128−cell
1k−cell
Dome
50%−epiboly
Shield
75%−epiboly
1−4−somites
14−19−somites
20−25−somites
Prim−5
Prim−15
Prim−25
Long−pec
Protruding−mouth
Day−4
Day−5
Transcript
ENSDART00000148548
ENSDART00000148879
ENSDART00000171123

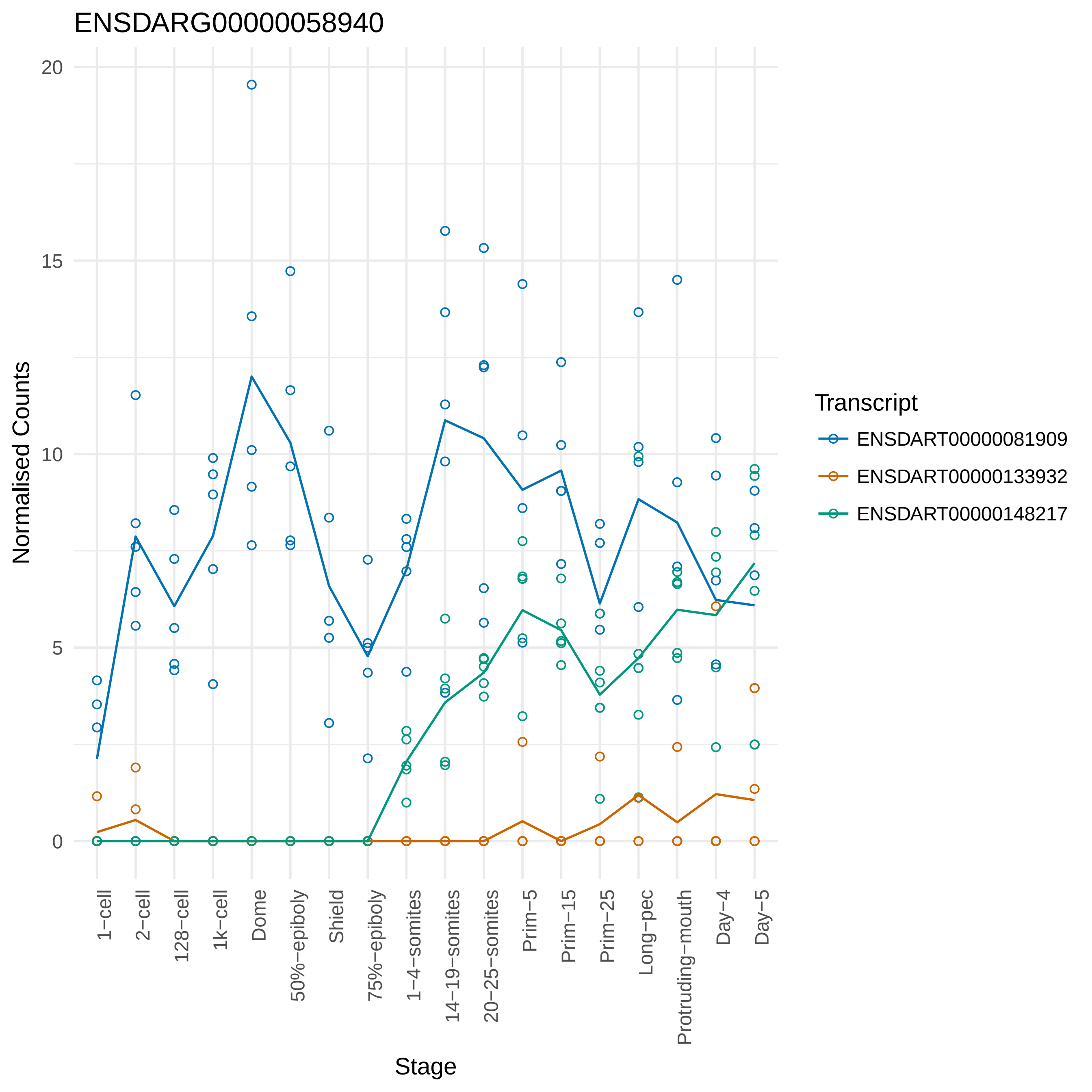
ENSDARG00000058940
Normalised Counts
0
5
10
15
20
1−cell
2−cell
128−cell
1k−cell
Dome
50%−epiboly
Shield
75%−epiboly
1−4−somites
14−19−somites
20−25−somites
Prim−5
Prim−15
Prim−25
Long−pec
Protruding−mouth
Day−4
Day−5
Stage
Transcript
ENSDART00000081909
ENSDART00000133932
ENSDART00000148217

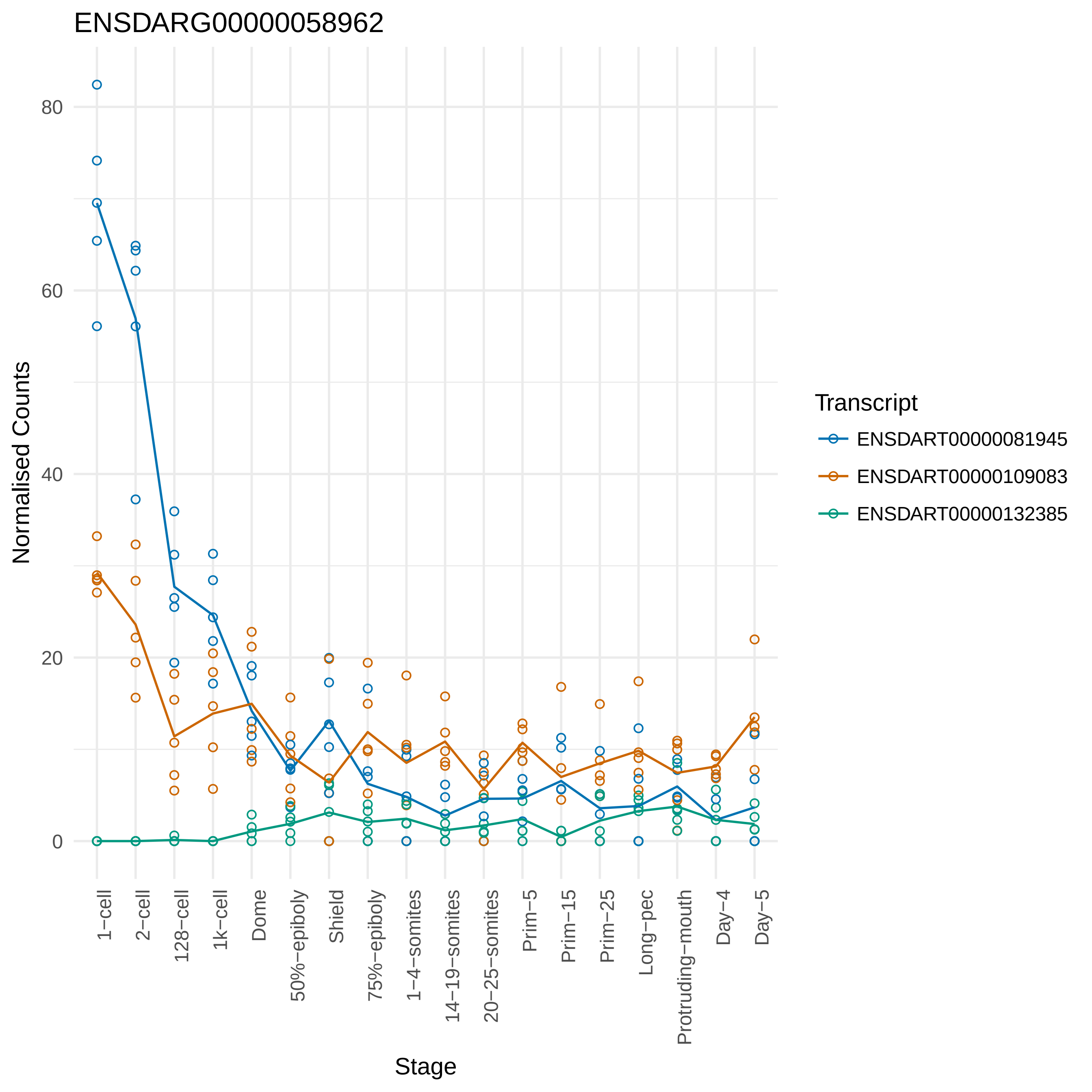
ENSDARG00000058962
Normalised Counts
80
60
40
20
0
1−cell
2−cell
128−cell
1k−cell
Dome
50%−epiboly
Shield
75%−epiboly
1−4−somites
14−19−somites
20−25−somites
Prim−5
Prim−15
Prim−25
Long−pec
Protruding−mouth
Day−4
Day−5
Stage
Transcript
ENSDART00000081945
ENSDART00000109083
ENSDART00000132385

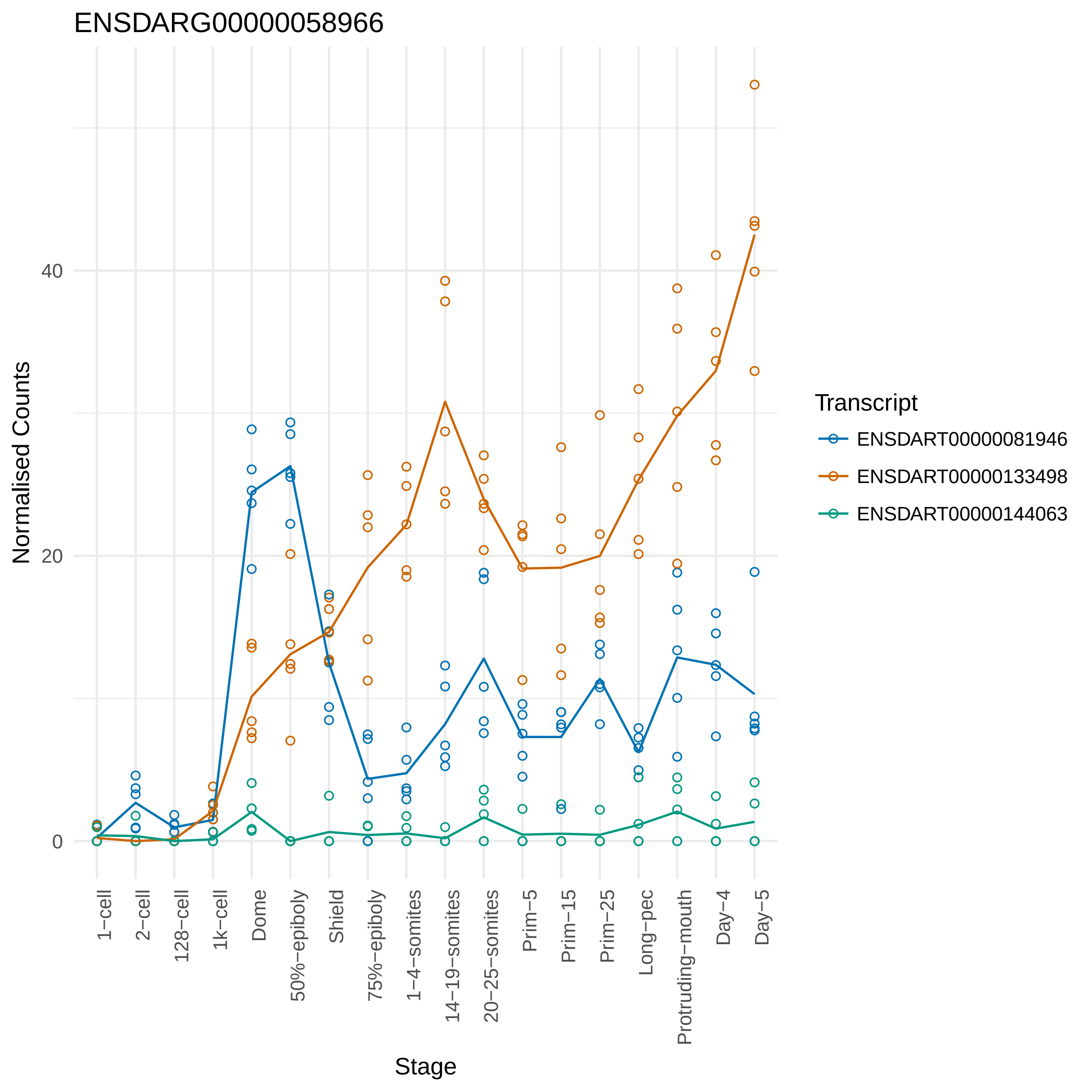

ENSDARG00000058966
Normalised Counts
40
20
0
1−cell
2−cell
128−cell
1k−cell
Dome
50%−epiboly
Shield
75%−epiboly
1−4−somites
14−19−somites
20−25−somites
Prim−5
Prim−15
Prim−25
Long−pec
Protruding−mouth
Day−4
Day−5
Stage
Transcript
ENSDART00000081946
ENSDART00000133498
ENSDART00000144063

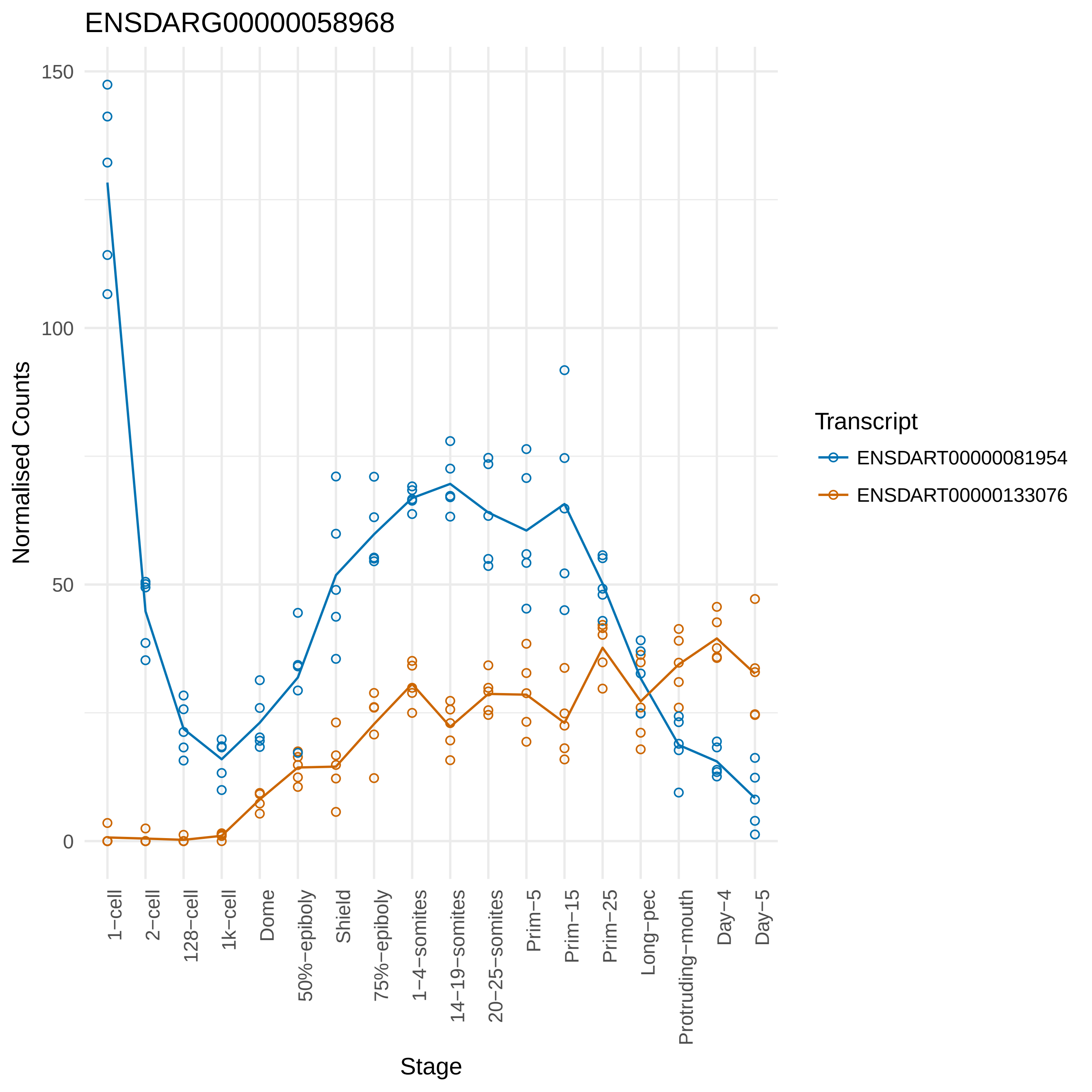

ENSDARG00000058968
Normalised Counts
150
100
50
0
1−cell
2−cell
128−cell
1k−cell
Dome
50%−epiboly
Shield
75%−epiboly
1−4−somites
14−19−somites
20−25−somites
Prim−5
Prim−15
Prim−25
Long−pec
Protruding−mouth
Day−4
Day−5
Stage
Transcript
ENSDART00000081954
ENSDART00000133076

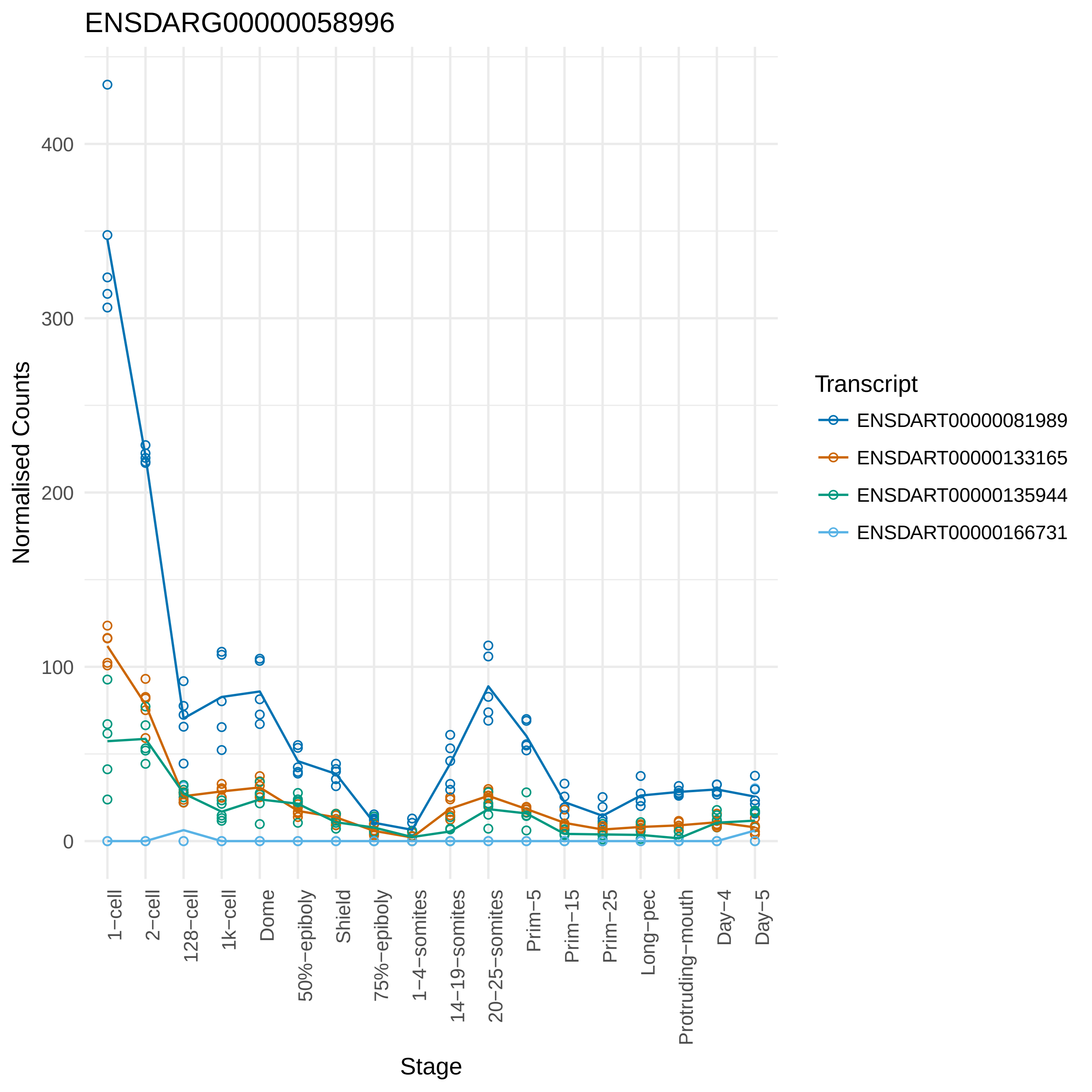
ENSDARG00000058996
Normalised Counts
0
100
200
300
400
Stage
1−cell
2−cell
128−cell
1k−cell
Dome
50%−epiboly
Shield
75%−epiboly
1−4−somites
14−19−somites
20−25−somites
Prim−5
Prim−15
Prim−25
Long−pec
Protruding−mouth
Day−4
Day−5
Transcript
ENSDART00000081989
ENSDART00000133165
ENSDART00000135944
ENSDART00000166731

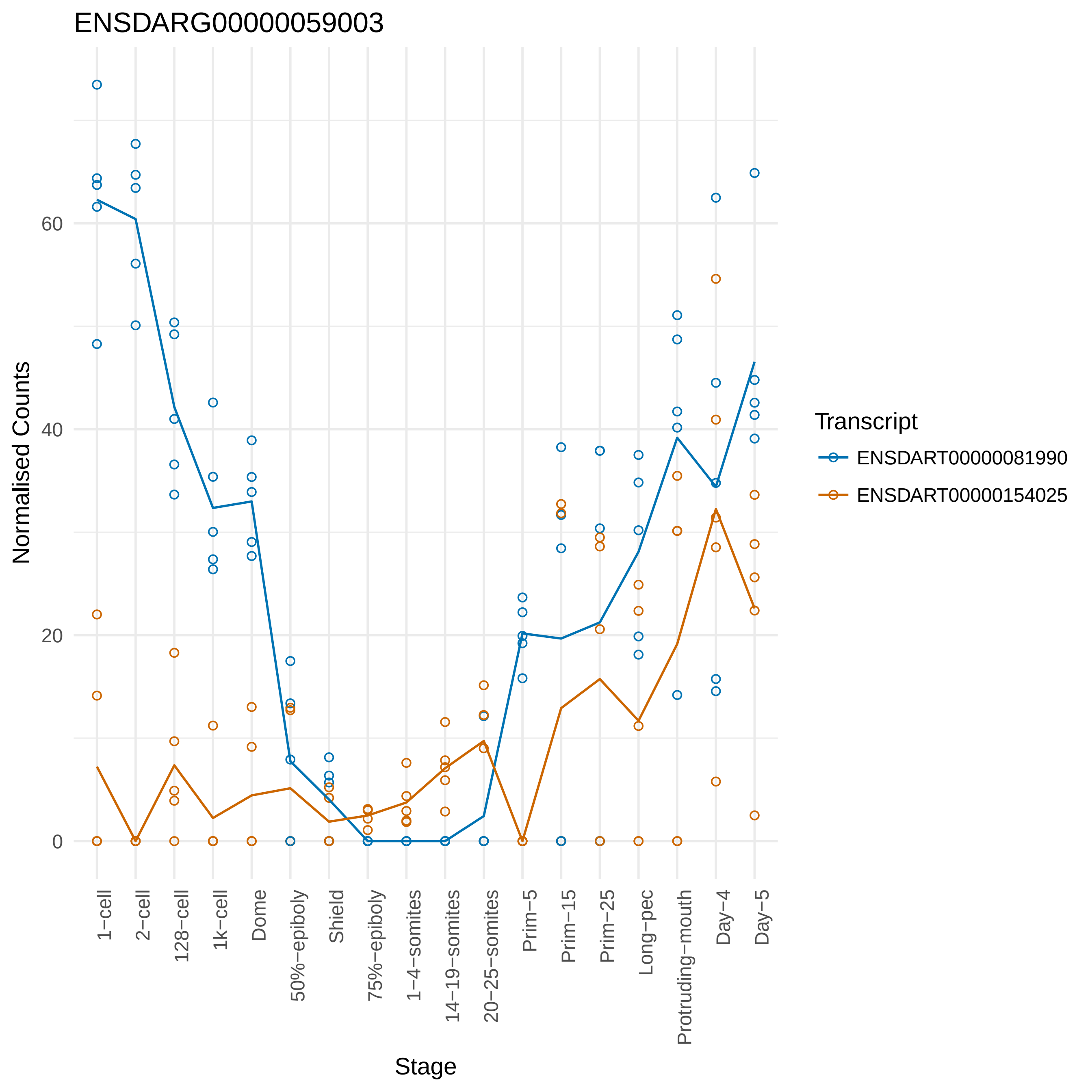

ENSDARG00000059003
Normalised Counts
60
40
20
0
1−cell
2−cell
128−cell
1k−cell
Dome
50%−epiboly
Shield
75%−epiboly
1−4−somites
14−19−somites
20−25−somites
Prim−5
Prim−15
Prim−25
Long−pec
Protruding−mouth
Day−4
Day−5
Stage
Transcript
ENSDART00000081990
ENSDART00000154025

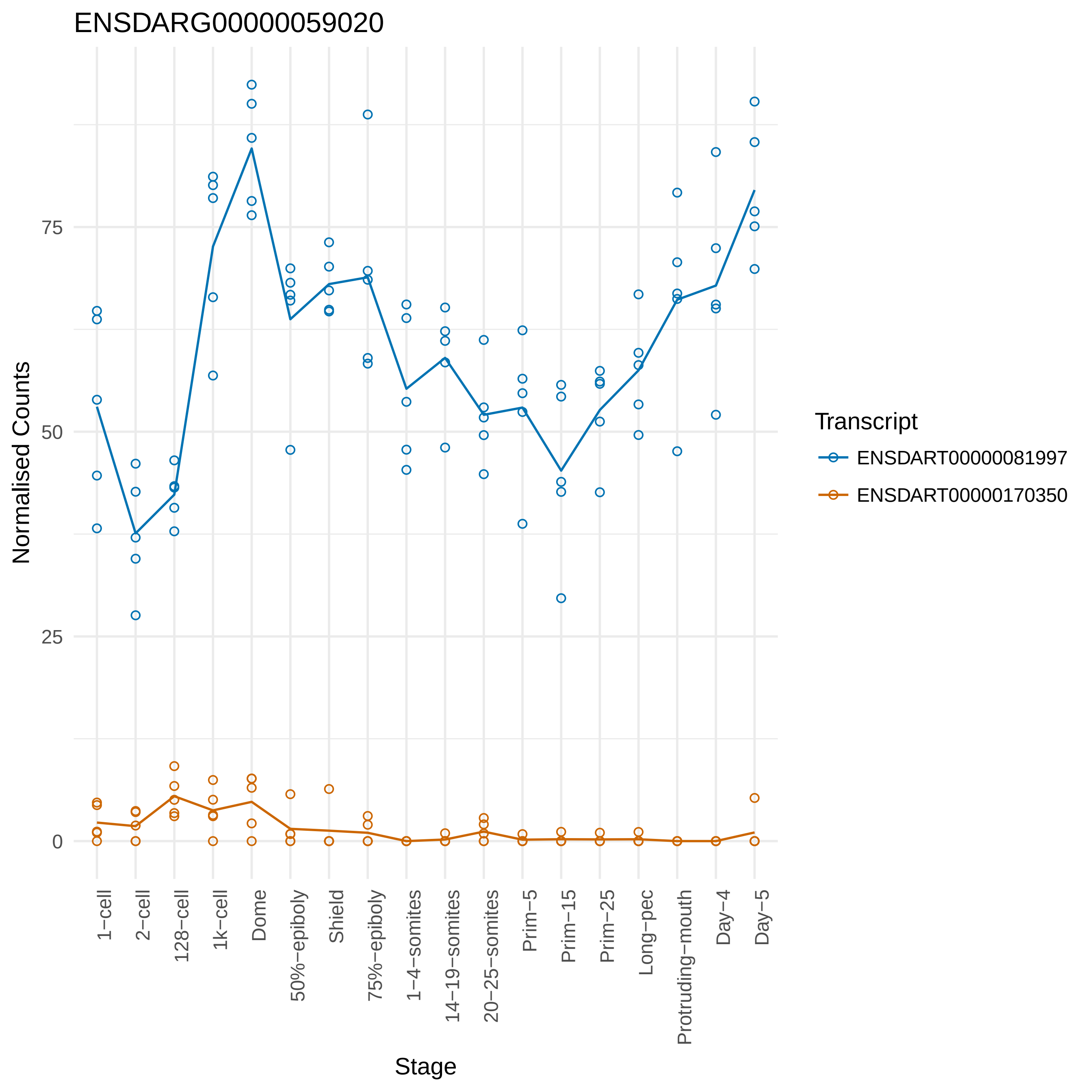

ENSDARG00000059020
Normalised Counts
0
25
50
75
1−cell
2−cell
128−cell
1k−cell
Dome
50%−epiboly
Shield
75%−epiboly
1−4−somites
14−19−somites
20−25−somites
Prim−5
Prim−15
Prim−25
Long−pec
Protruding−mouth
Day−4
Day−5
Stage
Transcript
ENSDART00000081997
ENSDART00000170350

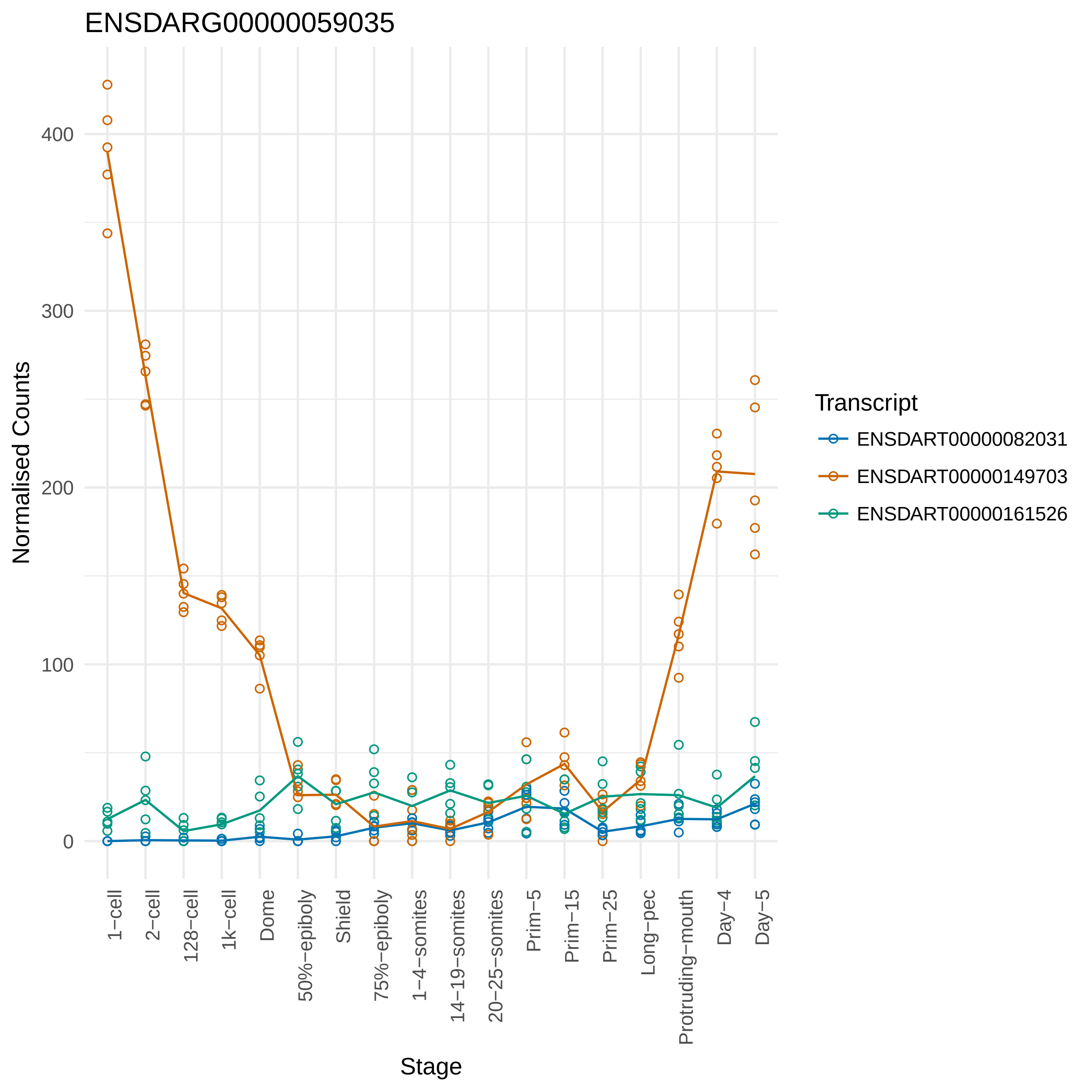

ENSDARG00000059035
Normalised Counts
400
300
200
100
0
1−cell
2−cell
128−cell
1k−cell
Dome
50%−epiboly
Shield
75%−epiboly
1−4−somites
14−19−somites
20−25−somites
Prim−5
Prim−15
Prim−25
Long−pec
Protruding−mouth
Day−4
Day−5
Stage
Transcript
ENSDART00000082031
ENSDART00000149703
ENSDART00000161526

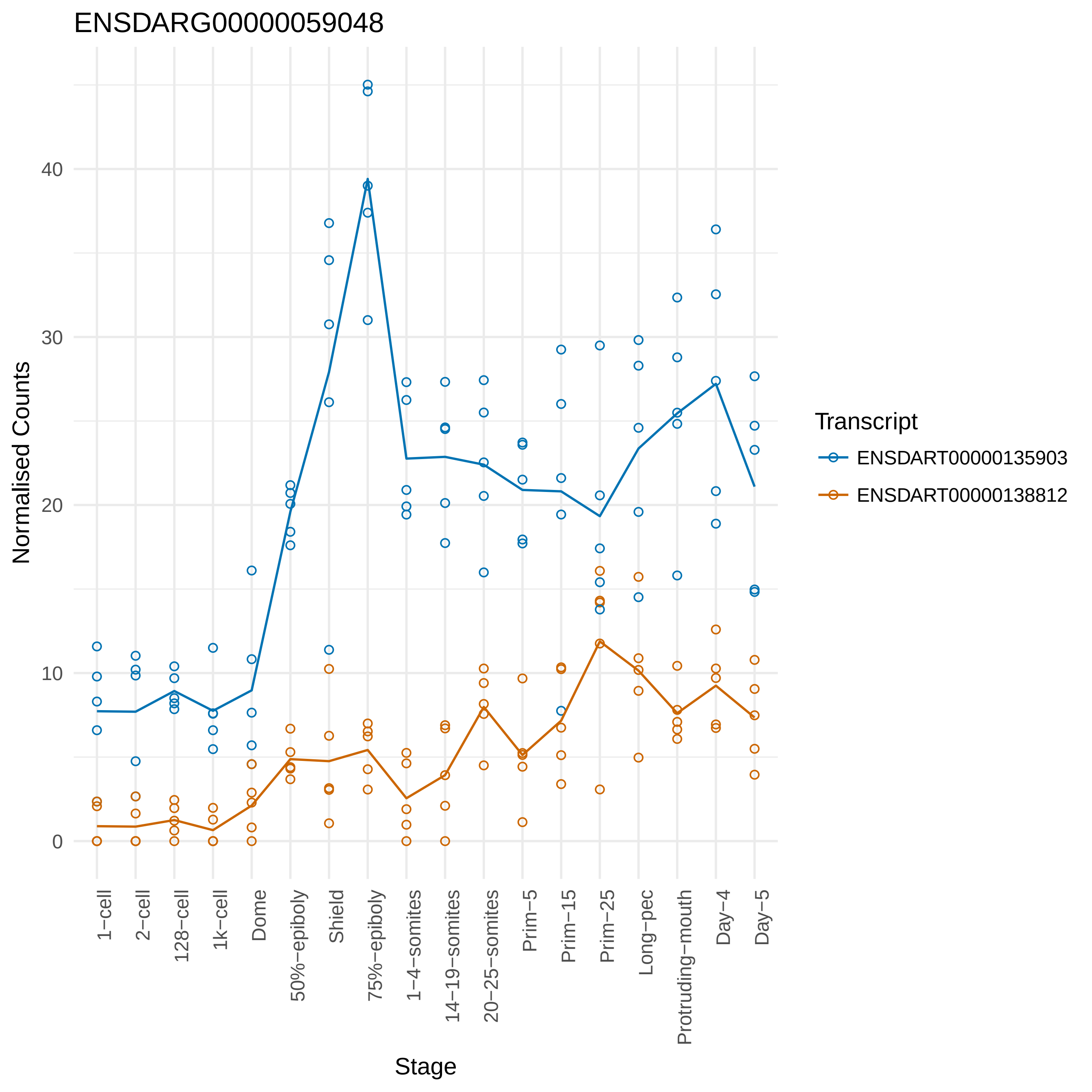

ENSDARG00000059048
Normalised Counts
0
10
20
30
40
Stage
1−cell
2−cell
128−cell
1k−cell
Dome
50%−epiboly
Shield
75%−epiboly
1−4−somites
14−19−somites
20−25−somites
Prim−5
Prim−15
Prim−25
Long−pec
Protruding−mouth
Day−4
Day−5
Transcript
ENSDART00000135903
ENSDART00000138812

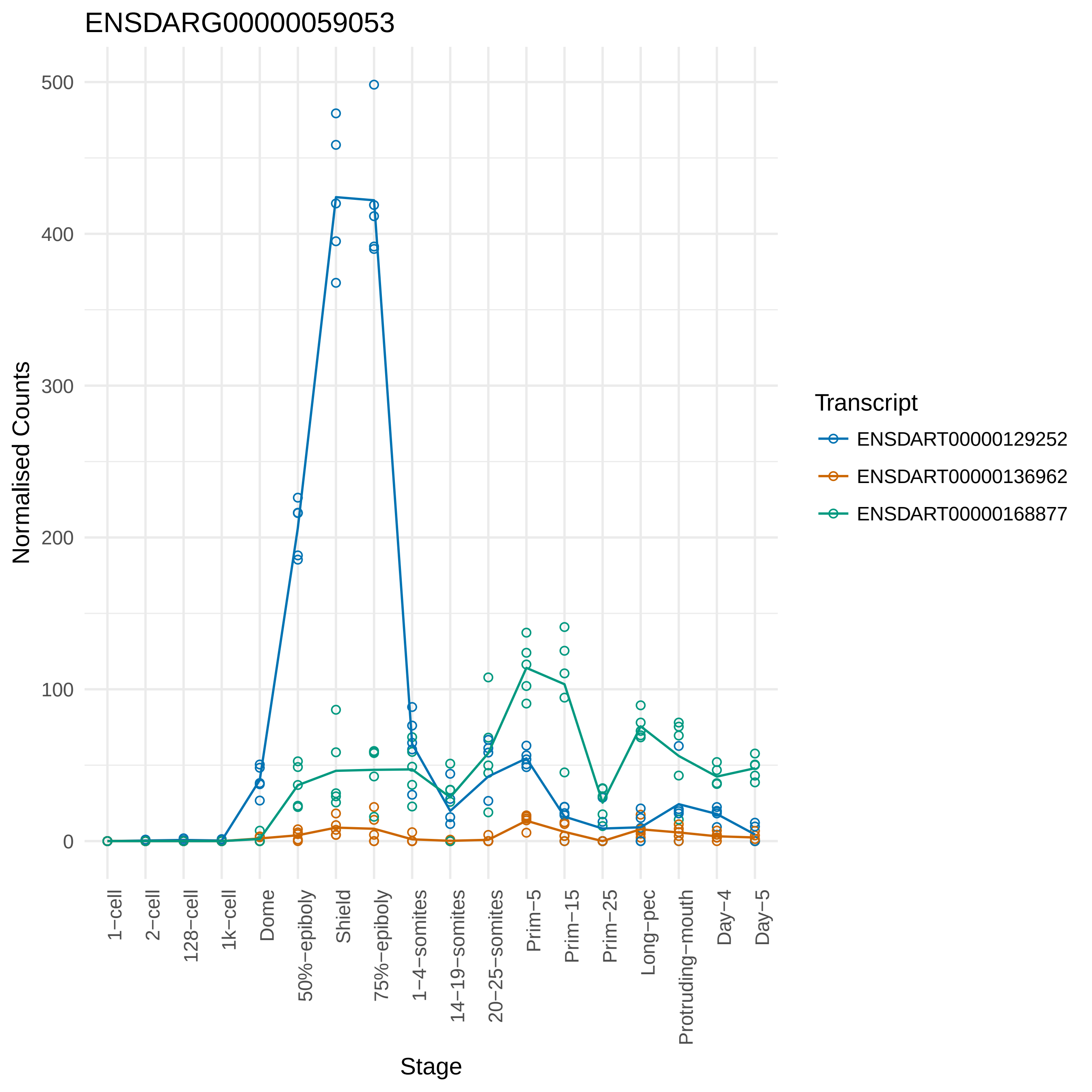

ENSDARG00000059053
Normalised Counts
500
400
300
200
100
0
1−cell
2−cell
128−cell
1k−cell
Dome
50%−epiboly
Shield
75%−epiboly
1−4−somites
14−19−somites
20−25−somites
Prim−5
Prim−15
Prim−25
Long−pec
Protruding−mouth
Day−4
Day−5
Stage
Transcript
ENSDART00000129252
ENSDART00000136962
ENSDART00000168877

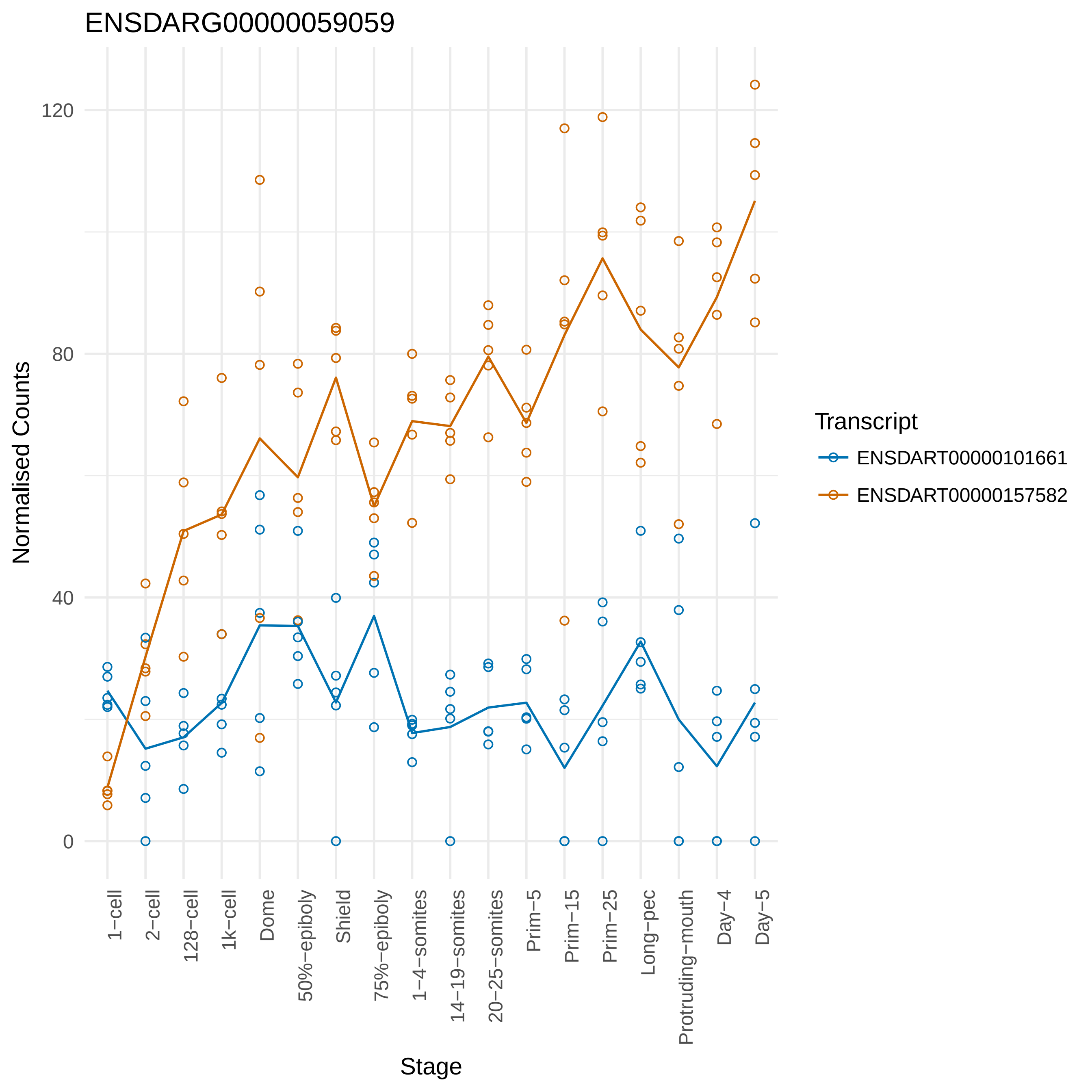

ENSDARG00000059059
Normalised Counts
120
80
40
0
1−cell
2−cell
128−cell
1k−cell
Dome
50%−epiboly
Shield
75%−epiboly
1−4−somites
14−19−somites
20−25−somites
Prim−5
Prim−15
Prim−25
Long−pec
Protruding−mouth
Day−4
Day−5
Stage
Transcript
ENSDART00000101661
ENSDART00000157582

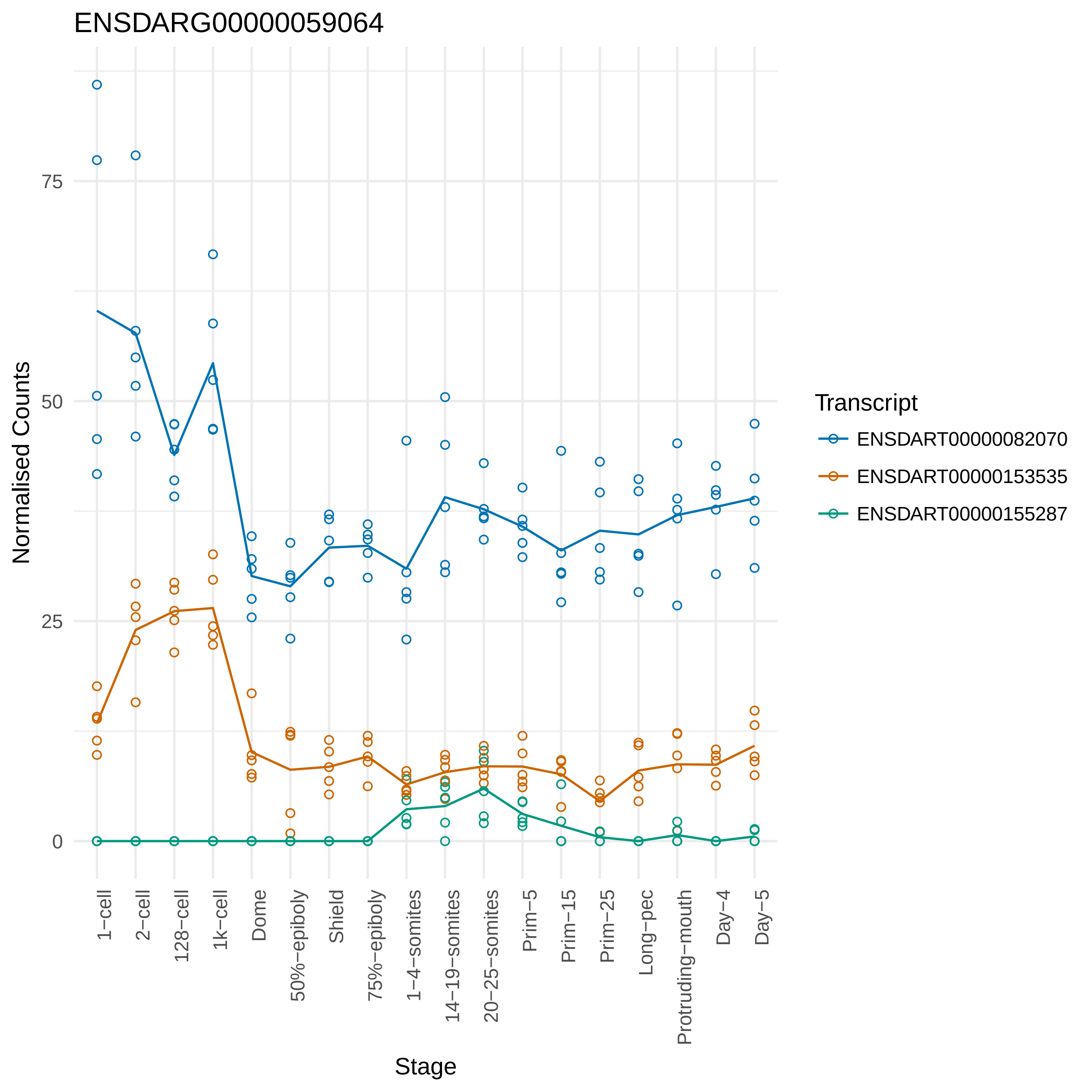

ENSDARG00000059064
Normalised Counts
75
50
25
0
1−cell
2−cell
128−cell
1k−cell
Dome
50%−epiboly
Shield
75%−epiboly
1−4−somites
14−19−somites
20−25−somites
Prim−5
Prim−15
Prim−25
Long−pec
Protruding−mouth
Day−4
Day−5
Stage
Transcript
ENSDART00000082070
ENSDART00000153535
ENSDART00000155287

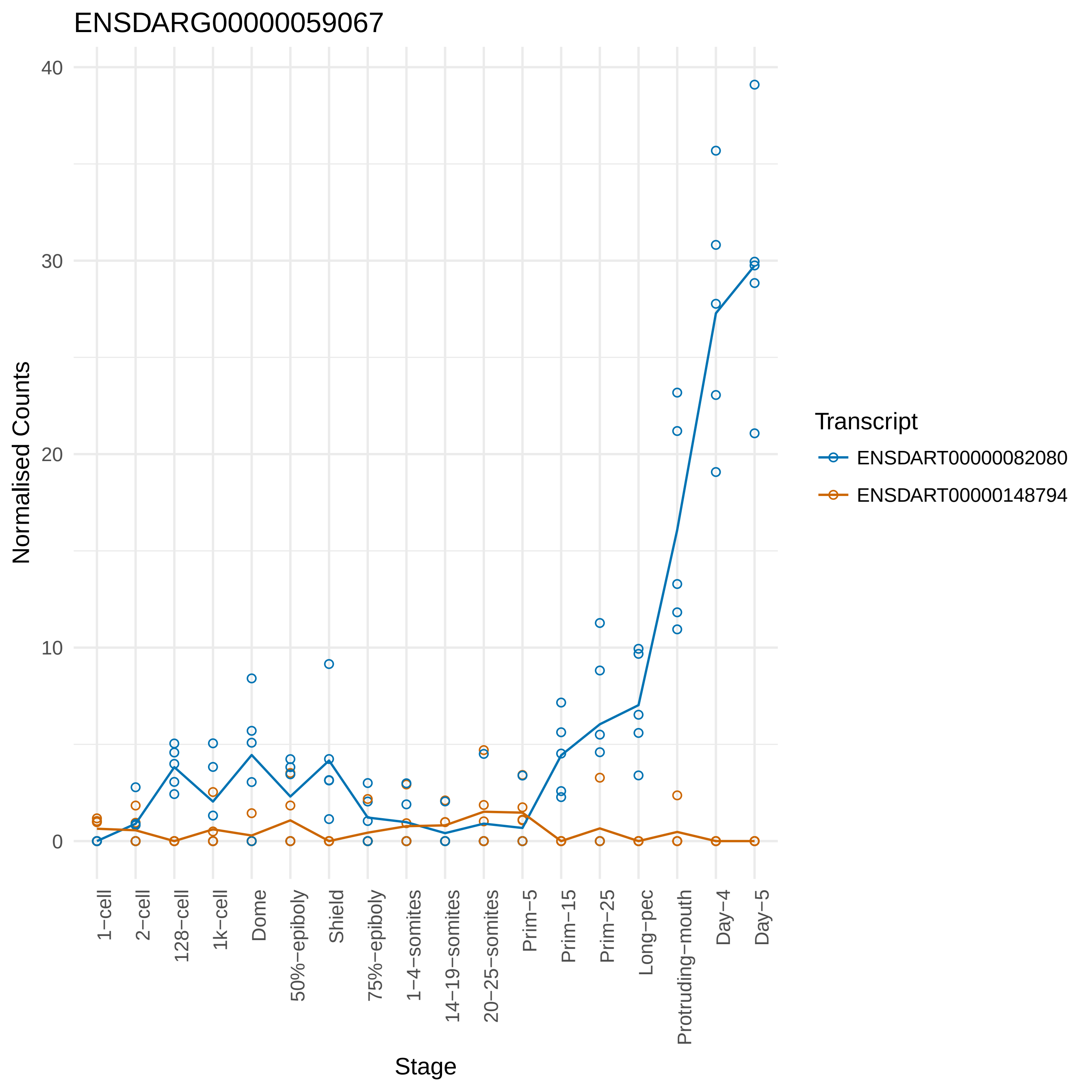
ENSDARG00000059067
Normalised Counts
0
10
20
30
40
1−cell
2−cell
128−cell
1k−cell
Dome
50%−epiboly
Shield
75%−epiboly
1−4−somites
14−19−somites
20−25−somites
Prim−5
Prim−15
Prim−25
Long−pec
Protruding−mouth
Day−4
Day−5
Stage
Transcript
ENSDART00000082080
ENSDART00000148794

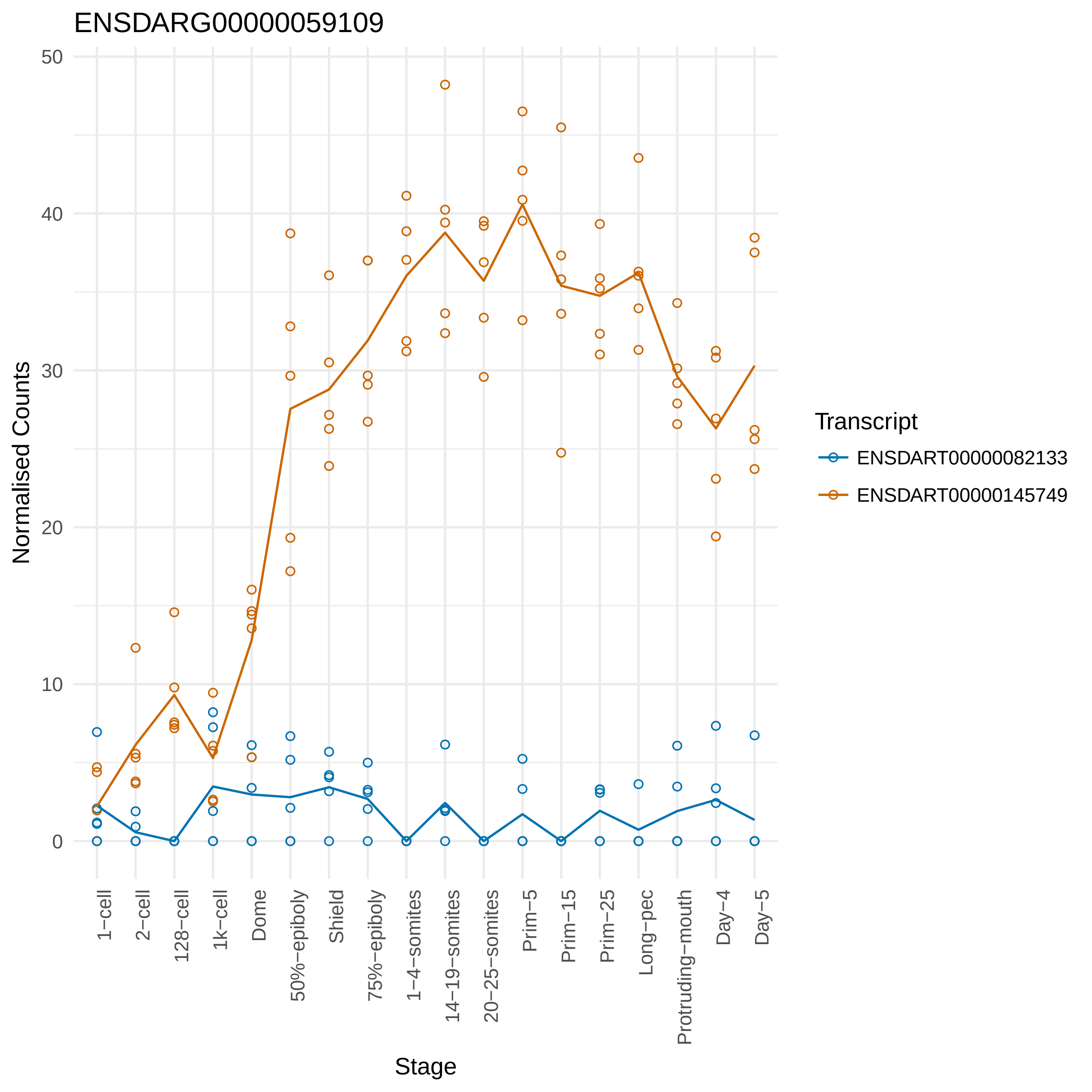
ENSDARG00000059109
Normalised Counts
0
10
20
30
40
50
Stage
1−cell
2−cell
128−cell
1k−cell
Dome
50%−epiboly
Shield
75%−epiboly
1−4−somites
14−19−somites
20−25−somites
Prim−5
Prim−15
Prim−25
Long−pec
Protruding−mouth
Day−4
Day−5
Transcript
ENSDART00000082133
ENSDART00000145749

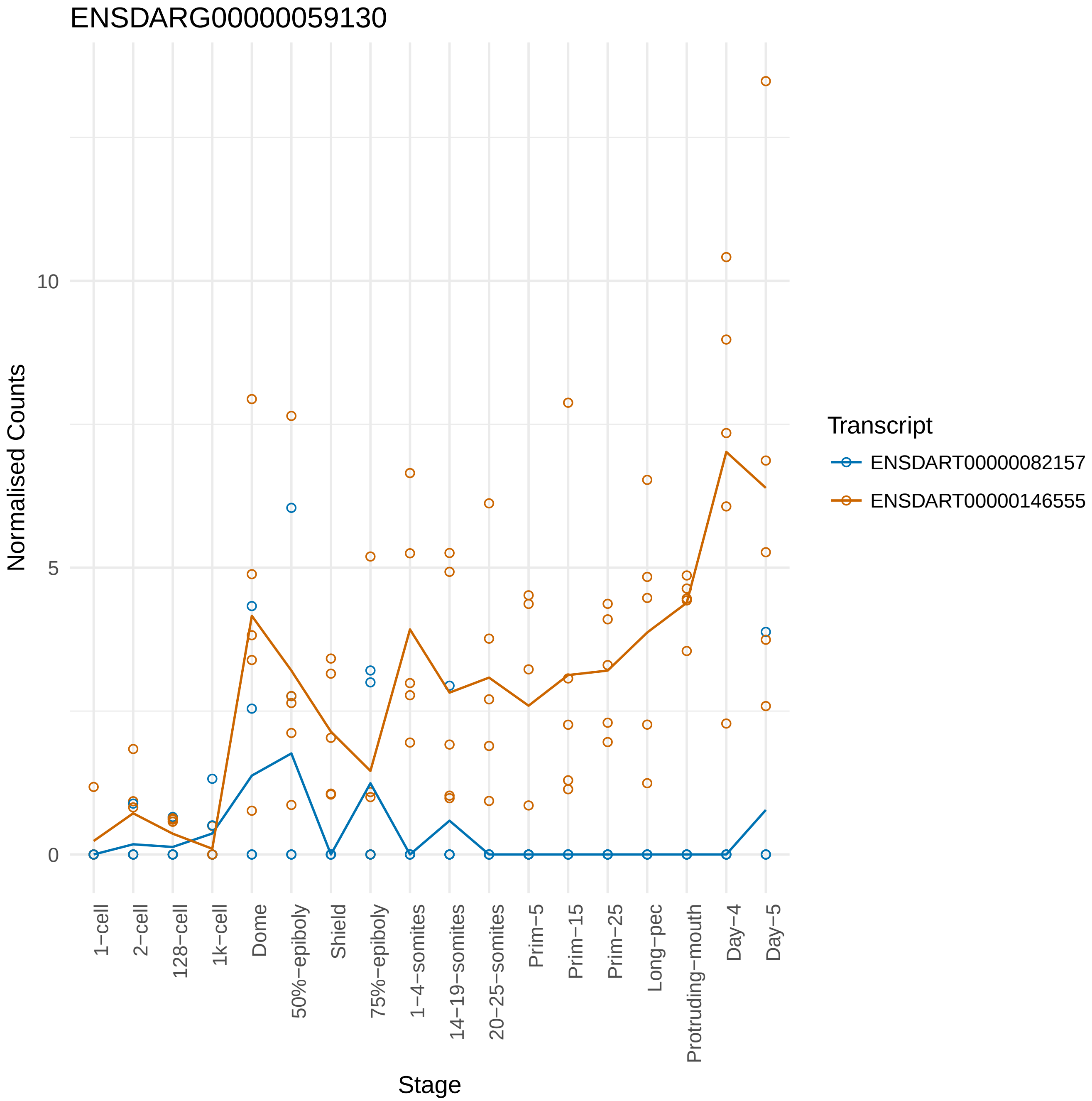
ENSDARG00000059130
Normalised Counts
10
5
0
1−cell
2−cell
128−cell
1k−cell
Dome
50%−epiboly
Shield
75%−epiboly
1−4−somites
14−19−somites
20−25−somites
Prim−5
Prim−15
Prim−25
Long−pec
Protruding−mouth
Day−4
Day−5
Stage
Transcript
ENSDART00000082157
ENSDART00000146555

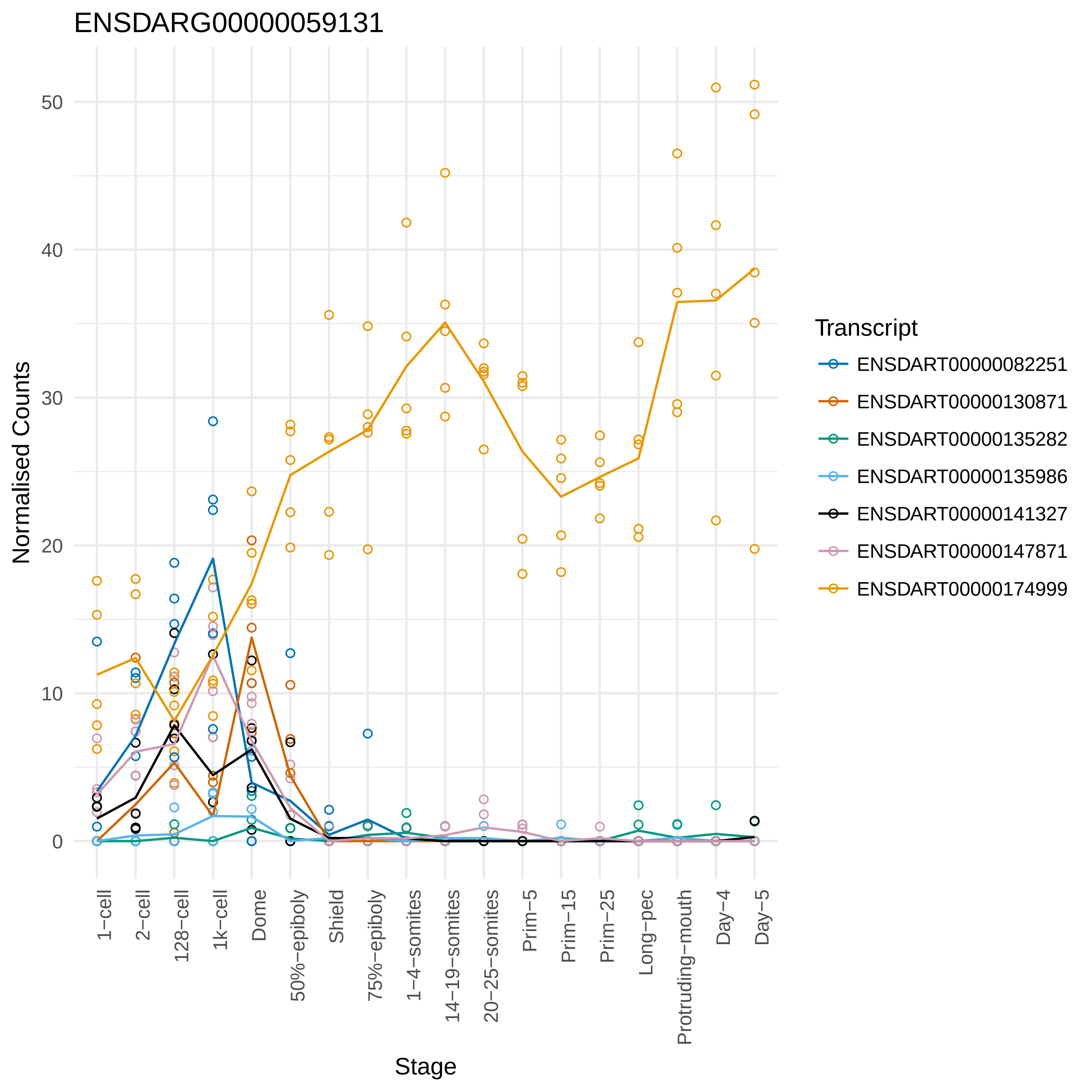

ENSDARG00000059131
Normalised Counts
0
10
20
30
40
50
1−cell
2−cell
128−cell
1k−cell
Dome
50%−epiboly
Shield
75%−epiboly
1−4−somites
14−19−somites
20−25−somites
Prim−5
Prim−15
Prim−25
Long−pec
Protruding−mouth
Day−4
Day−5
Stage
Transcript
ENSDART00000082251
ENSDART00000130871
ENSDART00000135282
ENSDART00000135986
ENSDART00000141327
ENSDART00000147871
ENSDART00000174999

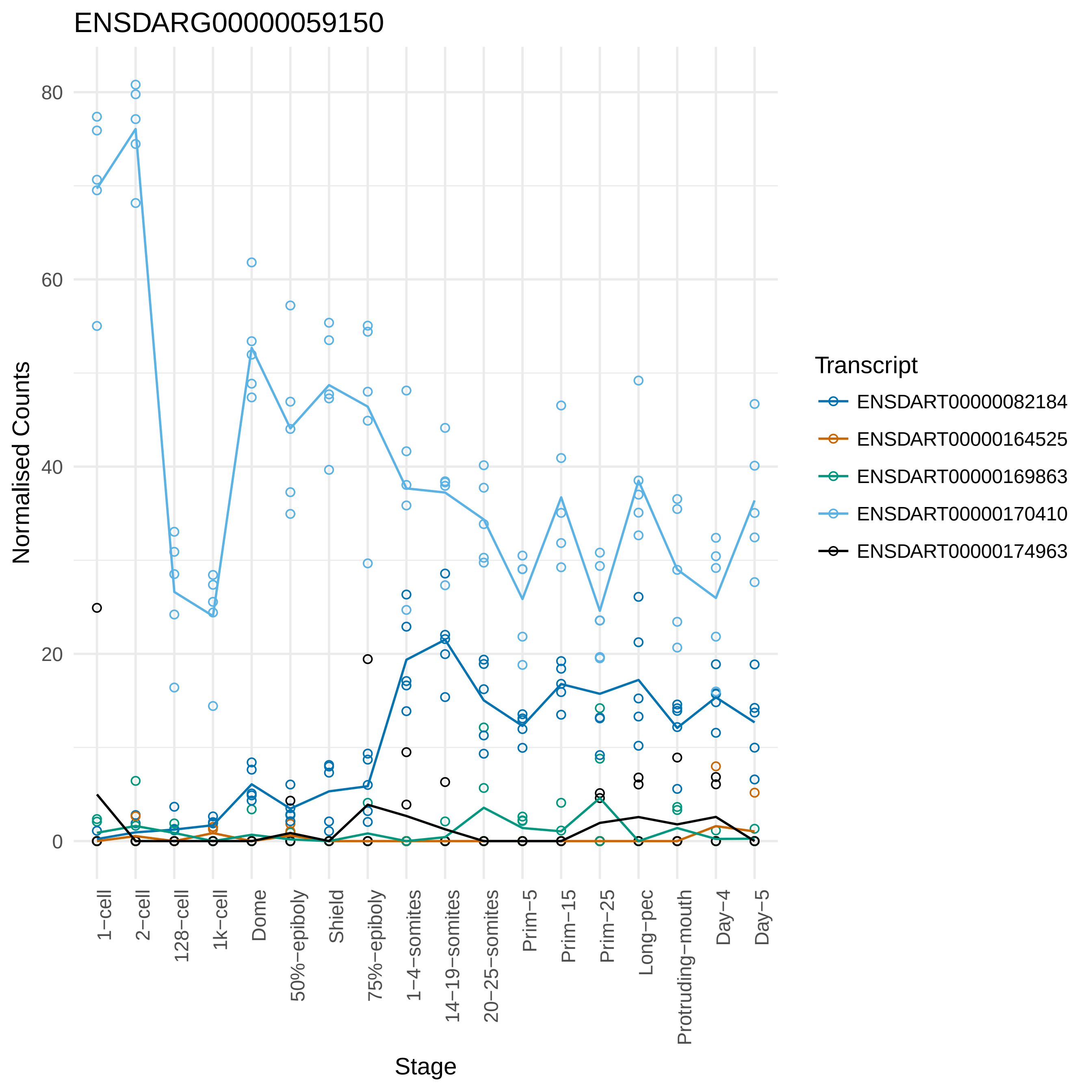

ENSDARG00000059150
Normalised Counts
0
20
40
60
80
Stage
1−cell
2−cell
128−cell
1k−cell
Dome
50%−epiboly
Shield
75%−epiboly
1−4−somites
14−19−somites
20−25−somites
Prim−5
Prim−15
Prim−25
Long−pec
Protruding−mouth
Day−4
Day−5
Transcript
ENSDART00000082184
ENSDART00000164525
ENSDART00000169863
ENSDART00000170410
ENSDART00000174963

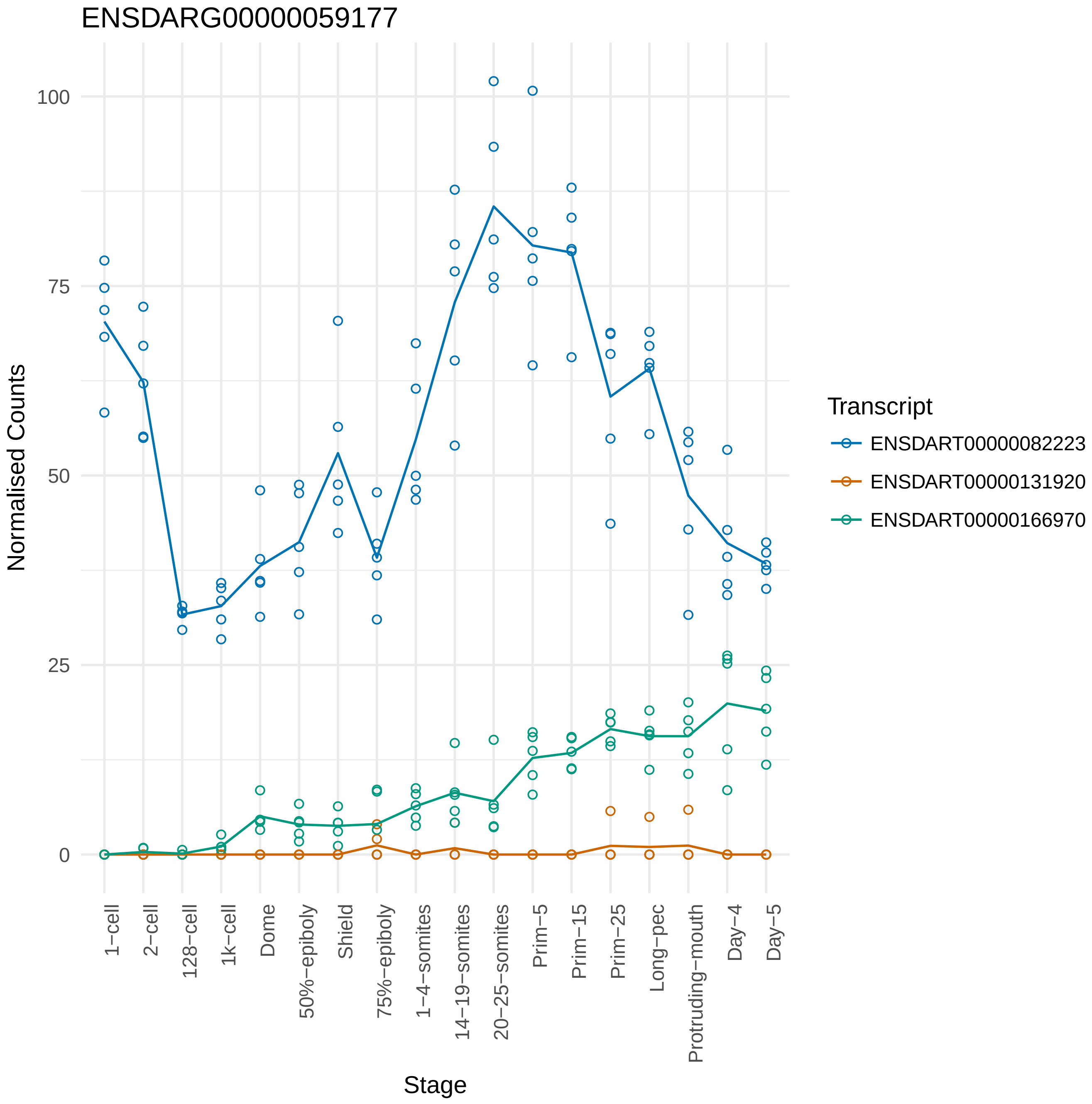
ENSDARG00000059177
Normalised Counts
100
75
50
25
0
1−cell
2−cell
128−cell
1k−cell
Dome
50%−epiboly
Shield
75%−epiboly
1−4−somites
14−19−somites
20−25−somites
Prim−5
Prim−15
Prim−25
Long−pec
Protruding−mouth
Day−4
Day−5
Stage
Transcript
ENSDART00000082223
ENSDART00000131920
ENSDART00000166970

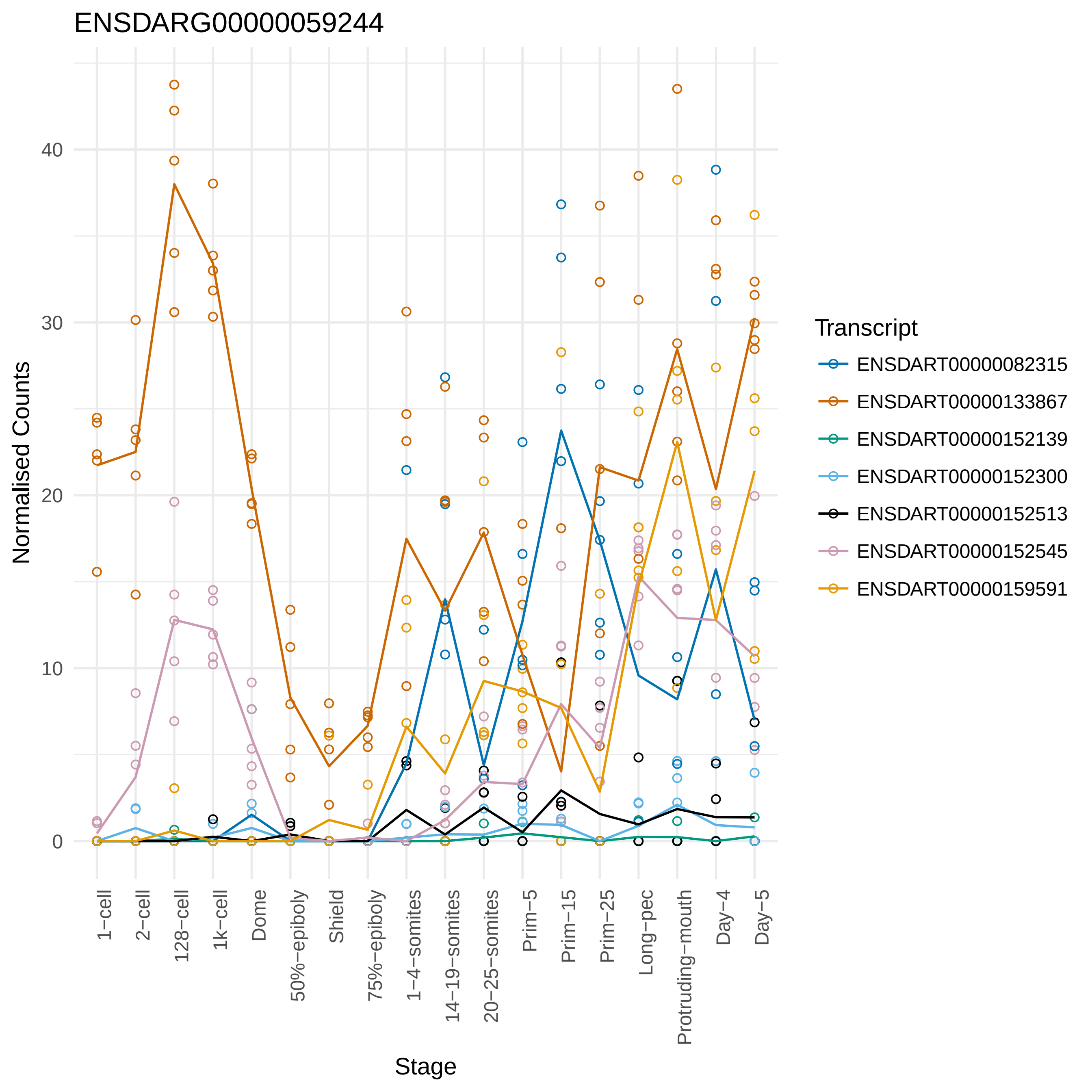

ENSDARG00000059244
Normalised Counts
0
10
20
30
40
1−cell
2−cell
128−cell
1k−cell
Dome
50%−epiboly
Shield
75%−epiboly
1−4−somites
14−19−somites
20−25−somites
Prim−5
Prim−15
Prim−25
Long−pec
Protruding−mouth
Day−4
Day−5
Stage
Transcript
ENSDART00000082315
ENSDART00000133867
ENSDART00000152139
ENSDART00000152300
ENSDART00000152513
ENSDART00000152545
ENSDART00000159591

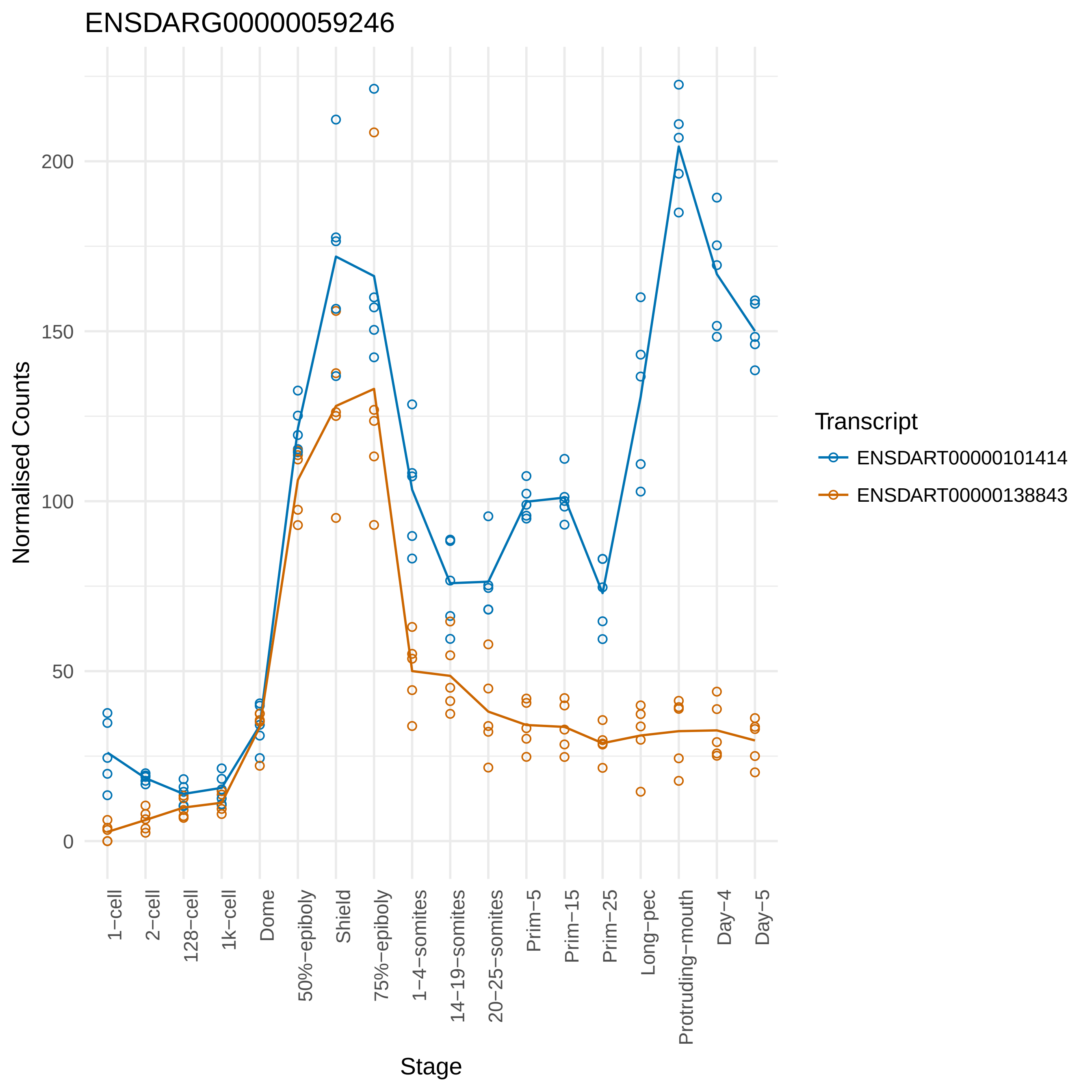
ENSDARG00000059246
Normalised Counts
0
50
100
150
200
1−cell
2−cell
128−cell
1k−cell
Dome
50%−epiboly
Shield
75%−epiboly
1−4−somites
14−19−somites
20−25−somites
Prim−5
Prim−15
Prim−25
Long−pec
Protruding−mouth
Day−4
Day−5
Stage
Transcript
ENSDART00000101414
ENSDART00000138843

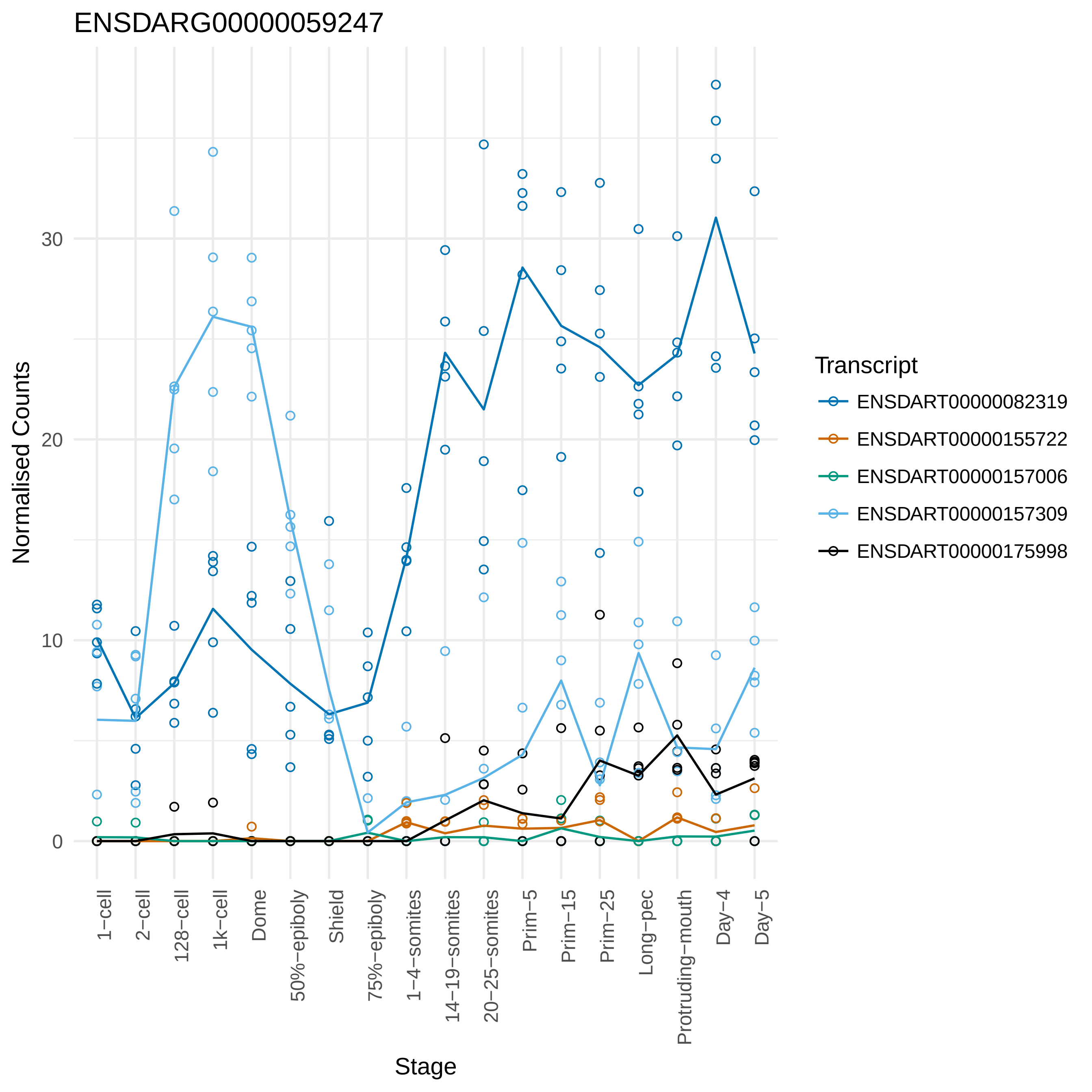
ENSDARG00000059247
Normalised Counts
0
10
20
30
1−cell
2−cell
128−cell
1k−cell
Dome
50%−epiboly
Shield
75%−epiboly
1−4−somites
14−19−somites
20−25−somites
Prim−5
Prim−15
Prim−25
Long−pec
Protruding−mouth
Day−4
Day−5
Stage
Transcript
ENSDART00000082319
ENSDART00000155722
ENSDART00000157006
ENSDART00000157309
ENSDART00000175998

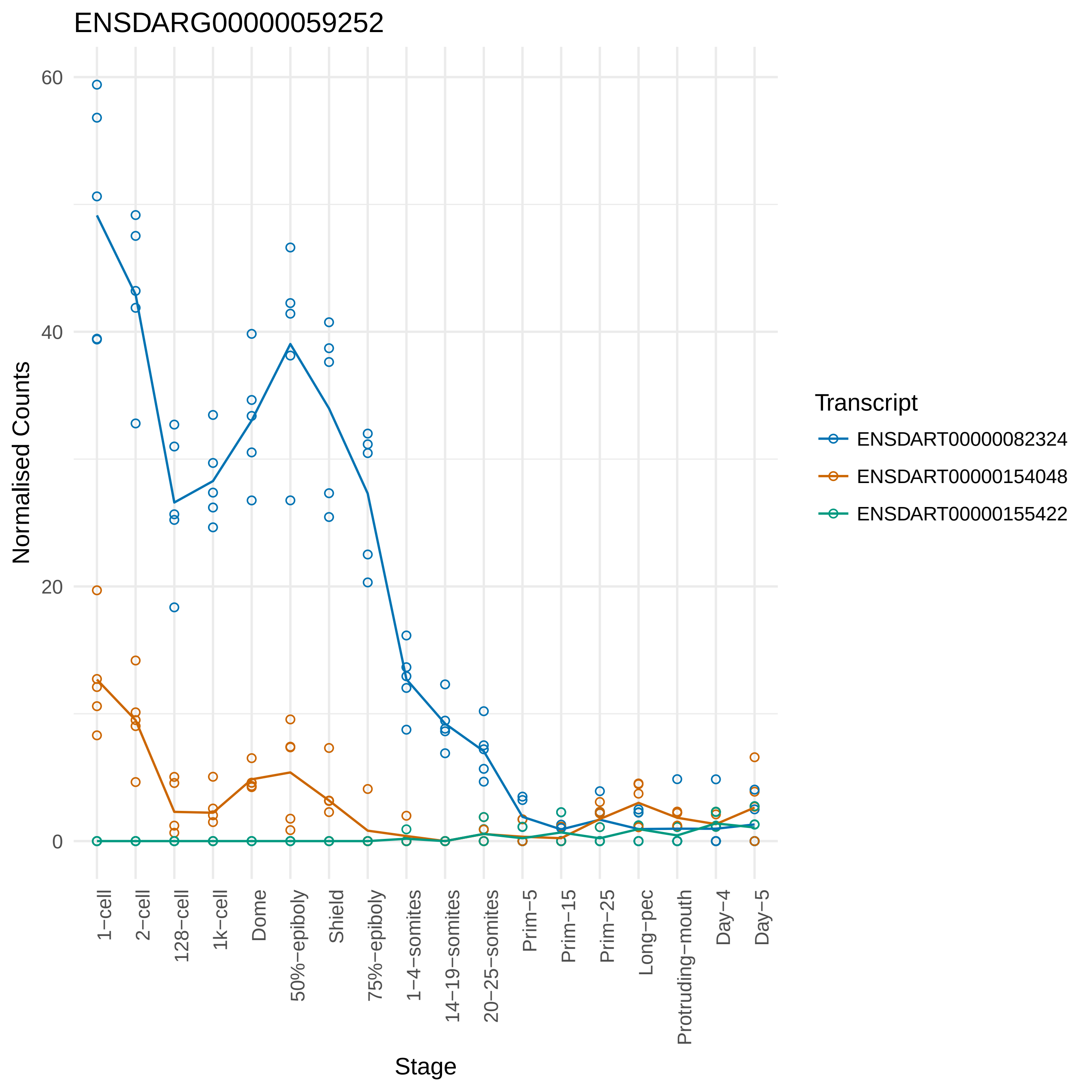

ENSDARG00000059252
Normalised Counts
60
40
20
0
1−cell
2−cell
128−cell
1k−cell
Dome
50%−epiboly
Shield
75%−epiboly
1−4−somites
14−19−somites
20−25−somites
Prim−5
Prim−15
Prim−25
Long−pec
Protruding−mouth
Day−4
Day−5
Stage
Transcript
ENSDART00000082324
ENSDART00000154048
ENSDART00000155422

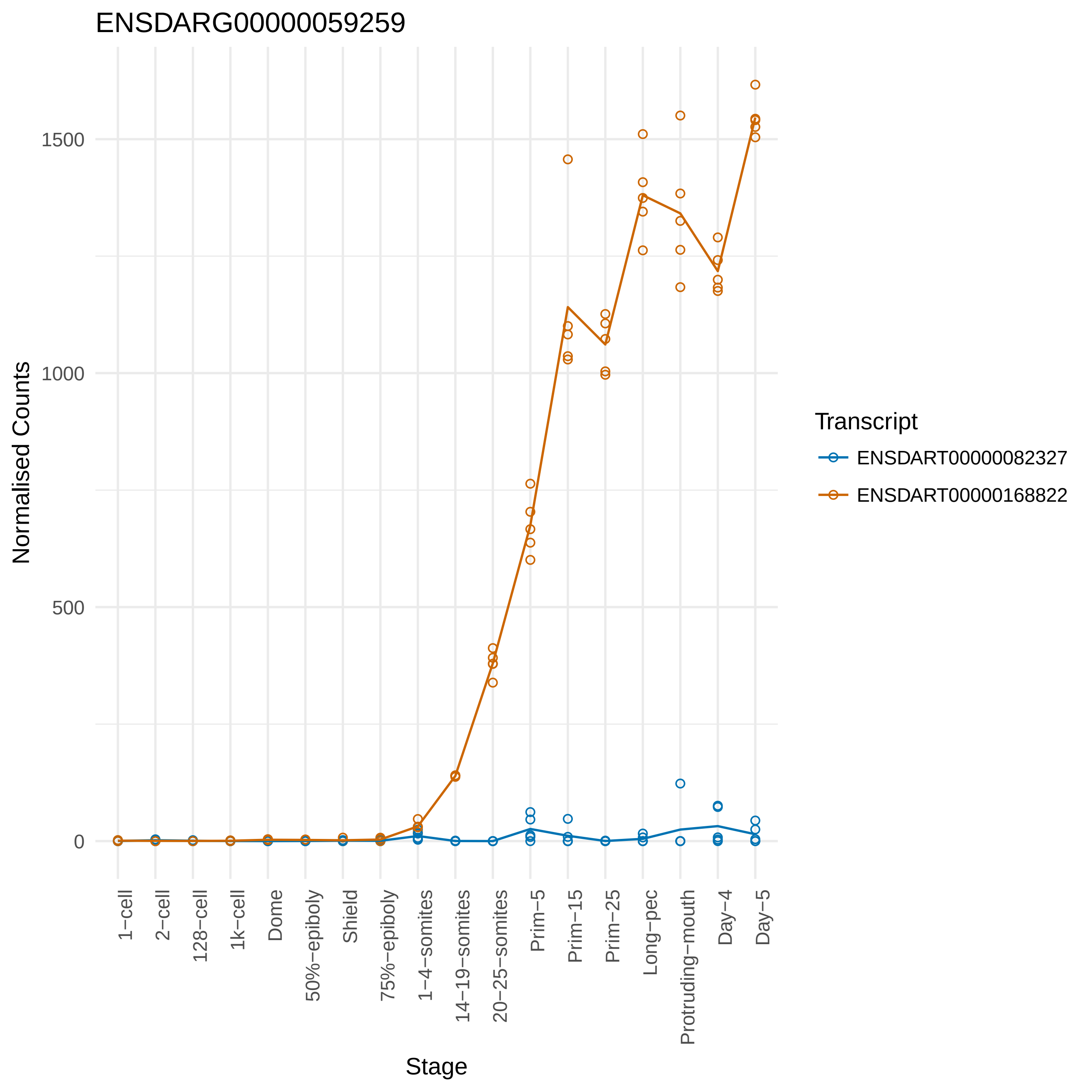
ENSDARG00000059259
Normalised Counts
1500
1000
500
0
1−cell
2−cell
128−cell
1k−cell
Dome
50%−epiboly
Shield
75%−epiboly
1−4−somites
14−19−somites
20−25−somites
Prim−5
Prim−15
Prim−25
Long−pec
Protruding−mouth
Day−4
Day−5
Stage
Transcript
ENSDART00000082327
ENSDART00000168822

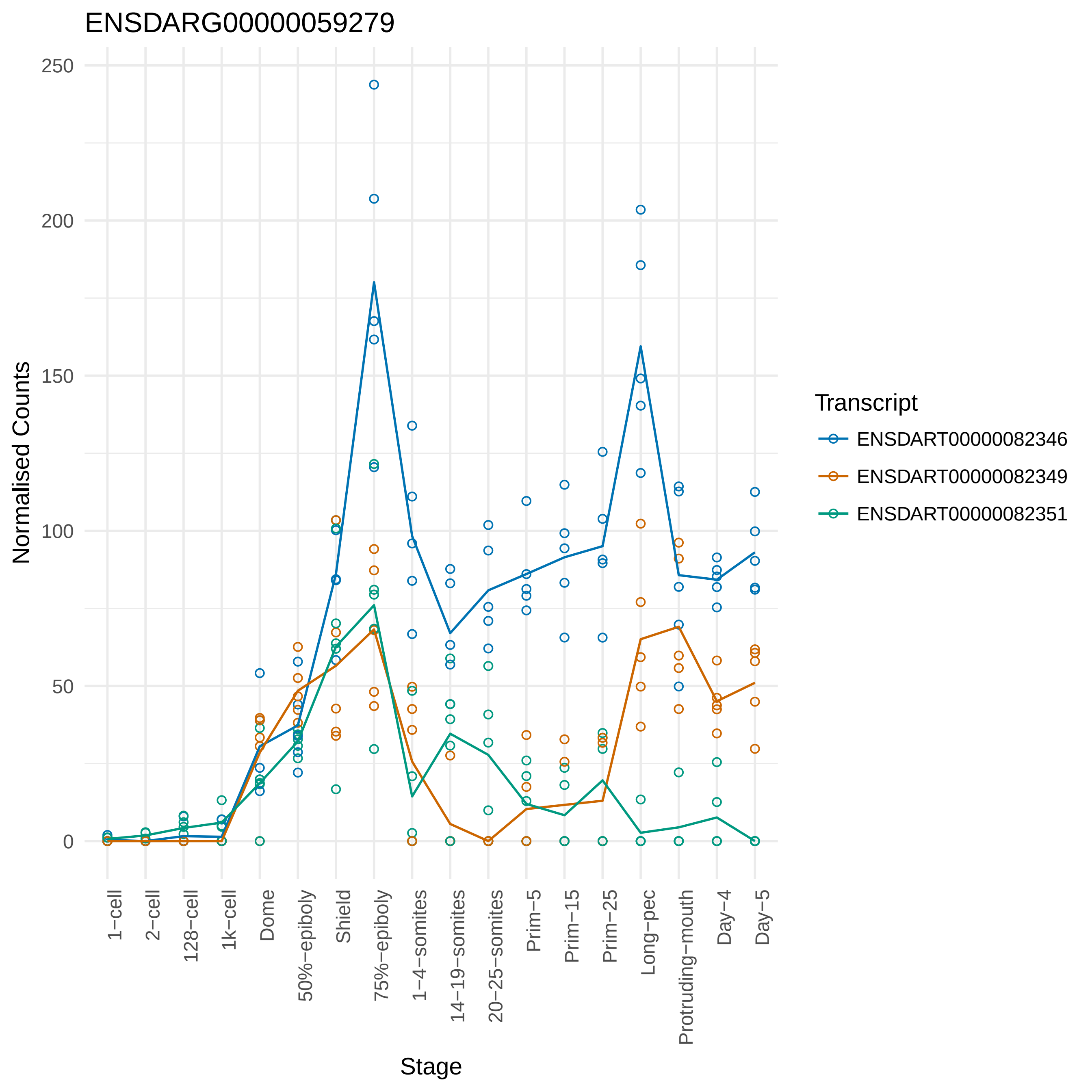

ENSDARG00000059279
Normalised Counts
0
50
100
150
200
250
1−cell
2−cell
128−cell
1k−cell
Dome
50%−epiboly
Shield
75%−epiboly
1−4−somites
14−19−somites
20−25−somites
Prim−5
Prim−15
Prim−25
Long−pec
Protruding−mouth
Day−4
Day−5
Stage
Transcript
ENSDART00000082346
ENSDART00000082349
ENSDART00000082351

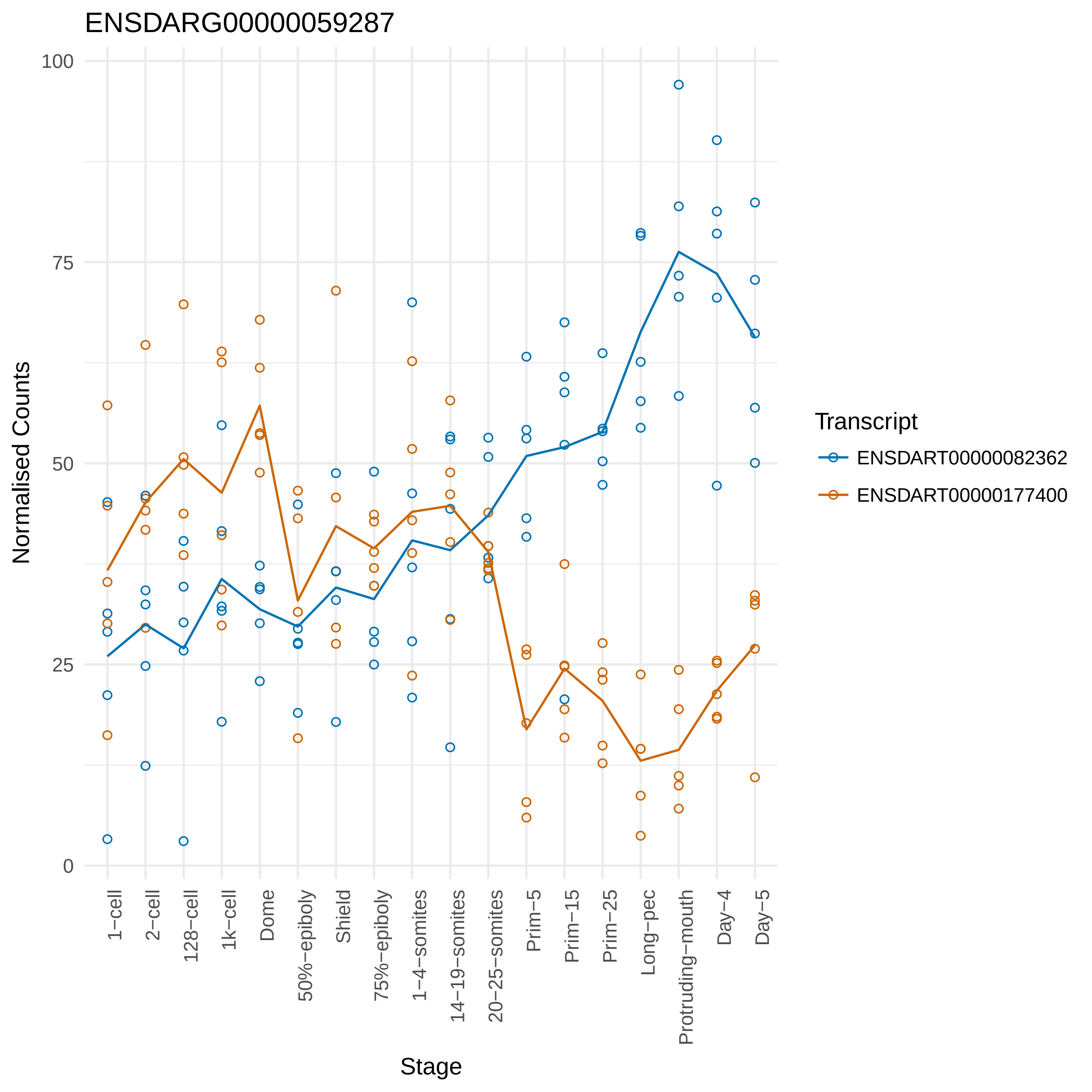
ENSDARG00000059287
Normalised Counts
100
75
50
25
0
1−cell
2−cell
128−cell
1k−cell
Dome
50%−epiboly
Shield
75%−epiboly
1−4−somites
14−19−somites
20−25−somites
Prim−5
Prim−15
Prim−25
Long−pec
Protruding−mouth
Day−4
Day−5
Stage
Transcript
ENSDART00000082362
ENSDART00000177400

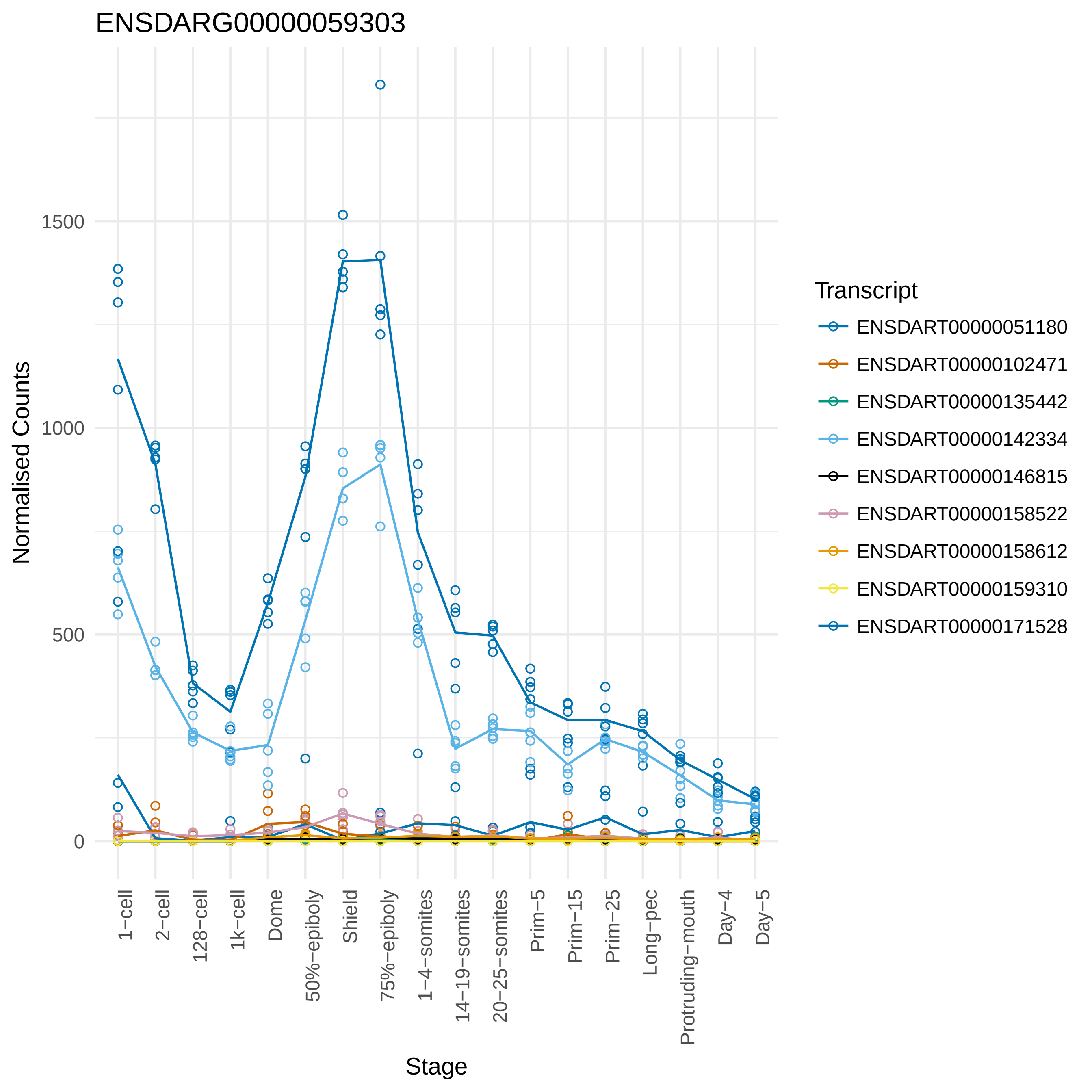
ENSDARG00000059303
Normalised Counts
0
500
1000
1500
Stage
1−cell
2−cell
128−cell
1k−cell
Dome
50%−epiboly
Shield
75%−epiboly
1−4−somites
14−19−somites
20−25−somites
Prim−5
Prim−15
Prim−25
Long−pec
Protruding−mouth
Day−4
Day−5
Transcript
ENSDART00000051180
ENSDART00000102471
ENSDART00000135442
ENSDART00000142334
ENSDART00000146815
ENSDART00000158522
ENSDART00000158612
ENSDART00000159310
ENSDART00000171528

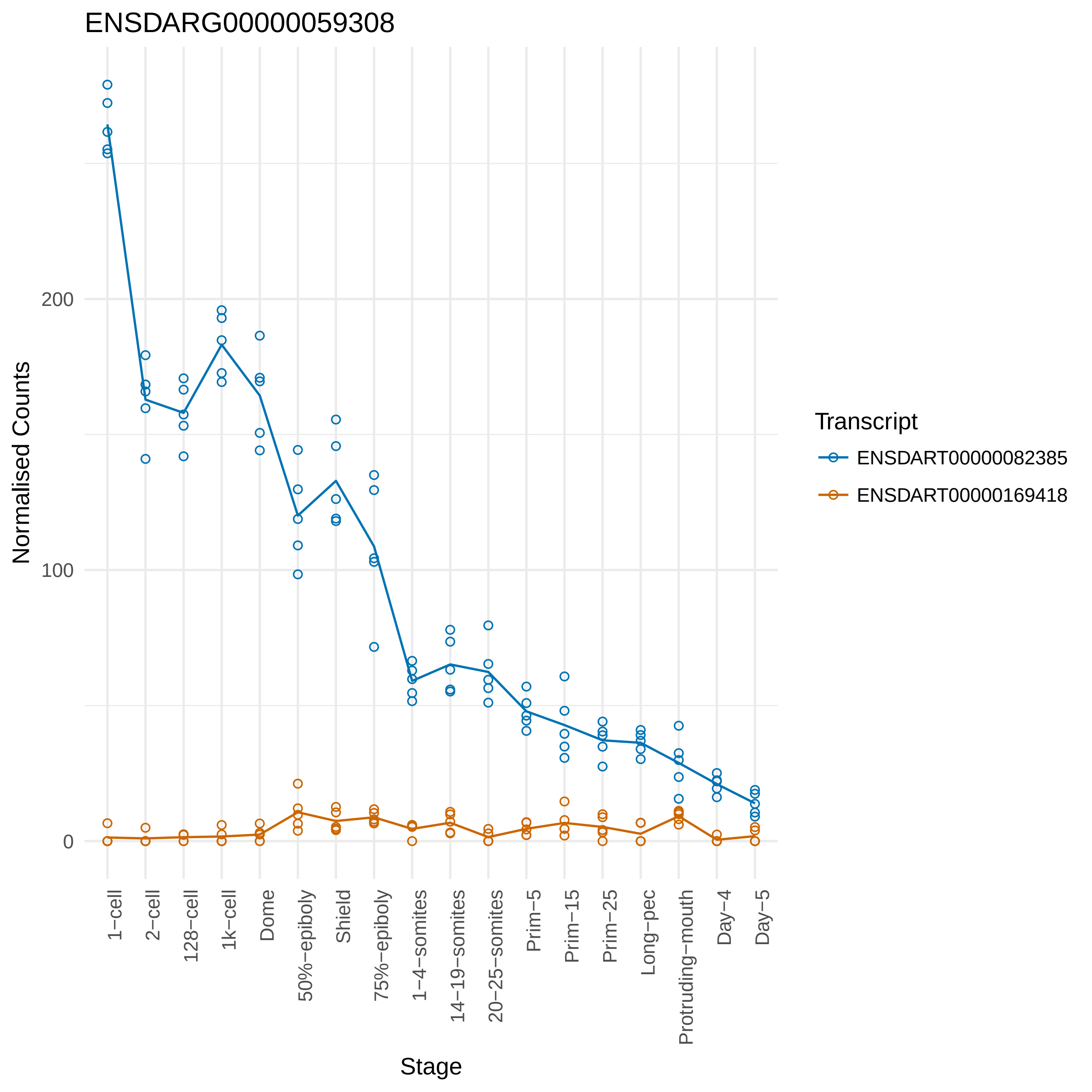
ENSDARG00000059308
Normalised Counts
200
100
0
1−cell
2−cell
128−cell
1k−cell
Dome
50%−epiboly
Shield
75%−epiboly
1−4−somites
14−19−somites
20−25−somites
Prim−5
Prim−15
Prim−25
Long−pec
Protruding−mouth
Day−4
Day−5
Stage
Transcript
ENSDART00000082385
ENSDART00000169418

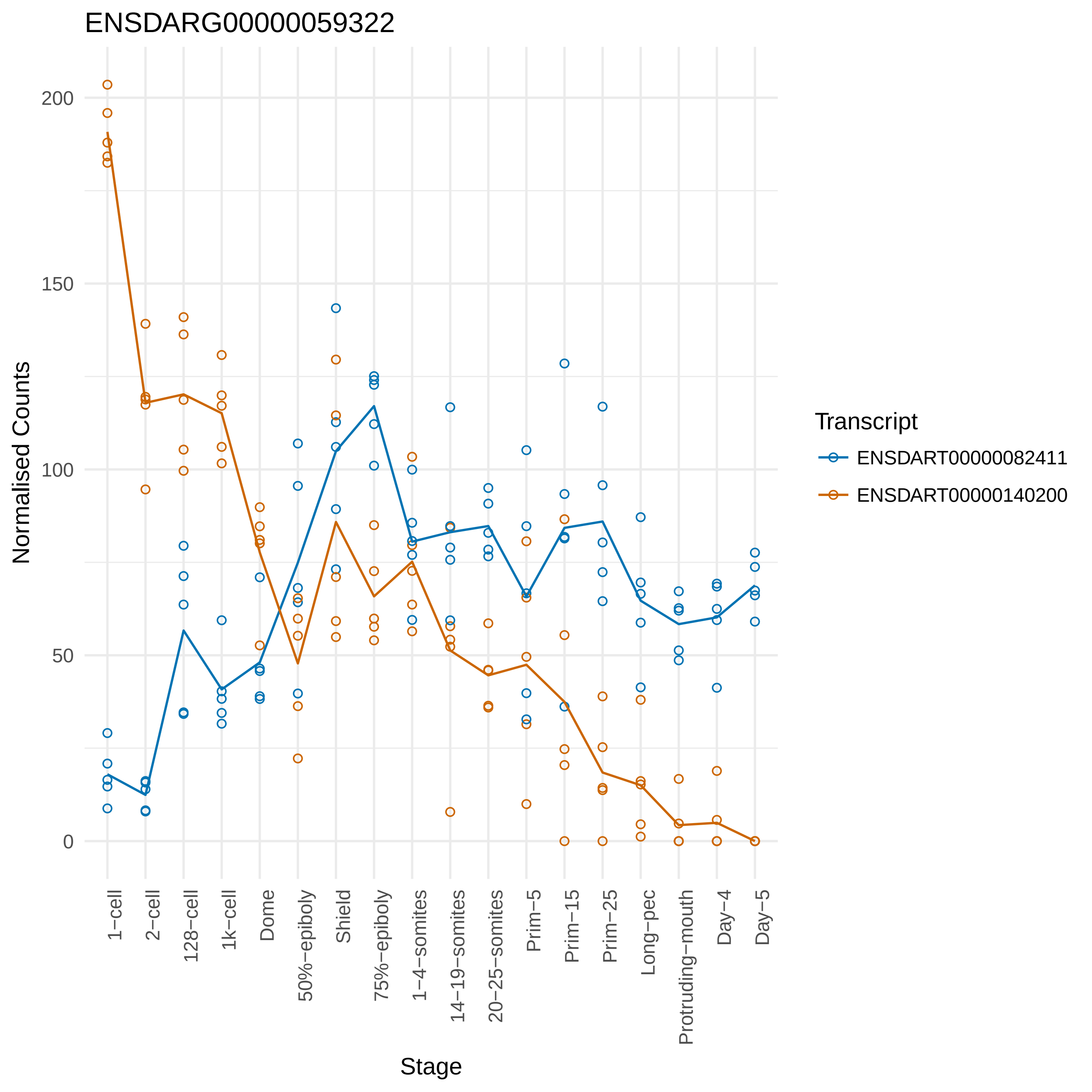

ENSDARG00000059322
Normalised Counts
200
150
100
50
0
1−cell
2−cell
128−cell
1k−cell
Dome
50%−epiboly
Shield
75%−epiboly
1−4−somites
14−19−somites
20−25−somites
Prim−5
Prim−15
Prim−25
Long−pec
Protruding−mouth
Day−4
Day−5
Stage
Transcript
ENSDART00000082411
ENSDART00000140200

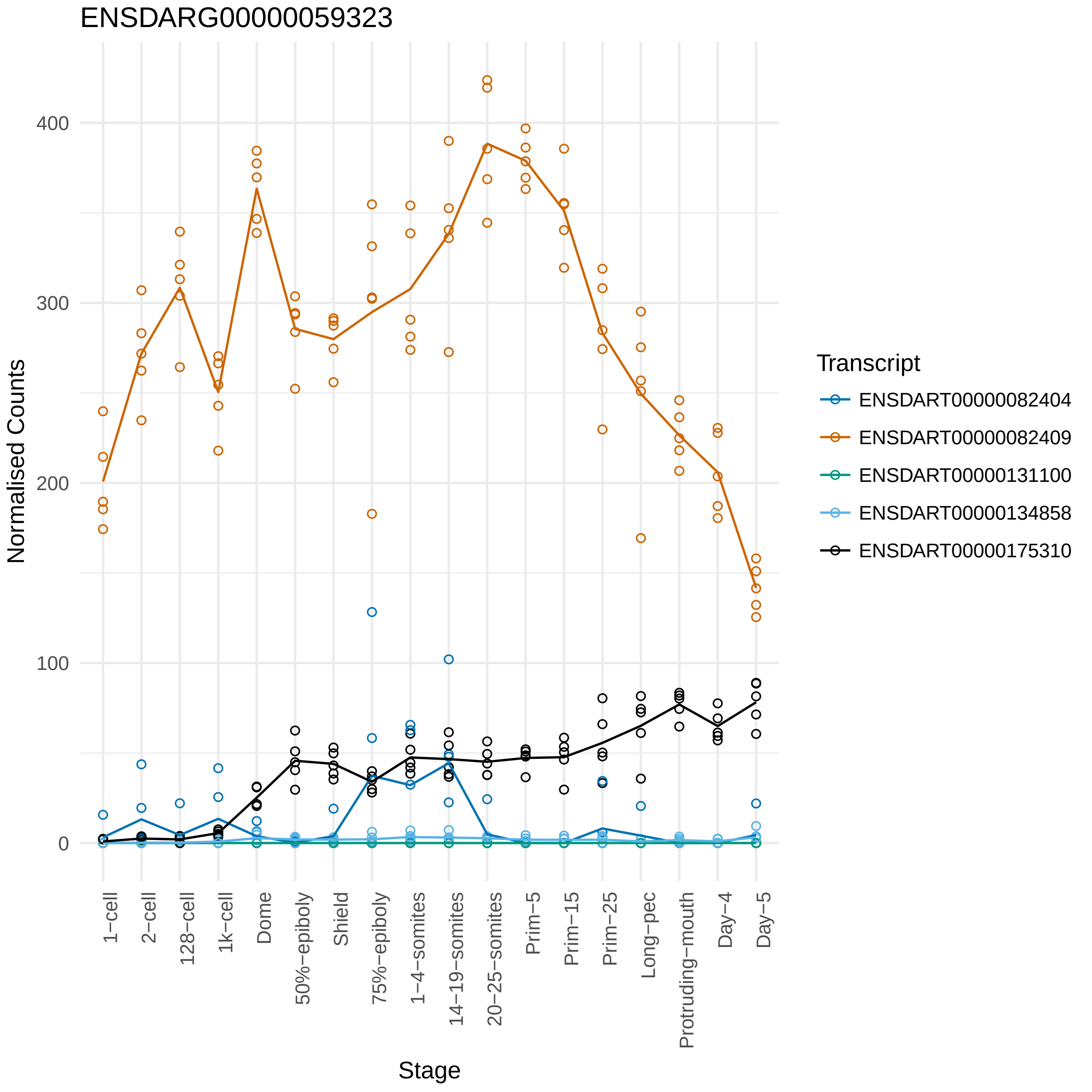
ENSDARG00000059323
Normalised Counts
0
100
200
300
400
Stage
1−cell
2−cell
128−cell
1k−cell
Dome
50%−epiboly
Shield
75%−epiboly
1−4−somites
14−19−somites
20−25−somites
Prim−5
Prim−15
Prim−25
Long−pec
Protruding−mouth
Day−4
Day−5
Transcript
ENSDART00000082404
ENSDART00000082409
ENSDART00000131100
ENSDART00000134858
ENSDART00000175310

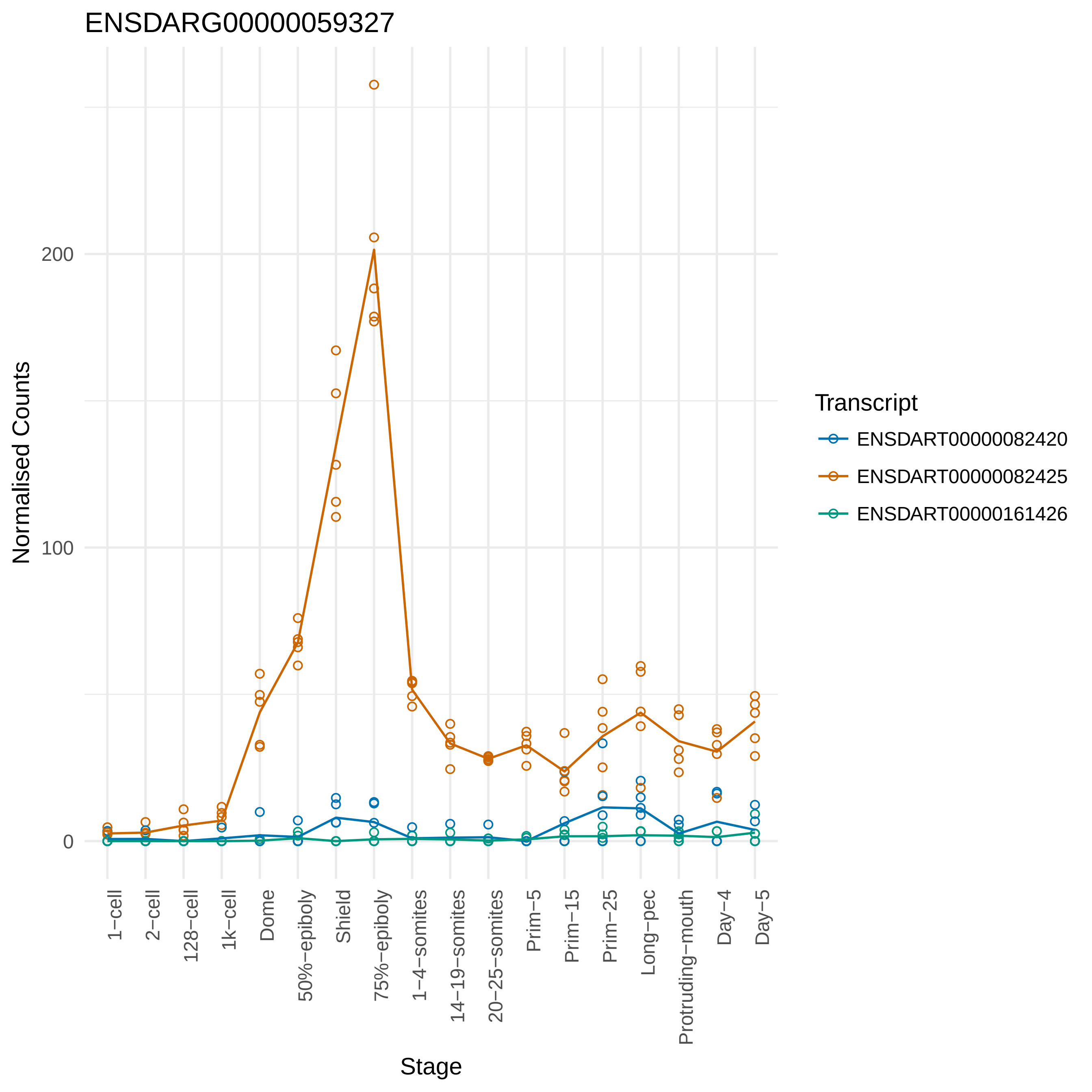

ENSDARG00000059327
Normalised Counts
200
100
0
1−cell
2−cell
128−cell
1k−cell
Dome
50%−epiboly
Shield
75%−epiboly
1−4−somites
14−19−somites
20−25−somites
Prim−5
Prim−15
Prim−25
Long−pec
Protruding−mouth
Day−4
Day−5
Stage
Transcript
ENSDART00000082420
ENSDART00000082425
ENSDART00000161426

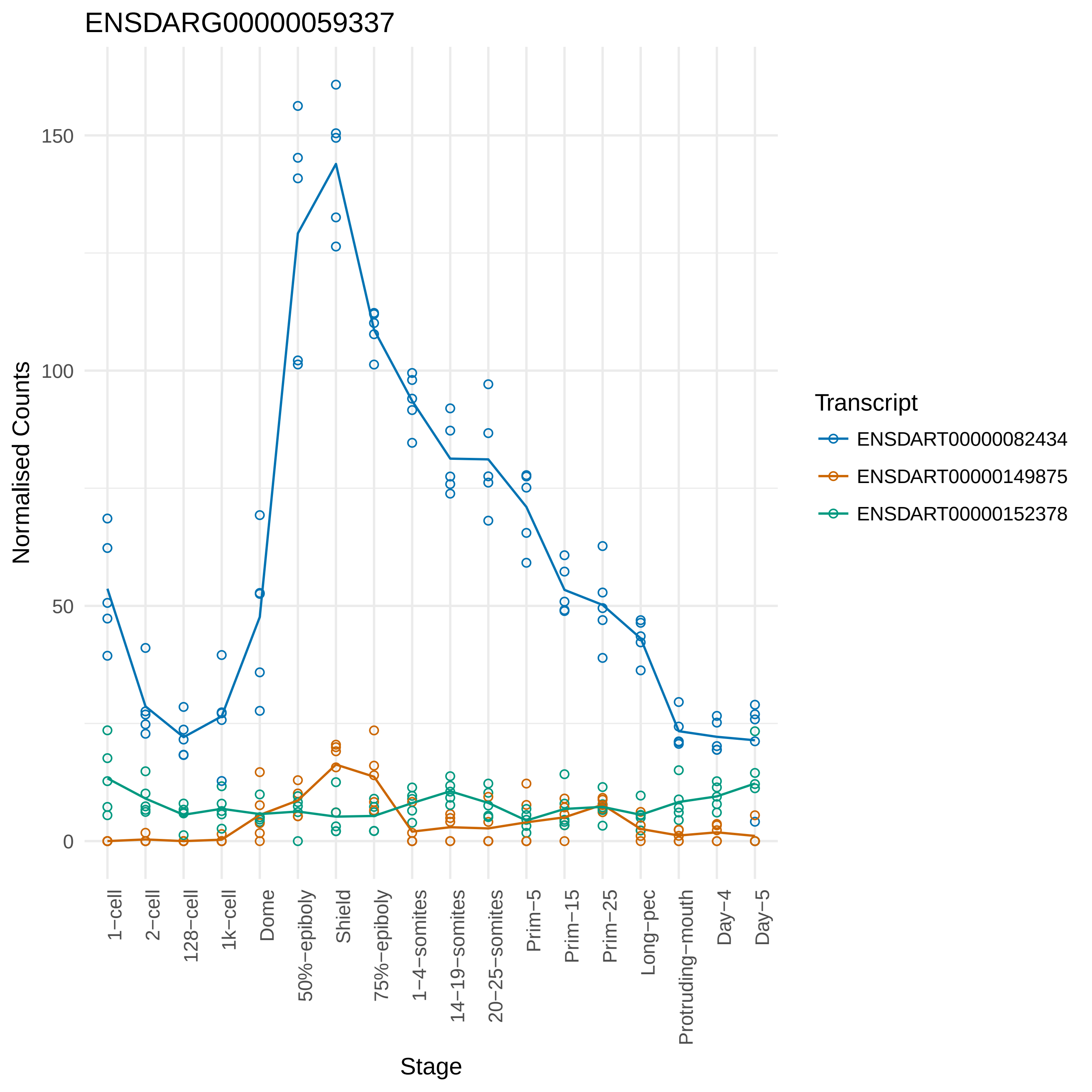

ENSDARG00000059337
Normalised Counts
150
100
50
0
1−cell
2−cell
128−cell
1k−cell
Dome
50%−epiboly
Shield
75%−epiboly
1−4−somites
14−19−somites
20−25−somites
Prim−5
Prim−15
Prim−25
Long−pec
Protruding−mouth
Day−4
Day−5
Stage
Transcript
ENSDART00000082434
ENSDART00000149875
ENSDART00000152378

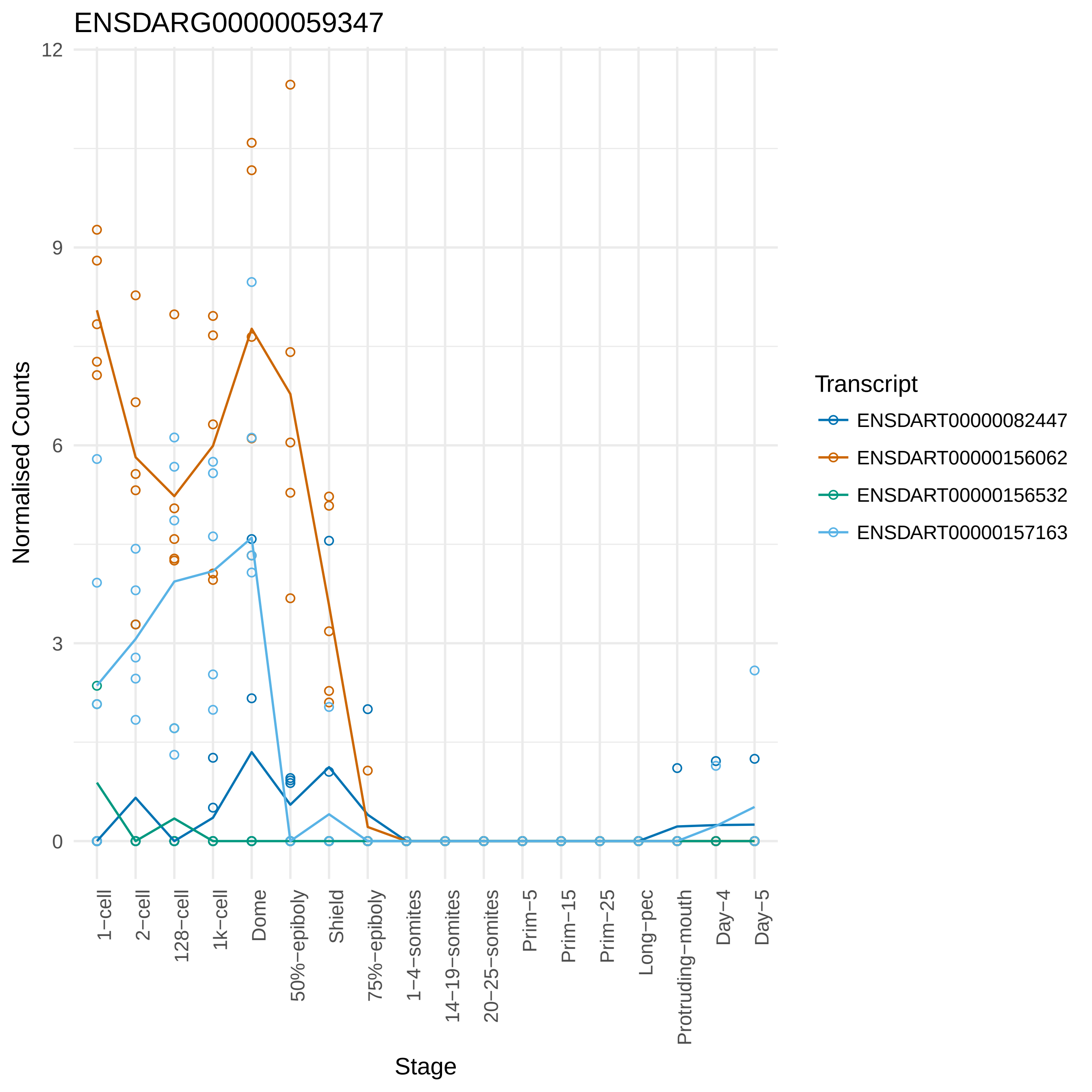
ENSDARG00000059347
Normalised Counts
12
9
6
3
0
1−cell
2−cell
128−cell
1k−cell
Dome
50%−epiboly
Shield
75%−epiboly
1−4−somites
14−19−somites
20−25−somites
Prim−5
Prim−15
Prim−25
Long−pec
Protruding−mouth
Day−4
Day−5
Stage
Transcript
ENSDART00000082447
ENSDART00000156062
ENSDART00000156532
ENSDART00000157163

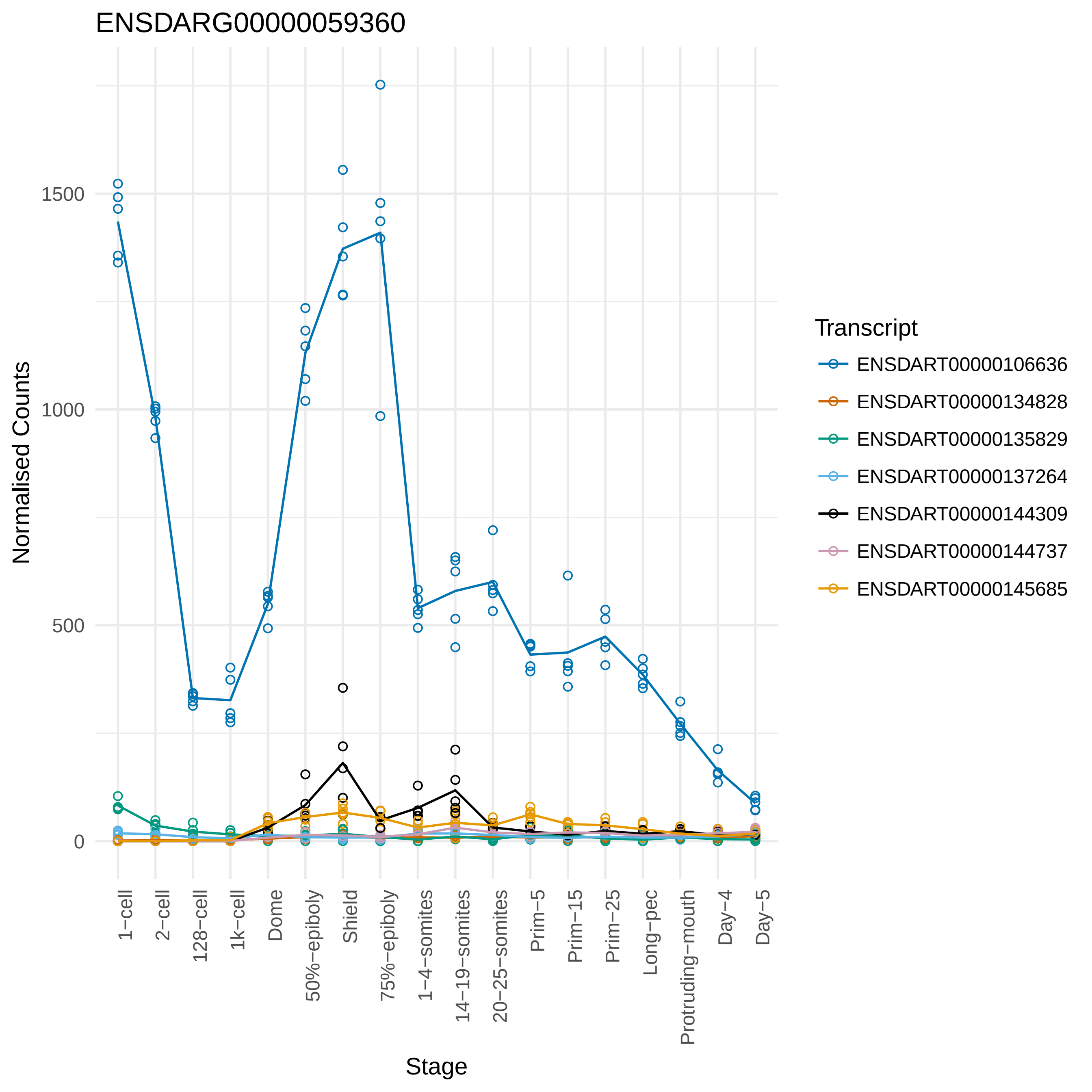
ENSDARG00000059360
Normalised Counts
1500
1000
500
0
1−cell
2−cell
128−cell
1k−cell
Dome
50%−epiboly
Shield
75%−epiboly
1−4−somites
14−19−somites
20−25−somites
Prim−5
Prim−15
Prim−25
Long−pec
Protruding−mouth
Day−4
Day−5
Stage
Transcript
ENSDART00000106636
ENSDART00000134828
ENSDART00000135829
ENSDART00000137264
ENSDART00000144309
ENSDART00000144737
ENSDART00000145685

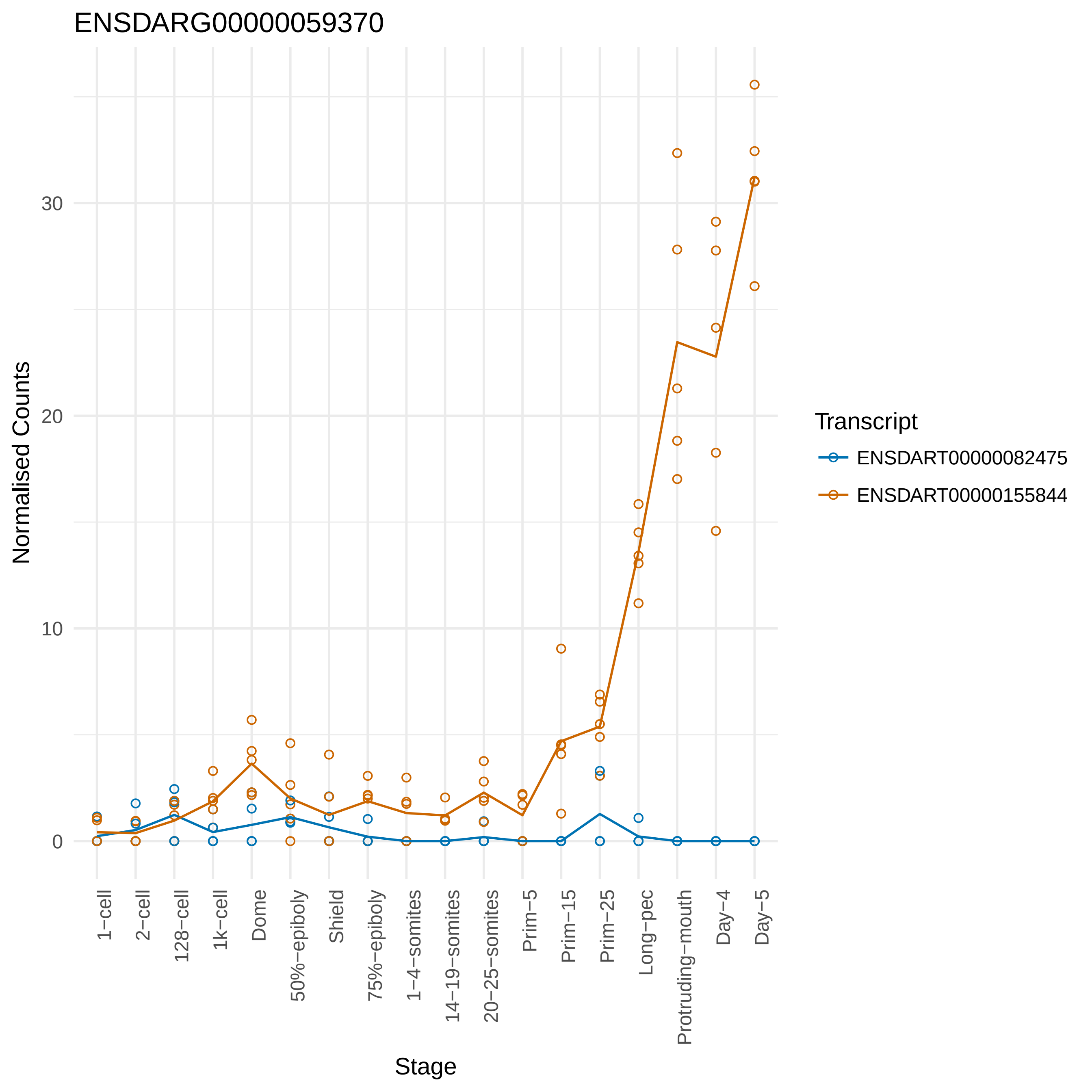
ENSDARG00000059370
Normalised Counts
30
20
10
0
1−cell
2−cell
128−cell
1k−cell
Dome
50%−epiboly
Shield
75%−epiboly
1−4−somites
14−19−somites
20−25−somites
Prim−5
Prim−15
Prim−25
Long−pec
Protruding−mouth
Day−4
Day−5
Stage
Transcript
ENSDART00000082475
ENSDART00000155844

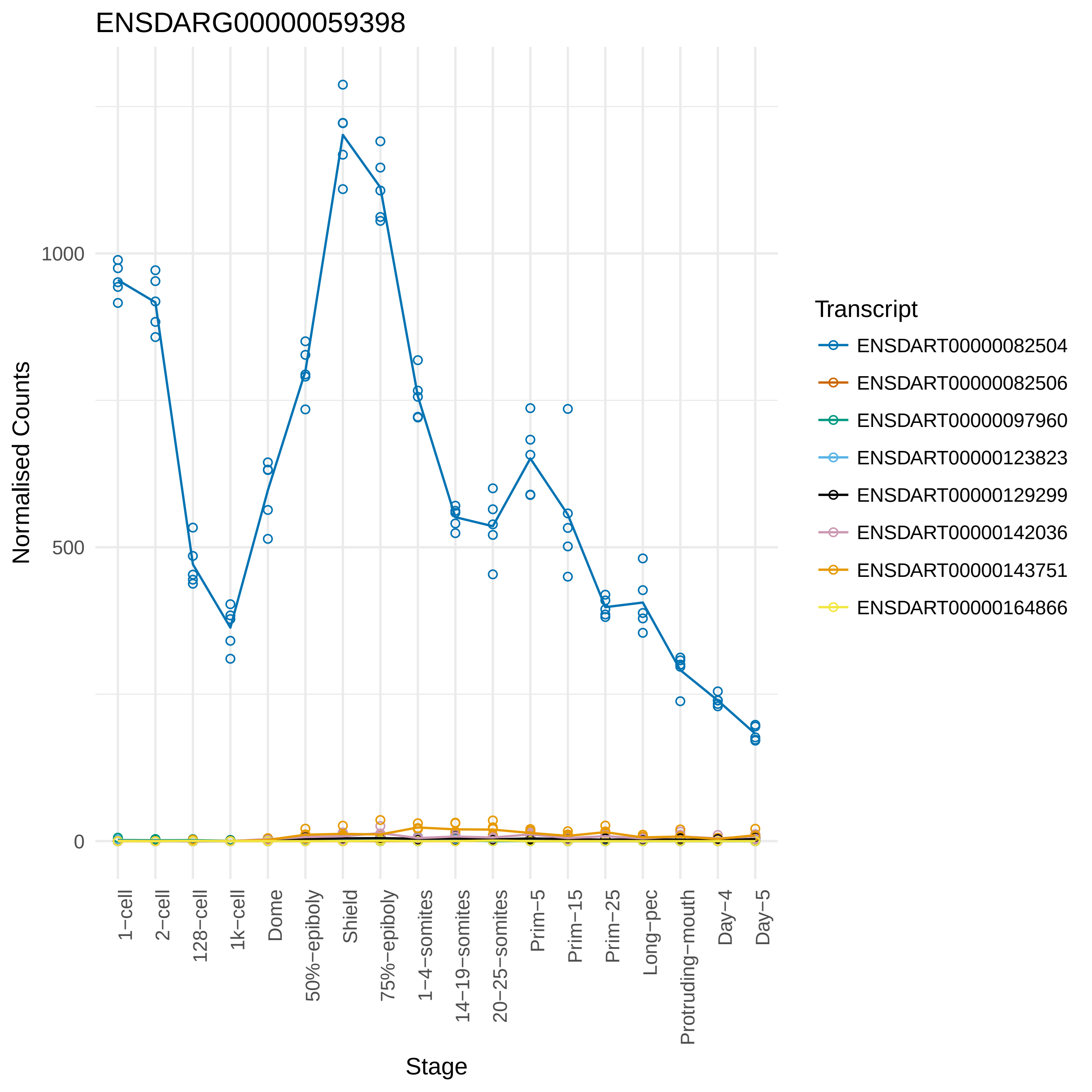

ENSDARG00000059398
Normalised Counts
1000
500
0
1−cell
2−cell
128−cell
1k−cell
Dome
50%−epiboly
Shield
75%−epiboly
1−4−somites
14−19−somites
20−25−somites
Prim−5
Prim−15
Prim−25
Long−pec
Protruding−mouth
Day−4
Day−5
Stage
Transcript
ENSDART00000082504
ENSDART00000082506
ENSDART00000097960
ENSDART00000123823
ENSDART00000129299
ENSDART00000142036
ENSDART00000143751
ENSDART00000164866

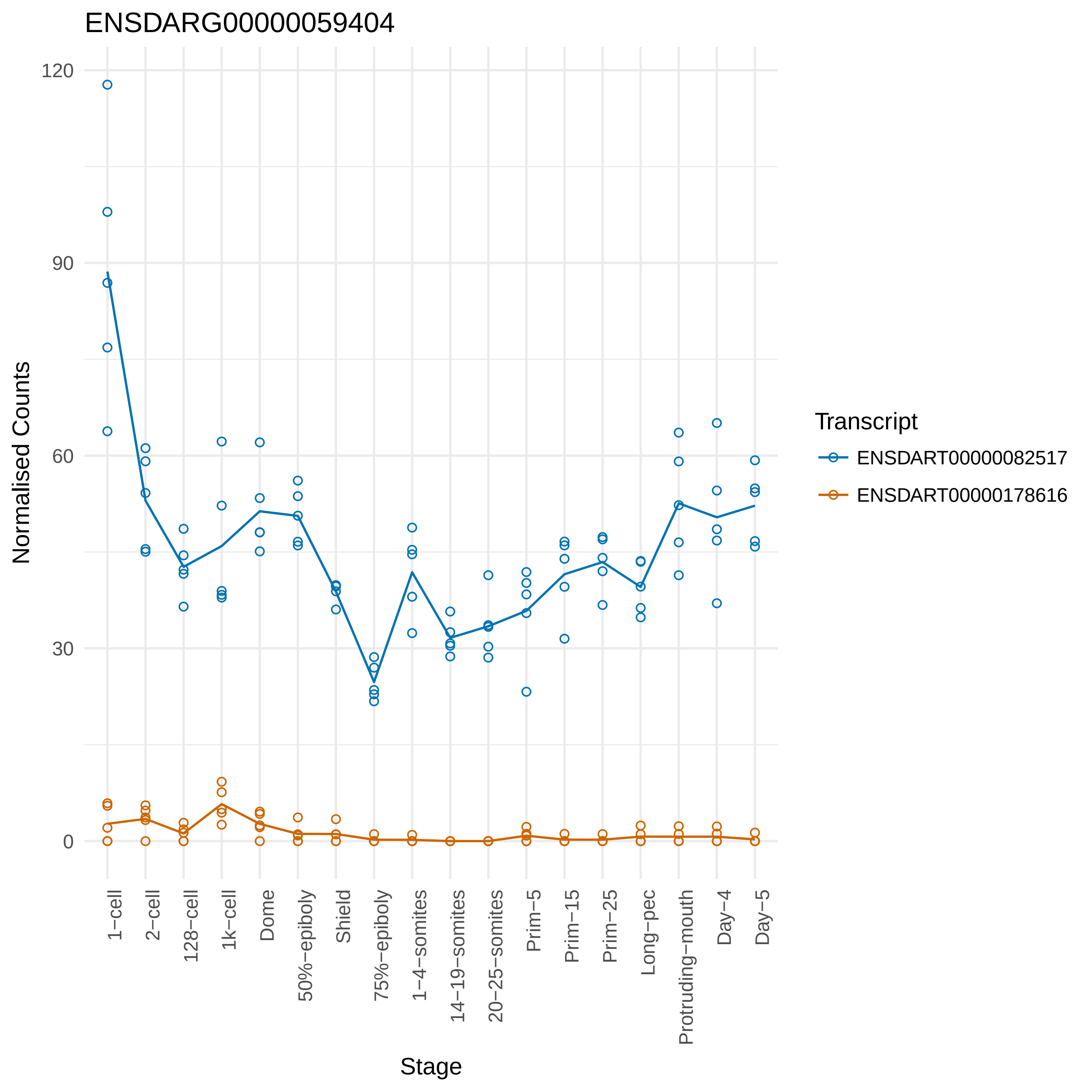

ENSDARG00000059404
Normalised Counts
0
30
60
90
120
Stage
1−cell
2−cell
128−cell
1k−cell
Dome
50%−epiboly
Shield
75%−epiboly
1−4−somites
14−19−somites
20−25−somites
Prim−5
Prim−15
Prim−25
Long−pec
Protruding−mouth
Day−4
Day−5
Transcript
ENSDART00000082517
ENSDART00000178616

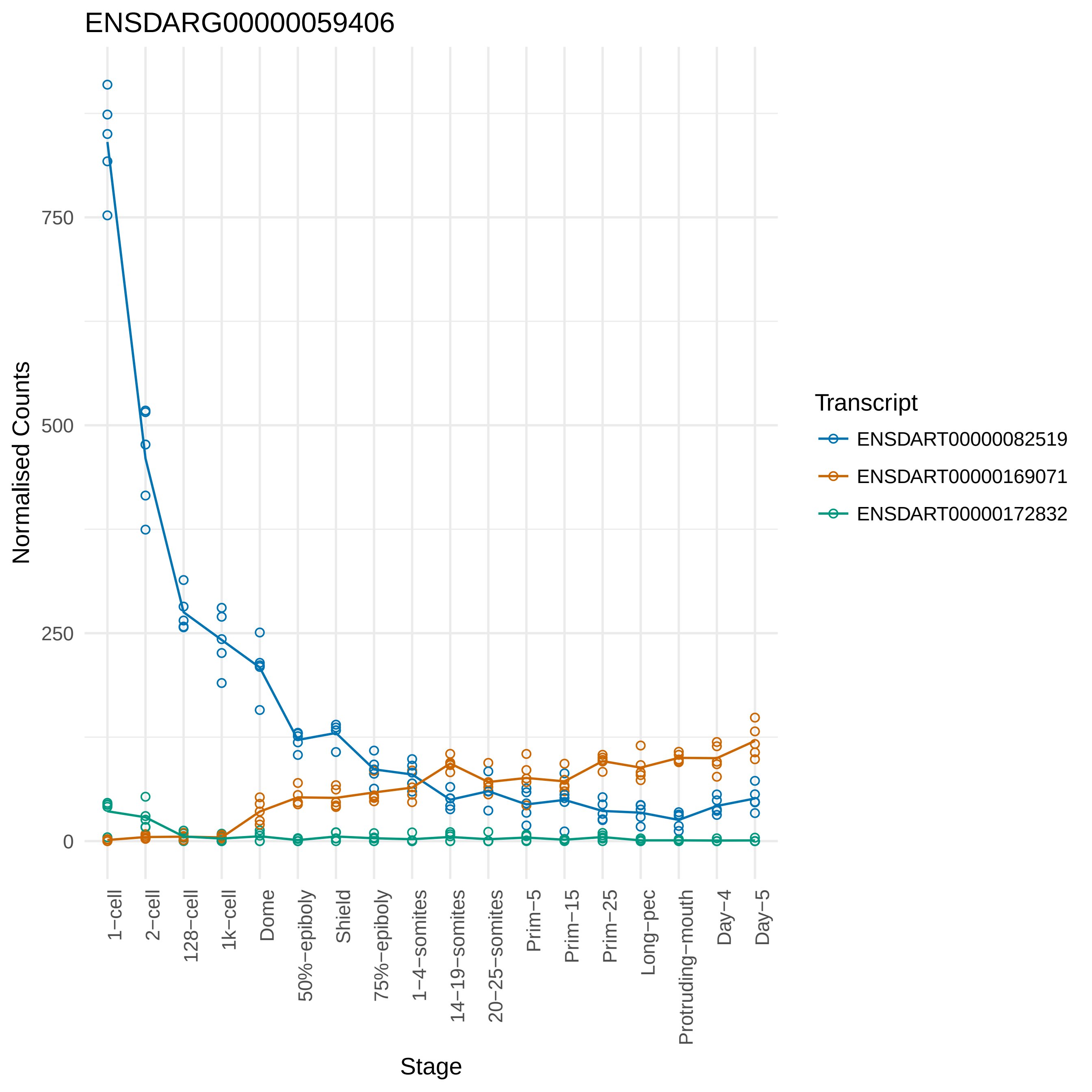
ENSDARG00000059406
Normalised Counts
750
500
250
0
1−cell
2−cell
128−cell
1k−cell
Dome
50%−epiboly
Shield
75%−epiboly
1−4−somites
14−19−somites
20−25−somites
Prim−5
Prim−15
Prim−25
Long−pec
Protruding−mouth
Day−4
Day−5
Stage
Transcript
ENSDART00000082519
ENSDART00000169071
ENSDART00000172832

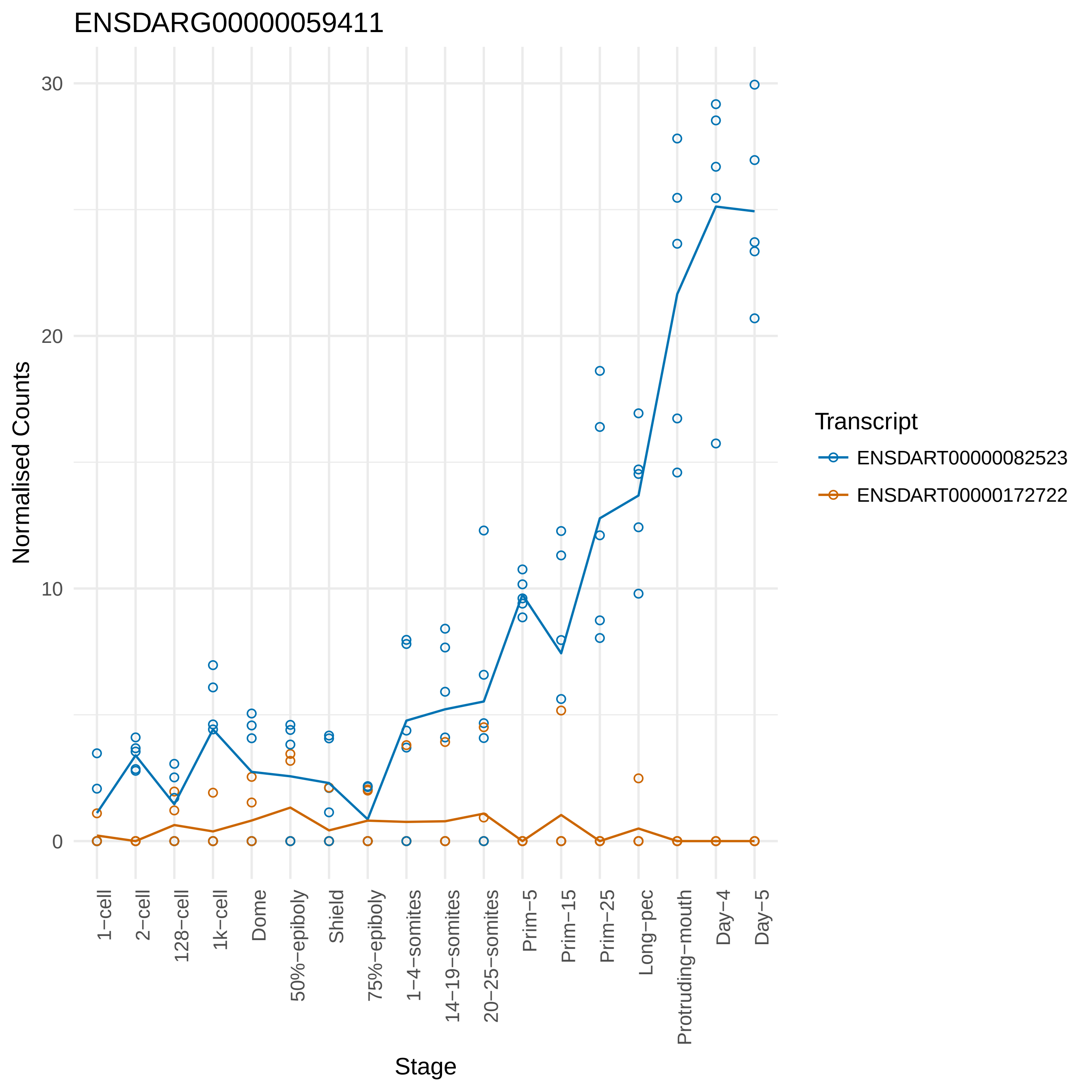

ENSDARG00000059411
Normalised Counts
30
20
10
0
1−cell
2−cell
128−cell
1k−cell
Dome
50%−epiboly
Shield
75%−epiboly
1−4−somites
14−19−somites
20−25−somites
Prim−5
Prim−15
Prim−25
Long−pec
Protruding−mouth
Day−4
Day−5
Stage
Transcript
ENSDART00000082523
ENSDART00000172722

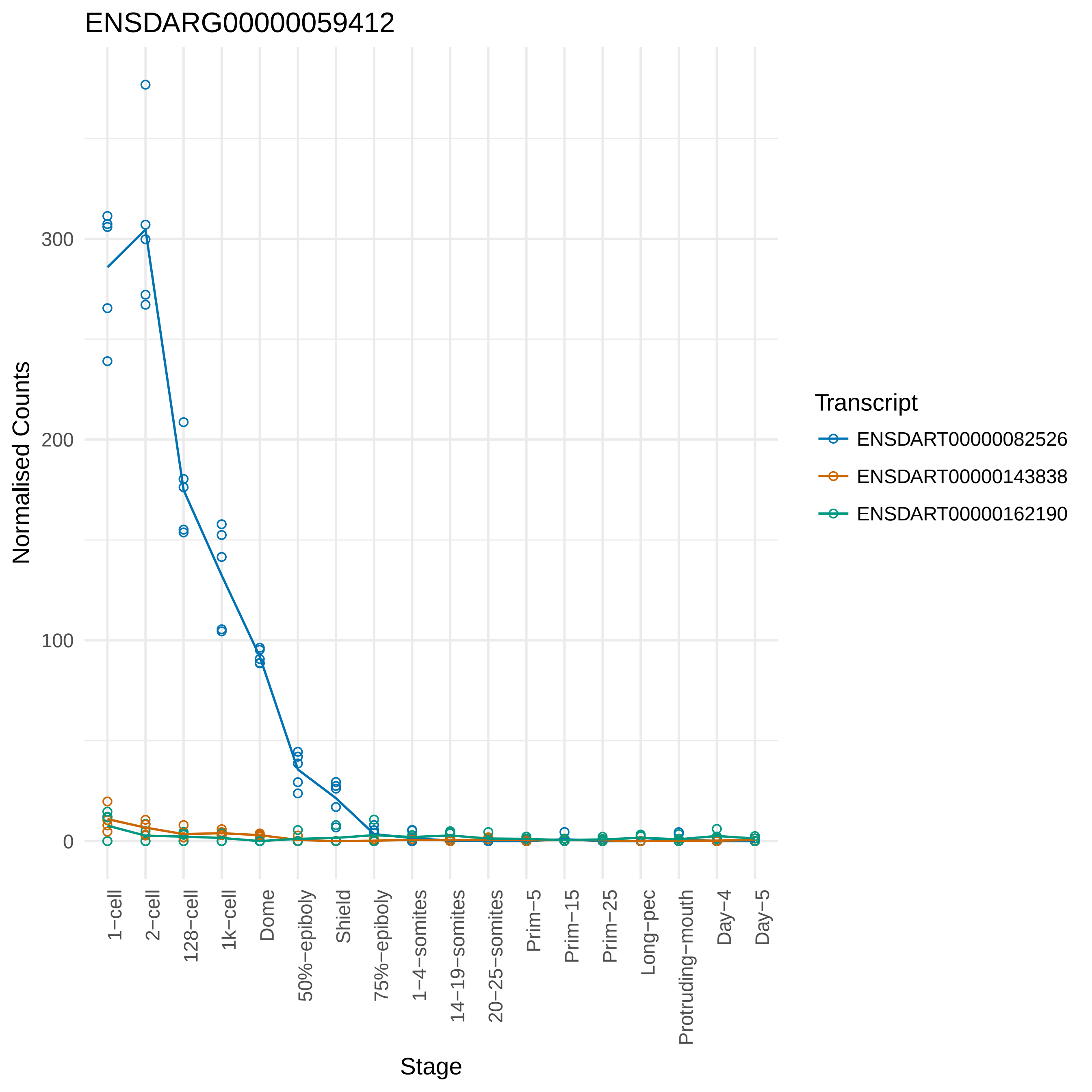
ENSDARG00000059412
Normalised Counts
300
200
100
0
1−cell
2−cell
128−cell
1k−cell
Dome
50%−epiboly
Shield
75%−epiboly
1−4−somites
14−19−somites
20−25−somites
Prim−5
Prim−15
Prim−25
Long−pec
Protruding−mouth
Day−4
Day−5
Stage
Transcript
ENSDART00000082526
ENSDART00000143838
ENSDART00000162190

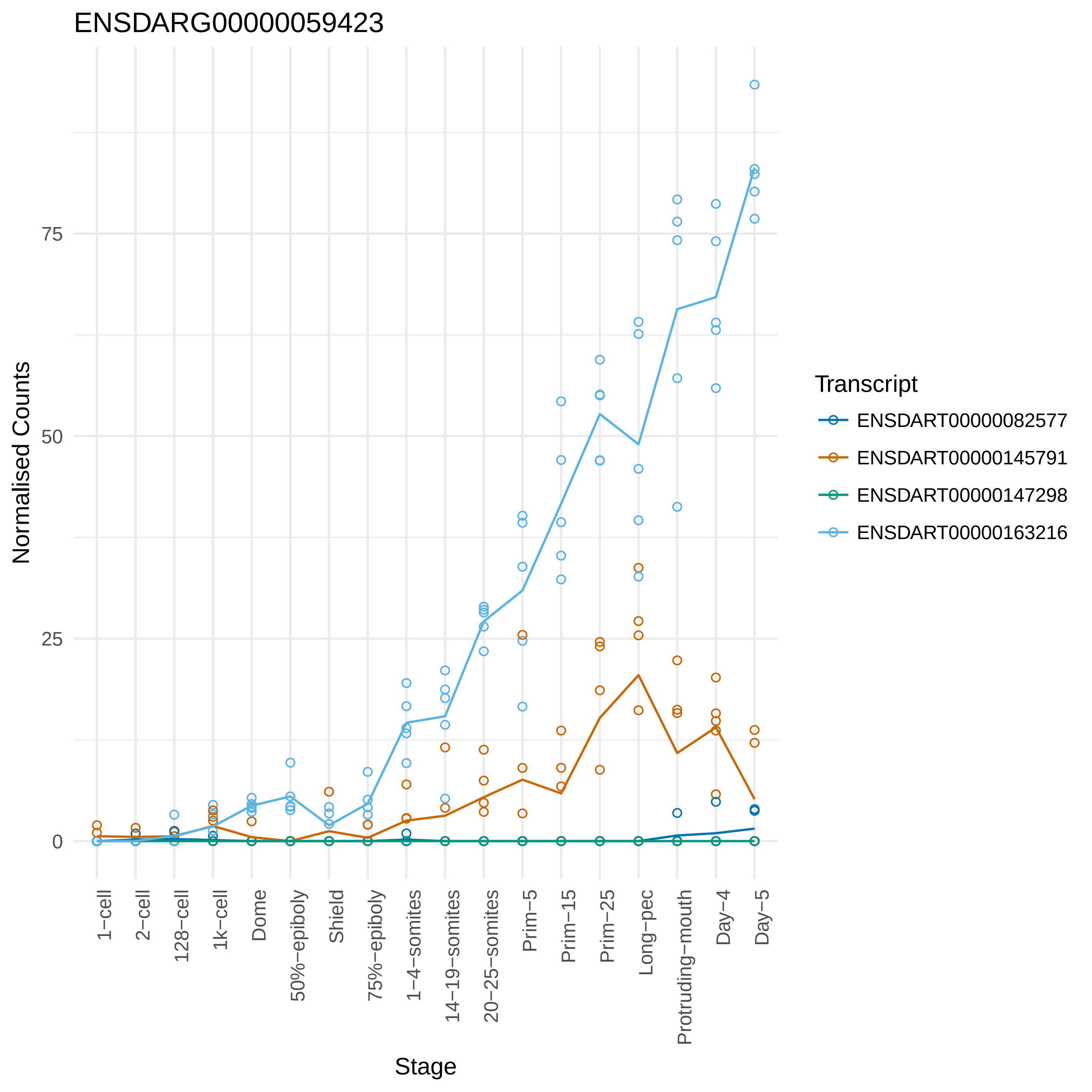
ENSDARG00000059423
Normalised Counts
0
25
50
75
Stage
1−cell
2−cell
128−cell
1k−cell
Dome
50%−epiboly
Shield
75%−epiboly
1−4−somites
14−19−somites
20−25−somites
Prim−5
Prim−15
Prim−25
Long−pec
Protruding−mouth
Day−4
Day−5
Transcript
ENSDART00000082577
ENSDART00000145791
ENSDART00000147298
ENSDART00000163216

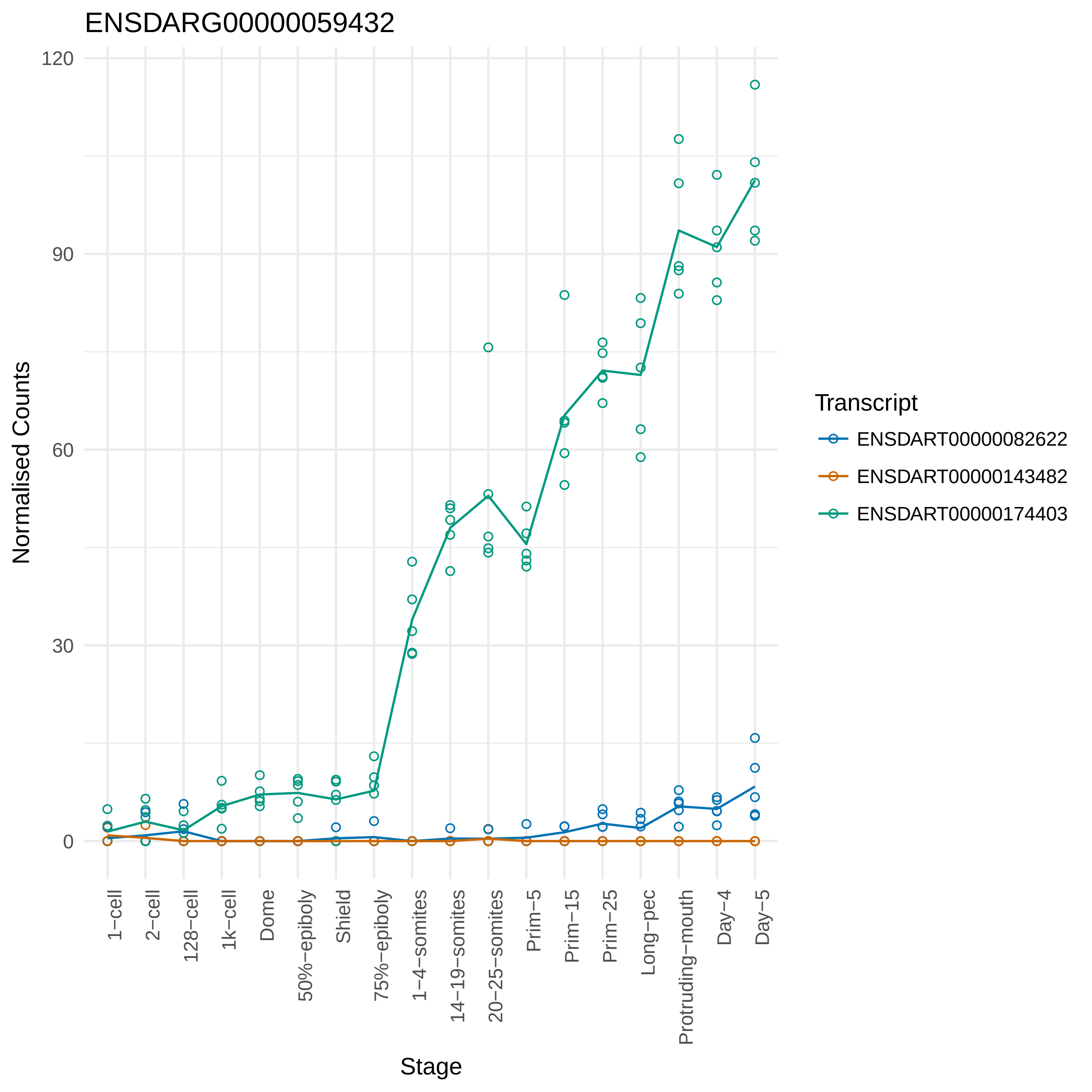
ENSDARG00000059432
Normalised Counts
120
90
60
30
0
1−cell
2−cell
128−cell
1k−cell
Dome
50%−epiboly
Shield
75%−epiboly
1−4−somites
14−19−somites
20−25−somites
Prim−5
Prim−15
Prim−25
Long−pec
Protruding−mouth
Day−4
Day−5
Stage
Transcript
ENSDART00000082622
ENSDART00000143482
ENSDART00000174403

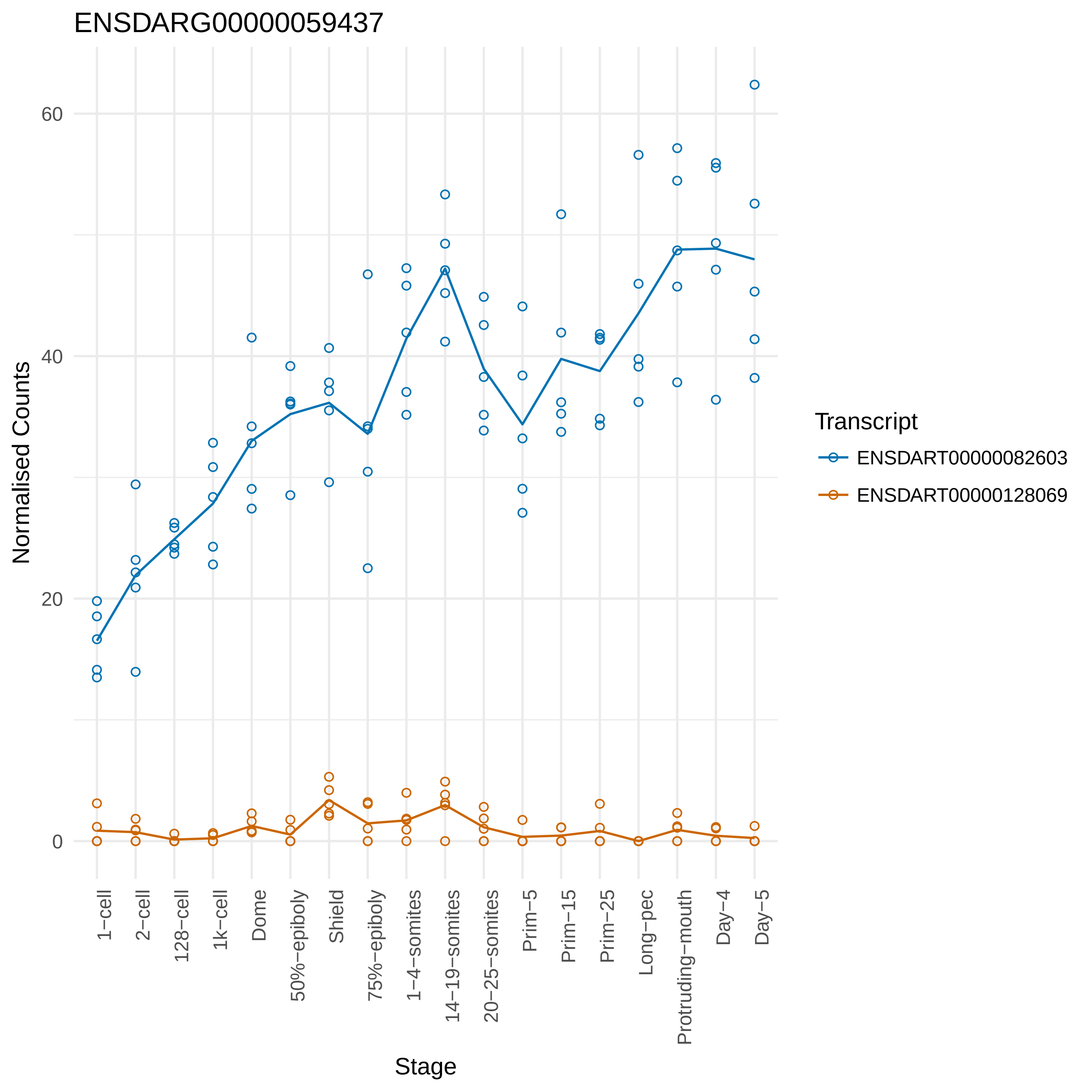
ENSDARG00000059437
Normalised Counts
0
20
40
60
Stage
1−cell
2−cell
128−cell
1k−cell
Dome
50%−epiboly
Shield
75%−epiboly
1−4−somites
14−19−somites
20−25−somites
Prim−5
Prim−15
Prim−25
Long−pec
Protruding−mouth
Day−4
Day−5
Transcript
ENSDART00000082603
ENSDART00000128069

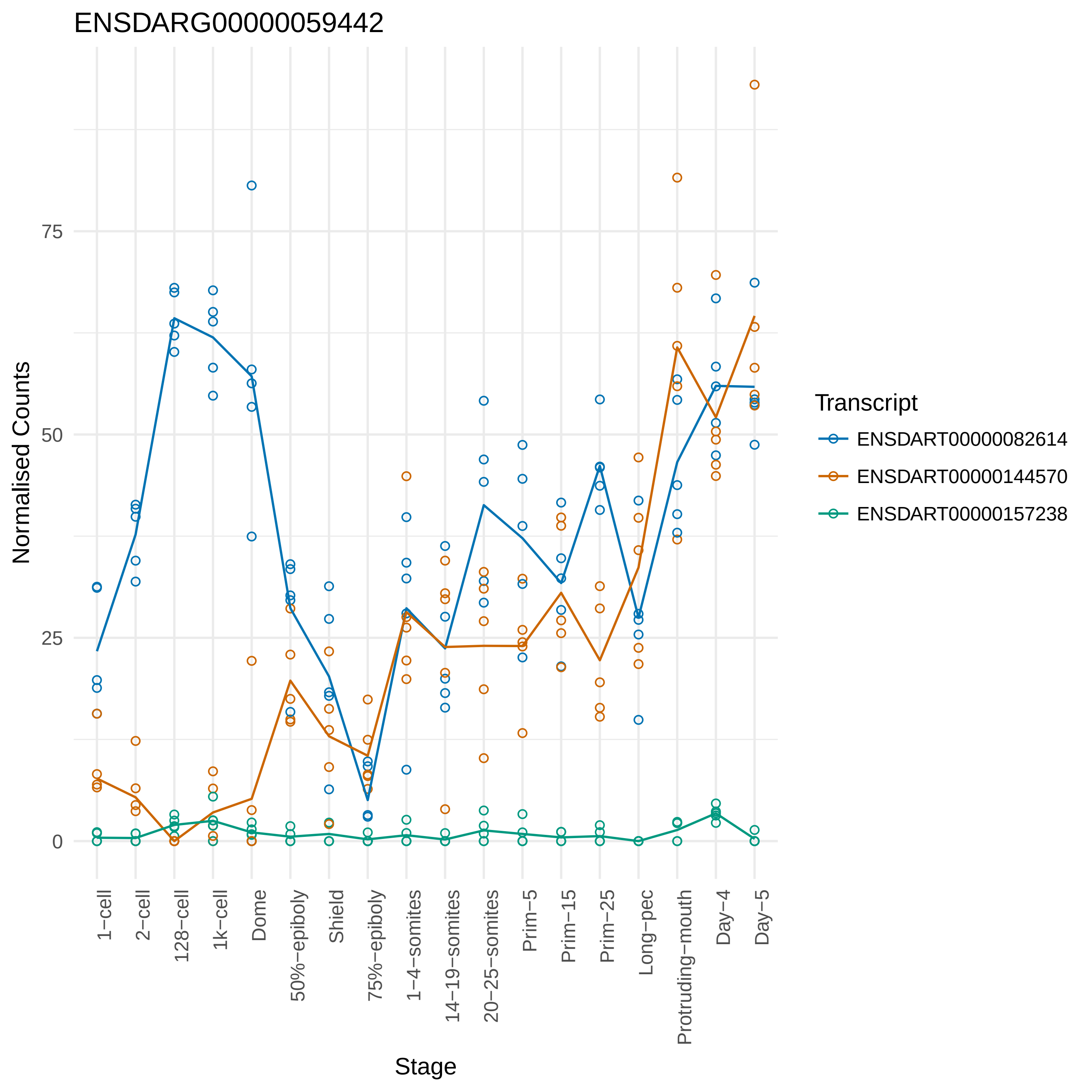
ENSDARG00000059442
Normalised Counts
0
25
50
75
Stage
1−cell
2−cell
128−cell
1k−cell
Dome
50%−epiboly
Shield
75%−epiboly
1−4−somites
14−19−somites
20−25−somites
Prim−5
Prim−15
Prim−25
Long−pec
Protruding−mouth
Day−4
Day−5
Transcript
ENSDART00000082614
ENSDART00000144570
ENSDART00000157238

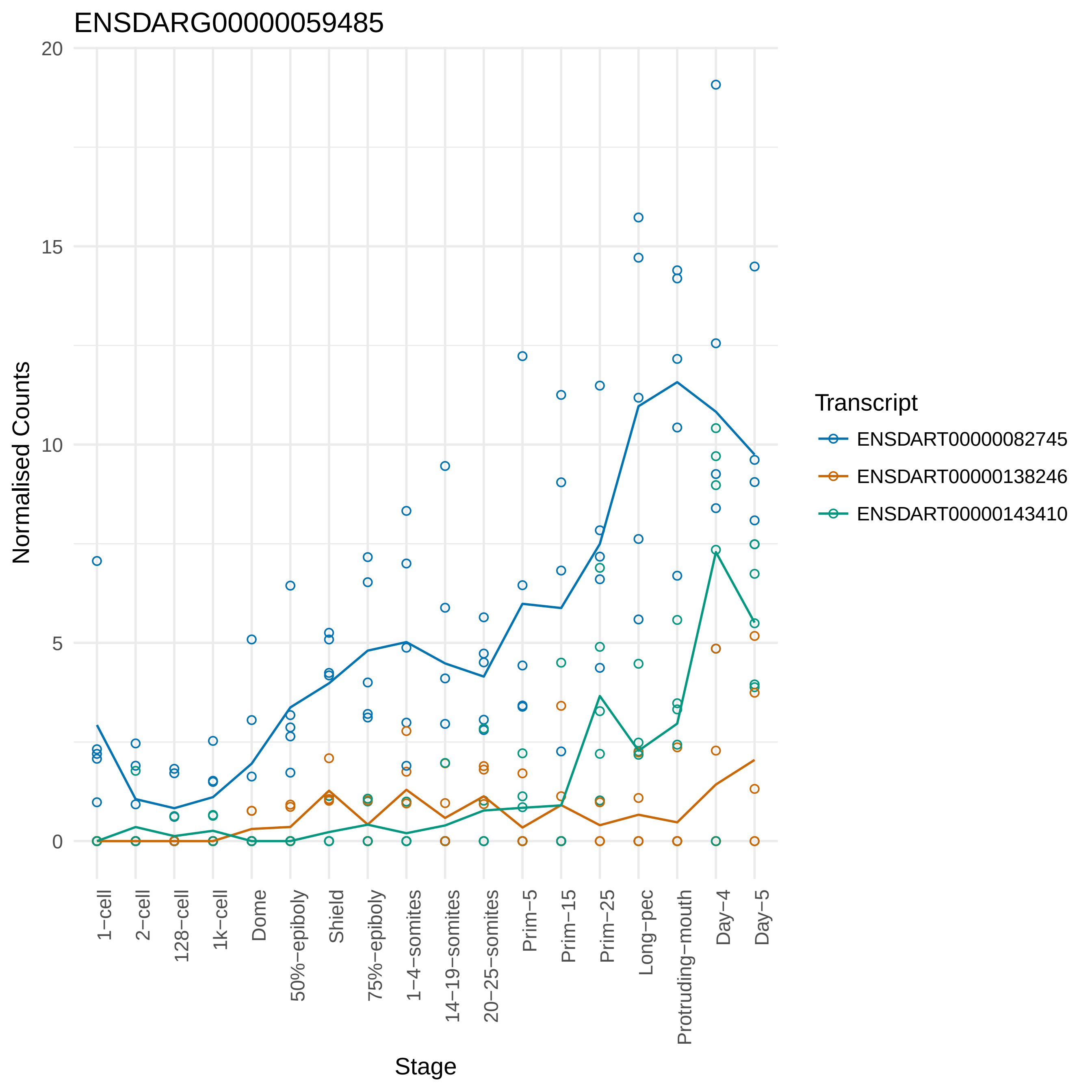
ENSDARG00000059485
Normalised Counts
Stage
Transcript
ENSDART00000082745
ENSDART00000138246
ENSDART00000143410
0
5
10
15
20
1−cell
2−cell
128−cell
1k−cell
Dome
50%−epiboly
Shield
75%−epiboly
1−4−somites
14−19−somites
20−25−somites
Prim−5
Prim−15
Prim−25
Long−pec
Protruding−mouth
Day−4
Day−5

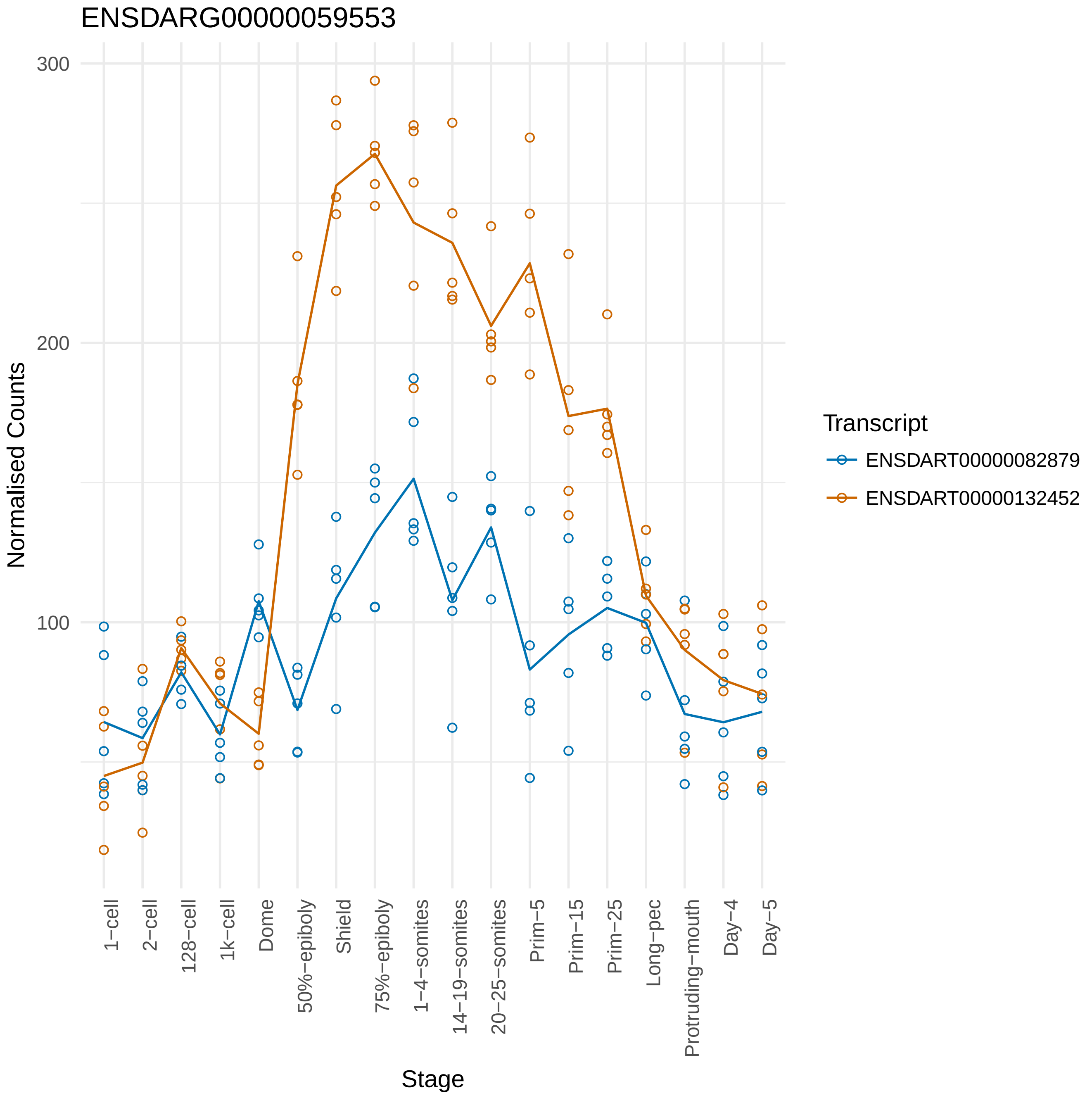

ENSDARG00000059553
Normalised Counts
300
200
100
1−cell
2−cell
128−cell
1k−cell
Dome
50%−epiboly
Shield
75%−epiboly
1−4−somites
14−19−somites
20−25−somites
Prim−5
Prim−15
Prim−25
Long−pec
Protruding−mouth
Day−4
Day−5
Stage
Transcript
ENSDART00000082879
ENSDART00000132452

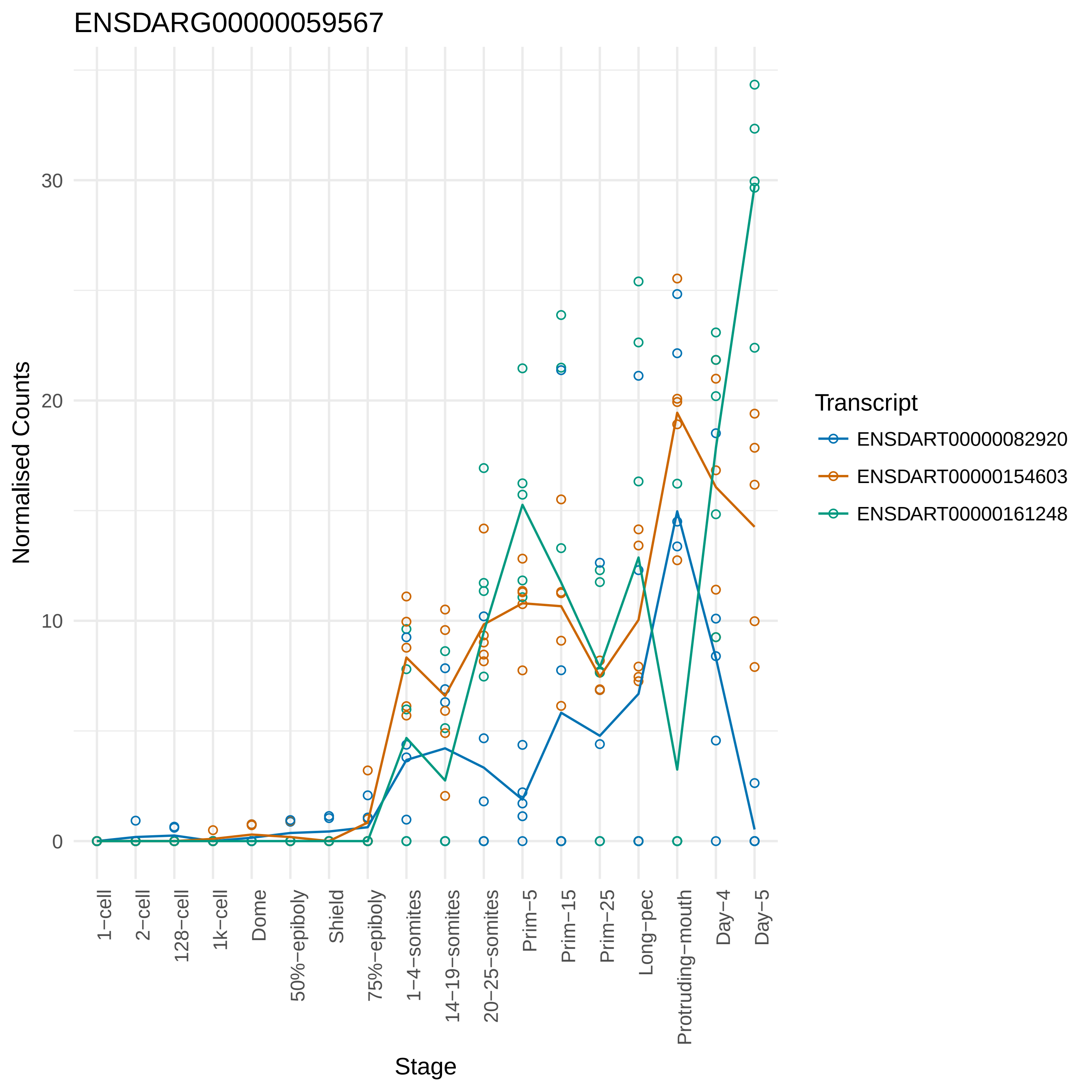

ENSDARG00000059567
Normalised Counts
0
10
20
30
1−cell
2−cell
128−cell
1k−cell
Dome
50%−epiboly
Shield
75%−epiboly
1−4−somites
14−19−somites
20−25−somites
Prim−5
Prim−15
Prim−25
Long−pec
Protruding−mouth
Day−4
Day−5
Stage
Transcript
ENSDART00000082920
ENSDART00000154603
ENSDART00000161248

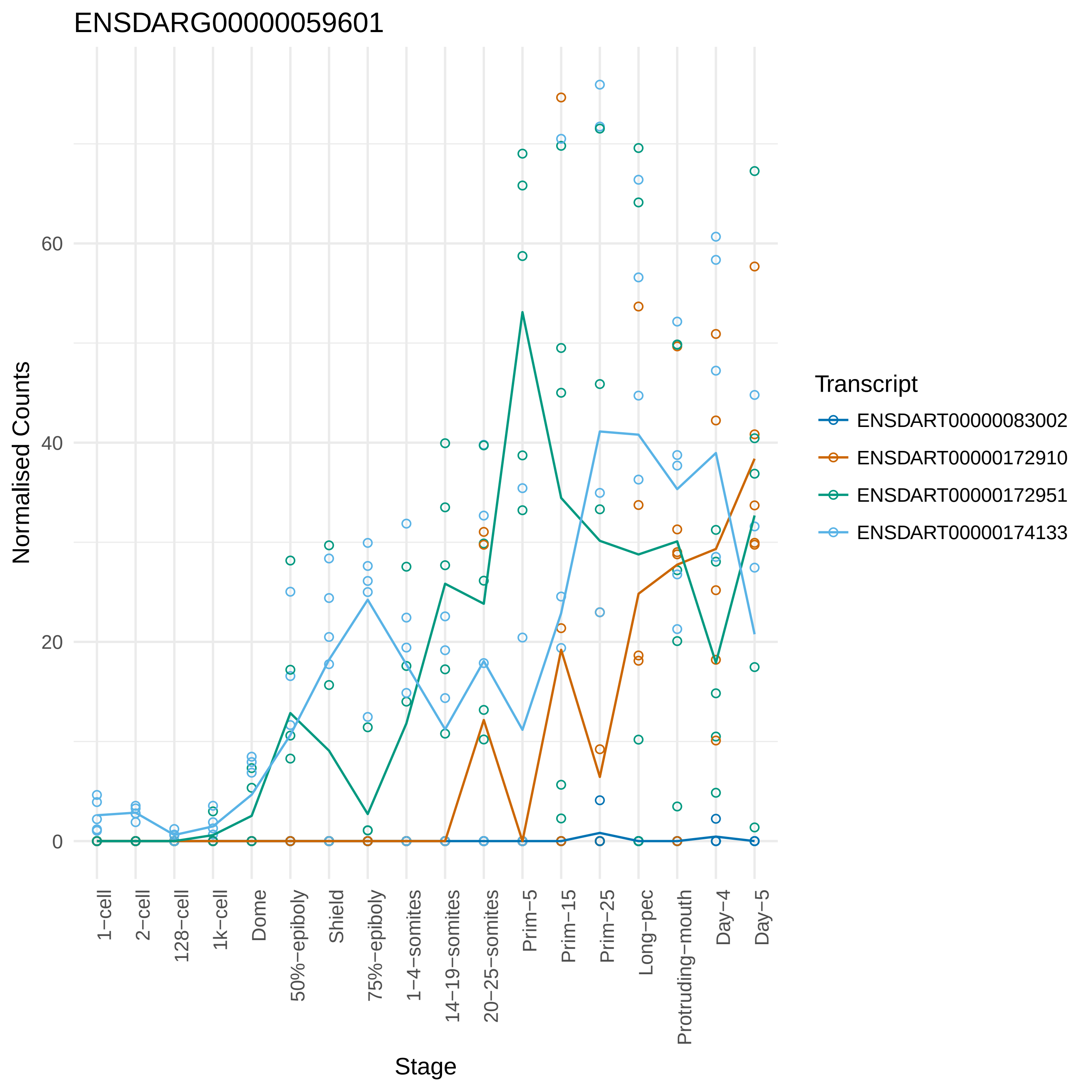

ENSDARG00000059601
Normalised Counts
0
20
40
60
Stage
1−cell
2−cell
128−cell
1k−cell
Dome
50%−epiboly
Shield
75%−epiboly
1−4−somites
14−19−somites
20−25−somites
Prim−5
Prim−15
Prim−25
Long−pec
Protruding−mouth
Day−4
Day−5
Transcript
ENSDART00000083002
ENSDART00000172910
ENSDART00000172951
ENSDART00000174133

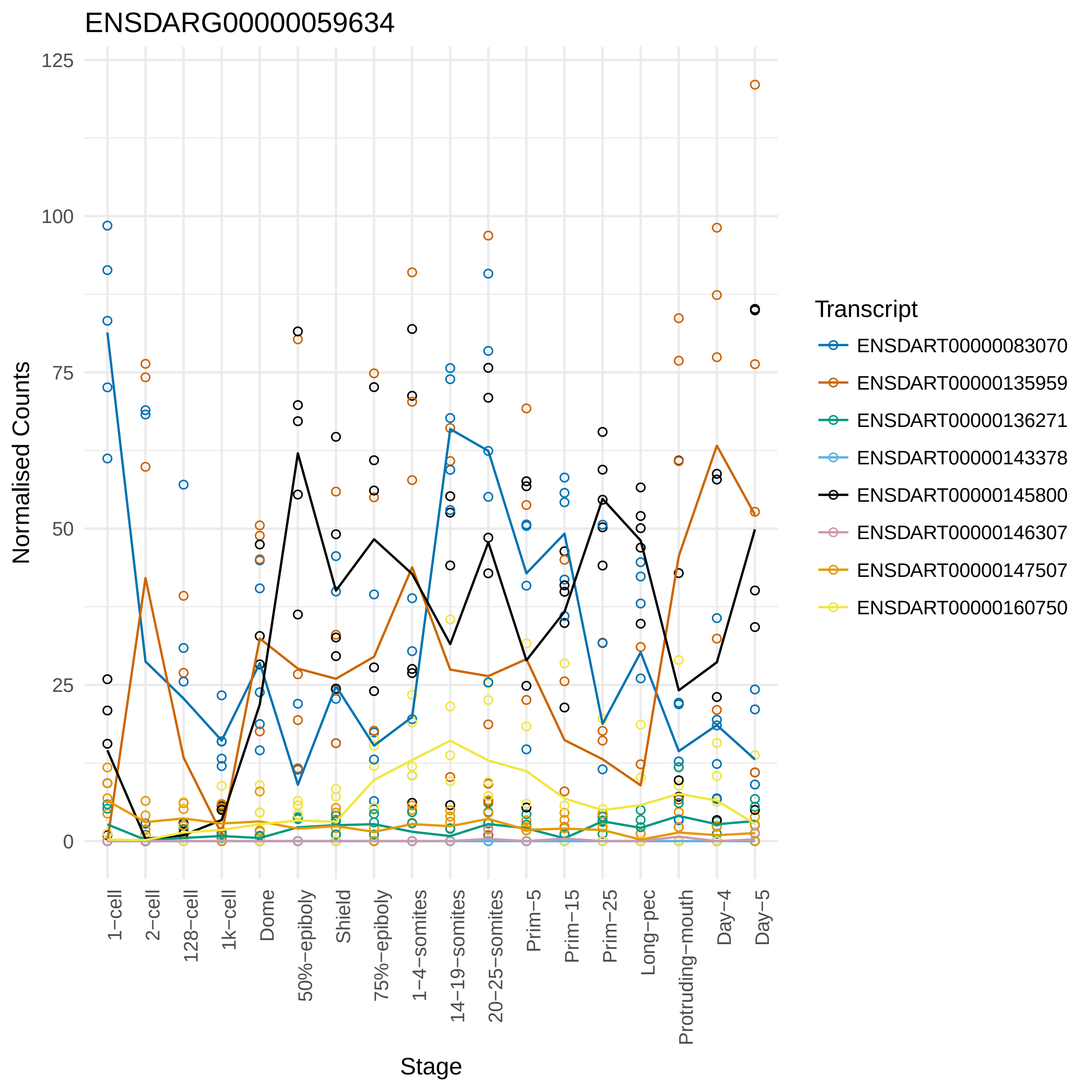

ENSDARG00000059634
Normalised Counts
125
100
75
50
25
0
1−cell
2−cell
128−cell
1k−cell
Dome
50%−epiboly
Shield
75%−epiboly
1−4−somites
14−19−somites
20−25−somites
Prim−5
Prim−15
Prim−25
Long−pec
Protruding−mouth
Day−4
Day−5
Stage
Transcript
ENSDART00000083070
ENSDART00000135959
ENSDART00000136271
ENSDART00000143378
ENSDART00000145800
ENSDART00000146307
ENSDART00000147507
ENSDART00000160750

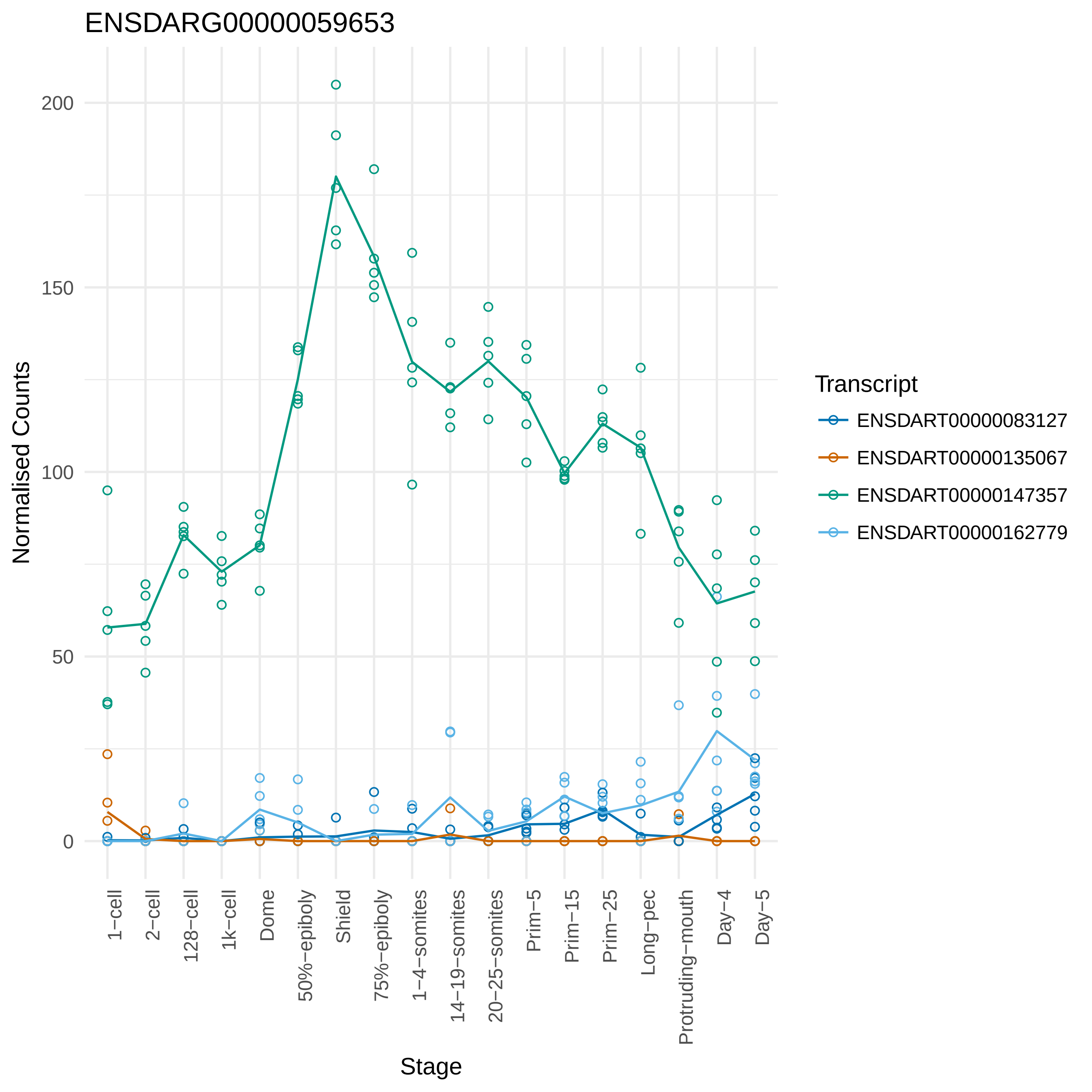

ENSDARG00000059653
Normalised Counts
200
150
100
50
0
1−cell
2−cell
128−cell
1k−cell
Dome
50%−epiboly
Shield
75%−epiboly
1−4−somites
14−19−somites
20−25−somites
Prim−5
Prim−15
Prim−25
Long−pec
Protruding−mouth
Day−4
Day−5
Stage
Transcript
ENSDART00000083127
ENSDART00000135067
ENSDART00000147357
ENSDART00000162779

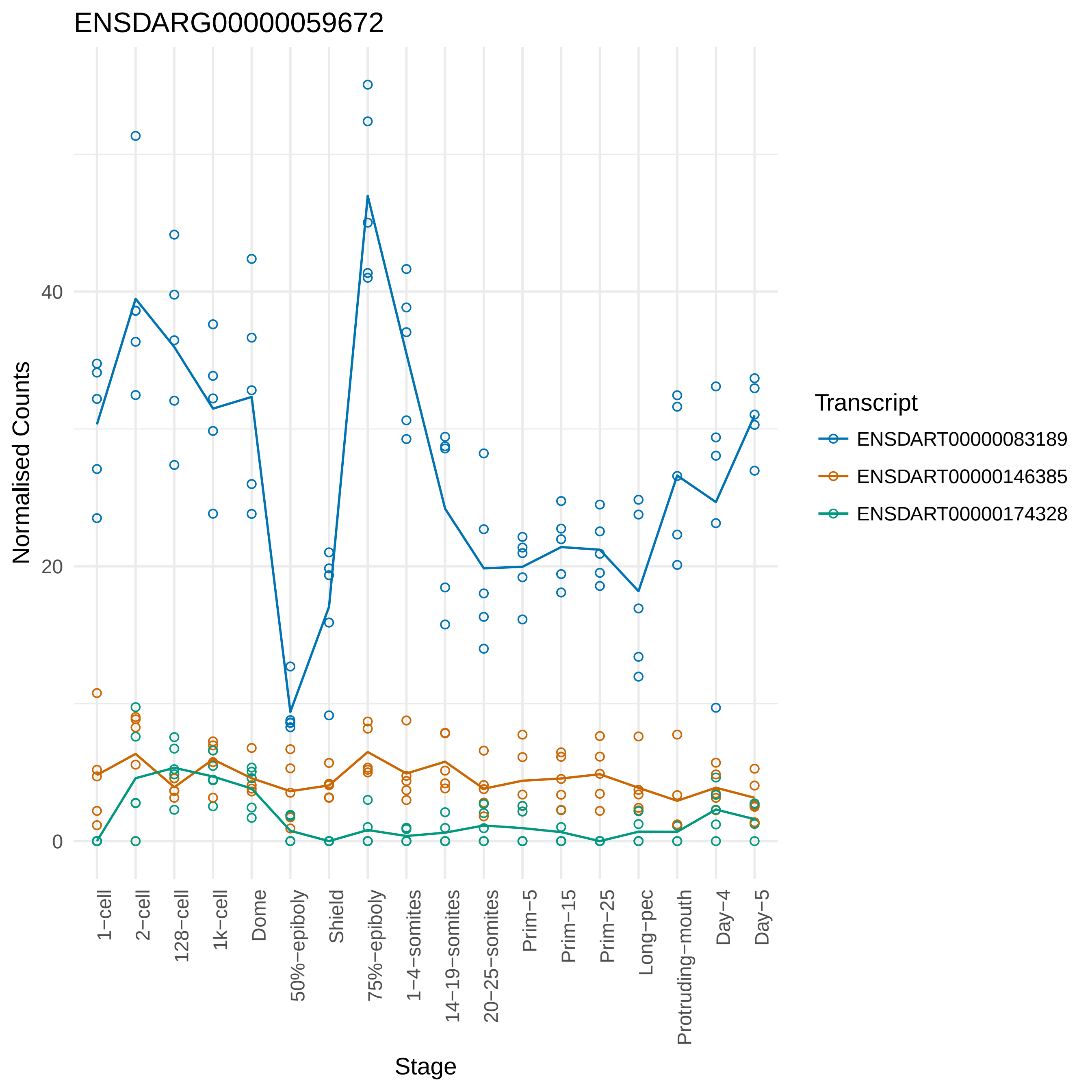

ENSDARG00000059672
Normalised Counts
0
20
40
Stage
1−cell
2−cell
128−cell
1k−cell
Dome
50%−epiboly
Shield
75%−epiboly
1−4−somites
14−19−somites
20−25−somites
Prim−5
Prim−15
Prim−25
Long−pec
Protruding−mouth
Day−4
Day−5
Transcript
ENSDART00000083189
ENSDART00000146385
ENSDART00000174328

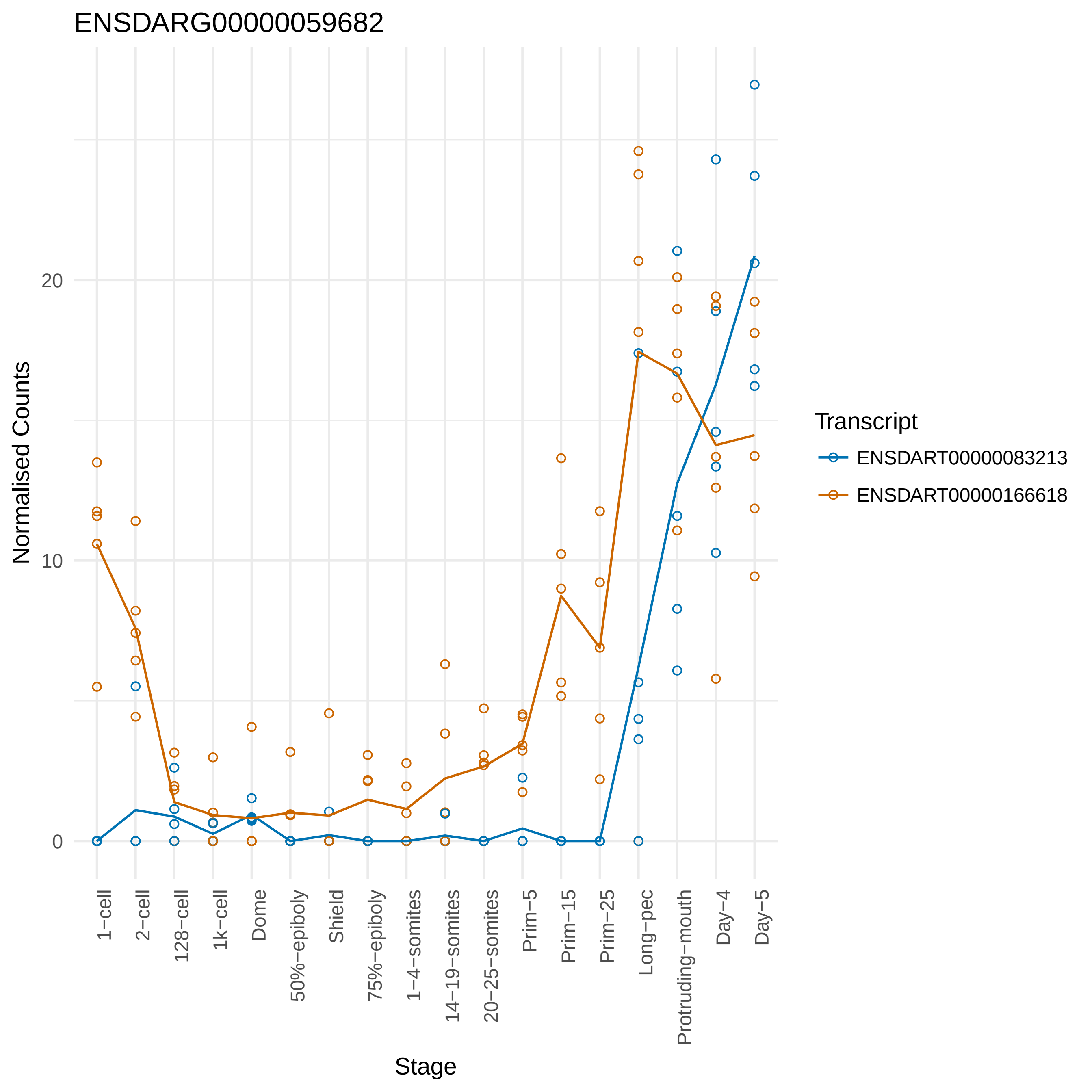

ENSDARG00000059682
Normalised Counts
0
10
20
1−cell
2−cell
128−cell
1k−cell
Dome
50%−epiboly
Shield
75%−epiboly
1−4−somites
14−19−somites
20−25−somites
Prim−5
Prim−15
Prim−25
Long−pec
Protruding−mouth
Day−4
Day−5
Stage
Transcript
ENSDART00000083213
ENSDART00000166618

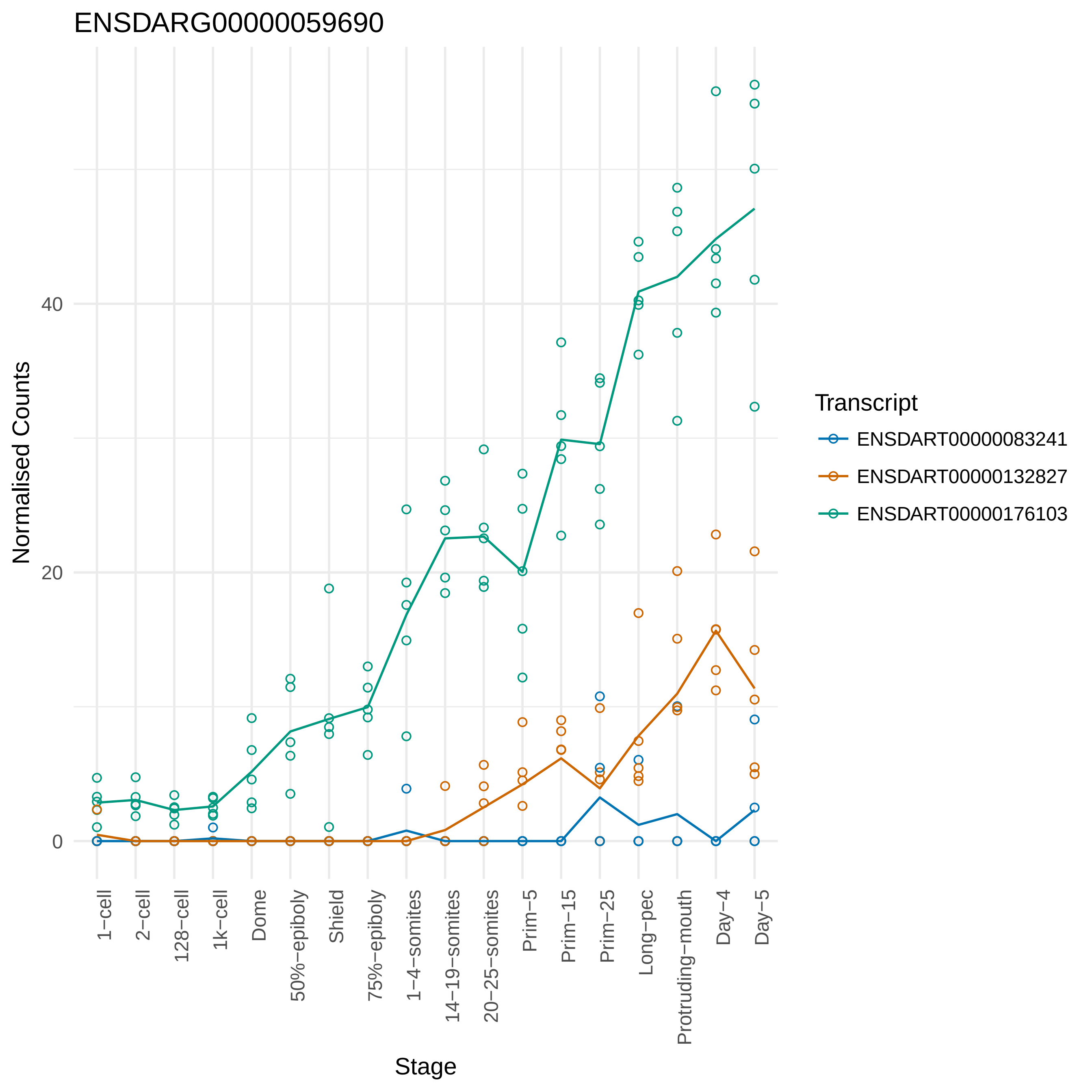
ENSDARG00000059690
Normalised Counts
0
20
40
Stage
1−cell
2−cell
128−cell
1k−cell
Dome
50%−epiboly
Shield
75%−epiboly
1−4−somites
14−19−somites
20−25−somites
Prim−5
Prim−15
Prim−25
Long−pec
Protruding−mouth
Day−4
Day−5
Transcript
ENSDART00000083241
ENSDART00000132827
ENSDART00000176103

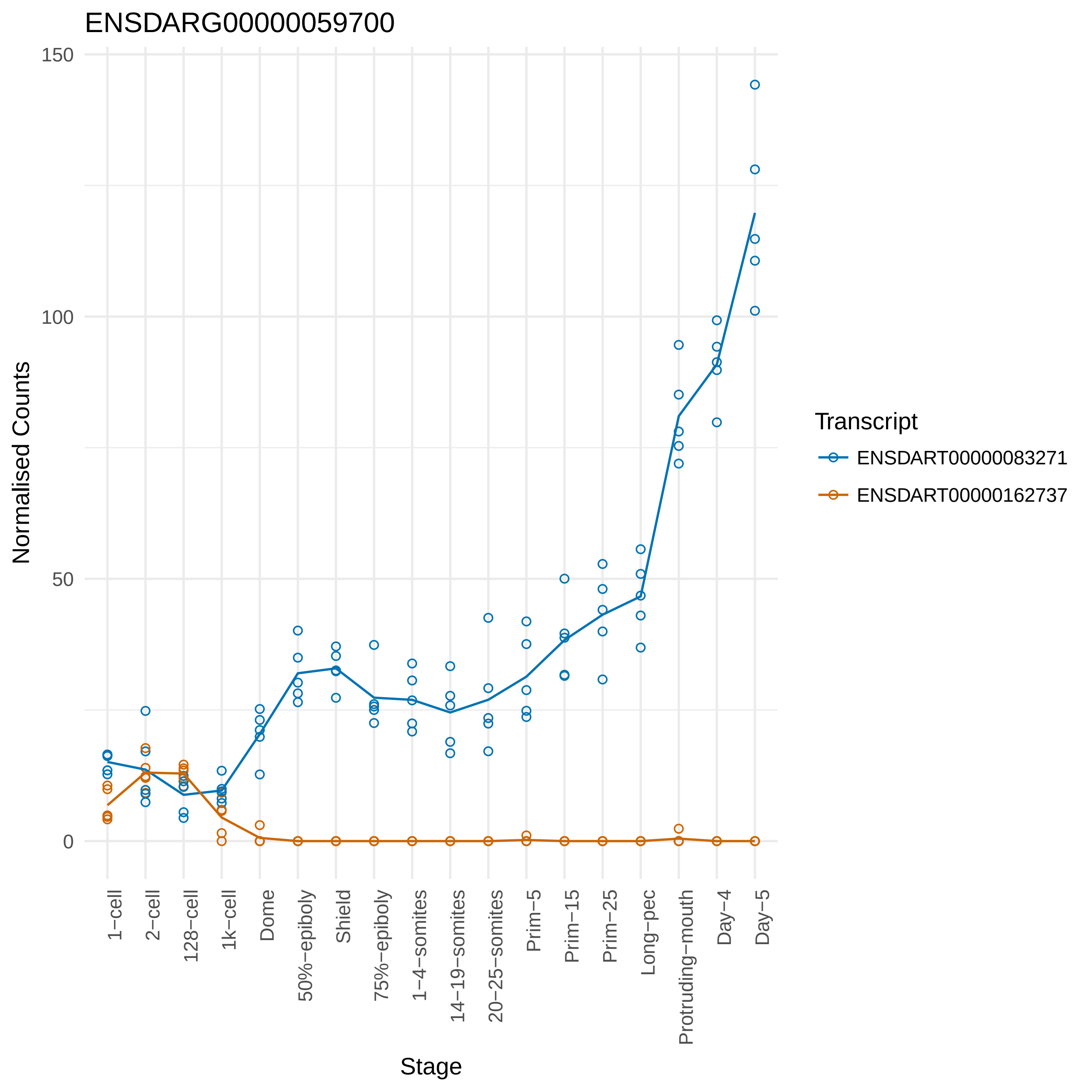
ENSDARG00000059700
Normalised Counts
150
100
50
0
1−cell
2−cell
128−cell
1k−cell
Dome
50%−epiboly
Shield
75%−epiboly
1−4−somites
14−19−somites
20−25−somites
Prim−5
Prim−15
Prim−25
Long−pec
Protruding−mouth
Day−4
Day−5
Stage
Transcript
ENSDART00000083271
ENSDART00000162737

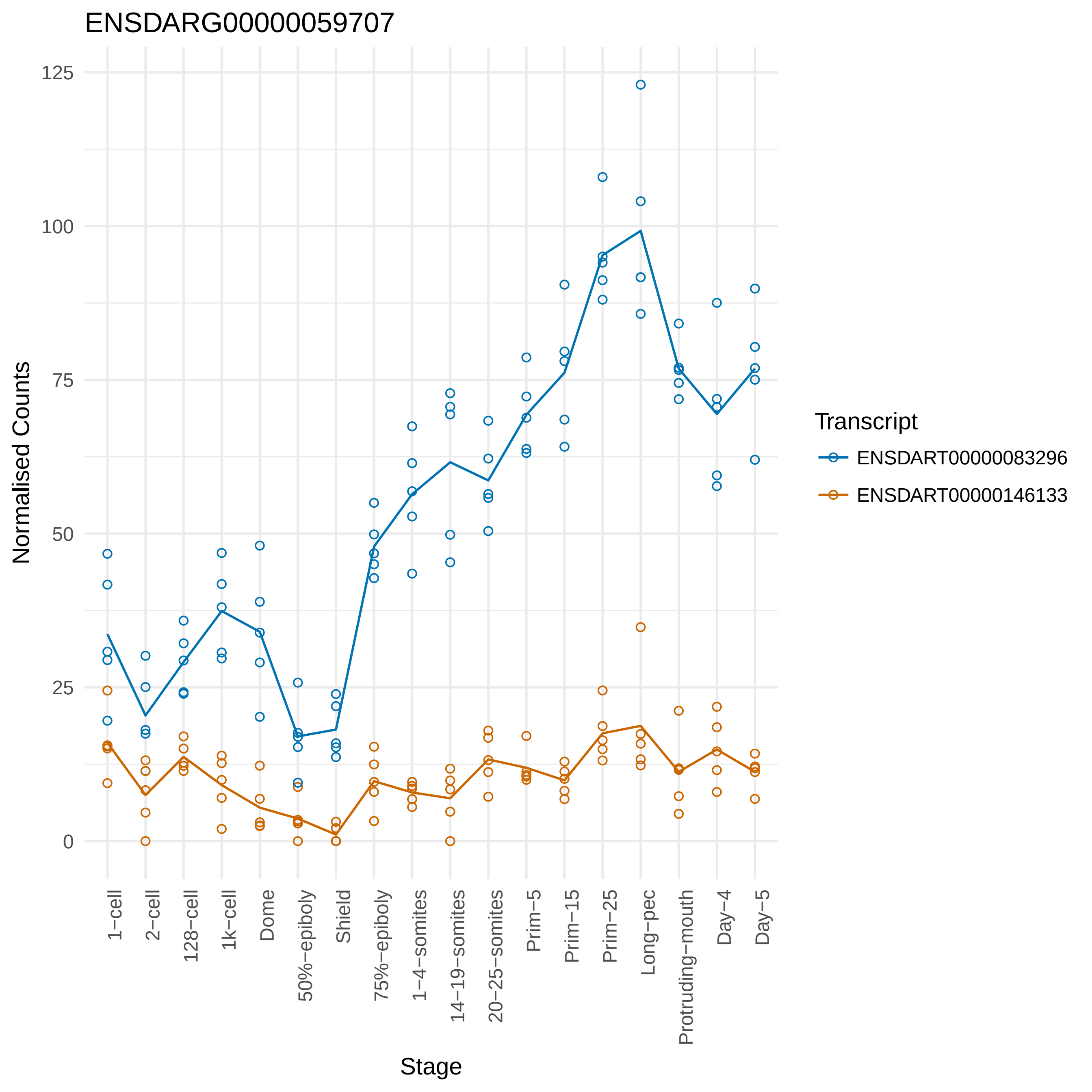

ENSDARG00000059707
Normalised Counts
125
100
75
50
25
0
1−cell
2−cell
128−cell
1k−cell
Dome
50%−epiboly
Shield
75%−epiboly
1−4−somites
14−19−somites
20−25−somites
Prim−5
Prim−15
Prim−25
Long−pec
Protruding−mouth
Day−4
Day−5
Stage
Transcript
ENSDART00000083296
ENSDART00000146133

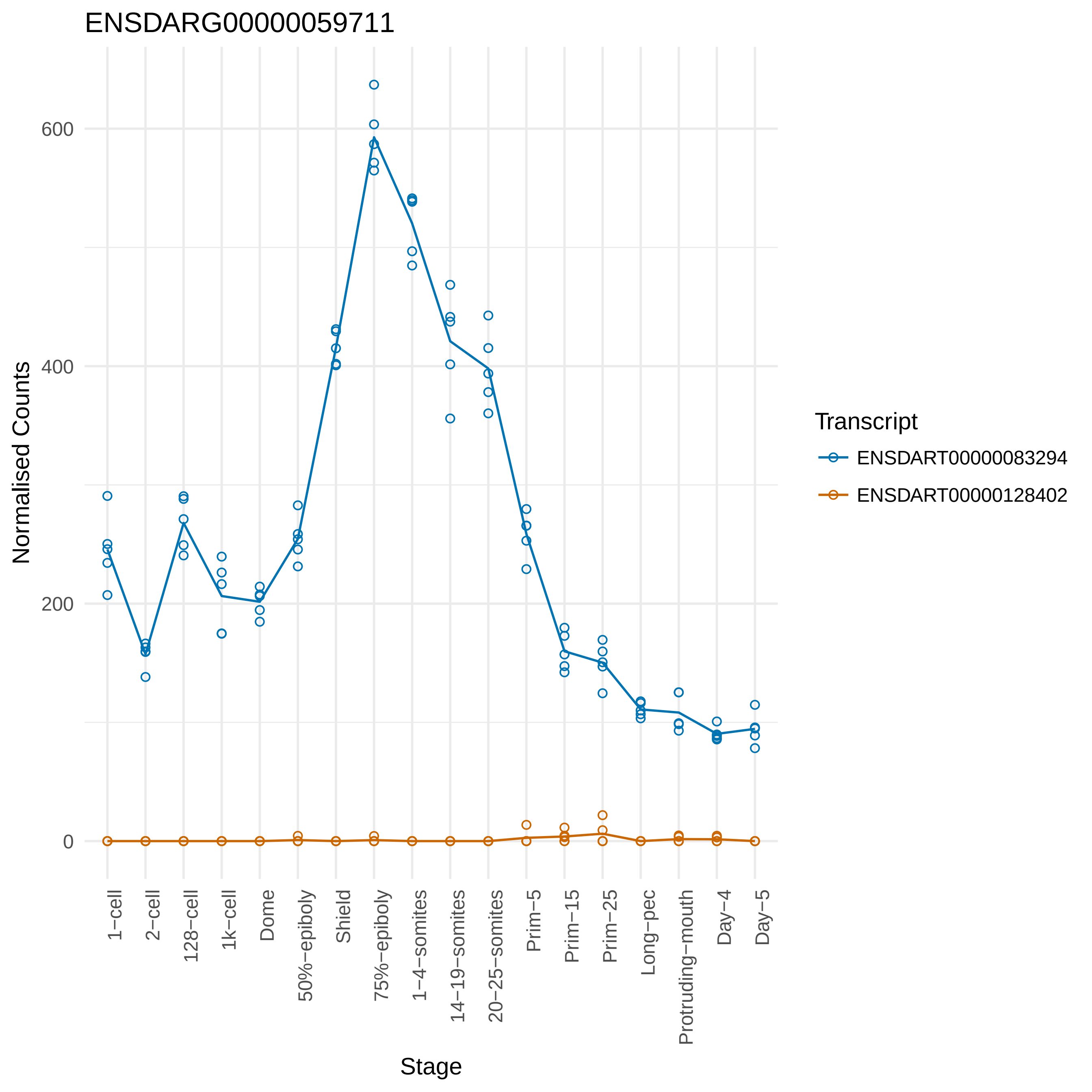

ENSDARG00000059711
Normalised Counts
600
400
200
0
Stage
1−cell
2−cell
128−cell
1k−cell
Dome
50%−epiboly
Shield
75%−epiboly
1−4−somites
14−19−somites
20−25−somites
Prim−5
Prim−15
Prim−25
Long−pec
Protruding−mouth
Day−4
Day−5
Transcript
ENSDART00000083294
ENSDART00000128402

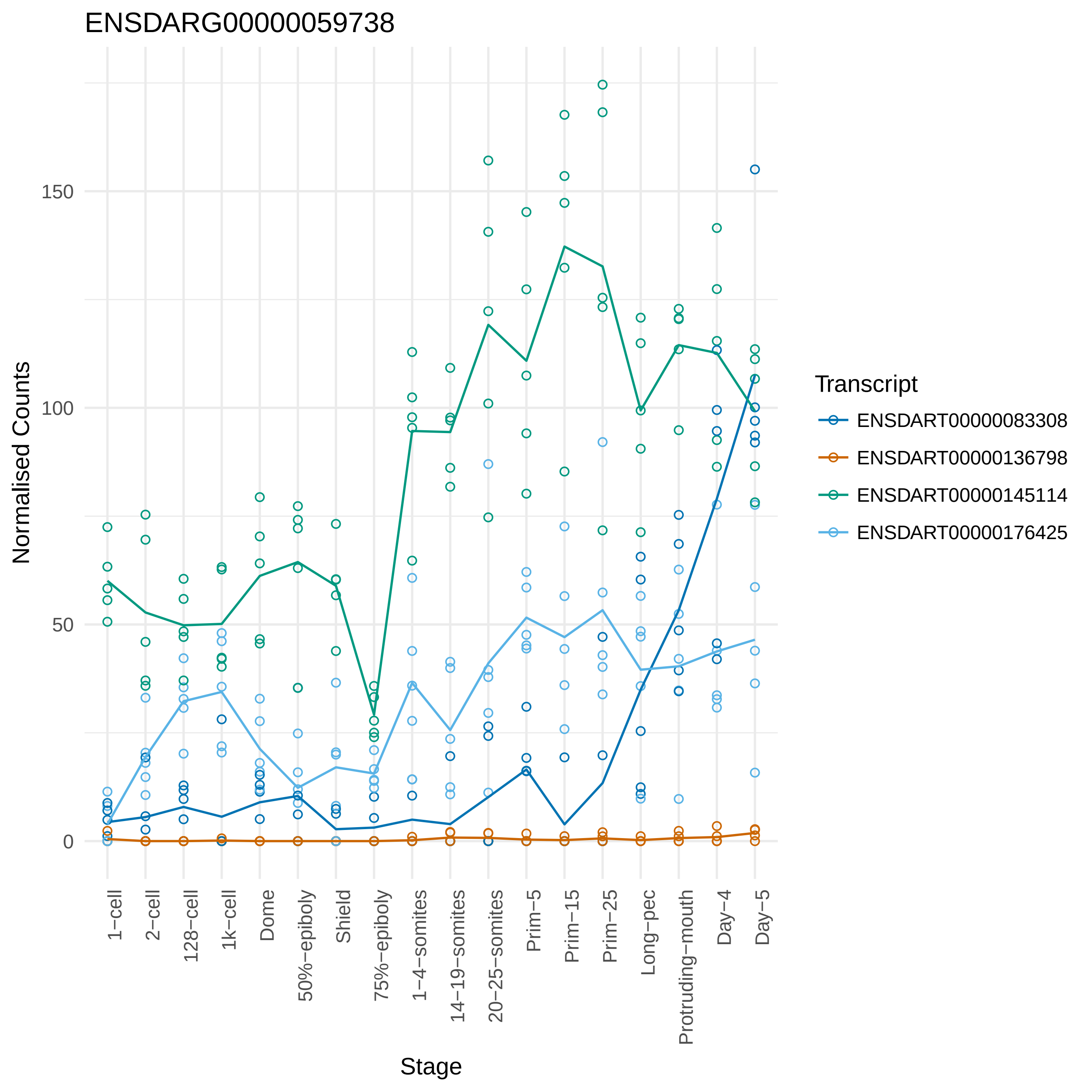
ENSDARG00000059738
Normalised Counts
150
100
50
0
1−cell
2−cell
128−cell
1k−cell
Dome
50%−epiboly
Shield
75%−epiboly
1−4−somites
14−19−somites
20−25−somites
Prim−5
Prim−15
Prim−25
Long−pec
Protruding−mouth
Day−4
Day−5
Stage
Transcript
ENSDART00000083308
ENSDART00000136798
ENSDART00000145114
ENSDART00000176425

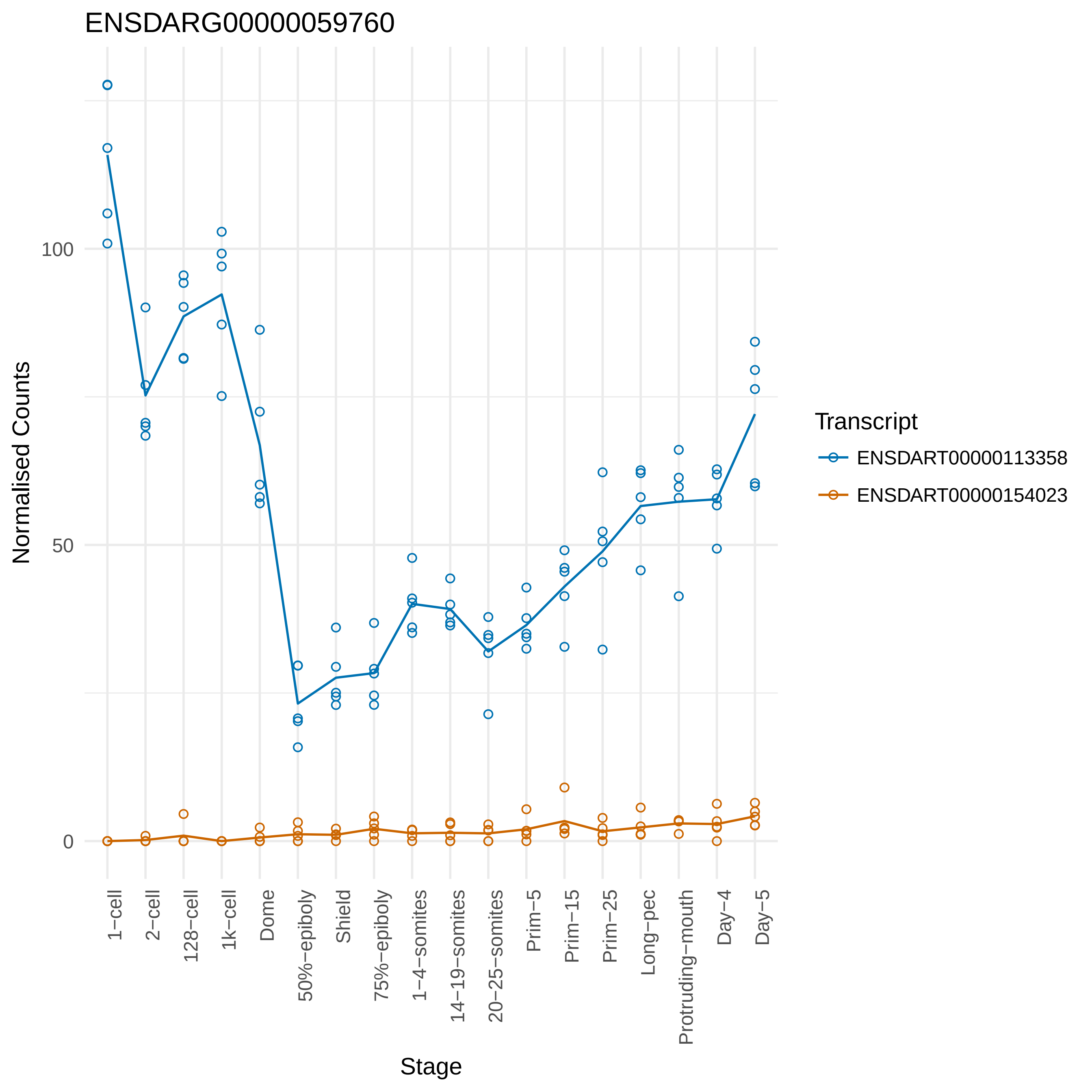

ENSDARG00000059760
Normalised Counts
100
50
0
1−cell
2−cell
128−cell
1k−cell
Dome
50%−epiboly
Shield
75%−epiboly
1−4−somites
14−19−somites
20−25−somites
Prim−5
Prim−15
Prim−25
Long−pec
Protruding−mouth
Day−4
Day−5
Stage
Transcript
ENSDART00000113358
ENSDART00000154023

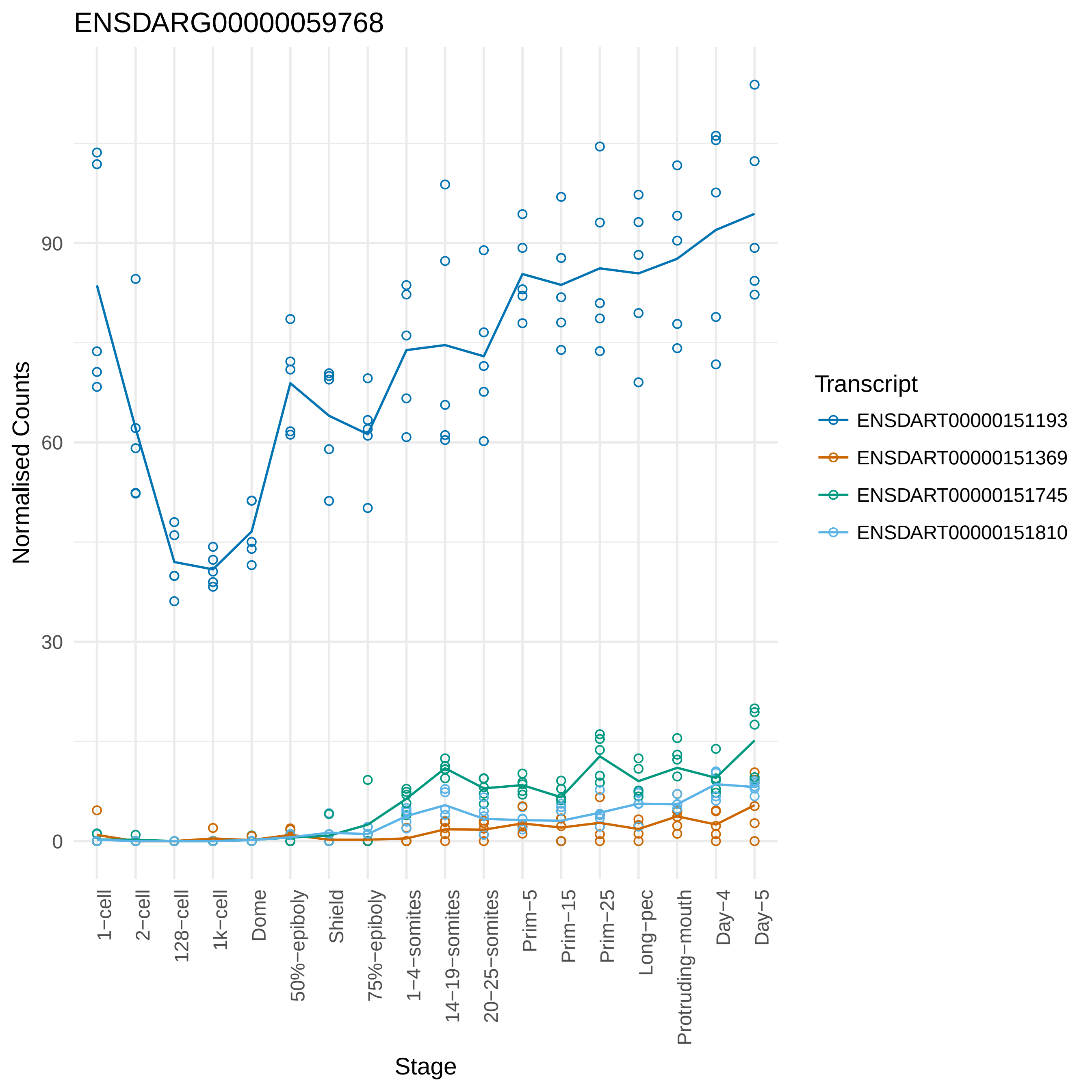

ENSDARG00000059768
Normalised Counts
0
30
60
90
Stage
1−cell
2−cell
128−cell
1k−cell
Dome
50%−epiboly
Shield
75%−epiboly
1−4−somites
14−19−somites
20−25−somites
Prim−5
Prim−15
Prim−25
Long−pec
Protruding−mouth
Day−4
Day−5
Transcript
ENSDART00000151193
ENSDART00000151369
ENSDART00000151745
ENSDART00000151810

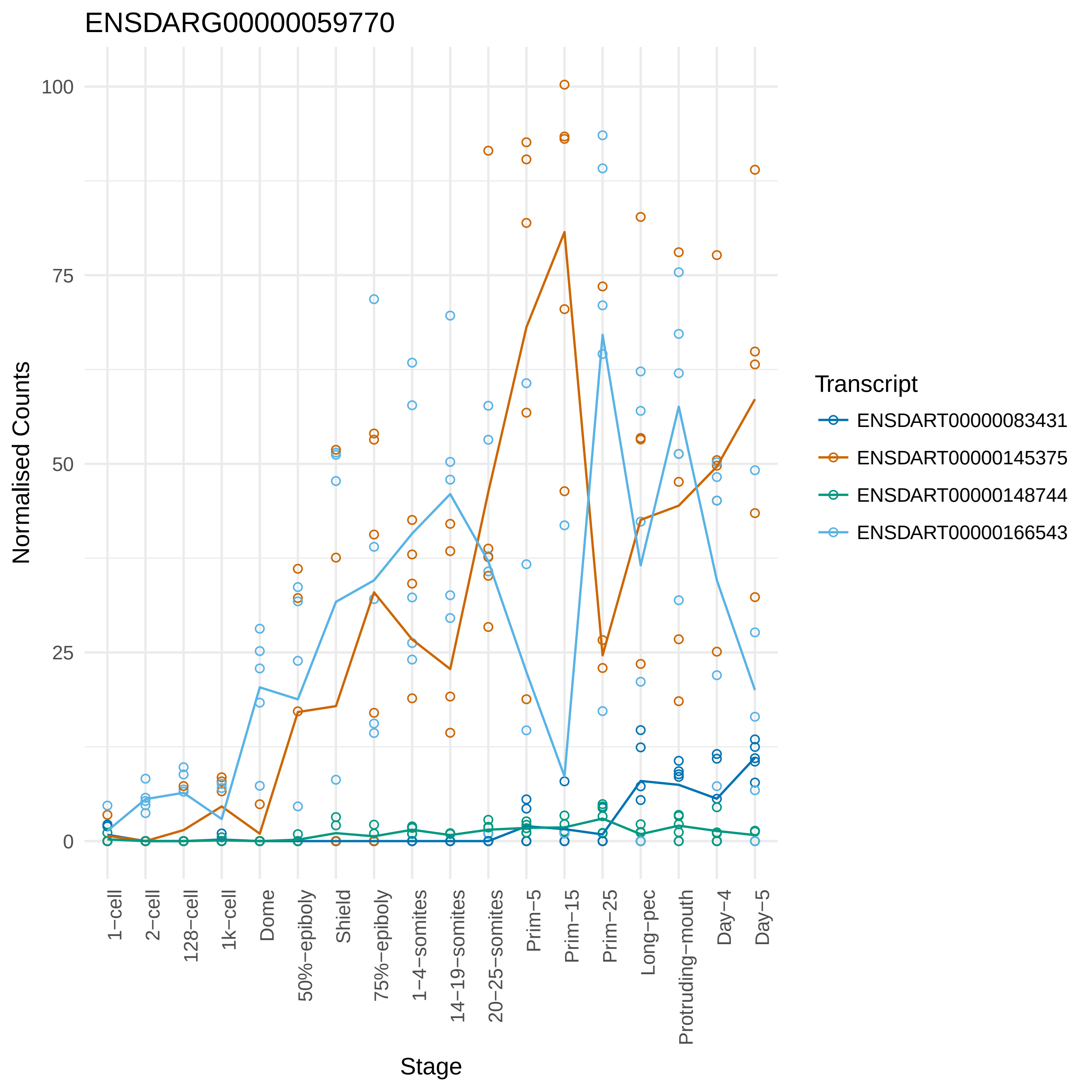
ENSDARG00000059770
Normalised Counts
0
25
50
75
100
1−cell
2−cell
128−cell
1k−cell
Dome
50%−epiboly
Shield
75%−epiboly
1−4−somites
14−19−somites
20−25−somites
Prim−5
Prim−15
Prim−25
Long−pec
Protruding−mouth
Day−4
Day−5
Stage
Transcript
ENSDART00000083431
ENSDART00000145375
ENSDART00000148744
ENSDART00000166543

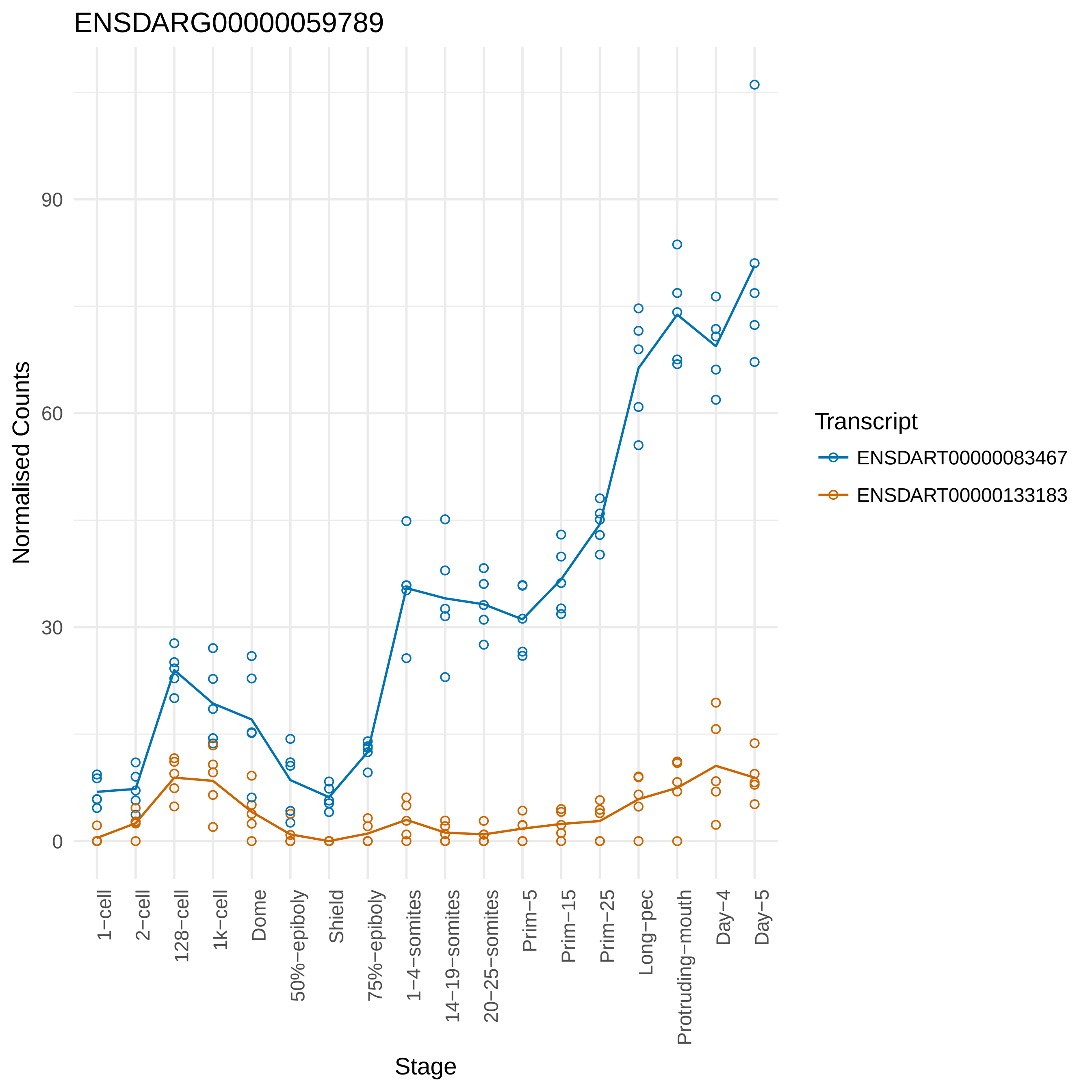
ENSDARG00000059789
Normalised Counts
90
60
30
0
1−cell
2−cell
128−cell
1k−cell
Dome
50%−epiboly
Shield
75%−epiboly
1−4−somites
14−19−somites
20−25−somites
Prim−5
Prim−15
Prim−25
Long−pec
Protruding−mouth
Day−4
Day−5
Stage
Transcript
ENSDART00000083467
ENSDART00000133183

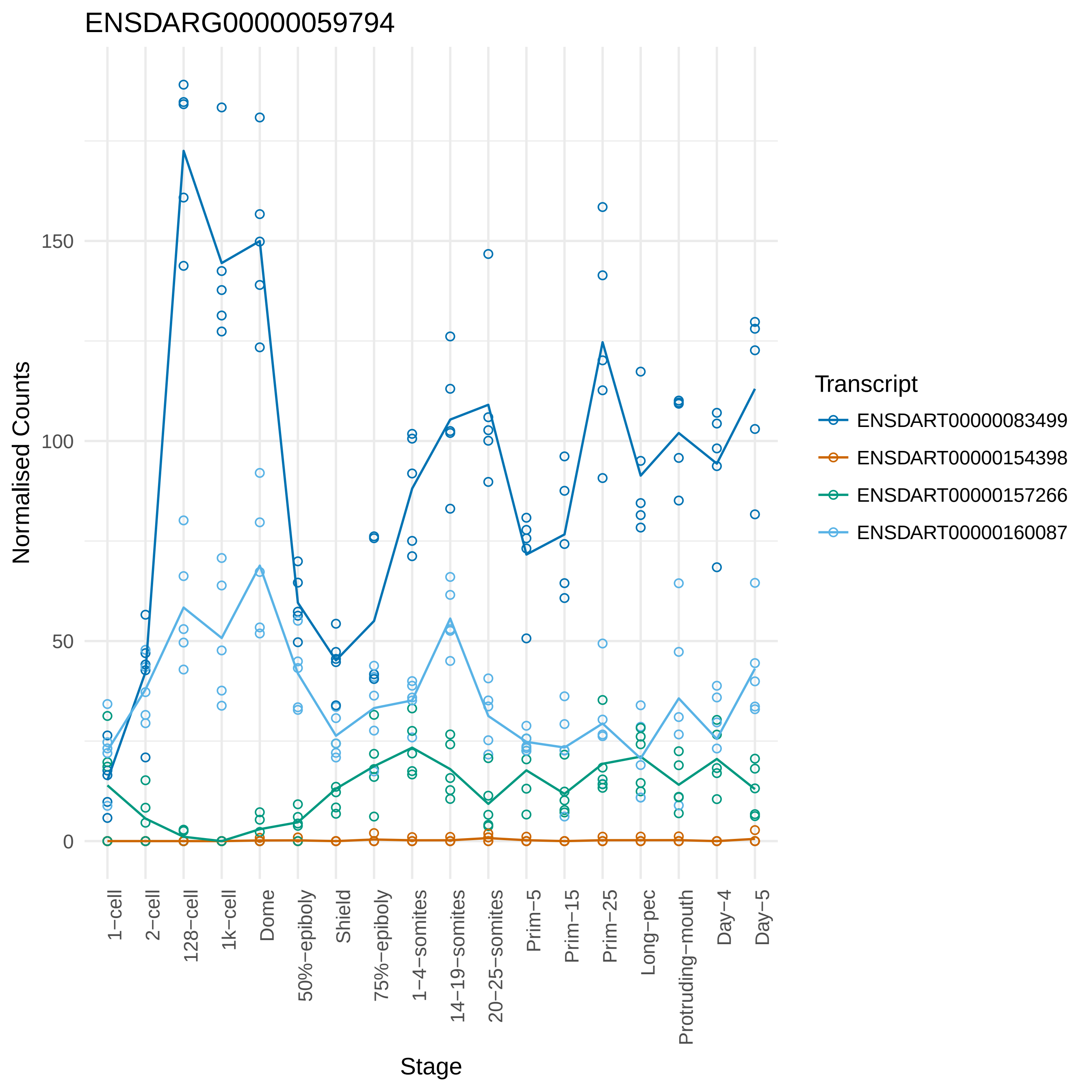

ENSDARG00000059794
Normalised Counts
150
100
50
0
1−cell
2−cell
128−cell
1k−cell
Dome
50%−epiboly
Shield
75%−epiboly
1−4−somites
14−19−somites
20−25−somites
Prim−5
Prim−15
Prim−25
Long−pec
Protruding−mouth
Day−4
Day−5
Stage
Transcript
ENSDART00000083499
ENSDART00000154398
ENSDART00000157266
ENSDART00000160087

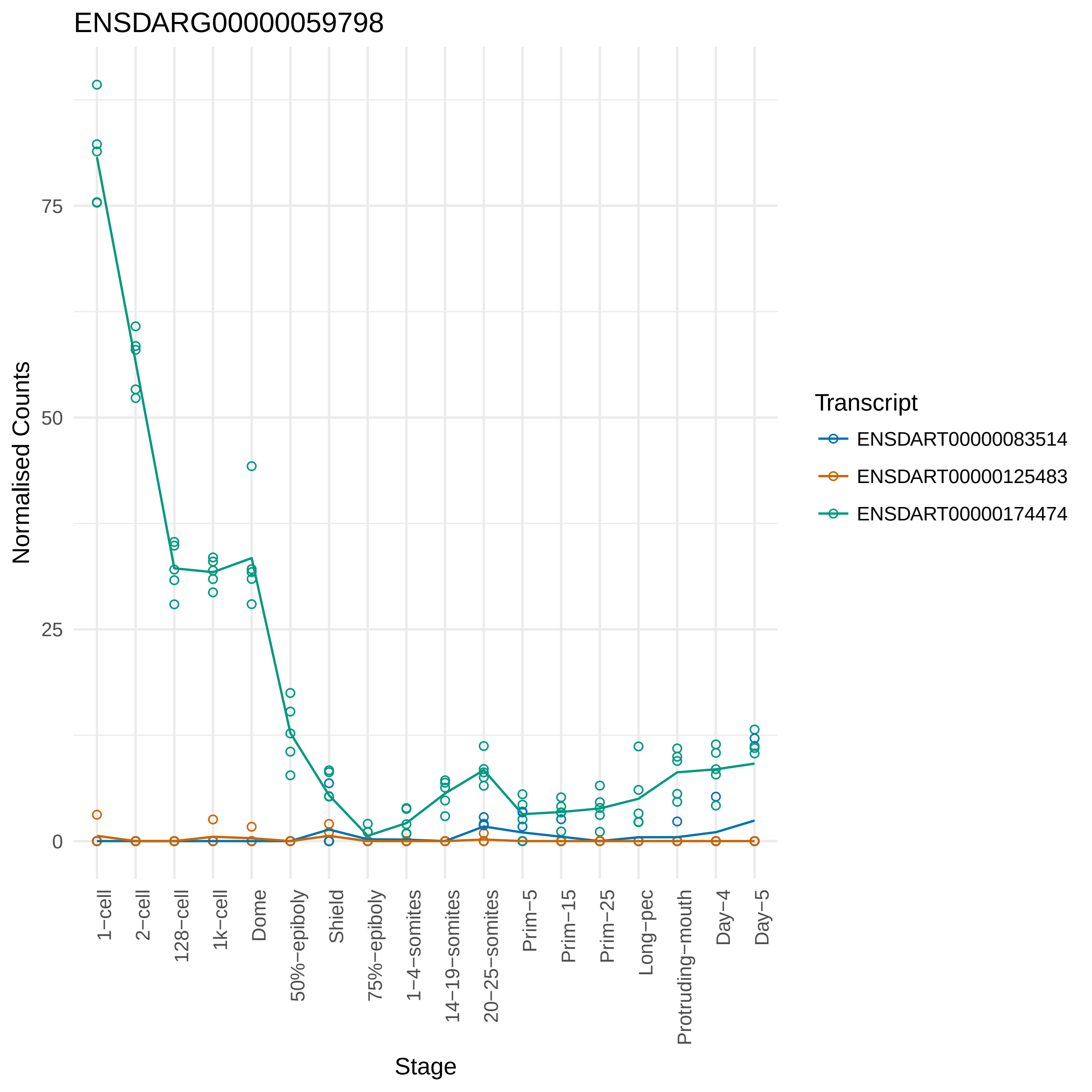

ENSDARG00000059798
Normalised Counts
75
50
25
0
1−cell
2−cell
128−cell
1k−cell
Dome
50%−epiboly
Shield
75%−epiboly
1−4−somites
14−19−somites
20−25−somites
Prim−5
Prim−15
Prim−25
Long−pec
Protruding−mouth
Day−4
Day−5
Stage
Transcript
ENSDART00000083514
ENSDART00000125483
ENSDART00000174474

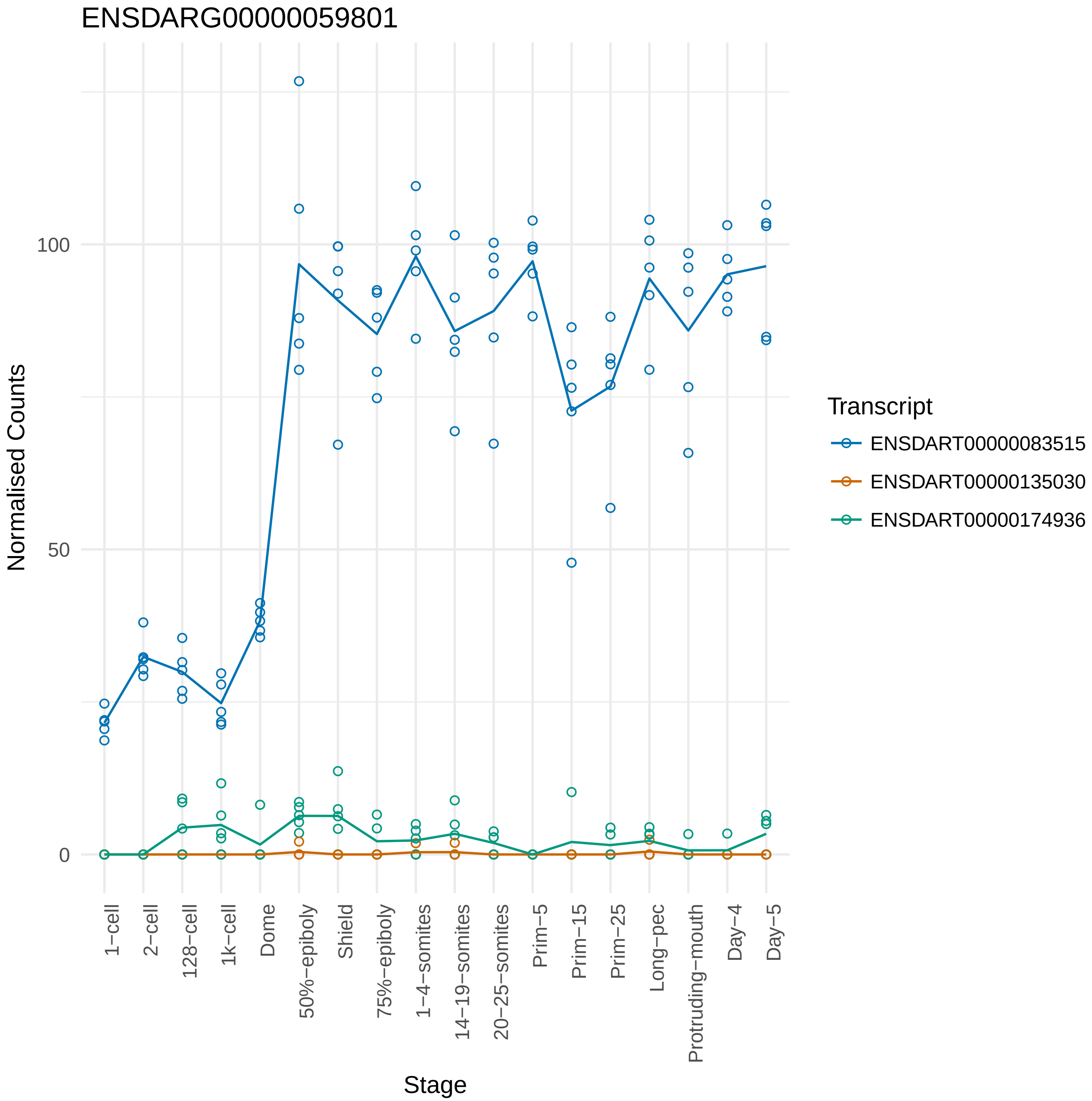
ENSDARG00000059801
Normalised Counts
100
50
0
1−cell
2−cell
128−cell
1k−cell
Dome
50%−epiboly
Shield
75%−epiboly
1−4−somites
14−19−somites
20−25−somites
Prim−5
Prim−15
Prim−25
Long−pec
Protruding−mouth
Day−4
Day−5
Stage
Transcript
ENSDART00000083515
ENSDART00000135030
ENSDART00000174936

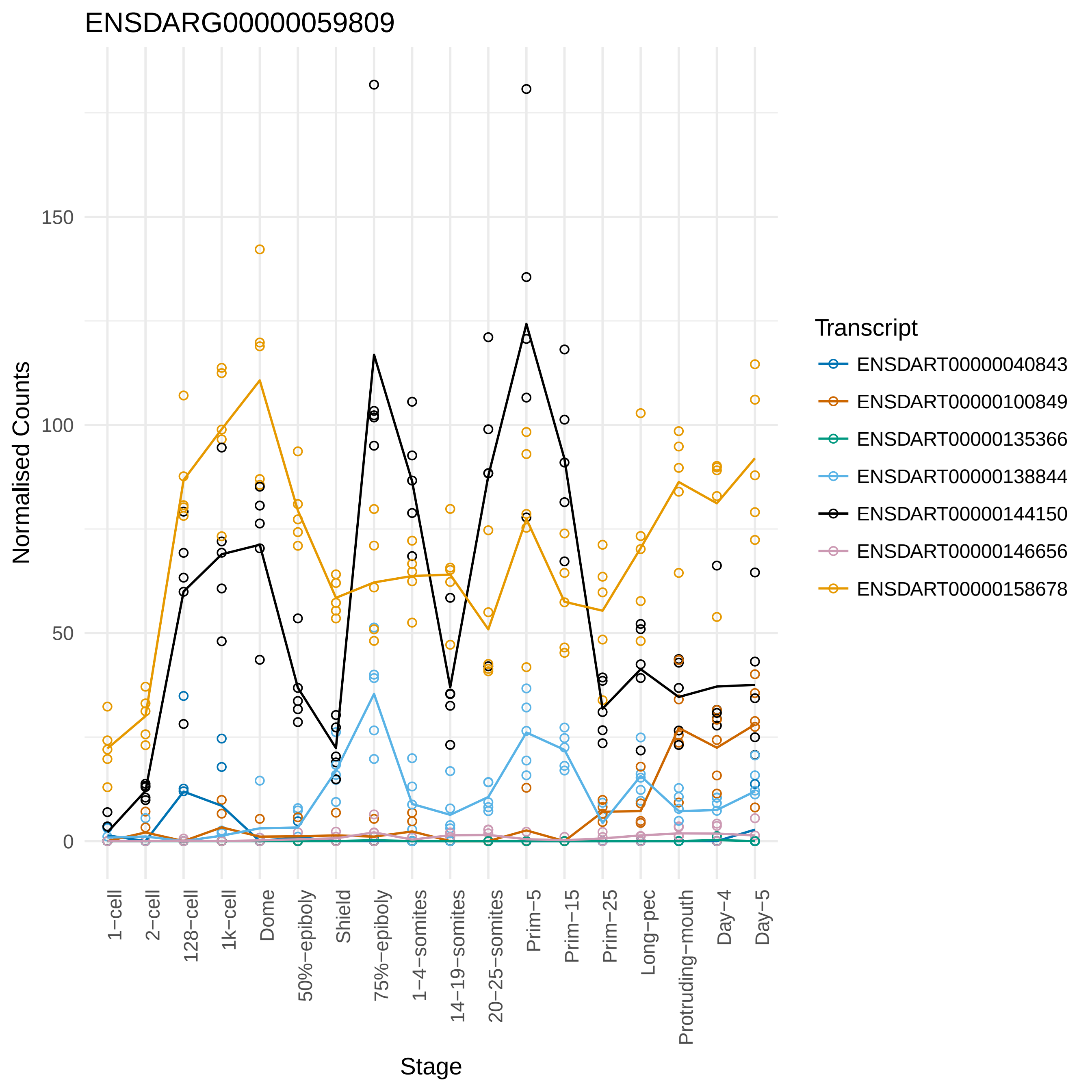
ENSDARG00000059809
Normalised Counts
0
50
100
150
Stage
1−cell
2−cell
128−cell
1k−cell
Dome
50%−epiboly
Shield
75%−epiboly
1−4−somites
14−19−somites
20−25−somites
Prim−5
Prim−15
Prim−25
Long−pec
Protruding−mouth
Day−4
Day−5
Transcript
ENSDART00000040843
ENSDART00000100849
ENSDART00000135366
ENSDART00000138844
ENSDART00000144150
ENSDART00000146656
ENSDART00000158678

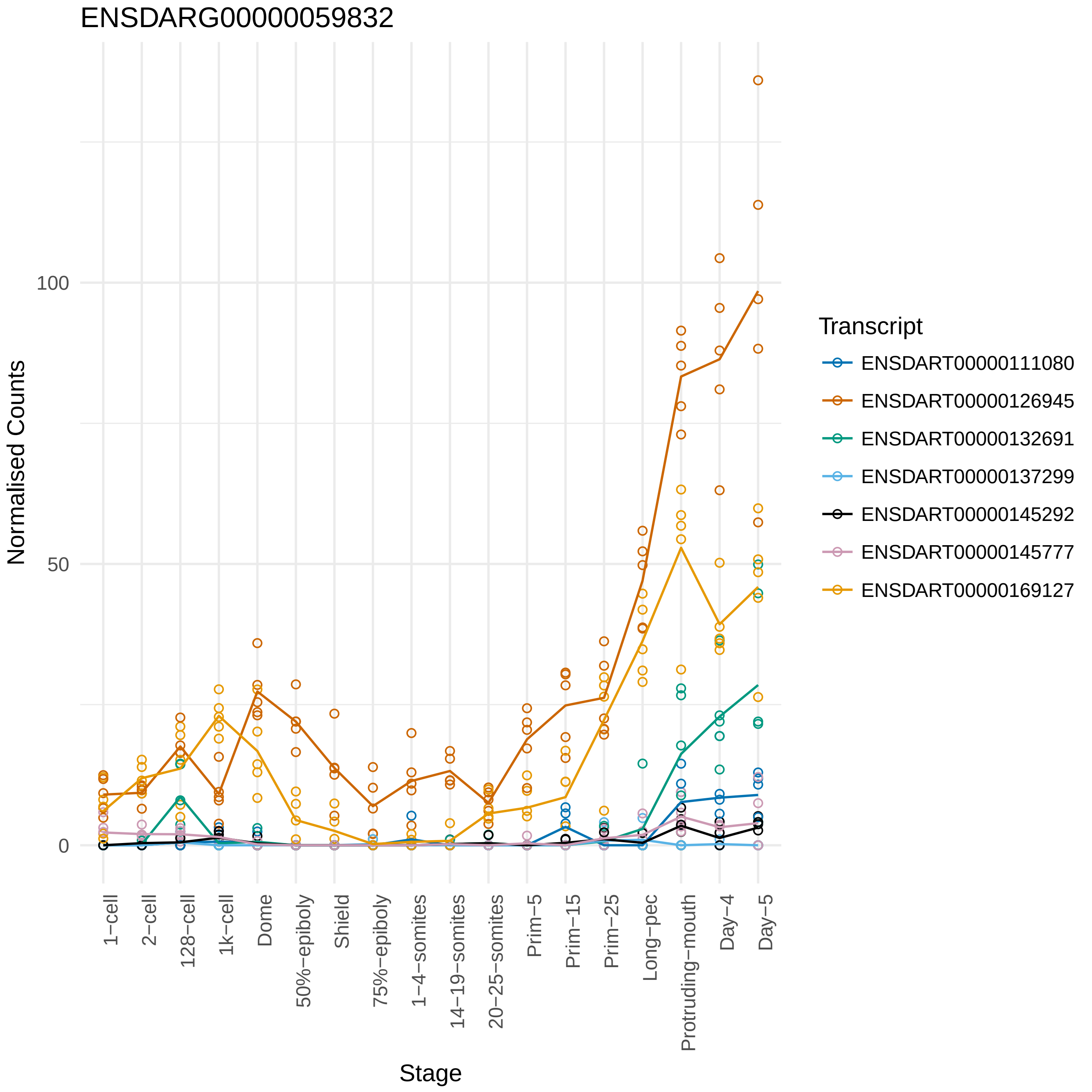

ENSDARG00000059832
Normalised Counts
100
50
0
1−cell
2−cell
128−cell
1k−cell
Dome
50%−epiboly
Shield
75%−epiboly
1−4−somites
14−19−somites
20−25−somites
Prim−5
Prim−15
Prim−25
Long−pec
Protruding−mouth
Day−4
Day−5
Stage
Transcript
ENSDART00000111080
ENSDART00000126945
ENSDART00000132691
ENSDART00000137299
ENSDART00000145292
ENSDART00000145777
ENSDART00000169127

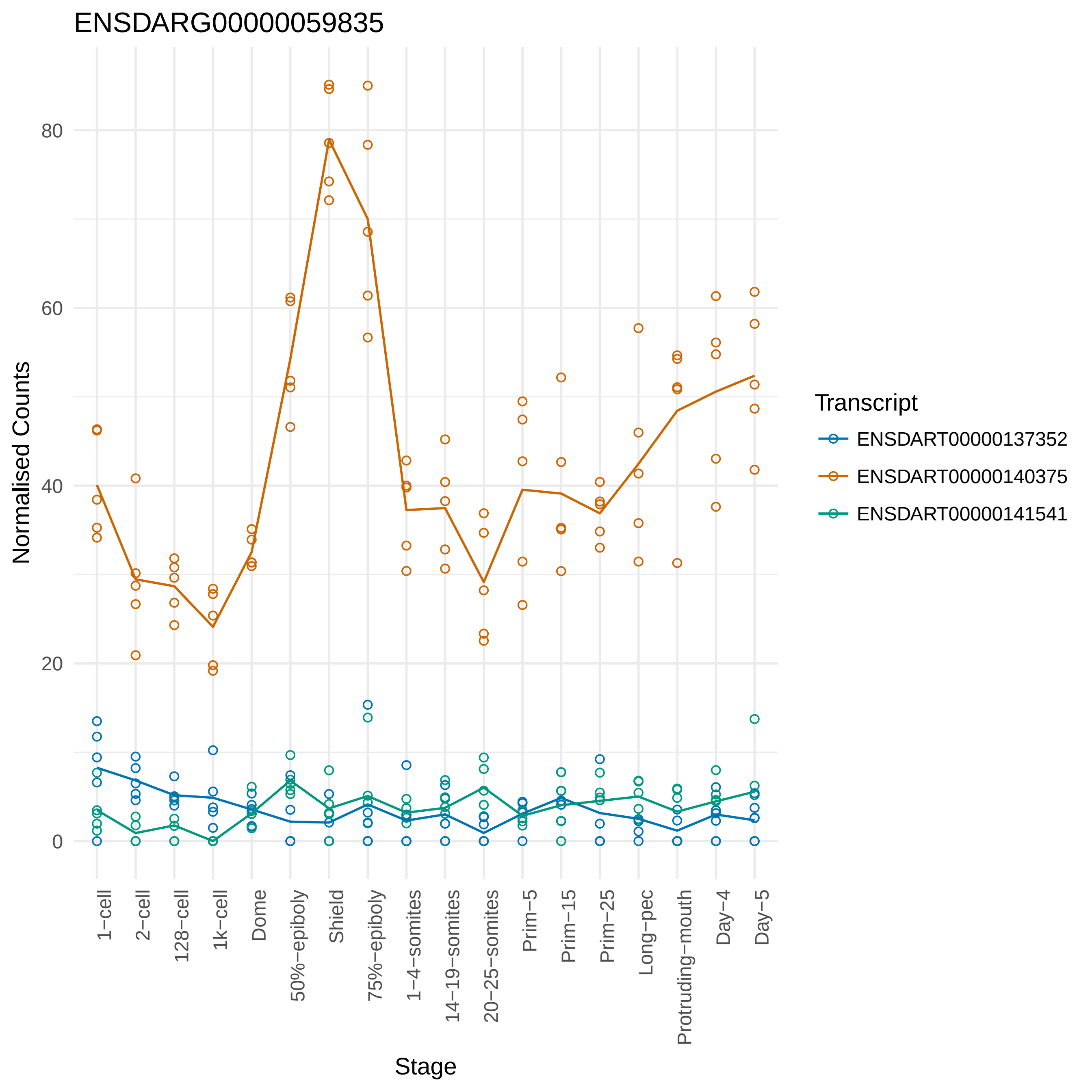

ENSDARG00000059835
Normalised Counts
0
20
40
60
80
1−cell
2−cell
128−cell
1k−cell
Dome
50%−epiboly
Shield
75%−epiboly
1−4−somites
14−19−somites
20−25−somites
Prim−5
Prim−15
Prim−25
Long−pec
Protruding−mouth
Day−4
Day−5
Stage
Transcript
ENSDART00000137352
ENSDART00000140375
ENSDART00000141541

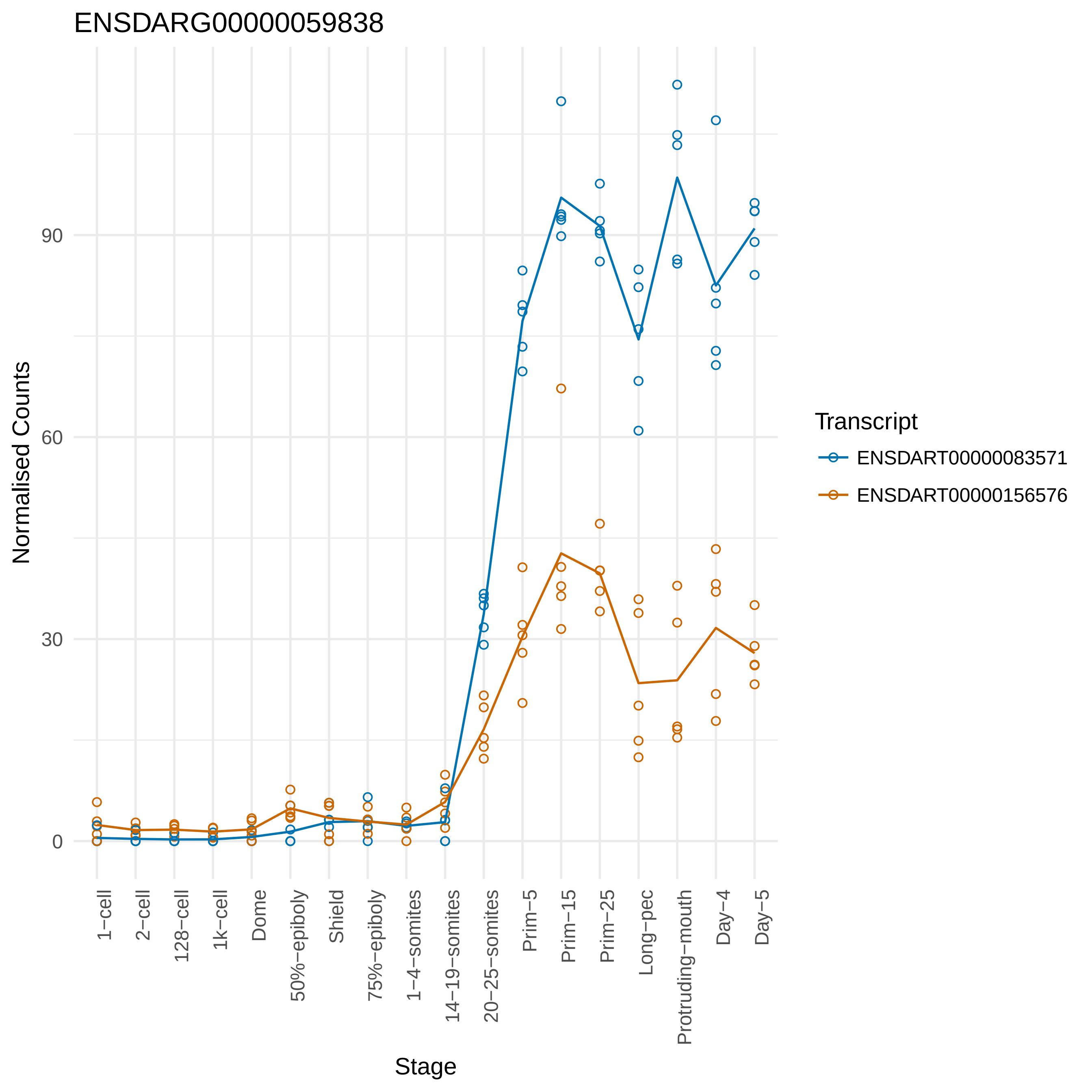

ENSDARG00000059838
Normalised Counts
90
60
30
0
1−cell
2−cell
128−cell
1k−cell
Dome
50%−epiboly
Shield
75%−epiboly
1−4−somites
14−19−somites
20−25−somites
Prim−5
Prim−15
Prim−25
Long−pec
Protruding−mouth
Day−4
Day−5
Stage
Transcript
ENSDART00000083571
ENSDART00000156576

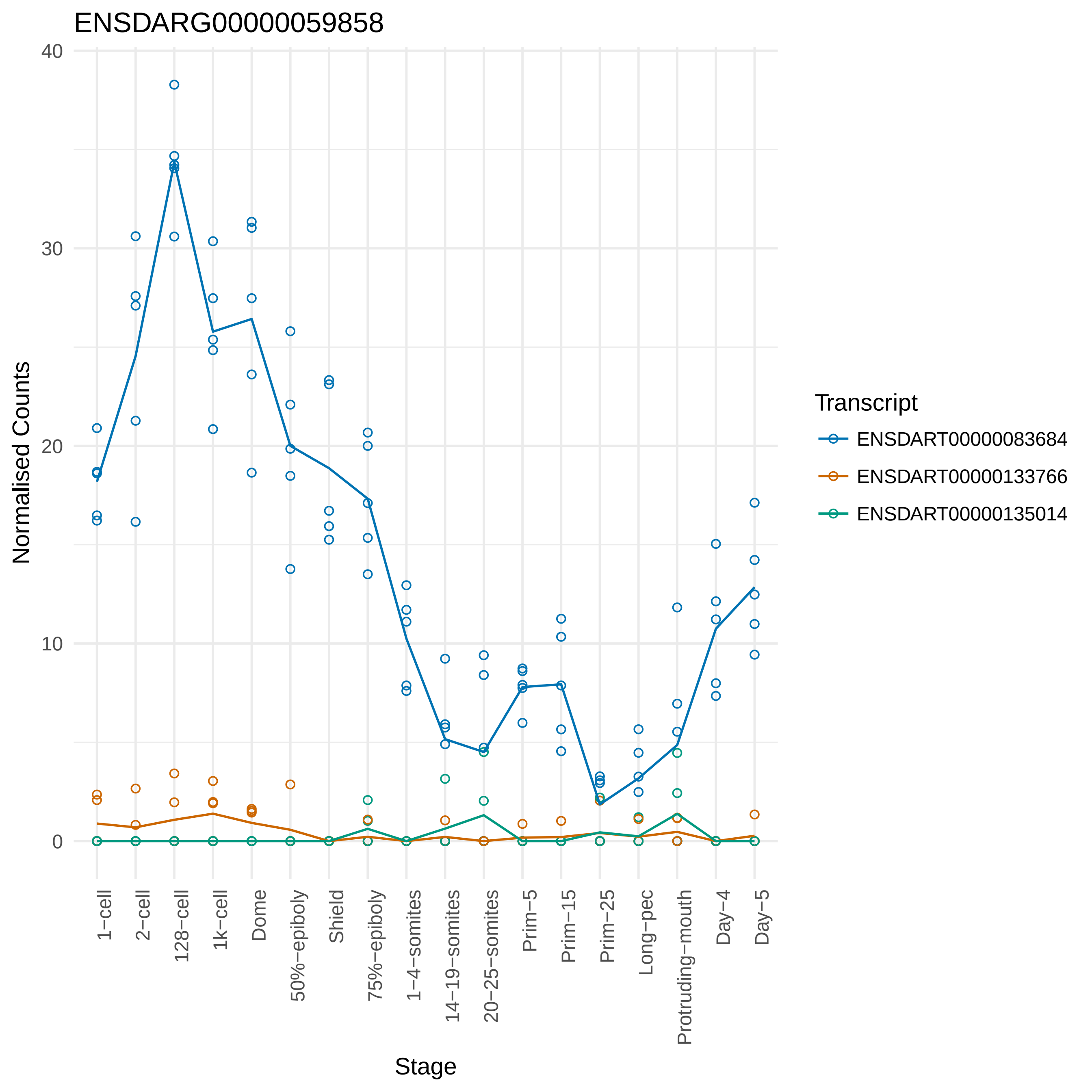
ENSDARG00000059858
Normalised Counts
40
30
20
10
0
Stage
1−cell
2−cell
128−cell
1k−cell
Dome
50%−epiboly
Shield
75%−epiboly
1−4−somites
14−19−somites
20−25−somites
Prim−5
Prim−15
Prim−25
Long−pec
Protruding−mouth
Day−4
Day−5
Transcript
ENSDART00000083684
ENSDART00000133766
ENSDART00000135014

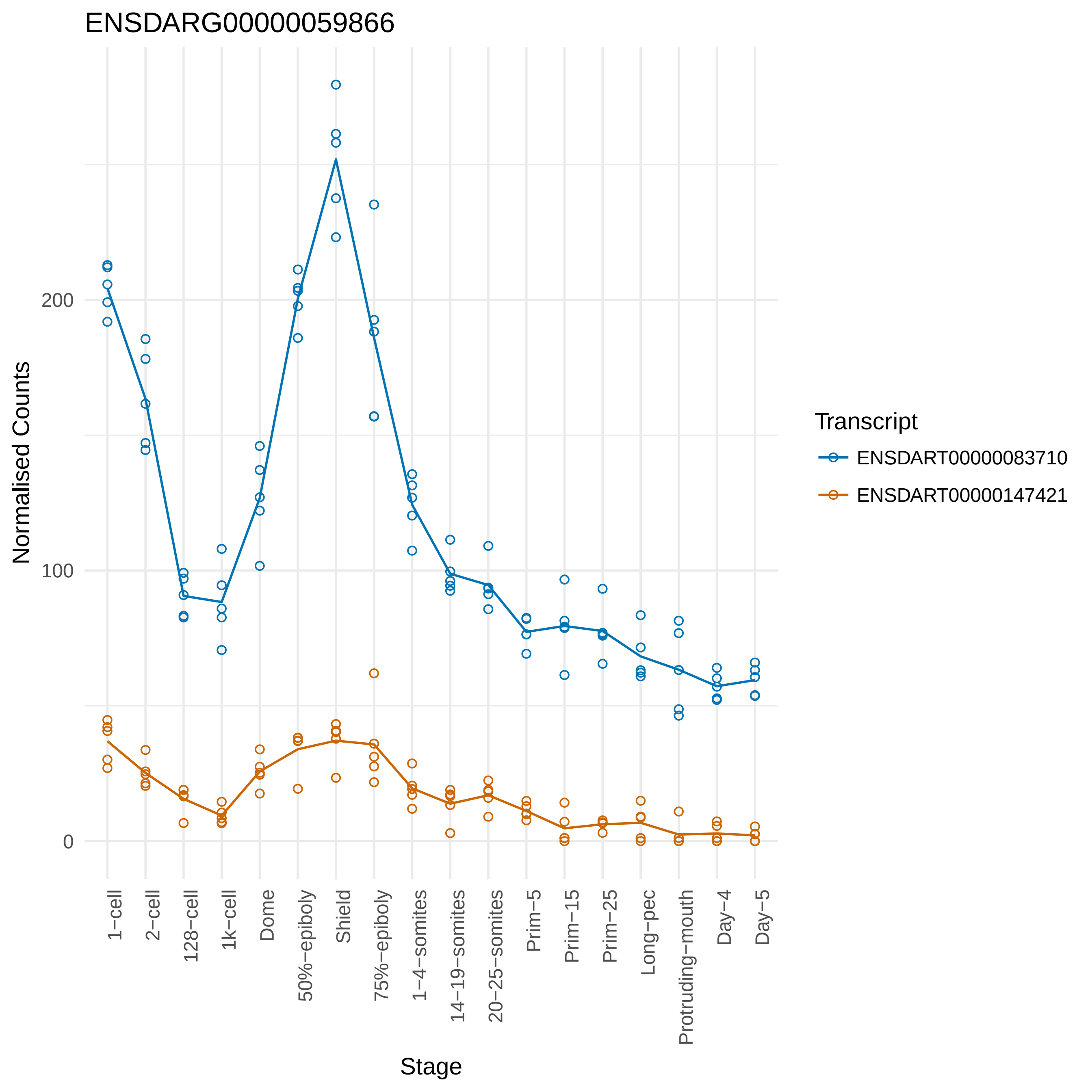

ENSDARG00000059866
Normalised Counts
200
100
0
1−cell
2−cell
128−cell
1k−cell
Dome
50%−epiboly
Shield
75%−epiboly
1−4−somites
14−19−somites
20−25−somites
Prim−5
Prim−15
Prim−25
Long−pec
Protruding−mouth
Day−4
Day−5
Stage
Transcript
ENSDART00000083710
ENSDART00000147421

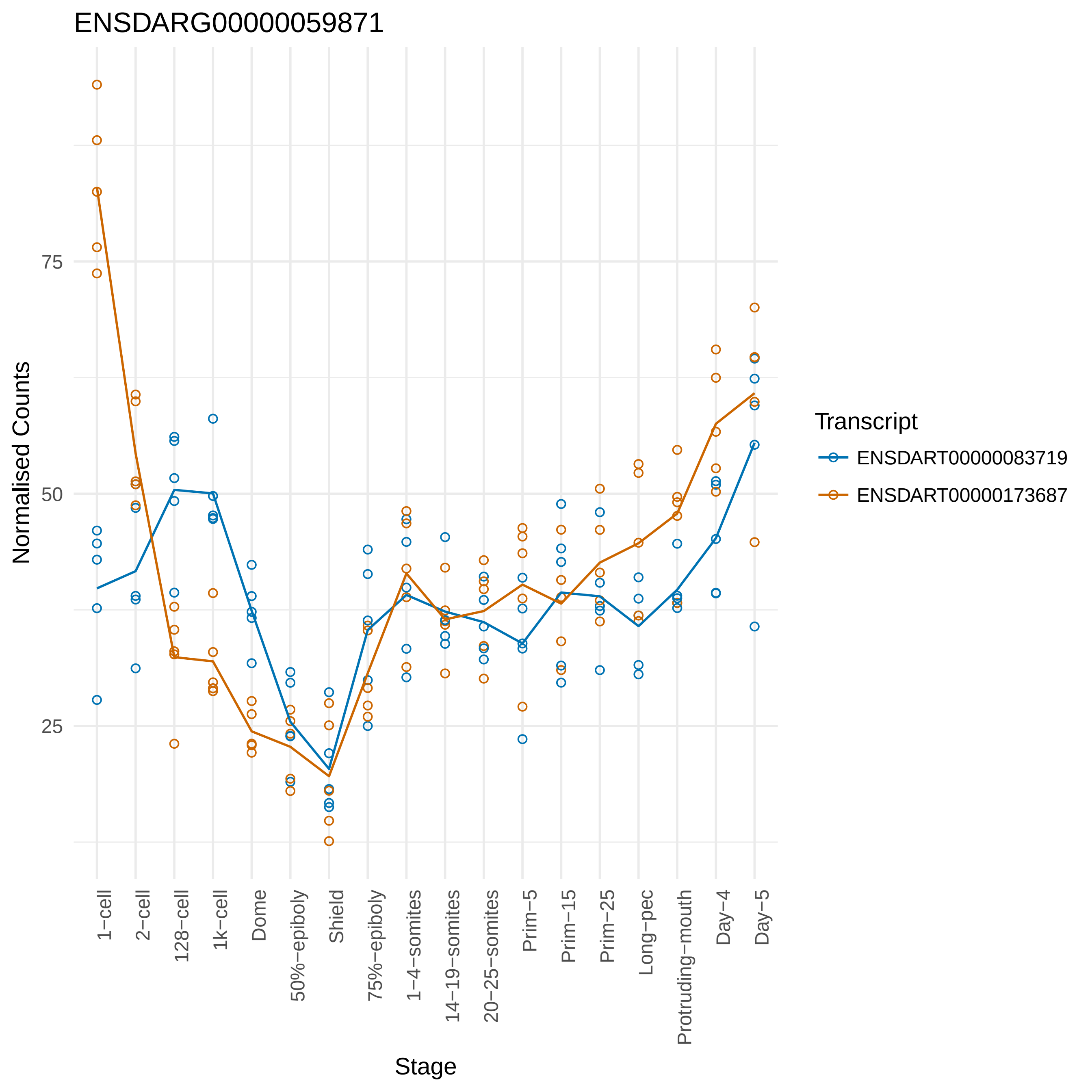
ENSDARG00000059871
Normalised Counts
75
50
25
Stage
1−cell
2−cell
128−cell
1k−cell
Dome
50%−epiboly
Shield
75%−epiboly
1−4−somites
14−19−somites
20−25−somites
Prim−5
Prim−15
Prim−25
Long−pec
Protruding−mouth
Day−4
Day−5
Transcript
ENSDART00000083719
ENSDART00000173687

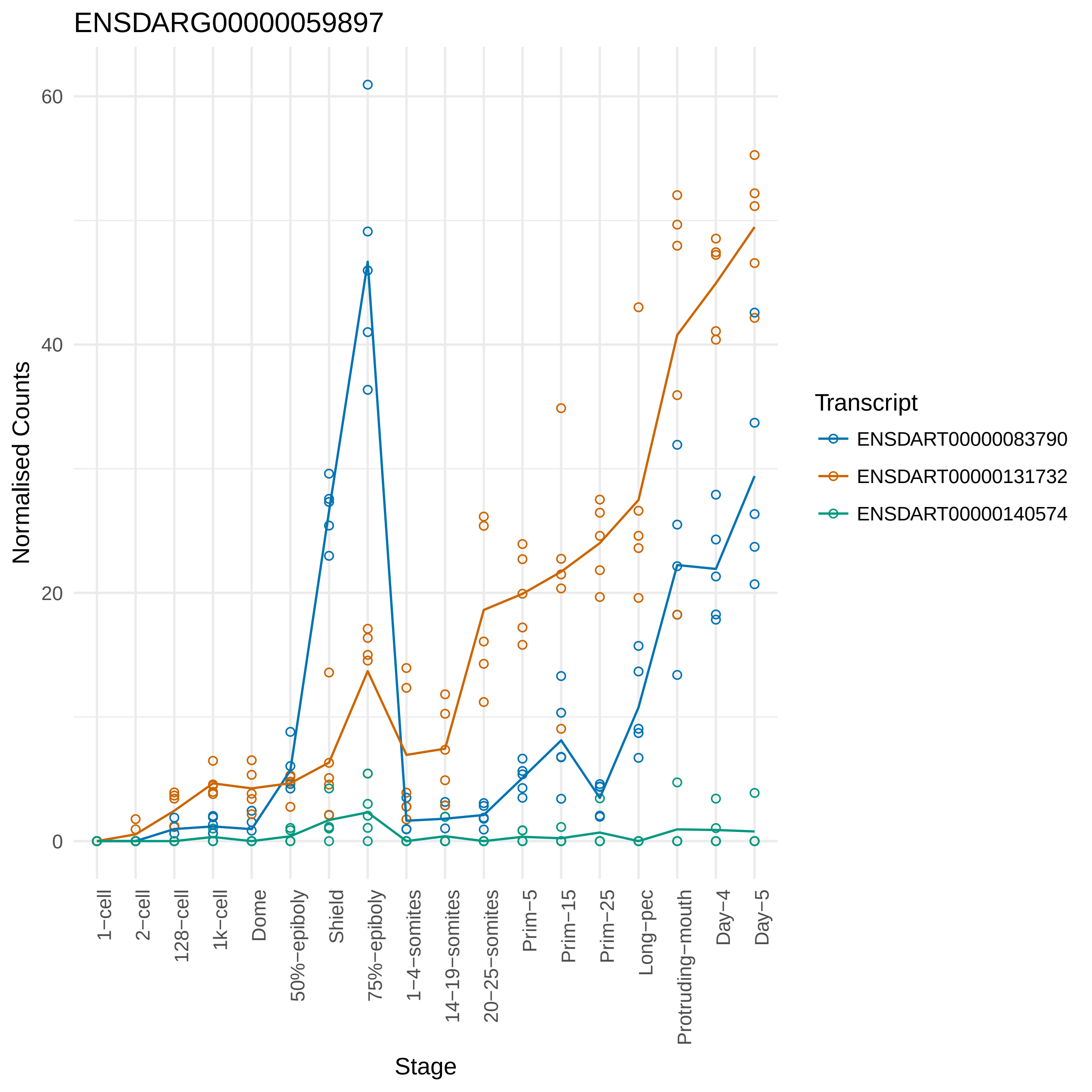
ENSDARG00000059897
Normalised Counts
0
20
40
60
Stage
1−cell
2−cell
128−cell
1k−cell
Dome
50%−epiboly
Shield
75%−epiboly
1−4−somites
14−19−somites
20−25−somites
Prim−5
Prim−15
Prim−25
Long−pec
Protruding−mouth
Day−4
Day−5
Transcript
ENSDART00000083790
ENSDART00000131732
ENSDART00000140574

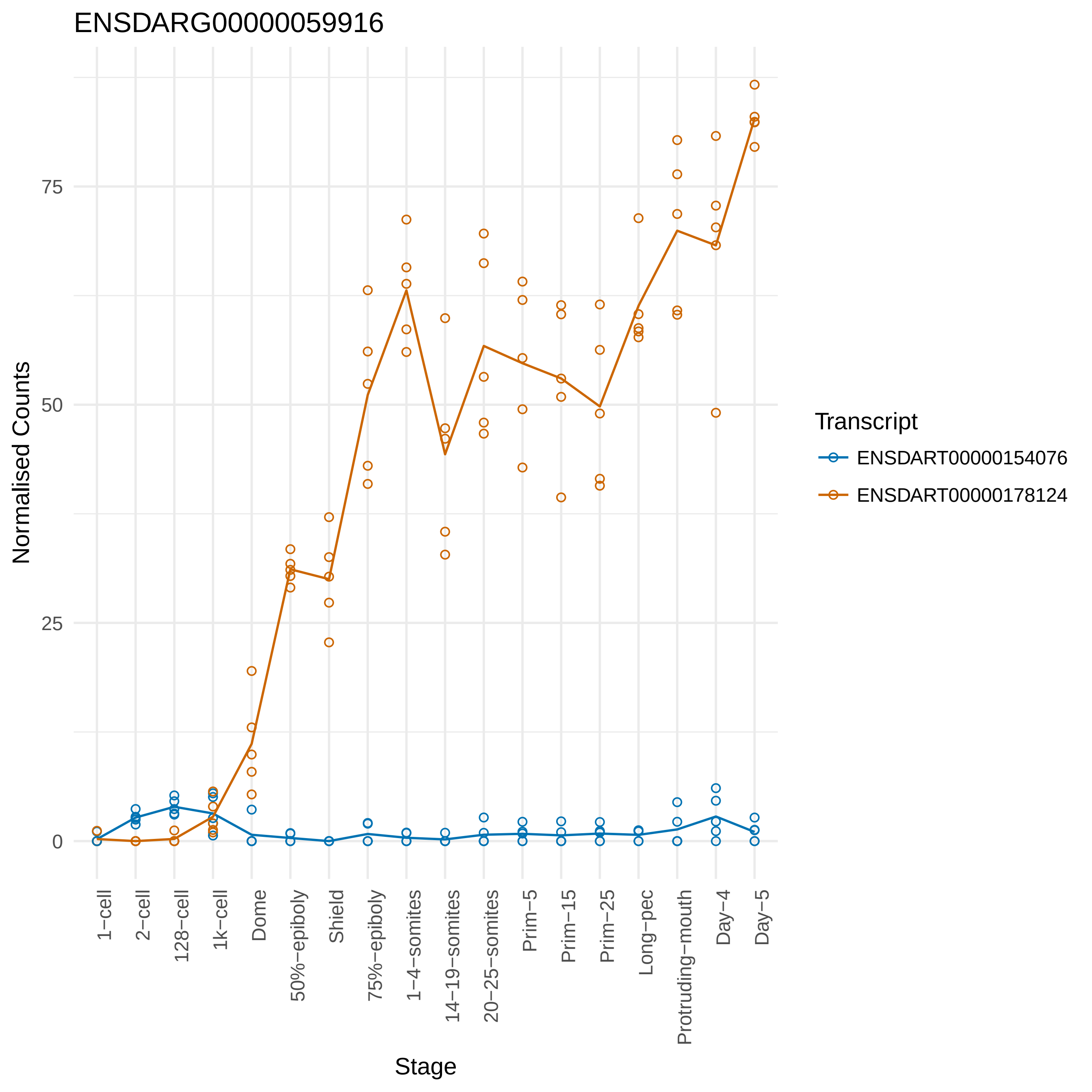
ENSDARG00000059916
Normalised Counts
0
25
50
75
Stage
1−cell
2−cell
128−cell
1k−cell
Dome
50%−epiboly
Shield
75%−epiboly
1−4−somites
14−19−somites
20−25−somites
Prim−5
Prim−15
Prim−25
Long−pec
Protruding−mouth
Day−4
Day−5
Transcript
ENSDART00000154076
ENSDART00000178124

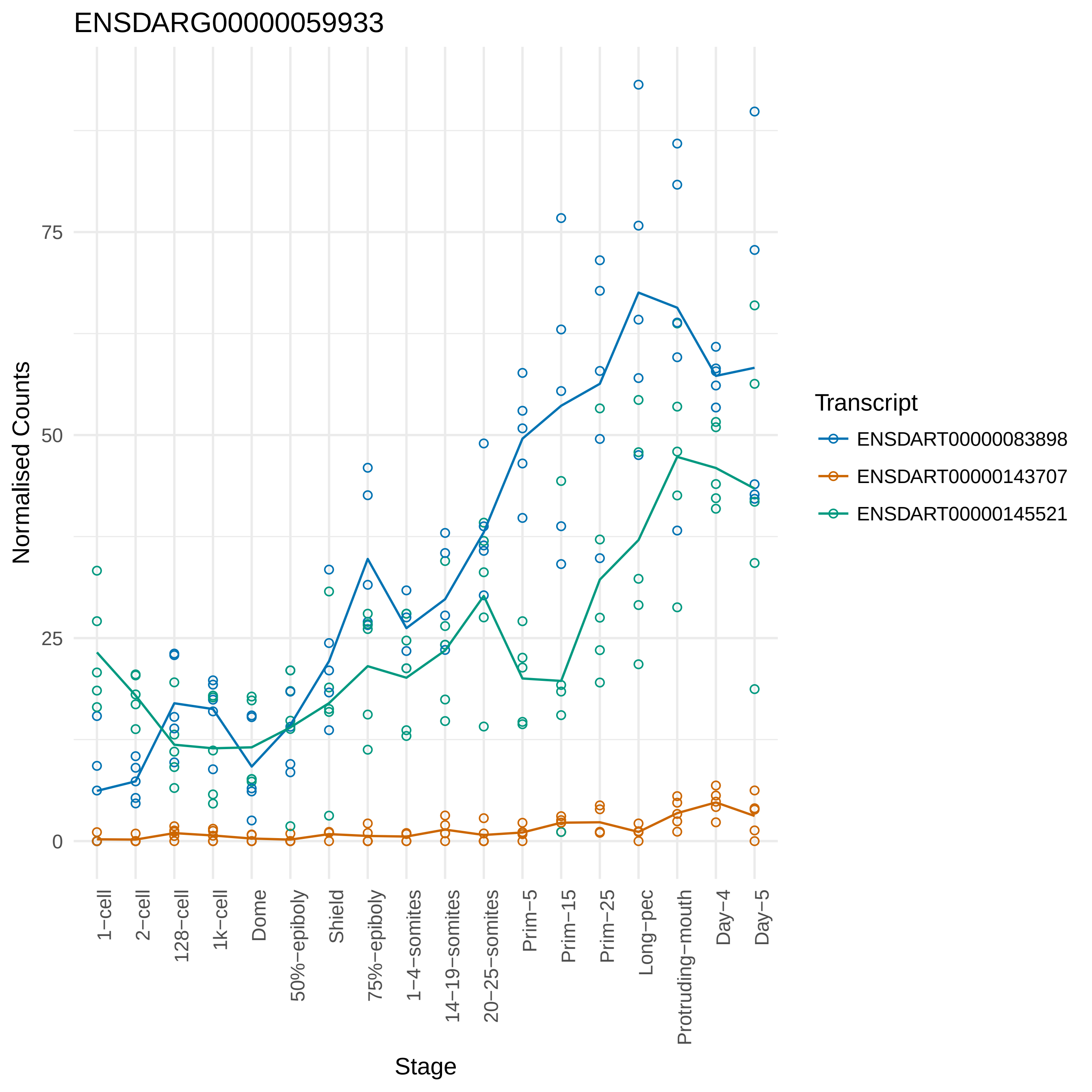

ENSDARG00000059933
Normalised Counts
0
25
50
75
Stage
1−cell
2−cell
128−cell
1k−cell
Dome
50%−epiboly
Shield
75%−epiboly
1−4−somites
14−19−somites
20−25−somites
Prim−5
Prim−15
Prim−25
Long−pec
Protruding−mouth
Day−4
Day−5
Transcript
ENSDART00000083898
ENSDART00000143707
ENSDART00000145521

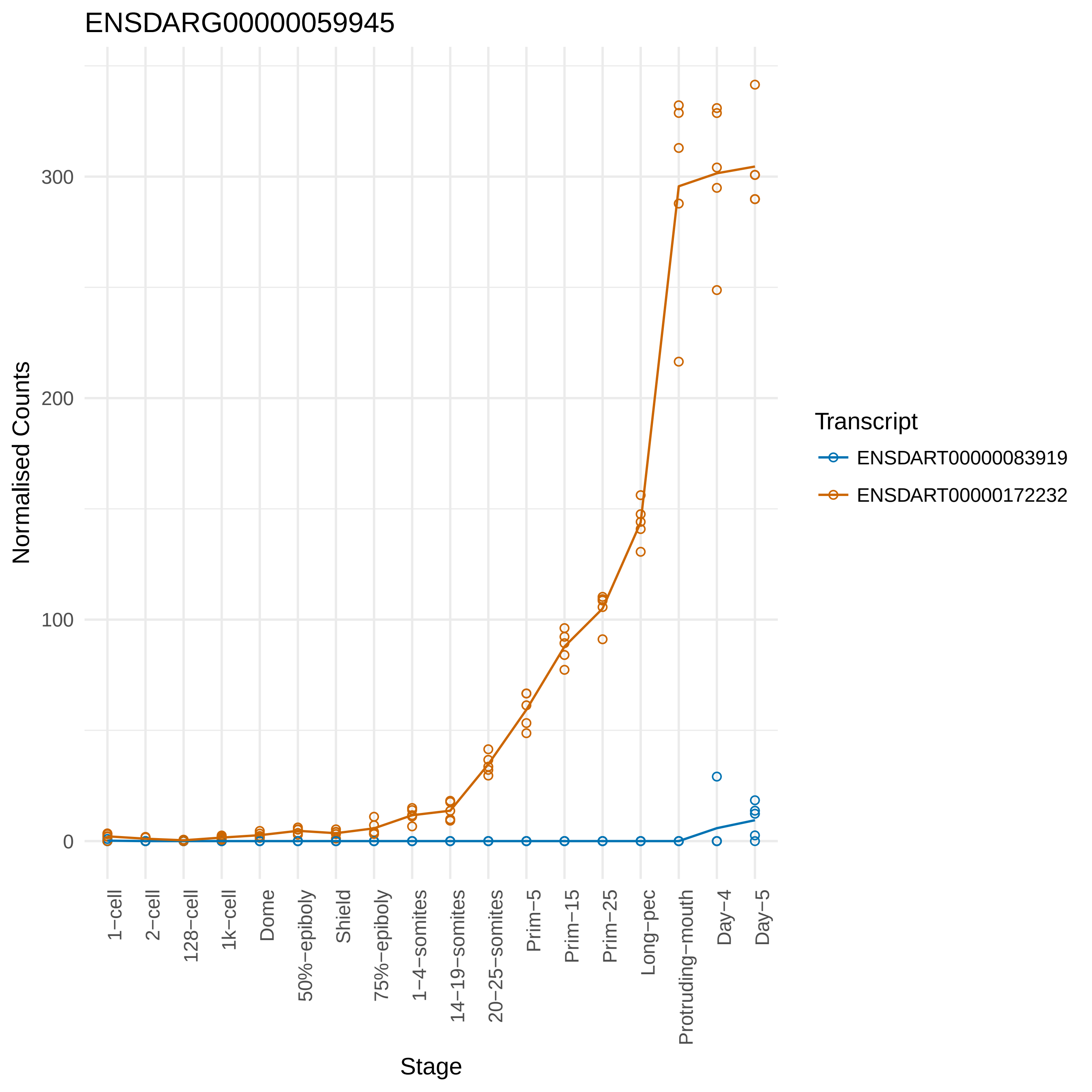

ENSDARG00000059945
Normalised Counts
300
200
100
0
1−cell
2−cell
128−cell
1k−cell
Dome
50%−epiboly
Shield
75%−epiboly
1−4−somites
14−19−somites
20−25−somites
Prim−5
Prim−15
Prim−25
Long−pec
Protruding−mouth
Day−4
Day−5
Stage
Transcript
ENSDART00000083919
ENSDART00000172232

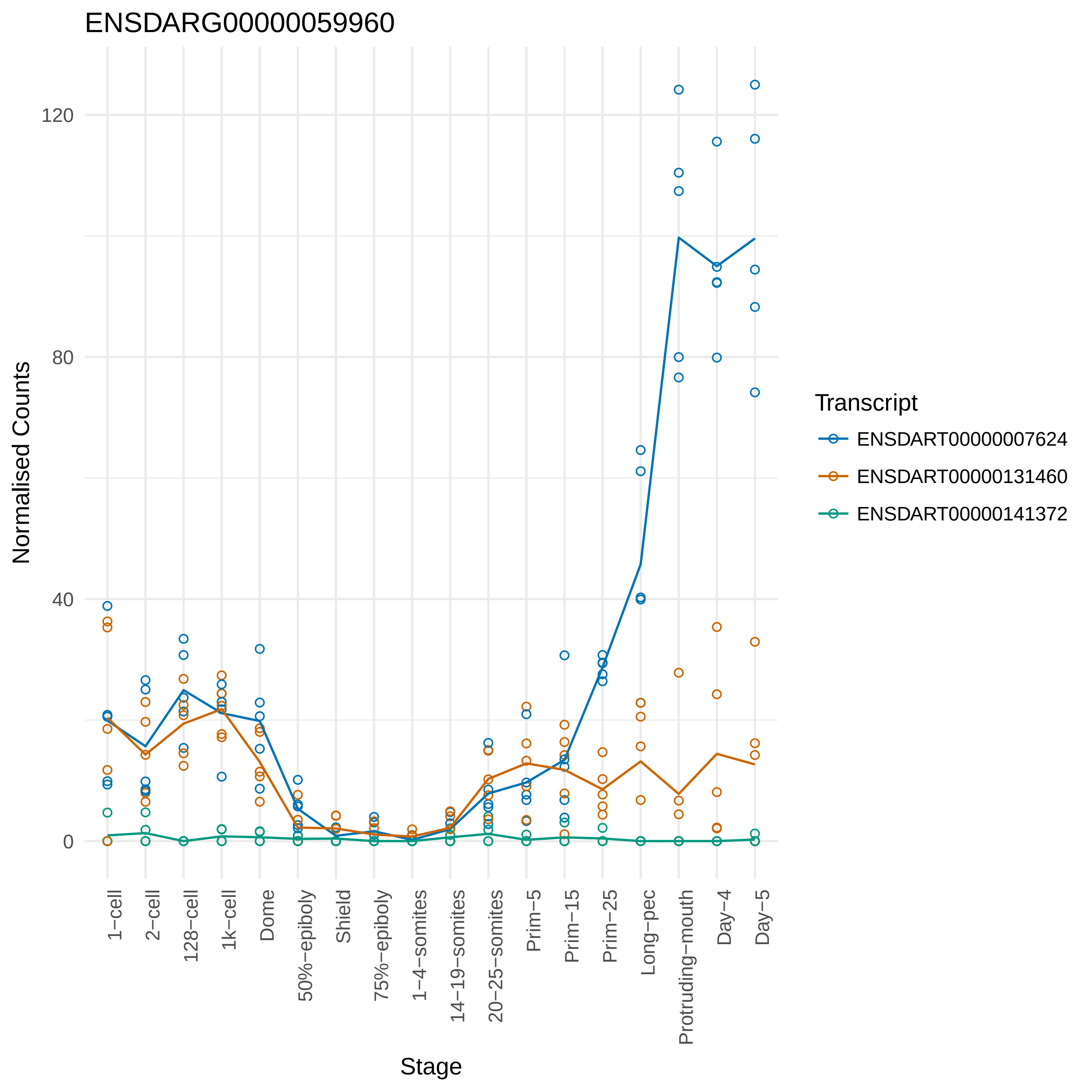

ENSDARG00000059960
Normalised Counts
120
80
40
0
1−cell
2−cell
128−cell
1k−cell
Dome
50%−epiboly
Shield
75%−epiboly
1−4−somites
14−19−somites
20−25−somites
Prim−5
Prim−15
Prim−25
Long−pec
Protruding−mouth
Day−4
Day−5
Stage
Transcript
ENSDART00000007624
ENSDART00000131460
ENSDART00000141372

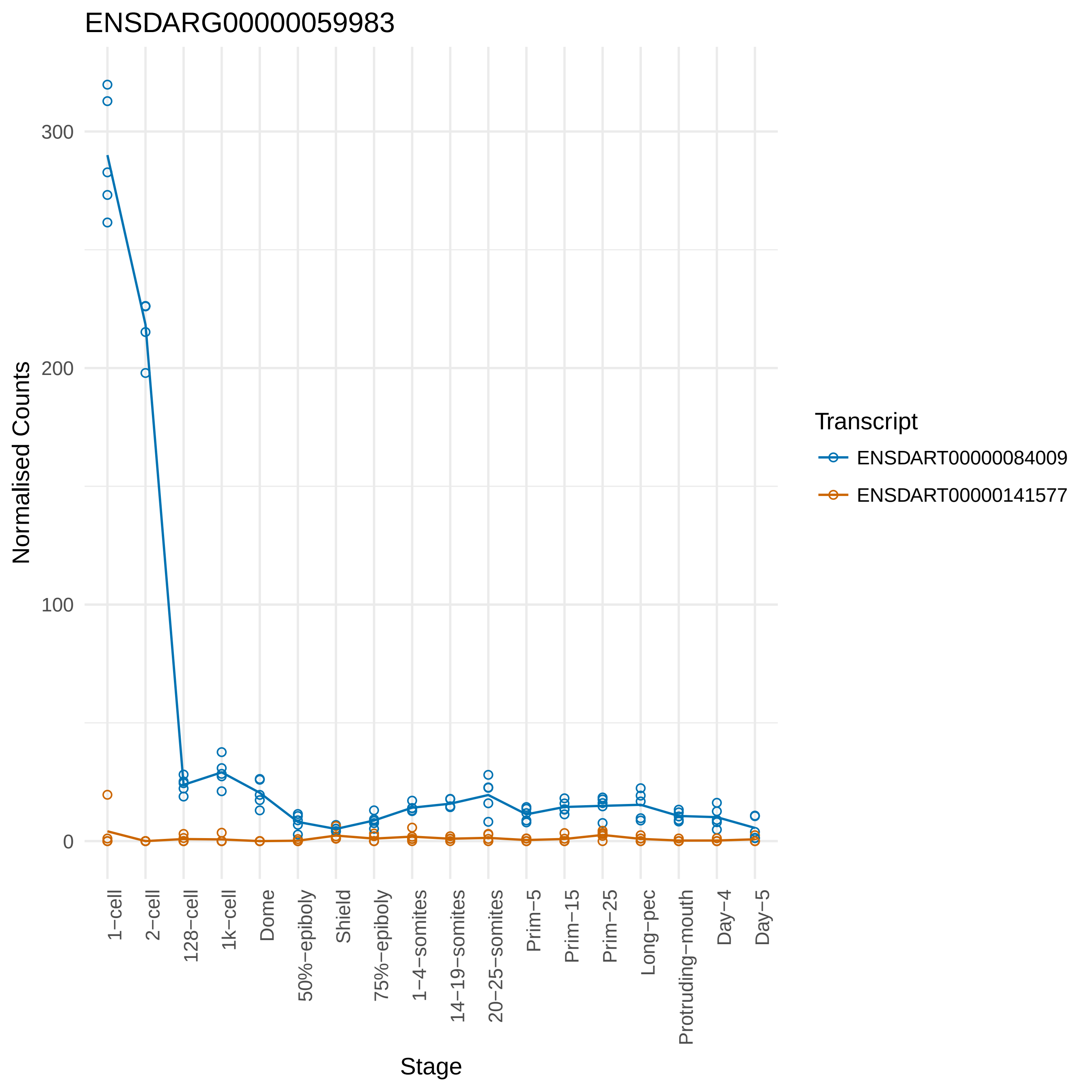

ENSDARG00000059983
Normalised Counts
300
200
100
0
1−cell
2−cell
128−cell
1k−cell
Dome
50%−epiboly
Shield
75%−epiboly
1−4−somites
14−19−somites
20−25−somites
Prim−5
Prim−15
Prim−25
Long−pec
Protruding−mouth
Day−4
Day−5
Stage
Transcript
ENSDART00000084009
ENSDART00000141577

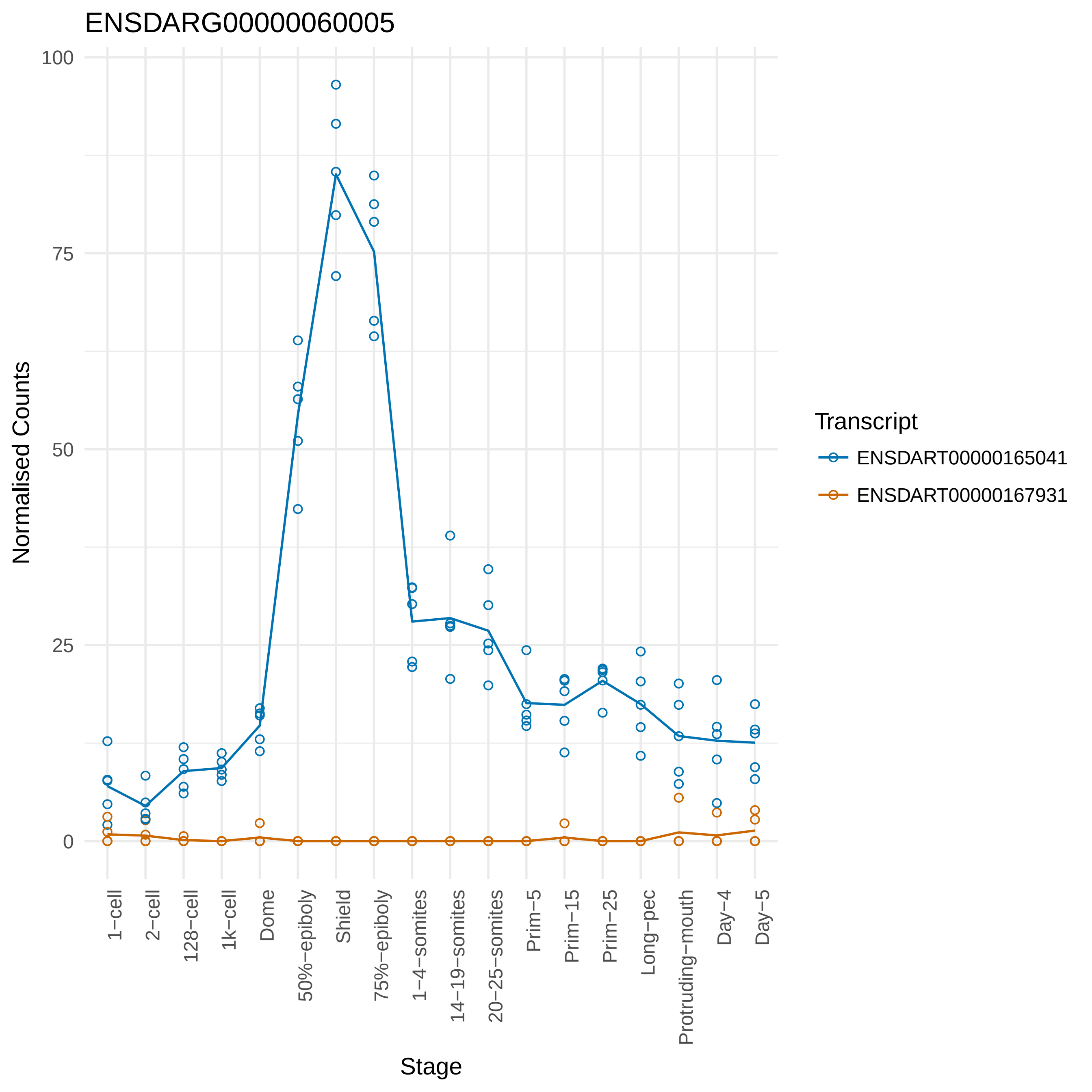
ENSDARG00000060005
Normalised Counts
100
75
50
25
0
1−cell
2−cell
128−cell
1k−cell
Dome
50%−epiboly
Shield
75%−epiboly
1−4−somites
14−19−somites
20−25−somites
Prim−5
Prim−15
Prim−25
Long−pec
Protruding−mouth
Day−4
Day−5
Stage
Transcript
ENSDART00000165041
ENSDART00000167931

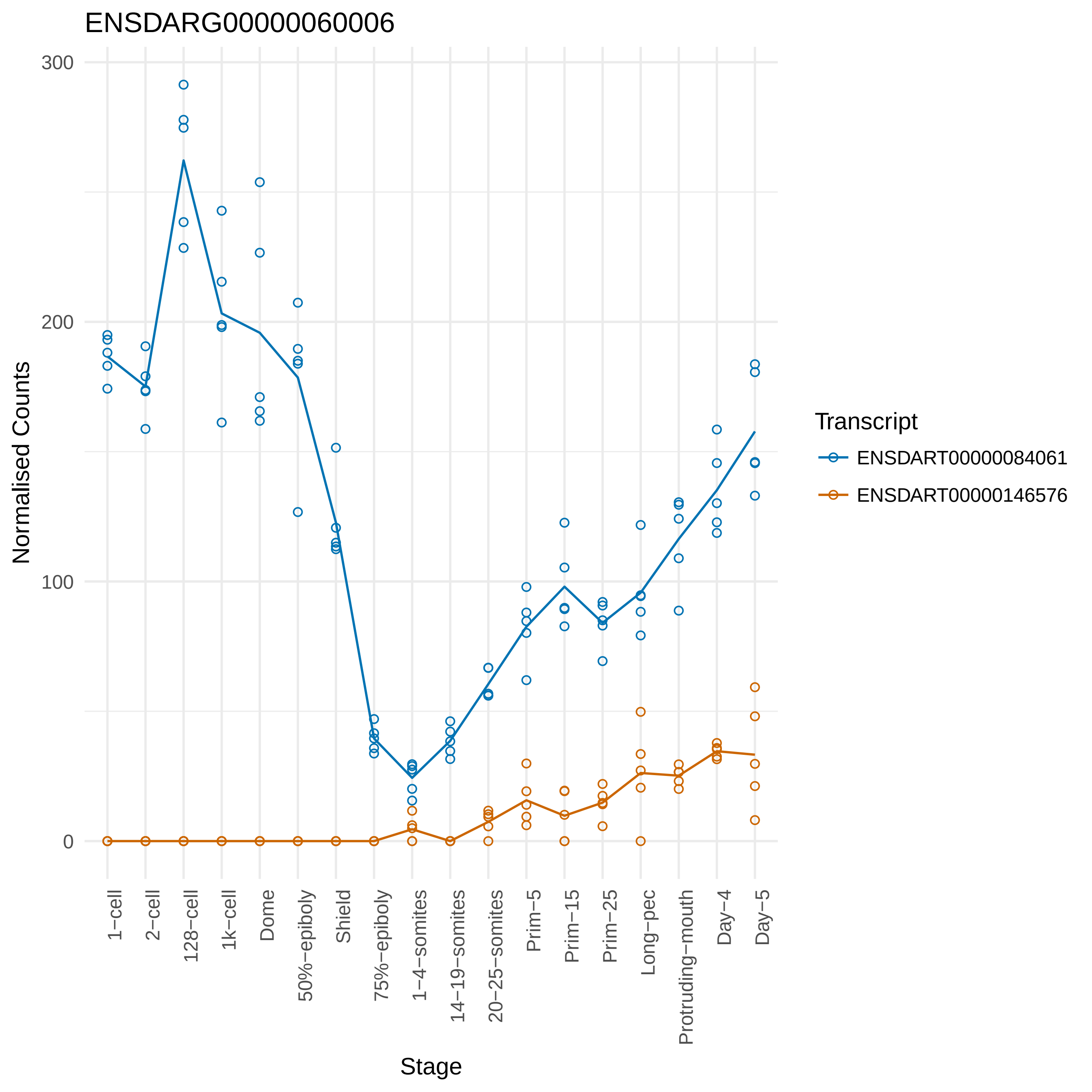
ENSDARG00000060006
300
200
100
0
Normalised Counts
1−cell
2−cell
128−cell
1k−cell
Dome
50%−epiboly
Shield
75%−epiboly
1−4−somites
14−19−somites
20−25−somites
Prim−5
Prim−15
Prim−25
Long−pec
Protruding−mouth
Day−4
Day−5
Stage
Transcript
ENSDART00000084061
ENSDART00000146576

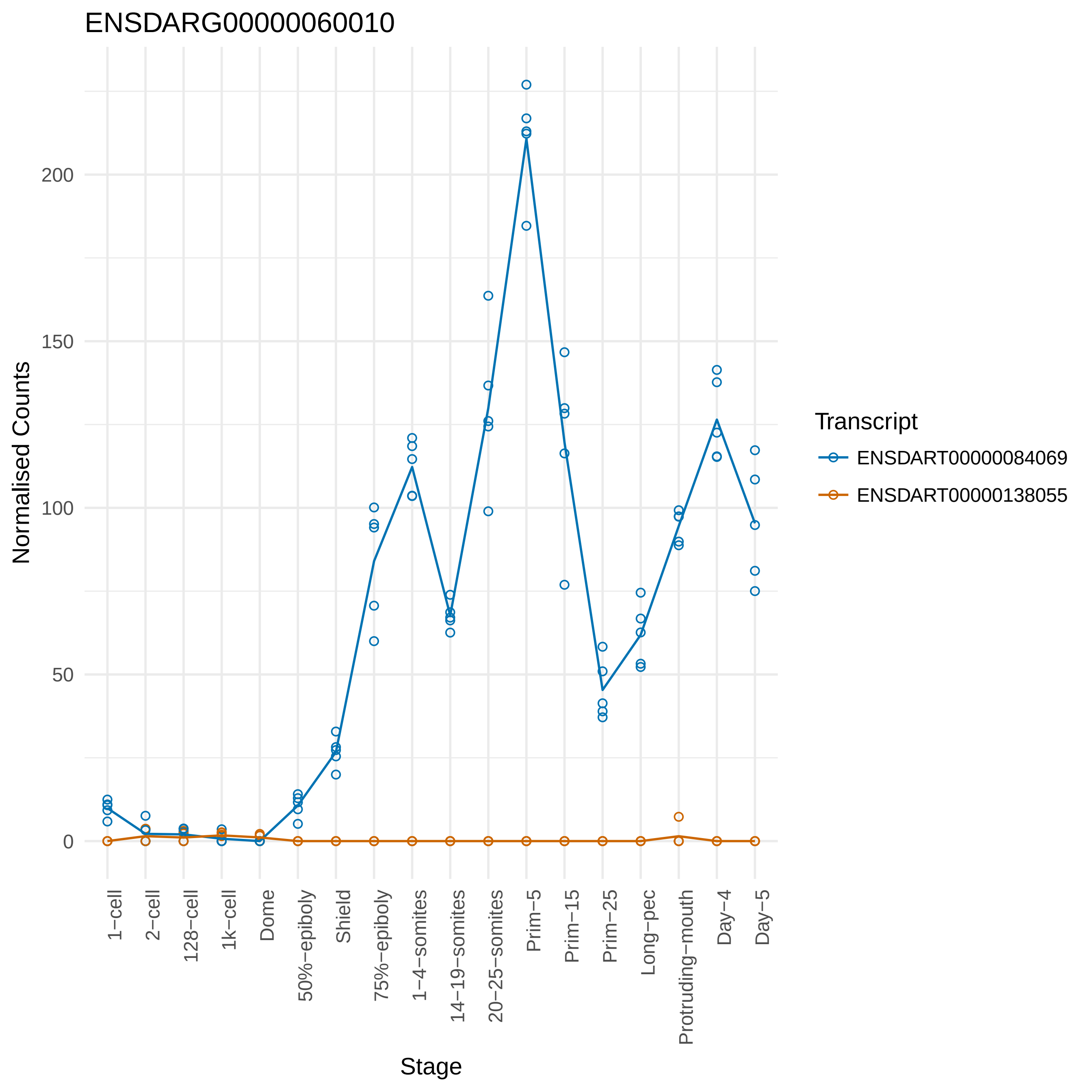

ENSDARG00000060010
Normalised Counts
0
50
100
150
200
Stage
1−cell
2−cell
128−cell
1k−cell
Dome
50%−epiboly
Shield
75%−epiboly
1−4−somites
14−19−somites
20−25−somites
Prim−5
Prim−15
Prim−25
Long−pec
Protruding−mouth
Day−4
Day−5
Transcript
ENSDART00000084069
ENSDART00000138055

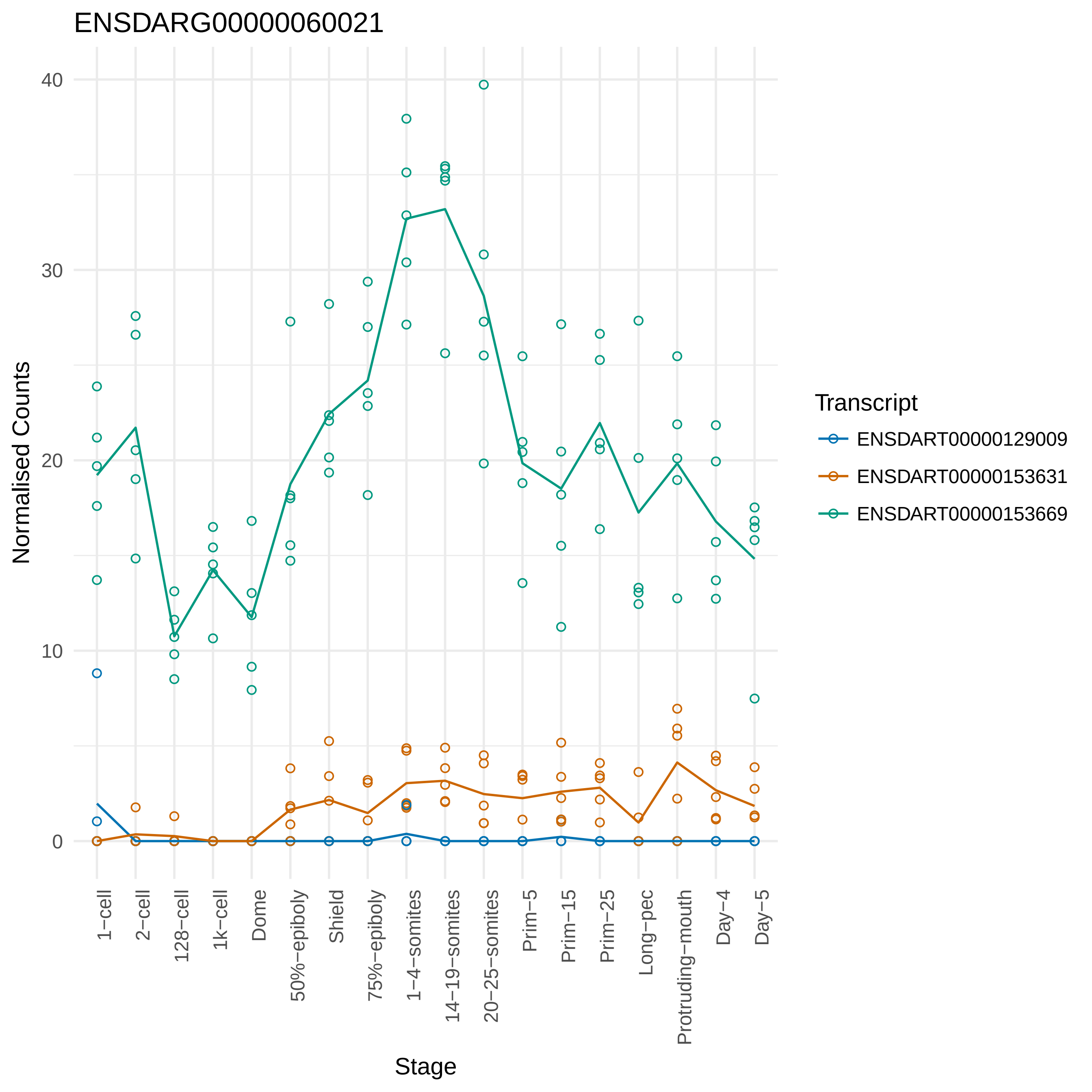

ENSDARG00000060021
Normalised Counts
0
10
20
30
40
1−cell
2−cell
128−cell
1k−cell
Dome
50%−epiboly
Shield
75%−epiboly
1−4−somites
14−19−somites
20−25−somites
Prim−5
Prim−15
Prim−25
Long−pec
Protruding−mouth
Day−4
Day−5
Stage
Transcript
ENSDART00000129009
ENSDART00000153631
ENSDART00000153669

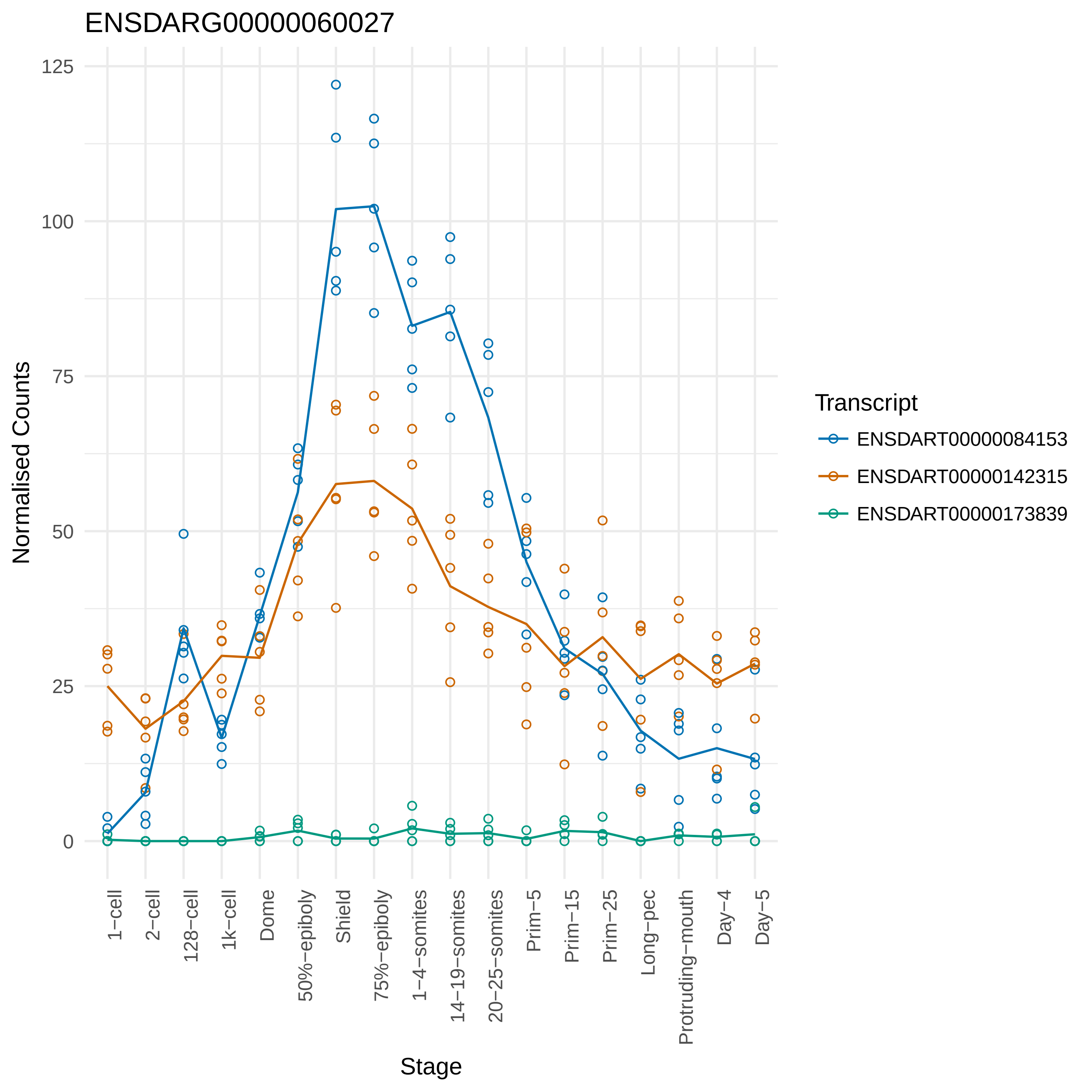

ENSDARG00000060027
Normalised Counts
125
100
75
50
25
0
1−cell
2−cell
128−cell
1k−cell
Dome
50%−epiboly
Shield
75%−epiboly
1−4−somites
14−19−somites
20−25−somites
Prim−5
Prim−15
Prim−25
Long−pec
Protruding−mouth
Day−4
Day−5
Stage
Transcript
ENSDART00000084153
ENSDART00000142315
ENSDART00000173839

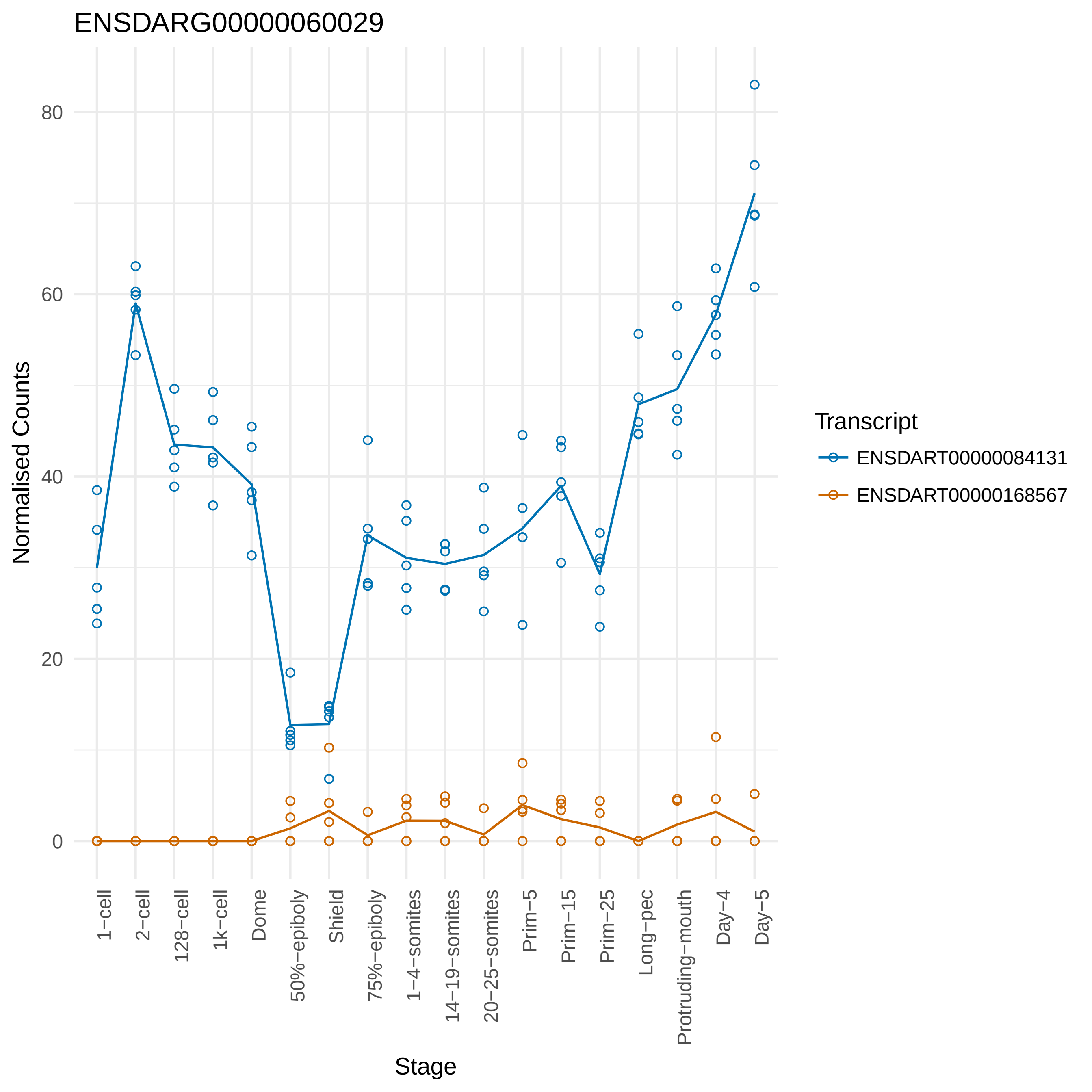

ENSDARG00000060029
Normalised Counts
0
20
40
60
80
1−cell
2−cell
128−cell
1k−cell
Dome
50%−epiboly
Shield
75%−epiboly
1−4−somites
14−19−somites
20−25−somites
Prim−5
Prim−15
Prim−25
Long−pec
Protruding−mouth
Day−4
Day−5
Stage
Transcript
ENSDART00000084131
ENSDART00000168567

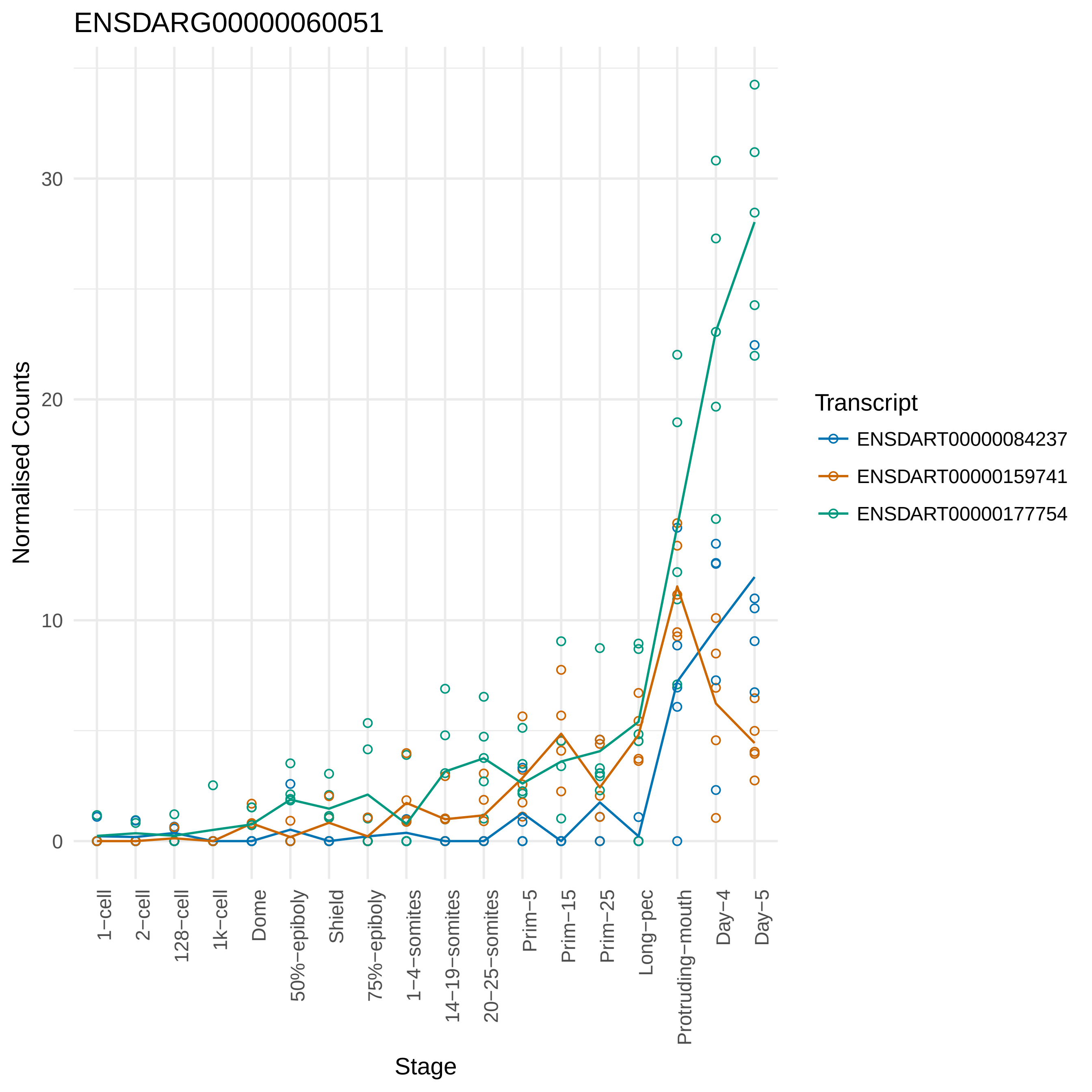
ENSDARG00000060051
Normalised Counts
30
20
10
0
1−cell
2−cell
128−cell
1k−cell
Dome
50%−epiboly
Shield
75%−epiboly
1−4−somites
14−19−somites
20−25−somites
Prim−5
Prim−15
Prim−25
Long−pec
Protruding−mouth
Day−4
Day−5
Stage
Transcript
ENSDART00000084237
ENSDART00000159741
ENSDART00000177754

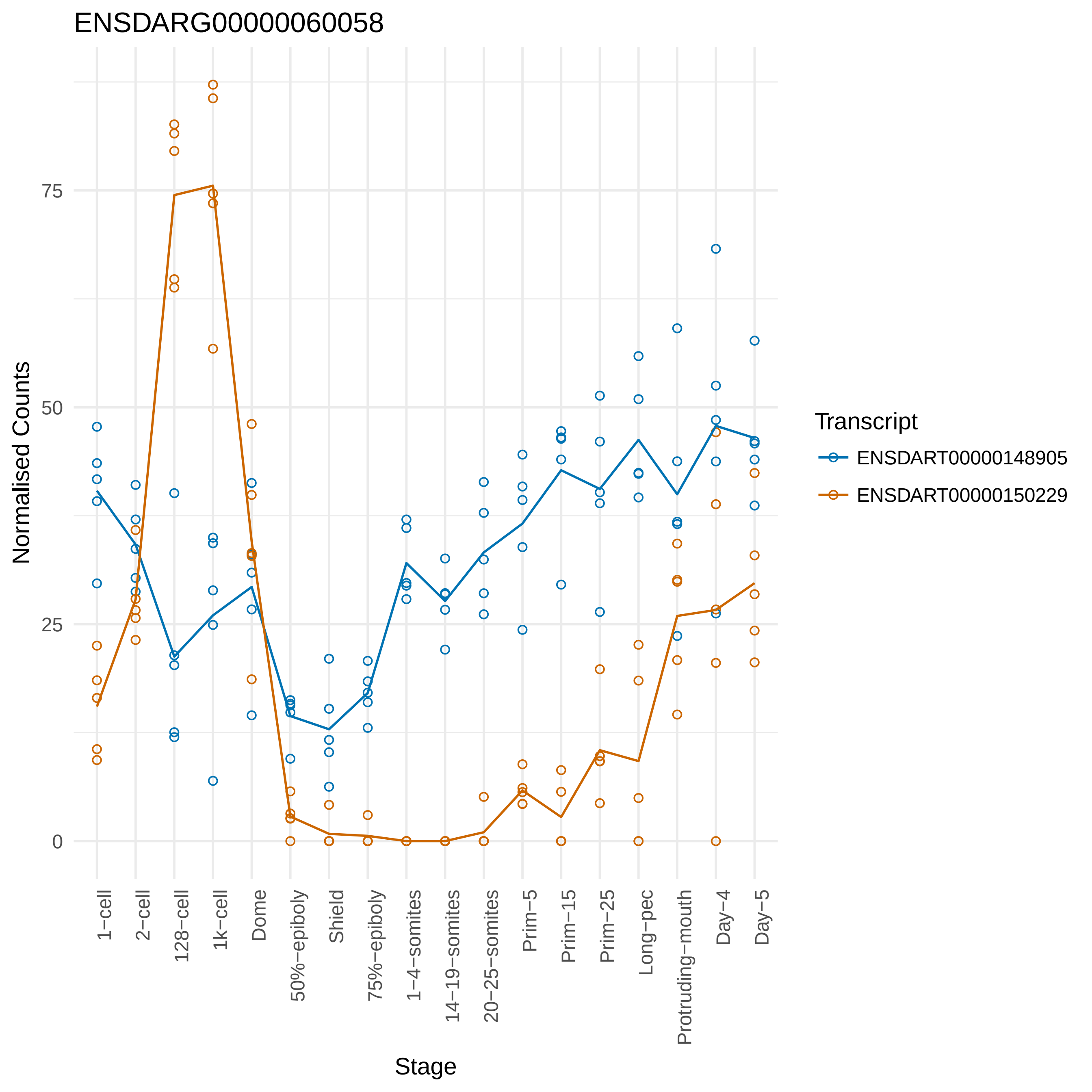

ENSDARG00000060058
Normalised Counts
75
50
25
0
1−cell
2−cell
128−cell
1k−cell
Dome
50%−epiboly
Shield
75%−epiboly
1−4−somites
14−19−somites
20−25−somites
Prim−5
Prim−15
Prim−25
Long−pec
Protruding−mouth
Day−4
Day−5
Stage
Transcript
ENSDART00000148905
ENSDART00000150229

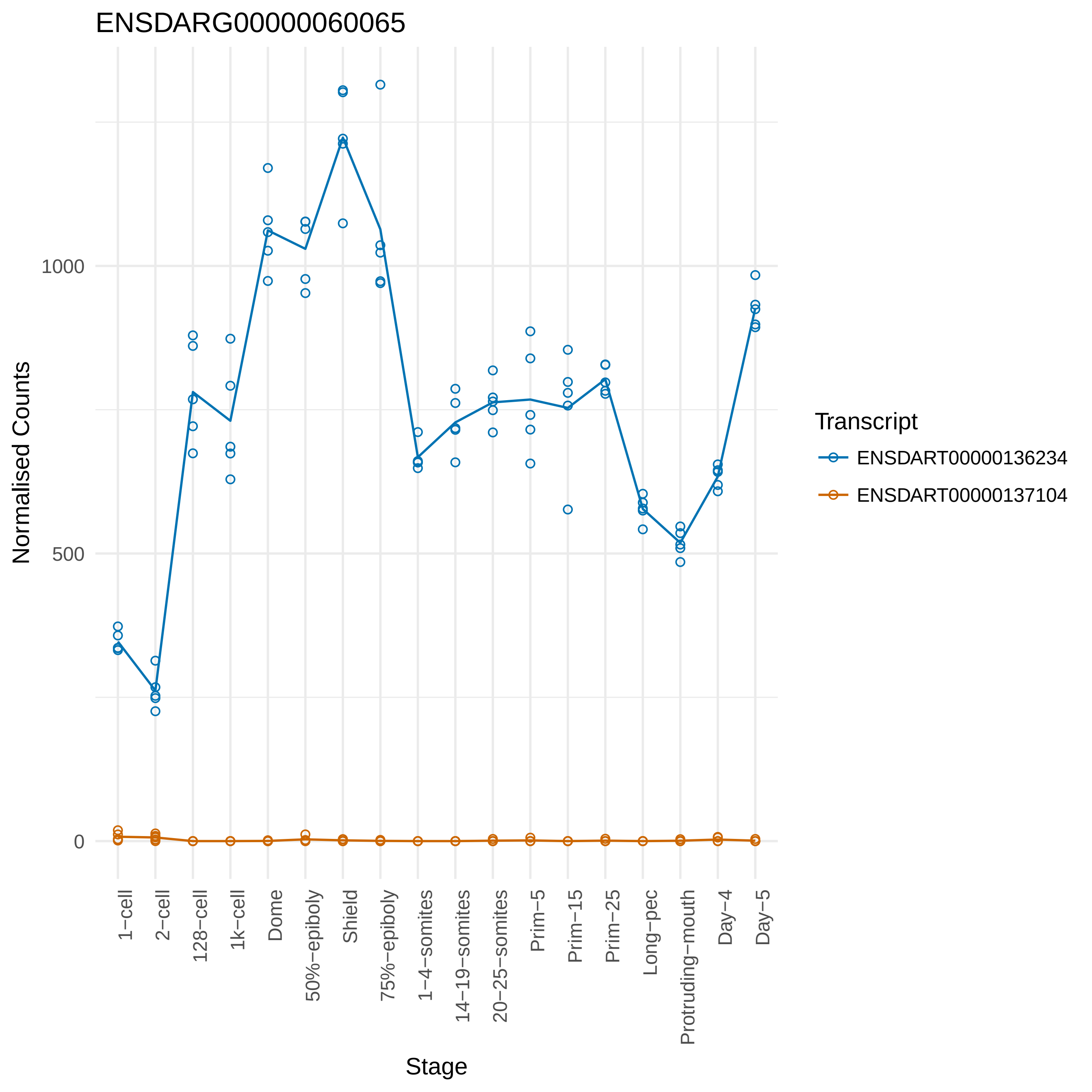

ENSDARG00000060065
Normalised Counts
1000
500
0
1−cell
2−cell
128−cell
1k−cell
Dome
50%−epiboly
Shield
75%−epiboly
1−4−somites
14−19−somites
20−25−somites
Prim−5
Prim−15
Prim−25
Long−pec
Protruding−mouth
Day−4
Day−5
Stage
Transcript
ENSDART00000136234
ENSDART00000137104

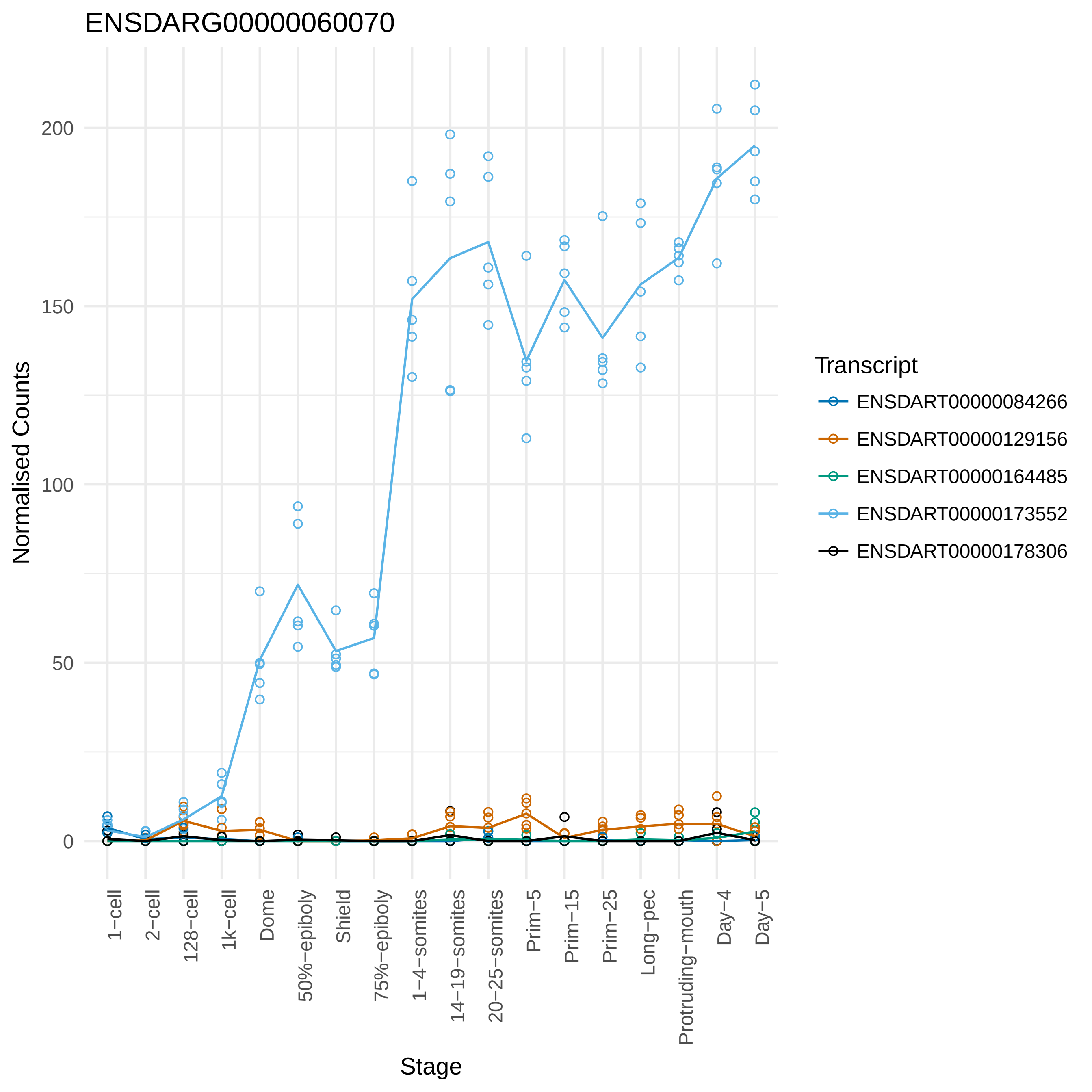
ENSDARG00000060070
Normalised Counts
0
50
100
150
200
Stage
1−cell
2−cell
128−cell
1k−cell
Dome
50%−epiboly
Shield
75%−epiboly
1−4−somites
14−19−somites
20−25−somites
Prim−5
Prim−15
Prim−25
Long−pec
Protruding−mouth
Day−4
Day−5
Transcript
ENSDART00000084266
ENSDART00000129156
ENSDART00000164485
ENSDART00000173552
ENSDART00000178306

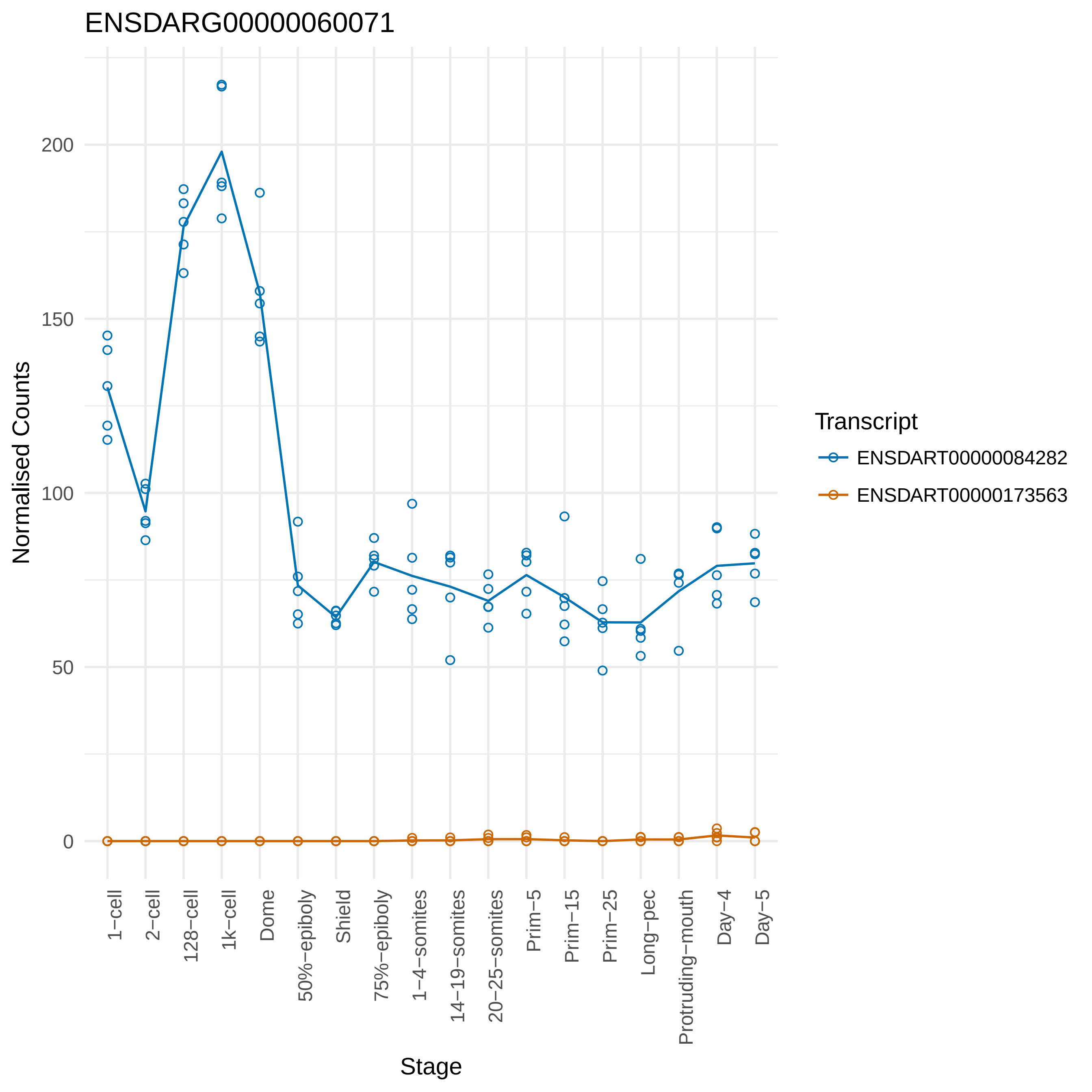

ENSDARG00000060071
Normalised Counts
200
150
100
50
0
1−cell
2−cell
128−cell
1k−cell
Dome
50%−epiboly
Shield
75%−epiboly
1−4−somites
14−19−somites
20−25−somites
Prim−5
Prim−15
Prim−25
Long−pec
Protruding−mouth
Day−4
Day−5
Stage
Transcript
ENSDART00000084282
ENSDART00000173563

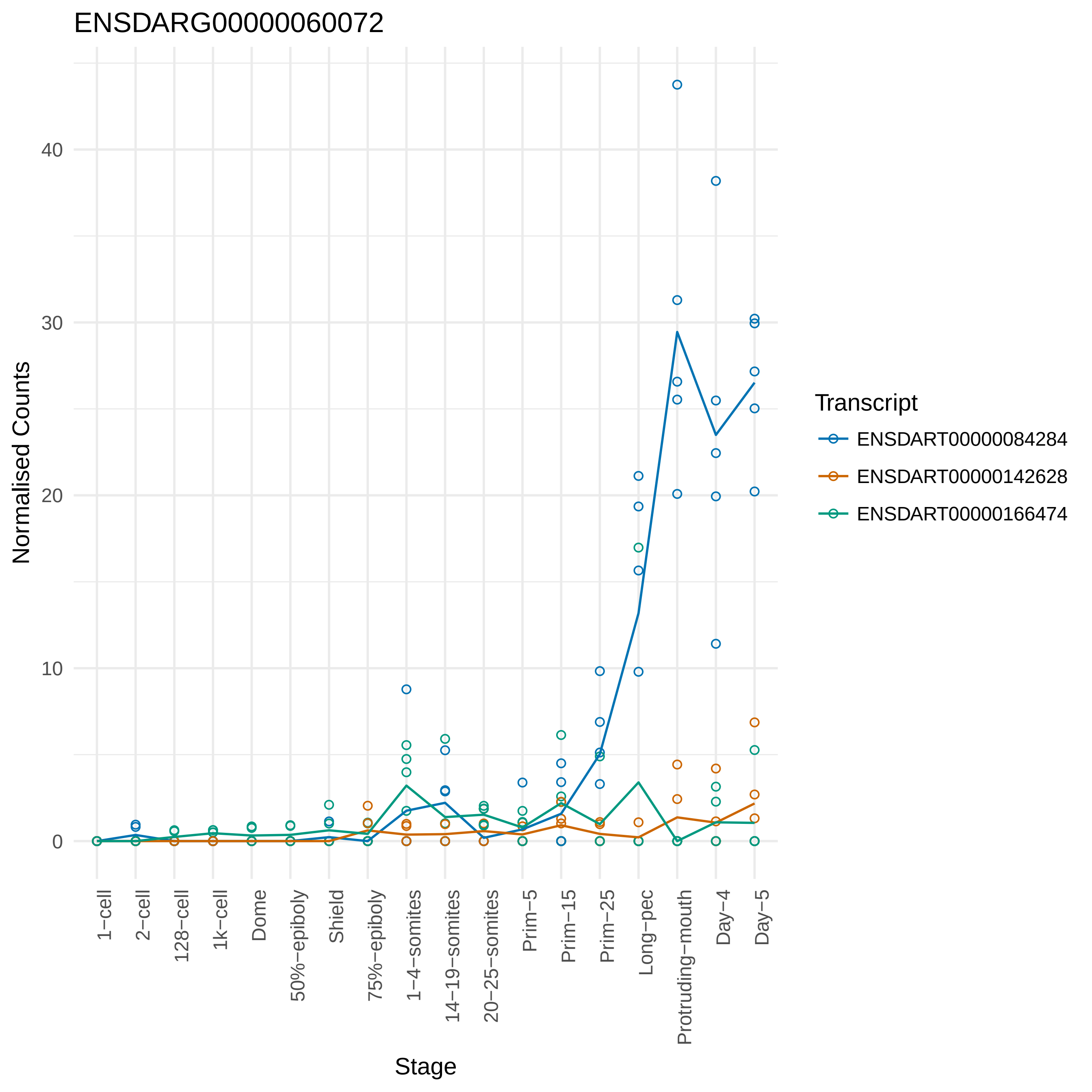

ENSDARG00000060072
Normalised Counts
0
10
20
30
40
Stage
1−cell
2−cell
128−cell
1k−cell
Dome
50%−epiboly
Shield
75%−epiboly
1−4−somites
14−19−somites
20−25−somites
Prim−5
Prim−15
Prim−25
Long−pec
Protruding−mouth
Day−4
Day−5
Transcript
ENSDART00000084284
ENSDART00000142628
ENSDART00000166474

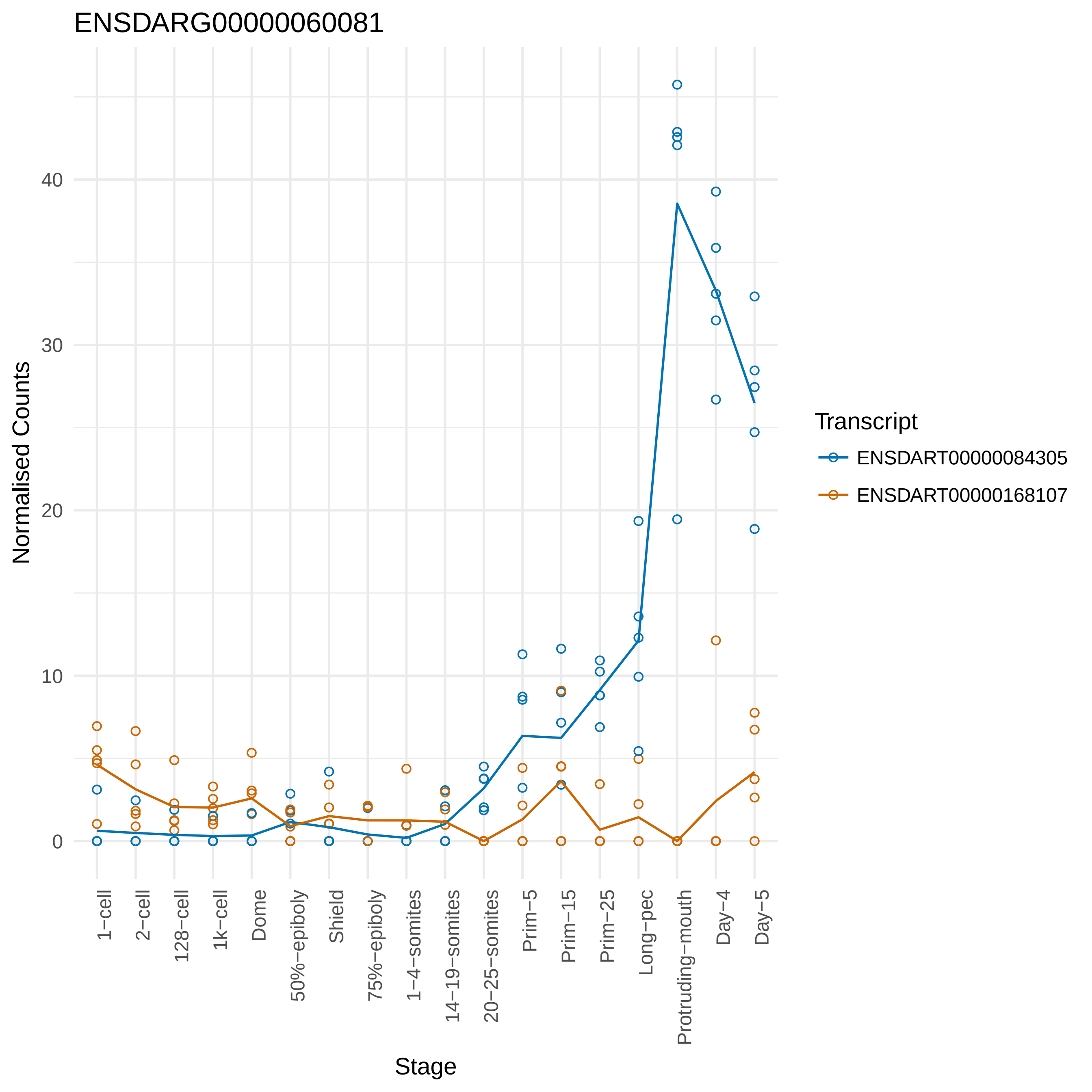
ENSDARG00000060081
Normalised Counts
0
10
20
30
40
Stage
1−cell
2−cell
128−cell
1k−cell
Dome
50%−epiboly
Shield
75%−epiboly
1−4−somites
14−19−somites
20−25−somites
Prim−5
Prim−15
Prim−25
Long−pec
Protruding−mouth
Day−4
Day−5
Transcript
ENSDART00000084305
ENSDART00000168107

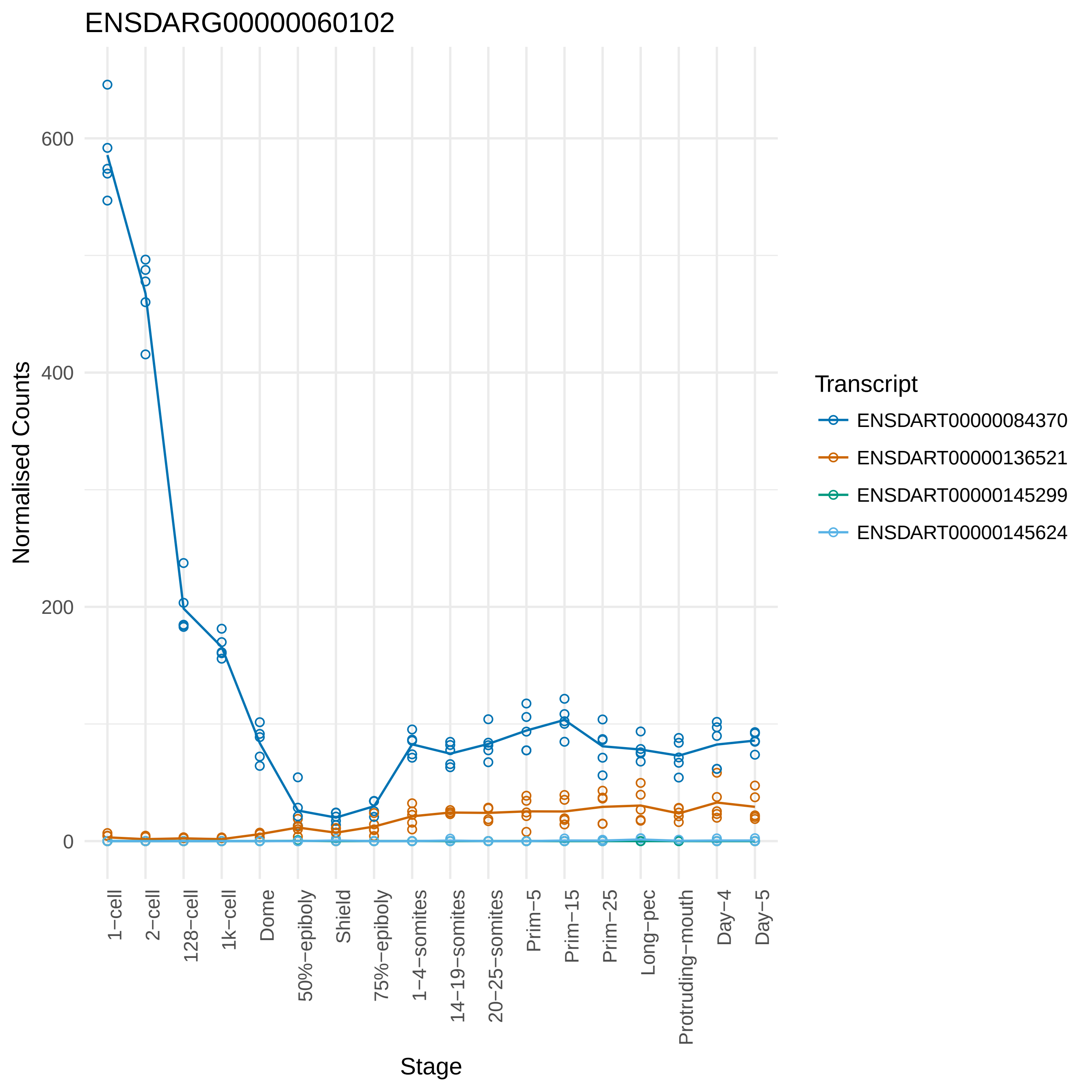

ENSDARG00000060102
Normalised Counts
600
400
200
0
1−cell
2−cell
128−cell
1k−cell
Dome
50%−epiboly
Shield
75%−epiboly
1−4−somites
14−19−somites
20−25−somites
Prim−5
Prim−15
Prim−25
Long−pec
Protruding−mouth
Day−4
Day−5
Stage
Transcript
ENSDART00000084370
ENSDART00000136521
ENSDART00000145299
ENSDART00000145624

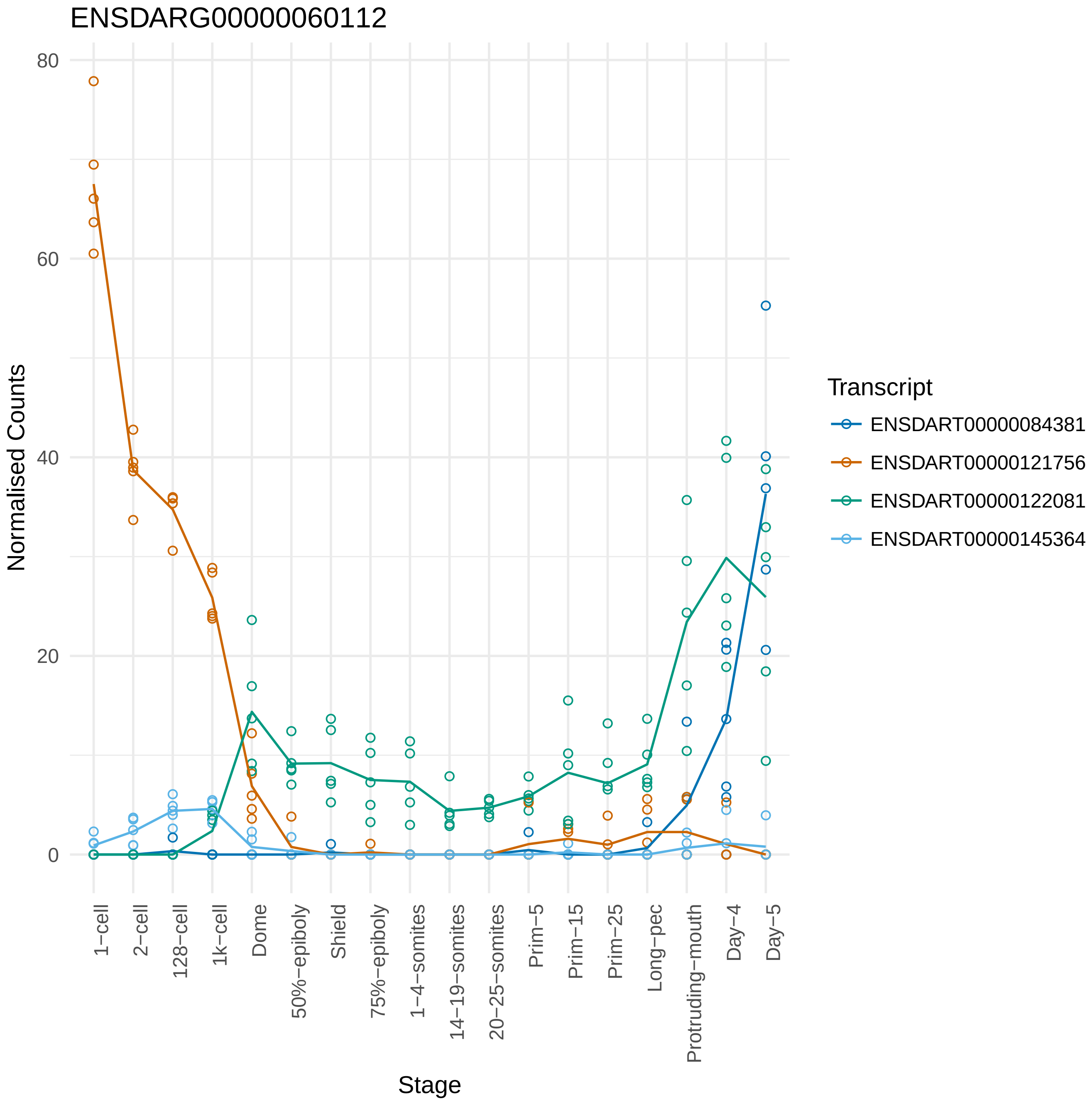
ENSDARG00000060112
Normalised Counts
80
60
40
20
0
1−cell
2−cell
128−cell
1k−cell
Dome
50%−epiboly
Shield
75%−epiboly
1−4−somites
14−19−somites
20−25−somites
Prim−5
Prim−15
Prim−25
Long−pec
Protruding−mouth
Day−4
Day−5
Stage
Transcript
ENSDART00000084381
ENSDART00000121756
ENSDART00000122081
ENSDART00000145364

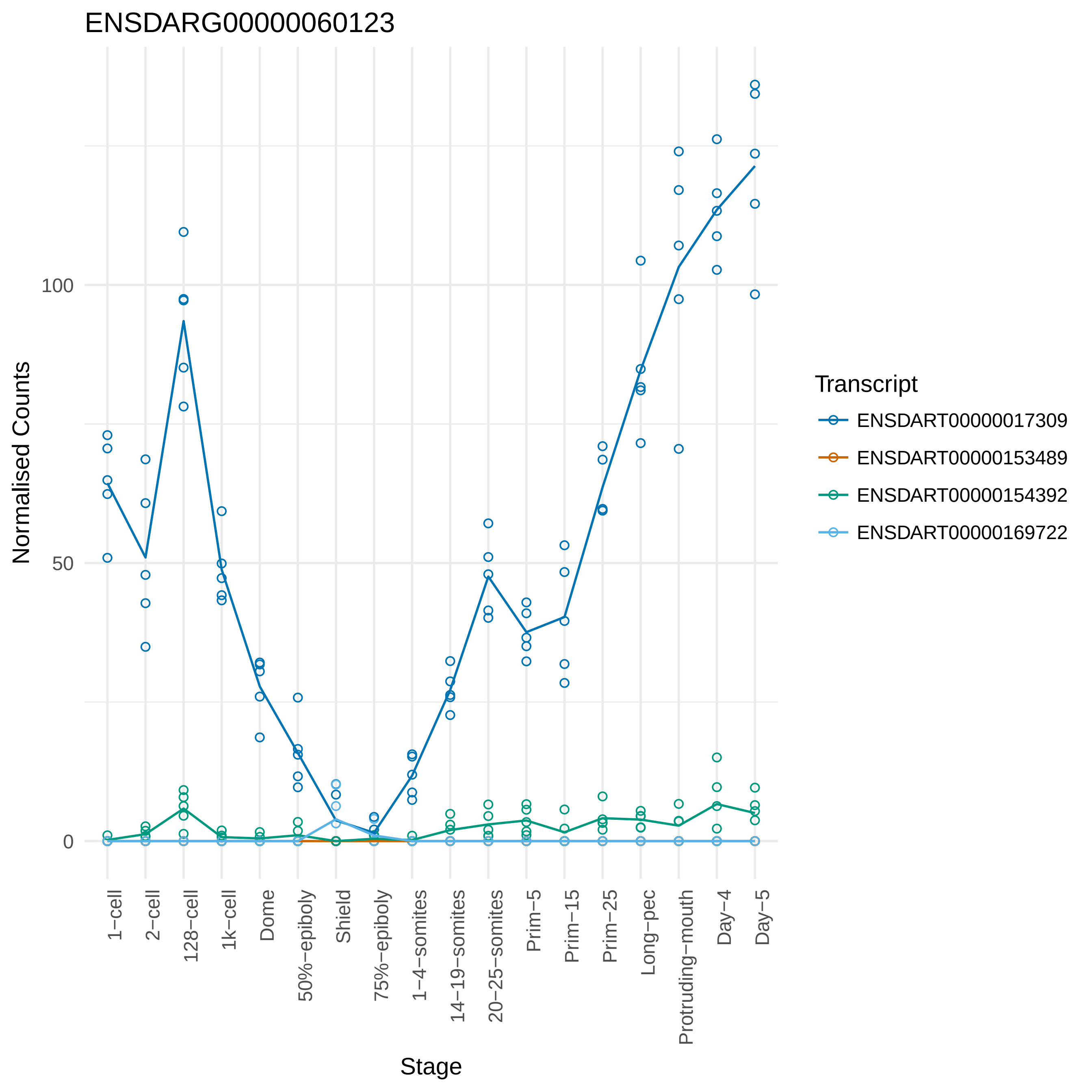

ENSDARG00000060123
Normalised Counts
100
50
0
1−cell
2−cell
128−cell
1k−cell
Dome
50%−epiboly
Shield
75%−epiboly
1−4−somites
14−19−somites
20−25−somites
Prim−5
Prim−15
Prim−25
Long−pec
Protruding−mouth
Day−4
Day−5
Stage
Transcript
ENSDART00000017309
ENSDART00000153489
ENSDART00000154392
ENSDART00000169722

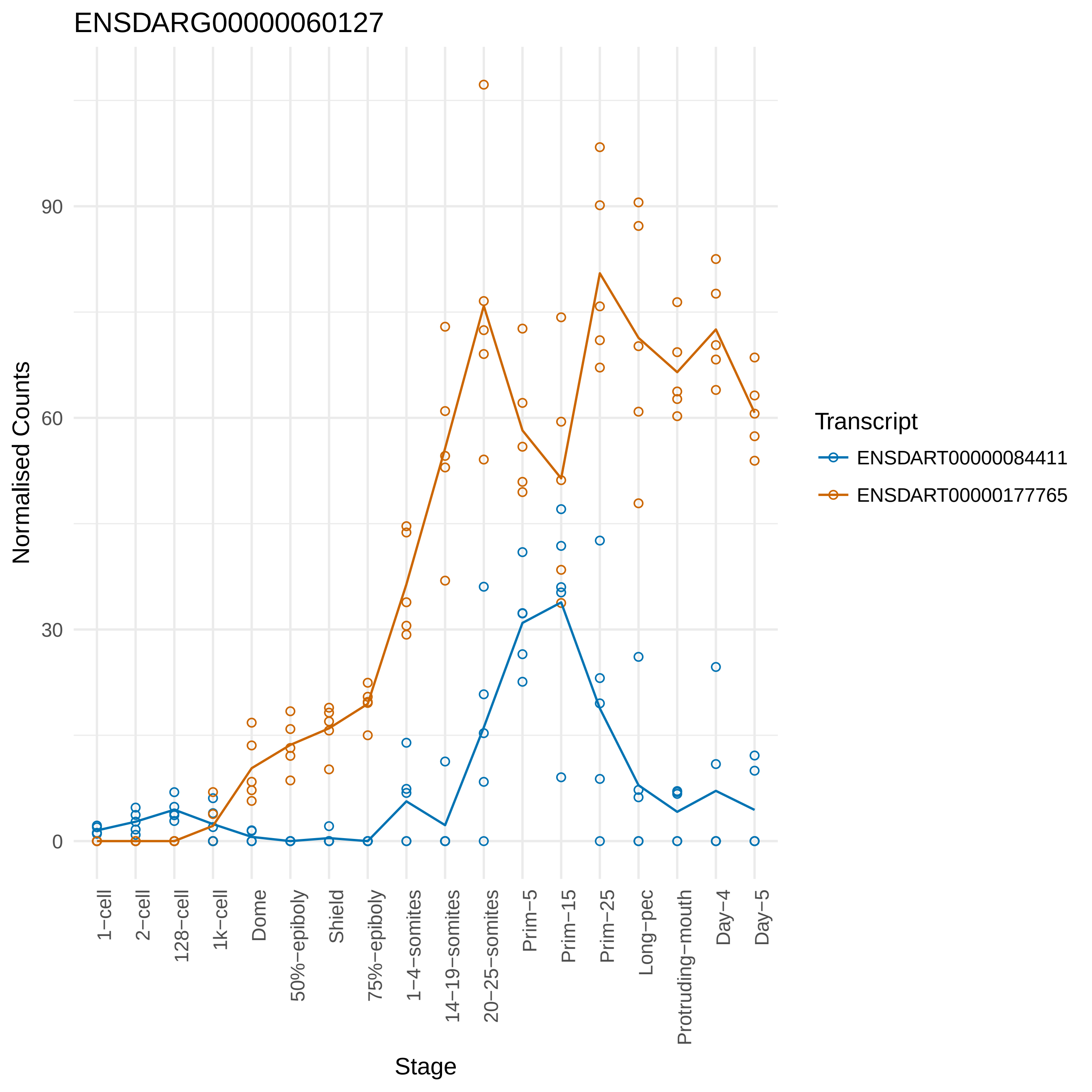
ENSDARG00000060127
Normalised Counts
0
30
60
90
Stage
1−cell
2−cell
128−cell
1k−cell
Dome
50%−epiboly
Shield
75%−epiboly
1−4−somites
14−19−somites
20−25−somites
Prim−5
Prim−15
Prim−25
Long−pec
Protruding−mouth
Day−4
Day−5
Transcript
ENSDART00000084411
ENSDART00000177765

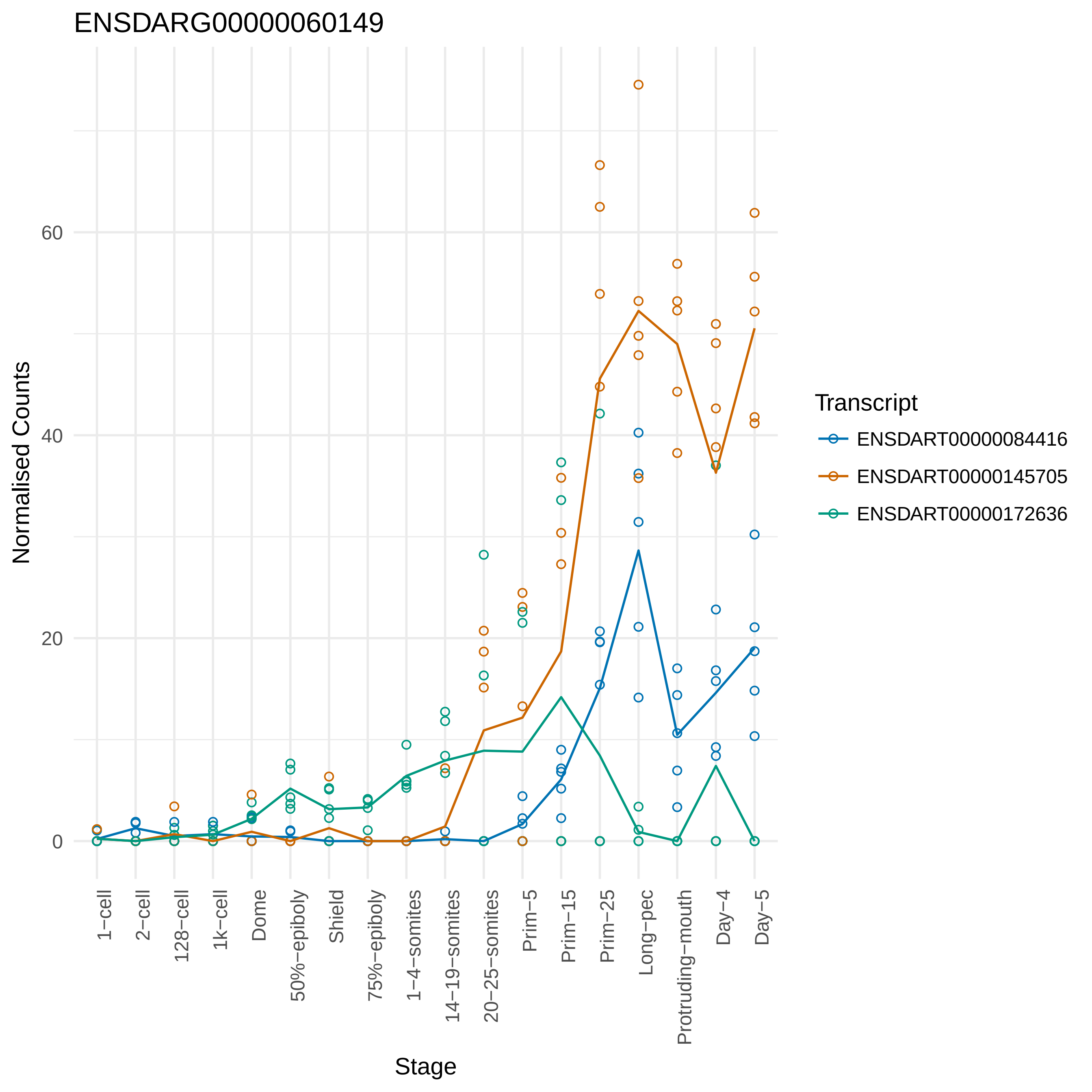

ENSDARG00000060149
Normalised Counts
0
20
40
60
1−cell
2−cell
128−cell
1k−cell
Dome
50%−epiboly
Shield
75%−epiboly
1−4−somites
14−19−somites
20−25−somites
Prim−5
Prim−15
Prim−25
Long−pec
Protruding−mouth
Day−4
Day−5
Stage
Transcript
ENSDART00000084416
ENSDART00000145705
ENSDART00000172636

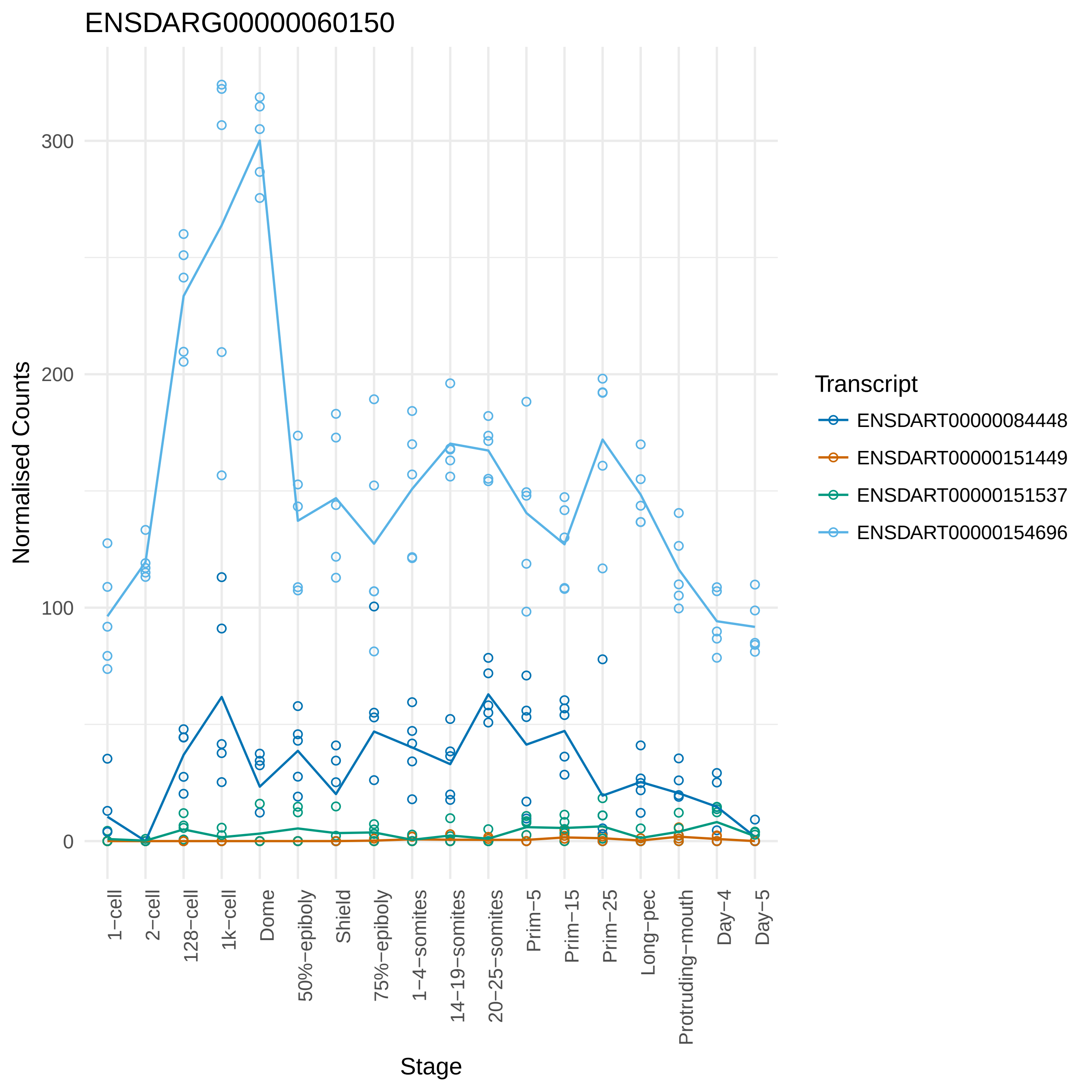
ENSDARG00000060150
Normalised Counts
300
200
100
0
1−cell
2−cell
128−cell
1k−cell
Dome
50%−epiboly
Shield
75%−epiboly
1−4−somites
14−19−somites
20−25−somites
Prim−5
Prim−15
Prim−25
Long−pec
Protruding−mouth
Day−4
Day−5
Stage
Transcript
ENSDART00000084448
ENSDART00000151449
ENSDART00000151537
ENSDART00000154696

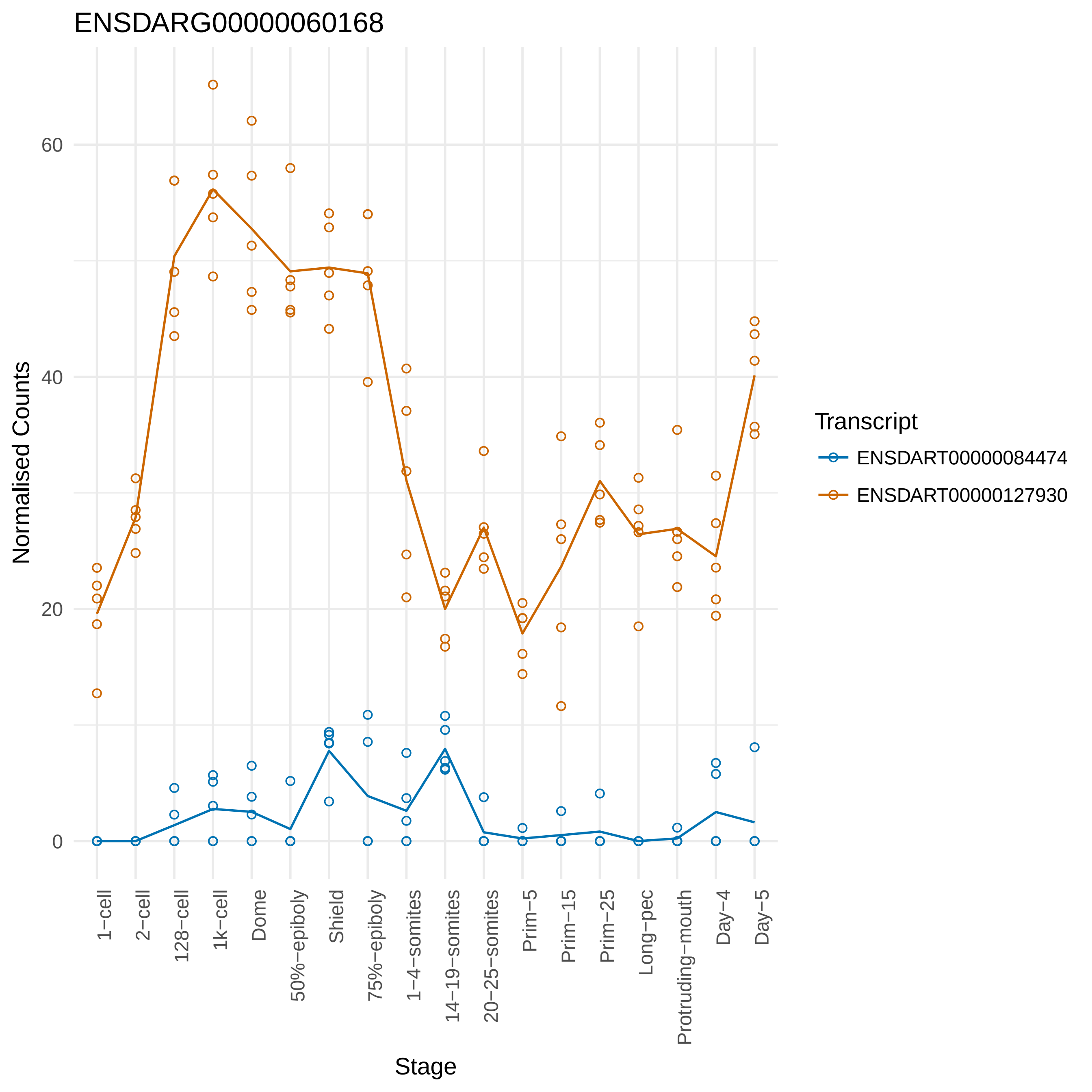

ENSDARG00000060168
Normalised Counts
60
40
20
0
1−cell
2−cell
128−cell
1k−cell
Dome
50%−epiboly
Shield
75%−epiboly
1−4−somites
14−19−somites
20−25−somites
Prim−5
Prim−15
Prim−25
Long−pec
Protruding−mouth
Day−4
Day−5
Stage
Transcript
ENSDART00000084474
ENSDART00000127930

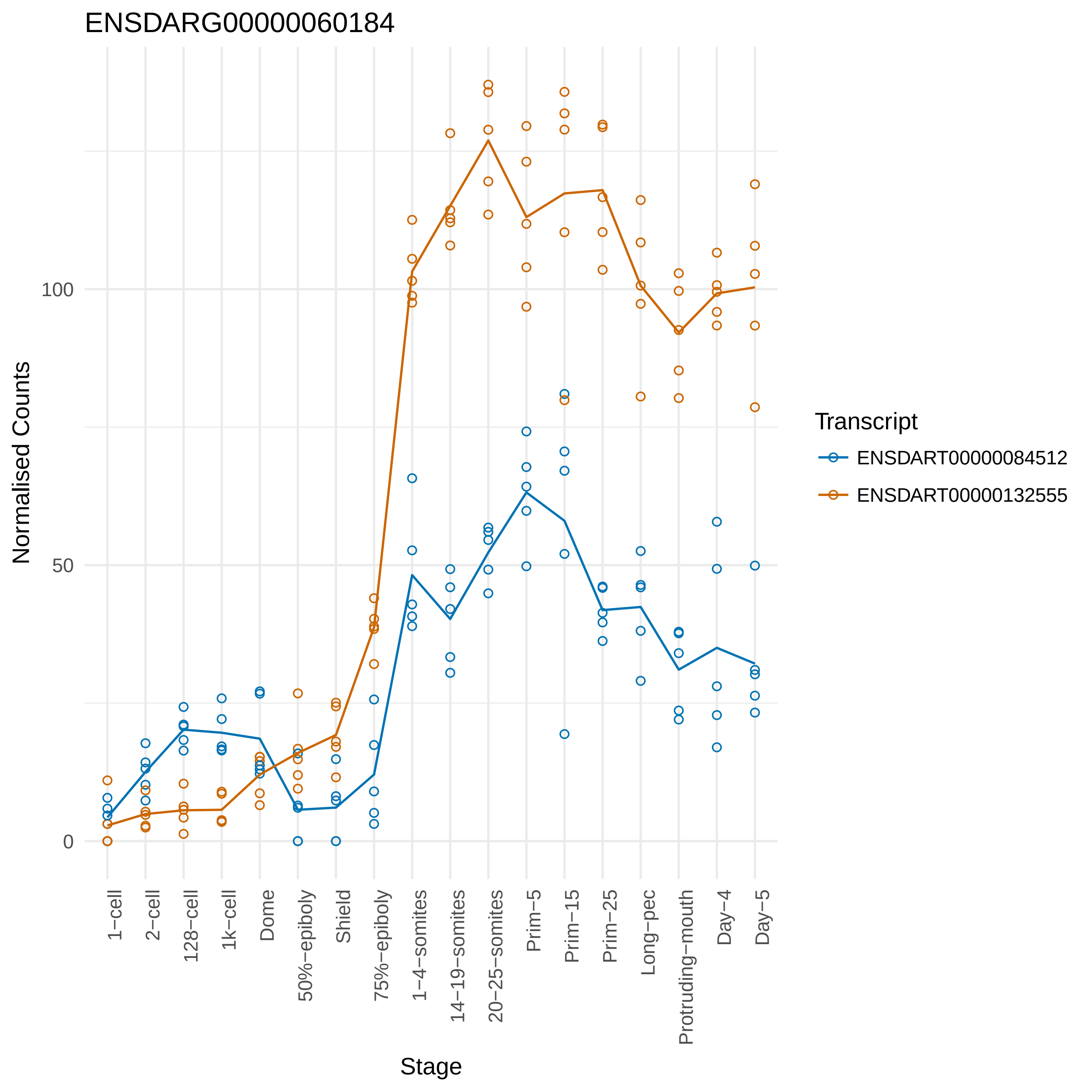

ENSDARG00000060184
Normalised Counts
0
50
100
1−cell
2−cell
128−cell
1k−cell
Dome
50%−epiboly
Shield
75%−epiboly
1−4−somites
14−19−somites
20−25−somites
Prim−5
Prim−15
Prim−25
Long−pec
Protruding−mouth
Day−4
Day−5
Stage
Transcript
ENSDART00000084512
ENSDART00000132555

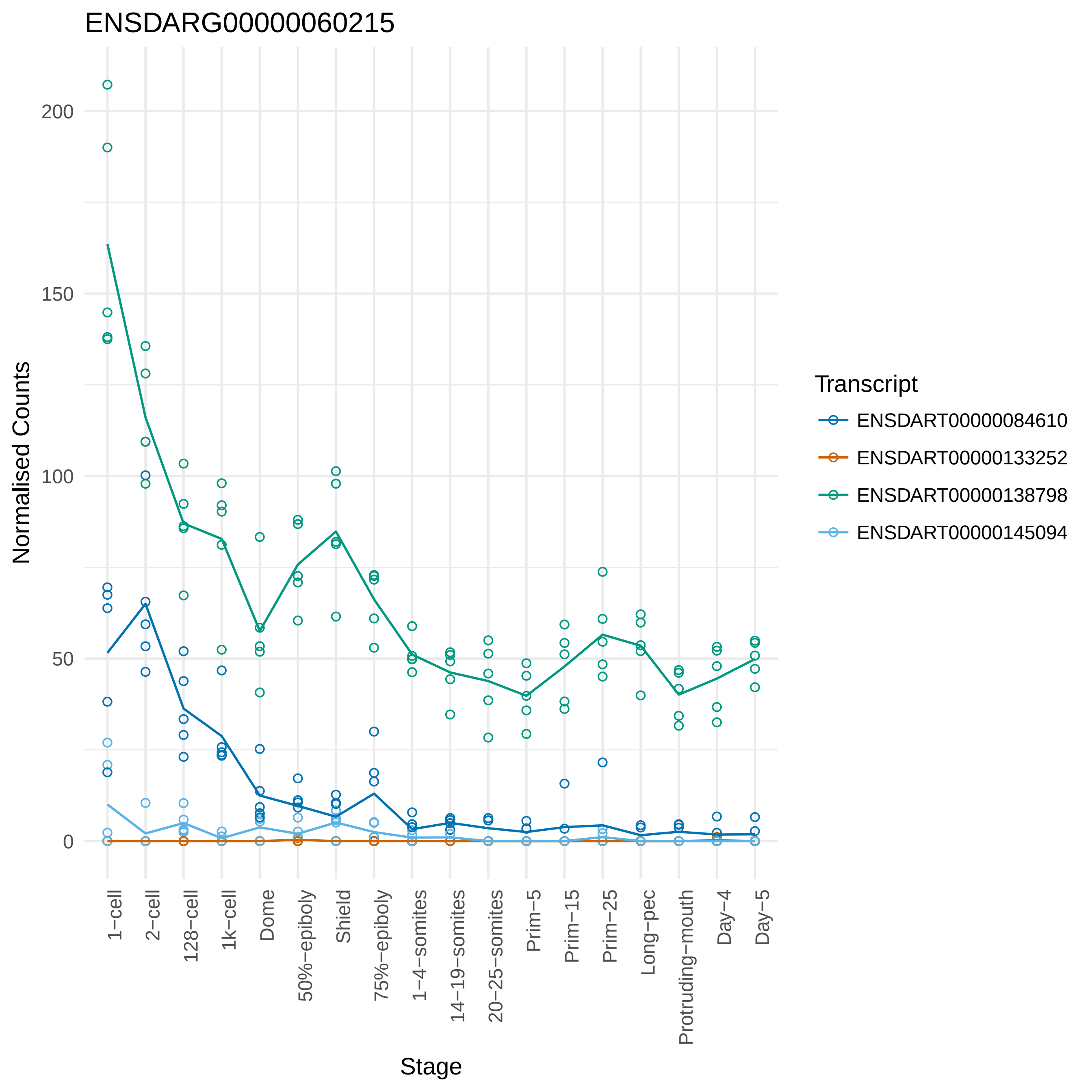

ENSDARG00000060215
Normalised Counts
0
50
100
150
200
1−cell
2−cell
128−cell
1k−cell
Dome
50%−epiboly
Shield
75%−epiboly
1−4−somites
14−19−somites
20−25−somites
Prim−5
Prim−15
Prim−25
Long−pec
Protruding−mouth
Day−4
Day−5
Stage
Transcript
ENSDART00000084610
ENSDART00000133252
ENSDART00000138798
ENSDART00000145094

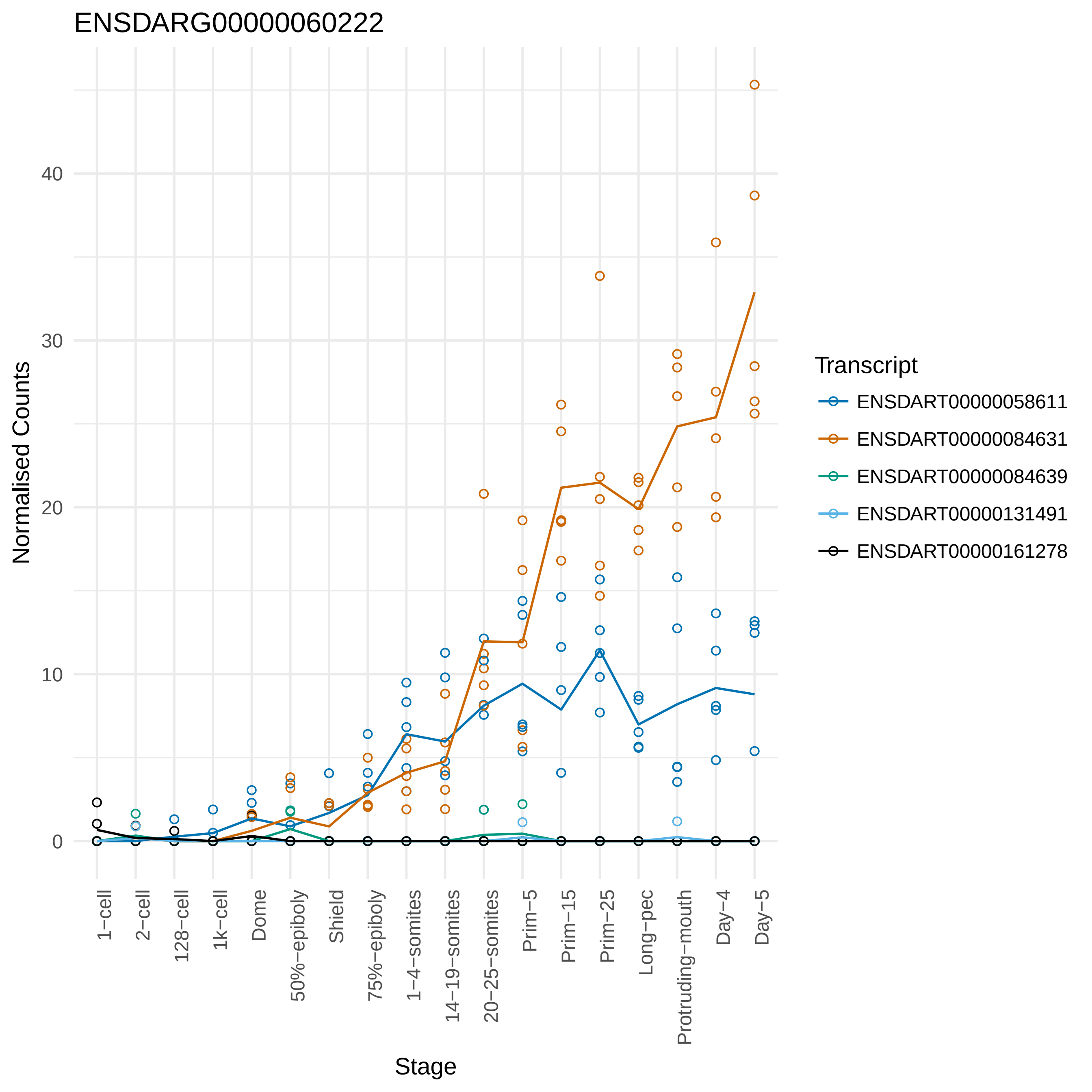

ENSDARG00000060222
Normalised Counts
0
10
20
30
40
1−cell
2−cell
128−cell
1k−cell
Dome
50%−epiboly
Shield
75%−epiboly
1−4−somites
14−19−somites
20−25−somites
Prim−5
Prim−15
Prim−25
Long−pec
Protruding−mouth
Day−4
Day−5
Stage
Transcript
ENSDART00000058611
ENSDART00000084631
ENSDART00000084639
ENSDART00000131491
ENSDART00000161278

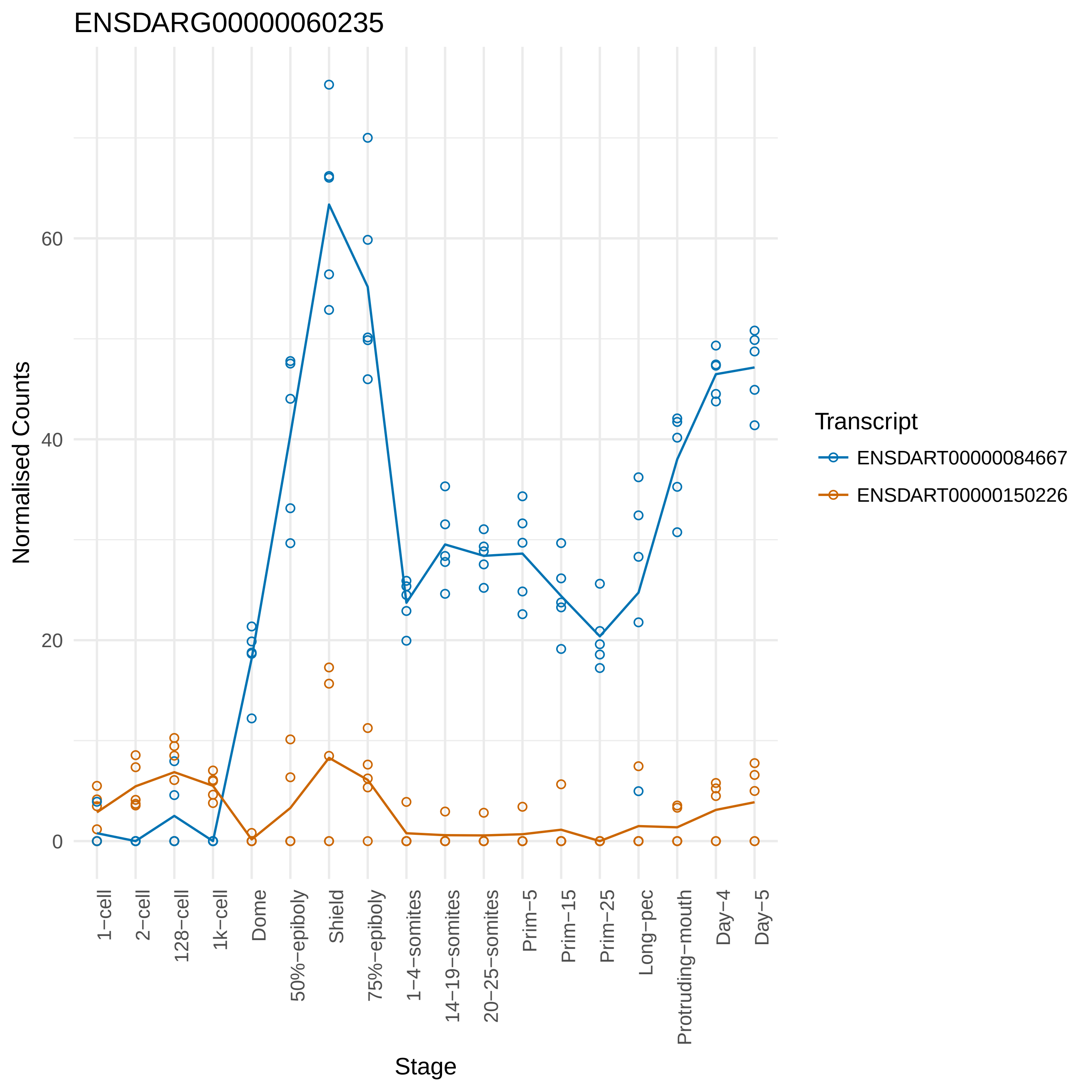

ENSDARG00000060235
Normalised Counts
0
20
40
60
Stage
1−cell
2−cell
128−cell
1k−cell
Dome
50%−epiboly
Shield
75%−epiboly
1−4−somites
14−19−somites
20−25−somites
Prim−5
Prim−15
Prim−25
Long−pec
Protruding−mouth
Day−4
Day−5
Transcript
ENSDART00000084667
ENSDART00000150226

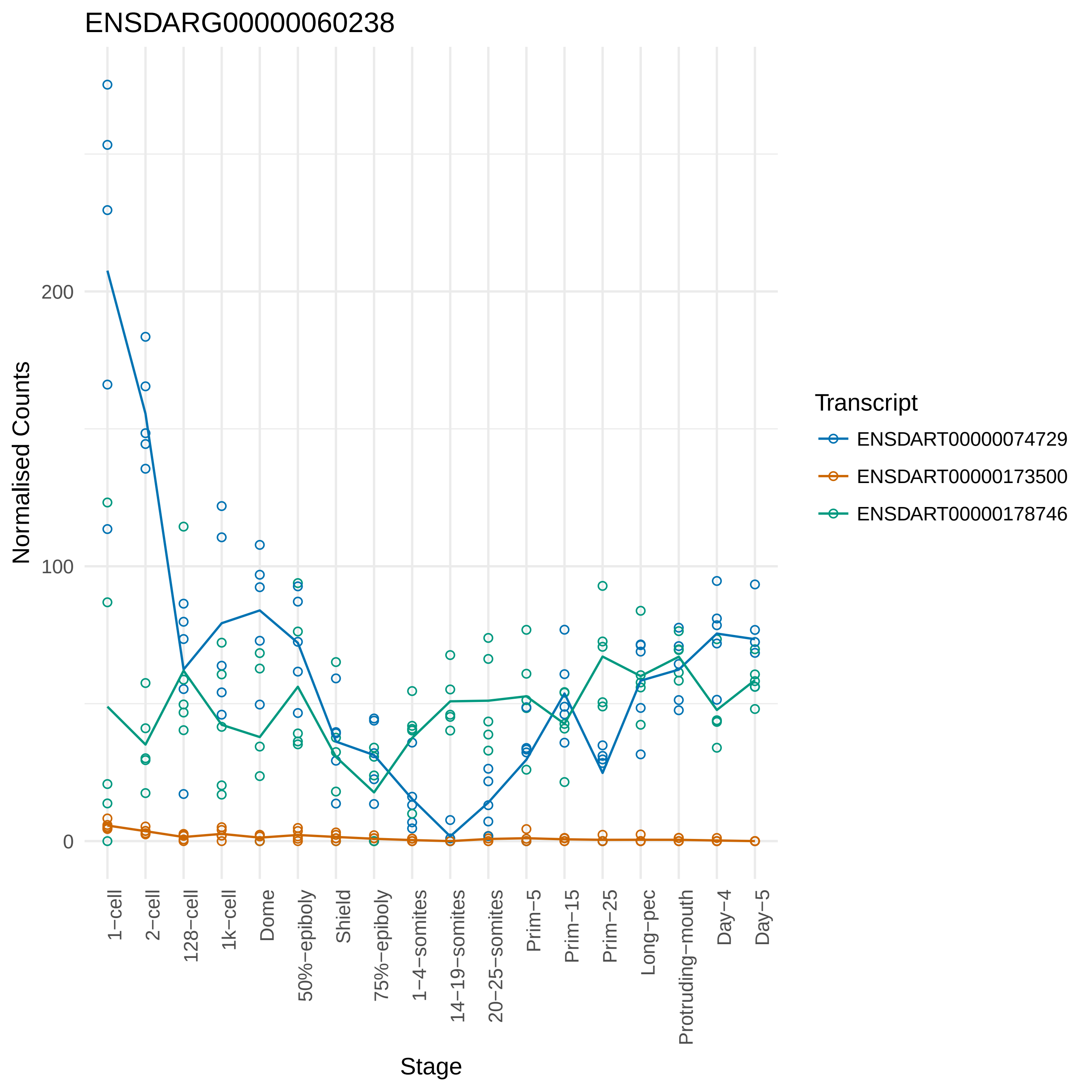

ENSDARG00000060238
Normalised Counts
200
100
0
1−cell
2−cell
128−cell
1k−cell
Dome
50%−epiboly
Shield
75%−epiboly
1−4−somites
14−19−somites
20−25−somites
Prim−5
Prim−15
Prim−25
Long−pec
Protruding−mouth
Day−4
Day−5
Stage
Transcript
ENSDART00000074729
ENSDART00000173500
ENSDART00000178746

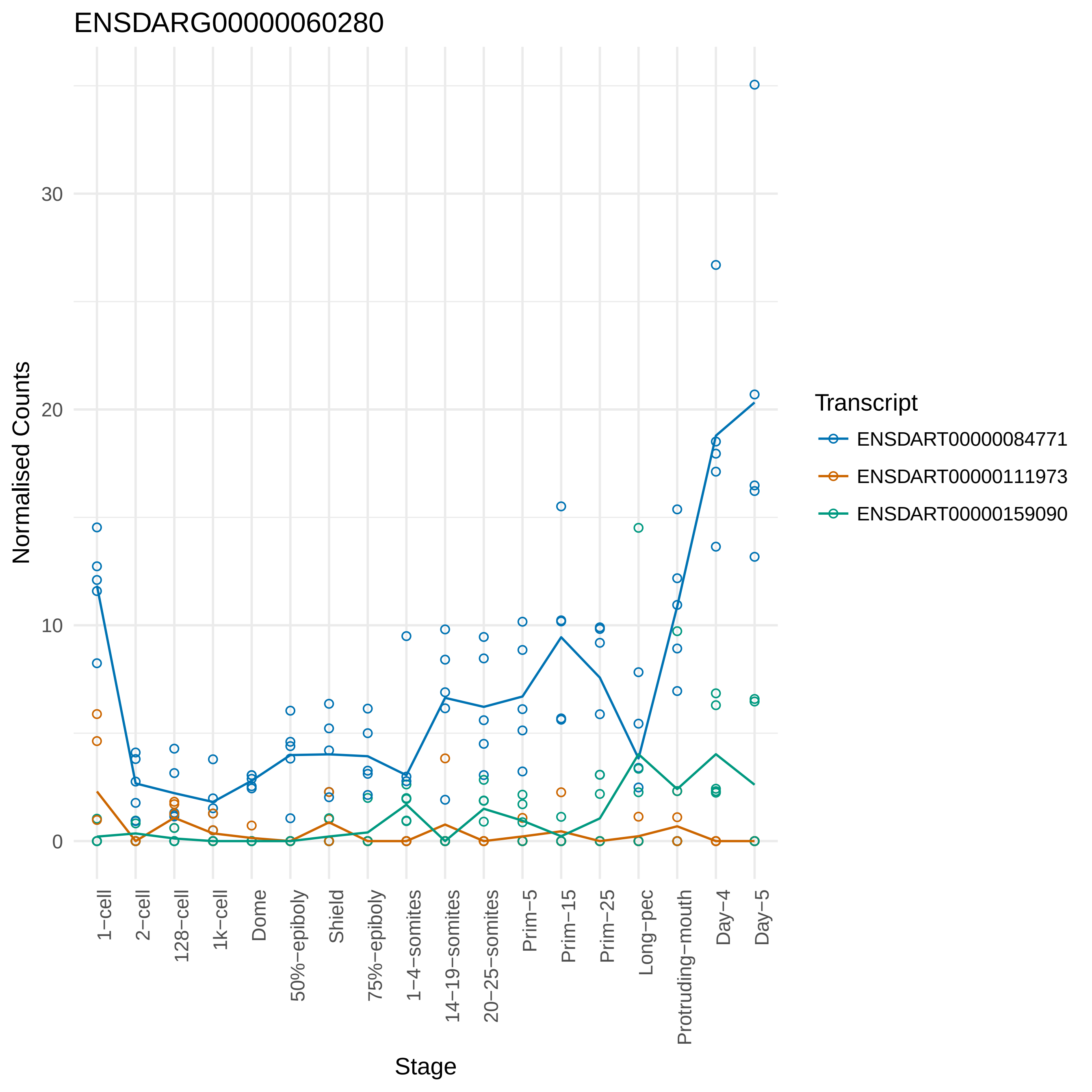

ENSDARG00000060280
Normalised Counts
0
10
20
30
Stage
1−cell
2−cell
128−cell
1k−cell
Dome
50%−epiboly
Shield
75%−epiboly
1−4−somites
14−19−somites
20−25−somites
Prim−5
Prim−15
Prim−25
Long−pec
Protruding−mouth
Day−4
Day−5
Transcript
ENSDART00000084771
ENSDART00000111973
ENSDART00000159090

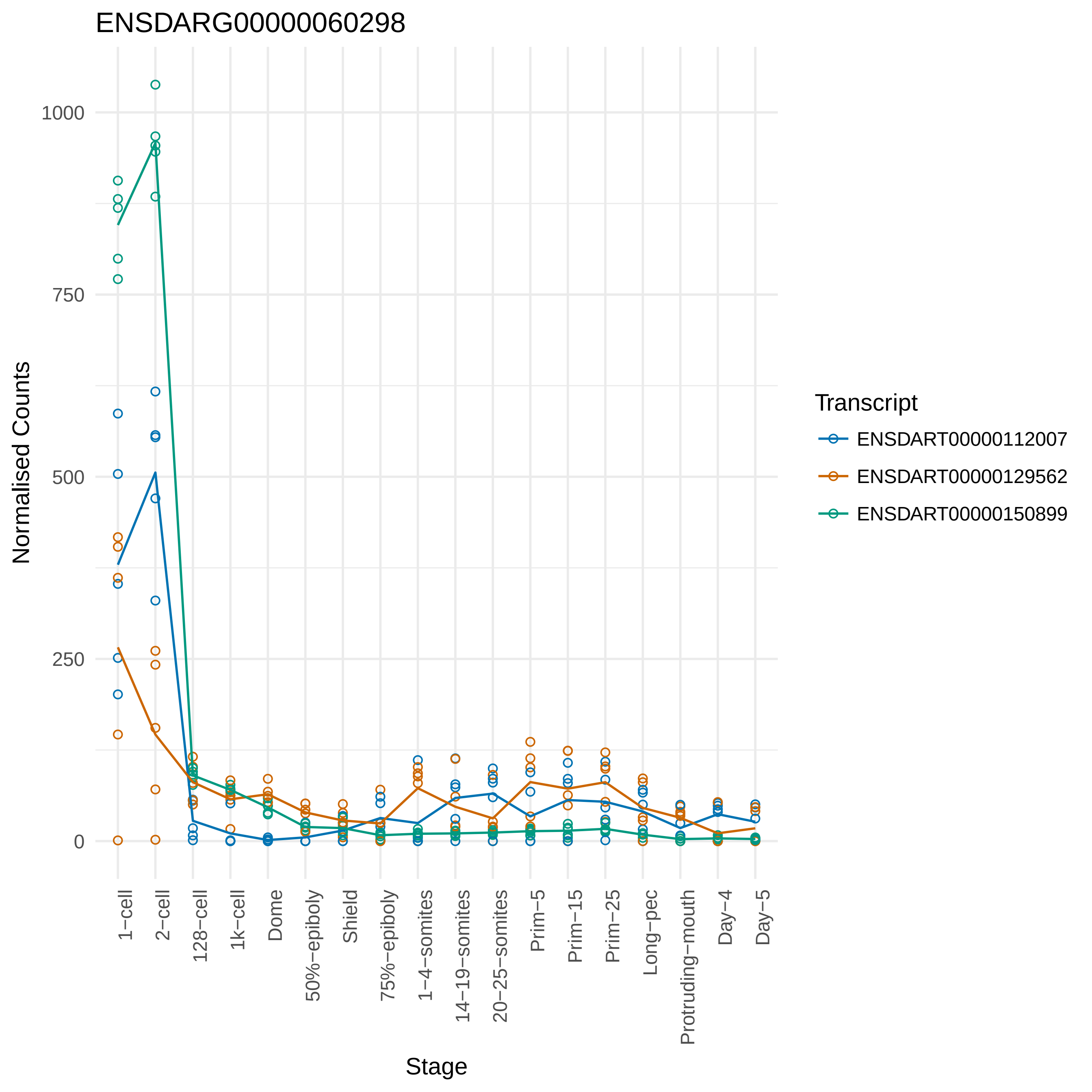
ENSDARG00000060298
Normalised Counts
1000
750
500
250
0
1−cell
2−cell
128−cell
1k−cell
Dome
50%−epiboly
Shield
75%−epiboly
1−4−somites
14−19−somites
20−25−somites
Prim−5
Prim−15
Prim−25
Long−pec
Protruding−mouth
Day−4
Day−5
Stage
Transcript
ENSDART00000112007
ENSDART00000129562
ENSDART00000150899

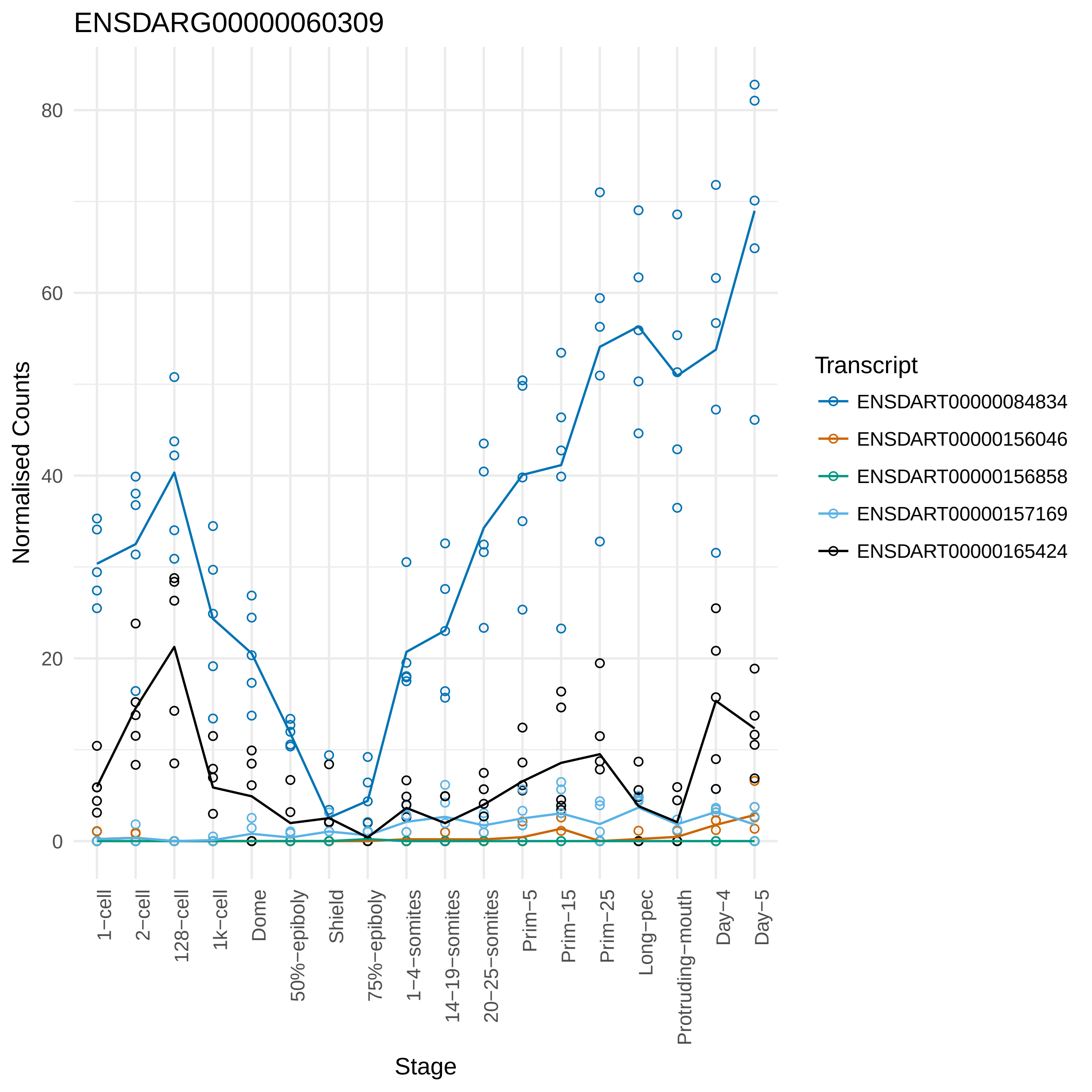

ENSDARG00000060309
Normalised Counts
0
20
40
60
80
1−cell
2−cell
128−cell
1k−cell
Dome
50%−epiboly
Shield
75%−epiboly
1−4−somites
14−19−somites
20−25−somites
Prim−5
Prim−15
Prim−25
Long−pec
Protruding−mouth
Day−4
Day−5
Stage
Transcript
ENSDART00000084834
ENSDART00000156046
ENSDART00000156858
ENSDART00000157169
ENSDART00000165424

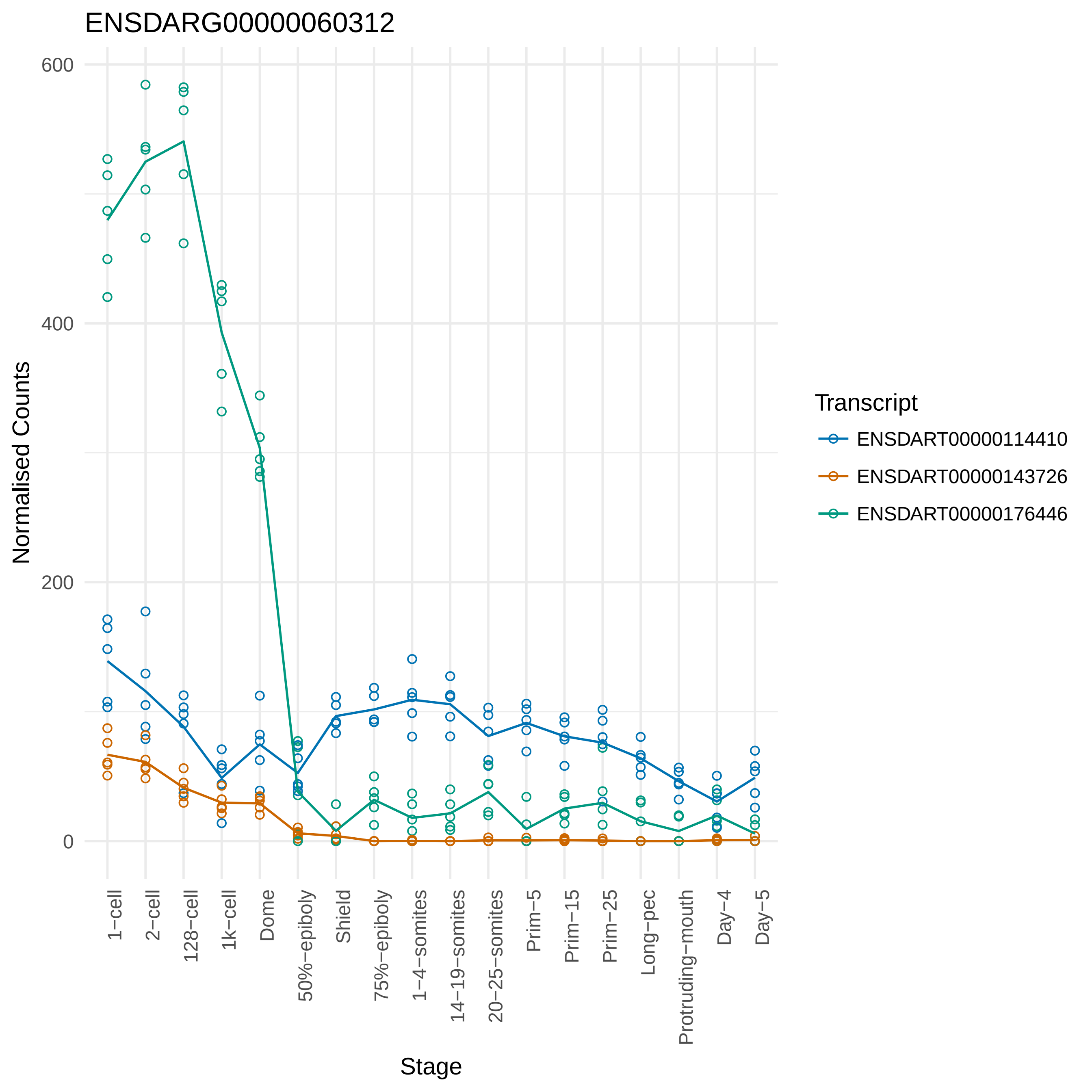
ENSDARG00000060312
Normalised Counts
600
400
200
0
1−cell
2−cell
128−cell
1k−cell
Dome
50%−epiboly
Shield
75%−epiboly
1−4−somites
14−19−somites
20−25−somites
Prim−5
Prim−15
Prim−25
Long−pec
Protruding−mouth
Day−4
Day−5
Stage
Transcript
ENSDART00000114410
ENSDART00000143726
ENSDART00000176446

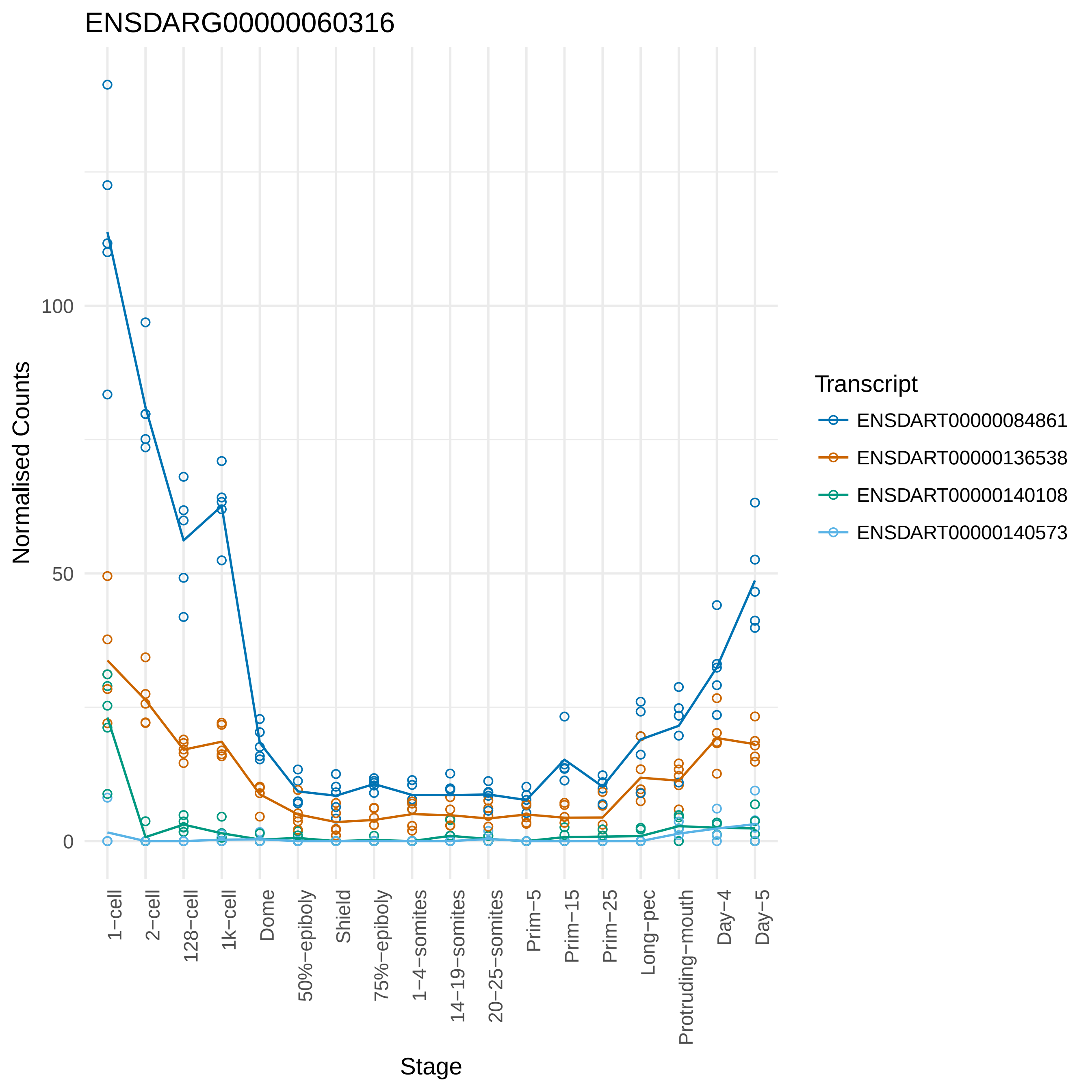
ENSDARG00000060316
Normalised Counts
100
50
0
1−cell
2−cell
128−cell
1k−cell
Dome
50%−epiboly
Shield
75%−epiboly
1−4−somites
14−19−somites
20−25−somites
Prim−5
Prim−15
Prim−25
Long−pec
Protruding−mouth
Day−4
Day−5
Stage
Transcript
ENSDART00000084861
ENSDART00000136538
ENSDART00000140108
ENSDART00000140573

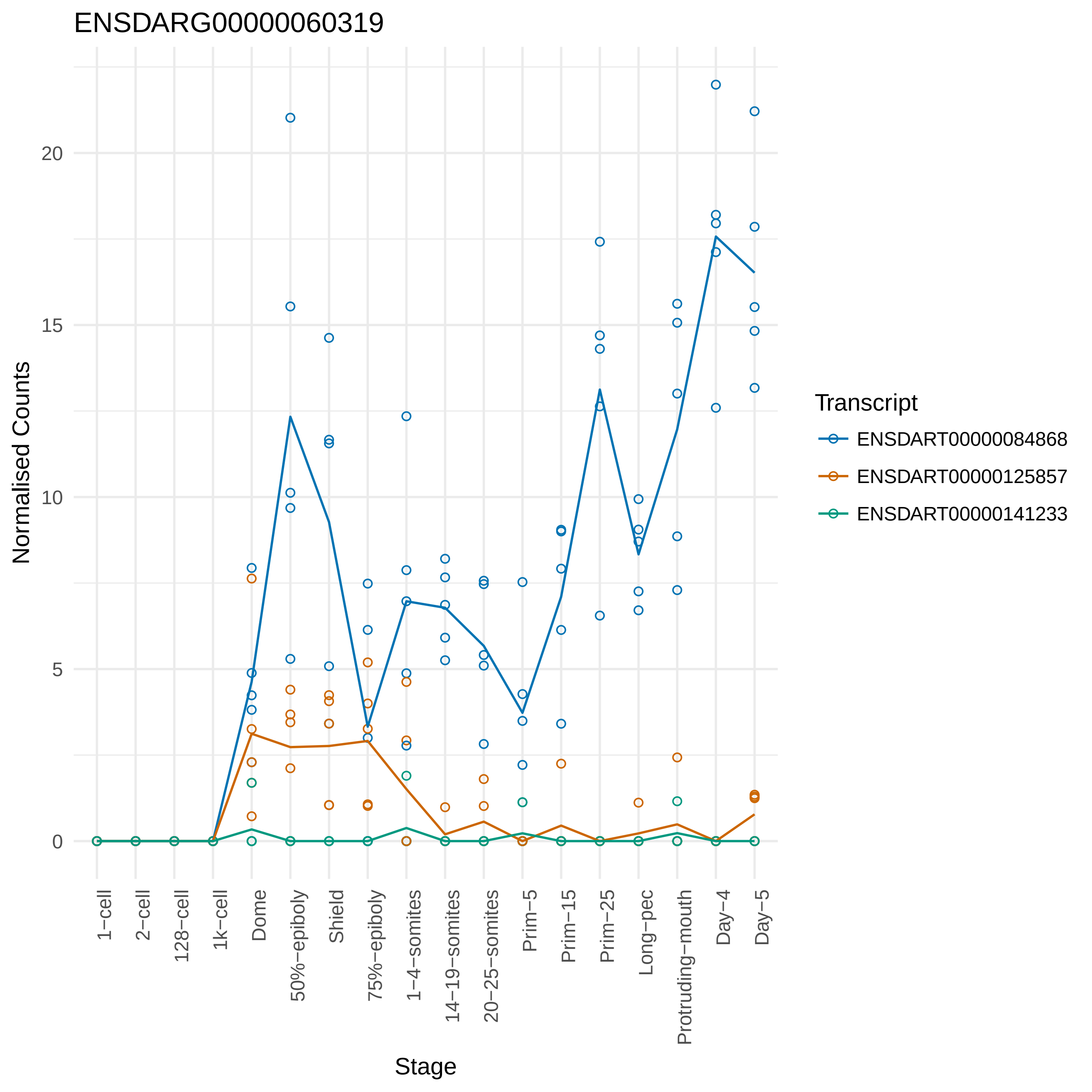

ENSDARG00000060319
Normalised Counts
0
5
10
15
20
1−cell
2−cell
128−cell
1k−cell
Dome
50%−epiboly
Shield
75%−epiboly
1−4−somites
14−19−somites
20−25−somites
Prim−5
Prim−15
Prim−25
Long−pec
Protruding−mouth
Day−4
Day−5
Stage
Transcript
ENSDART00000084868
ENSDART00000125857
ENSDART00000141233

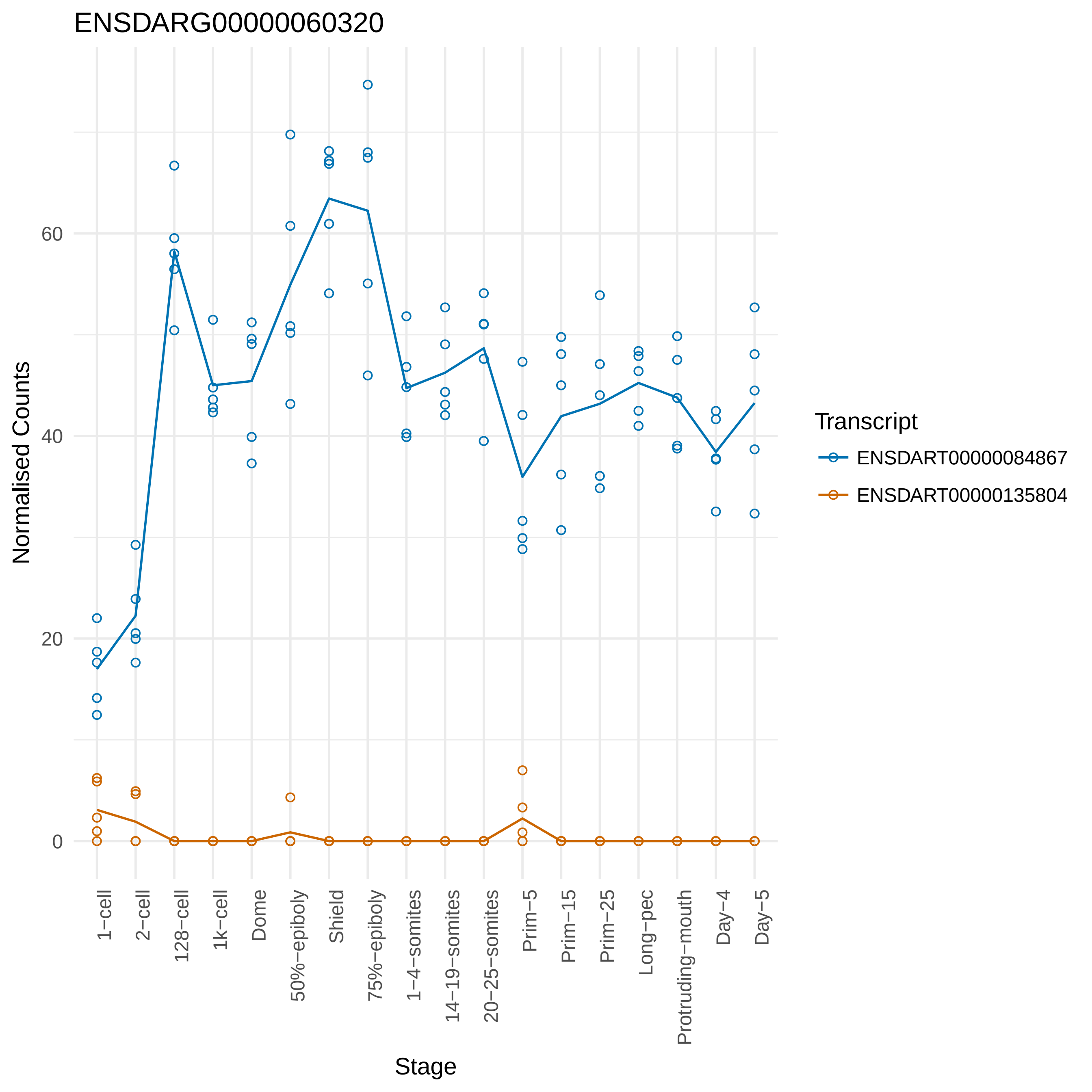

ENSDARG00000060320
Normalised Counts
0
20
40
60
Stage
1−cell
2−cell
128−cell
1k−cell
Dome
50%−epiboly
Shield
75%−epiboly
1−4−somites
14−19−somites
20−25−somites
Prim−5
Prim−15
Prim−25
Long−pec
Protruding−mouth
Day−4
Day−5
Transcript
ENSDART00000084867
ENSDART00000135804

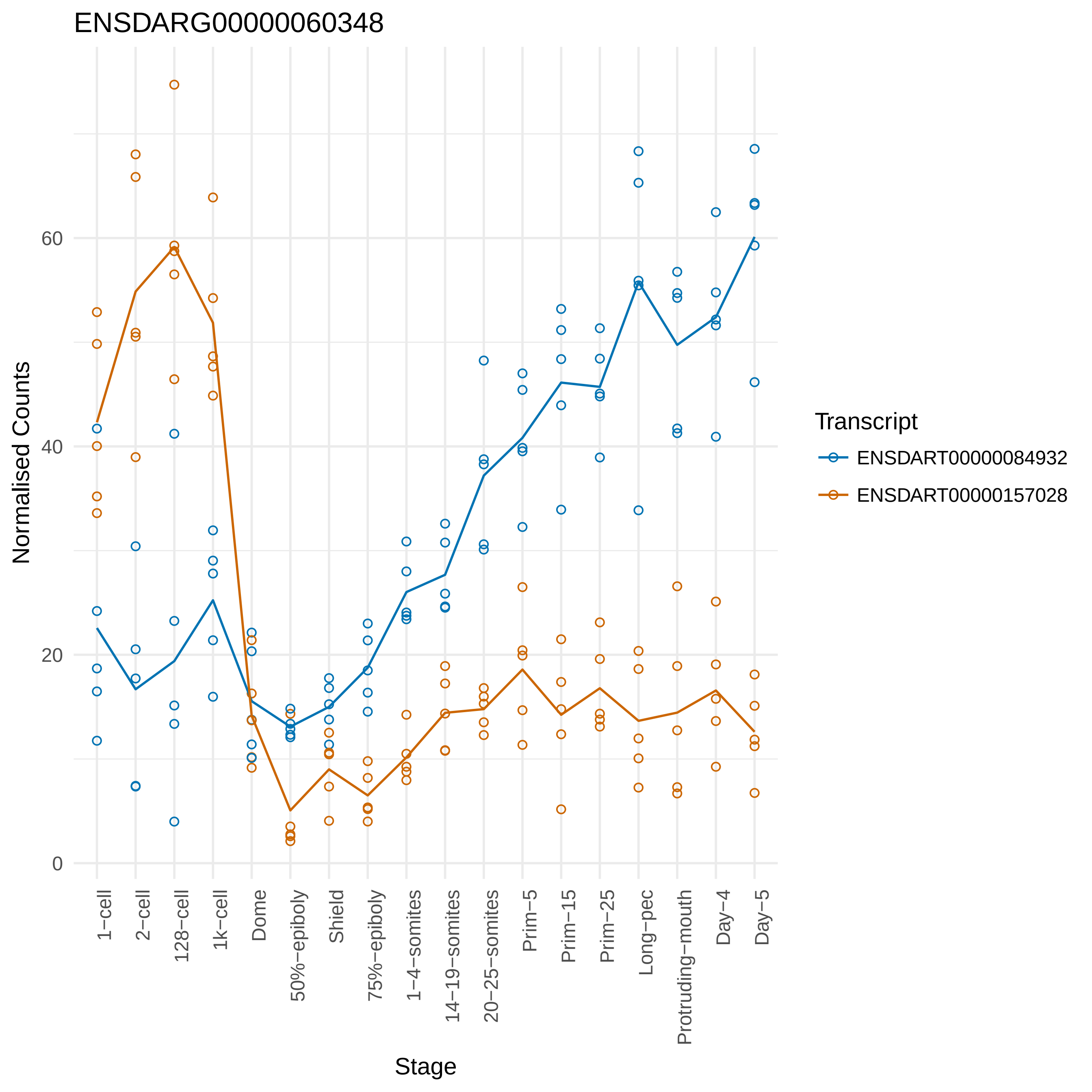

ENSDARG00000060348
Normalised Counts
0
20
40
60
1−cell
2−cell
128−cell
1k−cell
Dome
50%−epiboly
Shield
75%−epiboly
1−4−somites
14−19−somites
20−25−somites
Prim−5
Prim−15
Prim−25
Long−pec
Protruding−mouth
Day−4
Day−5
Stage
Transcript
ENSDART00000084932
ENSDART00000157028

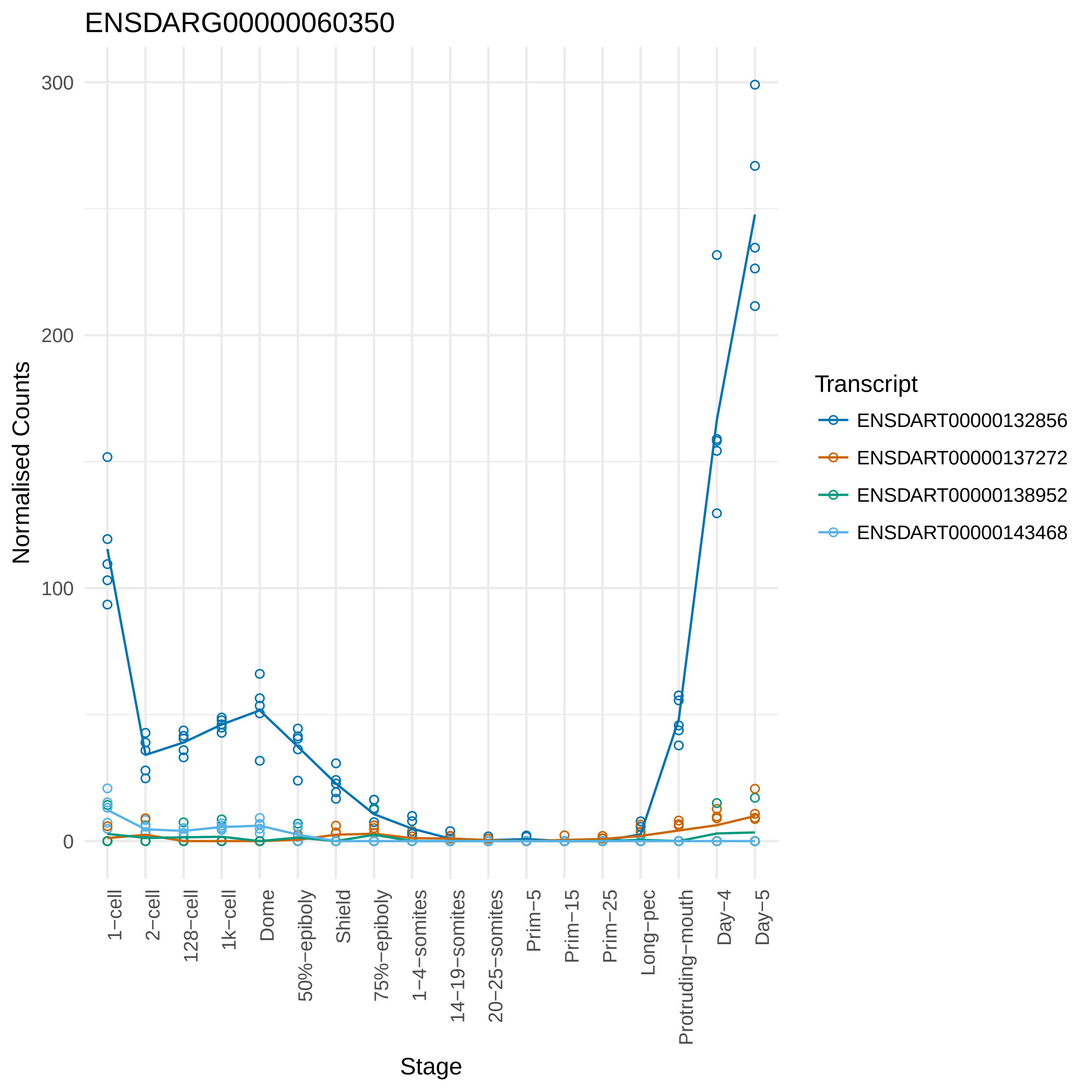

ENSDARG00000060350
Normalised Counts
300
200
100
0
1−cell
2−cell
128−cell
1k−cell
Dome
50%−epiboly
Shield
75%−epiboly
1−4−somites
14−19−somites
20−25−somites
Prim−5
Prim−15
Prim−25
Long−pec
Protruding−mouth
Day−4
Day−5
Stage
Transcript
ENSDART00000132856
ENSDART00000137272
ENSDART00000138952
ENSDART00000143468

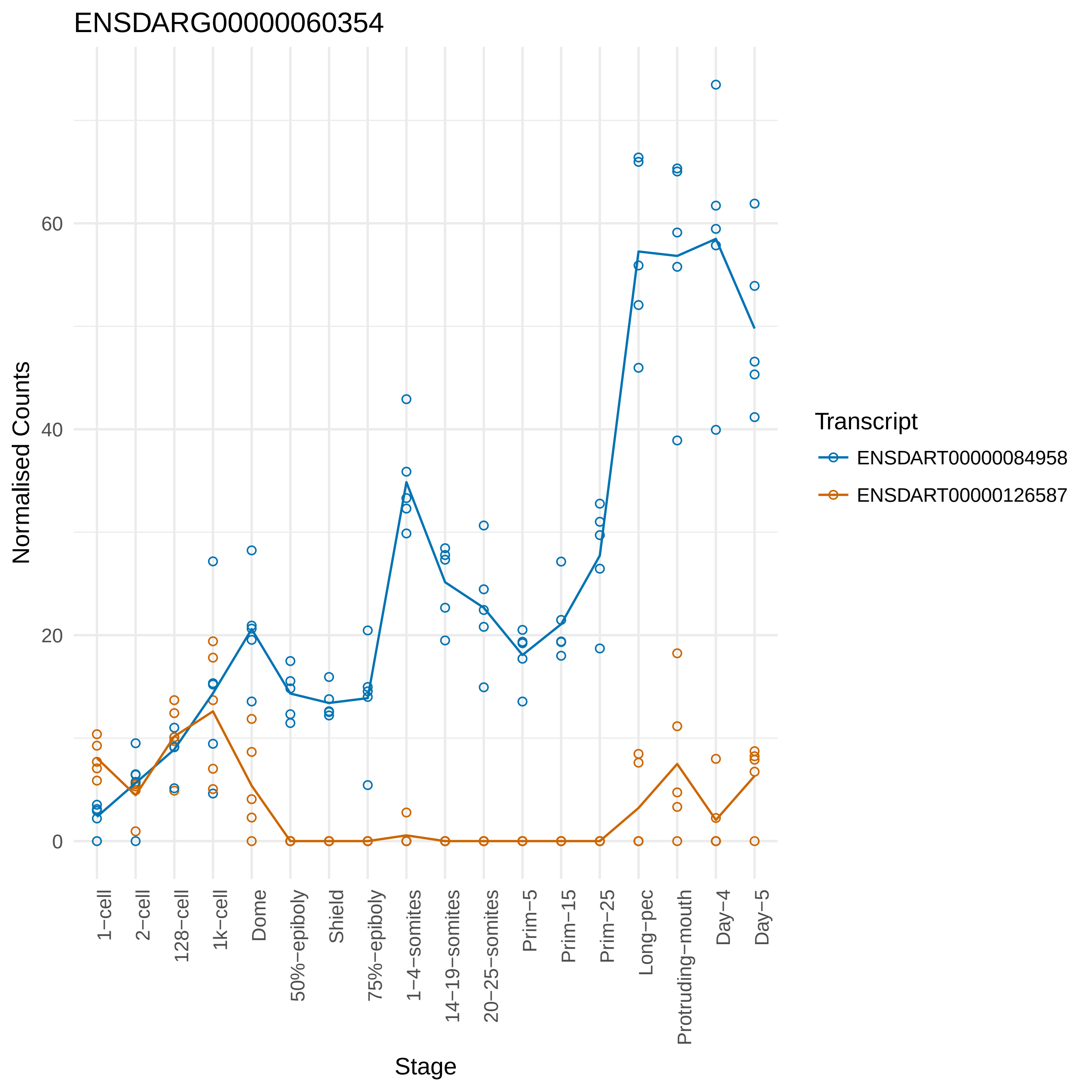

ENSDARG00000060354
Normalised Counts
0
20
40
60
Stage
1−cell
2−cell
128−cell
1k−cell
Dome
50%−epiboly
Shield
75%−epiboly
1−4−somites
14−19−somites
20−25−somites
Prim−5
Prim−15
Prim−25
Long−pec
Protruding−mouth
Day−4
Day−5
Transcript
ENSDART00000084958
ENSDART00000126587

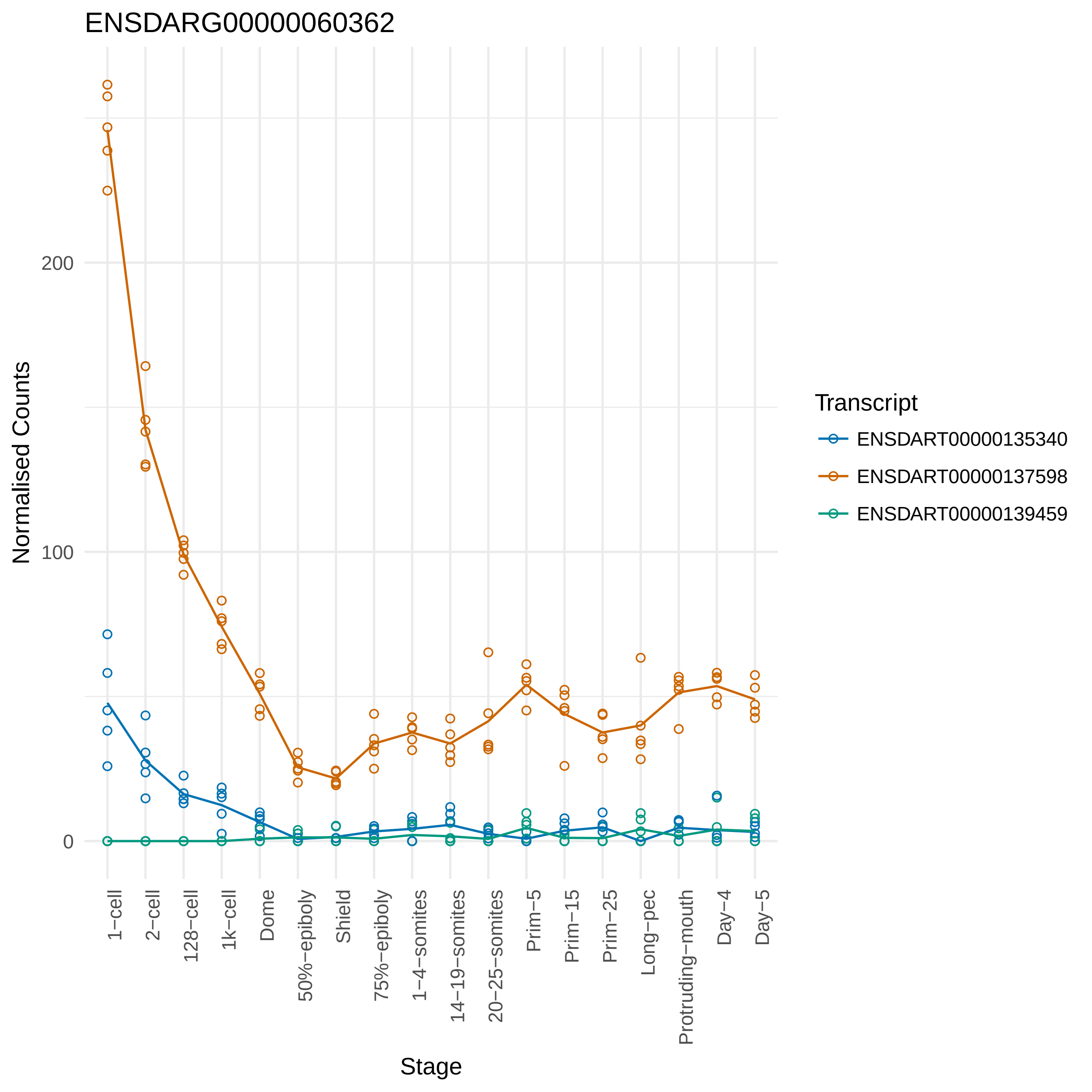
ENSDARG00000060362
Normalised Counts
200
100
0
1−cell
2−cell
128−cell
1k−cell
Dome
50%−epiboly
Shield
75%−epiboly
1−4−somites
14−19−somites
20−25−somites
Prim−5
Prim−15
Prim−25
Long−pec
Protruding−mouth
Day−4
Day−5
Stage
Transcript
ENSDART00000135340
ENSDART00000137598
ENSDART00000139459

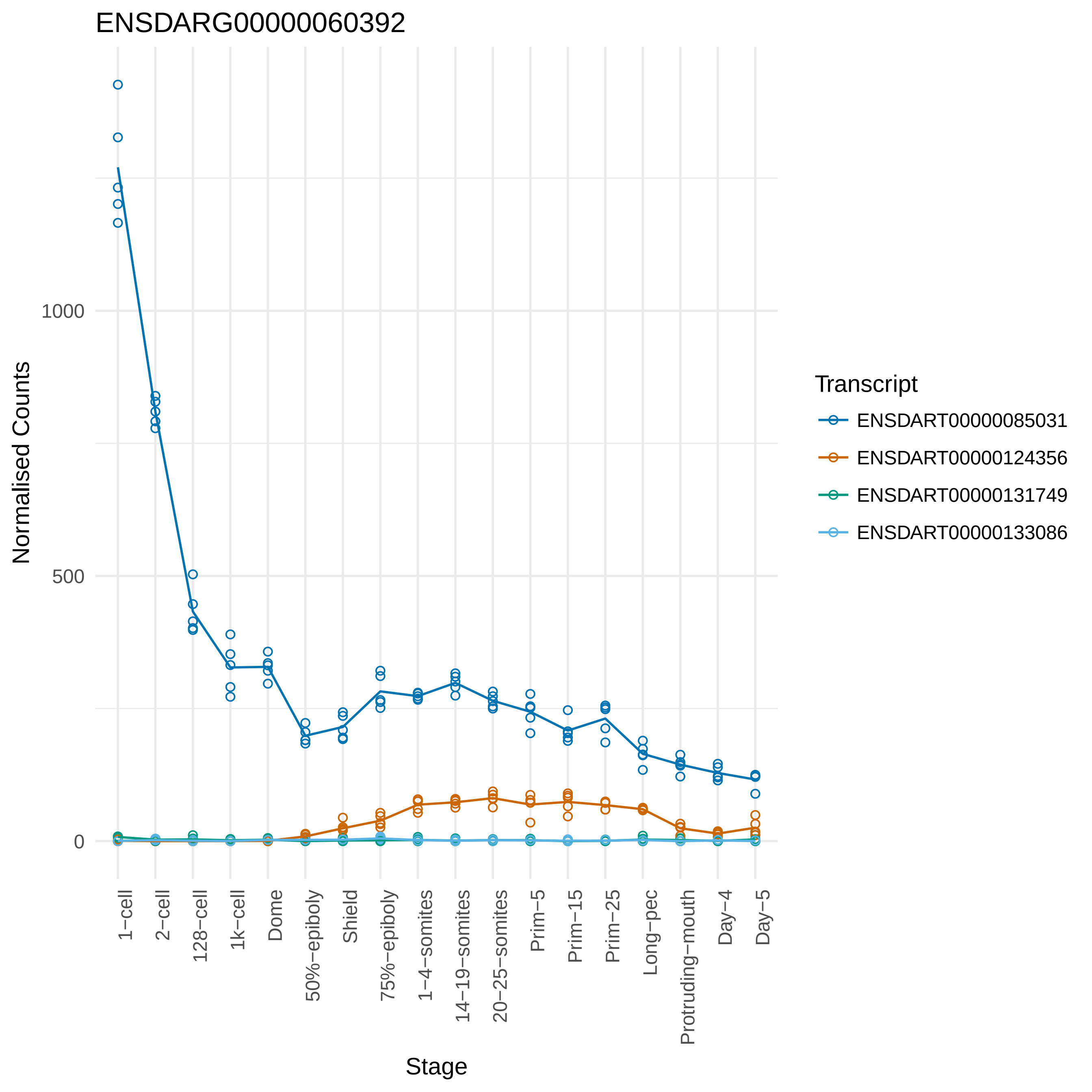

ENSDARG00000060392
Normalised Counts
1000
500
0
1−cell
2−cell
128−cell
1k−cell
Dome
50%−epiboly
Shield
75%−epiboly
1−4−somites
14−19−somites
20−25−somites
Prim−5
Prim−15
Prim−25
Long−pec
Protruding−mouth
Day−4
Day−5
Stage
Transcript
ENSDART00000085031
ENSDART00000124356
ENSDART00000131749
ENSDART00000133086

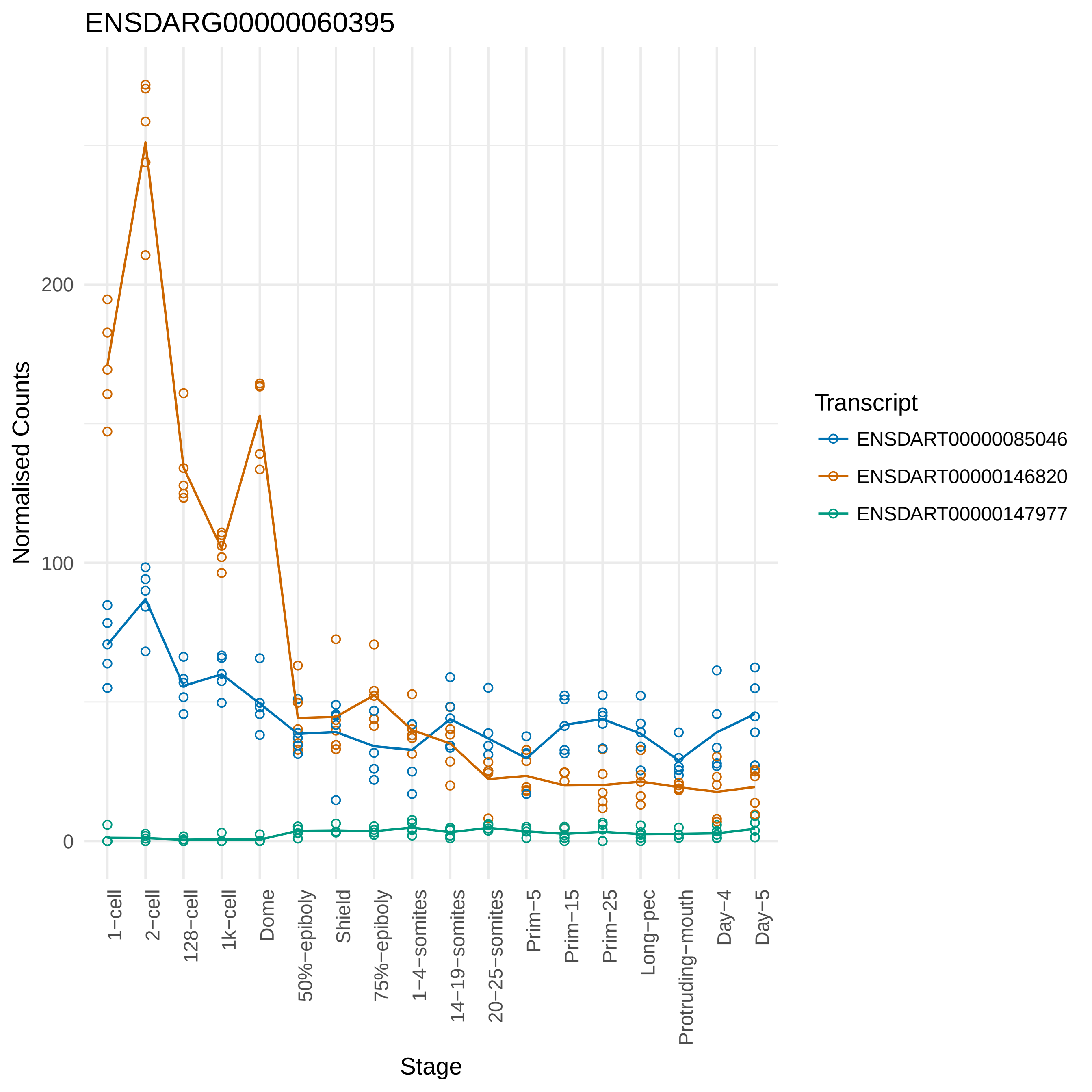
ENSDARG00000060395
Normalised Counts
0
100
200
Stage
1−cell
2−cell
128−cell
1k−cell
Dome
50%−epiboly
Shield
75%−epiboly
1−4−somites
14−19−somites
20−25−somites
Prim−5
Prim−15
Prim−25
Long−pec
Protruding−mouth
Day−4
Day−5
Transcript
ENSDART00000085046
ENSDART00000146820
ENSDART00000147977

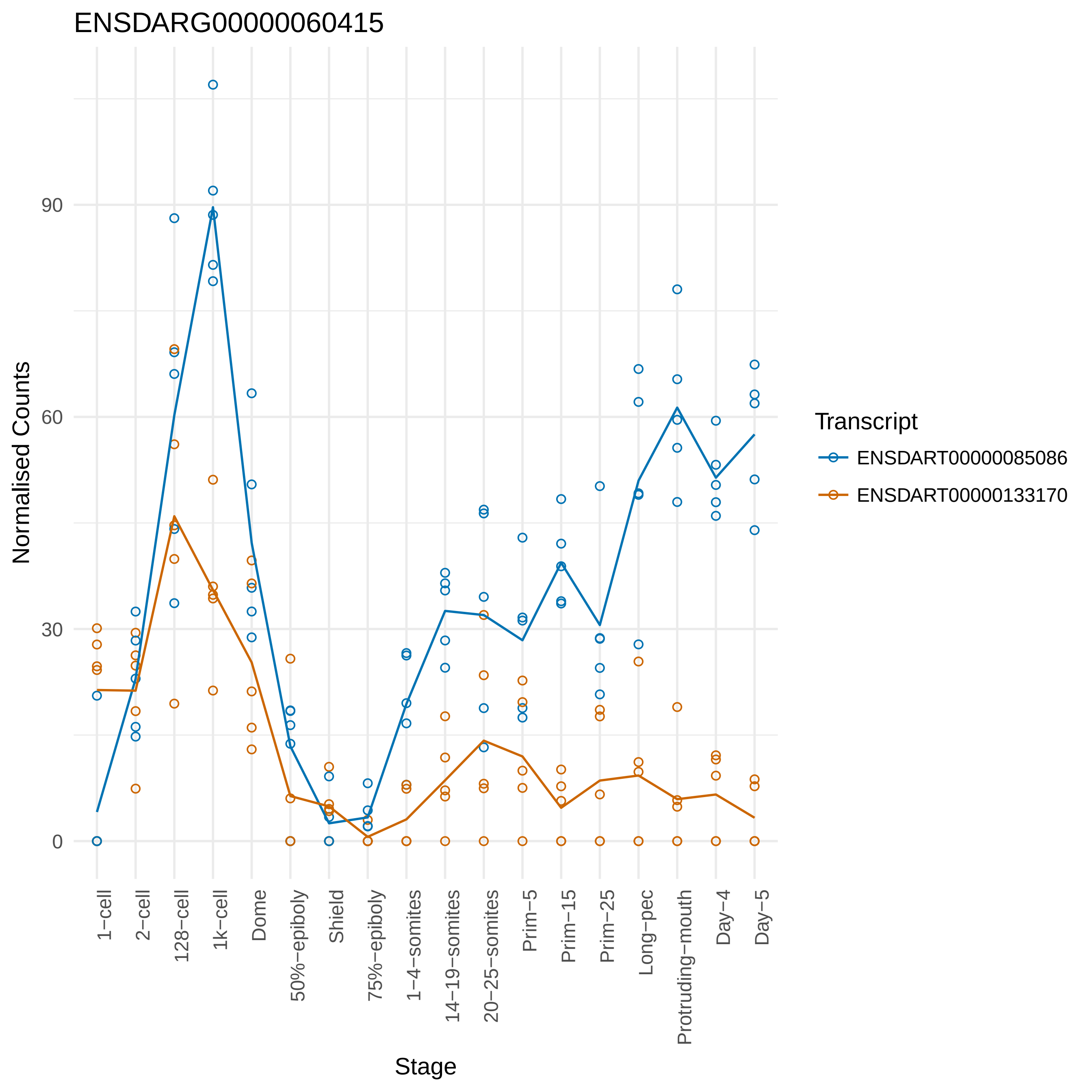
ENSDARG00000060415
Normalised Counts
0
30
60
90
1−cell
2−cell
128−cell
1k−cell
Dome
50%−epiboly
Shield
75%−epiboly
1−4−somites
14−19−somites
20−25−somites
Prim−5
Prim−15
Prim−25
Long−pec
Protruding−mouth
Day−4
Day−5
Stage
Transcript
ENSDART00000085086
ENSDART00000133170

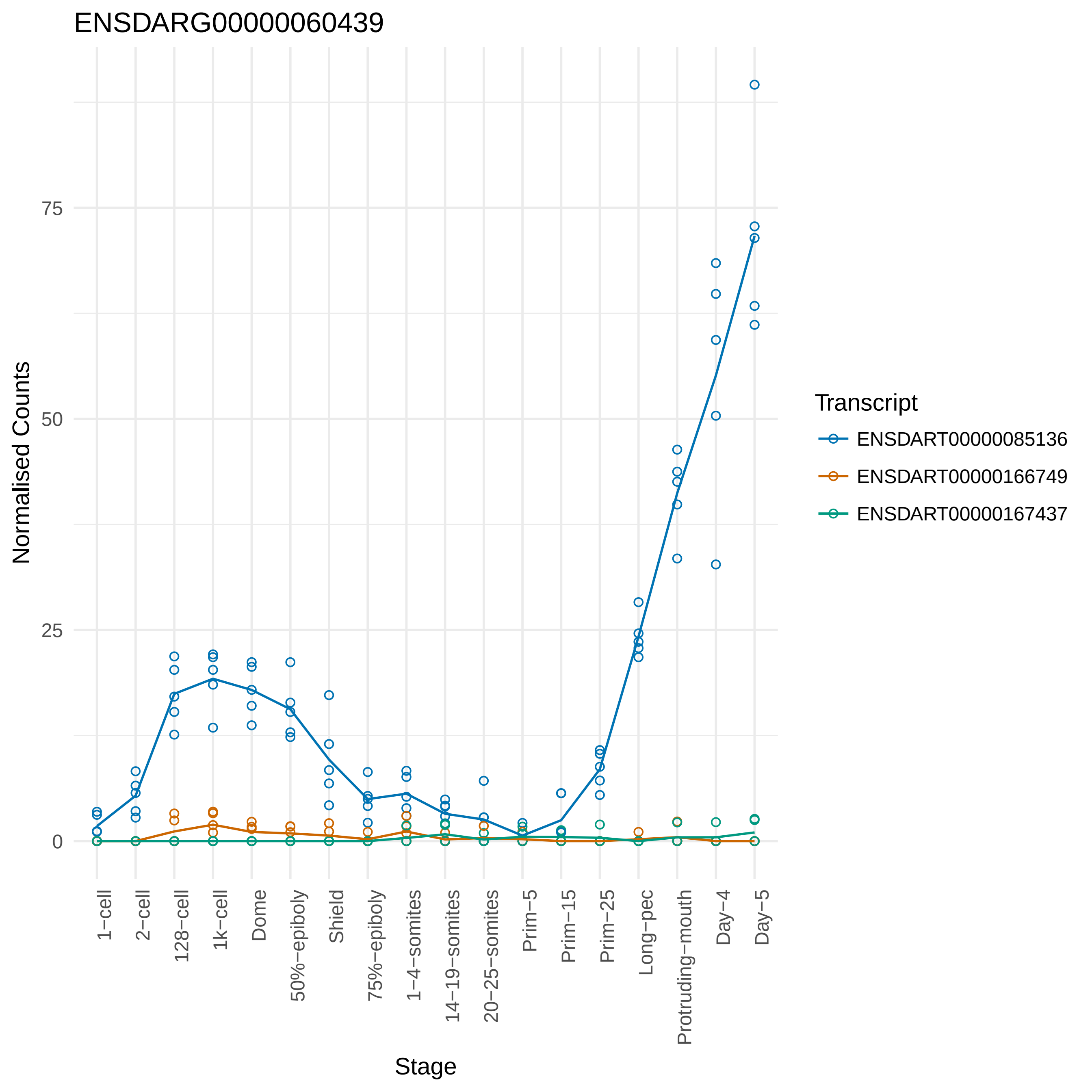

ENSDARG00000060439
Normalised Counts
75
50
25
0
1−cell
2−cell
128−cell
1k−cell
Dome
50%−epiboly
Shield
75%−epiboly
1−4−somites
14−19−somites
20−25−somites
Prim−5
Prim−15
Prim−25
Long−pec
Protruding−mouth
Day−4
Day−5
Stage
Transcript
ENSDART00000085136
ENSDART00000166749
ENSDART00000167437

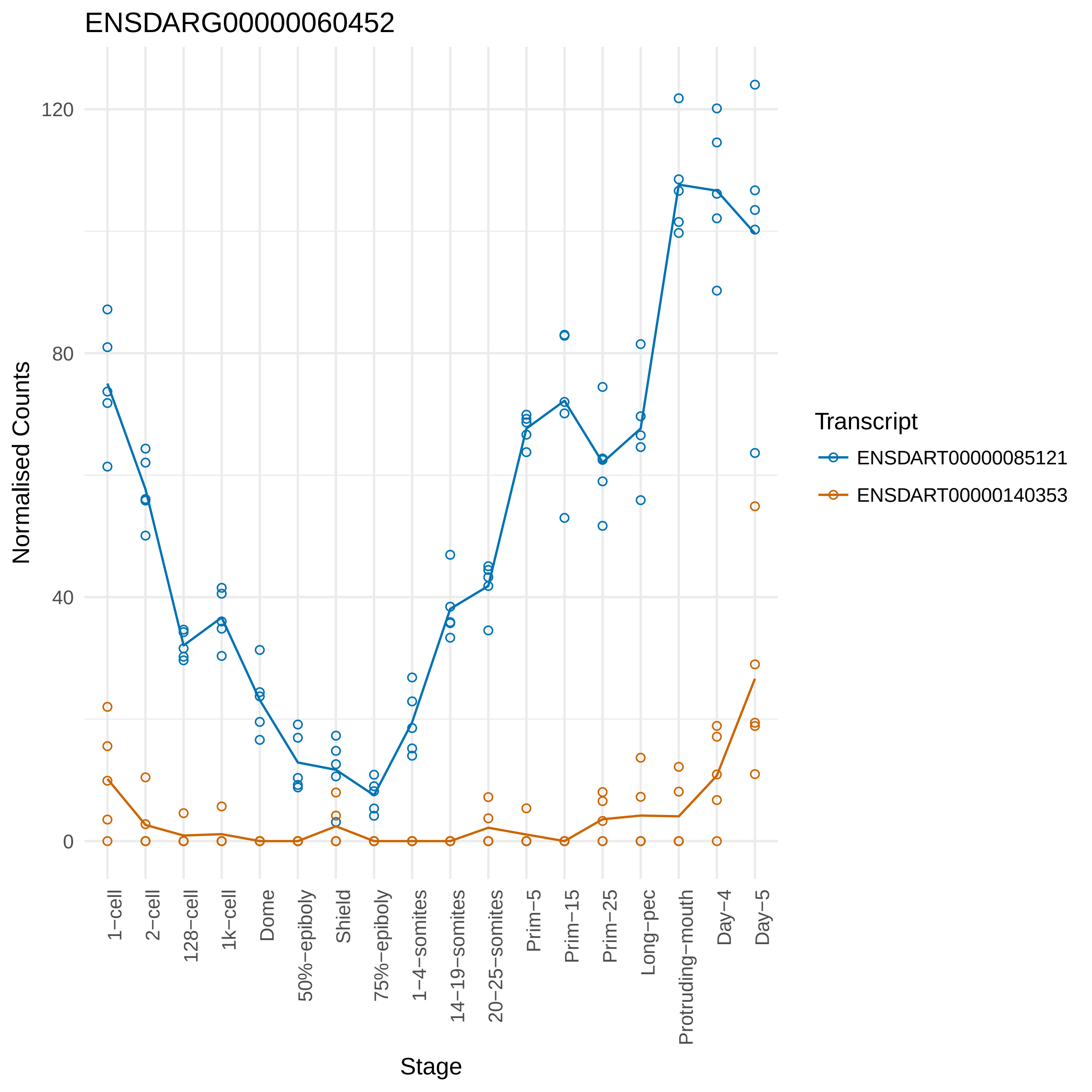

ENSDARG00000060452
Normalised Counts
120
80
40
0
1−cell
2−cell
128−cell
1k−cell
Dome
50%−epiboly
Shield
75%−epiboly
1−4−somites
14−19−somites
20−25−somites
Prim−5
Prim−15
Prim−25
Long−pec
Protruding−mouth
Day−4
Day−5
Stage
Transcript
ENSDART00000085121
ENSDART00000140353

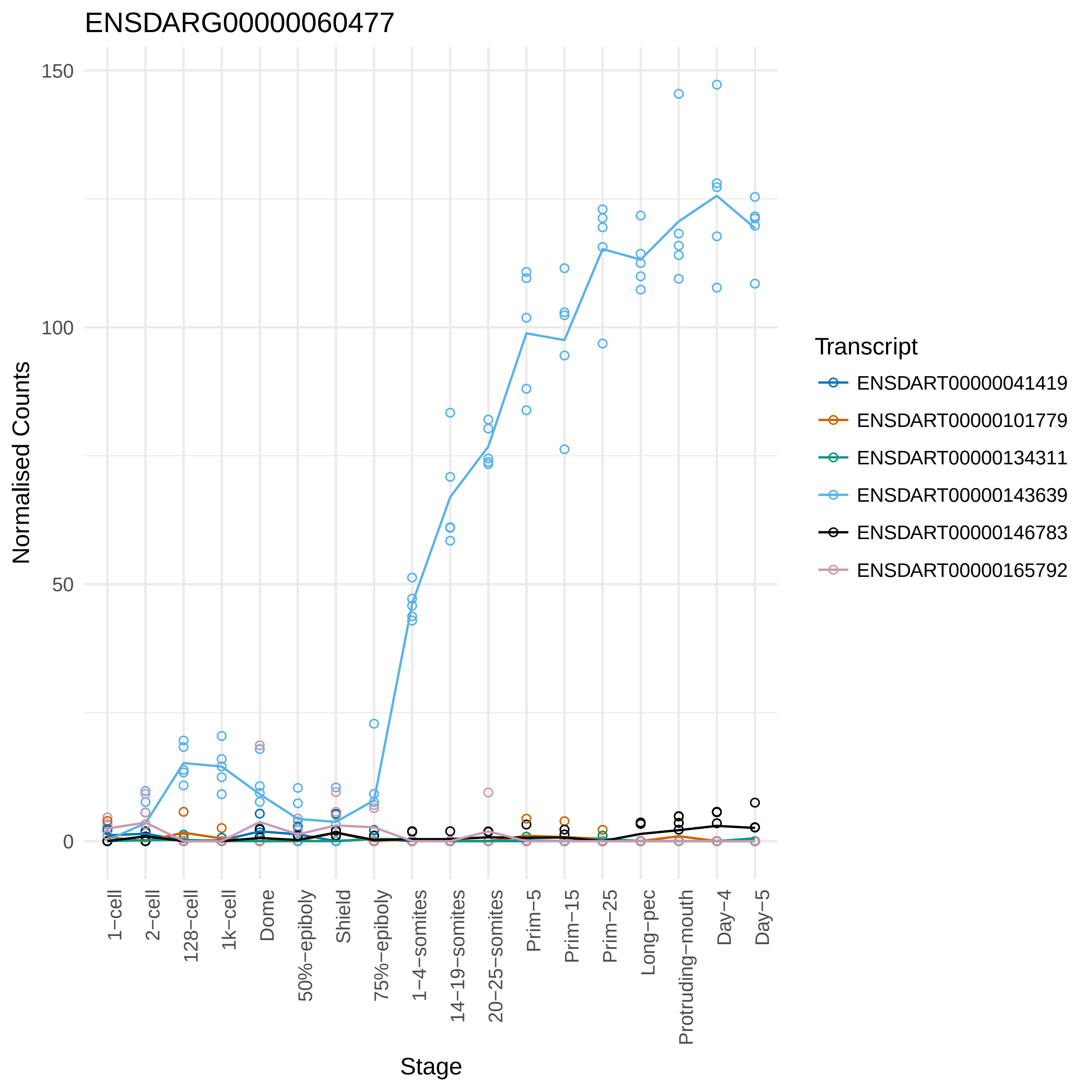
ENSDARG00000060477
Normalised Counts
150
100
50
0
Stage
1−cell
2−cell
128−cell
1k−cell
Dome
50%−epiboly
Shield
75%−epiboly
1−4−somites
14−19−somites
20−25−somites
Prim−5
Prim−15
Prim−25
Long−pec
Protruding−mouth
Day−4
Day−5
Transcript
ENSDART00000041419
ENSDART00000101779
ENSDART00000134311
ENSDART00000143639
ENSDART00000146783
ENSDART00000165792

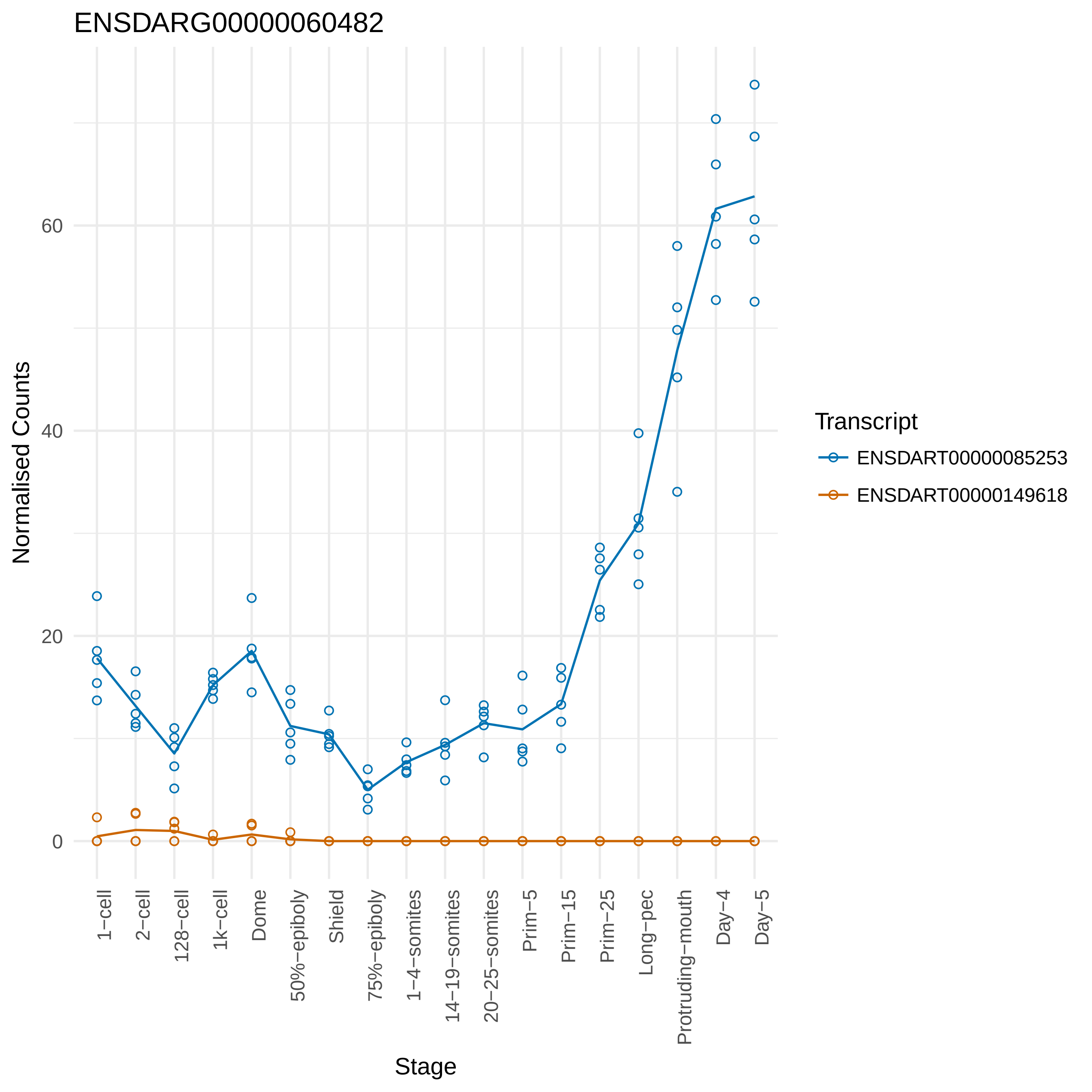

ENSDARG00000060482
Normalised Counts
0
20
40
60
1−cell
2−cell
128−cell
1k−cell
Dome
50%−epiboly
Shield
75%−epiboly
1−4−somites
14−19−somites
20−25−somites
Prim−5
Prim−15
Prim−25
Long−pec
Protruding−mouth
Day−4
Day−5
Stage
Transcript
ENSDART00000085253
ENSDART00000149618

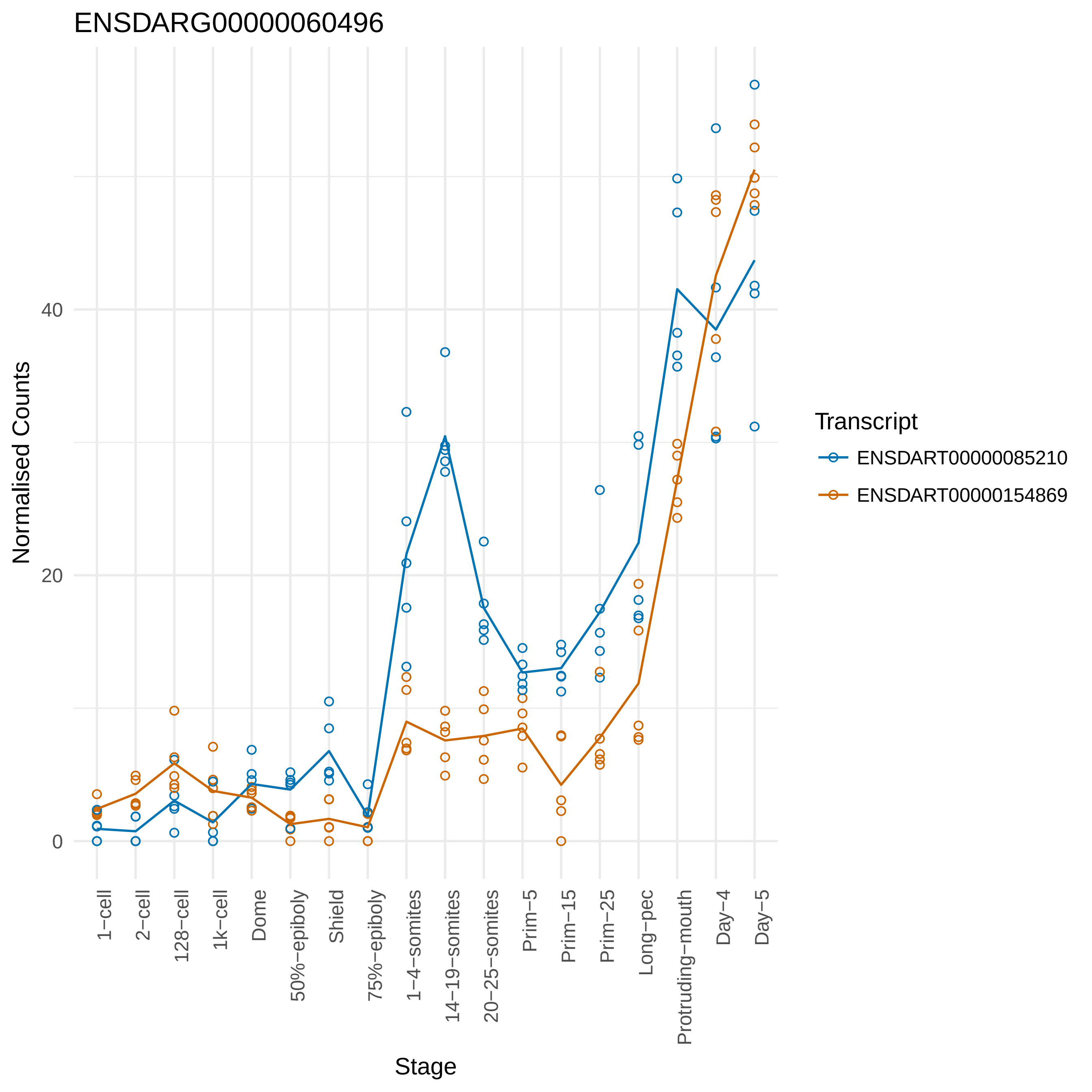
ENSDARG00000060496
Normalised Counts
0
20
40
1−cell
2−cell
128−cell
1k−cell
Dome
50%−epiboly
Shield
75%−epiboly
1−4−somites
14−19−somites
20−25−somites
Prim−5
Prim−15
Prim−25
Long−pec
Protruding−mouth
Day−4
Day−5
Stage
Transcript
ENSDART00000085210
ENSDART00000154869

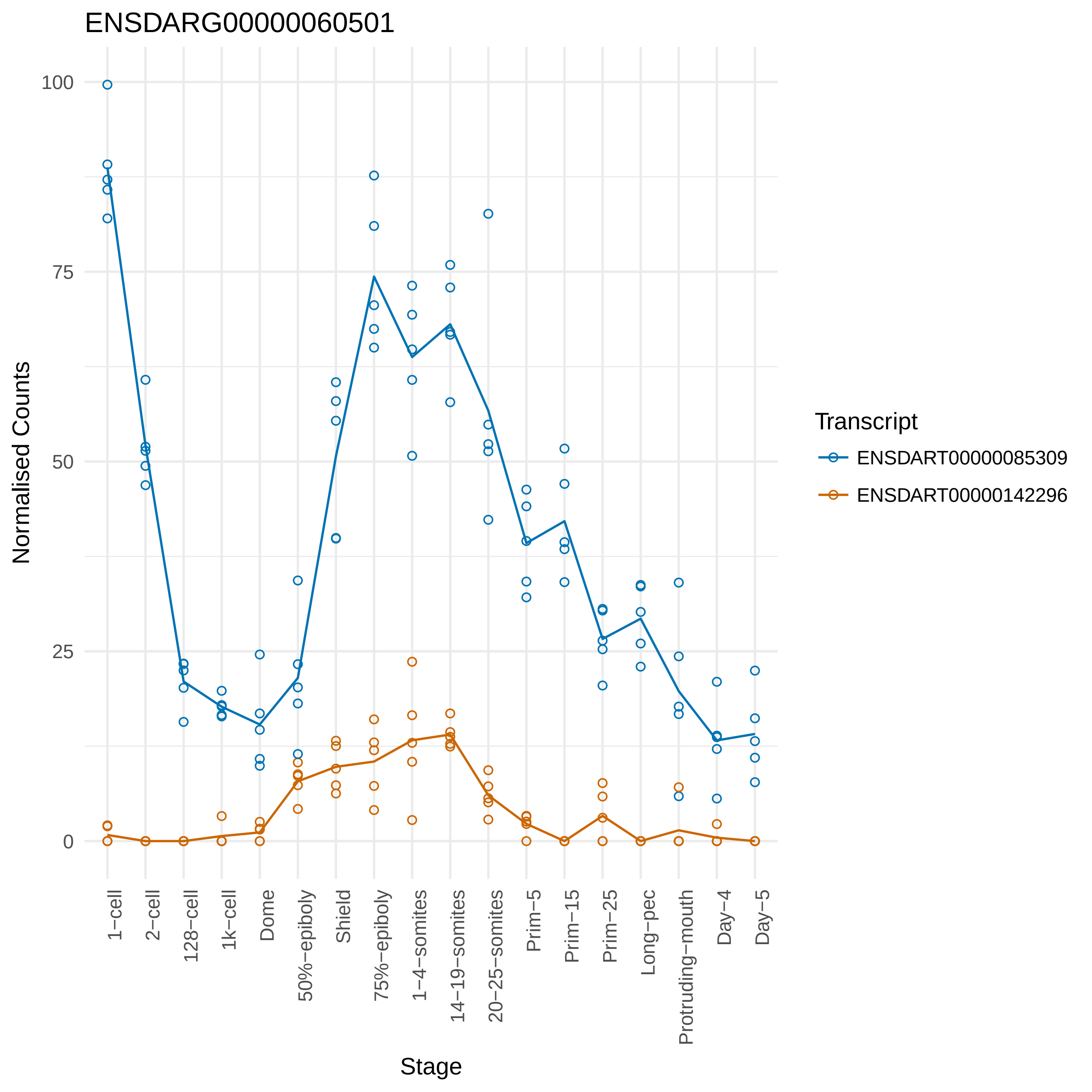
ENSDARG00000060501
Normalised Counts
100
75
50
25
0
Stage
1−cell
2−cell
128−cell
1k−cell
Dome
50%−epiboly
Shield
75%−epiboly
1−4−somites
14−19−somites
20−25−somites
Prim−5
Prim−15
Prim−25
Long−pec
Protruding−mouth
Day−4
Day−5
Transcript
ENSDART00000085309
ENSDART00000142296

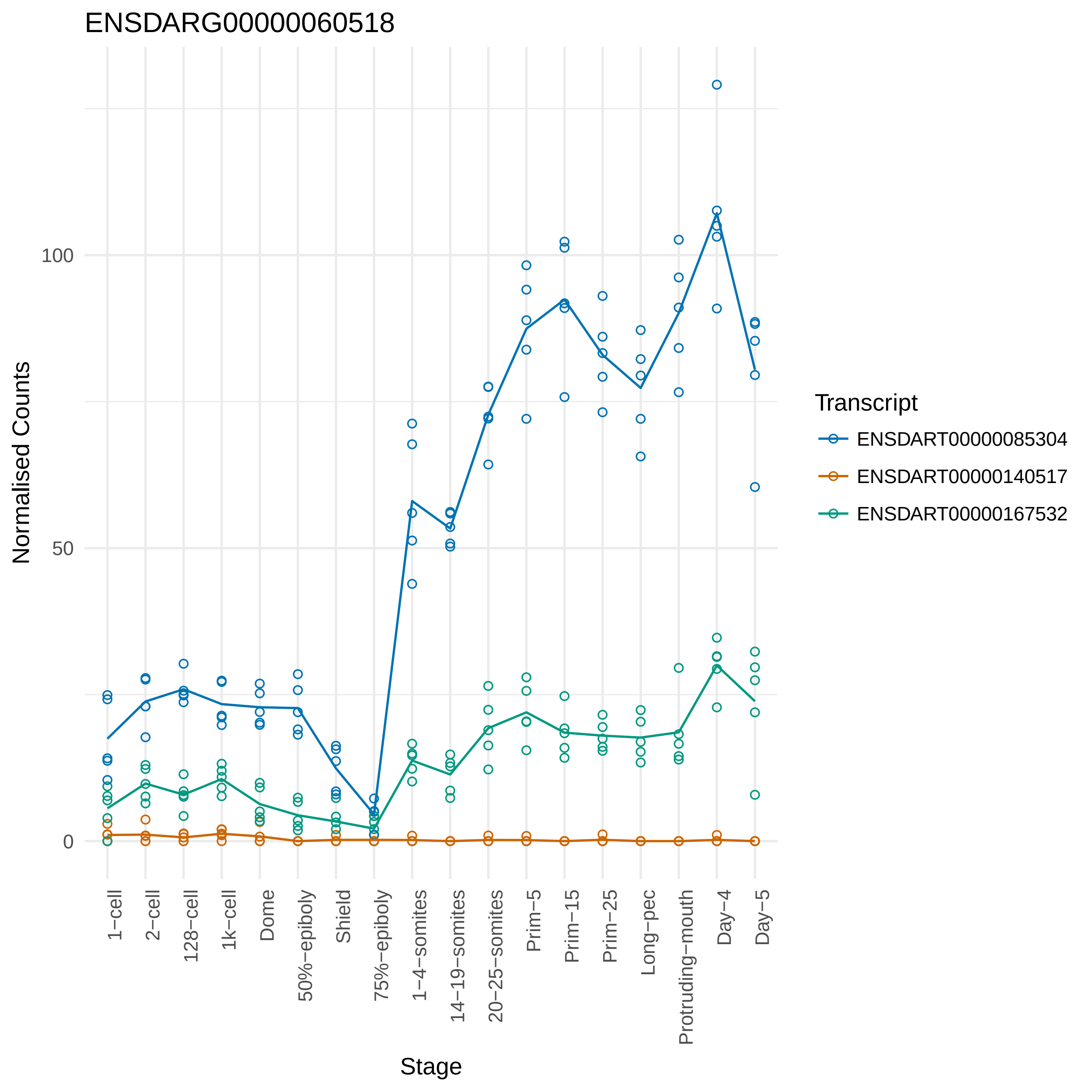

ENSDARG00000060518
Normalised Counts
100
50
0
1−cell
2−cell
128−cell
1k−cell
Dome
50%−epiboly
Shield
75%−epiboly
1−4−somites
14−19−somites
20−25−somites
Prim−5
Prim−15
Prim−25
Long−pec
Protruding−mouth
Day−4
Day−5
Stage
Transcript
ENSDART00000085304
ENSDART00000140517
ENSDART00000167532

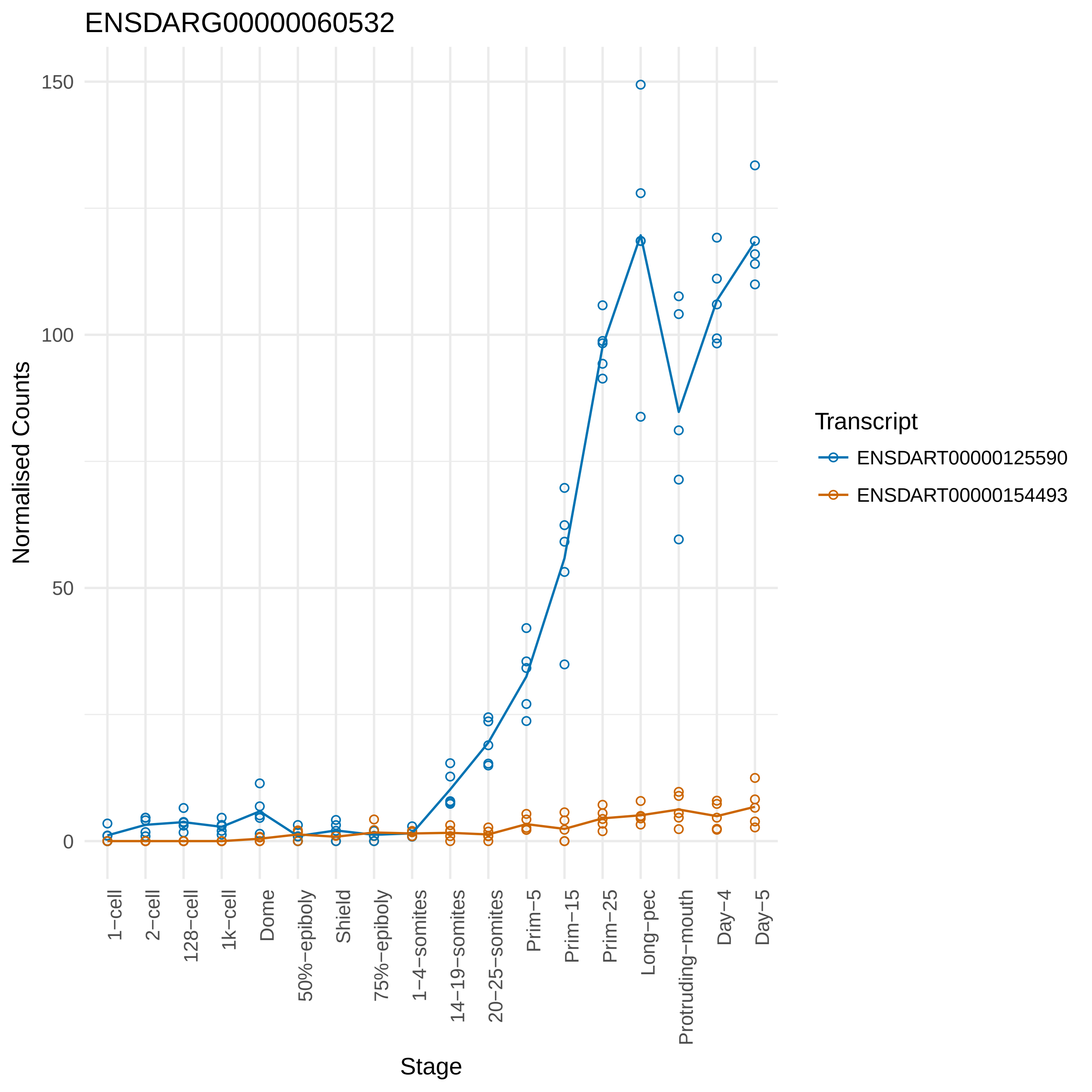

ENSDARG00000060532
Normalised Counts
150
100
50
0
1−cell
2−cell
128−cell
1k−cell
Dome
50%−epiboly
Shield
75%−epiboly
1−4−somites
14−19−somites
20−25−somites
Prim−5
Prim−15
Prim−25
Long−pec
Protruding−mouth
Day−4
Day−5
Stage
Transcript
ENSDART00000125590
ENSDART00000154493

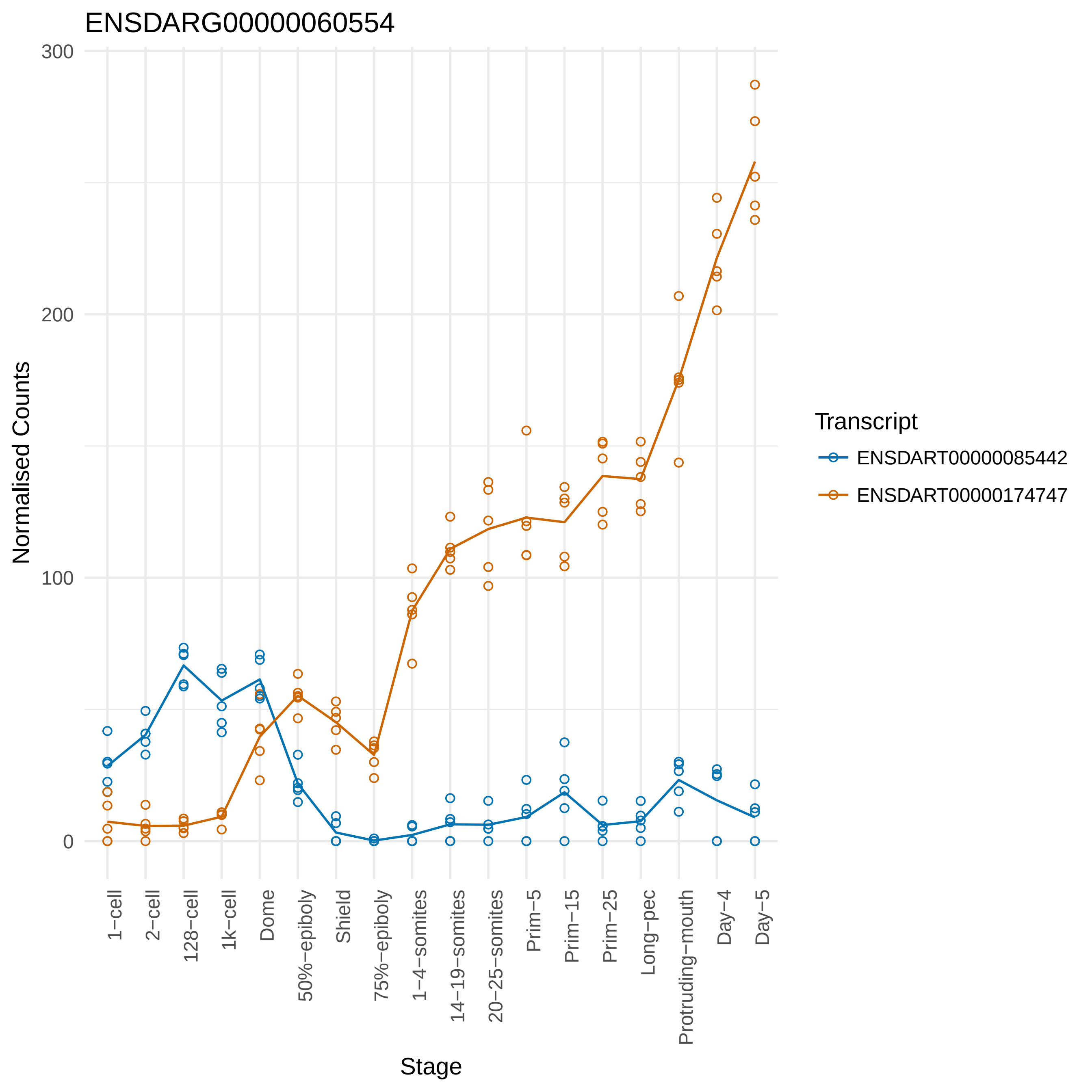

ENSDARG00000060554
Normalised Counts
300
200
100
0
1−cell
2−cell
128−cell
1k−cell
Dome
50%−epiboly
Shield
75%−epiboly
1−4−somites
14−19−somites
20−25−somites
Prim−5
Prim−15
Prim−25
Long−pec
Protruding−mouth
Day−4
Day−5
Stage
Transcript
ENSDART00000085442
ENSDART00000174747

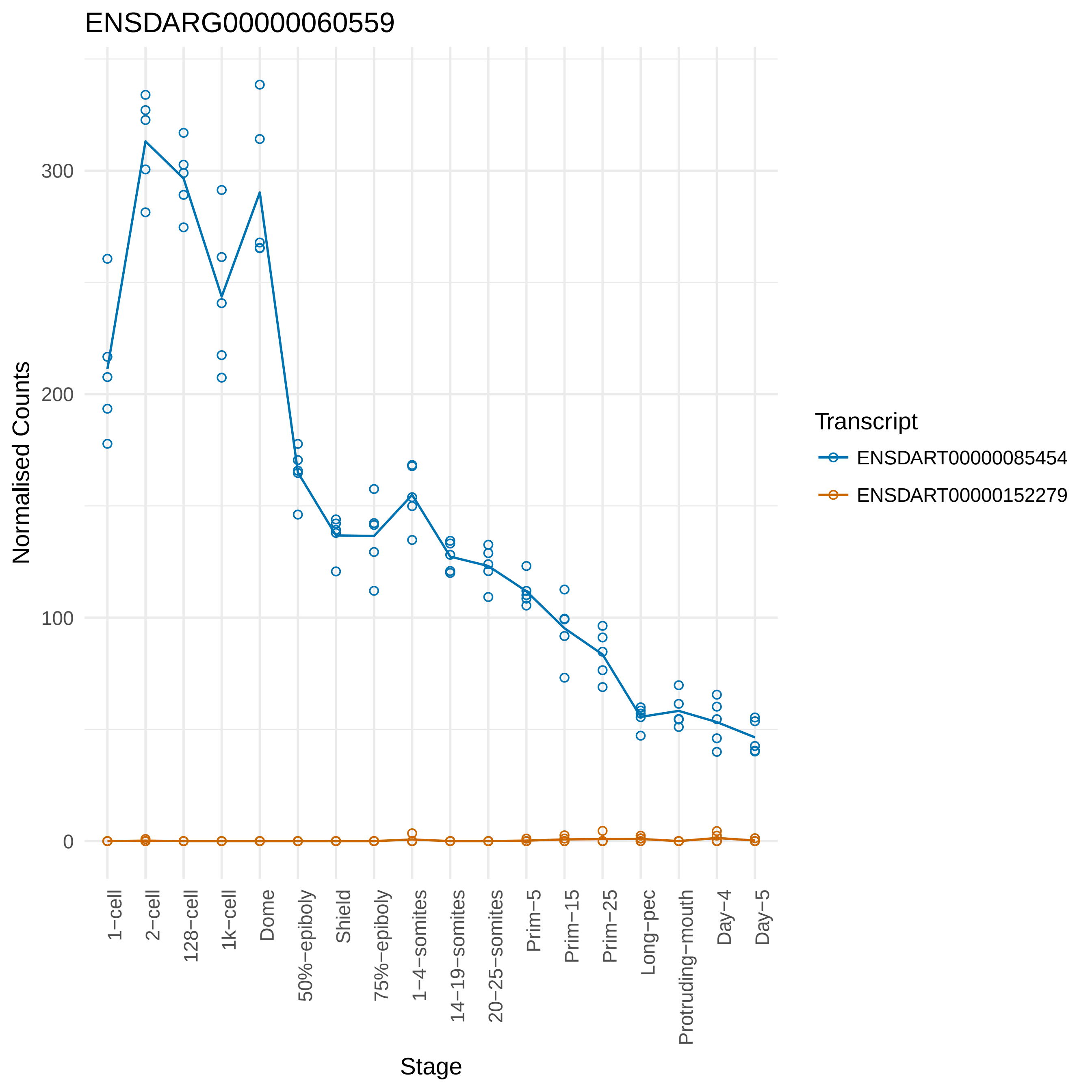

ENSDARG00000060559
Normalised Counts
300
200
100
0
1−cell
2−cell
128−cell
1k−cell
Dome
50%−epiboly
Shield
75%−epiboly
1−4−somites
14−19−somites
20−25−somites
Prim−5
Prim−15
Prim−25
Long−pec
Protruding−mouth
Day−4
Day−5
Stage
Transcript
ENSDART00000085454
ENSDART00000152279

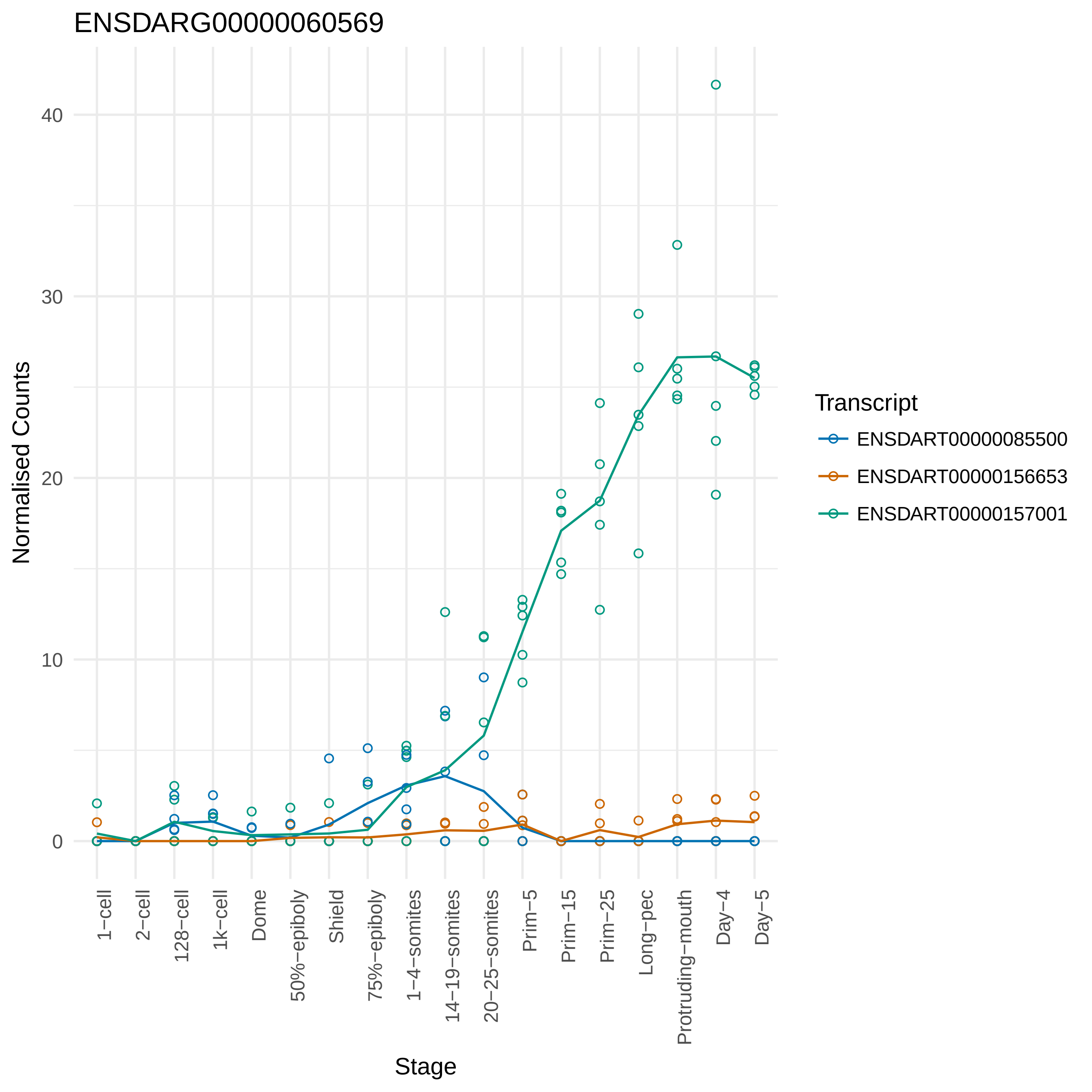
ENSDARG00000060569
Normalised Counts
0
10
20
30
40
1−cell
2−cell
128−cell
1k−cell
Dome
50%−epiboly
Shield
75%−epiboly
1−4−somites
14−19−somites
20−25−somites
Prim−5
Prim−15
Prim−25
Long−pec
Protruding−mouth
Day−4
Day−5
Stage
Transcript
ENSDART00000085500
ENSDART00000156653
ENSDART00000157001

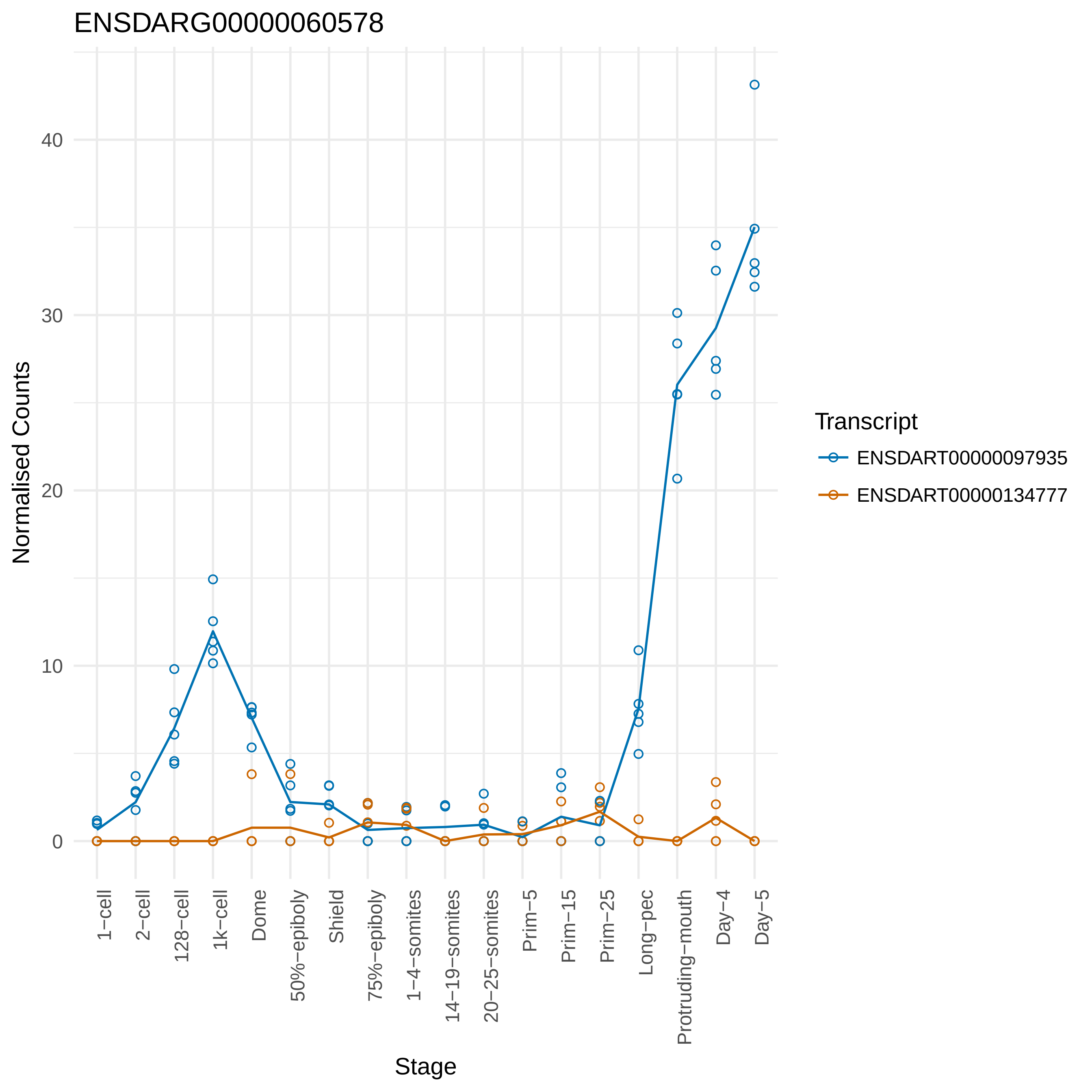
ENSDARG00000060578
Normalised Counts
0
10
20
30
40
1−cell
2−cell
128−cell
1k−cell
Dome
50%−epiboly
Shield
75%−epiboly
1−4−somites
14−19−somites
20−25−somites
Prim−5
Prim−15
Prim−25
Long−pec
Protruding−mouth
Day−4
Day−5
Stage
Transcript
ENSDART00000097935
ENSDART00000134777

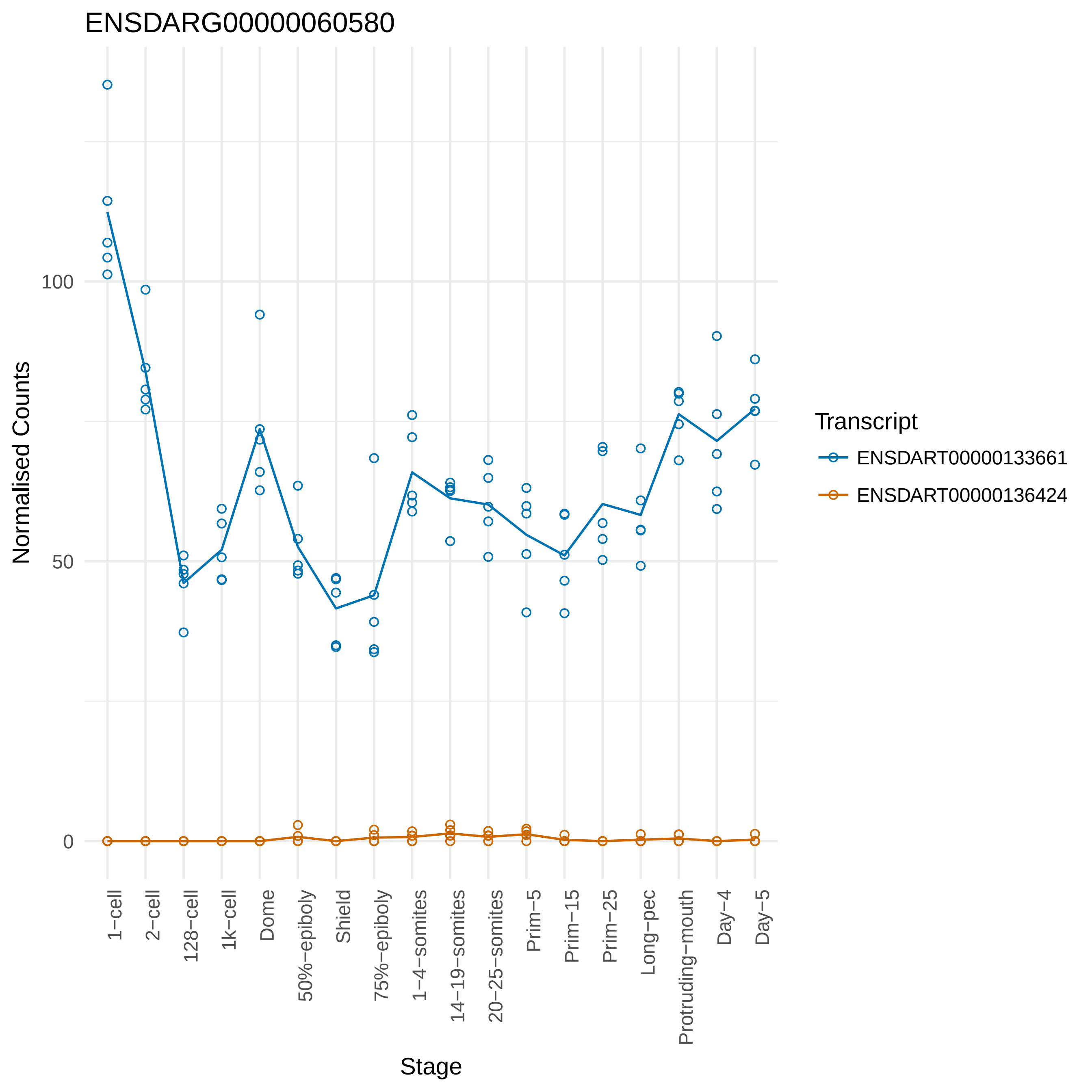

ENSDARG00000060580
Normalised Counts
100
50
0
Stage
1−cell
2−cell
128−cell
1k−cell
Dome
50%−epiboly
Shield
75%−epiboly
1−4−somites
14−19−somites
20−25−somites
Prim−5
Prim−15
Prim−25
Long−pec
Protruding−mouth
Day−4
Day−5
Transcript
ENSDART00000133661
ENSDART00000136424

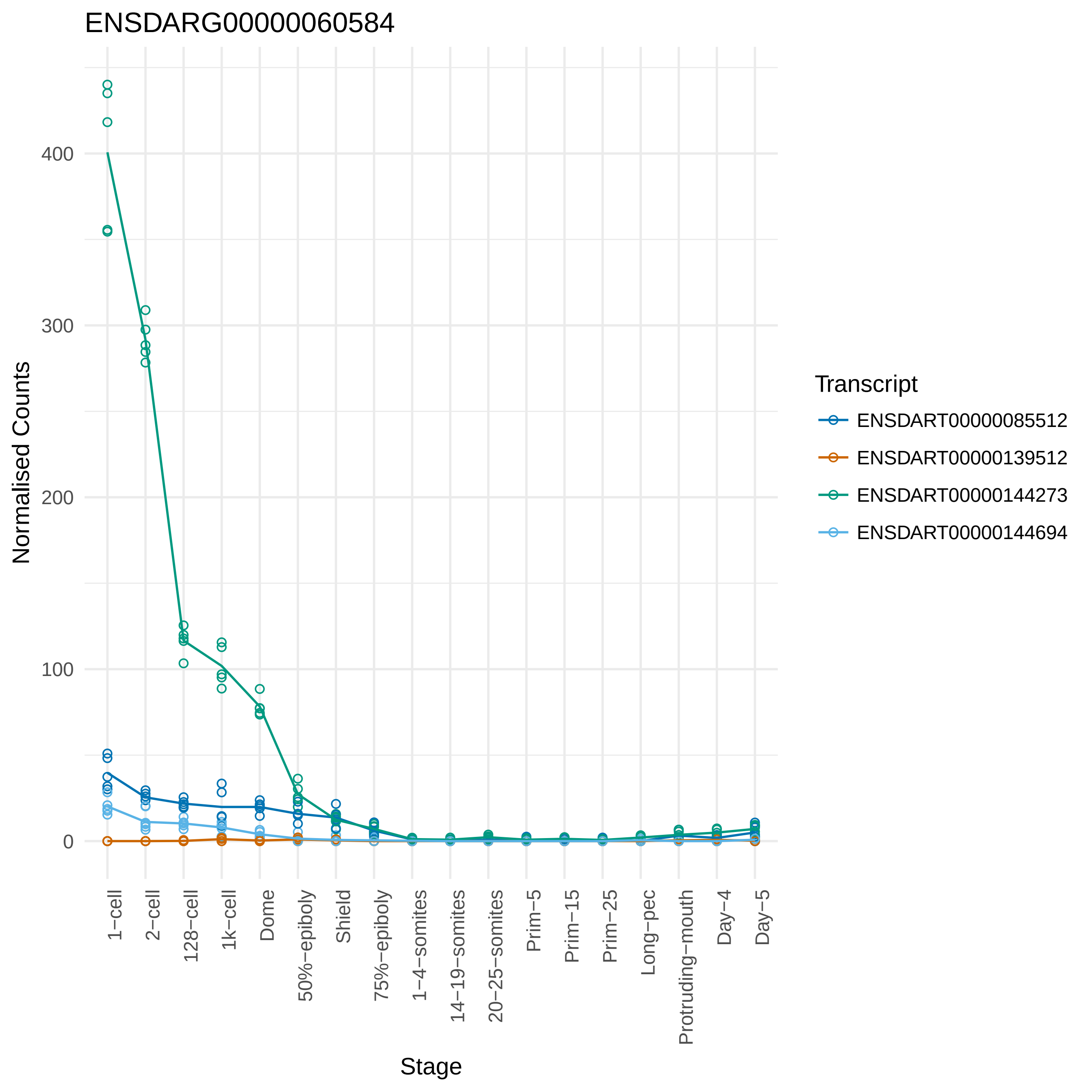

ENSDARG00000060584
Normalised Counts
0
100
200
300
400
Stage
1−cell
2−cell
128−cell
1k−cell
Dome
50%−epiboly
Shield
75%−epiboly
1−4−somites
14−19−somites
20−25−somites
Prim−5
Prim−15
Prim−25
Long−pec
Protruding−mouth
Day−4
Day−5
Transcript
ENSDART00000085512
ENSDART00000139512
ENSDART00000144273
ENSDART00000144694

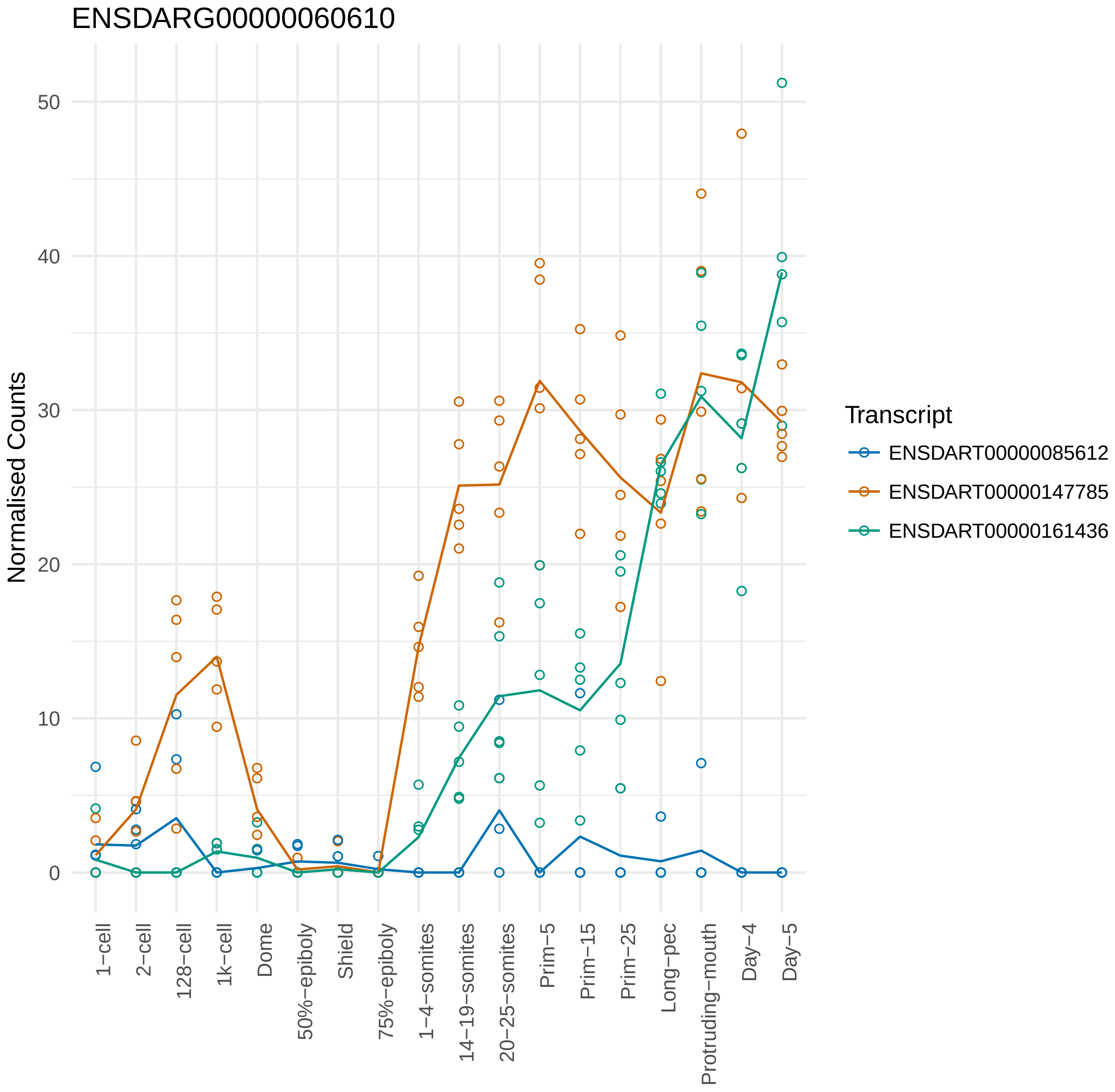
ENSDARG00000060610
Normalised Counts
0
10
20
30
40
50
1−cell
2−cell
128−cell
1k−cell
Dome
50%−epiboly
Shield
75%−epiboly
1−4−somites
14−19−somites
20−25−somites
Prim−5
Prim−15
Prim−25
Long−pec
Protruding−mouth
Day−4
Day−5
Stage
Transcript
ENSDART00000085612
ENSDART00000147785
ENSDART00000161436

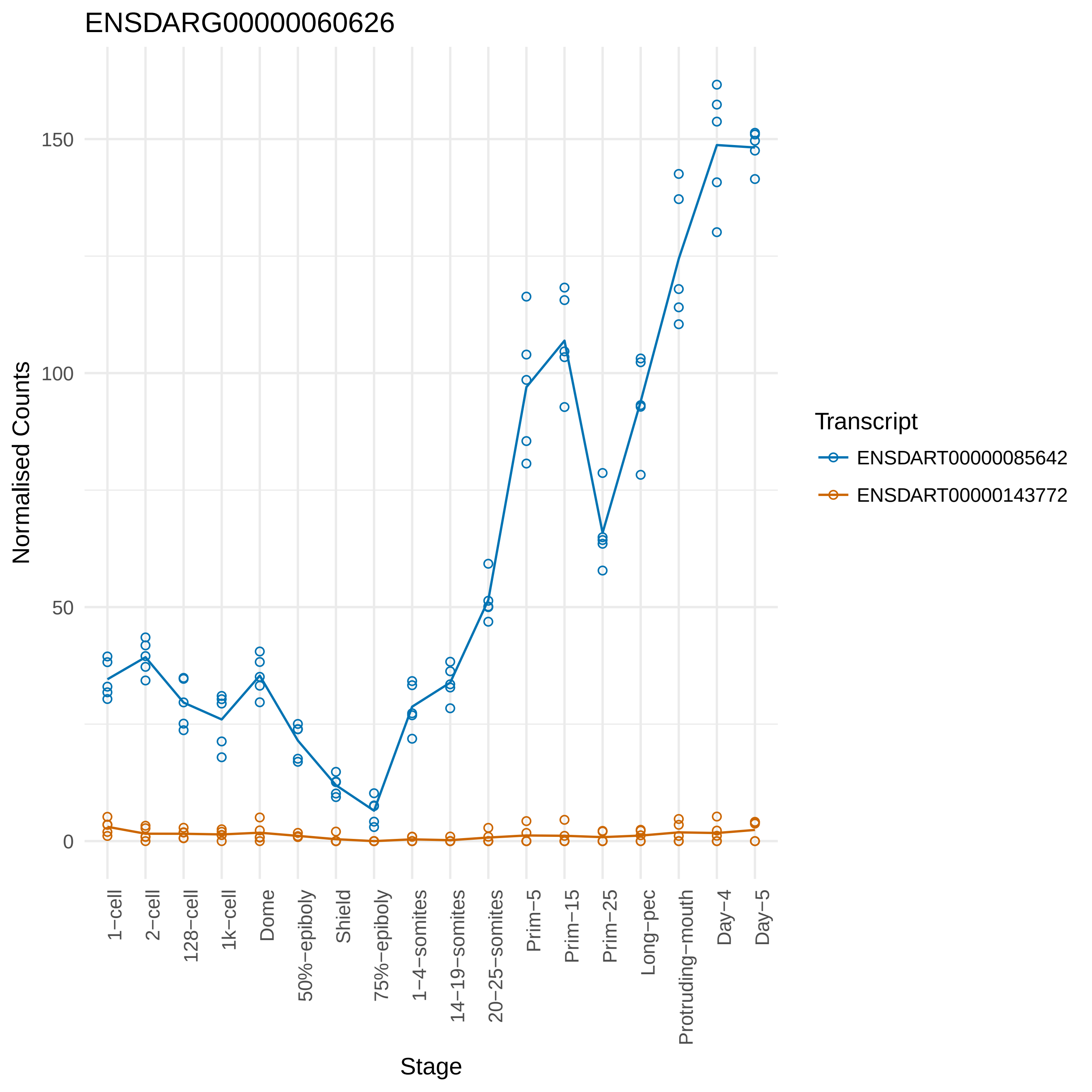
ENSDARG00000060626
Normalised Counts
150
100
50
0
1−cell
2−cell
128−cell
1k−cell
Dome
50%−epiboly
Shield
75%−epiboly
1−4−somites
14−19−somites
20−25−somites
Prim−5
Prim−15
Prim−25
Long−pec
Protruding−mouth
Day−4
Day−5
Stage
Transcript
ENSDART00000085642
ENSDART00000143772

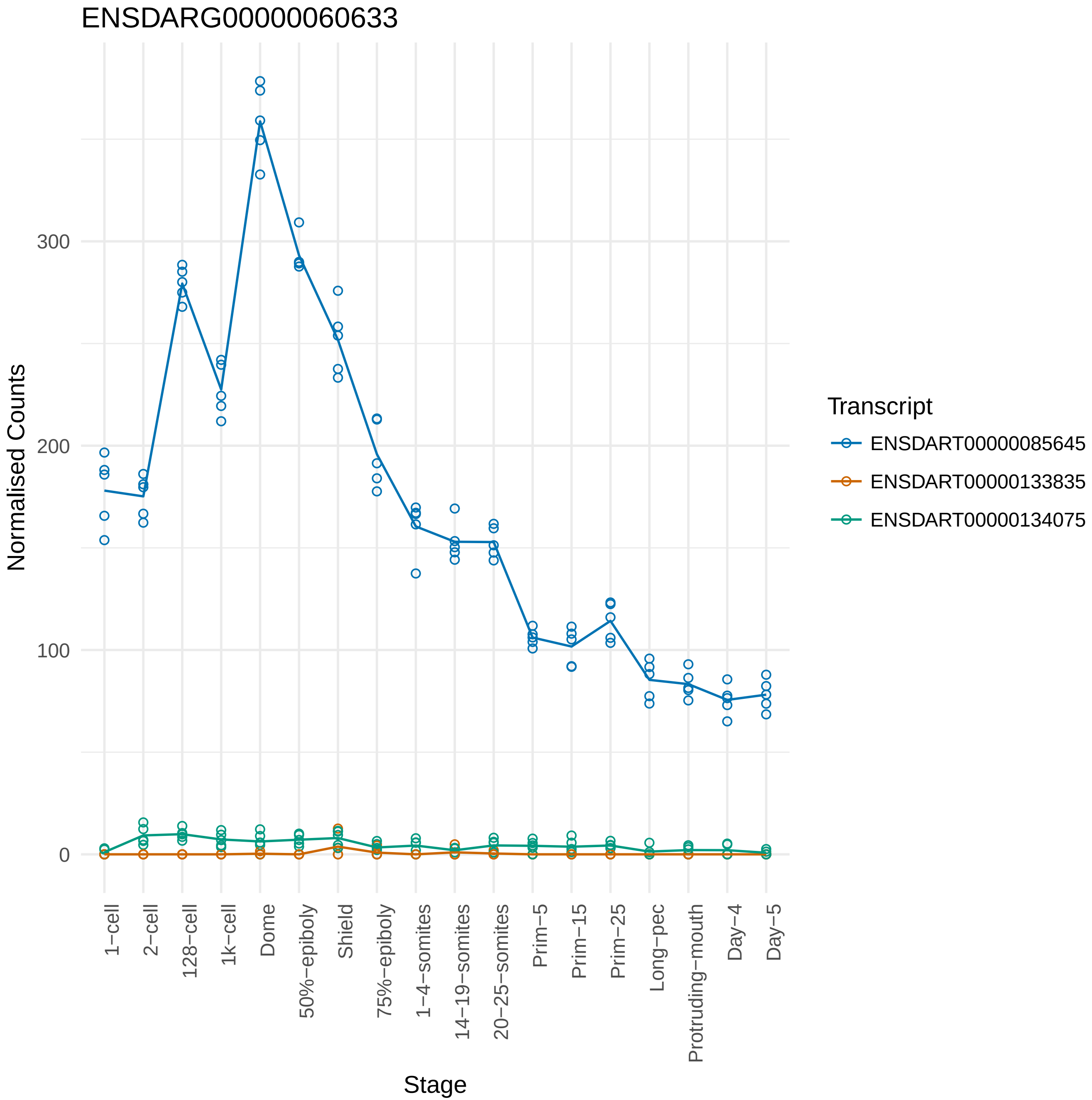
ENSDARG00000060633
Normalised Counts
0
100
200
300
1−cell
2−cell
128−cell
1k−cell
Dome
50%−epiboly
Shield
75%−epiboly
1−4−somites
14−19−somites
20−25−somites
Prim−5
Prim−15
Prim−25
Long−pec
Protruding−mouth
Day−4
Day−5
Stage
Transcript
ENSDART00000085645
ENSDART00000133835
ENSDART00000134075

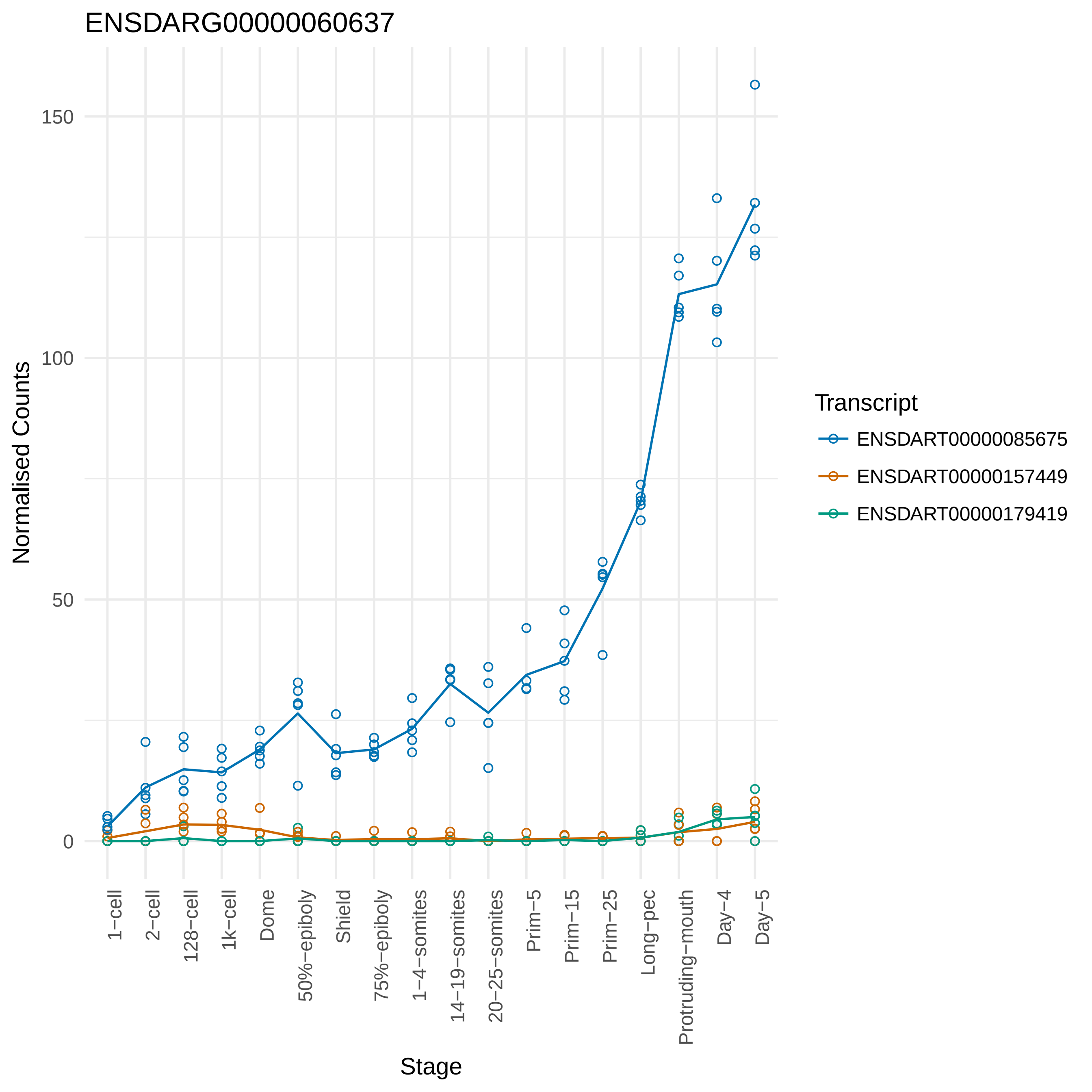

ENSDARG00000060637
Normalised Counts
150
100
50
0
1−cell
2−cell
128−cell
1k−cell
Dome
50%−epiboly
Shield
75%−epiboly
1−4−somites
14−19−somites
20−25−somites
Prim−5
Prim−15
Prim−25
Long−pec
Protruding−mouth
Day−4
Day−5
Stage
Transcript
ENSDART00000085675
ENSDART00000157449
ENSDART00000179419

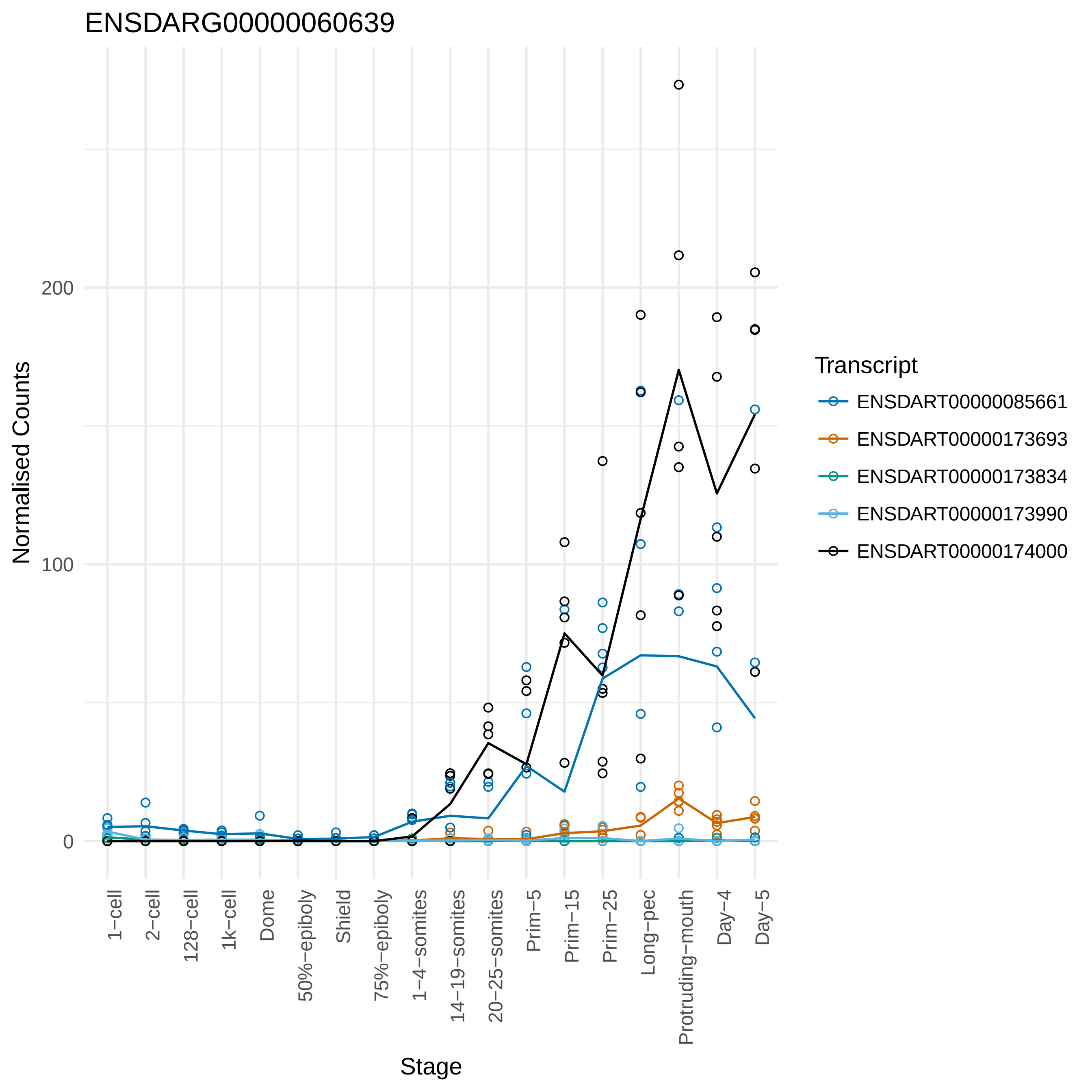

ENSDARG00000060639
Normalised Counts
0
100
200
Stage
1−cell
2−cell
128−cell
1k−cell
Dome
50%−epiboly
Shield
75%−epiboly
1−4−somites
14−19−somites
20−25−somites
Prim−5
Prim−15
Prim−25
Long−pec
Protruding−mouth
Day−4
Day−5
Transcript
ENSDART00000085661
ENSDART00000173693
ENSDART00000173834
ENSDART00000173990
ENSDART00000174000

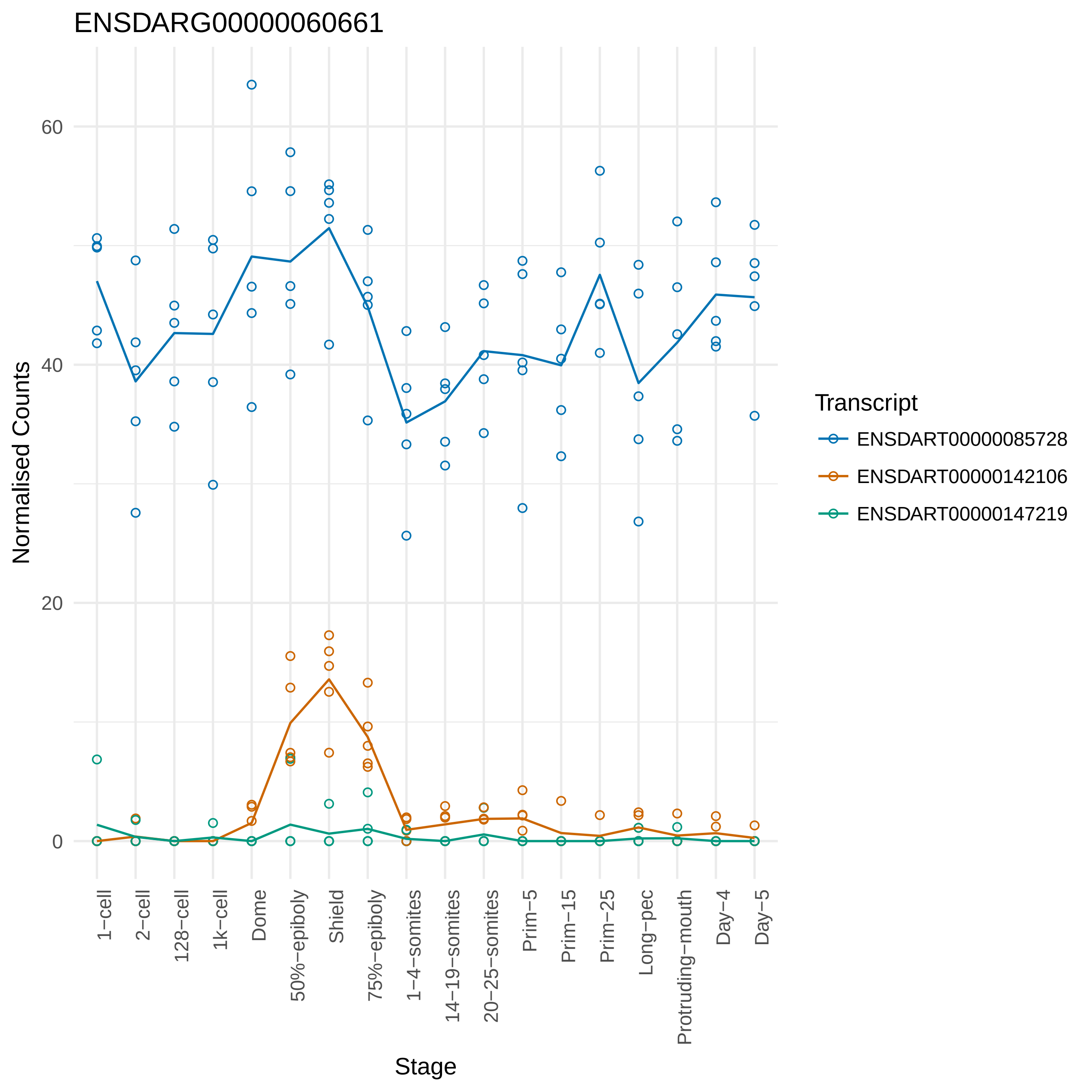

ENSDARG00000060661
Normalised Counts
0
20
40
60
1−cell
2−cell
128−cell
1k−cell
Dome
50%−epiboly
Shield
75%−epiboly
1−4−somites
14−19−somites
20−25−somites
Prim−5
Prim−15
Prim−25
Long−pec
Protruding−mouth
Day−4
Day−5
Stage
Transcript
ENSDART00000085728
ENSDART00000142106
ENSDART00000147219

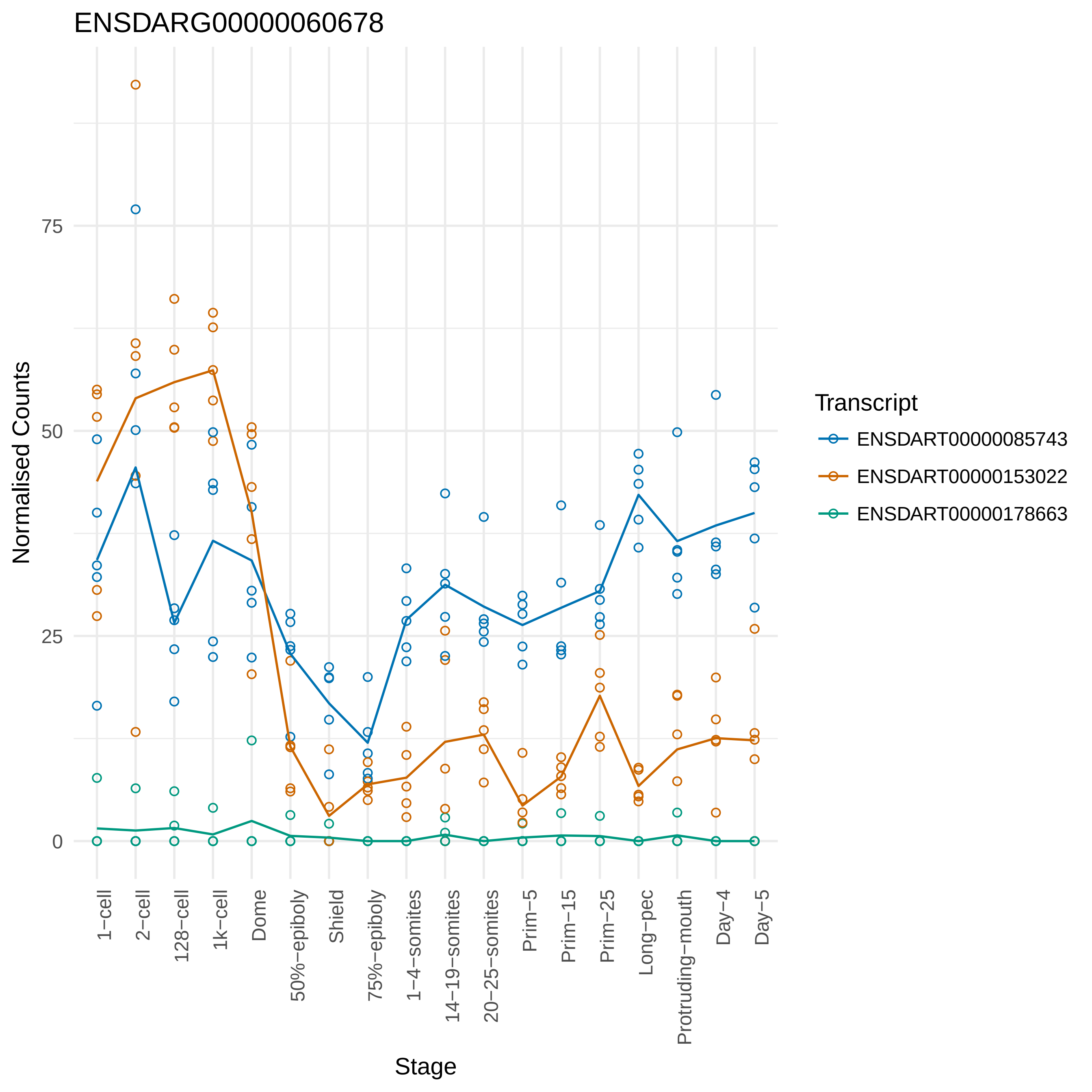

ENSDARG00000060678
Normalised Counts
0
25
50
75
Stage
1−cell
2−cell
128−cell
1k−cell
Dome
50%−epiboly
Shield
75%−epiboly
1−4−somites
14−19−somites
20−25−somites
Prim−5
Prim−15
Prim−25
Long−pec
Protruding−mouth
Day−4
Day−5
Transcript
ENSDART00000085743
ENSDART00000153022
ENSDART00000178663

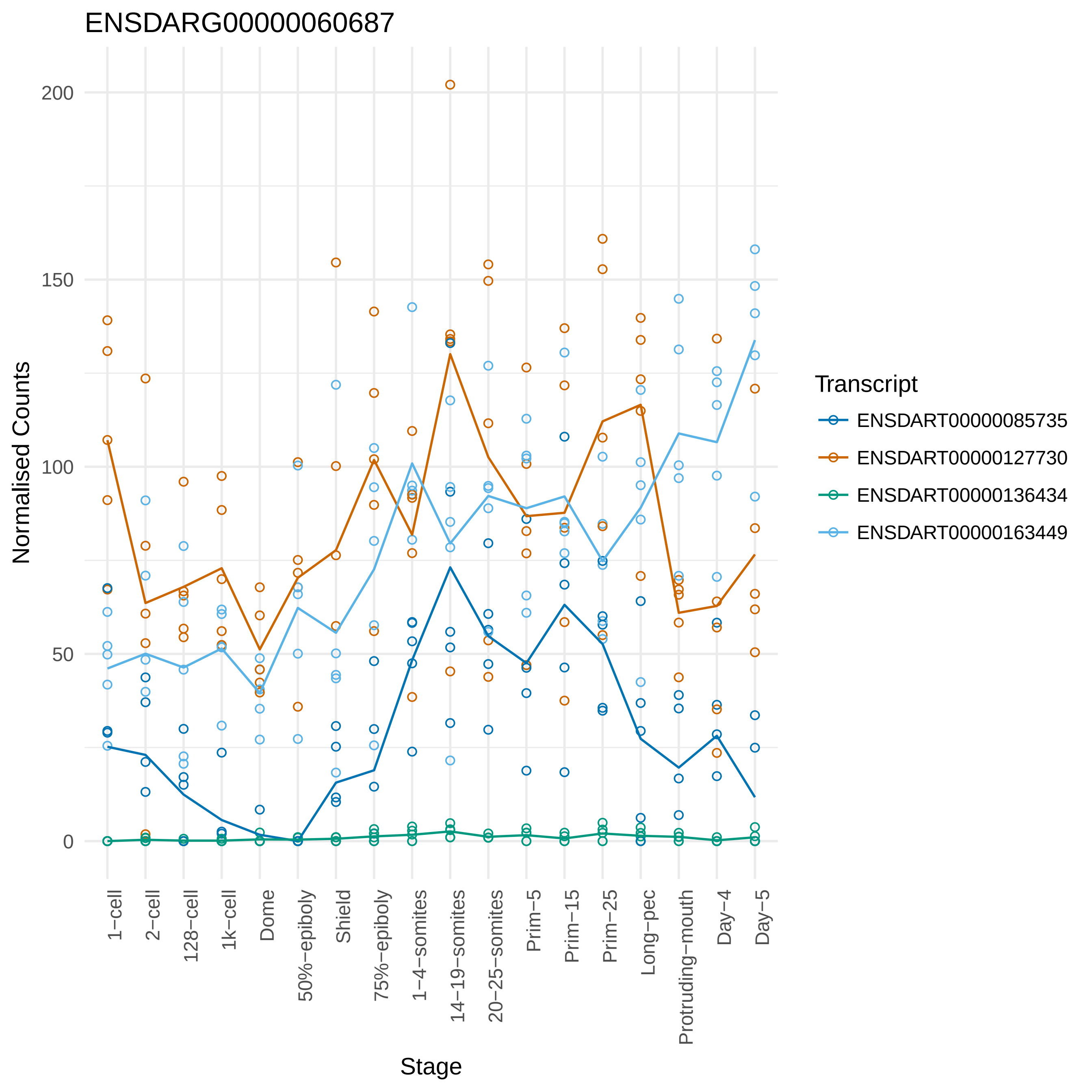

ENSDARG00000060687
Normalised Counts
0
50
100
150
200
1−cell
2−cell
128−cell
1k−cell
Dome
50%−epiboly
Shield
75%−epiboly
1−4−somites
14−19−somites
20−25−somites
Prim−5
Prim−15
Prim−25
Long−pec
Protruding−mouth
Day−4
Day−5
Stage
Transcript
ENSDART00000085735
ENSDART00000127730
ENSDART00000136434
ENSDART00000163449

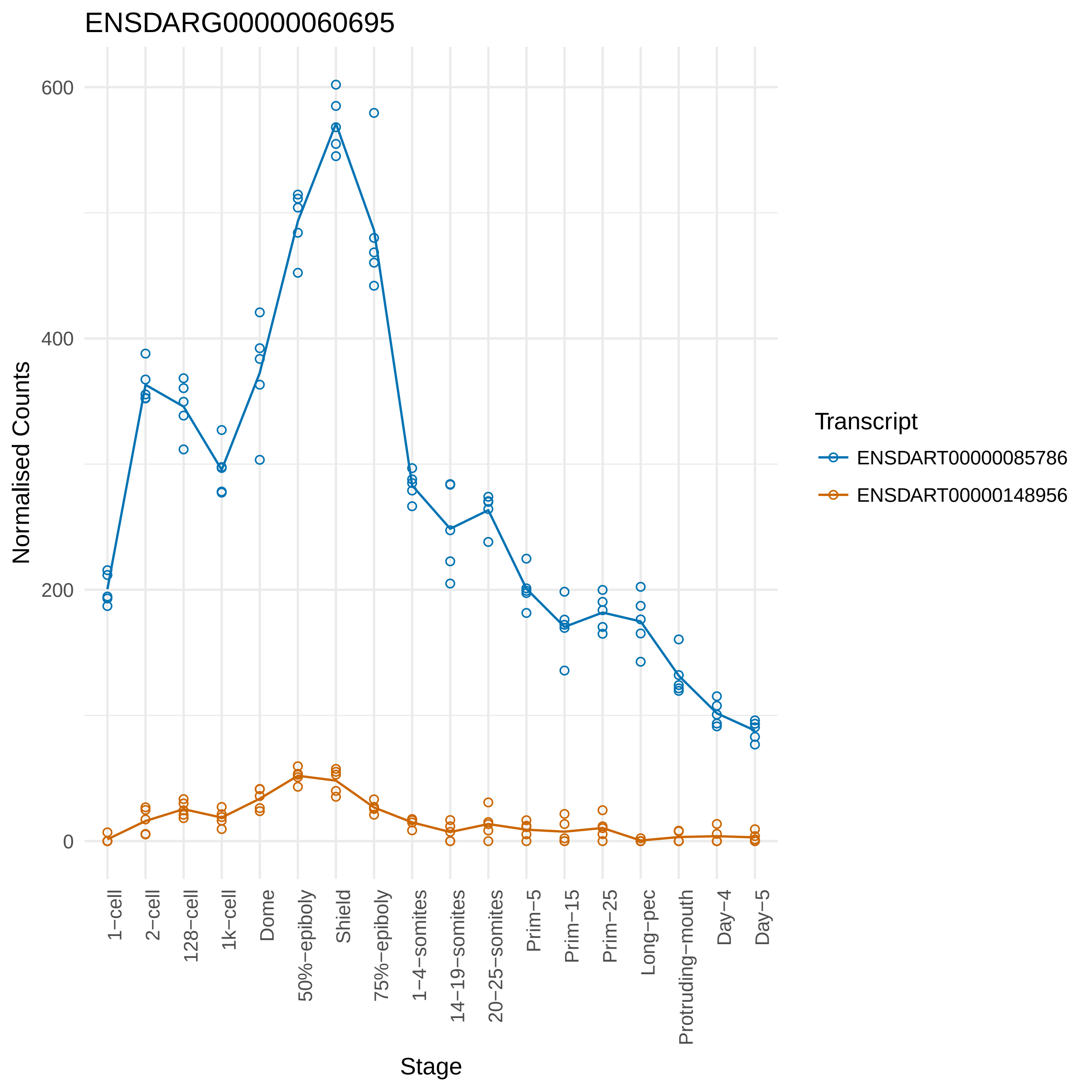

ENSDARG00000060695
Normalised Counts
600
400
200
0
1−cell
2−cell
128−cell
1k−cell
Dome
50%−epiboly
Shield
75%−epiboly
1−4−somites
14−19−somites
20−25−somites
Prim−5
Prim−15
Prim−25
Long−pec
Protruding−mouth
Day−4
Day−5
Stage
Transcript
ENSDART00000085786
ENSDART00000148956

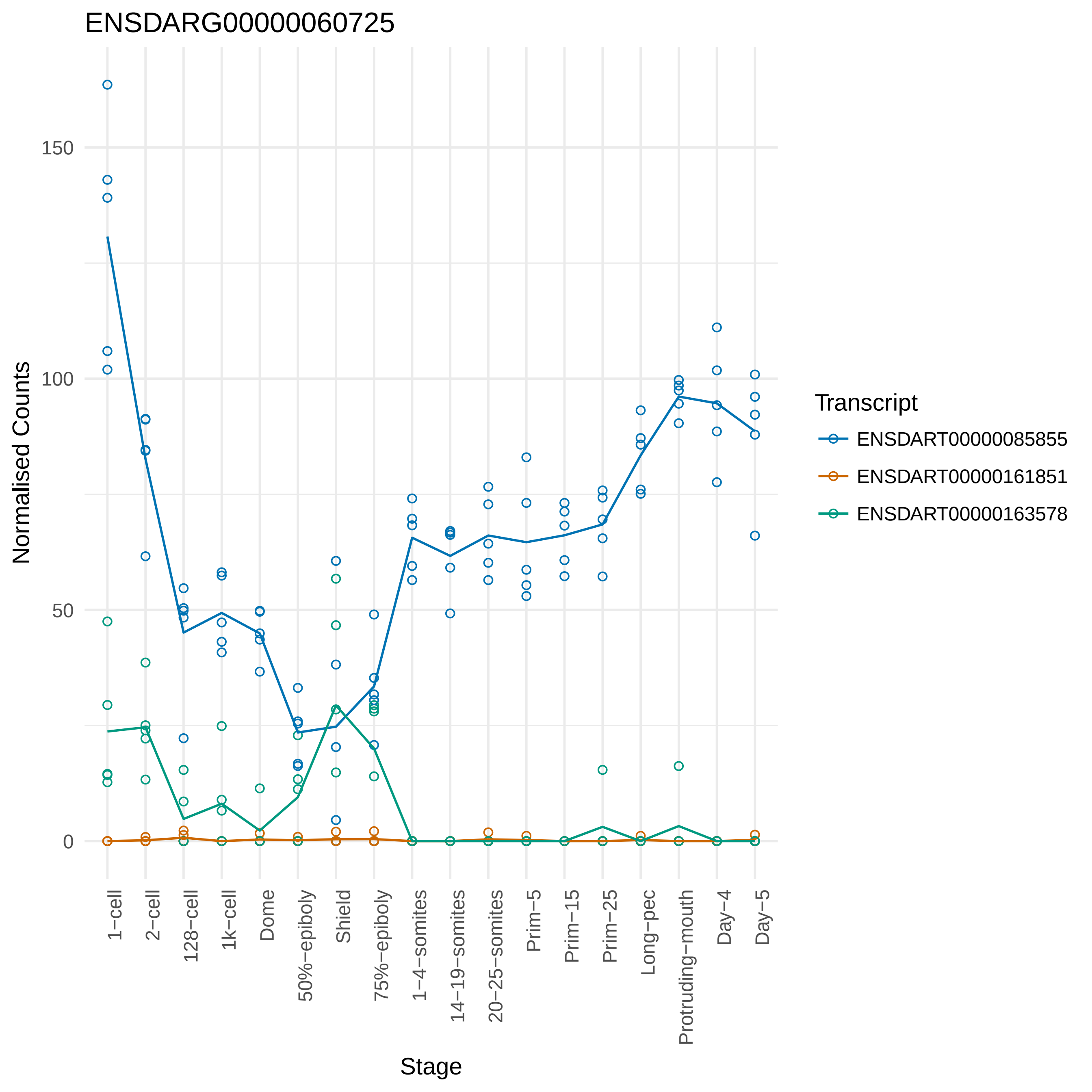

ENSDARG00000060725
Normalised Counts
150
100
50
0
1−cell
2−cell
128−cell
1k−cell
Dome
50%−epiboly
Shield
75%−epiboly
1−4−somites
14−19−somites
20−25−somites
Prim−5
Prim−15
Prim−25
Long−pec
Protruding−mouth
Day−4
Day−5
Stage
Transcript
ENSDART00000085855
ENSDART00000161851
ENSDART00000163578

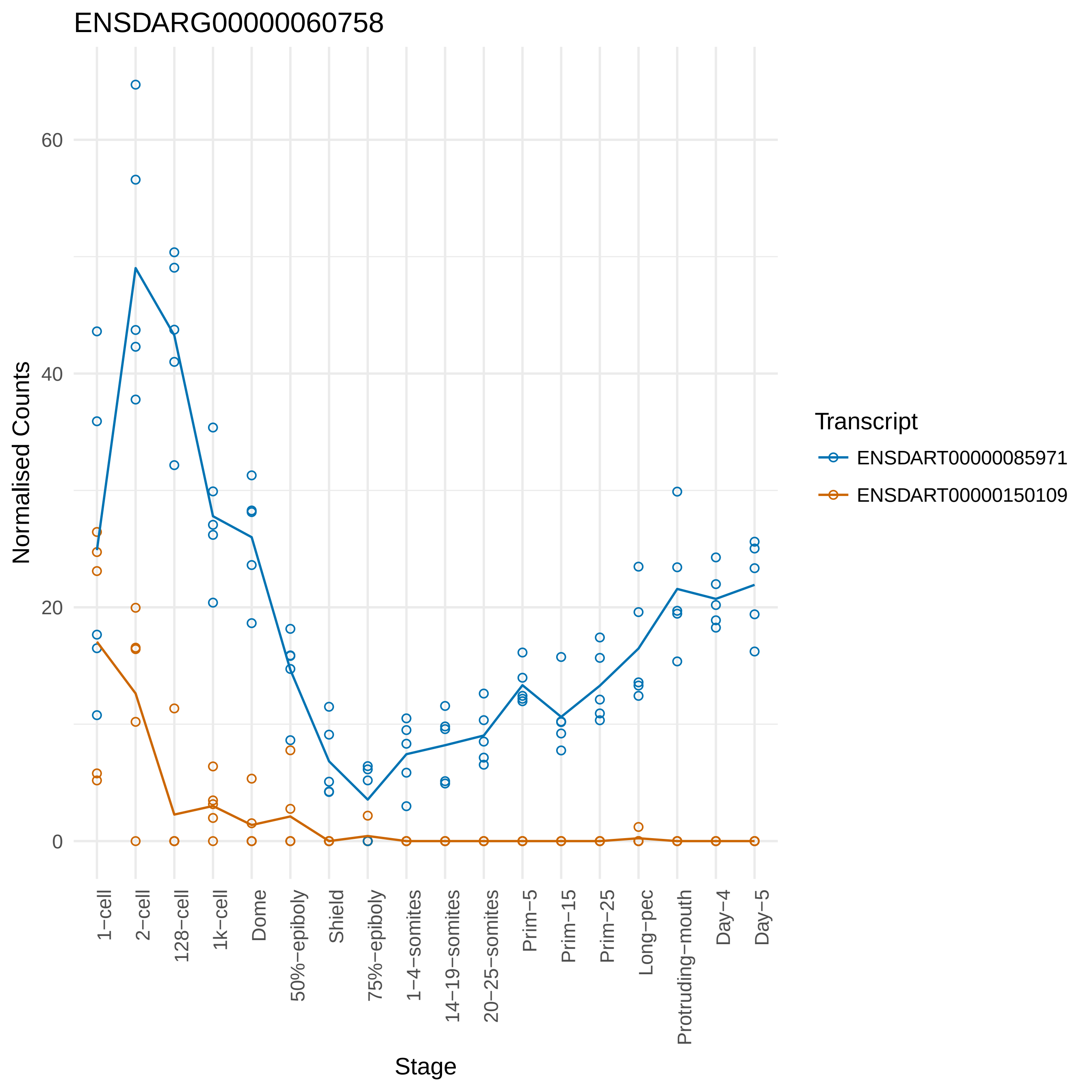

ENSDARG00000060758
Normalised Counts
60
40
20
0
1−cell
2−cell
128−cell
1k−cell
Dome
50%−epiboly
Shield
75%−epiboly
1−4−somites
14−19−somites
20−25−somites
Prim−5
Prim−15
Prim−25
Long−pec
Protruding−mouth
Day−4
Day−5
Stage
Transcript
ENSDART00000085971
ENSDART00000150109

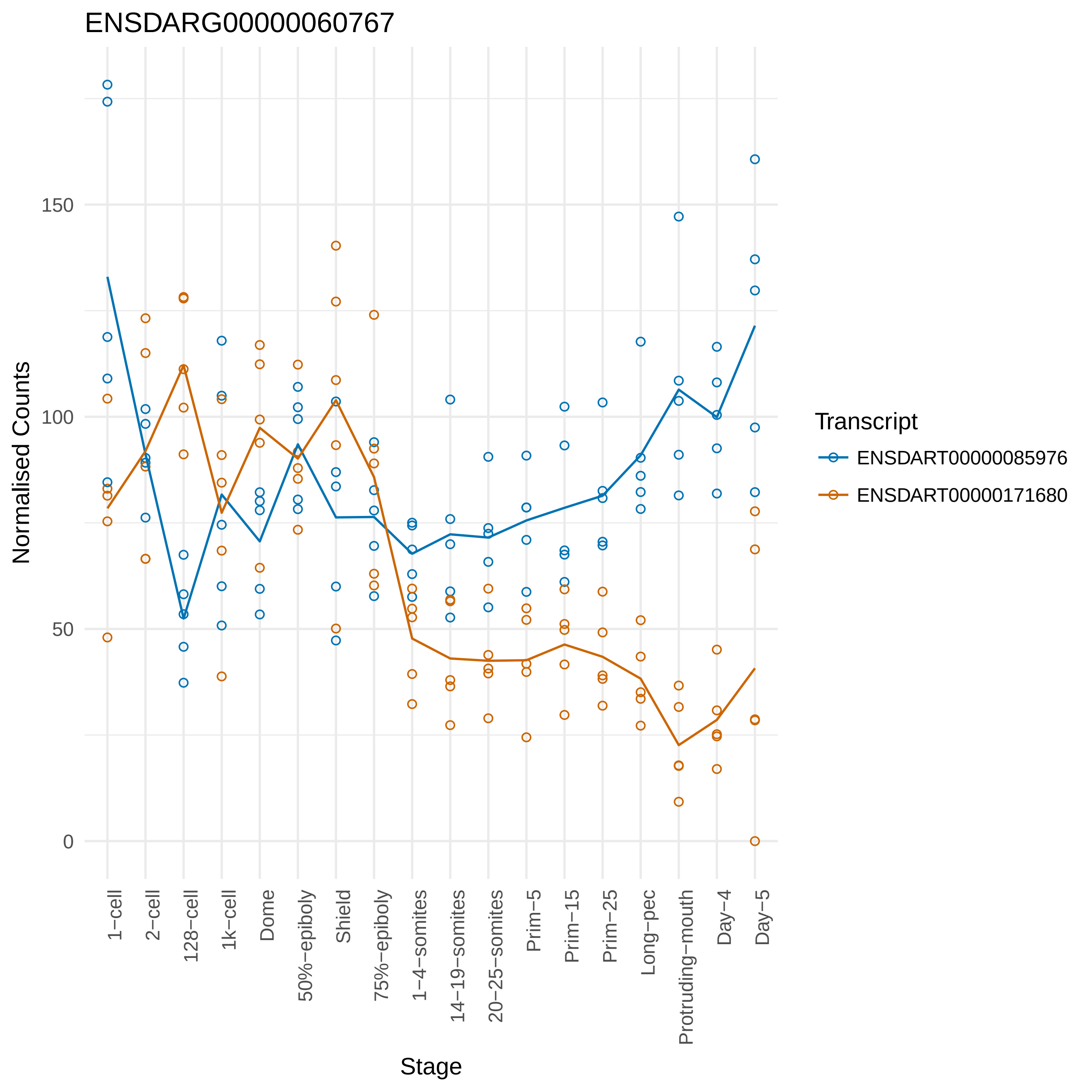
ENSDARG00000060767
Normalised Counts
150
100
50
0
1−cell
2−cell
128−cell
1k−cell
Dome
50%−epiboly
Shield
75%−epiboly
1−4−somites
14−19−somites
20−25−somites
Prim−5
Prim−15
Prim−25
Long−pec
Protruding−mouth
Day−4
Day−5
Stage
Transcript
ENSDART00000085976
ENSDART00000171680

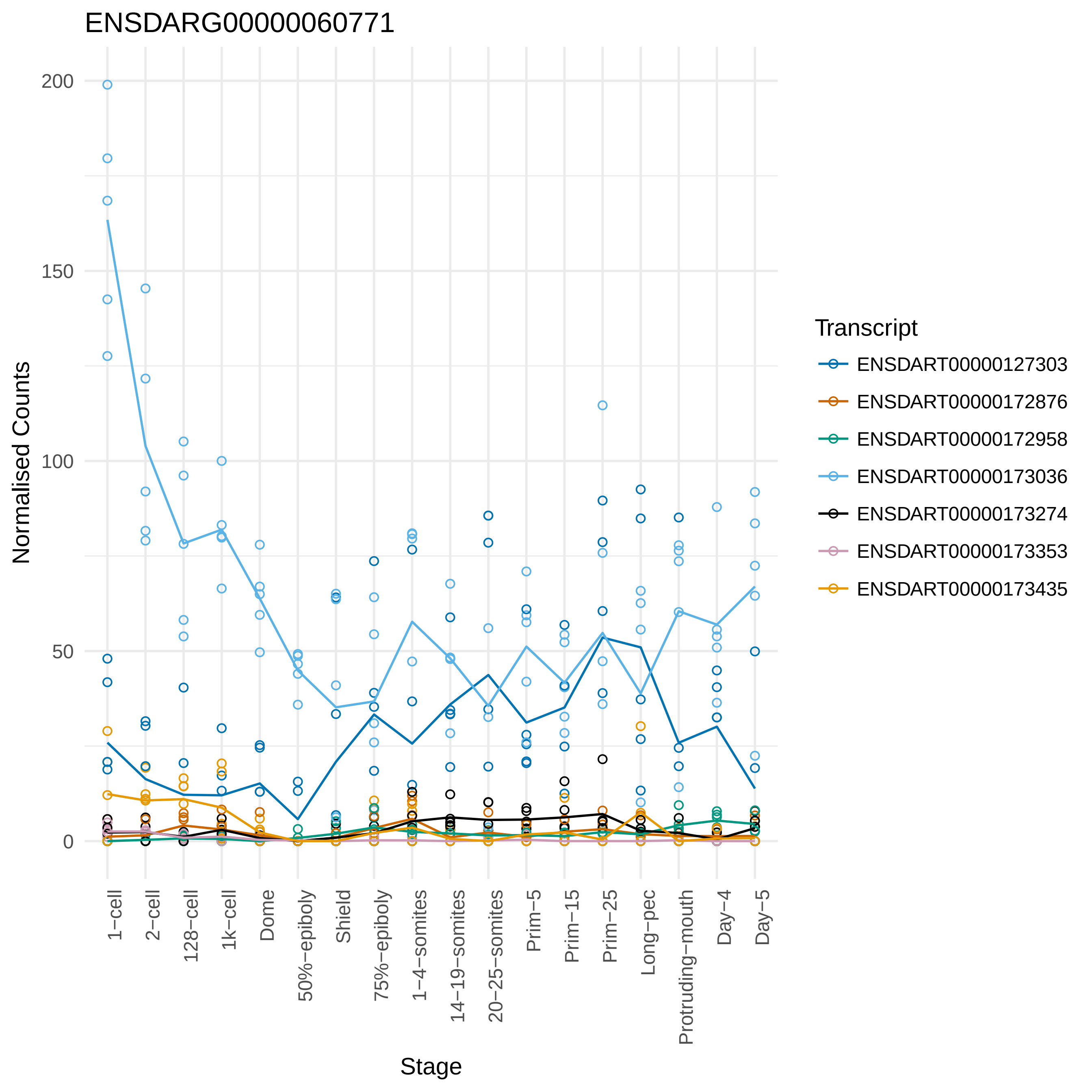

ENSDARG00000060771
Normalised Counts
200
150
100
50
0
1−cell
2−cell
128−cell
1k−cell
Dome
50%−epiboly
Shield
75%−epiboly
1−4−somites
14−19−somites
20−25−somites
Prim−5
Prim−15
Prim−25
Long−pec
Protruding−mouth
Day−4
Day−5
Stage
Transcript
ENSDART00000127303
ENSDART00000172876
ENSDART00000172958
ENSDART00000173036
ENSDART00000173274
ENSDART00000173353
ENSDART00000173435

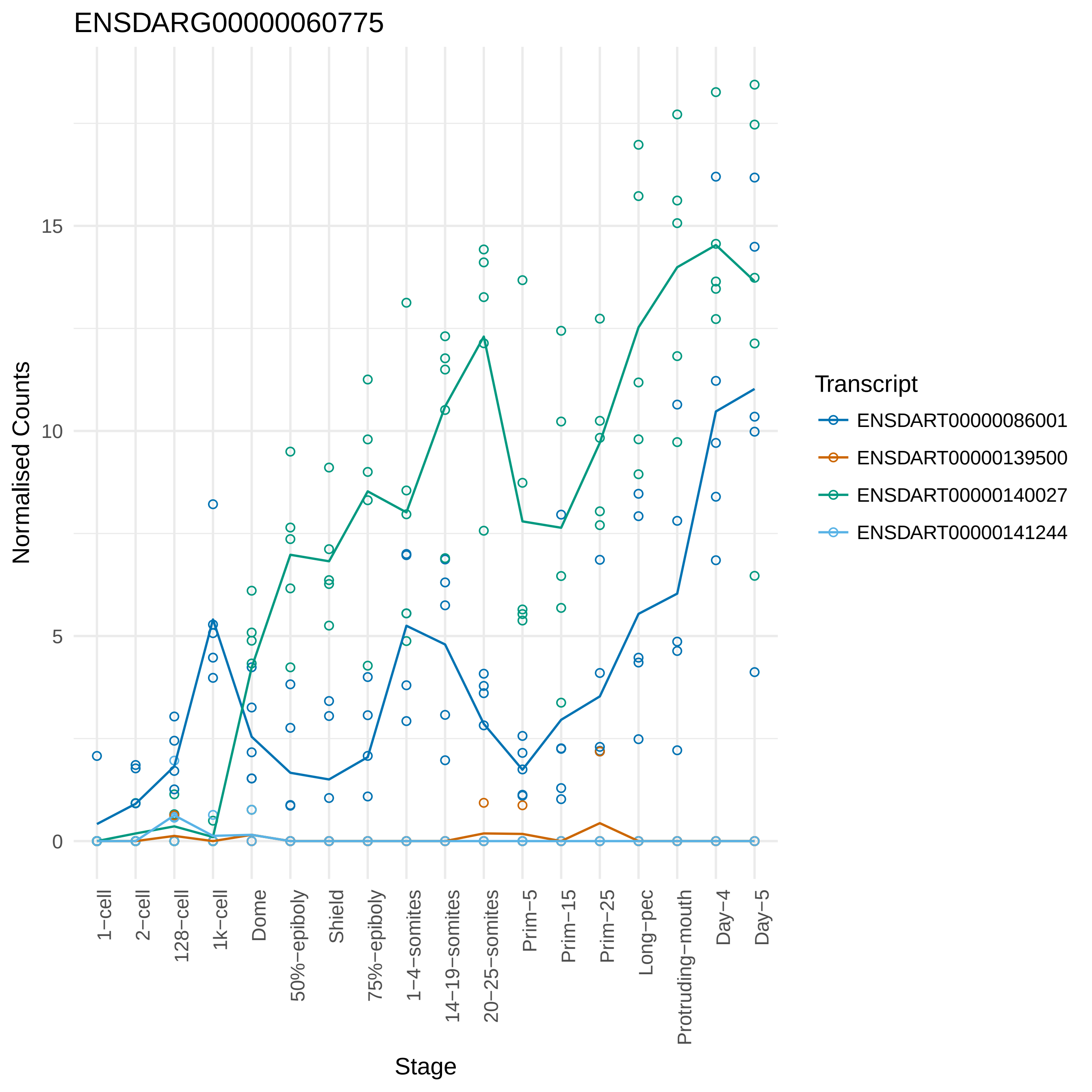
ENSDARG00000060775
Normalised Counts
0
5
10
15
Stage
1−cell
2−cell
128−cell
1k−cell
Dome
50%−epiboly
Shield
75%−epiboly
1−4−somites
14−19−somites
20−25−somites
Prim−5
Prim−15
Prim−25
Long−pec
Protruding−mouth
Day−4
Day−5
Transcript
ENSDART00000086001
ENSDART00000139500
ENSDART00000140027
ENSDART00000141244

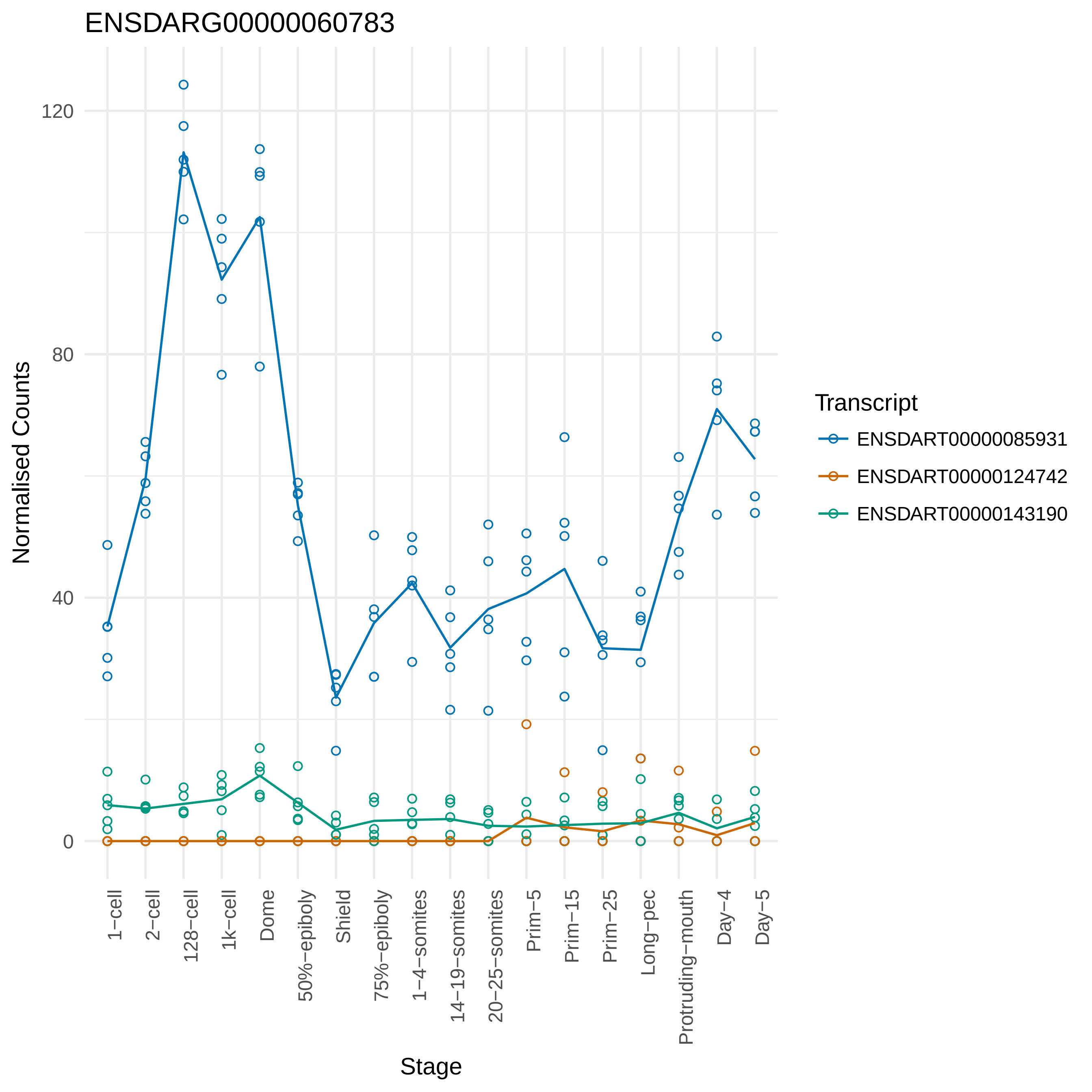
ENSDARG00000060783
Normalised Counts
120
80
40
0
1−cell
2−cell
128−cell
1k−cell
Dome
50%−epiboly
Shield
75%−epiboly
1−4−somites
14−19−somites
20−25−somites
Prim−5
Prim−15
Prim−25
Long−pec
Protruding−mouth
Day−4
Day−5
Stage
Transcript
ENSDART00000085931
ENSDART00000124742
ENSDART00000143190

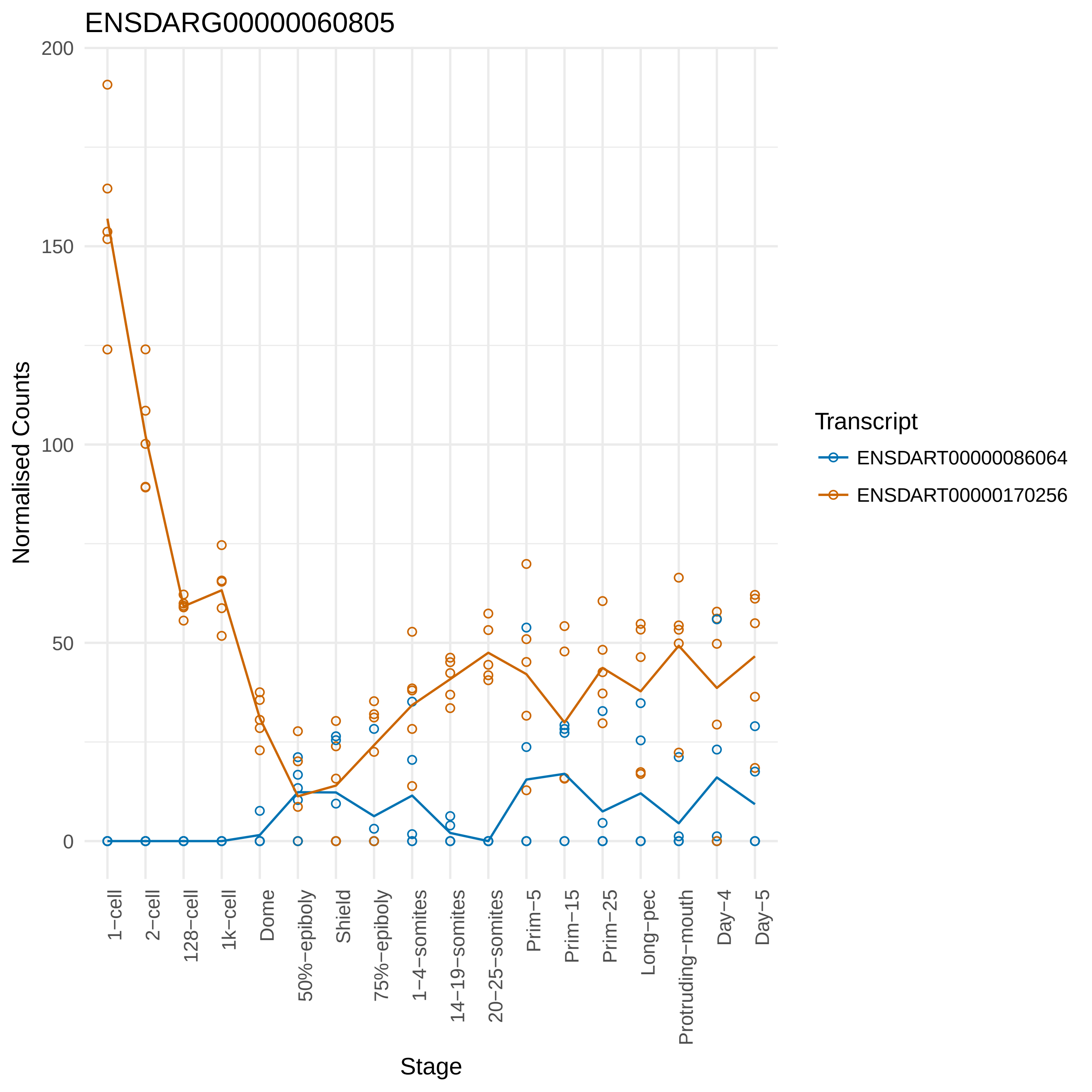

ENSDARG00000060805
Normalised Counts
200
150
100
50
0
1−cell
2−cell
128−cell
1k−cell
Dome
50%−epiboly
Shield
75%−epiboly
1−4−somites
14−19−somites
20−25−somites
Prim−5
Prim−15
Prim−25
Long−pec
Protruding−mouth
Day−4
Day−5
Stage
Transcript
ENSDART00000086064
ENSDART00000170256

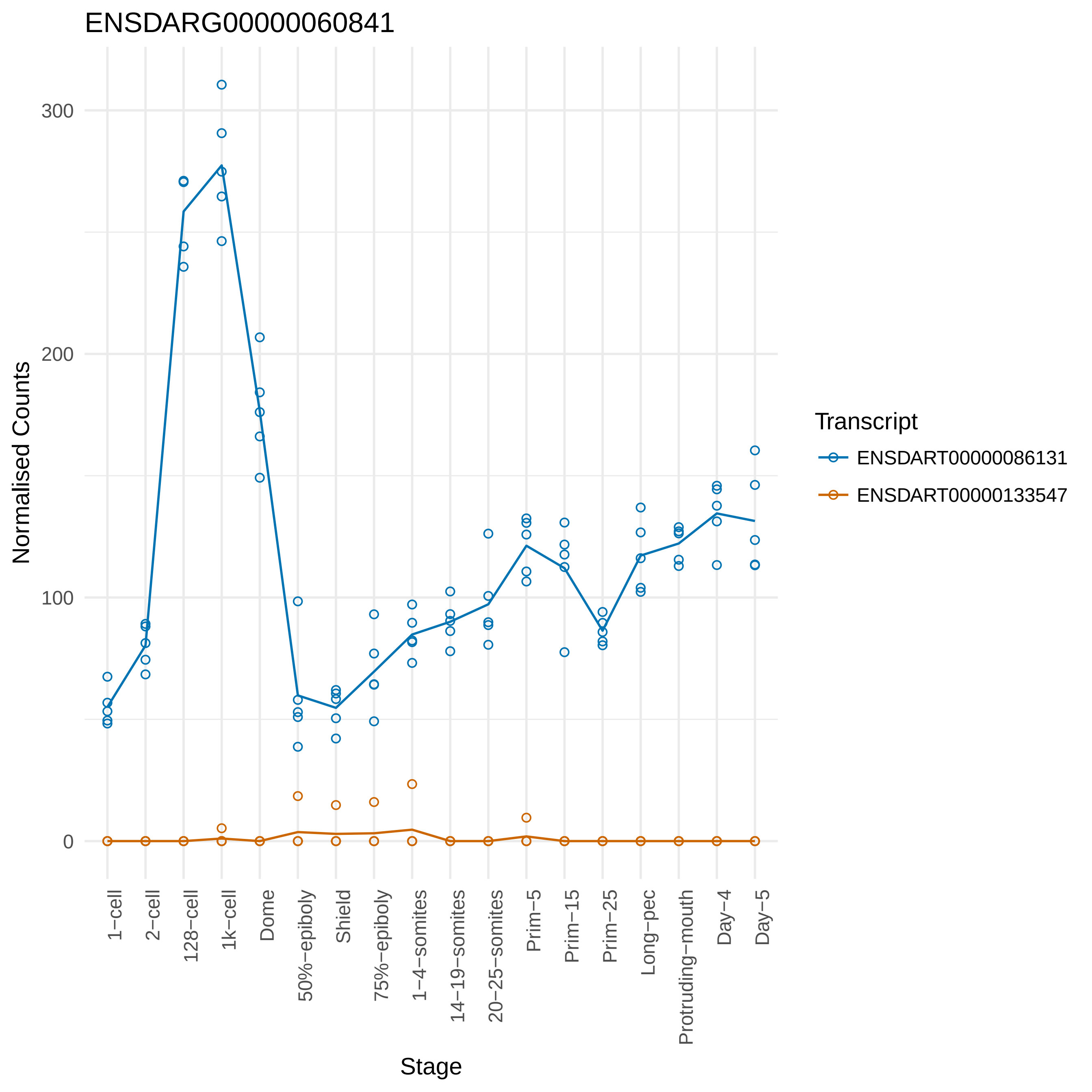

ENSDARG00000060841
Normalised Counts
300
200
100
0
1−cell
2−cell
128−cell
1k−cell
Dome
50%−epiboly
Shield
75%−epiboly
1−4−somites
14−19−somites
20−25−somites
Prim−5
Prim−15
Prim−25
Long−pec
Protruding−mouth
Day−4
Day−5
Stage
Transcript
ENSDART00000086131
ENSDART00000133547

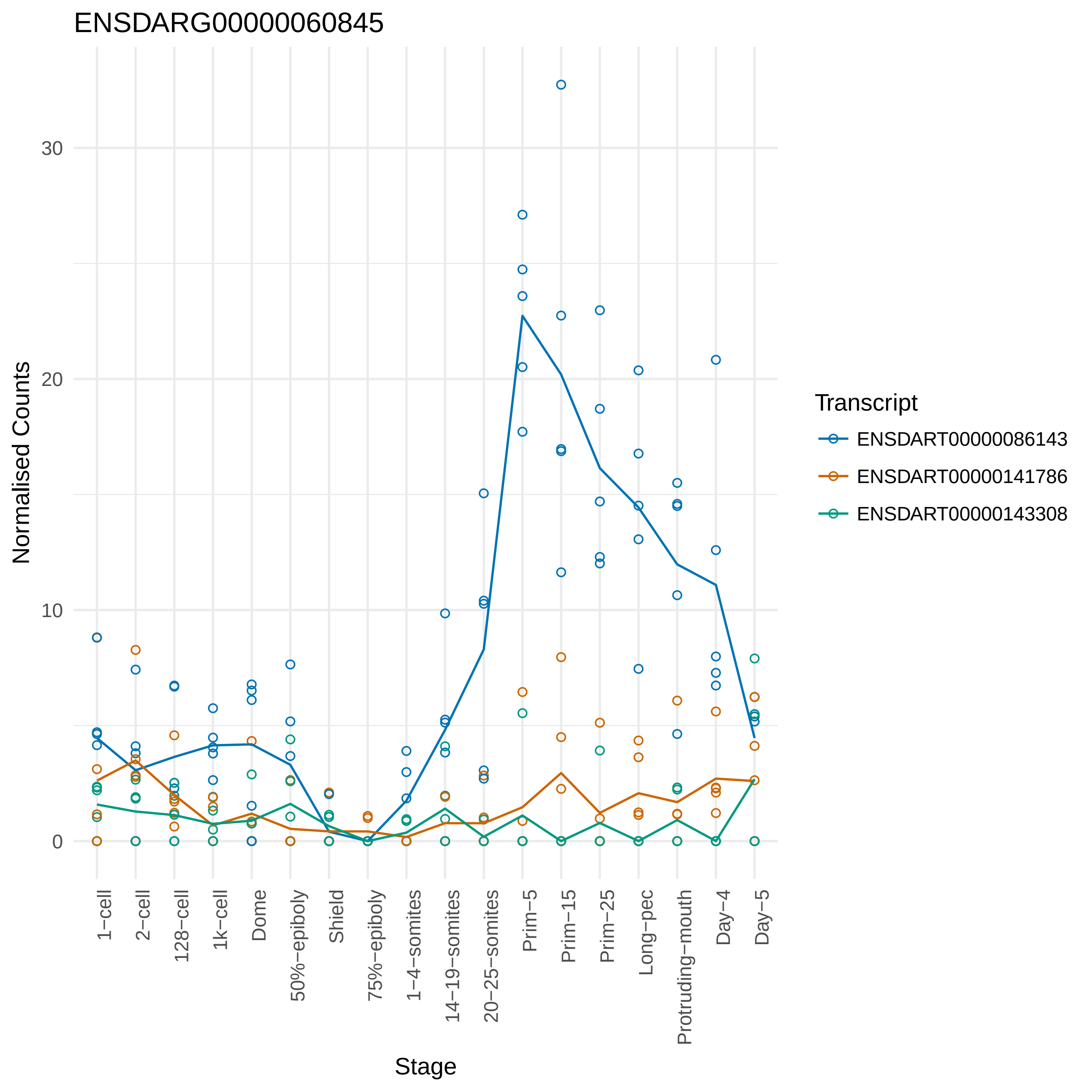
ENSDARG00000060845
Normalised Counts
30
20
10
0
1−cell
2−cell
128−cell
1k−cell
Dome
50%−epiboly
Shield
75%−epiboly
1−4−somites
14−19−somites
20−25−somites
Prim−5
Prim−15
Prim−25
Long−pec
Protruding−mouth
Day−4
Day−5
Stage
Transcript
ENSDART00000086143
ENSDART00000141786
ENSDART00000143308

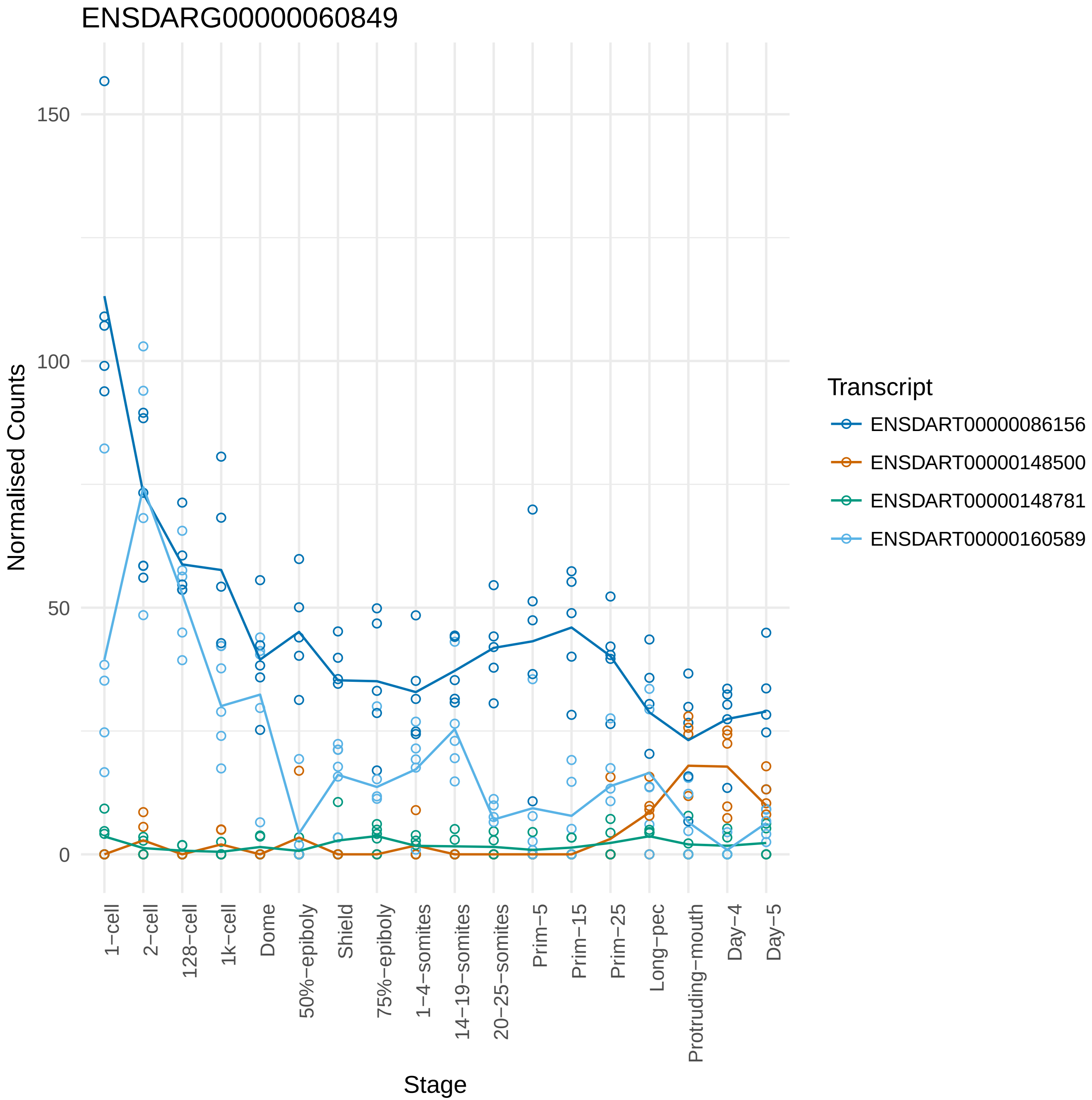

ENSDARG00000060849
Normalised Counts
150
100
50
0
1−cell
2−cell
128−cell
1k−cell
Dome
50%−epiboly
Shield
75%−epiboly
1−4−somites
14−19−somites
20−25−somites
Prim−5
Prim−15
Prim−25
Long−pec
Protruding−mouth
Day−4
Day−5
Stage
Transcript
ENSDART00000086156
ENSDART00000148500
ENSDART00000148781
ENSDART00000160589

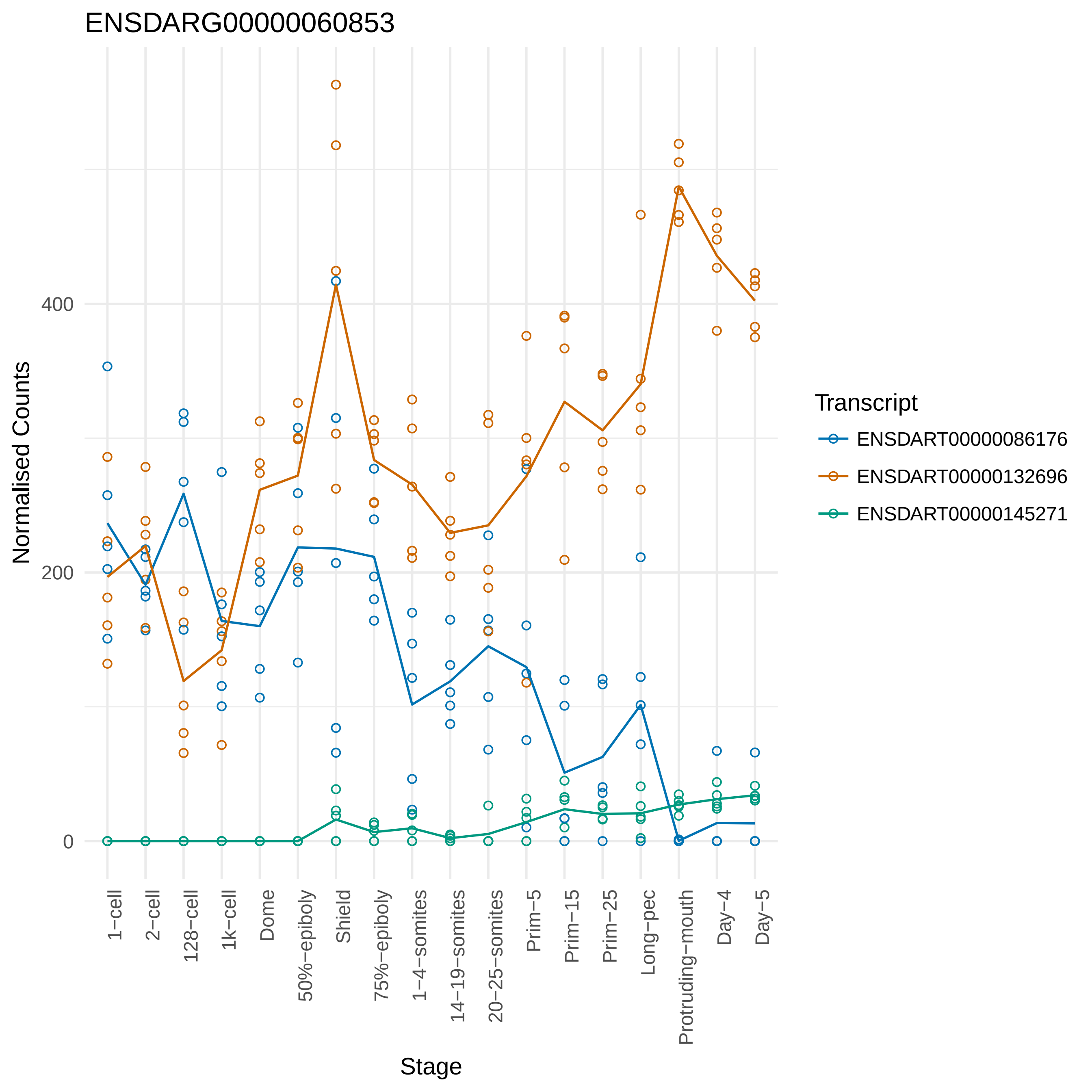
ENSDARG00000060853
Normalised Counts
0
200
400
Stage
1−cell
2−cell
128−cell
1k−cell
Dome
50%−epiboly
Shield
75%−epiboly
1−4−somites
14−19−somites
20−25−somites
Prim−5
Prim−15
Prim−25
Long−pec
Protruding−mouth
Day−4
Day−5
Transcript
ENSDART00000086176
ENSDART00000132696
ENSDART00000145271

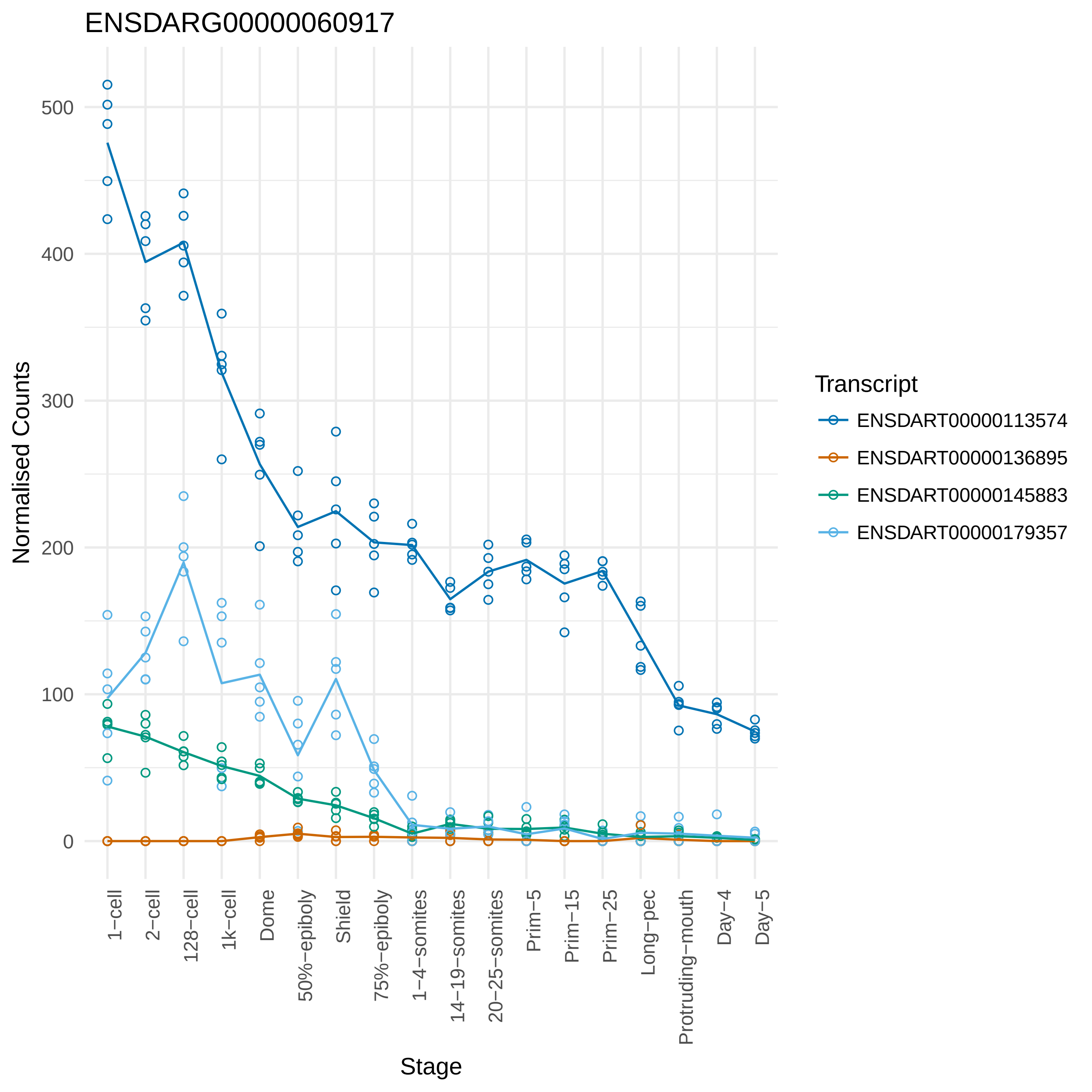
ENSDARG00000060917
Normalised Counts
0
100
200
300
400
500
1−cell
2−cell
128−cell
1k−cell
Dome
50%−epiboly
Shield
75%−epiboly
1−4−somites
14−19−somites
20−25−somites
Prim−5
Prim−15
Prim−25
Long−pec
Protruding−mouth
Day−4
Day−5
Stage
Transcript
ENSDART00000113574
ENSDART00000136895
ENSDART00000145883
ENSDART00000179357

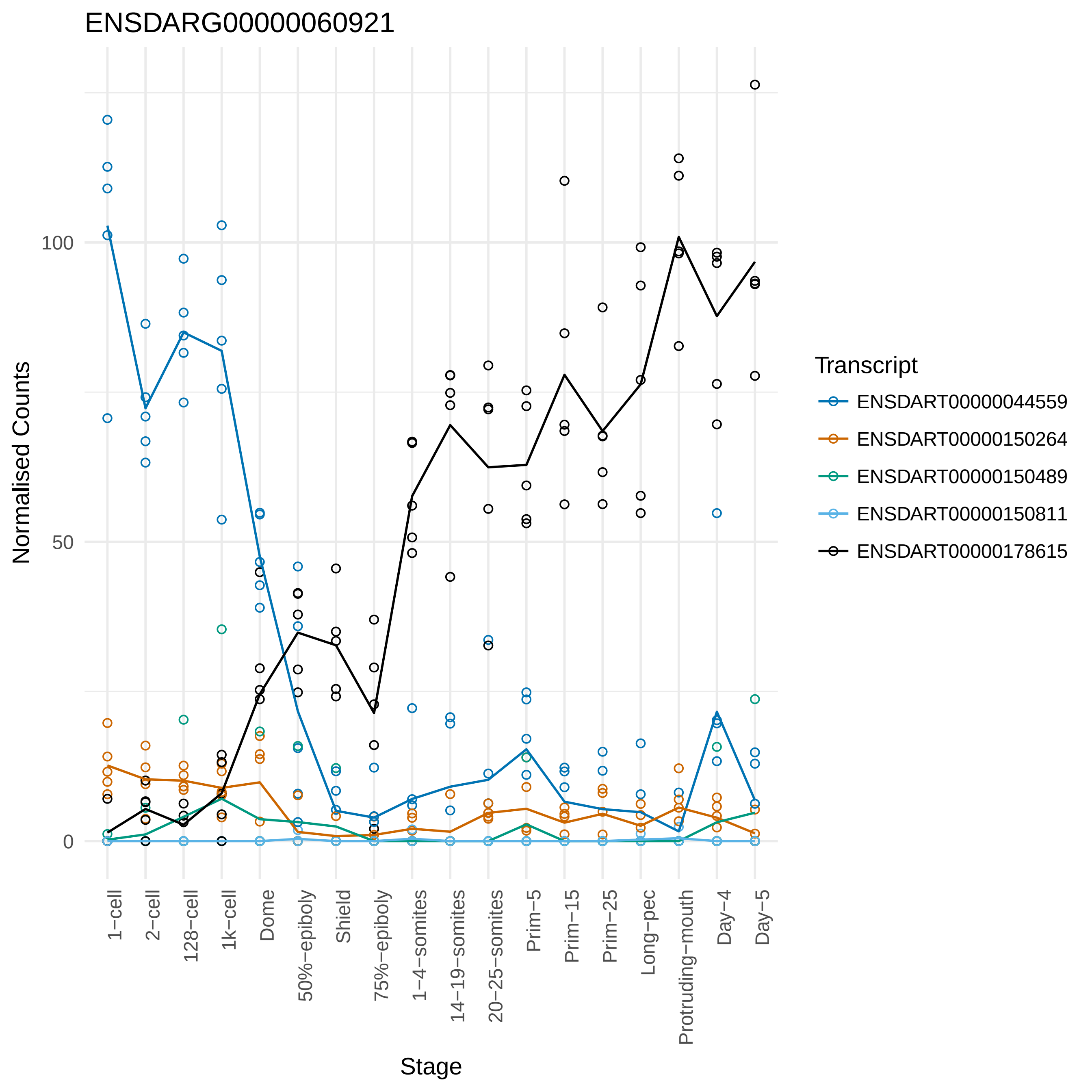

ENSDARG00000060921
Normalised Counts
100
50
0
1−cell
2−cell
128−cell
1k−cell
Dome
50%−epiboly
Shield
75%−epiboly
1−4−somites
14−19−somites
20−25−somites
Prim−5
Prim−15
Prim−25
Long−pec
Protruding−mouth
Day−4
Day−5
Stage
Transcript
ENSDART00000044559
ENSDART00000150264
ENSDART00000150489
ENSDART00000150811
ENSDART00000178615

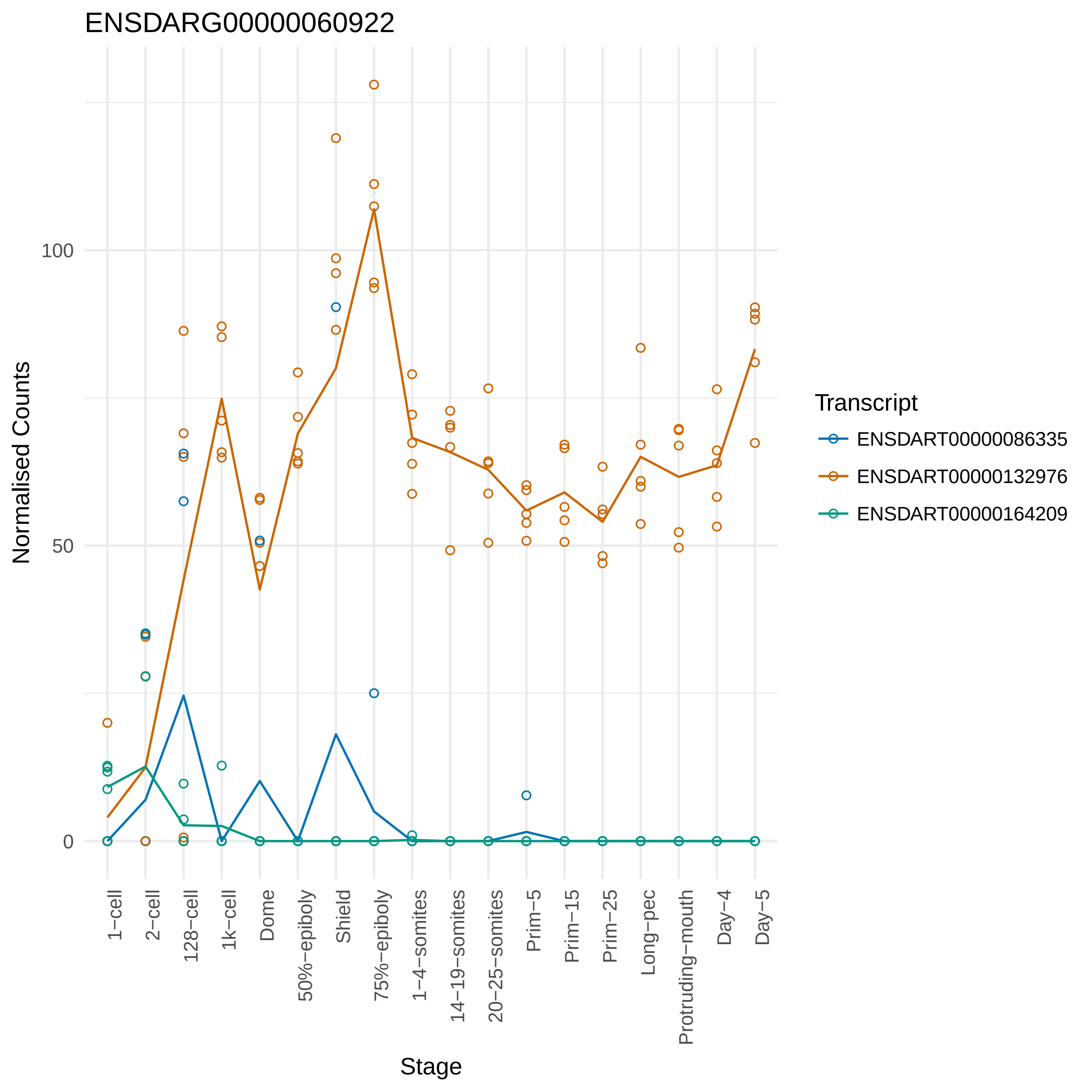
ENSDARG00000060922
Normalised Counts
100
50
0
1−cell
2−cell
128−cell
1k−cell
Dome
50%−epiboly
Shield
75%−epiboly
1−4−somites
14−19−somites
20−25−somites
Prim−5
Prim−15
Prim−25
Long−pec
Protruding−mouth
Day−4
Day−5
Stage
Transcript
ENSDART00000086335
ENSDART00000132976
ENSDART00000164209

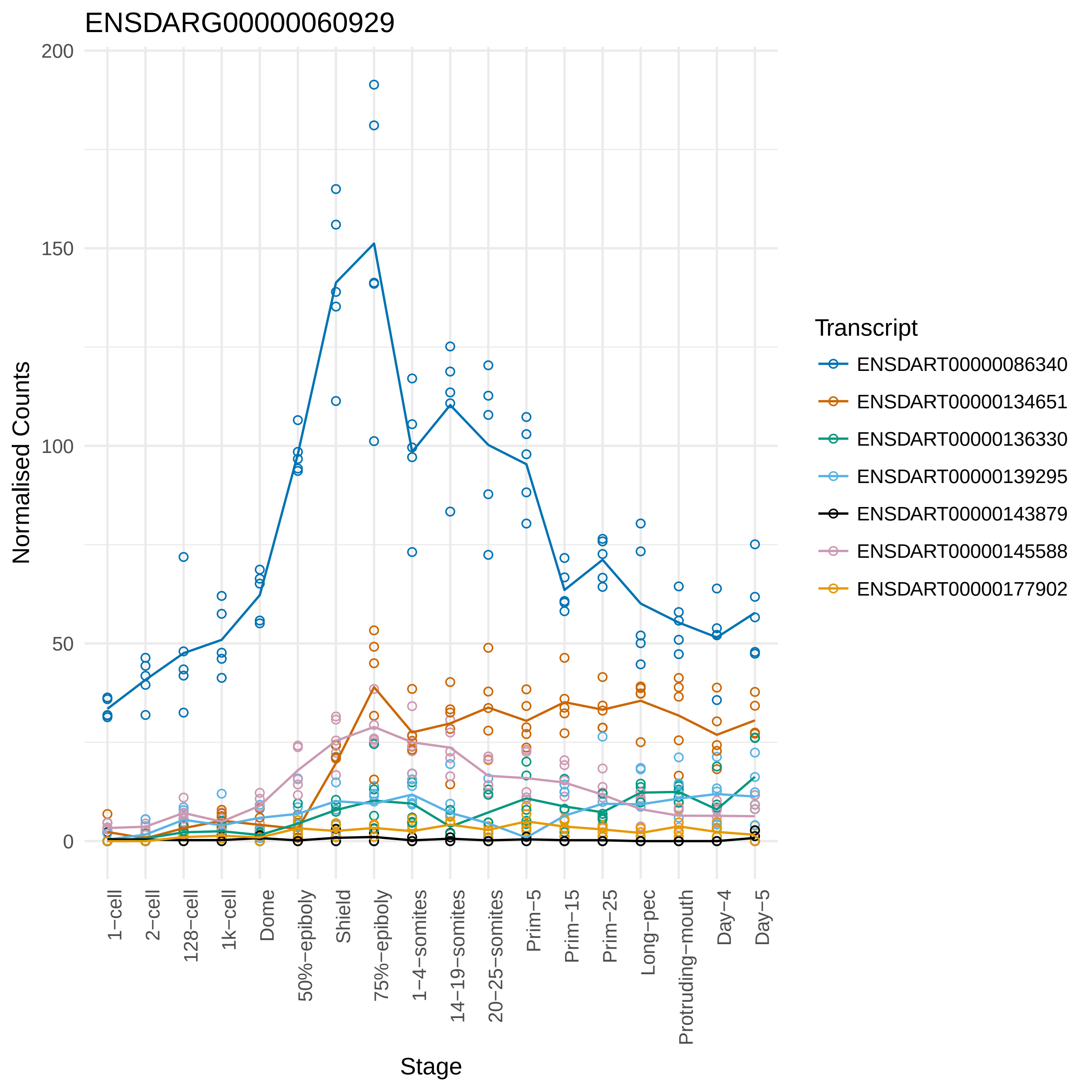

ENSDARG00000060929
Normalised Counts
0
50
100
150
200
Stage
1−cell
2−cell
128−cell
1k−cell
Dome
50%−epiboly
Shield
75%−epiboly
1−4−somites
14−19−somites
20−25−somites
Prim−5
Prim−15
Prim−25
Long−pec
Protruding−mouth
Day−4
Day−5
Transcript
ENSDART00000086340
ENSDART00000134651
ENSDART00000136330
ENSDART00000139295
ENSDART00000143879
ENSDART00000145588
ENSDART00000177902

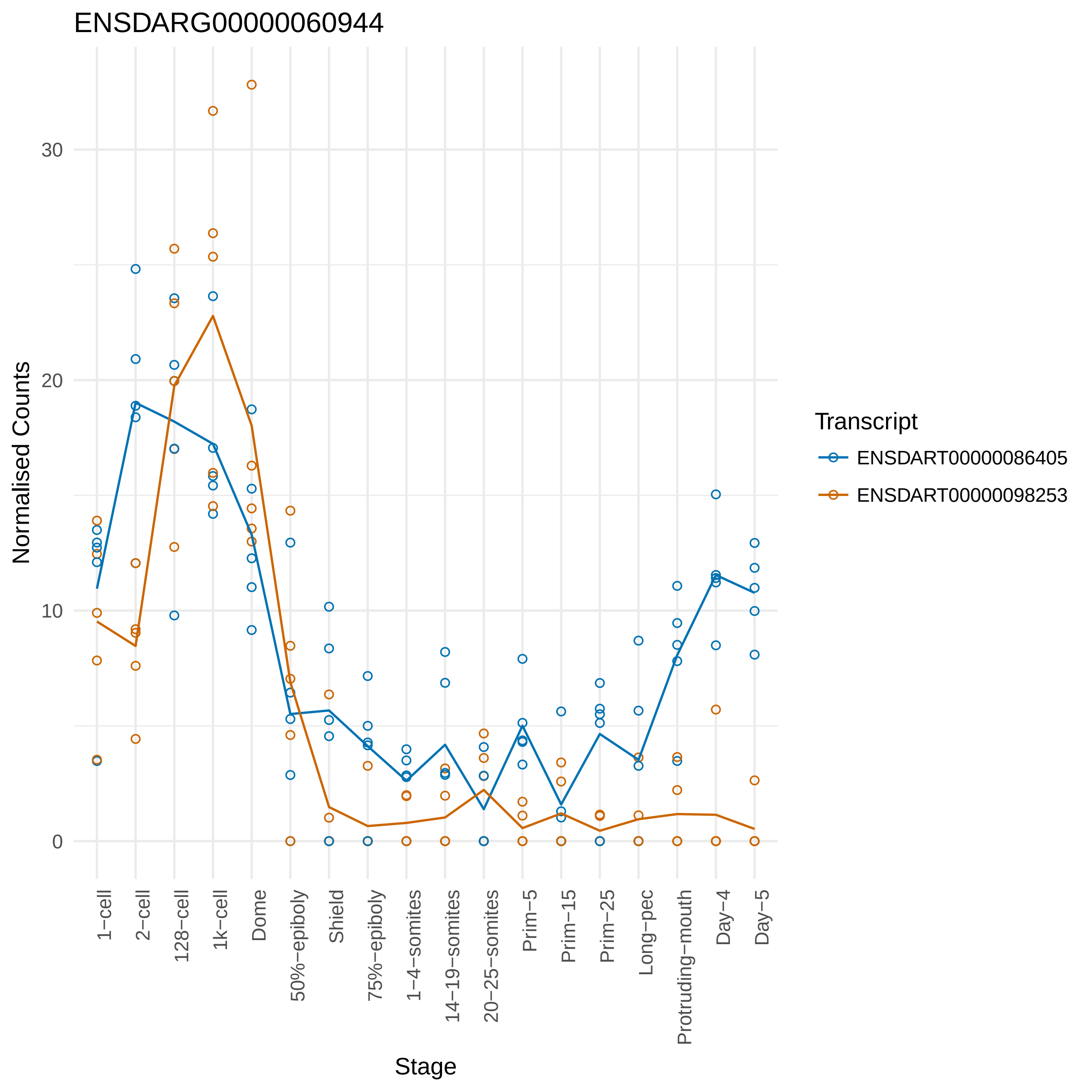

ENSDARG00000060944
Normalised Counts
30
20
10
0
1−cell
2−cell
128−cell
1k−cell
Dome
50%−epiboly
Shield
75%−epiboly
1−4−somites
14−19−somites
20−25−somites
Prim−5
Prim−15
Prim−25
Long−pec
Protruding−mouth
Day−4
Day−5
Stage
Transcript
ENSDART00000086405
ENSDART00000098253

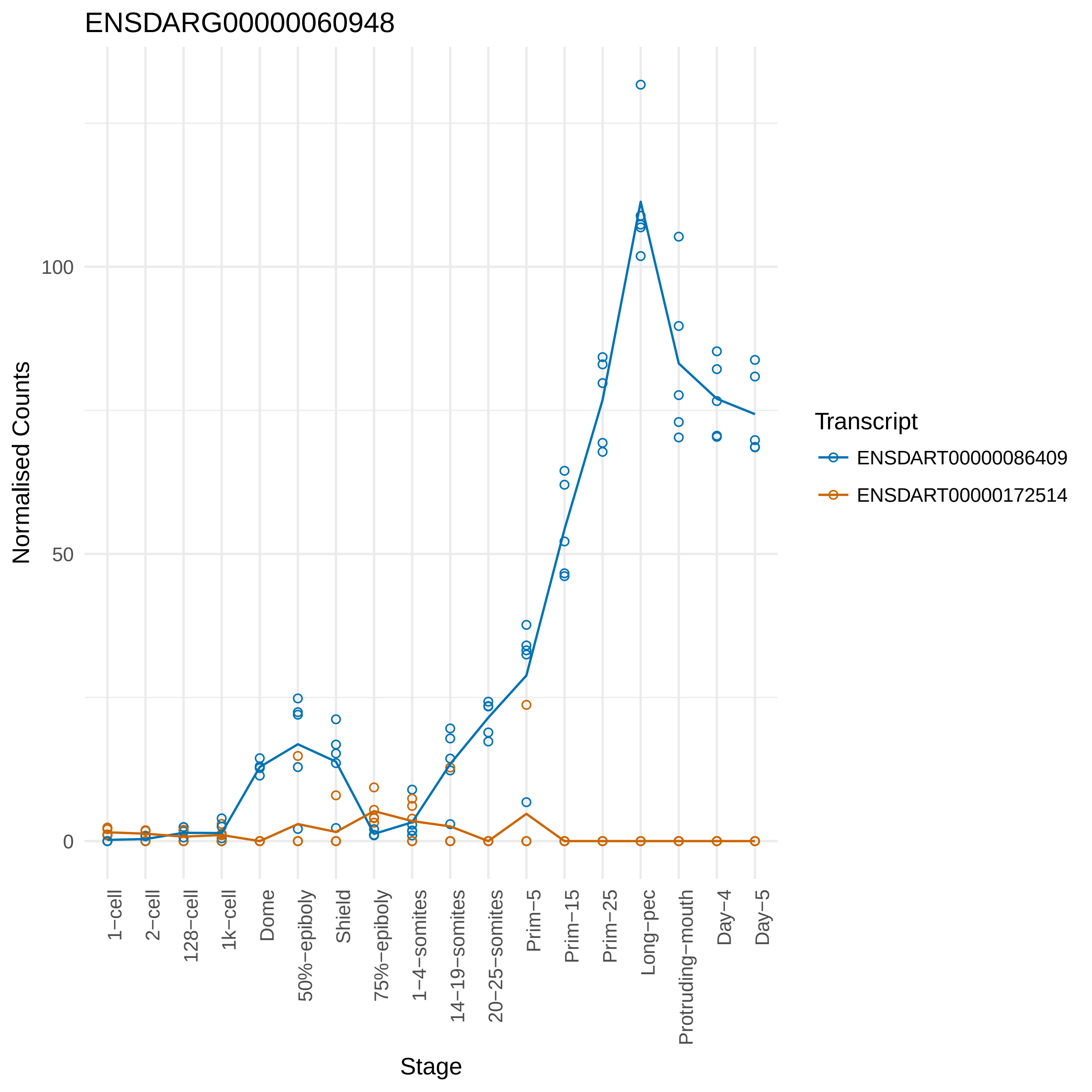
ENSDARG00000060948
Normalised Counts
100
50
0
1−cell
2−cell
128−cell
1k−cell
Dome
50%−epiboly
Shield
75%−epiboly
1−4−somites
14−19−somites
20−25−somites
Prim−5
Prim−15
Prim−25
Long−pec
Protruding−mouth
Day−4
Day−5
Stage
Transcript
ENSDART00000086409
ENSDART00000172514

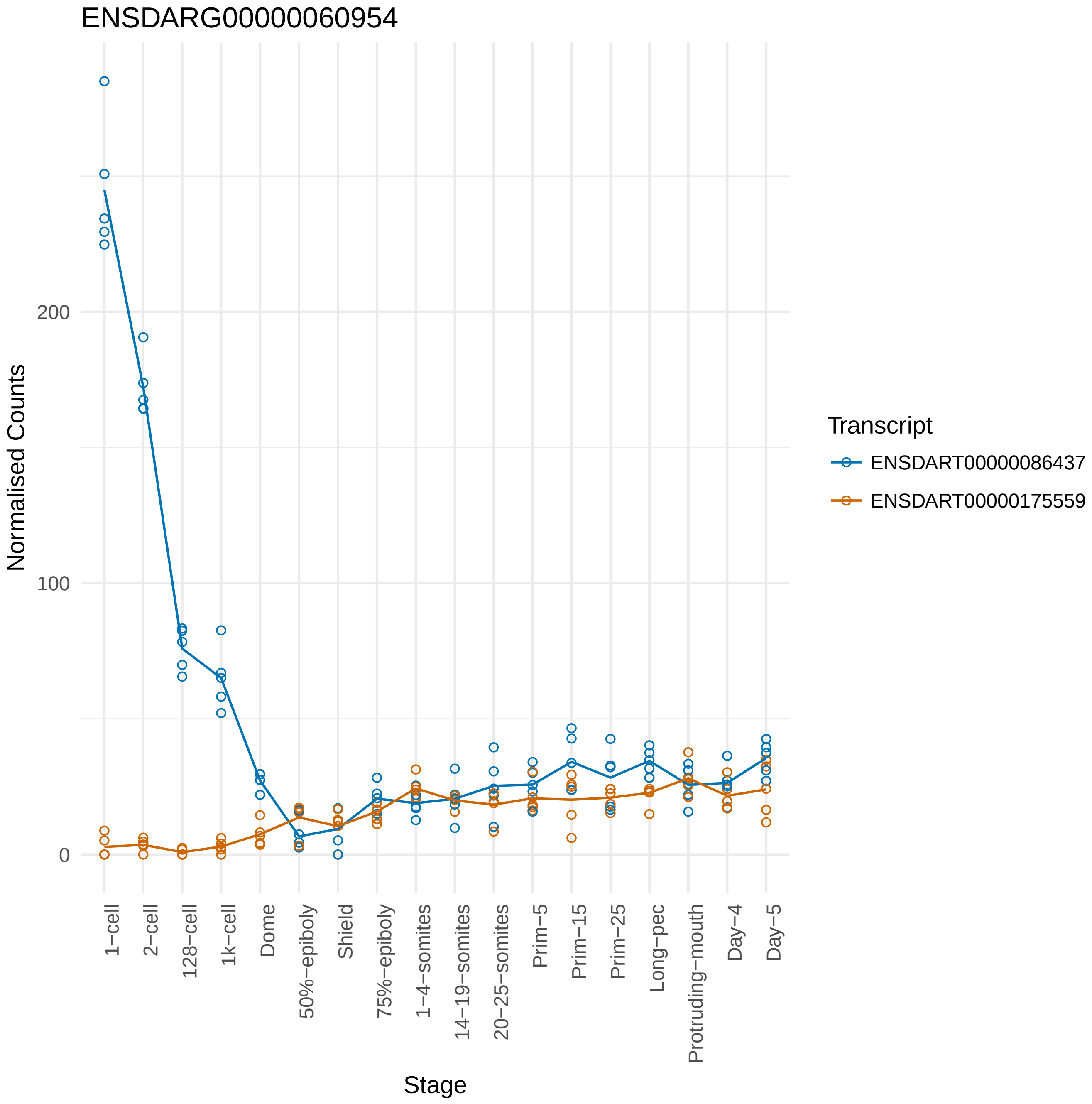

ENSDARG00000060954
Normalised Counts
200
100
0
1−cell
2−cell
128−cell
1k−cell
Dome
50%−epiboly
Shield
75%−epiboly
1−4−somites
14−19−somites
20−25−somites
Prim−5
Prim−15
Prim−25
Long−pec
Protruding−mouth
Day−4
Day−5
Stage
Transcript
ENSDART00000086437
ENSDART00000175559

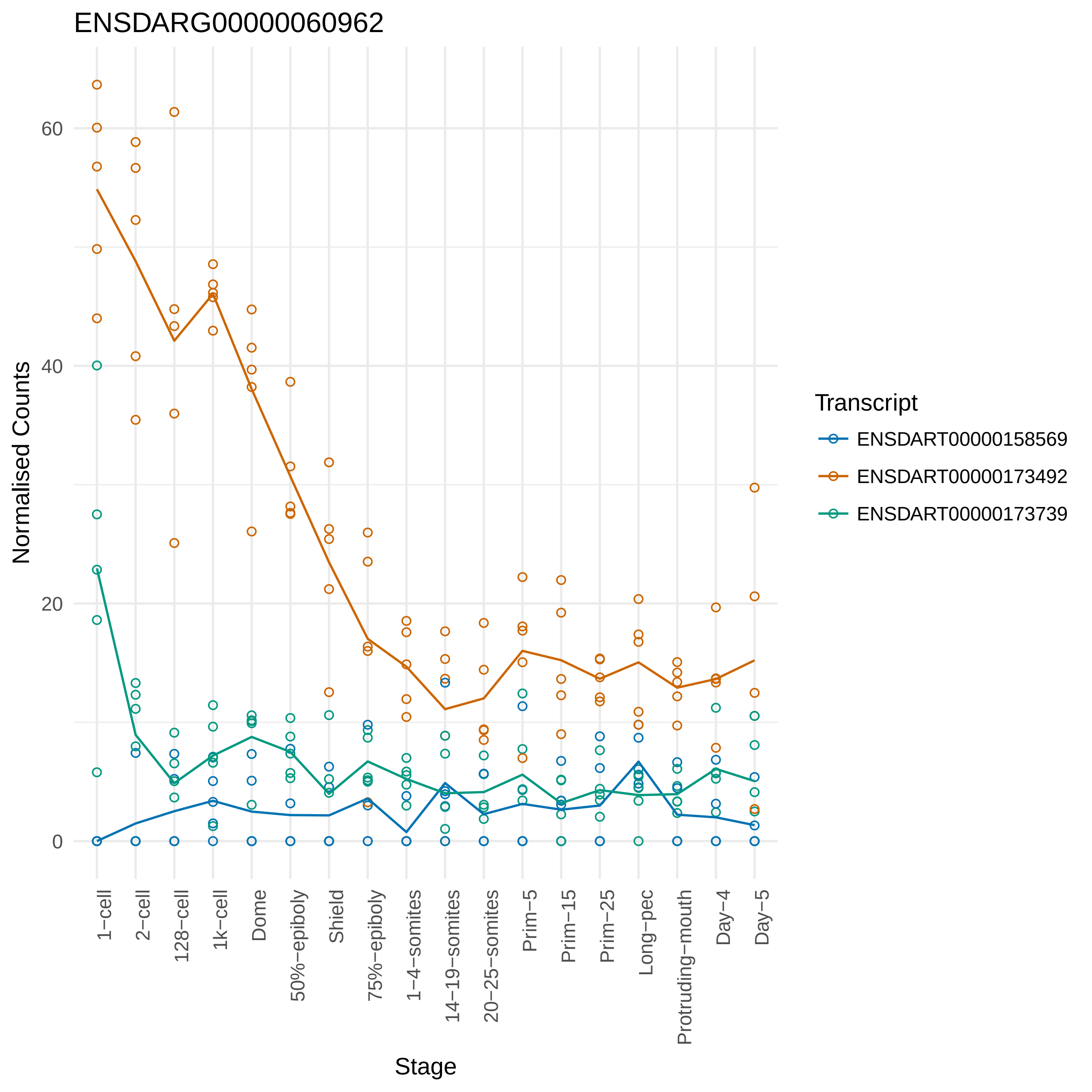
ENSDARG00000060962
Normalised Counts
0
20
40
60
Stage
1−cell
2−cell
128−cell
1k−cell
Dome
50%−epiboly
Shield
75%−epiboly
1−4−somites
14−19−somites
20−25−somites
Prim−5
Prim−15
Prim−25
Long−pec
Protruding−mouth
Day−4
Day−5
Transcript
ENSDART00000158569
ENSDART00000173492
ENSDART00000173739

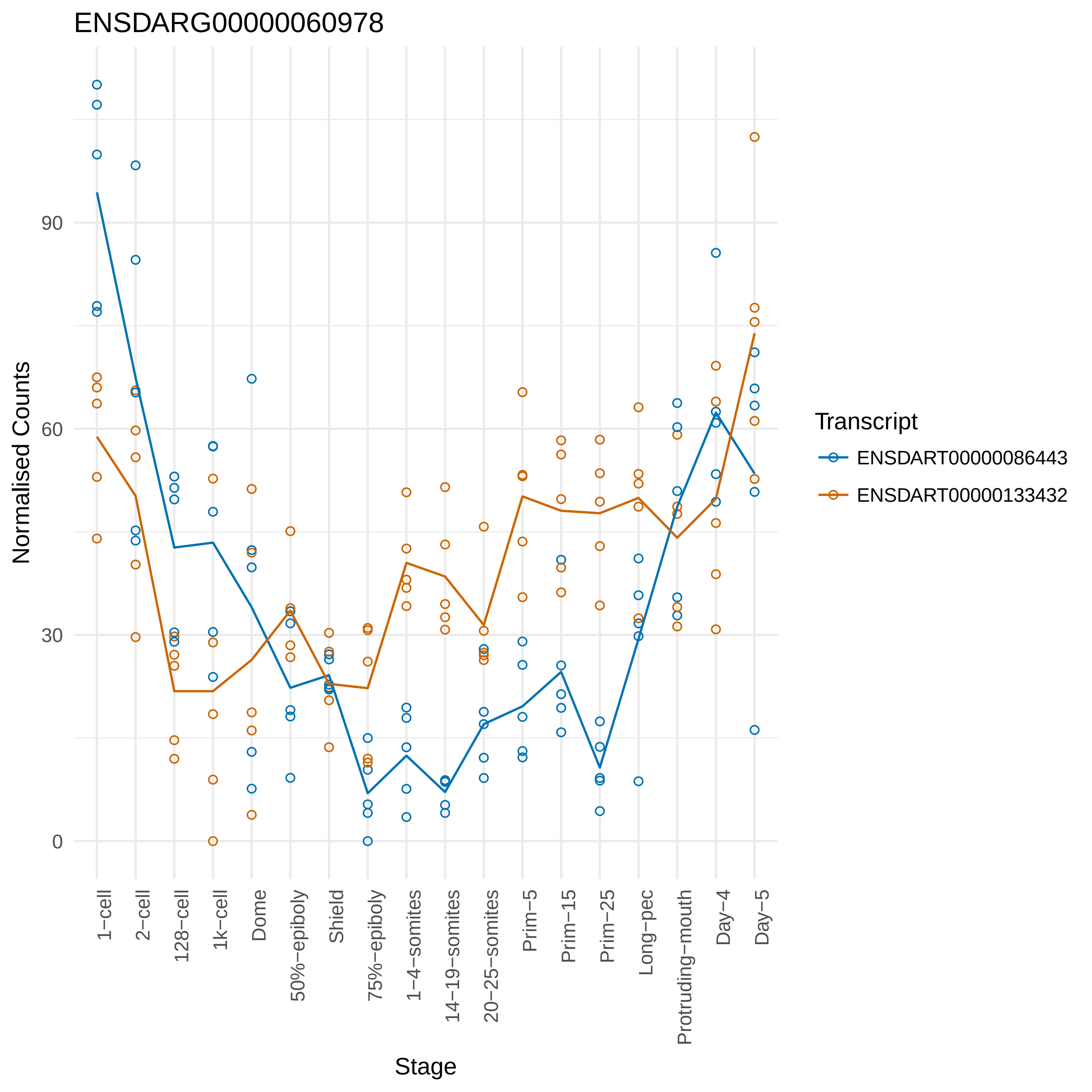
ENSDARG00000060978
Normalised Counts
90
60
30
0
1−cell
2−cell
128−cell
1k−cell
Dome
50%−epiboly
Shield
75%−epiboly
1−4−somites
14−19−somites
20−25−somites
Prim−5
Prim−15
Prim−25
Long−pec
Protruding−mouth
Day−4
Day−5
Stage
Transcript
ENSDART00000086443
ENSDART00000133432

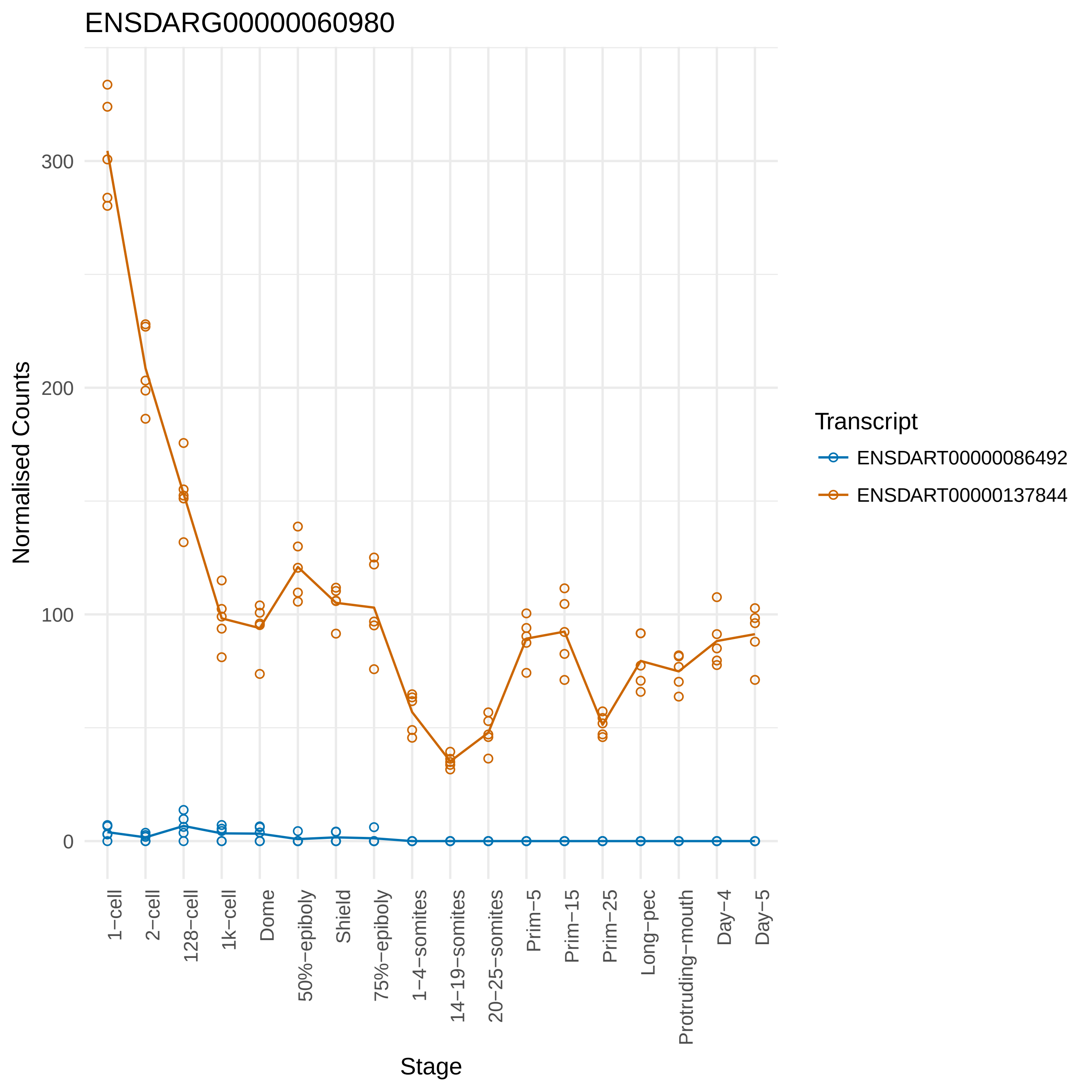

ENSDARG00000060980
Normalised Counts
300
200
100
0
1−cell
2−cell
128−cell
1k−cell
Dome
50%−epiboly
Shield
75%−epiboly
1−4−somites
14−19−somites
20−25−somites
Prim−5
Prim−15
Prim−25
Long−pec
Protruding−mouth
Day−4
Day−5
Stage
Transcript
ENSDART00000086492
ENSDART00000137844

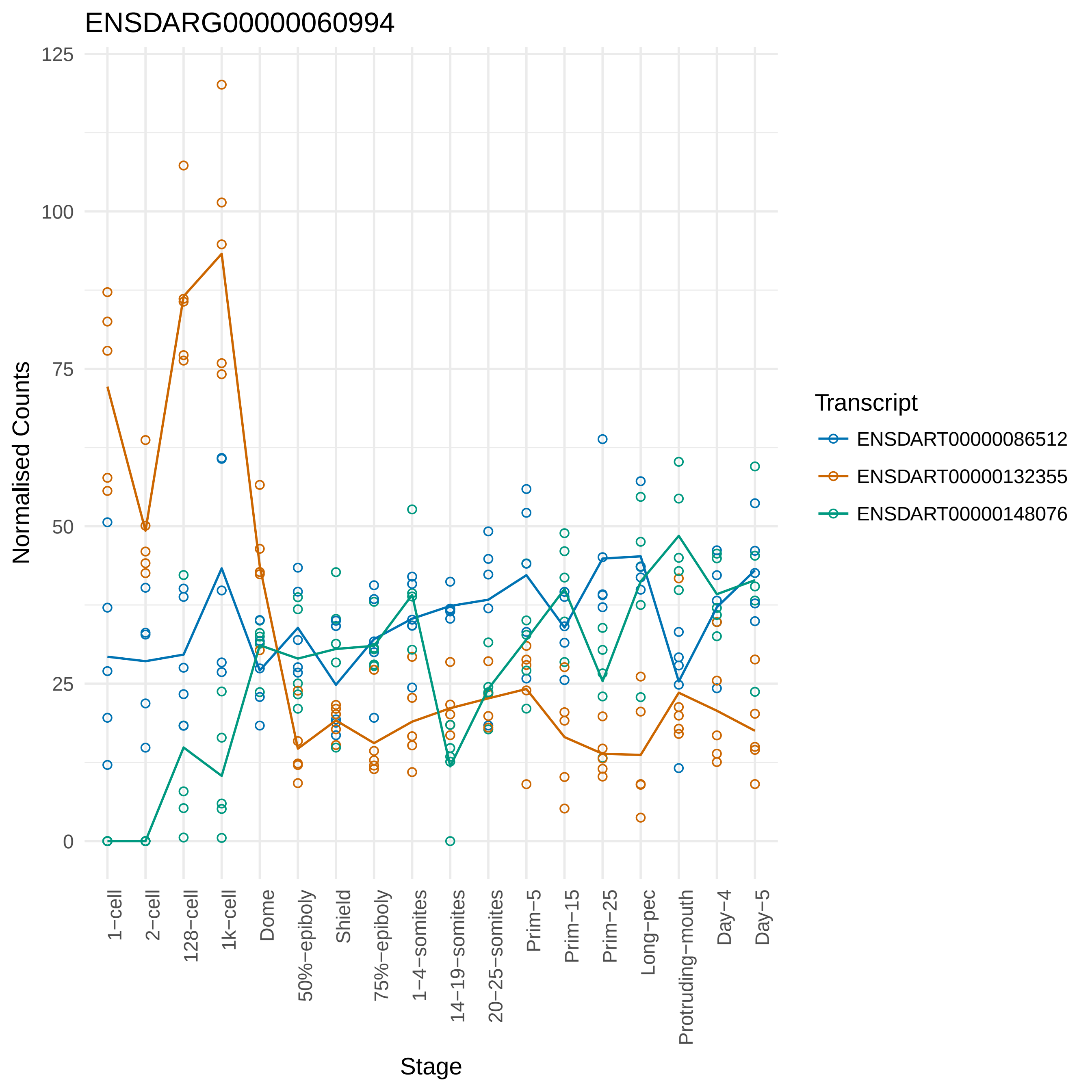
ENSDARG00000060994
Normalised Counts
125
100
75
50
25
0
1−cell
2−cell
128−cell
1k−cell
Dome
50%−epiboly
Shield
75%−epiboly
1−4−somites
14−19−somites
20−25−somites
Prim−5
Prim−15
Prim−25
Long−pec
Protruding−mouth
Day−4
Day−5
Stage
Transcript
ENSDART00000086512
ENSDART00000132355
ENSDART00000148076

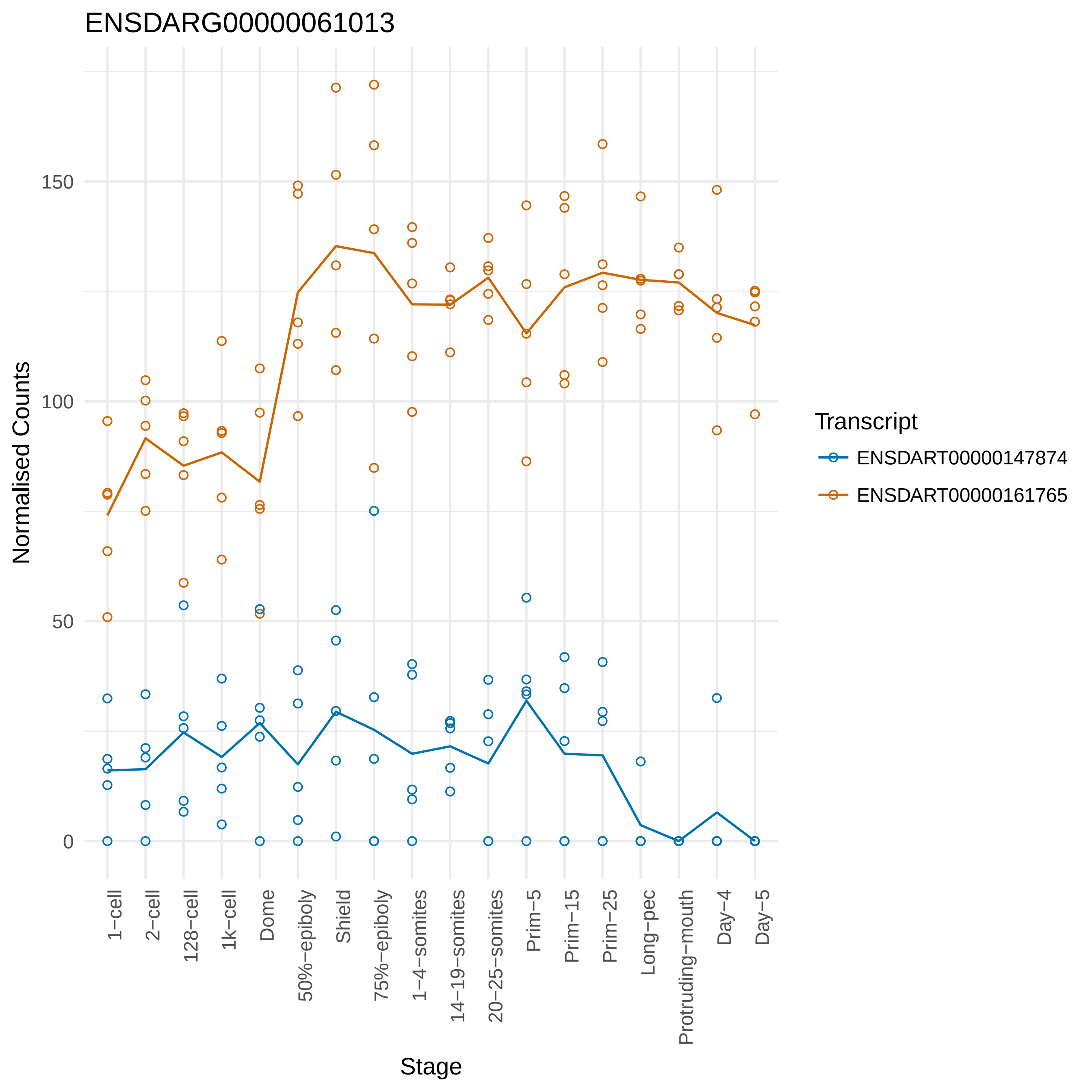
ENSDARG00000061013
Normalised Counts
150
100
50
0
1−cell
2−cell
128−cell
1k−cell
Dome
50%−epiboly
Shield
75%−epiboly
1−4−somites
14−19−somites
20−25−somites
Prim−5
Prim−15
Prim−25
Long−pec
Protruding−mouth
Day−4
Day−5
Stage
Transcript
ENSDART00000147874
ENSDART00000161765

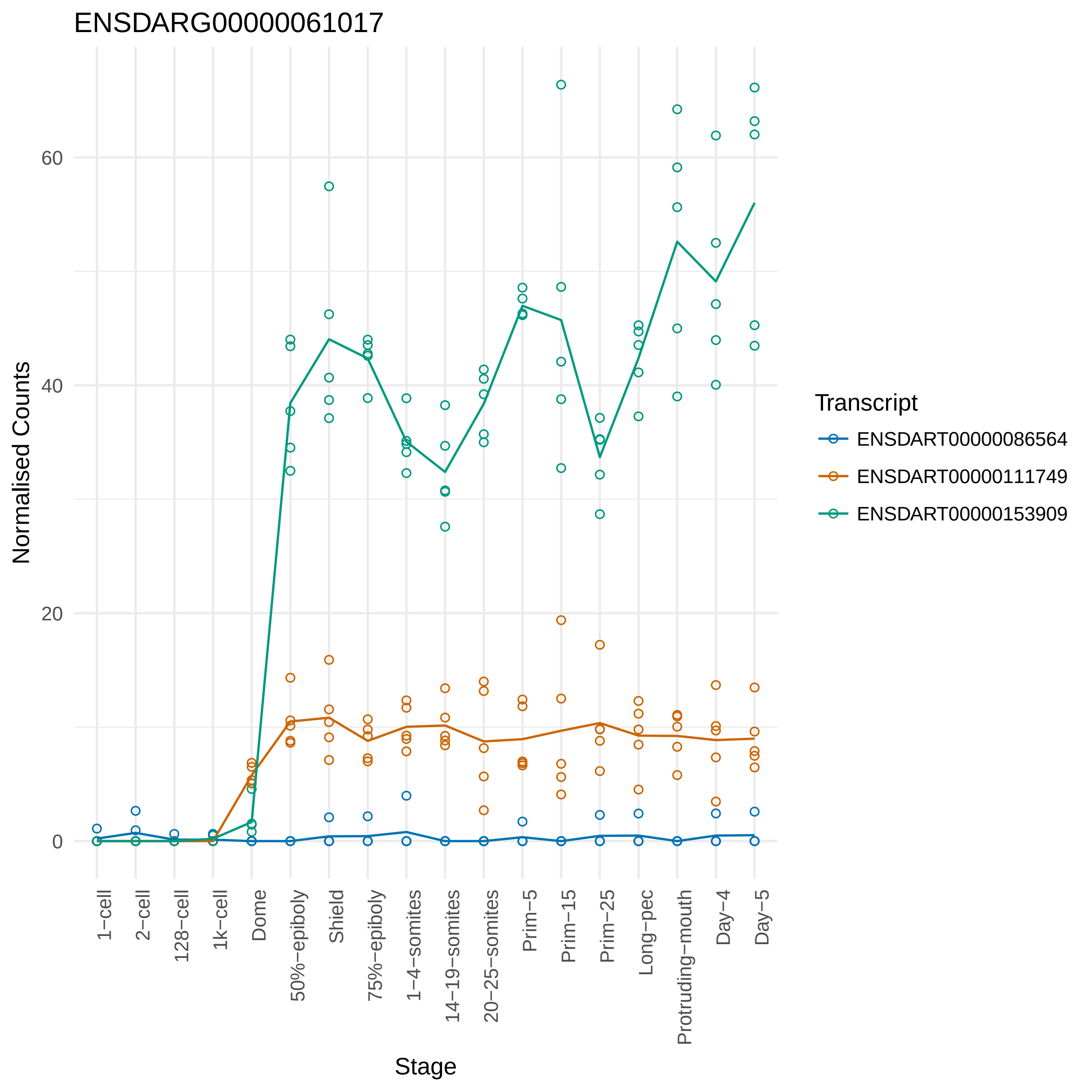
ENSDARG00000061017
Normalised Counts
0
20
40
60
1−cell
2−cell
128−cell
1k−cell
Dome
50%−epiboly
Shield
75%−epiboly
1−4−somites
14−19−somites
20−25−somites
Prim−5
Prim−15
Prim−25
Long−pec
Protruding−mouth
Day−4
Day−5
Stage
Transcript
ENSDART00000086564
ENSDART00000111749
ENSDART00000153909

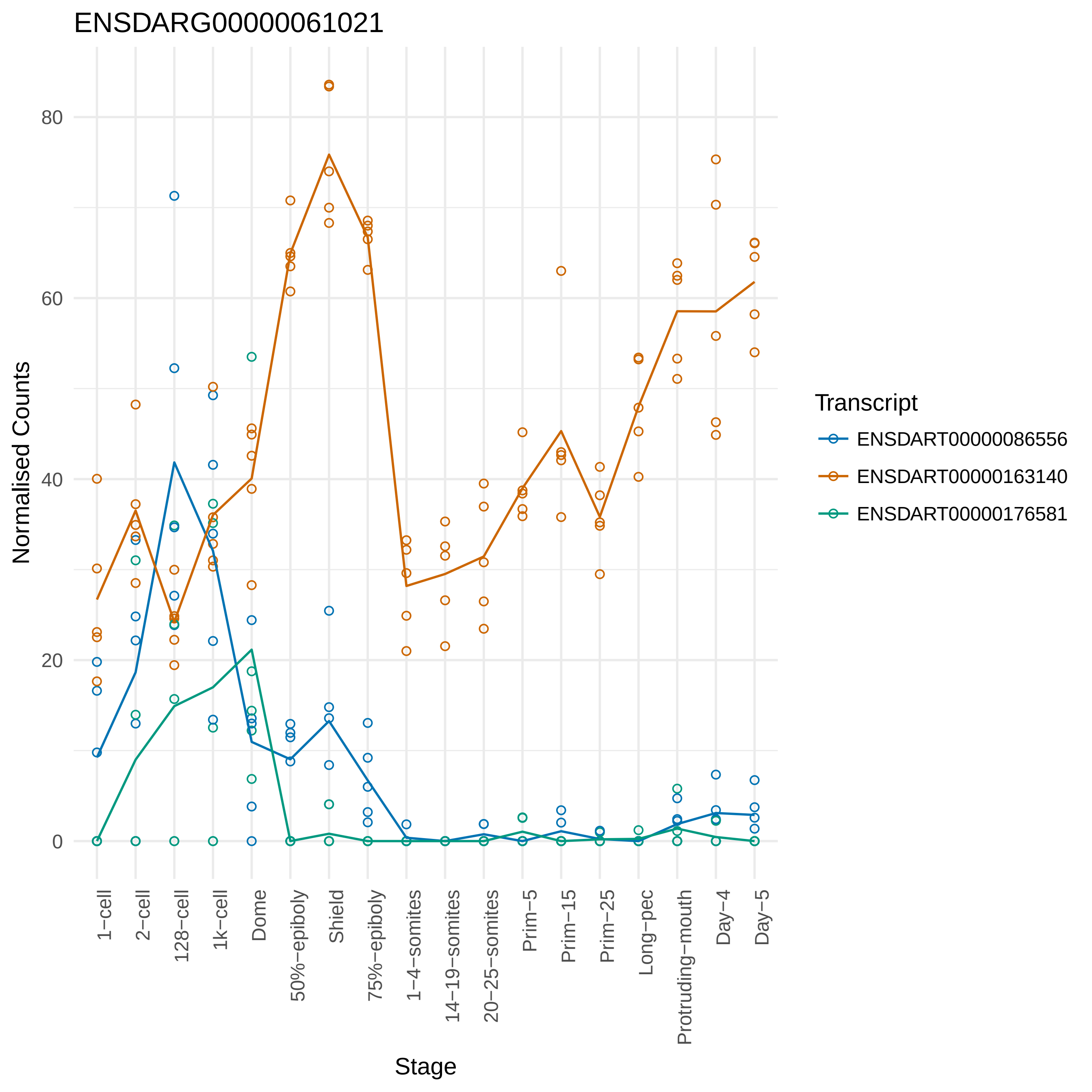
ENSDARG00000061021
Normalised Counts
80
60
40
20
0
1−cell
2−cell
128−cell
1k−cell
Dome
50%−epiboly
Shield
75%−epiboly
1−4−somites
14−19−somites
20−25−somites
Prim−5
Prim−15
Prim−25
Long−pec
Protruding−mouth
Day−4
Day−5
Stage
Transcript
ENSDART00000086556
ENSDART00000163140
ENSDART00000176581

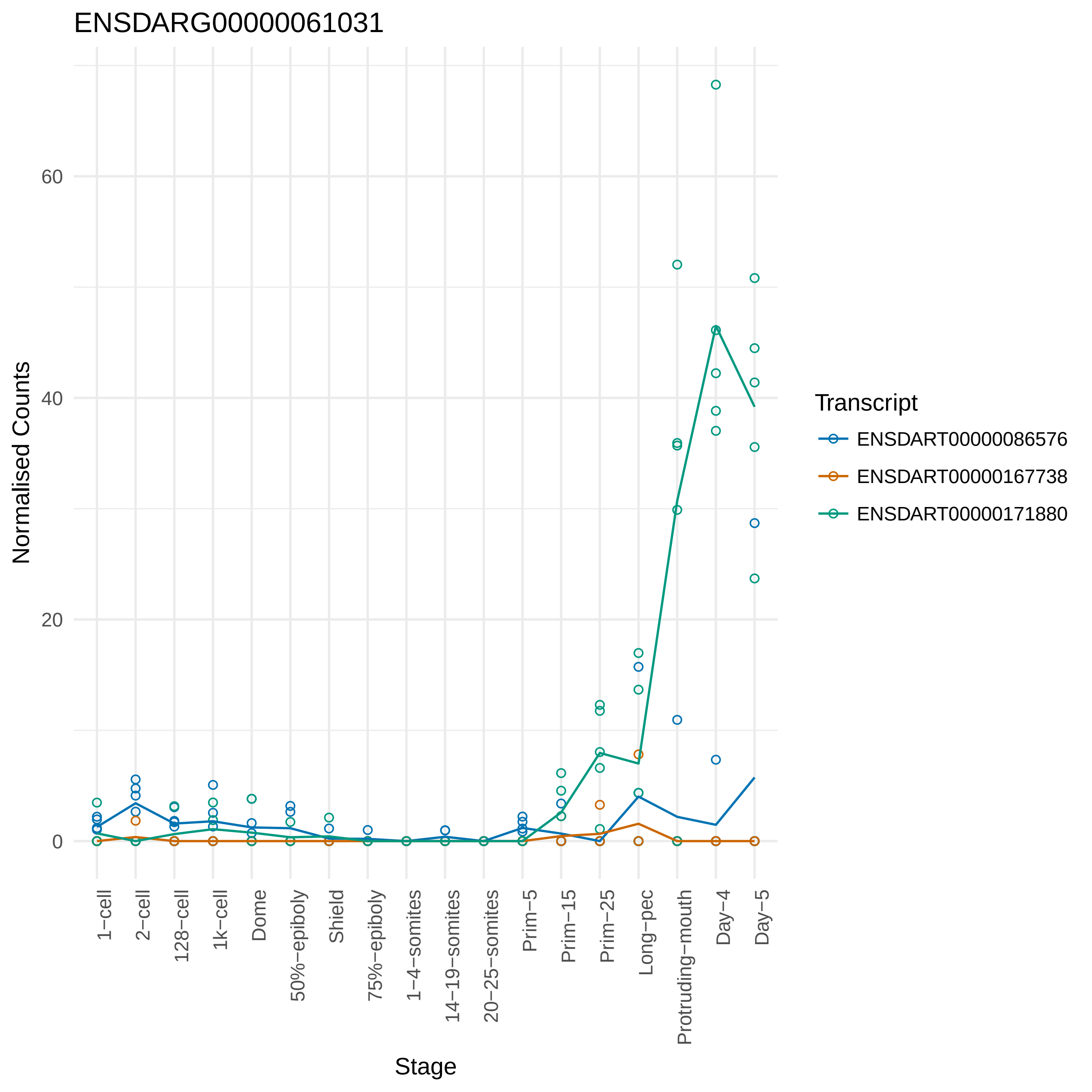

ENSDARG00000061031
Normalised Counts
60
40
20
0
1−cell
2−cell
128−cell
1k−cell
Dome
50%−epiboly
Shield
75%−epiboly
1−4−somites
14−19−somites
20−25−somites
Prim−5
Prim−15
Prim−25
Long−pec
Protruding−mouth
Day−4
Day−5
Stage
Transcript
ENSDART00000086576
ENSDART00000167738
ENSDART00000171880

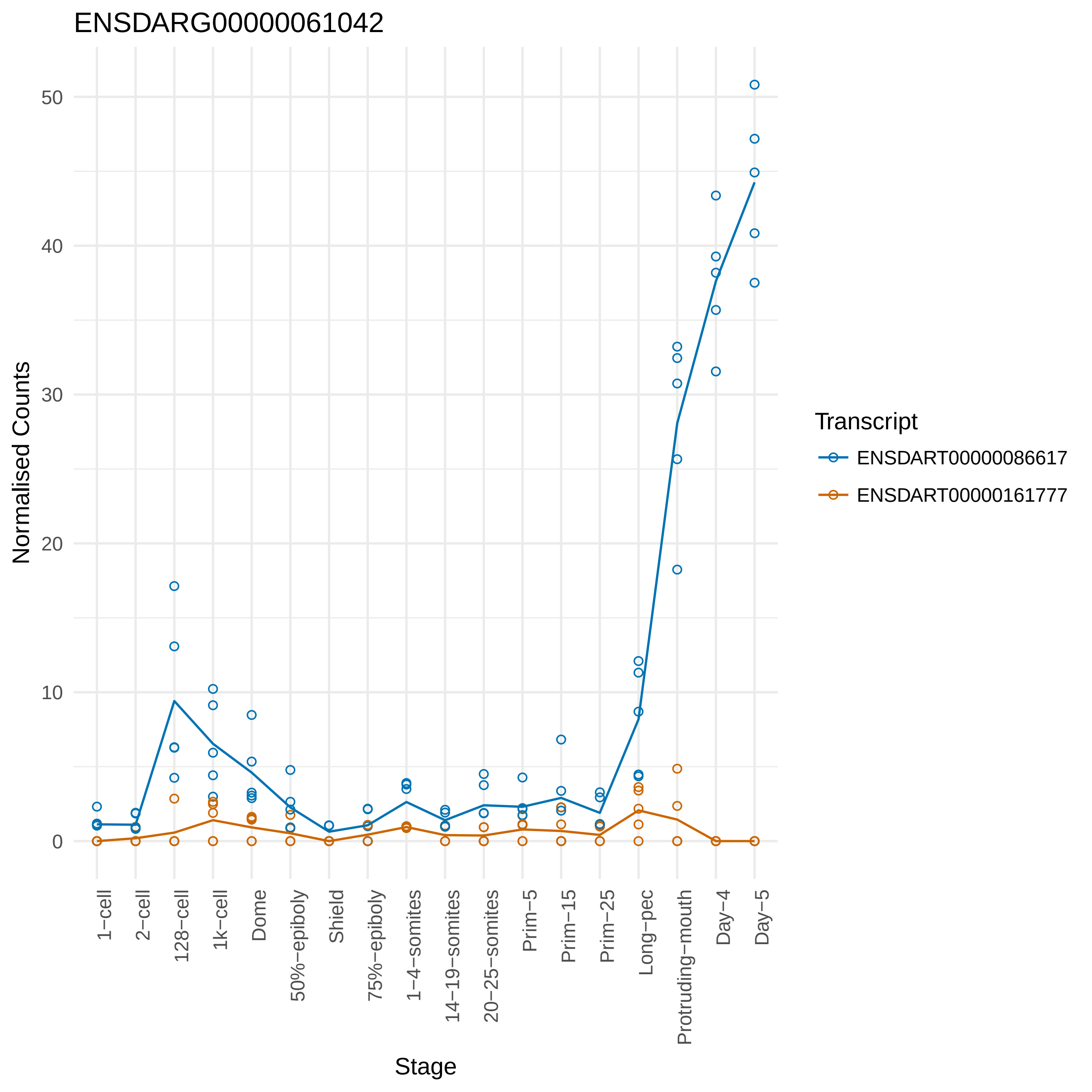
ENSDARG00000061042
Normalised Counts
50
40
30
20
10
0
1−cell
2−cell
128−cell
1k−cell
Dome
50%−epiboly
Shield
75%−epiboly
1−4−somites
14−19−somites
20−25−somites
Prim−5
Prim−15
Prim−25
Long−pec
Protruding−mouth
Day−4
Day−5
Stage
Transcript
ENSDART00000086617
ENSDART00000161777

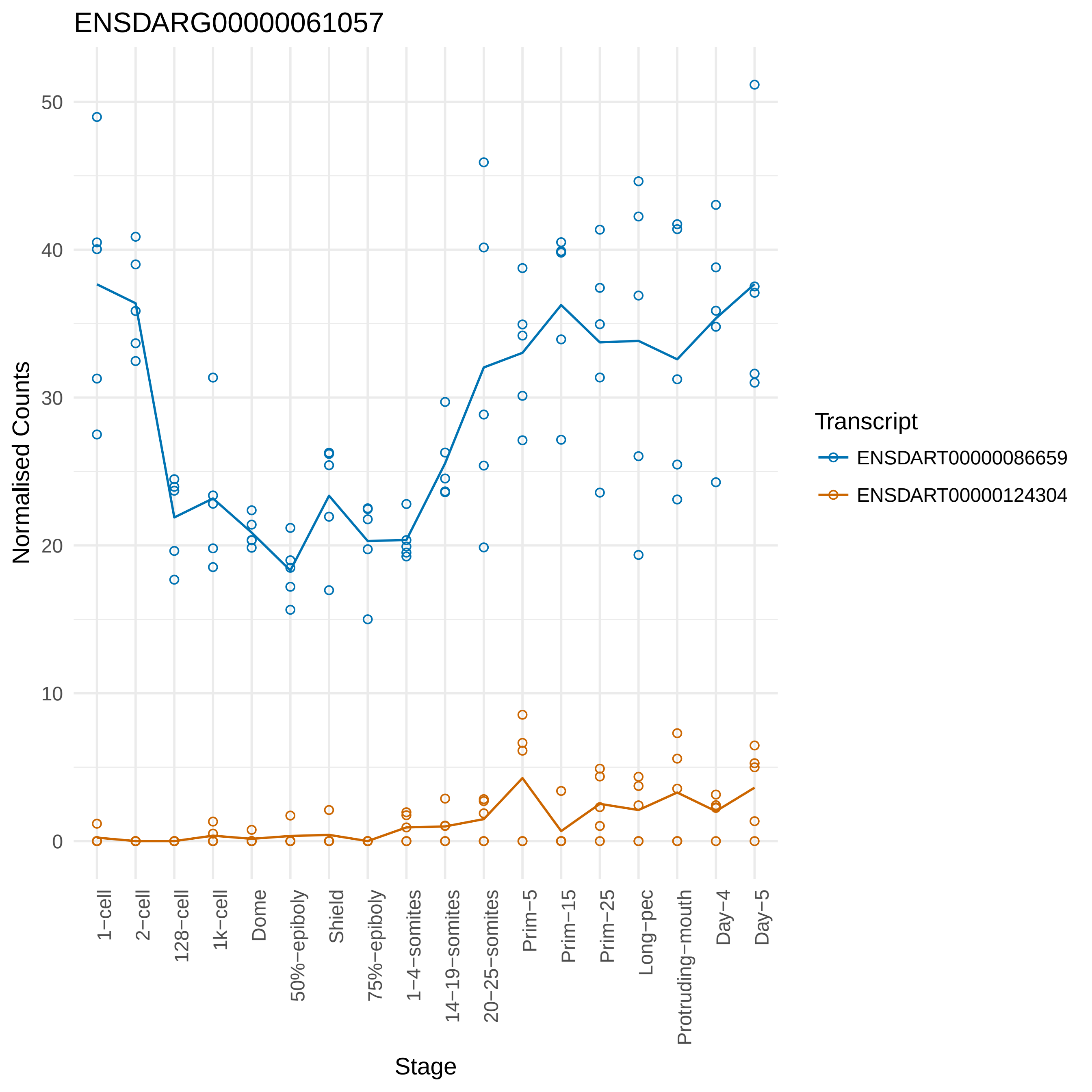

ENSDARG00000061057
Normalised Counts
0
10
20
30
40
50
1−cell
2−cell
128−cell
1k−cell
Dome
50%−epiboly
Shield
75%−epiboly
1−4−somites
14−19−somites
20−25−somites
Prim−5
Prim−15
Prim−25
Long−pec
Protruding−mouth
Day−4
Day−5
Stage
Transcript
ENSDART00000086659
ENSDART00000124304

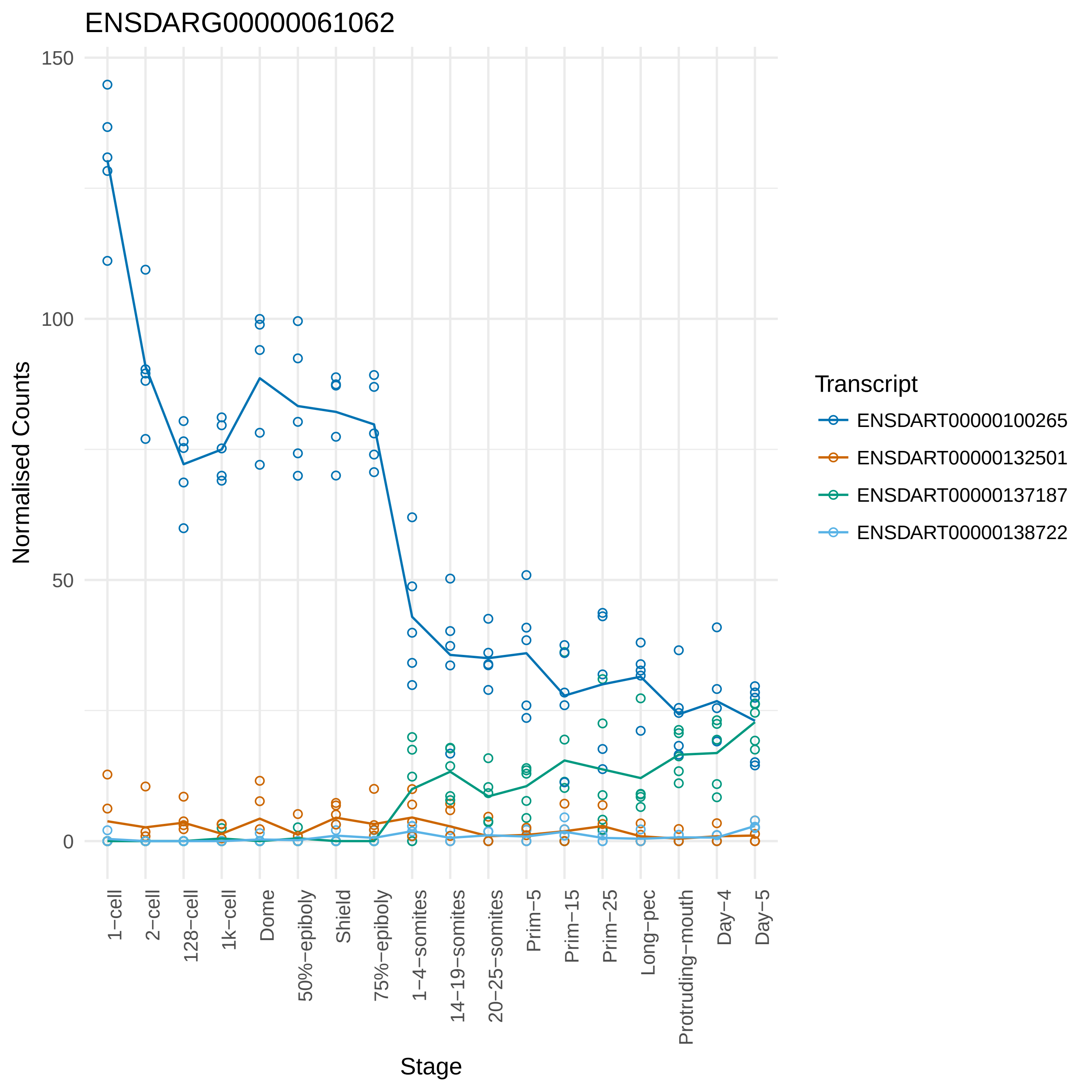

ENSDARG00000061062
Normalised Counts
150
100
50
0
Stage
1−cell
2−cell
128−cell
1k−cell
Dome
50%−epiboly
Shield
75%−epiboly
1−4−somites
14−19−somites
20−25−somites
Prim−5
Prim−15
Prim−25
Long−pec
Protruding−mouth
Day−4
Day−5
Transcript
ENSDART00000100265
ENSDART00000132501
ENSDART00000137187
ENSDART00000138722

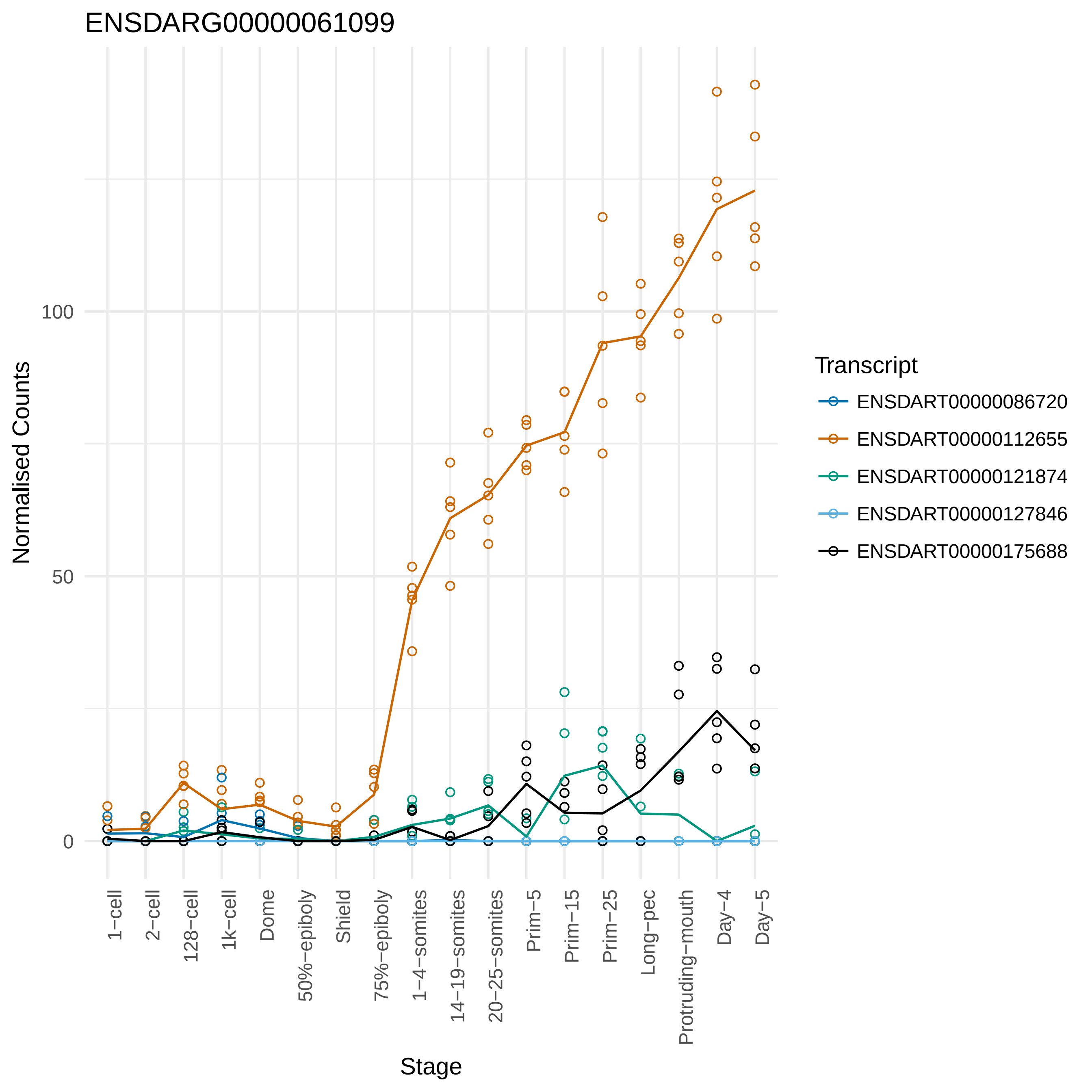

ENSDARG00000061099
Normalised Counts
0
50
100
Stage
1−cell
2−cell
128−cell
1k−cell
Dome
50%−epiboly
Shield
75%−epiboly
1−4−somites
14−19−somites
20−25−somites
Prim−5
Prim−15
Prim−25
Long−pec
Protruding−mouth
Day−4
Day−5
Transcript
ENSDART00000086720
ENSDART00000112655
ENSDART00000121874
ENSDART00000127846
ENSDART00000175688

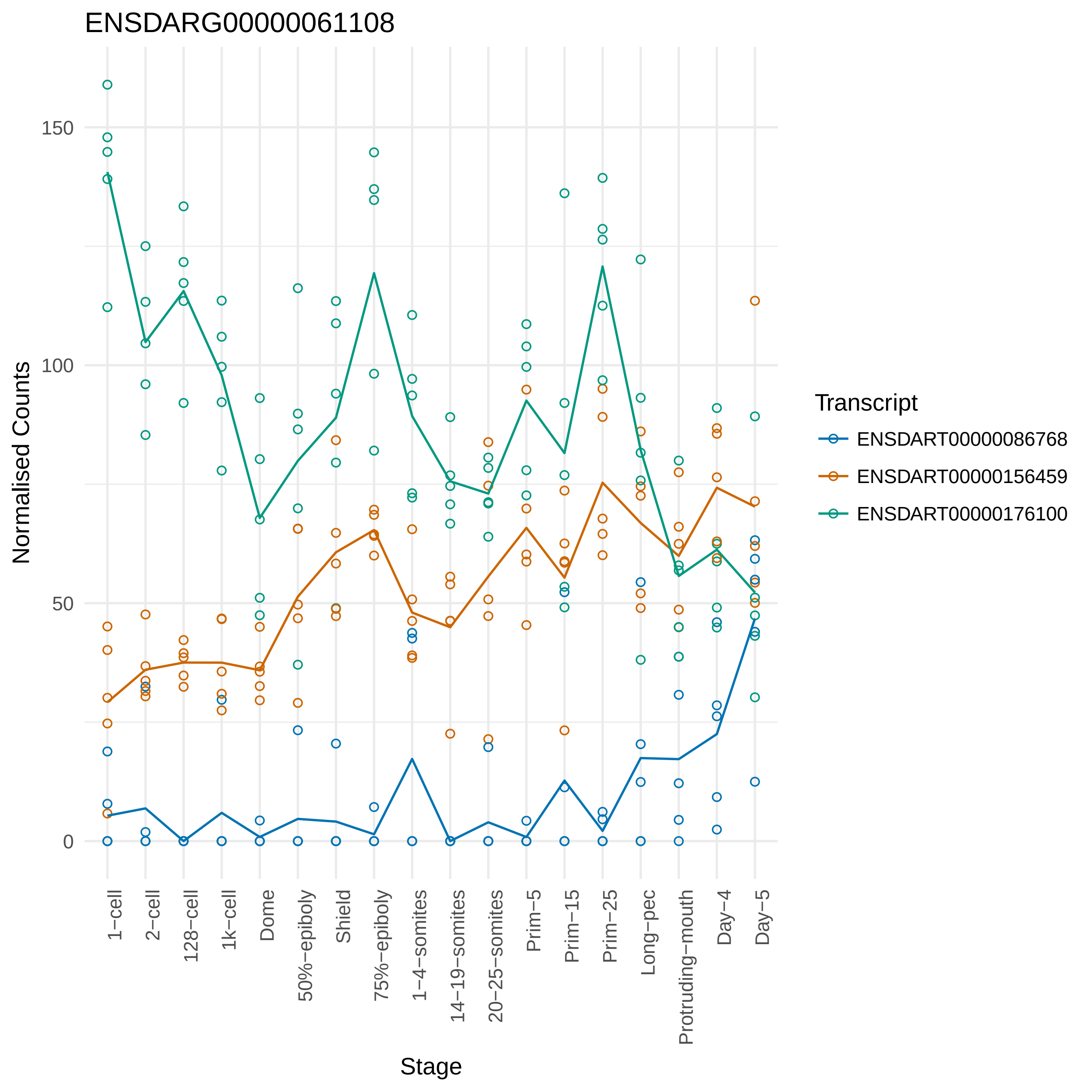
ENSDARG00000061108
Normalised Counts
0
50
100
150
Stage
1−cell
2−cell
128−cell
1k−cell
Dome
50%−epiboly
Shield
75%−epiboly
1−4−somites
14−19−somites
20−25−somites
Prim−5
Prim−15
Prim−25
Long−pec
Protruding−mouth
Day−4
Day−5
Transcript
ENSDART00000086768
ENSDART00000156459
ENSDART00000176100

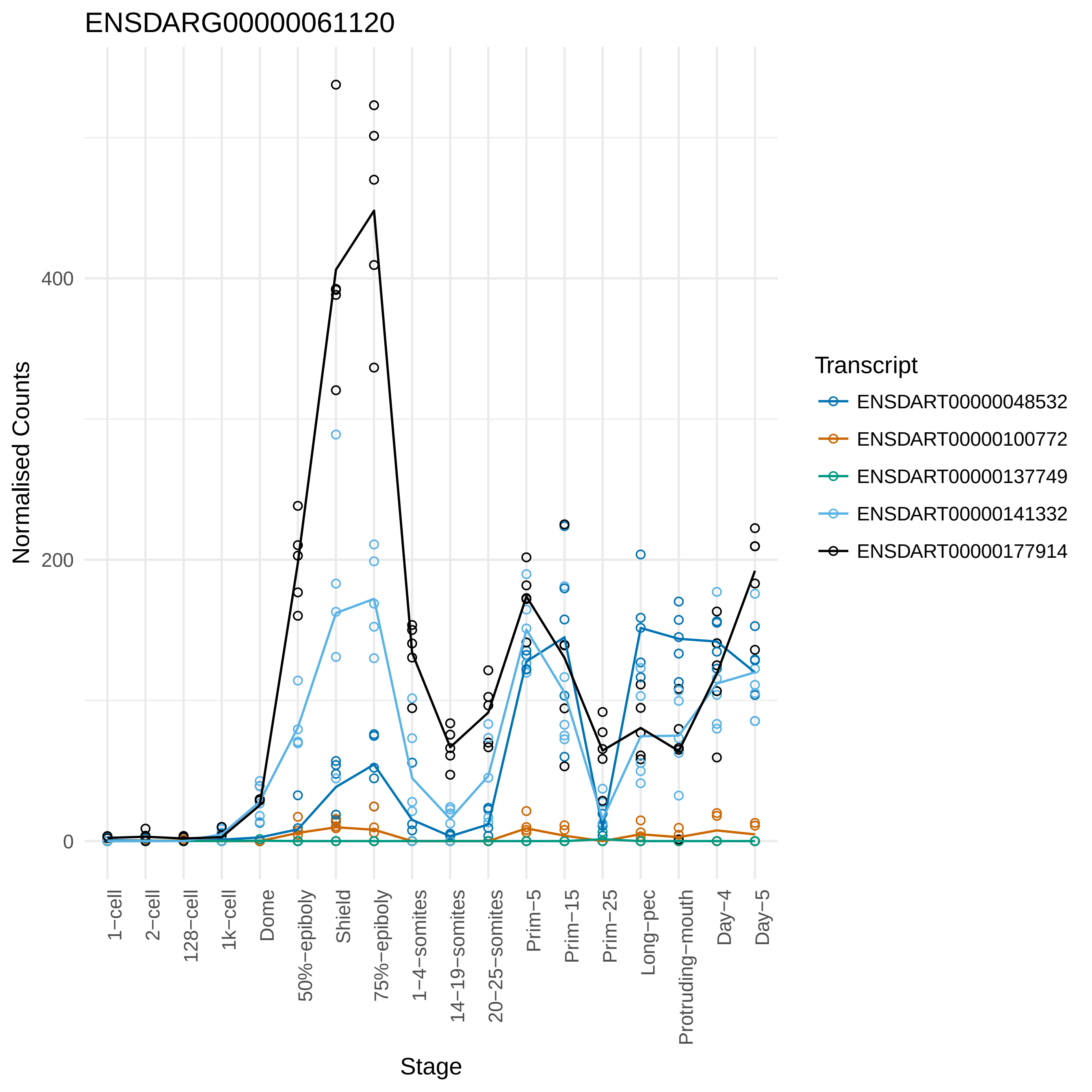
ENSDARG00000061120
Normalised Counts
400
200
0
Stage
1−cell
2−cell
128−cell
1k−cell
Dome
50%−epiboly
Shield
75%−epiboly
1−4−somites
14−19−somites
20−25−somites
Prim−5
Prim−15
Prim−25
Long−pec
Protruding−mouth
Day−4
Day−5
Transcript
ENSDART00000048532
ENSDART00000100772
ENSDART00000137749
ENSDART00000141332
ENSDART00000177914

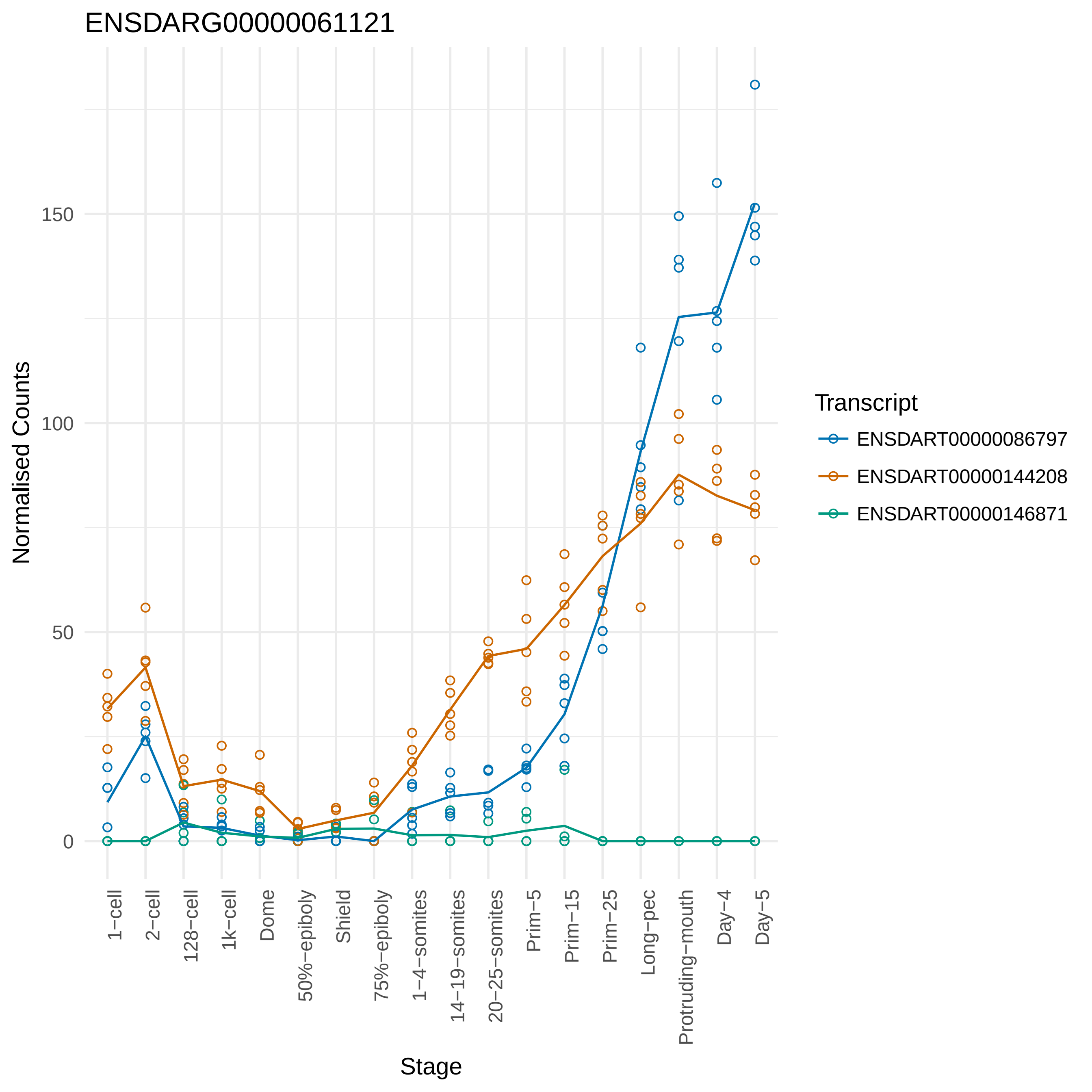

ENSDARG00000061121
Normalised Counts
150
100
50
0
1−cell
2−cell
128−cell
1k−cell
Dome
50%−epiboly
Shield
75%−epiboly
1−4−somites
14−19−somites
20−25−somites
Prim−5
Prim−15
Prim−25
Long−pec
Protruding−mouth
Day−4
Day−5
Stage
Transcript
ENSDART00000086797
ENSDART00000144208
ENSDART00000146871

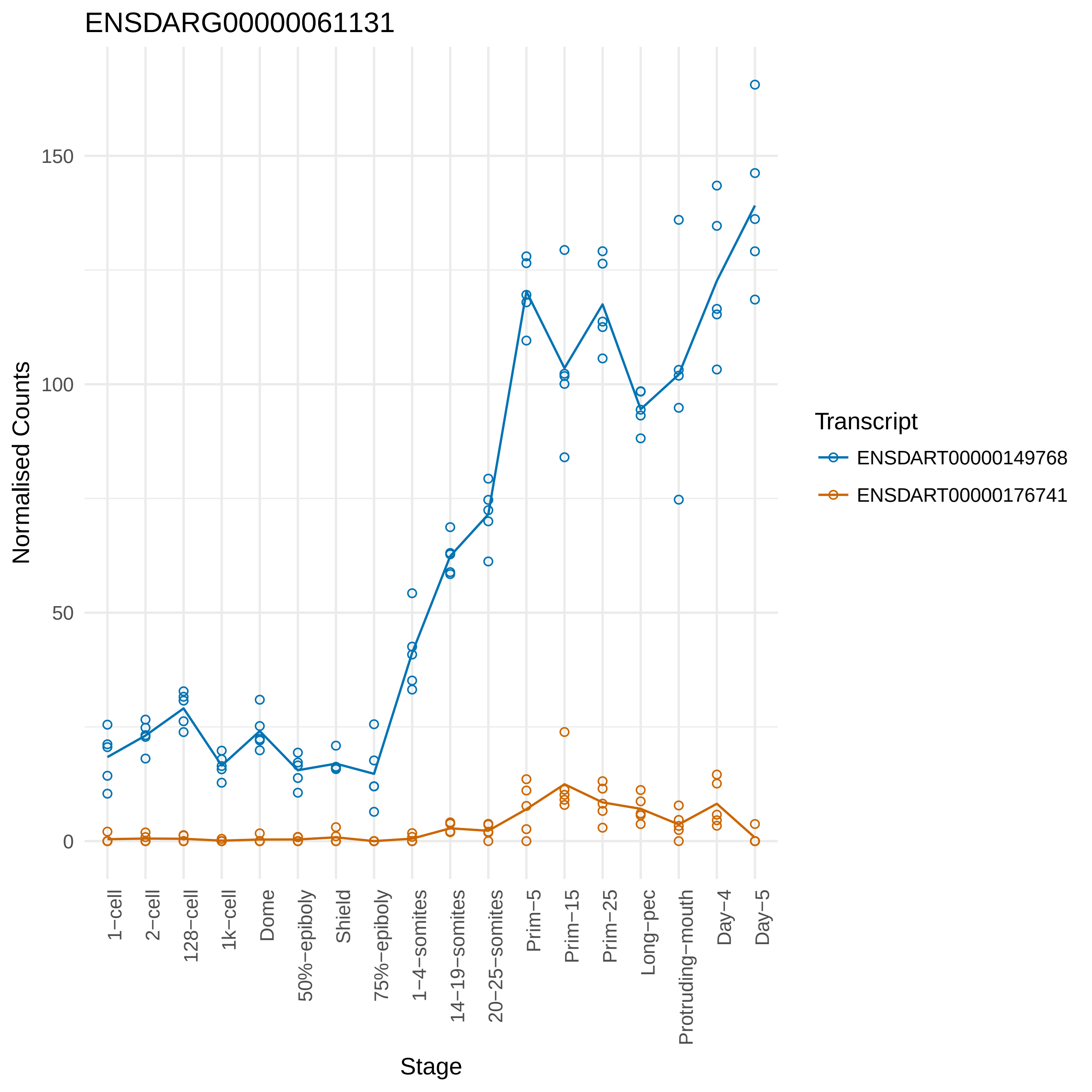

ENSDARG00000061131
Normalised Counts
150
100
50
0
1−cell
2−cell
128−cell
1k−cell
Dome
50%−epiboly
Shield
75%−epiboly
1−4−somites
14−19−somites
20−25−somites
Prim−5
Prim−15
Prim−25
Long−pec
Protruding−mouth
Day−4
Day−5
Stage
Transcript
ENSDART00000149768
ENSDART00000176741

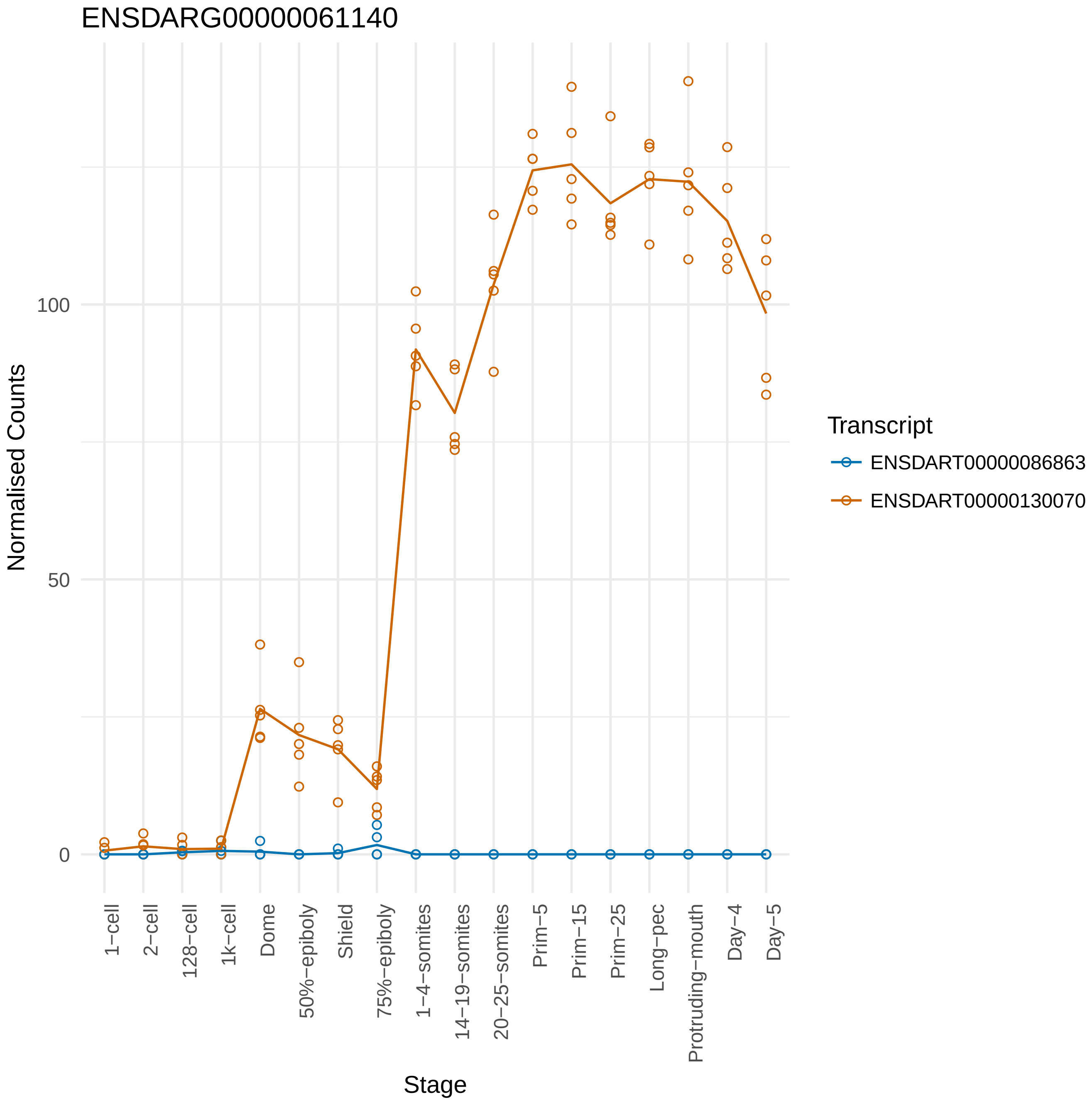

ENSDARG00000061140
Normalised Counts
100
50
0
1−cell
2−cell
128−cell
1k−cell
Dome
50%−epiboly
Shield
75%−epiboly
1−4−somites
14−19−somites
20−25−somites
Prim−5
Prim−15
Prim−25
Long−pec
Protruding−mouth
Day−4
Day−5
Stage
Transcript
ENSDART00000086863
ENSDART00000130070

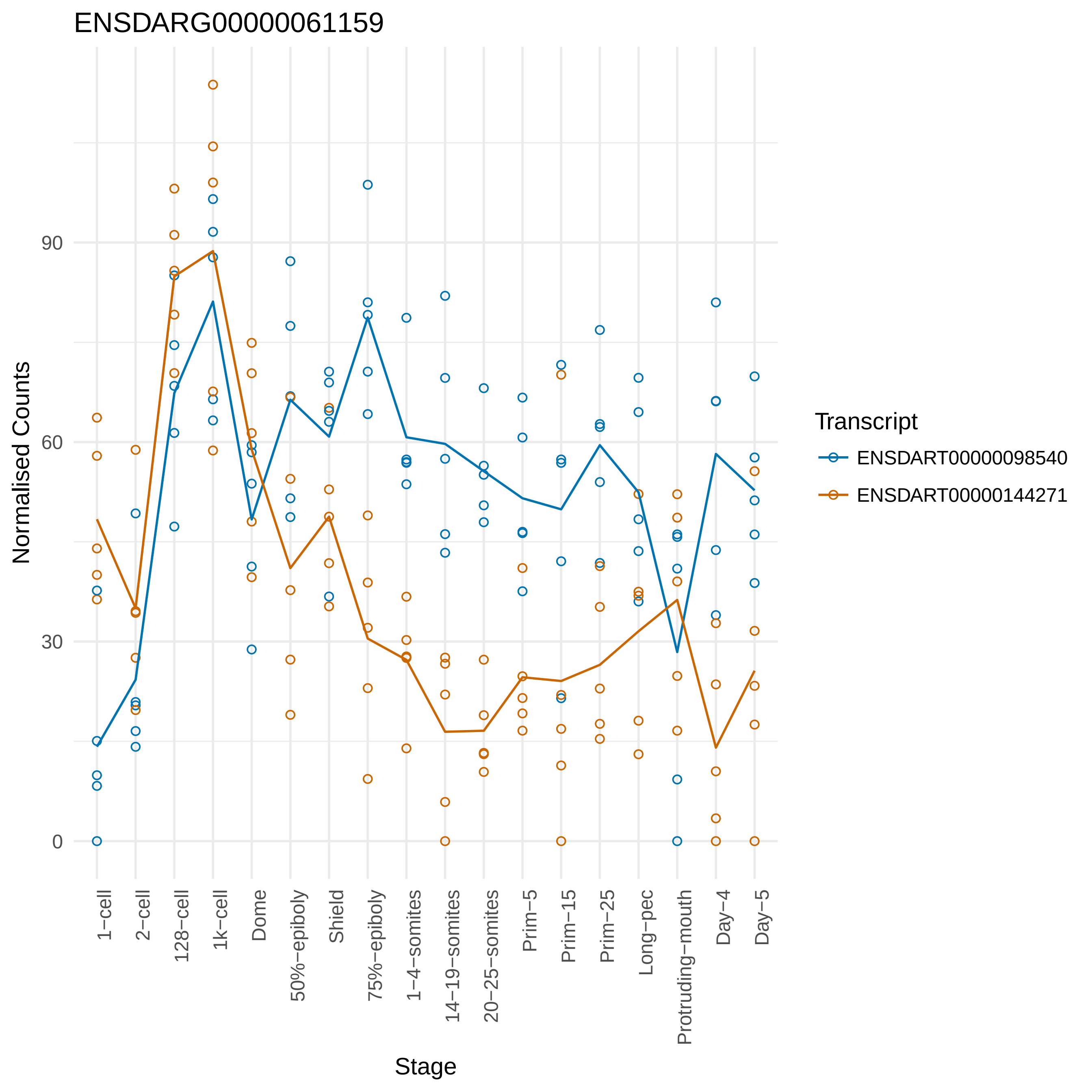

ENSDARG00000061159
Normalised Counts
0
30
60
90
Stage
1−cell
2−cell
128−cell
1k−cell
Dome
50%−epiboly
Shield
75%−epiboly
1−4−somites
14−19−somites
20−25−somites
Prim−5
Prim−15
Prim−25
Long−pec
Protruding−mouth
Day−4
Day−5
Transcript
ENSDART00000098540
ENSDART00000144271

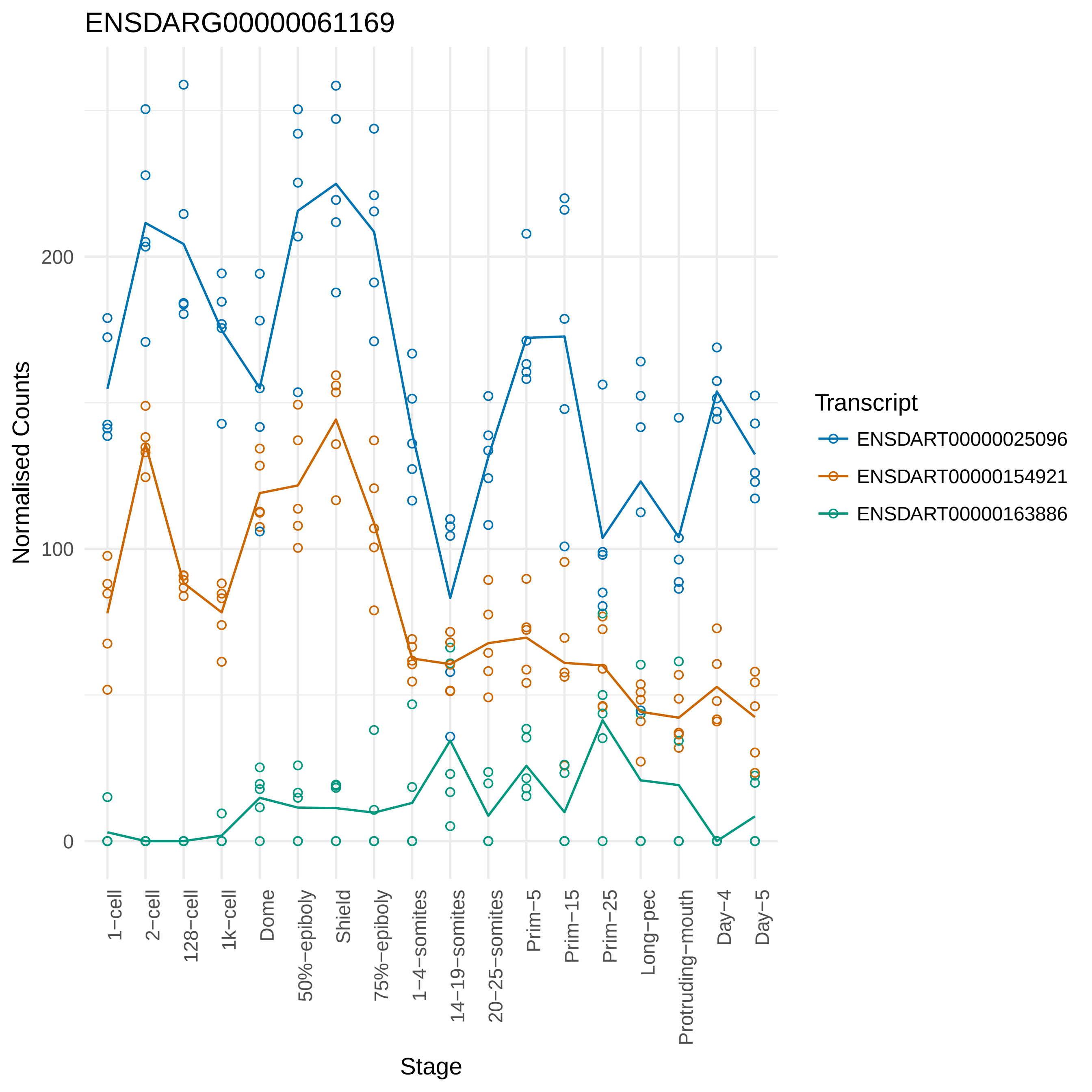
ENSDARG00000061169
Normalised Counts
200
100
0
Stage
1−cell
2−cell
128−cell
1k−cell
Dome
50%−epiboly
Shield
75%−epiboly
1−4−somites
14−19−somites
20−25−somites
Prim−5
Prim−15
Prim−25
Long−pec
Protruding−mouth
Day−4
Day−5
Transcript
ENSDART00000025096
ENSDART00000154921
ENSDART00000163886

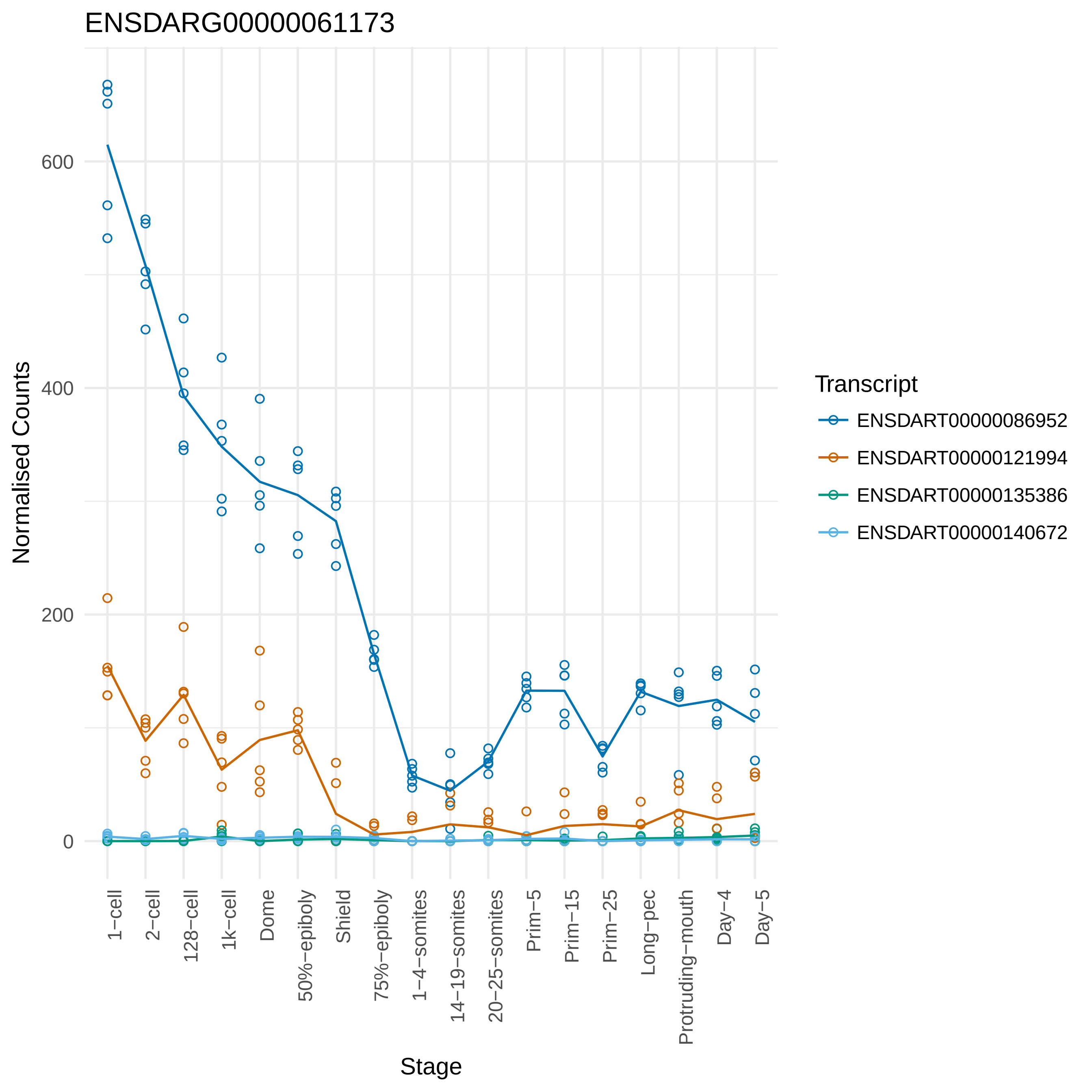

ENSDARG00000061173
Normalised Counts
600
400
200
0
1−cell
2−cell
128−cell
1k−cell
Dome
50%−epiboly
Shield
75%−epiboly
1−4−somites
14−19−somites
20−25−somites
Prim−5
Prim−15
Prim−25
Long−pec
Protruding−mouth
Day−4
Day−5
Stage
Transcript
ENSDART00000086952
ENSDART00000121994
ENSDART00000135386
ENSDART00000140672

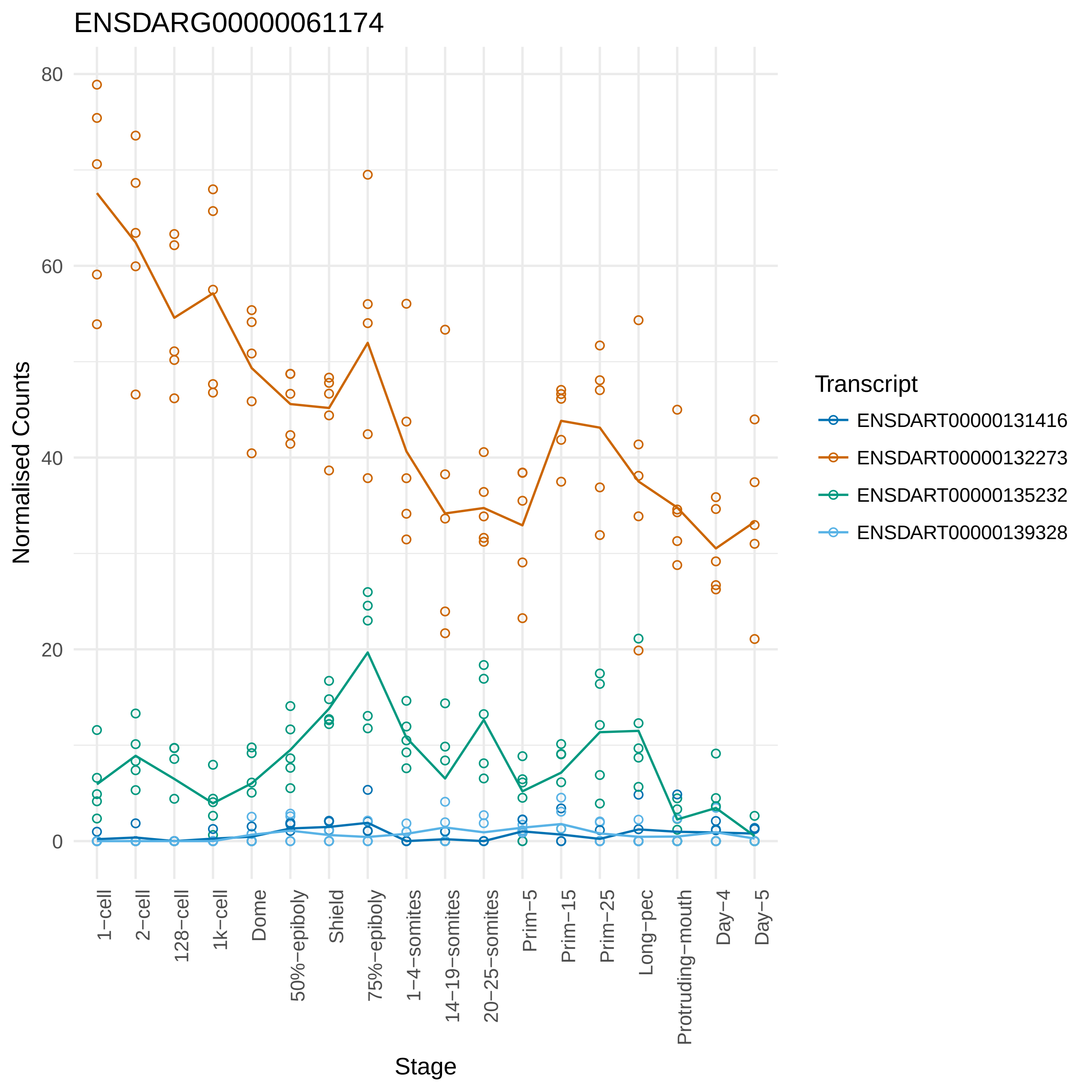
ENSDARG00000061174
Normalised Counts
0
20
40
60
80
1−cell
2−cell
128−cell
1k−cell
Dome
50%−epiboly
Shield
75%−epiboly
1−4−somites
14−19−somites
20−25−somites
Prim−5
Prim−15
Prim−25
Long−pec
Protruding−mouth
Day−4
Day−5
Stage
Transcript
ENSDART00000131416
ENSDART00000132273
ENSDART00000135232
ENSDART00000139328

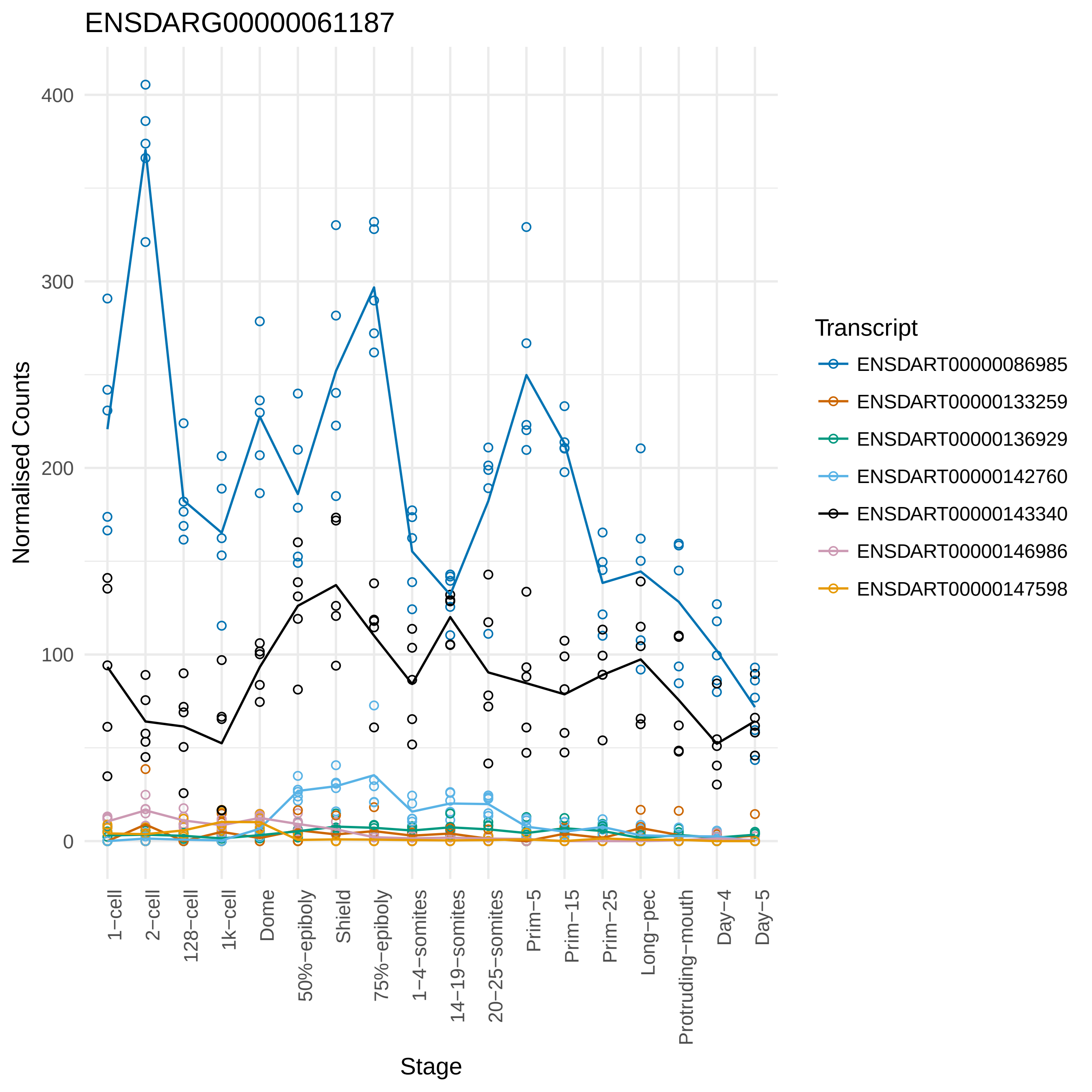
ENSDARG00000061187
Normalised Counts
400
300
200
100
0
1−cell
2−cell
128−cell
1k−cell
Dome
50%−epiboly
Shield
75%−epiboly
1−4−somites
14−19−somites
20−25−somites
Prim−5
Prim−15
Prim−25
Long−pec
Protruding−mouth
Day−4
Day−5
Stage
Transcript
ENSDART00000086985
ENSDART00000133259
ENSDART00000136929
ENSDART00000142760
ENSDART00000143340
ENSDART00000146986
ENSDART00000147598

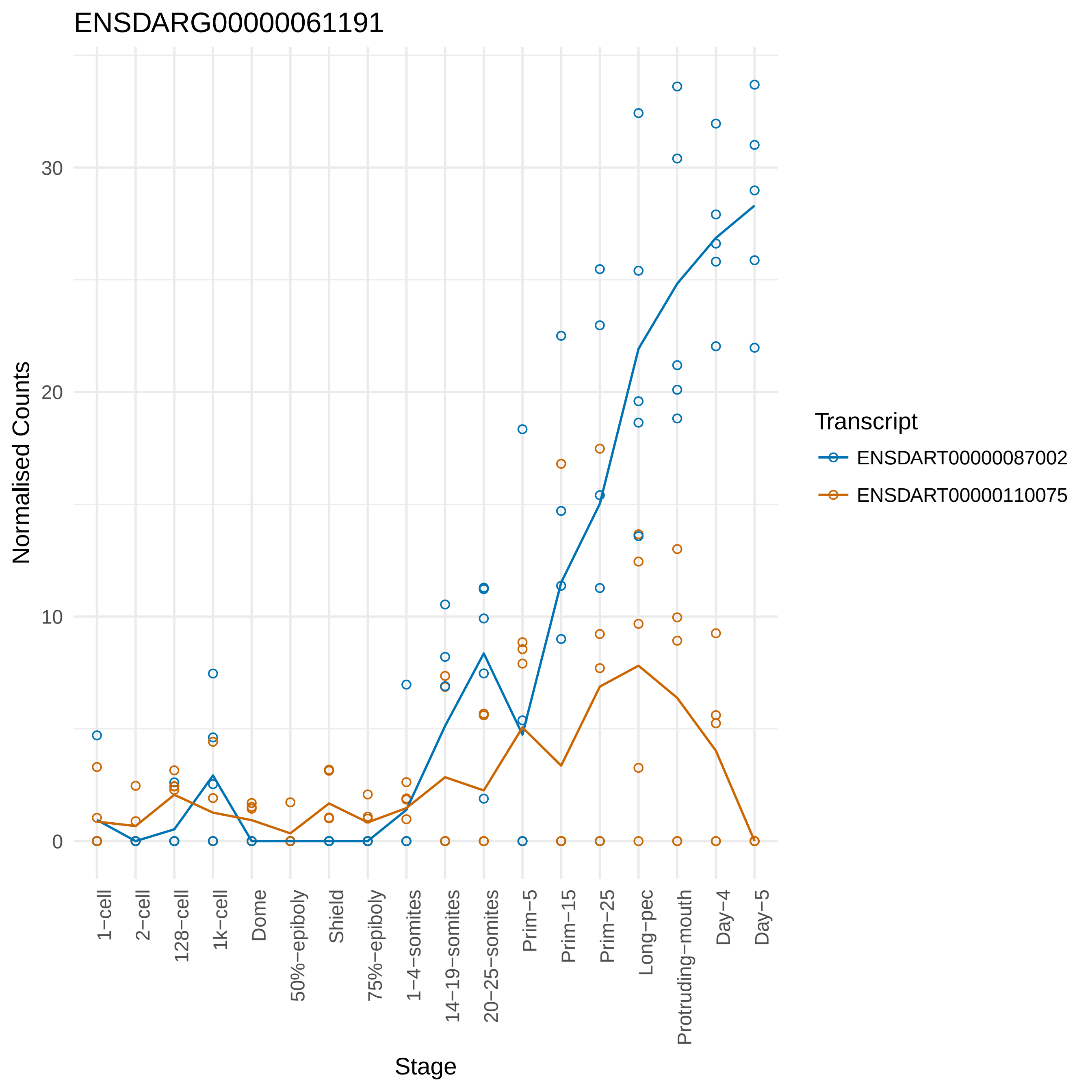
ENSDARG00000061191
Normalised Counts
0
10
20
30
Stage
1−cell
2−cell
128−cell
1k−cell
Dome
50%−epiboly
Shield
75%−epiboly
1−4−somites
14−19−somites
20−25−somites
Prim−5
Prim−15
Prim−25
Long−pec
Protruding−mouth
Day−4
Day−5
Transcript
ENSDART00000087002
ENSDART00000110075

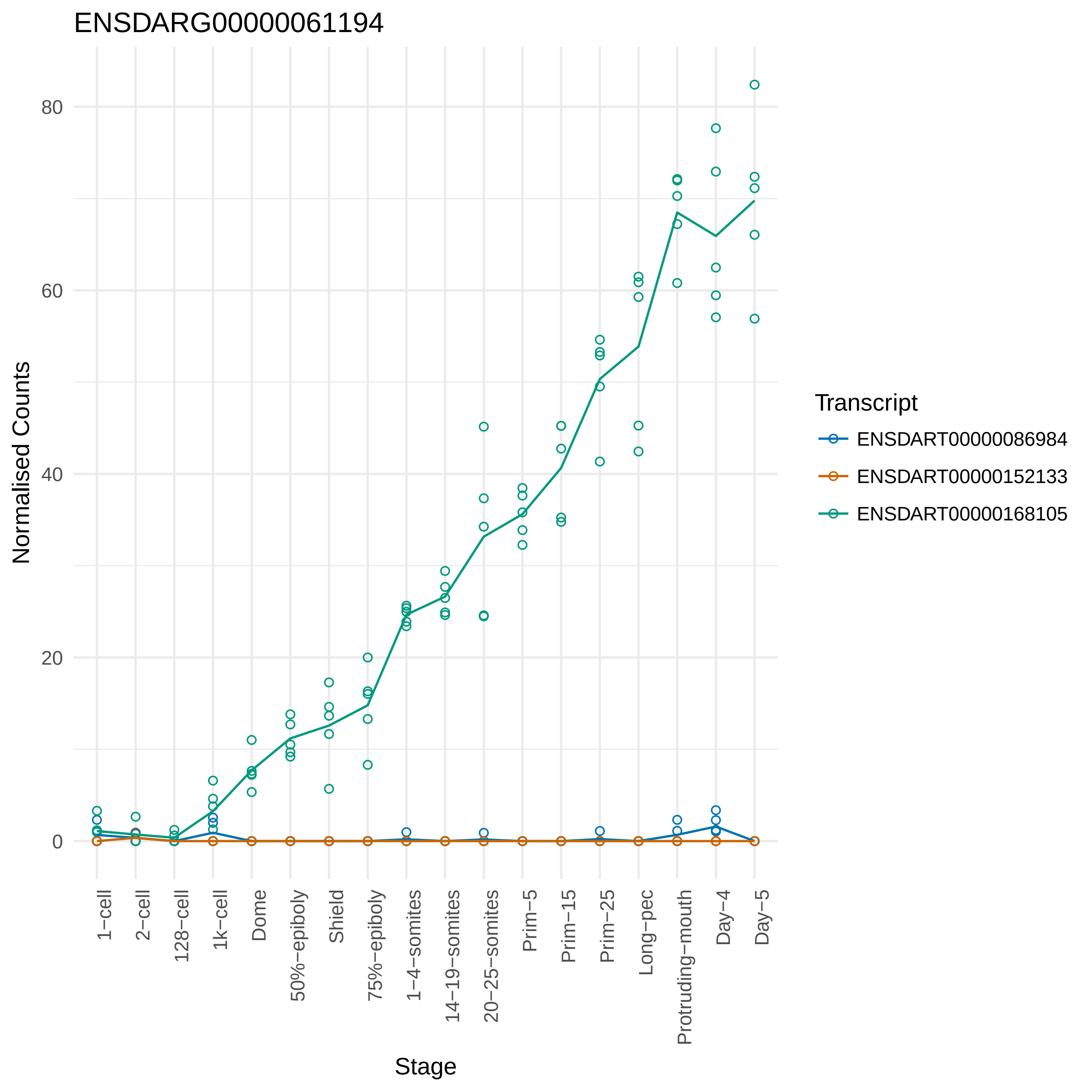

ENSDARG00000061194
Normalised Counts
0
20
40
60
80
1−cell
2−cell
128−cell
1k−cell
Dome
50%−epiboly
Shield
75%−epiboly
1−4−somites
14−19−somites
20−25−somites
Prim−5
Prim−15
Prim−25
Long−pec
Protruding−mouth
Day−4
Day−5
Stage
Transcript
ENSDART00000086984
ENSDART00000152133
ENSDART00000168105

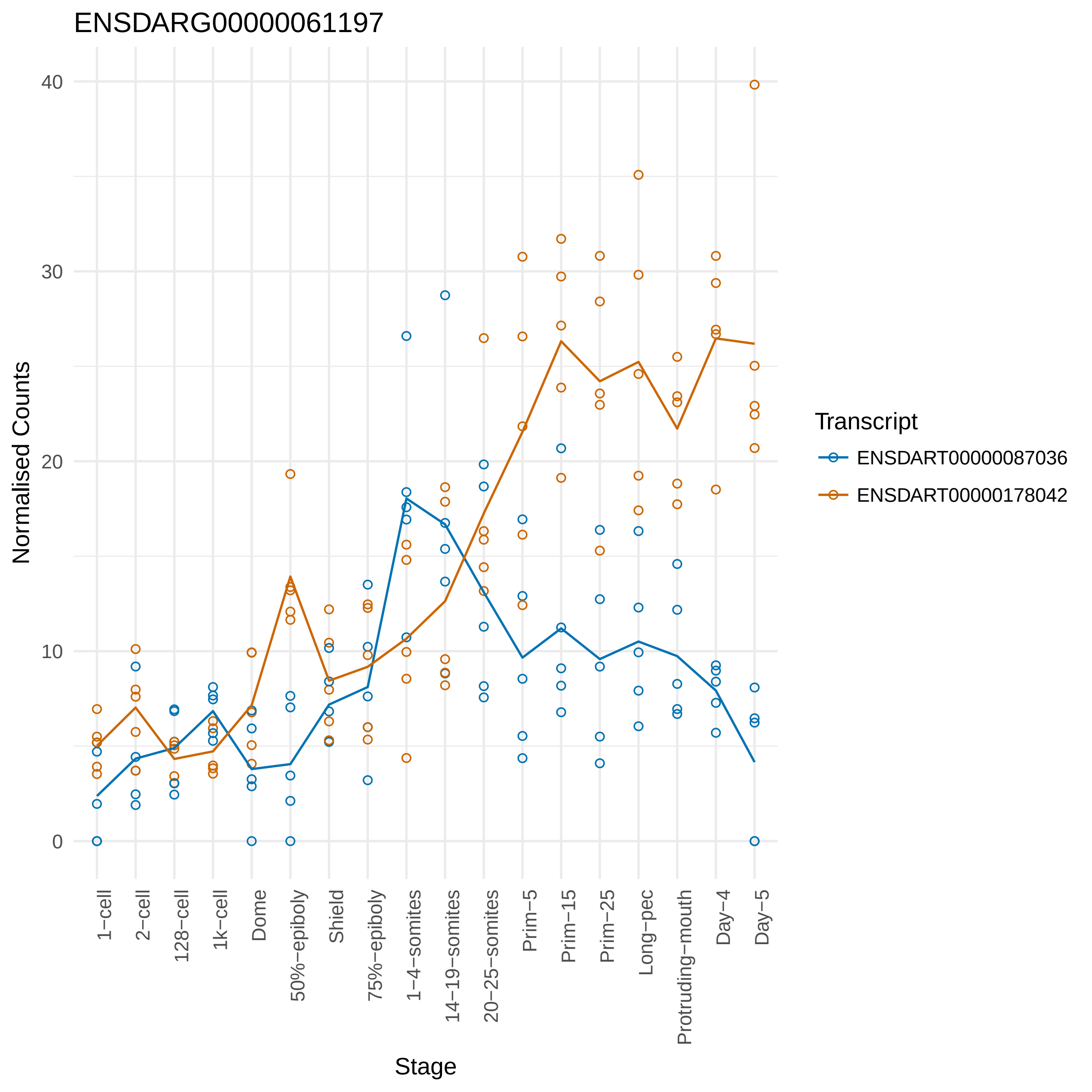
ENSDARG00000061197
Normalised Counts
0
10
20
30
40
1−cell
2−cell
128−cell
1k−cell
Dome
50%−epiboly
Shield
75%−epiboly
1−4−somites
14−19−somites
20−25−somites
Prim−5
Prim−15
Prim−25
Long−pec
Protruding−mouth
Day−4
Day−5
Stage
Transcript
ENSDART00000087036
ENSDART00000178042

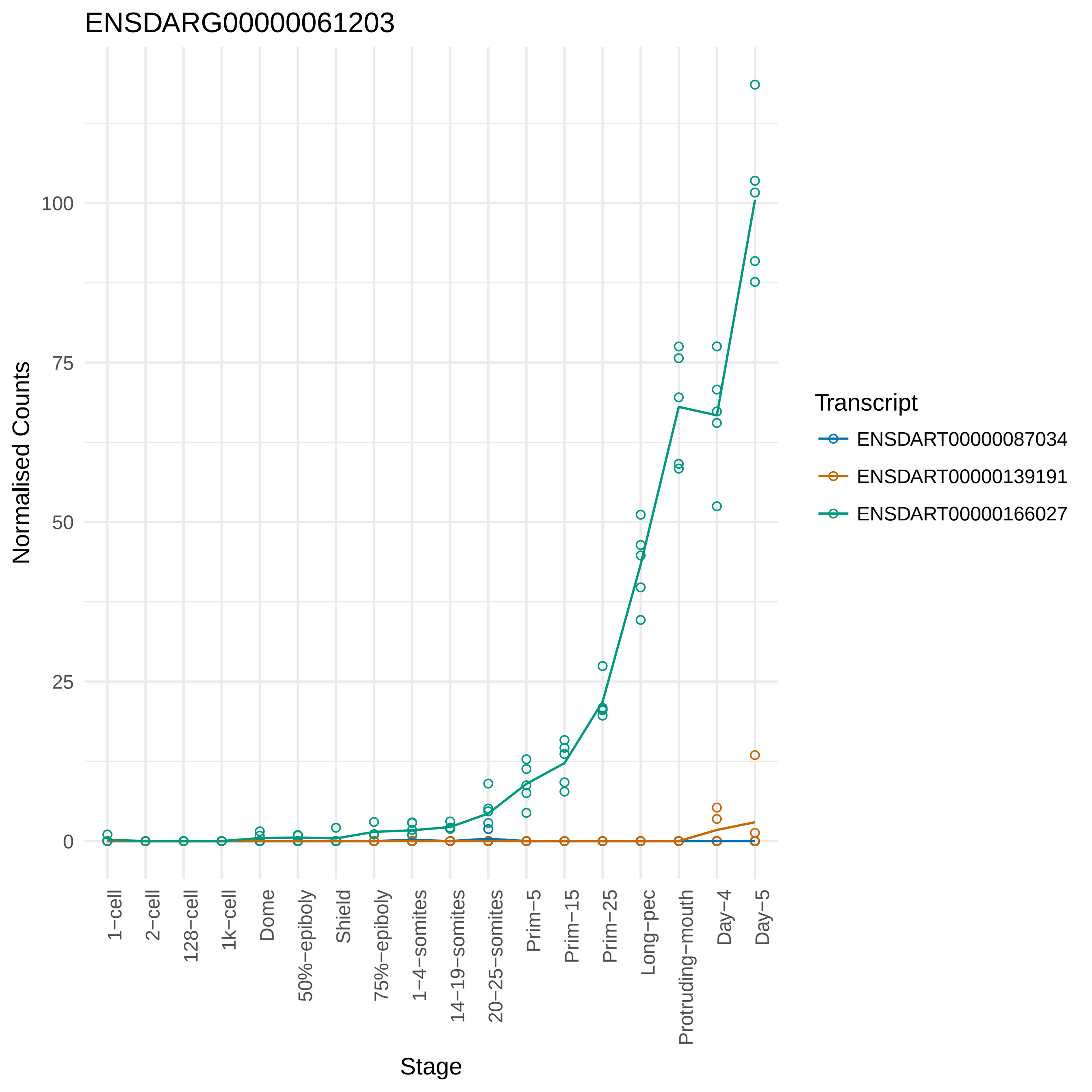
ENSDARG00000061203
Normalised Counts
0
25
50
75
100
1−cell
2−cell
128−cell
1k−cell
Dome
50%−epiboly
Shield
75%−epiboly
1−4−somites
14−19−somites
20−25−somites
Prim−5
Prim−15
Prim−25
Long−pec
Protruding−mouth
Day−4
Day−5
Stage
Transcript
ENSDART00000087034
ENSDART00000139191
ENSDART00000166027

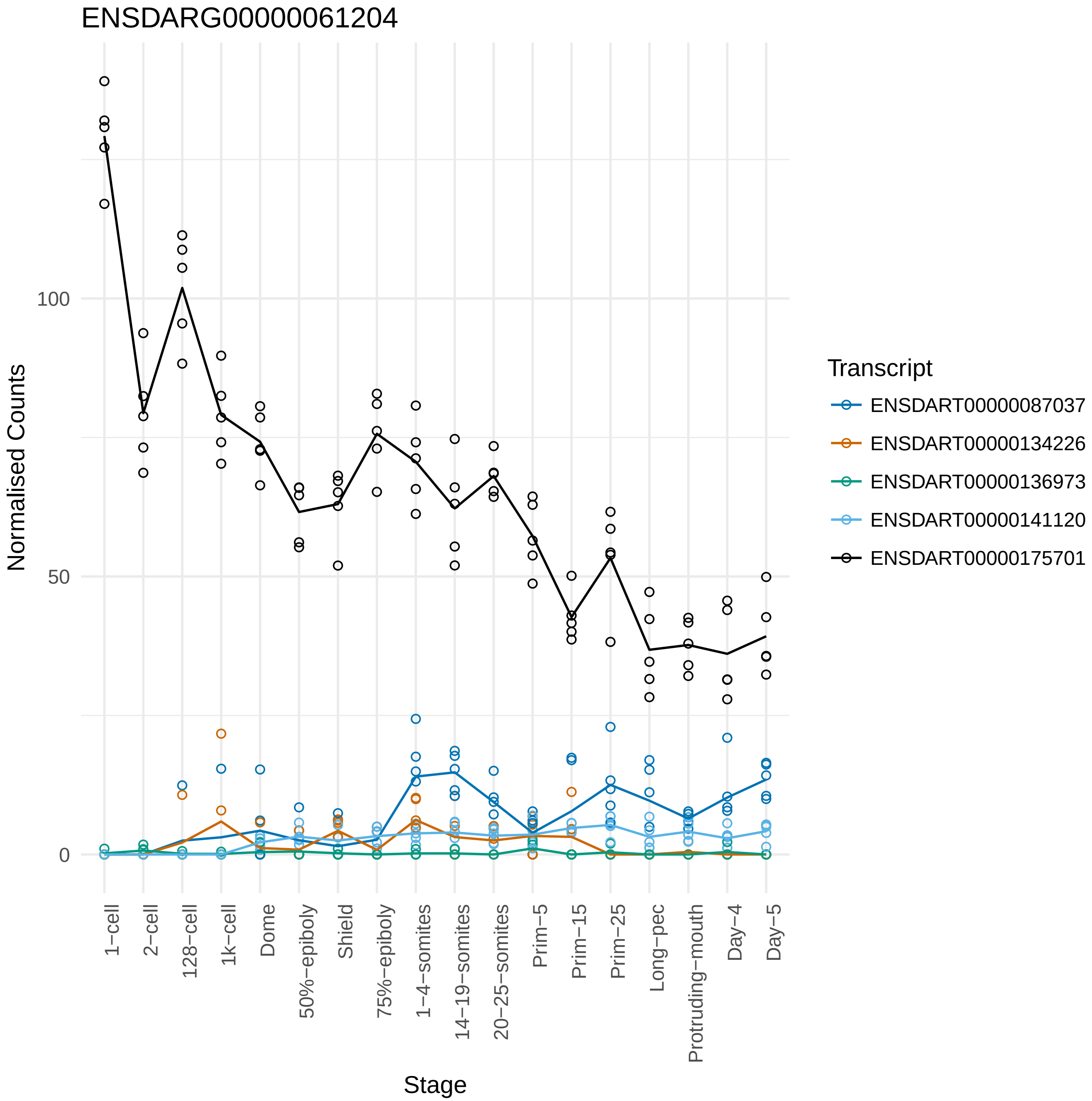
ENSDARG00000061204
Normalised Counts
100
50
0
Stage
1−cell
2−cell
128−cell
1k−cell
Dome
50%−epiboly
Shield
75%−epiboly
1−4−somites
14−19−somites
20−25−somites
Prim−5
Prim−15
Prim−25
Long−pec
Protruding−mouth
Day−4
Day−5
Transcript
ENSDART00000087037
ENSDART00000134226
ENSDART00000136973
ENSDART00000141120
ENSDART00000175701

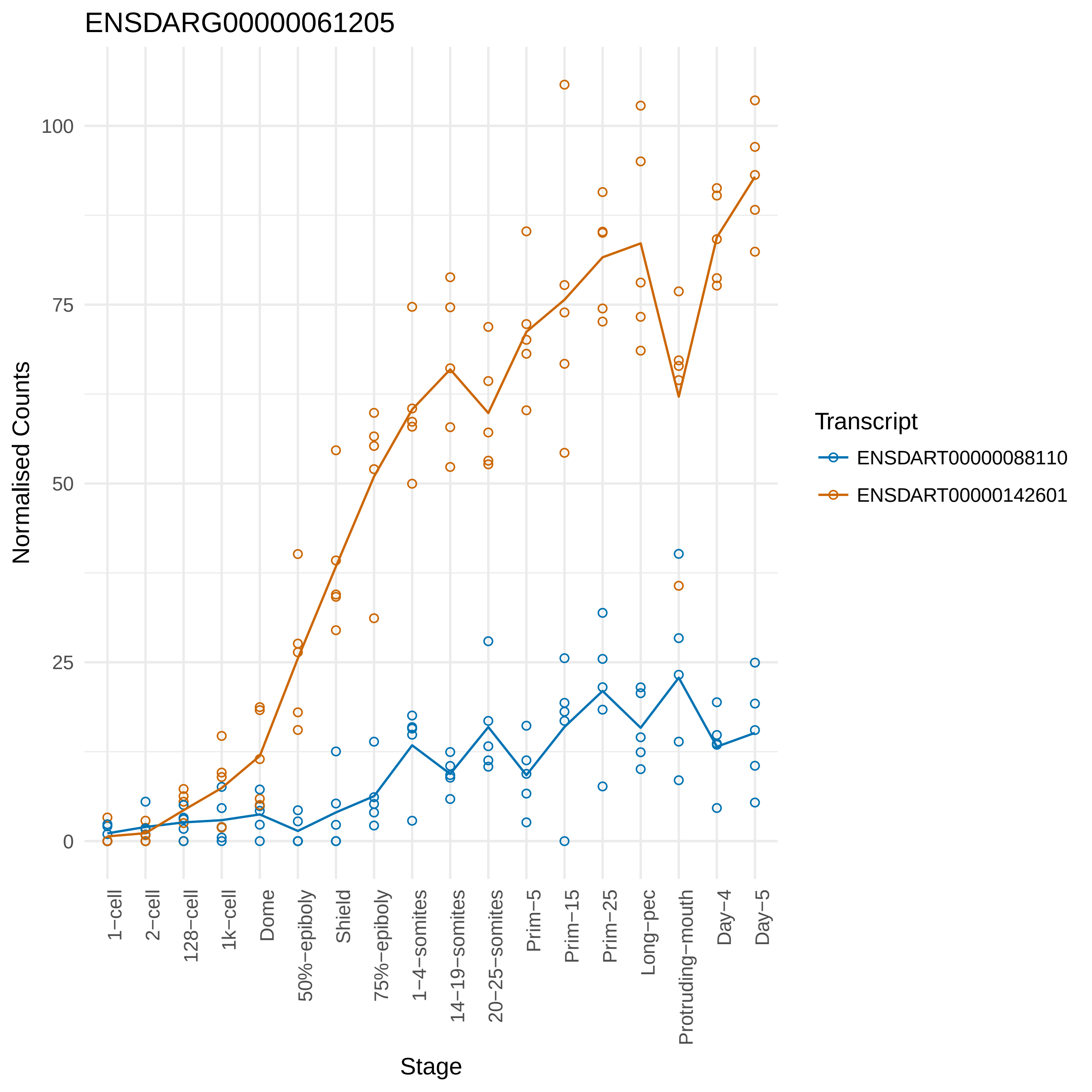

ENSDARG00000061205
Normalised Counts
100
75
50
25
0
1−cell
2−cell
128−cell
1k−cell
Dome
50%−epiboly
Shield
75%−epiboly
1−4−somites
14−19−somites
20−25−somites
Prim−5
Prim−15
Prim−25
Long−pec
Protruding−mouth
Day−4
Day−5
Stage
Transcript
ENSDART00000088110
ENSDART00000142601

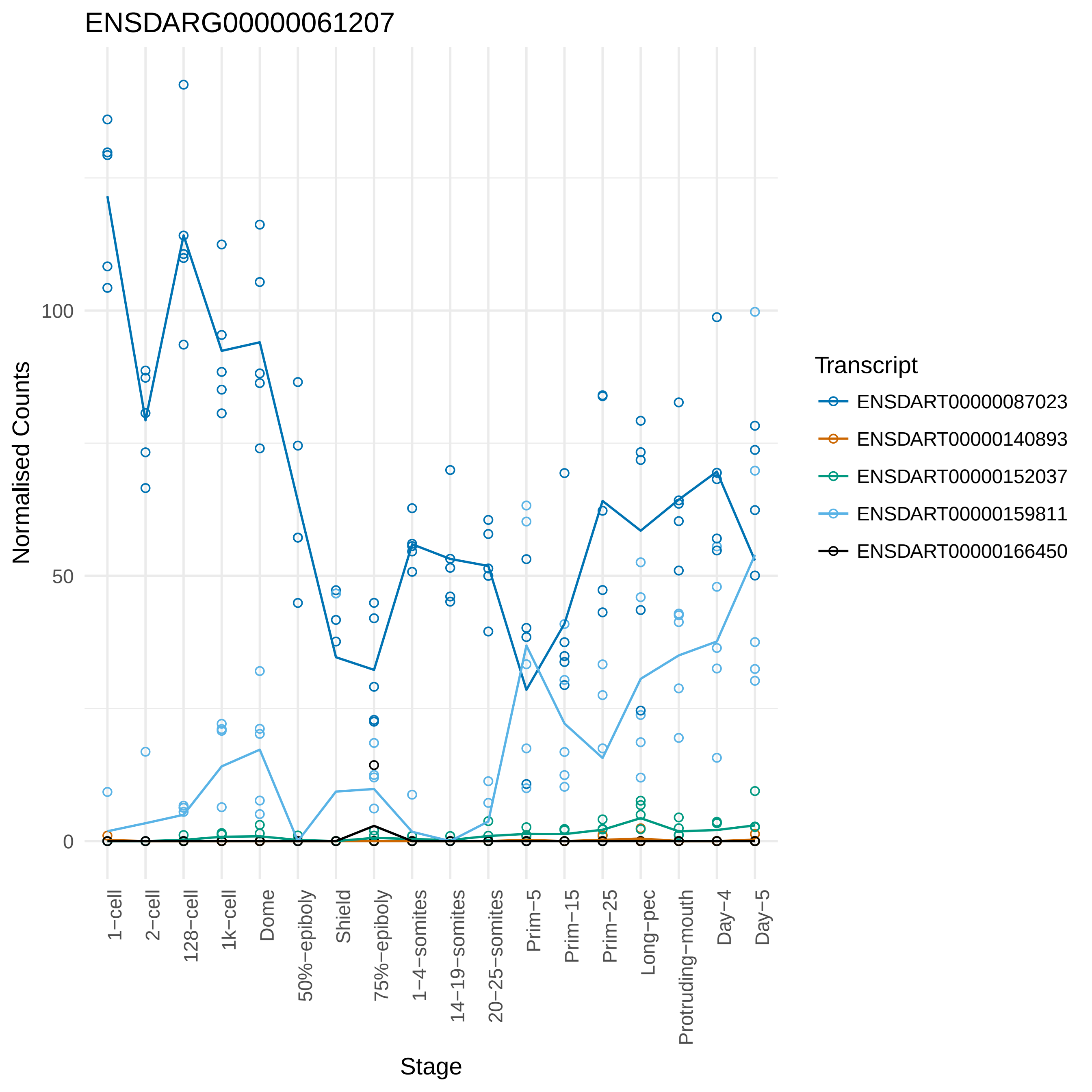

ENSDARG00000061207
Normalised Counts
100
50
0
Stage
1−cell
2−cell
128−cell
1k−cell
Dome
50%−epiboly
Shield
75%−epiboly
1−4−somites
14−19−somites
20−25−somites
Prim−5
Prim−15
Prim−25
Long−pec
Protruding−mouth
Day−4
Day−5
Transcript
ENSDART00000087023
ENSDART00000140893
ENSDART00000152037
ENSDART00000159811
ENSDART00000166450

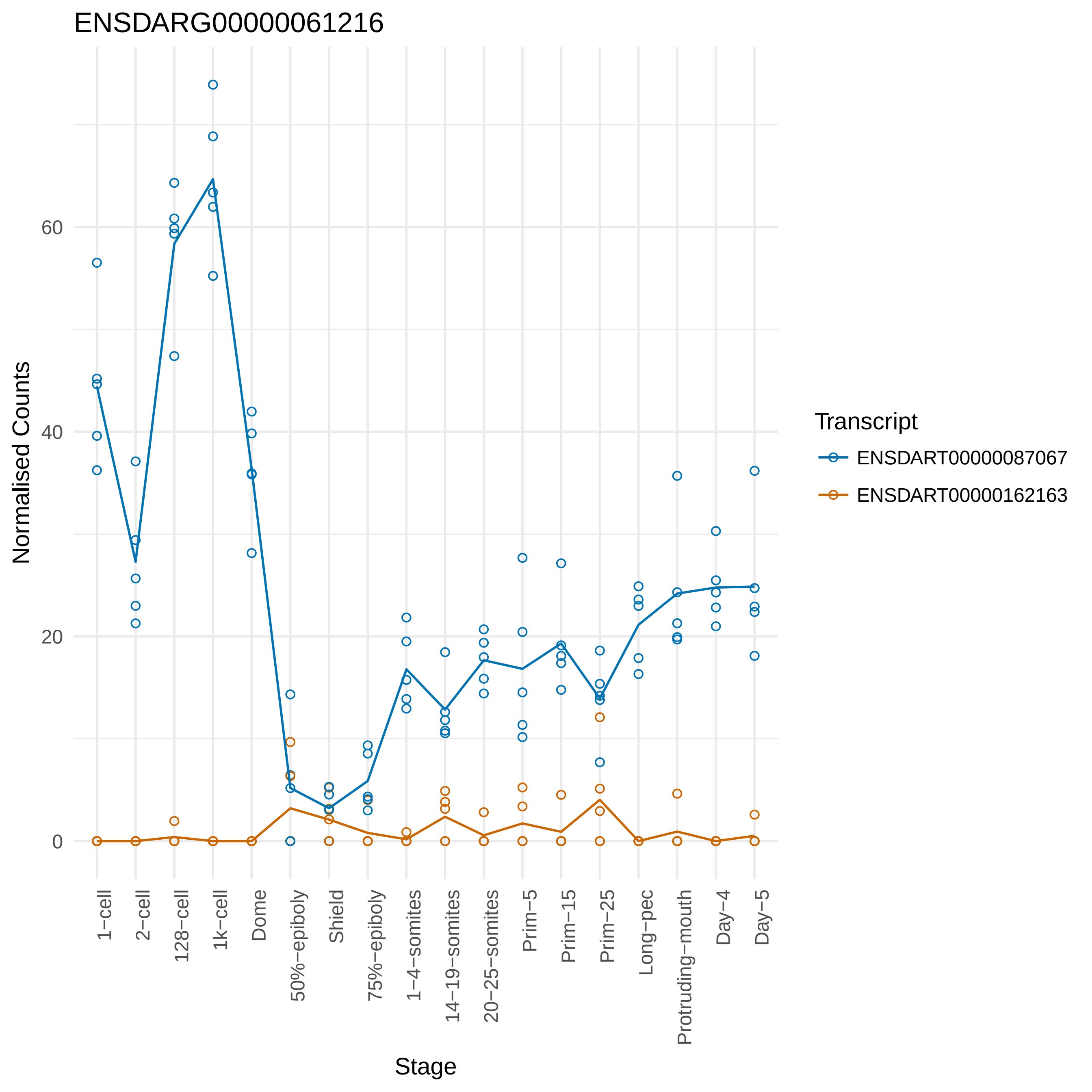
ENSDARG00000061216
Normalised Counts
0
20
40
60
Stage
1−cell
2−cell
128−cell
1k−cell
Dome
50%−epiboly
Shield
75%−epiboly
1−4−somites
14−19−somites
20−25−somites
Prim−5
Prim−15
Prim−25
Long−pec
Protruding−mouth
Day−4
Day−5
Transcript
ENSDART00000087067
ENSDART00000162163

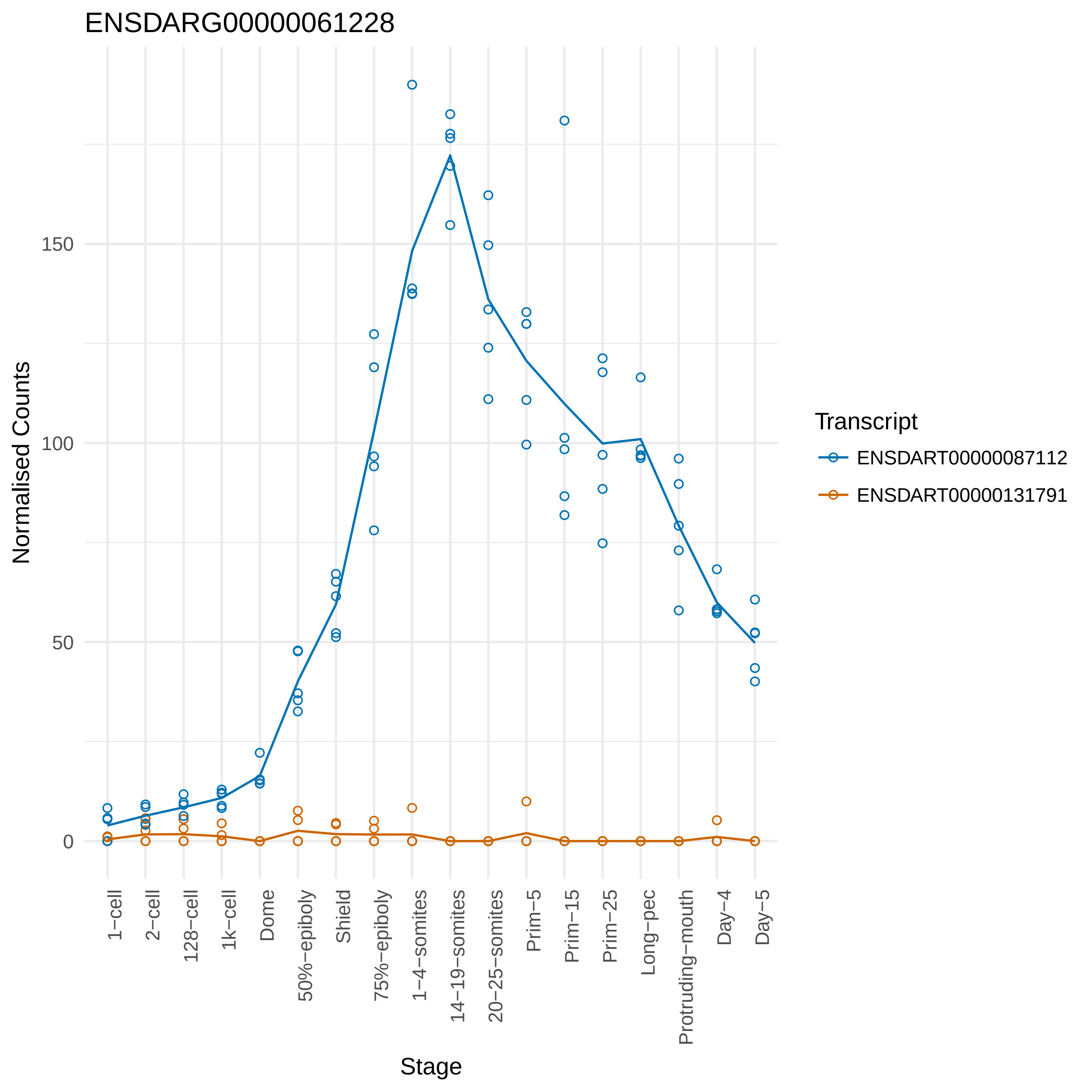
ENSDARG00000061228
Normalised Counts
0
50
100
150
Stage
1−cell
2−cell
128−cell
1k−cell
Dome
50%−epiboly
Shield
75%−epiboly
1−4−somites
14−19−somites
20−25−somites
Prim−5
Prim−15
Prim−25
Long−pec
Protruding−mouth
Day−4
Day−5
Transcript
ENSDART00000087112
ENSDART00000131791

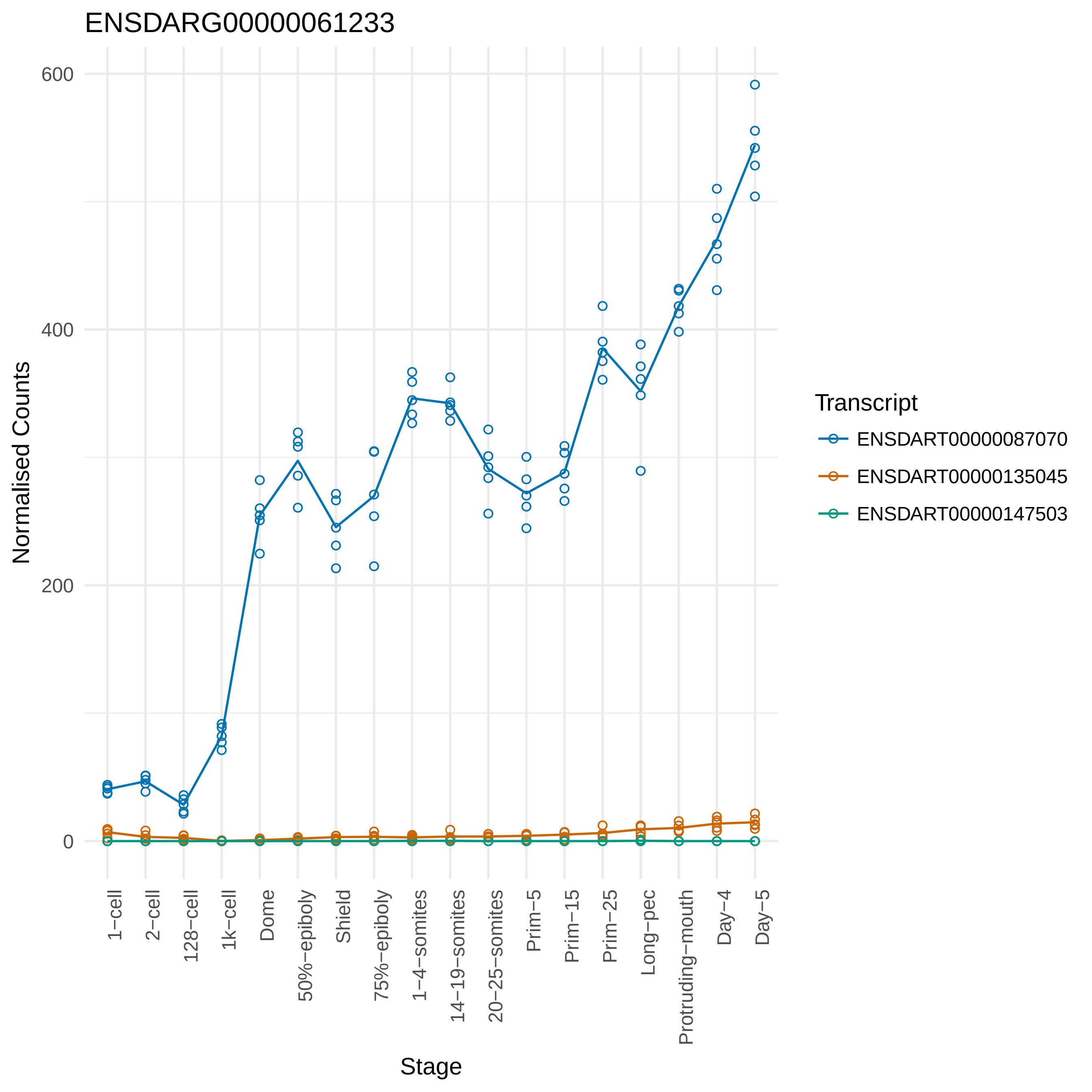

ENSDARG00000061233
Normalised Counts
600
400
200
0
1−cell
2−cell
128−cell
1k−cell
Dome
50%−epiboly
Shield
75%−epiboly
1−4−somites
14−19−somites
20−25−somites
Prim−5
Prim−15
Prim−25
Long−pec
Protruding−mouth
Day−4
Day−5
Stage
Transcript
ENSDART00000087070
ENSDART00000135045
ENSDART00000147503

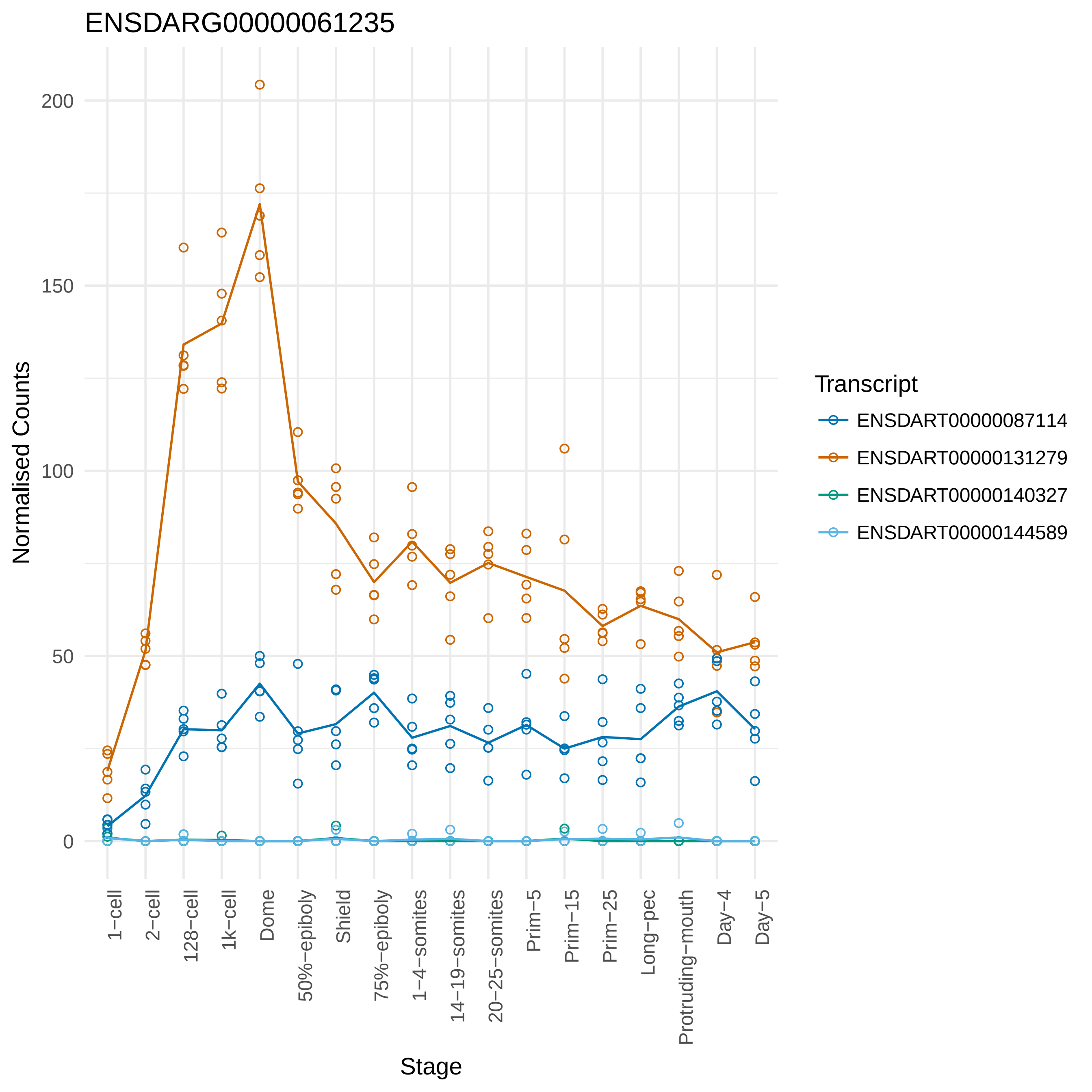

ENSDARG00000061235
Normalised Counts
200
150
100
50
0
1−cell
2−cell
128−cell
1k−cell
Dome
50%−epiboly
Shield
75%−epiboly
1−4−somites
14−19−somites
20−25−somites
Prim−5
Prim−15
Prim−25
Long−pec
Protruding−mouth
Day−4
Day−5
Stage
Transcript
ENSDART00000087114
ENSDART00000131279
ENSDART00000140327
ENSDART00000144589

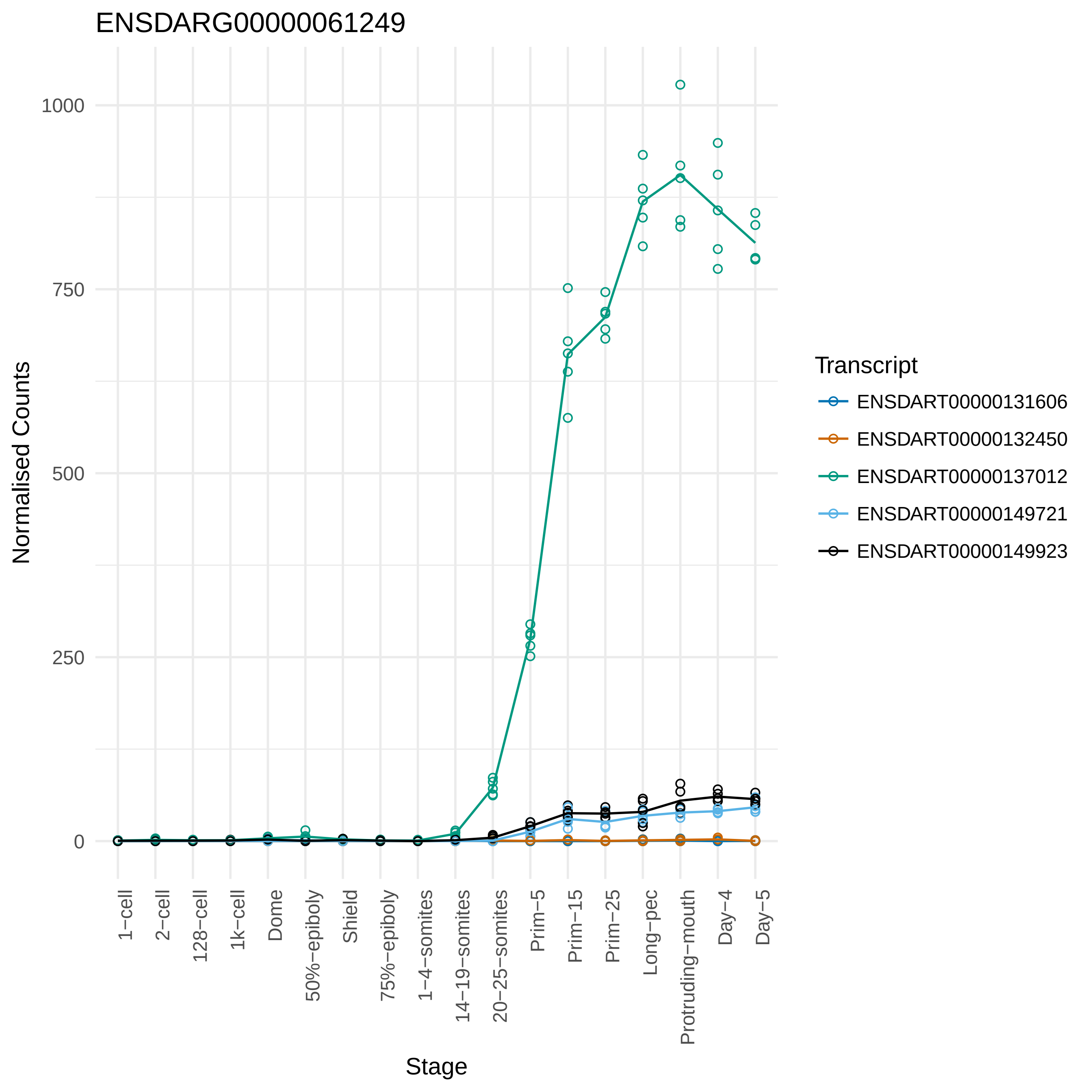
ENSDARG00000061249
Normalised Counts
1000
750
500
250
0
1−cell
2−cell
128−cell
1k−cell
Dome
50%−epiboly
Shield
75%−epiboly
1−4−somites
14−19−somites
20−25−somites
Prim−5
Prim−15
Prim−25
Long−pec
Protruding−mouth
Day−4
Day−5
Stage
Transcript
ENSDART00000131606
ENSDART00000132450
ENSDART00000137012
ENSDART00000149721
ENSDART00000149923

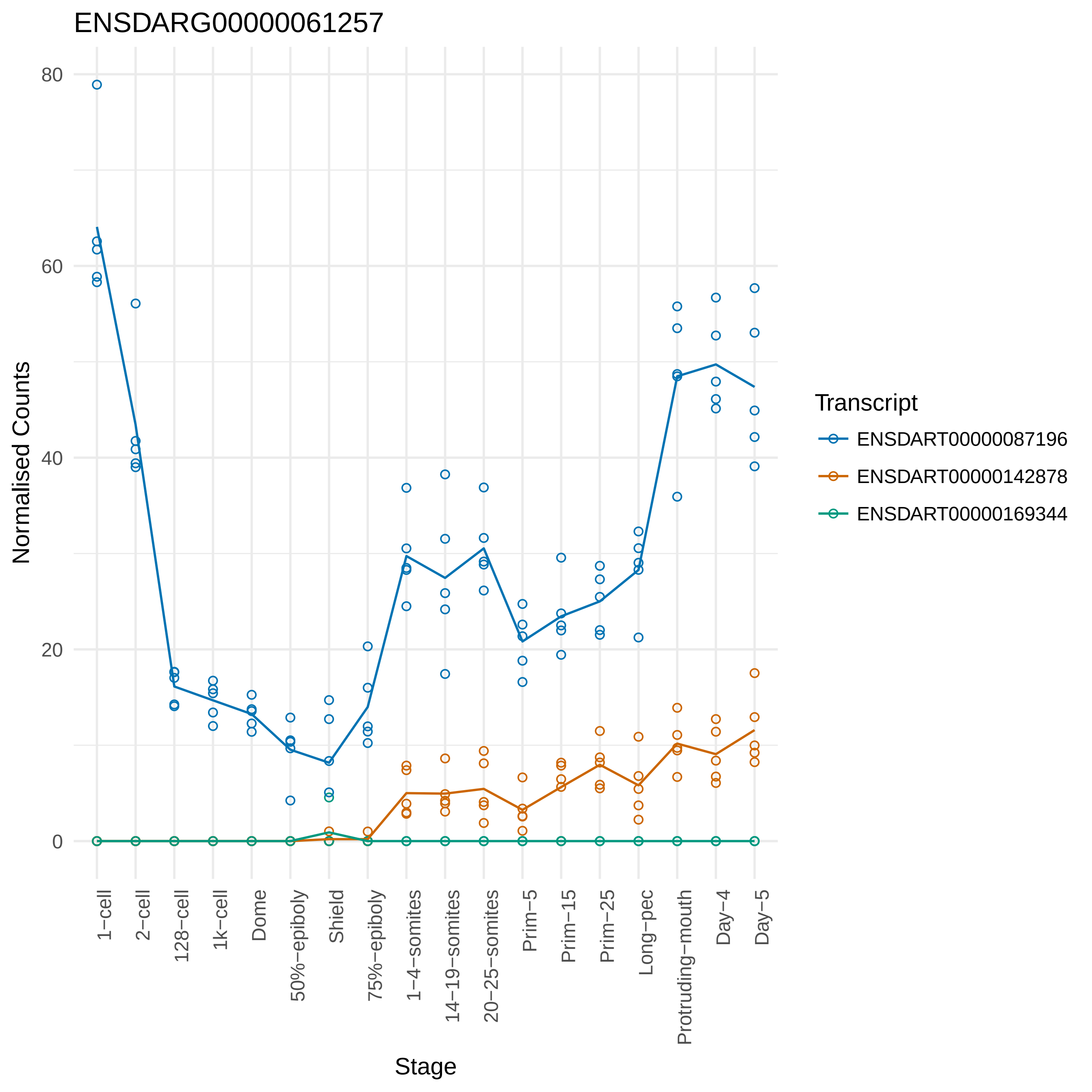

ENSDARG00000061257
Normalised Counts
0
20
40
60
80
1−cell
2−cell
128−cell
1k−cell
Dome
50%−epiboly
Shield
75%−epiboly
1−4−somites
14−19−somites
20−25−somites
Prim−5
Prim−15
Prim−25
Long−pec
Protruding−mouth
Day−4
Day−5
Stage
Transcript
ENSDART00000087196
ENSDART00000142878
ENSDART00000169344

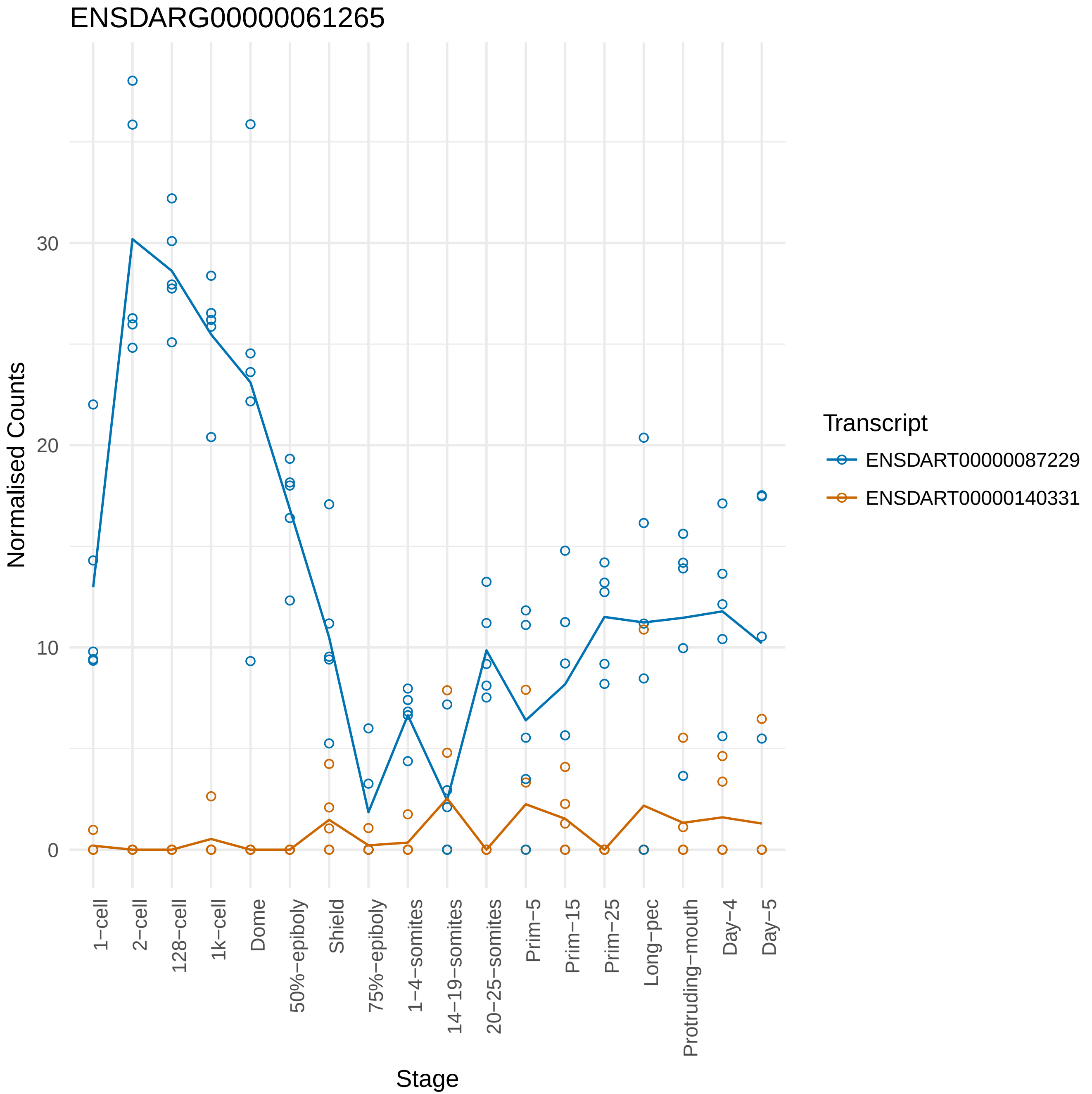

ENSDARG00000061265
Normalised Counts
30
20
10
0
Stage
1−cell
2−cell
128−cell
1k−cell
Dome
50%−epiboly
Shield
75%−epiboly
1−4−somites
14−19−somites
20−25−somites
Prim−5
Prim−15
Prim−25
Long−pec
Protruding−mouth
Day−4
Day−5
Transcript
ENSDART00000087229
ENSDART00000140331

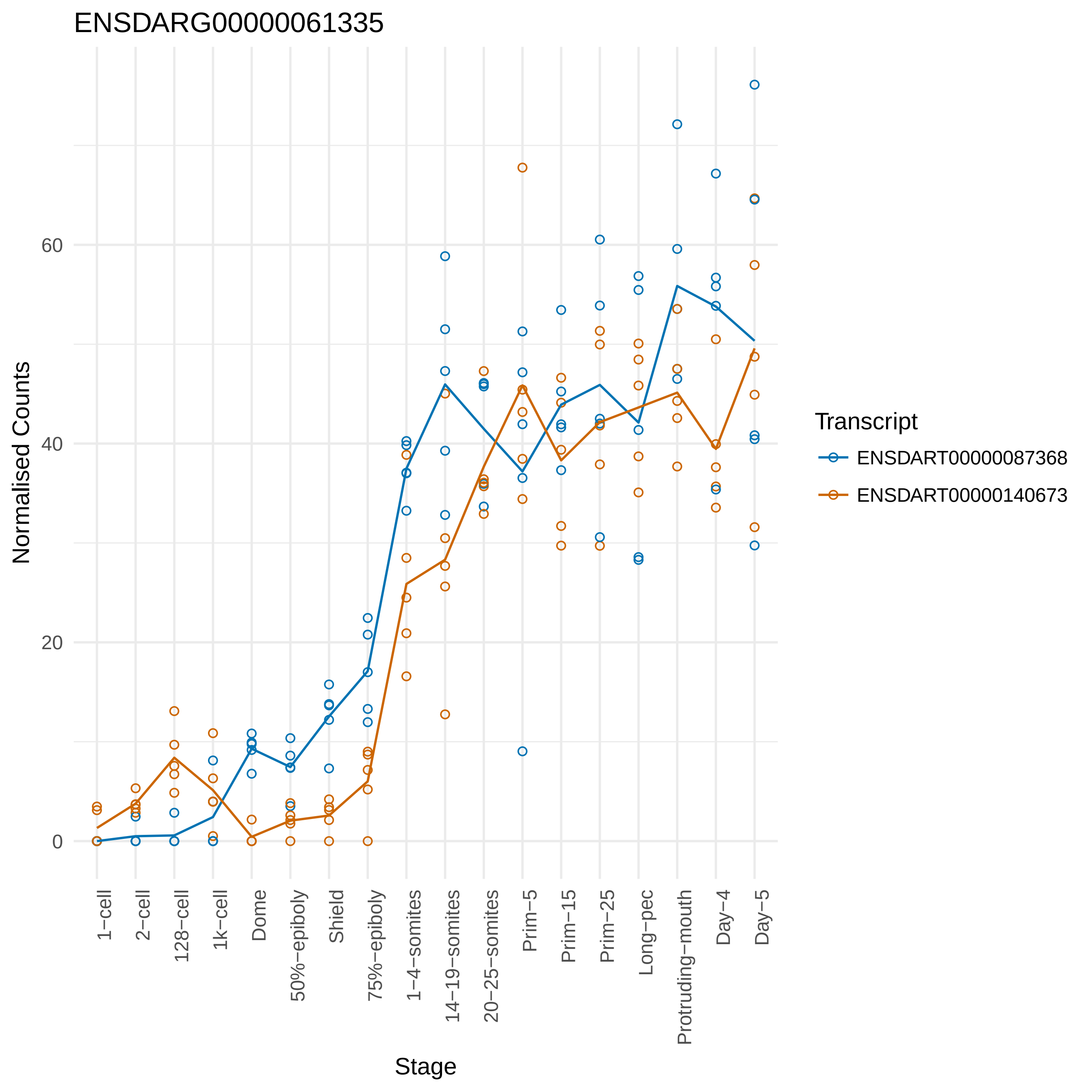

ENSDARG00000061335
Normalised Counts
0
20
40
60
Stage
1−cell
2−cell
128−cell
1k−cell
Dome
50%−epiboly
Shield
75%−epiboly
1−4−somites
14−19−somites
20−25−somites
Prim−5
Prim−15
Prim−25
Long−pec
Protruding−mouth
Day−4
Day−5
Transcript
ENSDART00000087368
ENSDART00000140673

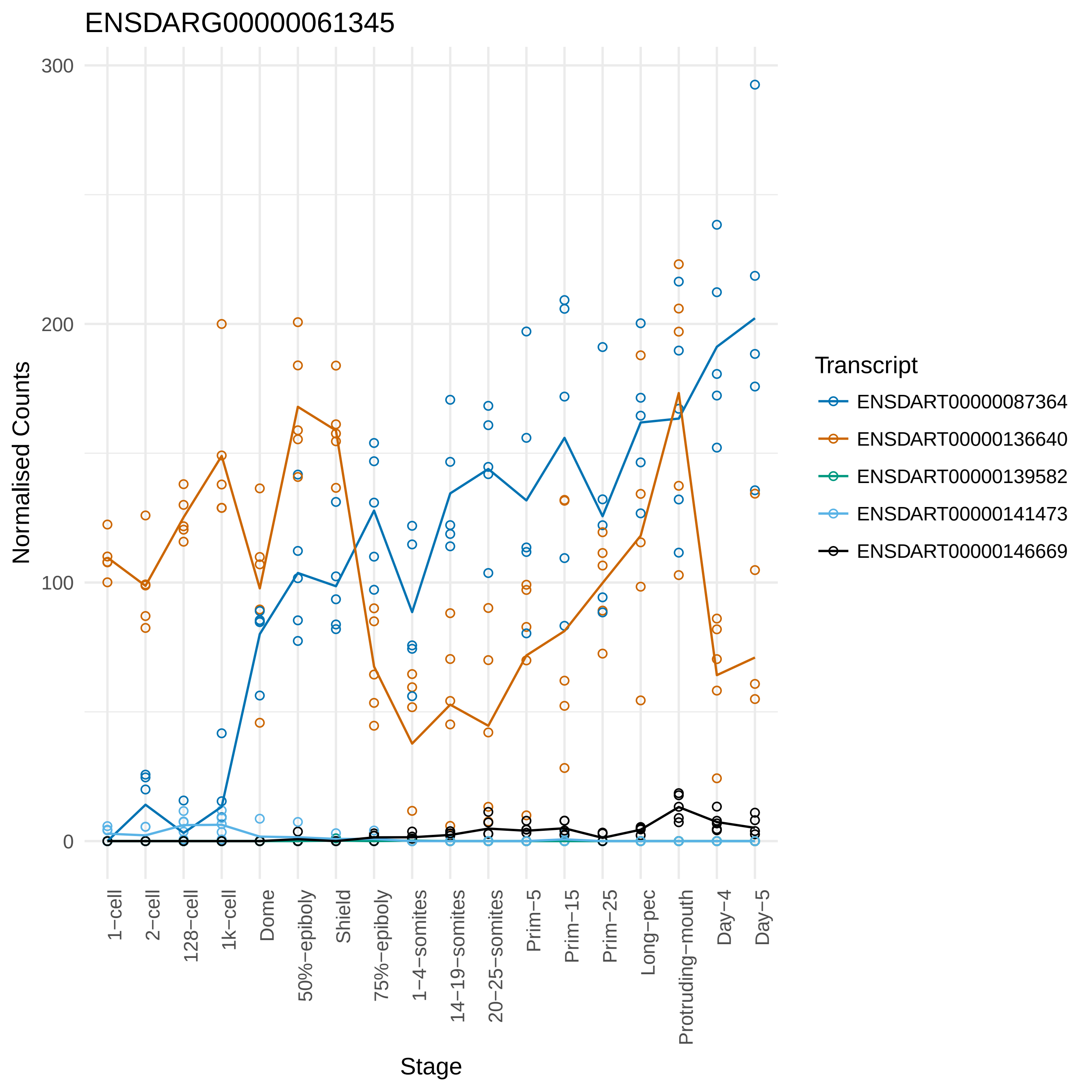

ENSDARG00000061345
Normalised Counts
300
200
100
0
1−cell
2−cell
128−cell
1k−cell
Dome
50%−epiboly
Shield
75%−epiboly
1−4−somites
14−19−somites
20−25−somites
Prim−5
Prim−15
Prim−25
Long−pec
Protruding−mouth
Day−4
Day−5
Stage
Transcript
ENSDART00000087364
ENSDART00000136640
ENSDART00000139582
ENSDART00000141473
ENSDART00000146669

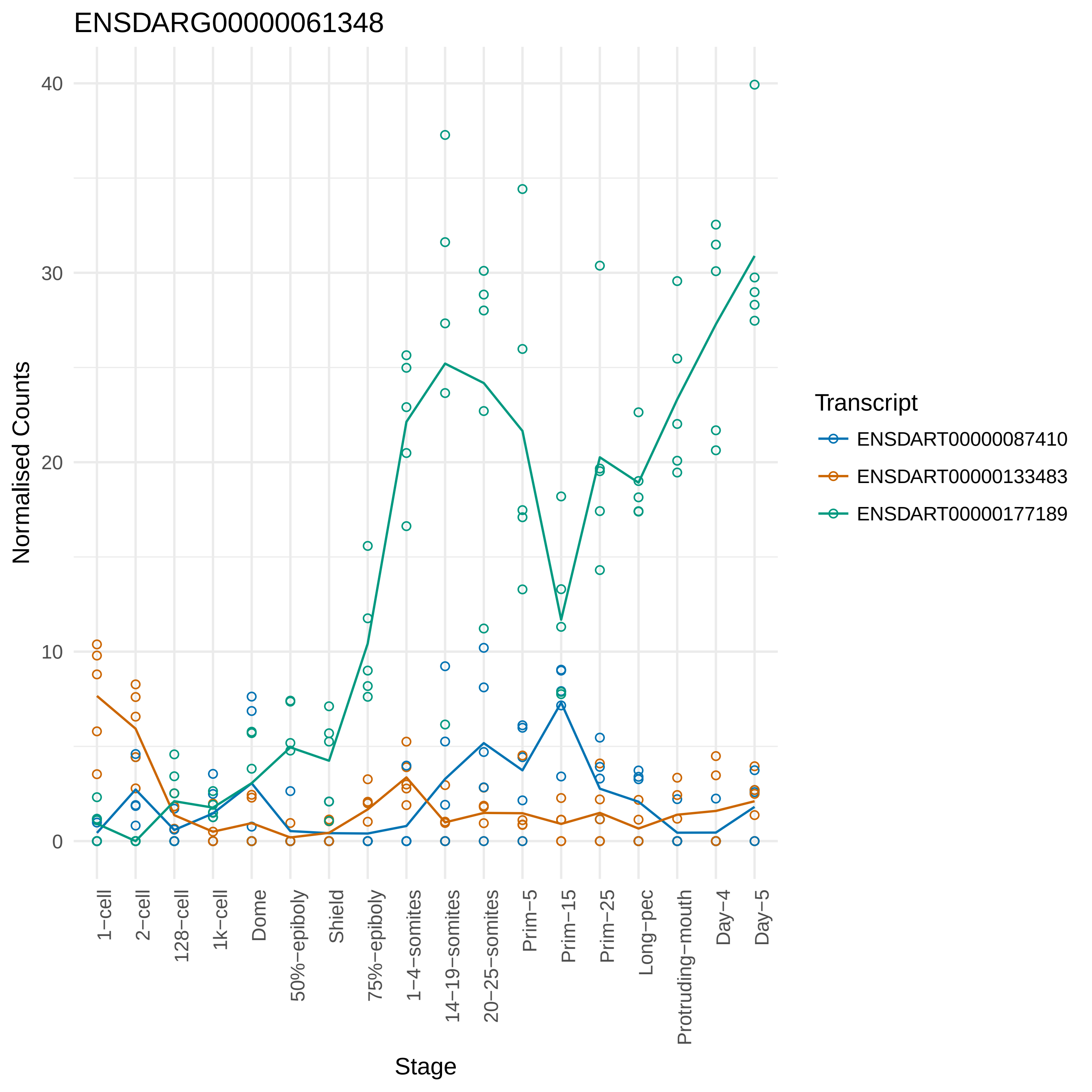

ENSDARG00000061348
Normalised Counts
0
10
20
30
40
Stage
1−cell
2−cell
128−cell
1k−cell
Dome
50%−epiboly
Shield
75%−epiboly
1−4−somites
14−19−somites
20−25−somites
Prim−5
Prim−15
Prim−25
Long−pec
Protruding−mouth
Day−4
Day−5
Transcript
ENSDART00000087410
ENSDART00000133483
ENSDART00000177189

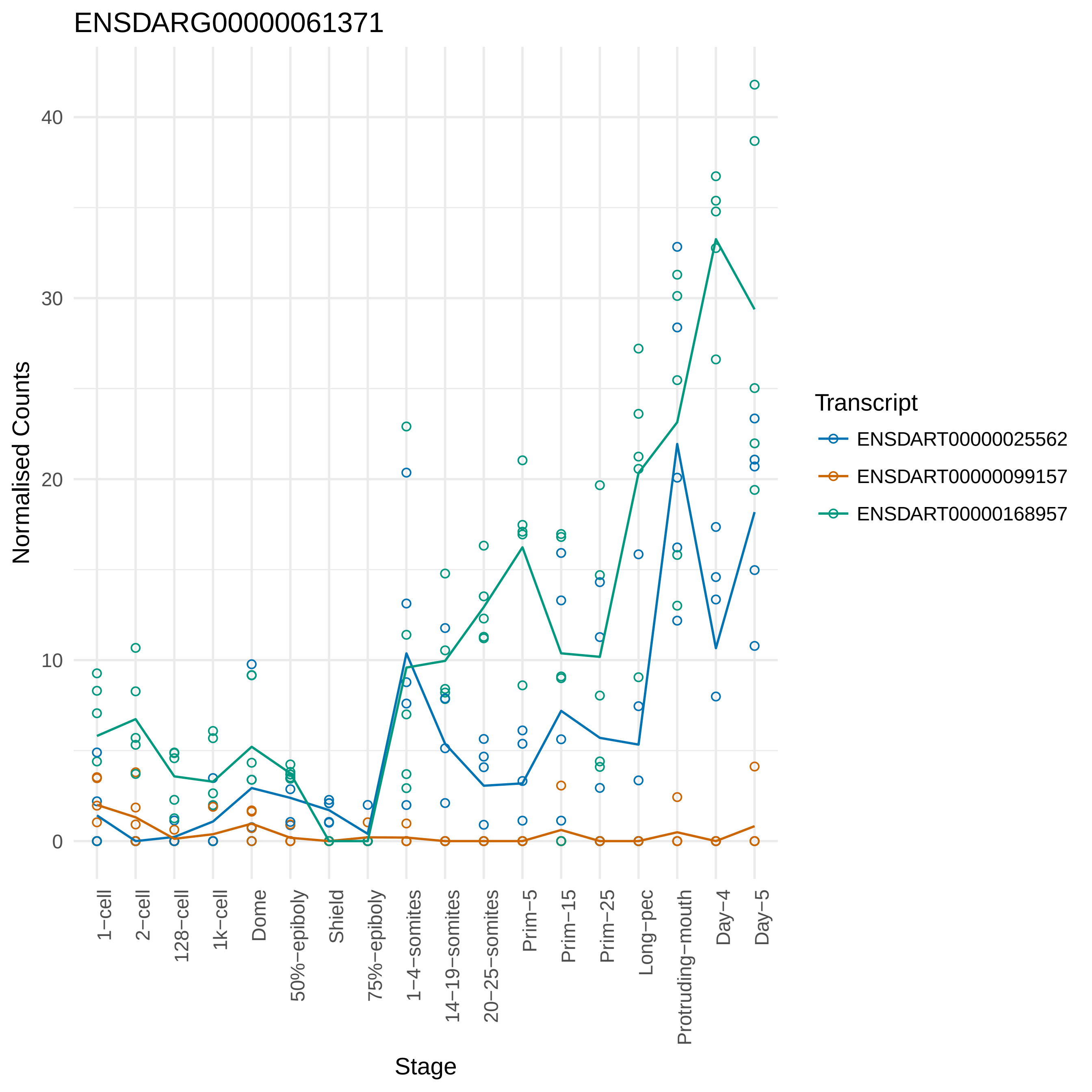

ENSDARG00000061371
Normalised Counts
0
10
20
30
40
Stage
1−cell
2−cell
128−cell
1k−cell
Dome
50%−epiboly
Shield
75%−epiboly
1−4−somites
14−19−somites
20−25−somites
Prim−5
Prim−15
Prim−25
Long−pec
Protruding−mouth
Day−4
Day−5
Transcript
ENSDART00000025562
ENSDART00000099157
ENSDART00000168957

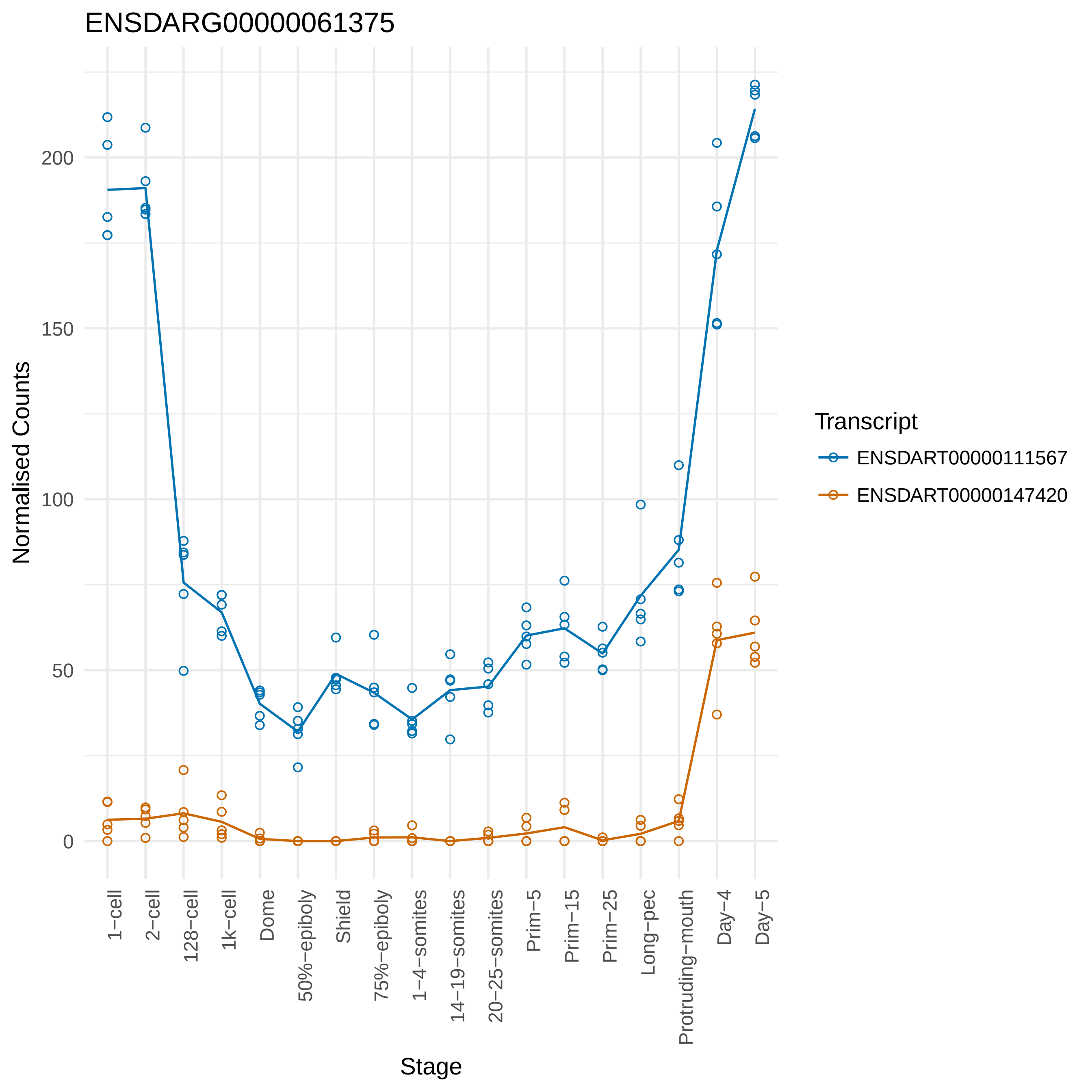

ENSDARG00000061375
Normalised Counts
200
150
100
50
0
1−cell
2−cell
128−cell
1k−cell
Dome
50%−epiboly
Shield
75%−epiboly
1−4−somites
14−19−somites
20−25−somites
Prim−5
Prim−15
Prim−25
Long−pec
Protruding−mouth
Day−4
Day−5
Stage
Transcript
ENSDART00000111567
ENSDART00000147420

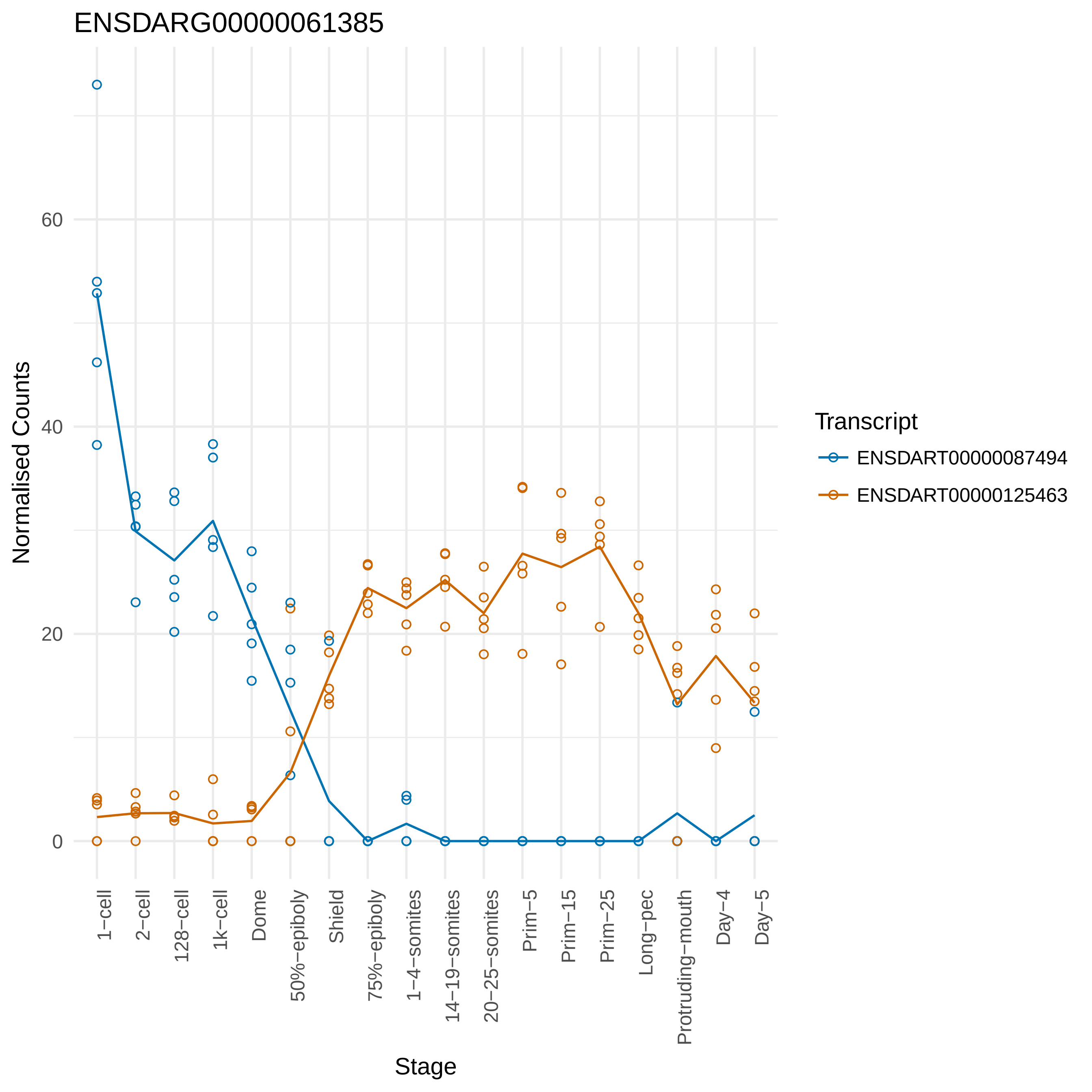
ENSDARG00000061385
Normalised Counts
60
40
20
0
1−cell
2−cell
128−cell
1k−cell
Dome
50%−epiboly
Shield
75%−epiboly
1−4−somites
14−19−somites
20−25−somites
Prim−5
Prim−15
Prim−25
Long−pec
Protruding−mouth
Day−4
Day−5
Stage
Transcript
ENSDART00000087494
ENSDART00000125463

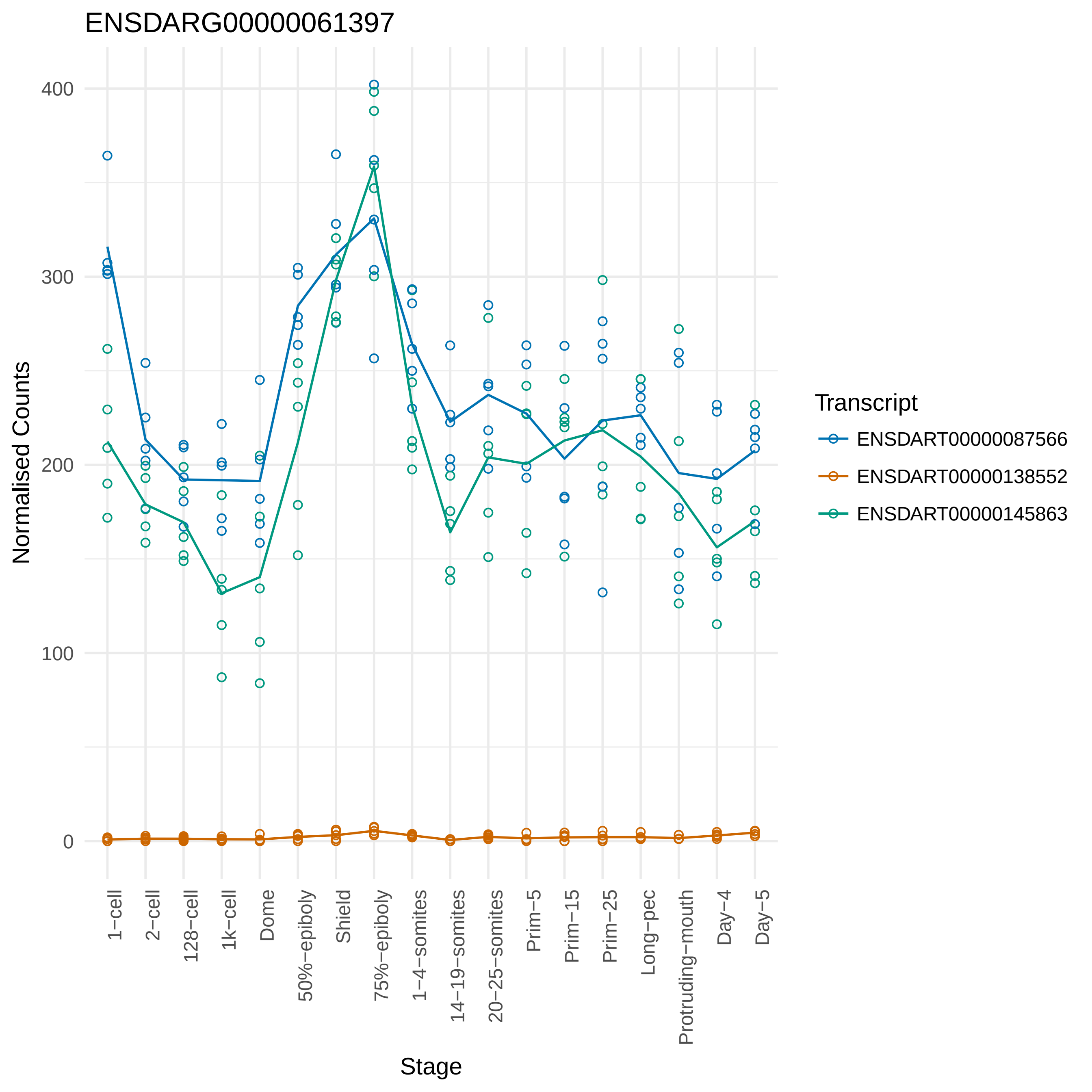
ENSDARG00000061397
Normalised Counts
400
300
200
100
0
1−cell
2−cell
128−cell
1k−cell
Dome
50%−epiboly
Shield
75%−epiboly
1−4−somites
14−19−somites
20−25−somites
Prim−5
Prim−15
Prim−25
Long−pec
Protruding−mouth
Day−4
Day−5
Stage
Transcript
ENSDART00000087566
ENSDART00000138552
ENSDART00000145863

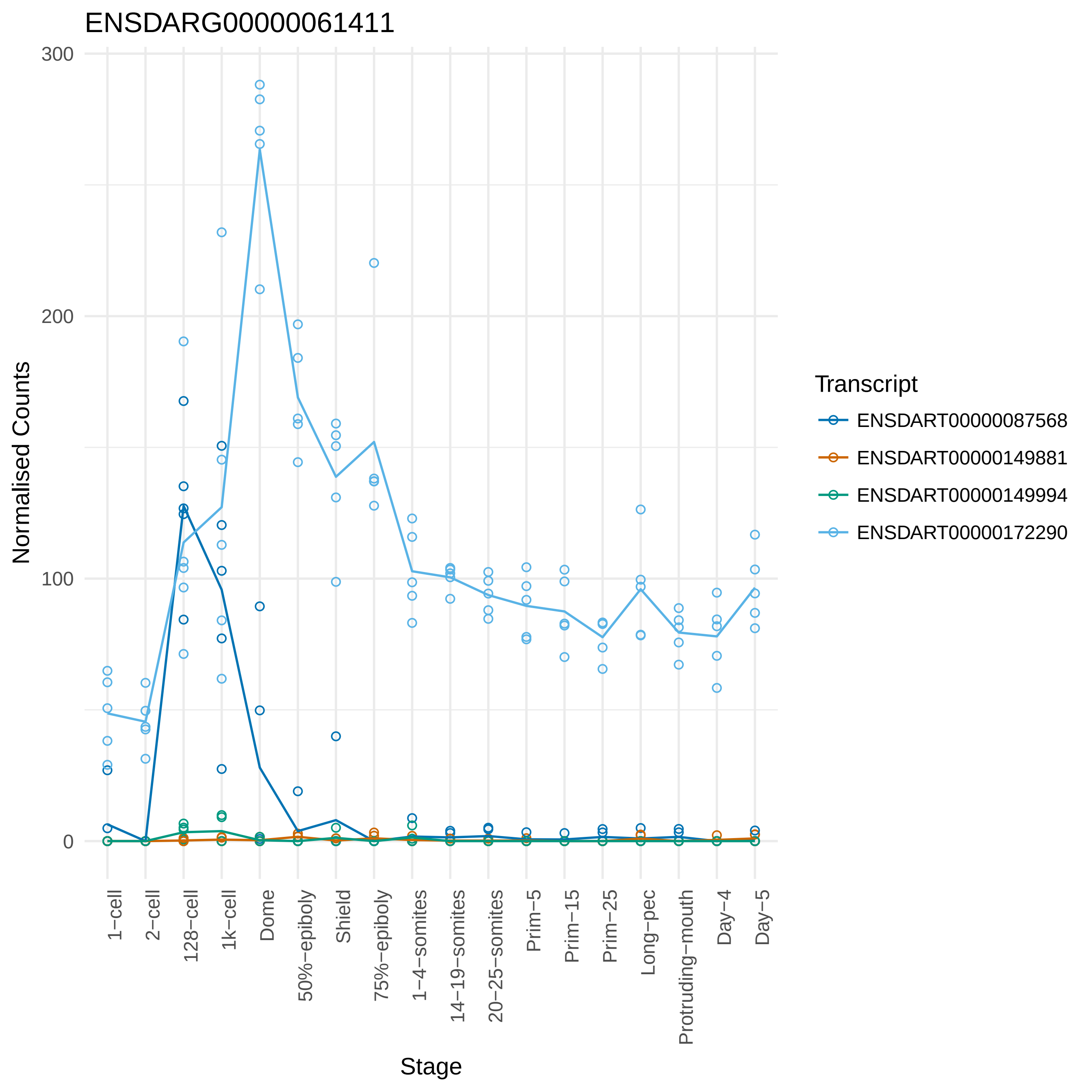
ENSDARG00000061411
Normalised Counts
300
200
100
0
1−cell
2−cell
128−cell
1k−cell
Dome
50%−epiboly
Shield
75%−epiboly
1−4−somites
14−19−somites
20−25−somites
Prim−5
Prim−15
Prim−25
Long−pec
Protruding−mouth
Day−4
Day−5
Stage
Transcript
ENSDART00000087568
ENSDART00000149881
ENSDART00000149994
ENSDART00000172290

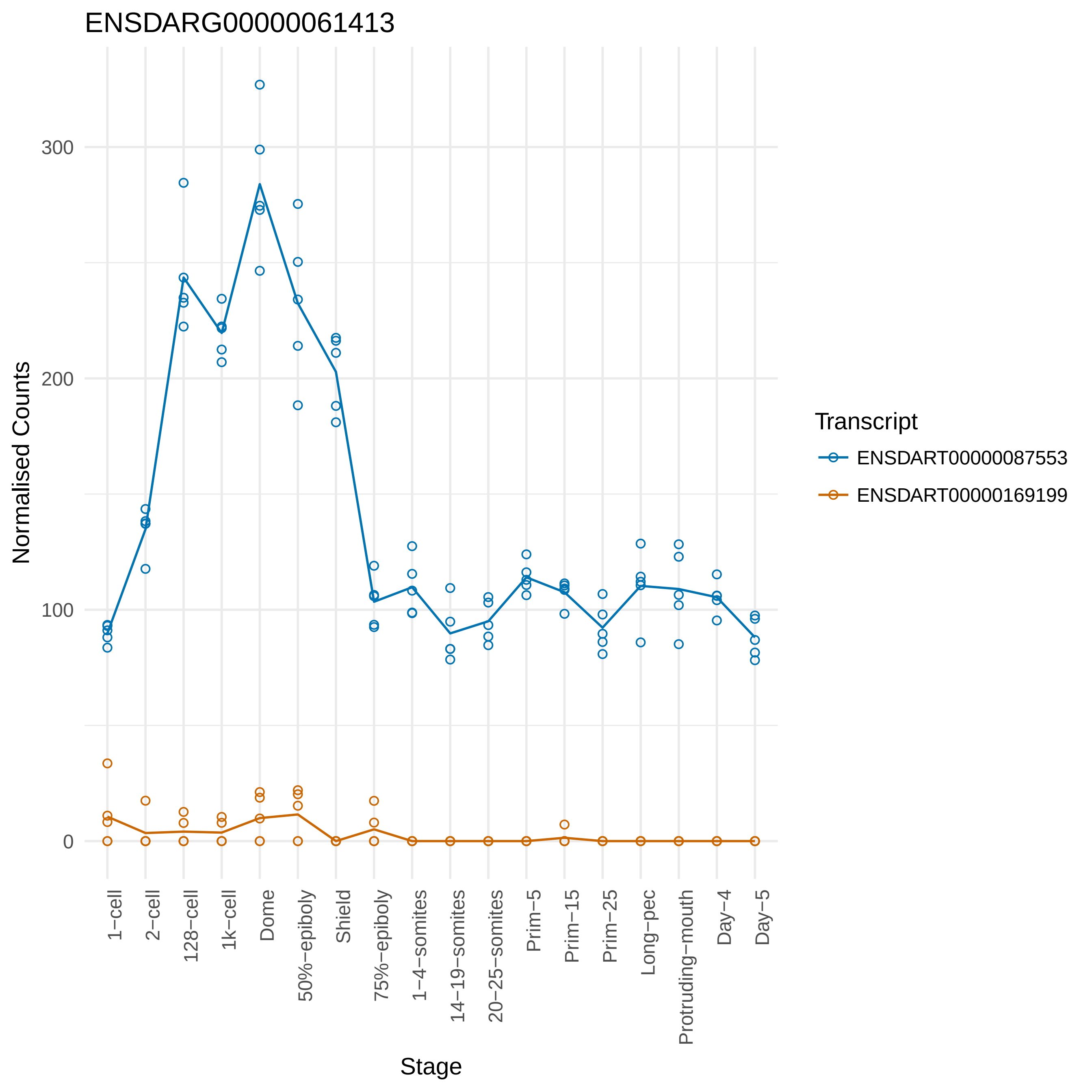
ENSDARG00000061413
Normalised Counts
300
200
100
0
1−cell
2−cell
128−cell
1k−cell
Dome
50%−epiboly
Shield
75%−epiboly
1−4−somites
14−19−somites
20−25−somites
Prim−5
Prim−15
Prim−25
Long−pec
Protruding−mouth
Day−4
Day−5
Stage
Transcript
ENSDART00000087553
ENSDART00000169199

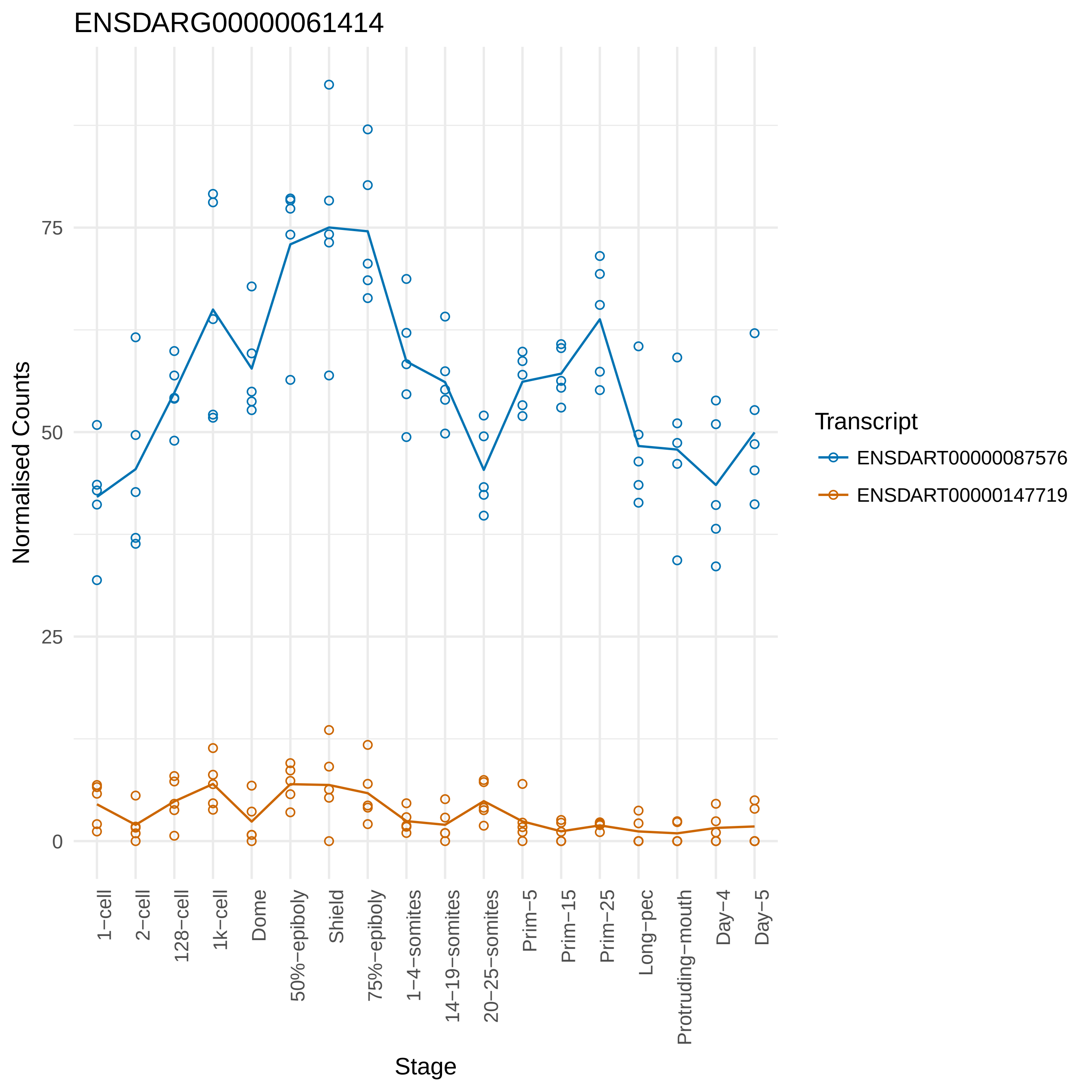

ENSDARG00000061414
Normalised Counts
0
25
50
75
Stage
1−cell
2−cell
128−cell
1k−cell
Dome
50%−epiboly
Shield
75%−epiboly
1−4−somites
14−19−somites
20−25−somites
Prim−5
Prim−15
Prim−25
Long−pec
Protruding−mouth
Day−4
Day−5
Transcript
ENSDART00000087576
ENSDART00000147719

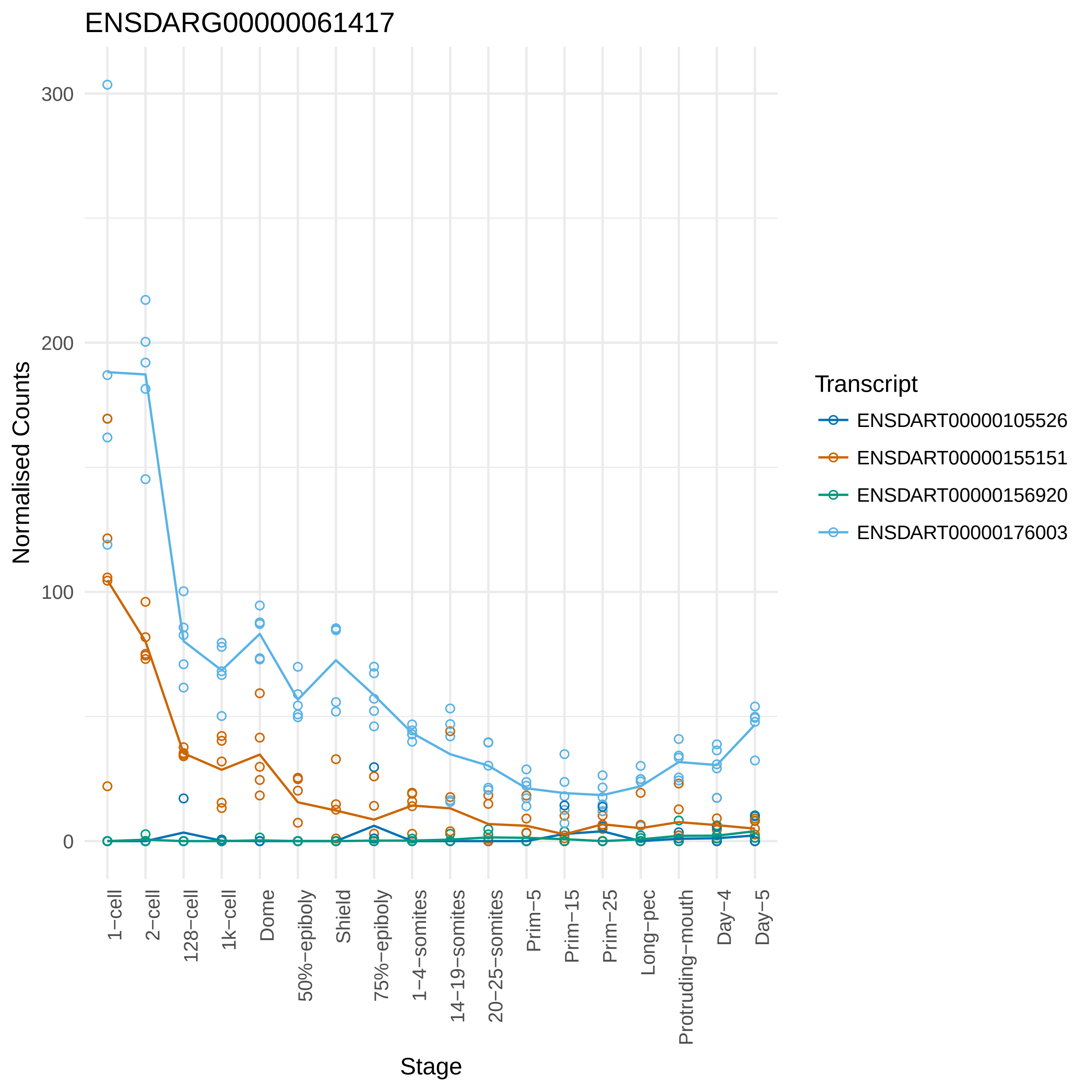
ENSDARG00000061417
Normalised Counts
300
200
100
0
1−cell
2−cell
128−cell
1k−cell
Dome
50%−epiboly
Shield
75%−epiboly
1−4−somites
14−19−somites
20−25−somites
Prim−5
Prim−15
Prim−25
Long−pec
Protruding−mouth
Day−4
Day−5
Stage
Transcript
ENSDART00000105526
ENSDART00000155151
ENSDART00000156920
ENSDART00000176003

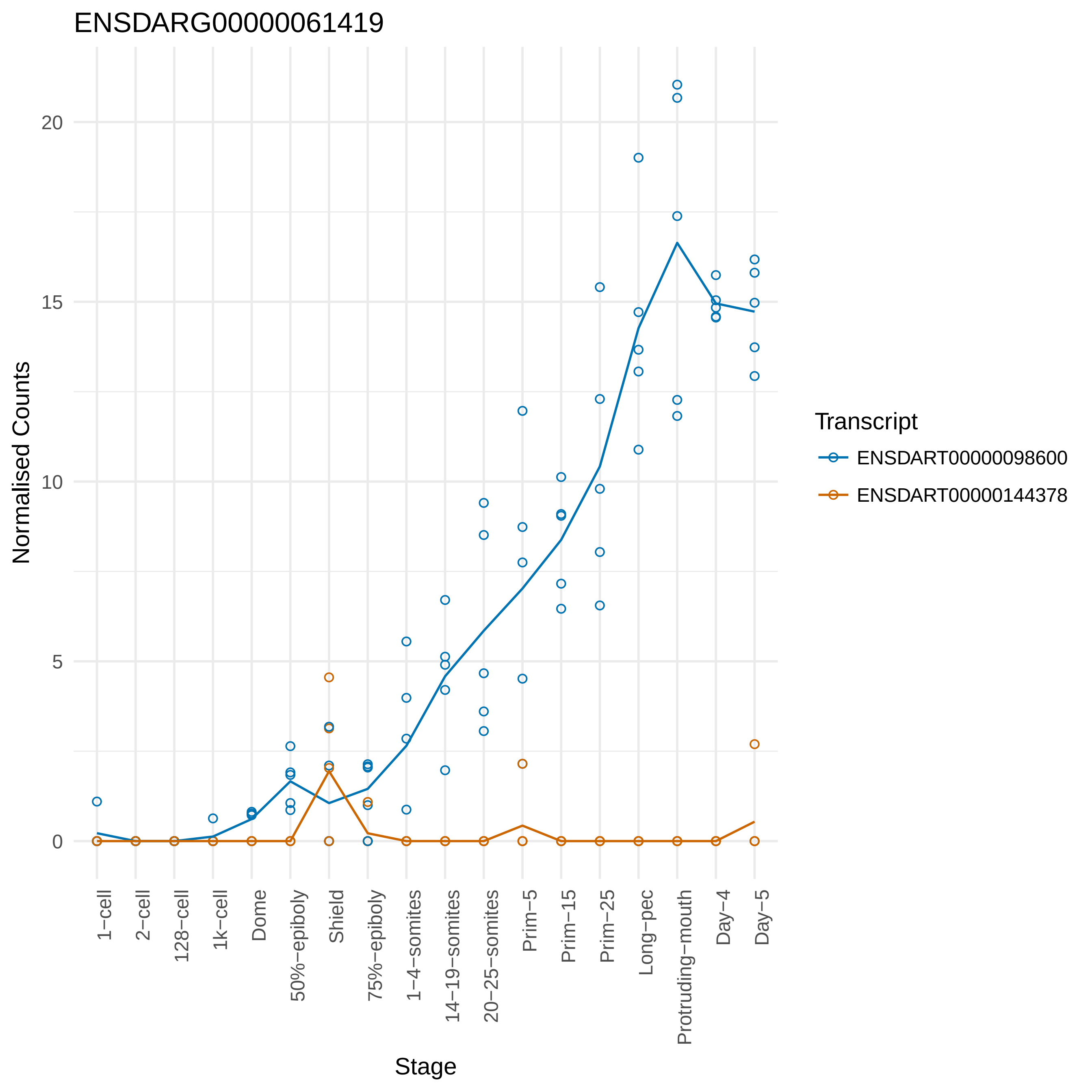

ENSDARG00000061419
Normalised Counts
20
15
10
5
0
1−cell
2−cell
128−cell
1k−cell
Dome
50%−epiboly
Shield
75%−epiboly
1−4−somites
14−19−somites
20−25−somites
Prim−5
Prim−15
Prim−25
Long−pec
Protruding−mouth
Day−4
Day−5
Stage
Transcript
ENSDART00000098600
ENSDART00000144378

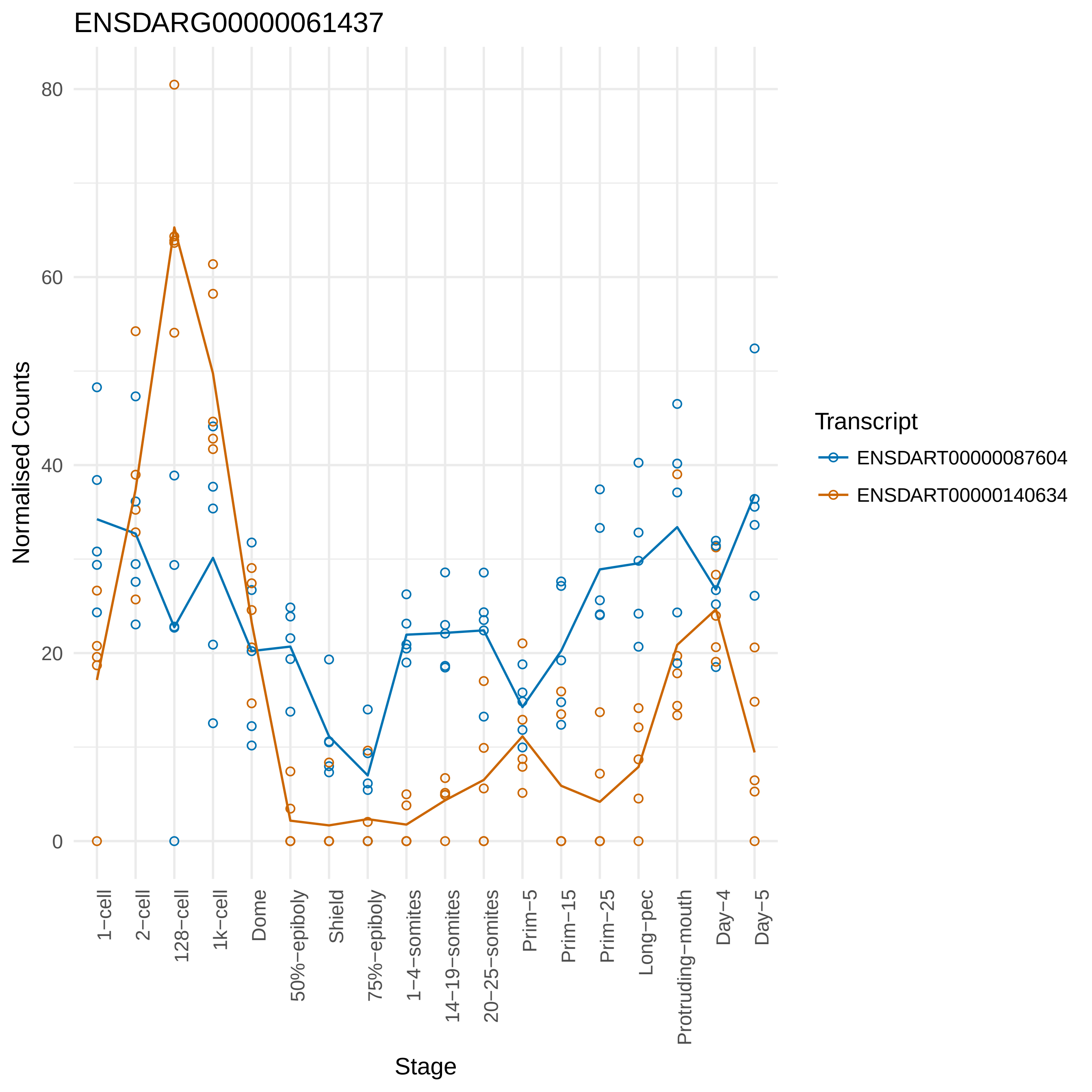

ENSDARG00000061437
Normalised Counts
80
60
40
20
0
1−cell
2−cell
128−cell
1k−cell
Dome
50%−epiboly
Shield
75%−epiboly
1−4−somites
14−19−somites
20−25−somites
Prim−5
Prim−15
Prim−25
Long−pec
Protruding−mouth
Day−4
Day−5
Stage
Transcript
ENSDART00000087604
ENSDART00000140634

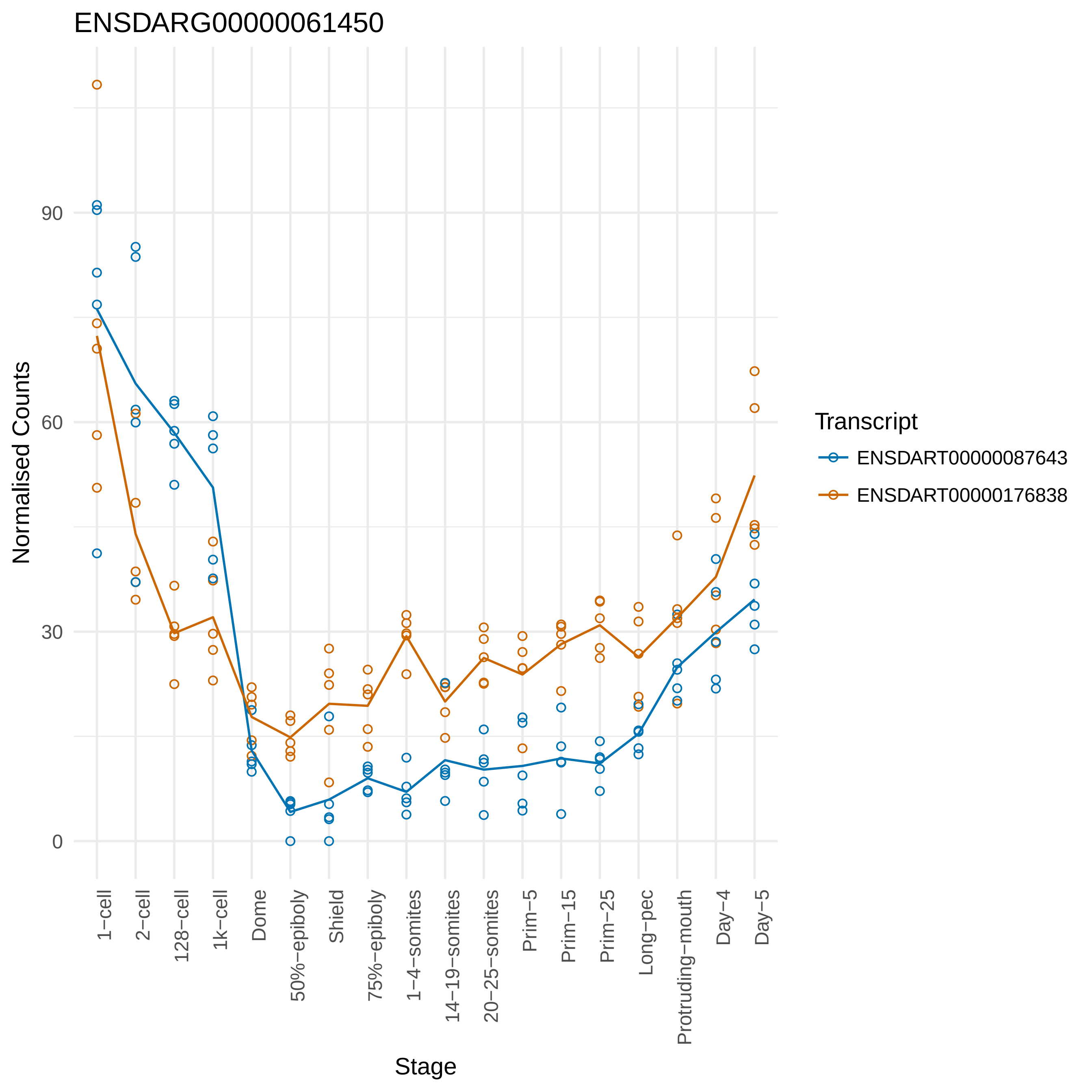

ENSDARG00000061450
Normalised Counts
0
30
60
90
Stage
1−cell
2−cell
128−cell
1k−cell
Dome
50%−epiboly
Shield
75%−epiboly
1−4−somites
14−19−somites
20−25−somites
Prim−5
Prim−15
Prim−25
Long−pec
Protruding−mouth
Day−4
Day−5
Transcript
ENSDART00000087643
ENSDART00000176838

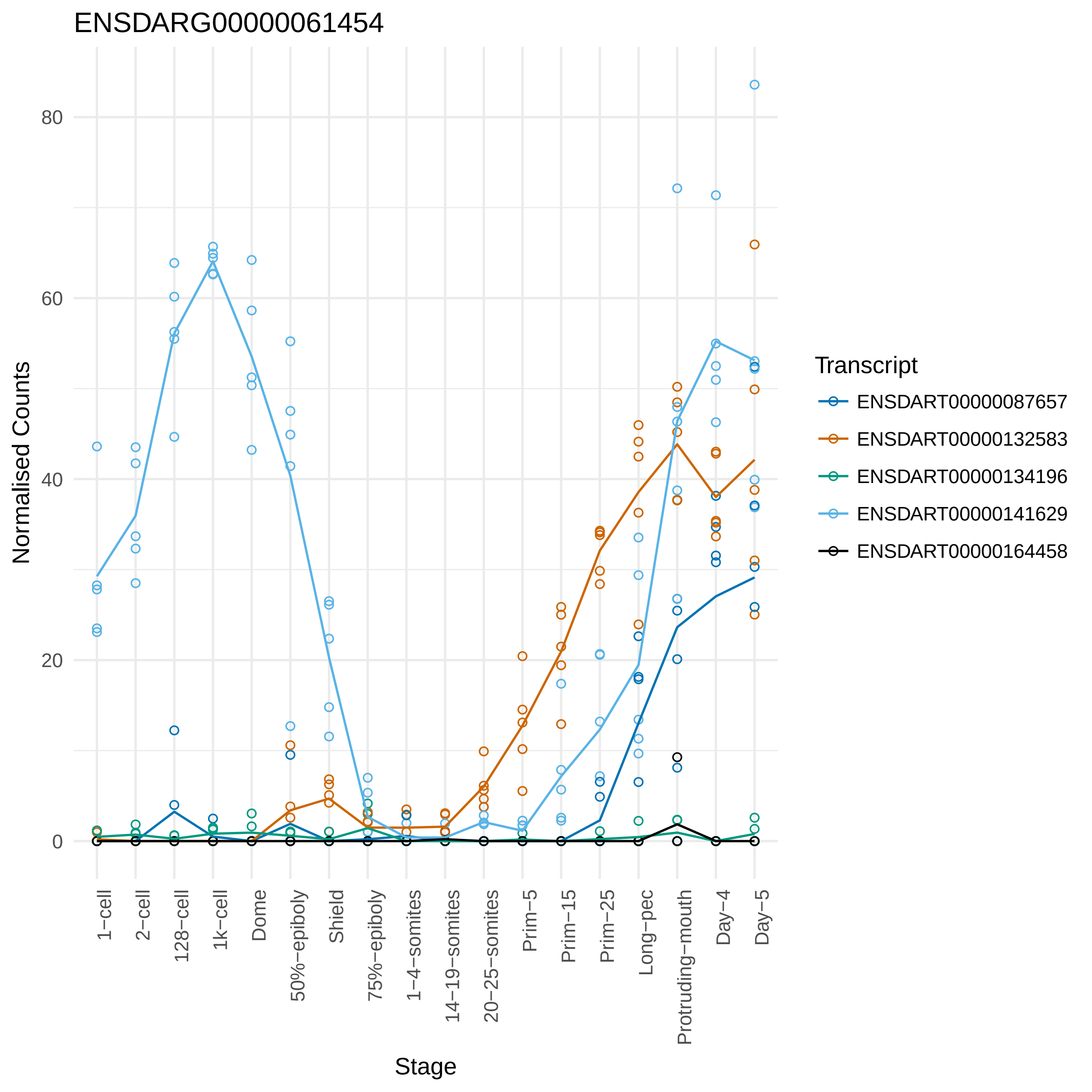

ENSDARG00000061454
Normalised Counts
0
20
40
60
80
1−cell
2−cell
128−cell
1k−cell
Dome
50%−epiboly
Shield
75%−epiboly
1−4−somites
14−19−somites
20−25−somites
Prim−5
Prim−15
Prim−25
Long−pec
Protruding−mouth
Day−4
Day−5
Stage
Transcript
ENSDART00000087657
ENSDART00000132583
ENSDART00000134196
ENSDART00000141629
ENSDART00000164458

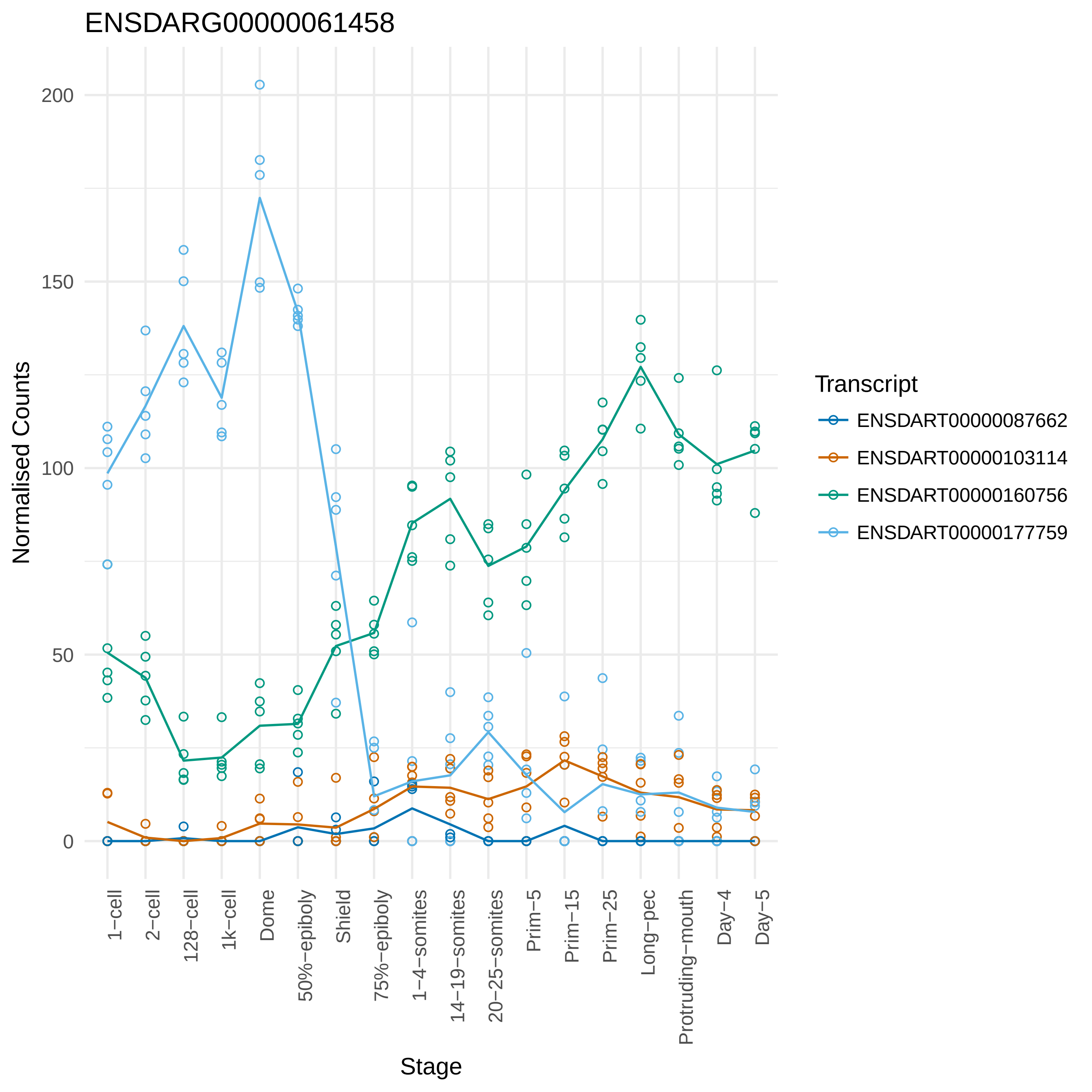

ENSDARG00000061458
Normalised Counts
200
150
100
50
0
1−cell
2−cell
128−cell
1k−cell
Dome
50%−epiboly
Shield
75%−epiboly
1−4−somites
14−19−somites
20−25−somites
Prim−5
Prim−15
Prim−25
Long−pec
Protruding−mouth
Day−4
Day−5
Stage
Transcript
ENSDART00000087662
ENSDART00000103114
ENSDART00000160756
ENSDART00000177759

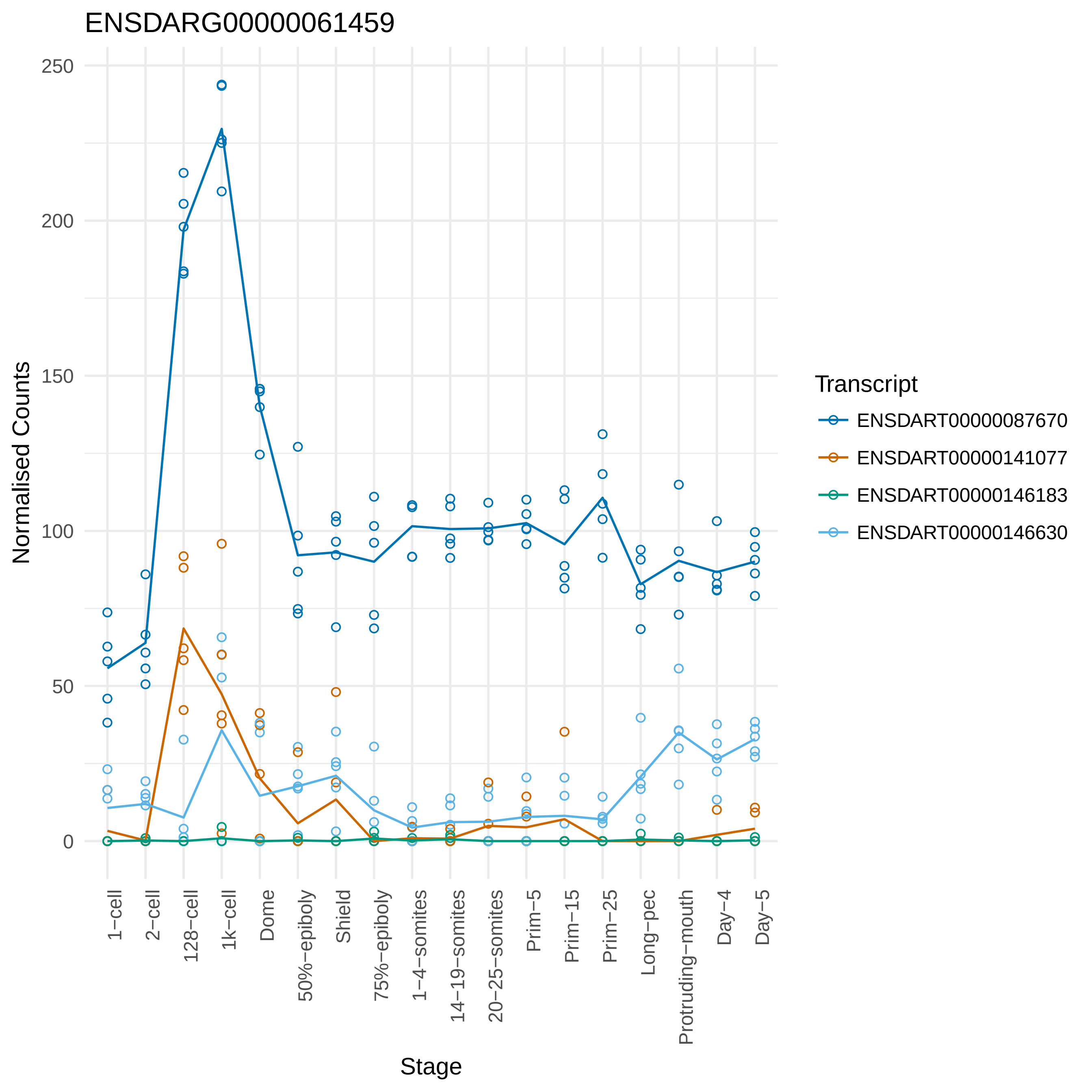
ENSDARG00000061459
Normalised Counts
250
200
150
100
50
0
1−cell
2−cell
128−cell
1k−cell
Dome
50%−epiboly
Shield
75%−epiboly
1−4−somites
14−19−somites
20−25−somites
Prim−5
Prim−15
Prim−25
Long−pec
Protruding−mouth
Day−4
Day−5
Stage
Transcript
ENSDART00000087670
ENSDART00000141077
ENSDART00000146183
ENSDART00000146630

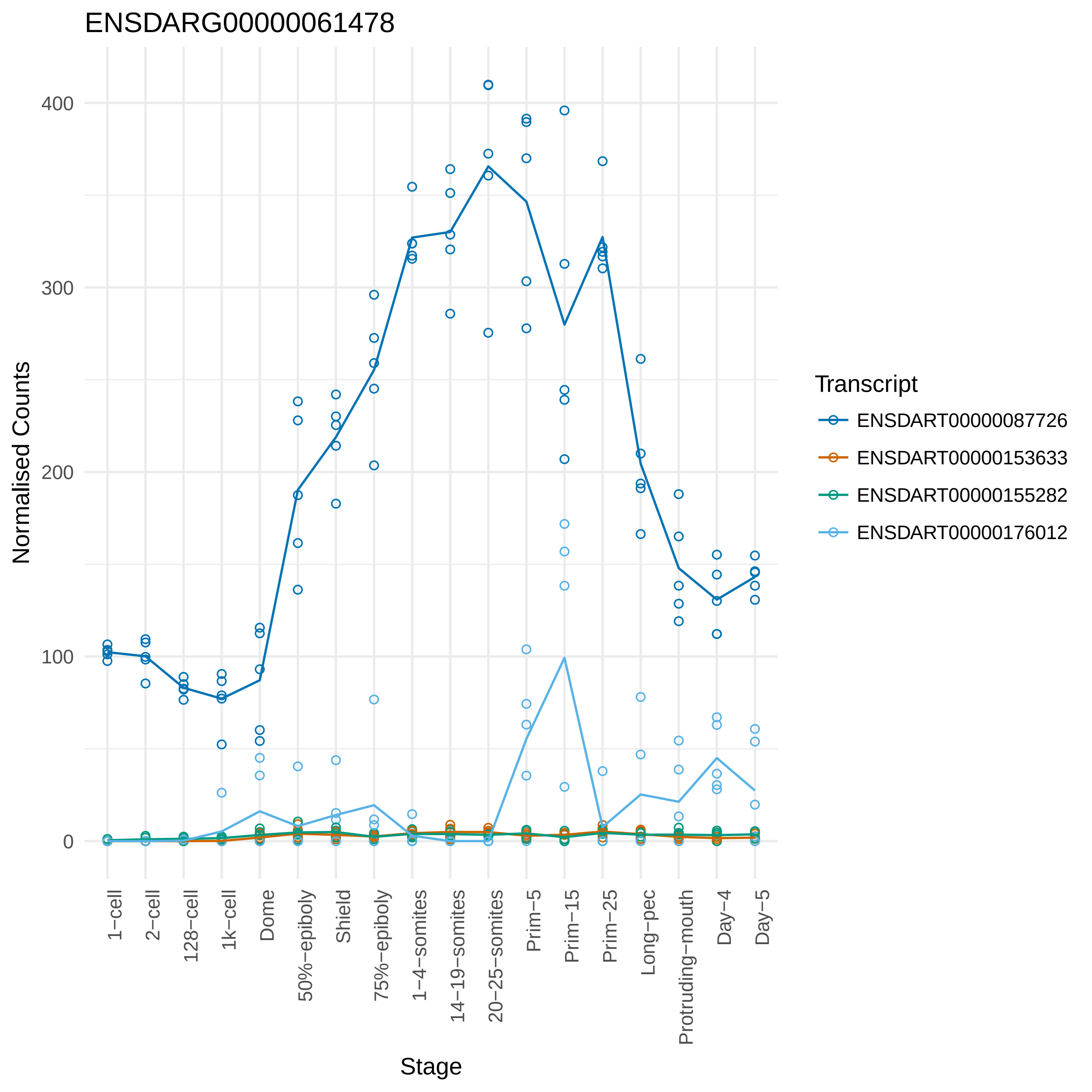

ENSDARG00000061478
Normalised Counts
400
300
200
100
0
Stage
1−cell
2−cell
128−cell
1k−cell
Dome
50%−epiboly
Shield
75%−epiboly
1−4−somites
14−19−somites
20−25−somites
Prim−5
Prim−15
Prim−25
Long−pec
Protruding−mouth
Day−4
Day−5
Transcript
ENSDART00000087726
ENSDART00000153633
ENSDART00000155282
ENSDART00000176012

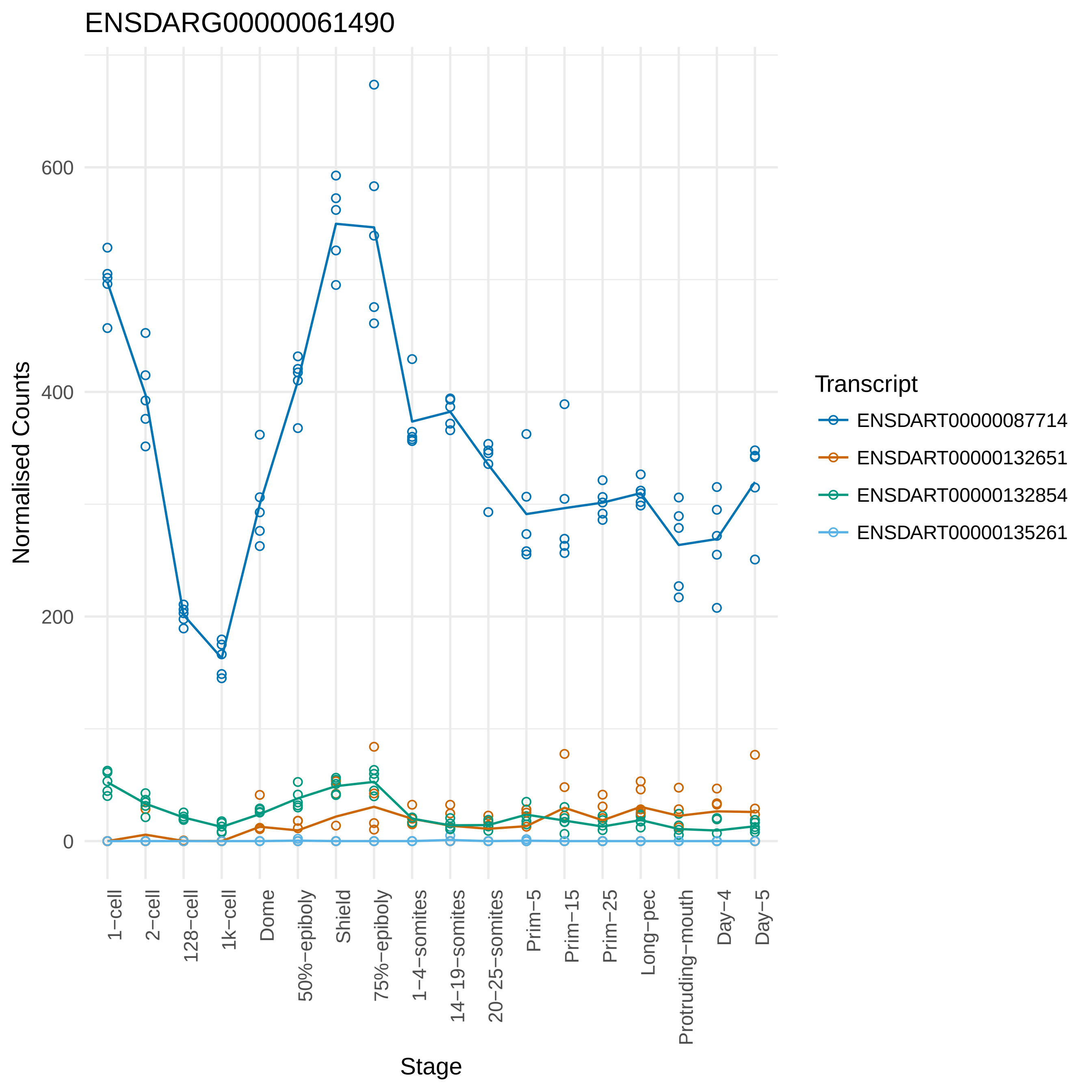
ENSDARG00000061490
Normalised Counts
600
400
200
0
1−cell
2−cell
128−cell
1k−cell
Dome
50%−epiboly
Shield
75%−epiboly
1−4−somites
14−19−somites
20−25−somites
Prim−5
Prim−15
Prim−25
Long−pec
Protruding−mouth
Day−4
Day−5
Stage
Transcript
ENSDART00000087714
ENSDART00000132651
ENSDART00000132854
ENSDART00000135261

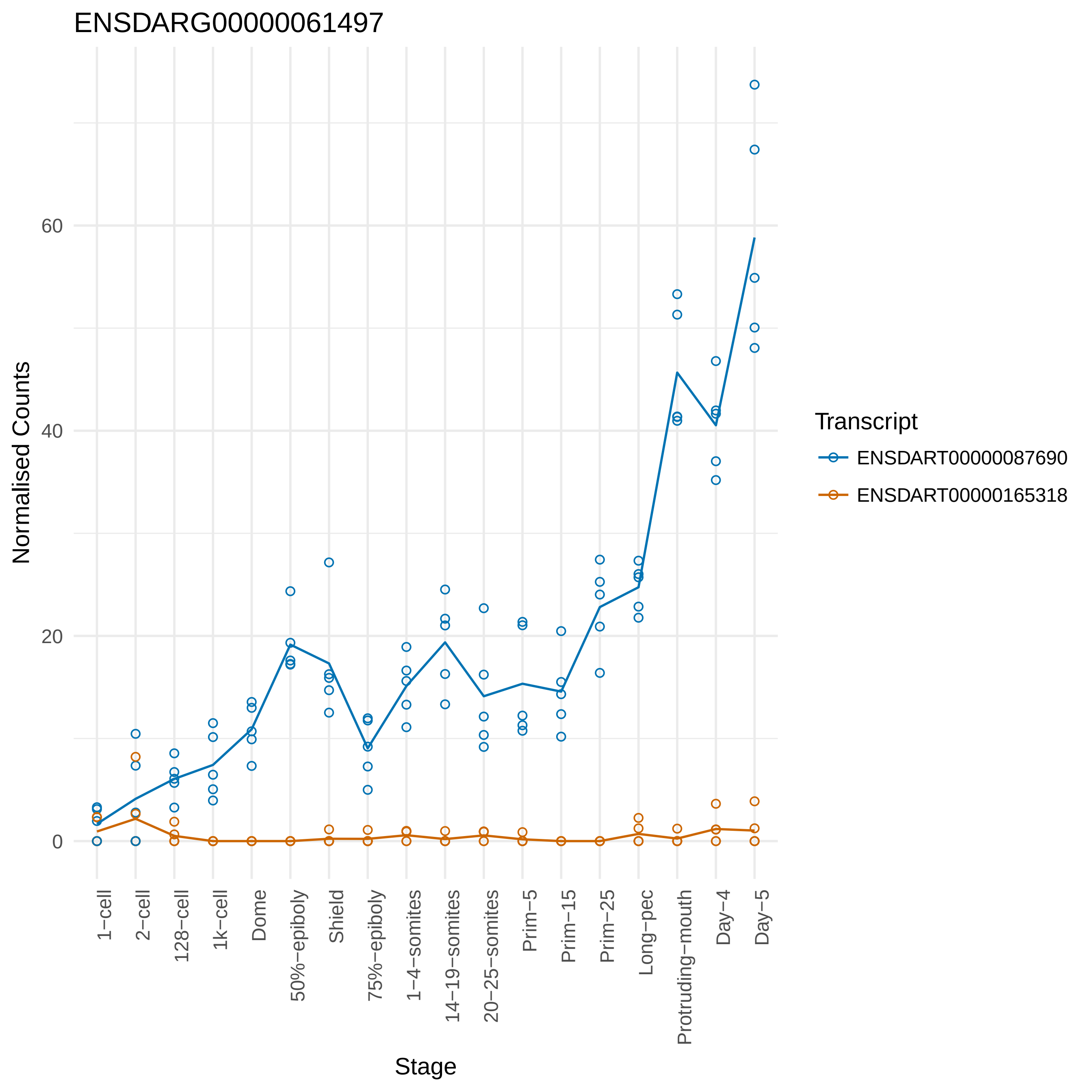

ENSDARG00000061497
Normalised Counts
60
40
20
0
1−cell
2−cell
128−cell
1k−cell
Dome
50%−epiboly
Shield
75%−epiboly
1−4−somites
14−19−somites
20−25−somites
Prim−5
Prim−15
Prim−25
Long−pec
Protruding−mouth
Day−4
Day−5
Stage
Transcript
ENSDART00000087690
ENSDART00000165318

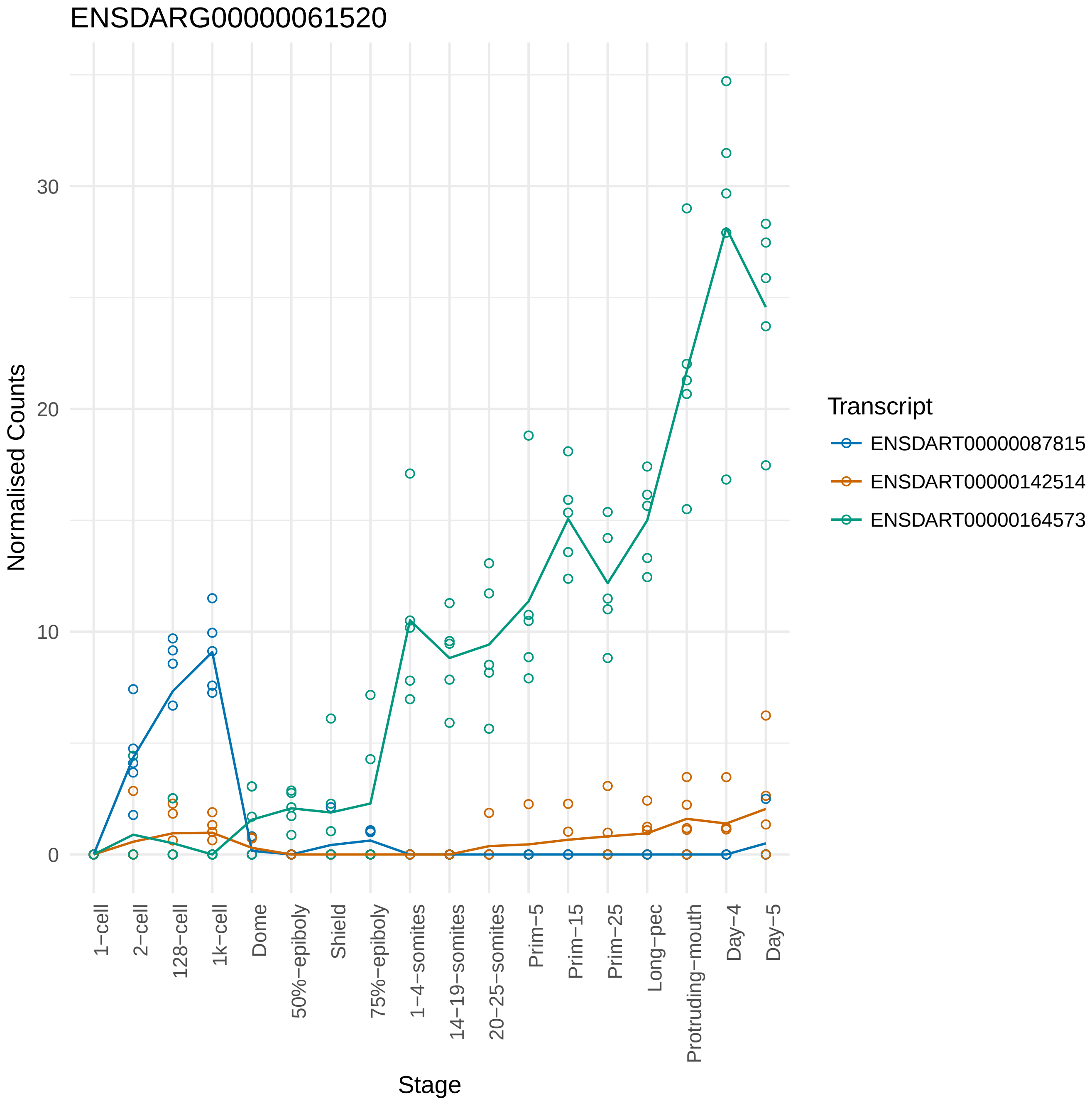

ENSDARG00000061520
Normalised Counts
0
10
20
30
1−cell
2−cell
128−cell
1k−cell
Dome
50%−epiboly
Shield
75%−epiboly
1−4−somites
14−19−somites
20−25−somites
Prim−5
Prim−15
Prim−25
Long−pec
Protruding−mouth
Day−4
Day−5
Stage
Transcript
ENSDART00000087815
ENSDART00000142514
ENSDART00000164573

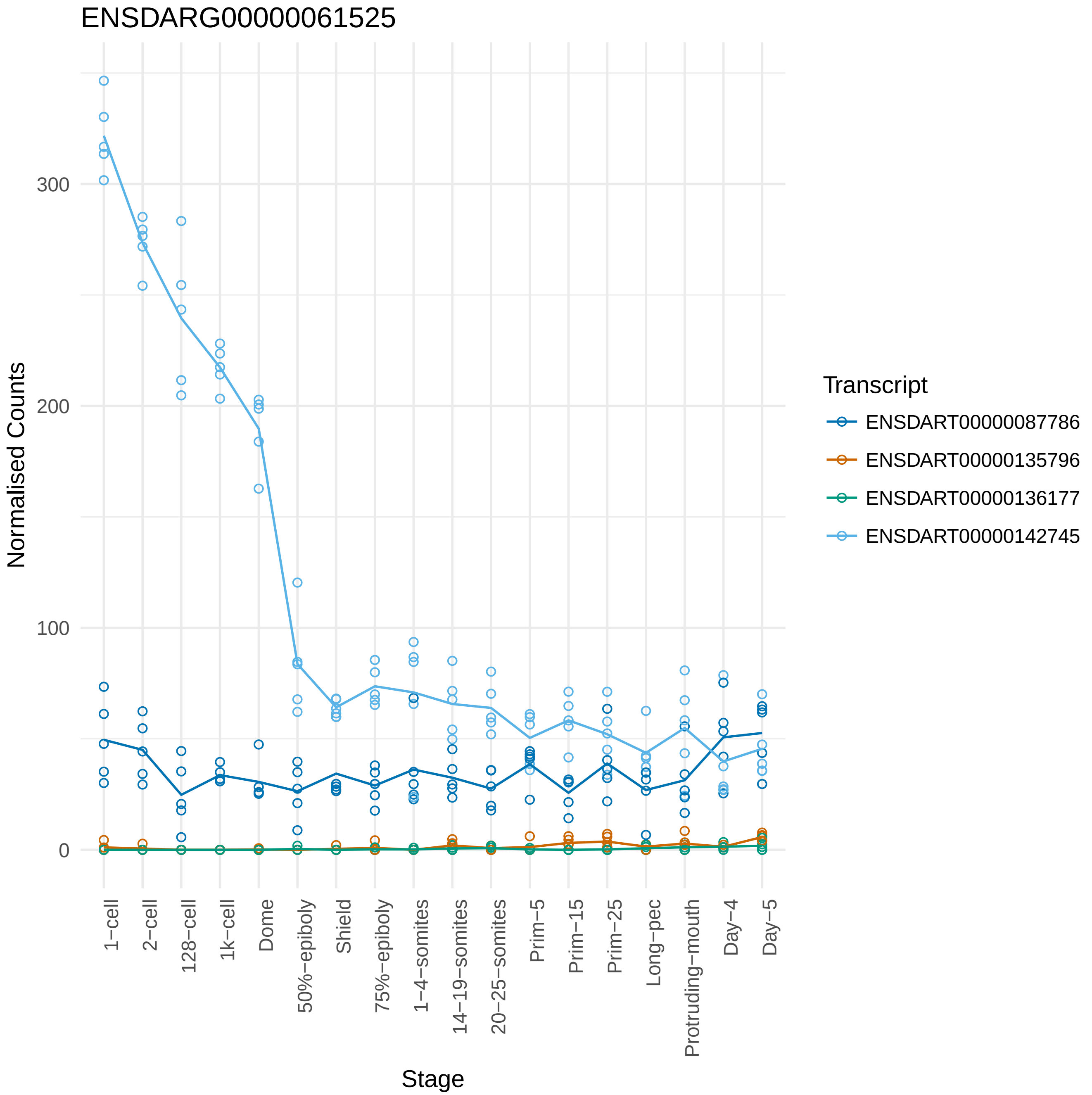

ENSDARG00000061525
Normalised Counts
300
200
100
0
1−cell
2−cell
128−cell
1k−cell
Dome
50%−epiboly
Shield
75%−epiboly
1−4−somites
14−19−somites
20−25−somites
Prim−5
Prim−15
Prim−25
Long−pec
Protruding−mouth
Day−4
Day−5
Stage
Transcript
ENSDART00000087786
ENSDART00000135796
ENSDART00000136177
ENSDART00000142745

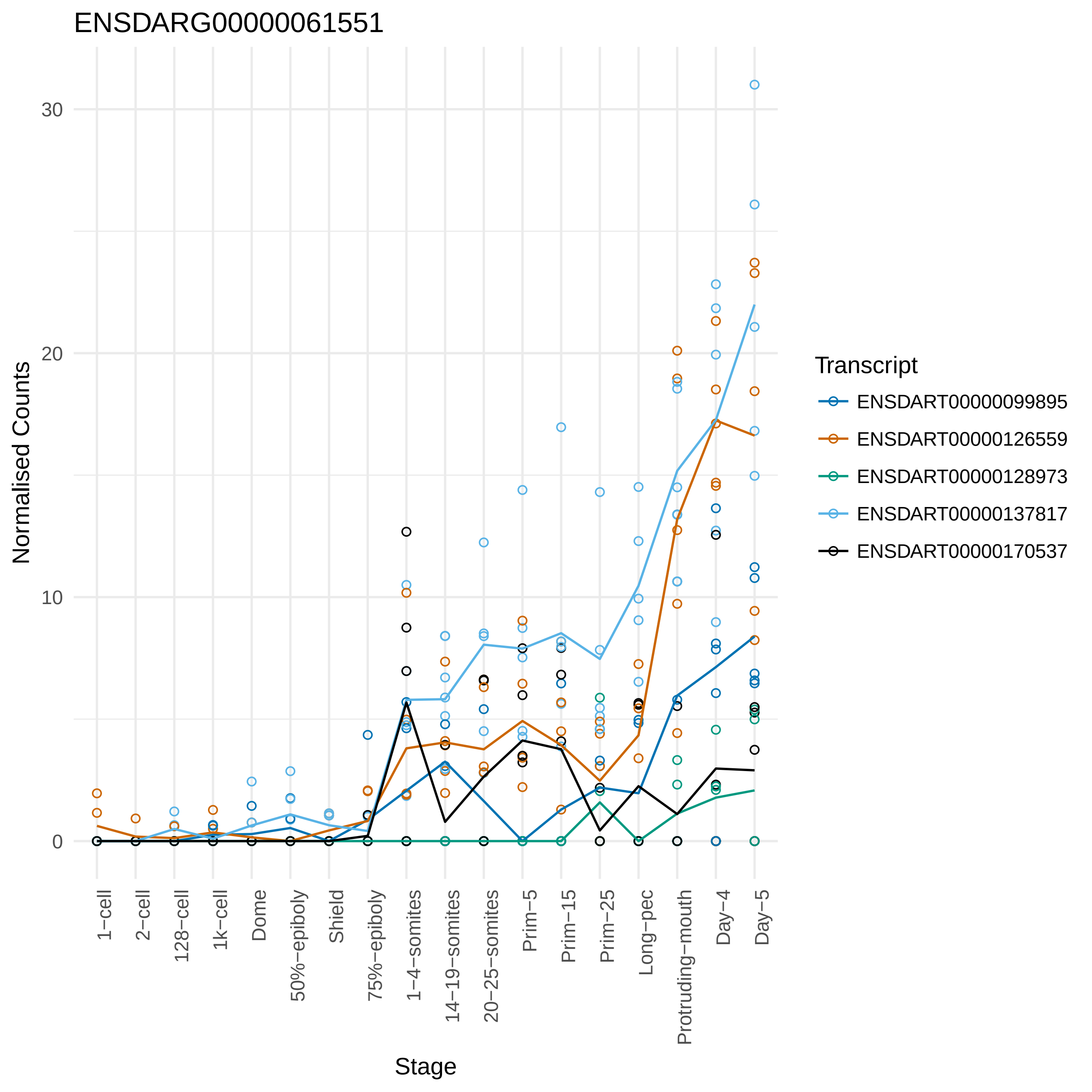
ENSDARG00000061551
Normalised Counts
0
10
20
30
1−cell
2−cell
128−cell
1k−cell
Dome
50%−epiboly
Shield
75%−epiboly
1−4−somites
14−19−somites
20−25−somites
Prim−5
Prim−15
Prim−25
Long−pec
Protruding−mouth
Day−4
Day−5
Stage
Transcript
ENSDART00000099895
ENSDART00000126559
ENSDART00000128973
ENSDART00000137817
ENSDART00000170537

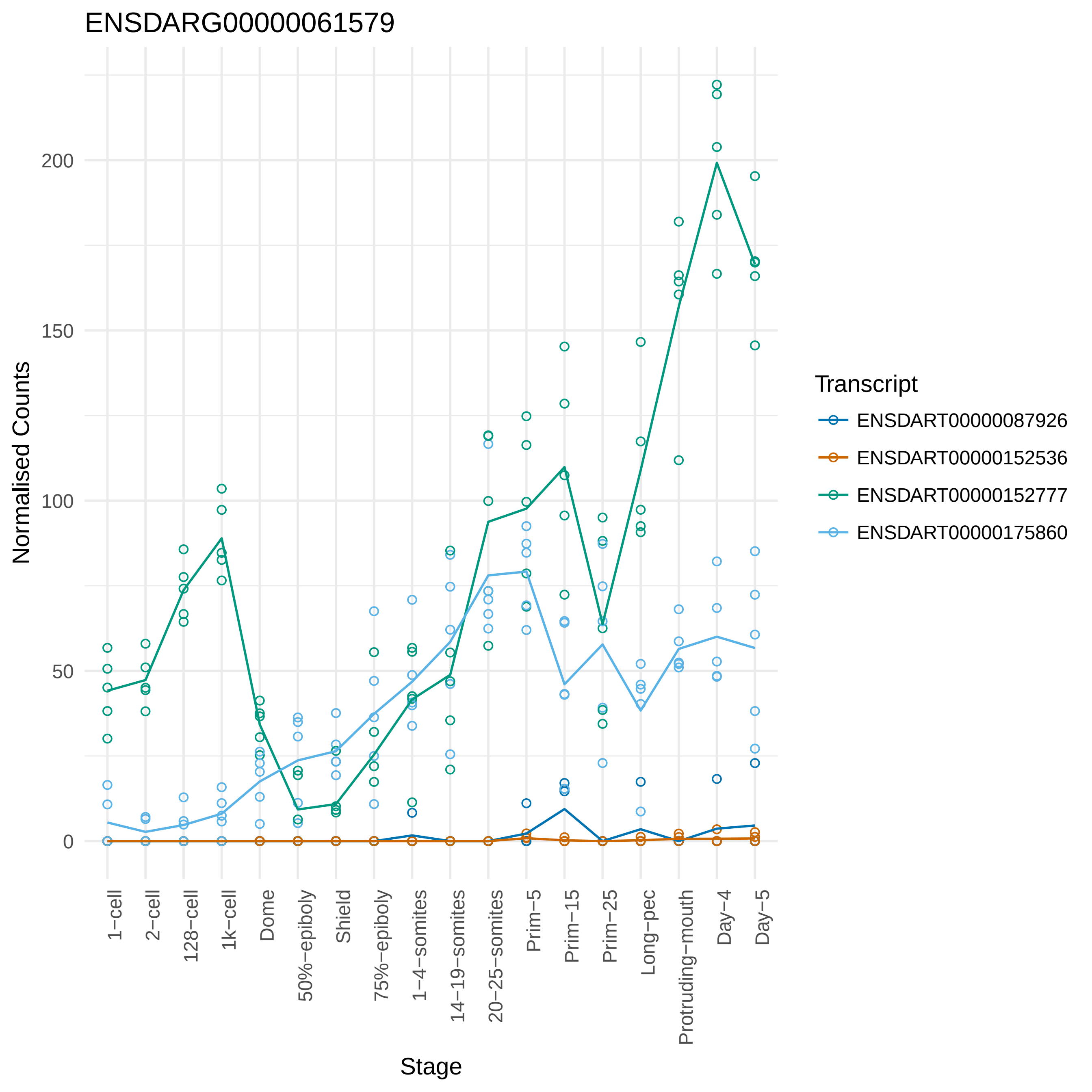

ENSDARG00000061579
Normalised Counts
200
150
100
50
0
1−cell
2−cell
128−cell
1k−cell
Dome
50%−epiboly
Shield
75%−epiboly
1−4−somites
14−19−somites
20−25−somites
Prim−5
Prim−15
Prim−25
Long−pec
Protruding−mouth
Day−4
Day−5
Stage
Transcript
ENSDART00000087926
ENSDART00000152536
ENSDART00000152777
ENSDART00000175860

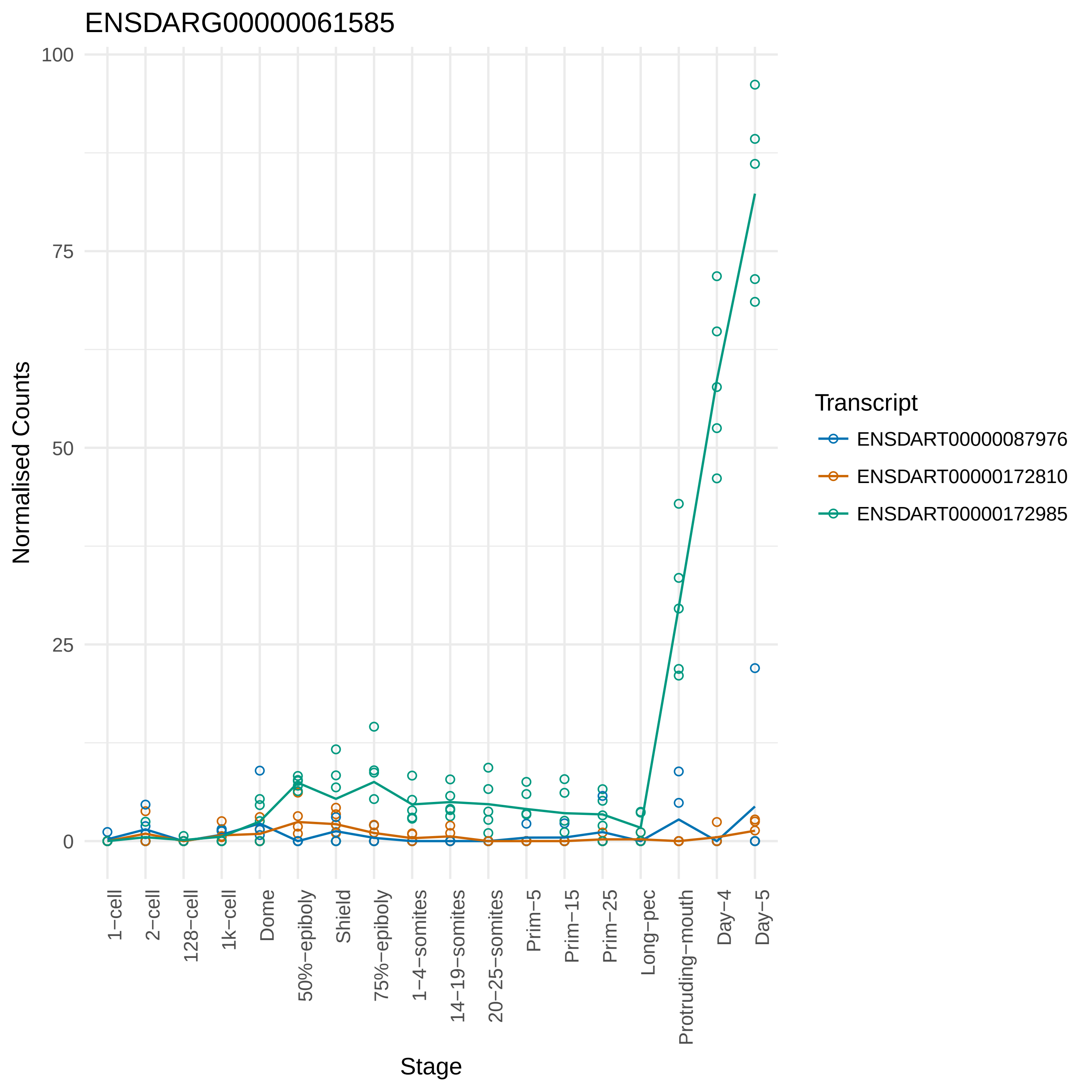

ENSDARG00000061585
100
75
50
25
0
Normalised Counts
1−cell
2−cell
128−cell
1k−cell
Dome
50%−epiboly
Shield
75%−epiboly
1−4−somites
14−19−somites
20−25−somites
Prim−5
Prim−15
Prim−25
Long−pec
Protruding−mouth
Day−4
Day−5
Stage
Transcript
ENSDART00000087976
ENSDART00000172810
ENSDART00000172985

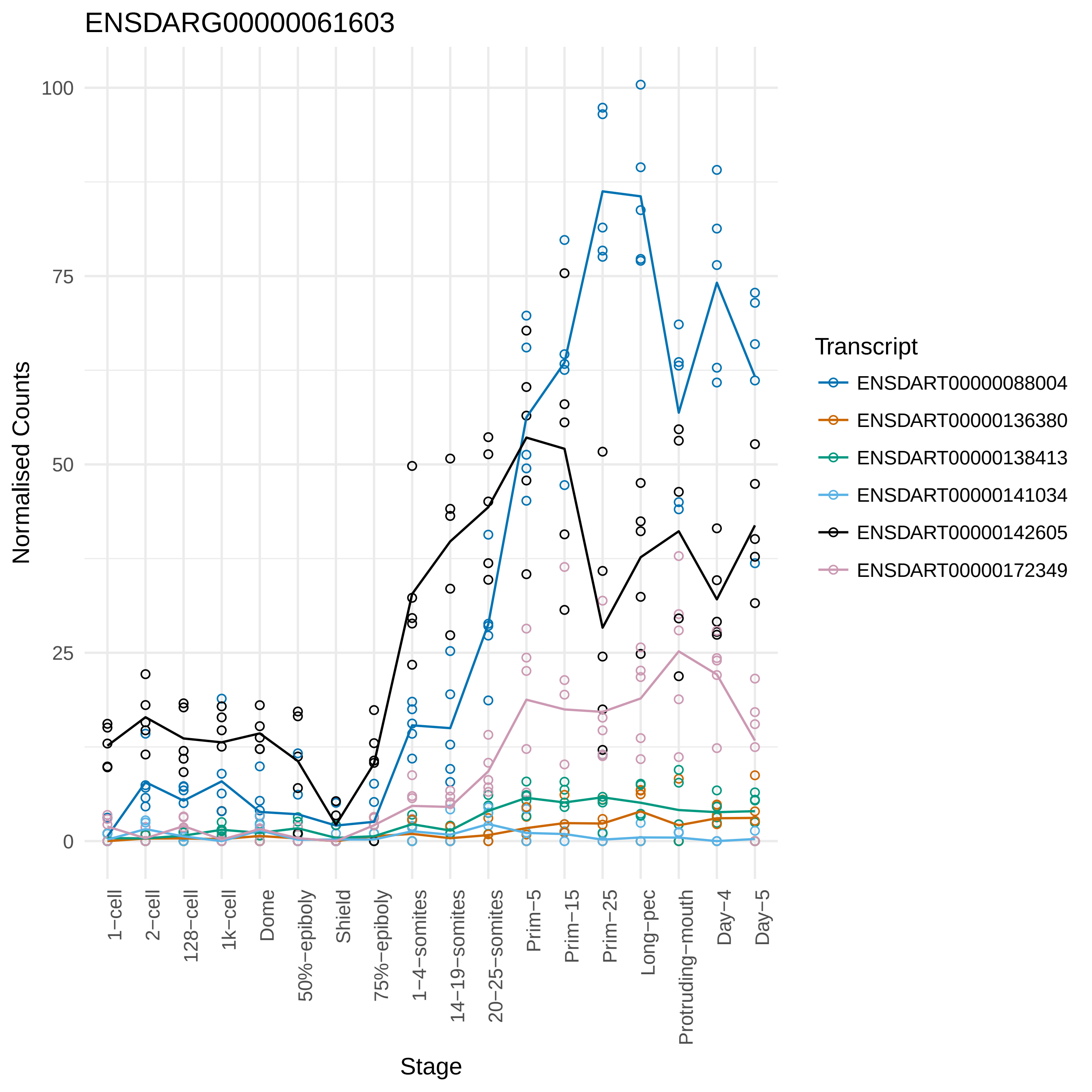

ENSDARG00000061603
Normalised Counts
100
75
50
25
0
1−cell
2−cell
128−cell
1k−cell
Dome
50%−epiboly
Shield
75%−epiboly
1−4−somites
14−19−somites
20−25−somites
Prim−5
Prim−15
Prim−25
Long−pec
Protruding−mouth
Day−4
Day−5
Stage
Transcript
ENSDART00000088004
ENSDART00000136380
ENSDART00000138413
ENSDART00000141034
ENSDART00000142605
ENSDART00000172349

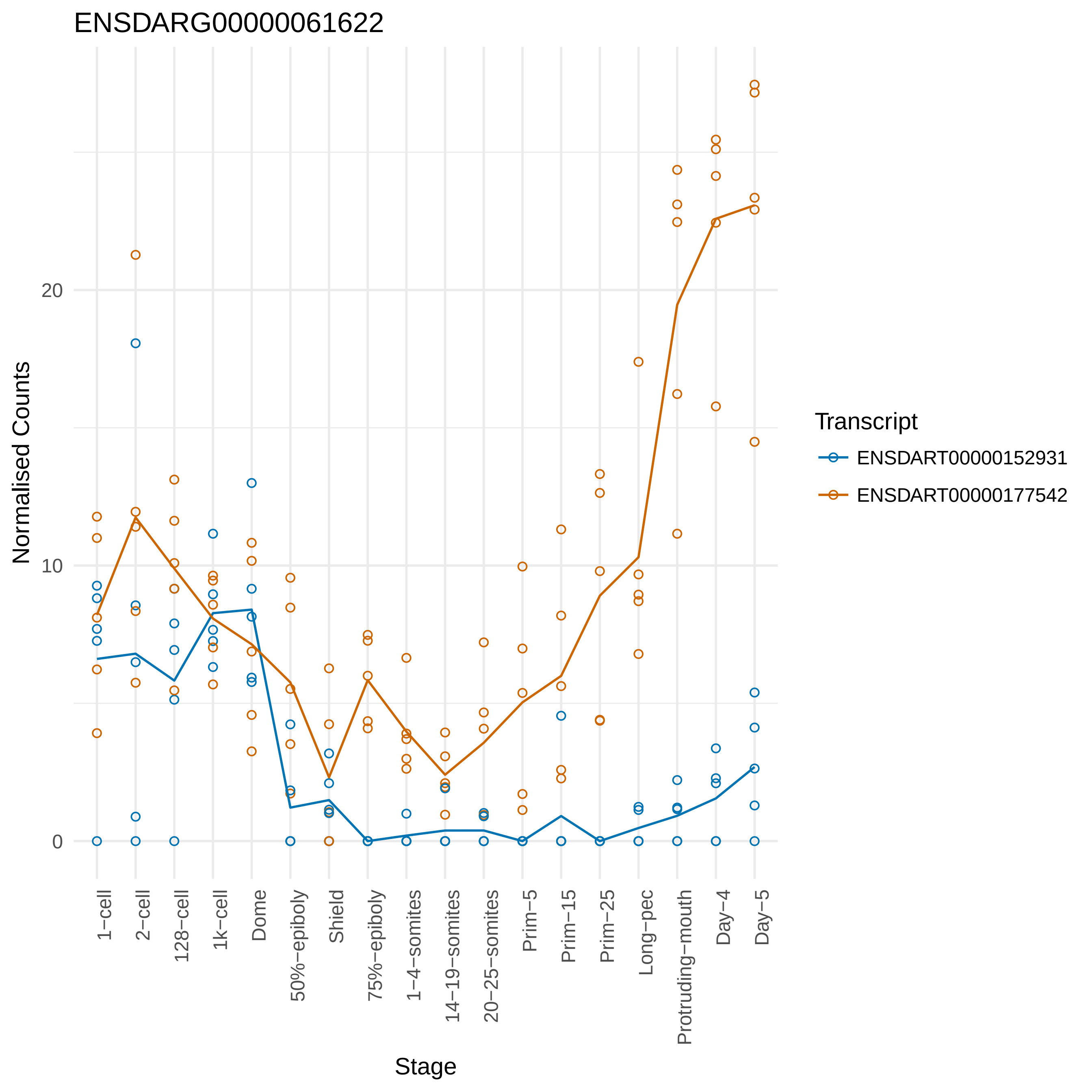
ENSDARG00000061622
Normalised Counts
0
10
20
1−cell
2−cell
128−cell
1k−cell
Dome
50%−epiboly
Shield
75%−epiboly
1−4−somites
14−19−somites
20−25−somites
Prim−5
Prim−15
Prim−25
Long−pec
Protruding−mouth
Day−4
Day−5
Stage
Transcript
ENSDART00000152931
ENSDART00000177542

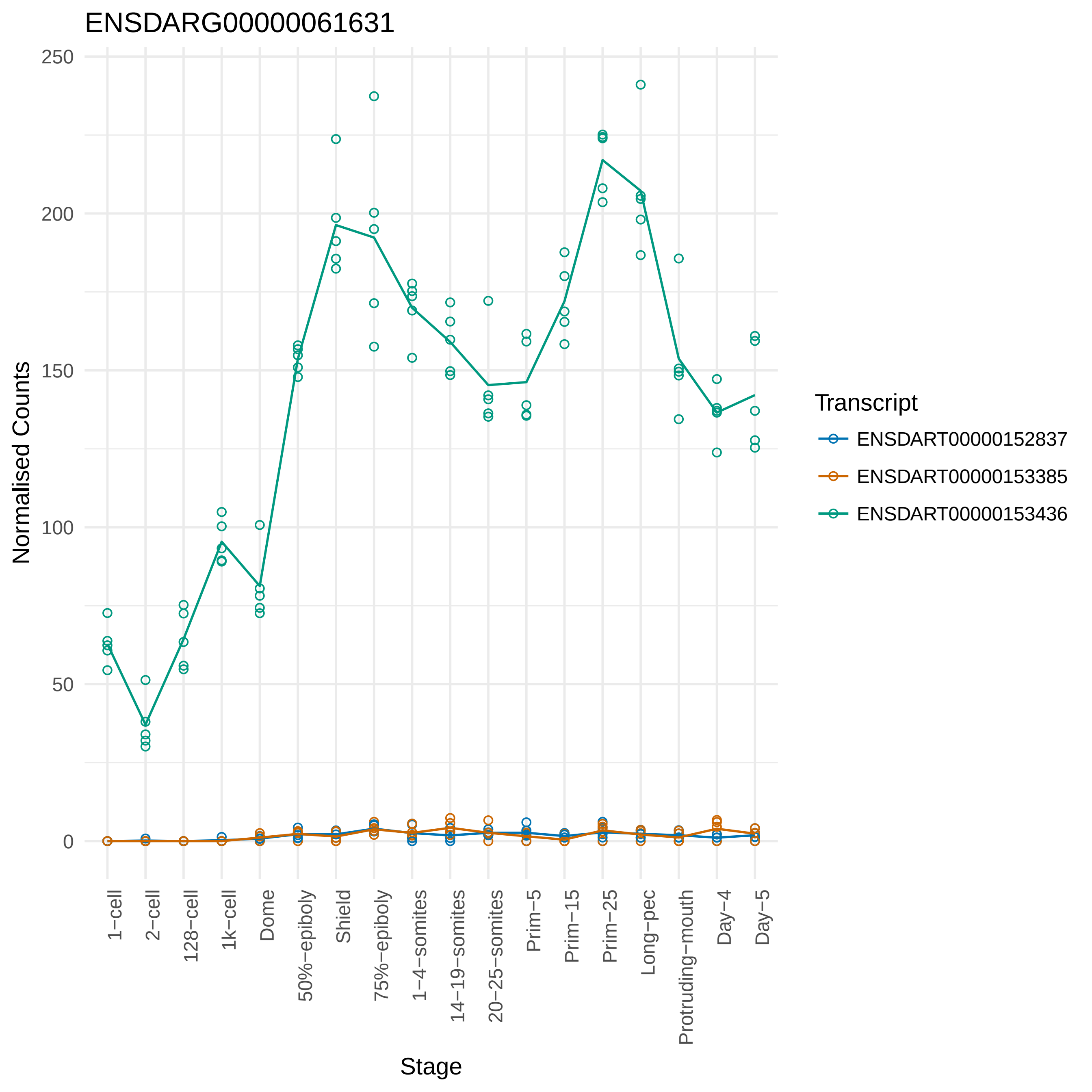
ENSDARG00000061631
Normalised Counts
250
200
150
100
50
0
1−cell
2−cell
128−cell
1k−cell
Dome
50%−epiboly
Shield
75%−epiboly
1−4−somites
14−19−somites
20−25−somites
Prim−5
Prim−15
Prim−25
Long−pec
Protruding−mouth
Day−4
Day−5
Stage
Transcript
ENSDART00000152837
ENSDART00000153385
ENSDART00000153436

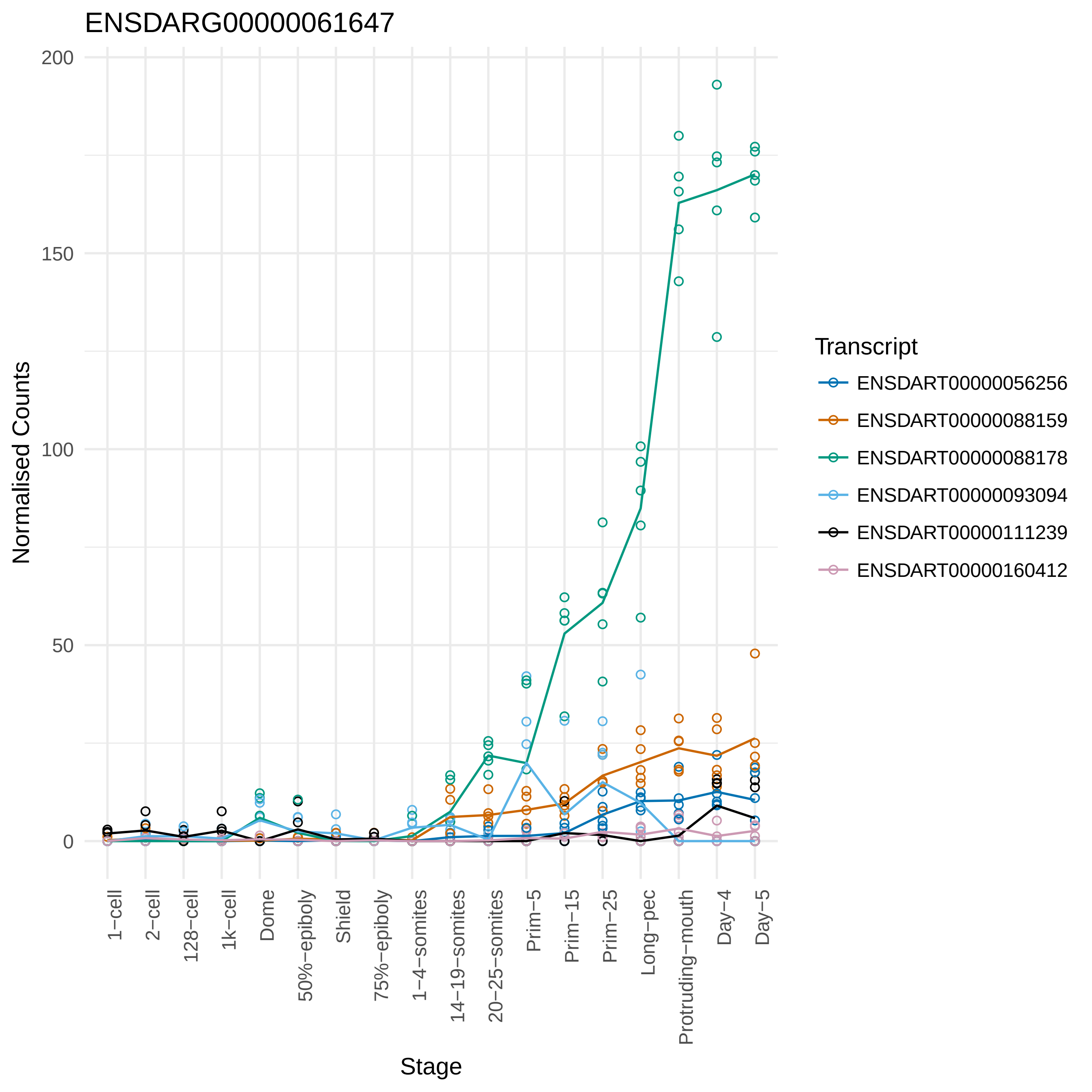

ENSDARG00000061647
Normalised Counts
200
150
100
50
0
1−cell
2−cell
128−cell
1k−cell
Dome
50%−epiboly
Shield
75%−epiboly
1−4−somites
14−19−somites
20−25−somites
Prim−5
Prim−15
Prim−25
Long−pec
Protruding−mouth
Day−4
Day−5
Stage
Transcript
ENSDART00000056256
ENSDART00000088159
ENSDART00000088178
ENSDART00000093094
ENSDART00000111239
ENSDART00000160412

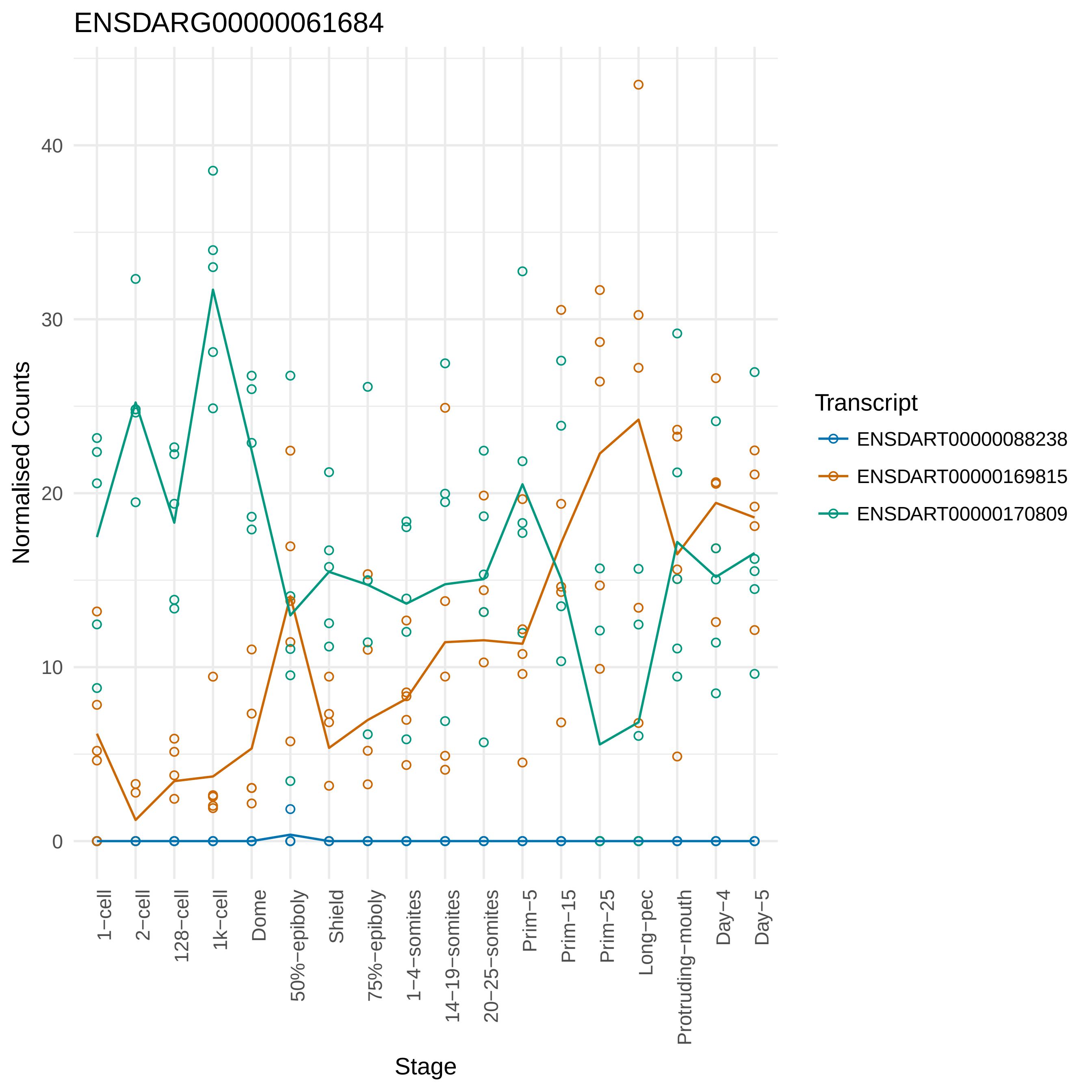

ENSDARG00000061684
Normalised Counts
0
10
20
30
40
1−cell
2−cell
128−cell
1k−cell
Dome
50%−epiboly
Shield
75%−epiboly
1−4−somites
14−19−somites
20−25−somites
Prim−5
Prim−15
Prim−25
Long−pec
Protruding−mouth
Day−4
Day−5
Stage
Transcript
ENSDART00000088238
ENSDART00000169815
ENSDART00000170809

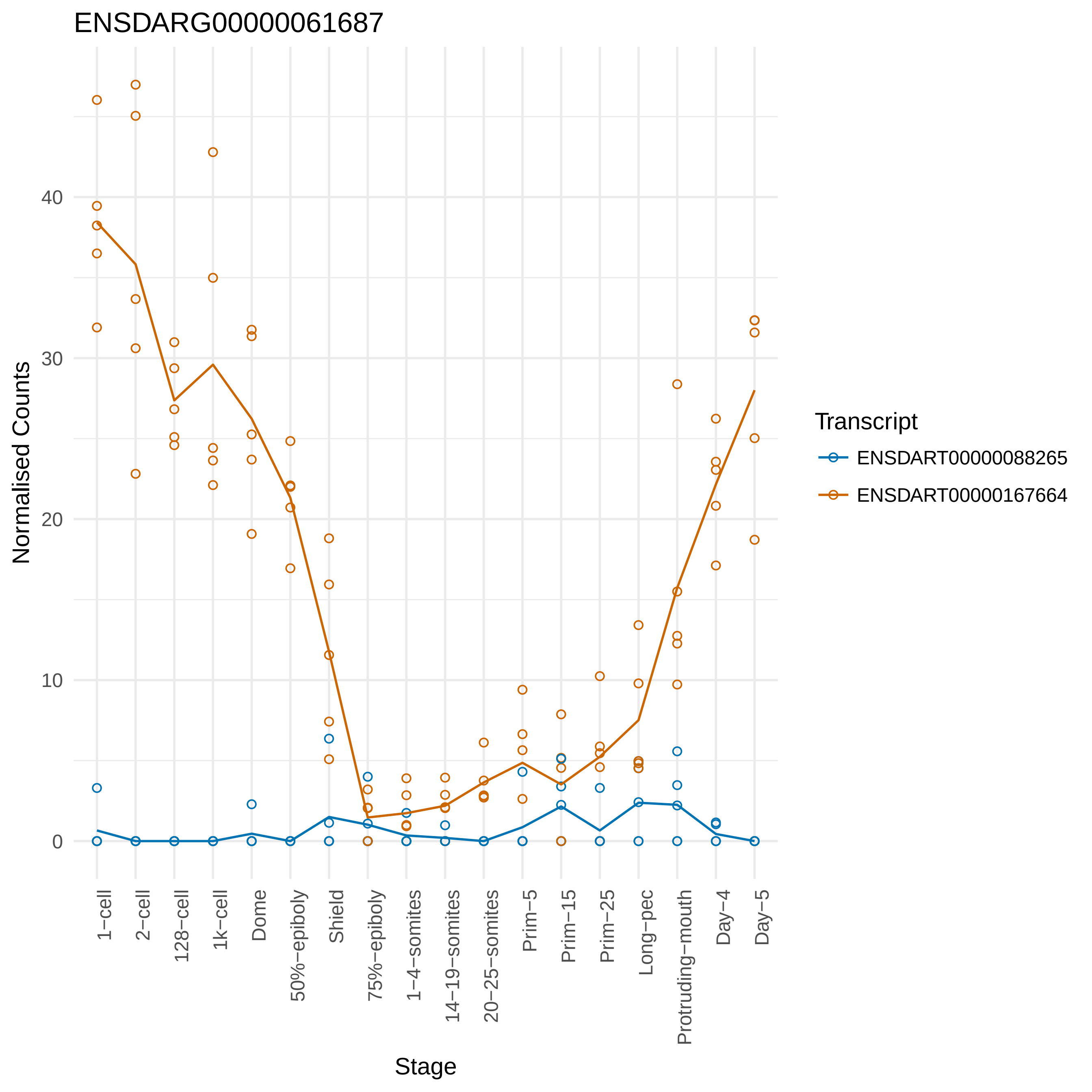
ENSDARG00000061687
Normalised Counts
0
10
20
30
40
Stage
1−cell
2−cell
128−cell
1k−cell
Dome
50%−epiboly
Shield
75%−epiboly
1−4−somites
14−19−somites
20−25−somites
Prim−5
Prim−15
Prim−25
Long−pec
Protruding−mouth
Day−4
Day−5
Transcript
ENSDART00000088265
ENSDART00000167664

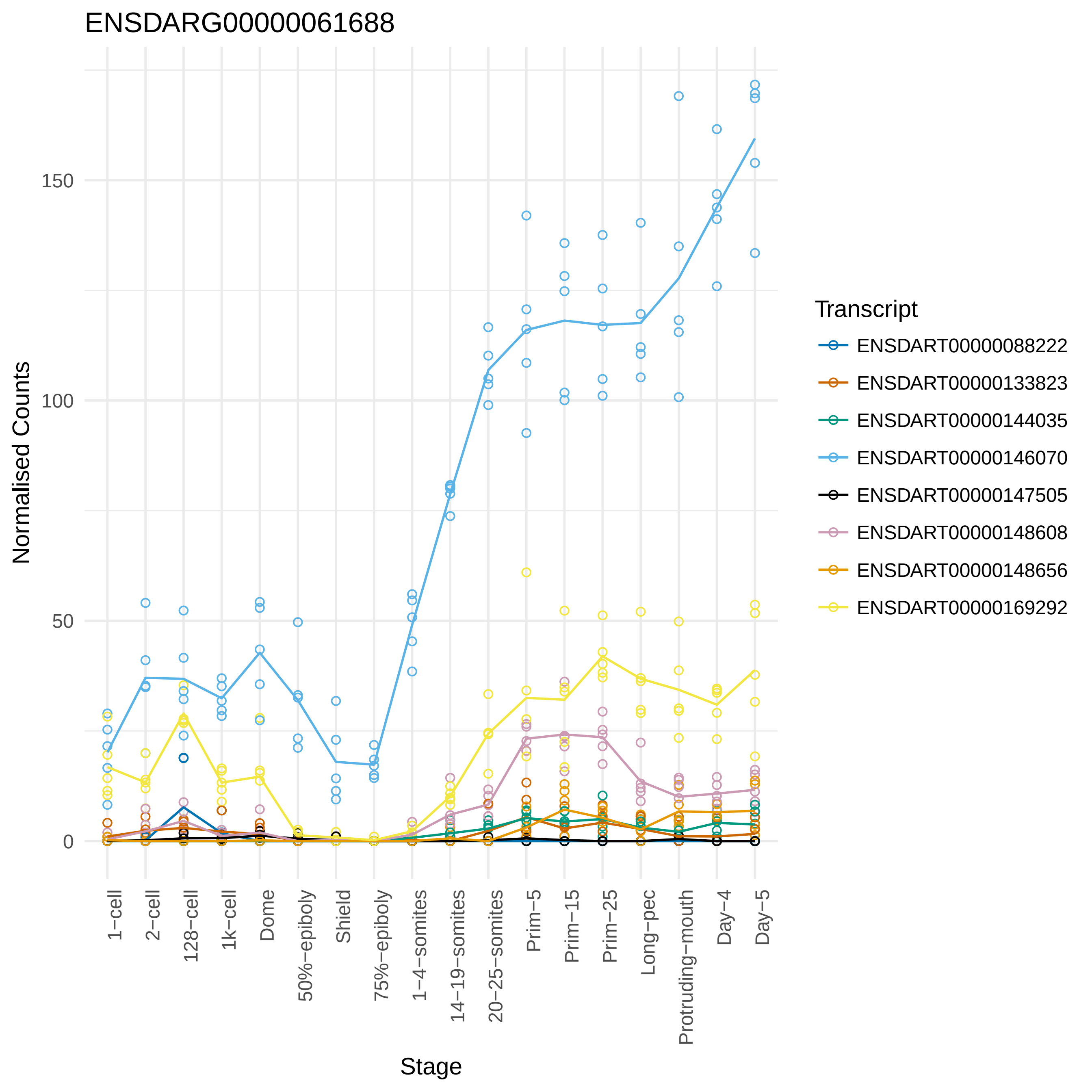

ENSDARG00000061688
Normalised Counts
150
100
50
0
1−cell
2−cell
128−cell
1k−cell
Dome
50%−epiboly
Shield
75%−epiboly
1−4−somites
14−19−somites
20−25−somites
Prim−5
Prim−15
Prim−25
Long−pec
Protruding−mouth
Day−4
Day−5
Stage
Transcript
ENSDART00000088222
ENSDART00000133823
ENSDART00000144035
ENSDART00000146070
ENSDART00000147505
ENSDART00000148608
ENSDART00000148656
ENSDART00000169292

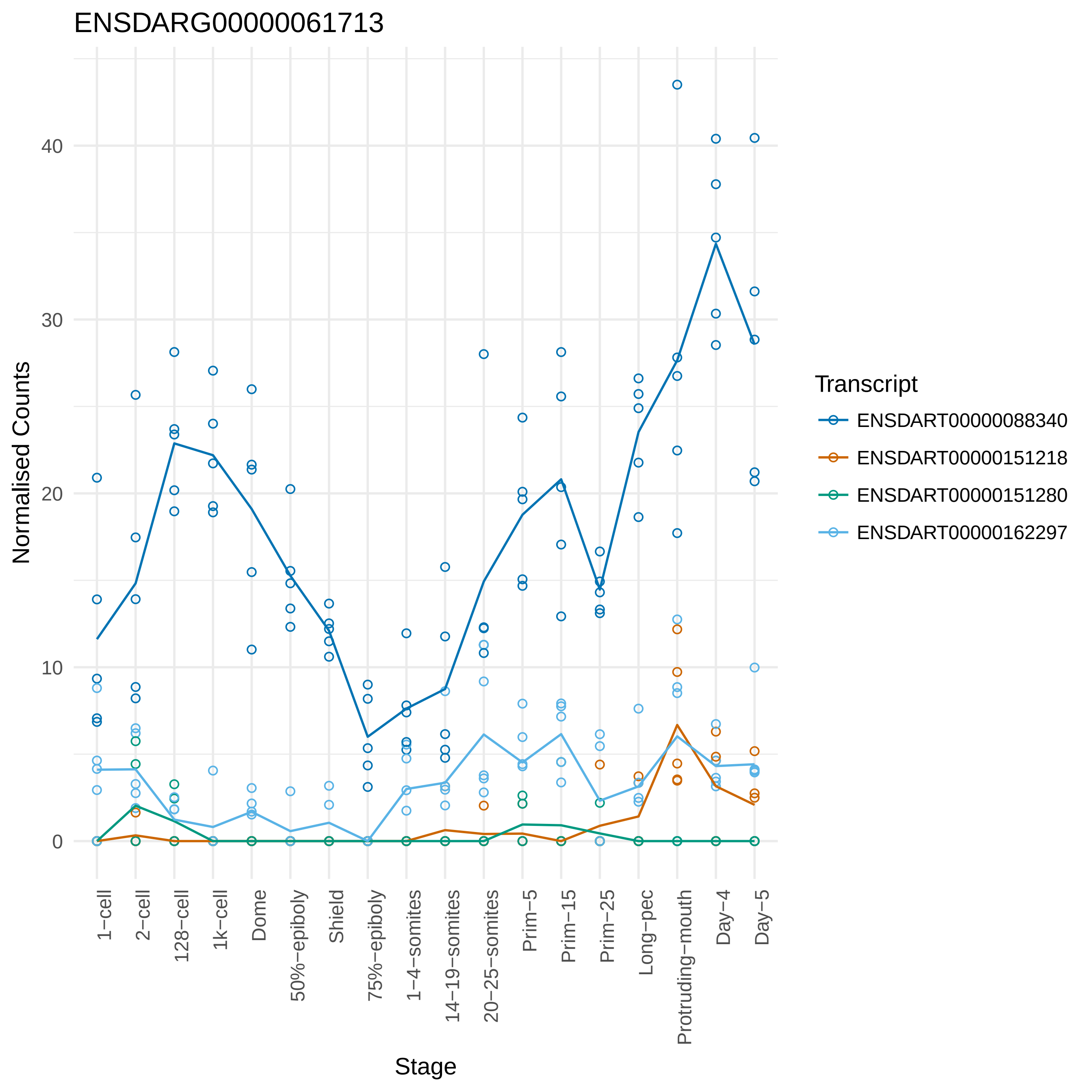

ENSDARG00000061713
Normalised Counts
0
10
20
30
40
Stage
1−cell
2−cell
128−cell
1k−cell
Dome
50%−epiboly
Shield
75%−epiboly
1−4−somites
14−19−somites
20−25−somites
Prim−5
Prim−15
Prim−25
Long−pec
Protruding−mouth
Day−4
Day−5
Transcript
ENSDART00000088340
ENSDART00000151218
ENSDART00000151280
ENSDART00000162297

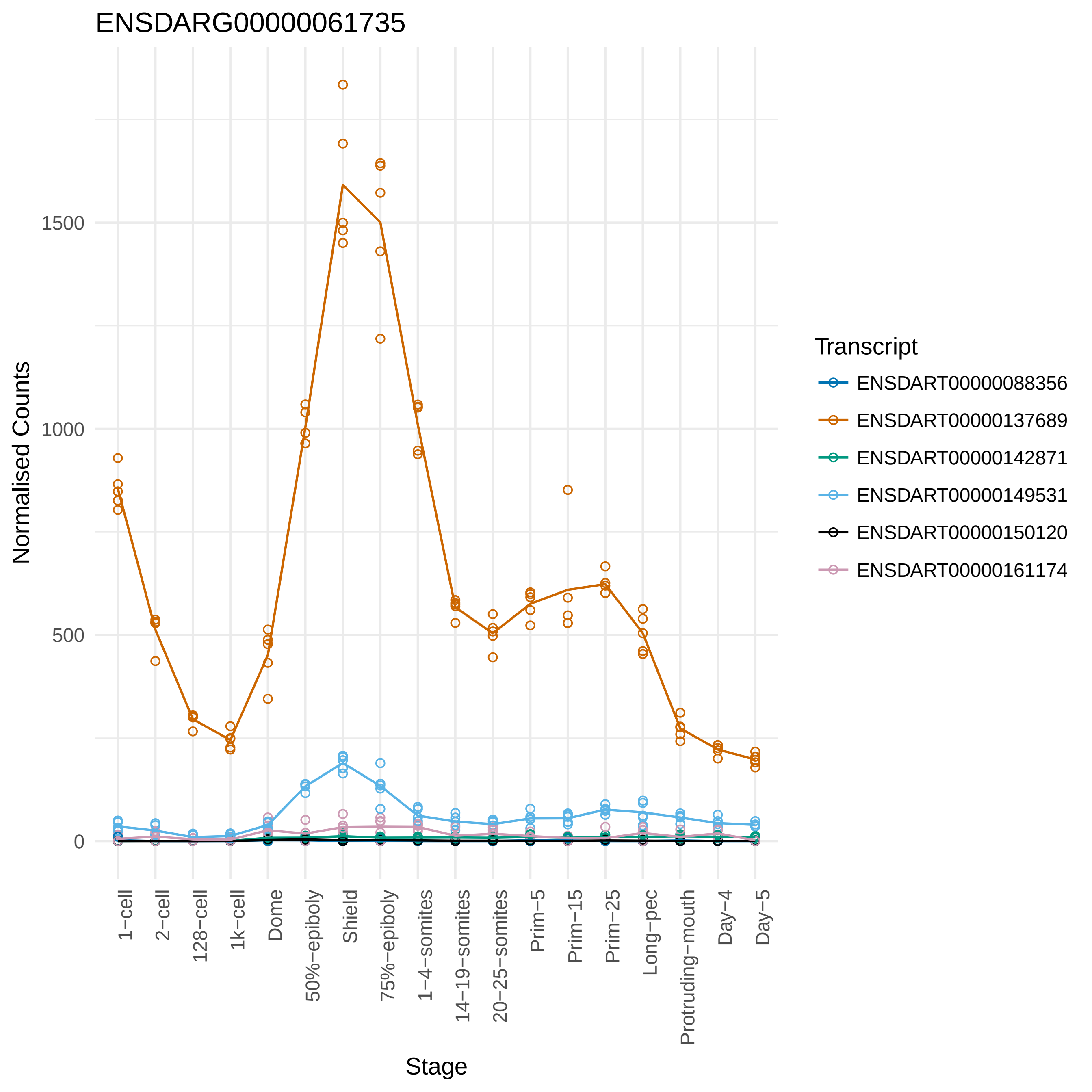

ENSDARG00000061735
Normalised Counts
0
500
1000
1500
Stage
1−cell
2−cell
128−cell
1k−cell
Dome
50%−epiboly
Shield
75%−epiboly
1−4−somites
14−19−somites
20−25−somites
Prim−5
Prim−15
Prim−25
Long−pec
Protruding−mouth
Day−4
Day−5
Transcript
ENSDART00000088356
ENSDART00000137689
ENSDART00000142871
ENSDART00000149531
ENSDART00000150120
ENSDART00000161174

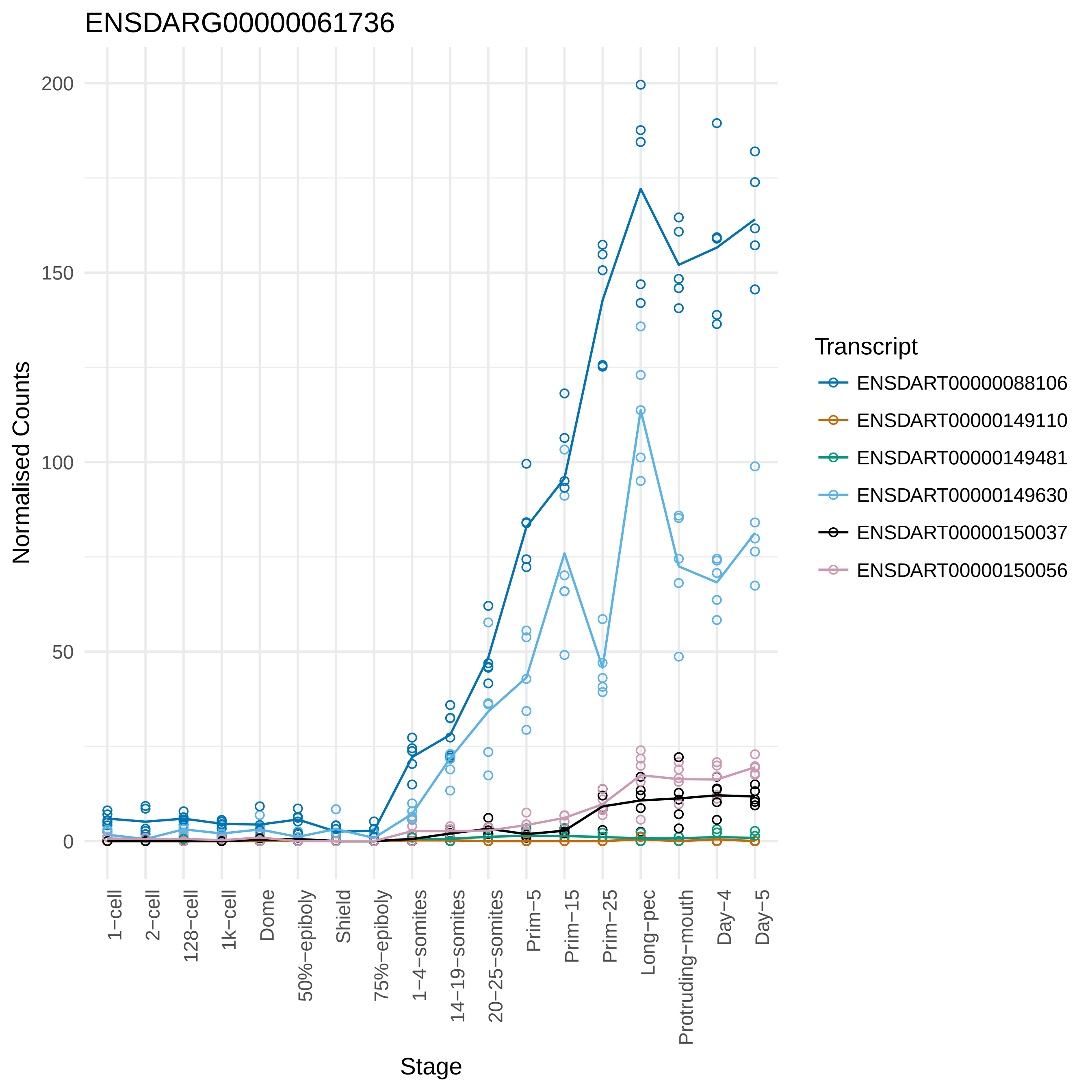
ENSDARG00000061736
Normalised Counts
200
150
100
50
0
1−cell
2−cell
128−cell
1k−cell
Dome
50%−epiboly
Shield
75%−epiboly
1−4−somites
14−19−somites
20−25−somites
Prim−5
Prim−15
Prim−25
Long−pec
Protruding−mouth
Day−4
Day−5
Stage
Transcript
ENSDART00000088106
ENSDART00000149110
ENSDART00000149481
ENSDART00000149630
ENSDART00000150037
ENSDART00000150056

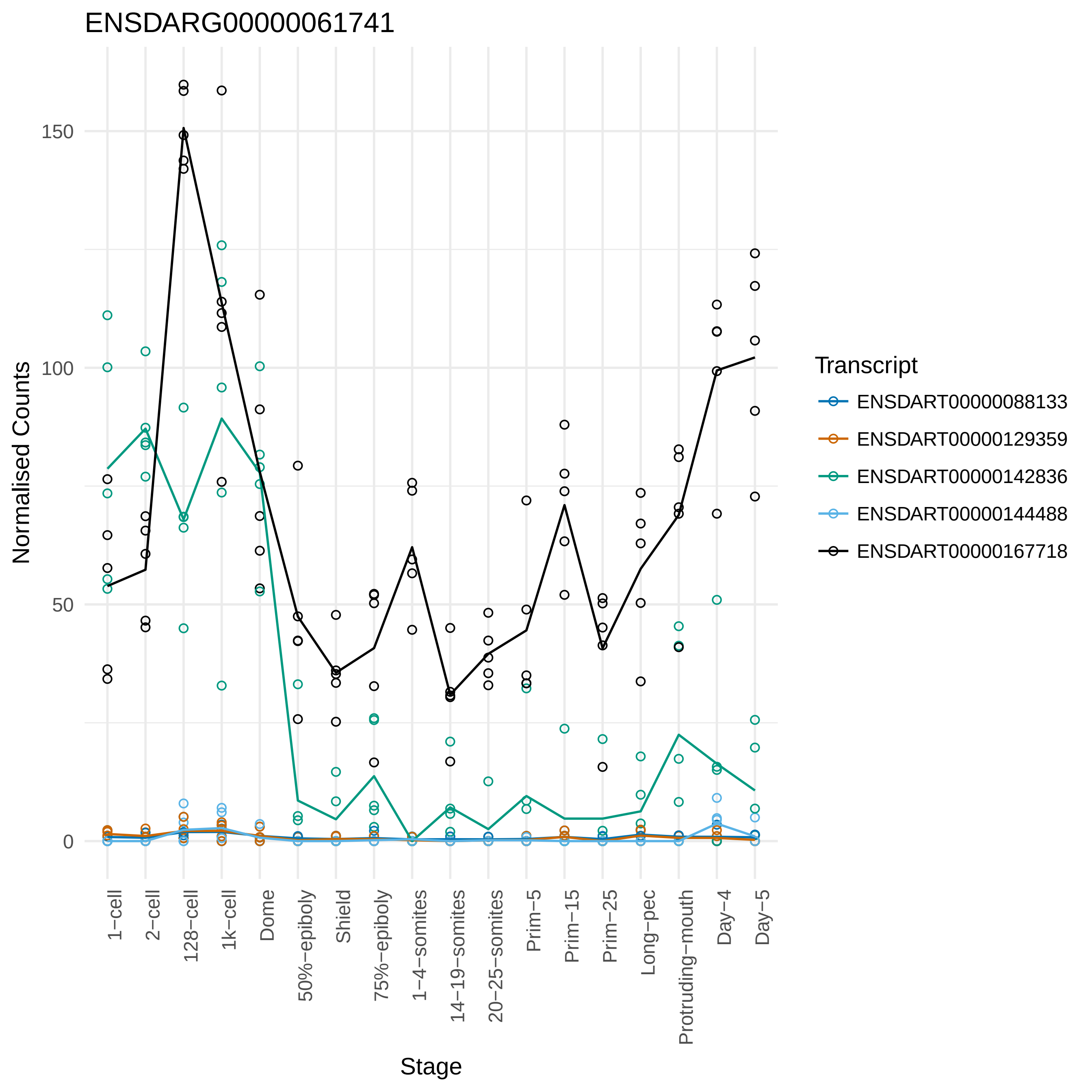
ENSDARG00000061741
Normalised Counts
150
100
50
0
1−cell
2−cell
128−cell
1k−cell
Dome
50%−epiboly
Shield
75%−epiboly
1−4−somites
14−19−somites
20−25−somites
Prim−5
Prim−15
Prim−25
Long−pec
Protruding−mouth
Day−4
Day−5
Stage
Transcript
ENSDART00000088133
ENSDART00000129359
ENSDART00000142836
ENSDART00000144488
ENSDART00000167718

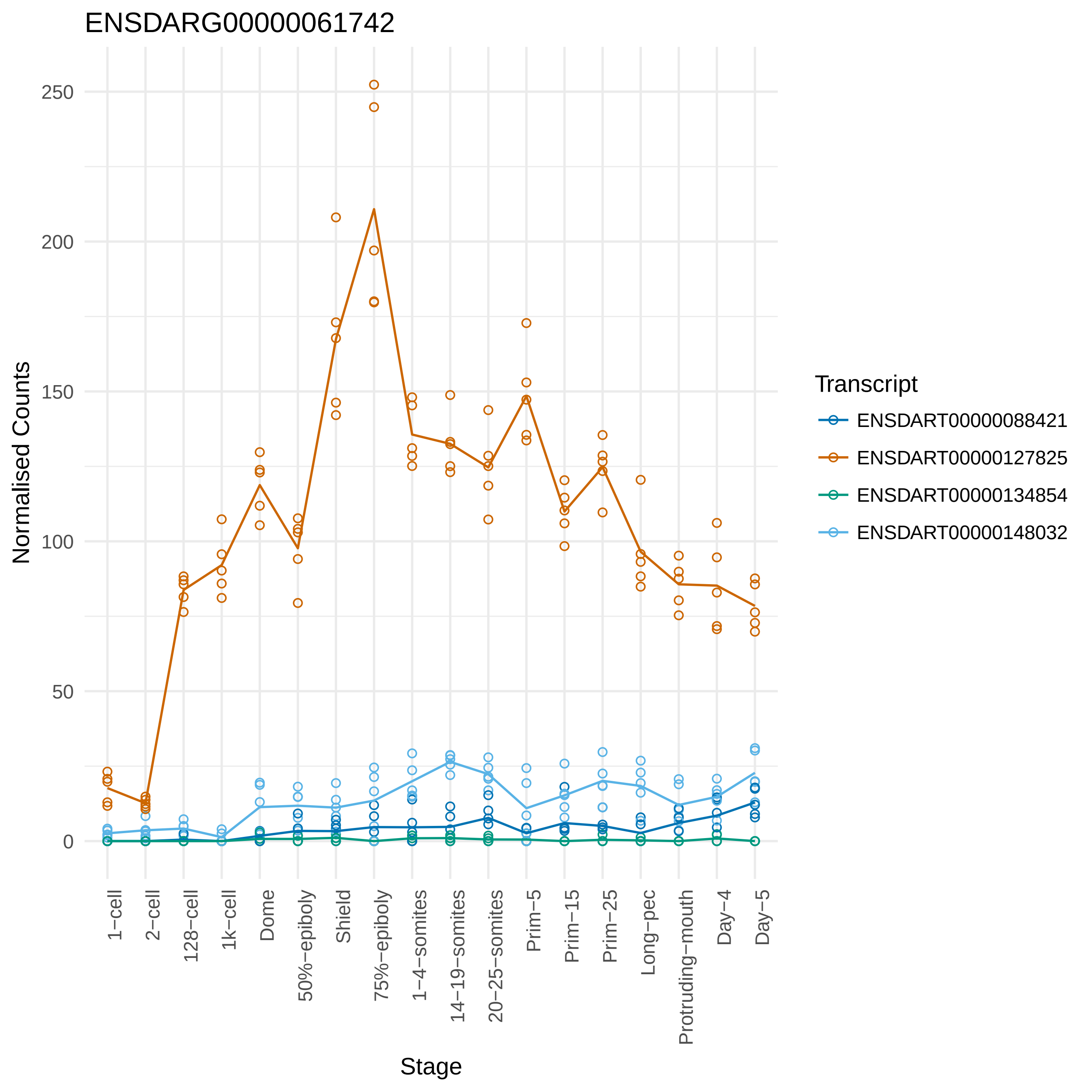

ENSDARG00000061742
Normalised Counts
250
200
150
100
50
0
1−cell
2−cell
128−cell
1k−cell
Dome
50%−epiboly
Shield
75%−epiboly
1−4−somites
14−19−somites
20−25−somites
Prim−5
Prim−15
Prim−25
Long−pec
Protruding−mouth
Day−4
Day−5
Stage
Transcript
ENSDART00000088421
ENSDART00000127825
ENSDART00000134854
ENSDART00000148032

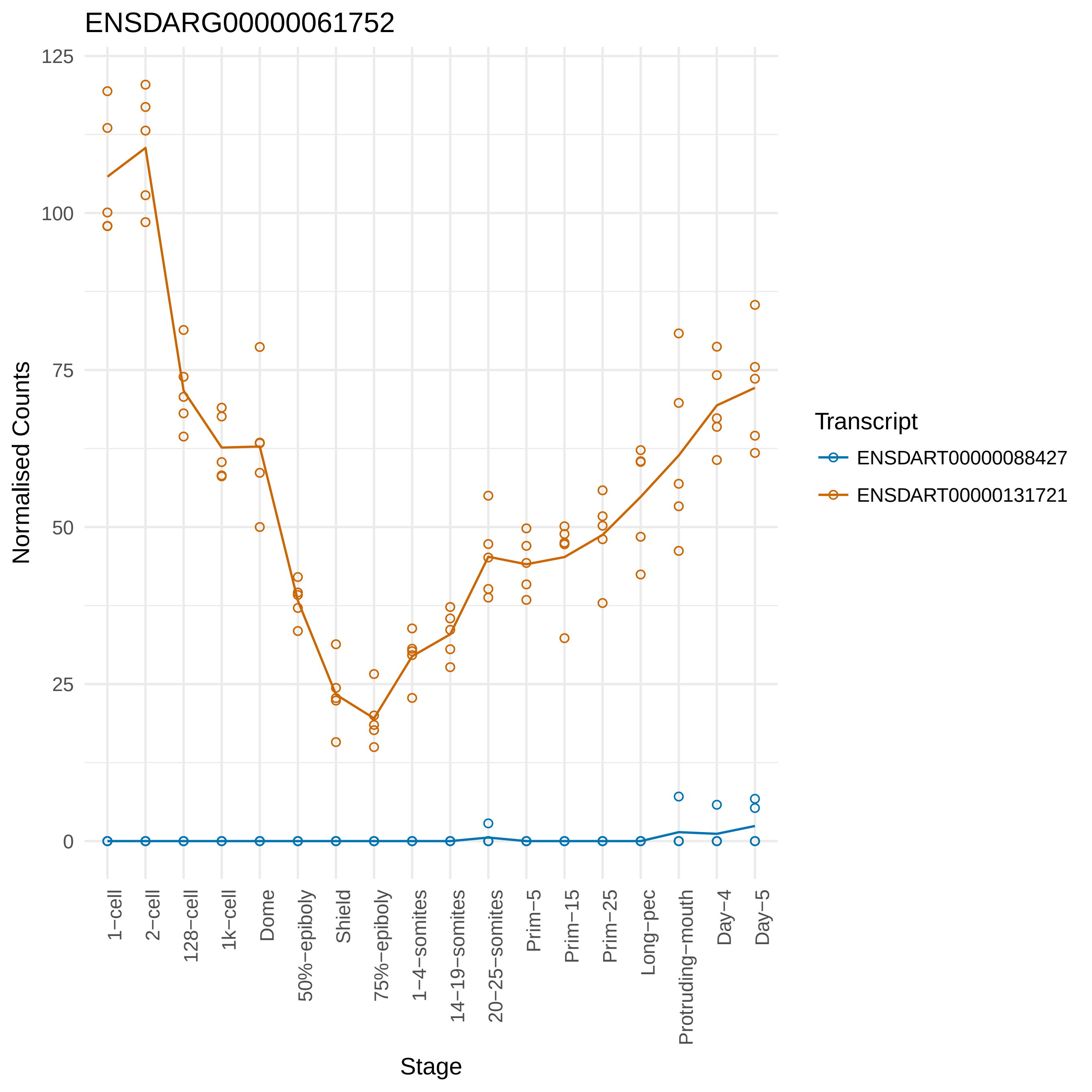
ENSDARG00000061752
Normalised Counts
125
100
75
50
25
0
1−cell
2−cell
128−cell
1k−cell
Dome
50%−epiboly
Shield
75%−epiboly
1−4−somites
14−19−somites
20−25−somites
Prim−5
Prim−15
Prim−25
Long−pec
Protruding−mouth
Day−4
Day−5
Stage
Transcript
ENSDART00000088427
ENSDART00000131721

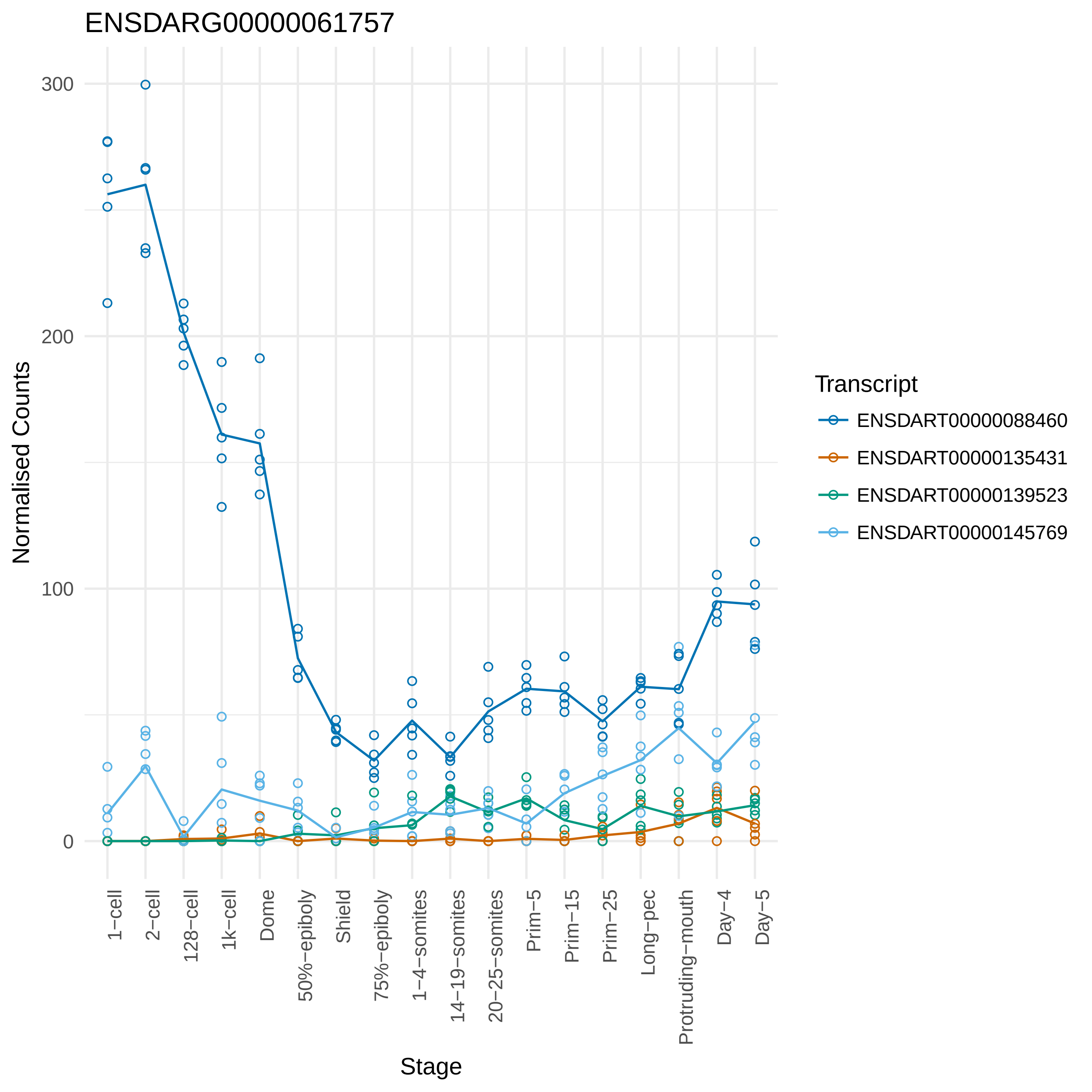

ENSDARG00000061757
Normalised Counts
300
200
100
0
1−cell
2−cell
128−cell
1k−cell
Dome
50%−epiboly
Shield
75%−epiboly
1−4−somites
14−19−somites
20−25−somites
Prim−5
Prim−15
Prim−25
Long−pec
Protruding−mouth
Day−4
Day−5
Stage
Transcript
ENSDART00000088460
ENSDART00000135431
ENSDART00000139523
ENSDART00000145769

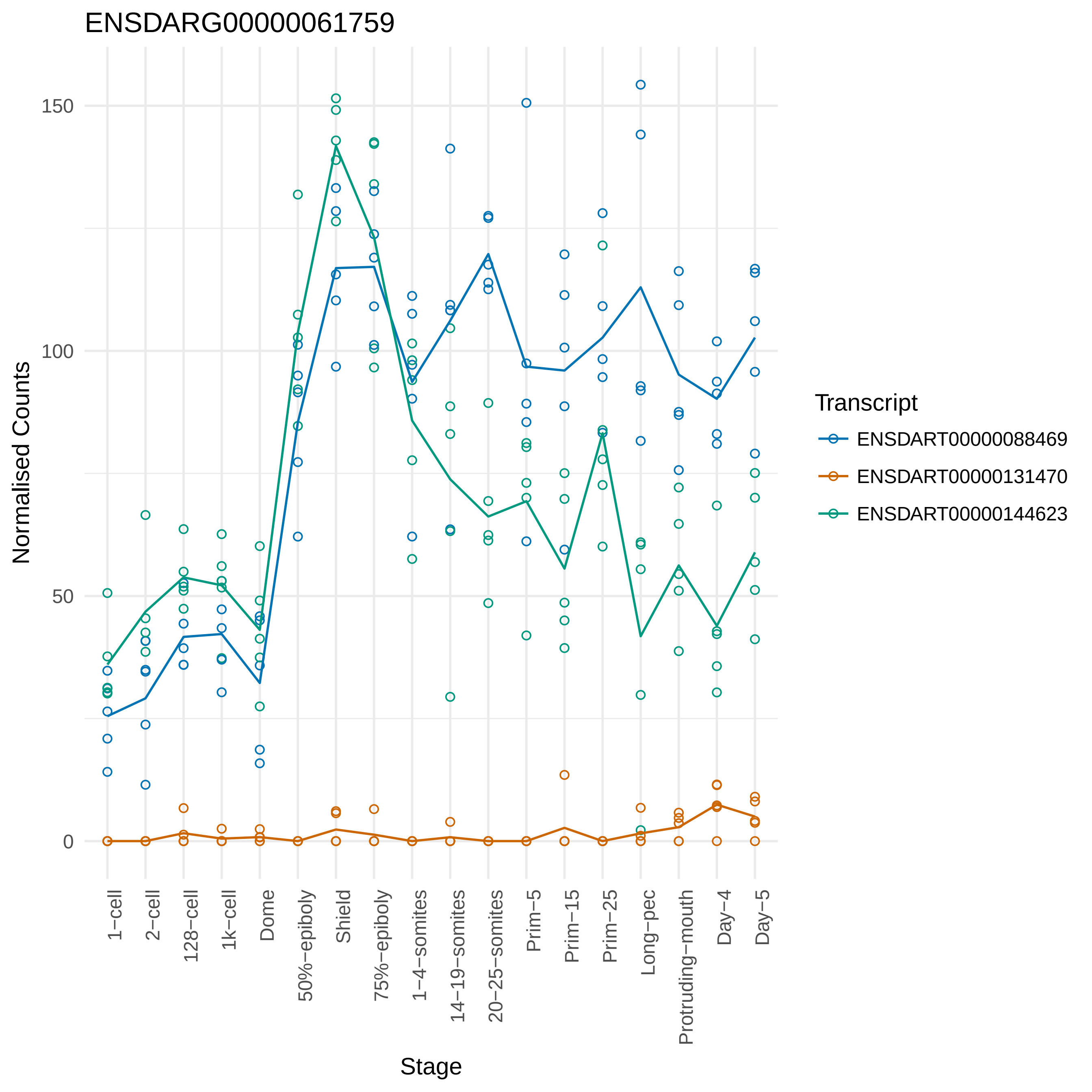
ENSDARG00000061759
Normalised Counts
150
100
50
0
1−cell
2−cell
128−cell
1k−cell
Dome
50%−epiboly
Shield
75%−epiboly
1−4−somites
14−19−somites
20−25−somites
Prim−5
Prim−15
Prim−25
Long−pec
Protruding−mouth
Day−4
Day−5
Stage
Transcript
ENSDART00000088469
ENSDART00000131470
ENSDART00000144623

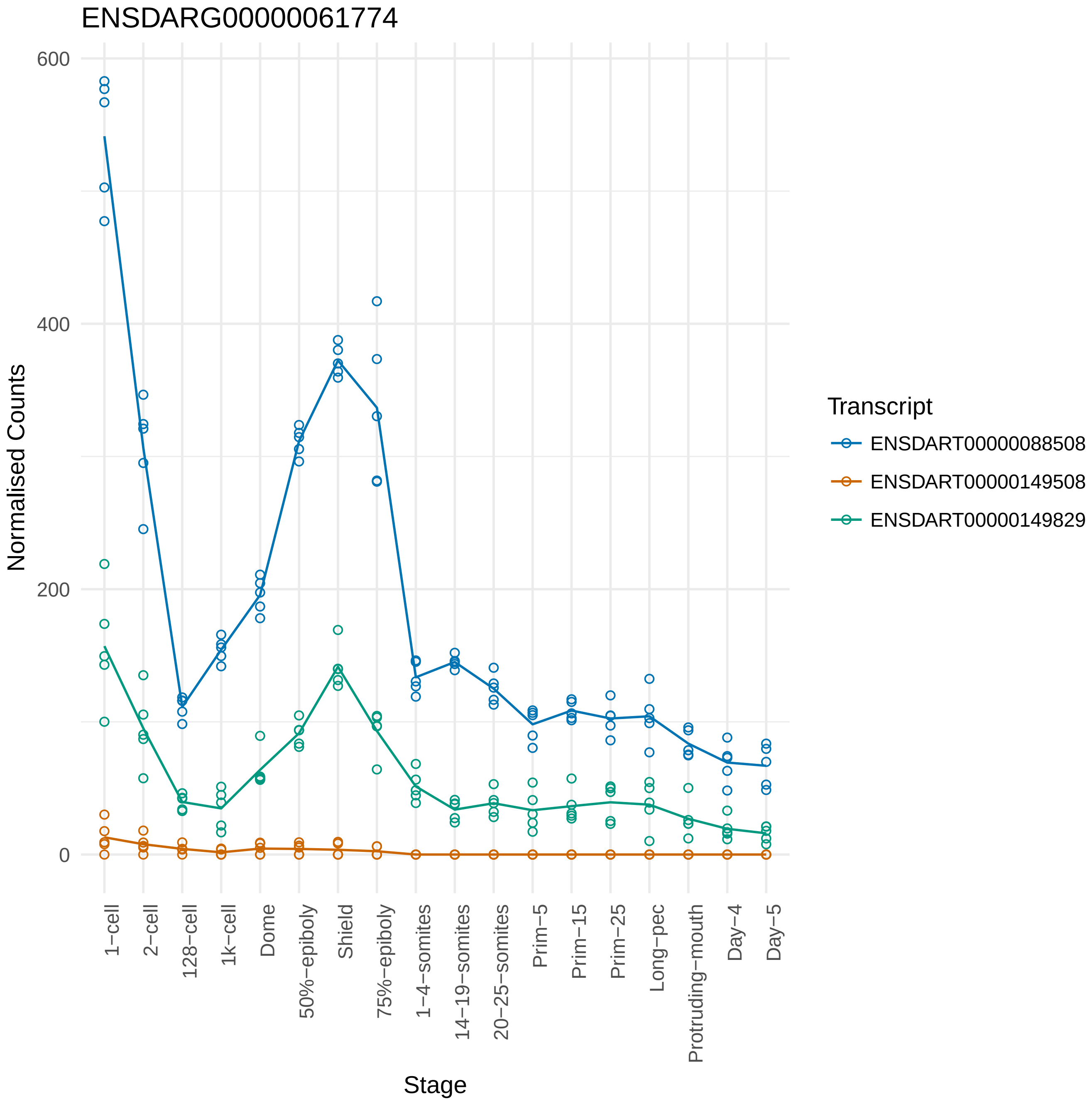

ENSDARG00000061774
Normalised Counts
600
400
200
0
1−cell
2−cell
128−cell
1k−cell
Dome
50%−epiboly
Shield
75%−epiboly
1−4−somites
14−19−somites
20−25−somites
Prim−5
Prim−15
Prim−25
Long−pec
Protruding−mouth
Day−4
Day−5
Stage
Transcript
ENSDART00000088508
ENSDART00000149508
ENSDART00000149829

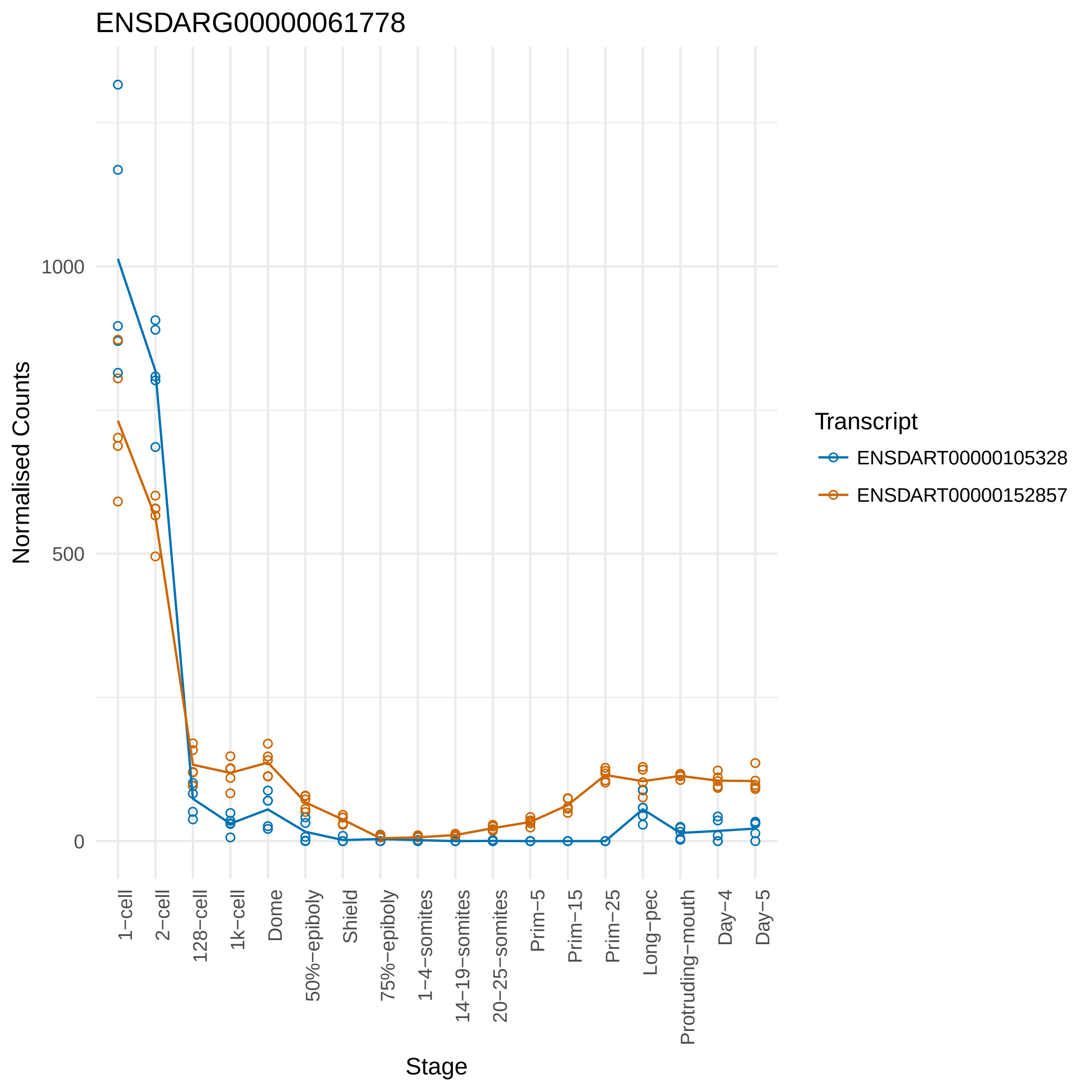
ENSDARG00000061778
Normalised Counts
1000
500
0
1−cell
2−cell
128−cell
1k−cell
Dome
50%−epiboly
Shield
75%−epiboly
1−4−somites
14−19−somites
20−25−somites
Prim−5
Prim−15
Prim−25
Long−pec
Protruding−mouth
Day−4
Day−5
Stage
Transcript
ENSDART00000105328
ENSDART00000152857

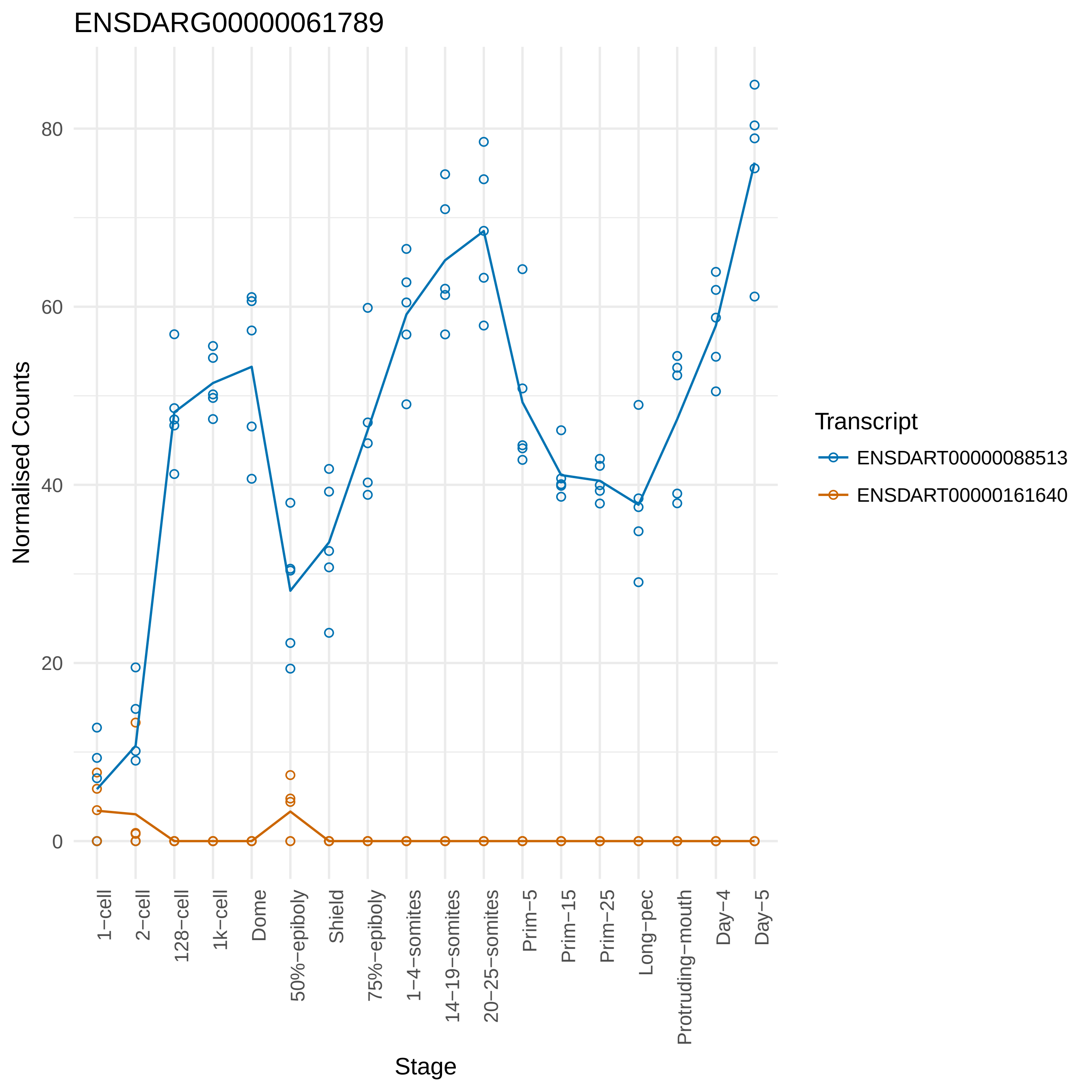
ENSDARG00000061789
Normalised Counts
0
20
40
60
80
Stage
1−cell
2−cell
128−cell
1k−cell
Dome
50%−epiboly
Shield
75%−epiboly
1−4−somites
14−19−somites
20−25−somites
Prim−5
Prim−15
Prim−25
Long−pec
Protruding−mouth
Day−4
Day−5
Transcript
ENSDART00000088513
ENSDART00000161640

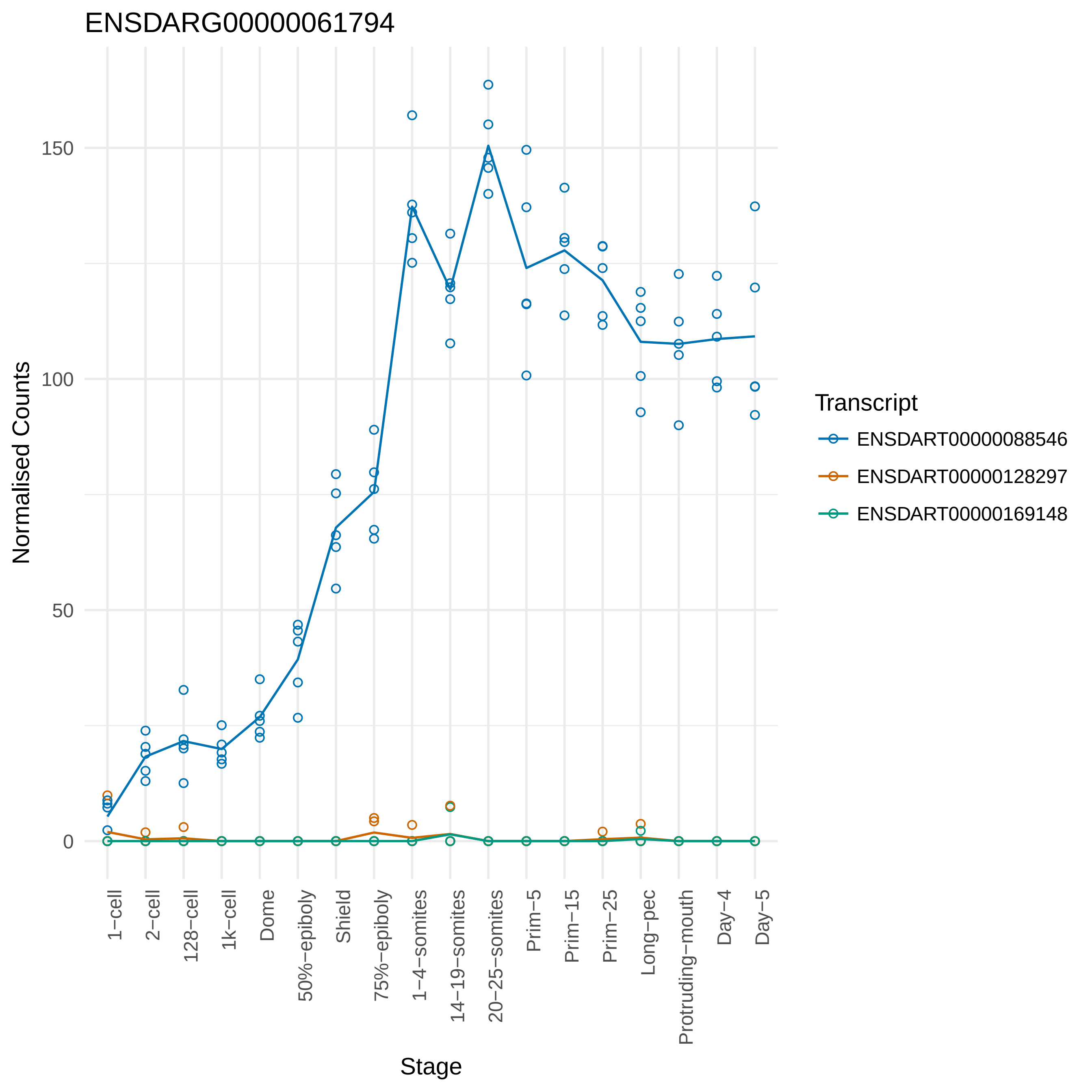

ENSDARG00000061794
Normalised Counts
150
100
50
0
1−cell
2−cell
128−cell
1k−cell
Dome
50%−epiboly
Shield
75%−epiboly
1−4−somites
14−19−somites
20−25−somites
Prim−5
Prim−15
Prim−25
Long−pec
Protruding−mouth
Day−4
Day−5
Stage
Transcript
ENSDART00000088546
ENSDART00000128297
ENSDART00000169148

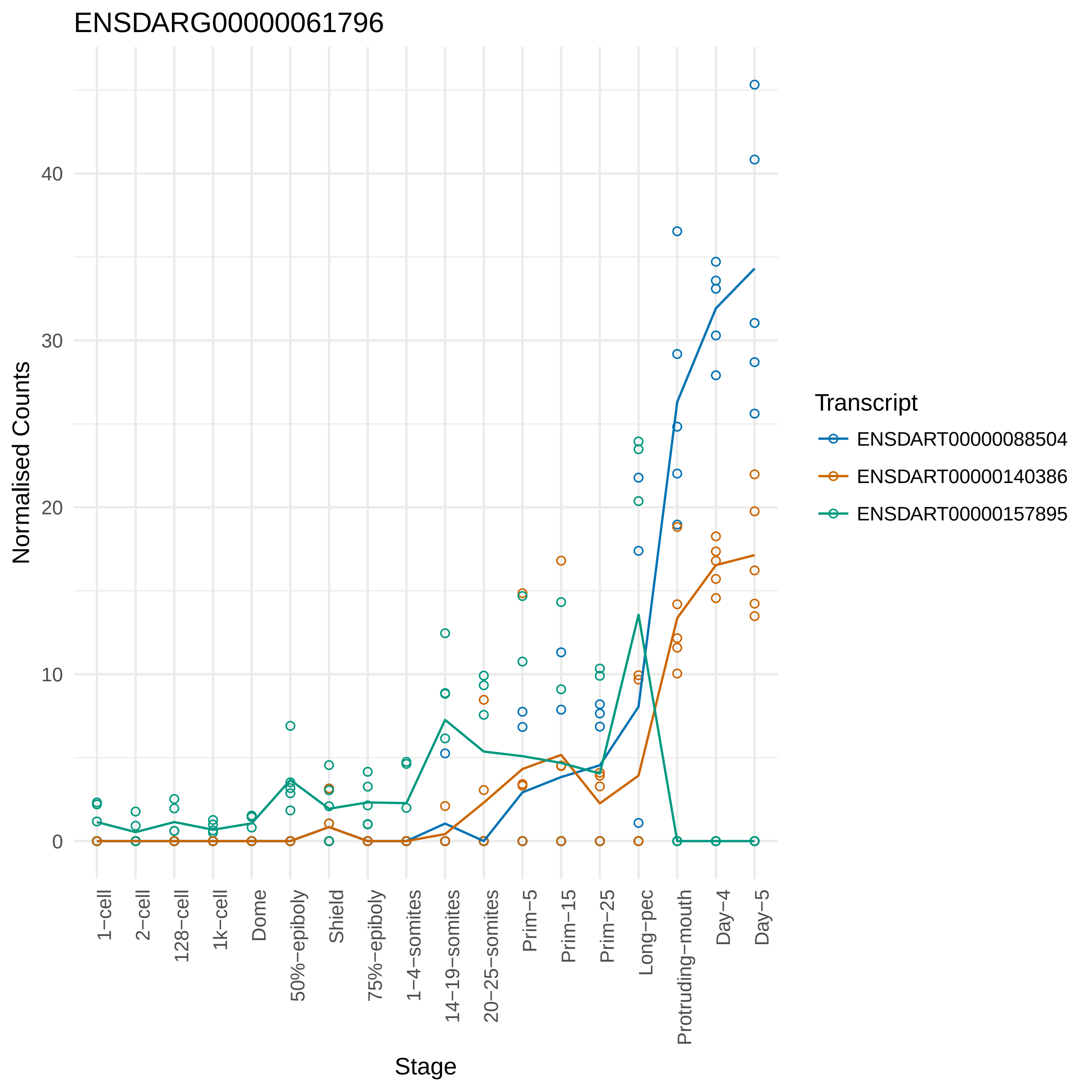

ENSDARG00000061796
Normalised Counts
0
10
20
30
40
Stage
1−cell
2−cell
128−cell
1k−cell
Dome
50%−epiboly
Shield
75%−epiboly
1−4−somites
14−19−somites
20−25−somites
Prim−5
Prim−15
Prim−25
Long−pec
Protruding−mouth
Day−4
Day−5
Transcript
ENSDART00000088504
ENSDART00000140386
ENSDART00000157895

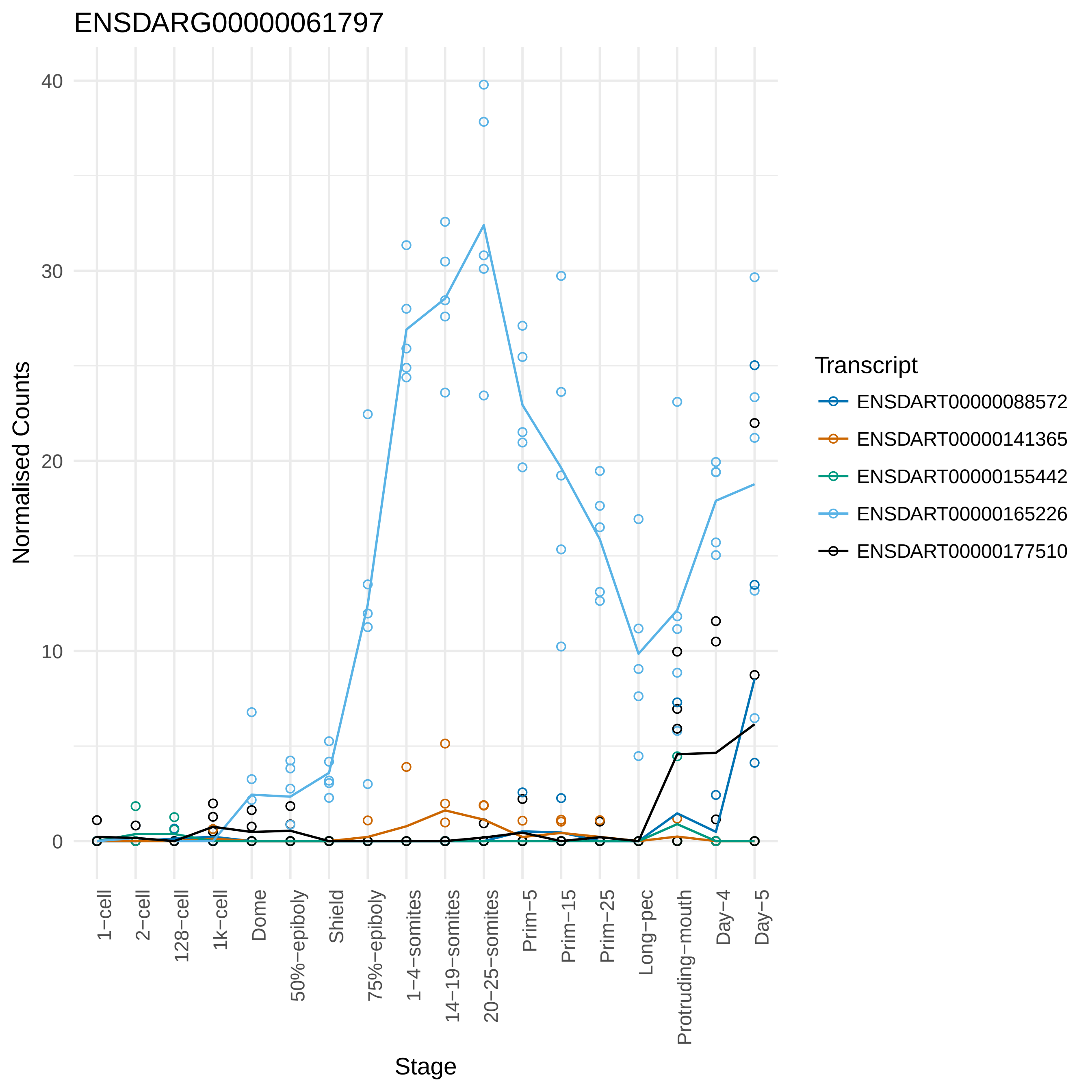

ENSDARG00000061797
Normalised Counts
40
30
20
10
0
1−cell
2−cell
128−cell
1k−cell
Dome
50%−epiboly
Shield
75%−epiboly
1−4−somites
14−19−somites
20−25−somites
Prim−5
Prim−15
Prim−25
Long−pec
Protruding−mouth
Day−4
Day−5
Stage
Transcript
ENSDART00000088572
ENSDART00000141365
ENSDART00000155442
ENSDART00000165226
ENSDART00000177510

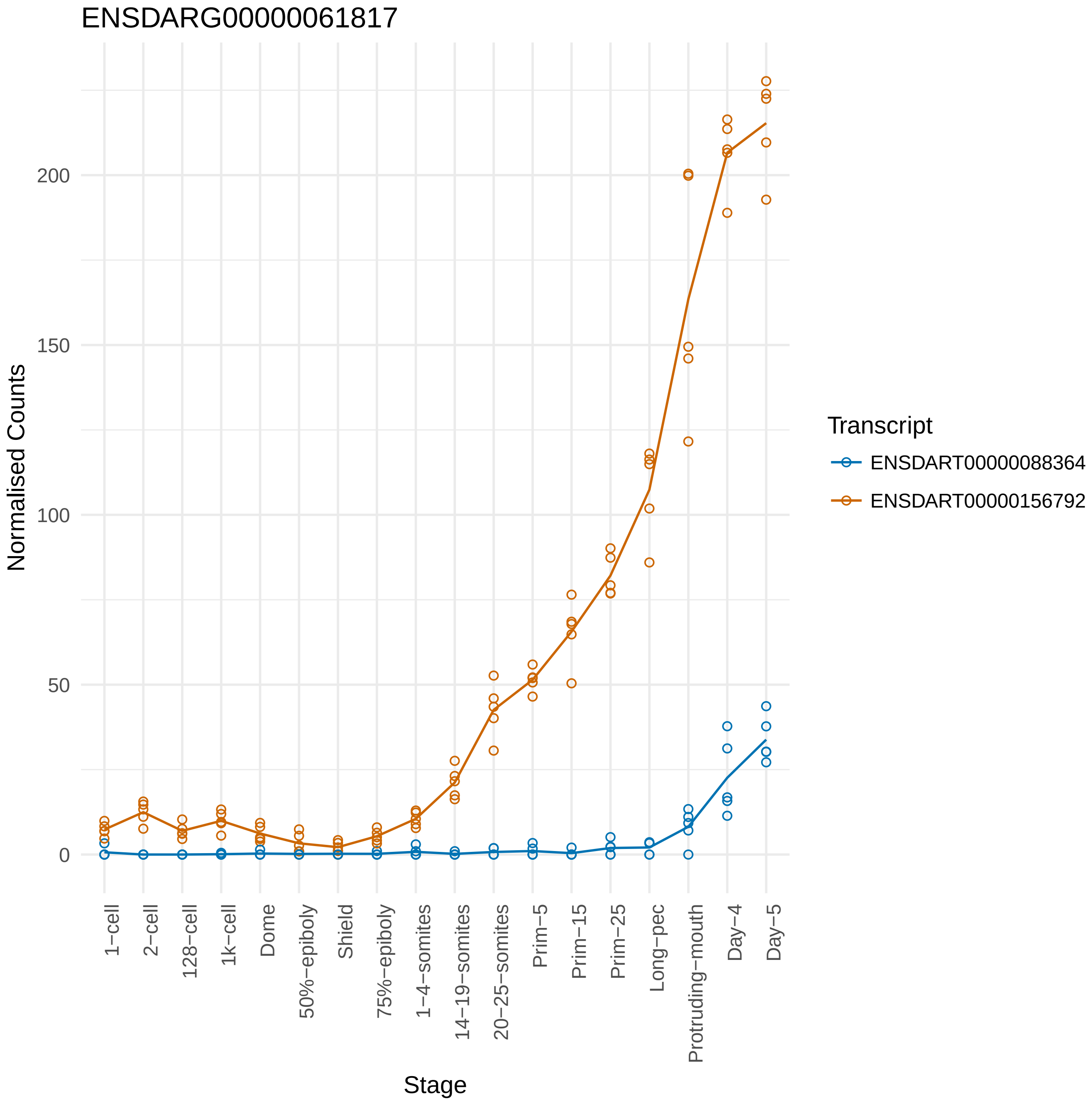

ENSDARG00000061817
Normalised Counts
0
50
100
150
200
Stage
1−cell
2−cell
128−cell
1k−cell
Dome
50%−epiboly
Shield
75%−epiboly
1−4−somites
14−19−somites
20−25−somites
Prim−5
Prim−15
Prim−25
Long−pec
Protruding−mouth
Day−4
Day−5
Transcript
ENSDART00000088364
ENSDART00000156792

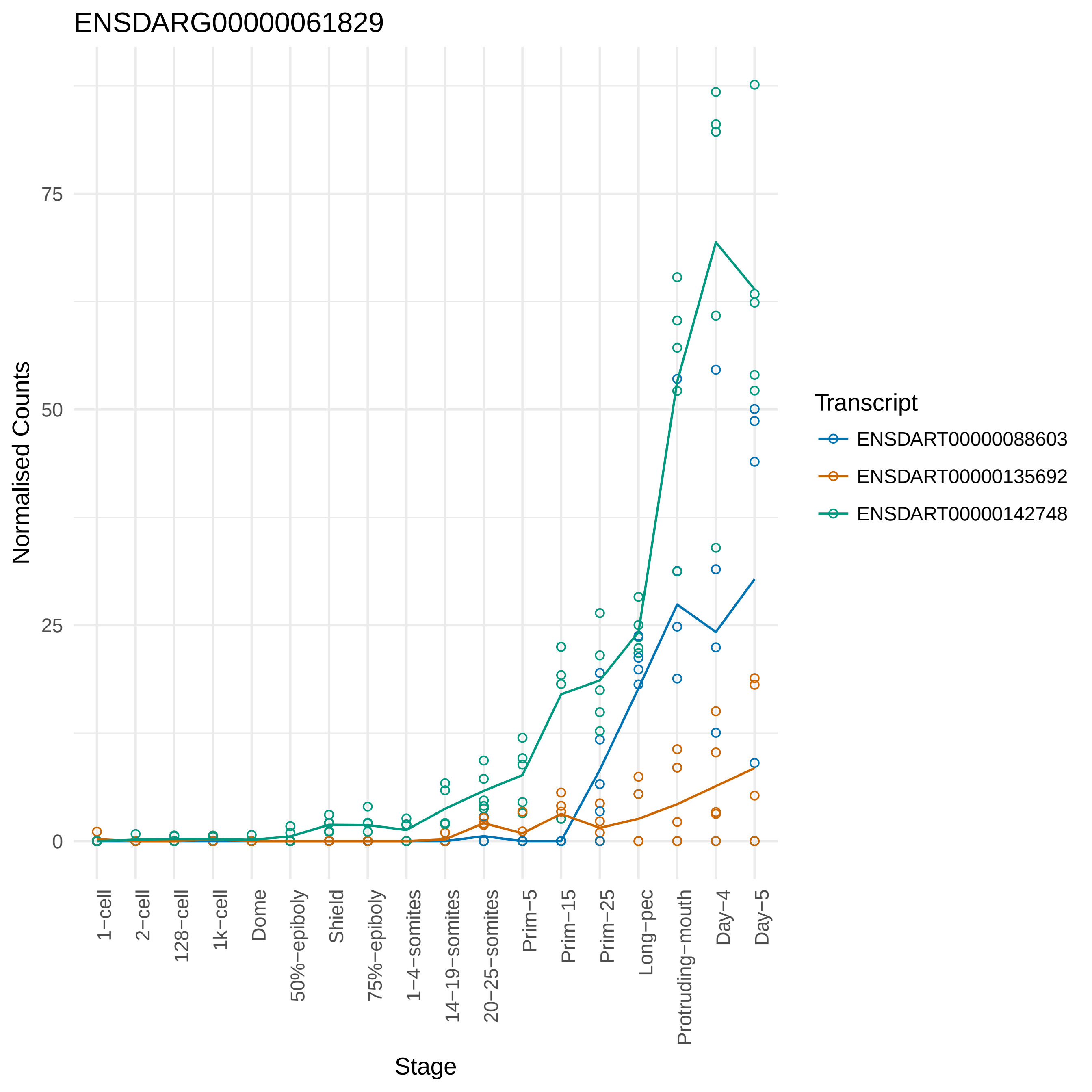
ENSDARG00000061829
Normalised Counts
75
50
25
0
1−cell
2−cell
128−cell
1k−cell
Dome
50%−epiboly
Shield
75%−epiboly
1−4−somites
14−19−somites
20−25−somites
Prim−5
Prim−15
Prim−25
Long−pec
Protruding−mouth
Day−4
Day−5
Stage
Transcript
ENSDART00000088603
ENSDART00000135692
ENSDART00000142748

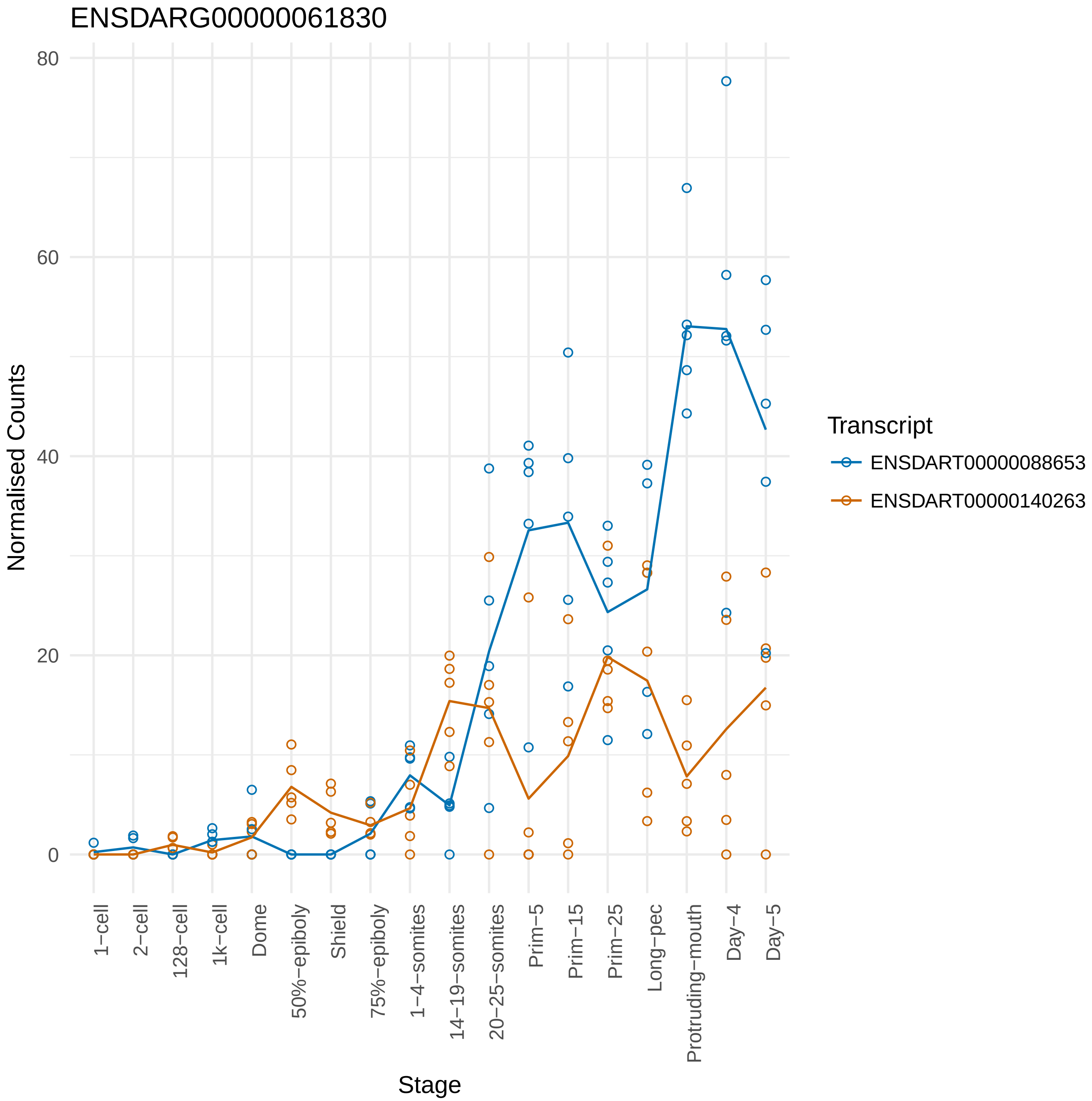

ENSDARG00000061830
Normalised Counts
0
20
40
60
80
Stage
1−cell
2−cell
128−cell
1k−cell
Dome
50%−epiboly
Shield
75%−epiboly
1−4−somites
14−19−somites
20−25−somites
Prim−5
Prim−15
Prim−25
Long−pec
Protruding−mouth
Day−4
Day−5
Transcript
ENSDART00000088653
ENSDART00000140263

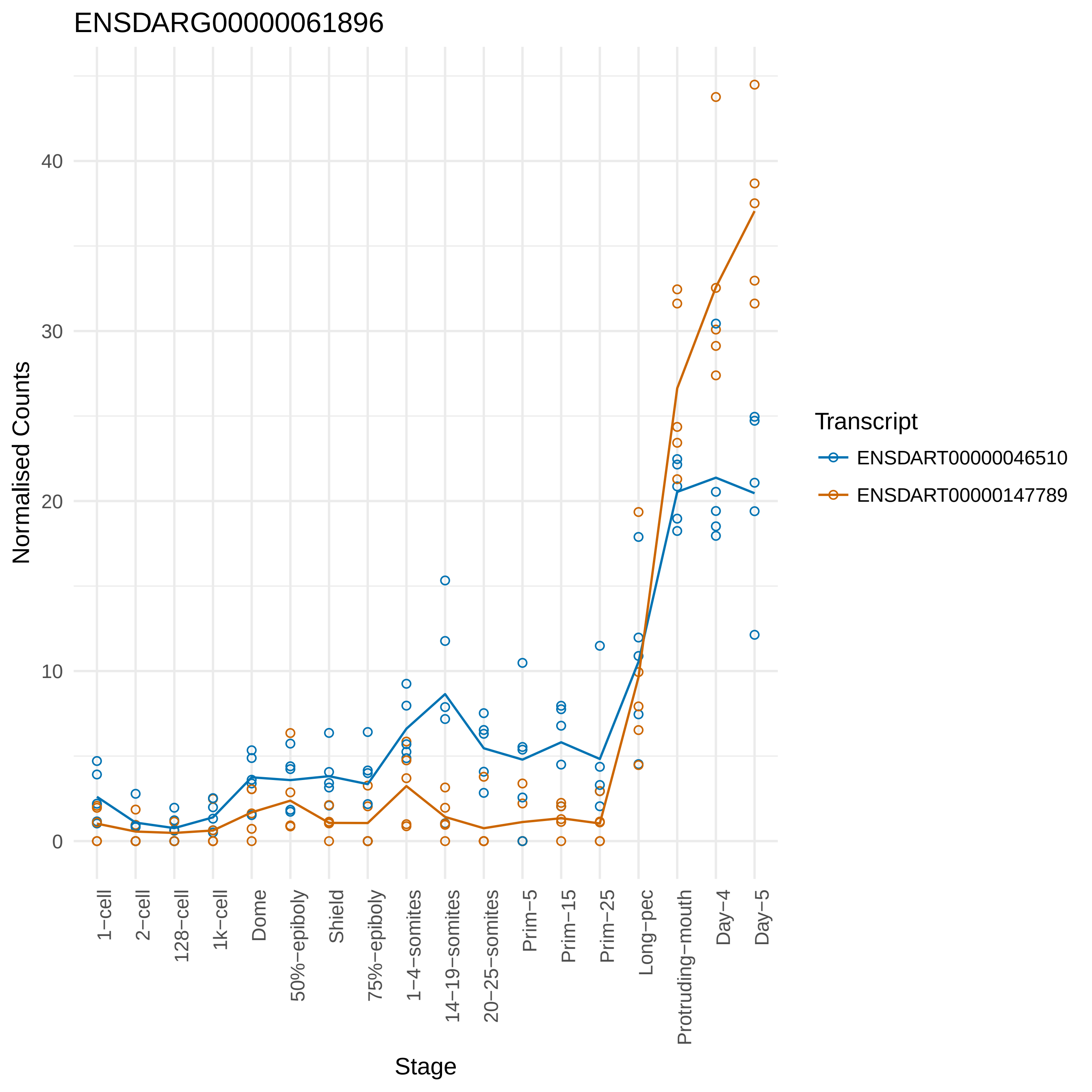
ENSDARG00000061896
Normalised Counts
0
10
20
30
40
1−cell
2−cell
128−cell
1k−cell
Dome
50%−epiboly
Shield
75%−epiboly
1−4−somites
14−19−somites
20−25−somites
Prim−5
Prim−15
Prim−25
Long−pec
Protruding−mouth
Day−4
Day−5
Stage
Transcript
ENSDART00000046510
ENSDART00000147789

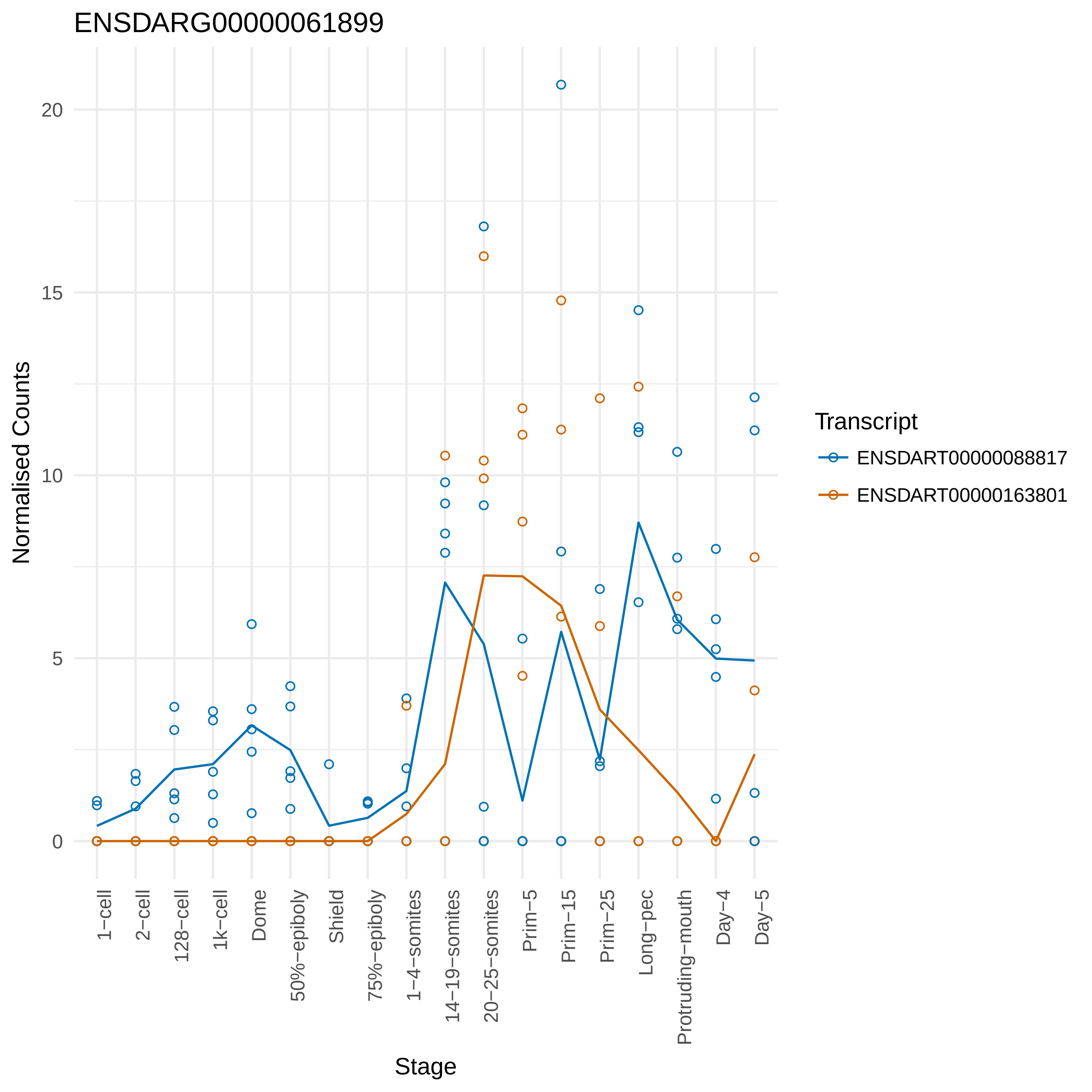
ENSDARG00000061899
Normalised Counts
0
5
10
15
20
1−cell
2−cell
128−cell
1k−cell
Dome
50%−epiboly
Shield
75%−epiboly
1−4−somites
14−19−somites
20−25−somites
Prim−5
Prim−15
Prim−25
Long−pec
Protruding−mouth
Day−4
Day−5
Stage
Transcript
ENSDART00000088817
ENSDART00000163801

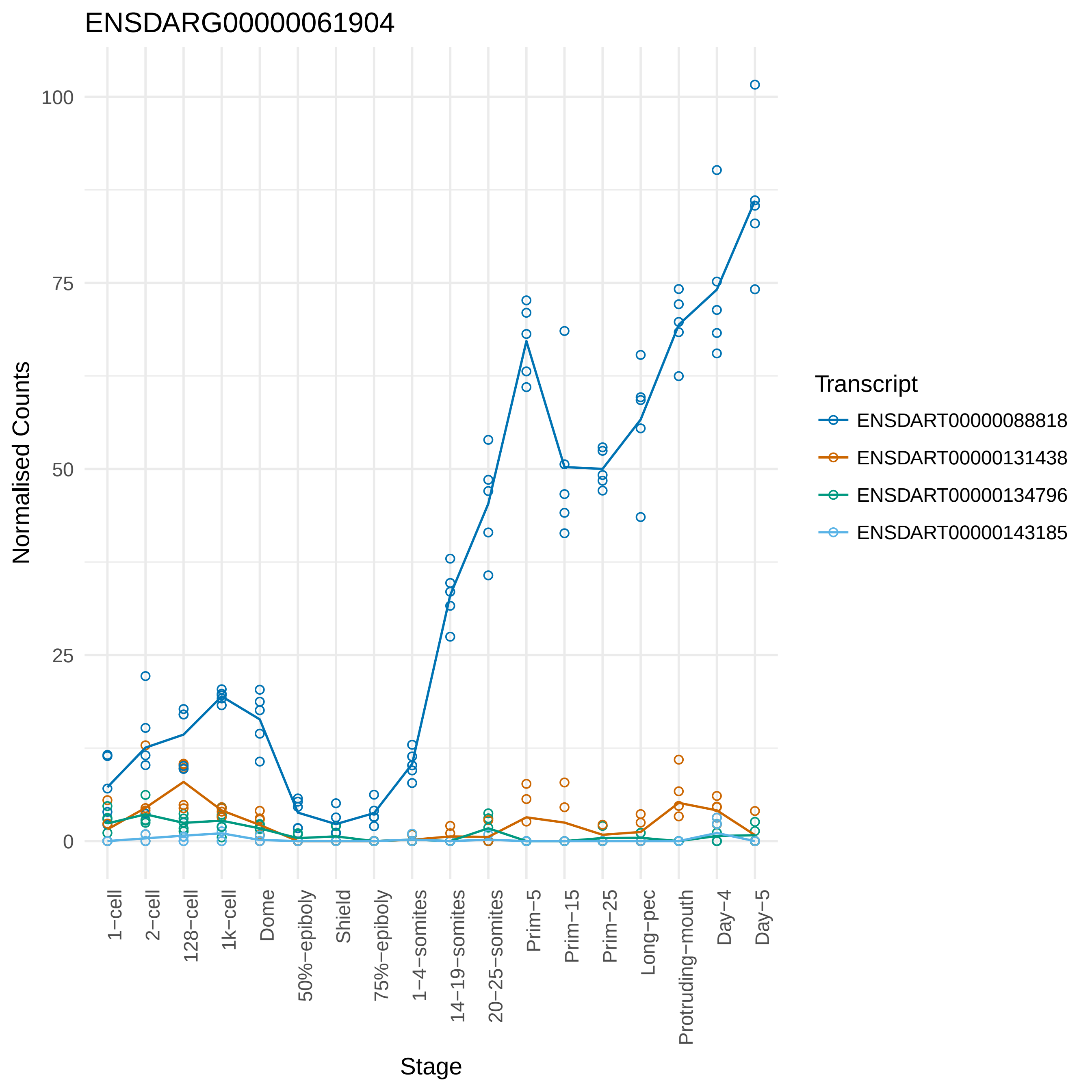
ENSDARG00000061904
Normalised Counts
100
75
50
25
0
1−cell
2−cell
128−cell
1k−cell
Dome
50%−epiboly
Shield
75%−epiboly
1−4−somites
14−19−somites
20−25−somites
Prim−5
Prim−15
Prim−25
Long−pec
Protruding−mouth
Day−4
Day−5
Stage
Transcript
ENSDART00000088818
ENSDART00000131438
ENSDART00000134796
ENSDART00000143185

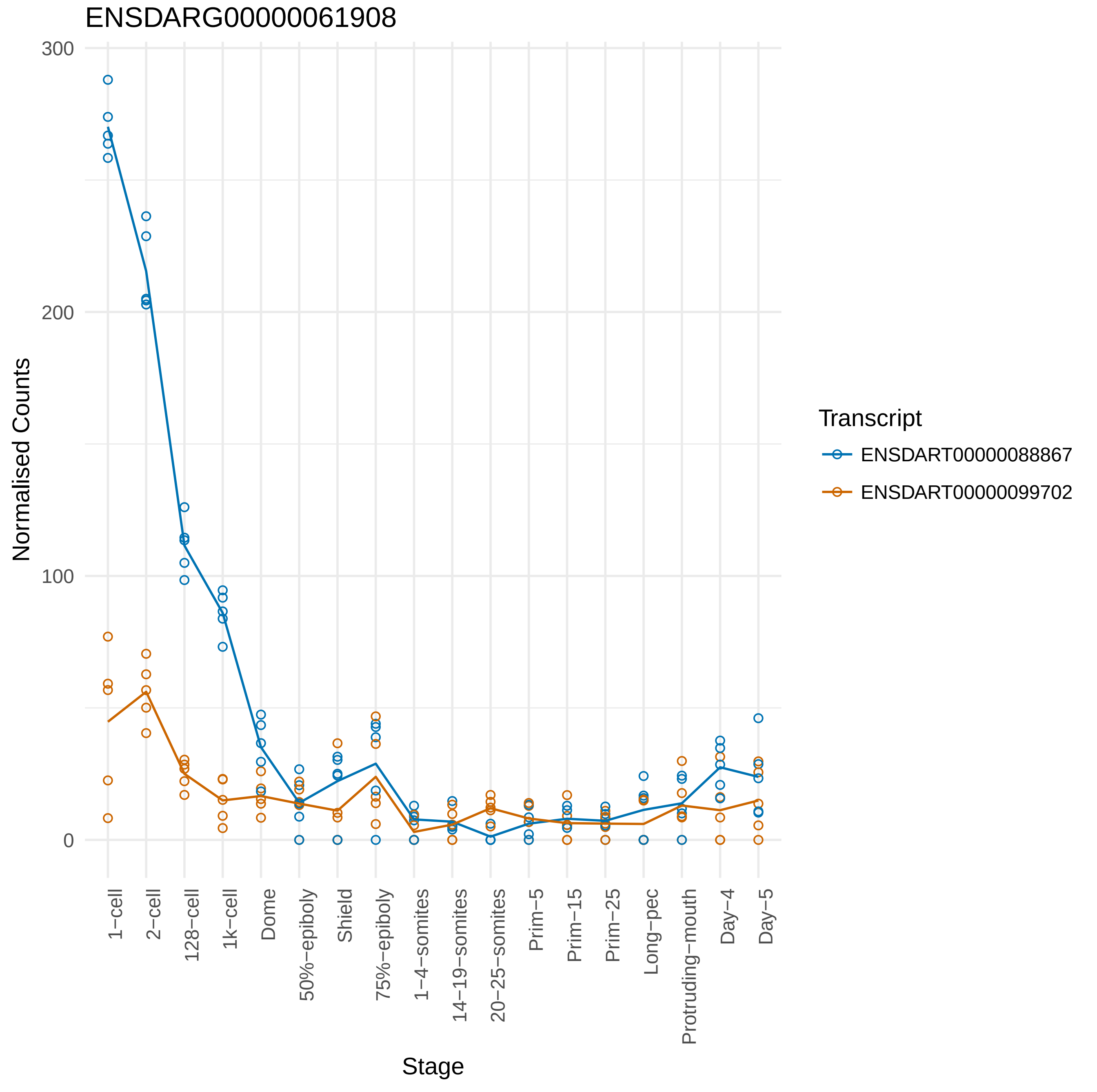
ENSDARG00000061908
Normalised Counts
300
200
100
0
1−cell
2−cell
128−cell
1k−cell
Dome
50%−epiboly
Shield
75%−epiboly
1−4−somites
14−19−somites
20−25−somites
Prim−5
Prim−15
Prim−25
Long−pec
Protruding−mouth
Day−4
Day−5
Stage
Transcript
ENSDART00000088867
ENSDART00000099702

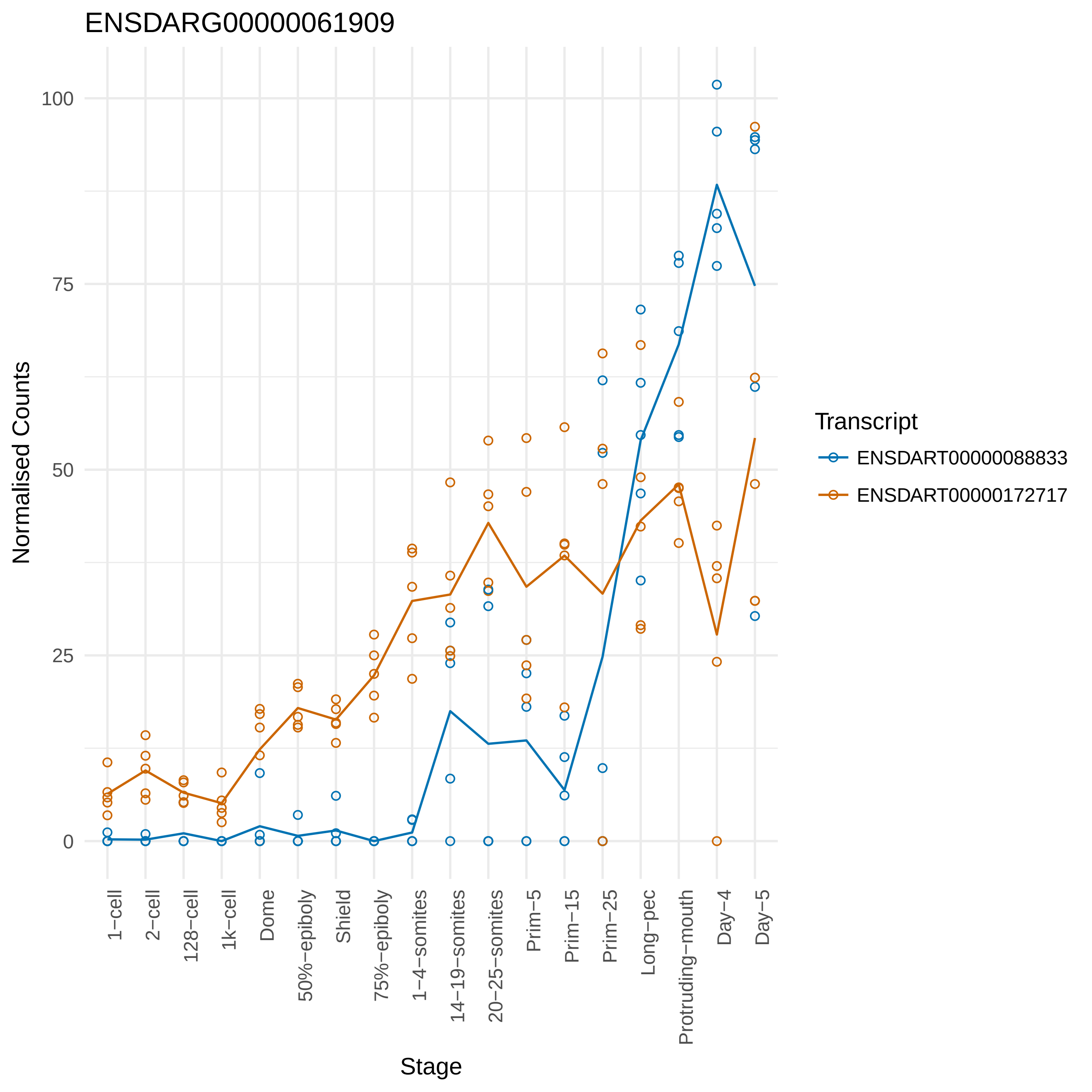

ENSDARG00000061909
Normalised Counts
0
25
50
75
100
Stage
1−cell
2−cell
128−cell
1k−cell
Dome
50%−epiboly
Shield
75%−epiboly
1−4−somites
14−19−somites
20−25−somites
Prim−5
Prim−15
Prim−25
Long−pec
Protruding−mouth
Day−4
Day−5
Transcript
ENSDART00000088833
ENSDART00000172717

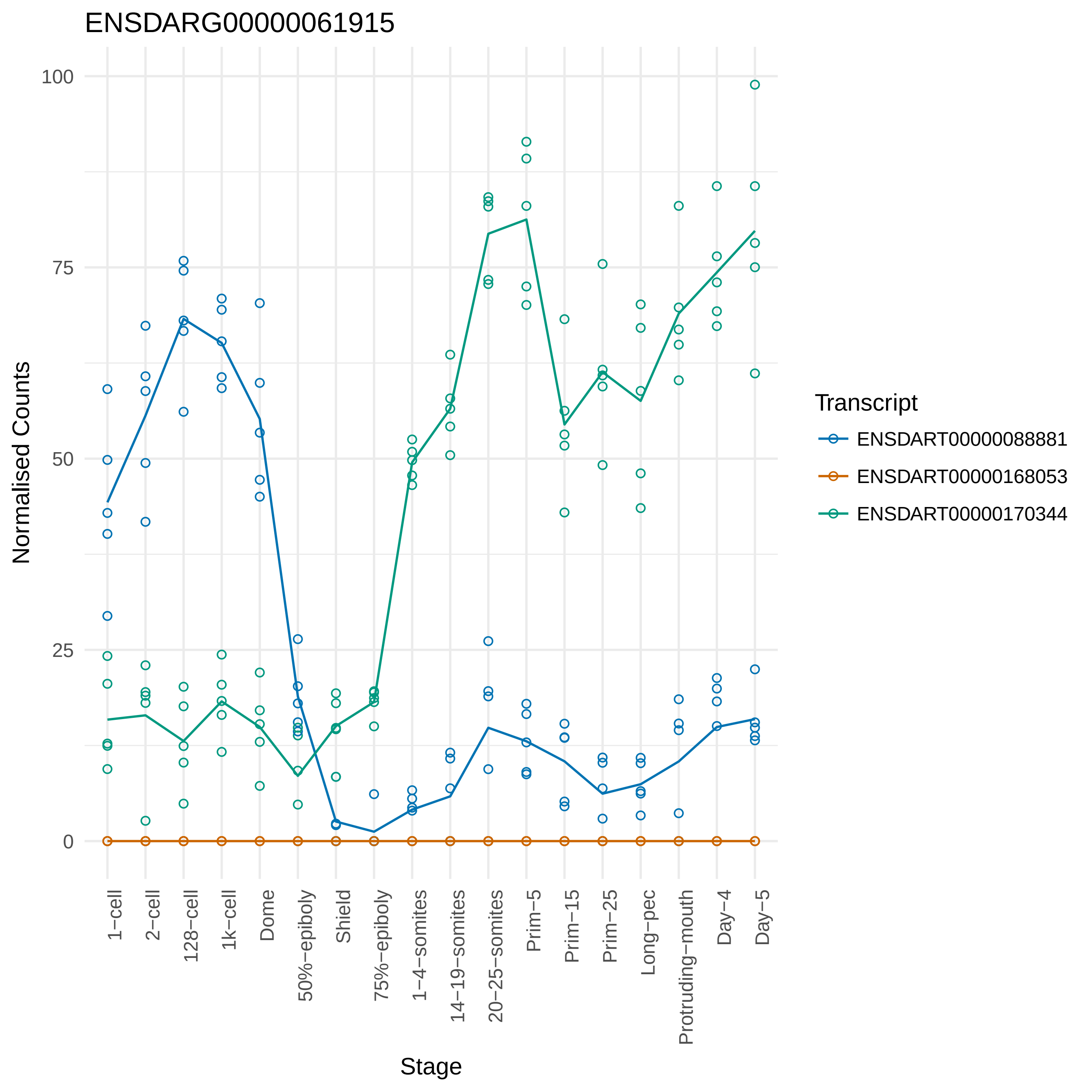
ENSDARG00000061915
Normalised Counts
100
75
50
25
0
1−cell
2−cell
128−cell
1k−cell
Dome
50%−epiboly
Shield
75%−epiboly
1−4−somites
14−19−somites
20−25−somites
Prim−5
Prim−15
Prim−25
Long−pec
Protruding−mouth
Day−4
Day−5
Stage
Transcript
ENSDART00000088881
ENSDART00000168053
ENSDART00000170344

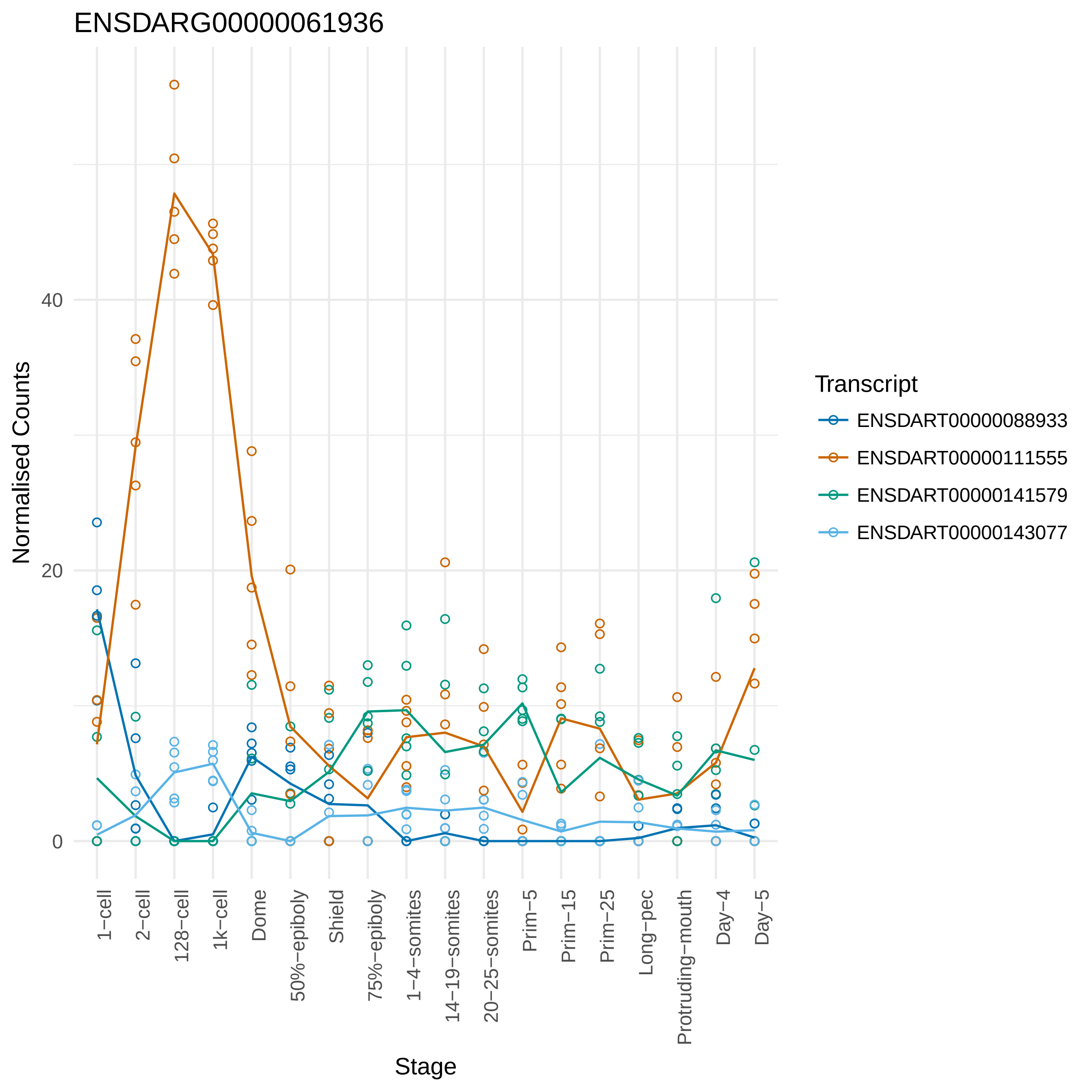

ENSDARG00000061936
Normalised Counts
40
20
0
1−cell
2−cell
128−cell
1k−cell
Dome
50%−epiboly
Shield
75%−epiboly
1−4−somites
14−19−somites
20−25−somites
Prim−5
Prim−15
Prim−25
Long−pec
Protruding−mouth
Day−4
Day−5
Stage
Transcript
ENSDART00000088933
ENSDART00000111555
ENSDART00000141579
ENSDART00000143077

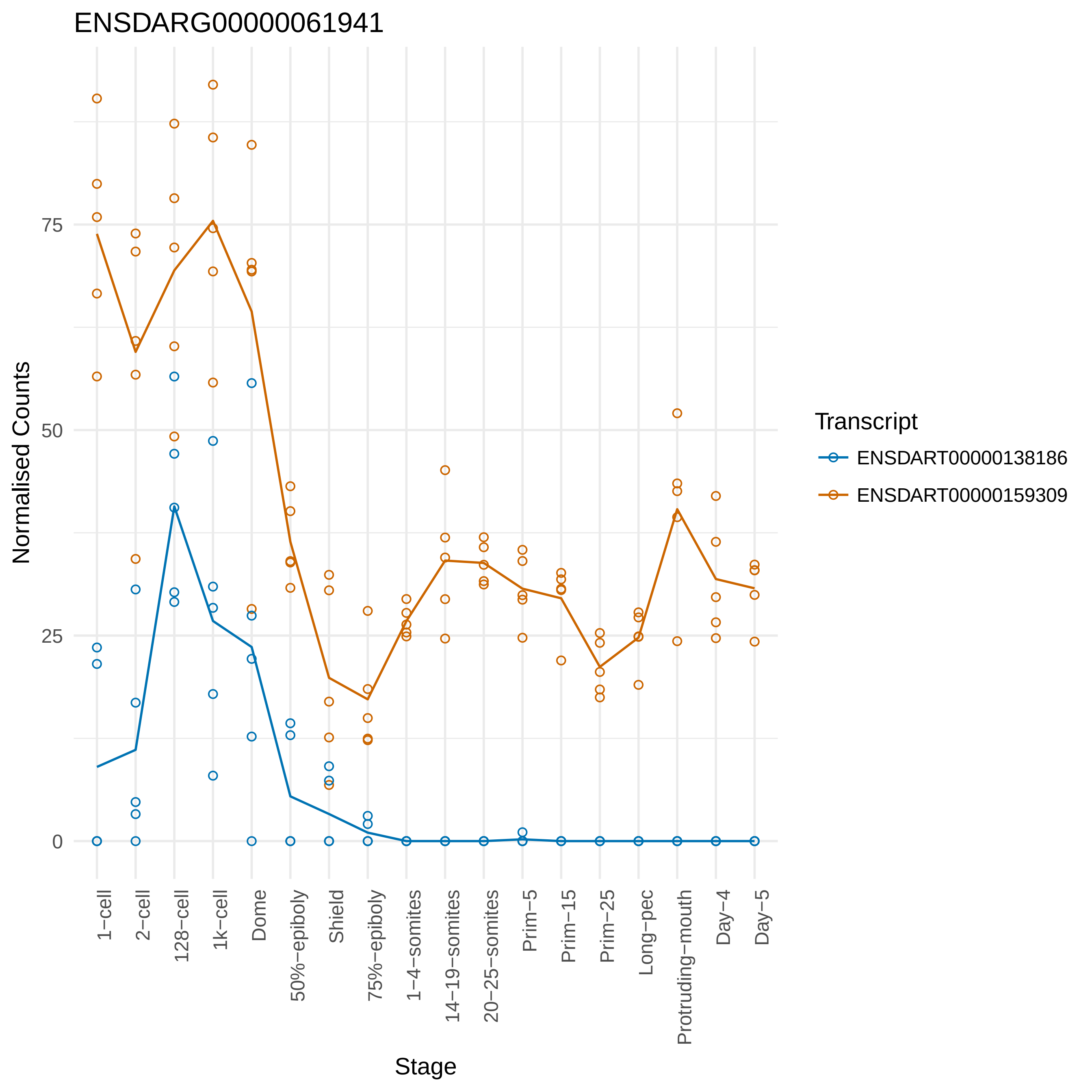
ENSDARG00000061941
Normalised Counts
0
25
50
75
Stage
1−cell
2−cell
128−cell
1k−cell
Dome
50%−epiboly
Shield
75%−epiboly
1−4−somites
14−19−somites
20−25−somites
Prim−5
Prim−15
Prim−25
Long−pec
Protruding−mouth
Day−4
Day−5
Transcript
ENSDART00000138186
ENSDART00000159309

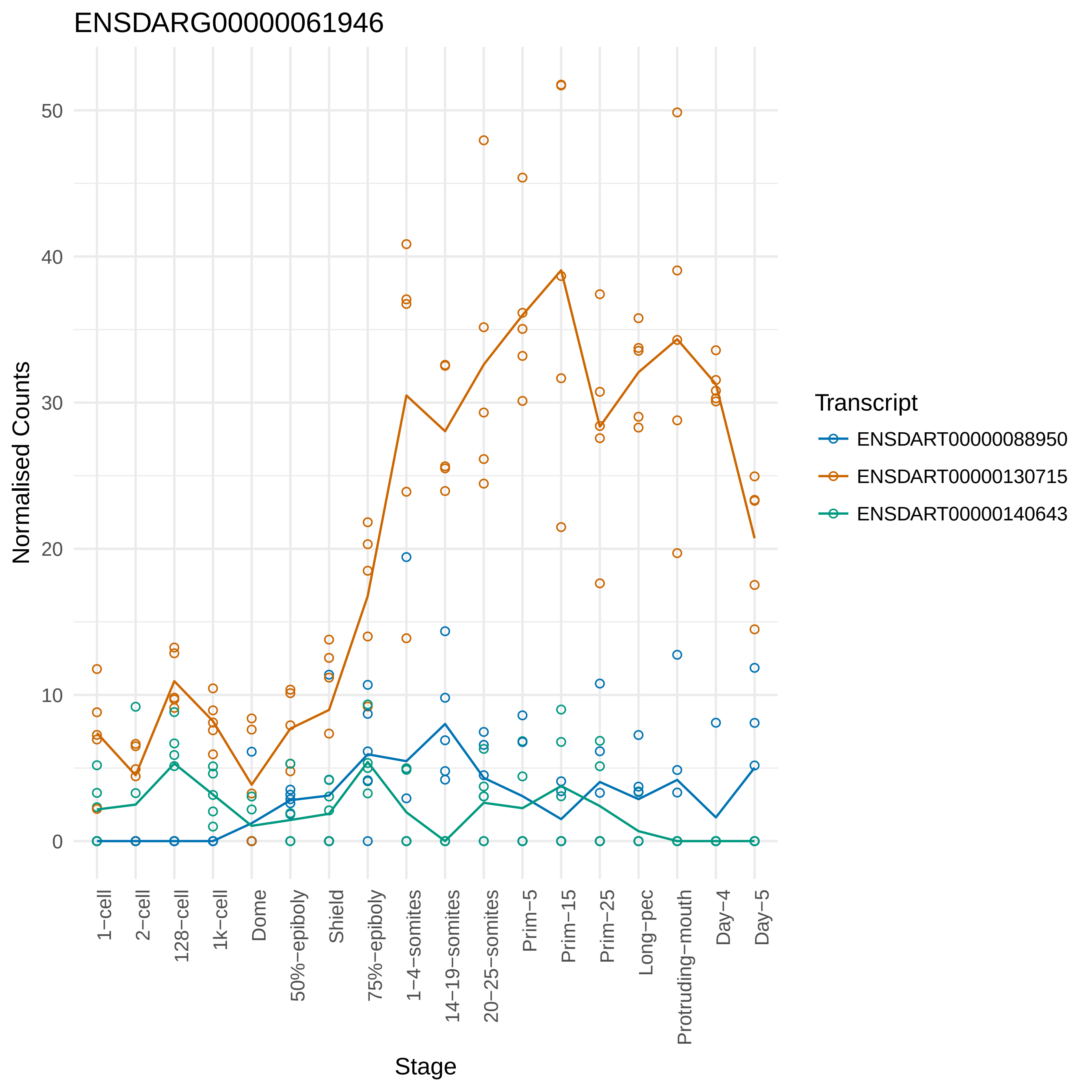

ENSDARG00000061946
Normalised Counts
0
10
20
30
40
50
1−cell
2−cell
128−cell
1k−cell
Dome
50%−epiboly
Shield
75%−epiboly
1−4−somites
14−19−somites
20−25−somites
Prim−5
Prim−15
Prim−25
Long−pec
Protruding−mouth
Day−4
Day−5
Stage
Transcript
ENSDART00000088950
ENSDART00000130715
ENSDART00000140643

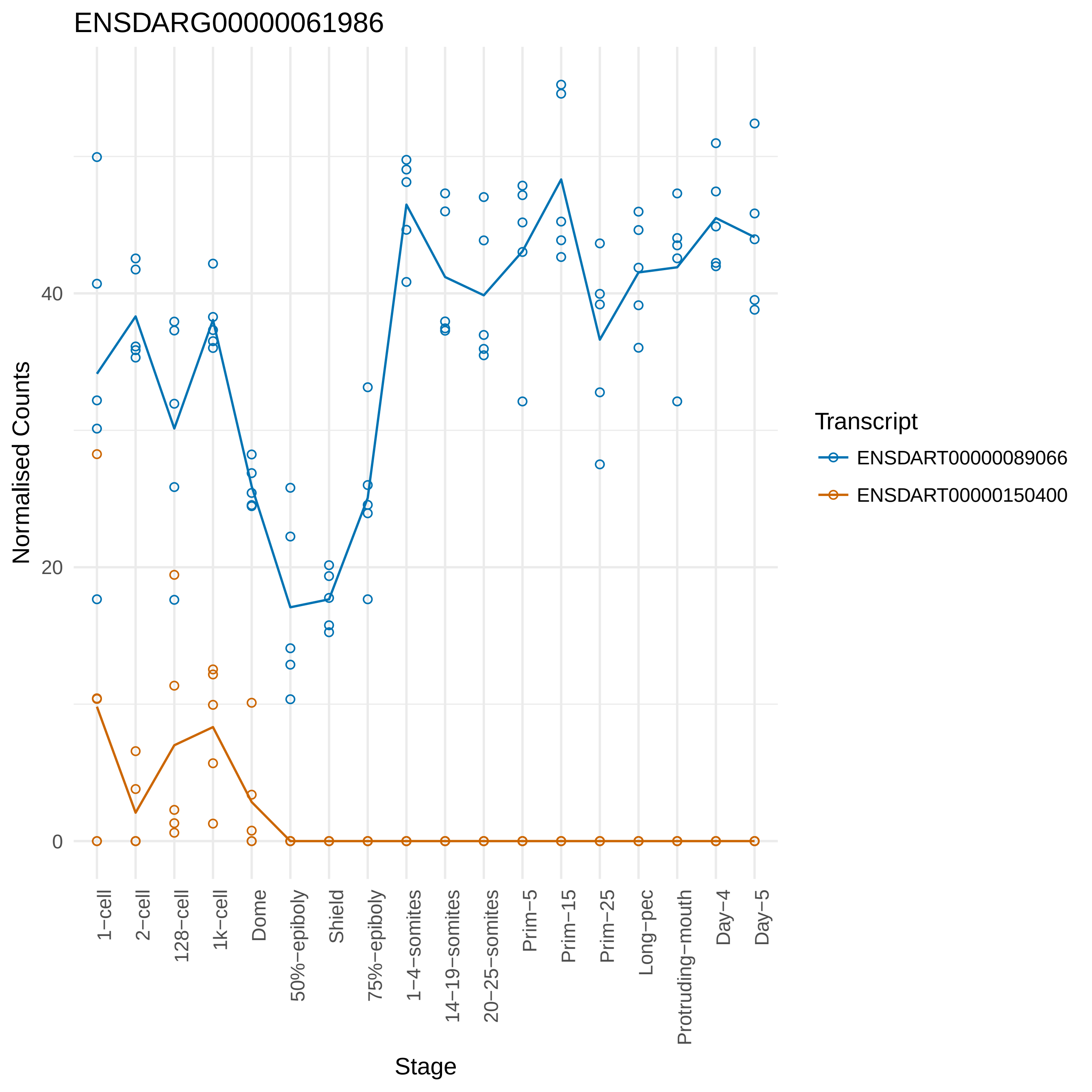

ENSDARG00000061986
Normalised Counts
0
20
40
Stage
1−cell
2−cell
128−cell
1k−cell
Dome
50%−epiboly
Shield
75%−epiboly
1−4−somites
14−19−somites
20−25−somites
Prim−5
Prim−15
Prim−25
Long−pec
Protruding−mouth
Day−4
Day−5
Transcript
ENSDART00000089066
ENSDART00000150400

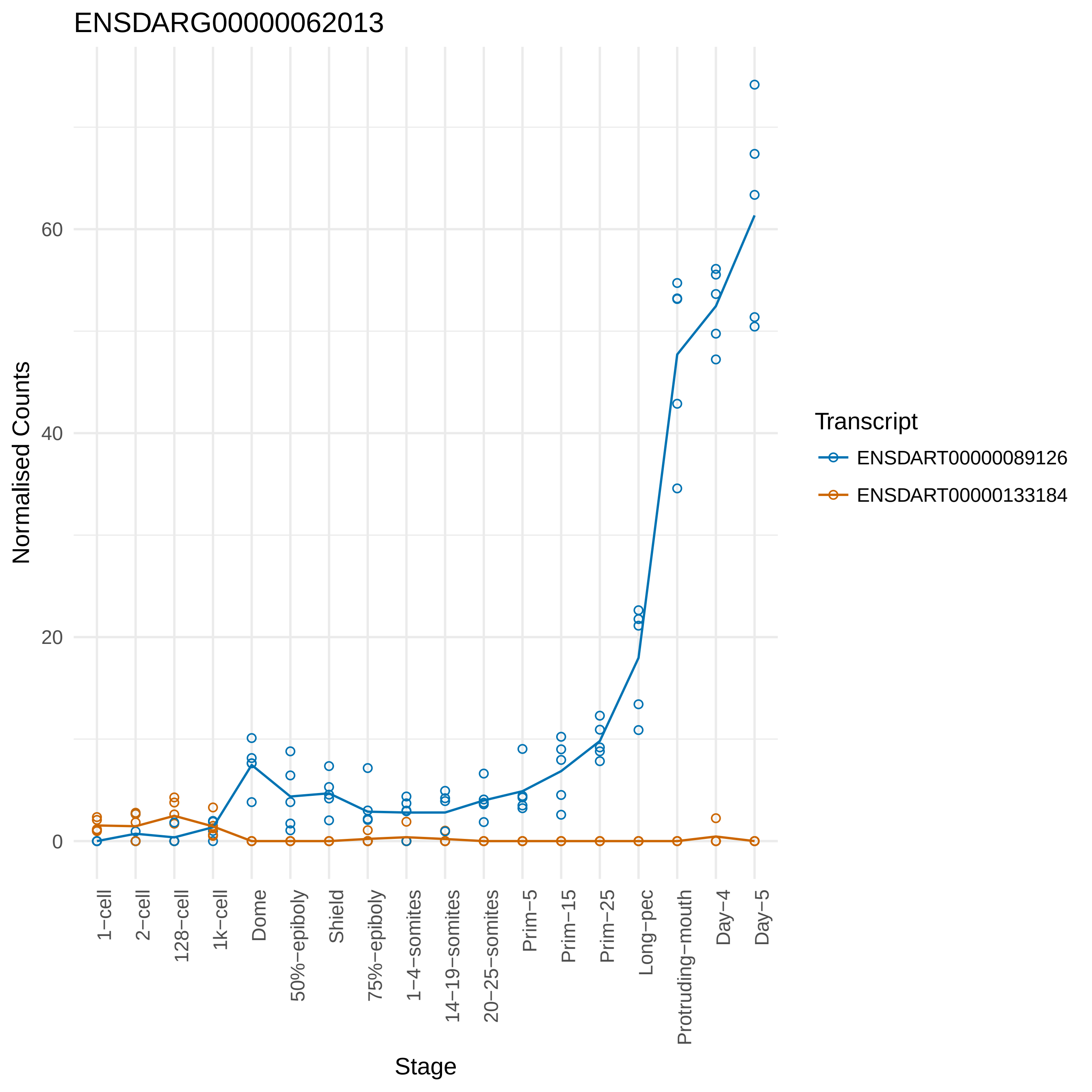

ENSDARG00000062013
Normalised Counts
60
40
20
0
1−cell
2−cell
128−cell
1k−cell
Dome
50%−epiboly
Shield
75%−epiboly
1−4−somites
14−19−somites
20−25−somites
Prim−5
Prim−15
Prim−25
Long−pec
Protruding−mouth
Day−4
Day−5
Stage
Transcript
ENSDART00000089126
ENSDART00000133184

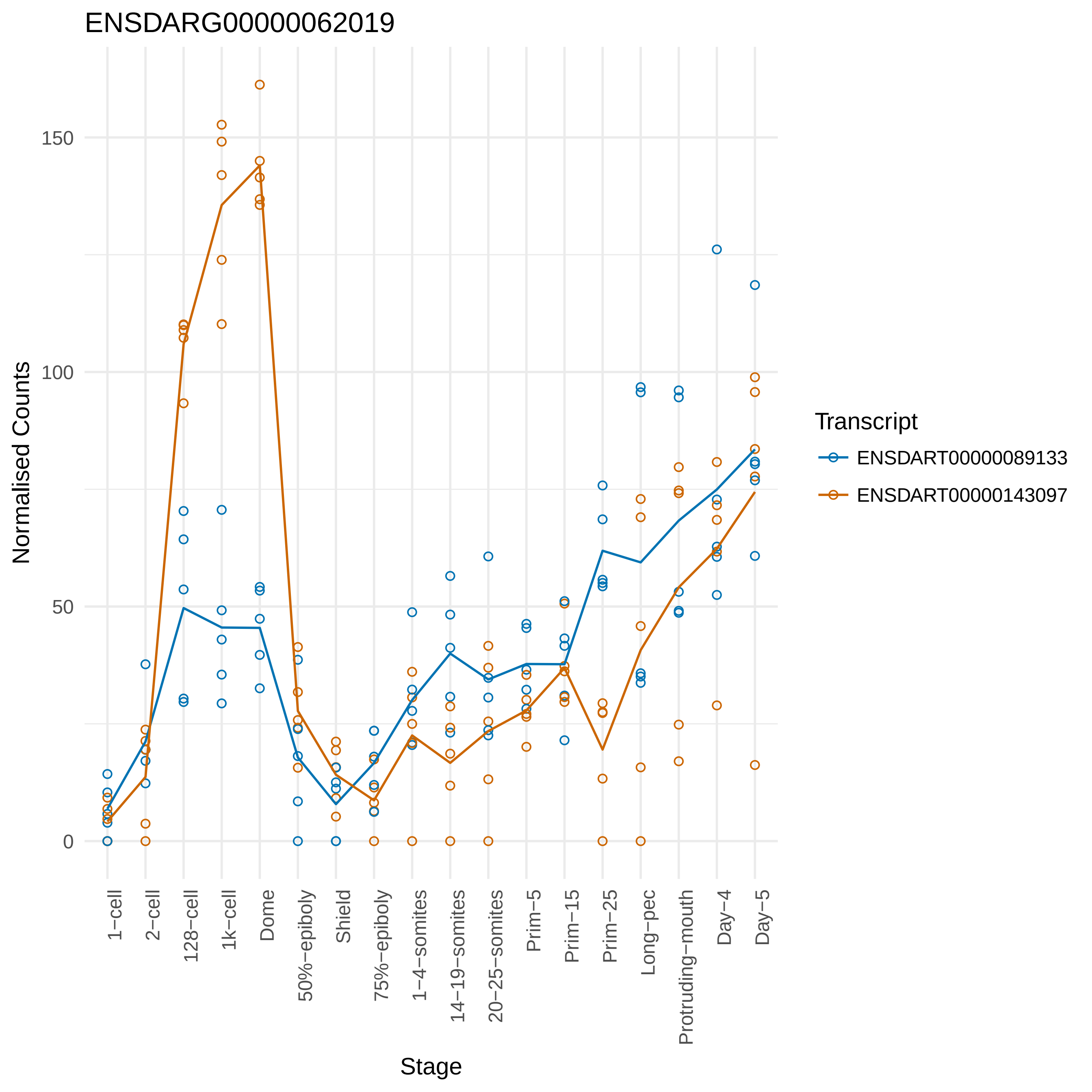

ENSDARG00000062019
Normalised Counts
0
50
100
150
1−cell
2−cell
128−cell
1k−cell
Dome
50%−epiboly
Shield
75%−epiboly
1−4−somites
14−19−somites
20−25−somites
Prim−5
Prim−15
Prim−25
Long−pec
Protruding−mouth
Day−4
Day−5
Stage
Transcript
ENSDART00000089133
ENSDART00000143097

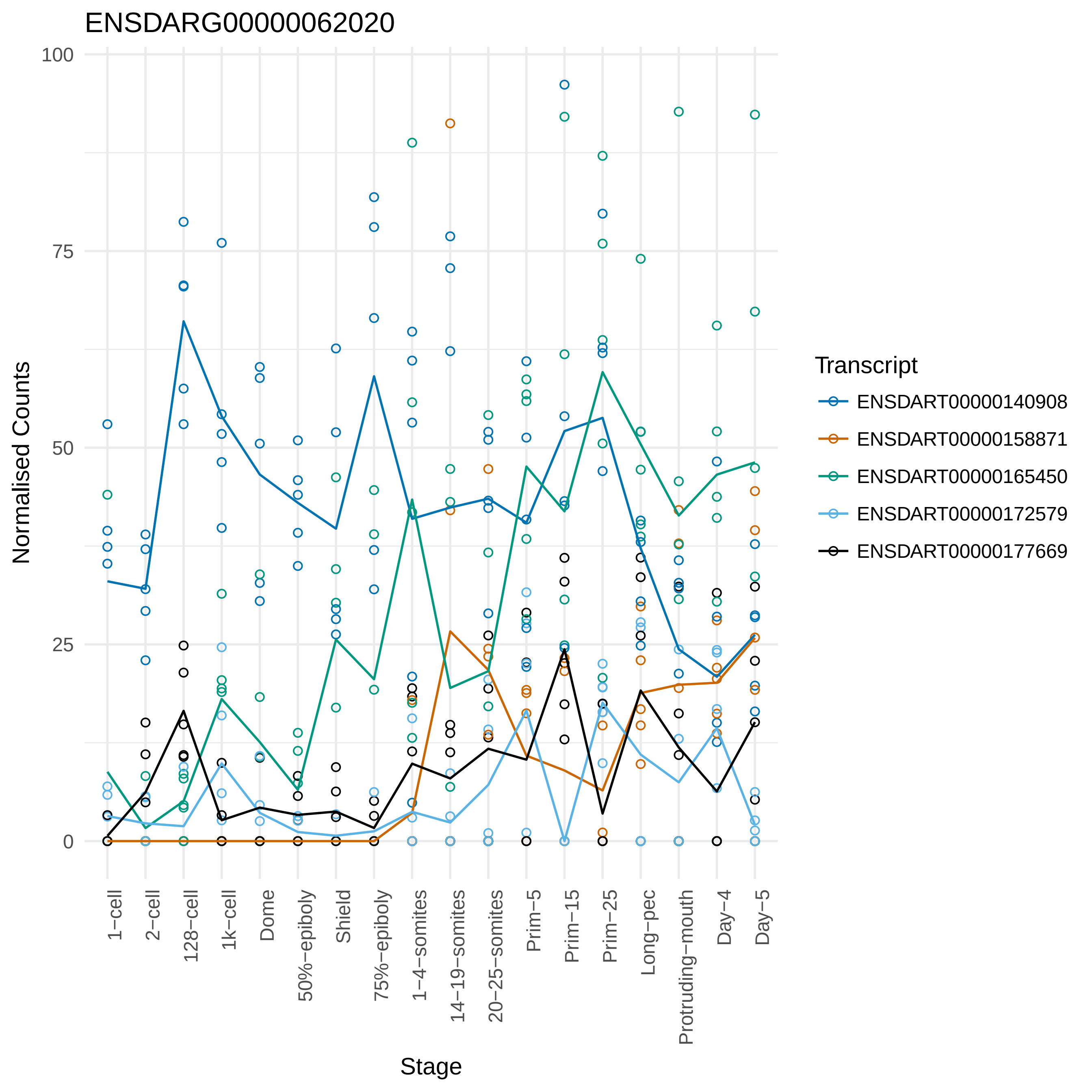

ENSDARG00000062020
Normalised Counts
100
75
50
25
0
1−cell
2−cell
128−cell
1k−cell
Dome
50%−epiboly
Shield
75%−epiboly
1−4−somites
14−19−somites
20−25−somites
Prim−5
Prim−15
Prim−25
Long−pec
Protruding−mouth
Day−4
Day−5
Stage
Transcript
ENSDART00000140908
ENSDART00000158871
ENSDART00000165450
ENSDART00000172579
ENSDART00000177669

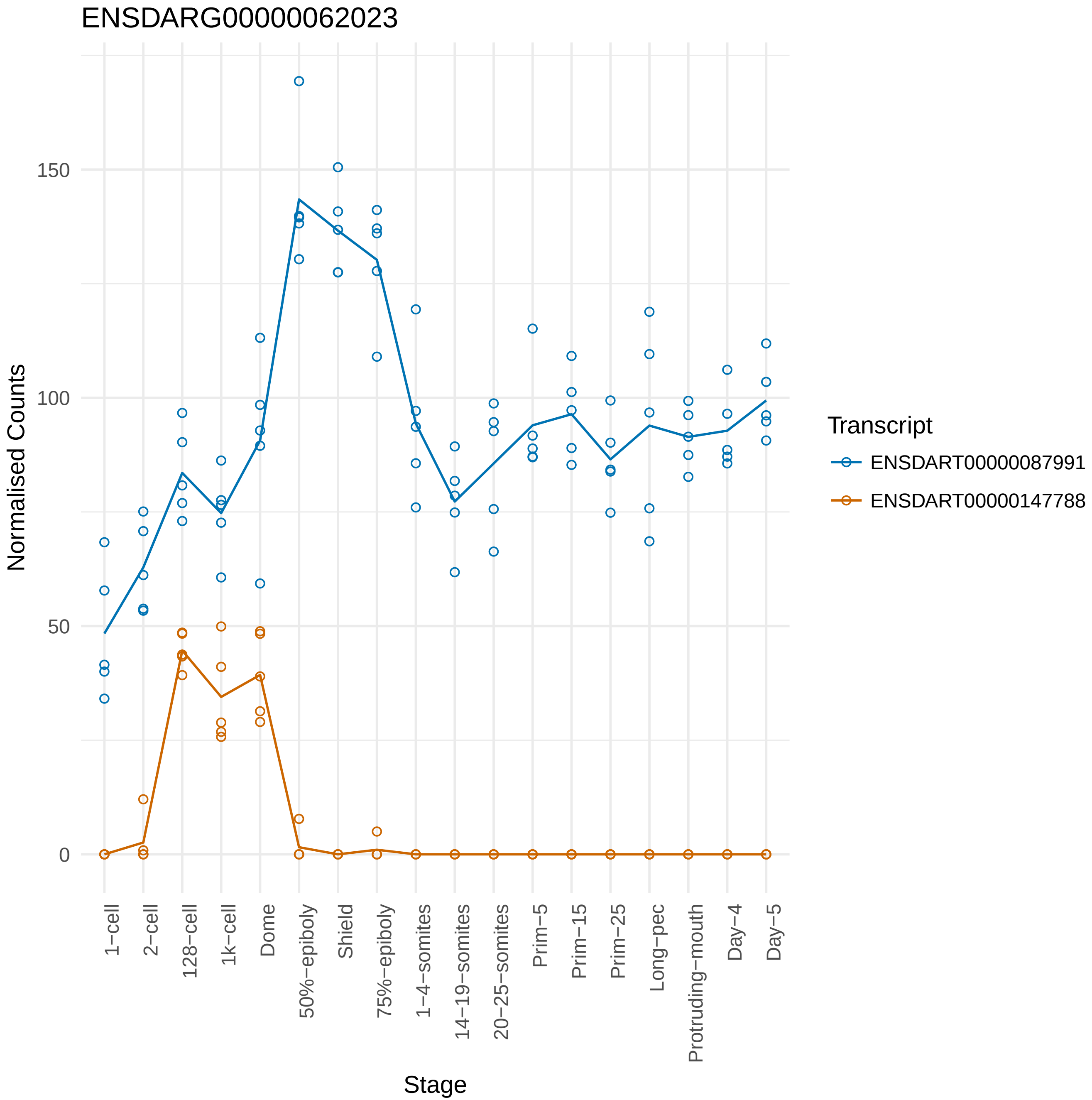

ENSDARG00000062023
Normalised Counts
150
100
50
0
1−cell
2−cell
128−cell
1k−cell
Dome
50%−epiboly
Shield
75%−epiboly
1−4−somites
14−19−somites
20−25−somites
Prim−5
Prim−15
Prim−25
Long−pec
Protruding−mouth
Day−4
Day−5
Stage
Transcript
ENSDART00000087991
ENSDART00000147788

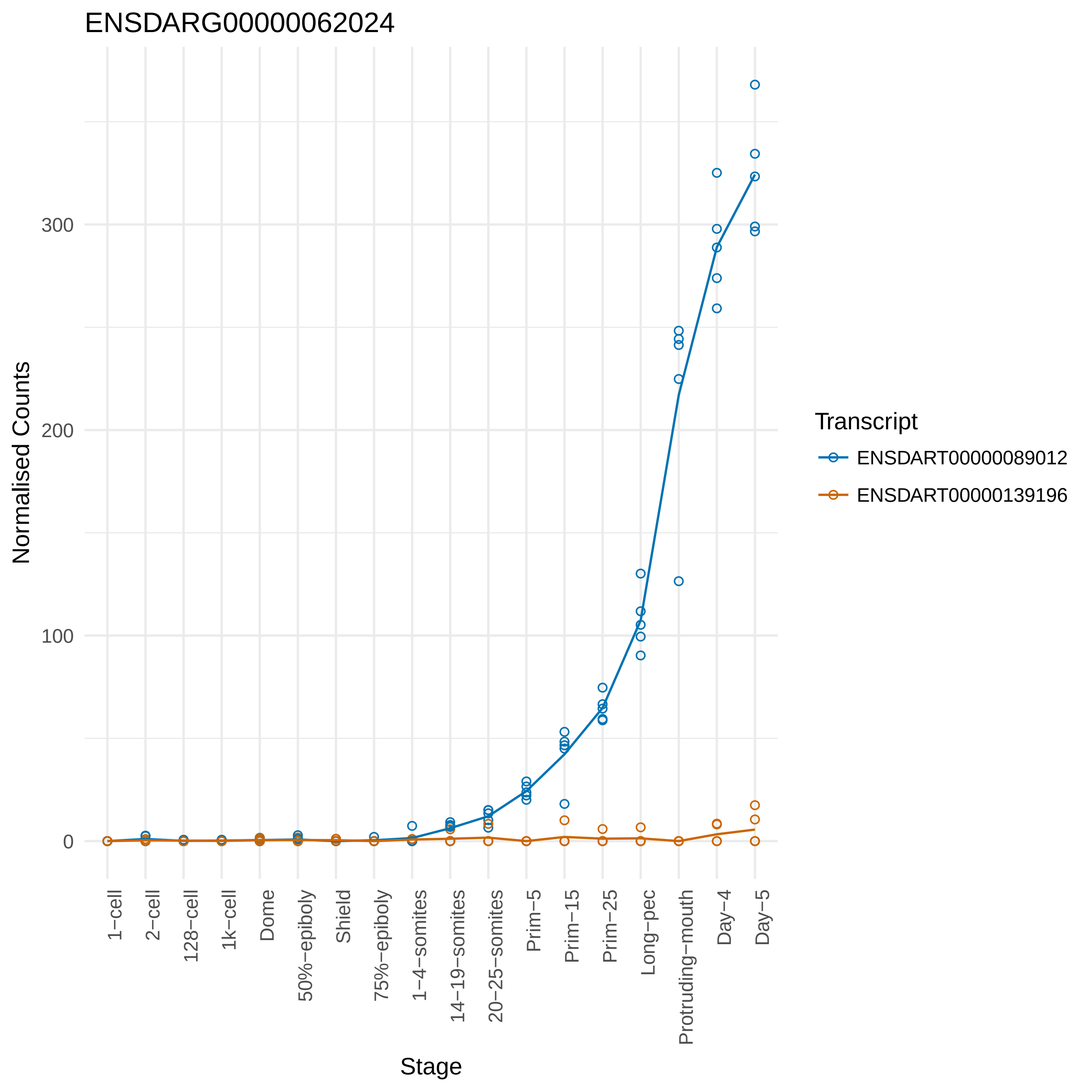

ENSDARG00000062024
Normalised Counts
300
200
100
0
1−cell
2−cell
128−cell
1k−cell
Dome
50%−epiboly
Shield
75%−epiboly
1−4−somites
14−19−somites
20−25−somites
Prim−5
Prim−15
Prim−25
Long−pec
Protruding−mouth
Day−4
Day−5
Stage
Transcript
ENSDART00000089012
ENSDART00000139196

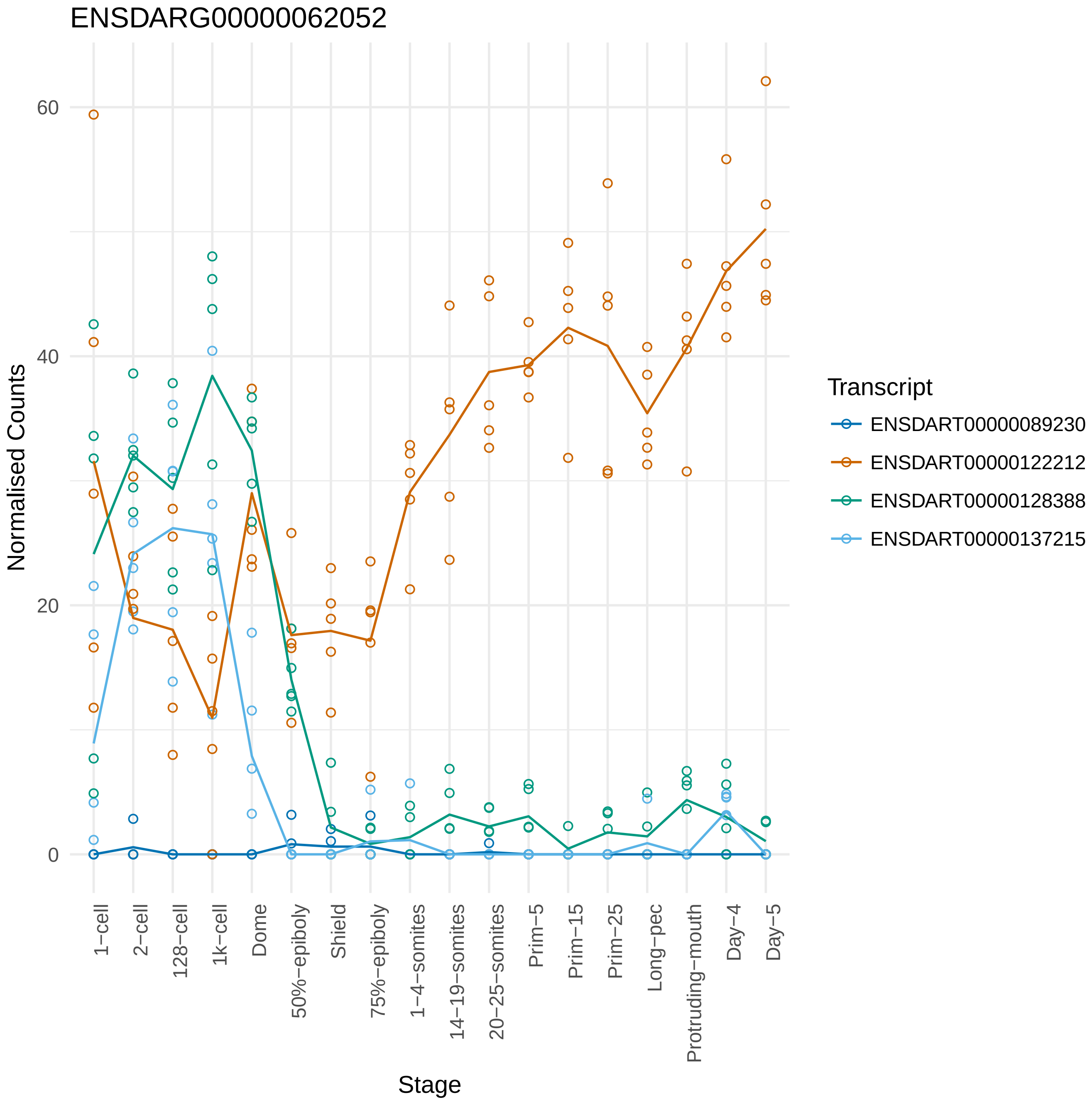

ENSDARG00000062052
Normalised Counts
60
40
20
0
1−cell
2−cell
128−cell
1k−cell
Dome
50%−epiboly
Shield
75%−epiboly
1−4−somites
14−19−somites
20−25−somites
Prim−5
Prim−15
Prim−25
Long−pec
Protruding−mouth
Day−4
Day−5
Stage
Transcript
ENSDART00000089230
ENSDART00000122212
ENSDART00000128388
ENSDART00000137215

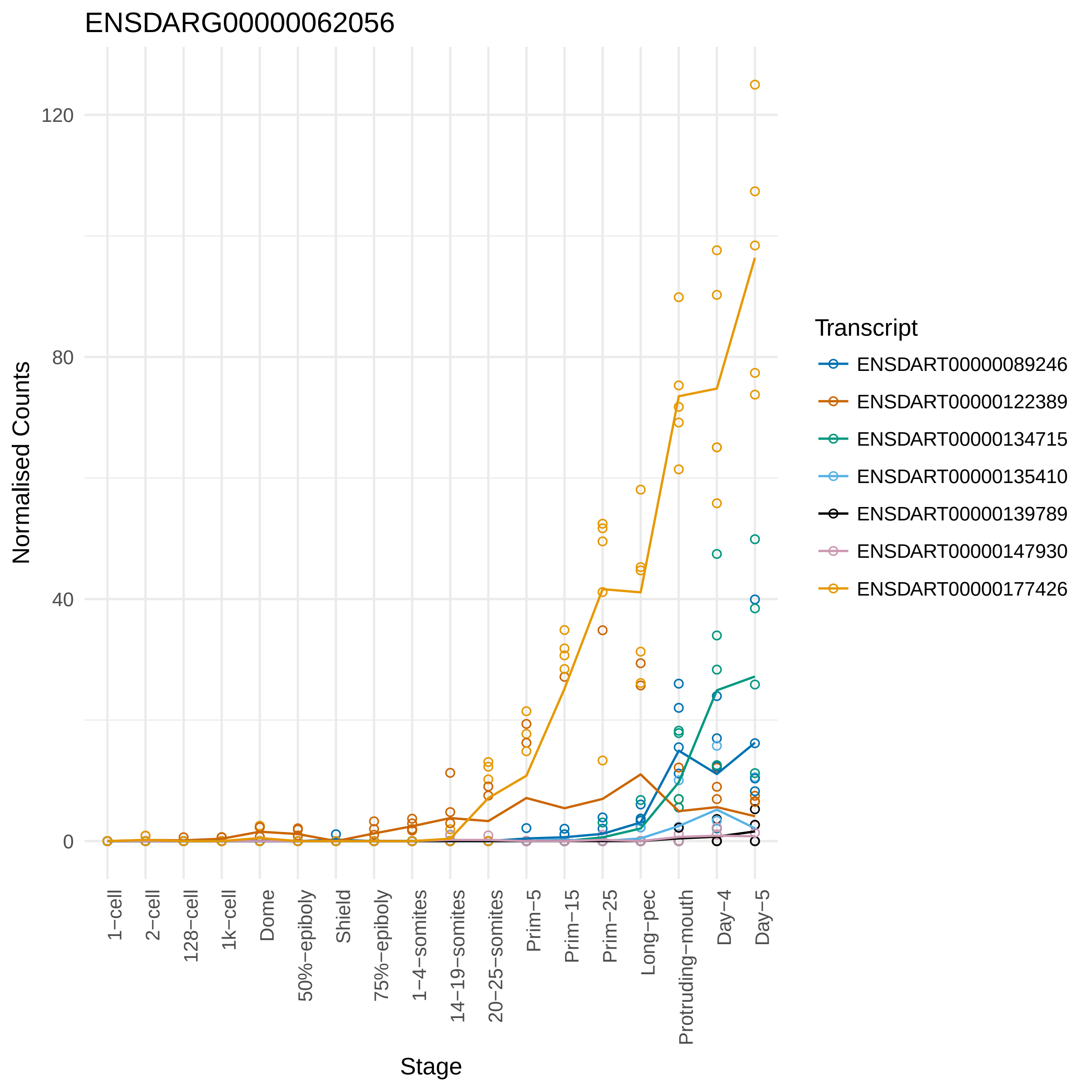
ENSDARG00000062056
Normalised Counts
120
80
40
0
1−cell
2−cell
128−cell
1k−cell
Dome
50%−epiboly
Shield
75%−epiboly
1−4−somites
14−19−somites
20−25−somites
Prim−5
Prim−15
Prim−25
Long−pec
Protruding−mouth
Day−4
Day−5
Stage
Transcript
ENSDART00000089246
ENSDART00000122389
ENSDART00000134715
ENSDART00000135410
ENSDART00000139789
ENSDART00000147930
ENSDART00000177426

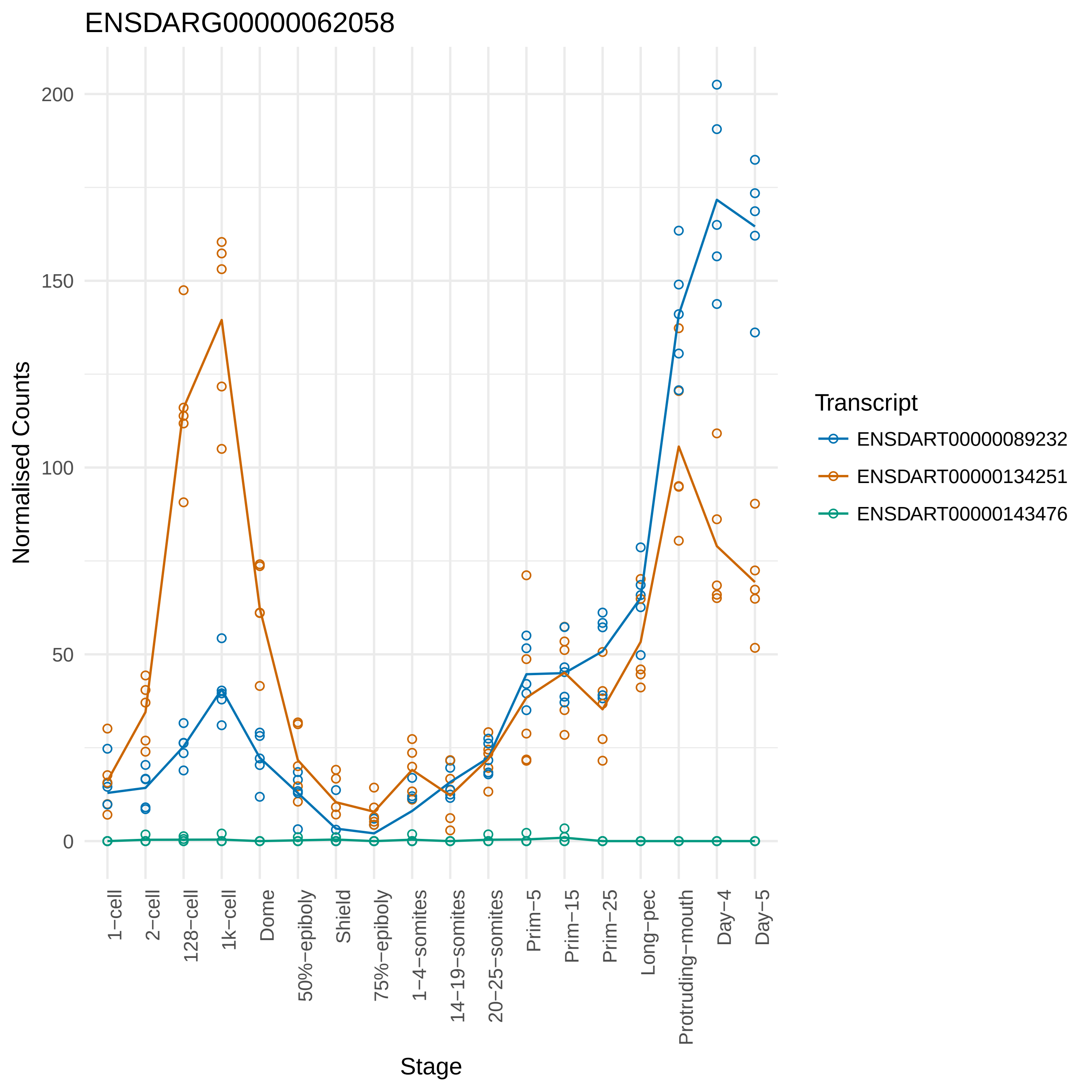

ENSDARG00000062058
Normalised Counts
200
150
100
50
0
1−cell
2−cell
128−cell
1k−cell
Dome
50%−epiboly
Shield
75%−epiboly
1−4−somites
14−19−somites
20−25−somites
Prim−5
Prim−15
Prim−25
Long−pec
Protruding−mouth
Day−4
Day−5
Stage
Transcript
ENSDART00000089232
ENSDART00000134251
ENSDART00000143476

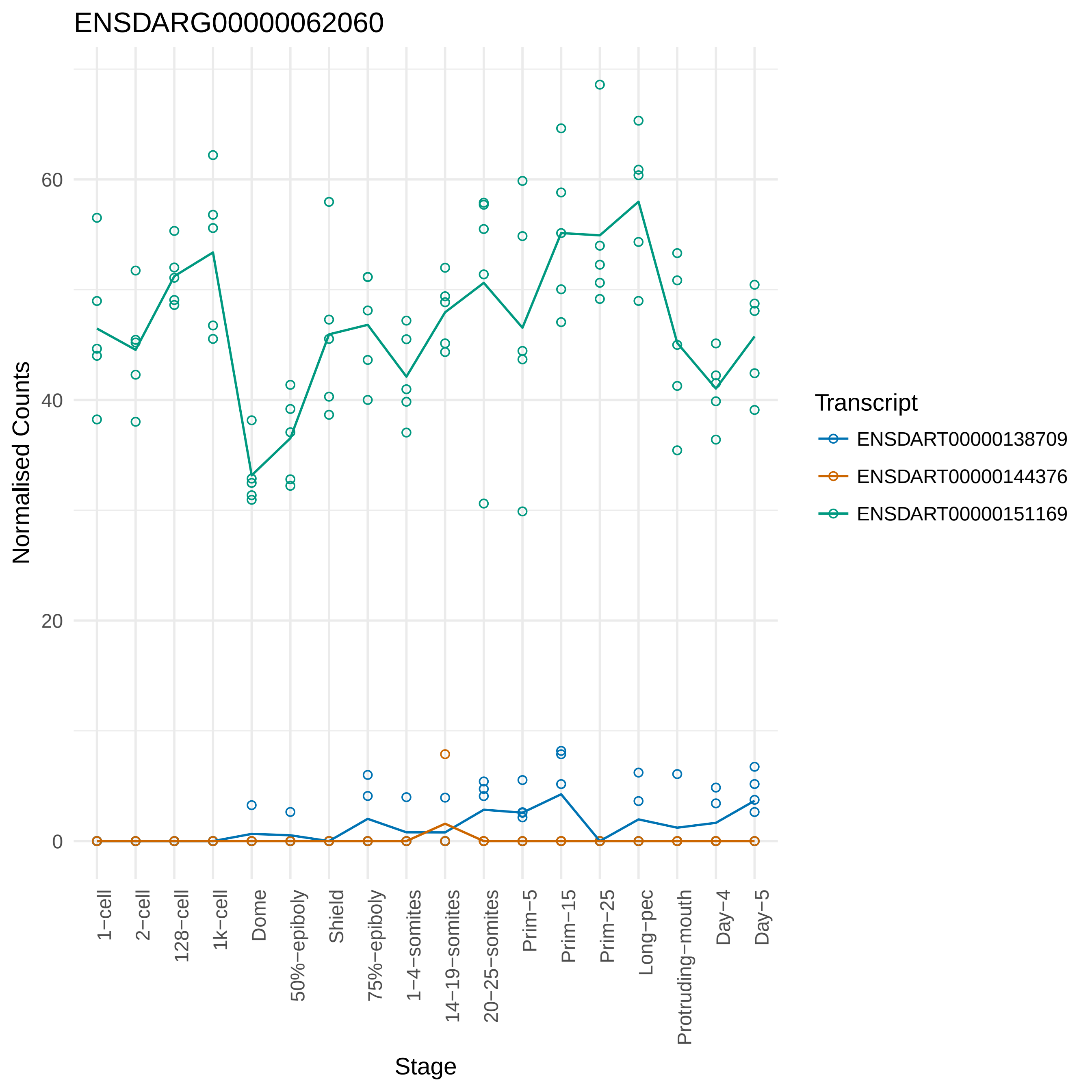

ENSDARG00000062060
Normalised Counts
60
40
20
0
1−cell
2−cell
128−cell
1k−cell
Dome
50%−epiboly
Shield
75%−epiboly
1−4−somites
14−19−somites
20−25−somites
Prim−5
Prim−15
Prim−25
Long−pec
Protruding−mouth
Day−4
Day−5
Stage
Transcript
ENSDART00000138709
ENSDART00000144376
ENSDART00000151169

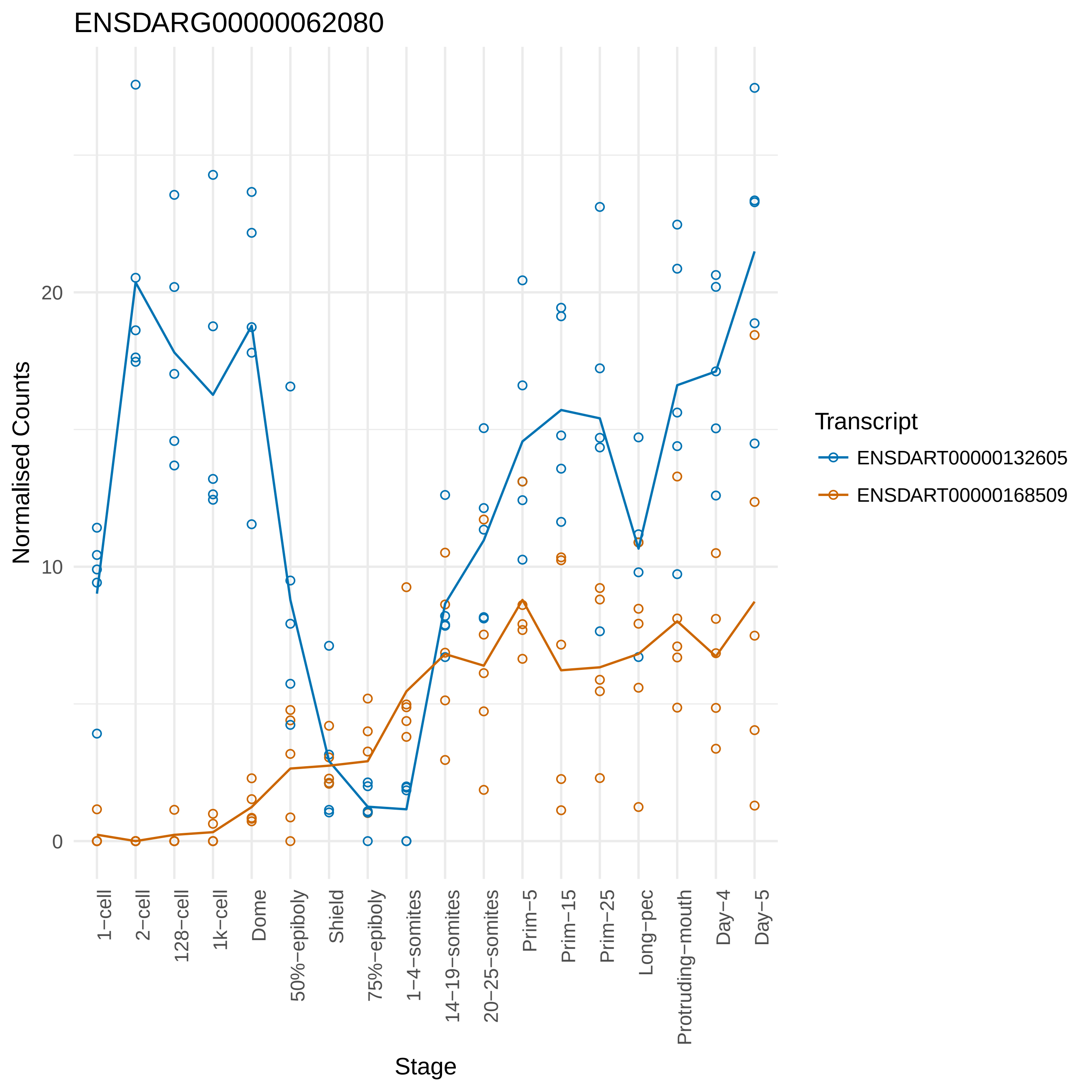
ENSDARG00000062080
Normalised Counts
0
10
20
1−cell
2−cell
128−cell
1k−cell
Dome
50%−epiboly
Shield
75%−epiboly
1−4−somites
14−19−somites
20−25−somites
Prim−5
Prim−15
Prim−25
Long−pec
Protruding−mouth
Day−4
Day−5
Stage
Transcript
ENSDART00000132605
ENSDART00000168509

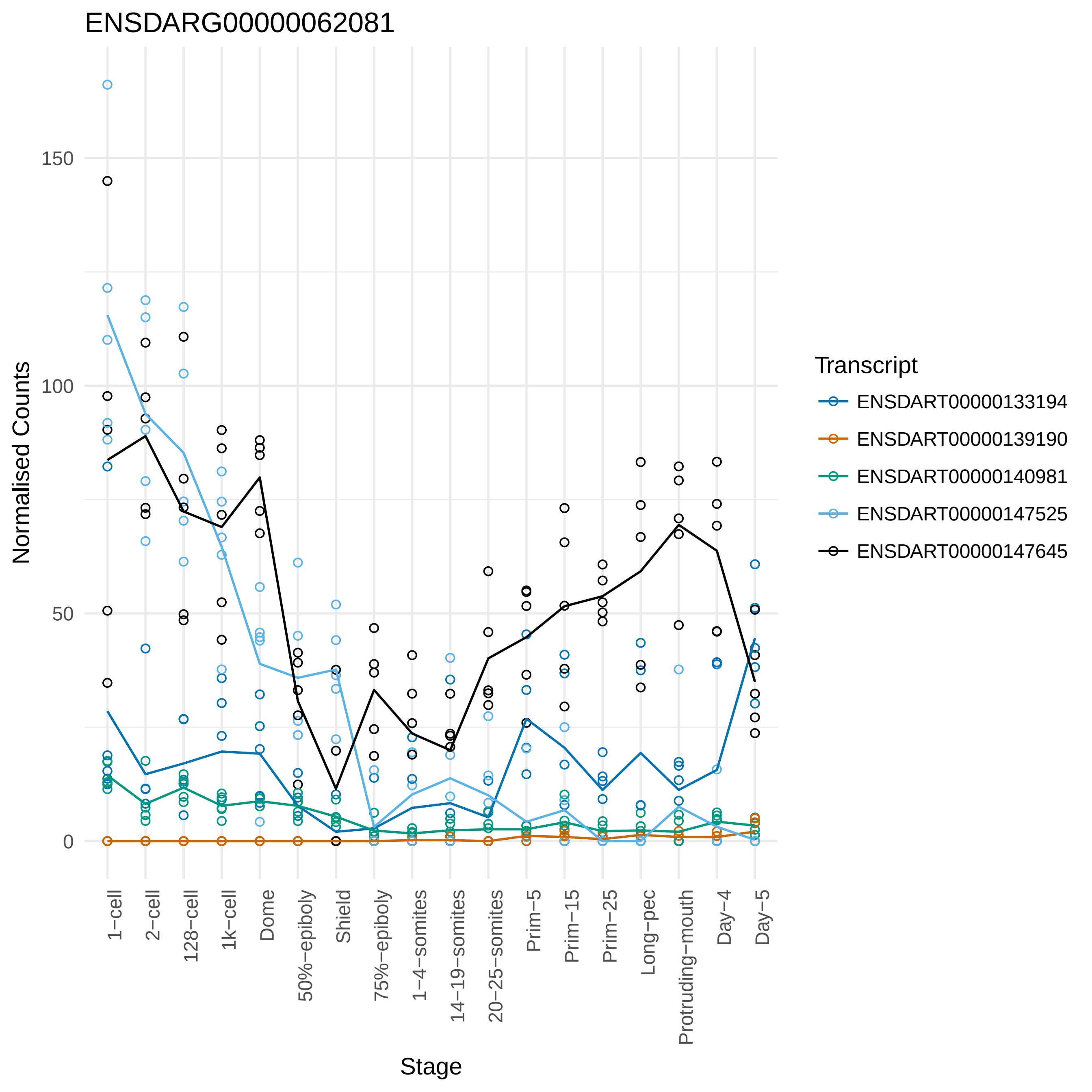
ENSDARG00000062081
Normalised Counts
150
100
50
0
1−cell
2−cell
128−cell
1k−cell
Dome
50%−epiboly
Shield
75%−epiboly
1−4−somites
14−19−somites
20−25−somites
Prim−5
Prim−15
Prim−25
Long−pec
Protruding−mouth
Day−4
Day−5
Stage
Transcript
ENSDART00000133194
ENSDART00000139190
ENSDART00000140981
ENSDART00000147525
ENSDART00000147645

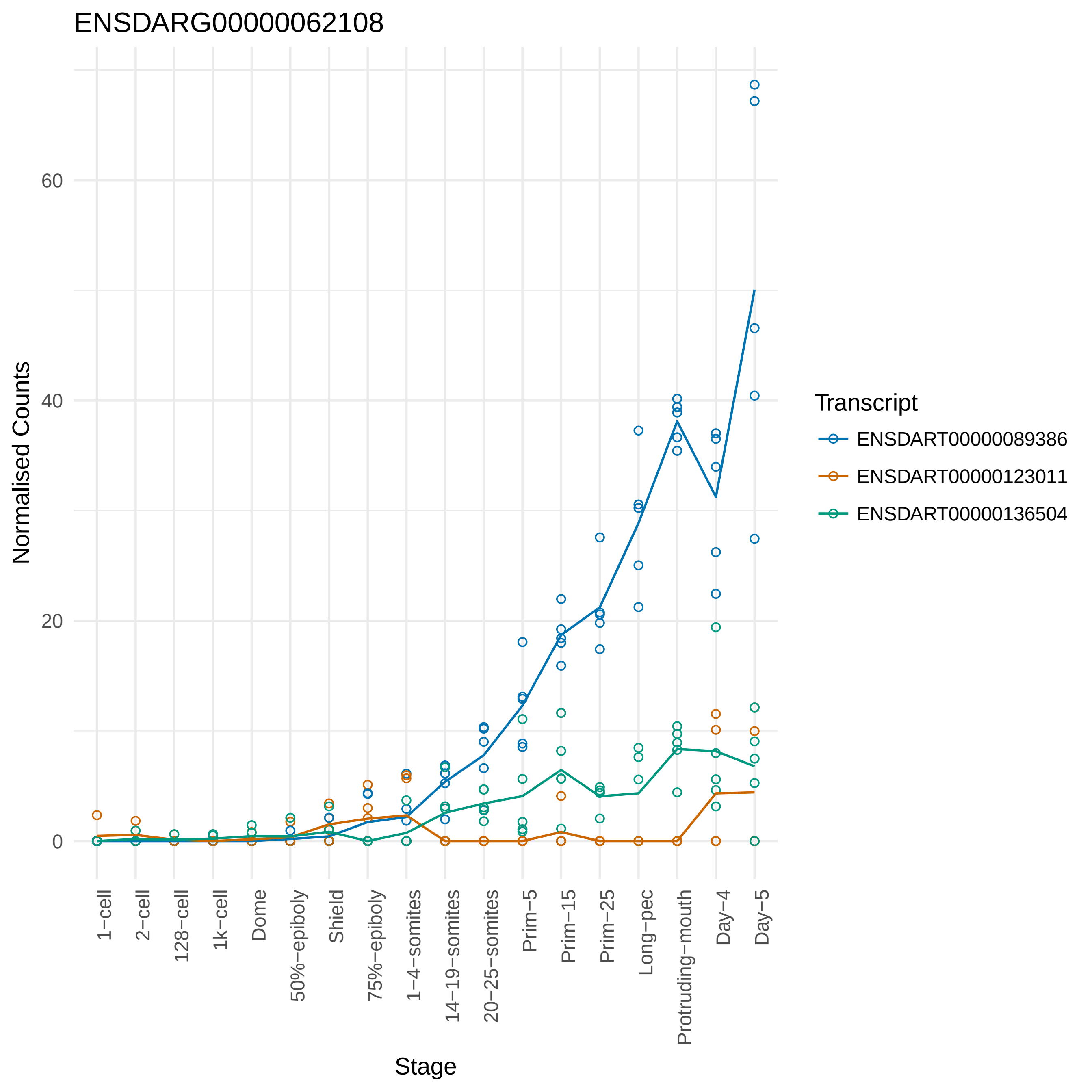

ENSDARG00000062108
Normalised Counts
60
40
20
0
Stage
1−cell
2−cell
128−cell
1k−cell
Dome
50%−epiboly
Shield
75%−epiboly
1−4−somites
14−19−somites
20−25−somites
Prim−5
Prim−15
Prim−25
Long−pec
Protruding−mouth
Day−4
Day−5
Transcript
ENSDART00000089386
ENSDART00000123011
ENSDART00000136504

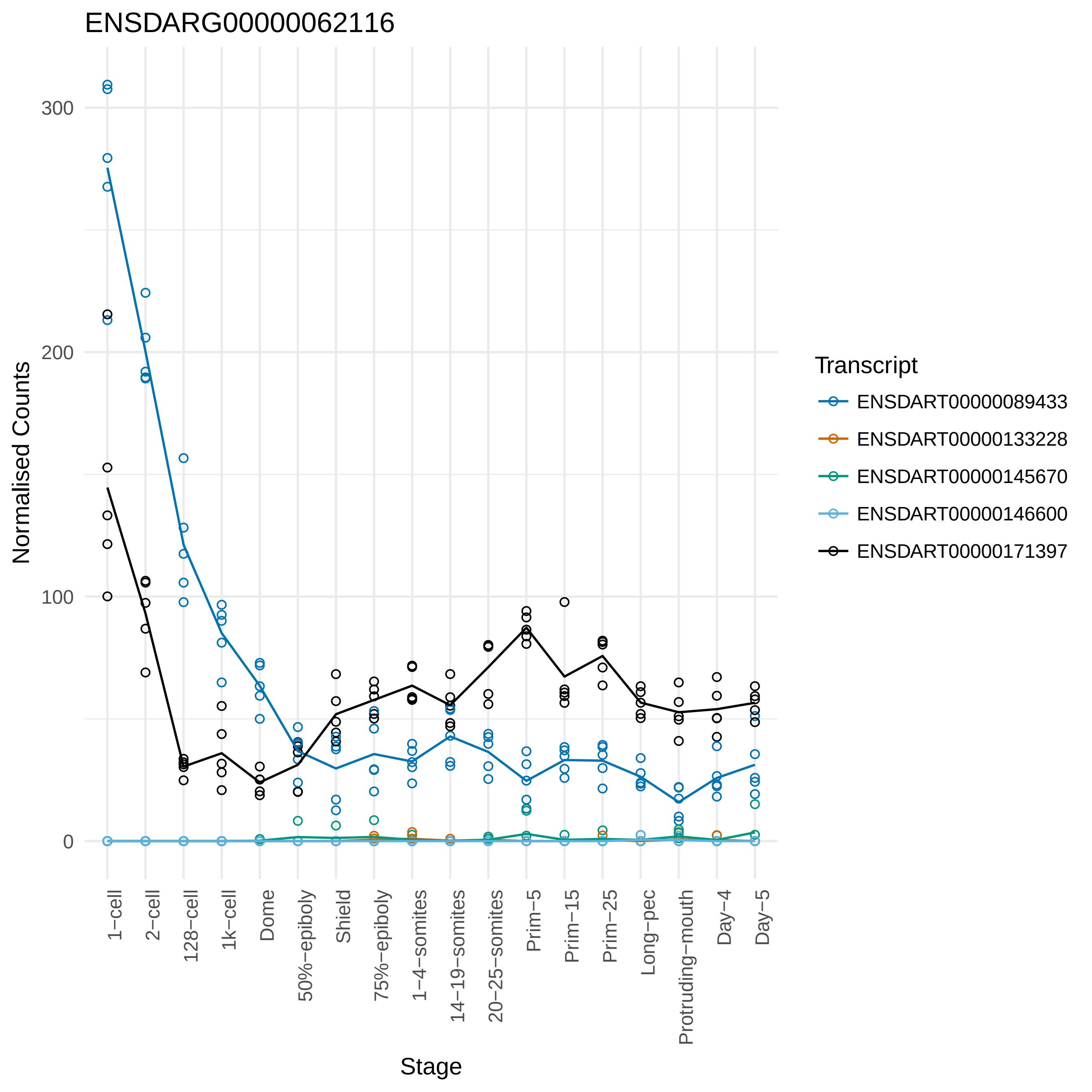
ENSDARG00000062116
Normalised Counts
300
200
100
0
1−cell
2−cell
128−cell
1k−cell
Dome
50%−epiboly
Shield
75%−epiboly
1−4−somites
14−19−somites
20−25−somites
Prim−5
Prim−15
Prim−25
Long−pec
Protruding−mouth
Day−4
Day−5
Stage
Transcript
ENSDART00000089433
ENSDART00000133228
ENSDART00000145670
ENSDART00000146600
ENSDART00000171397

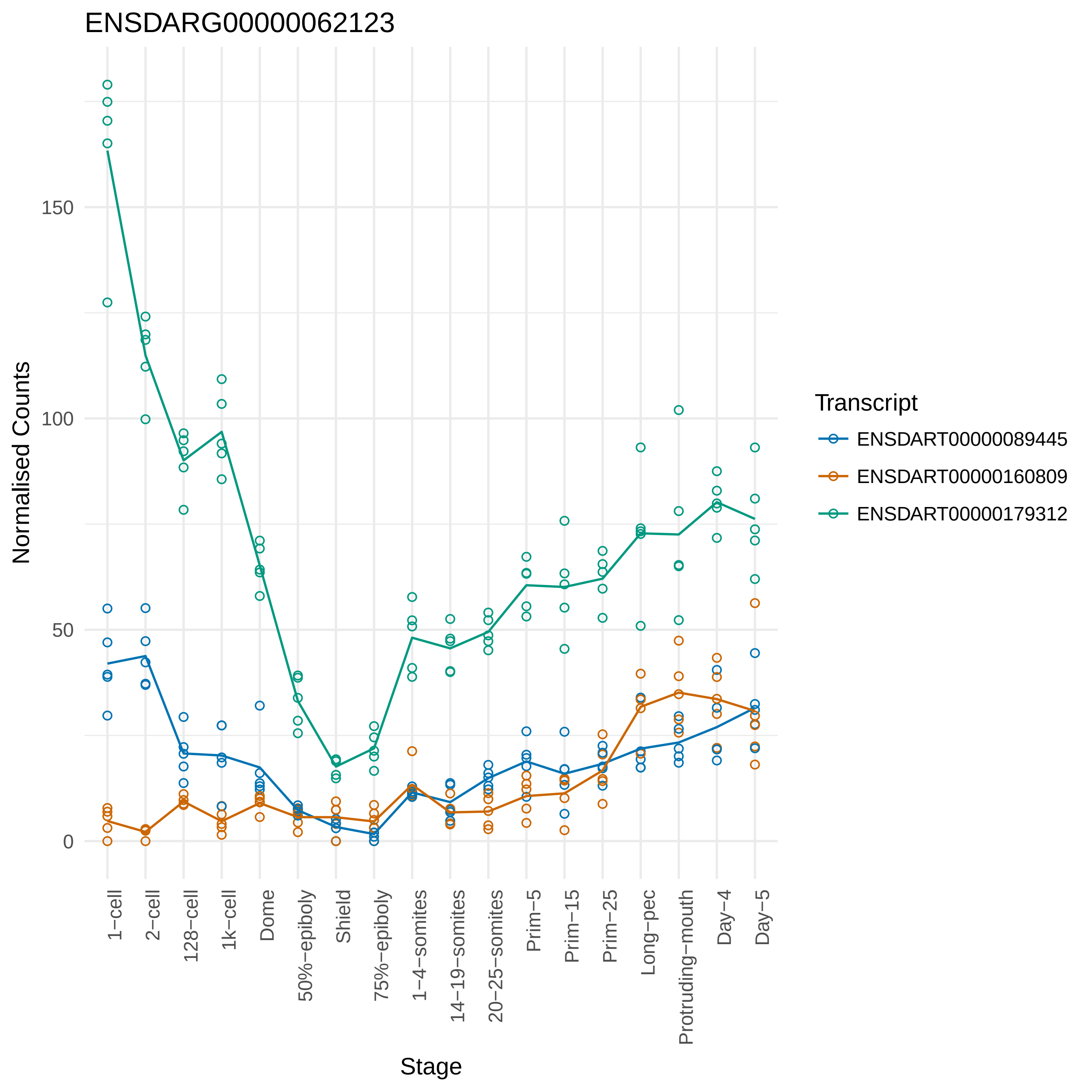

ENSDARG00000062123
Normalised Counts
0
50
100
150
Stage
1−cell
2−cell
128−cell
1k−cell
Dome
50%−epiboly
Shield
75%−epiboly
1−4−somites
14−19−somites
20−25−somites
Prim−5
Prim−15
Prim−25
Long−pec
Protruding−mouth
Day−4
Day−5
Transcript
ENSDART00000089445
ENSDART00000160809
ENSDART00000179312

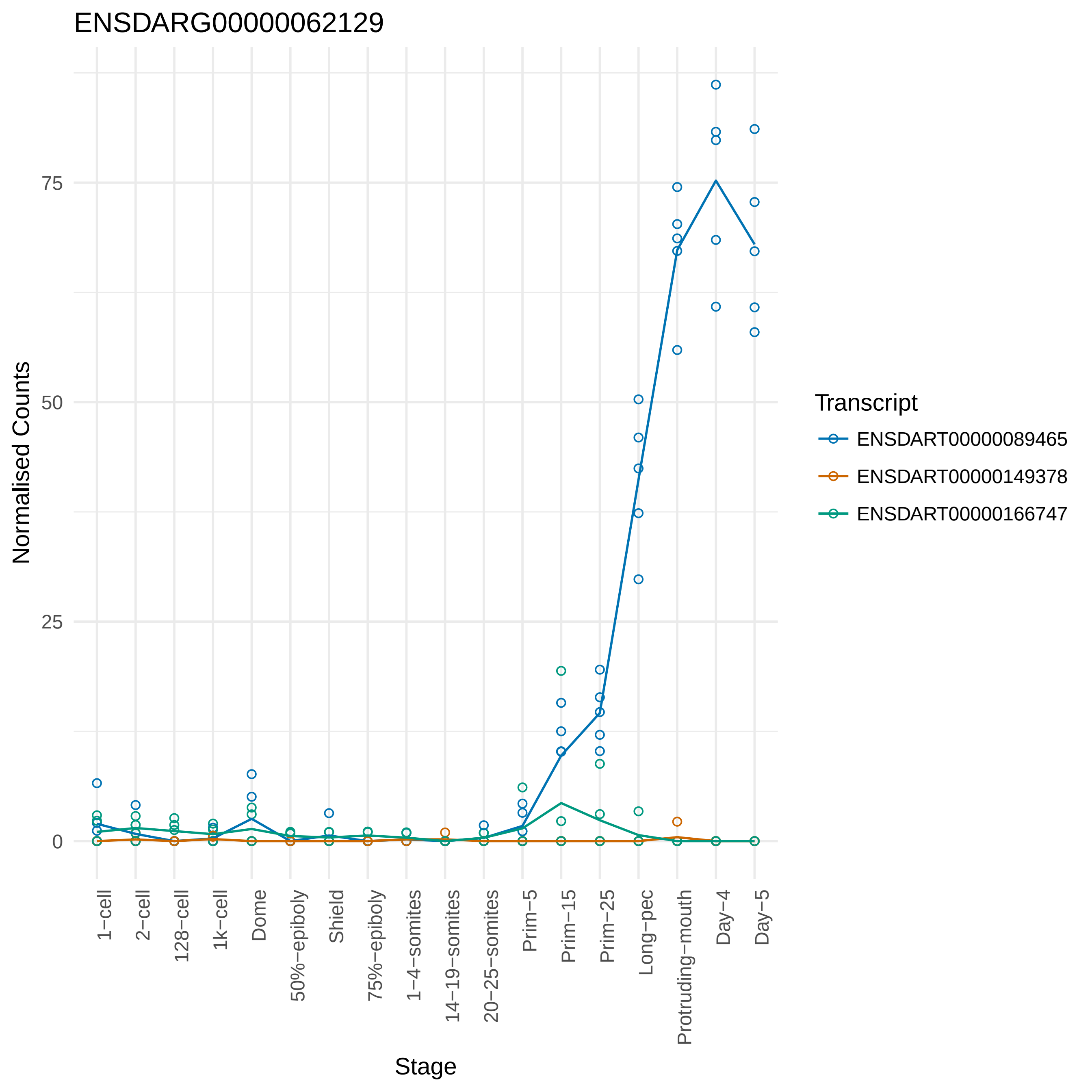

ENSDARG00000062129
Normalised Counts
0
25
50
75
Stage
1−cell
2−cell
128−cell
1k−cell
Dome
50%−epiboly
Shield
75%−epiboly
1−4−somites
14−19−somites
20−25−somites
Prim−5
Prim−15
Prim−25
Long−pec
Protruding−mouth
Day−4
Day−5
Transcript
ENSDART00000089465
ENSDART00000149378
ENSDART00000166747

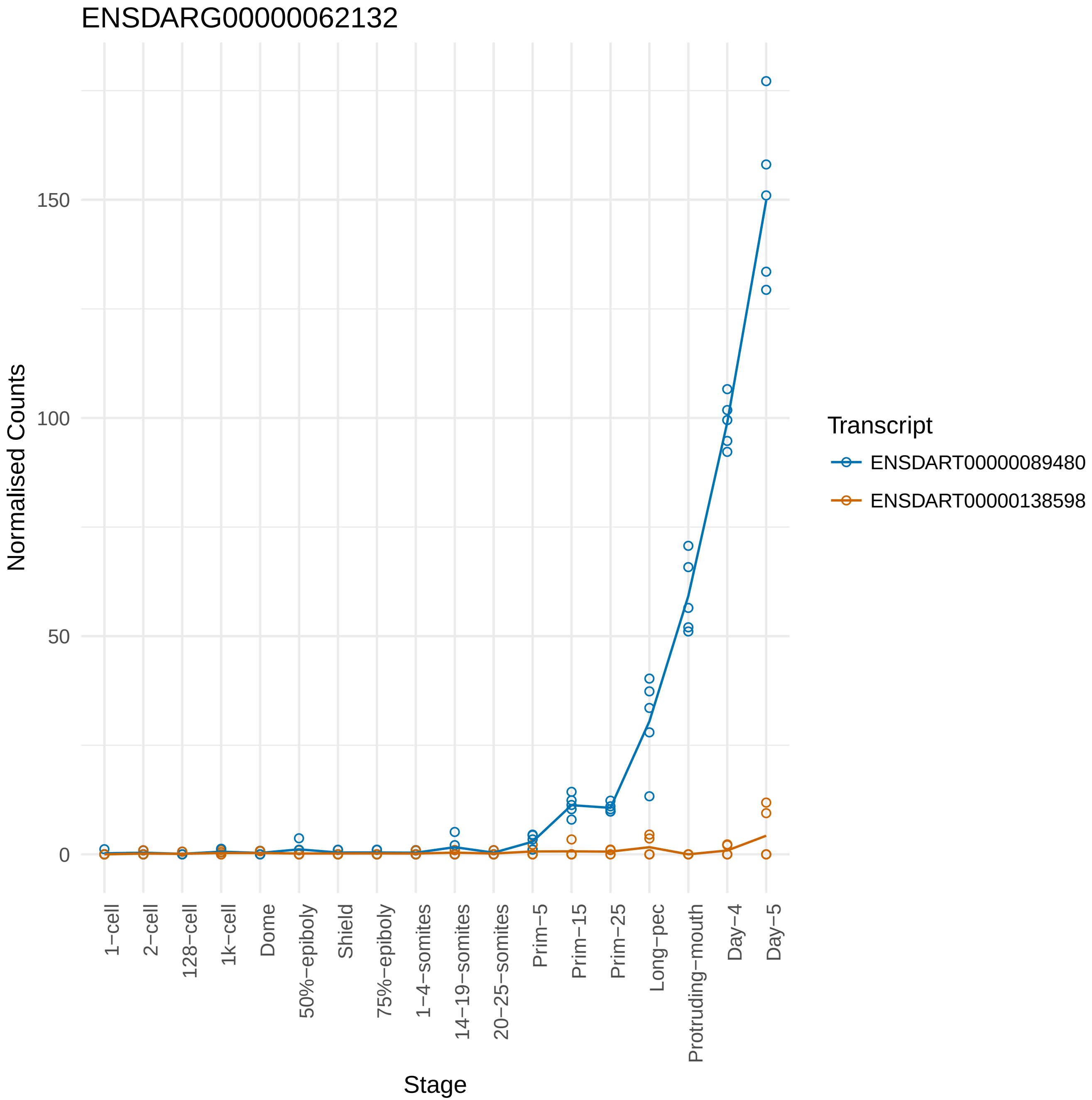
ENSDARG00000062132
Normalised Counts
150
100
50
0
1−cell
2−cell
128−cell
1k−cell
Dome
50%−epiboly
Shield
75%−epiboly
1−4−somites
14−19−somites
20−25−somites
Prim−5
Prim−15
Prim−25
Long−pec
Protruding−mouth
Day−4
Day−5
Stage
Transcript
ENSDART00000089480
ENSDART00000138598

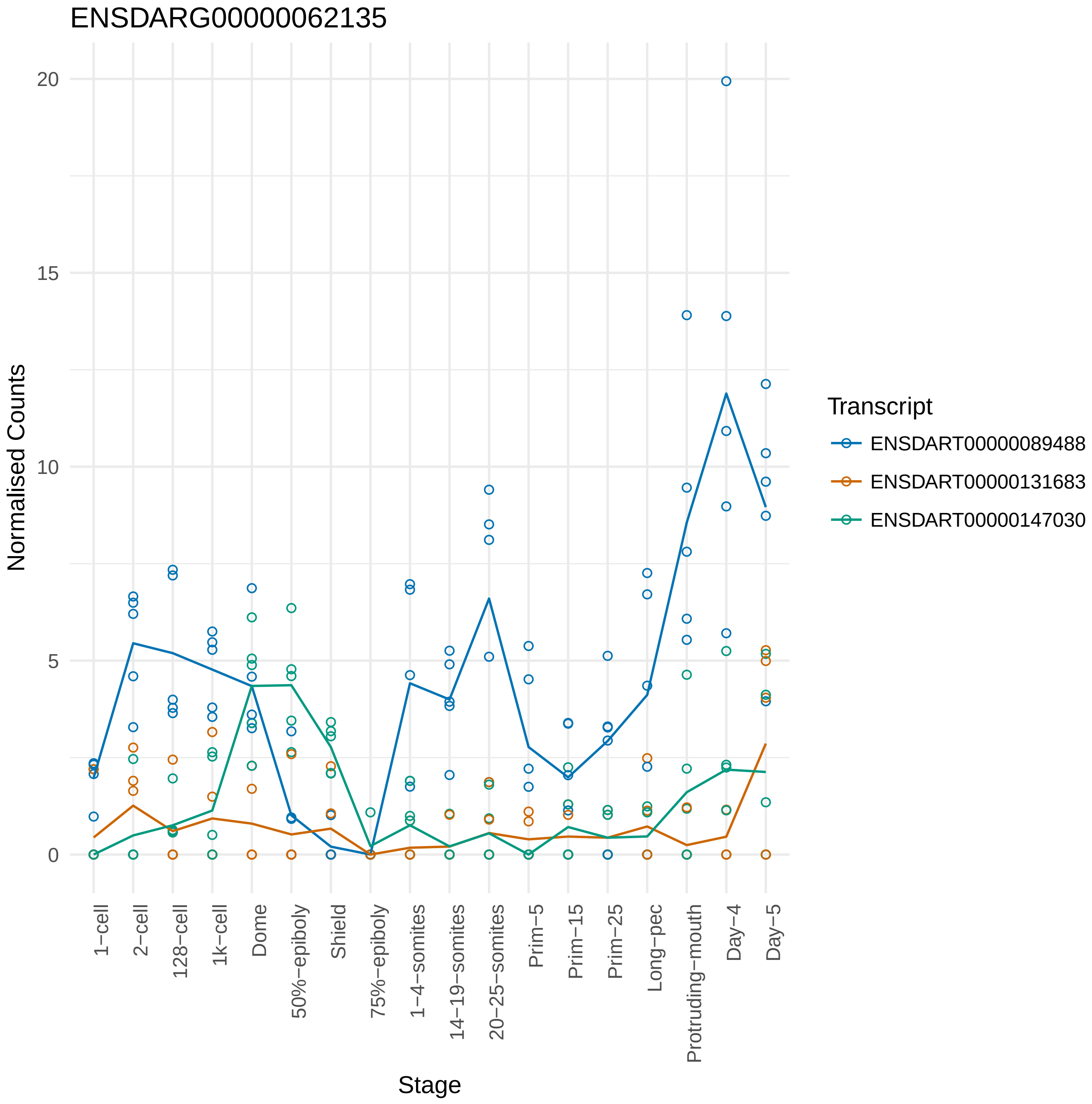

ENSDARG00000062135
Normalised Counts
20
15
10
5
0
1−cell
2−cell
128−cell
1k−cell
Dome
50%−epiboly
Shield
75%−epiboly
1−4−somites
14−19−somites
20−25−somites
Prim−5
Prim−15
Prim−25
Long−pec
Protruding−mouth
Day−4
Day−5
Stage
Transcript
ENSDART00000089488
ENSDART00000131683
ENSDART00000147030

ENSDARG00000062139

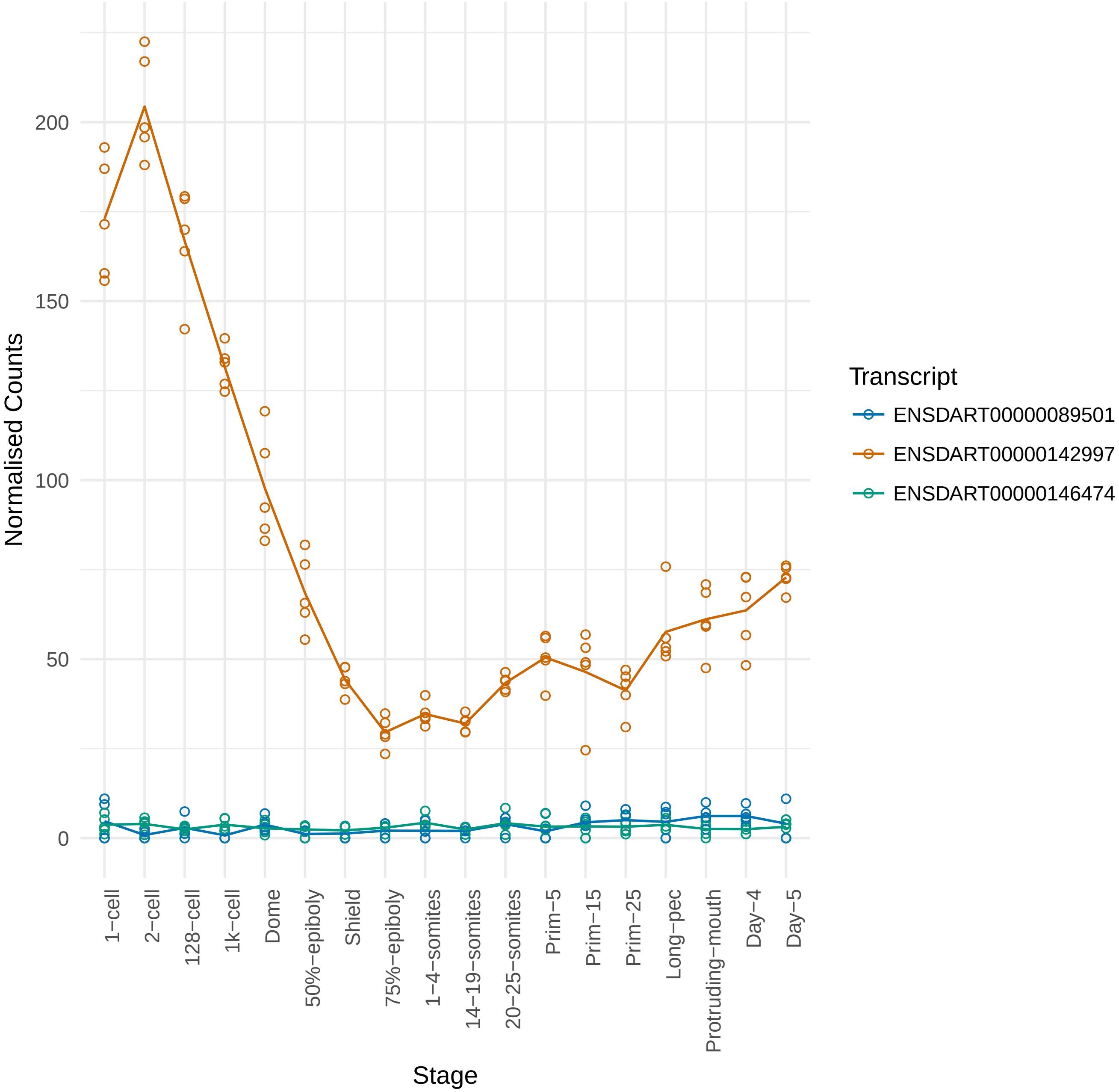

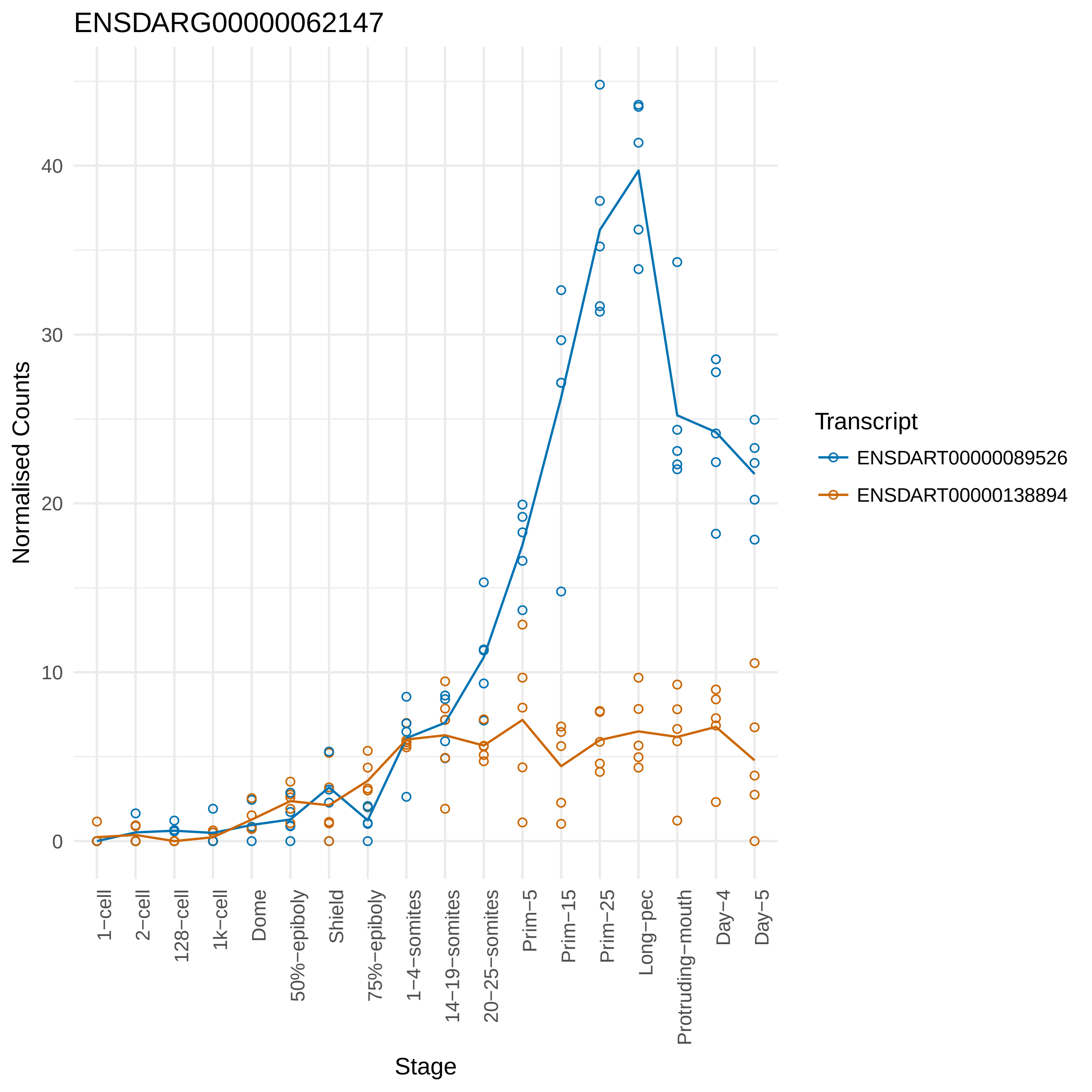
ENSDARG00000062147
Normalised Counts
0
10
20
30
40
Stage
1−cell
2−cell
128−cell
1k−cell
Dome
50%−epiboly
Shield
75%−epiboly
1−4−somites
14−19−somites
20−25−somites
Prim−5
Prim−15
Prim−25
Long−pec
Protruding−mouth
Day−4
Day−5
Transcript
ENSDART00000089526
ENSDART00000138894

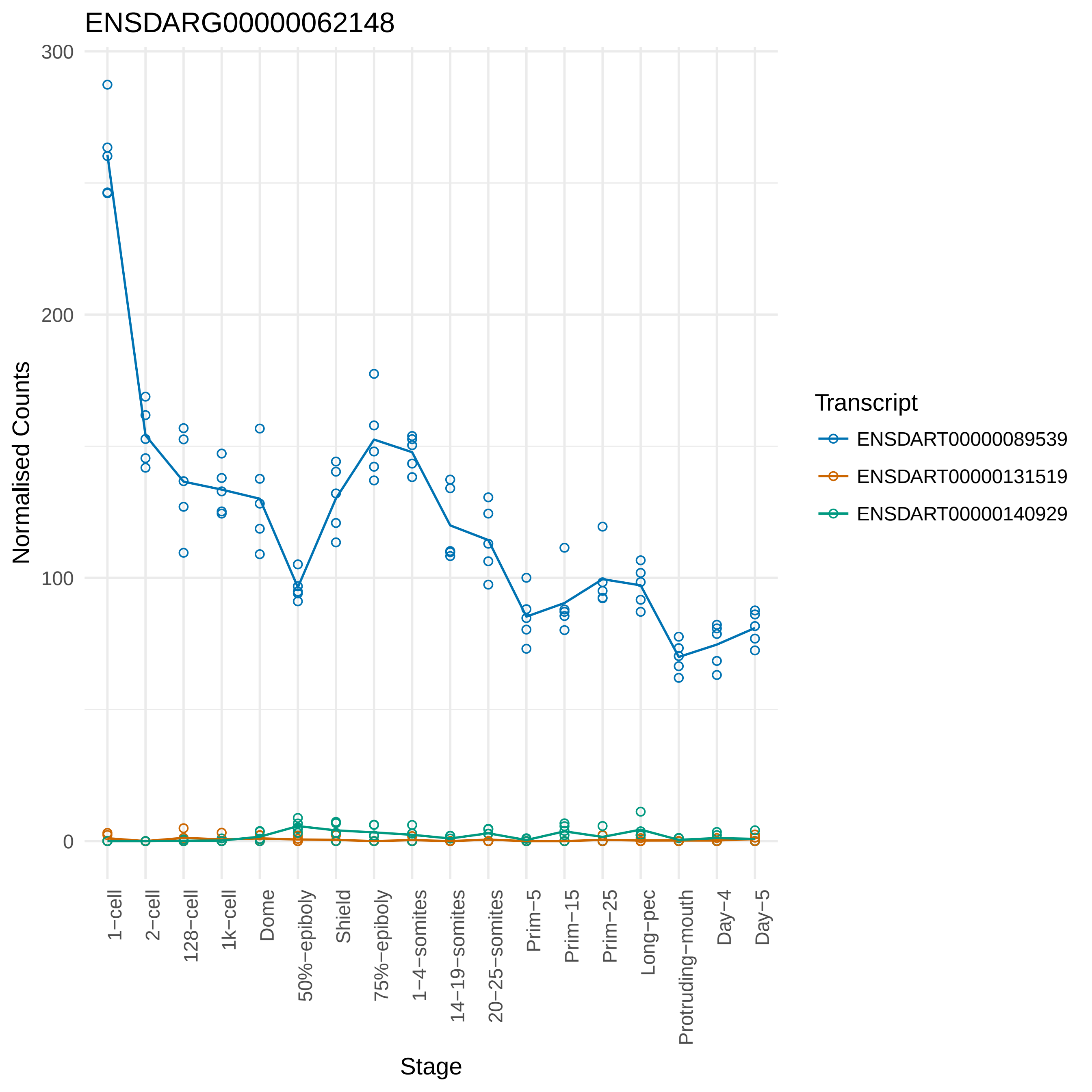
ENSDARG00000062148
Normalised Counts
300
200
100
0
1−cell
2−cell
128−cell
1k−cell
Dome
50%−epiboly
Shield
75%−epiboly
1−4−somites
14−19−somites
20−25−somites
Prim−5
Prim−15
Prim−25
Long−pec
Protruding−mouth
Day−4
Day−5
Stage
Transcript
ENSDART00000089539
ENSDART00000131519
ENSDART00000140929

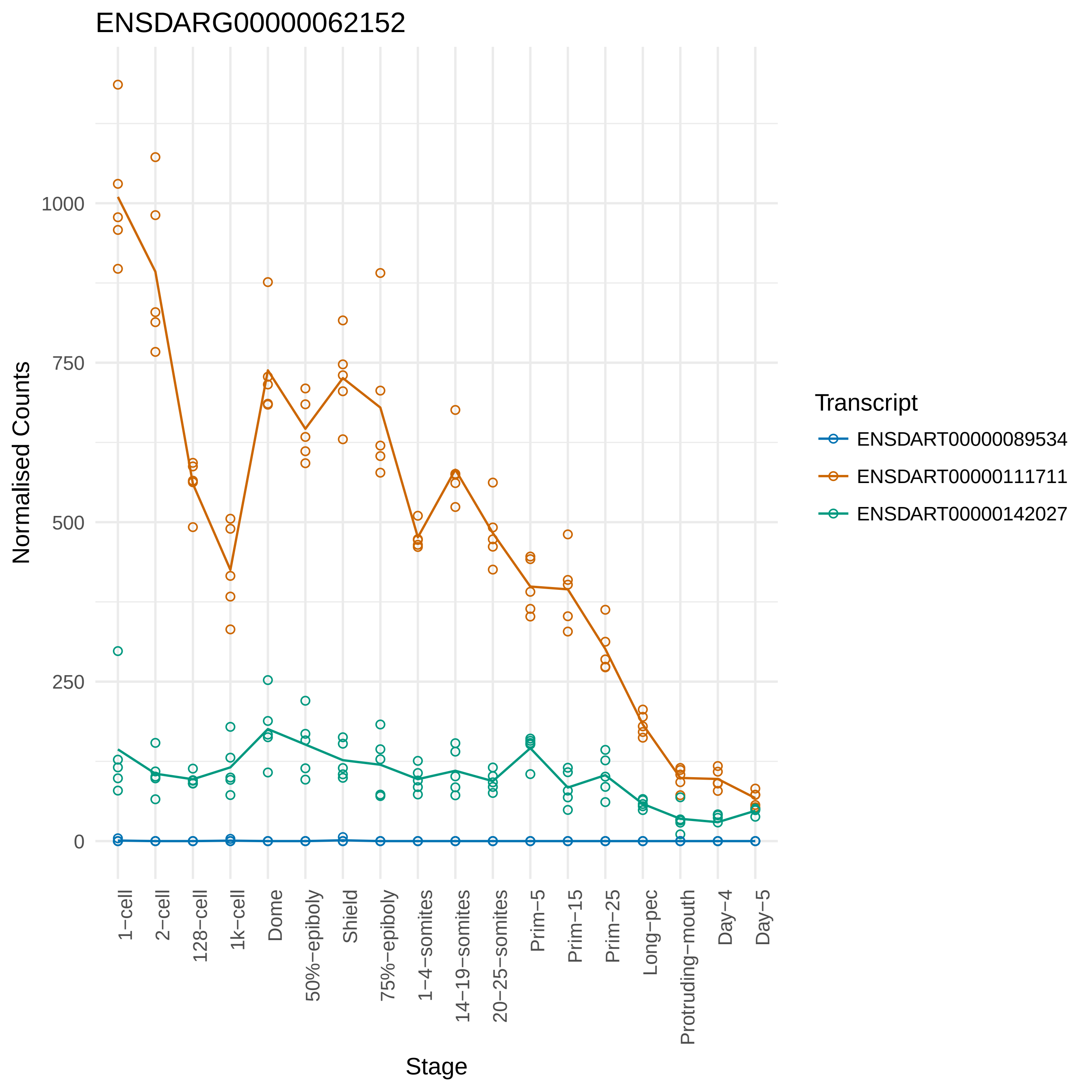

ENSDARG00000062152
Normalised Counts
0
250
500
750
1000
Stage
1−cell
2−cell
128−cell
1k−cell
Dome
50%−epiboly
Shield
75%−epiboly
1−4−somites
14−19−somites
20−25−somites
Prim−5
Prim−15
Prim−25
Long−pec
Protruding−mouth
Day−4
Day−5
Transcript
ENSDART00000089534
ENSDART00000111711
ENSDART00000142027

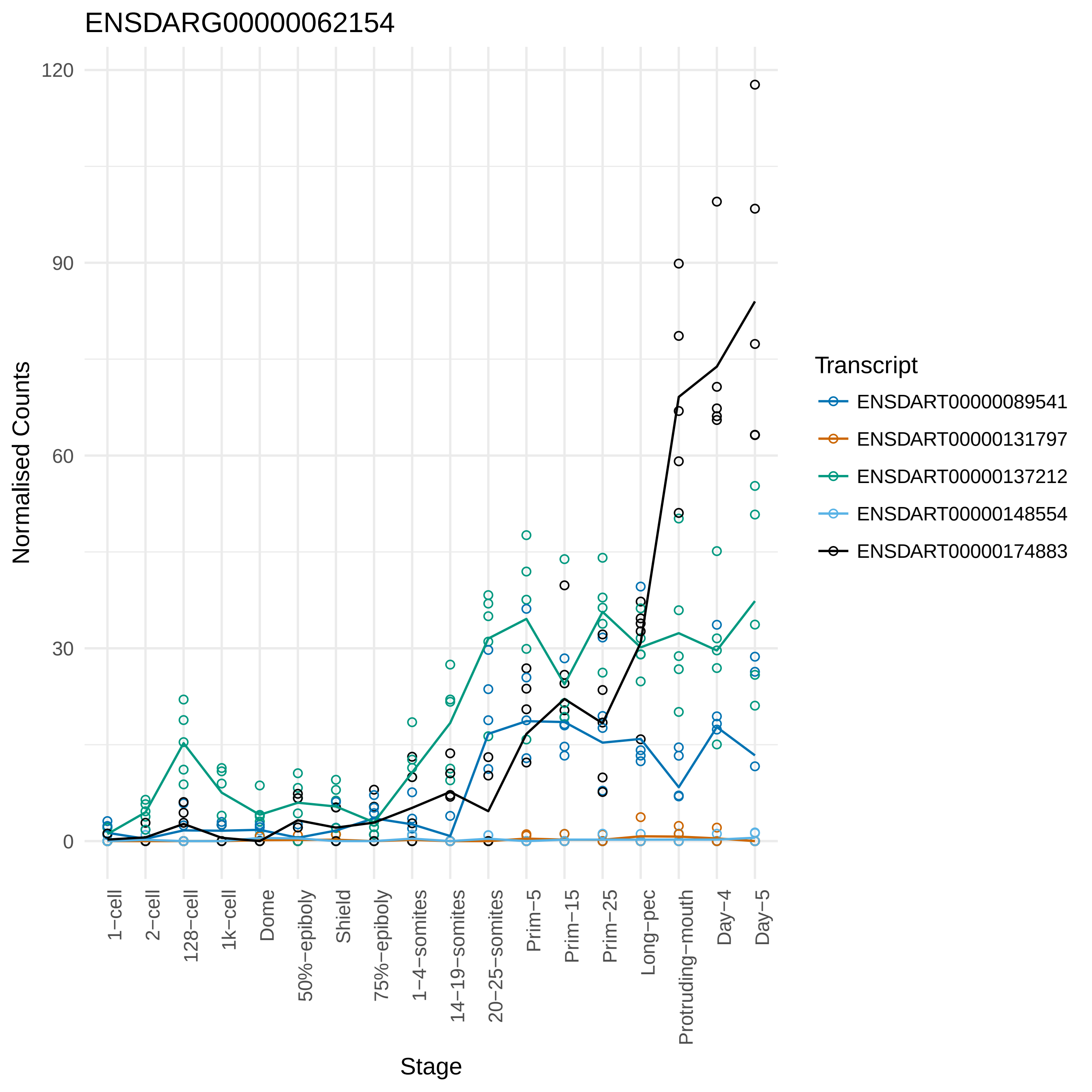

ENSDARG00000062154
Normalised Counts
120
90
60
30
0
1−cell
2−cell
128−cell
1k−cell
Dome
50%−epiboly
Shield
75%−epiboly
1−4−somites
14−19−somites
20−25−somites
Prim−5
Prim−15
Prim−25
Long−pec
Protruding−mouth
Day−4
Day−5
Stage
Transcript
ENSDART00000089541
ENSDART00000131797
ENSDART00000137212
ENSDART00000148554
ENSDART00000174883

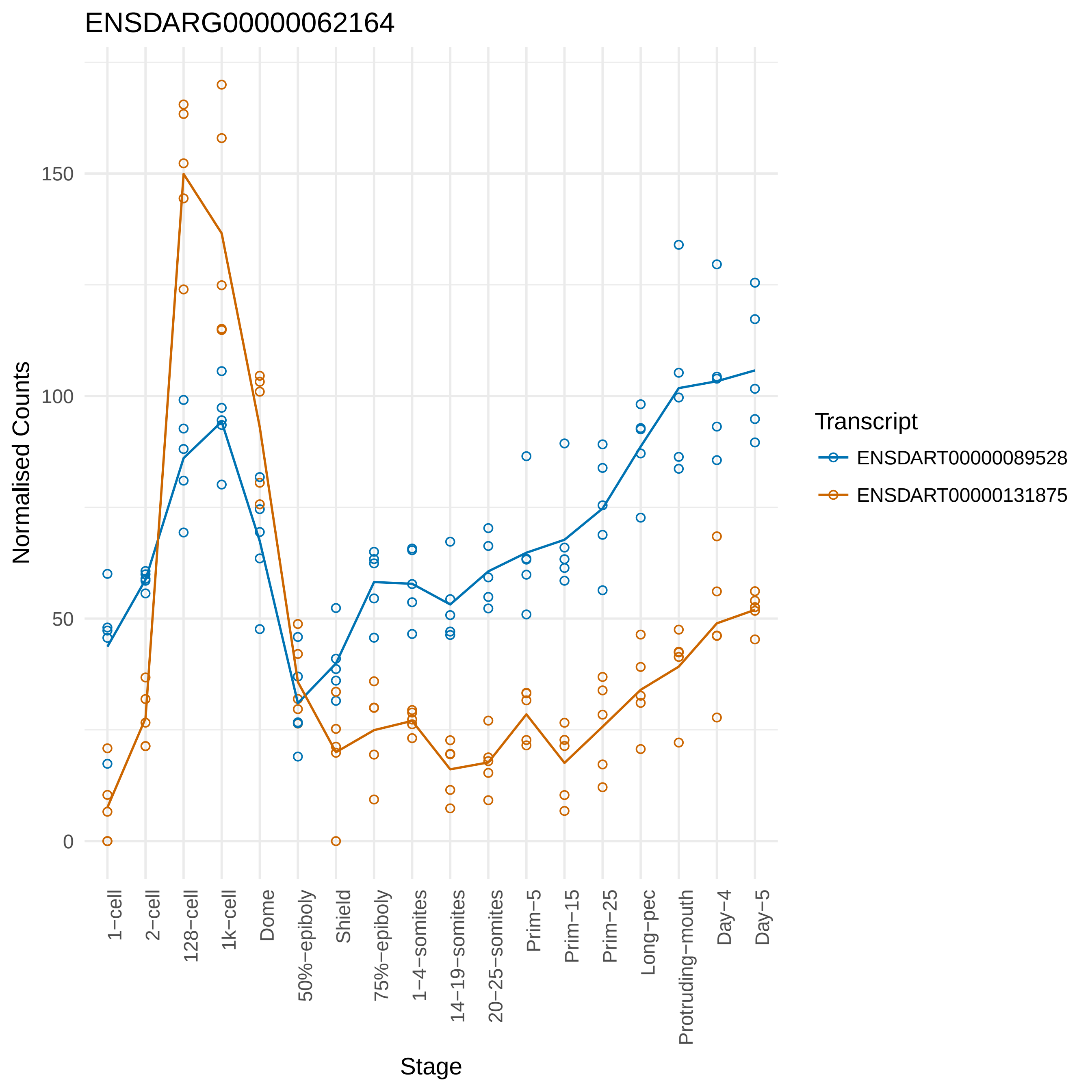

ENSDARG00000062164
Normalised Counts
150
100
50
0
1−cell
2−cell
128−cell
1k−cell
Dome
50%−epiboly
Shield
75%−epiboly
1−4−somites
14−19−somites
20−25−somites
Prim−5
Prim−15
Prim−25
Long−pec
Protruding−mouth
Day−4
Day−5
Stage
Transcript
ENSDART00000089528
ENSDART00000131875

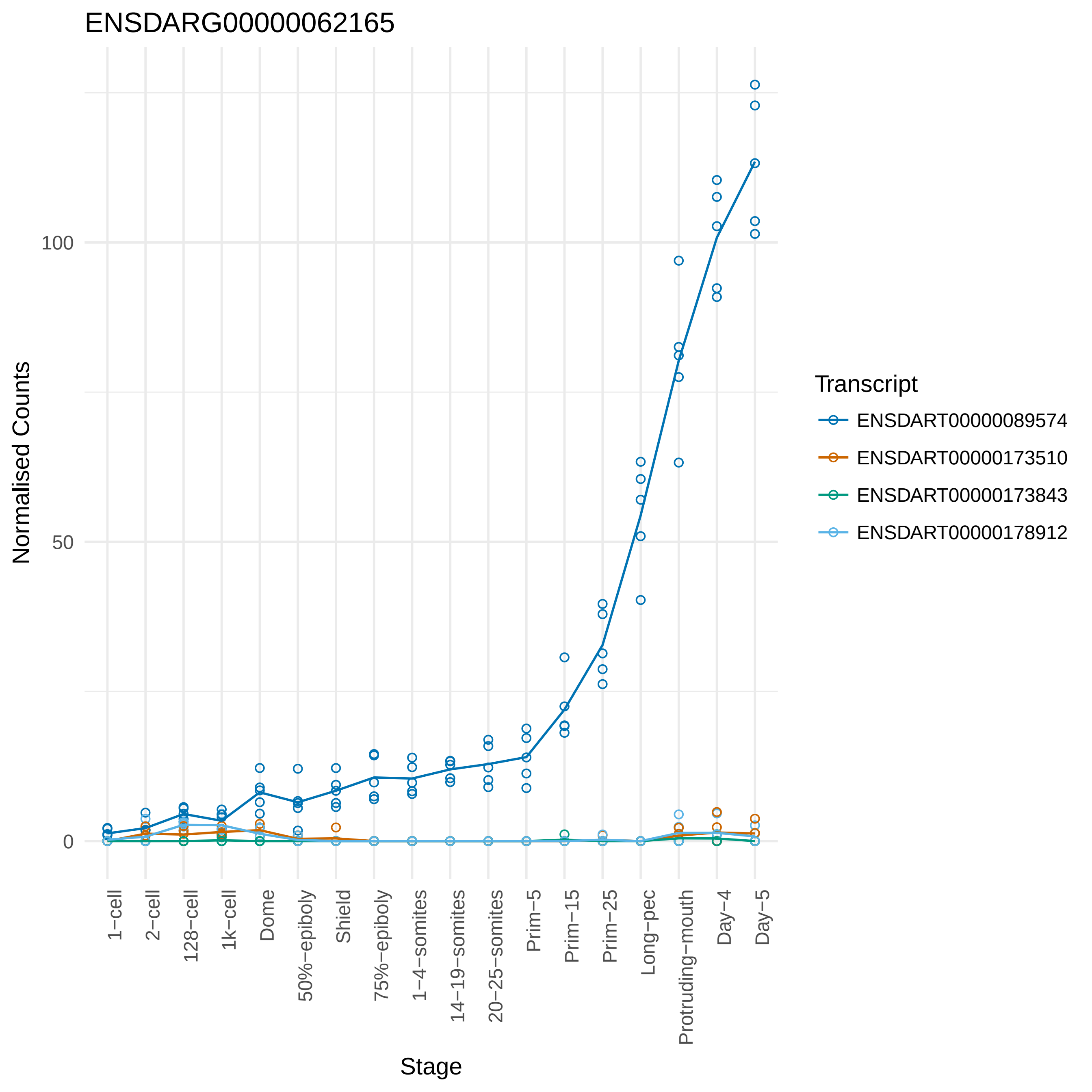
ENSDARG00000062165
Normalised Counts
0
50
100
1−cell
2−cell
128−cell
1k−cell
Dome
50%−epiboly
Shield
75%−epiboly
1−4−somites
14−19−somites
20−25−somites
Prim−5
Prim−15
Prim−25
Long−pec
Protruding−mouth
Day−4
Day−5
Stage
Transcript
ENSDART00000089574
ENSDART00000173510
ENSDART00000173843
ENSDART00000178912

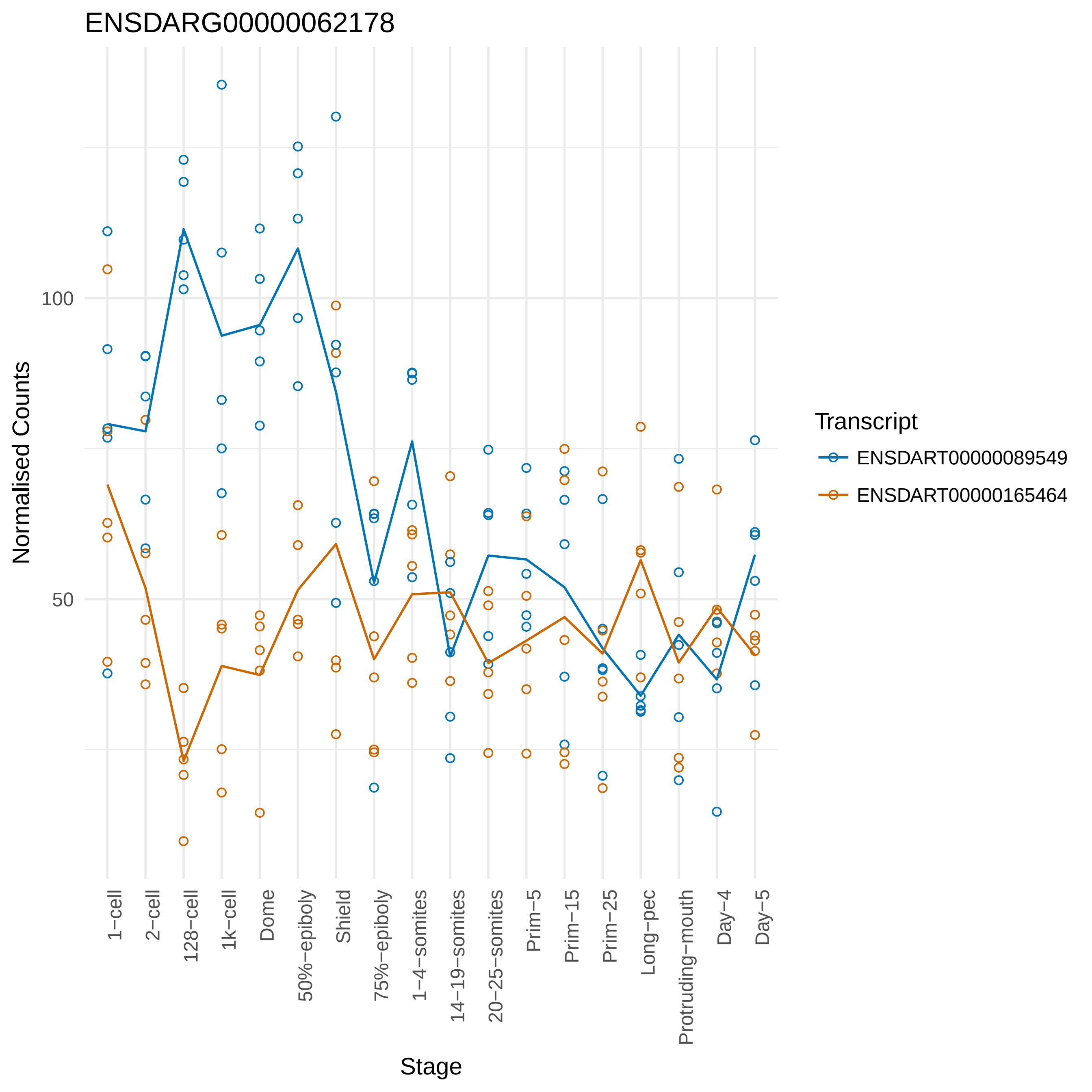
ENSDARG00000062178
Normalised Counts
100
50
1−cell
2−cell
128−cell
1k−cell
Dome
50%−epiboly
Shield
75%−epiboly
1−4−somites
14−19−somites
20−25−somites
Prim−5
Prim−15
Prim−25
Long−pec
Protruding−mouth
Day−4
Day−5
Stage
Transcript
ENSDART00000089549
ENSDART00000165464

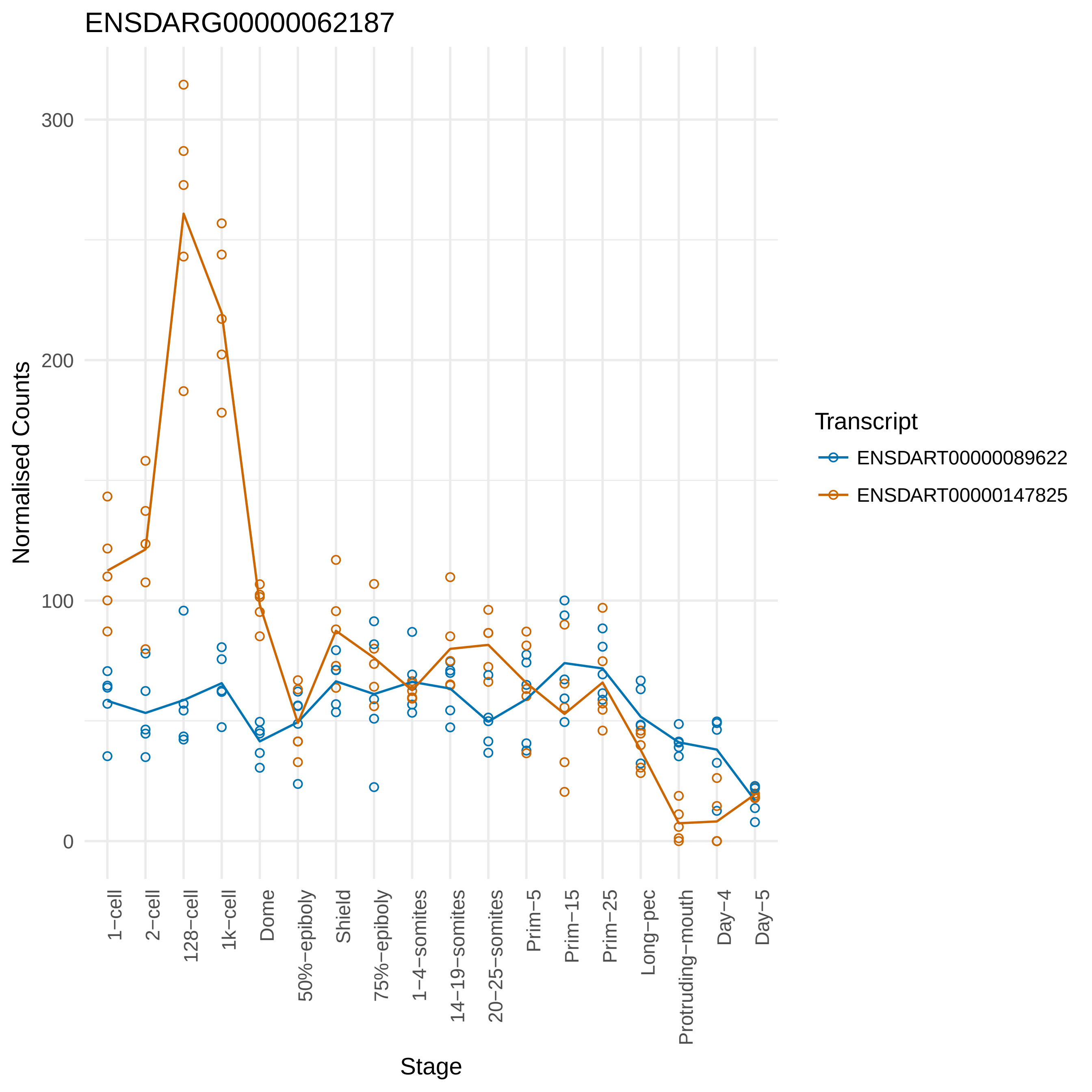

ENSDARG00000062187
Normalised Counts
300
200
100
0
1−cell
2−cell
128−cell
1k−cell
Dome
50%−epiboly
Shield
75%−epiboly
1−4−somites
14−19−somites
20−25−somites
Prim−5
Prim−15
Prim−25
Long−pec
Protruding−mouth
Day−4
Day−5
Stage
Transcript
ENSDART00000089622
ENSDART00000147825

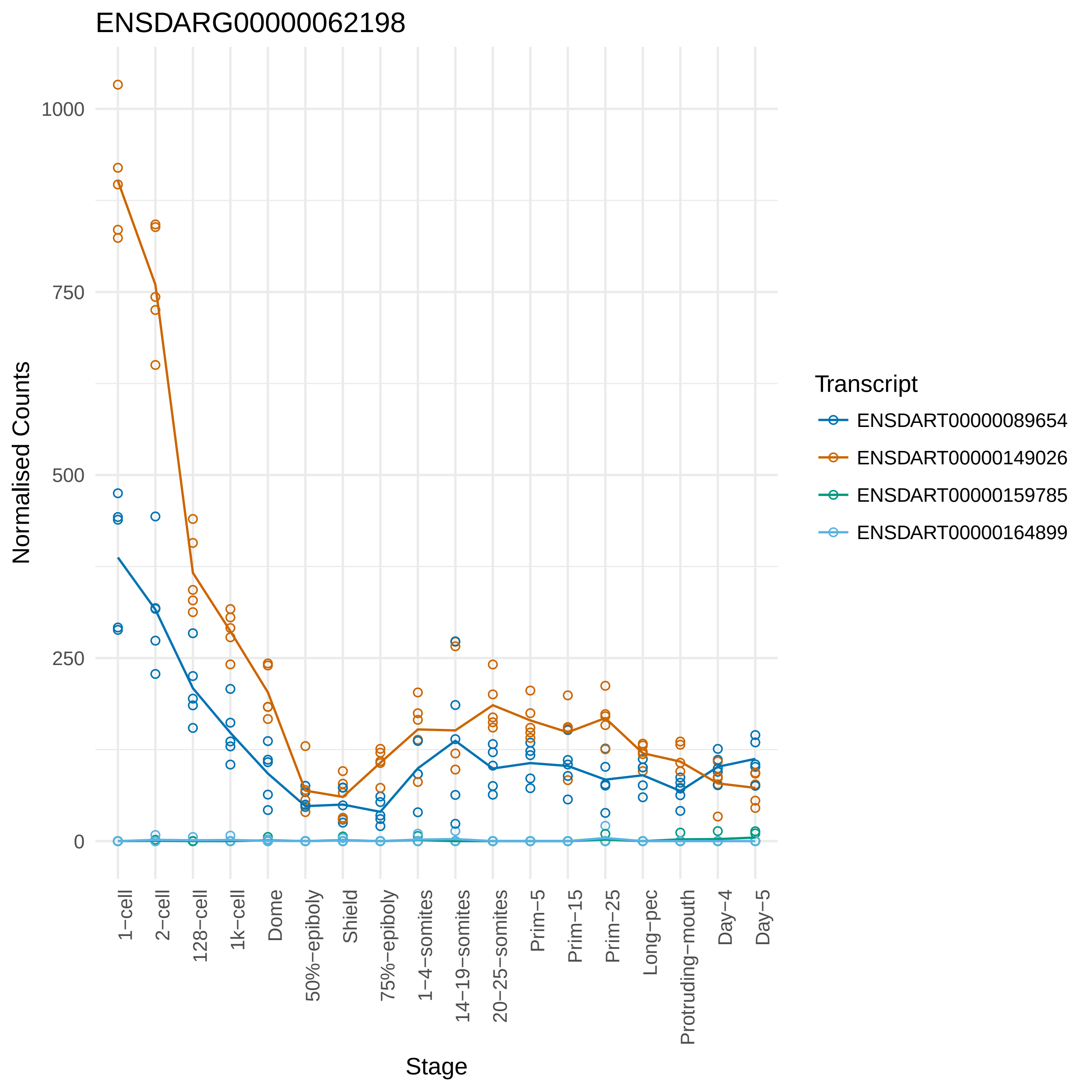

ENSDARG00000062198
Normalised Counts
1000
750
500
250
0
1−cell
2−cell
128−cell
1k−cell
Dome
50%−epiboly
Shield
75%−epiboly
1−4−somites
14−19−somites
20−25−somites
Prim−5
Prim−15
Prim−25
Long−pec
Protruding−mouth
Day−4
Day−5
Stage
Transcript
ENSDART00000089654
ENSDART00000149026
ENSDART00000159785
ENSDART00000164899

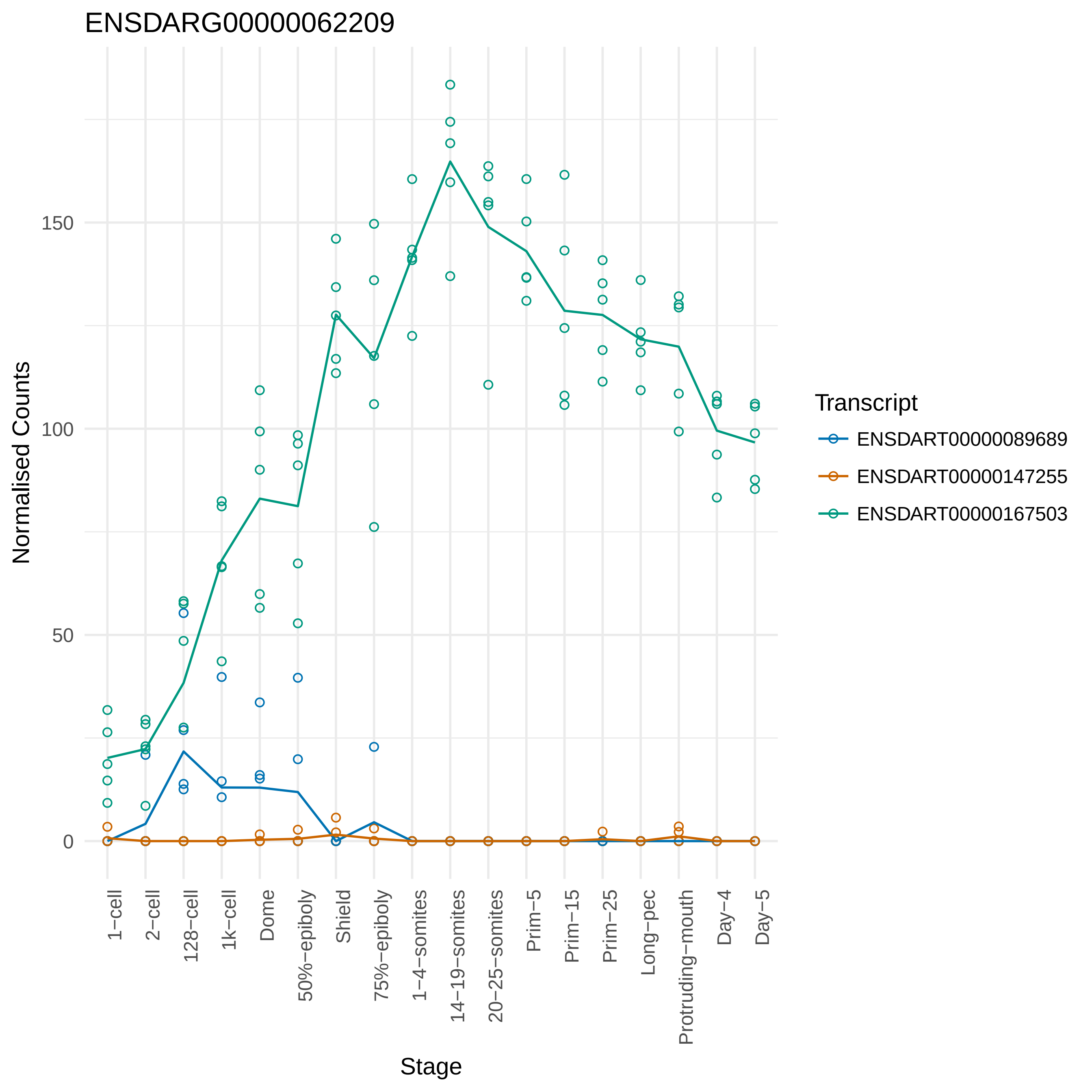

ENSDARG00000062209
Normalised Counts
0
50
100
150
1−cell
2−cell
128−cell
1k−cell
Dome
50%−epiboly
Shield
75%−epiboly
1−4−somites
14−19−somites
20−25−somites
Prim−5
Prim−15
Prim−25
Long−pec
Protruding−mouth
Day−4
Day−5
Stage
Transcript
ENSDART00000089689
ENSDART00000147255
ENSDART00000167503

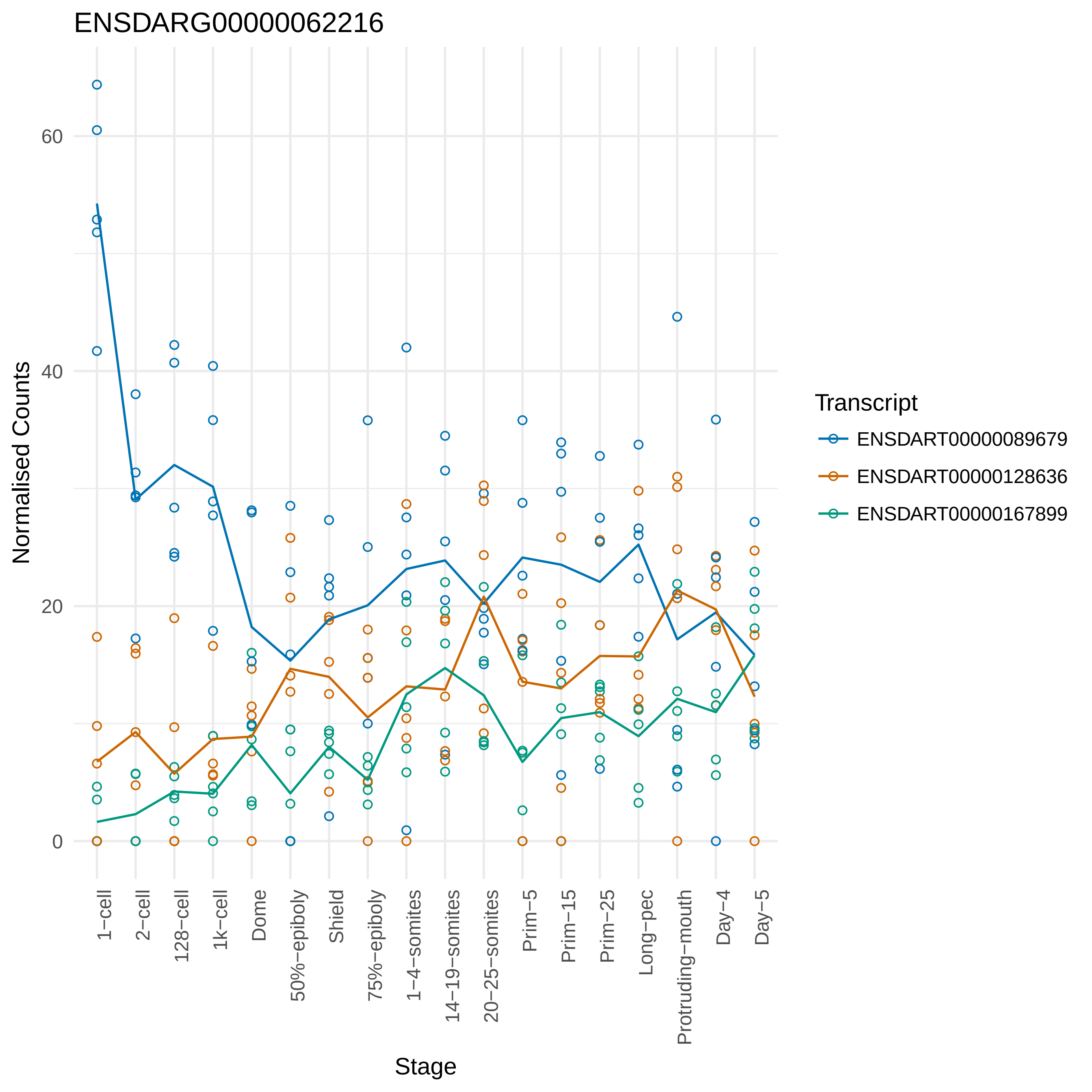

ENSDARG00000062216
Normalised Counts
0
20
40
60
Stage
1−cell
2−cell
128−cell
1k−cell
Dome
50%−epiboly
Shield
75%−epiboly
1−4−somites
14−19−somites
20−25−somites
Prim−5
Prim−15
Prim−25
Long−pec
Protruding−mouth
Day−4
Day−5
Transcript
ENSDART00000089679
ENSDART00000128636
ENSDART00000167899

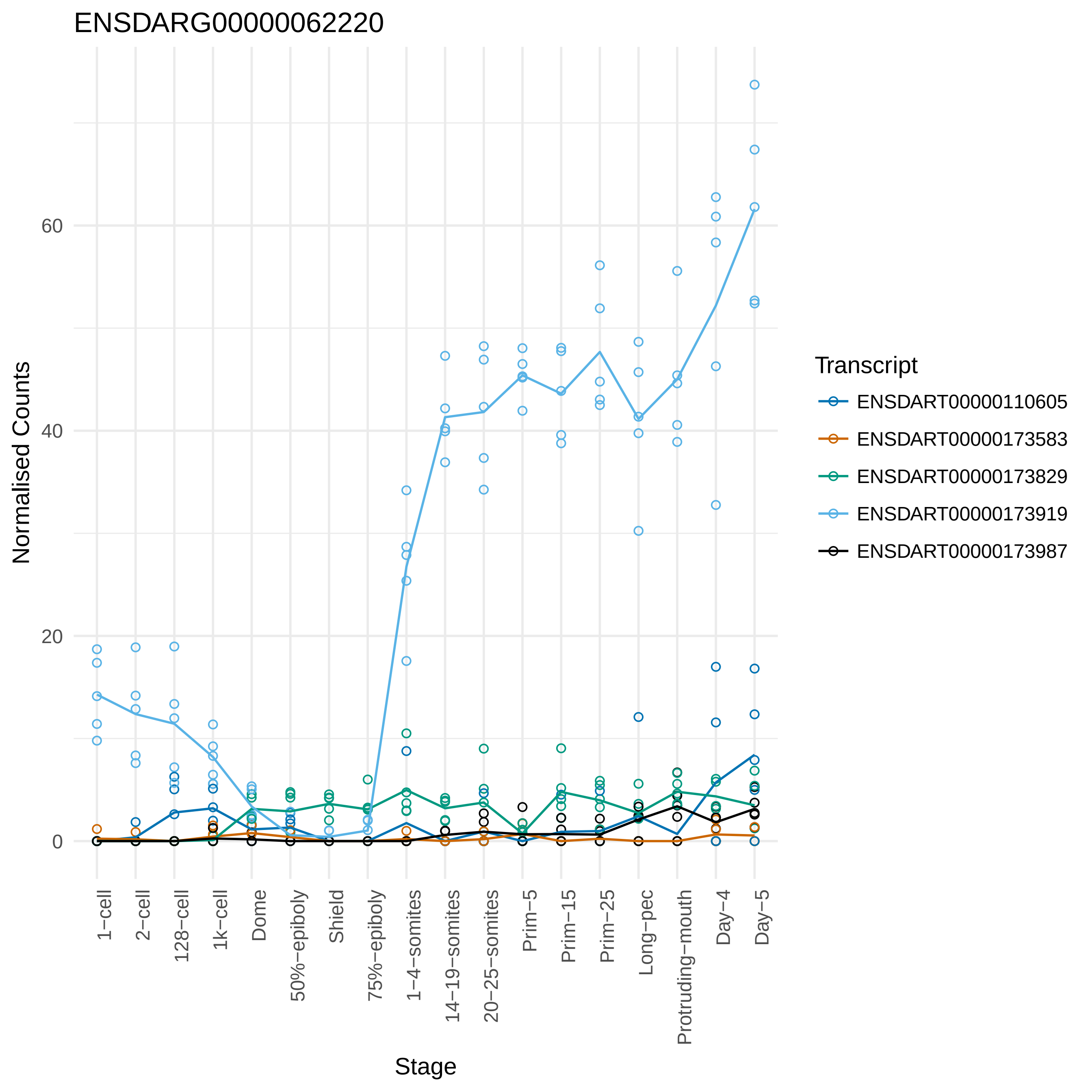
ENSDARG00000062220
Normalised Counts
0
20
40
60
Stage
1−cell
2−cell
128−cell
1k−cell
Dome
50%−epiboly
Shield
75%−epiboly
1−4−somites
14−19−somites
20−25−somites
Prim−5
Prim−15
Prim−25
Long−pec
Protruding−mouth
Day−4
Day−5
Transcript
ENSDART00000110605
ENSDART00000173583
ENSDART00000173829
ENSDART00000173919
ENSDART00000173987

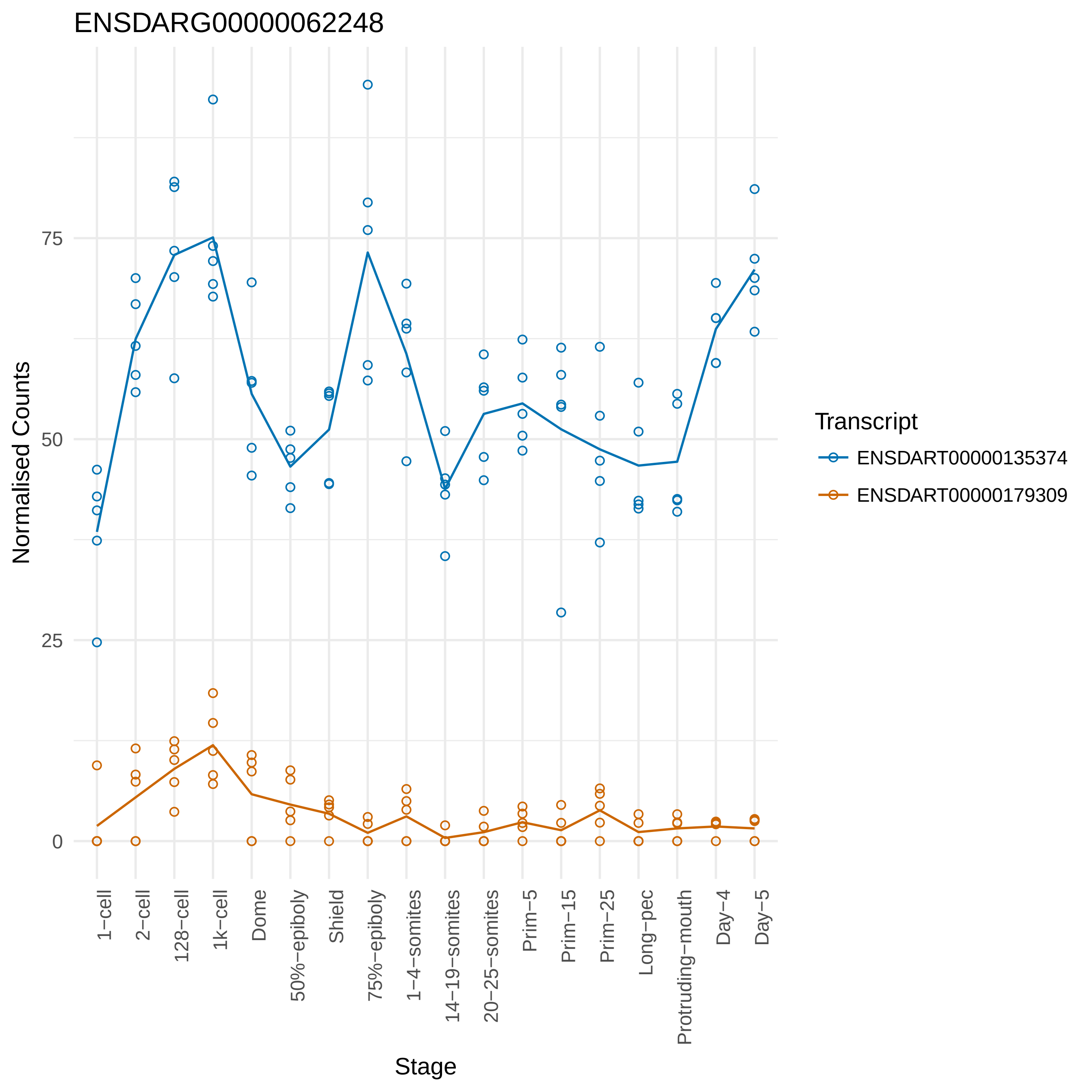

ENSDARG00000062248
Normalised Counts
0
25
50
75
1−cell
2−cell
128−cell
1k−cell
Dome
50%−epiboly
Shield
75%−epiboly
1−4−somites
14−19−somites
20−25−somites
Prim−5
Prim−15
Prim−25
Long−pec
Protruding−mouth
Day−4
Day−5
Stage
Transcript
ENSDART00000135374
ENSDART00000179309

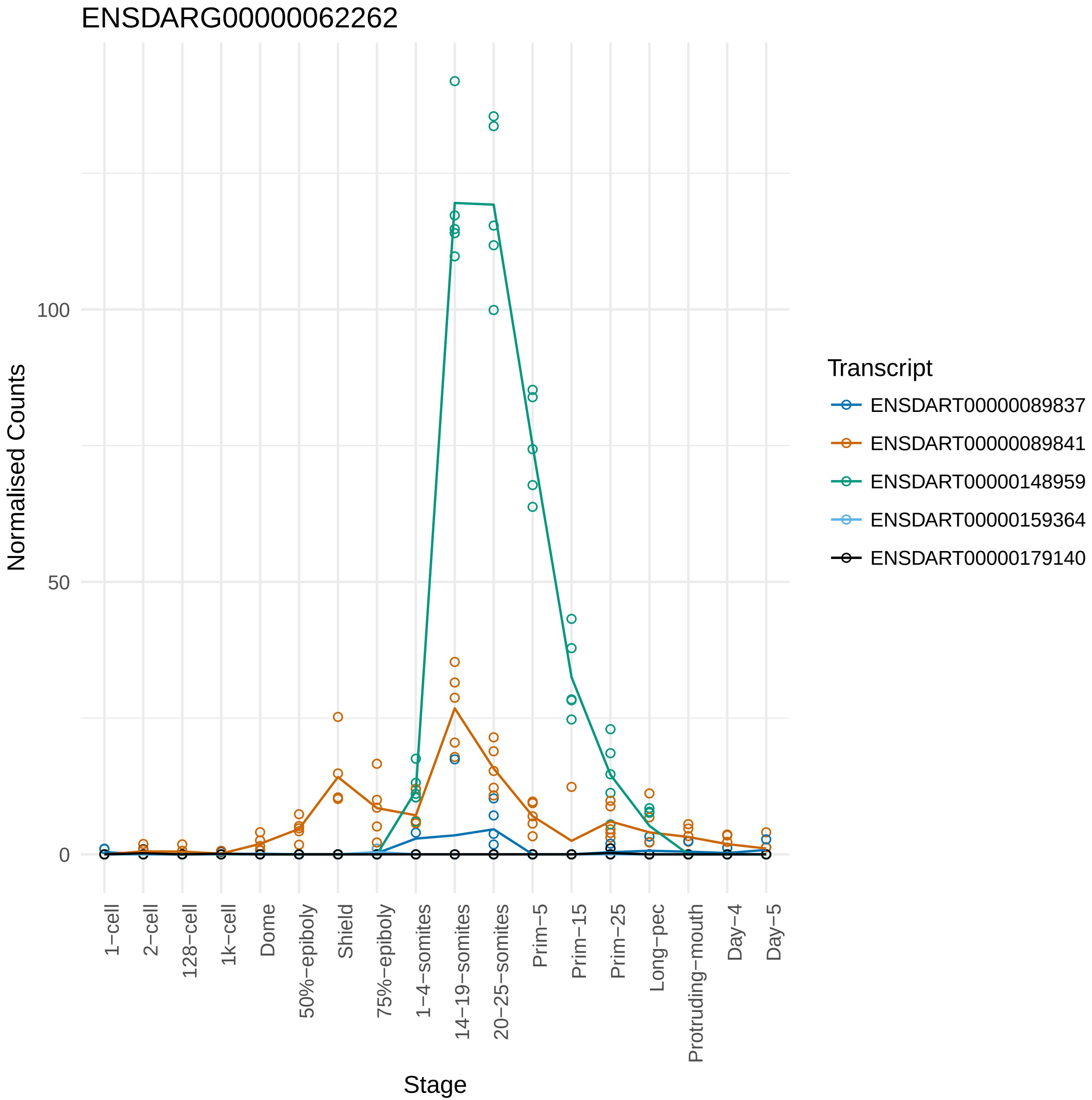
ENSDARG00000062262
Normalised Counts
100
50
0
1−cell
2−cell
128−cell
1k−cell
Dome
50%−epiboly
Shield
75%−epiboly
1−4−somites
14−19−somites
20−25−somites
Prim−5
Prim−15
Prim−25
Long−pec
Protruding−mouth
Day−4
Day−5
Stage
Transcript
ENSDART00000089837
ENSDART00000089841
ENSDART00000148959
ENSDART00000159364
ENSDART00000179140

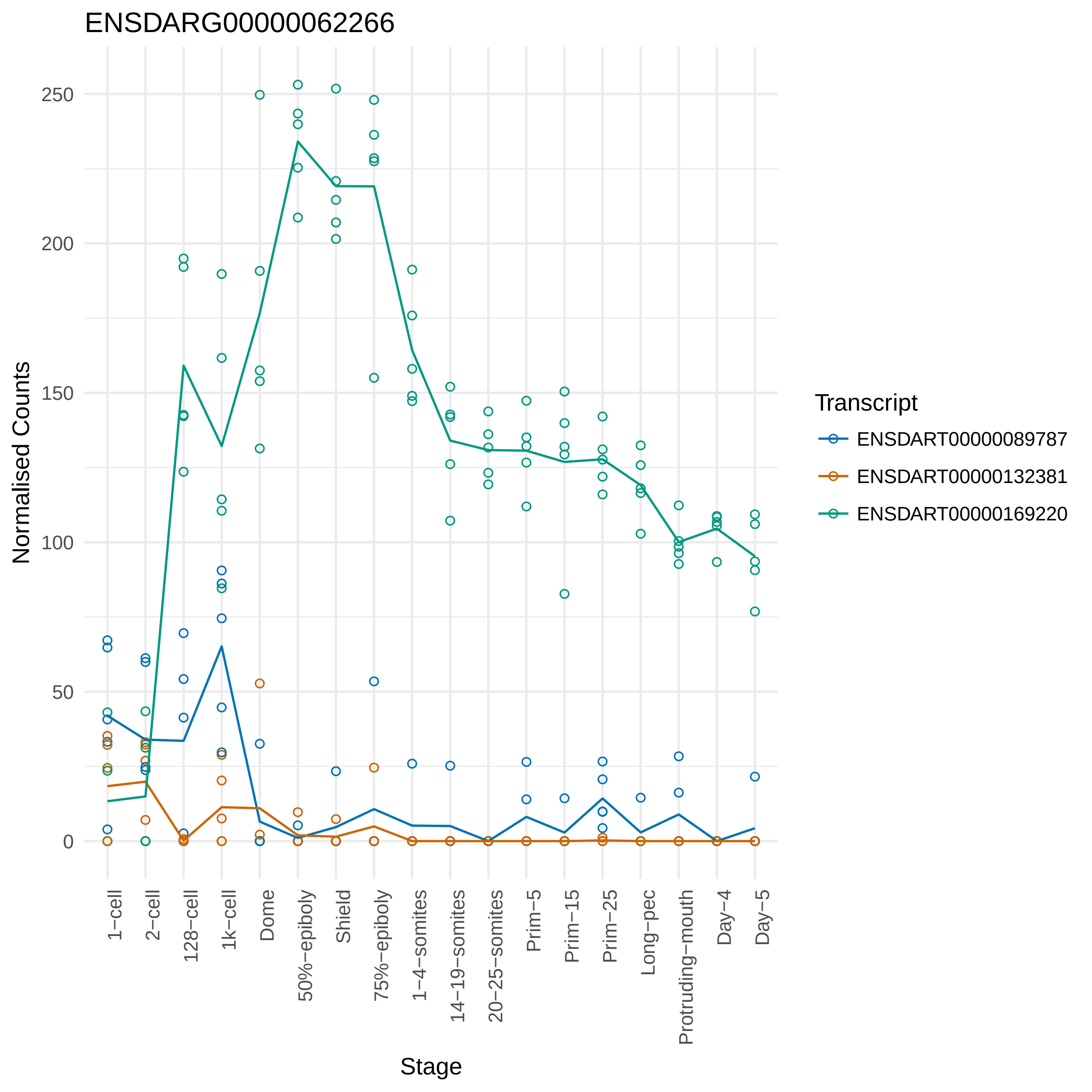
ENSDARG00000062266
Normalised Counts
0
50
100
150
200
250
Stage
1−cell
2−cell
128−cell
1k−cell
Dome
50%−epiboly
Shield
75%−epiboly
1−4−somites
14−19−somites
20−25−somites
Prim−5
Prim−15
Prim−25
Long−pec
Protruding−mouth
Day−4
Day−5
Transcript
ENSDART00000089787
ENSDART00000132381
ENSDART00000169220

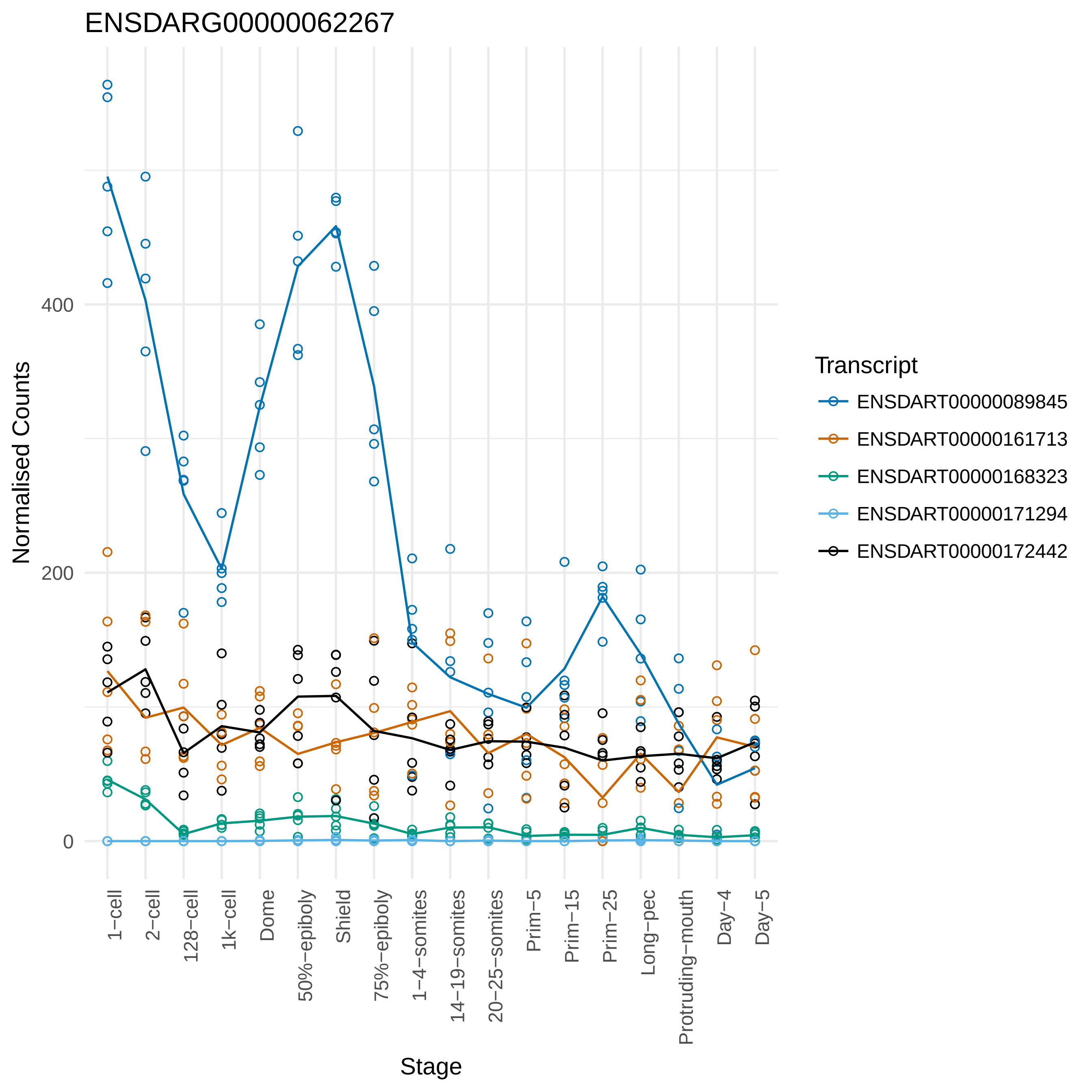

ENSDARG00000062267
Normalised Counts
400
200
0
1−cell
2−cell
128−cell
1k−cell
Dome
50%−epiboly
Shield
75%−epiboly
1−4−somites
14−19−somites
20−25−somites
Prim−5
Prim−15
Prim−25
Long−pec
Protruding−mouth
Day−4
Day−5
Stage
Transcript
ENSDART00000089845
ENSDART00000161713
ENSDART00000168323
ENSDART00000171294
ENSDART00000172442

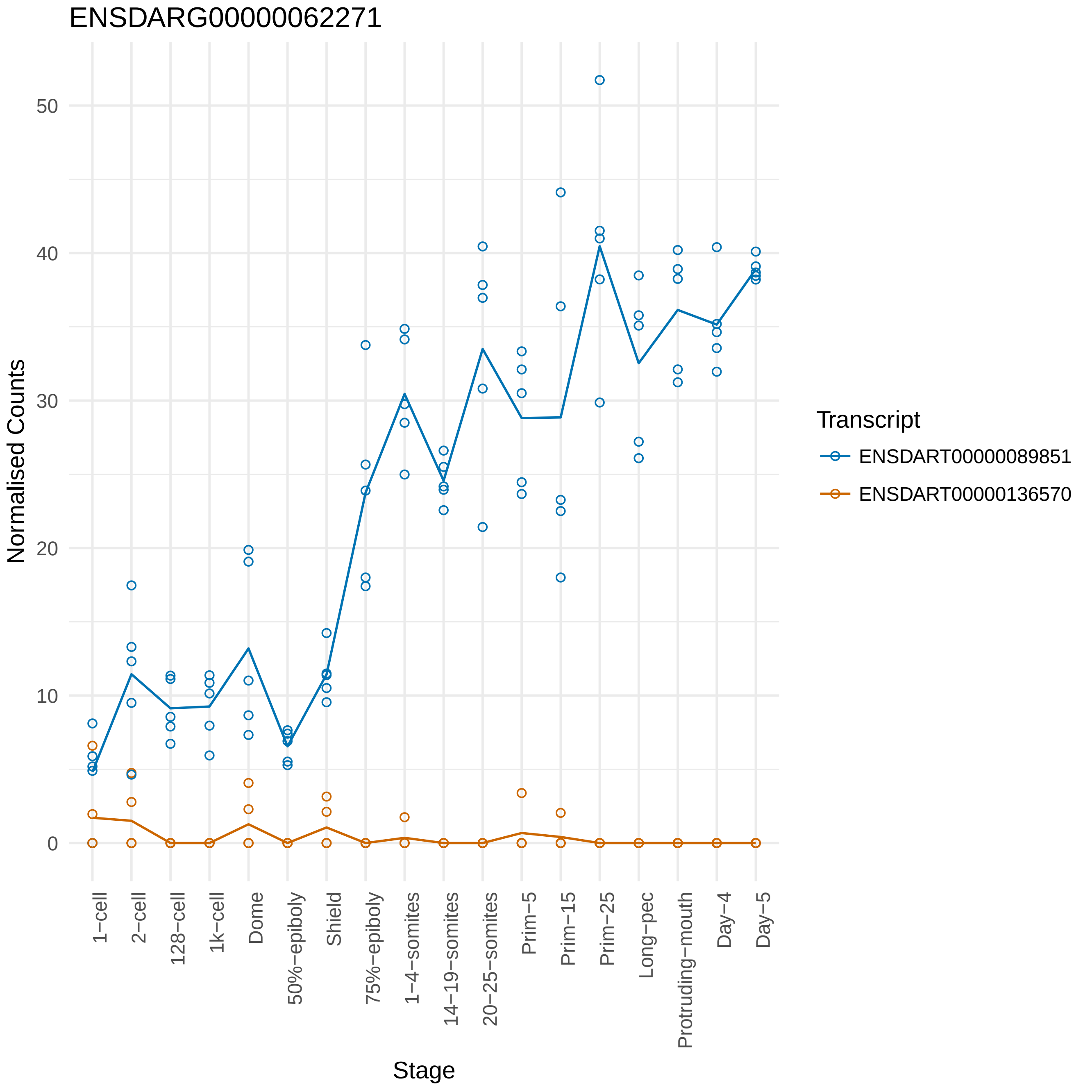

ENSDARG00000062271
Normalised Counts
0
10
20
30
40
50
Stage
1−cell
2−cell
128−cell
1k−cell
Dome
50%−epiboly
Shield
75%−epiboly
1−4−somites
14−19−somites
20−25−somites
Prim−5
Prim−15
Prim−25
Long−pec
Protruding−mouth
Day−4
Day−5
Transcript
ENSDART00000089851
ENSDART00000136570

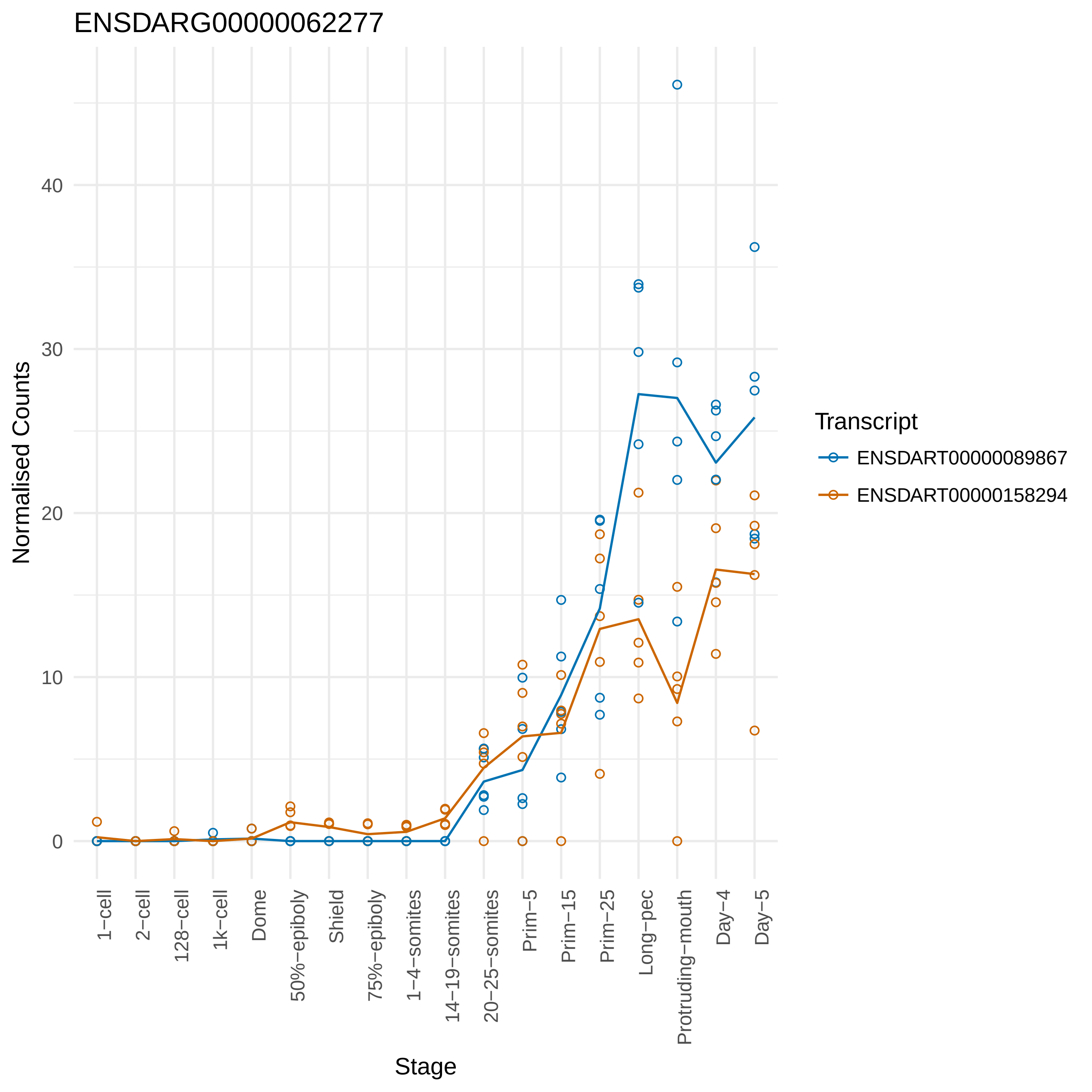
ENSDARG00000062277
Normalised Counts
0
10
20
30
40
Stage
1−cell
2−cell
128−cell
1k−cell
Dome
50%−epiboly
Shield
75%−epiboly
1−4−somites
14−19−somites
20−25−somites
Prim−5
Prim−15
Prim−25
Long−pec
Protruding−mouth
Day−4
Day−5
Transcript
ENSDART00000089867
ENSDART00000158294

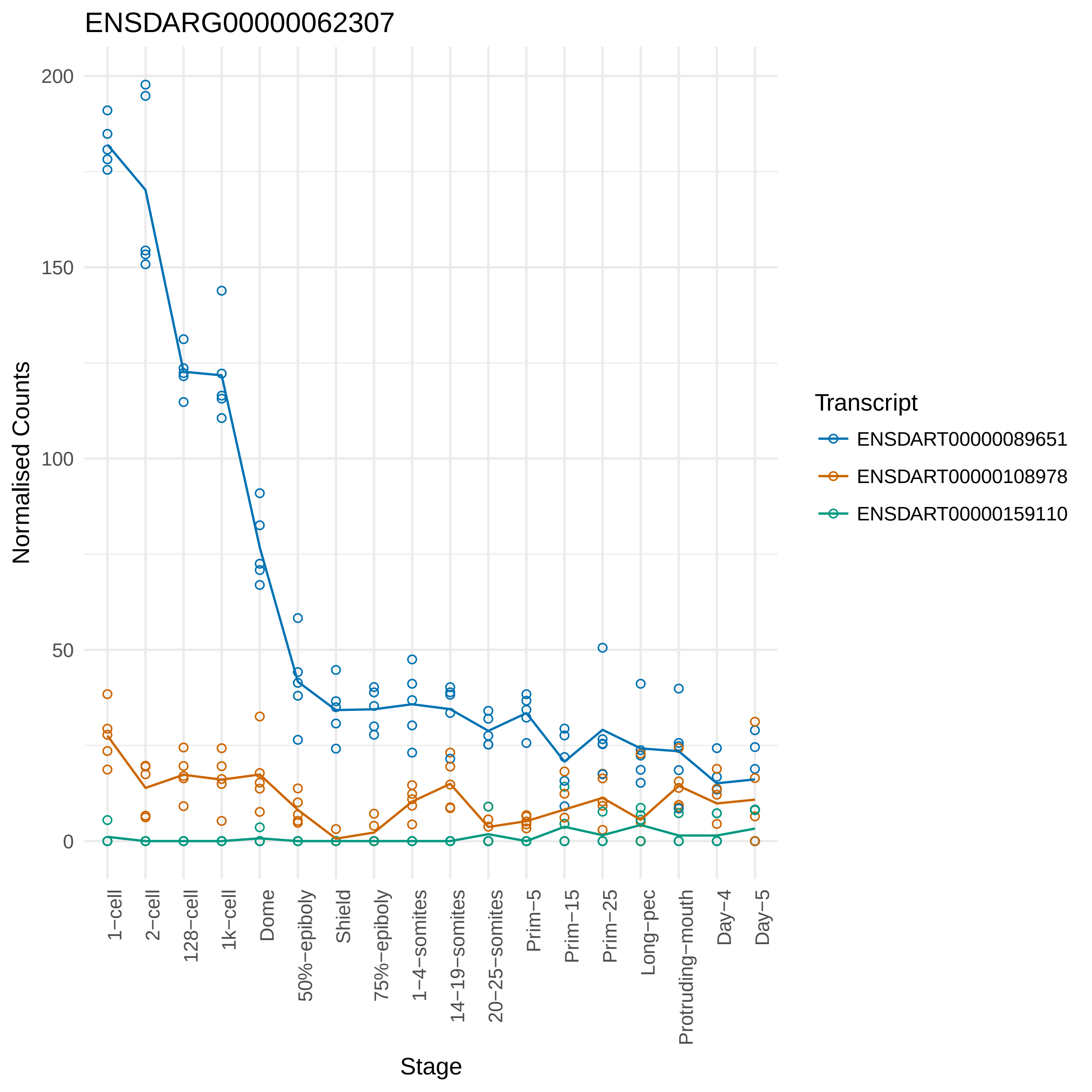

ENSDARG00000062307
Normalised Counts
200
150
100
50
0
1−cell
2−cell
128−cell
1k−cell
Dome
50%−epiboly
Shield
75%−epiboly
1−4−somites
14−19−somites
20−25−somites
Prim−5
Prim−15
Prim−25
Long−pec
Protruding−mouth
Day−4
Day−5
Stage
Transcript
ENSDART00000089651
ENSDART00000108978
ENSDART00000159110

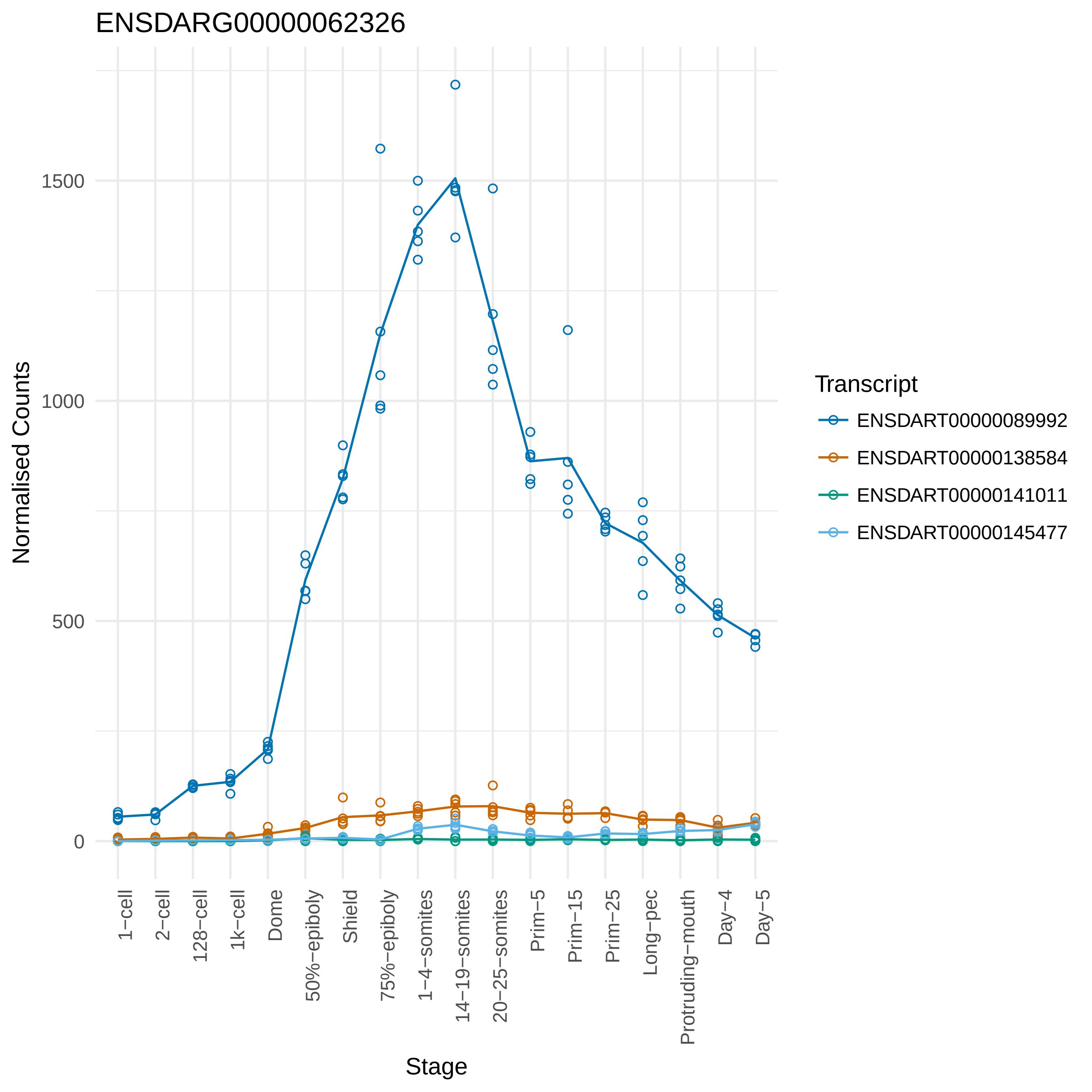
ENSDARG00000062326
Normalised Counts
0
500
1000
1500
Stage
1−cell
2−cell
128−cell
1k−cell
Dome
50%−epiboly
Shield
75%−epiboly
1−4−somites
14−19−somites
20−25−somites
Prim−5
Prim−15
Prim−25
Long−pec
Protruding−mouth
Day−4
Day−5
Transcript
ENSDART00000089992
ENSDART00000138584
ENSDART00000141011
ENSDART00000145477

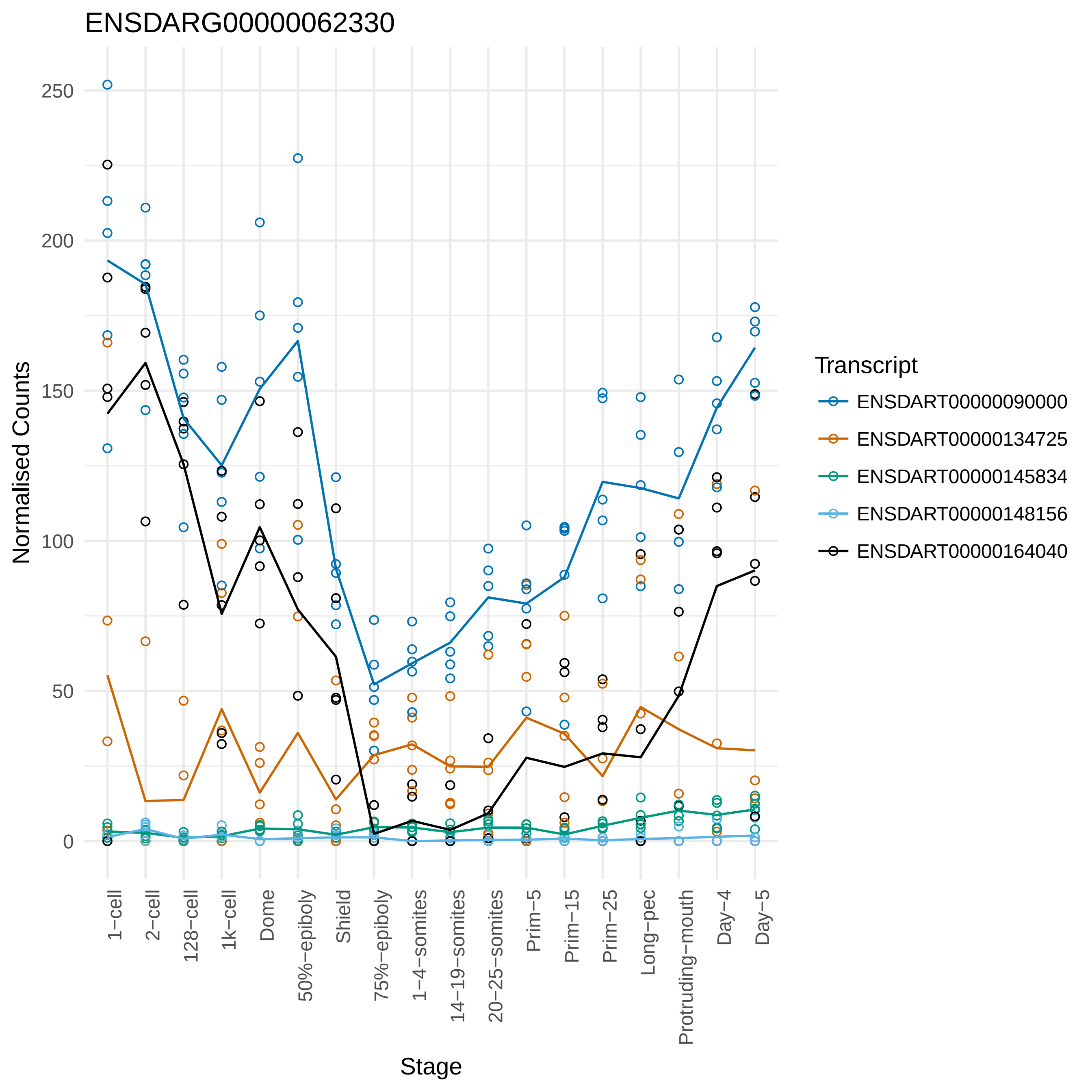
ENSDARG00000062330
Normalised Counts
0
50
100
150
200
250
Stage
1−cell
2−cell
128−cell
1k−cell
Dome
50%−epiboly
Shield
75%−epiboly
1−4−somites
14−19−somites
20−25−somites
Prim−5
Prim−15
Prim−25
Long−pec
Protruding−mouth
Day−4
Day−5
Transcript
ENSDART00000090000
ENSDART00000134725
ENSDART00000145834
ENSDART00000148156
ENSDART00000164040

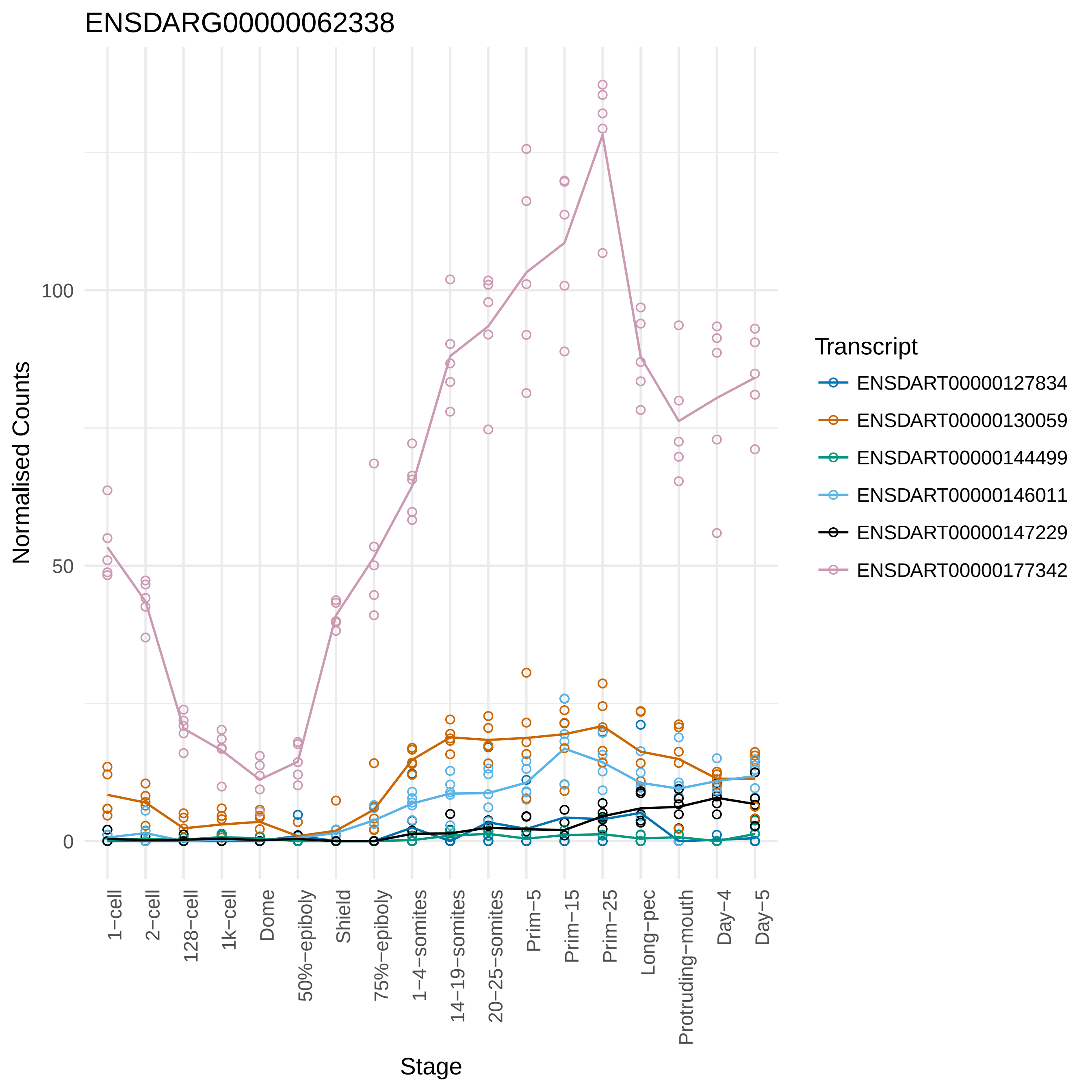

ENSDARG00000062338
Normalised Counts
100
50
0
1−cell
2−cell
128−cell
1k−cell
Dome
50%−epiboly
Shield
75%−epiboly
1−4−somites
14−19−somites
20−25−somites
Prim−5
Prim−15
Prim−25
Long−pec
Protruding−mouth
Day−4
Day−5
Stage
Transcript
ENSDART00000127834
ENSDART00000130059
ENSDART00000144499
ENSDART00000146011
ENSDART00000147229
ENSDART00000177342

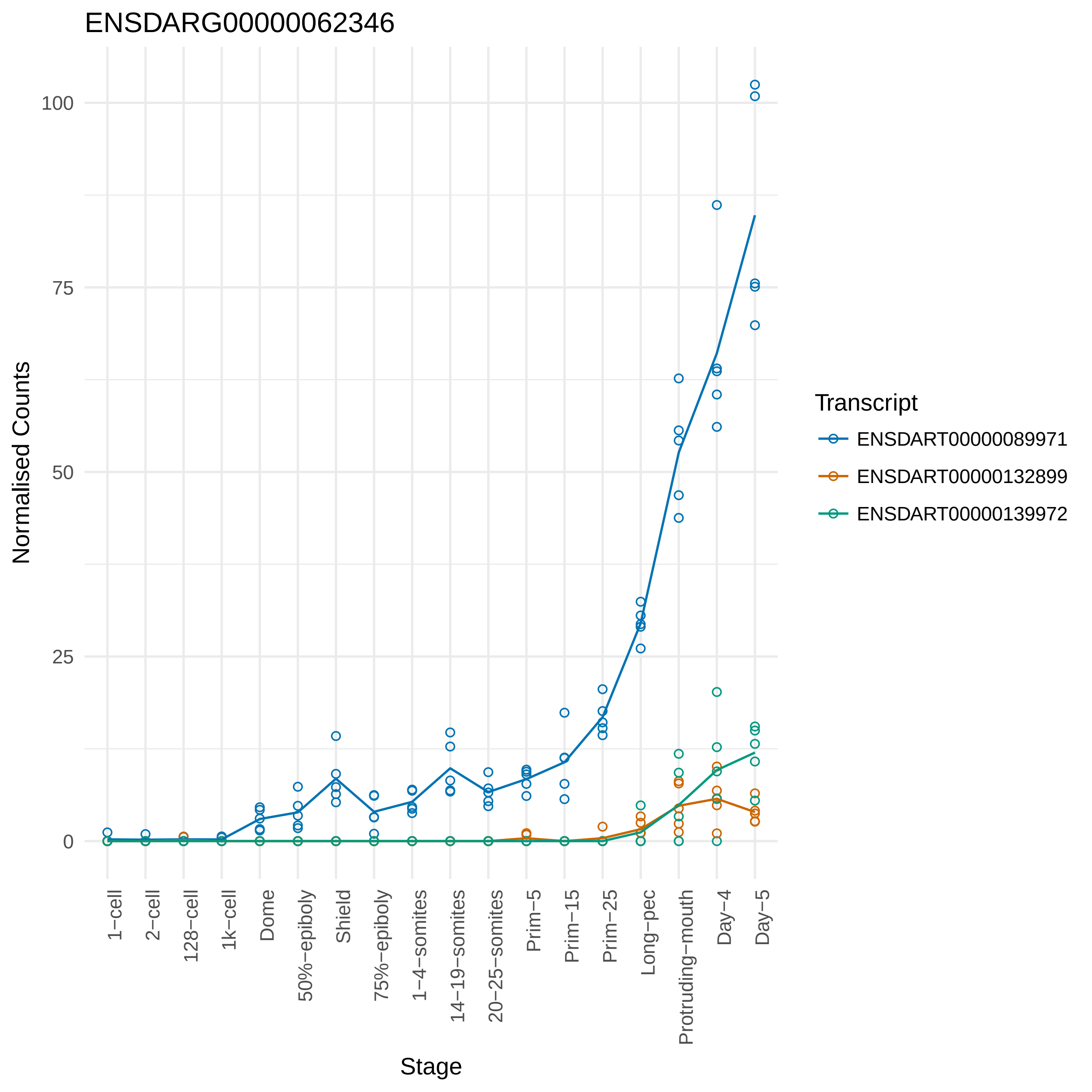

ENSDARG00000062346
Normalised Counts
100
75
50
25
0
1−cell
2−cell
128−cell
1k−cell
Dome
50%−epiboly
Shield
75%−epiboly
1−4−somites
14−19−somites
20−25−somites
Prim−5
Prim−15
Prim−25
Long−pec
Protruding−mouth
Day−4
Day−5
Stage
Transcript
ENSDART00000089971
ENSDART00000132899
ENSDART00000139972

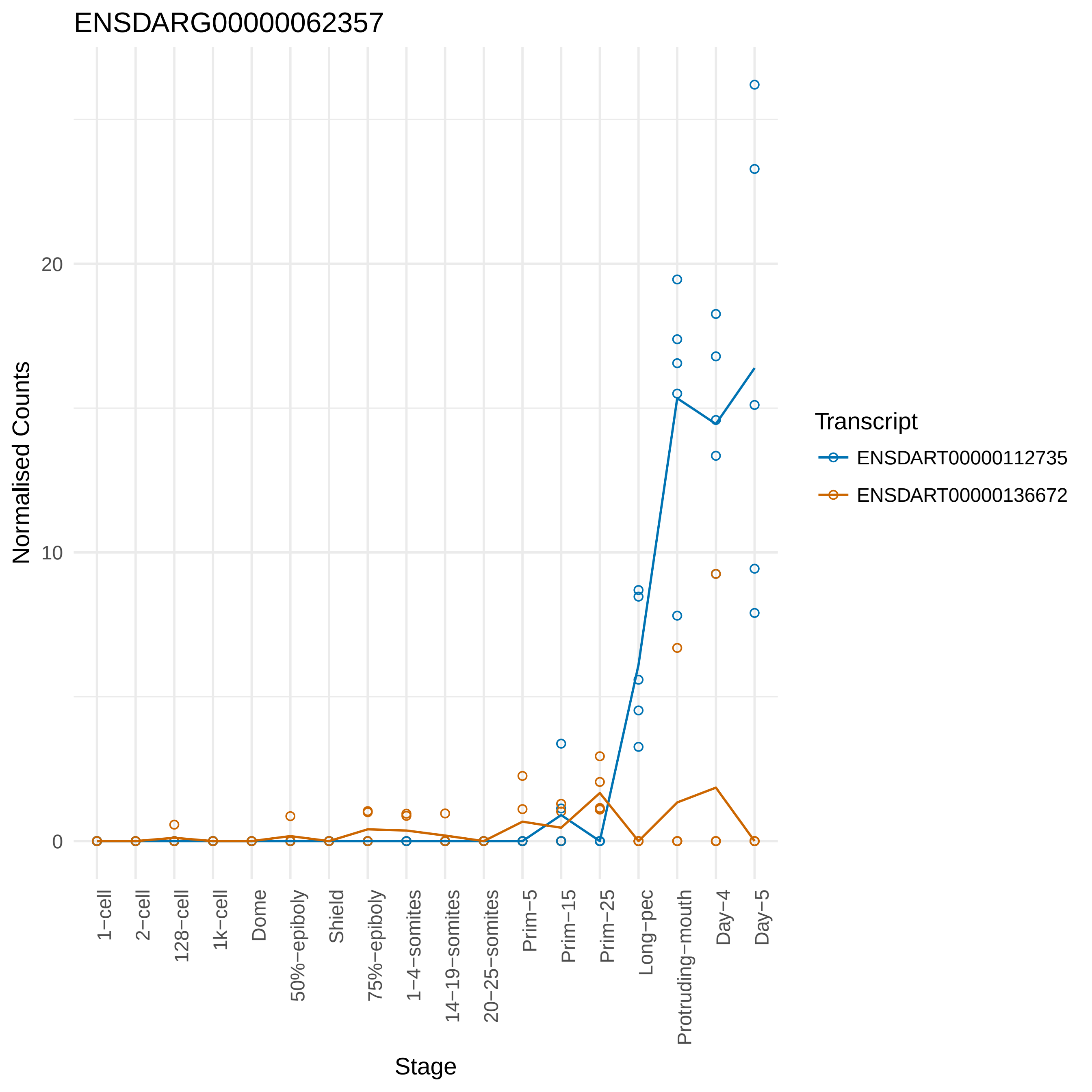
ENSDARG00000062357
Normalised Counts
0
10
20
1−cell
2−cell
128−cell
1k−cell
Dome
50%−epiboly
Shield
75%−epiboly
1−4−somites
14−19−somites
20−25−somites
Prim−5
Prim−15
Prim−25
Long−pec
Protruding−mouth
Day−4
Day−5
Stage
Transcript
ENSDART00000112735
ENSDART00000136672

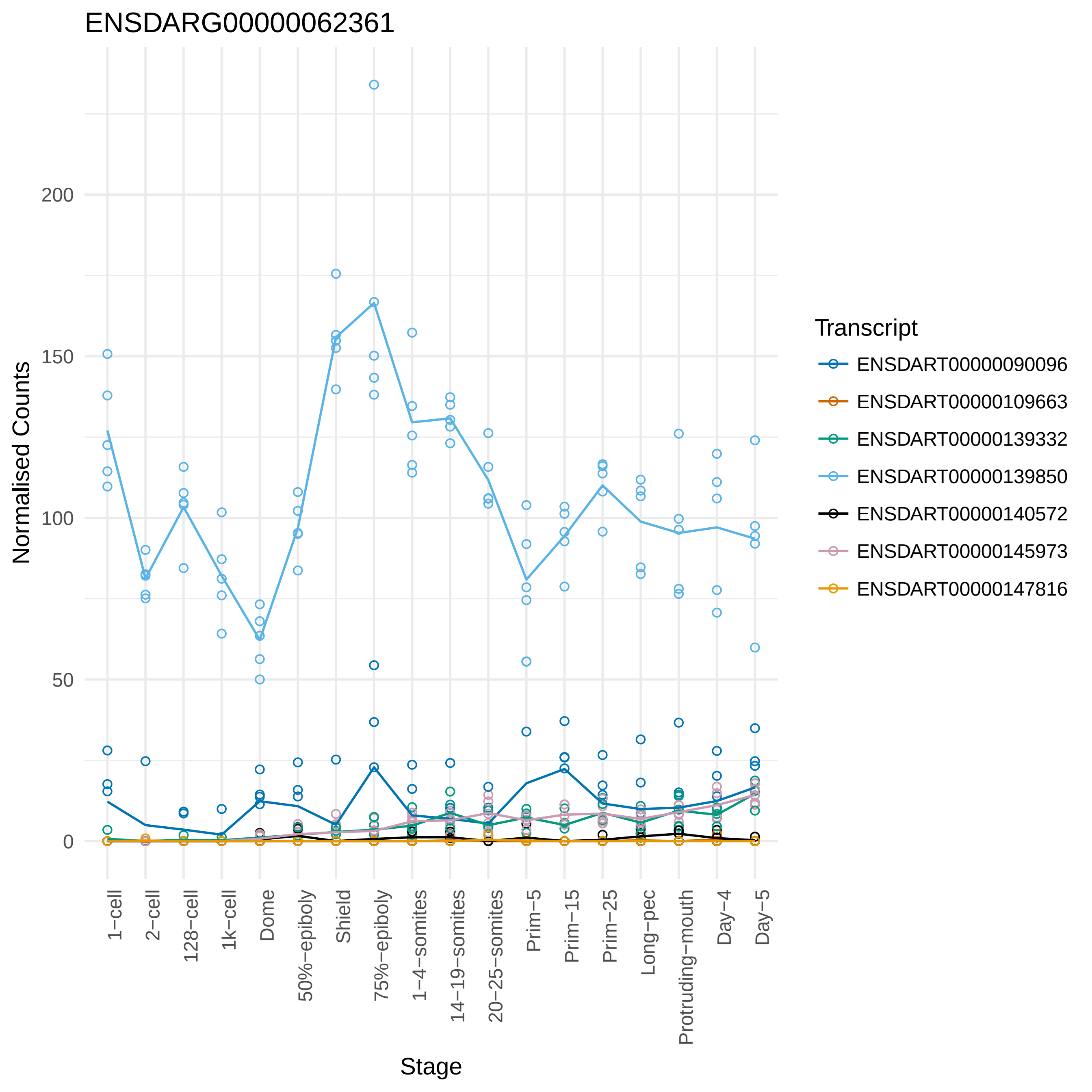

ENSDARG00000062361
Normalised Counts
0
50
100
150
200
1−cell
2−cell
128−cell
1k−cell
Dome
50%−epiboly
Shield
75%−epiboly
1−4−somites
14−19−somites
20−25−somites
Prim−5
Prim−15
Prim−25
Long−pec
Protruding−mouth
Day−4
Day−5
Stage
Transcript
ENSDART00000090096
ENSDART00000109663
ENSDART00000139332
ENSDART00000139850
ENSDART00000140572
ENSDART00000145973
ENSDART00000147816

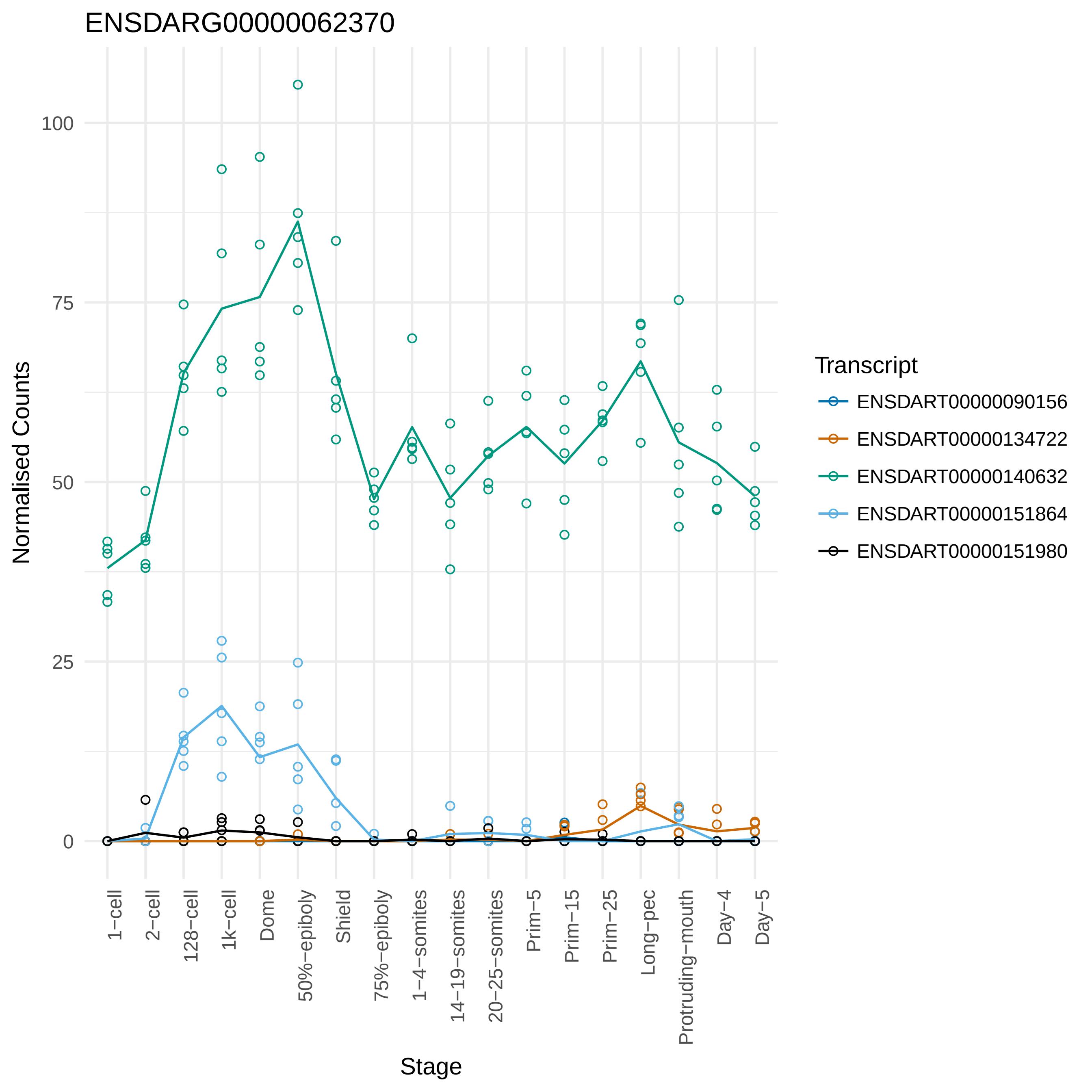

ENSDARG00000062370
Normalised Counts
100
75
50
25
0
1−cell
2−cell
128−cell
1k−cell
Dome
50%−epiboly
Shield
75%−epiboly
1−4−somites
14−19−somites
20−25−somites
Prim−5
Prim−15
Prim−25
Long−pec
Protruding−mouth
Day−4
Day−5
Stage
Transcript
ENSDART00000090156
ENSDART00000134722
ENSDART00000140632
ENSDART00000151864
ENSDART00000151980

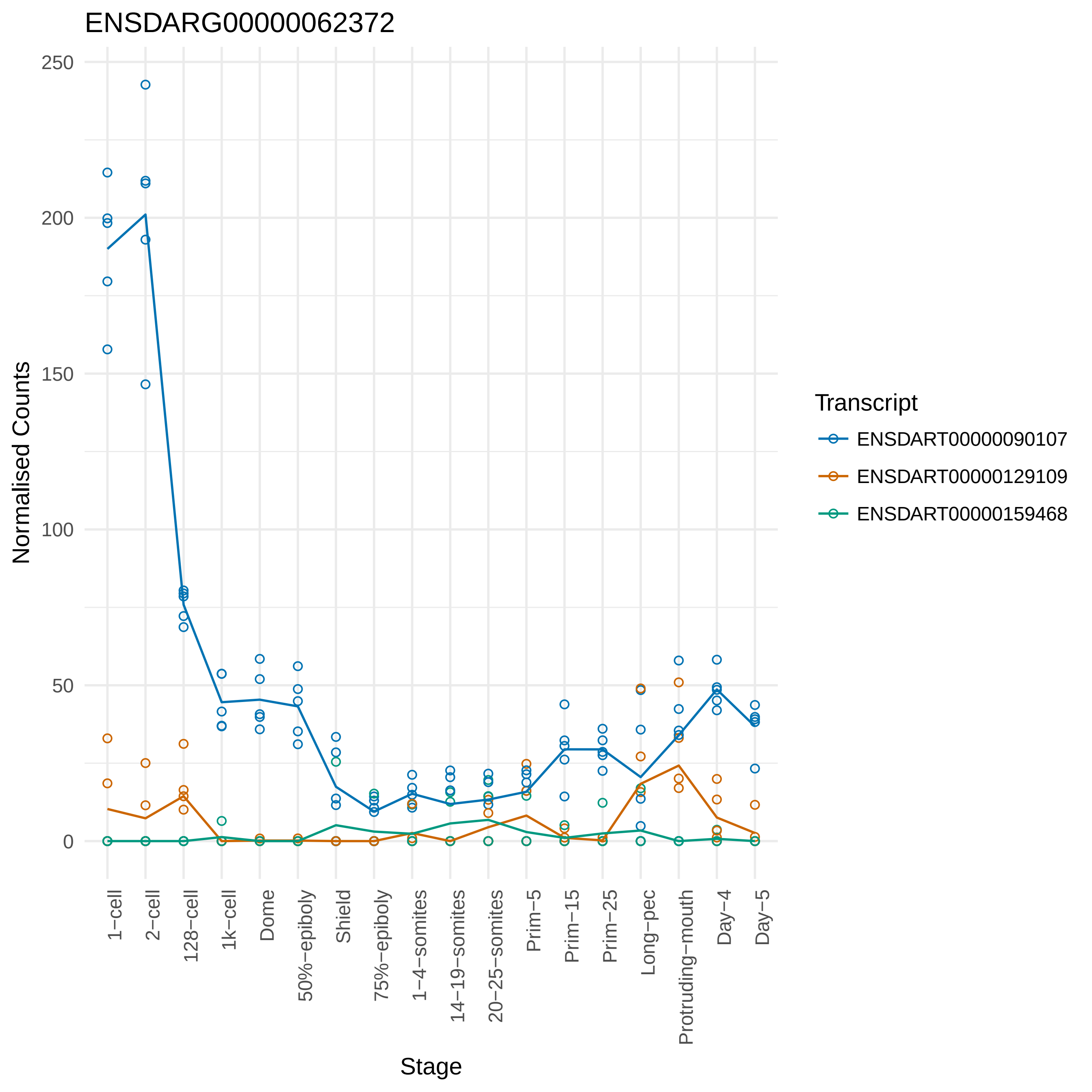

ENSDARG00000062372
Normalised Counts
250
200
150
100
50
0
1−cell
2−cell
128−cell
1k−cell
Dome
50%−epiboly
Shield
75%−epiboly
1−4−somites
14−19−somites
20−25−somites
Prim−5
Prim−15
Prim−25
Long−pec
Protruding−mouth
Day−4
Day−5
Stage
Transcript
ENSDART00000090107
ENSDART00000129109
ENSDART00000159468

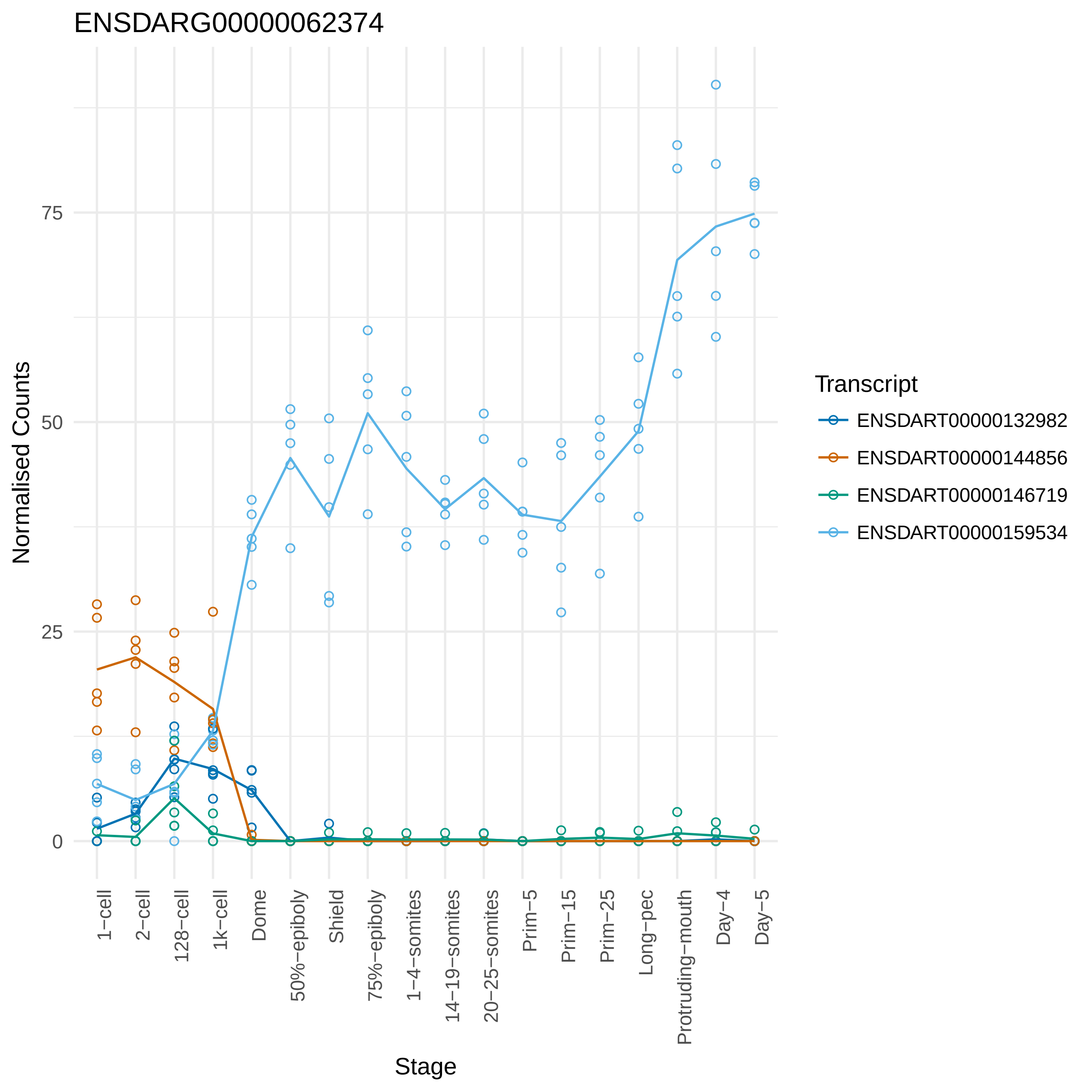
ENSDARG00000062374
Normalised Counts
0
25
50
75
Stage
1−cell
2−cell
128−cell
1k−cell
Dome
50%−epiboly
Shield
75%−epiboly
1−4−somites
14−19−somites
20−25−somites
Prim−5
Prim−15
Prim−25
Long−pec
Protruding−mouth
Day−4
Day−5
Transcript
ENSDART00000132982
ENSDART00000144856
ENSDART00000146719
ENSDART00000159534

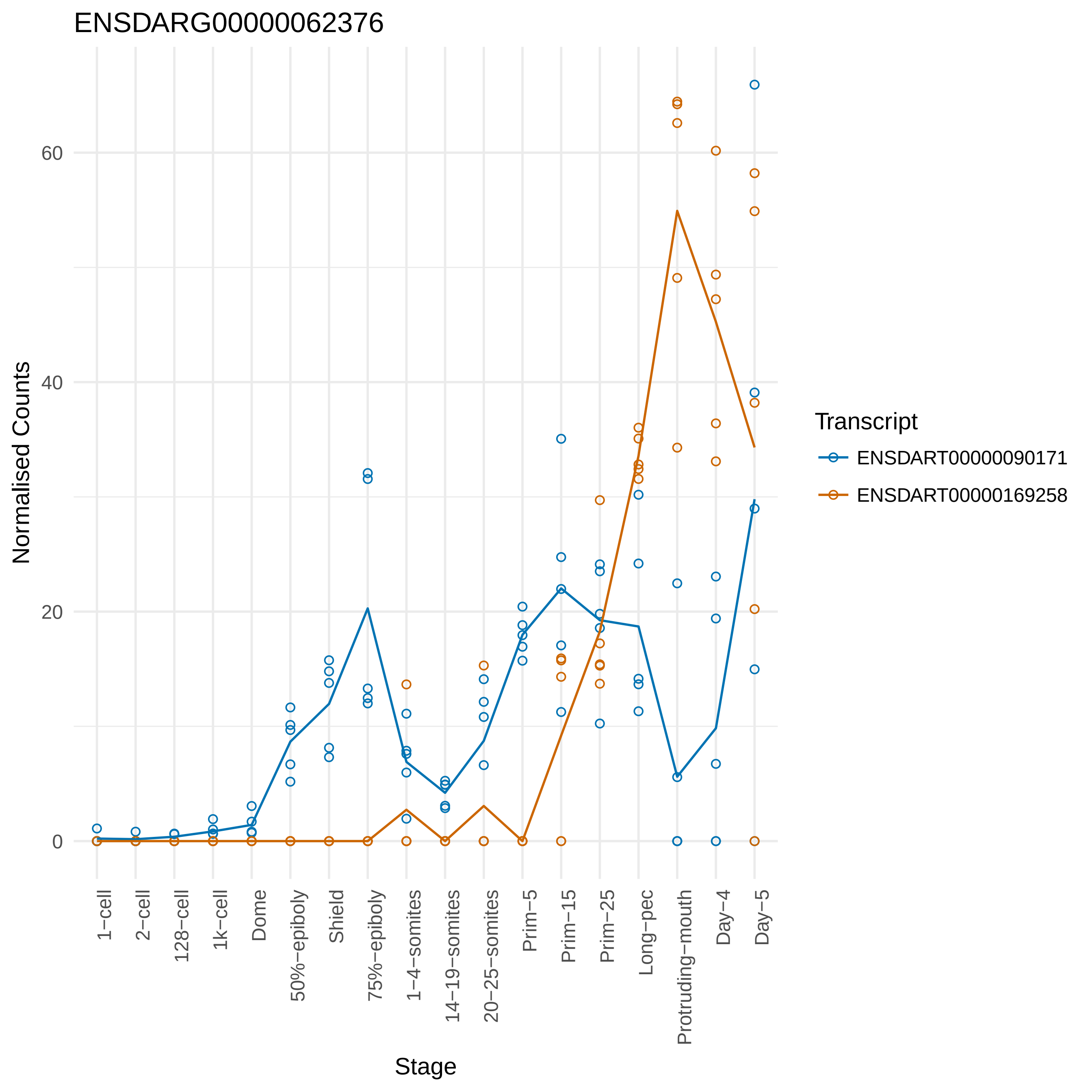
ENSDARG00000062376
Normalised Counts
0
20
40
60
1−cell
2−cell
128−cell
1k−cell
Dome
50%−epiboly
Shield
75%−epiboly
1−4−somites
14−19−somites
20−25−somites
Prim−5
Prim−15
Prim−25
Long−pec
Protruding−mouth
Day−4
Day−5
Stage
Transcript
ENSDART00000090171
ENSDART00000169258

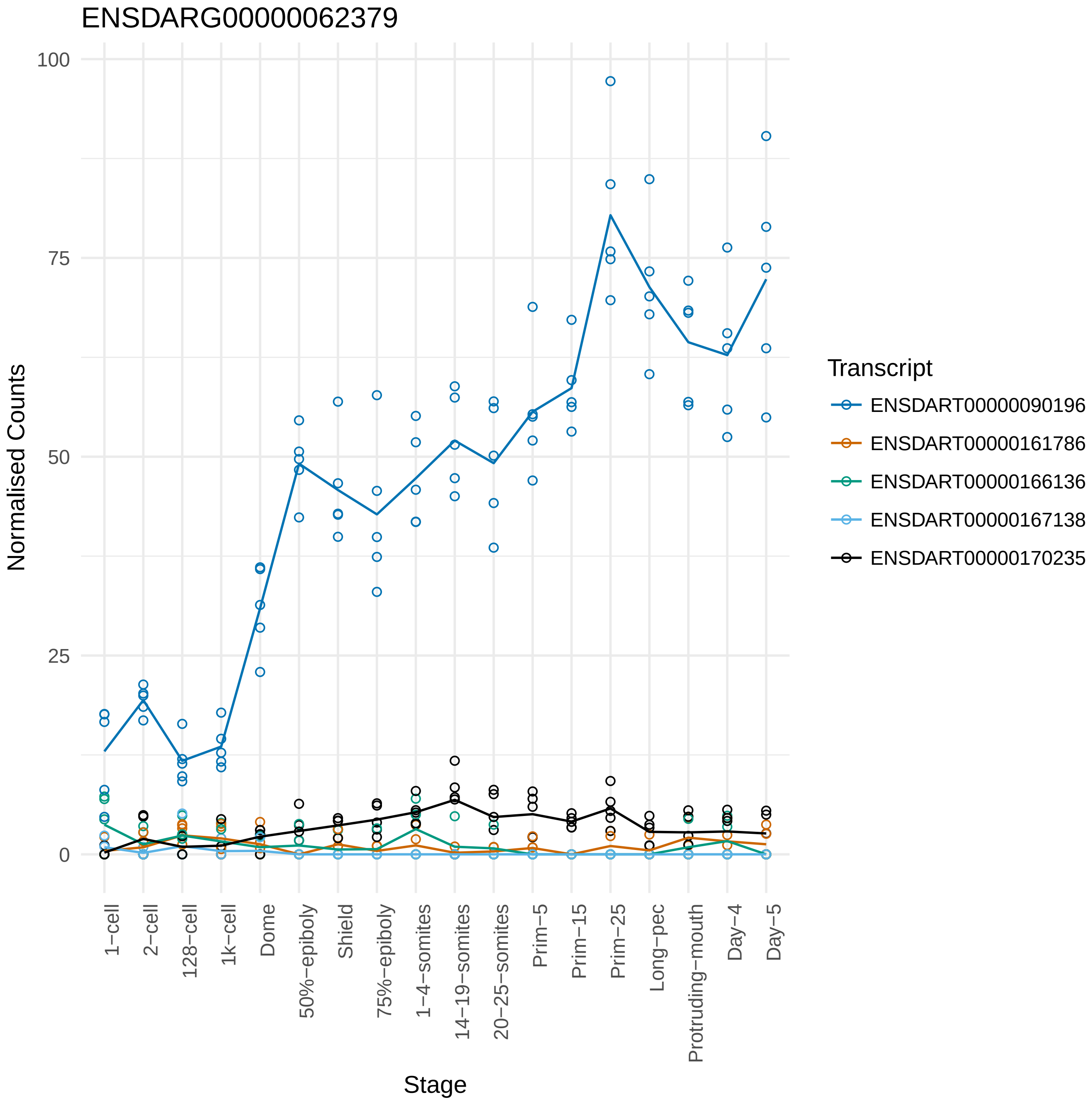
ENSDARG00000062379
Normalised Counts
100
75
50
25
0
1−cell
2−cell
128−cell
1k−cell
Dome
50%−epiboly
Shield
75%−epiboly
1−4−somites
14−19−somites
20−25−somites
Prim−5
Prim−15
Prim−25
Long−pec
Protruding−mouth
Day−4
Day−5
Stage
Transcript
ENSDART00000090196
ENSDART00000161786
ENSDART00000166136
ENSDART00000167138
ENSDART00000170235

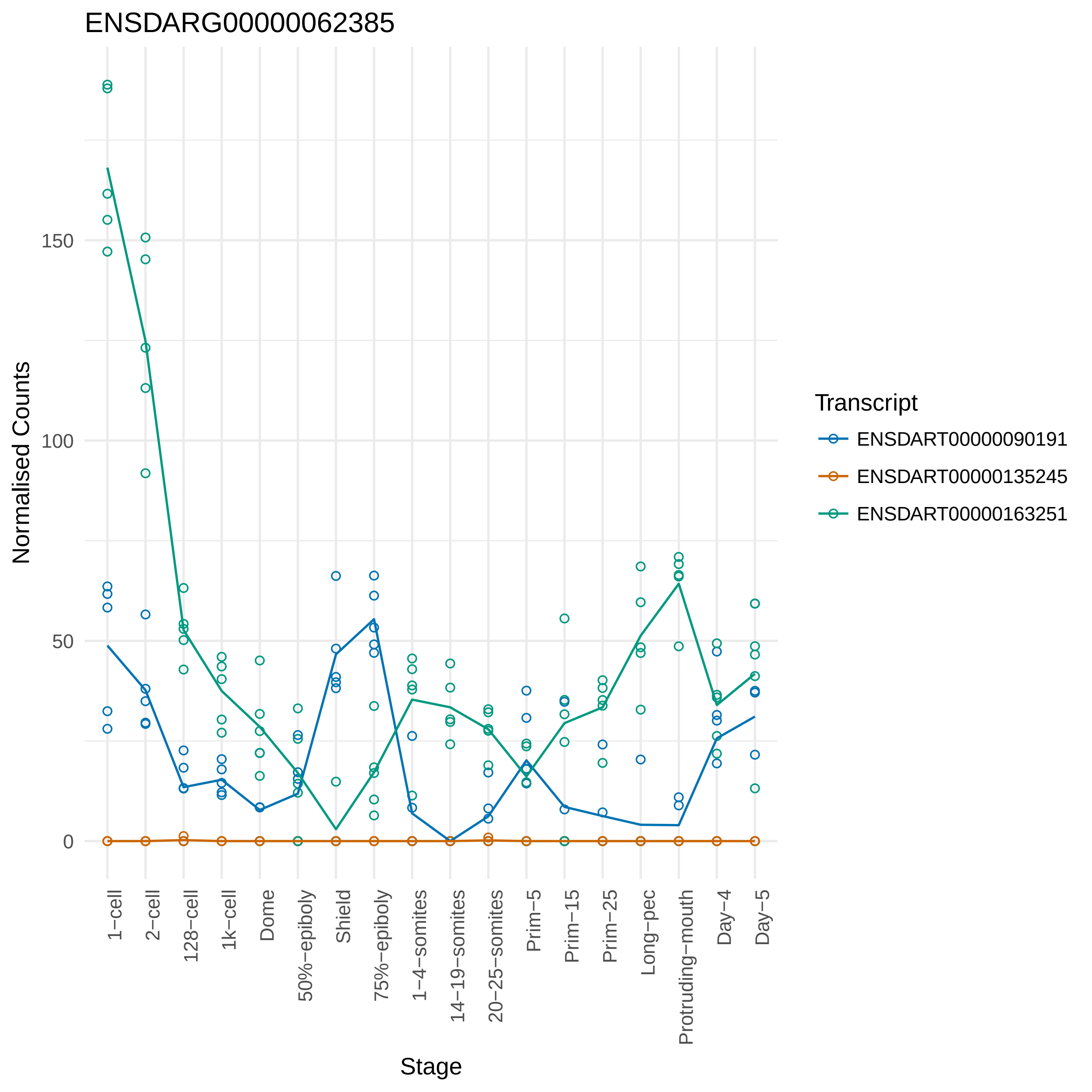

ENSDARG00000062385
Normalised Counts
0
50
100
150
Stage
1−cell
2−cell
128−cell
1k−cell
Dome
50%−epiboly
Shield
75%−epiboly
1−4−somites
14−19−somites
20−25−somites
Prim−5
Prim−15
Prim−25
Long−pec
Protruding−mouth
Day−4
Day−5
Transcript
ENSDART00000090191
ENSDART00000135245
ENSDART00000163251

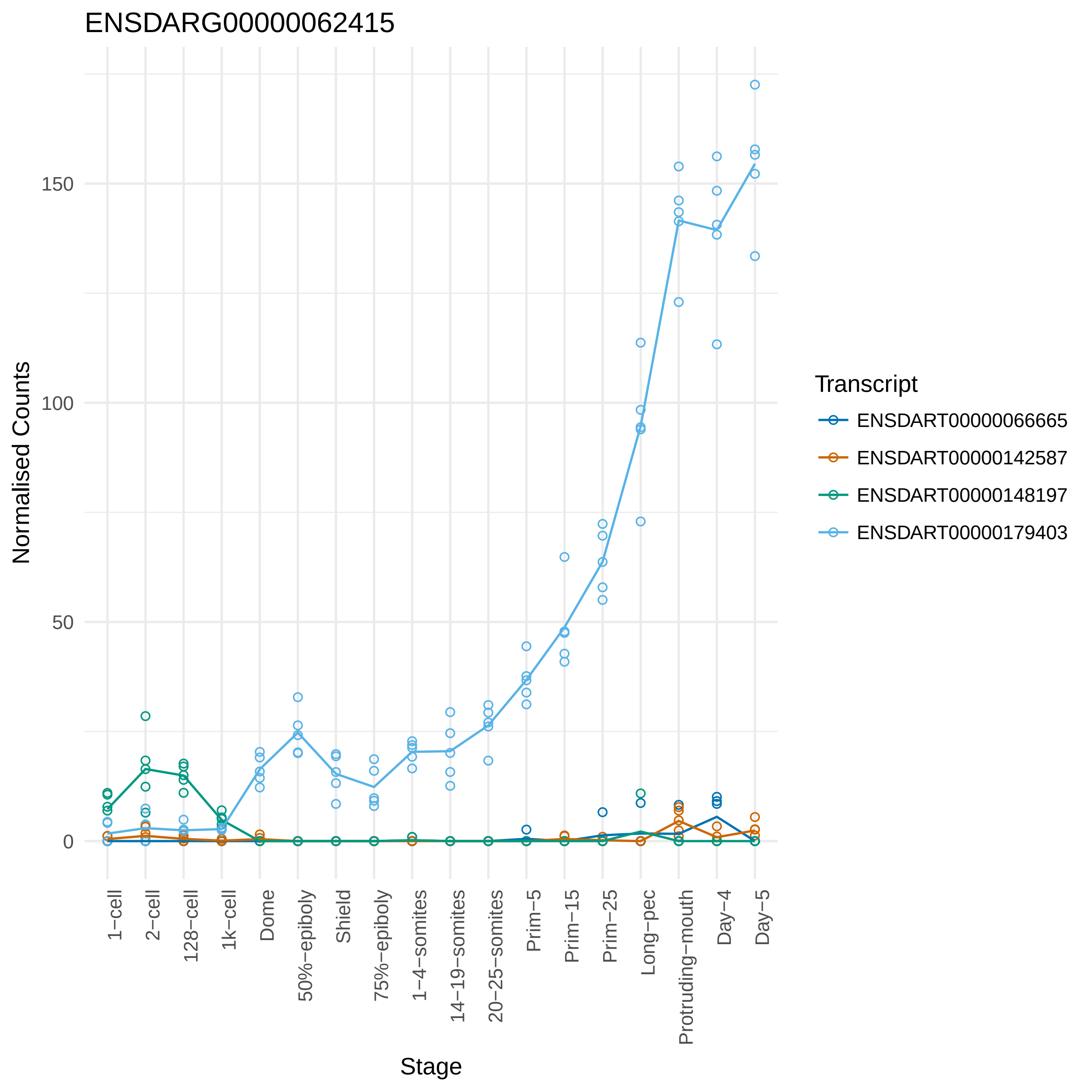

ENSDARG00000062415
Normalised Counts
150
100
50
0
1−cell
2−cell
128−cell
1k−cell
Dome
50%−epiboly
Shield
75%−epiboly
1−4−somites
14−19−somites
20−25−somites
Prim−5
Prim−15
Prim−25
Long−pec
Protruding−mouth
Day−4
Day−5
Stage
Transcript
ENSDART00000066665
ENSDART00000142587
ENSDART00000148197
ENSDART00000179403

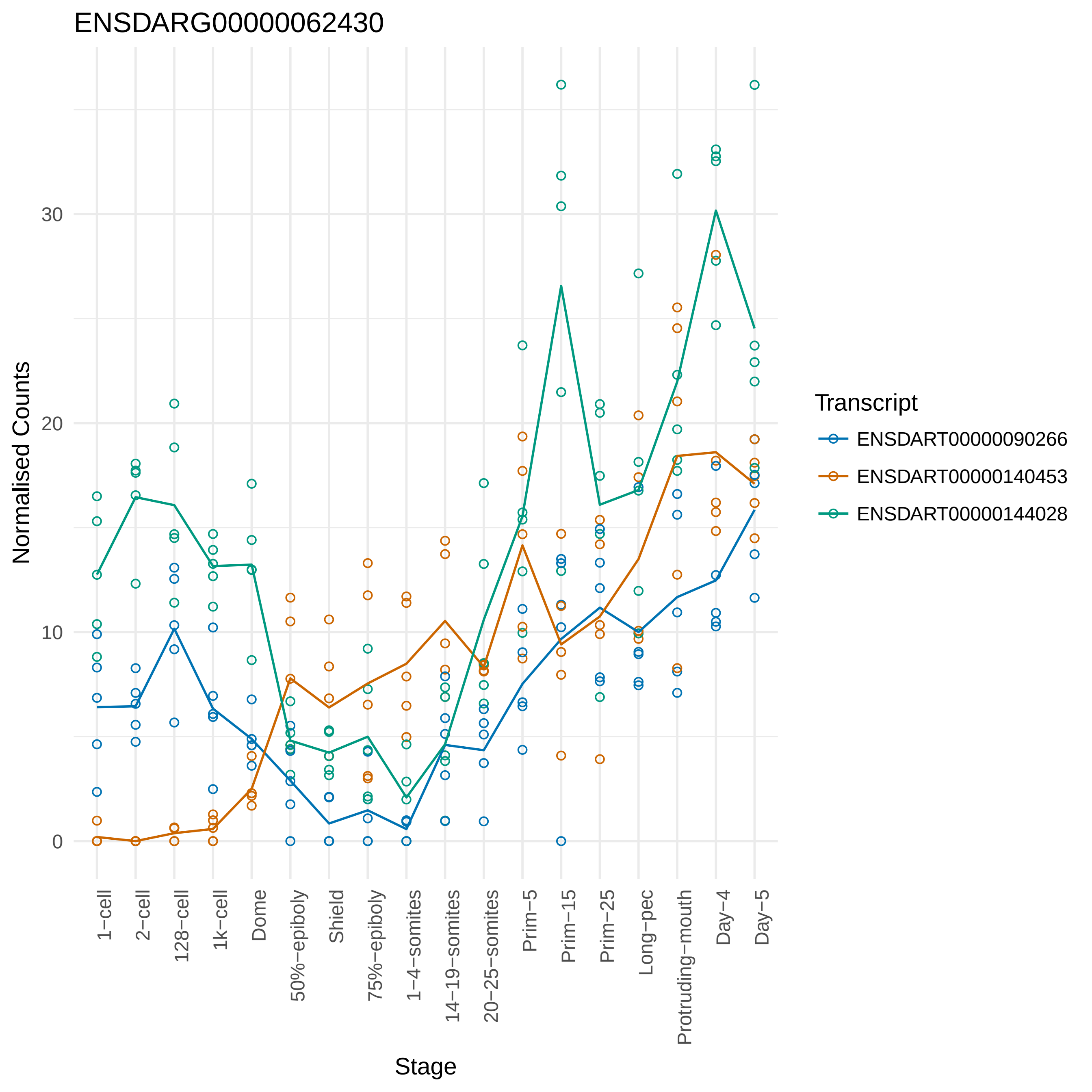

ENSDARG00000062430
Normalised Counts
0
10
20
30
1−cell
2−cell
128−cell
1k−cell
Dome
50%−epiboly
Shield
75%−epiboly
1−4−somites
14−19−somites
20−25−somites
Prim−5
Prim−15
Prim−25
Long−pec
Protruding−mouth
Day−4
Day−5
Stage
Transcript
ENSDART00000090266
ENSDART00000140453
ENSDART00000144028

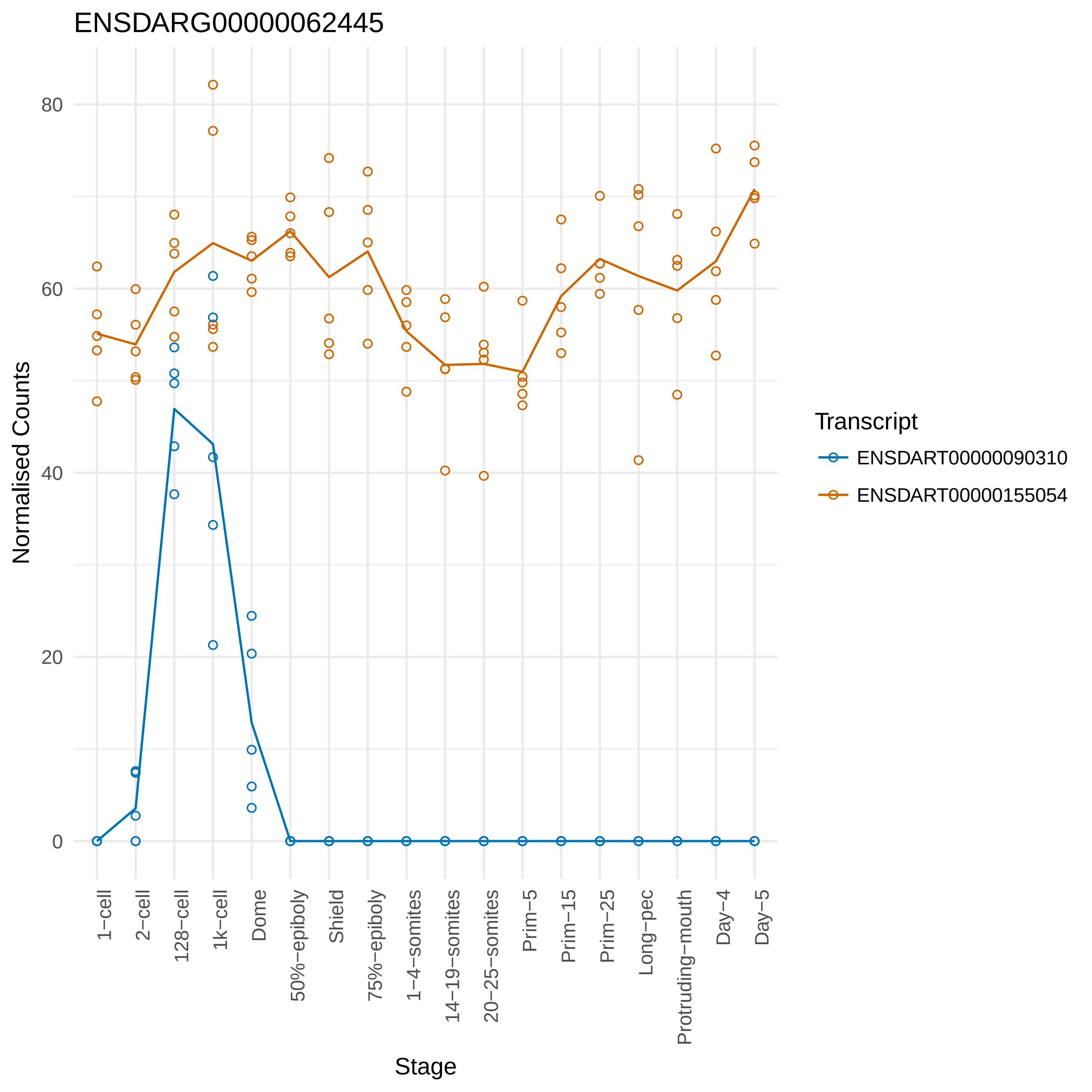
ENSDARG00000062445
Normalised Counts
80
60
40
20
0
1−cell
2−cell
128−cell
1k−cell
Dome
50%−epiboly
Shield
75%−epiboly
1−4−somites
14−19−somites
20−25−somites
Prim−5
Prim−15
Prim−25
Long−pec
Protruding−mouth
Day−4
Day−5
Stage
Transcript
ENSDART00000090310
ENSDART00000155054

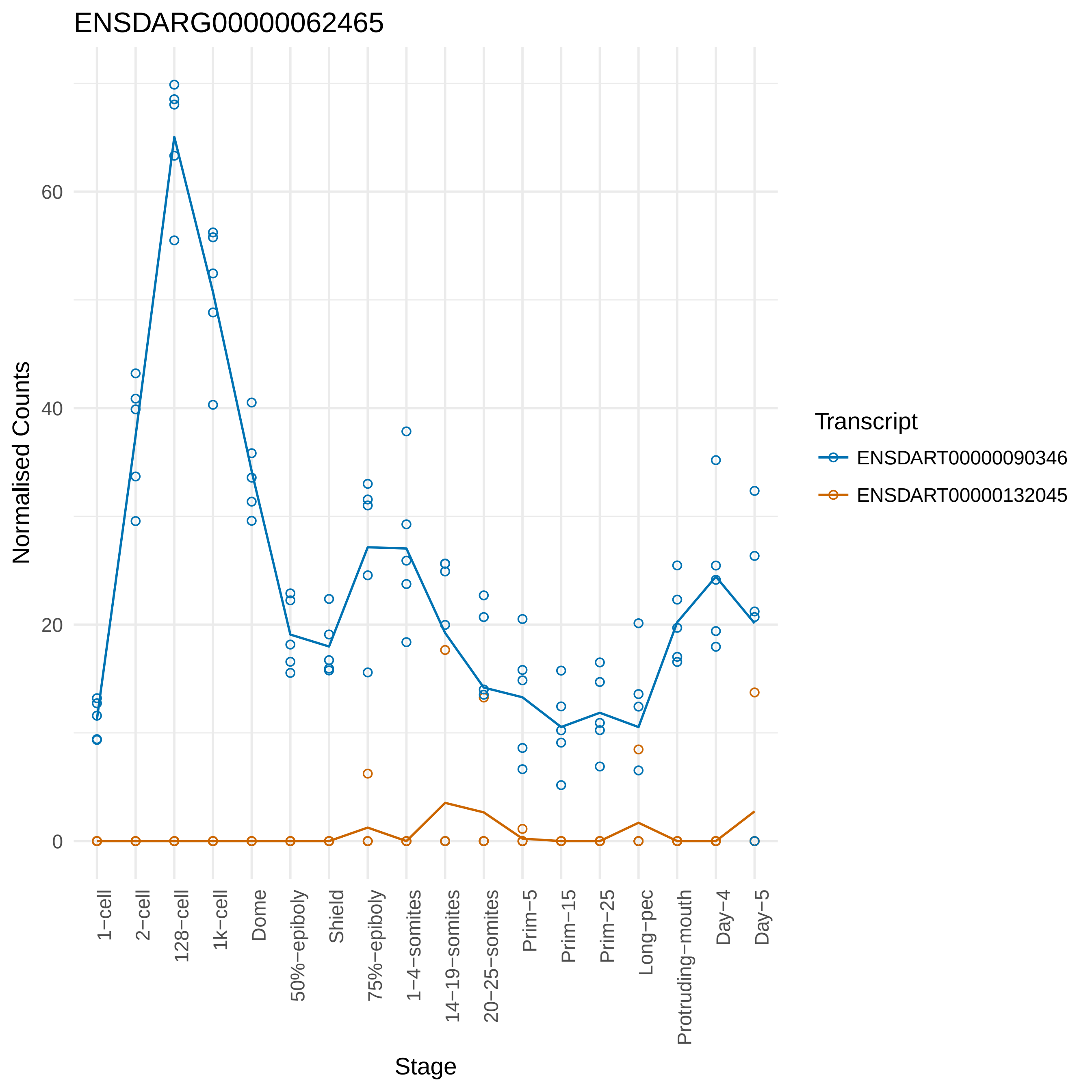
ENSDARG00000062465
Normalised Counts
0
20
40
60
1−cell
2−cell
128−cell
1k−cell
Dome
50%−epiboly
Shield
75%−epiboly
1−4−somites
14−19−somites
20−25−somites
Prim−5
Prim−15
Prim−25
Long−pec
Protruding−mouth
Day−4
Day−5
Stage
Transcript
ENSDART00000090346
ENSDART00000132045

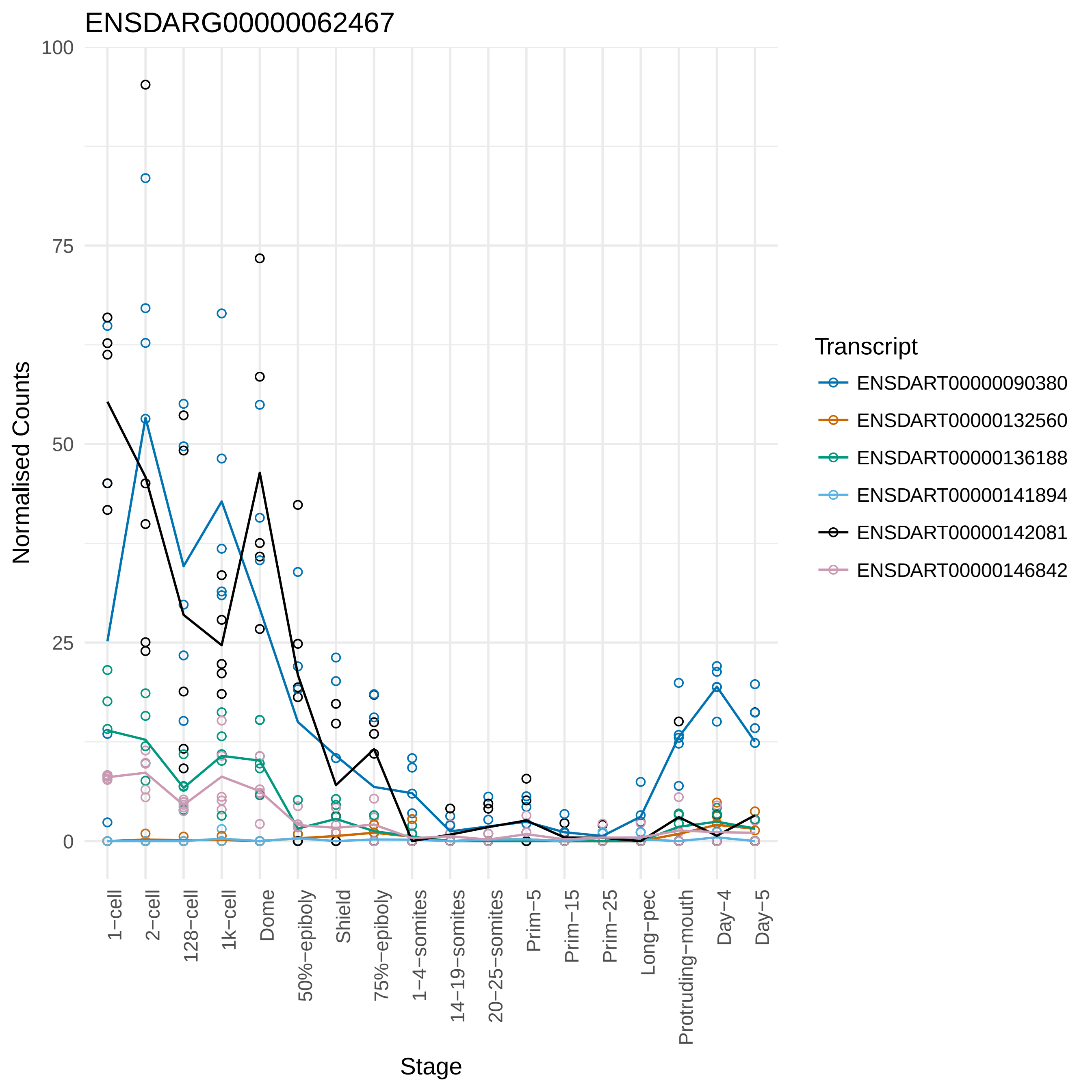
ENSDARG00000062467
Normalised Counts
100
75
50
25
0
1−cell
2−cell
128−cell
1k−cell
Dome
50%−epiboly
Shield
75%−epiboly
1−4−somites
14−19−somites
20−25−somites
Prim−5
Prim−15
Prim−25
Long−pec
Protruding−mouth
Day−4
Day−5
Stage
Transcript
ENSDART00000090380
ENSDART00000132560
ENSDART00000136188
ENSDART00000141894
ENSDART00000142081
ENSDART00000146842

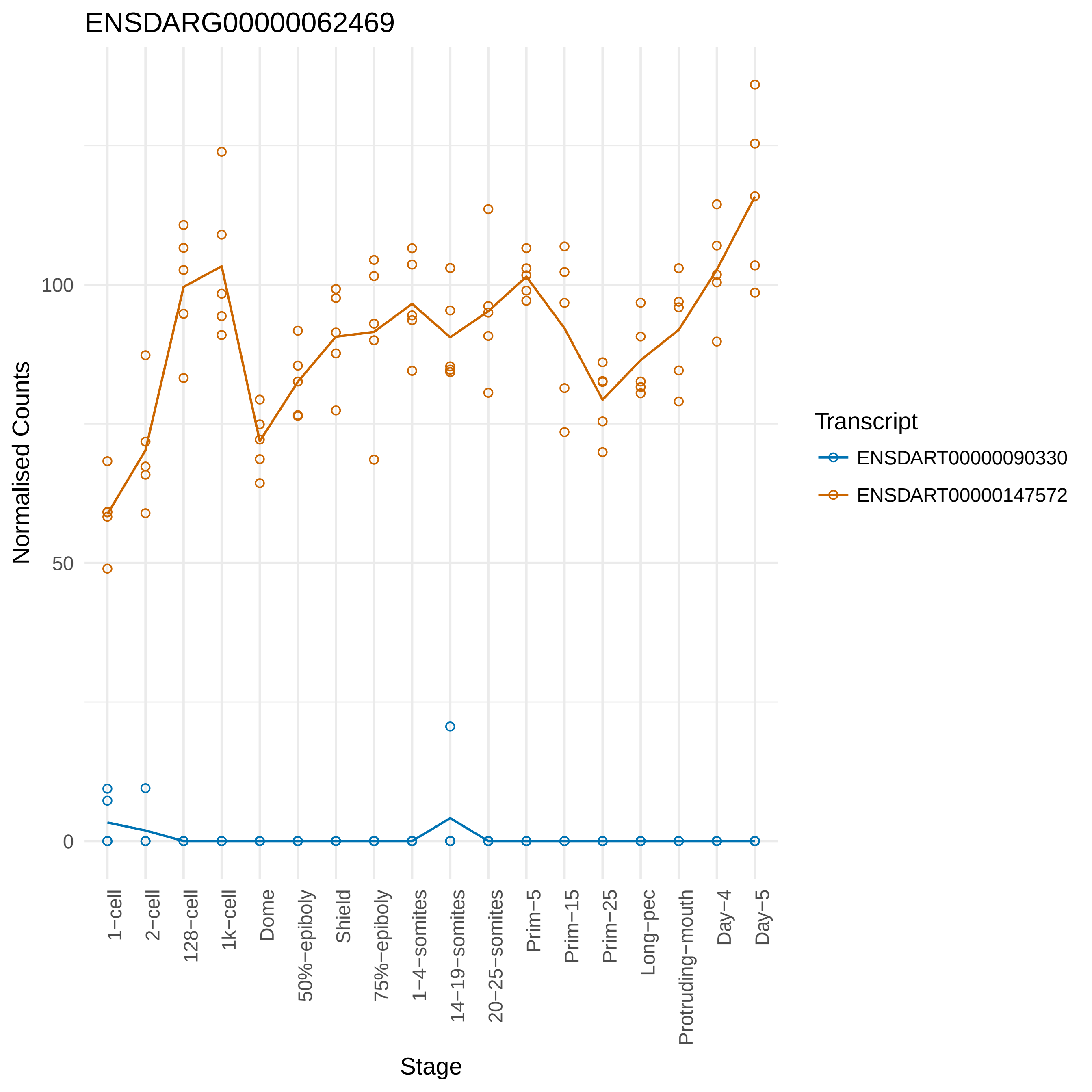

ENSDARG00000062469
Normalised Counts
100
50
0
1−cell
2−cell
128−cell
1k−cell
Dome
50%−epiboly
Shield
75%−epiboly
1−4−somites
14−19−somites
20−25−somites
Prim−5
Prim−15
Prim−25
Long−pec
Protruding−mouth
Day−4
Day−5
Stage
Transcript
ENSDART00000090330
ENSDART00000147572

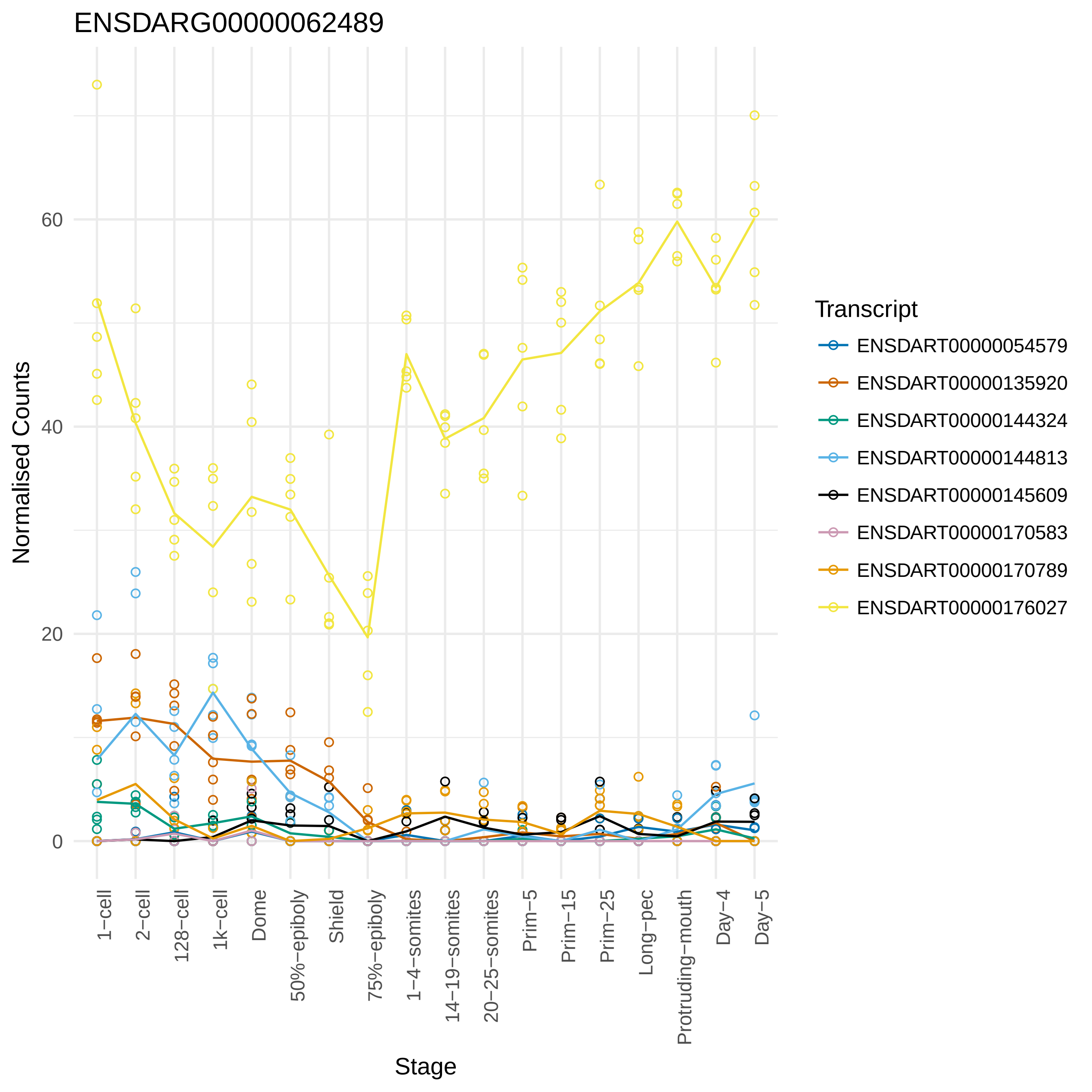

ENSDARG00000062489
Normalised Counts
0
20
40
60
1−cell
2−cell
128−cell
1k−cell
Dome
50%−epiboly
Shield
75%−epiboly
1−4−somites
14−19−somites
20−25−somites
Prim−5
Prim−15
Prim−25
Long−pec
Protruding−mouth
Day−4
Day−5
Stage
Transcript
ENSDART00000054579
ENSDART00000135920
ENSDART00000144324
ENSDART00000144813
ENSDART00000145609
ENSDART00000170583
ENSDART00000170789
ENSDART00000176027

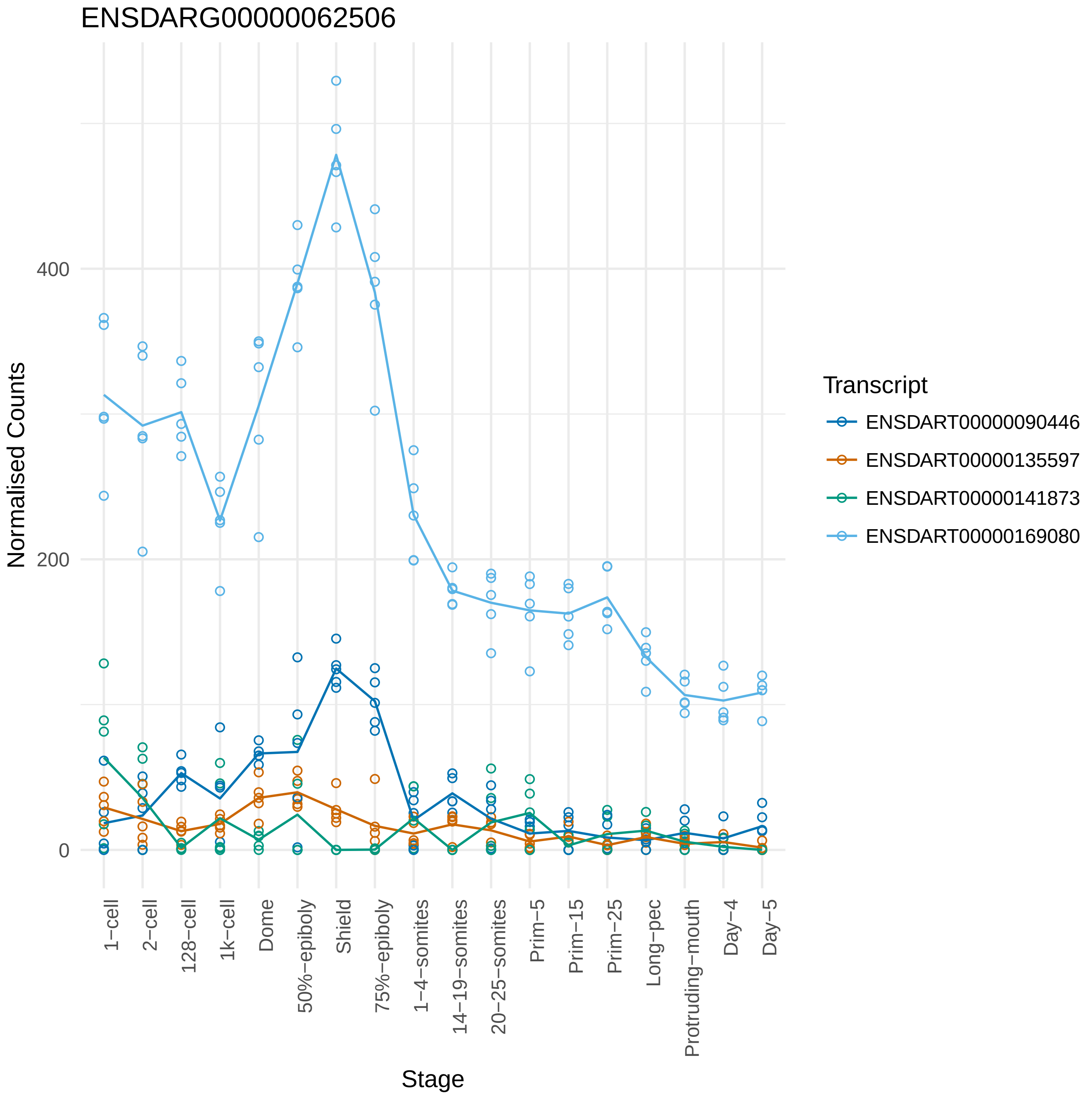

ENSDARG00000062506
Normalised Counts
400
200
0
1−cell
2−cell
128−cell
1k−cell
Dome
50%−epiboly
Shield
75%−epiboly
1−4−somites
14−19−somites
20−25−somites
Prim−5
Prim−15
Prim−25
Long−pec
Protruding−mouth
Day−4
Day−5
Stage
Transcript
ENSDART00000090446
ENSDART00000135597
ENSDART00000141873
ENSDART00000169080

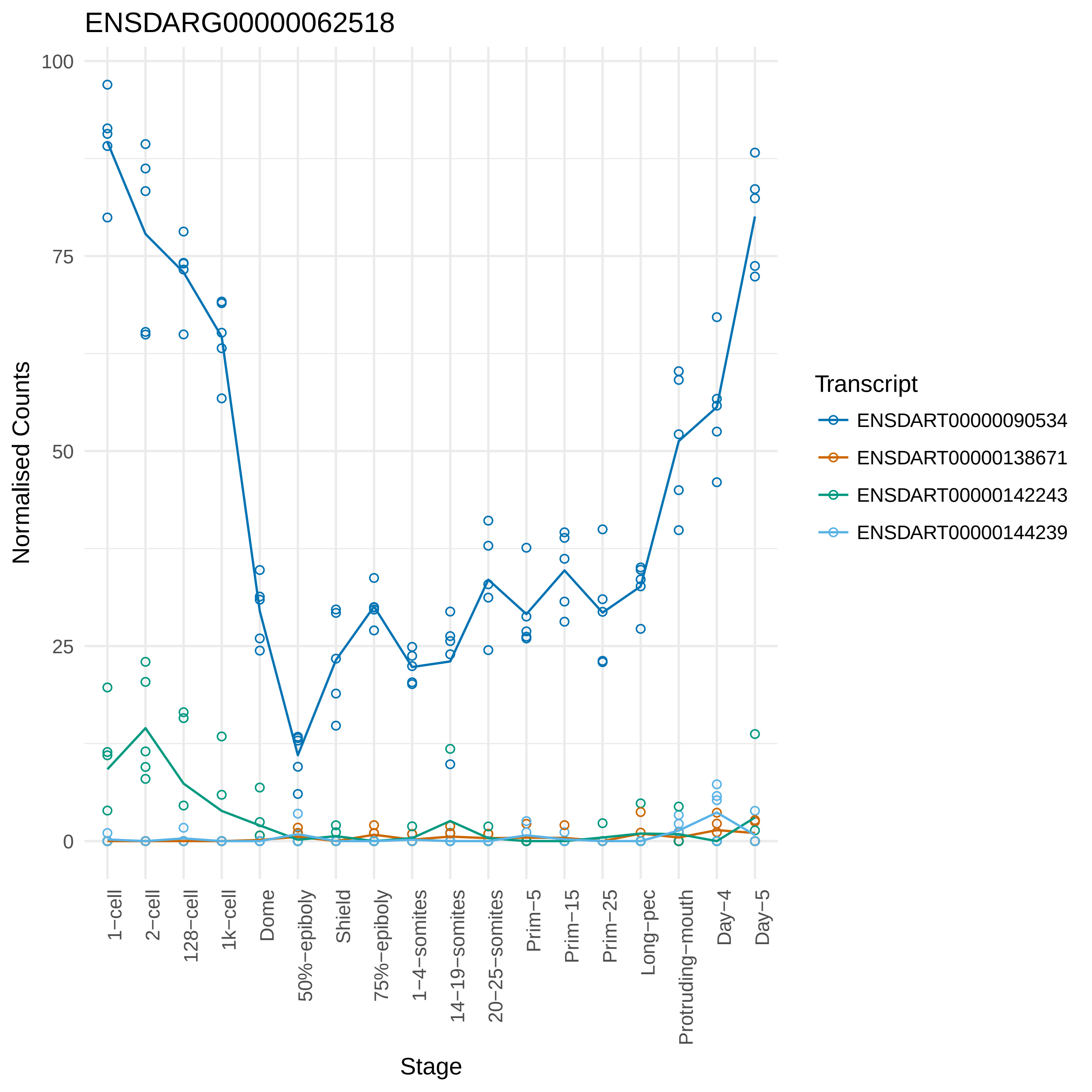

ENSDARG00000062518
Normalised Counts
100
75
50
25
0
1−cell
2−cell
128−cell
1k−cell
Dome
50%−epiboly
Shield
75%−epiboly
1−4−somites
14−19−somites
20−25−somites
Prim−5
Prim−15
Prim−25
Long−pec
Protruding−mouth
Day−4
Day−5
Stage
Transcript
ENSDART00000090534
ENSDART00000138671
ENSDART00000142243
ENSDART00000144239

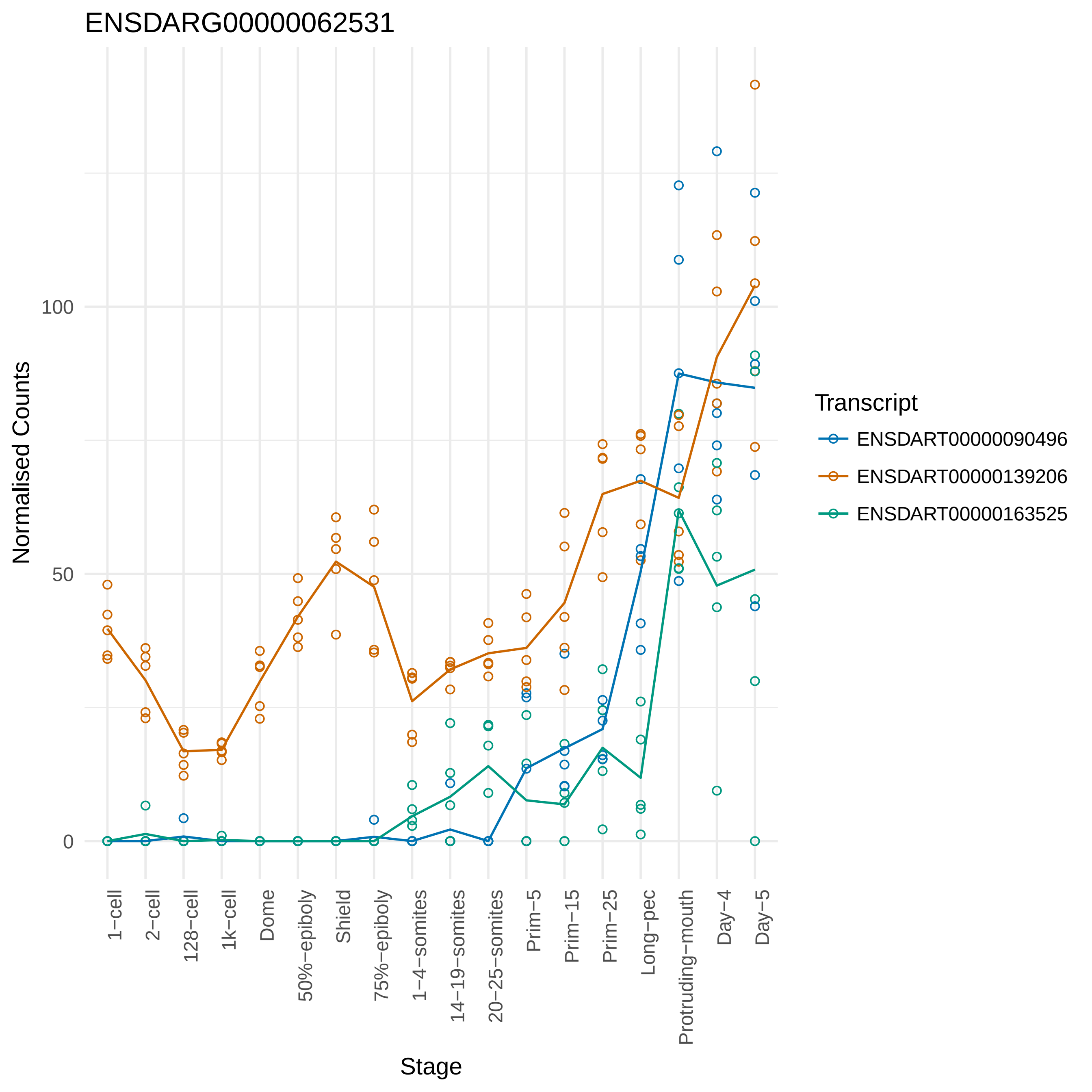
ENSDARG00000062531
Normalised Counts
100
50
0
1−cell
2−cell
128−cell
1k−cell
Dome
50%−epiboly
Shield
75%−epiboly
1−4−somites
14−19−somites
20−25−somites
Prim−5
Prim−15
Prim−25
Long−pec
Protruding−mouth
Day−4
Day−5
Stage
Transcript
ENSDART00000090496
ENSDART00000139206
ENSDART00000163525

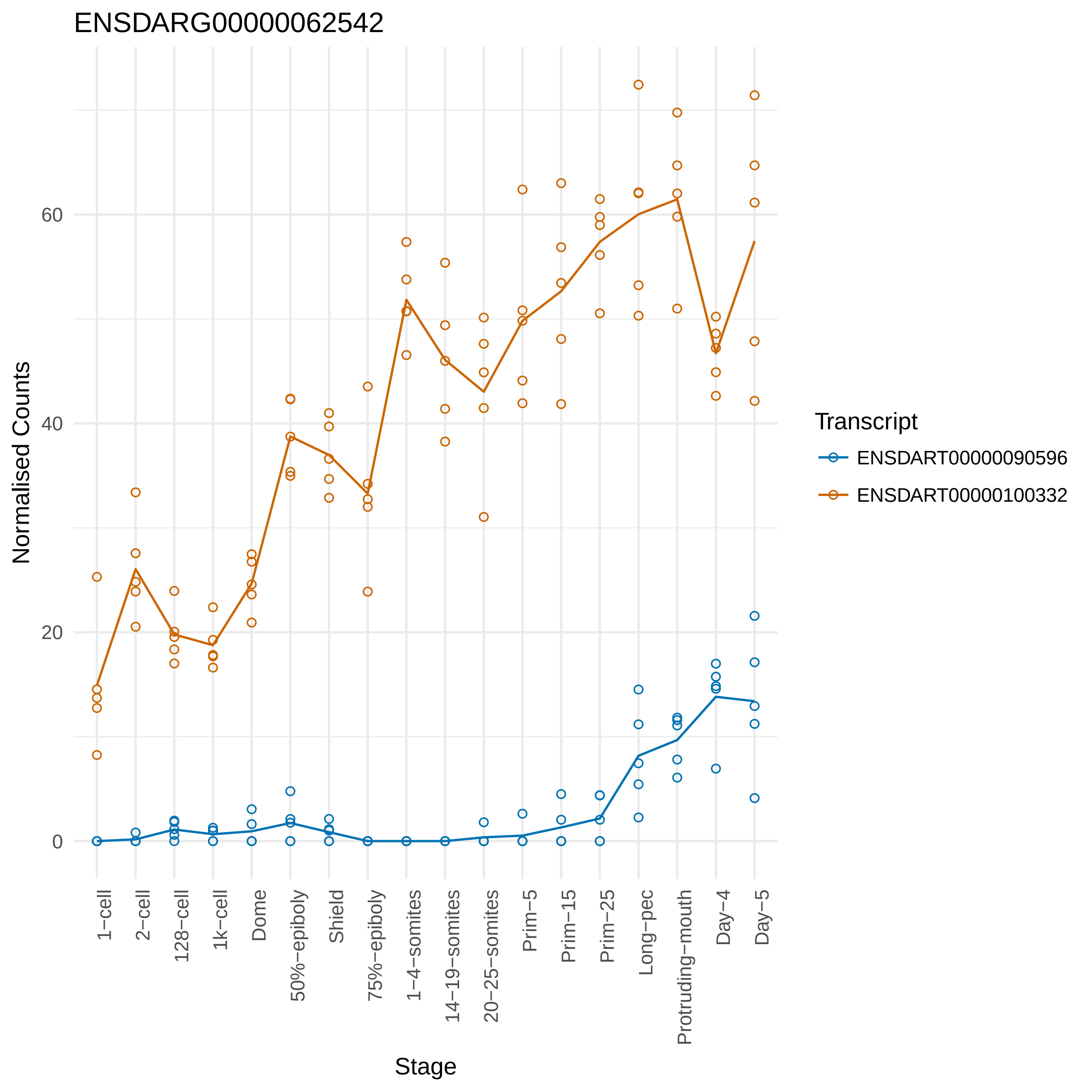
ENSDARG00000062542
Normalised Counts
0
20
40
60
Stage
1−cell
2−cell
128−cell
1k−cell
Dome
50%−epiboly
Shield
75%−epiboly
1−4−somites
14−19−somites
20−25−somites
Prim−5
Prim−15
Prim−25
Long−pec
Protruding−mouth
Day−4
Day−5
Transcript
ENSDART00000090596
ENSDART00000100332

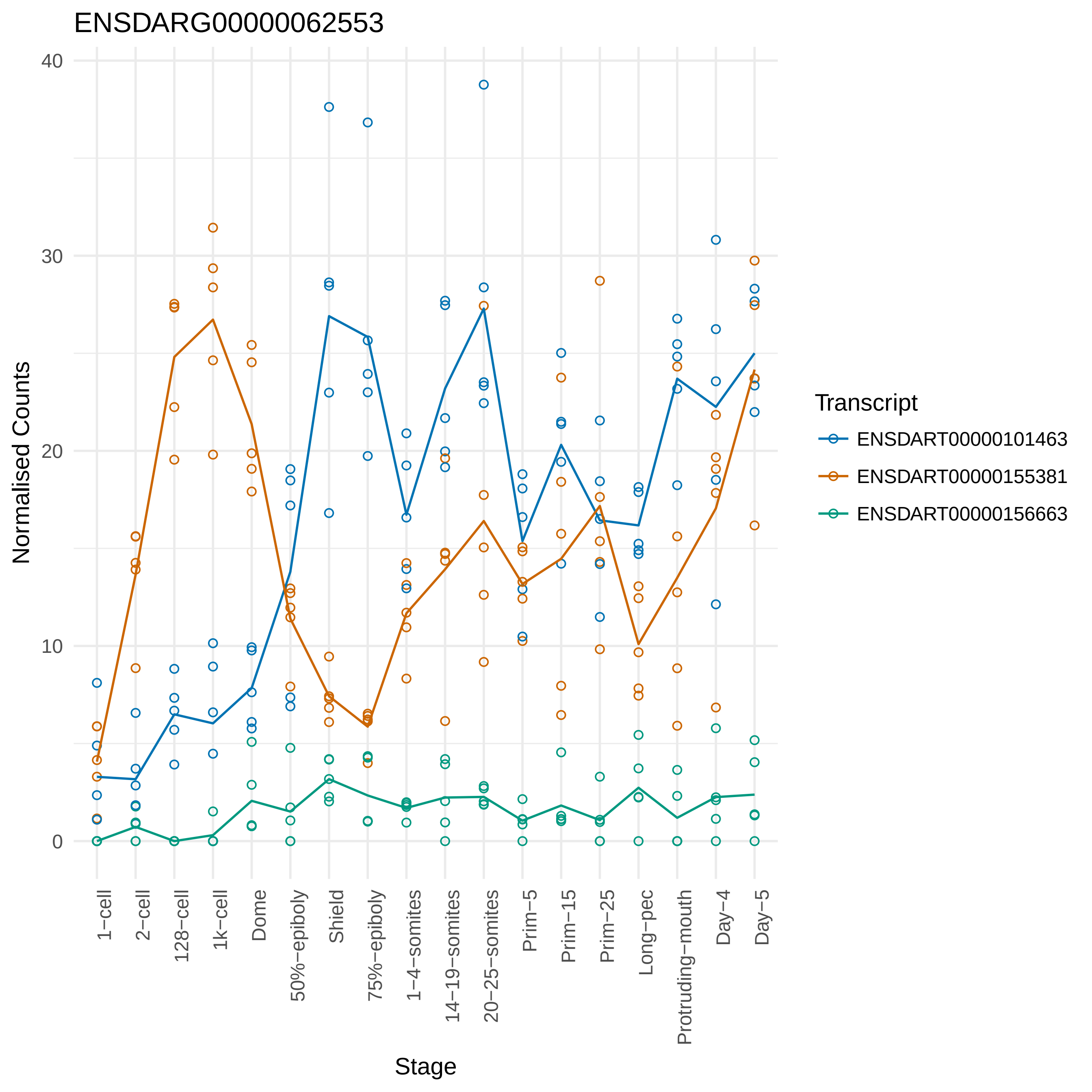
ENSDARG00000062553
Normalised Counts
0
10
20
30
40
Stage
1−cell
2−cell
128−cell
1k−cell
Dome
50%−epiboly
Shield
75%−epiboly
1−4−somites
14−19−somites
20−25−somites
Prim−5
Prim−15
Prim−25
Long−pec
Protruding−mouth
Day−4
Day−5
Transcript
ENSDART00000101463
ENSDART00000155381
ENSDART00000156663

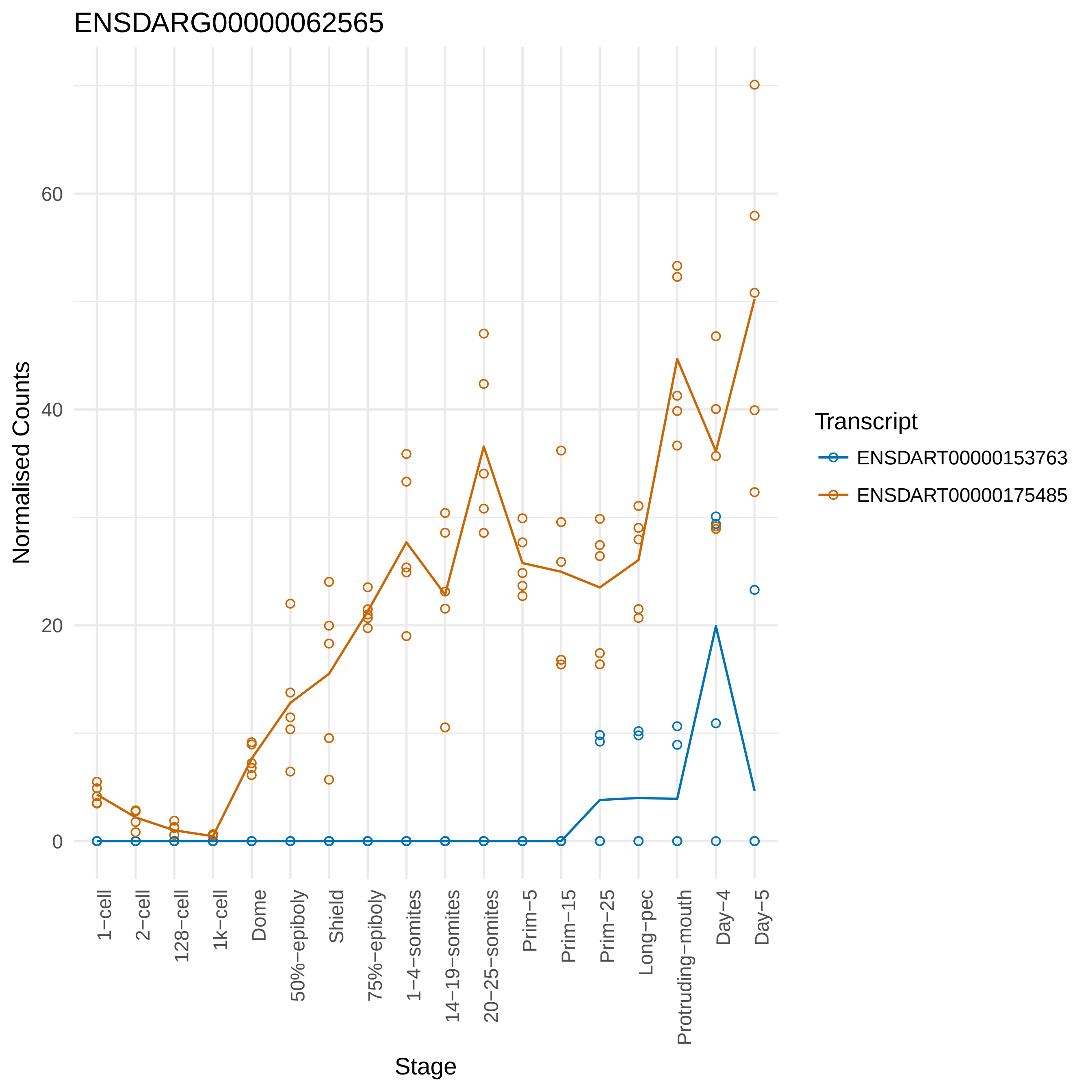

ENSDARG00000062565
Normalised Counts
60
40
20
0
1−cell
2−cell
128−cell
1k−cell
Dome
50%−epiboly
Shield
75%−epiboly
1−4−somites
14−19−somites
20−25−somites
Prim−5
Prim−15
Prim−25
Long−pec
Protruding−mouth
Day−4
Day−5
Stage
Transcript
ENSDART00000153763
ENSDART00000175485

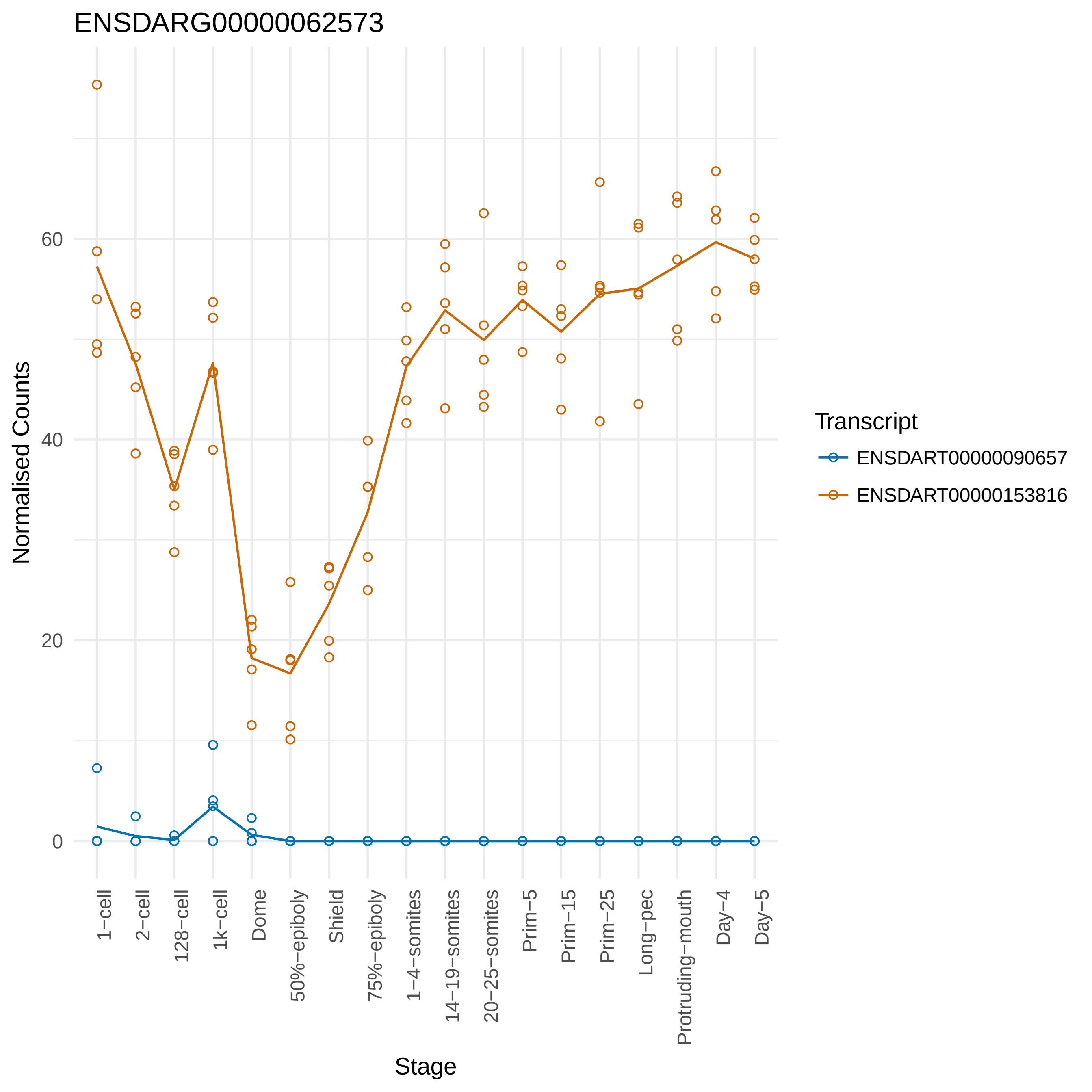

ENSDARG00000062573
Normalised Counts
0
20
40
60
Stage
1−cell
2−cell
128−cell
1k−cell
Dome
50%−epiboly
Shield
75%−epiboly
1−4−somites
14−19−somites
20−25−somites
Prim−5
Prim−15
Prim−25
Long−pec
Protruding−mouth
Day−4
Day−5
Transcript
ENSDART00000090657
ENSDART00000153816

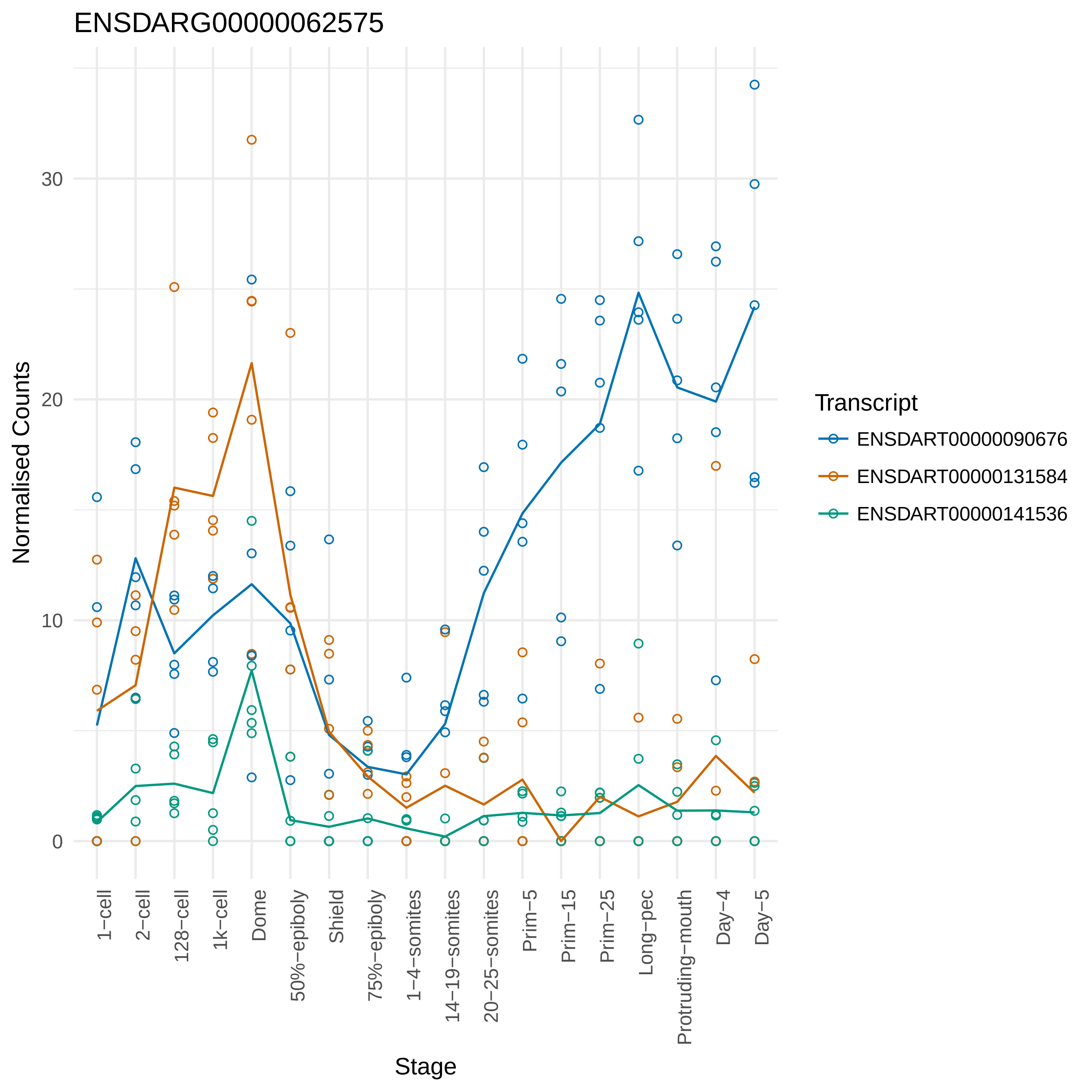

ENSDARG00000062575
Normalised Counts
0
10
20
30
1−cell
2−cell
128−cell
1k−cell
Dome
50%−epiboly
Shield
75%−epiboly
1−4−somites
14−19−somites
20−25−somites
Prim−5
Prim−15
Prim−25
Long−pec
Protruding−mouth
Day−4
Day−5
Stage
Transcript
ENSDART00000090676
ENSDART00000131584
ENSDART00000141536

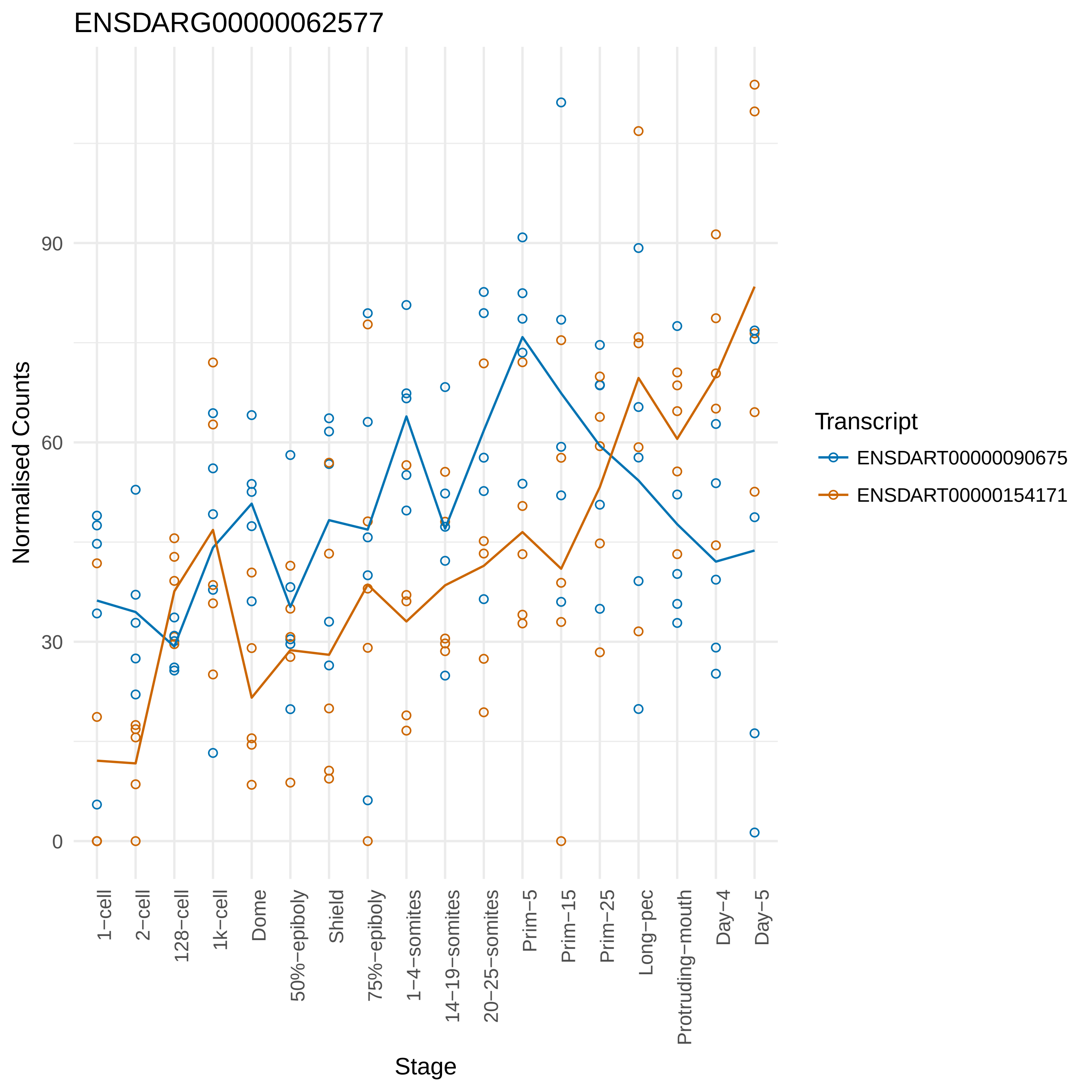

ENSDARG00000062577
Normalised Counts
0
30
60
90
Stage
1−cell
2−cell
128−cell
1k−cell
Dome
50%−epiboly
Shield
75%−epiboly
1−4−somites
14−19−somites
20−25−somites
Prim−5
Prim−15
Prim−25
Long−pec
Protruding−mouth
Day−4
Day−5
Transcript
ENSDART00000090675
ENSDART00000154171

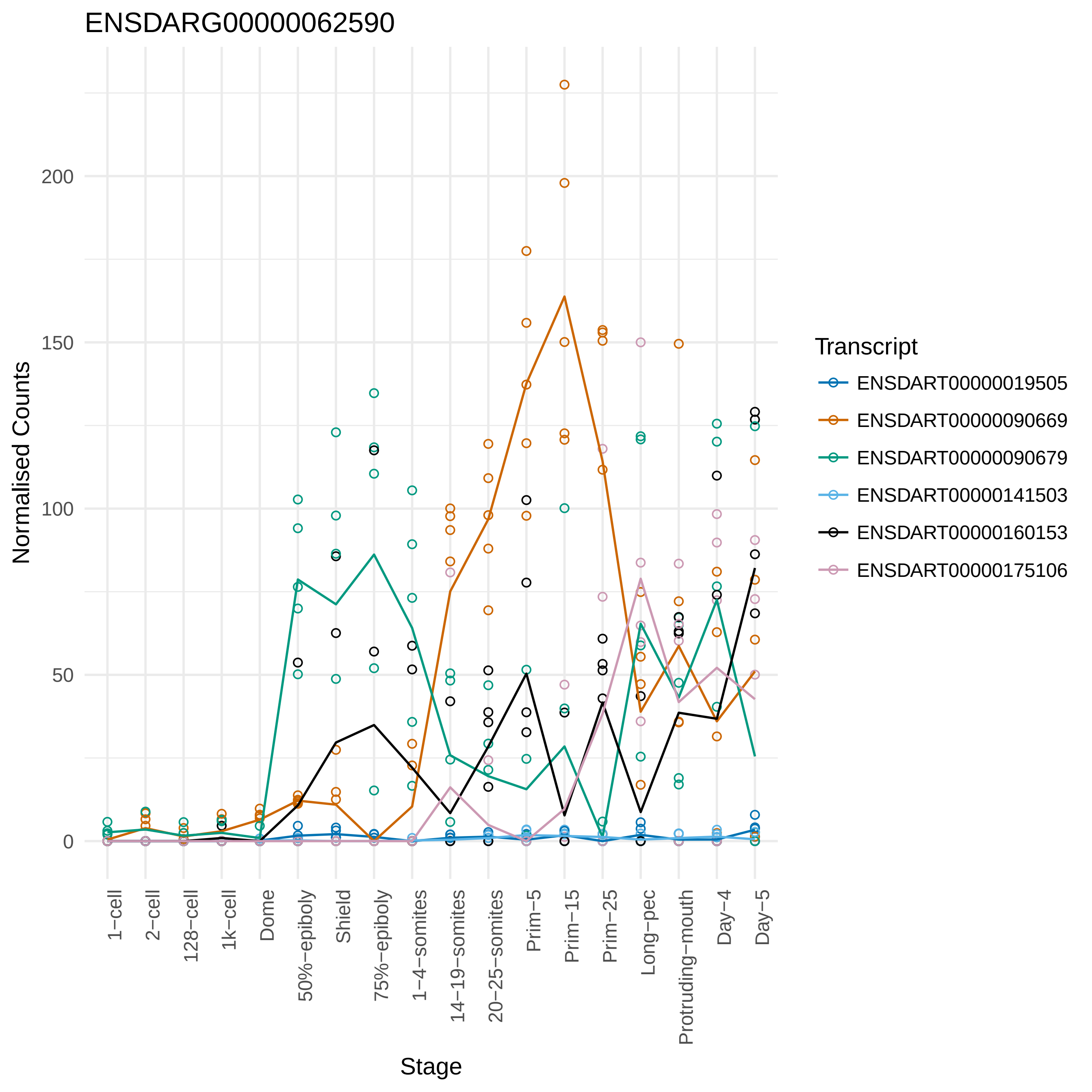

ENSDARG00000062590
Normalised Counts
0
50
100
150
200
Stage
1−cell
2−cell
128−cell
1k−cell
Dome
50%−epiboly
Shield
75%−epiboly
1−4−somites
14−19−somites
20−25−somites
Prim−5
Prim−15
Prim−25
Long−pec
Protruding−mouth
Day−4
Day−5
Transcript
ENSDART00000019505
ENSDART00000090669
ENSDART00000090679
ENSDART00000141503
ENSDART00000160153
ENSDART00000175106

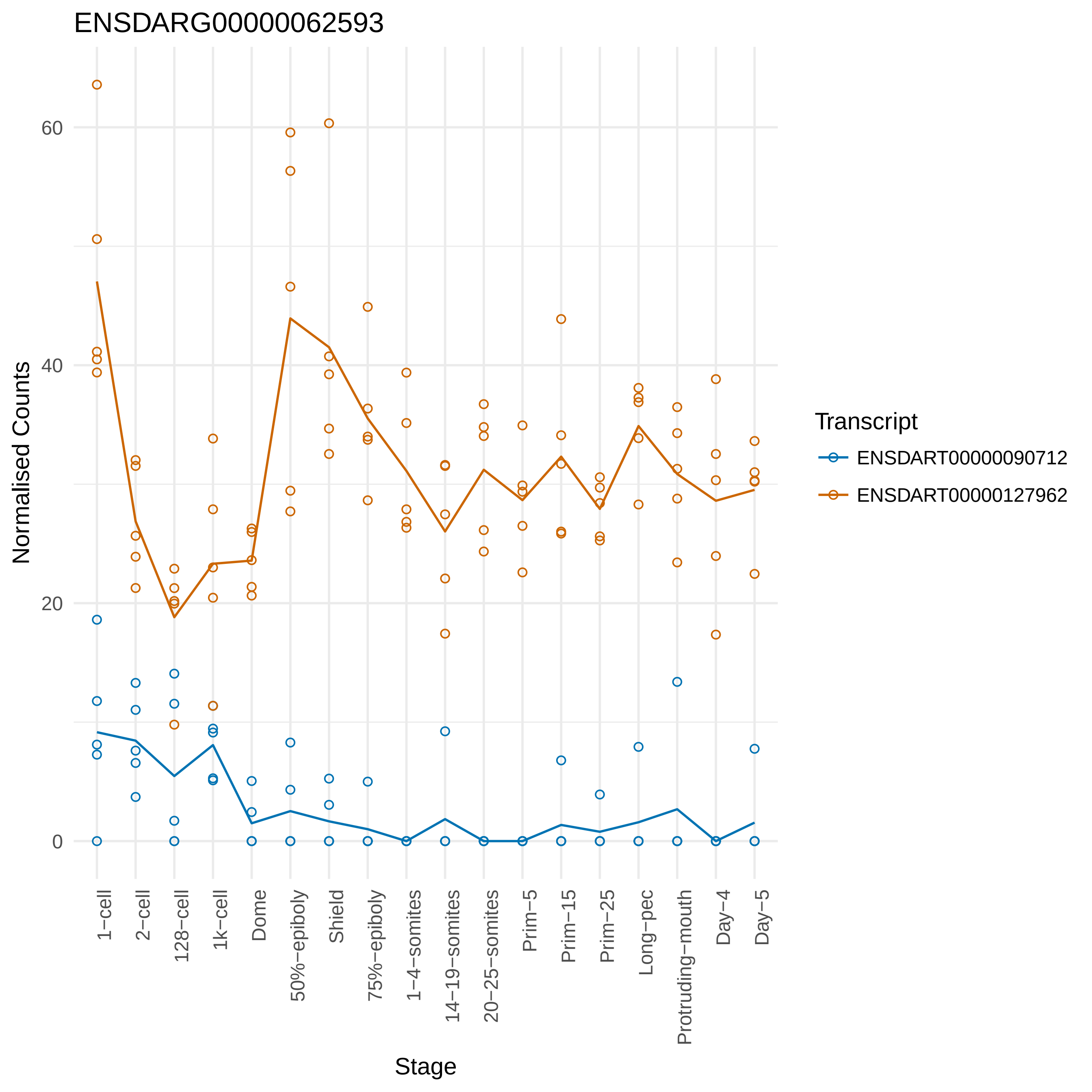

ENSDARG00000062593
Normalised Counts
0
20
40
60
1−cell
2−cell
128−cell
1k−cell
Dome
50%−epiboly
Shield
75%−epiboly
1−4−somites
14−19−somites
20−25−somites
Prim−5
Prim−15
Prim−25
Long−pec
Protruding−mouth
Day−4
Day−5
Stage
Transcript
ENSDART00000090712
ENSDART00000127962

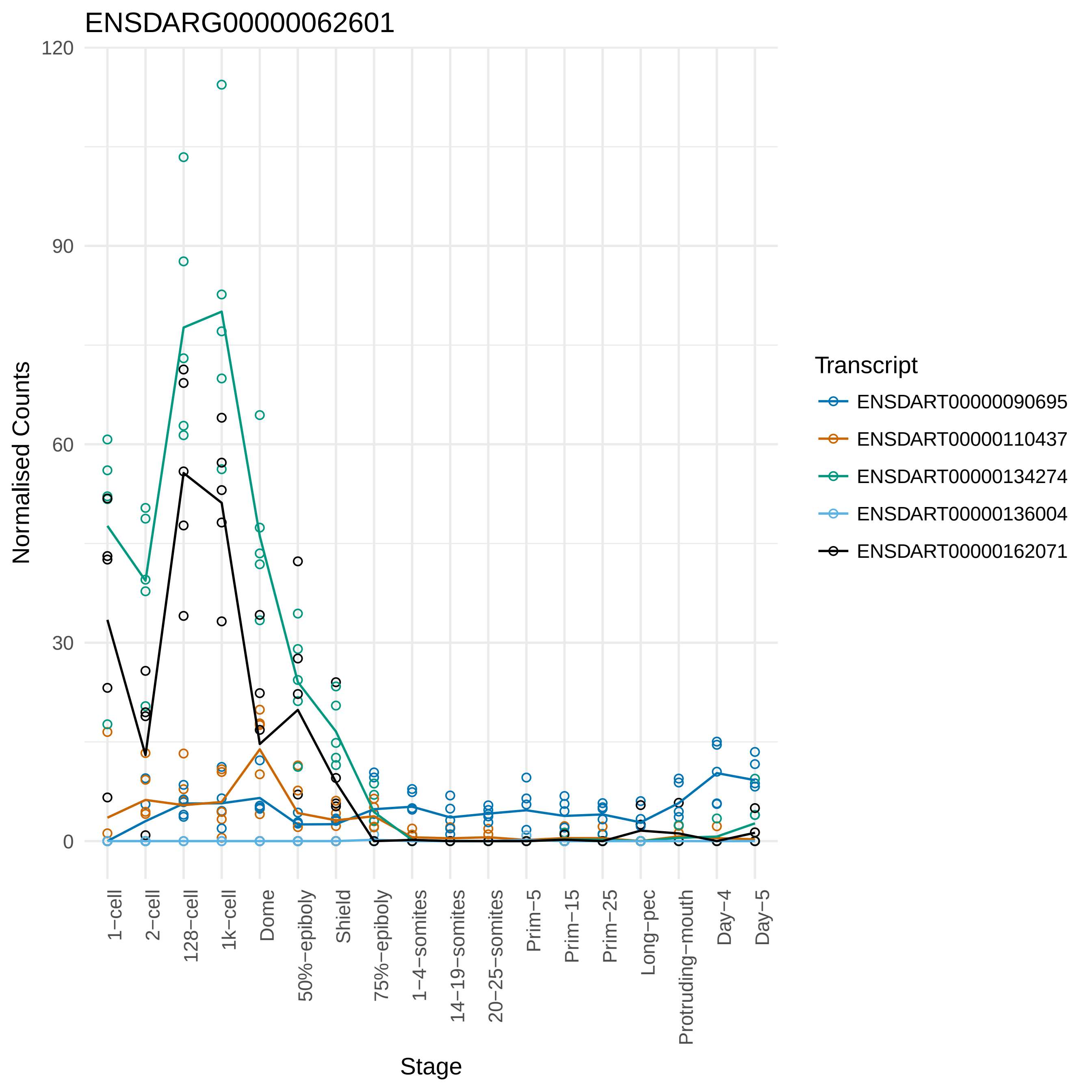

ENSDARG00000062601
Normalised Counts
120
90
60
30
0
1−cell
2−cell
128−cell
1k−cell
Dome
50%−epiboly
Shield
75%−epiboly
1−4−somites
14−19−somites
20−25−somites
Prim−5
Prim−15
Prim−25
Long−pec
Protruding−mouth
Day−4
Day−5
Stage
Transcript
ENSDART00000090695
ENSDART00000110437
ENSDART00000134274
ENSDART00000136004
ENSDART00000162071

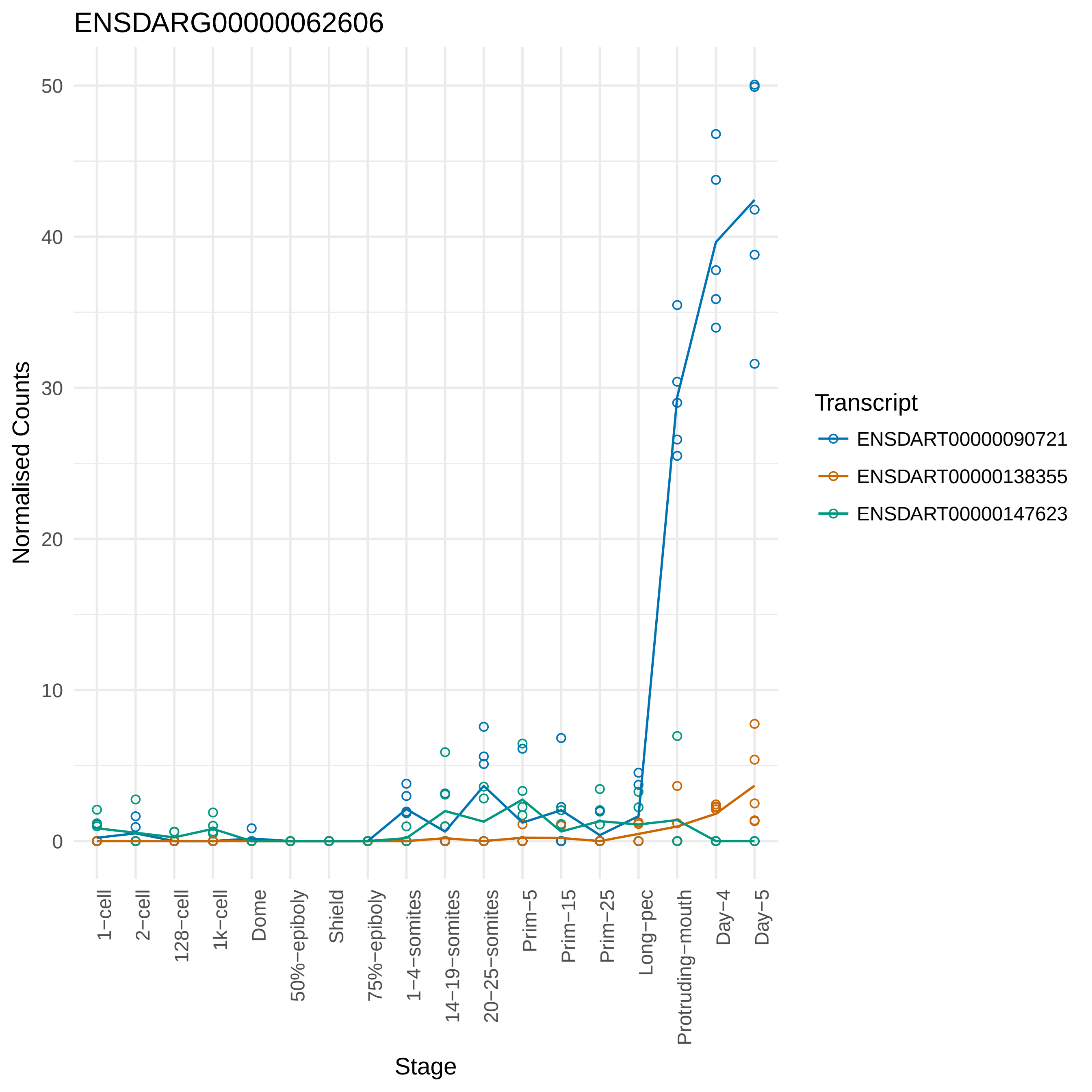

ENSDARG00000062606
Normalised Counts
50
40
30
20
10
0
1−cell
2−cell
128−cell
1k−cell
Dome
50%−epiboly
Shield
75%−epiboly
1−4−somites
14−19−somites
20−25−somites
Prim−5
Prim−15
Prim−25
Long−pec
Protruding−mouth
Day−4
Day−5
Stage
Transcript
ENSDART00000090721
ENSDART00000138355
ENSDART00000147623

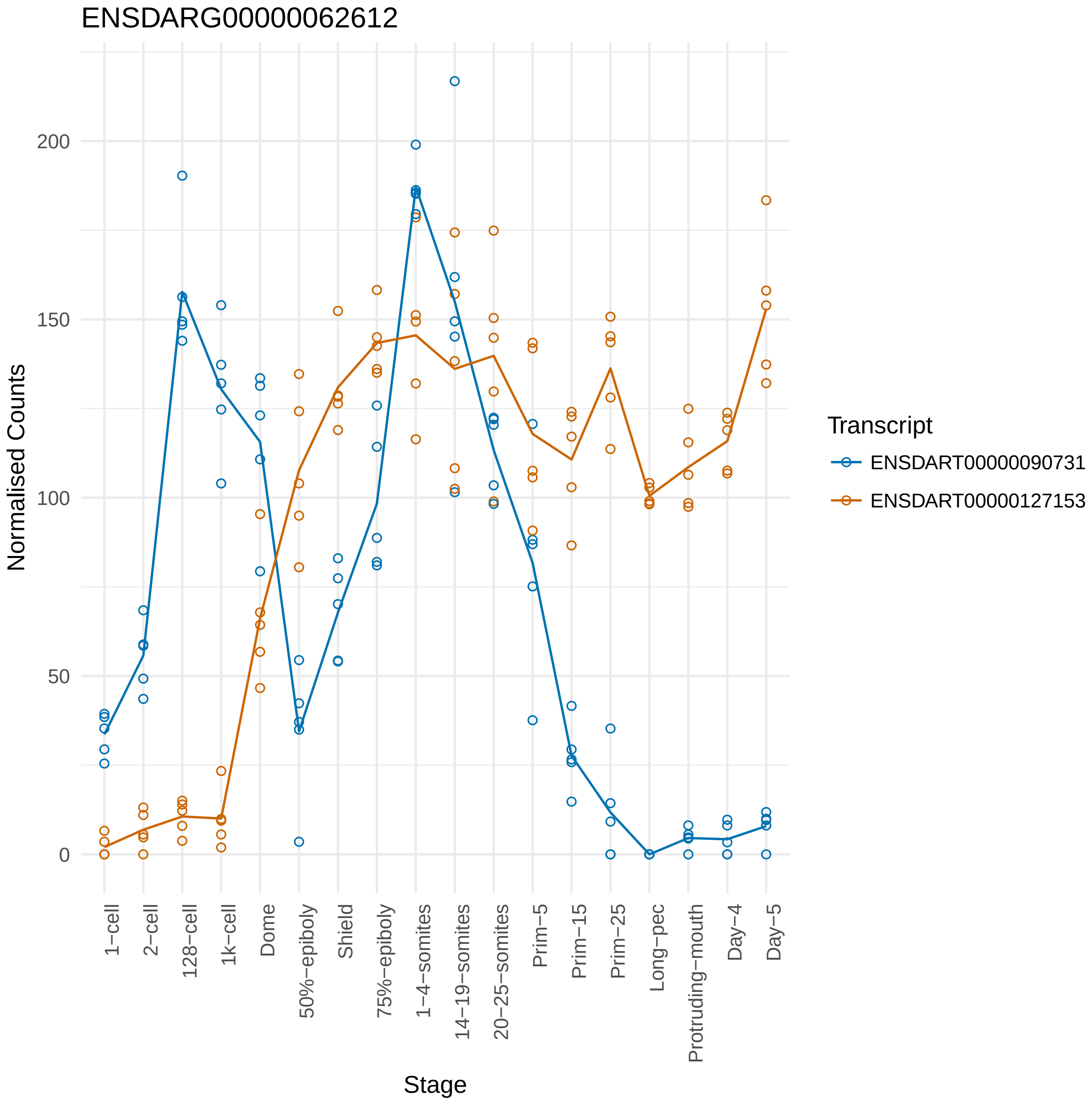

ENSDARG00000062612
Normalised Counts
0
50
100
150
200
1−cell
2−cell
128−cell
1k−cell
Dome
50%−epiboly
Shield
75%−epiboly
1−4−somites
14−19−somites
20−25−somites
Prim−5
Prim−15
Prim−25
Long−pec
Protruding−mouth
Day−4
Day−5
Stage
Transcript
ENSDART00000090731
ENSDART00000127153

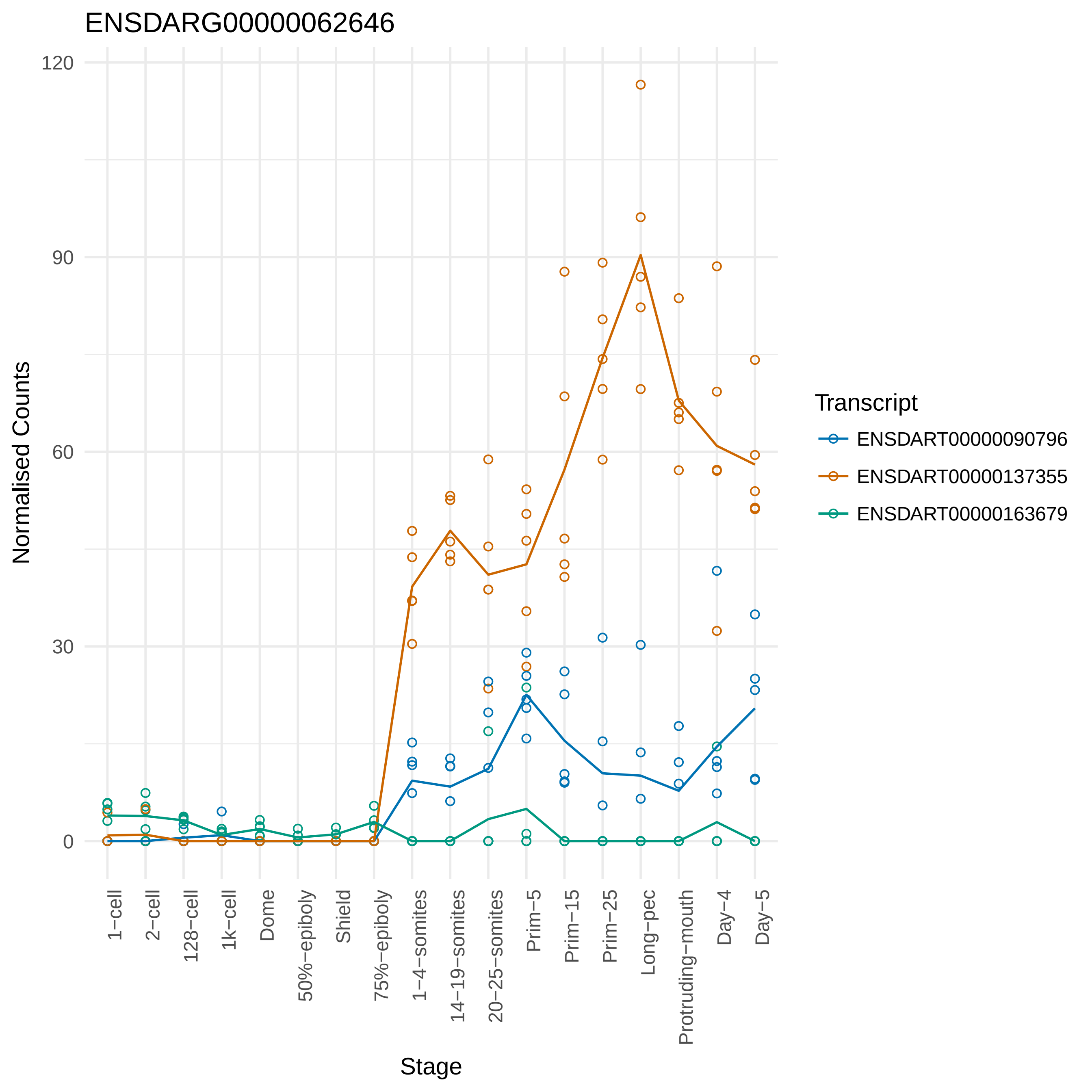
ENSDARG00000062646
Normalised Counts
120
90
60
30
0
Stage
1−cell
2−cell
128−cell
1k−cell
Dome
50%−epiboly
Shield
75%−epiboly
1−4−somites
14−19−somites
20−25−somites
Prim−5
Prim−15
Prim−25
Long−pec
Protruding−mouth
Day−4
Day−5
Transcript
ENSDART00000090796
ENSDART00000137355
ENSDART00000163679

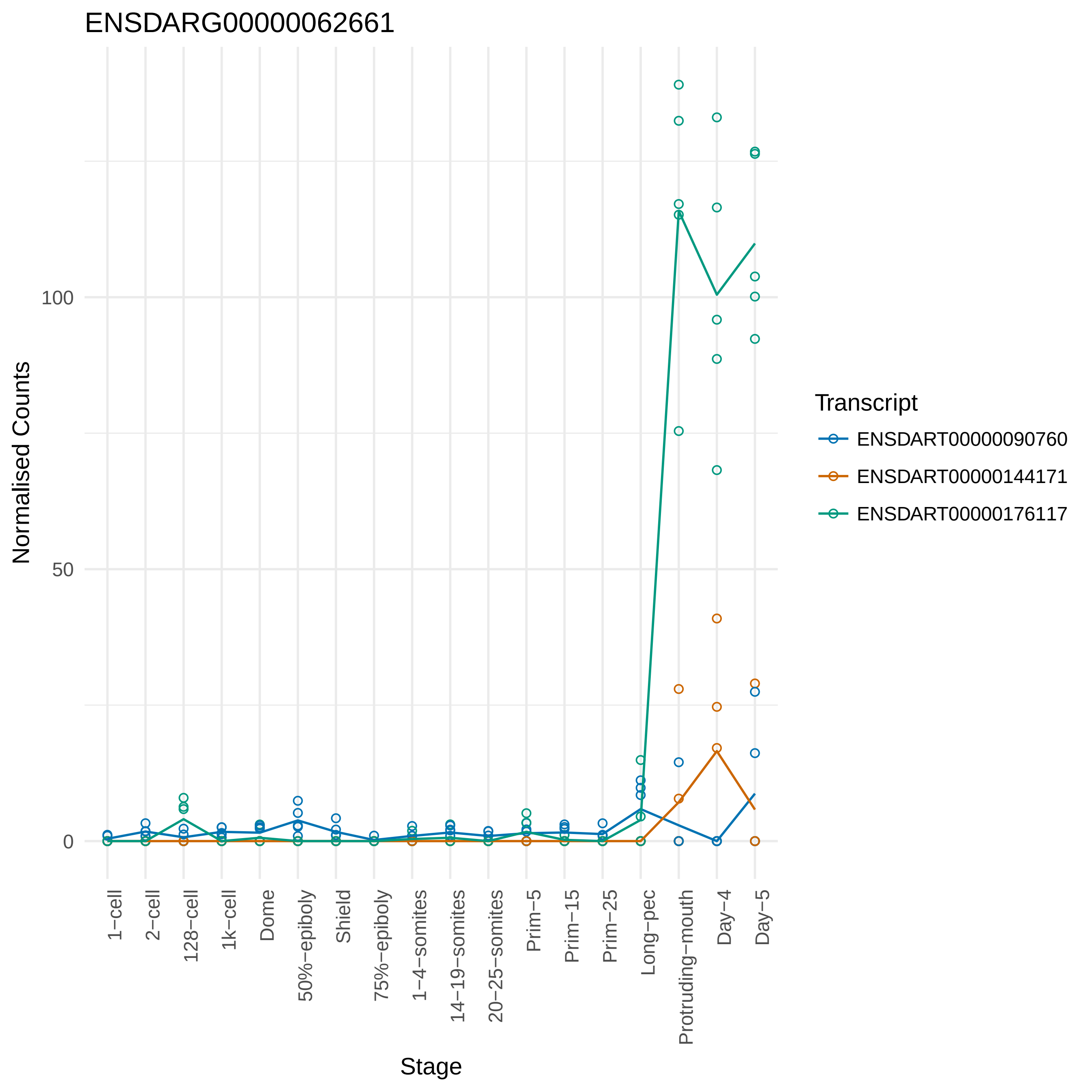

ENSDARG00000062661
Normalised Counts
100
50
0
1−cell
2−cell
128−cell
1k−cell
Dome
50%−epiboly
Shield
75%−epiboly
1−4−somites
14−19−somites
20−25−somites
Prim−5
Prim−15
Prim−25
Long−pec
Protruding−mouth
Day−4
Day−5
Stage
Transcript
ENSDART00000090760
ENSDART00000144171
ENSDART00000176117

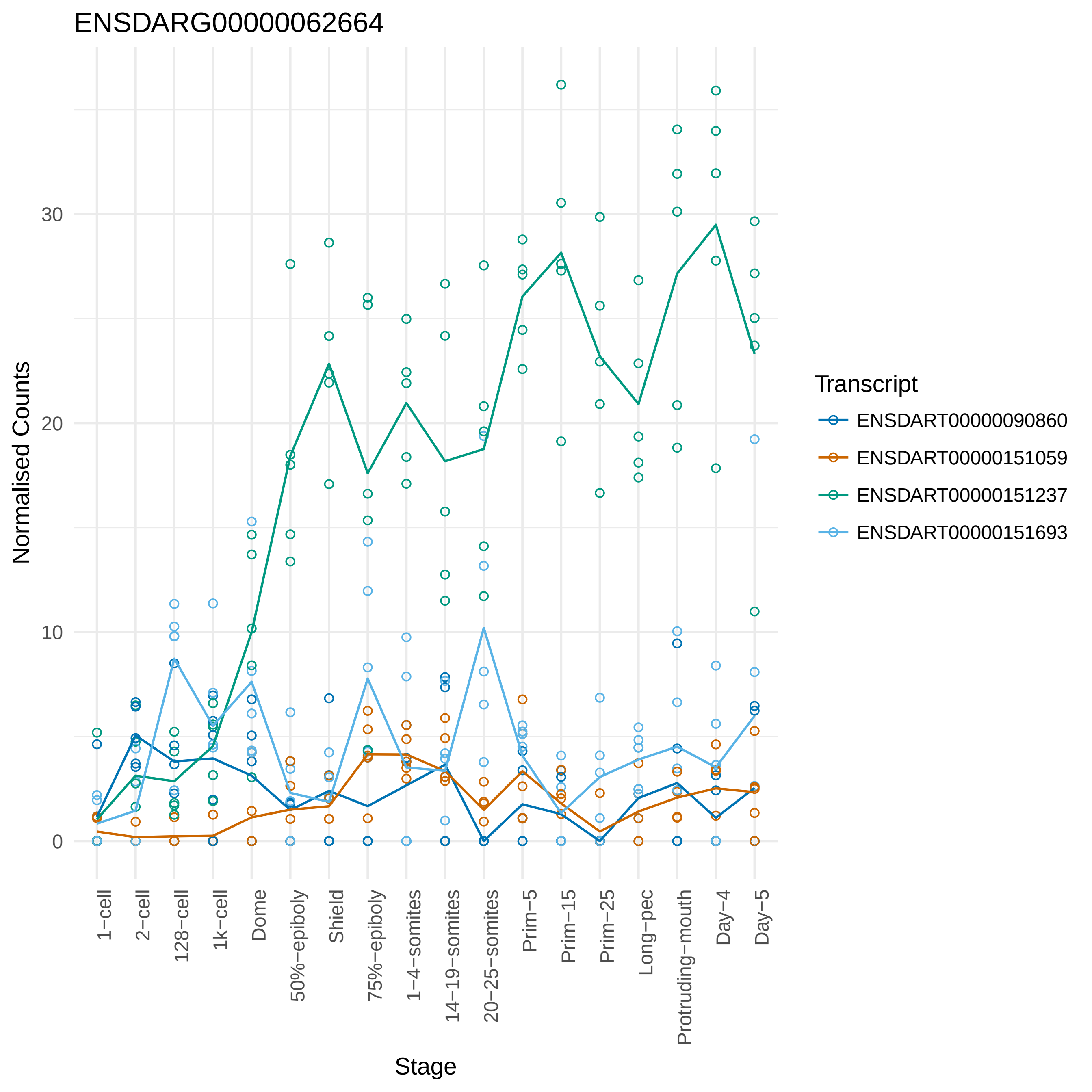

ENSDARG00000062664
Normalised Counts
0
10
20
30
Stage
1−cell
2−cell
128−cell
1k−cell
Dome
50%−epiboly
Shield
75%−epiboly
1−4−somites
14−19−somites
20−25−somites
Prim−5
Prim−15
Prim−25
Long−pec
Protruding−mouth
Day−4
Day−5
Transcript
ENSDART00000090860
ENSDART00000151059
ENSDART00000151237
ENSDART00000151693

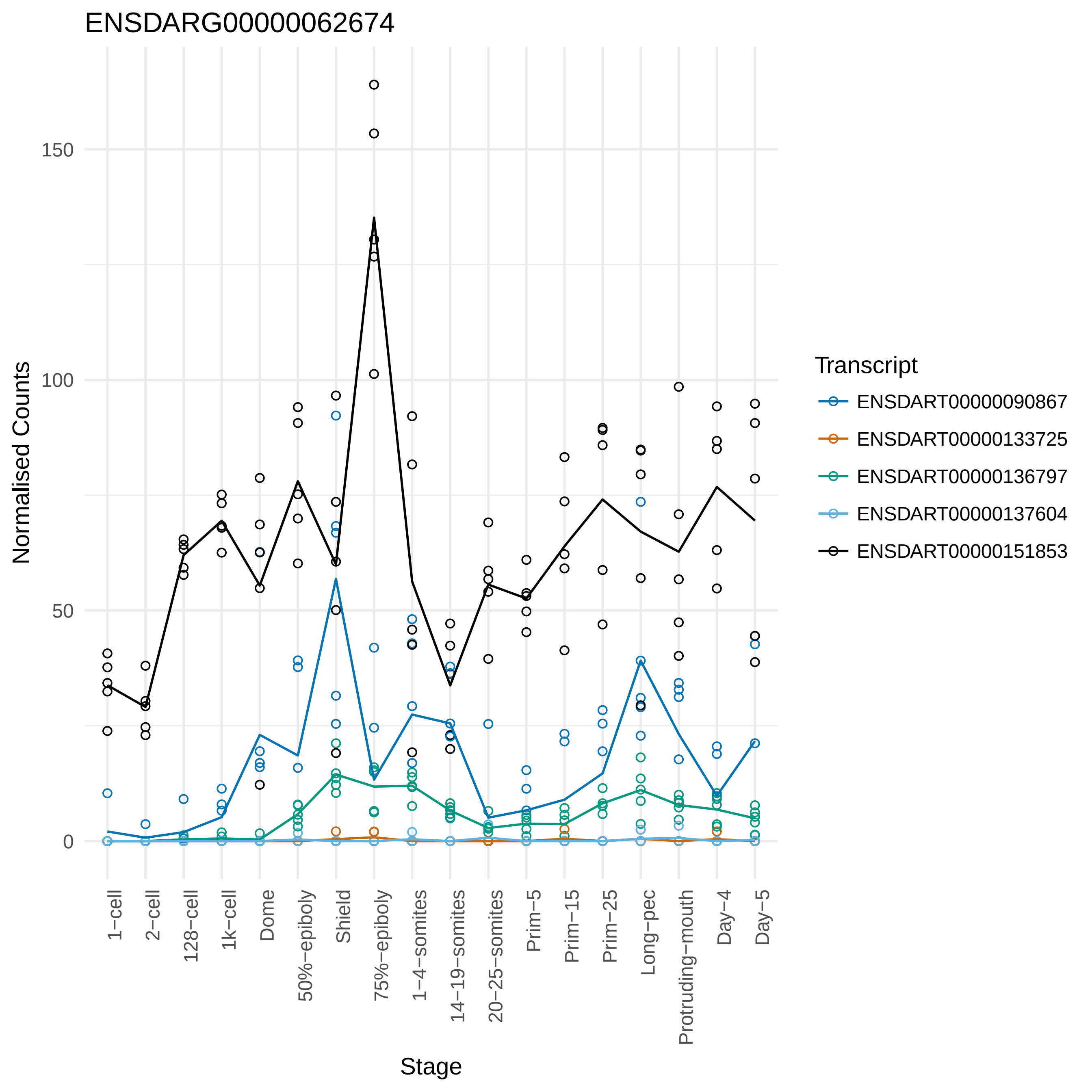

ENSDARG00000062674
Normalised Counts
150
100
50
0
1−cell
2−cell
128−cell
1k−cell
Dome
50%−epiboly
Shield
75%−epiboly
1−4−somites
14−19−somites
20−25−somites
Prim−5
Prim−15
Prim−25
Long−pec
Protruding−mouth
Day−4
Day−5
Stage
Transcript
ENSDART00000090867
ENSDART00000133725
ENSDART00000136797
ENSDART00000137604
ENSDART00000151853

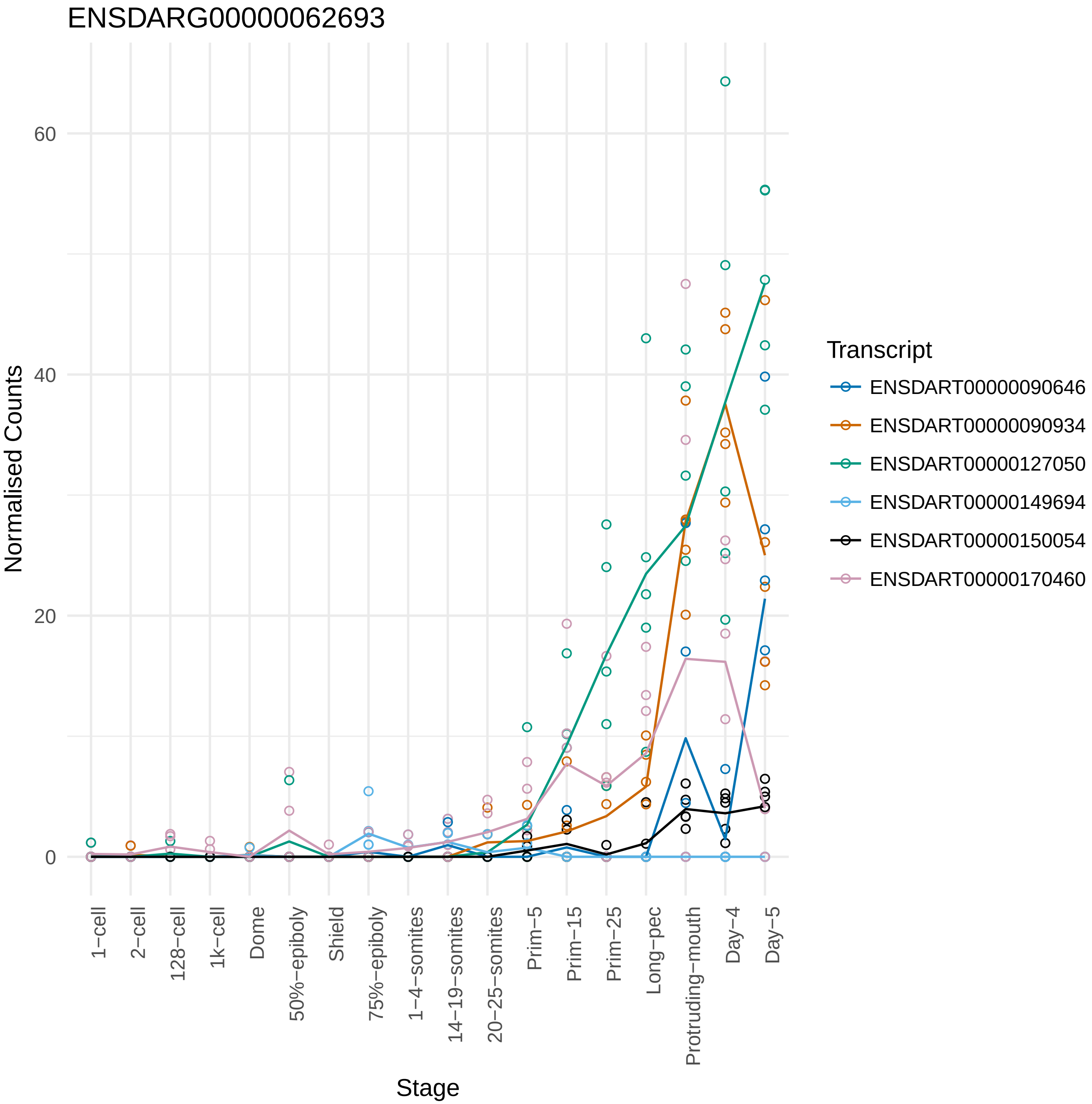
ENSDARG00000062693
Normalised Counts
0
20
40
60
1−cell
2−cell
128−cell
1k−cell
Dome
50%−epiboly
Shield
75%−epiboly
1−4−somites
14−19−somites
20−25−somites
Prim−5
Prim−15
Prim−25
Long−pec
Protruding−mouth
Day−4
Day−5
Stage
Transcript
ENSDART00000090646
ENSDART00000090934
ENSDART00000127050
ENSDART00000149694
ENSDART00000150054
ENSDART00000170460

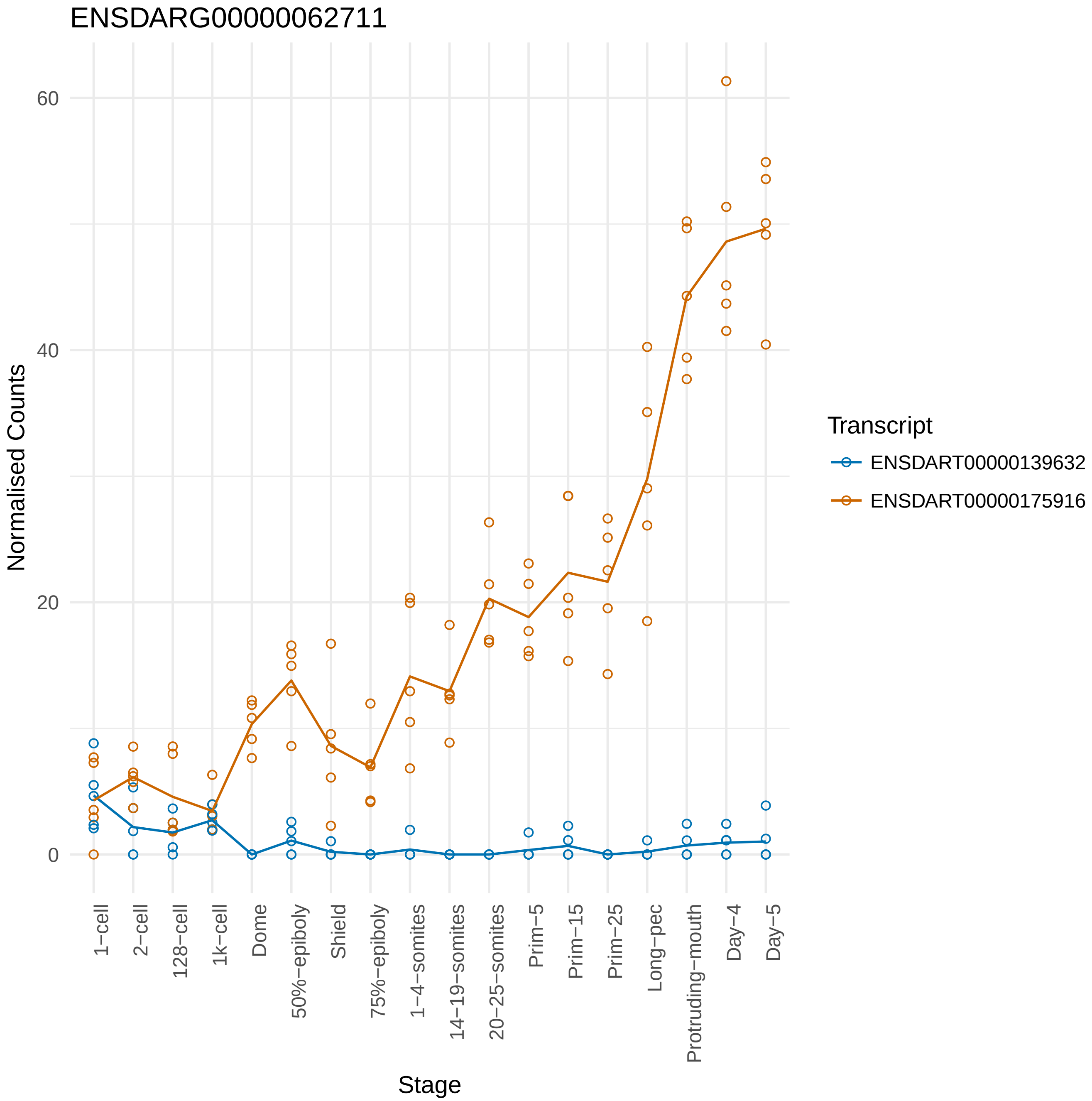

ENSDARG00000062711
Normalised Counts
60
40
20
0
1−cell
2−cell
128−cell
1k−cell
Dome
50%−epiboly
Shield
75%−epiboly
1−4−somites
14−19−somites
20−25−somites
Prim−5
Prim−15
Prim−25
Long−pec
Protruding−mouth
Day−4
Day−5
Stage
Transcript
ENSDART00000139632
ENSDART00000175916

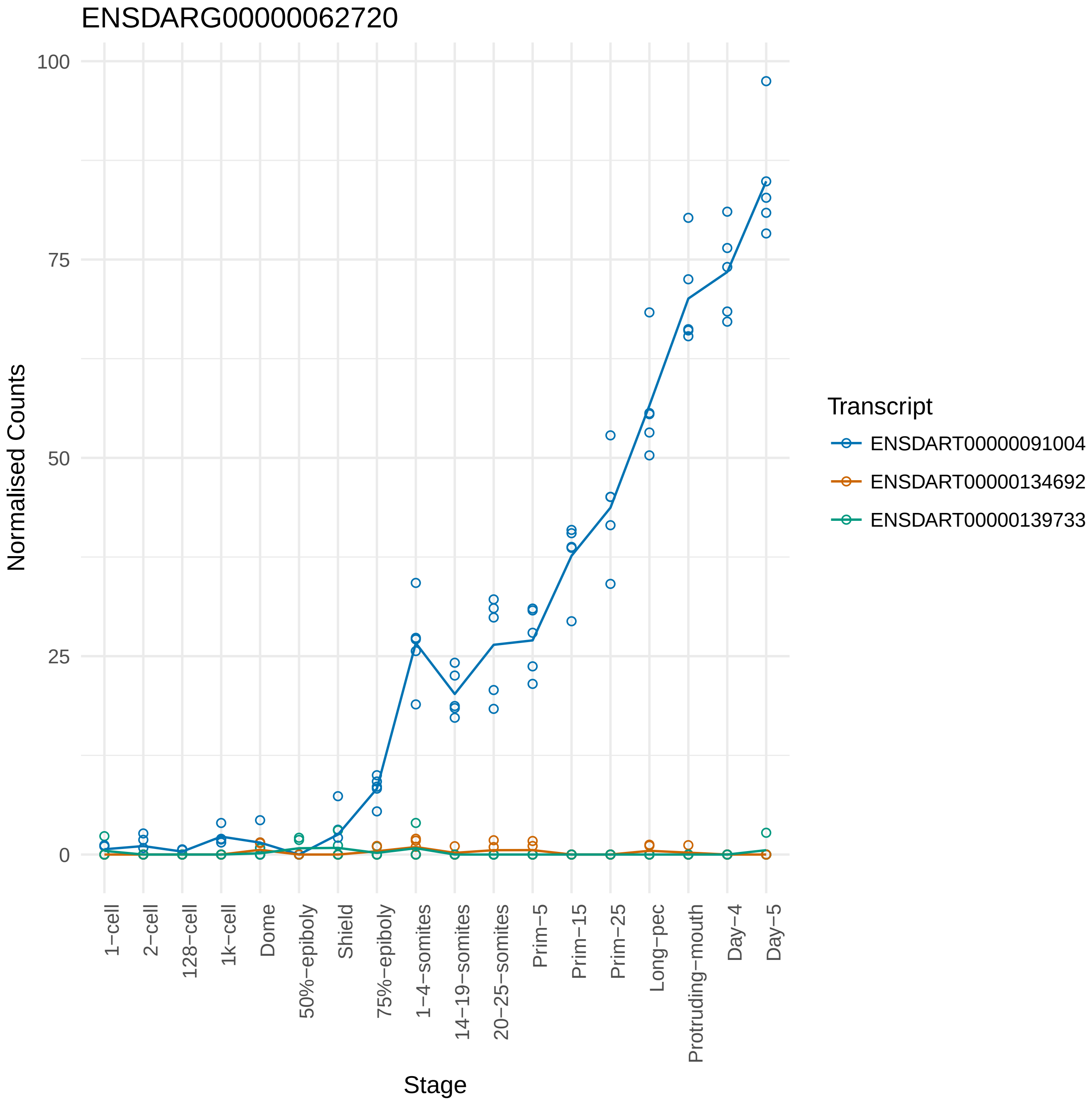

ENSDARG00000062720
Normalised Counts
100
75
50
25
0
1−cell
2−cell
128−cell
1k−cell
Dome
50%−epiboly
Shield
75%−epiboly
1−4−somites
14−19−somites
20−25−somites
Prim−5
Prim−15
Prim−25
Long−pec
Protruding−mouth
Day−4
Day−5
Stage
Transcript
ENSDART00000091004
ENSDART00000134692
ENSDART00000139733

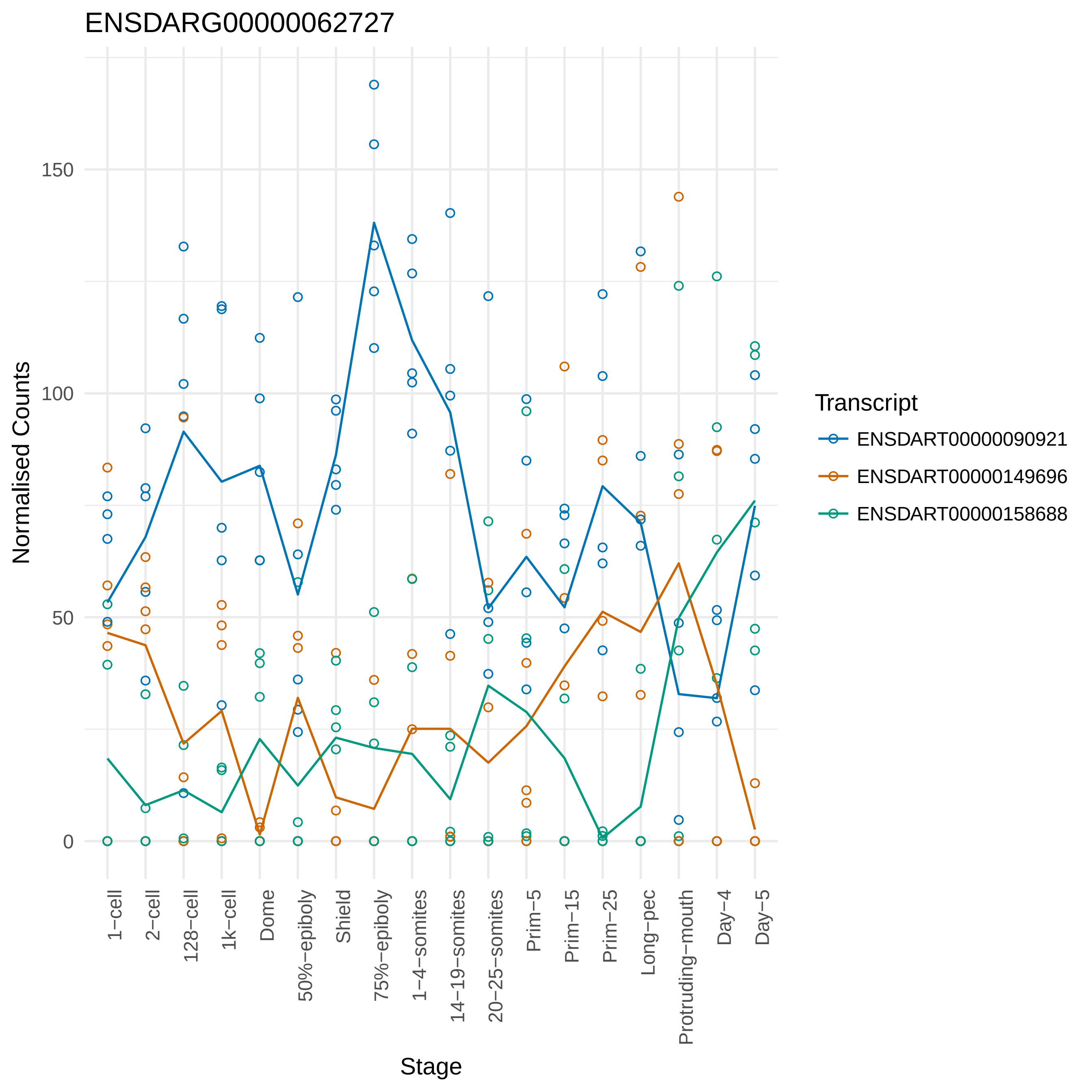

ENSDARG00000062727
Normalised Counts
150
100
50
0
1−cell
2−cell
128−cell
1k−cell
Dome
50%−epiboly
Shield
75%−epiboly
1−4−somites
14−19−somites
20−25−somites
Prim−5
Prim−15
Prim−25
Long−pec
Protruding−mouth
Day−4
Day−5
Stage
Transcript
ENSDART00000090921
ENSDART00000149696
ENSDART00000158688

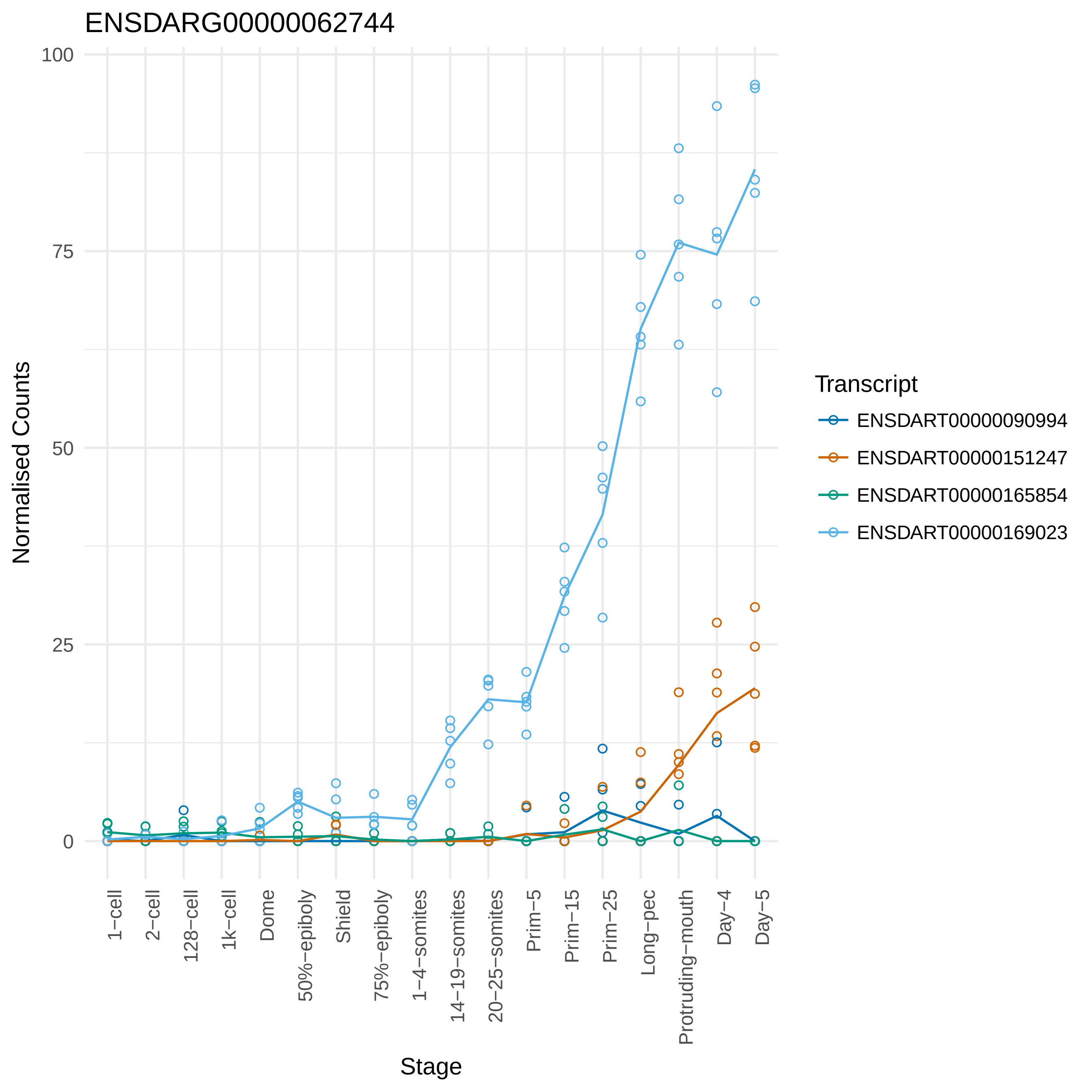
ENSDARG00000062744
Normalised Counts
100
75
50
25
0
1−cell
2−cell
128−cell
1k−cell
Dome
50%−epiboly
Shield
75%−epiboly
1−4−somites
14−19−somites
20−25−somites
Prim−5
Prim−15
Prim−25
Long−pec
Protruding−mouth
Day−4
Day−5
Stage
Transcript
ENSDART00000090994
ENSDART00000151247
ENSDART00000165854
ENSDART00000169023

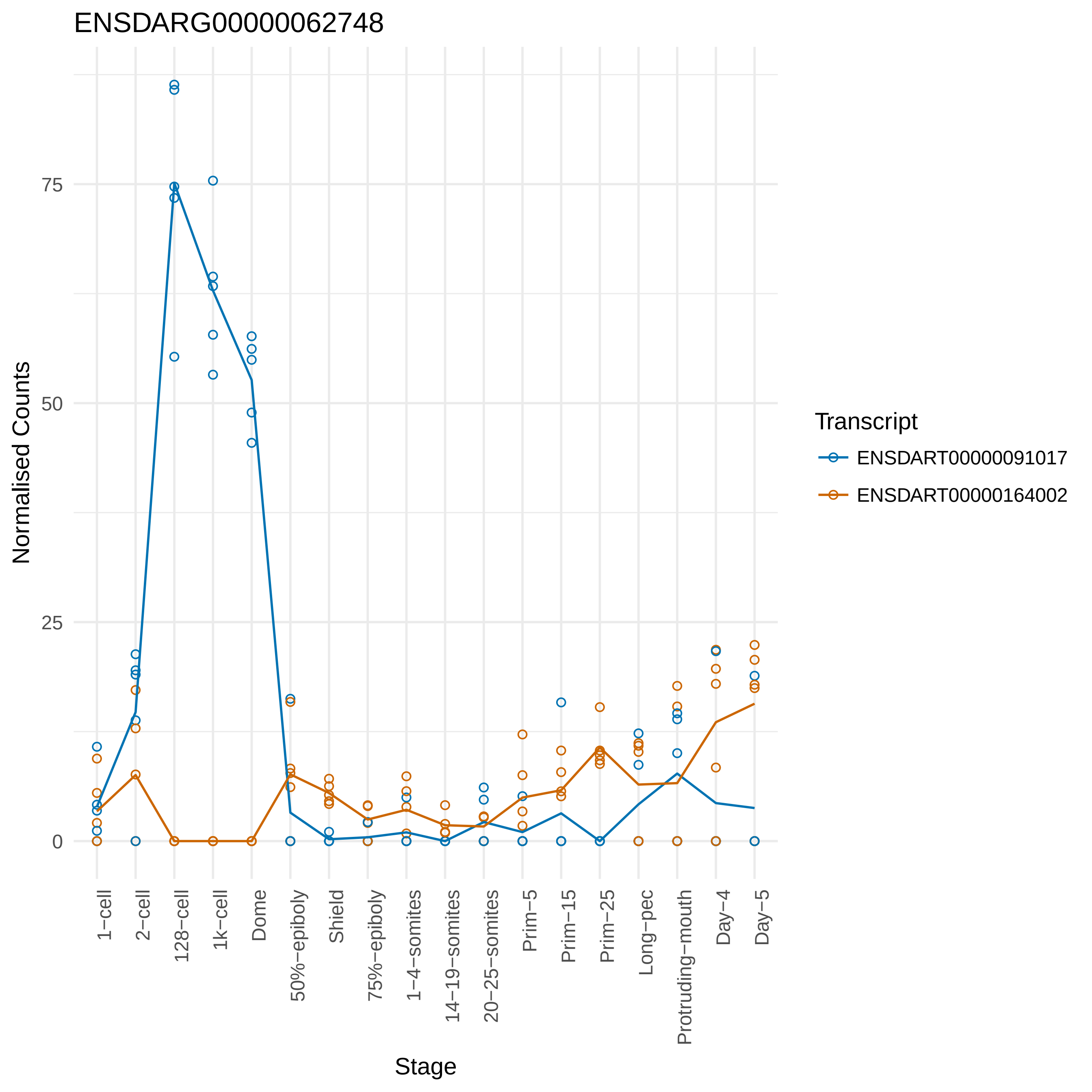

ENSDARG00000062748
Normalised Counts
75
50
25
0
1−cell
2−cell
128−cell
1k−cell
Dome
50%−epiboly
Shield
75%−epiboly
1−4−somites
14−19−somites
20−25−somites
Prim−5
Prim−15
Prim−25
Long−pec
Protruding−mouth
Day−4
Day−5
Stage
Transcript
ENSDART00000091017
ENSDART00000164002

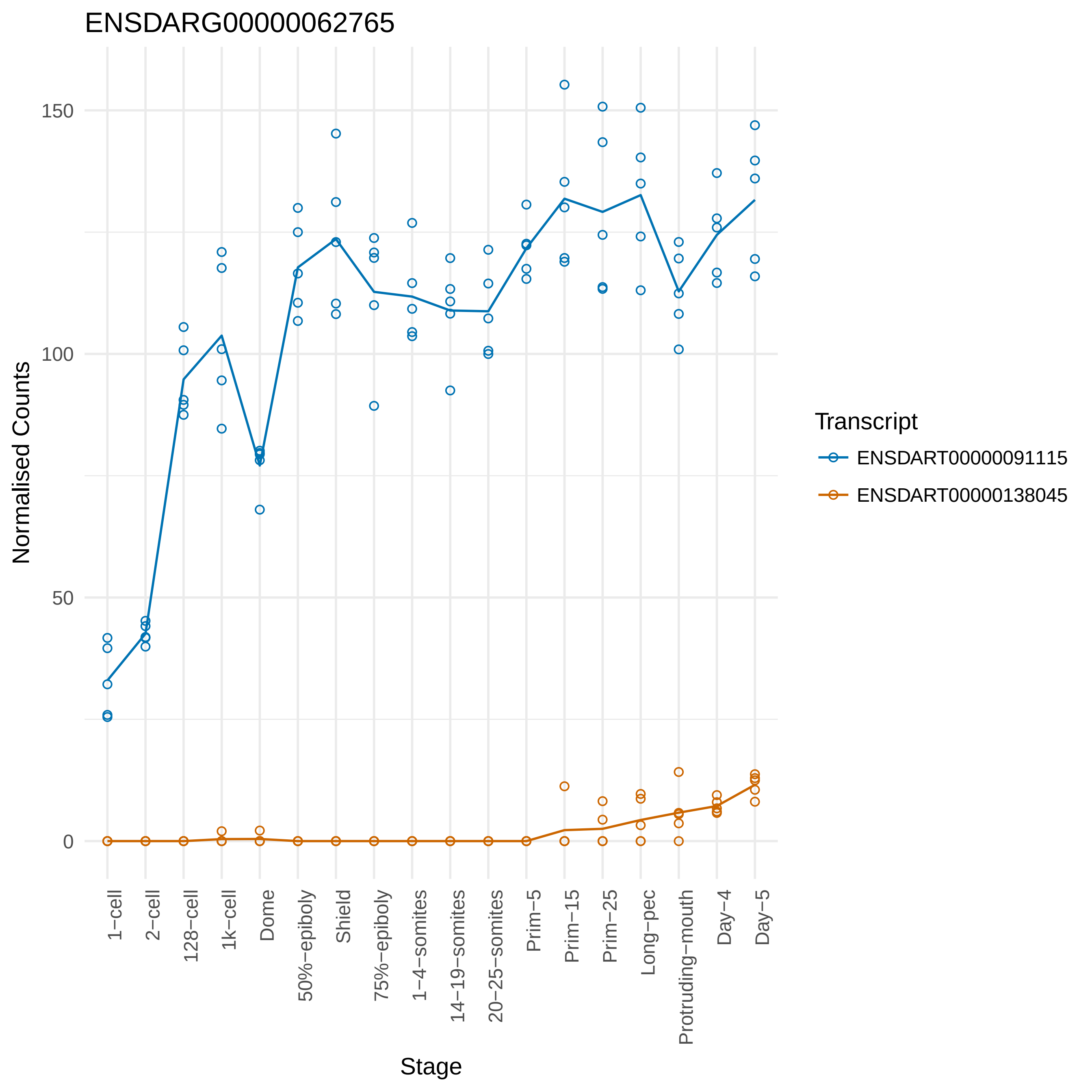
ENSDARG00000062765
Normalised Counts
150
100
50
0
Stage
1−cell
2−cell
128−cell
1k−cell
Dome
50%−epiboly
Shield
75%−epiboly
1−4−somites
14−19−somites
20−25−somites
Prim−5
Prim−15
Prim−25
Long−pec
Protruding−mouth
Day−4
Day−5
Transcript
ENSDART00000091115
ENSDART00000138045

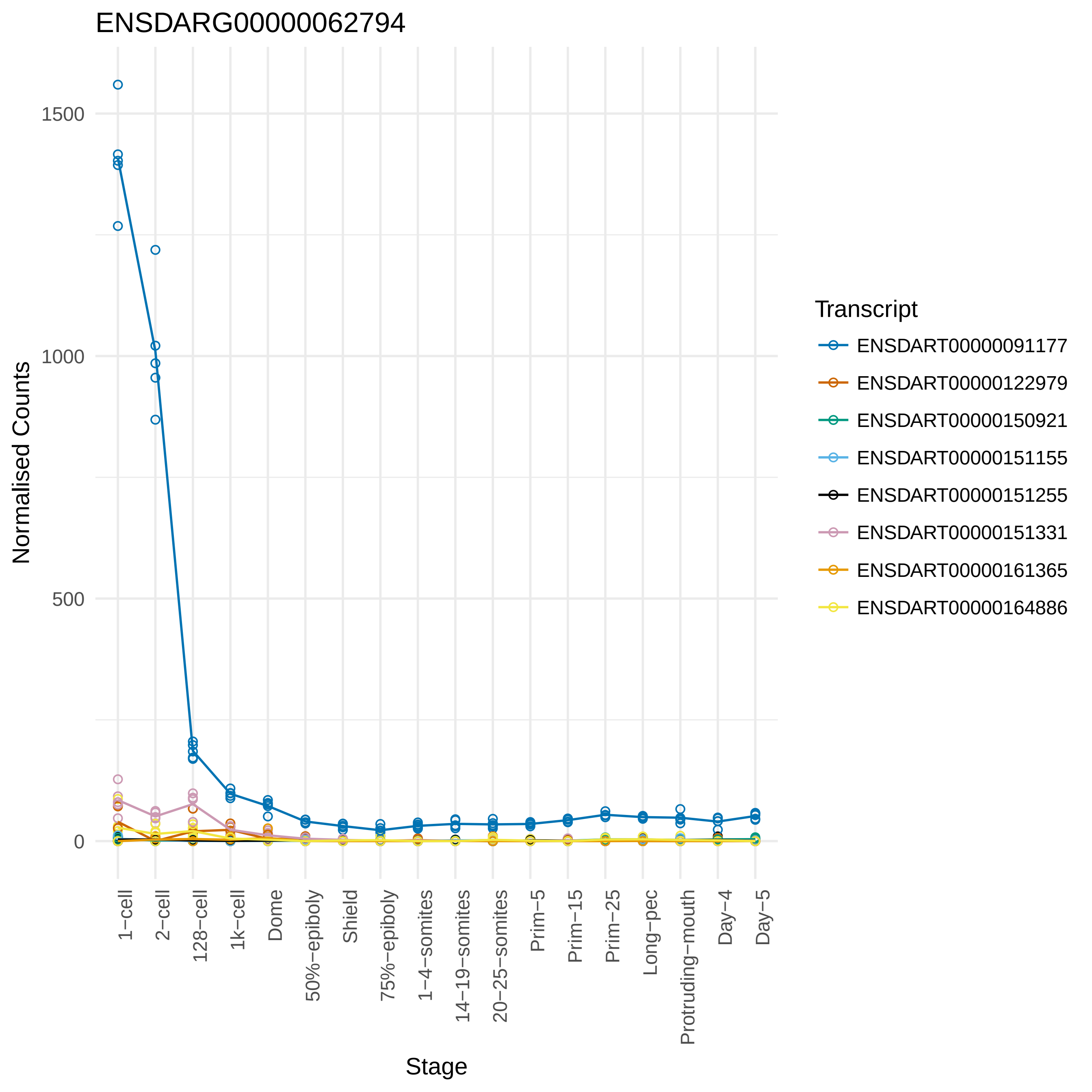
ENSDARG00000062794
Normalised Counts
1500
1000
500
0
Stage
1−cell
2−cell
128−cell
1k−cell
Dome
50%−epiboly
Shield
75%−epiboly
1−4−somites
14−19−somites
20−25−somites
Prim−5
Prim−15
Prim−25
Long−pec
Protruding−mouth
Day−4
Day−5
Transcript
ENSDART00000091177
ENSDART00000122979
ENSDART00000150921
ENSDART00000151155
ENSDART00000151255
ENSDART00000151331
ENSDART00000161365
ENSDART00000164886

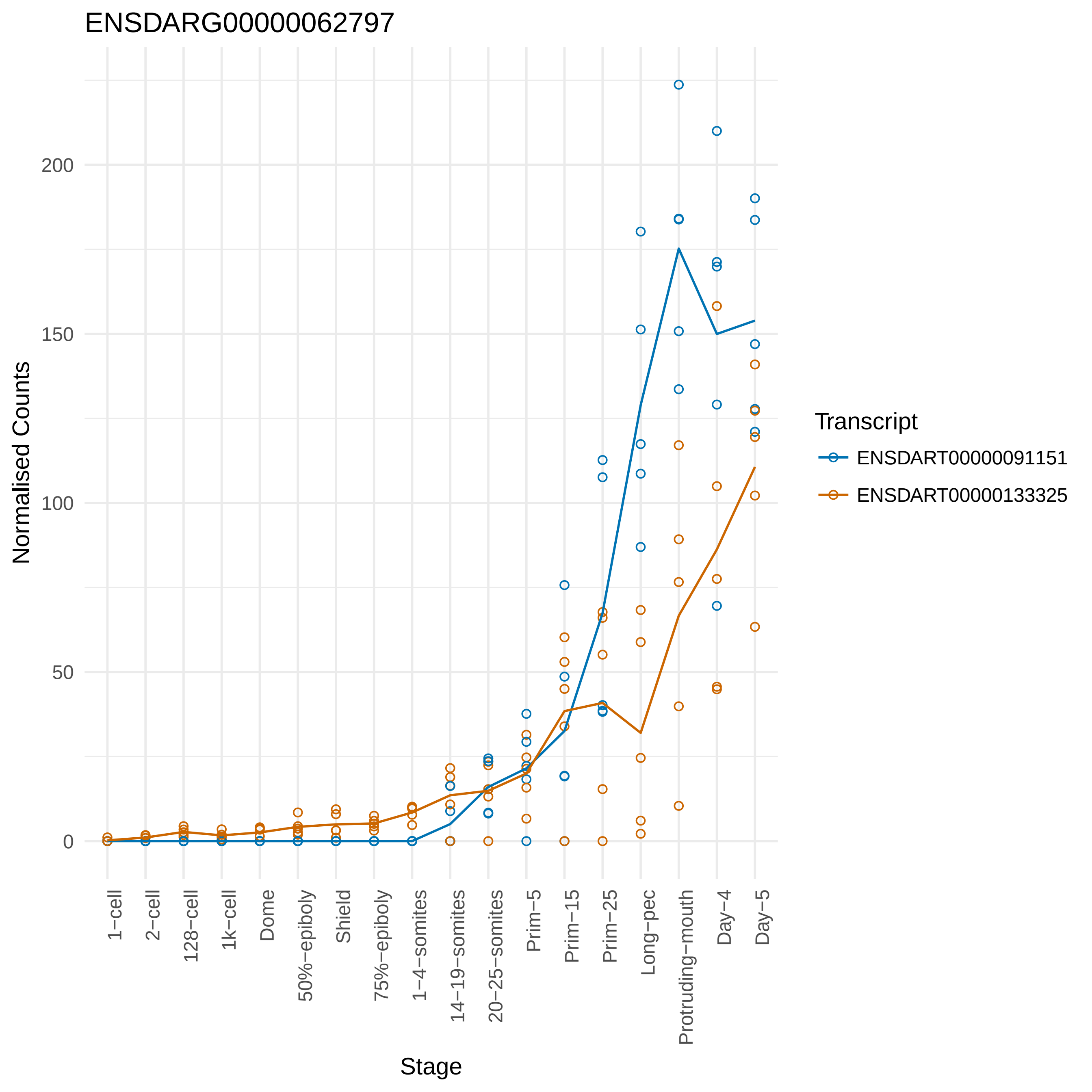

ENSDARG00000062797
Normalised Counts
200
150
100
50
0
1−cell
2−cell
128−cell
1k−cell
Dome
50%−epiboly
Shield
75%−epiboly
1−4−somites
14−19−somites
20−25−somites
Prim−5
Prim−15
Prim−25
Long−pec
Protruding−mouth
Day−4
Day−5
Stage
Transcript
ENSDART00000091151
ENSDART00000133325

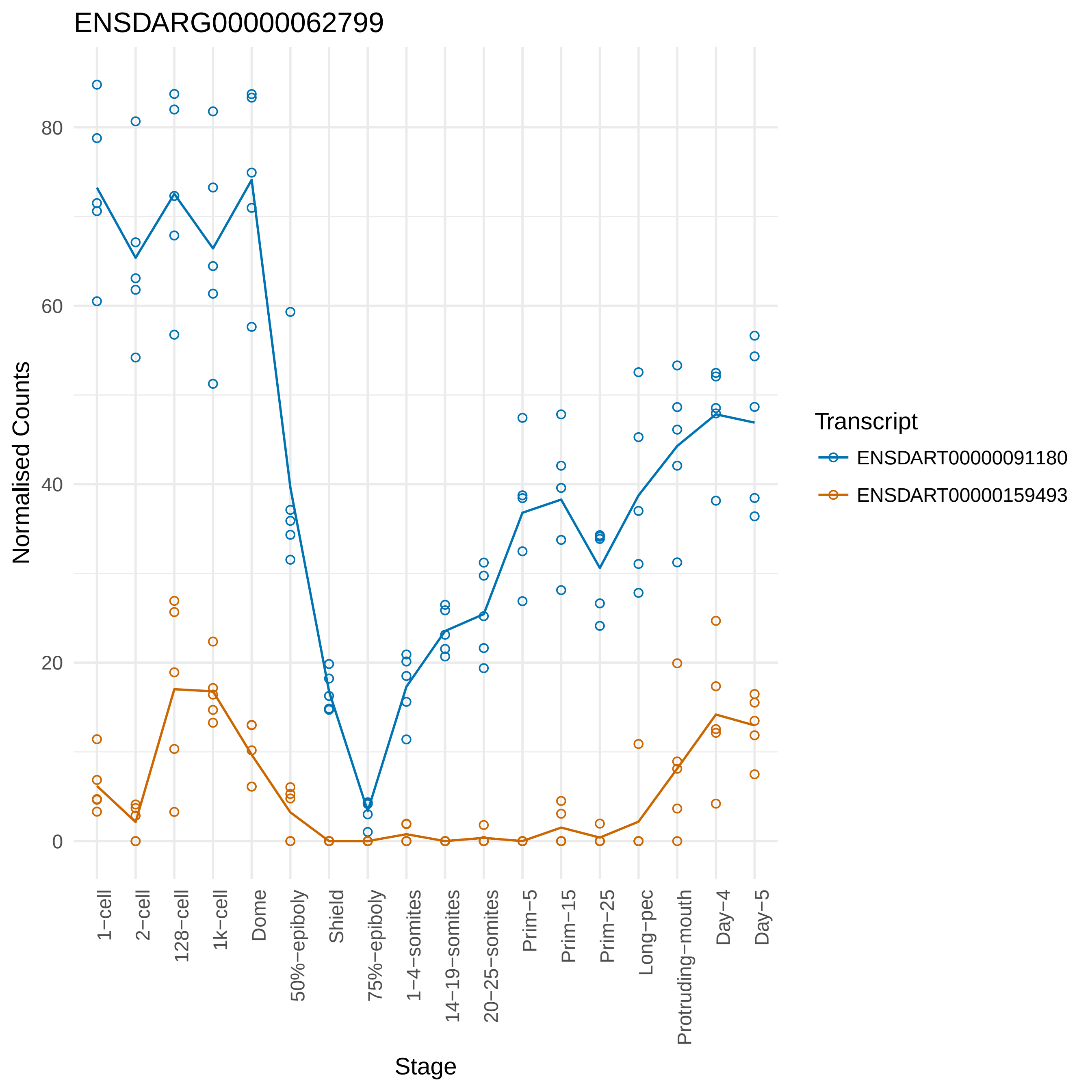
ENSDARG00000062799
Normalised Counts
0
20
40
60
80
Stage
1−cell
2−cell
128−cell
1k−cell
Dome
50%−epiboly
Shield
75%−epiboly
1−4−somites
14−19−somites
20−25−somites
Prim−5
Prim−15
Prim−25
Long−pec
Protruding−mouth
Day−4
Day−5
Transcript
ENSDART00000091180
ENSDART00000159493

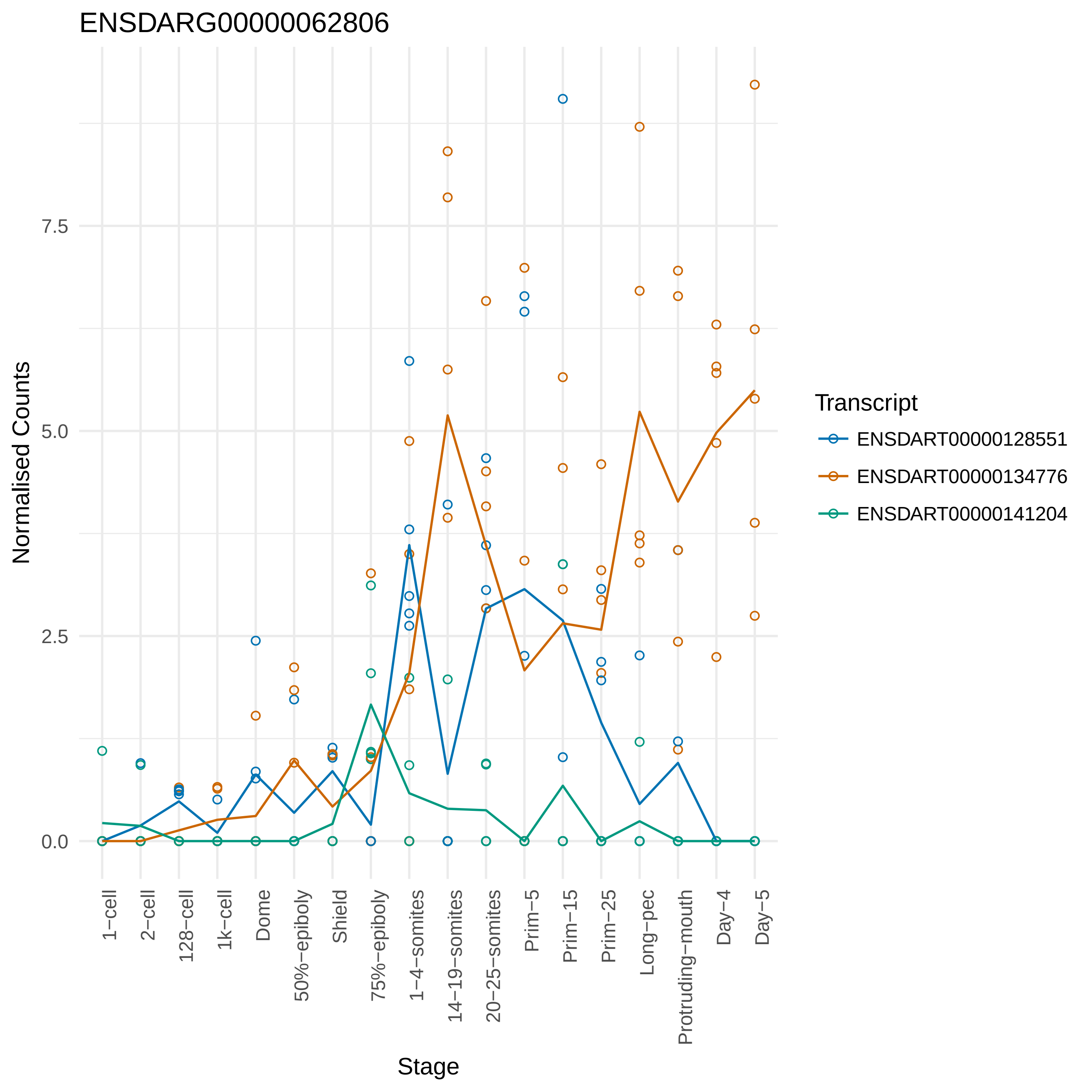

ENSDARG00000062806
Normalised Counts
7.5
5.0
2.5
0.0
1−cell
2−cell
128−cell
1k−cell
Dome
50%−epiboly
Shield
75%−epiboly
1−4−somites
14−19−somites
20−25−somites
Prim−5
Prim−15
Prim−25
Long−pec
Protruding−mouth
Day−4
Day−5
Stage
Transcript
ENSDART00000128551
ENSDART00000134776
ENSDART00000141204

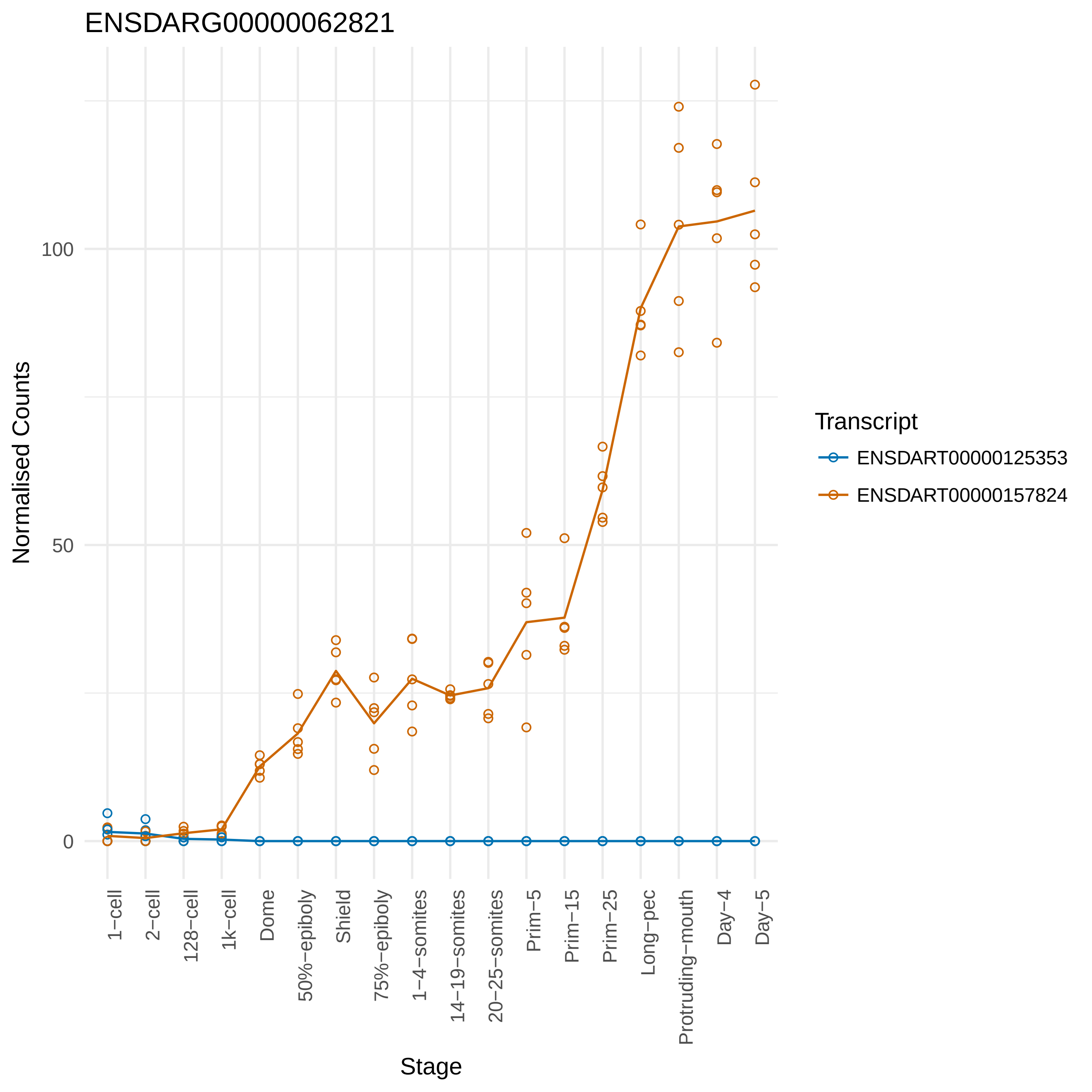

ENSDARG00000062821
Normalised Counts
100
50
0
1−cell
2−cell
128−cell
1k−cell
Dome
50%−epiboly
Shield
75%−epiboly
1−4−somites
14−19−somites
20−25−somites
Prim−5
Prim−15
Prim−25
Long−pec
Protruding−mouth
Day−4
Day−5
Stage
Transcript
ENSDART00000125353
ENSDART00000157824

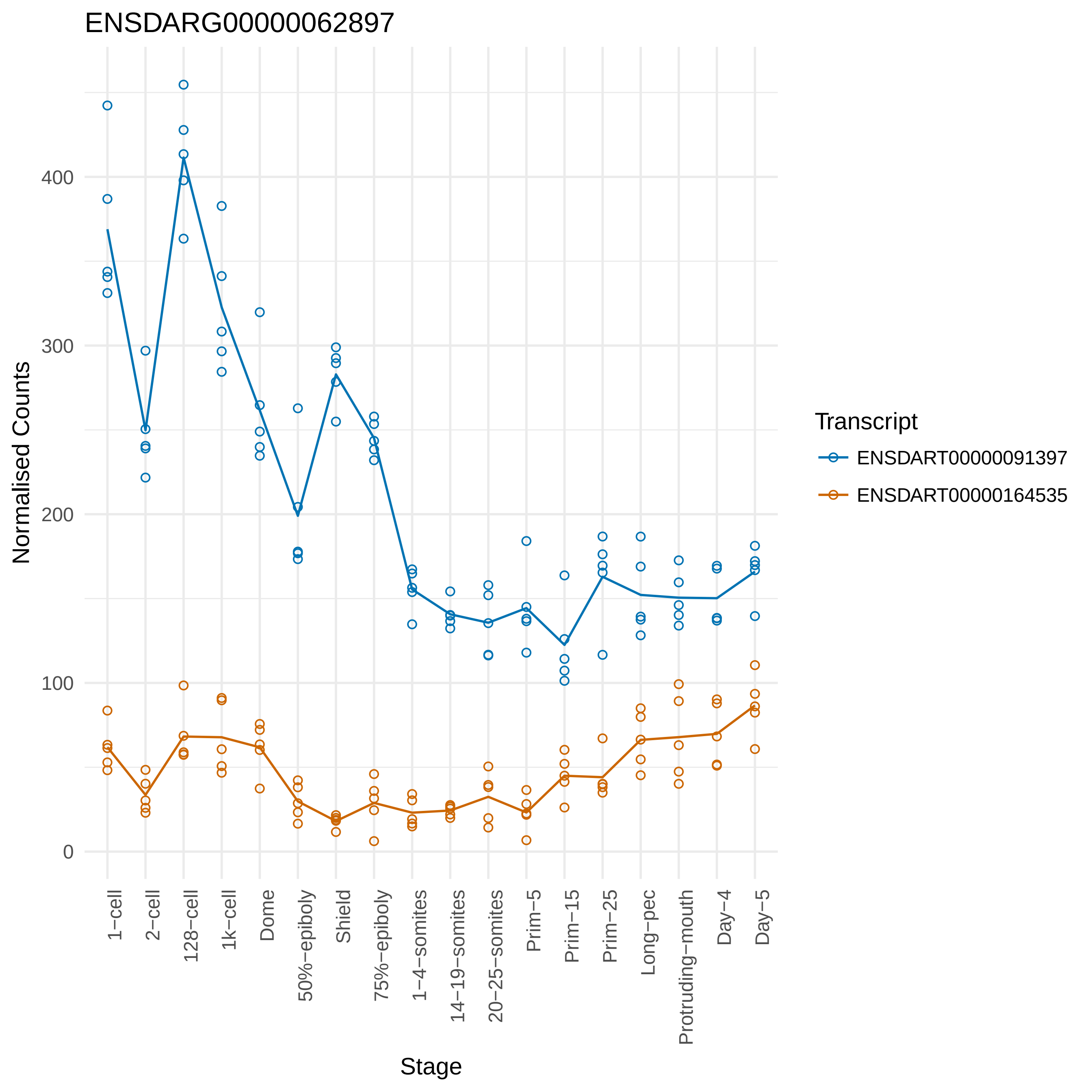

ENSDARG00000062897
Normalised Counts
400
300
200
100
0
1−cell
2−cell
128−cell
1k−cell
Dome
50%−epiboly
Shield
75%−epiboly
1−4−somites
14−19−somites
20−25−somites
Prim−5
Prim−15
Prim−25
Long−pec
Protruding−mouth
Day−4
Day−5
Stage
Transcript
ENSDART00000091397
ENSDART00000164535

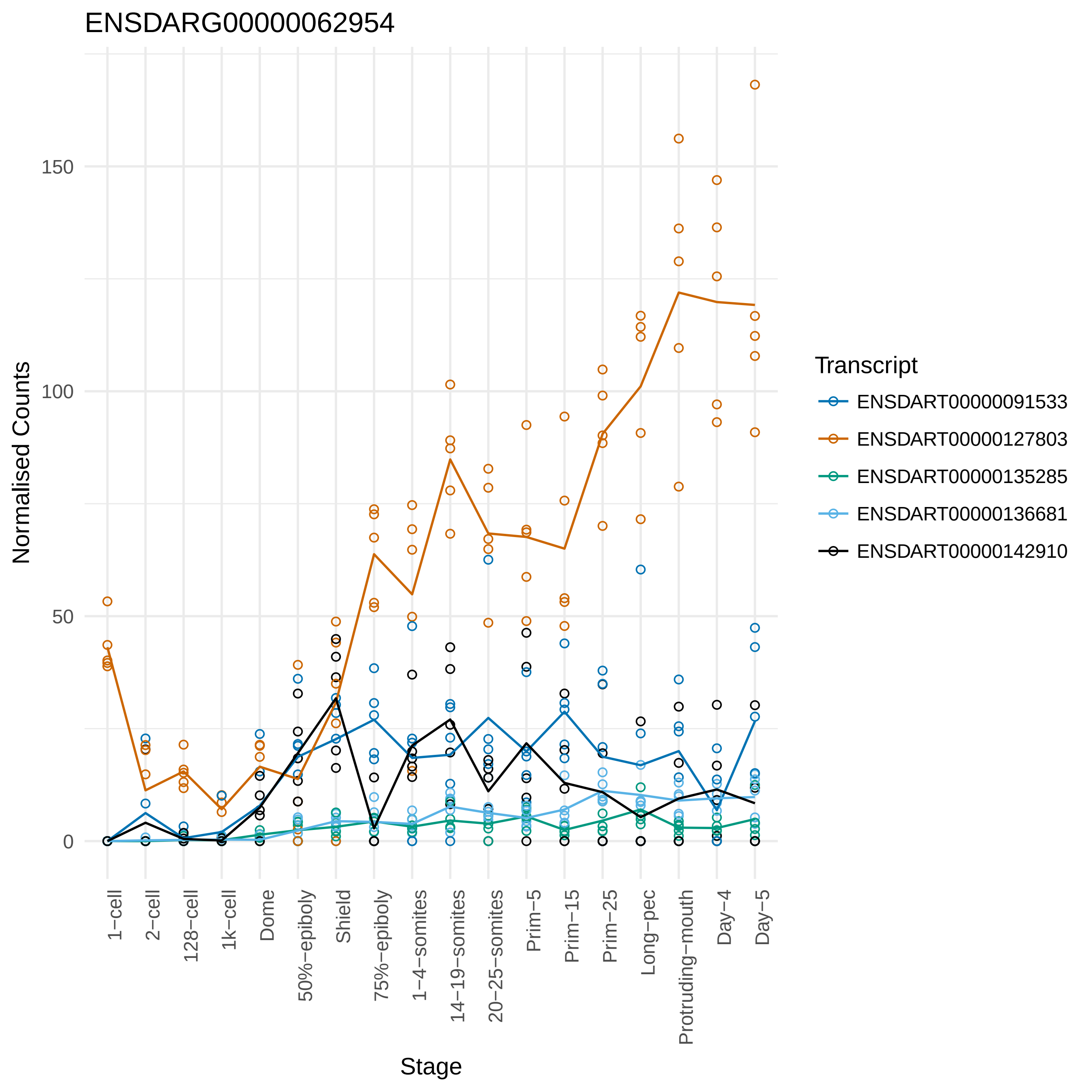

ENSDARG00000062954
Normalised Counts
150
100
50
0
Stage
1−cell
2−cell
128−cell
1k−cell
Dome
50%−epiboly
Shield
75%−epiboly
1−4−somites
14−19−somites
20−25−somites
Prim−5
Prim−15
Prim−25
Long−pec
Protruding−mouth
Day−4
Day−5
Transcript
ENSDART00000091533
ENSDART00000127803
ENSDART00000135285
ENSDART00000136681
ENSDART00000142910

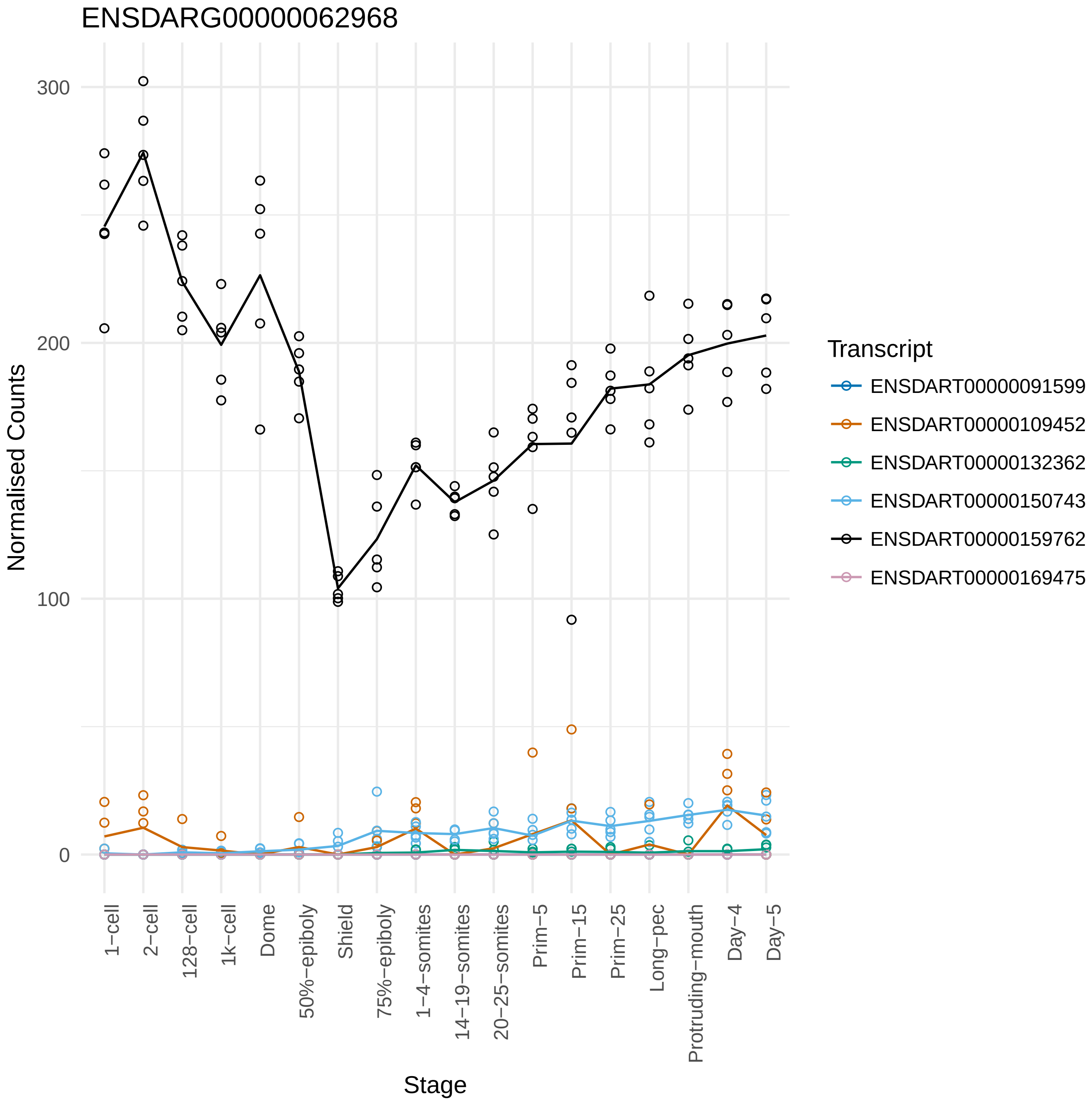

ENSDARG00000062968
Normalised Counts
300
200
100
0
1−cell
2−cell
128−cell
1k−cell
Dome
50%−epiboly
Shield
75%−epiboly
1−4−somites
14−19−somites
20−25−somites
Prim−5
Prim−15
Prim−25
Long−pec
Protruding−mouth
Day−4
Day−5
Stage
Transcript
ENSDART00000091599
ENSDART00000109452
ENSDART00000132362
ENSDART00000150743
ENSDART00000159762
ENSDART00000169475

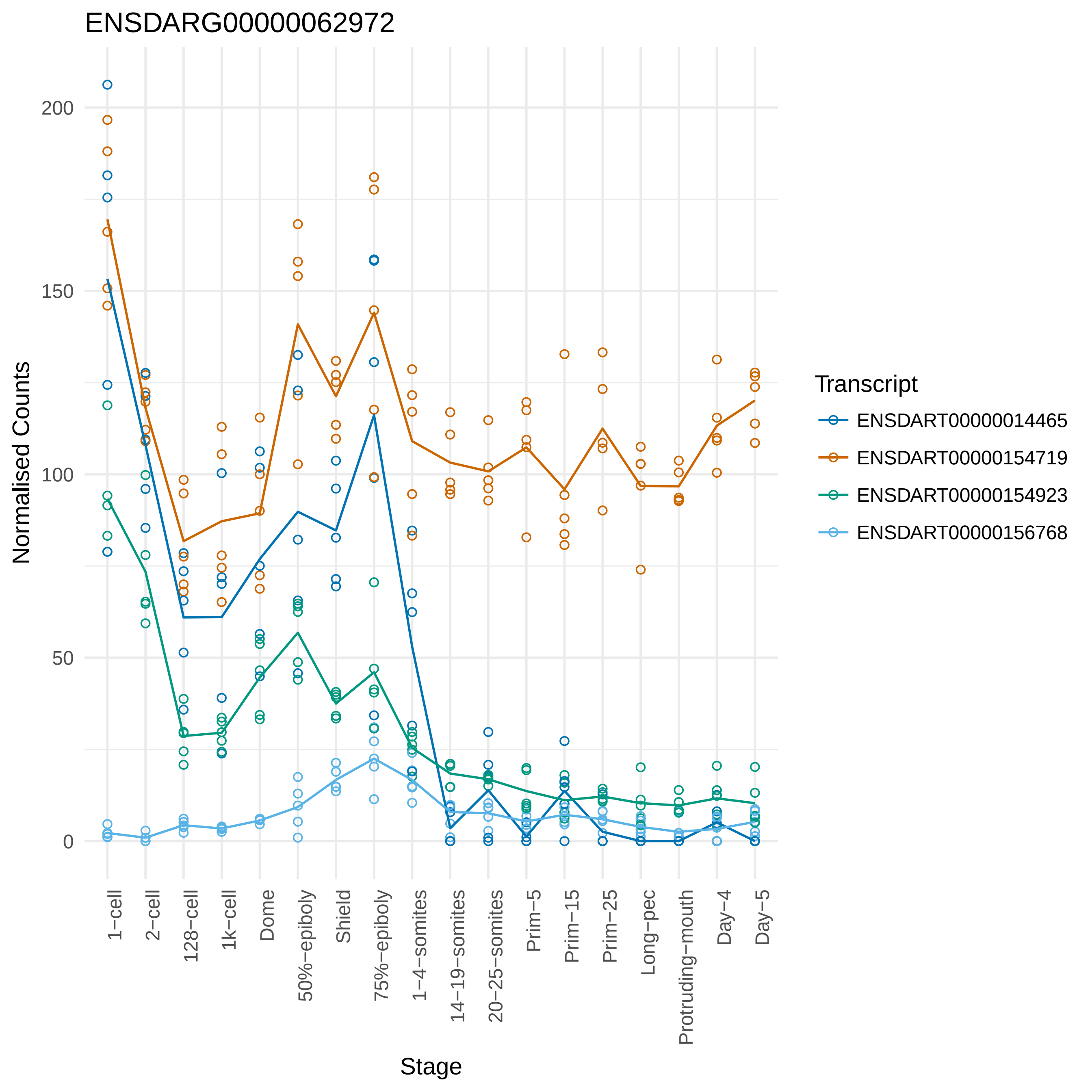

ENSDARG00000062972
Normalised Counts
200
150
100
50
0
1−cell
2−cell
128−cell
1k−cell
Dome
50%−epiboly
Shield
75%−epiboly
1−4−somites
14−19−somites
20−25−somites
Prim−5
Prim−15
Prim−25
Long−pec
Protruding−mouth
Day−4
Day−5
Stage
Transcript
ENSDART00000014465
ENSDART00000154719
ENSDART00000154923
ENSDART00000156768

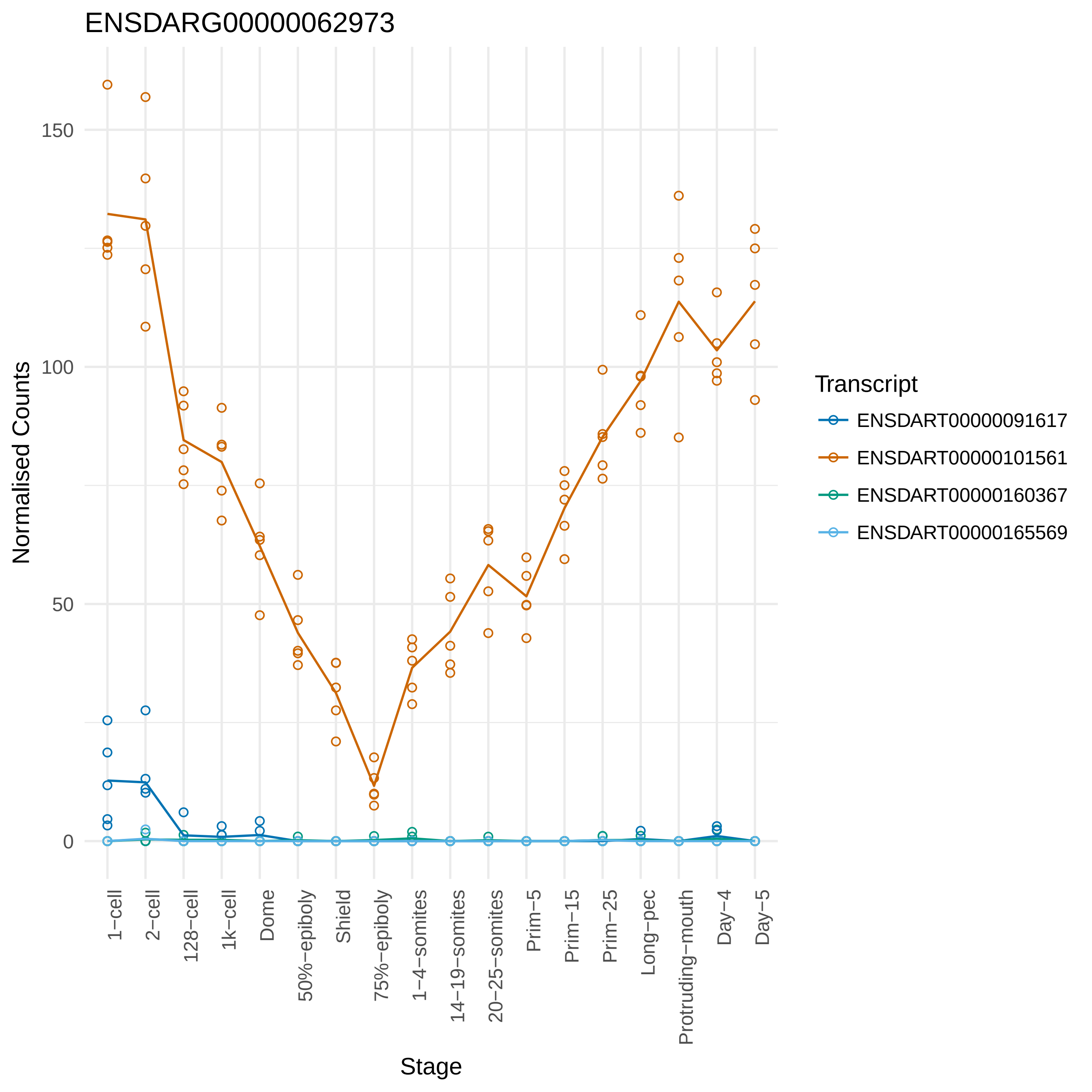

ENSDARG00000062973
Normalised Counts
150
100
50
0
1−cell
2−cell
128−cell
1k−cell
Dome
50%−epiboly
Shield
75%−epiboly
1−4−somites
14−19−somites
20−25−somites
Prim−5
Prim−15
Prim−25
Long−pec
Protruding−mouth
Day−4
Day−5
Stage
Transcript
ENSDART00000091617
ENSDART00000101561
ENSDART00000160367
ENSDART00000165569

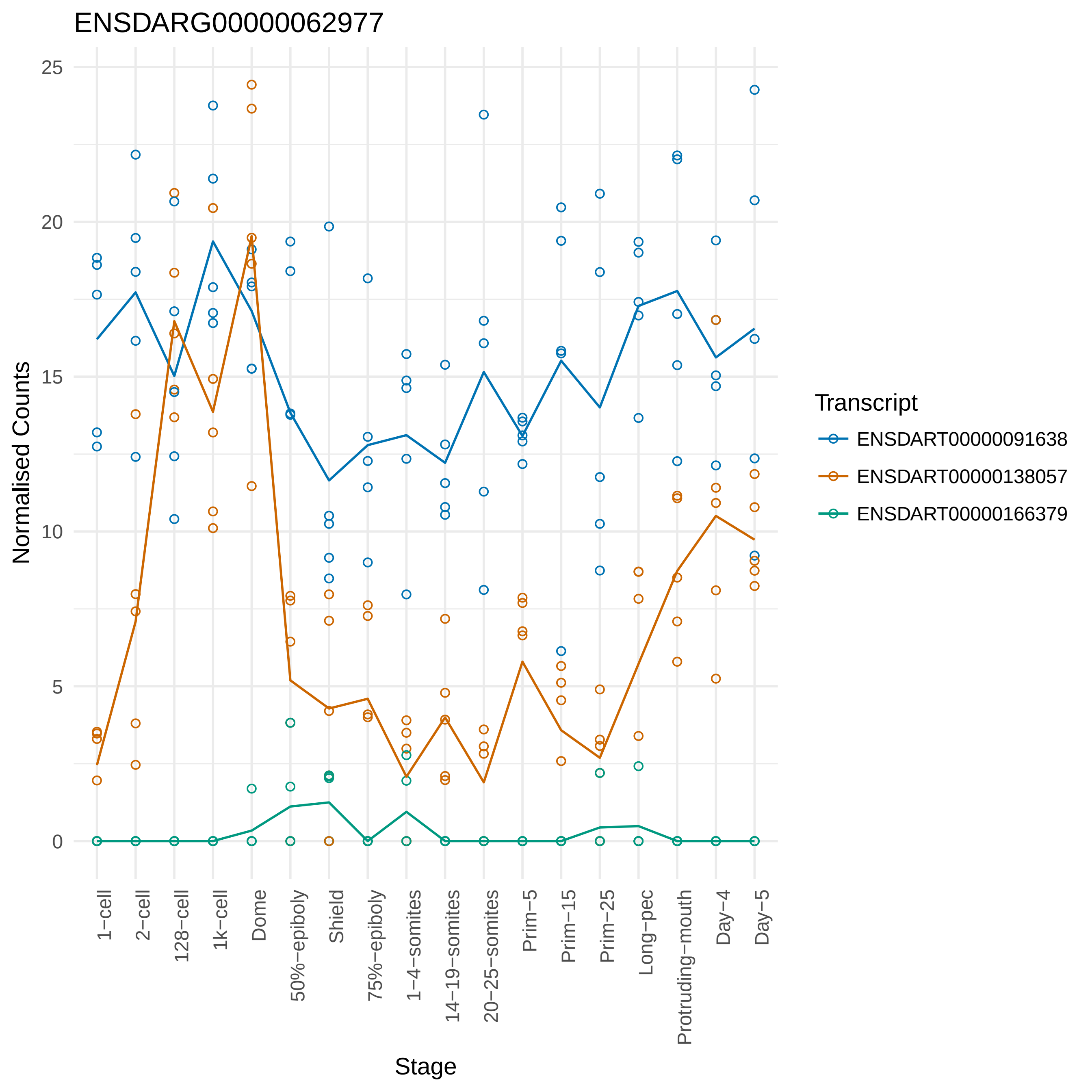

ENSDARG00000062977
Normalised Counts
25
20
15
10
5
0
1−cell
2−cell
128−cell
1k−cell
Dome
50%−epiboly
Shield
75%−epiboly
1−4−somites
14−19−somites
20−25−somites
Prim−5
Prim−15
Prim−25
Long−pec
Protruding−mouth
Day−4
Day−5
Stage
Transcript
ENSDART00000091638
ENSDART00000138057
ENSDART00000166379

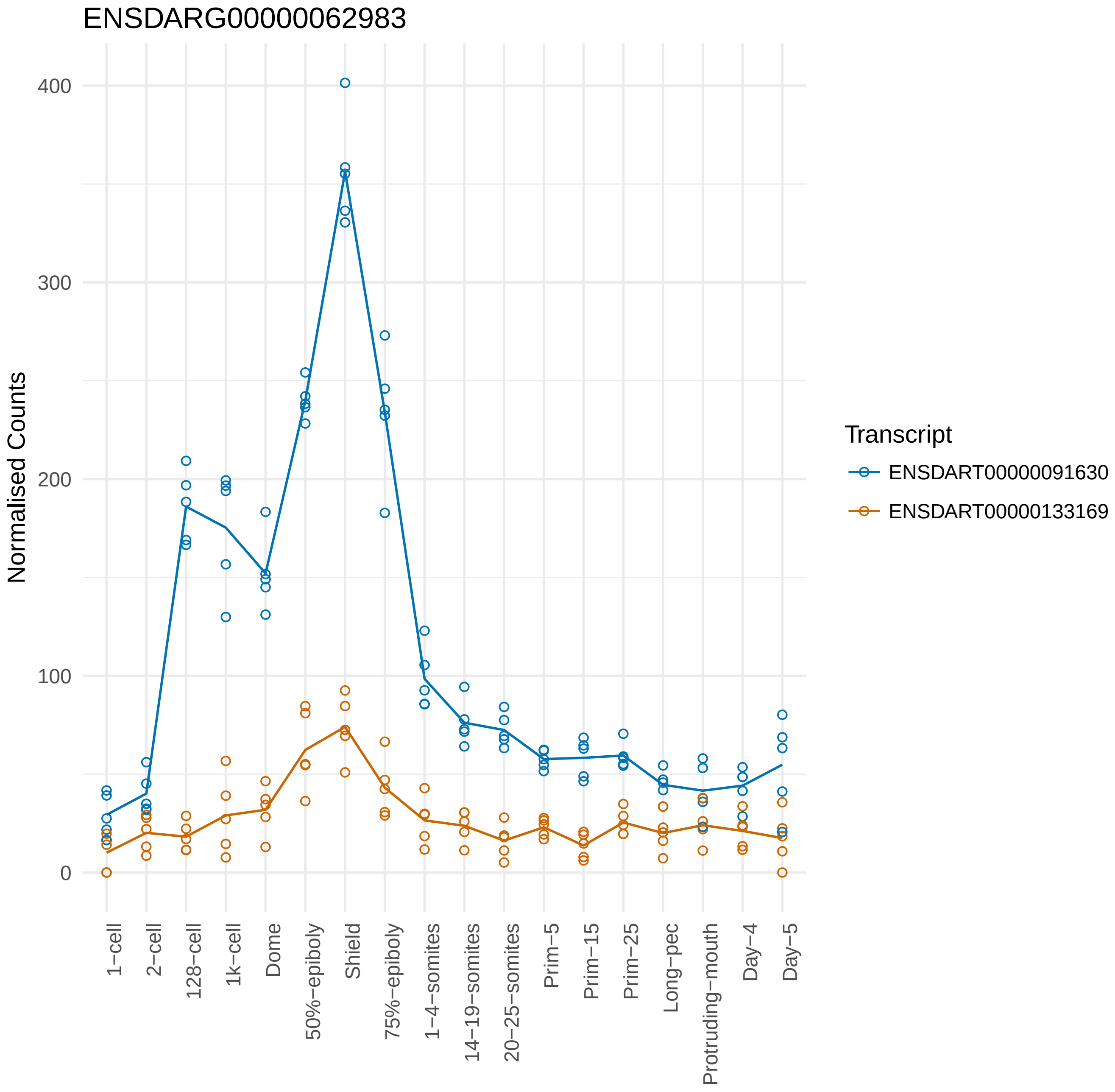

ENSDARG00000062983
Normalised Counts
400
300
200
100
0
1−cell
2−cell
128−cell
1k−cell
Dome
50%−epiboly
Shield
75%−epiboly
1−4−somites
14−19−somites
20−25−somites
Prim−5
Prim−15
Prim−25
Long−pec
Protruding−mouth
Day−4
Day−5
Stage
Transcript
ENSDART00000091630
ENSDART00000133169

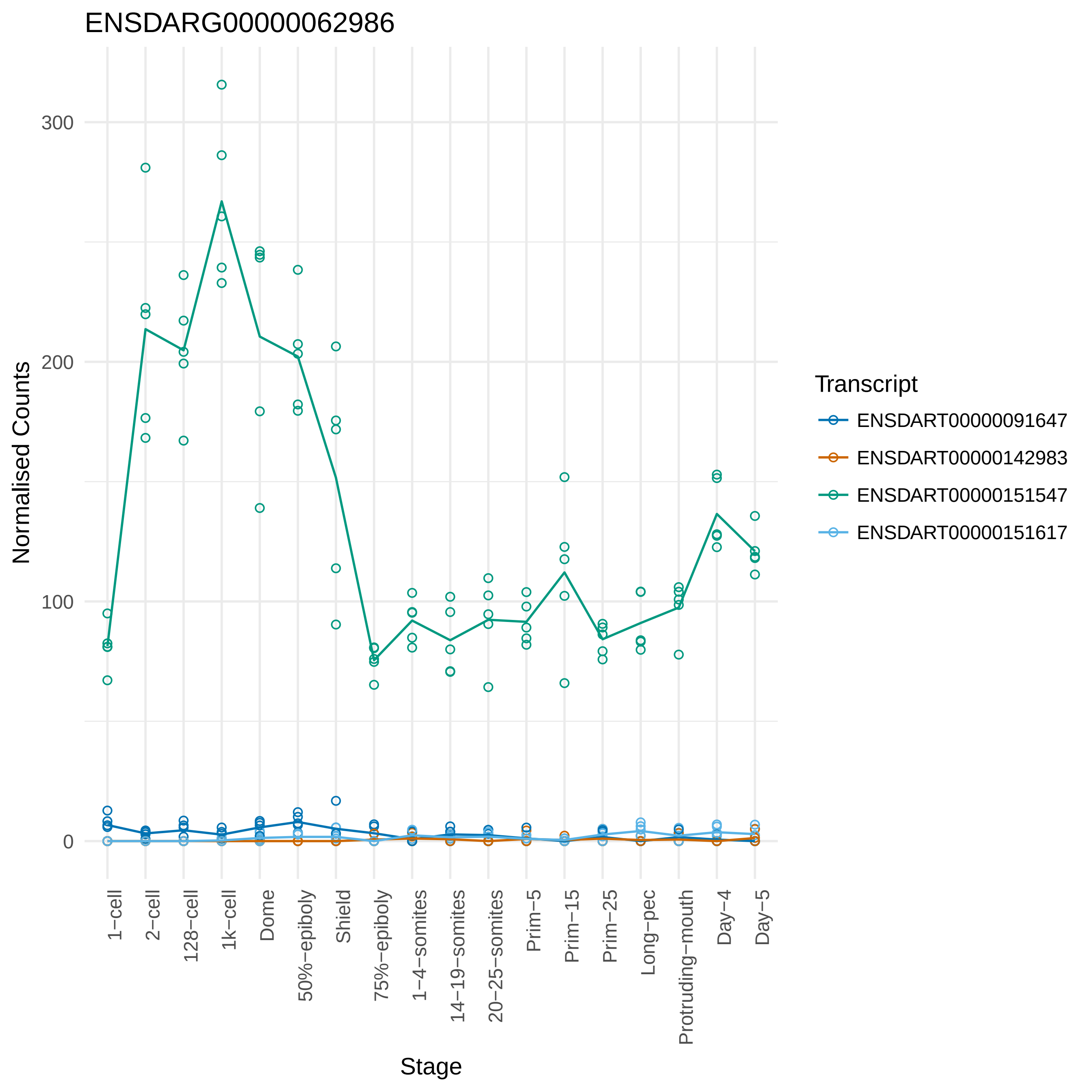
ENSDARG00000062986
Normalised Counts
300
200
100
0
1−cell
2−cell
128−cell
1k−cell
Dome
50%−epiboly
Shield
75%−epiboly
1−4−somites
14−19−somites
20−25−somites
Prim−5
Prim−15
Prim−25
Long−pec
Protruding−mouth
Day−4
Day−5
Stage
Transcript
ENSDART00000091647
ENSDART00000142983
ENSDART00000151547
ENSDART00000151617

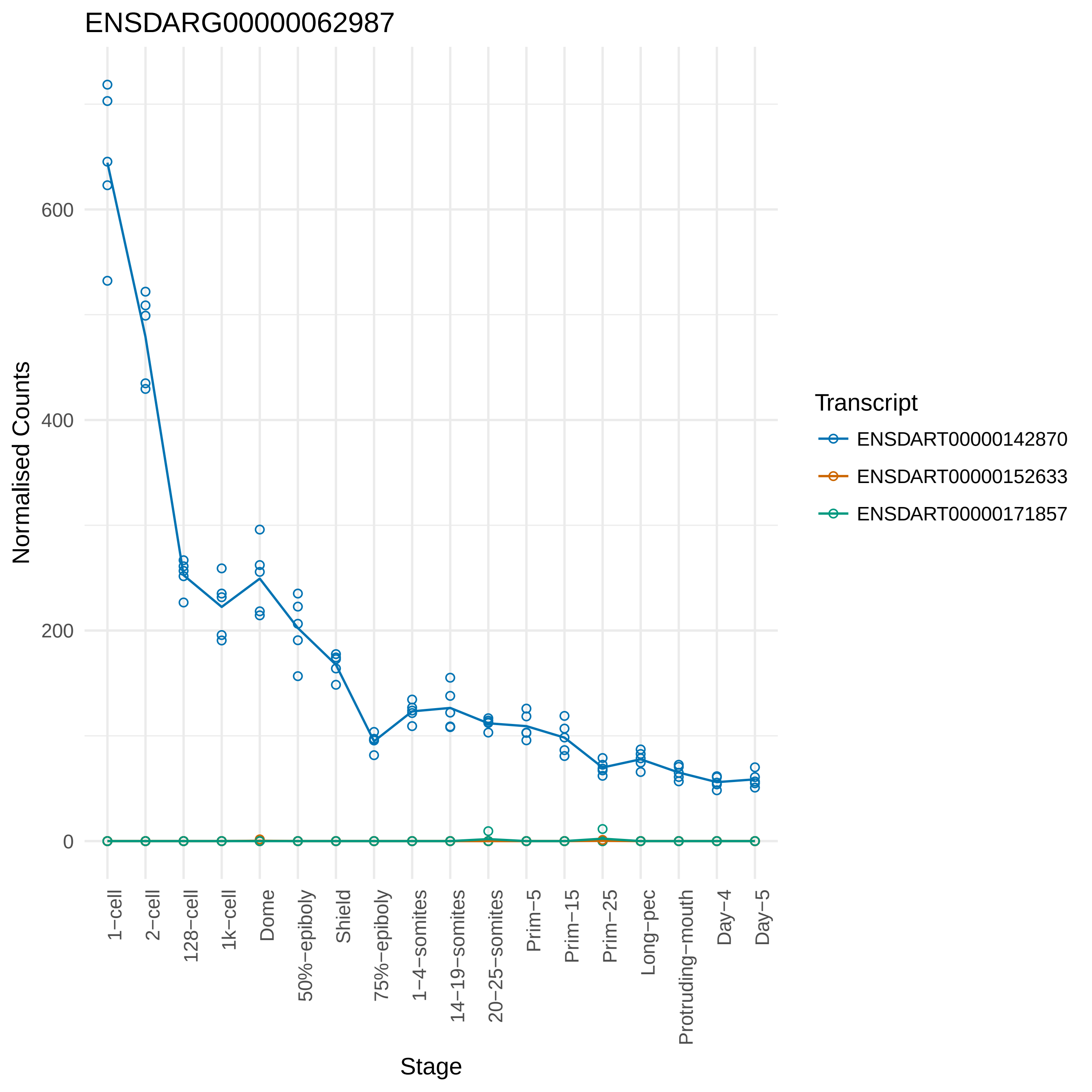

ENSDARG00000062987
Normalised Counts
600
400
200
0
1−cell
2−cell
128−cell
1k−cell
Dome
50%−epiboly
Shield
75%−epiboly
1−4−somites
14−19−somites
20−25−somites
Prim−5
Prim−15
Prim−25
Long−pec
Protruding−mouth
Day−4
Day−5
Stage
Transcript
ENSDART00000142870
ENSDART00000152633
ENSDART00000171857

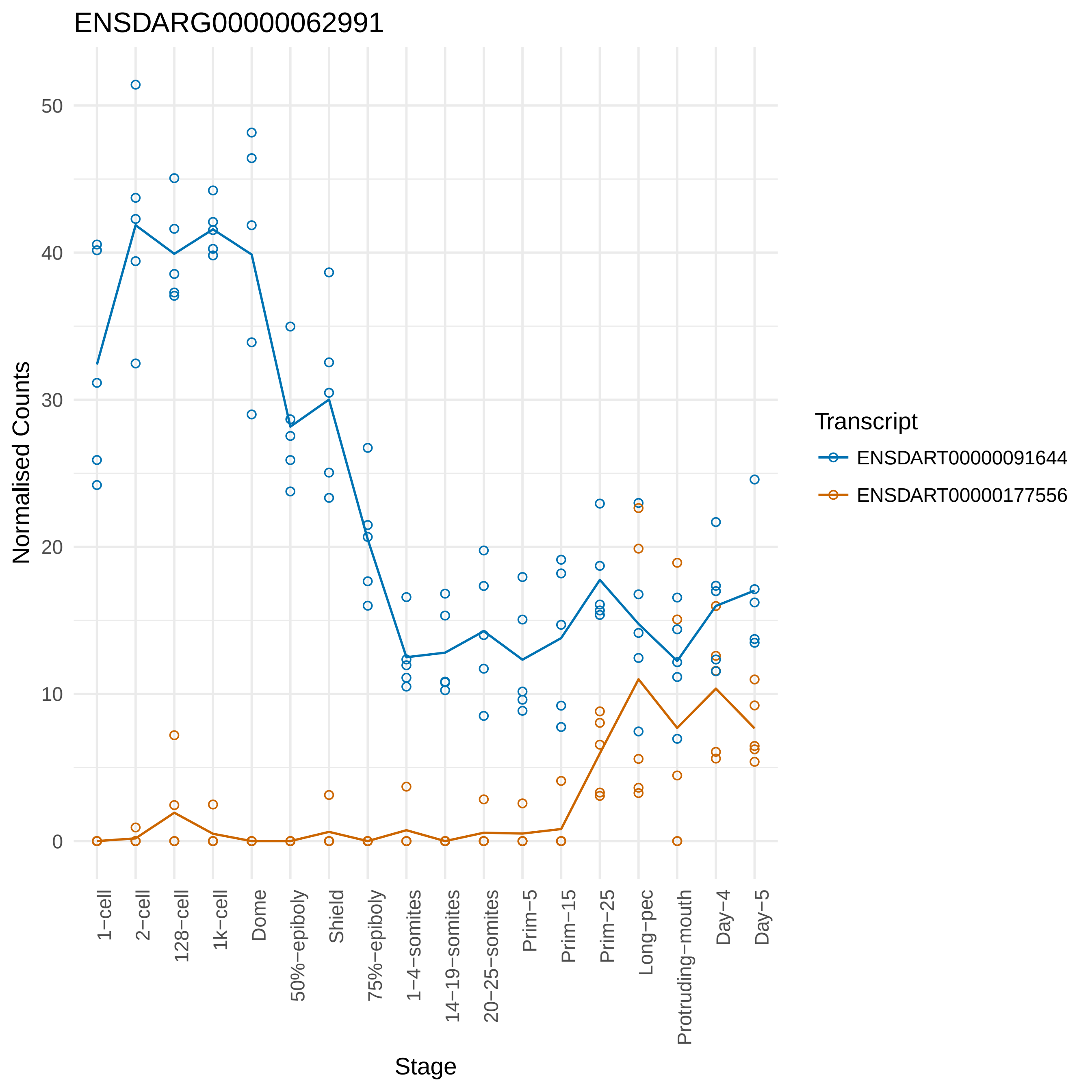
ENSDARG00000062991
Normalised Counts
50
40
30
20
10
0
1−cell
2−cell
128−cell
1k−cell
Dome
50%−epiboly
Shield
75%−epiboly
1−4−somites
14−19−somites
20−25−somites
Prim−5
Prim−15
Prim−25
Long−pec
Protruding−mouth
Day−4
Day−5
Stage
Transcript
ENSDART00000091644
ENSDART00000177556

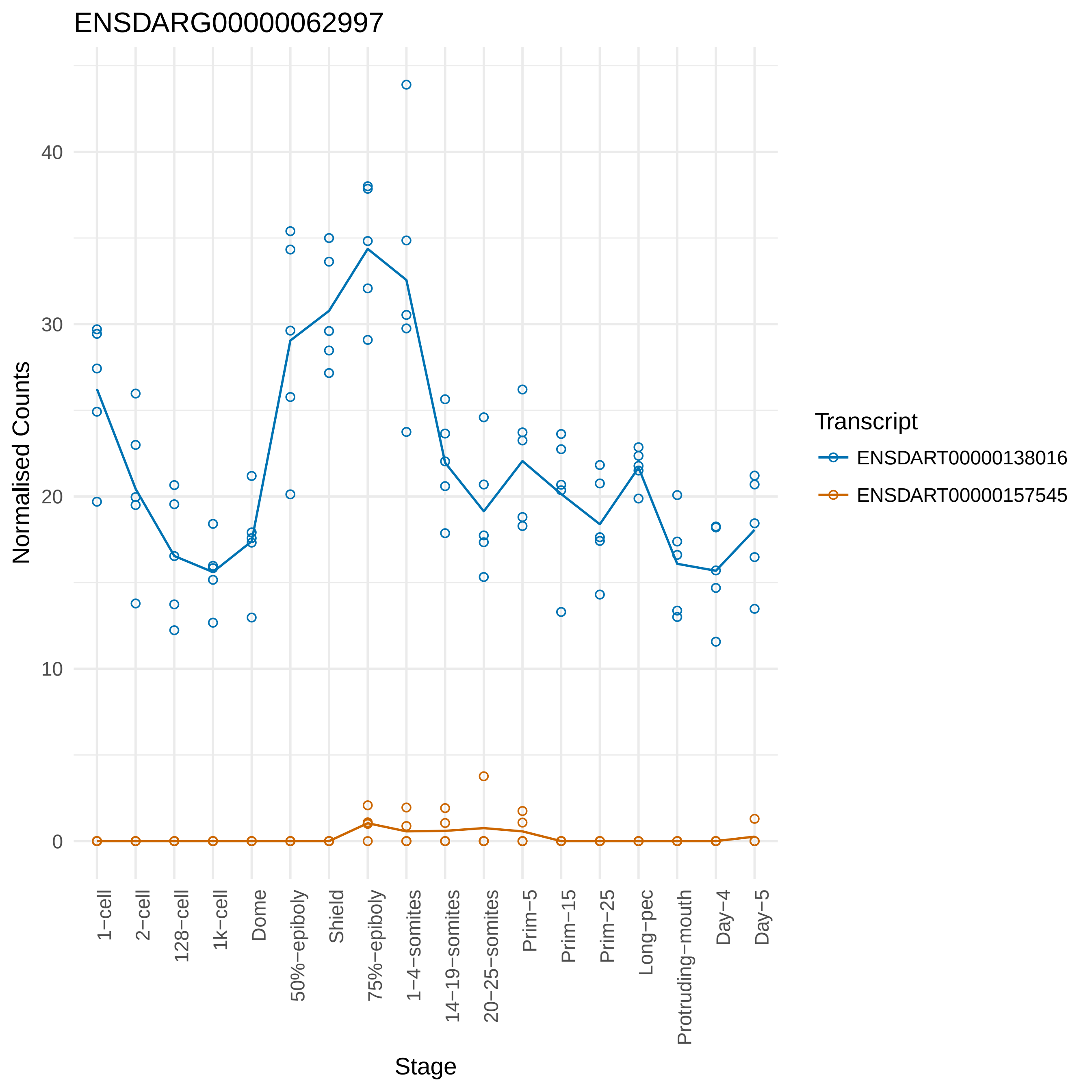

ENSDARG00000062997
Normalised Counts
40
30
20
10
0
1−cell
2−cell
128−cell
1k−cell
Dome
50%−epiboly
Shield
75%−epiboly
1−4−somites
14−19−somites
20−25−somites
Prim−5
Prim−15
Prim−25
Long−pec
Protruding−mouth
Day−4
Day−5
Stage
Transcript
ENSDART00000138016
ENSDART00000157545

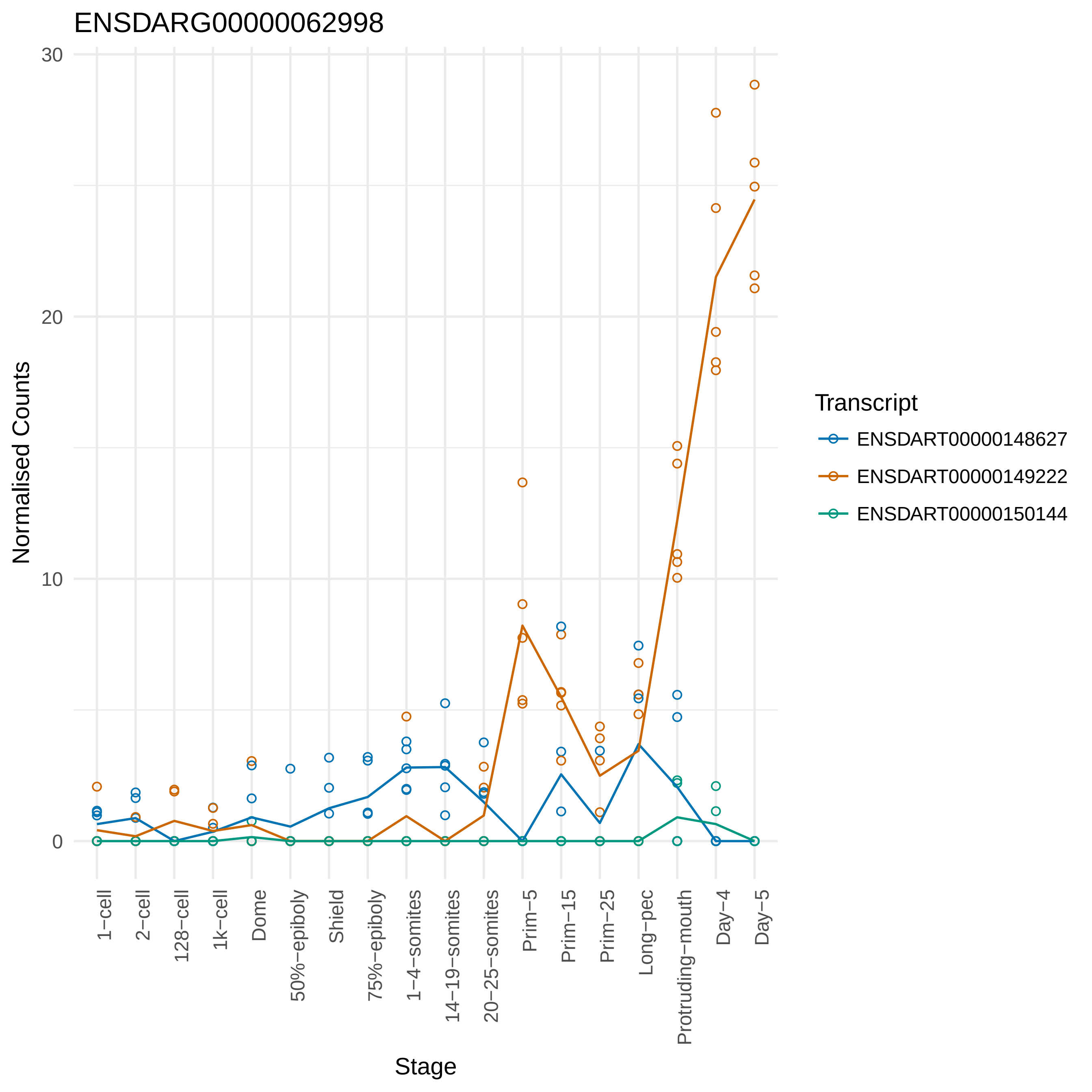

ENSDARG00000062998
Normalised Counts
0
10
20
30
1−cell
2−cell
128−cell
1k−cell
Dome
50%−epiboly
Shield
75%−epiboly
1−4−somites
14−19−somites
20−25−somites
Prim−5
Prim−15
Prim−25
Long−pec
Protruding−mouth
Day−4
Day−5
Stage
Transcript
ENSDART00000148627
ENSDART00000149222
ENSDART00000150144

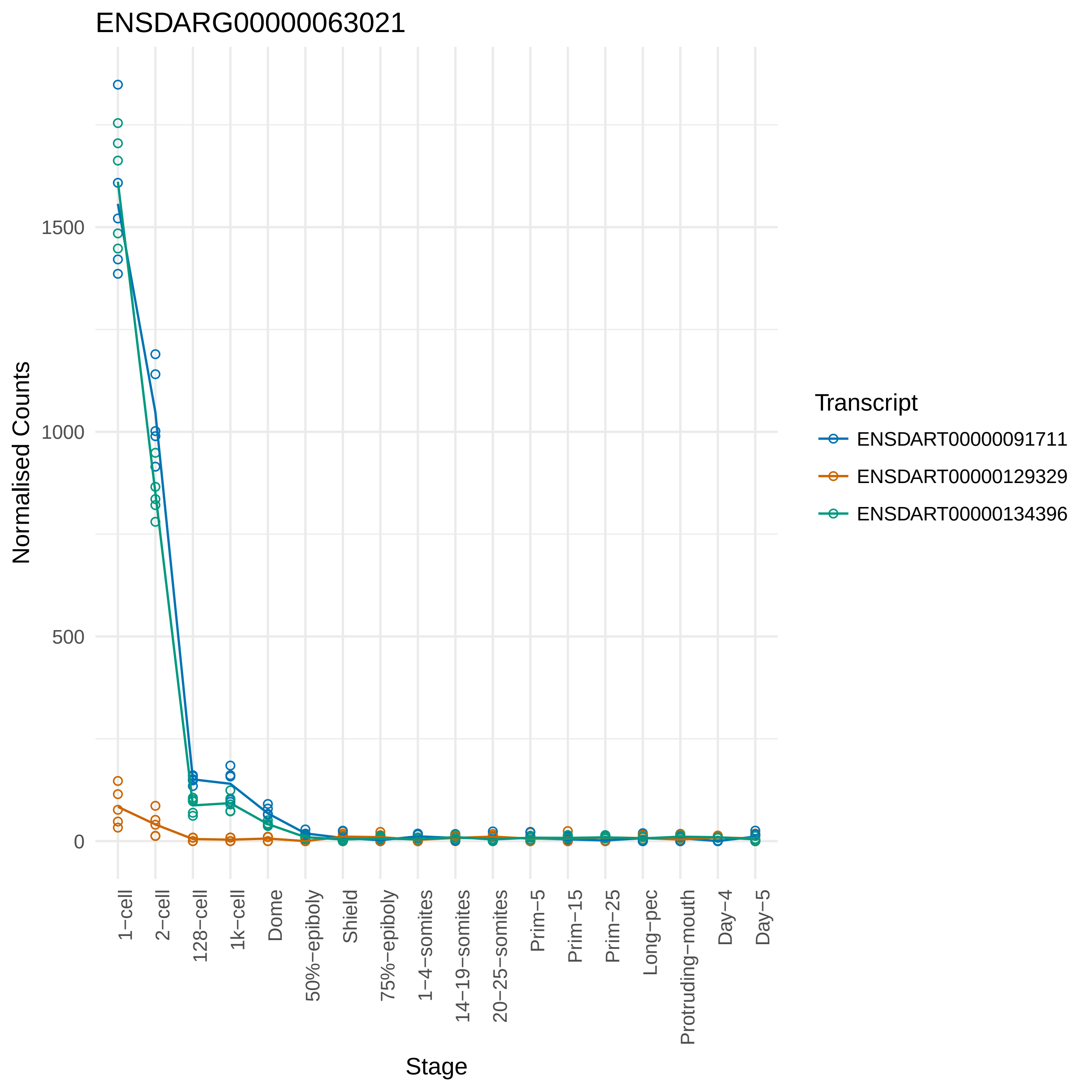
ENSDARG00000063021
Normalised Counts
0
500
1000
1500
Stage
1−cell
2−cell
128−cell
1k−cell
Dome
50%−epiboly
Shield
75%−epiboly
1−4−somites
14−19−somites
20−25−somites
Prim−5
Prim−15
Prim−25
Long−pec
Protruding−mouth
Day−4
Day−5
Transcript
ENSDART00000091711
ENSDART00000129329
ENSDART00000134396

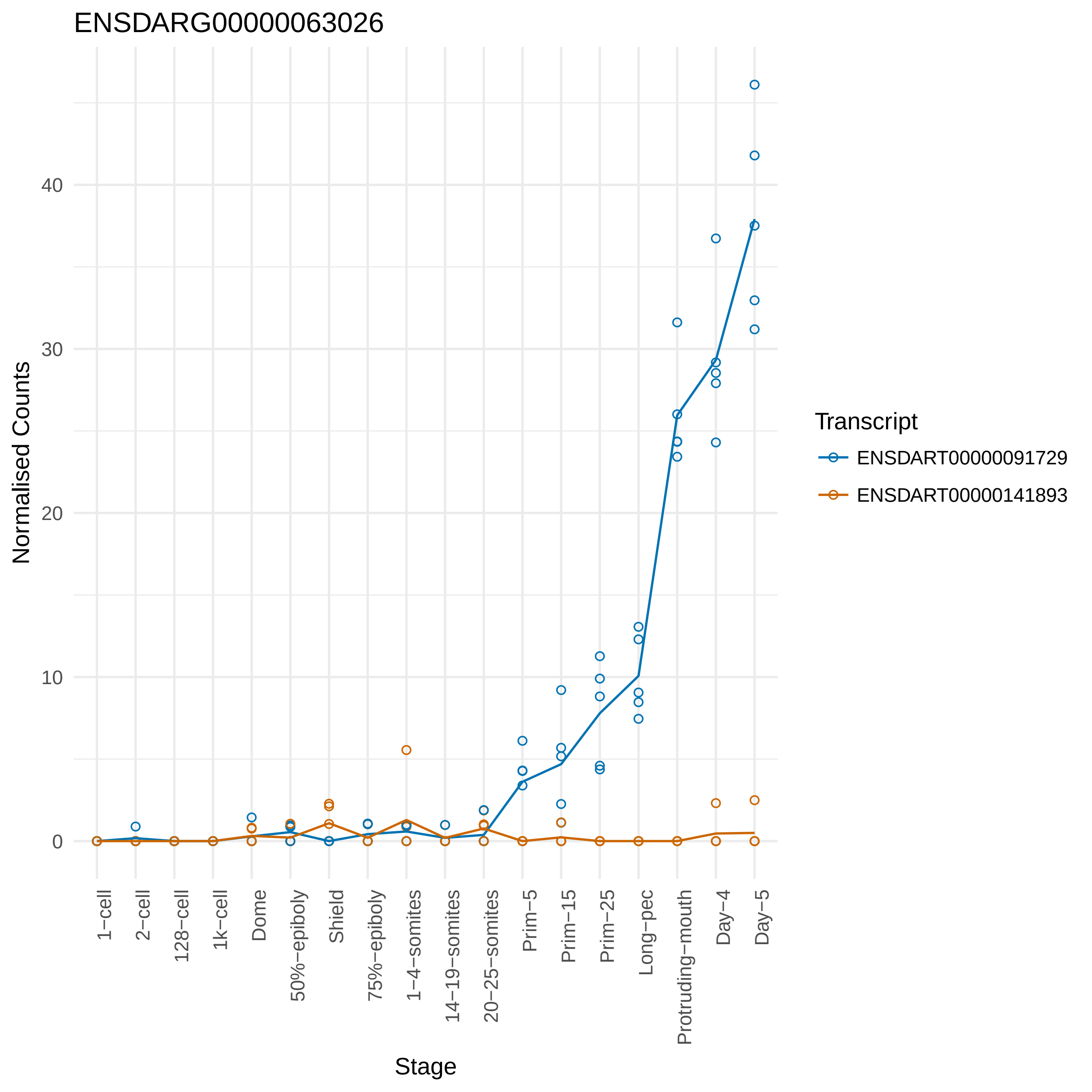

ENSDARG00000063026
Normalised Counts
0
10
20
30
40
1−cell
2−cell
128−cell
1k−cell
Dome
50%−epiboly
Shield
75%−epiboly
1−4−somites
14−19−somites
20−25−somites
Prim−5
Prim−15
Prim−25
Long−pec
Protruding−mouth
Day−4
Day−5
Stage
Transcript
ENSDART00000091729
ENSDART00000141893

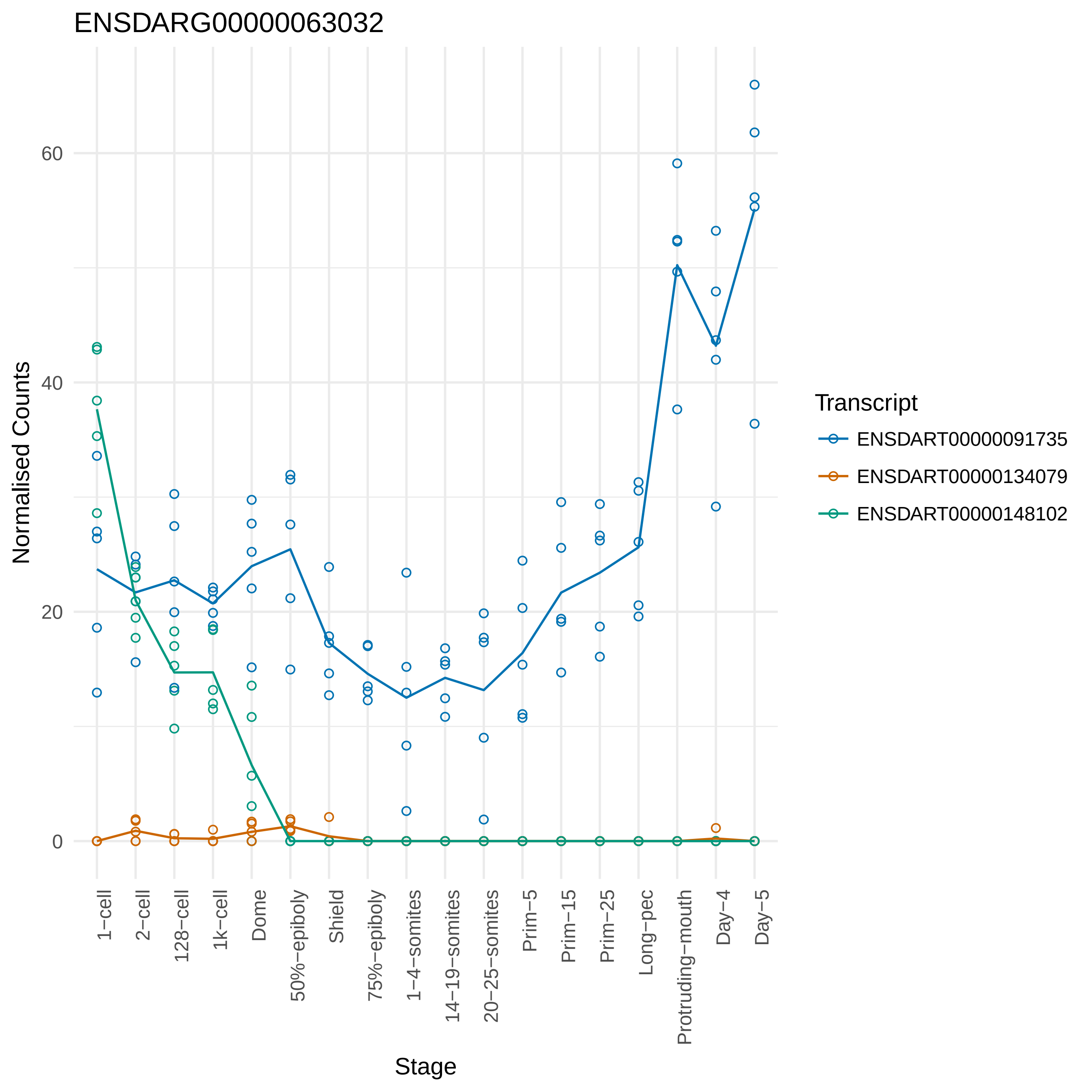

ENSDARG00000063032
Normalised Counts
0
20
40
60
Stage
1−cell
2−cell
128−cell
1k−cell
Dome
50%−epiboly
Shield
75%−epiboly
1−4−somites
14−19−somites
20−25−somites
Prim−5
Prim−15
Prim−25
Long−pec
Protruding−mouth
Day−4
Day−5
Transcript
ENSDART00000091735
ENSDART00000134079
ENSDART00000148102

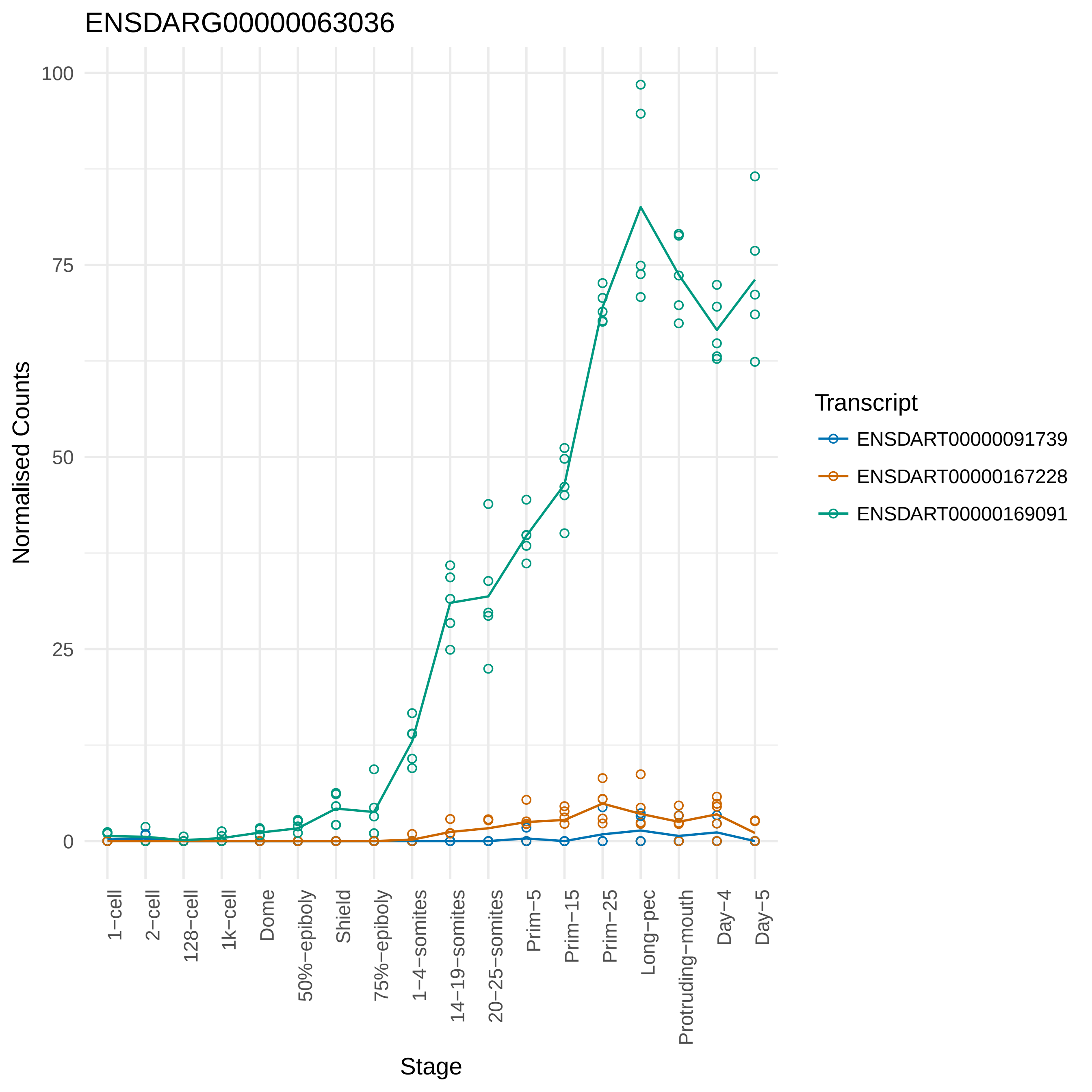
ENSDARG00000063036
Normalised Counts
100
75
50
25
0
1−cell
2−cell
128−cell
1k−cell
Dome
50%−epiboly
Shield
75%−epiboly
1−4−somites
14−19−somites
20−25−somites
Prim−5
Prim−15
Prim−25
Long−pec
Protruding−mouth
Day−4
Day−5
Stage
Transcript
ENSDART00000091739
ENSDART00000167228
ENSDART00000169091

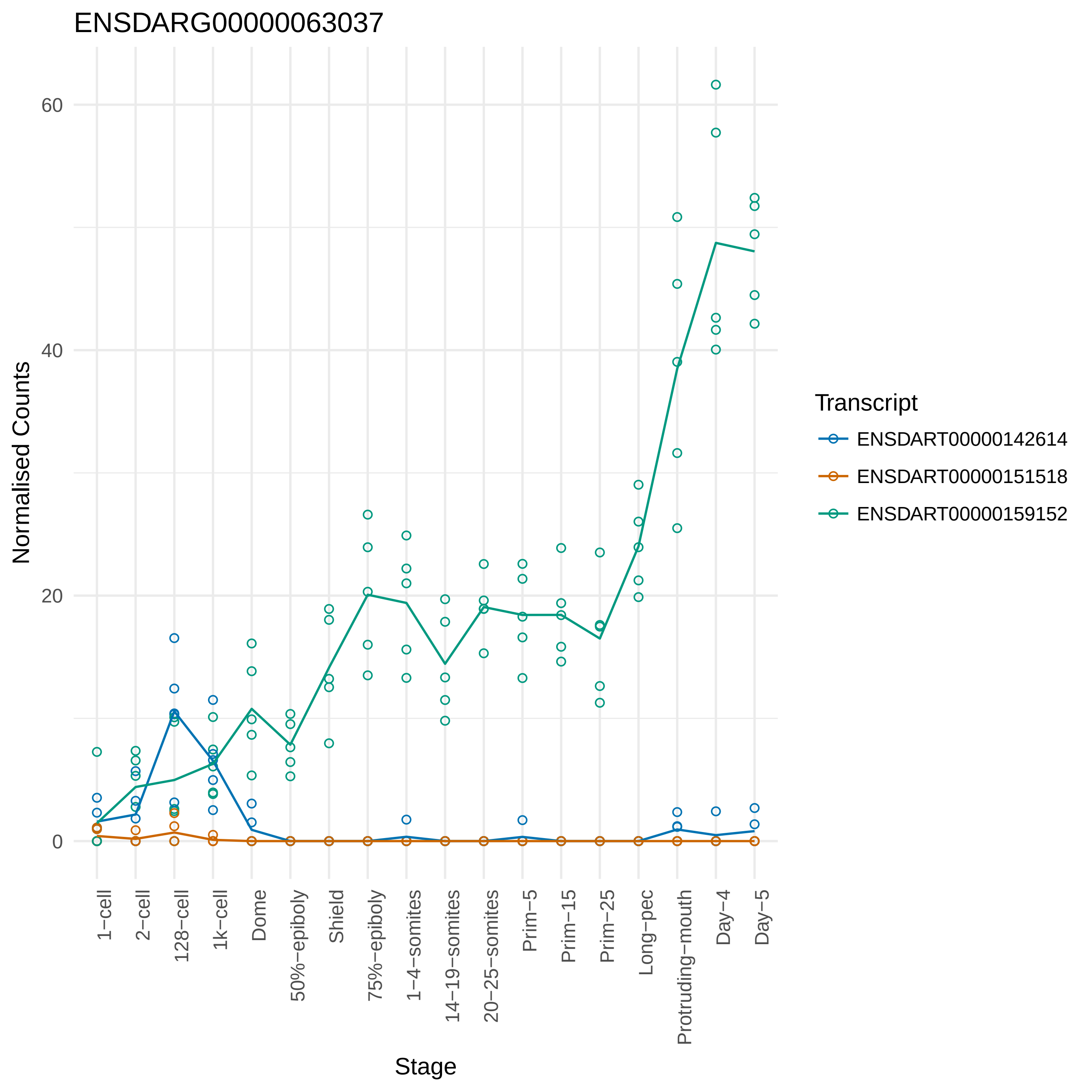

ENSDARG00000063037
Normalised Counts
60
40
20
0
1−cell
2−cell
128−cell
1k−cell
Dome
50%−epiboly
Shield
75%−epiboly
1−4−somites
14−19−somites
20−25−somites
Prim−5
Prim−15
Prim−25
Long−pec
Protruding−mouth
Day−4
Day−5
Stage
Transcript
ENSDART00000142614
ENSDART00000151518
ENSDART00000159152

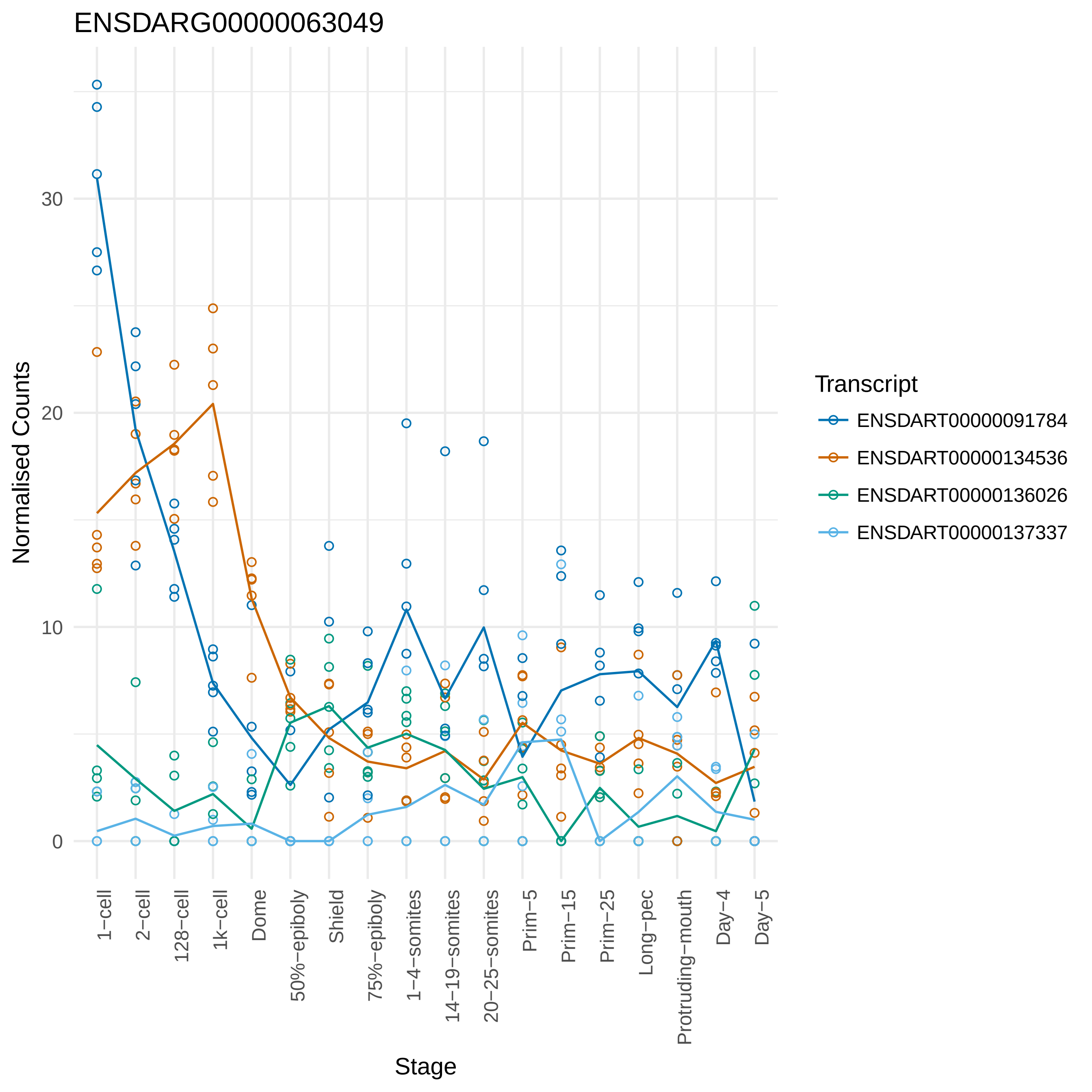

ENSDARG00000063049
Normalised Counts
0
10
20
30
1−cell
2−cell
128−cell
1k−cell
Dome
50%−epiboly
Shield
75%−epiboly
1−4−somites
14−19−somites
20−25−somites
Prim−5
Prim−15
Prim−25
Long−pec
Protruding−mouth
Day−4
Day−5
Stage
Transcript
ENSDART00000091784
ENSDART00000134536
ENSDART00000136026
ENSDART00000137337

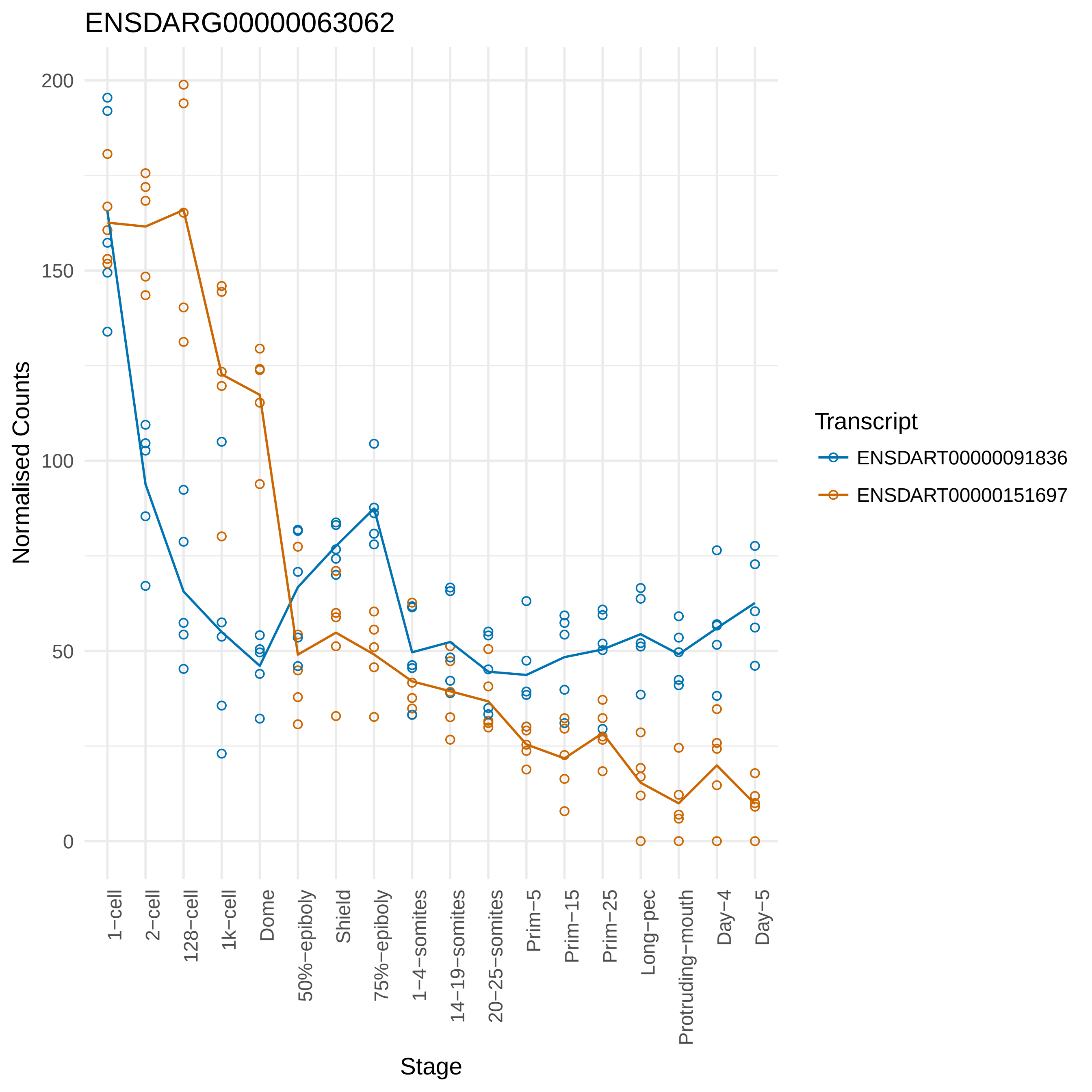

ENSDARG00000063062
Normalised Counts
200
150
100
50
0
1−cell
2−cell
128−cell
1k−cell
Dome
50%−epiboly
Shield
75%−epiboly
1−4−somites
14−19−somites
20−25−somites
Prim−5
Prim−15
Prim−25
Long−pec
Protruding−mouth
Day−4
Day−5
Stage
Transcript
ENSDART00000091836
ENSDART00000151697

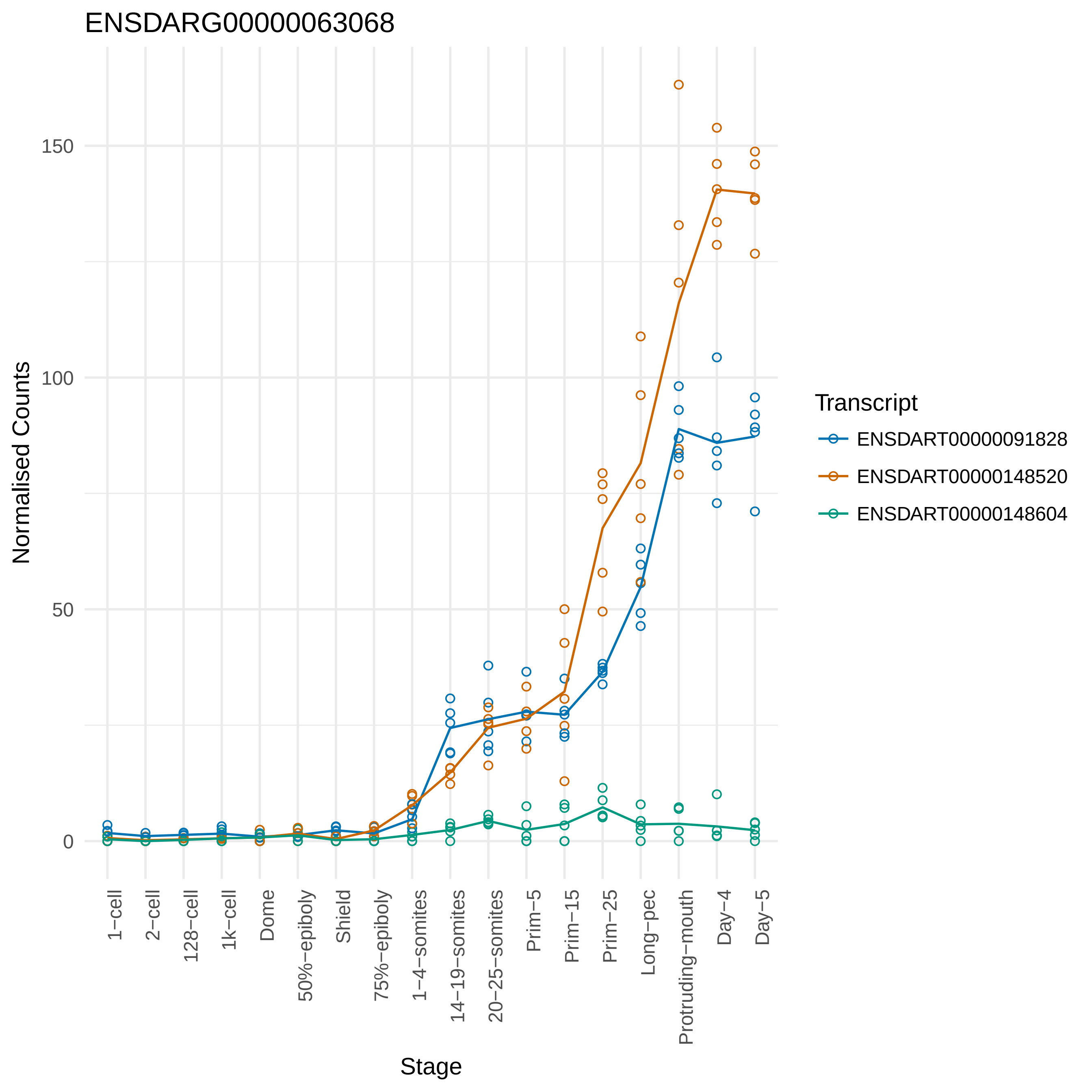

ENSDARG00000063068
Normalised Counts
150
100
50
0
1−cell
2−cell
128−cell
1k−cell
Dome
50%−epiboly
Shield
75%−epiboly
1−4−somites
14−19−somites
20−25−somites
Prim−5
Prim−15
Prim−25
Long−pec
Protruding−mouth
Day−4
Day−5
Stage
Transcript
ENSDART00000091828
ENSDART00000148520
ENSDART00000148604

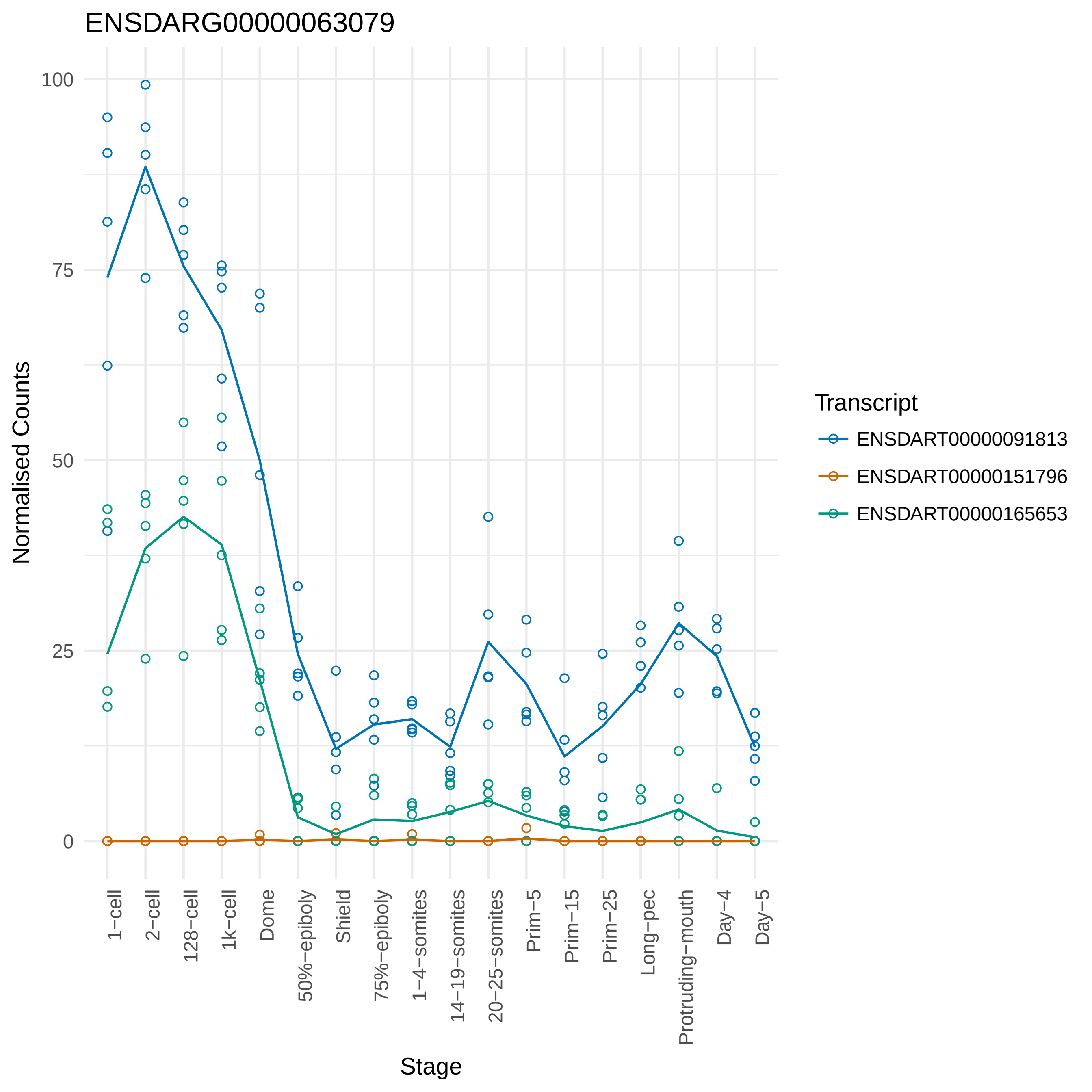
ENSDARG00000063079
Normalised Counts
100
75
50
25
0
1−cell
2−cell
128−cell
1k−cell
Dome
50%−epiboly
Shield
75%−epiboly
1−4−somites
14−19−somites
20−25−somites
Prim−5
Prim−15
Prim−25
Long−pec
Protruding−mouth
Day−4
Day−5
Stage
Transcript
ENSDART00000091813
ENSDART00000151796
ENSDART00000165653

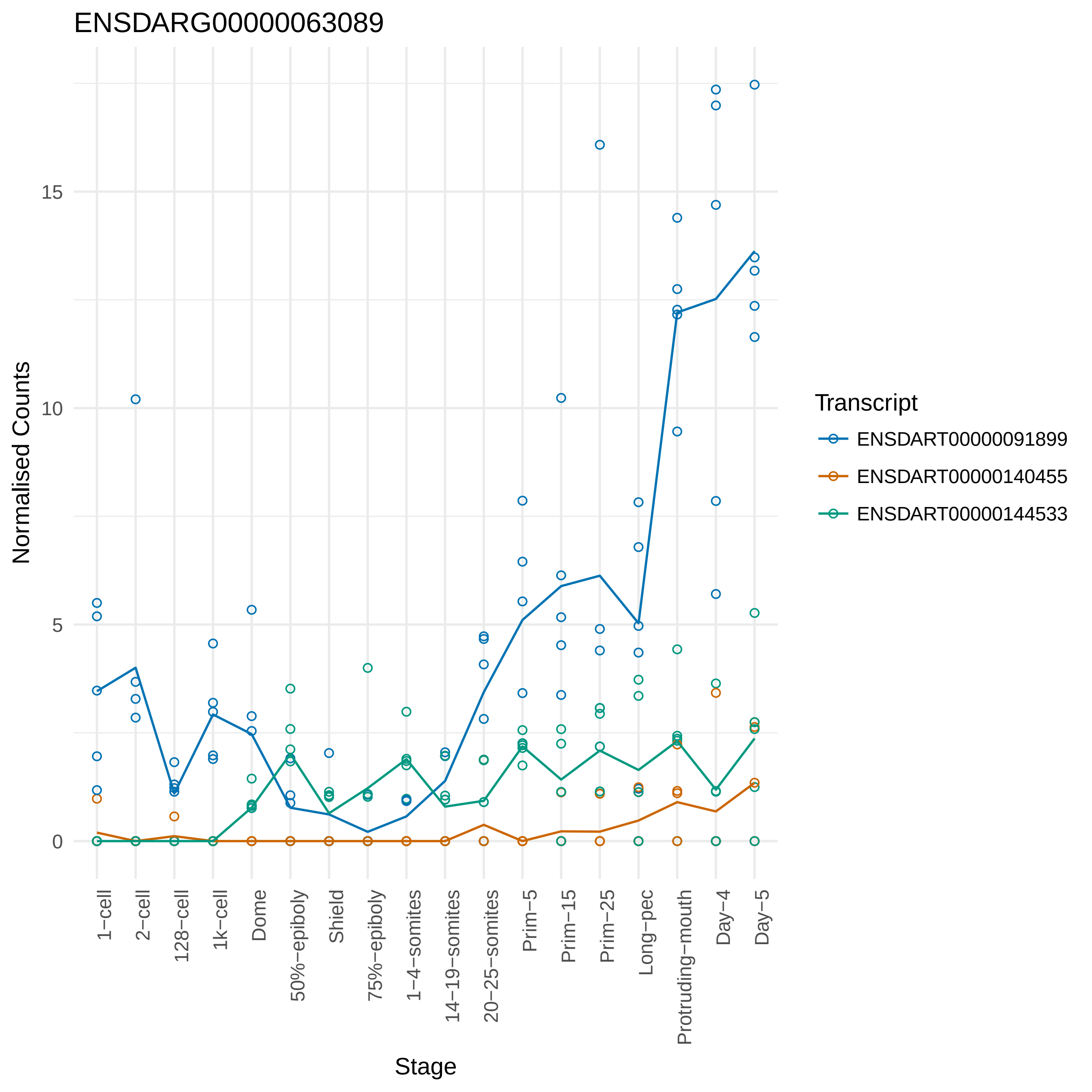

ENSDARG00000063089
Normalised Counts
0
5
10
15
Stage
1−cell
2−cell
128−cell
1k−cell
Dome
50%−epiboly
Shield
75%−epiboly
1−4−somites
14−19−somites
20−25−somites
Prim−5
Prim−15
Prim−25
Long−pec
Protruding−mouth
Day−4
Day−5
Transcript
ENSDART00000091899
ENSDART00000140455
ENSDART00000144533

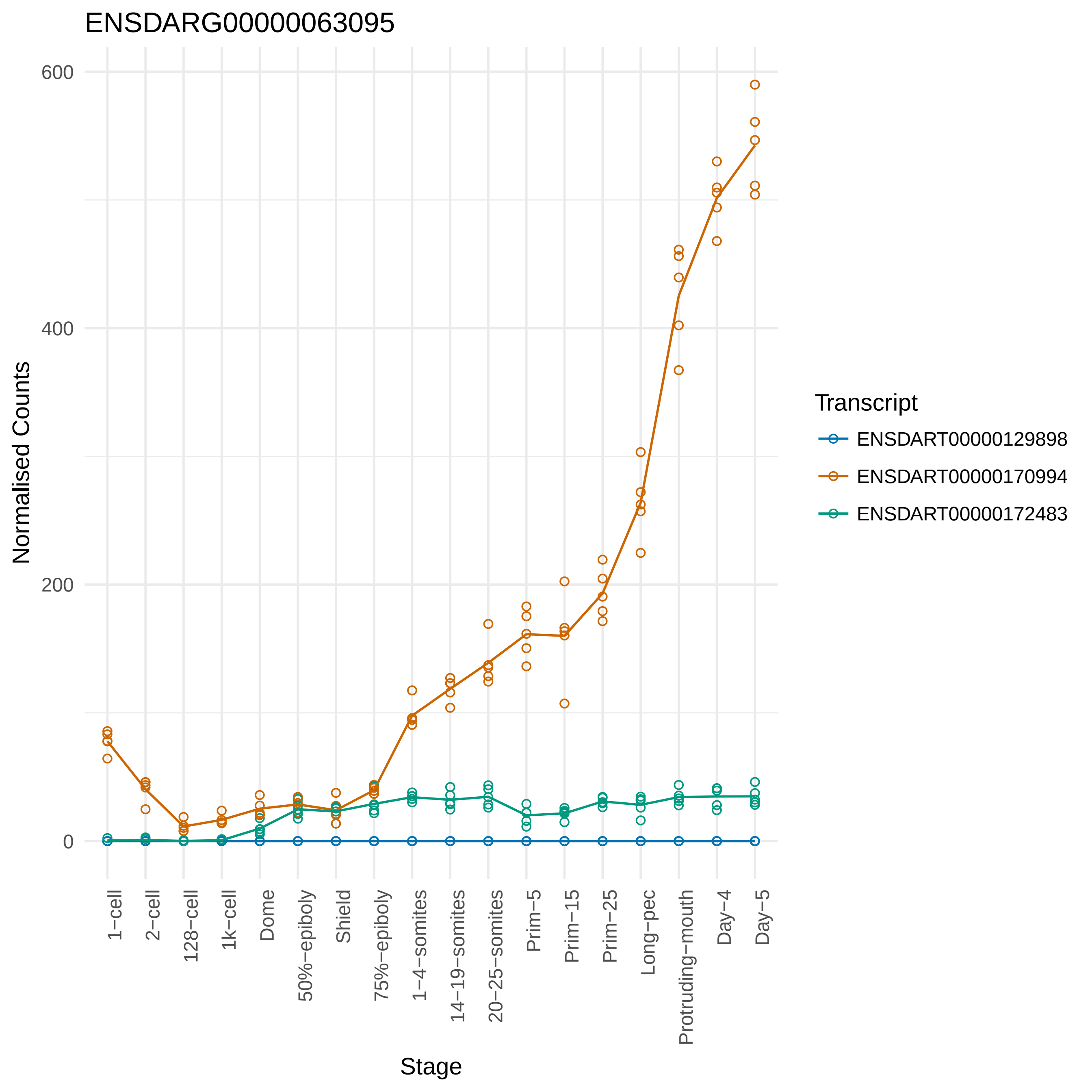

ENSDARG00000063095
Normalised Counts
600
400
200
0
1−cell
2−cell
128−cell
1k−cell
Dome
50%−epiboly
Shield
75%−epiboly
1−4−somites
14−19−somites
20−25−somites
Prim−5
Prim−15
Prim−25
Long−pec
Protruding−mouth
Day−4
Day−5
Stage
Transcript
ENSDART00000129898
ENSDART00000170994
ENSDART00000172483

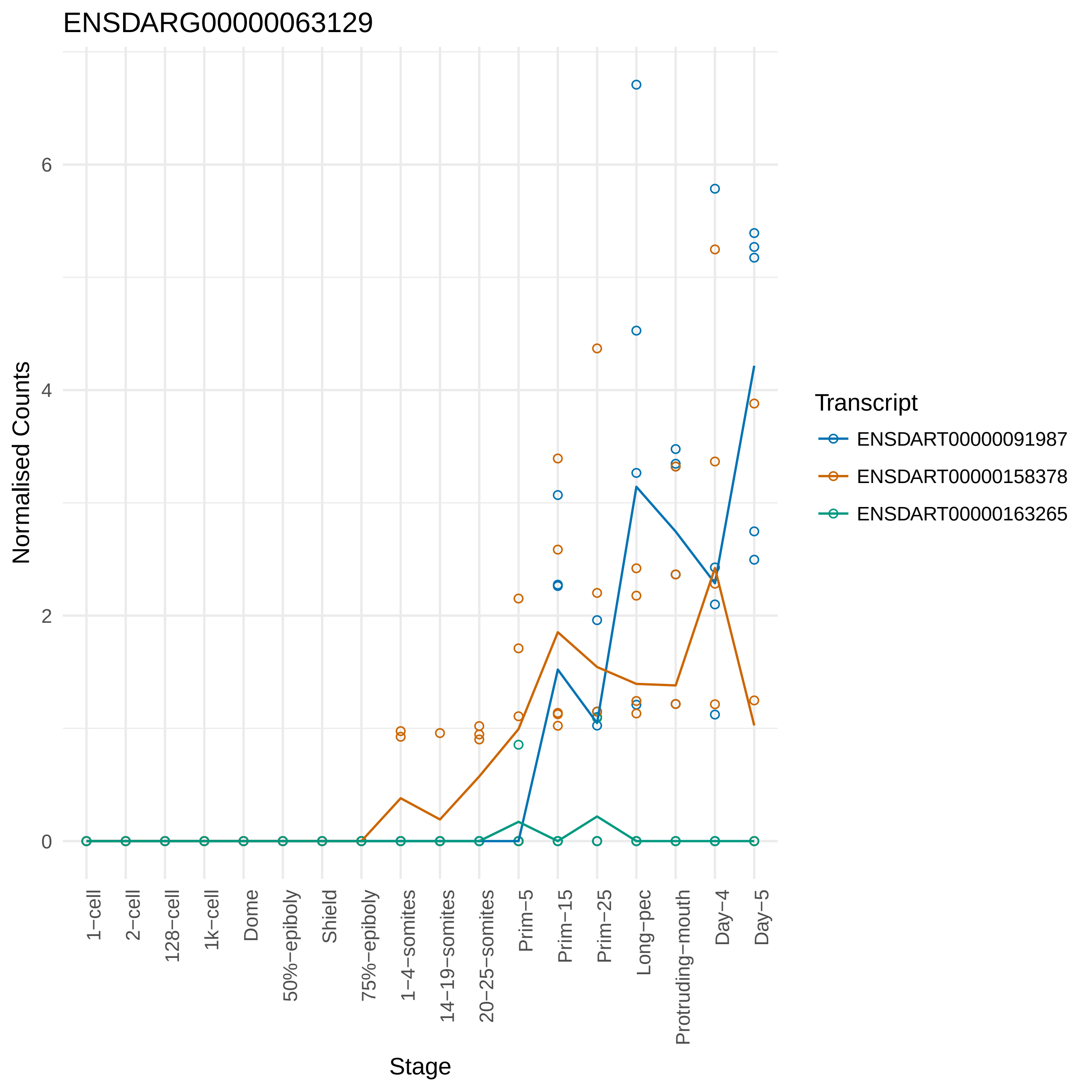

ENSDARG00000063129
Normalised Counts
6
4
2
0
1−cell
2−cell
128−cell
1k−cell
Dome
50%−epiboly
Shield
75%−epiboly
1−4−somites
14−19−somites
20−25−somites
Prim−5
Prim−15
Prim−25
Long−pec
Protruding−mouth
Day−4
Day−5
Stage
Transcript
ENSDART00000091987
ENSDART00000158378
ENSDART00000163265

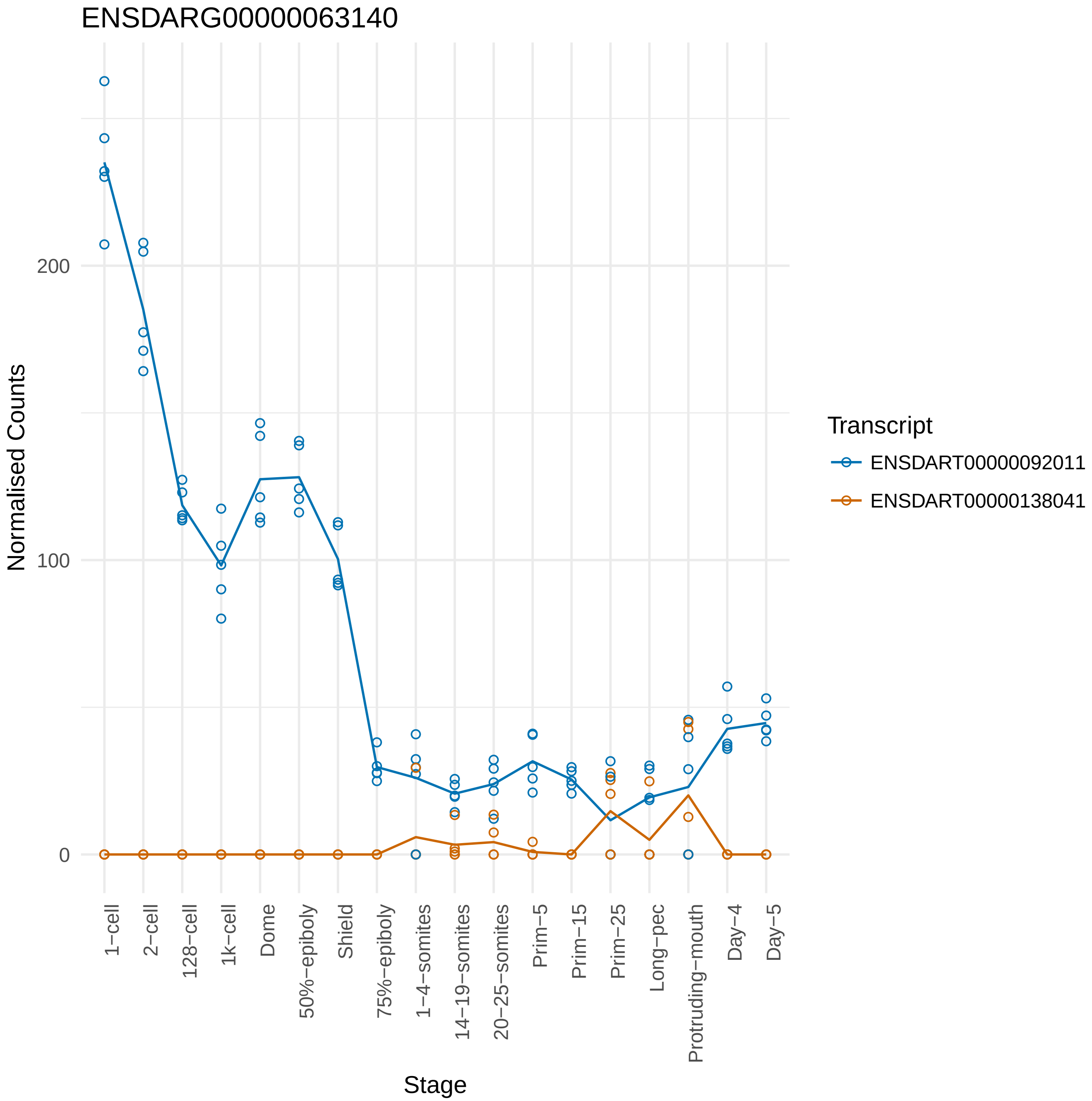

ENSDARG00000063140
Normalised Counts
200
100
0
1−cell
2−cell
128−cell
1k−cell
Dome
50%−epiboly
Shield
75%−epiboly
1−4−somites
14−19−somites
20−25−somites
Prim−5
Prim−15
Prim−25
Long−pec
Protruding−mouth
Day−4
Day−5
Stage
Transcript
ENSDART00000092011
ENSDART00000138041

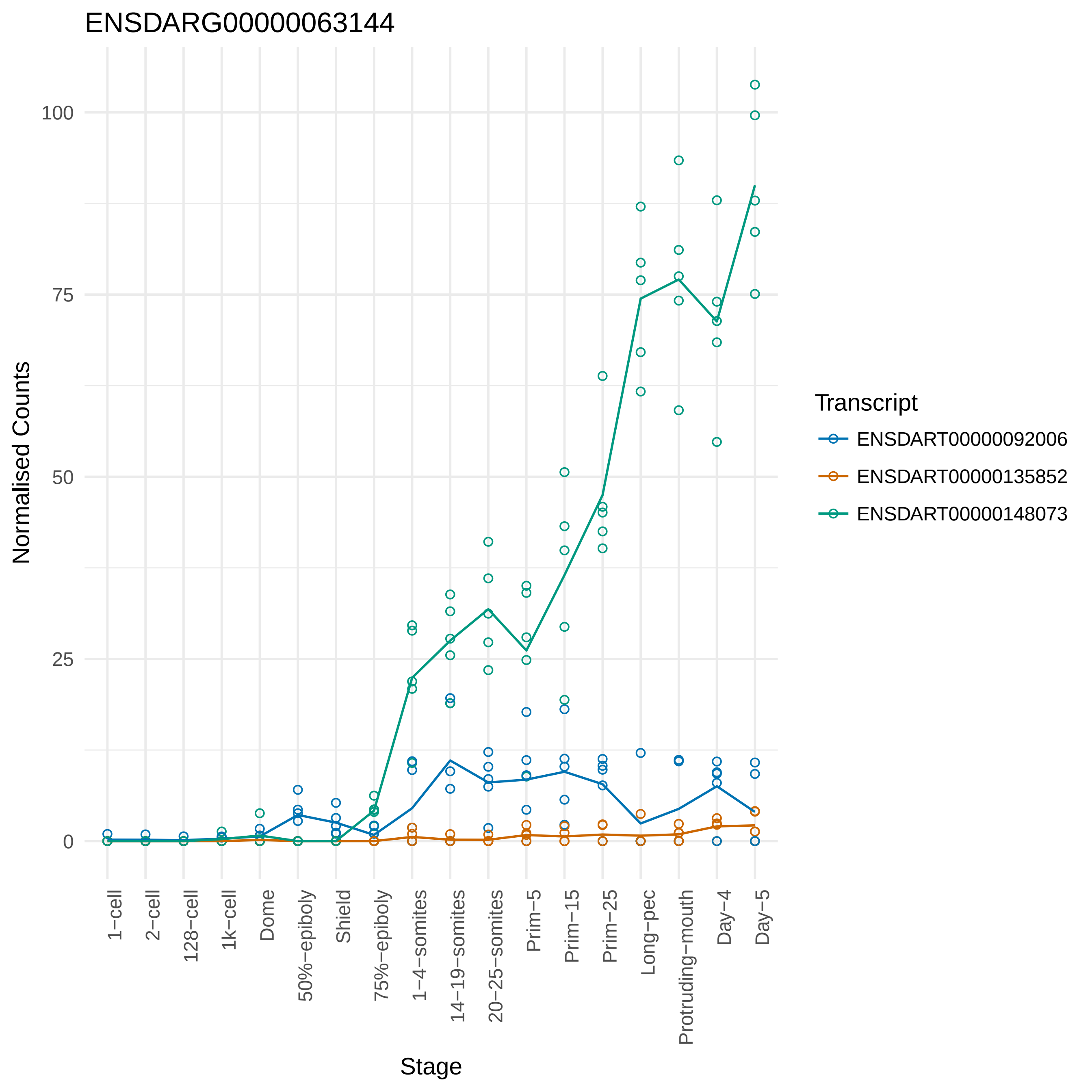

ENSDARG00000063144
Normalised Counts
100
75
50
25
0
1−cell
2−cell
128−cell
1k−cell
Dome
50%−epiboly
Shield
75%−epiboly
1−4−somites
14−19−somites
20−25−somites
Prim−5
Prim−15
Prim−25
Long−pec
Protruding−mouth
Day−4
Day−5
Stage
Transcript
ENSDART00000092006
ENSDART00000135852
ENSDART00000148073

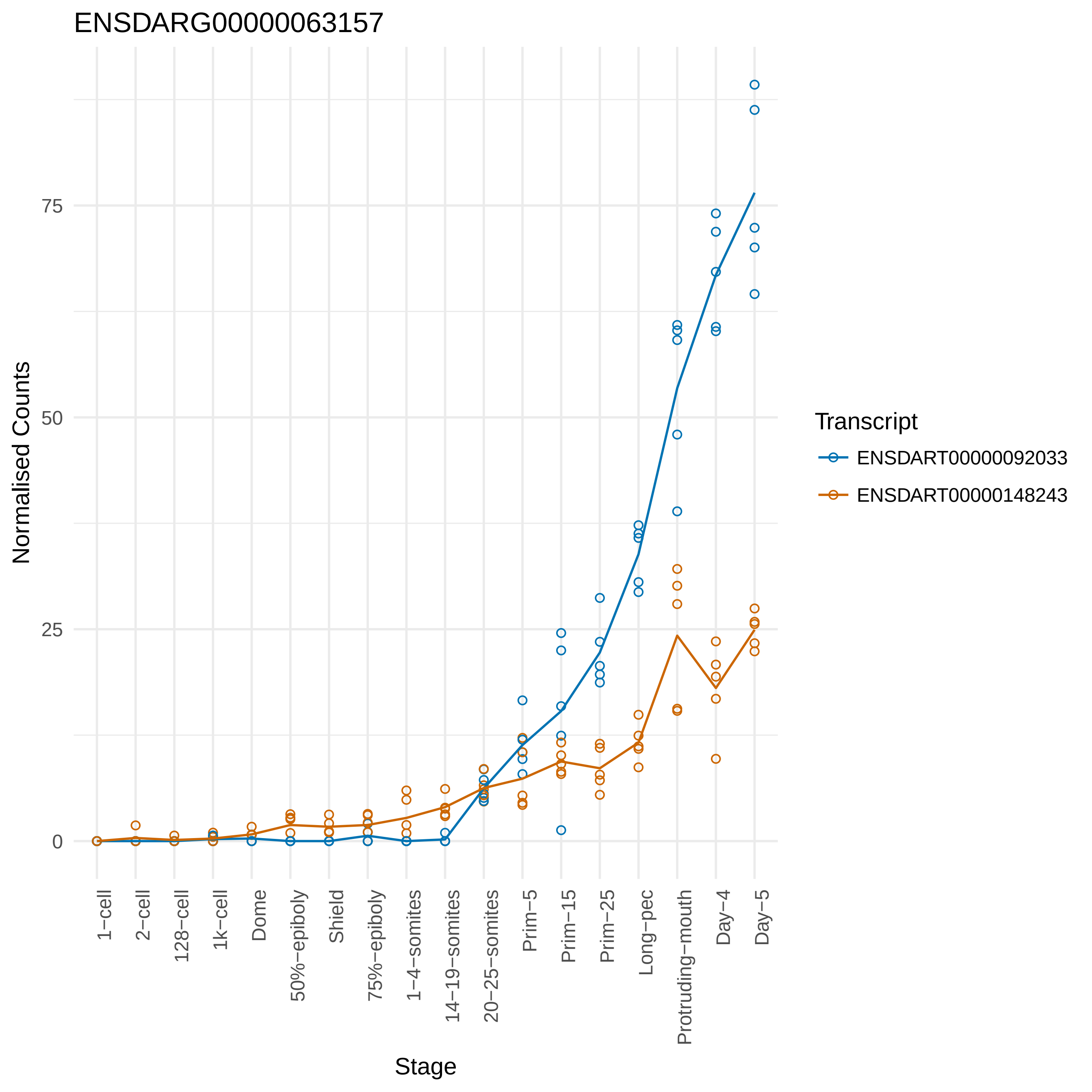

ENSDARG00000063157
Normalised Counts
0
25
50
75
1−cell
2−cell
128−cell
1k−cell
Dome
50%−epiboly
Shield
75%−epiboly
1−4−somites
14−19−somites
20−25−somites
Prim−5
Prim−15
Prim−25
Long−pec
Protruding−mouth
Day−4
Day−5
Stage
Transcript
ENSDART00000092033
ENSDART00000148243

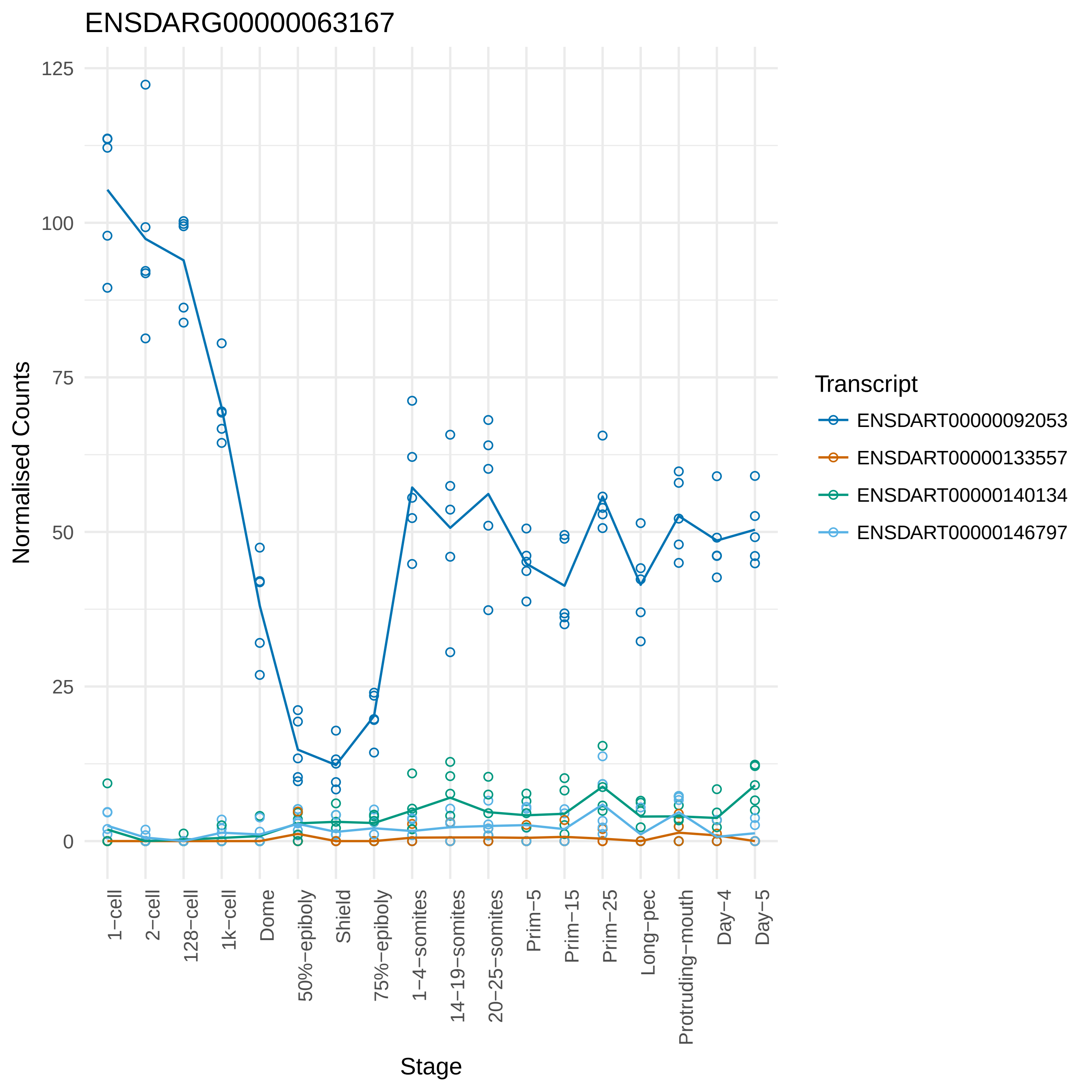

ENSDARG00000063167
Normalised Counts
125
100
75
50
25
0
1−cell
2−cell
128−cell
1k−cell
Dome
50%−epiboly
Shield
75%−epiboly
1−4−somites
14−19−somites
20−25−somites
Prim−5
Prim−15
Prim−25
Long−pec
Protruding−mouth
Day−4
Day−5
Stage
Transcript
ENSDART00000092053
ENSDART00000133557
ENSDART00000140134
ENSDART00000146797

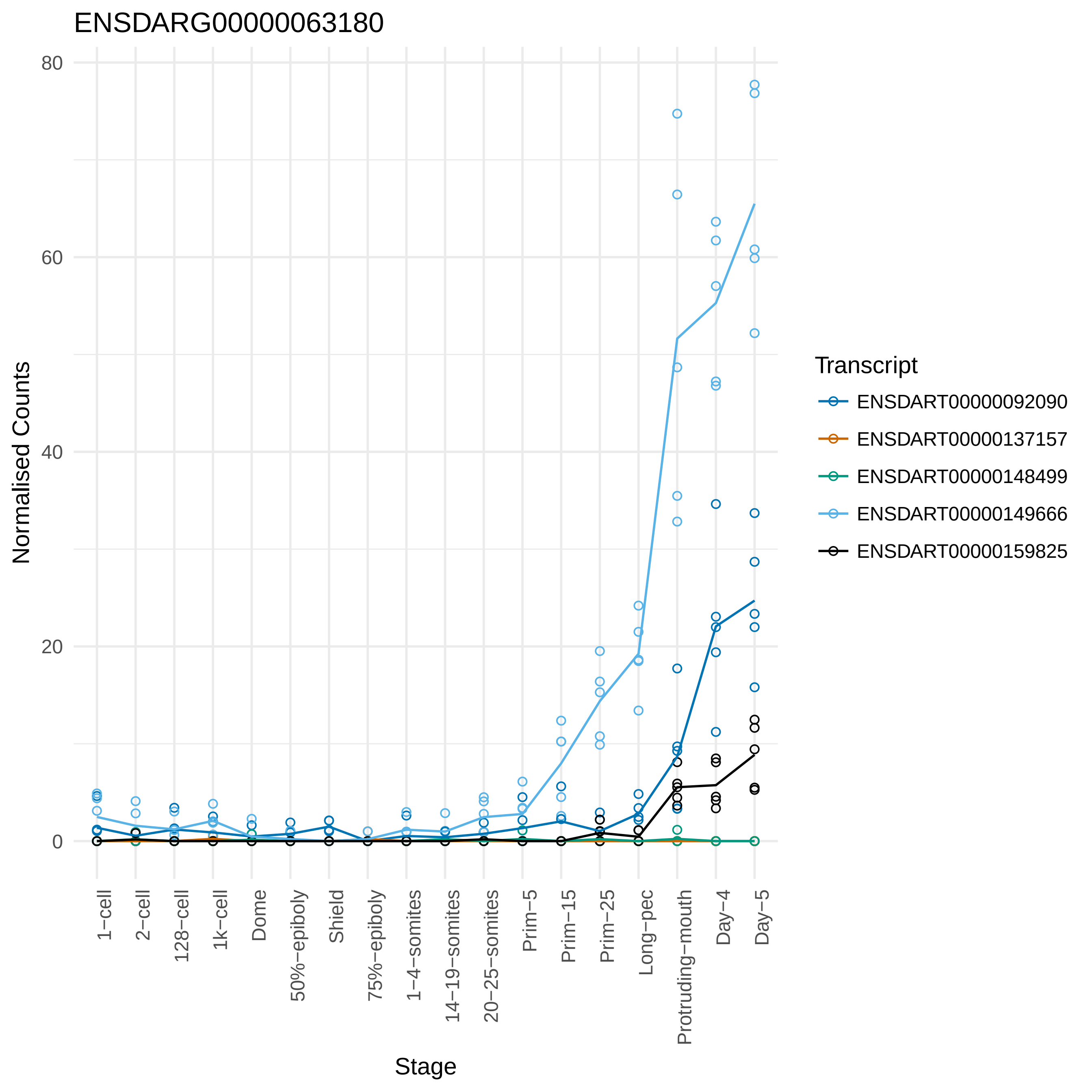

ENSDARG00000063180
Normalised Counts
0
20
40
60
80
1−cell
2−cell
128−cell
1k−cell
Dome
50%−epiboly
Shield
75%−epiboly
1−4−somites
14−19−somites
20−25−somites
Prim−5
Prim−15
Prim−25
Long−pec
Protruding−mouth
Day−4
Day−5
Stage
Transcript
ENSDART00000092090
ENSDART00000137157
ENSDART00000148499
ENSDART00000149666
ENSDART00000159825

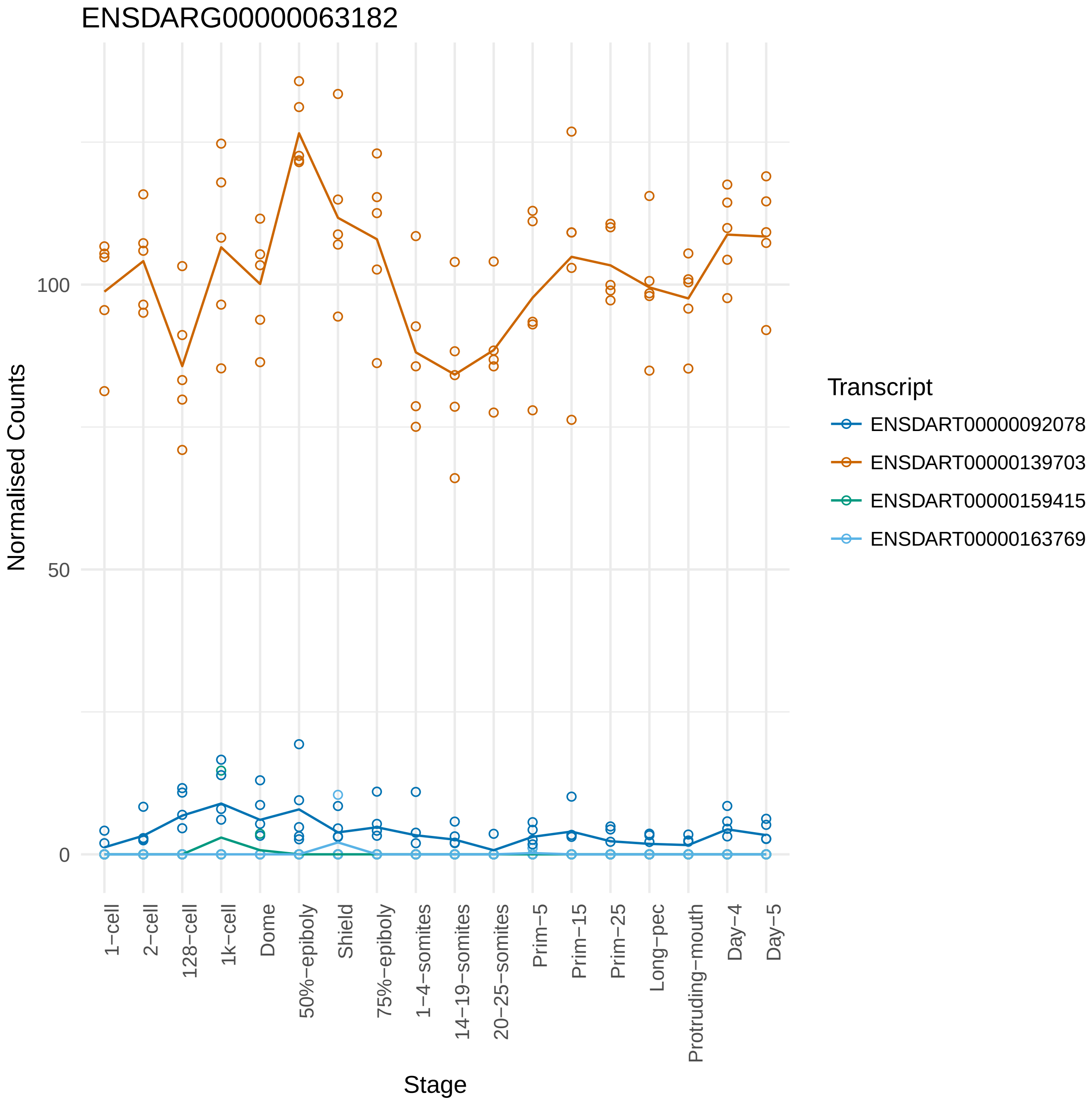

ENSDARG00000063182
Normalised Counts
0
50
100
Stage
1−cell
2−cell
128−cell
1k−cell
Dome
50%−epiboly
Shield
75%−epiboly
1−4−somites
14−19−somites
20−25−somites
Prim−5
Prim−15
Prim−25
Long−pec
Protruding−mouth
Day−4
Day−5
Transcript
ENSDART00000092078
ENSDART00000139703
ENSDART00000159415
ENSDART00000163769

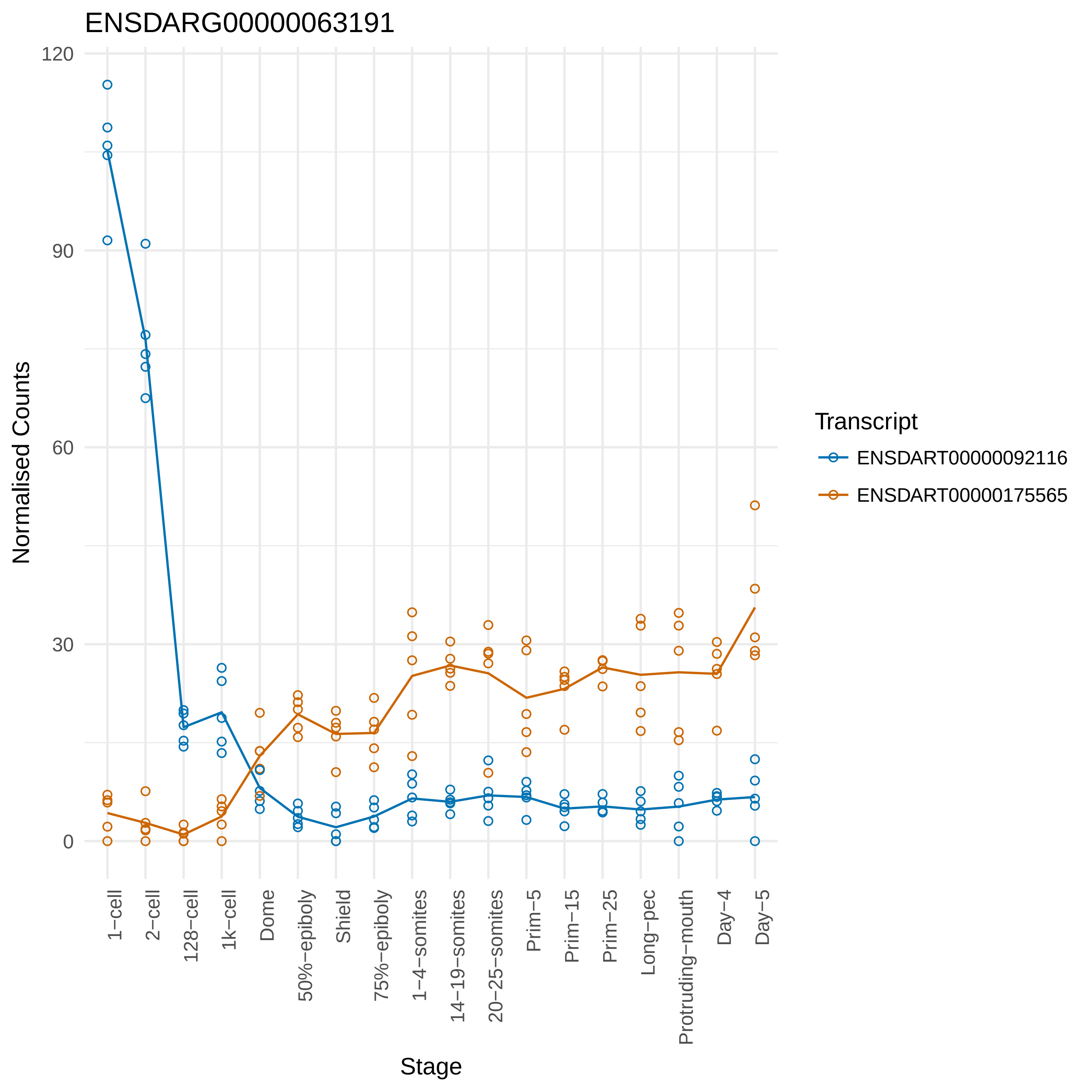

ENSDARG00000063191
Normalised Counts
120
90
60
30
0
1−cell
2−cell
128−cell
1k−cell
Dome
50%−epiboly
Shield
75%−epiboly
1−4−somites
14−19−somites
20−25−somites
Prim−5
Prim−15
Prim−25
Long−pec
Protruding−mouth
Day−4
Day−5
Stage
Transcript
ENSDART00000092116
ENSDART00000175565

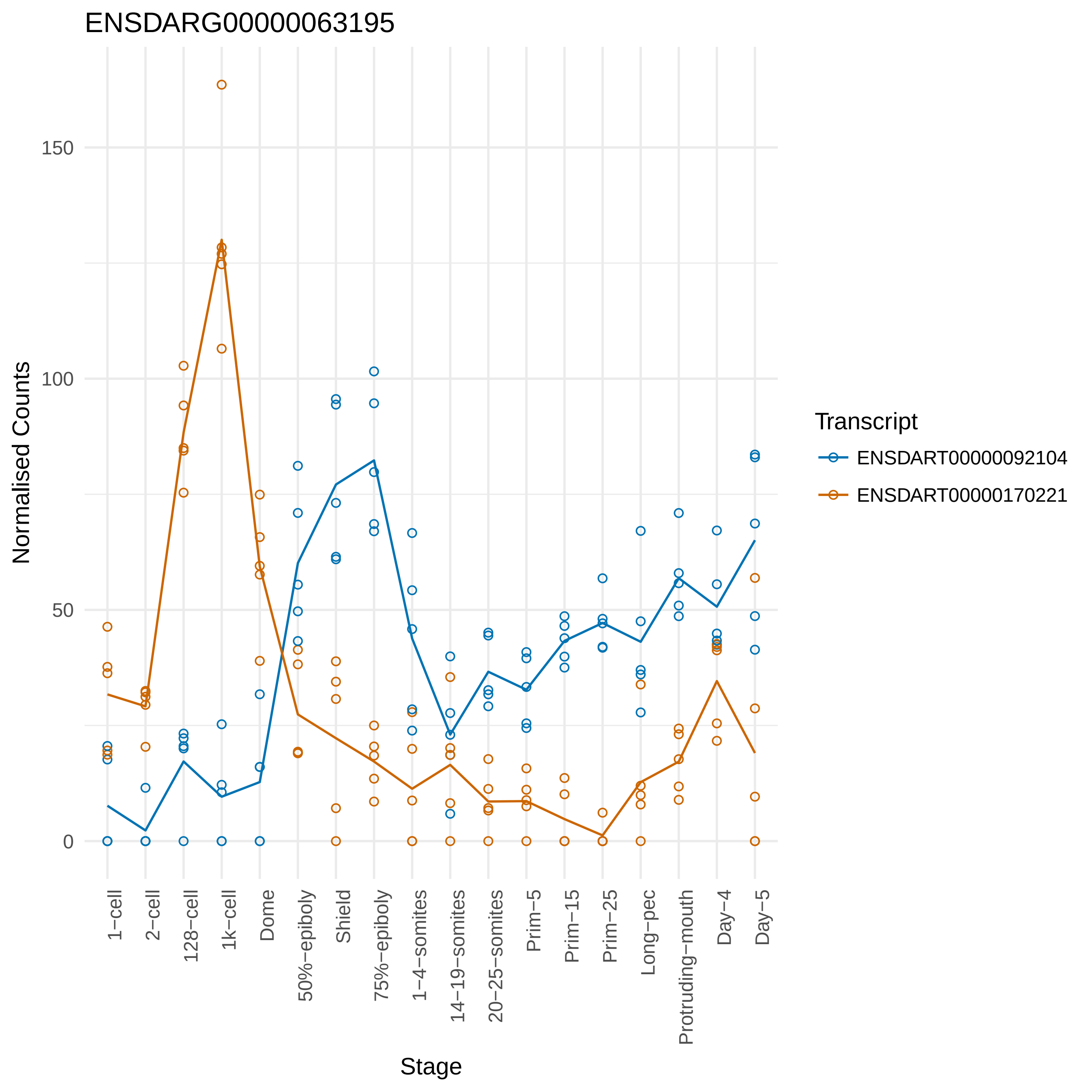
ENSDARG00000063195
Normalised Counts
150
100
50
0
1−cell
2−cell
128−cell
1k−cell
Dome
50%−epiboly
Shield
75%−epiboly
1−4−somites
14−19−somites
20−25−somites
Prim−5
Prim−15
Prim−25
Long−pec
Protruding−mouth
Day−4
Day−5
Stage
Transcript
ENSDART00000092104
ENSDART00000170221

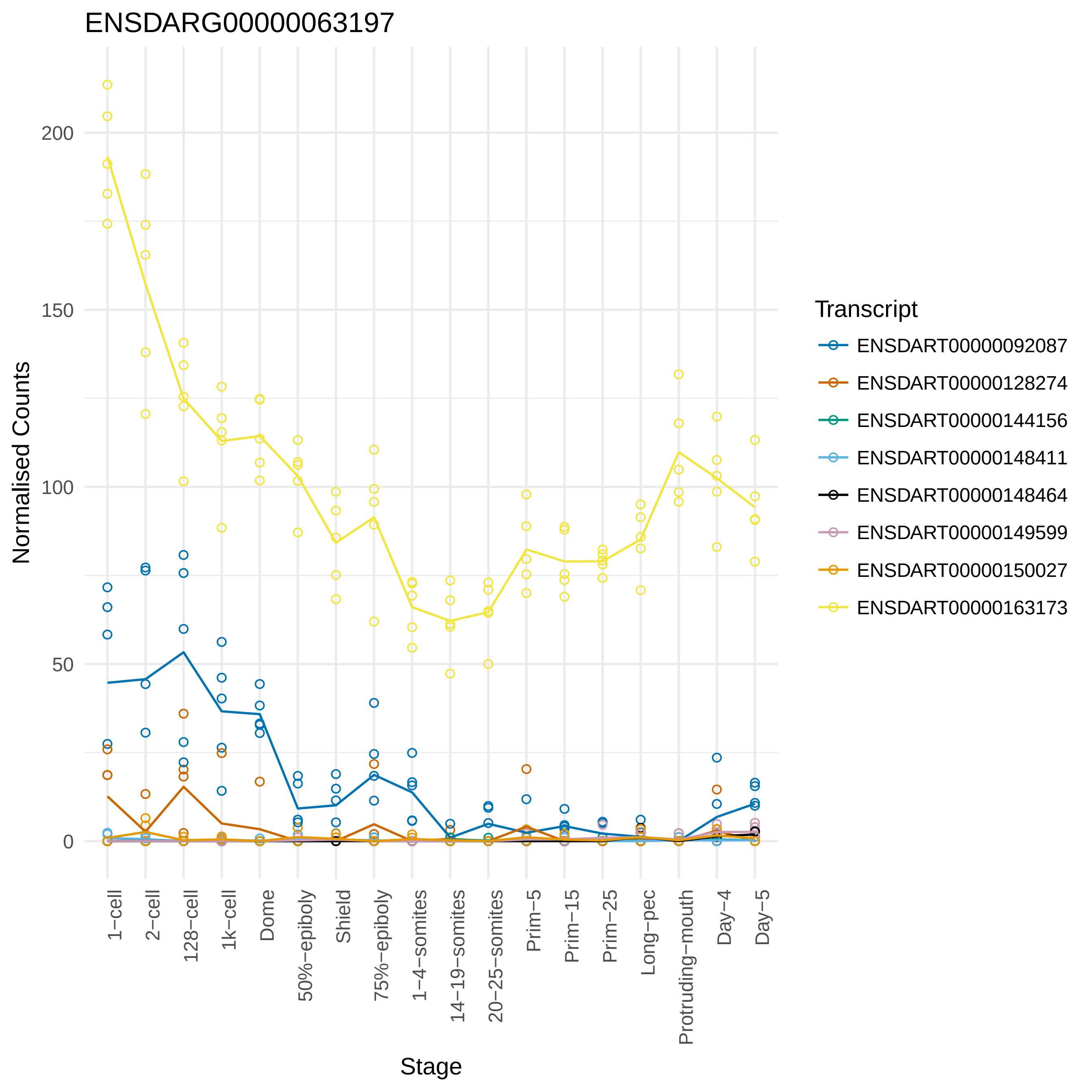
ENSDARG00000063197
Normalised Counts
200
150
100
50
0
1−cell
2−cell
128−cell
1k−cell
Dome
50%−epiboly
Shield
75%−epiboly
1−4−somites
14−19−somites
20−25−somites
Prim−5
Prim−15
Prim−25
Long−pec
Protruding−mouth
Day−4
Day−5
Stage
Transcript
ENSDART00000092087
ENSDART00000128274
ENSDART00000144156
ENSDART00000148411
ENSDART00000148464
ENSDART00000149599
ENSDART00000150027
ENSDART00000163173

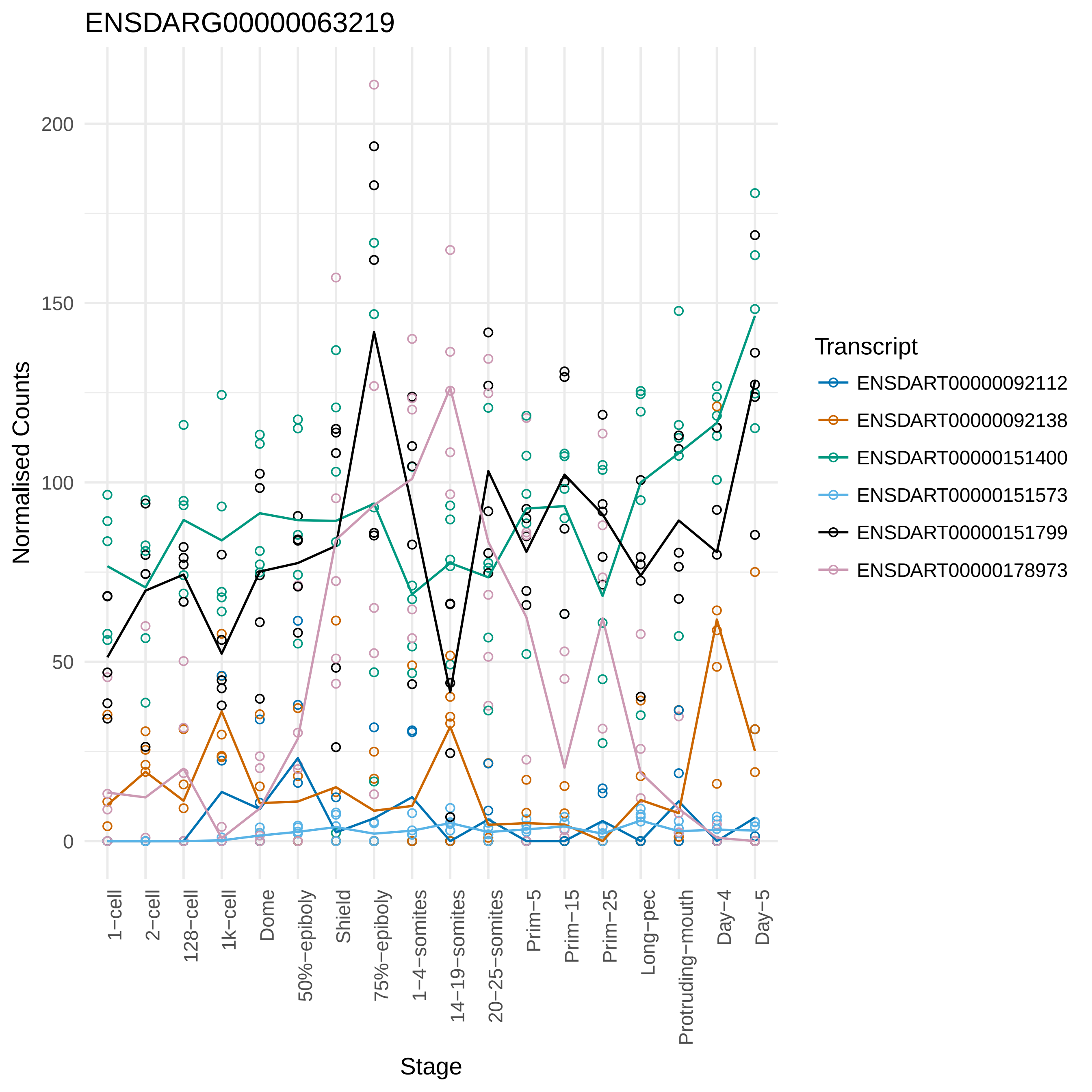
ENSDARG00000063219
Normalised Counts
0
50
100
150
200
Stage
1−cell
2−cell
128−cell
1k−cell
Dome
50%−epiboly
Shield
75%−epiboly
1−4−somites
14−19−somites
20−25−somites
Prim−5
Prim−15
Prim−25
Long−pec
Protruding−mouth
Day−4
Day−5
Transcript
ENSDART00000092112
ENSDART00000092138
ENSDART00000151400
ENSDART00000151573
ENSDART00000151799
ENSDART00000178973

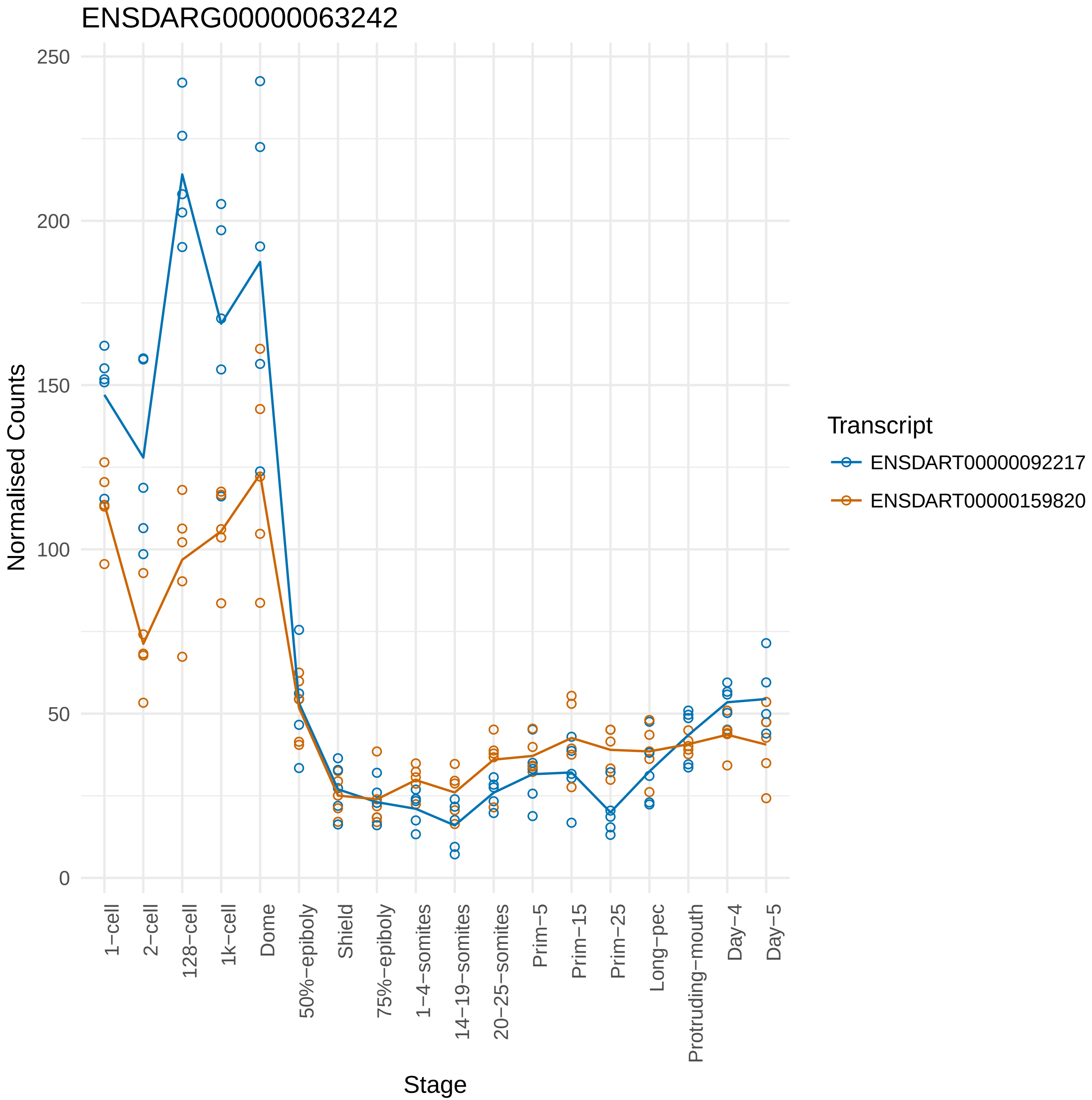

ENSDARG00000063242
Normalised Counts
250
200
150
100
50
0
1−cell
2−cell
128−cell
1k−cell
Dome
50%−epiboly
Shield
75%−epiboly
1−4−somites
14−19−somites
20−25−somites
Prim−5
Prim−15
Prim−25
Long−pec
Protruding−mouth
Day−4
Day−5
Stage
Transcript
ENSDART00000092217
ENSDART00000159820

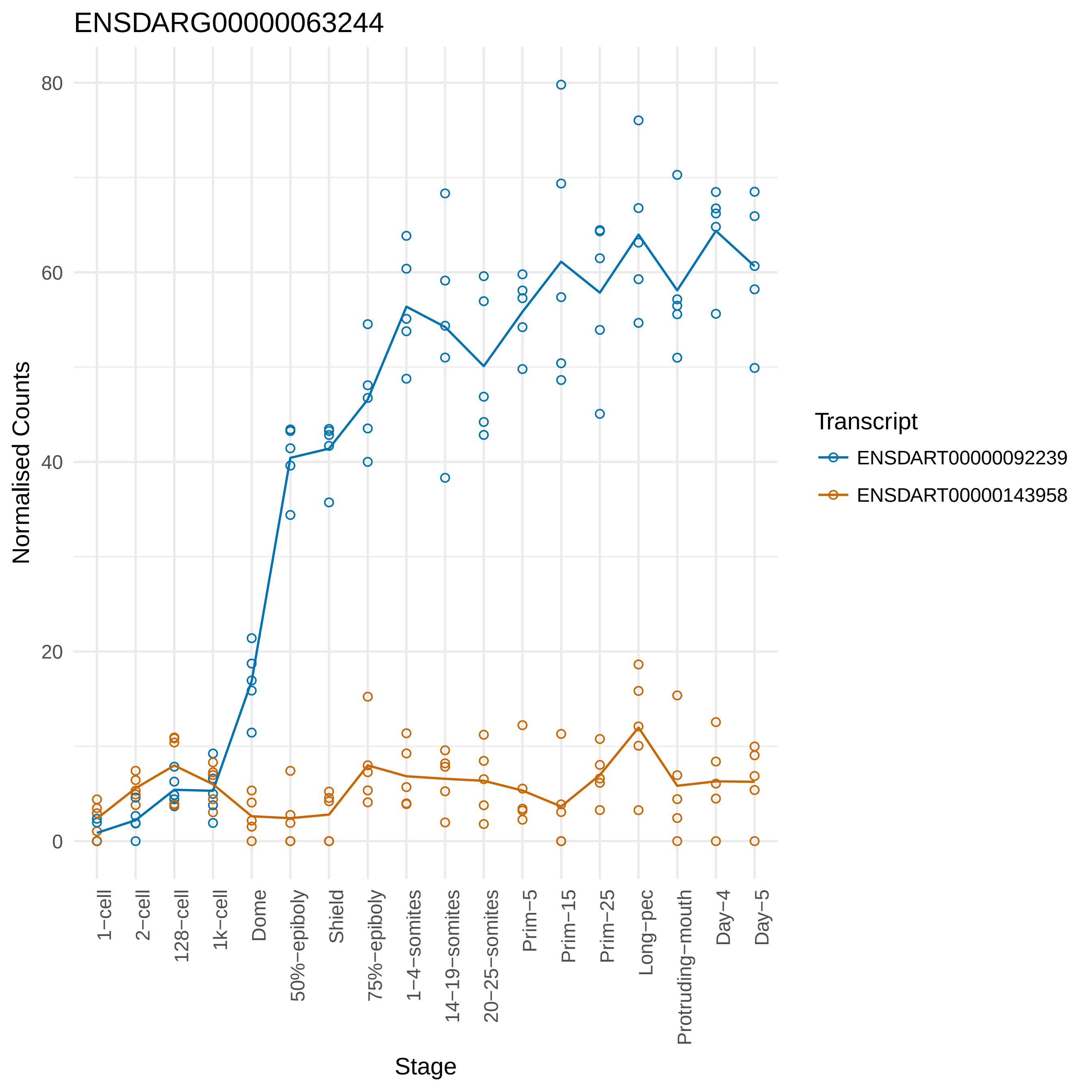

ENSDARG00000063244
Normalised Counts
80
60
40
20
0
1−cell
2−cell
128−cell
1k−cell
Dome
50%−epiboly
Shield
75%−epiboly
1−4−somites
14−19−somites
20−25−somites
Prim−5
Prim−15
Prim−25
Long−pec
Protruding−mouth
Day−4
Day−5
Stage
Transcript
ENSDART00000092239
ENSDART00000143958

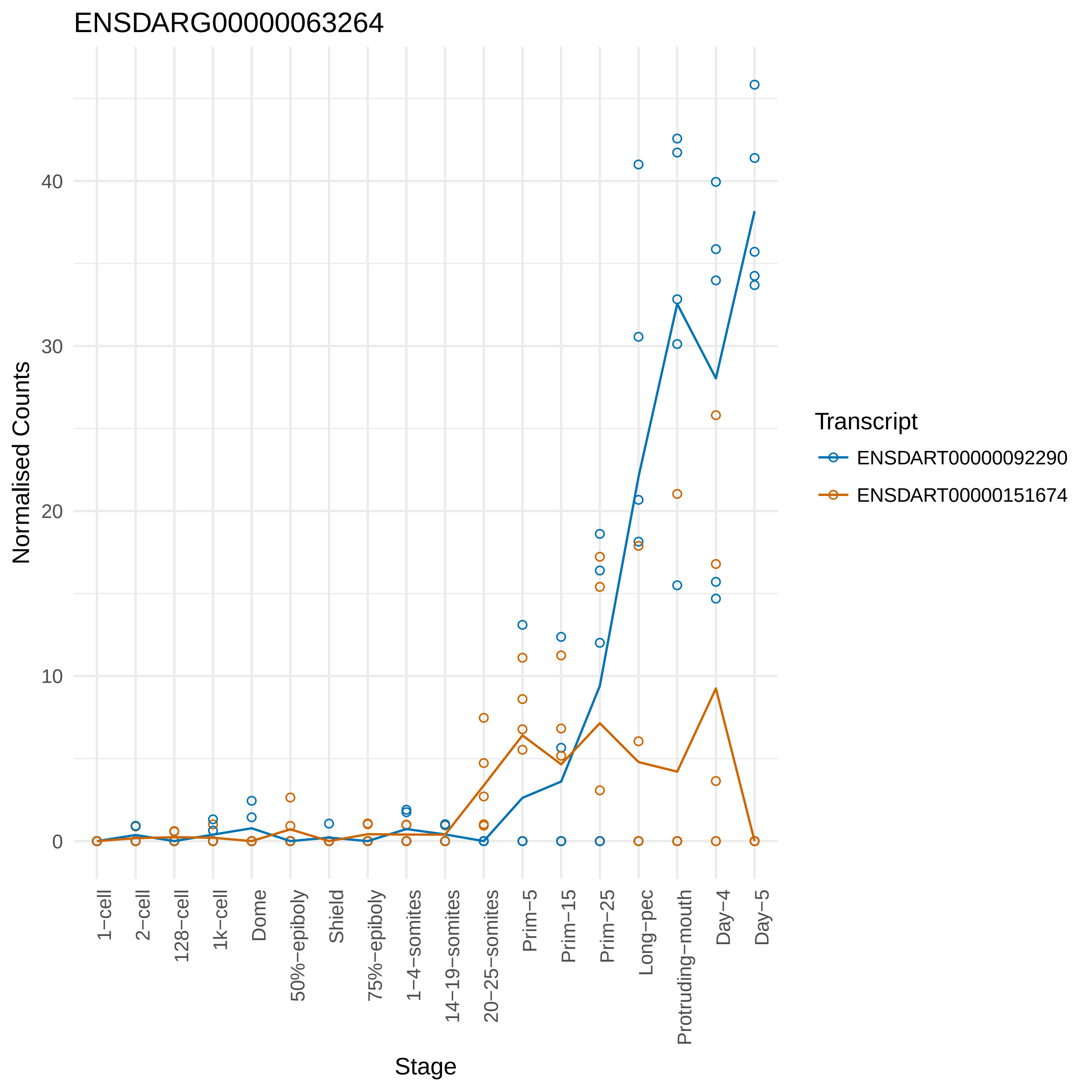
ENSDARG00000063264
Normalised Counts
0
10
20
30
40
Stage
1−cell
2−cell
128−cell
1k−cell
Dome
50%−epiboly
Shield
75%−epiboly
1−4−somites
14−19−somites
20−25−somites
Prim−5
Prim−15
Prim−25
Long−pec
Protruding−mouth
Day−4
Day−5
Transcript
ENSDART00000092290
ENSDART00000151674

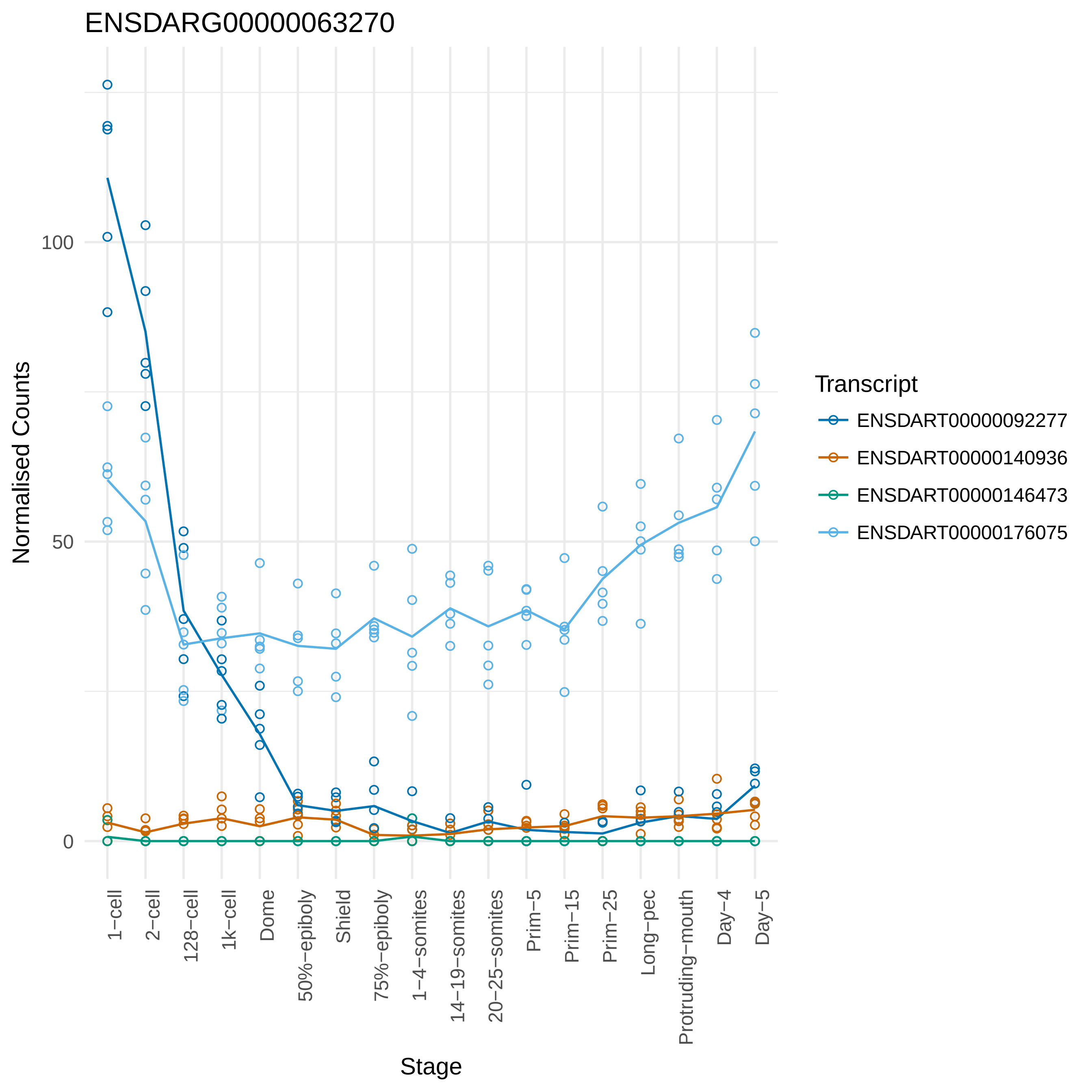
ENSDARG00000063270
Normalised Counts
100
50
0
Stage
1−cell
2−cell
128−cell
1k−cell
Dome
50%−epiboly
Shield
75%−epiboly
1−4−somites
14−19−somites
20−25−somites
Prim−5
Prim−15
Prim−25
Long−pec
Protruding−mouth
Day−4
Day−5
Transcript
ENSDART00000092277
ENSDART00000140936
ENSDART00000146473
ENSDART00000176075

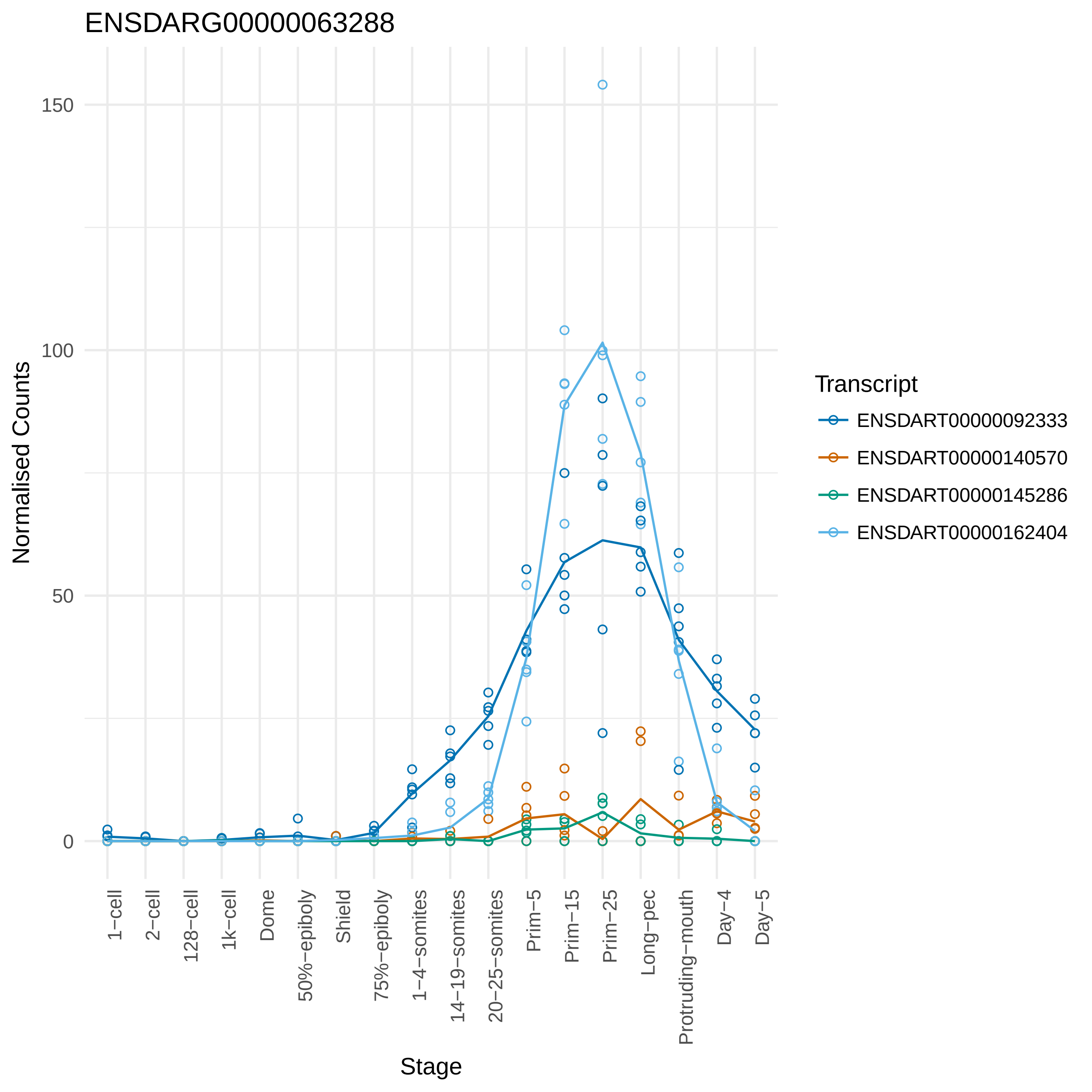

ENSDARG00000063288
Normalised Counts
150
100
50
0
1−cell
2−cell
128−cell
1k−cell
Dome
50%−epiboly
Shield
75%−epiboly
1−4−somites
14−19−somites
20−25−somites
Prim−5
Prim−15
Prim−25
Long−pec
Protruding−mouth
Day−4
Day−5
Stage
Transcript
ENSDART00000092333
ENSDART00000140570
ENSDART00000145286
ENSDART00000162404

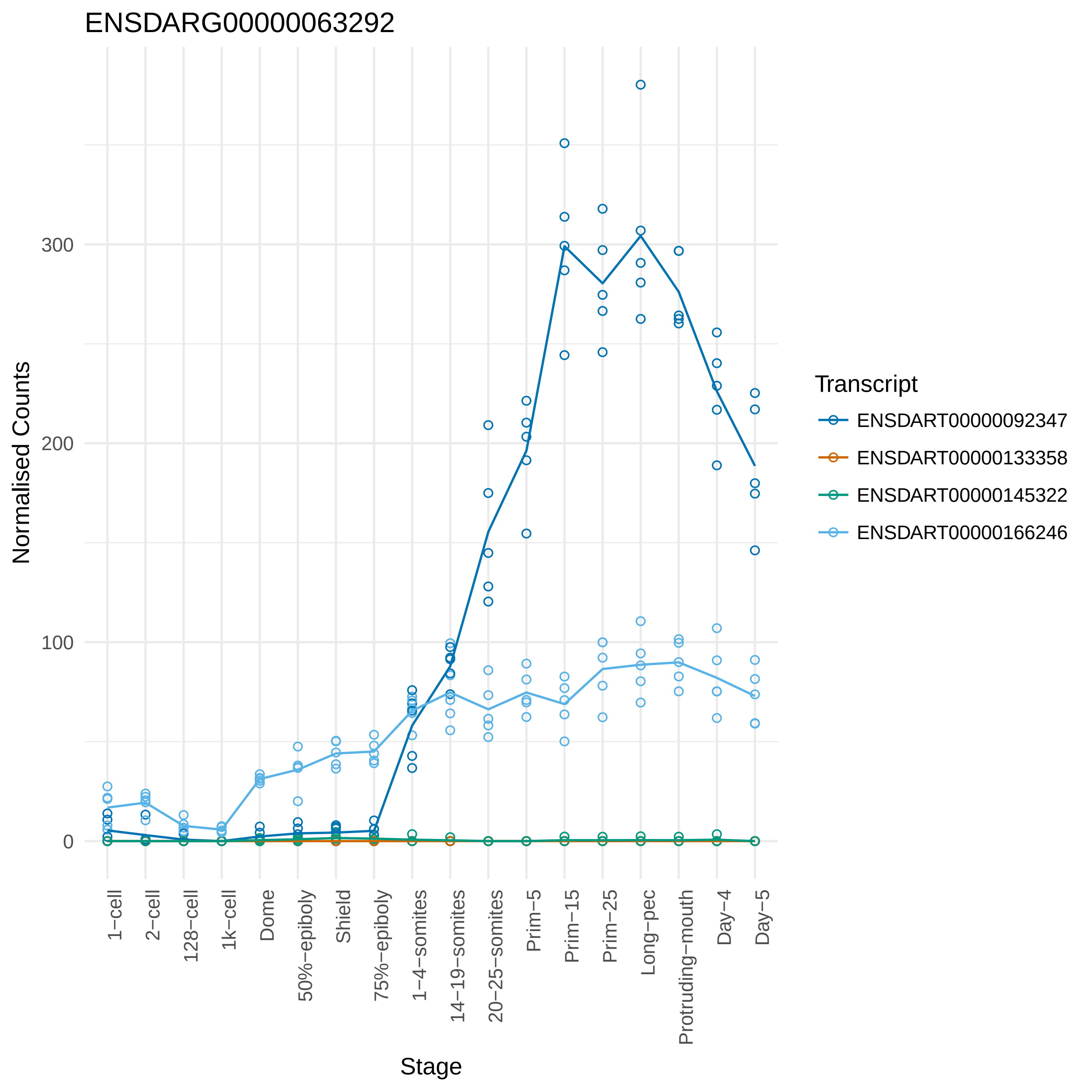

ENSDARG00000063292
Normalised Counts
300
200
100
0
1−cell
2−cell
128−cell
1k−cell
Dome
50%−epiboly
Shield
75%−epiboly
1−4−somites
14−19−somites
20−25−somites
Prim−5
Prim−15
Prim−25
Long−pec
Protruding−mouth
Day−4
Day−5
Stage
Transcript
ENSDART00000092347
ENSDART00000133358
ENSDART00000145322
ENSDART00000166246

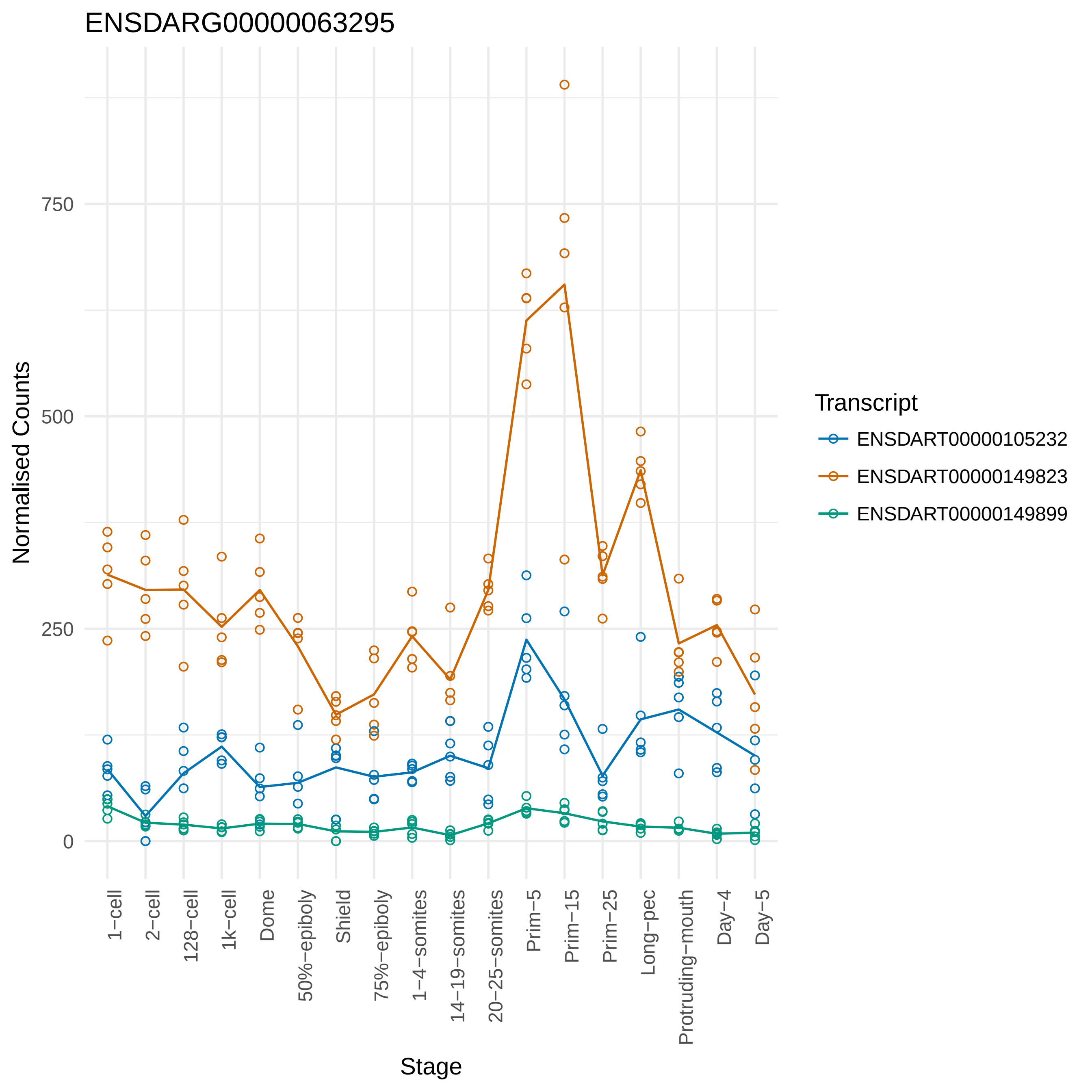
ENSDARG00000063295
Normalised Counts
0
250
500
750
Stage
1−cell
2−cell
128−cell
1k−cell
Dome
50%−epiboly
Shield
75%−epiboly
1−4−somites
14−19−somites
20−25−somites
Prim−5
Prim−15
Prim−25
Long−pec
Protruding−mouth
Day−4
Day−5
Transcript
ENSDART00000105232
ENSDART00000149823
ENSDART00000149899

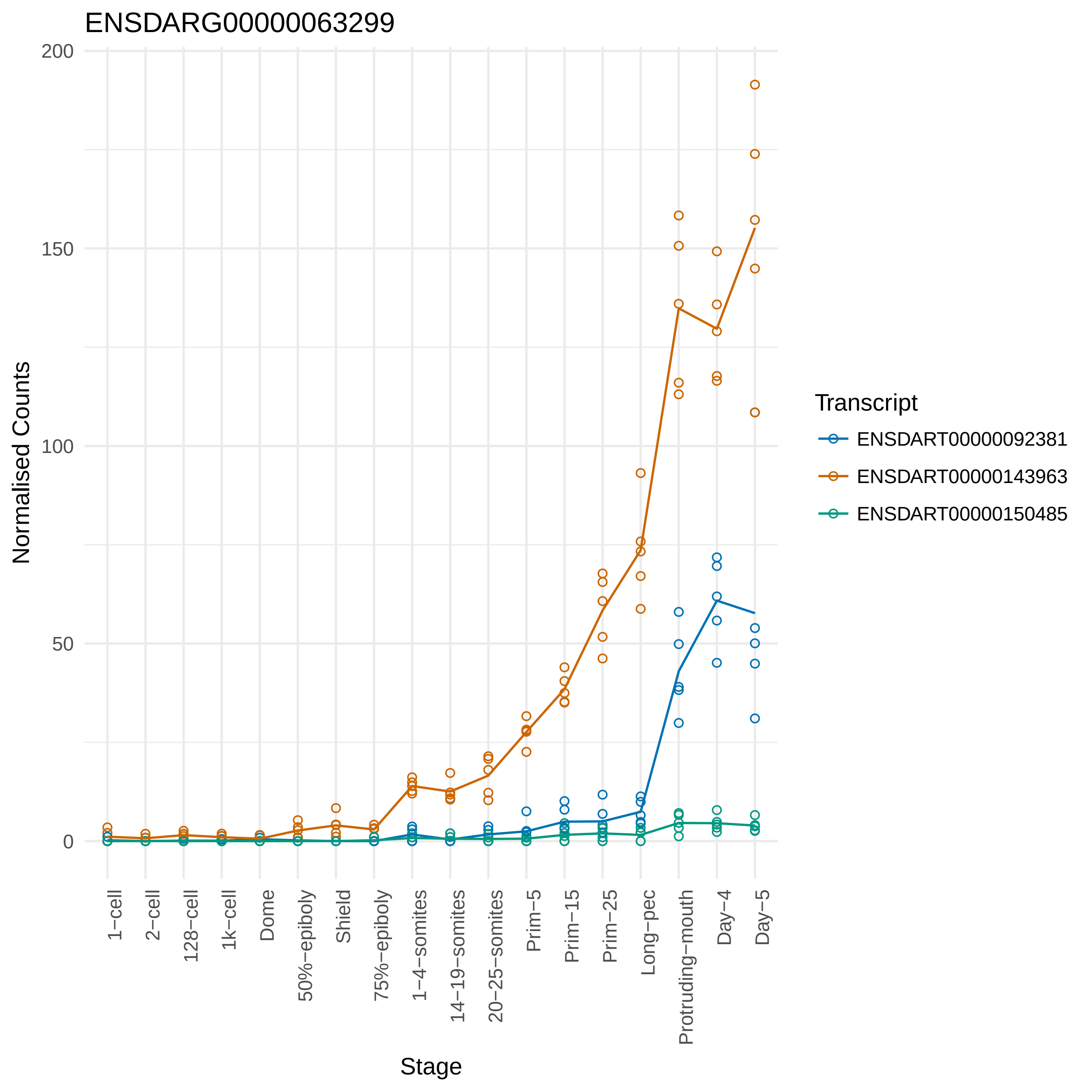

ENSDARG00000063299
Normalised Counts
200
150
100
50
0
1−cell
2−cell
128−cell
1k−cell
Dome
50%−epiboly
Shield
75%−epiboly
1−4−somites
14−19−somites
20−25−somites
Prim−5
Prim−15
Prim−25
Long−pec
Protruding−mouth
Day−4
Day−5
Stage
Transcript
ENSDART00000092381
ENSDART00000143963
ENSDART00000150485

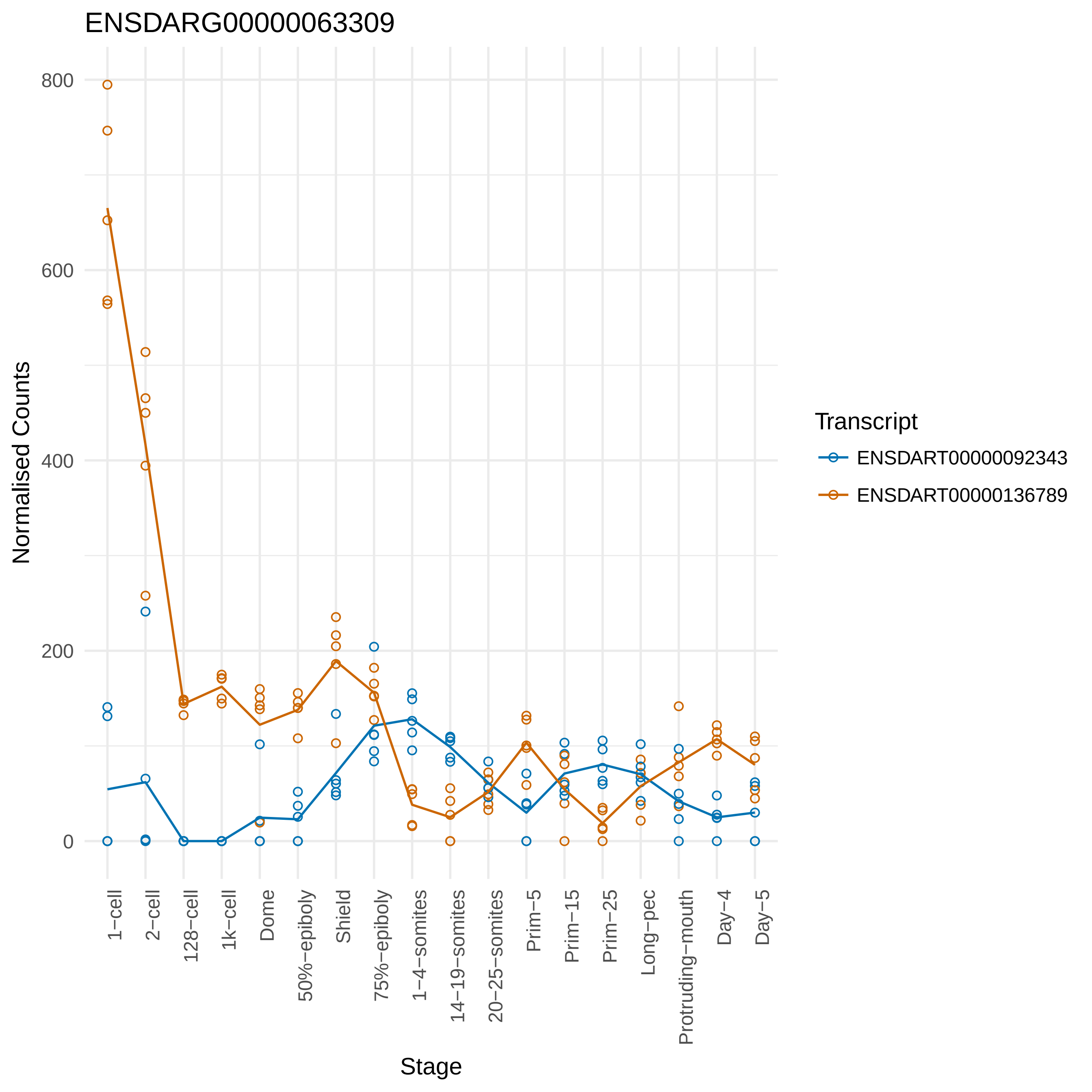
ENSDARG00000063309
Normalised Counts
800
600
400
200
0
1−cell
2−cell
128−cell
1k−cell
Dome
50%−epiboly
Shield
75%−epiboly
1−4−somites
14−19−somites
20−25−somites
Prim−5
Prim−15
Prim−25
Long−pec
Protruding−mouth
Day−4
Day−5
Stage
Transcript
ENSDART00000092343
ENSDART00000136789

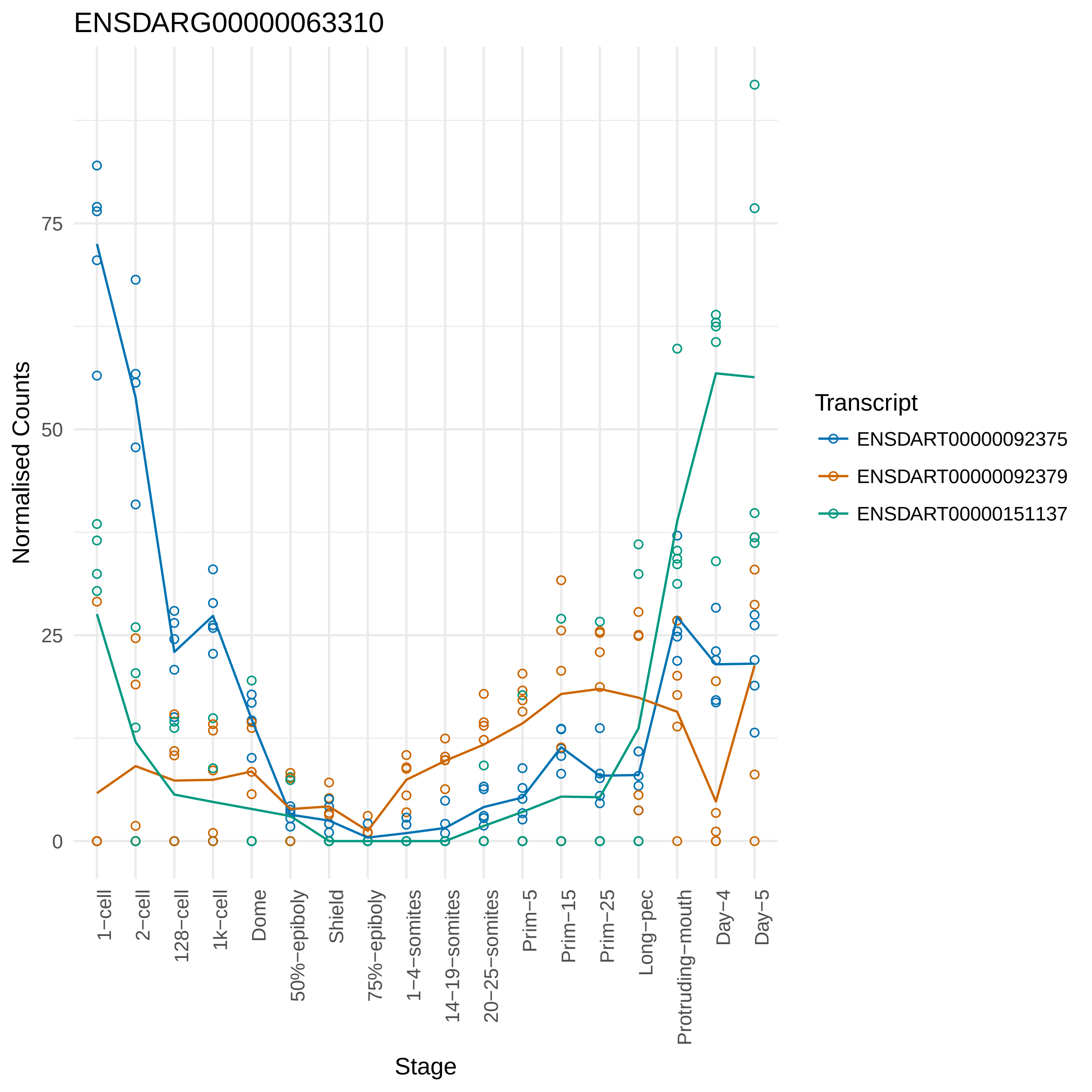

ENSDARG00000063310
Normalised Counts
0
25
50
75
Stage
1−cell
2−cell
128−cell
1k−cell
Dome
50%−epiboly
Shield
75%−epiboly
1−4−somites
14−19−somites
20−25−somites
Prim−5
Prim−15
Prim−25
Long−pec
Protruding−mouth
Day−4
Day−5
Transcript
ENSDART00000092375
ENSDART00000092379
ENSDART00000151137

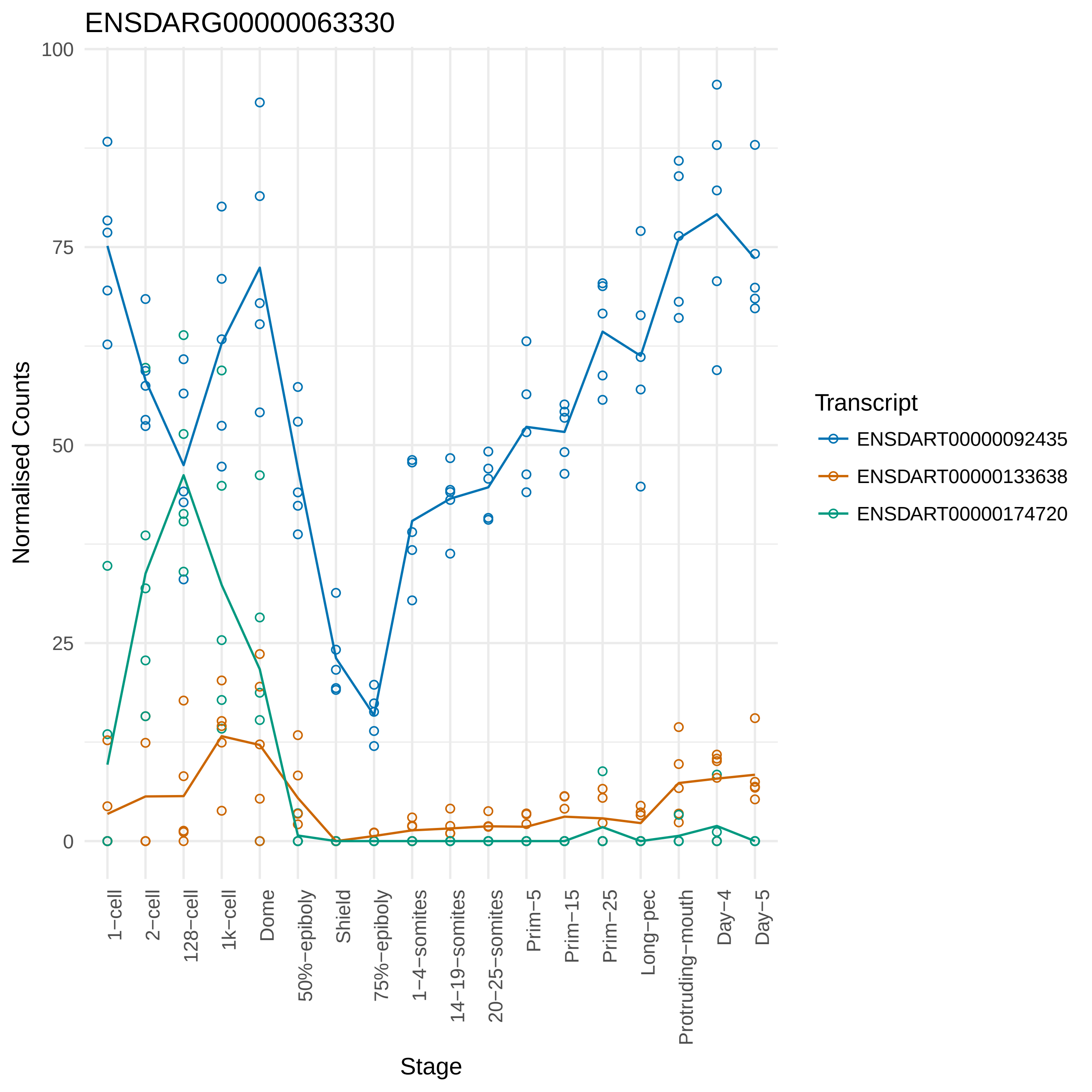
ENSDARG00000063330
Normalised Counts
100
75
50
25
0
1−cell
2−cell
128−cell
1k−cell
Dome
50%−epiboly
Shield
75%−epiboly
1−4−somites
14−19−somites
20−25−somites
Prim−5
Prim−15
Prim−25
Long−pec
Protruding−mouth
Day−4
Day−5
Stage
Transcript
ENSDART00000092435
ENSDART00000133638
ENSDART00000174720

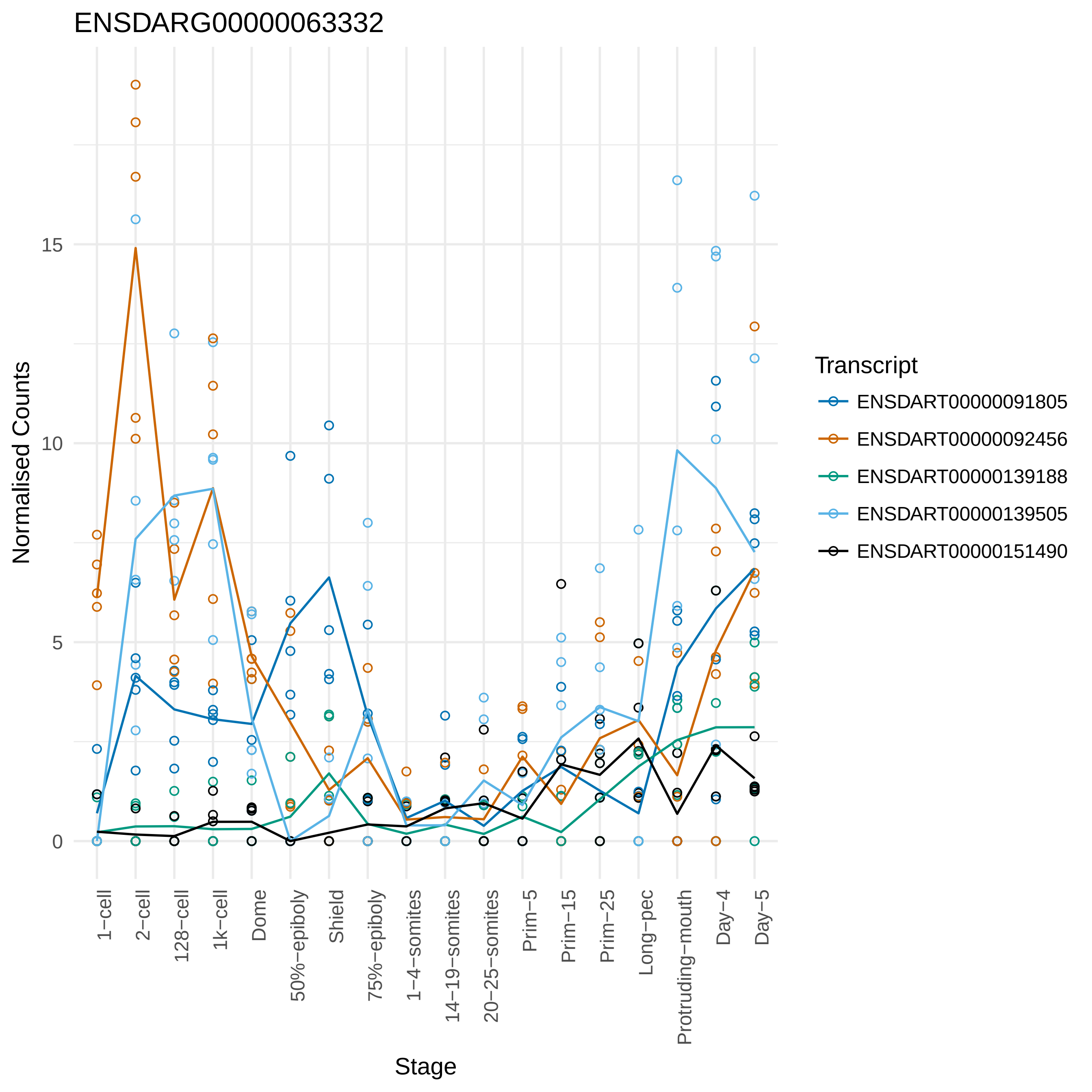
ENSDARG00000063332
Normalised Counts
Stage
15
10
5
0
1−cell
2−cell
128−cell
1k−cell
Dome
50%−epiboly
Shield
75%−epiboly
1−4−somites
14−19−somites
20−25−somites
Prim−5
Prim−15
Prim−25
Long−pec
Protruding−mouth
Day−4
Day−5
Transcript
ENSDART00000091805
ENSDART00000092456
ENSDART00000139188
ENSDART00000139505
ENSDART00000151490

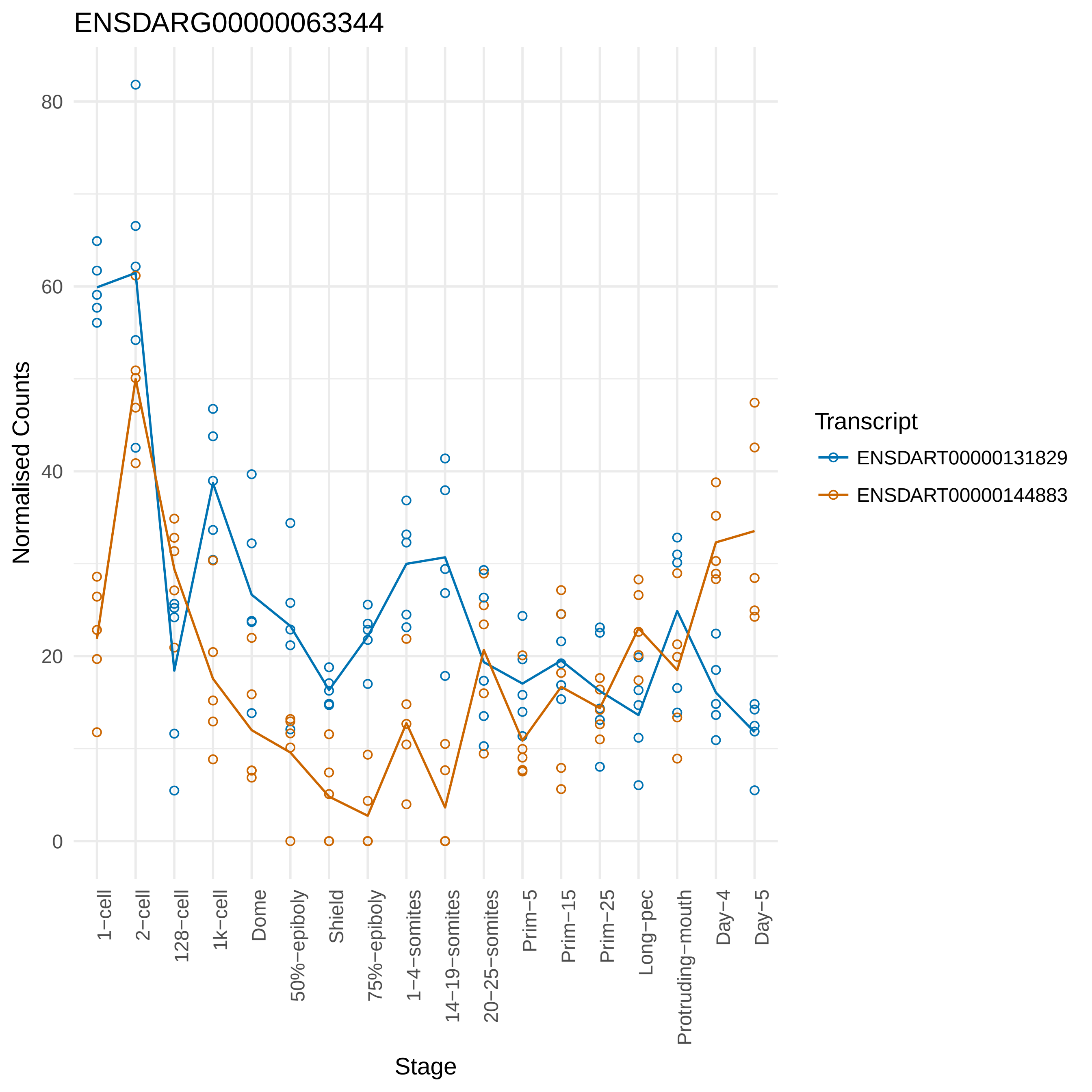

ENSDARG00000063344
Normalised Counts
0
20
40
60
80
Stage
1−cell
2−cell
128−cell
1k−cell
Dome
50%−epiboly
Shield
75%−epiboly
1−4−somites
14−19−somites
20−25−somites
Prim−5
Prim−15
Prim−25
Long−pec
Protruding−mouth
Day−4
Day−5
Transcript
ENSDART00000131829
ENSDART00000144883

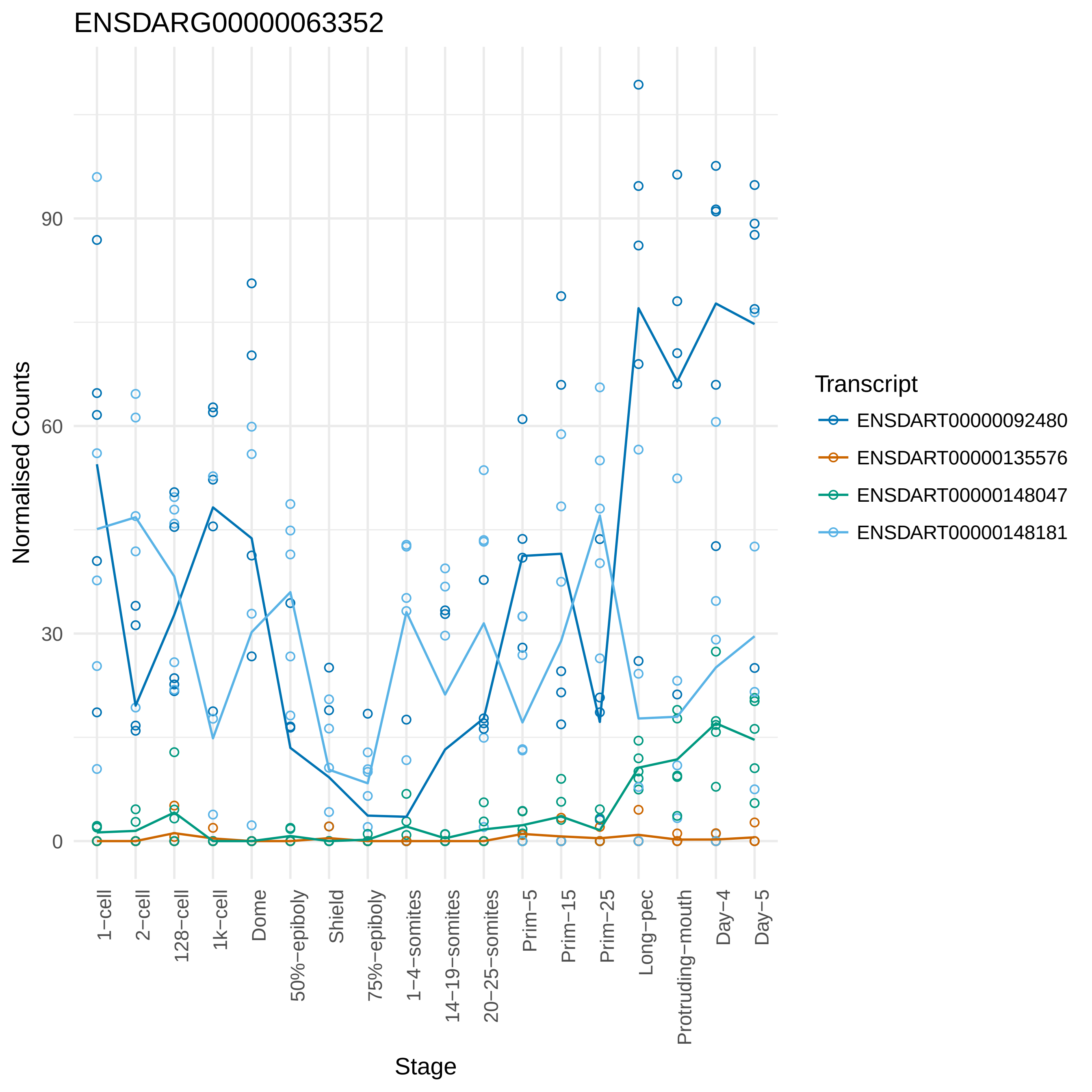
ENSDARG00000063352
Normalised Counts
0
30
60
90
1−cell
2−cell
128−cell
1k−cell
Dome
50%−epiboly
Shield
75%−epiboly
1−4−somites
14−19−somites
20−25−somites
Prim−5
Prim−15
Prim−25
Long−pec
Protruding−mouth
Day−4
Day−5
Stage
Transcript
ENSDART00000092480
ENSDART00000135576
ENSDART00000148047
ENSDART00000148181

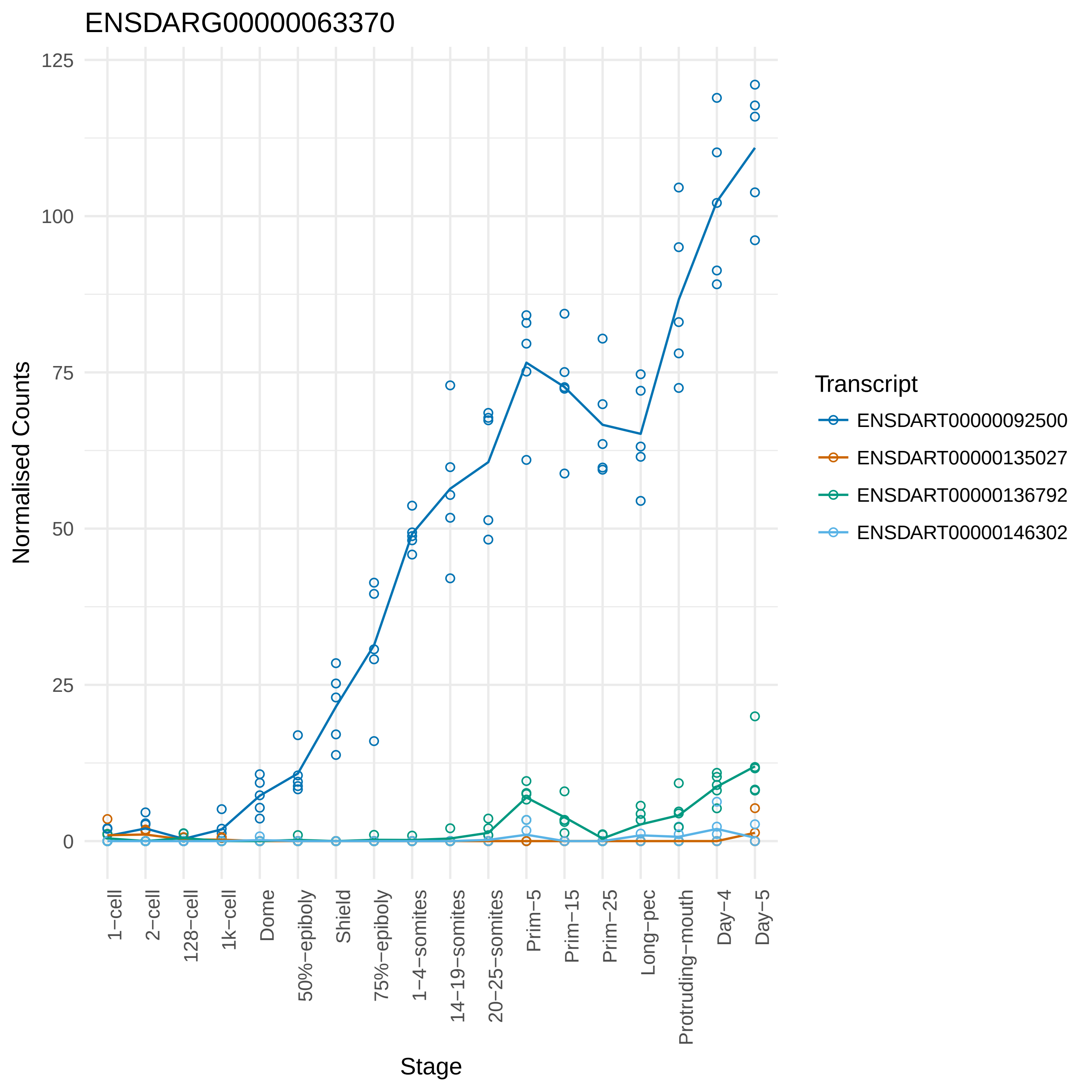
ENSDARG00000063370
Normalised Counts
125
100
75
50
25
0
Stage
1−cell
2−cell
128−cell
1k−cell
Dome
50%−epiboly
Shield
75%−epiboly
1−4−somites
14−19−somites
20−25−somites
Prim−5
Prim−15
Prim−25
Long−pec
Protruding−mouth
Day−4
Day−5
Transcript
ENSDART00000092500
ENSDART00000135027
ENSDART00000136792
ENSDART00000146302

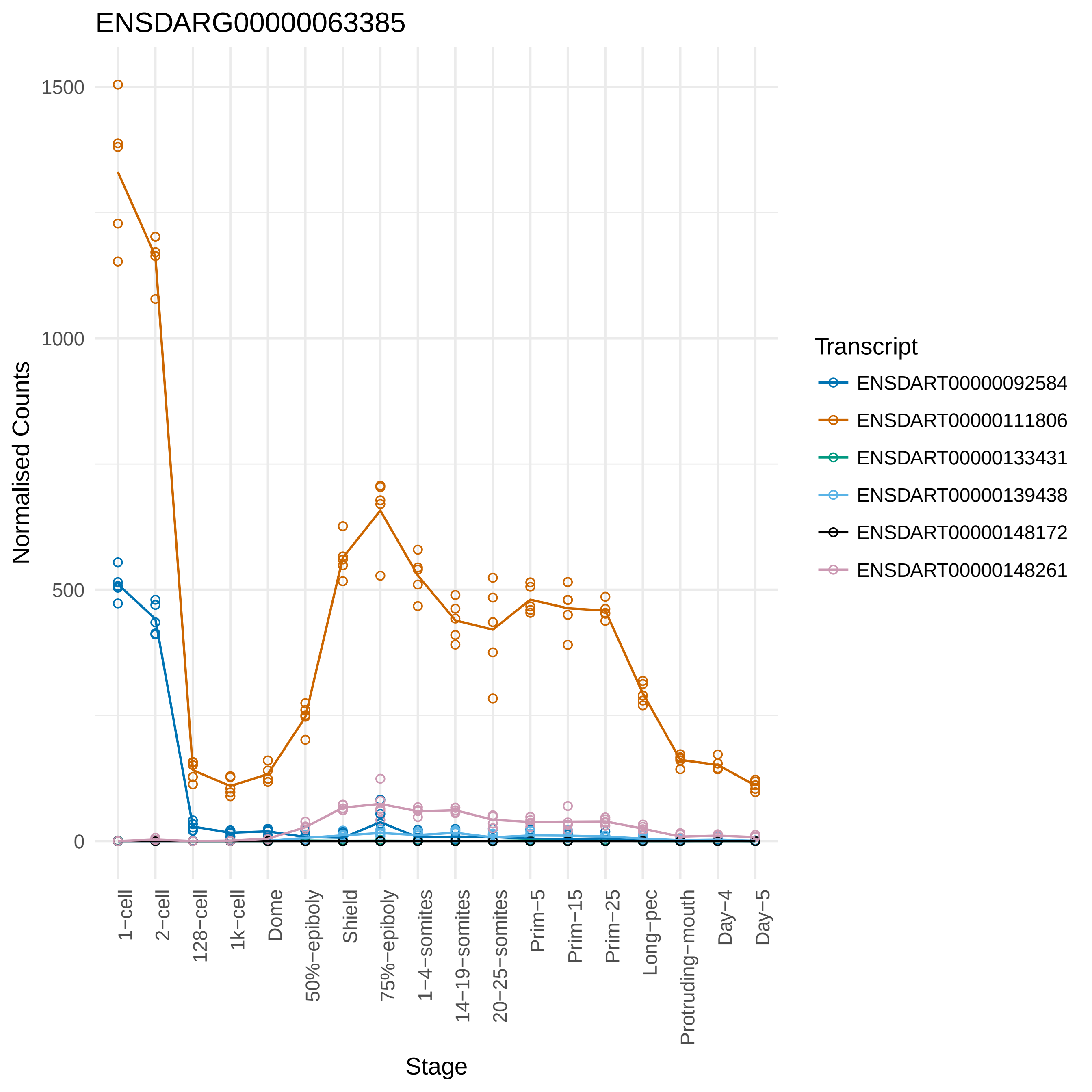
ENSDARG00000063385
Normalised Counts
1500
1000
500
0
1−cell
2−cell
128−cell
1k−cell
Dome
50%−epiboly
Shield
75%−epiboly
1−4−somites
14−19−somites
20−25−somites
Prim−5
Prim−15
Prim−25
Long−pec
Protruding−mouth
Day−4
Day−5
Stage
Transcript
ENSDART00000092584
ENSDART00000111806
ENSDART00000133431
ENSDART00000139438
ENSDART00000148172
ENSDART00000148261

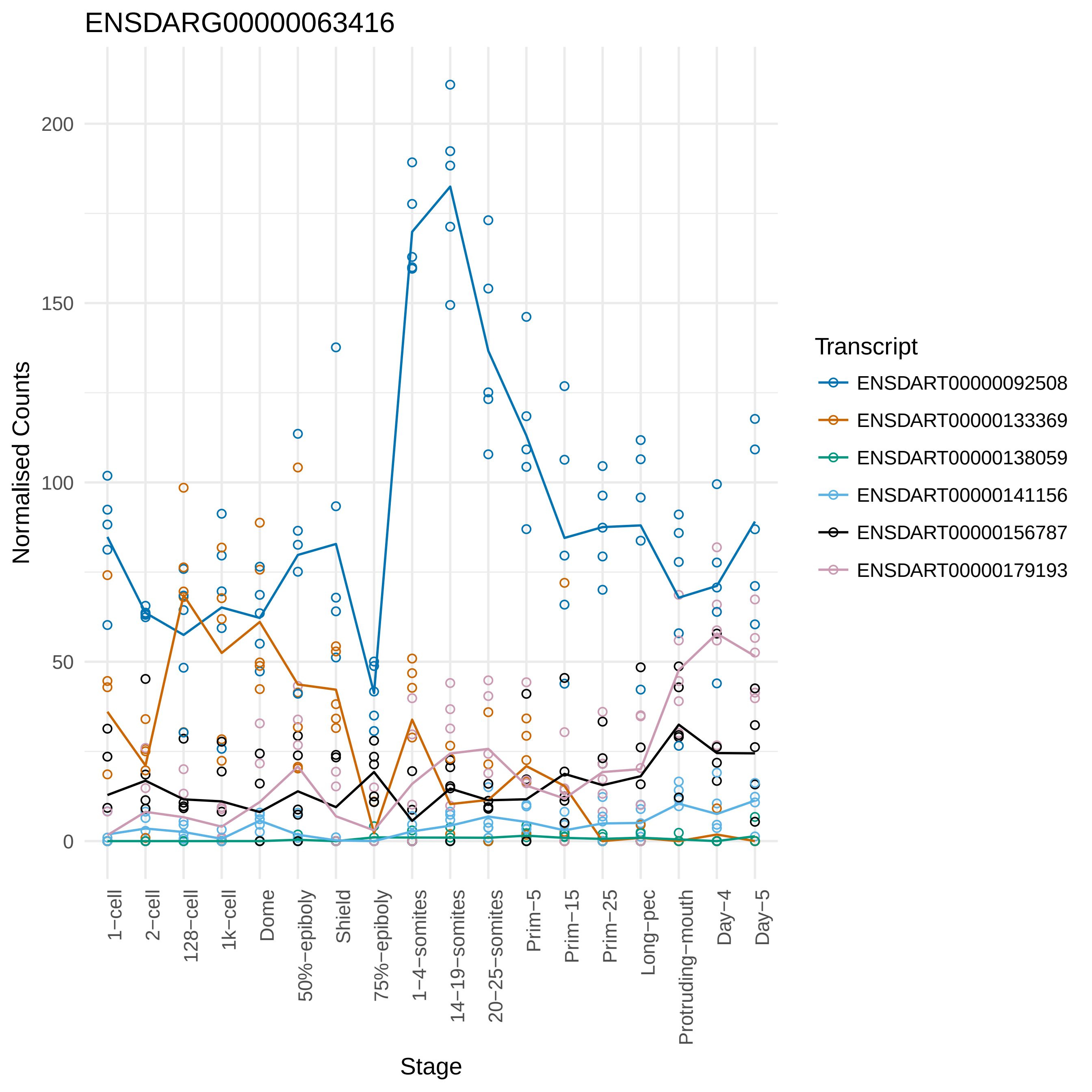

ENSDARG00000063416
Normalised Counts
0
50
100
150
200
1−cell
2−cell
128−cell
1k−cell
Dome
50%−epiboly
Shield
75%−epiboly
1−4−somites
14−19−somites
20−25−somites
Prim−5
Prim−15
Prim−25
Long−pec
Protruding−mouth
Day−4
Day−5
Stage
Transcript
ENSDART00000092508
ENSDART00000133369
ENSDART00000138059
ENSDART00000141156
ENSDART00000156787
ENSDART00000179193

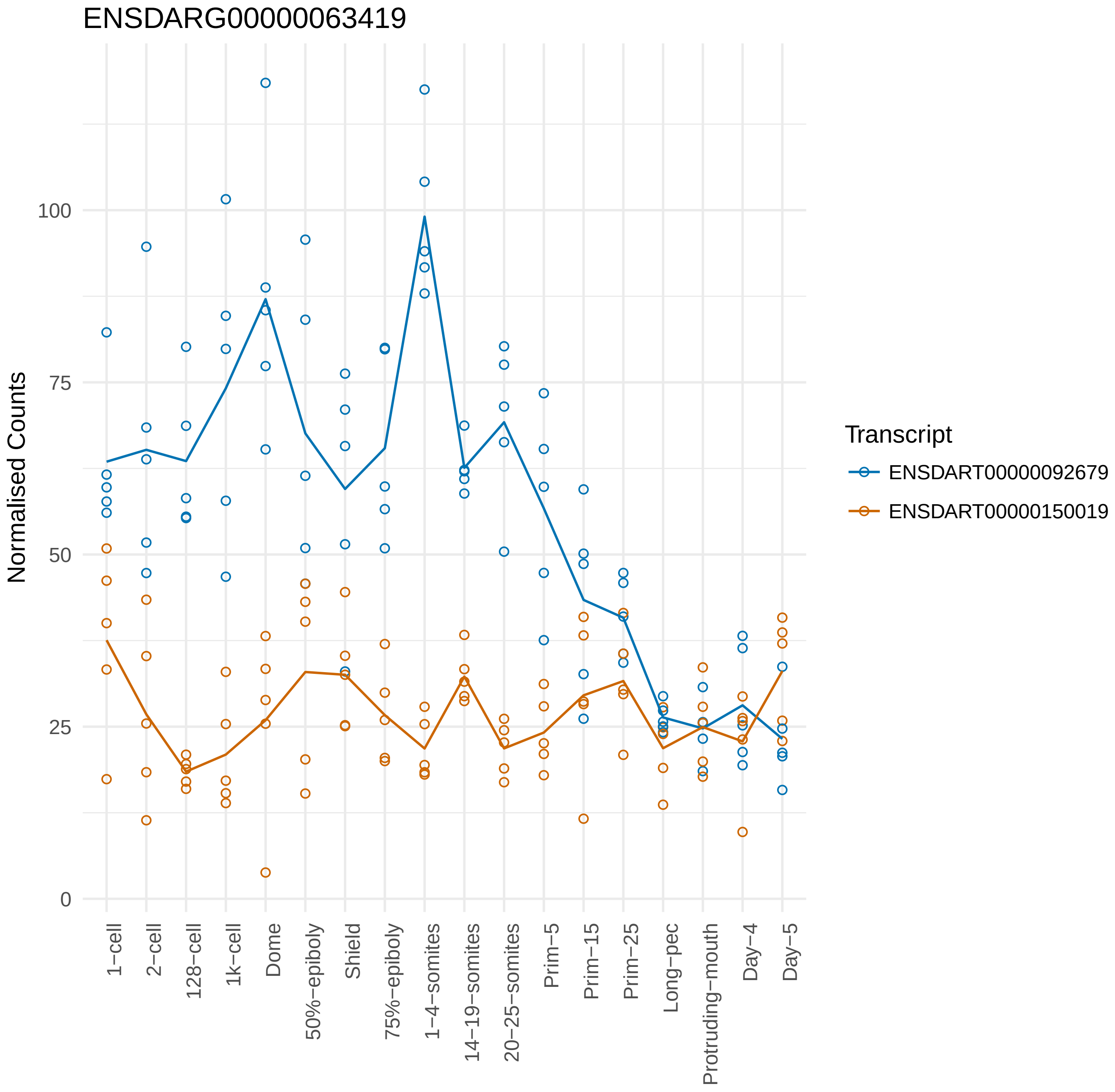

ENSDARG00000063419
Normalised Counts
0
25
50
75
100
1−cell
2−cell
128−cell
1k−cell
Dome
50%−epiboly
Shield
75%−epiboly
1−4−somites
14−19−somites
20−25−somites
Prim−5
Prim−15
Prim−25
Long−pec
Protruding−mouth
Day−4
Day−5
Stage
Transcript
ENSDART00000092679
ENSDART00000150019

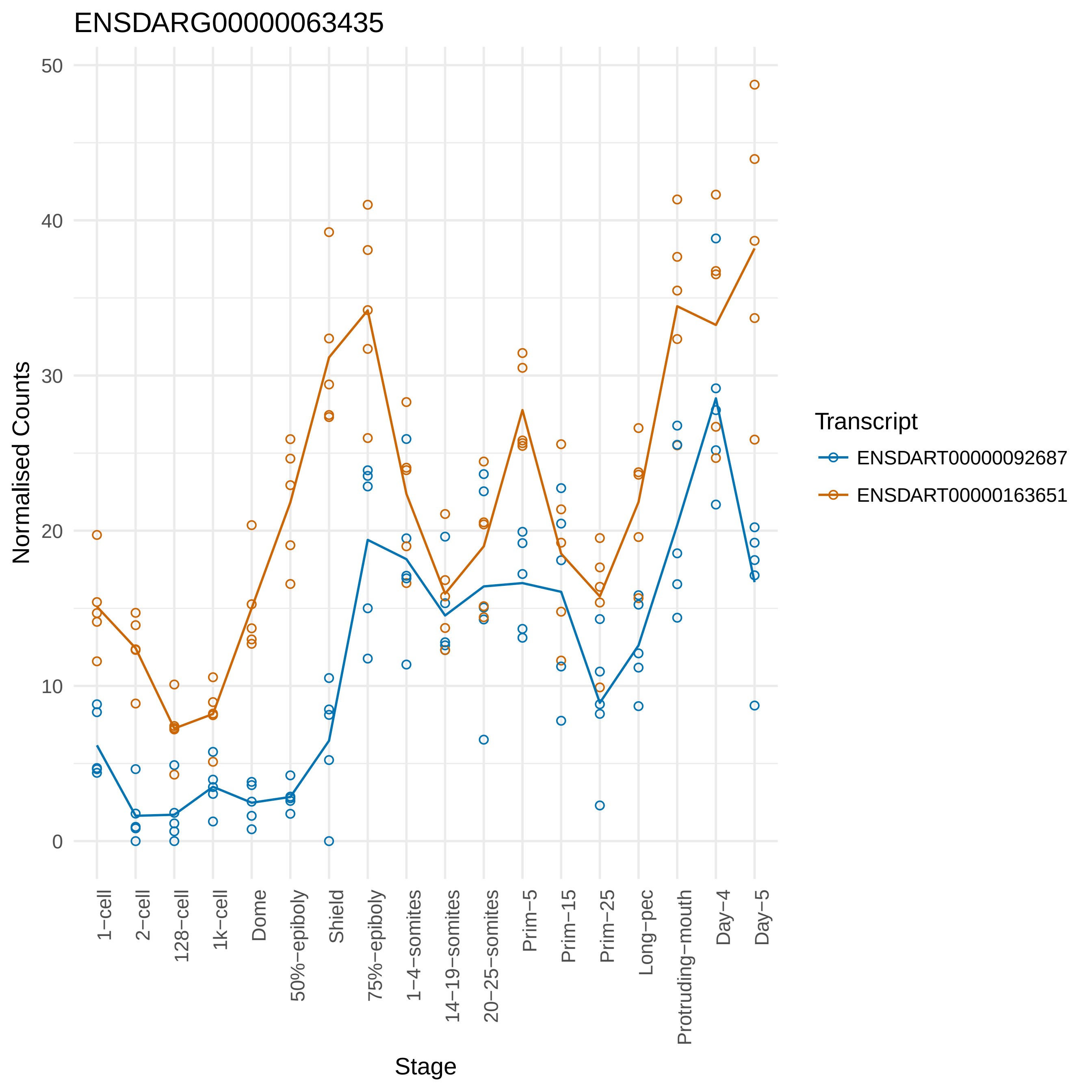

ENSDARG00000063435
Normalised Counts
0
10
20
30
40
50
Stage
1−cell
2−cell
128−cell
1k−cell
Dome
50%−epiboly
Shield
75%−epiboly
1−4−somites
14−19−somites
20−25−somites
Prim−5
Prim−15
Prim−25
Long−pec
Protruding−mouth
Day−4
Day−5
Transcript
ENSDART00000092687
ENSDART00000163651

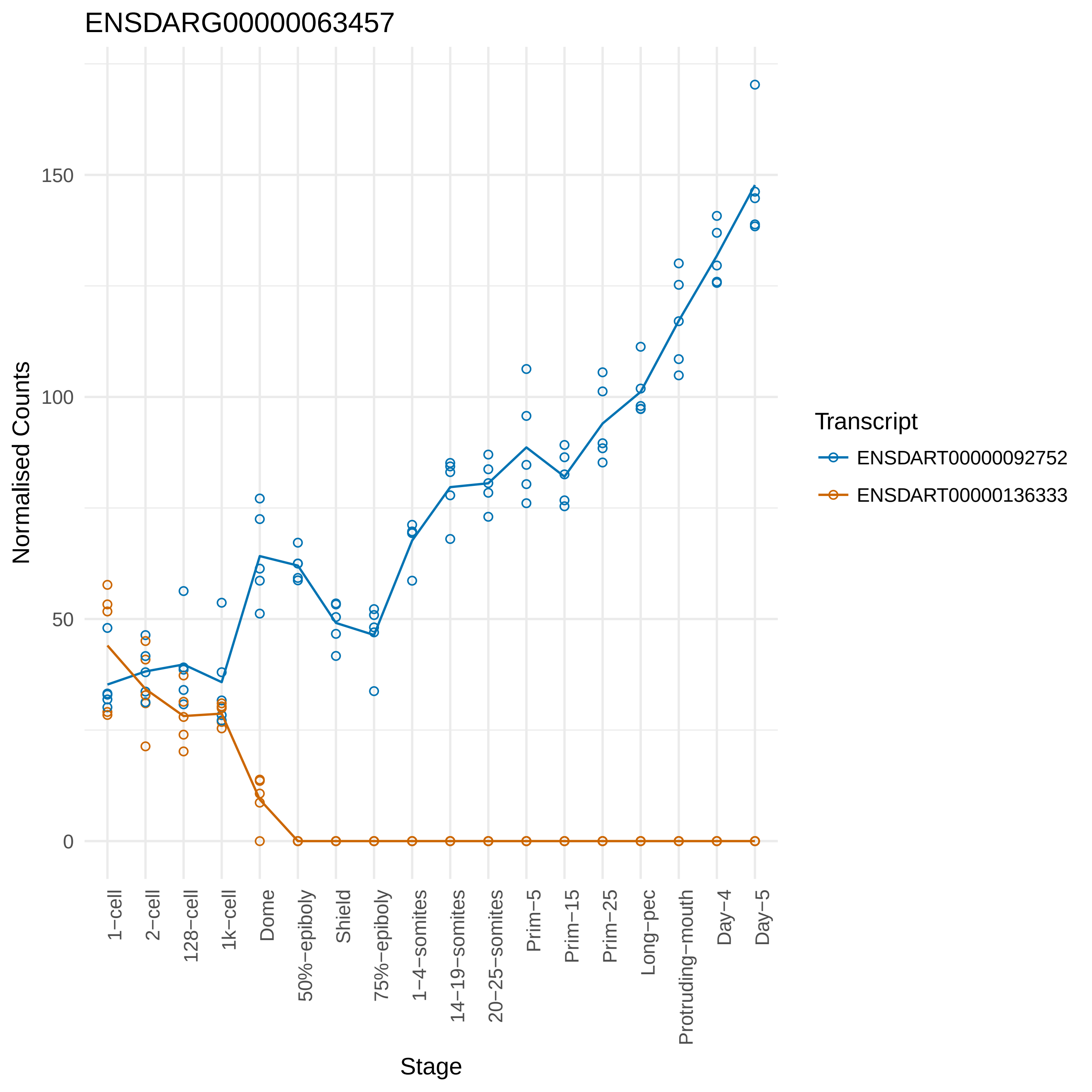

ENSDARG00000063457
Normalised Counts
150
100
50
0
1−cell
2−cell
128−cell
1k−cell
Dome
50%−epiboly
Shield
75%−epiboly
1−4−somites
14−19−somites
20−25−somites
Prim−5
Prim−15
Prim−25
Long−pec
Protruding−mouth
Day−4
Day−5
Stage
Transcript
ENSDART00000092752
ENSDART00000136333

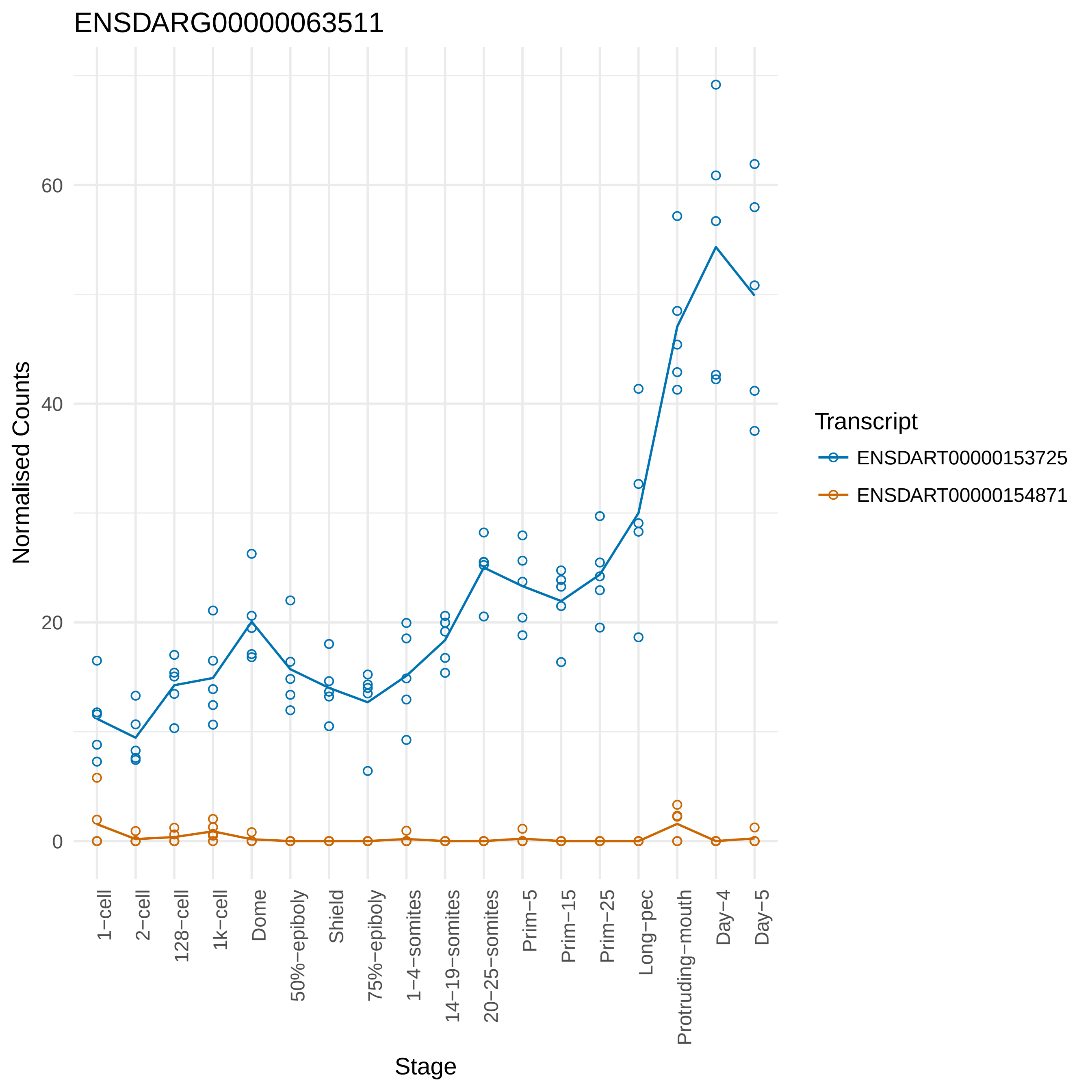

ENSDARG00000063511
Normalised Counts
0
20
40
60
Stage
1−cell
2−cell
128−cell
1k−cell
Dome
50%−epiboly
Shield
75%−epiboly
1−4−somites
14−19−somites
20−25−somites
Prim−5
Prim−15
Prim−25
Long−pec
Protruding−mouth
Day−4
Day−5
Transcript
ENSDART00000153725
ENSDART00000154871

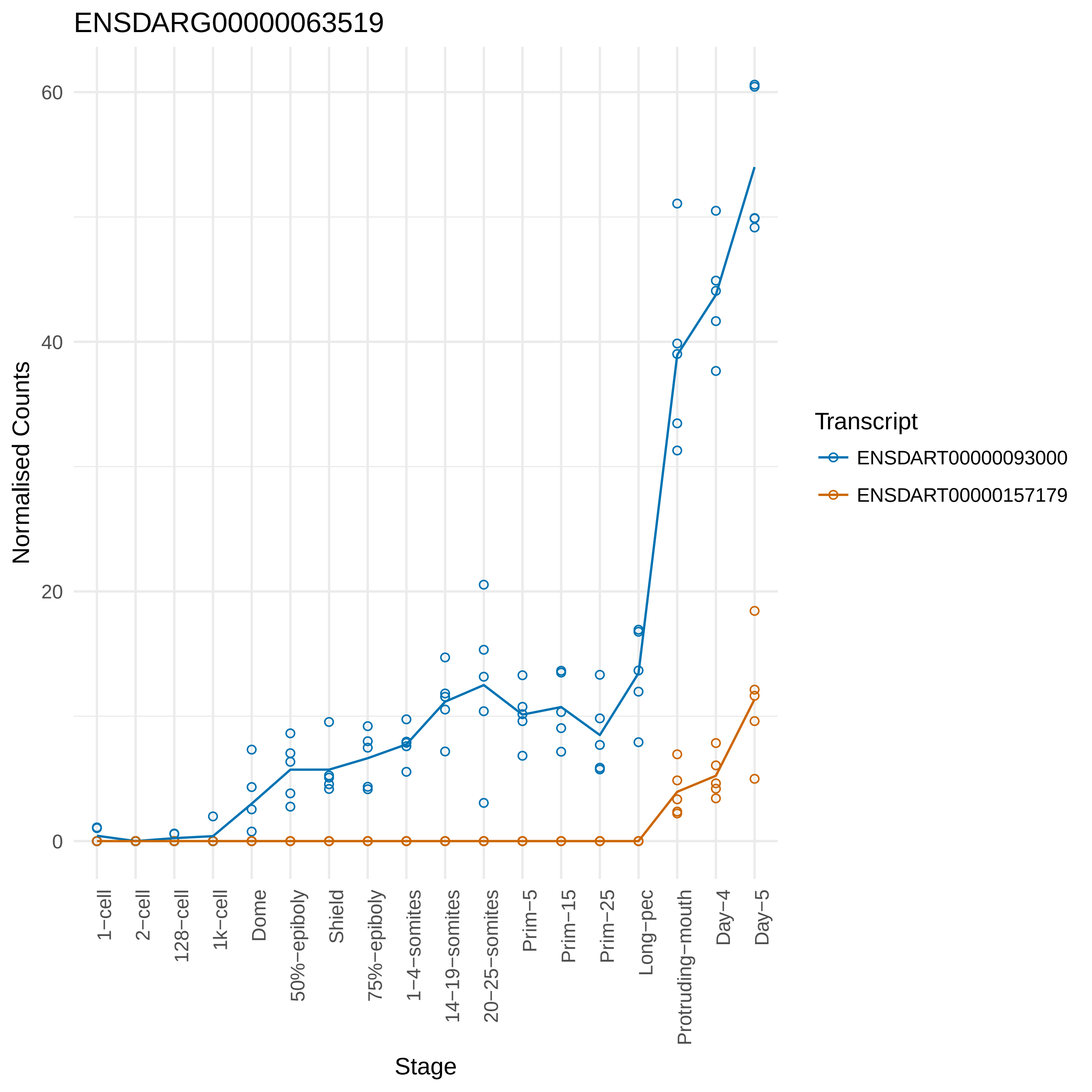

ENSDARG00000063519
Normalised Counts
60
40
20
0
1−cell
2−cell
128−cell
1k−cell
Dome
50%−epiboly
Shield
75%−epiboly
1−4−somites
14−19−somites
20−25−somites
Prim−5
Prim−15
Prim−25
Long−pec
Protruding−mouth
Day−4
Day−5
Stage
Transcript
ENSDART00000093000
ENSDART00000157179

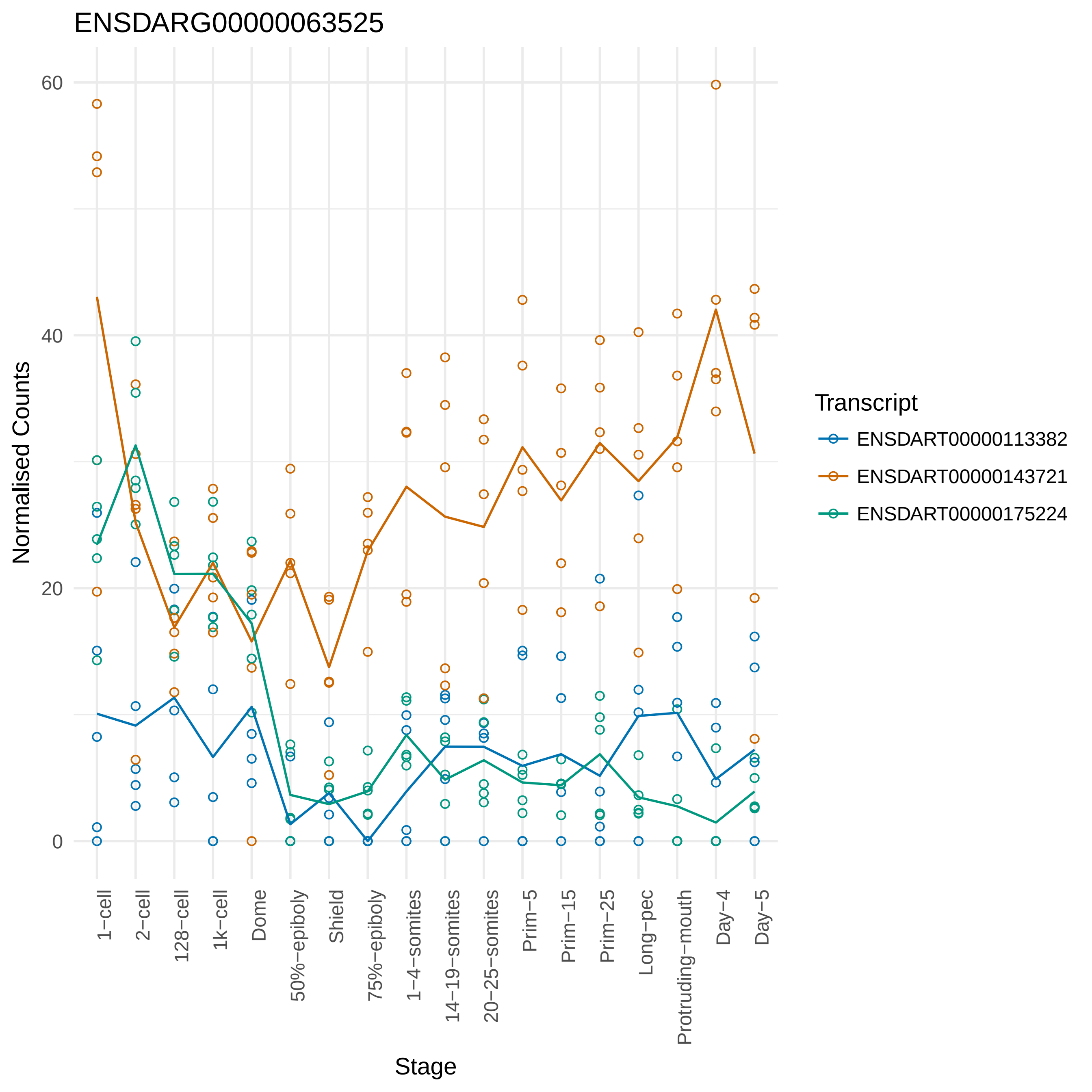

ENSDARG00000063525
Normalised Counts
0
20
40
60
Stage
1−cell
2−cell
128−cell
1k−cell
Dome
50%−epiboly
Shield
75%−epiboly
1−4−somites
14−19−somites
20−25−somites
Prim−5
Prim−15
Prim−25
Long−pec
Protruding−mouth
Day−4
Day−5
Transcript
ENSDART00000113382
ENSDART00000143721
ENSDART00000175224

ENSDARG00000063527

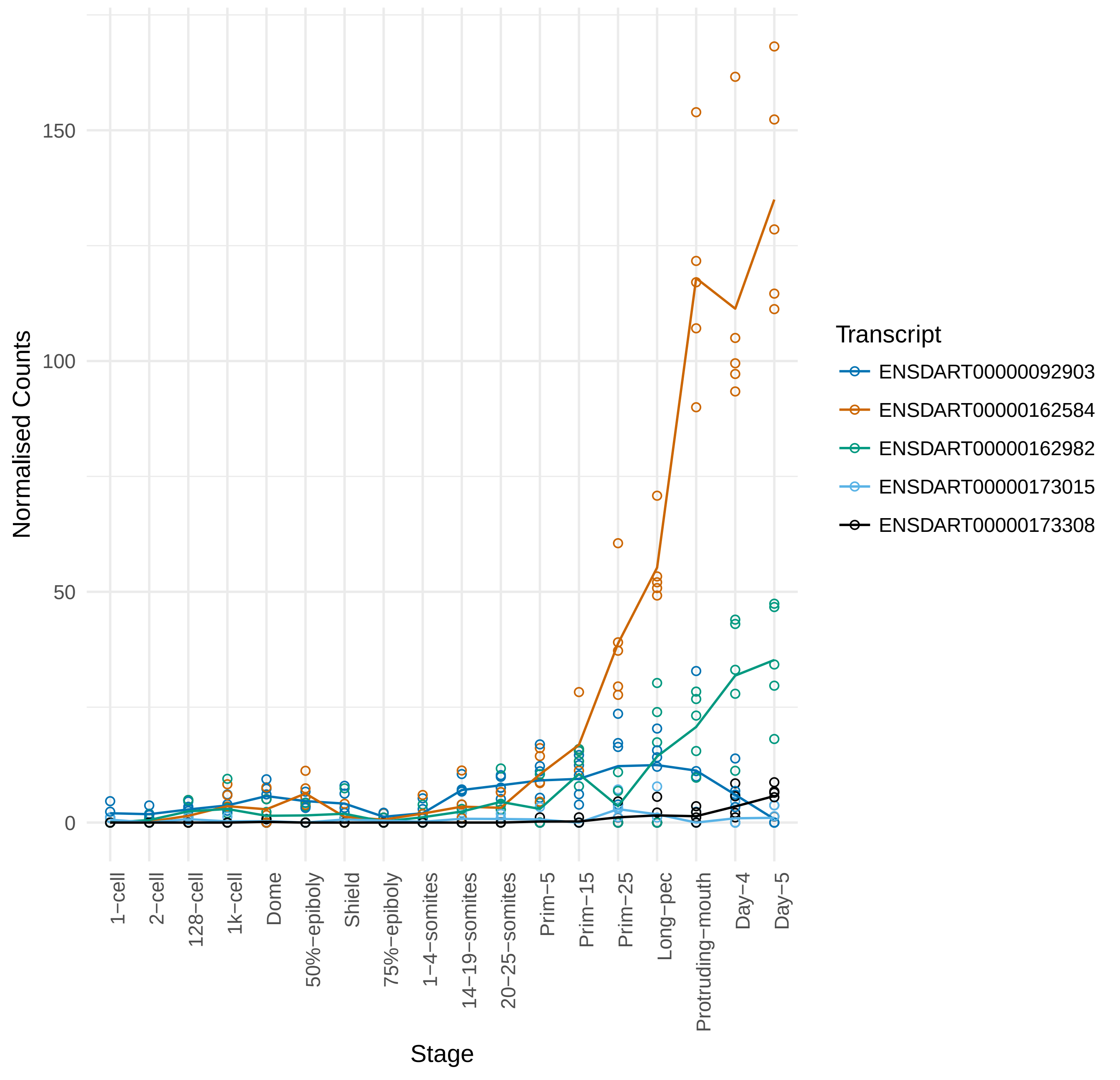

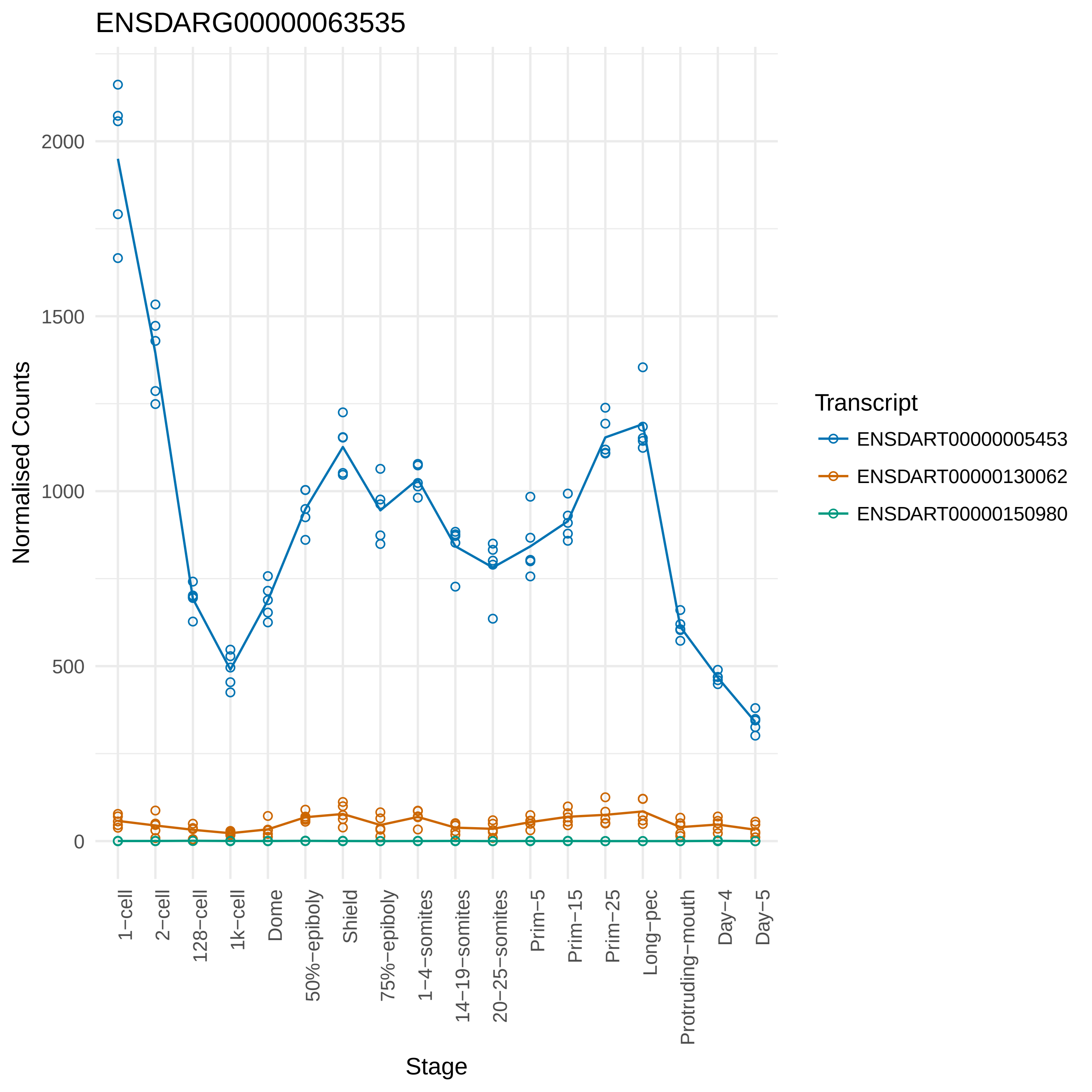

ENSDARG00000063535
Normalised Counts
2000
1500
1000
500
0
Stage
1−cell
2−cell
128−cell
1k−cell
Dome
50%−epiboly
Shield
75%−epiboly
1−4−somites
14−19−somites
20−25−somites
Prim−5
Prim−15
Prim−25
Long−pec
Protruding−mouth
Day−4
Day−5
Transcript
ENSDART00000005453
ENSDART00000130062
ENSDART00000150980

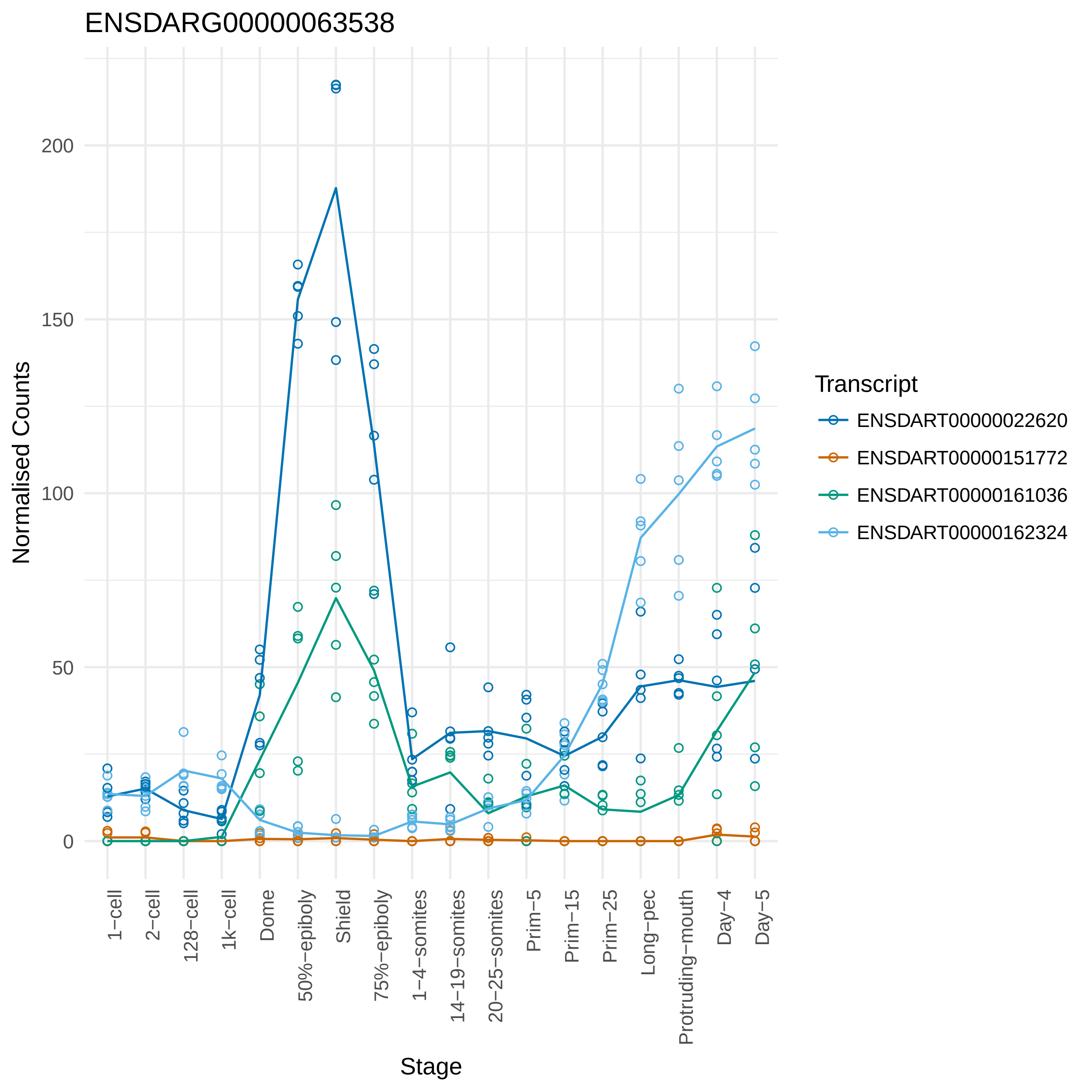

ENSDARG00000063538
Normalised Counts
0
50
100
150
200
1−cell
2−cell
128−cell
1k−cell
Dome
50%−epiboly
Shield
75%−epiboly
1−4−somites
14−19−somites
20−25−somites
Prim−5
Prim−15
Prim−25
Long−pec
Protruding−mouth
Day−4
Day−5
Stage
Transcript
ENSDART00000022620
ENSDART00000151772
ENSDART00000161036
ENSDART00000162324

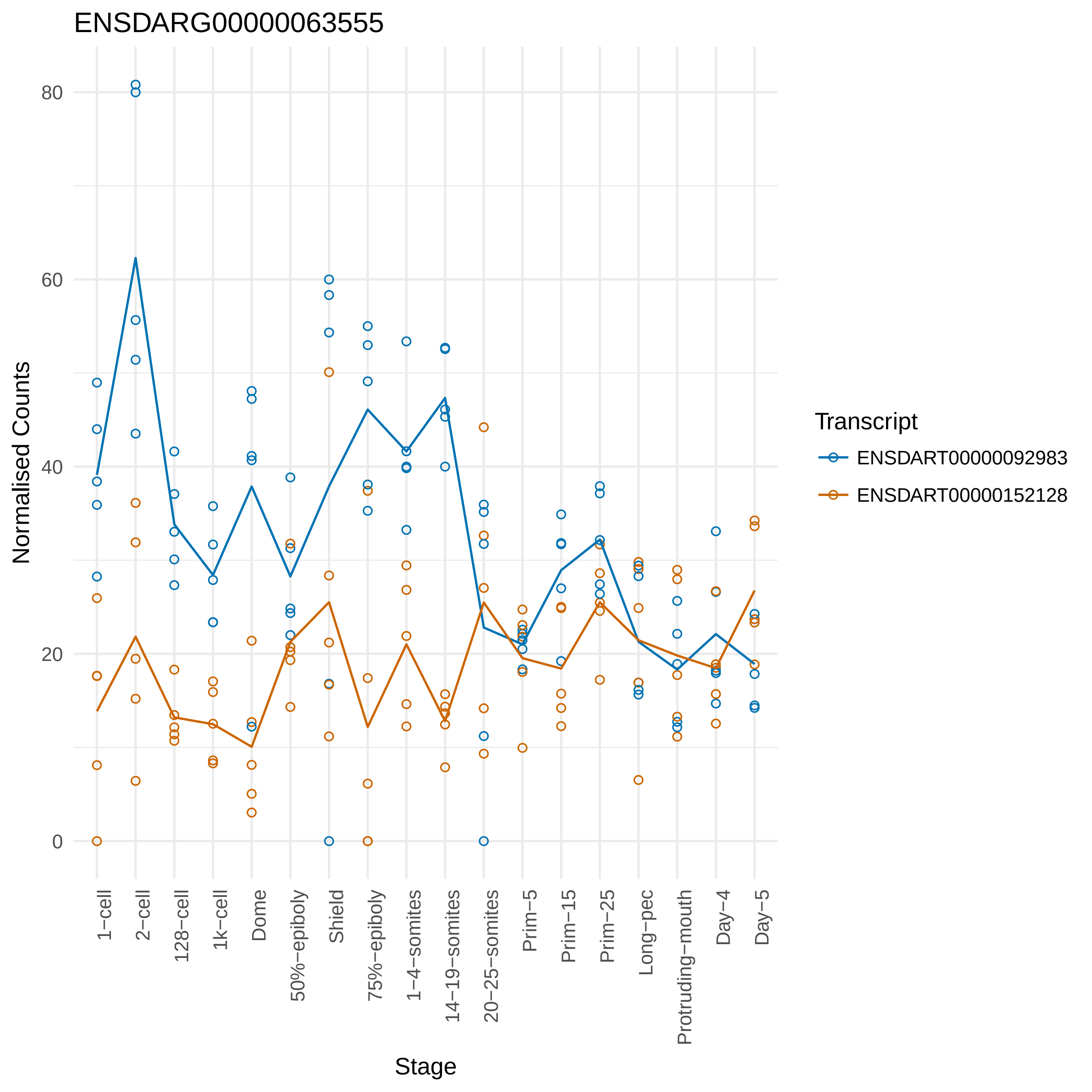
ENSDARG00000063555
Normalised Counts
80
60
40
20
0
1−cell
2−cell
128−cell
1k−cell
Dome
50%−epiboly
Shield
75%−epiboly
1−4−somites
14−19−somites
20−25−somites
Prim−5
Prim−15
Prim−25
Long−pec
Protruding−mouth
Day−4
Day−5
Stage
Transcript
ENSDART00000092983
ENSDART00000152128

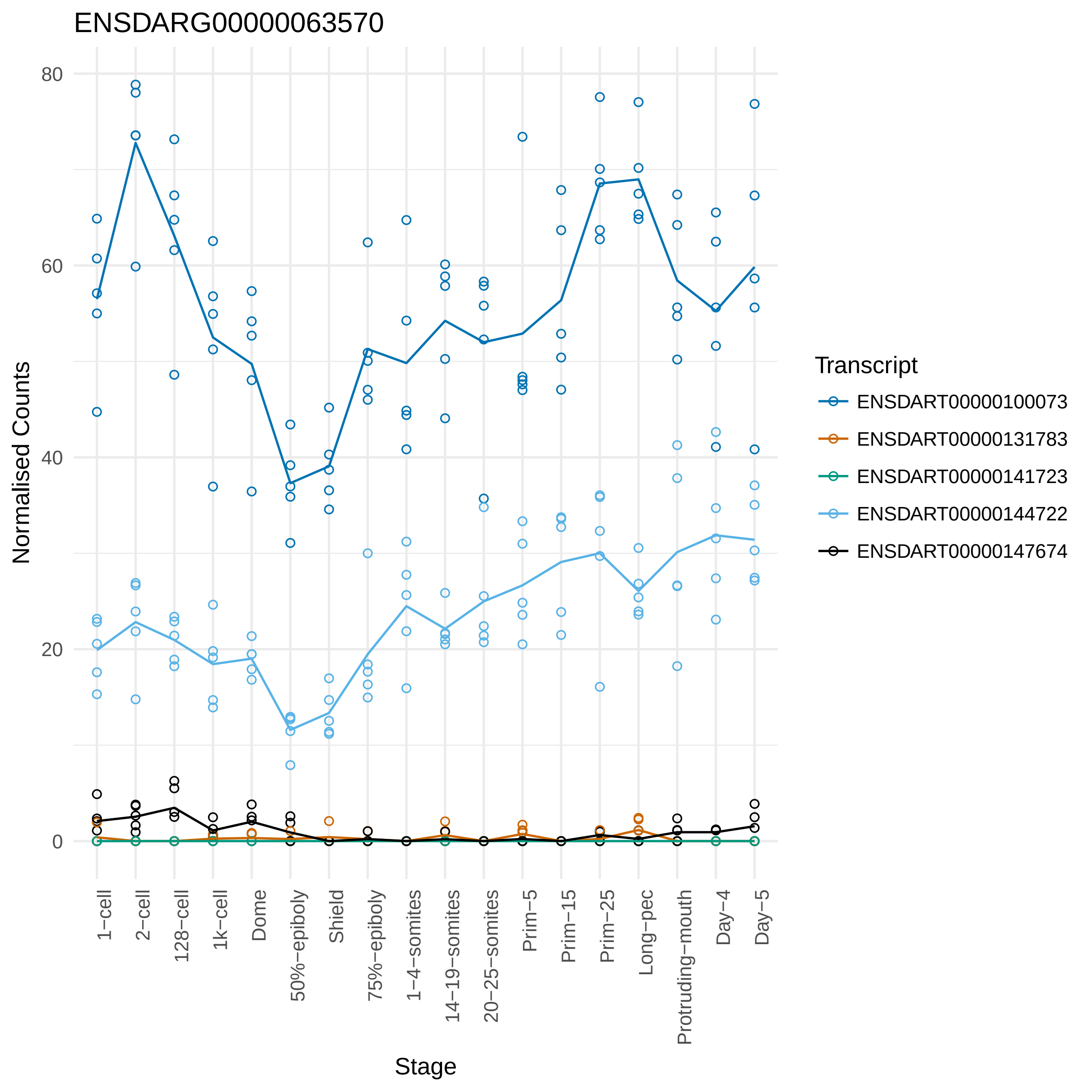
ENSDARG00000063570
Normalised Counts
0
20
40
60
80
Stage
1−cell
2−cell
128−cell
1k−cell
Dome
50%−epiboly
Shield
75%−epiboly
1−4−somites
14−19−somites
20−25−somites
Prim−5
Prim−15
Prim−25
Long−pec
Protruding−mouth
Day−4
Day−5
Transcript
ENSDART00000100073
ENSDART00000131783
ENSDART00000141723
ENSDART00000144722
ENSDART00000147674

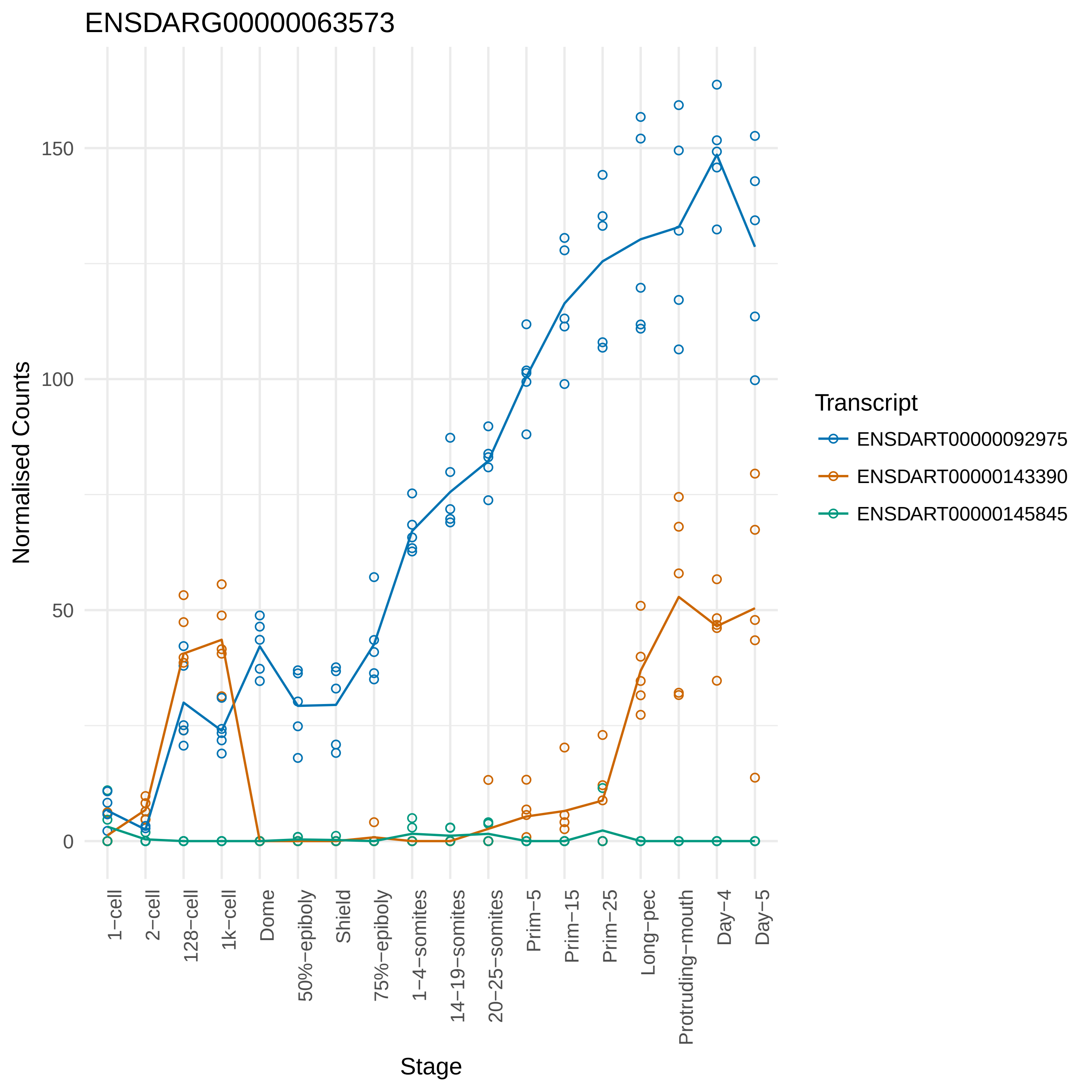

ENSDARG00000063573
Normalised Counts
150
100
50
0
1−cell
2−cell
128−cell
1k−cell
Dome
50%−epiboly
Shield
75%−epiboly
1−4−somites
14−19−somites
20−25−somites
Prim−5
Prim−15
Prim−25
Long−pec
Protruding−mouth
Day−4
Day−5
Stage
Transcript
ENSDART00000092975
ENSDART00000143390
ENSDART00000145845

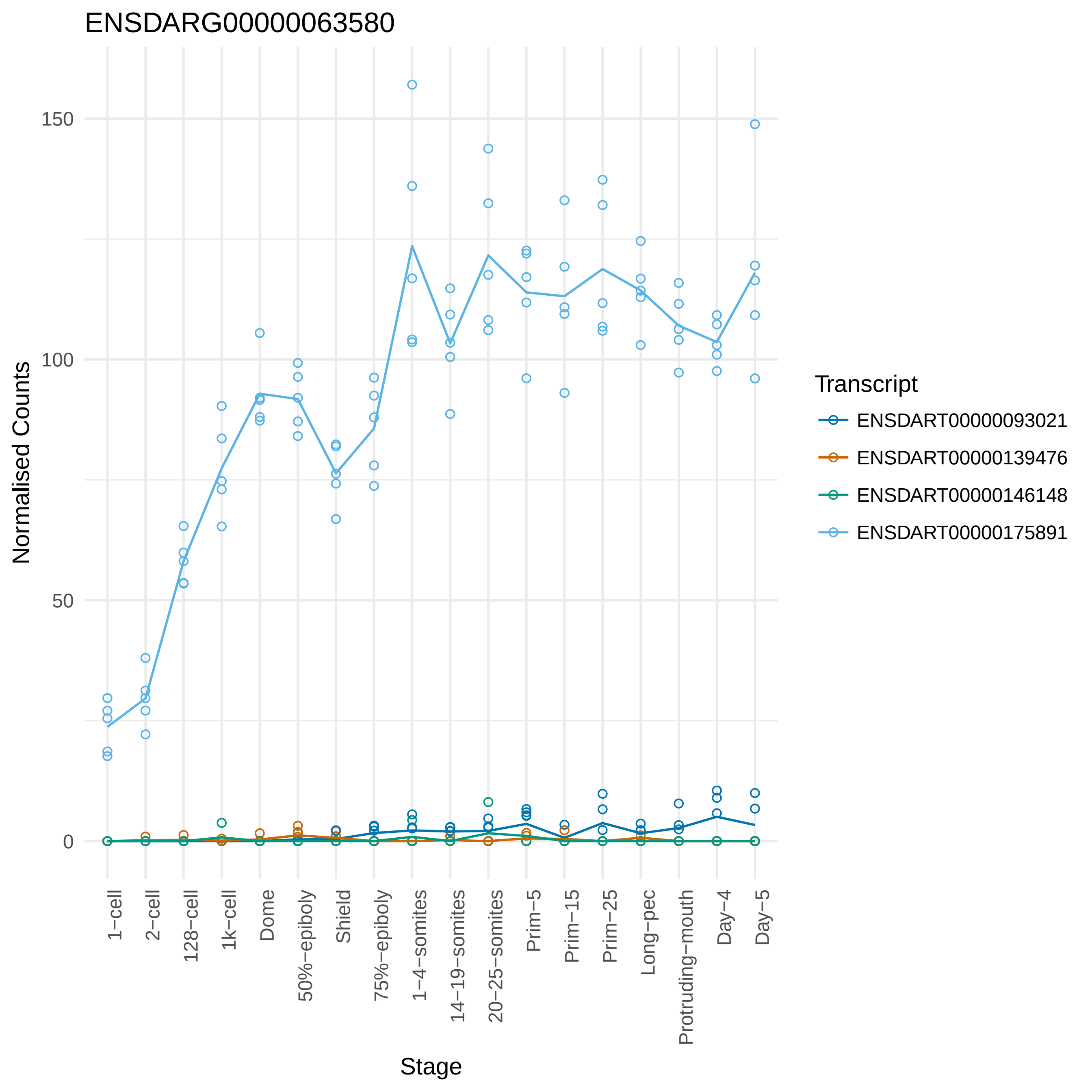
ENSDARG00000063580
Normalised Counts
150
100
50
0
1−cell
2−cell
128−cell
1k−cell
Dome
50%−epiboly
Shield
75%−epiboly
1−4−somites
14−19−somites
20−25−somites
Prim−5
Prim−15
Prim−25
Long−pec
Protruding−mouth
Day−4
Day−5
Stage
Transcript
ENSDART00000093021
ENSDART00000139476
ENSDART00000146148
ENSDART00000175891

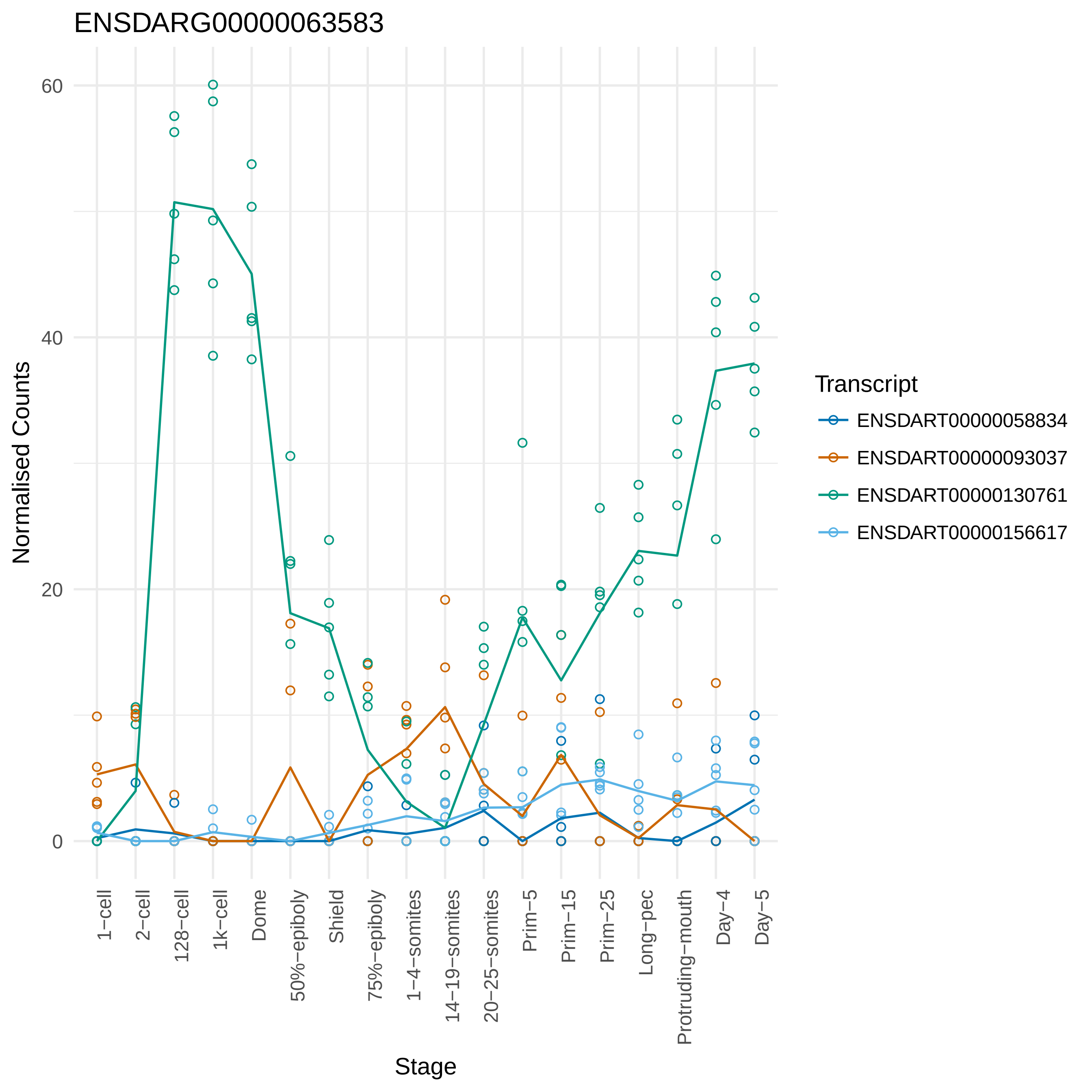
ENSDARG00000063583
Normalised Counts
60
40
20
0
1−cell
2−cell
128−cell
1k−cell
Dome
50%−epiboly
Shield
75%−epiboly
1−4−somites
14−19−somites
20−25−somites
Prim−5
Prim−15
Prim−25
Long−pec
Protruding−mouth
Day−4
Day−5
Stage
Transcript
ENSDART00000058834
ENSDART00000093037
ENSDART00000130761
ENSDART00000156617

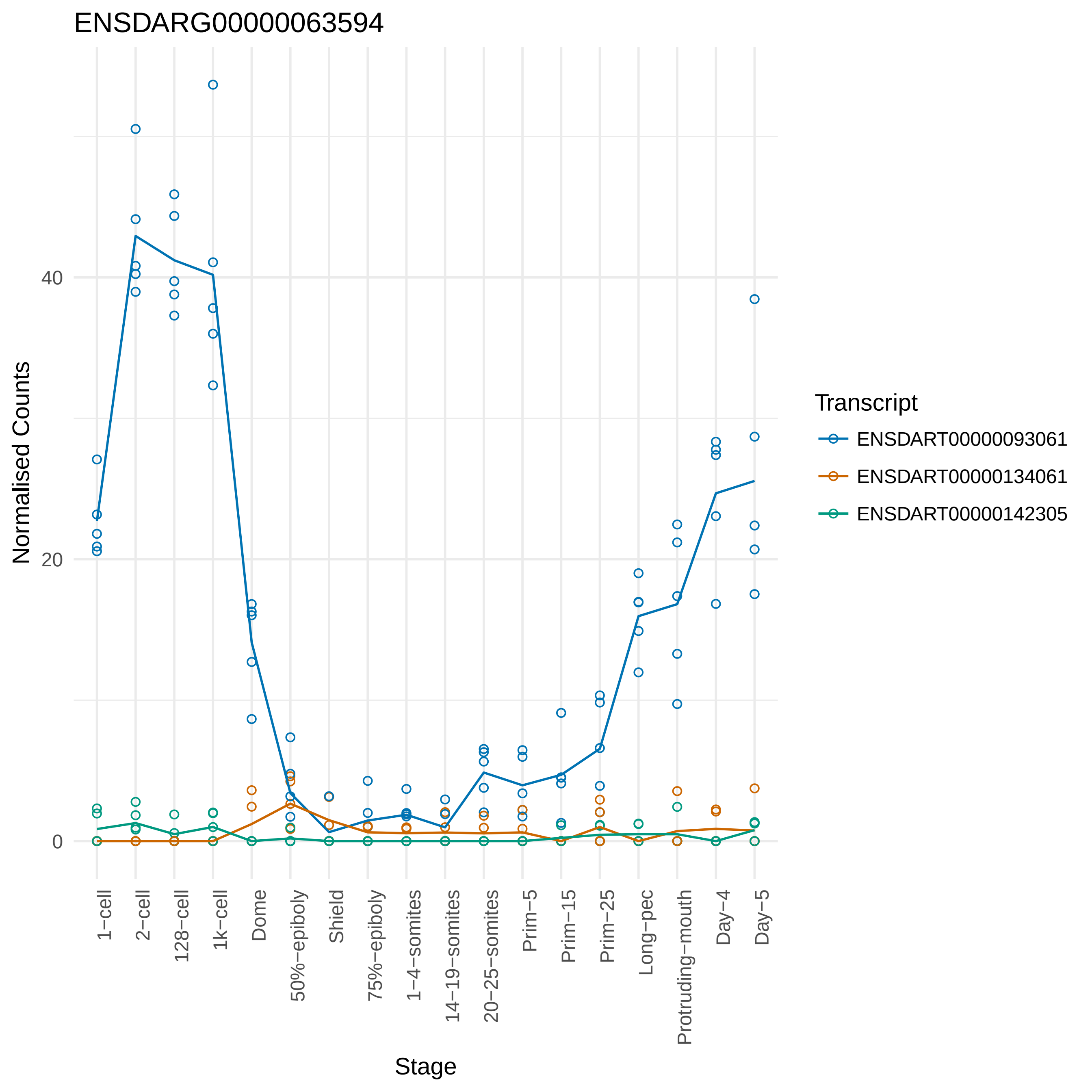
ENSDARG00000063594
Normalised Counts
0
20
40
1−cell
2−cell
128−cell
1k−cell
Dome
50%−epiboly
Shield
75%−epiboly
1−4−somites
14−19−somites
20−25−somites
Prim−5
Prim−15
Prim−25
Long−pec
Protruding−mouth
Day−4
Day−5
Stage
Transcript
ENSDART00000093061
ENSDART00000134061
ENSDART00000142305

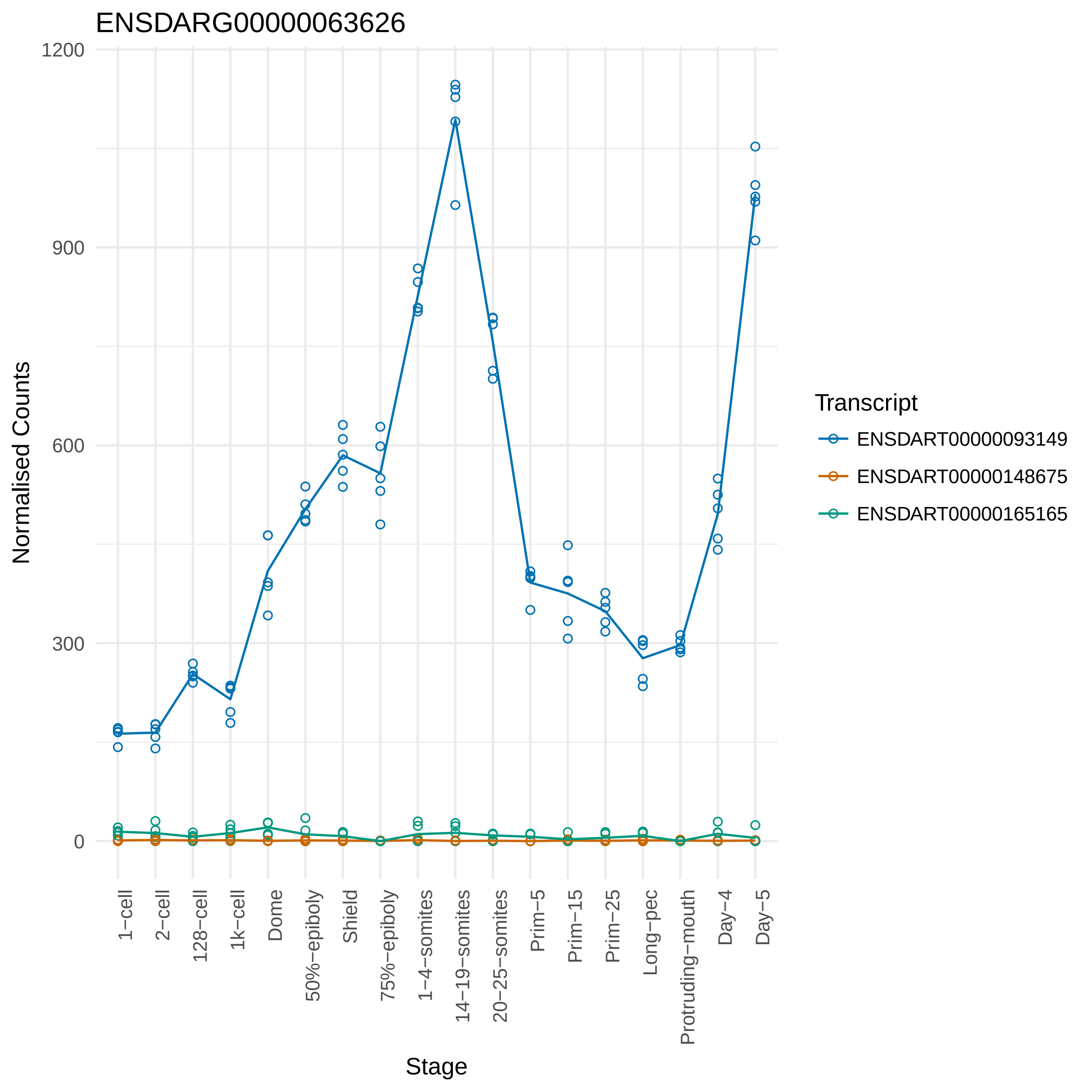
ENSDARG00000063626
Normalised Counts
1200
900
600
300
0
1−cell
2−cell
128−cell
1k−cell
Dome
50%−epiboly
Shield
75%−epiboly
1−4−somites
14−19−somites
20−25−somites
Prim−5
Prim−15
Prim−25
Long−pec
Protruding−mouth
Day−4
Day−5
Stage
Transcript
ENSDART00000093149
ENSDART00000148675
ENSDART00000165165

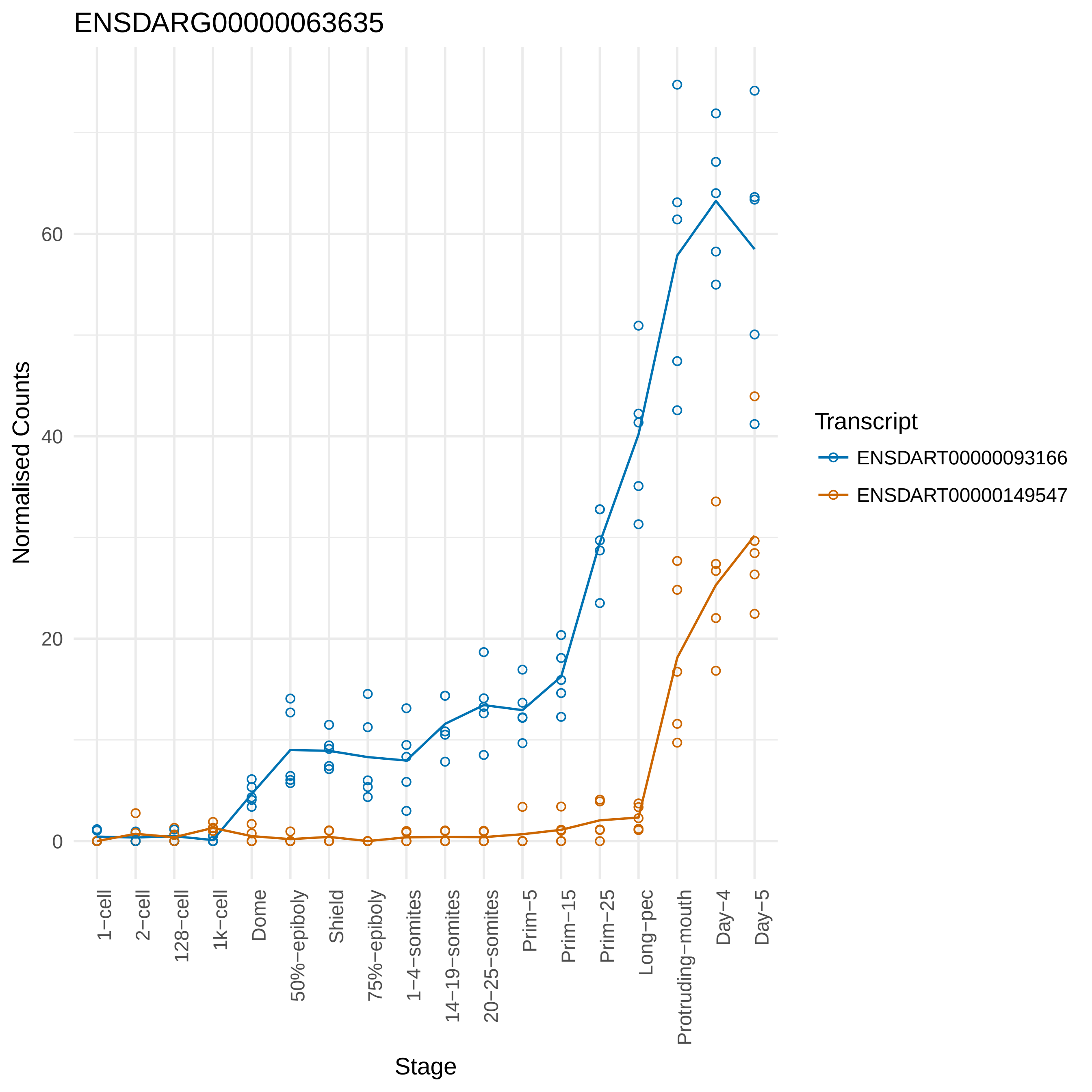
ENSDARG00000063635
Normalised Counts
0
20
40
60
Stage
1−cell
2−cell
128−cell
1k−cell
Dome
50%−epiboly
Shield
75%−epiboly
1−4−somites
14−19−somites
20−25−somites
Prim−5
Prim−15
Prim−25
Long−pec
Protruding−mouth
Day−4
Day−5
Transcript
ENSDART00000093166
ENSDART00000149547

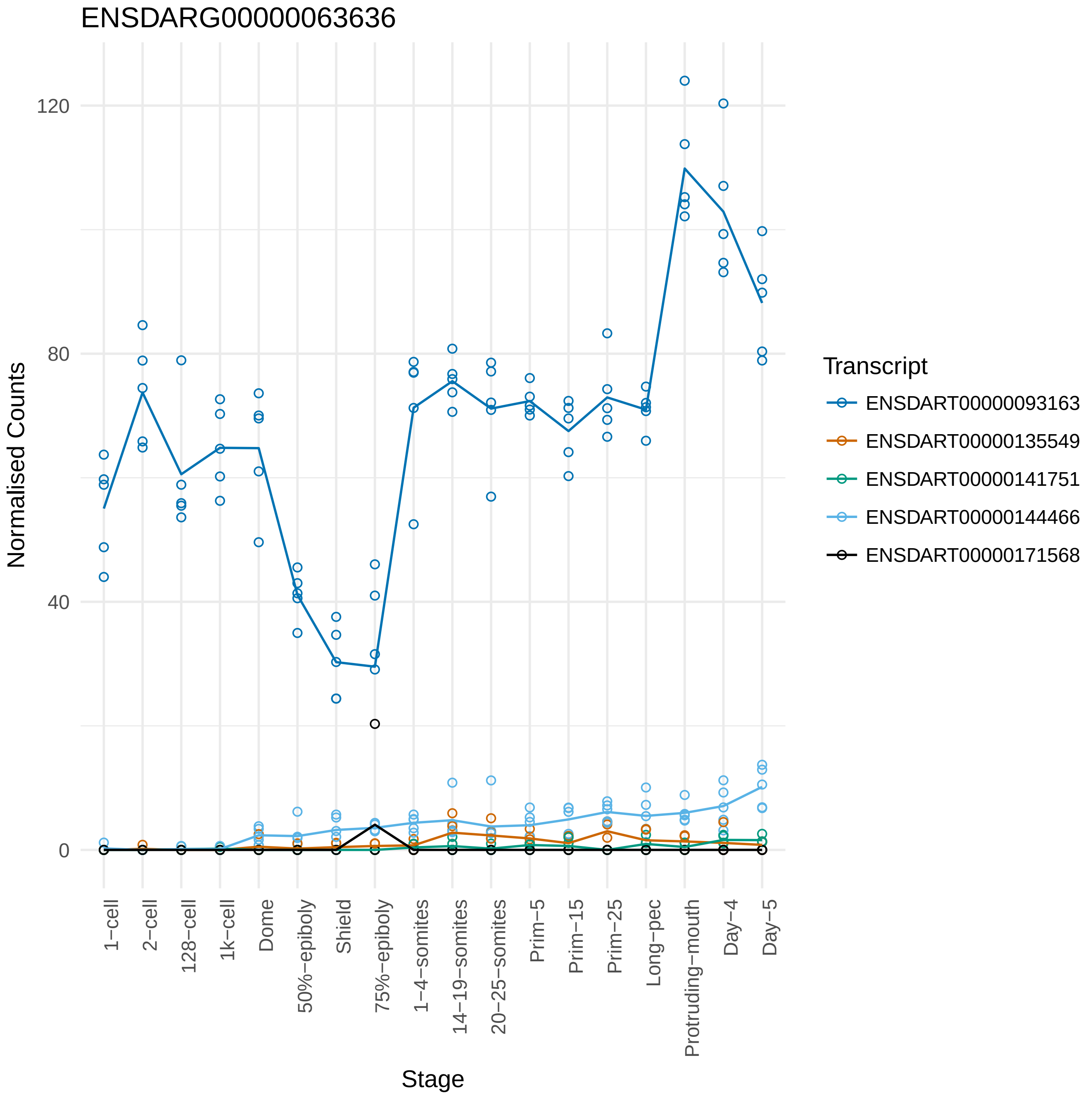

ENSDARG00000063636
Normalised Counts
120
80
40
0
1−cell
2−cell
128−cell
1k−cell
Dome
50%−epiboly
Shield
75%−epiboly
1−4−somites
14−19−somites
20−25−somites
Prim−5
Prim−15
Prim−25
Long−pec
Protruding−mouth
Day−4
Day−5
Stage
Transcript
ENSDART00000093163
ENSDART00000135549
ENSDART00000141751
ENSDART00000144466
ENSDART00000171568

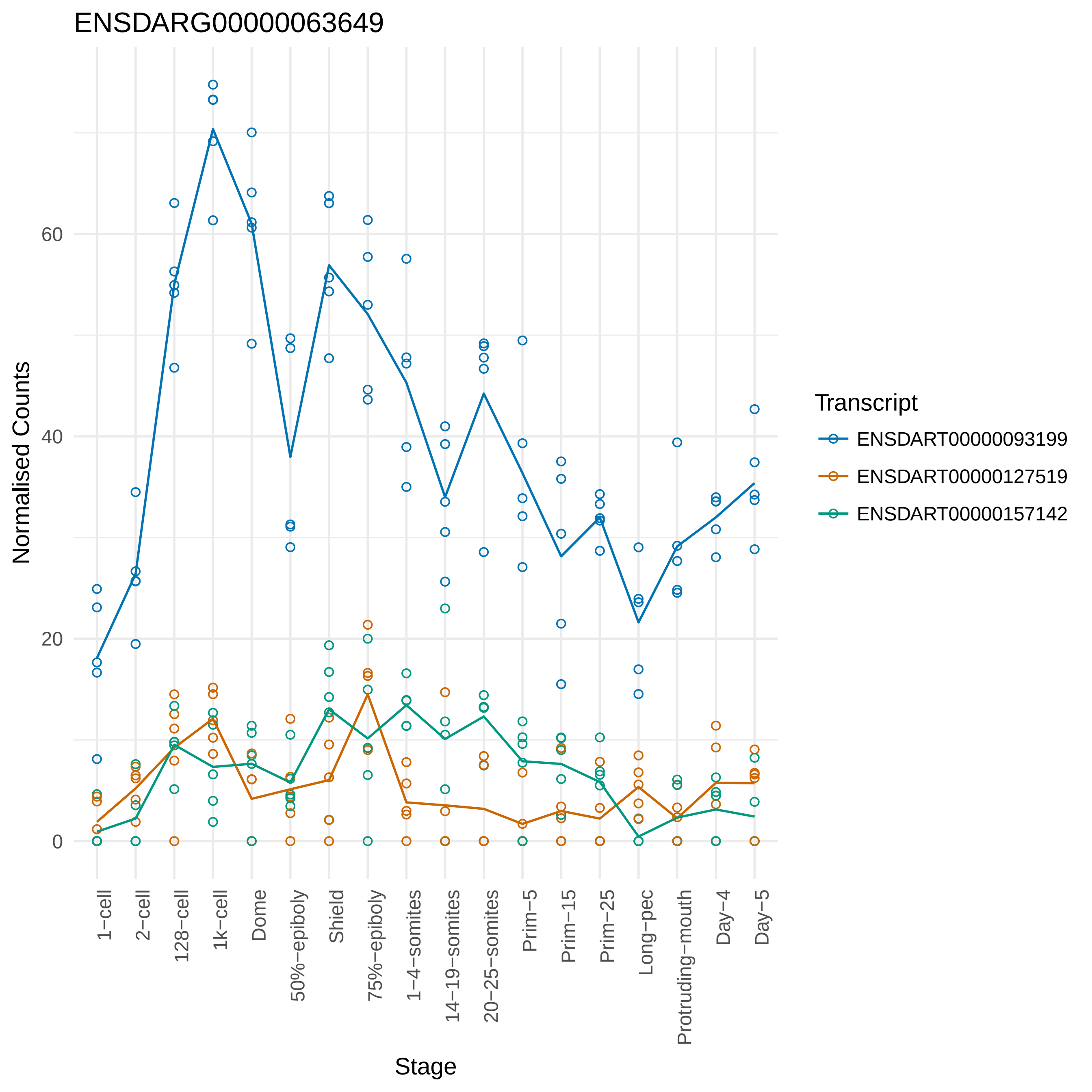

ENSDARG00000063649
Normalised Counts
60
40
20
0
1−cell
2−cell
128−cell
1k−cell
Dome
50%−epiboly
Shield
75%−epiboly
1−4−somites
14−19−somites
20−25−somites
Prim−5
Prim−15
Prim−25
Long−pec
Protruding−mouth
Day−4
Day−5
Stage
Transcript
ENSDART00000093199
ENSDART00000127519
ENSDART00000157142

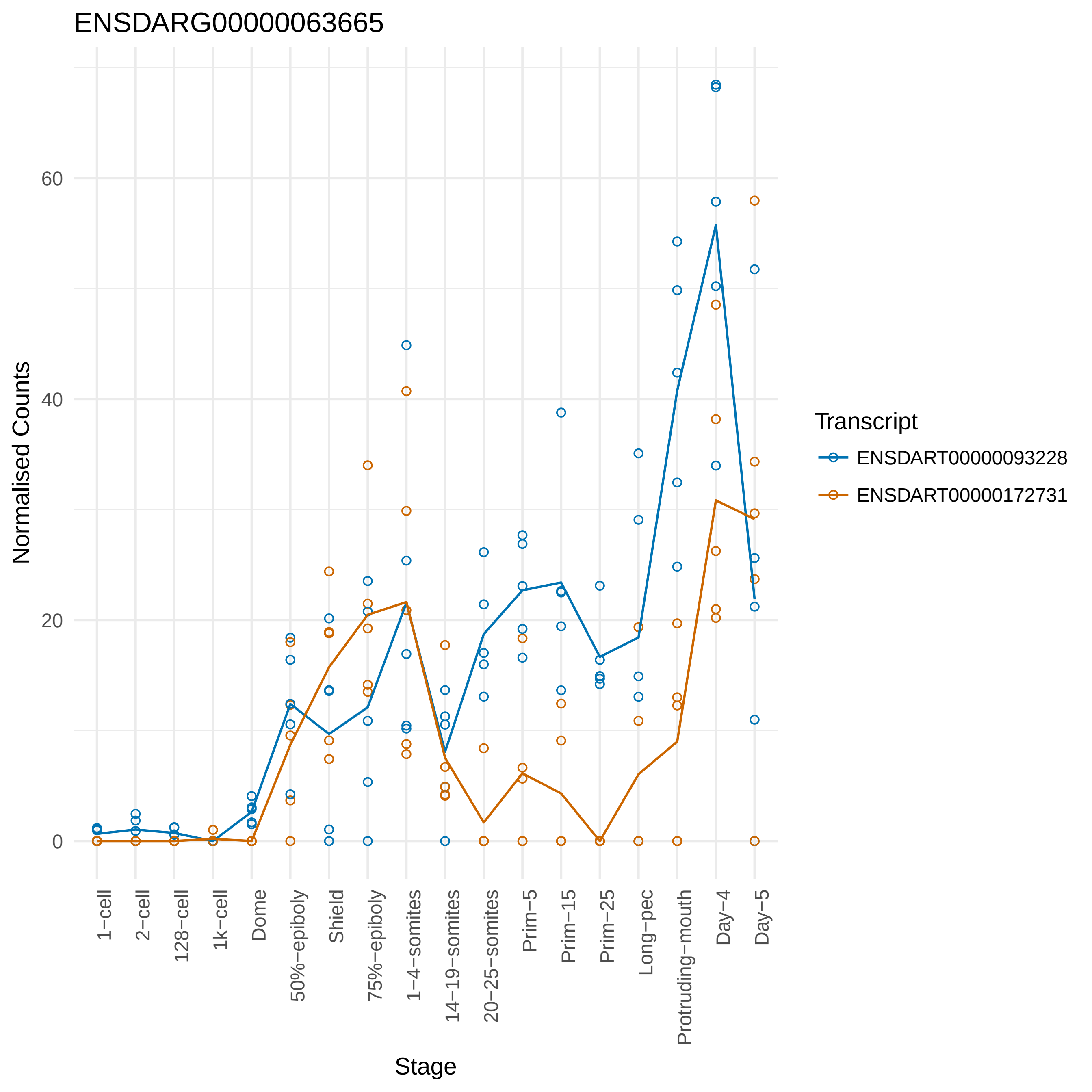

ENSDARG00000063665
Normalised Counts
0
20
40
60
1−cell
2−cell
128−cell
1k−cell
Dome
50%−epiboly
Shield
75%−epiboly
1−4−somites
14−19−somites
20−25−somites
Prim−5
Prim−15
Prim−25
Long−pec
Protruding−mouth
Day−4
Day−5
Stage
Transcript
ENSDART00000093228
ENSDART00000172731

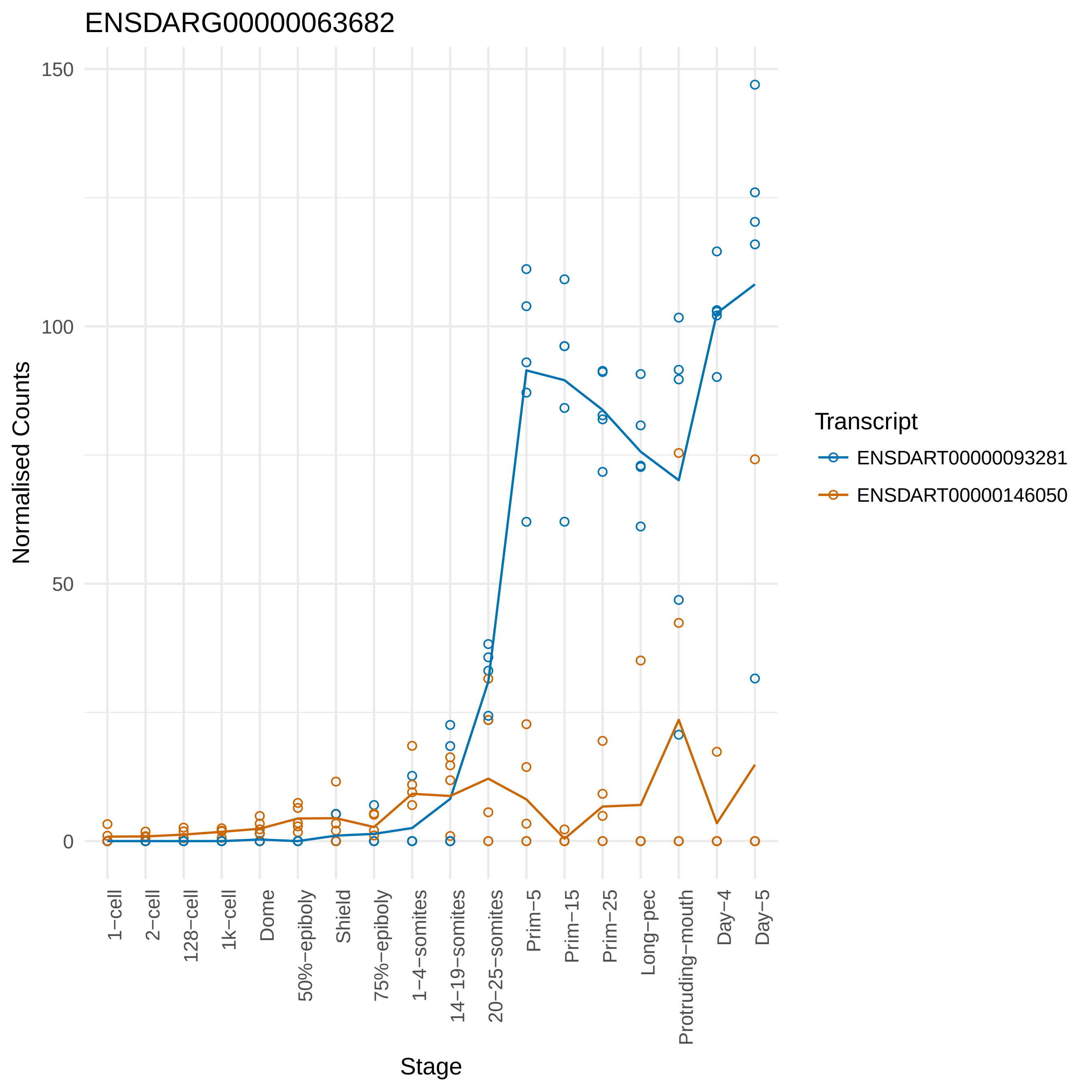

ENSDARG00000063682
Normalised Counts
150
100
50
0
1−cell
2−cell
128−cell
1k−cell
Dome
50%−epiboly
Shield
75%−epiboly
1−4−somites
14−19−somites
20−25−somites
Prim−5
Prim−15
Prim−25
Long−pec
Protruding−mouth
Day−4
Day−5
Stage
Transcript
ENSDART00000093281
ENSDART00000146050

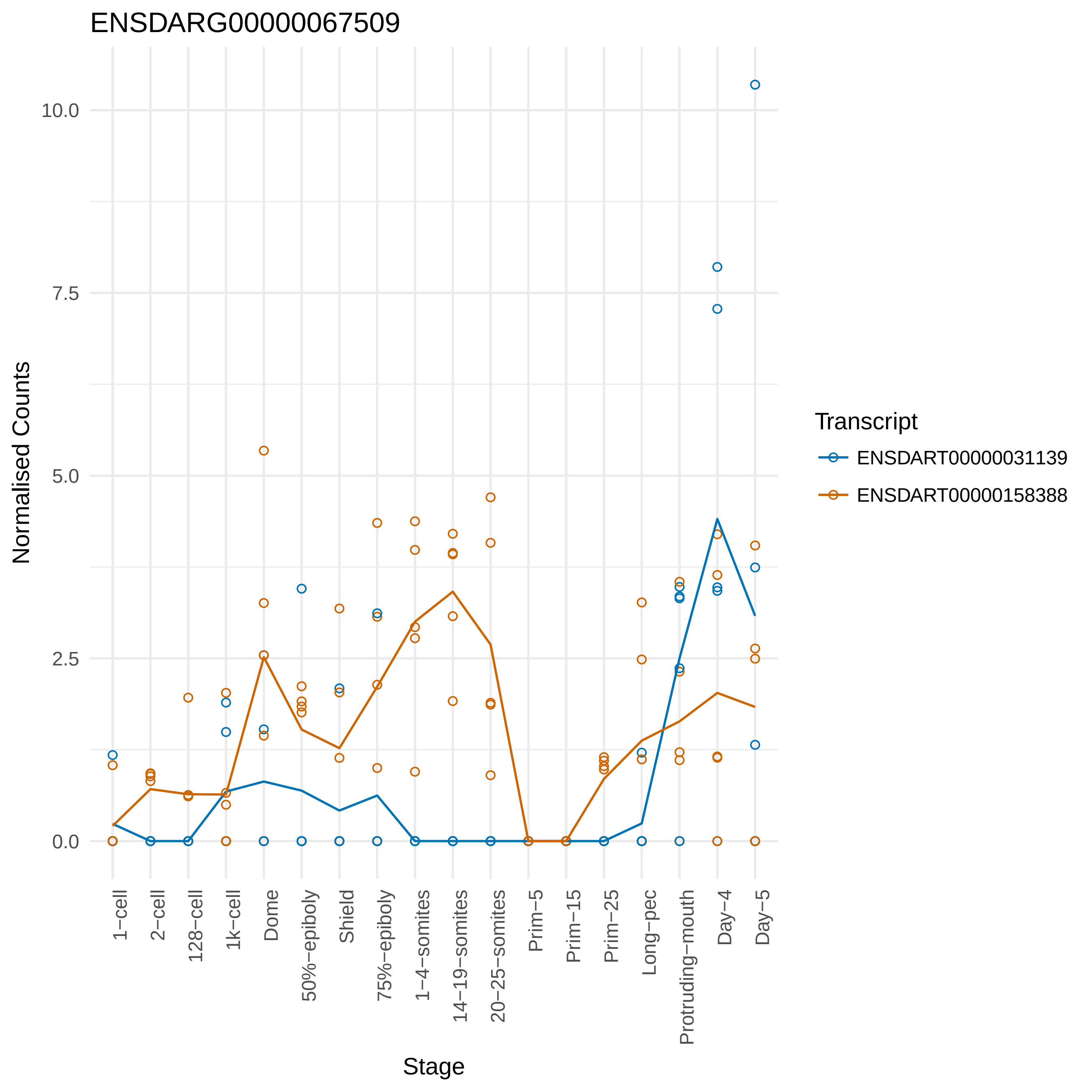

ENSDARG00000067509
Normalised Counts
10.0
7.5
5.0
2.5
0.0
Stage
1−cell
2−cell
128−cell
1k−cell
Dome
50%−epiboly
Shield
75%−epiboly
1−4−somites
14−19−somites
20−25−somites
Prim−5
Prim−15
Prim−25
Long−pec
Protruding−mouth
Day−4
Day−5
Transcript
ENSDART00000031139
ENSDART00000158388

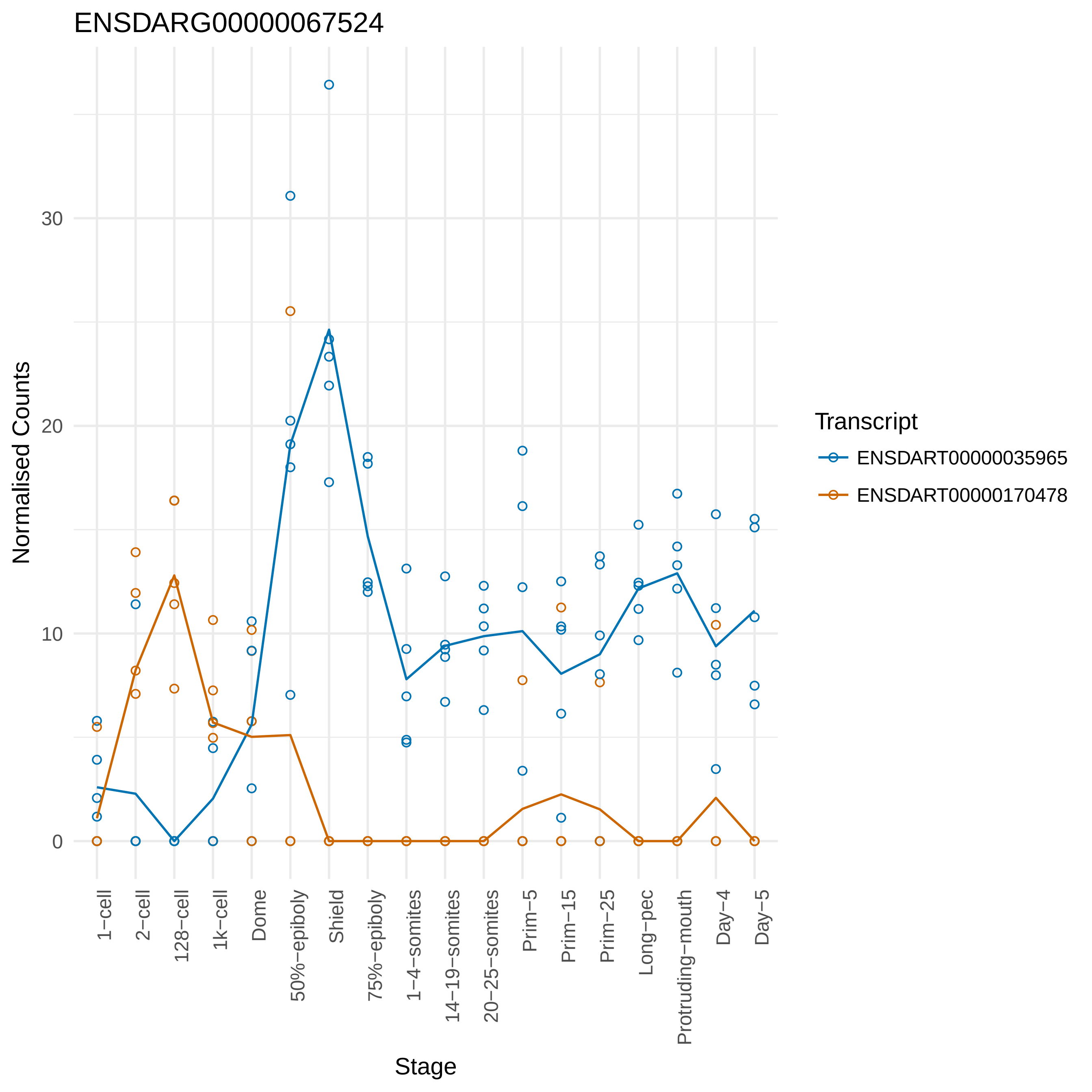
ENSDARG00000067524
Normalised Counts
0
10
20
30
Stage
1−cell
2−cell
128−cell
1k−cell
Dome
50%−epiboly
Shield
75%−epiboly
1−4−somites
14−19−somites
20−25−somites
Prim−5
Prim−15
Prim−25
Long−pec
Protruding−mouth
Day−4
Day−5
Transcript
ENSDART00000035965
ENSDART00000170478

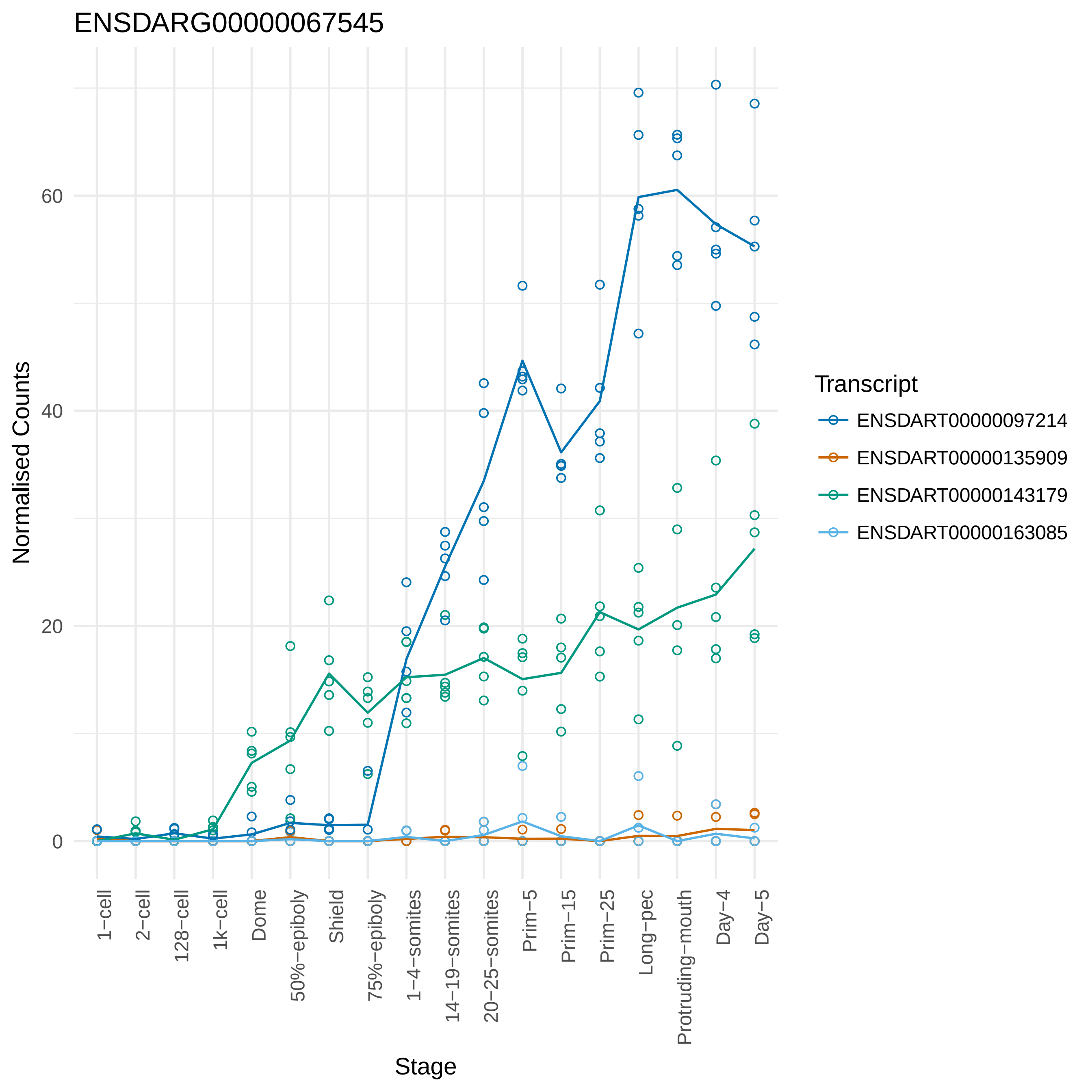
ENSDARG00000067545
Normalised Counts
0
20
40
60
Stage
1−cell
2−cell
128−cell
1k−cell
Dome
50%−epiboly
Shield
75%−epiboly
1−4−somites
14−19−somites
20−25−somites
Prim−5
Prim−15
Prim−25
Long−pec
Protruding−mouth
Day−4
Day−5
Transcript
ENSDART00000097214
ENSDART00000135909
ENSDART00000143179
ENSDART00000163085

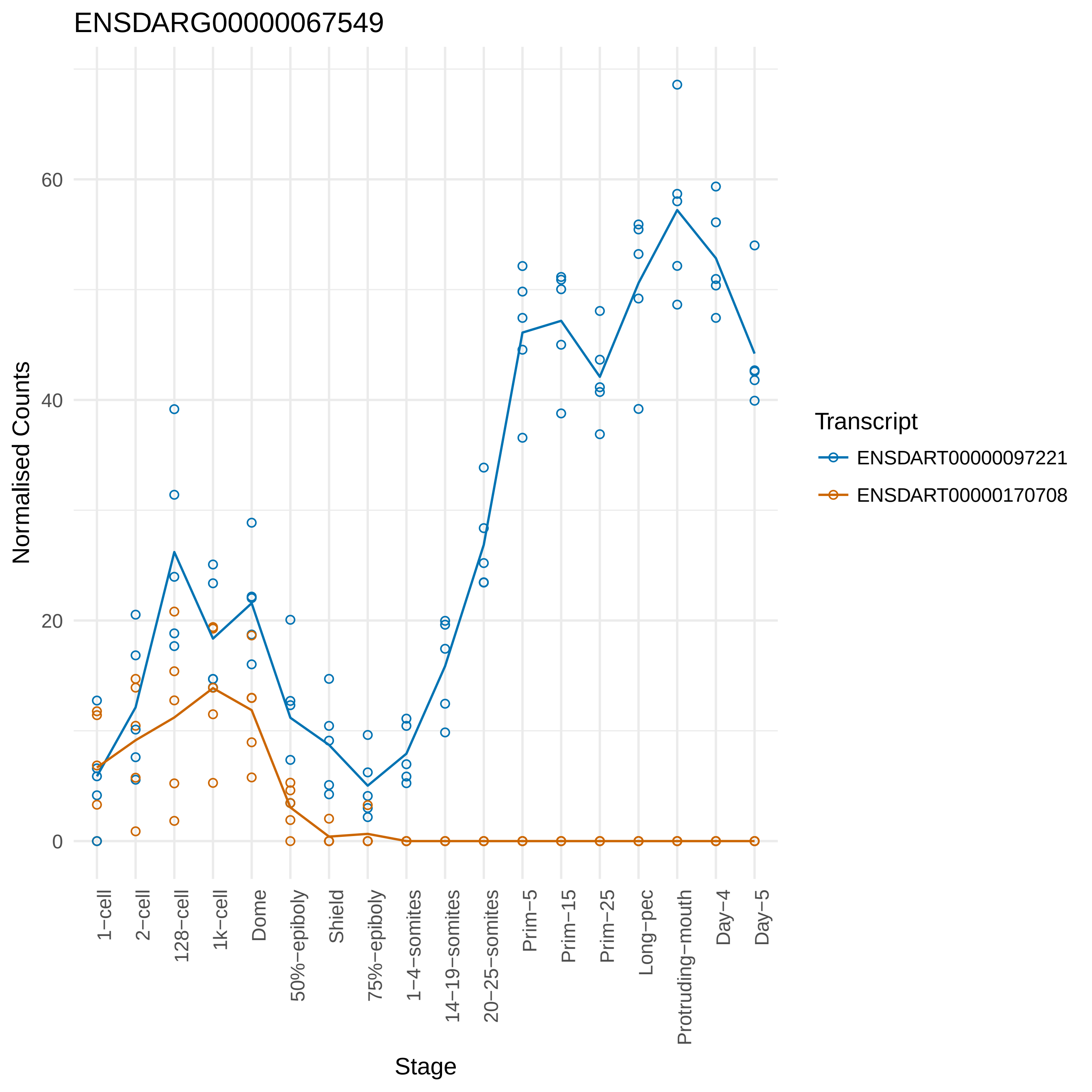

ENSDARG00000067549
Normalised Counts
0
20
40
60
Stage
1−cell
2−cell
128−cell
1k−cell
Dome
50%−epiboly
Shield
75%−epiboly
1−4−somites
14−19−somites
20−25−somites
Prim−5
Prim−15
Prim−25
Long−pec
Protruding−mouth
Day−4
Day−5
Transcript
ENSDART00000097221
ENSDART00000170708

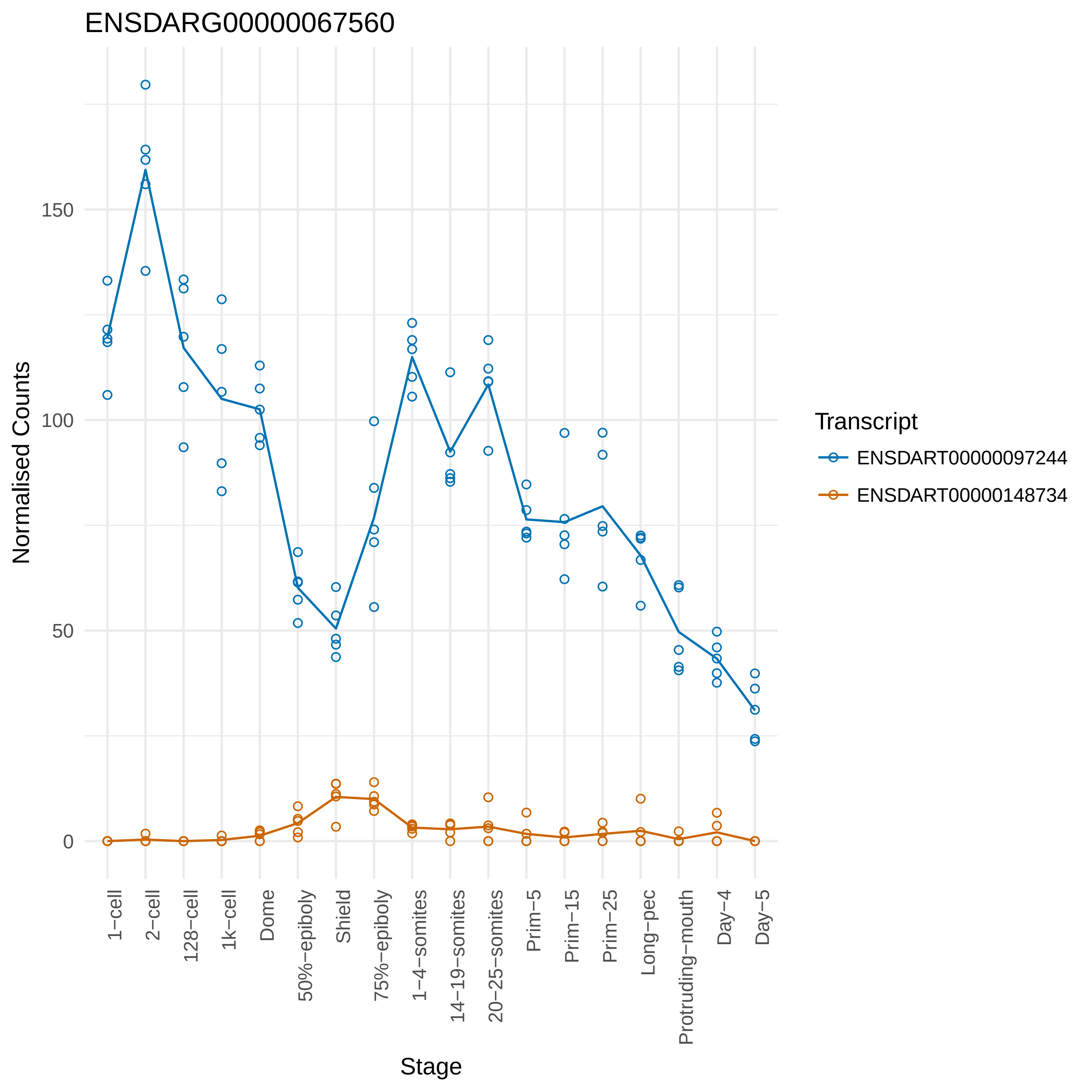

ENSDARG00000067560
Normalised Counts
150
100
50
0
1−cell
2−cell
128−cell
1k−cell
Dome
50%−epiboly
Shield
75%−epiboly
1−4−somites
14−19−somites
20−25−somites
Prim−5
Prim−15
Prim−25
Long−pec
Protruding−mouth
Day−4
Day−5
Stage
Transcript
ENSDART00000097244
ENSDART00000148734

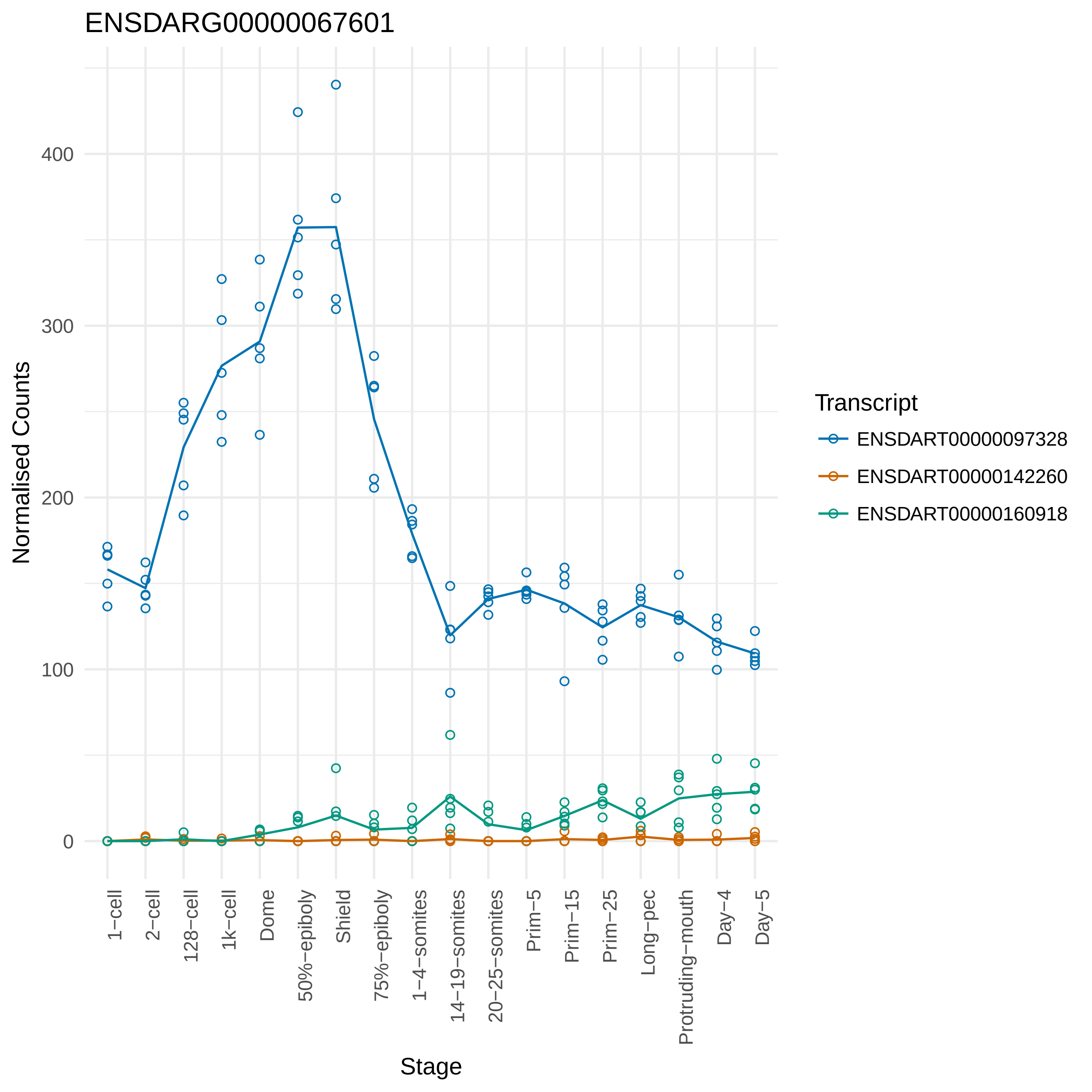

ENSDARG00000067601
Normalised Counts
400
300
200
100
0
1−cell
2−cell
128−cell
1k−cell
Dome
50%−epiboly
Shield
75%−epiboly
1−4−somites
14−19−somites
20−25−somites
Prim−5
Prim−15
Prim−25
Long−pec
Protruding−mouth
Day−4
Day−5
Stage
Transcript
ENSDART00000097328
ENSDART00000142260
ENSDART00000160918

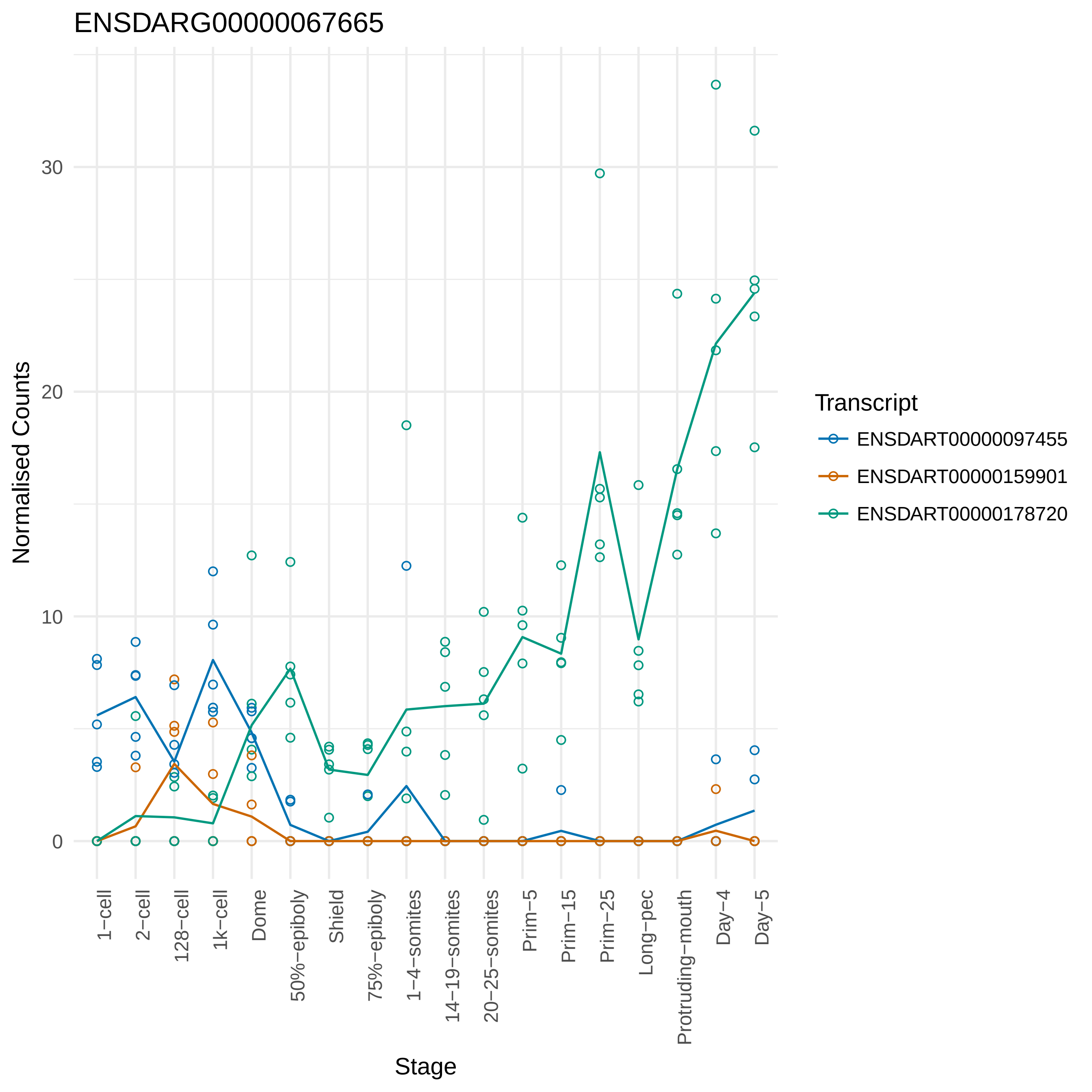

ENSDARG00000067665
Normalised Counts
30
20
10
0
1−cell
2−cell
128−cell
1k−cell
Dome
50%−epiboly
Shield
75%−epiboly
1−4−somites
14−19−somites
20−25−somites
Prim−5
Prim−15
Prim−25
Long−pec
Protruding−mouth
Day−4
Day−5
Stage
Transcript
ENSDART00000097455
ENSDART00000159901
ENSDART00000178720

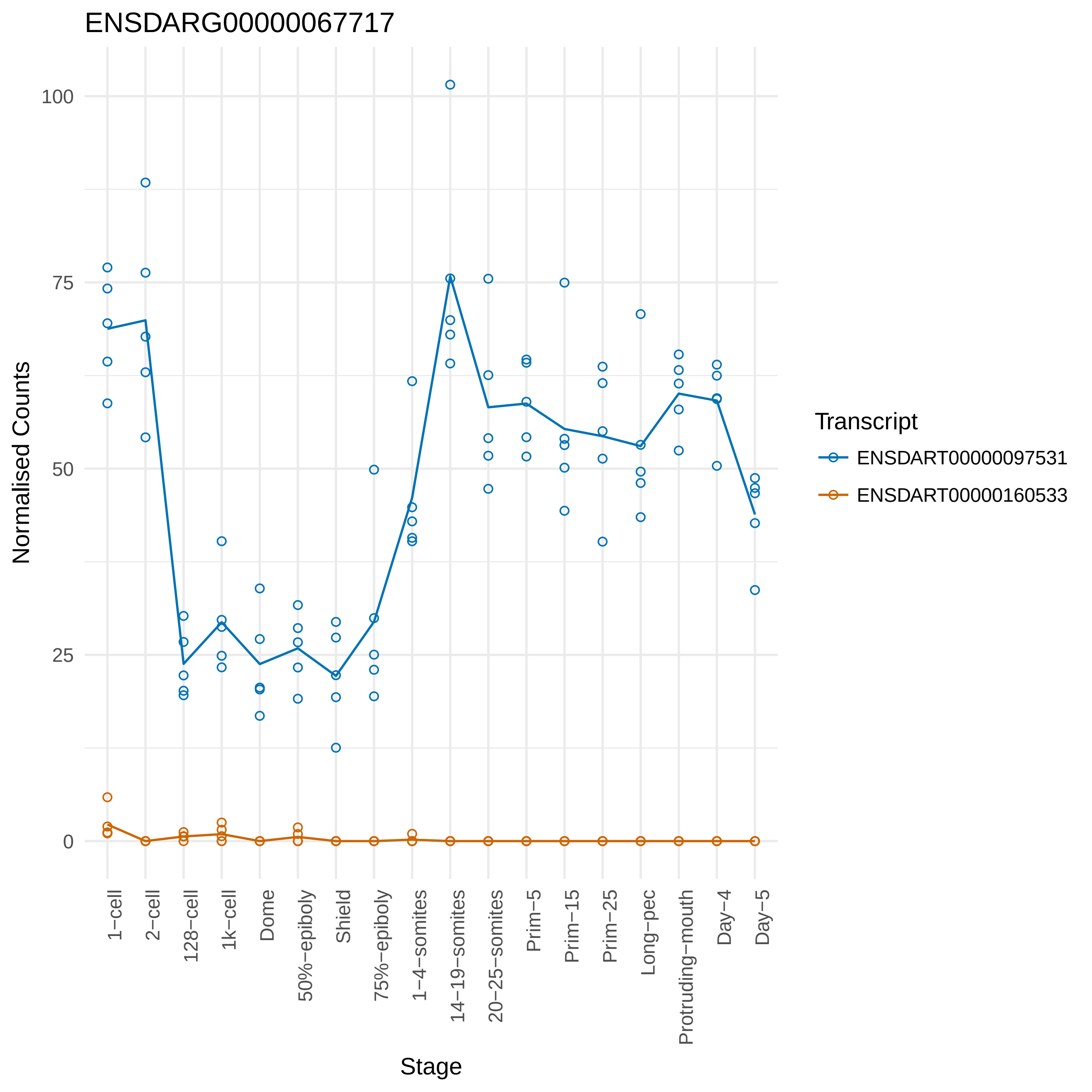

ENSDARG00000067717
Normalised Counts
100
75
50
25
0
1−cell
2−cell
128−cell
1k−cell
Dome
50%−epiboly
Shield
75%−epiboly
1−4−somites
14−19−somites
20−25−somites
Prim−5
Prim−15
Prim−25
Long−pec
Protruding−mouth
Day−4
Day−5
Stage
Transcript
ENSDART00000097531
ENSDART00000160533

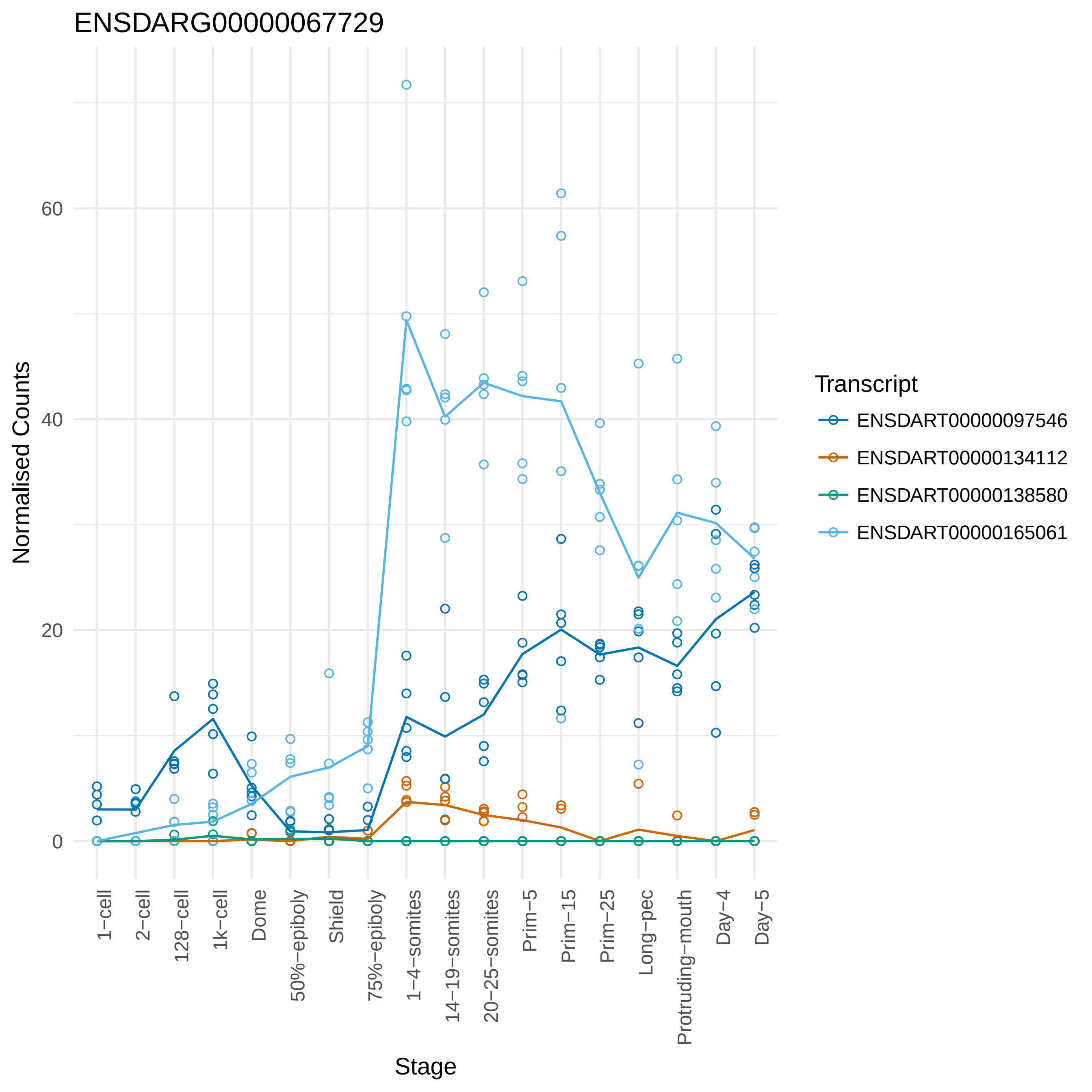
ENSDARG00000067729
Normalised Counts
0
20
40
60
Stage
1−cell
2−cell
128−cell
1k−cell
Dome
50%−epiboly
Shield
75%−epiboly
1−4−somites
14−19−somites
20−25−somites
Prim−5
Prim−15
Prim−25
Long−pec
Protruding−mouth
Day−4
Day−5
Transcript
ENSDART00000097546
ENSDART00000134112
ENSDART00000138580
ENSDART00000165061

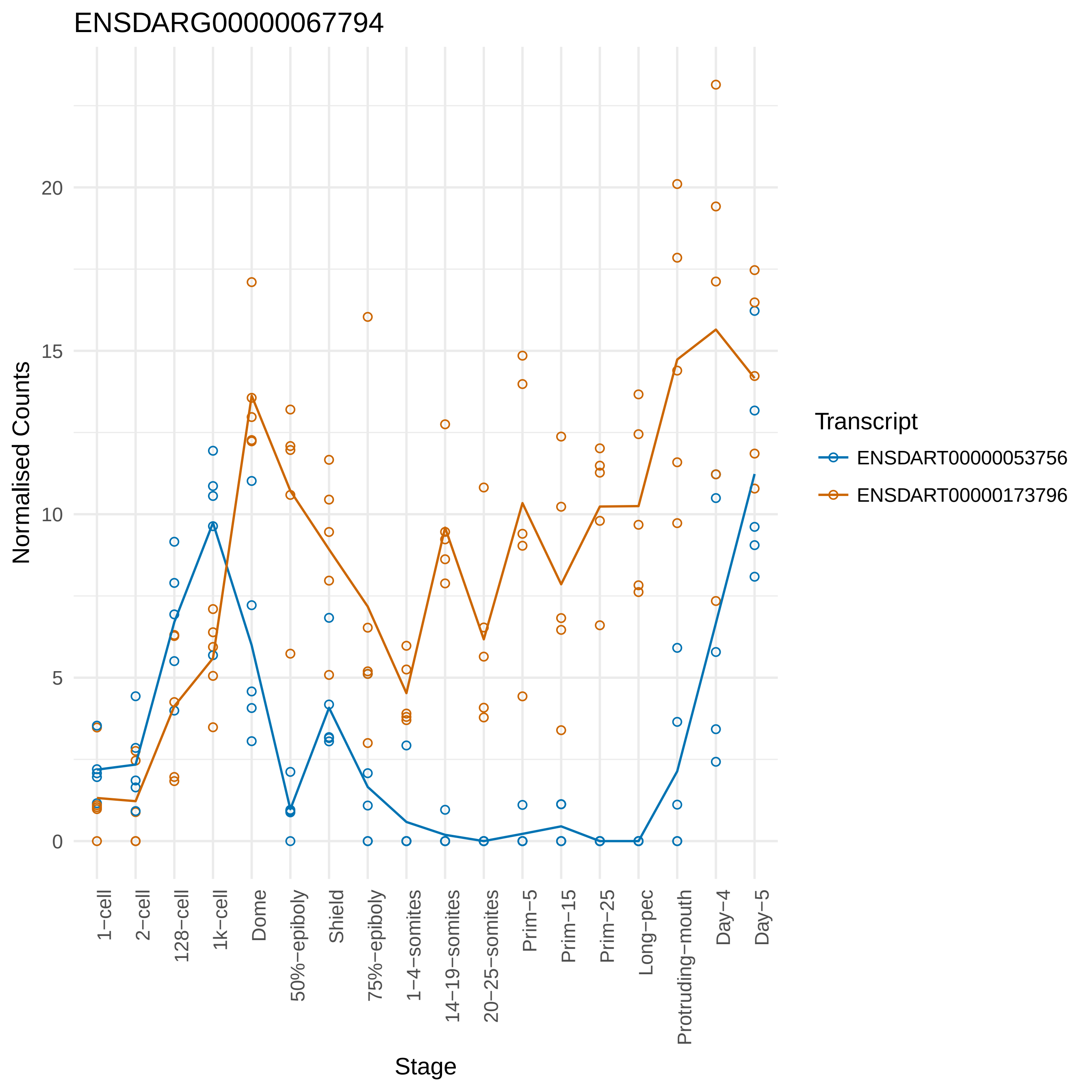
ENSDARG00000067794
Normalised Counts
0
5
10
15
20
Stage
1−cell
2−cell
128−cell
1k−cell
Dome
50%−epiboly
Shield
75%−epiboly
1−4−somites
14−19−somites
20−25−somites
Prim−5
Prim−15
Prim−25
Long−pec
Protruding−mouth
Day−4
Day−5
Transcript
ENSDART00000053756
ENSDART00000173796

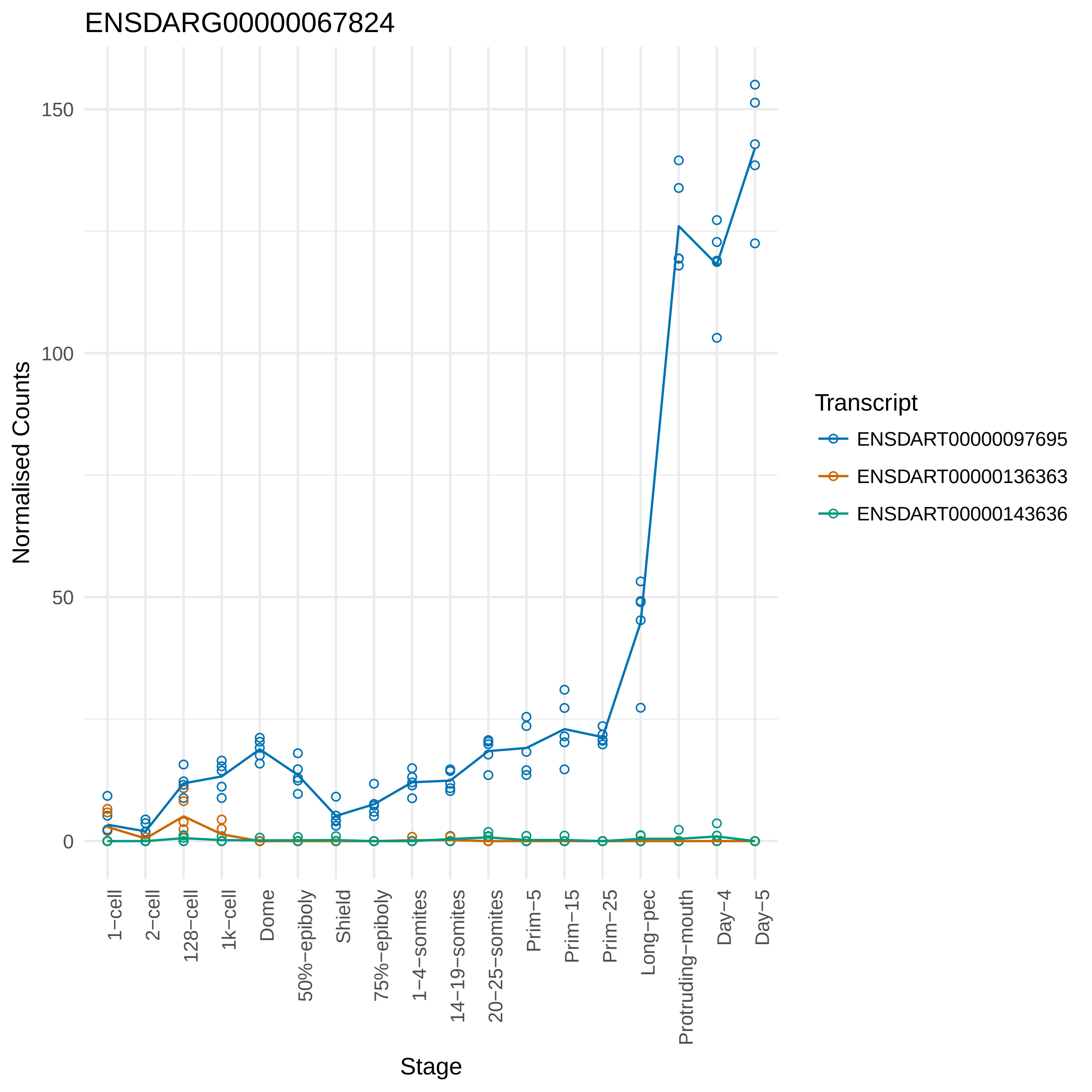
ENSDARG00000067824
Normalised Counts
0
50
100
150
1−cell
2−cell
128−cell
1k−cell
Dome
50%−epiboly
Shield
75%−epiboly
1−4−somites
14−19−somites
20−25−somites
Prim−5
Prim−15
Prim−25
Long−pec
Protruding−mouth
Day−4
Day−5
Stage
Transcript
ENSDART00000097695
ENSDART00000136363
ENSDART00000143636

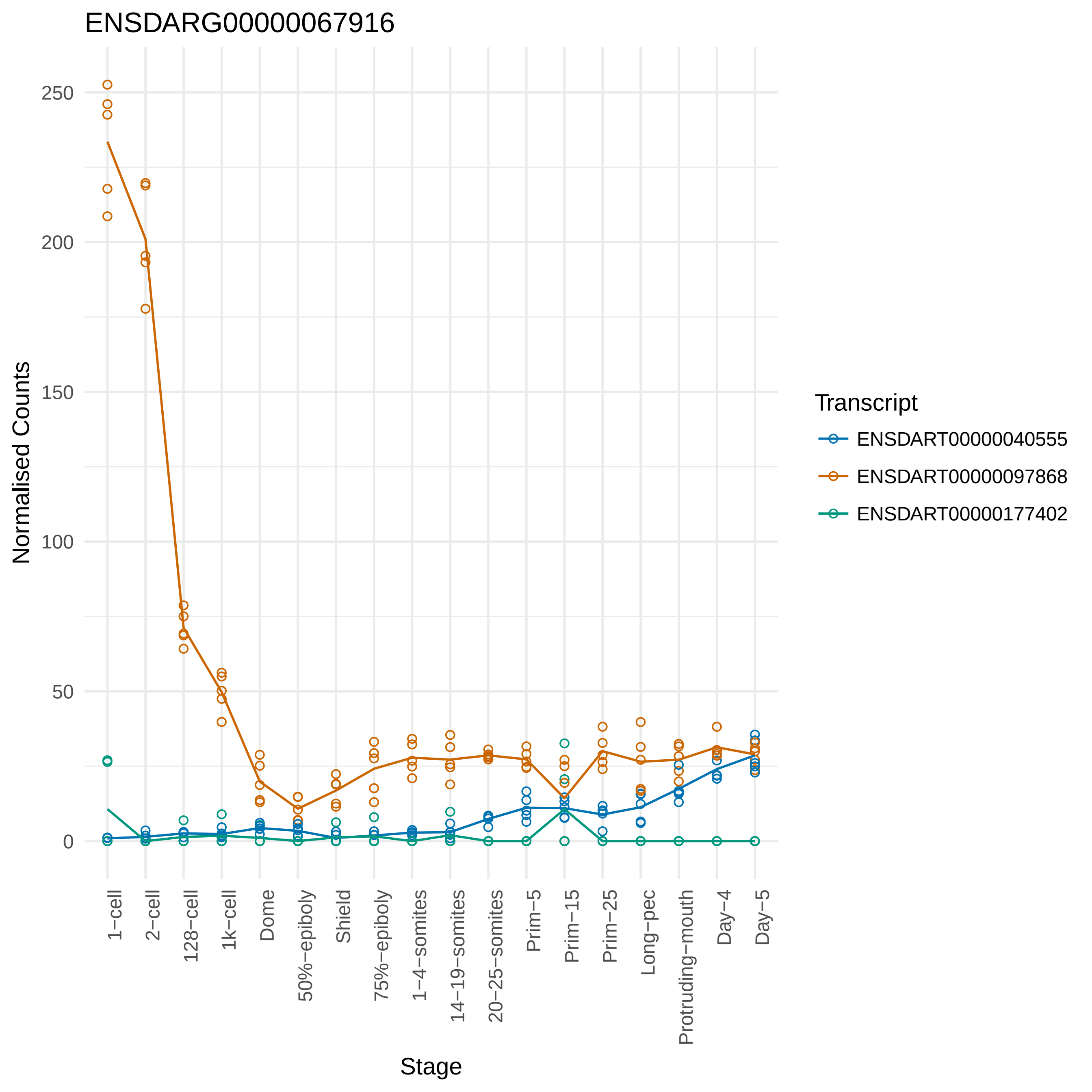

ENSDARG00000067916
Normalised Counts
250
200
150
100
50
0
1−cell
2−cell
128−cell
1k−cell
Dome
50%−epiboly
Shield
75%−epiboly
1−4−somites
14−19−somites
20−25−somites
Prim−5
Prim−15
Prim−25
Long−pec
Protruding−mouth
Day−4
Day−5
Stage
Transcript
ENSDART00000040555
ENSDART00000097868
ENSDART00000177402

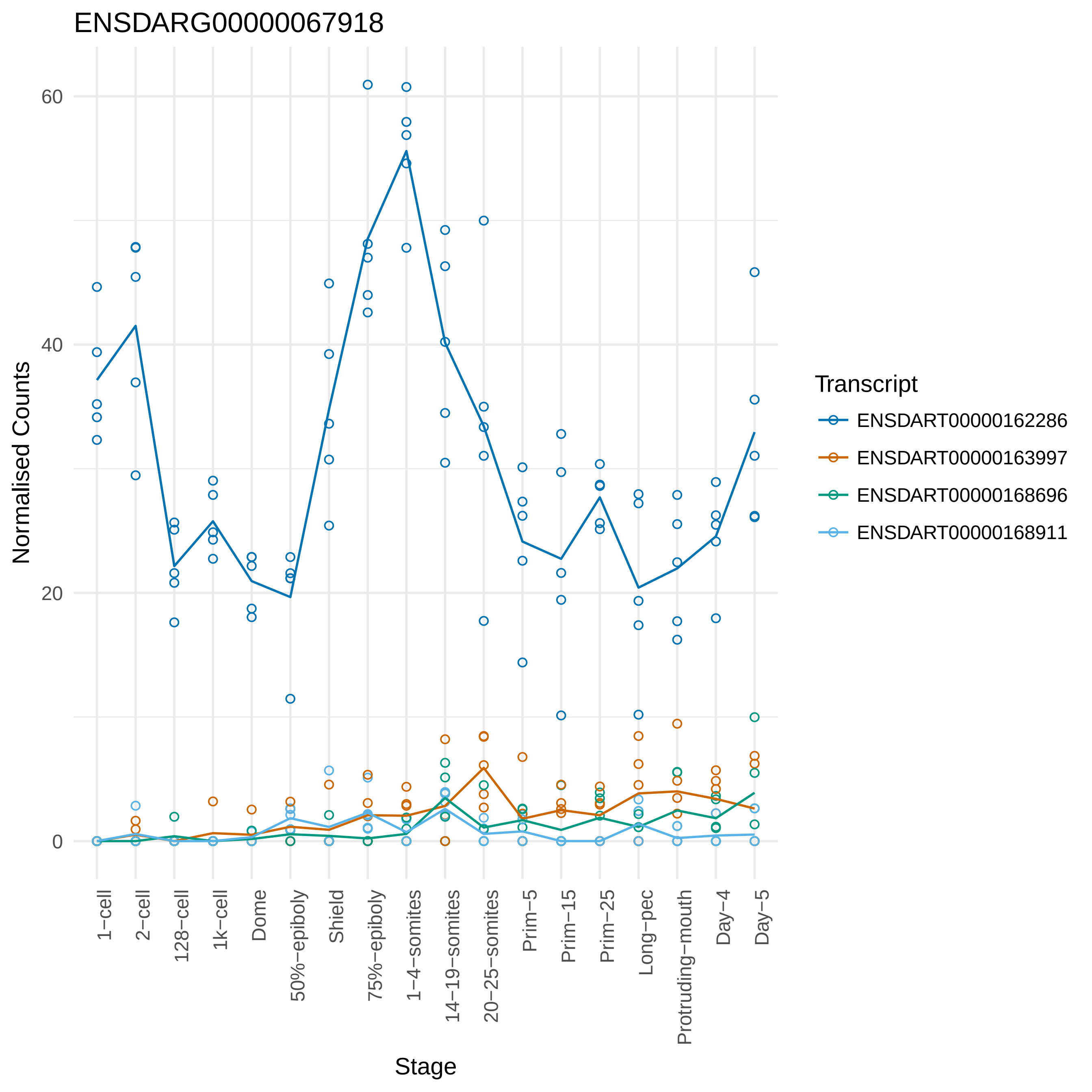
ENSDARG00000067918
Normalised Counts
0
20
40
60
1−cell
2−cell
128−cell
1k−cell
Dome
50%−epiboly
Shield
75%−epiboly
1−4−somites
14−19−somites
20−25−somites
Prim−5
Prim−15
Prim−25
Long−pec
Protruding−mouth
Day−4
Day−5
Stage
Transcript
ENSDART00000162286
ENSDART00000163997
ENSDART00000168696
ENSDART00000168911

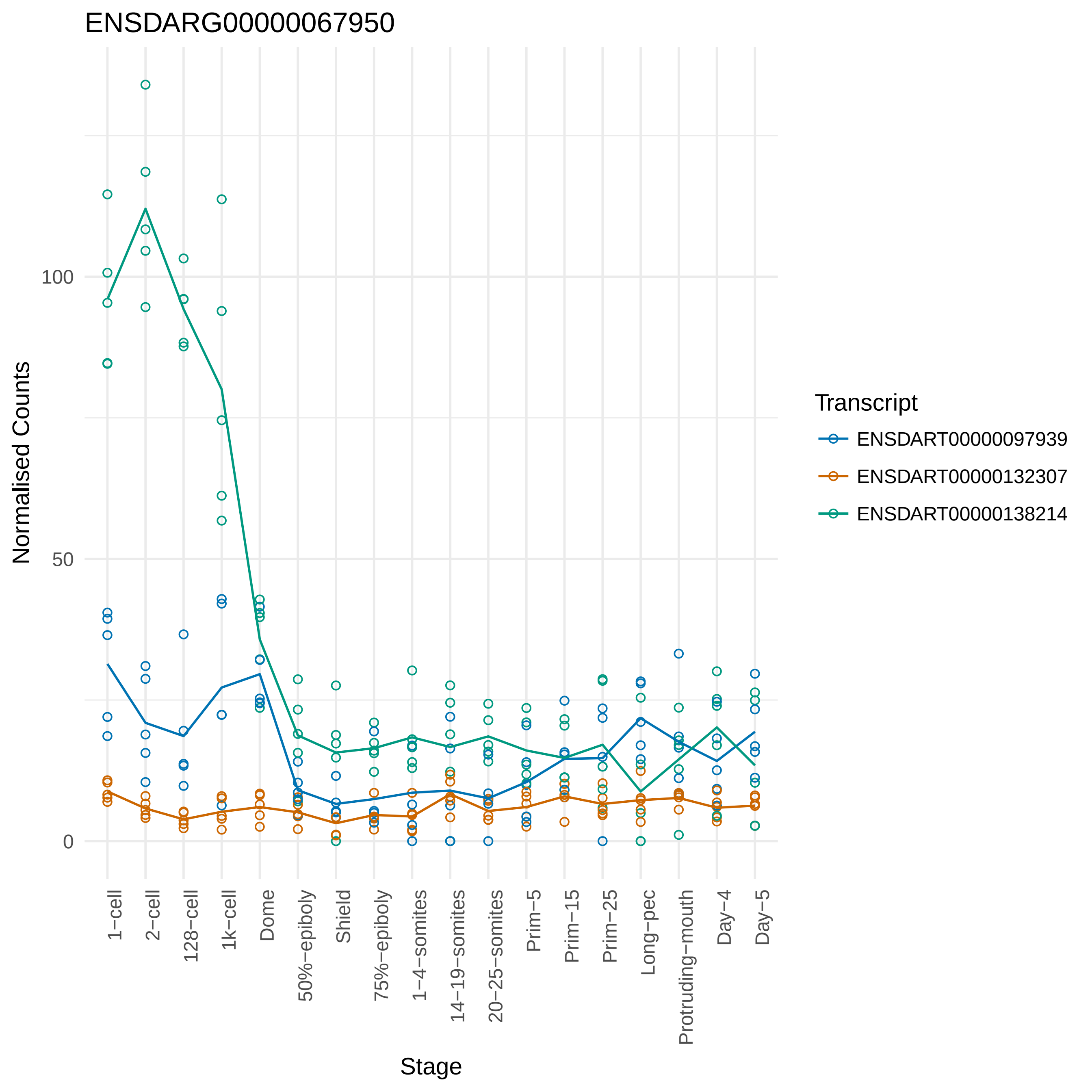
ENSDARG00000067950
Normalised Counts
100
50
0
Stage
1−cell
2−cell
128−cell
1k−cell
Dome
50%−epiboly
Shield
75%−epiboly
1−4−somites
14−19−somites
20−25−somites
Prim−5
Prim−15
Prim−25
Long−pec
Protruding−mouth
Day−4
Day−5
Transcript
ENSDART00000097939
ENSDART00000132307
ENSDART00000138214

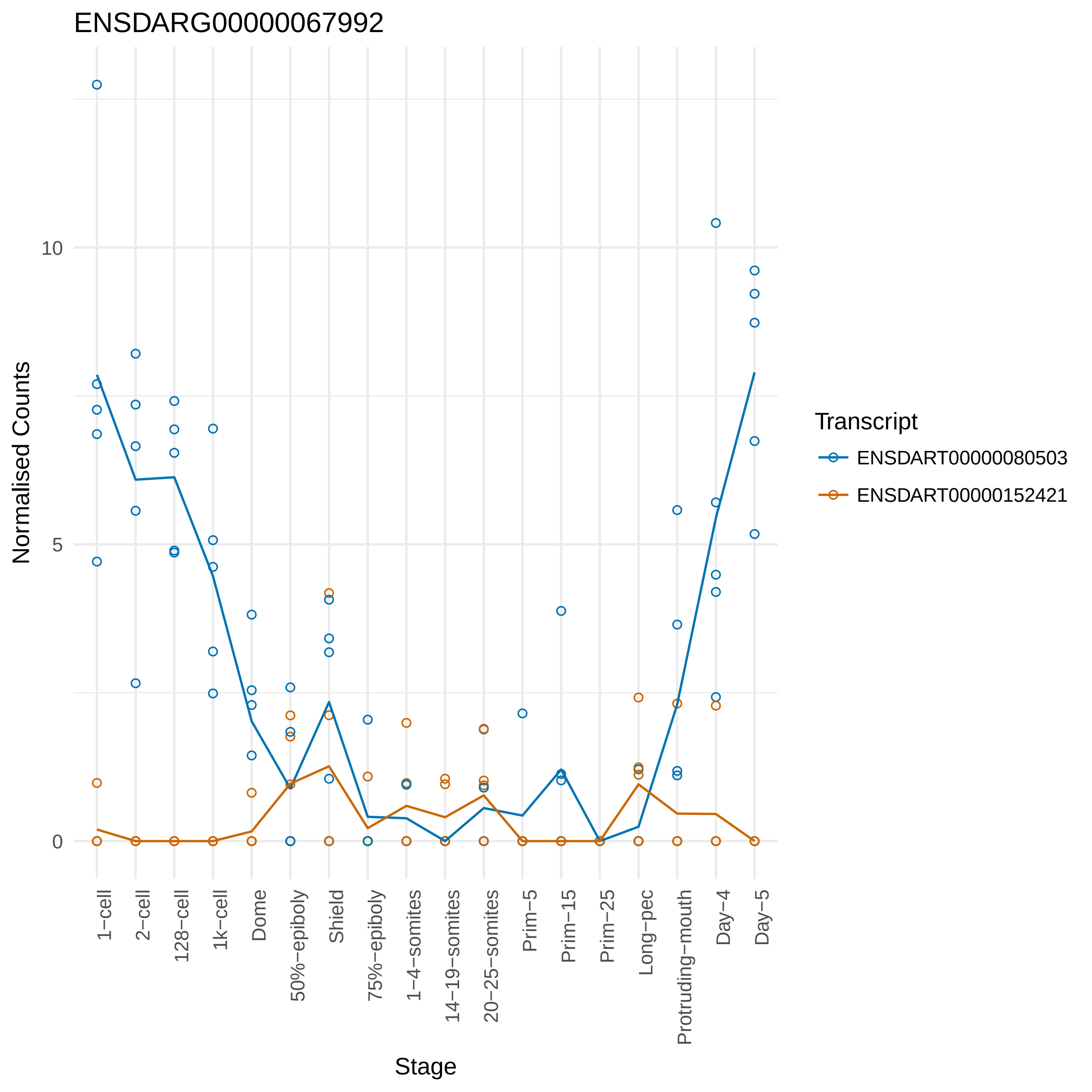

ENSDARG00000067992
Normalised Counts
10
5
0
1−cell
2−cell
128−cell
1k−cell
Dome
50%−epiboly
Shield
75%−epiboly
1−4−somites
14−19−somites
20−25−somites
Prim−5
Prim−15
Prim−25
Long−pec
Protruding−mouth
Day−4
Day−5
Stage
Transcript
ENSDART00000080503
ENSDART00000152421

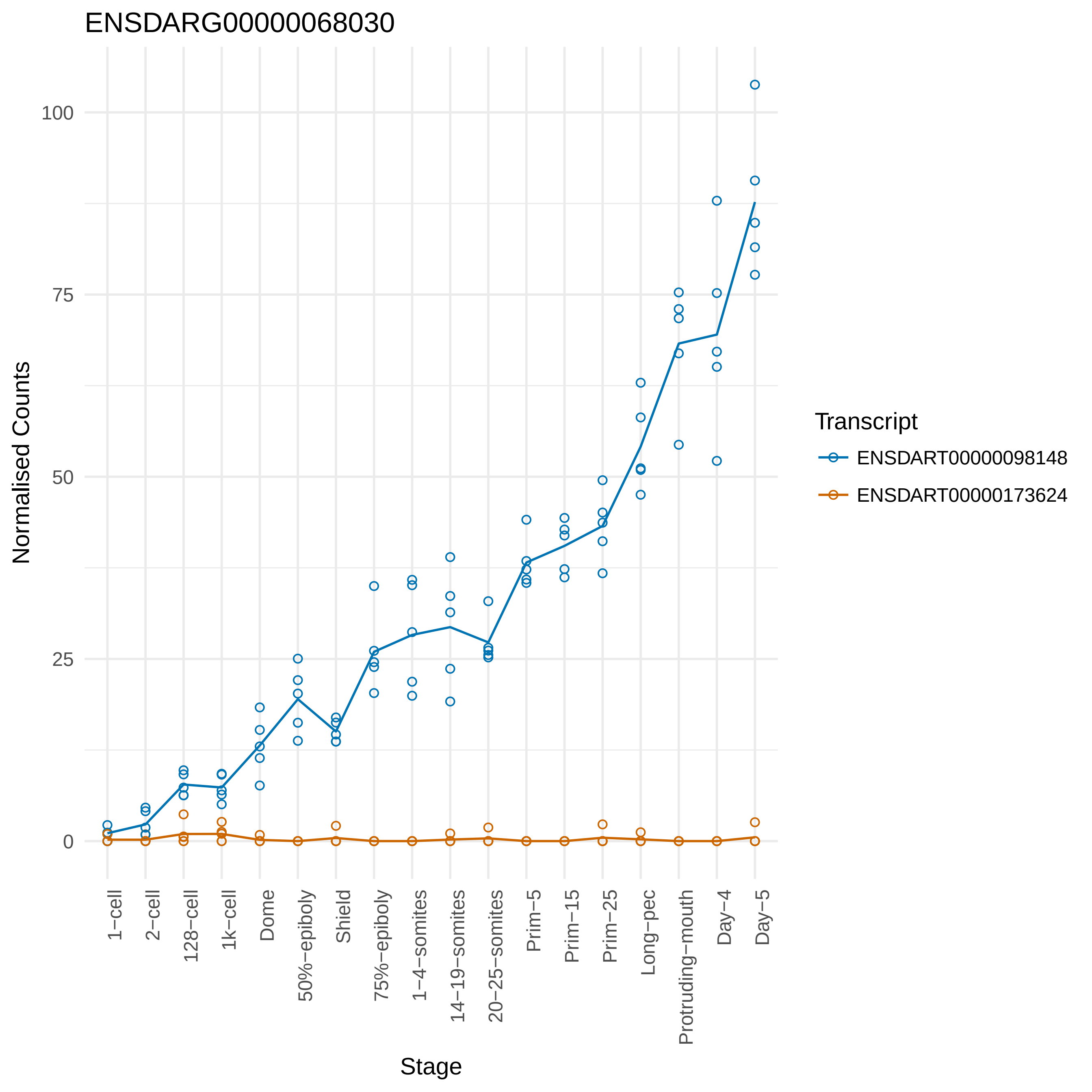
ENSDARG00000068030
Normalised Counts
100
75
50
25
0
1−cell
2−cell
128−cell
1k−cell
Dome
50%−epiboly
Shield
75%−epiboly
1−4−somites
14−19−somites
20−25−somites
Prim−5
Prim−15
Prim−25
Long−pec
Protruding−mouth
Day−4
Day−5
Stage
Transcript
ENSDART00000098148
ENSDART00000173624

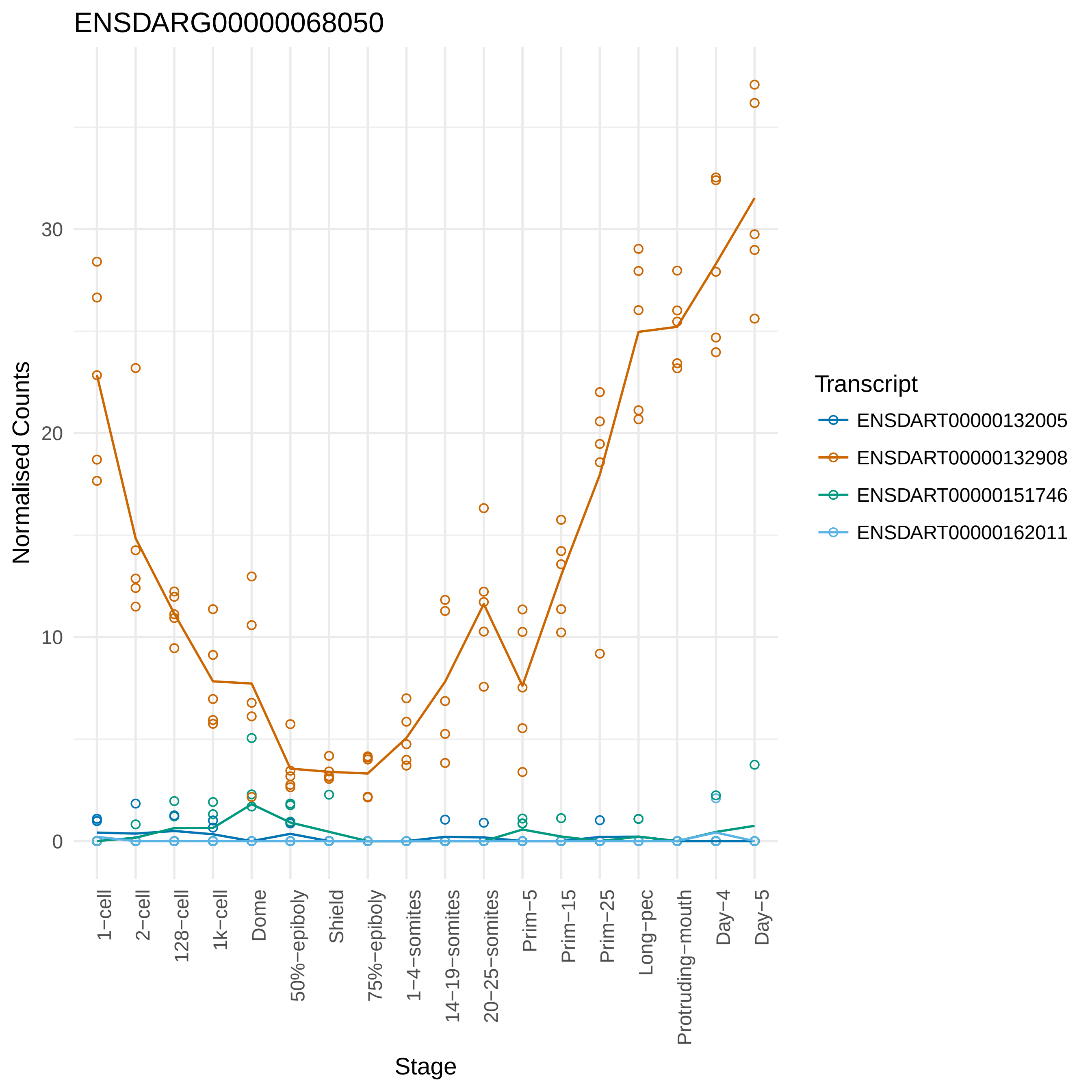
ENSDARG00000068050
Normalised Counts
0
10
20
30
1−cell
2−cell
128−cell
1k−cell
Dome
50%−epiboly
Shield
75%−epiboly
1−4−somites
14−19−somites
20−25−somites
Prim−5
Prim−15
Prim−25
Long−pec
Protruding−mouth
Day−4
Day−5
Stage
Transcript
ENSDART00000132005
ENSDART00000132908
ENSDART00000151746
ENSDART00000162011

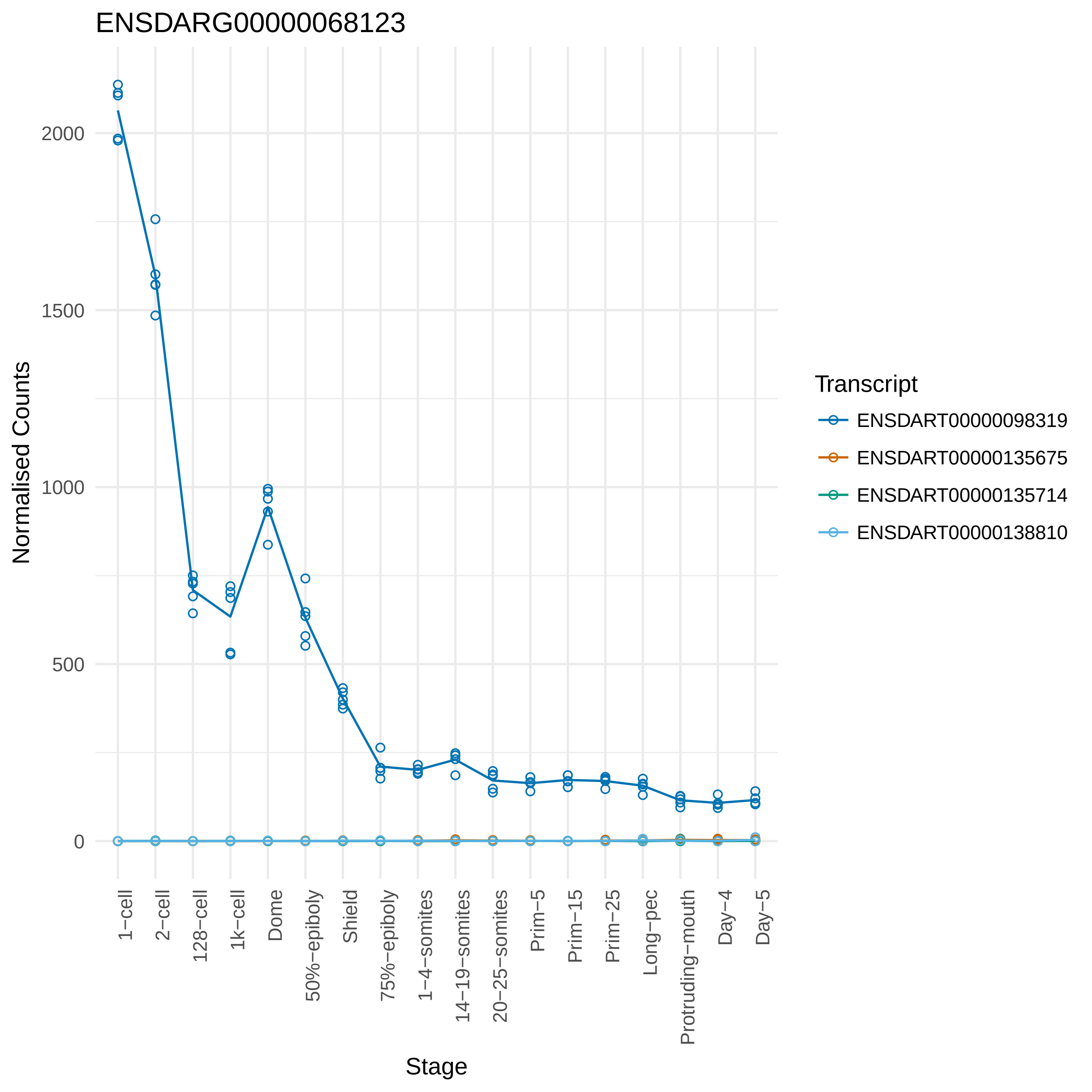

ENSDARG00000068123
Normalised Counts
2000
1500
1000
500
0
1−cell
2−cell
128−cell
1k−cell
Dome
50%−epiboly
Shield
75%−epiboly
1−4−somites
14−19−somites
20−25−somites
Prim−5
Prim−15
Prim−25
Long−pec
Protruding−mouth
Day−4
Day−5
Stage
Transcript
ENSDART00000098319
ENSDART00000135675
ENSDART00000135714
ENSDART00000138810

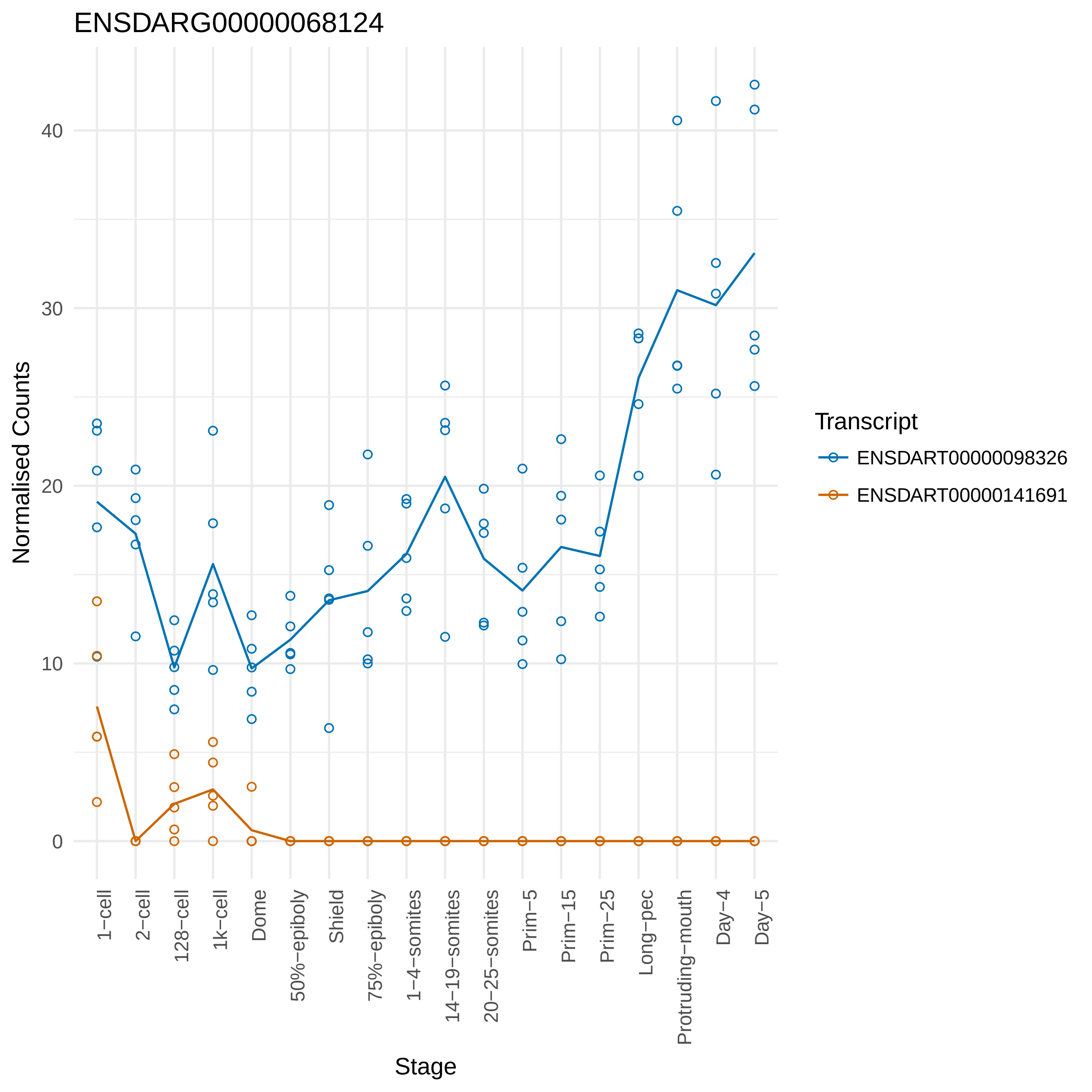

ENSDARG00000068124
Normalised Counts
0
10
20
30
40
Stage
1−cell
2−cell
128−cell
1k−cell
Dome
50%−epiboly
Shield
75%−epiboly
1−4−somites
14−19−somites
20−25−somites
Prim−5
Prim−15
Prim−25
Long−pec
Protruding−mouth
Day−4
Day−5
Transcript
ENSDART00000098326
ENSDART00000141691

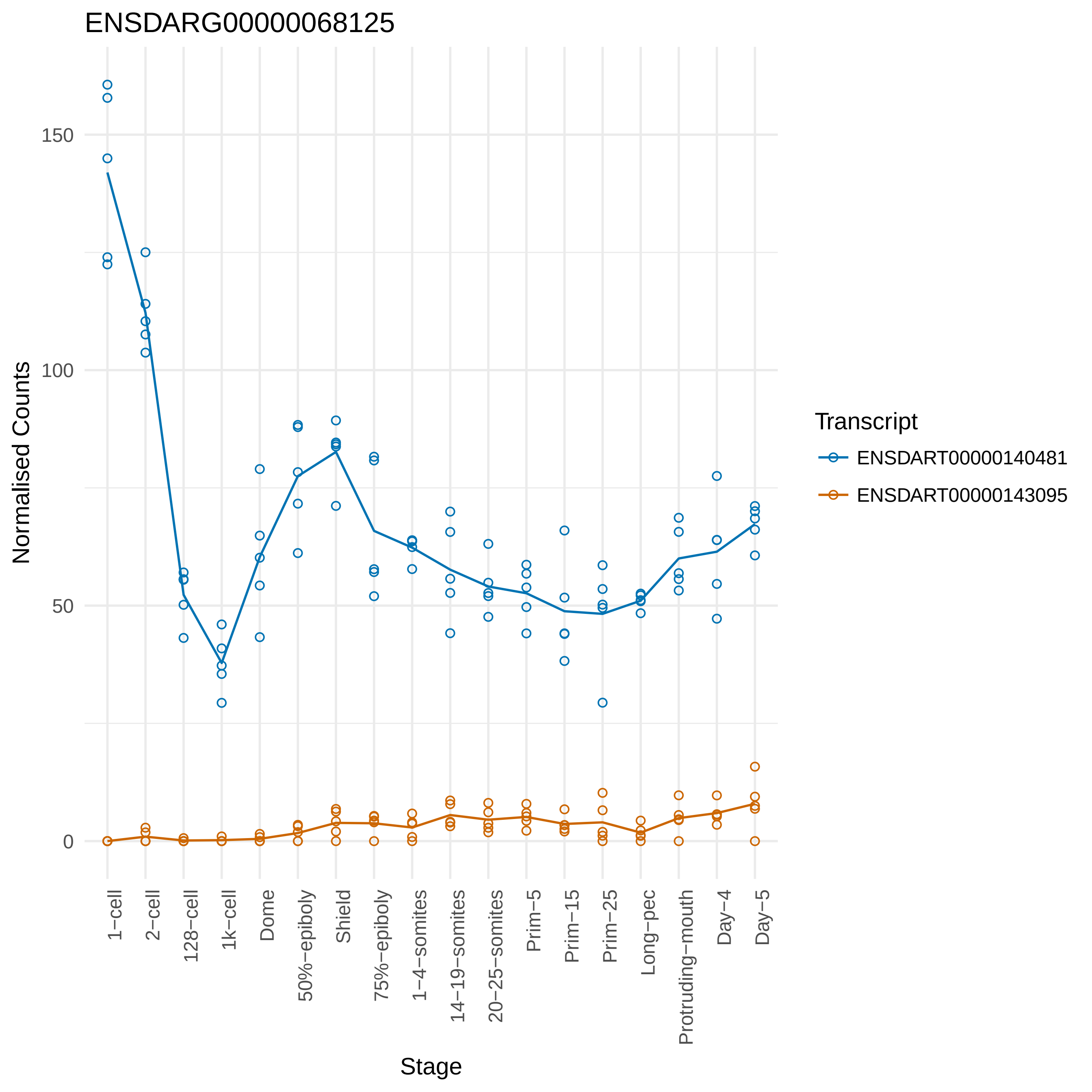
ENSDARG00000068125
Normalised Counts
150
100
50
0
Stage
1−cell
2−cell
128−cell
1k−cell
Dome
50%−epiboly
Shield
75%−epiboly
1−4−somites
14−19−somites
20−25−somites
Prim−5
Prim−15
Prim−25
Long−pec
Protruding−mouth
Day−4
Day−5
Transcript
ENSDART00000140481
ENSDART00000143095

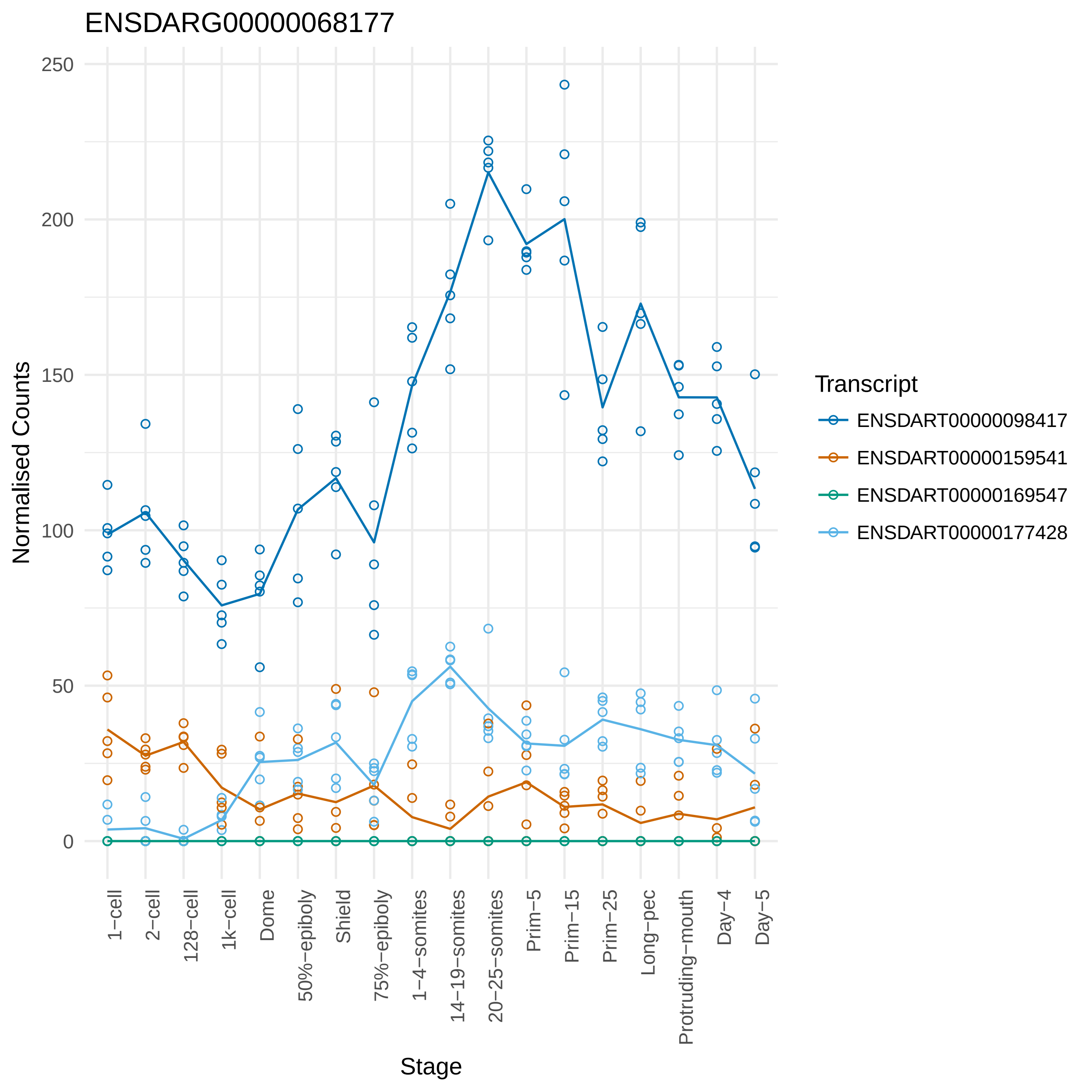

ENSDARG00000068177
Normalised Counts
250
200
150
100
50
0
1−cell
2−cell
128−cell
1k−cell
Dome
50%−epiboly
Shield
75%−epiboly
1−4−somites
14−19−somites
20−25−somites
Prim−5
Prim−15
Prim−25
Long−pec
Protruding−mouth
Day−4
Day−5
Stage
Transcript
ENSDART00000098417
ENSDART00000159541
ENSDART00000169547
ENSDART00000177428

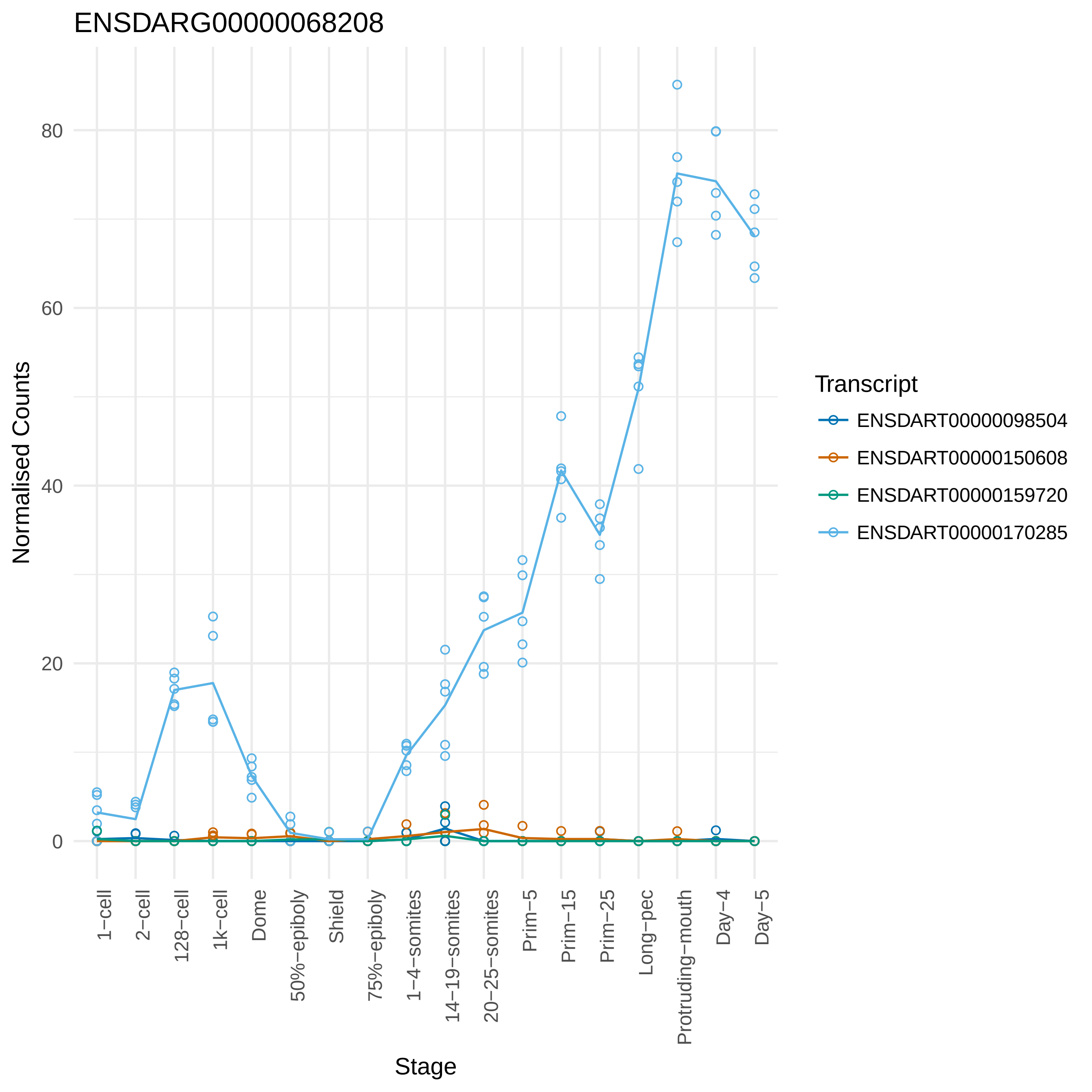

ENSDARG00000068208
Normalised Counts
0
20
40
60
80
1−cell
2−cell
128−cell
1k−cell
Dome
50%−epiboly
Shield
75%−epiboly
1−4−somites
14−19−somites
20−25−somites
Prim−5
Prim−15
Prim−25
Long−pec
Protruding−mouth
Day−4
Day−5
Stage
Transcript
ENSDART00000098504
ENSDART00000150608
ENSDART00000159720
ENSDART00000170285

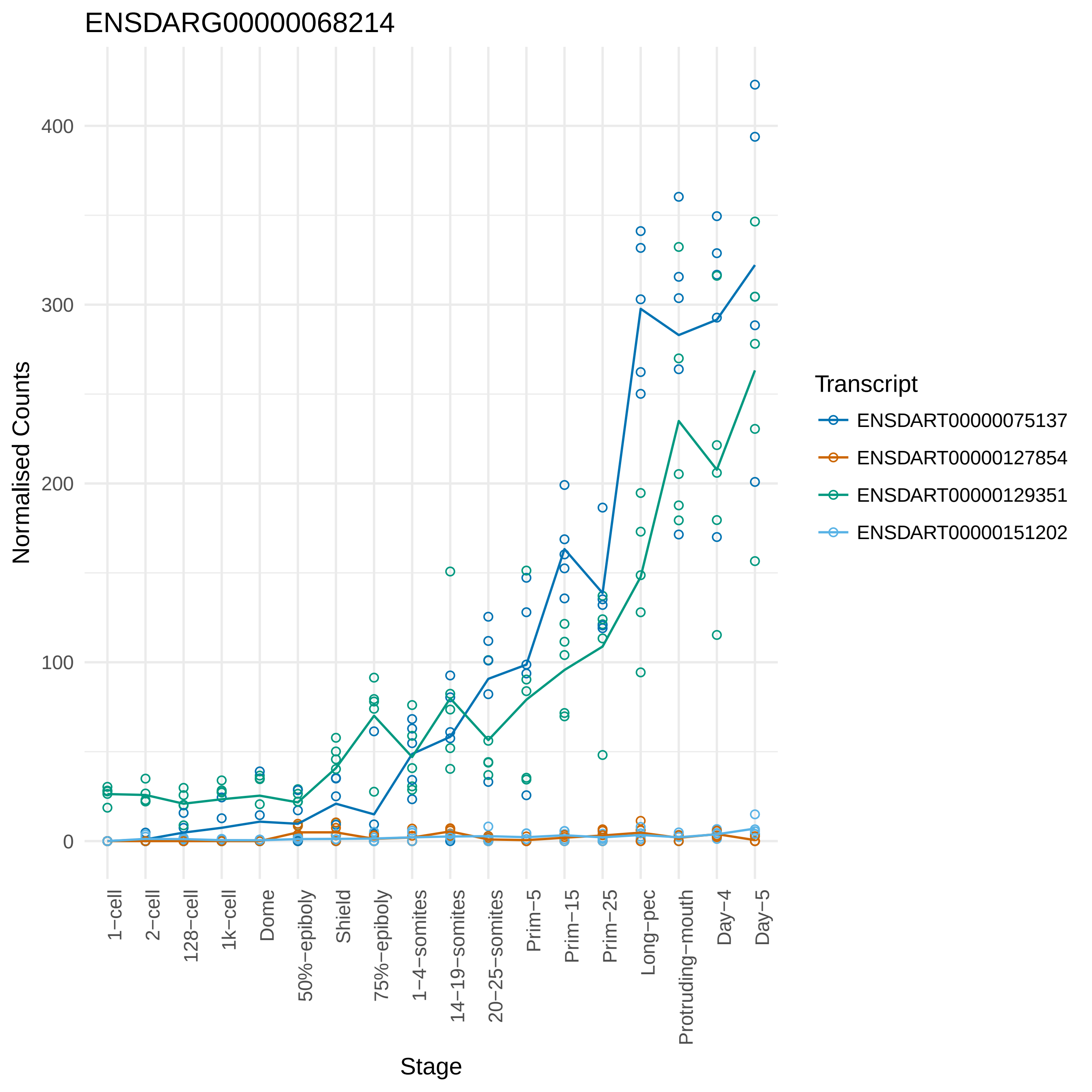
ENSDARG00000068214
Normalised Counts
0
100
200
300
400
1−cell
2−cell
128−cell
1k−cell
Dome
50%−epiboly
Shield
75%−epiboly
1−4−somites
14−19−somites
20−25−somites
Prim−5
Prim−15
Prim−25
Long−pec
Protruding−mouth
Day−4
Day−5
Stage
Transcript
ENSDART00000075137
ENSDART00000127854
ENSDART00000129351
ENSDART00000151202

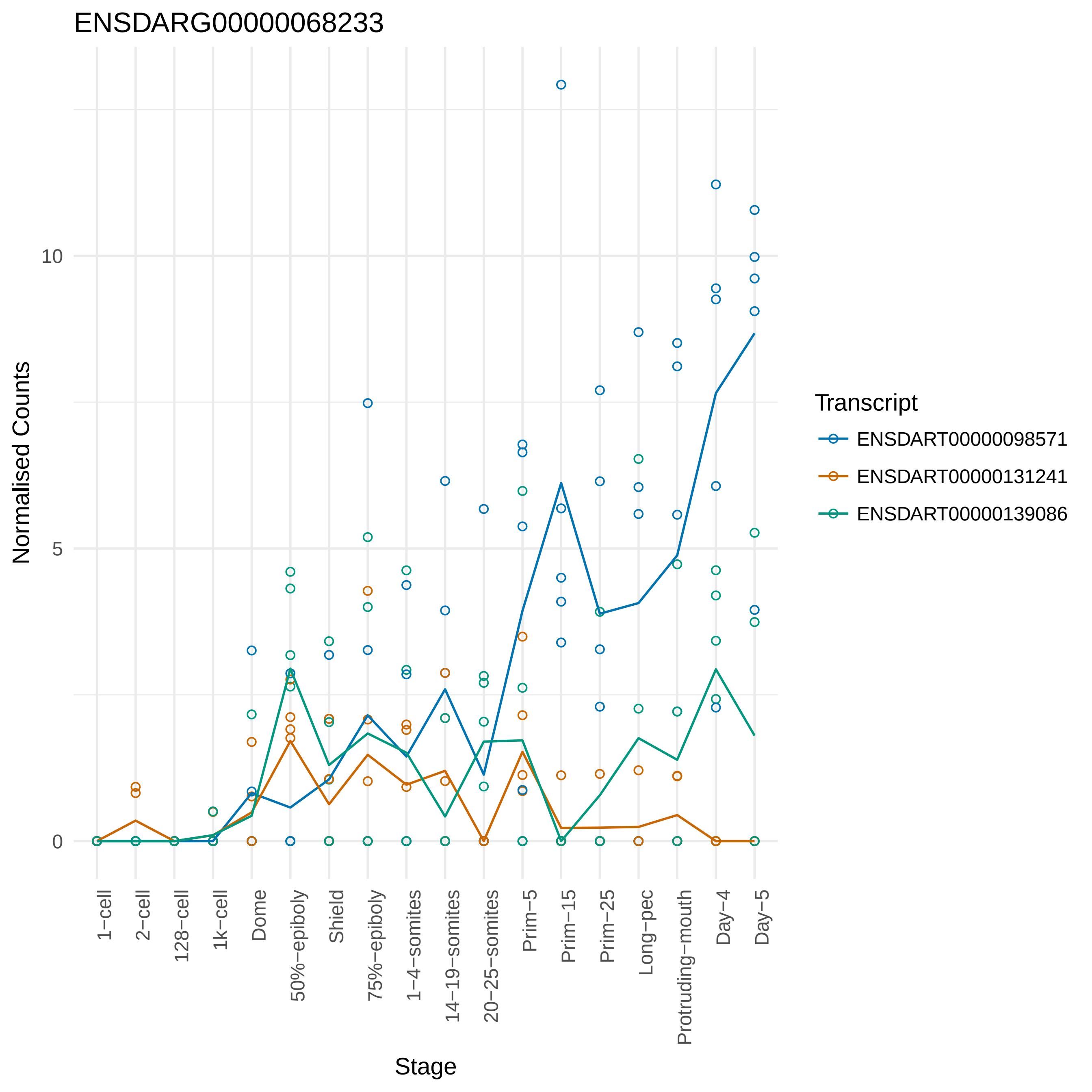

ENSDARG00000068233
Normalised Counts
10
5
0
1−cell
2−cell
128−cell
1k−cell
Dome
50%−epiboly
Shield
75%−epiboly
1−4−somites
14−19−somites
20−25−somites
Prim−5
Prim−15
Prim−25
Long−pec
Protruding−mouth
Day−4
Day−5
Stage
Transcript
ENSDART00000098571
ENSDART00000131241
ENSDART00000139086

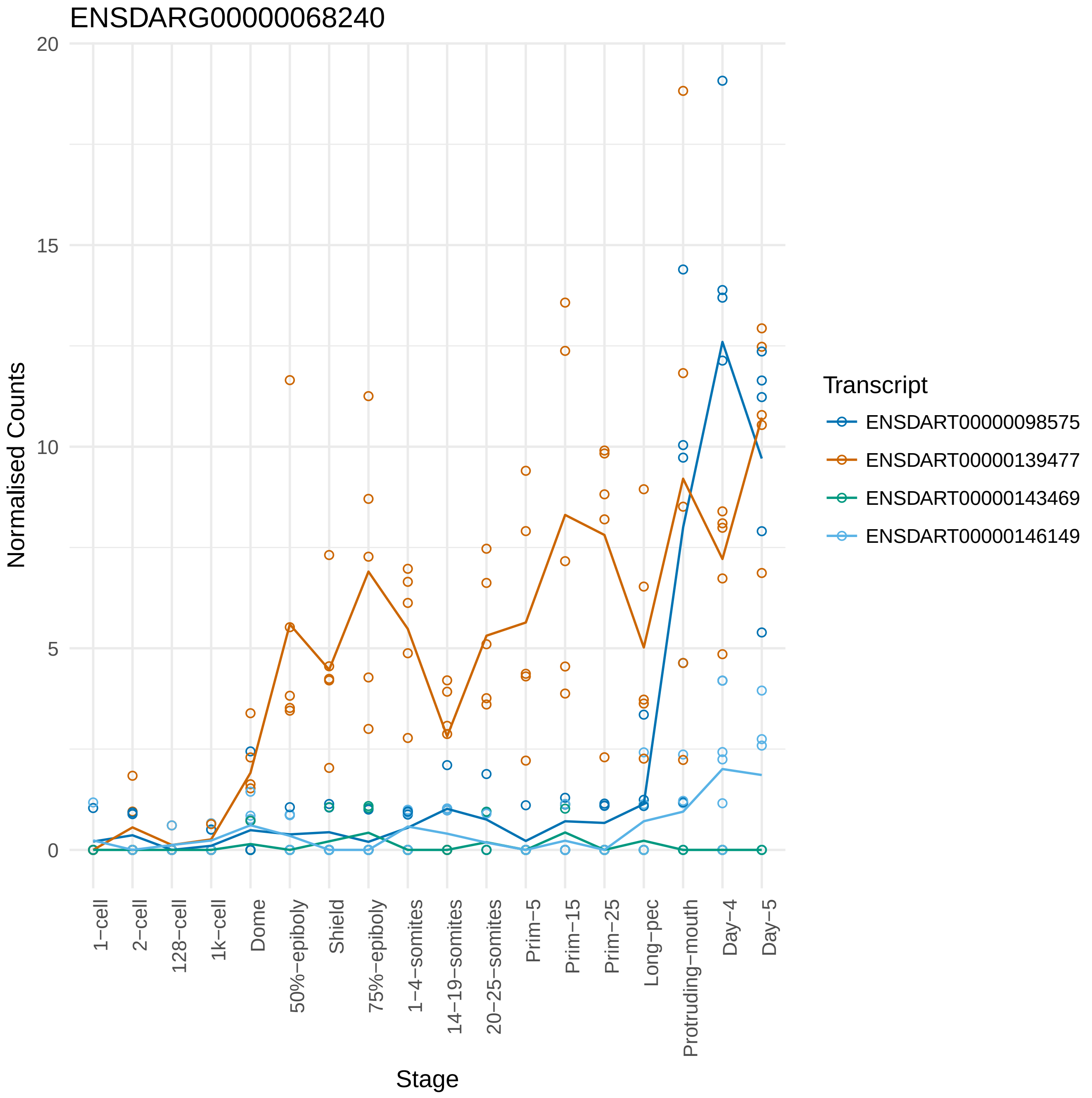

ENSDARG00000068240
Normalised Counts
20
15
10
5
0
1−cell
2−cell
128−cell
1k−cell
Dome
50%−epiboly
Shield
75%−epiboly
1−4−somites
14−19−somites
20−25−somites
Prim−5
Prim−15
Prim−25
Long−pec
Protruding−mouth
Day−4
Day−5
Stage
Transcript
ENSDART00000098575
ENSDART00000139477
ENSDART00000143469
ENSDART00000146149

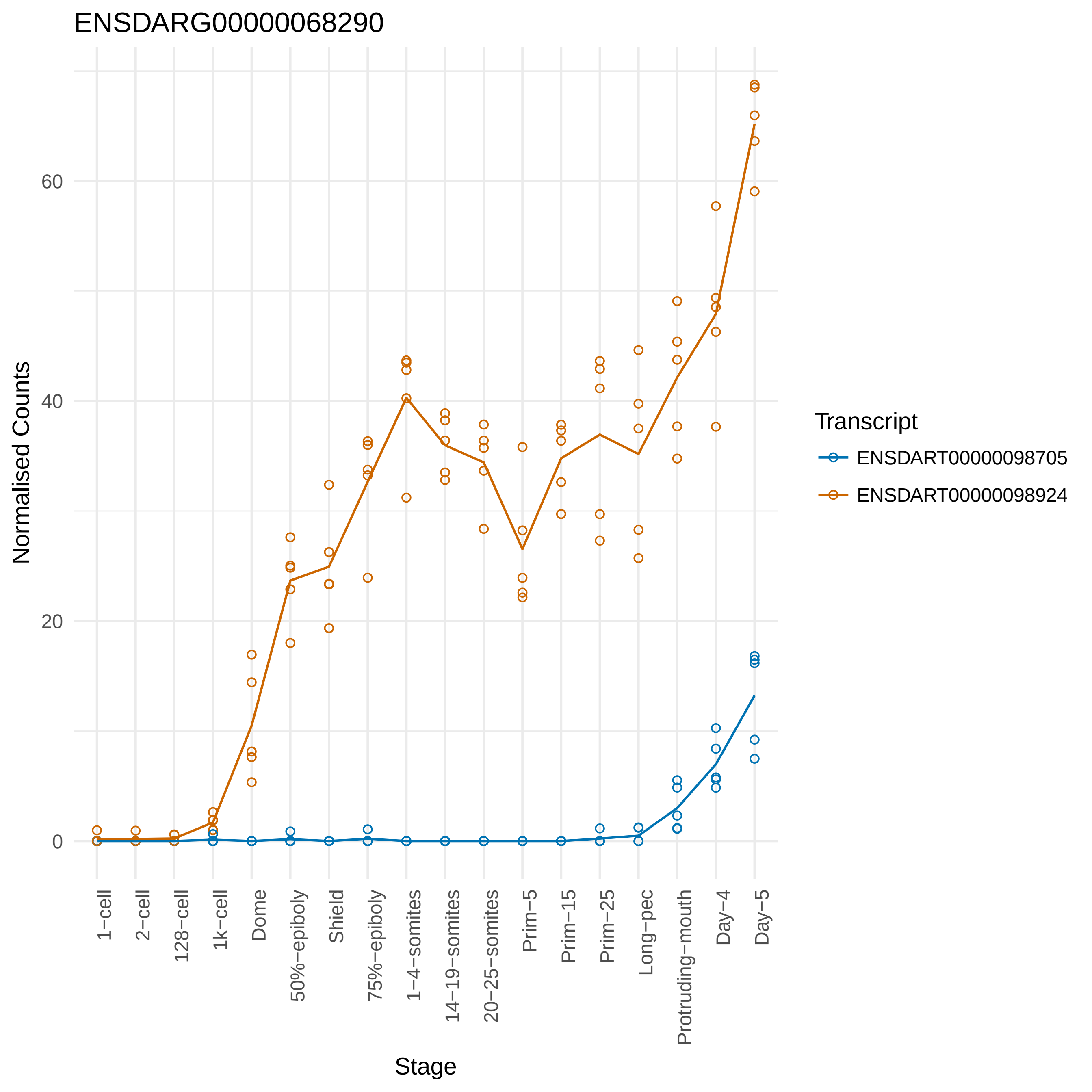
ENSDARG00000068290
Normalised Counts
60
40
20
0
1−cell
2−cell
128−cell
1k−cell
Dome
50%−epiboly
Shield
75%−epiboly
1−4−somites
14−19−somites
20−25−somites
Prim−5
Prim−15
Prim−25
Long−pec
Protruding−mouth
Day−4
Day−5
Stage
Transcript
ENSDART00000098705
ENSDART00000098924

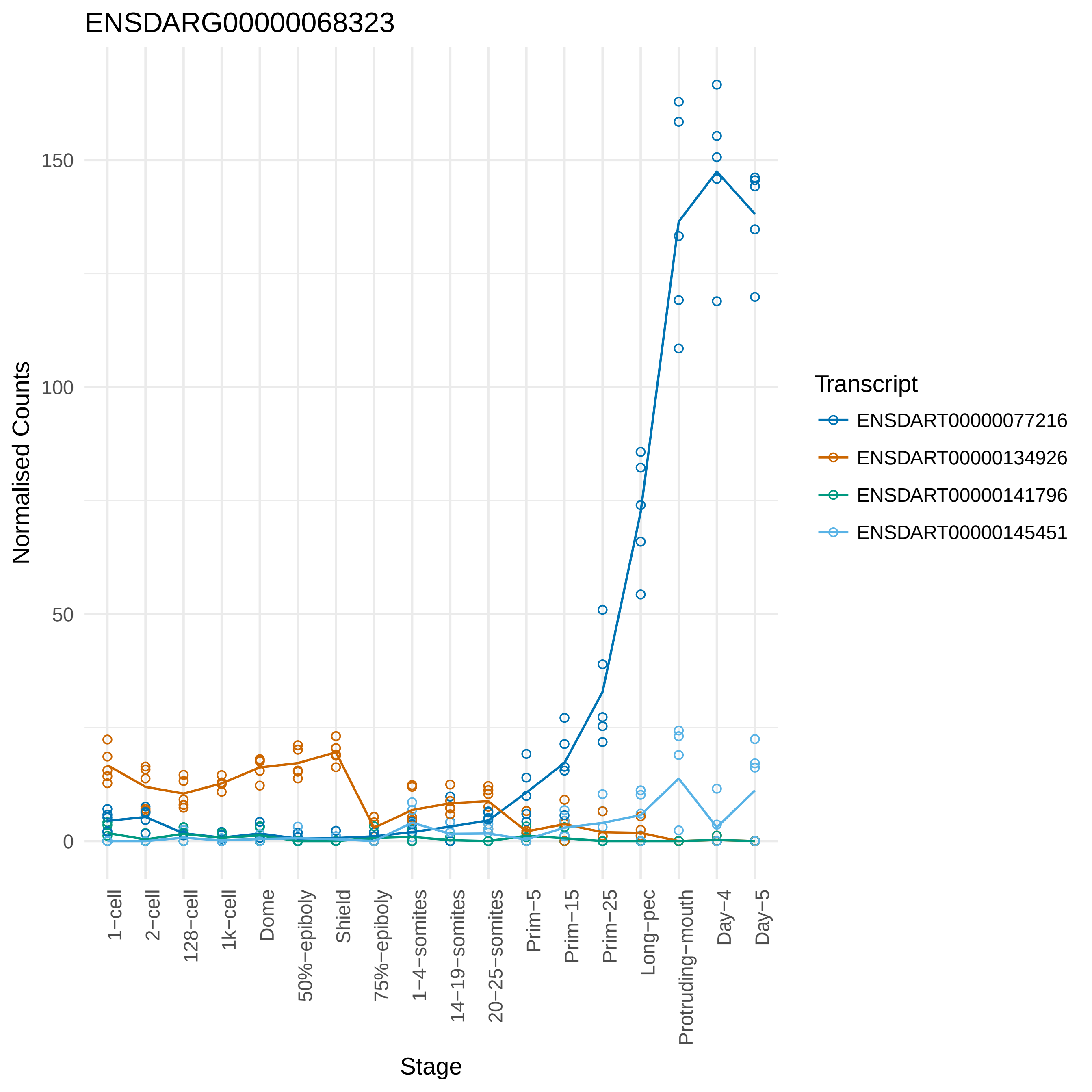
ENSDARG00000068323
Normalised Counts
150
100
50
0
1−cell
2−cell
128−cell
1k−cell
Dome
50%−epiboly
Shield
75%−epiboly
1−4−somites
14−19−somites
20−25−somites
Prim−5
Prim−15
Prim−25
Long−pec
Protruding−mouth
Day−4
Day−5
Stage
Transcript
ENSDART00000077216
ENSDART00000134926
ENSDART00000141796
ENSDART00000145451

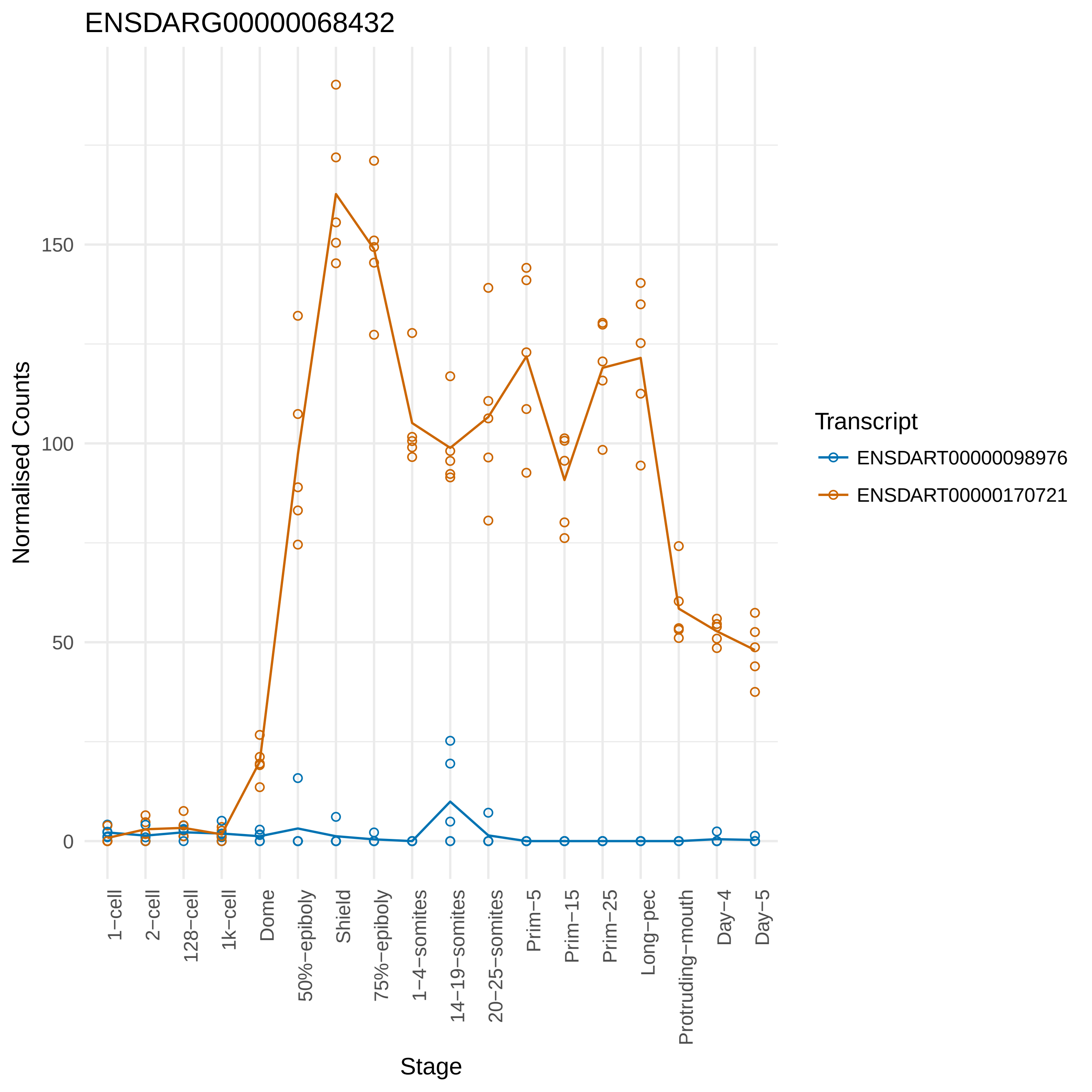
ENSDARG00000068432
Normalised Counts
0
50
100
150
Stage
1−cell
2−cell
128−cell
1k−cell
Dome
50%−epiboly
Shield
75%−epiboly
1−4−somites
14−19−somites
20−25−somites
Prim−5
Prim−15
Prim−25
Long−pec
Protruding−mouth
Day−4
Day−5
Transcript
ENSDART00000098976
ENSDART00000170721

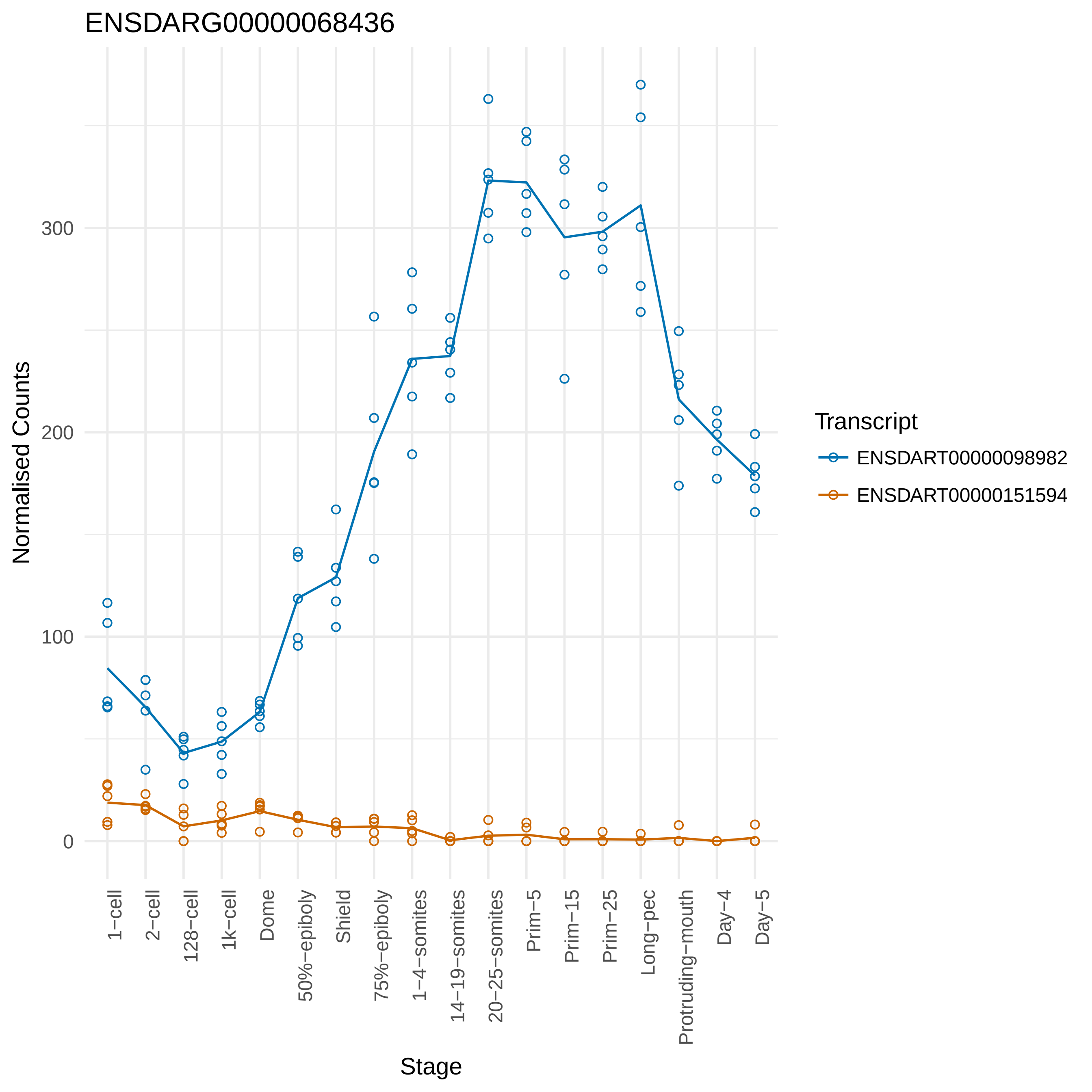
ENSDARG00000068436
Normalised Counts
0
100
200
300
Stage
1−cell
2−cell
128−cell
1k−cell
Dome
50%−epiboly
Shield
75%−epiboly
1−4−somites
14−19−somites
20−25−somites
Prim−5
Prim−15
Prim−25
Long−pec
Protruding−mouth
Day−4
Day−5
Transcript
ENSDART00000098982
ENSDART00000151594

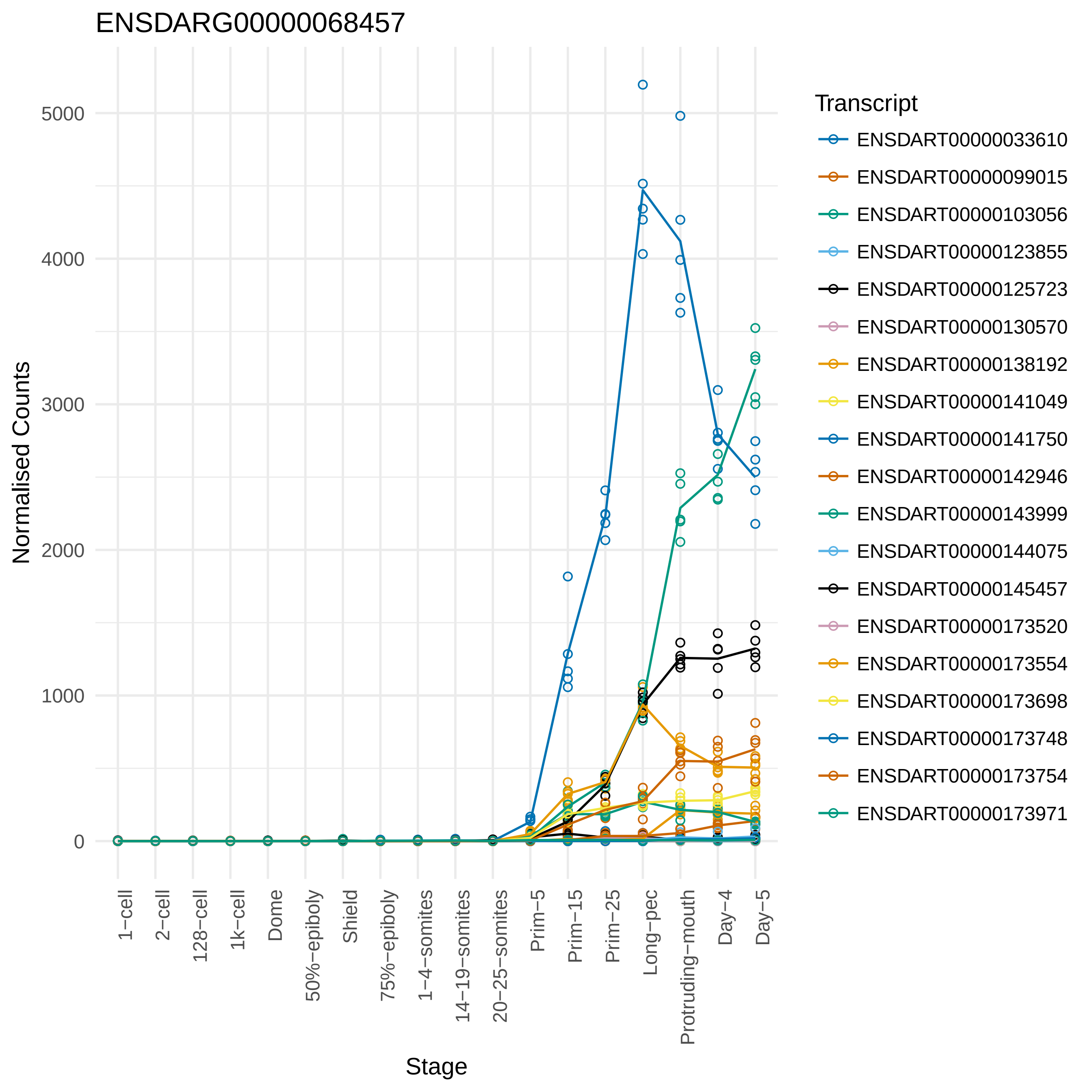

ENSDARG00000068457
Normalised Counts
0
1000
2000
3000
4000
5000
1−cell
2−cell
128−cell
1k−cell
Dome
50%−epiboly
Shield
75%−epiboly
1−4−somites
14−19−somites
20−25−somites
Prim−5
Prim−15
Prim−25
Long−pec
Protruding−mouth
Day−4
Day−5
Stage
Transcript
ENSDART00000033610
ENSDART00000099015
ENSDART00000103056
ENSDART00000123855
ENSDART00000125723
ENSDART00000130570
ENSDART00000138192
ENSDART00000141049
ENSDART00000141750
ENSDART00000142946
ENSDART00000143999
ENSDART00000144075
ENSDART00000145457
ENSDART00000173520
ENSDART00000173554
ENSDART00000173698
ENSDART00000173748
ENSDART00000173754
ENSDART00000173971

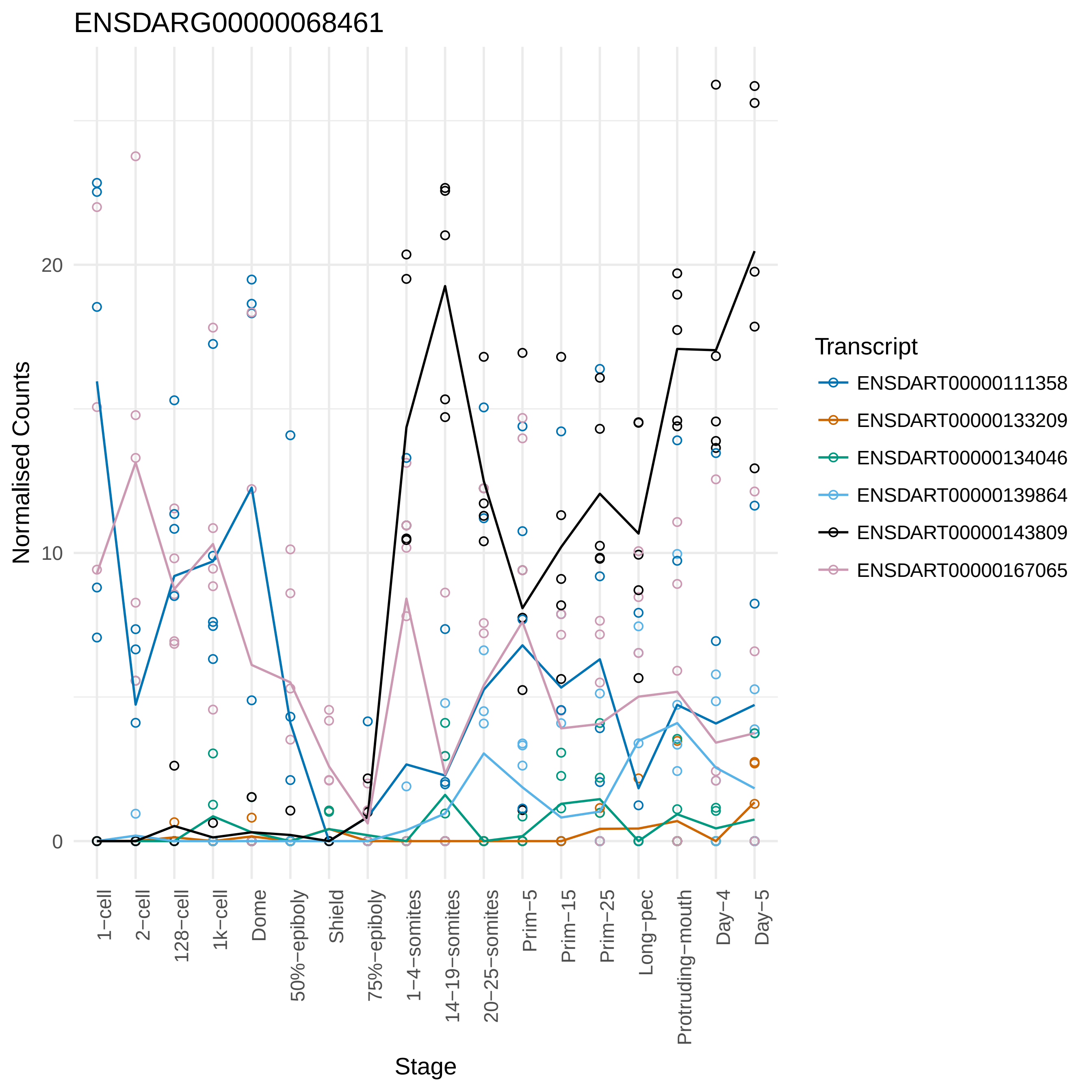

ENSDARG00000068461
Normalised Counts
20
10
0
1−cell
2−cell
128−cell
1k−cell
Dome
50%−epiboly
Shield
75%−epiboly
1−4−somites
14−19−somites
20−25−somites
Prim−5
Prim−15
Prim−25
Long−pec
Protruding−mouth
Day−4
Day−5
Stage
Transcript
ENSDART00000111358
ENSDART00000133209
ENSDART00000134046
ENSDART00000139864
ENSDART00000143809
ENSDART00000167065

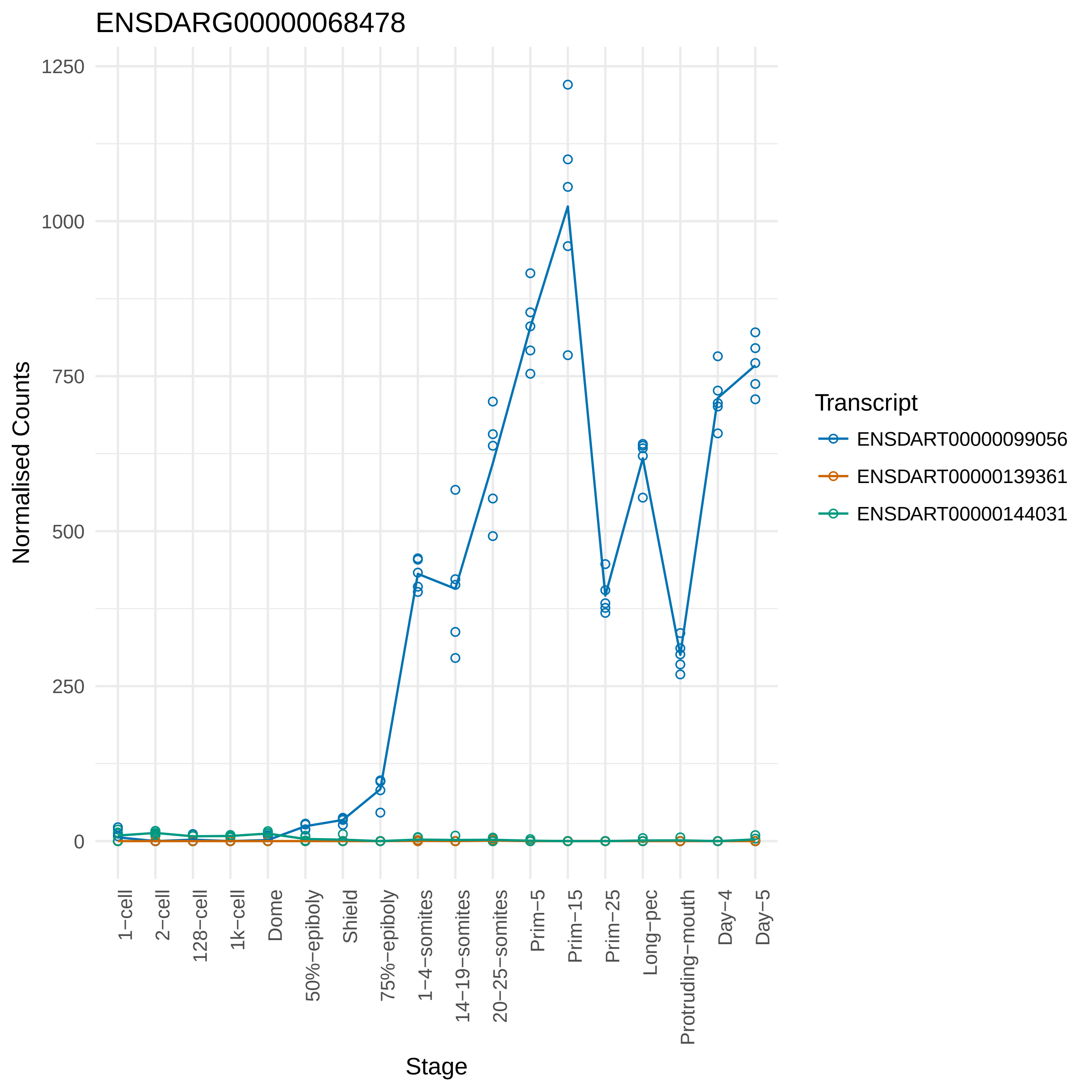

ENSDARG00000068478
Normalised Counts
1250
1000
750
500
250
0
1−cell
2−cell
128−cell
1k−cell
Dome
50%−epiboly
Shield
75%−epiboly
1−4−somites
14−19−somites
20−25−somites
Prim−5
Prim−15
Prim−25
Long−pec
Protruding−mouth
Day−4
Day−5
Stage
Transcript
ENSDART00000099056
ENSDART00000139361
ENSDART00000144031

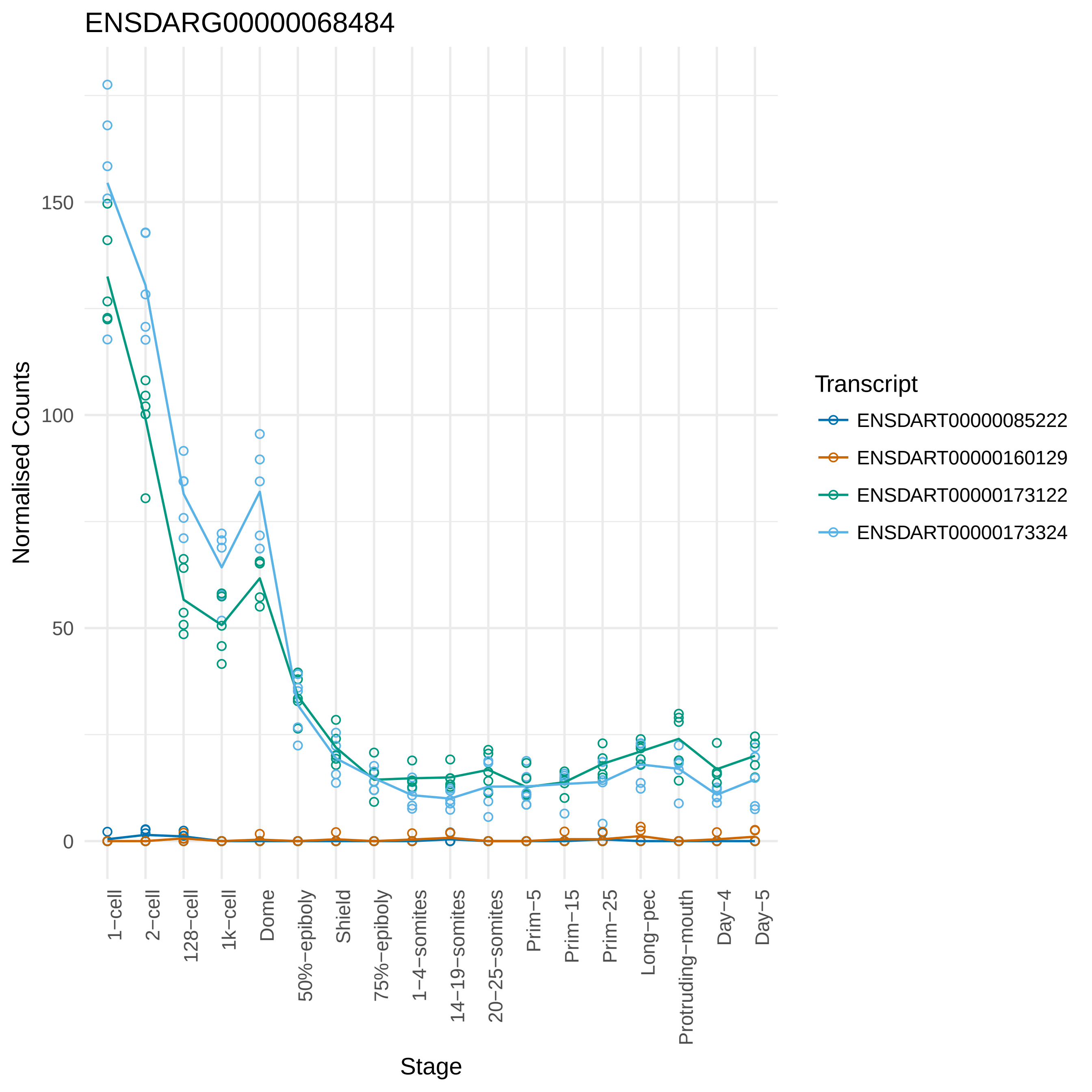

ENSDARG00000068484
Normalised Counts
150
100
50
0
1−cell
2−cell
128−cell
1k−cell
Dome
50%−epiboly
Shield
75%−epiboly
1−4−somites
14−19−somites
20−25−somites
Prim−5
Prim−15
Prim−25
Long−pec
Protruding−mouth
Day−4
Day−5
Stage
Transcript
ENSDART00000085222
ENSDART00000160129
ENSDART00000173122
ENSDART00000173324

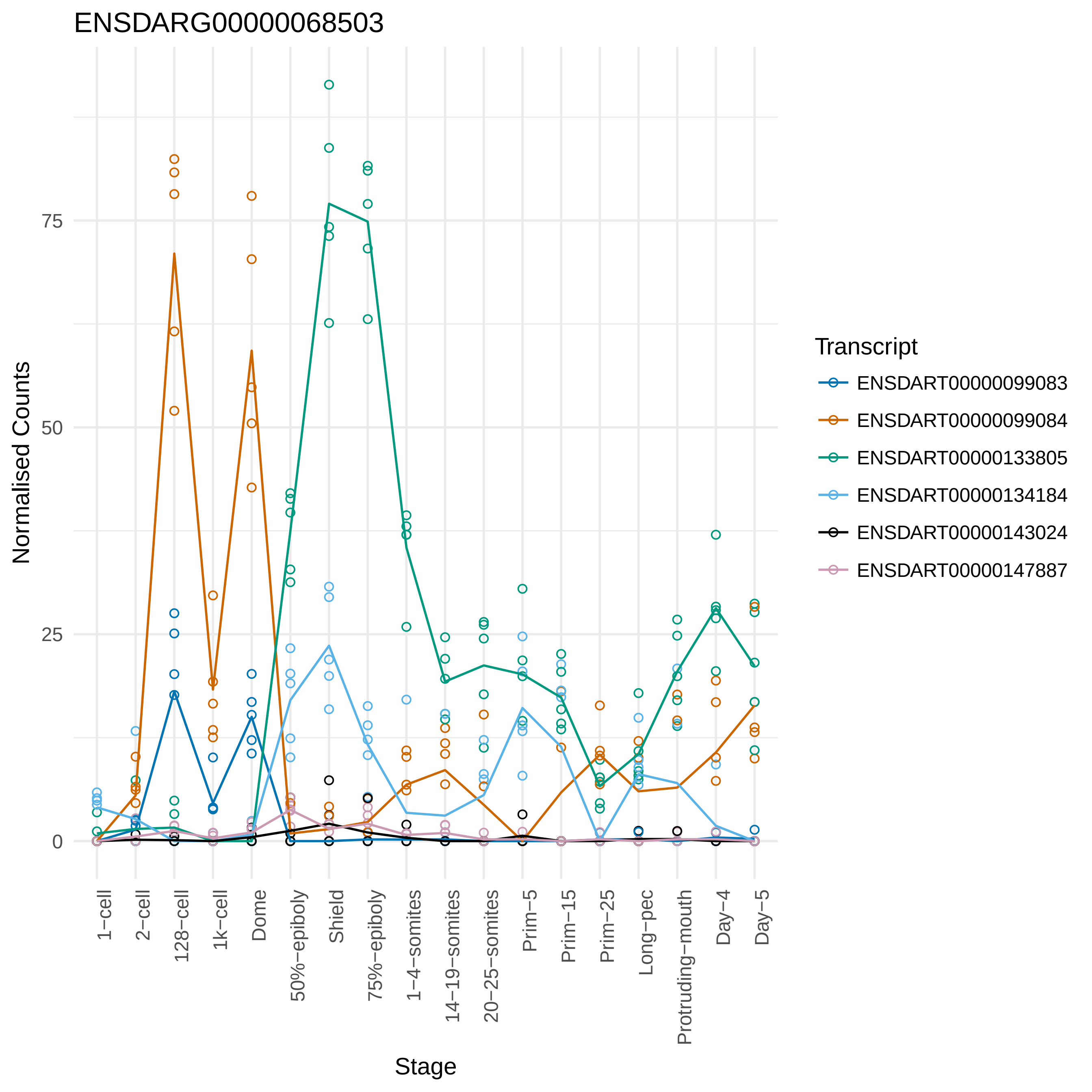

ENSDARG00000068503
Normalised Counts
0
25
50
75
1−cell
2−cell
128−cell
1k−cell
Dome
50%−epiboly
Shield
75%−epiboly
1−4−somites
14−19−somites
20−25−somites
Prim−5
Prim−15
Prim−25
Long−pec
Protruding−mouth
Day−4
Day−5
Stage
Transcript
ENSDART00000099083
ENSDART00000099084
ENSDART00000133805
ENSDART00000134184
ENSDART00000143024
ENSDART00000147887

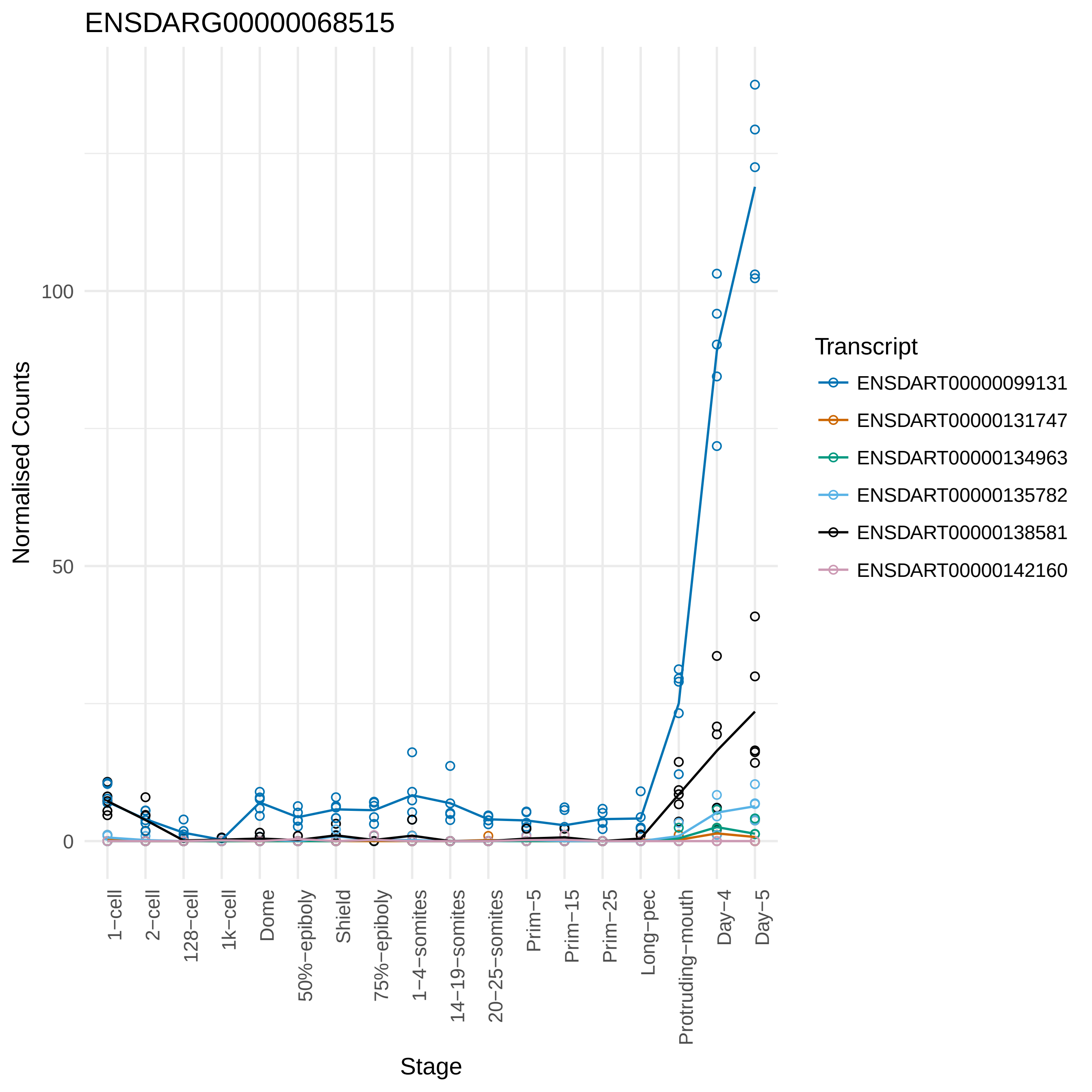

ENSDARG00000068515
Normalised Counts
100
50
0
1−cell
2−cell
128−cell
1k−cell
Dome
50%−epiboly
Shield
75%−epiboly
1−4−somites
14−19−somites
20−25−somites
Prim−5
Prim−15
Prim−25
Long−pec
Protruding−mouth
Day−4
Day−5
Stage
Transcript
ENSDART00000099131
ENSDART00000131747
ENSDART00000134963
ENSDART00000135782
ENSDART00000138581
ENSDART00000142160

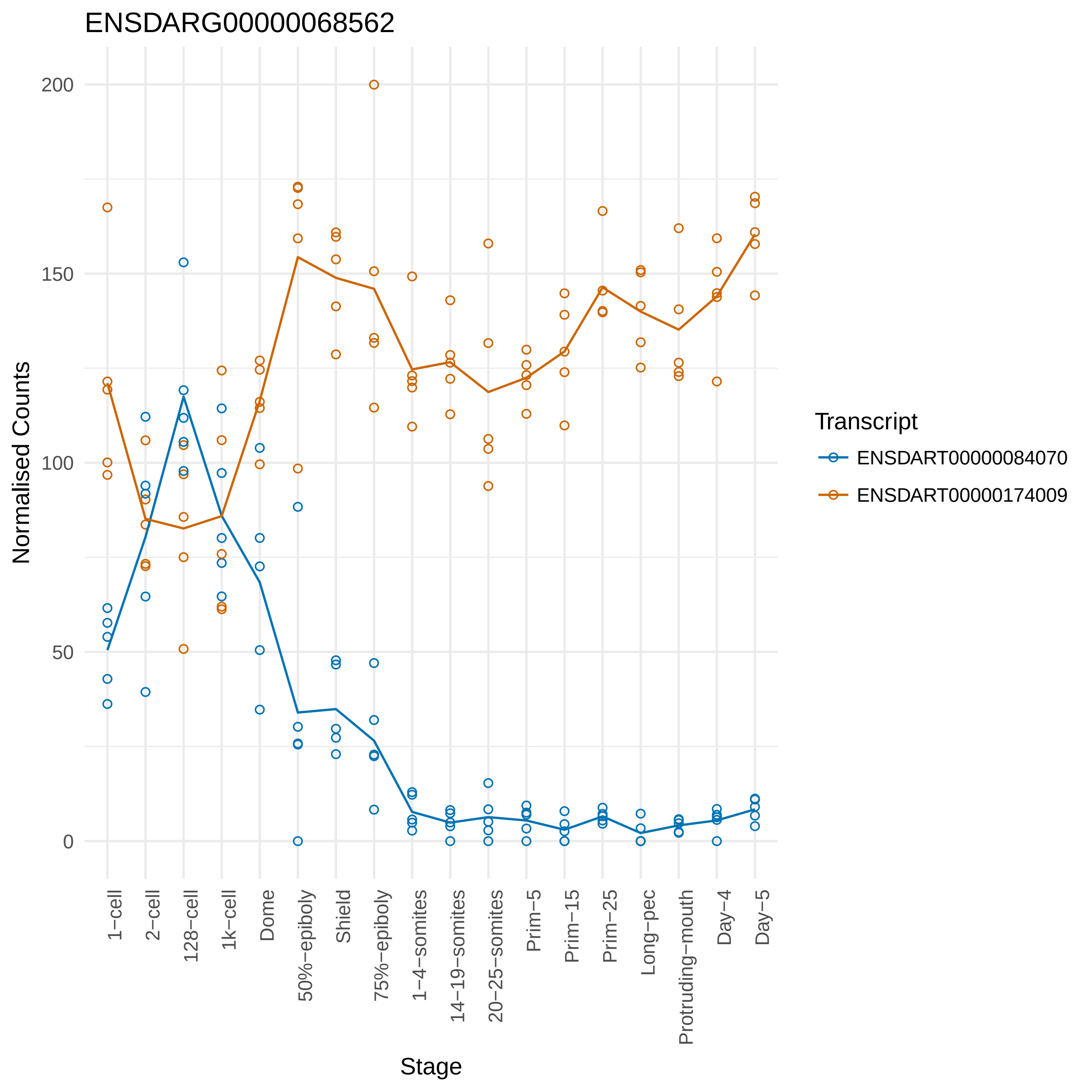

ENSDARG00000068562
Normalised Counts
200
150
100
50
0
1−cell
2−cell
128−cell
1k−cell
Dome
50%−epiboly
Shield
75%−epiboly
1−4−somites
14−19−somites
20−25−somites
Prim−5
Prim−15
Prim−25
Long−pec
Protruding−mouth
Day−4
Day−5
Stage
Transcript
ENSDART00000084070
ENSDART00000174009

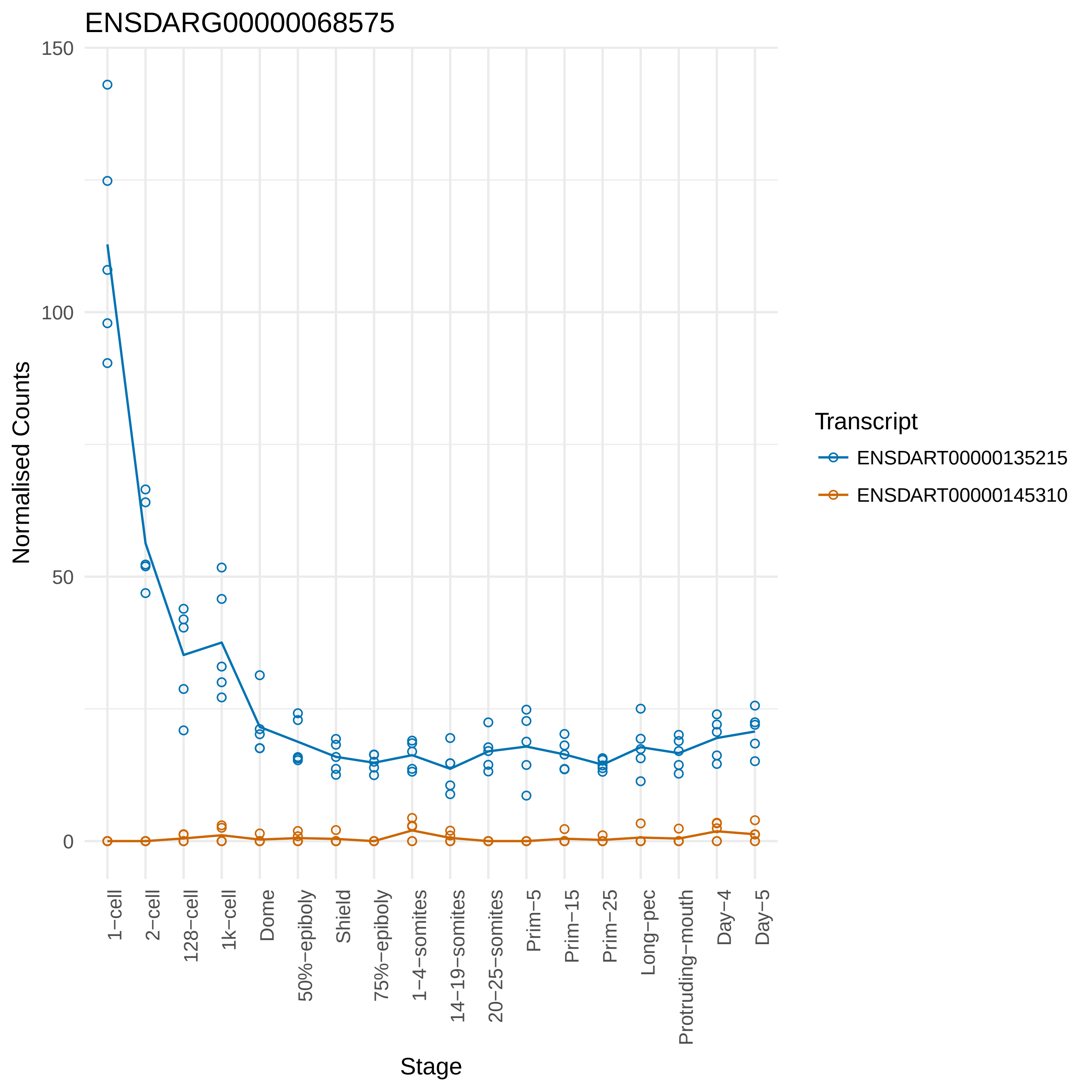

ENSDARG00000068575
150
100
50
0
Normalised Counts
1−cell
2−cell
128−cell
1k−cell
Dome
50%−epiboly
Shield
75%−epiboly
1−4−somites
14−19−somites
20−25−somites
Prim−5
Prim−15
Prim−25
Long−pec
Protruding−mouth
Day−4
Day−5
Stage
Transcript
ENSDART00000135215
ENSDART00000145310

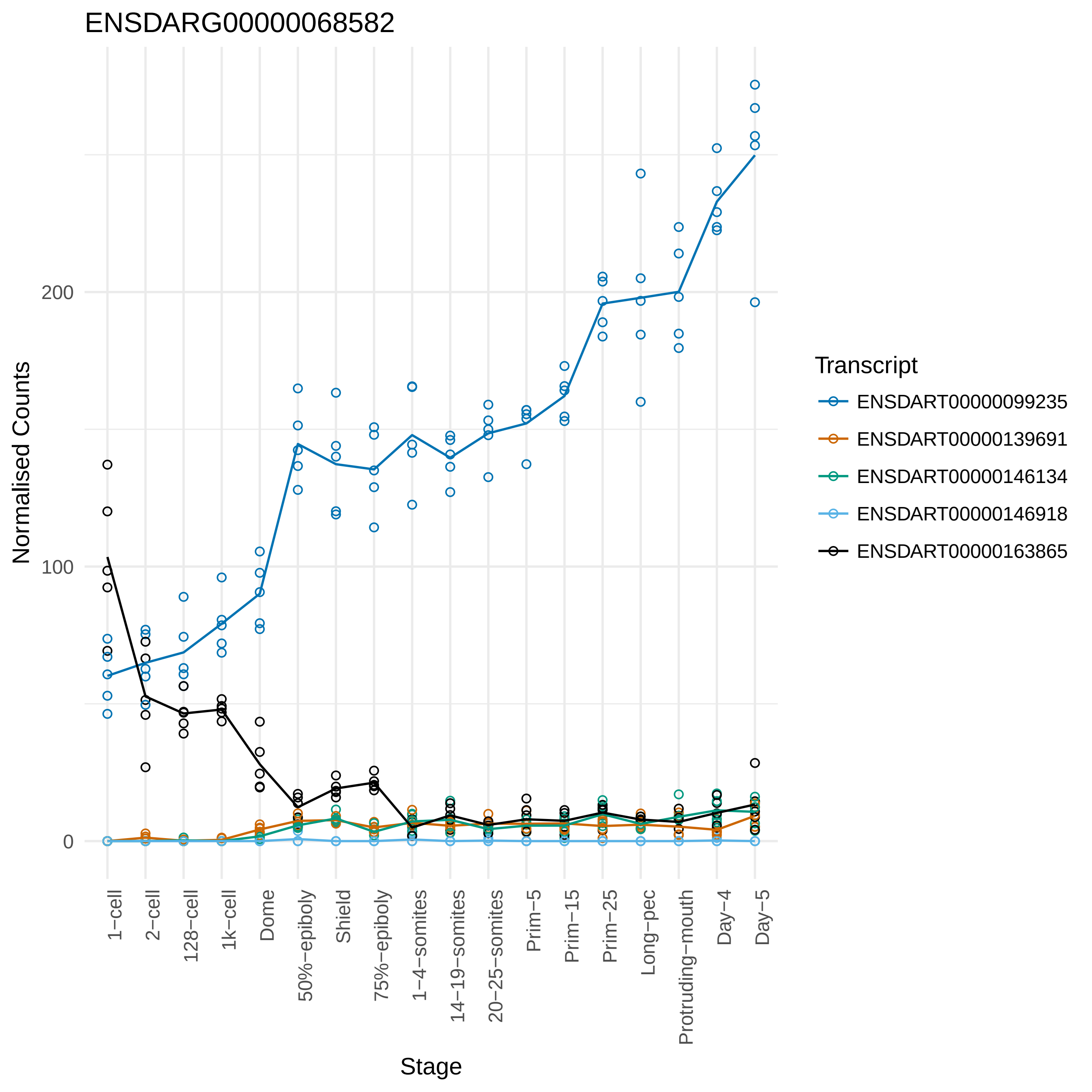
ENSDARG00000068582
Normalised Counts
0
100
200
Stage
1−cell
2−cell
128−cell
1k−cell
Dome
50%−epiboly
Shield
75%−epiboly
1−4−somites
14−19−somites
20−25−somites
Prim−5
Prim−15
Prim−25
Long−pec
Protruding−mouth
Day−4
Day−5
Transcript
ENSDART00000099235
ENSDART00000139691
ENSDART00000146134
ENSDART00000146918
ENSDART00000163865

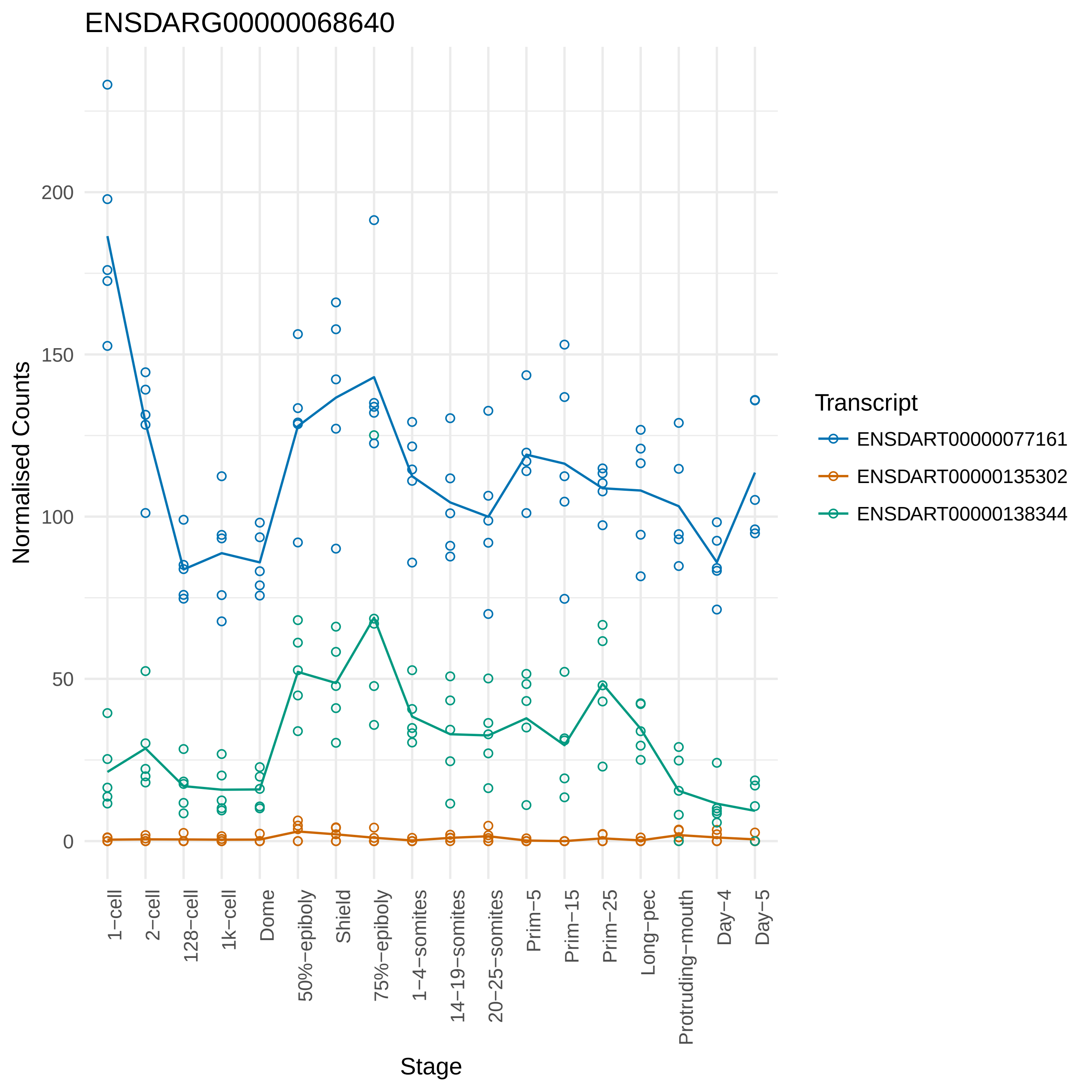
ENSDARG00000068640
Normalised Counts
200
150
100
50
0
Stage
1−cell
2−cell
128−cell
1k−cell
Dome
50%−epiboly
Shield
75%−epiboly
1−4−somites
14−19−somites
20−25−somites
Prim−5
Prim−15
Prim−25
Long−pec
Protruding−mouth
Day−4
Day−5
Transcript
ENSDART00000077161
ENSDART00000135302
ENSDART00000138344

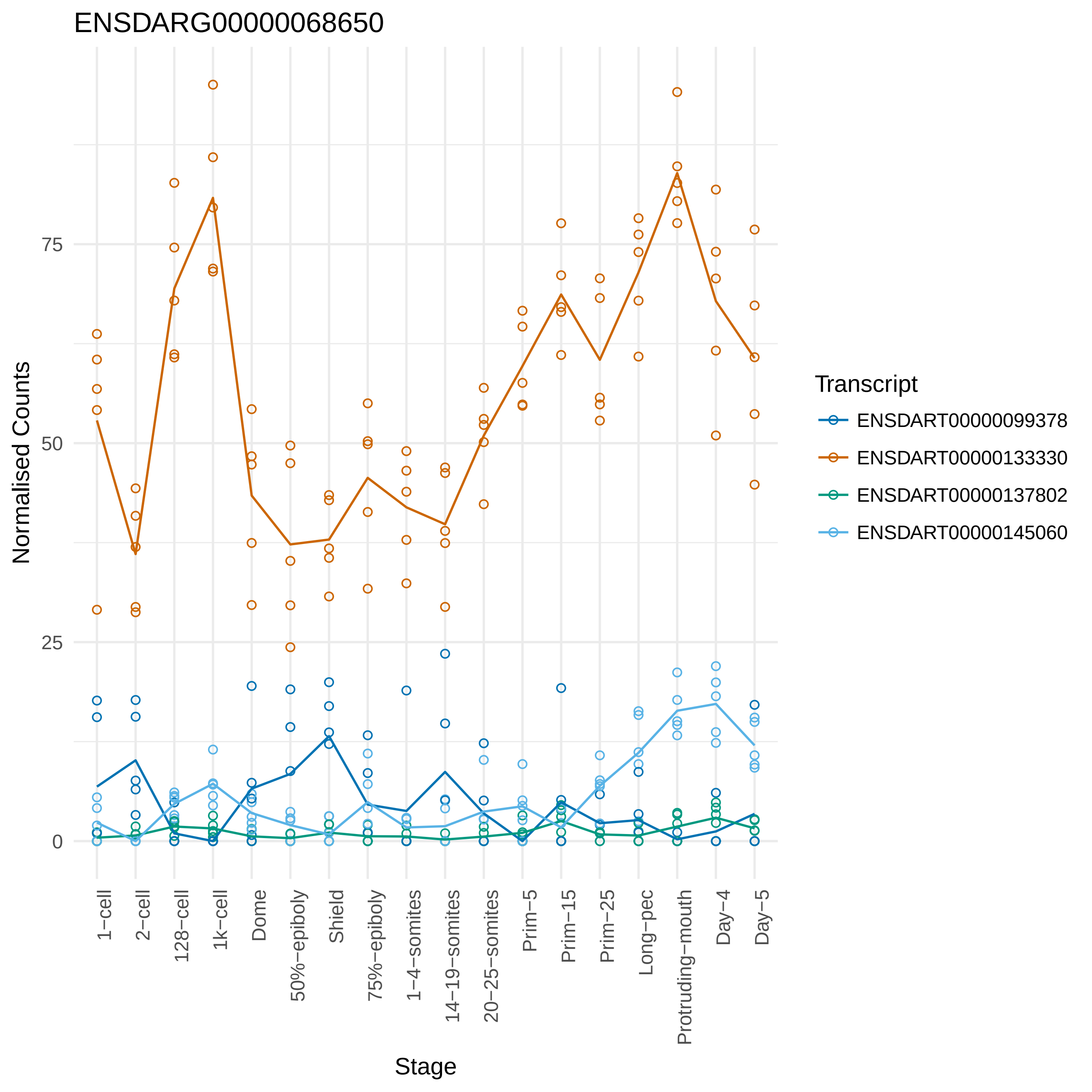
ENSDARG00000068650
Normalised Counts
0
25
50
75
1−cell
2−cell
128−cell
1k−cell
Dome
50%−epiboly
Shield
75%−epiboly
1−4−somites
14−19−somites
20−25−somites
Prim−5
Prim−15
Prim−25
Long−pec
Protruding−mouth
Day−4
Day−5
Stage
Transcript
ENSDART00000099378
ENSDART00000133330
ENSDART00000137802
ENSDART00000145060

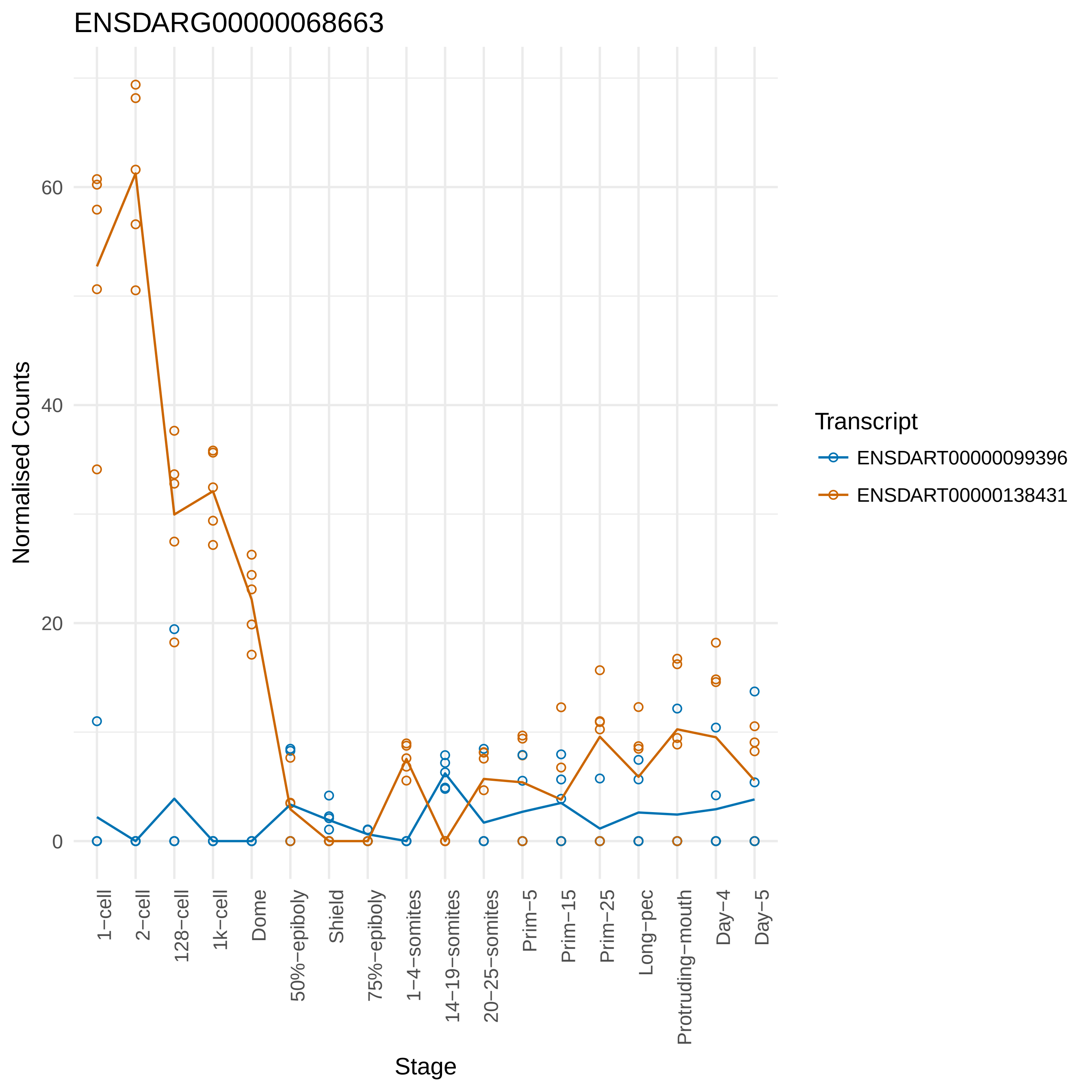
ENSDARG00000068663
Normalised Counts
60
40
20
0
1−cell
2−cell
128−cell
1k−cell
Dome
50%−epiboly
Shield
75%−epiboly
1−4−somites
14−19−somites
20−25−somites
Prim−5
Prim−15
Prim−25
Long−pec
Protruding−mouth
Day−4
Day−5
Stage
Transcript
ENSDART00000099396
ENSDART00000138431

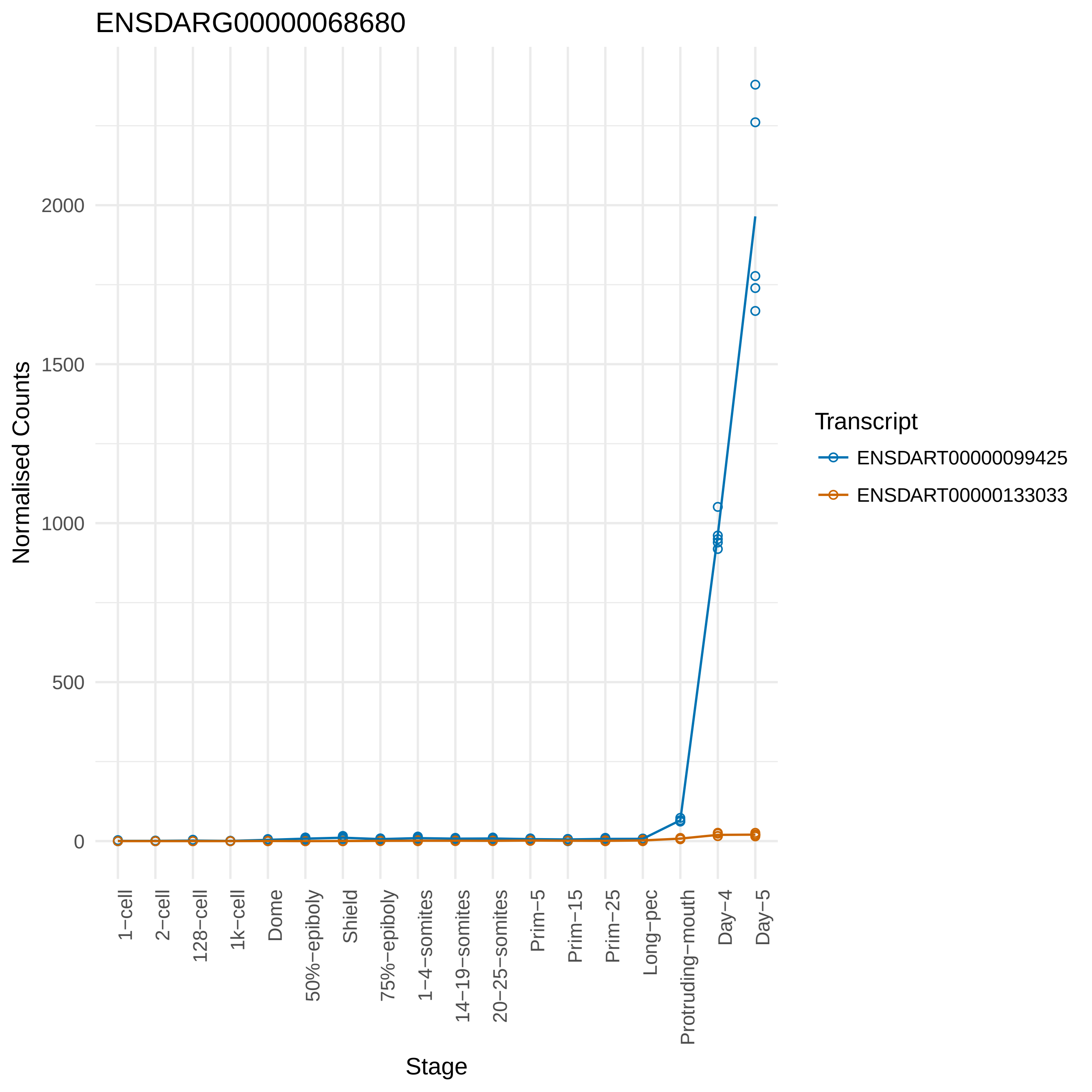

ENSDARG00000068680
Normalised Counts
0
500
1000
1500
2000
Stage
1−cell
2−cell
128−cell
1k−cell
Dome
50%−epiboly
Shield
75%−epiboly
1−4−somites
14−19−somites
20−25−somites
Prim−5
Prim−15
Prim−25
Long−pec
Protruding−mouth
Day−4
Day−5
Transcript
ENSDART00000099425
ENSDART00000133033

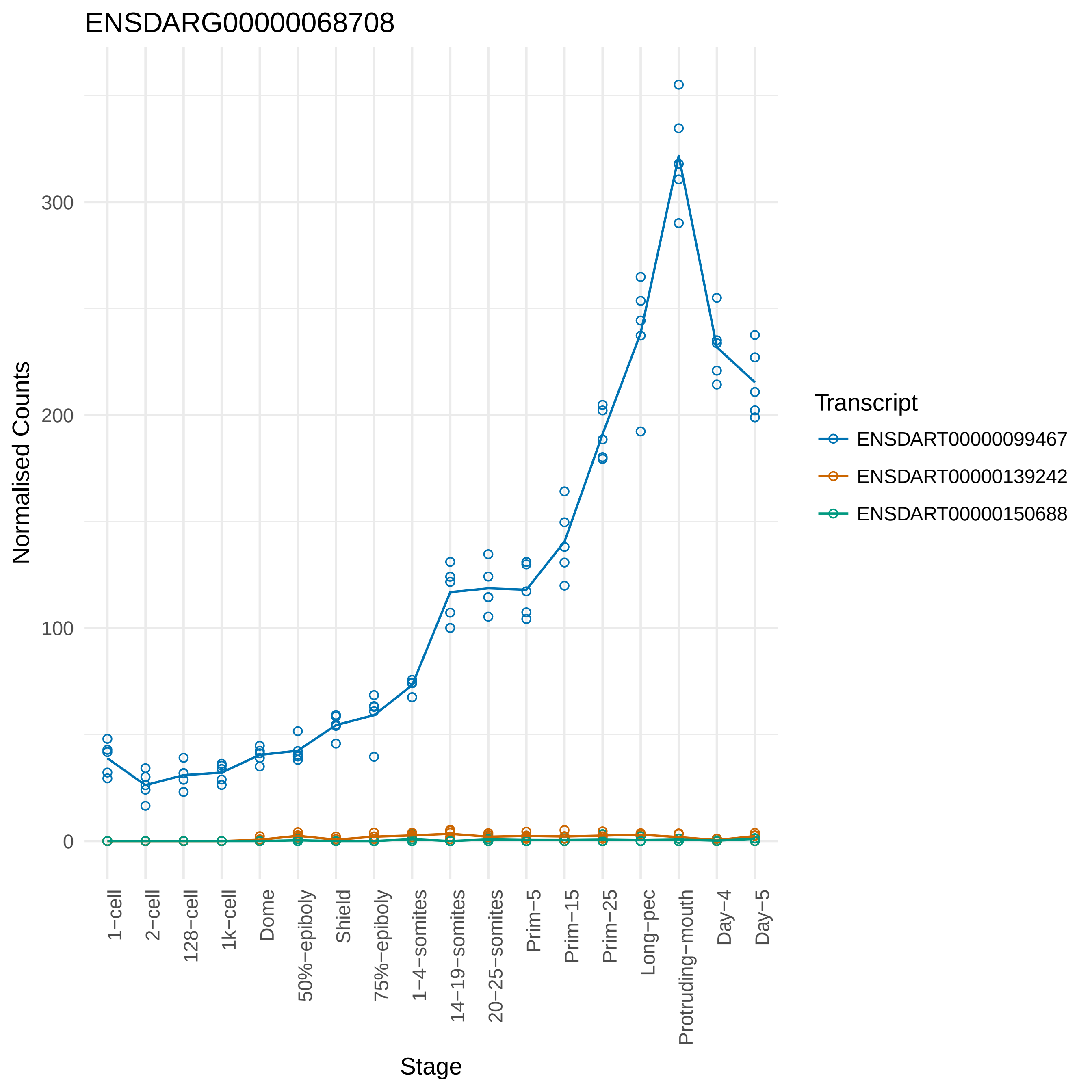
ENSDARG00000068708
Normalised Counts
300
200
100
0
1−cell
2−cell
128−cell
1k−cell
Dome
50%−epiboly
Shield
75%−epiboly
1−4−somites
14−19−somites
20−25−somites
Prim−5
Prim−15
Prim−25
Long−pec
Protruding−mouth
Day−4
Day−5
Stage
Transcript
ENSDART00000099467
ENSDART00000139242
ENSDART00000150688

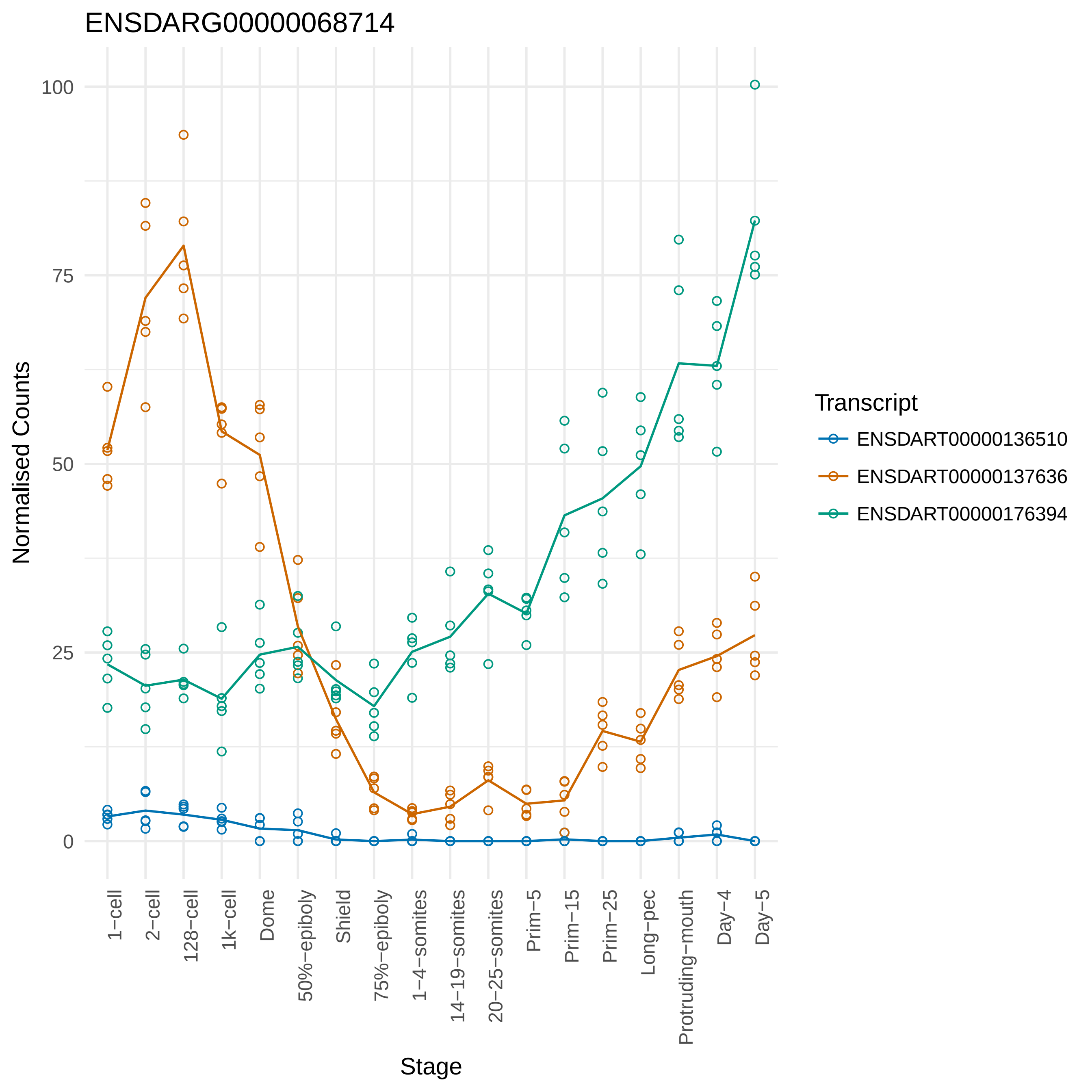
ENSDARG00000068714
Normalised Counts
100
75
50
25
0
1−cell
2−cell
128−cell
1k−cell
Dome
50%−epiboly
Shield
75%−epiboly
1−4−somites
14−19−somites
20−25−somites
Prim−5
Prim−15
Prim−25
Long−pec
Protruding−mouth
Day−4
Day−5
Stage
Transcript
ENSDART00000136510
ENSDART00000137636
ENSDART00000176394

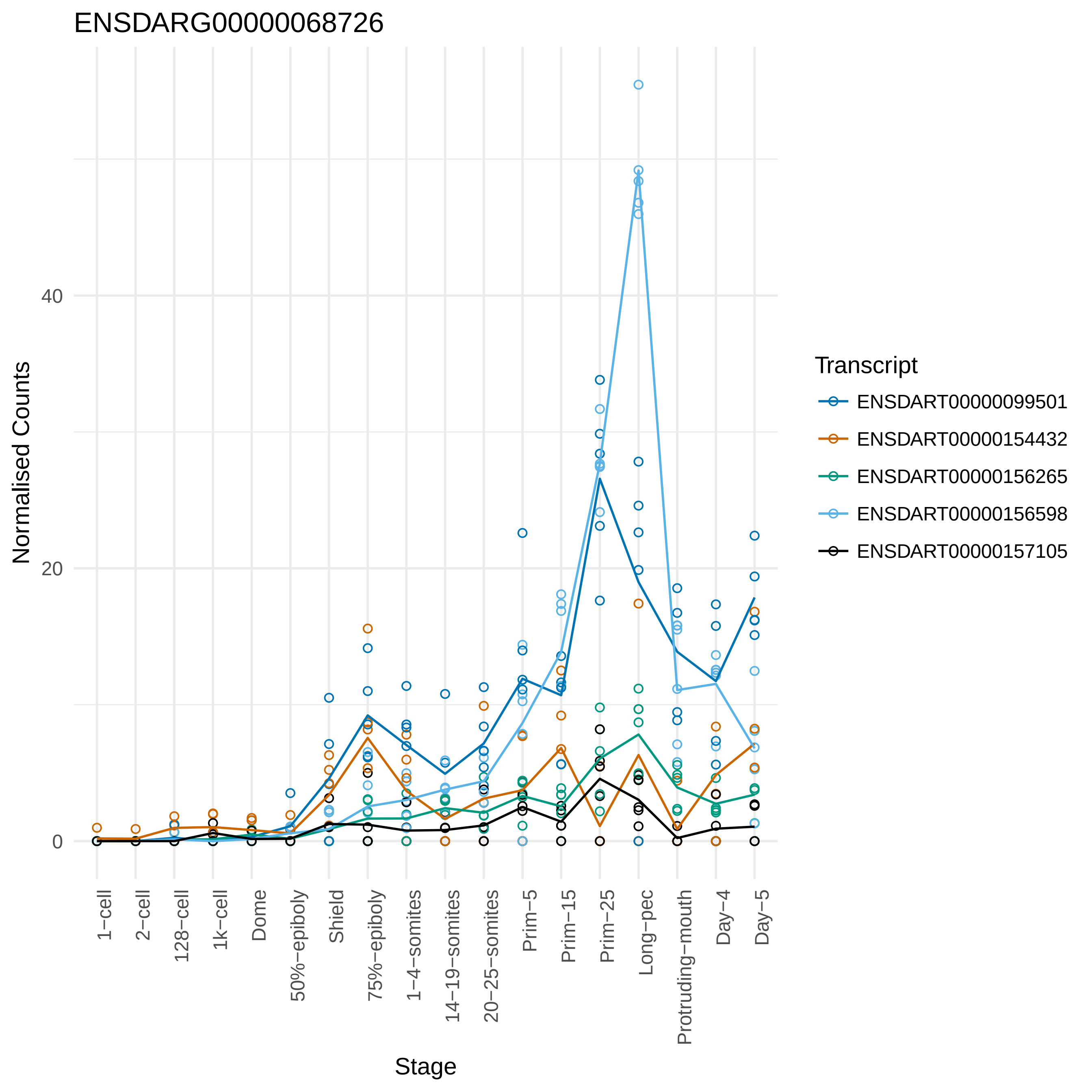

ENSDARG00000068726
Normalised Counts
0
20
40
Stage
1−cell
2−cell
128−cell
1k−cell
Dome
50%−epiboly
Shield
75%−epiboly
1−4−somites
14−19−somites
20−25−somites
Prim−5
Prim−15
Prim−25
Long−pec
Protruding−mouth
Day−4
Day−5
Transcript
ENSDART00000099501
ENSDART00000154432
ENSDART00000156265
ENSDART00000156598
ENSDART00000157105

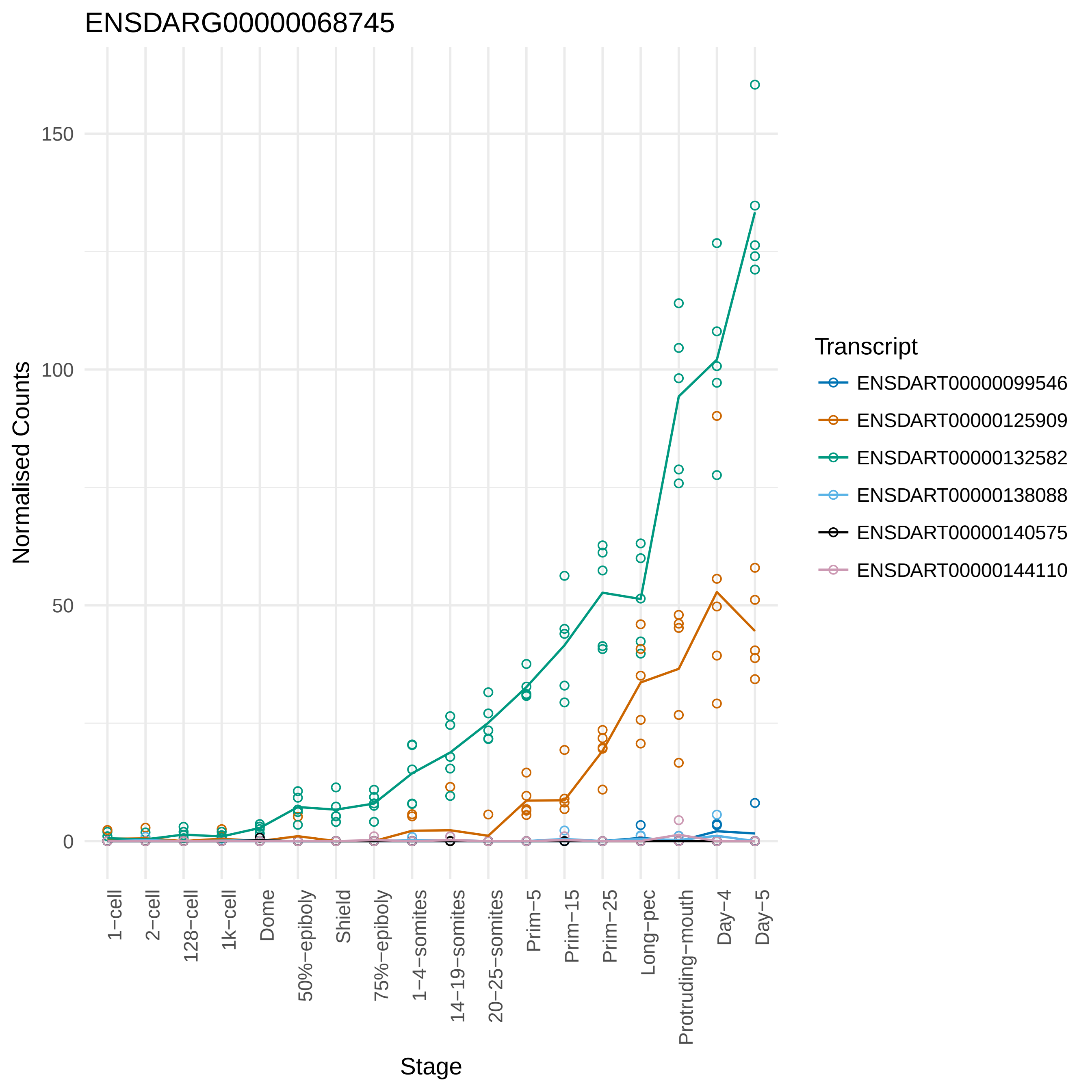

ENSDARG00000068745
Normalised Counts
150
100
50
0
1−cell
2−cell
128−cell
1k−cell
Dome
50%−epiboly
Shield
75%−epiboly
1−4−somites
14−19−somites
20−25−somites
Prim−5
Prim−15
Prim−25
Long−pec
Protruding−mouth
Day−4
Day−5
Stage
Transcript
ENSDART00000099546
ENSDART00000125909
ENSDART00000132582
ENSDART00000138088
ENSDART00000140575
ENSDART00000144110

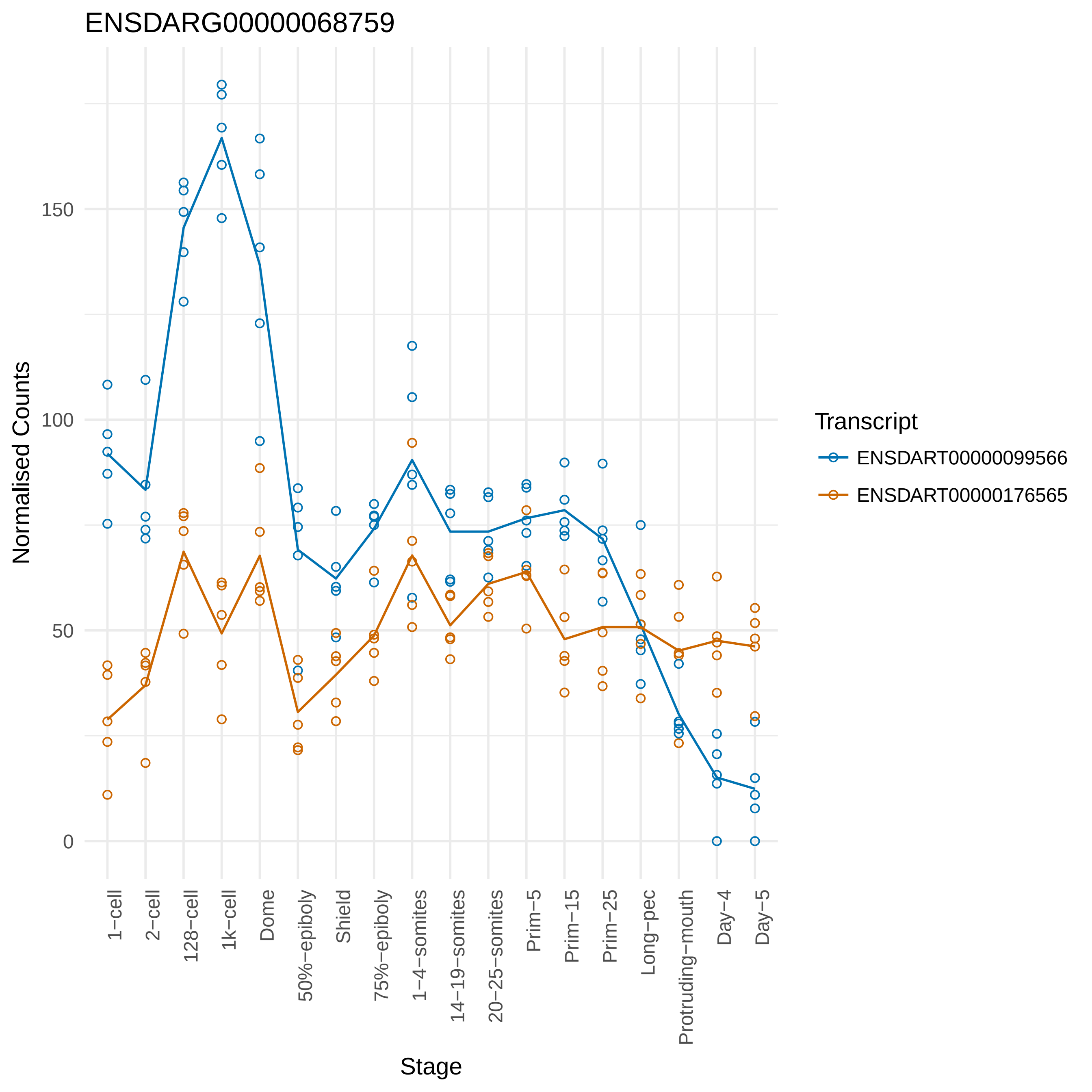

ENSDARG00000068759
Normalised Counts
150
100
50
0
1−cell
2−cell
128−cell
1k−cell
Dome
50%−epiboly
Shield
75%−epiboly
1−4−somites
14−19−somites
20−25−somites
Prim−5
Prim−15
Prim−25
Long−pec
Protruding−mouth
Day−4
Day−5
Stage
Transcript
ENSDART00000099566
ENSDART00000176565

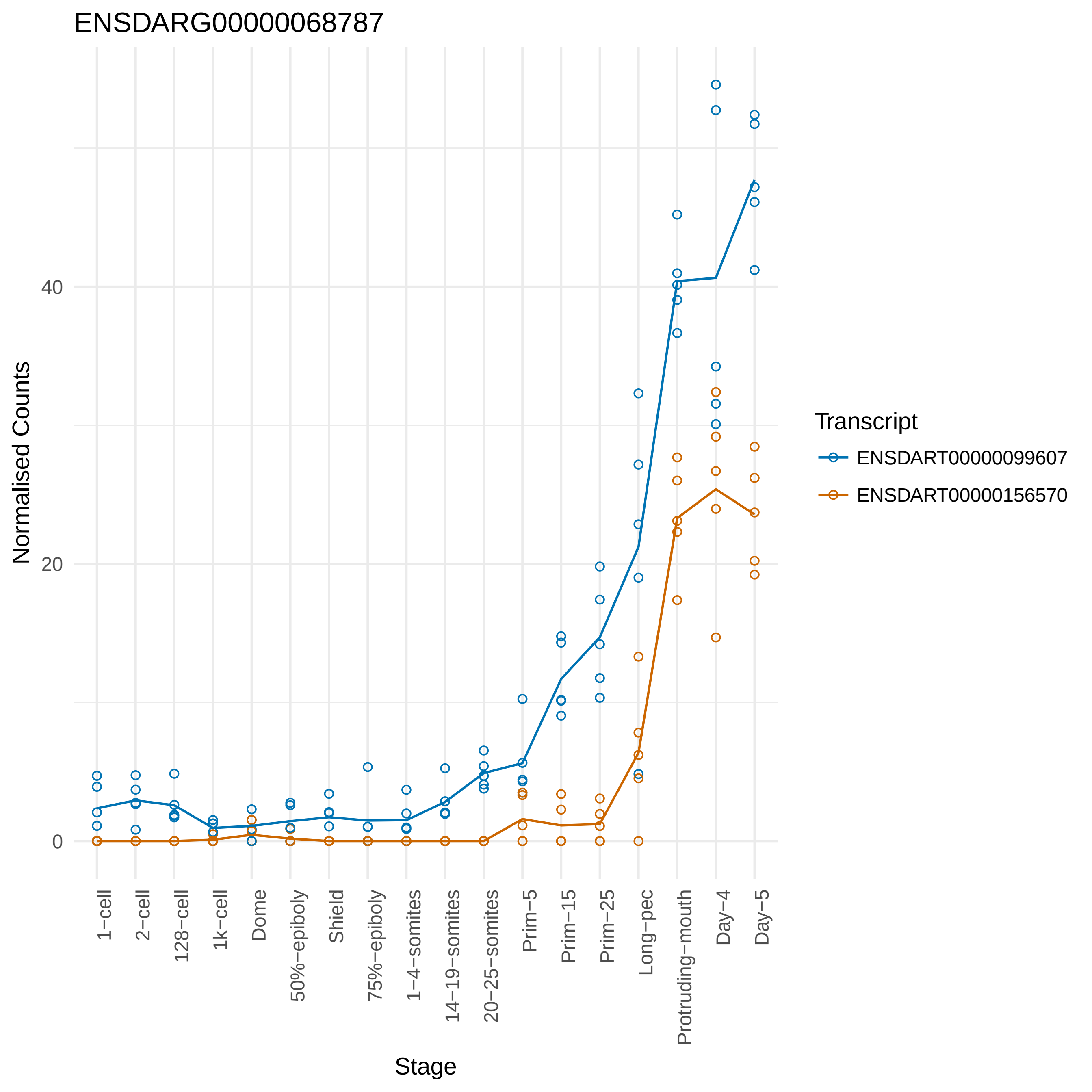

ENSDARG00000068787
Normalised Counts
0
20
40
1−cell
2−cell
128−cell
1k−cell
Dome
50%−epiboly
Shield
75%−epiboly
1−4−somites
14−19−somites
20−25−somites
Prim−5
Prim−15
Prim−25
Long−pec
Protruding−mouth
Day−4
Day−5
Stage
Transcript
ENSDART00000099607
ENSDART00000156570

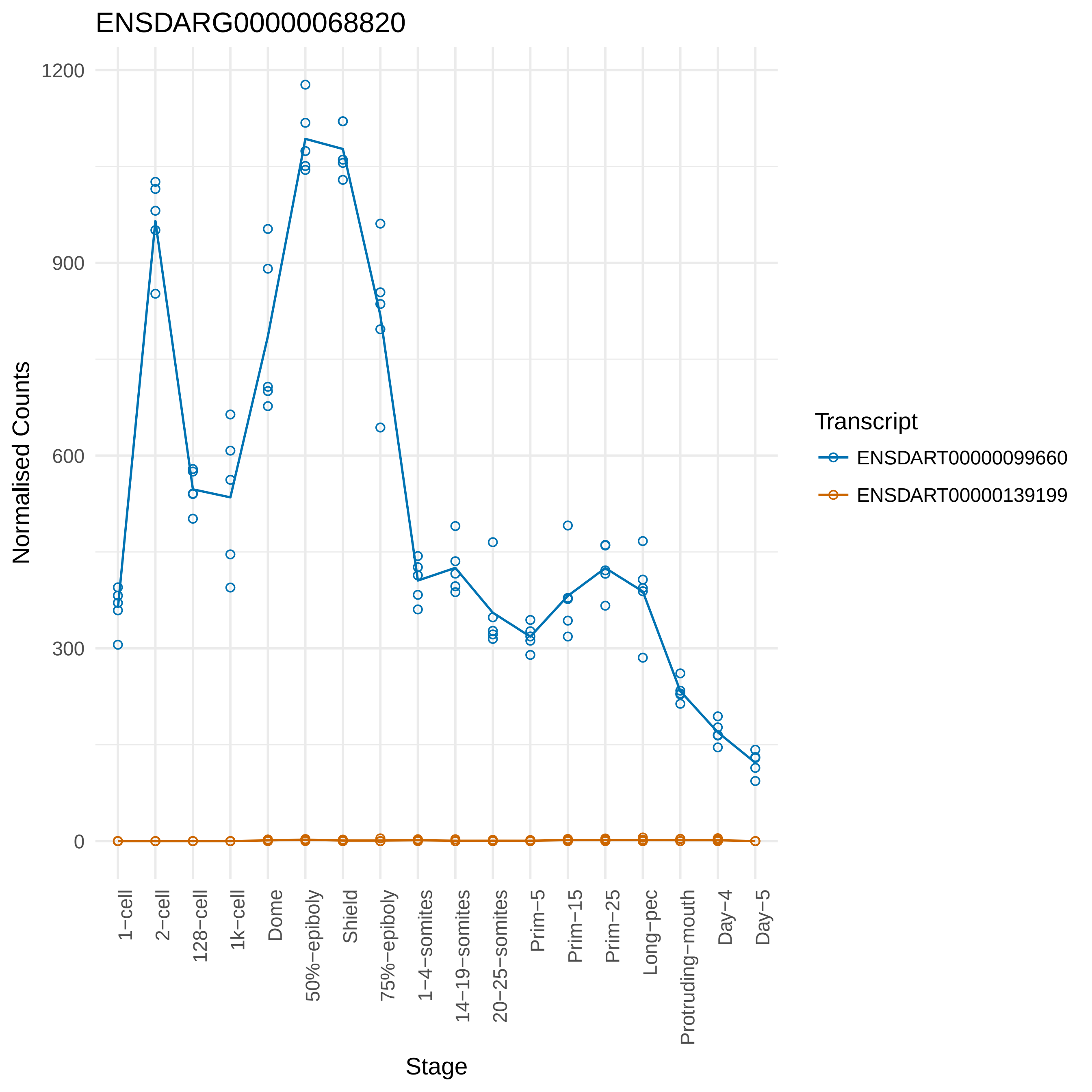

ENSDARG00000068820
Normalised Counts
1200
900
600
300
0
1−cell
2−cell
128−cell
1k−cell
Dome
50%−epiboly
Shield
75%−epiboly
1−4−somites
14−19−somites
20−25−somites
Prim−5
Prim−15
Prim−25
Long−pec
Protruding−mouth
Day−4
Day−5
Stage
Transcript
ENSDART00000099660
ENSDART00000139199

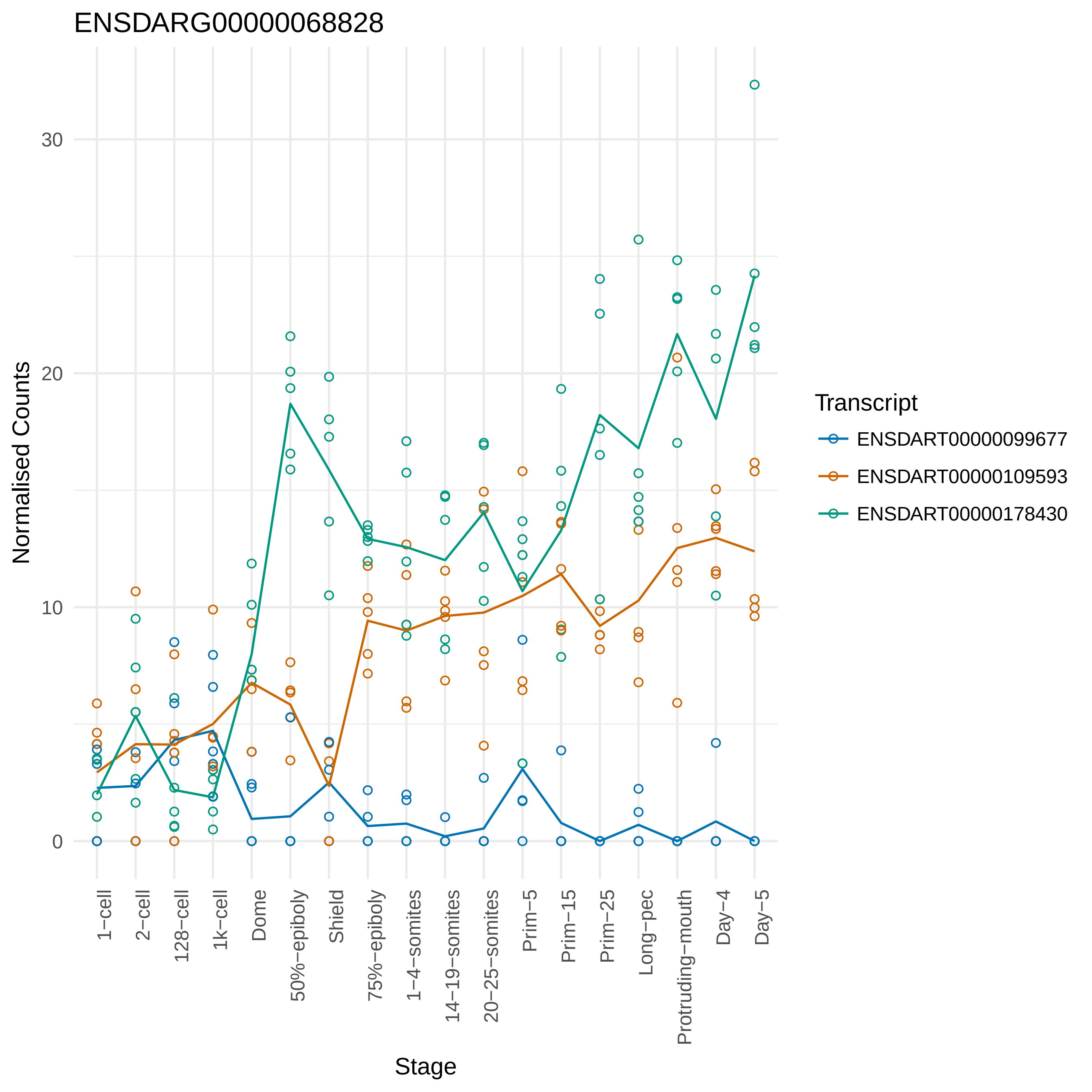

ENSDARG00000068828
Normalised Counts
30
20
10
0
1−cell
2−cell
128−cell
1k−cell
Dome
50%−epiboly
Shield
75%−epiboly
1−4−somites
14−19−somites
20−25−somites
Prim−5
Prim−15
Prim−25
Long−pec
Protruding−mouth
Day−4
Day−5
Stage
Transcript
ENSDART00000099677
ENSDART00000109593
ENSDART00000178430

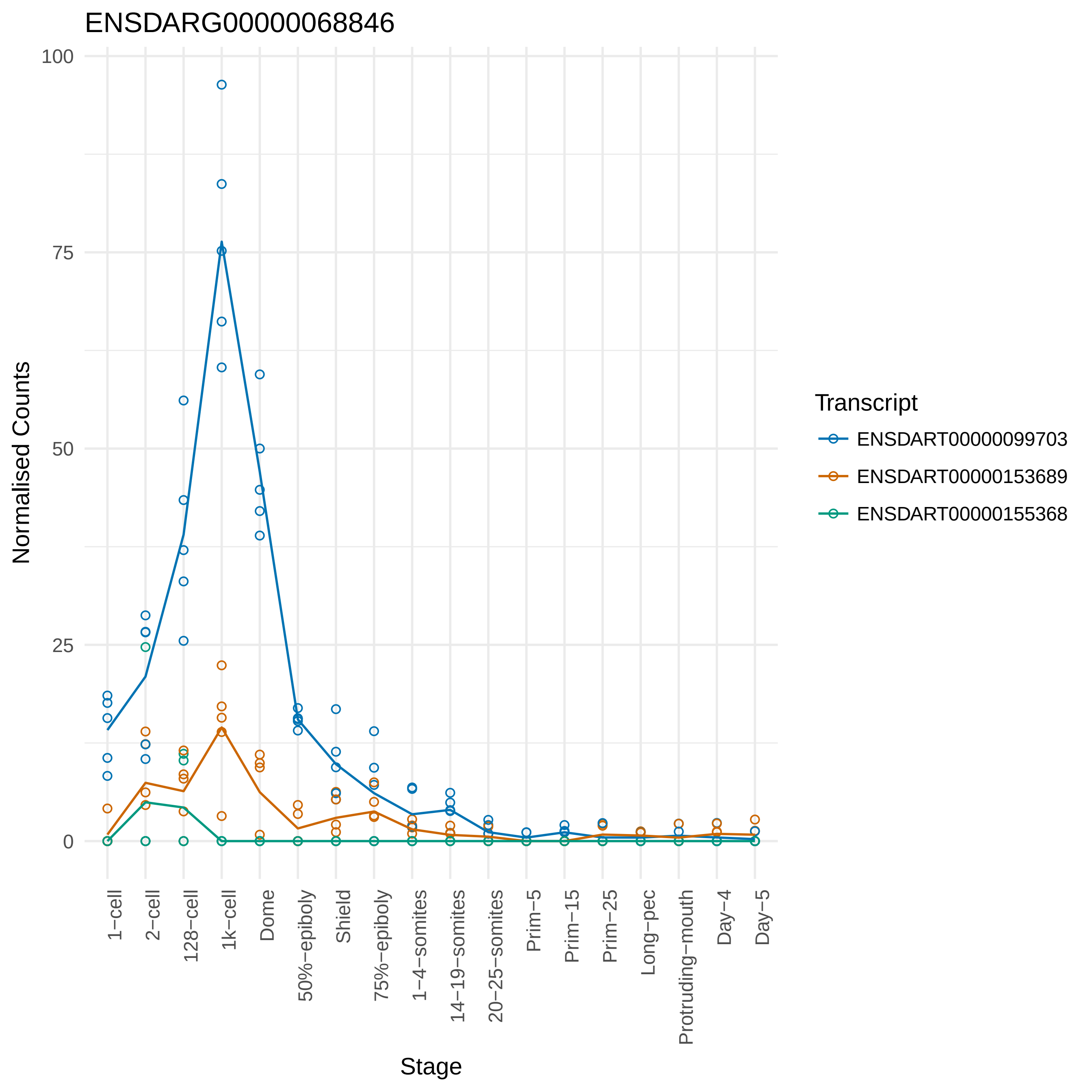

ENSDARG00000068846
Normalised Counts
100
75
50
25
0
1−cell
2−cell
128−cell
1k−cell
Dome
50%−epiboly
Shield
75%−epiboly
1−4−somites
14−19−somites
20−25−somites
Prim−5
Prim−15
Prim−25
Long−pec
Protruding−mouth
Day−4
Day−5
Stage
Transcript
ENSDART00000099703
ENSDART00000153689
ENSDART00000155368

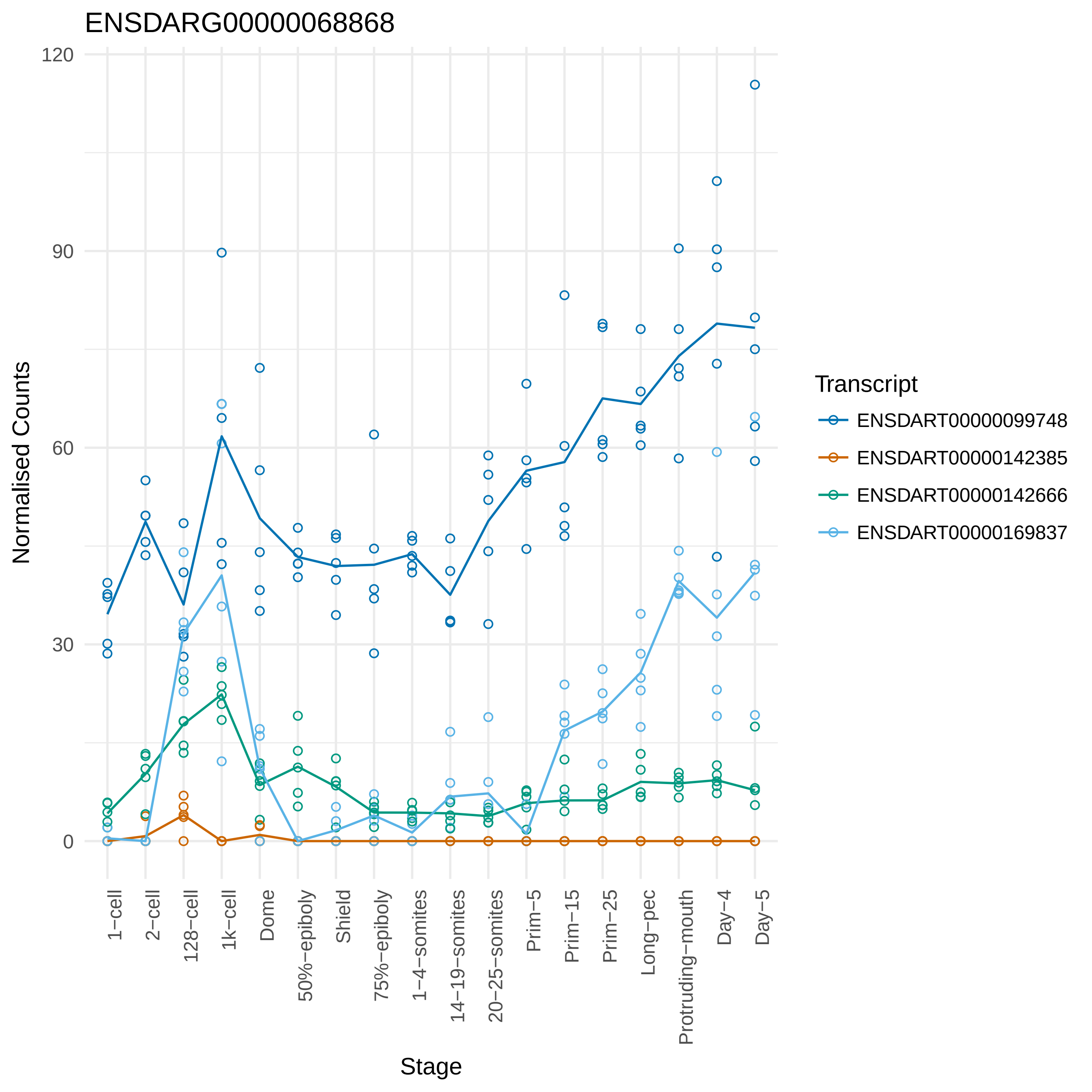

ENSDARG00000068868
Normalised Counts
120
90
60
30
0
1−cell
2−cell
128−cell
1k−cell
Dome
50%−epiboly
Shield
75%−epiboly
1−4−somites
14−19−somites
20−25−somites
Prim−5
Prim−15
Prim−25
Long−pec
Protruding−mouth
Day−4
Day−5
Stage
Transcript
ENSDART00000099748
ENSDART00000142385
ENSDART00000142666
ENSDART00000169837

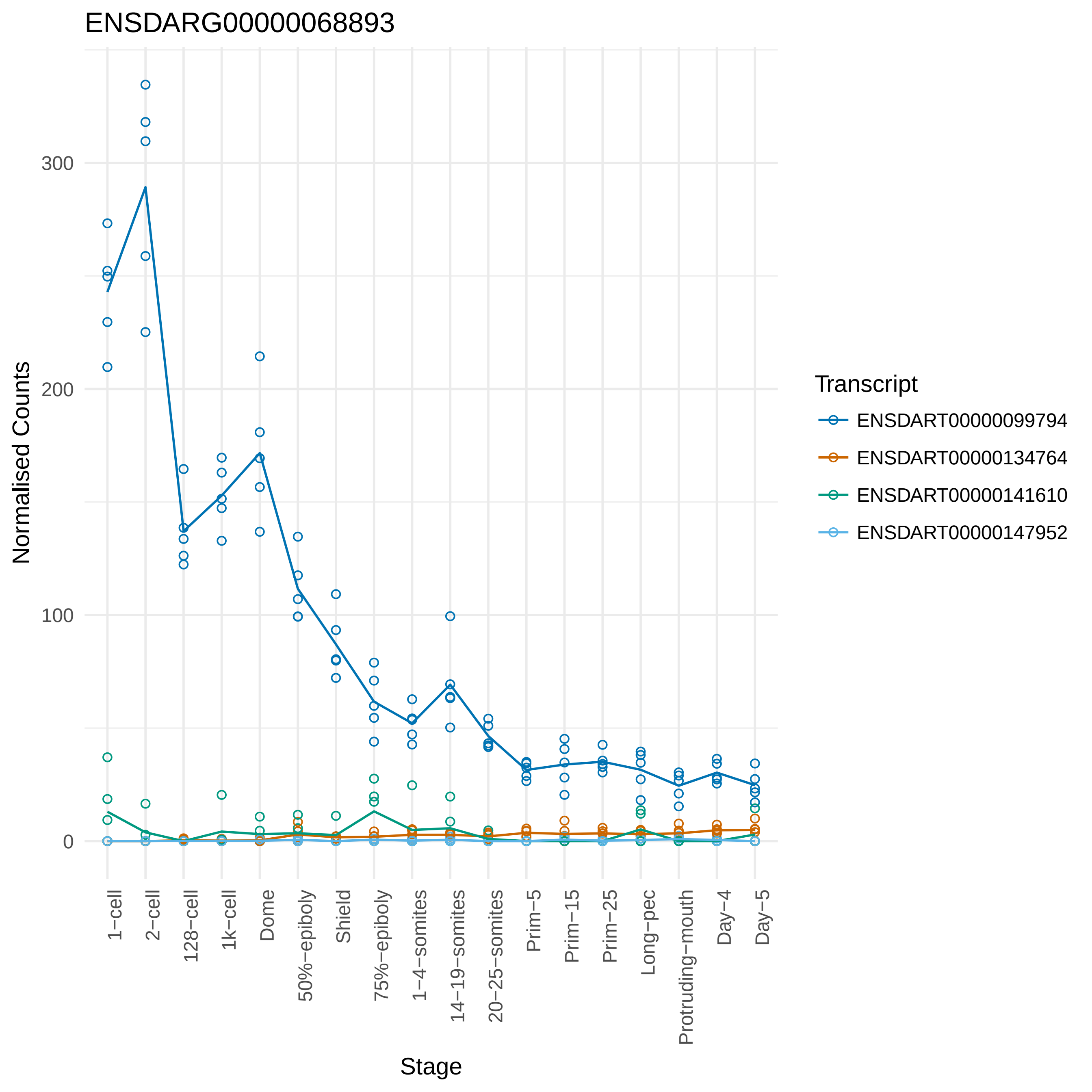

ENSDARG00000068893
Normalised Counts
300
200
100
0
1−cell
2−cell
128−cell
1k−cell
Dome
50%−epiboly
Shield
75%−epiboly
1−4−somites
14−19−somites
20−25−somites
Prim−5
Prim−15
Prim−25
Long−pec
Protruding−mouth
Day−4
Day−5
Stage
Transcript
ENSDART00000099794
ENSDART00000134764
ENSDART00000141610
ENSDART00000147952

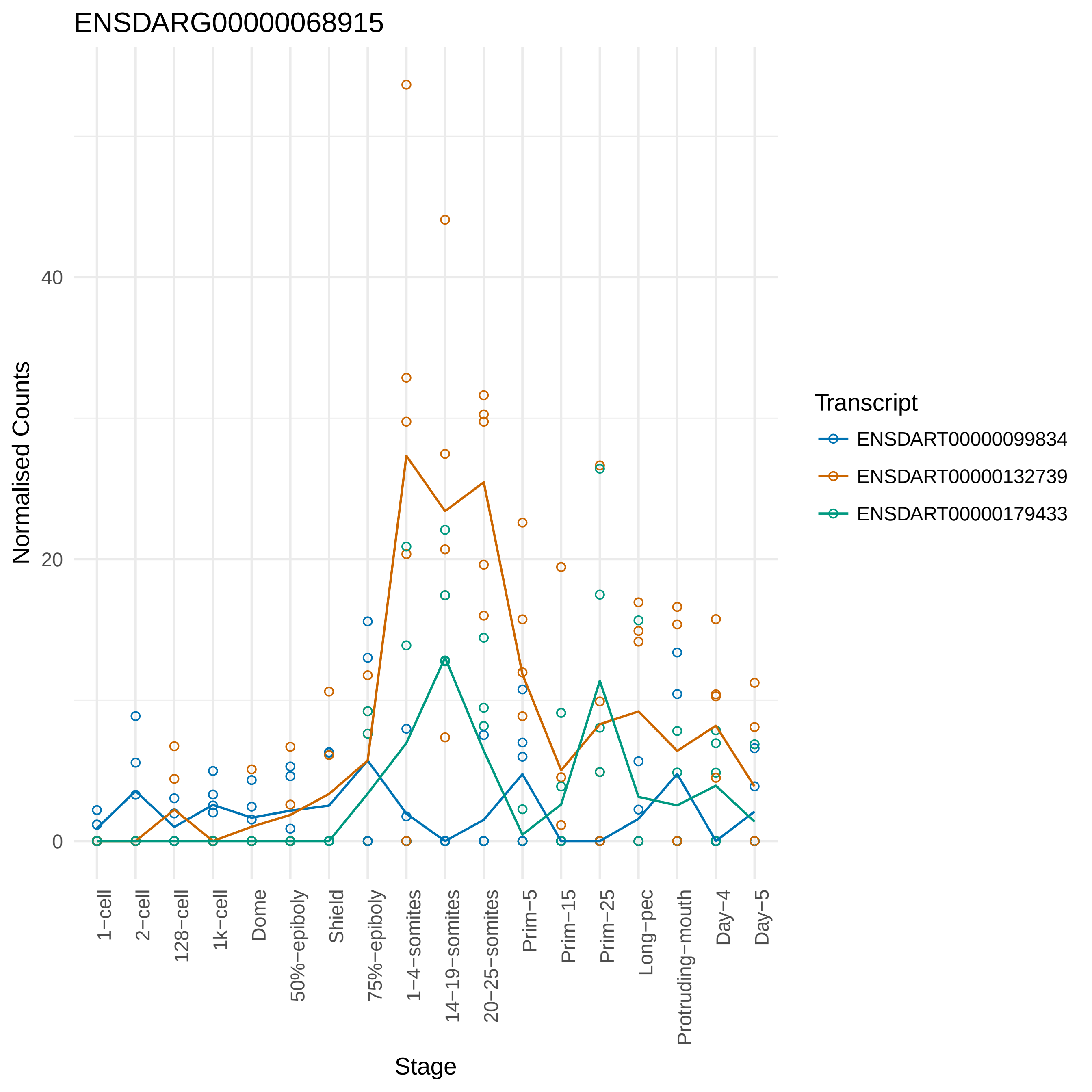

ENSDARG00000068915
Normalised Counts
0
20
40
Stage
1−cell
2−cell
128−cell
1k−cell
Dome
50%−epiboly
Shield
75%−epiboly
1−4−somites
14−19−somites
20−25−somites
Prim−5
Prim−15
Prim−25
Long−pec
Protruding−mouth
Day−4
Day−5
Transcript
ENSDART00000099834
ENSDART00000132739
ENSDART00000179433

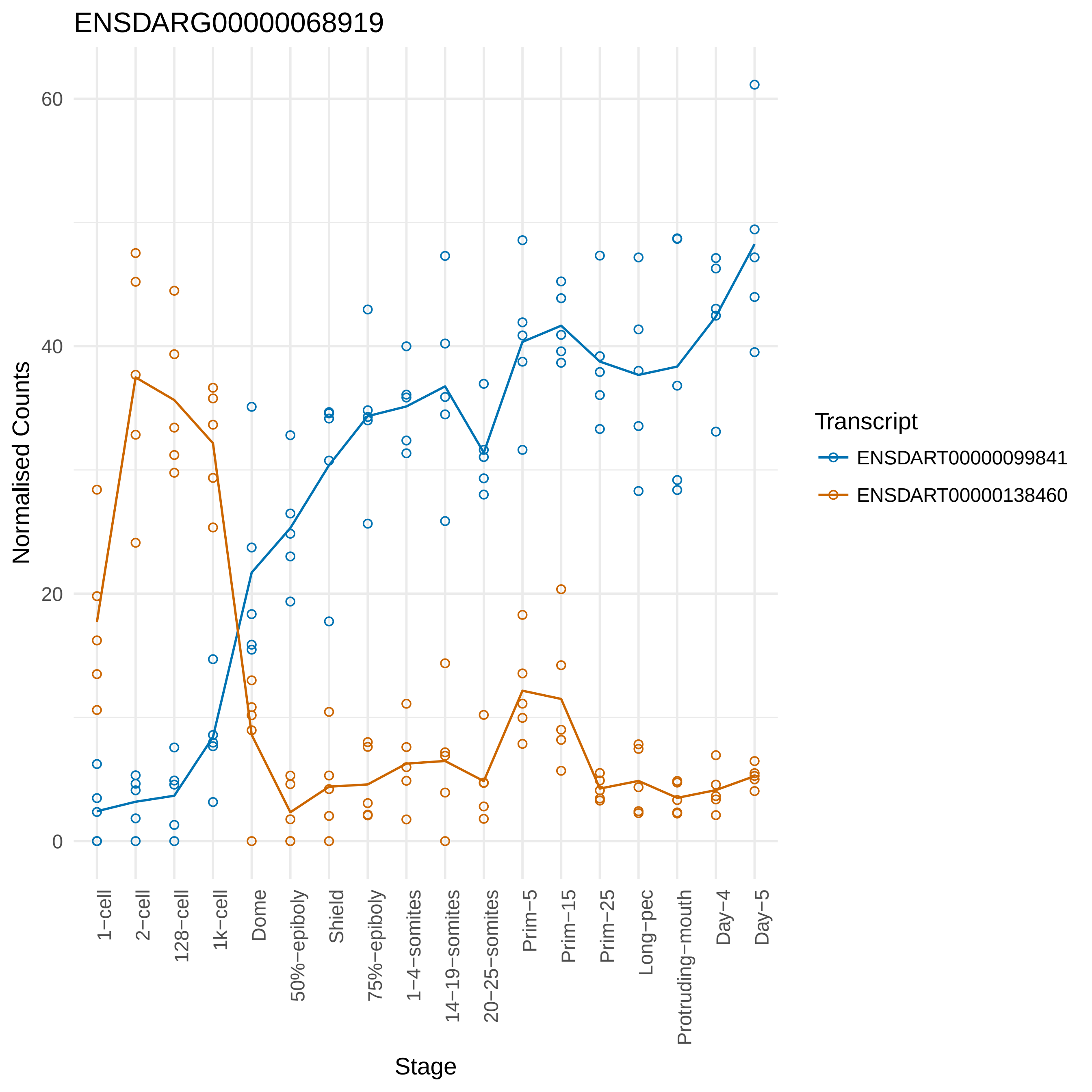

ENSDARG00000068919
Normalised Counts
0
20
40
60
1−cell
2−cell
128−cell
1k−cell
Dome
50%−epiboly
Shield
75%−epiboly
1−4−somites
14−19−somites
20−25−somites
Prim−5
Prim−15
Prim−25
Long−pec
Protruding−mouth
Day−4
Day−5
Stage
Transcript
ENSDART00000099841
ENSDART00000138460

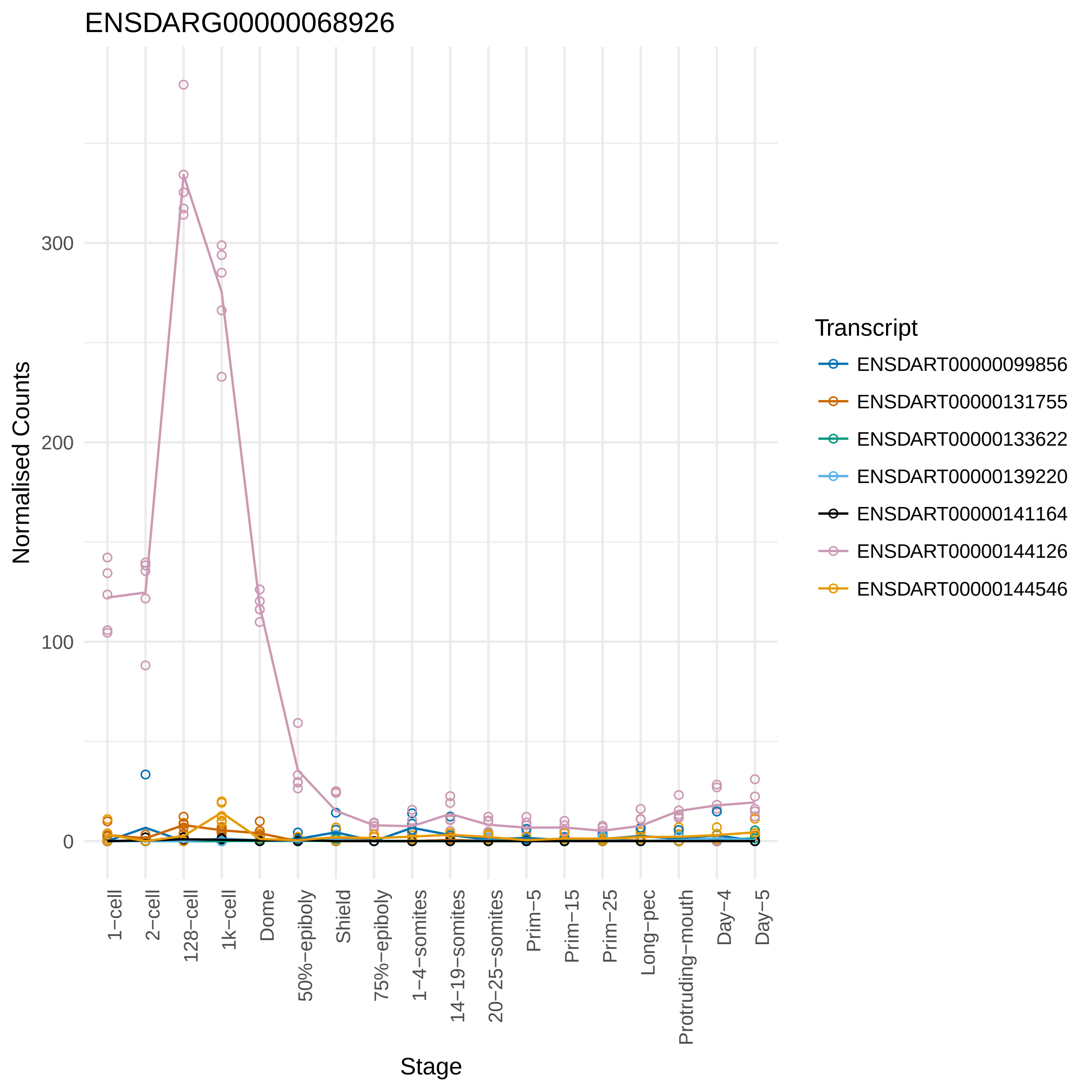

ENSDARG00000068926
Normalised Counts
0
100
200
300
Stage
1−cell
2−cell
128−cell
1k−cell
Dome
50%−epiboly
Shield
75%−epiboly
1−4−somites
14−19−somites
20−25−somites
Prim−5
Prim−15
Prim−25
Long−pec
Protruding−mouth
Day−4
Day−5
Transcript
ENSDART00000099856
ENSDART00000131755
ENSDART00000133622
ENSDART00000139220
ENSDART00000141164
ENSDART00000144126
ENSDART00000144546

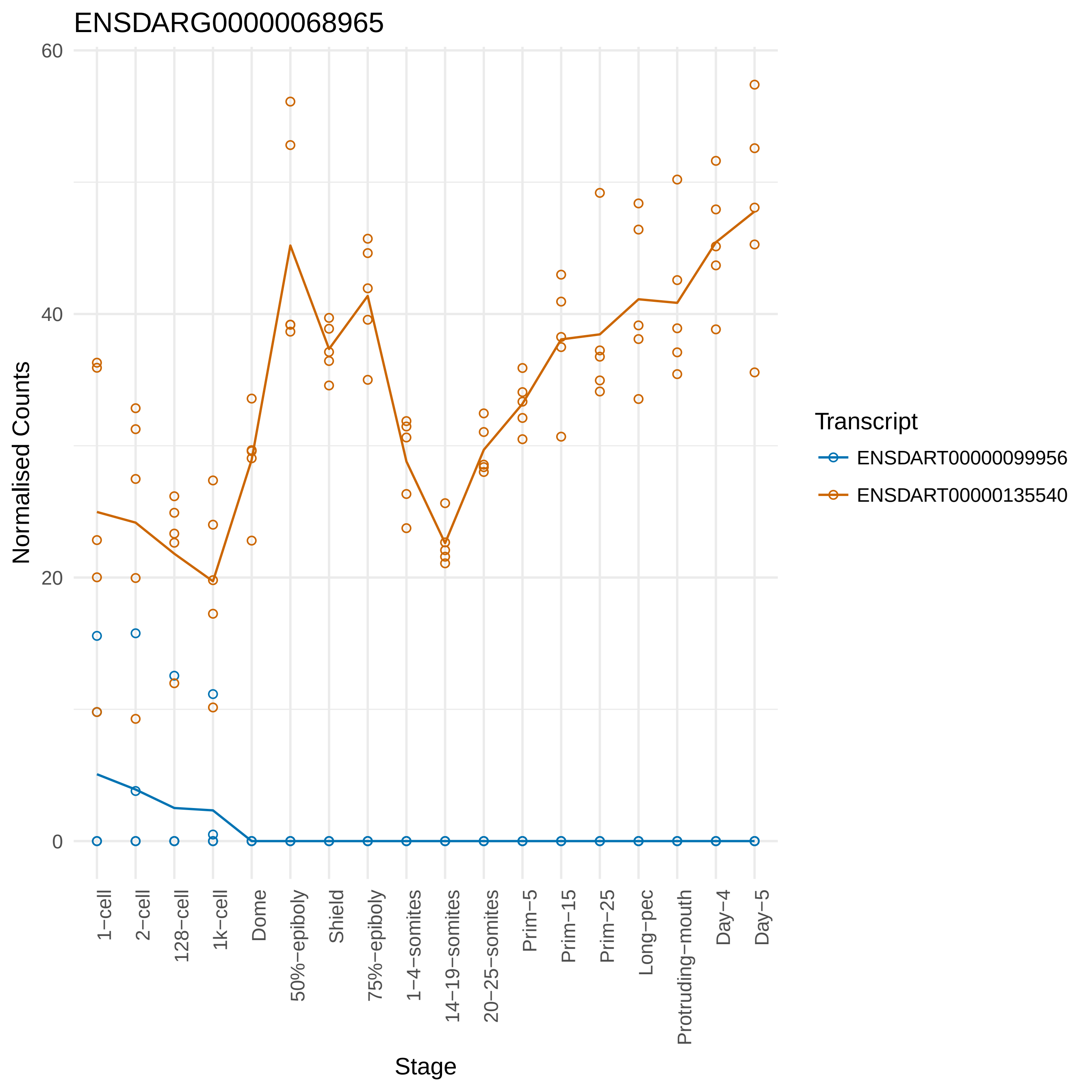

ENSDARG00000068965
Normalised Counts
60
40
20
0
Stage
1−cell
2−cell
128−cell
1k−cell
Dome
50%−epiboly
Shield
75%−epiboly
1−4−somites
14−19−somites
20−25−somites
Prim−5
Prim−15
Prim−25
Long−pec
Protruding−mouth
Day−4
Day−5
Transcript
ENSDART00000099956
ENSDART00000135540

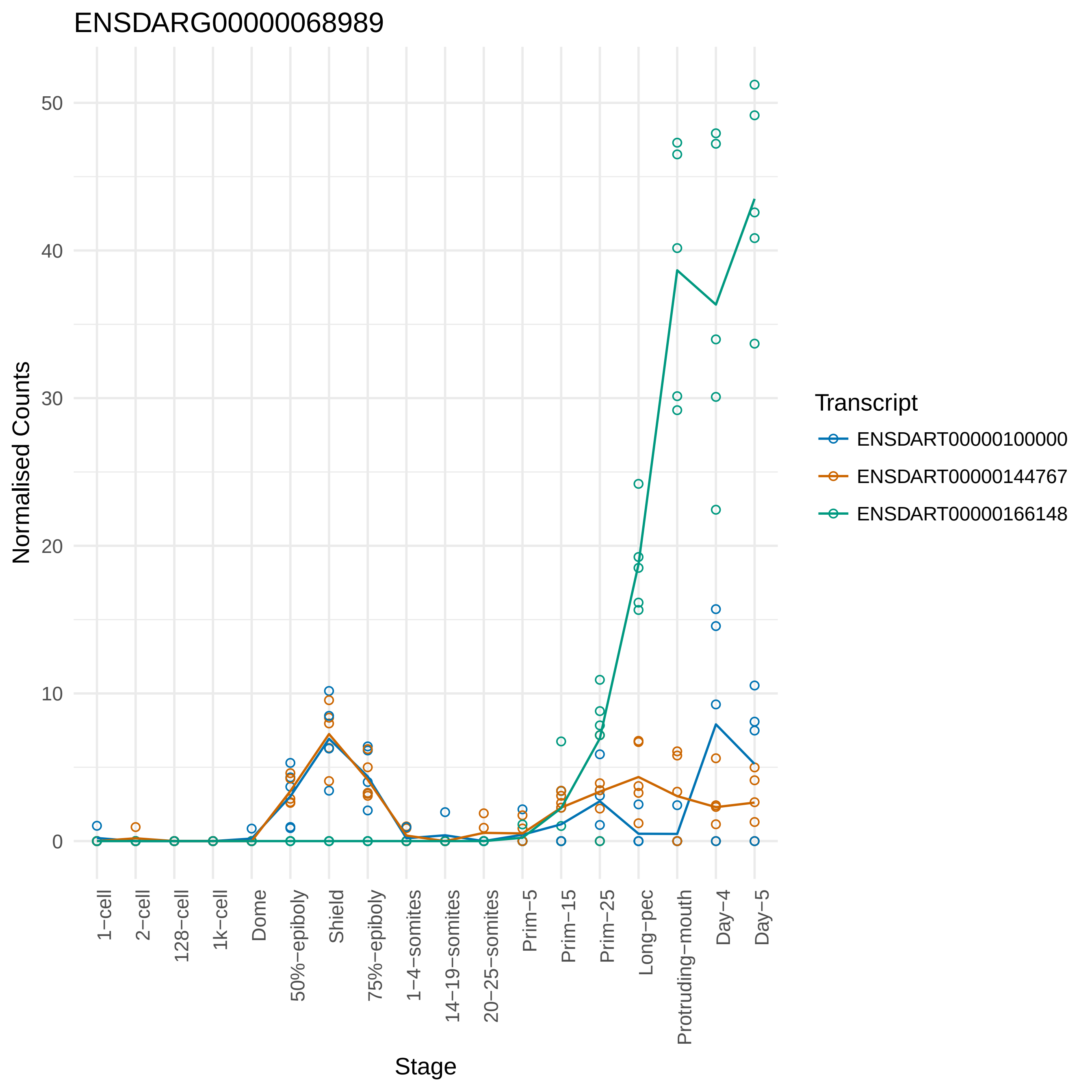
ENSDARG00000068989
Normalised Counts
0
10
20
30
40
50
1−cell
2−cell
128−cell
1k−cell
Dome
50%−epiboly
Shield
75%−epiboly
1−4−somites
14−19−somites
20−25−somites
Prim−5
Prim−15
Prim−25
Long−pec
Protruding−mouth
Day−4
Day−5
Stage
Transcript
ENSDART00000100000
ENSDART00000144767
ENSDART00000166148

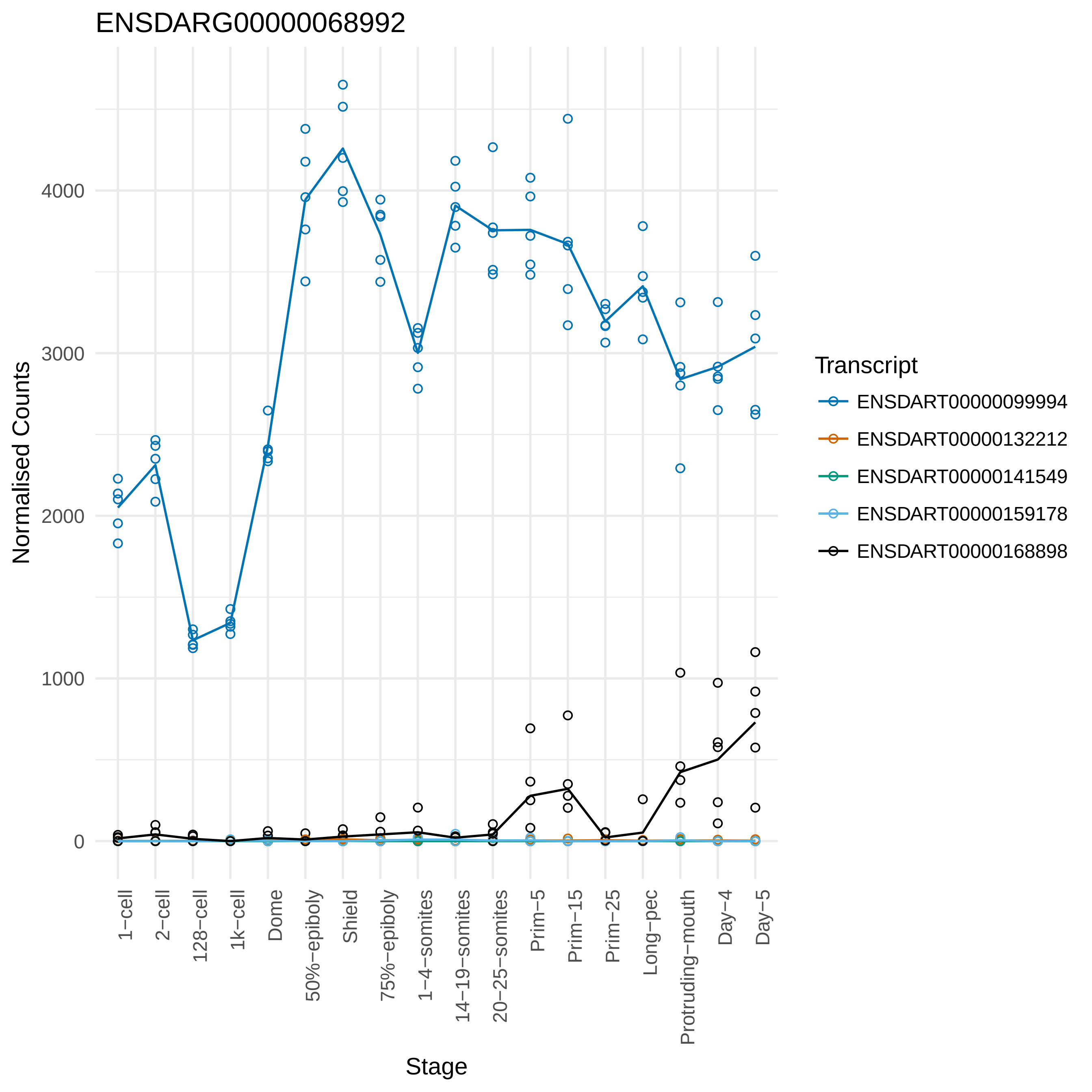

ENSDARG00000068992
Normalised Counts
4000
3000
2000
1000
0
1−cell
2−cell
128−cell
1k−cell
Dome
50%−epiboly
Shield
75%−epiboly
1−4−somites
14−19−somites
20−25−somites
Prim−5
Prim−15
Prim−25
Long−pec
Protruding−mouth
Day−4
Day−5
Stage
Transcript
ENSDART00000099994
ENSDART00000132212
ENSDART00000141549
ENSDART00000159178
ENSDART00000168898

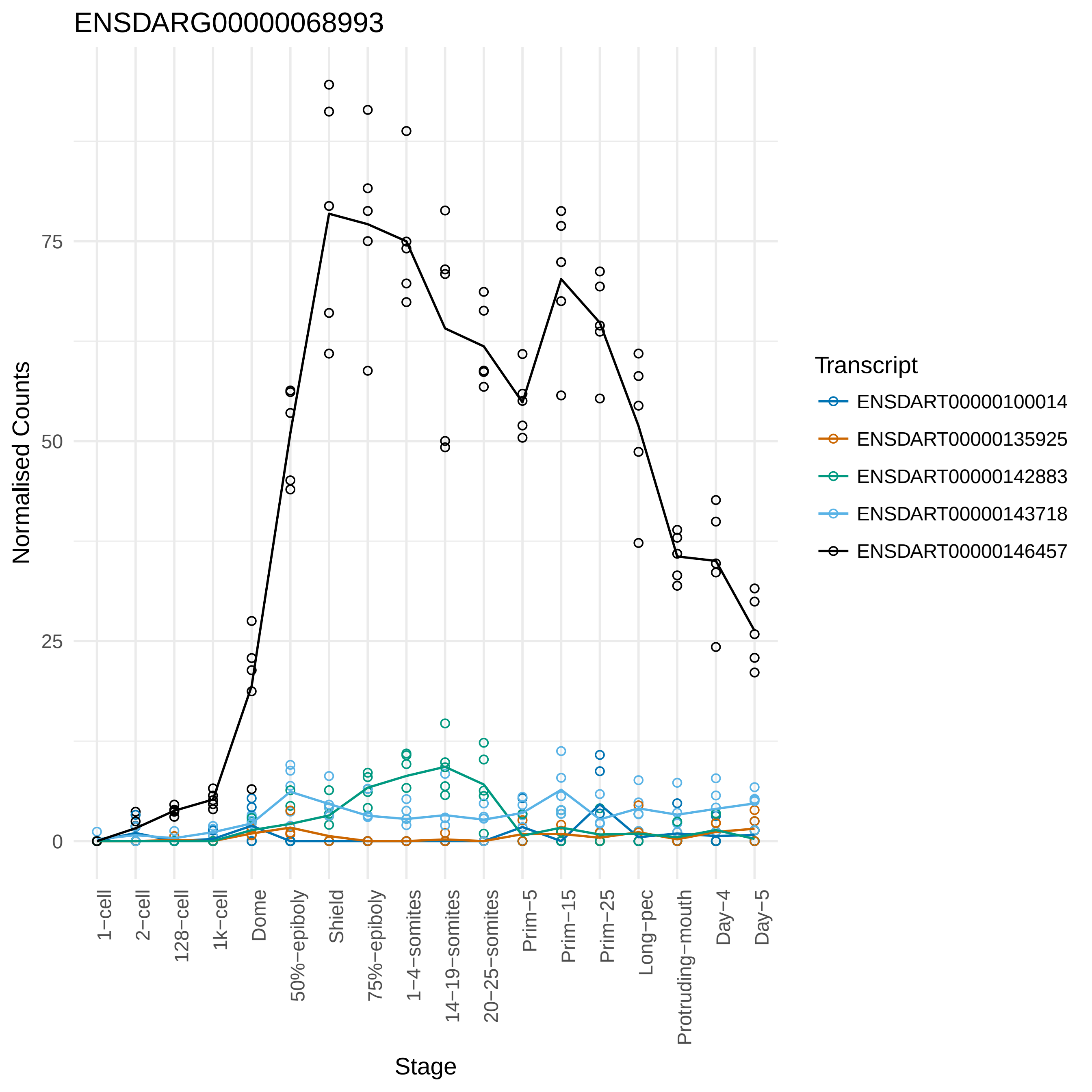

ENSDARG00000068993
Normalised Counts
0
25
50
75
1−cell
2−cell
128−cell
1k−cell
Dome
50%−epiboly
Shield
75%−epiboly
1−4−somites
14−19−somites
20−25−somites
Prim−5
Prim−15
Prim−25
Long−pec
Protruding−mouth
Day−4
Day−5
Stage
Transcript
ENSDART00000100014
ENSDART00000135925
ENSDART00000142883
ENSDART00000143718
ENSDART00000146457

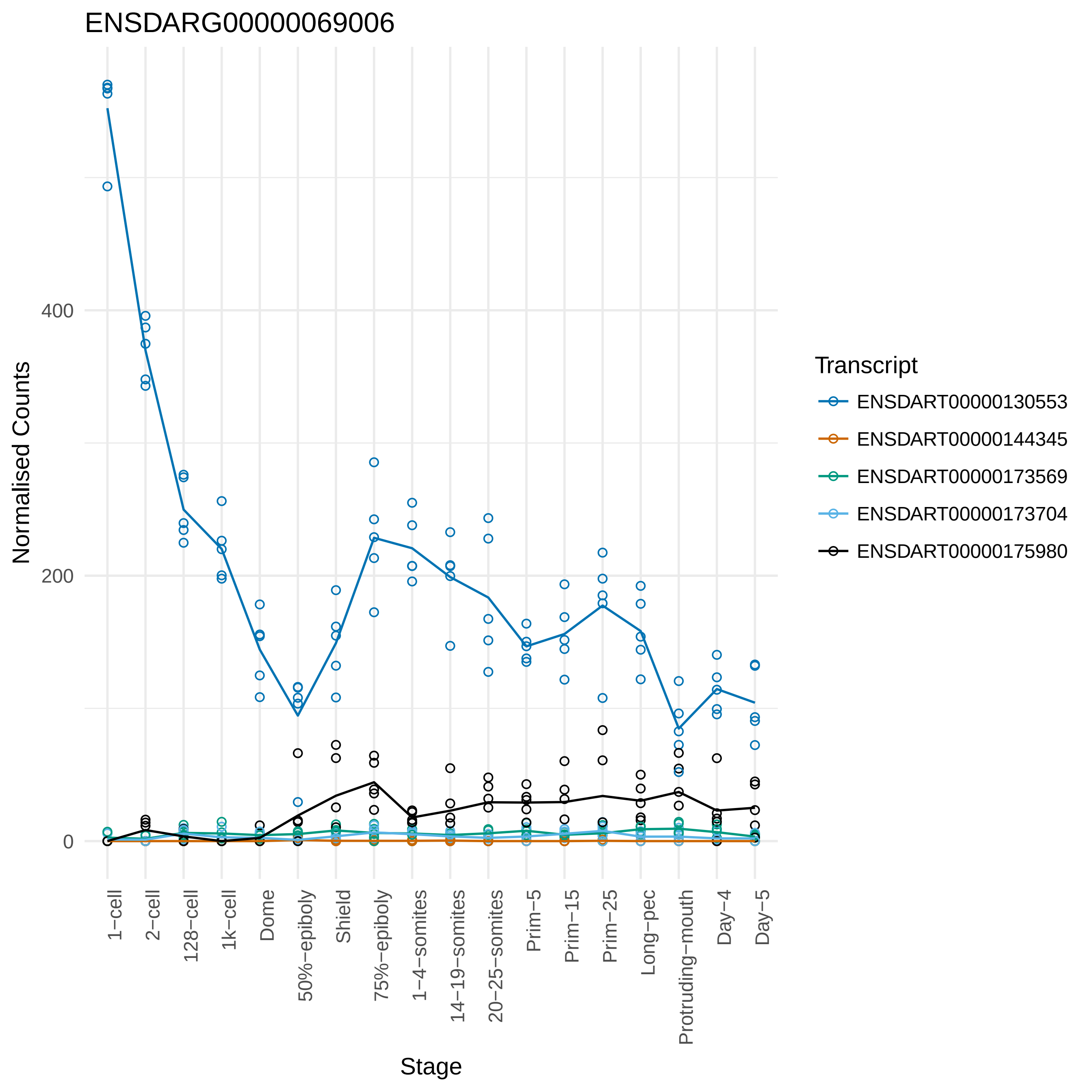

ENSDARG00000069006
Normalised Counts
400
200
0
1−cell
2−cell
128−cell
1k−cell
Dome
50%−epiboly
Shield
75%−epiboly
1−4−somites
14−19−somites
20−25−somites
Prim−5
Prim−15
Prim−25
Long−pec
Protruding−mouth
Day−4
Day−5
Stage
Transcript
ENSDART00000130553
ENSDART00000144345
ENSDART00000173569
ENSDART00000173704
ENSDART00000175980

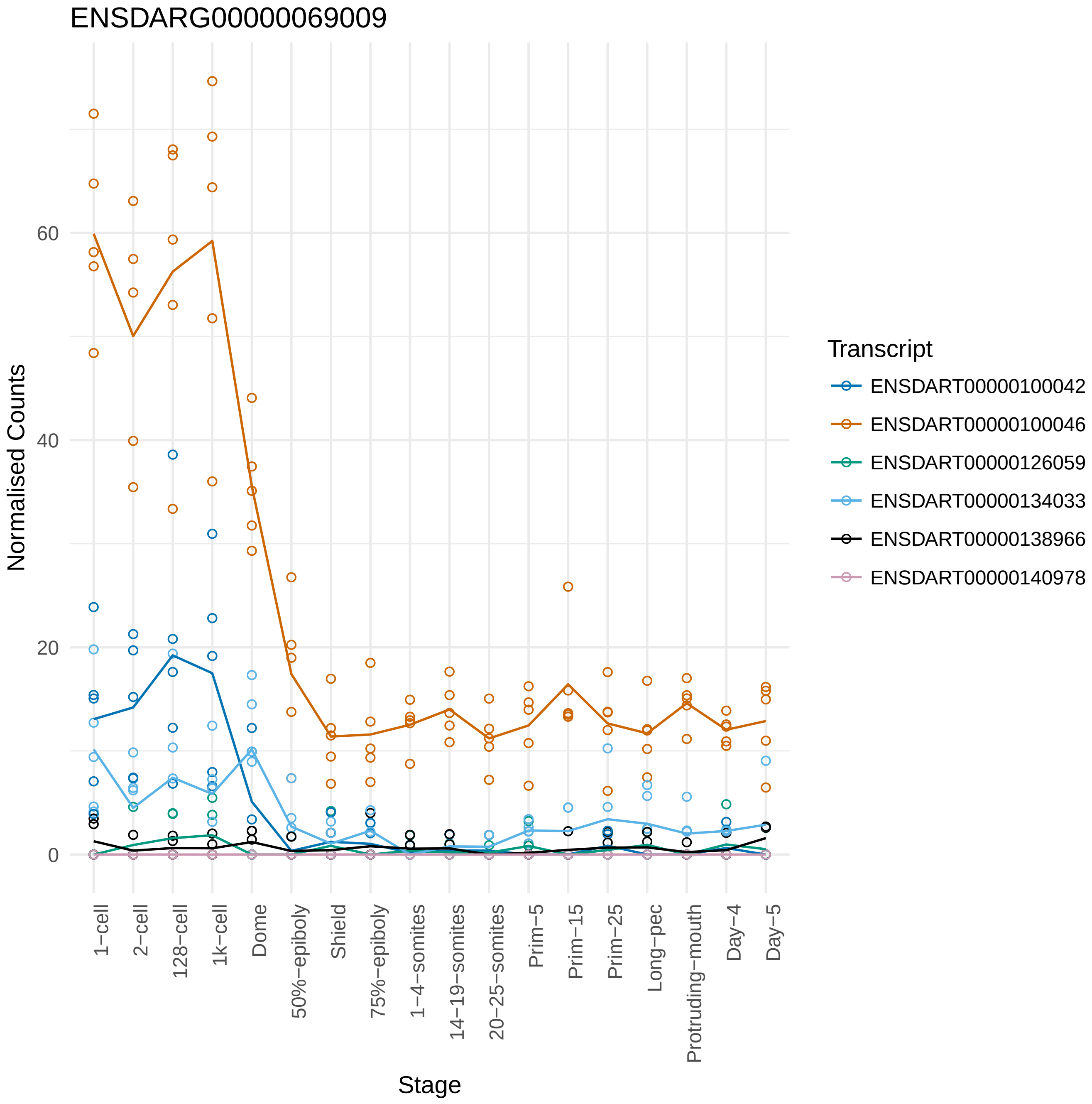

ENSDARG00000069009
Normalised Counts
0
20
40
60
Stage
1−cell
2−cell
128−cell
1k−cell
Dome
50%−epiboly
Shield
75%−epiboly
1−4−somites
14−19−somites
20−25−somites
Prim−5
Prim−15
Prim−25
Long−pec
Protruding−mouth
Day−4
Day−5
Transcript
ENSDART00000100042
ENSDART00000100046
ENSDART00000126059
ENSDART00000134033
ENSDART00000138966
ENSDART00000140978

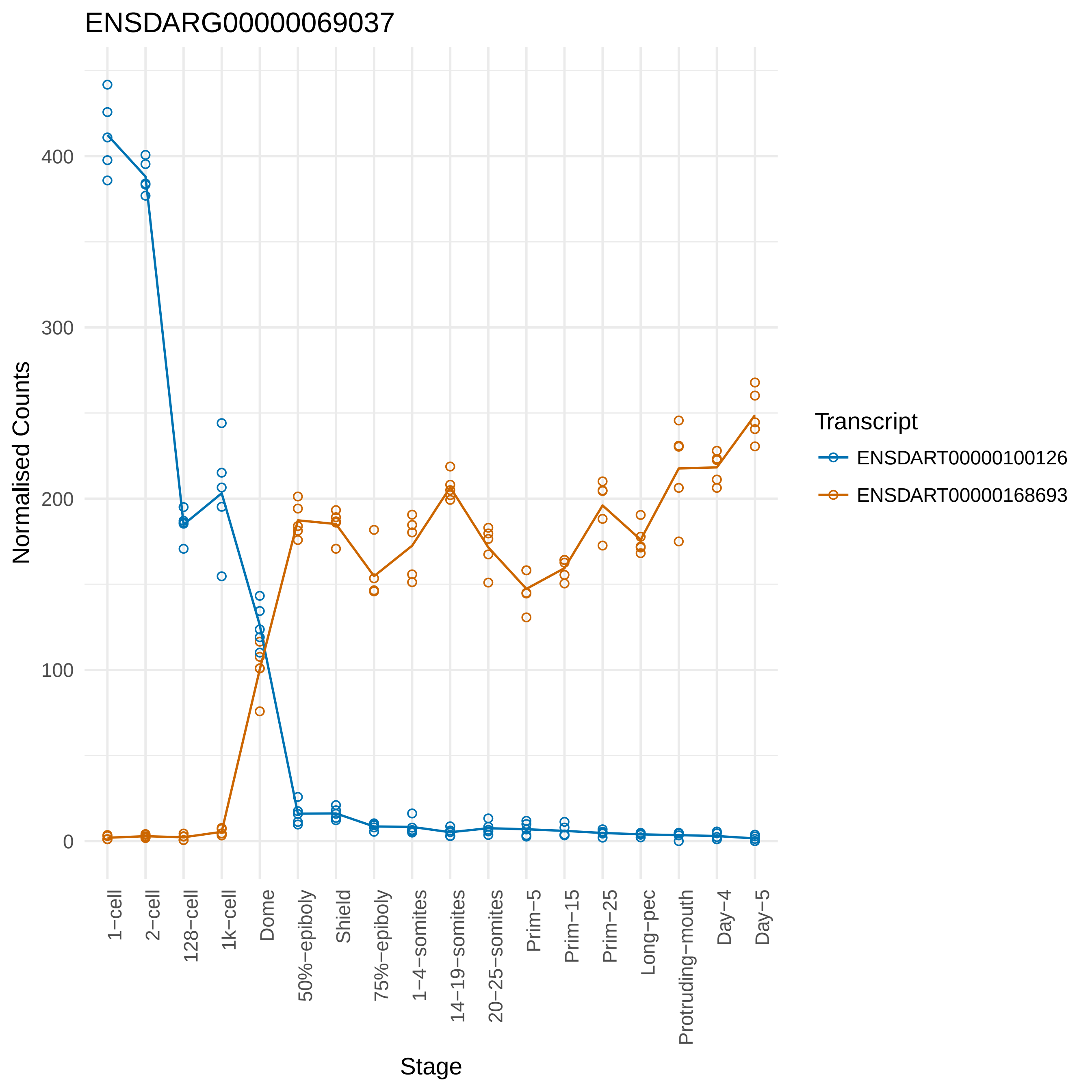

ENSDARG00000069037
Normalised Counts
400
300
200
100
0
1−cell
2−cell
128−cell
1k−cell
Dome
50%−epiboly
Shield
75%−epiboly
1−4−somites
14−19−somites
20−25−somites
Prim−5
Prim−15
Prim−25
Long−pec
Protruding−mouth
Day−4
Day−5
Stage
Transcript
ENSDART00000100126
ENSDART00000168693

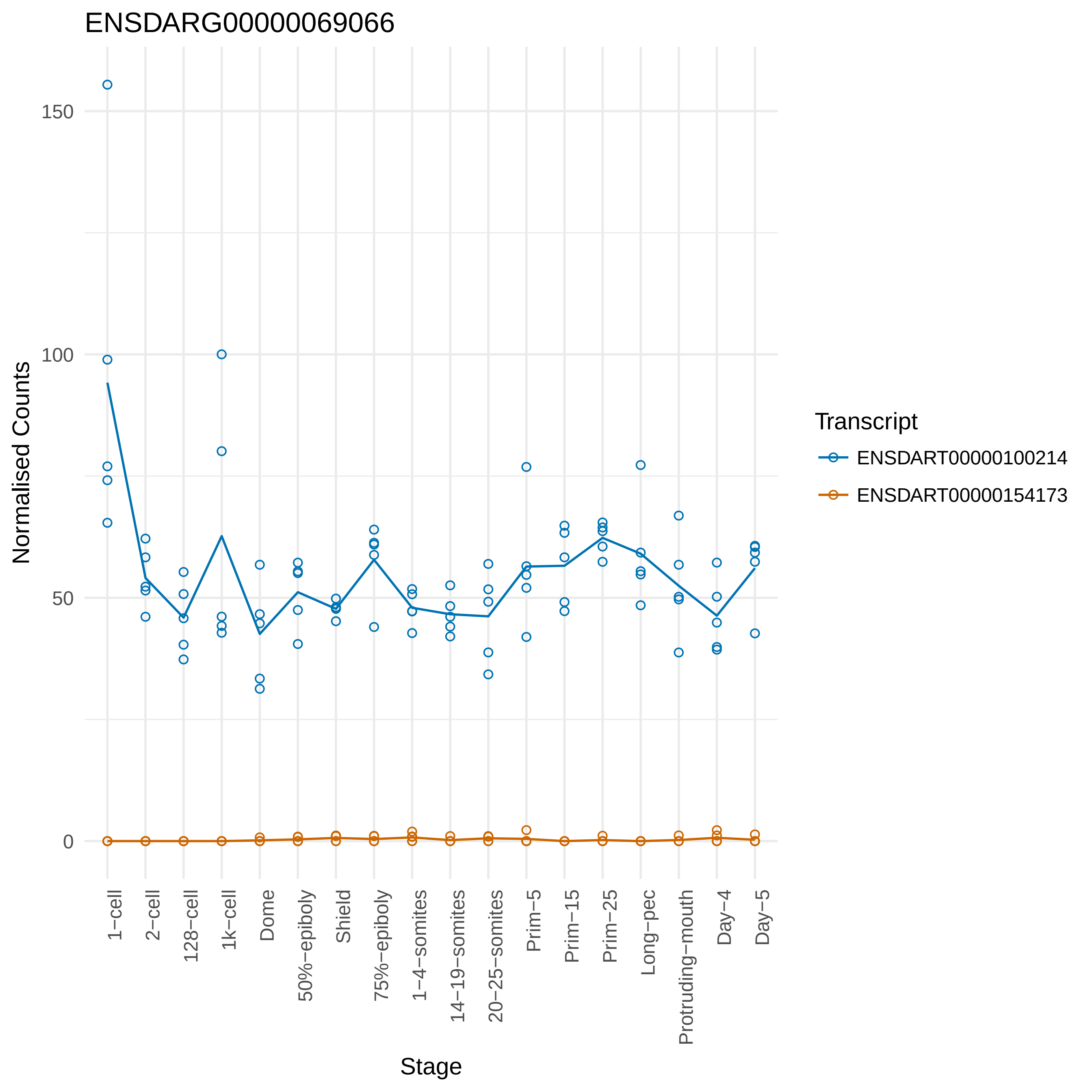

ENSDARG00000069066
Normalised Counts
150
100
50
0
1−cell
2−cell
128−cell
1k−cell
Dome
50%−epiboly
Shield
75%−epiboly
1−4−somites
14−19−somites
20−25−somites
Prim−5
Prim−15
Prim−25
Long−pec
Protruding−mouth
Day−4
Day−5
Stage
Transcript
ENSDART00000100214
ENSDART00000154173

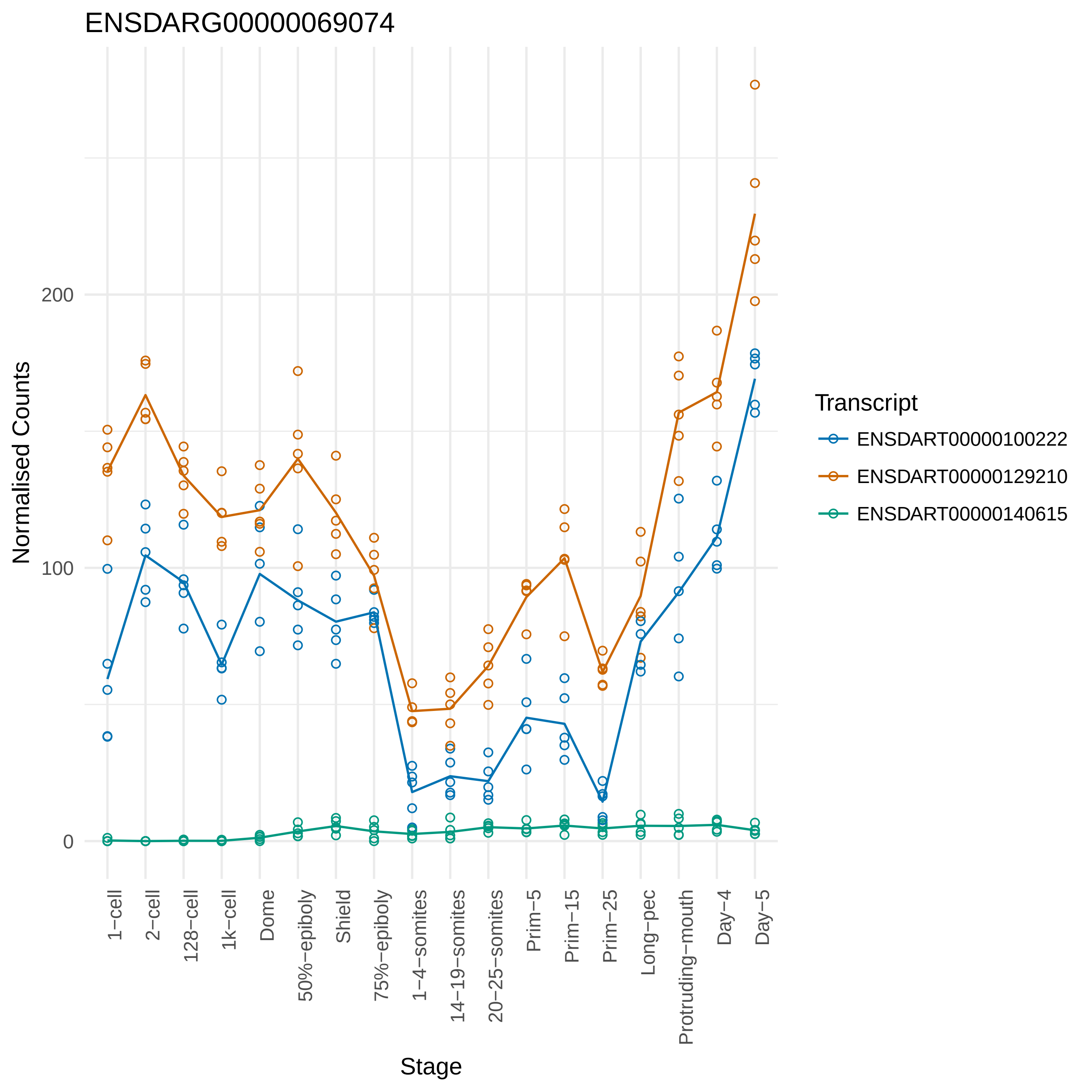
ENSDARG00000069074
Normalised Counts
0
100
200
Stage
1−cell
2−cell
128−cell
1k−cell
Dome
50%−epiboly
Shield
75%−epiboly
1−4−somites
14−19−somites
20−25−somites
Prim−5
Prim−15
Prim−25
Long−pec
Protruding−mouth
Day−4
Day−5
Transcript
ENSDART00000100222
ENSDART00000129210
ENSDART00000140615

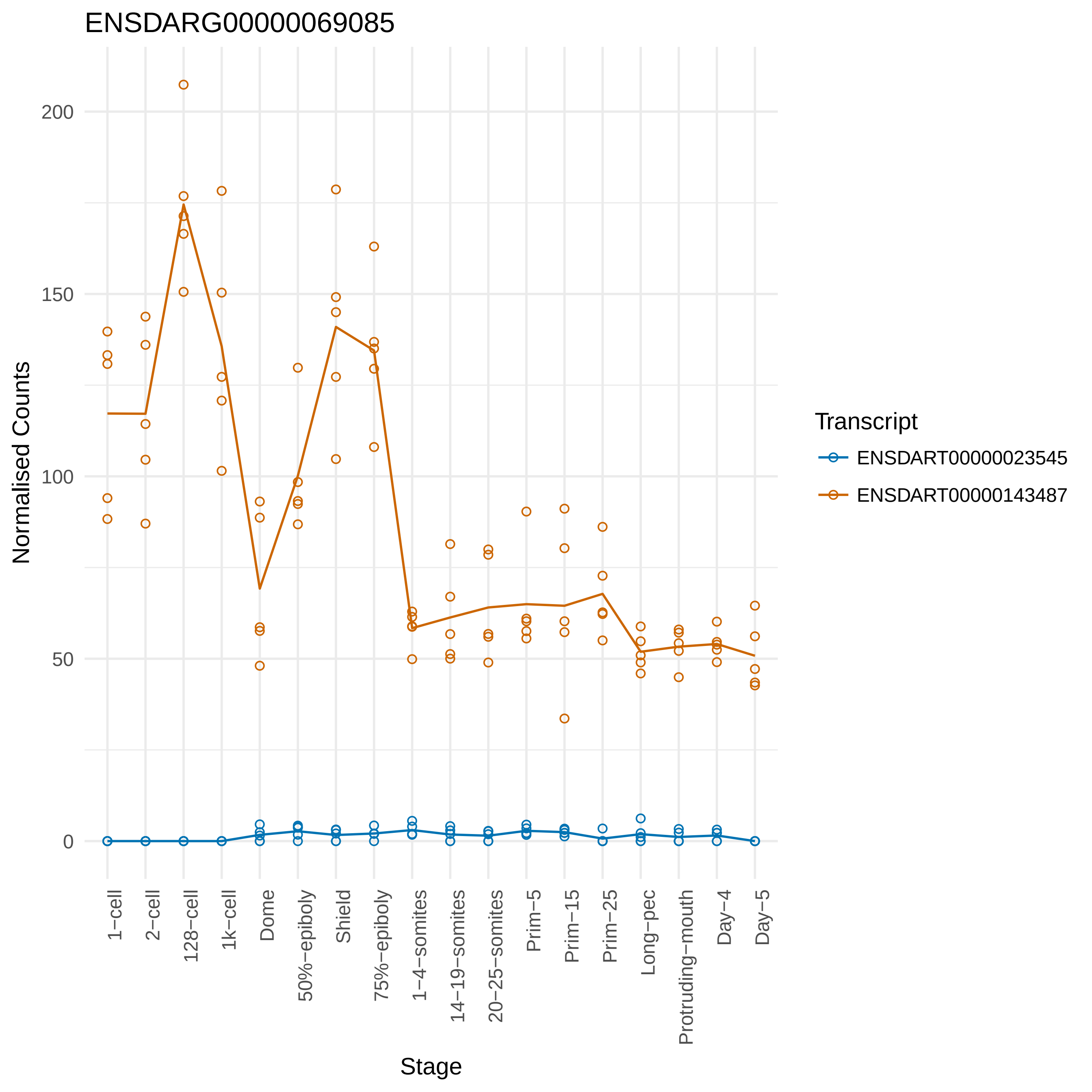

ENSDARG00000069085
Normalised Counts
200
150
100
50
0
1−cell
2−cell
128−cell
1k−cell
Dome
50%−epiboly
Shield
75%−epiboly
1−4−somites
14−19−somites
20−25−somites
Prim−5
Prim−15
Prim−25
Long−pec
Protruding−mouth
Day−4
Day−5
Stage
Transcript
ENSDART00000023545
ENSDART00000143487

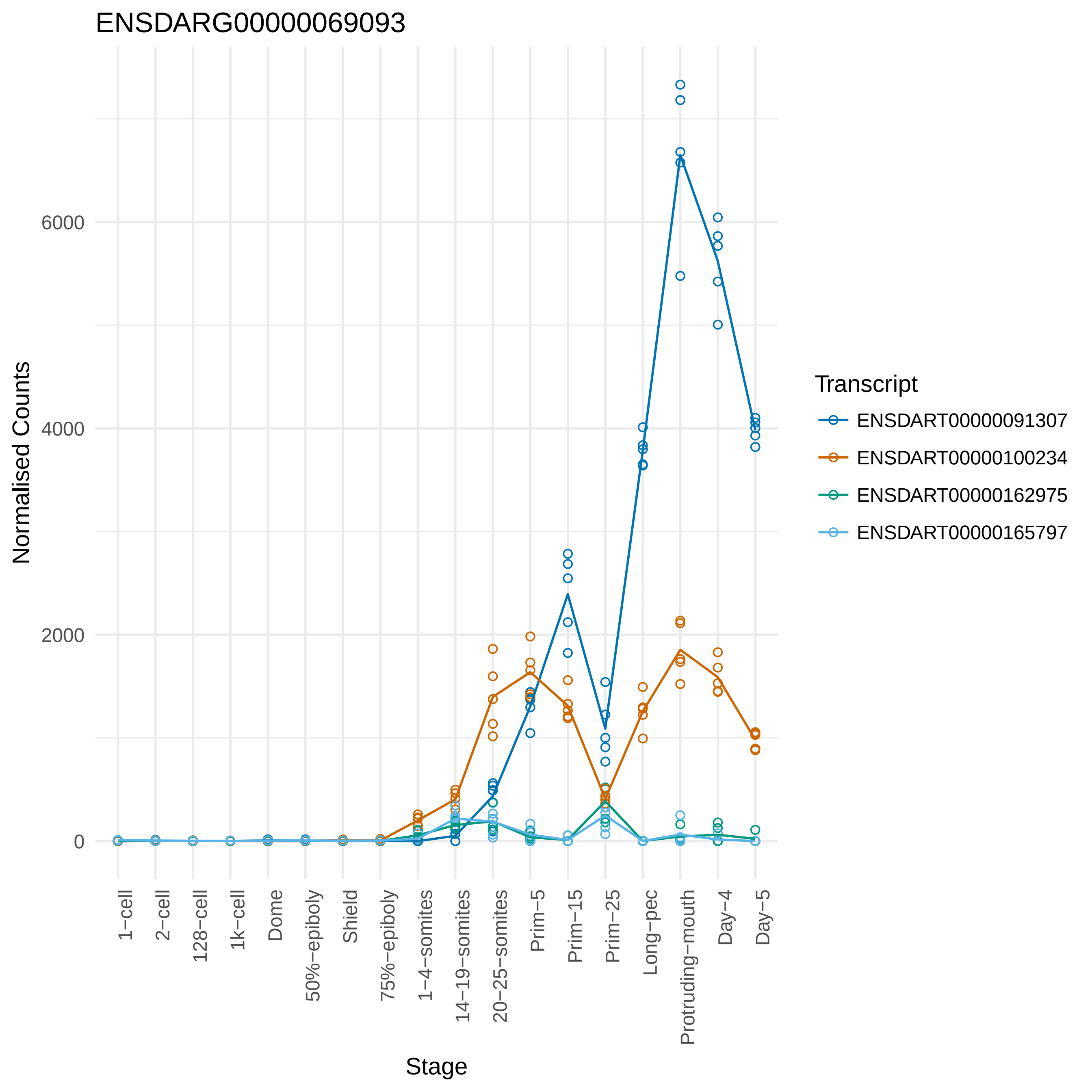

ENSDARG00000069093
Normalised Counts
0
2000
4000
6000
Stage
1−cell
2−cell
128−cell
1k−cell
Dome
50%−epiboly
Shield
75%−epiboly
1−4−somites
14−19−somites
20−25−somites
Prim−5
Prim−15
Prim−25
Long−pec
Protruding−mouth
Day−4
Day−5
Transcript
ENSDART00000091307
ENSDART00000100234
ENSDART00000162975
ENSDART00000165797

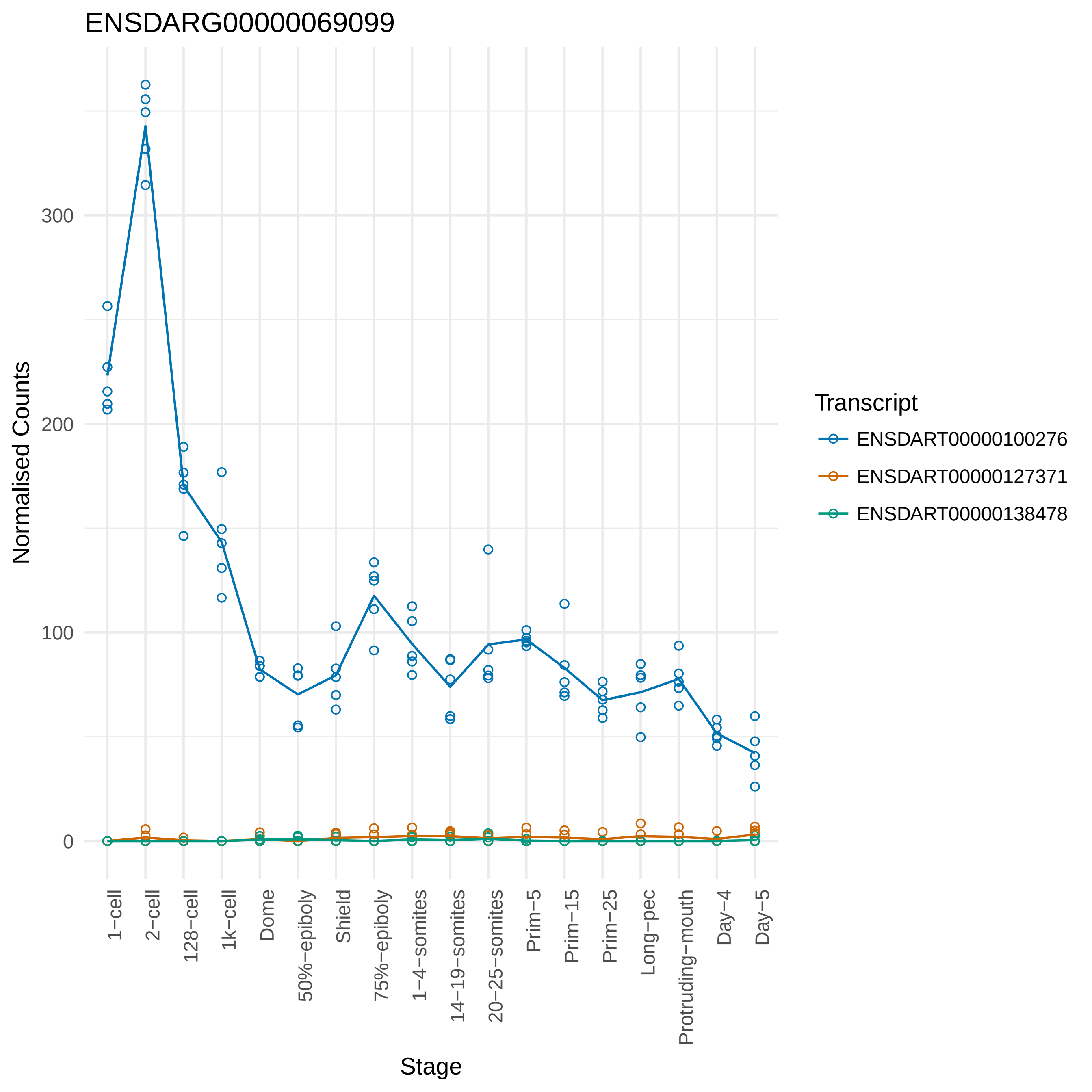

ENSDARG00000069099
Normalised Counts
300
200
100
0
1−cell
2−cell
128−cell
1k−cell
Dome
50%−epiboly
Shield
75%−epiboly
1−4−somites
14−19−somites
20−25−somites
Prim−5
Prim−15
Prim−25
Long−pec
Protruding−mouth
Day−4
Day−5
Stage
Transcript
ENSDART00000100276
ENSDART00000127371
ENSDART00000138478

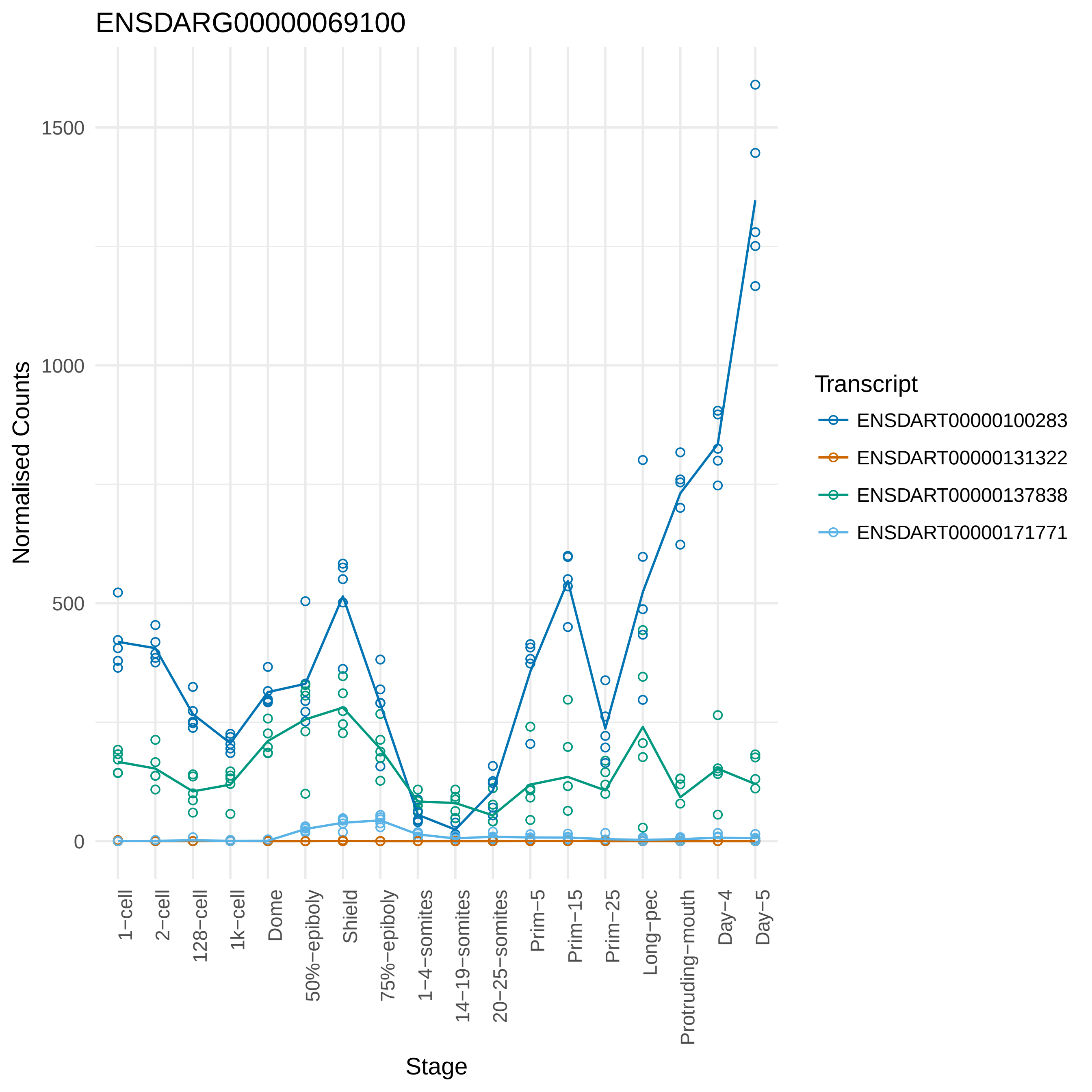

ENSDARG00000069100
Normalised Counts
1500
1000
500
0
1−cell
2−cell
128−cell
1k−cell
Dome
50%−epiboly
Shield
75%−epiboly
1−4−somites
14−19−somites
20−25−somites
Prim−5
Prim−15
Prim−25
Long−pec
Protruding−mouth
Day−4
Day−5
Stage
Transcript
ENSDART00000100283
ENSDART00000131322
ENSDART00000137838
ENSDART00000171771

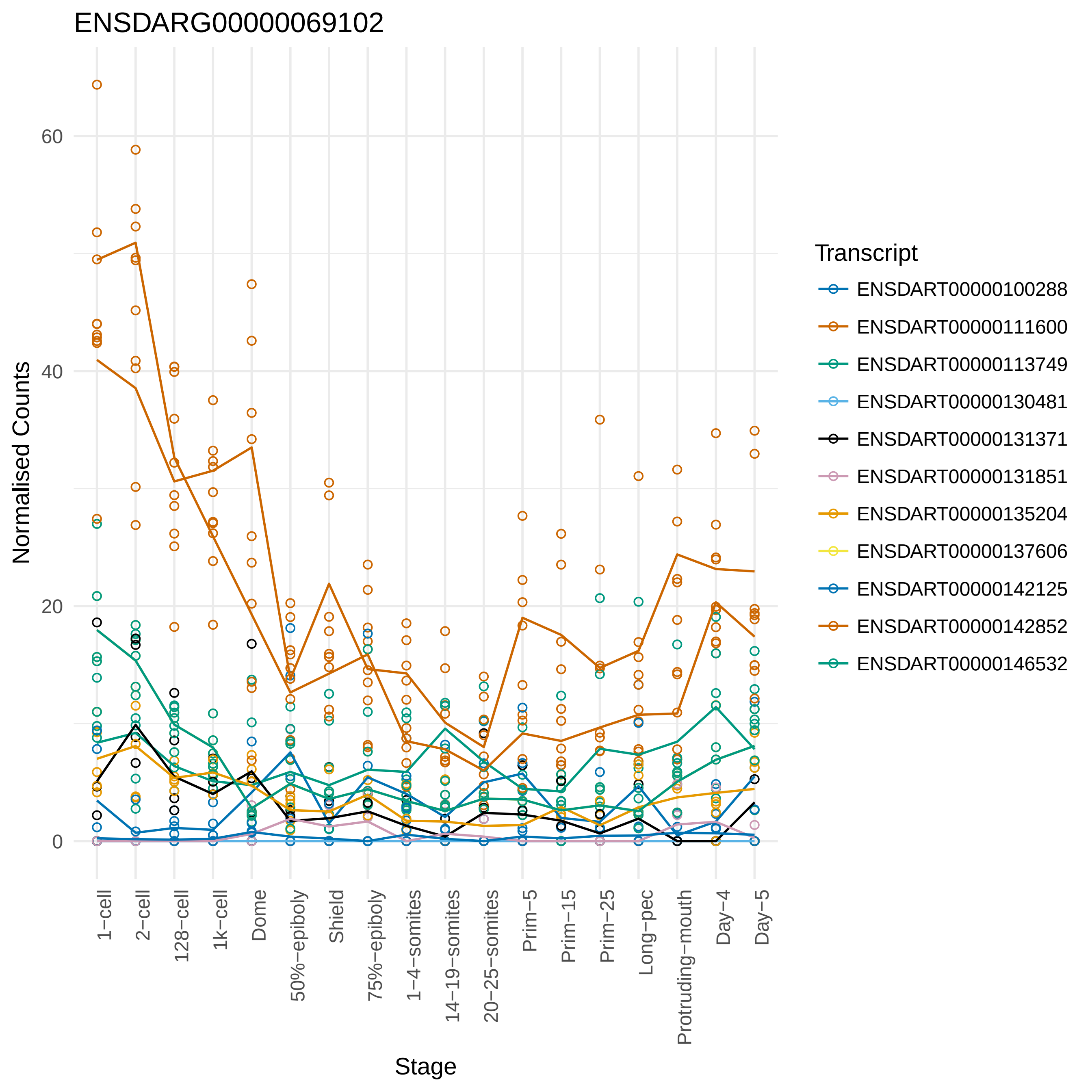

ENSDARG00000069102
Normalised Counts
0
20
40
60
1−cell
2−cell
128−cell
1k−cell
Dome
50%−epiboly
Shield
75%−epiboly
1−4−somites
14−19−somites
20−25−somites
Prim−5
Prim−15
Prim−25
Long−pec
Protruding−mouth
Day−4
Day−5
Stage
Transcript
ENSDART00000100288
ENSDART00000111600
ENSDART00000113749
ENSDART00000130481
ENSDART00000131371
ENSDART00000131851
ENSDART00000135204
ENSDART00000137606
ENSDART00000142125
ENSDART00000142852
ENSDART00000146532

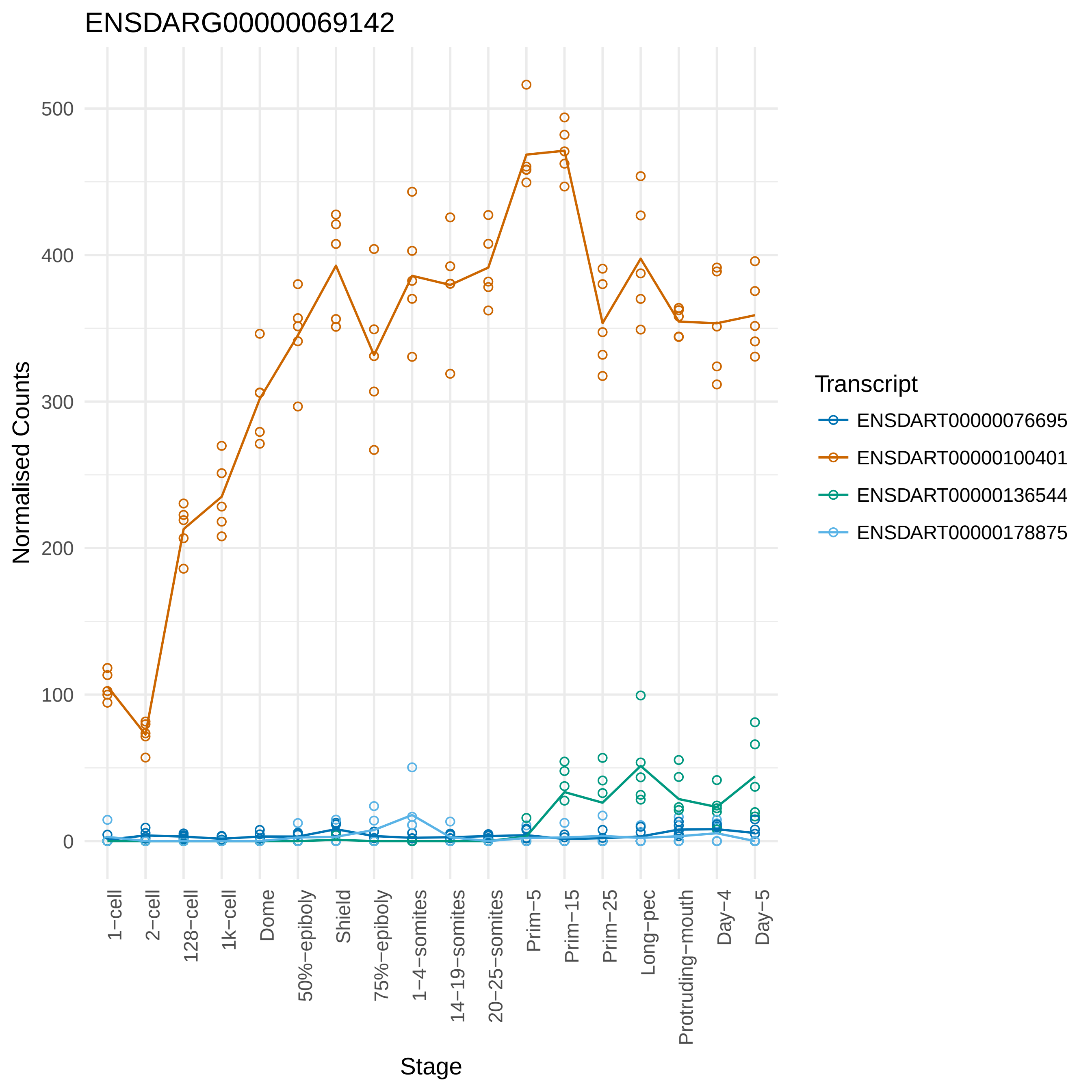

ENSDARG00000069142
Normalised Counts
500
400
300
200
100
0
1−cell
2−cell
128−cell
1k−cell
Dome
50%−epiboly
Shield
75%−epiboly
1−4−somites
14−19−somites
20−25−somites
Prim−5
Prim−15
Prim−25
Long−pec
Protruding−mouth
Day−4
Day−5
Stage
Transcript
ENSDART00000076695
ENSDART00000100401
ENSDART00000136544
ENSDART00000178875

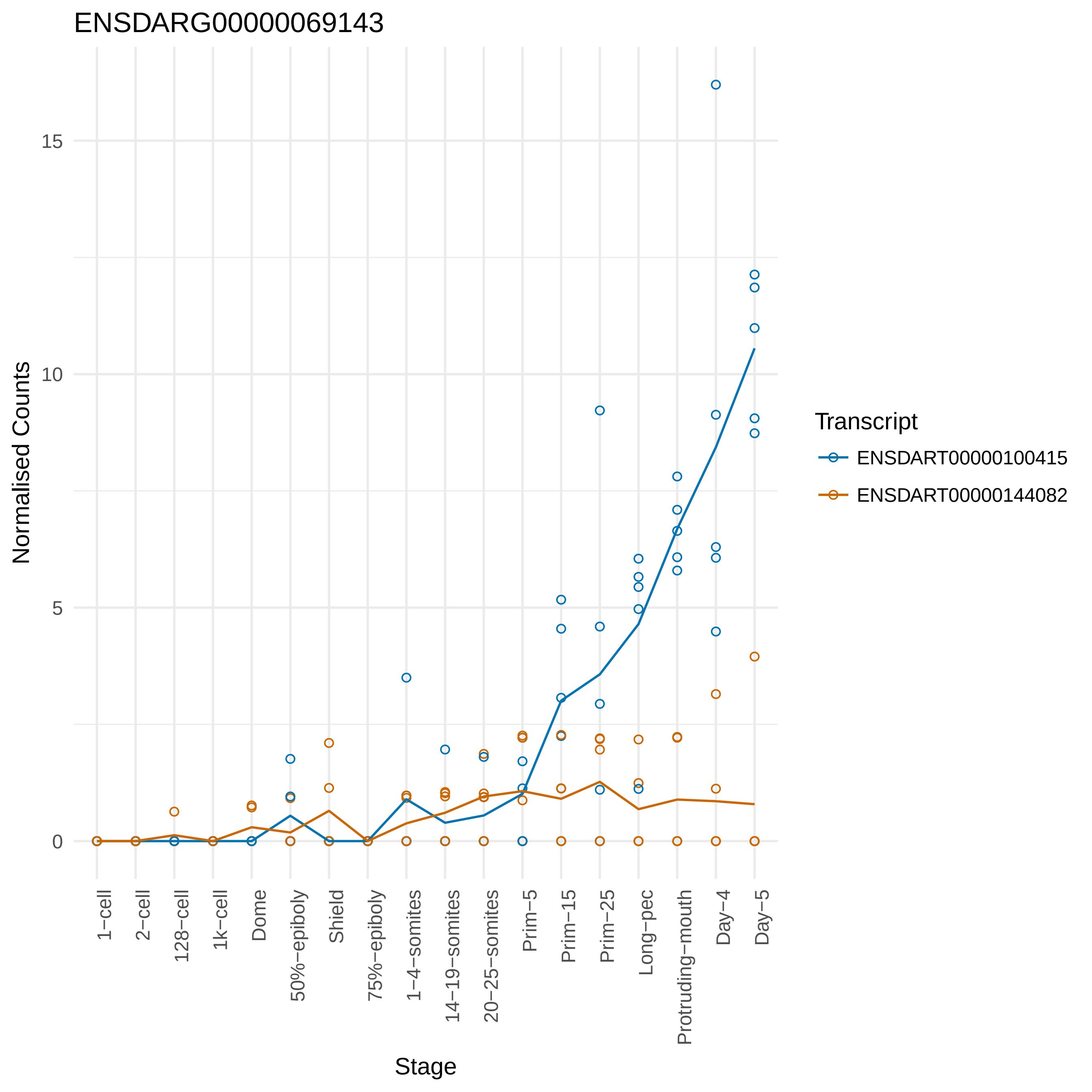
ENSDARG00000069143
Normalised Counts
0
5
10
15
Stage
1−cell
2−cell
128−cell
1k−cell
Dome
50%−epiboly
Shield
75%−epiboly
1−4−somites
14−19−somites
20−25−somites
Prim−5
Prim−15
Prim−25
Long−pec
Protruding−mouth
Day−4
Day−5
Transcript
ENSDART00000100415
ENSDART00000144082

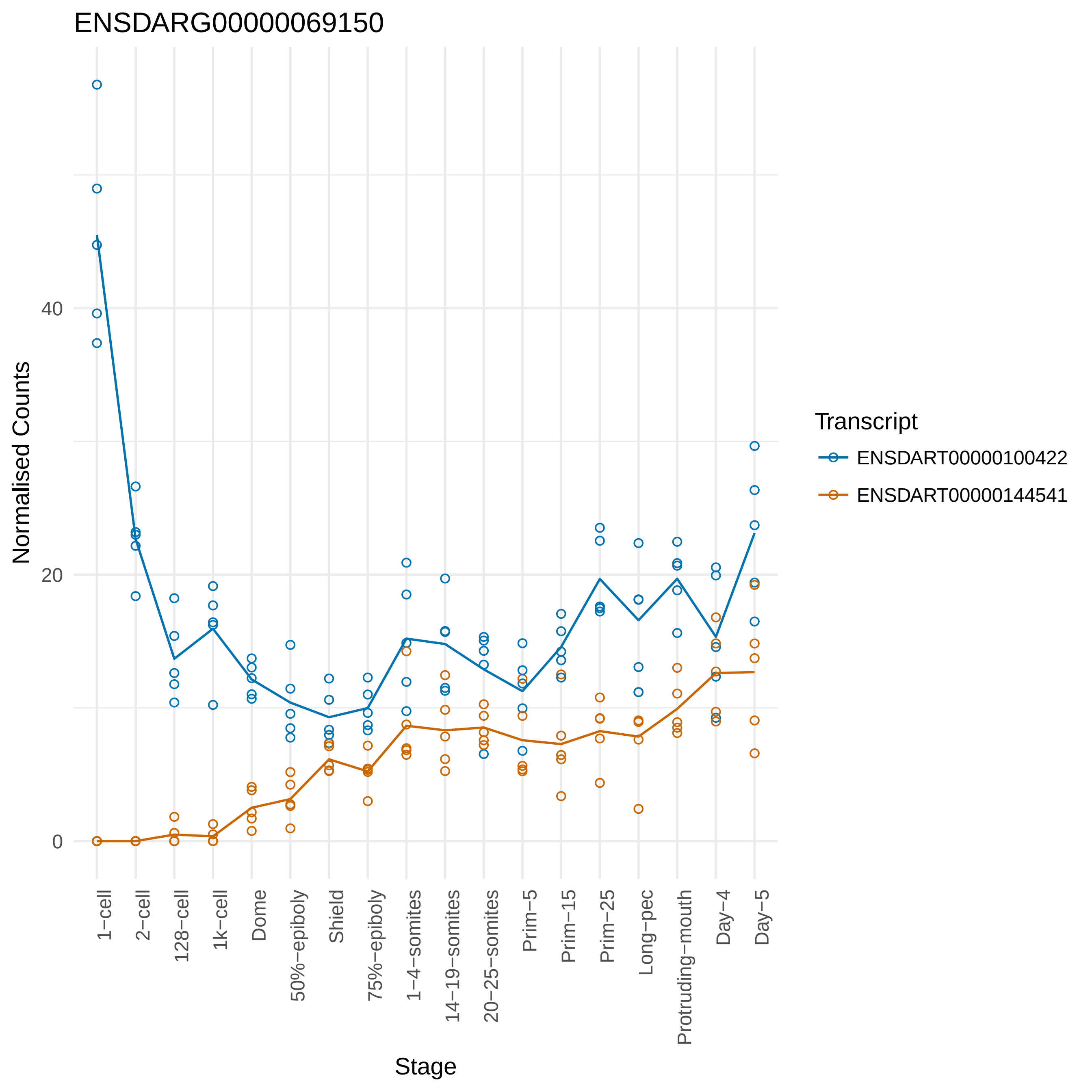
ENSDARG00000069150
Normalised Counts
0
20
40
Stage
1−cell
2−cell
128−cell
1k−cell
Dome
50%−epiboly
Shield
75%−epiboly
1−4−somites
14−19−somites
20−25−somites
Prim−5
Prim−15
Prim−25
Long−pec
Protruding−mouth
Day−4
Day−5
Transcript
ENSDART00000100422
ENSDART00000144541

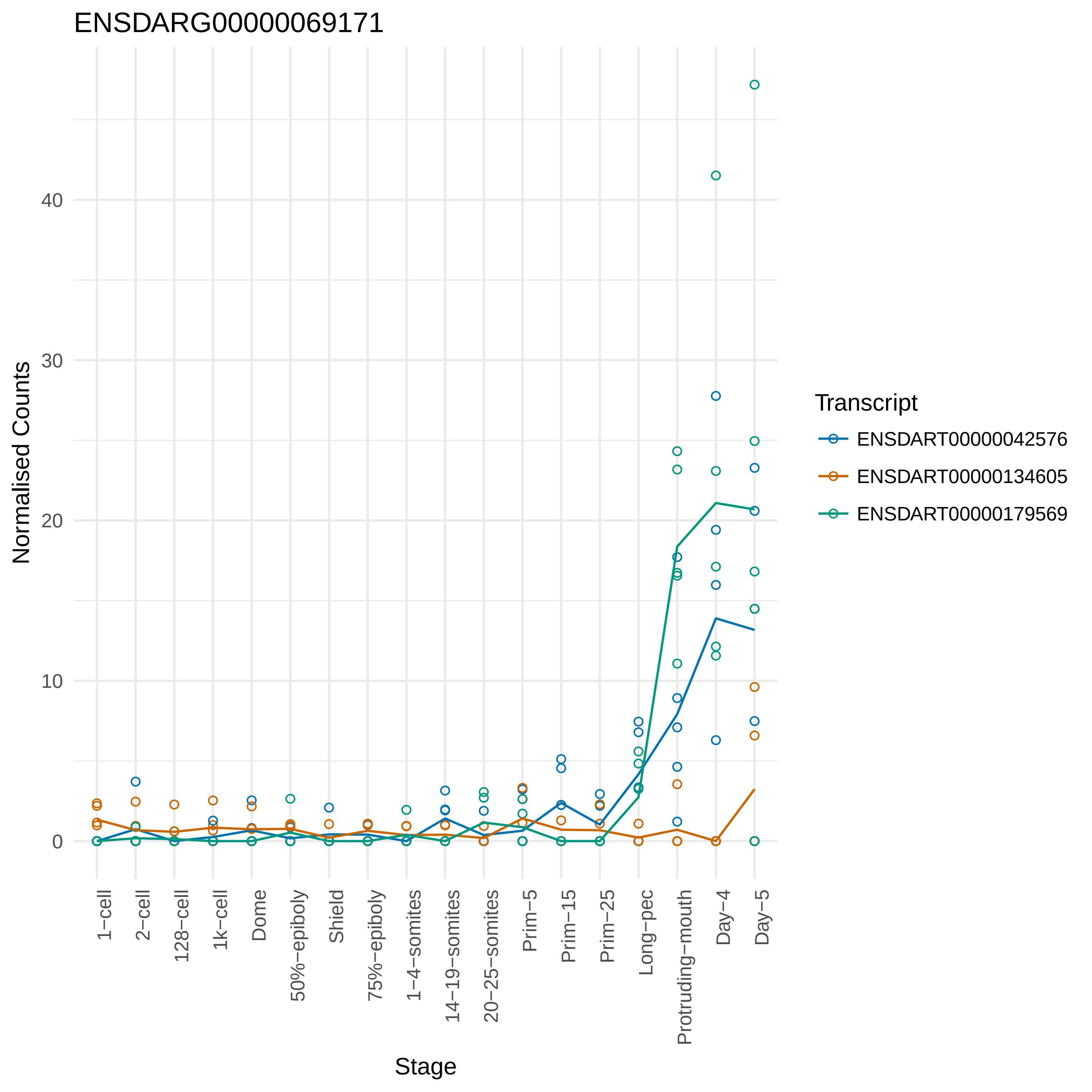

ENSDARG00000069171
Normalised Counts
0
10
20
30
40
1−cell
2−cell
128−cell
1k−cell
Dome
50%−epiboly
Shield
75%−epiboly
1−4−somites
14−19−somites
20−25−somites
Prim−5
Prim−15
Prim−25
Long−pec
Protruding−mouth
Day−4
Day−5
Stage
Transcript
ENSDART00000042576
ENSDART00000134605
ENSDART00000179569

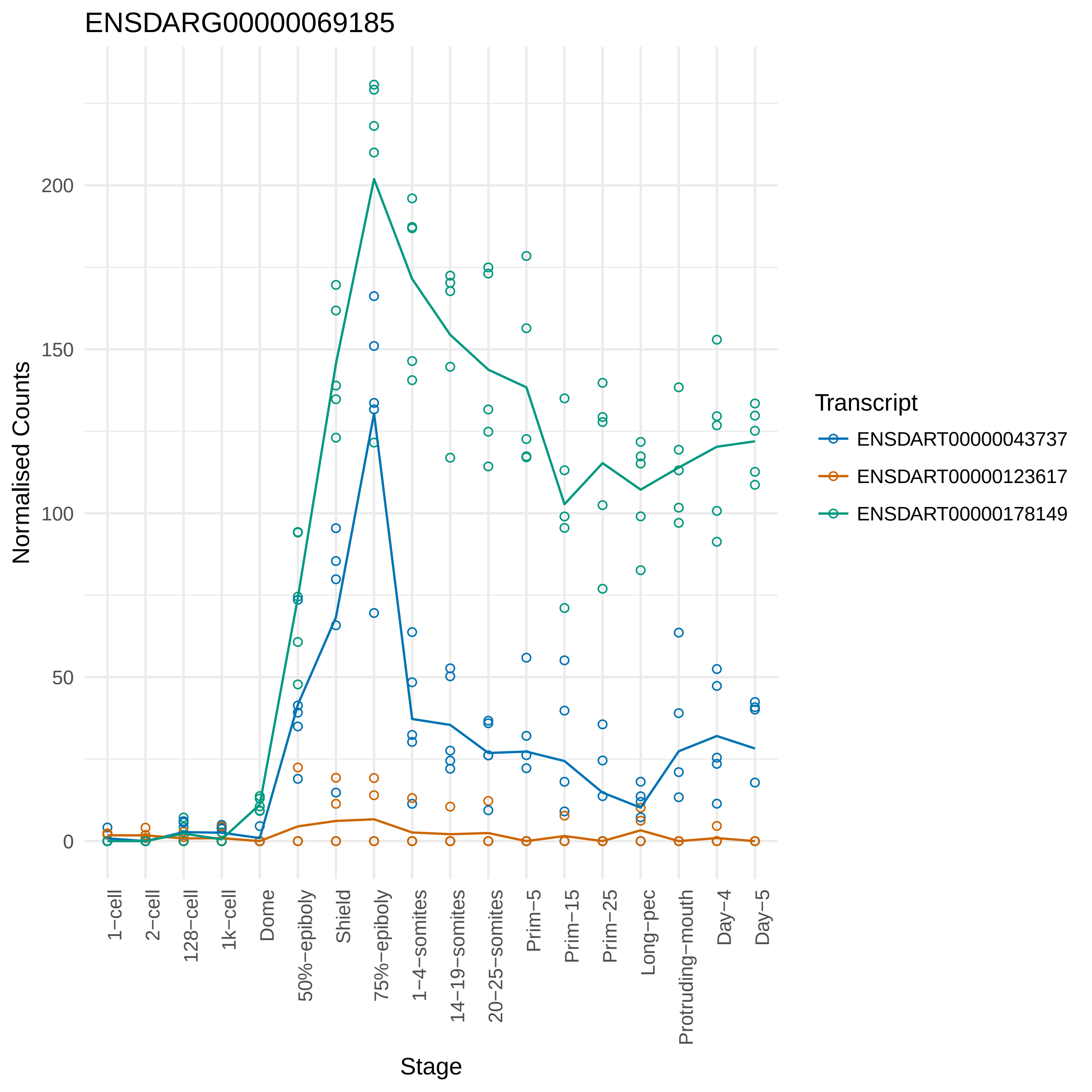

ENSDARG00000069185
Normalised Counts
200
150
100
50
0
1−cell
2−cell
128−cell
1k−cell
Dome
50%−epiboly
Shield
75%−epiboly
1−4−somites
14−19−somites
20−25−somites
Prim−5
Prim−15
Prim−25
Long−pec
Protruding−mouth
Day−4
Day−5
Stage
Transcript
ENSDART00000043737
ENSDART00000123617
ENSDART00000178149

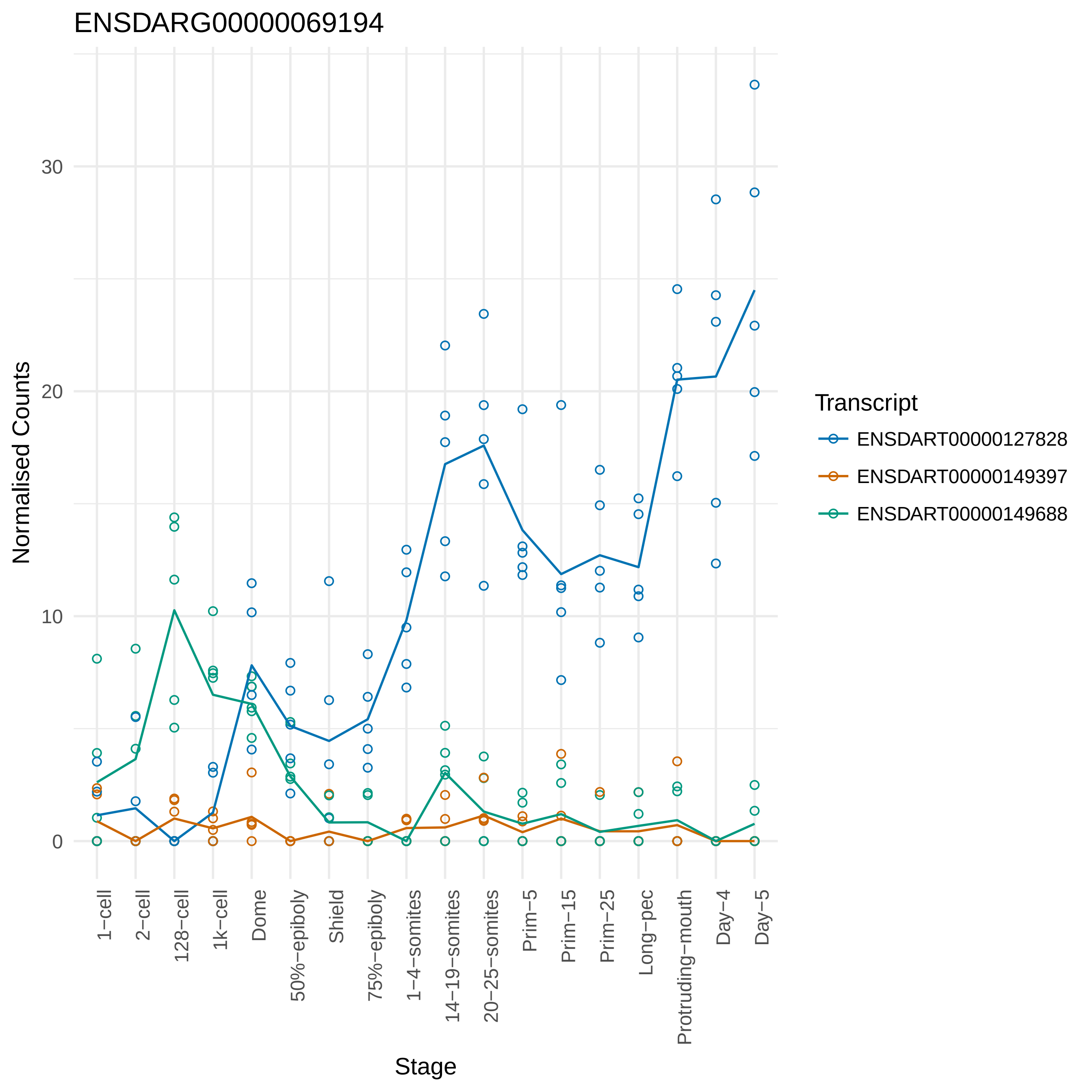

ENSDARG00000069194
Normalised Counts
0
10
20
30
Stage
1−cell
2−cell
128−cell
1k−cell
Dome
50%−epiboly
Shield
75%−epiboly
1−4−somites
14−19−somites
20−25−somites
Prim−5
Prim−15
Prim−25
Long−pec
Protruding−mouth
Day−4
Day−5
Transcript
ENSDART00000127828
ENSDART00000149397
ENSDART00000149688

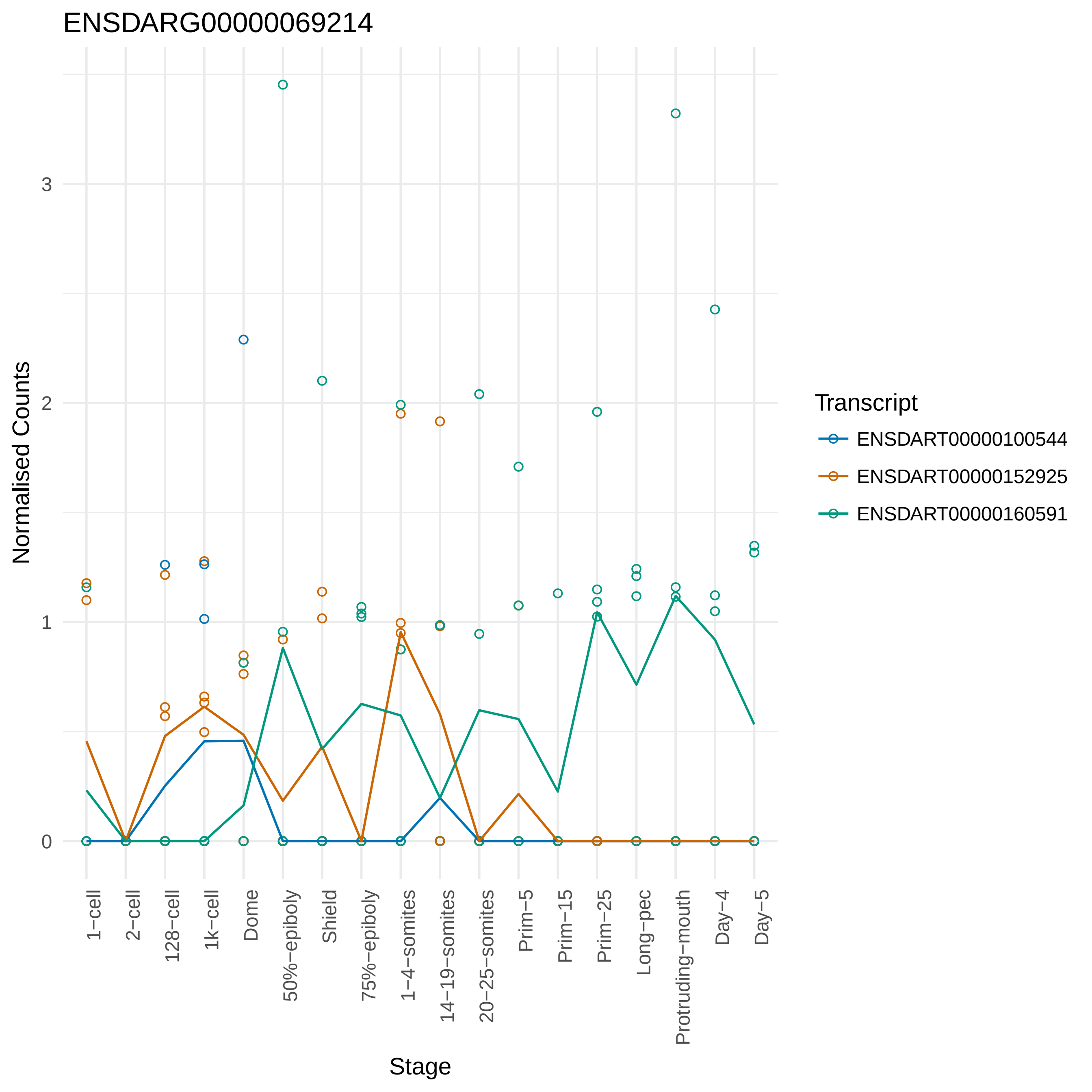
ENSDARG00000069214
Normalised Counts
0
1
2
3
1−cell
2−cell
128−cell
1k−cell
Dome
50%−epiboly
Shield
75%−epiboly
1−4−somites
14−19−somites
20−25−somites
Prim−5
Prim−15
Prim−25
Long−pec
Protruding−mouth
Day−4
Day−5
Stage
Transcript
ENSDART00000100544
ENSDART00000152925
ENSDART00000160591

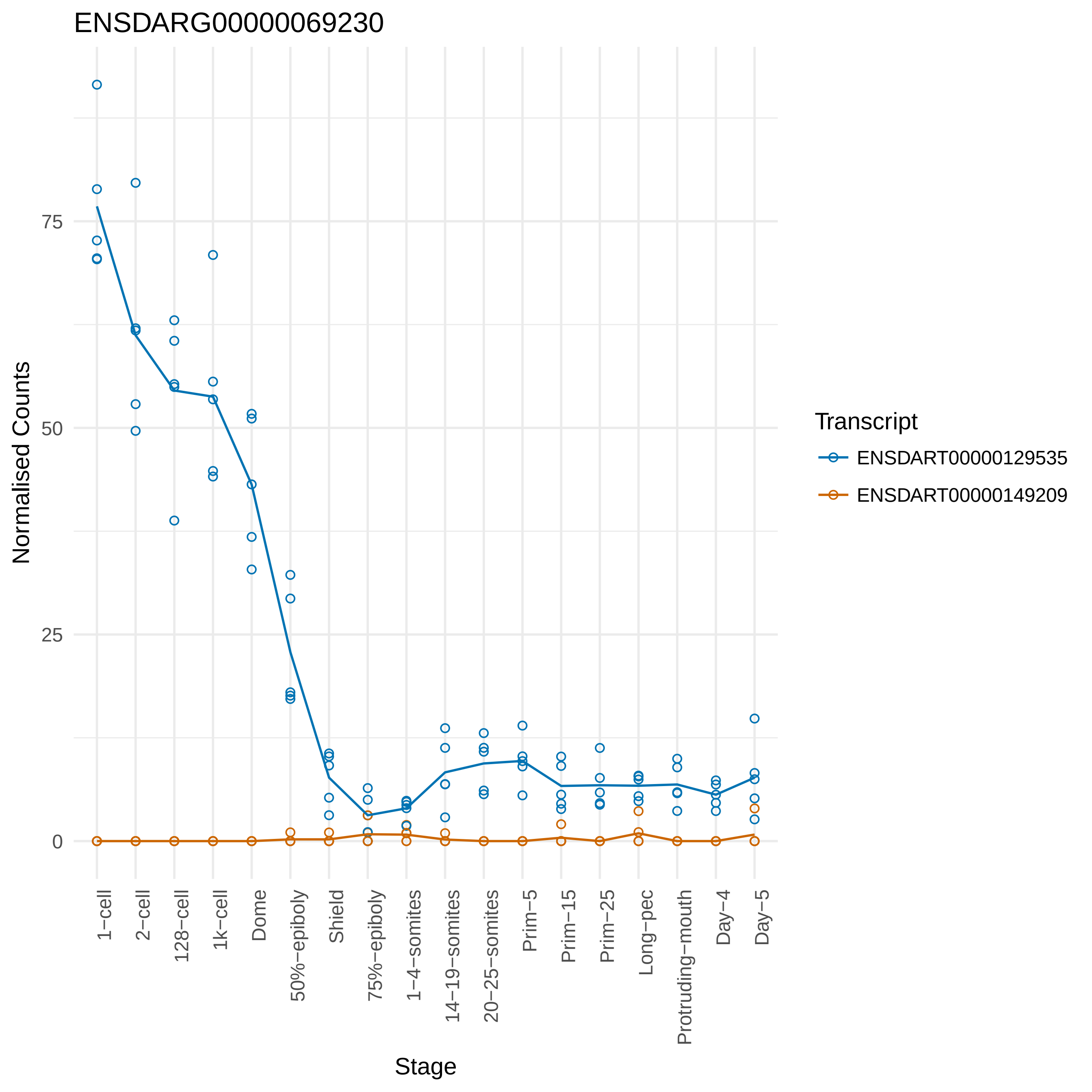
ENSDARG00000069230
Normalised Counts
75
50
25
0
1−cell
2−cell
128−cell
1k−cell
Dome
50%−epiboly
Shield
75%−epiboly
1−4−somites
14−19−somites
20−25−somites
Prim−5
Prim−15
Prim−25
Long−pec
Protruding−mouth
Day−4
Day−5
Stage
Transcript
ENSDART00000129535
ENSDART00000149209

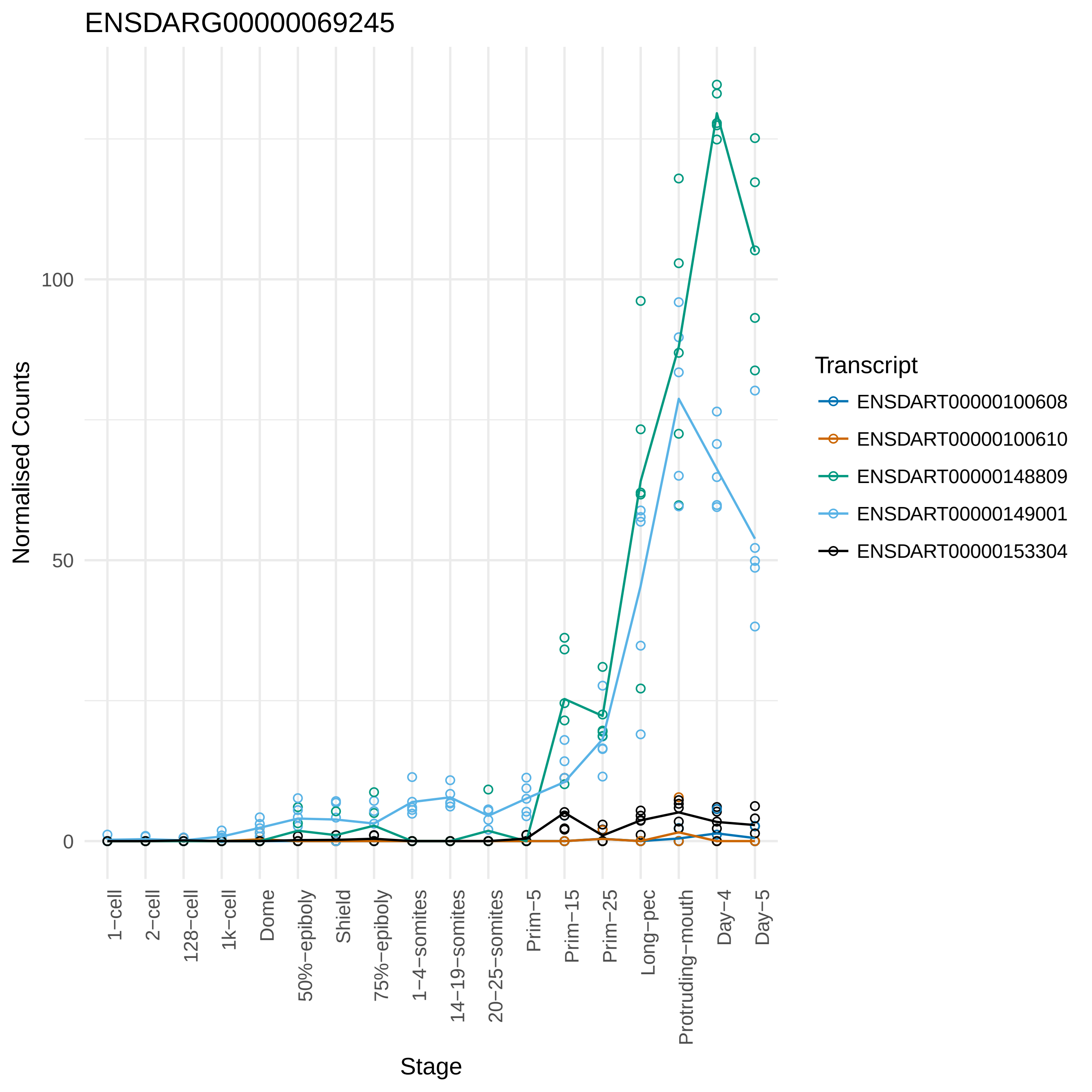

ENSDARG00000069245
Normalised Counts
100
50
0
1−cell
2−cell
128−cell
1k−cell
Dome
50%−epiboly
Shield
75%−epiboly
1−4−somites
14−19−somites
20−25−somites
Prim−5
Prim−15
Prim−25
Long−pec
Protruding−mouth
Day−4
Day−5
Stage
Transcript
ENSDART00000100608
ENSDART00000100610
ENSDART00000148809
ENSDART00000149001
ENSDART00000153304

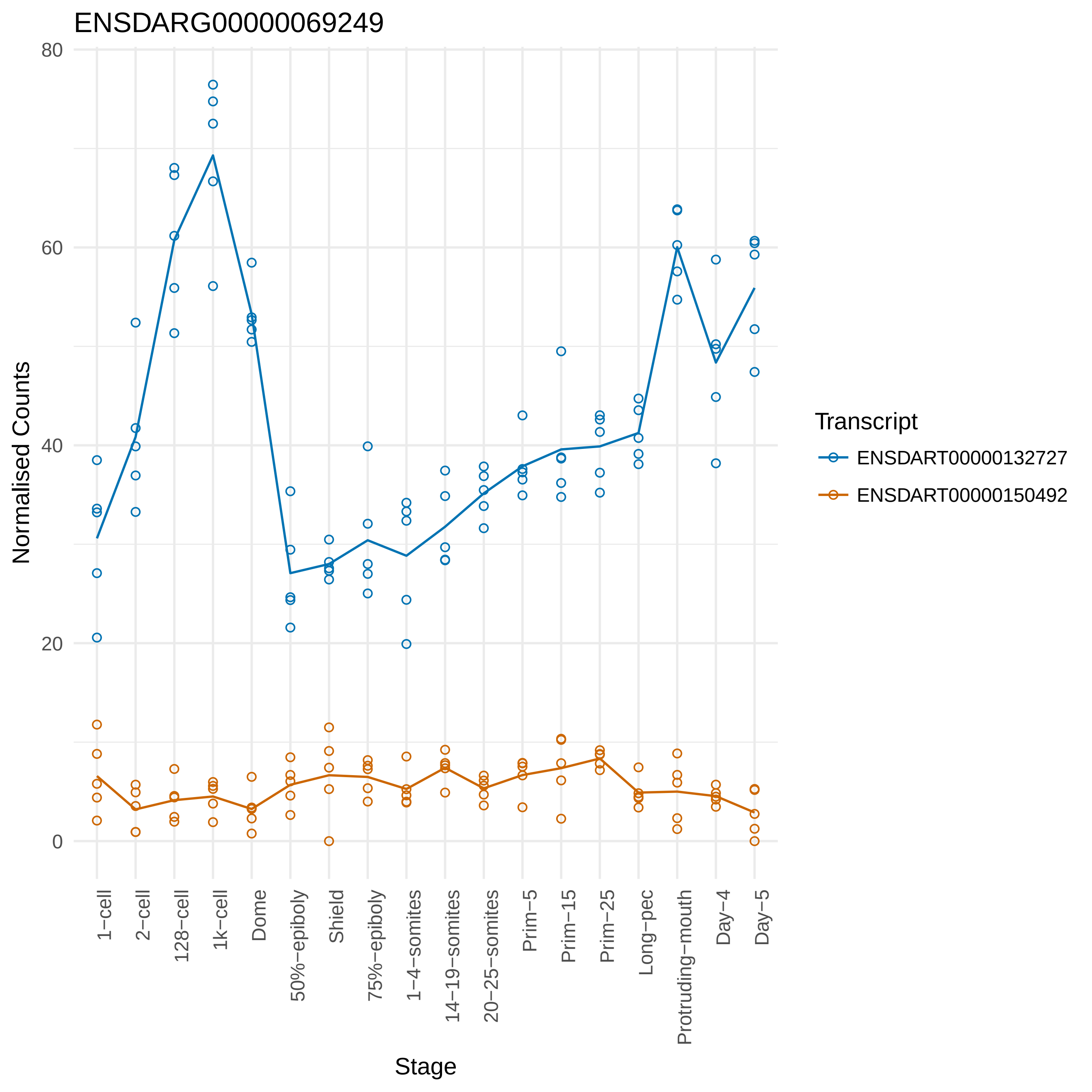
ENSDARG00000069249
Normalised Counts
80
60
40
20
0
1−cell
2−cell
128−cell
1k−cell
Dome
50%−epiboly
Shield
75%−epiboly
1−4−somites
14−19−somites
20−25−somites
Prim−5
Prim−15
Prim−25
Long−pec
Protruding−mouth
Day−4
Day−5
Stage
Transcript
ENSDART00000132727
ENSDART00000150492

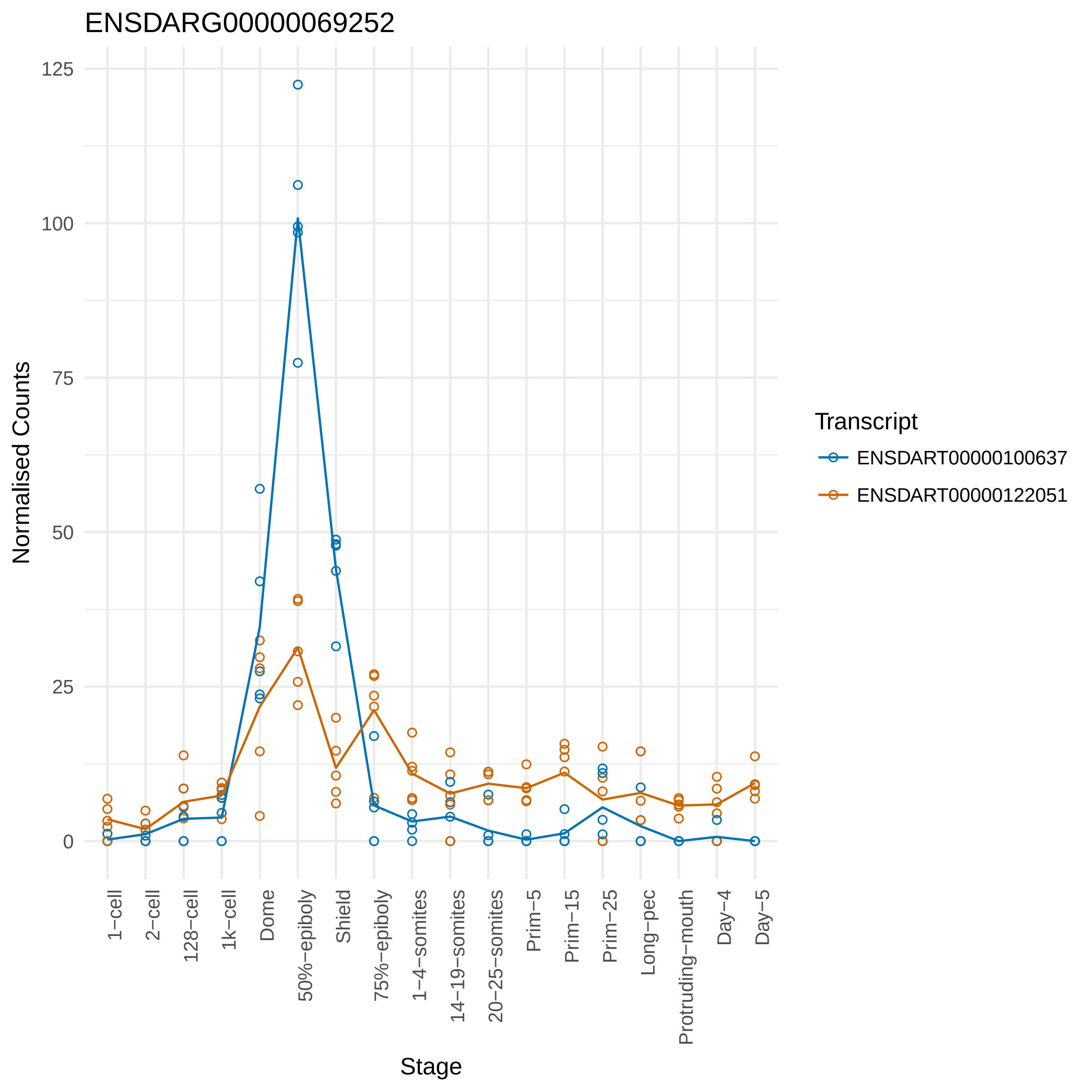
ENSDARG00000069252
Normalised Counts
125
100
75
50
25
0
1−cell
2−cell
128−cell
1k−cell
Dome
50%−epiboly
Shield
75%−epiboly
1−4−somites
14−19−somites
20−25−somites
Prim−5
Prim−15
Prim−25
Long−pec
Protruding−mouth
Day−4
Day−5
Stage
Transcript
ENSDART00000100637
ENSDART00000122051

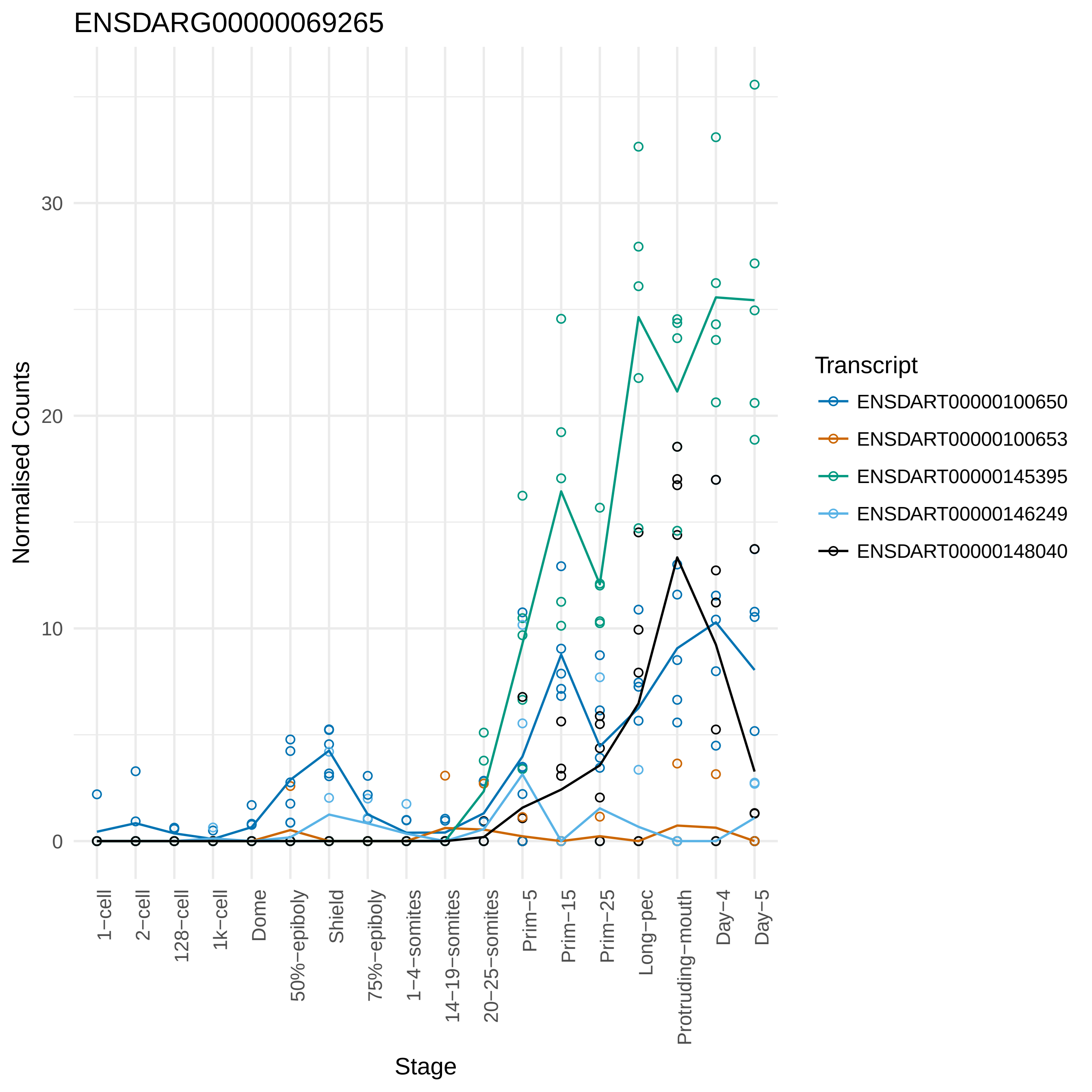

ENSDARG00000069265
Normalised Counts
0
10
20
30
Stage
1−cell
2−cell
128−cell
1k−cell
Dome
50%−epiboly
Shield
75%−epiboly
1−4−somites
14−19−somites
20−25−somites
Prim−5
Prim−15
Prim−25
Long−pec
Protruding−mouth
Day−4
Day−5
Transcript
ENSDART00000100650
ENSDART00000100653
ENSDART00000145395
ENSDART00000146249
ENSDART00000148040

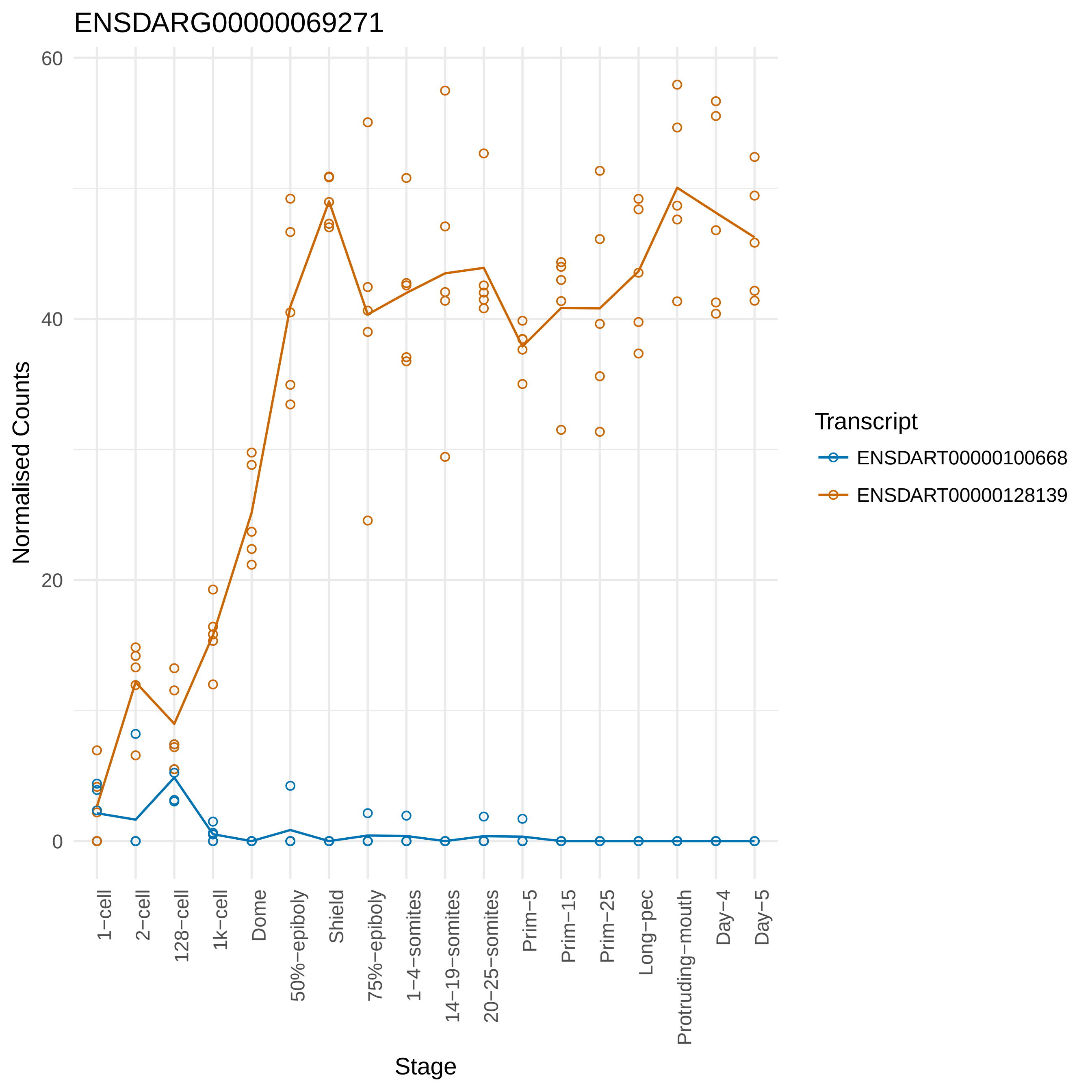

ENSDARG00000069271
Normalised Counts
60
40
20
0
1−cell
2−cell
128−cell
1k−cell
Dome
50%−epiboly
Shield
75%−epiboly
1−4−somites
14−19−somites
20−25−somites
Prim−5
Prim−15
Prim−25
Long−pec
Protruding−mouth
Day−4
Day−5
Stage
Transcript
ENSDART00000100668
ENSDART00000128139

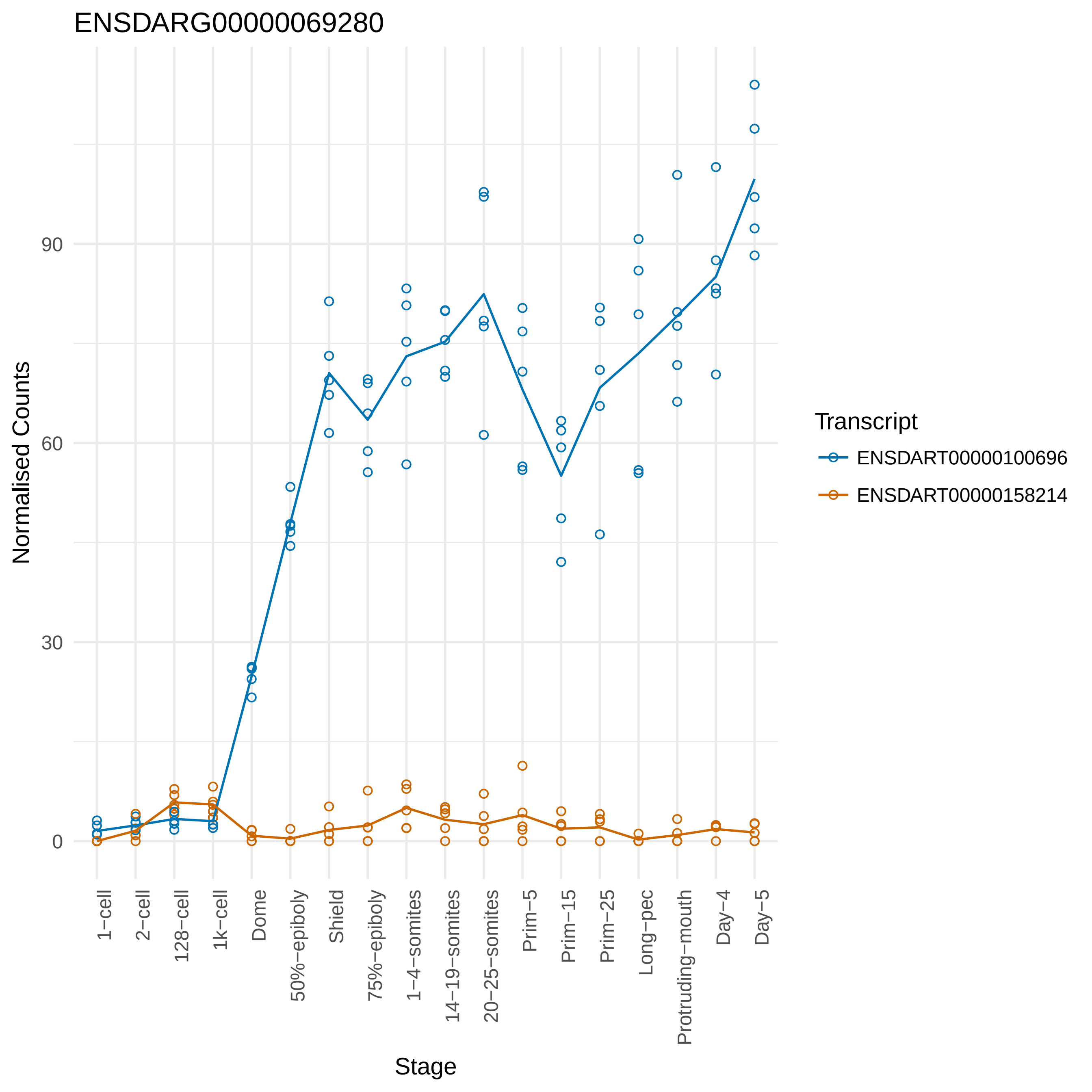

ENSDARG00000069280
Normalised Counts
90
60
30
0
1−cell
2−cell
128−cell
1k−cell
Dome
50%−epiboly
Shield
75%−epiboly
1−4−somites
14−19−somites
20−25−somites
Prim−5
Prim−15
Prim−25
Long−pec
Protruding−mouth
Day−4
Day−5
Stage
Transcript
ENSDART00000100696
ENSDART00000158214

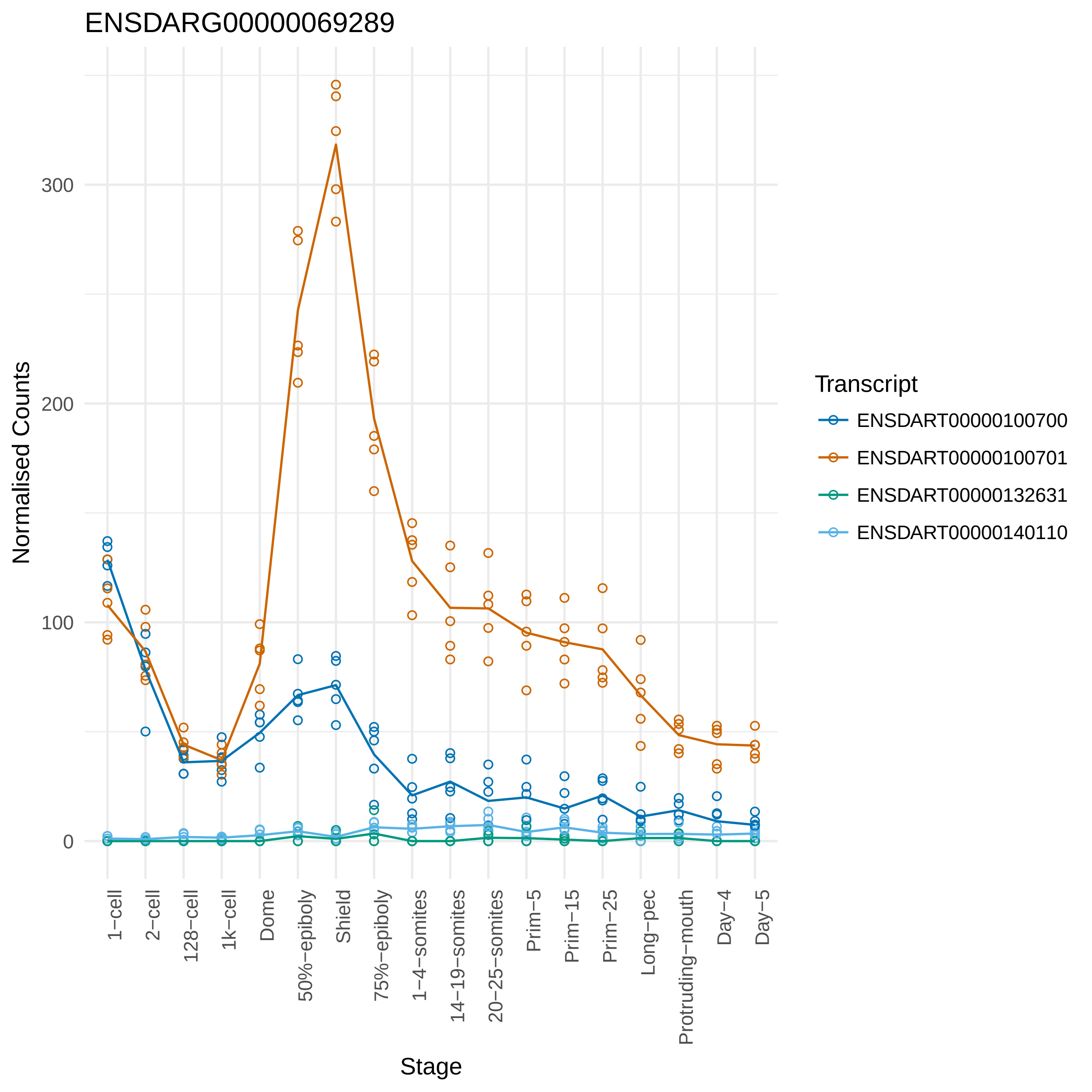

ENSDARG00000069289
Normalised Counts
0
100
200
300
Stage
1−cell
2−cell
128−cell
1k−cell
Dome
50%−epiboly
Shield
75%−epiboly
1−4−somites
14−19−somites
20−25−somites
Prim−5
Prim−15
Prim−25
Long−pec
Protruding−mouth
Day−4
Day−5
Transcript
ENSDART00000100700
ENSDART00000100701
ENSDART00000132631
ENSDART00000140110

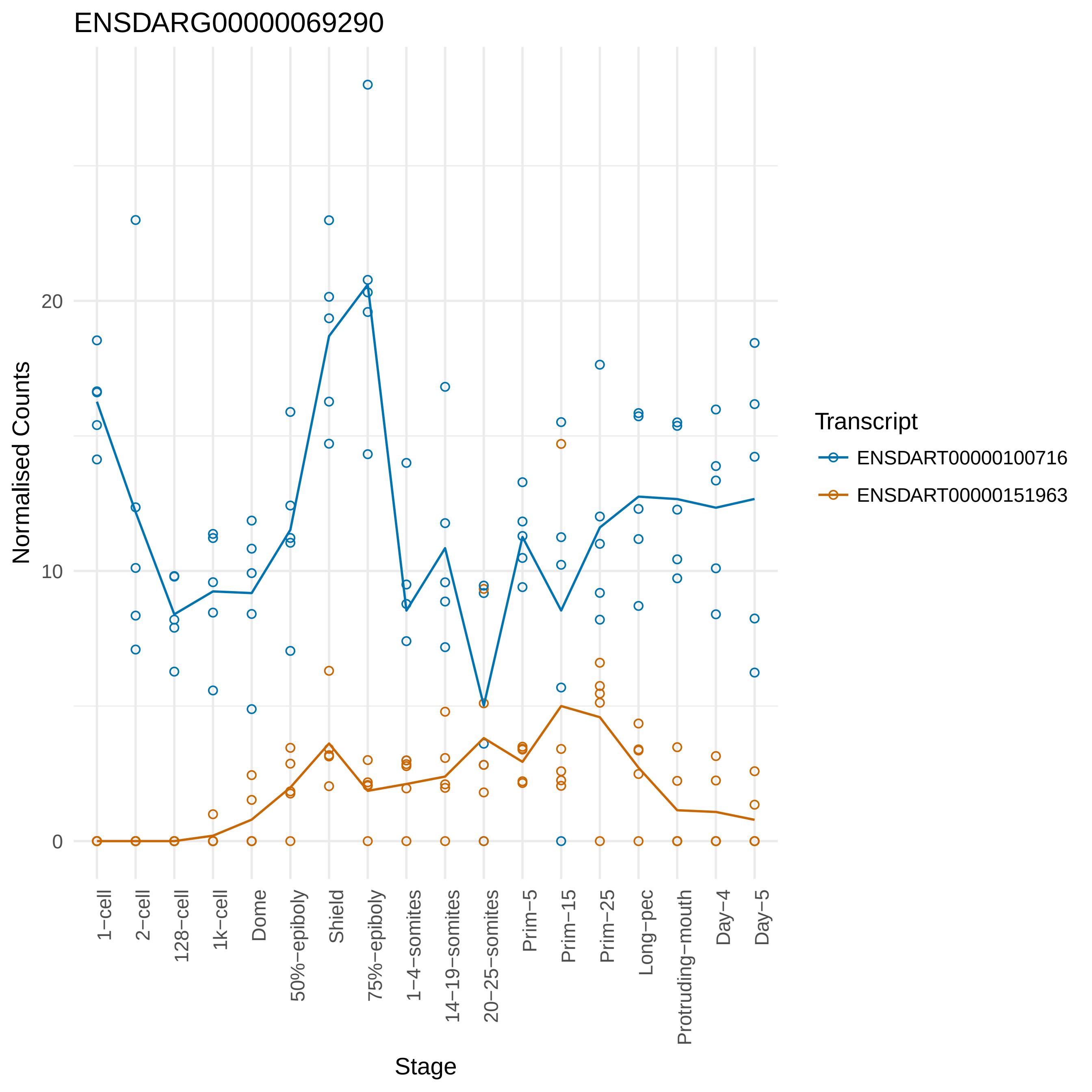

ENSDARG00000069290
Normalised Counts
20
10
0
1−cell
2−cell
128−cell
1k−cell
Dome
50%−epiboly
Shield
75%−epiboly
1−4−somites
14−19−somites
20−25−somites
Prim−5
Prim−15
Prim−25
Long−pec
Protruding−mouth
Day−4
Day−5
Stage
Transcript
ENSDART00000100716
ENSDART00000151963

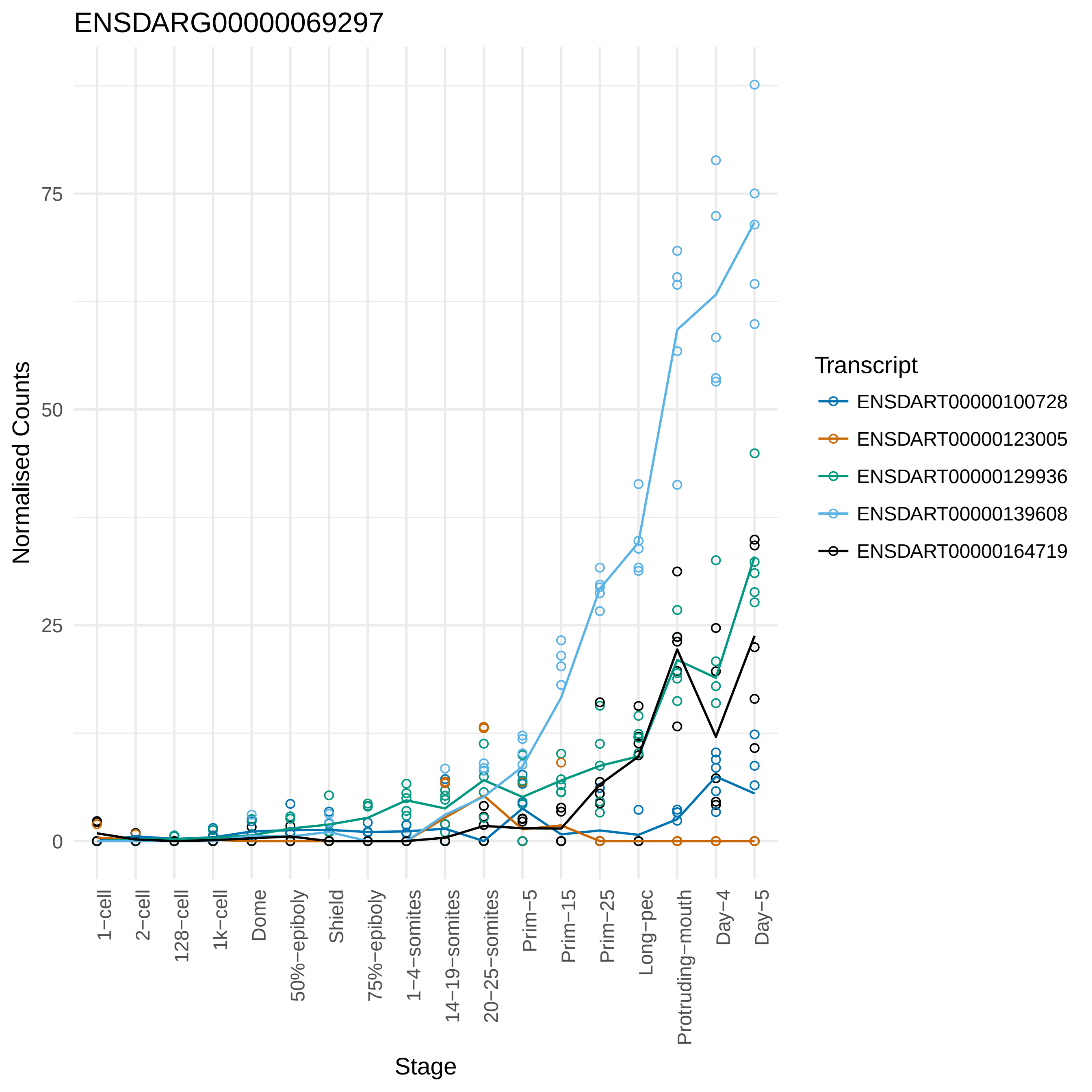

ENSDARG00000069297
Normalised Counts
75
50
25
0
1−cell
2−cell
128−cell
1k−cell
Dome
50%−epiboly
Shield
75%−epiboly
1−4−somites
14−19−somites
20−25−somites
Prim−5
Prim−15
Prim−25
Long−pec
Protruding−mouth
Day−4
Day−5
Stage
Transcript
ENSDART00000100728
ENSDART00000123005
ENSDART00000129936
ENSDART00000139608
ENSDART00000164719

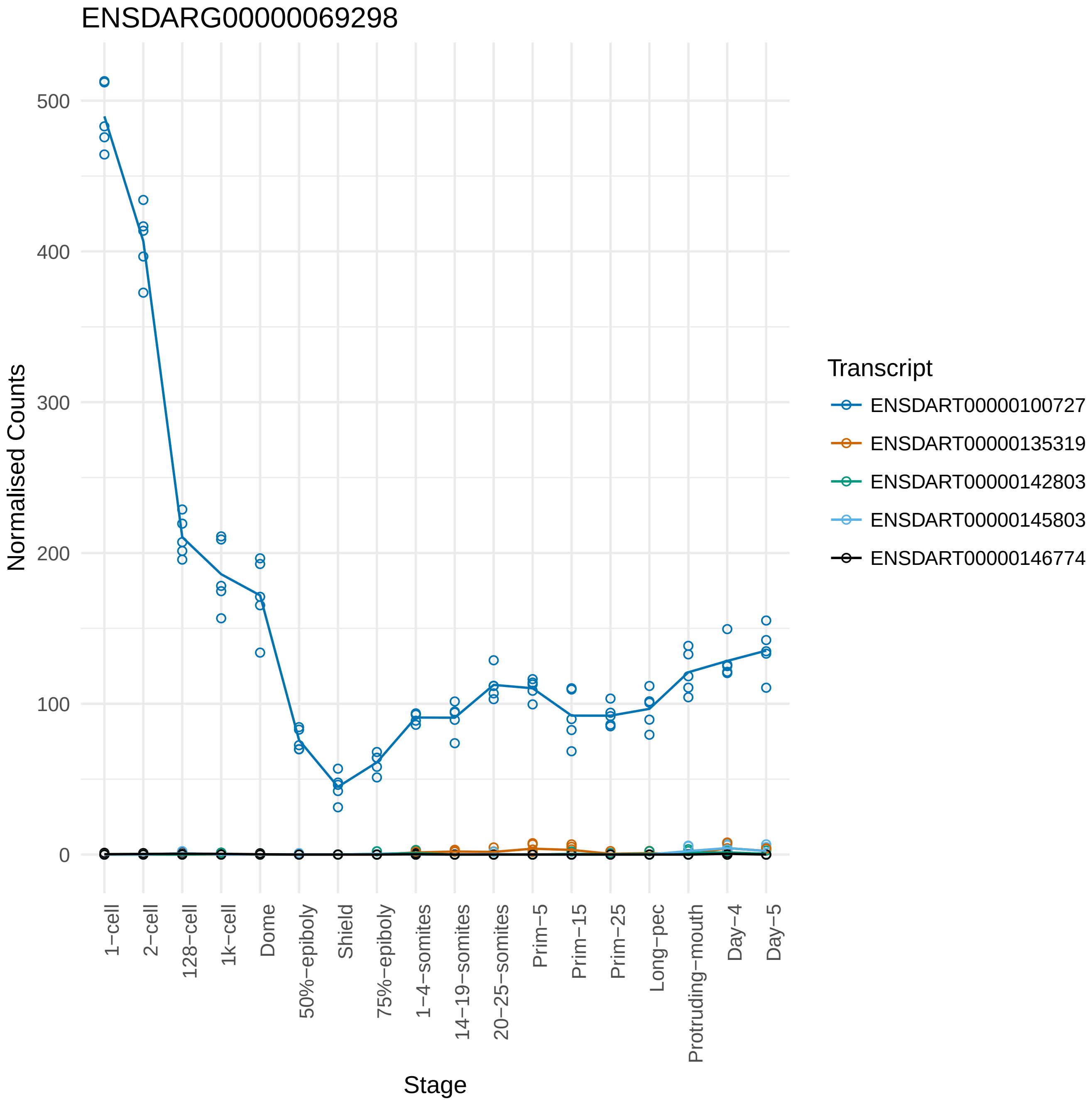

ENSDARG00000069298
Normalised Counts
500
400
300
200
100
0
1−cell
2−cell
128−cell
1k−cell
Dome
50%−epiboly
Shield
75%−epiboly
1−4−somites
14−19−somites
20−25−somites
Prim−5
Prim−15
Prim−25
Long−pec
Protruding−mouth
Day−4
Day−5
Stage
Transcript
ENSDART00000100727
ENSDART00000135319
ENSDART00000142803
ENSDART00000145803
ENSDART00000146774

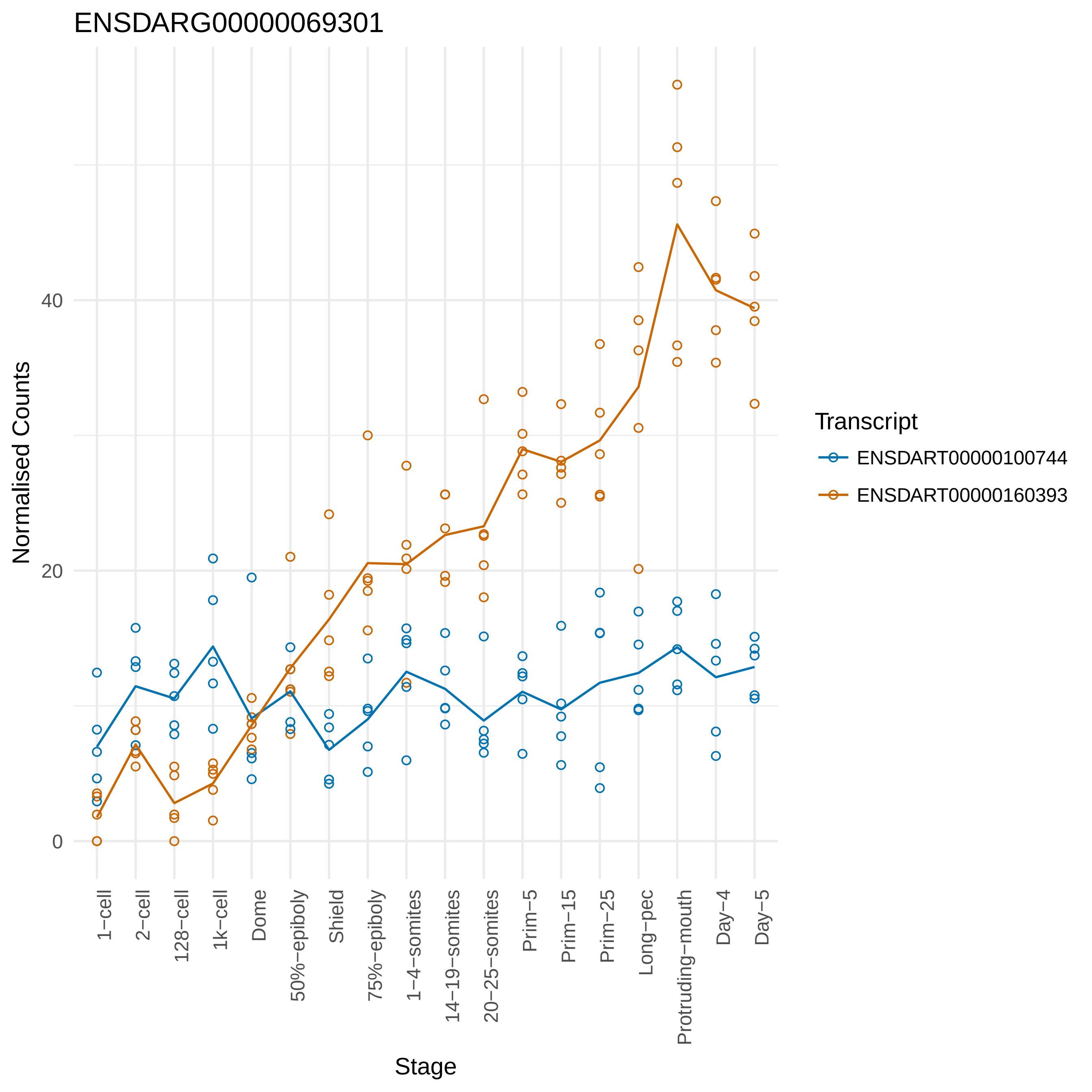

ENSDARG00000069301
Normalised Counts
0
20
40
Stage
1−cell
2−cell
128−cell
1k−cell
Dome
50%−epiboly
Shield
75%−epiboly
1−4−somites
14−19−somites
20−25−somites
Prim−5
Prim−15
Prim−25
Long−pec
Protruding−mouth
Day−4
Day−5
Transcript
ENSDART00000100744
ENSDART00000160393

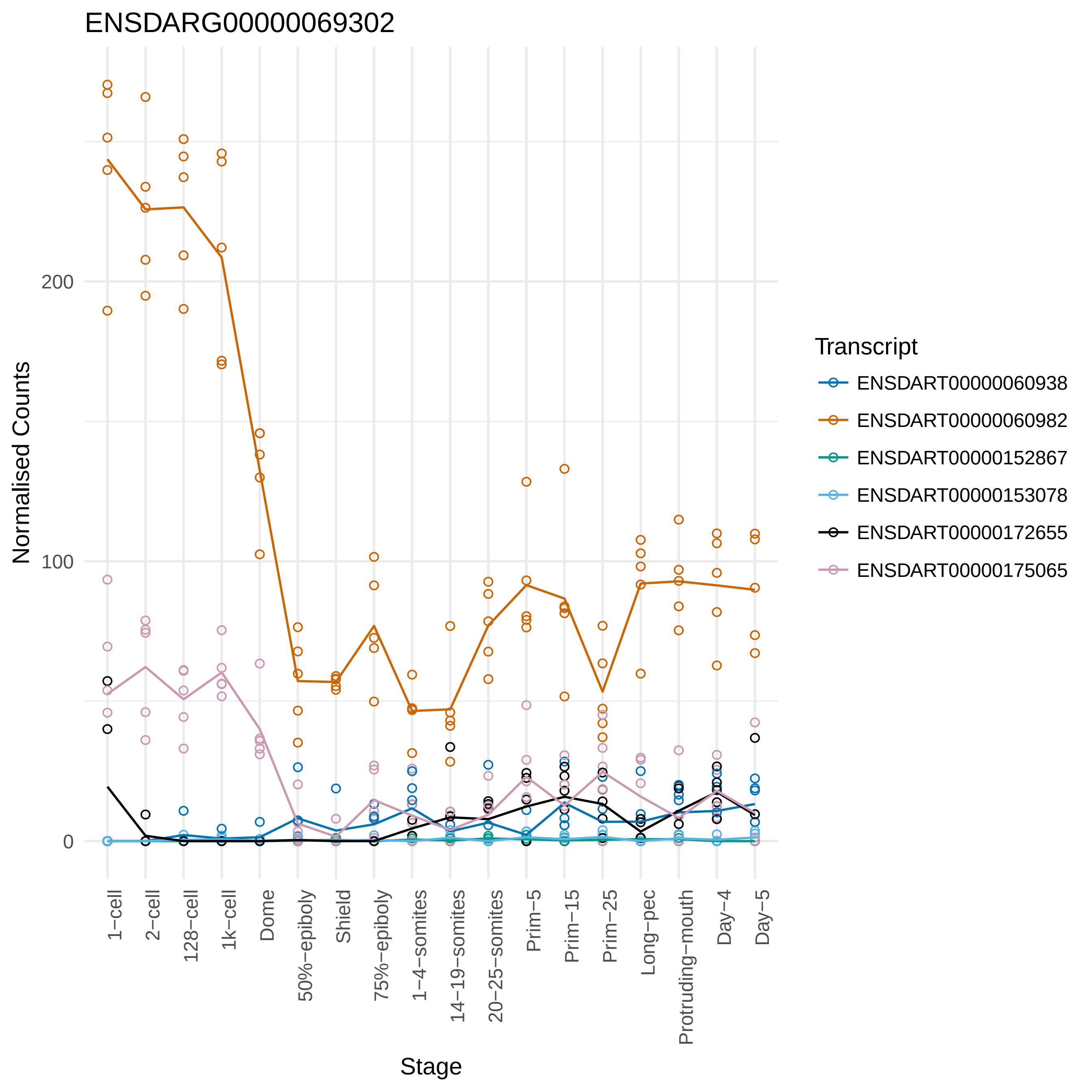

ENSDARG00000069302
Normalised Counts
200
100
0
Stage
1−cell
2−cell
128−cell
1k−cell
Dome
50%−epiboly
Shield
75%−epiboly
1−4−somites
14−19−somites
20−25−somites
Prim−5
Prim−15
Prim−25
Long−pec
Protruding−mouth
Day−4
Day−5
Transcript
ENSDART00000060938
ENSDART00000060982
ENSDART00000152867
ENSDART00000153078
ENSDART00000172655
ENSDART00000175065

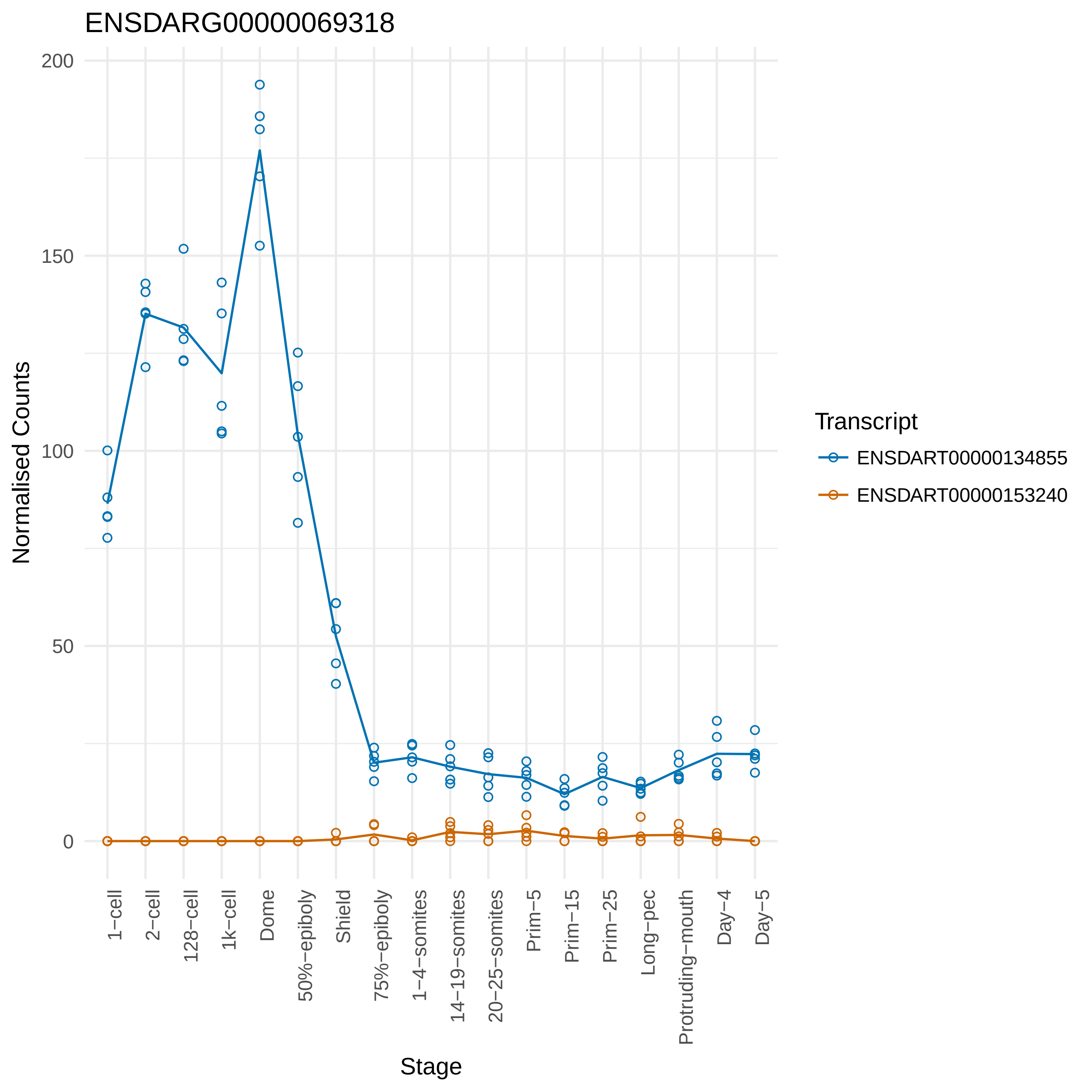

ENSDARG00000069318
Normalised Counts
200
150
100
50
0
1−cell
2−cell
128−cell
1k−cell
Dome
50%−epiboly
Shield
75%−epiboly
1−4−somites
14−19−somites
20−25−somites
Prim−5
Prim−15
Prim−25
Long−pec
Protruding−mouth
Day−4
Day−5
Stage
Transcript
ENSDART00000134855
ENSDART00000153240

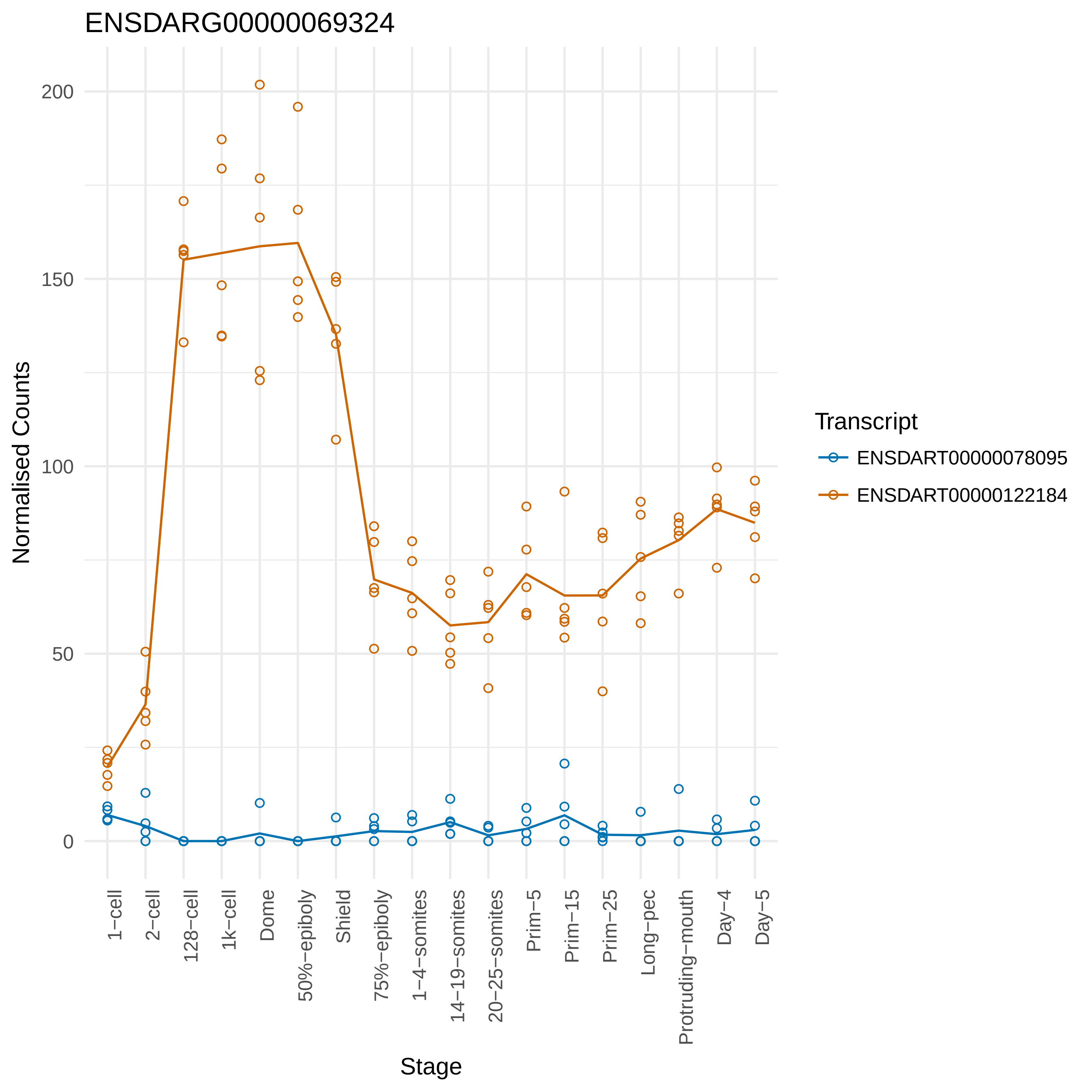
ENSDARG00000069324
Normalised Counts
200
150
100
50
0
1−cell
2−cell
128−cell
1k−cell
Dome
50%−epiboly
Shield
75%−epiboly
1−4−somites
14−19−somites
20−25−somites
Prim−5
Prim−15
Prim−25
Long−pec
Protruding−mouth
Day−4
Day−5
Stage
Transcript
ENSDART00000078095
ENSDART00000122184

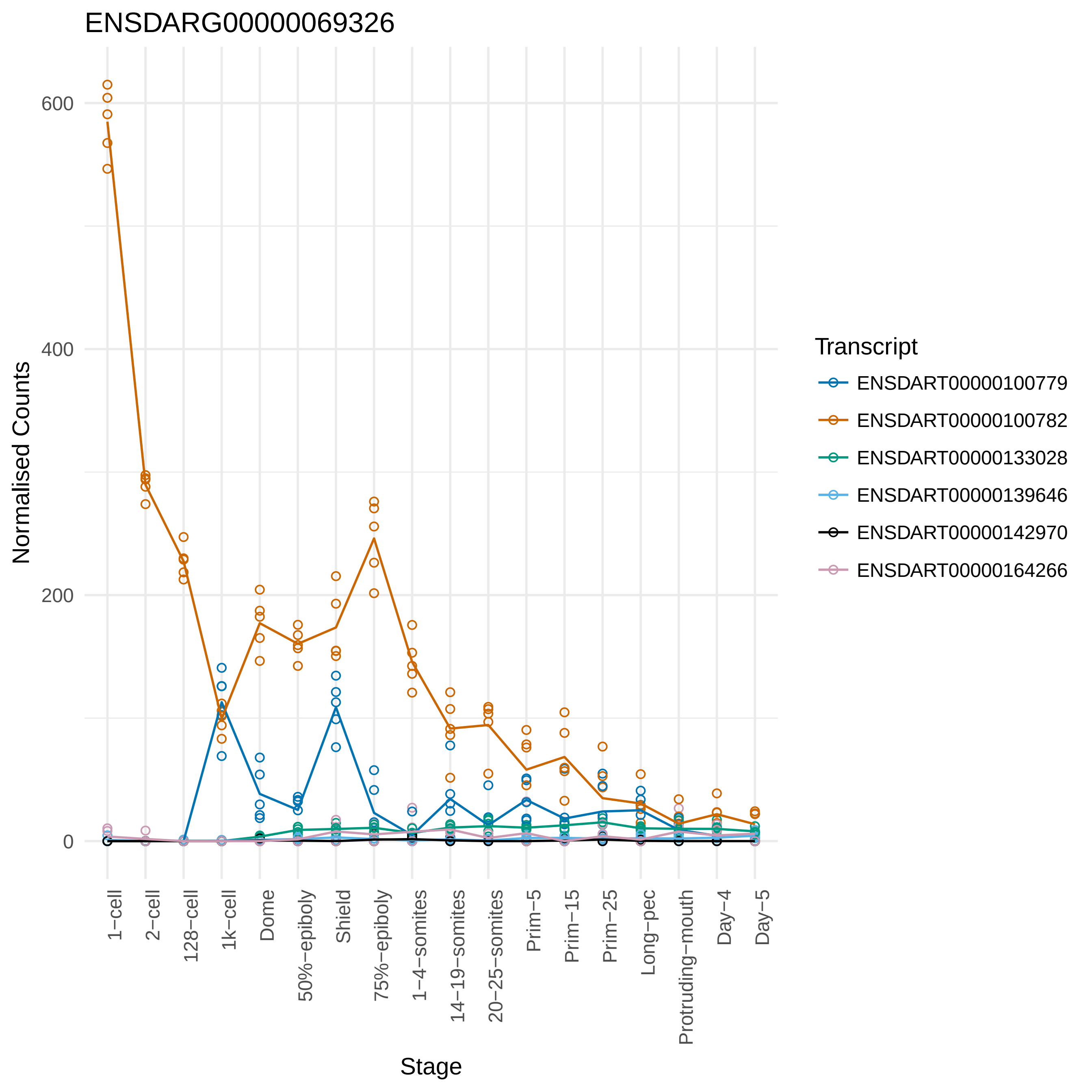
ENSDARG00000069326
Normalised Counts
600
400
200
0
Stage
1−cell
2−cell
128−cell
1k−cell
Dome
50%−epiboly
Shield
75%−epiboly
1−4−somites
14−19−somites
20−25−somites
Prim−5
Prim−15
Prim−25
Long−pec
Protruding−mouth
Day−4
Day−5
Transcript
ENSDART00000100779
ENSDART00000100782
ENSDART00000133028
ENSDART00000139646
ENSDART00000142970
ENSDART00000164266

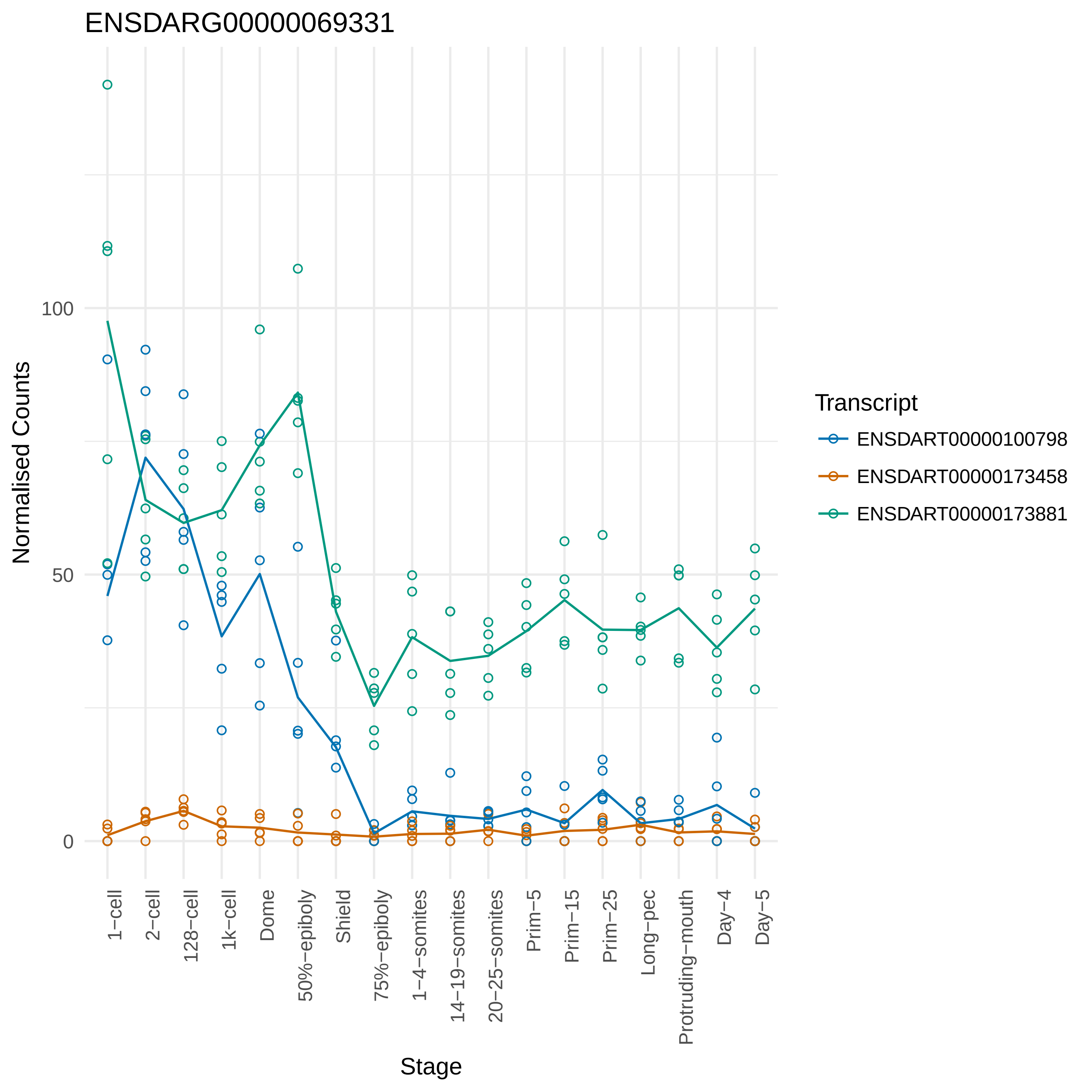

ENSDARG00000069331
Normalised Counts
100
50
0
1−cell
2−cell
128−cell
1k−cell
Dome
50%−epiboly
Shield
75%−epiboly
1−4−somites
14−19−somites
20−25−somites
Prim−5
Prim−15
Prim−25
Long−pec
Protruding−mouth
Day−4
Day−5
Stage
Transcript
ENSDART00000100798
ENSDART00000173458
ENSDART00000173881

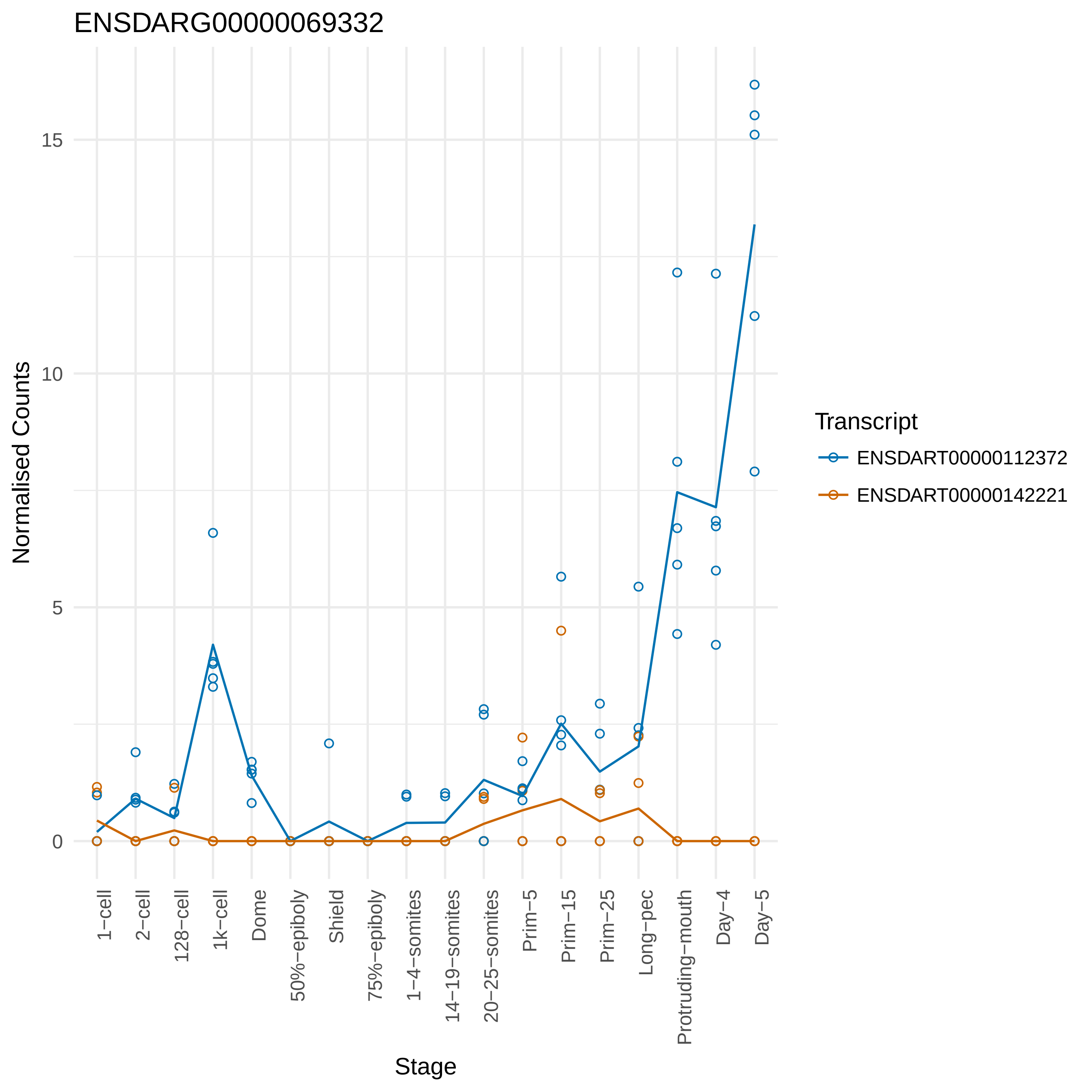
ENSDARG00000069332
Normalised Counts
0
5
10
15
Stage
1−cell
2−cell
128−cell
1k−cell
Dome
50%−epiboly
Shield
75%−epiboly
1−4−somites
14−19−somites
20−25−somites
Prim−5
Prim−15
Prim−25
Long−pec
Protruding−mouth
Day−4
Day−5
Transcript
ENSDART00000112372
ENSDART00000142221

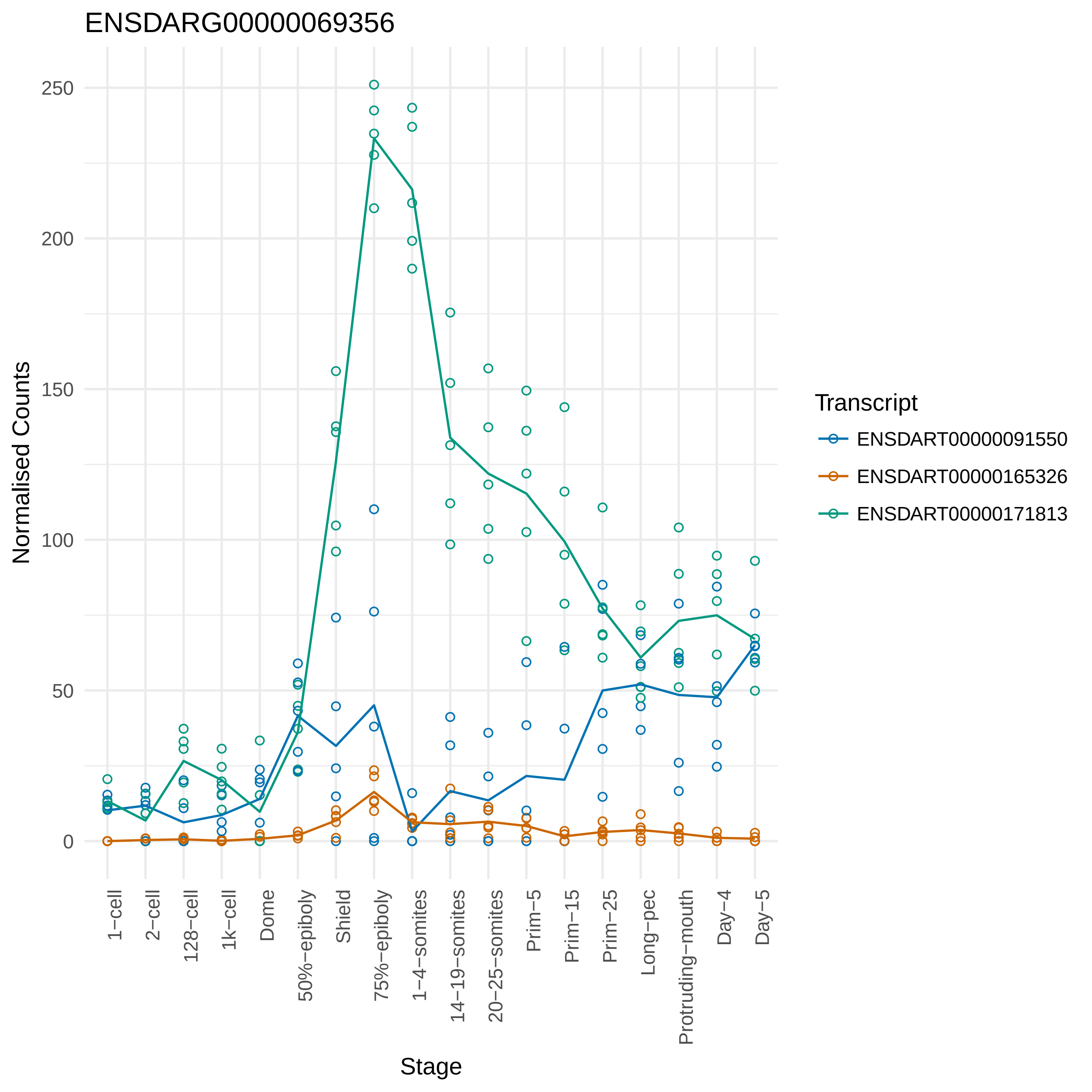

ENSDARG00000069356
Normalised Counts
250
200
150
100
50
0
1−cell
2−cell
128−cell
1k−cell
Dome
50%−epiboly
Shield
75%−epiboly
1−4−somites
14−19−somites
20−25−somites
Prim−5
Prim−15
Prim−25
Long−pec
Protruding−mouth
Day−4
Day−5
Stage
Transcript
ENSDART00000091550
ENSDART00000165326
ENSDART00000171813

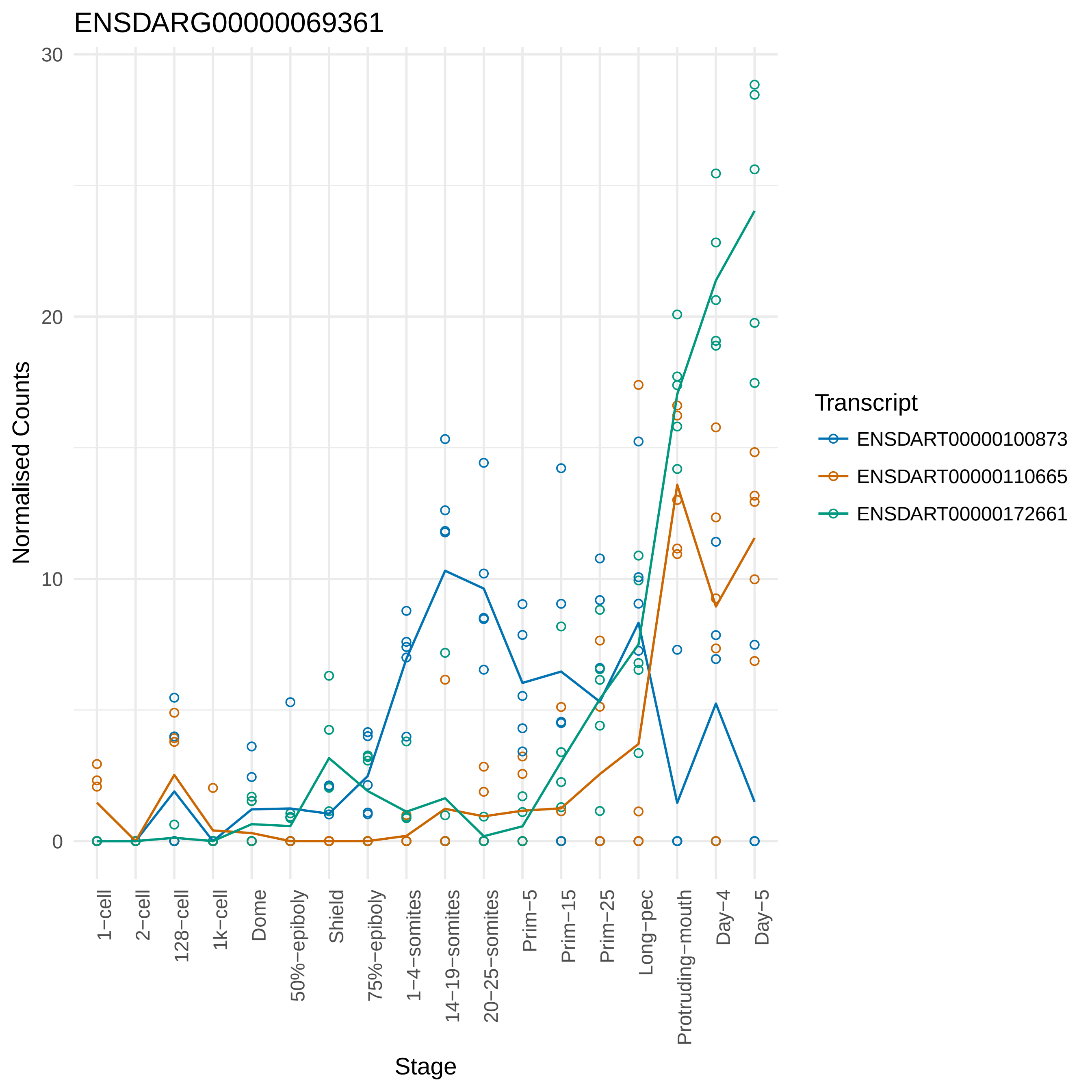

ENSDARG00000069361
Normalised Counts
Stage
30
20
10
0
1−cell
2−cell
128−cell
1k−cell
Dome
50%−epiboly
Shield
75%−epiboly
1−4−somites
14−19−somites
20−25−somites
Prim−5
Prim−15
Prim−25
Long−pec
Protruding−mouth
Day−4
Day−5
Transcript
ENSDART00000100873
ENSDART00000110665
ENSDART00000172661

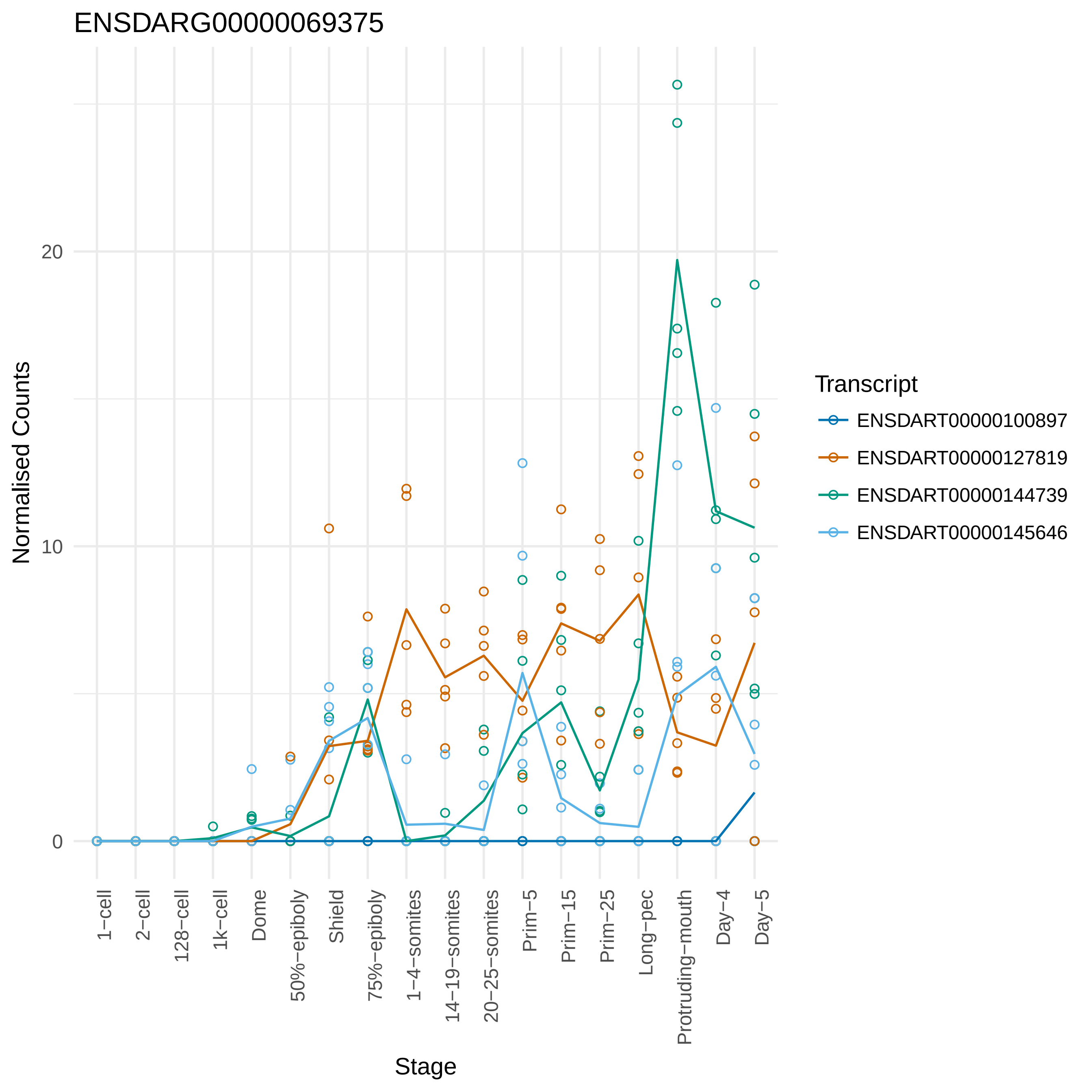
ENSDARG00000069375
Normalised Counts
20
10
0
Stage
1−cell
2−cell
128−cell
1k−cell
Dome
50%−epiboly
Shield
75%−epiboly
1−4−somites
14−19−somites
20−25−somites
Prim−5
Prim−15
Prim−25
Long−pec
Protruding−mouth
Day−4
Day−5
Transcript
ENSDART00000100897
ENSDART00000127819
ENSDART00000144739
ENSDART00000145646

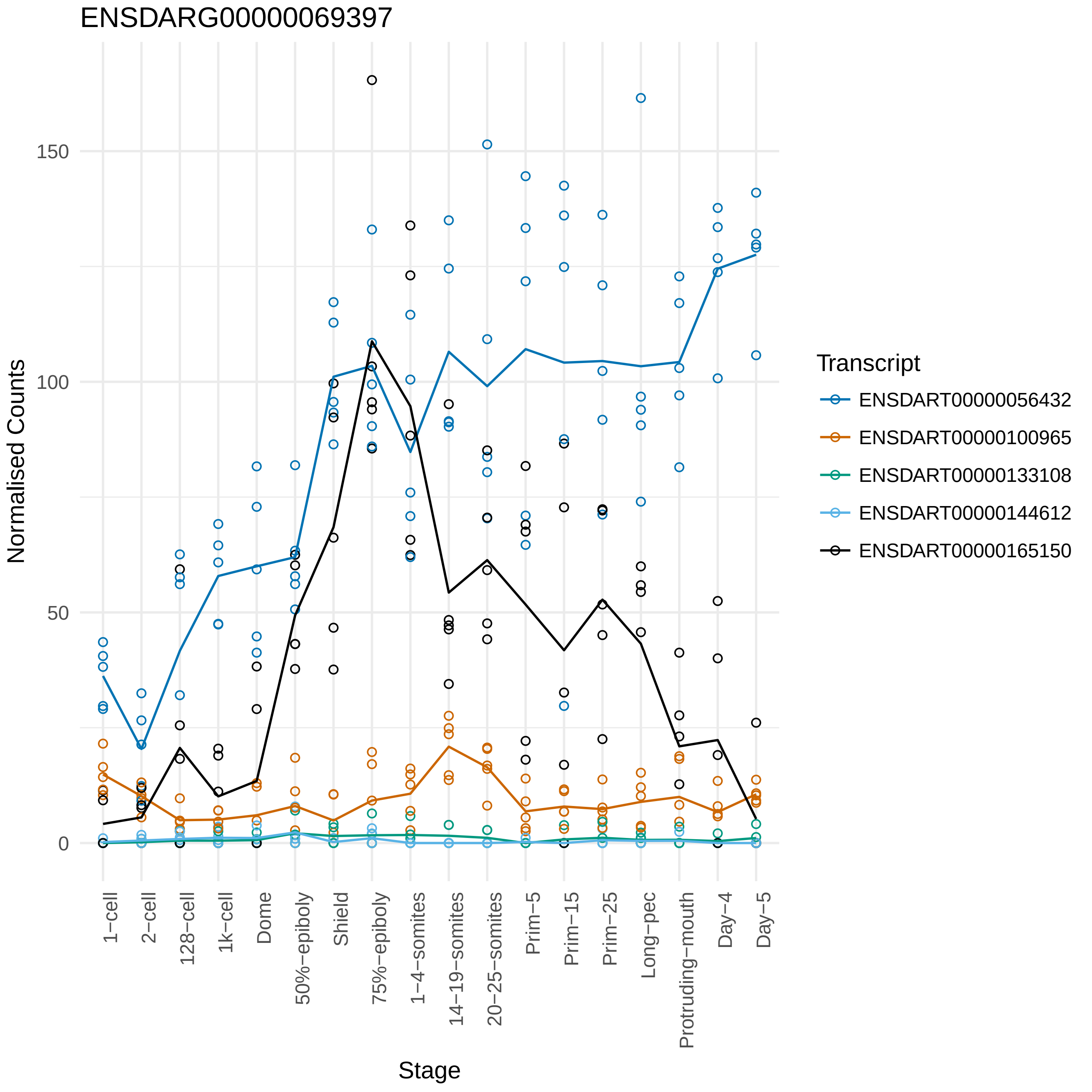
ENSDARG00000069397
Normalised Counts
150
100
50
0
1−cell
2−cell
128−cell
1k−cell
Dome
50%−epiboly
Shield
75%−epiboly
1−4−somites
14−19−somites
20−25−somites
Prim−5
Prim−15
Prim−25
Long−pec
Protruding−mouth
Day−4
Day−5
Stage
Transcript
ENSDART00000056432
ENSDART00000100965
ENSDART00000133108
ENSDART00000144612
ENSDART00000165150

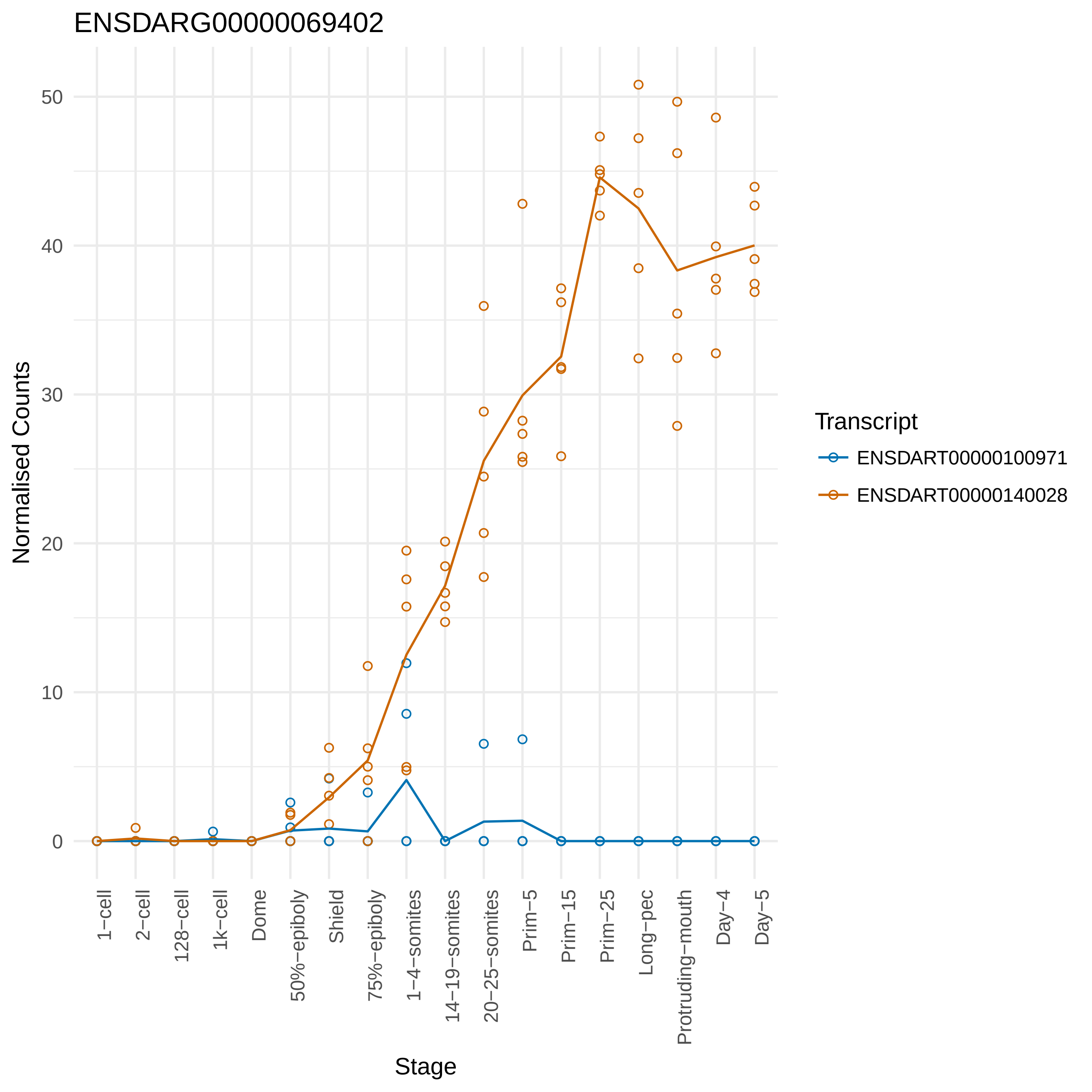

ENSDARG00000069402
Normalised Counts
0
10
20
30
40
50
1−cell
2−cell
128−cell
1k−cell
Dome
50%−epiboly
Shield
75%−epiboly
1−4−somites
14−19−somites
20−25−somites
Prim−5
Prim−15
Prim−25
Long−pec
Protruding−mouth
Day−4
Day−5
Stage
Transcript
ENSDART00000100971
ENSDART00000140028

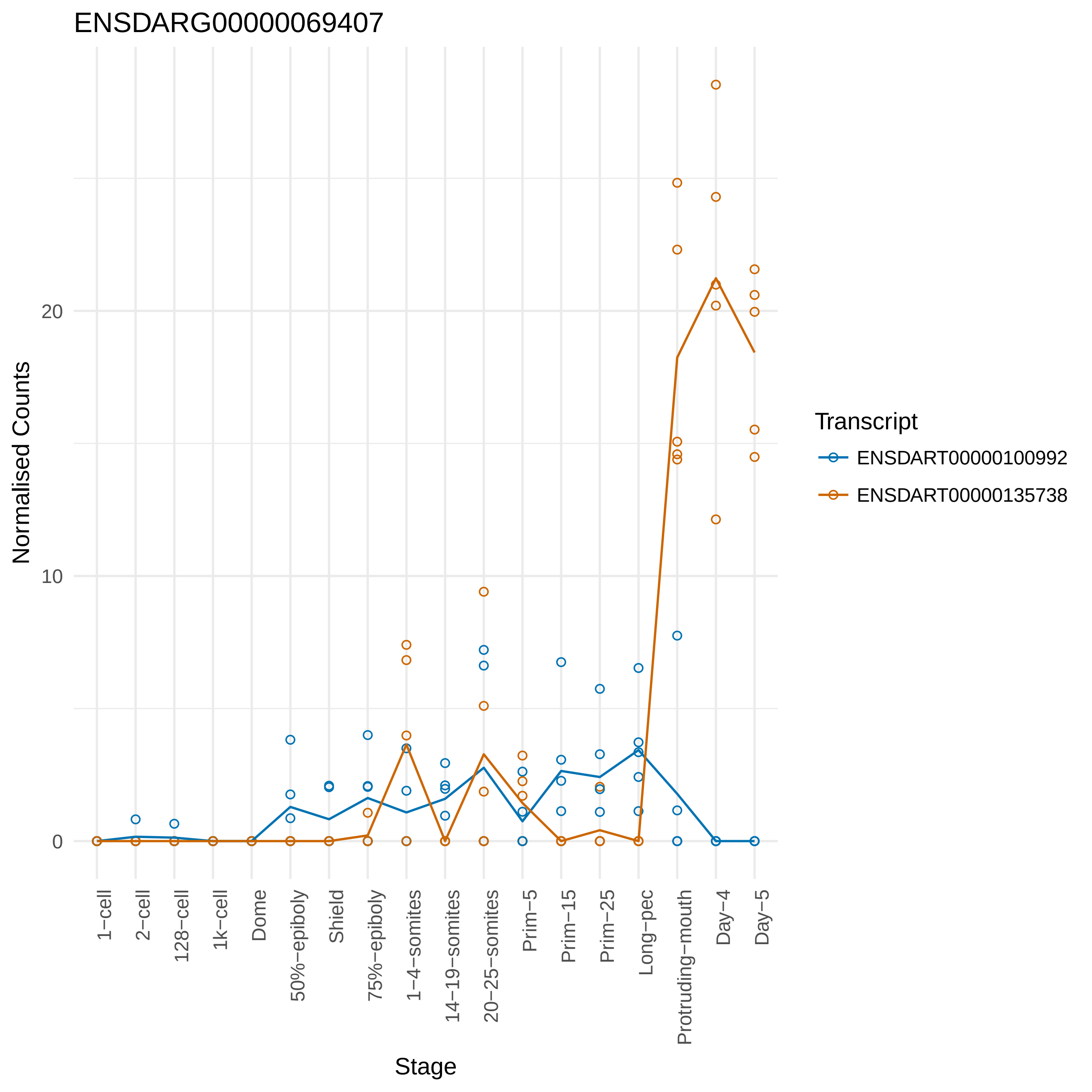

ENSDARG00000069407
Normalised Counts
0
10
20
1−cell
2−cell
128−cell
1k−cell
Dome
50%−epiboly
Shield
75%−epiboly
1−4−somites
14−19−somites
20−25−somites
Prim−5
Prim−15
Prim−25
Long−pec
Protruding−mouth
Day−4
Day−5
Stage
Transcript
ENSDART00000100992
ENSDART00000135738

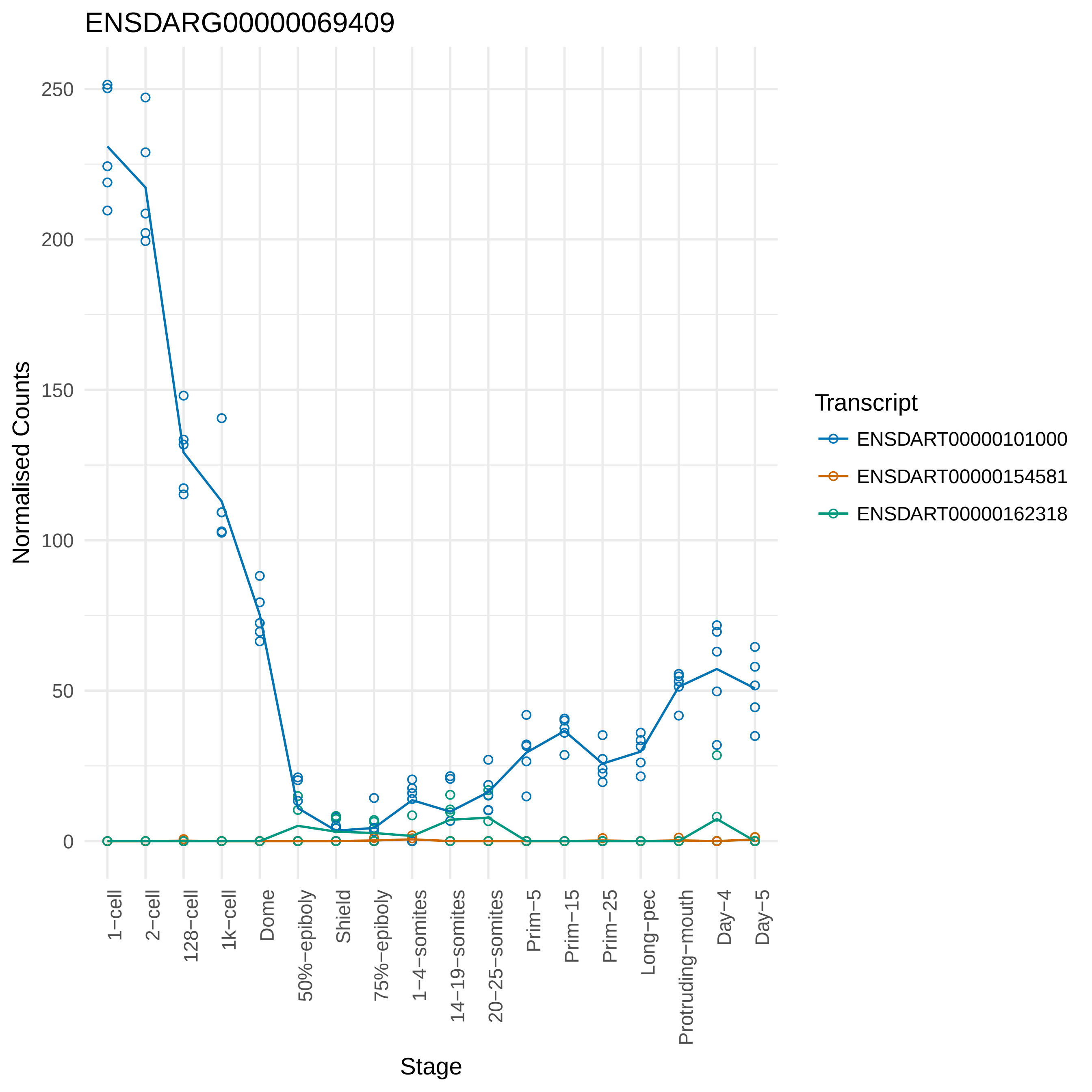
ENSDARG00000069409
Normalised Counts
250
200
150
100
50
0
1−cell
2−cell
128−cell
1k−cell
Dome
50%−epiboly
Shield
75%−epiboly
1−4−somites
14−19−somites
20−25−somites
Prim−5
Prim−15
Prim−25
Long−pec
Protruding−mouth
Day−4
Day−5
Stage
Transcript
ENSDART00000101000
ENSDART00000154581
ENSDART00000162318

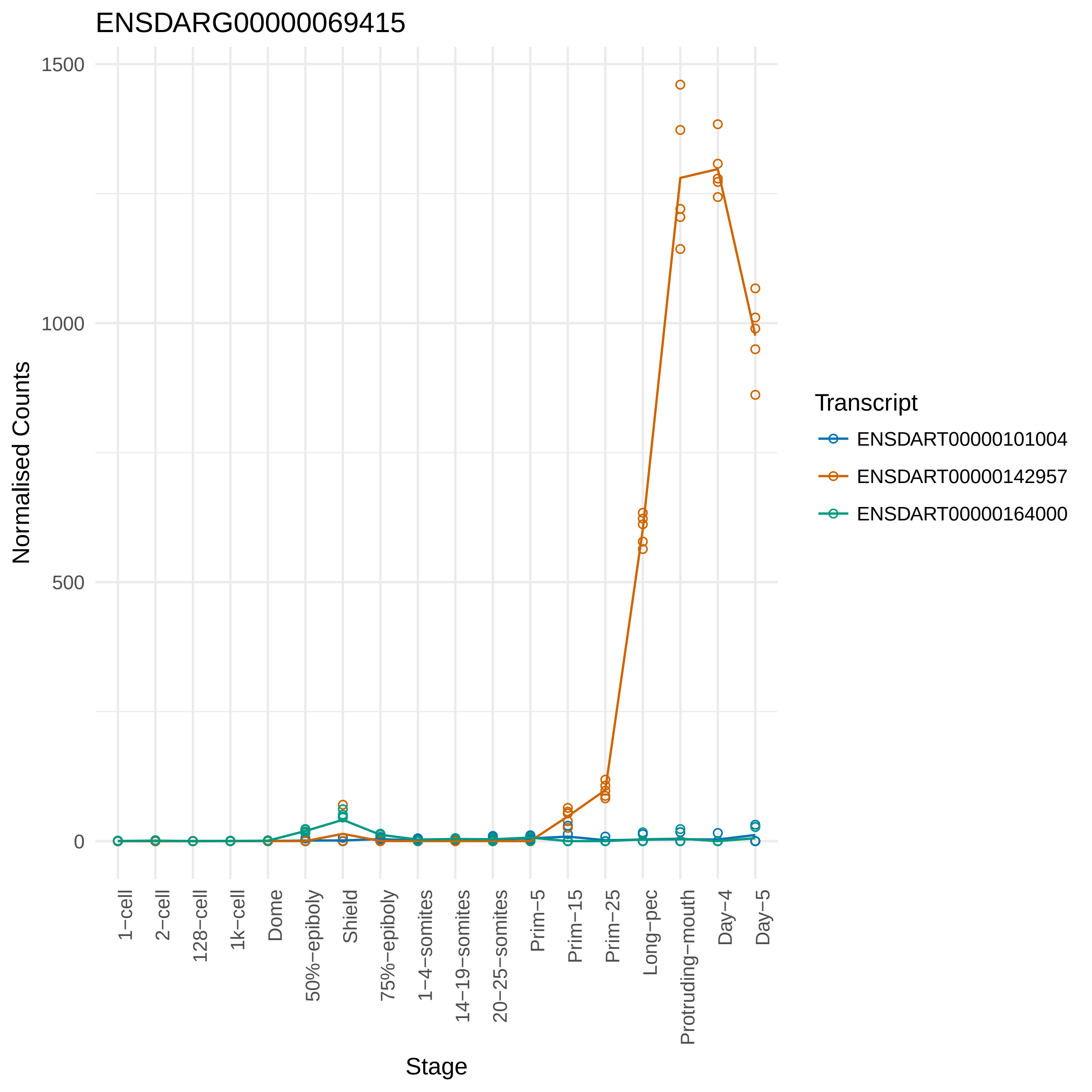

ENSDARG00000069415
Normalised Counts
1500
1000
500
0
1−cell
2−cell
128−cell
1k−cell
Dome
50%−epiboly
Shield
75%−epiboly
1−4−somites
14−19−somites
20−25−somites
Prim−5
Prim−15
Prim−25
Long−pec
Protruding−mouth
Day−4
Day−5
Stage
Transcript
ENSDART00000101004
ENSDART00000142957
ENSDART00000164000

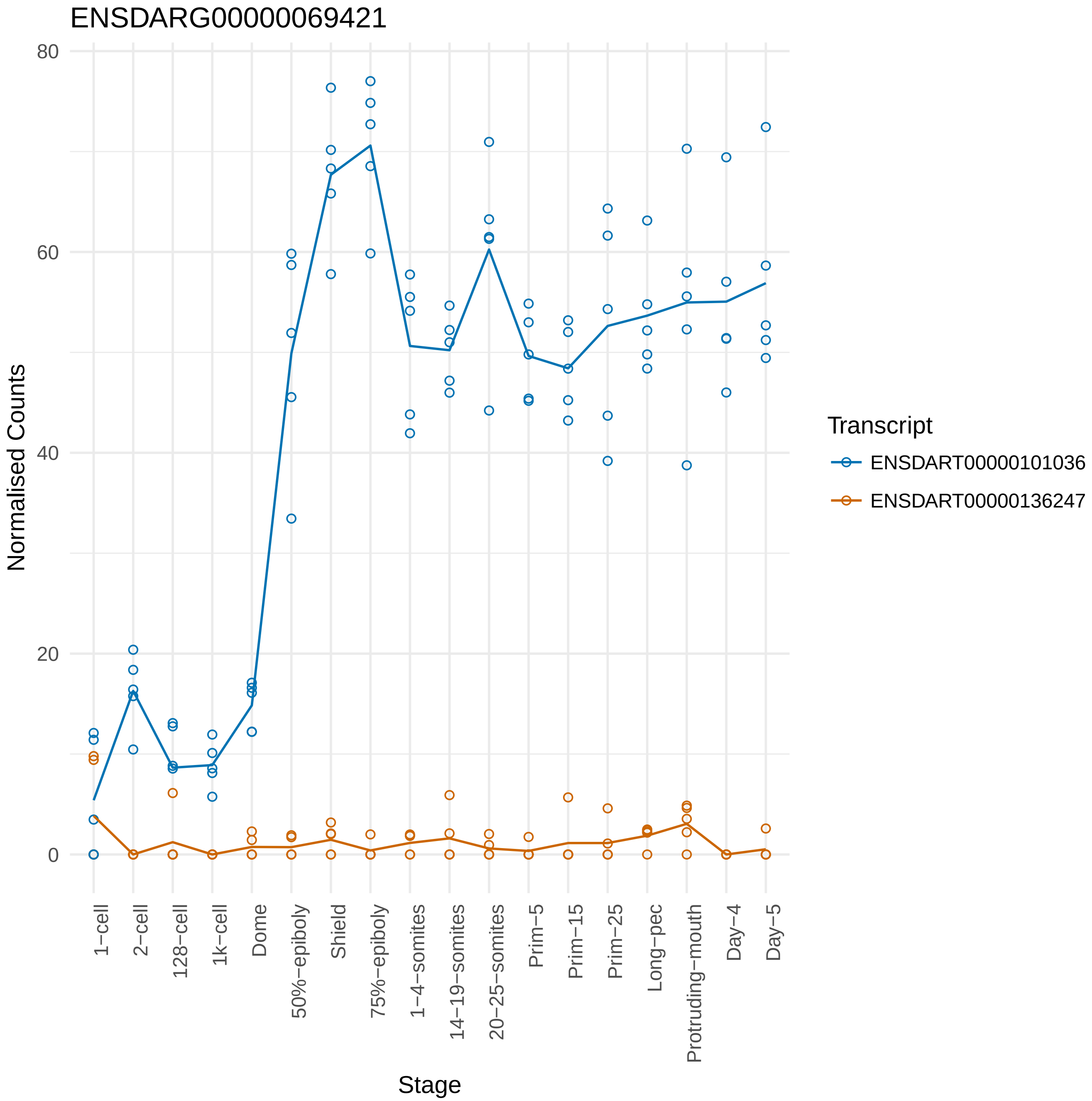
ENSDARG00000069421
Normalised Counts
80
60
40
20
0
1−cell
2−cell
128−cell
1k−cell
Dome
50%−epiboly
Shield
75%−epiboly
1−4−somites
14−19−somites
20−25−somites
Prim−5
Prim−15
Prim−25
Long−pec
Protruding−mouth
Day−4
Day−5
Stage
Transcript
ENSDART00000101036
ENSDART00000136247

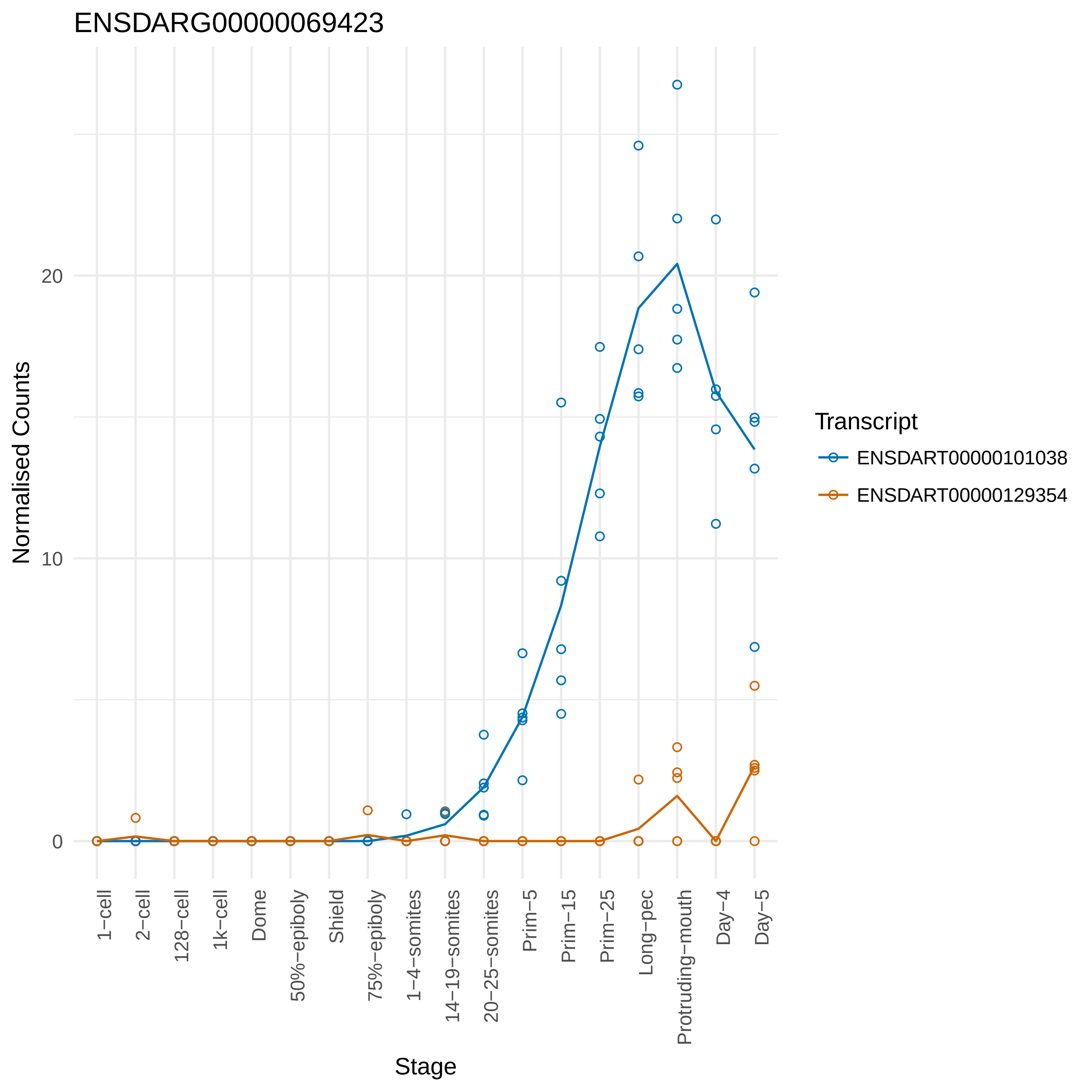
ENSDARG00000069423
Normalised Counts
20
10
0
1−cell
2−cell
128−cell
1k−cell
Dome
50%−epiboly
Shield
75%−epiboly
1−4−somites
14−19−somites
20−25−somites
Prim−5
Prim−15
Prim−25
Long−pec
Protruding−mouth
Day−4
Day−5
Stage
Transcript
ENSDART00000101038
ENSDART00000129354

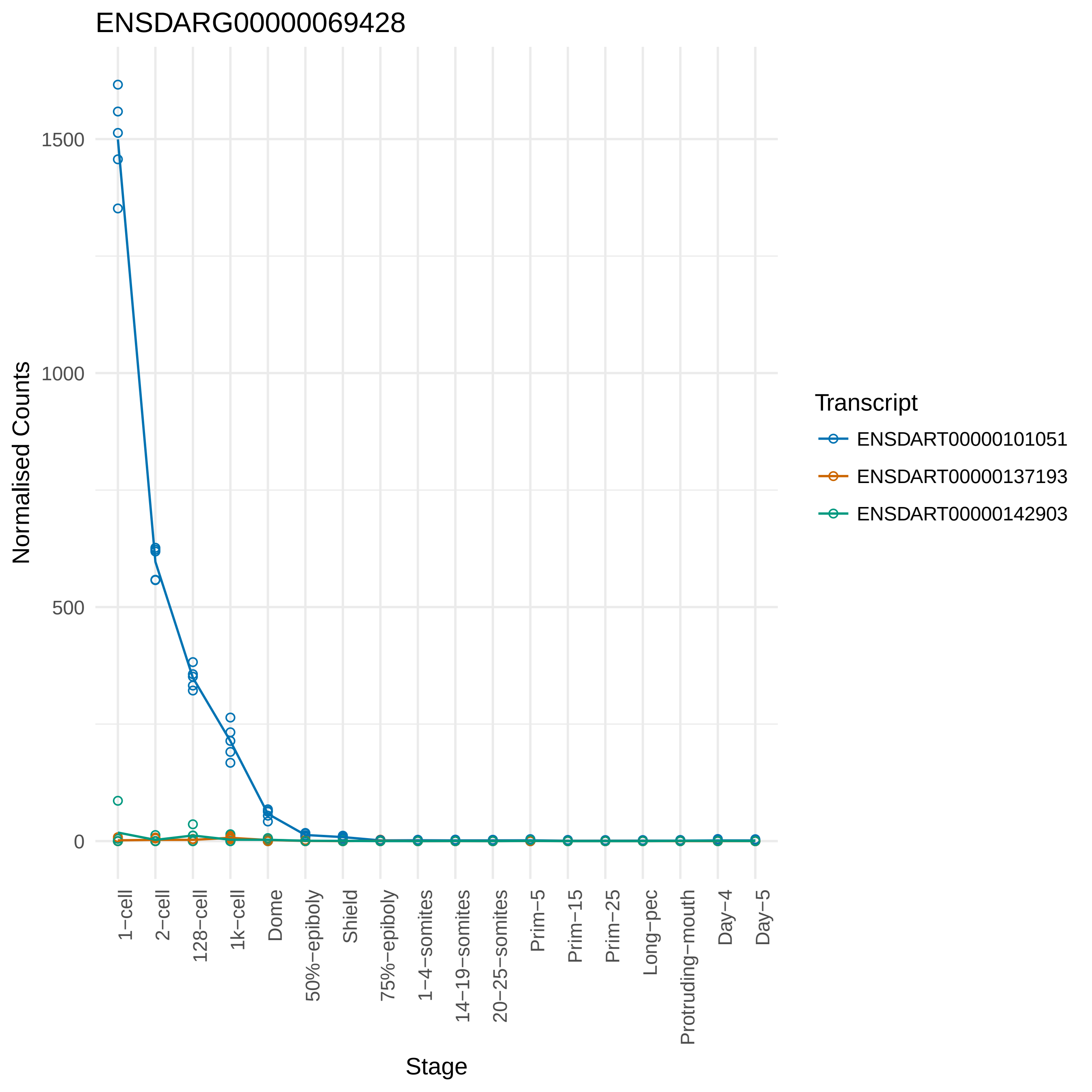
ENSDARG00000069428
Normalised Counts
1500
1000
500
0
Stage
1−cell
2−cell
128−cell
1k−cell
Dome
50%−epiboly
Shield
75%−epiboly
1−4−somites
14−19−somites
20−25−somites
Prim−5
Prim−15
Prim−25
Long−pec
Protruding−mouth
Day−4
Day−5
Transcript
ENSDART00000101051
ENSDART00000137193
ENSDART00000142903

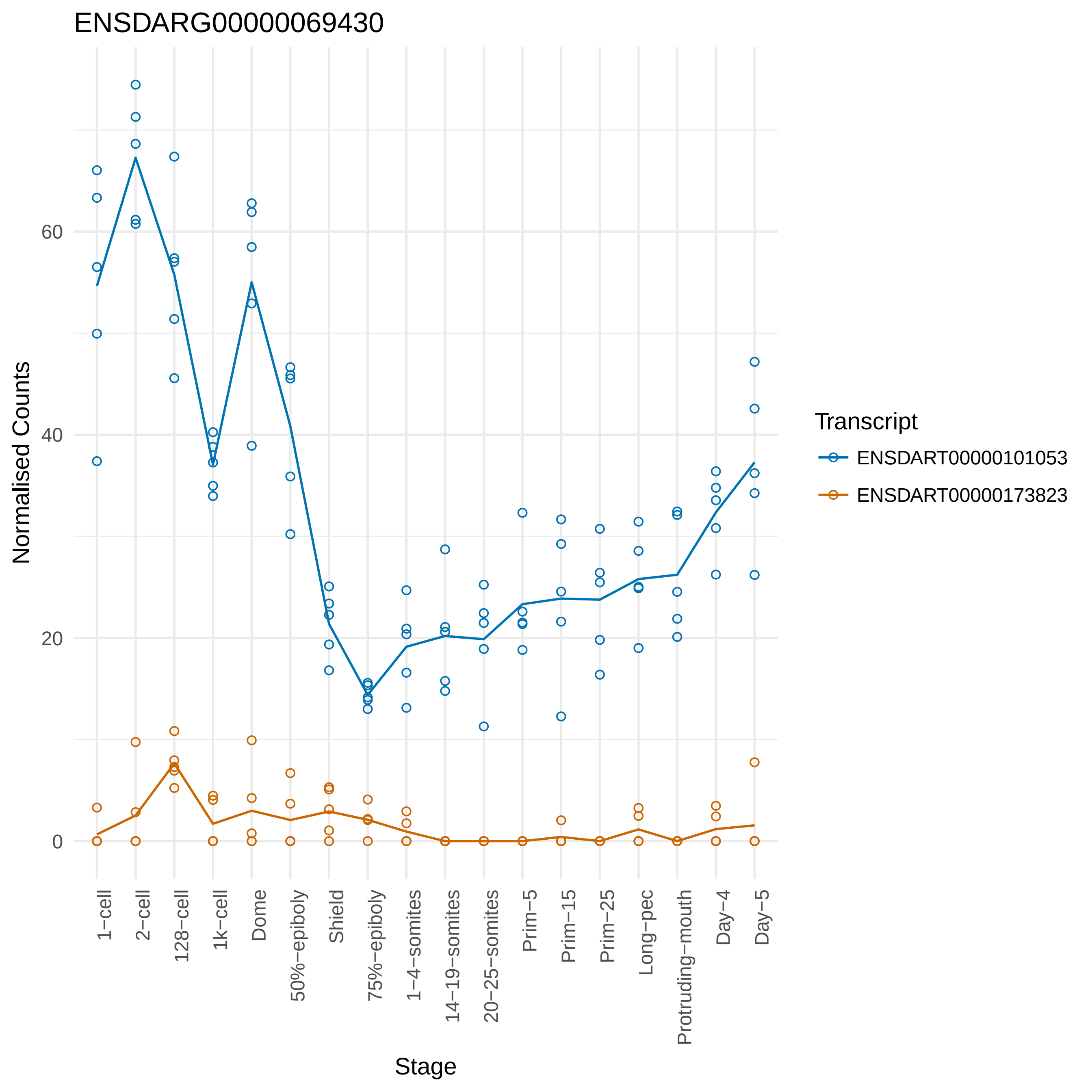
ENSDARG00000069430
Normalised Counts
0
20
40
60
Stage
1−cell
2−cell
128−cell
1k−cell
Dome
50%−epiboly
Shield
75%−epiboly
1−4−somites
14−19−somites
20−25−somites
Prim−5
Prim−15
Prim−25
Long−pec
Protruding−mouth
Day−4
Day−5
Transcript
ENSDART00000101053
ENSDART00000173823

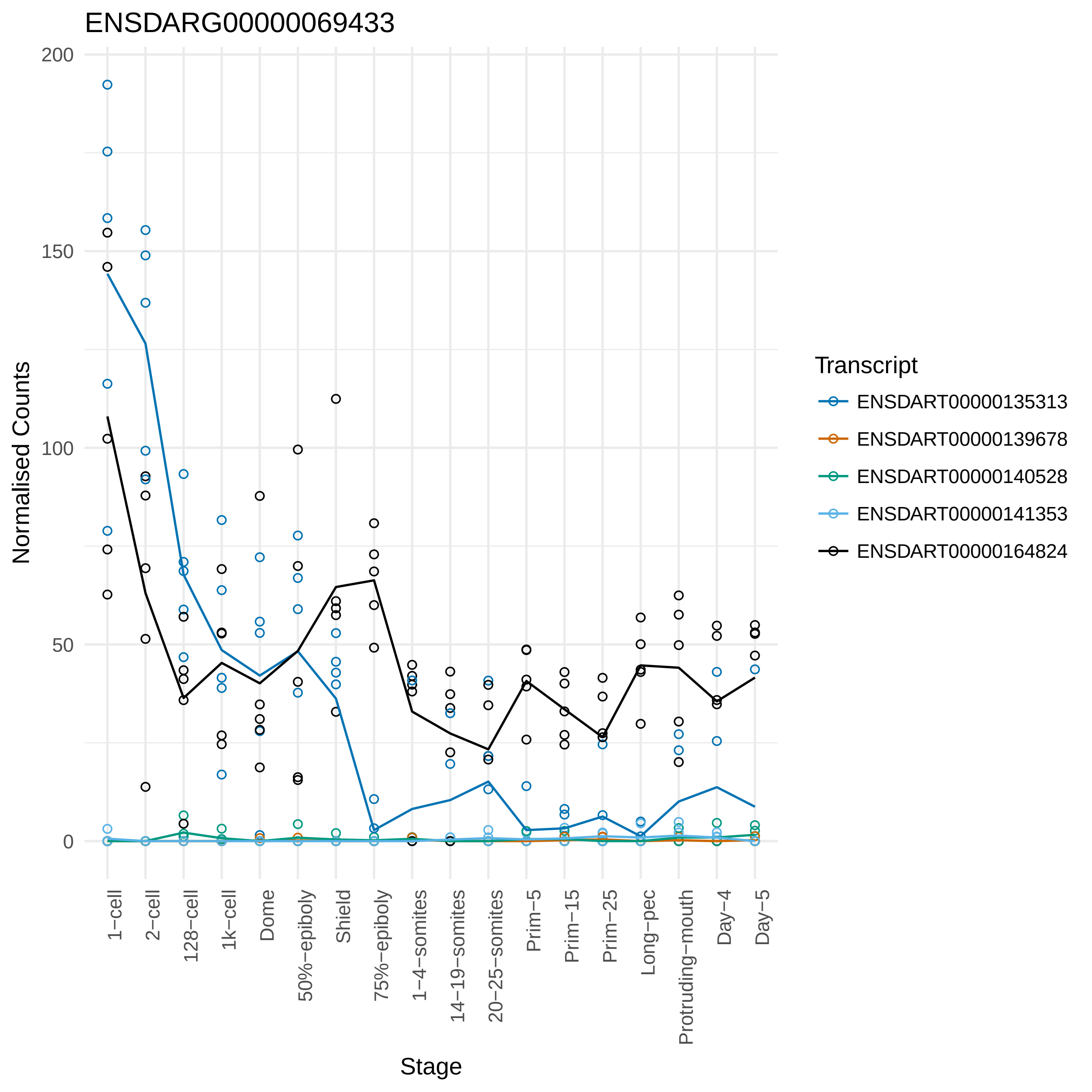

ENSDARG00000069433
Normalised Counts
200
150
100
50
0
1−cell
2−cell
128−cell
1k−cell
Dome
50%−epiboly
Shield
75%−epiboly
1−4−somites
14−19−somites
20−25−somites
Prim−5
Prim−15
Prim−25
Long−pec
Protruding−mouth
Day−4
Day−5
Stage
Transcript
ENSDART00000135313
ENSDART00000139678
ENSDART00000140528
ENSDART00000141353
ENSDART00000164824

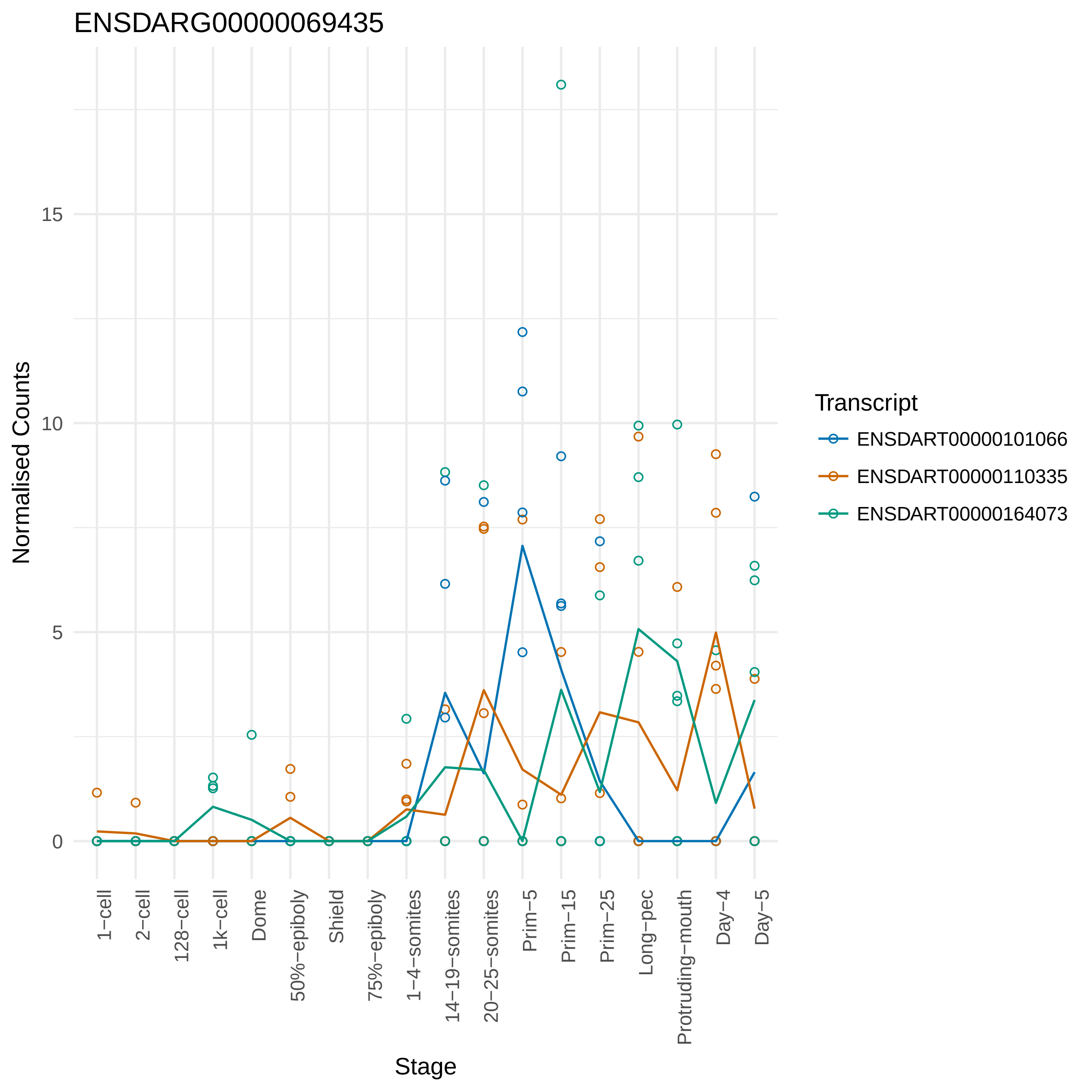
ENSDARG00000069435
Normalised Counts
15
10
5
0
Stage
1−cell
2−cell
128−cell
1k−cell
Dome
50%−epiboly
Shield
75%−epiboly
1−4−somites
14−19−somites
20−25−somites
Prim−5
Prim−15
Prim−25
Long−pec
Protruding−mouth
Day−4
Day−5
Transcript
ENSDART00000101066
ENSDART00000110335
ENSDART00000164073

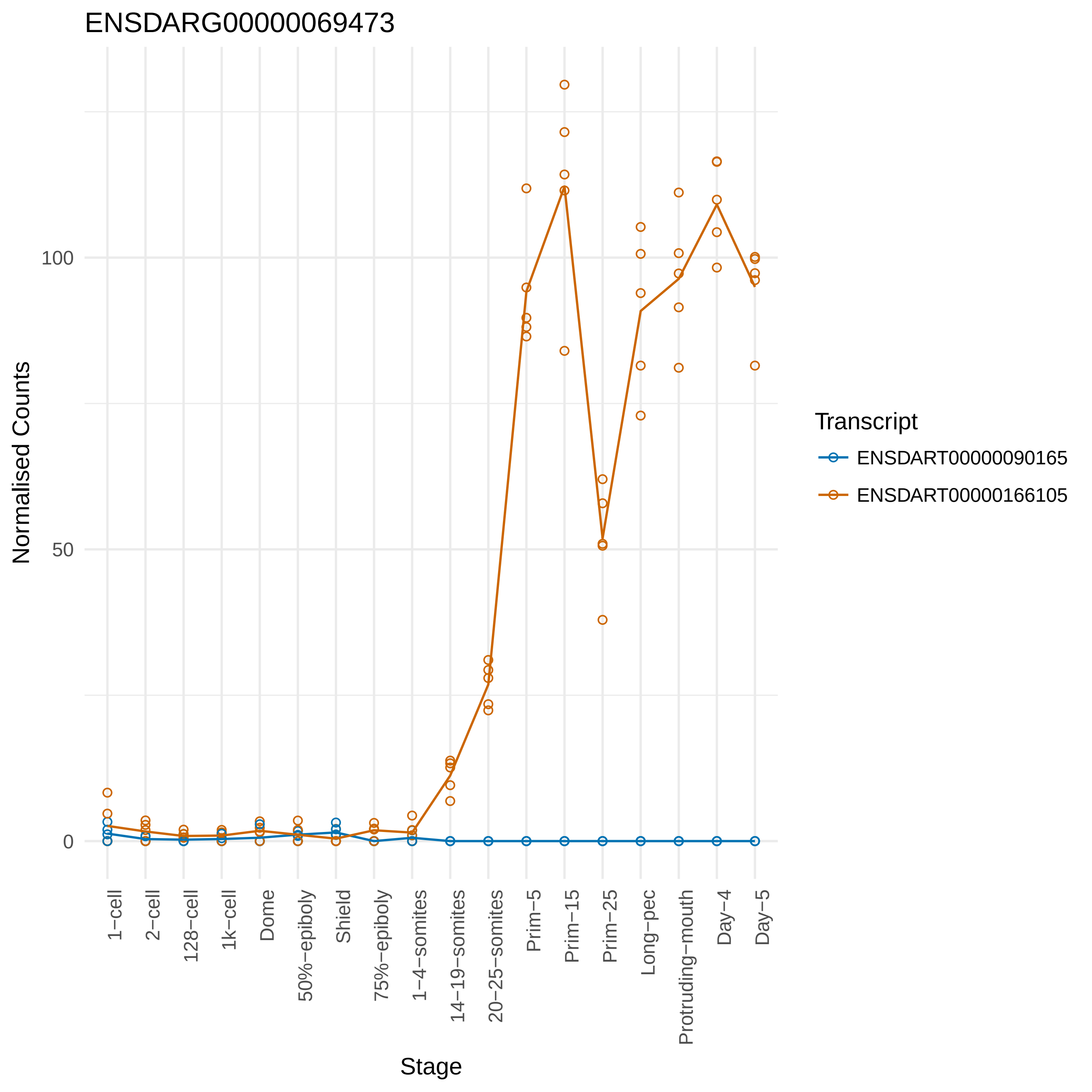

ENSDARG00000069473
Normalised Counts
100
50
0
1−cell
2−cell
128−cell
1k−cell
Dome
50%−epiboly
Shield
75%−epiboly
1−4−somites
14−19−somites
20−25−somites
Prim−5
Prim−15
Prim−25
Long−pec
Protruding−mouth
Day−4
Day−5
Stage
Transcript
ENSDART00000090165
ENSDART00000166105

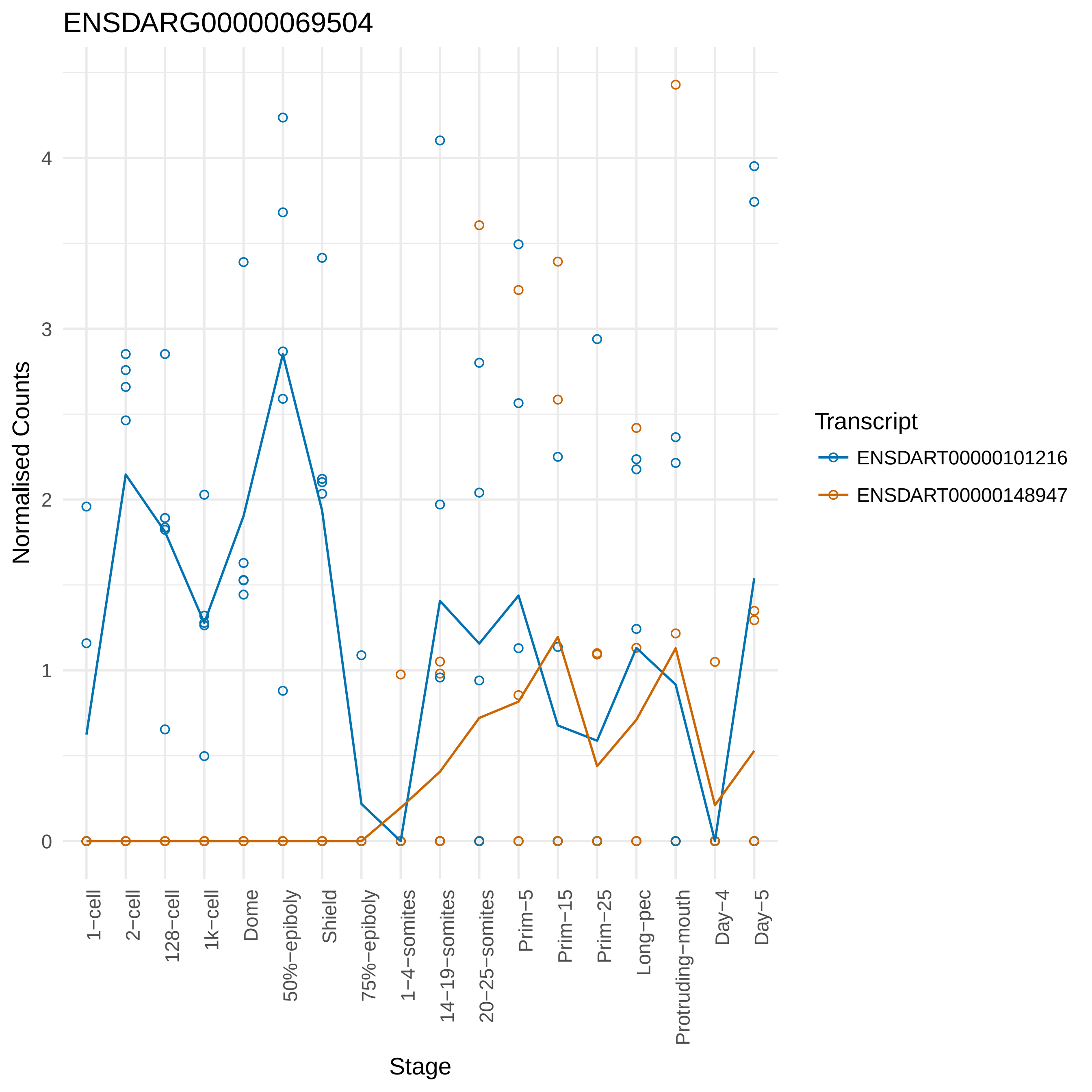
ENSDARG00000069504
Normalised Counts
0
1
2
3
4
Stage
1−cell
2−cell
128−cell
1k−cell
Dome
50%−epiboly
Shield
75%−epiboly
1−4−somites
14−19−somites
20−25−somites
Prim−5
Prim−15
Prim−25
Long−pec
Protruding−mouth
Day−4
Day−5
Transcript
ENSDART00000101216
ENSDART00000148947

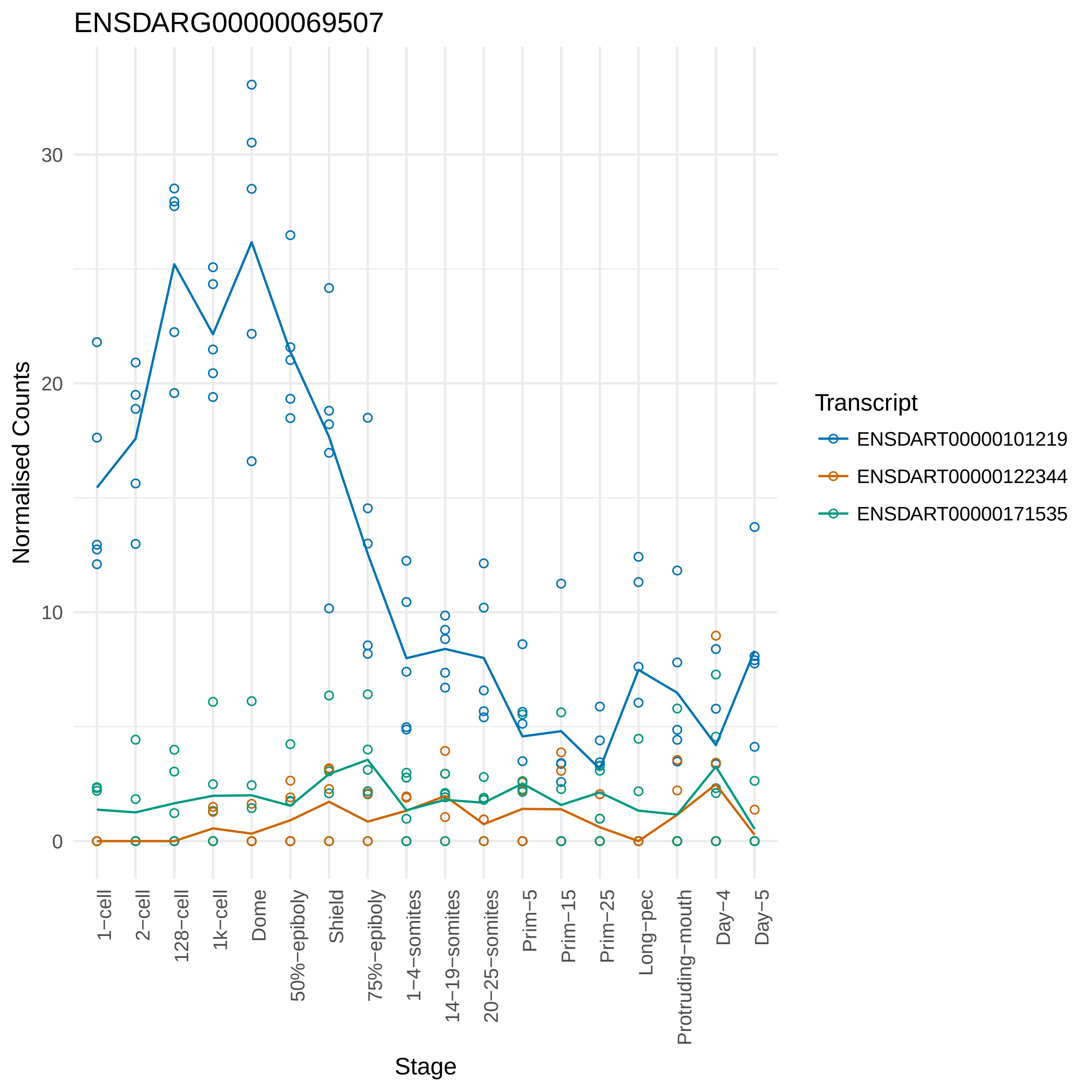

ENSDARG00000069507
Normalised Counts
30
20
10
0
1−cell
2−cell
128−cell
1k−cell
Dome
50%−epiboly
Shield
75%−epiboly
1−4−somites
14−19−somites
20−25−somites
Prim−5
Prim−15
Prim−25
Long−pec
Protruding−mouth
Day−4
Day−5
Stage
Transcript
ENSDART00000101219
ENSDART00000122344
ENSDART00000171535

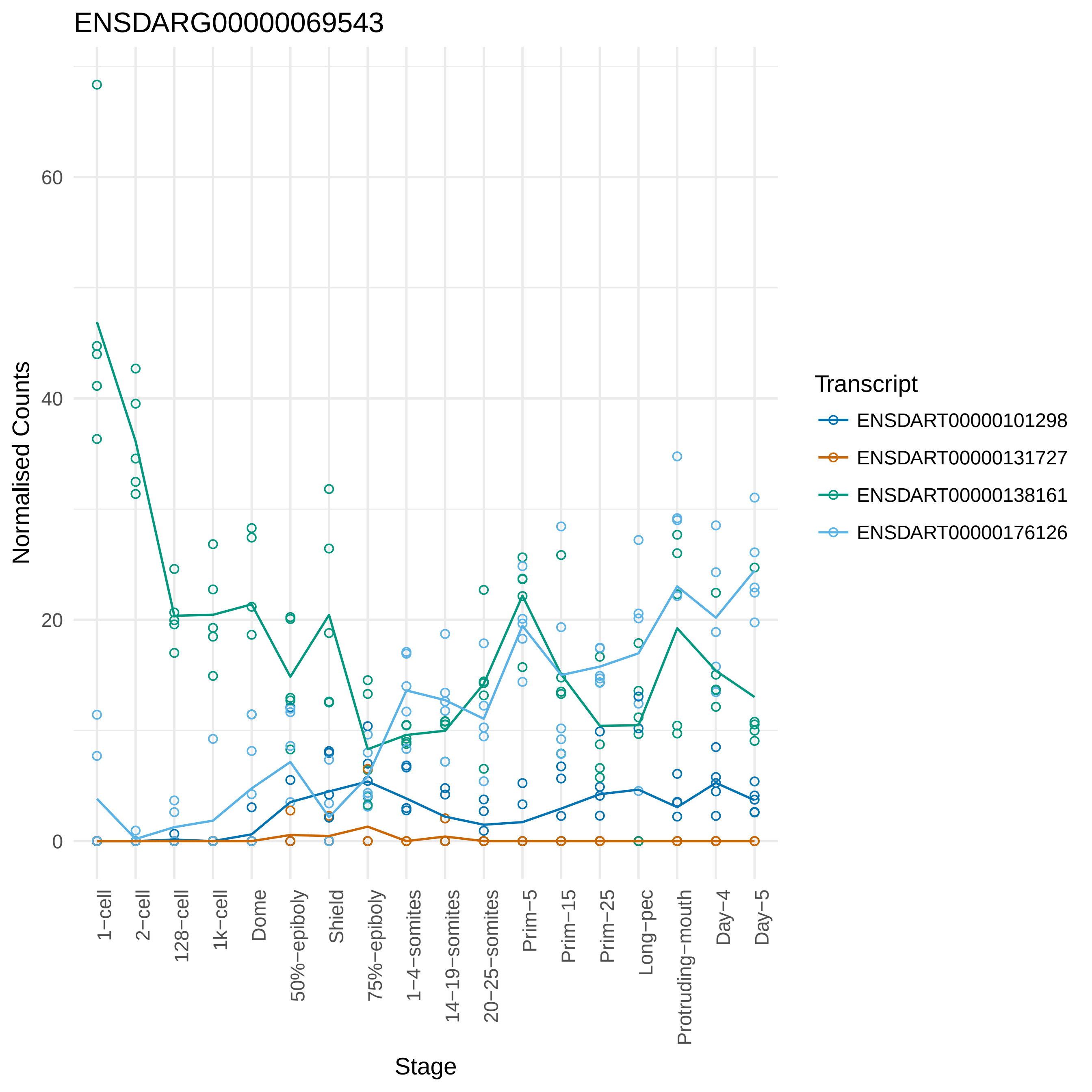

ENSDARG00000069543
Normalised Counts
0
20
40
60
Stage
1−cell
2−cell
128−cell
1k−cell
Dome
50%−epiboly
Shield
75%−epiboly
1−4−somites
14−19−somites
20−25−somites
Prim−5
Prim−15
Prim−25
Long−pec
Protruding−mouth
Day−4
Day−5
Transcript
ENSDART00000101298
ENSDART00000131727
ENSDART00000138161
ENSDART00000176126

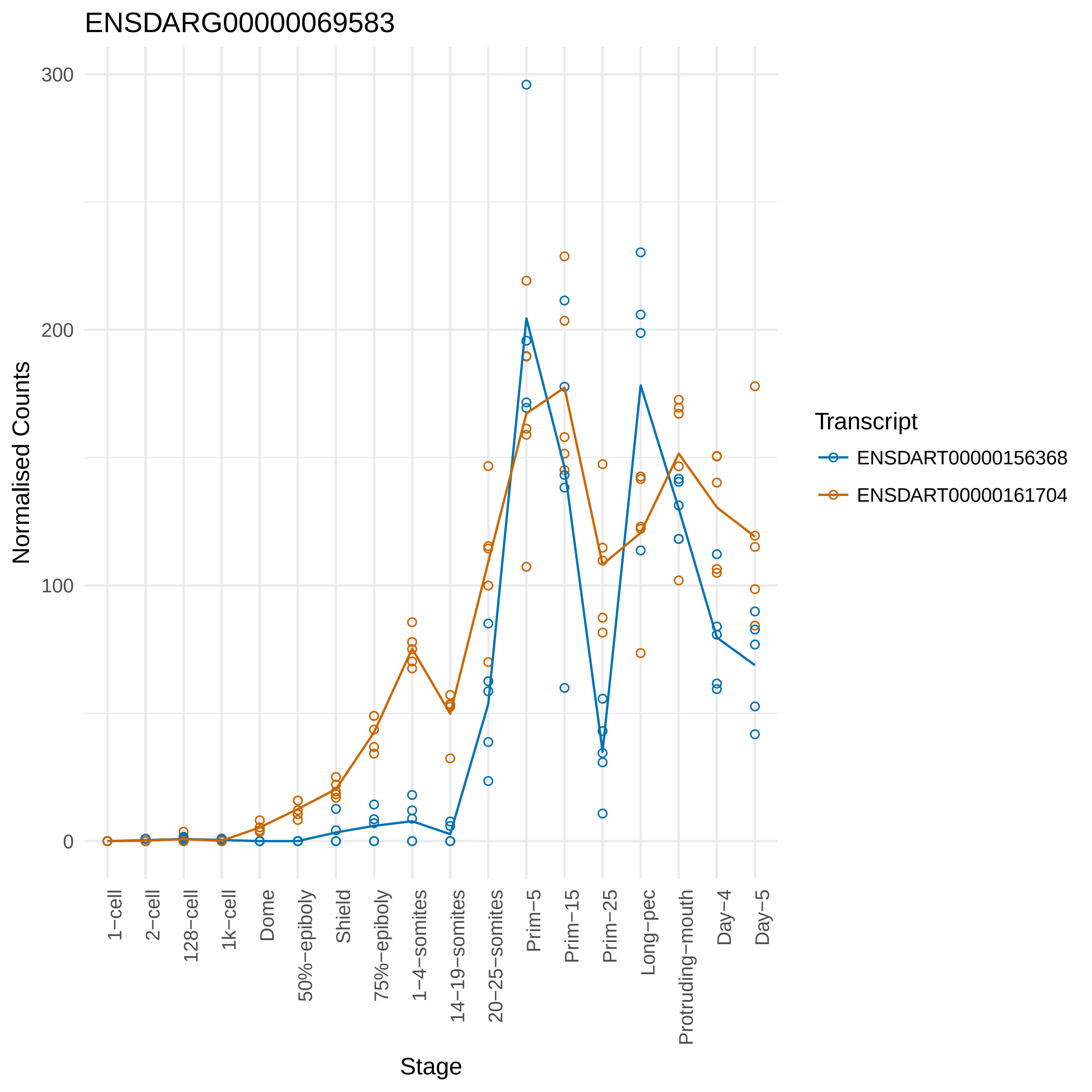

ENSDARG00000069583
Normalised Counts
300
200
100
0
1−cell
2−cell
128−cell
1k−cell
Dome
50%−epiboly
Shield
75%−epiboly
1−4−somites
14−19−somites
20−25−somites
Prim−5
Prim−15
Prim−25
Long−pec
Protruding−mouth
Day−4
Day−5
Stage
Transcript
ENSDART00000156368
ENSDART00000161704

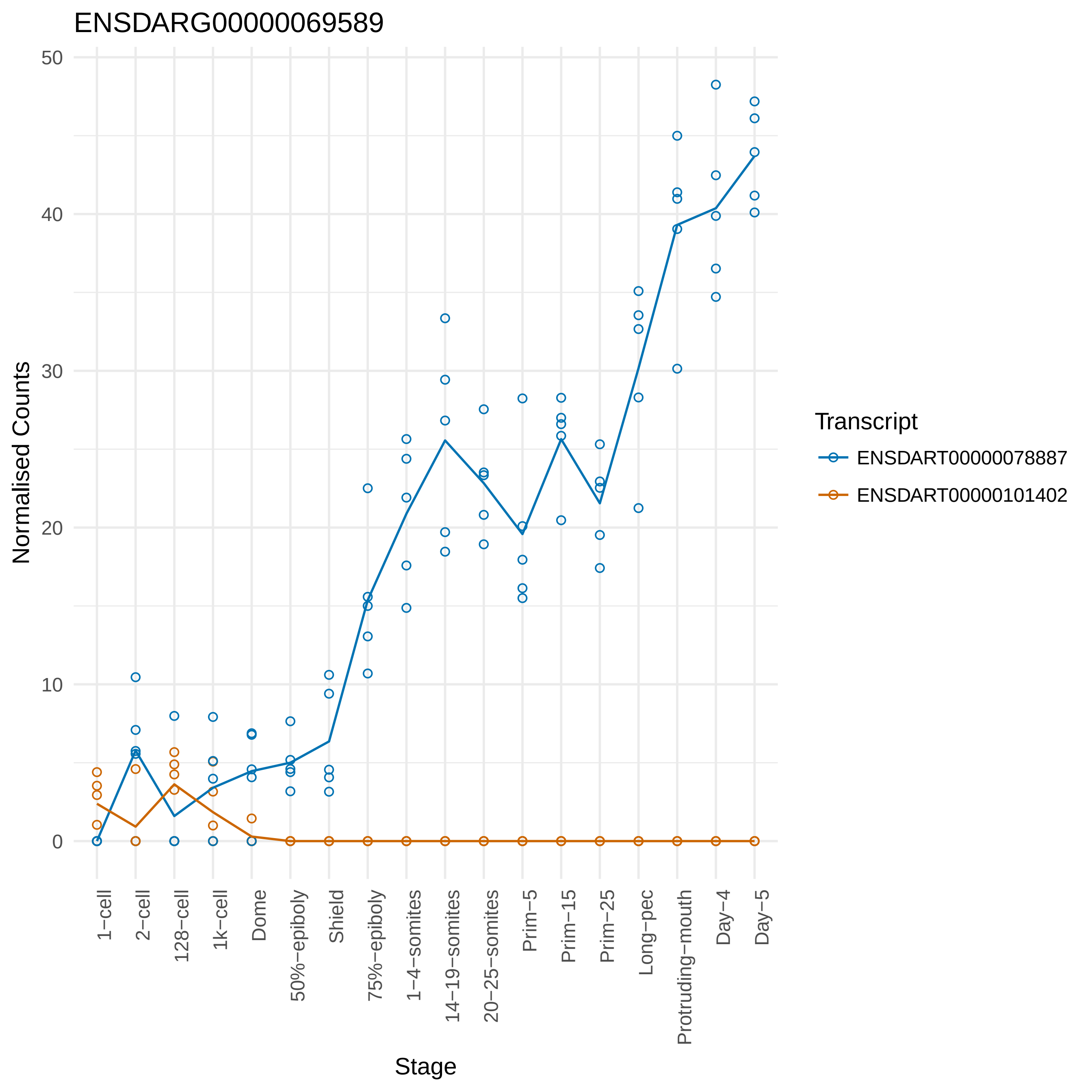
ENSDARG00000069589
Normalised Counts
50
40
30
20
10
0
1−cell
2−cell
128−cell
1k−cell
Dome
50%−epiboly
Shield
75%−epiboly
1−4−somites
14−19−somites
20−25−somites
Prim−5
Prim−15
Prim−25
Long−pec
Protruding−mouth
Day−4
Day−5
Stage
Transcript
ENSDART00000078887
ENSDART00000101402

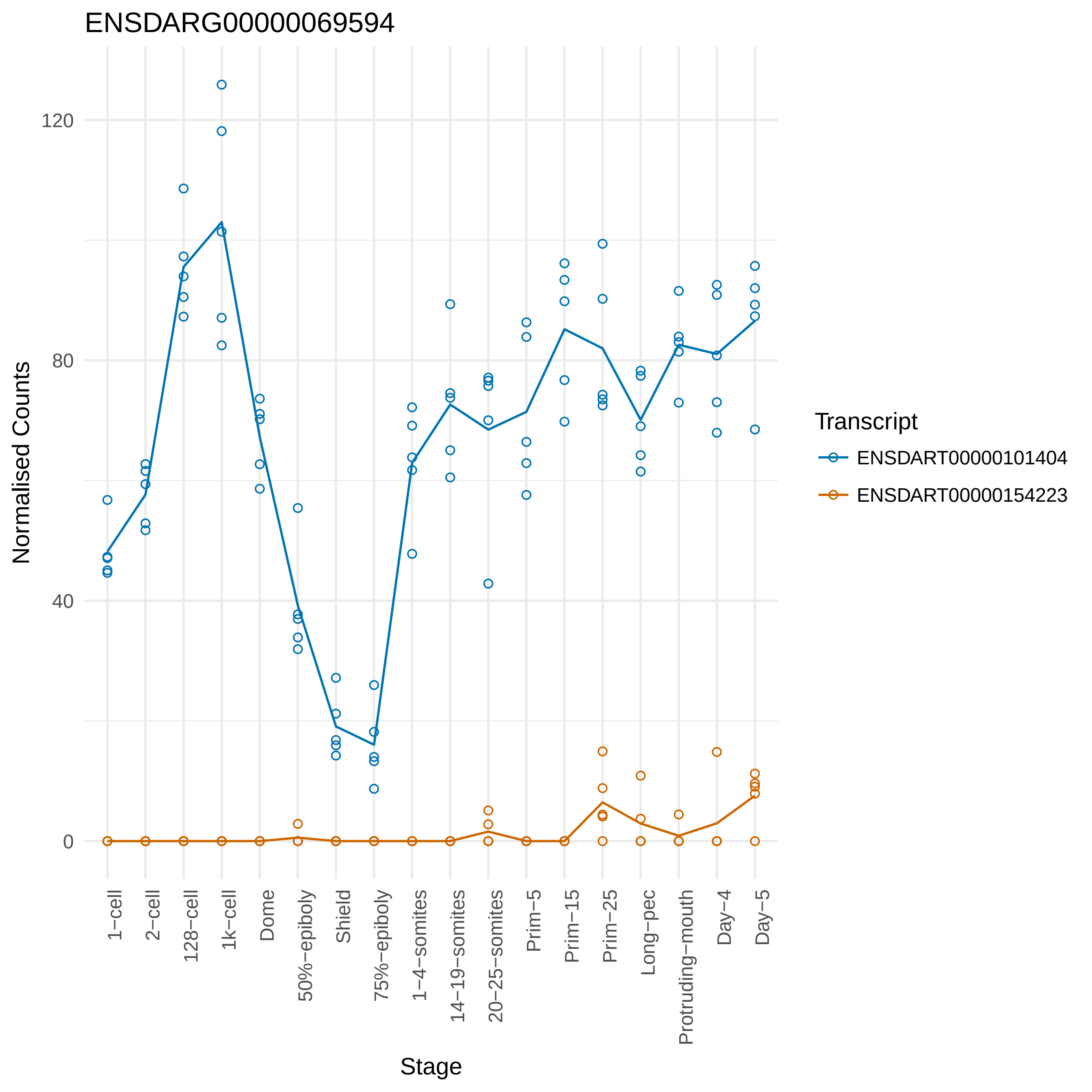

ENSDARG00000069594
Normalised Counts
0
40
80
120
1−cell
2−cell
128−cell
1k−cell
Dome
50%−epiboly
Shield
75%−epiboly
1−4−somites
14−19−somites
20−25−somites
Prim−5
Prim−15
Prim−25
Long−pec
Protruding−mouth
Day−4
Day−5
Stage
Transcript
ENSDART00000101404
ENSDART00000154223

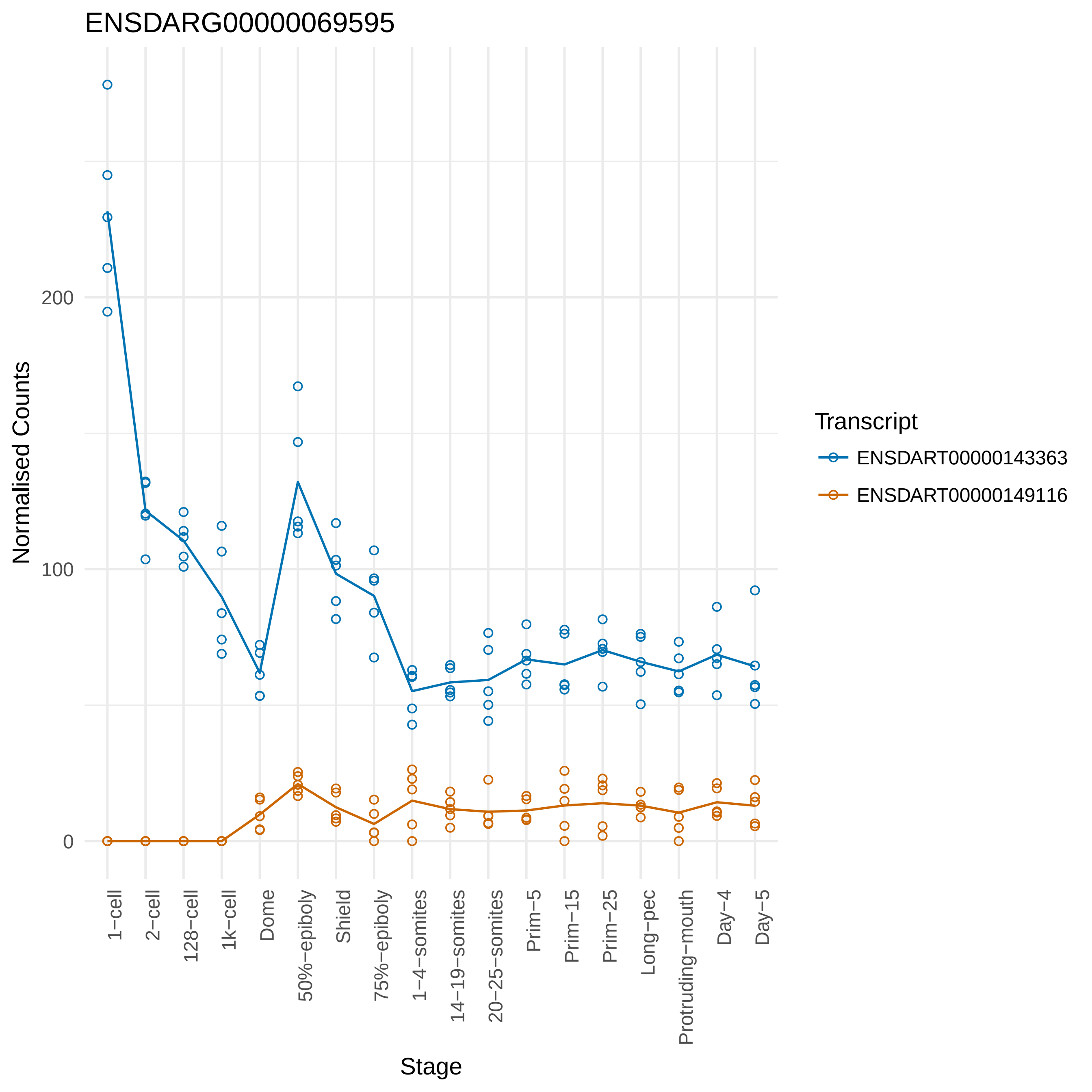

ENSDARG00000069595
Normalised Counts
200
100
0
1−cell
2−cell
128−cell
1k−cell
Dome
50%−epiboly
Shield
75%−epiboly
1−4−somites
14−19−somites
20−25−somites
Prim−5
Prim−15
Prim−25
Long−pec
Protruding−mouth
Day−4
Day−5
Stage
Transcript
ENSDART00000143363
ENSDART00000149116

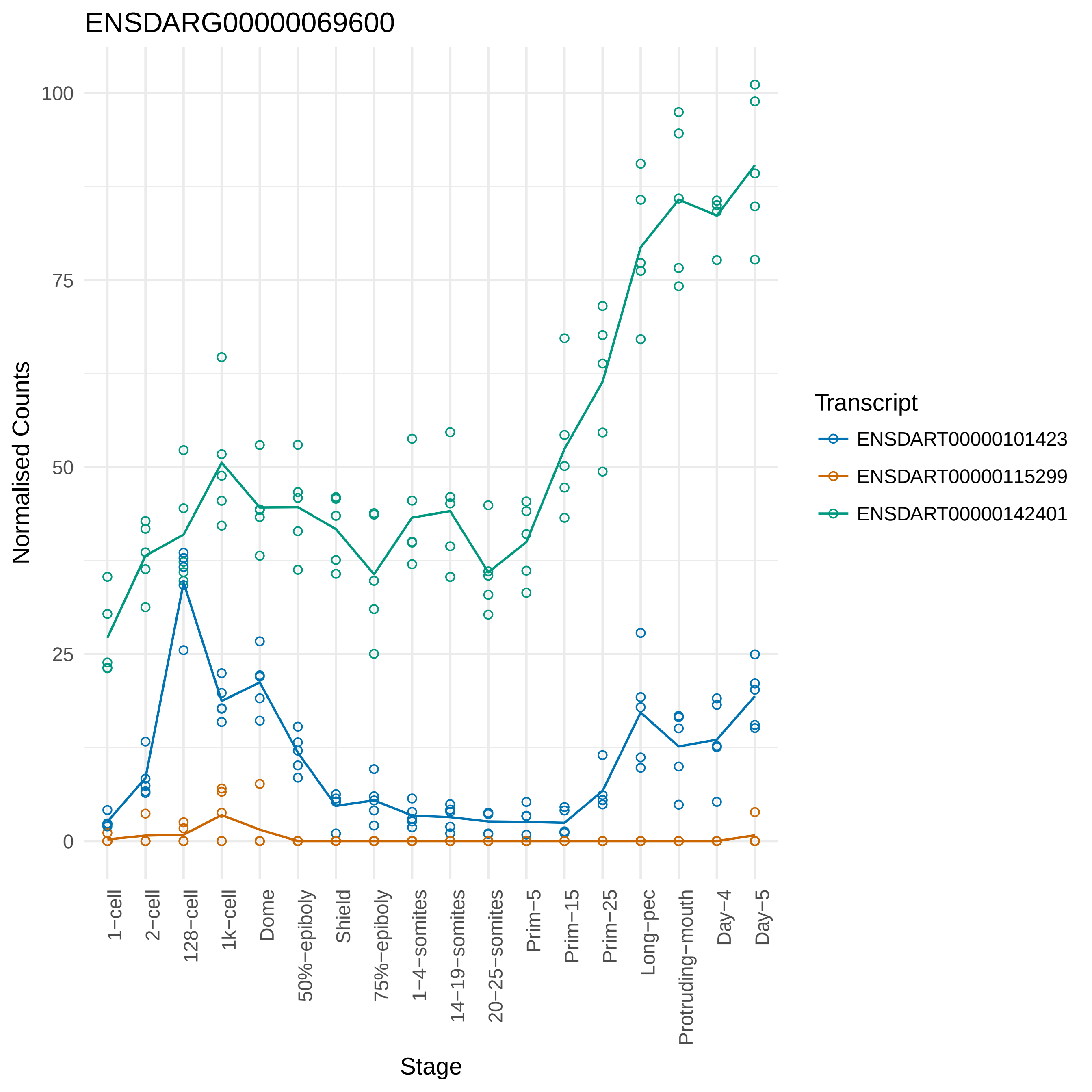
ENSDARG00000069600
Normalised Counts
100
75
50
25
0
1−cell
2−cell
128−cell
1k−cell
Dome
50%−epiboly
Shield
75%−epiboly
1−4−somites
14−19−somites
20−25−somites
Prim−5
Prim−15
Prim−25
Long−pec
Protruding−mouth
Day−4
Day−5
Stage
Transcript
ENSDART00000101423
ENSDART00000115299
ENSDART00000142401

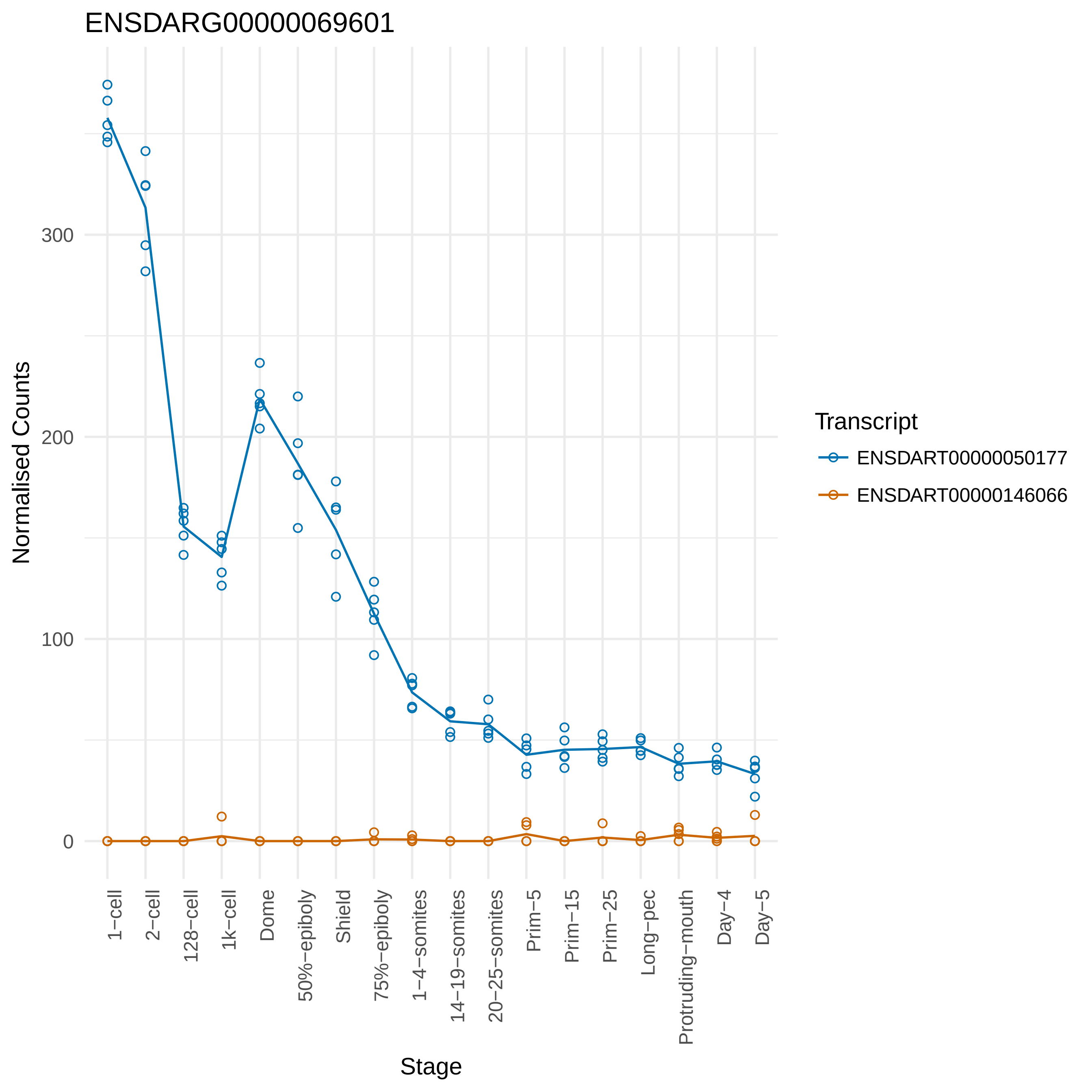

ENSDARG00000069601
Normalised Counts
300
200
100
0
1−cell
2−cell
128−cell
1k−cell
Dome
50%−epiboly
Shield
75%−epiboly
1−4−somites
14−19−somites
20−25−somites
Prim−5
Prim−15
Prim−25
Long−pec
Protruding−mouth
Day−4
Day−5
Stage
Transcript
ENSDART00000050177
ENSDART00000146066

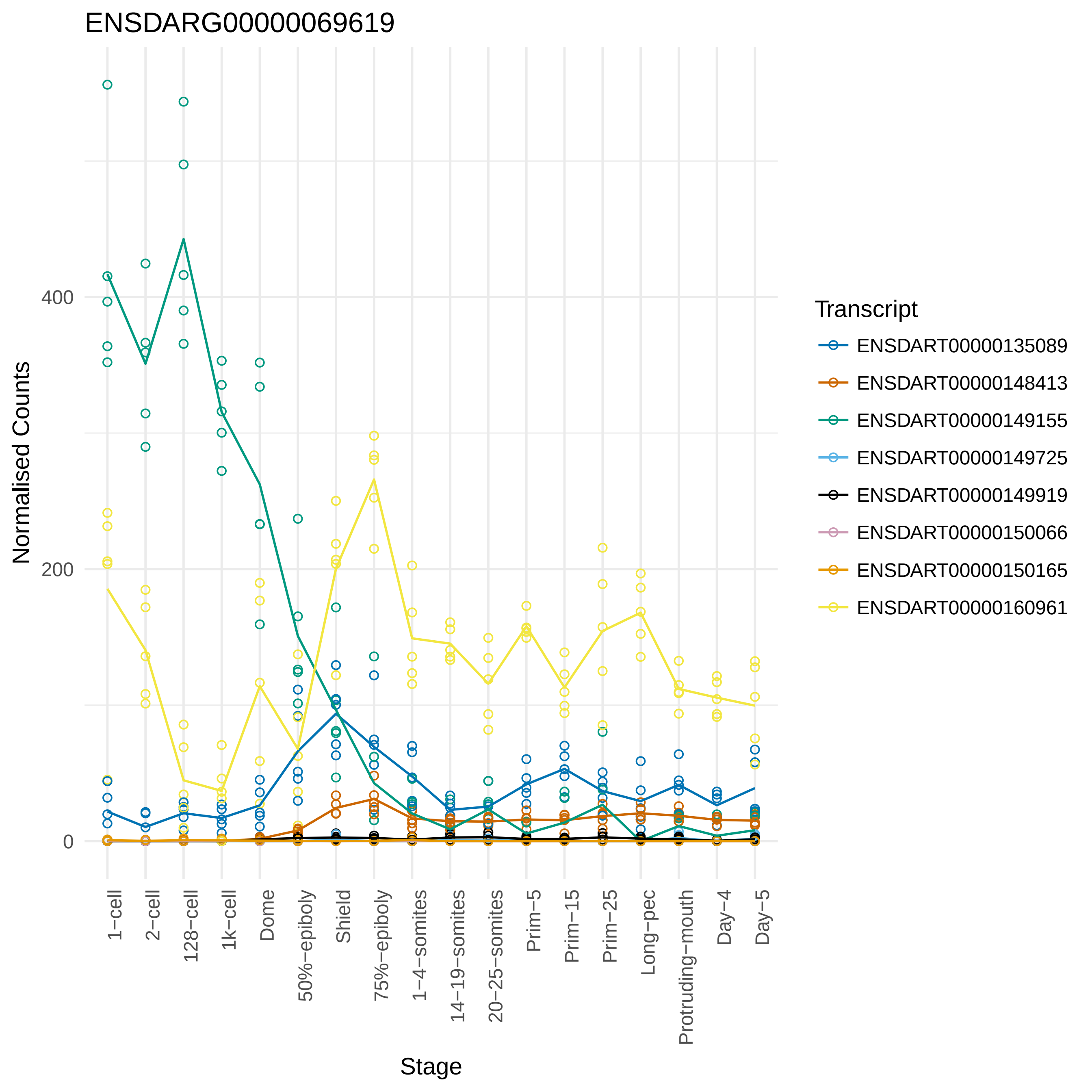

ENSDARG00000069619
Normalised Counts
0
200
400
1−cell
2−cell
128−cell
1k−cell
Dome
50%−epiboly
Shield
75%−epiboly
1−4−somites
14−19−somites
20−25−somites
Prim−5
Prim−15
Prim−25
Long−pec
Protruding−mouth
Day−4
Day−5
Stage
Transcript
ENSDART00000135089
ENSDART00000148413
ENSDART00000149155
ENSDART00000149725
ENSDART00000149919
ENSDART00000150066
ENSDART00000150165
ENSDART00000160961

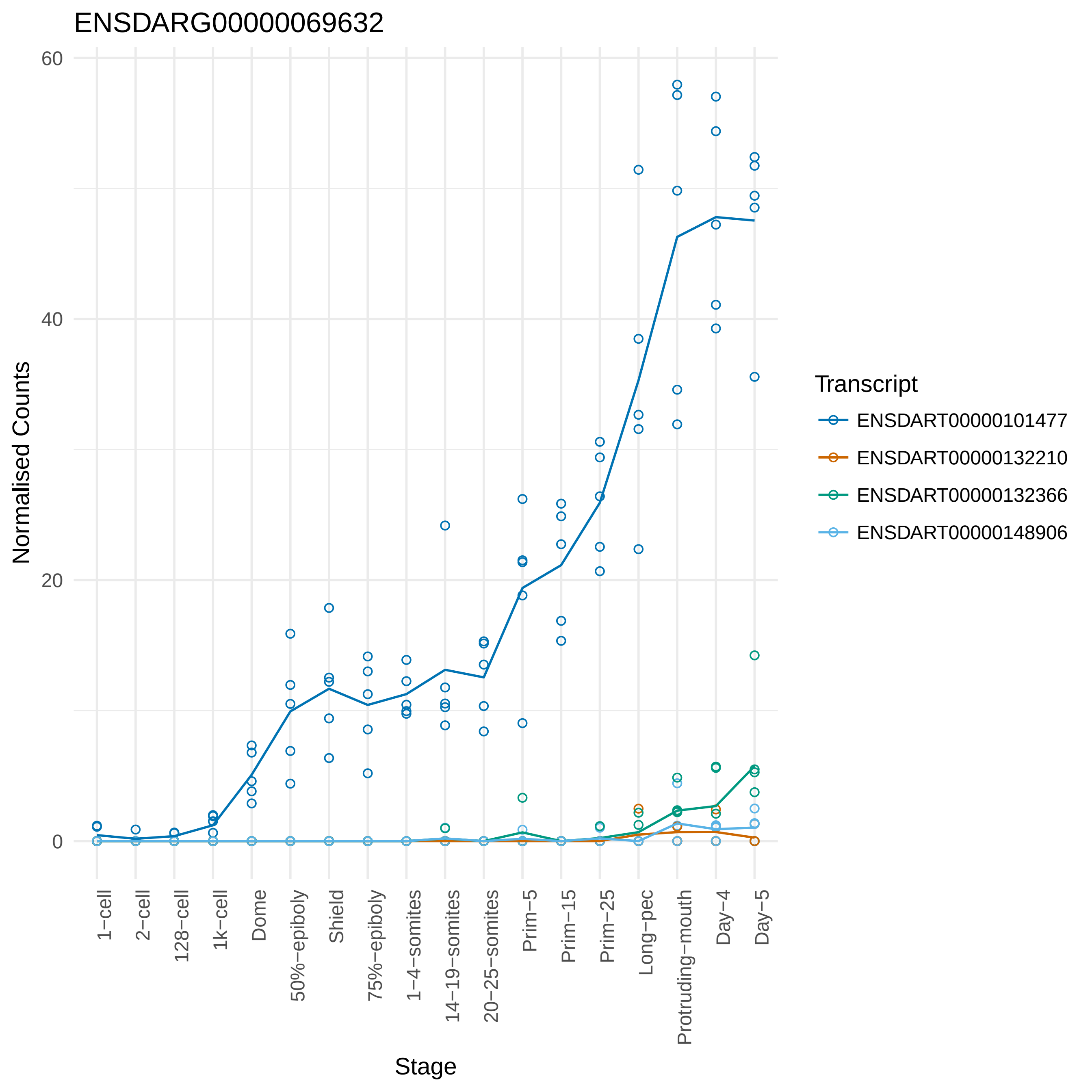
ENSDARG00000069632
Normalised Counts
Stage
60
40
20
0
1−cell
2−cell
128−cell
1k−cell
Dome
50%−epiboly
Shield
75%−epiboly
1−4−somites
14−19−somites
20−25−somites
Prim−5
Prim−15
Prim−25
Long−pec
Protruding−mouth
Day−4
Day−5
Transcript
ENSDART00000101477
ENSDART00000132210
ENSDART00000132366
ENSDART00000148906

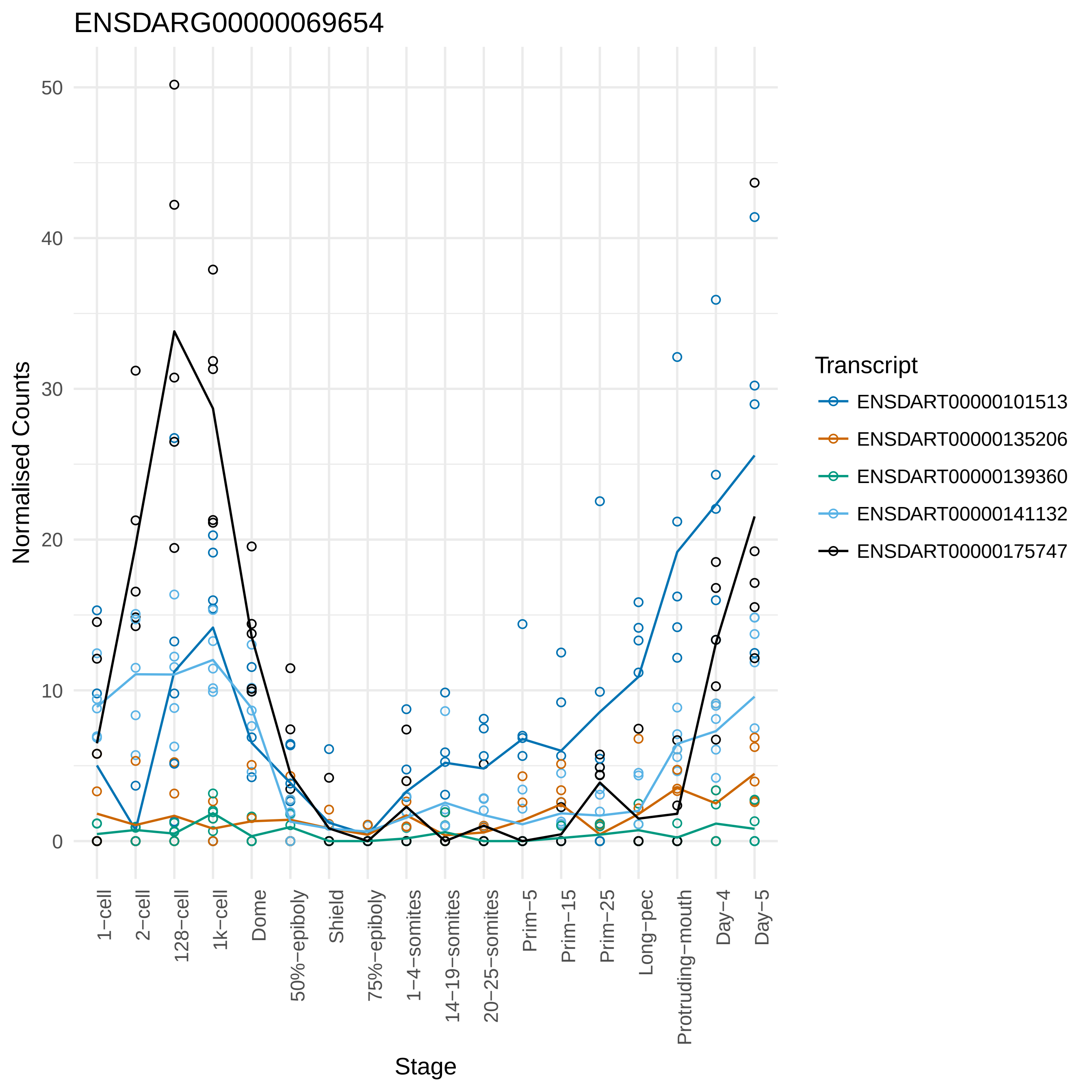
ENSDARG00000069654
Normalised Counts
0
10
20
30
40
50
1−cell
2−cell
128−cell
1k−cell
Dome
50%−epiboly
Shield
75%−epiboly
1−4−somites
14−19−somites
20−25−somites
Prim−5
Prim−15
Prim−25
Long−pec
Protruding−mouth
Day−4
Day−5
Stage
Transcript
ENSDART00000101513
ENSDART00000135206
ENSDART00000139360
ENSDART00000141132
ENSDART00000175747

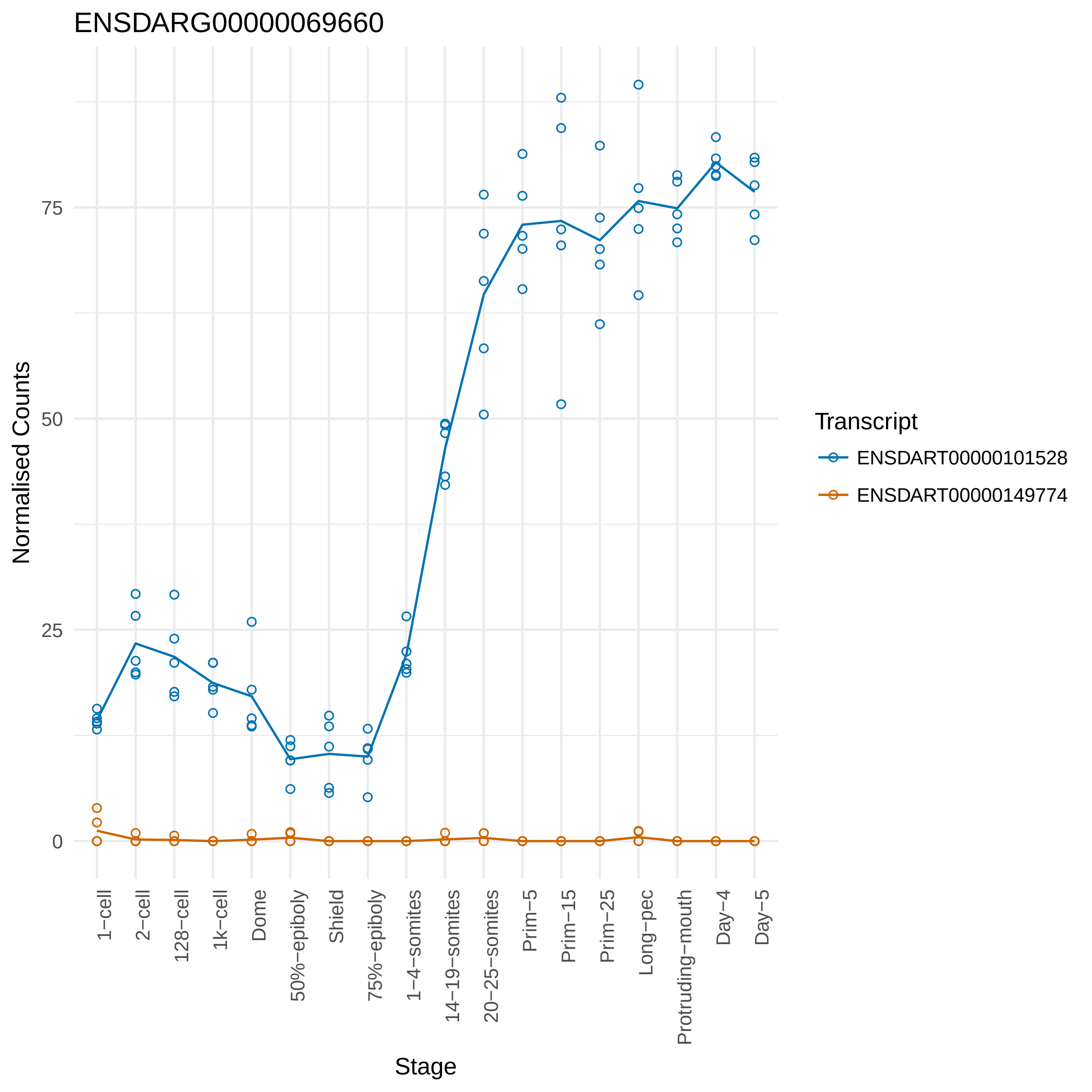

ENSDARG00000069660
Normalised Counts
0
25
50
75
1−cell
2−cell
128−cell
1k−cell
Dome
50%−epiboly
Shield
75%−epiboly
1−4−somites
14−19−somites
20−25−somites
Prim−5
Prim−15
Prim−25
Long−pec
Protruding−mouth
Day−4
Day−5
Stage
Transcript
ENSDART00000101528
ENSDART00000149774

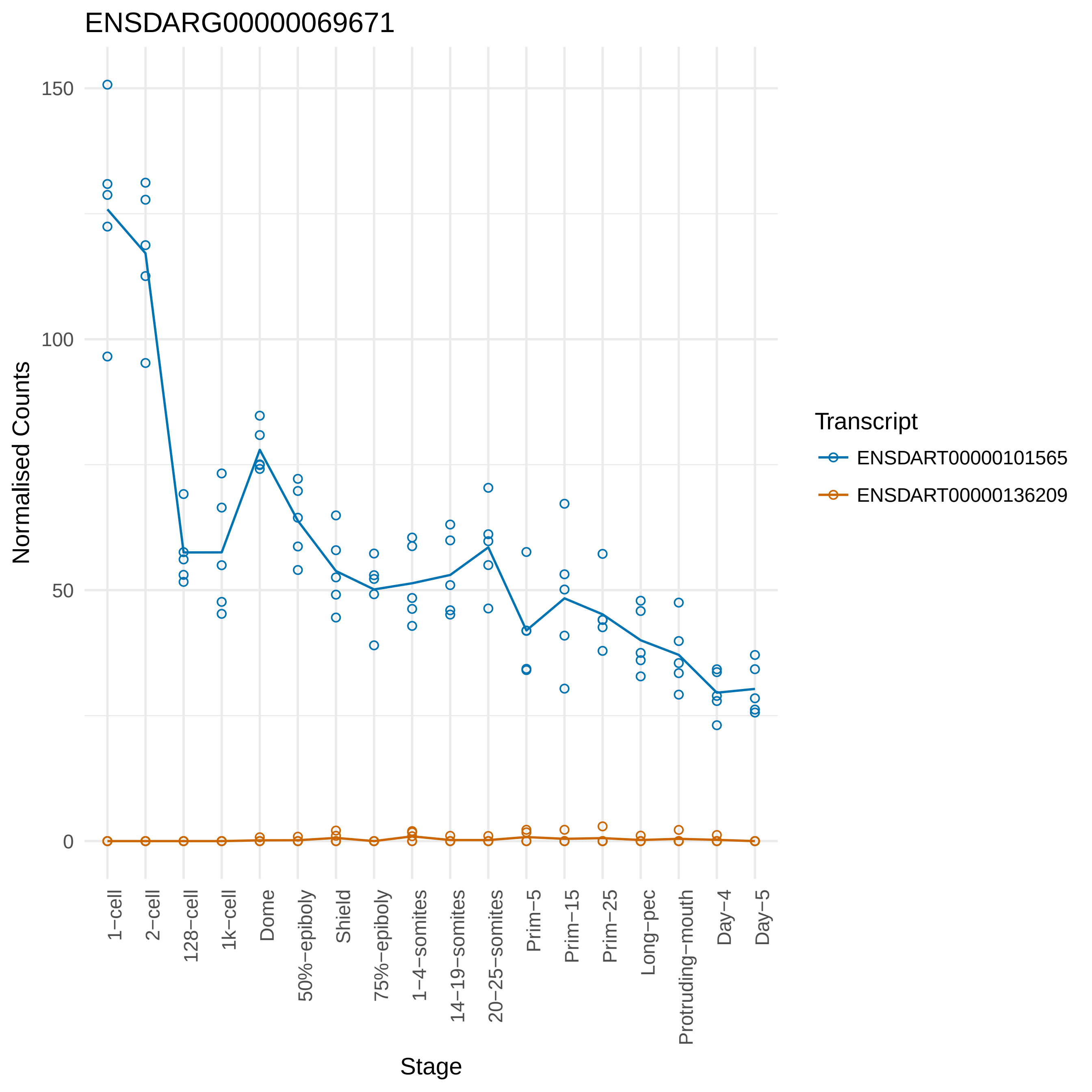

ENSDARG00000069671
Normalised Counts
150
100
50
0
Stage
1−cell
2−cell
128−cell
1k−cell
Dome
50%−epiboly
Shield
75%−epiboly
1−4−somites
14−19−somites
20−25−somites
Prim−5
Prim−15
Prim−25
Long−pec
Protruding−mouth
Day−4
Day−5
Transcript
ENSDART00000101565
ENSDART00000136209

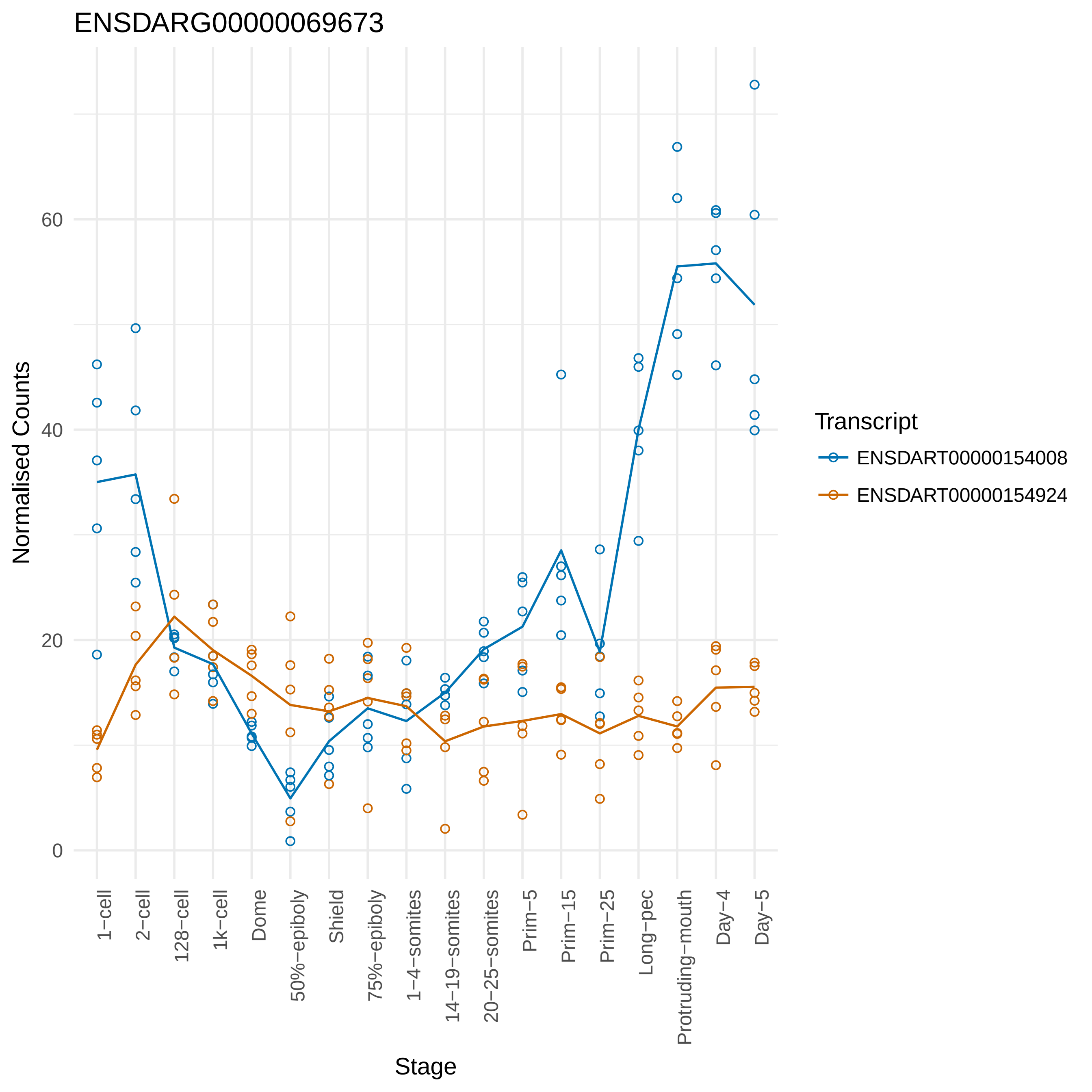

ENSDARG00000069673
Normalised Counts
0
20
40
60
Stage
1−cell
2−cell
128−cell
1k−cell
Dome
50%−epiboly
Shield
75%−epiboly
1−4−somites
14−19−somites
20−25−somites
Prim−5
Prim−15
Prim−25
Long−pec
Protruding−mouth
Day−4
Day−5
Transcript
ENSDART00000154008
ENSDART00000154924

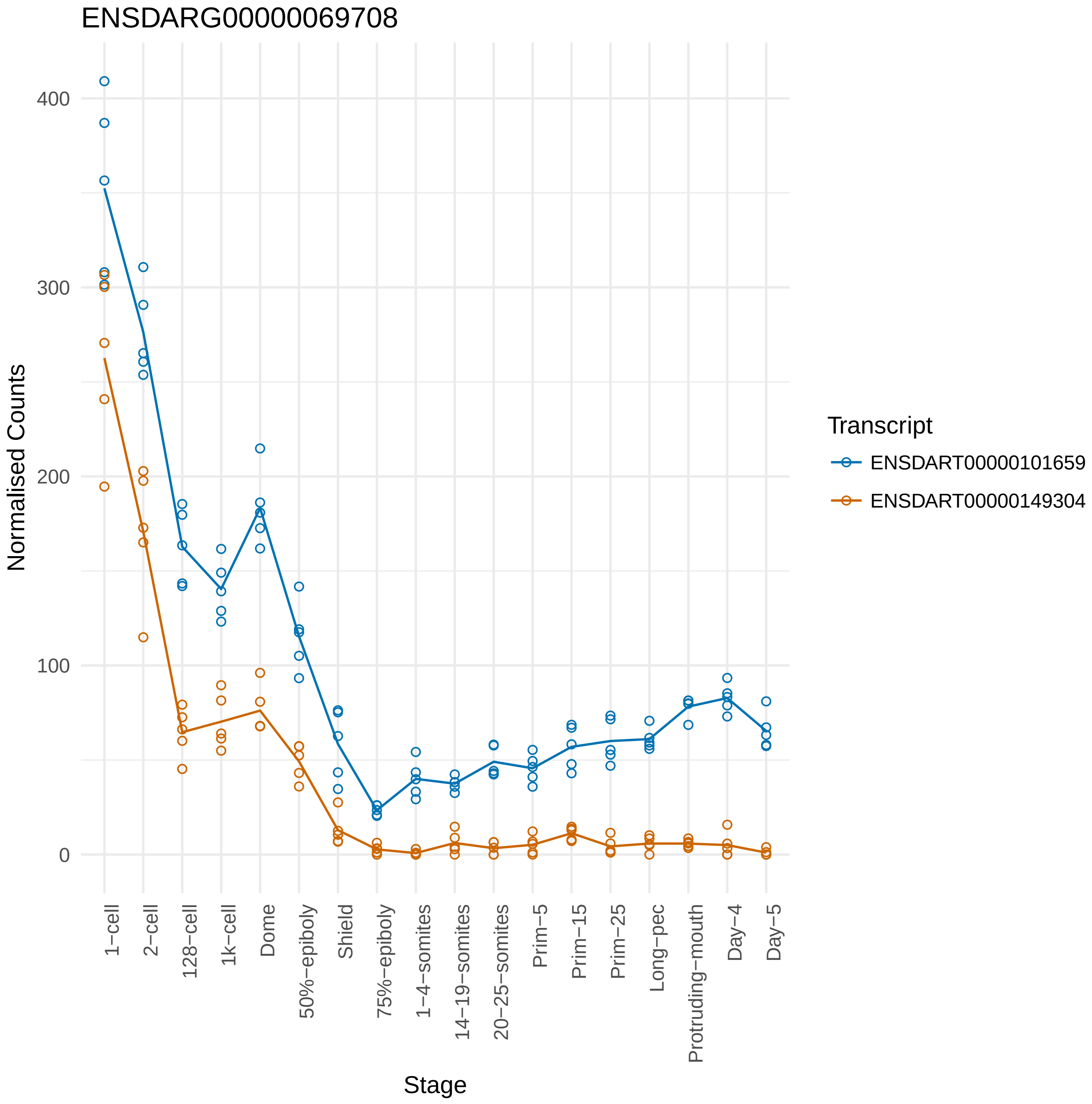
ENSDARG00000069708
Normalised Counts
400
300
200
100
0
1−cell
2−cell
128−cell
1k−cell
Dome
50%−epiboly
Shield
75%−epiboly
1−4−somites
14−19−somites
20−25−somites
Prim−5
Prim−15
Prim−25
Long−pec
Protruding−mouth
Day−4
Day−5
Stage
Transcript
ENSDART00000101659
ENSDART00000149304

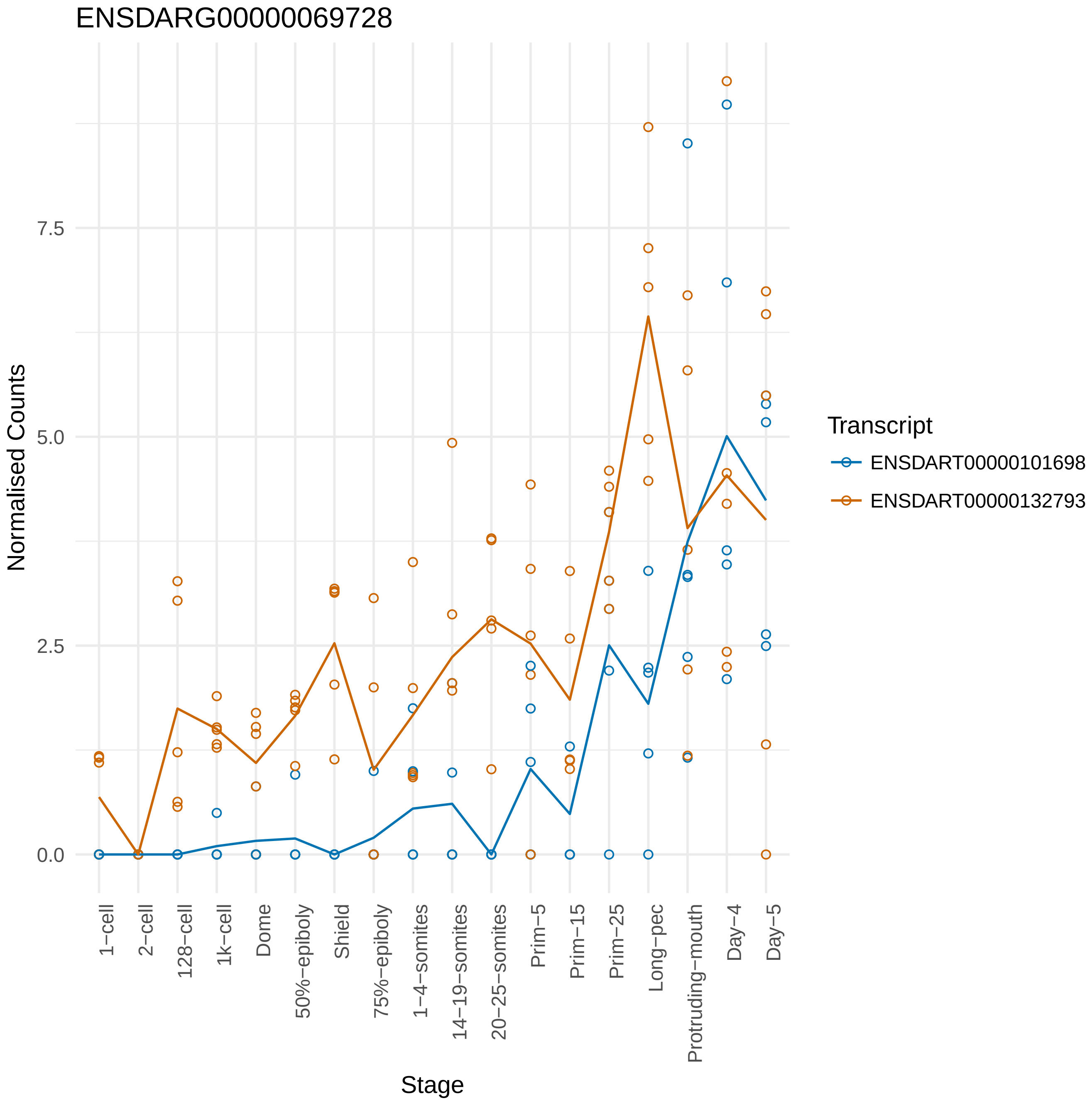
ENSDARG00000069728
Normalised Counts
7.5
5.0
2.5
0.0
Stage
1−cell
2−cell
128−cell
1k−cell
Dome
50%−epiboly
Shield
75%−epiboly
1−4−somites
14−19−somites
20−25−somites
Prim−5
Prim−15
Prim−25
Long−pec
Protruding−mouth
Day−4
Day−5
Transcript
ENSDART00000101698
ENSDART00000132793

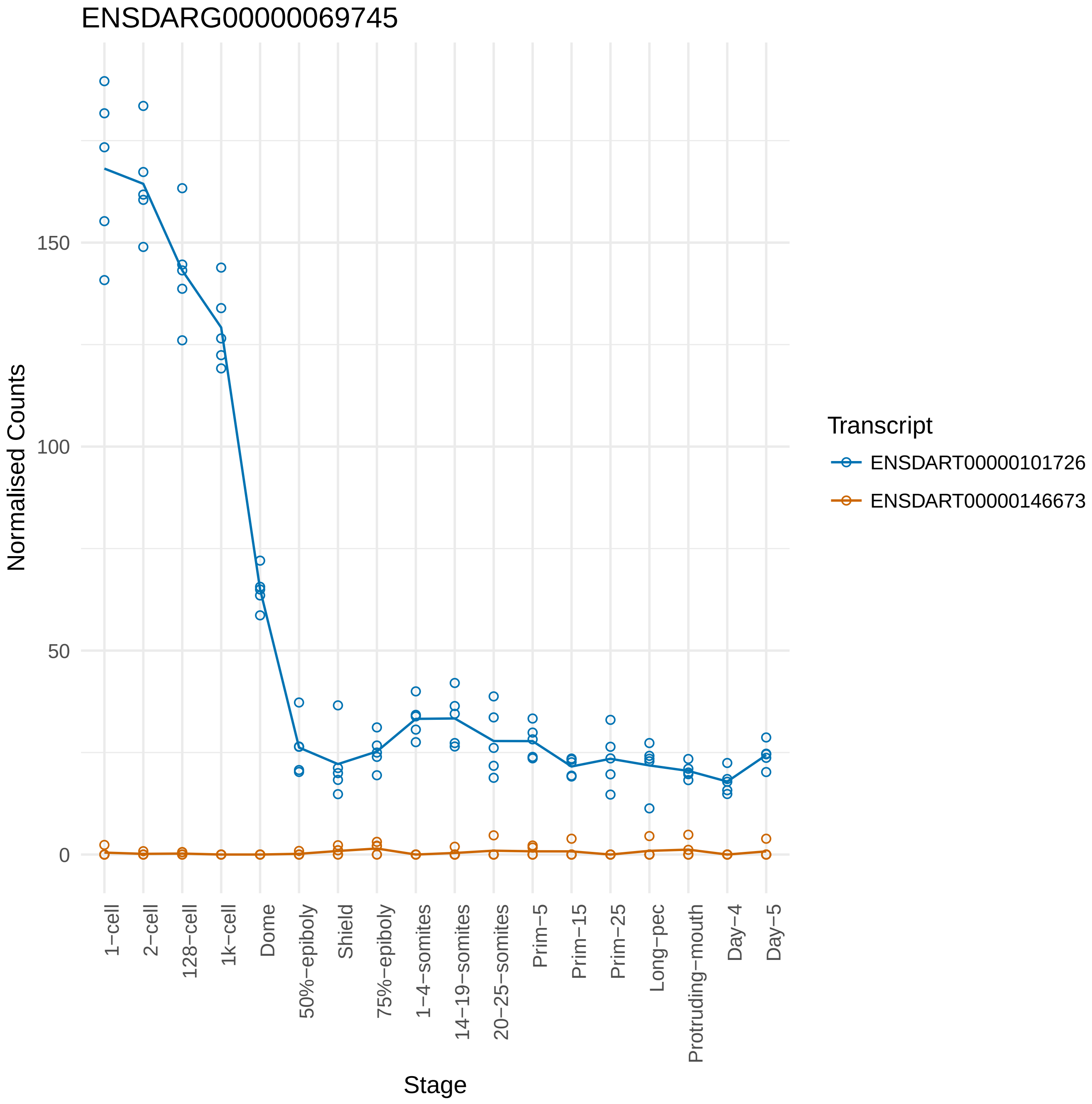

ENSDARG00000069745
Normalised Counts
150
100
50
0
1−cell
2−cell
128−cell
1k−cell
Dome
50%−epiboly
Shield
75%−epiboly
1−4−somites
14−19−somites
20−25−somites
Prim−5
Prim−15
Prim−25
Long−pec
Protruding−mouth
Day−4
Day−5
Stage
Transcript
ENSDART00000101726
ENSDART00000146673

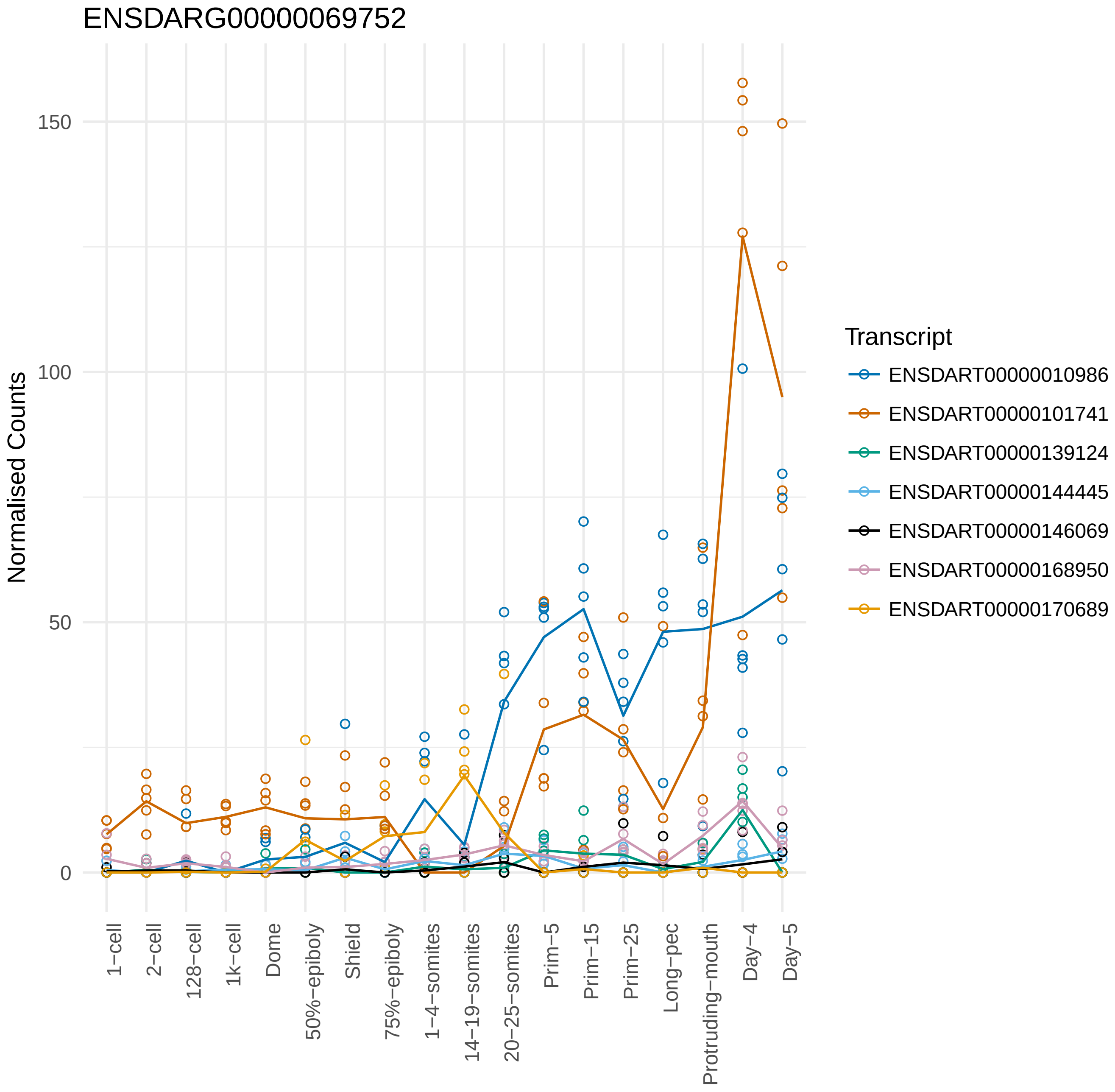

ENSDARG00000069752
Normalised Counts
150
100
50
0
1−cell
2−cell
128−cell
1k−cell
Dome
50%−epiboly
Shield
75%−epiboly
1−4−somites
14−19−somites
20−25−somites
Prim−5
Prim−15
Prim−25
Long−pec
Protruding−mouth
Day−4
Day−5
Stage
Transcript
ENSDART00000010986
ENSDART00000101741
ENSDART00000139124
ENSDART00000144445
ENSDART00000146069
ENSDART00000168950
ENSDART00000170689

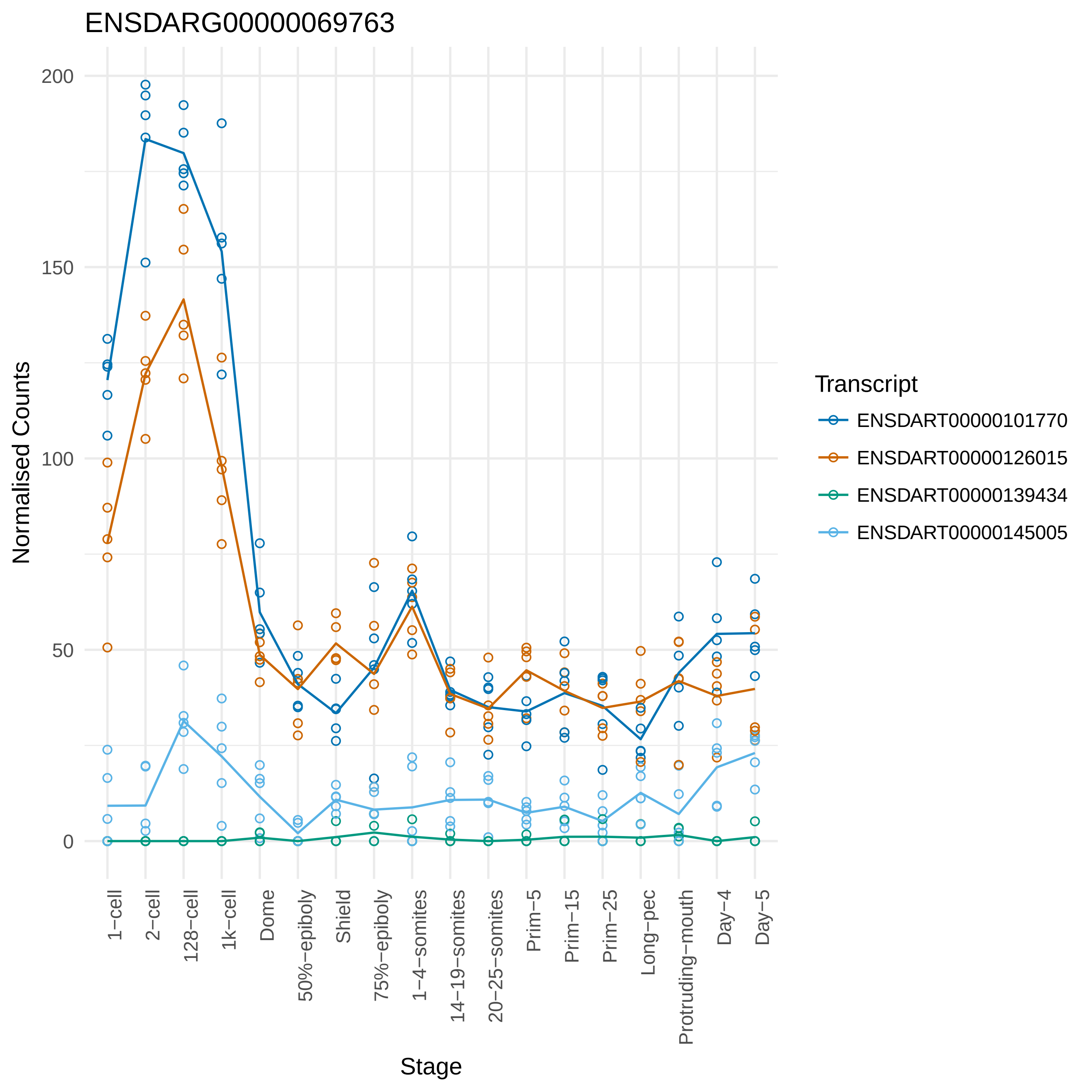
ENSDARG00000069763
Normalised Counts
200
150
100
50
0
1−cell
2−cell
128−cell
1k−cell
Dome
50%−epiboly
Shield
75%−epiboly
1−4−somites
14−19−somites
20−25−somites
Prim−5
Prim−15
Prim−25
Long−pec
Protruding−mouth
Day−4
Day−5
Stage
Transcript
ENSDART00000101770
ENSDART00000126015
ENSDART00000139434
ENSDART00000145005

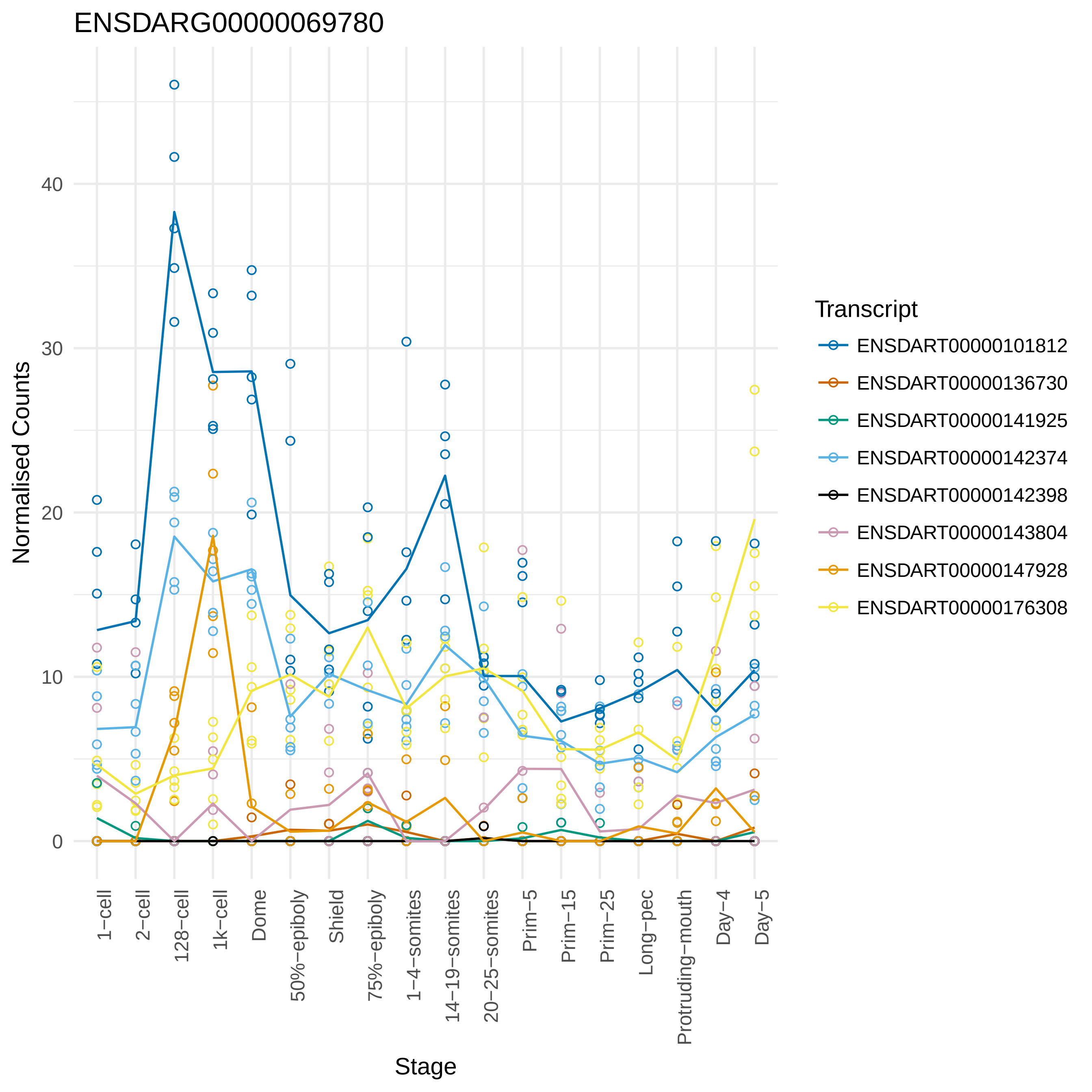

ENSDARG00000069780
Normalised Counts
0
10
20
30
40
1−cell
2−cell
128−cell
1k−cell
Dome
50%−epiboly
Shield
75%−epiboly
1−4−somites
14−19−somites
20−25−somites
Prim−5
Prim−15
Prim−25
Long−pec
Protruding−mouth
Day−4
Day−5
Stage
Transcript
ENSDART00000101812
ENSDART00000136730
ENSDART00000141925
ENSDART00000142374
ENSDART00000142398
ENSDART00000143804
ENSDART00000147928
ENSDART00000176308

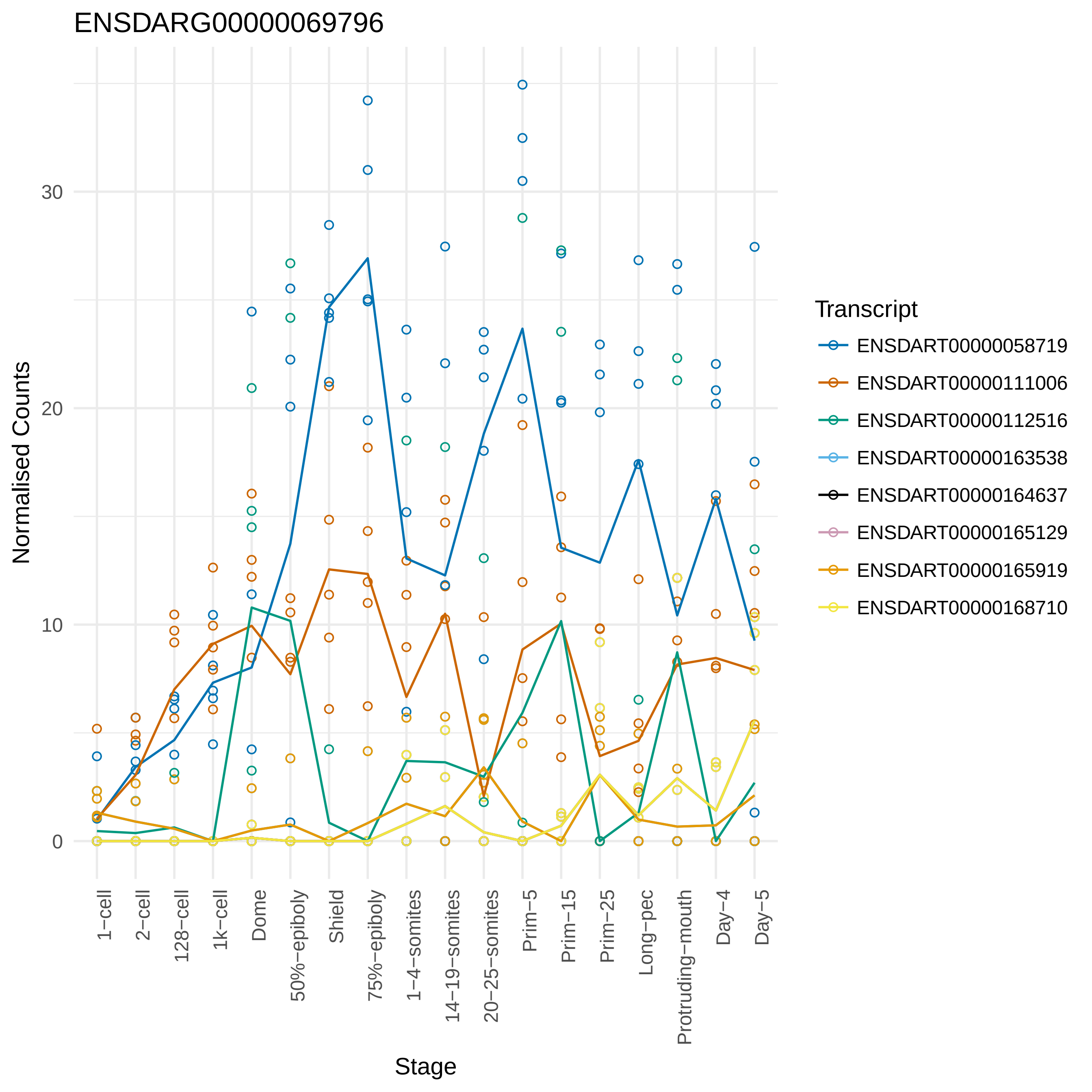

ENSDARG00000069796
Normalised Counts
0
10
20
30
1−cell
2−cell
128−cell
1k−cell
Dome
50%−epiboly
Shield
75%−epiboly
1−4−somites
14−19−somites
20−25−somites
Prim−5
Prim−15
Prim−25
Long−pec
Protruding−mouth
Day−4
Day−5
Stage
Transcript
ENSDART00000058719
ENSDART00000111006
ENSDART00000112516
ENSDART00000163538
ENSDART00000164637
ENSDART00000165129
ENSDART00000165919
ENSDART00000168710

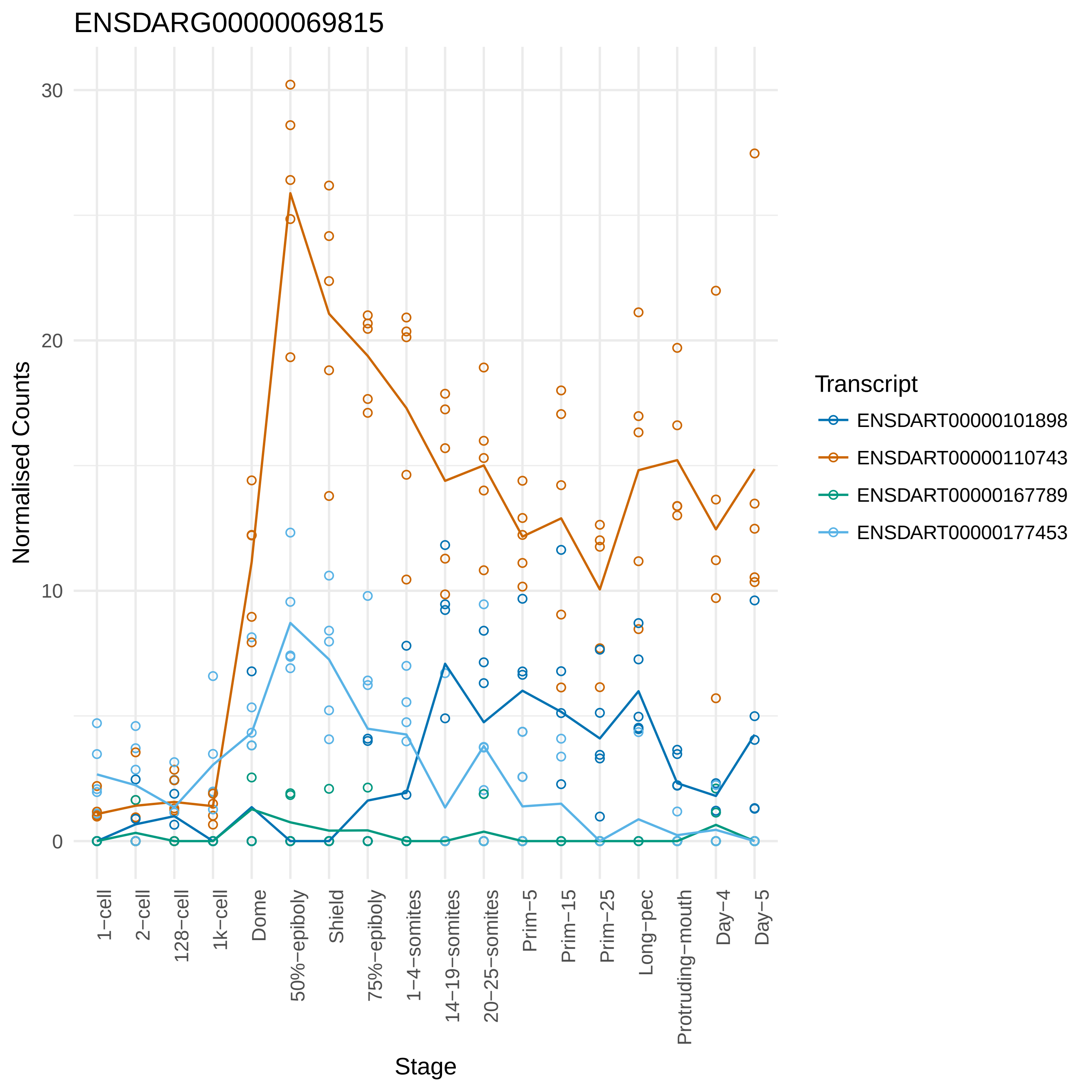
ENSDARG00000069815
Normalised Counts
30
20
10
0
1−cell
2−cell
128−cell
1k−cell
Dome
50%−epiboly
Shield
75%−epiboly
1−4−somites
14−19−somites
20−25−somites
Prim−5
Prim−15
Prim−25
Long−pec
Protruding−mouth
Day−4
Day−5
Stage
Transcript
ENSDART00000101898
ENSDART00000110743
ENSDART00000167789
ENSDART00000177453

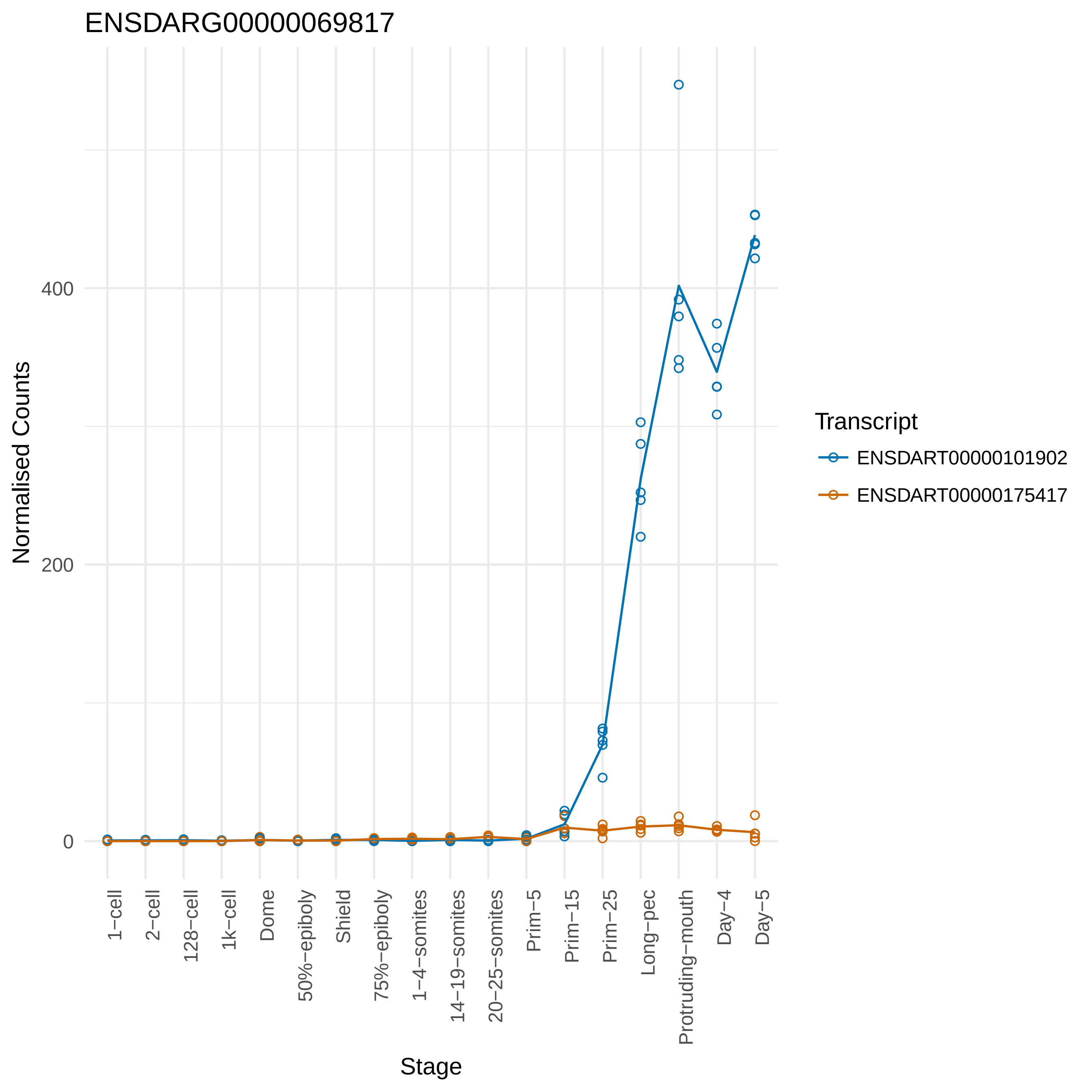

ENSDARG00000069817
Normalised Counts
400
200
0
1−cell
2−cell
128−cell
1k−cell
Dome
50%−epiboly
Shield
75%−epiboly
1−4−somites
14−19−somites
20−25−somites
Prim−5
Prim−15
Prim−25
Long−pec
Protruding−mouth
Day−4
Day−5
Stage
Transcript
ENSDART00000101902
ENSDART00000175417

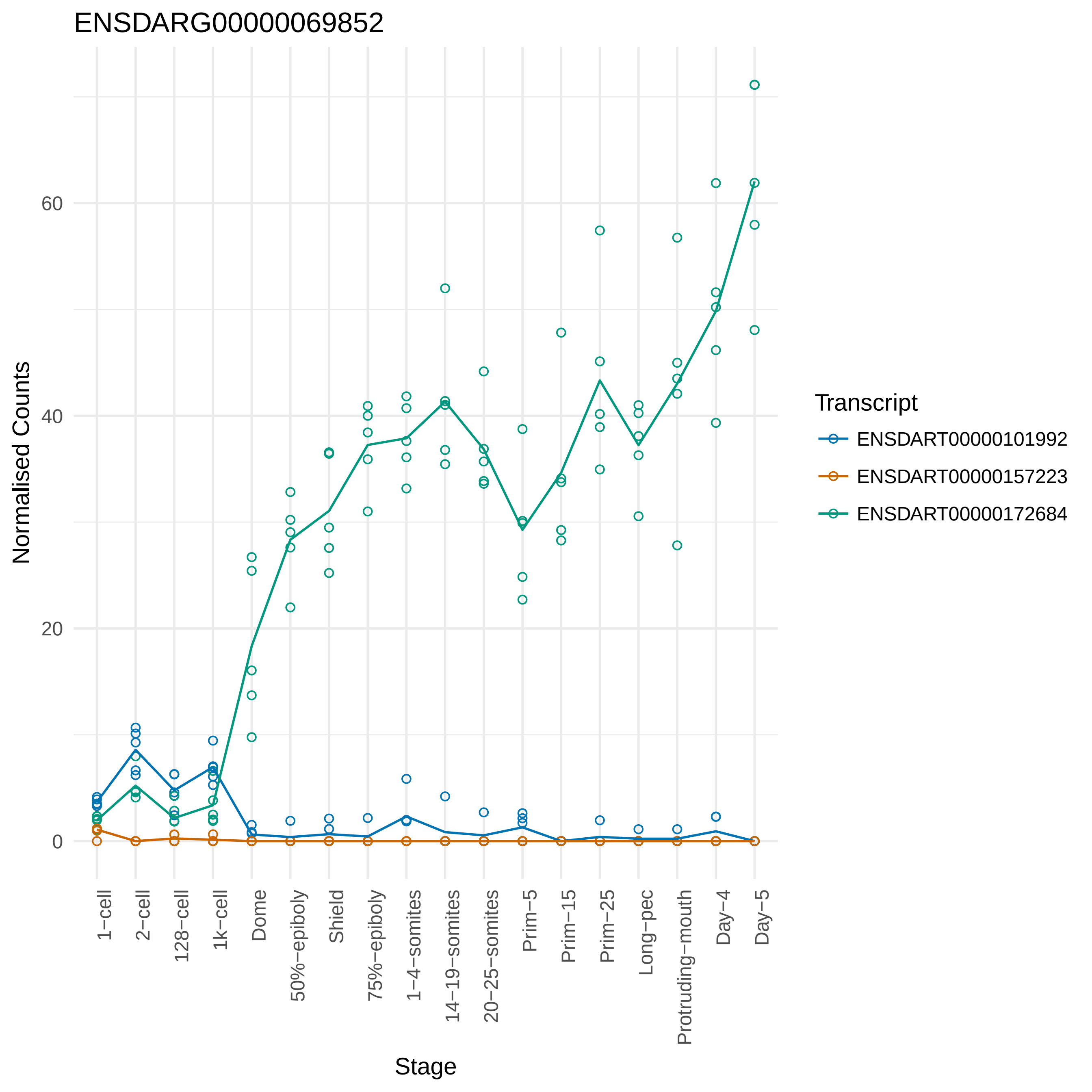
ENSDARG00000069852
Normalised Counts
0
20
40
60
Stage
1−cell
2−cell
128−cell
1k−cell
Dome
50%−epiboly
Shield
75%−epiboly
1−4−somites
14−19−somites
20−25−somites
Prim−5
Prim−15
Prim−25
Long−pec
Protruding−mouth
Day−4
Day−5
Transcript
ENSDART00000101992
ENSDART00000157223
ENSDART00000172684

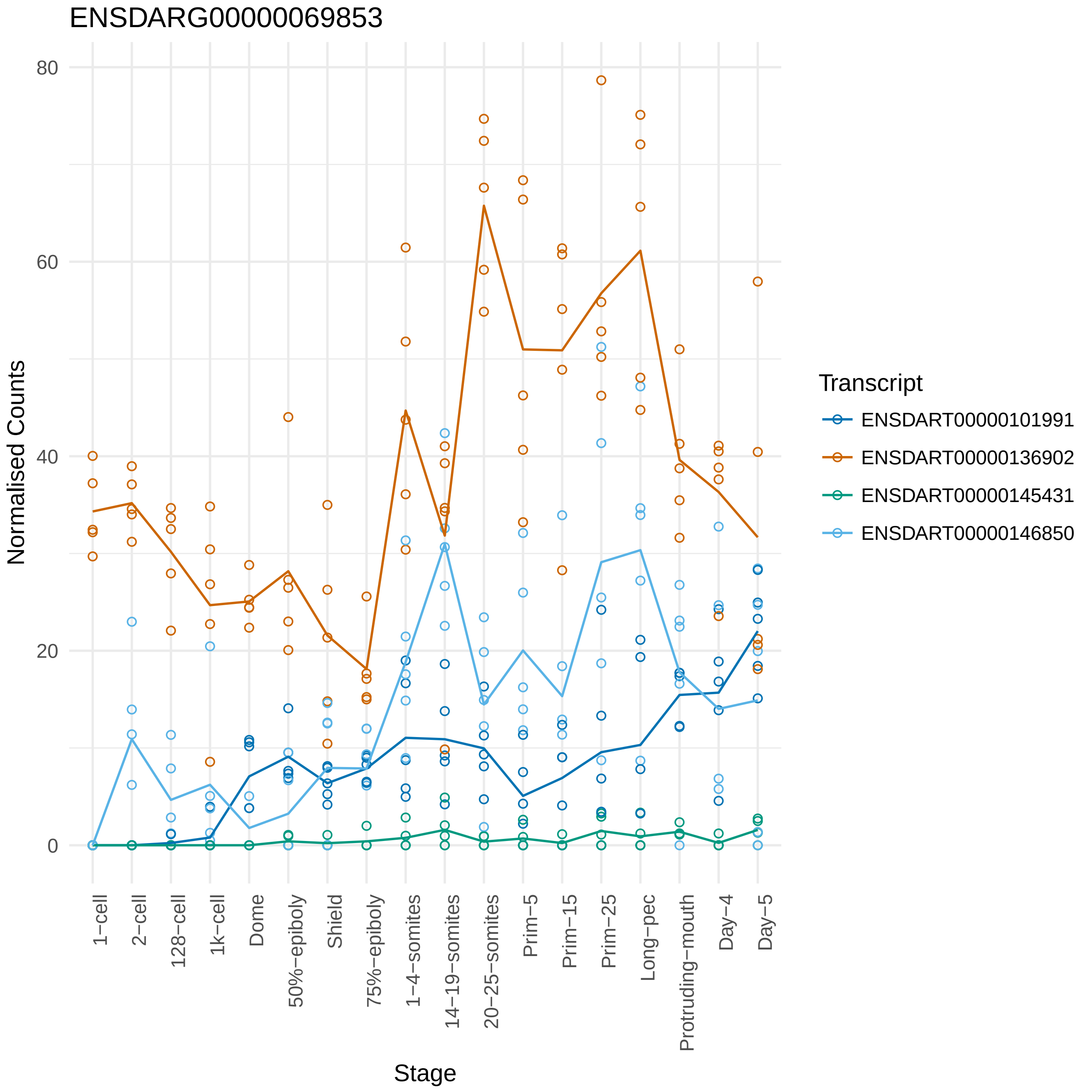

ENSDARG00000069853
Normalised Counts
80
60
40
20
0
1−cell
2−cell
128−cell
1k−cell
Dome
50%−epiboly
Shield
75%−epiboly
1−4−somites
14−19−somites
20−25−somites
Prim−5
Prim−15
Prim−25
Long−pec
Protruding−mouth
Day−4
Day−5
Stage
Transcript
ENSDART00000101991
ENSDART00000136902
ENSDART00000145431
ENSDART00000146850

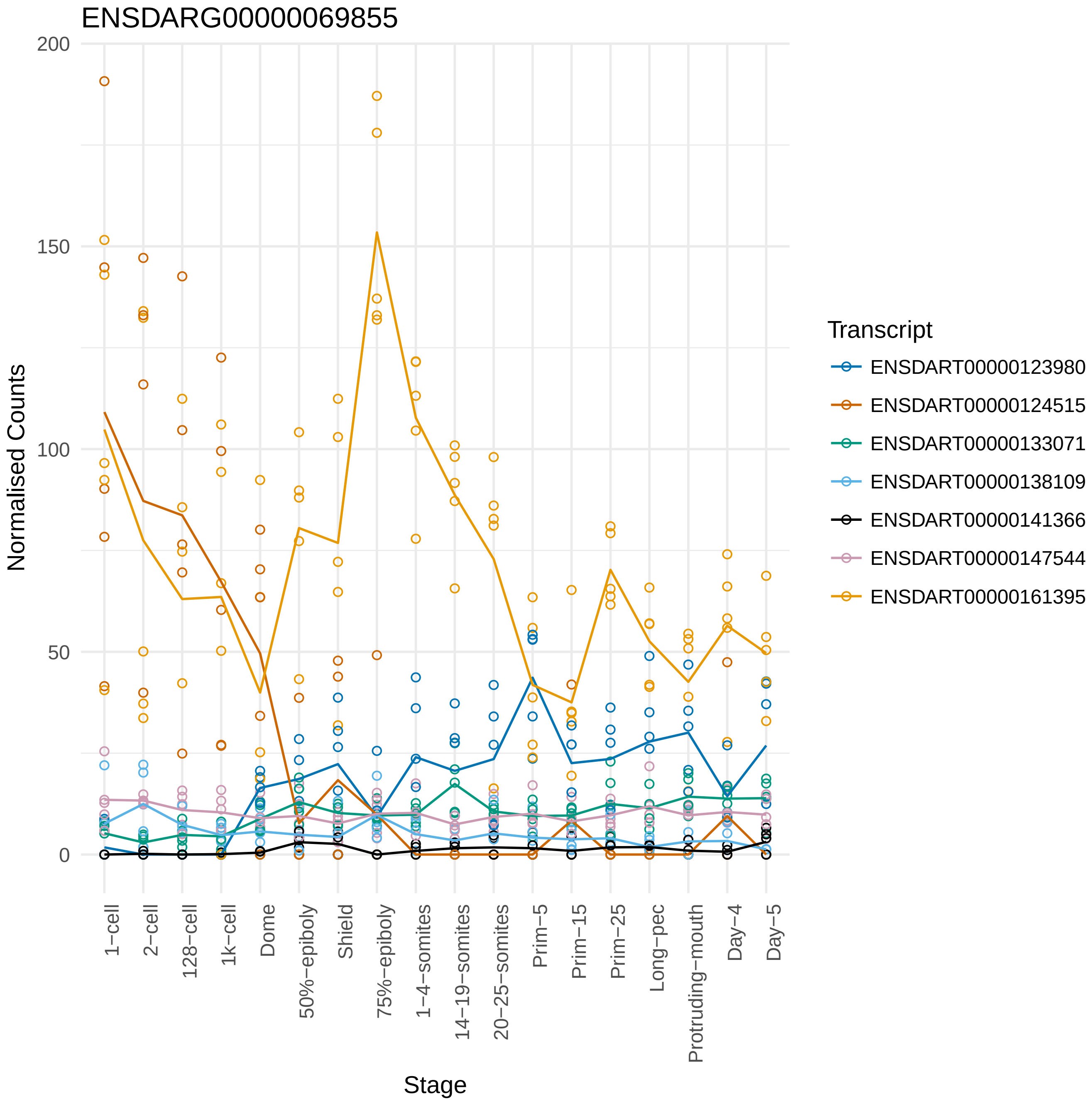

ENSDARG00000069855
Normalised Counts
200
150
100
50
0
1−cell
2−cell
128−cell
1k−cell
Dome
50%−epiboly
Shield
75%−epiboly
1−4−somites
14−19−somites
20−25−somites
Prim−5
Prim−15
Prim−25
Long−pec
Protruding−mouth
Day−4
Day−5
Stage
Transcript
ENSDART00000123980
ENSDART00000124515
ENSDART00000133071
ENSDART00000138109
ENSDART00000141366
ENSDART00000147544
ENSDART00000161395

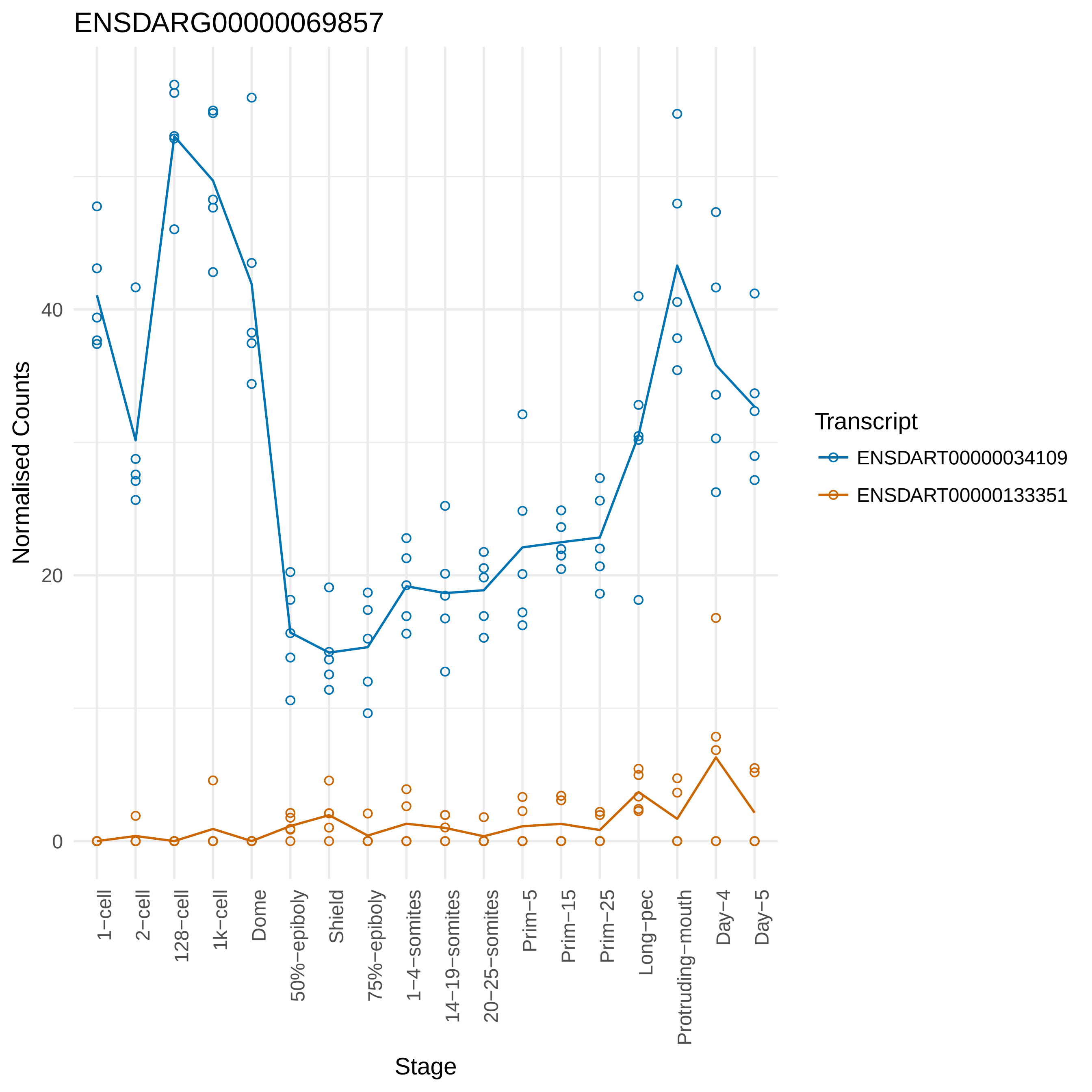
ENSDARG00000069857
Normalised Counts
0
20
40
Stage
1−cell
2−cell
128−cell
1k−cell
Dome
50%−epiboly
Shield
75%−epiboly
1−4−somites
14−19−somites
20−25−somites
Prim−5
Prim−15
Prim−25
Long−pec
Protruding−mouth
Day−4
Day−5
Transcript
ENSDART00000034109
ENSDART00000133351

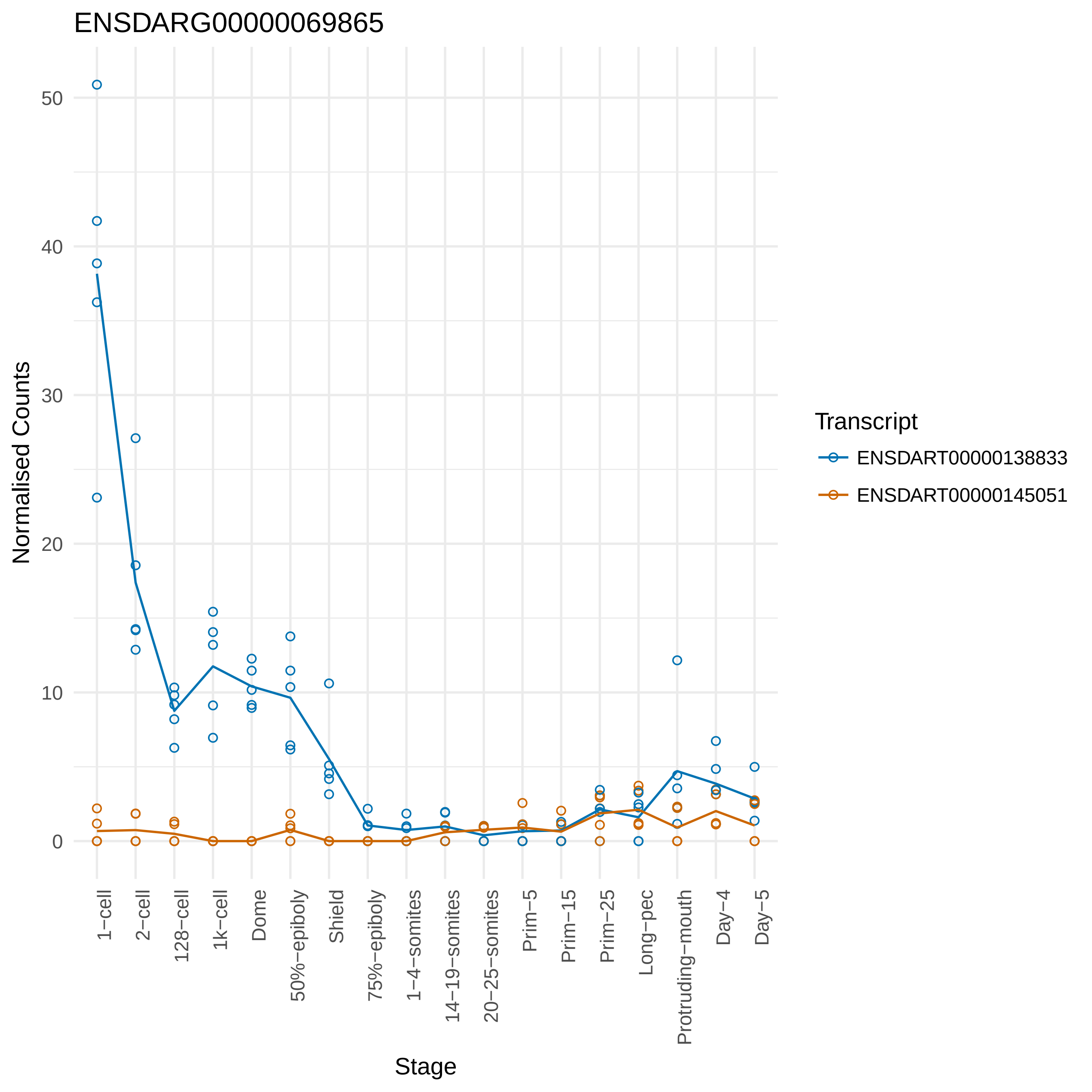

ENSDARG00000069865
Normalised Counts
50
40
30
20
10
0
1−cell
2−cell
128−cell
1k−cell
Dome
50%−epiboly
Shield
75%−epiboly
1−4−somites
14−19−somites
20−25−somites
Prim−5
Prim−15
Prim−25
Long−pec
Protruding−mouth
Day−4
Day−5
Stage
Transcript
ENSDART00000138833
ENSDART00000145051

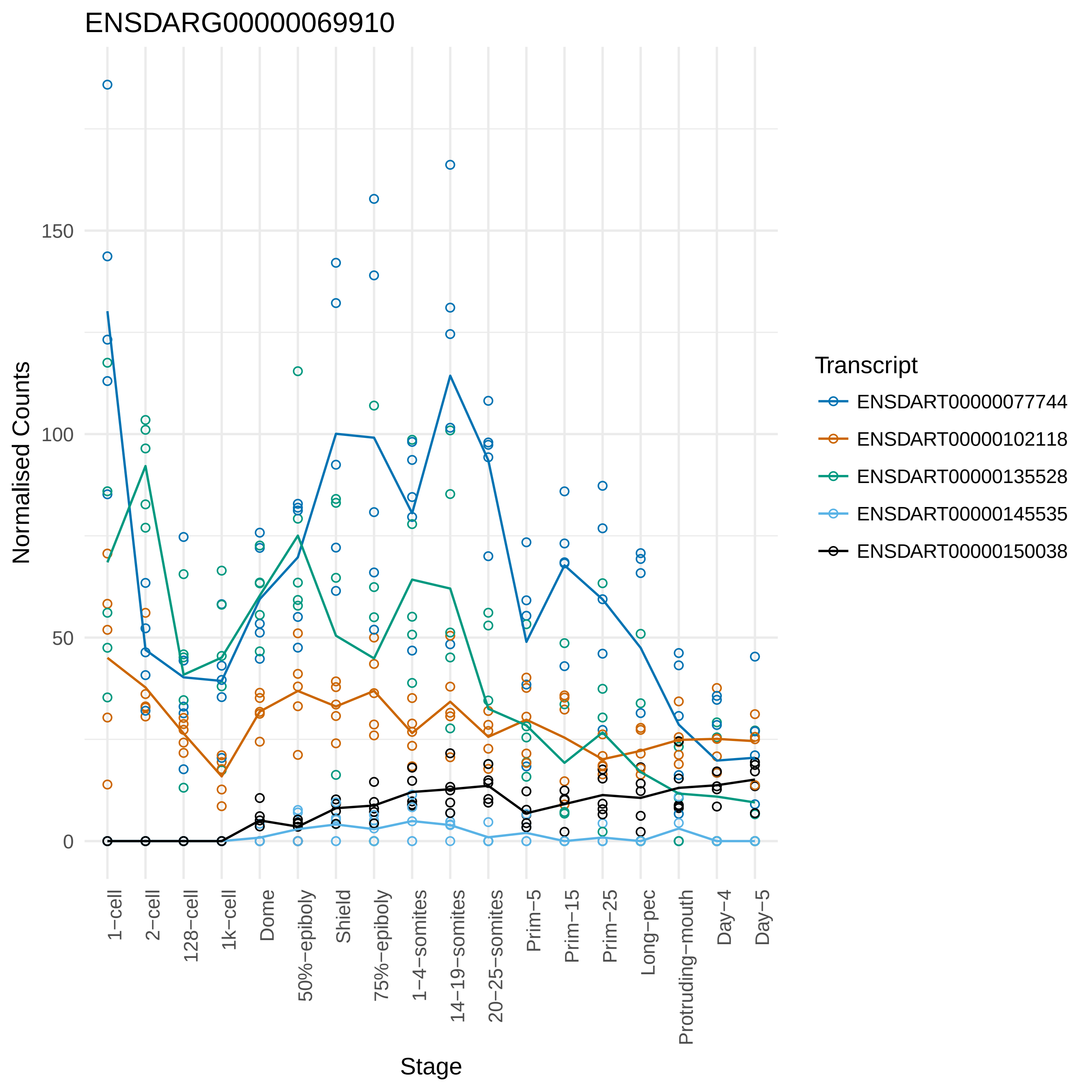

ENSDARG00000069910
Normalised Counts
150
100
50
0
1−cell
2−cell
128−cell
1k−cell
Dome
50%−epiboly
Shield
75%−epiboly
1−4−somites
14−19−somites
20−25−somites
Prim−5
Prim−15
Prim−25
Long−pec
Protruding−mouth
Day−4
Day−5
Stage
Transcript
ENSDART00000077744
ENSDART00000102118
ENSDART00000135528
ENSDART00000145535
ENSDART00000150038

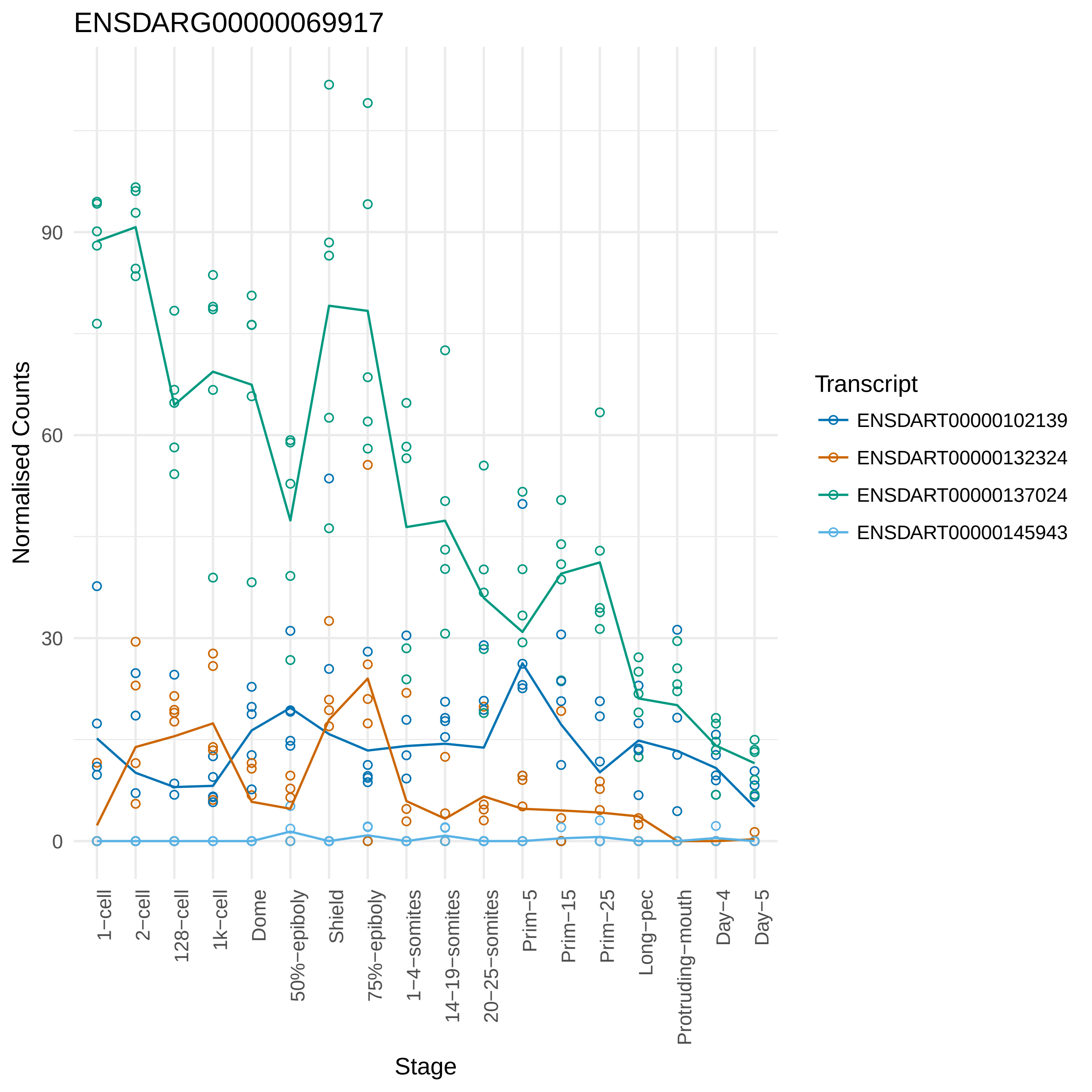
ENSDARG00000069917
Normalised Counts
0
30
60
90
Stage
1−cell
2−cell
128−cell
1k−cell
Dome
50%−epiboly
Shield
75%−epiboly
1−4−somites
14−19−somites
20−25−somites
Prim−5
Prim−15
Prim−25
Long−pec
Protruding−mouth
Day−4
Day−5
Transcript
ENSDART00000102139
ENSDART00000132324
ENSDART00000137024
ENSDART00000145943

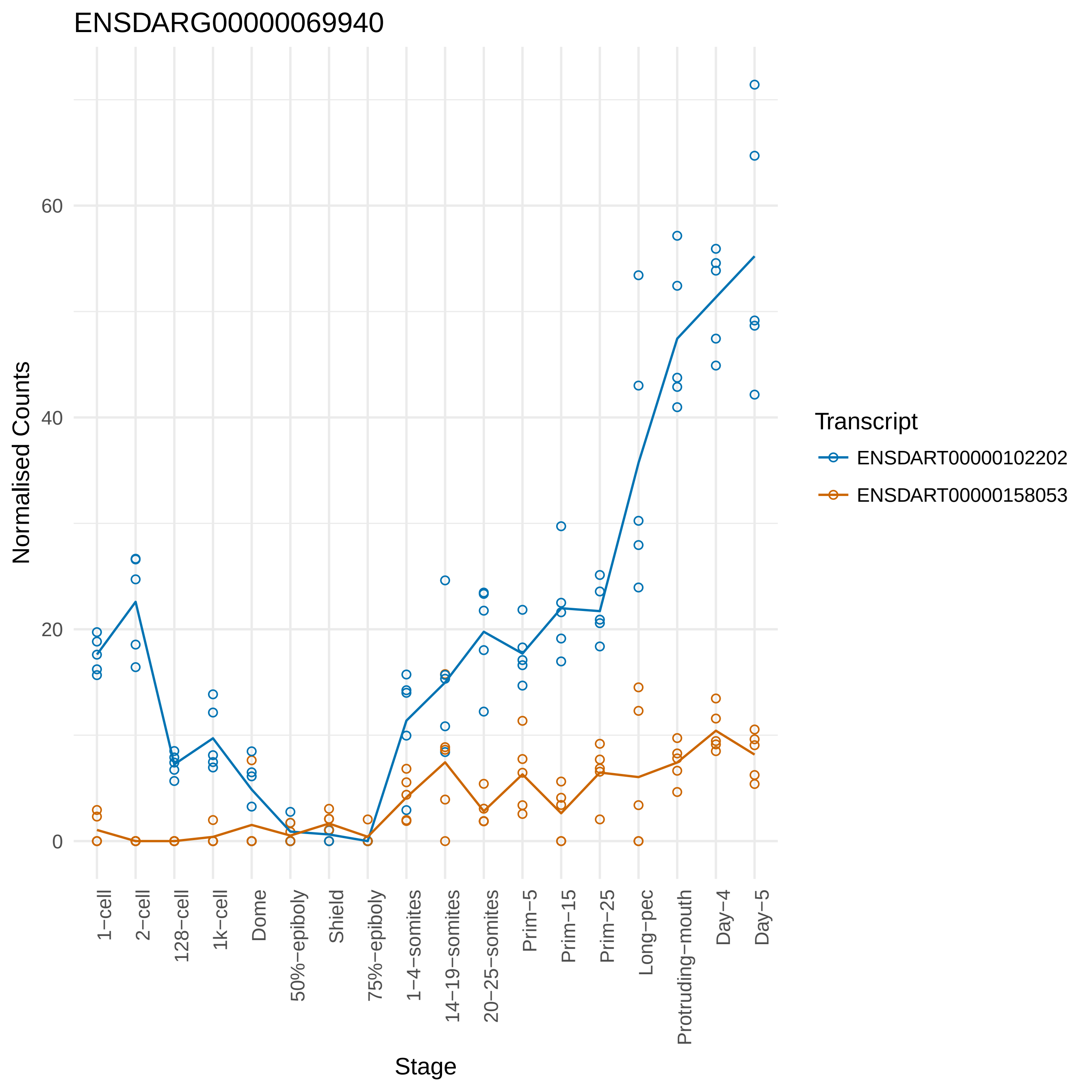
ENSDARG00000069940
Normalised Counts
0
20
40
60
1−cell
2−cell
128−cell
1k−cell
Dome
50%−epiboly
Shield
75%−epiboly
1−4−somites
14−19−somites
20−25−somites
Prim−5
Prim−15
Prim−25
Long−pec
Protruding−mouth
Day−4
Day−5
Stage
Transcript
ENSDART00000102202
ENSDART00000158053

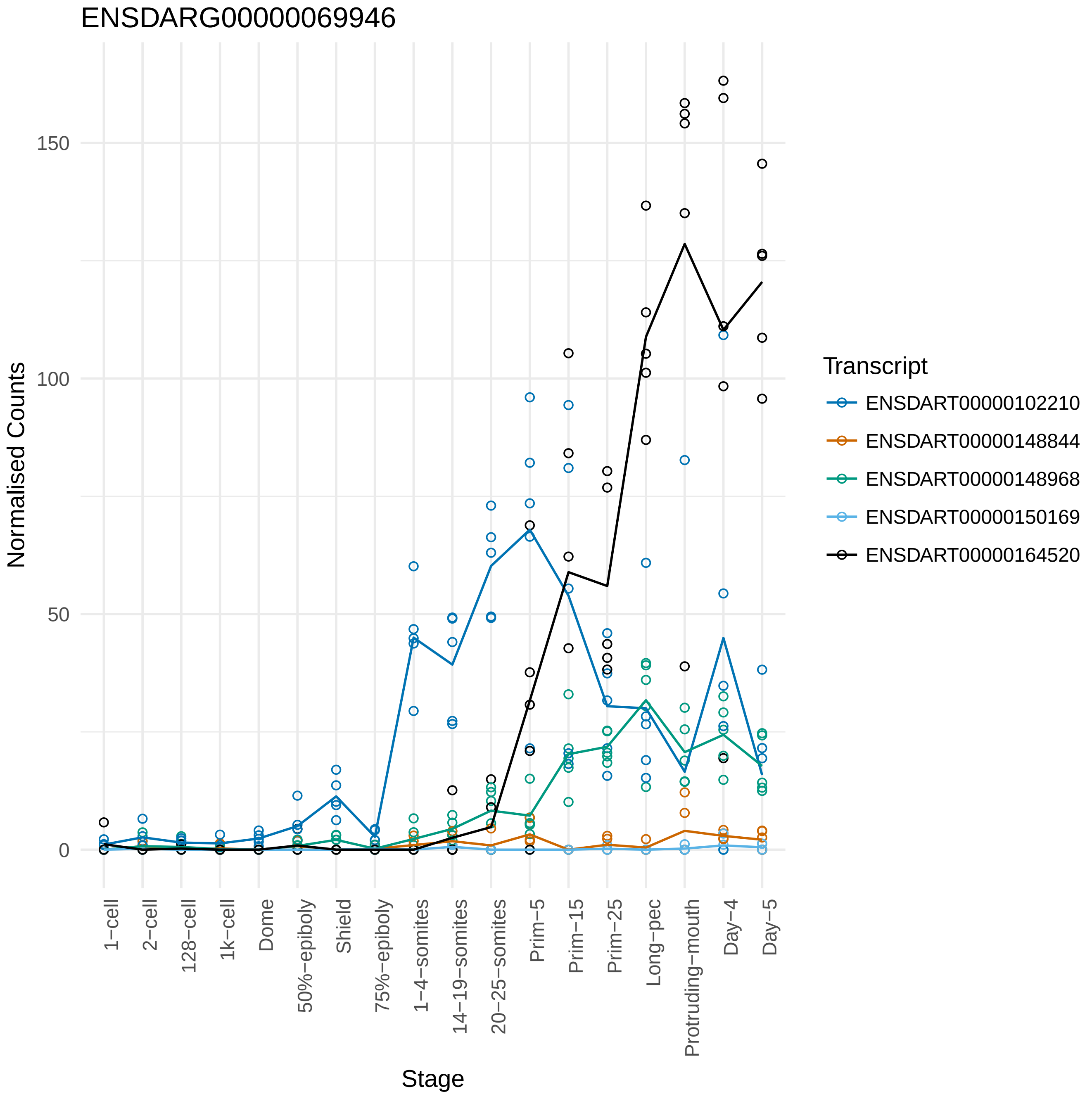

ENSDARG00000069946
Normalised Counts
150
100
50
0
1−cell
2−cell
128−cell
1k−cell
Dome
50%−epiboly
Shield
75%−epiboly
1−4−somites
14−19−somites
20−25−somites
Prim−5
Prim−15
Prim−25
Long−pec
Protruding−mouth
Day−4
Day−5
Stage
Transcript
ENSDART00000102210
ENSDART00000148844
ENSDART00000148968
ENSDART00000150169
ENSDART00000164520

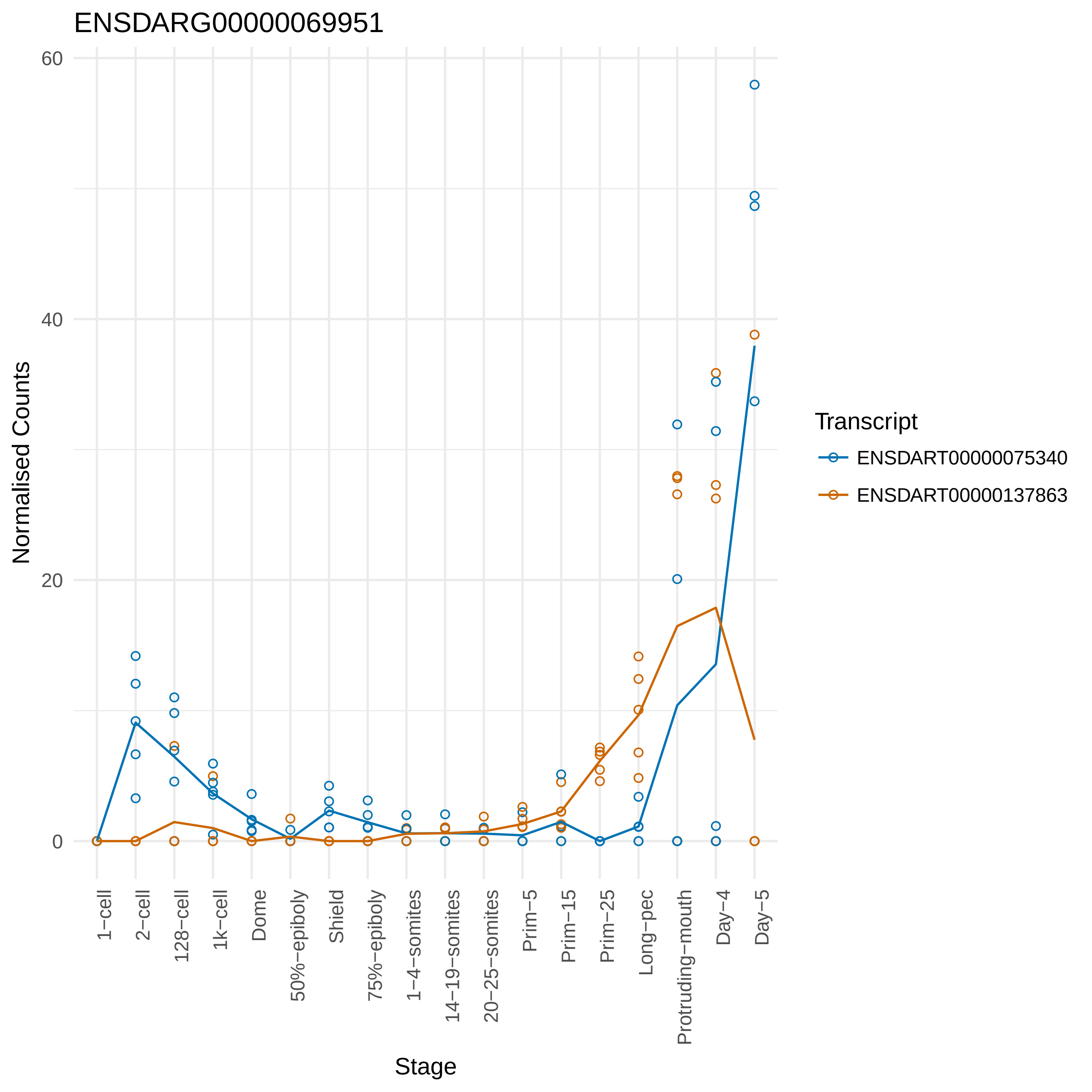
ENSDARG00000069951
Normalised Counts
60
40
20
0
Stage
1−cell
2−cell
128−cell
1k−cell
Dome
50%−epiboly
Shield
75%−epiboly
1−4−somites
14−19−somites
20−25−somites
Prim−5
Prim−15
Prim−25
Long−pec
Protruding−mouth
Day−4
Day−5
Transcript
ENSDART00000075340
ENSDART00000137863

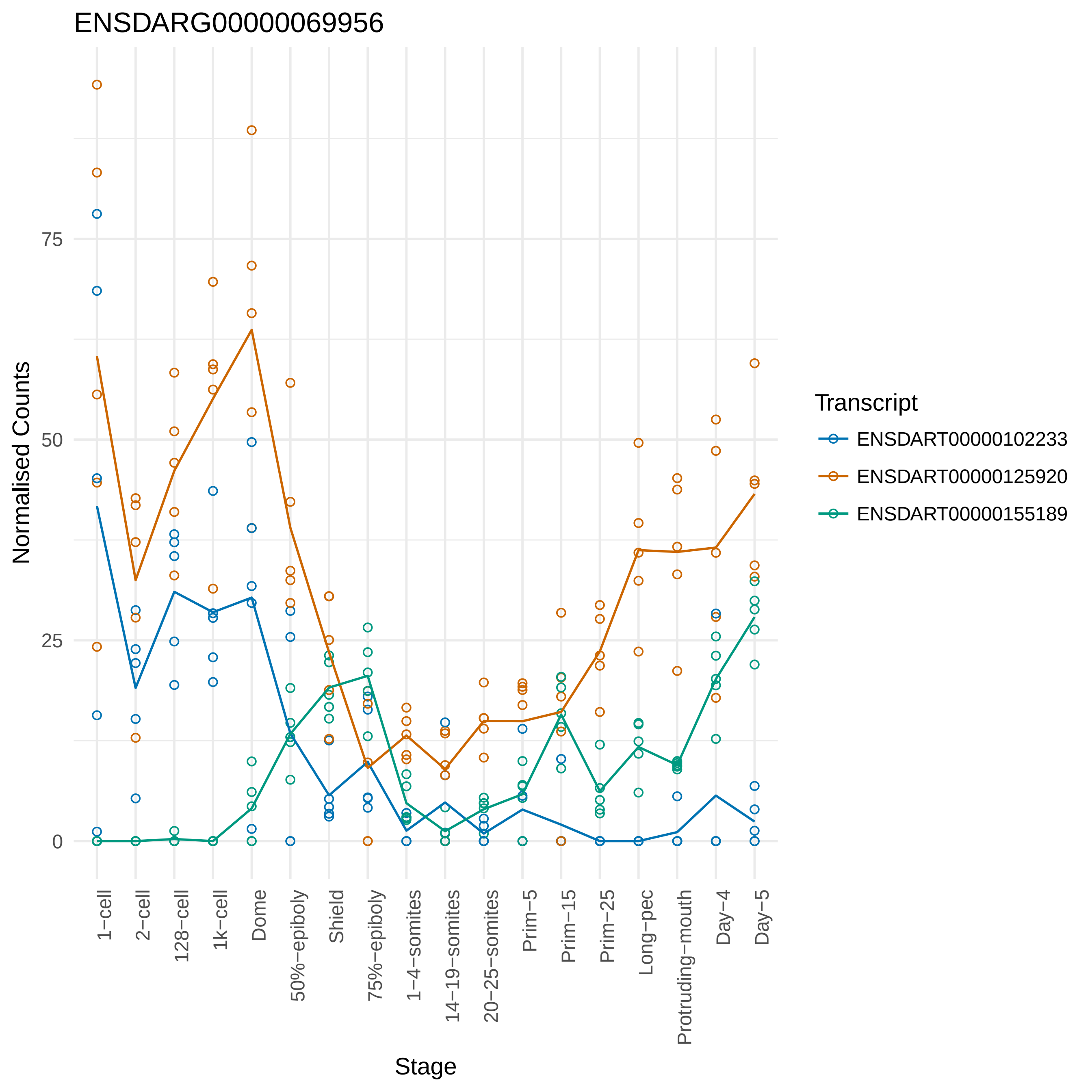

ENSDARG00000069956
Normalised Counts
0
25
50
75
Stage
1−cell
2−cell
128−cell
1k−cell
Dome
50%−epiboly
Shield
75%−epiboly
1−4−somites
14−19−somites
20−25−somites
Prim−5
Prim−15
Prim−25
Long−pec
Protruding−mouth
Day−4
Day−5
Transcript
ENSDART00000102233
ENSDART00000125920
ENSDART00000155189

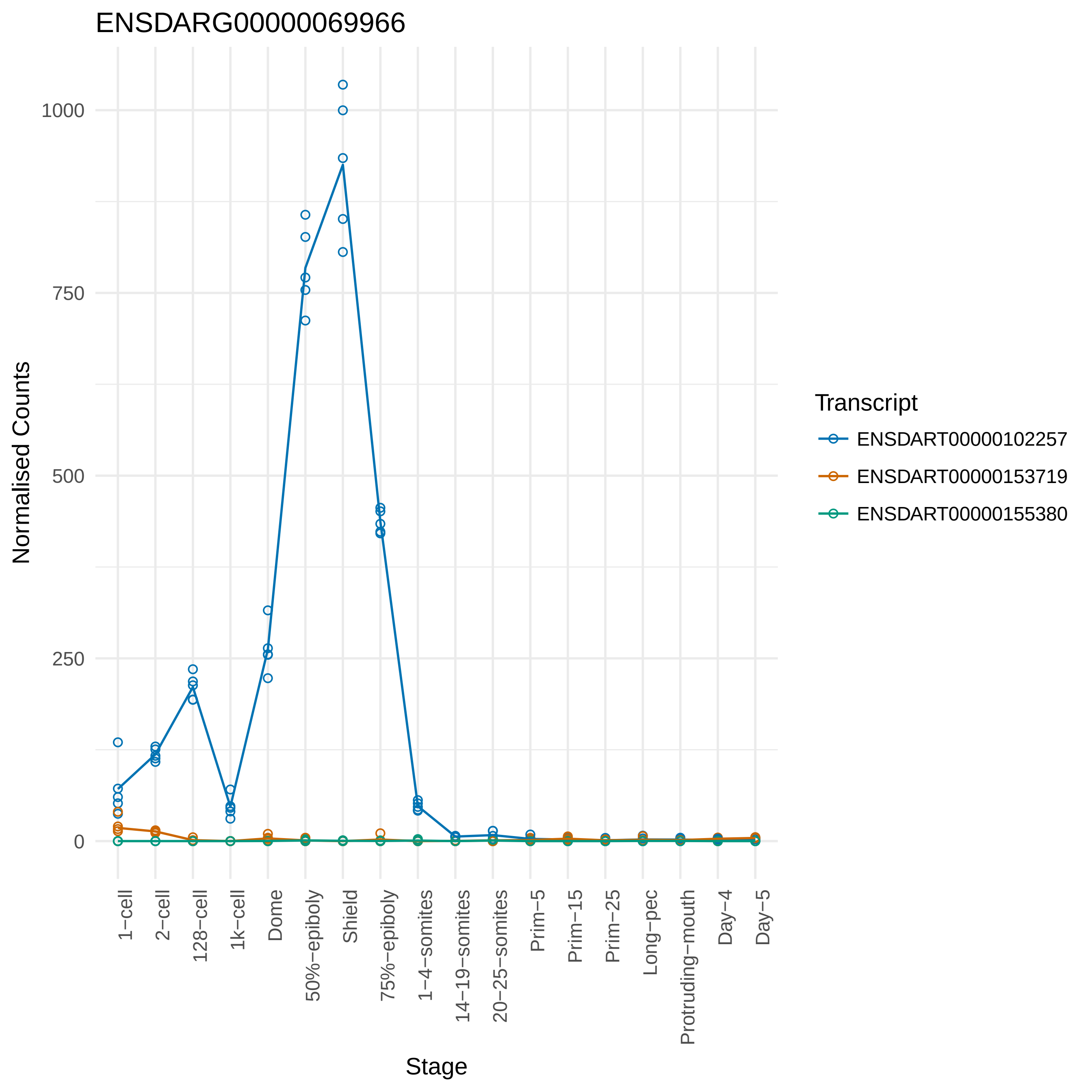
ENSDARG00000069966
Normalised Counts
1000
750
500
250
0
1−cell
2−cell
128−cell
1k−cell
Dome
50%−epiboly
Shield
75%−epiboly
1−4−somites
14−19−somites
20−25−somites
Prim−5
Prim−15
Prim−25
Long−pec
Protruding−mouth
Day−4
Day−5
Stage
Transcript
ENSDART00000102257
ENSDART00000153719
ENSDART00000155380

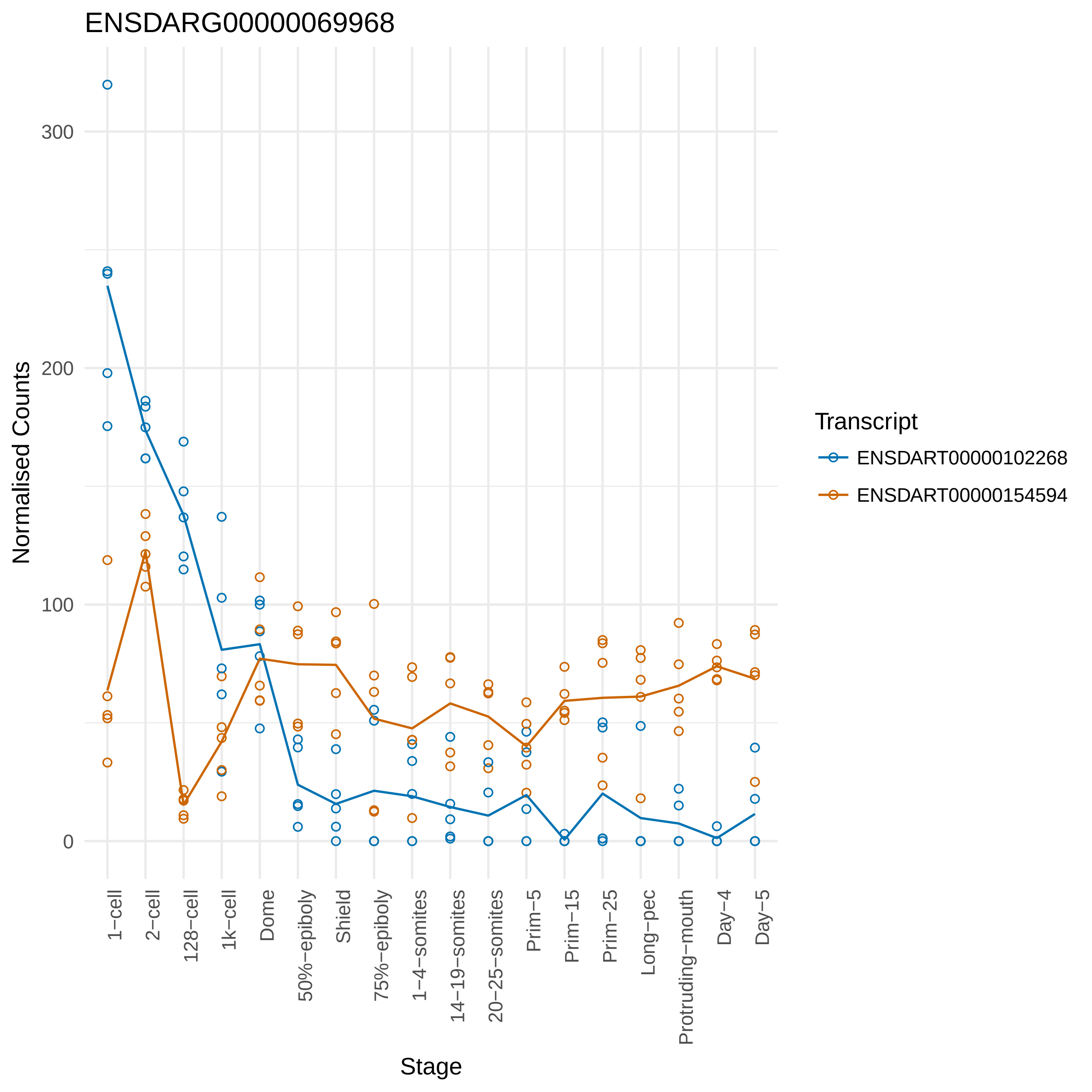

ENSDARG00000069968
Normalised Counts
300
200
100
0
1−cell
2−cell
128−cell
1k−cell
Dome
50%−epiboly
Shield
75%−epiboly
1−4−somites
14−19−somites
20−25−somites
Prim−5
Prim−15
Prim−25
Long−pec
Protruding−mouth
Day−4
Day−5
Stage
Transcript
ENSDART00000102268
ENSDART00000154594

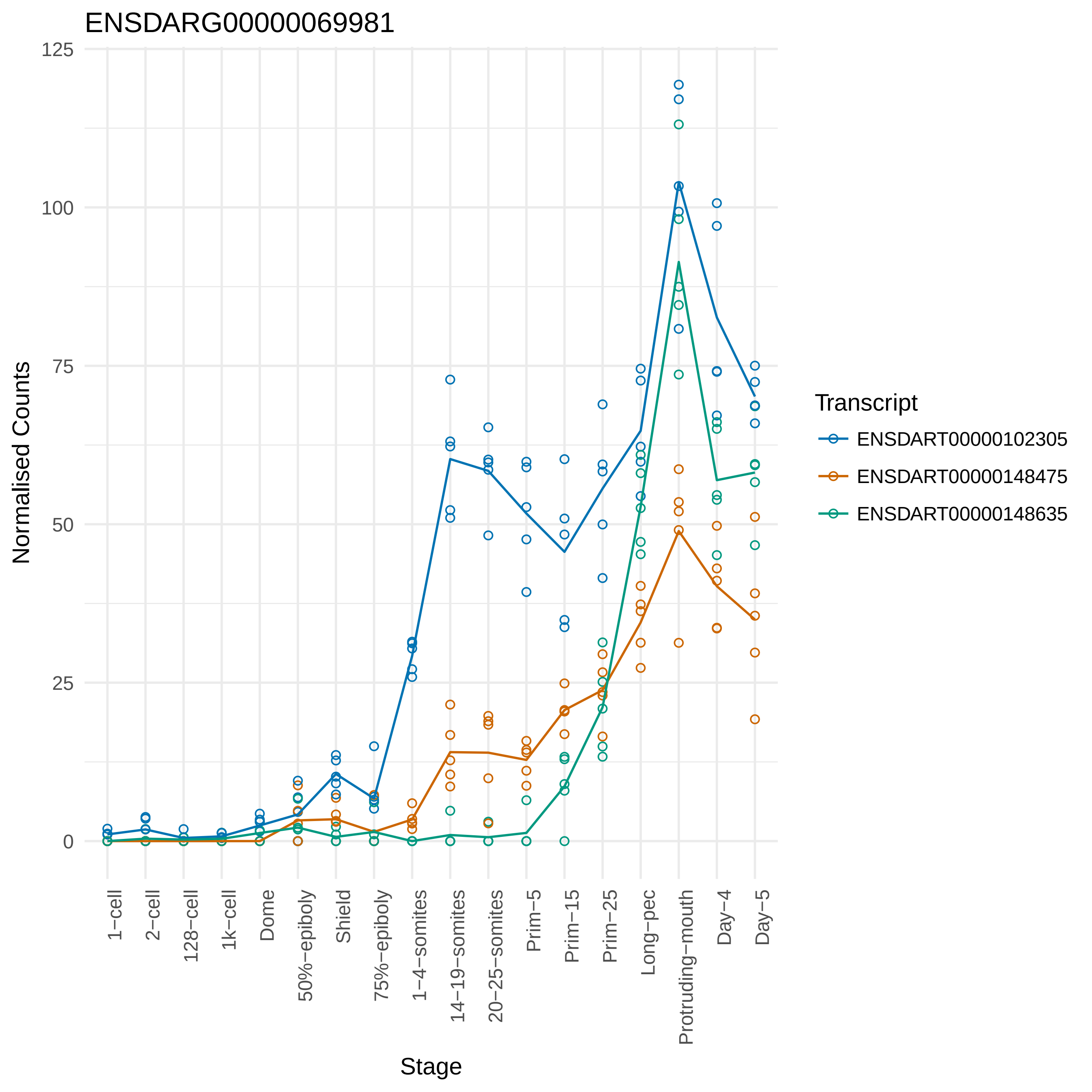

ENSDARG00000069981
Normalised Counts
125
100
75
50
25
0
1−cell
2−cell
128−cell
1k−cell
Dome
50%−epiboly
Shield
75%−epiboly
1−4−somites
14−19−somites
20−25−somites
Prim−5
Prim−15
Prim−25
Long−pec
Protruding−mouth
Day−4
Day−5
Stage
Transcript
ENSDART00000102305
ENSDART00000148475
ENSDART00000148635

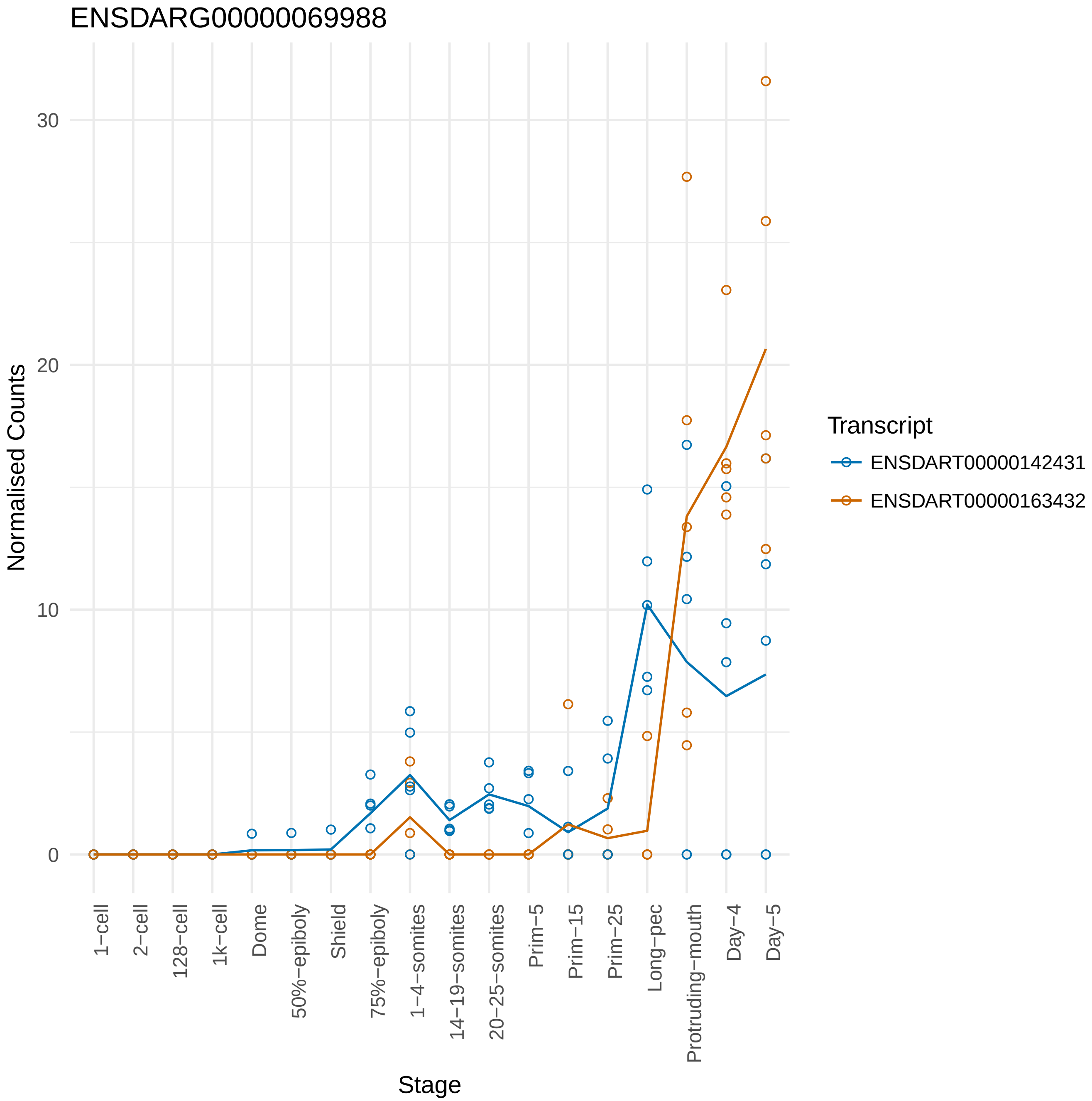

ENSDARG00000069988
Normalised Counts
30
20
10
0
1−cell
2−cell
128−cell
1k−cell
Dome
50%−epiboly
Shield
75%−epiboly
1−4−somites
14−19−somites
20−25−somites
Prim−5
Prim−15
Prim−25
Long−pec
Protruding−mouth
Day−4
Day−5
Stage
Transcript
ENSDART00000142431
ENSDART00000163432

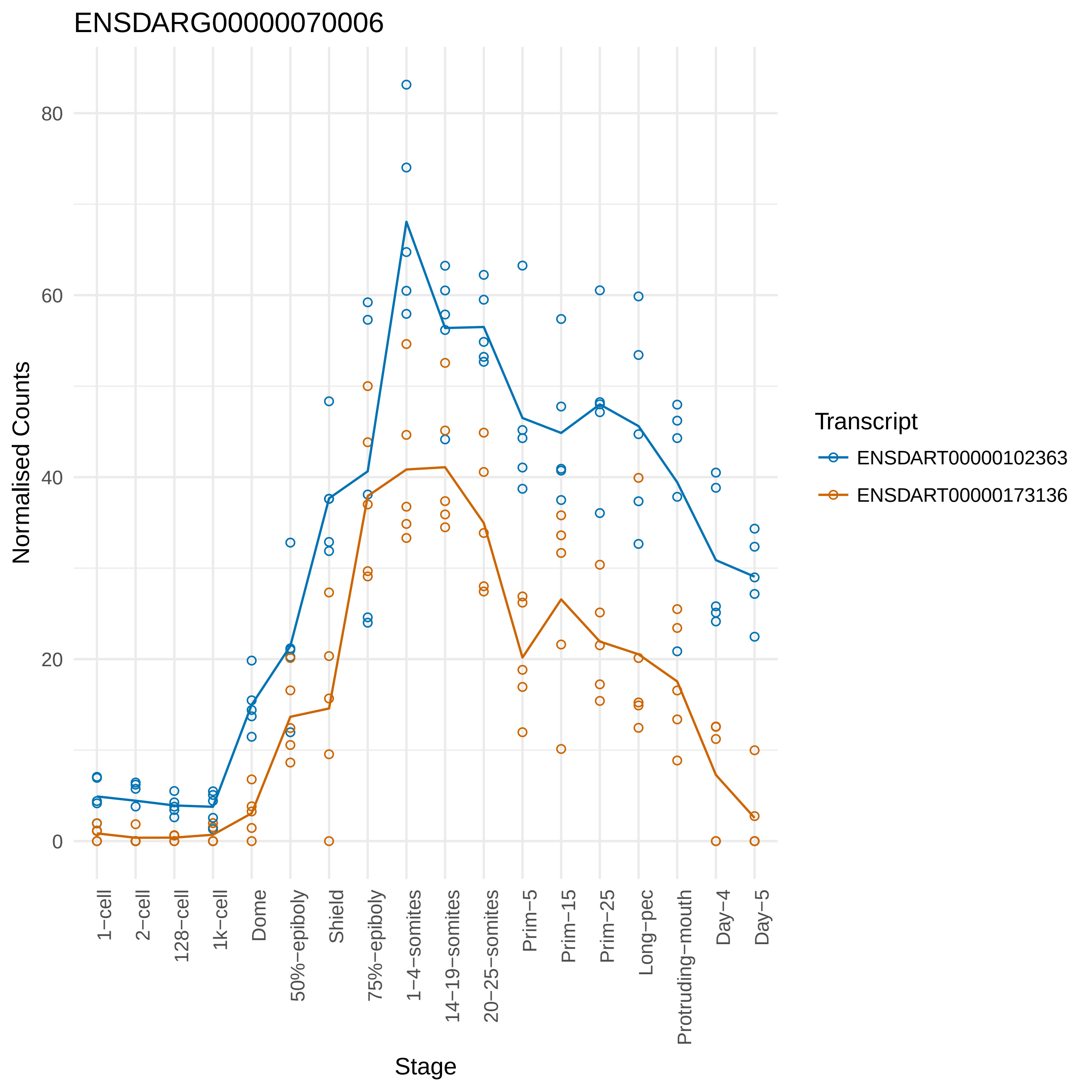

ENSDARG00000070006
Normalised Counts
0
20
40
60
80
Stage
1−cell
2−cell
128−cell
1k−cell
Dome
50%−epiboly
Shield
75%−epiboly
1−4−somites
14−19−somites
20−25−somites
Prim−5
Prim−15
Prim−25
Long−pec
Protruding−mouth
Day−4
Day−5
Transcript
ENSDART00000102363
ENSDART00000173136

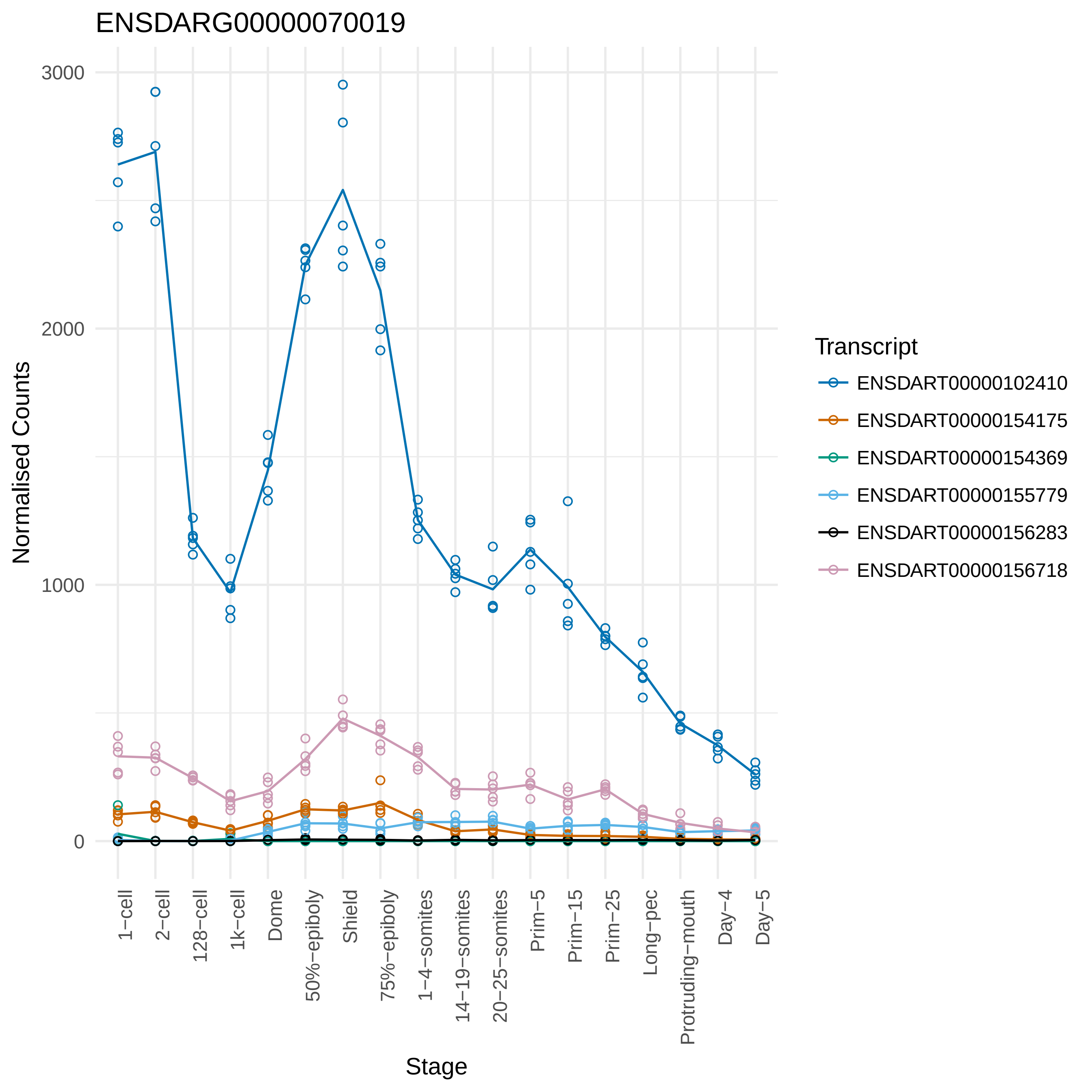

ENSDARG00000070019
Normalised Counts
3000
2000
1000
0
1−cell
2−cell
128−cell
1k−cell
Dome
50%−epiboly
Shield
75%−epiboly
1−4−somites
14−19−somites
20−25−somites
Prim−5
Prim−15
Prim−25
Long−pec
Protruding−mouth
Day−4
Day−5
Stage
Transcript
ENSDART00000102410
ENSDART00000154175
ENSDART00000154369
ENSDART00000155779
ENSDART00000156283
ENSDART00000156718

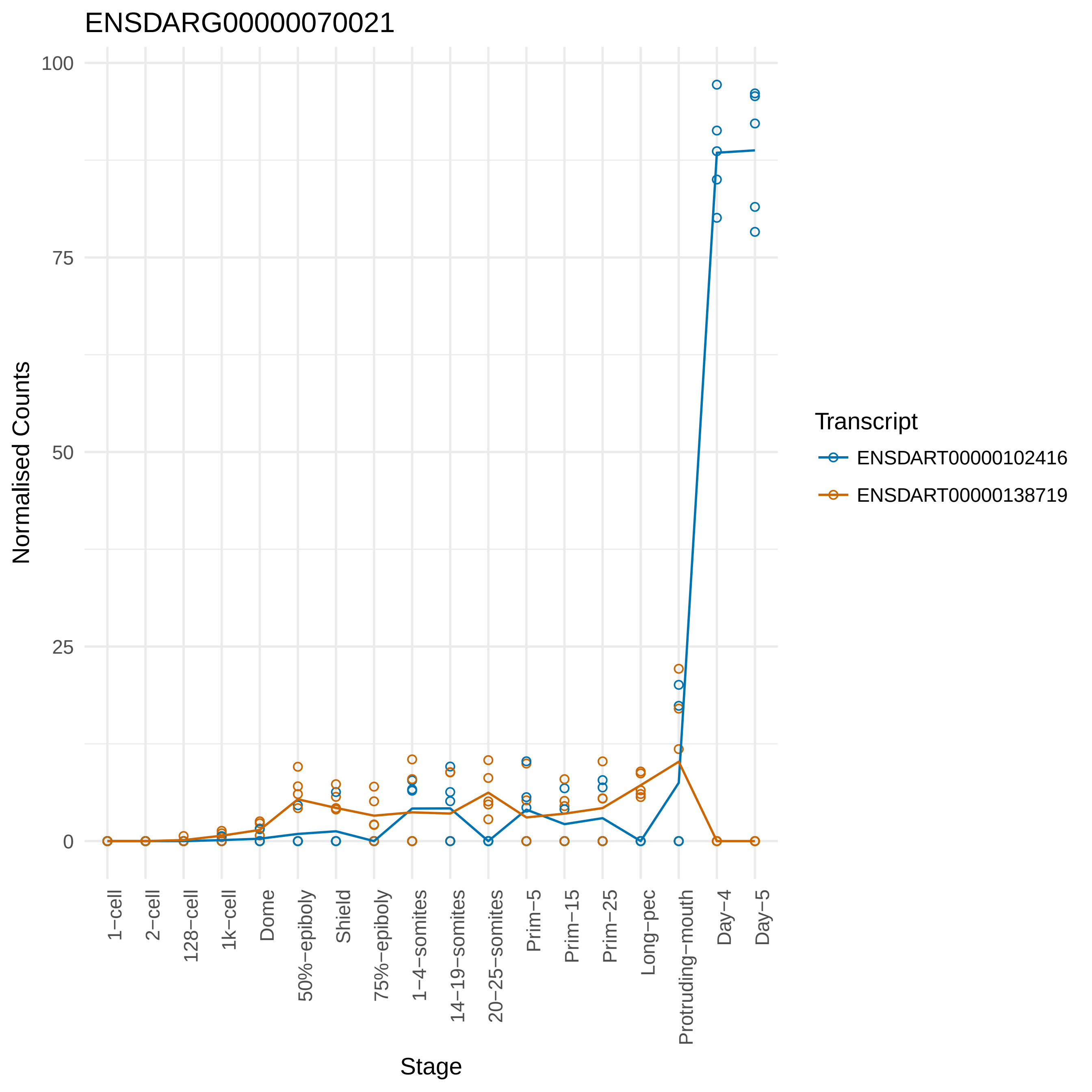

ENSDARG00000070021
Normalised Counts
100
75
50
25
0
1−cell
2−cell
128−cell
1k−cell
Dome
50%−epiboly
Shield
75%−epiboly
1−4−somites
14−19−somites
20−25−somites
Prim−5
Prim−15
Prim−25
Long−pec
Protruding−mouth
Day−4
Day−5
Stage
Transcript
ENSDART00000102416
ENSDART00000138719

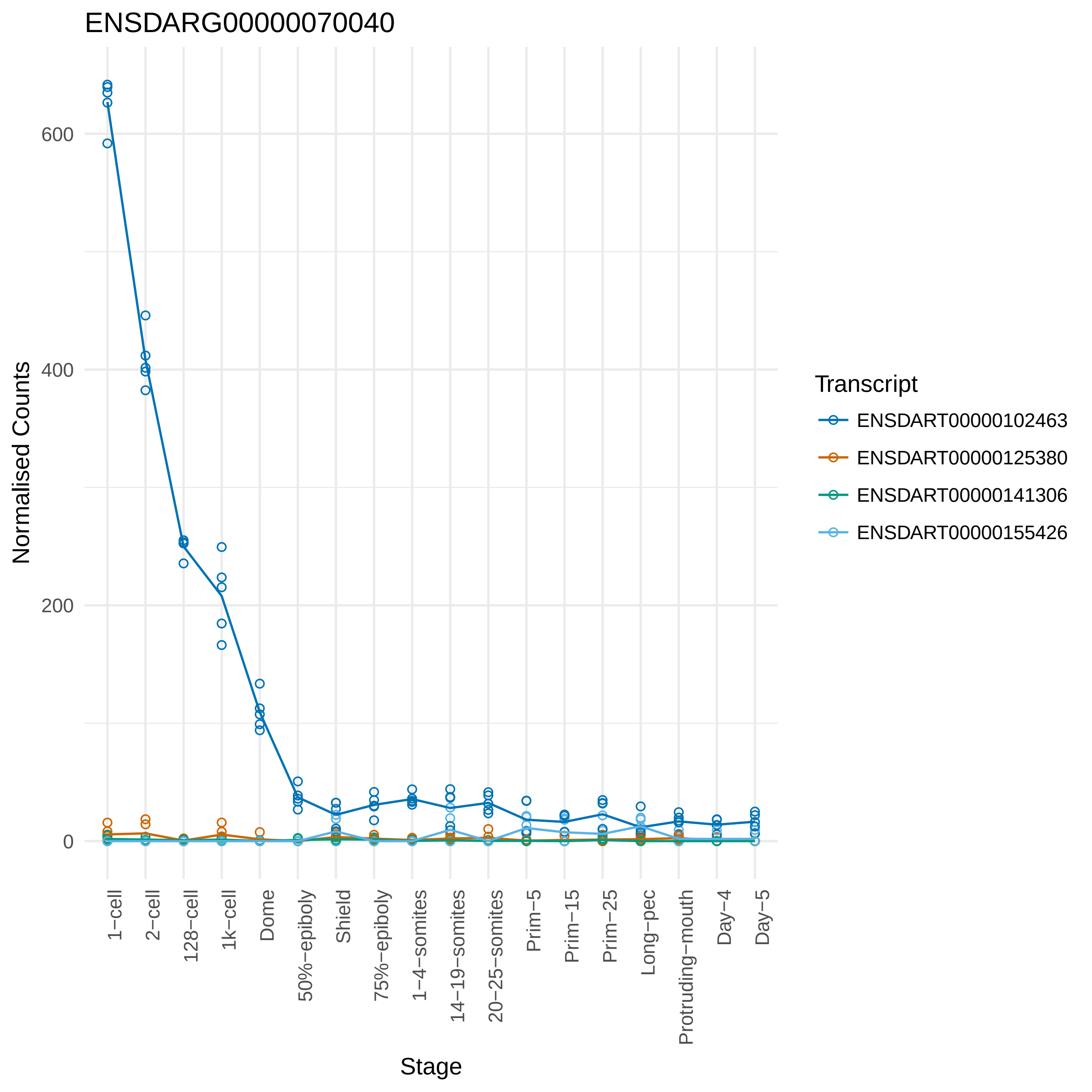

ENSDARG00000070040
Normalised Counts
600
400
200
0
1−cell
2−cell
128−cell
1k−cell
Dome
50%−epiboly
Shield
75%−epiboly
1−4−somites
14−19−somites
20−25−somites
Prim−5
Prim−15
Prim−25
Long−pec
Protruding−mouth
Day−4
Day−5
Stage
Transcript
ENSDART00000102463
ENSDART00000125380
ENSDART00000141306
ENSDART00000155426

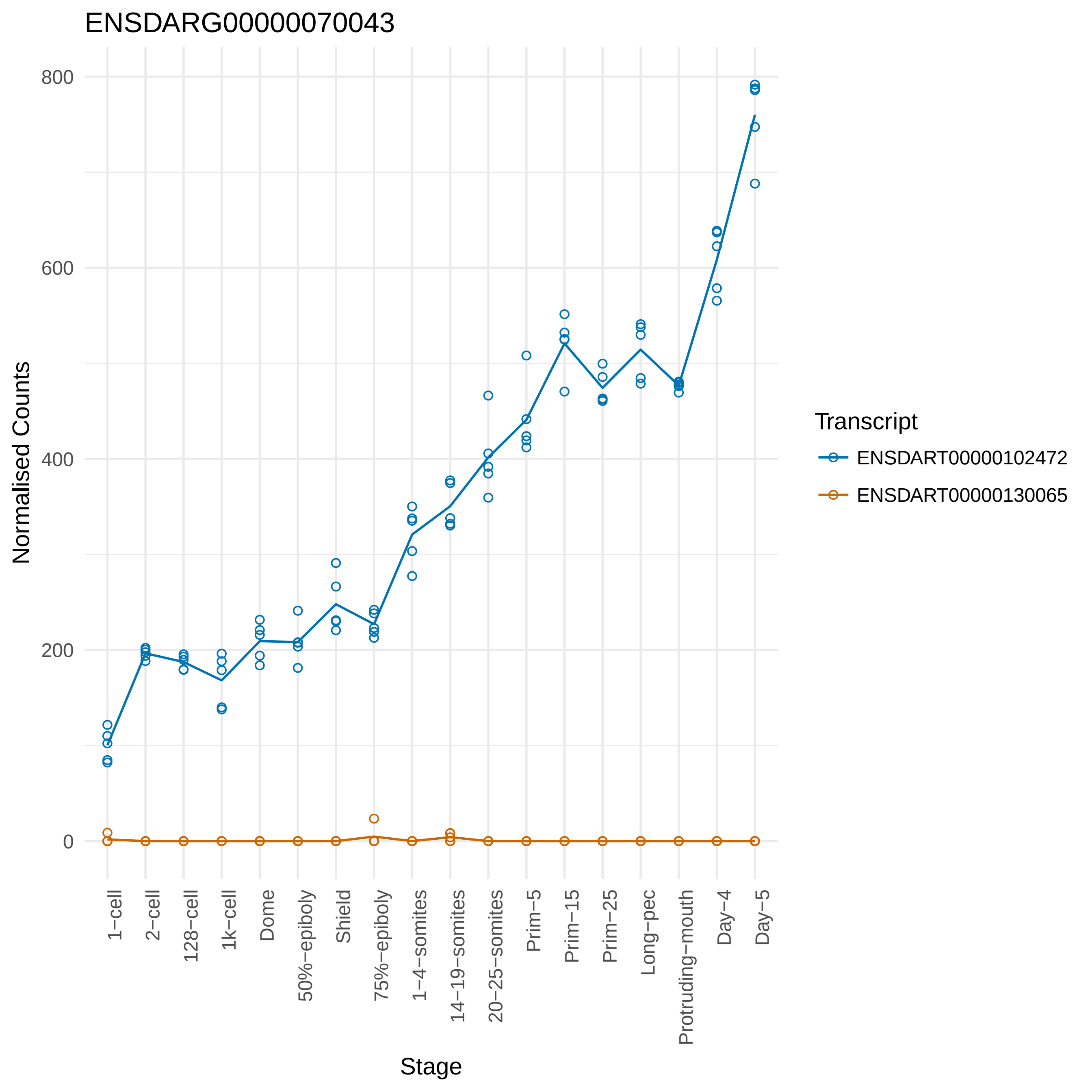

ENSDARG00000070043
Normalised Counts
800
600
400
200
0
1−cell
2−cell
128−cell
1k−cell
Dome
50%−epiboly
Shield
75%−epiboly
1−4−somites
14−19−somites
20−25−somites
Prim−5
Prim−15
Prim−25
Long−pec
Protruding−mouth
Day−4
Day−5
Stage
Transcript
ENSDART00000102472
ENSDART00000130065

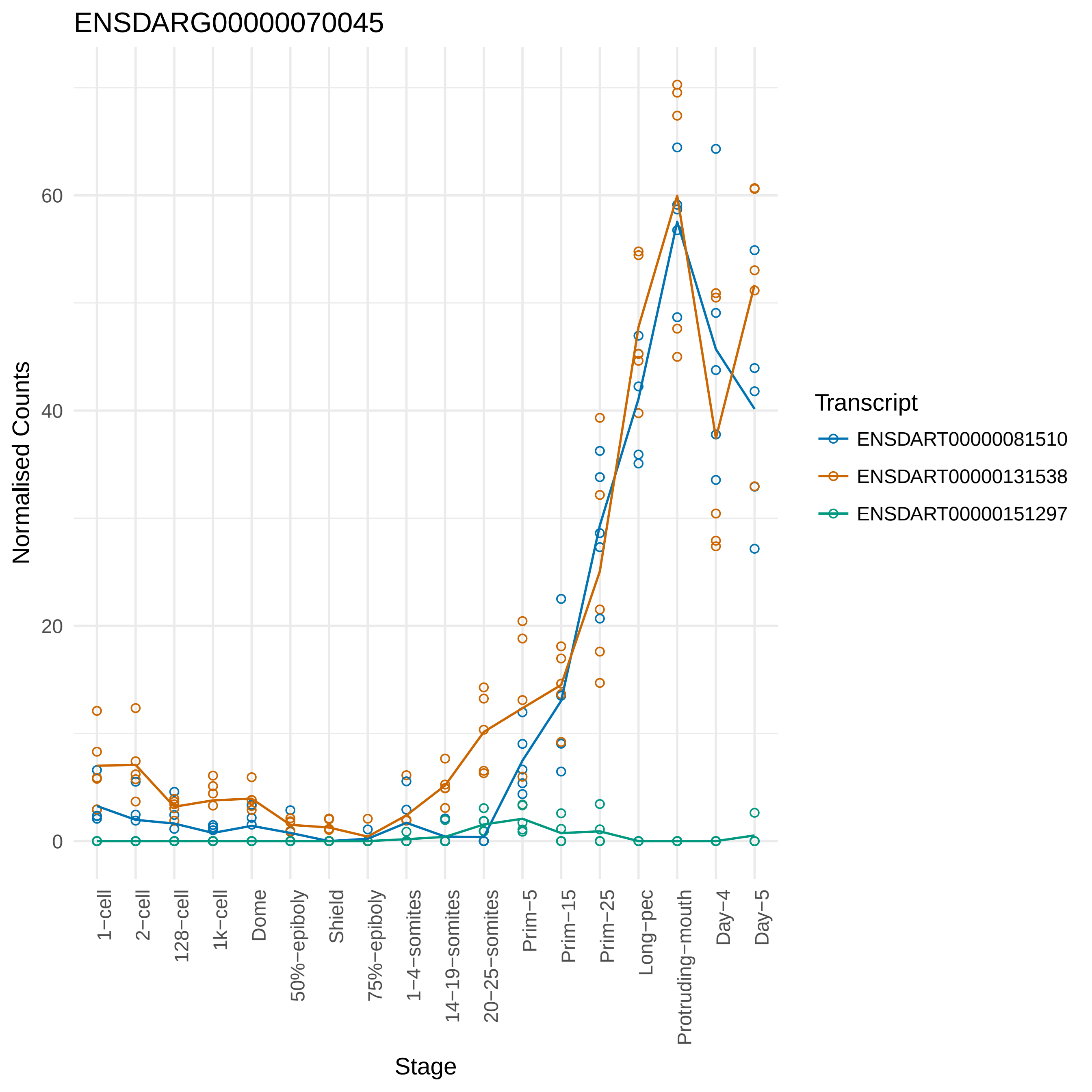

ENSDARG00000070045
Normalised Counts
0
20
40
60
Stage
1−cell
2−cell
128−cell
1k−cell
Dome
50%−epiboly
Shield
75%−epiboly
1−4−somites
14−19−somites
20−25−somites
Prim−5
Prim−15
Prim−25
Long−pec
Protruding−mouth
Day−4
Day−5
Transcript
ENSDART00000081510
ENSDART00000131538
ENSDART00000151297

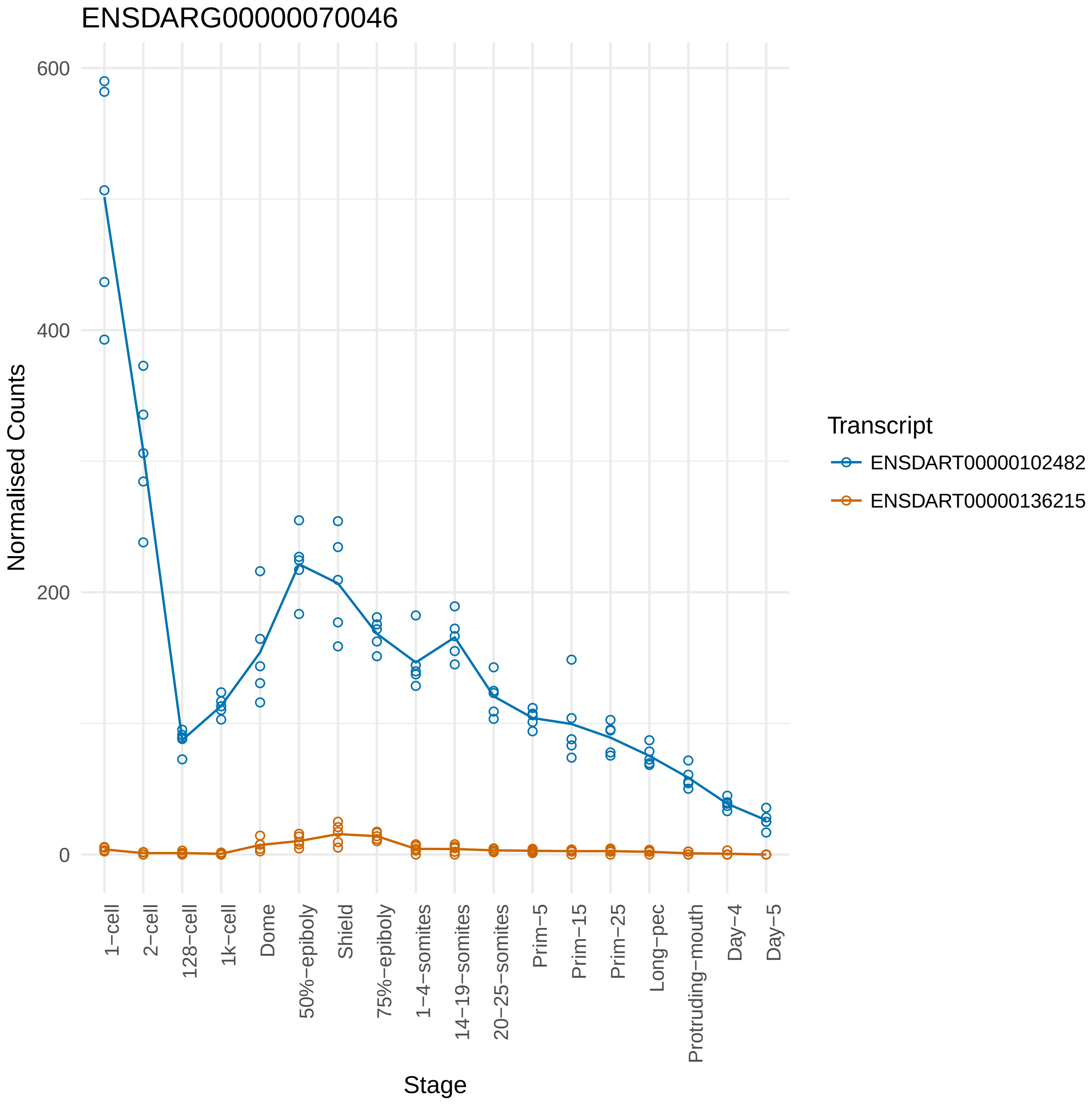
ENSDARG00000070046
Normalised Counts
600
400
200
0
1−cell
2−cell
128−cell
1k−cell
Dome
50%−epiboly
Shield
75%−epiboly
1−4−somites
14−19−somites
20−25−somites
Prim−5
Prim−15
Prim−25
Long−pec
Protruding−mouth
Day−4
Day−5
Stage
Transcript
ENSDART00000102482
ENSDART00000136215

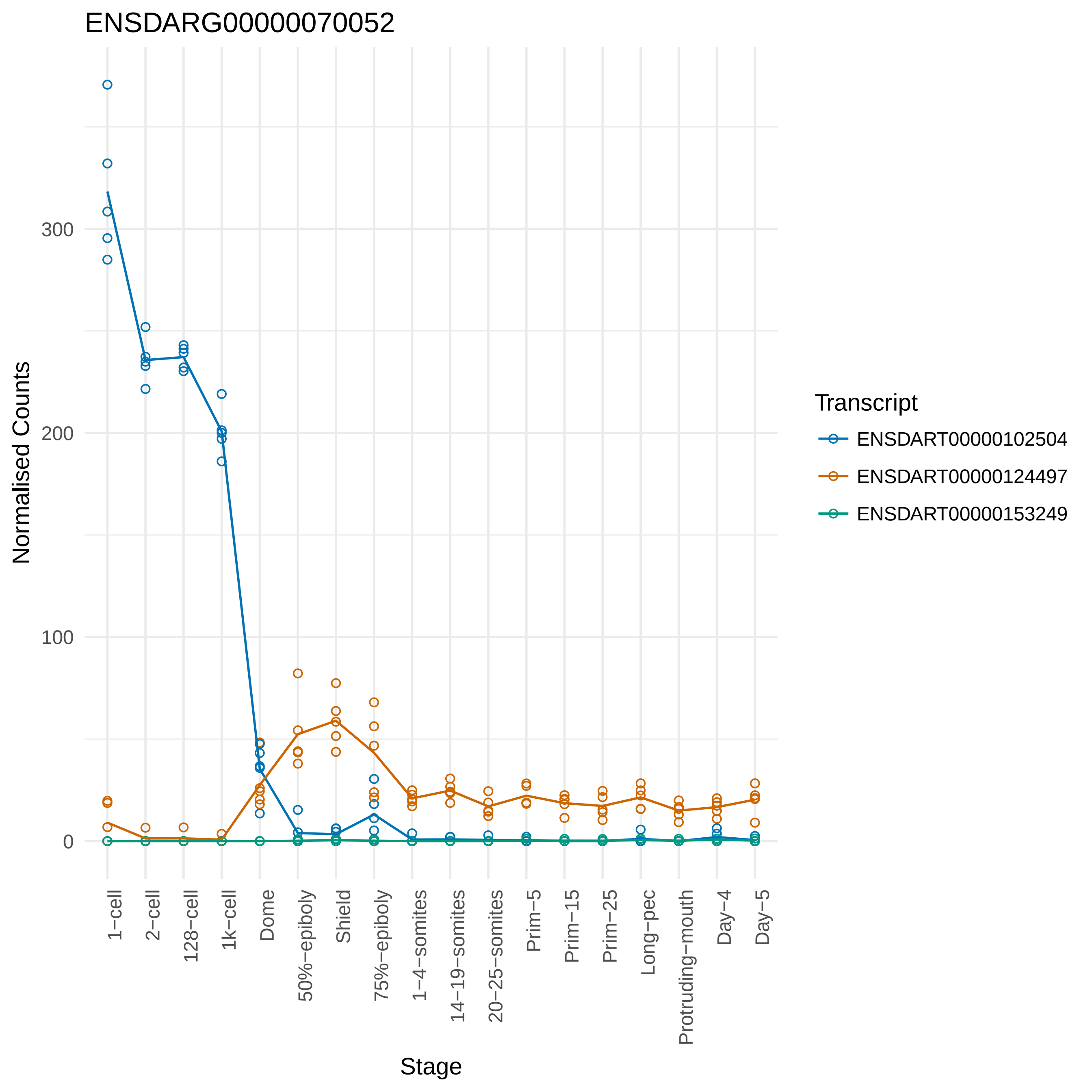

ENSDARG00000070052
Normalised Counts
300
200
100
0
1−cell
2−cell
128−cell
1k−cell
Dome
50%−epiboly
Shield
75%−epiboly
1−4−somites
14−19−somites
20−25−somites
Prim−5
Prim−15
Prim−25
Long−pec
Protruding−mouth
Day−4
Day−5
Stage
Transcript
ENSDART00000102504
ENSDART00000124497
ENSDART00000153249

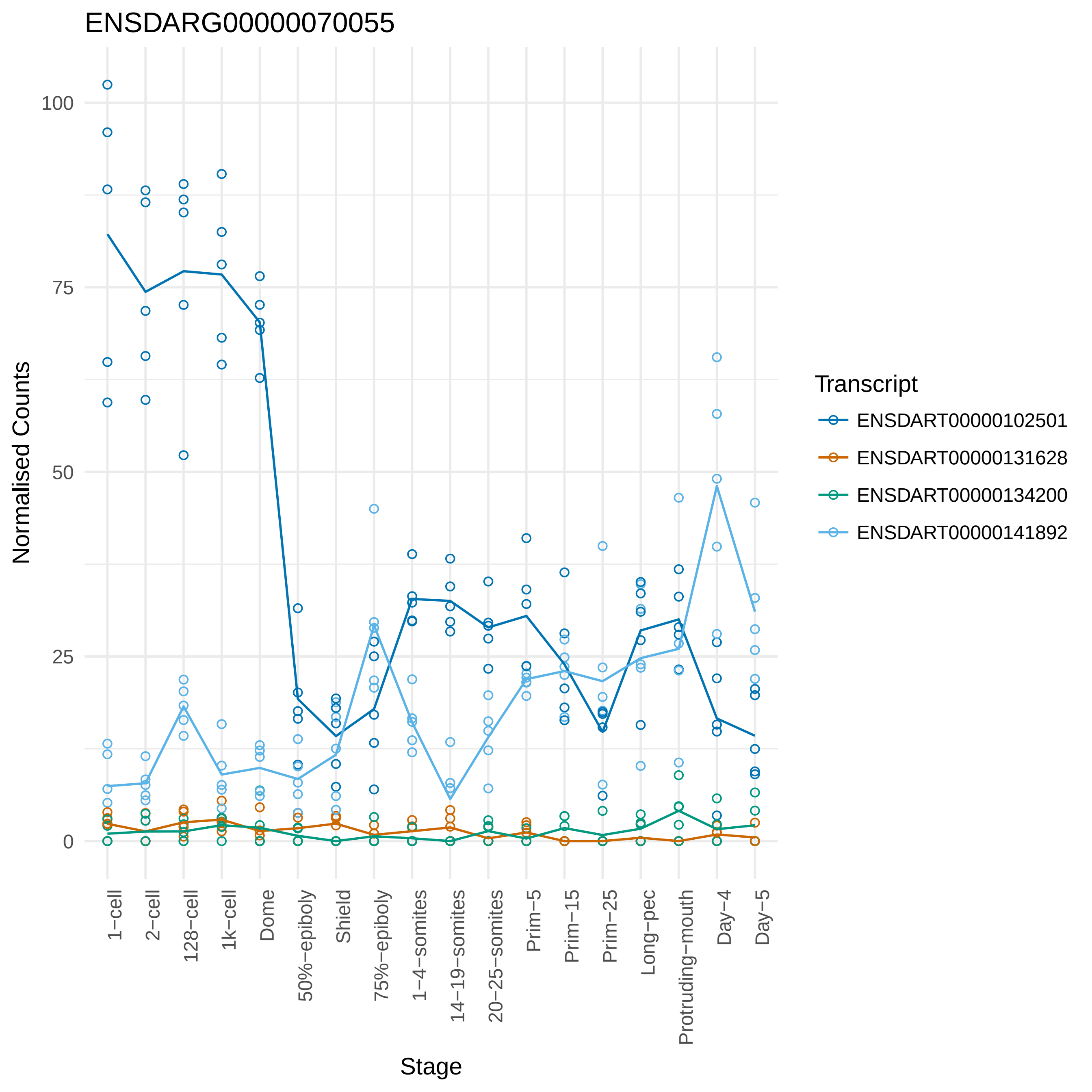

ENSDARG00000070055
Normalised Counts
100
75
50
25
0
1−cell
2−cell
128−cell
1k−cell
Dome
50%−epiboly
Shield
75%−epiboly
1−4−somites
14−19−somites
20−25−somites
Prim−5
Prim−15
Prim−25
Long−pec
Protruding−mouth
Day−4
Day−5
Stage
Transcript
ENSDART00000102501
ENSDART00000131628
ENSDART00000134200
ENSDART00000141892

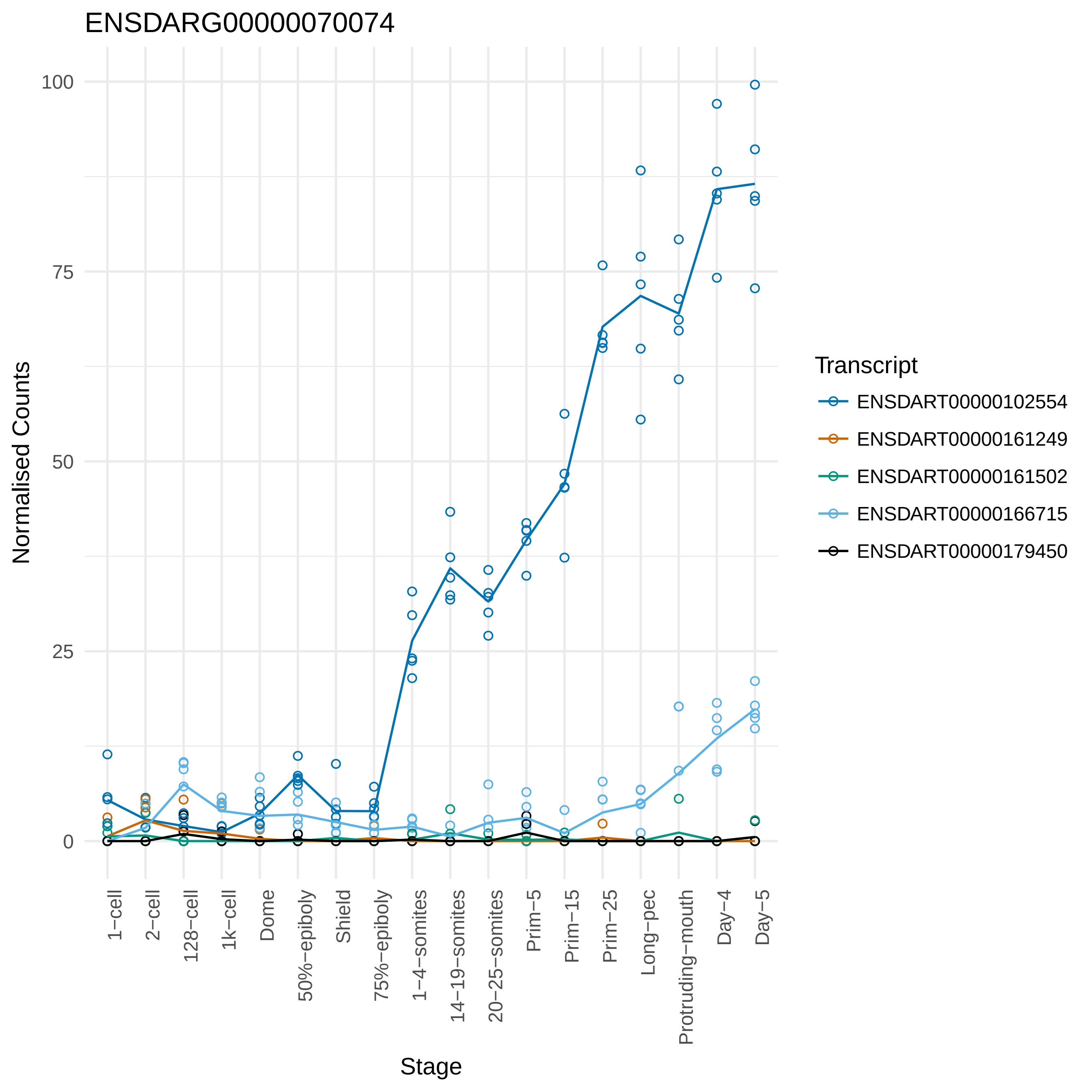

ENSDARG00000070074
Normalised Counts
0
25
50
75
100
1−cell
2−cell
128−cell
1k−cell
Dome
50%−epiboly
Shield
75%−epiboly
1−4−somites
14−19−somites
20−25−somites
Prim−5
Prim−15
Prim−25
Long−pec
Protruding−mouth
Day−4
Day−5
Stage
Transcript
ENSDART00000102554
ENSDART00000161249
ENSDART00000161502
ENSDART00000166715
ENSDART00000179450

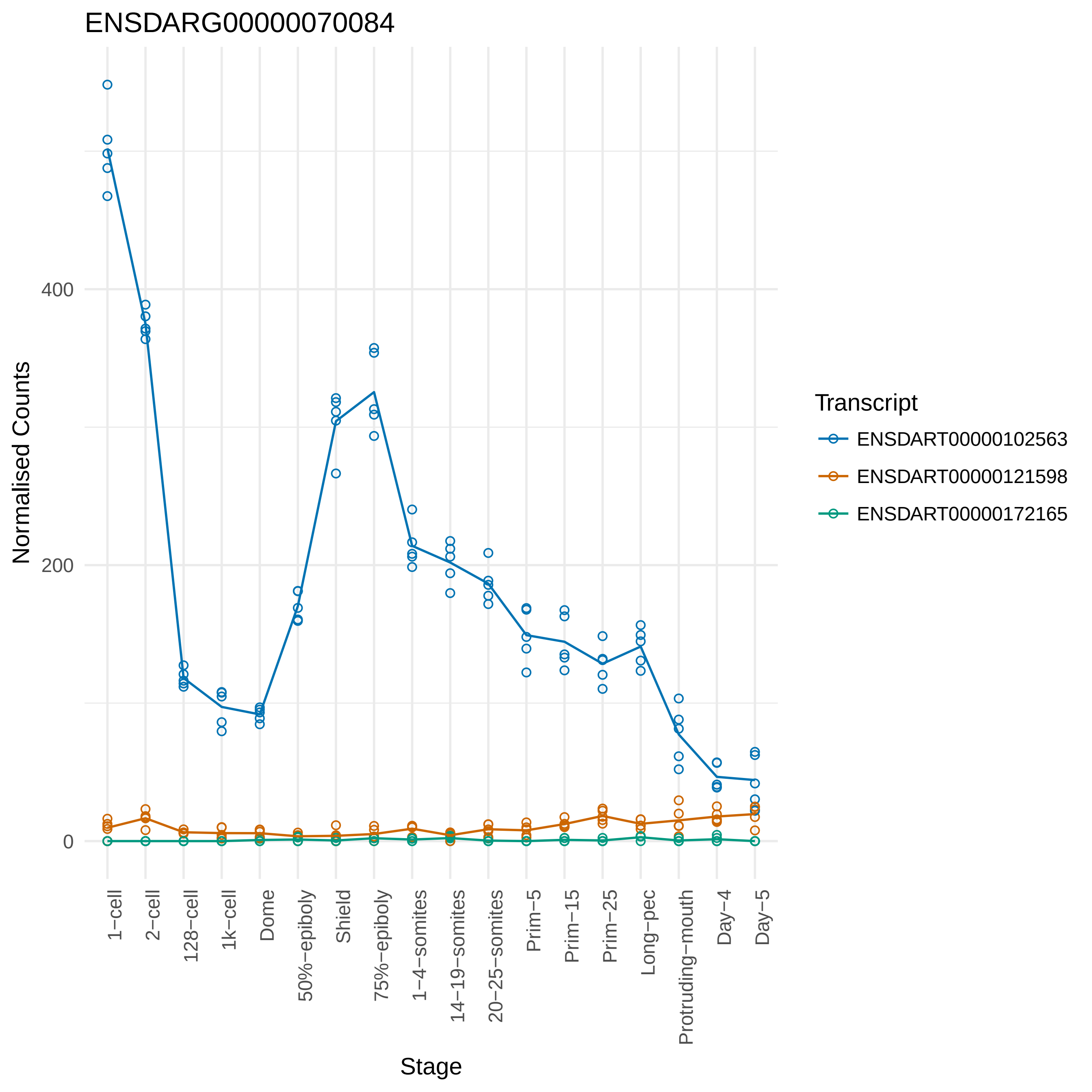
ENSDARG00000070084
Normalised Counts
400
200
0
1−cell
2−cell
128−cell
1k−cell
Dome
50%−epiboly
Shield
75%−epiboly
1−4−somites
14−19−somites
20−25−somites
Prim−5
Prim−15
Prim−25
Long−pec
Protruding−mouth
Day−4
Day−5
Stage
Transcript
ENSDART00000102563
ENSDART00000121598
ENSDART00000172165

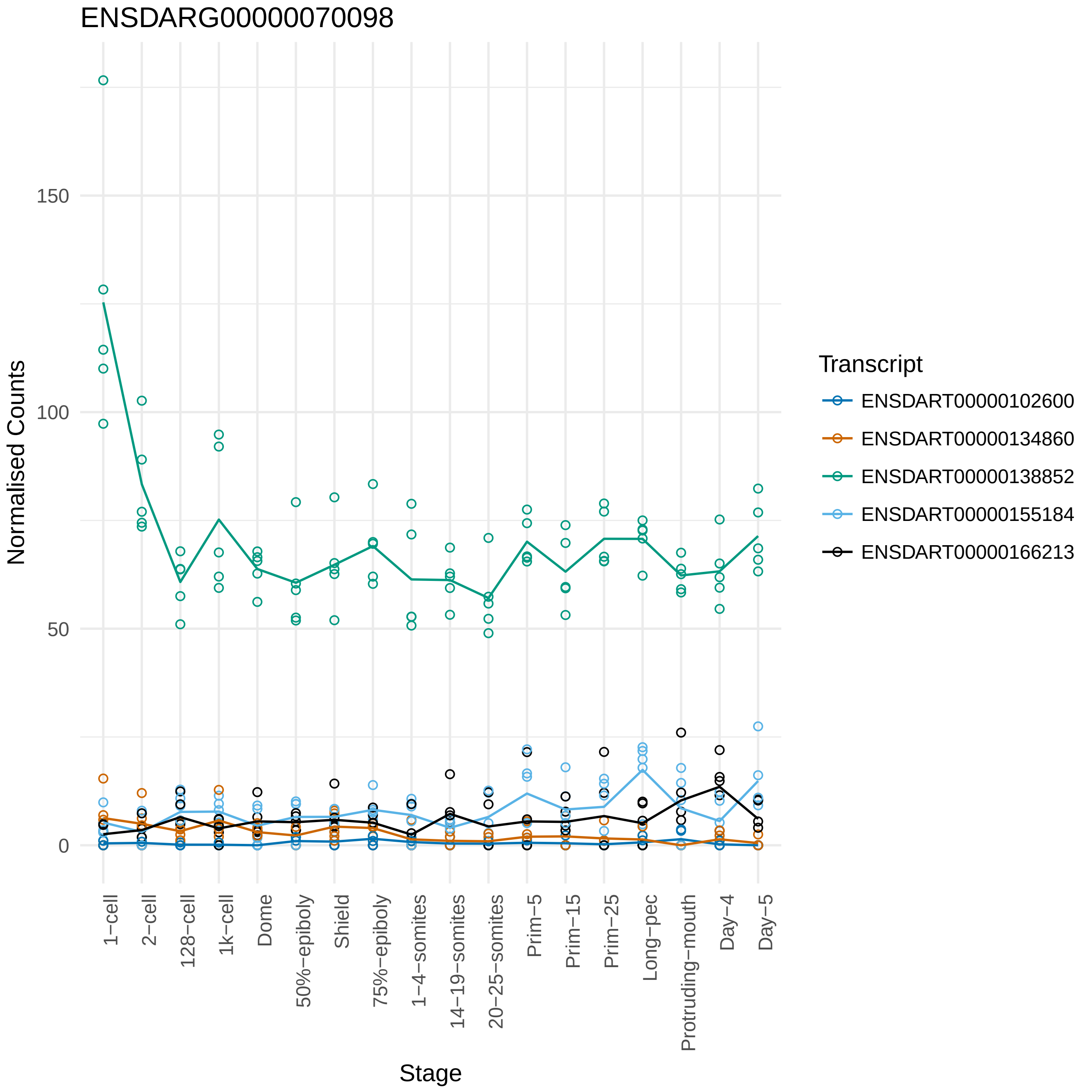

ENSDARG00000070098
Normalised Counts
150
100
50
0
1−cell
2−cell
128−cell
1k−cell
Dome
50%−epiboly
Shield
75%−epiboly
1−4−somites
14−19−somites
20−25−somites
Prim−5
Prim−15
Prim−25
Long−pec
Protruding−mouth
Day−4
Day−5
Stage
Transcript
ENSDART00000102600
ENSDART00000134860
ENSDART00000138852
ENSDART00000155184
ENSDART00000166213

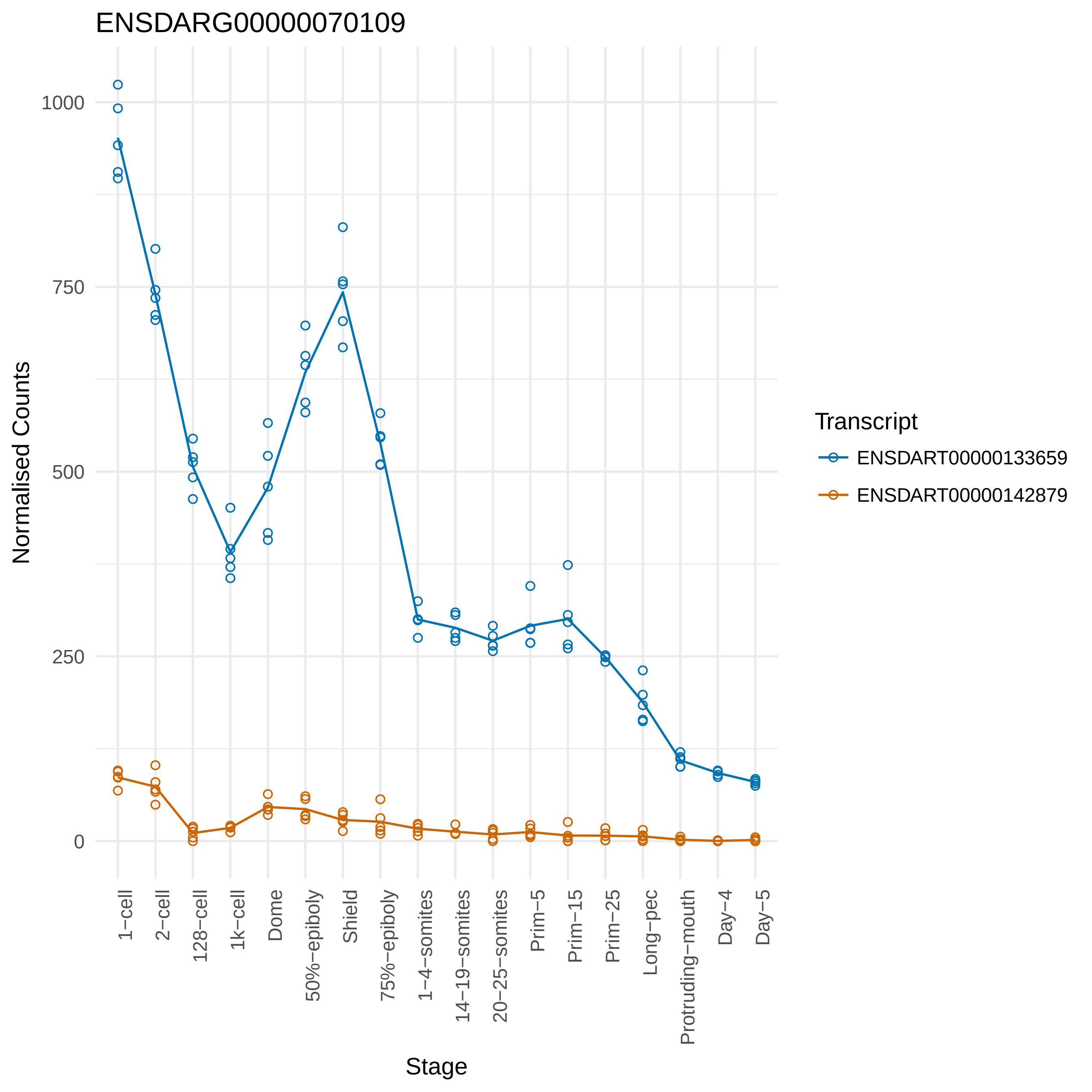
ENSDARG00000070109
Normalised Counts
1000
750
500
250
0
1−cell
2−cell
128−cell
1k−cell
Dome
50%−epiboly
Shield
75%−epiboly
1−4−somites
14−19−somites
20−25−somites
Prim−5
Prim−15
Prim−25
Long−pec
Protruding−mouth
Day−4
Day−5
Stage
Transcript
ENSDART00000133659
ENSDART00000142879

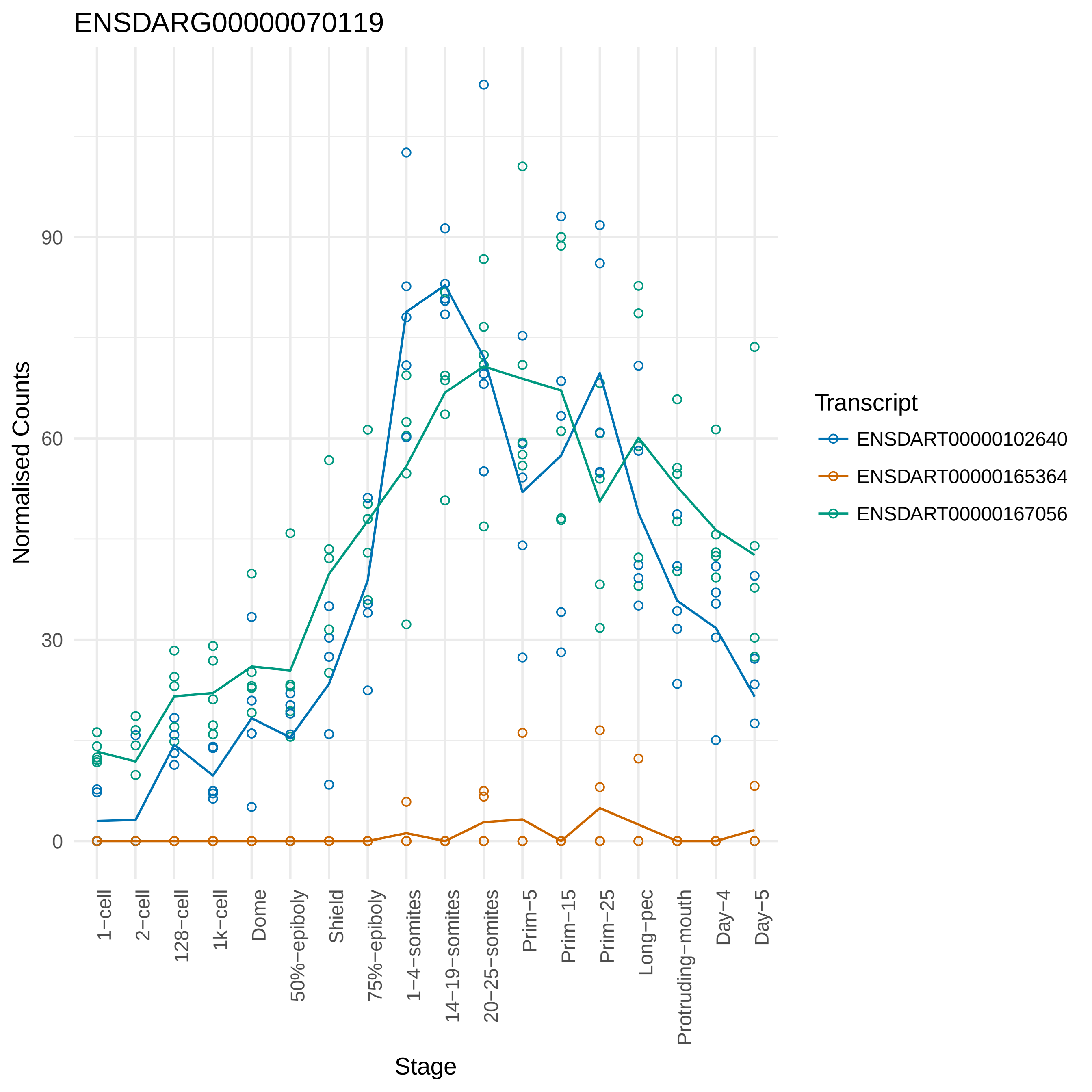
ENSDARG00000070119
Normalised Counts
0
30
60
90
Stage
1−cell
2−cell
128−cell
1k−cell
Dome
50%−epiboly
Shield
75%−epiboly
1−4−somites
14−19−somites
20−25−somites
Prim−5
Prim−15
Prim−25
Long−pec
Protruding−mouth
Day−4
Day−5
Transcript
ENSDART00000102640
ENSDART00000165364
ENSDART00000167056

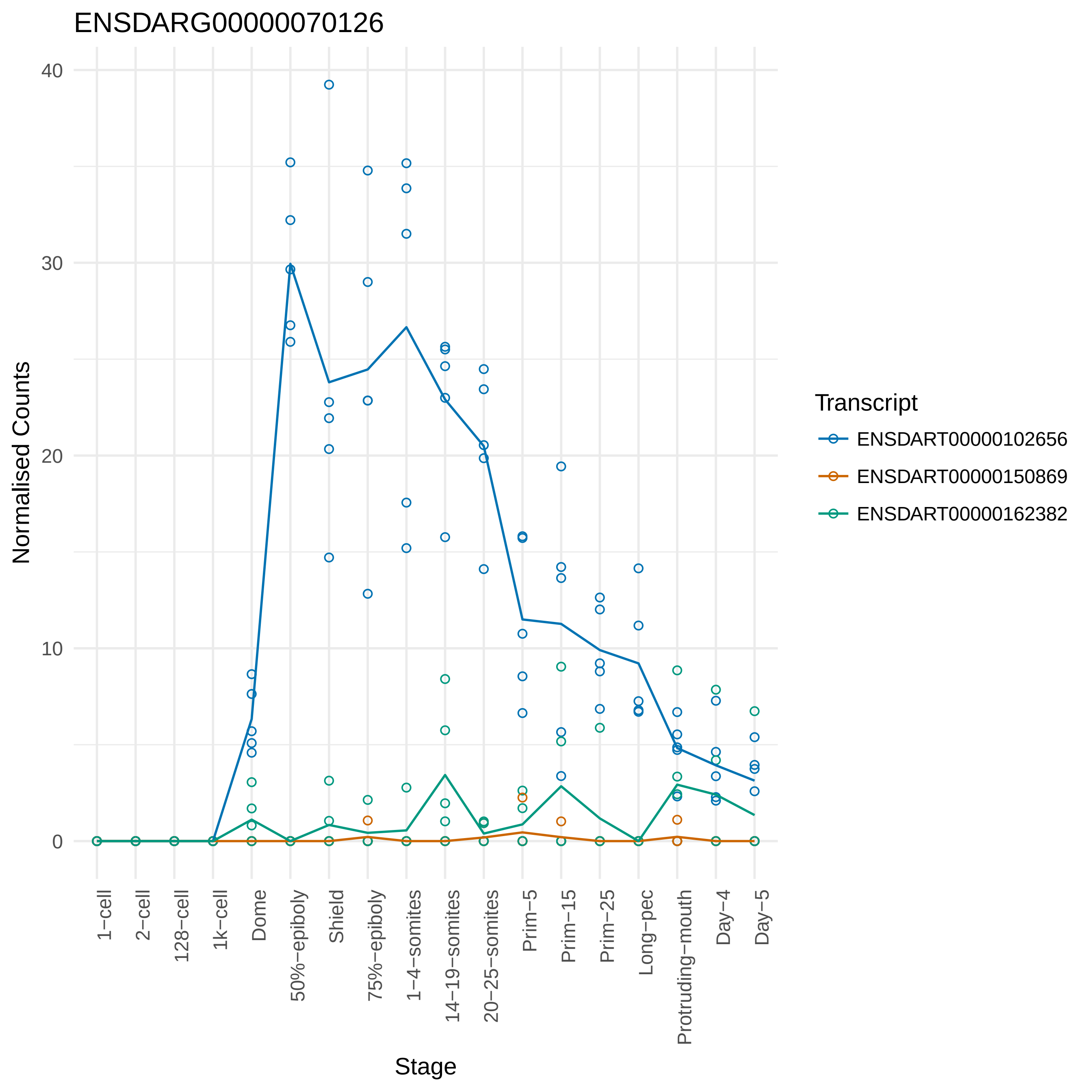

ENSDARG00000070126
Normalised Counts
40
30
20
10
0
1−cell
2−cell
128−cell
1k−cell
Dome
50%−epiboly
Shield
75%−epiboly
1−4−somites
14−19−somites
20−25−somites
Prim−5
Prim−15
Prim−25
Long−pec
Protruding−mouth
Day−4
Day−5
Stage
Transcript
ENSDART00000102656
ENSDART00000150869
ENSDART00000162382

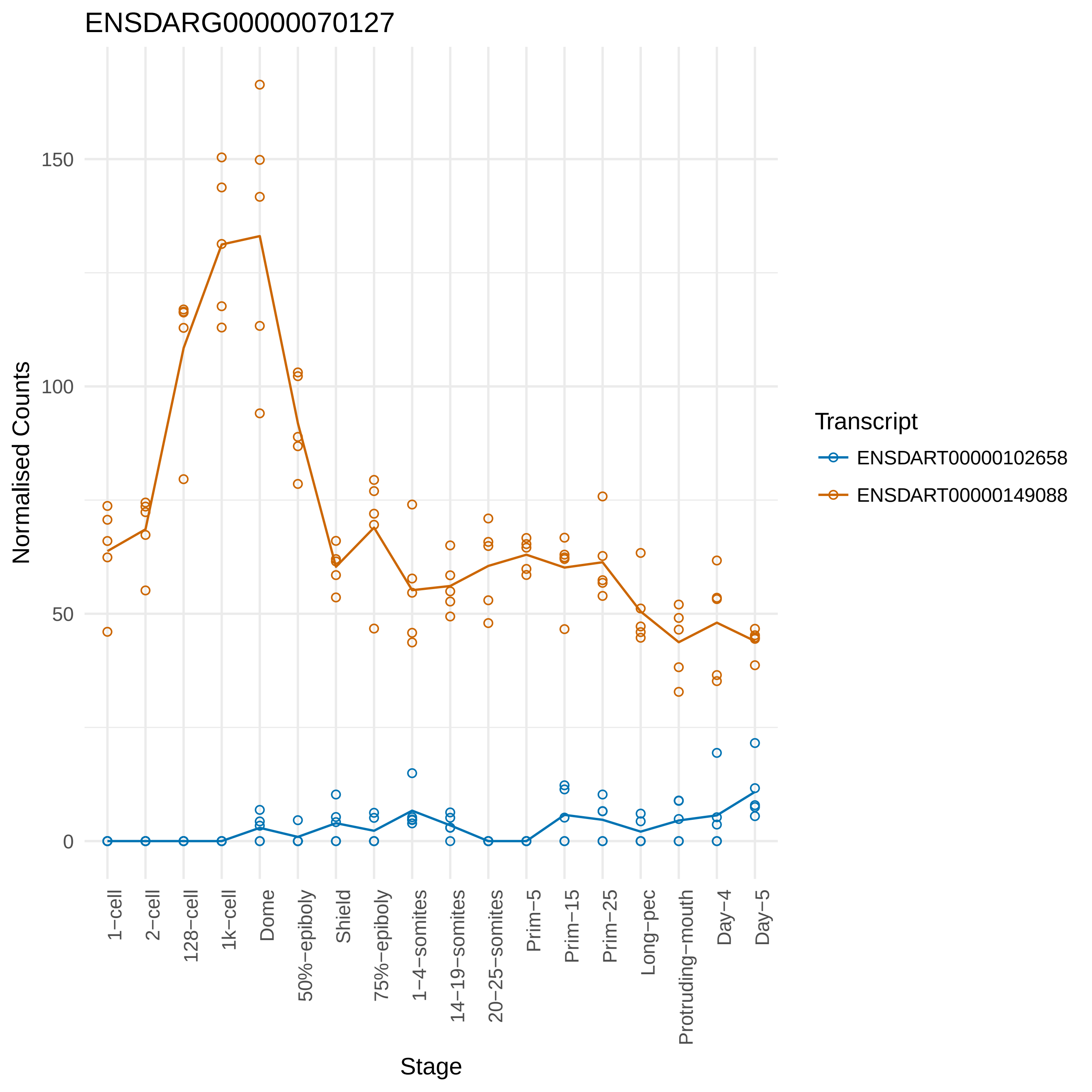

ENSDARG00000070127
Normalised Counts
150
100
50
0
1−cell
2−cell
128−cell
1k−cell
Dome
50%−epiboly
Shield
75%−epiboly
1−4−somites
14−19−somites
20−25−somites
Prim−5
Prim−15
Prim−25
Long−pec
Protruding−mouth
Day−4
Day−5
Stage
Transcript
ENSDART00000102658
ENSDART00000149088

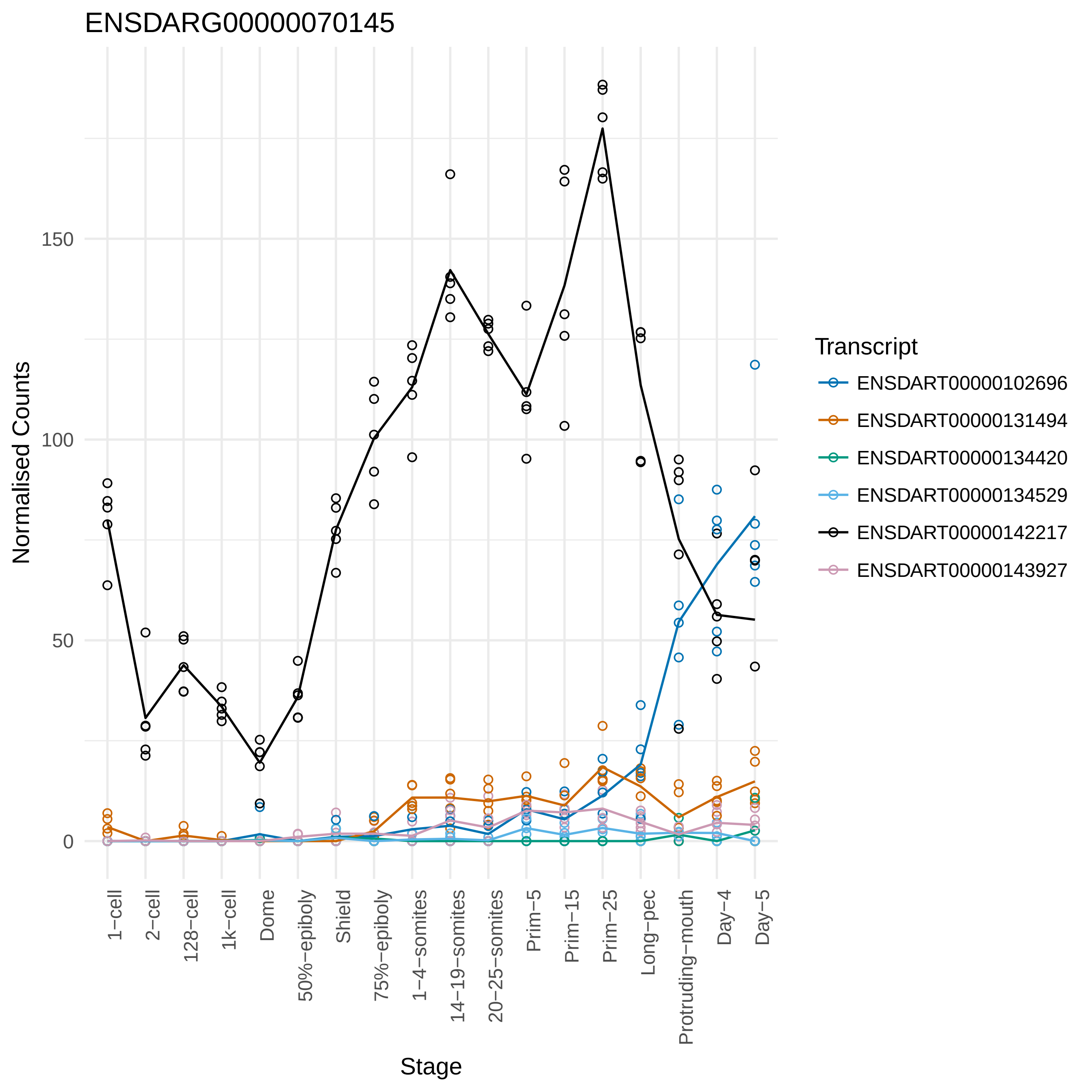

ENSDARG00000070145
Normalised Counts
150
100
50
0
1−cell
2−cell
128−cell
1k−cell
Dome
50%−epiboly
Shield
75%−epiboly
1−4−somites
14−19−somites
20−25−somites
Prim−5
Prim−15
Prim−25
Long−pec
Protruding−mouth
Day−4
Day−5
Stage
Transcript
ENSDART00000102696
ENSDART00000131494
ENSDART00000134420
ENSDART00000134529
ENSDART00000142217
ENSDART00000143927

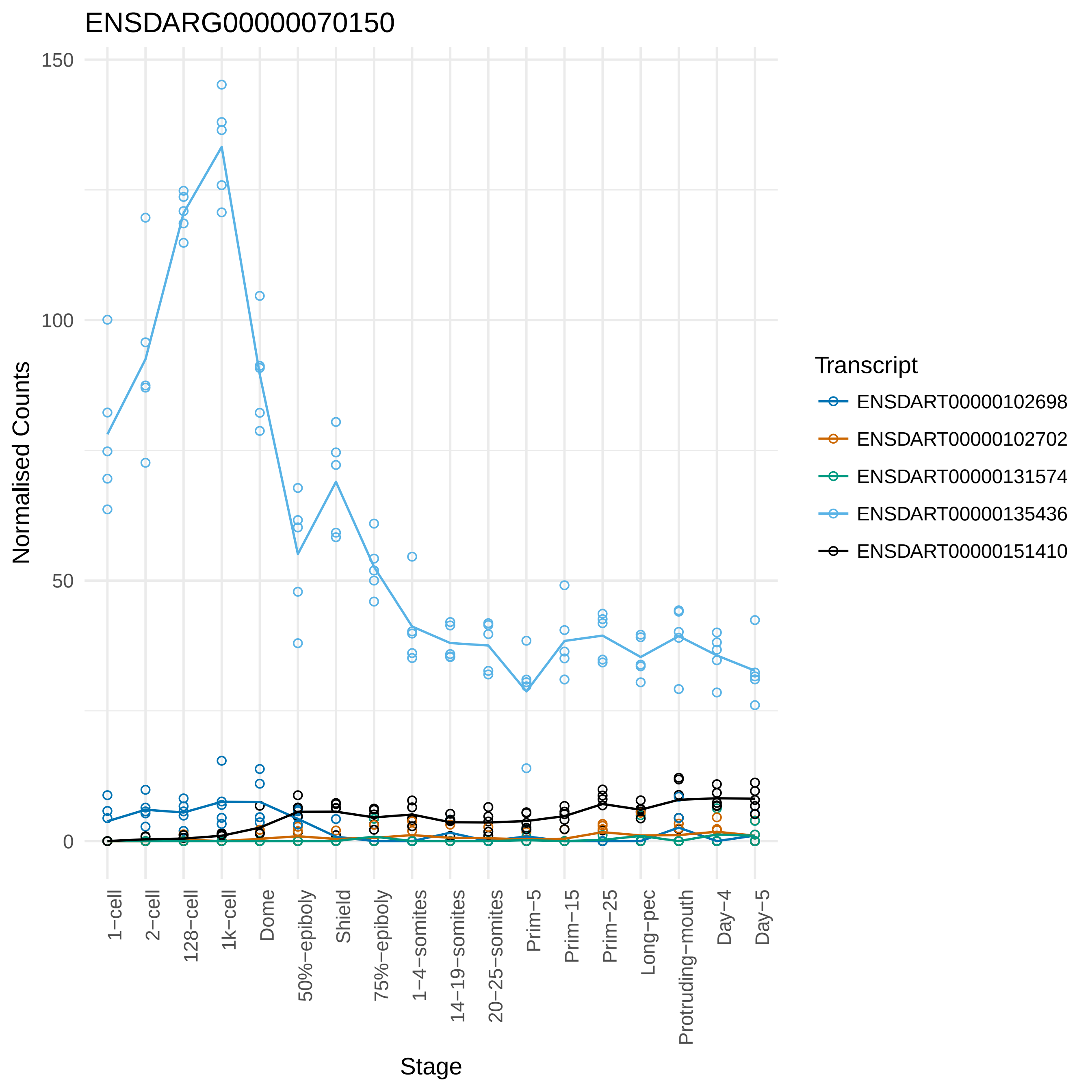

ENSDARG00000070150
Normalised Counts
150
100
50
0
1−cell
2−cell
128−cell
1k−cell
Dome
50%−epiboly
Shield
75%−epiboly
1−4−somites
14−19−somites
20−25−somites
Prim−5
Prim−15
Prim−25
Long−pec
Protruding−mouth
Day−4
Day−5
Stage
Transcript
ENSDART00000102698
ENSDART00000102702
ENSDART00000131574
ENSDART00000135436
ENSDART00000151410

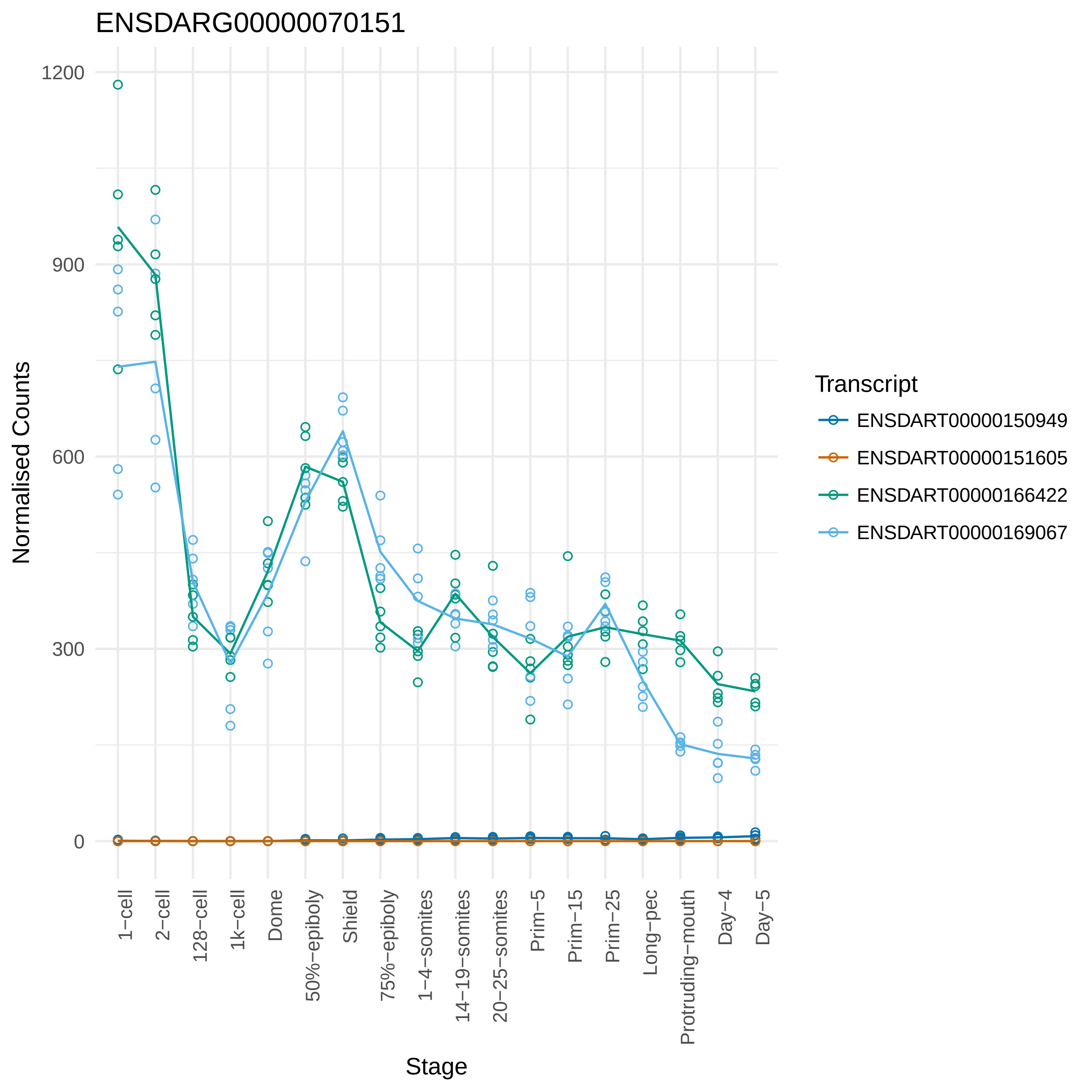

ENSDARG00000070151
1200
900
600
300
0
Normalised Counts
1−cell
2−cell
128−cell
1k−cell
Dome
50%−epiboly
Shield
75%−epiboly
1−4−somites
14−19−somites
20−25−somites
Prim−5
Prim−15
Prim−25
Long−pec
Protruding−mouth
Day−4
Day−5
Stage
Transcript
ENSDART00000150949
ENSDART00000151605
ENSDART00000166422
ENSDART00000169067

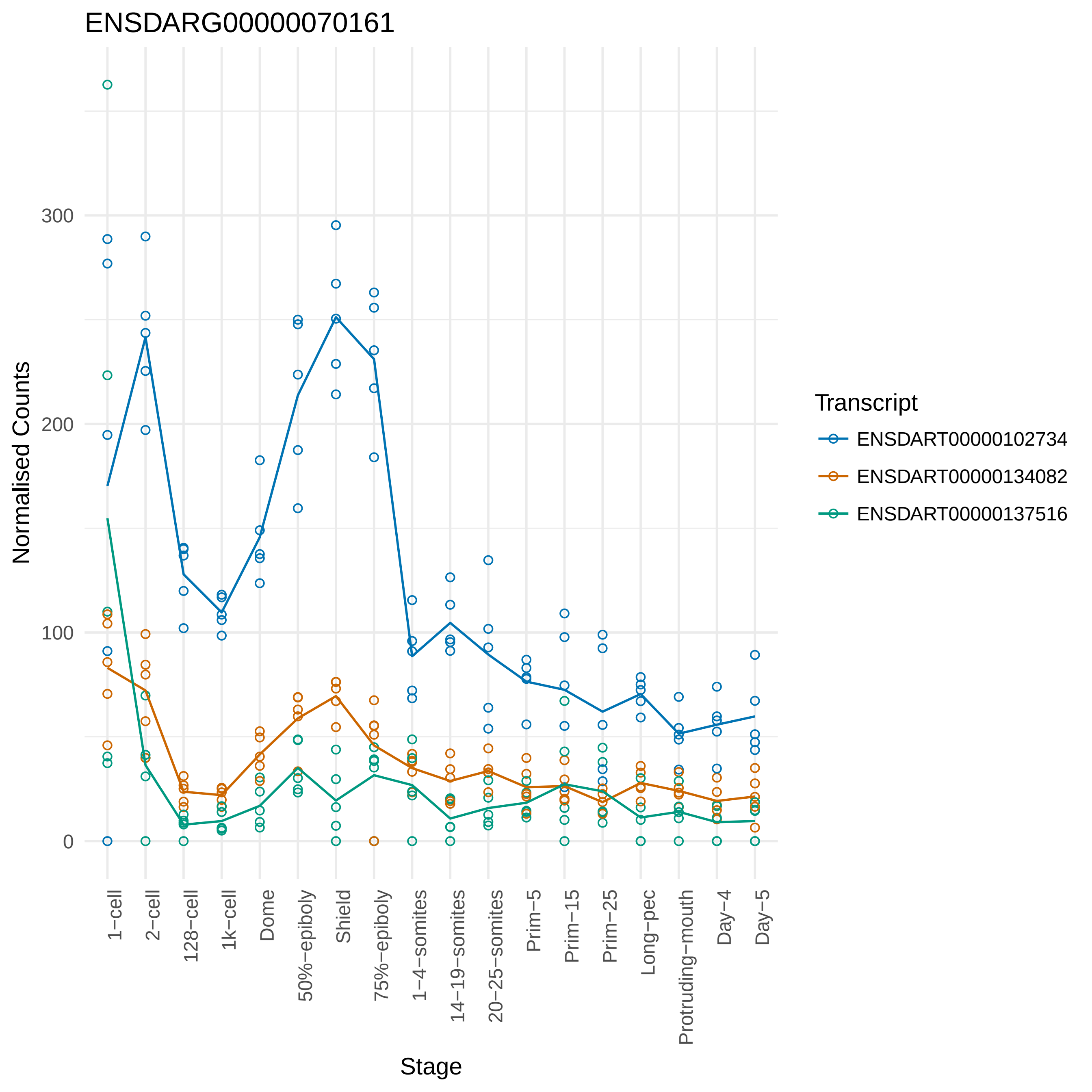

ENSDARG00000070161
Normalised Counts
300
200
100
0
1−cell
2−cell
128−cell
1k−cell
Dome
50%−epiboly
Shield
75%−epiboly
1−4−somites
14−19−somites
20−25−somites
Prim−5
Prim−15
Prim−25
Long−pec
Protruding−mouth
Day−4
Day−5
Stage
Transcript
ENSDART00000102734
ENSDART00000134082
ENSDART00000137516

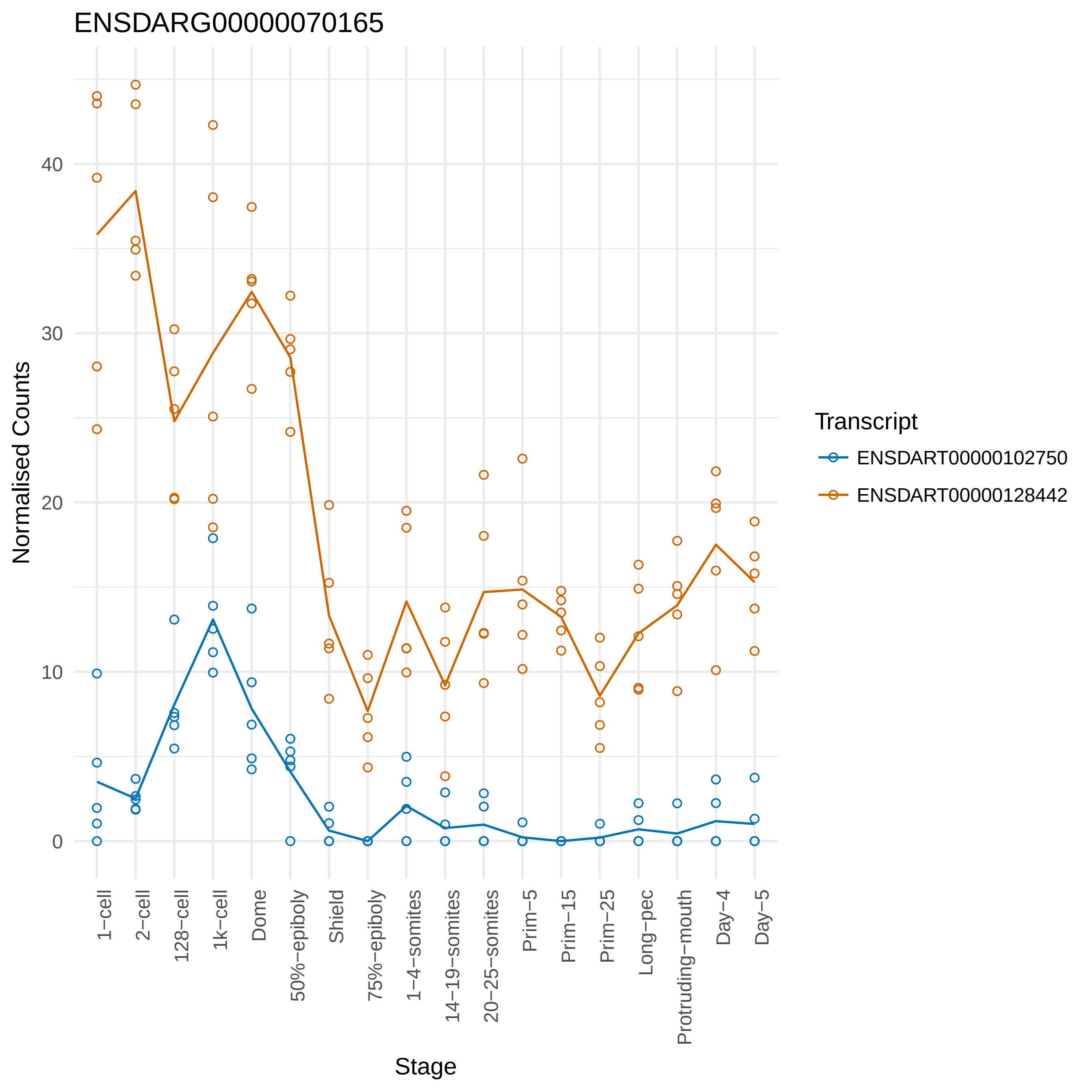

ENSDARG00000070165
Normalised Counts
0
10
20
30
40
1−cell
2−cell
128−cell
1k−cell
Dome
50%−epiboly
Shield
75%−epiboly
1−4−somites
14−19−somites
20−25−somites
Prim−5
Prim−15
Prim−25
Long−pec
Protruding−mouth
Day−4
Day−5
Stage
Transcript
ENSDART00000102750
ENSDART00000128442

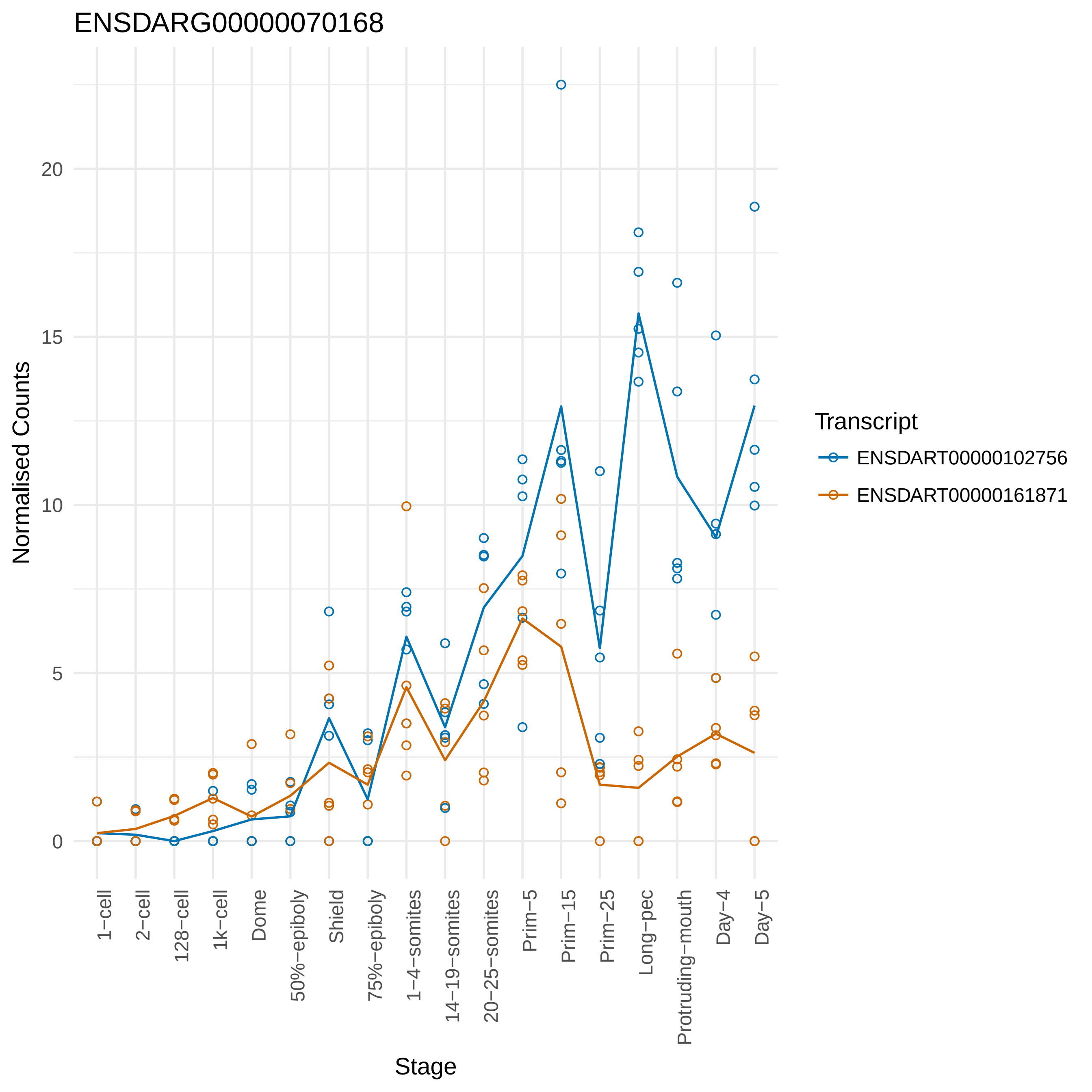

ENSDARG00000070168
Normalised Counts
0
5
10
15
20
Stage
1−cell
2−cell
128−cell
1k−cell
Dome
50%−epiboly
Shield
75%−epiboly
1−4−somites
14−19−somites
20−25−somites
Prim−5
Prim−15
Prim−25
Long−pec
Protruding−mouth
Day−4
Day−5
Transcript
ENSDART00000102756
ENSDART00000161871

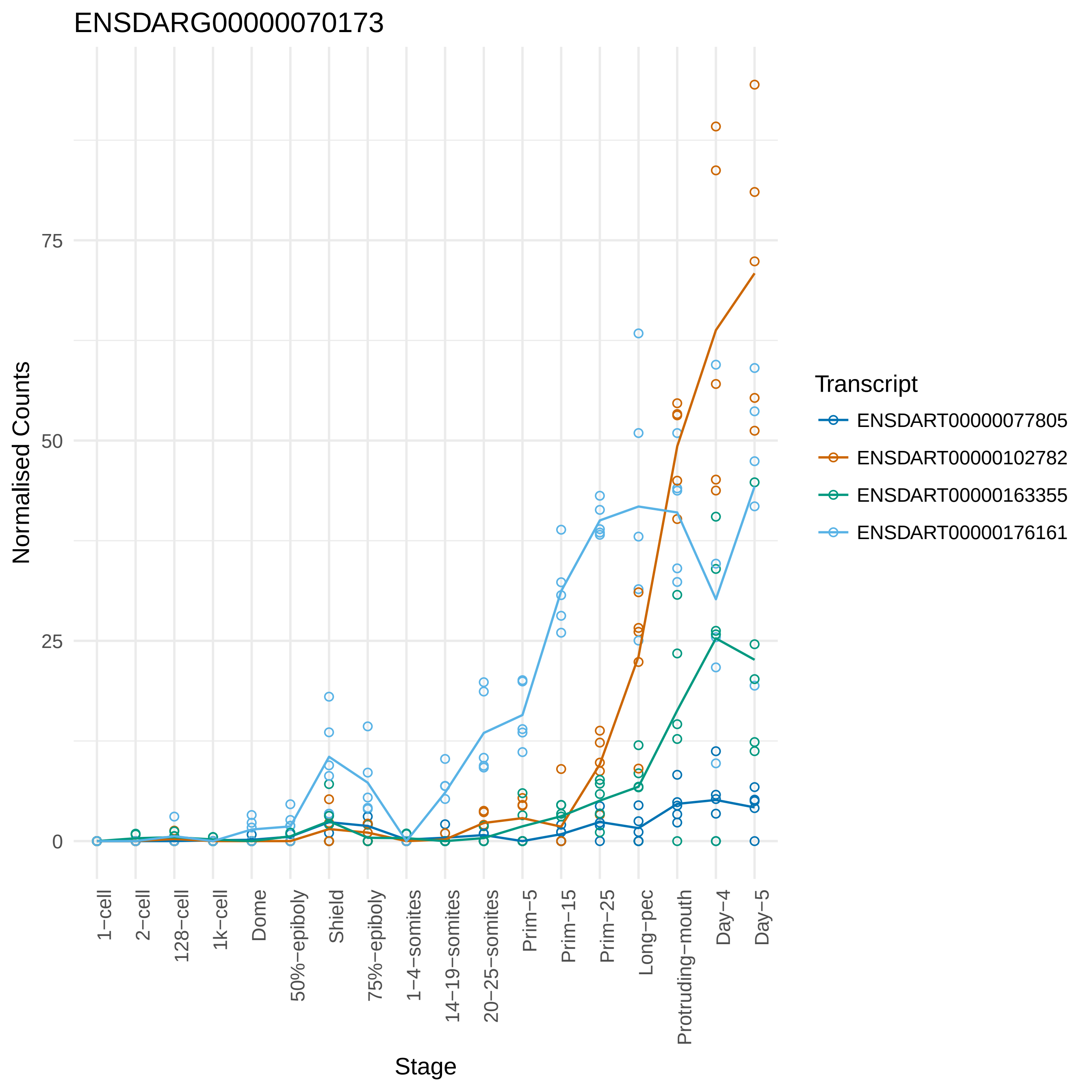
ENSDARG00000070173
Normalised Counts
0
25
50
75
Stage
1−cell
2−cell
128−cell
1k−cell
Dome
50%−epiboly
Shield
75%−epiboly
1−4−somites
14−19−somites
20−25−somites
Prim−5
Prim−15
Prim−25
Long−pec
Protruding−mouth
Day−4
Day−5
Transcript
ENSDART00000077805
ENSDART00000102782
ENSDART00000163355
ENSDART00000176161

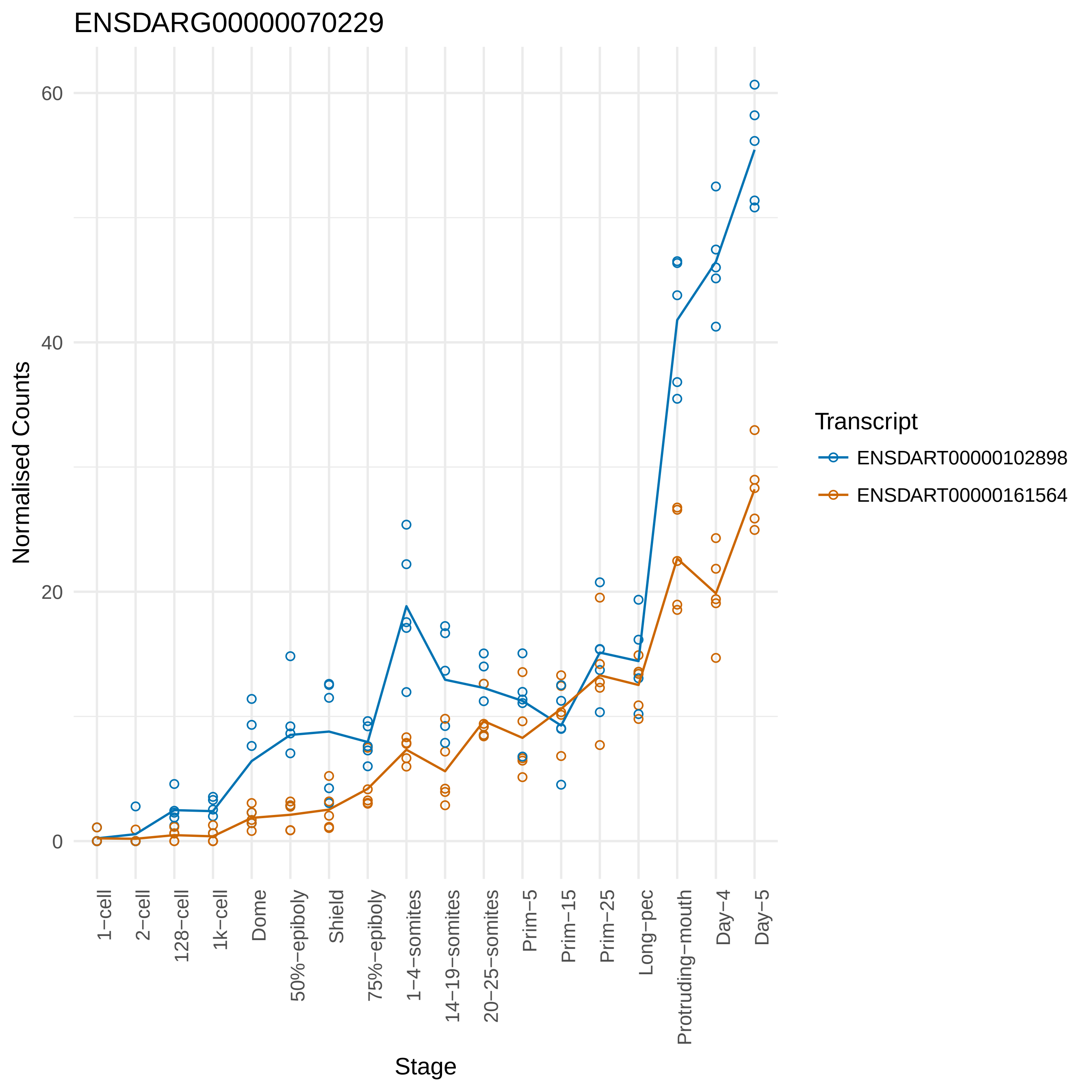

ENSDARG00000070229
Normalised Counts
60
40
20
0
1−cell
2−cell
128−cell
1k−cell
Dome
50%−epiboly
Shield
75%−epiboly
1−4−somites
14−19−somites
20−25−somites
Prim−5
Prim−15
Prim−25
Long−pec
Protruding−mouth
Day−4
Day−5
Stage
Transcript
ENSDART00000102898
ENSDART00000161564

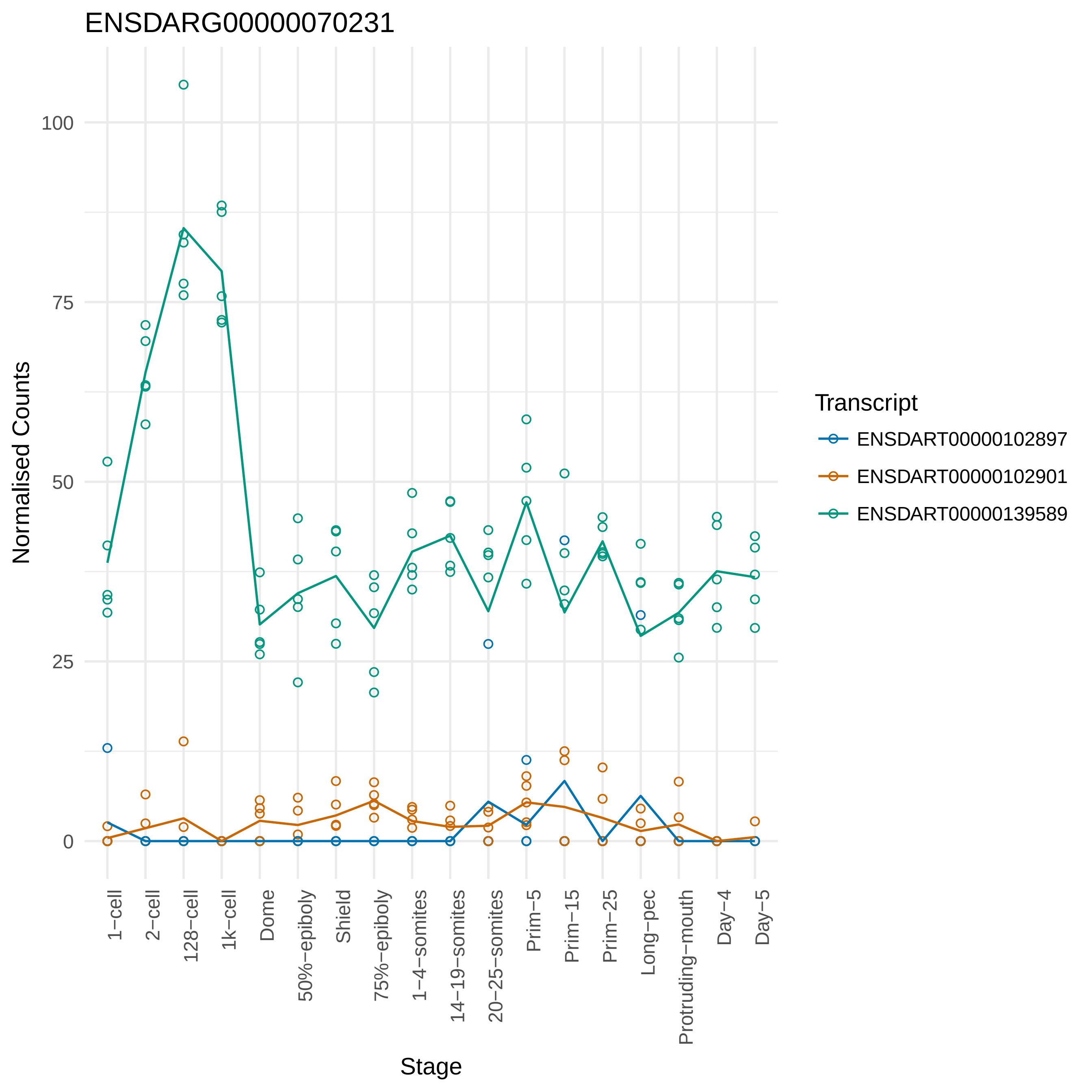
ENSDARG00000070231
Normalised Counts
100
75
50
25
0
1−cell
2−cell
128−cell
1k−cell
Dome
50%−epiboly
Shield
75%−epiboly
1−4−somites
14−19−somites
20−25−somites
Prim−5
Prim−15
Prim−25
Long−pec
Protruding−mouth
Day−4
Day−5
Stage
Transcript
ENSDART00000102897
ENSDART00000102901
ENSDART00000139589

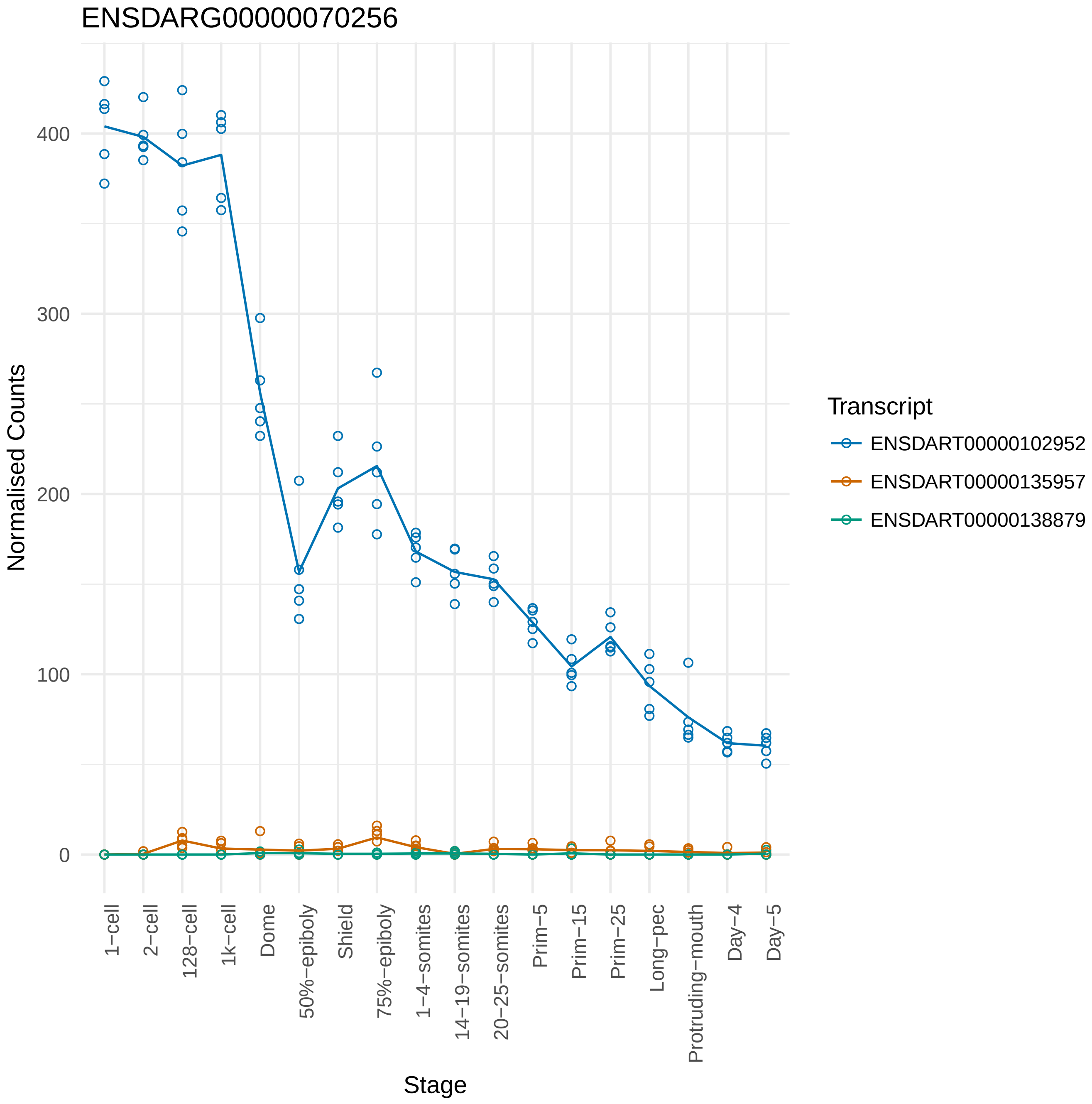
ENSDARG00000070256
Normalised Counts
0
100
200
300
400
1−cell
2−cell
128−cell
1k−cell
Dome
50%−epiboly
Shield
75%−epiboly
1−4−somites
14−19−somites
20−25−somites
Prim−5
Prim−15
Prim−25
Long−pec
Protruding−mouth
Day−4
Day−5
Stage
Transcript
ENSDART00000102952
ENSDART00000135957
ENSDART00000138879

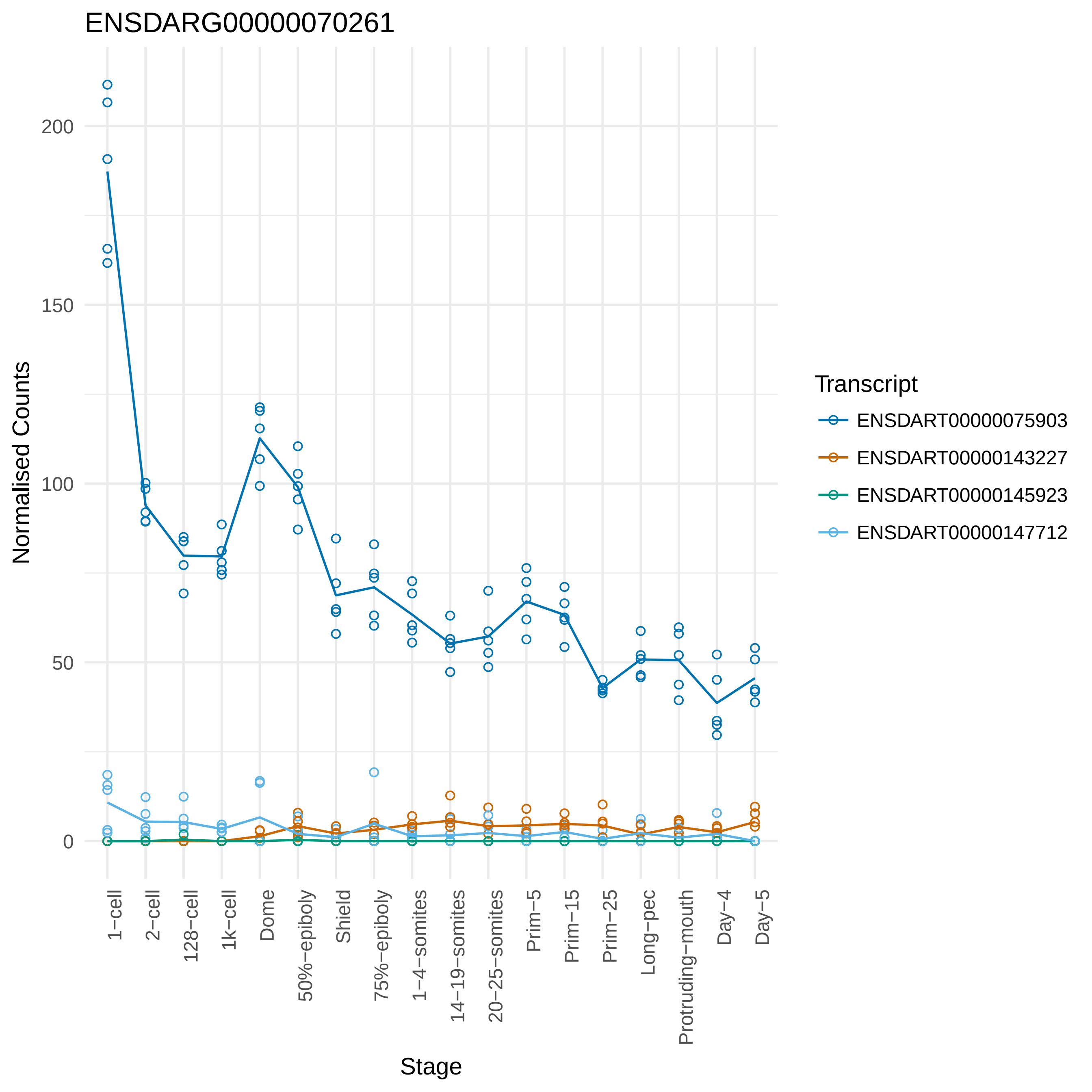
ENSDARG00000070261
Normalised Counts
200
150
100
50
0
1−cell
2−cell
128−cell
1k−cell
Dome
50%−epiboly
Shield
75%−epiboly
1−4−somites
14−19−somites
20−25−somites
Prim−5
Prim−15
Prim−25
Long−pec
Protruding−mouth
Day−4
Day−5
Stage
Transcript
ENSDART00000075903
ENSDART00000143227
ENSDART00000145923
ENSDART00000147712

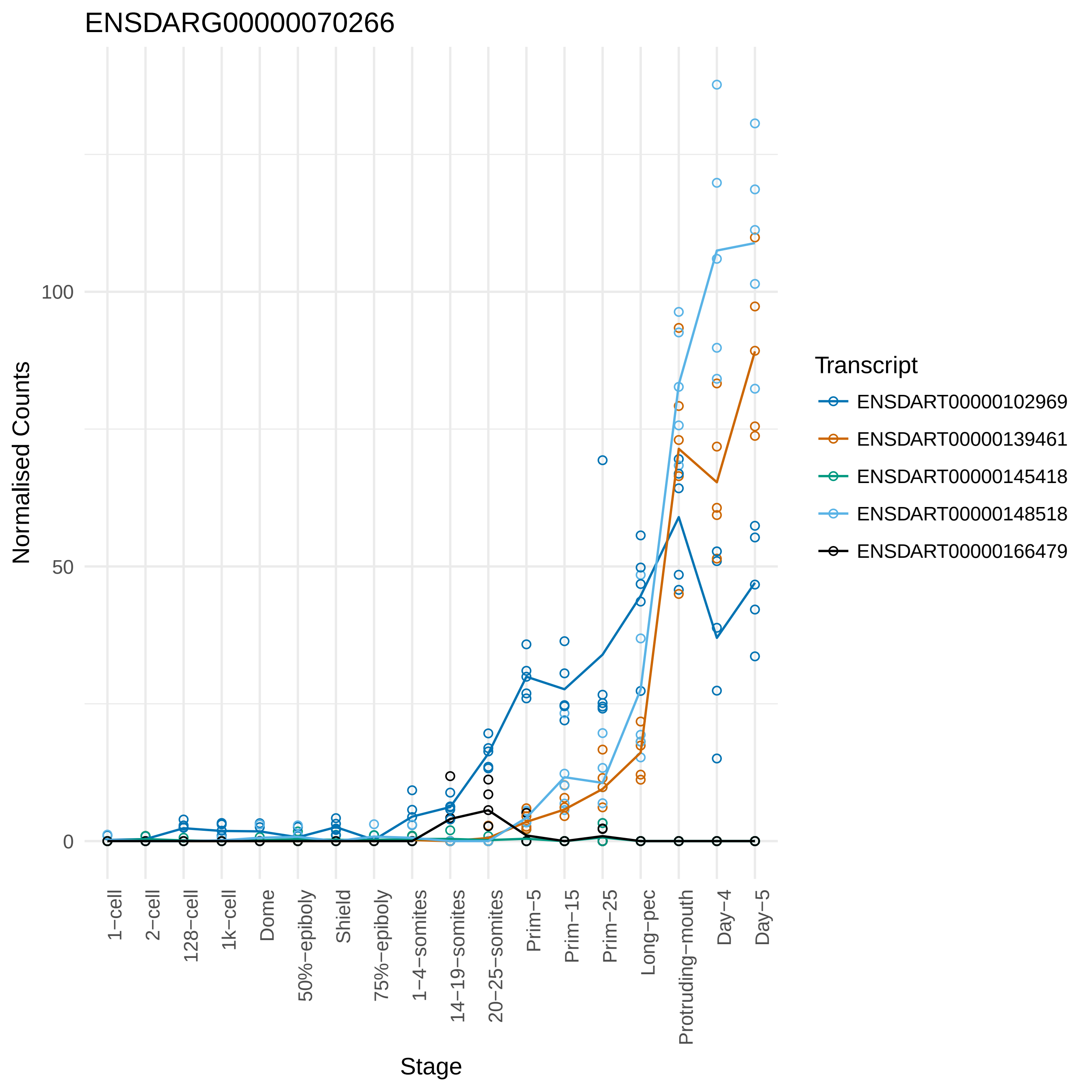

ENSDARG00000070266
Normalised Counts
100
50
0
1−cell
2−cell
128−cell
1k−cell
Dome
50%−epiboly
Shield
75%−epiboly
1−4−somites
14−19−somites
20−25−somites
Prim−5
Prim−15
Prim−25
Long−pec
Protruding−mouth
Day−4
Day−5
Stage
Transcript
ENSDART00000102969
ENSDART00000139461
ENSDART00000145418
ENSDART00000148518
ENSDART00000166479

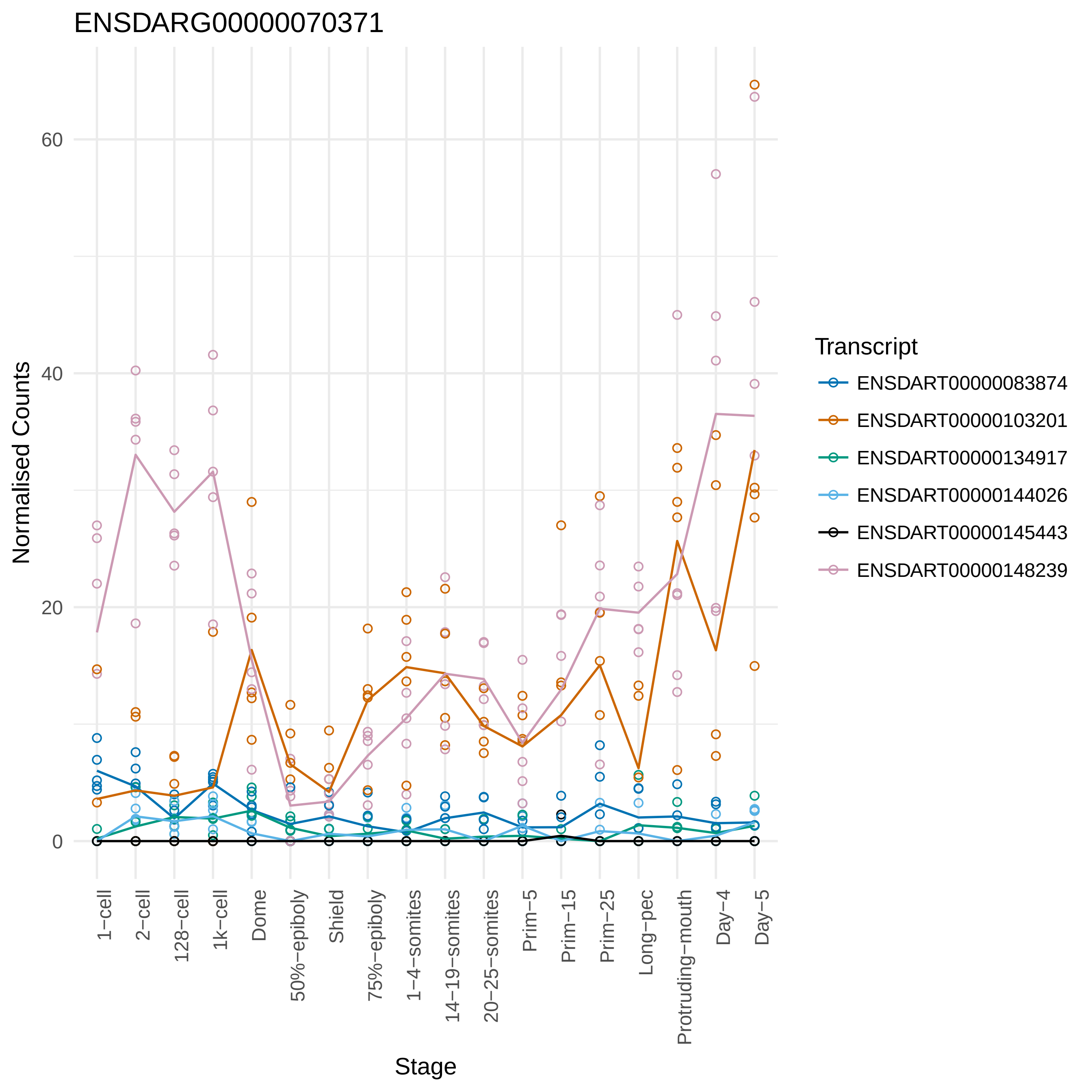

ENSDARG00000070371
Normalised Counts
0
20
40
60
1−cell
2−cell
128−cell
1k−cell
Dome
50%−epiboly
Shield
75%−epiboly
1−4−somites
14−19−somites
20−25−somites
Prim−5
Prim−15
Prim−25
Long−pec
Protruding−mouth
Day−4
Day−5
Stage
Transcript
ENSDART00000083874
ENSDART00000103201
ENSDART00000134917
ENSDART00000144026
ENSDART00000145443
ENSDART00000148239

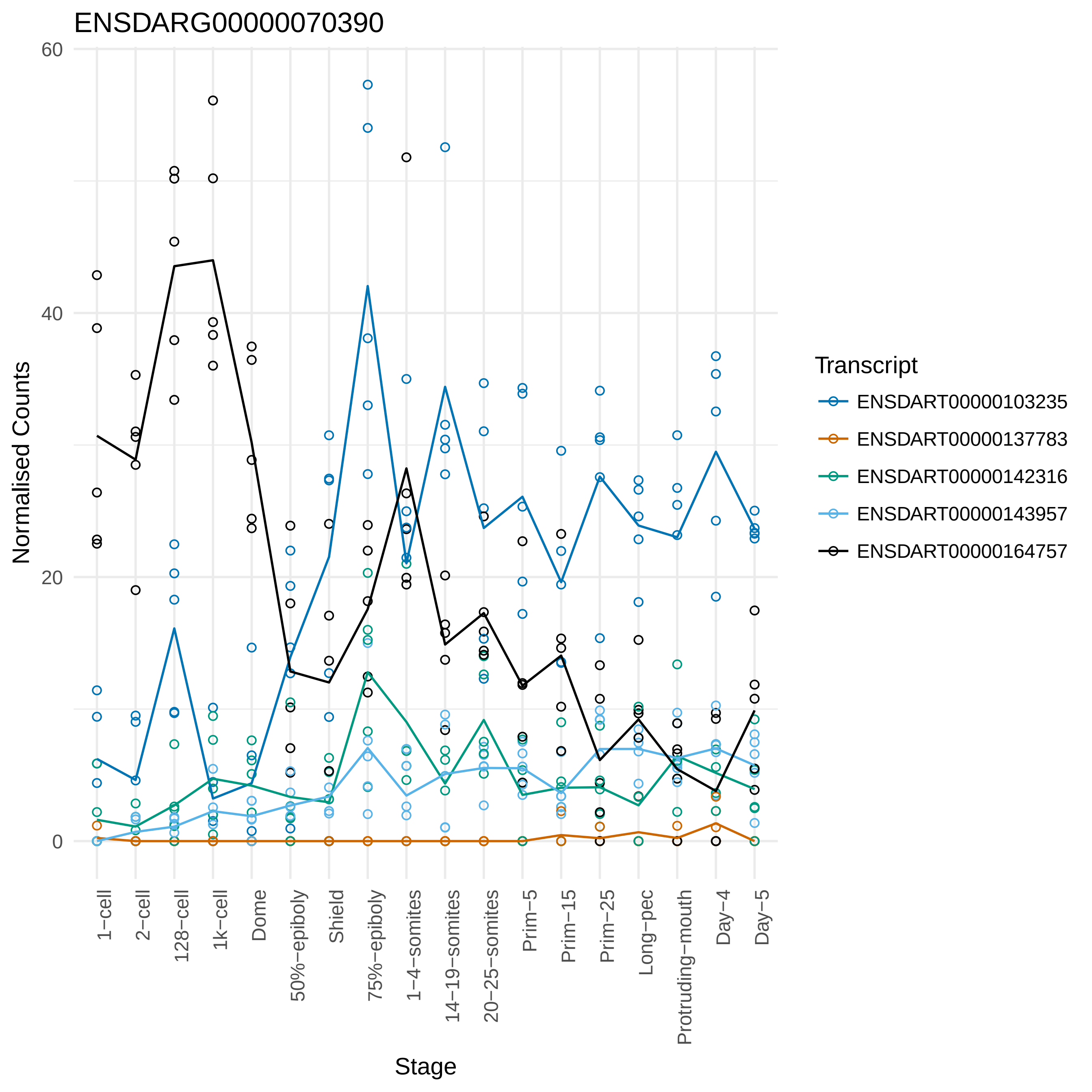

ENSDARG00000070390
Normalised Counts
60
40
20
0
1−cell
2−cell
128−cell
1k−cell
Dome
50%−epiboly
Shield
75%−epiboly
1−4−somites
14−19−somites
20−25−somites
Prim−5
Prim−15
Prim−25
Long−pec
Protruding−mouth
Day−4
Day−5
Stage
Transcript
ENSDART00000103235
ENSDART00000137783
ENSDART00000142316
ENSDART00000143957
ENSDART00000164757

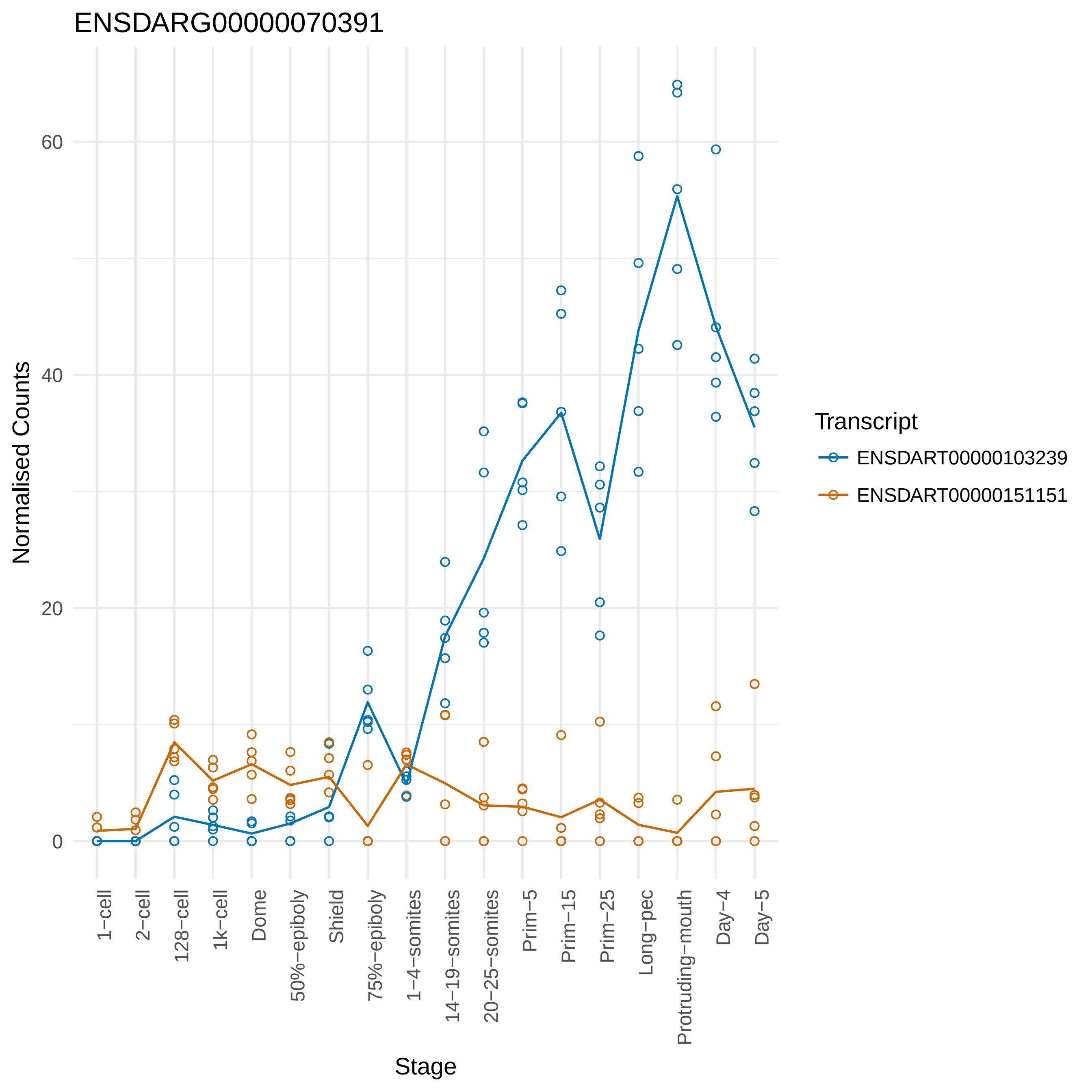
ENSDARG00000070391
Normalised Counts
0
20
40
60
1−cell
2−cell
128−cell
1k−cell
Dome
50%−epiboly
Shield
75%−epiboly
1−4−somites
14−19−somites
20−25−somites
Prim−5
Prim−15
Prim−25
Long−pec
Protruding−mouth
Day−4
Day−5
Stage
Transcript
ENSDART00000103239
ENSDART00000151151

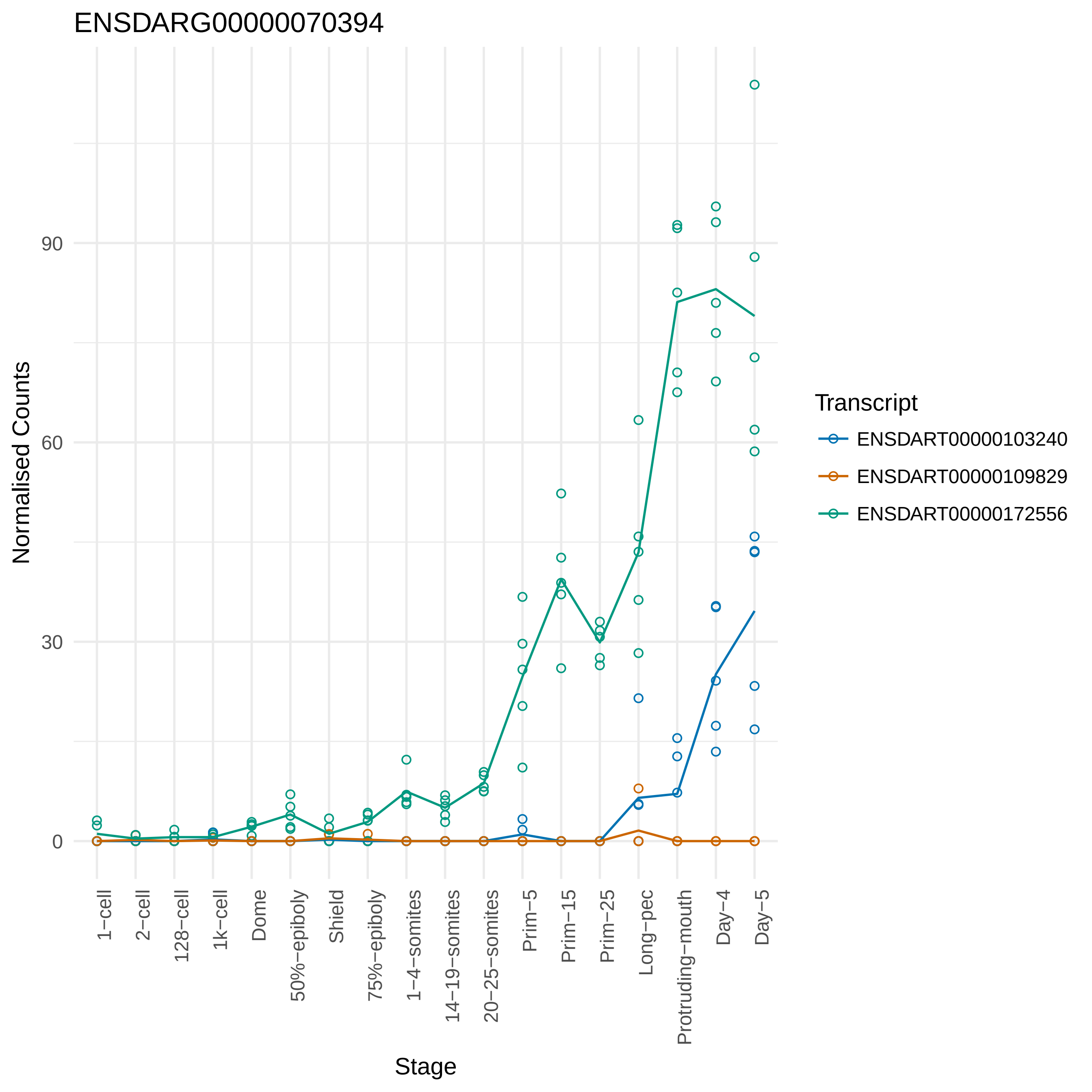

ENSDARG00000070394
Normalised Counts
0
30
60
90
Stage
1−cell
2−cell
128−cell
1k−cell
Dome
50%−epiboly
Shield
75%−epiboly
1−4−somites
14−19−somites
20−25−somites
Prim−5
Prim−15
Prim−25
Long−pec
Protruding−mouth
Day−4
Day−5
Transcript
ENSDART00000103240
ENSDART00000109829
ENSDART00000172556

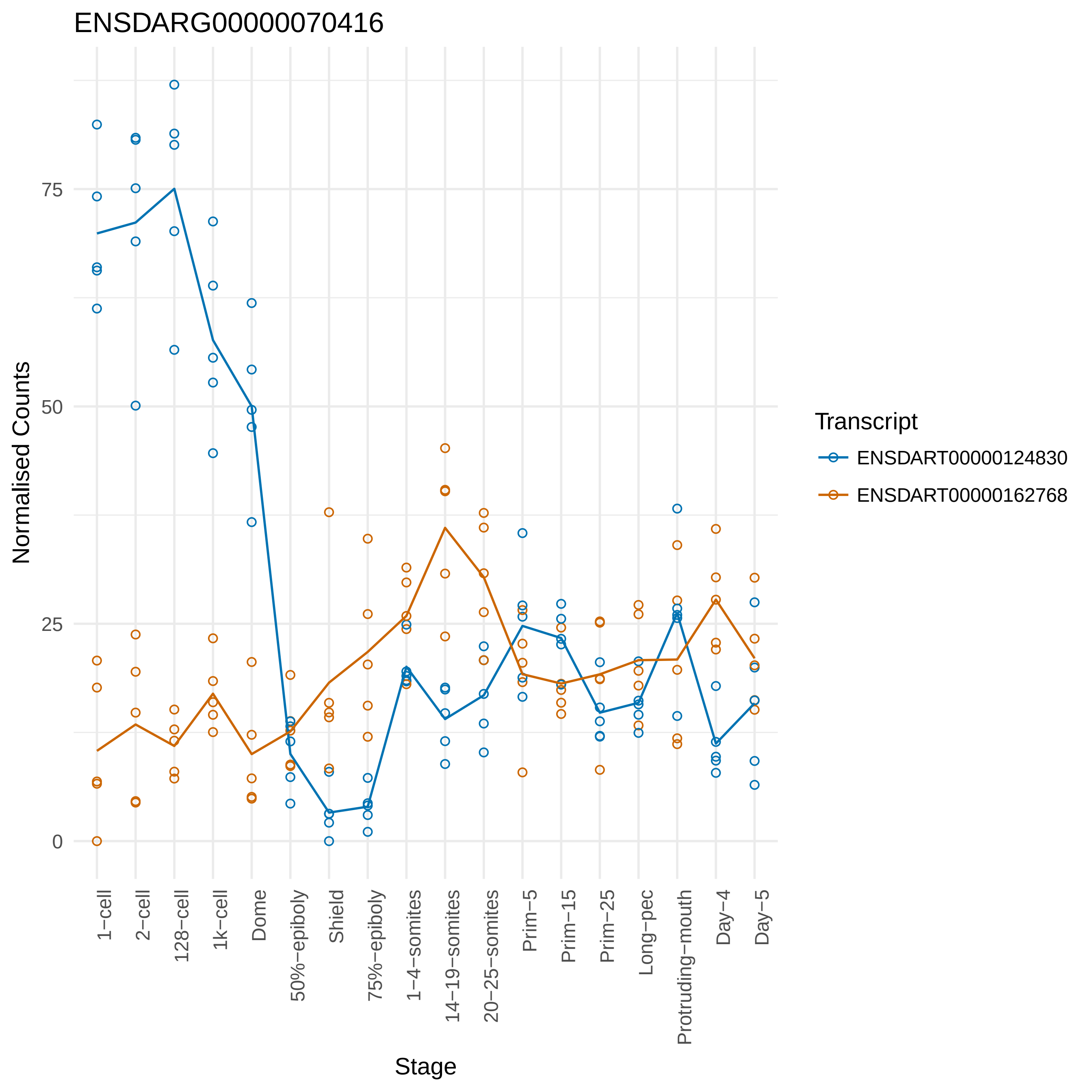

ENSDARG00000070416
Normalised Counts
75
50
25
0
1−cell
2−cell
128−cell
1k−cell
Dome
50%−epiboly
Shield
75%−epiboly
1−4−somites
14−19−somites
20−25−somites
Prim−5
Prim−15
Prim−25
Long−pec
Protruding−mouth
Day−4
Day−5
Stage
Transcript
ENSDART00000124830
ENSDART00000162768

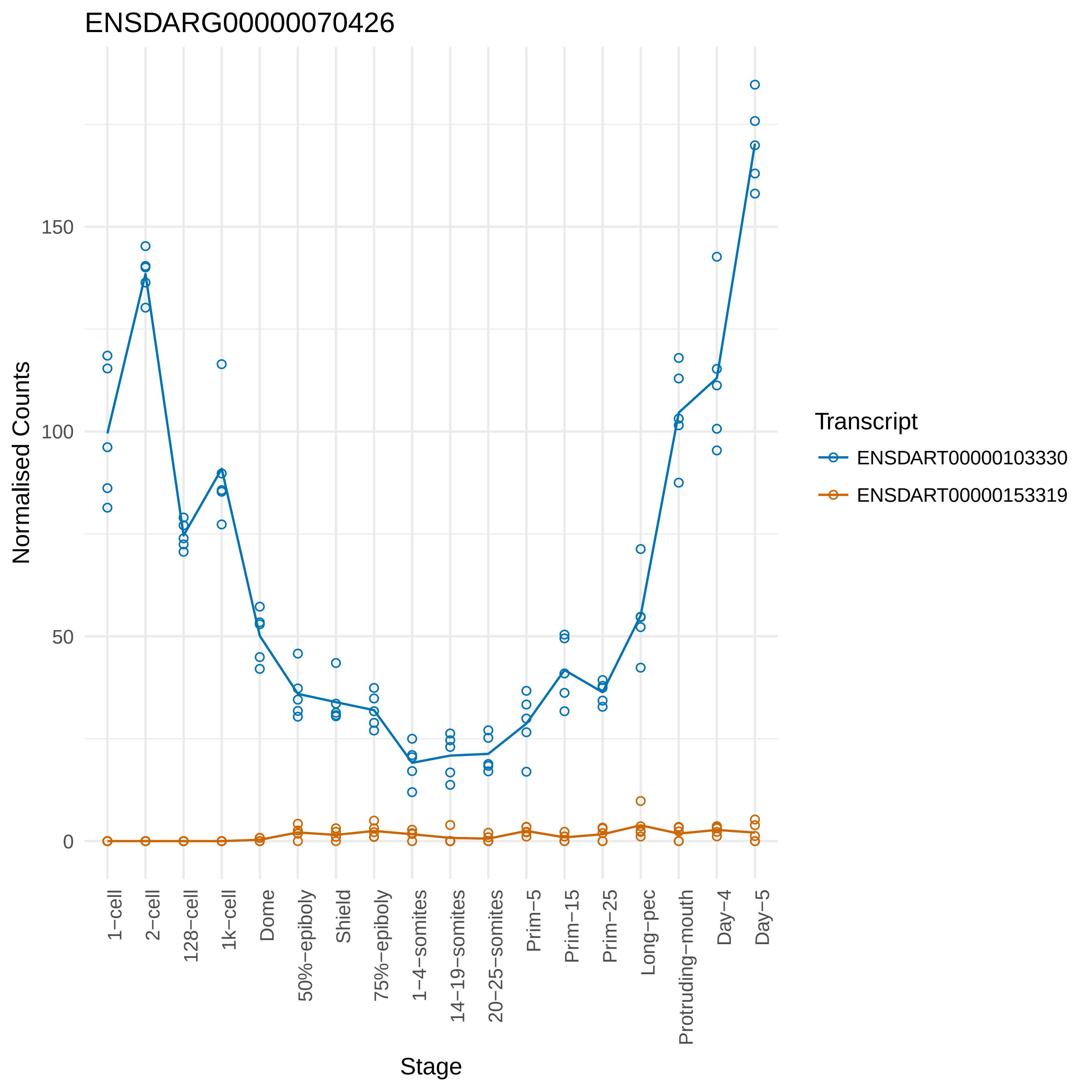
ENSDARG00000070426
Normalised Counts
0
50
100
150
Stage
1−cell
2−cell
128−cell
1k−cell
Dome
50%−epiboly
Shield
75%−epiboly
1−4−somites
14−19−somites
20−25−somites
Prim−5
Prim−15
Prim−25
Long−pec
Protruding−mouth
Day−4
Day−5
Transcript
ENSDART00000103330
ENSDART00000153319

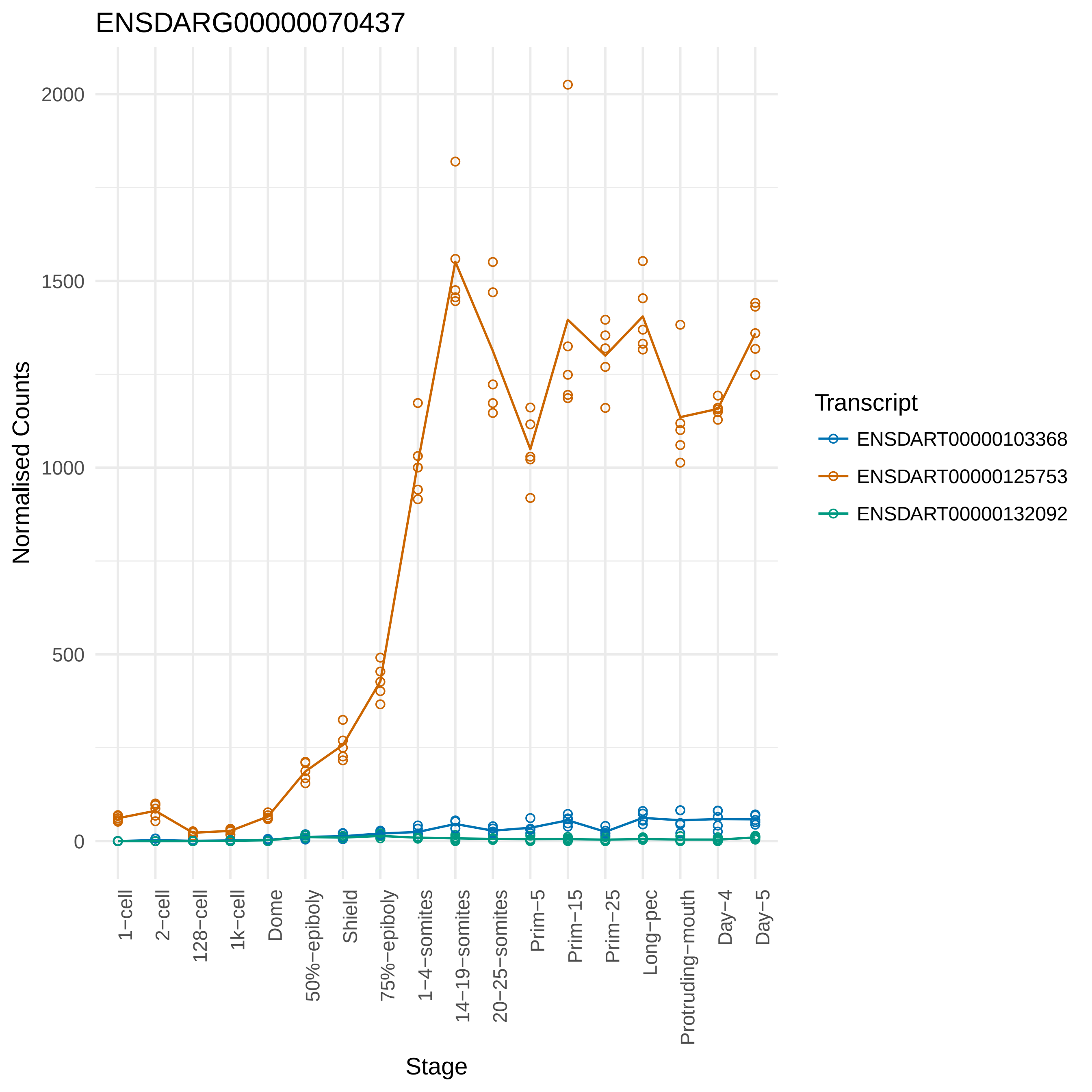

ENSDARG00000070437
Normalised Counts
2000
1500
1000
500
0
Stage
1−cell
2−cell
128−cell
1k−cell
Dome
50%−epiboly
Shield
75%−epiboly
1−4−somites
14−19−somites
20−25−somites
Prim−5
Prim−15
Prim−25
Long−pec
Protruding−mouth
Day−4
Day−5
Transcript
ENSDART00000103368
ENSDART00000125753
ENSDART00000132092

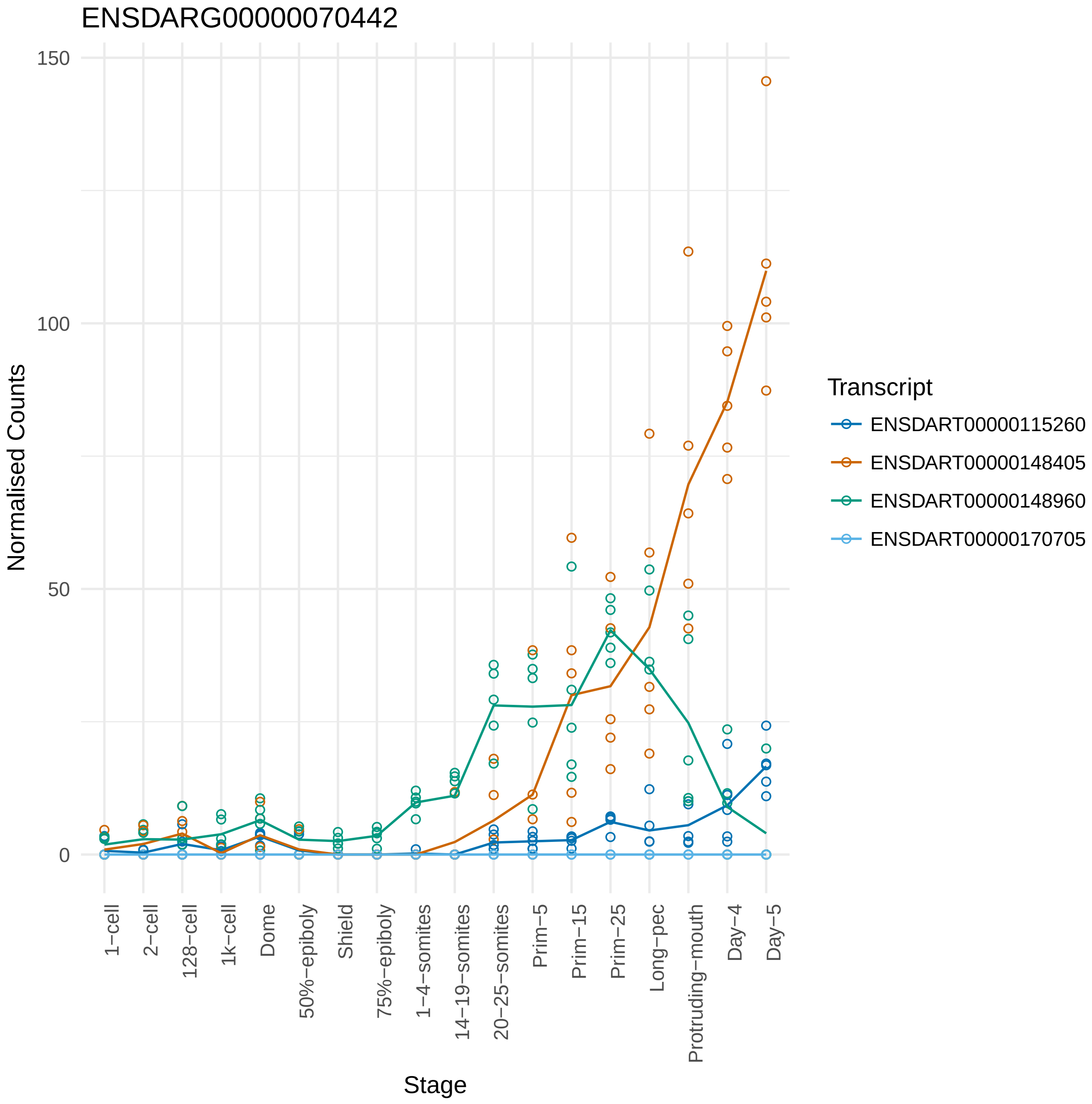
ENSDARG00000070442
Normalised Counts
150
100
50
0
1−cell
2−cell
128−cell
1k−cell
Dome
50%−epiboly
Shield
75%−epiboly
1−4−somites
14−19−somites
20−25−somites
Prim−5
Prim−15
Prim−25
Long−pec
Protruding−mouth
Day−4
Day−5
Stage
Transcript
ENSDART00000115260
ENSDART00000148405
ENSDART00000148960
ENSDART00000170705

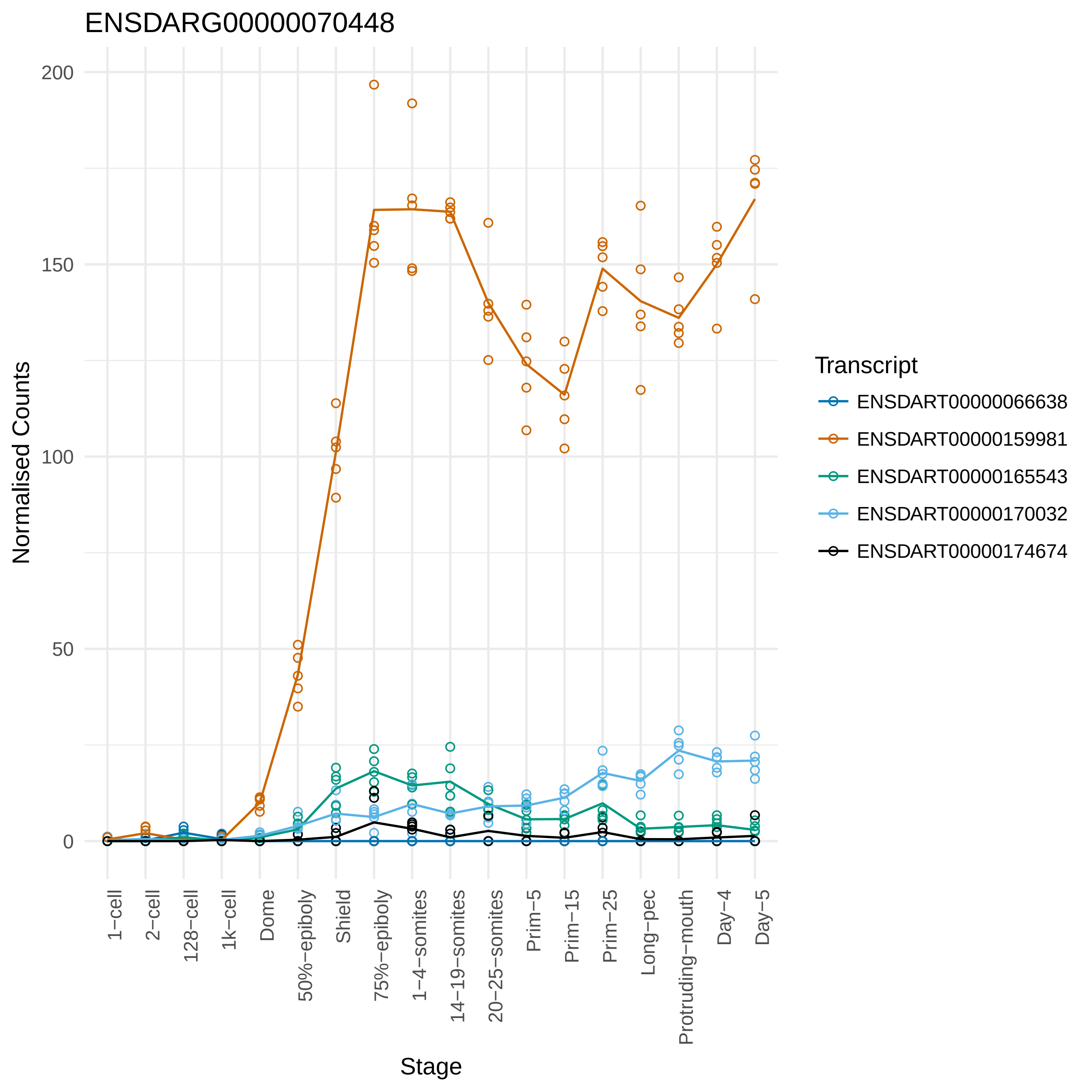

ENSDARG00000070448
Normalised Counts
200
150
100
50
0
1−cell
2−cell
128−cell
1k−cell
Dome
50%−epiboly
Shield
75%−epiboly
1−4−somites
14−19−somites
20−25−somites
Prim−5
Prim−15
Prim−25
Long−pec
Protruding−mouth
Day−4
Day−5
Stage
Transcript
ENSDART00000066638
ENSDART00000159981
ENSDART00000165543
ENSDART00000170032
ENSDART00000174674

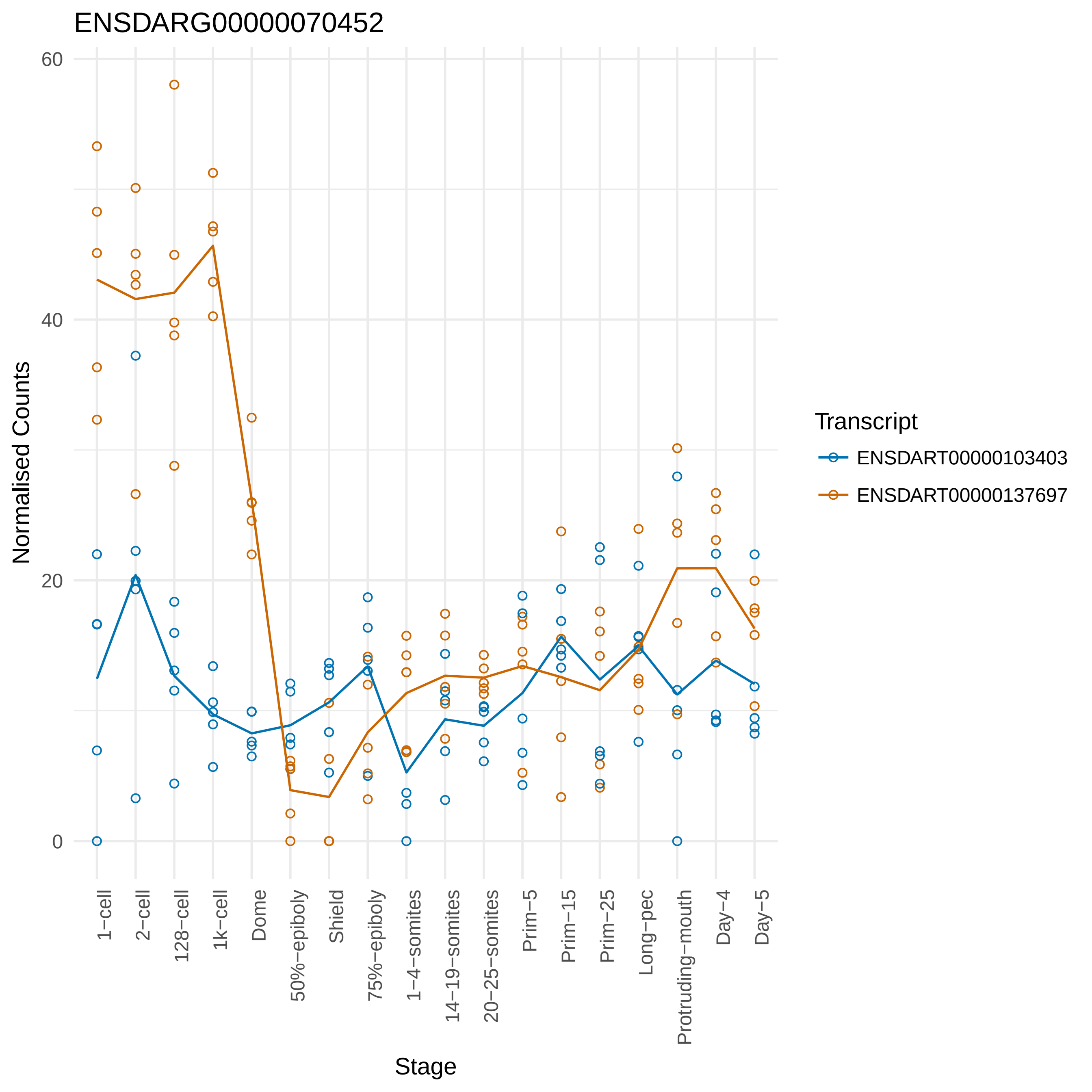

ENSDARG00000070452
Normalised Counts
0
20
40
60
1−cell
2−cell
128−cell
1k−cell
Dome
50%−epiboly
Shield
75%−epiboly
1−4−somites
14−19−somites
20−25−somites
Prim−5
Prim−15
Prim−25
Long−pec
Protruding−mouth
Day−4
Day−5
Stage
Transcript
ENSDART00000103403
ENSDART00000137697

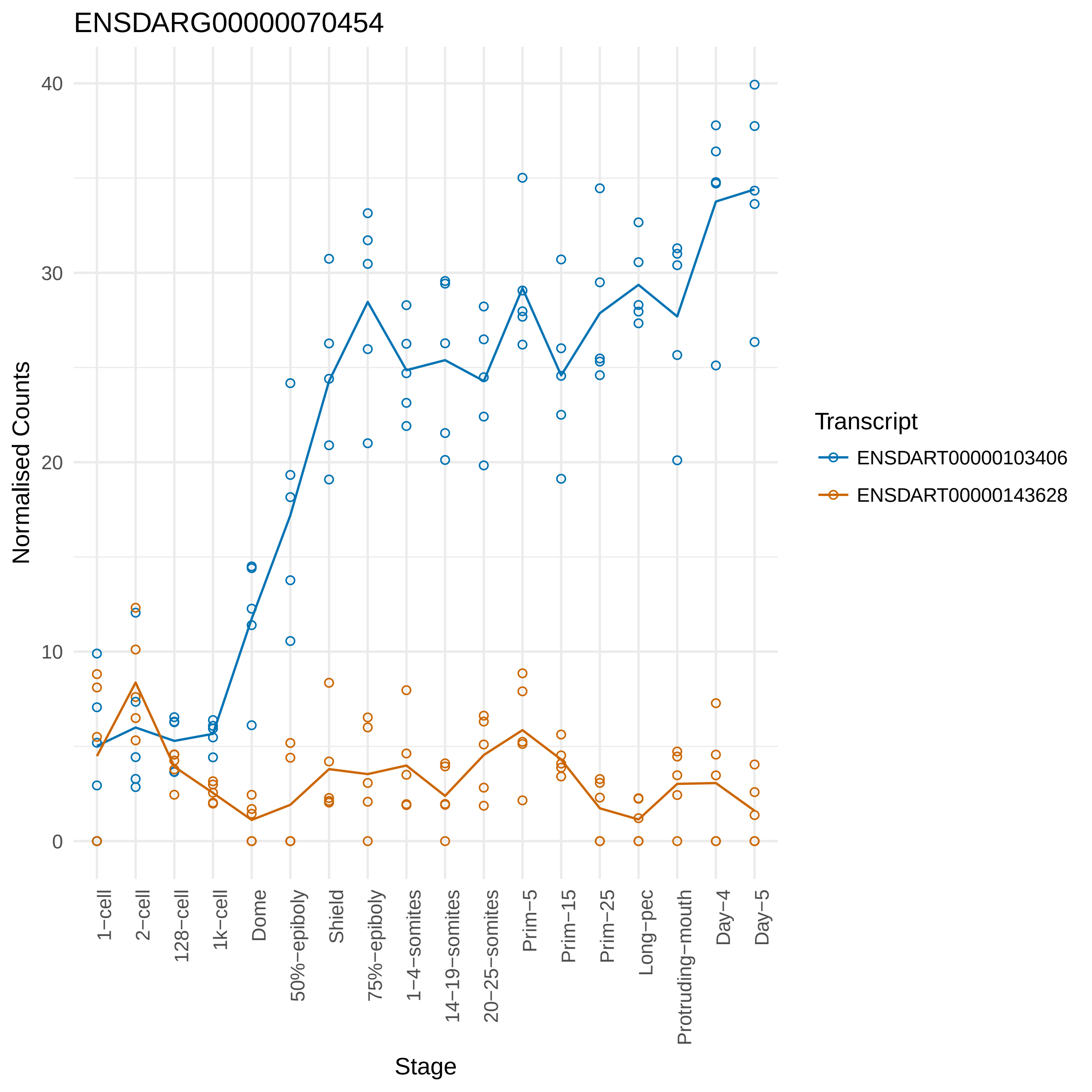
ENSDARG00000070454
Normalised Counts
40
30
20
10
0
1−cell
2−cell
128−cell
1k−cell
Dome
50%−epiboly
Shield
75%−epiboly
1−4−somites
14−19−somites
20−25−somites
Prim−5
Prim−15
Prim−25
Long−pec
Protruding−mouth
Day−4
Day−5
Stage
Transcript
ENSDART00000103406
ENSDART00000143628

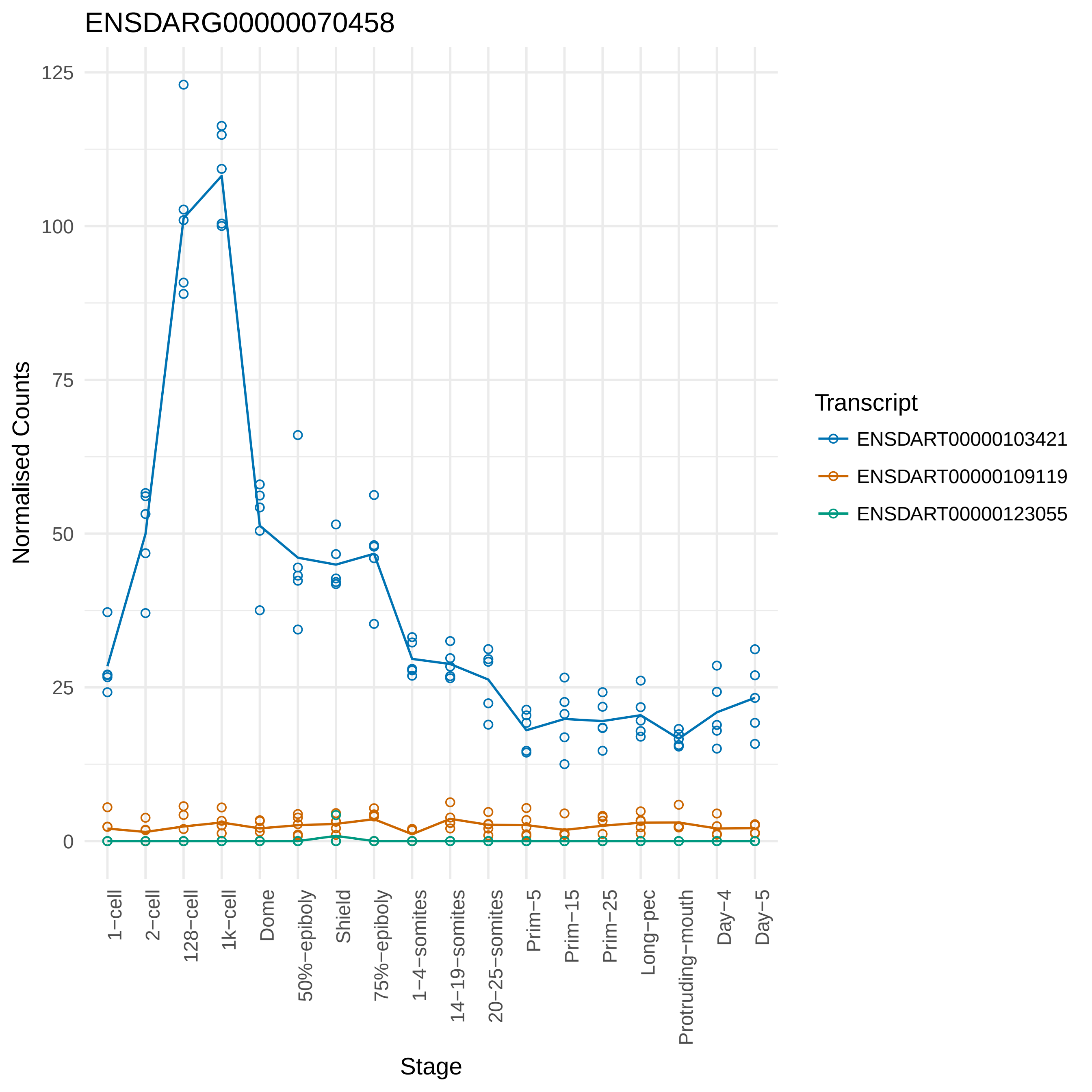
ENSDARG00000070458
Normalised Counts
125
100
75
50
25
0
1−cell
2−cell
128−cell
1k−cell
Dome
50%−epiboly
Shield
75%−epiboly
1−4−somites
14−19−somites
20−25−somites
Prim−5
Prim−15
Prim−25
Long−pec
Protruding−mouth
Day−4
Day−5
Stage
Transcript
ENSDART00000103421
ENSDART00000109119
ENSDART00000123055

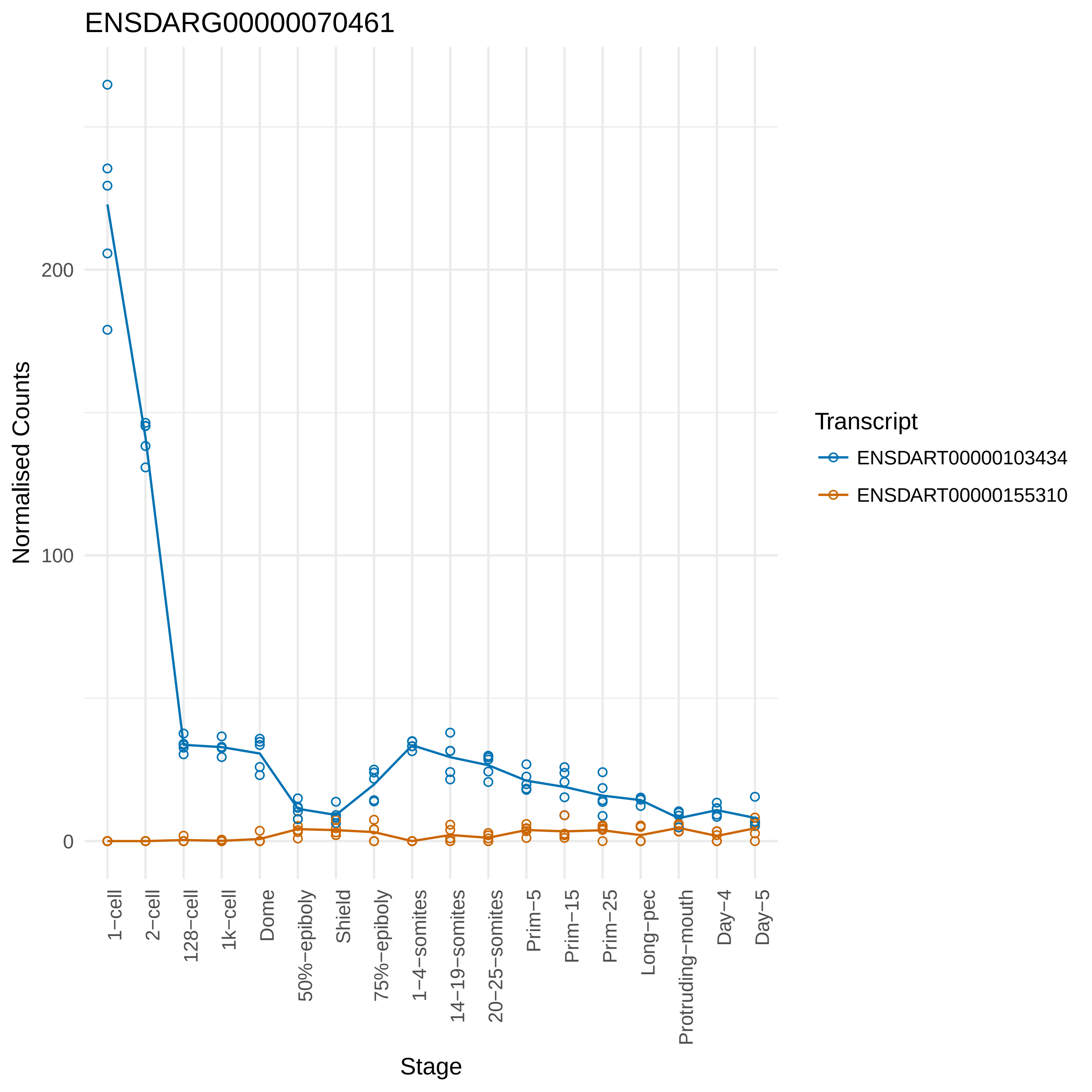

ENSDARG00000070461
Normalised Counts
200
100
0
1−cell
2−cell
128−cell
1k−cell
Dome
50%−epiboly
Shield
75%−epiboly
1−4−somites
14−19−somites
20−25−somites
Prim−5
Prim−15
Prim−25
Long−pec
Protruding−mouth
Day−4
Day−5
Stage
Transcript
ENSDART00000103434
ENSDART00000155310

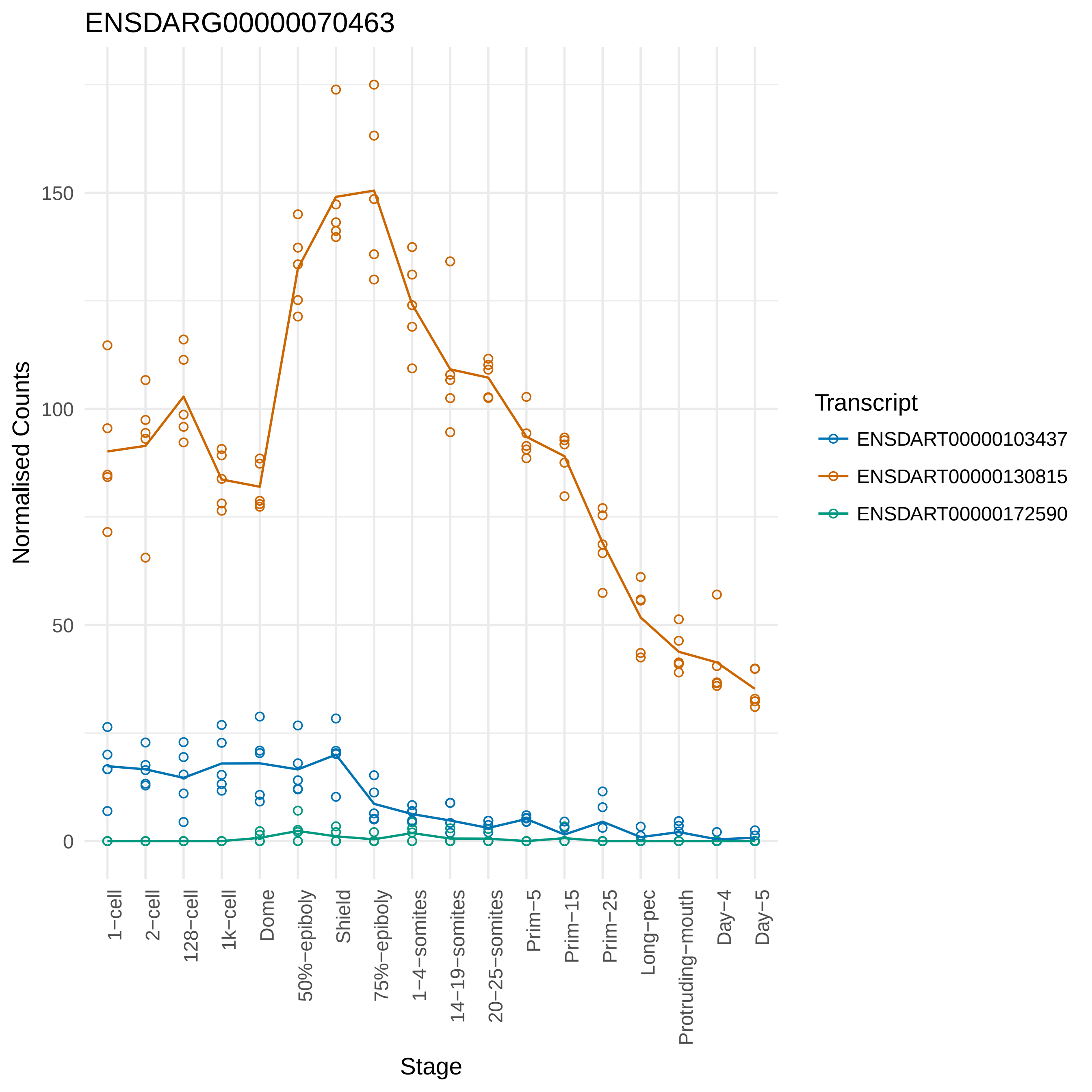

ENSDARG00000070463
Normalised Counts
150
100
50
0
1−cell
2−cell
128−cell
1k−cell
Dome
50%−epiboly
Shield
75%−epiboly
1−4−somites
14−19−somites
20−25−somites
Prim−5
Prim−15
Prim−25
Long−pec
Protruding−mouth
Day−4
Day−5
Stage
Transcript
ENSDART00000103437
ENSDART00000130815
ENSDART00000172590

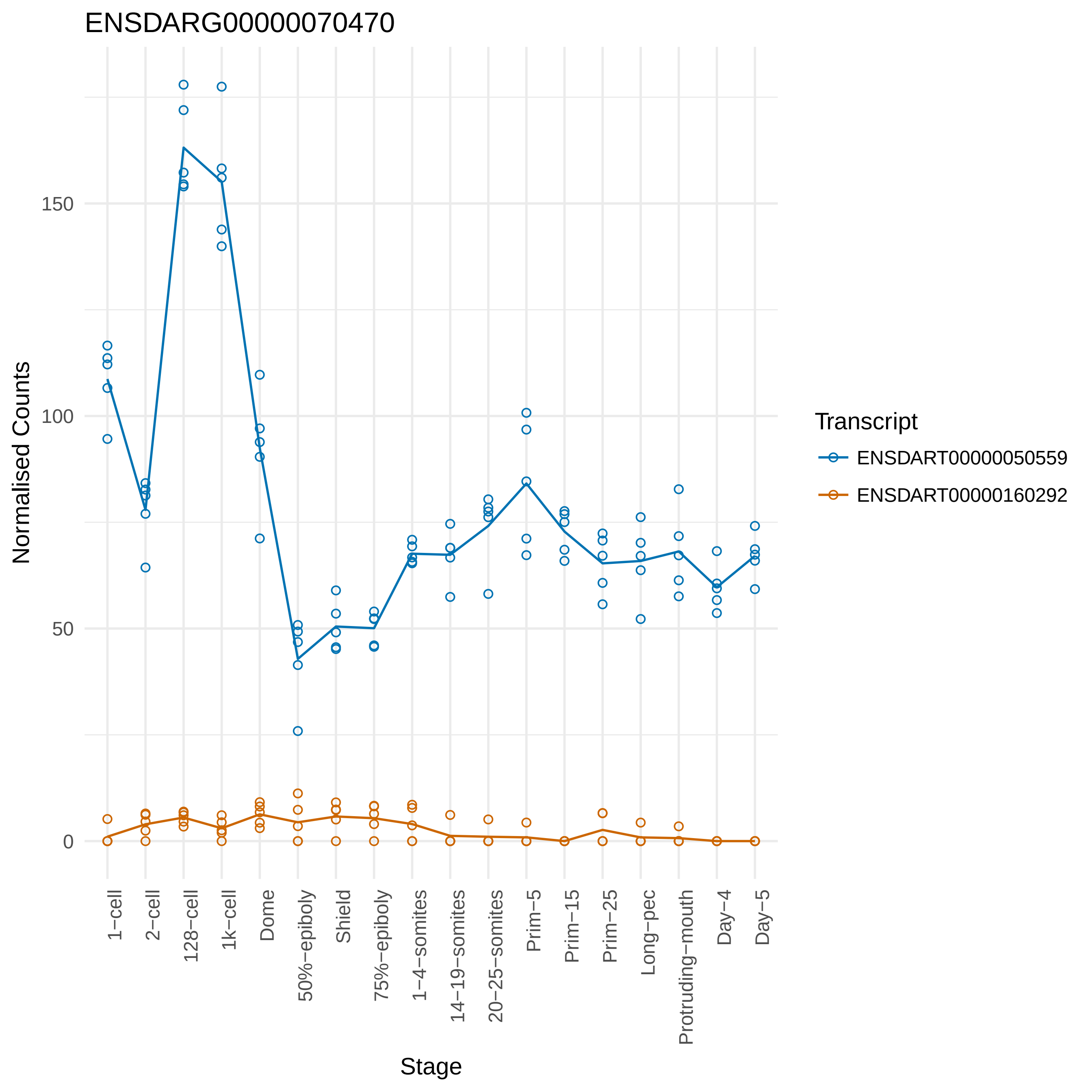

ENSDARG00000070470
Normalised Counts
150
100
50
0
1−cell
2−cell
128−cell
1k−cell
Dome
50%−epiboly
Shield
75%−epiboly
1−4−somites
14−19−somites
20−25−somites
Prim−5
Prim−15
Prim−25
Long−pec
Protruding−mouth
Day−4
Day−5
Stage
Transcript
ENSDART00000050559
ENSDART00000160292

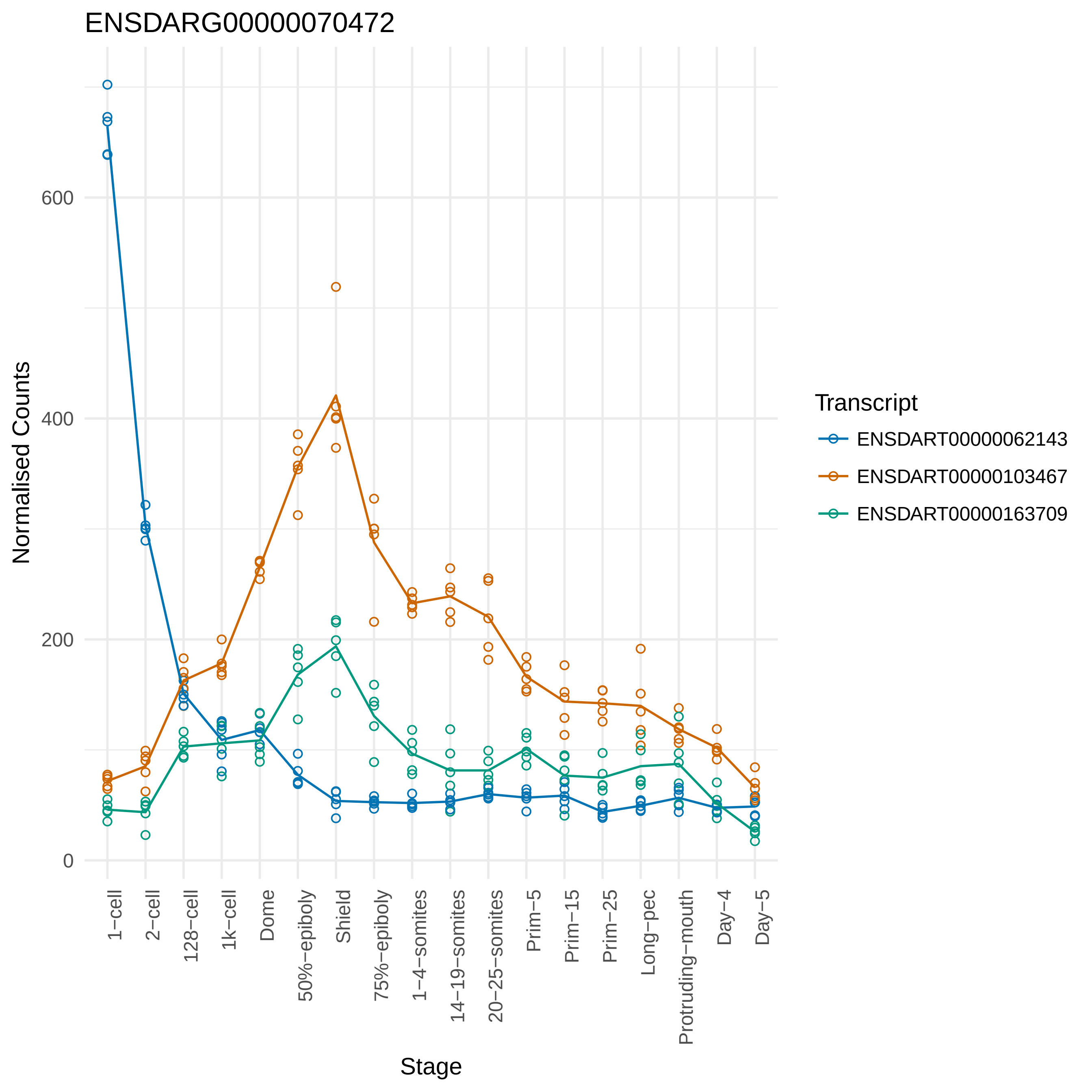
ENSDARG00000070472
Normalised Counts
600
400
200
0
1−cell
2−cell
128−cell
1k−cell
Dome
50%−epiboly
Shield
75%−epiboly
1−4−somites
14−19−somites
20−25−somites
Prim−5
Prim−15
Prim−25
Long−pec
Protruding−mouth
Day−4
Day−5
Stage
Transcript
ENSDART00000062143
ENSDART00000103467
ENSDART00000163709

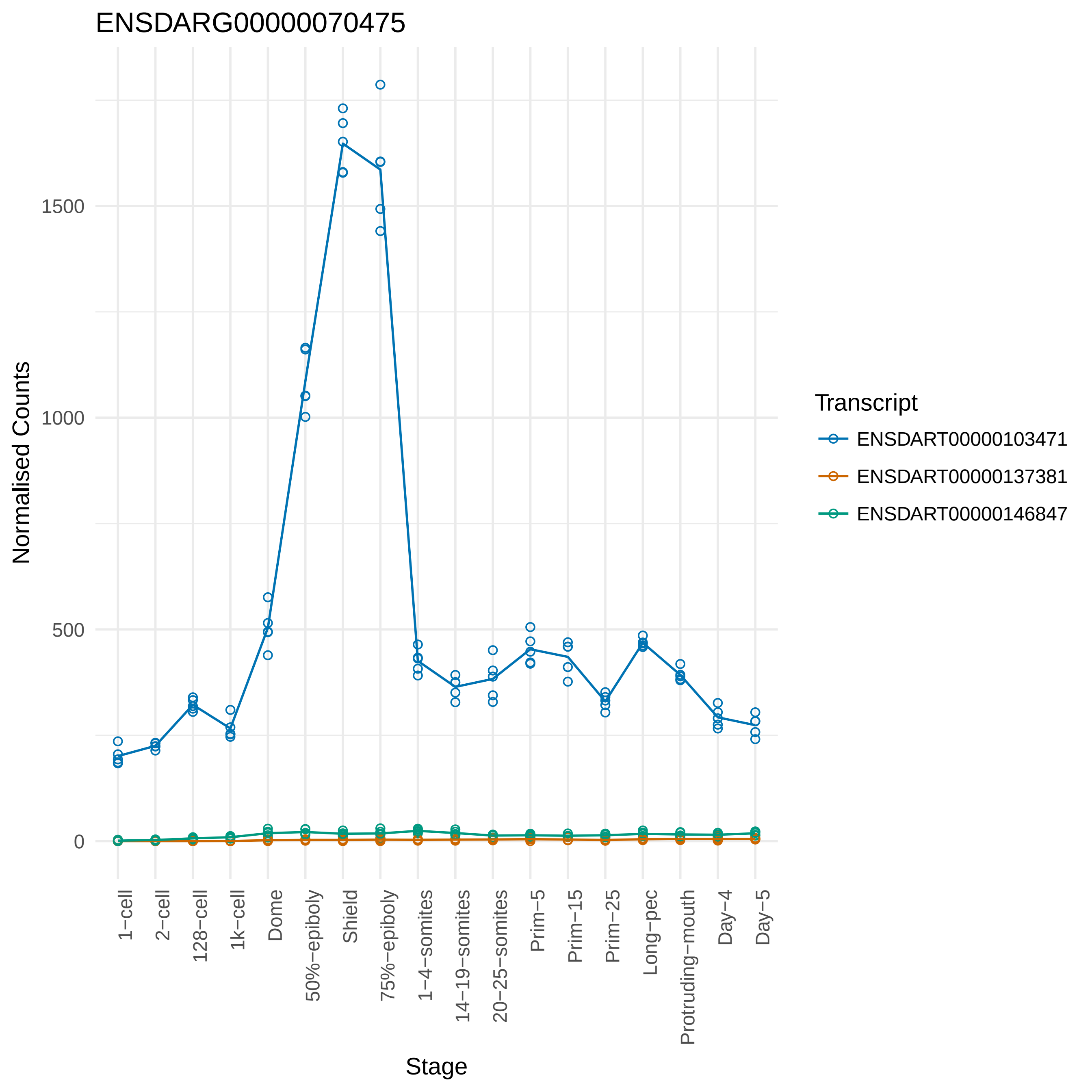

ENSDARG00000070475
Normalised Counts
1500
1000
500
0
1−cell
2−cell
128−cell
1k−cell
Dome
50%−epiboly
Shield
75%−epiboly
1−4−somites
14−19−somites
20−25−somites
Prim−5
Prim−15
Prim−25
Long−pec
Protruding−mouth
Day−4
Day−5
Stage
Transcript
ENSDART00000103471
ENSDART00000137381
ENSDART00000146847

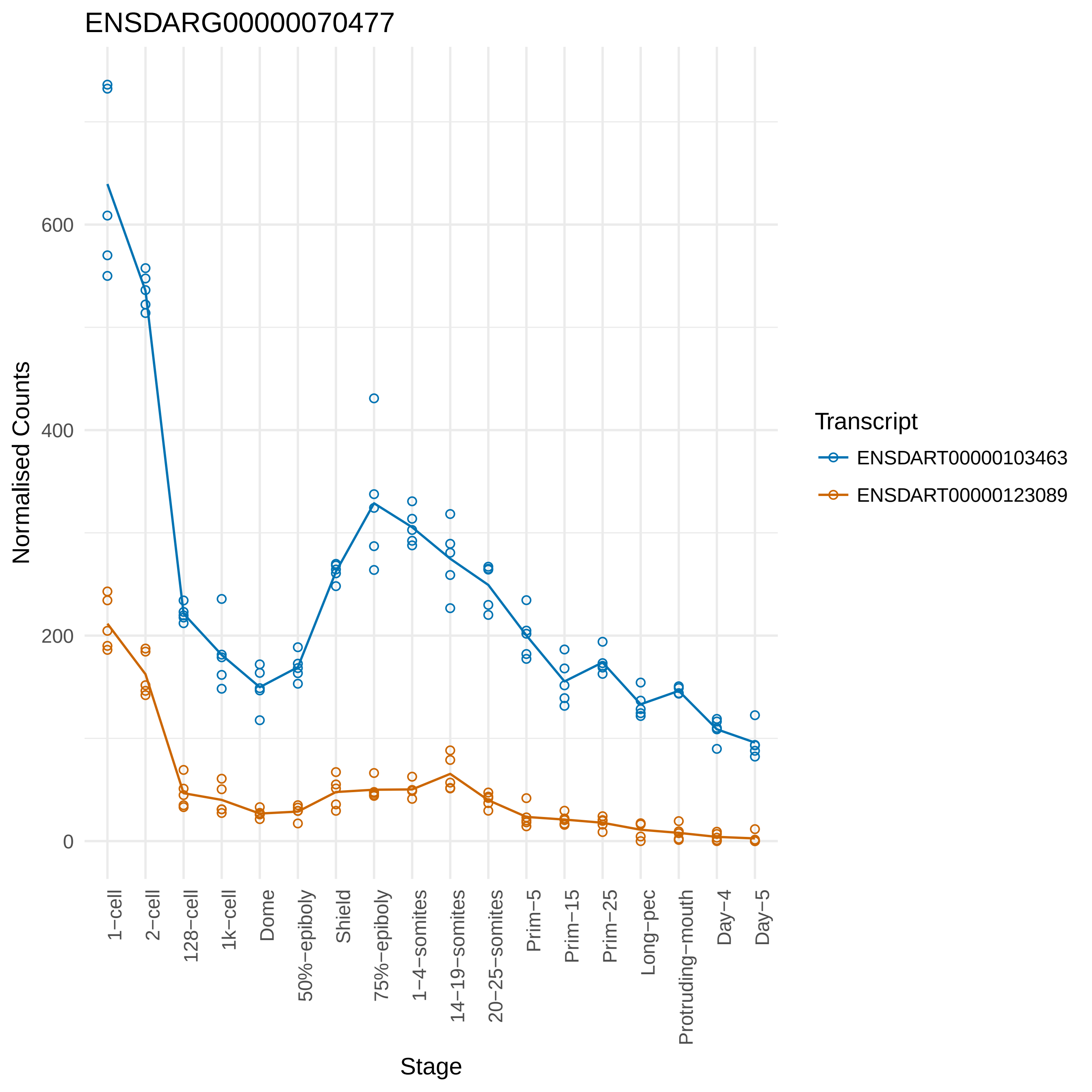

ENSDARG00000070477
Normalised Counts
600
400
200
0
1−cell
2−cell
128−cell
1k−cell
Dome
50%−epiboly
Shield
75%−epiboly
1−4−somites
14−19−somites
20−25−somites
Prim−5
Prim−15
Prim−25
Long−pec
Protruding−mouth
Day−4
Day−5
Stage
Transcript
ENSDART00000103463
ENSDART00000123089

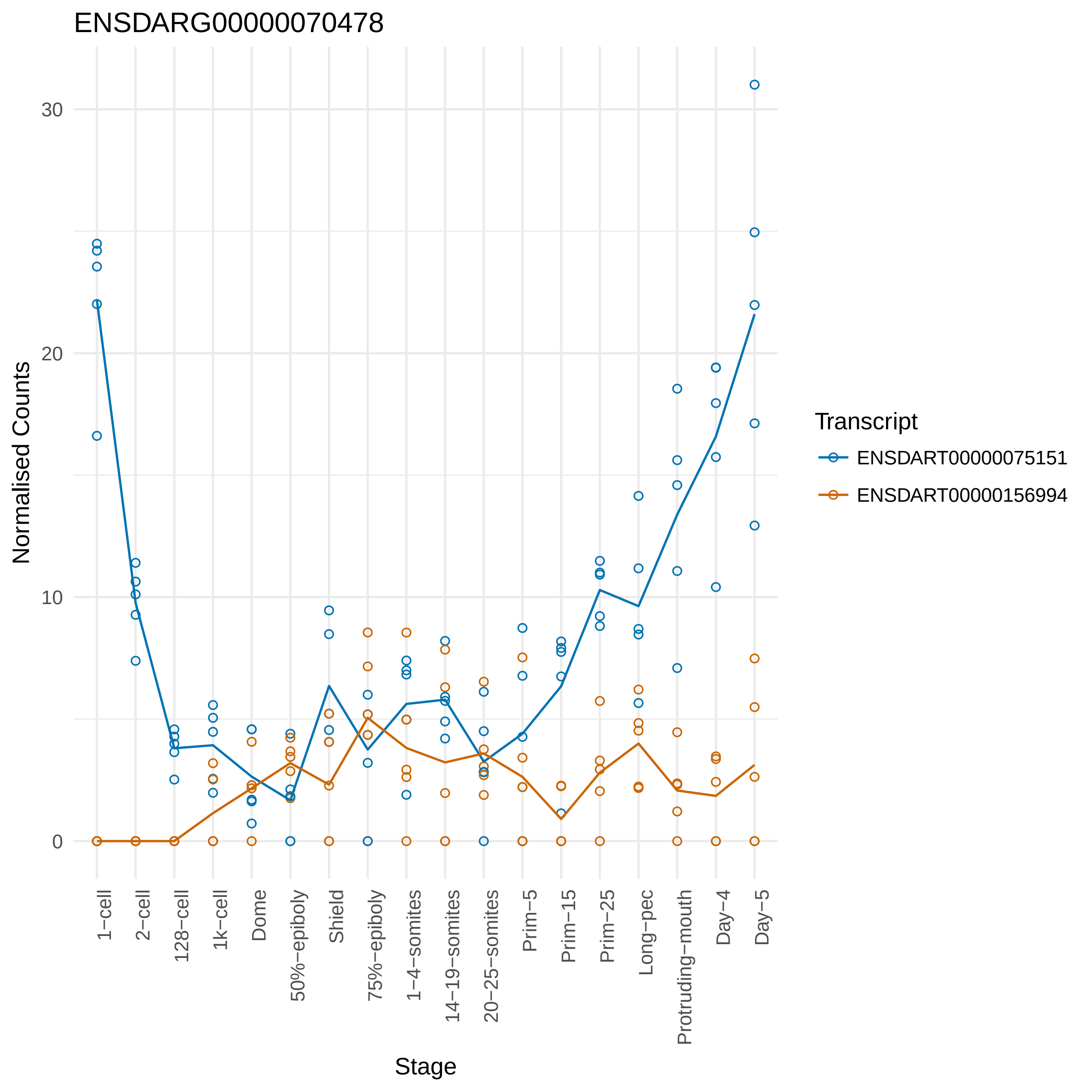
ENSDARG00000070478
Normalised Counts
0
10
20
30
1−cell
2−cell
128−cell
1k−cell
Dome
50%−epiboly
Shield
75%−epiboly
1−4−somites
14−19−somites
20−25−somites
Prim−5
Prim−15
Prim−25
Long−pec
Protruding−mouth
Day−4
Day−5
Stage
Transcript
ENSDART00000075151
ENSDART00000156994

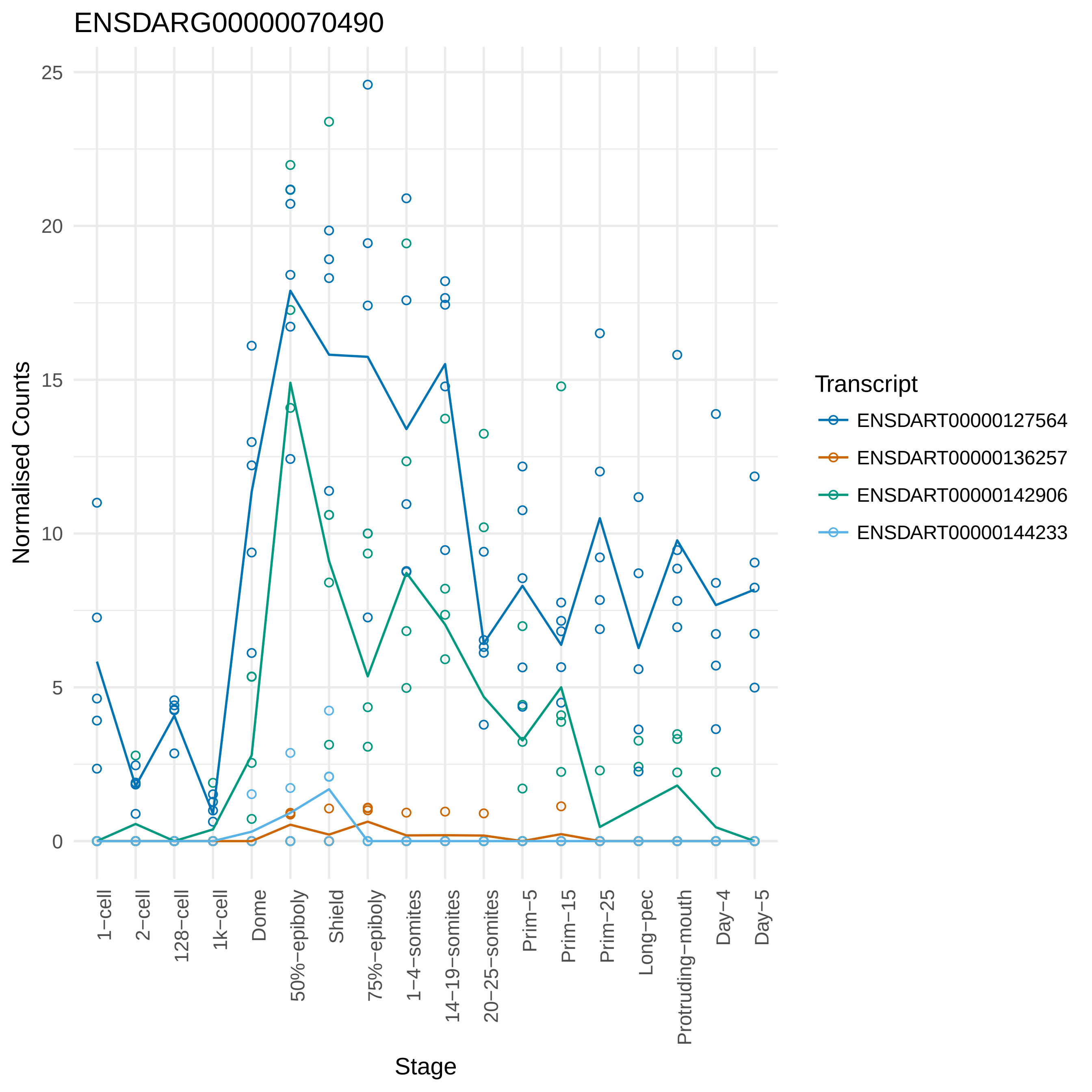
ENSDARG00000070490
Normalised Counts
25
20
15
10
5
0
1−cell
2−cell
128−cell
1k−cell
Dome
50%−epiboly
Shield
75%−epiboly
1−4−somites
14−19−somites
20−25−somites
Prim−5
Prim−15
Prim−25
Long−pec
Protruding−mouth
Day−4
Day−5
Stage
Transcript
ENSDART00000127564
ENSDART00000136257
ENSDART00000142906
ENSDART00000144233

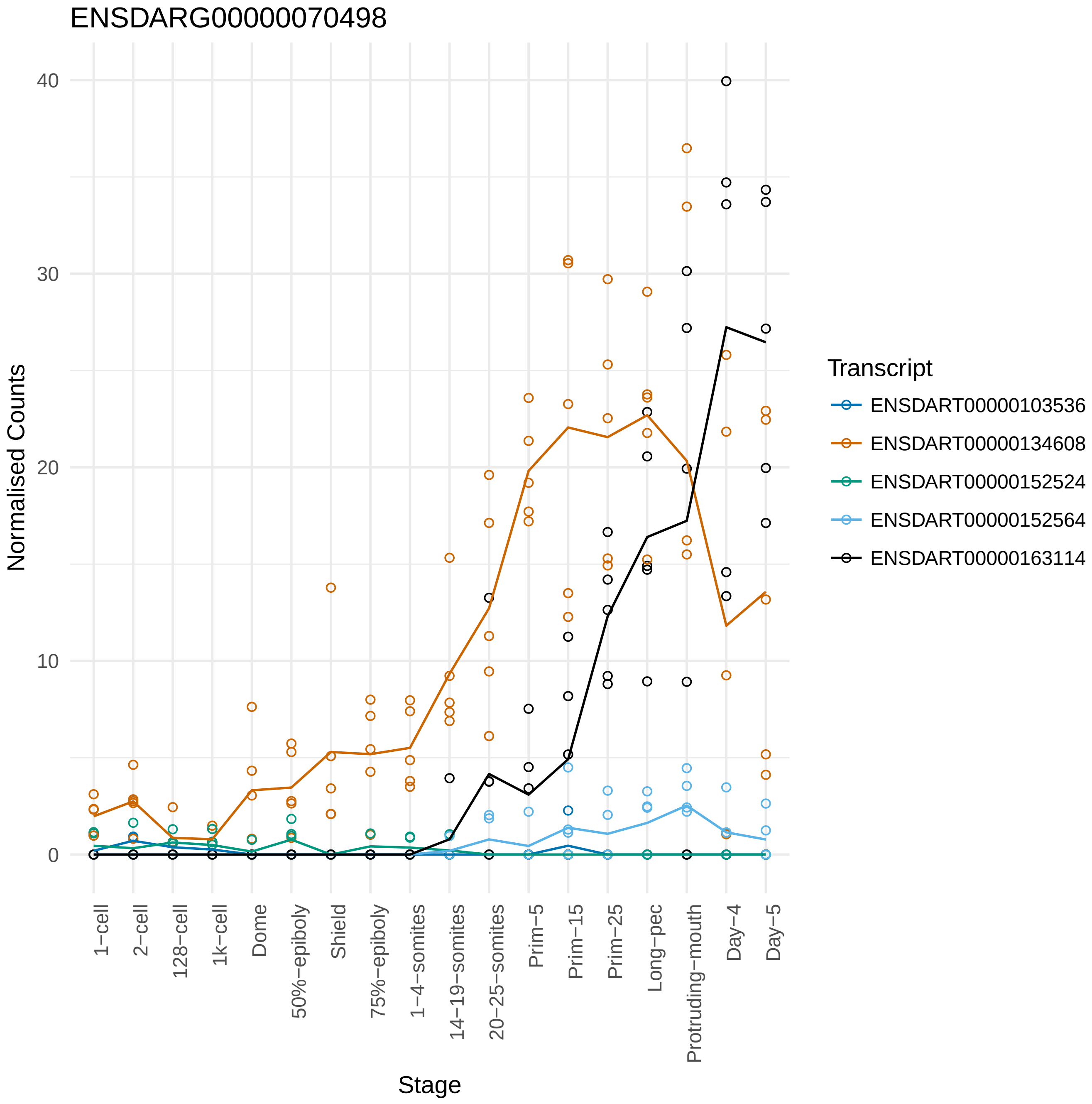

ENSDARG00000070498
Normalised Counts
0
10
20
30
40
1−cell
2−cell
128−cell
1k−cell
Dome
50%−epiboly
Shield
75%−epiboly
1−4−somites
14−19−somites
20−25−somites
Prim−5
Prim−15
Prim−25
Long−pec
Protruding−mouth
Day−4
Day−5
Stage
Transcript
ENSDART00000103536
ENSDART00000134608
ENSDART00000152524
ENSDART00000152564
ENSDART00000163114

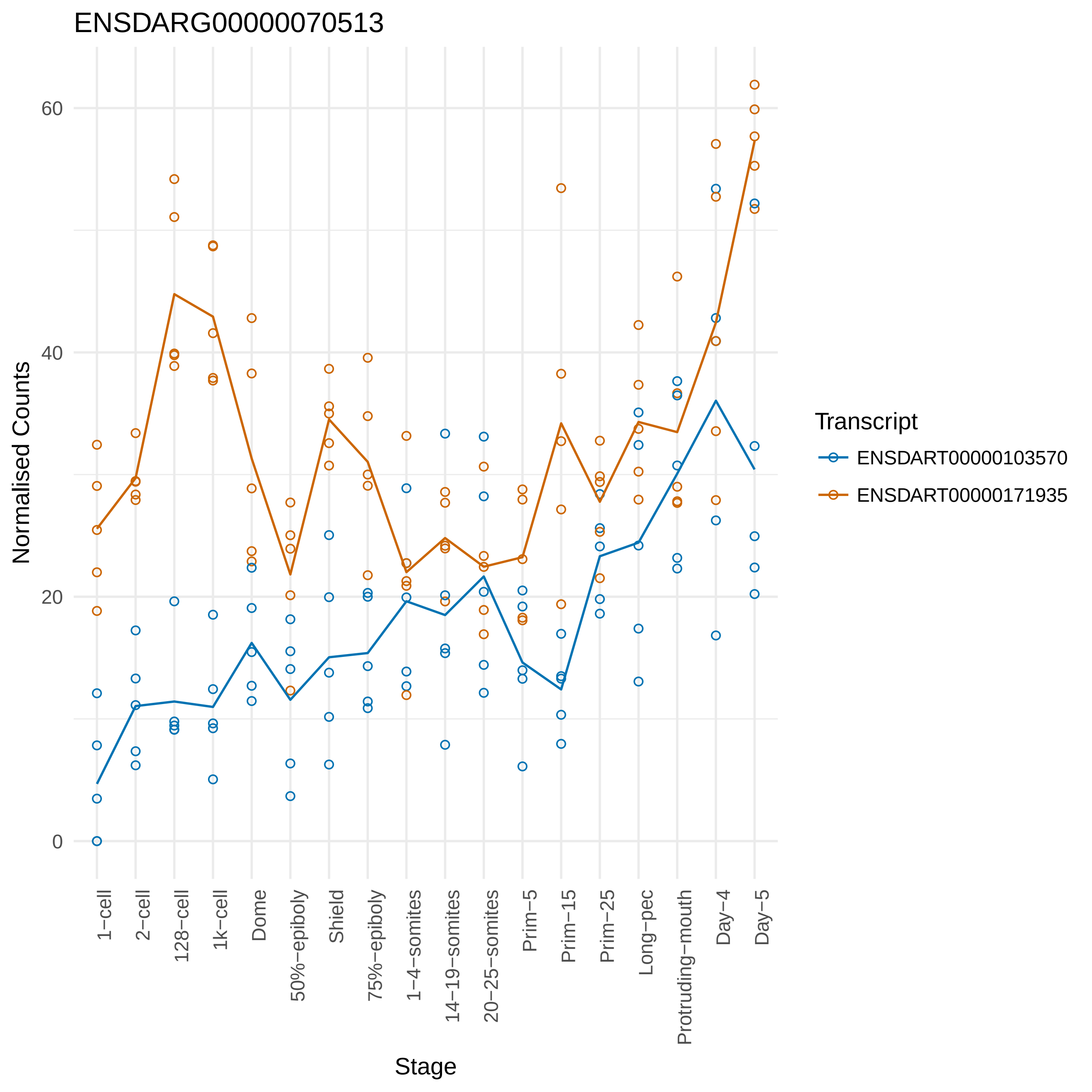
ENSDARG00000070513
Normalised Counts
0
20
40
60
1−cell
2−cell
128−cell
1k−cell
Dome
50%−epiboly
Shield
75%−epiboly
1−4−somites
14−19−somites
20−25−somites
Prim−5
Prim−15
Prim−25
Long−pec
Protruding−mouth
Day−4
Day−5
Stage
Transcript
ENSDART00000103570
ENSDART00000171935

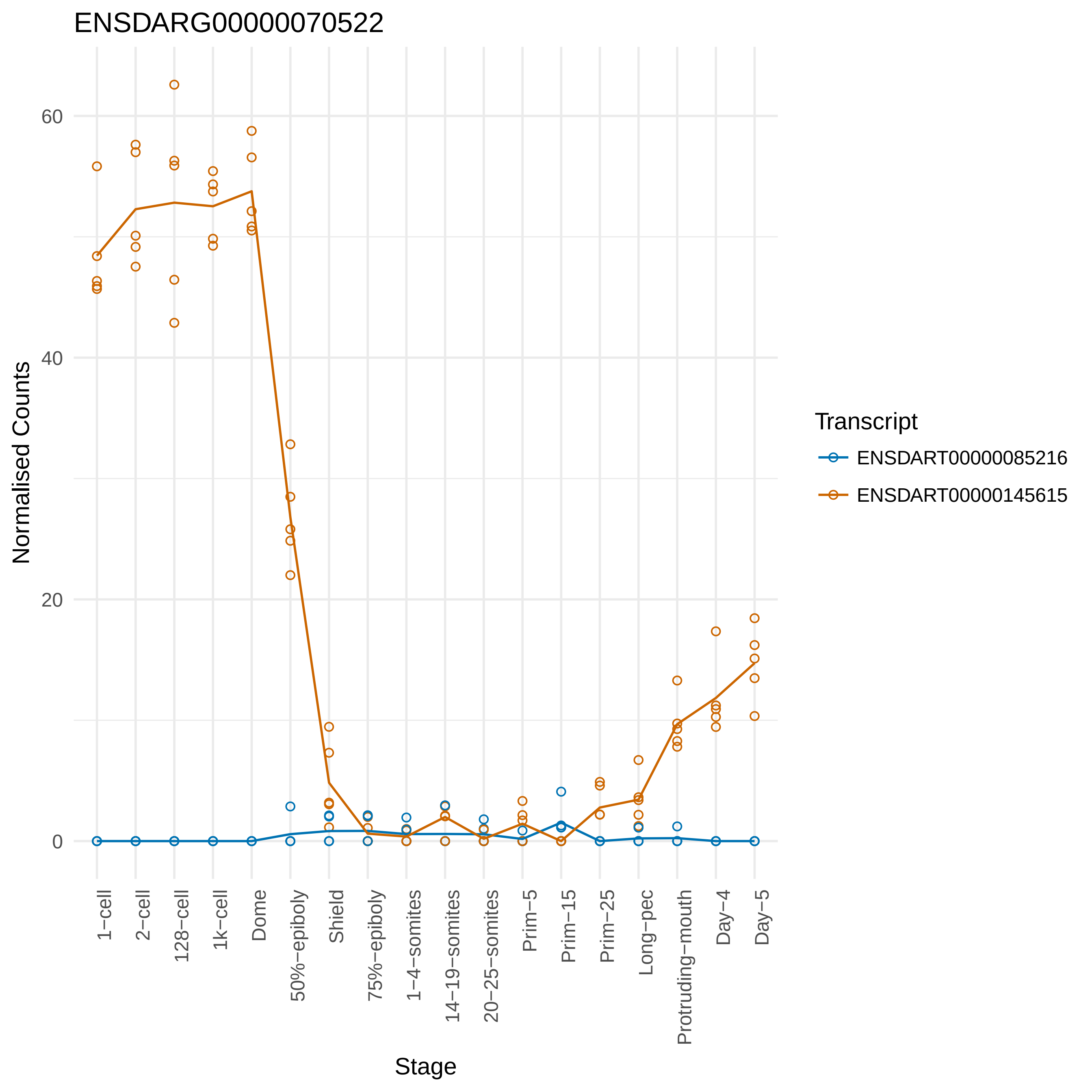

ENSDARG00000070522
Normalised Counts
60
40
20
0
Stage
1−cell
2−cell
128−cell
1k−cell
Dome
50%−epiboly
Shield
75%−epiboly
1−4−somites
14−19−somites
20−25−somites
Prim−5
Prim−15
Prim−25
Long−pec
Protruding−mouth
Day−4
Day−5
Transcript
ENSDART00000085216
ENSDART00000145615

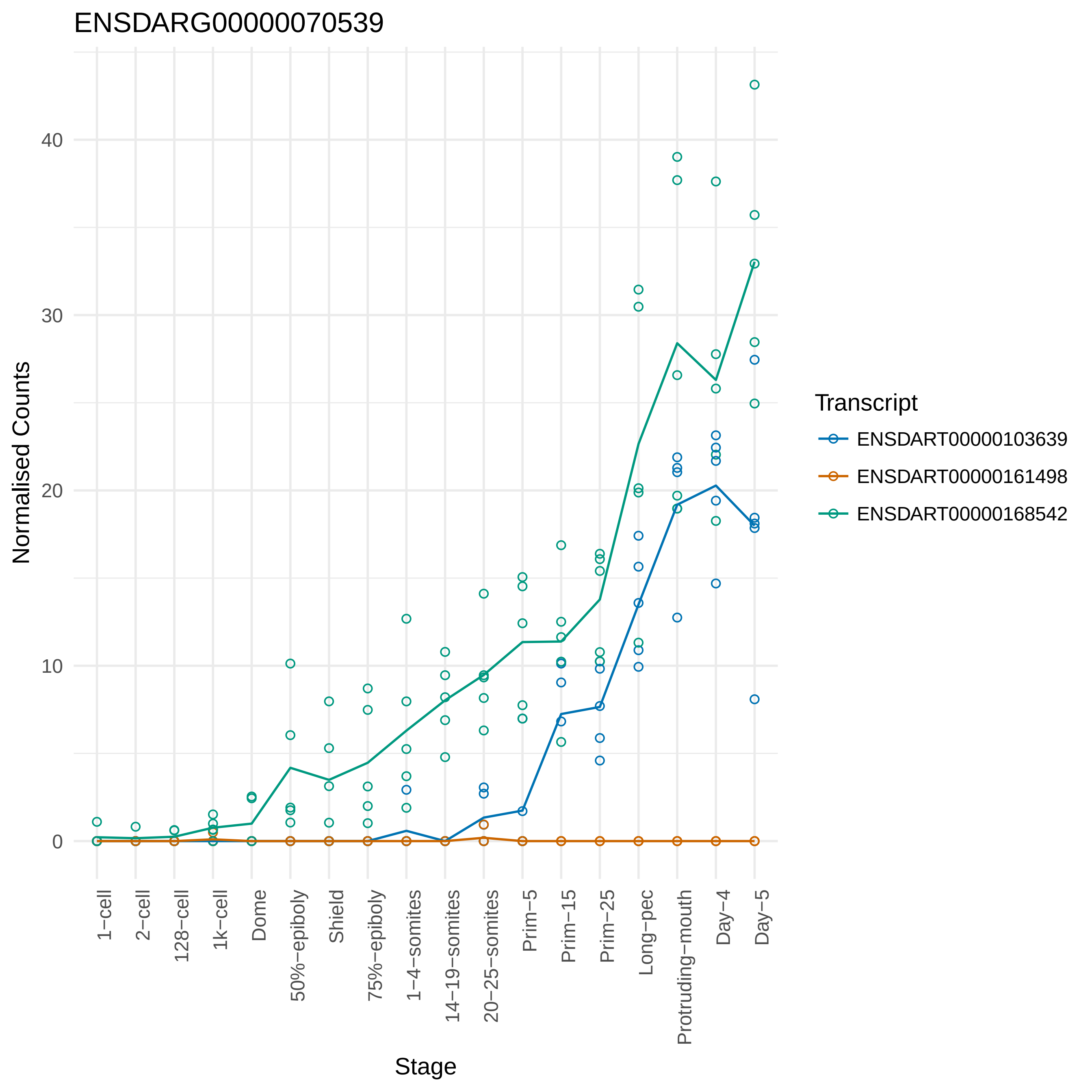
ENSDARG00000070539
Normalised Counts
0
10
20
30
40
Stage
1−cell
2−cell
128−cell
1k−cell
Dome
50%−epiboly
Shield
75%−epiboly
1−4−somites
14−19−somites
20−25−somites
Prim−5
Prim−15
Prim−25
Long−pec
Protruding−mouth
Day−4
Day−5
Transcript
ENSDART00000103639
ENSDART00000161498
ENSDART00000168542

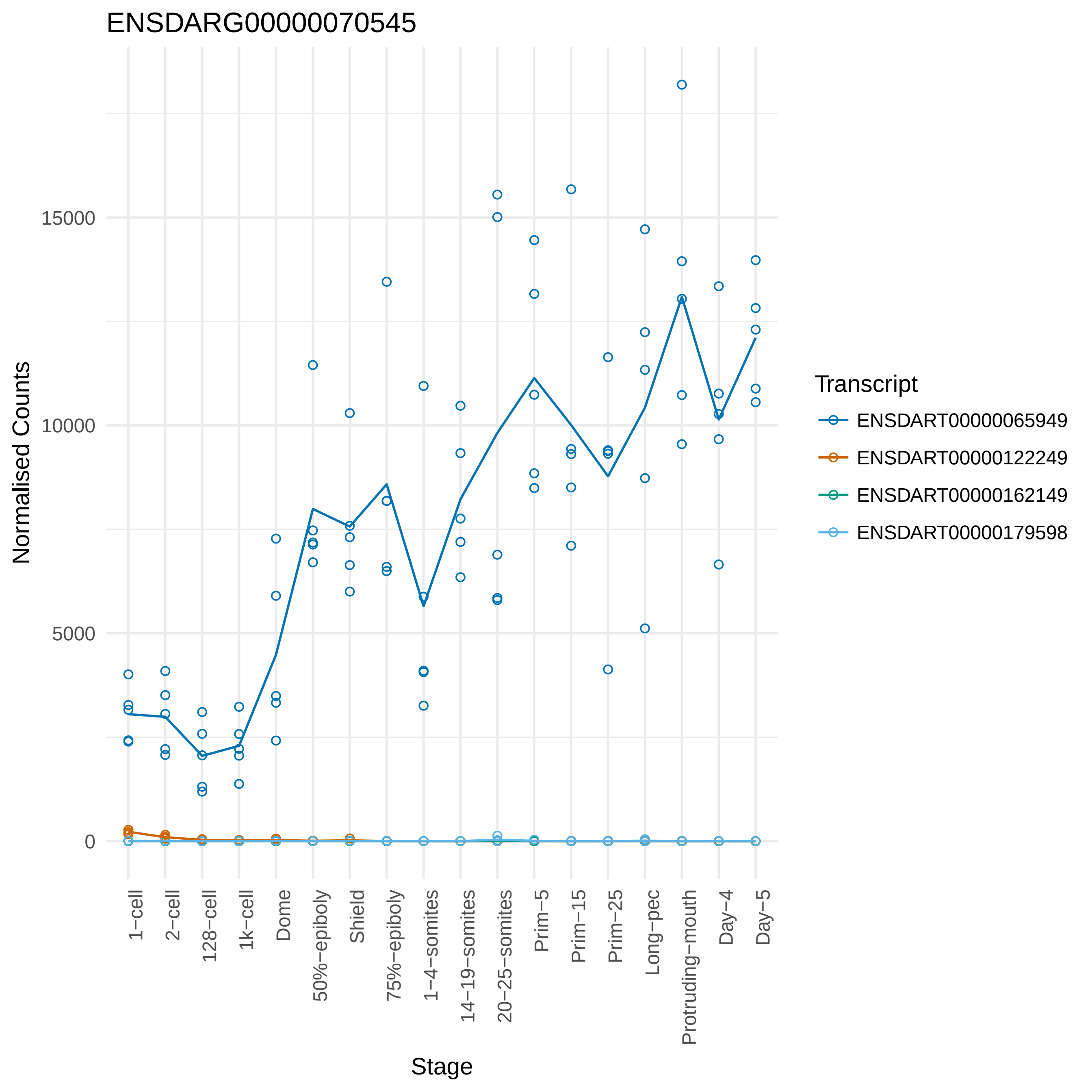
ENSDARG00000070545
Normalised Counts
15000
10000
5000
0
1−cell
2−cell
128−cell
1k−cell
Dome
50%−epiboly
Shield
75%−epiboly
1−4−somites
14−19−somites
20−25−somites
Prim−5
Prim−15
Prim−25
Long−pec
Protruding−mouth
Day−4
Day−5
Stage
Transcript
ENSDART00000065949
ENSDART00000122249
ENSDART00000162149
ENSDART00000179598

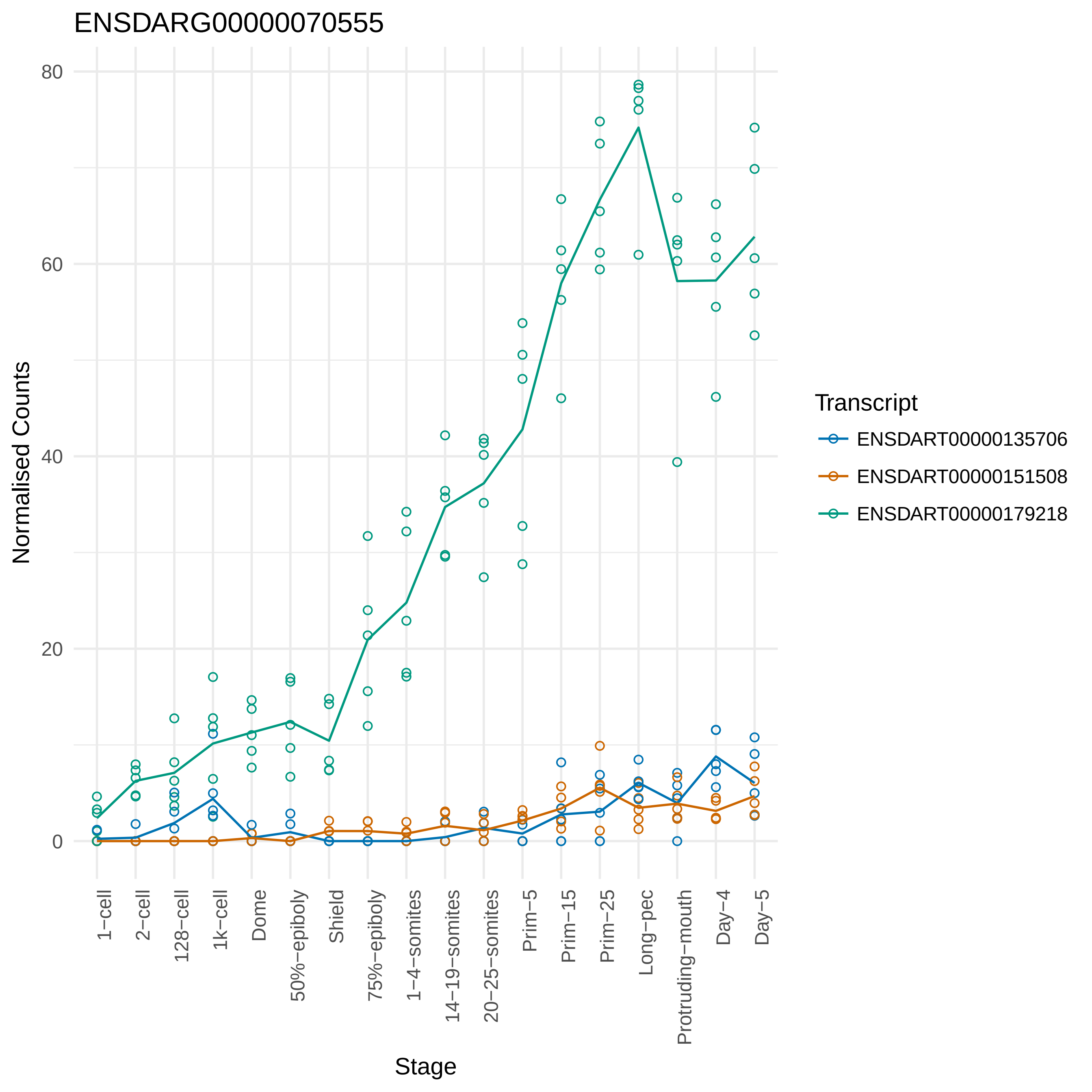

ENSDARG00000070555
Normalised Counts
80
60
40
20
0
1−cell
2−cell
128−cell
1k−cell
Dome
50%−epiboly
Shield
75%−epiboly
1−4−somites
14−19−somites
20−25−somites
Prim−5
Prim−15
Prim−25
Long−pec
Protruding−mouth
Day−4
Day−5
Stage
Transcript
ENSDART00000135706
ENSDART00000151508
ENSDART00000179218

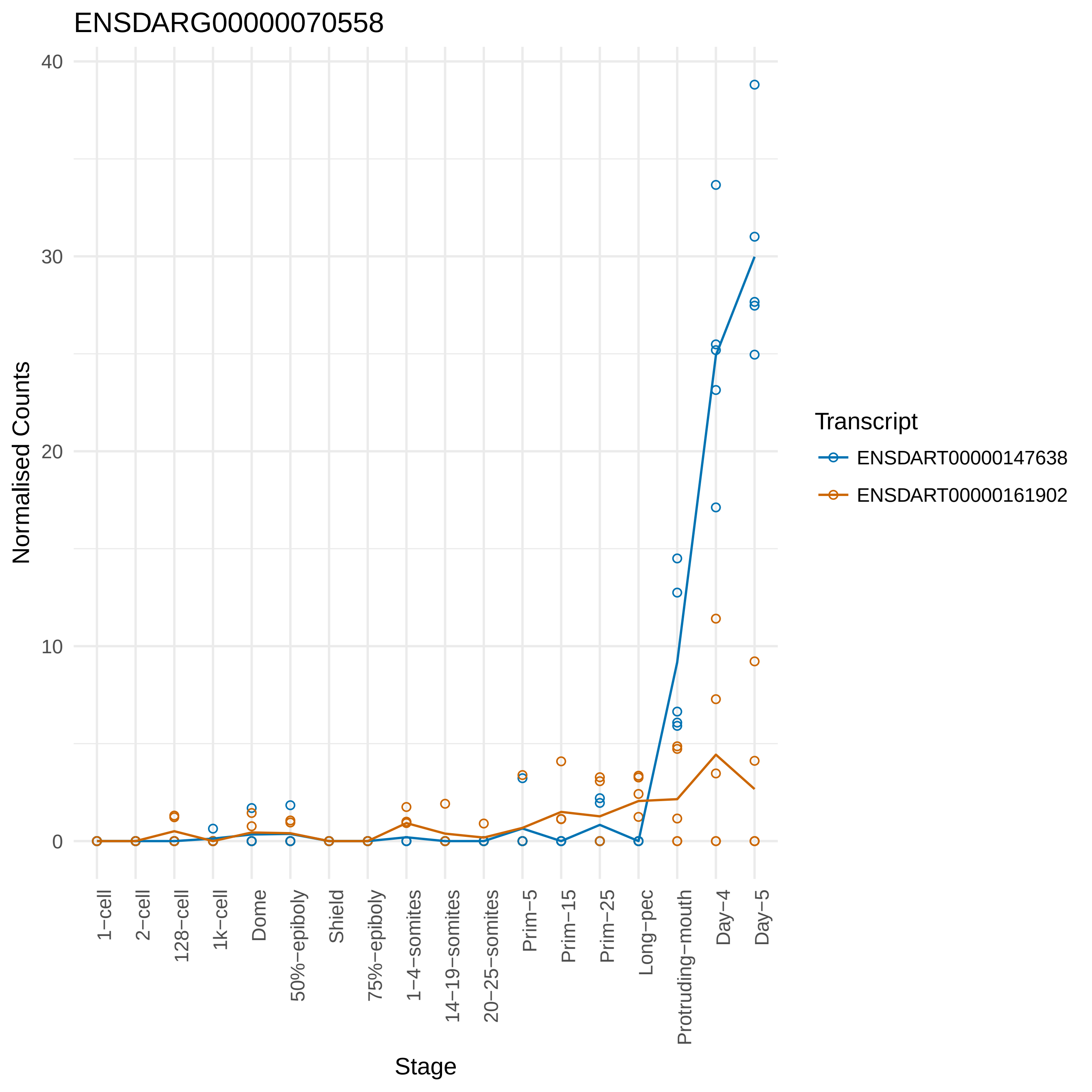

ENSDARG00000070558
Normalised Counts
40
30
20
10
0
1−cell
2−cell
128−cell
1k−cell
Dome
50%−epiboly
Shield
75%−epiboly
1−4−somites
14−19−somites
20−25−somites
Prim−5
Prim−15
Prim−25
Long−pec
Protruding−mouth
Day−4
Day−5
Stage
Transcript
ENSDART00000147638
ENSDART00000161902

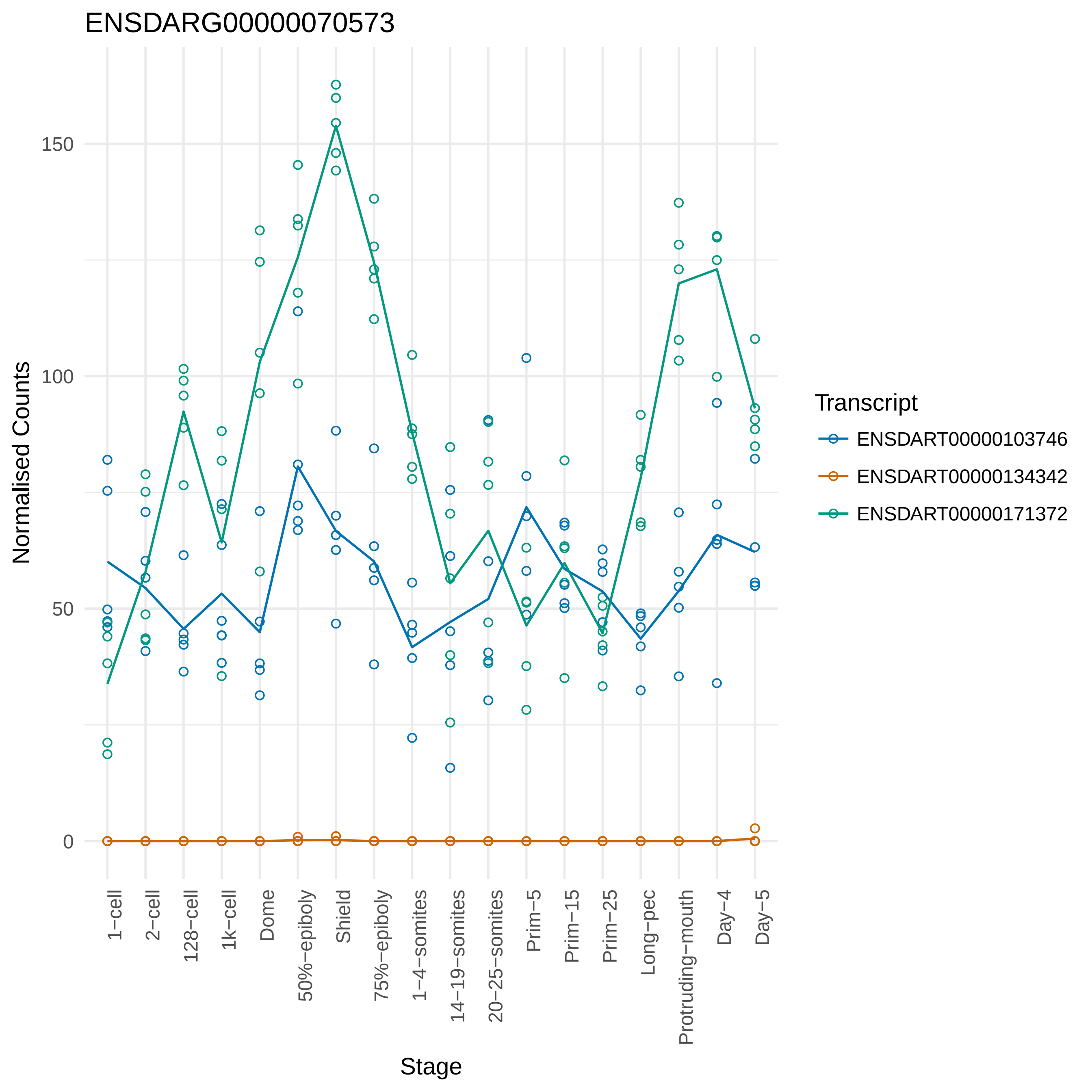

ENSDARG00000070573
Normalised Counts
150
100
50
0
1−cell
2−cell
128−cell
1k−cell
Dome
50%−epiboly
Shield
75%−epiboly
1−4−somites
14−19−somites
20−25−somites
Prim−5
Prim−15
Prim−25
Long−pec
Protruding−mouth
Day−4
Day−5
Stage
Transcript
ENSDART00000103746
ENSDART00000134342
ENSDART00000171372

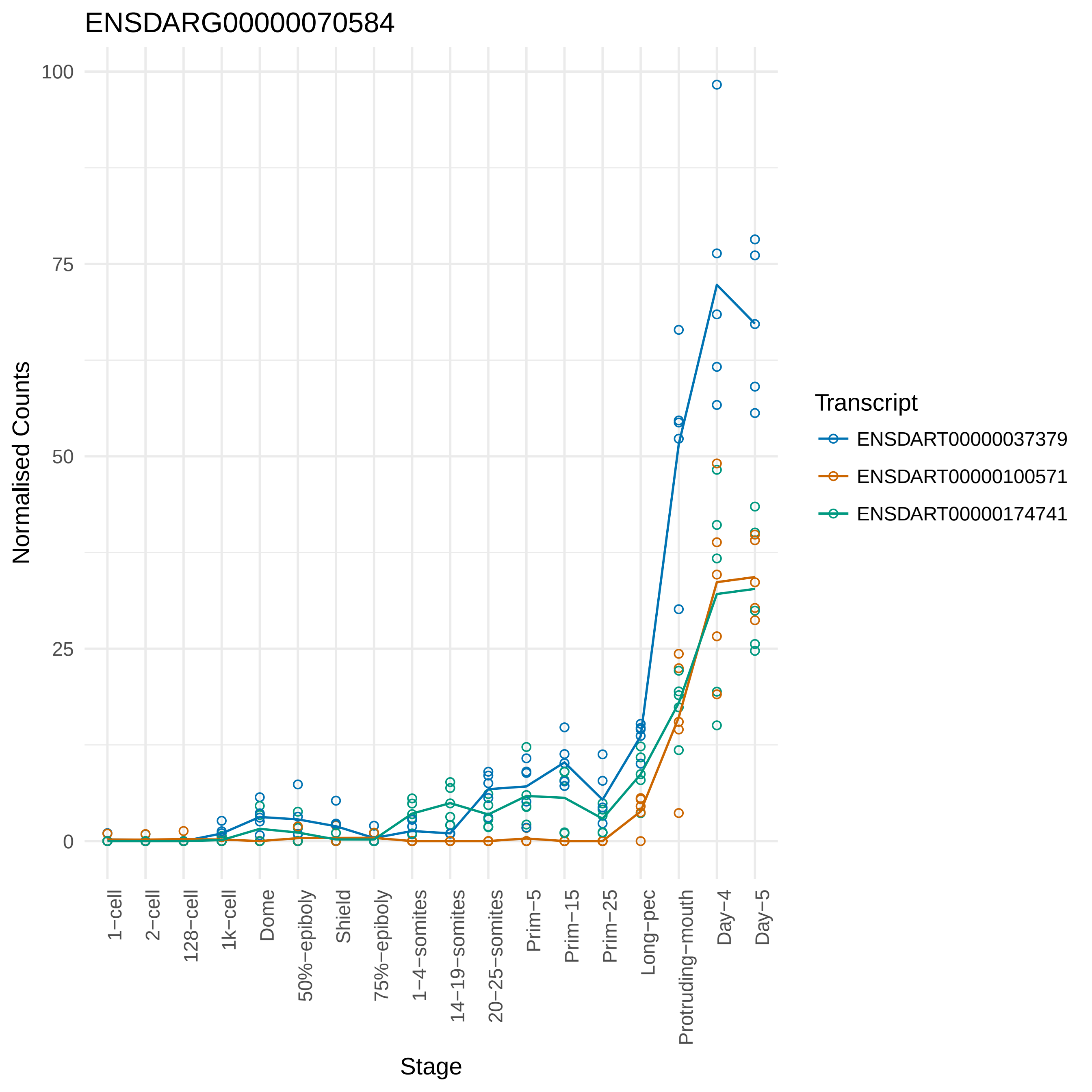

ENSDARG00000070584
Normalised Counts
100
75
50
25
0
1−cell
2−cell
128−cell
1k−cell
Dome
50%−epiboly
Shield
75%−epiboly
1−4−somites
14−19−somites
20−25−somites
Prim−5
Prim−15
Prim−25
Long−pec
Protruding−mouth
Day−4
Day−5
Stage
Transcript
ENSDART00000037379
ENSDART00000100571
ENSDART00000174741

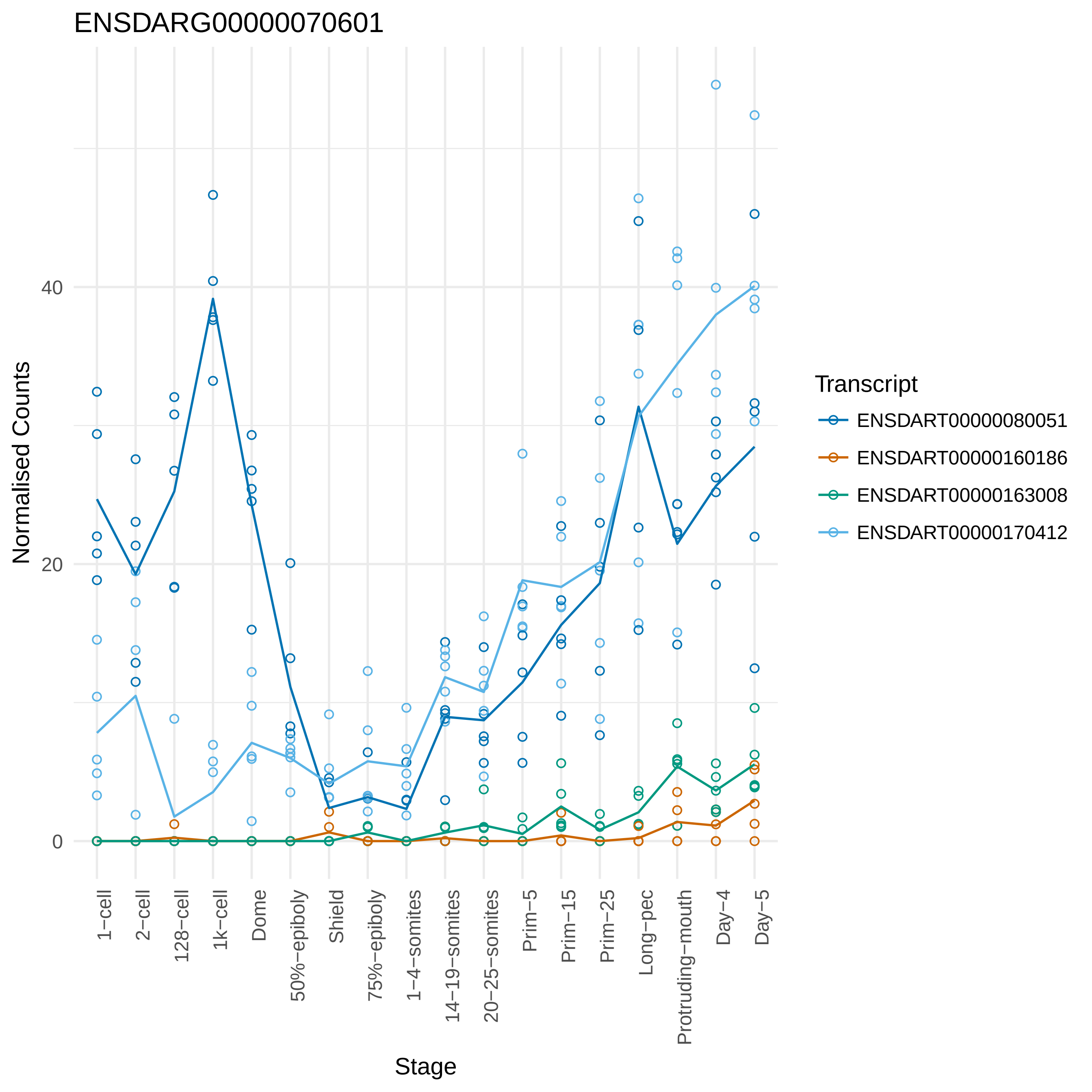

ENSDARG00000070601
Normalised Counts
0
20
40
Stage
1−cell
2−cell
128−cell
1k−cell
Dome
50%−epiboly
Shield
75%−epiboly
1−4−somites
14−19−somites
20−25−somites
Prim−5
Prim−15
Prim−25
Long−pec
Protruding−mouth
Day−4
Day−5
Transcript
ENSDART00000080051
ENSDART00000160186
ENSDART00000163008
ENSDART00000170412

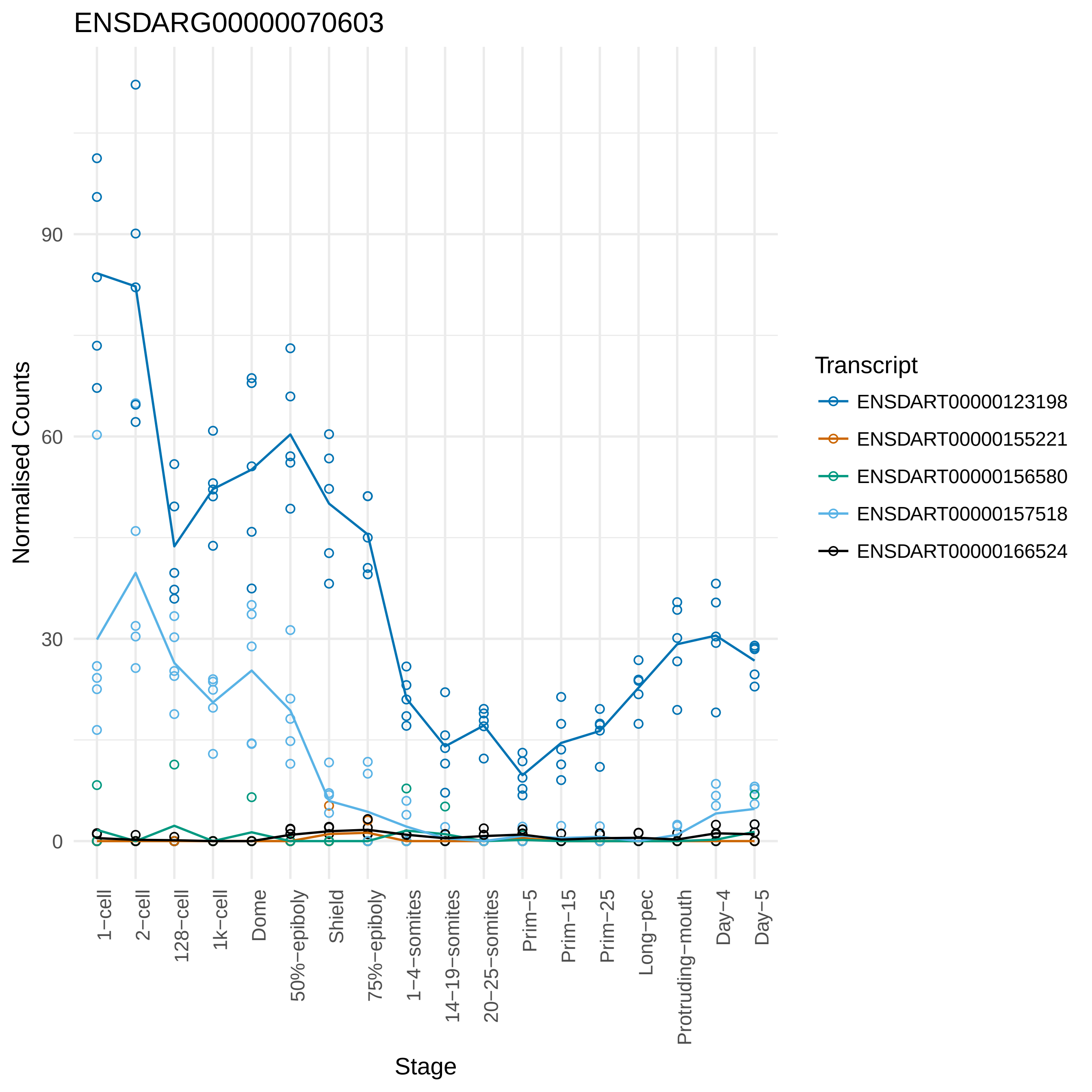

ENSDARG00000070603
Normalised Counts
0
30
60
90
Stage
1−cell
2−cell
128−cell
1k−cell
Dome
50%−epiboly
Shield
75%−epiboly
1−4−somites
14−19−somites
20−25−somites
Prim−5
Prim−15
Prim−25
Long−pec
Protruding−mouth
Day−4
Day−5
Transcript
ENSDART00000123198
ENSDART00000155221
ENSDART00000156580
ENSDART00000157518
ENSDART00000166524

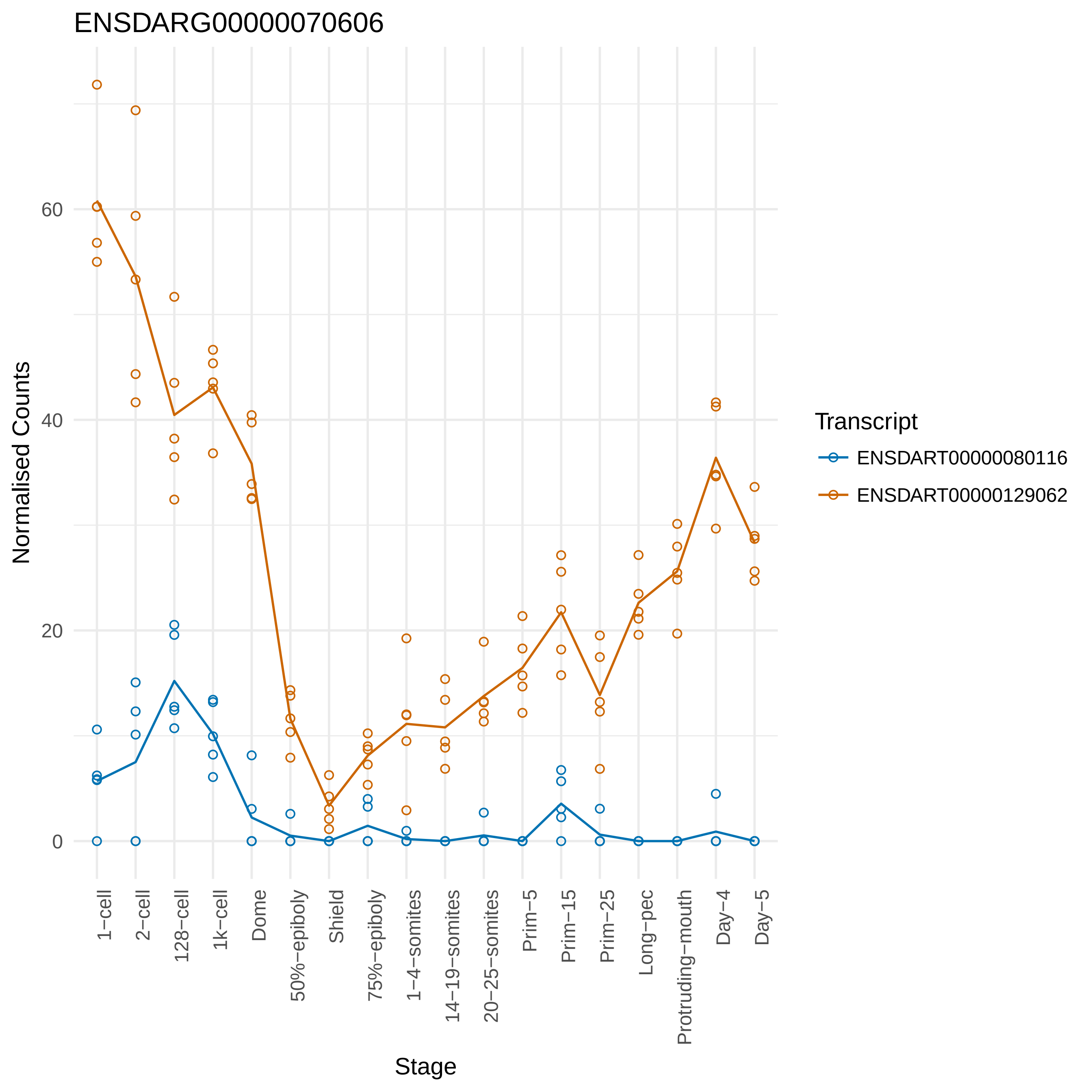

ENSDARG00000070606
Normalised Counts
0
20
40
60
Stage
1−cell
2−cell
128−cell
1k−cell
Dome
50%−epiboly
Shield
75%−epiboly
1−4−somites
14−19−somites
20−25−somites
Prim−5
Prim−15
Prim−25
Long−pec
Protruding−mouth
Day−4
Day−5
Transcript
ENSDART00000080116
ENSDART00000129062

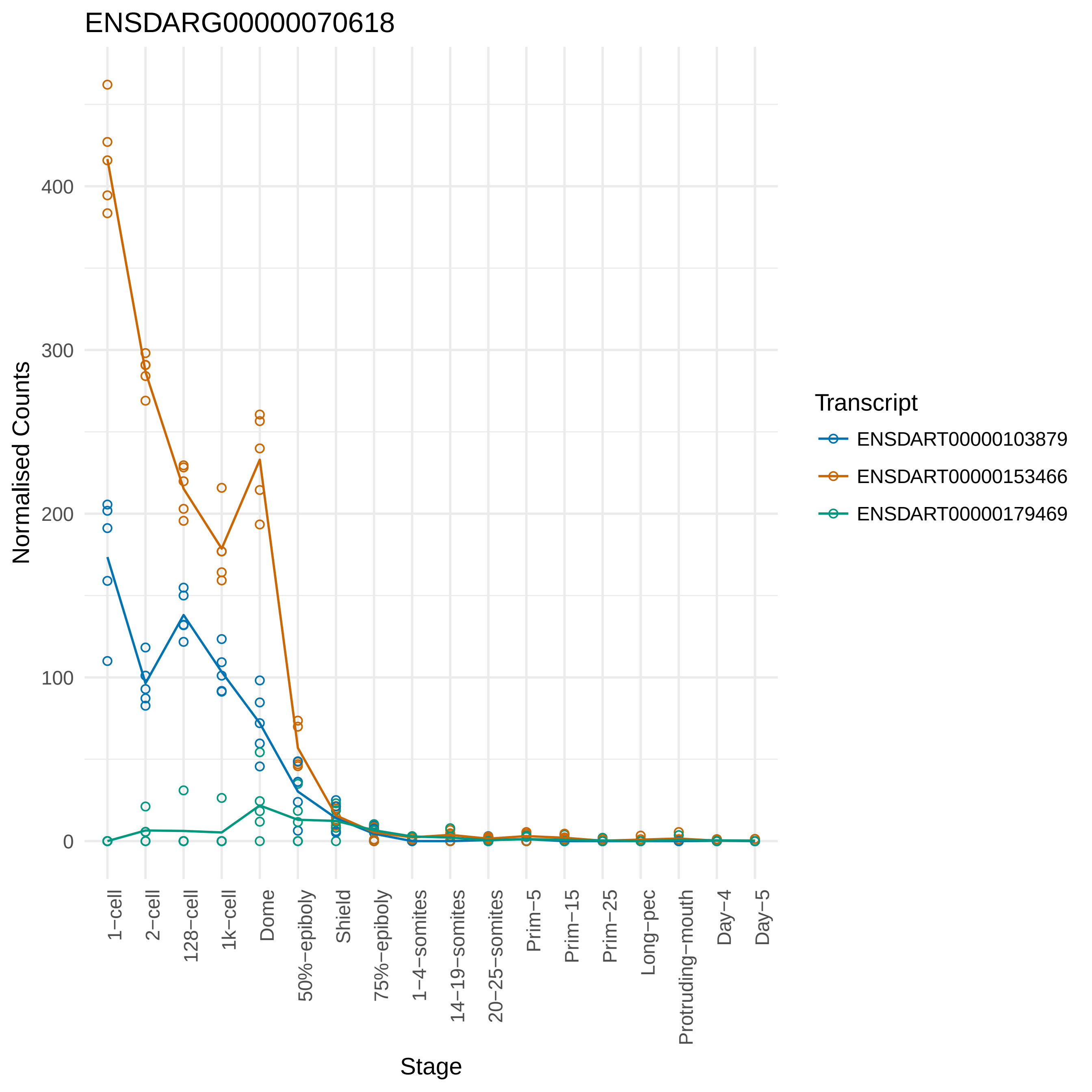

ENSDARG00000070618
Normalised Counts
400
300
200
100
0
1−cell
2−cell
128−cell
1k−cell
Dome
50%−epiboly
Shield
75%−epiboly
1−4−somites
14−19−somites
20−25−somites
Prim−5
Prim−15
Prim−25
Long−pec
Protruding−mouth
Day−4
Day−5
Stage
Transcript
ENSDART00000103879
ENSDART00000153466
ENSDART00000179469

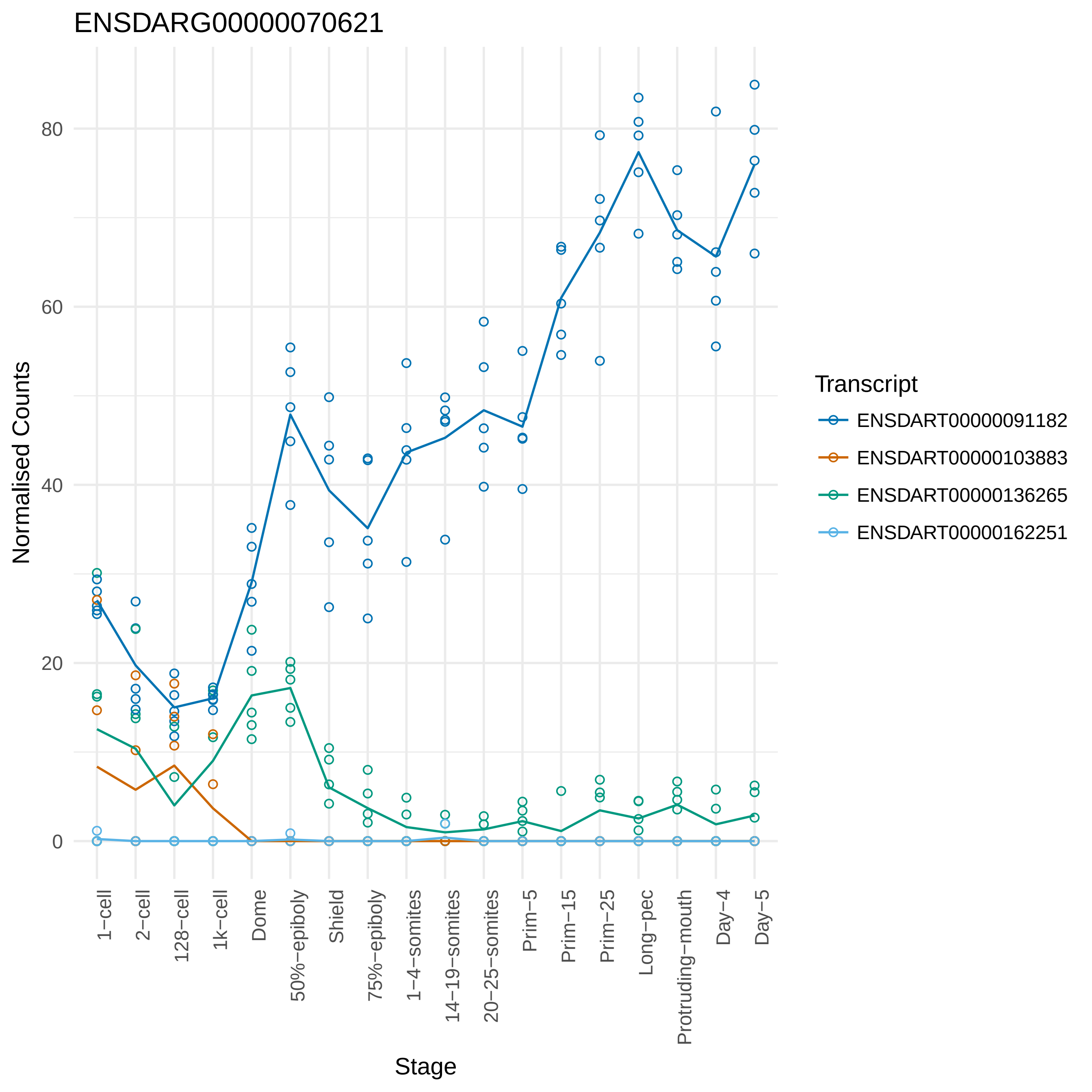

ENSDARG00000070621
Normalised Counts
0
20
40
60
80
1−cell
2−cell
128−cell
1k−cell
Dome
50%−epiboly
Shield
75%−epiboly
1−4−somites
14−19−somites
20−25−somites
Prim−5
Prim−15
Prim−25
Long−pec
Protruding−mouth
Day−4
Day−5
Stage
Transcript
ENSDART00000091182
ENSDART00000103883
ENSDART00000136265
ENSDART00000162251

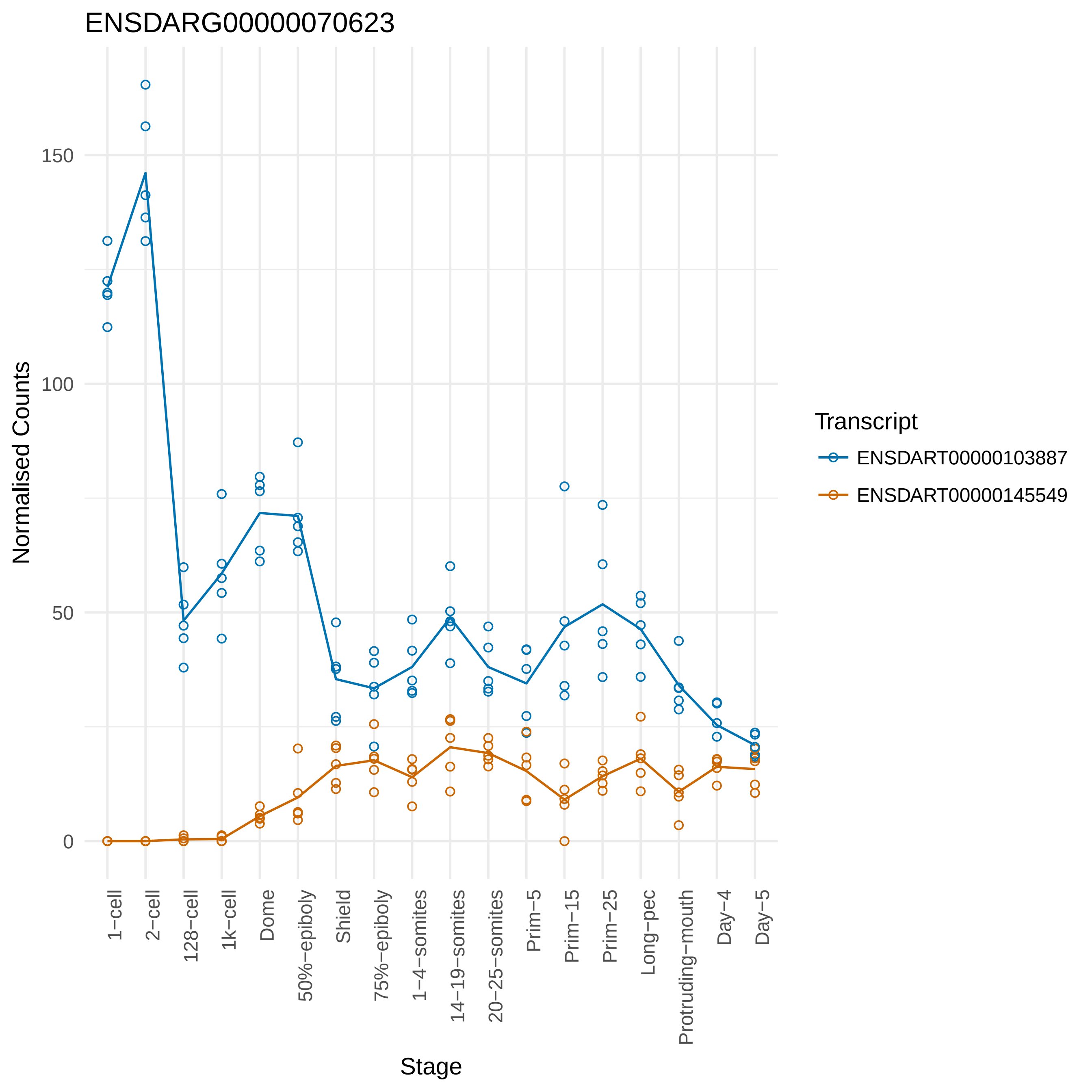

ENSDARG00000070623
Normalised Counts
150
100
50
0
1−cell
2−cell
128−cell
1k−cell
Dome
50%−epiboly
Shield
75%−epiboly
1−4−somites
14−19−somites
20−25−somites
Prim−5
Prim−15
Prim−25
Long−pec
Protruding−mouth
Day−4
Day−5
Stage
Transcript
ENSDART00000103887
ENSDART00000145549

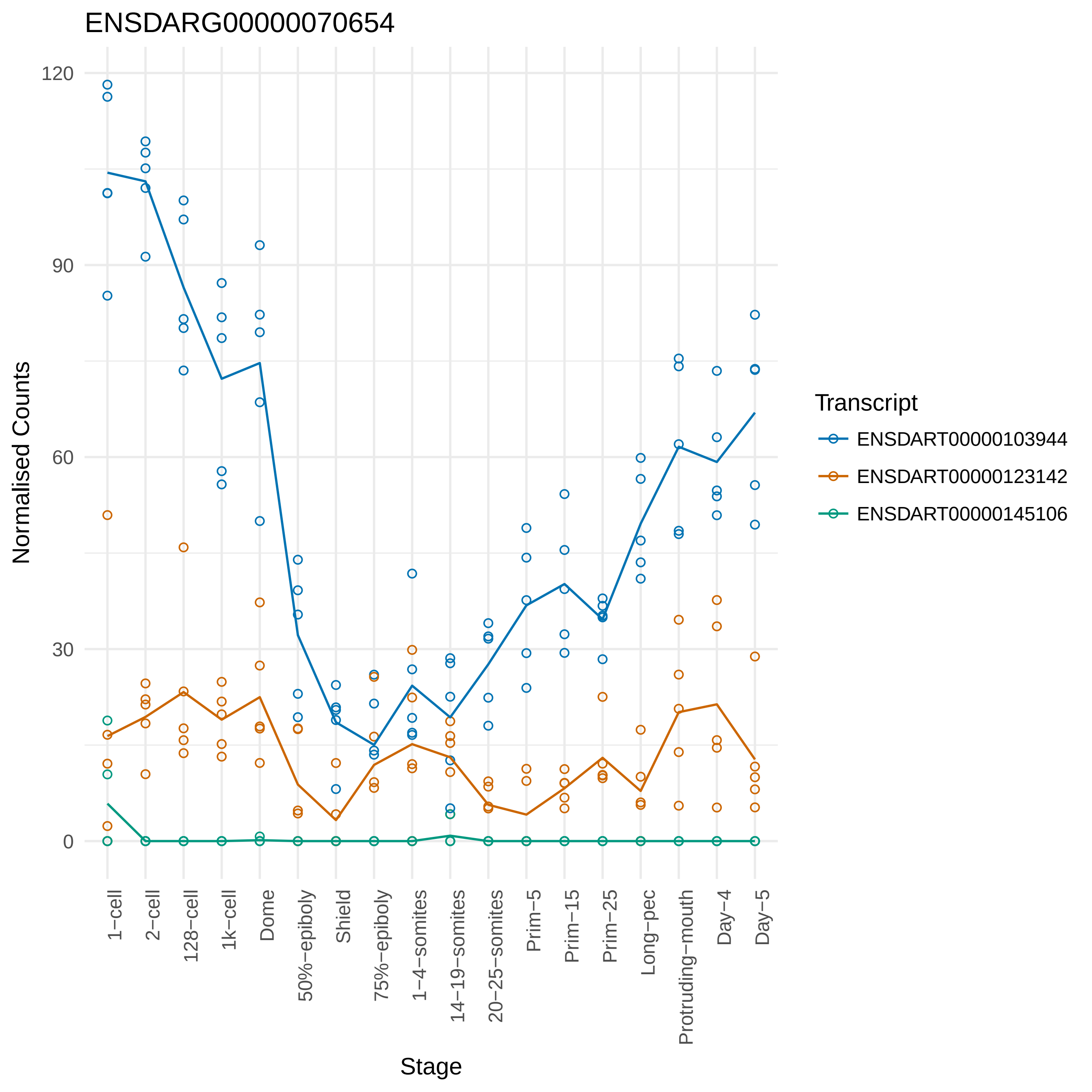

ENSDARG00000070654
Normalised Counts
120
90
60
30
0
1−cell
2−cell
128−cell
1k−cell
Dome
50%−epiboly
Shield
75%−epiboly
1−4−somites
14−19−somites
20−25−somites
Prim−5
Prim−15
Prim−25
Long−pec
Protruding−mouth
Day−4
Day−5
Stage
Transcript
ENSDART00000103944
ENSDART00000123142
ENSDART00000145106

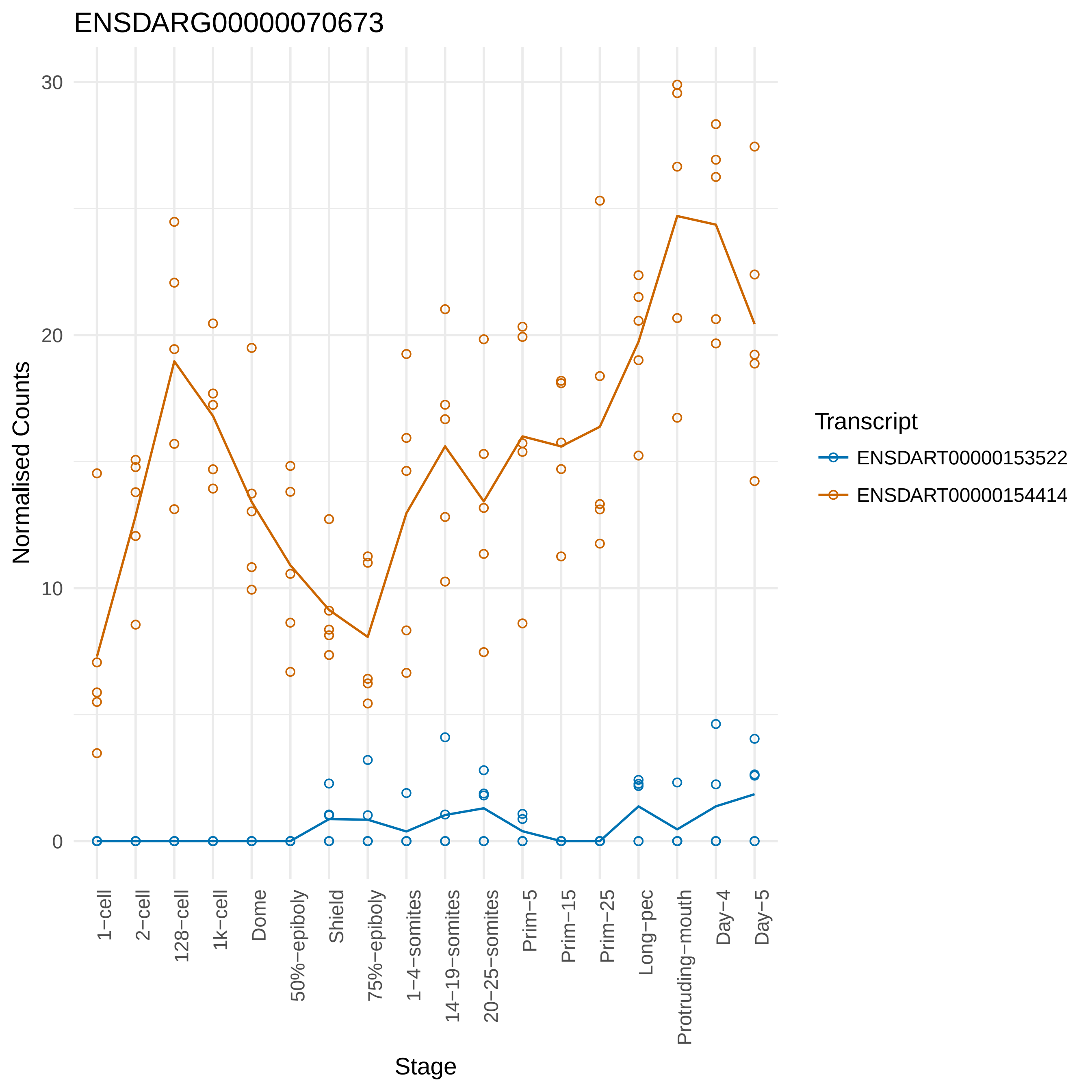

ENSDARG00000070673
Normalised Counts
30
20
10
0
1−cell
2−cell
128−cell
1k−cell
Dome
50%−epiboly
Shield
75%−epiboly
1−4−somites
14−19−somites
20−25−somites
Prim−5
Prim−15
Prim−25
Long−pec
Protruding−mouth
Day−4
Day−5
Stage
Transcript
ENSDART00000153522
ENSDART00000154414

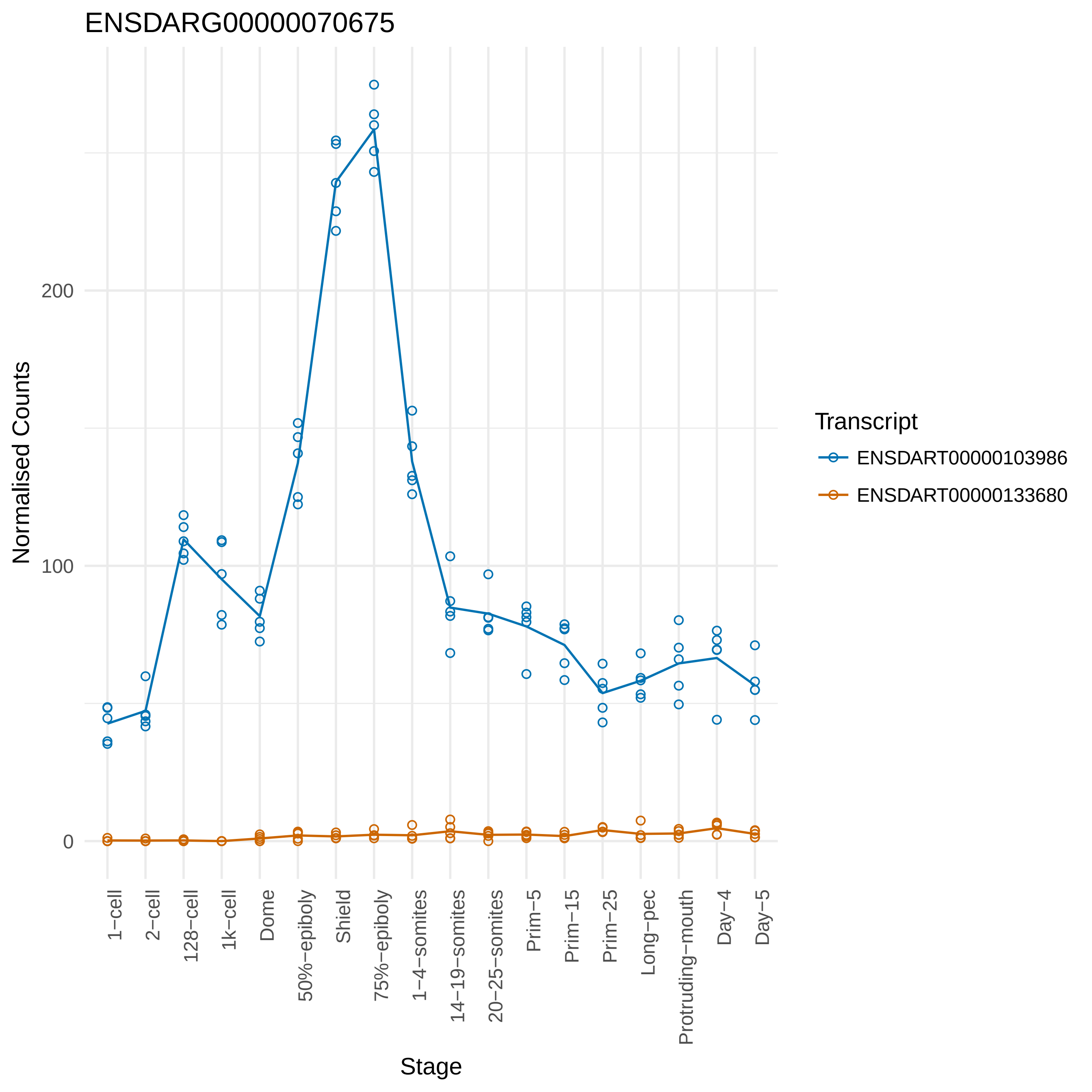
ENSDARG00000070675
Normalised Counts
200
100
0
1−cell
2−cell
128−cell
1k−cell
Dome
50%−epiboly
Shield
75%−epiboly
1−4−somites
14−19−somites
20−25−somites
Prim−5
Prim−15
Prim−25
Long−pec
Protruding−mouth
Day−4
Day−5
Stage
Transcript
ENSDART00000103986
ENSDART00000133680

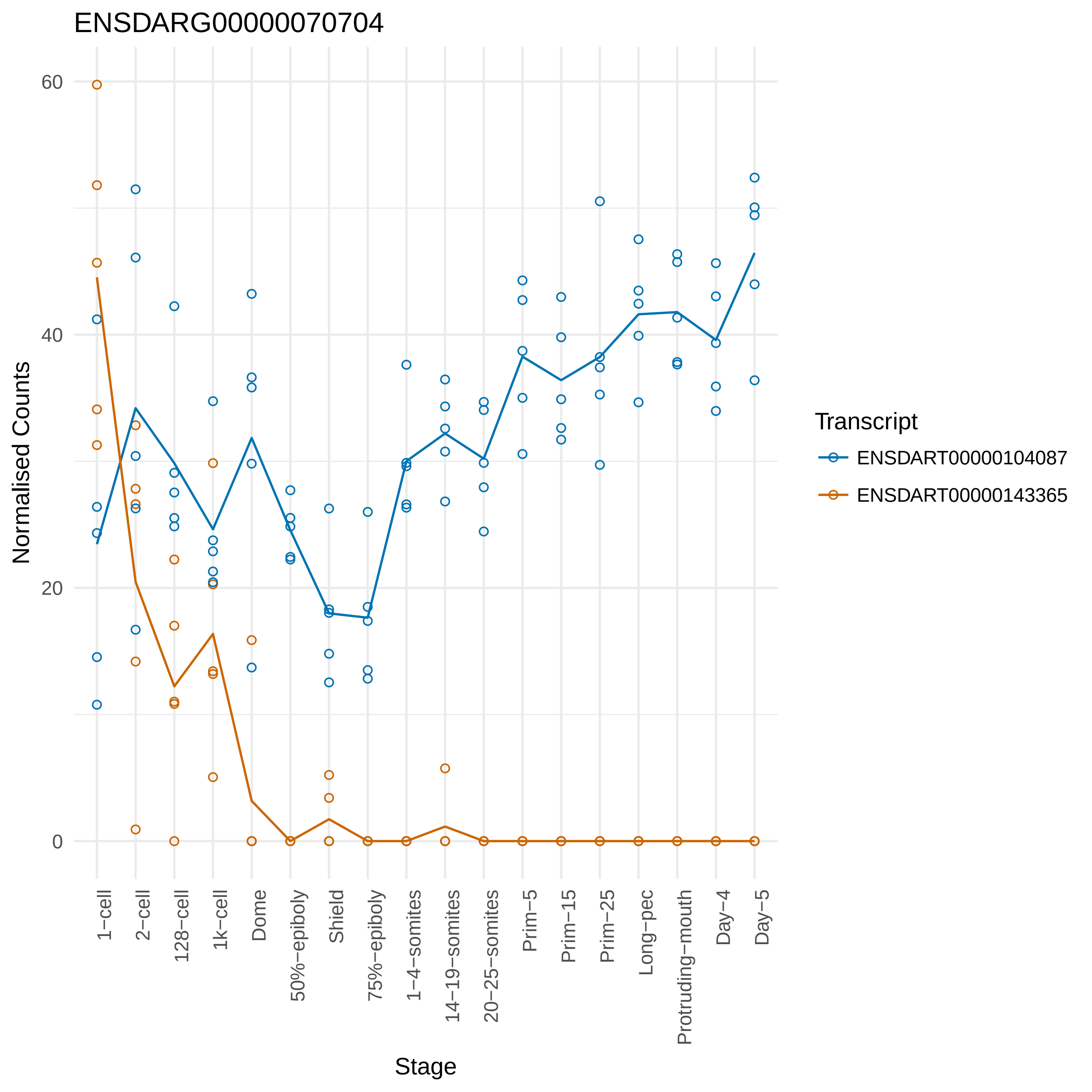

ENSDARG00000070704
Normalised Counts
0
20
40
60
Stage
1−cell
2−cell
128−cell
1k−cell
Dome
50%−epiboly
Shield
75%−epiboly
1−4−somites
14−19−somites
20−25−somites
Prim−5
Prim−15
Prim−25
Long−pec
Protruding−mouth
Day−4
Day−5
Transcript
ENSDART00000104087
ENSDART00000143365

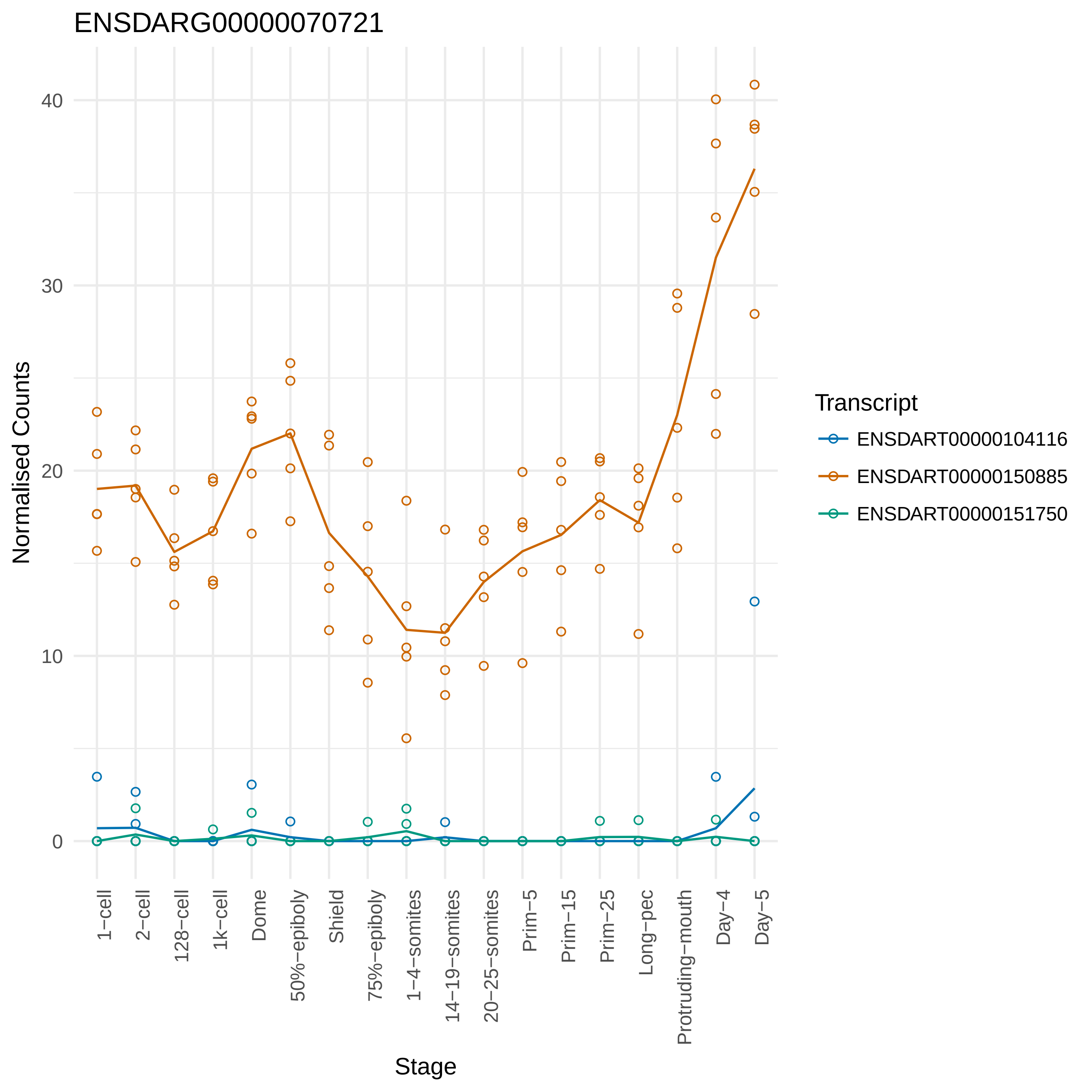
ENSDARG00000070721
Normalised Counts
0
10
20
30
40
1−cell
2−cell
128−cell
1k−cell
Dome
50%−epiboly
Shield
75%−epiboly
1−4−somites
14−19−somites
20−25−somites
Prim−5
Prim−15
Prim−25
Long−pec
Protruding−mouth
Day−4
Day−5
Stage
Transcript
ENSDART00000104116
ENSDART00000150885
ENSDART00000151750

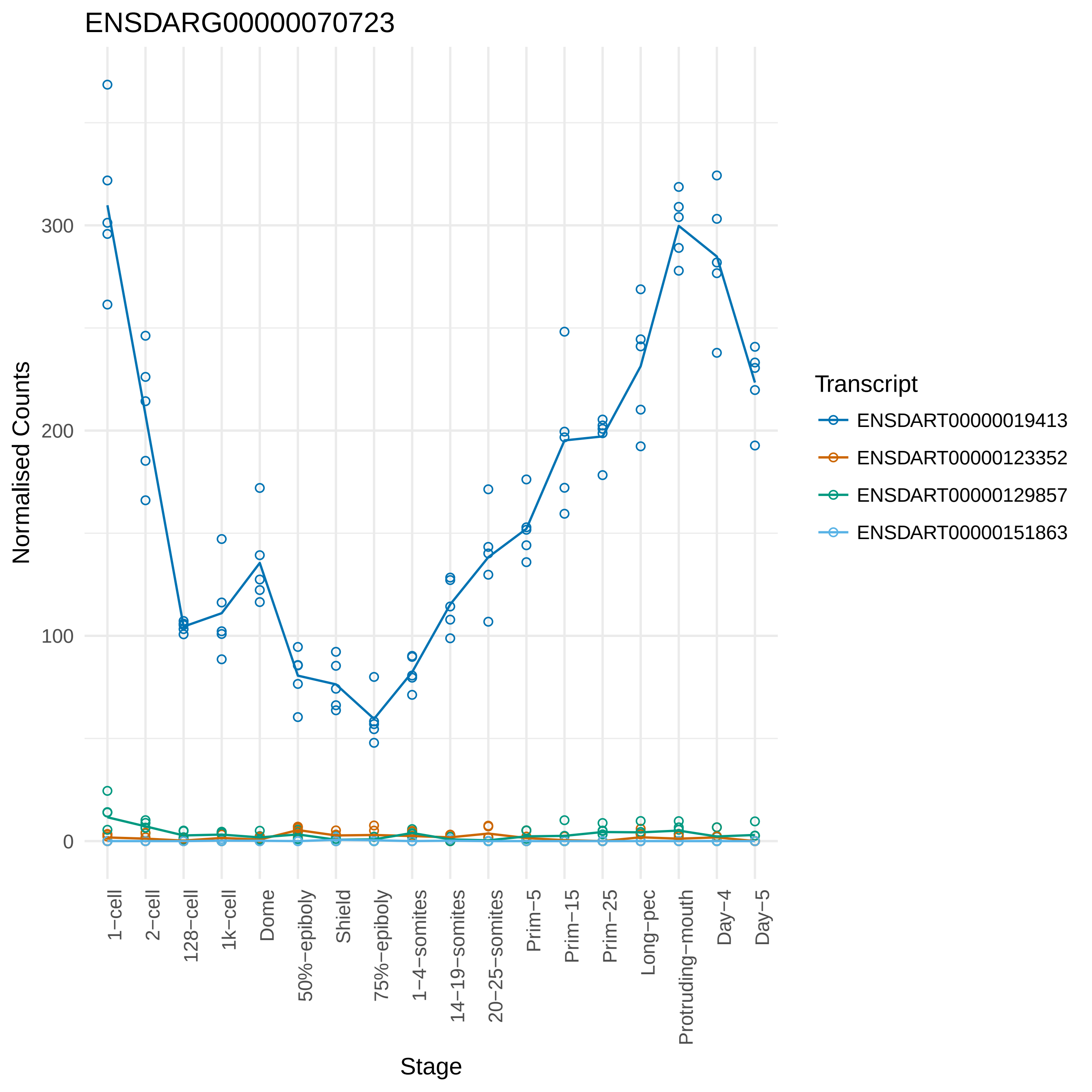
ENSDARG00000070723
Normalised Counts
300
200
100
0
1−cell
2−cell
128−cell
1k−cell
Dome
50%−epiboly
Shield
75%−epiboly
1−4−somites
14−19−somites
20−25−somites
Prim−5
Prim−15
Prim−25
Long−pec
Protruding−mouth
Day−4
Day−5
Stage
Transcript
ENSDART00000019413
ENSDART00000123352
ENSDART00000129857
ENSDART00000151863

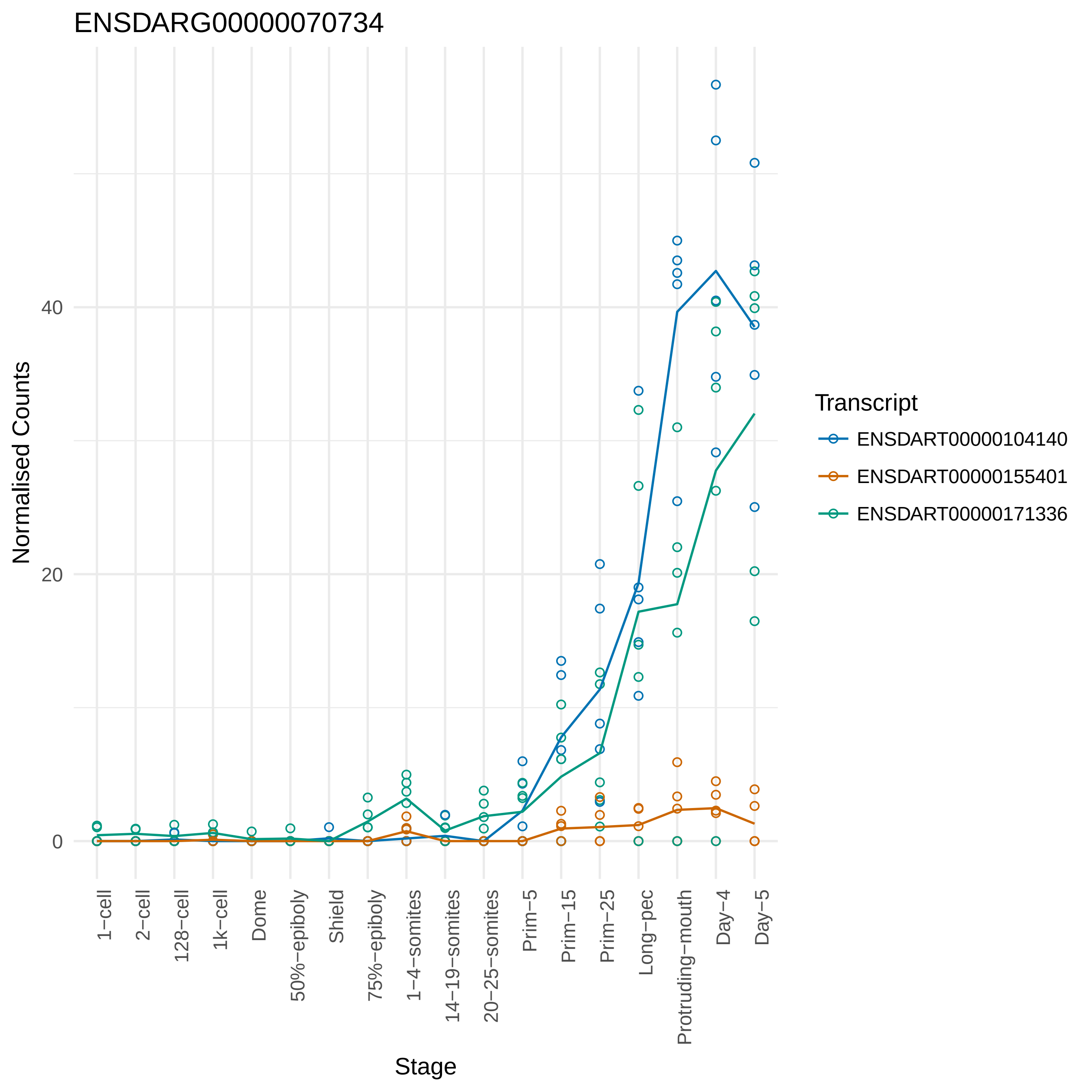
ENSDARG00000070734
Normalised Counts
0
20
40
Stage
1−cell
2−cell
128−cell
1k−cell
Dome
50%−epiboly
Shield
75%−epiboly
1−4−somites
14−19−somites
20−25−somites
Prim−5
Prim−15
Prim−25
Long−pec
Protruding−mouth
Day−4
Day−5
Transcript
ENSDART00000104140
ENSDART00000155401
ENSDART00000171336

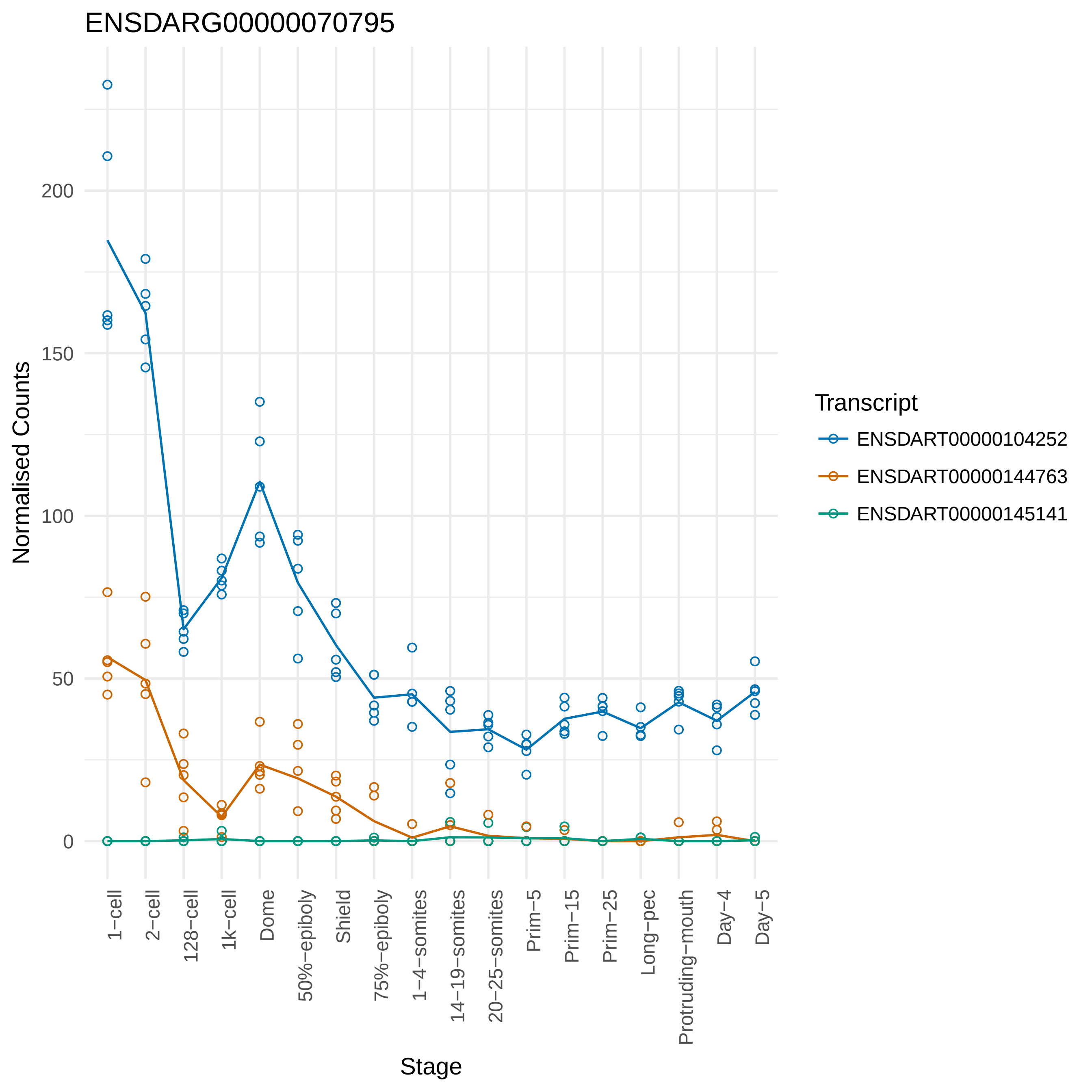

ENSDARG00000070795
Normalised Counts
200
150
100
50
0
1−cell
2−cell
128−cell
1k−cell
Dome
50%−epiboly
Shield
75%−epiboly
1−4−somites
14−19−somites
20−25−somites
Prim−5
Prim−15
Prim−25
Long−pec
Protruding−mouth
Day−4
Day−5
Stage
Transcript
ENSDART00000104252
ENSDART00000144763
ENSDART00000145141

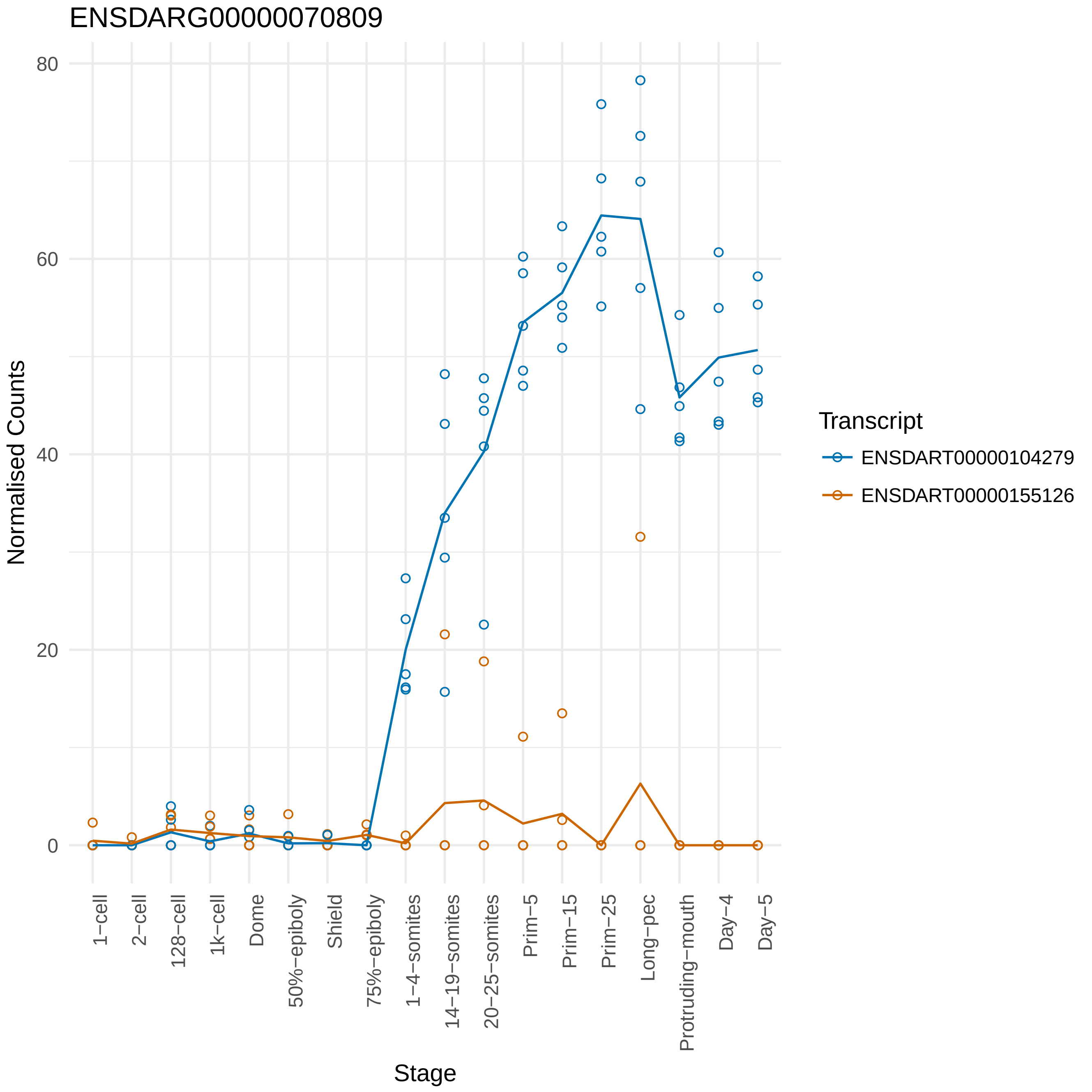
ENSDARG00000070809
Normalised Counts
80
60
40
20
0
1−cell
2−cell
128−cell
1k−cell
Dome
50%−epiboly
Shield
75%−epiboly
1−4−somites
14−19−somites
20−25−somites
Prim−5
Prim−15
Prim−25
Long−pec
Protruding−mouth
Day−4
Day−5
Stage
Transcript
ENSDART00000104279
ENSDART00000155126

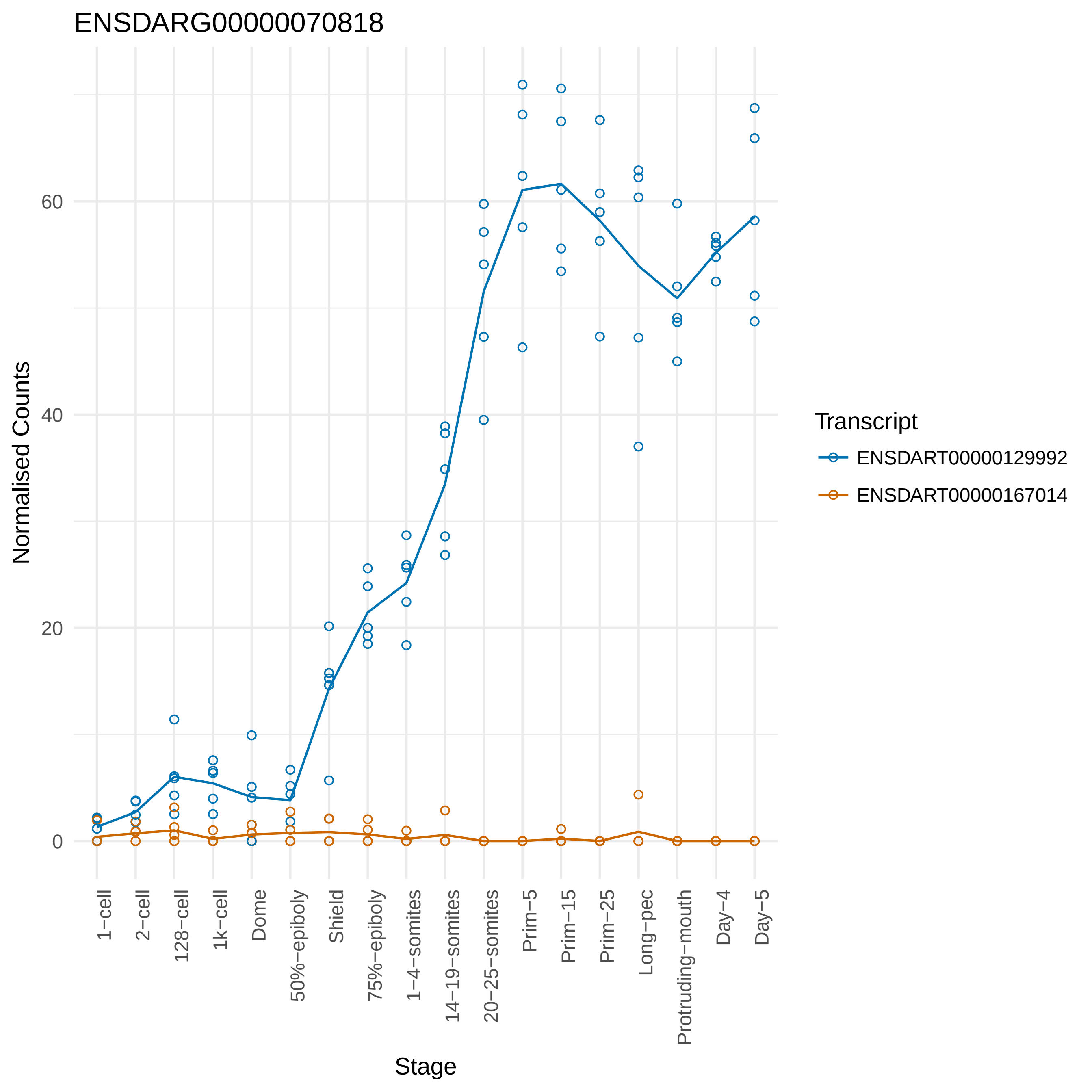
ENSDARG00000070818
Normalised Counts
60
40
20
0
1−cell
2−cell
128−cell
1k−cell
Dome
50%−epiboly
Shield
75%−epiboly
1−4−somites
14−19−somites
20−25−somites
Prim−5
Prim−15
Prim−25
Long−pec
Protruding−mouth
Day−4
Day−5
Stage
Transcript
ENSDART00000129992
ENSDART00000167014

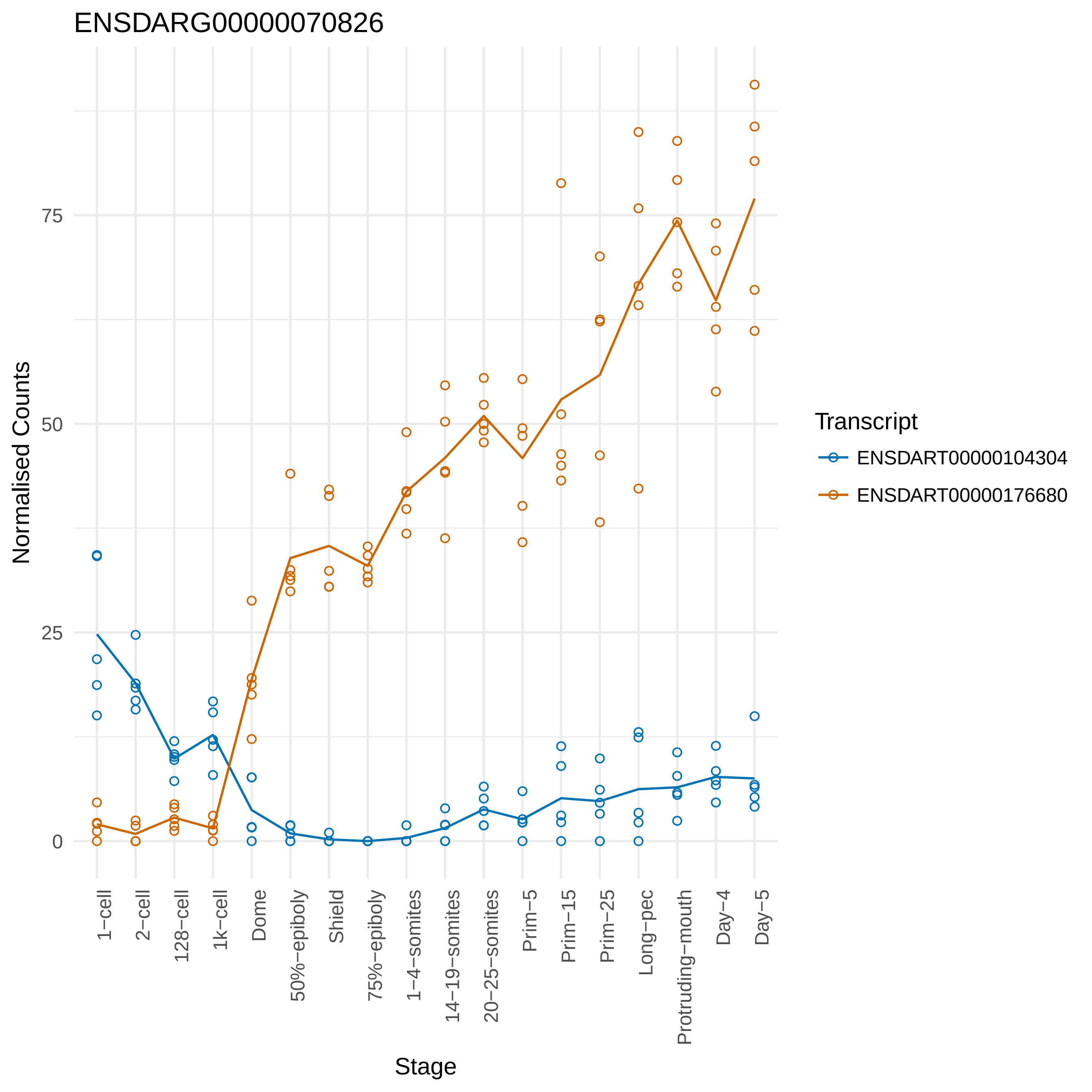
ENSDARG00000070826
Normalised Counts
0
25
50
75
1−cell
2−cell
128−cell
1k−cell
Dome
50%−epiboly
Shield
75%−epiboly
1−4−somites
14−19−somites
20−25−somites
Prim−5
Prim−15
Prim−25
Long−pec
Protruding−mouth
Day−4
Day−5
Stage
Transcript
ENSDART00000104304
ENSDART00000176680

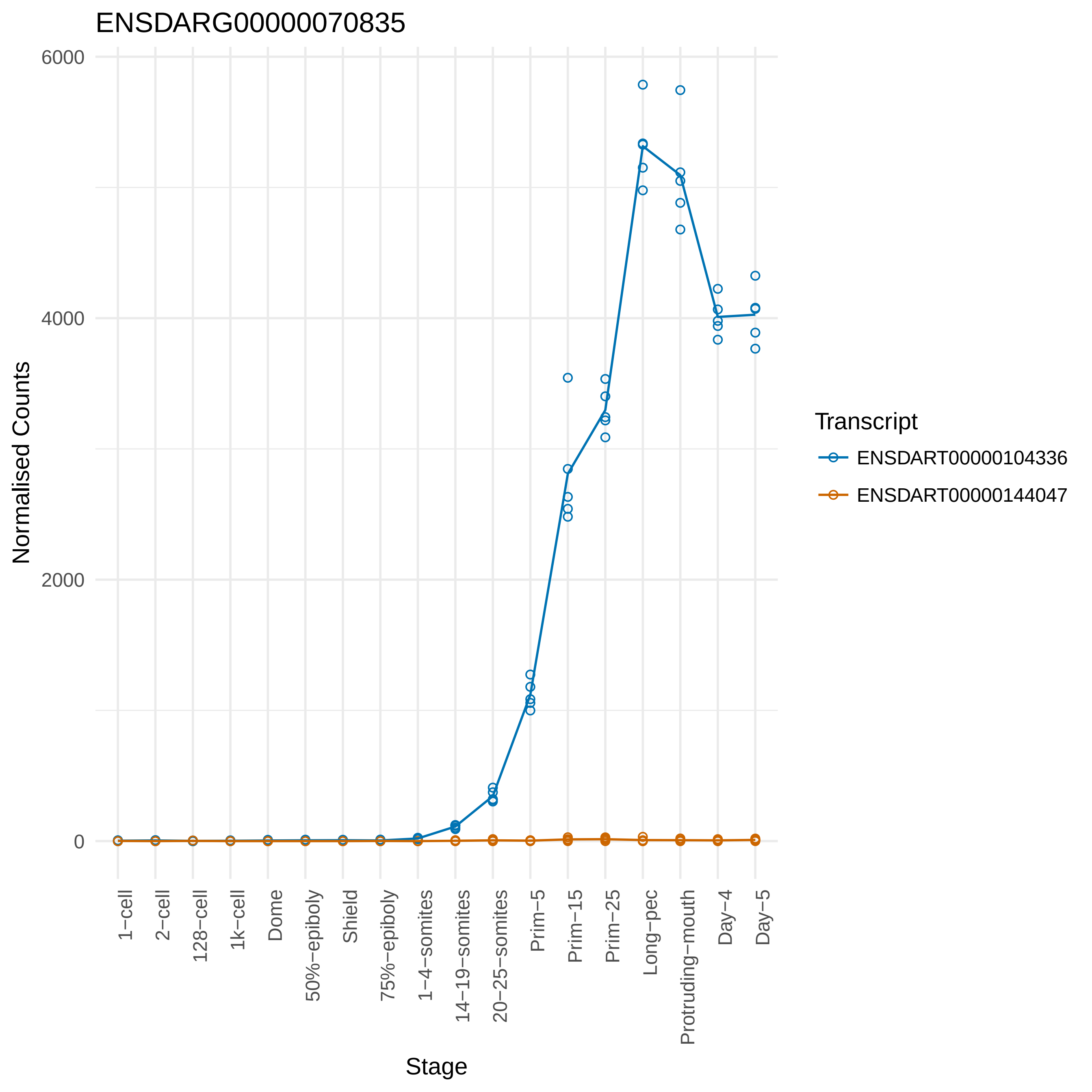
ENSDARG00000070835
Normalised Counts
6000
4000
2000
0
1−cell
2−cell
128−cell
1k−cell
Dome
50%−epiboly
Shield
75%−epiboly
1−4−somites
14−19−somites
20−25−somites
Prim−5
Prim−15
Prim−25
Long−pec
Protruding−mouth
Day−4
Day−5
Stage
Transcript
ENSDART00000104336
ENSDART00000144047

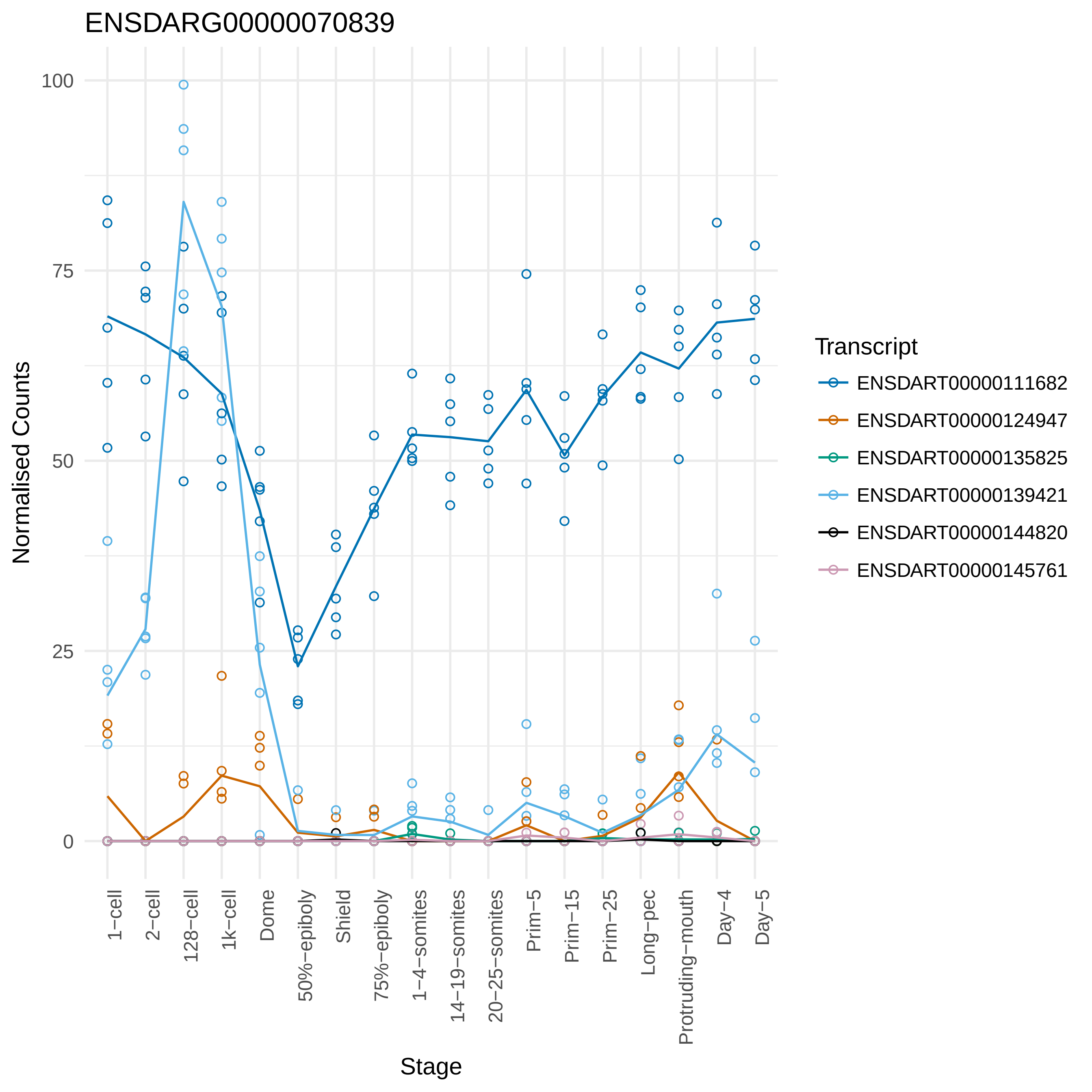

ENSDARG00000070839
Normalised Counts
100
75
50
25
0
1−cell
2−cell
128−cell
1k−cell
Dome
50%−epiboly
Shield
75%−epiboly
1−4−somites
14−19−somites
20−25−somites
Prim−5
Prim−15
Prim−25
Long−pec
Protruding−mouth
Day−4
Day−5
Stage
Transcript
ENSDART00000111682
ENSDART00000124947
ENSDART00000135825
ENSDART00000139421
ENSDART00000144820
ENSDART00000145761

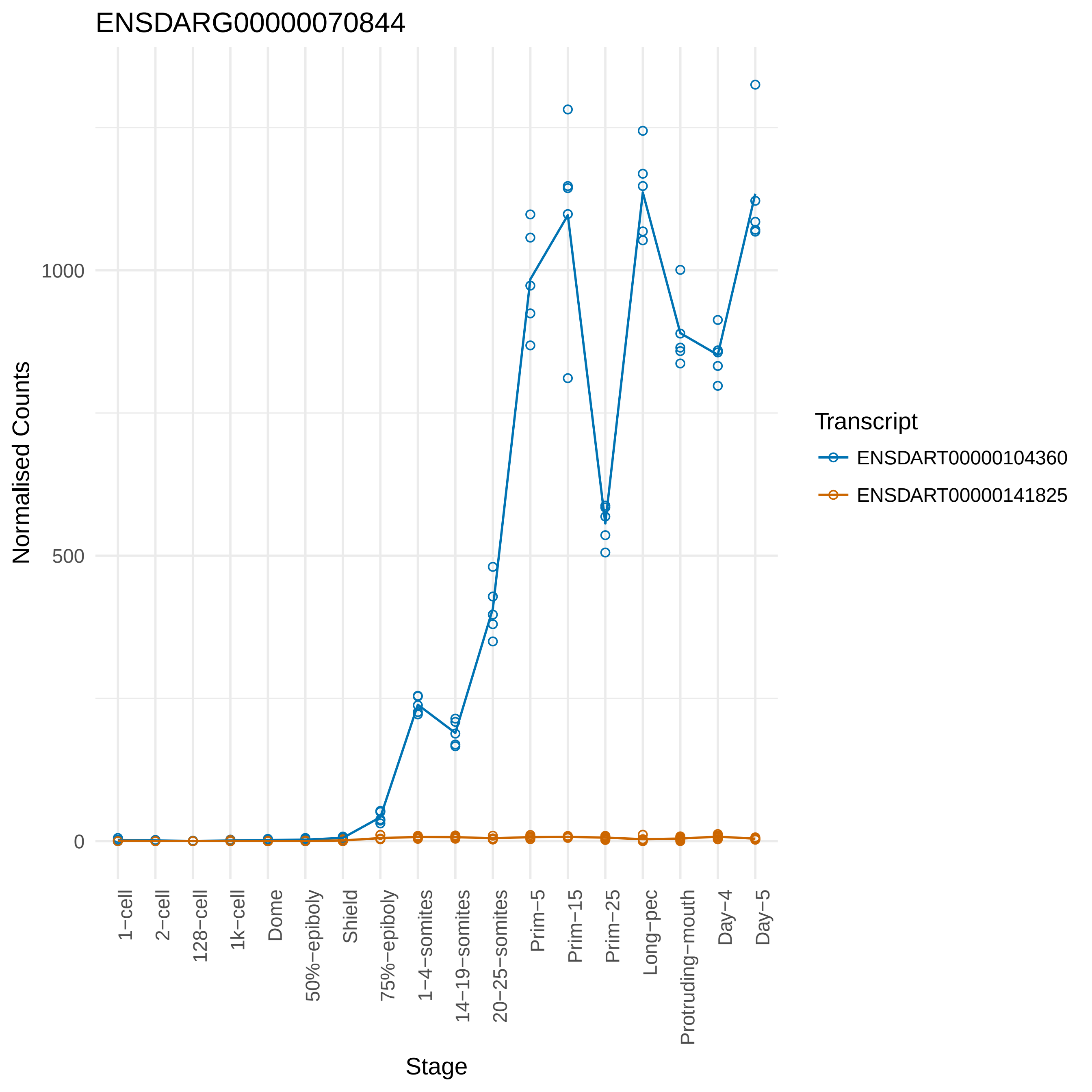

ENSDARG00000070844
Normalised Counts
1000
500
0
1−cell
2−cell
128−cell
1k−cell
Dome
50%−epiboly
Shield
75%−epiboly
1−4−somites
14−19−somites
20−25−somites
Prim−5
Prim−15
Prim−25
Long−pec
Protruding−mouth
Day−4
Day−5
Stage
Transcript
ENSDART00000104360
ENSDART00000141825

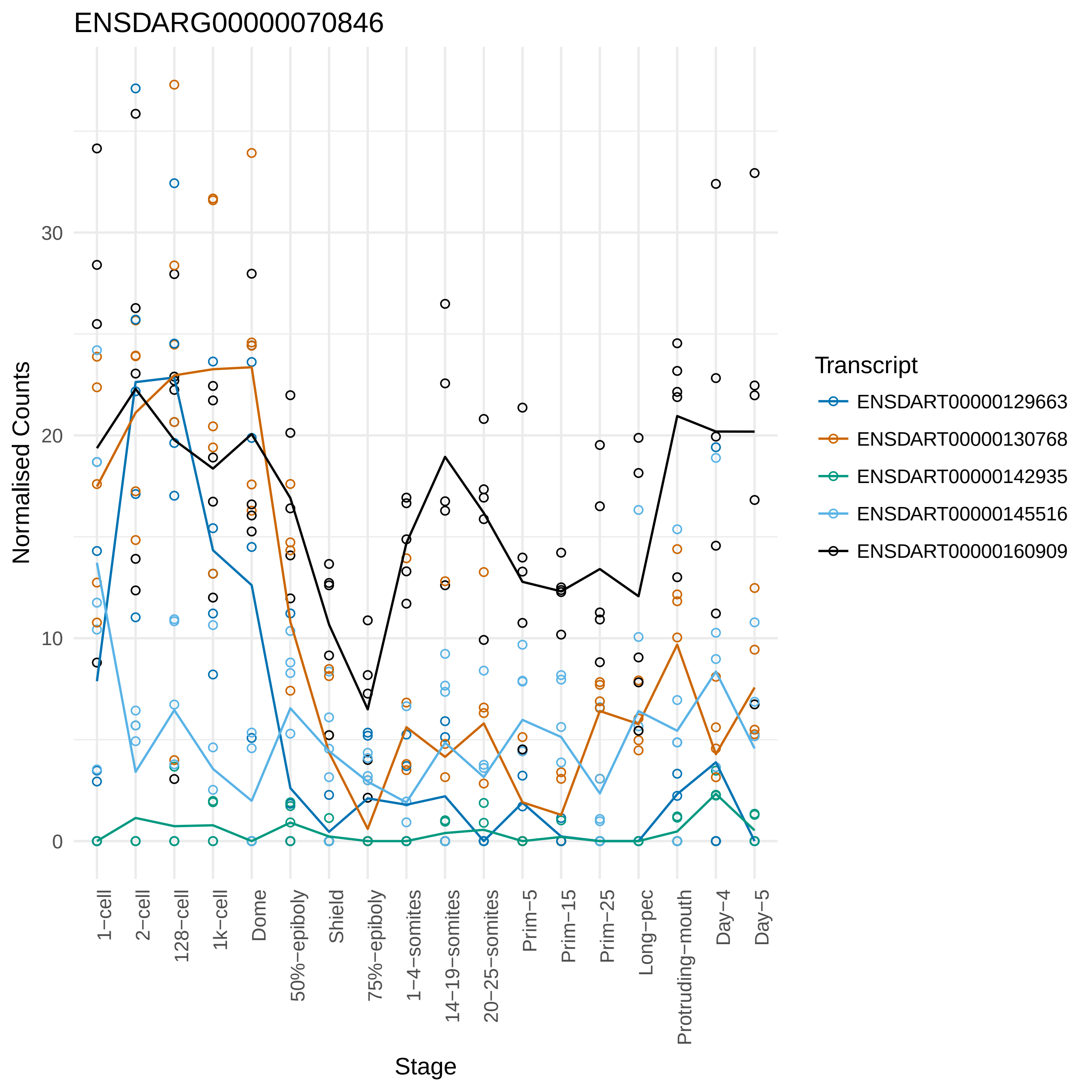

ENSDARG00000070846
Normalised Counts
Stage
30
20
10
0
1−cell
2−cell
128−cell
1k−cell
Dome
50%−epiboly
Shield
75%−epiboly
1−4−somites
14−19−somites
20−25−somites
Prim−5
Prim−15
Prim−25
Long−pec
Protruding−mouth
Day−4
Day−5
Transcript
ENSDART00000129663
ENSDART00000130768
ENSDART00000142935
ENSDART00000145516
ENSDART00000160909

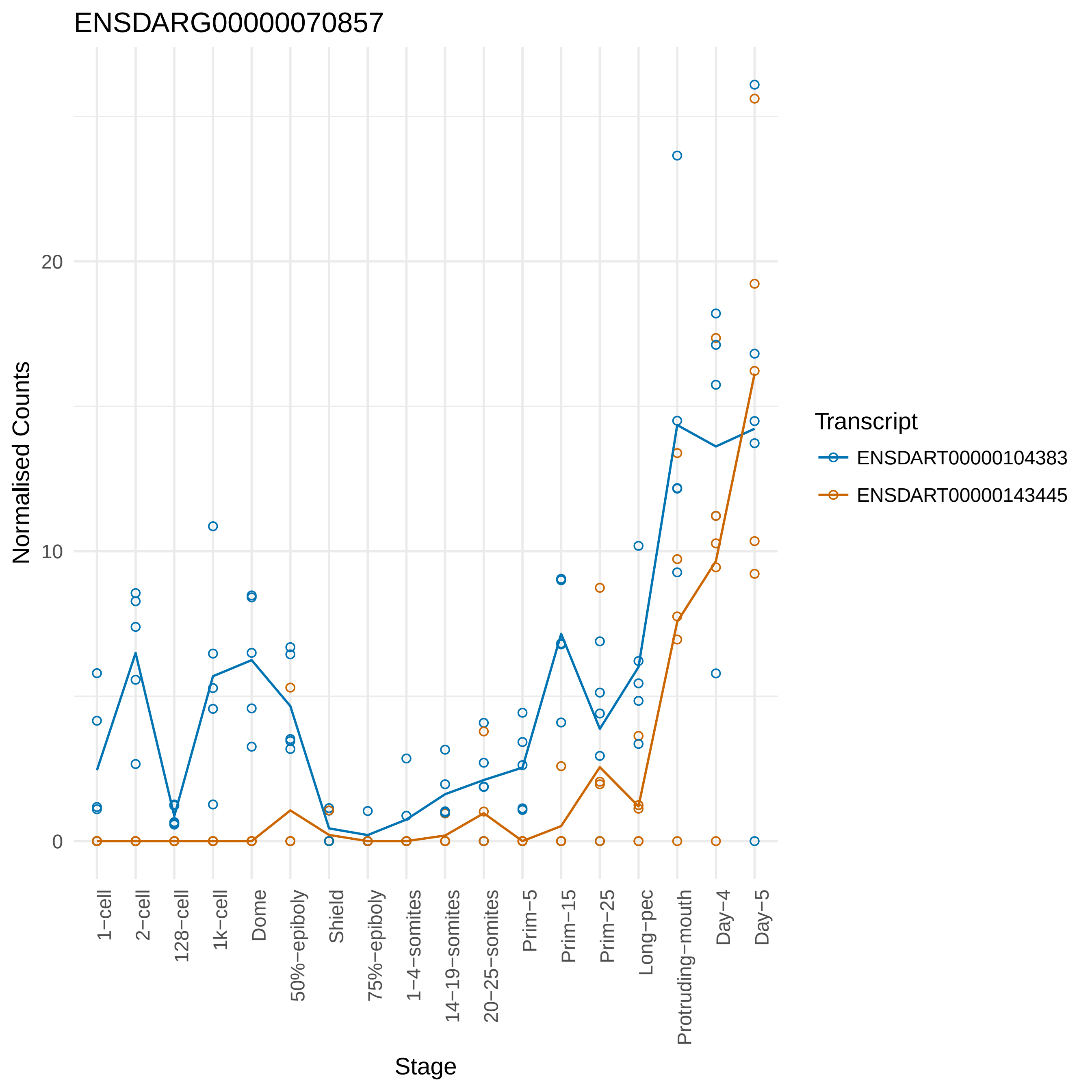

ENSDARG00000070857
Normalised Counts
20
10
0
1−cell
2−cell
128−cell
1k−cell
Dome
50%−epiboly
Shield
75%−epiboly
1−4−somites
14−19−somites
20−25−somites
Prim−5
Prim−15
Prim−25
Long−pec
Protruding−mouth
Day−4
Day−5
Stage
Transcript
ENSDART00000104383
ENSDART00000143445

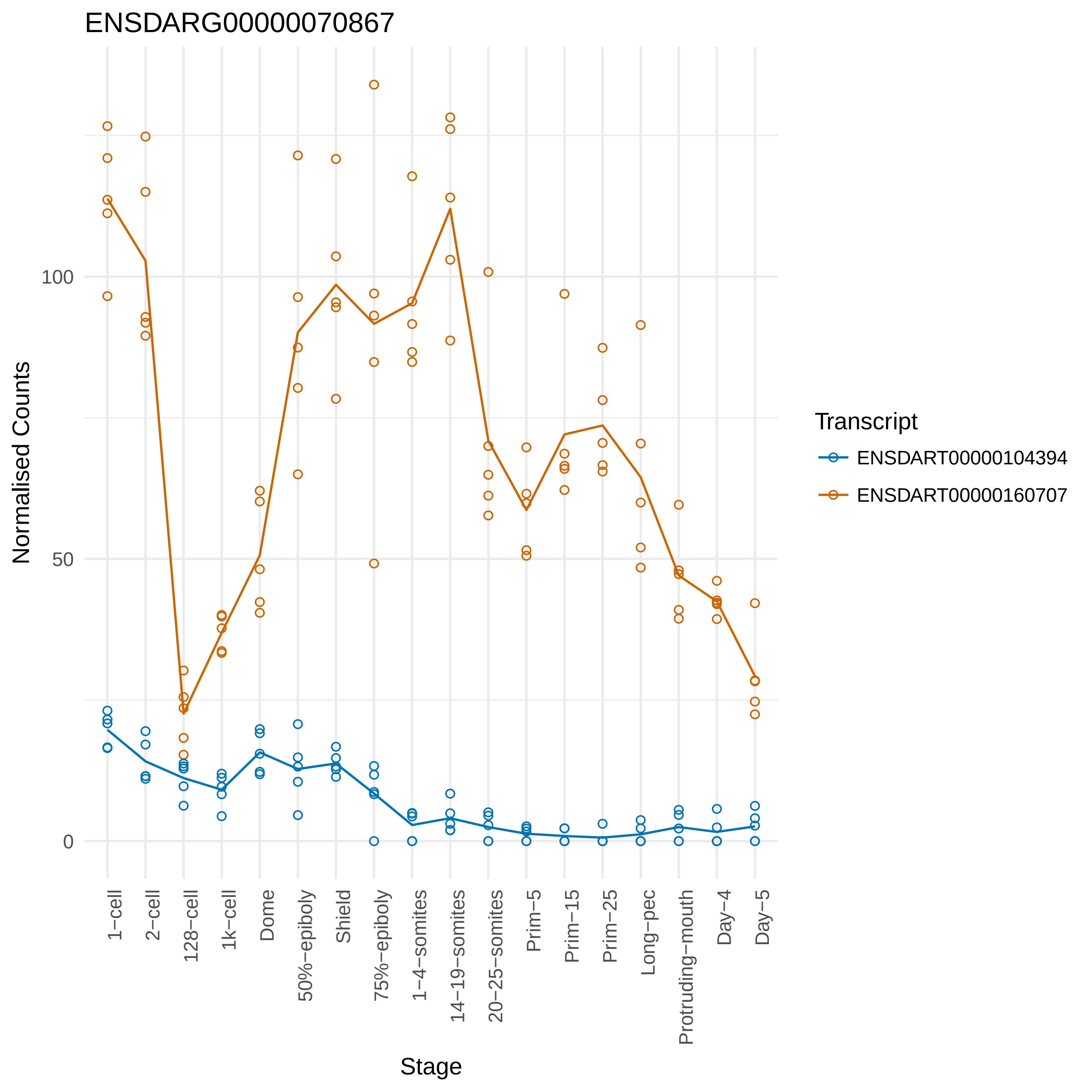

ENSDARG00000070867
Normalised Counts
100
50
0
1−cell
2−cell
128−cell
1k−cell
Dome
50%−epiboly
Shield
75%−epiboly
1−4−somites
14−19−somites
20−25−somites
Prim−5
Prim−15
Prim−25
Long−pec
Protruding−mouth
Day−4
Day−5
Stage
Transcript
ENSDART00000104394
ENSDART00000160707

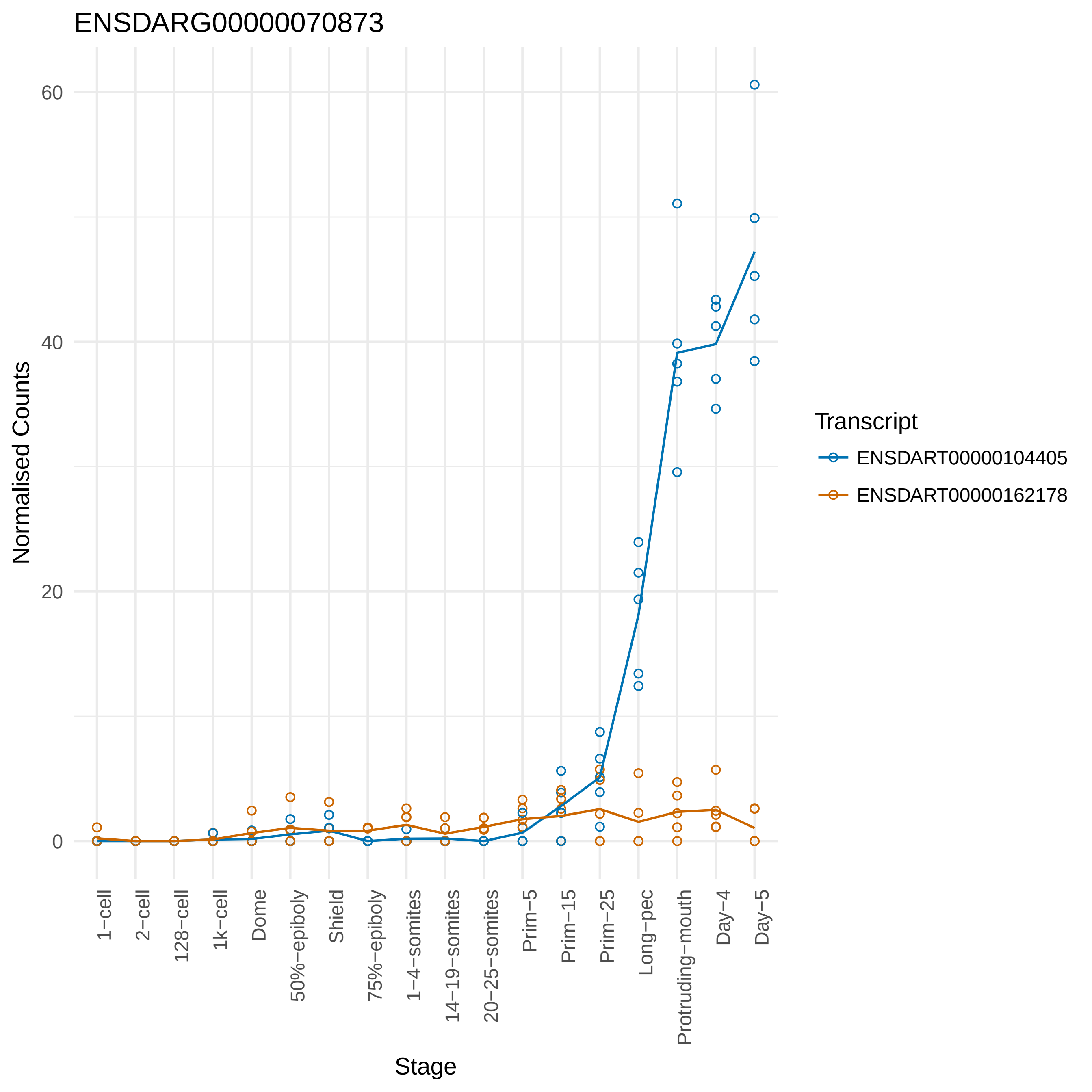

ENSDARG00000070873
Normalised Counts
60
40
20
0
1−cell
2−cell
128−cell
1k−cell
Dome
50%−epiboly
Shield
75%−epiboly
1−4−somites
14−19−somites
20−25−somites
Prim−5
Prim−15
Prim−25
Long−pec
Protruding−mouth
Day−4
Day−5
Stage
Transcript
ENSDART00000104405
ENSDART00000162178

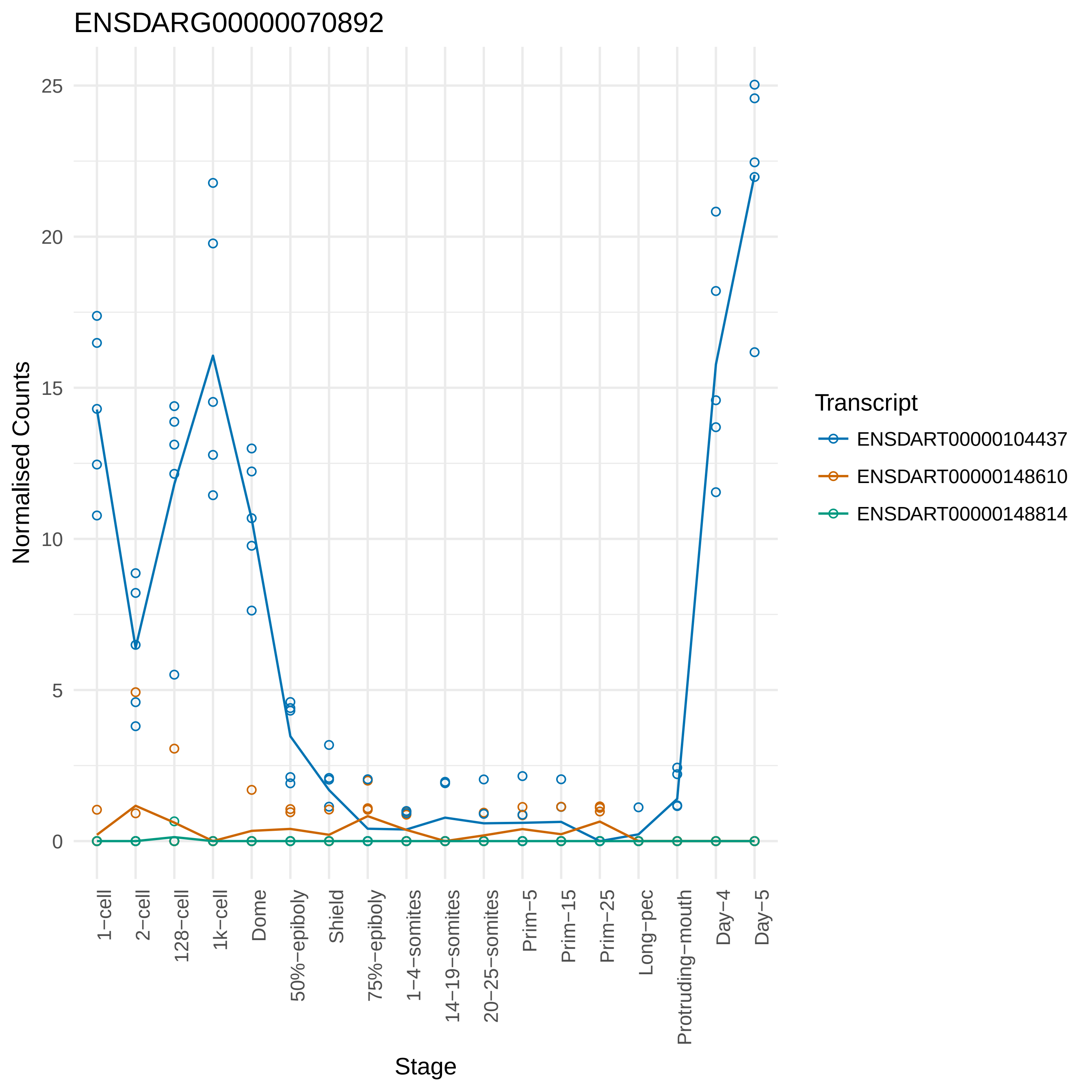

ENSDARG00000070892
Normalised Counts
25
20
15
10
5
0
1−cell
2−cell
128−cell
1k−cell
Dome
50%−epiboly
Shield
75%−epiboly
1−4−somites
14−19−somites
20−25−somites
Prim−5
Prim−15
Prim−25
Long−pec
Protruding−mouth
Day−4
Day−5
Stage
Transcript
ENSDART00000104437
ENSDART00000148610
ENSDART00000148814

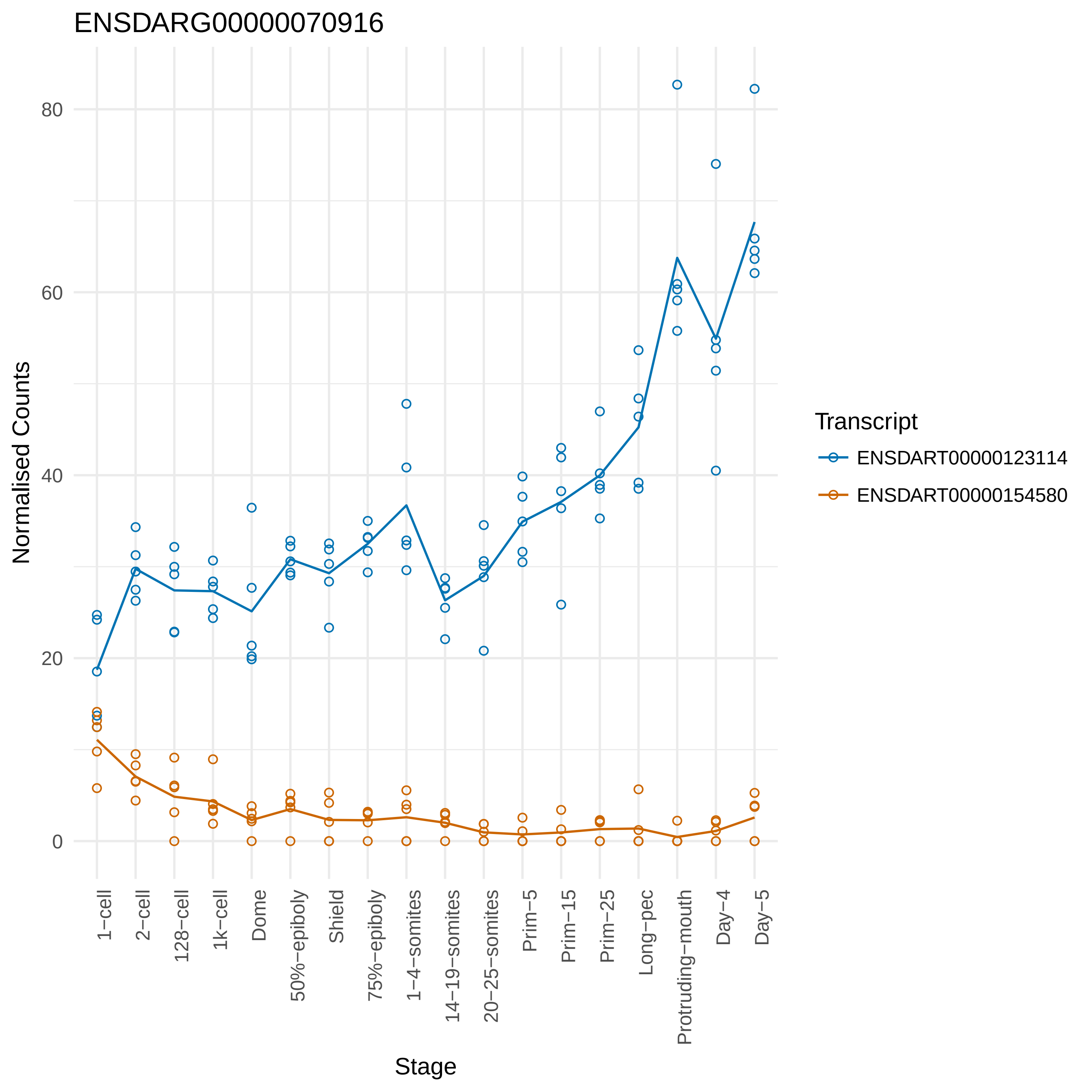
ENSDARG00000070916
Normalised Counts
0
20
40
60
80
Stage
1−cell
2−cell
128−cell
1k−cell
Dome
50%−epiboly
Shield
75%−epiboly
1−4−somites
14−19−somites
20−25−somites
Prim−5
Prim−15
Prim−25
Long−pec
Protruding−mouth
Day−4
Day−5
Transcript
ENSDART00000123114
ENSDART00000154580

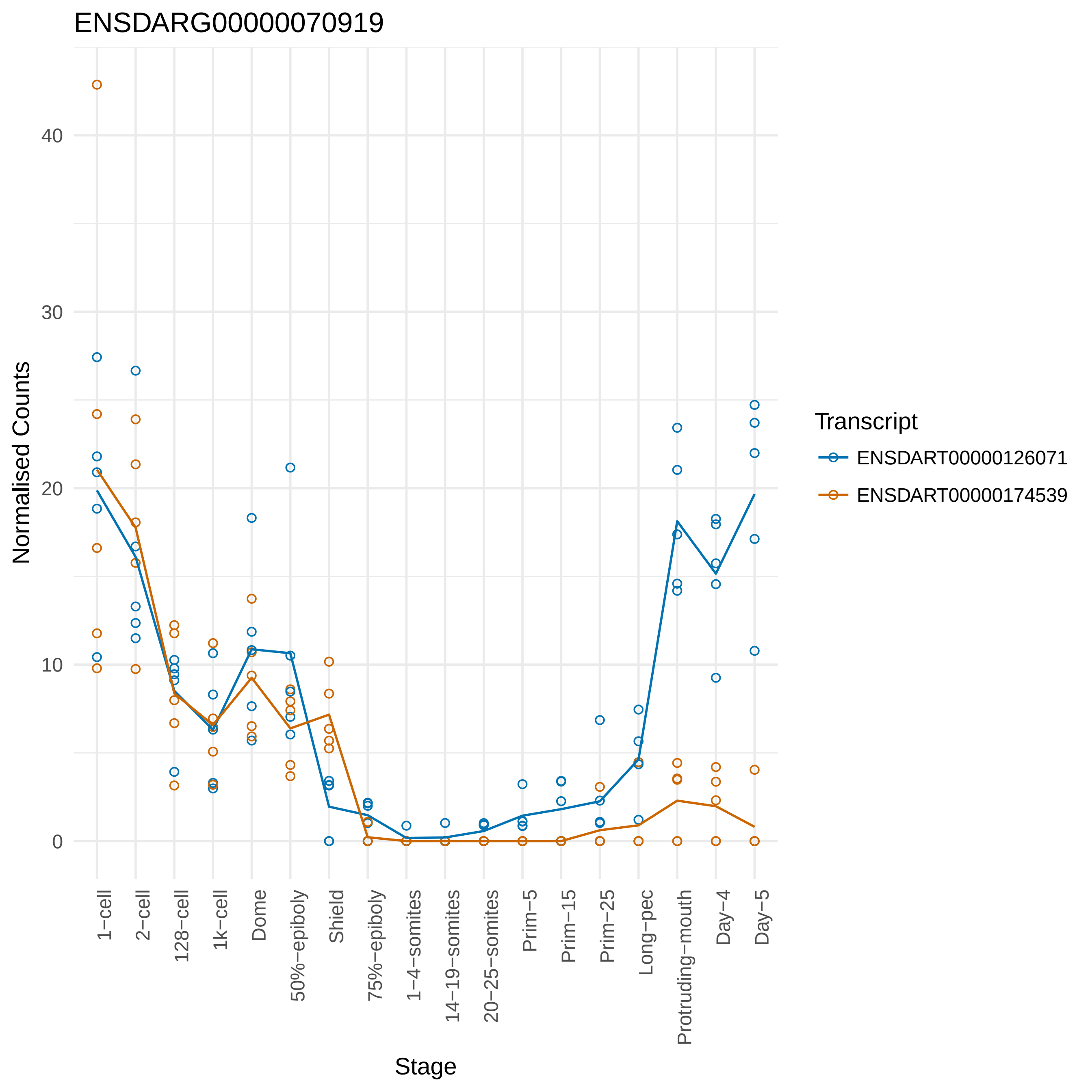

ENSDARG00000070919
Normalised Counts
40
30
20
10
0
1−cell
2−cell
128−cell
1k−cell
Dome
50%−epiboly
Shield
75%−epiboly
1−4−somites
14−19−somites
20−25−somites
Prim−5
Prim−15
Prim−25
Long−pec
Protruding−mouth
Day−4
Day−5
Stage
Transcript
ENSDART00000126071
ENSDART00000174539

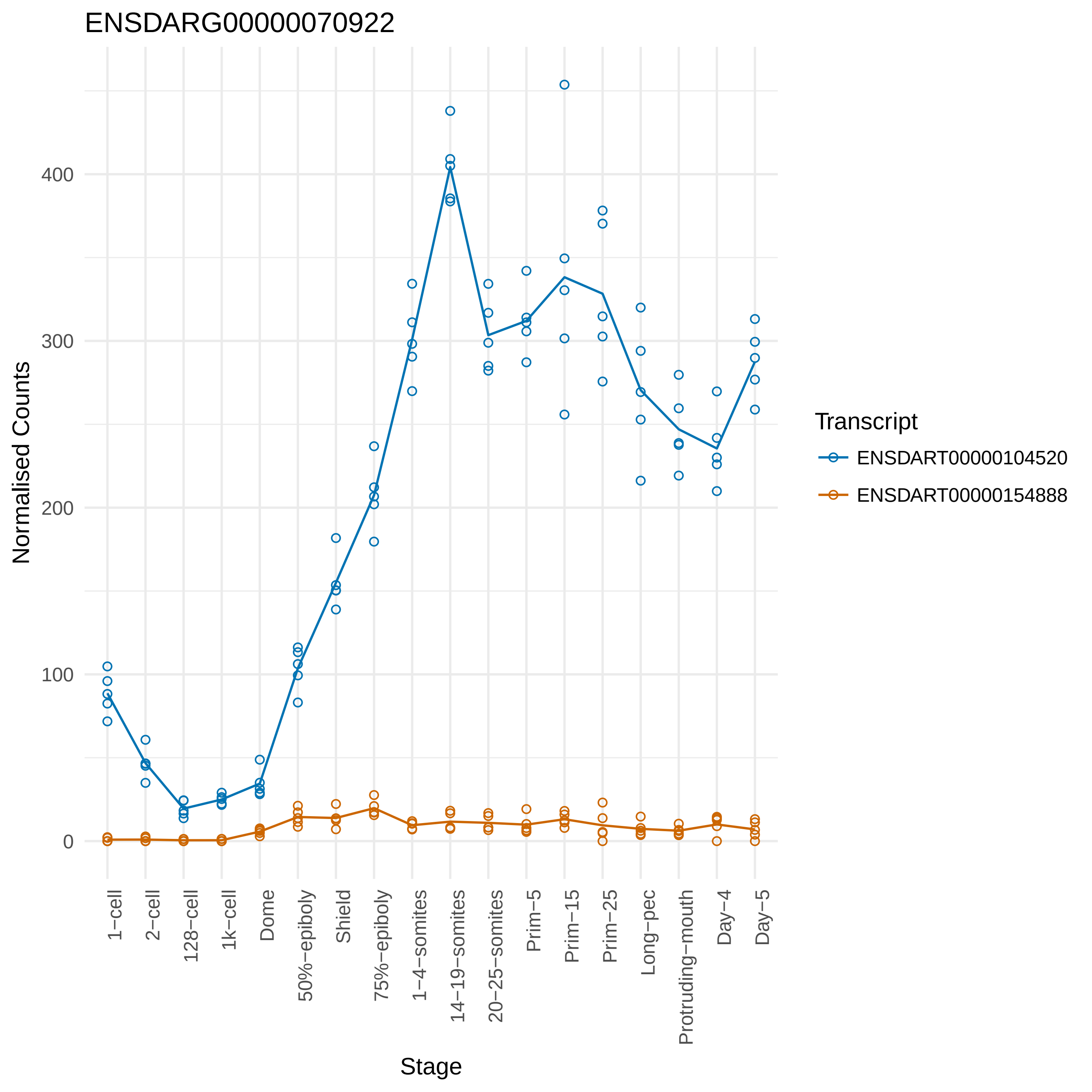

ENSDARG00000070922
Normalised Counts
0
100
200
300
400
Stage
1−cell
2−cell
128−cell
1k−cell
Dome
50%−epiboly
Shield
75%−epiboly
1−4−somites
14−19−somites
20−25−somites
Prim−5
Prim−15
Prim−25
Long−pec
Protruding−mouth
Day−4
Day−5
Transcript
ENSDART00000104520
ENSDART00000154888

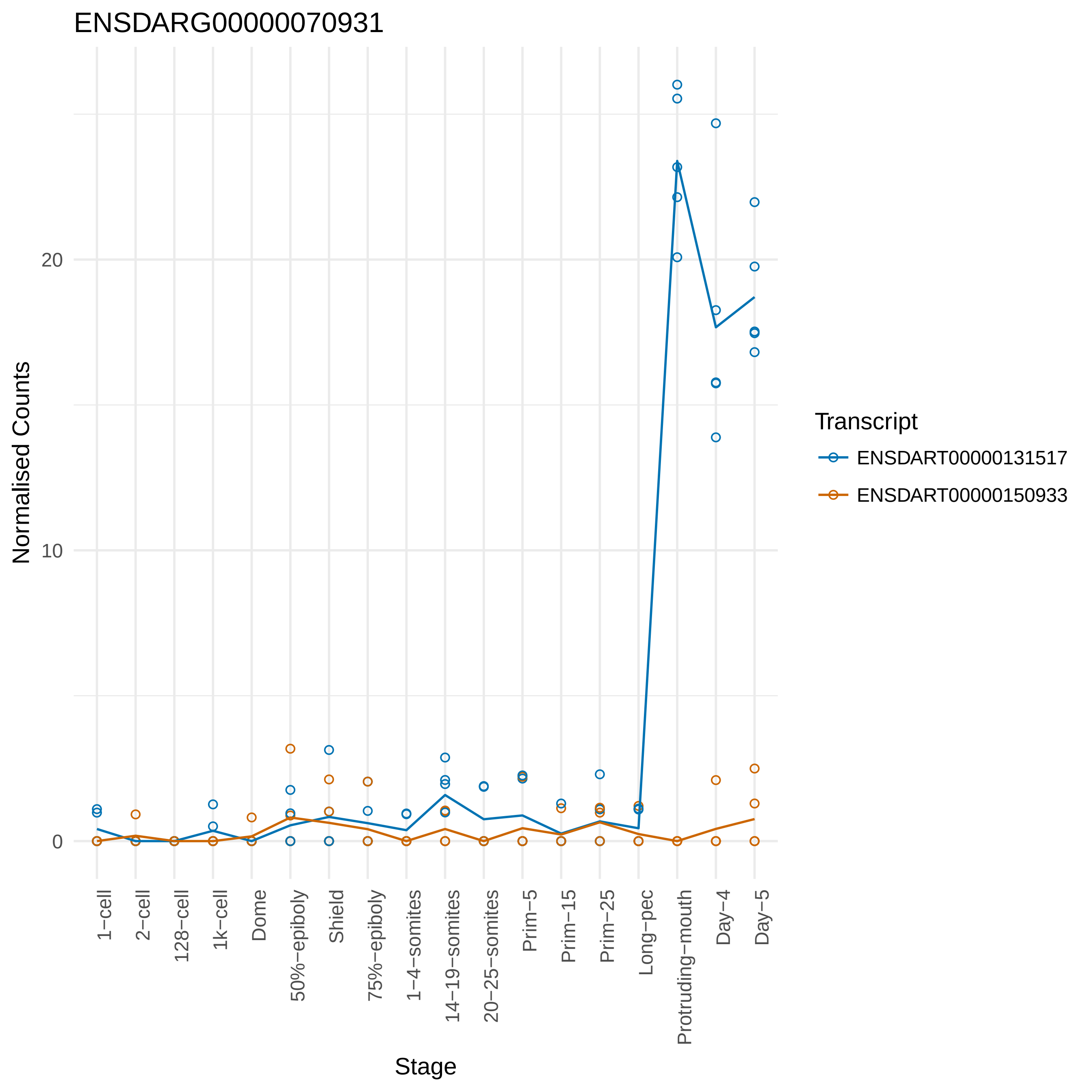

ENSDARG00000070931
Normalised Counts
0
10
20
Stage
1−cell
2−cell
128−cell
1k−cell
Dome
50%−epiboly
Shield
75%−epiboly
1−4−somites
14−19−somites
20−25−somites
Prim−5
Prim−15
Prim−25
Long−pec
Protruding−mouth
Day−4
Day−5
Transcript
ENSDART00000131517
ENSDART00000150933

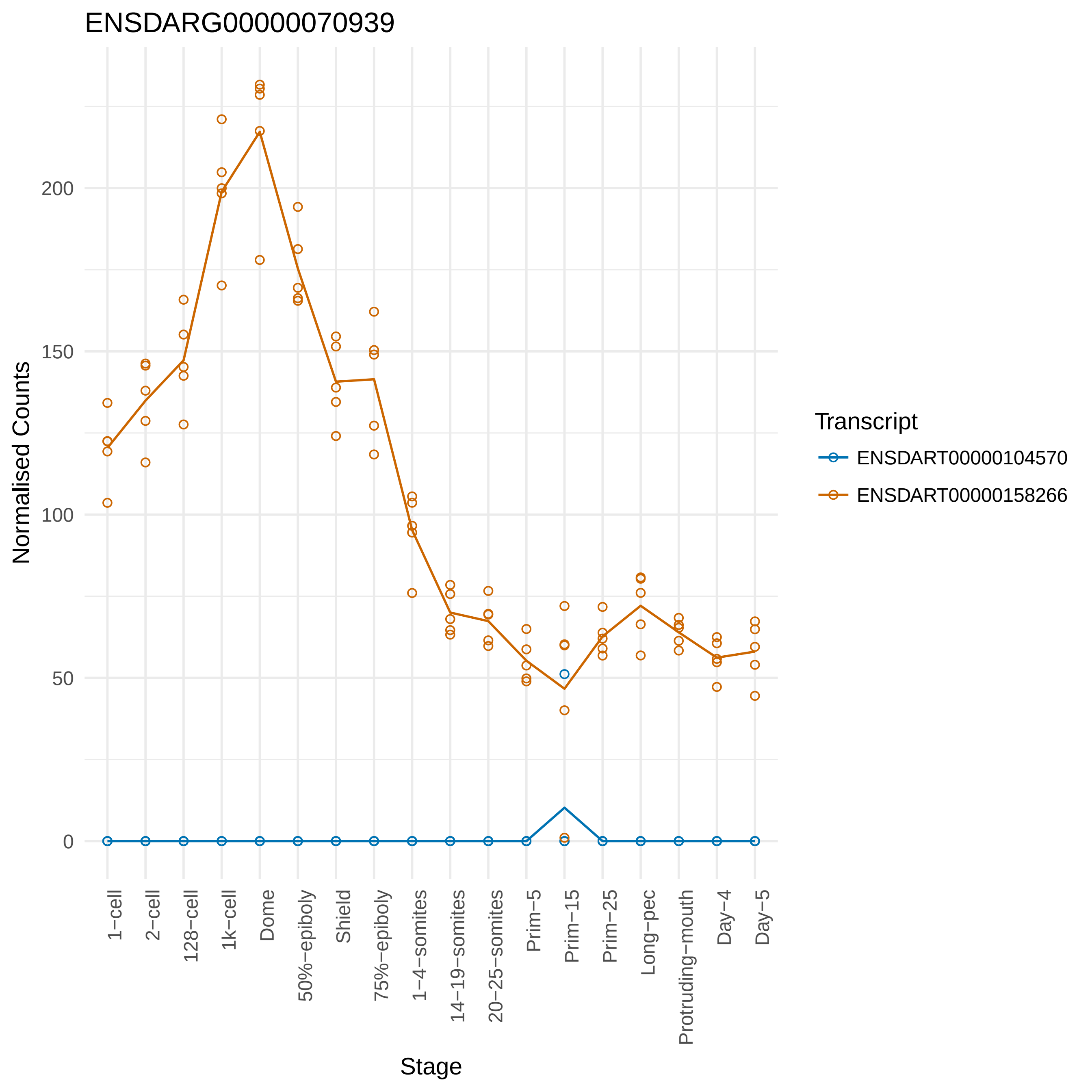

ENSDARG00000070939
Normalised Counts
200
150
100
50
0
1−cell
2−cell
128−cell
1k−cell
Dome
50%−epiboly
Shield
75%−epiboly
1−4−somites
14−19−somites
20−25−somites
Prim−5
Prim−15
Prim−25
Long−pec
Protruding−mouth
Day−4
Day−5
Stage
Transcript
ENSDART00000104570
ENSDART00000158266

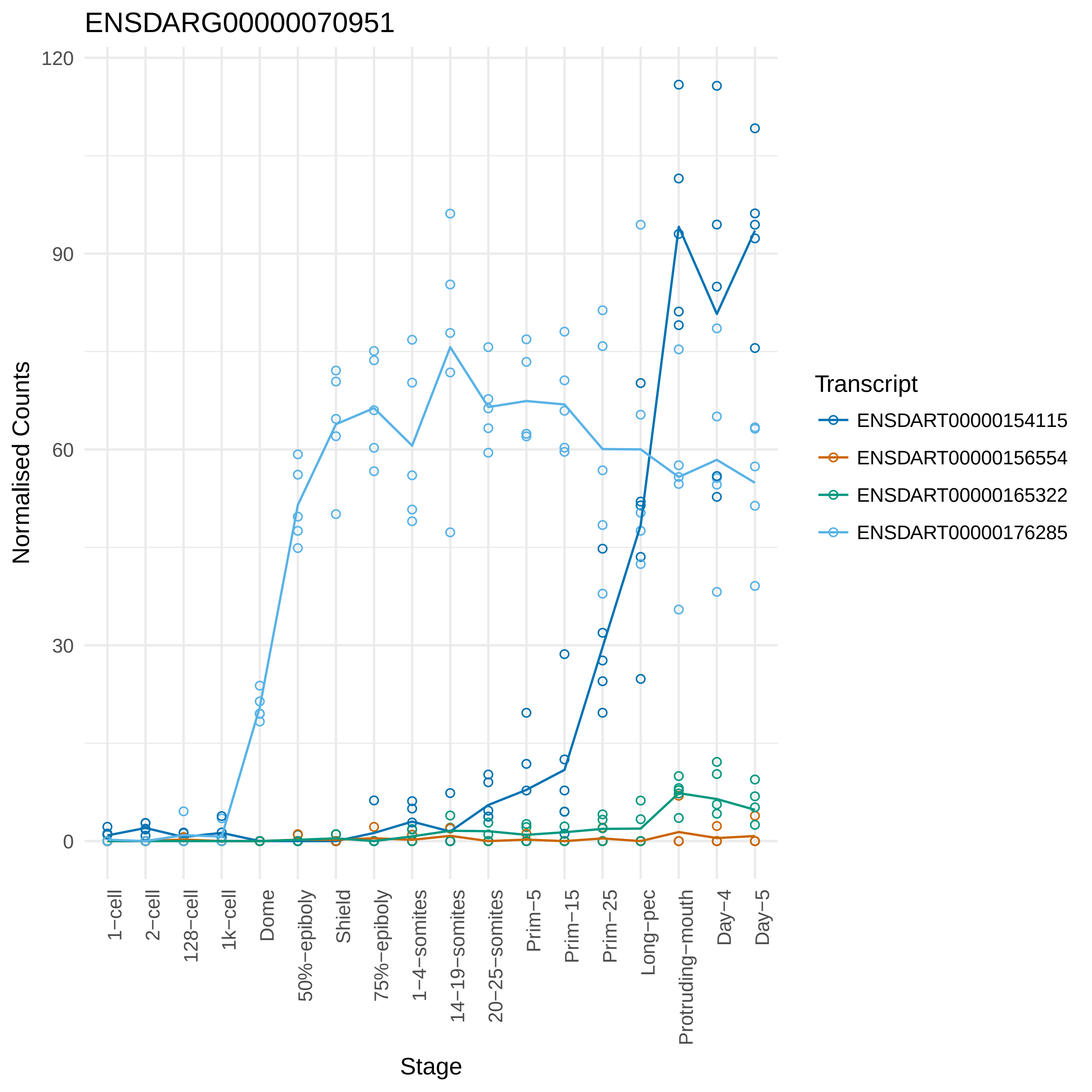
ENSDARG00000070951
Normalised Counts
120
90
60
30
0
1−cell
2−cell
128−cell
1k−cell
Dome
50%−epiboly
Shield
75%−epiboly
1−4−somites
14−19−somites
20−25−somites
Prim−5
Prim−15
Prim−25
Long−pec
Protruding−mouth
Day−4
Day−5
Stage
Transcript
ENSDART00000154115
ENSDART00000156554
ENSDART00000165322
ENSDART00000176285

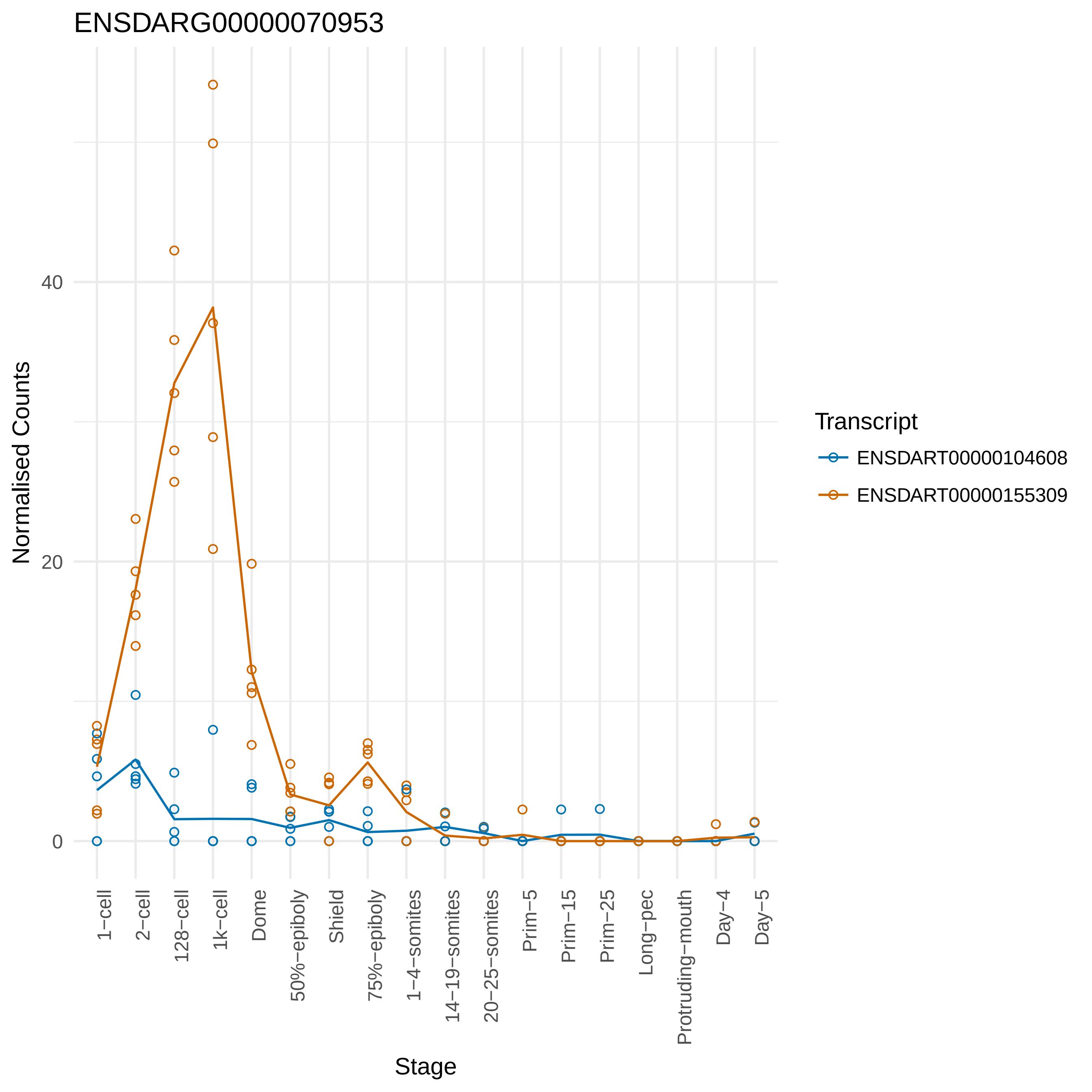

ENSDARG00000070953
Normalised Counts
40
20
0
1−cell
2−cell
128−cell
1k−cell
Dome
50%−epiboly
Shield
75%−epiboly
1−4−somites
14−19−somites
20−25−somites
Prim−5
Prim−15
Prim−25
Long−pec
Protruding−mouth
Day−4
Day−5
Stage
Transcript
ENSDART00000104608
ENSDART00000155309

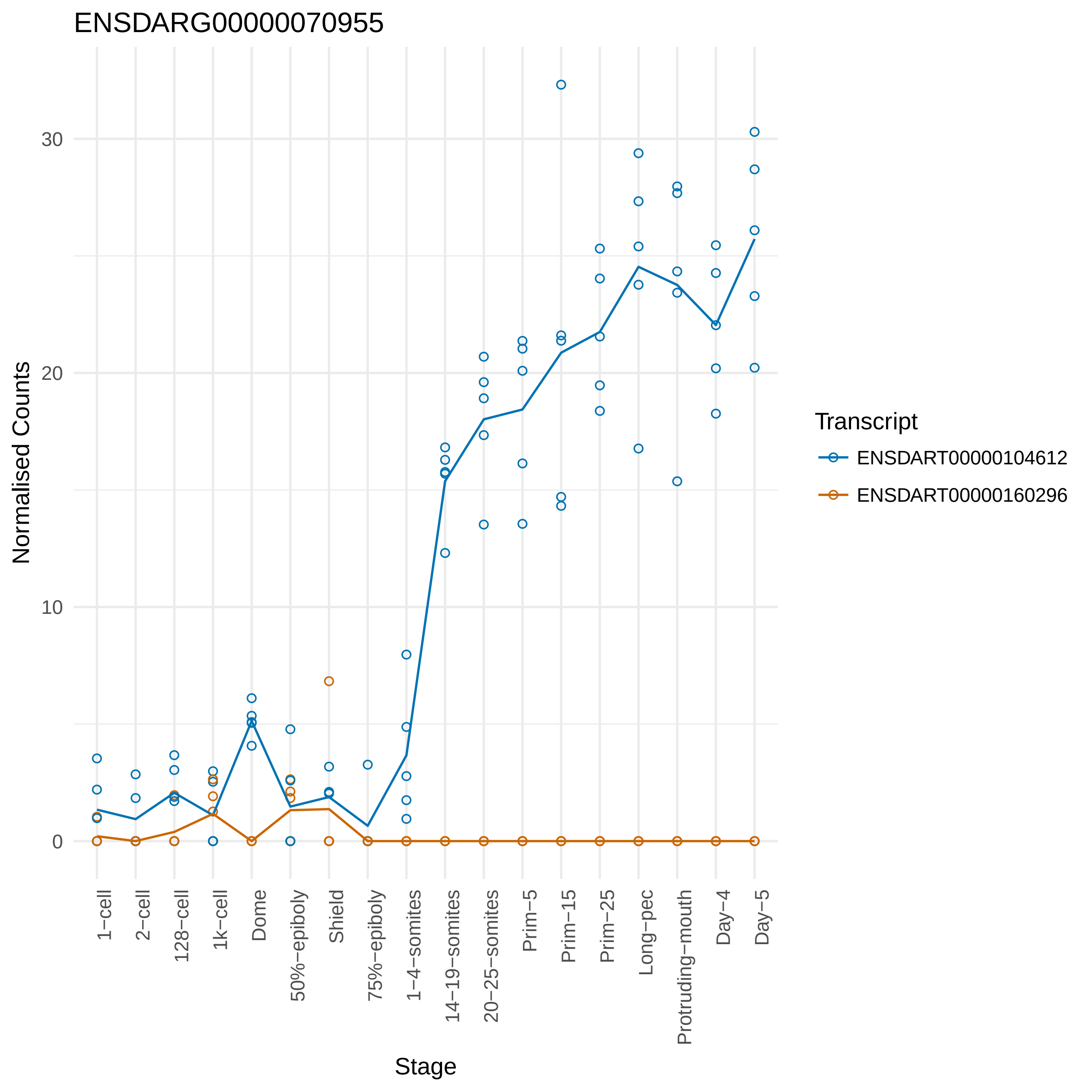
ENSDARG00000070955
Normalised Counts
0
10
20
30
1−cell
2−cell
128−cell
1k−cell
Dome
50%−epiboly
Shield
75%−epiboly
1−4−somites
14−19−somites
20−25−somites
Prim−5
Prim−15
Prim−25
Long−pec
Protruding−mouth
Day−4
Day−5
Stage
Transcript
ENSDART00000104612
ENSDART00000160296

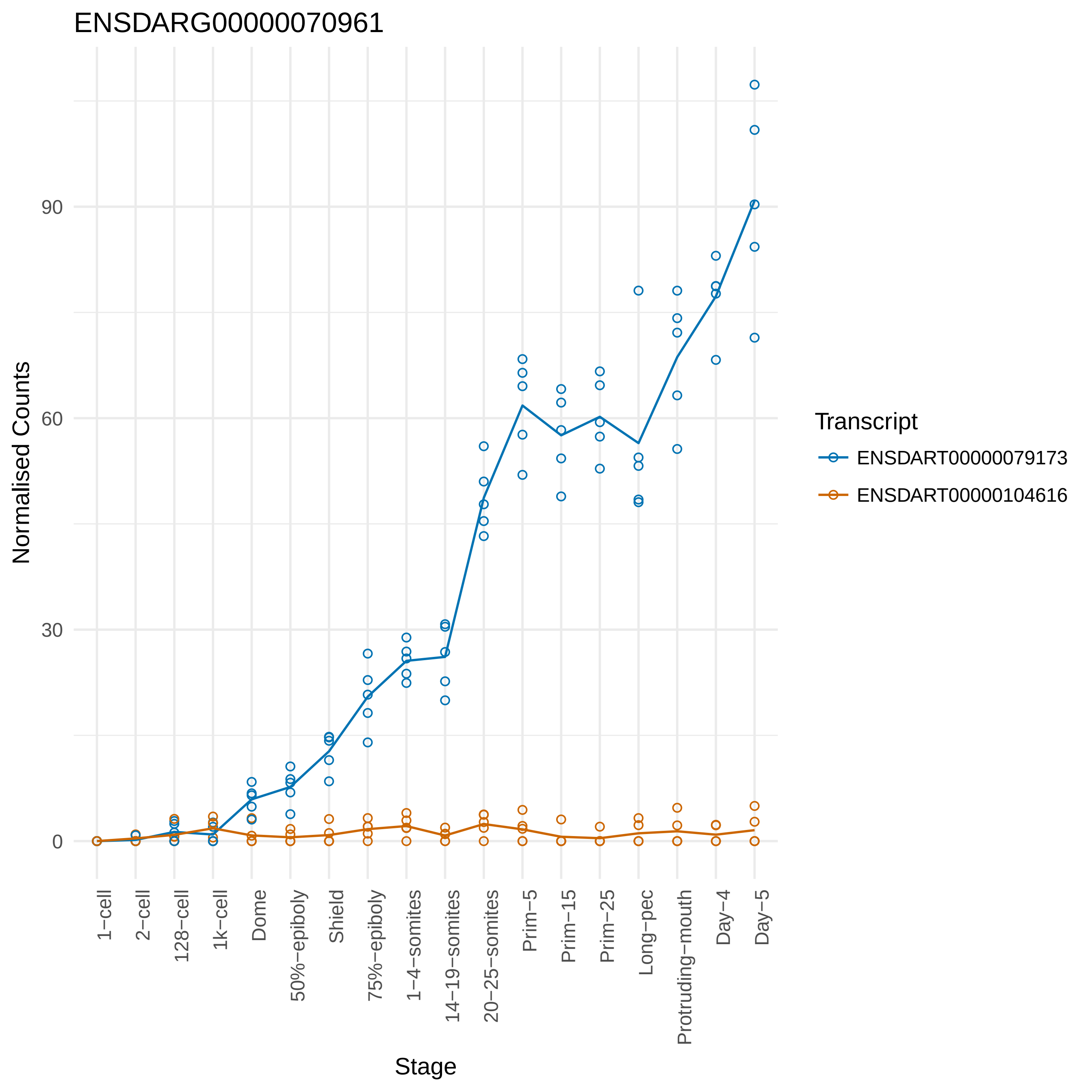
ENSDARG00000070961
Normalised Counts
90
60
30
0
1−cell
2−cell
128−cell
1k−cell
Dome
50%−epiboly
Shield
75%−epiboly
1−4−somites
14−19−somites
20−25−somites
Prim−5
Prim−15
Prim−25
Long−pec
Protruding−mouth
Day−4
Day−5
Stage
Transcript
ENSDART00000079173
ENSDART00000104616

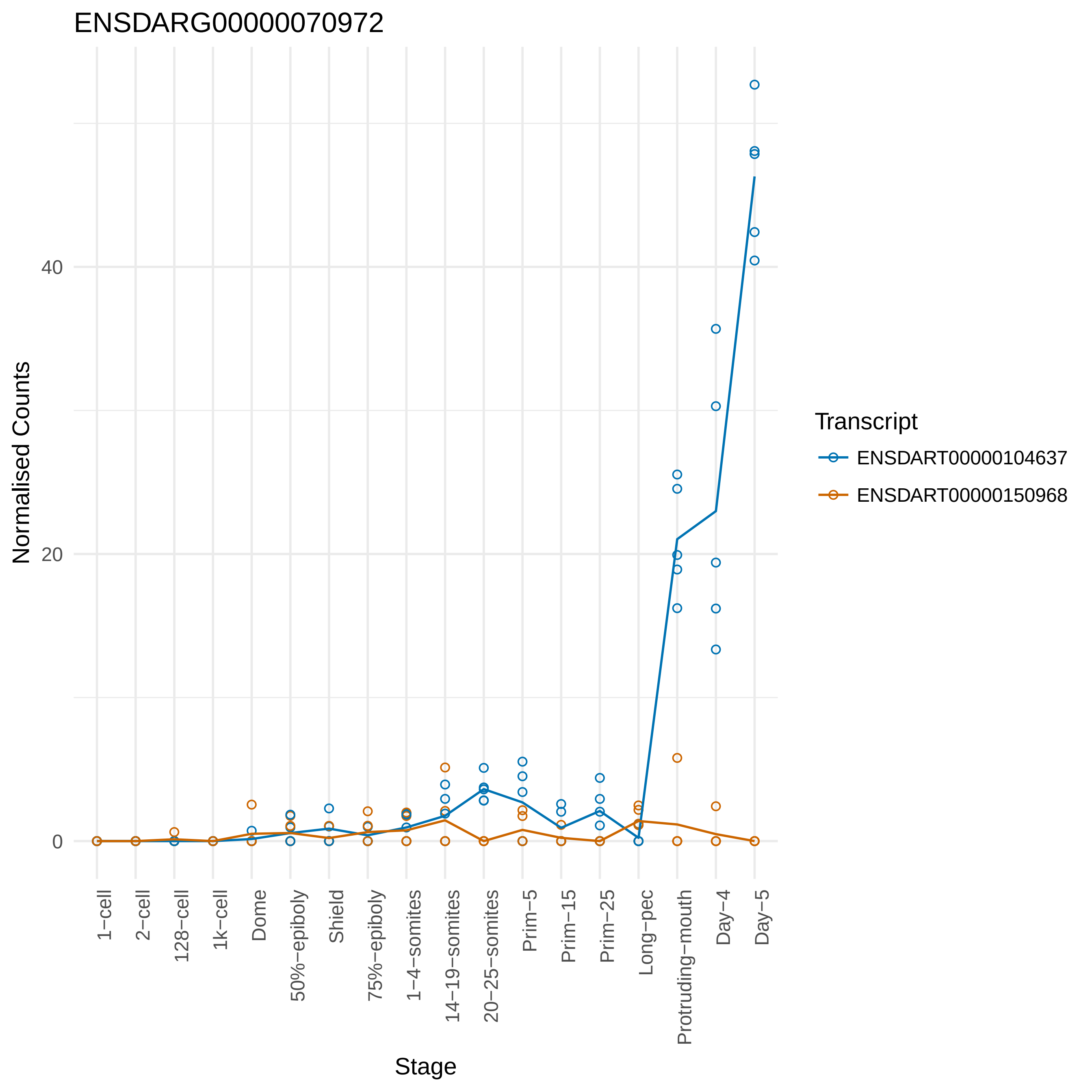

ENSDARG00000070972
Normalised Counts
0
20
40
Stage
1−cell
2−cell
128−cell
1k−cell
Dome
50%−epiboly
Shield
75%−epiboly
1−4−somites
14−19−somites
20−25−somites
Prim−5
Prim−15
Prim−25
Long−pec
Protruding−mouth
Day−4
Day−5
Transcript
ENSDART00000104637
ENSDART00000150968

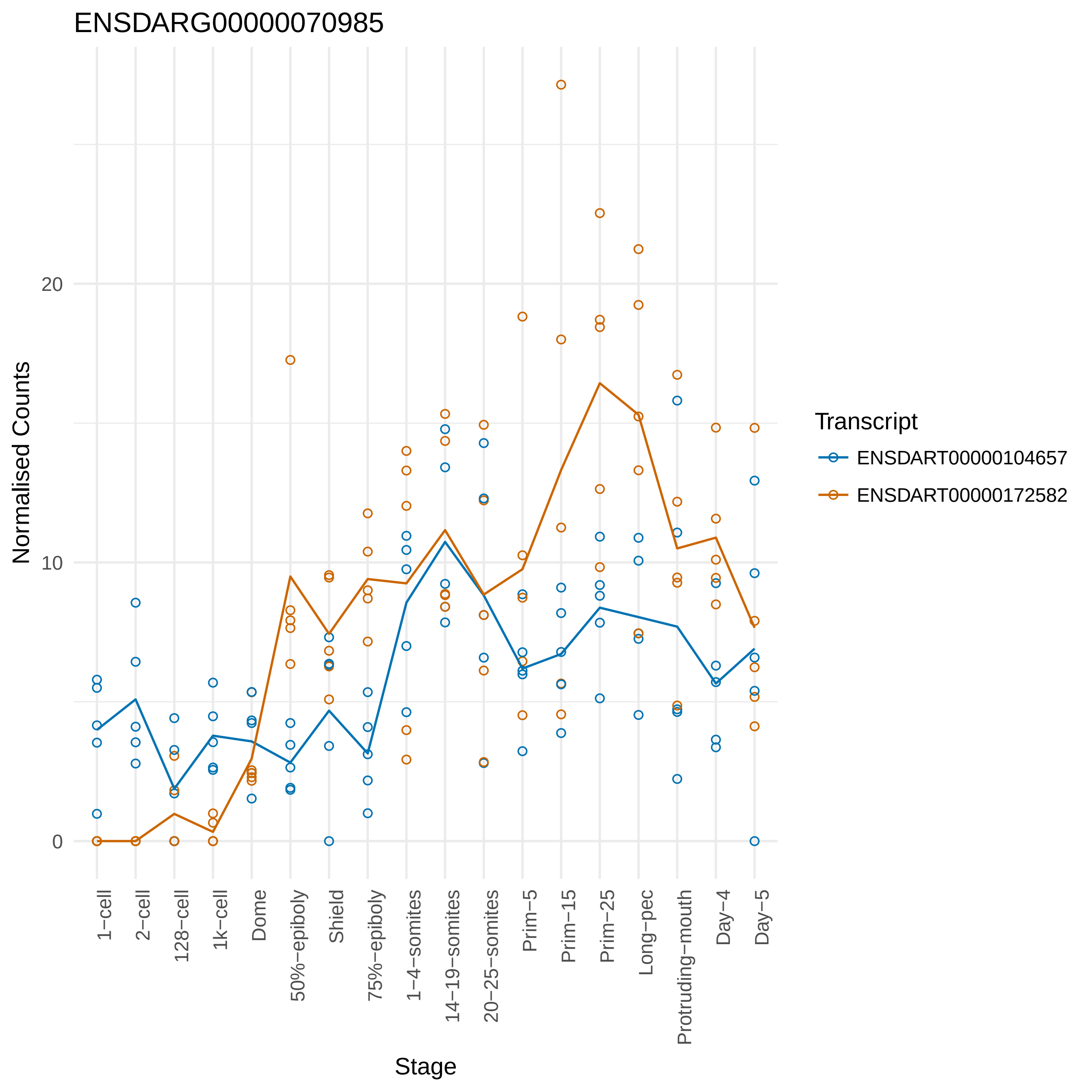

ENSDARG00000070985
Normalised Counts
0
10
20
Stage
1−cell
2−cell
128−cell
1k−cell
Dome
50%−epiboly
Shield
75%−epiboly
1−4−somites
14−19−somites
20−25−somites
Prim−5
Prim−15
Prim−25
Long−pec
Protruding−mouth
Day−4
Day−5
Transcript
ENSDART00000104657
ENSDART00000172582

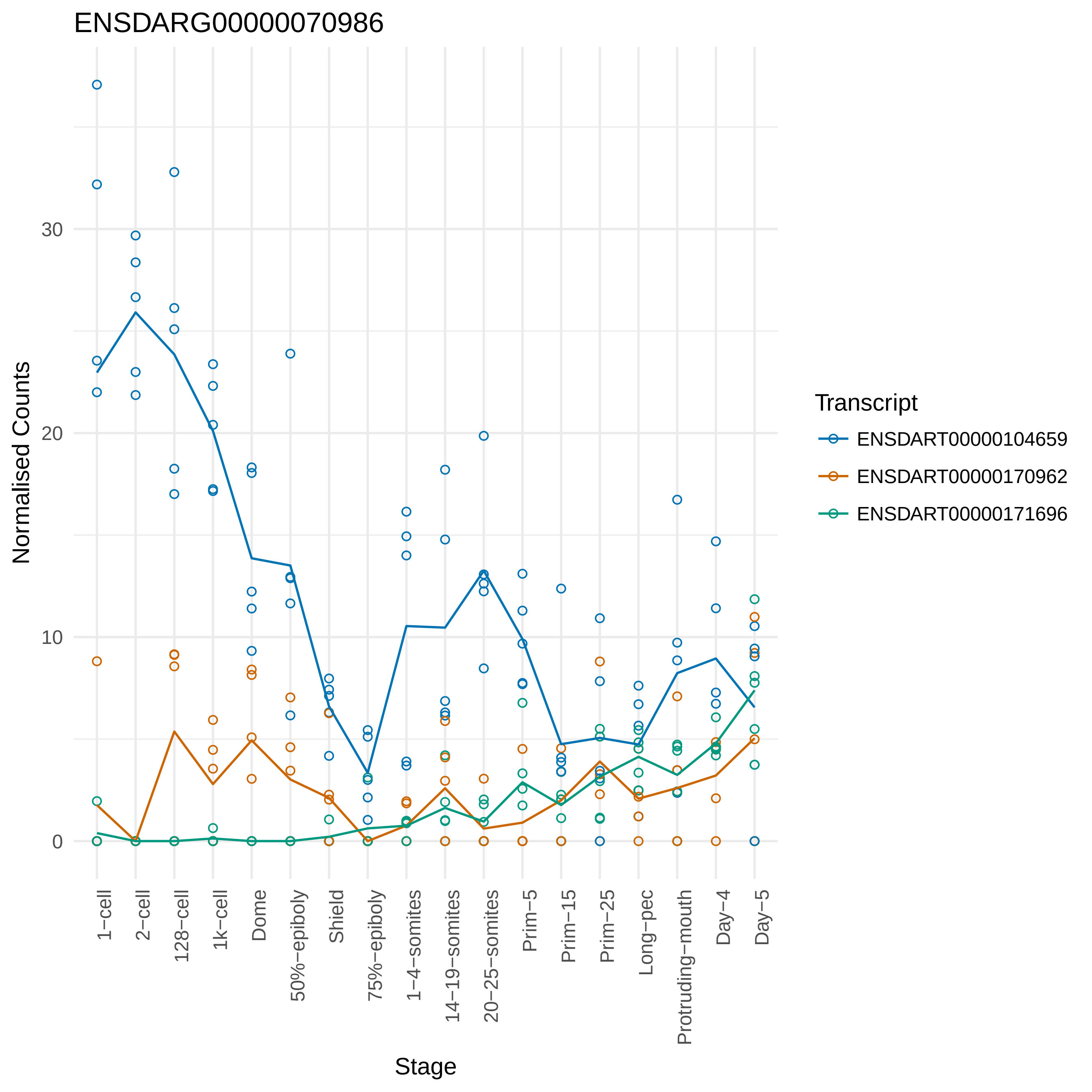

ENSDARG00000070986
Normalised Counts
0
10
20
30
Stage
1−cell
2−cell
128−cell
1k−cell
Dome
50%−epiboly
Shield
75%−epiboly
1−4−somites
14−19−somites
20−25−somites
Prim−5
Prim−15
Prim−25
Long−pec
Protruding−mouth
Day−4
Day−5
Transcript
ENSDART00000104659
ENSDART00000170962
ENSDART00000171696

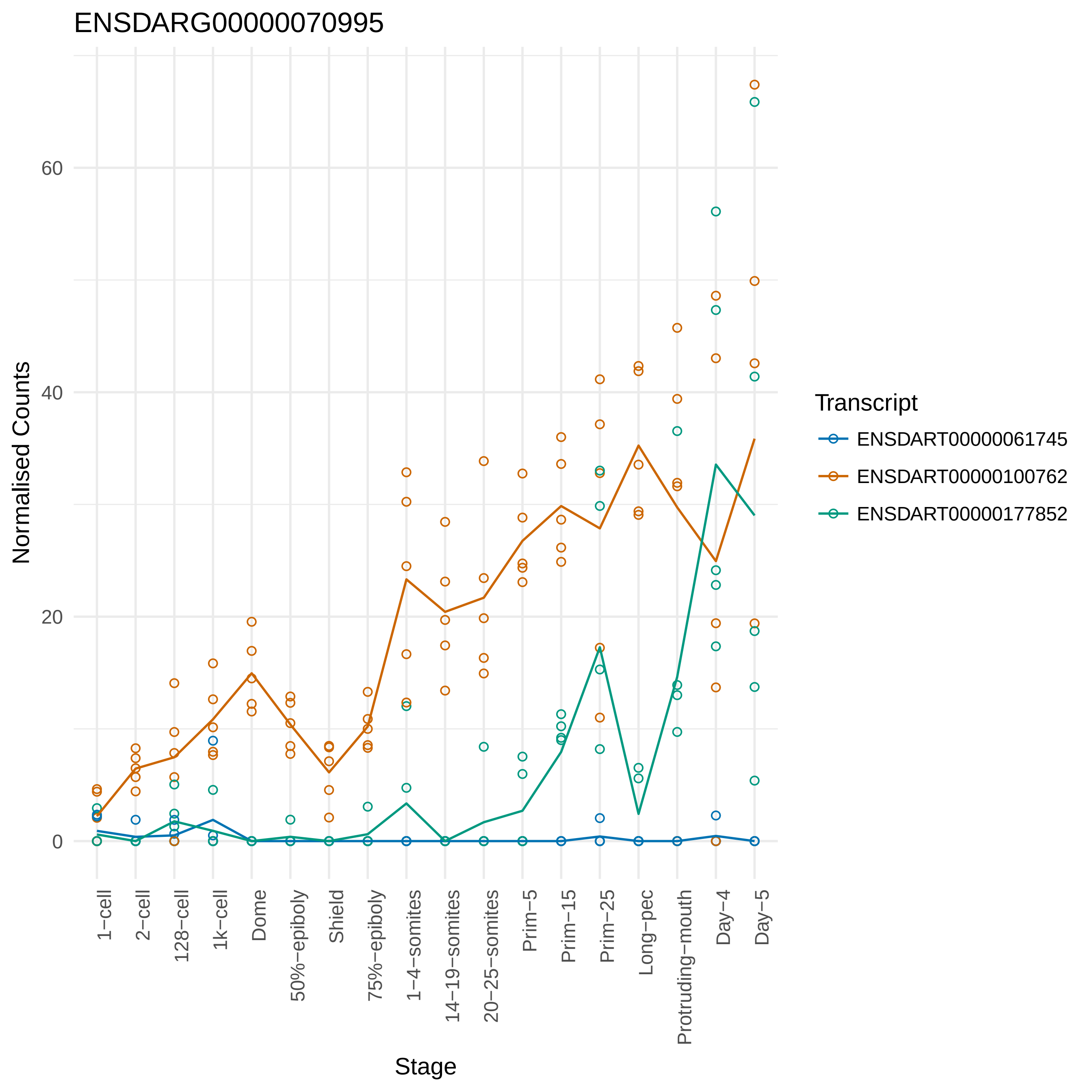

ENSDARG00000070995
Normalised Counts
0
20
40
60
Stage
1−cell
2−cell
128−cell
1k−cell
Dome
50%−epiboly
Shield
75%−epiboly
1−4−somites
14−19−somites
20−25−somites
Prim−5
Prim−15
Prim−25
Long−pec
Protruding−mouth
Day−4
Day−5
Transcript
ENSDART00000061745
ENSDART00000100762
ENSDART00000177852

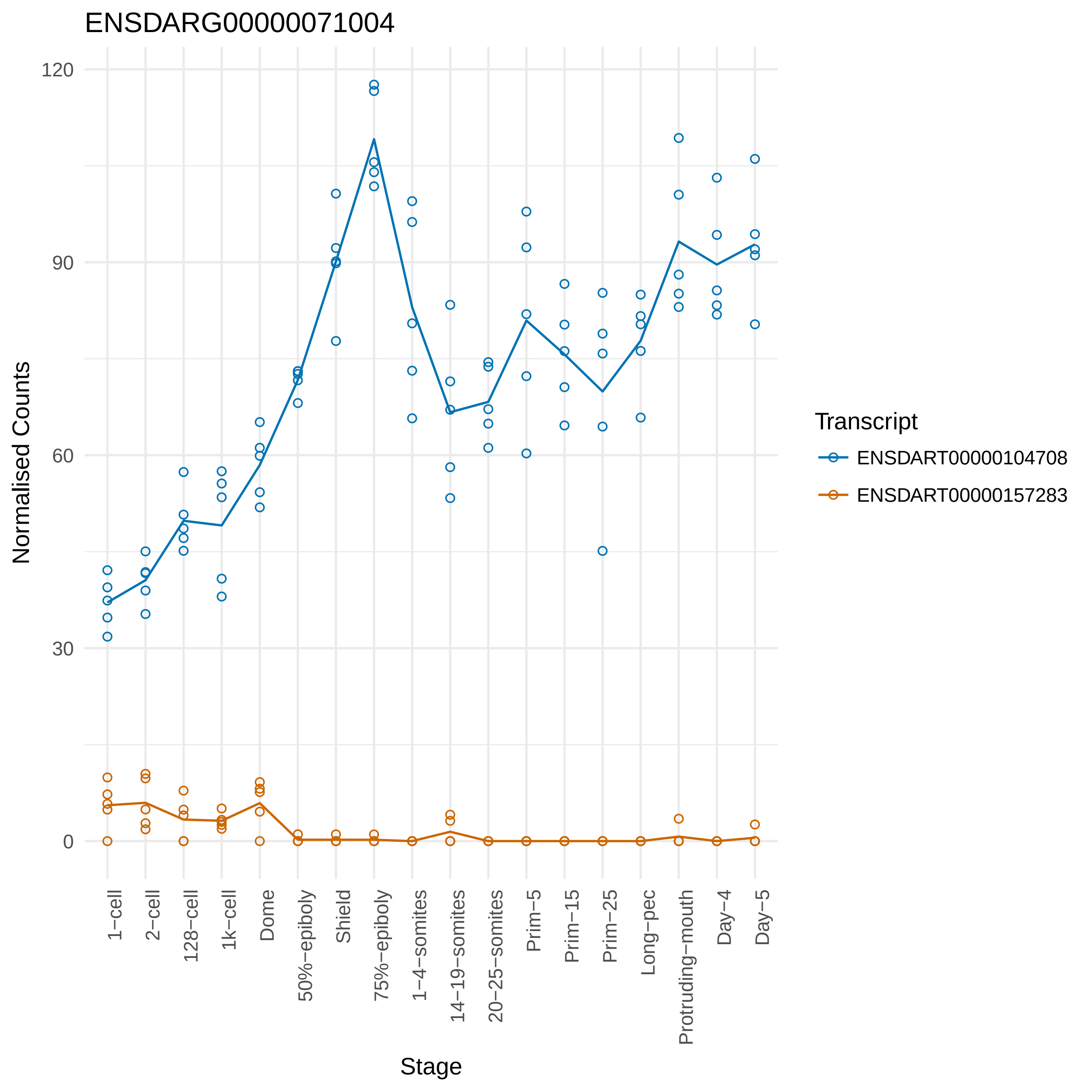

ENSDARG00000071004
Normalised Counts
120
90
60
30
0
1−cell
2−cell
128−cell
1k−cell
Dome
50%−epiboly
Shield
75%−epiboly
1−4−somites
14−19−somites
20−25−somites
Prim−5
Prim−15
Prim−25
Long−pec
Protruding−mouth
Day−4
Day−5
Stage
Transcript
ENSDART00000104708
ENSDART00000157283

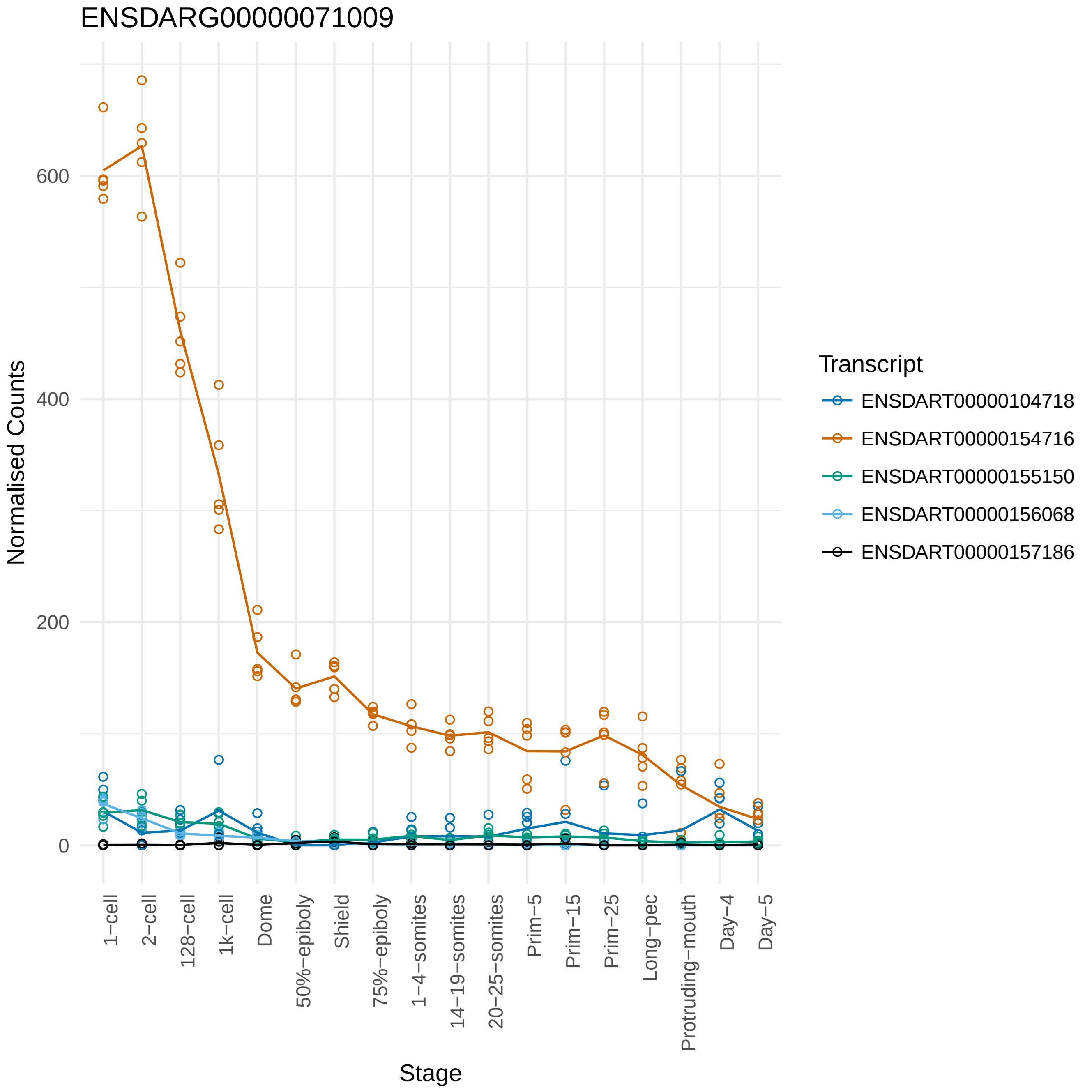

ENSDARG00000071009
Normalised Counts
600
400
200
0
1−cell
2−cell
128−cell
1k−cell
Dome
50%−epiboly
Shield
75%−epiboly
1−4−somites
14−19−somites
20−25−somites
Prim−5
Prim−15
Prim−25
Long−pec
Protruding−mouth
Day−4
Day−5
Stage
Transcript
ENSDART00000104718
ENSDART00000154716
ENSDART00000155150
ENSDART00000156068
ENSDART00000157186

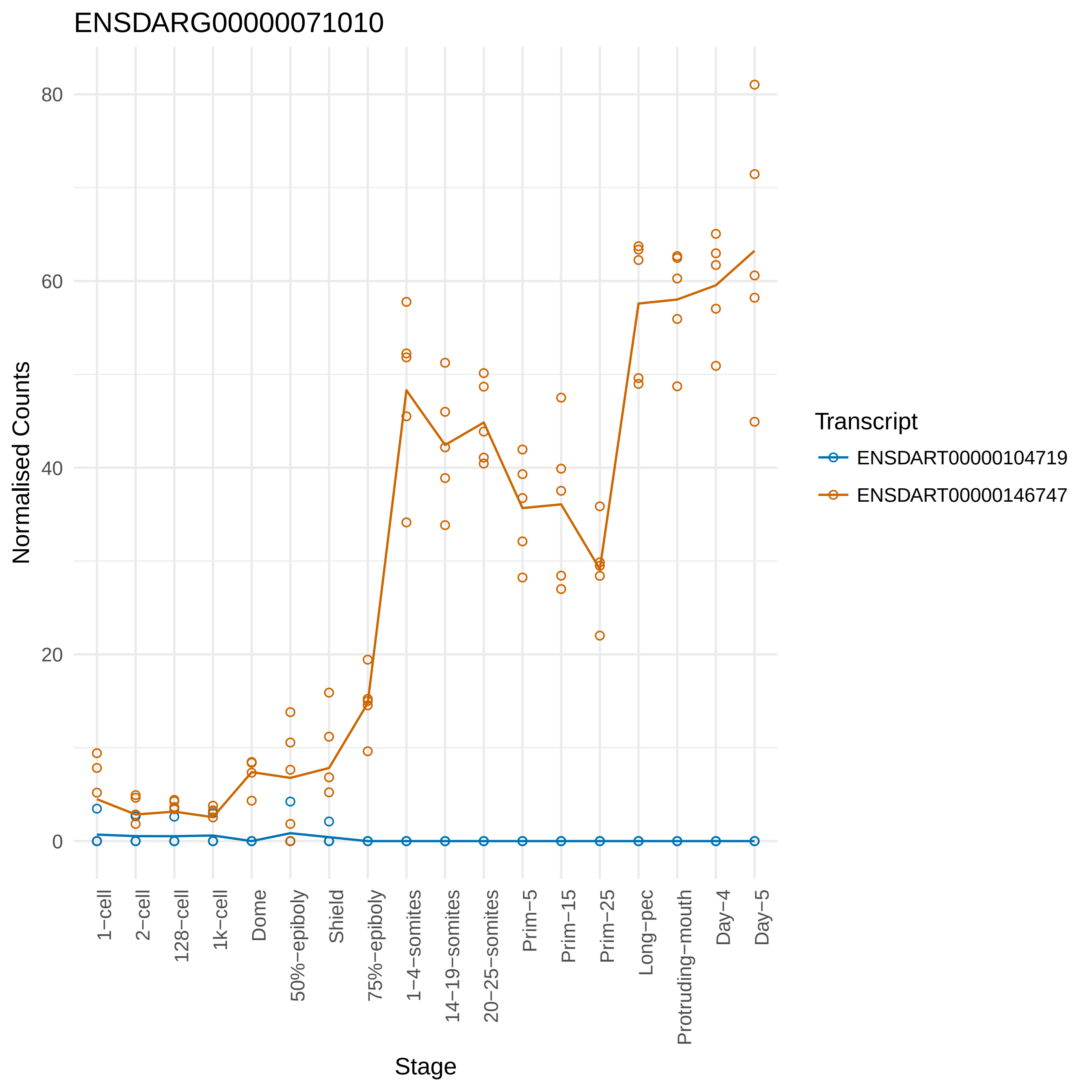
ENSDARG00000071010
Normalised Counts
0
20
40
60
80
1−cell
2−cell
128−cell
1k−cell
Dome
50%−epiboly
Shield
75%−epiboly
1−4−somites
14−19−somites
20−25−somites
Prim−5
Prim−15
Prim−25
Long−pec
Protruding−mouth
Day−4
Day−5
Stage
Transcript
ENSDART00000104719
ENSDART00000146747

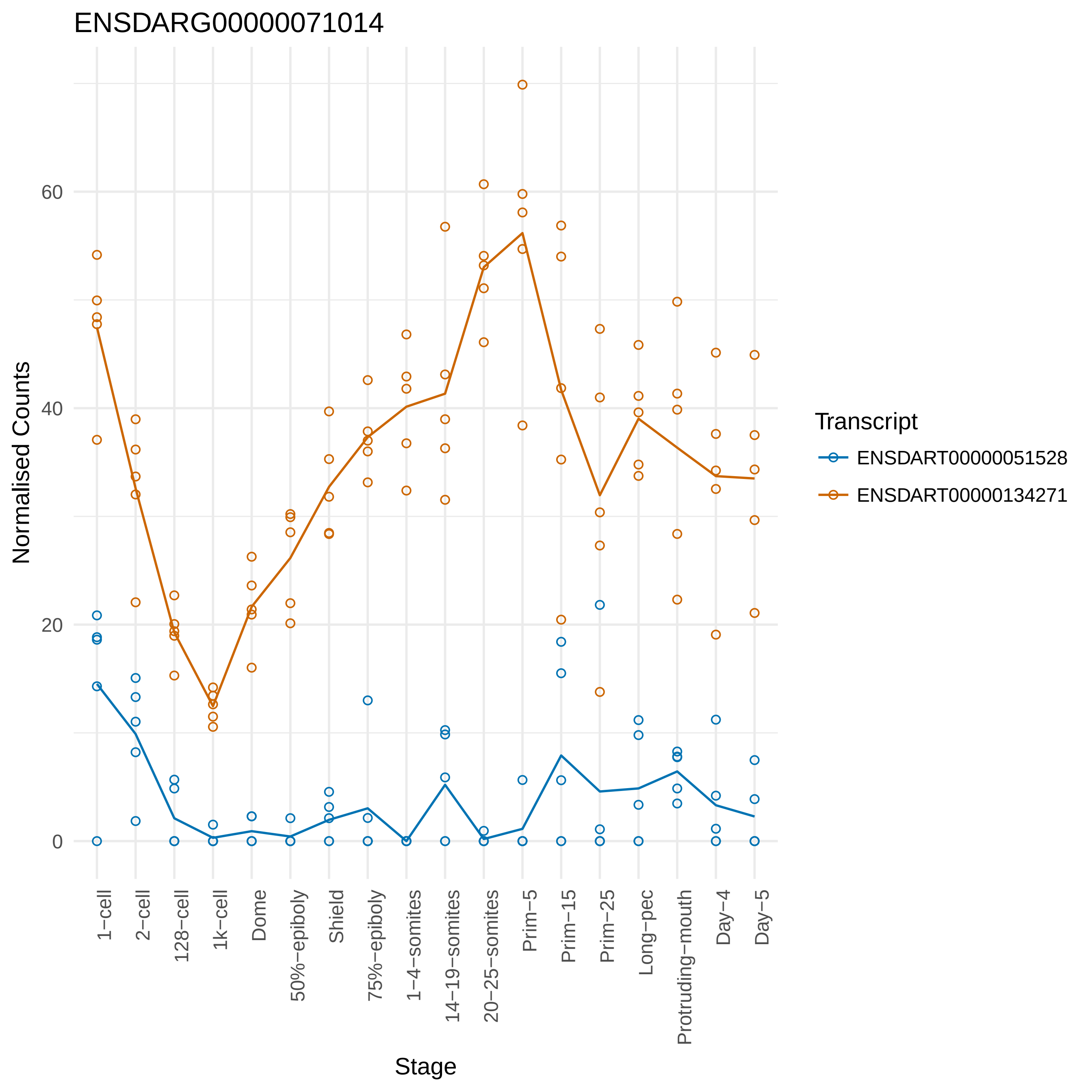

ENSDARG00000071014
Normalised Counts
0
20
40
60
Stage
1−cell
2−cell
128−cell
1k−cell
Dome
50%−epiboly
Shield
75%−epiboly
1−4−somites
14−19−somites
20−25−somites
Prim−5
Prim−15
Prim−25
Long−pec
Protruding−mouth
Day−4
Day−5
Transcript
ENSDART00000051528
ENSDART00000134271

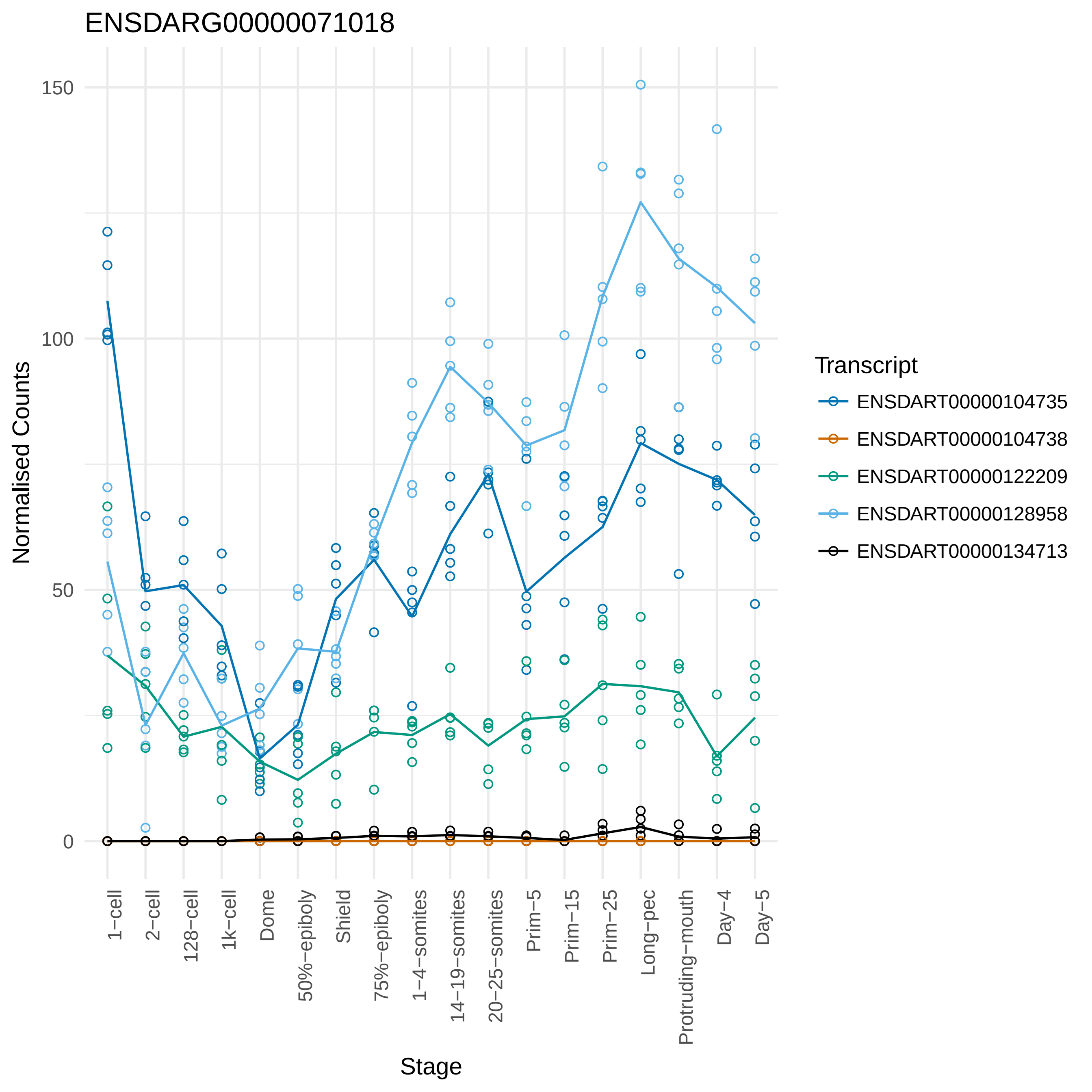

ENSDARG00000071018
Normalised Counts
150
100
50
0
1−cell
2−cell
128−cell
1k−cell
Dome
50%−epiboly
Shield
75%−epiboly
1−4−somites
14−19−somites
20−25−somites
Prim−5
Prim−15
Prim−25
Long−pec
Protruding−mouth
Day−4
Day−5
Stage
Transcript
ENSDART00000104735
ENSDART00000104738
ENSDART00000122209
ENSDART00000128958
ENSDART00000134713

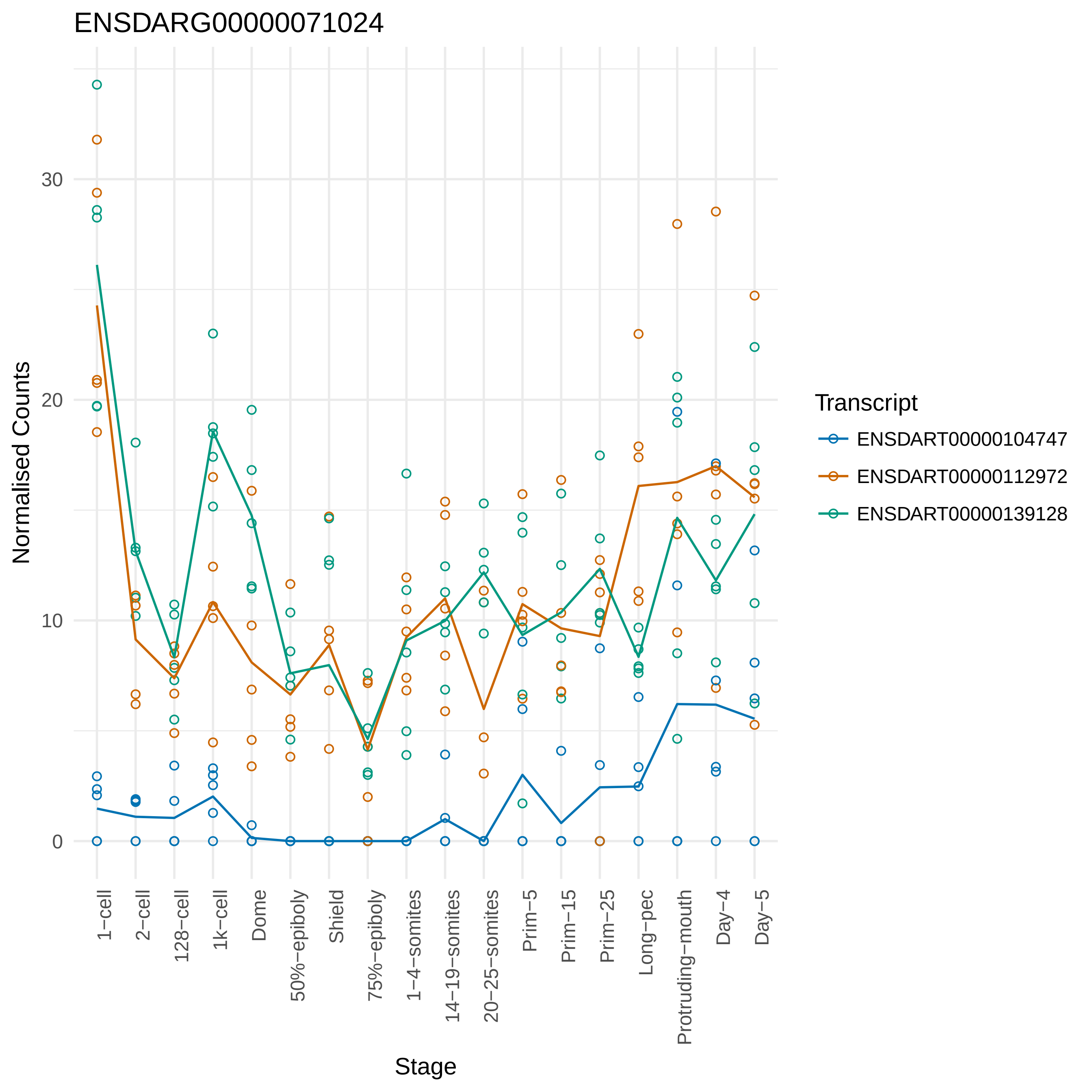

ENSDARG00000071024
Normalised Counts
0
10
20
30
1−cell
2−cell
128−cell
1k−cell
Dome
50%−epiboly
Shield
75%−epiboly
1−4−somites
14−19−somites
20−25−somites
Prim−5
Prim−15
Prim−25
Long−pec
Protruding−mouth
Day−4
Day−5
Stage
Transcript
ENSDART00000104747
ENSDART00000112972
ENSDART00000139128

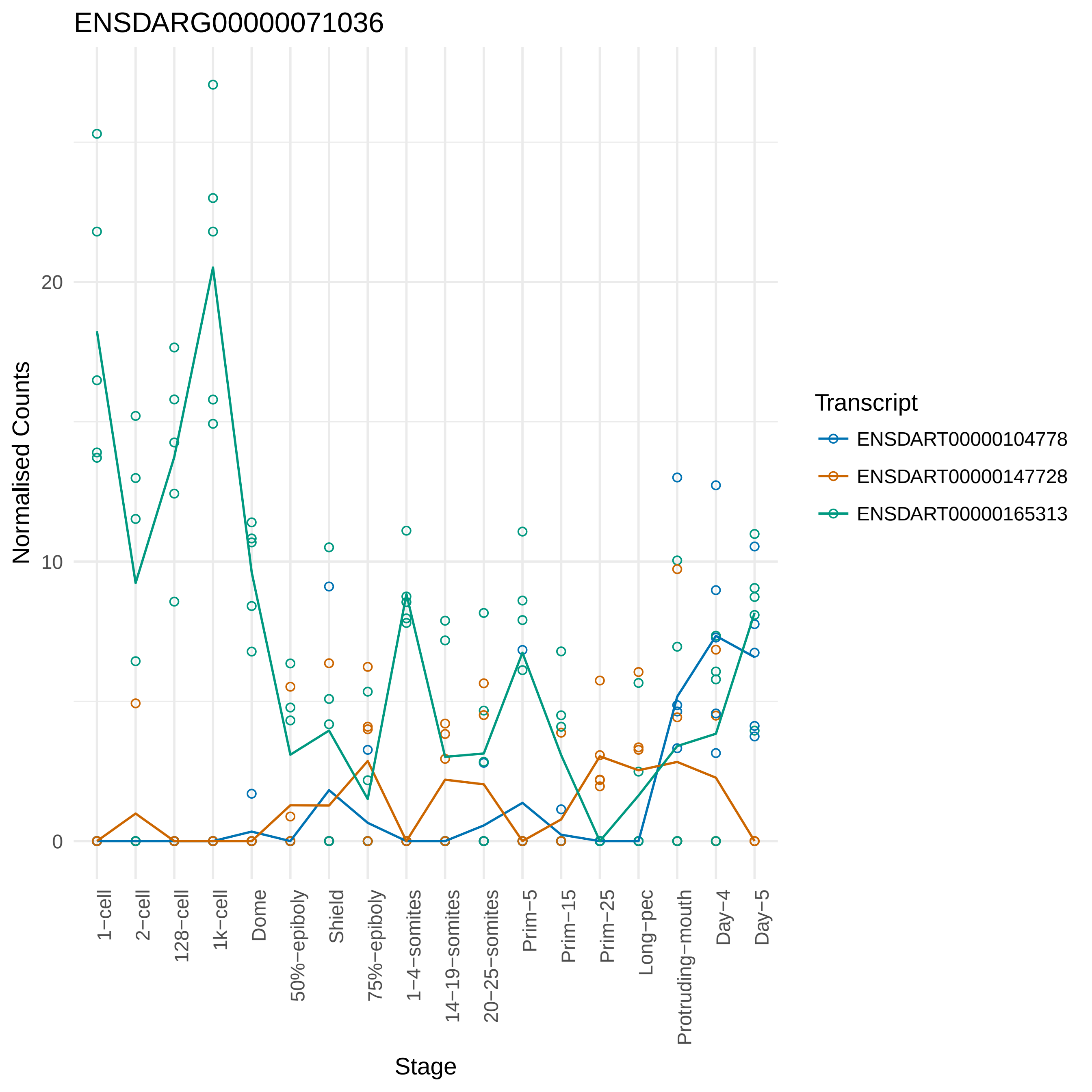
ENSDARG00000071036
Normalised Counts
20
10
0
1−cell
2−cell
128−cell
1k−cell
Dome
50%−epiboly
Shield
75%−epiboly
1−4−somites
14−19−somites
20−25−somites
Prim−5
Prim−15
Prim−25
Long−pec
Protruding−mouth
Day−4
Day−5
Stage
Transcript
ENSDART00000104778
ENSDART00000147728
ENSDART00000165313

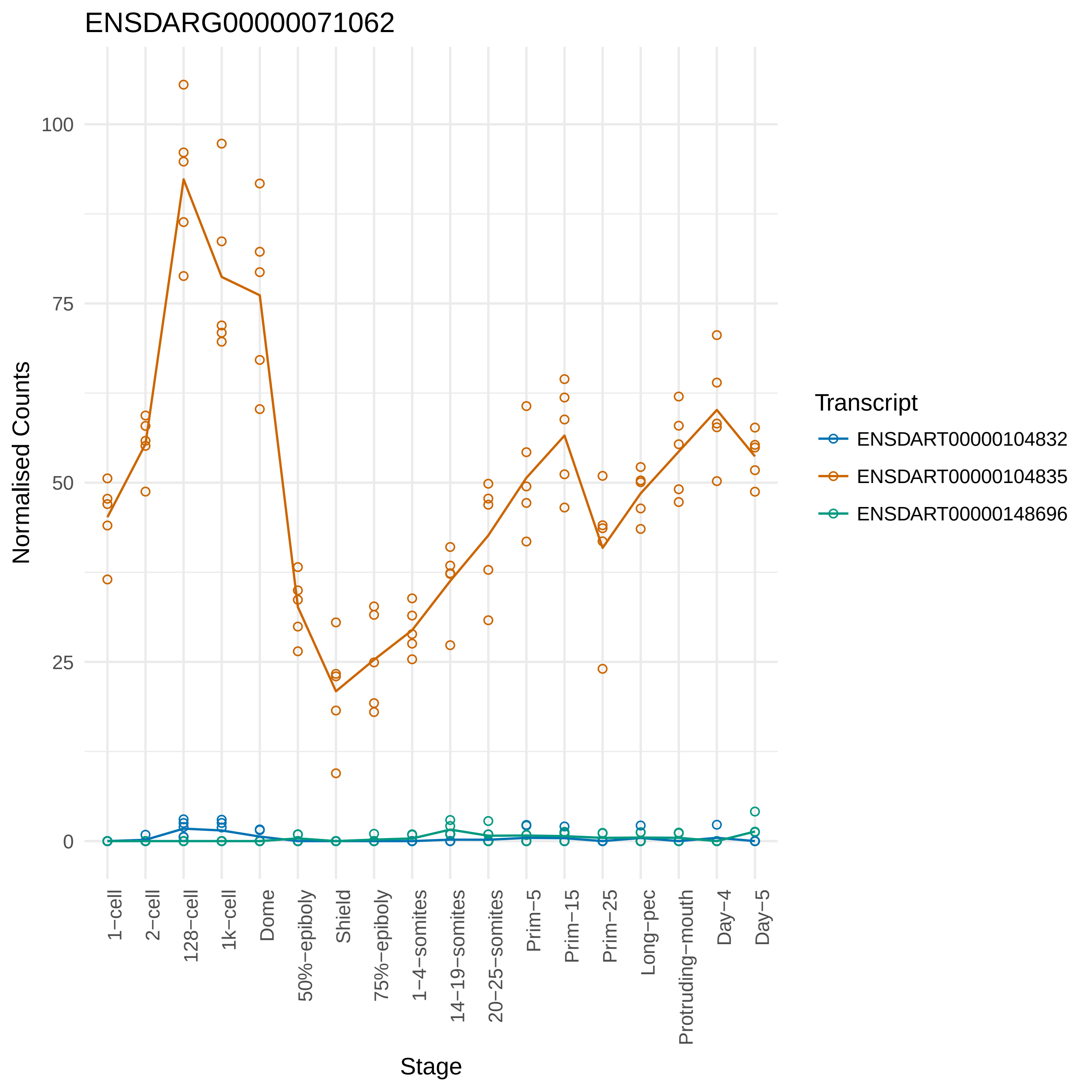

ENSDARG00000071062
Normalised Counts
100
75
50
25
0
1−cell
2−cell
128−cell
1k−cell
Dome
50%−epiboly
Shield
75%−epiboly
1−4−somites
14−19−somites
20−25−somites
Prim−5
Prim−15
Prim−25
Long−pec
Protruding−mouth
Day−4
Day−5
Stage
Transcript
ENSDART00000104832
ENSDART00000104835
ENSDART00000148696

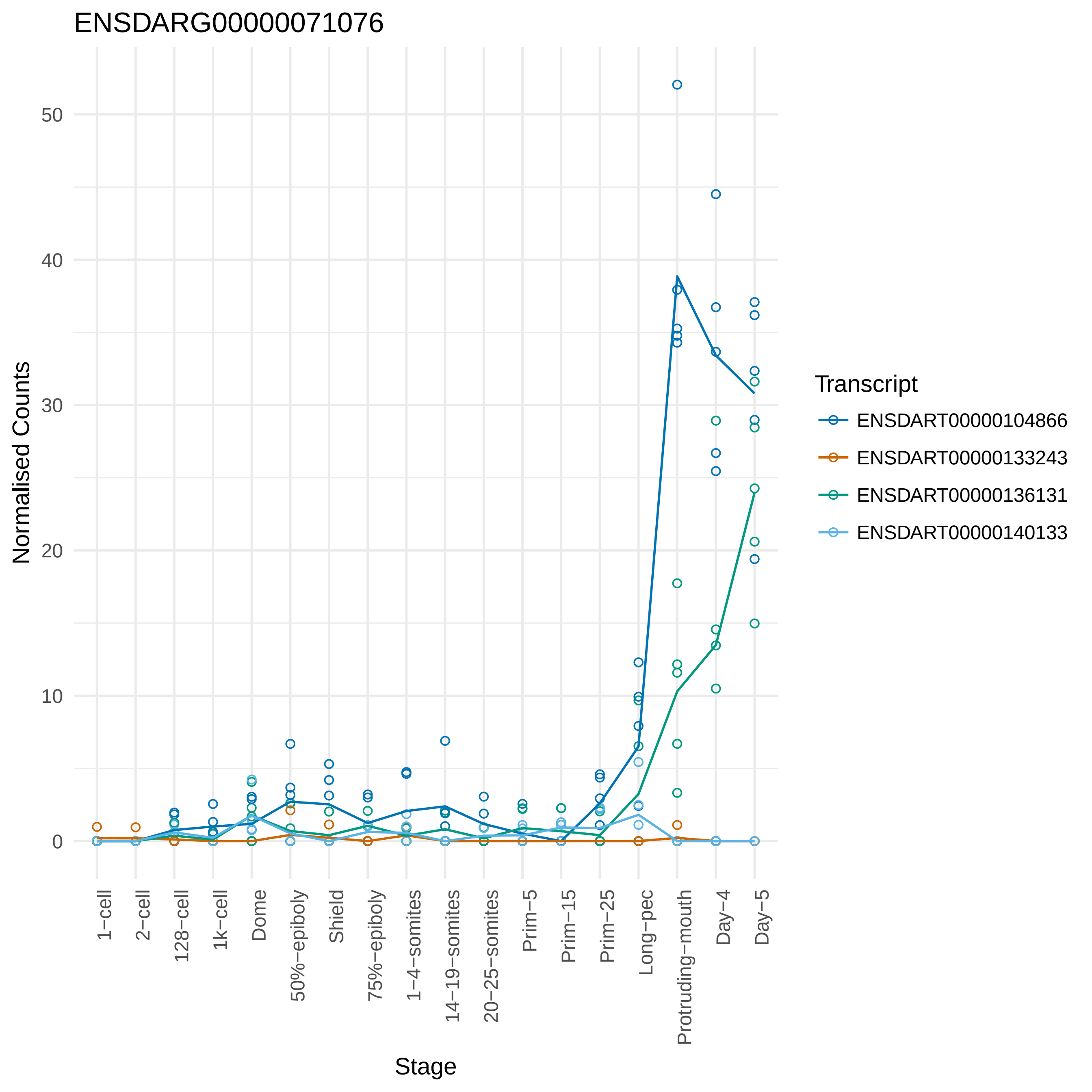

ENSDARG00000071076
Normalised Counts
50
40
30
20
10
0
1−cell
2−cell
128−cell
1k−cell
Dome
50%−epiboly
Shield
75%−epiboly
1−4−somites
14−19−somites
20−25−somites
Prim−5
Prim−15
Prim−25
Long−pec
Protruding−mouth
Day−4
Day−5
Stage
Transcript
ENSDART00000104866
ENSDART00000133243
ENSDART00000136131
ENSDART00000140133

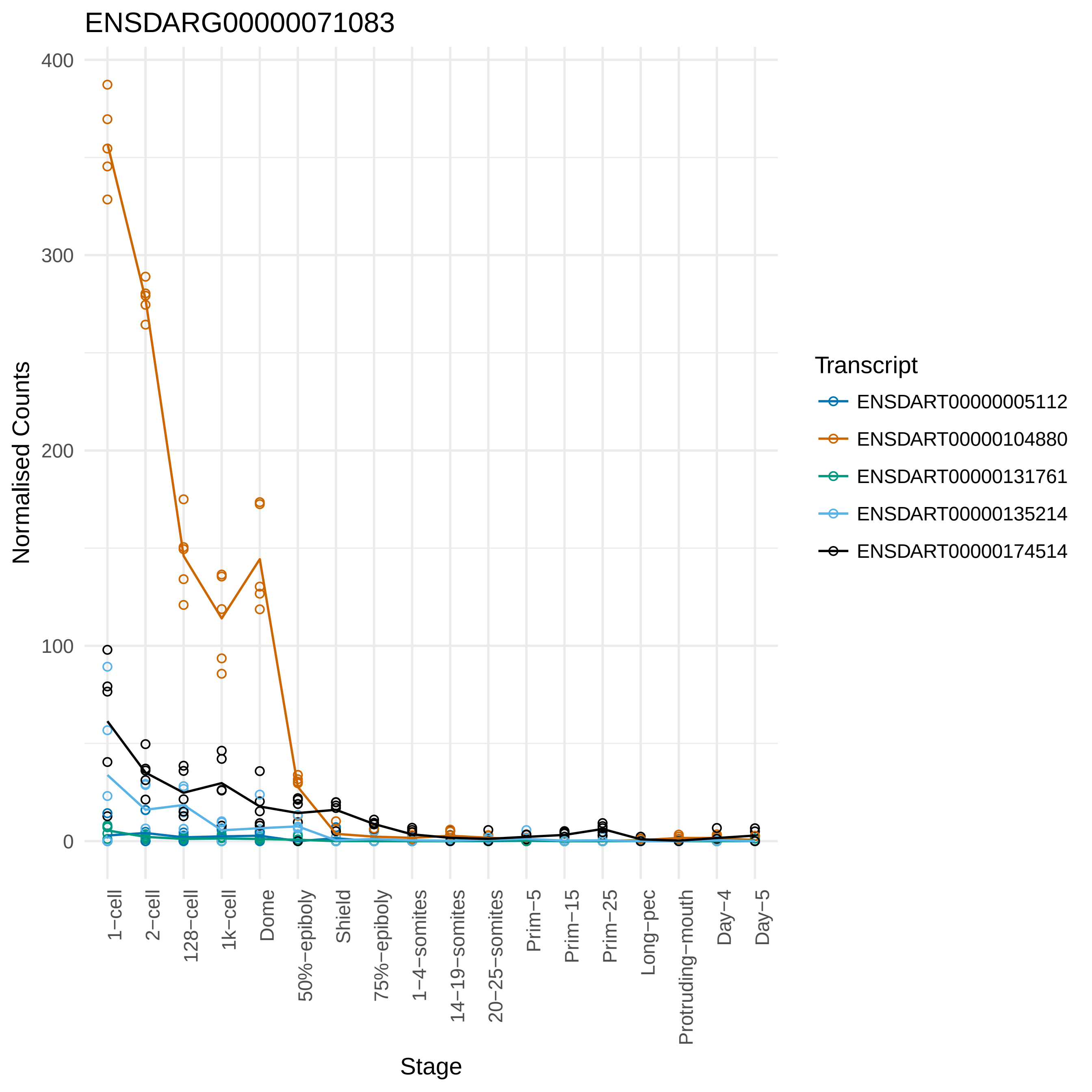

ENSDARG00000071083
Normalised Counts
400
300
200
100
0
1−cell
2−cell
128−cell
1k−cell
Dome
50%−epiboly
Shield
75%−epiboly
1−4−somites
14−19−somites
20−25−somites
Prim−5
Prim−15
Prim−25
Long−pec
Protruding−mouth
Day−4
Day−5
Stage
Transcript
ENSDART00000005112
ENSDART00000104880
ENSDART00000131761
ENSDART00000135214
ENSDART00000174514

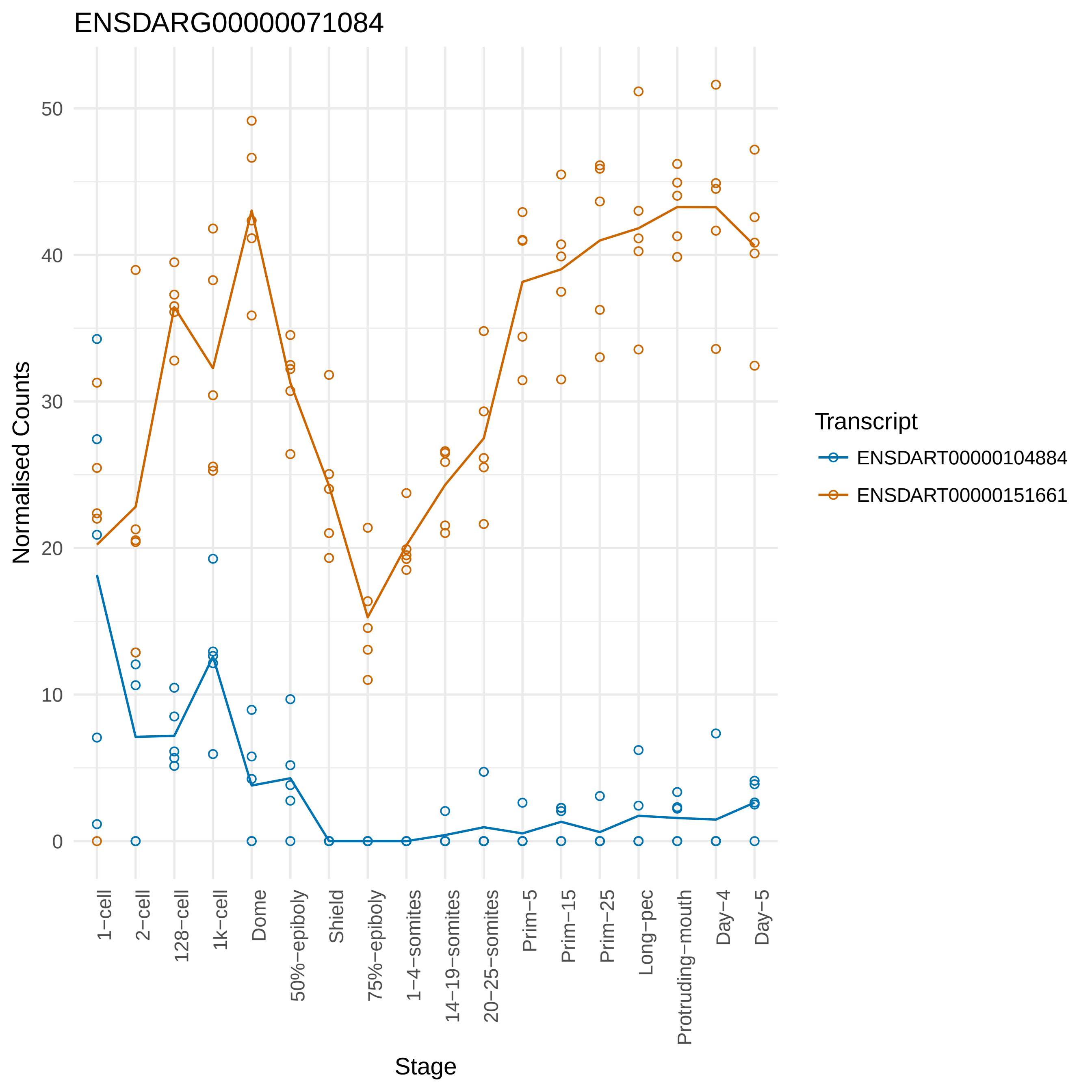

ENSDARG00000071084
Normalised Counts
0
10
20
30
40
50
1−cell
2−cell
128−cell
1k−cell
Dome
50%−epiboly
Shield
75%−epiboly
1−4−somites
14−19−somites
20−25−somites
Prim−5
Prim−15
Prim−25
Long−pec
Protruding−mouth
Day−4
Day−5
Stage
Transcript
ENSDART00000104884
ENSDART00000151661

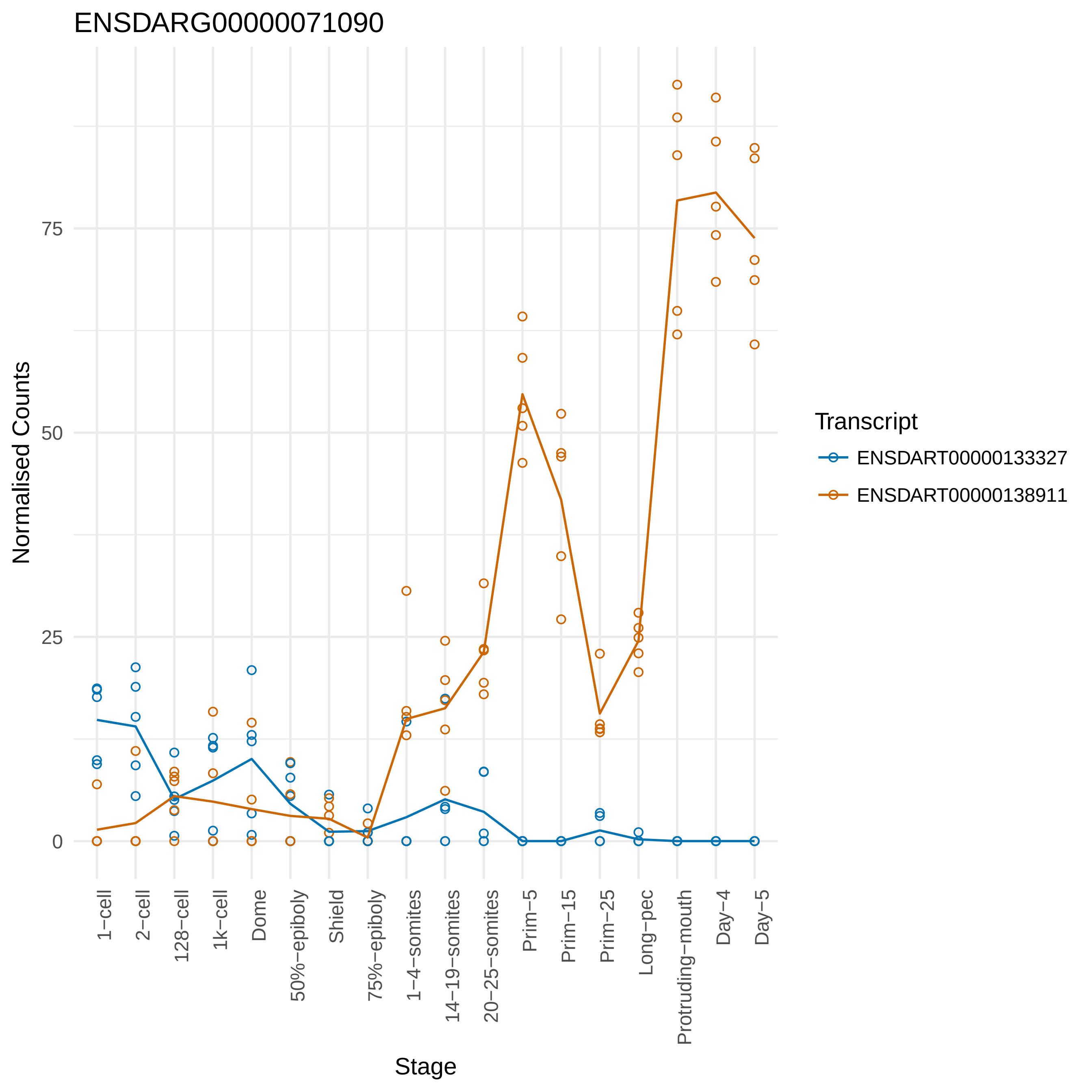
ENSDARG00000071090
Normalised Counts
75
50
25
0
1−cell
2−cell
128−cell
1k−cell
Dome
50%−epiboly
Shield
75%−epiboly
1−4−somites
14−19−somites
20−25−somites
Prim−5
Prim−15
Prim−25
Long−pec
Protruding−mouth
Day−4
Day−5
Stage
Transcript
ENSDART00000133327
ENSDART00000138911

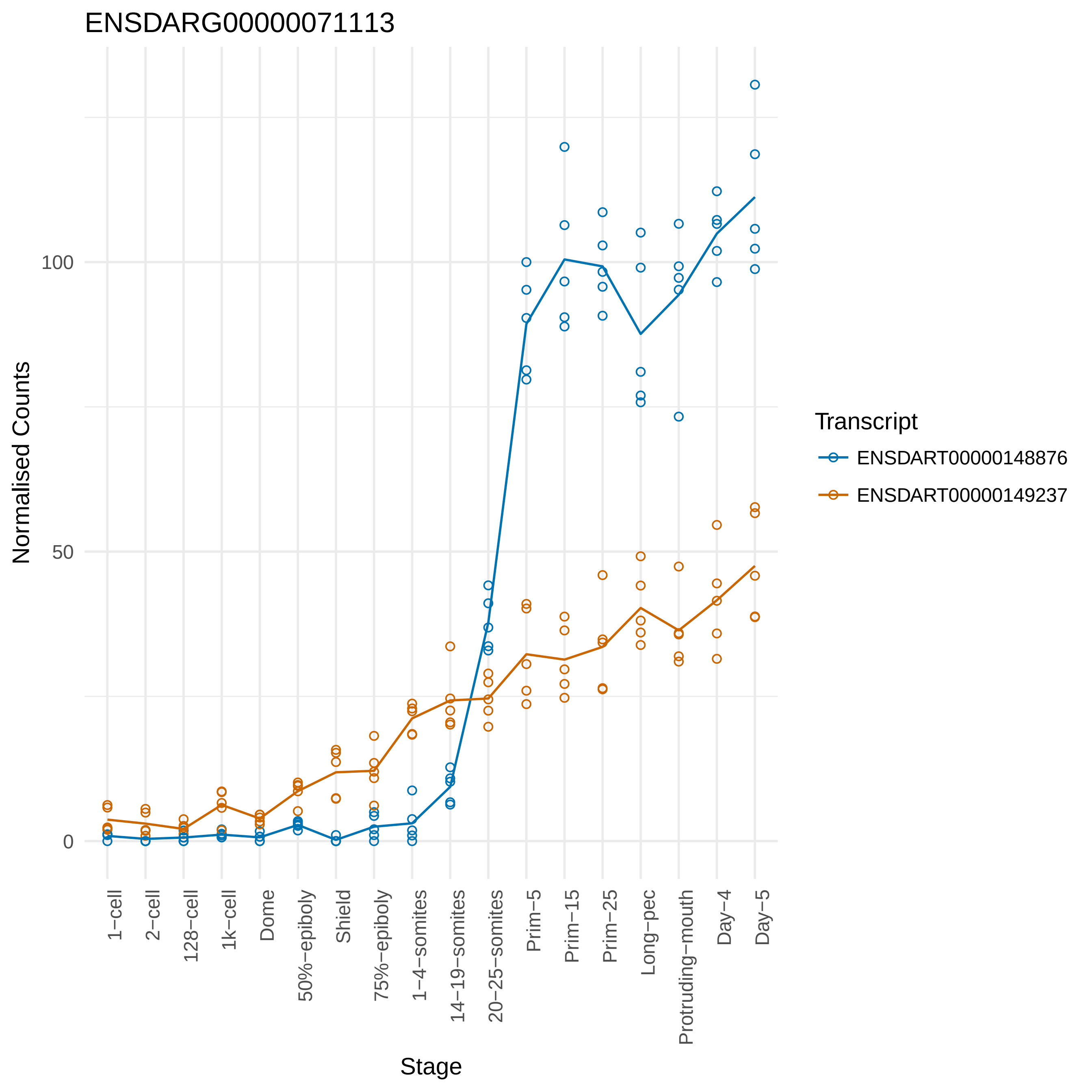
ENSDARG00000071113
Normalised Counts
100
50
0
1−cell
2−cell
128−cell
1k−cell
Dome
50%−epiboly
Shield
75%−epiboly
1−4−somites
14−19−somites
20−25−somites
Prim−5
Prim−15
Prim−25
Long−pec
Protruding−mouth
Day−4
Day−5
Stage
Transcript
ENSDART00000148876
ENSDART00000149237

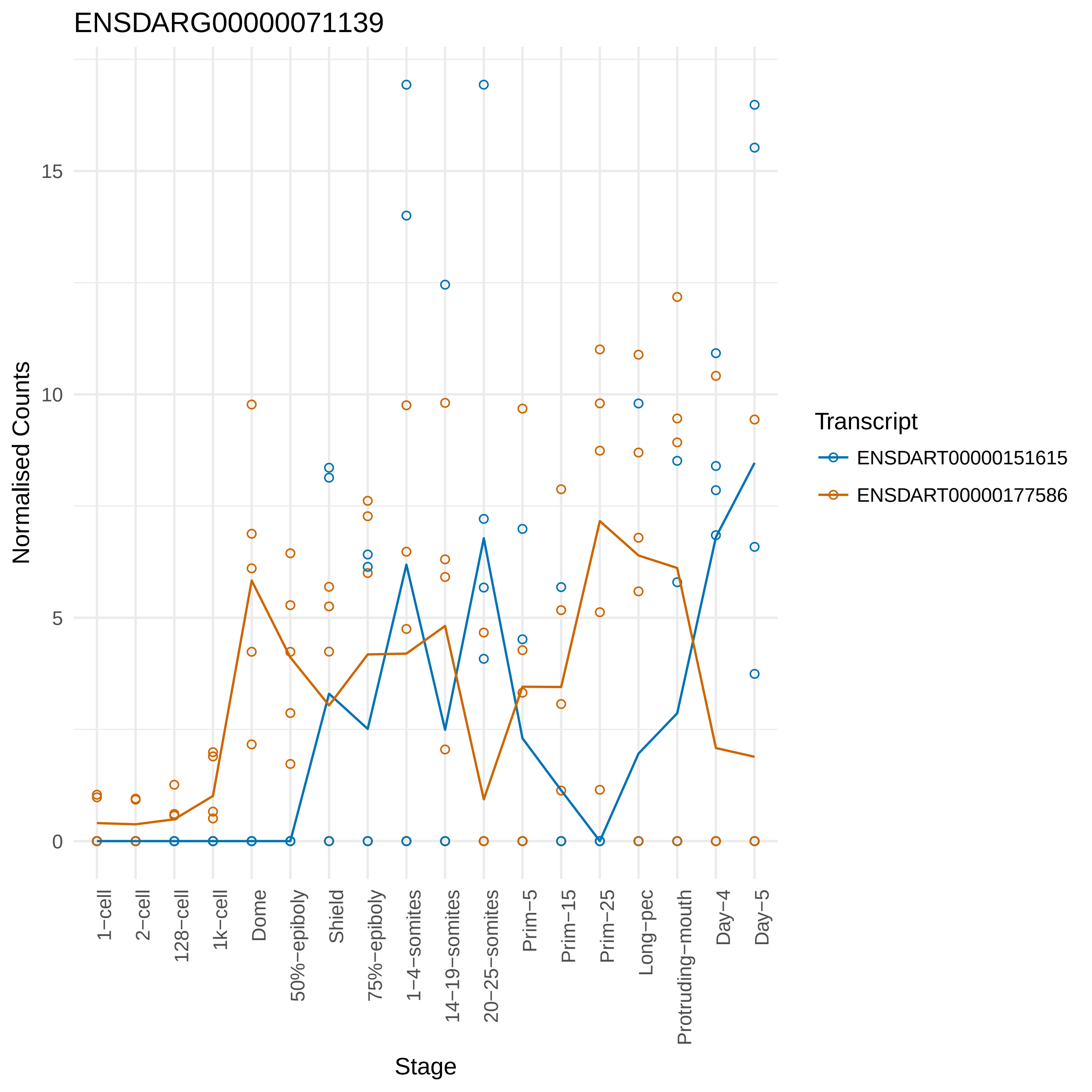
ENSDARG00000071139
Normalised Counts
0
5
10
15
1−cell
2−cell
128−cell
1k−cell
Dome
50%−epiboly
Shield
75%−epiboly
1−4−somites
14−19−somites
20−25−somites
Prim−5
Prim−15
Prim−25
Long−pec
Protruding−mouth
Day−4
Day−5
Stage
Transcript
ENSDART00000151615
ENSDART00000177586

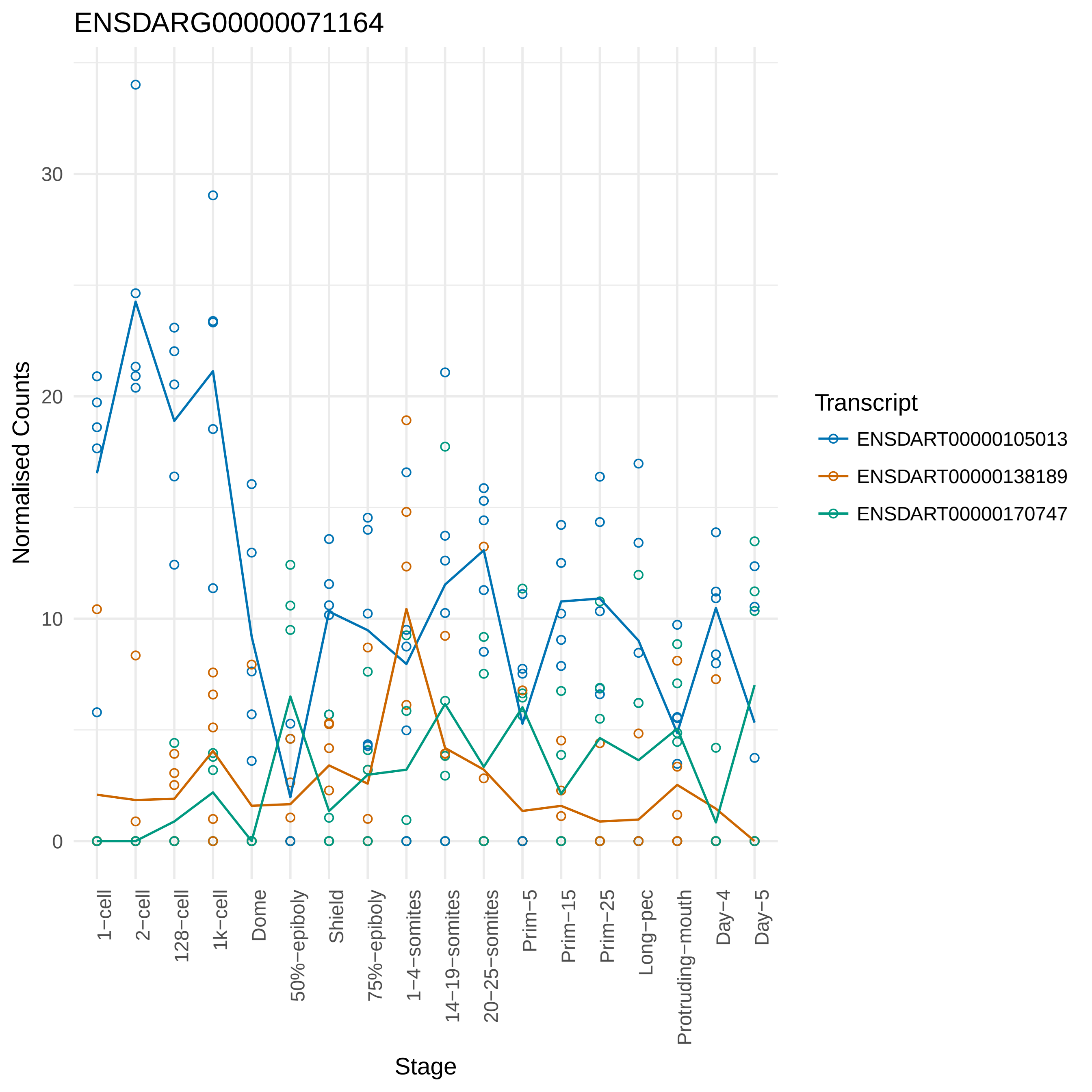
ENSDARG00000071164
Normalised Counts
30
20
10
0
1−cell
2−cell
128−cell
1k−cell
Dome
50%−epiboly
Shield
75%−epiboly
1−4−somites
14−19−somites
20−25−somites
Prim−5
Prim−15
Prim−25
Long−pec
Protruding−mouth
Day−4
Day−5
Stage
Transcript
ENSDART00000105013
ENSDART00000138189
ENSDART00000170747

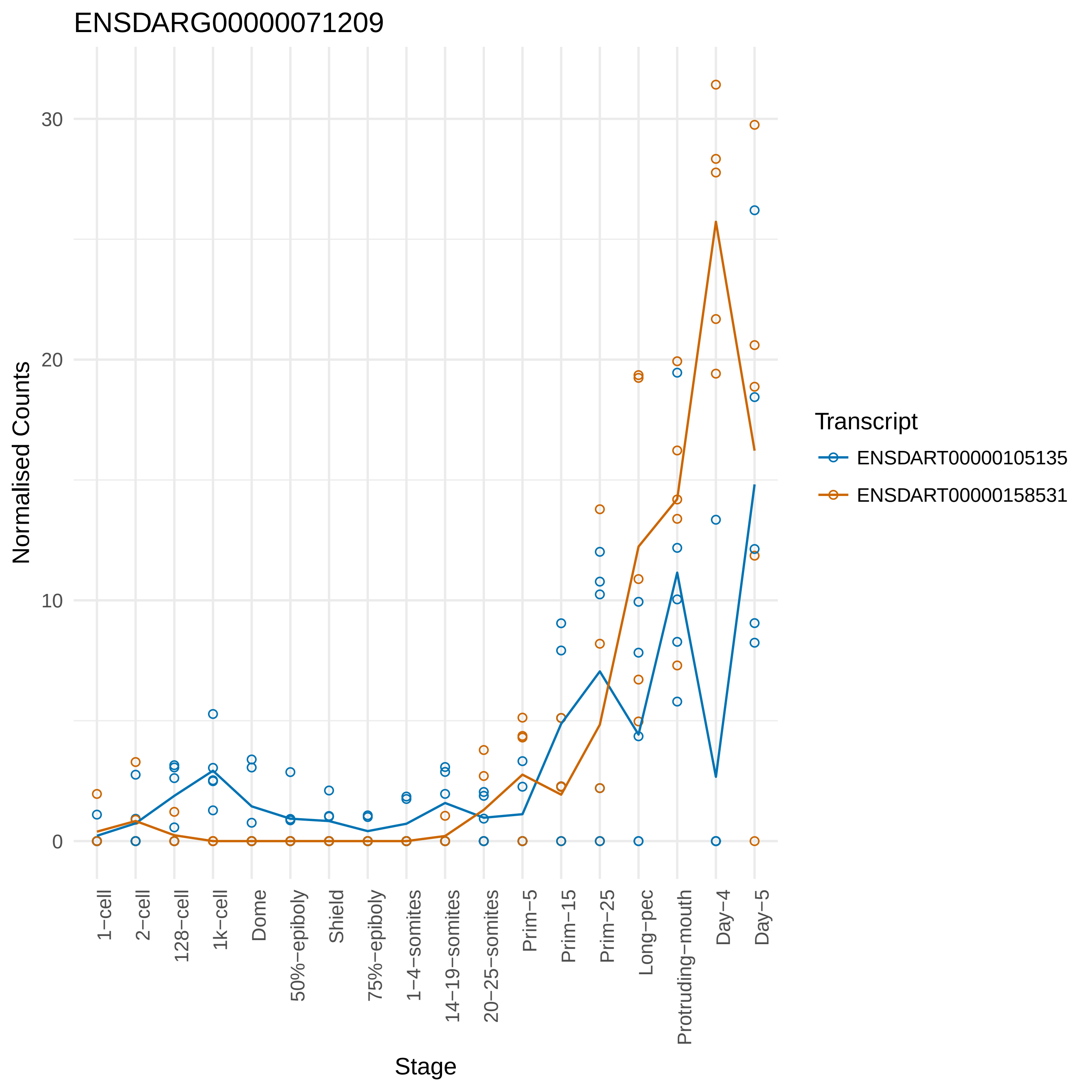

ENSDARG00000071209
Normalised Counts
30
20
10
0
1−cell
2−cell
128−cell
1k−cell
Dome
50%−epiboly
Shield
75%−epiboly
1−4−somites
14−19−somites
20−25−somites
Prim−5
Prim−15
Prim−25
Long−pec
Protruding−mouth
Day−4
Day−5
Stage
Transcript
ENSDART00000105135
ENSDART00000158531

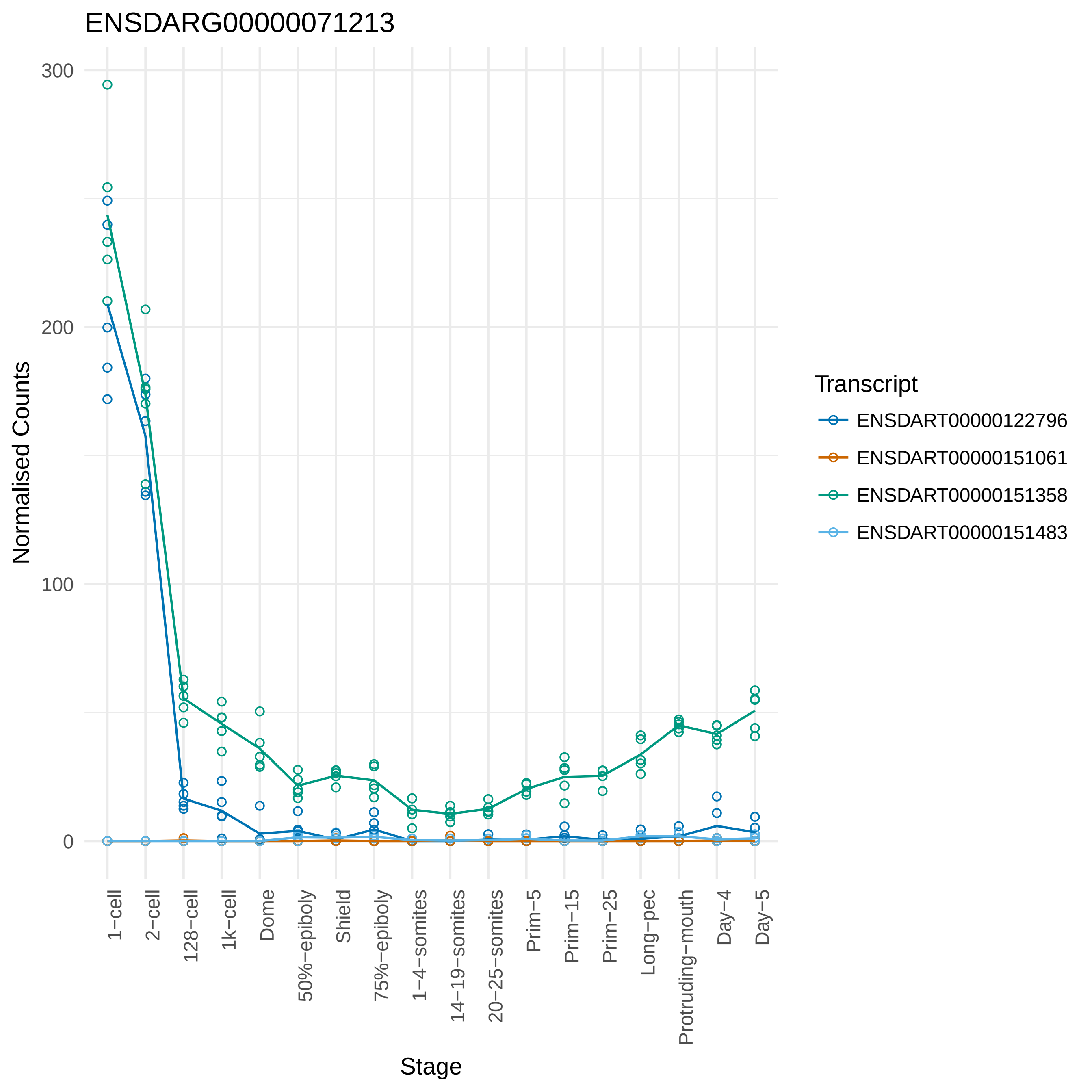
ENSDARG00000071213
Normalised Counts
300
200
100
0
1−cell
2−cell
128−cell
1k−cell
Dome
50%−epiboly
Shield
75%−epiboly
1−4−somites
14−19−somites
20−25−somites
Prim−5
Prim−15
Prim−25
Long−pec
Protruding−mouth
Day−4
Day−5
Stage
Transcript
ENSDART00000122796
ENSDART00000151061
ENSDART00000151358
ENSDART00000151483

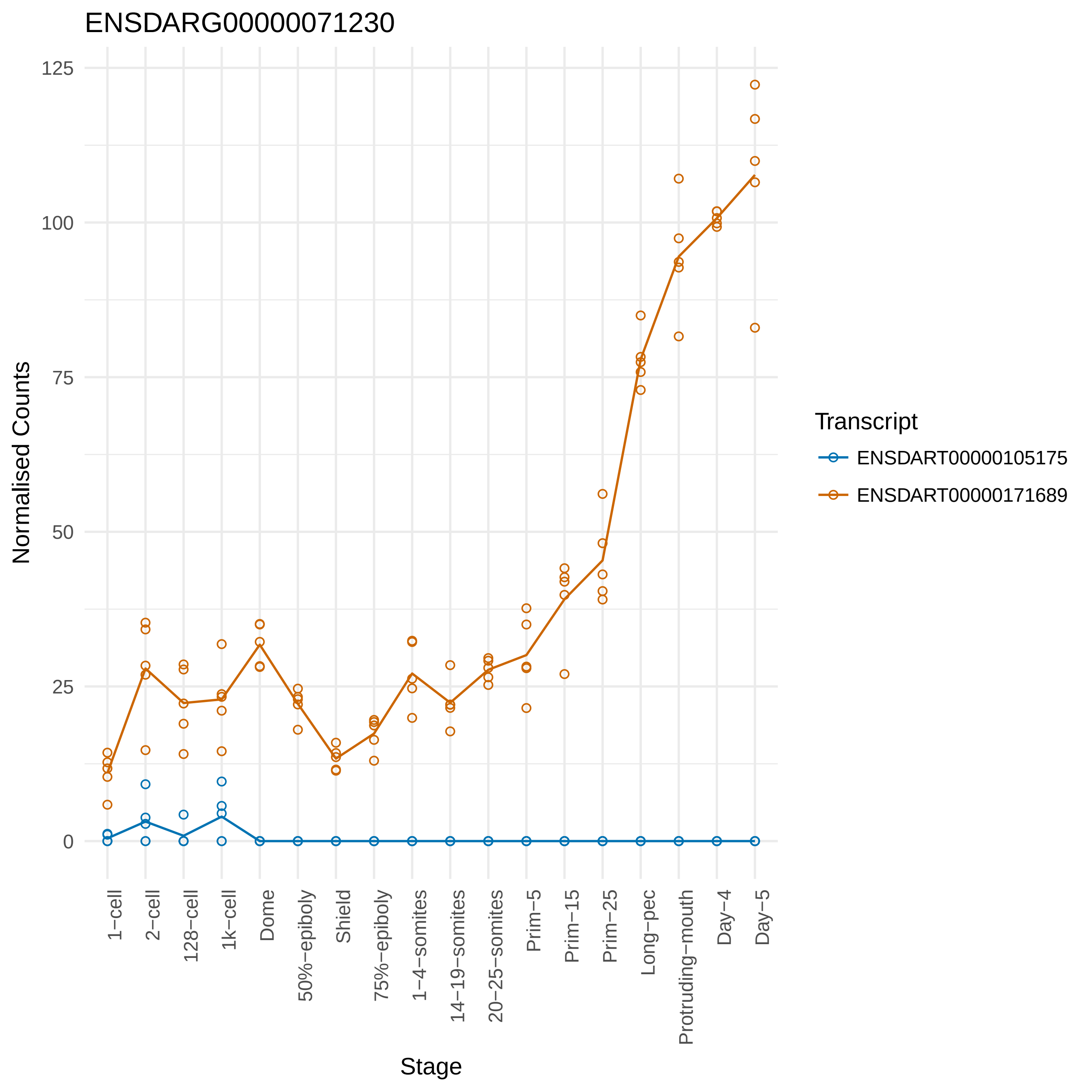
ENSDARG00000071230
Normalised Counts
125
100
75
50
25
0
1−cell
2−cell
128−cell
1k−cell
Dome
50%−epiboly
Shield
75%−epiboly
1−4−somites
14−19−somites
20−25−somites
Prim−5
Prim−15
Prim−25
Long−pec
Protruding−mouth
Day−4
Day−5
Stage
Transcript
ENSDART00000105175
ENSDART00000171689

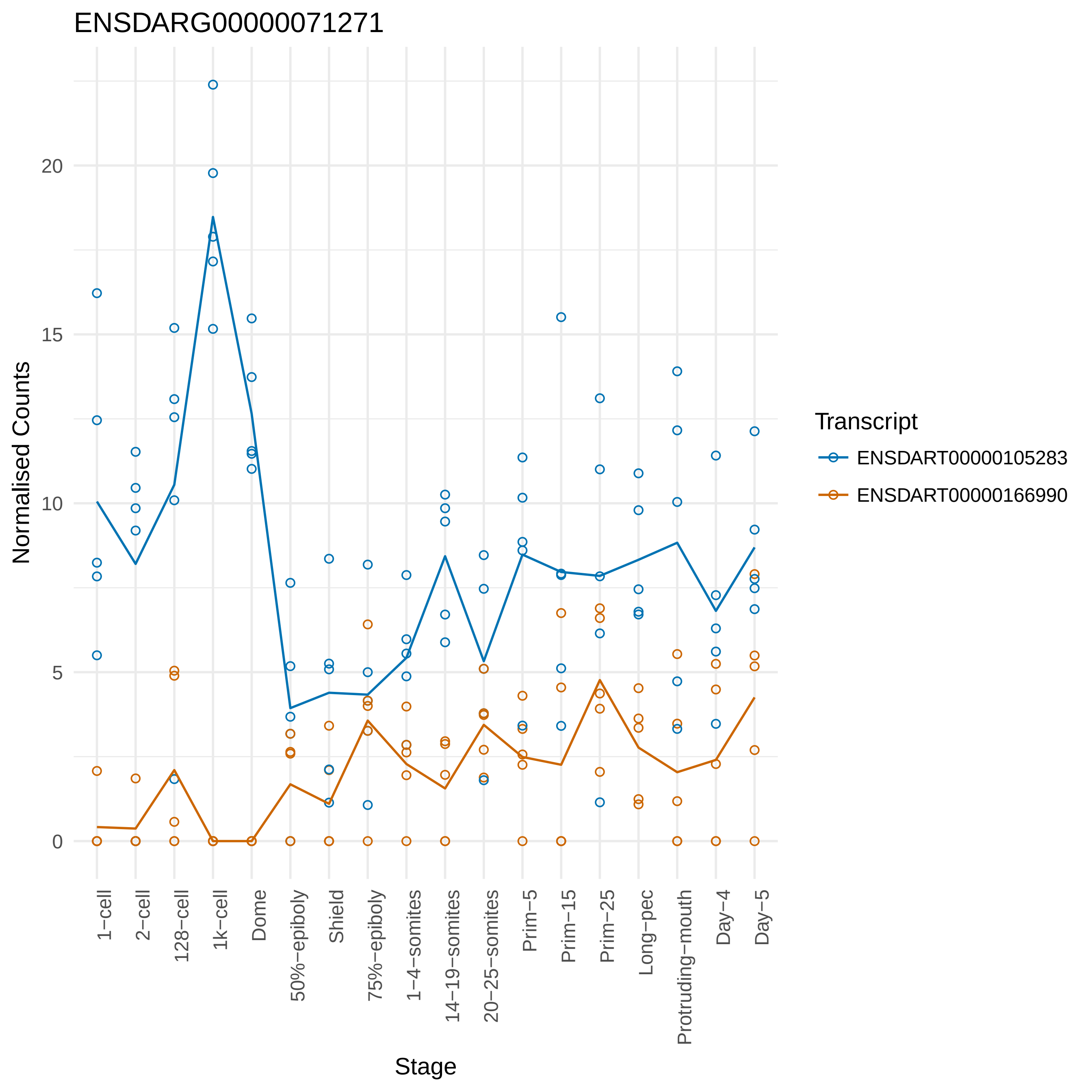

ENSDARG00000071271
Normalised Counts
0
5
10
15
20
Stage
1−cell
2−cell
128−cell
1k−cell
Dome
50%−epiboly
Shield
75%−epiboly
1−4−somites
14−19−somites
20−25−somites
Prim−5
Prim−15
Prim−25
Long−pec
Protruding−mouth
Day−4
Day−5
Transcript
ENSDART00000105283
ENSDART00000166990

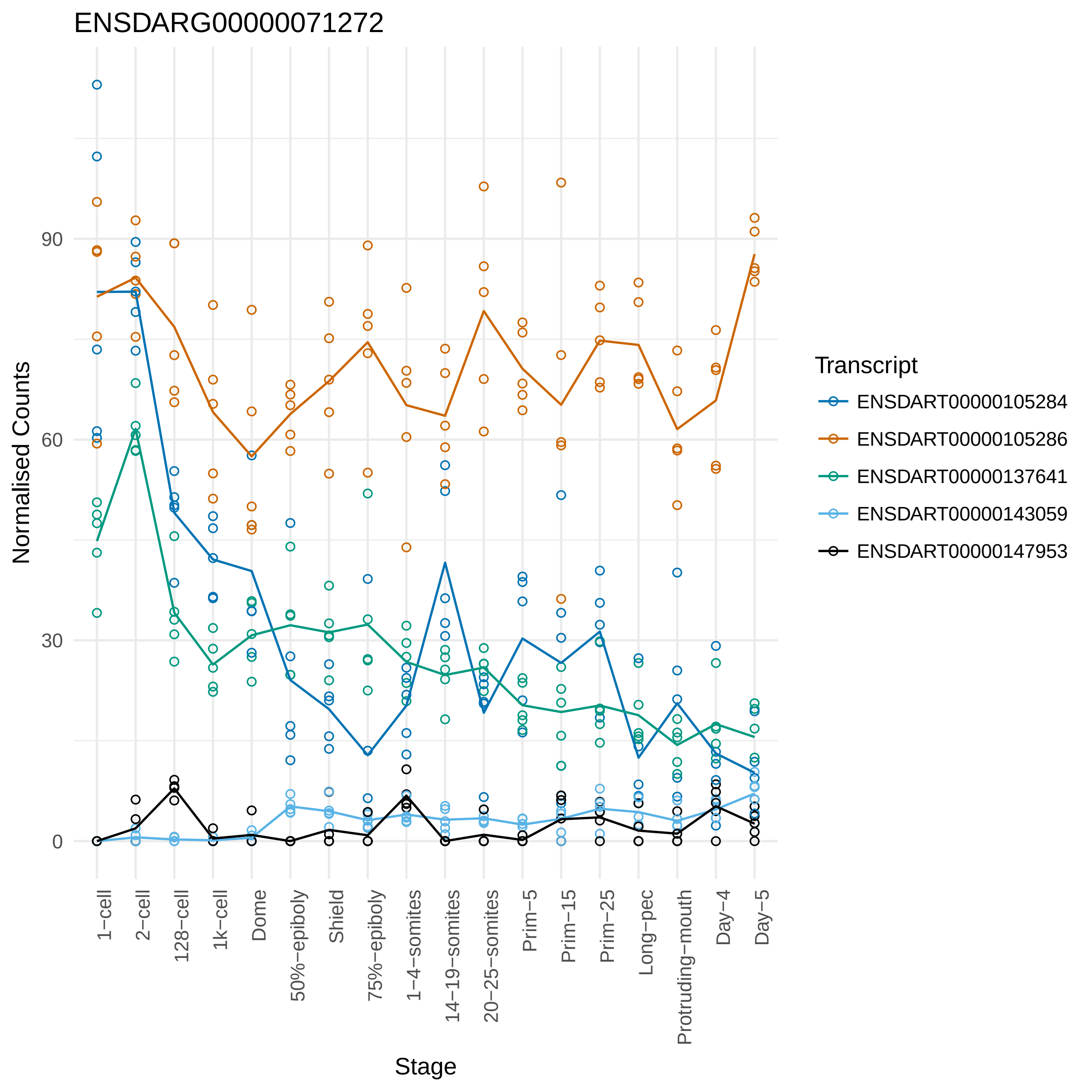
ENSDARG00000071272
Normalised Counts
0
30
60
90
1−cell
2−cell
128−cell
1k−cell
Dome
50%−epiboly
Shield
75%−epiboly
1−4−somites
14−19−somites
20−25−somites
Prim−5
Prim−15
Prim−25
Long−pec
Protruding−mouth
Day−4
Day−5
Stage
Transcript
ENSDART00000105284
ENSDART00000105286
ENSDART00000137641
ENSDART00000143059
ENSDART00000147953

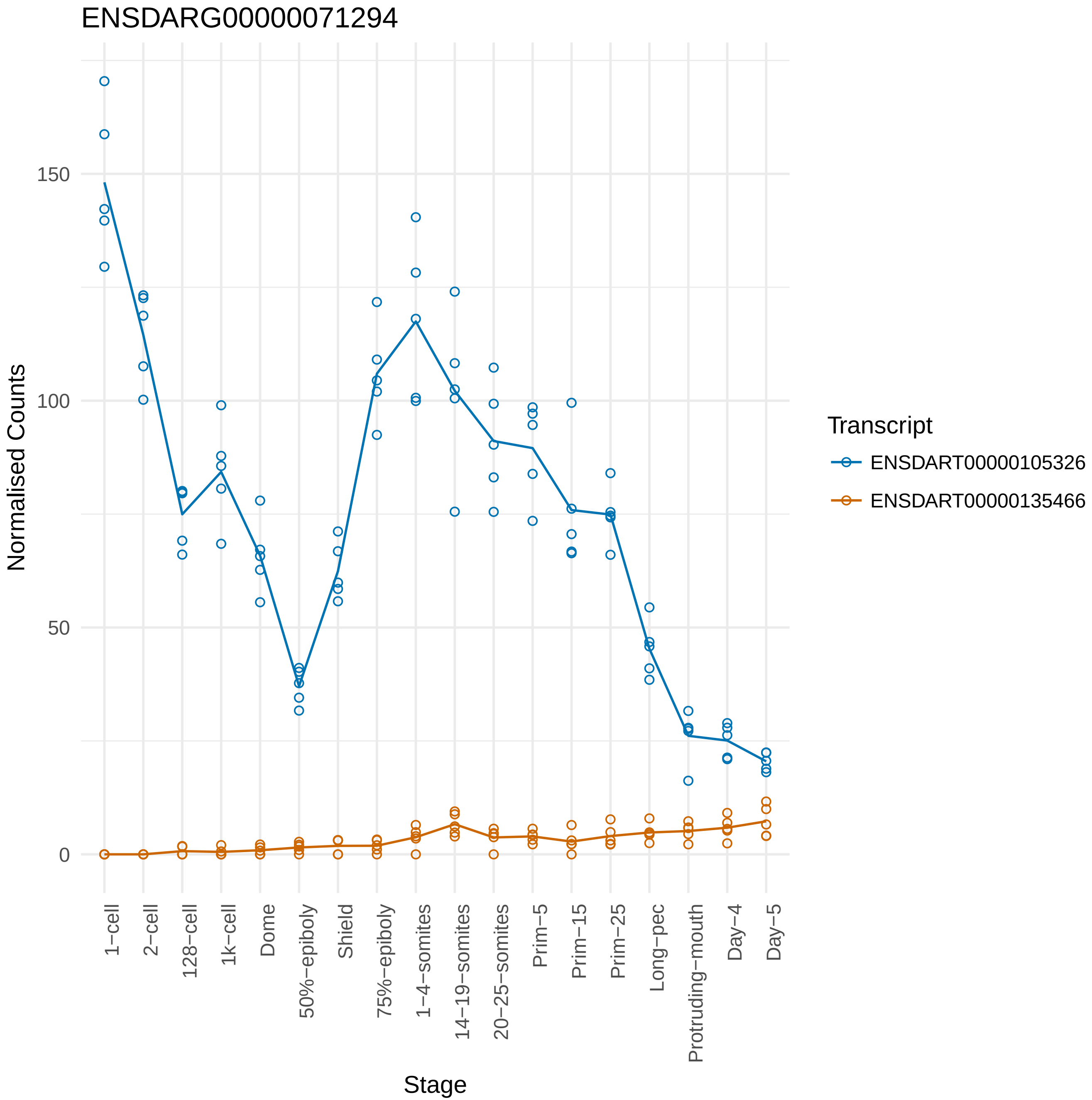

ENSDARG00000071294
Normalised Counts
150
100
50
0
Stage
1−cell
2−cell
128−cell
1k−cell
Dome
50%−epiboly
Shield
75%−epiboly
1−4−somites
14−19−somites
20−25−somites
Prim−5
Prim−15
Prim−25
Long−pec
Protruding−mouth
Day−4
Day−5
Transcript
ENSDART00000105326
ENSDART00000135466

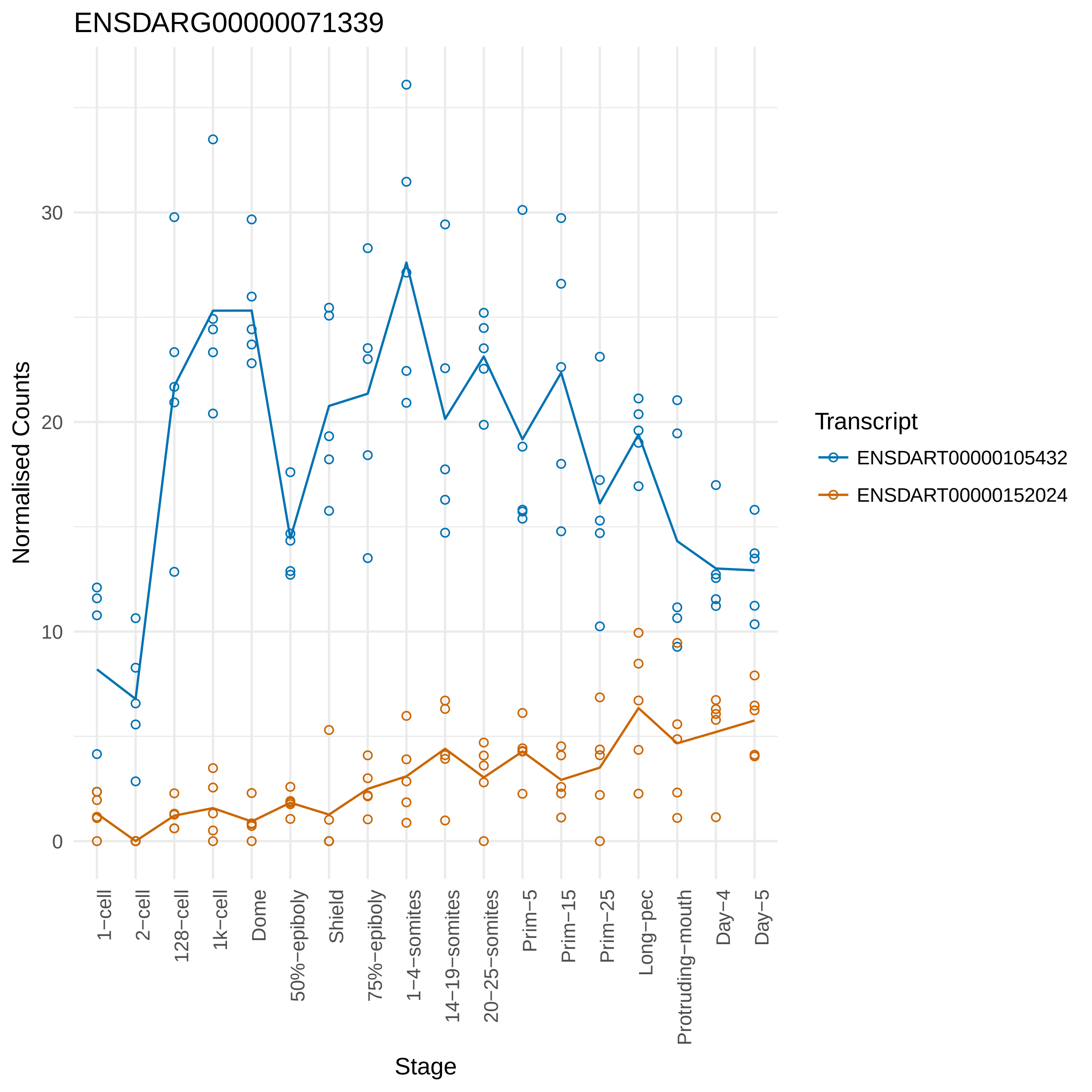
ENSDARG00000071339
Normalised Counts
0
10
20
30
1−cell
2−cell
128−cell
1k−cell
Dome
50%−epiboly
Shield
75%−epiboly
1−4−somites
14−19−somites
20−25−somites
Prim−5
Prim−15
Prim−25
Long−pec
Protruding−mouth
Day−4
Day−5
Stage
Transcript
ENSDART00000105432
ENSDART00000152024

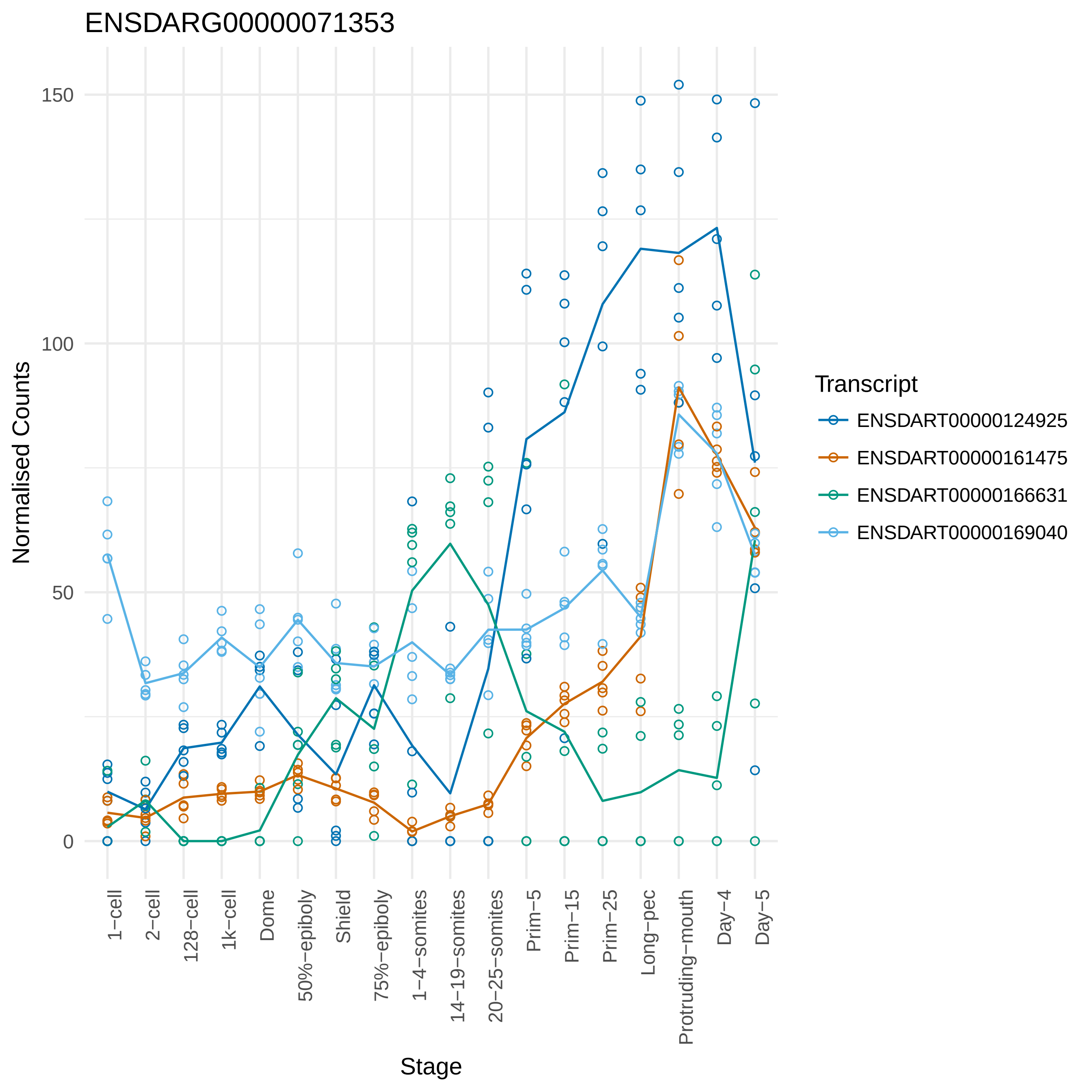

ENSDARG00000071353
Normalised Counts
0
50
100
150
Stage
1−cell
2−cell
128−cell
1k−cell
Dome
50%−epiboly
Shield
75%−epiboly
1−4−somites
14−19−somites
20−25−somites
Prim−5
Prim−15
Prim−25
Long−pec
Protruding−mouth
Day−4
Day−5
Transcript
ENSDART00000124925
ENSDART00000161475
ENSDART00000166631
ENSDART00000169040

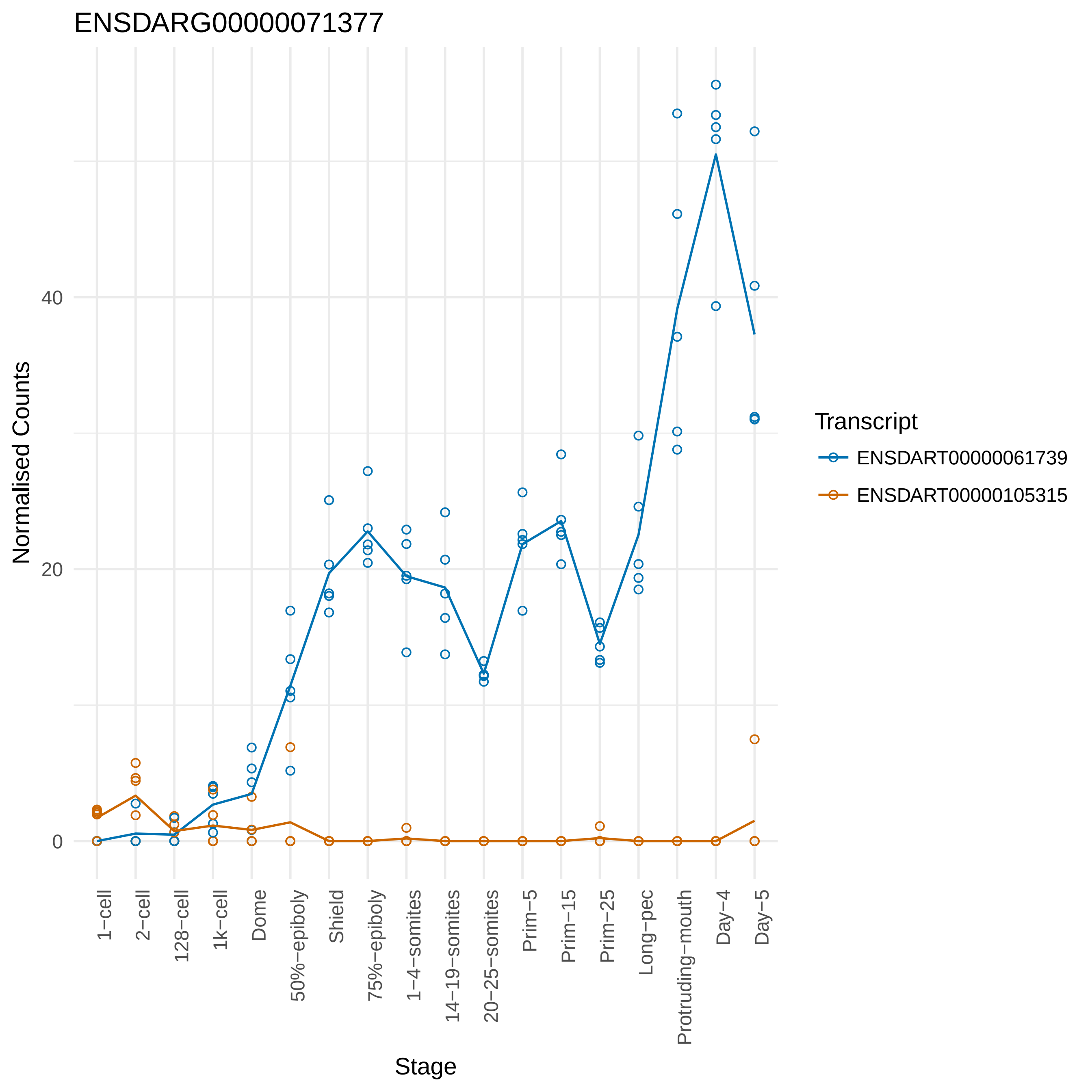

ENSDARG00000071377
Normalised Counts
0
20
40
Stage
1−cell
2−cell
128−cell
1k−cell
Dome
50%−epiboly
Shield
75%−epiboly
1−4−somites
14−19−somites
20−25−somites
Prim−5
Prim−15
Prim−25
Long−pec
Protruding−mouth
Day−4
Day−5
Transcript
ENSDART00000061739
ENSDART00000105315

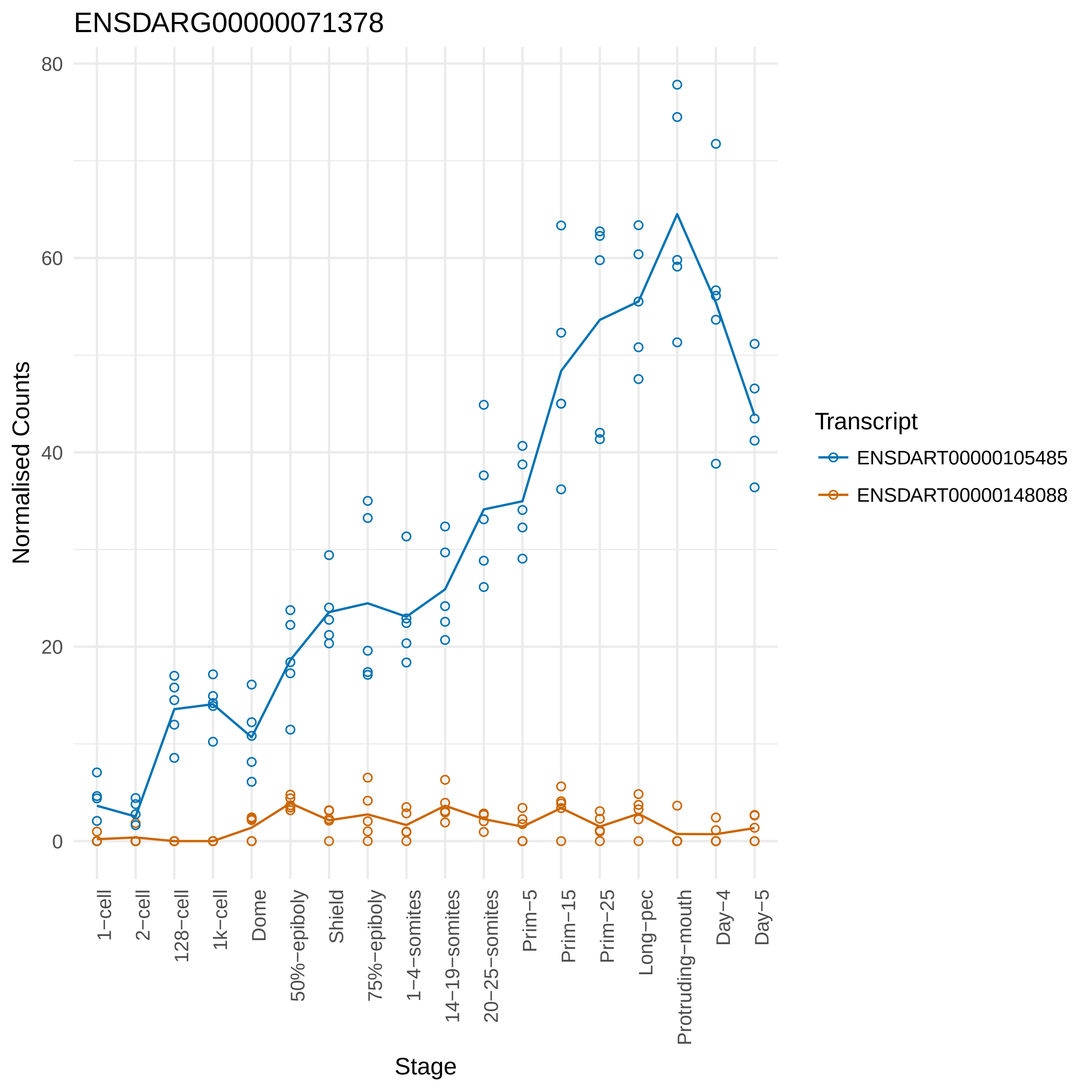

ENSDARG00000071378
Normalised Counts
0
20
40
60
80
Stage
1−cell
2−cell
128−cell
1k−cell
Dome
50%−epiboly
Shield
75%−epiboly
1−4−somites
14−19−somites
20−25−somites
Prim−5
Prim−15
Prim−25
Long−pec
Protruding−mouth
Day−4
Day−5
Transcript
ENSDART00000105485
ENSDART00000148088

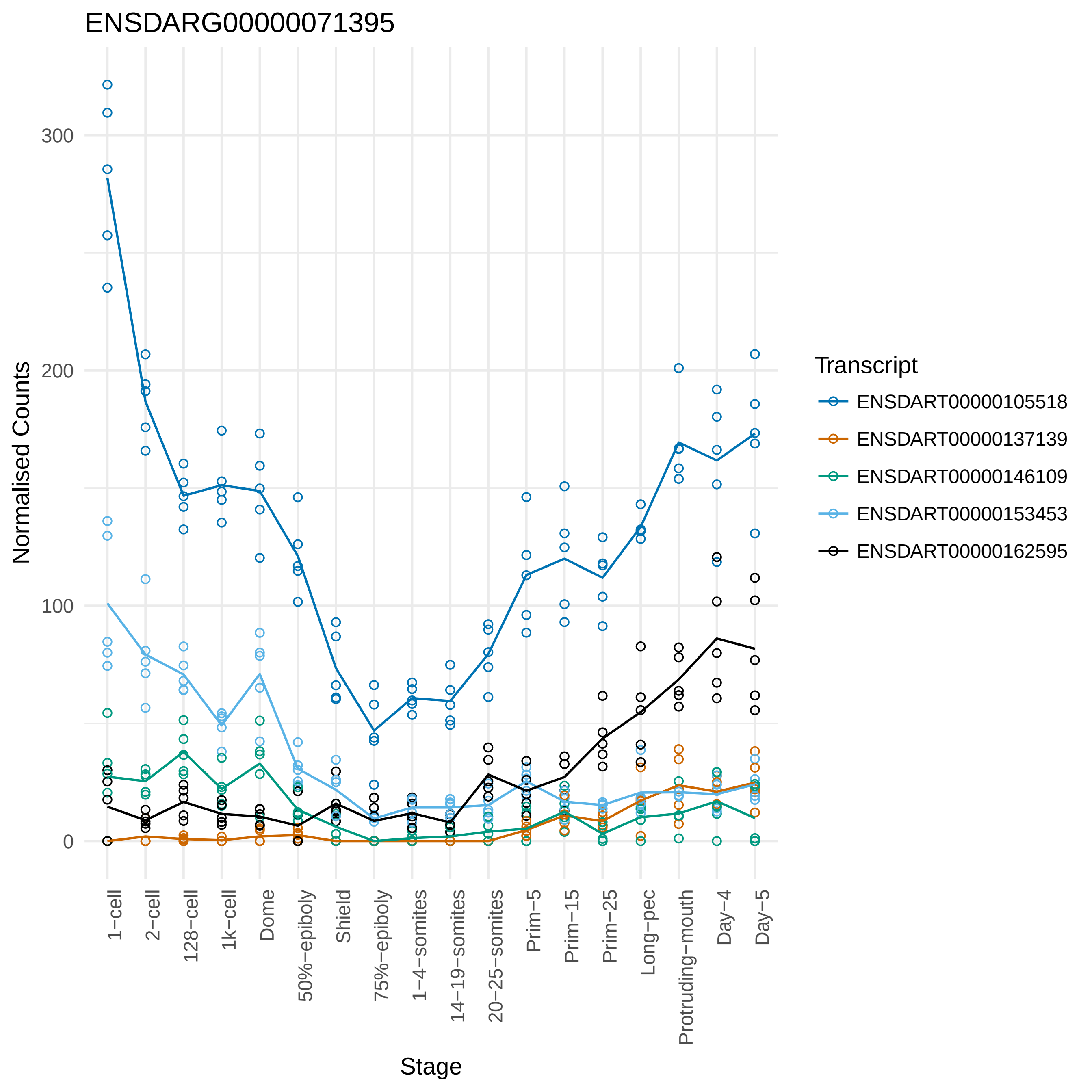
ENSDARG00000071395
Normalised Counts
300
200
100
0
1−cell
2−cell
128−cell
1k−cell
Dome
50%−epiboly
Shield
75%−epiboly
1−4−somites
14−19−somites
20−25−somites
Prim−5
Prim−15
Prim−25
Long−pec
Protruding−mouth
Day−4
Day−5
Stage
Transcript
ENSDART00000105518
ENSDART00000137139
ENSDART00000146109
ENSDART00000153453
ENSDART00000162595

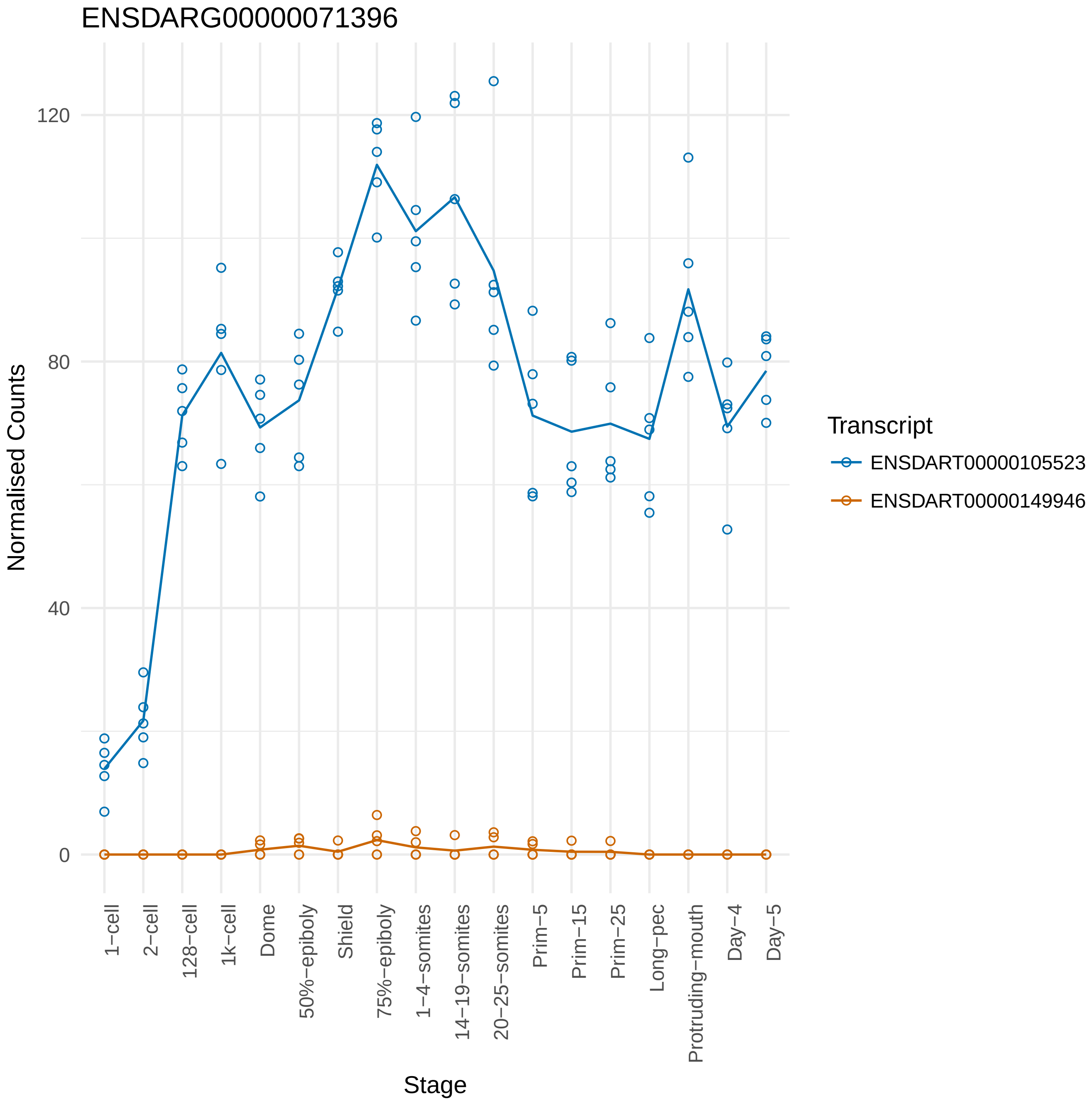

ENSDARG00000071396
Normalised Counts
120
80
40
0
Stage
1−cell
2−cell
128−cell
1k−cell
Dome
50%−epiboly
Shield
75%−epiboly
1−4−somites
14−19−somites
20−25−somites
Prim−5
Prim−15
Prim−25
Long−pec
Protruding−mouth
Day−4
Day−5
Transcript
ENSDART00000105523
ENSDART00000149946

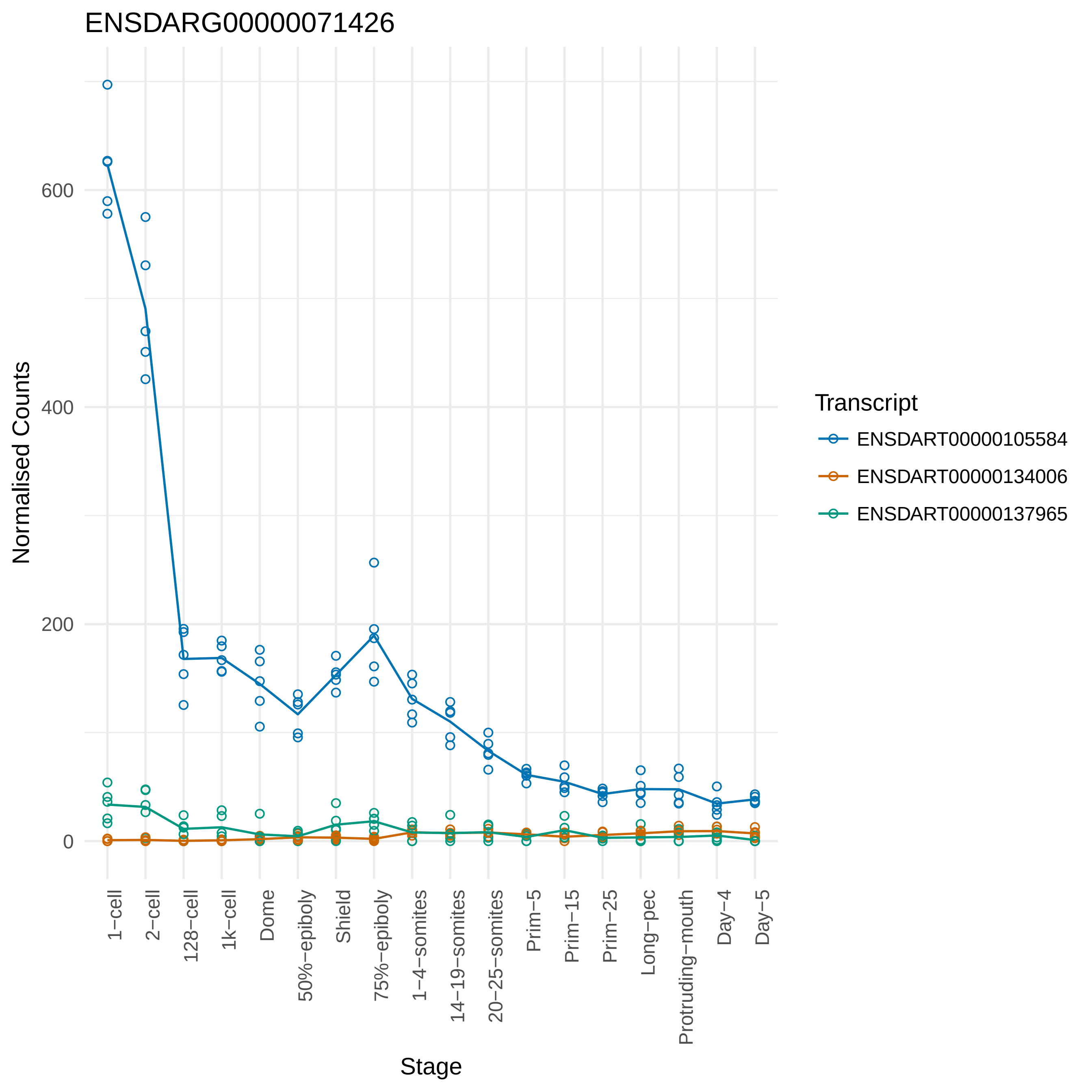
ENSDARG00000071426
Normalised Counts
600
400
200
0
1−cell
2−cell
128−cell
1k−cell
Dome
50%−epiboly
Shield
75%−epiboly
1−4−somites
14−19−somites
20−25−somites
Prim−5
Prim−15
Prim−25
Long−pec
Protruding−mouth
Day−4
Day−5
Stage
Transcript
ENSDART00000105584
ENSDART00000134006
ENSDART00000137965

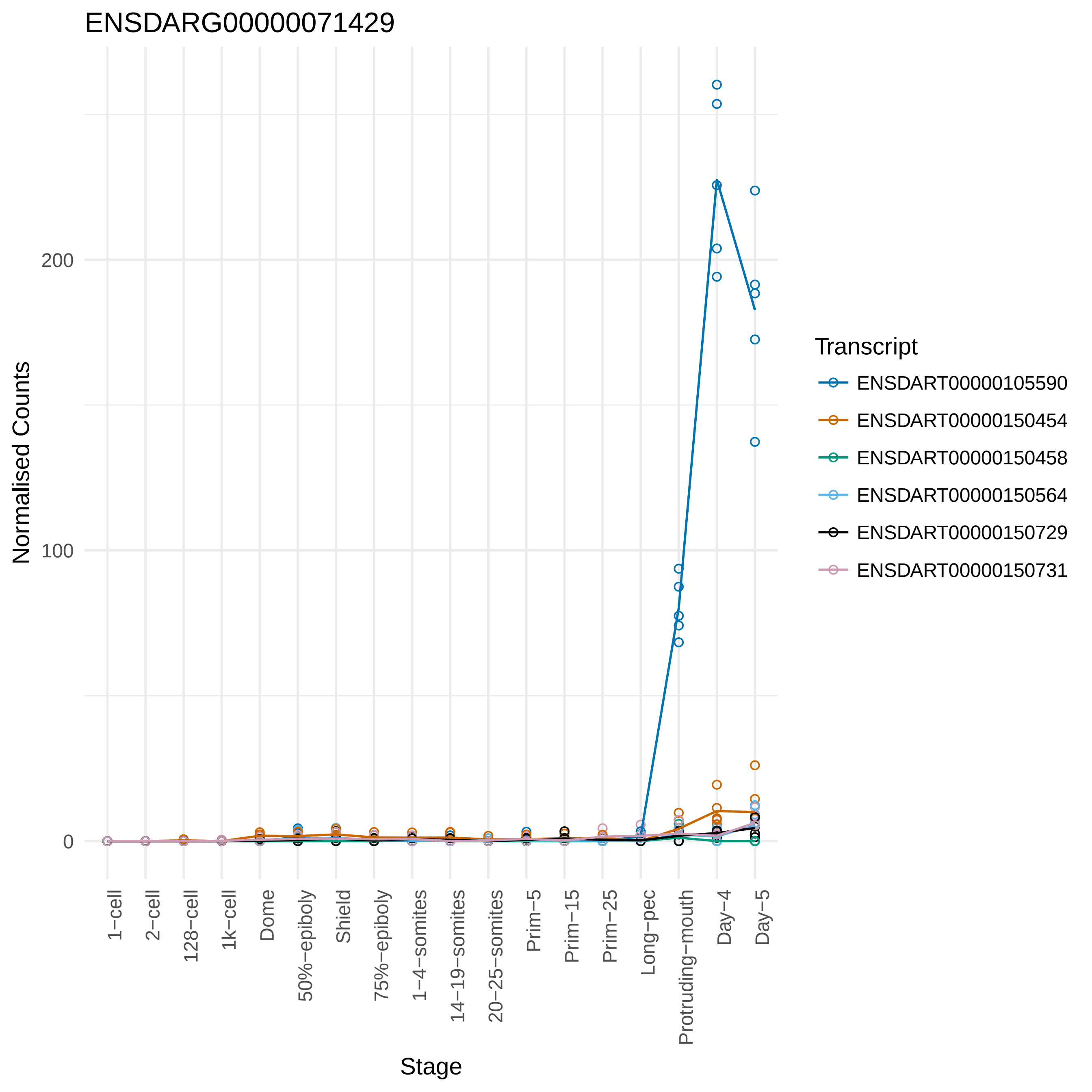
ENSDARG00000071429
Normalised Counts
200
100
0
1−cell
2−cell
128−cell
1k−cell
Dome
50%−epiboly
Shield
75%−epiboly
1−4−somites
14−19−somites
20−25−somites
Prim−5
Prim−15
Prim−25
Long−pec
Protruding−mouth
Day−4
Day−5
Stage
Transcript
ENSDART00000105590
ENSDART00000150454
ENSDART00000150458
ENSDART00000150564
ENSDART00000150729
ENSDART00000150731

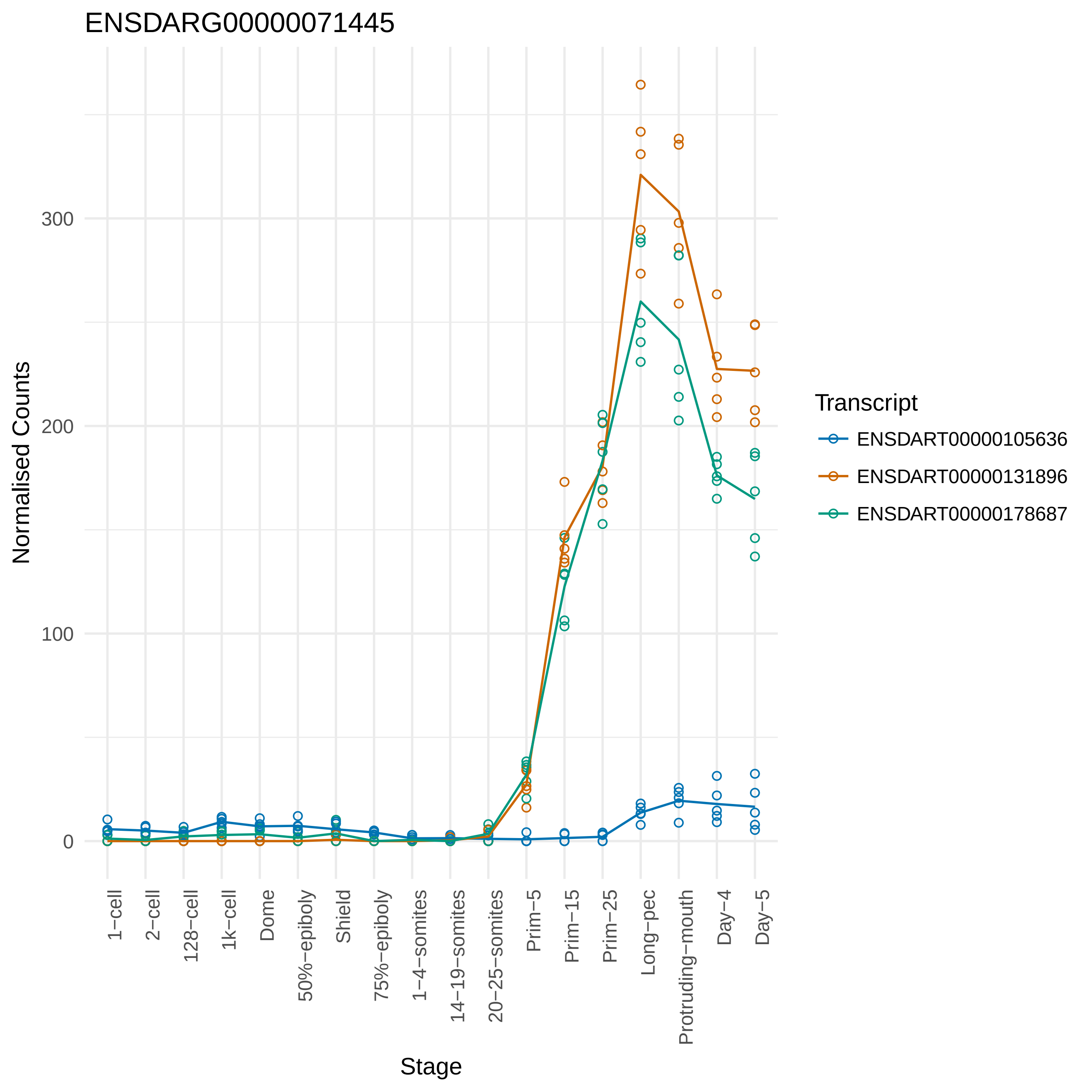
ENSDARG00000071445
Normalised Counts
300
200
100
0
1−cell
2−cell
128−cell
1k−cell
Dome
50%−epiboly
Shield
75%−epiboly
1−4−somites
14−19−somites
20−25−somites
Prim−5
Prim−15
Prim−25
Long−pec
Protruding−mouth
Day−4
Day−5
Stage
Transcript
ENSDART00000105636
ENSDART00000131896
ENSDART00000178687

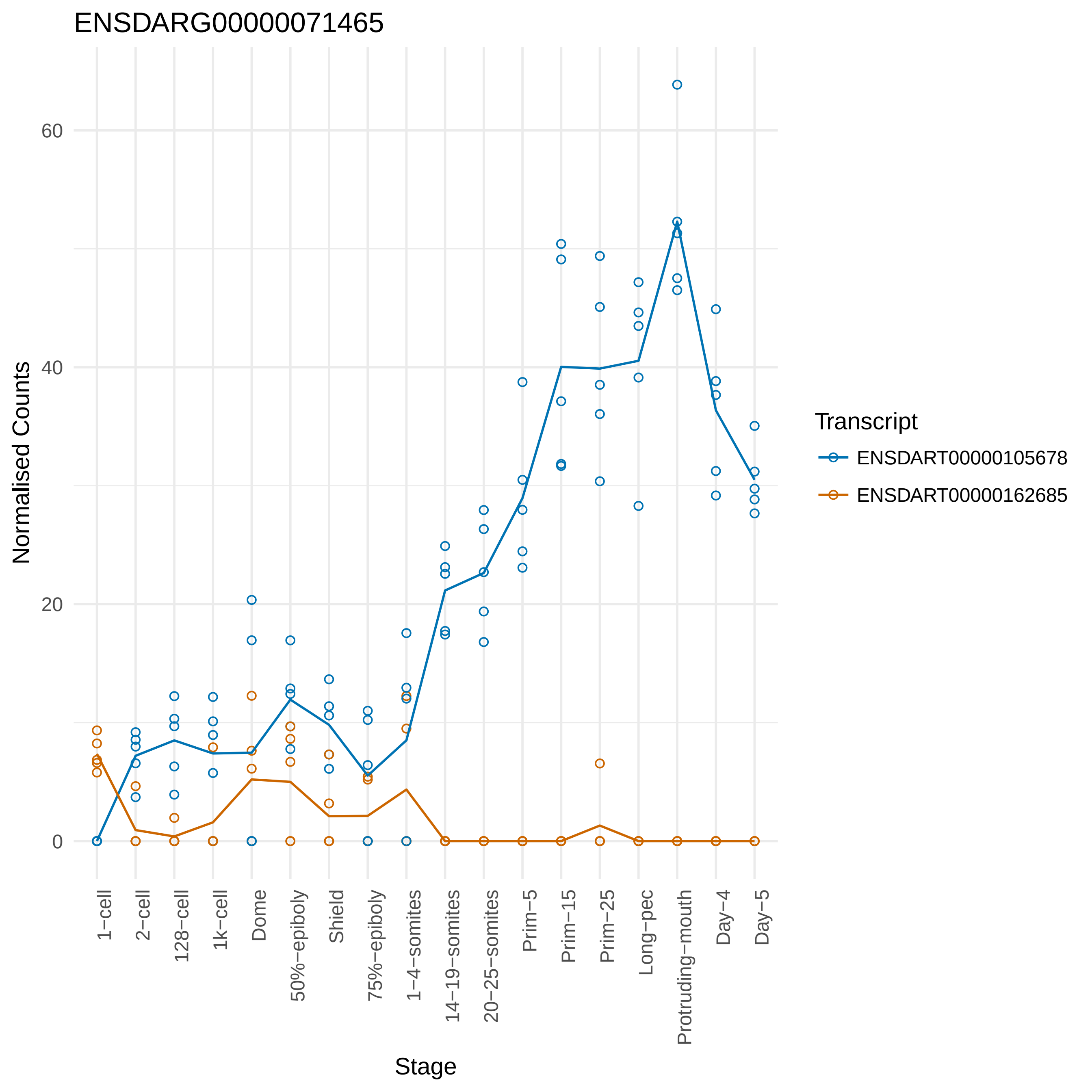

ENSDARG00000071465
Normalised Counts
0
20
40
60
Stage
1−cell
2−cell
128−cell
1k−cell
Dome
50%−epiboly
Shield
75%−epiboly
1−4−somites
14−19−somites
20−25−somites
Prim−5
Prim−15
Prim−25
Long−pec
Protruding−mouth
Day−4
Day−5
Transcript
ENSDART00000105678
ENSDART00000162685

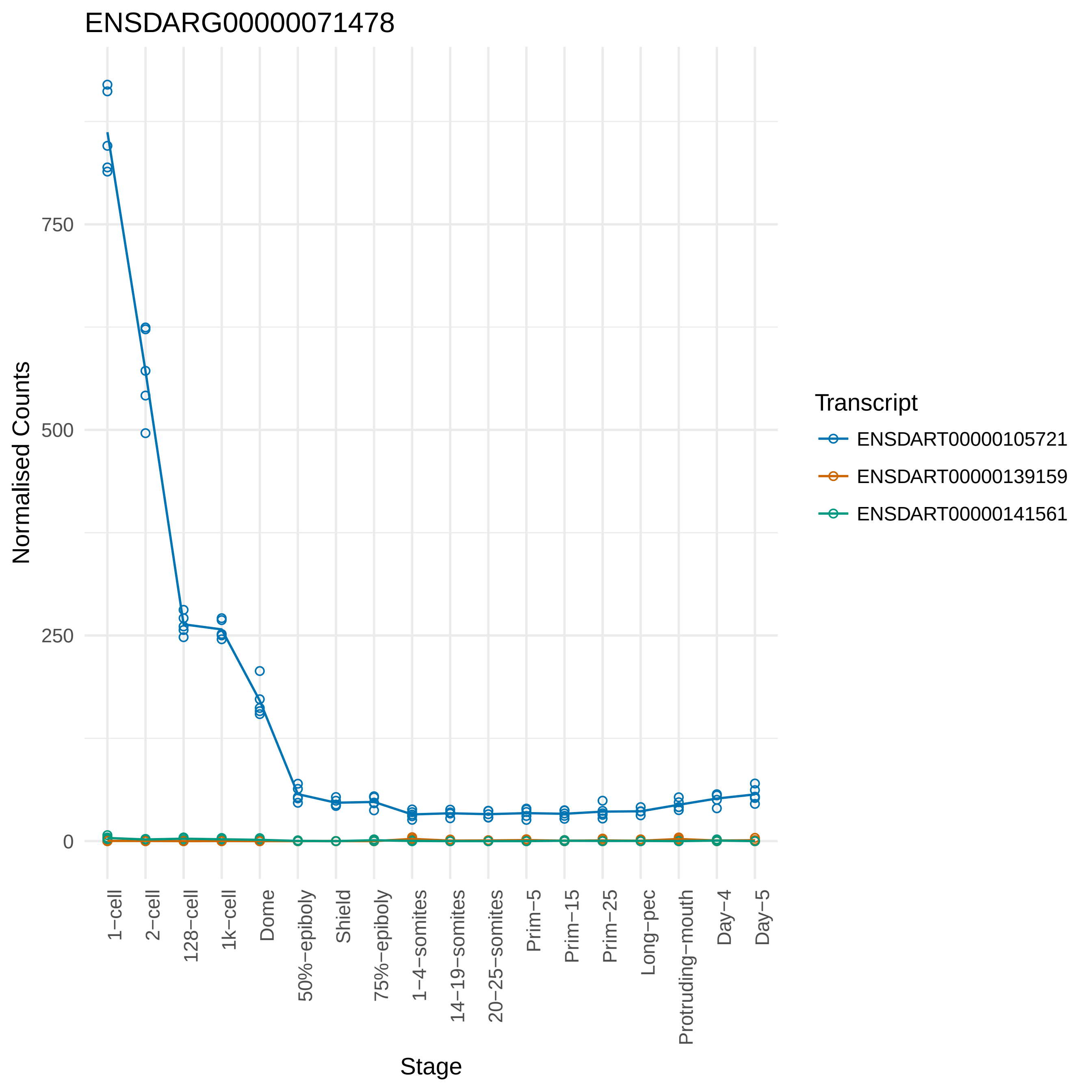

ENSDARG00000071478
Normalised Counts
750
500
250
0
1−cell
2−cell
128−cell
1k−cell
Dome
50%−epiboly
Shield
75%−epiboly
1−4−somites
14−19−somites
20−25−somites
Prim−5
Prim−15
Prim−25
Long−pec
Protruding−mouth
Day−4
Day−5
Stage
Transcript
ENSDART00000105721
ENSDART00000139159
ENSDART00000141561

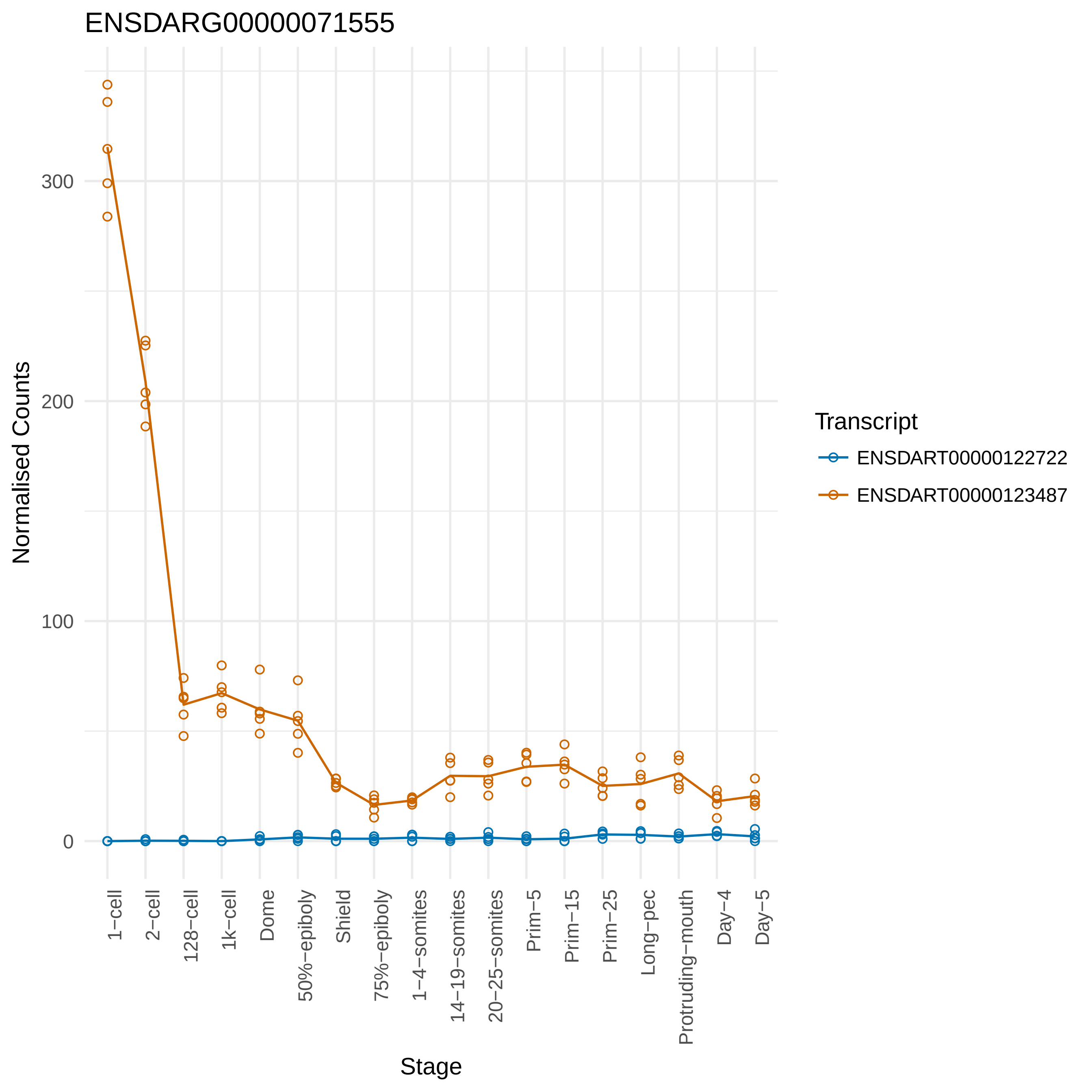
ENSDARG00000071555
Normalised Counts
300
200
100
0
1−cell
2−cell
128−cell
1k−cell
Dome
50%−epiboly
Shield
75%−epiboly
1−4−somites
14−19−somites
20−25−somites
Prim−5
Prim−15
Prim−25
Long−pec
Protruding−mouth
Day−4
Day−5
Stage
Transcript
ENSDART00000122722
ENSDART00000123487

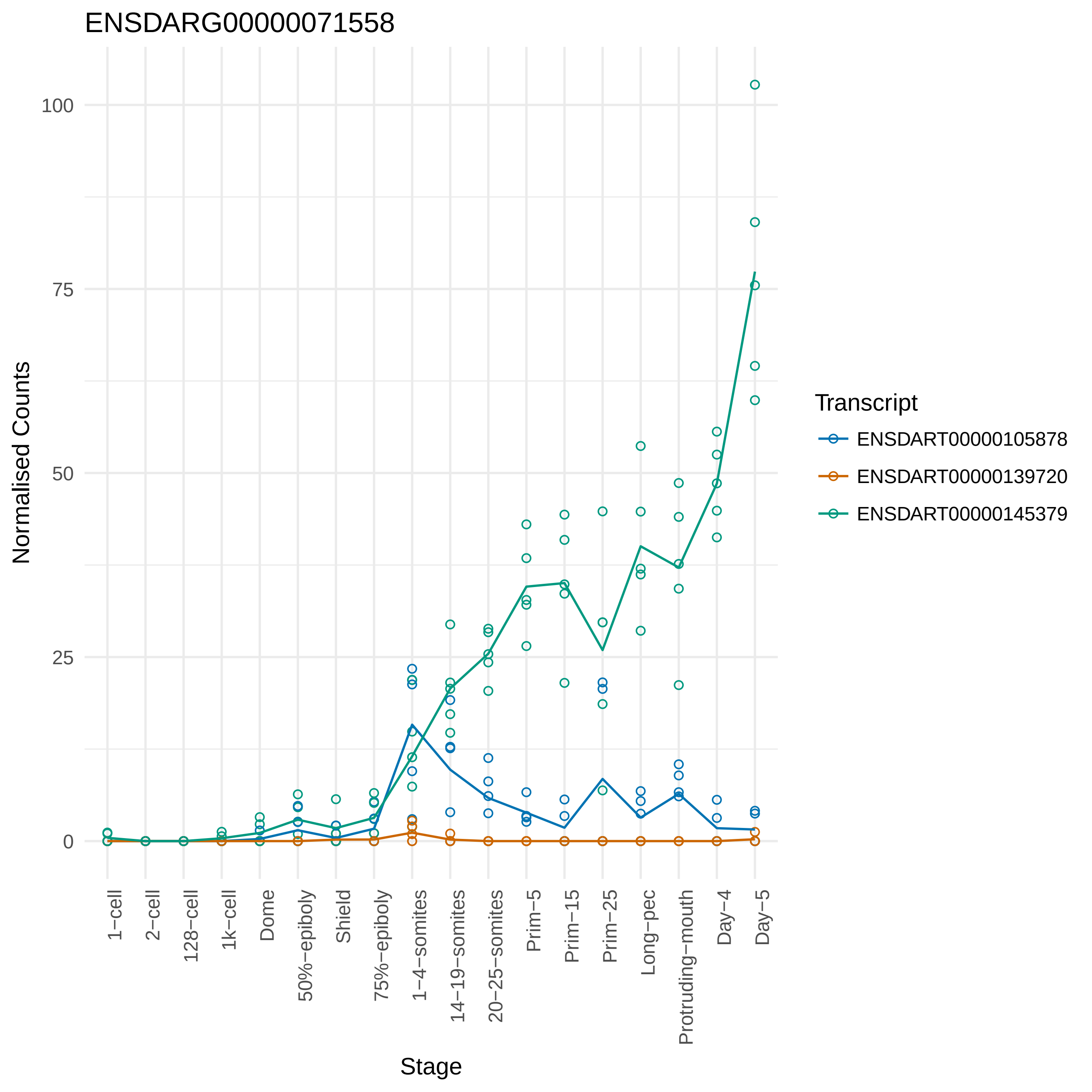
ENSDARG00000071558
Normalised Counts
100
75
50
25
0
1−cell
2−cell
128−cell
1k−cell
Dome
50%−epiboly
Shield
75%−epiboly
1−4−somites
14−19−somites
20−25−somites
Prim−5
Prim−15
Prim−25
Long−pec
Protruding−mouth
Day−4
Day−5
Stage
Transcript
ENSDART00000105878
ENSDART00000139720
ENSDART00000145379

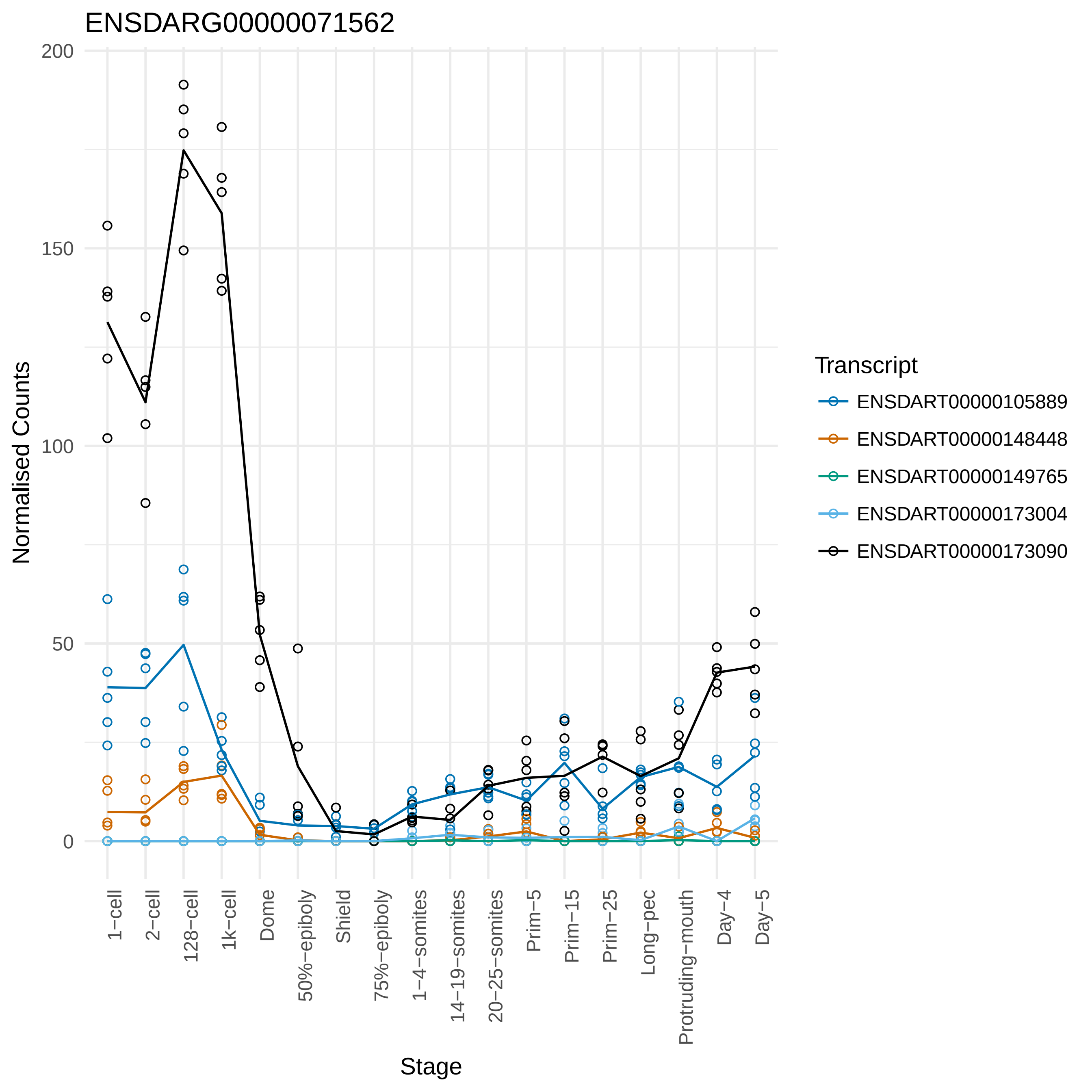

ENSDARG00000071562
Normalised Counts
200
150
100
50
0
1−cell
2−cell
128−cell
1k−cell
Dome
50%−epiboly
Shield
75%−epiboly
1−4−somites
14−19−somites
20−25−somites
Prim−5
Prim−15
Prim−25
Long−pec
Protruding−mouth
Day−4
Day−5
Stage
Transcript
ENSDART00000105889
ENSDART00000148448
ENSDART00000149765
ENSDART00000173004
ENSDART00000173090

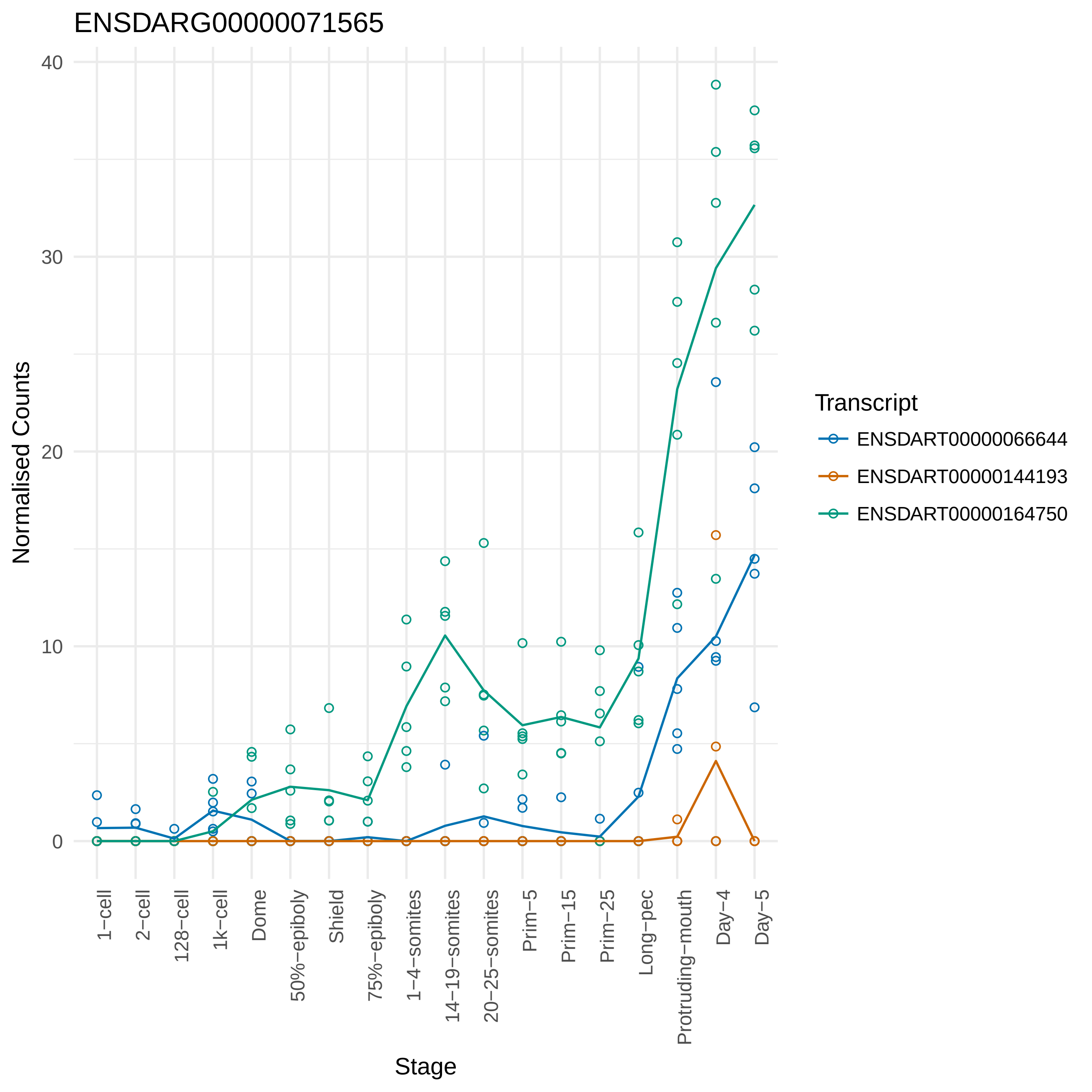
ENSDARG00000071565
Normalised Counts
40
30
20
10
0
1−cell
2−cell
128−cell
1k−cell
Dome
50%−epiboly
Shield
75%−epiboly
1−4−somites
14−19−somites
20−25−somites
Prim−5
Prim−15
Prim−25
Long−pec
Protruding−mouth
Day−4
Day−5
Stage
Transcript
ENSDART00000066644
ENSDART00000144193
ENSDART00000164750

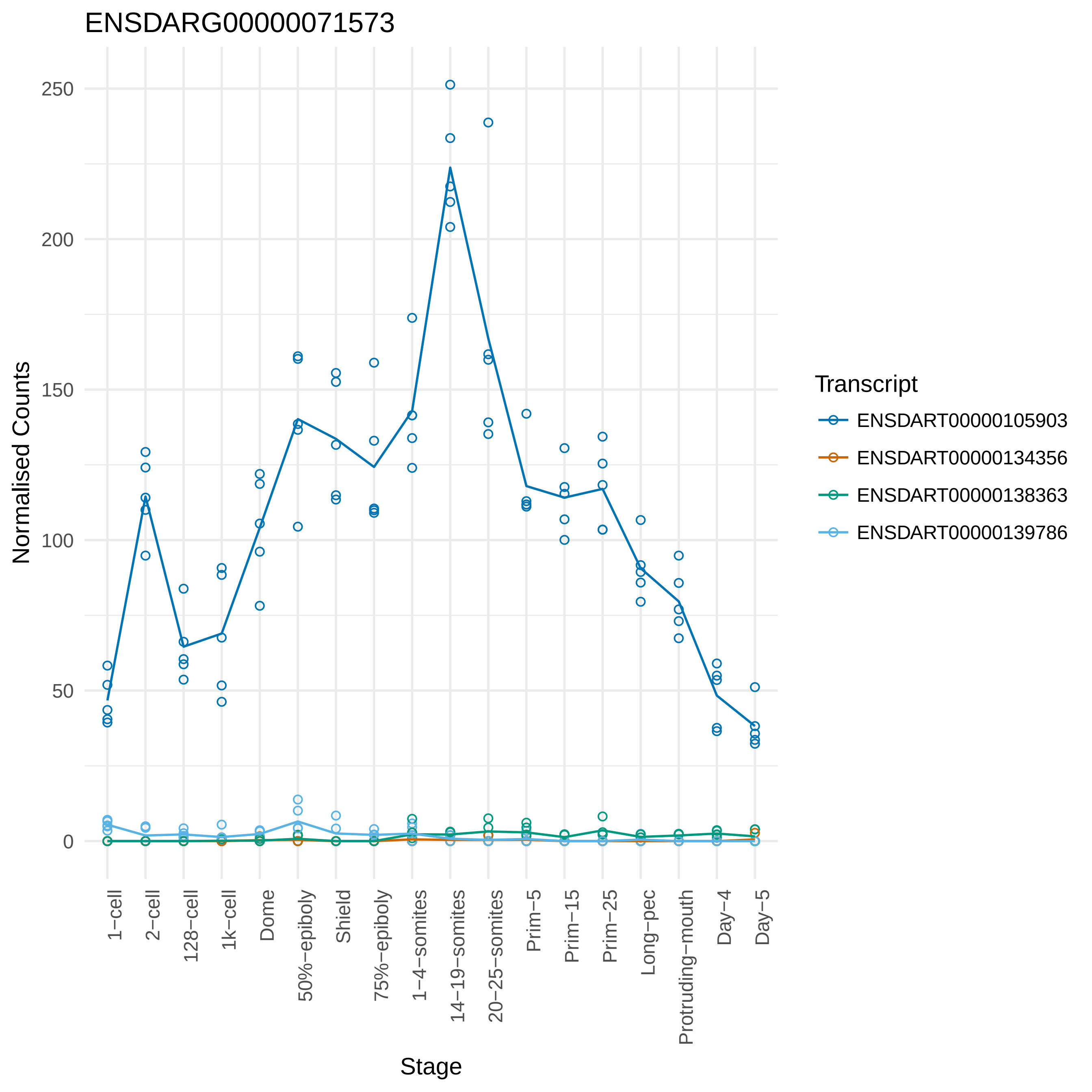

ENSDARG00000071573
Normalised Counts
250
200
150
100
50
0
1−cell
2−cell
128−cell
1k−cell
Dome
50%−epiboly
Shield
75%−epiboly
1−4−somites
14−19−somites
20−25−somites
Prim−5
Prim−15
Prim−25
Long−pec
Protruding−mouth
Day−4
Day−5
Stage
Transcript
ENSDART00000105903
ENSDART00000134356
ENSDART00000138363
ENSDART00000139786

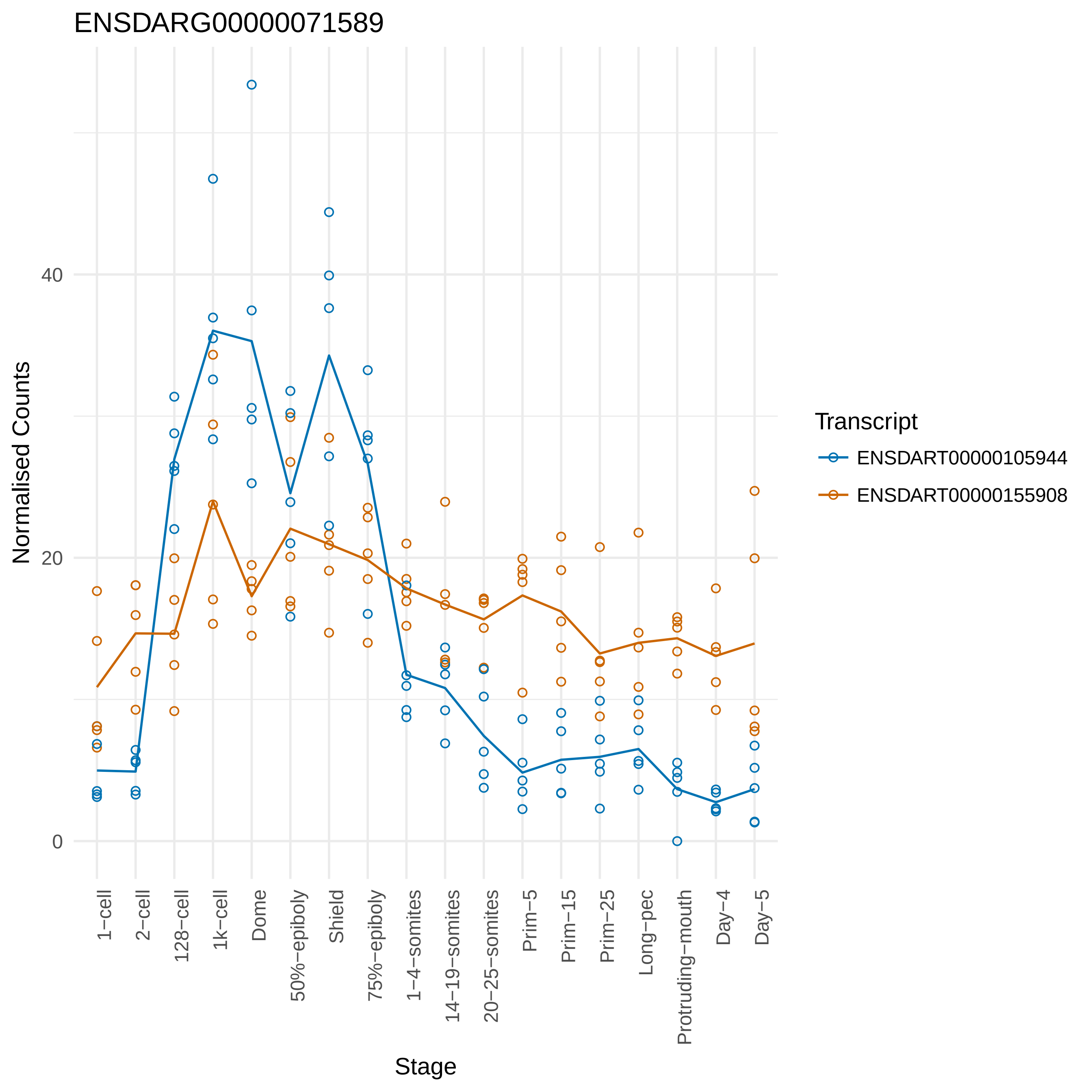
ENSDARG00000071589
Normalised Counts
0
20
40
1−cell
2−cell
128−cell
1k−cell
Dome
50%−epiboly
Shield
75%−epiboly
1−4−somites
14−19−somites
20−25−somites
Prim−5
Prim−15
Prim−25
Long−pec
Protruding−mouth
Day−4
Day−5
Stage
Transcript
ENSDART00000105944
ENSDART00000155908

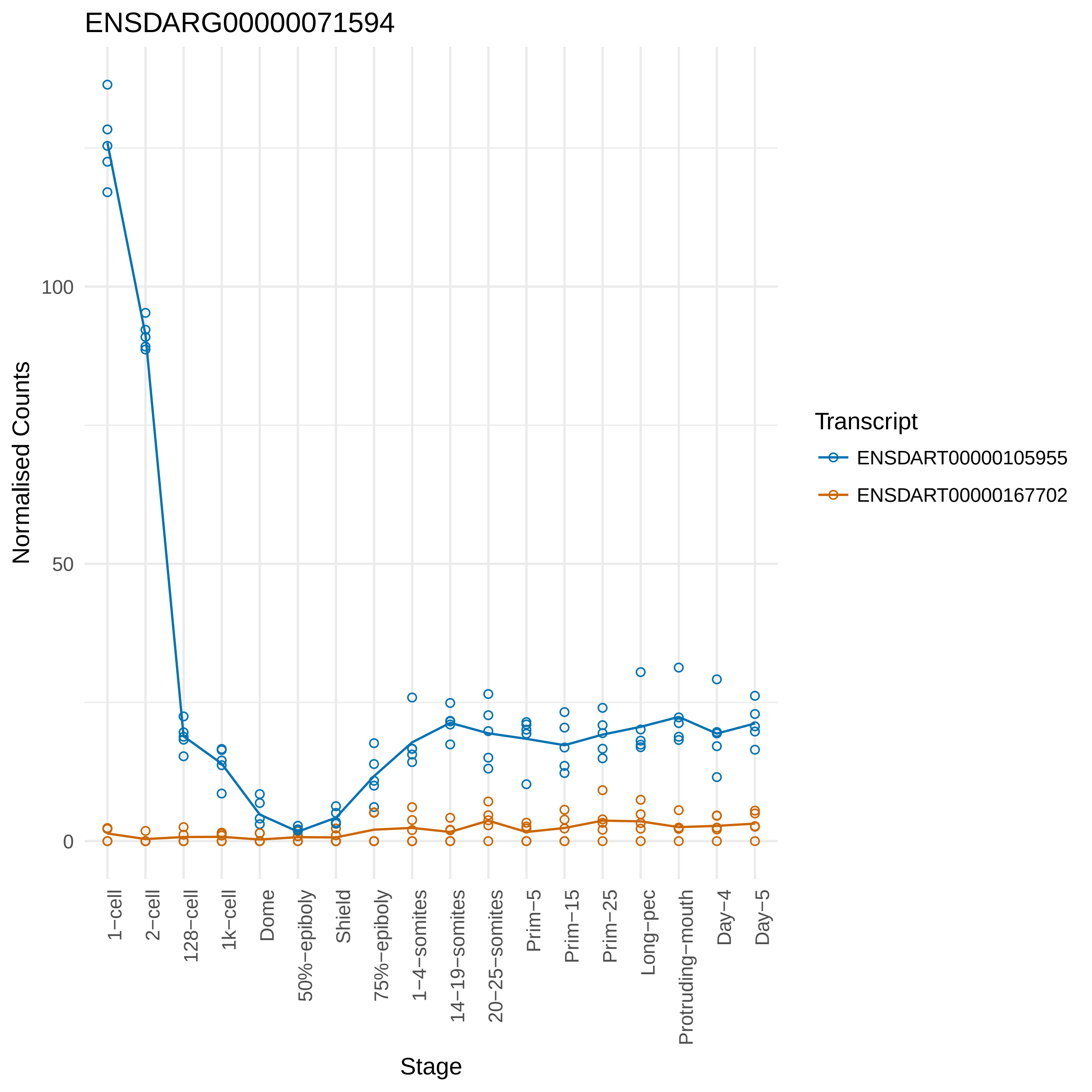
ENSDARG00000071594
Normalised Counts
0
50
100
Stage
1−cell
2−cell
128−cell
1k−cell
Dome
50%−epiboly
Shield
75%−epiboly
1−4−somites
14−19−somites
20−25−somites
Prim−5
Prim−15
Prim−25
Long−pec
Protruding−mouth
Day−4
Day−5
Transcript
ENSDART00000105955
ENSDART00000167702

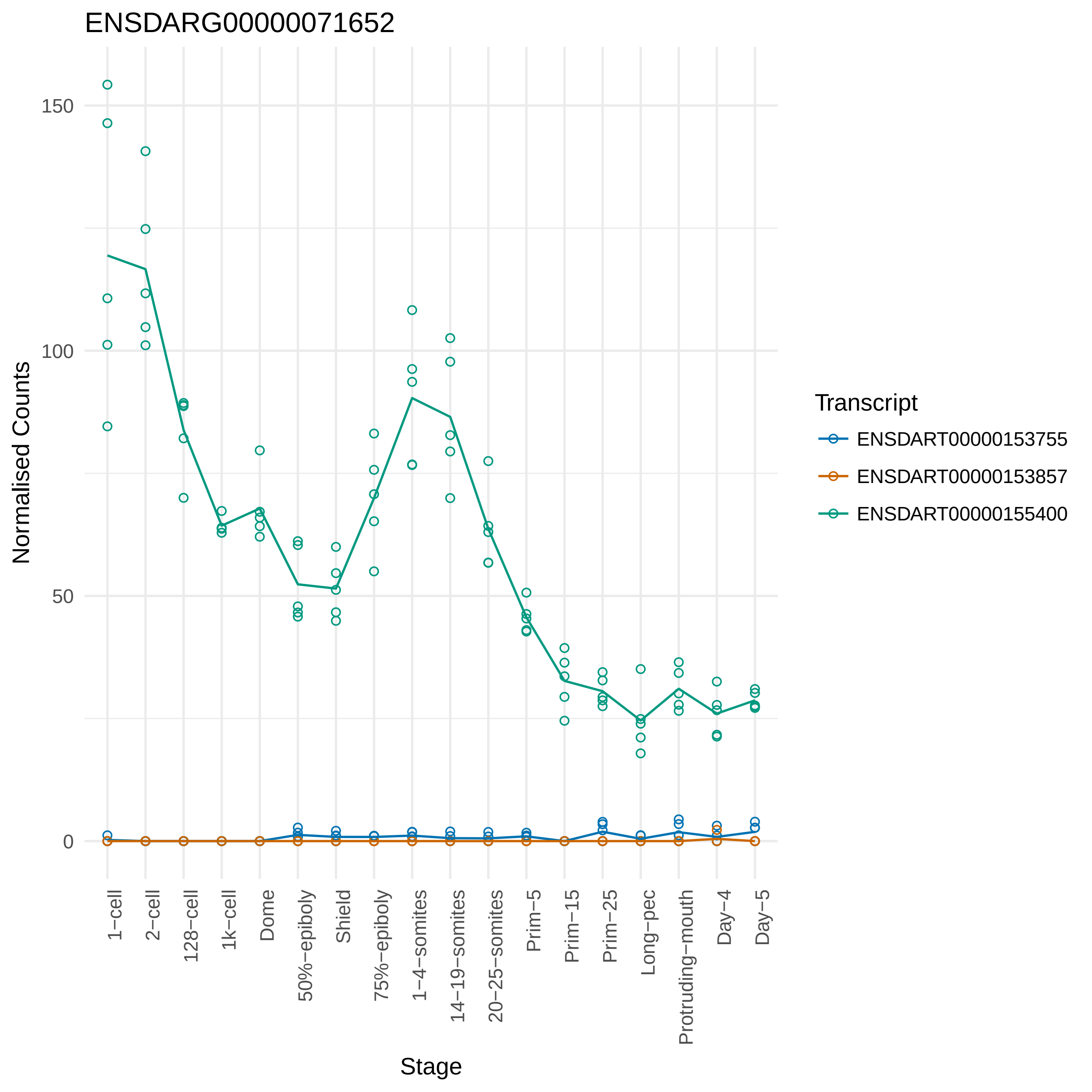

ENSDARG00000071652
Normalised Counts
150
100
50
0
1−cell
2−cell
128−cell
1k−cell
Dome
50%−epiboly
Shield
75%−epiboly
1−4−somites
14−19−somites
20−25−somites
Prim−5
Prim−15
Prim−25
Long−pec
Protruding−mouth
Day−4
Day−5
Stage
Transcript
ENSDART00000153755
ENSDART00000153857
ENSDART00000155400

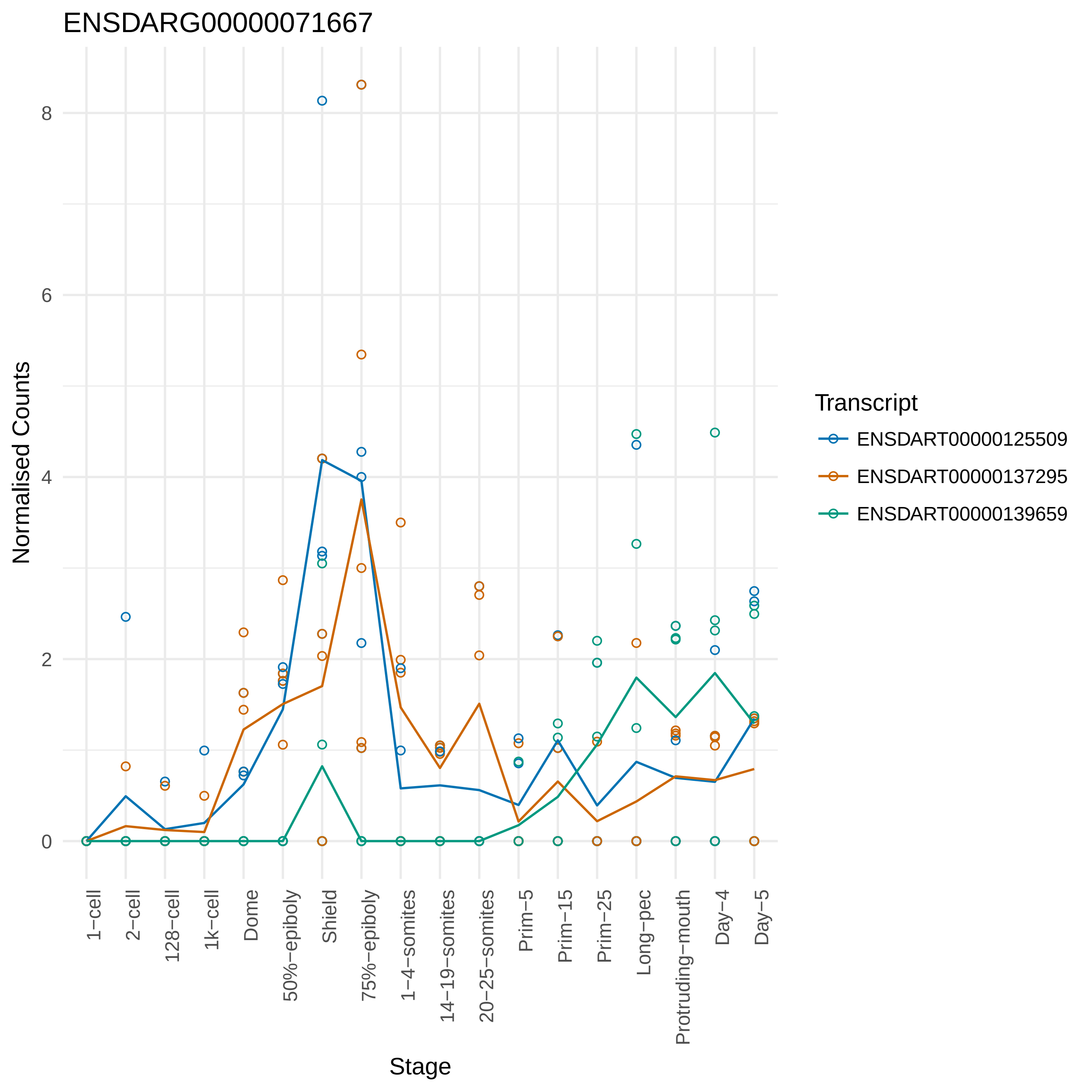

ENSDARG00000071667
Normalised Counts
0
2
4
6
8
Stage
1−cell
2−cell
128−cell
1k−cell
Dome
50%−epiboly
Shield
75%−epiboly
1−4−somites
14−19−somites
20−25−somites
Prim−5
Prim−15
Prim−25
Long−pec
Protruding−mouth
Day−4
Day−5
Transcript
ENSDART00000125509
ENSDART00000137295
ENSDART00000139659

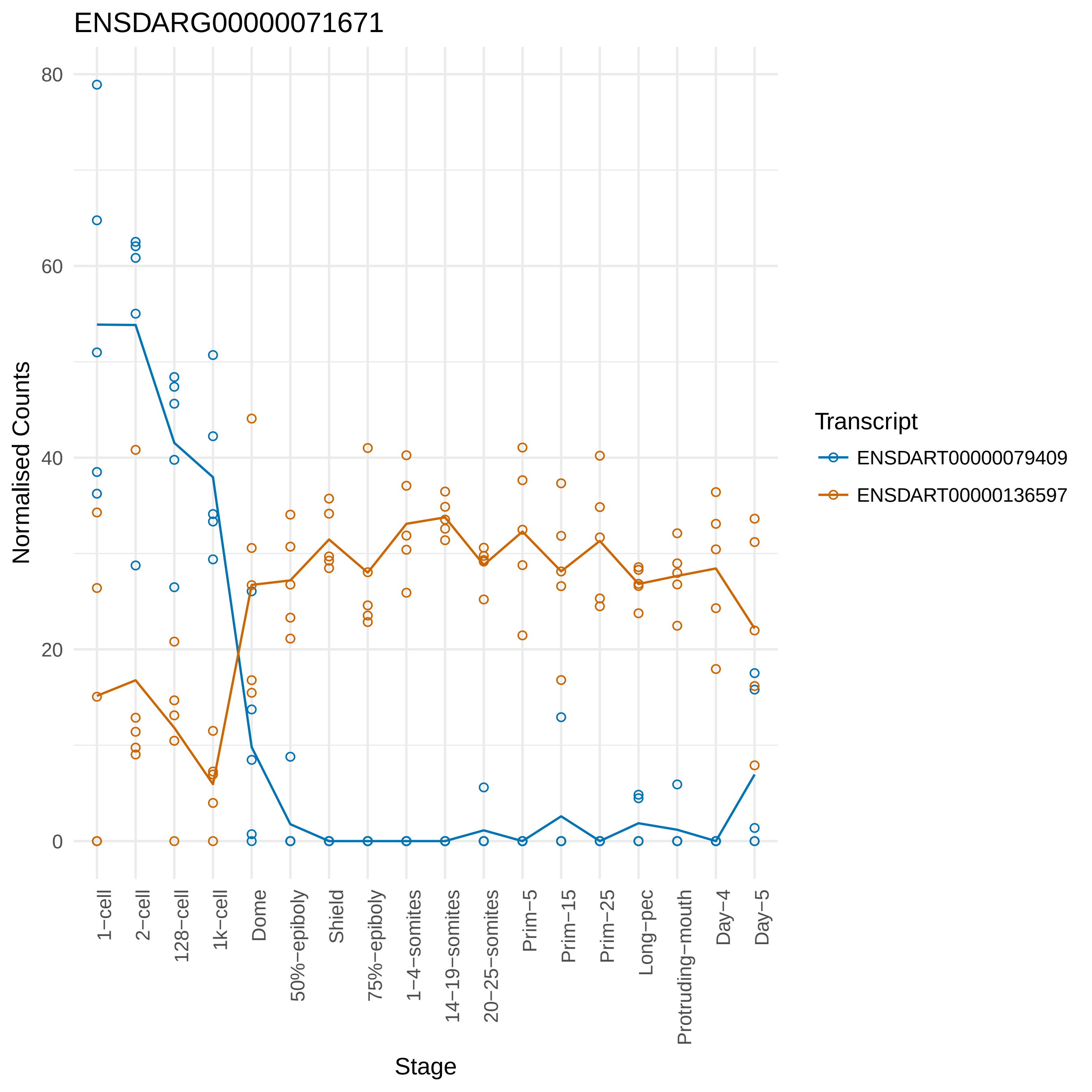
ENSDARG00000071671
Normalised Counts
0
20
40
60
80
1−cell
2−cell
128−cell
1k−cell
Dome
50%−epiboly
Shield
75%−epiboly
1−4−somites
14−19−somites
20−25−somites
Prim−5
Prim−15
Prim−25
Long−pec
Protruding−mouth
Day−4
Day−5
Stage
Transcript
ENSDART00000079409
ENSDART00000136597

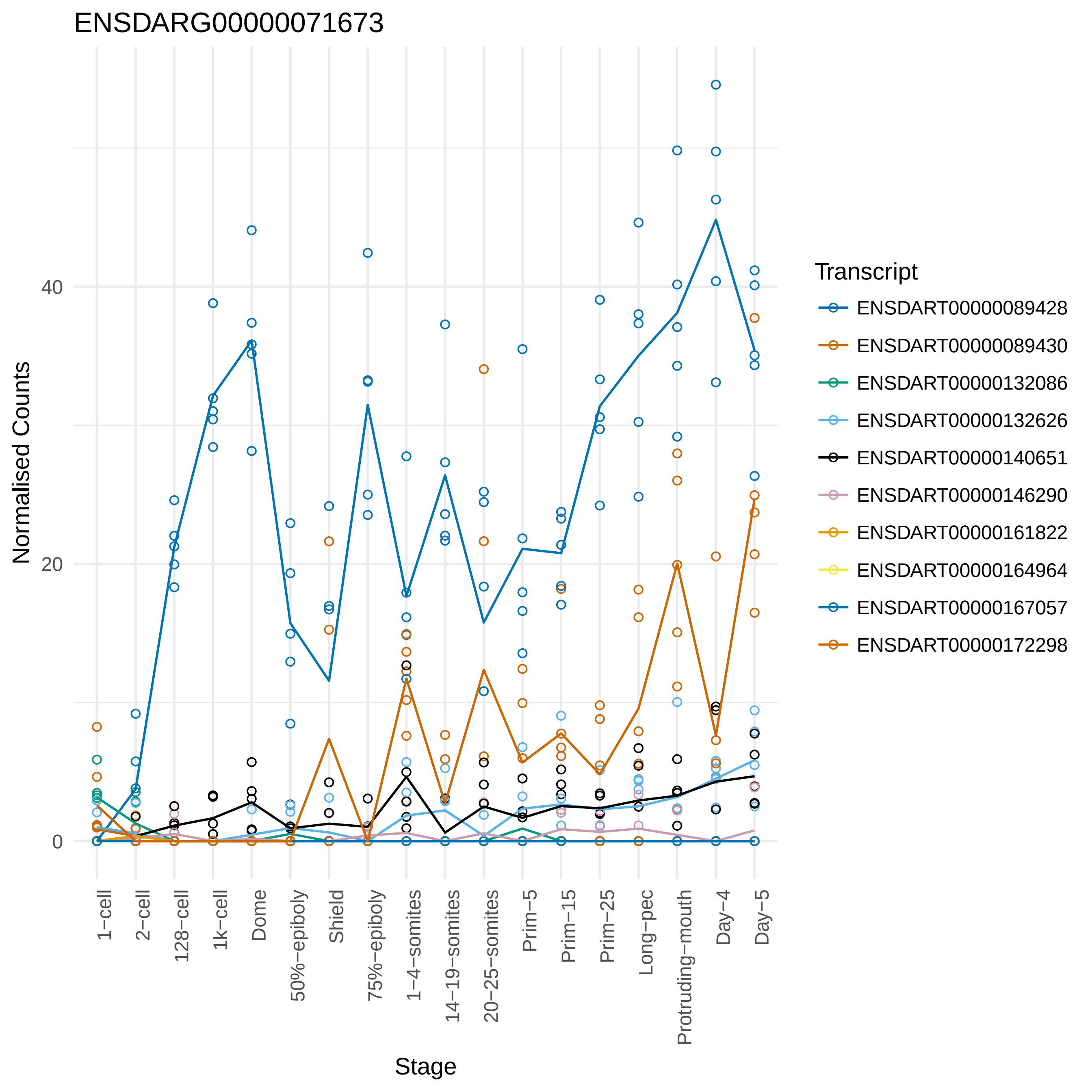

ENSDARG00000071673
Normalised Counts
0
20
40
1−cell
2−cell
128−cell
1k−cell
Dome
50%−epiboly
Shield
75%−epiboly
1−4−somites
14−19−somites
20−25−somites
Prim−5
Prim−15
Prim−25
Long−pec
Protruding−mouth
Day−4
Day−5
Stage
Transcript
ENSDART00000089428
ENSDART00000089430
ENSDART00000132086
ENSDART00000132626
ENSDART00000140651
ENSDART00000146290
ENSDART00000161822
ENSDART00000164964
ENSDART00000167057
ENSDART00000172298

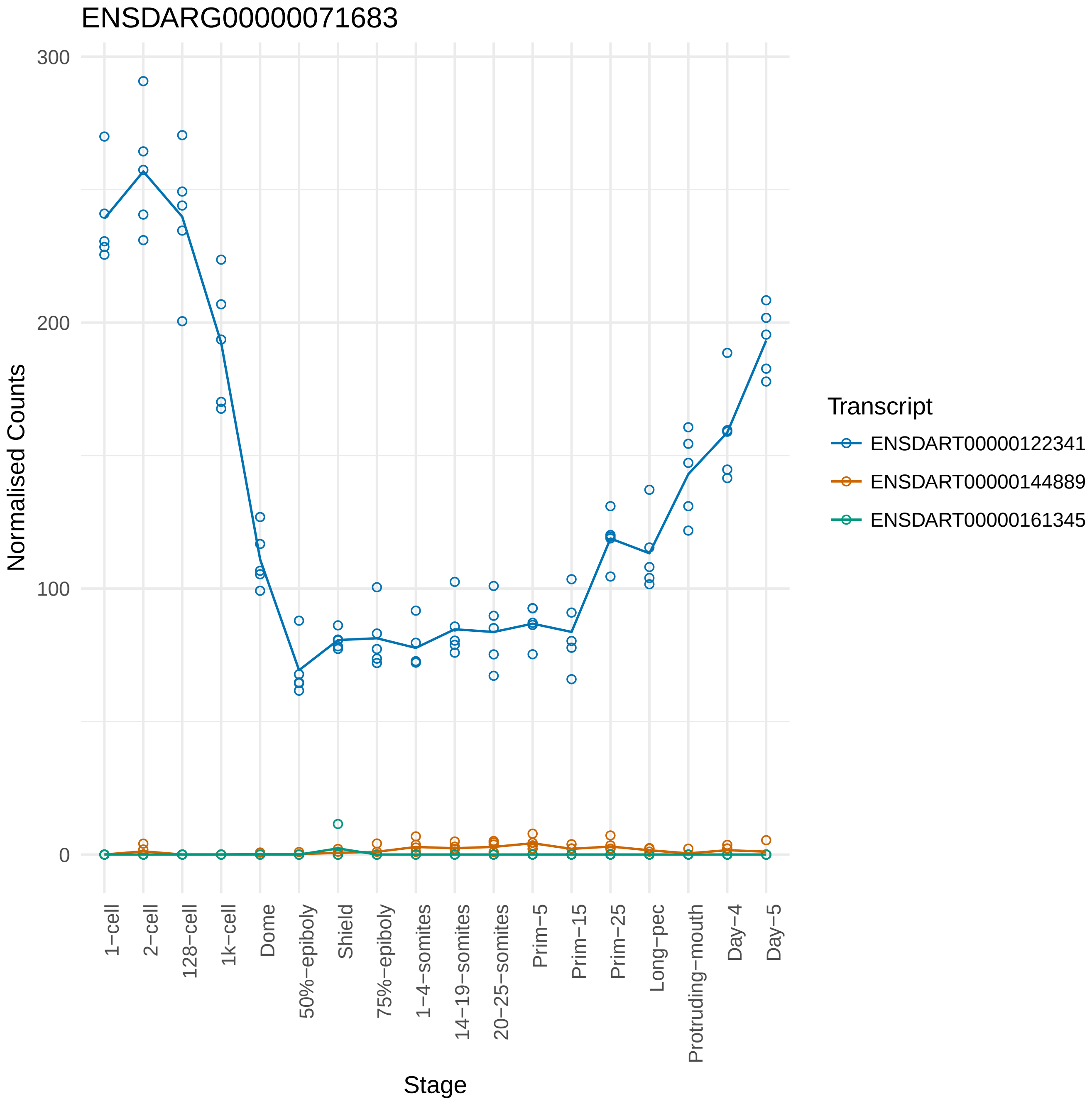
ENSDARG00000071683
Normalised Counts
300
200
100
0
1−cell
2−cell
128−cell
1k−cell
Dome
50%−epiboly
Shield
75%−epiboly
1−4−somites
14−19−somites
20−25−somites
Prim−5
Prim−15
Prim−25
Long−pec
Protruding−mouth
Day−4
Day−5
Stage
Transcript
ENSDART00000122341
ENSDART00000144889
ENSDART00000161345

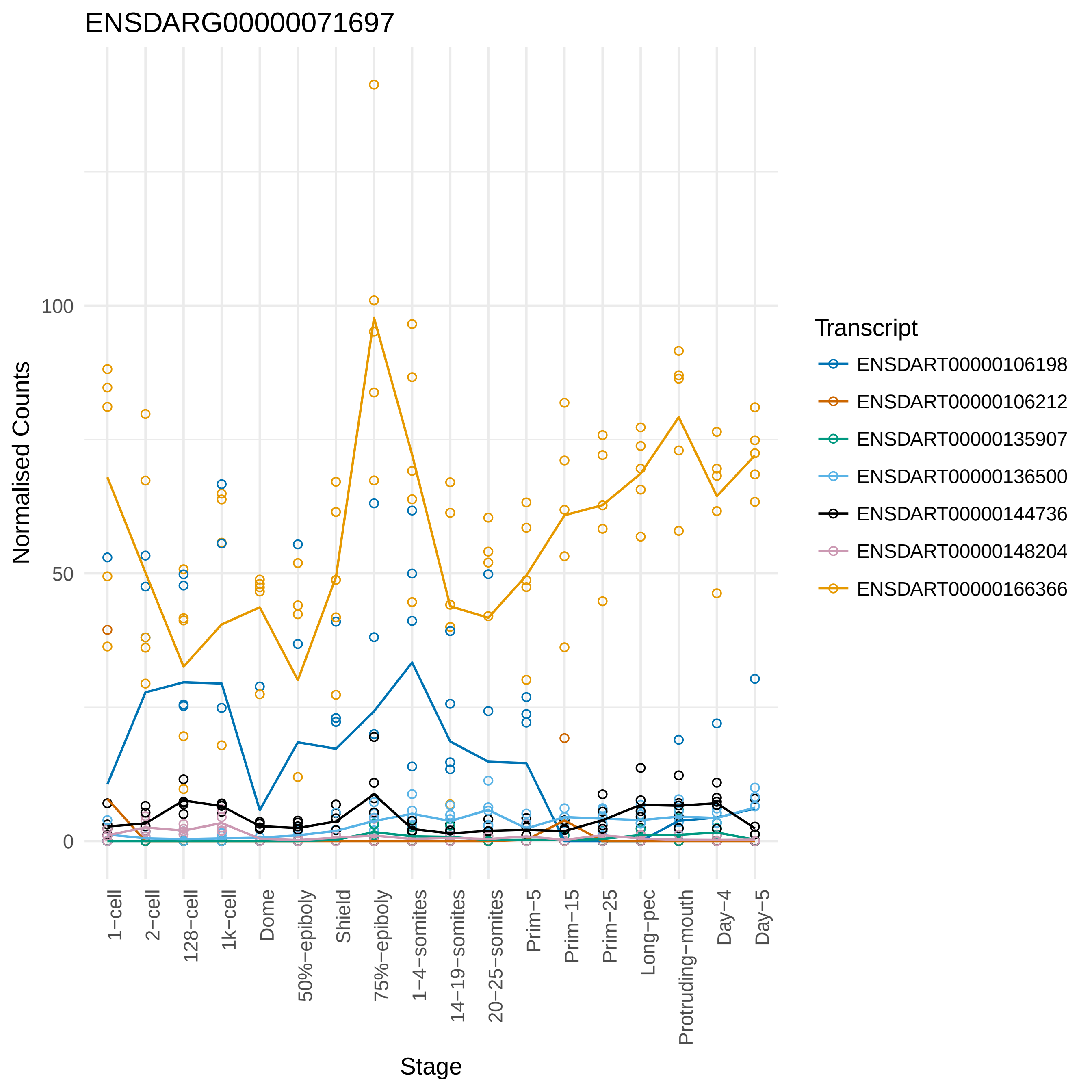

ENSDARG00000071697
Normalised Counts
100
50
0
1−cell
2−cell
128−cell
1k−cell
Dome
50%−epiboly
Shield
75%−epiboly
1−4−somites
14−19−somites
20−25−somites
Prim−5
Prim−15
Prim−25
Long−pec
Protruding−mouth
Day−4
Day−5
Stage
Transcript
ENSDART00000106198
ENSDART00000106212
ENSDART00000135907
ENSDART00000136500
ENSDART00000144736
ENSDART00000148204
ENSDART00000166366

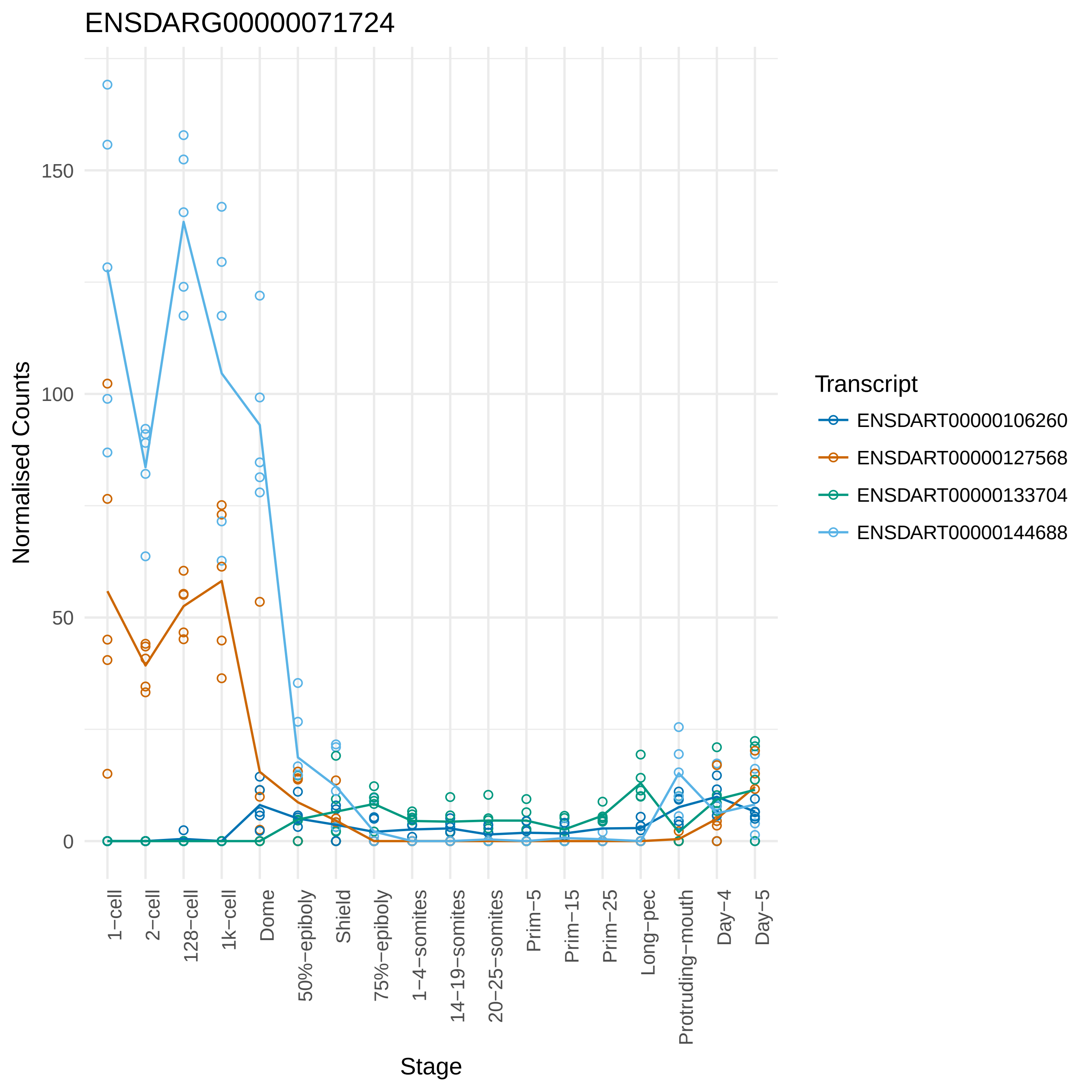
ENSDARG00000071724
Normalised Counts
150
100
50
0
1−cell
2−cell
128−cell
1k−cell
Dome
50%−epiboly
Shield
75%−epiboly
1−4−somites
14−19−somites
20−25−somites
Prim−5
Prim−15
Prim−25
Long−pec
Protruding−mouth
Day−4
Day−5
Stage
Transcript
ENSDART00000106260
ENSDART00000127568
ENSDART00000133704
ENSDART00000144688

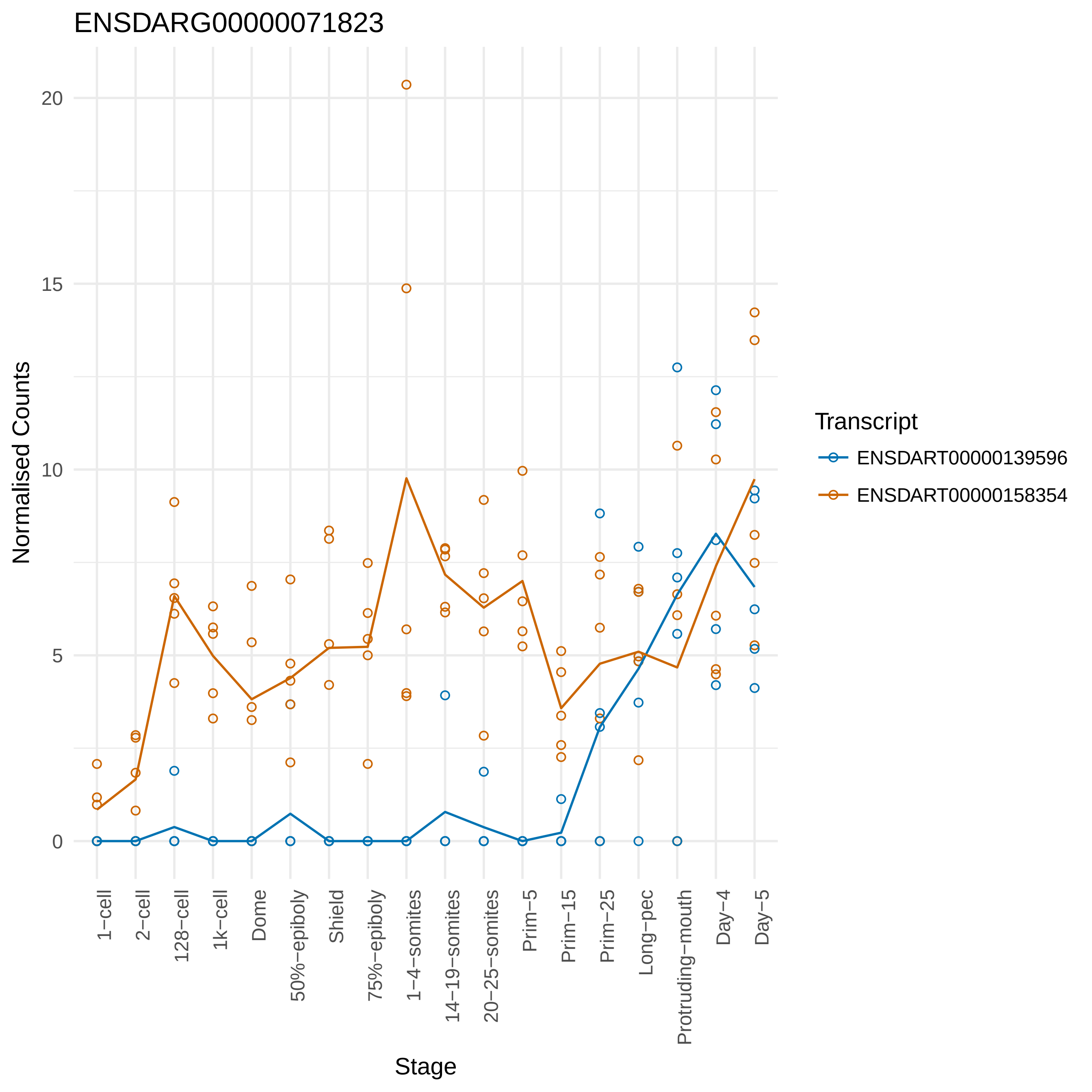
ENSDARG00000071823
Normalised Counts
0
5
10
15
20
Stage
1−cell
2−cell
128−cell
1k−cell
Dome
50%−epiboly
Shield
75%−epiboly
1−4−somites
14−19−somites
20−25−somites
Prim−5
Prim−15
Prim−25
Long−pec
Protruding−mouth
Day−4
Day−5
Transcript
ENSDART00000139596
ENSDART00000158354

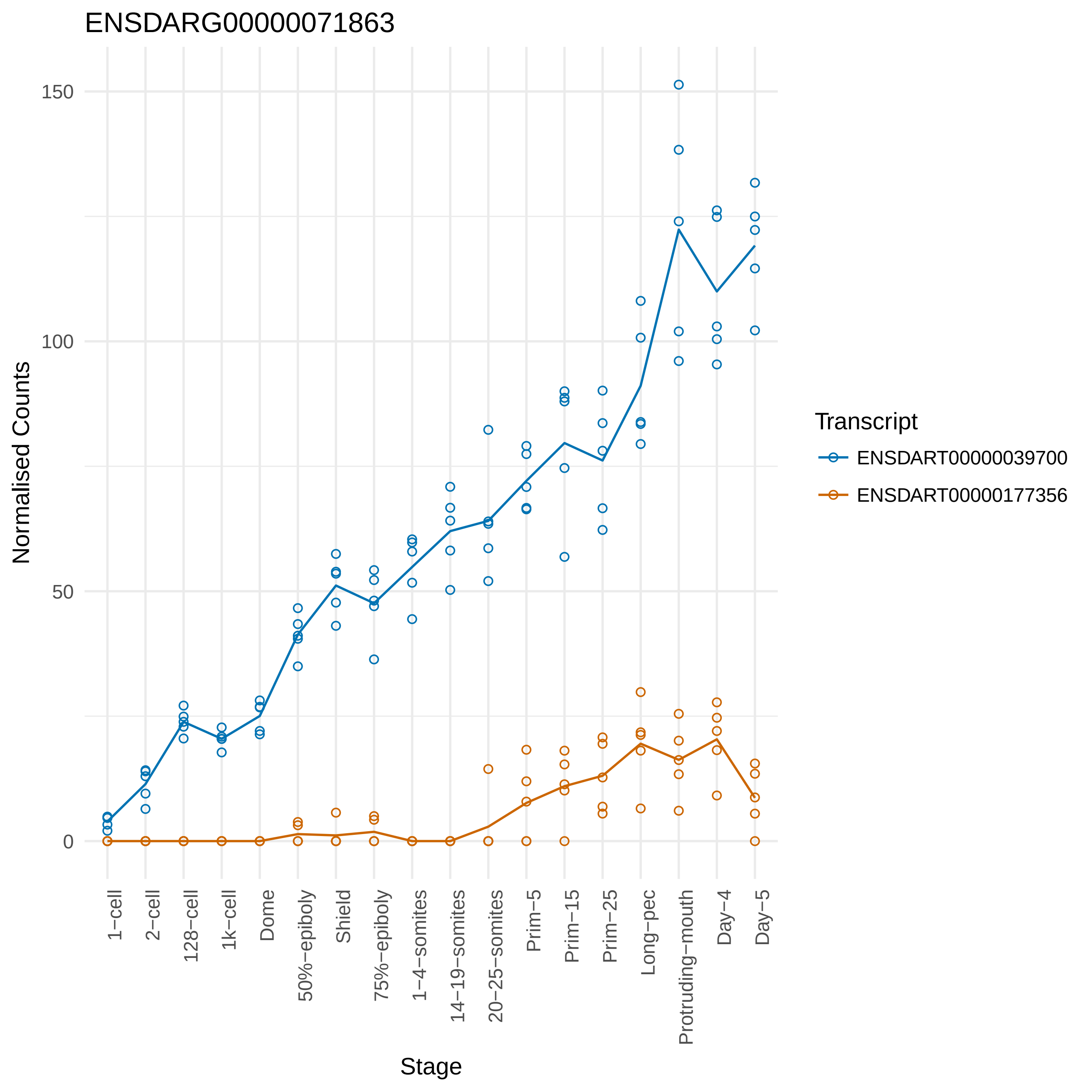

ENSDARG00000071863
Normalised Counts
150
100
50
0
1−cell
2−cell
128−cell
1k−cell
Dome
50%−epiboly
Shield
75%−epiboly
1−4−somites
14−19−somites
20−25−somites
Prim−5
Prim−15
Prim−25
Long−pec
Protruding−mouth
Day−4
Day−5
Stage
Transcript
ENSDART00000039700
ENSDART00000177356

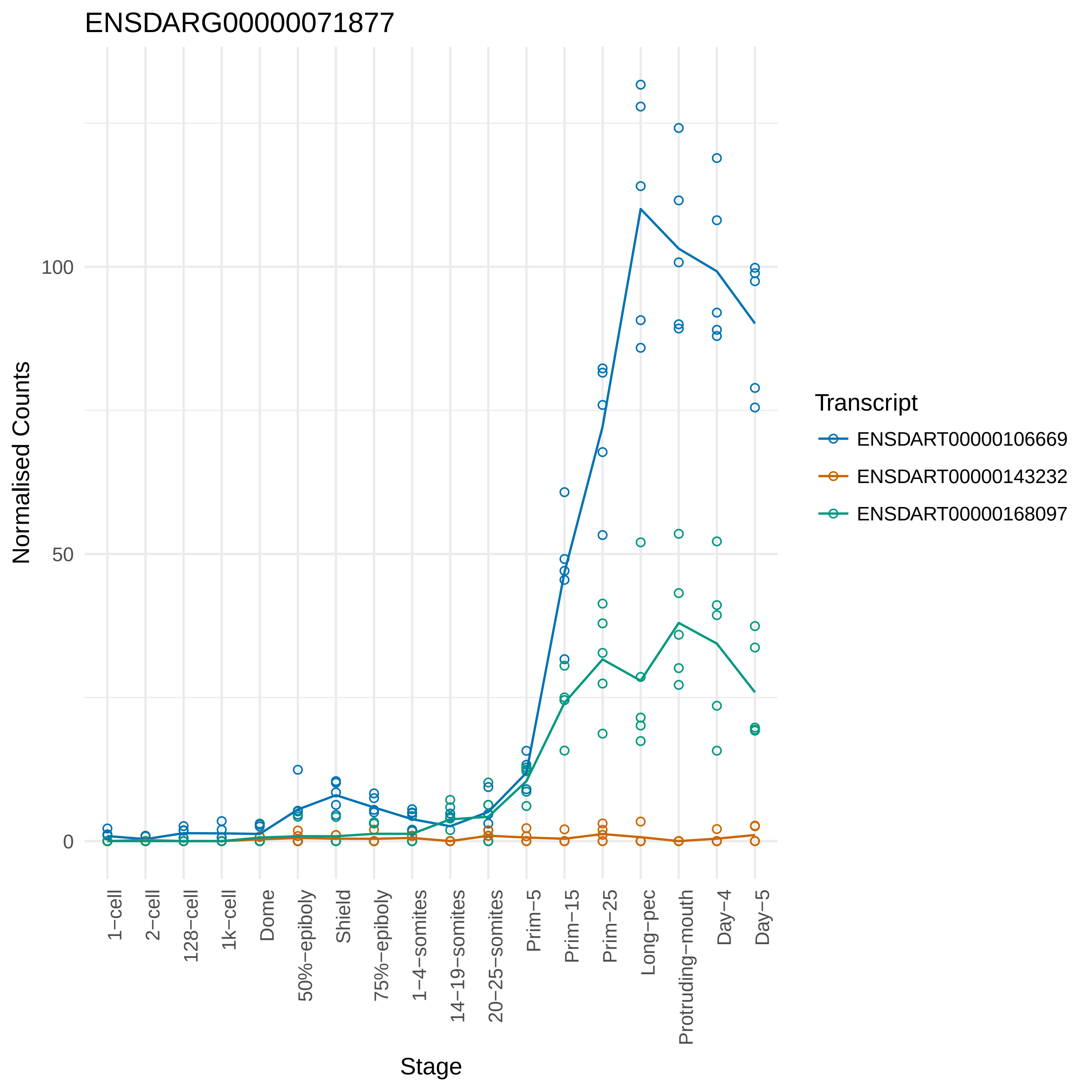

ENSDARG00000071877
Normalised Counts
100
50
0
1−cell
2−cell
128−cell
1k−cell
Dome
50%−epiboly
Shield
75%−epiboly
1−4−somites
14−19−somites
20−25−somites
Prim−5
Prim−15
Prim−25
Long−pec
Protruding−mouth
Day−4
Day−5
Stage
Transcript
ENSDART00000106669
ENSDART00000143232
ENSDART00000168097

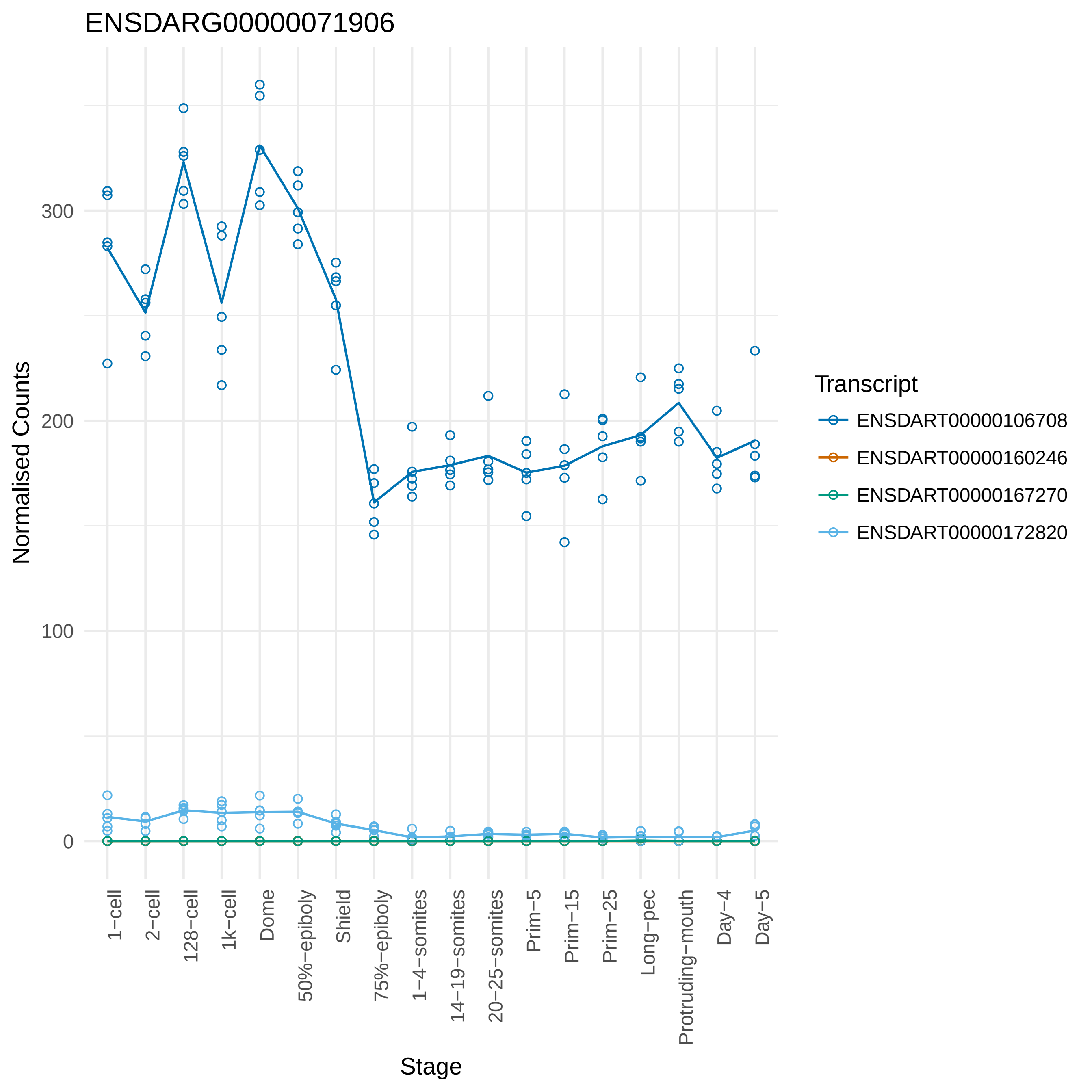

ENSDARG00000071906
Normalised Counts
300
200
100
0
1−cell
2−cell
128−cell
1k−cell
Dome
50%−epiboly
Shield
75%−epiboly
1−4−somites
14−19−somites
20−25−somites
Prim−5
Prim−15
Prim−25
Long−pec
Protruding−mouth
Day−4
Day−5
Stage
Transcript
ENSDART00000106708
ENSDART00000160246
ENSDART00000167270
ENSDART00000172820

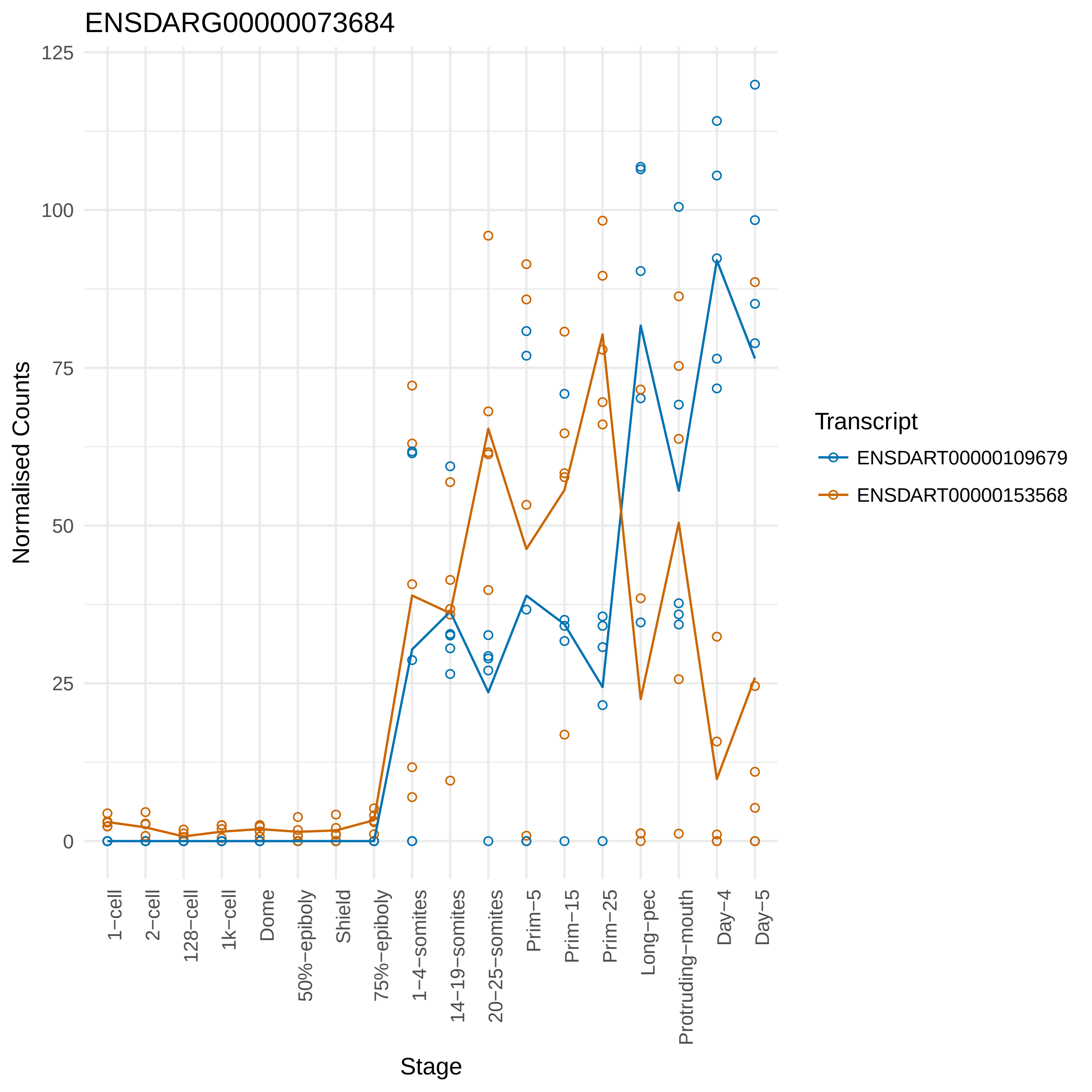

ENSDARG00000073684
Normalised Counts
125
100
75
50
25
0
1−cell
2−cell
128−cell
1k−cell
Dome
50%−epiboly
Shield
75%−epiboly
1−4−somites
14−19−somites
20−25−somites
Prim−5
Prim−15
Prim−25
Long−pec
Protruding−mouth
Day−4
Day−5
Stage
Transcript
ENSDART00000109679
ENSDART00000153568

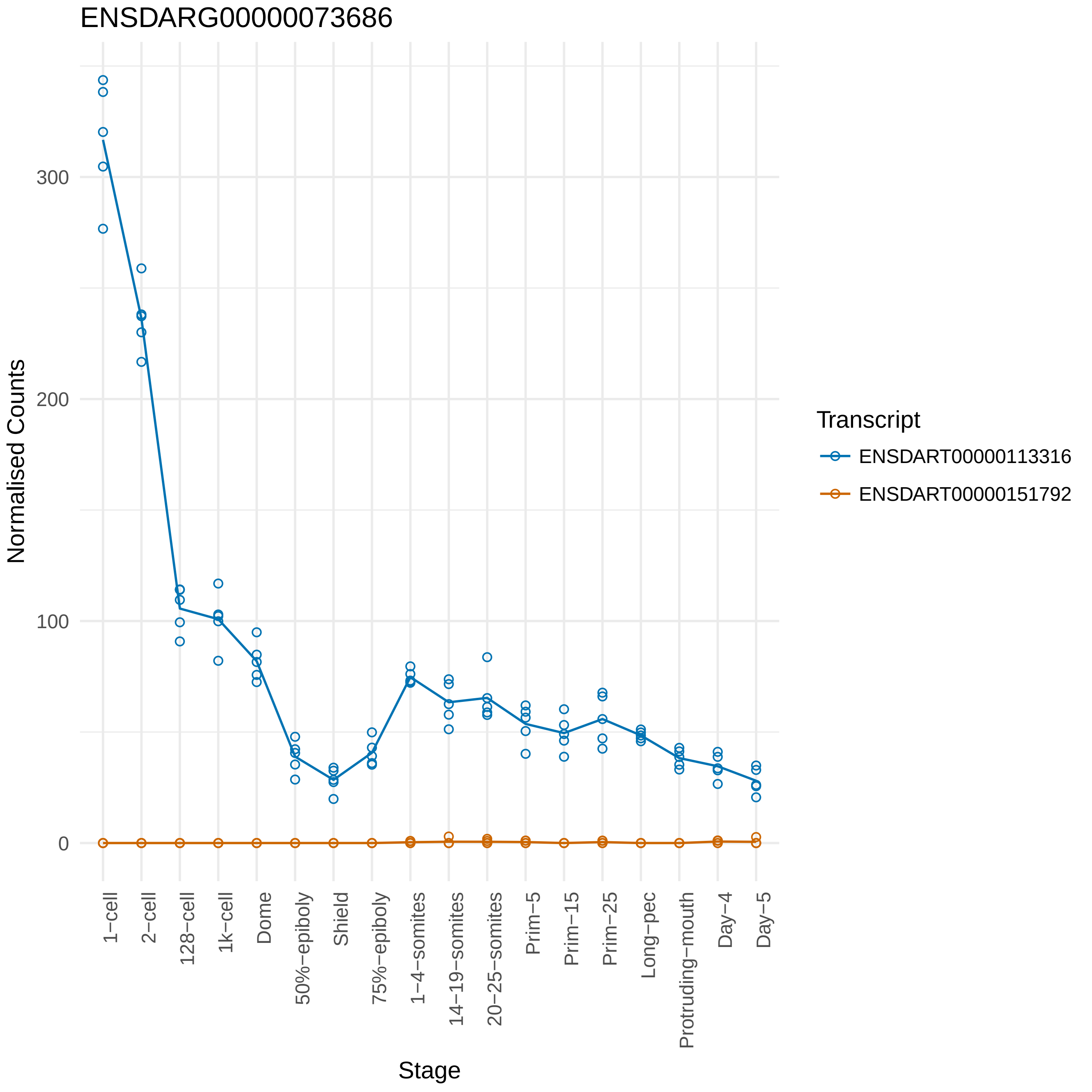

ENSDARG00000073686
Normalised Counts
300
200
100
0
1−cell
2−cell
128−cell
1k−cell
Dome
50%−epiboly
Shield
75%−epiboly
1−4−somites
14−19−somites
20−25−somites
Prim−5
Prim−15
Prim−25
Long−pec
Protruding−mouth
Day−4
Day−5
Stage
Transcript
ENSDART00000113316
ENSDART00000151792

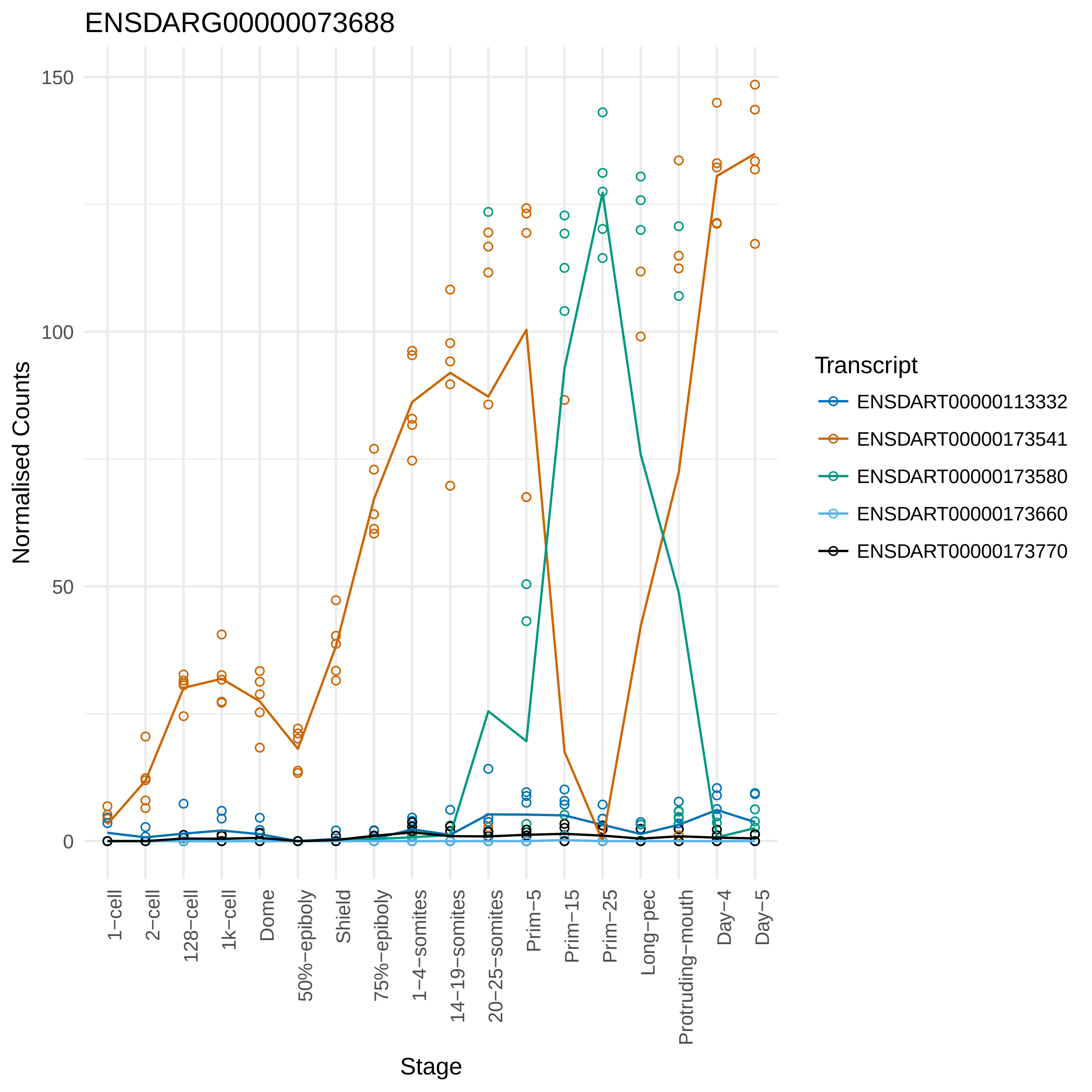
ENSDARG00000073688
Normalised Counts
150
100
50
0
1−cell
2−cell
128−cell
1k−cell
Dome
50%−epiboly
Shield
75%−epiboly
1−4−somites
14−19−somites
20−25−somites
Prim−5
Prim−15
Prim−25
Long−pec
Protruding−mouth
Day−4
Day−5
Stage
Transcript
ENSDART00000113332
ENSDART00000173541
ENSDART00000173580
ENSDART00000173660
ENSDART00000173770

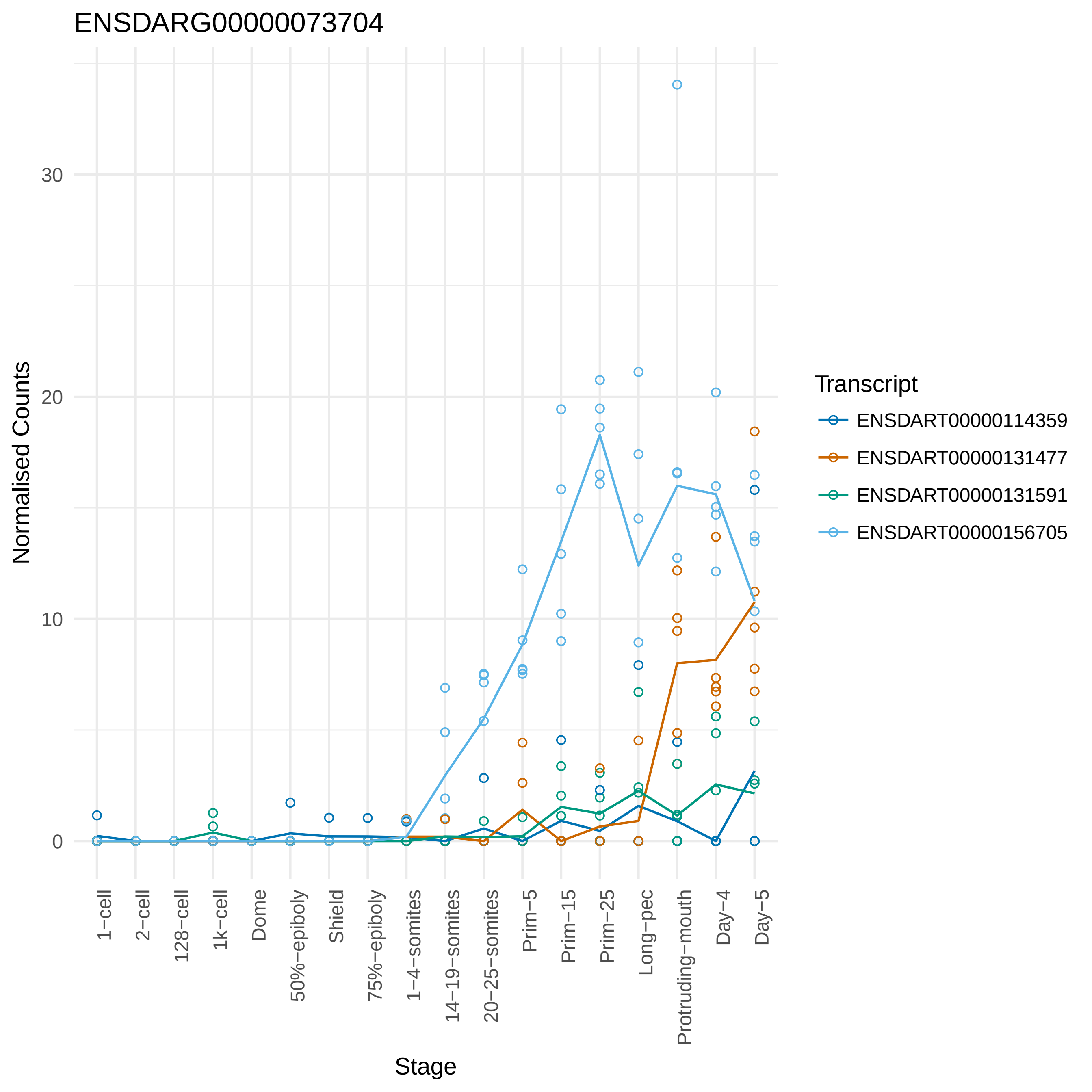

ENSDARG00000073704
Normalised Counts
0
10
20
30
1−cell
2−cell
128−cell
1k−cell
Dome
50%−epiboly
Shield
75%−epiboly
1−4−somites
14−19−somites
20−25−somites
Prim−5
Prim−15
Prim−25
Long−pec
Protruding−mouth
Day−4
Day−5
Stage
Transcript
ENSDART00000114359
ENSDART00000131477
ENSDART00000131591
ENSDART00000156705

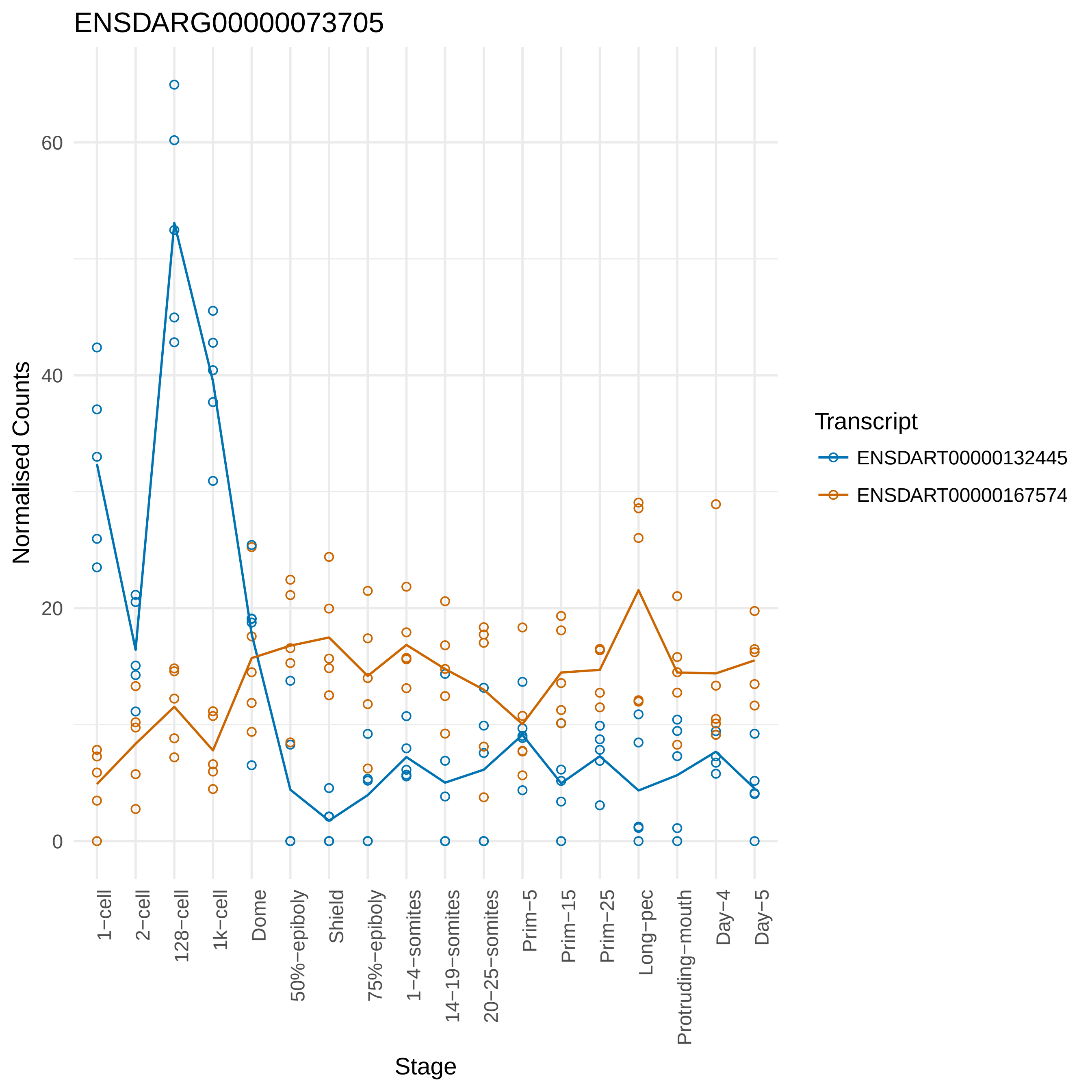

ENSDARG00000073705
Normalised Counts
0
20
40
60
Stage
1−cell
2−cell
128−cell
1k−cell
Dome
50%−epiboly
Shield
75%−epiboly
1−4−somites
14−19−somites
20−25−somites
Prim−5
Prim−15
Prim−25
Long−pec
Protruding−mouth
Day−4
Day−5
Transcript
ENSDART00000132445
ENSDART00000167574

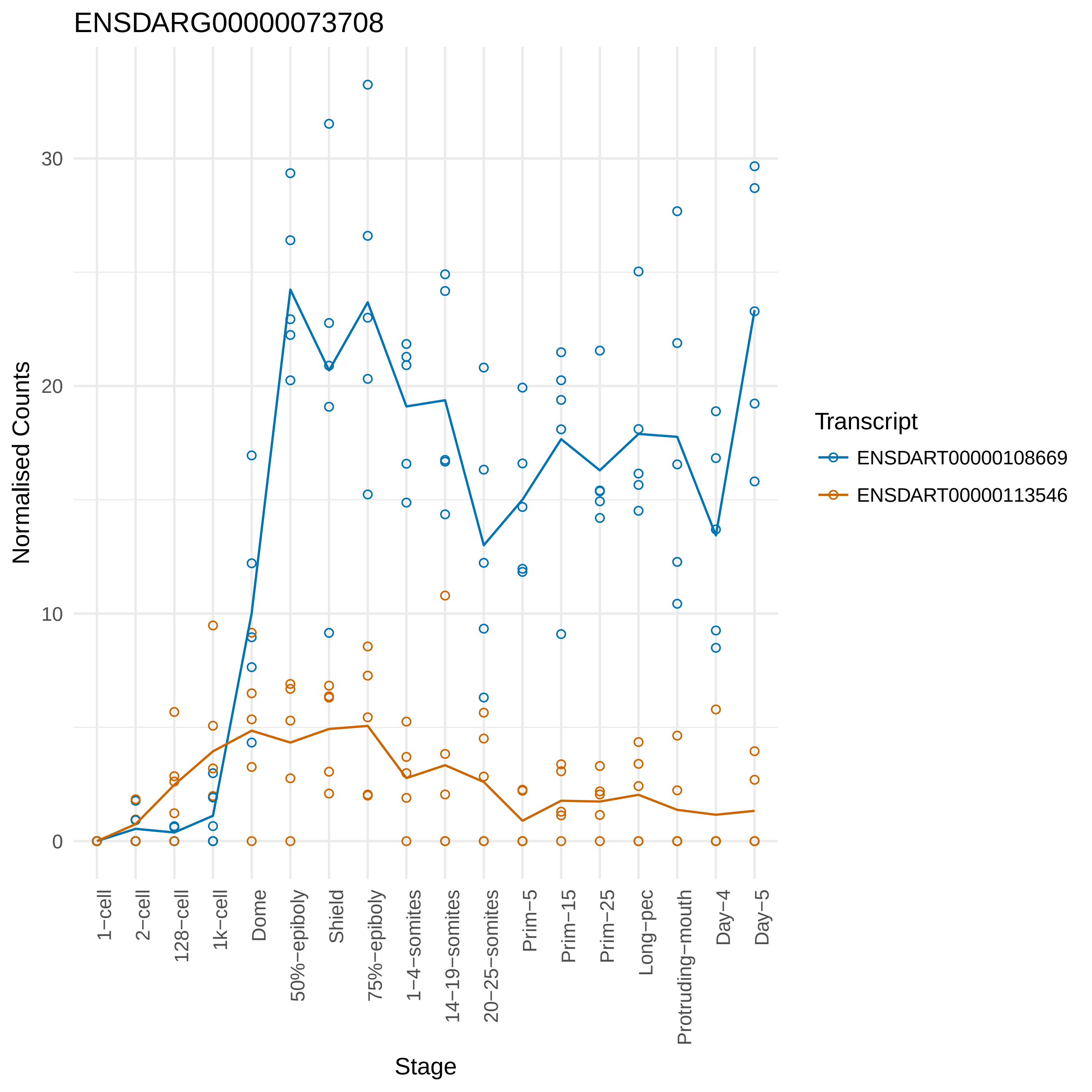
ENSDARG00000073708
Normalised Counts
30
20
10
0
1−cell
2−cell
128−cell
1k−cell
Dome
50%−epiboly
Shield
75%−epiboly
1−4−somites
14−19−somites
20−25−somites
Prim−5
Prim−15
Prim−25
Long−pec
Protruding−mouth
Day−4
Day−5
Stage
Transcript
ENSDART00000108669
ENSDART00000113546

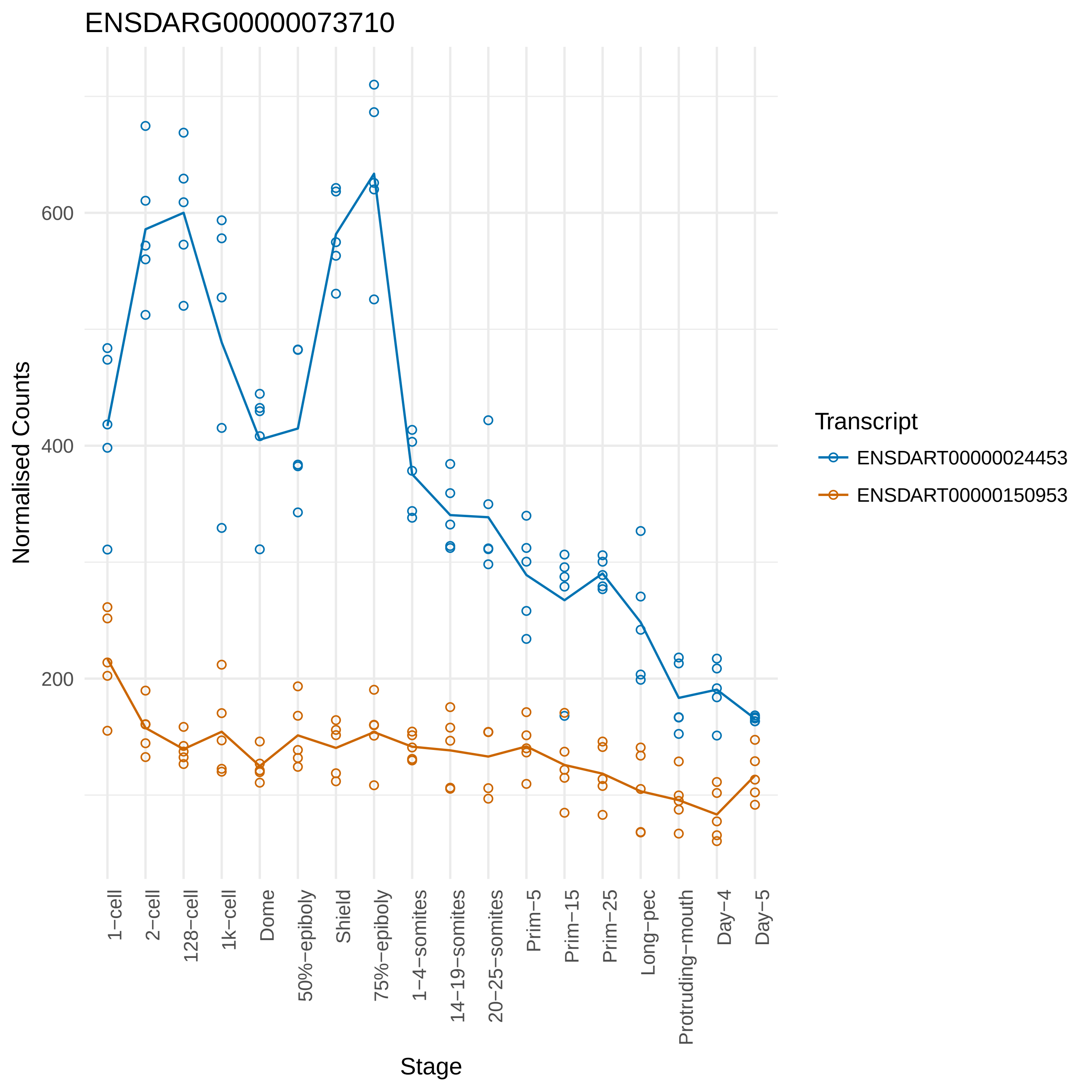

ENSDARG00000073710
Normalised Counts
600
400
200
Stage
1−cell
2−cell
128−cell
1k−cell
Dome
50%−epiboly
Shield
75%−epiboly
1−4−somites
14−19−somites
20−25−somites
Prim−5
Prim−15
Prim−25
Long−pec
Protruding−mouth
Day−4
Day−5
Transcript
ENSDART00000024453
ENSDART00000150953

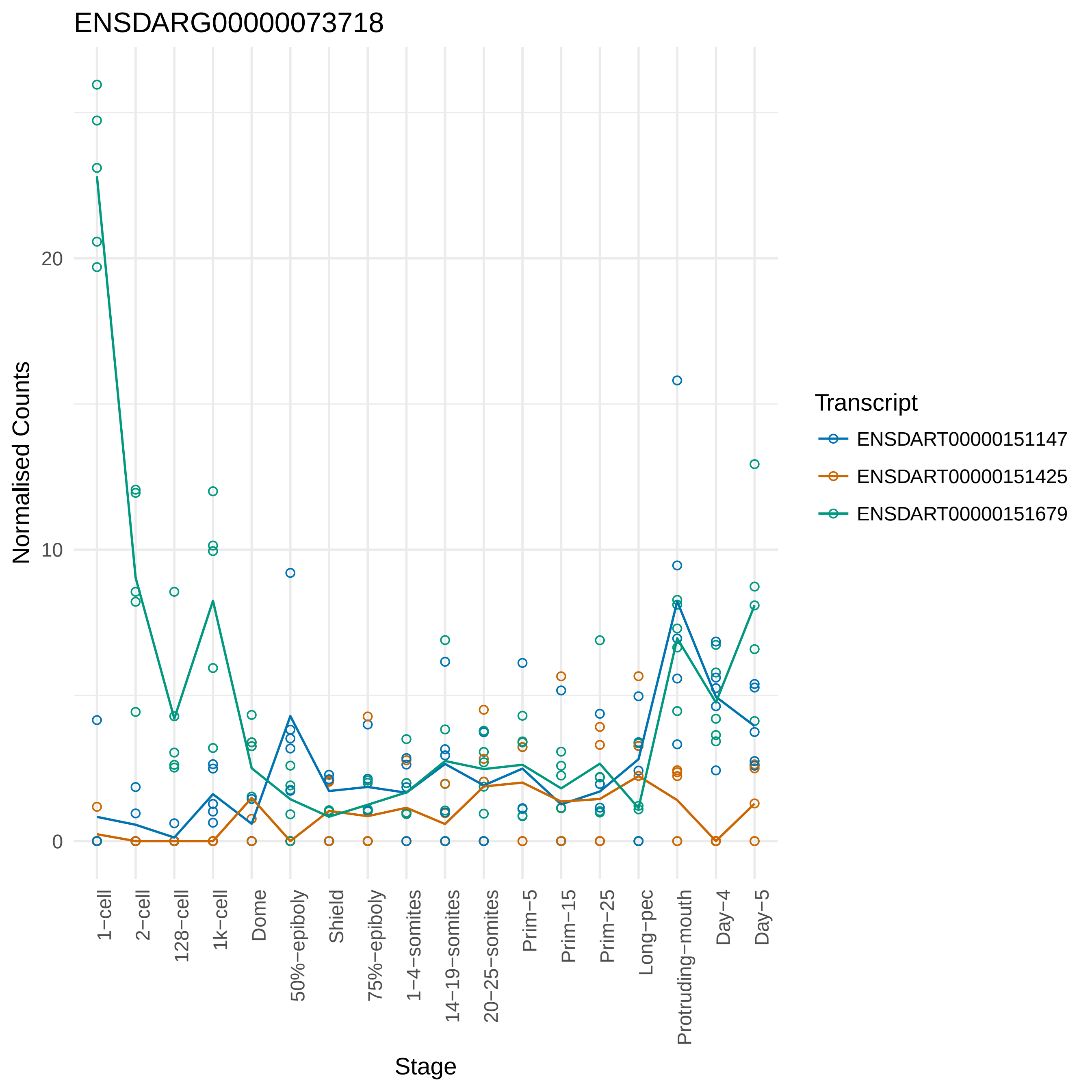

ENSDARG00000073718
Normalised Counts
20
10
0
1−cell
2−cell
128−cell
1k−cell
Dome
50%−epiboly
Shield
75%−epiboly
1−4−somites
14−19−somites
20−25−somites
Prim−5
Prim−15
Prim−25
Long−pec
Protruding−mouth
Day−4
Day−5
Stage
Transcript
ENSDART00000151147
ENSDART00000151425
ENSDART00000151679

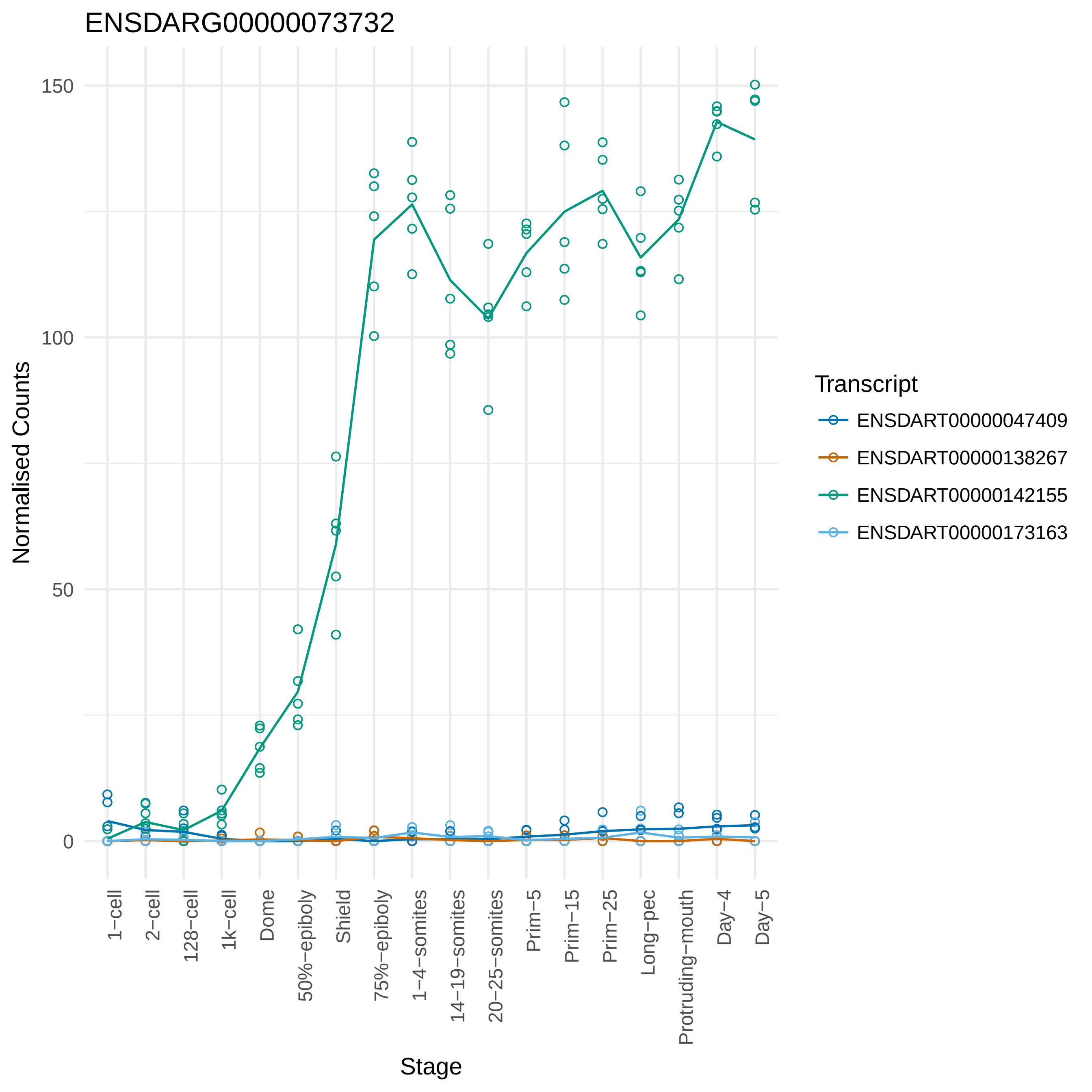

ENSDARG00000073732
Normalised Counts
150
100
50
0
1−cell
2−cell
128−cell
1k−cell
Dome
50%−epiboly
Shield
75%−epiboly
1−4−somites
14−19−somites
20−25−somites
Prim−5
Prim−15
Prim−25
Long−pec
Protruding−mouth
Day−4
Day−5
Stage
Transcript
ENSDART00000047409
ENSDART00000138267
ENSDART00000142155
ENSDART00000173163

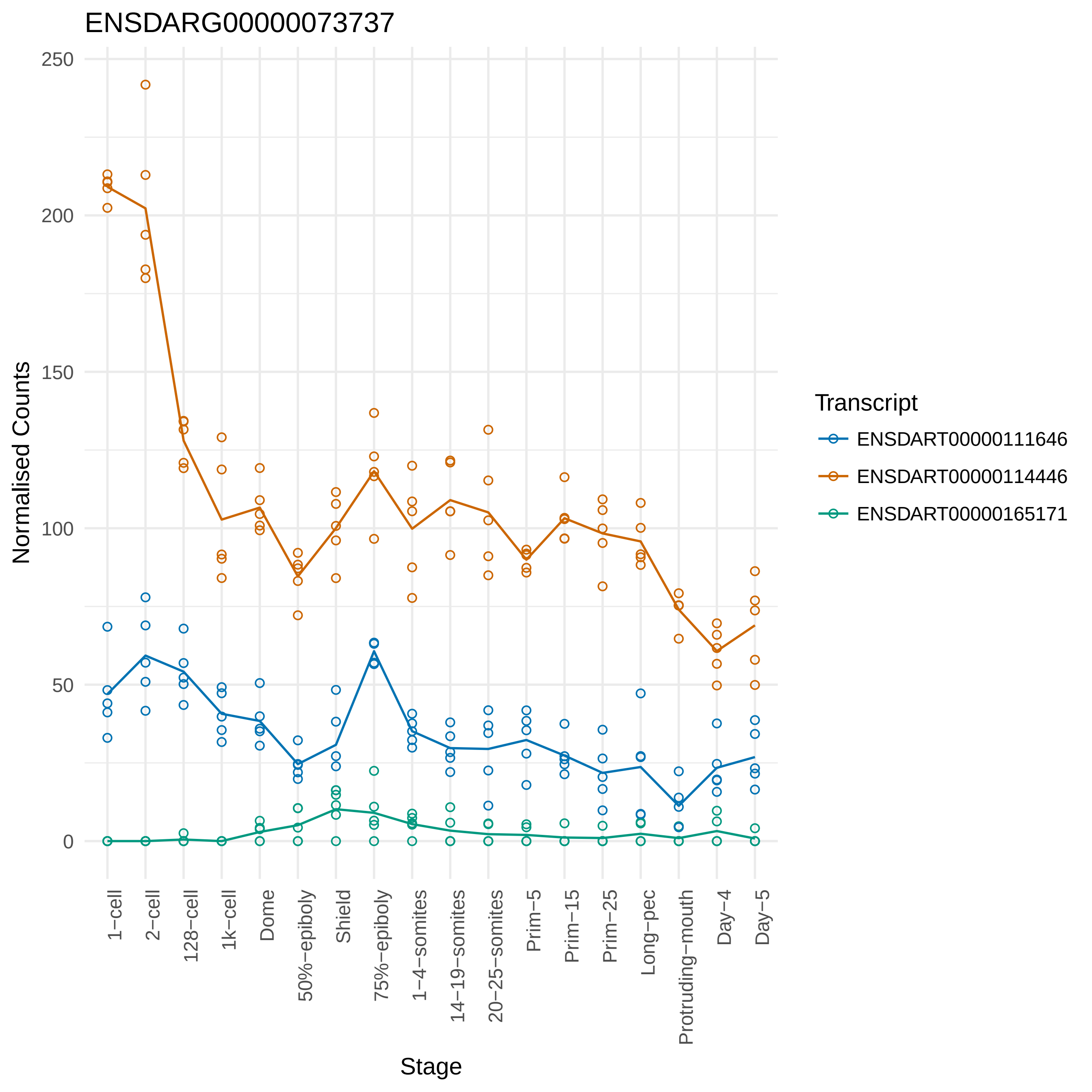

ENSDARG00000073737
Normalised Counts
0
50
100
150
200
250
Stage
1−cell
2−cell
128−cell
1k−cell
Dome
50%−epiboly
Shield
75%−epiboly
1−4−somites
14−19−somites
20−25−somites
Prim−5
Prim−15
Prim−25
Long−pec
Protruding−mouth
Day−4
Day−5
Transcript
ENSDART00000111646
ENSDART00000114446
ENSDART00000165171

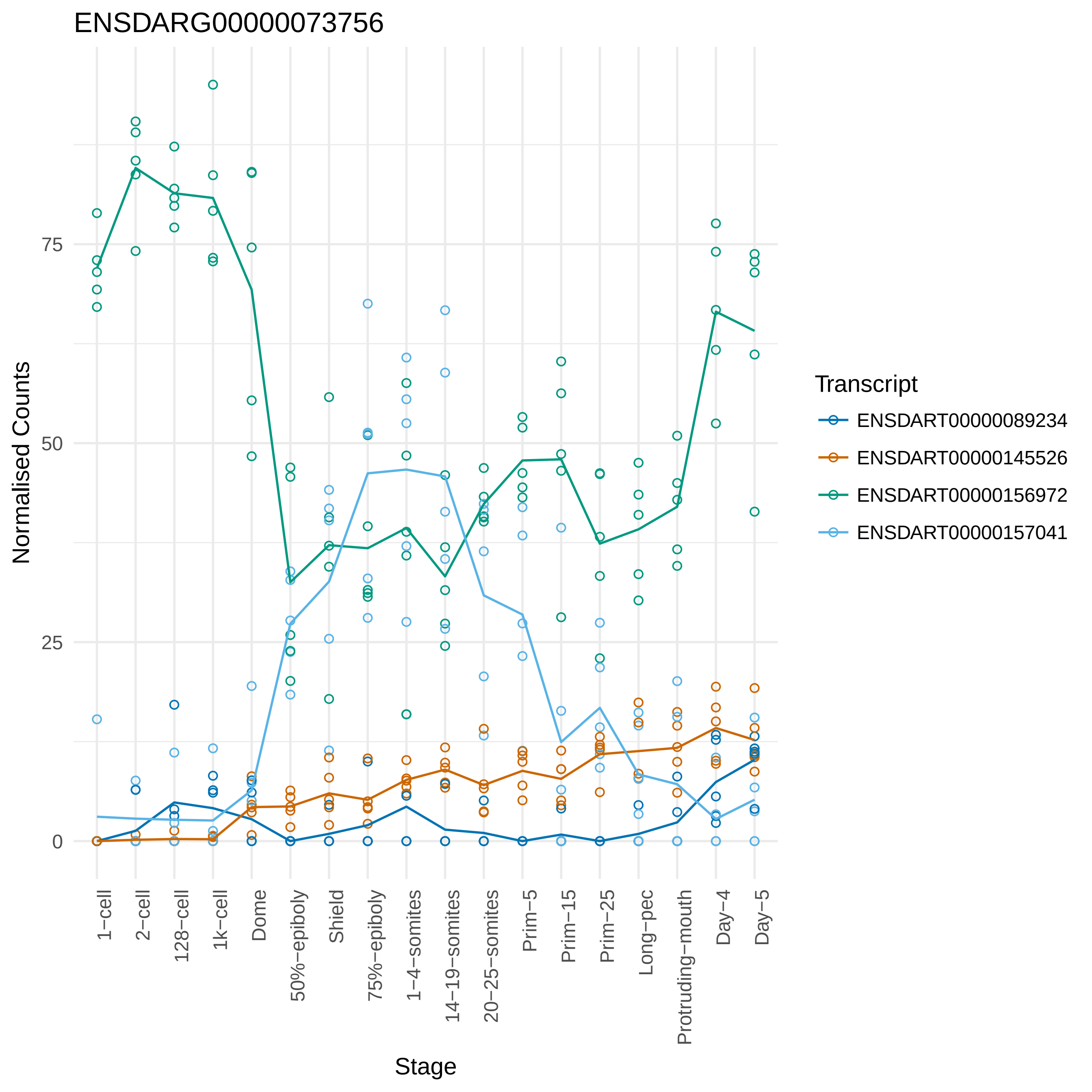
ENSDARG00000073756
Normalised Counts
0
25
50
75
Stage
1−cell
2−cell
128−cell
1k−cell
Dome
50%−epiboly
Shield
75%−epiboly
1−4−somites
14−19−somites
20−25−somites
Prim−5
Prim−15
Prim−25
Long−pec
Protruding−mouth
Day−4
Day−5
Transcript
ENSDART00000089234
ENSDART00000145526
ENSDART00000156972
ENSDART00000157041

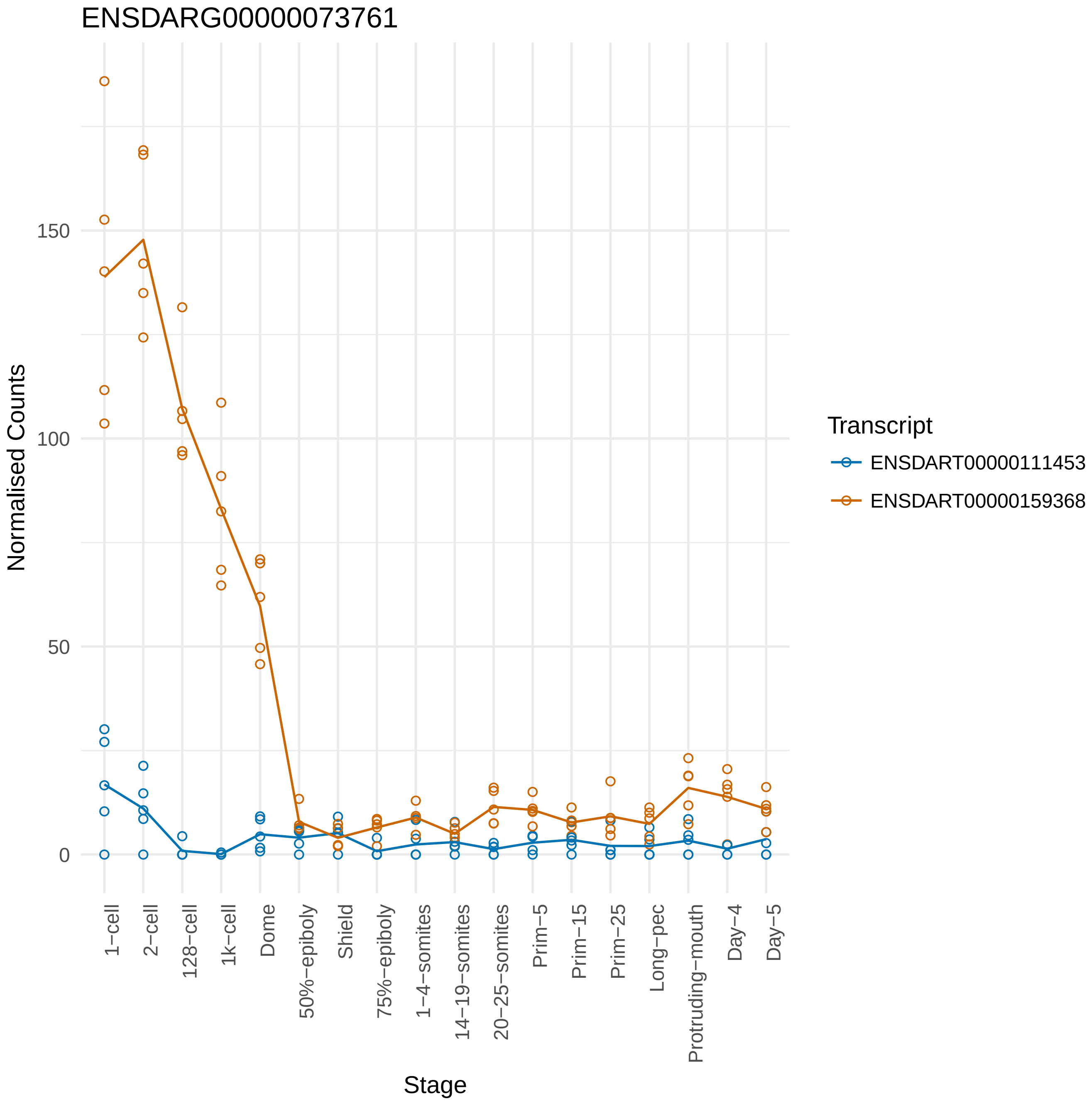
ENSDARG00000073761
Normalised Counts
150
100
50
0
1−cell
2−cell
128−cell
1k−cell
Dome
50%−epiboly
Shield
75%−epiboly
1−4−somites
14−19−somites
20−25−somites
Prim−5
Prim−15
Prim−25
Long−pec
Protruding−mouth
Day−4
Day−5
Stage
Transcript
ENSDART00000111453
ENSDART00000159368

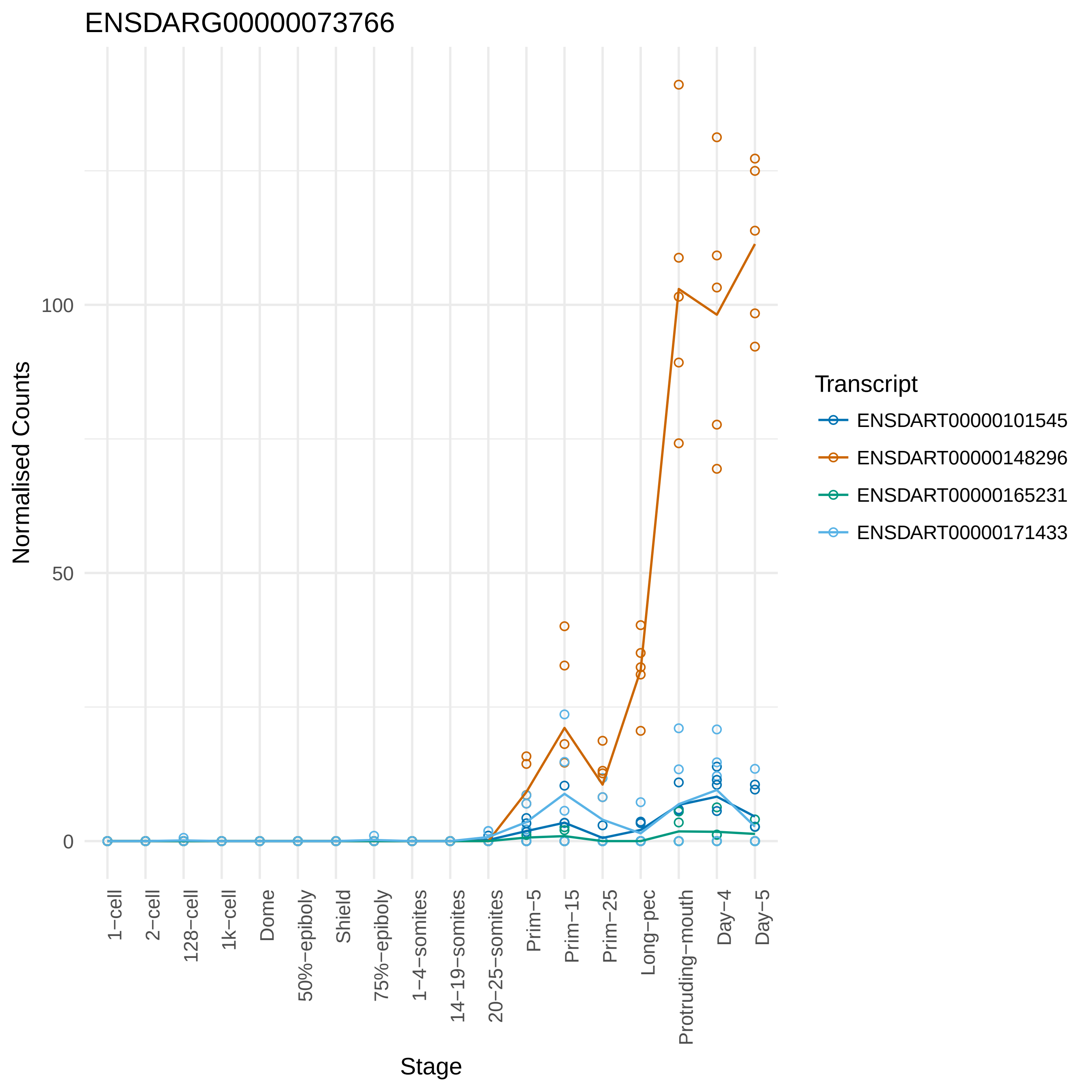

ENSDARG00000073766
Normalised Counts
100
50
0
1−cell
2−cell
128−cell
1k−cell
Dome
50%−epiboly
Shield
75%−epiboly
1−4−somites
14−19−somites
20−25−somites
Prim−5
Prim−15
Prim−25
Long−pec
Protruding−mouth
Day−4
Day−5
Stage
Transcript
ENSDART00000101545
ENSDART00000148296
ENSDART00000165231
ENSDART00000171433

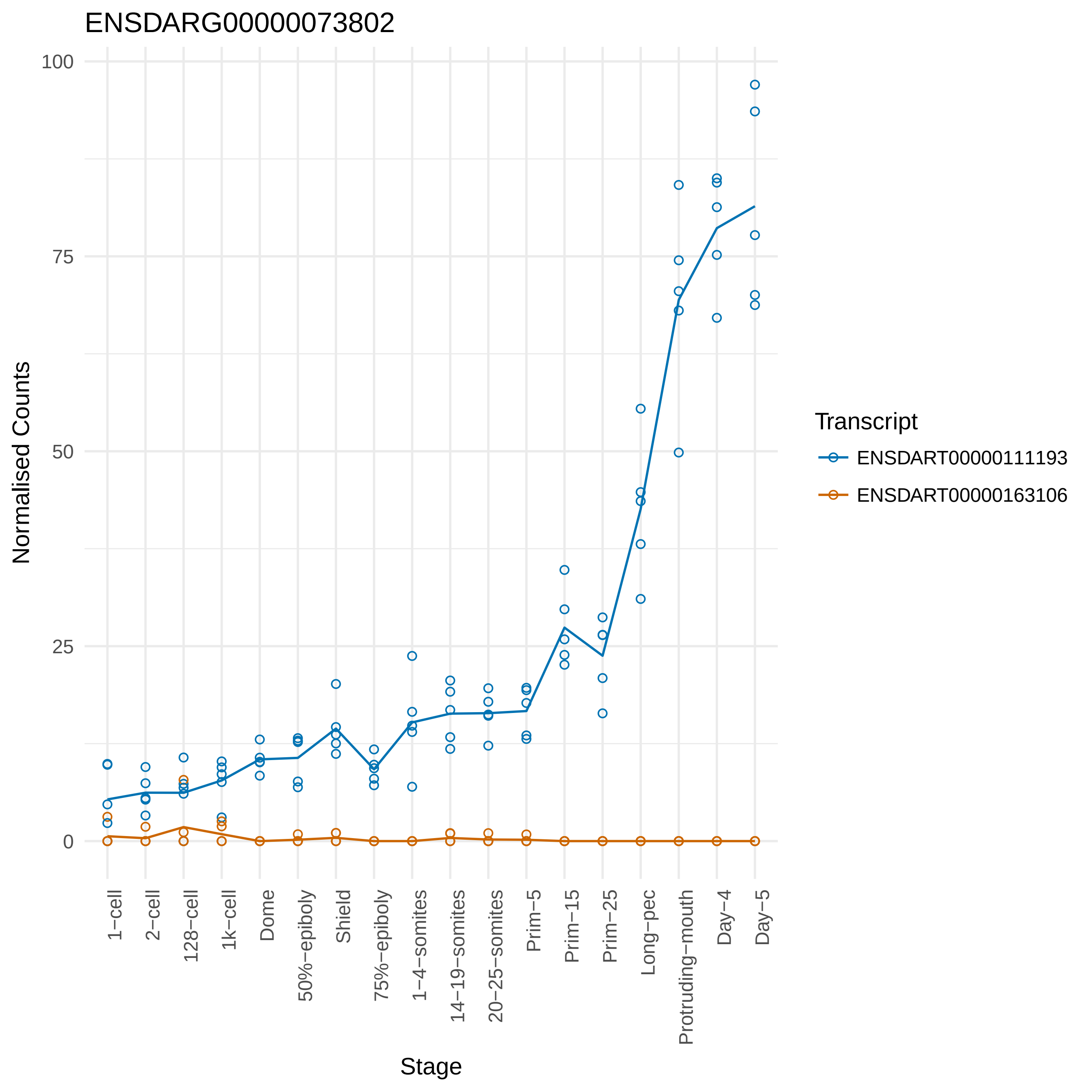
ENSDARG00000073802
Normalised Counts
100
75
50
25
0
1−cell
2−cell
128−cell
1k−cell
Dome
50%−epiboly
Shield
75%−epiboly
1−4−somites
14−19−somites
20−25−somites
Prim−5
Prim−15
Prim−25
Long−pec
Protruding−mouth
Day−4
Day−5
Stage
Transcript
ENSDART00000111193
ENSDART00000163106

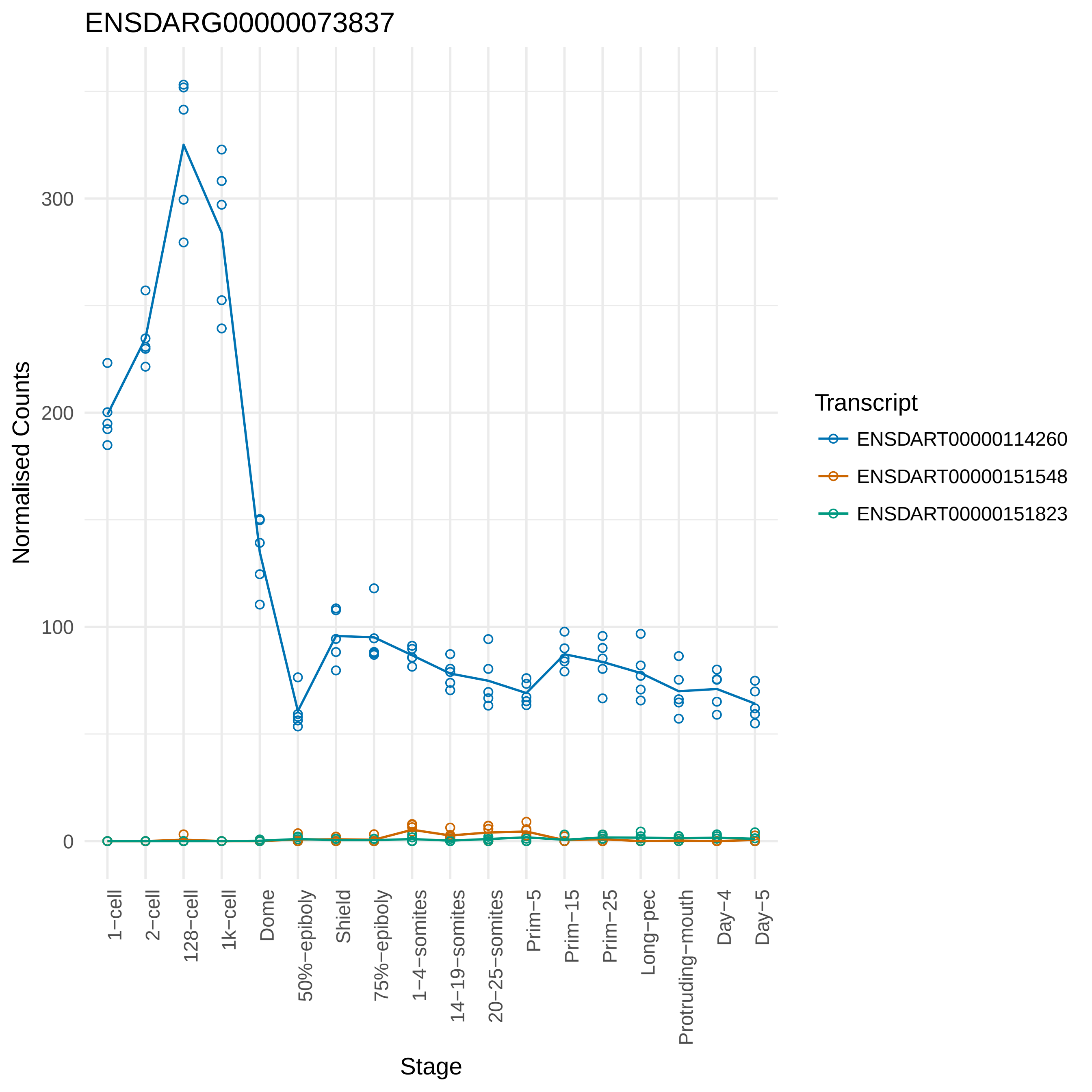

ENSDARG00000073837
Normalised Counts
300
200
100
0
1−cell
2−cell
128−cell
1k−cell
Dome
50%−epiboly
Shield
75%−epiboly
1−4−somites
14−19−somites
20−25−somites
Prim−5
Prim−15
Prim−25
Long−pec
Protruding−mouth
Day−4
Day−5
Stage
Transcript
ENSDART00000114260
ENSDART00000151548
ENSDART00000151823

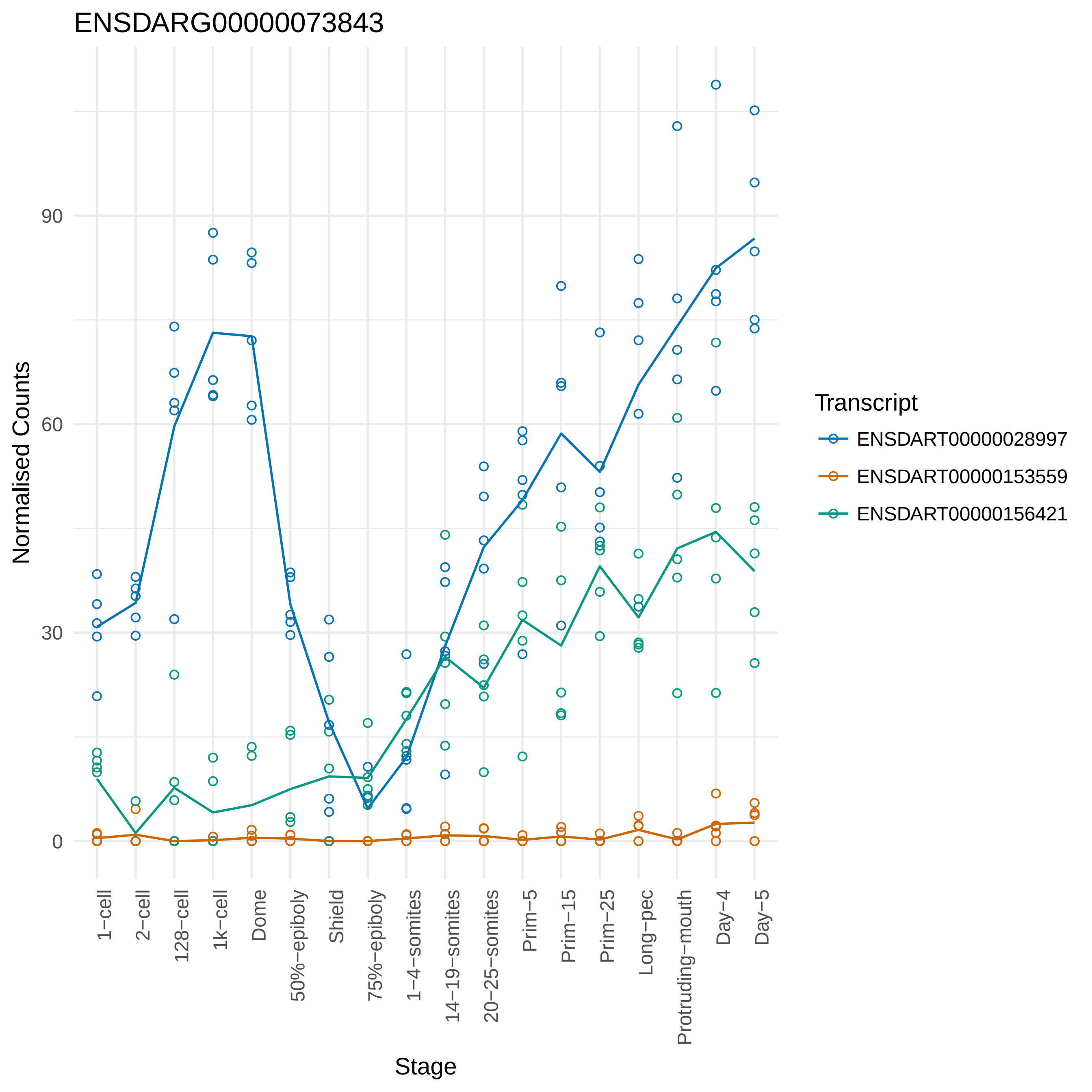

ENSDARG00000073843
Normalised Counts
0
30
60
90
1−cell
2−cell
128−cell
1k−cell
Dome
50%−epiboly
Shield
75%−epiboly
1−4−somites
14−19−somites
20−25−somites
Prim−5
Prim−15
Prim−25
Long−pec
Protruding−mouth
Day−4
Day−5
Stage
Transcript
ENSDART00000028997
ENSDART00000153559
ENSDART00000156421

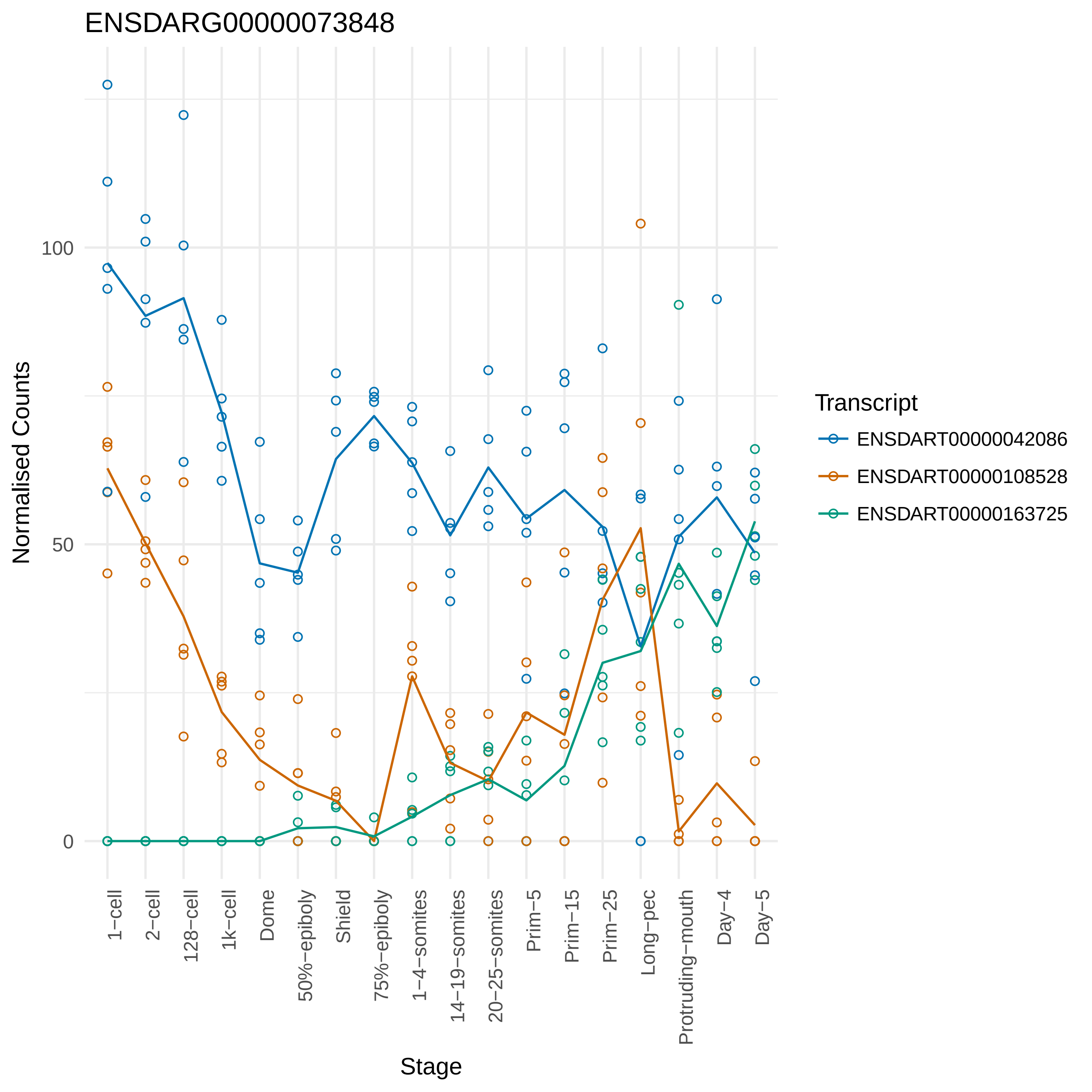
ENSDARG00000073848
Normalised Counts
100
50
0
1−cell
2−cell
128−cell
1k−cell
Dome
50%−epiboly
Shield
75%−epiboly
1−4−somites
14−19−somites
20−25−somites
Prim−5
Prim−15
Prim−25
Long−pec
Protruding−mouth
Day−4
Day−5
Stage
Transcript
ENSDART00000042086
ENSDART00000108528
ENSDART00000163725

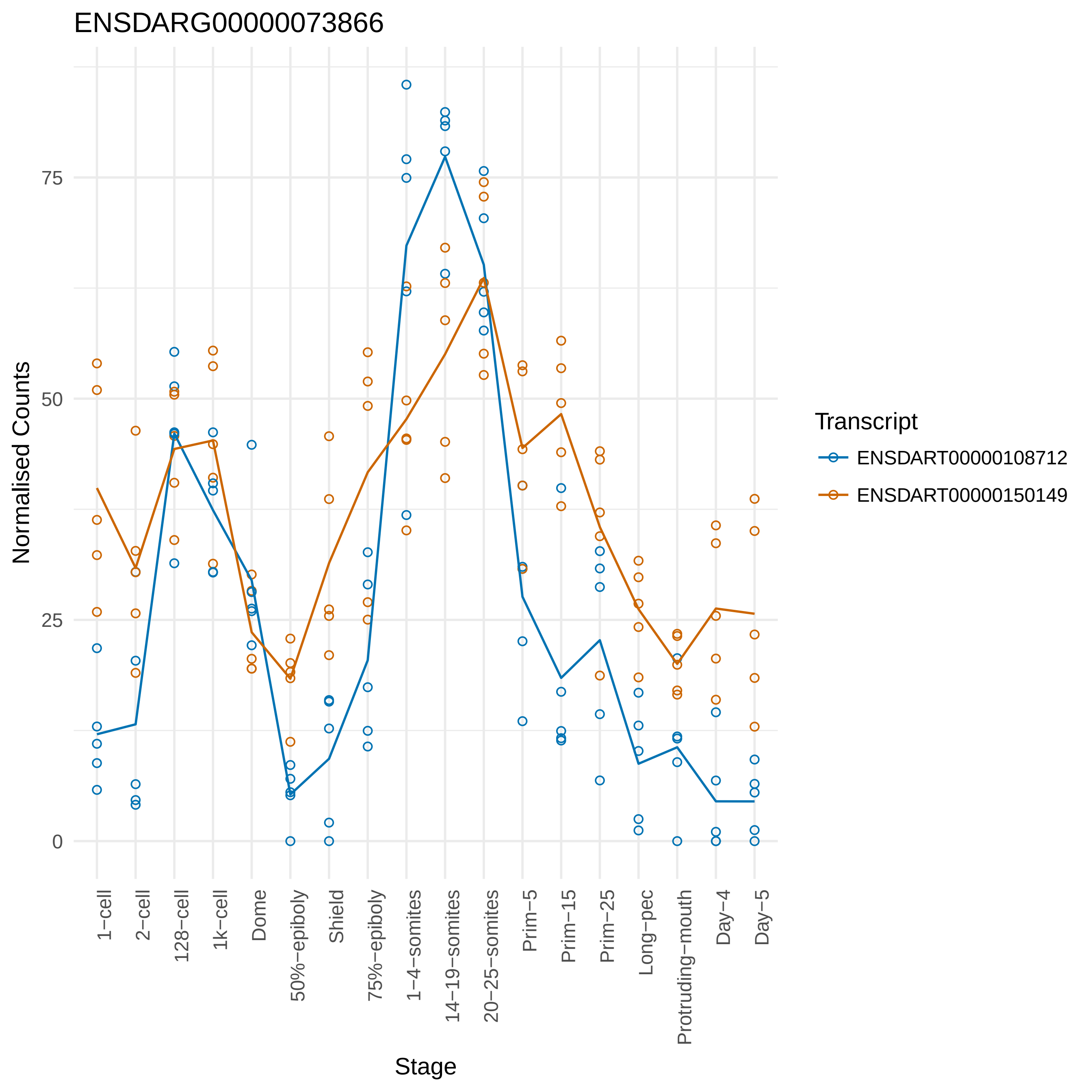
ENSDARG00000073866
Normalised Counts
0
25
50
75
1−cell
2−cell
128−cell
1k−cell
Dome
50%−epiboly
Shield
75%−epiboly
1−4−somites
14−19−somites
20−25−somites
Prim−5
Prim−15
Prim−25
Long−pec
Protruding−mouth
Day−4
Day−5
Stage
Transcript
ENSDART00000108712
ENSDART00000150149

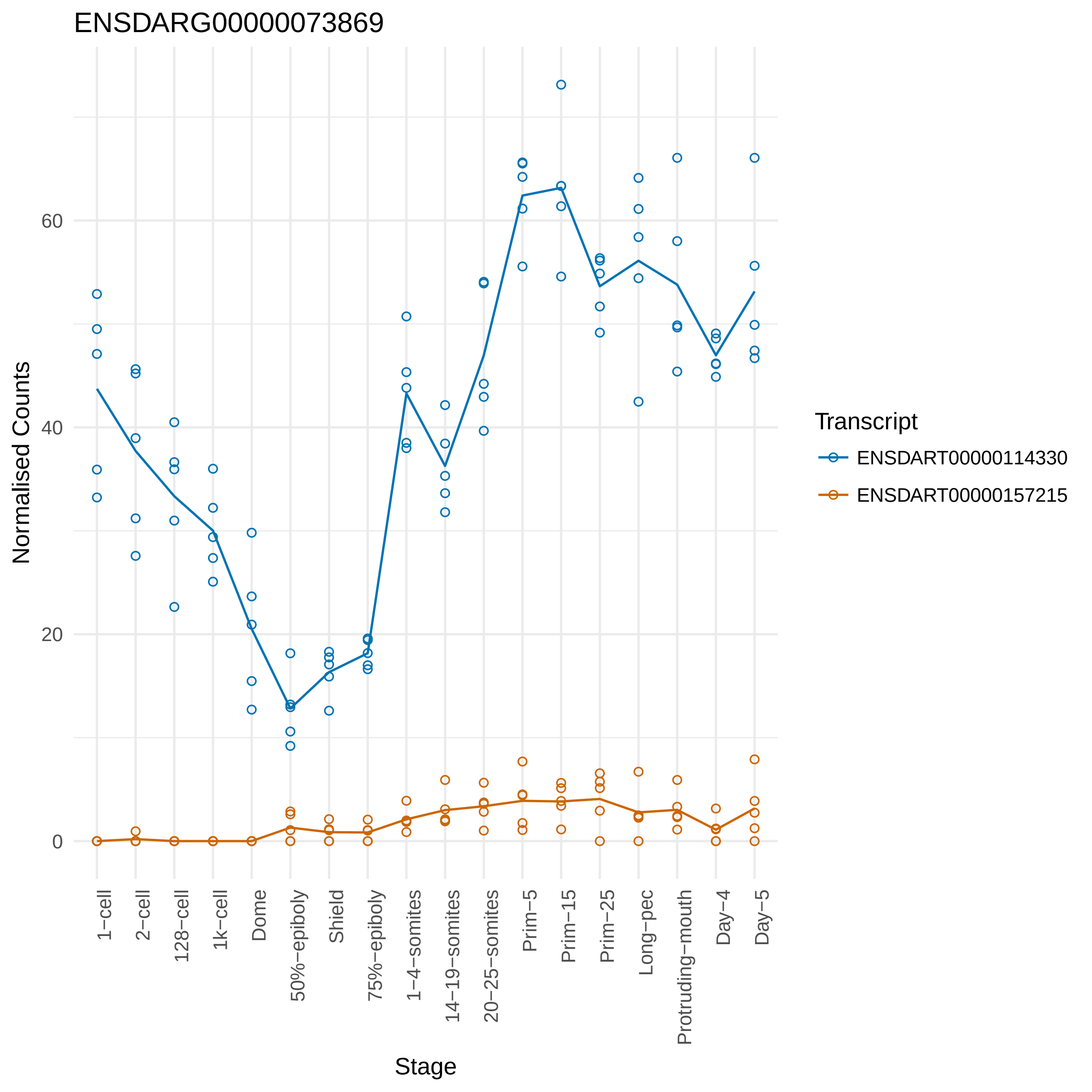
ENSDARG00000073869
Normalised Counts
60
40
20
0
1−cell
2−cell
128−cell
1k−cell
Dome
50%−epiboly
Shield
75%−epiboly
1−4−somites
14−19−somites
20−25−somites
Prim−5
Prim−15
Prim−25
Long−pec
Protruding−mouth
Day−4
Day−5
Stage
Transcript
ENSDART00000114330
ENSDART00000157215

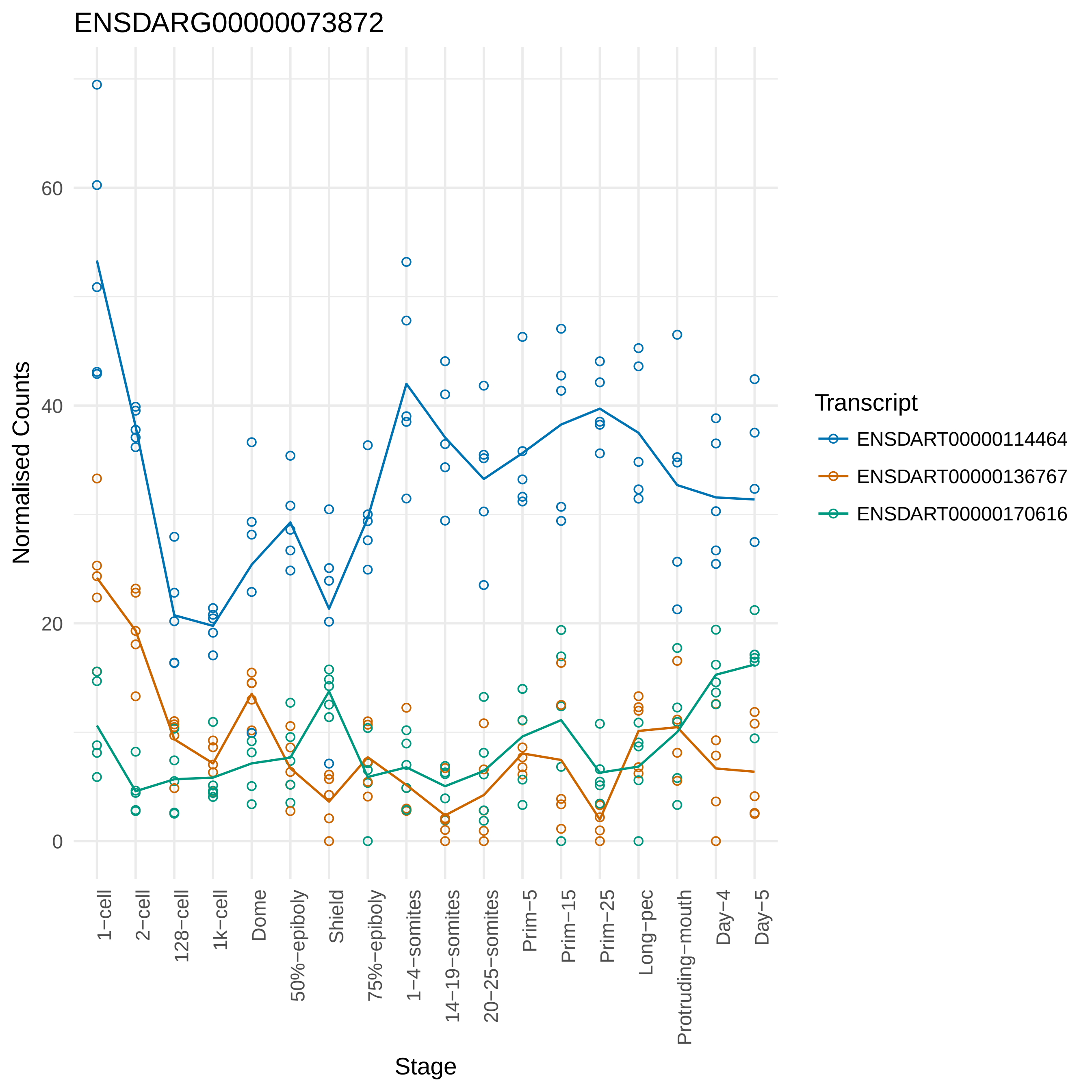

ENSDARG00000073872
Normalised Counts
0
20
40
60
Stage
1−cell
2−cell
128−cell
1k−cell
Dome
50%−epiboly
Shield
75%−epiboly
1−4−somites
14−19−somites
20−25−somites
Prim−5
Prim−15
Prim−25
Long−pec
Protruding−mouth
Day−4
Day−5
Transcript
ENSDART00000114464
ENSDART00000136767
ENSDART00000170616

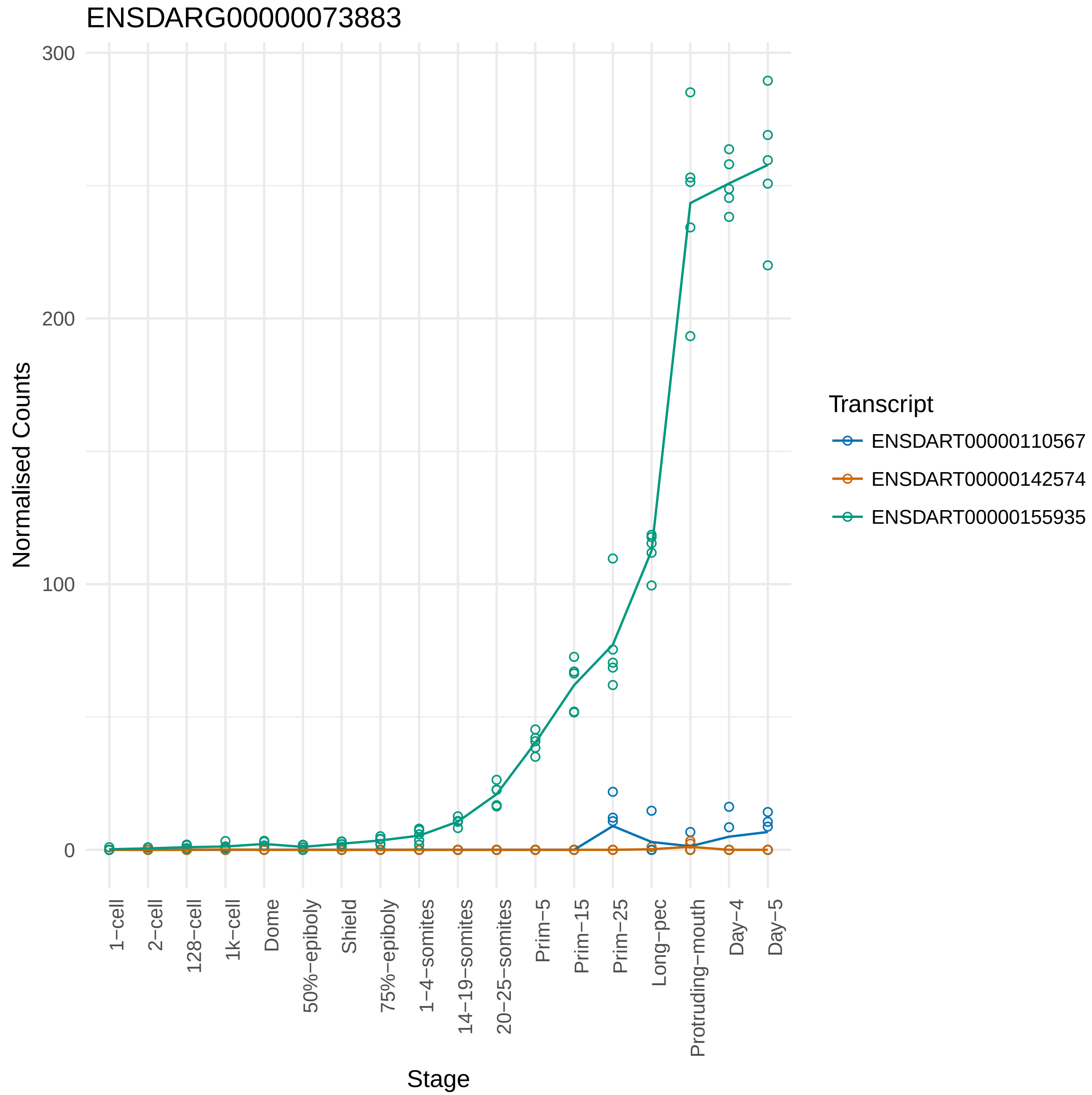

ENSDARG00000073883
Normalised Counts
300
200
100
0
1−cell
2−cell
128−cell
1k−cell
Dome
50%−epiboly
Shield
75%−epiboly
1−4−somites
14−19−somites
20−25−somites
Prim−5
Prim−15
Prim−25
Long−pec
Protruding−mouth
Day−4
Day−5
Stage
Transcript
ENSDART00000110567
ENSDART00000142574
ENSDART00000155935

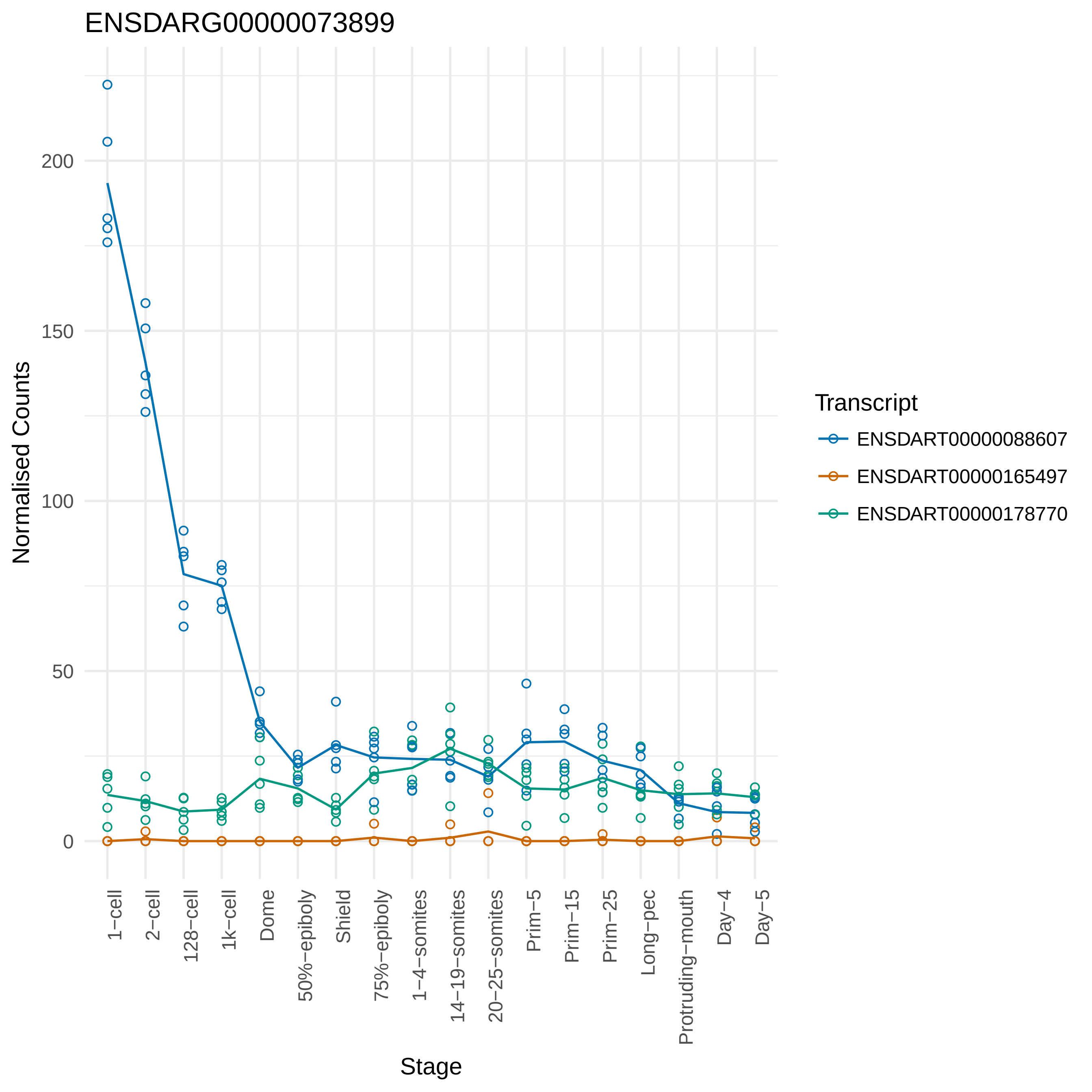

ENSDARG00000073899
Normalised Counts
200
150
100
50
0
1−cell
2−cell
128−cell
1k−cell
Dome
50%−epiboly
Shield
75%−epiboly
1−4−somites
14−19−somites
20−25−somites
Prim−5
Prim−15
Prim−25
Long−pec
Protruding−mouth
Day−4
Day−5
Stage
Transcript
ENSDART00000088607
ENSDART00000165497
ENSDART00000178770

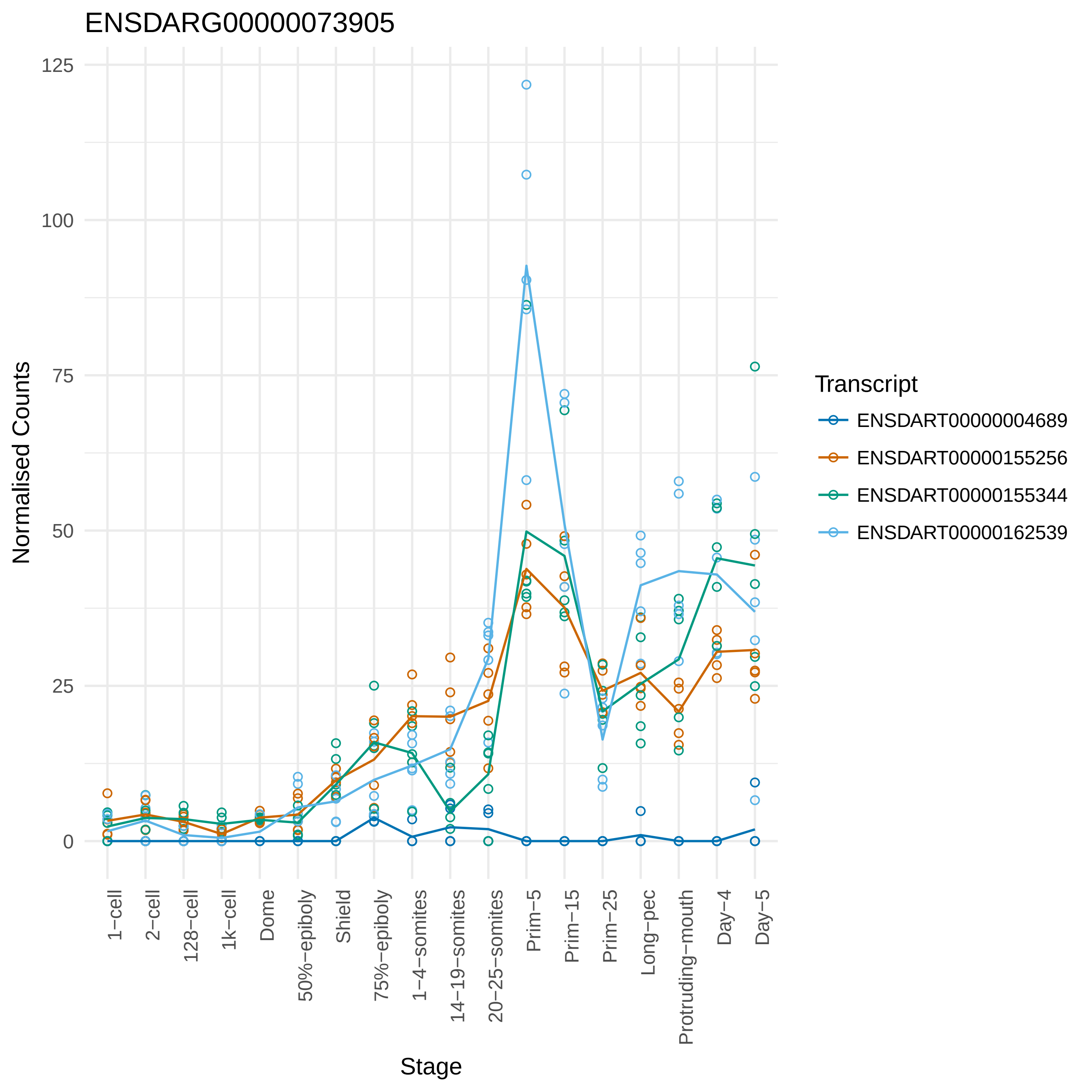

ENSDARG00000073905
Normalised Counts
125
100
75
50
25
0
1−cell
2−cell
128−cell
1k−cell
Dome
50%−epiboly
Shield
75%−epiboly
1−4−somites
14−19−somites
20−25−somites
Prim−5
Prim−15
Prim−25
Long−pec
Protruding−mouth
Day−4
Day−5
Stage
Transcript
ENSDART00000004689
ENSDART00000155256
ENSDART00000155344
ENSDART00000162539

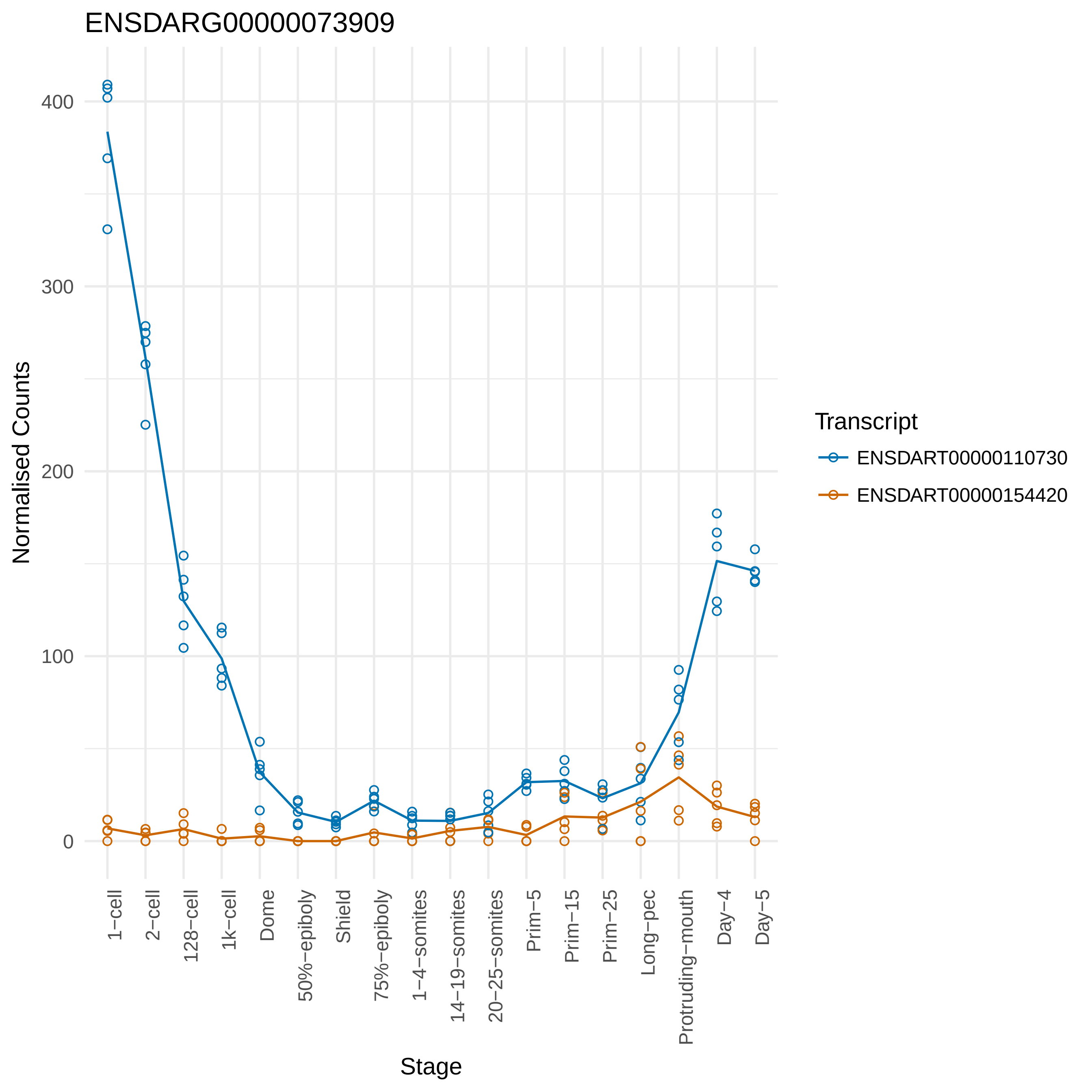

ENSDARG00000073909
Normalised Counts
0
100
200
300
400
Stage
1−cell
2−cell
128−cell
1k−cell
Dome
50%−epiboly
Shield
75%−epiboly
1−4−somites
14−19−somites
20−25−somites
Prim−5
Prim−15
Prim−25
Long−pec
Protruding−mouth
Day−4
Day−5
Transcript
ENSDART00000110730
ENSDART00000154420

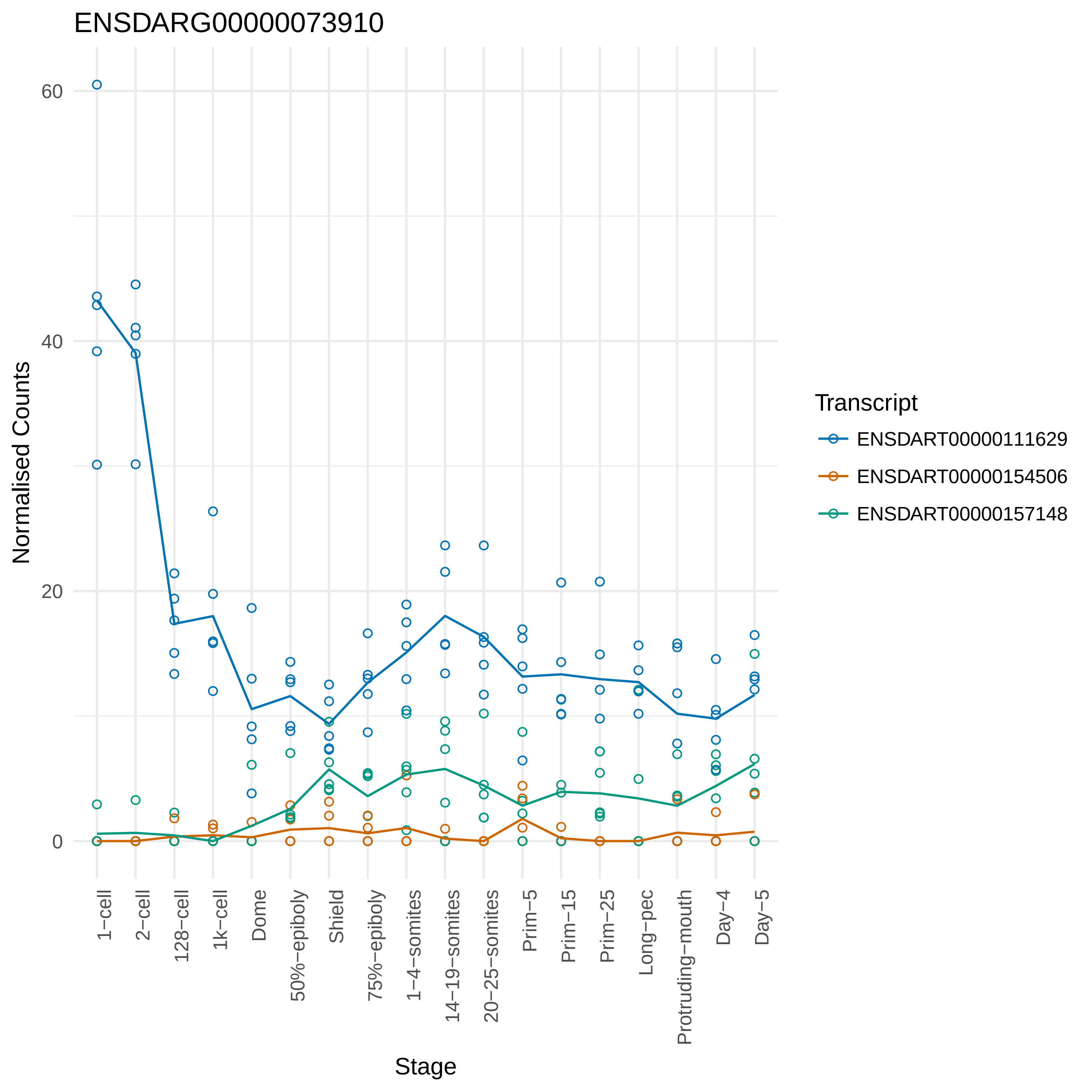

ENSDARG00000073910
Normalised Counts
60
40
20
0
1−cell
2−cell
128−cell
1k−cell
Dome
50%−epiboly
Shield
75%−epiboly
1−4−somites
14−19−somites
20−25−somites
Prim−5
Prim−15
Prim−25
Long−pec
Protruding−mouth
Day−4
Day−5
Stage
Transcript
ENSDART00000111629
ENSDART00000154506
ENSDART00000157148

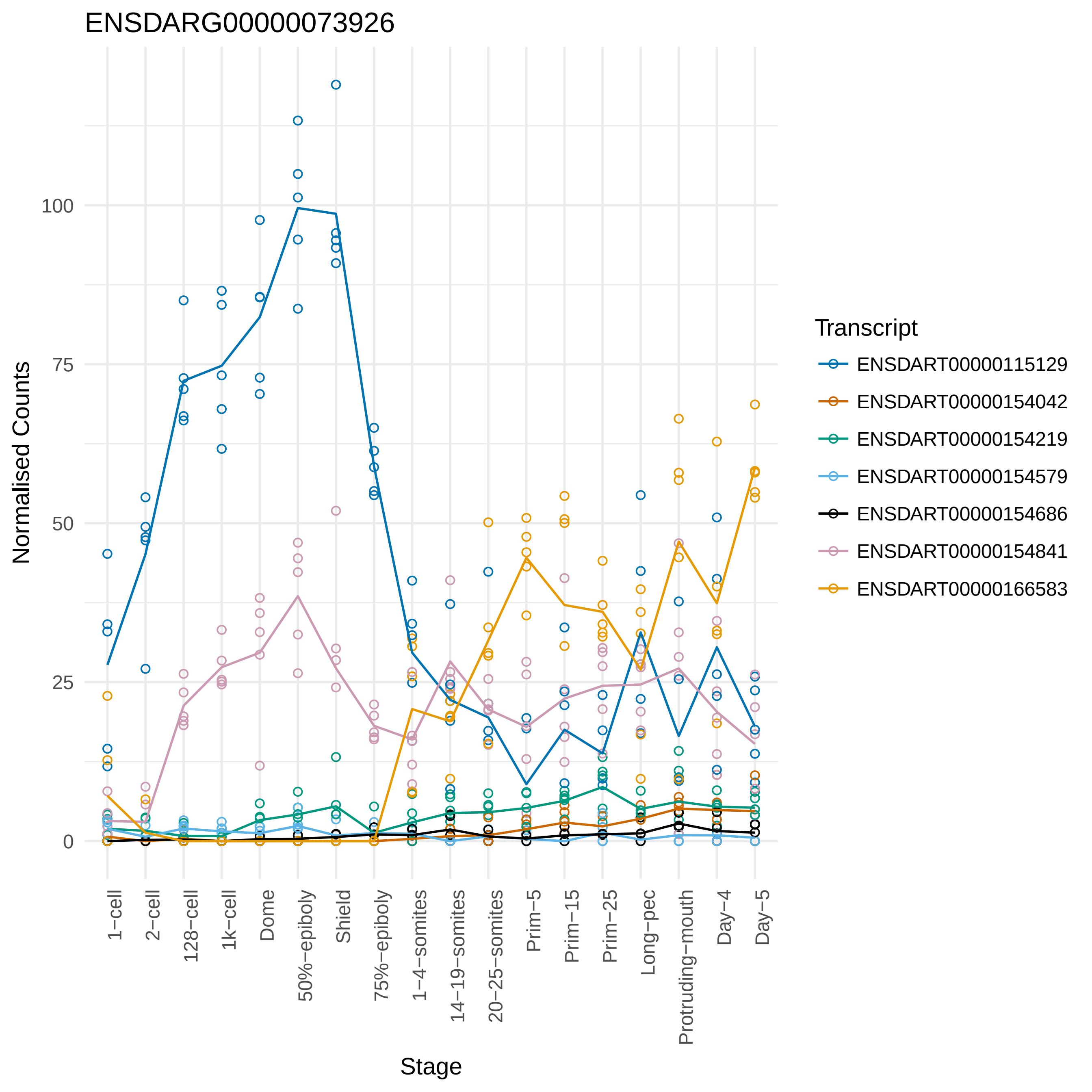
ENSDARG00000073926
Normalised Counts
0
25
50
75
100
1−cell
2−cell
128−cell
1k−cell
Dome
50%−epiboly
Shield
75%−epiboly
1−4−somites
14−19−somites
20−25−somites
Prim−5
Prim−15
Prim−25
Long−pec
Protruding−mouth
Day−4
Day−5
Stage
Transcript
ENSDART00000115129
ENSDART00000154042
ENSDART00000154219
ENSDART00000154579
ENSDART00000154686
ENSDART00000154841
ENSDART00000166583

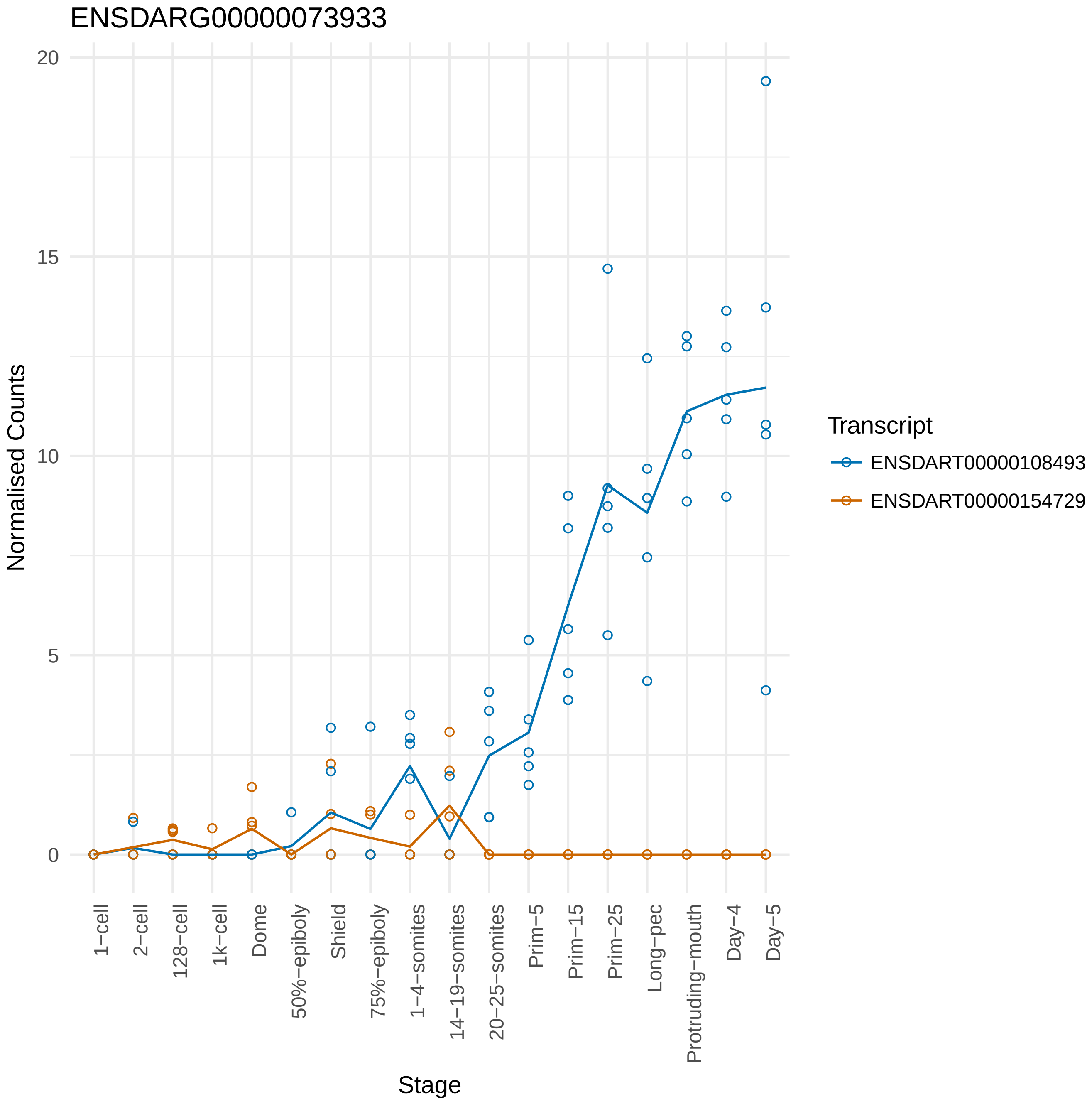

ENSDARG00000073933
Normalised Counts
0
5
10
15
20
1−cell
2−cell
128−cell
1k−cell
Dome
50%−epiboly
Shield
75%−epiboly
1−4−somites
14−19−somites
20−25−somites
Prim−5
Prim−15
Prim−25
Long−pec
Protruding−mouth
Day−4
Day−5
Stage
Transcript
ENSDART00000108493
ENSDART00000154729

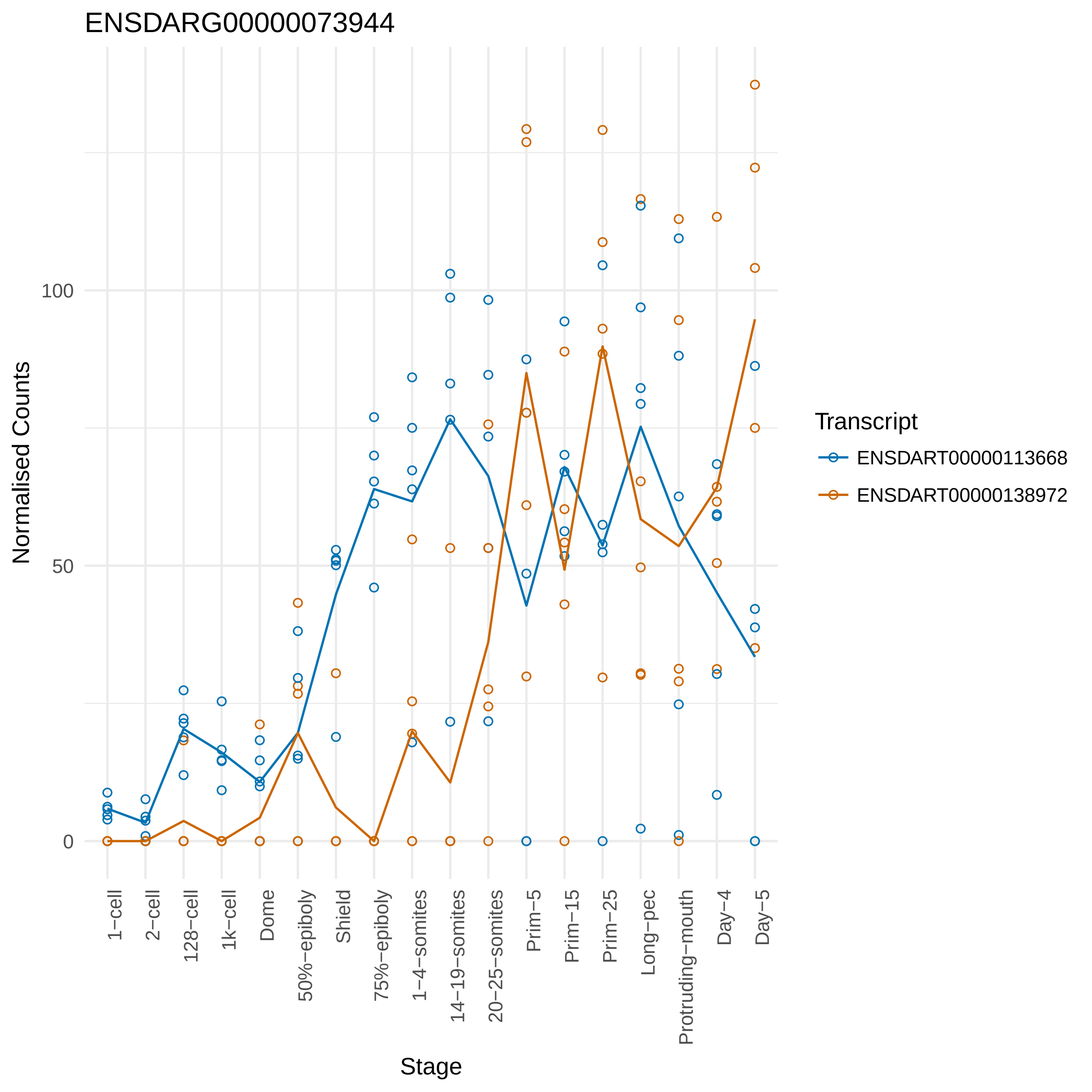
ENSDARG00000073944
Normalised Counts
100
50
0
Stage
1−cell
2−cell
128−cell
1k−cell
Dome
50%−epiboly
Shield
75%−epiboly
1−4−somites
14−19−somites
20−25−somites
Prim−5
Prim−15
Prim−25
Long−pec
Protruding−mouth
Day−4
Day−5
Transcript
ENSDART00000113668
ENSDART00000138972

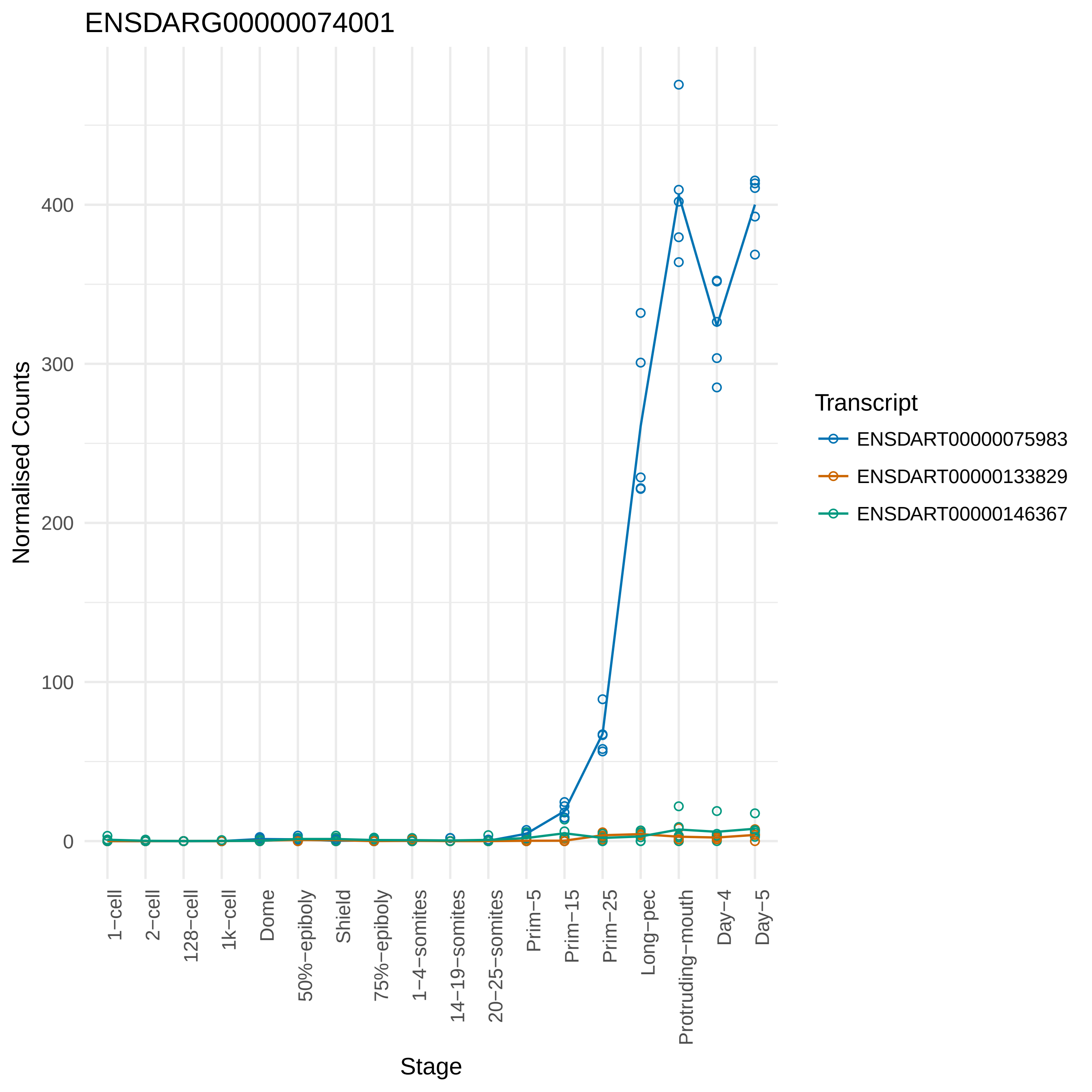
ENSDARG00000074001
Normalised Counts
400
300
200
100
0
1−cell
2−cell
128−cell
1k−cell
Dome
50%−epiboly
Shield
75%−epiboly
1−4−somites
14−19−somites
20−25−somites
Prim−5
Prim−15
Prim−25
Long−pec
Protruding−mouth
Day−4
Day−5
Stage
Transcript
ENSDART00000075983
ENSDART00000133829
ENSDART00000146367

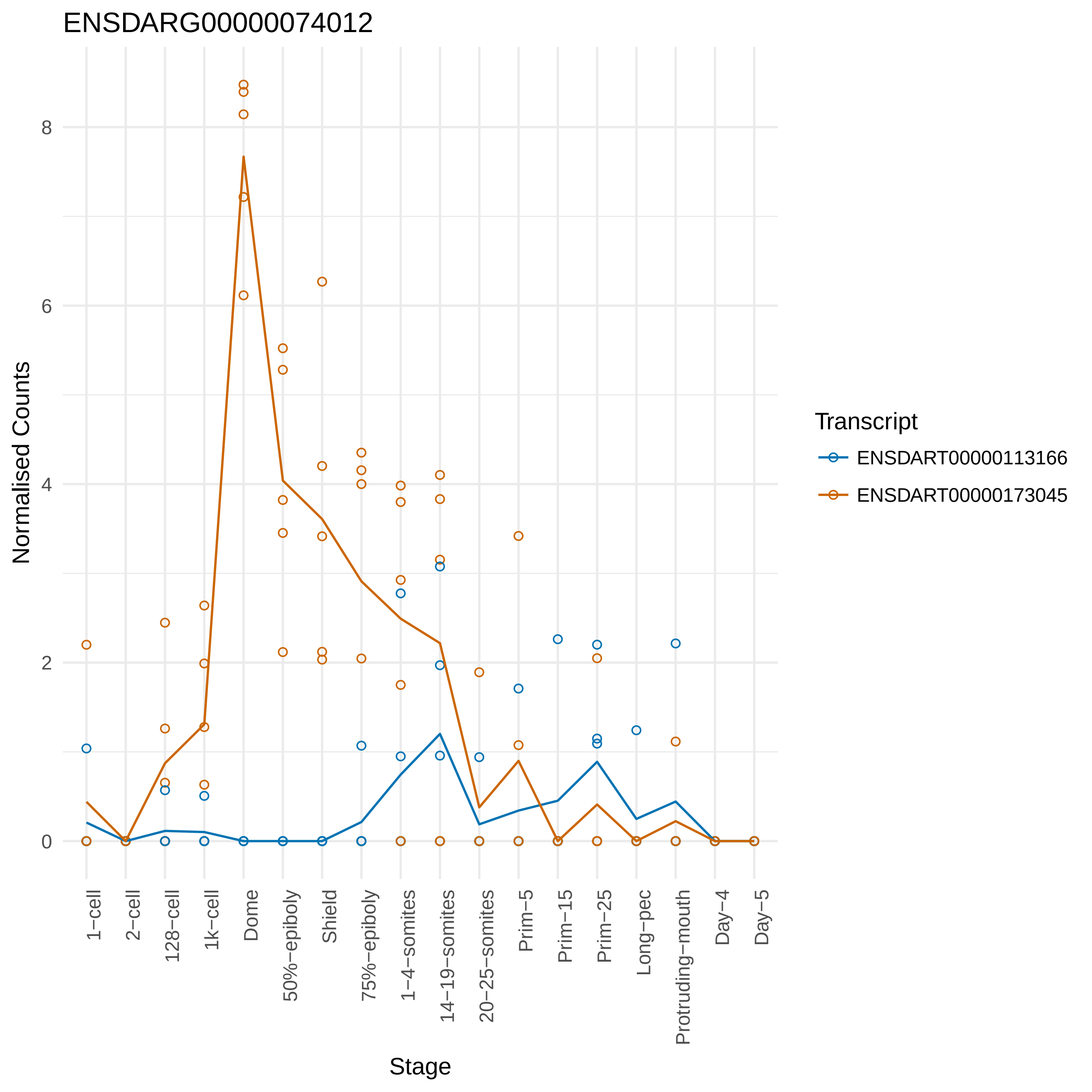

ENSDARG00000074012
Normalised Counts
8
6
4
2
0
Stage
1−cell
2−cell
128−cell
1k−cell
Dome
50%−epiboly
Shield
75%−epiboly
1−4−somites
14−19−somites
20−25−somites
Prim−5
Prim−15
Prim−25
Long−pec
Protruding−mouth
Day−4
Day−5
Transcript
ENSDART00000113166
ENSDART00000173045

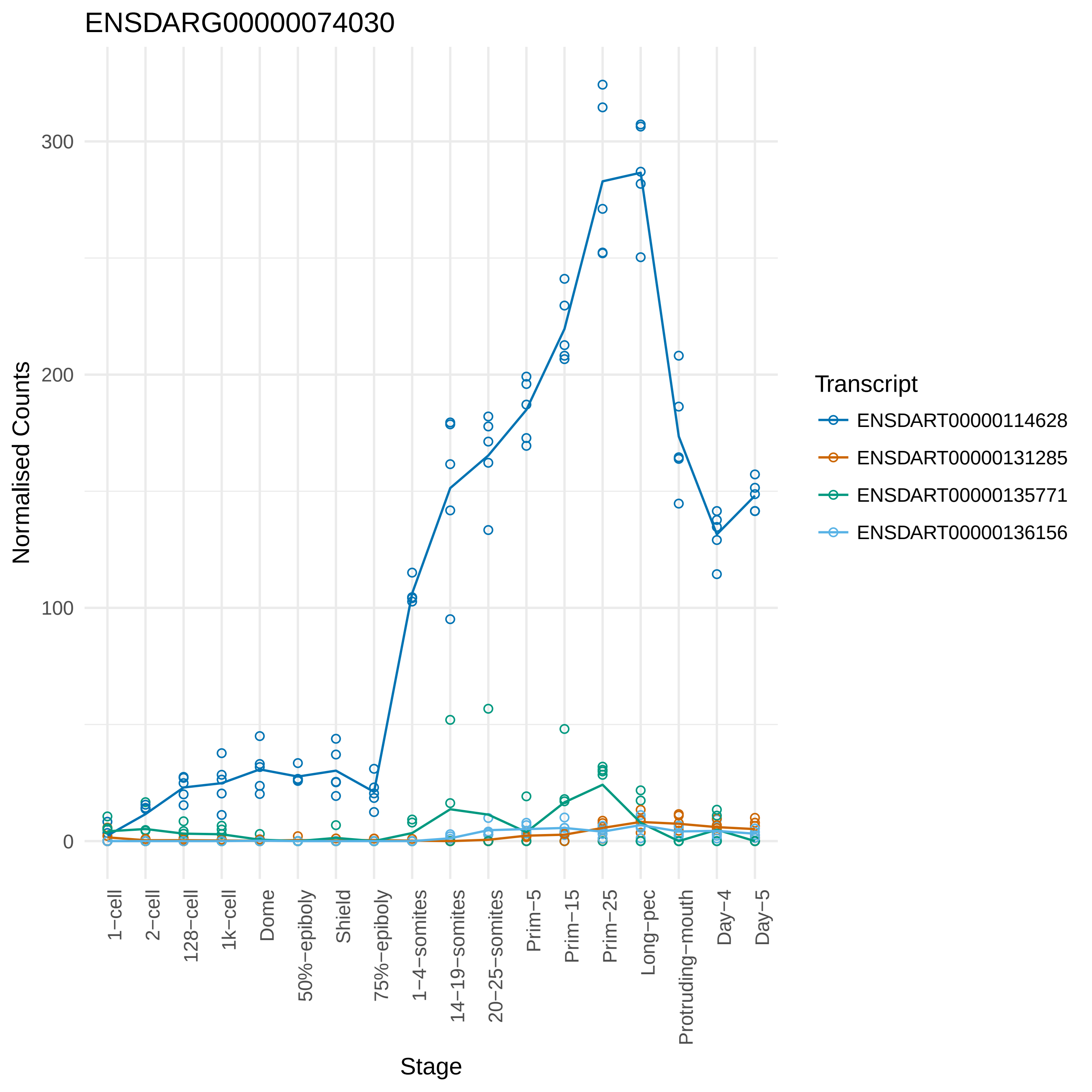
ENSDARG00000074030
Normalised Counts
300
200
100
0
1−cell
2−cell
128−cell
1k−cell
Dome
50%−epiboly
Shield
75%−epiboly
1−4−somites
14−19−somites
20−25−somites
Prim−5
Prim−15
Prim−25
Long−pec
Protruding−mouth
Day−4
Day−5
Stage
Transcript
ENSDART00000114628
ENSDART00000131285
ENSDART00000135771
ENSDART00000136156

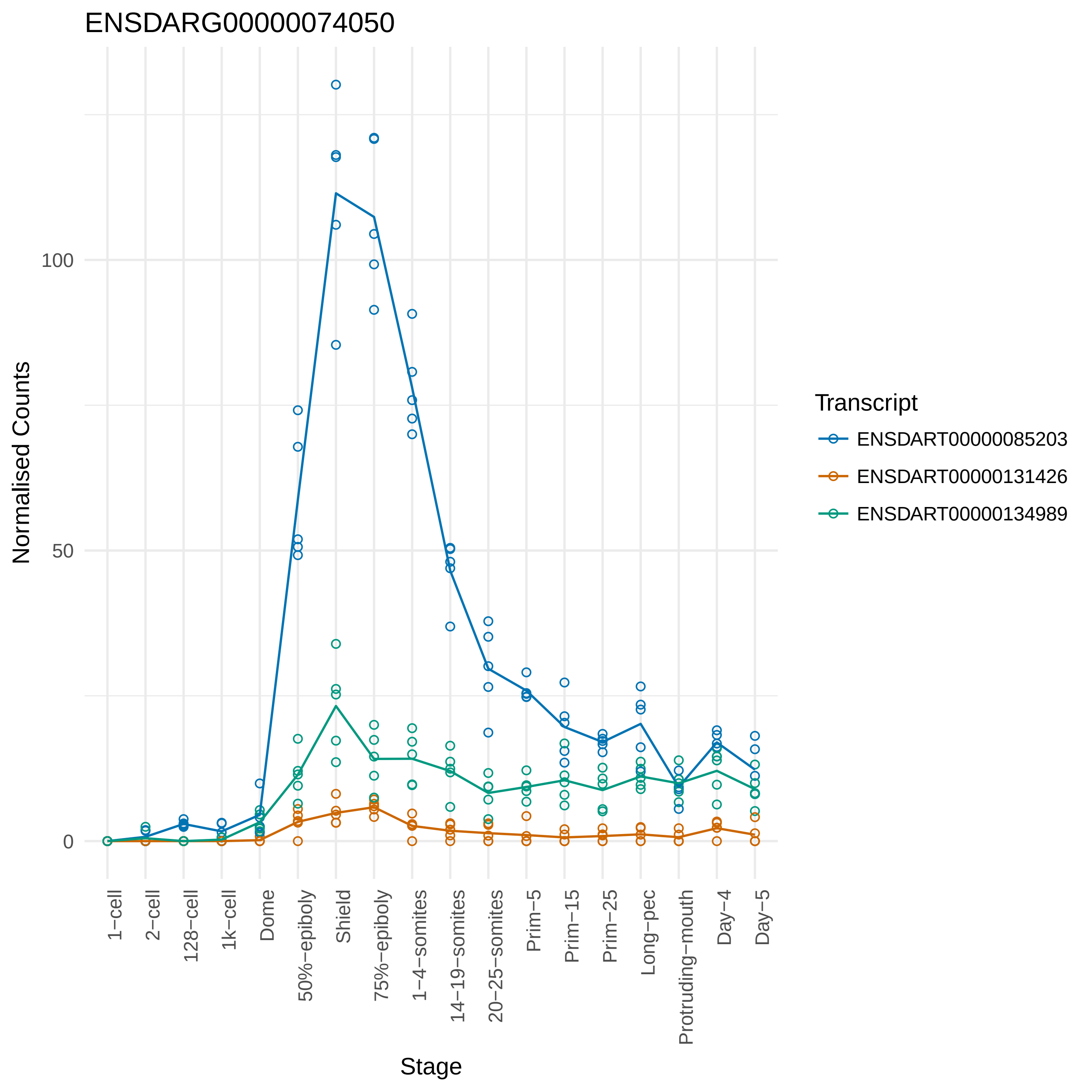

ENSDARG00000074050
Normalised Counts
100
50
0
1−cell
2−cell
128−cell
1k−cell
Dome
50%−epiboly
Shield
75%−epiboly
1−4−somites
14−19−somites
20−25−somites
Prim−5
Prim−15
Prim−25
Long−pec
Protruding−mouth
Day−4
Day−5
Stage
Transcript
ENSDART00000085203
ENSDART00000131426
ENSDART00000134989

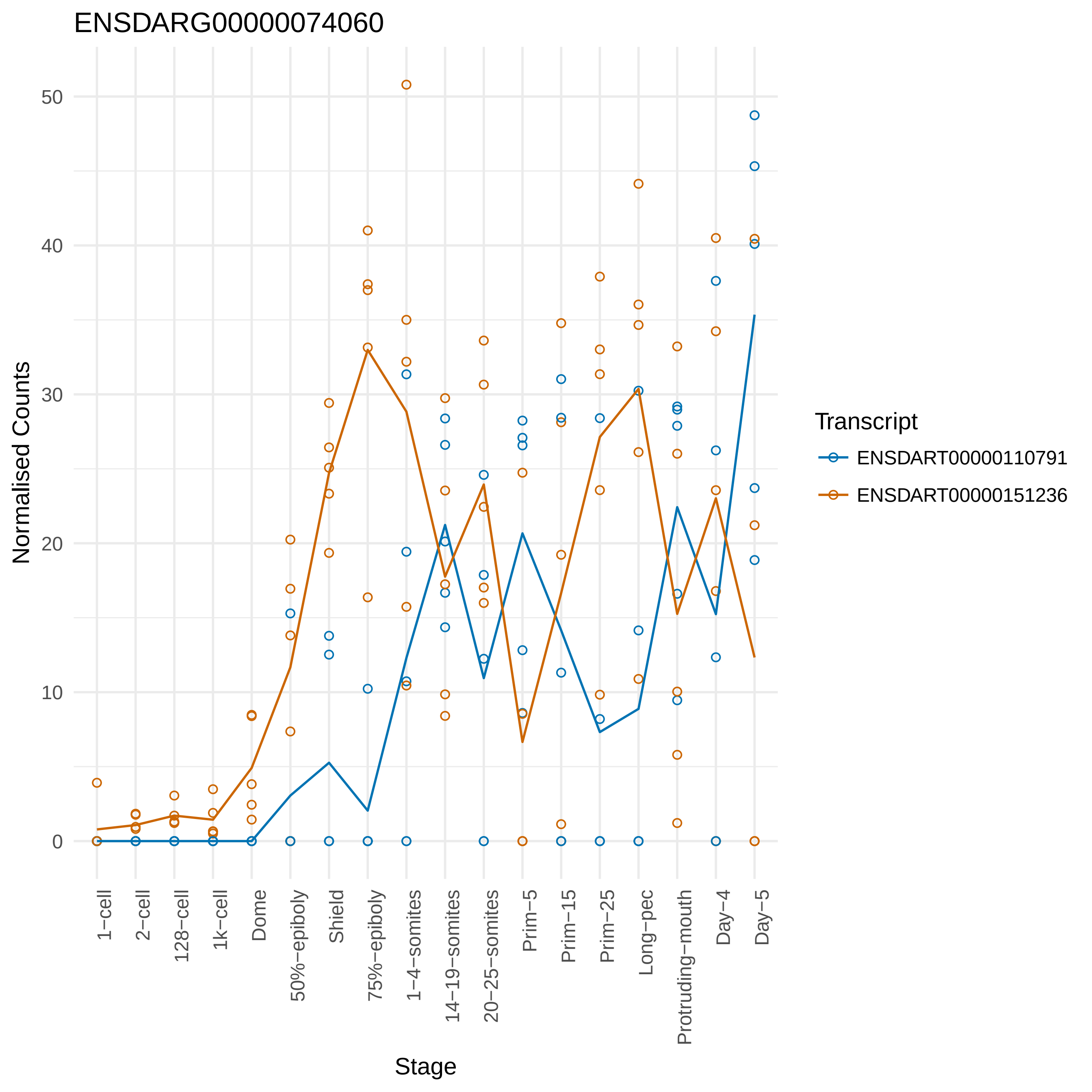
ENSDARG00000074060
Normalised Counts
0
10
20
30
40
50
1−cell
2−cell
128−cell
1k−cell
Dome
50%−epiboly
Shield
75%−epiboly
1−4−somites
14−19−somites
20−25−somites
Prim−5
Prim−15
Prim−25
Long−pec
Protruding−mouth
Day−4
Day−5
Stage
Transcript
ENSDART00000110791
ENSDART00000151236

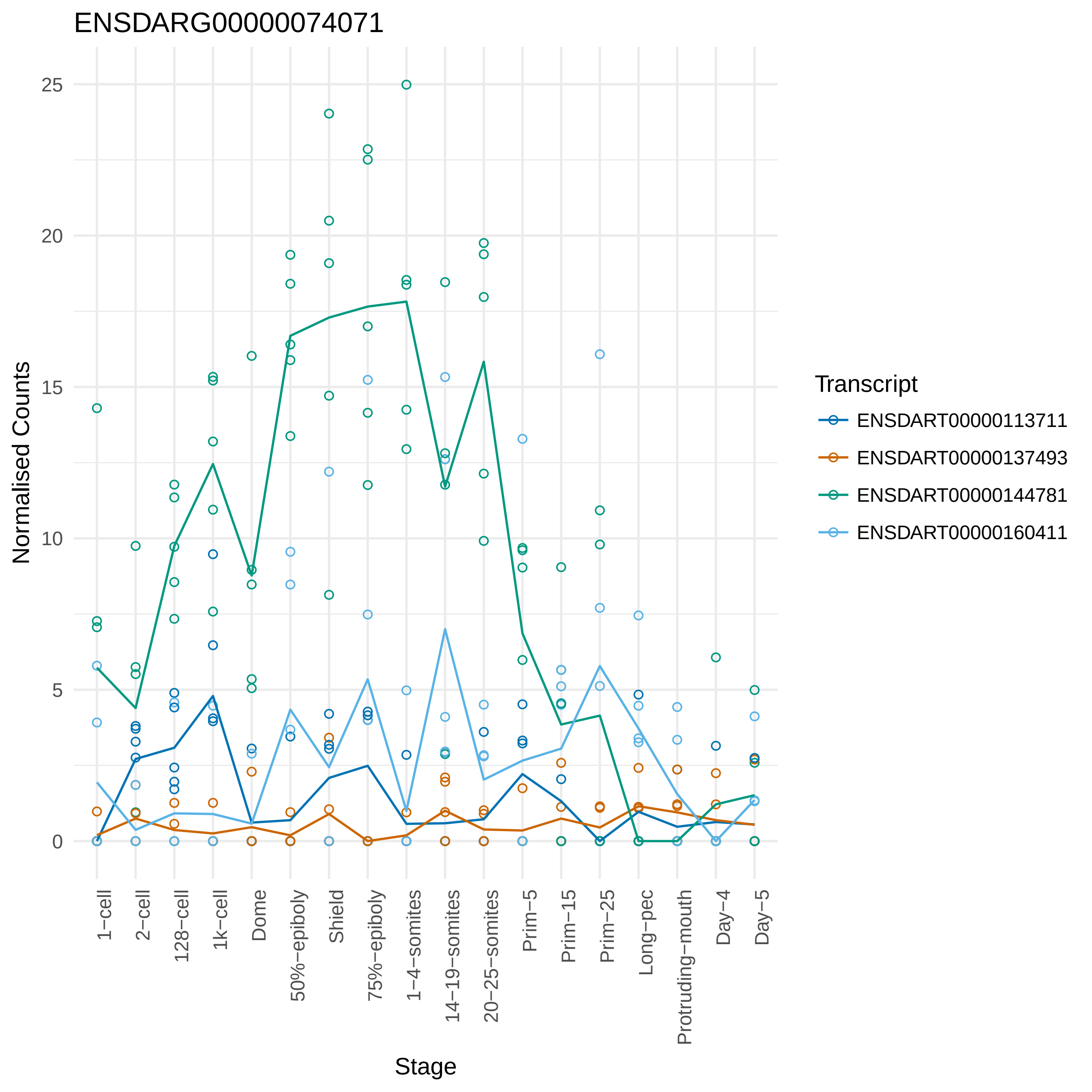

ENSDARG00000074071
Normalised Counts
0
5
10
15
20
25
Stage
1−cell
2−cell
128−cell
1k−cell
Dome
50%−epiboly
Shield
75%−epiboly
1−4−somites
14−19−somites
20−25−somites
Prim−5
Prim−15
Prim−25
Long−pec
Protruding−mouth
Day−4
Day−5
Transcript
ENSDART00000113711
ENSDART00000137493
ENSDART00000144781
ENSDART00000160411

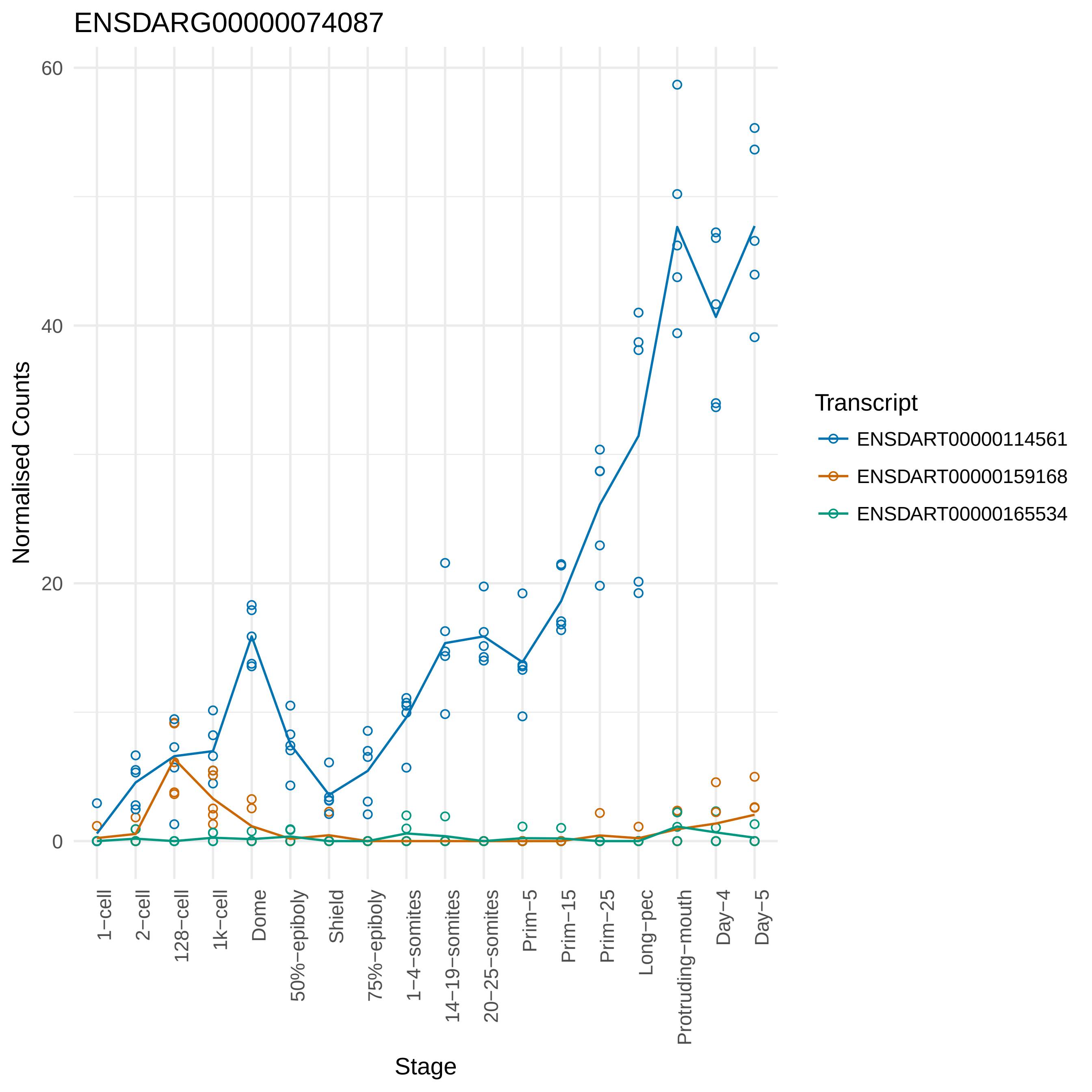
ENSDARG00000074087
Normalised Counts
60
40
20
0
1−cell
2−cell
128−cell
1k−cell
Dome
50%−epiboly
Shield
75%−epiboly
1−4−somites
14−19−somites
20−25−somites
Prim−5
Prim−15
Prim−25
Long−pec
Protruding−mouth
Day−4
Day−5
Stage
Transcript
ENSDART00000114561
ENSDART00000159168
ENSDART00000165534

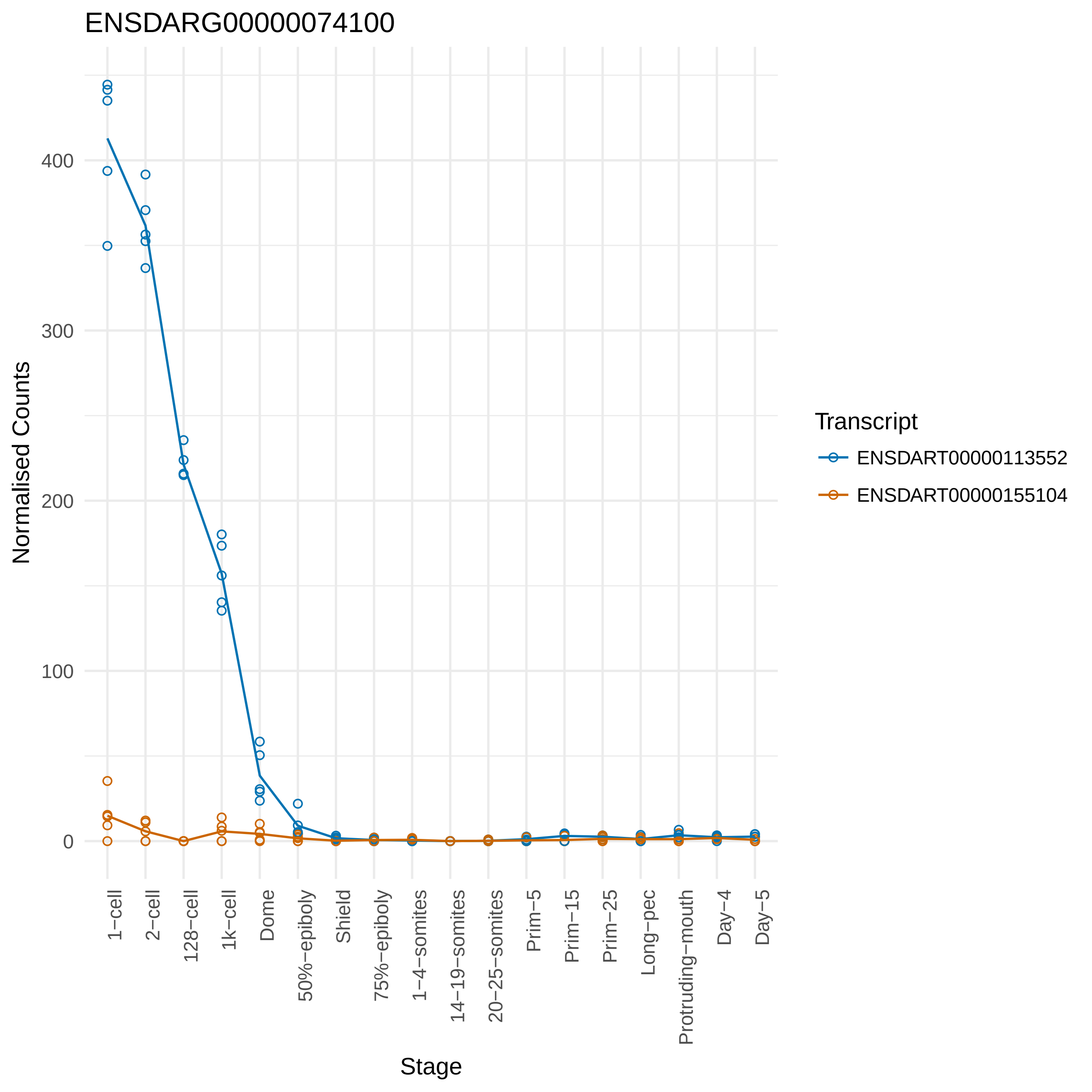
ENSDARG00000074100
Normalised Counts
0
100
200
300
400
Stage
1−cell
2−cell
128−cell
1k−cell
Dome
50%−epiboly
Shield
75%−epiboly
1−4−somites
14−19−somites
20−25−somites
Prim−5
Prim−15
Prim−25
Long−pec
Protruding−mouth
Day−4
Day−5
Transcript
ENSDART00000113552
ENSDART00000155104

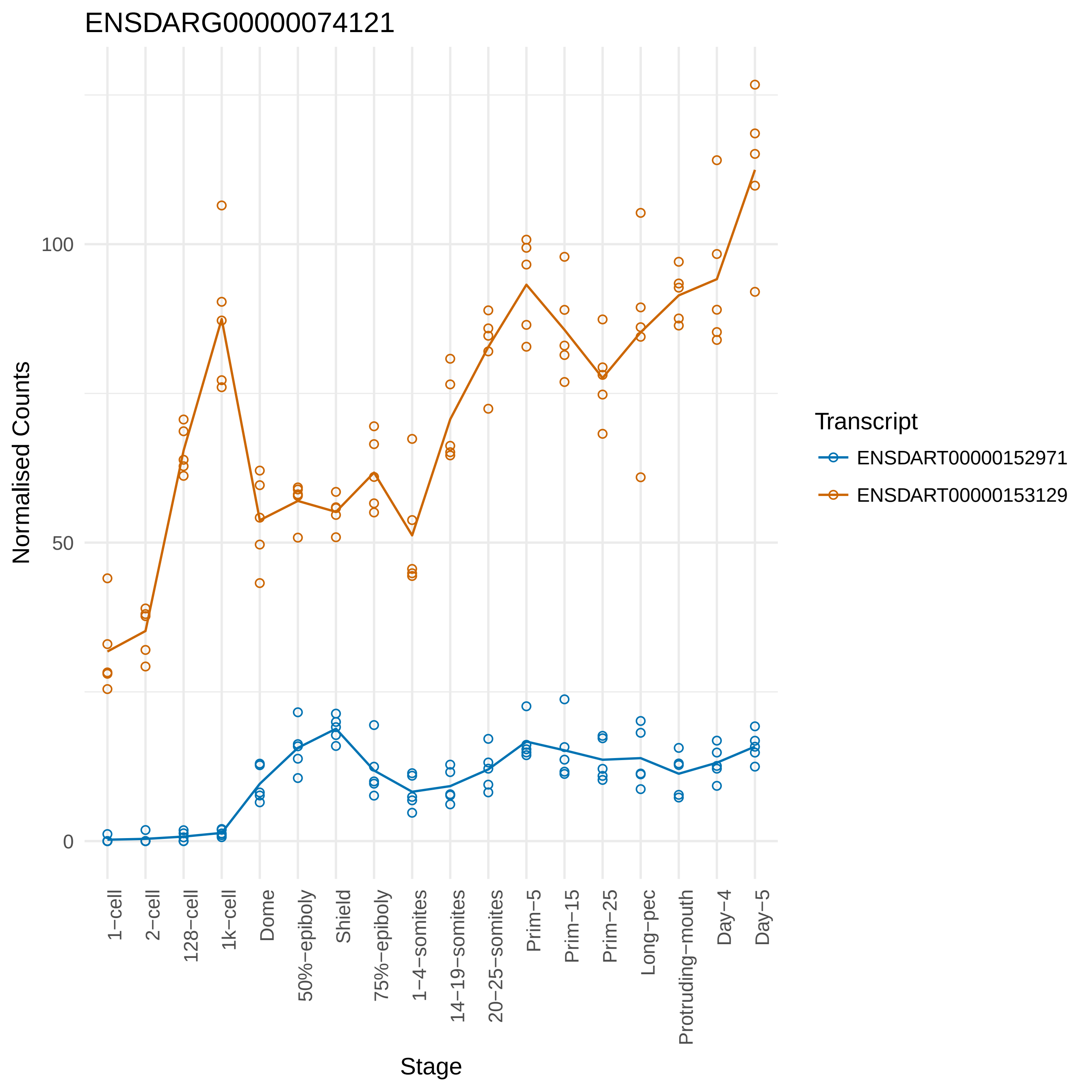

ENSDARG00000074121
Normalised Counts
100
50
0
1−cell
2−cell
128−cell
1k−cell
Dome
50%−epiboly
Shield
75%−epiboly
1−4−somites
14−19−somites
20−25−somites
Prim−5
Prim−15
Prim−25
Long−pec
Protruding−mouth
Day−4
Day−5
Stage
Transcript
ENSDART00000152971
ENSDART00000153129

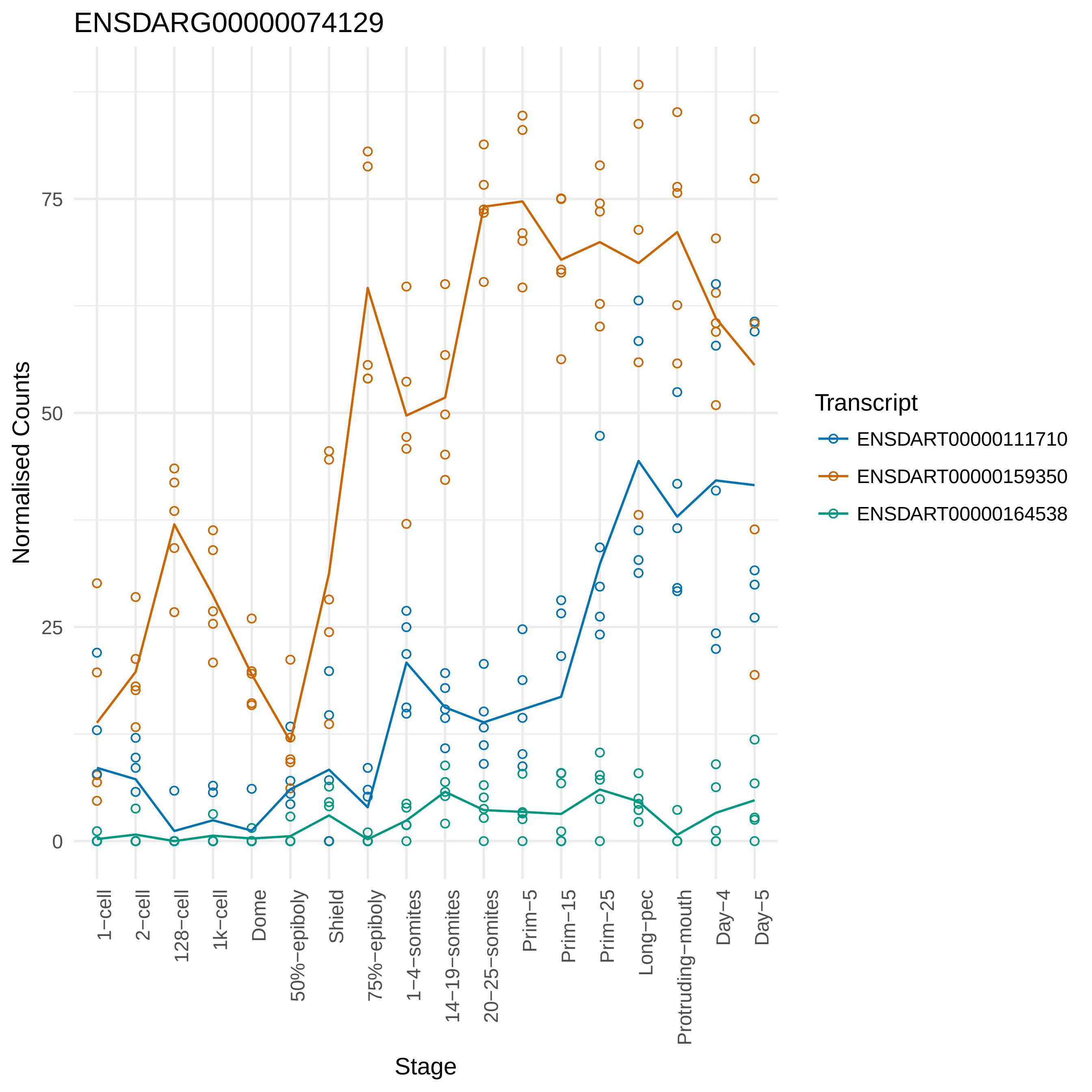
ENSDARG00000074129
Normalised Counts
0
25
50
75
1−cell
2−cell
128−cell
1k−cell
Dome
50%−epiboly
Shield
75%−epiboly
1−4−somites
14−19−somites
20−25−somites
Prim−5
Prim−15
Prim−25
Long−pec
Protruding−mouth
Day−4
Day−5
Stage
Transcript
ENSDART00000111710
ENSDART00000159350
ENSDART00000164538

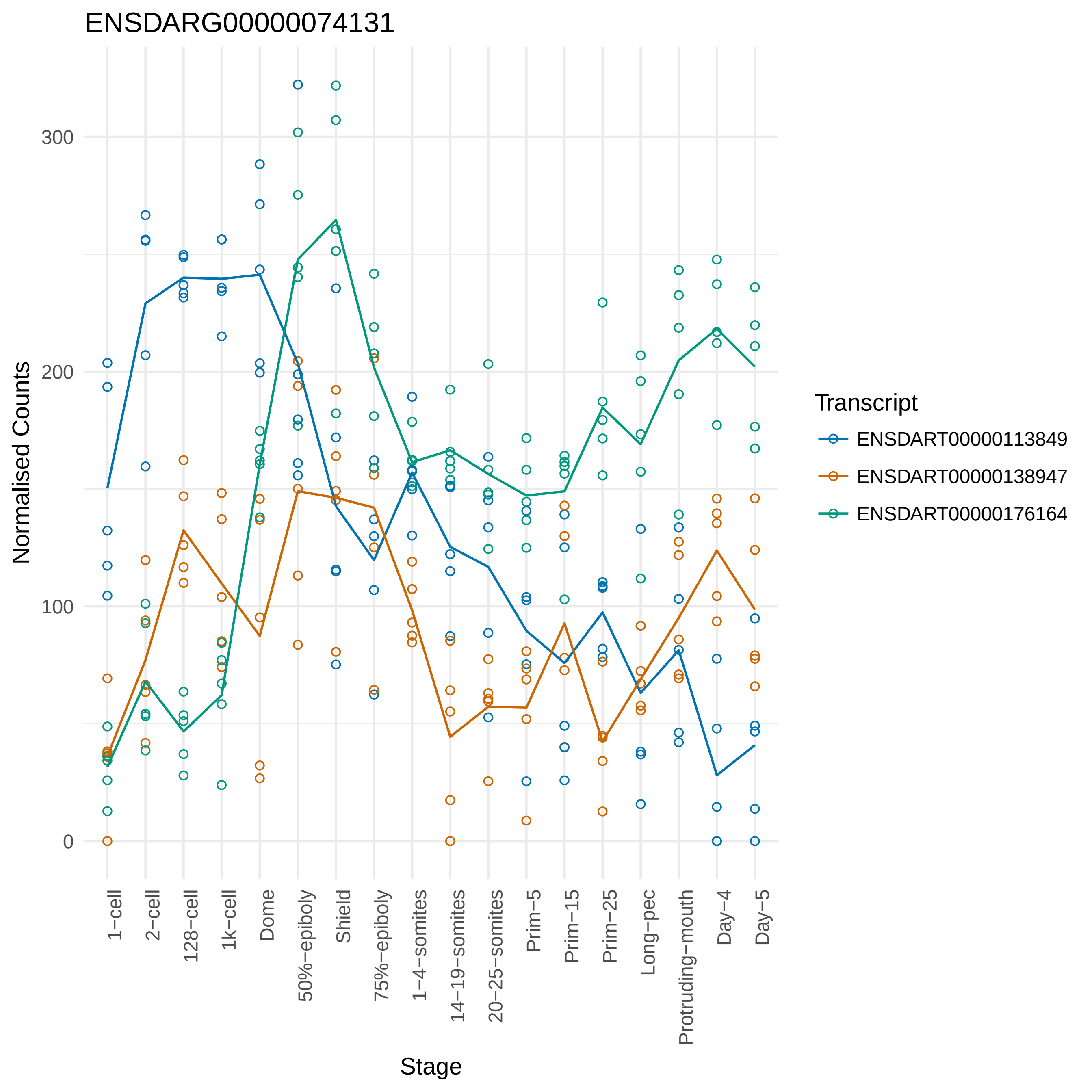

ENSDARG00000074131
Normalised Counts
300
200
100
0
1−cell
2−cell
128−cell
1k−cell
Dome
50%−epiboly
Shield
75%−epiboly
1−4−somites
14−19−somites
20−25−somites
Prim−5
Prim−15
Prim−25
Long−pec
Protruding−mouth
Day−4
Day−5
Stage
Transcript
ENSDART00000113849
ENSDART00000138947
ENSDART00000176164

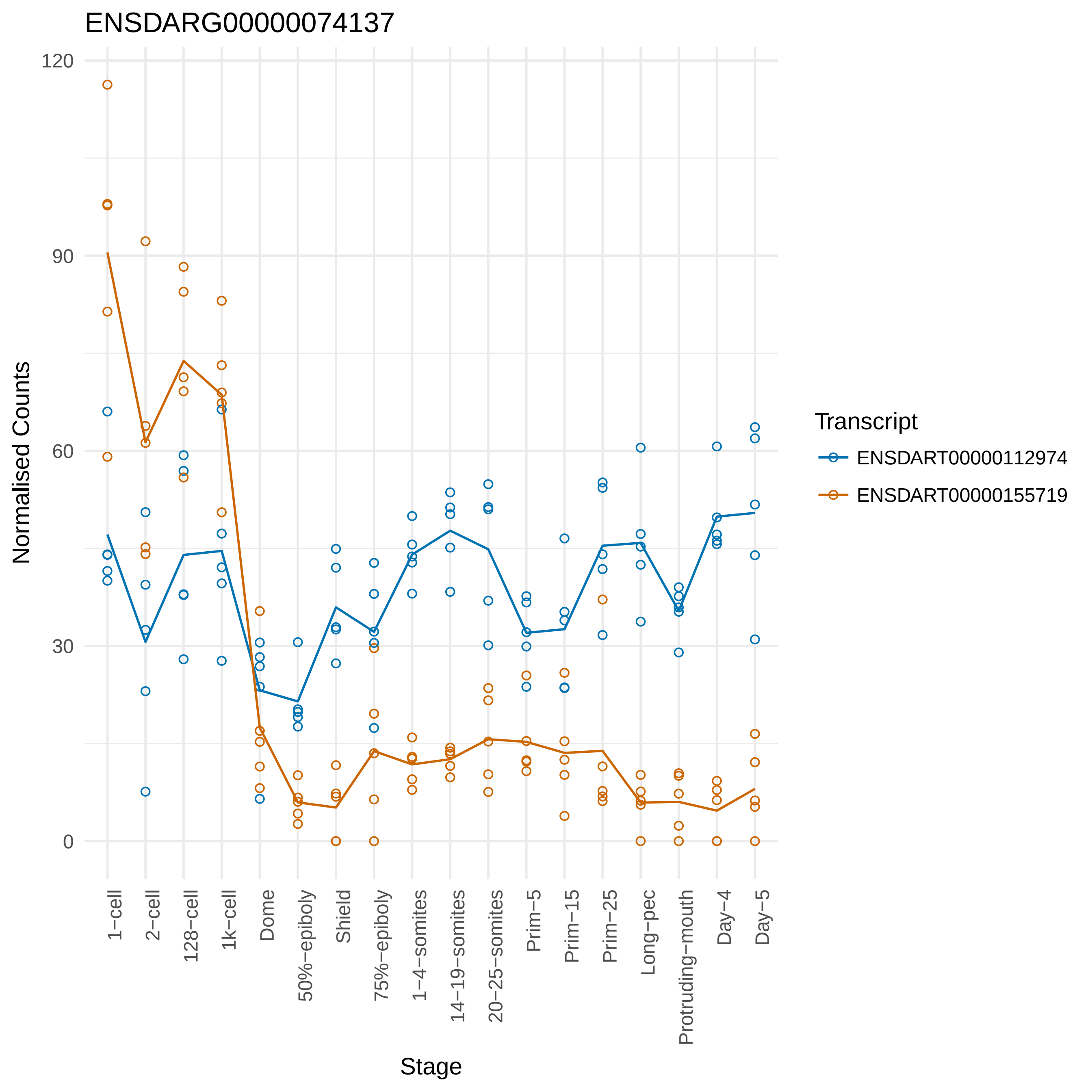

ENSDARG00000074137
Normalised Counts
120
90
60
30
0
1−cell
2−cell
128−cell
1k−cell
Dome
50%−epiboly
Shield
75%−epiboly
1−4−somites
14−19−somites
20−25−somites
Prim−5
Prim−15
Prim−25
Long−pec
Protruding−mouth
Day−4
Day−5
Stage
Transcript
ENSDART00000112974
ENSDART00000155719

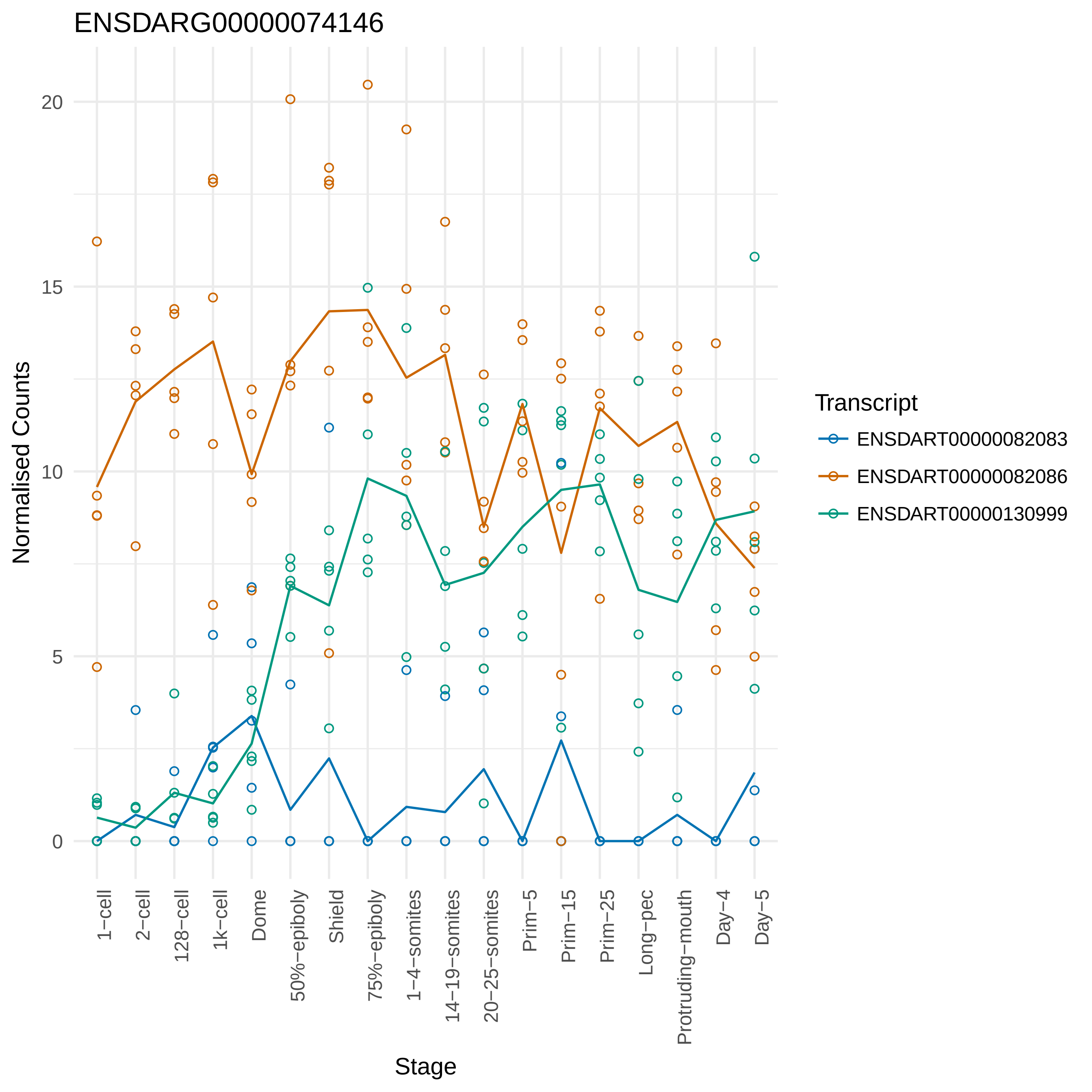

ENSDARG00000074146
Normalised Counts
20
15
10
5
0
1−cell
2−cell
128−cell
1k−cell
Dome
50%−epiboly
Shield
75%−epiboly
1−4−somites
14−19−somites
20−25−somites
Prim−5
Prim−15
Prim−25
Long−pec
Protruding−mouth
Day−4
Day−5
Stage
Transcript
ENSDART00000082083
ENSDART00000082086
ENSDART00000130999

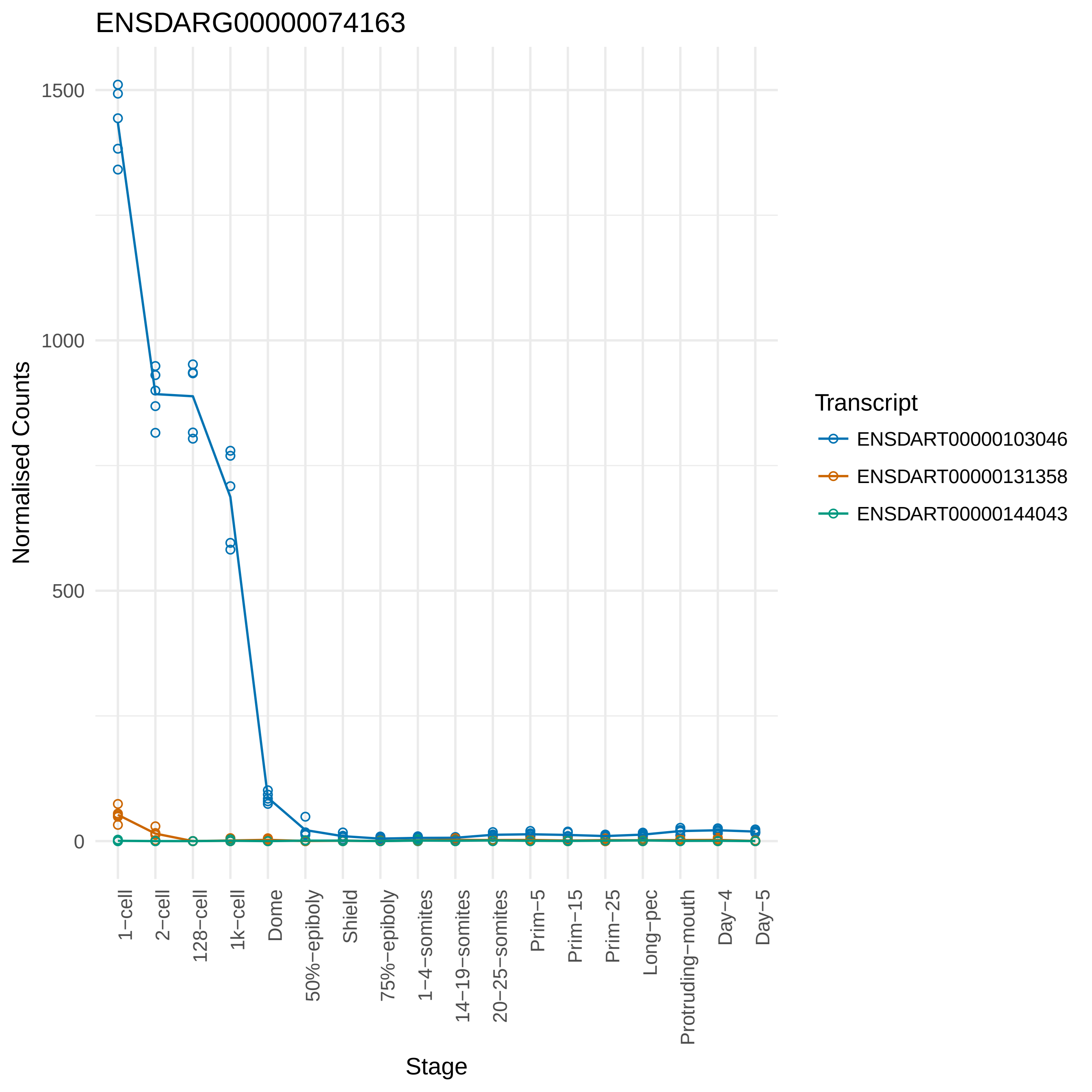
ENSDARG00000074163
Normalised Counts
1500
1000
500
0
1−cell
2−cell
128−cell
1k−cell
Dome
50%−epiboly
Shield
75%−epiboly
1−4−somites
14−19−somites
20−25−somites
Prim−5
Prim−15
Prim−25
Long−pec
Protruding−mouth
Day−4
Day−5
Stage
Transcript
ENSDART00000103046
ENSDART00000131358
ENSDART00000144043

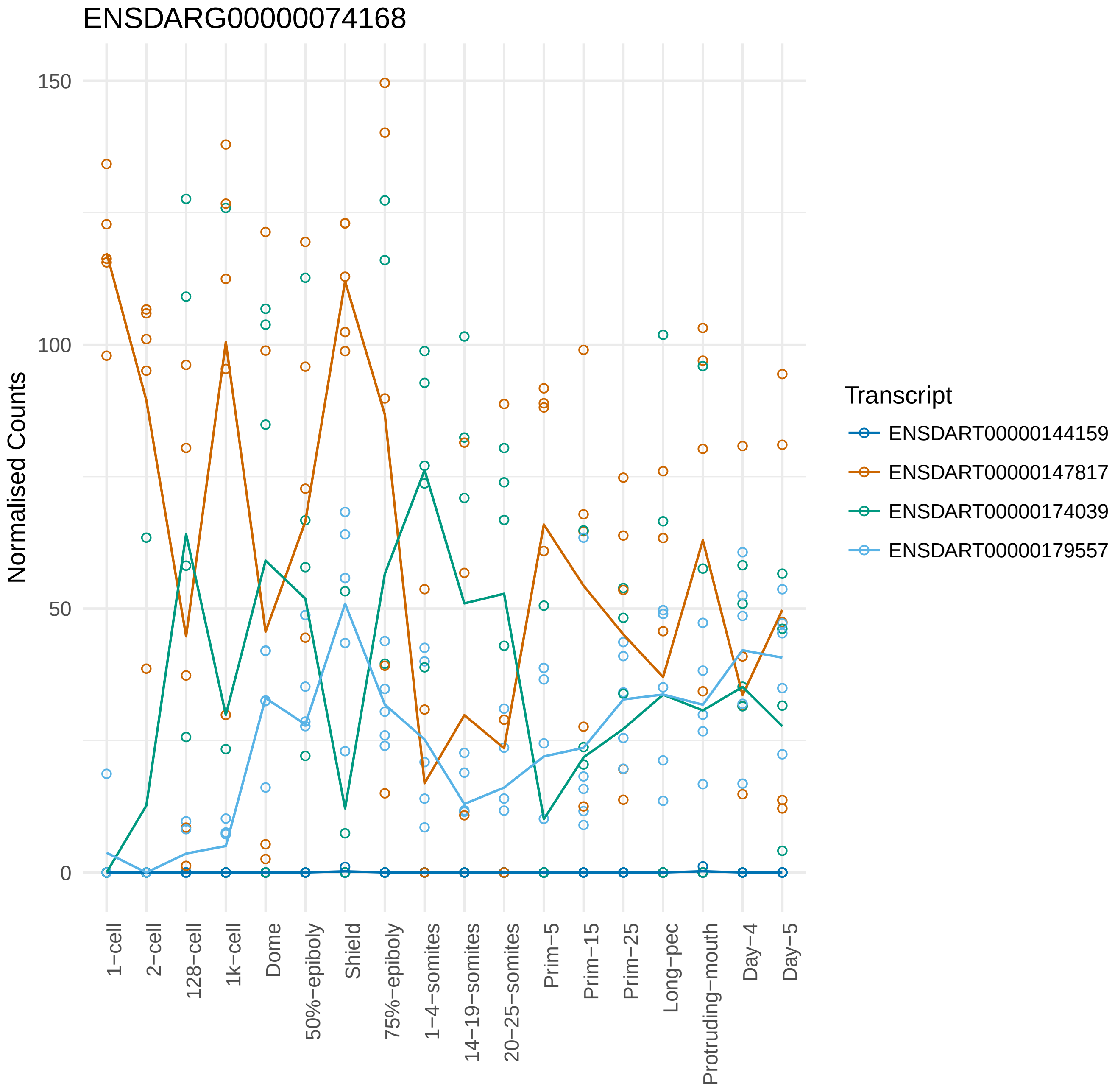

ENSDARG00000074168
Normalised Counts
150
100
50
0
Stage
1−cell
2−cell
128−cell
1k−cell
Dome
50%−epiboly
Shield
75%−epiboly
1−4−somites
14−19−somites
20−25−somites
Prim−5
Prim−15
Prim−25
Long−pec
Protruding−mouth
Day−4
Day−5
Transcript
ENSDART00000144159
ENSDART00000147817
ENSDART00000174039
ENSDART00000179557

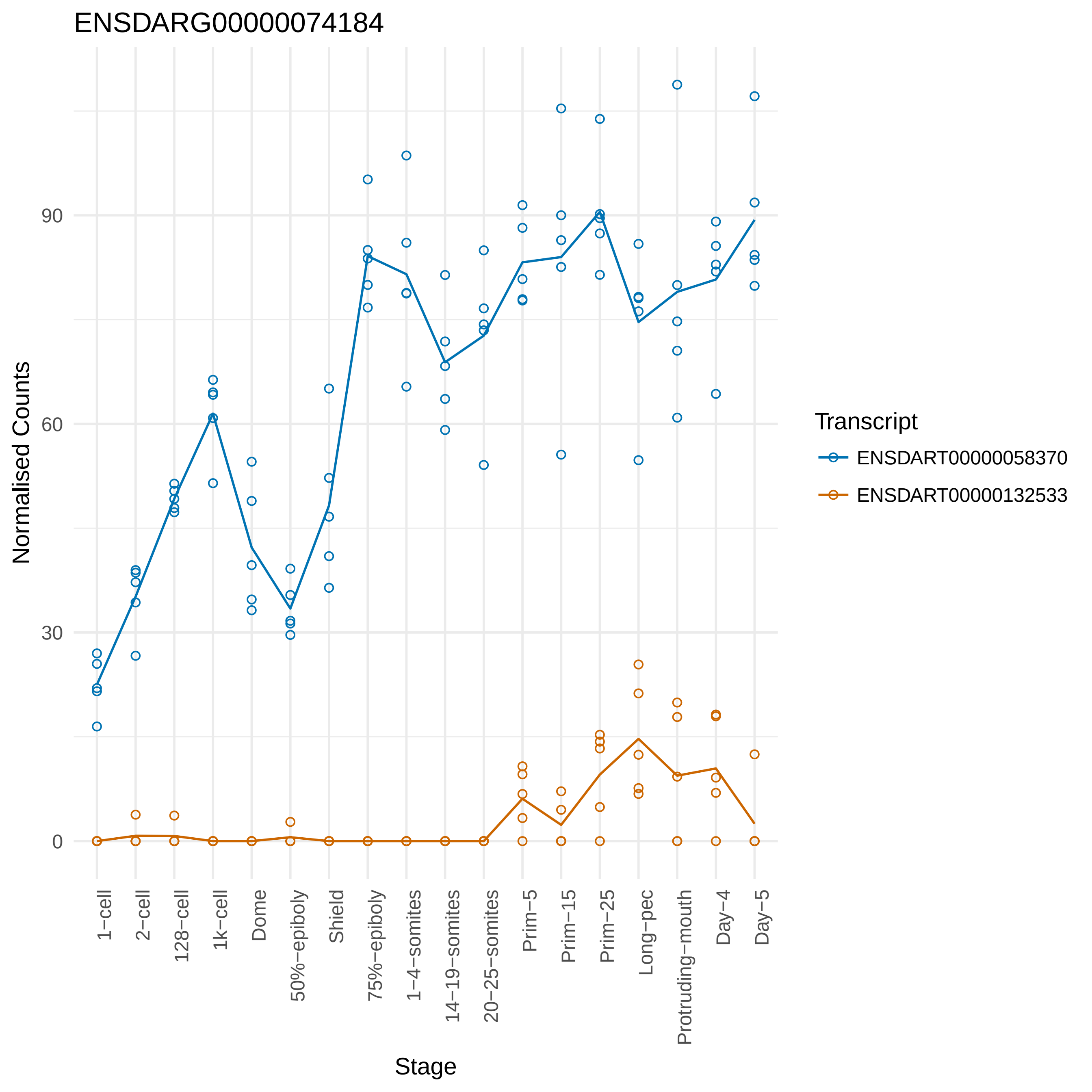
ENSDARG00000074184
Normalised Counts
0
30
60
90
1−cell
2−cell
128−cell
1k−cell
Dome
50%−epiboly
Shield
75%−epiboly
1−4−somites
14−19−somites
20−25−somites
Prim−5
Prim−15
Prim−25
Long−pec
Protruding−mouth
Day−4
Day−5
Stage
Transcript
ENSDART00000058370
ENSDART00000132533

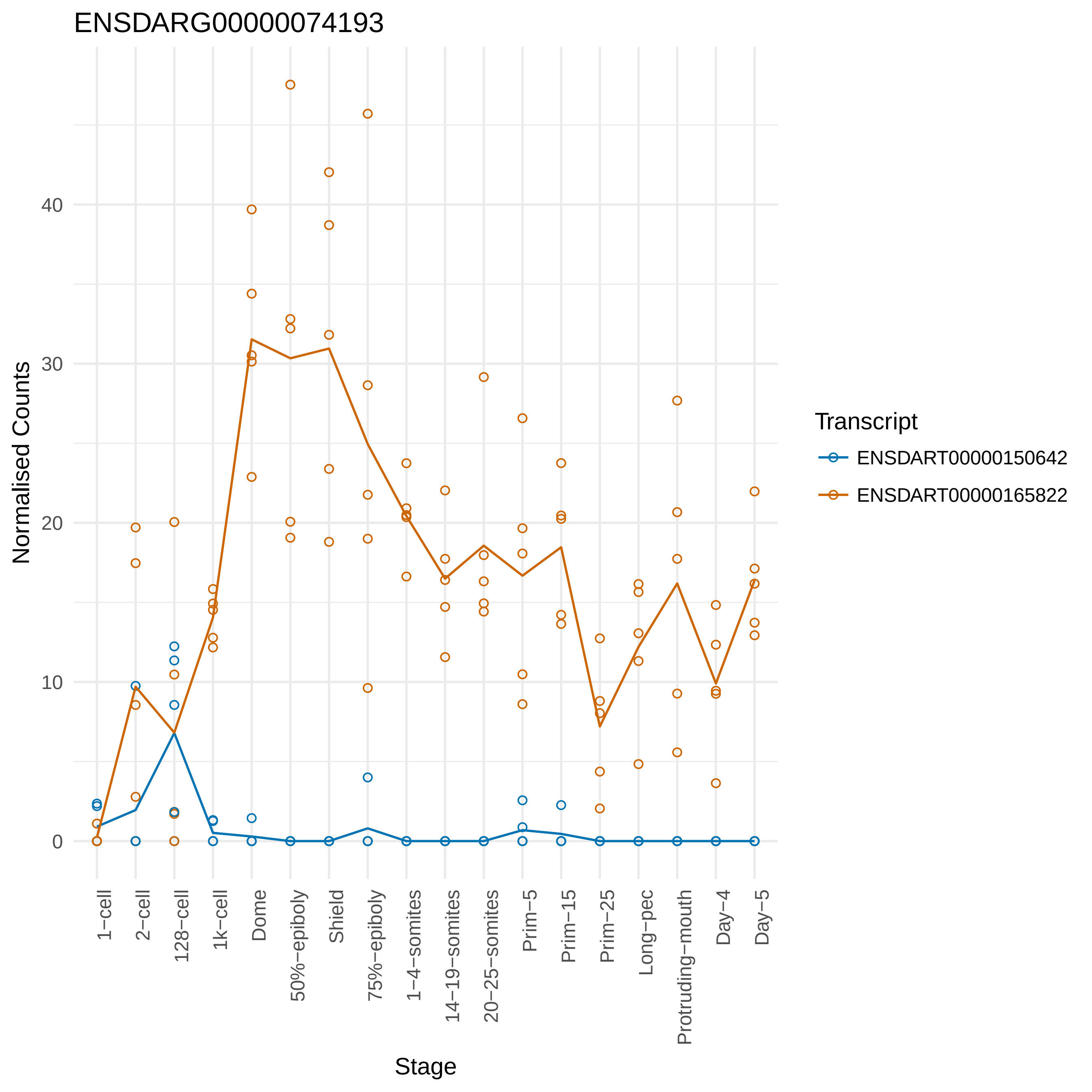
ENSDARG00000074193
Normalised Counts
0
10
20
30
40
1−cell
2−cell
128−cell
1k−cell
Dome
50%−epiboly
Shield
75%−epiboly
1−4−somites
14−19−somites
20−25−somites
Prim−5
Prim−15
Prim−25
Long−pec
Protruding−mouth
Day−4
Day−5
Stage
Transcript
ENSDART00000150642
ENSDART00000165822

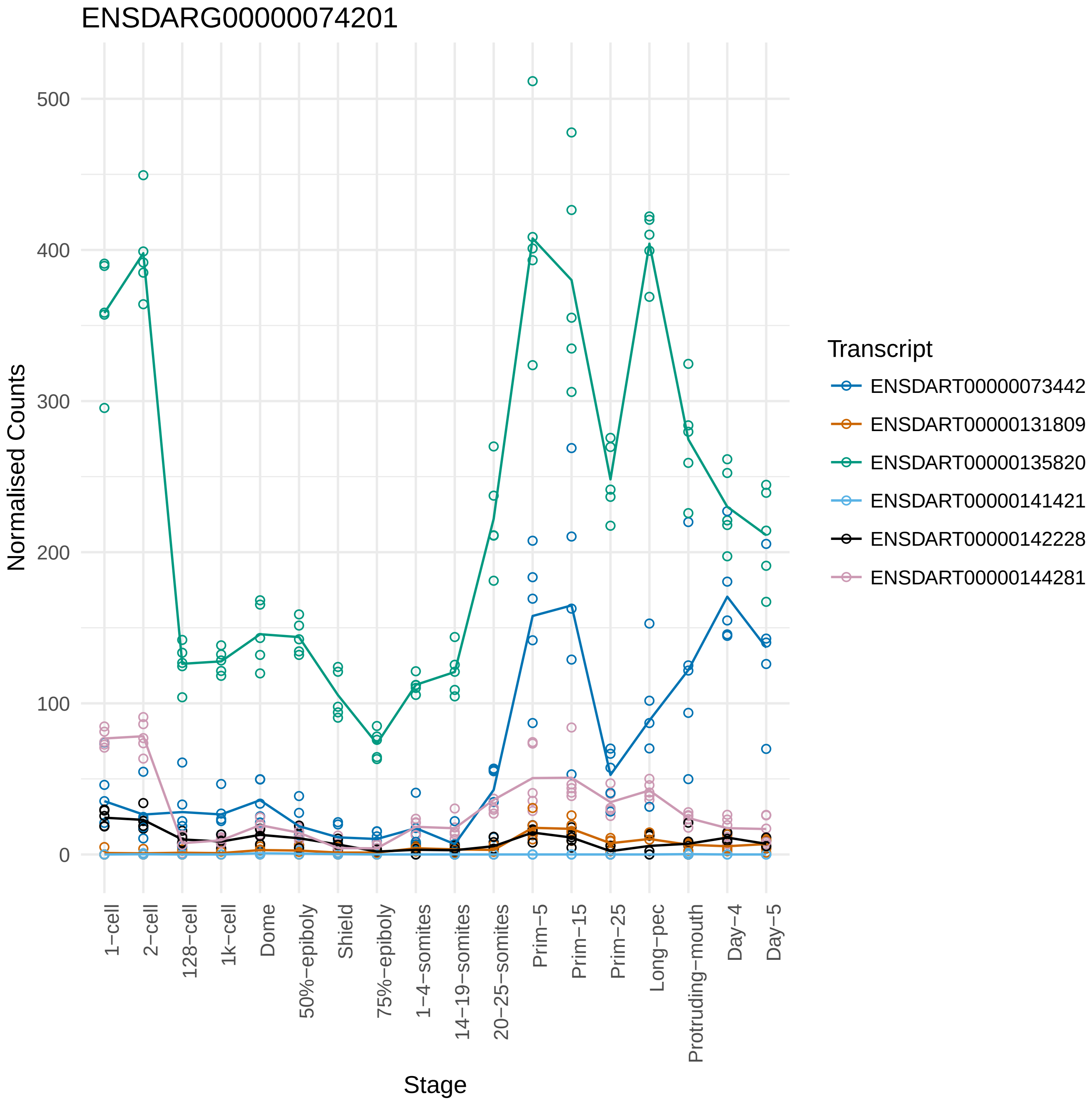
ENSDARG00000074201
Normalised Counts
500
400
300
200
100
0
Stage
1−cell
2−cell
128−cell
1k−cell
Dome
50%−epiboly
Shield
75%−epiboly
1−4−somites
14−19−somites
20−25−somites
Prim−5
Prim−15
Prim−25
Long−pec
Protruding−mouth
Day−4
Day−5
Transcript
ENSDART00000073442
ENSDART00000131809
ENSDART00000135820
ENSDART00000141421
ENSDART00000142228
ENSDART00000144281

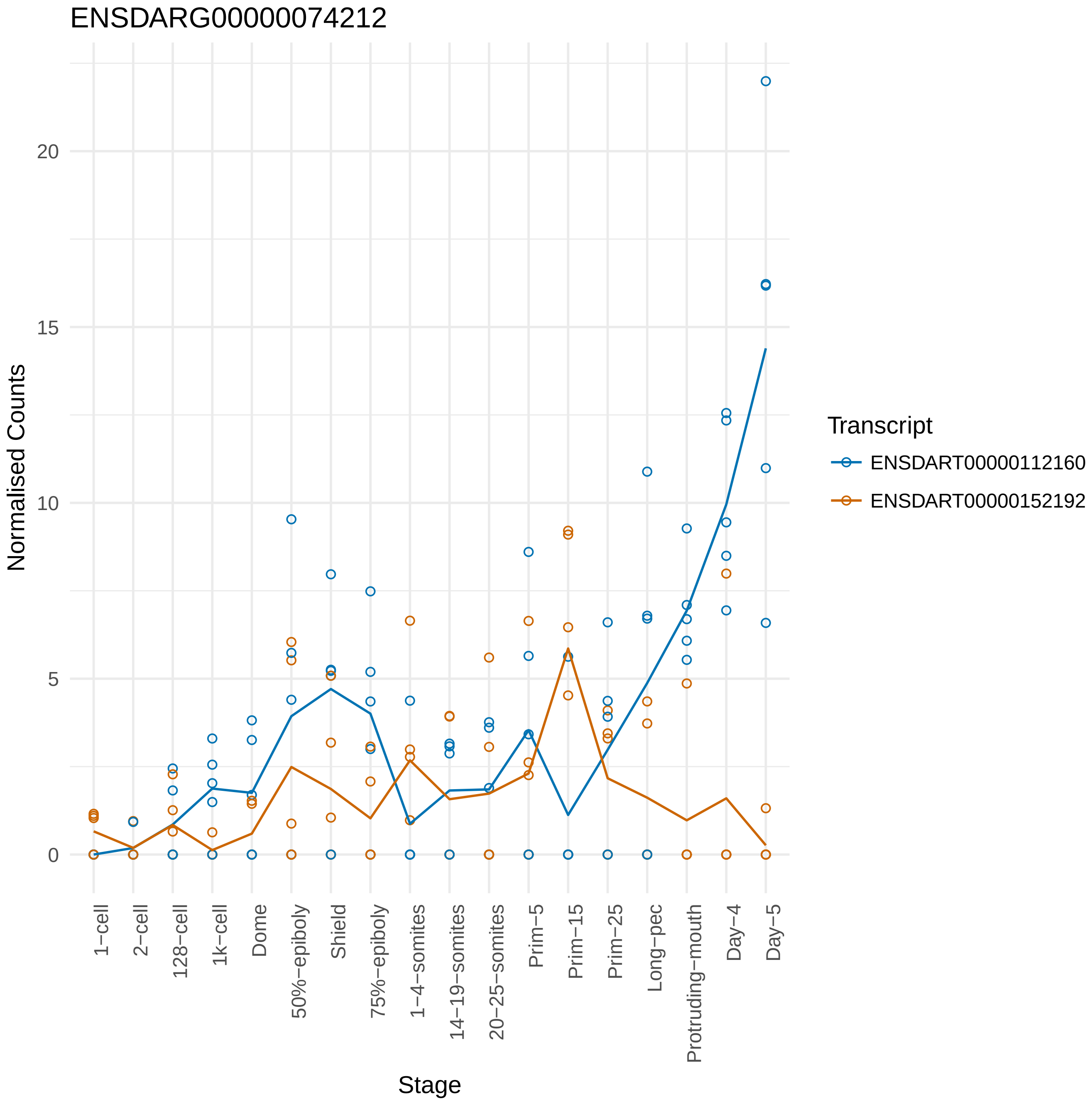

ENSDARG00000074212
Normalised Counts
0
5
10
15
20
1−cell
2−cell
128−cell
1k−cell
Dome
50%−epiboly
Shield
75%−epiboly
1−4−somites
14−19−somites
20−25−somites
Prim−5
Prim−15
Prim−25
Long−pec
Protruding−mouth
Day−4
Day−5
Stage
Transcript
ENSDART00000112160
ENSDART00000152192

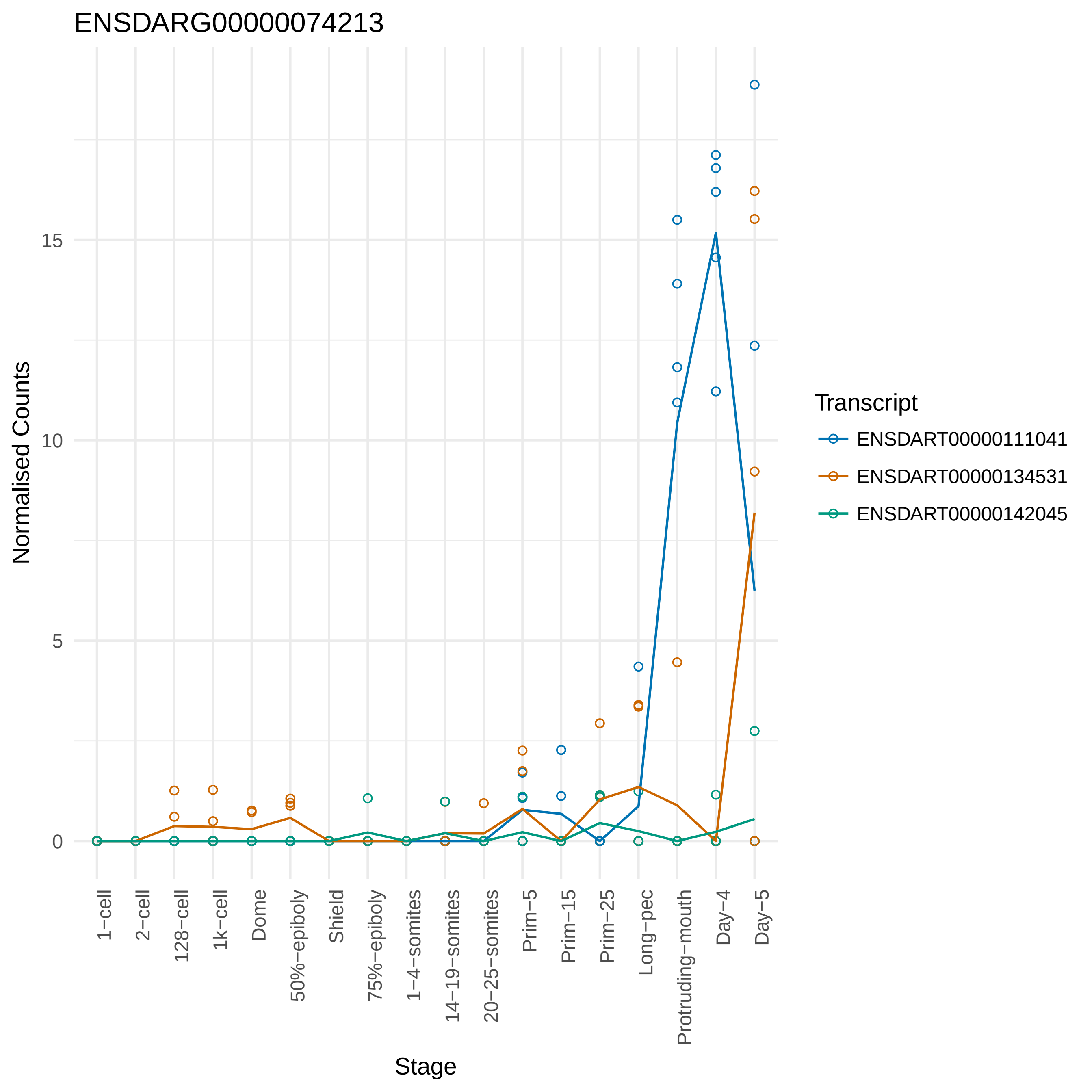
ENSDARG00000074213
Normalised Counts
15
10
5
0
1−cell
2−cell
128−cell
1k−cell
Dome
50%−epiboly
Shield
75%−epiboly
1−4−somites
14−19−somites
20−25−somites
Prim−5
Prim−15
Prim−25
Long−pec
Protruding−mouth
Day−4
Day−5
Stage
Transcript
ENSDART00000111041
ENSDART00000134531
ENSDART00000142045

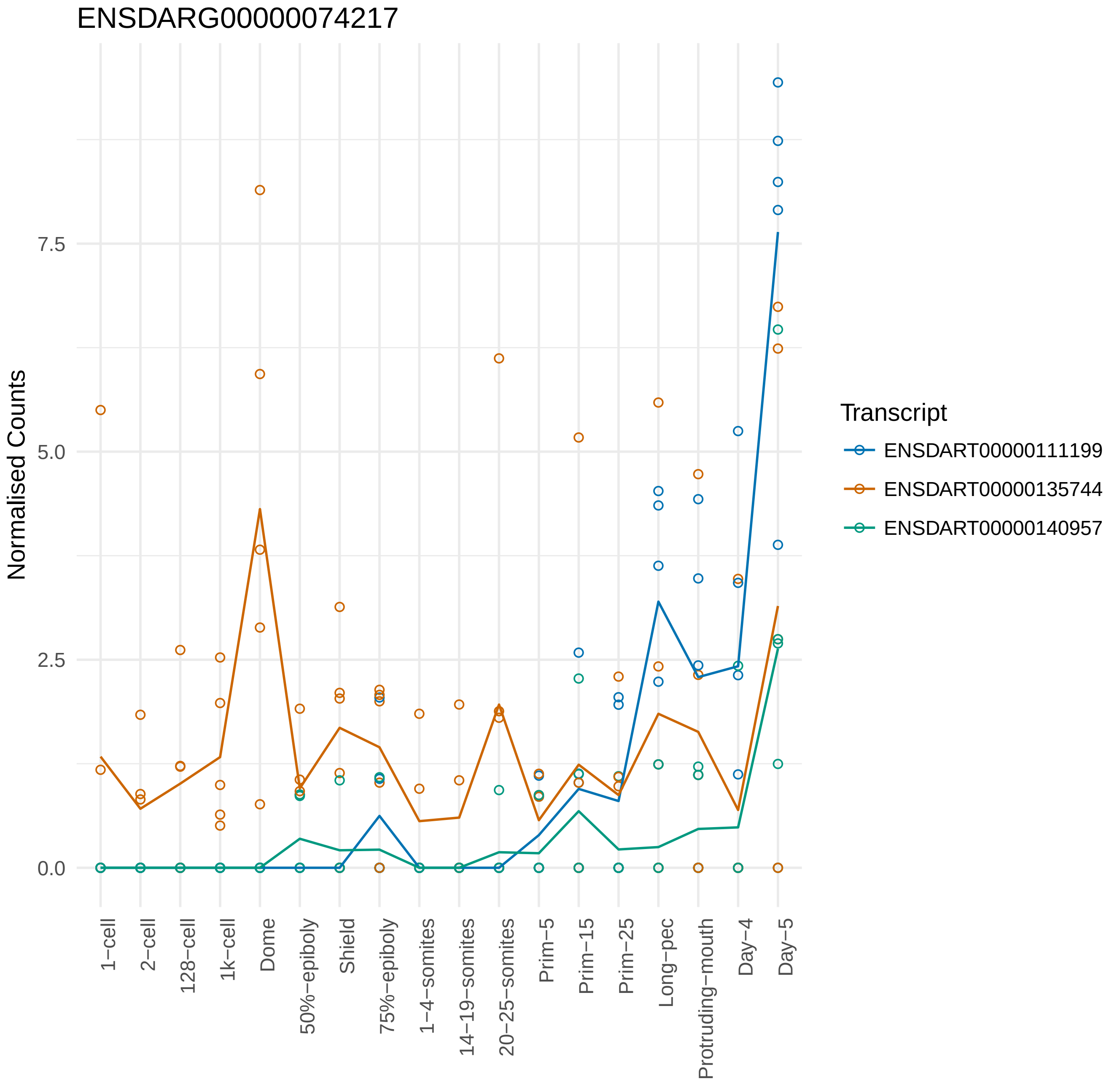

ENSDARG00000074217
Normalised Counts
0.0
2.5
5.0
7.5
Stage
1−cell
2−cell
128−cell
1k−cell
Dome
50%−epiboly
Shield
75%−epiboly
1−4−somites
14−19−somites
20−25−somites
Prim−5
Prim−15
Prim−25
Long−pec
Protruding−mouth
Day−4
Day−5
Transcript
ENSDART00000111199
ENSDART00000135744
ENSDART00000140957

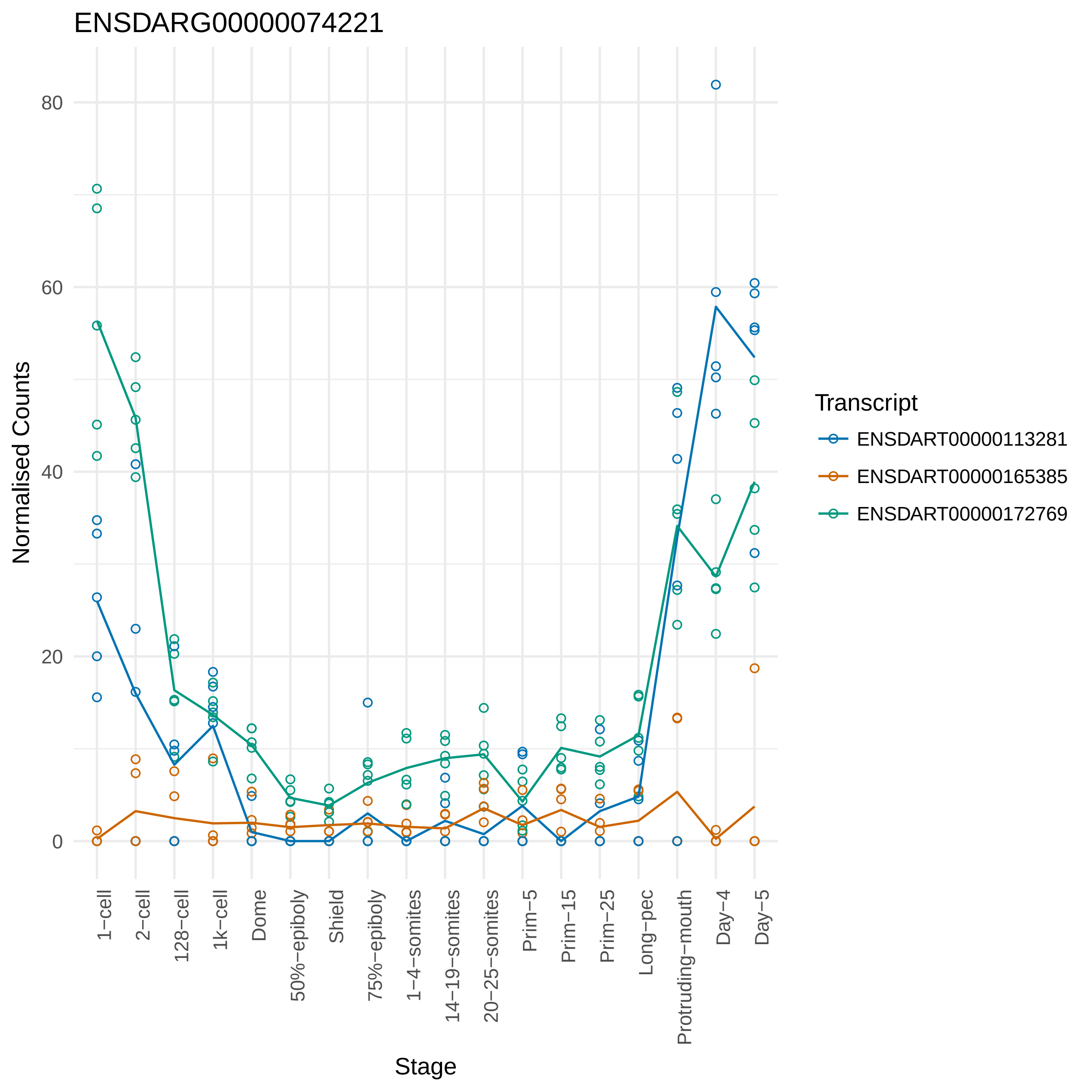

ENSDARG00000074221
Normalised Counts
0
20
40
60
80
1−cell
2−cell
128−cell
1k−cell
Dome
50%−epiboly
Shield
75%−epiboly
1−4−somites
14−19−somites
20−25−somites
Prim−5
Prim−15
Prim−25
Long−pec
Protruding−mouth
Day−4
Day−5
Stage
Transcript
ENSDART00000113281
ENSDART00000165385
ENSDART00000172769

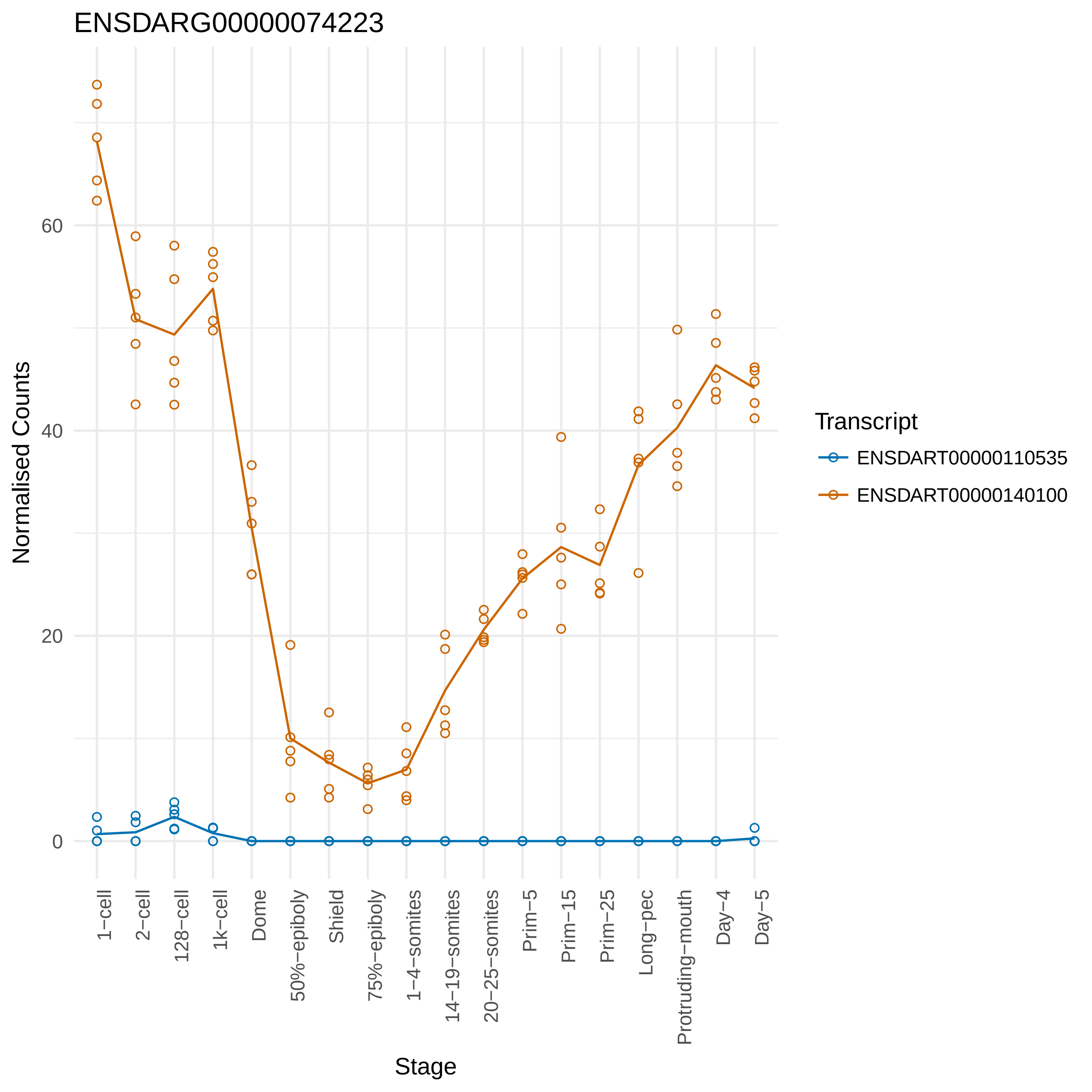

ENSDARG00000074223
Normalised Counts
60
40
20
0
1−cell
2−cell
128−cell
1k−cell
Dome
50%−epiboly
Shield
75%−epiboly
1−4−somites
14−19−somites
20−25−somites
Prim−5
Prim−15
Prim−25
Long−pec
Protruding−mouth
Day−4
Day−5
Stage
Transcript
ENSDART00000110535
ENSDART00000140100

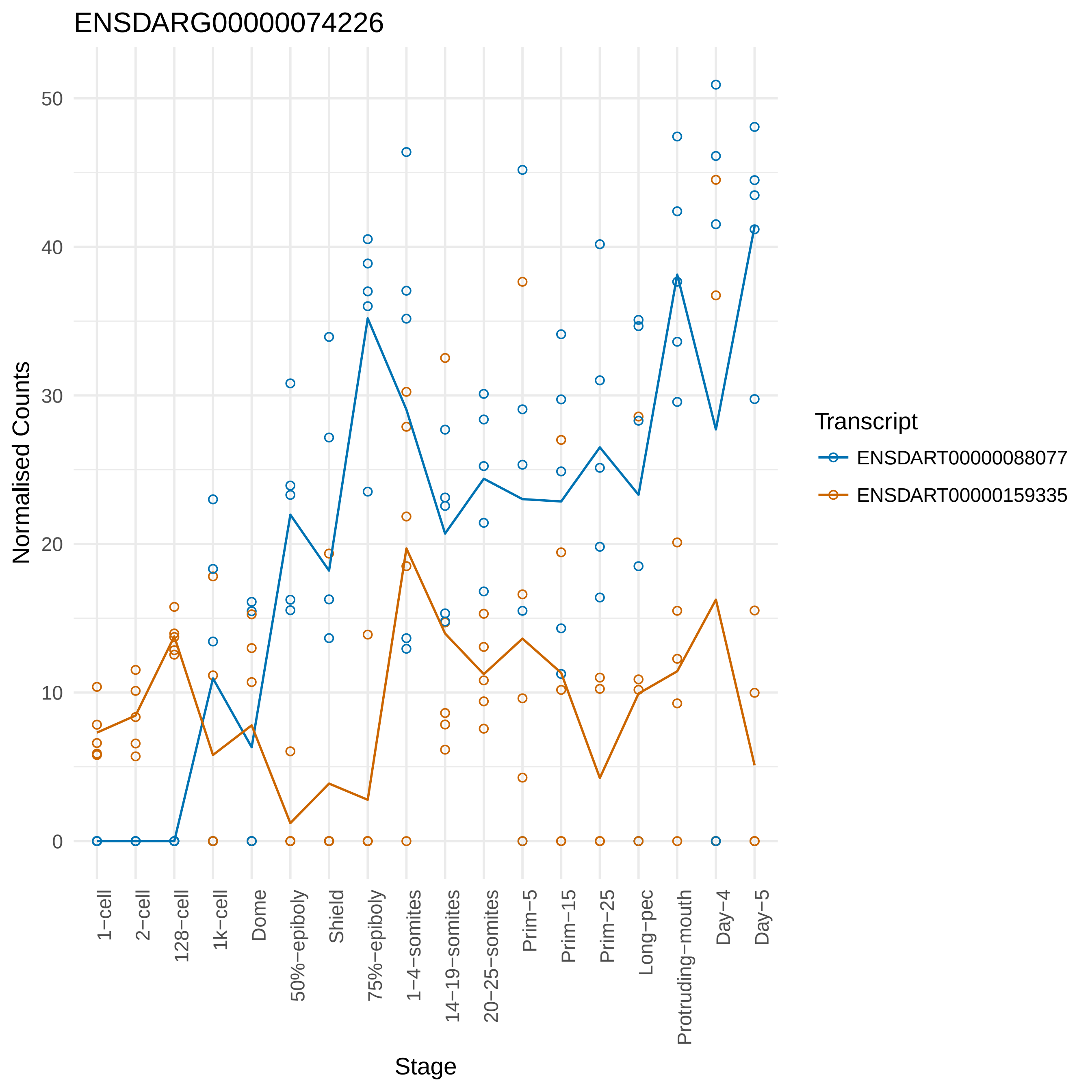
ENSDARG00000074226
Normalised Counts
Stage
Transcript
ENSDART00000088077
ENSDART00000159335
0
10
20
30
40
50
1−cell
2−cell
128−cell
1k−cell
Dome
50%−epiboly
Shield
75%−epiboly
1−4−somites
14−19−somites
20−25−somites
Prim−5
Prim−15
Prim−25
Long−pec
Protruding−mouth
Day−4
Day−5

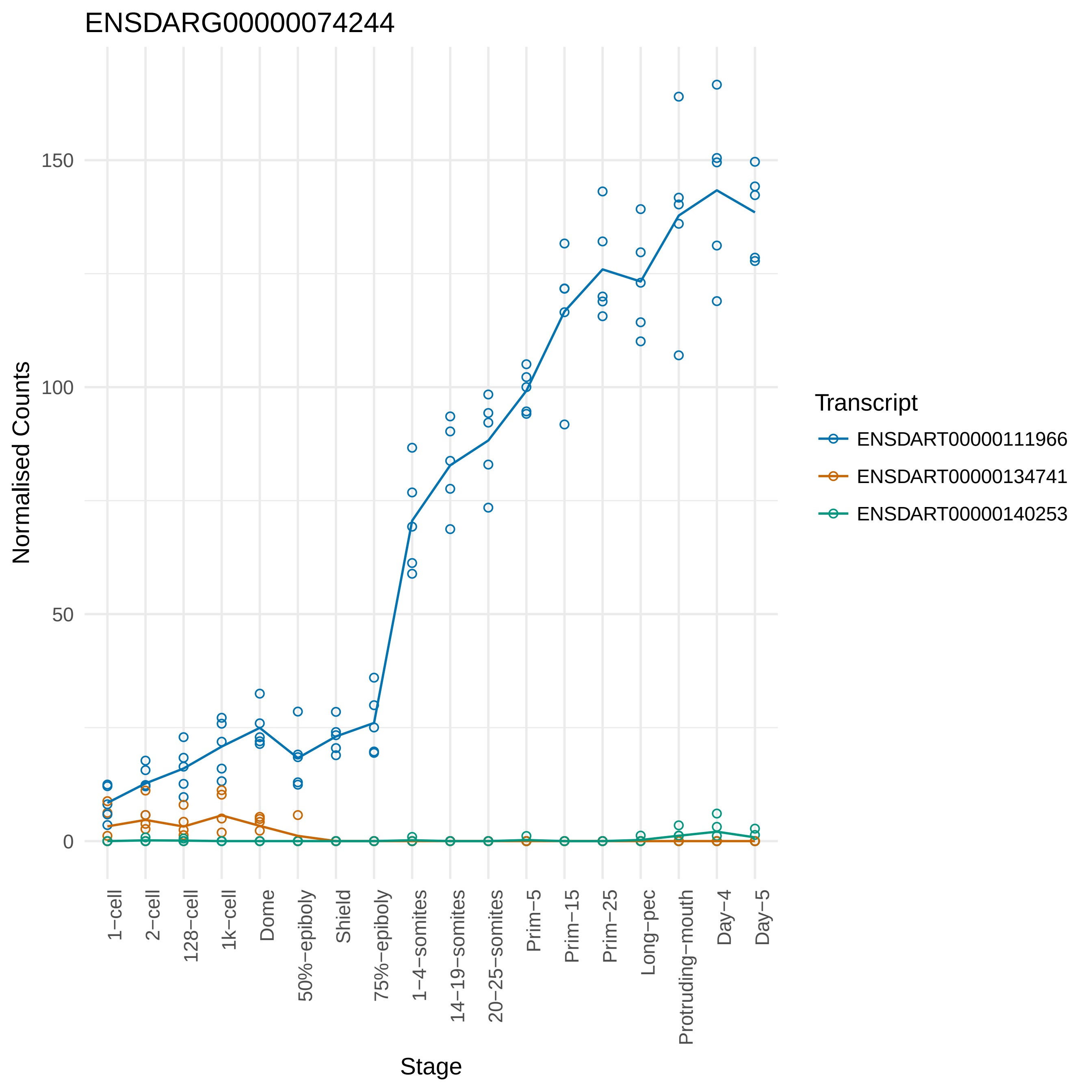

ENSDARG00000074244
Normalised Counts
0
50
100
150
Stage
1−cell
2−cell
128−cell
1k−cell
Dome
50%−epiboly
Shield
75%−epiboly
1−4−somites
14−19−somites
20−25−somites
Prim−5
Prim−15
Prim−25
Long−pec
Protruding−mouth
Day−4
Day−5
Transcript
ENSDART00000111966
ENSDART00000134741
ENSDART00000140253

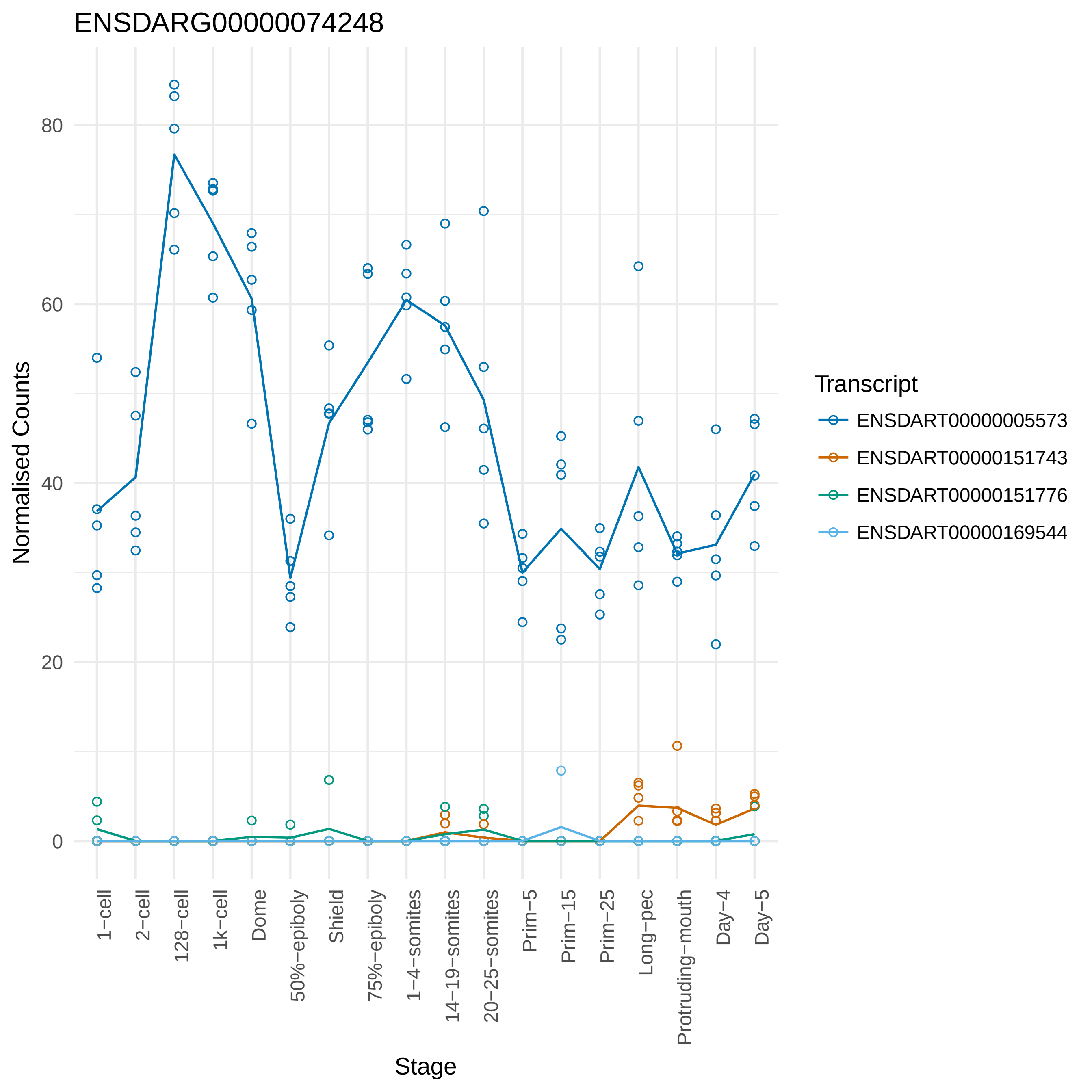

ENSDARG00000074248
Normalised Counts
80
60
40
20
0
1−cell
2−cell
128−cell
1k−cell
Dome
50%−epiboly
Shield
75%−epiboly
1−4−somites
14−19−somites
20−25−somites
Prim−5
Prim−15
Prim−25
Long−pec
Protruding−mouth
Day−4
Day−5
Stage
Transcript
ENSDART00000005573
ENSDART00000151743
ENSDART00000151776
ENSDART00000169544

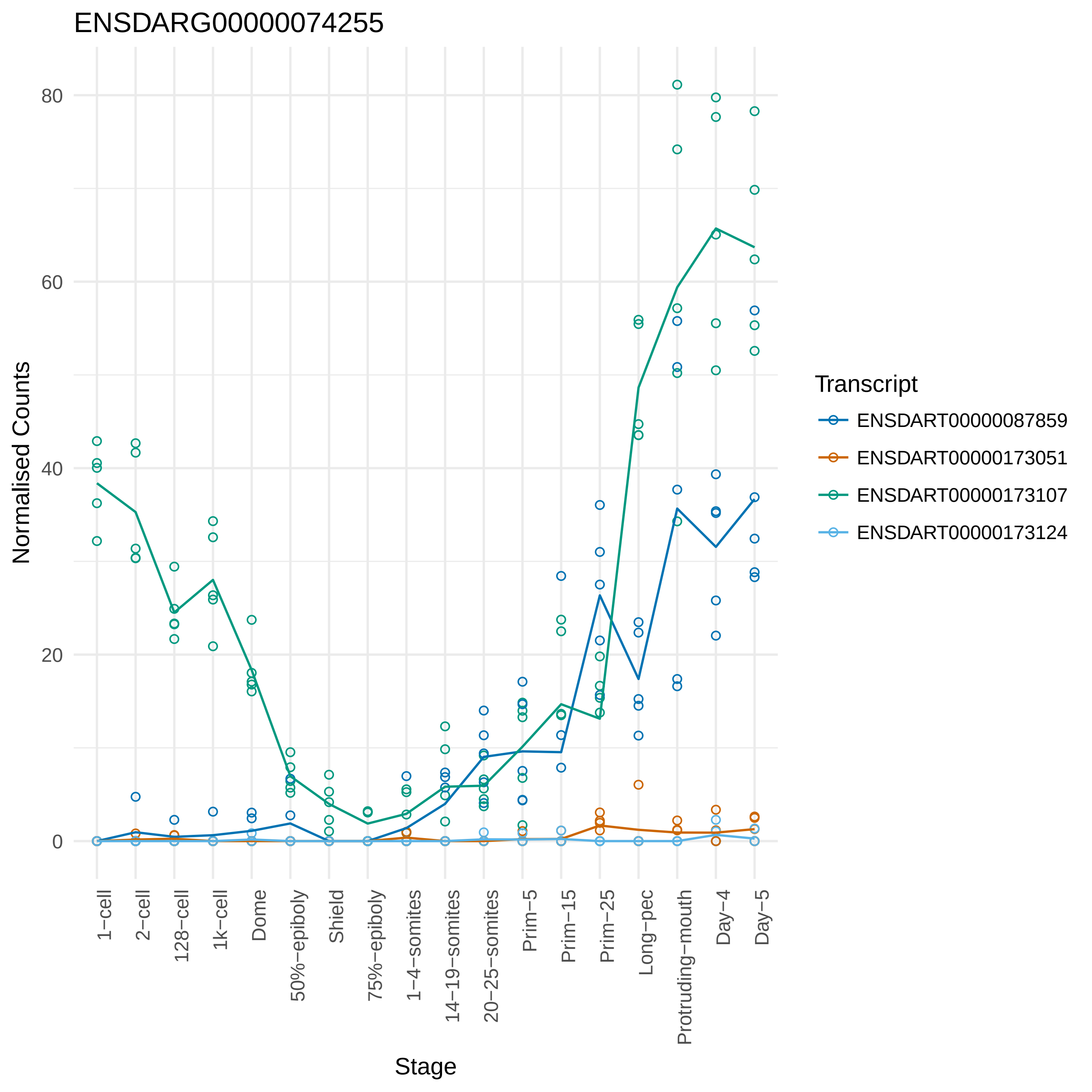
ENSDARG00000074255
Normalised Counts
0
20
40
60
80
1−cell
2−cell
128−cell
1k−cell
Dome
50%−epiboly
Shield
75%−epiboly
1−4−somites
14−19−somites
20−25−somites
Prim−5
Prim−15
Prim−25
Long−pec
Protruding−mouth
Day−4
Day−5
Stage
Transcript
ENSDART00000087859
ENSDART00000173051
ENSDART00000173107
ENSDART00000173124

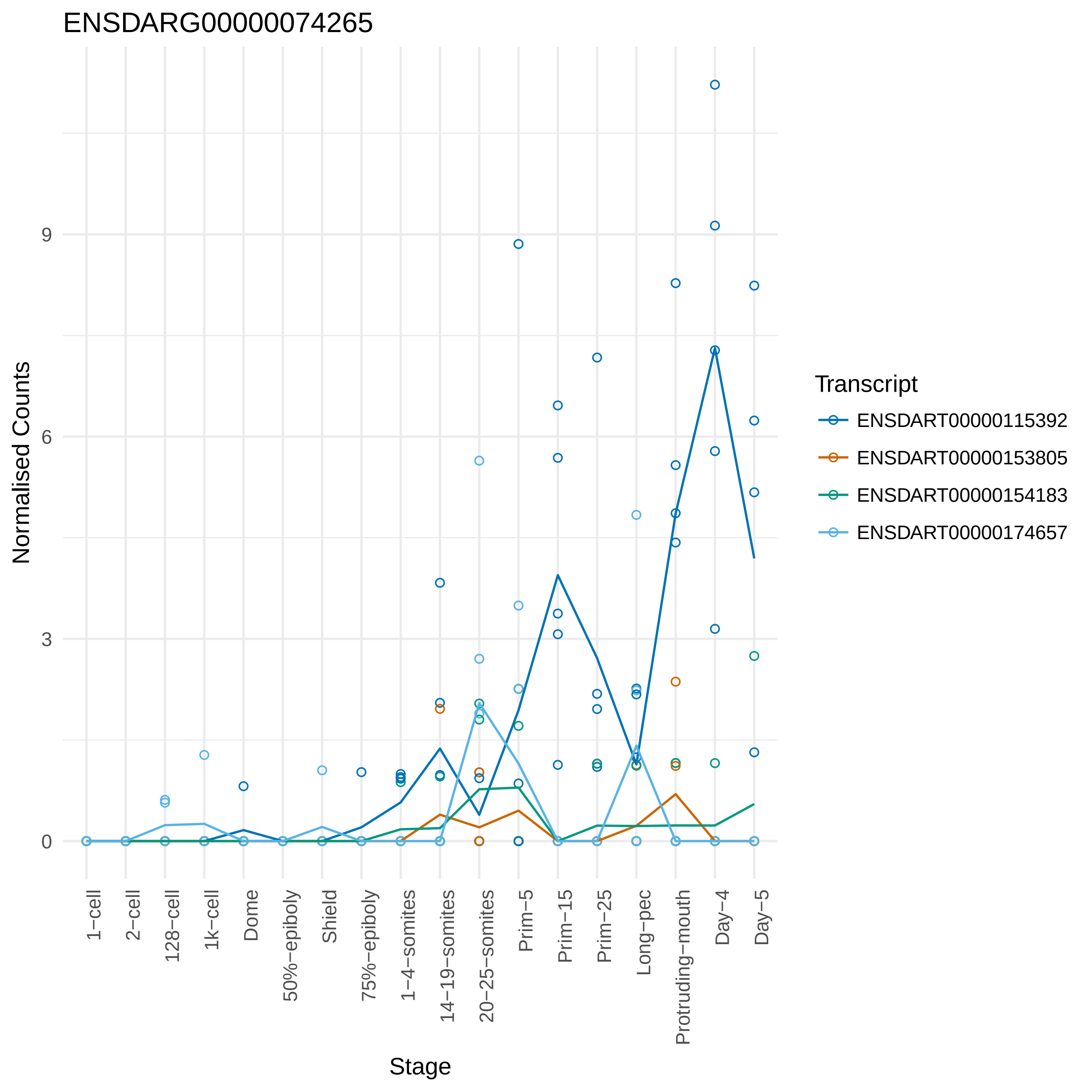

ENSDARG00000074265
Normalised Counts
0
3
6
9
1−cell
2−cell
128−cell
1k−cell
Dome
50%−epiboly
Shield
75%−epiboly
1−4−somites
14−19−somites
20−25−somites
Prim−5
Prim−15
Prim−25
Long−pec
Protruding−mouth
Day−4
Day−5
Stage
Transcript
ENSDART00000115392
ENSDART00000153805
ENSDART00000154183
ENSDART00000174657

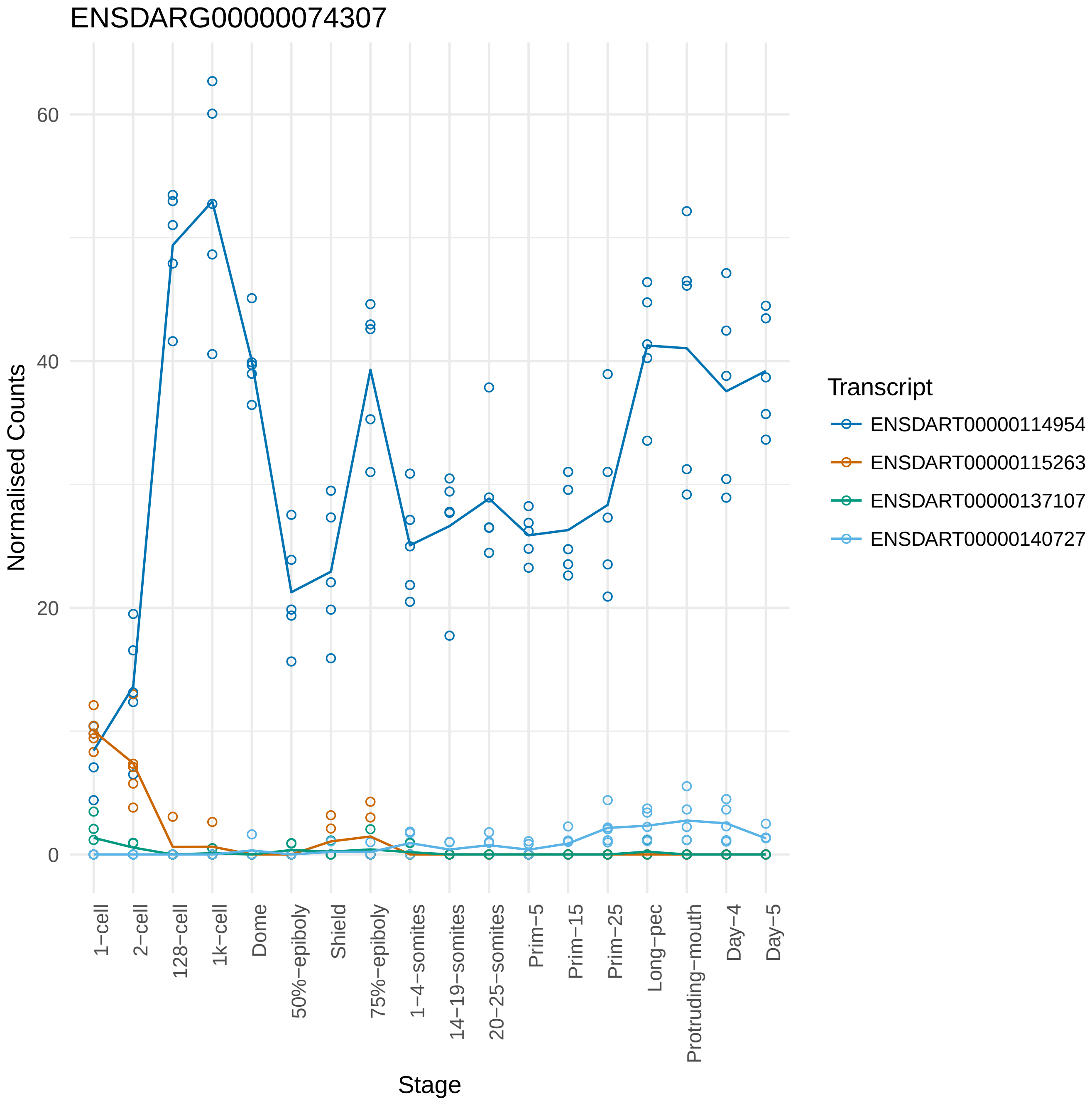
ENSDARG00000074307
Normalised Counts
0
20
40
60
1−cell
2−cell
128−cell
1k−cell
Dome
50%−epiboly
Shield
75%−epiboly
1−4−somites
14−19−somites
20−25−somites
Prim−5
Prim−15
Prim−25
Long−pec
Protruding−mouth
Day−4
Day−5
Stage
Transcript
ENSDART00000114954
ENSDART00000115263
ENSDART00000137107
ENSDART00000140727

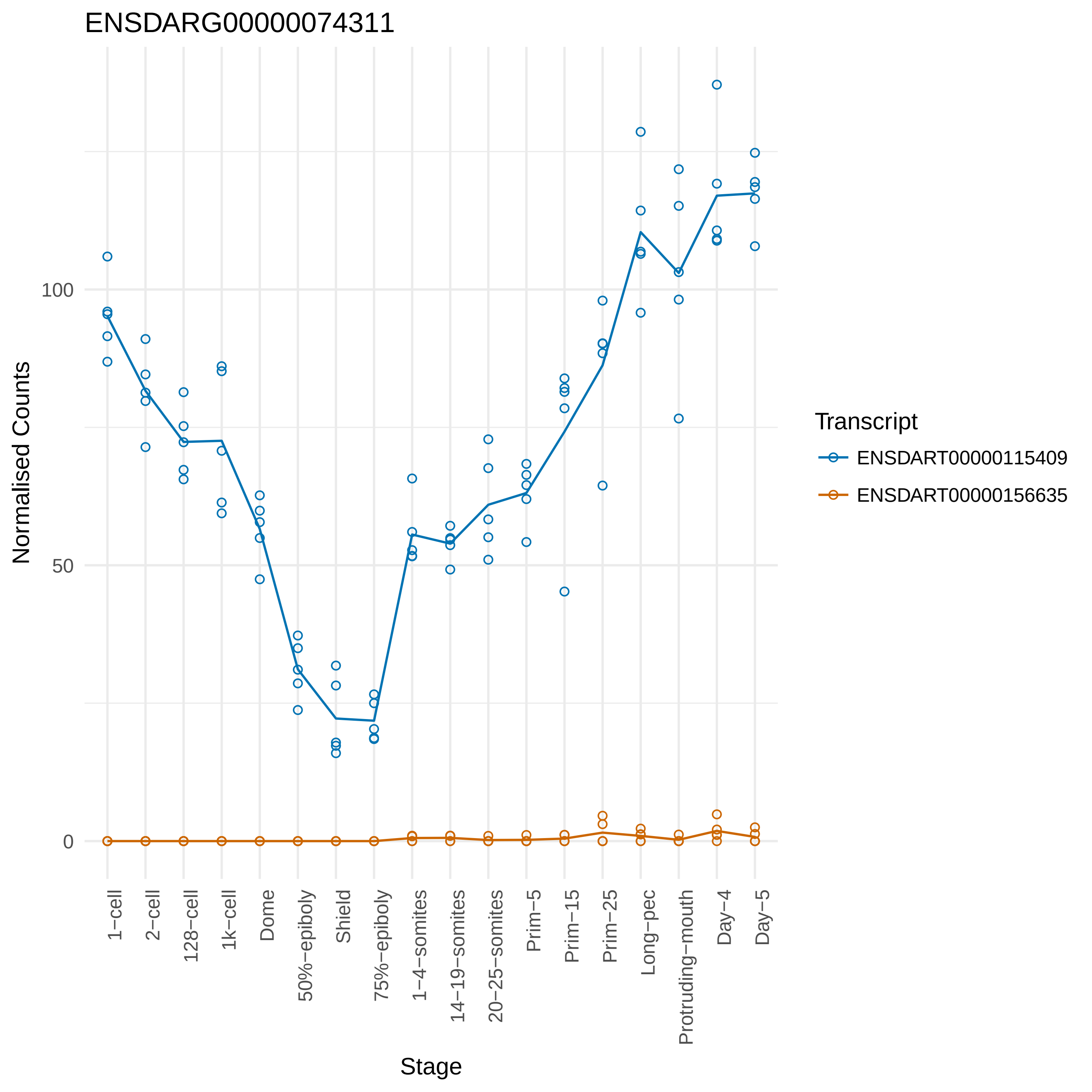
ENSDARG00000074311
Normalised Counts
100
50
0
1−cell
2−cell
128−cell
1k−cell
Dome
50%−epiboly
Shield
75%−epiboly
1−4−somites
14−19−somites
20−25−somites
Prim−5
Prim−15
Prim−25
Long−pec
Protruding−mouth
Day−4
Day−5
Stage
Transcript
ENSDART00000115409
ENSDART00000156635

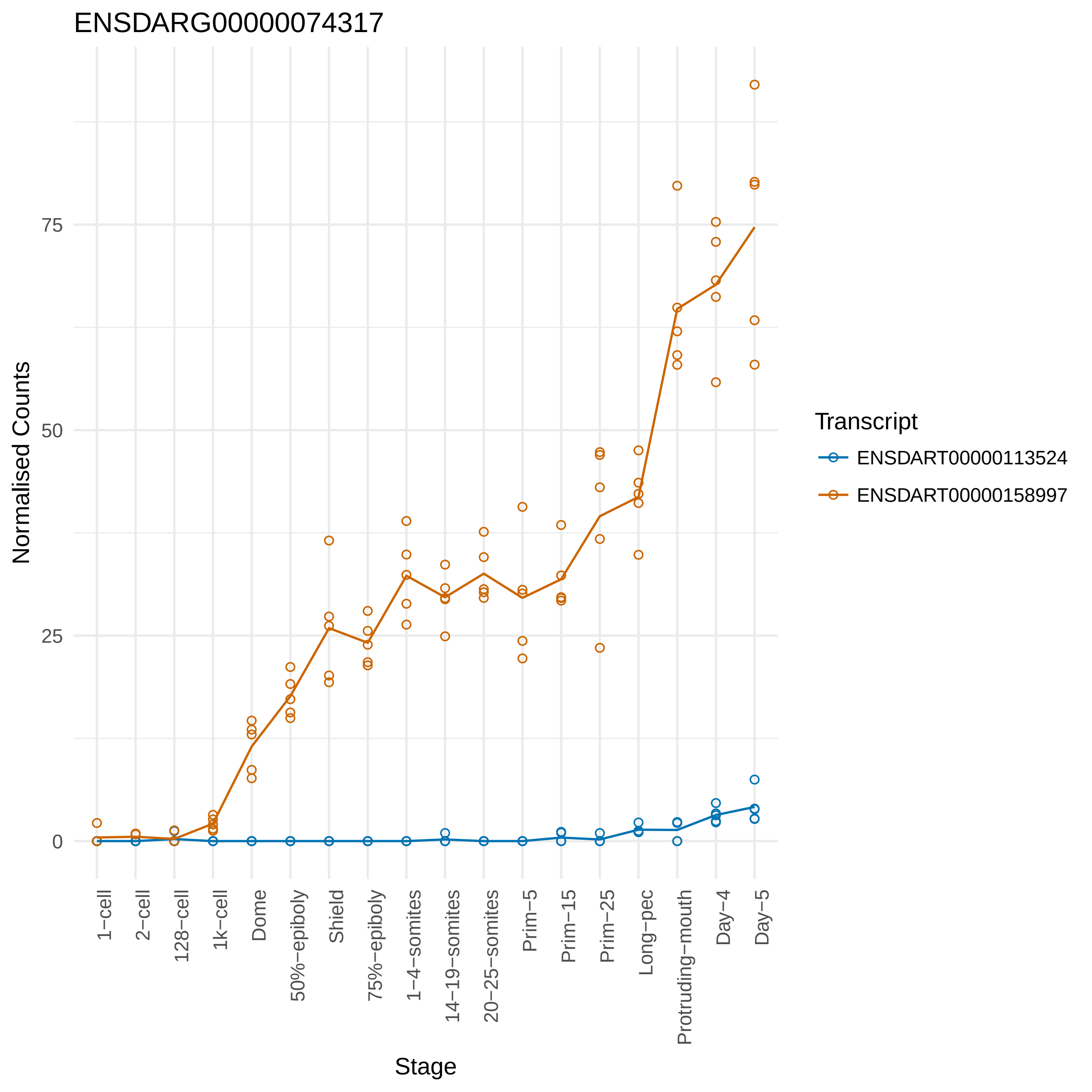
ENSDARG00000074317
Normalised Counts
75
50
25
0
1−cell
2−cell
128−cell
1k−cell
Dome
50%−epiboly
Shield
75%−epiboly
1−4−somites
14−19−somites
20−25−somites
Prim−5
Prim−15
Prim−25
Long−pec
Protruding−mouth
Day−4
Day−5
Stage
Transcript
ENSDART00000113524
ENSDART00000158997

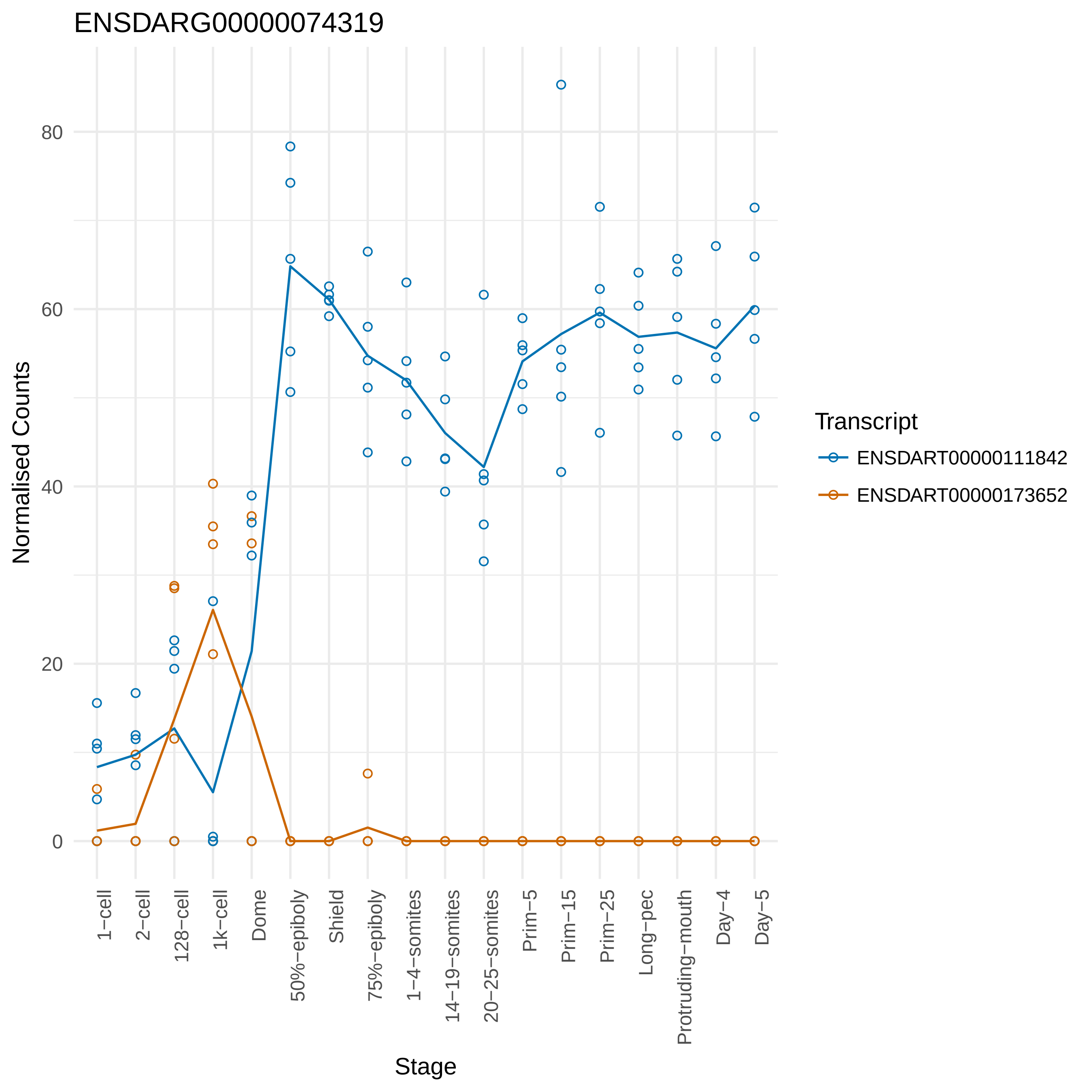

ENSDARG00000074319
Normalised Counts
80
60
40
20
0
1−cell
2−cell
128−cell
1k−cell
Dome
50%−epiboly
Shield
75%−epiboly
1−4−somites
14−19−somites
20−25−somites
Prim−5
Prim−15
Prim−25
Long−pec
Protruding−mouth
Day−4
Day−5
Stage
Transcript
ENSDART00000111842
ENSDART00000173652

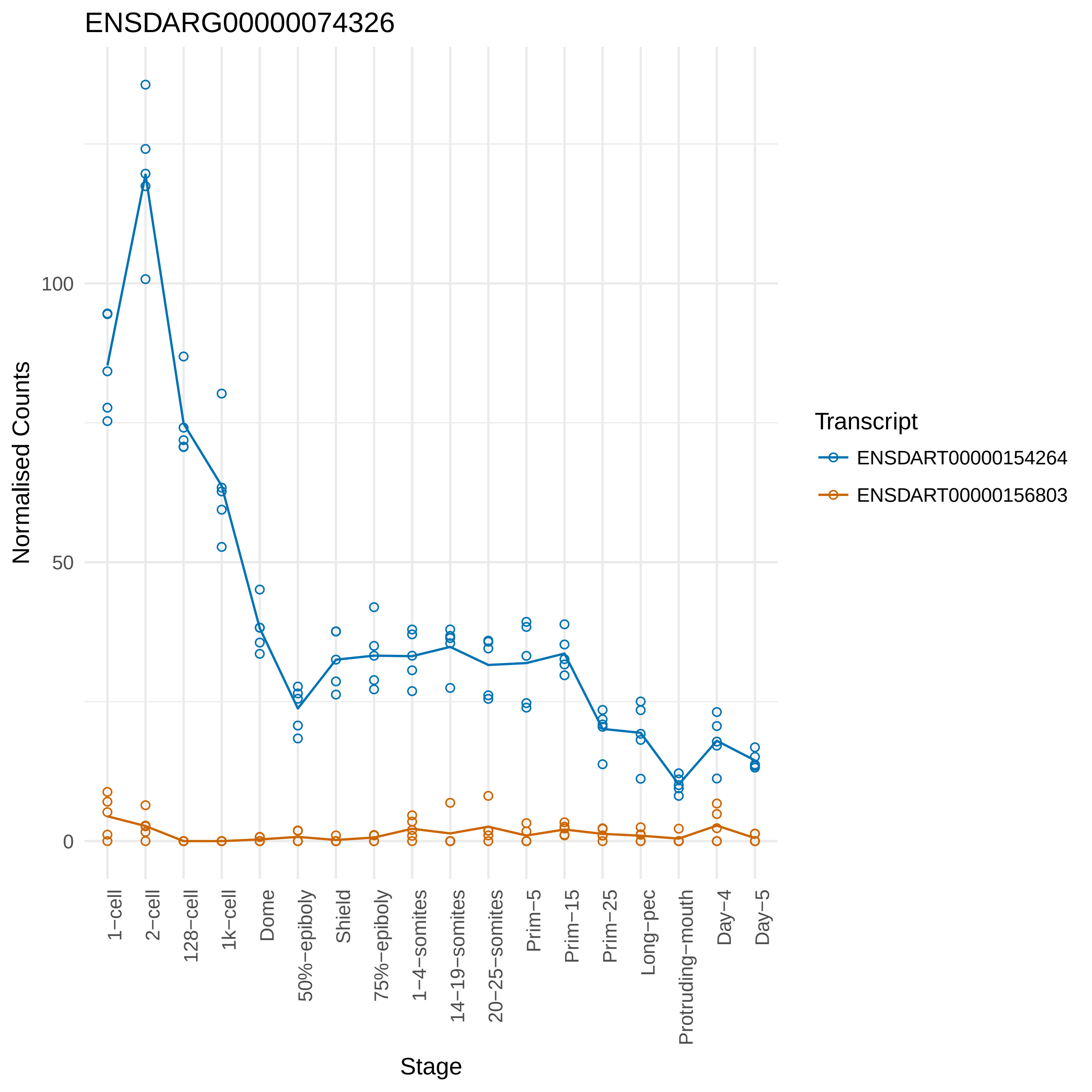
ENSDARG00000074326
Normalised Counts
100
50
0
1−cell
2−cell
128−cell
1k−cell
Dome
50%−epiboly
Shield
75%−epiboly
1−4−somites
14−19−somites
20−25−somites
Prim−5
Prim−15
Prim−25
Long−pec
Protruding−mouth
Day−4
Day−5
Stage
Transcript
ENSDART00000154264
ENSDART00000156803

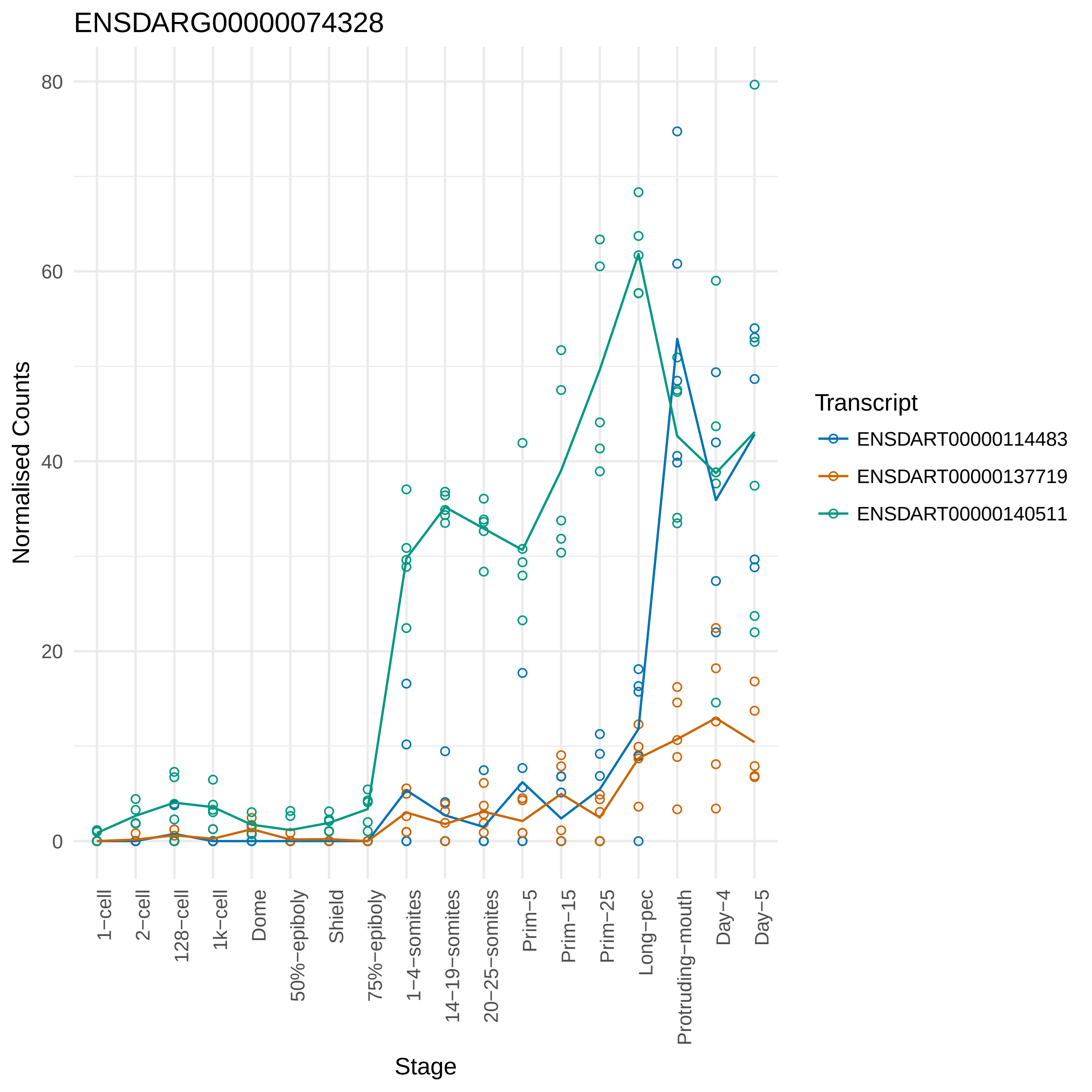

ENSDARG00000074328
Normalised Counts
0
20
40
60
80
1−cell
2−cell
128−cell
1k−cell
Dome
50%−epiboly
Shield
75%−epiboly
1−4−somites
14−19−somites
20−25−somites
Prim−5
Prim−15
Prim−25
Long−pec
Protruding−mouth
Day−4
Day−5
Stage
Transcript
ENSDART00000114483
ENSDART00000137719
ENSDART00000140511

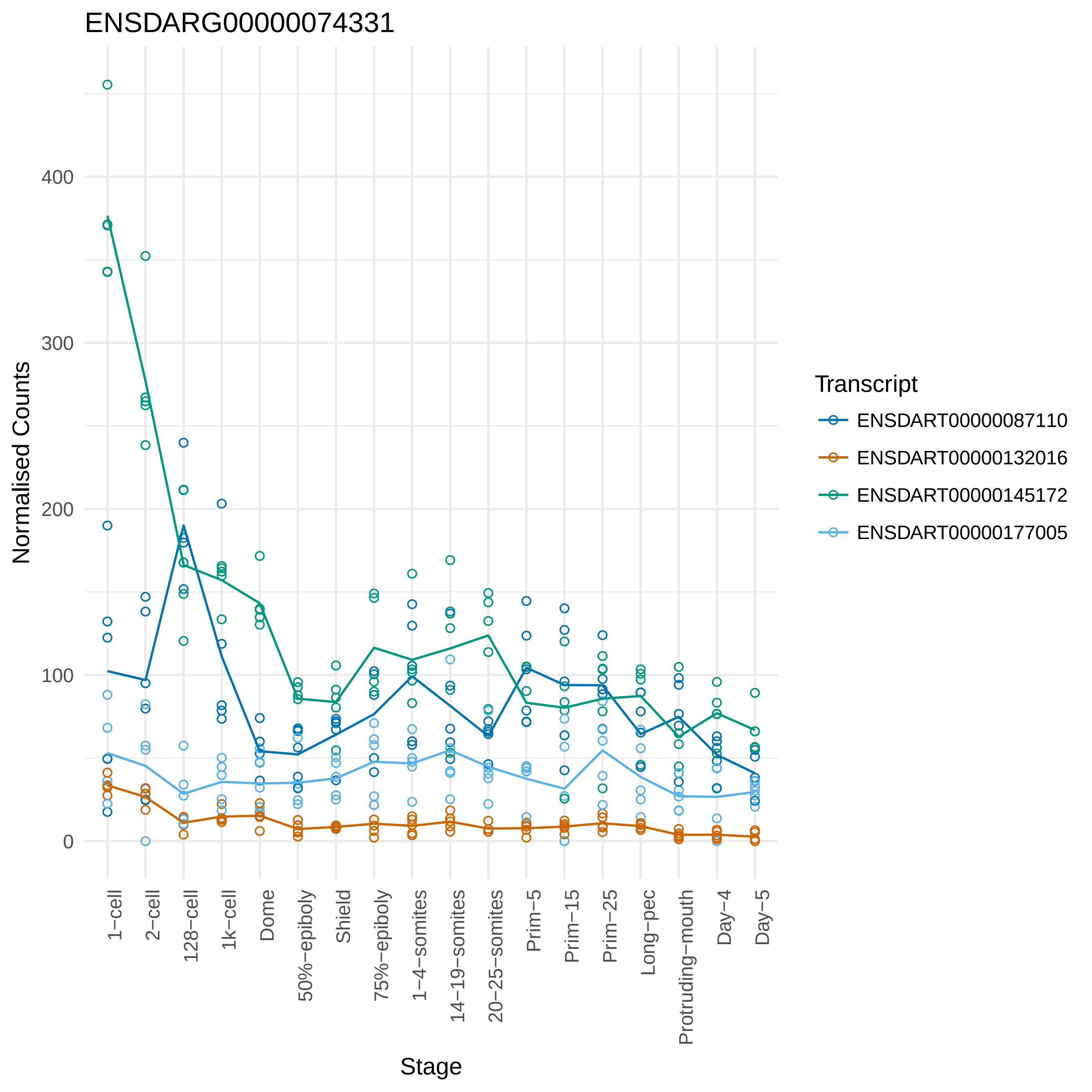

ENSDARG00000074331
Normalised Counts
0
100
200
300
400
Stage
1−cell
2−cell
128−cell
1k−cell
Dome
50%−epiboly
Shield
75%−epiboly
1−4−somites
14−19−somites
20−25−somites
Prim−5
Prim−15
Prim−25
Long−pec
Protruding−mouth
Day−4
Day−5
Transcript
ENSDART00000087110
ENSDART00000132016
ENSDART00000145172
ENSDART00000177005

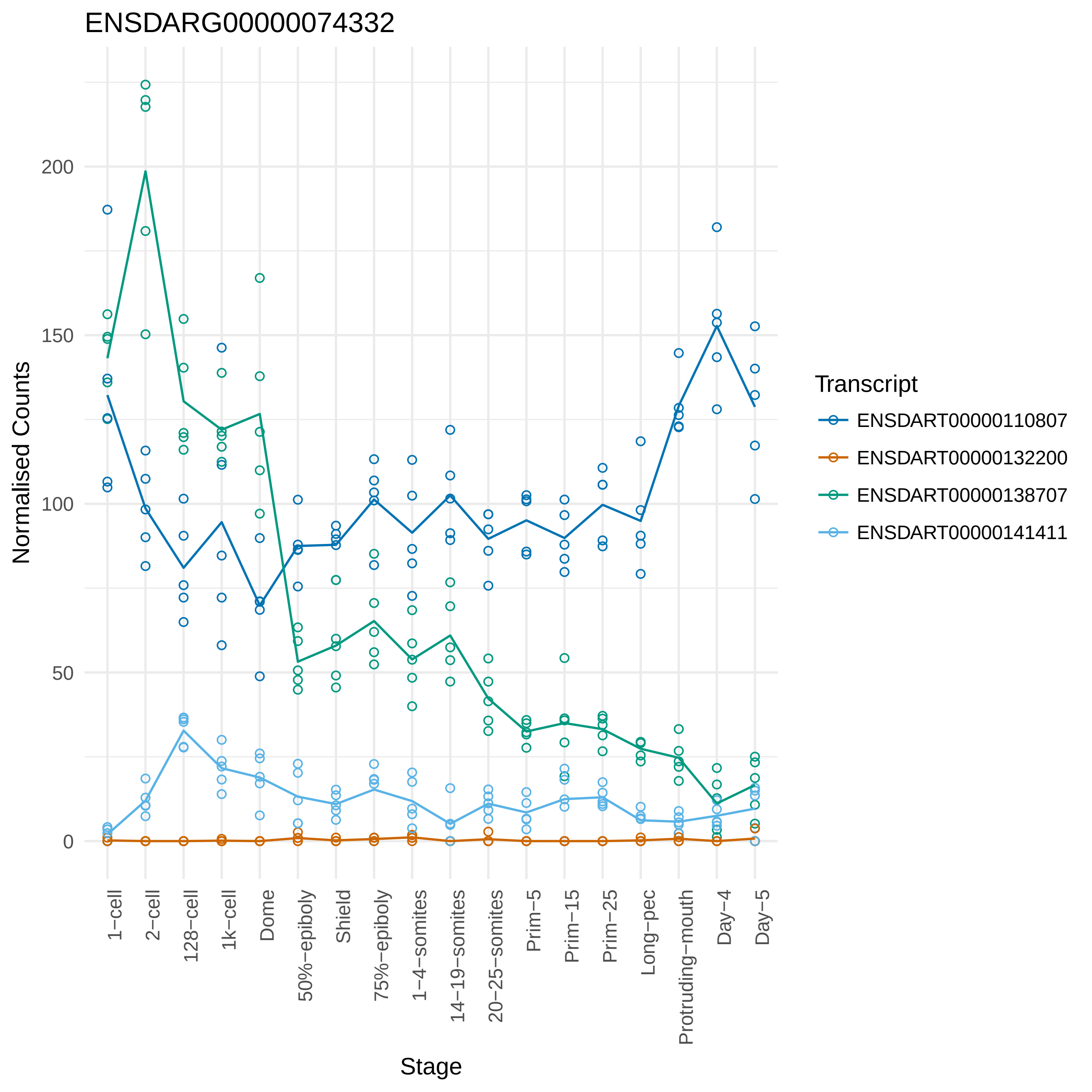

ENSDARG00000074332
Normalised Counts
0
50
100
150
200
Stage
1−cell
2−cell
128−cell
1k−cell
Dome
50%−epiboly
Shield
75%−epiboly
1−4−somites
14−19−somites
20−25−somites
Prim−5
Prim−15
Prim−25
Long−pec
Protruding−mouth
Day−4
Day−5
Transcript
ENSDART00000110807
ENSDART00000132200
ENSDART00000138707
ENSDART00000141411

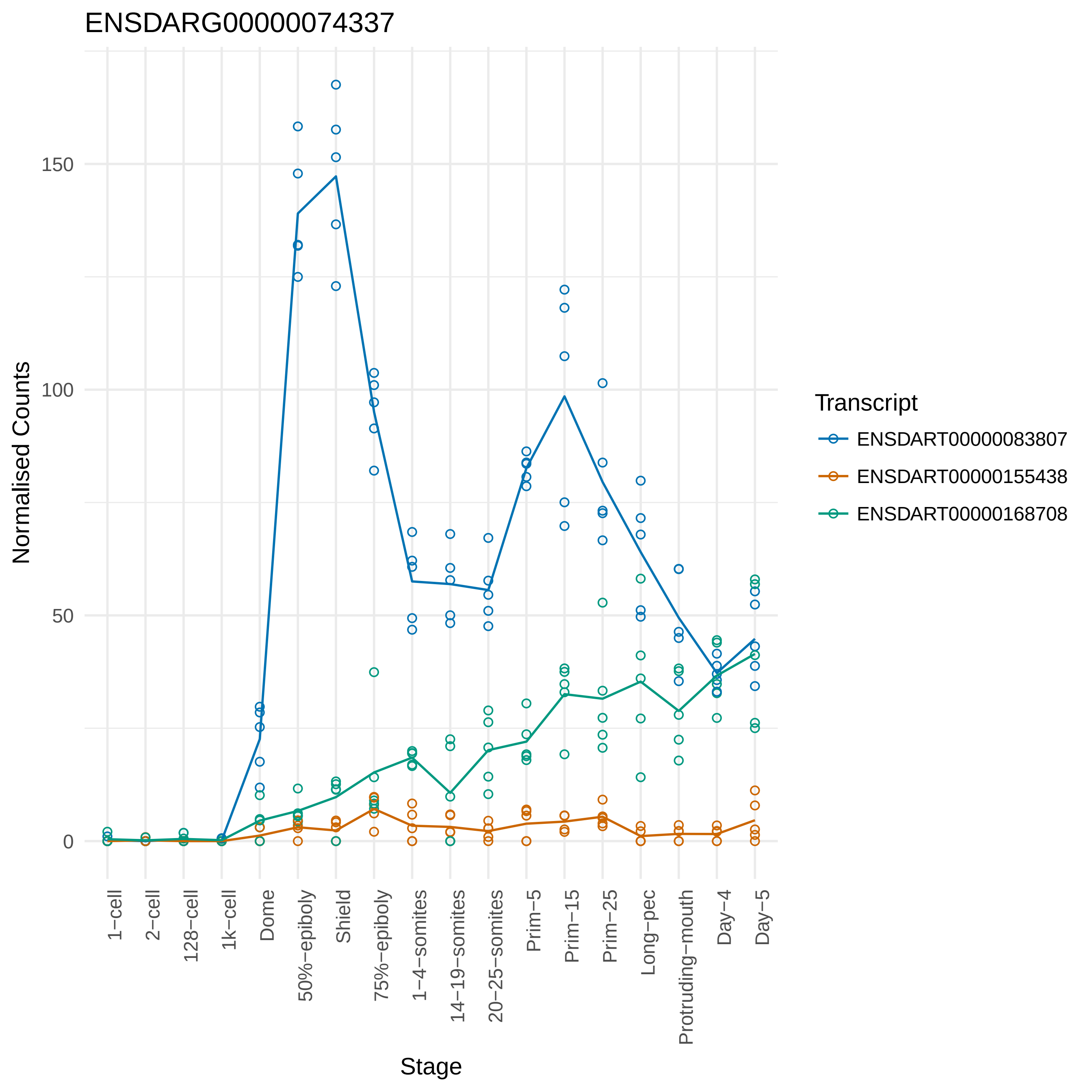

ENSDARG00000074337
Normalised Counts
150
100
50
0
1−cell
2−cell
128−cell
1k−cell
Dome
50%−epiboly
Shield
75%−epiboly
1−4−somites
14−19−somites
20−25−somites
Prim−5
Prim−15
Prim−25
Long−pec
Protruding−mouth
Day−4
Day−5
Stage
Transcript
ENSDART00000083807
ENSDART00000155438
ENSDART00000168708

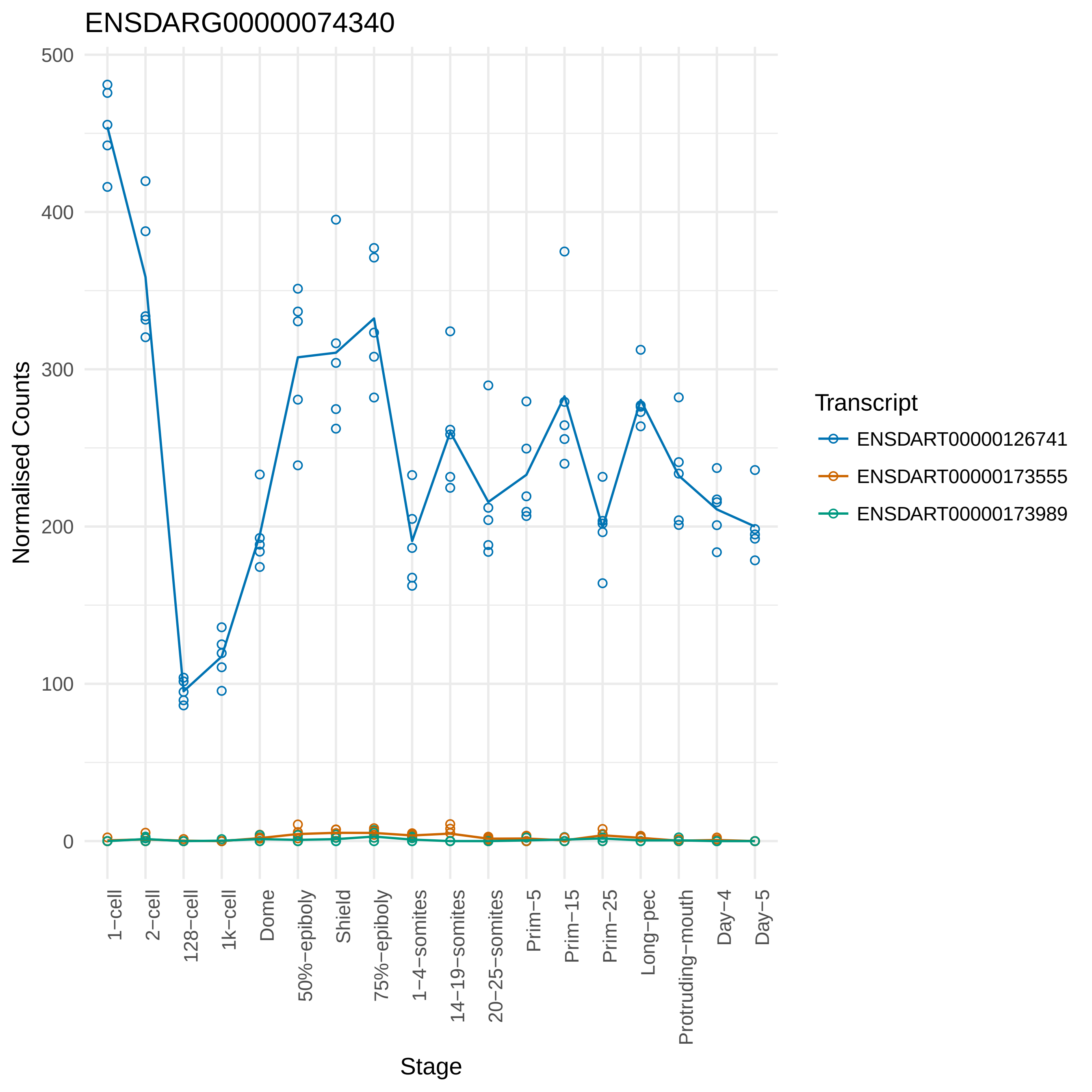

ENSDARG00000074340
Normalised Counts
500
400
300
200
100
0
1−cell
2−cell
128−cell
1k−cell
Dome
50%−epiboly
Shield
75%−epiboly
1−4−somites
14−19−somites
20−25−somites
Prim−5
Prim−15
Prim−25
Long−pec
Protruding−mouth
Day−4
Day−5
Stage
Transcript
ENSDART00000126741
ENSDART00000173555
ENSDART00000173989

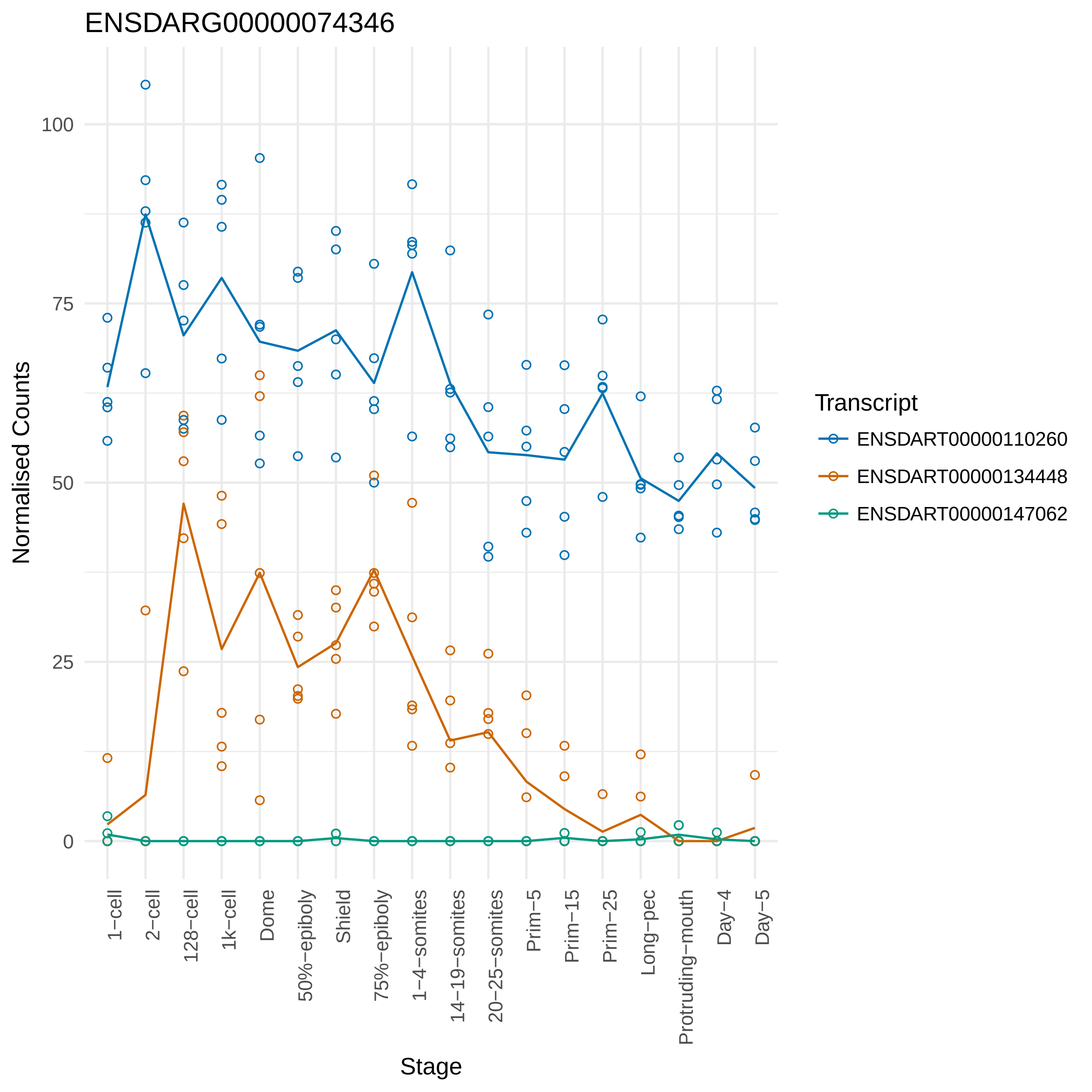
ENSDARG00000074346
Normalised Counts
100
75
50
25
0
1−cell
2−cell
128−cell
1k−cell
Dome
50%−epiboly
Shield
75%−epiboly
1−4−somites
14−19−somites
20−25−somites
Prim−5
Prim−15
Prim−25
Long−pec
Protruding−mouth
Day−4
Day−5
Stage
Transcript
ENSDART00000110260
ENSDART00000134448
ENSDART00000147062

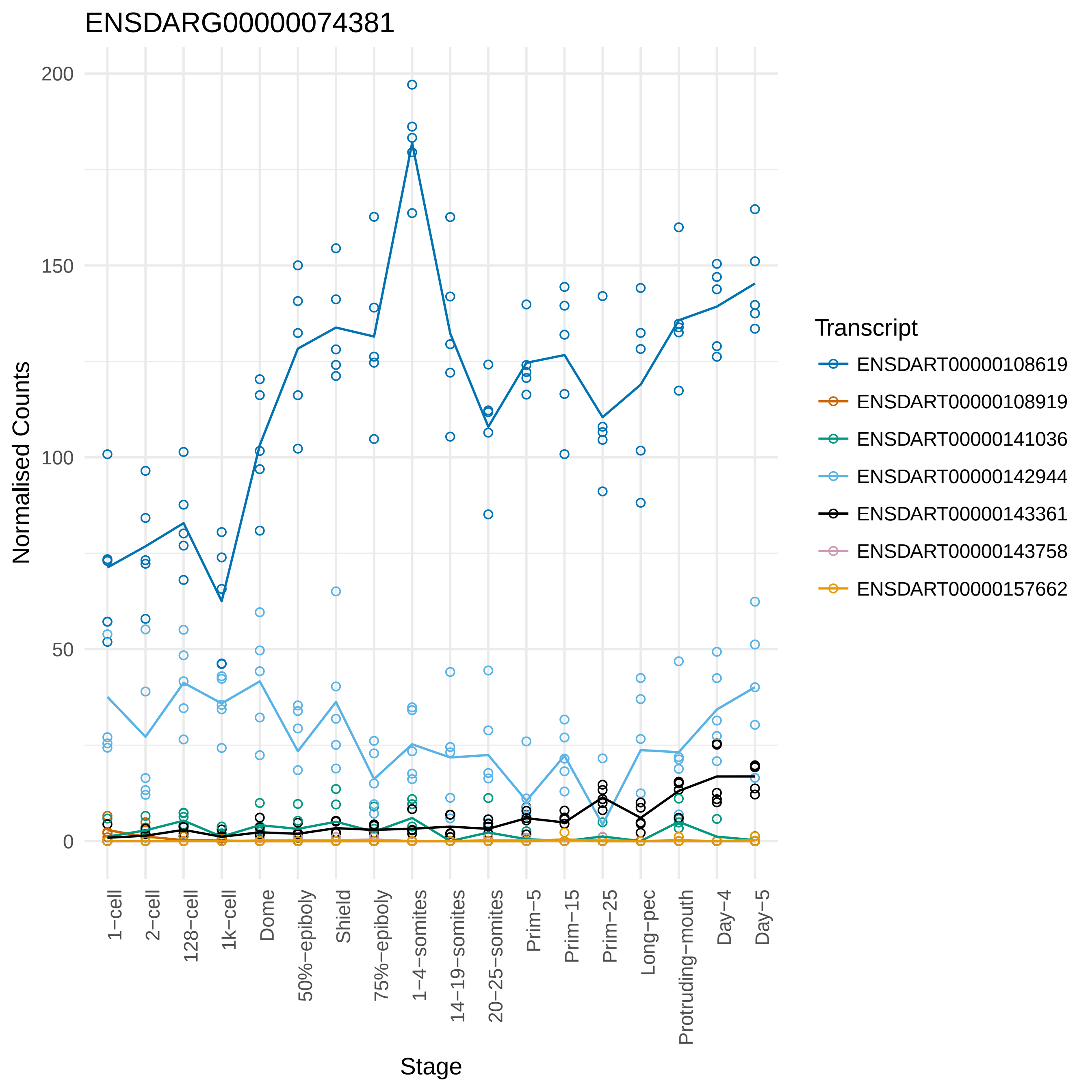

ENSDARG00000074381
Normalised Counts
200
150
100
50
0
1−cell
2−cell
128−cell
1k−cell
Dome
50%−epiboly
Shield
75%−epiboly
1−4−somites
14−19−somites
20−25−somites
Prim−5
Prim−15
Prim−25
Long−pec
Protruding−mouth
Day−4
Day−5
Stage
Transcript
ENSDART00000108619
ENSDART00000108919
ENSDART00000141036
ENSDART00000142944
ENSDART00000143361
ENSDART00000143758
ENSDART00000157662

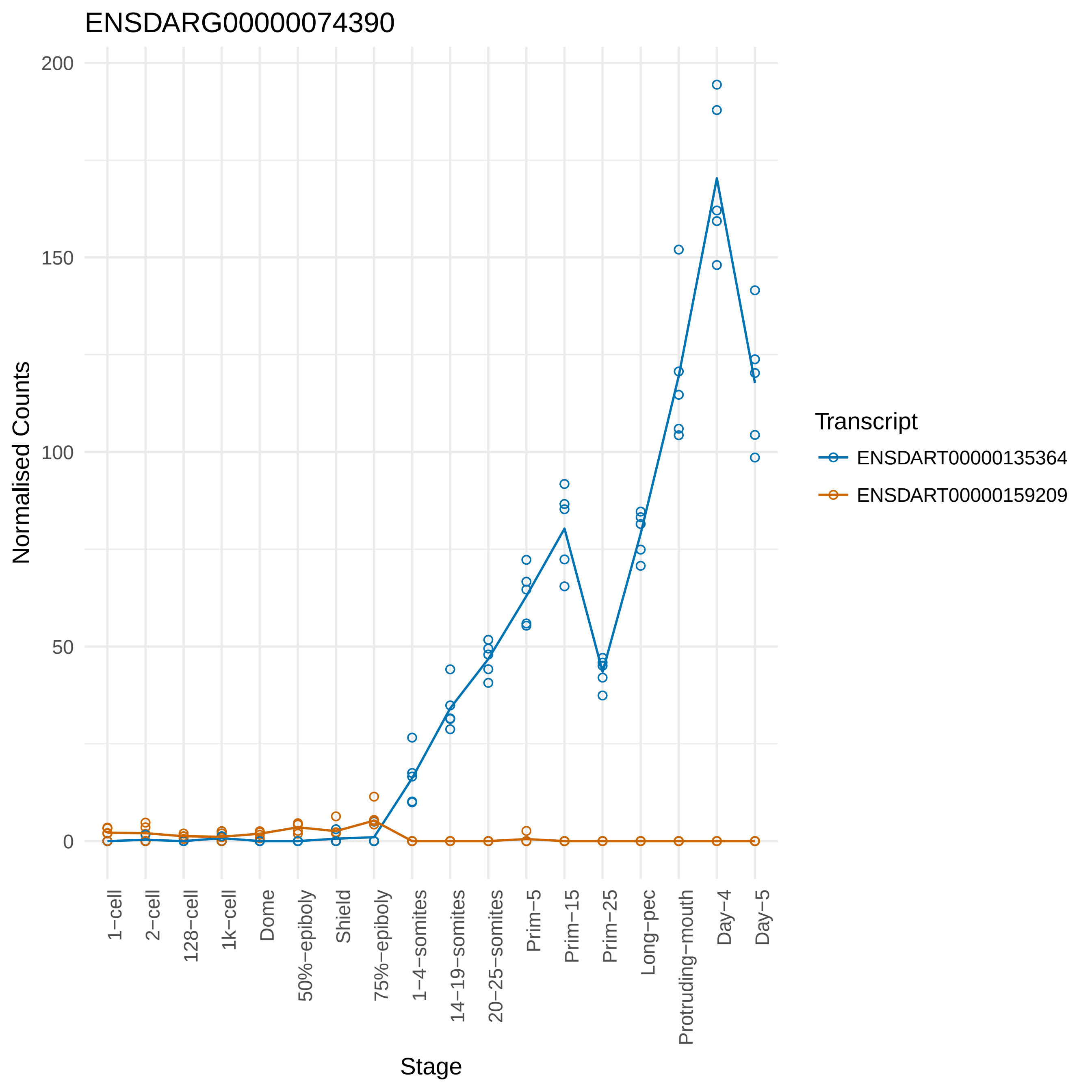

ENSDARG00000074390
Normalised Counts
200
150
100
50
0
1−cell
2−cell
128−cell
1k−cell
Dome
50%−epiboly
Shield
75%−epiboly
1−4−somites
14−19−somites
20−25−somites
Prim−5
Prim−15
Prim−25
Long−pec
Protruding−mouth
Day−4
Day−5
Stage
Transcript
ENSDART00000135364
ENSDART00000159209

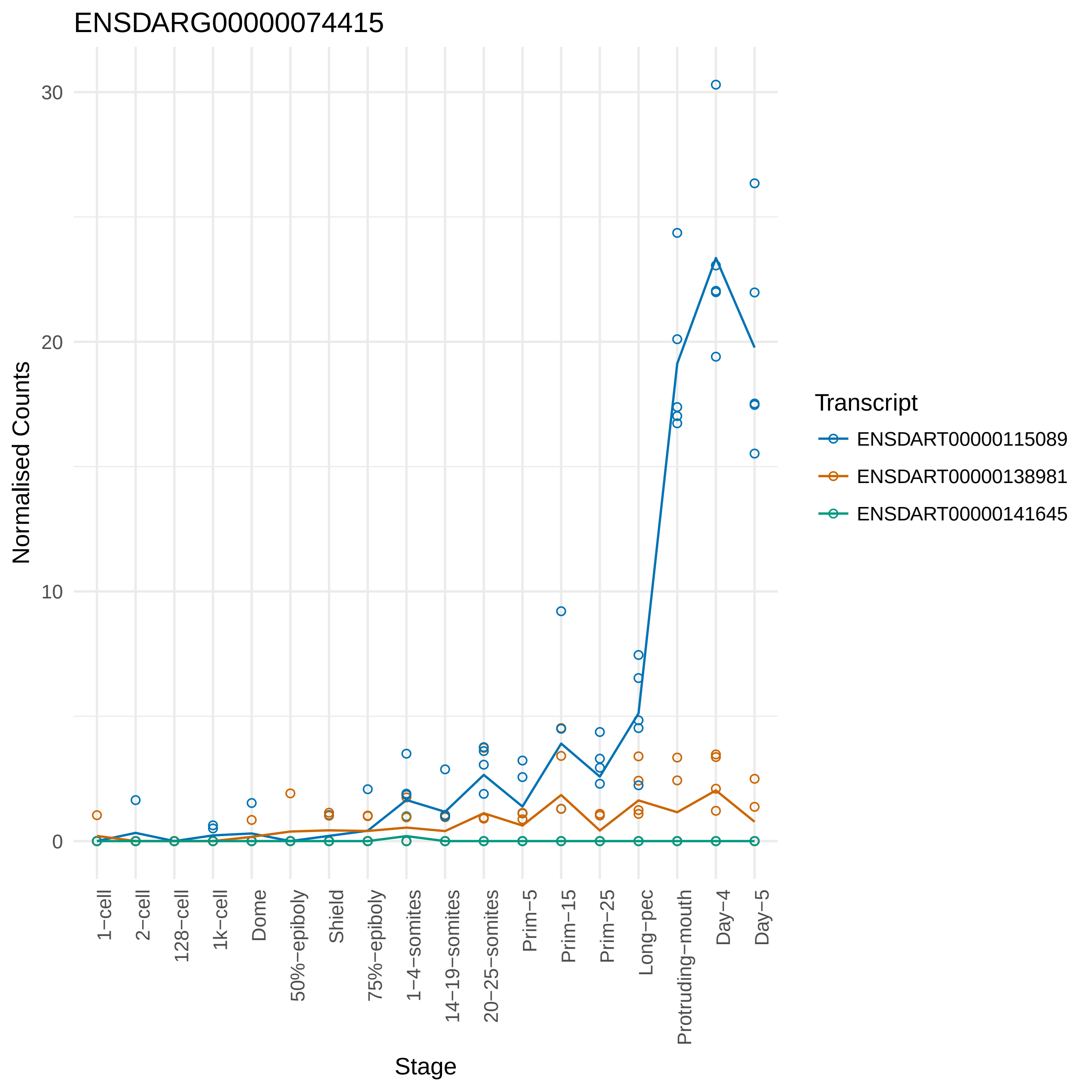

ENSDARG00000074415
Normalised Counts
30
20
10
0
Stage
1−cell
2−cell
128−cell
1k−cell
Dome
50%−epiboly
Shield
75%−epiboly
1−4−somites
14−19−somites
20−25−somites
Prim−5
Prim−15
Prim−25
Long−pec
Protruding−mouth
Day−4
Day−5
Transcript
ENSDART00000115089
ENSDART00000138981
ENSDART00000141645

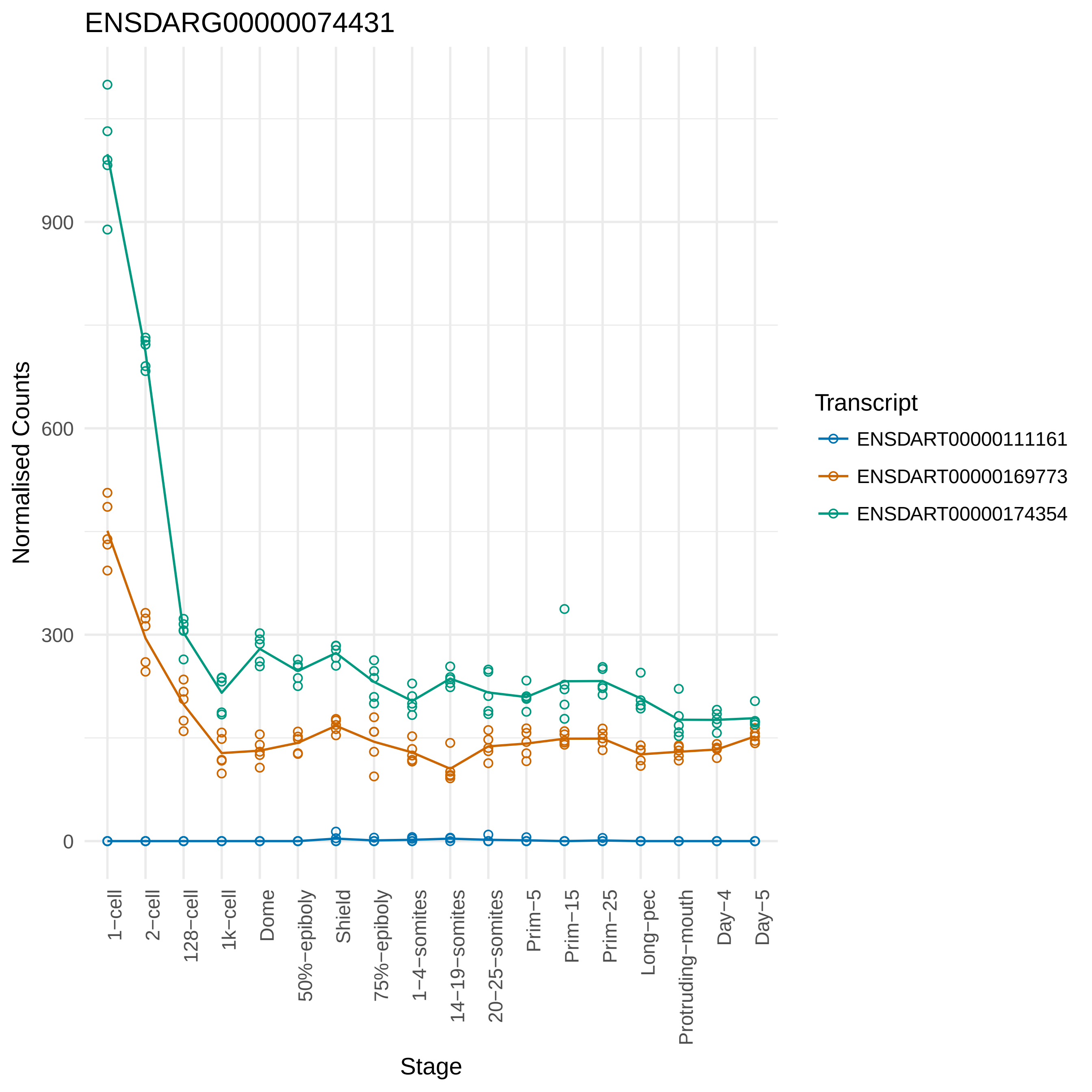
ENSDARG00000074431
Normalised Counts
900
600
300
0
1−cell
2−cell
128−cell
1k−cell
Dome
50%−epiboly
Shield
75%−epiboly
1−4−somites
14−19−somites
20−25−somites
Prim−5
Prim−15
Prim−25
Long−pec
Protruding−mouth
Day−4
Day−5
Stage
Transcript
ENSDART00000111161
ENSDART00000169773
ENSDART00000174354

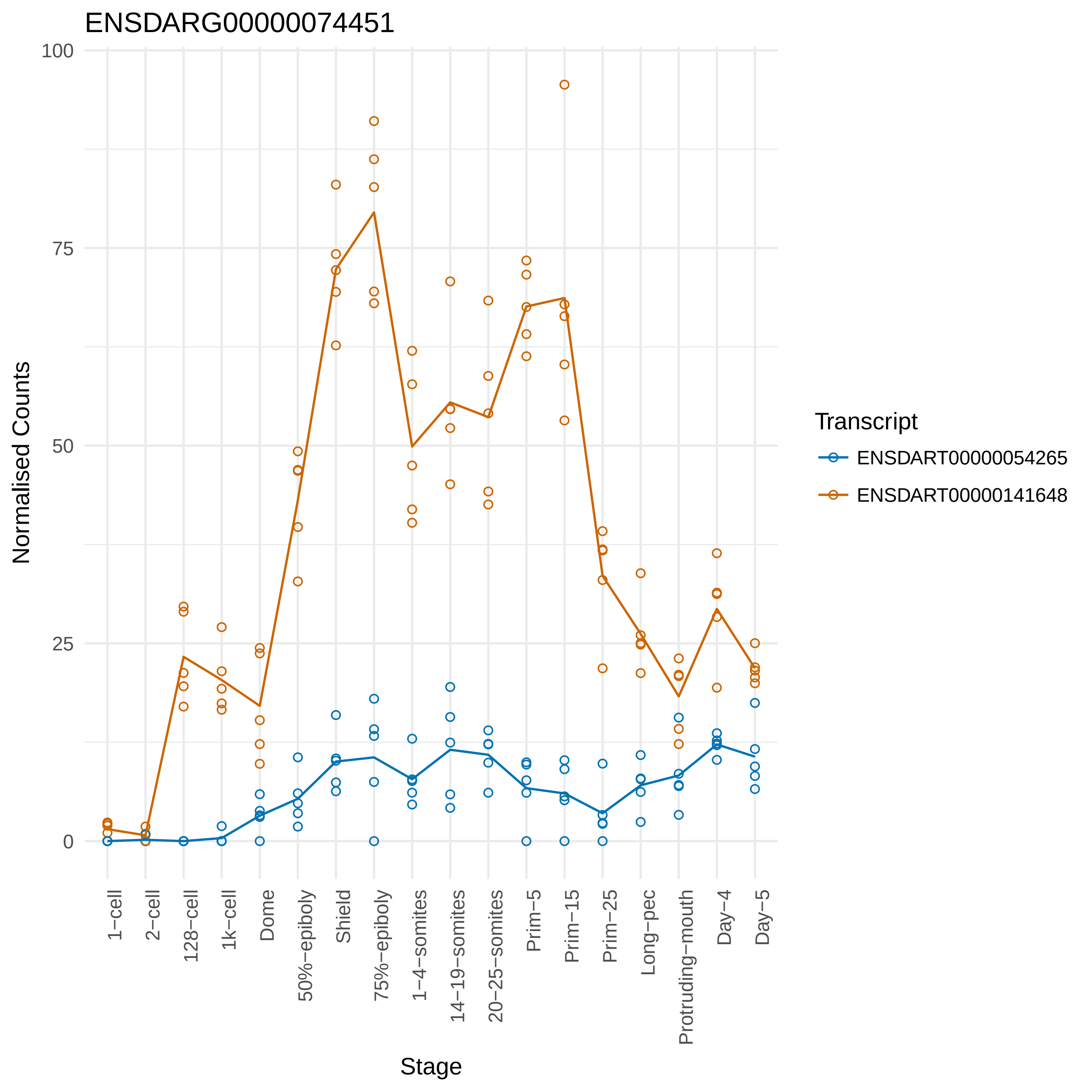

ENSDARG00000074451
Normalised Counts
100
75
50
25
0
1−cell
2−cell
128−cell
1k−cell
Dome
50%−epiboly
Shield
75%−epiboly
1−4−somites
14−19−somites
20−25−somites
Prim−5
Prim−15
Prim−25
Long−pec
Protruding−mouth
Day−4
Day−5
Stage
Transcript
ENSDART00000054265
ENSDART00000141648

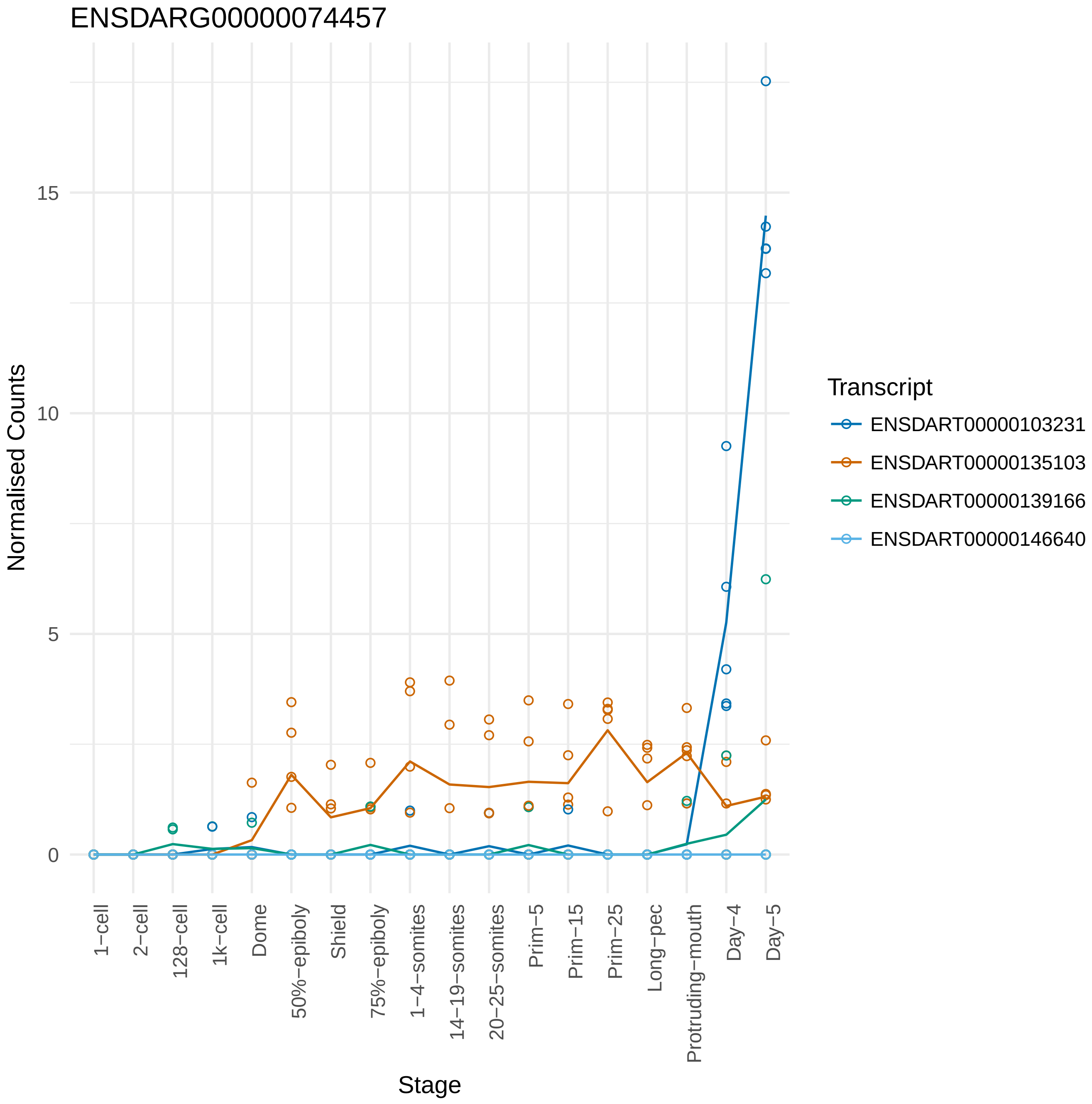

ENSDARG00000074457
Normalised Counts
0
5
10
15
1−cell
2−cell
128−cell
1k−cell
Dome
50%−epiboly
Shield
75%−epiboly
1−4−somites
14−19−somites
20−25−somites
Prim−5
Prim−15
Prim−25
Long−pec
Protruding−mouth
Day−4
Day−5
Stage
Transcript
ENSDART00000103231
ENSDART00000135103
ENSDART00000139166
ENSDART00000146640

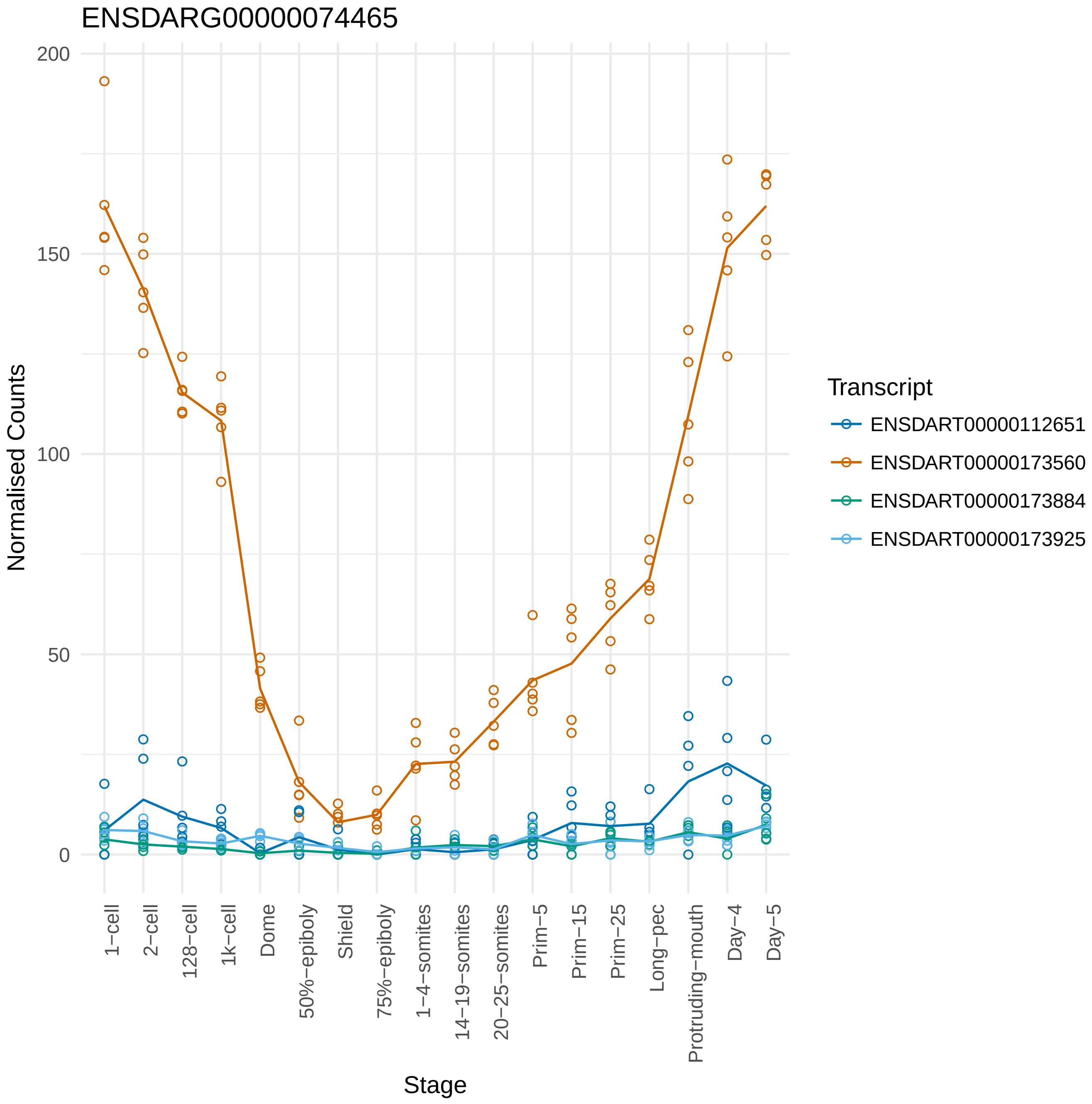

ENSDARG00000074465
Normalised Counts
200
150
100
50
0
1−cell
2−cell
128−cell
1k−cell
Dome
50%−epiboly
Shield
75%−epiboly
1−4−somites
14−19−somites
20−25−somites
Prim−5
Prim−15
Prim−25
Long−pec
Protruding−mouth
Day−4
Day−5
Stage
Transcript
ENSDART00000112651
ENSDART00000173560
ENSDART00000173884
ENSDART00000173925

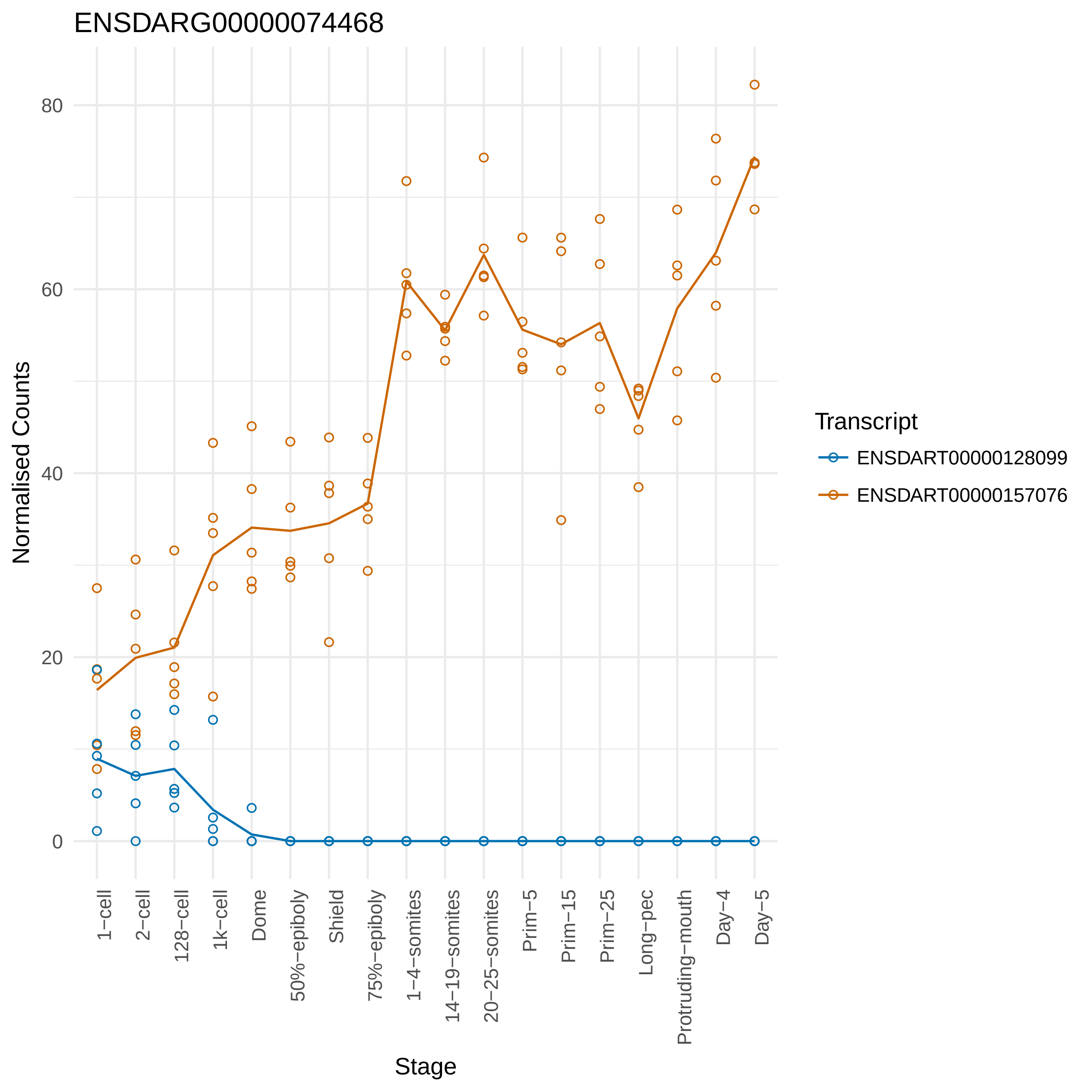
ENSDARG00000074468
Normalised Counts
0
20
40
60
80
Stage
1−cell
2−cell
128−cell
1k−cell
Dome
50%−epiboly
Shield
75%−epiboly
1−4−somites
14−19−somites
20−25−somites
Prim−5
Prim−15
Prim−25
Long−pec
Protruding−mouth
Day−4
Day−5
Transcript
ENSDART00000128099
ENSDART00000157076

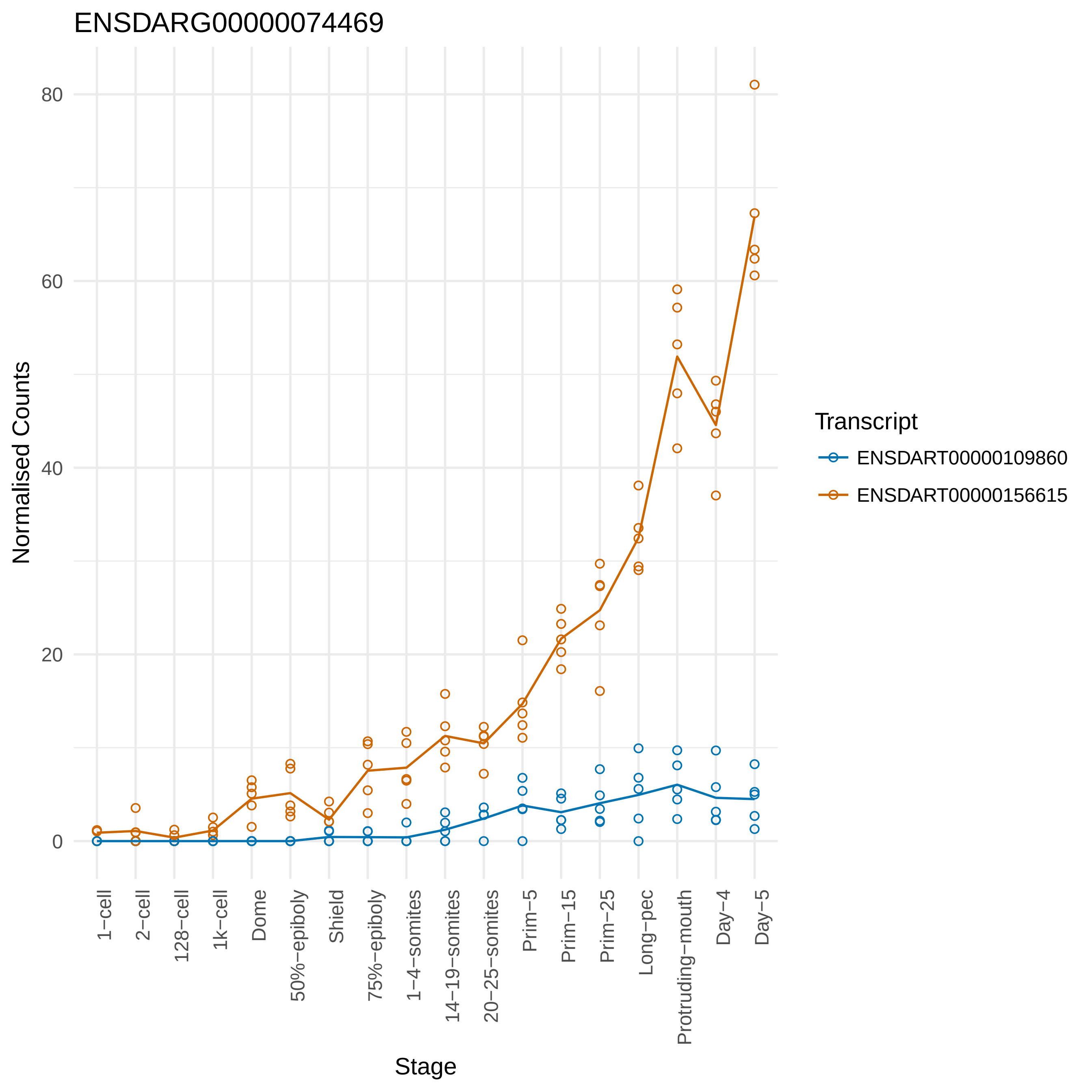

ENSDARG00000074469
Normalised Counts
0
20
40
60
80
Stage
1−cell
2−cell
128−cell
1k−cell
Dome
50%−epiboly
Shield
75%−epiboly
1−4−somites
14−19−somites
20−25−somites
Prim−5
Prim−15
Prim−25
Long−pec
Protruding−mouth
Day−4
Day−5
Transcript
ENSDART00000109860
ENSDART00000156615

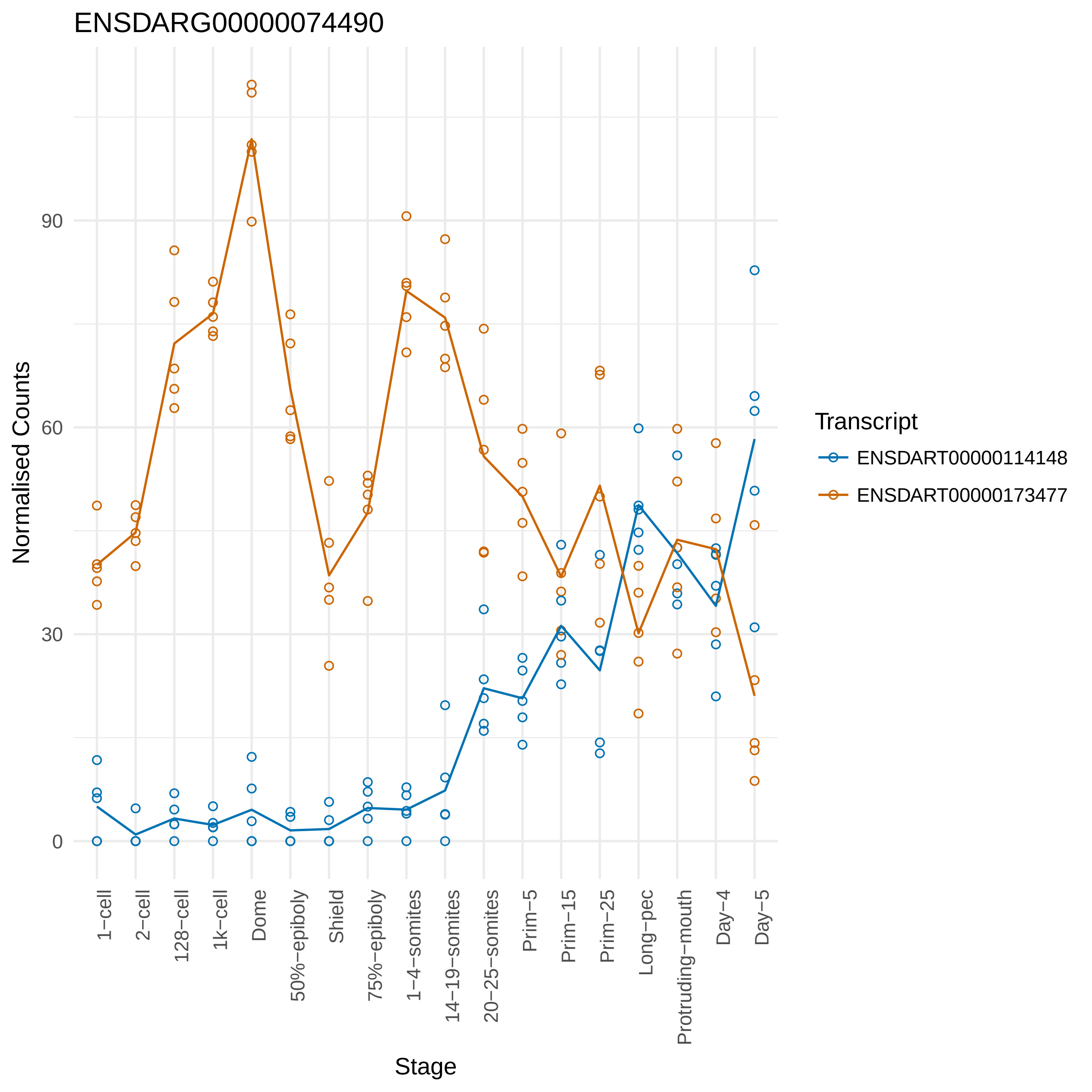

ENSDARG00000074490
Normalised Counts
0
30
60
90
Stage
1−cell
2−cell
128−cell
1k−cell
Dome
50%−epiboly
Shield
75%−epiboly
1−4−somites
14−19−somites
20−25−somites
Prim−5
Prim−15
Prim−25
Long−pec
Protruding−mouth
Day−4
Day−5
Transcript
ENSDART00000114148
ENSDART00000173477

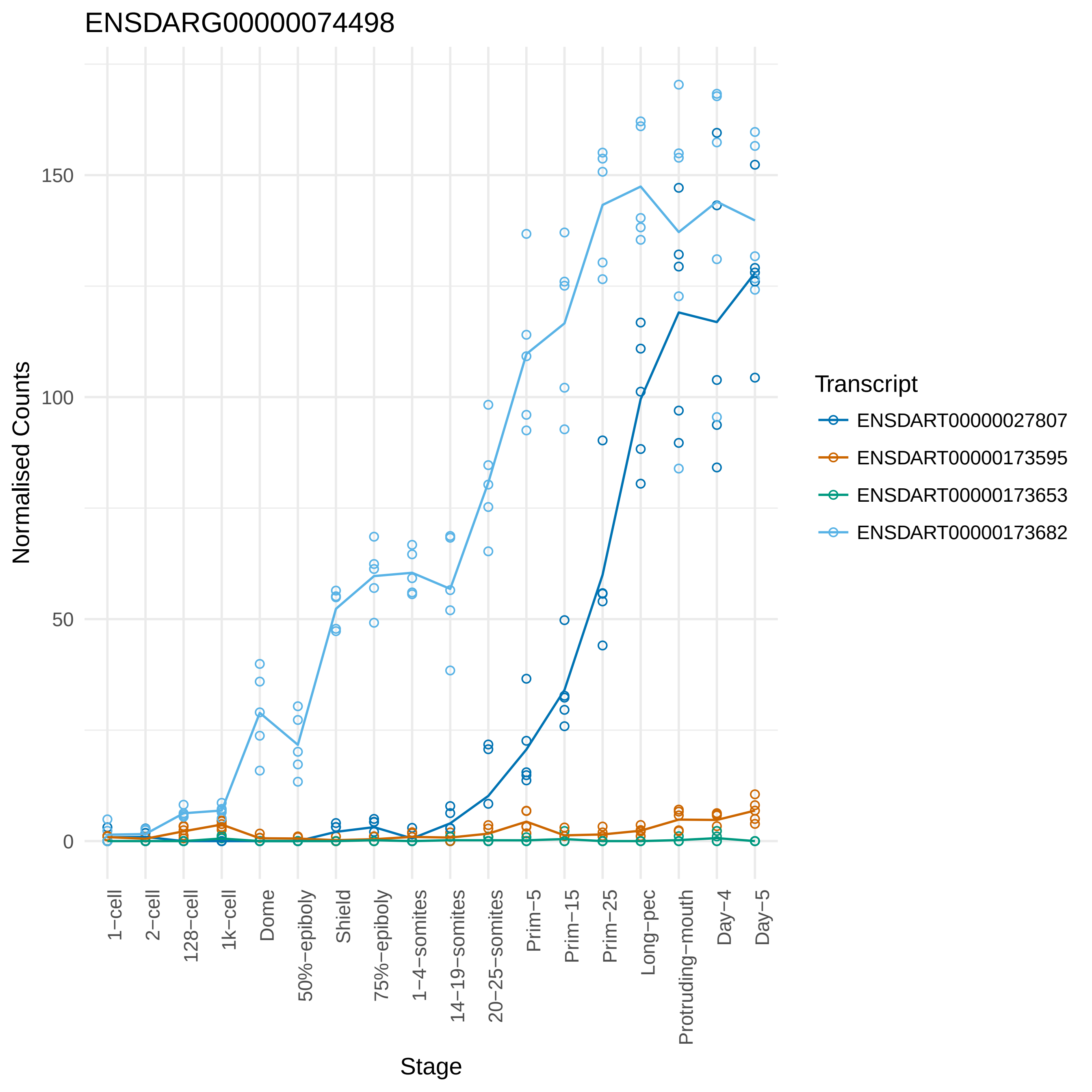

ENSDARG00000074498
Normalised Counts
0
50
100
150
Stage
1−cell
2−cell
128−cell
1k−cell
Dome
50%−epiboly
Shield
75%−epiboly
1−4−somites
14−19−somites
20−25−somites
Prim−5
Prim−15
Prim−25
Long−pec
Protruding−mouth
Day−4
Day−5
Transcript
ENSDART00000027807
ENSDART00000173595
ENSDART00000173653
ENSDART00000173682

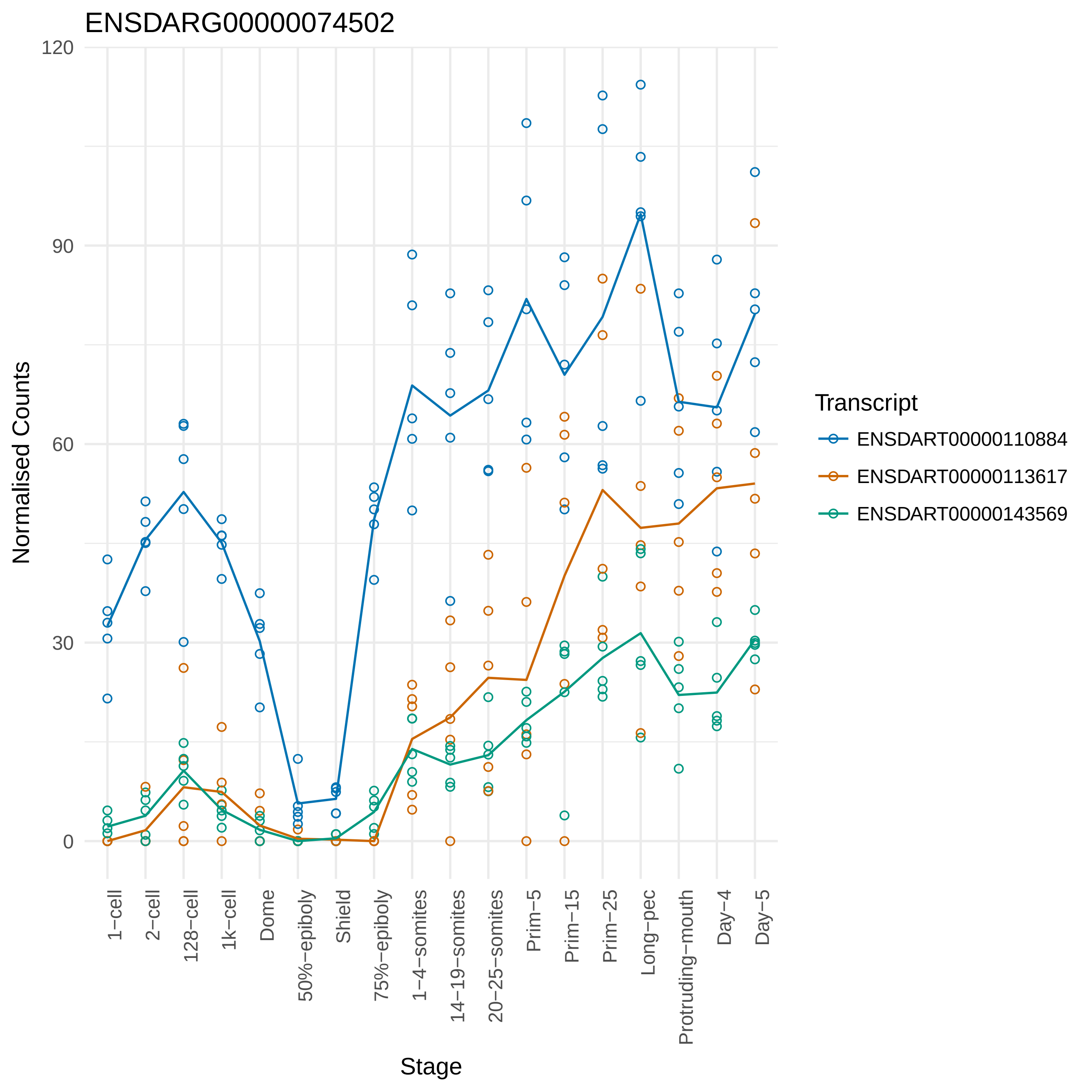
ENSDARG00000074502
Normalised Counts
120
90
60
30
0
Stage
1−cell
2−cell
128−cell
1k−cell
Dome
50%−epiboly
Shield
75%−epiboly
1−4−somites
14−19−somites
20−25−somites
Prim−5
Prim−15
Prim−25
Long−pec
Protruding−mouth
Day−4
Day−5
Transcript
ENSDART00000110884
ENSDART00000113617
ENSDART00000143569

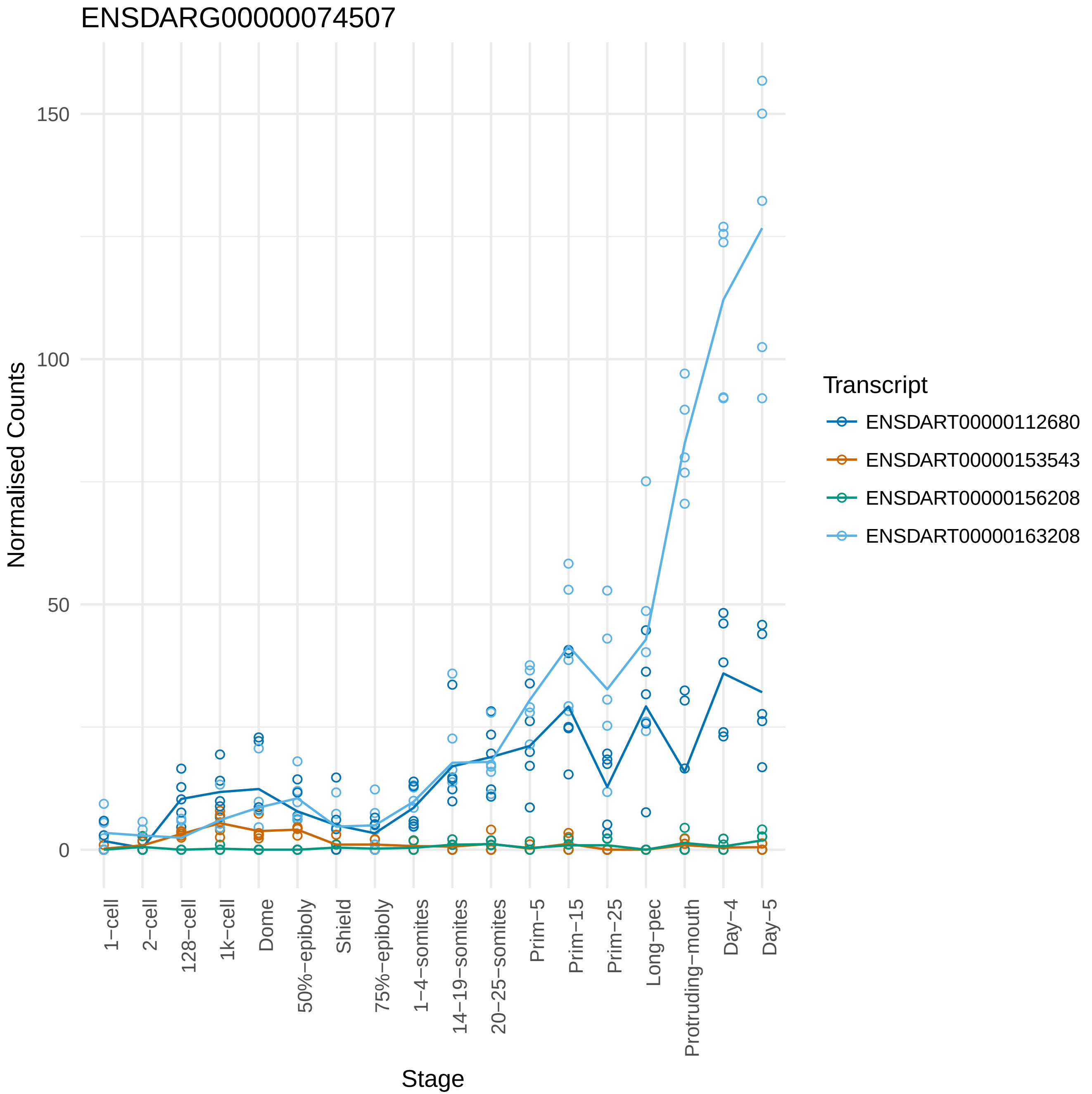
ENSDARG00000074507
Normalised Counts
150
100
50
0
1−cell
2−cell
128−cell
1k−cell
Dome
50%−epiboly
Shield
75%−epiboly
1−4−somites
14−19−somites
20−25−somites
Prim−5
Prim−15
Prim−25
Long−pec
Protruding−mouth
Day−4
Day−5
Stage
Transcript
ENSDART00000112680
ENSDART00000153543
ENSDART00000156208
ENSDART00000163208

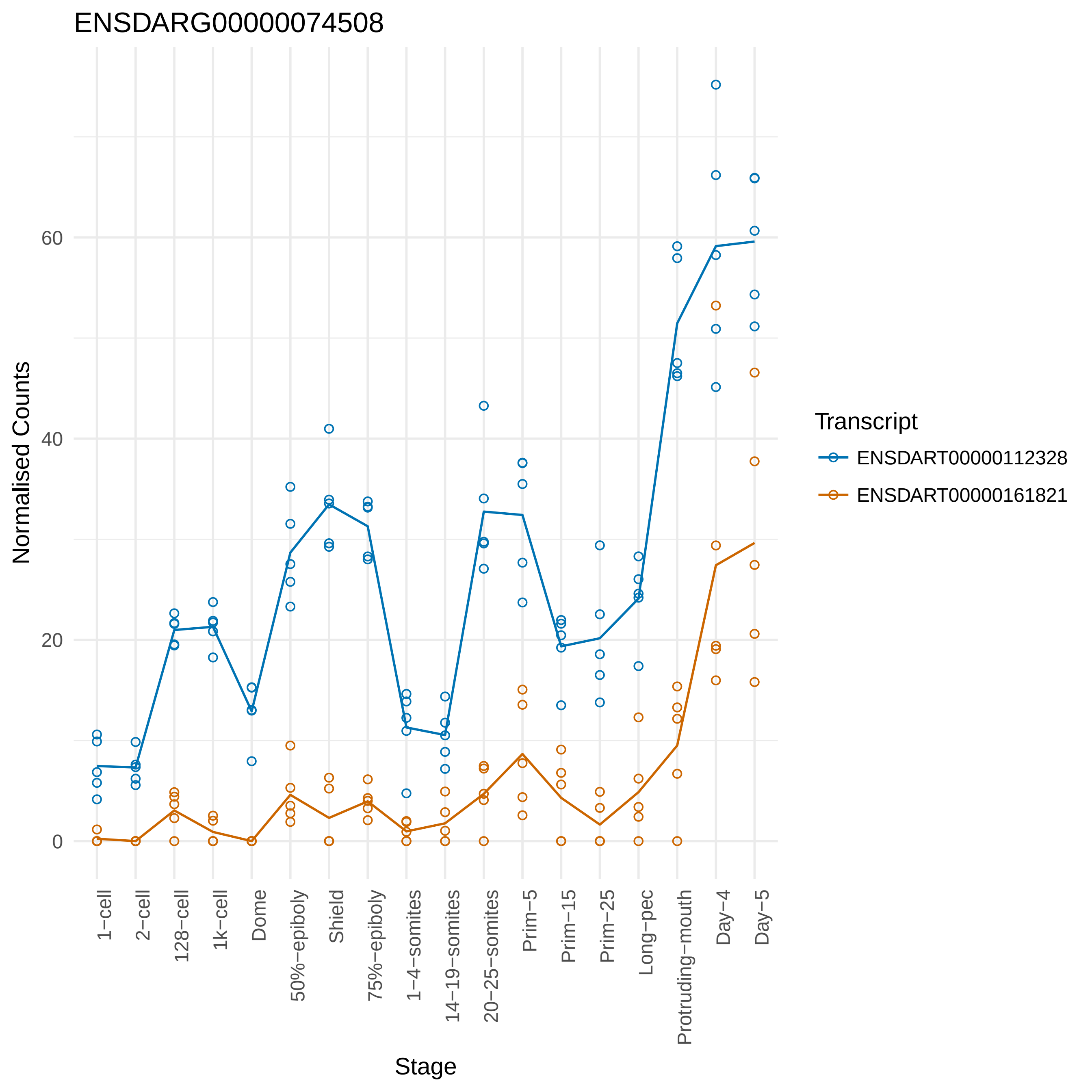
ENSDARG00000074508
Normalised Counts
0
20
40
60
Stage
1−cell
2−cell
128−cell
1k−cell
Dome
50%−epiboly
Shield
75%−epiboly
1−4−somites
14−19−somites
20−25−somites
Prim−5
Prim−15
Prim−25
Long−pec
Protruding−mouth
Day−4
Day−5
Transcript
ENSDART00000112328
ENSDART00000161821

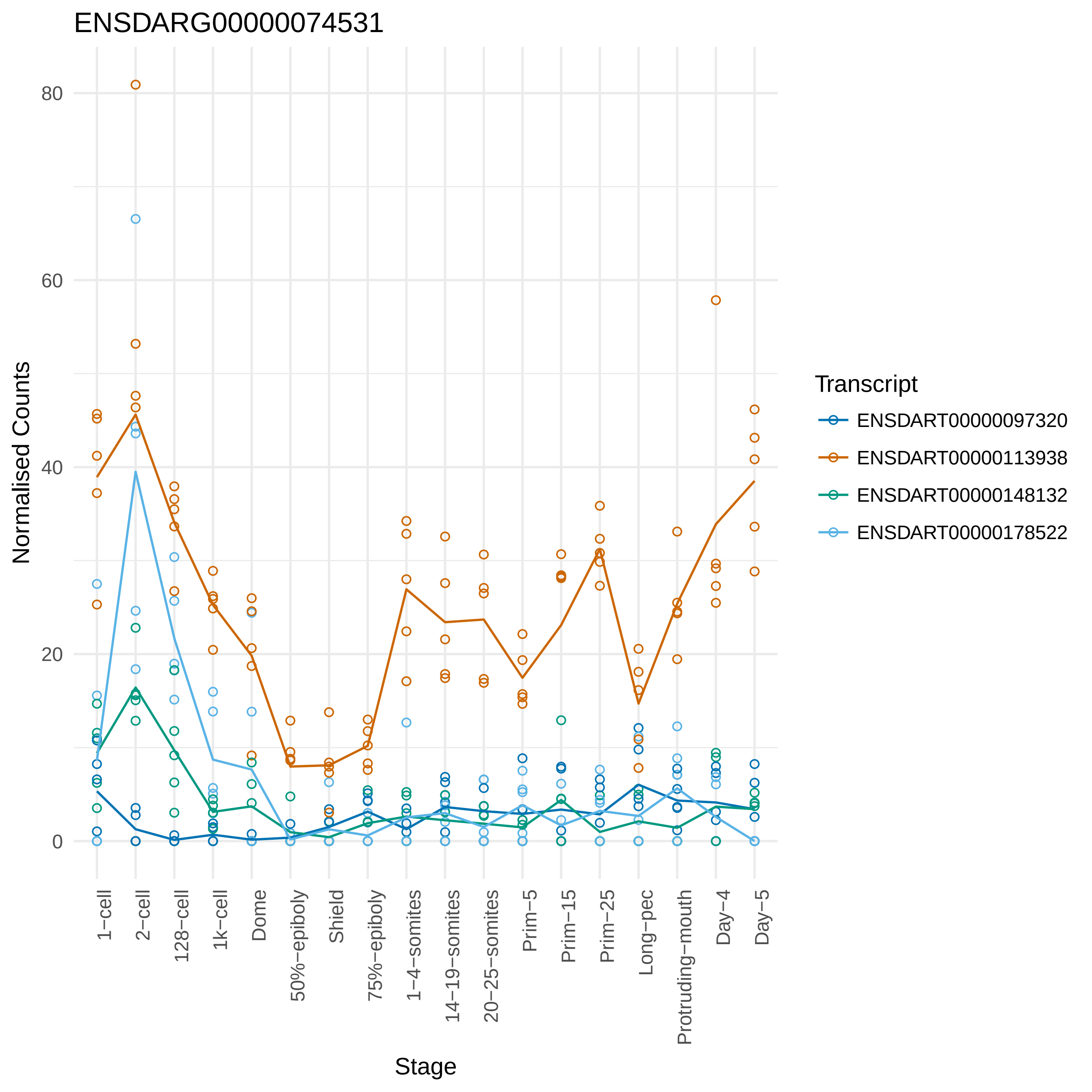
ENSDARG00000074531
Normalised Counts
0
20
40
60
80
Stage
1−cell
2−cell
128−cell
1k−cell
Dome
50%−epiboly
Shield
75%−epiboly
1−4−somites
14−19−somites
20−25−somites
Prim−5
Prim−15
Prim−25
Long−pec
Protruding−mouth
Day−4
Day−5
Transcript
ENSDART00000097320
ENSDART00000113938
ENSDART00000148132
ENSDART00000178522

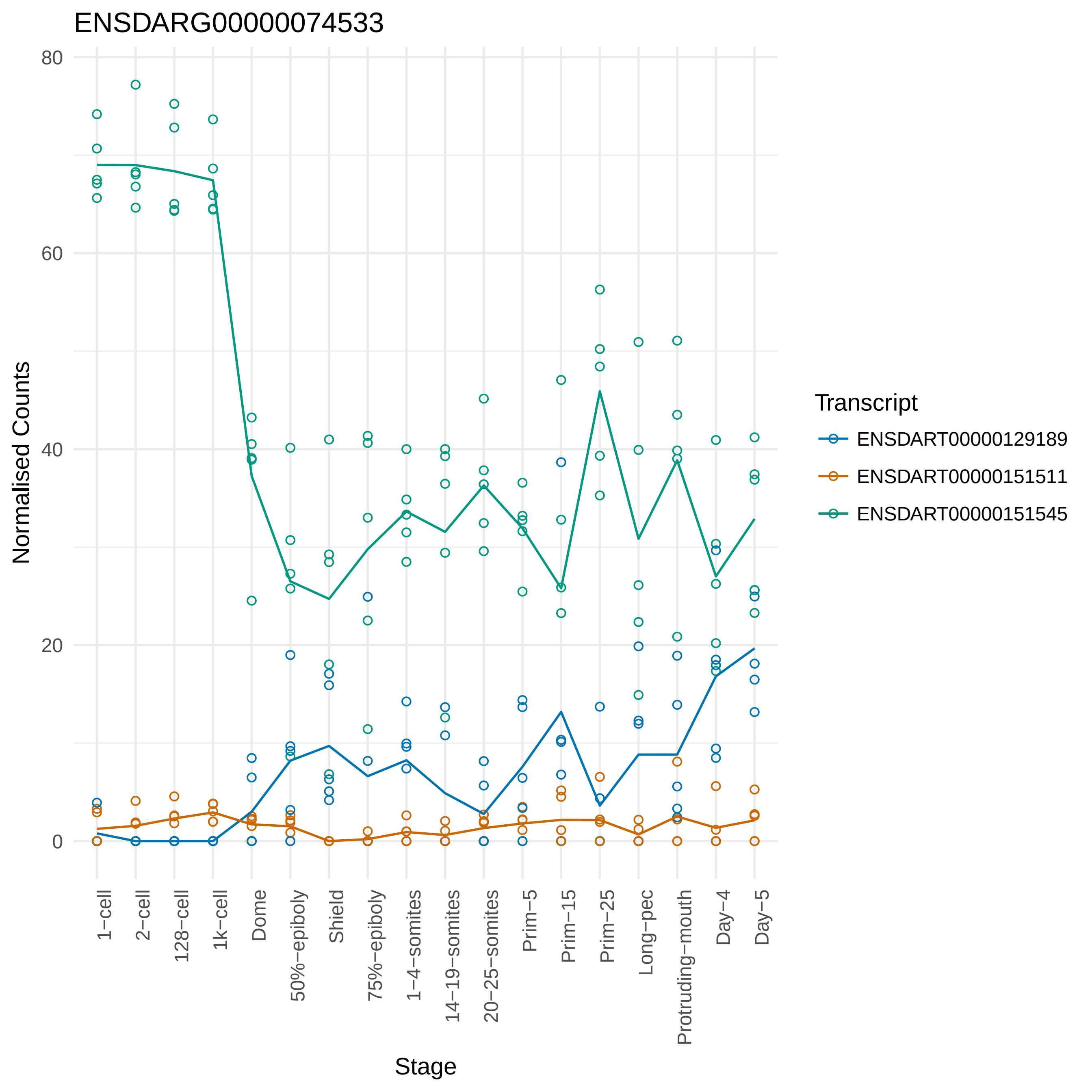
ENSDARG00000074533
Normalised Counts
80
60
40
20
0
1−cell
2−cell
128−cell
1k−cell
Dome
50%−epiboly
Shield
75%−epiboly
1−4−somites
14−19−somites
20−25−somites
Prim−5
Prim−15
Prim−25
Long−pec
Protruding−mouth
Day−4
Day−5
Stage
Transcript
ENSDART00000129189
ENSDART00000151511
ENSDART00000151545

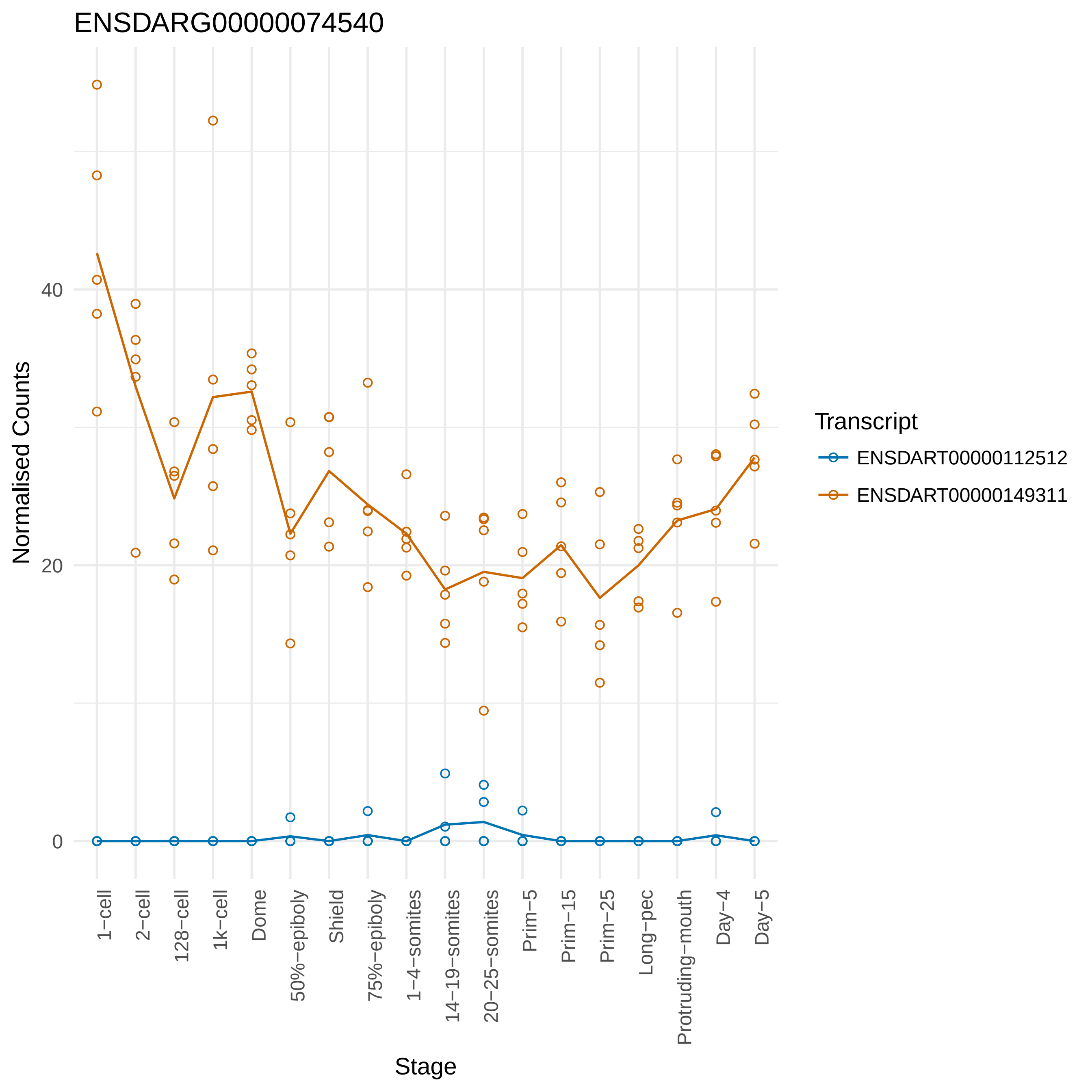
ENSDARG00000074540
Normalised Counts
0
20
40
Stage
1−cell
2−cell
128−cell
1k−cell
Dome
50%−epiboly
Shield
75%−epiboly
1−4−somites
14−19−somites
20−25−somites
Prim−5
Prim−15
Prim−25
Long−pec
Protruding−mouth
Day−4
Day−5
Transcript
ENSDART00000112512
ENSDART00000149311

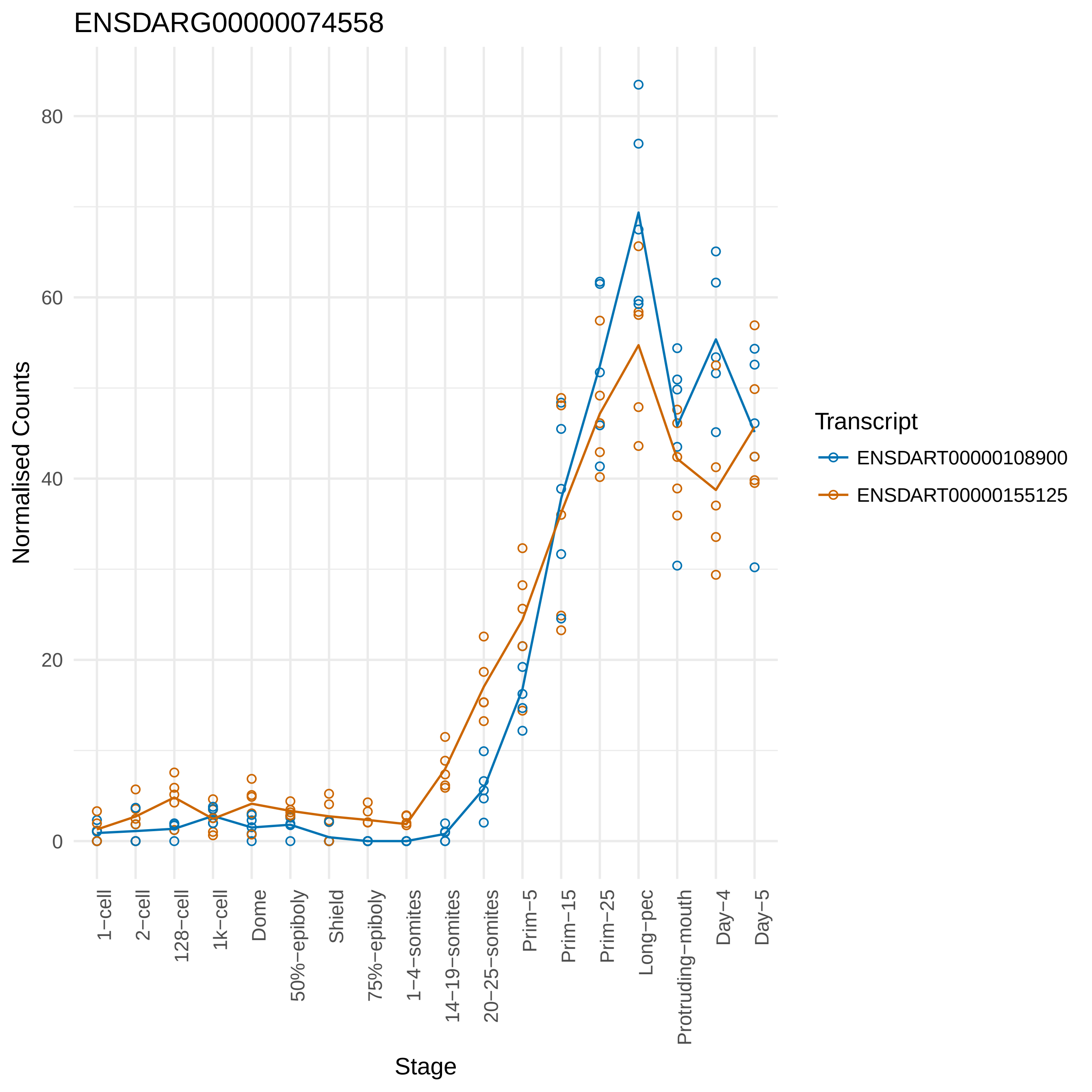

ENSDARG00000074558
Normalised Counts
80
60
40
20
0
1−cell
2−cell
128−cell
1k−cell
Dome
50%−epiboly
Shield
75%−epiboly
1−4−somites
14−19−somites
20−25−somites
Prim−5
Prim−15
Prim−25
Long−pec
Protruding−mouth
Day−4
Day−5
Stage
Transcript
ENSDART00000108900
ENSDART00000155125

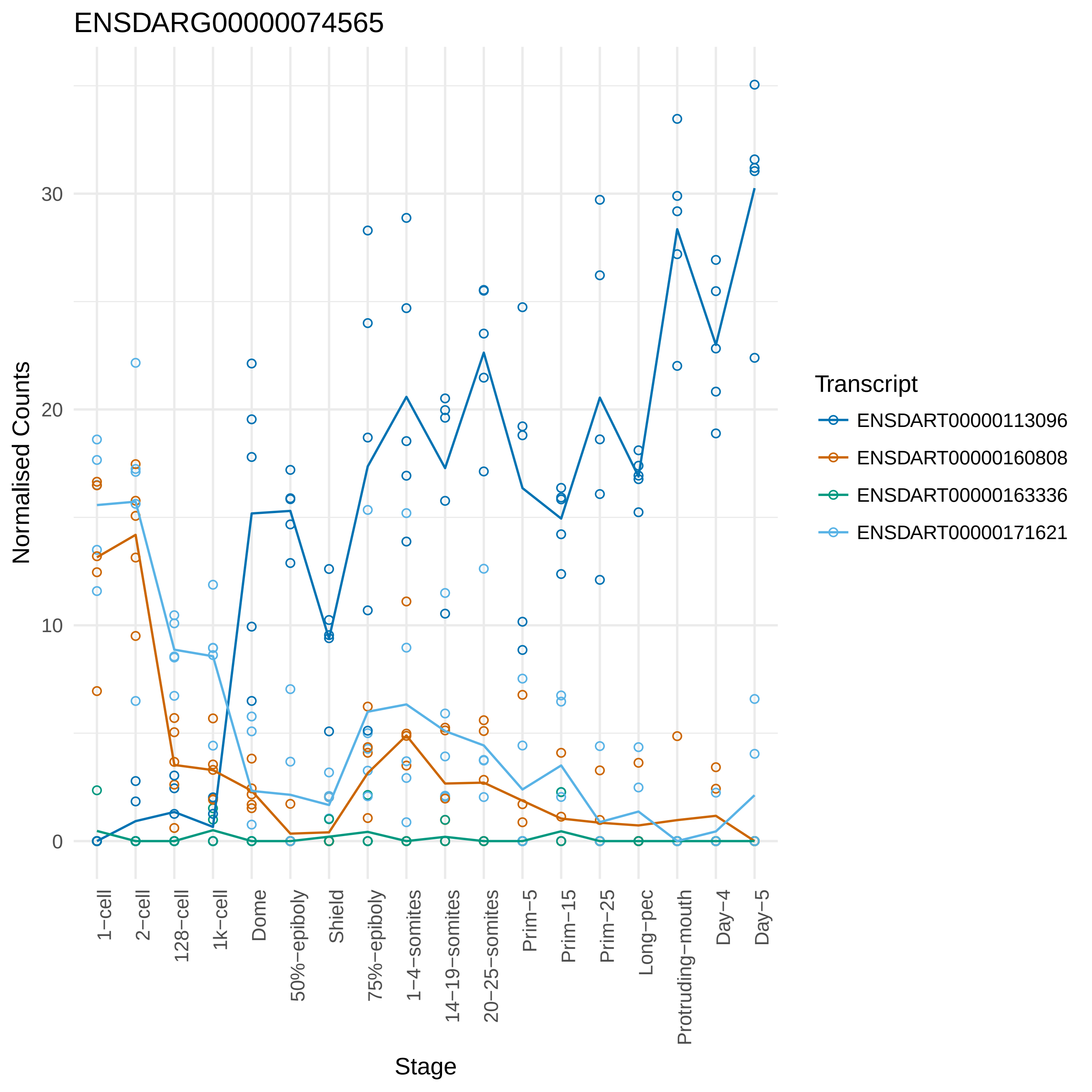
ENSDARG00000074565
Normalised Counts
0
10
20
30
1−cell
2−cell
128−cell
1k−cell
Dome
50%−epiboly
Shield
75%−epiboly
1−4−somites
14−19−somites
20−25−somites
Prim−5
Prim−15
Prim−25
Long−pec
Protruding−mouth
Day−4
Day−5
Stage
Transcript
ENSDART00000113096
ENSDART00000160808
ENSDART00000163336
ENSDART00000171621

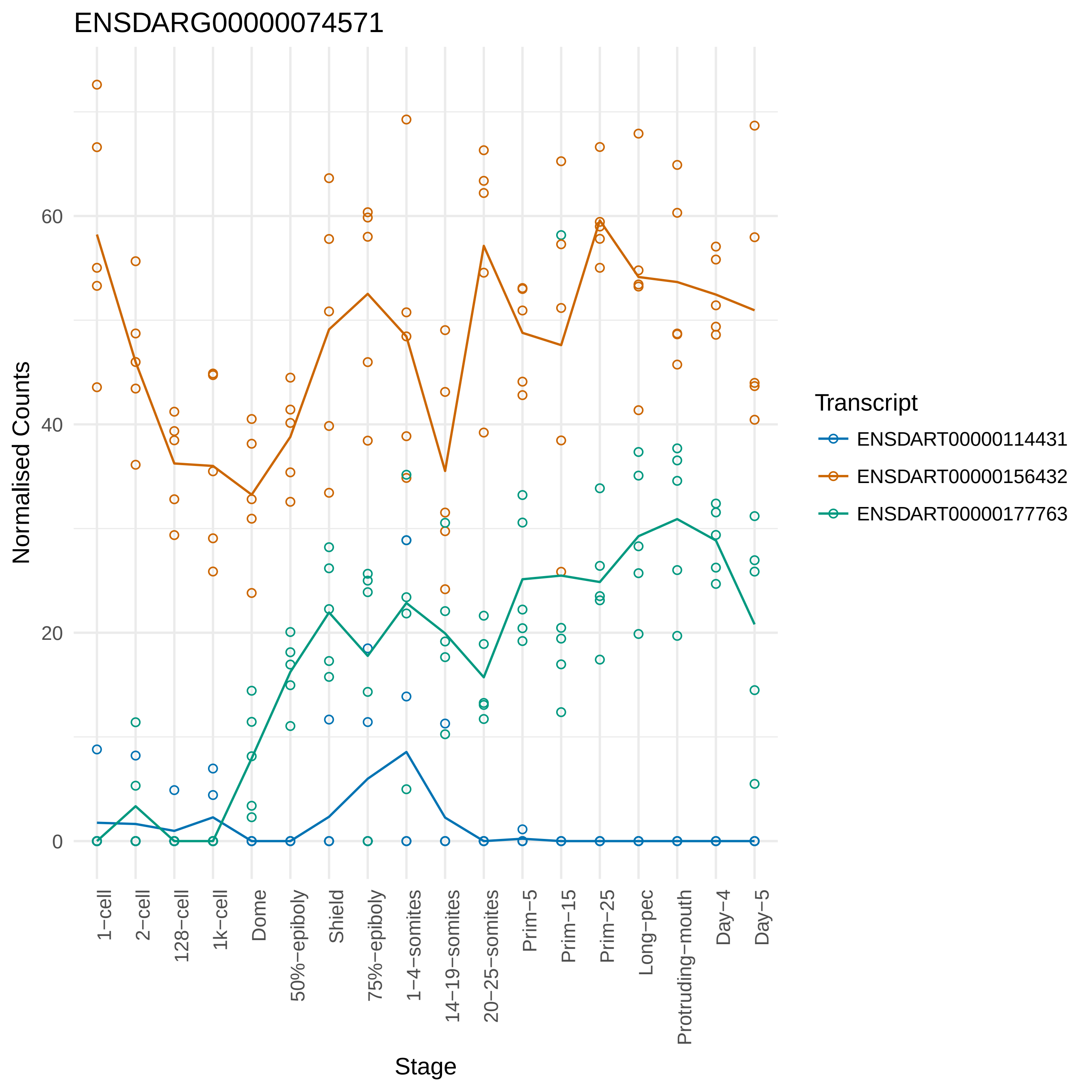

ENSDARG00000074571
Normalised Counts
0
20
40
60
1−cell
2−cell
128−cell
1k−cell
Dome
50%−epiboly
Shield
75%−epiboly
1−4−somites
14−19−somites
20−25−somites
Prim−5
Prim−15
Prim−25
Long−pec
Protruding−mouth
Day−4
Day−5
Stage
Transcript
ENSDART00000114431
ENSDART00000156432
ENSDART00000177763

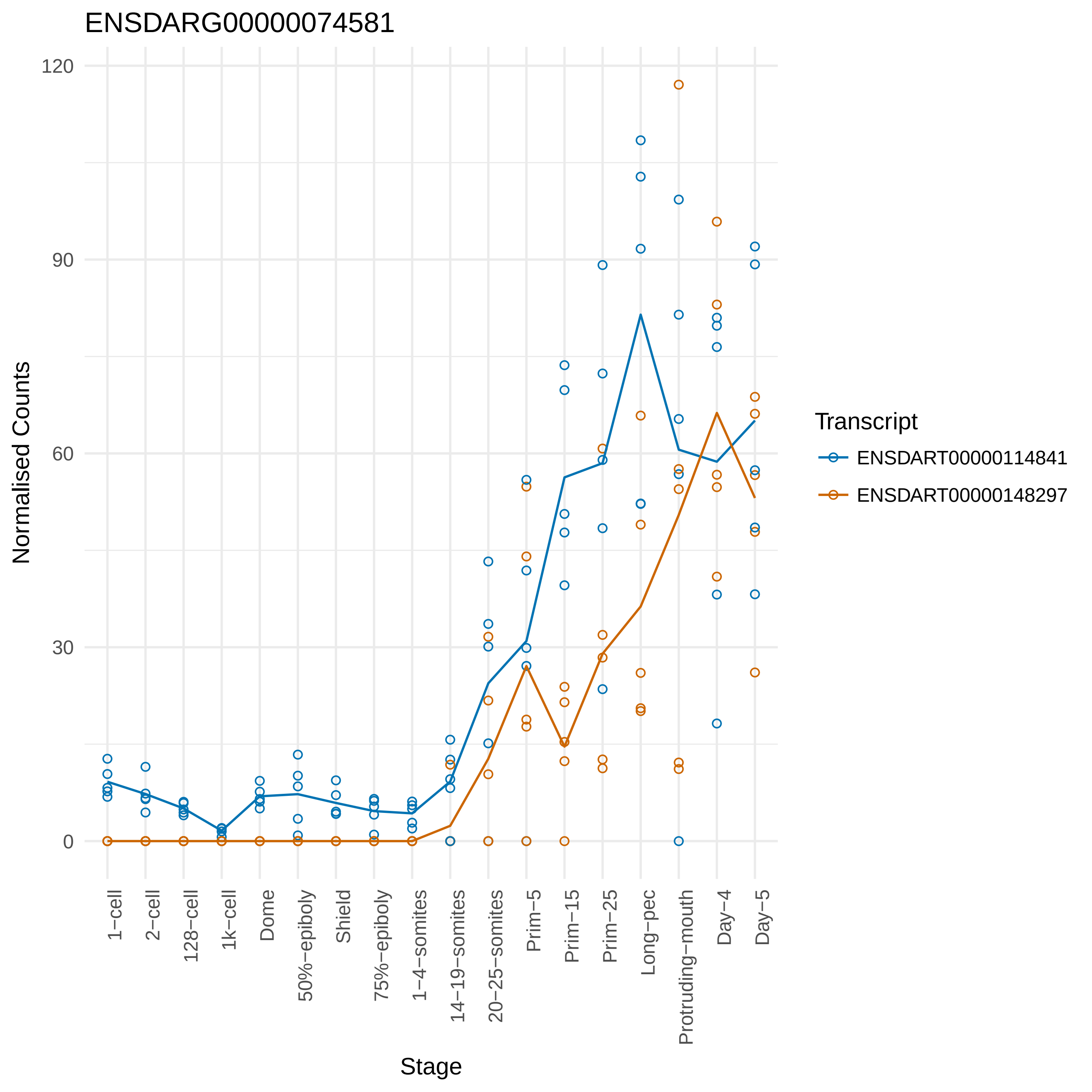
ENSDARG00000074581
Normalised Counts
120
90
60
30
0
1−cell
2−cell
128−cell
1k−cell
Dome
50%−epiboly
Shield
75%−epiboly
1−4−somites
14−19−somites
20−25−somites
Prim−5
Prim−15
Prim−25
Long−pec
Protruding−mouth
Day−4
Day−5
Stage
Transcript
ENSDART00000114841
ENSDART00000148297

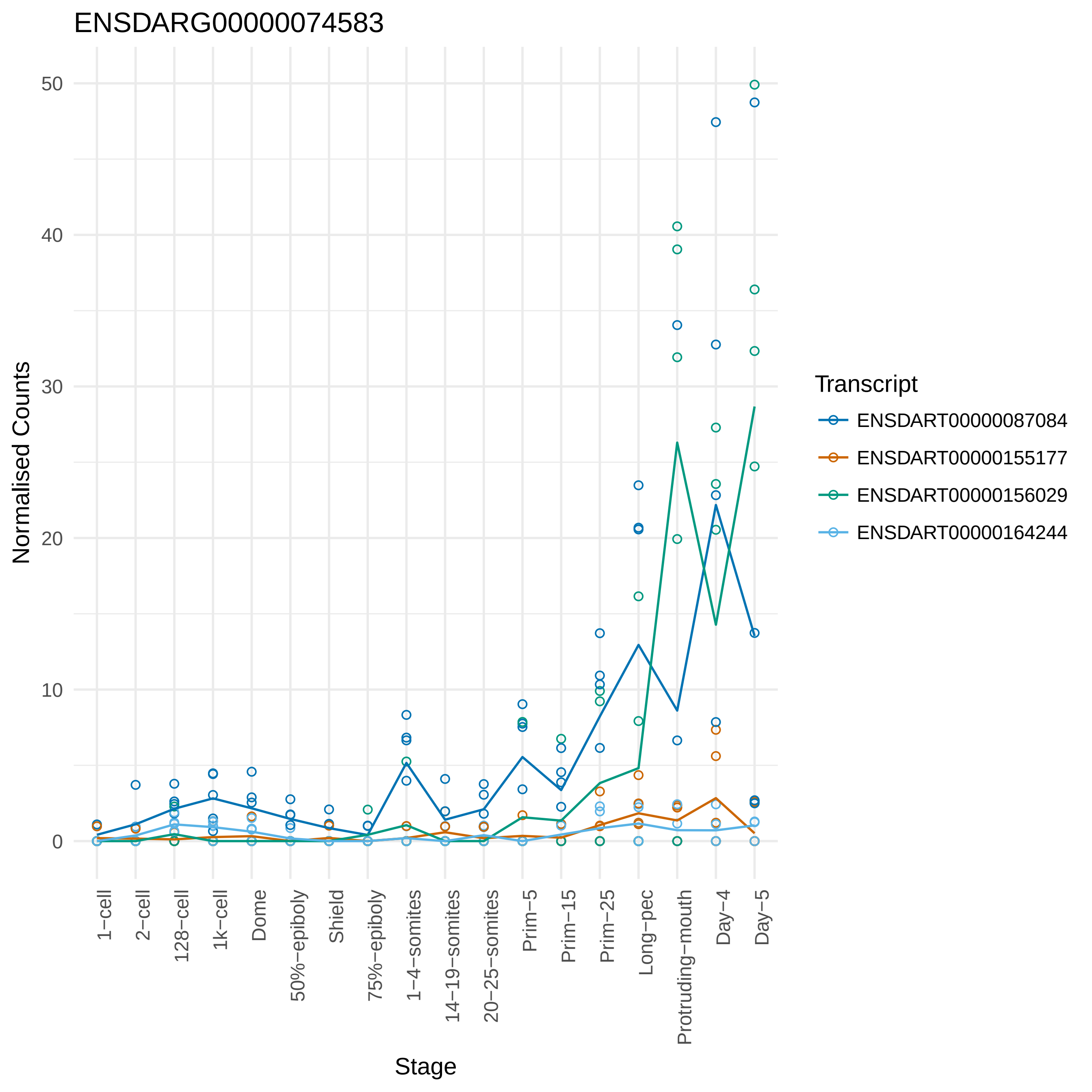

ENSDARG00000074583
Normalised Counts
0
10
20
30
40
50
1−cell
2−cell
128−cell
1k−cell
Dome
50%−epiboly
Shield
75%−epiboly
1−4−somites
14−19−somites
20−25−somites
Prim−5
Prim−15
Prim−25
Long−pec
Protruding−mouth
Day−4
Day−5
Stage
Transcript
ENSDART00000087084
ENSDART00000155177
ENSDART00000156029
ENSDART00000164244

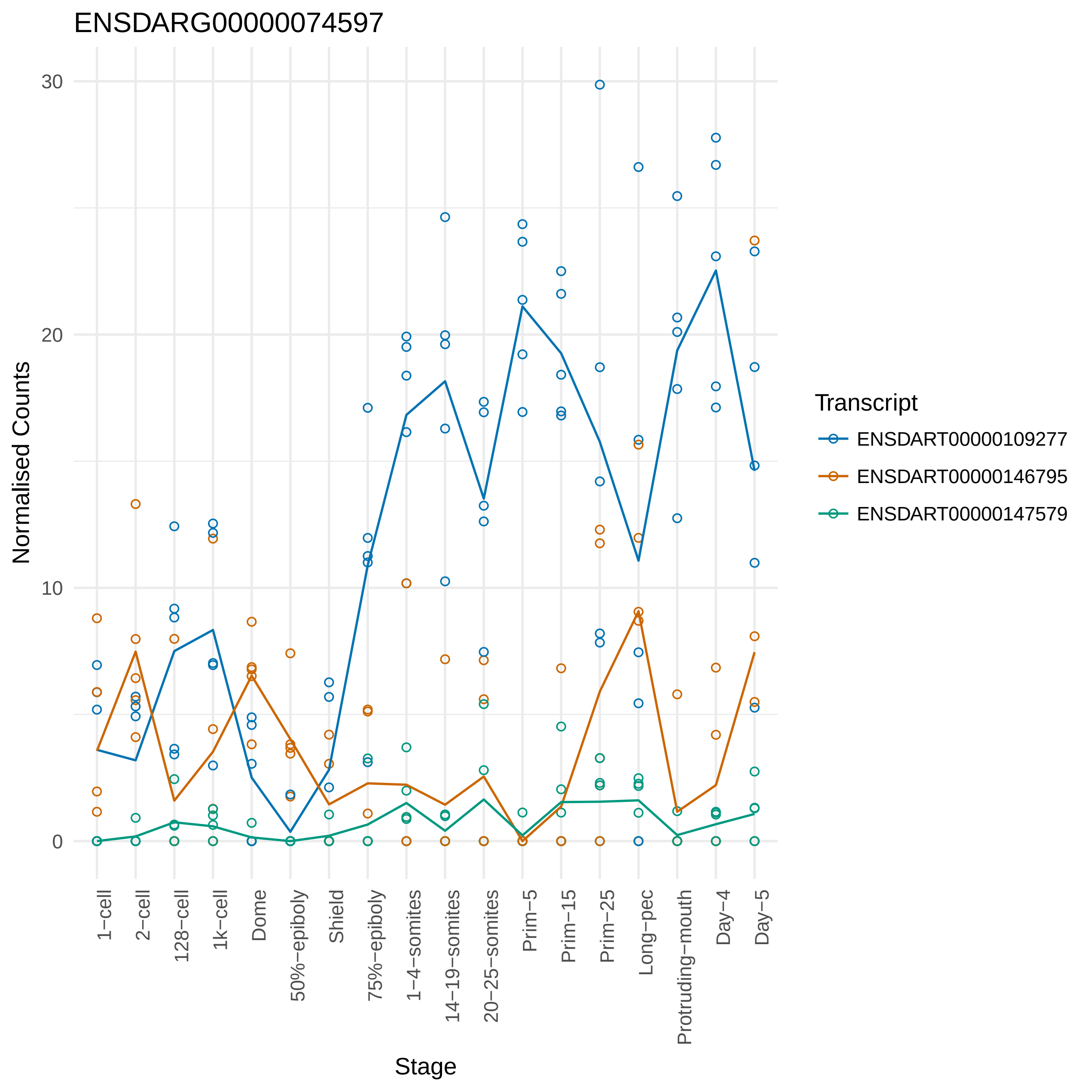

ENSDARG00000074597
Normalised Counts
30
20
10
0
1−cell
2−cell
128−cell
1k−cell
Dome
50%−epiboly
Shield
75%−epiboly
1−4−somites
14−19−somites
20−25−somites
Prim−5
Prim−15
Prim−25
Long−pec
Protruding−mouth
Day−4
Day−5
Stage
Transcript
ENSDART00000109277
ENSDART00000146795
ENSDART00000147579

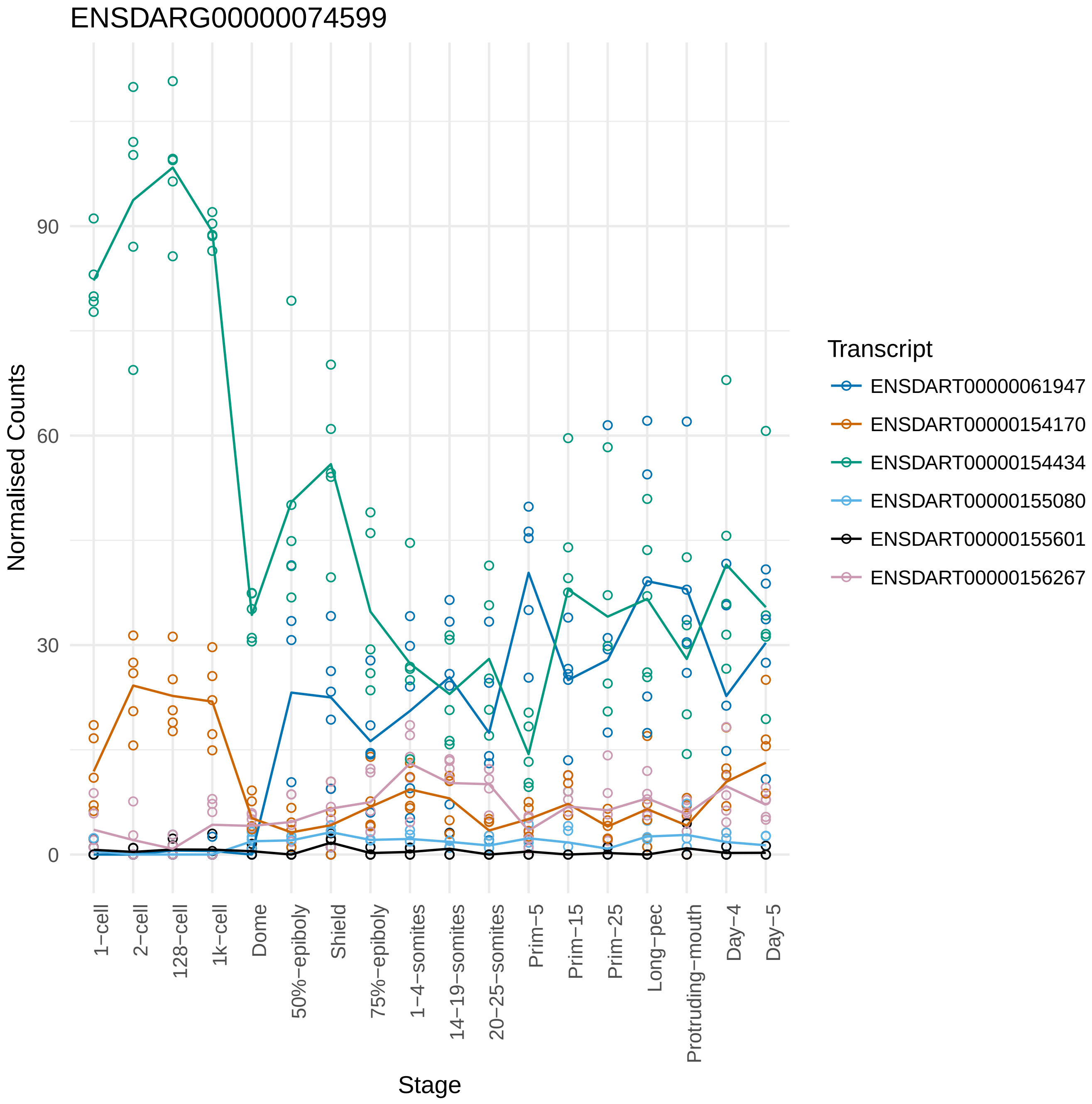

ENSDARG00000074599
Normalised Counts
90
60
30
0
1−cell
2−cell
128−cell
1k−cell
Dome
50%−epiboly
Shield
75%−epiboly
1−4−somites
14−19−somites
20−25−somites
Prim−5
Prim−15
Prim−25
Long−pec
Protruding−mouth
Day−4
Day−5
Stage
Transcript
ENSDART00000061947
ENSDART00000154170
ENSDART00000154434
ENSDART00000155080
ENSDART00000155601
ENSDART00000156267

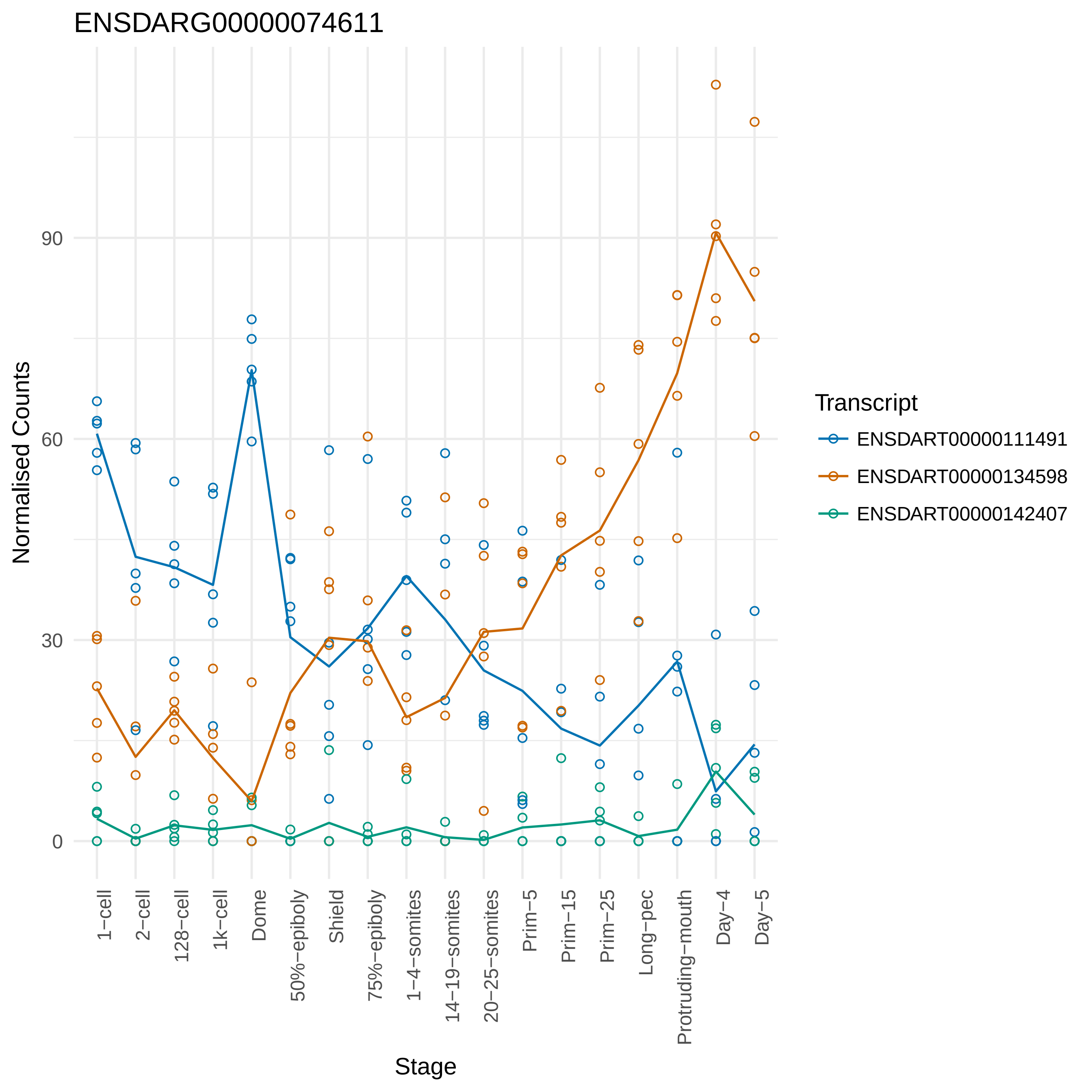

ENSDARG00000074611
Normalised Counts
0
30
60
90
1−cell
2−cell
128−cell
1k−cell
Dome
50%−epiboly
Shield
75%−epiboly
1−4−somites
14−19−somites
20−25−somites
Prim−5
Prim−15
Prim−25
Long−pec
Protruding−mouth
Day−4
Day−5
Stage
Transcript
ENSDART00000111491
ENSDART00000134598
ENSDART00000142407

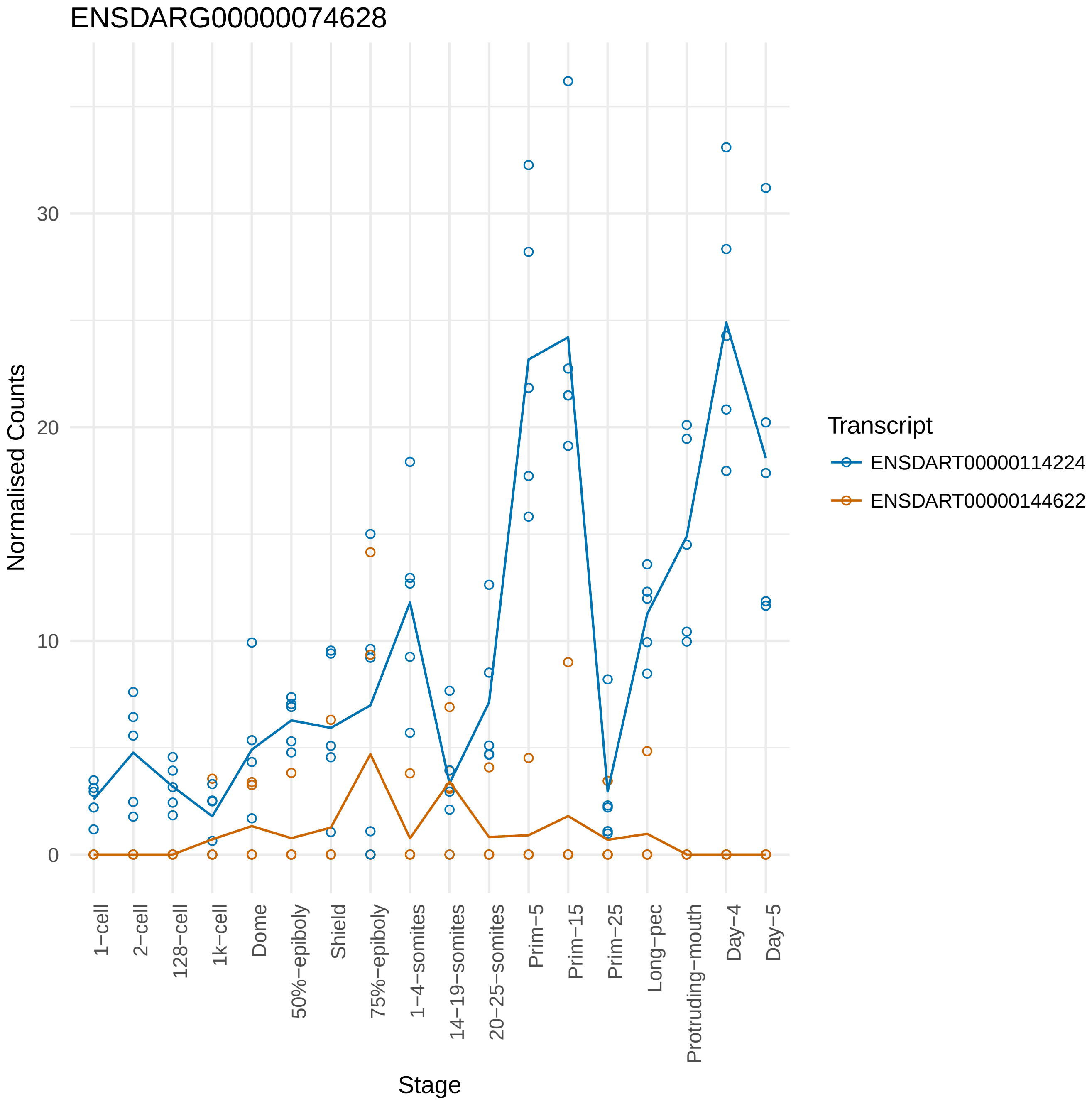
ENSDARG00000074628
Normalised Counts
0
10
20
30
1−cell
2−cell
128−cell
1k−cell
Dome
50%−epiboly
Shield
75%−epiboly
1−4−somites
14−19−somites
20−25−somites
Prim−5
Prim−15
Prim−25
Long−pec
Protruding−mouth
Day−4
Day−5
Stage
Transcript
ENSDART00000114224
ENSDART00000144622

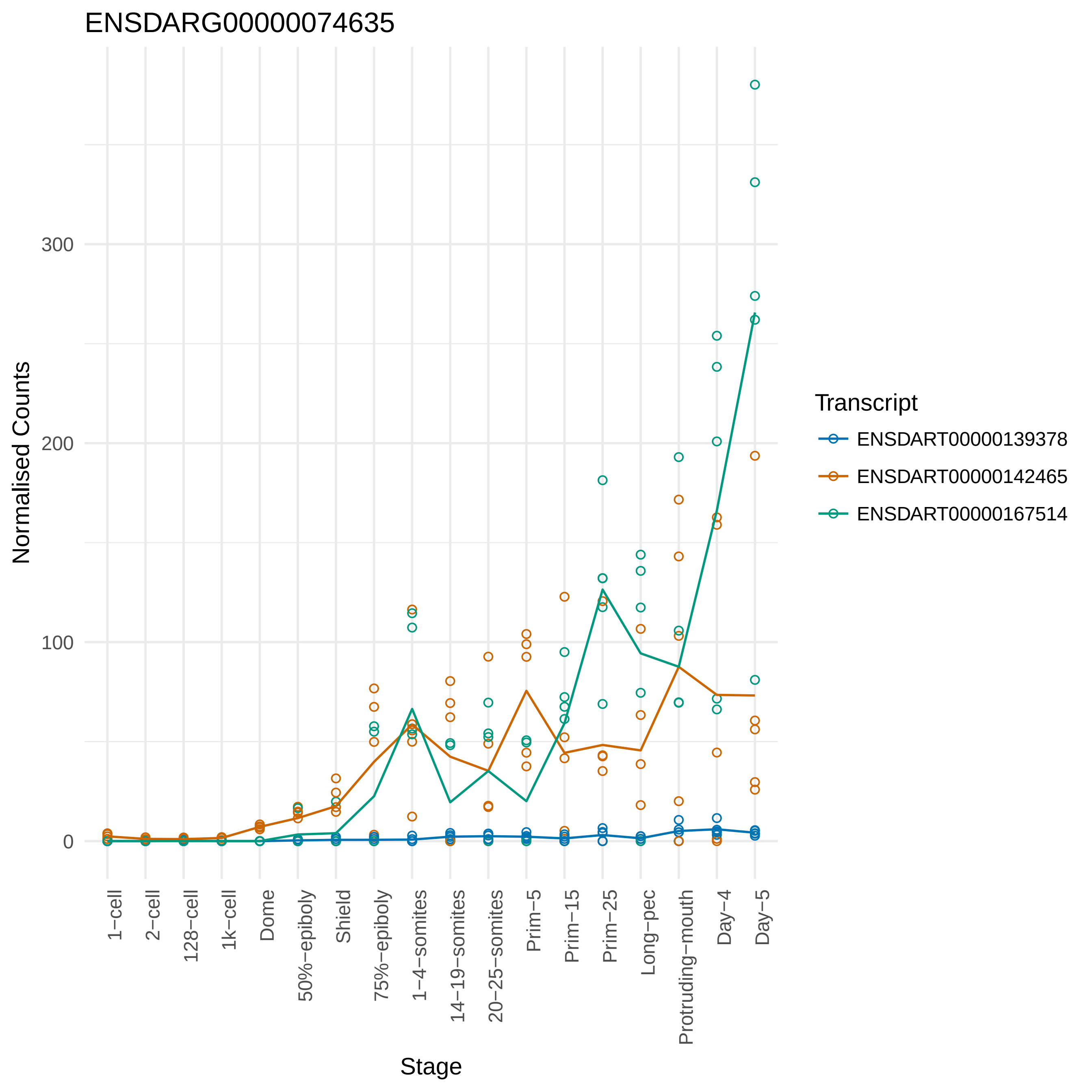

ENSDARG00000074635
Normalised Counts
300
200
100
0
1−cell
2−cell
128−cell
1k−cell
Dome
50%−epiboly
Shield
75%−epiboly
1−4−somites
14−19−somites
20−25−somites
Prim−5
Prim−15
Prim−25
Long−pec
Protruding−mouth
Day−4
Day−5
Stage
Transcript
ENSDART00000139378
ENSDART00000142465
ENSDART00000167514

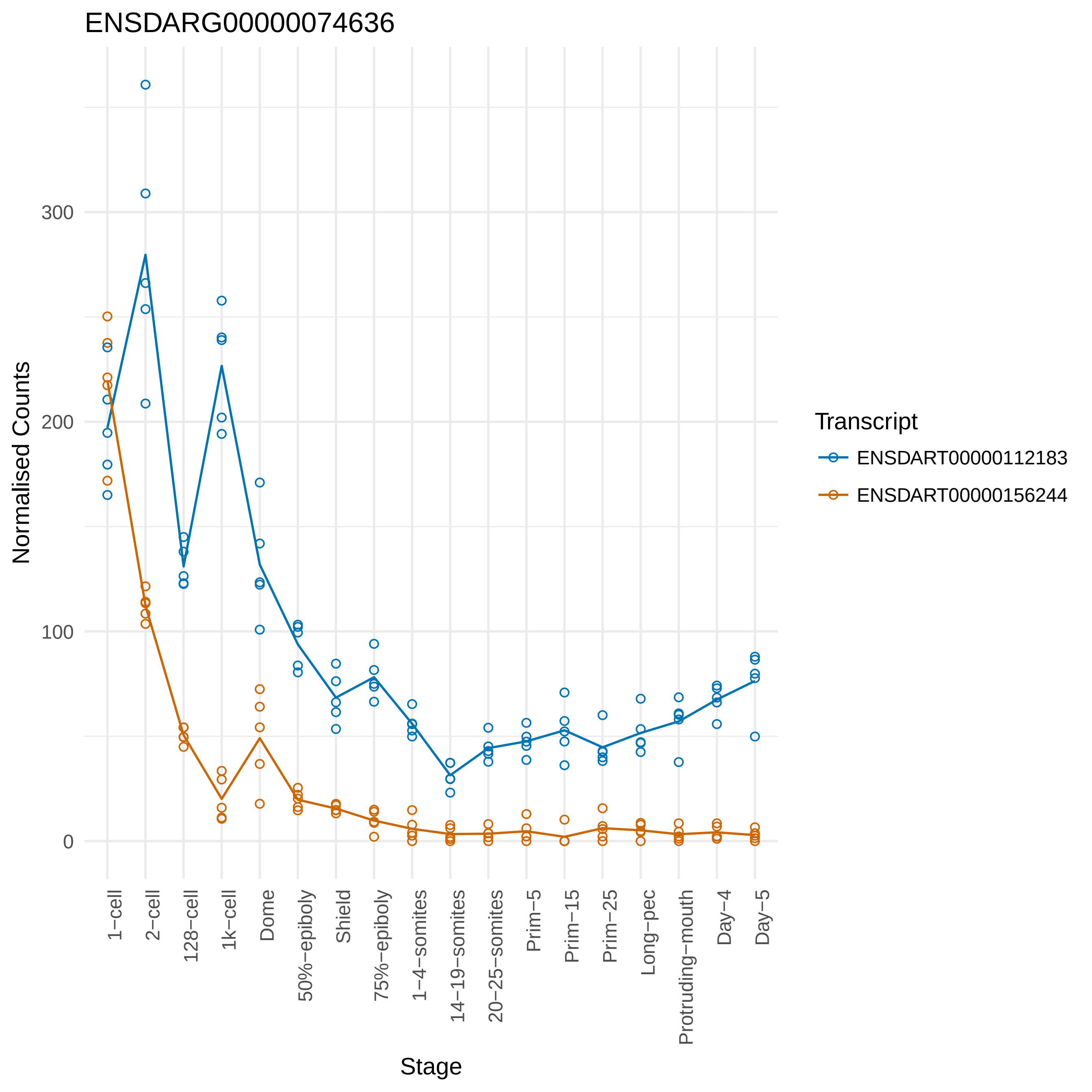

ENSDARG00000074636
Normalised Counts
300
200
100
0
1−cell
2−cell
128−cell
1k−cell
Dome
50%−epiboly
Shield
75%−epiboly
1−4−somites
14−19−somites
20−25−somites
Prim−5
Prim−15
Prim−25
Long−pec
Protruding−mouth
Day−4
Day−5
Stage
Transcript
ENSDART00000112183
ENSDART00000156244

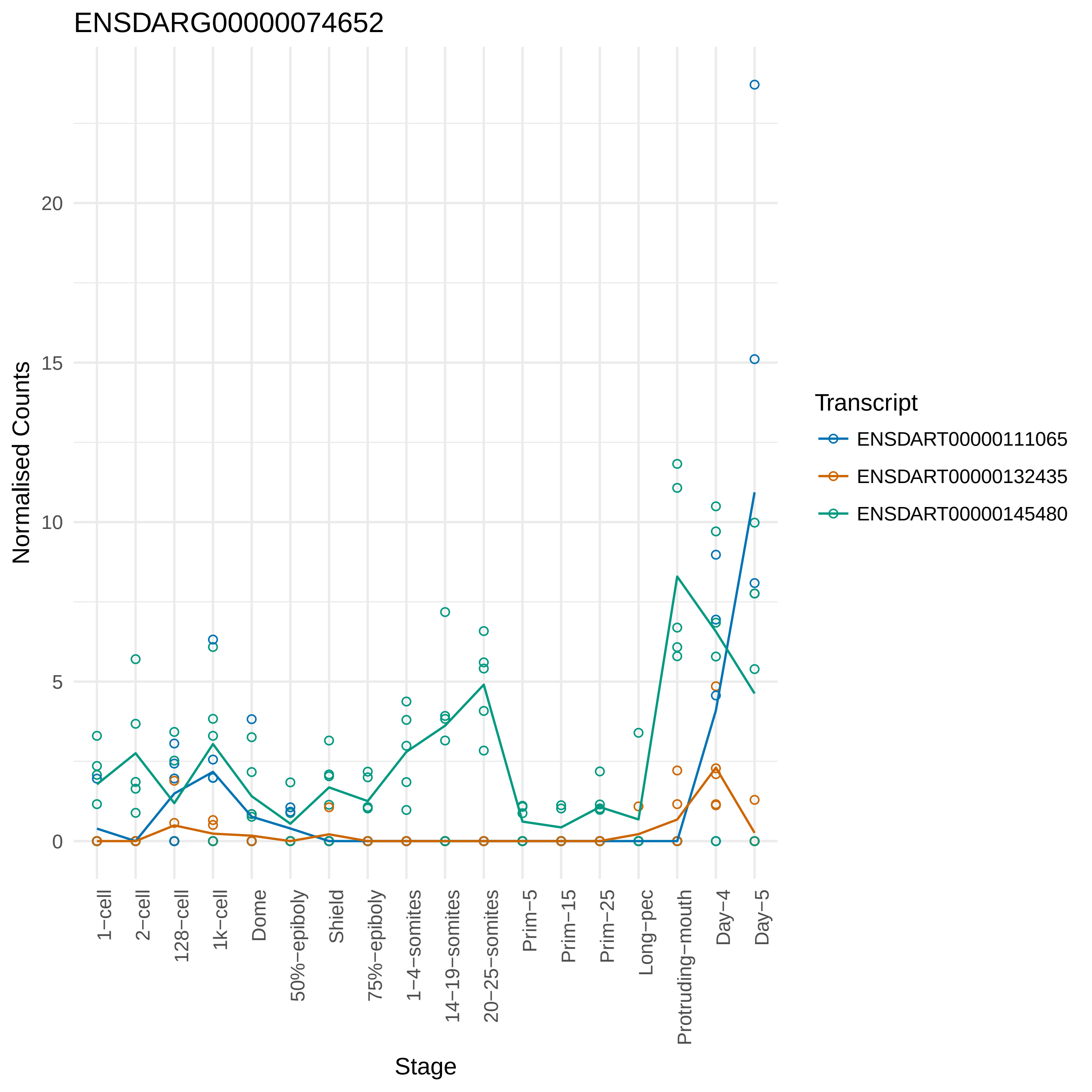
ENSDARG00000074652
Normalised Counts
0
5
10
15
20
1−cell
2−cell
128−cell
1k−cell
Dome
50%−epiboly
Shield
75%−epiboly
1−4−somites
14−19−somites
20−25−somites
Prim−5
Prim−15
Prim−25
Long−pec
Protruding−mouth
Day−4
Day−5
Stage
Transcript
ENSDART00000111065
ENSDART00000132435
ENSDART00000145480

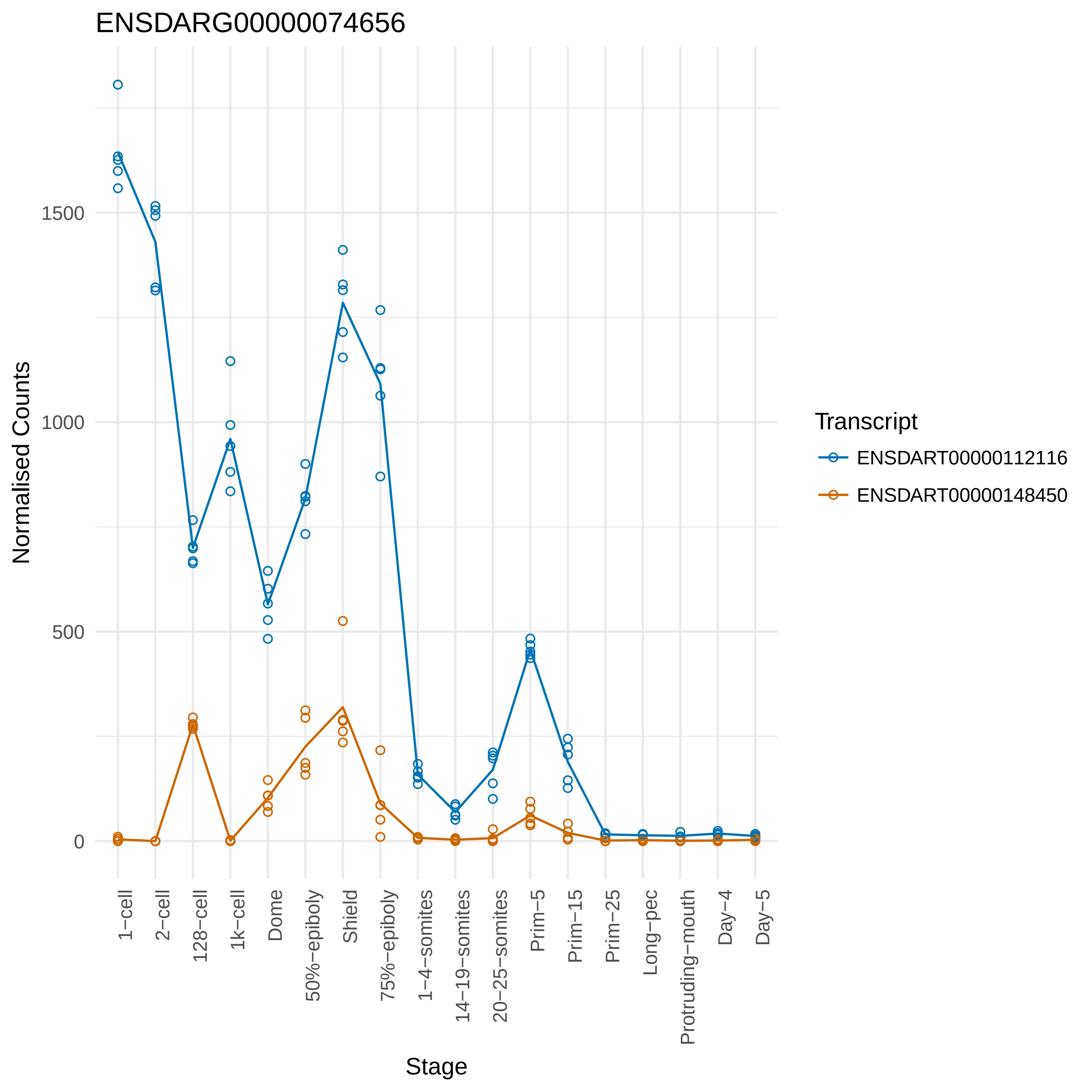

ENSDARG00000074656
Normalised Counts
0
500
1000
1500
Stage
1−cell
2−cell
128−cell
1k−cell
Dome
50%−epiboly
Shield
75%−epiboly
1−4−somites
14−19−somites
20−25−somites
Prim−5
Prim−15
Prim−25
Long−pec
Protruding−mouth
Day−4
Day−5
Transcript
ENSDART00000112116
ENSDART00000148450

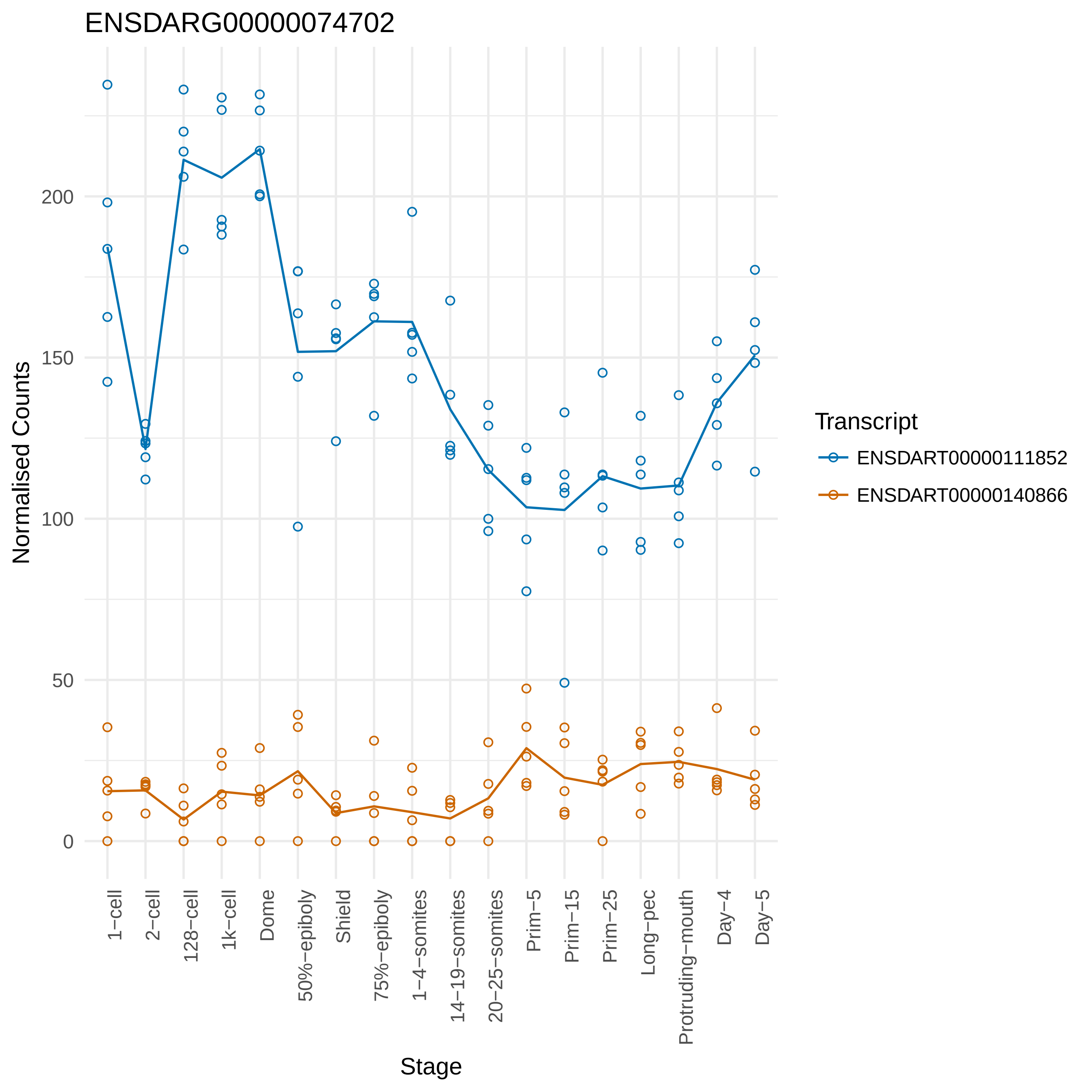
ENSDARG00000074702
Normalised Counts
200
150
100
50
0
1−cell
2−cell
128−cell
1k−cell
Dome
50%−epiboly
Shield
75%−epiboly
1−4−somites
14−19−somites
20−25−somites
Prim−5
Prim−15
Prim−25
Long−pec
Protruding−mouth
Day−4
Day−5
Stage
Transcript
ENSDART00000111852
ENSDART00000140866

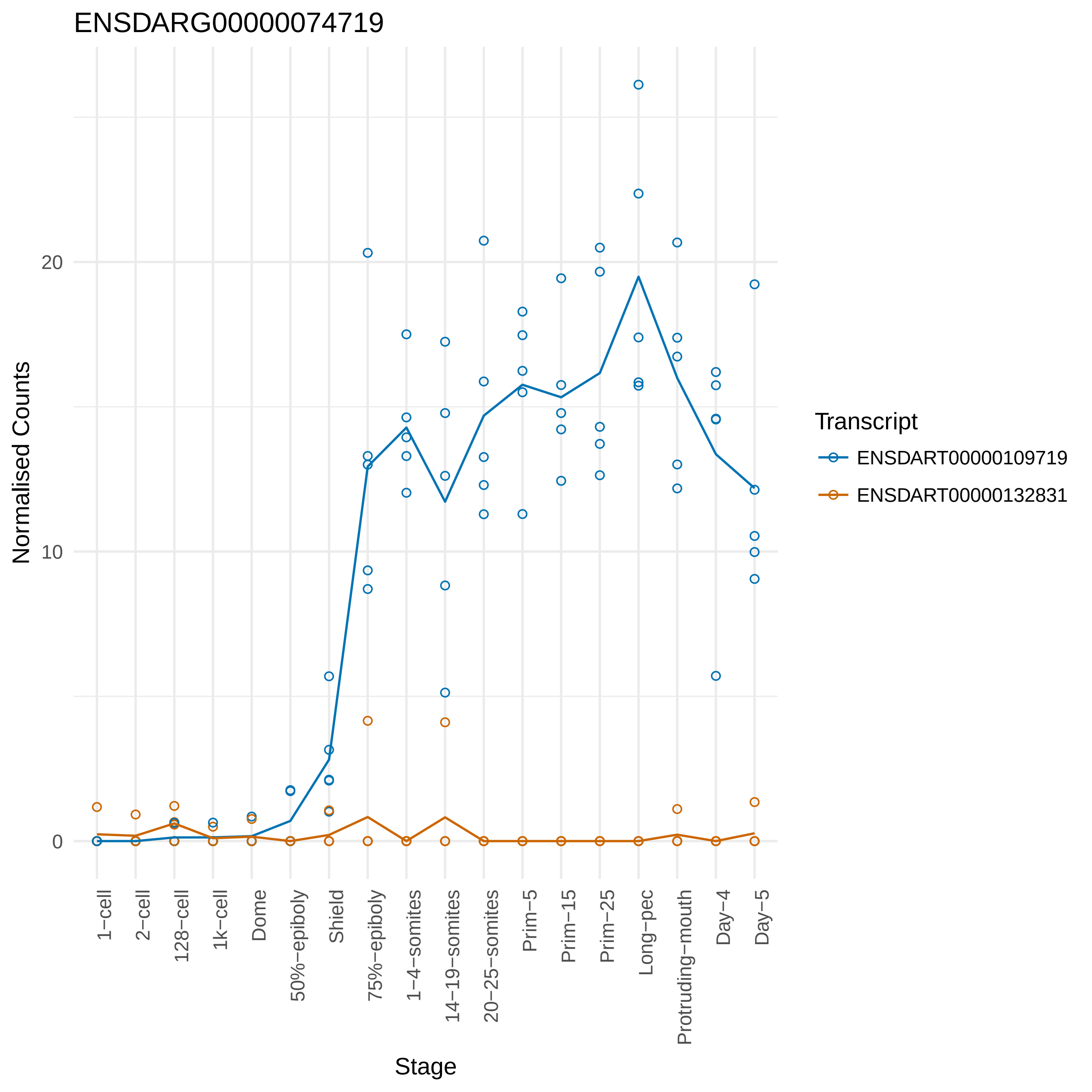
ENSDARG00000074719
Normalised Counts
0
10
20
Stage
1−cell
2−cell
128−cell
1k−cell
Dome
50%−epiboly
Shield
75%−epiboly
1−4−somites
14−19−somites
20−25−somites
Prim−5
Prim−15
Prim−25
Long−pec
Protruding−mouth
Day−4
Day−5
Transcript
ENSDART00000109719
ENSDART00000132831

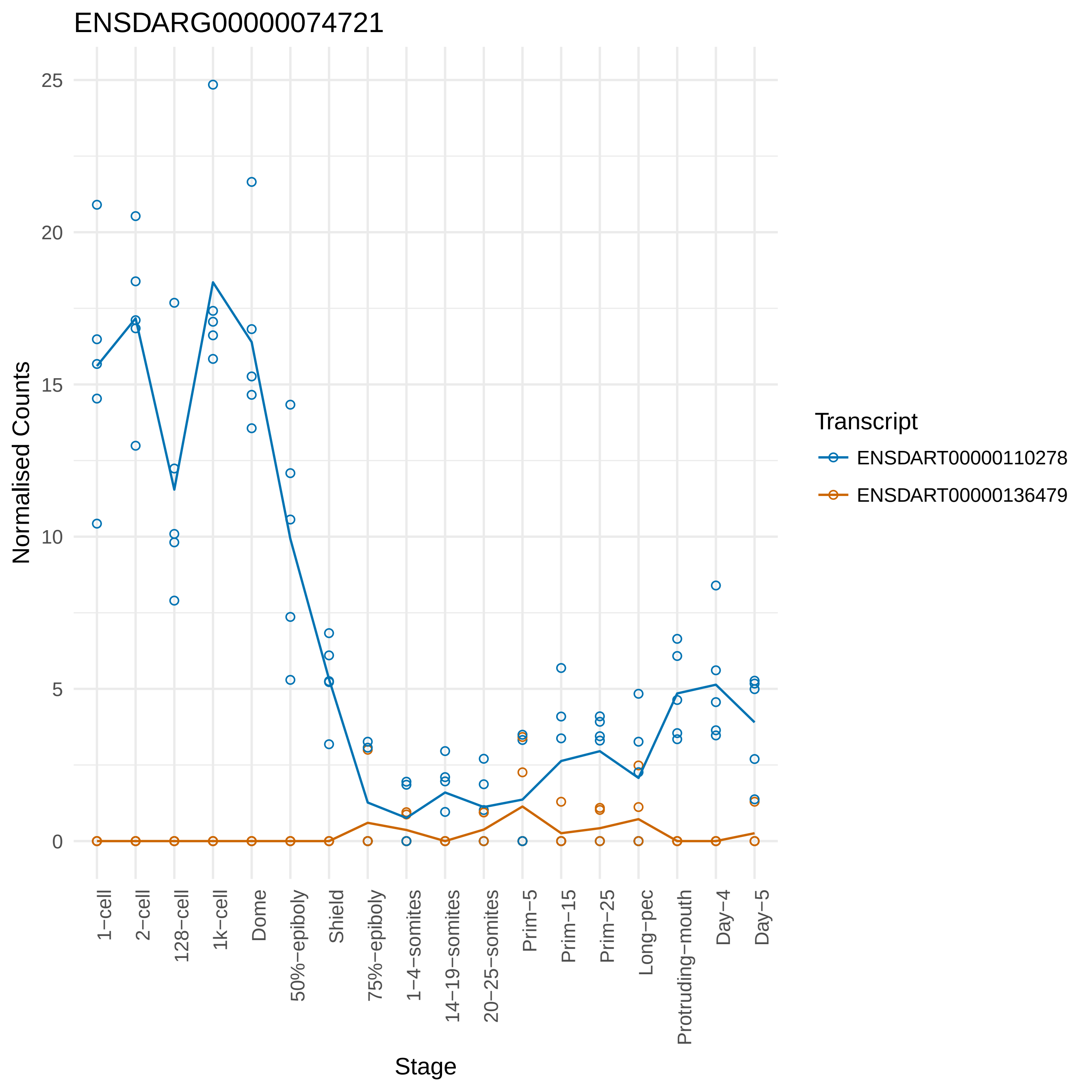
ENSDARG00000074721
Normalised Counts
0
5
10
15
20
25
1−cell
2−cell
128−cell
1k−cell
Dome
50%−epiboly
Shield
75%−epiboly
1−4−somites
14−19−somites
20−25−somites
Prim−5
Prim−15
Prim−25
Long−pec
Protruding−mouth
Day−4
Day−5
Stage
Transcript
ENSDART00000110278
ENSDART00000136479

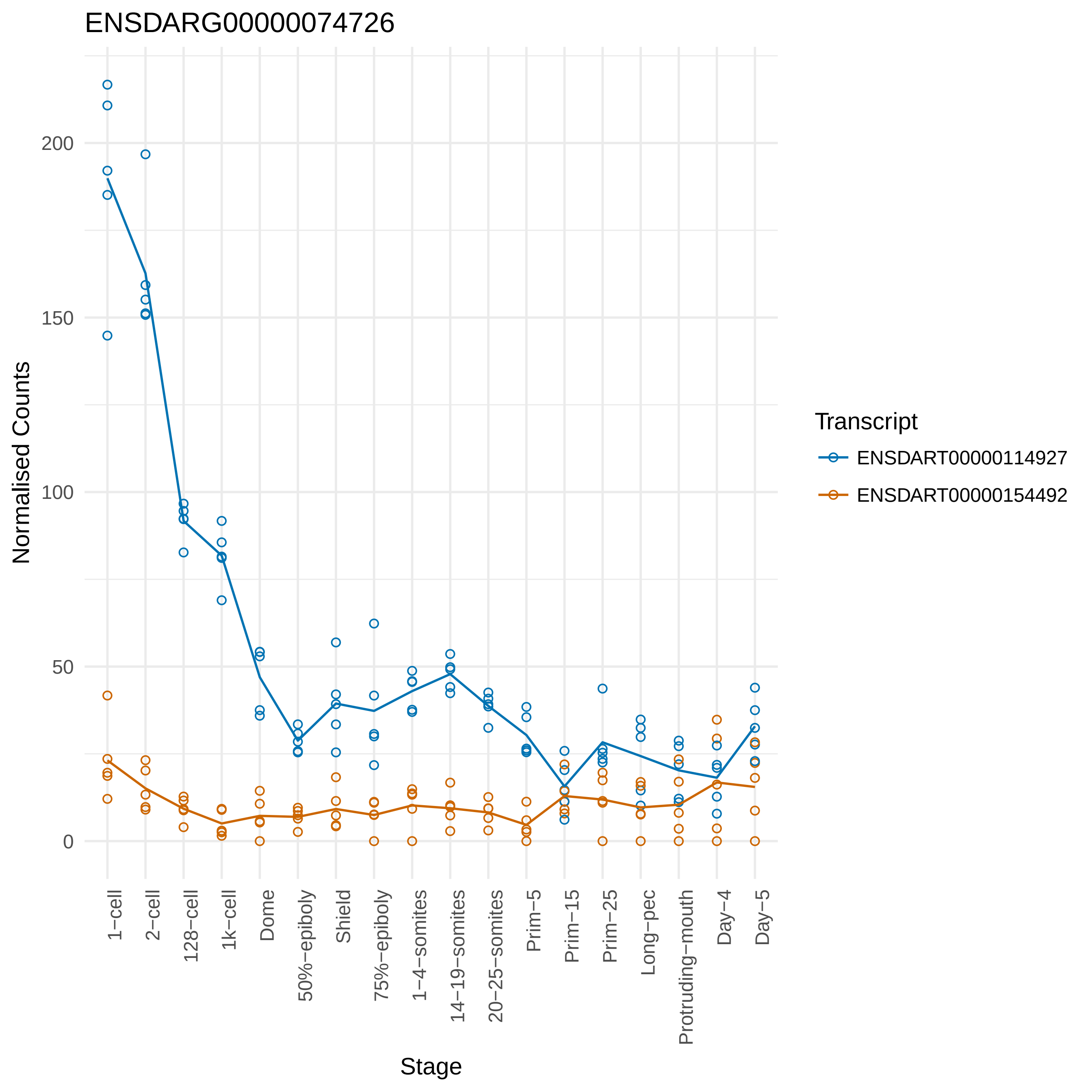
ENSDARG00000074726
Normalised Counts
0
50
100
150
200
Stage
1−cell
2−cell
128−cell
1k−cell
Dome
50%−epiboly
Shield
75%−epiboly
1−4−somites
14−19−somites
20−25−somites
Prim−5
Prim−15
Prim−25
Long−pec
Protruding−mouth
Day−4
Day−5
Transcript
ENSDART00000114927
ENSDART00000154492

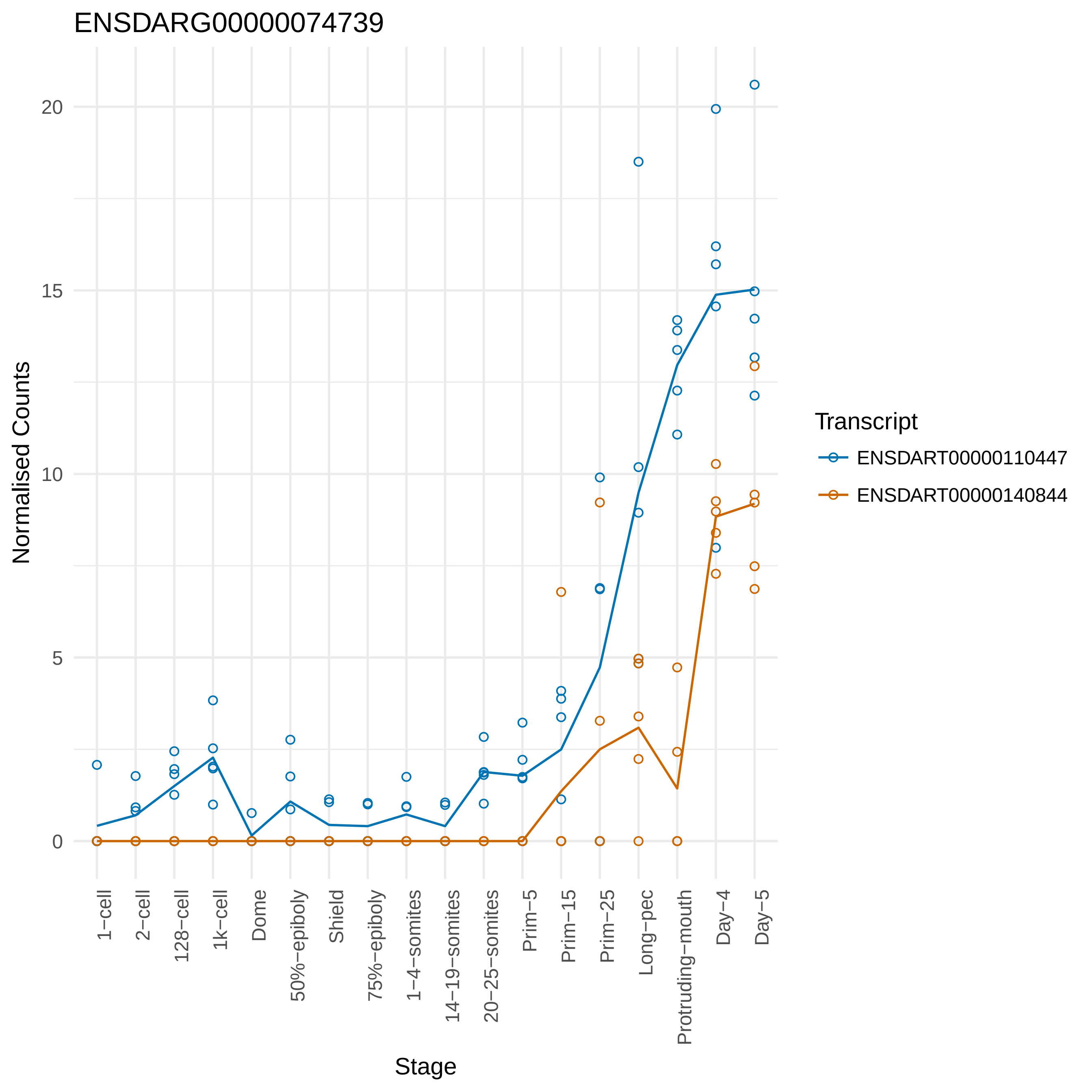

ENSDARG00000074739
Normalised Counts
Stage
Transcript
ENSDART00000110447
ENSDART00000140844
0
5
10
15
20
1−cell
2−cell
128−cell
1k−cell
Dome
50%−epiboly
Shield
75%−epiboly
1−4−somites
14−19−somites
20−25−somites
Prim−5
Prim−15
Prim−25
Long−pec
Protruding−mouth
Day−4
Day−5

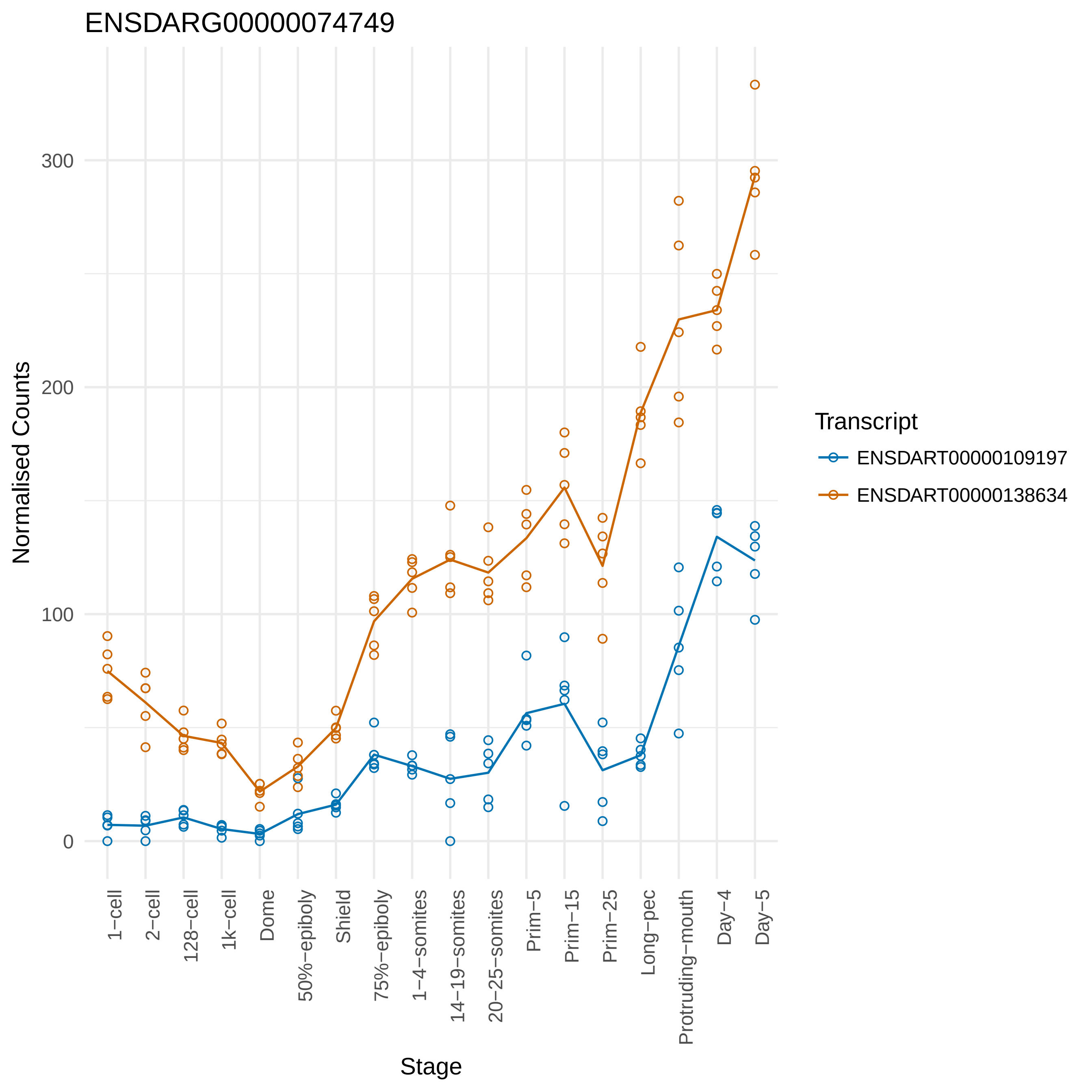

ENSDARG00000074749
Normalised Counts
300
200
100
0
1−cell
2−cell
128−cell
1k−cell
Dome
50%−epiboly
Shield
75%−epiboly
1−4−somites
14−19−somites
20−25−somites
Prim−5
Prim−15
Prim−25
Long−pec
Protruding−mouth
Day−4
Day−5
Stage
Transcript
ENSDART00000109197
ENSDART00000138634

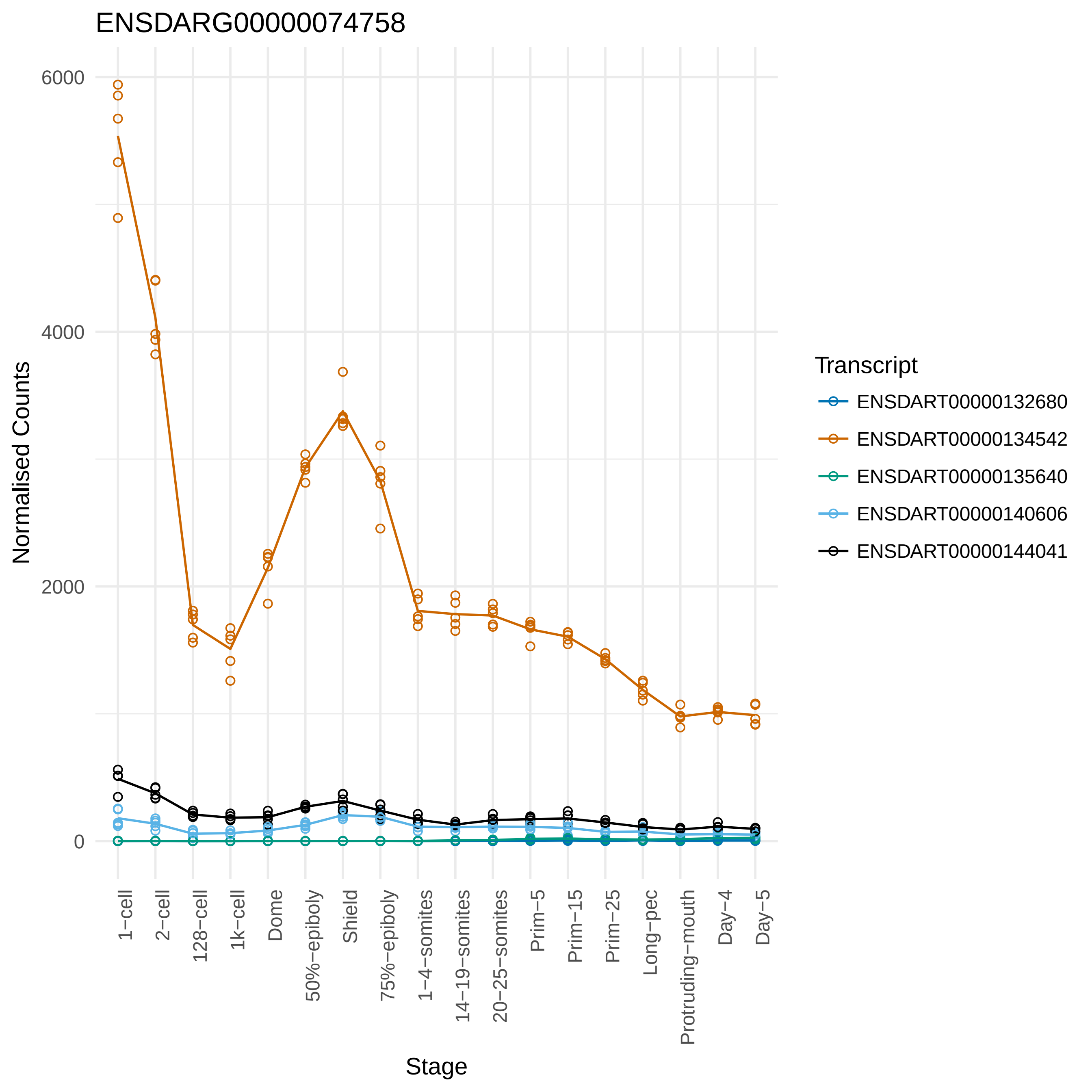

ENSDARG00000074758
Normalised Counts
6000
4000
2000
0
1−cell
2−cell
128−cell
1k−cell
Dome
50%−epiboly
Shield
75%−epiboly
1−4−somites
14−19−somites
20−25−somites
Prim−5
Prim−15
Prim−25
Long−pec
Protruding−mouth
Day−4
Day−5
Stage
Transcript
ENSDART00000132680
ENSDART00000134542
ENSDART00000135640
ENSDART00000140606
ENSDART00000144041

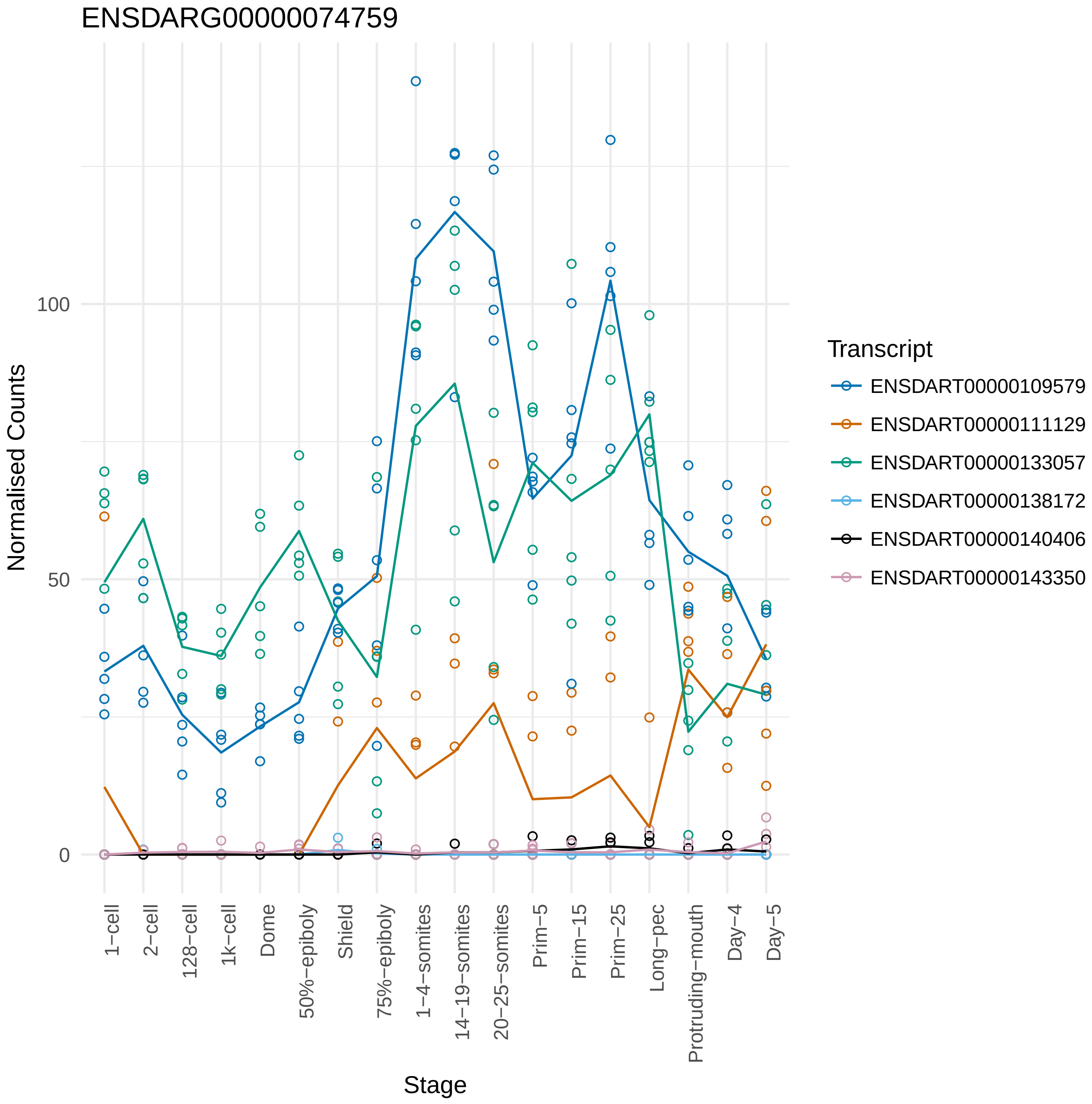
ENSDARG00000074759
Normalised Counts
100
50
0
1−cell
2−cell
128−cell
1k−cell
Dome
50%−epiboly
Shield
75%−epiboly
1−4−somites
14−19−somites
20−25−somites
Prim−5
Prim−15
Prim−25
Long−pec
Protruding−mouth
Day−4
Day−5
Stage
Transcript
ENSDART00000109579
ENSDART00000111129
ENSDART00000133057
ENSDART00000138172
ENSDART00000140406
ENSDART00000143350

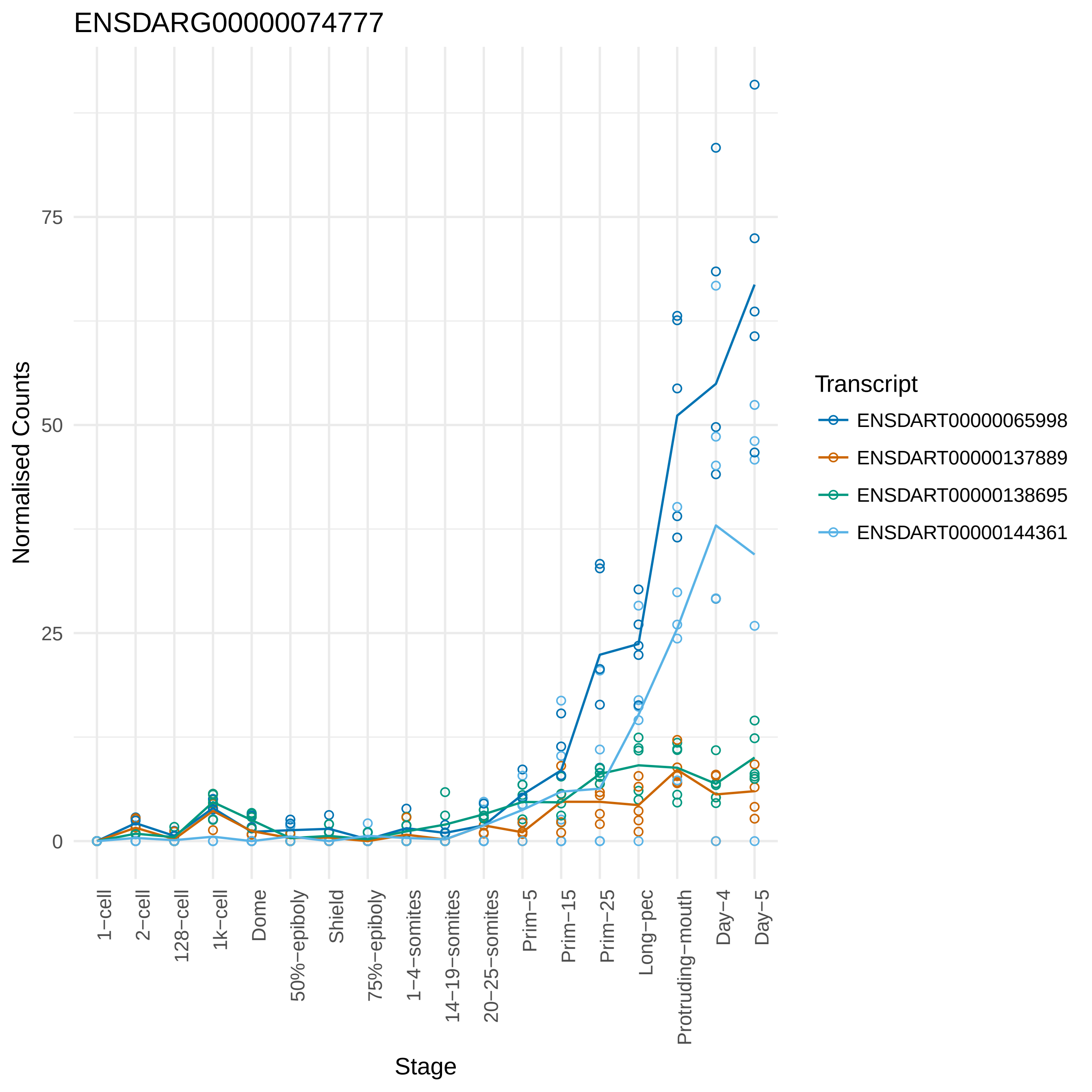
ENSDARG00000074777
Normalised Counts
0
25
50
75
1−cell
2−cell
128−cell
1k−cell
Dome
50%−epiboly
Shield
75%−epiboly
1−4−somites
14−19−somites
20−25−somites
Prim−5
Prim−15
Prim−25
Long−pec
Protruding−mouth
Day−4
Day−5
Stage
Transcript
ENSDART00000065998
ENSDART00000137889
ENSDART00000138695
ENSDART00000144361

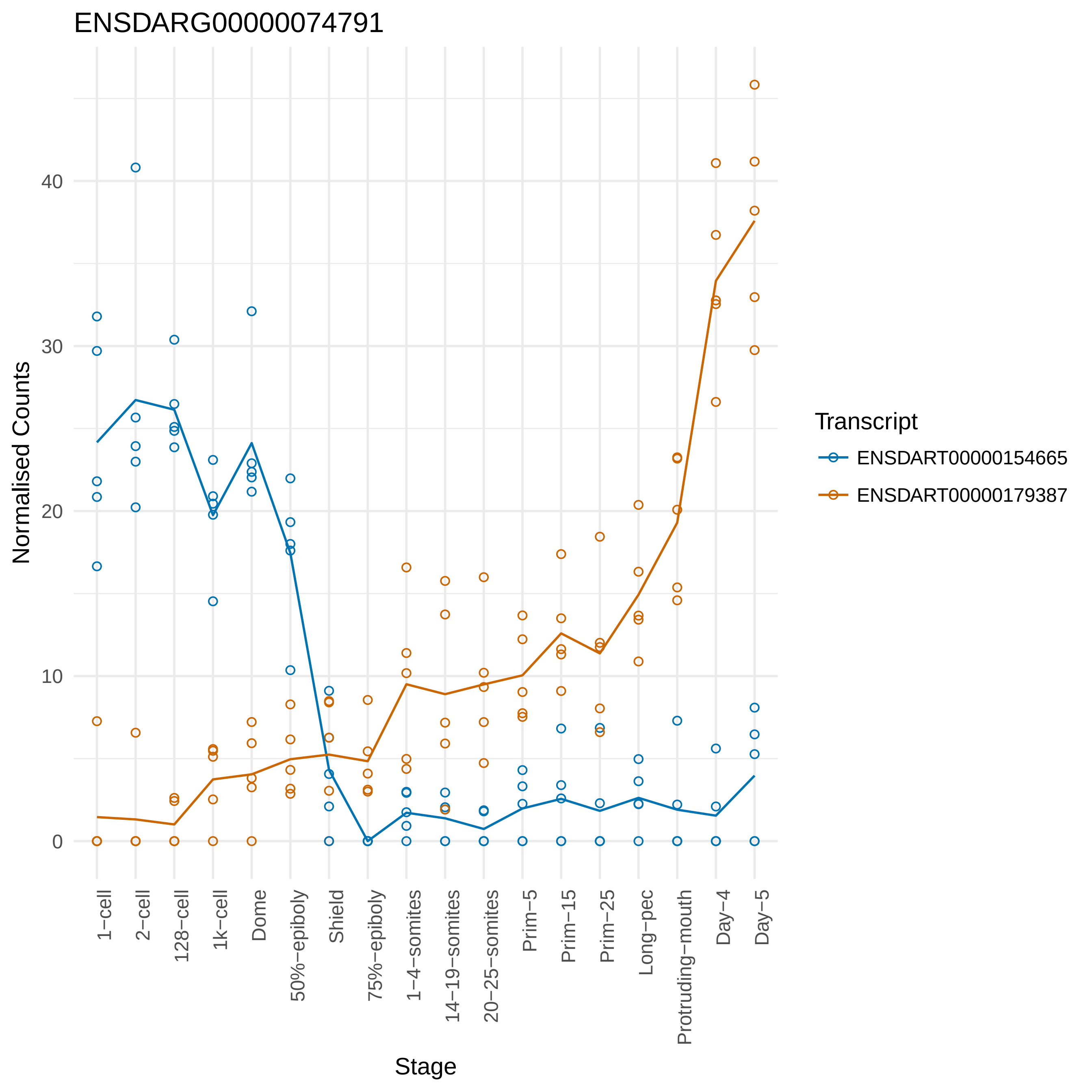
ENSDARG00000074791
Normalised Counts
0
10
20
30
40
Stage
1−cell
2−cell
128−cell
1k−cell
Dome
50%−epiboly
Shield
75%−epiboly
1−4−somites
14−19−somites
20−25−somites
Prim−5
Prim−15
Prim−25
Long−pec
Protruding−mouth
Day−4
Day−5
Transcript
ENSDART00000154665
ENSDART00000179387

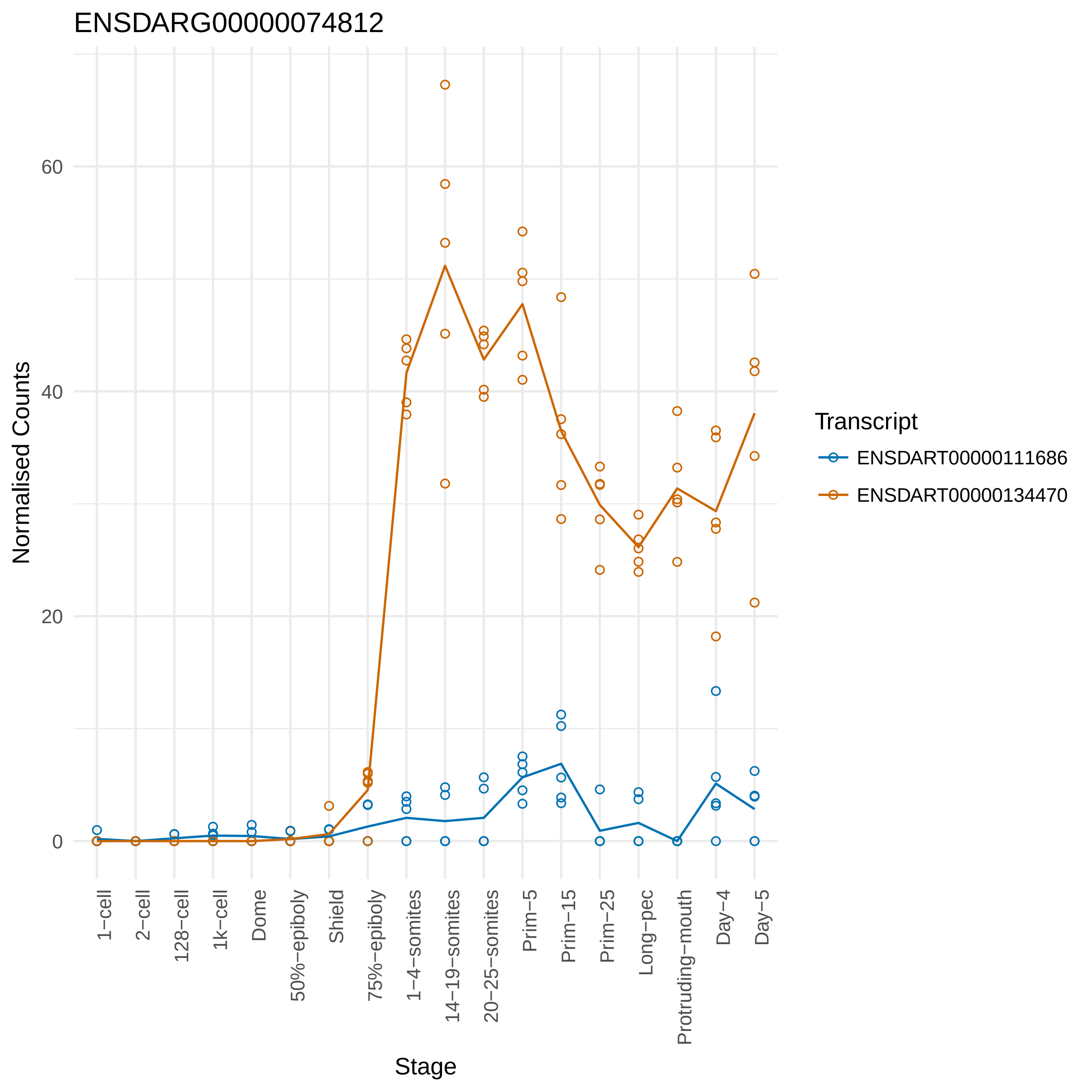

ENSDARG00000074812
Normalised Counts
60
40
20
0
1−cell
2−cell
128−cell
1k−cell
Dome
50%−epiboly
Shield
75%−epiboly
1−4−somites
14−19−somites
20−25−somites
Prim−5
Prim−15
Prim−25
Long−pec
Protruding−mouth
Day−4
Day−5
Stage
Transcript
ENSDART00000111686
ENSDART00000134470

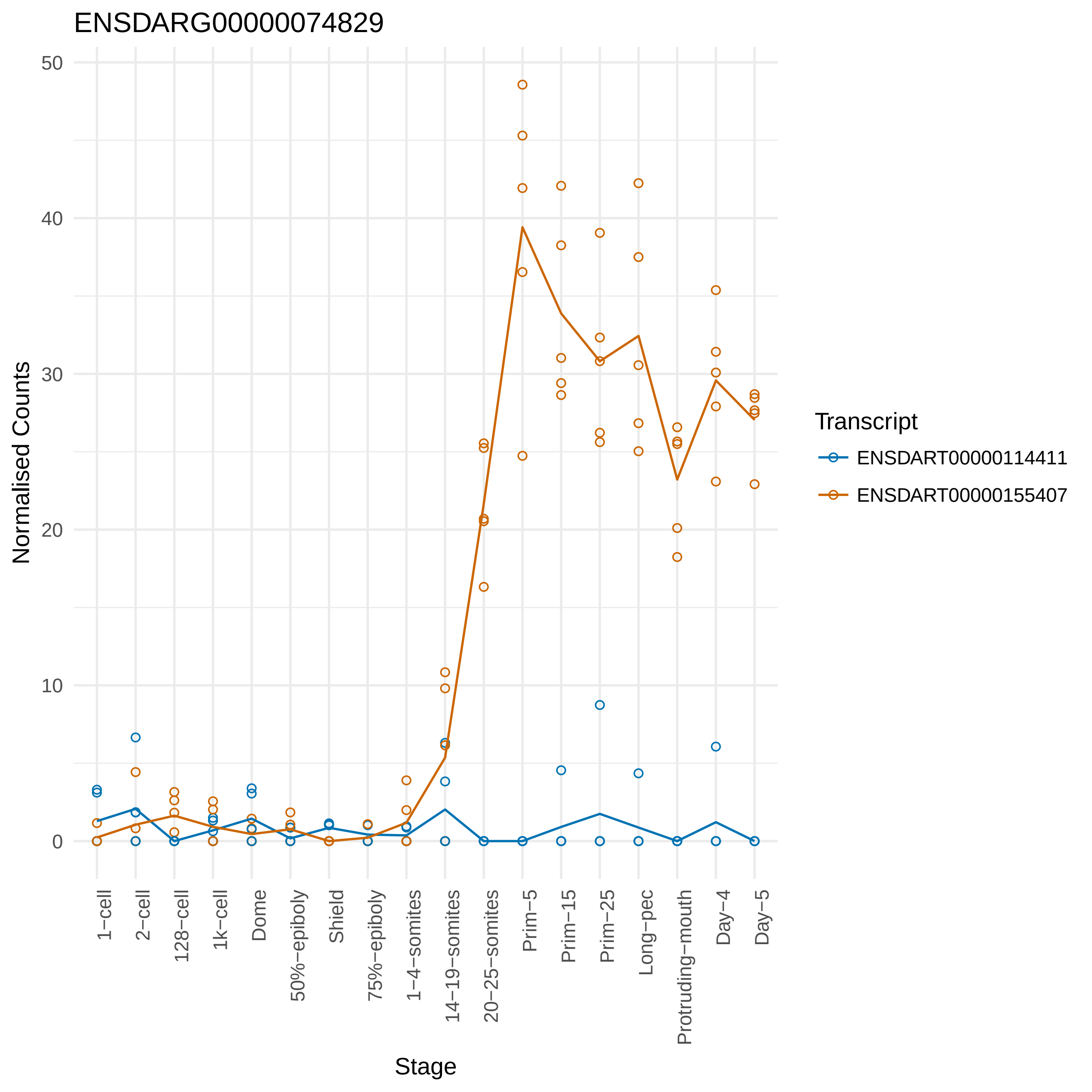
ENSDARG00000074829
Normalised Counts
50
40
30
20
10
0
Stage
1−cell
2−cell
128−cell
1k−cell
Dome
50%−epiboly
Shield
75%−epiboly
1−4−somites
14−19−somites
20−25−somites
Prim−5
Prim−15
Prim−25
Long−pec
Protruding−mouth
Day−4
Day−5
Transcript
ENSDART00000114411
ENSDART00000155407

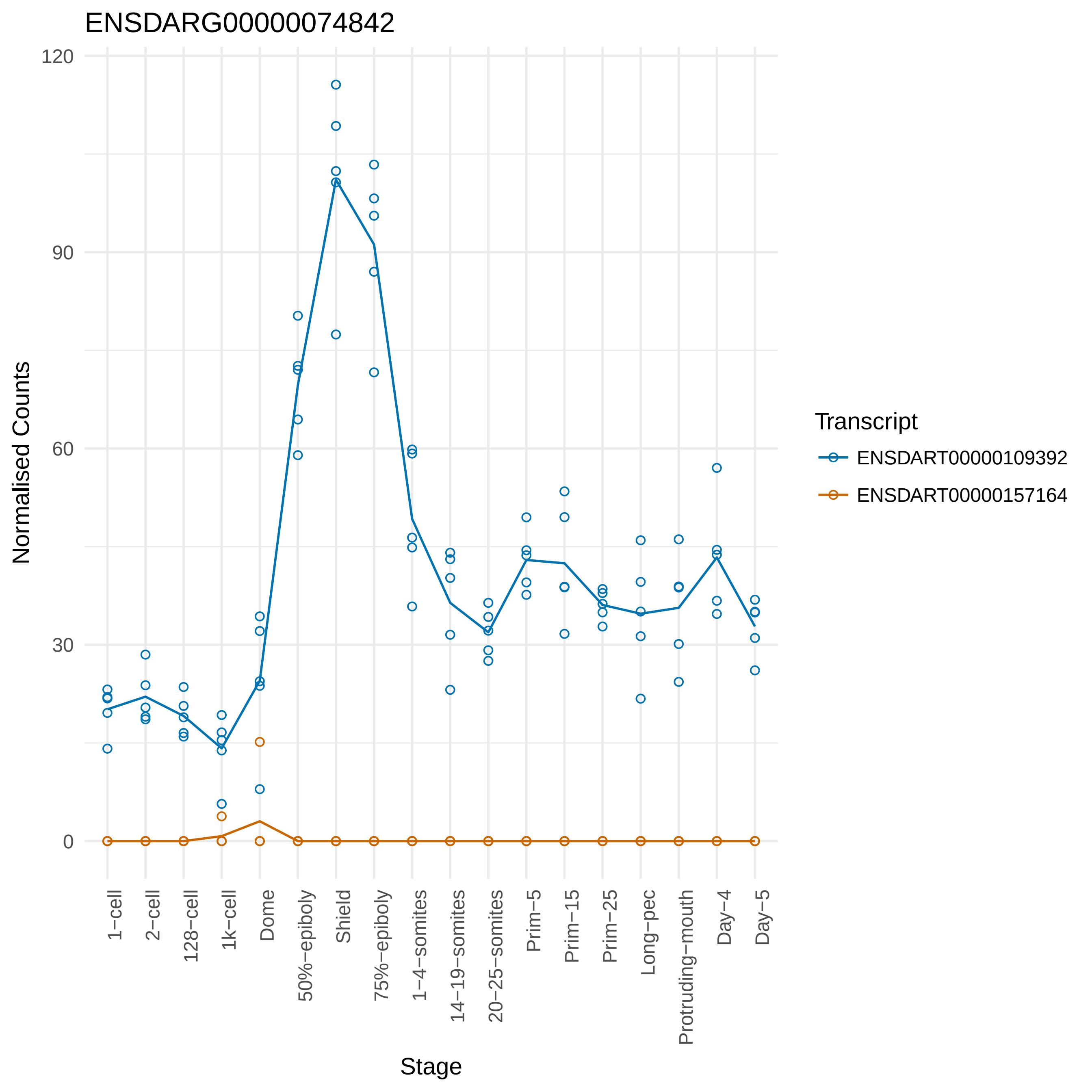
ENSDARG00000074842
Normalised Counts
120
90
60
30
0
1−cell
2−cell
128−cell
1k−cell
Dome
50%−epiboly
Shield
75%−epiboly
1−4−somites
14−19−somites
20−25−somites
Prim−5
Prim−15
Prim−25
Long−pec
Protruding−mouth
Day−4
Day−5
Stage
Transcript
ENSDART00000109392
ENSDART00000157164

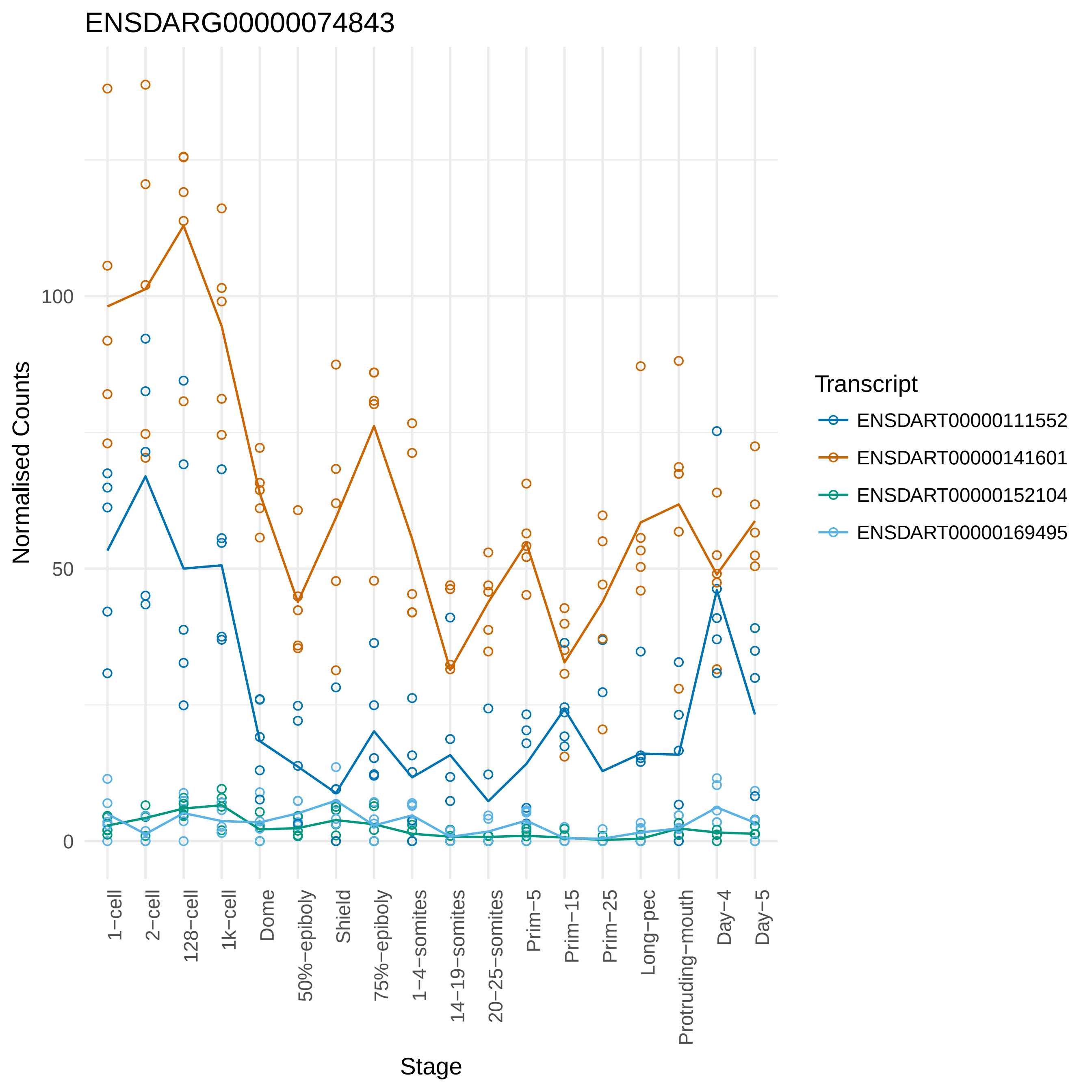

ENSDARG00000074843
Normalised Counts
100
50
0
1−cell
2−cell
128−cell
1k−cell
Dome
50%−epiboly
Shield
75%−epiboly
1−4−somites
14−19−somites
20−25−somites
Prim−5
Prim−15
Prim−25
Long−pec
Protruding−mouth
Day−4
Day−5
Stage
Transcript
ENSDART00000111552
ENSDART00000141601
ENSDART00000152104
ENSDART00000169495

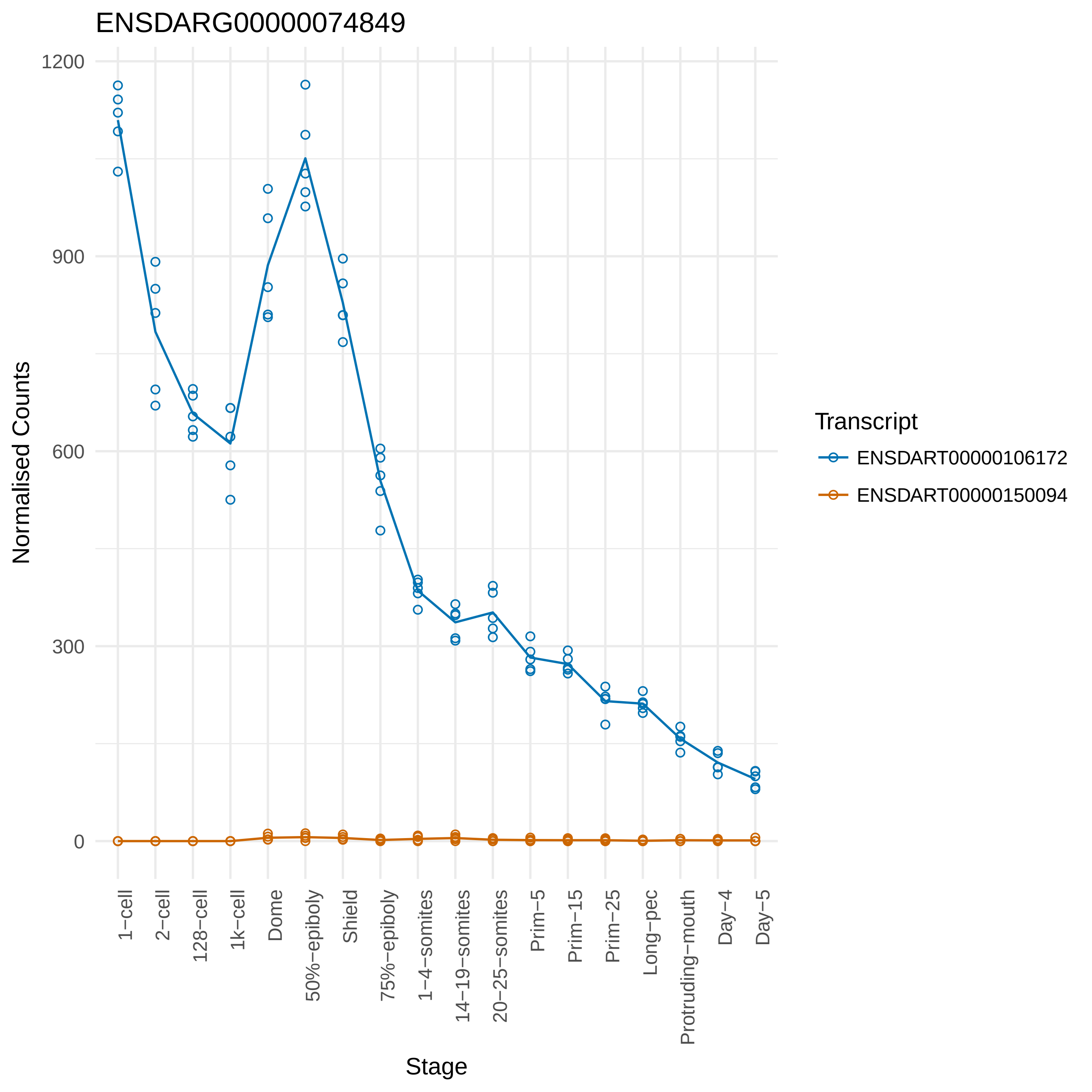

ENSDARG00000074849
1200
900
600
300
0
Normalised Counts
1−cell
2−cell
128−cell
1k−cell
Dome
50%−epiboly
Shield
75%−epiboly
1−4−somites
14−19−somites
20−25−somites
Prim−5
Prim−15
Prim−25
Long−pec
Protruding−mouth
Day−4
Day−5
Stage
Transcript
ENSDART00000106172
ENSDART00000150094

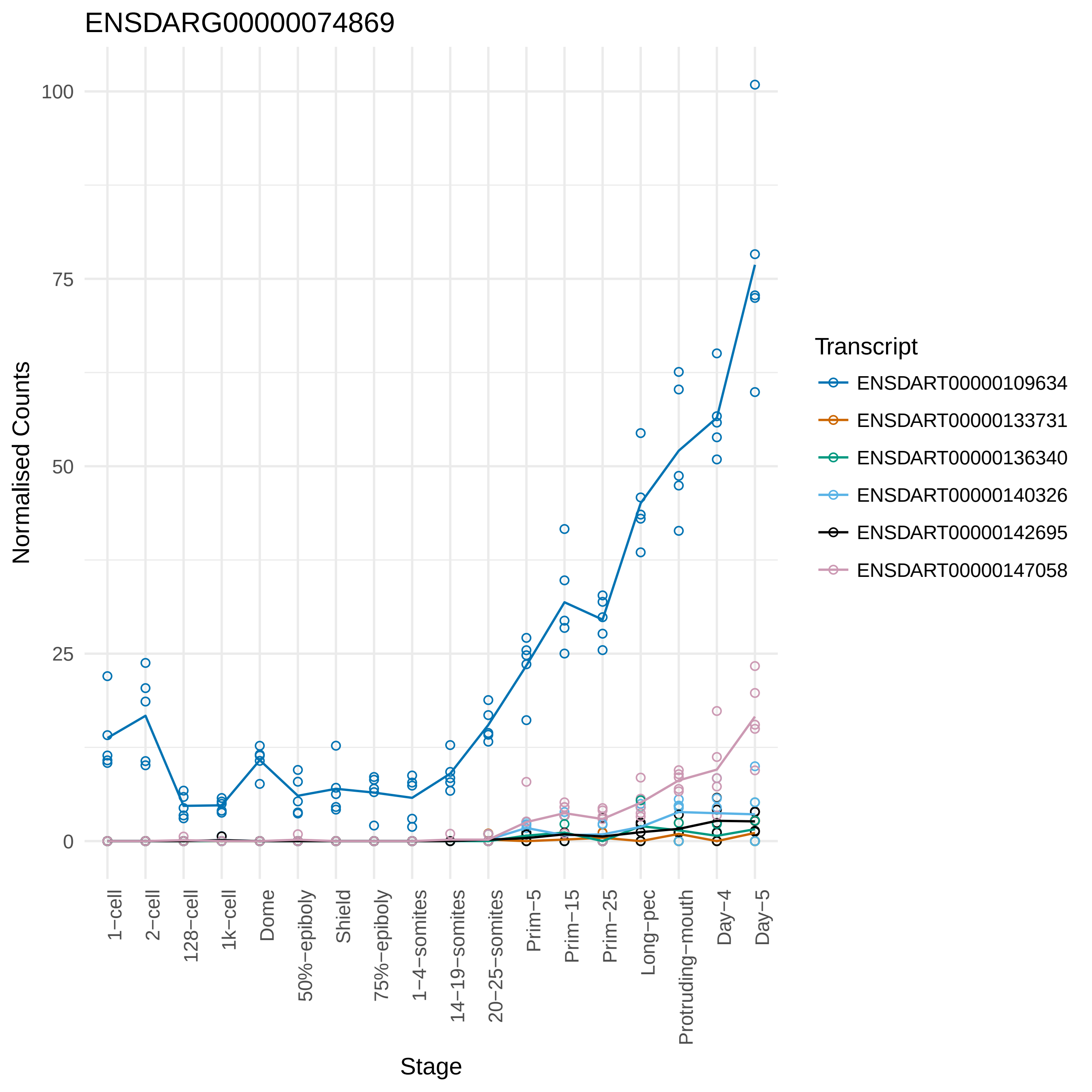
ENSDARG00000074869
Normalised Counts
0
25
50
75
100
Stage
1−cell
2−cell
128−cell
1k−cell
Dome
50%−epiboly
Shield
75%−epiboly
1−4−somites
14−19−somites
20−25−somites
Prim−5
Prim−15
Prim−25
Long−pec
Protruding−mouth
Day−4
Day−5
Transcript
ENSDART00000109634
ENSDART00000133731
ENSDART00000136340
ENSDART00000140326
ENSDART00000142695
ENSDART00000147058

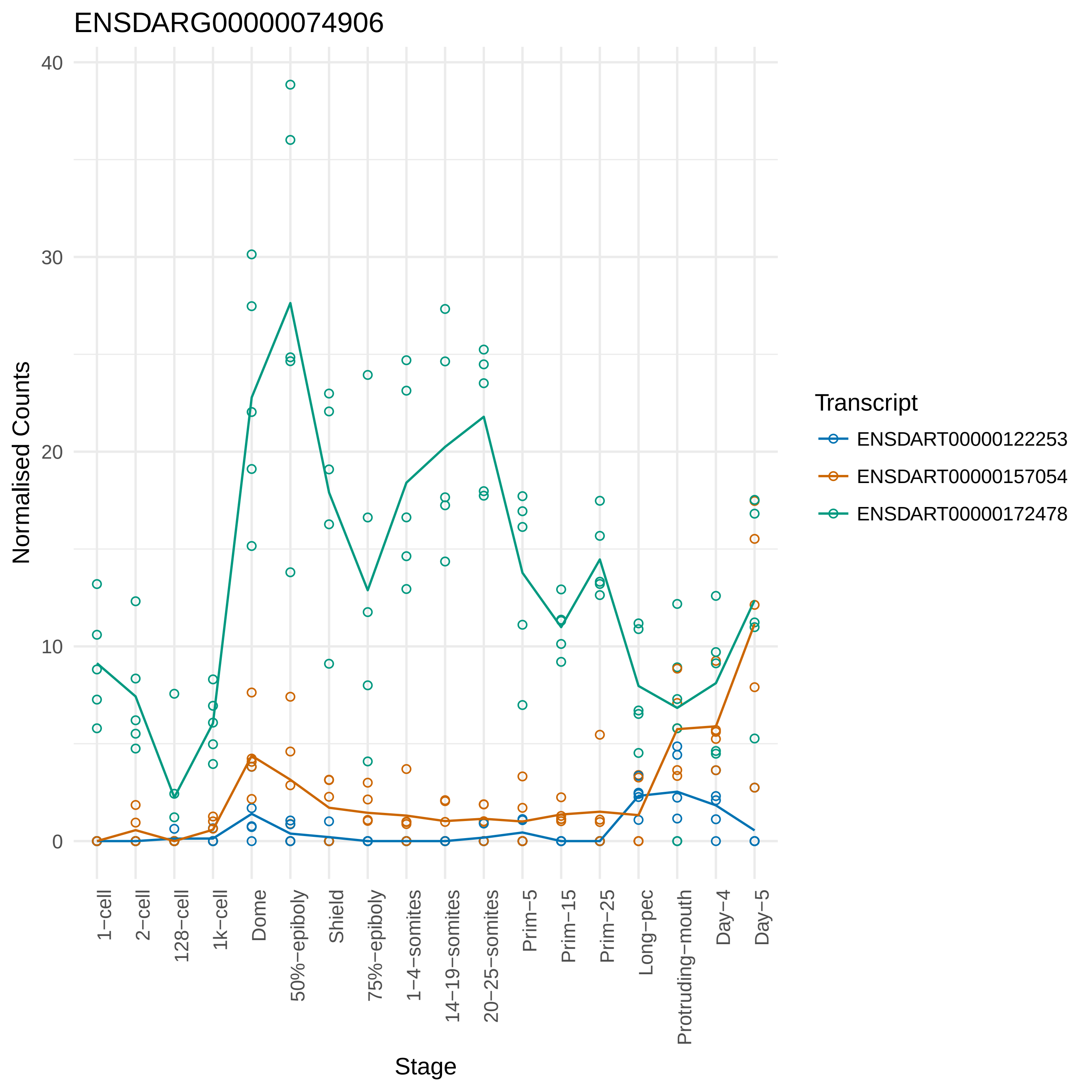
ENSDARG00000074906
Normalised Counts
40
30
20
10
0
1−cell
2−cell
128−cell
1k−cell
Dome
50%−epiboly
Shield
75%−epiboly
1−4−somites
14−19−somites
20−25−somites
Prim−5
Prim−15
Prim−25
Long−pec
Protruding−mouth
Day−4
Day−5
Stage
Transcript
ENSDART00000122253
ENSDART00000157054
ENSDART00000172478

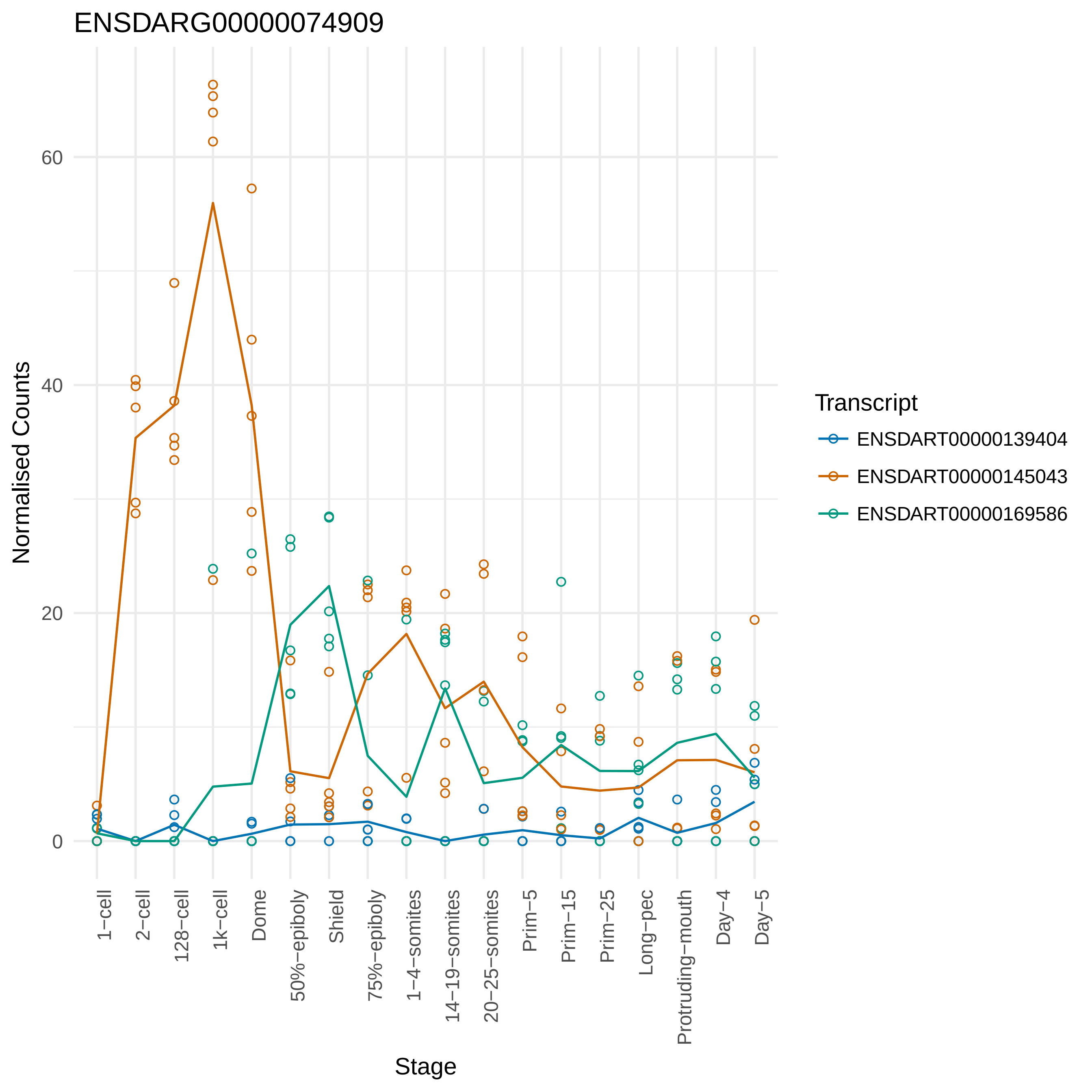

ENSDARG00000074909
Normalised Counts
60
40
20
0
1−cell
2−cell
128−cell
1k−cell
Dome
50%−epiboly
Shield
75%−epiboly
1−4−somites
14−19−somites
20−25−somites
Prim−5
Prim−15
Prim−25
Long−pec
Protruding−mouth
Day−4
Day−5
Stage
Transcript
ENSDART00000139404
ENSDART00000145043
ENSDART00000169586

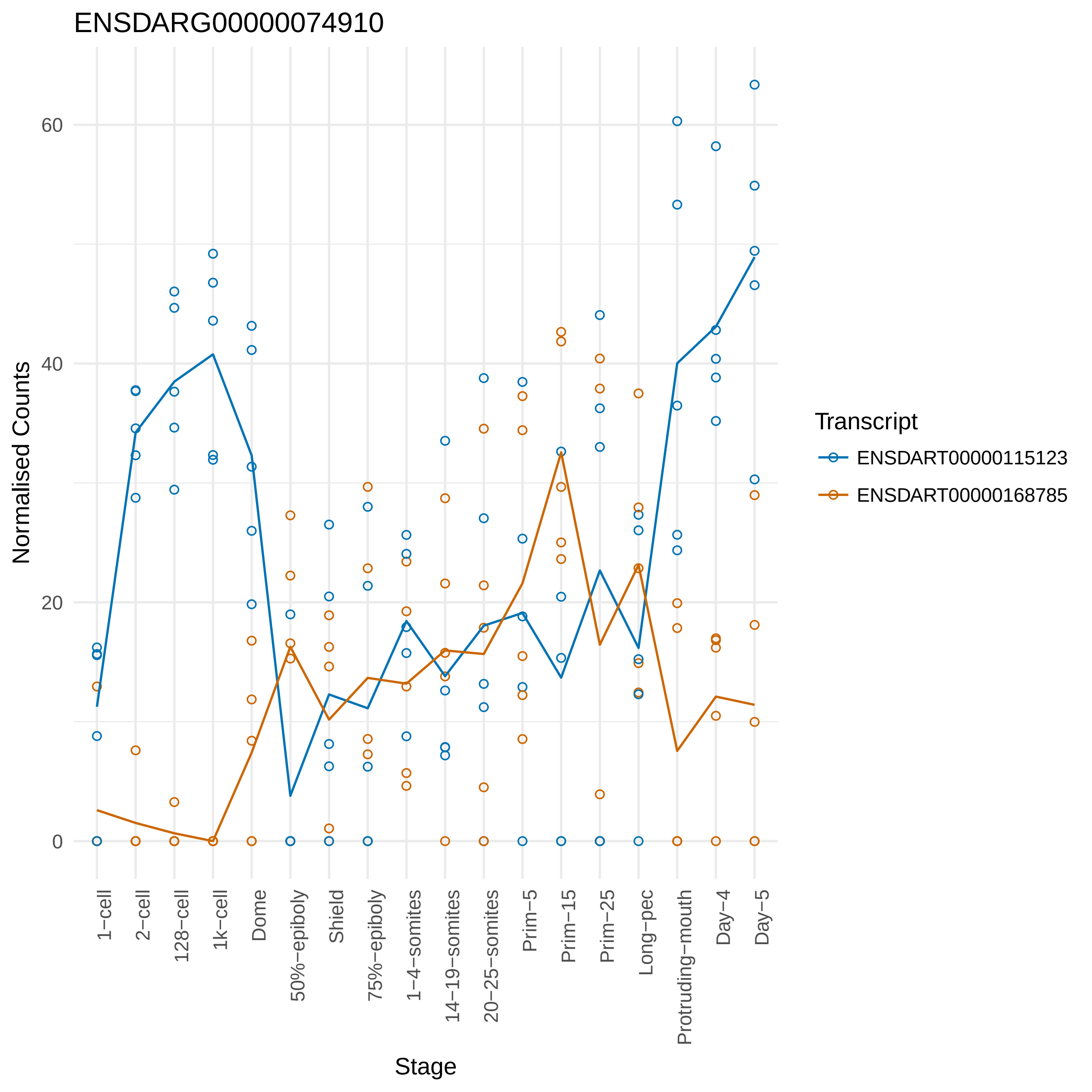

ENSDARG00000074910
Normalised Counts
0
20
40
60
1−cell
2−cell
128−cell
1k−cell
Dome
50%−epiboly
Shield
75%−epiboly
1−4−somites
14−19−somites
20−25−somites
Prim−5
Prim−15
Prim−25
Long−pec
Protruding−mouth
Day−4
Day−5
Stage
Transcript
ENSDART00000115123
ENSDART00000168785

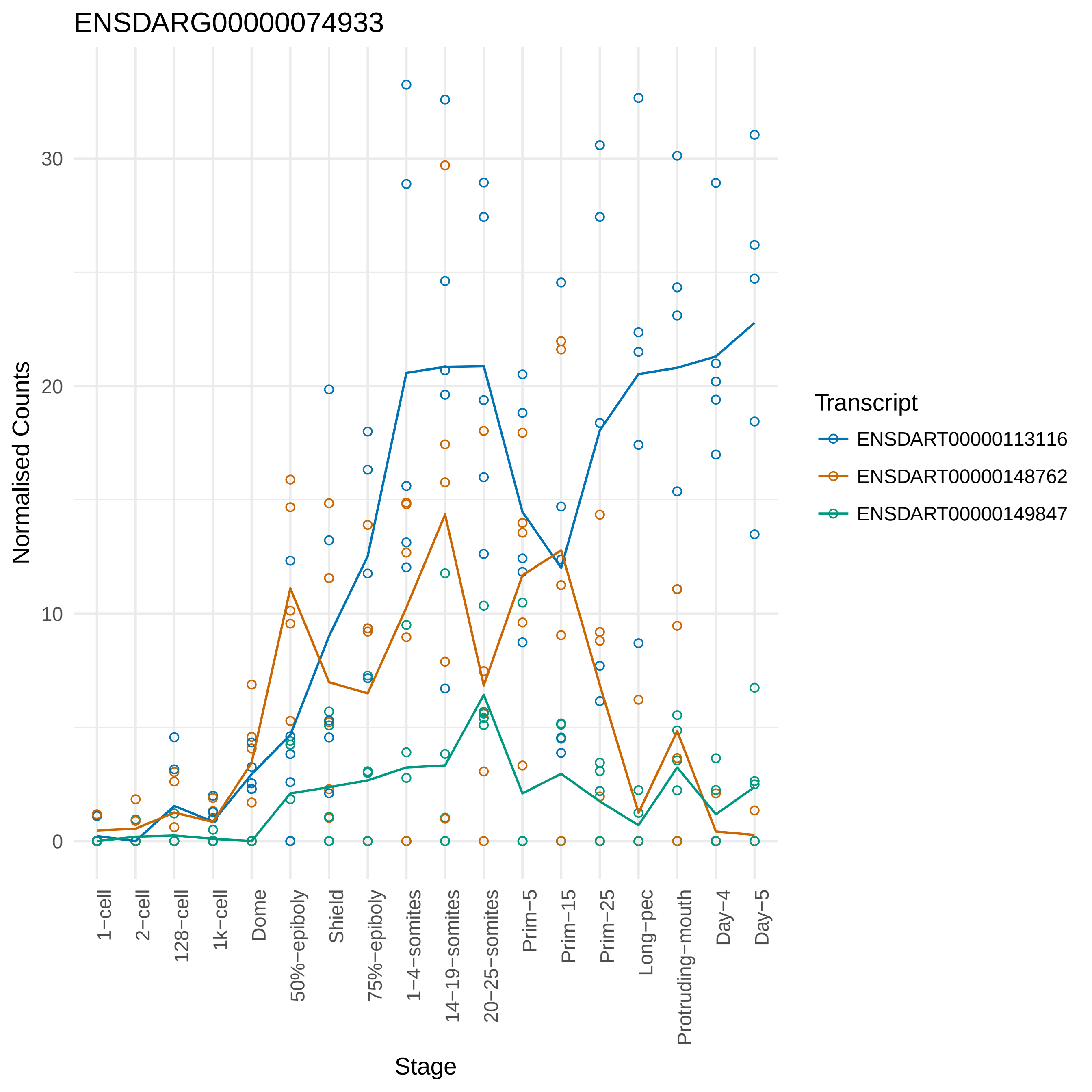

ENSDARG00000074933
Normalised Counts
30
20
10
0
1−cell
2−cell
128−cell
1k−cell
Dome
50%−epiboly
Shield
75%−epiboly
1−4−somites
14−19−somites
20−25−somites
Prim−5
Prim−15
Prim−25
Long−pec
Protruding−mouth
Day−4
Day−5
Stage
Transcript
ENSDART00000113116
ENSDART00000148762
ENSDART00000149847

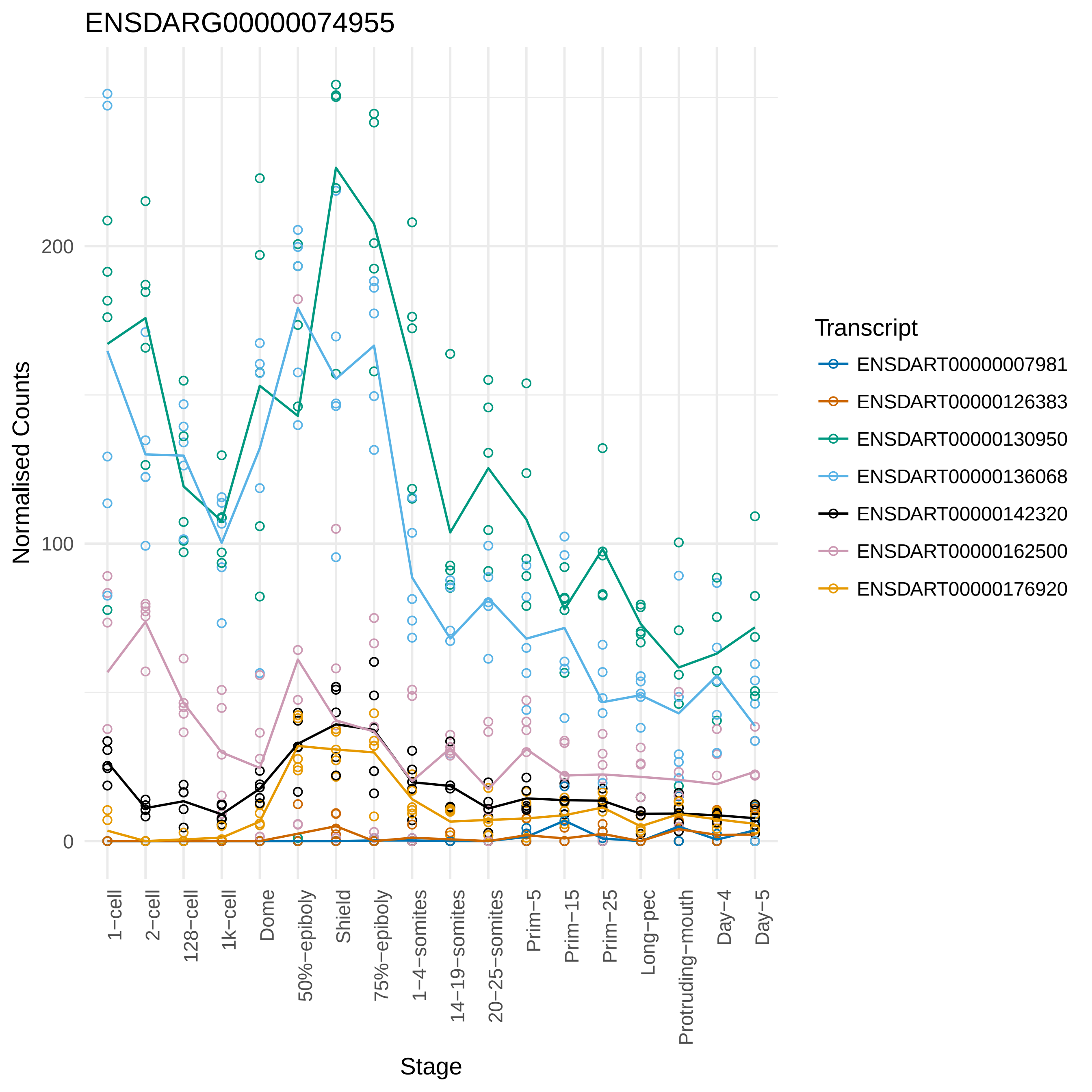

ENSDARG00000074955
Normalised Counts
200
100
0
1−cell
2−cell
128−cell
1k−cell
Dome
50%−epiboly
Shield
75%−epiboly
1−4−somites
14−19−somites
20−25−somites
Prim−5
Prim−15
Prim−25
Long−pec
Protruding−mouth
Day−4
Day−5
Stage
Transcript
ENSDART00000007981
ENSDART00000126383
ENSDART00000130950
ENSDART00000136068
ENSDART00000142320
ENSDART00000162500
ENSDART00000176920

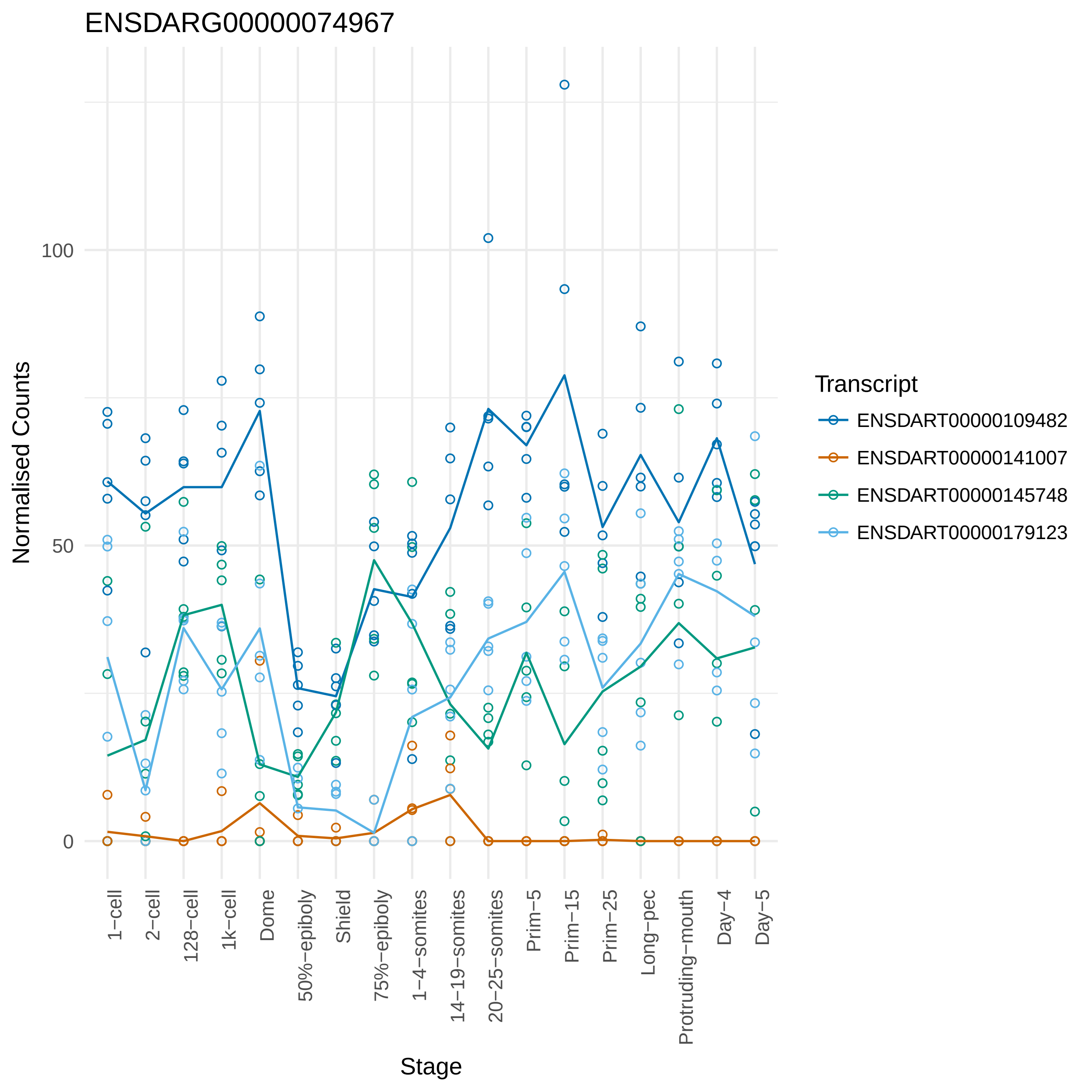

ENSDARG00000074967
Normalised Counts
100
50
0
1−cell
2−cell
128−cell
1k−cell
Dome
50%−epiboly
Shield
75%−epiboly
1−4−somites
14−19−somites
20−25−somites
Prim−5
Prim−15
Prim−25
Long−pec
Protruding−mouth
Day−4
Day−5
Stage
Transcript
ENSDART00000109482
ENSDART00000141007
ENSDART00000145748
ENSDART00000179123

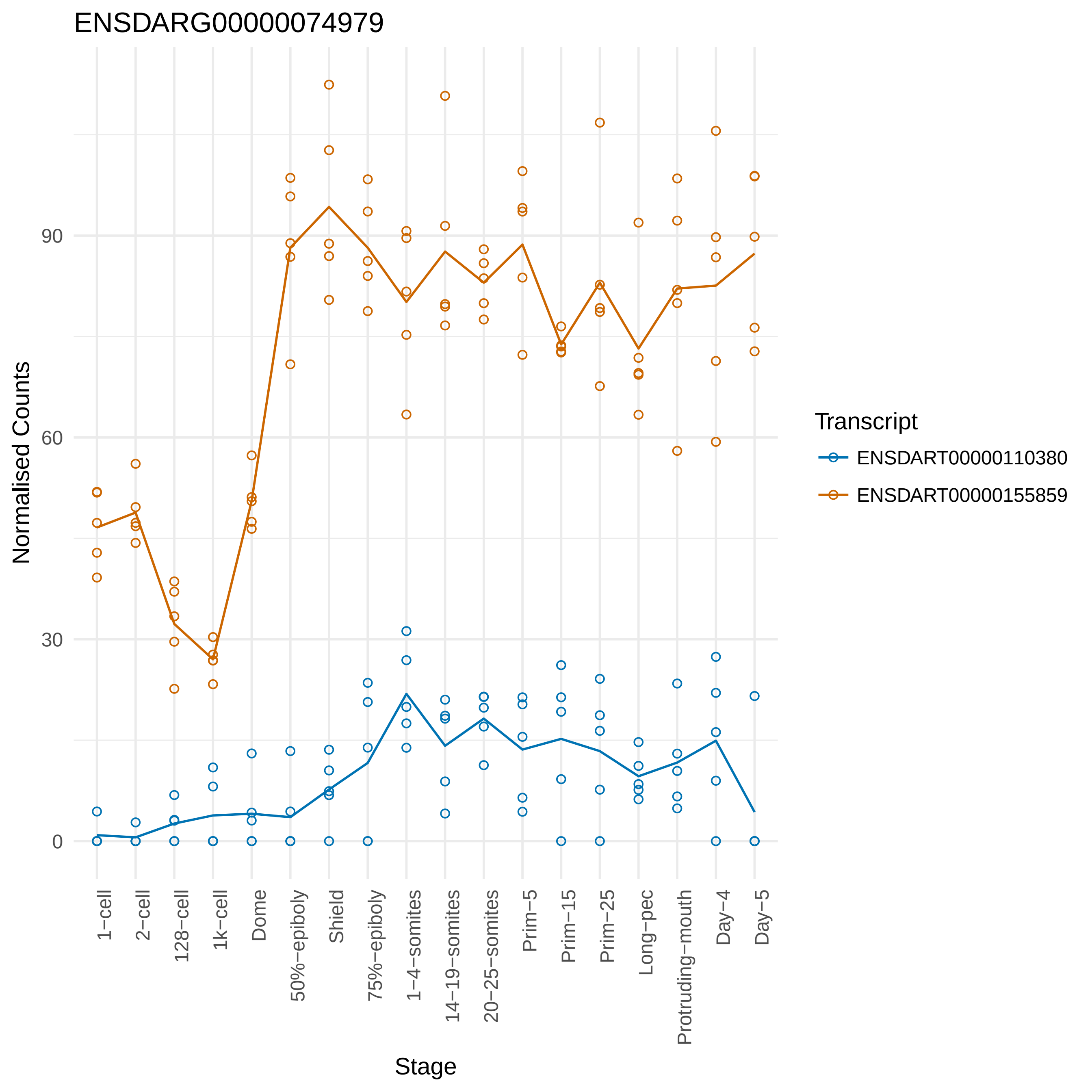

ENSDARG00000074979
Normalised Counts
0
30
60
90
1−cell
2−cell
128−cell
1k−cell
Dome
50%−epiboly
Shield
75%−epiboly
1−4−somites
14−19−somites
20−25−somites
Prim−5
Prim−15
Prim−25
Long−pec
Protruding−mouth
Day−4
Day−5
Stage
Transcript
ENSDART00000110380
ENSDART00000155859

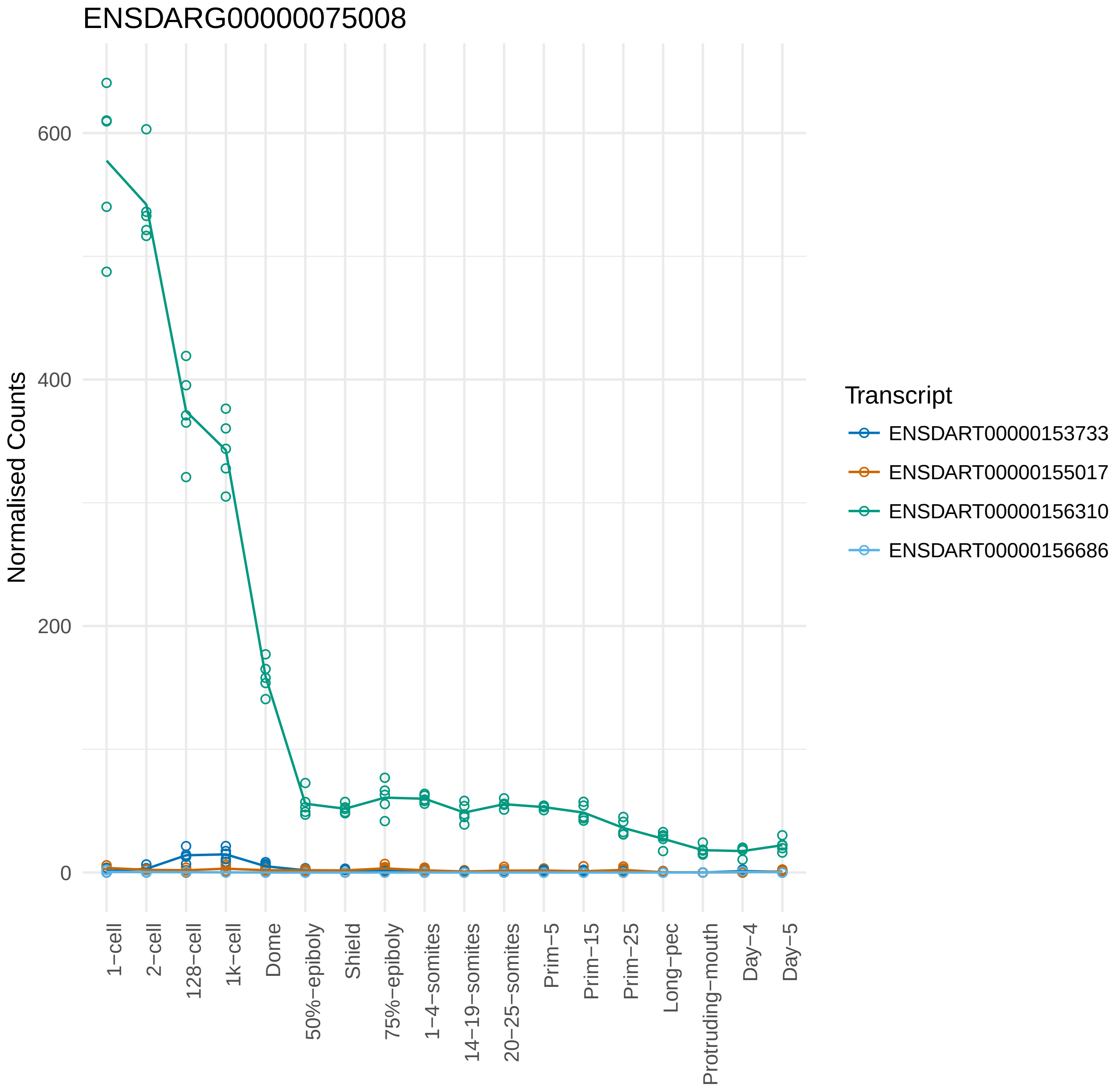
ENSDARG00000075008
Normalised Counts
600
400
200
0
Stage
1−cell
2−cell
128−cell
1k−cell
Dome
50%−epiboly
Shield
75%−epiboly
1−4−somites
14−19−somites
20−25−somites
Prim−5
Prim−15
Prim−25
Long−pec
Protruding−mouth
Day−4
Day−5
Transcript
ENSDART00000153733
ENSDART00000155017
ENSDART00000156310
ENSDART00000156686

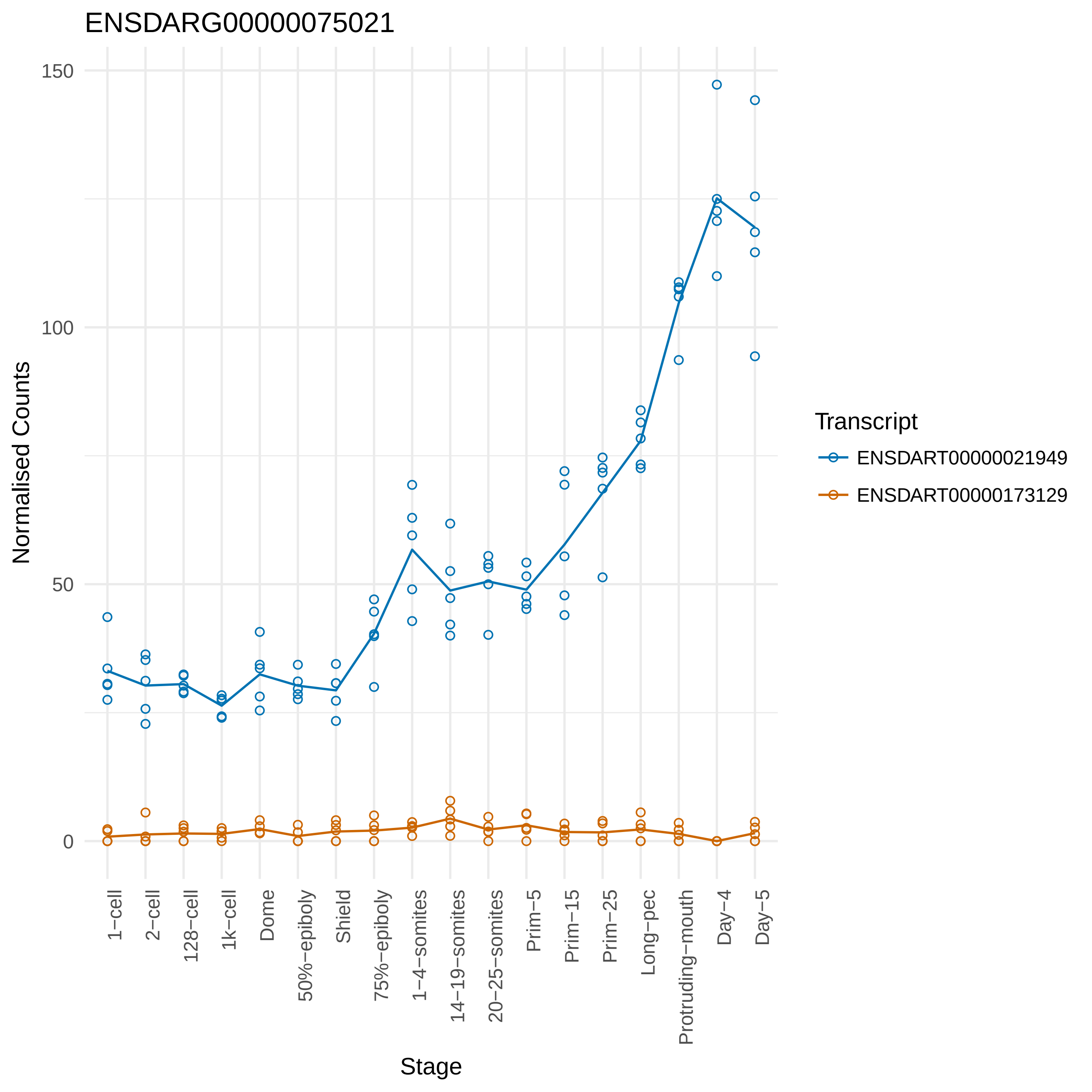
ENSDARG00000075021
Normalised Counts
150
100
50
0
Stage
1−cell
2−cell
128−cell
1k−cell
Dome
50%−epiboly
Shield
75%−epiboly
1−4−somites
14−19−somites
20−25−somites
Prim−5
Prim−15
Prim−25
Long−pec
Protruding−mouth
Day−4
Day−5
Transcript
ENSDART00000021949
ENSDART00000173129

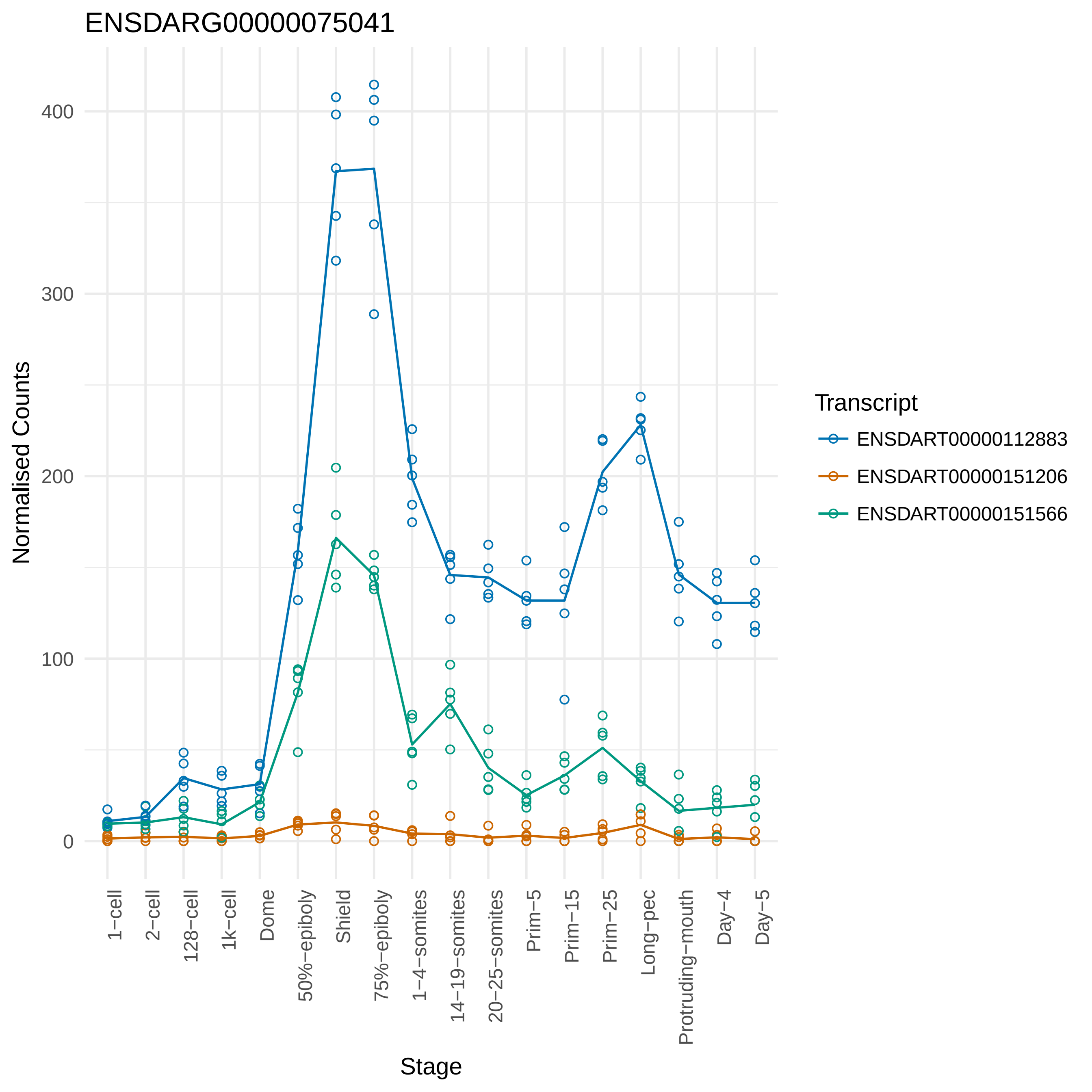
ENSDARG00000075041
Normalised Counts
0
100
200
300
400
1−cell
2−cell
128−cell
1k−cell
Dome
50%−epiboly
Shield
75%−epiboly
1−4−somites
14−19−somites
20−25−somites
Prim−5
Prim−15
Prim−25
Long−pec
Protruding−mouth
Day−4
Day−5
Stage
Transcript
ENSDART00000112883
ENSDART00000151206
ENSDART00000151566

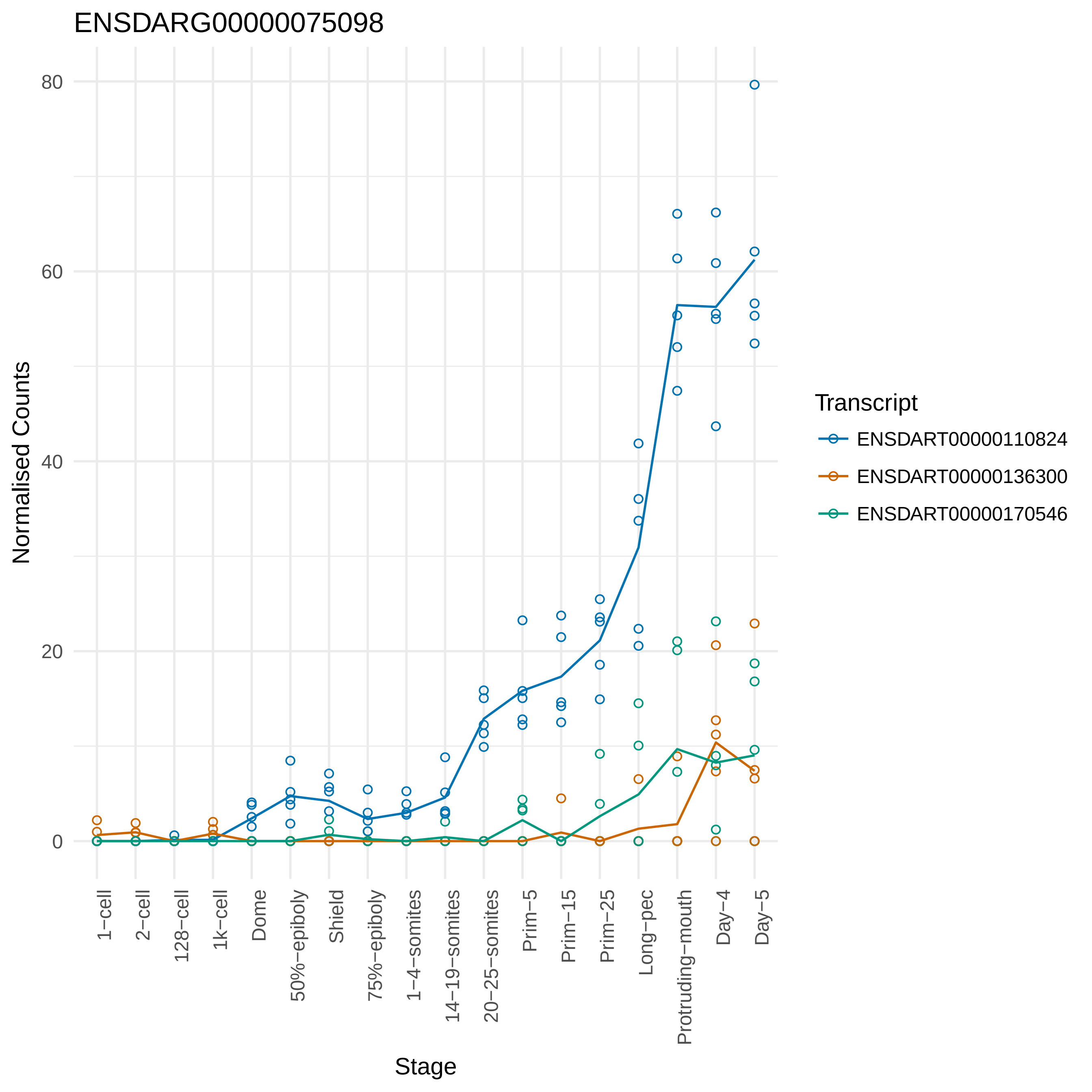
ENSDARG00000075098
Normalised Counts
80
60
40
20
0
1−cell
2−cell
128−cell
1k−cell
Dome
50%−epiboly
Shield
75%−epiboly
1−4−somites
14−19−somites
20−25−somites
Prim−5
Prim−15
Prim−25
Long−pec
Protruding−mouth
Day−4
Day−5
Stage
Transcript
ENSDART00000110824
ENSDART00000136300
ENSDART00000170546

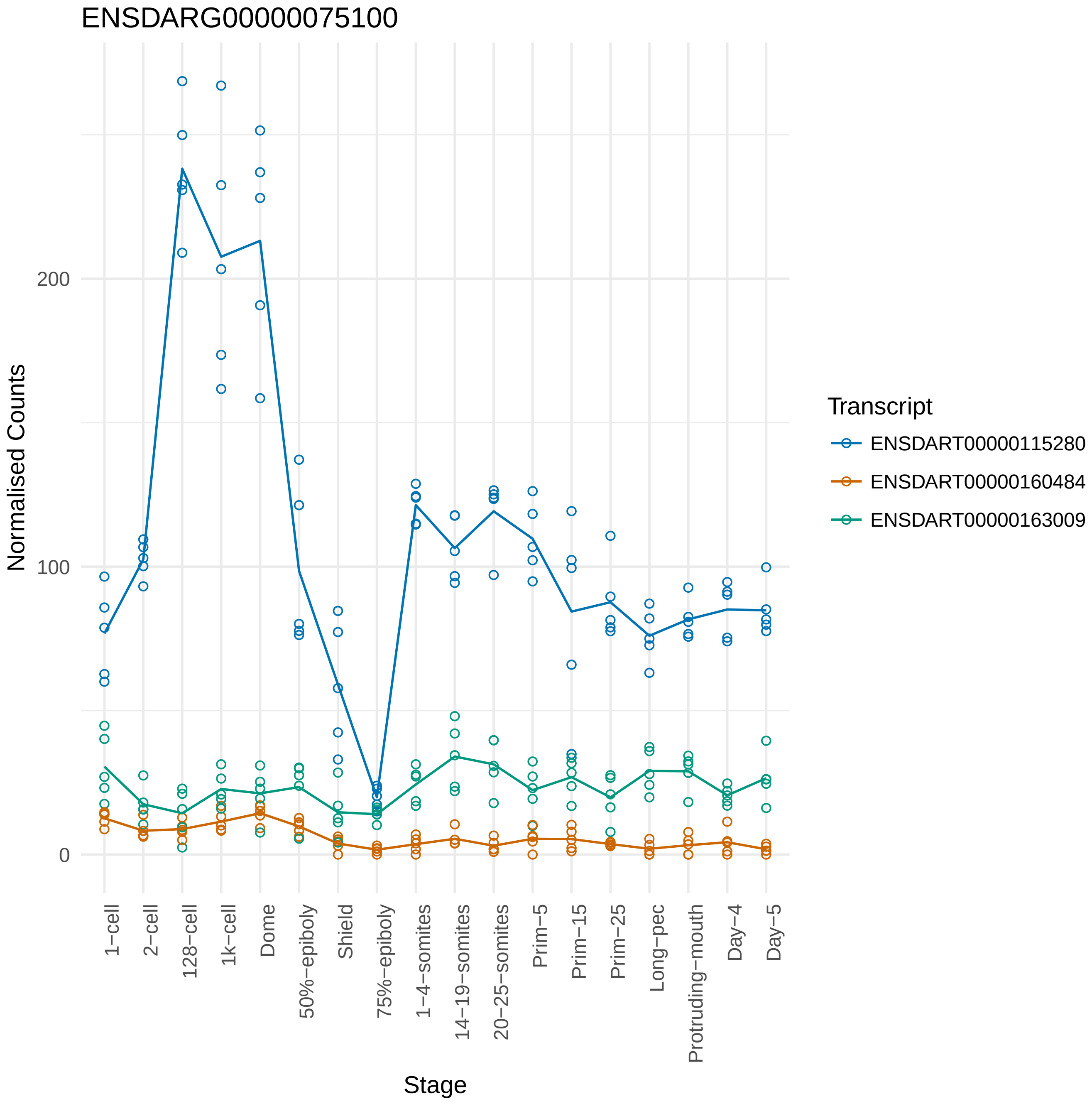

ENSDARG00000075100
Normalised Counts
200
100
0
1−cell
2−cell
128−cell
1k−cell
Dome
50%−epiboly
Shield
75%−epiboly
1−4−somites
14−19−somites
20−25−somites
Prim−5
Prim−15
Prim−25
Long−pec
Protruding−mouth
Day−4
Day−5
Stage
Transcript
ENSDART00000115280
ENSDART00000160484
ENSDART00000163009

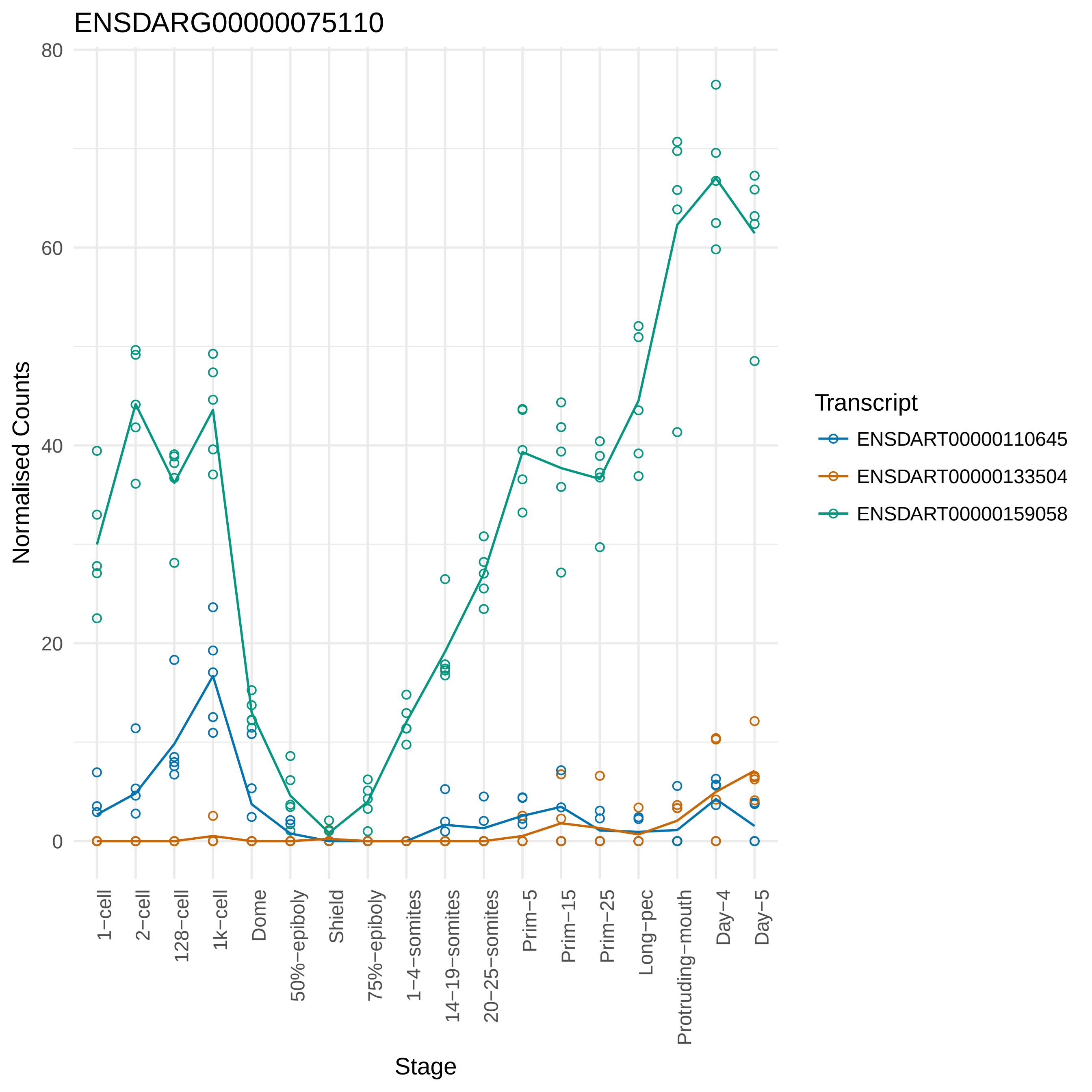
ENSDARG00000075110
Normalised Counts
80
60
40
20
0
1−cell
2−cell
128−cell
1k−cell
Dome
50%−epiboly
Shield
75%−epiboly
1−4−somites
14−19−somites
20−25−somites
Prim−5
Prim−15
Prim−25
Long−pec
Protruding−mouth
Day−4
Day−5
Stage
Transcript
ENSDART00000110645
ENSDART00000133504
ENSDART00000159058

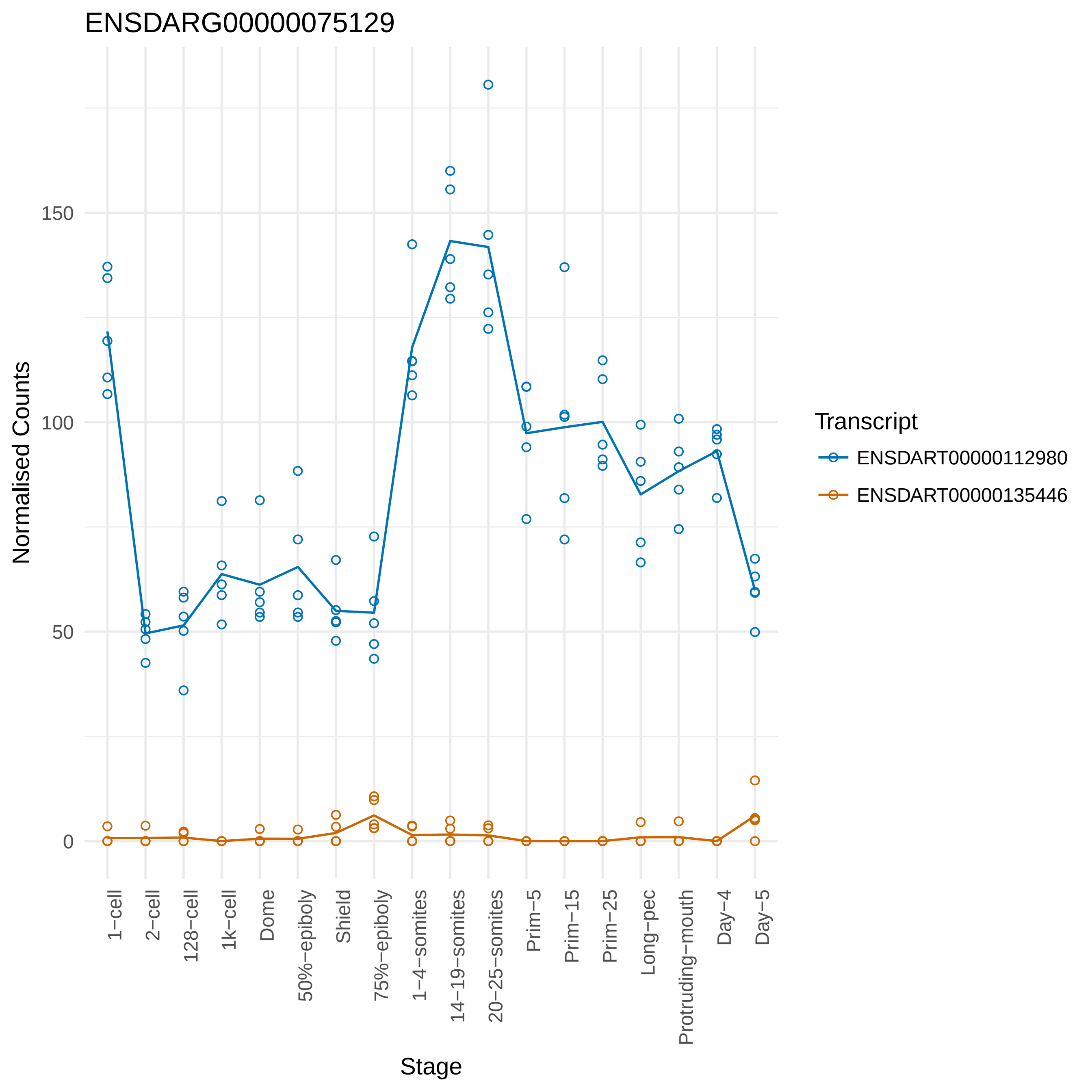
ENSDARG00000075129
Normalised Counts
150
100
50
0
1−cell
2−cell
128−cell
1k−cell
Dome
50%−epiboly
Shield
75%−epiboly
1−4−somites
14−19−somites
20−25−somites
Prim−5
Prim−15
Prim−25
Long−pec
Protruding−mouth
Day−4
Day−5
Stage
Transcript
ENSDART00000112980
ENSDART00000135446

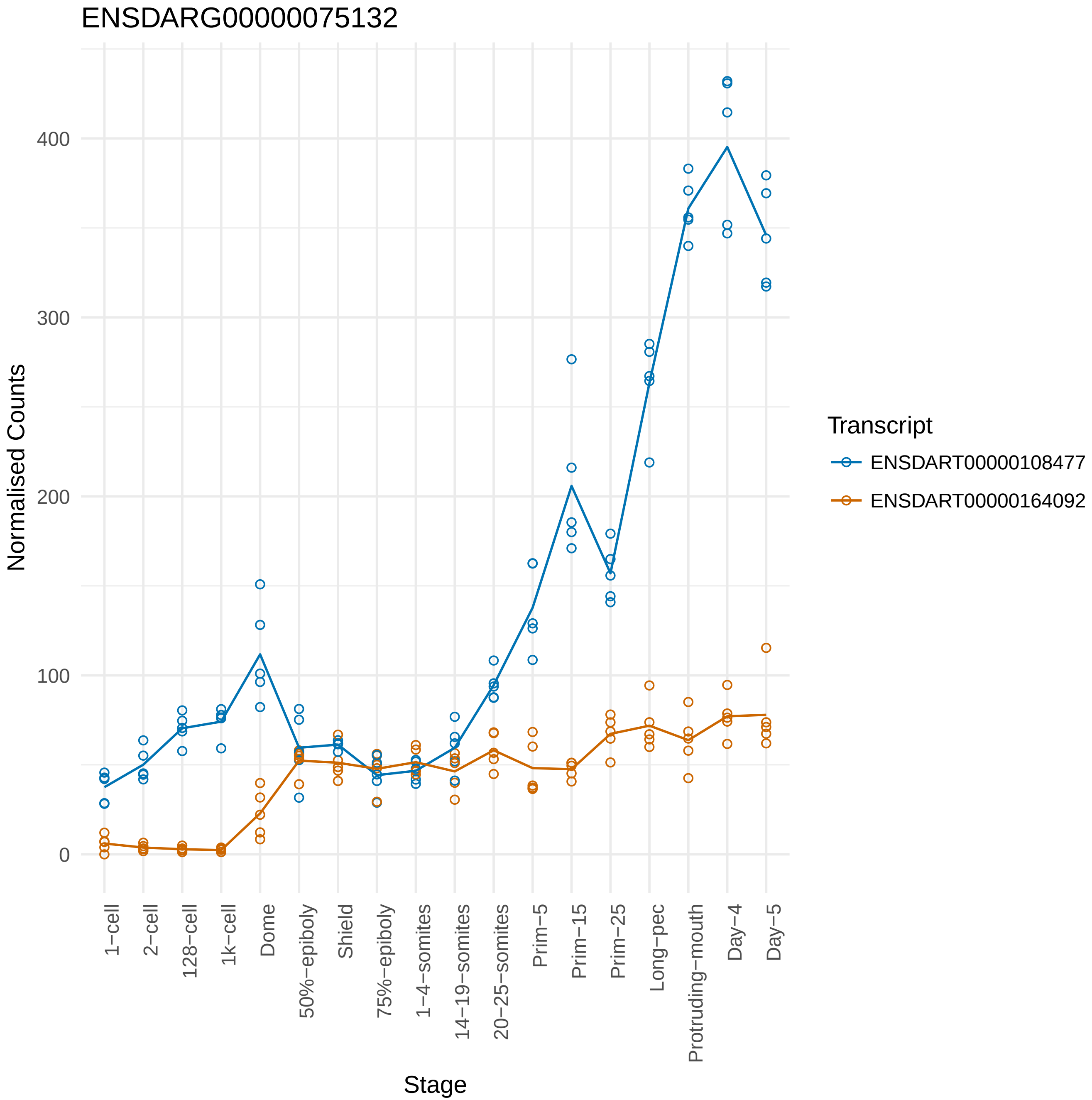
ENSDARG00000075132
Normalised Counts
0
100
200
300
400
Stage
1−cell
2−cell
128−cell
1k−cell
Dome
50%−epiboly
Shield
75%−epiboly
1−4−somites
14−19−somites
20−25−somites
Prim−5
Prim−15
Prim−25
Long−pec
Protruding−mouth
Day−4
Day−5
Transcript
ENSDART00000108477
ENSDART00000164092

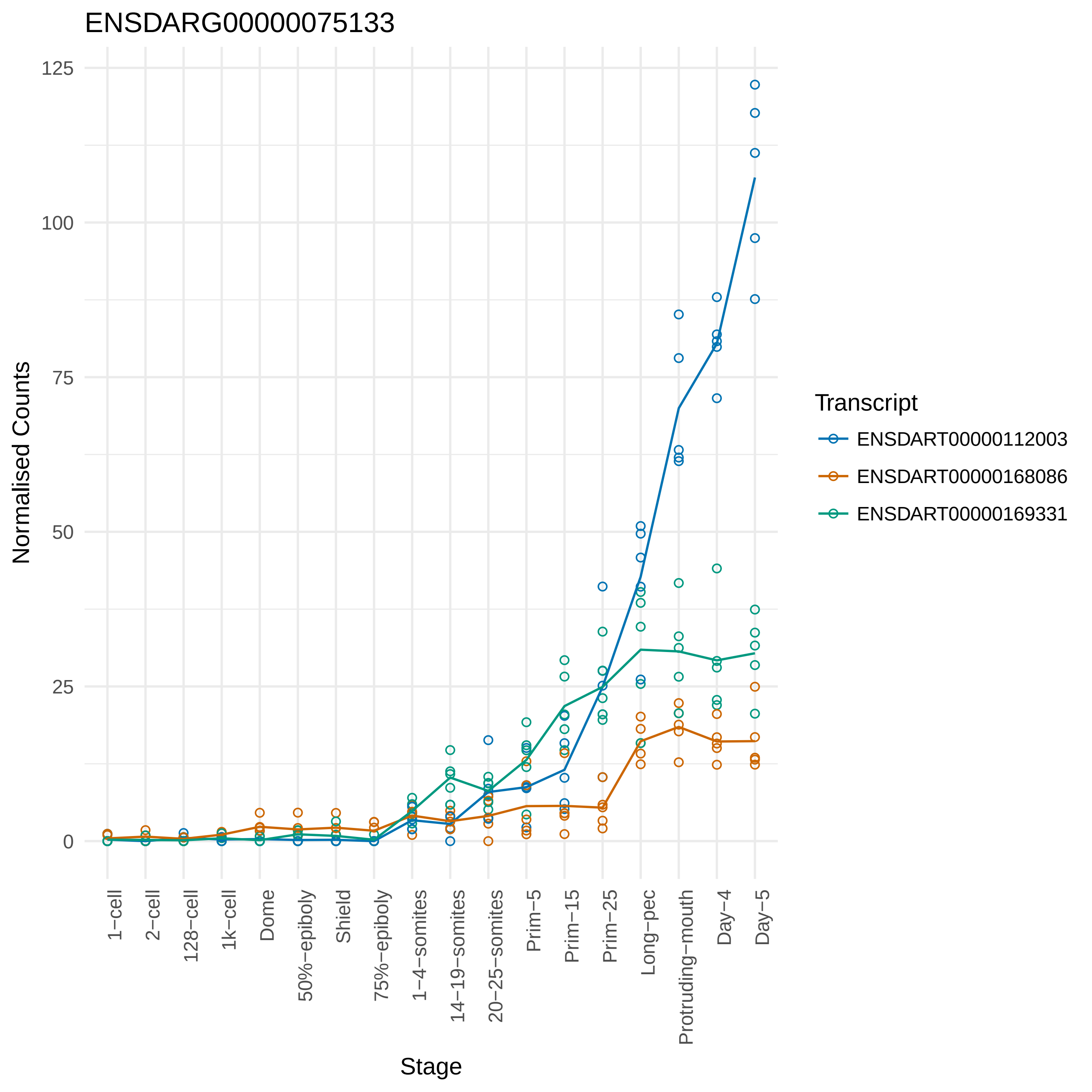
ENSDARG00000075133
Normalised Counts
125
100
75
50
25
0
1−cell
2−cell
128−cell
1k−cell
Dome
50%−epiboly
Shield
75%−epiboly
1−4−somites
14−19−somites
20−25−somites
Prim−5
Prim−15
Prim−25
Long−pec
Protruding−mouth
Day−4
Day−5
Stage
Transcript
ENSDART00000112003
ENSDART00000168086
ENSDART00000169331

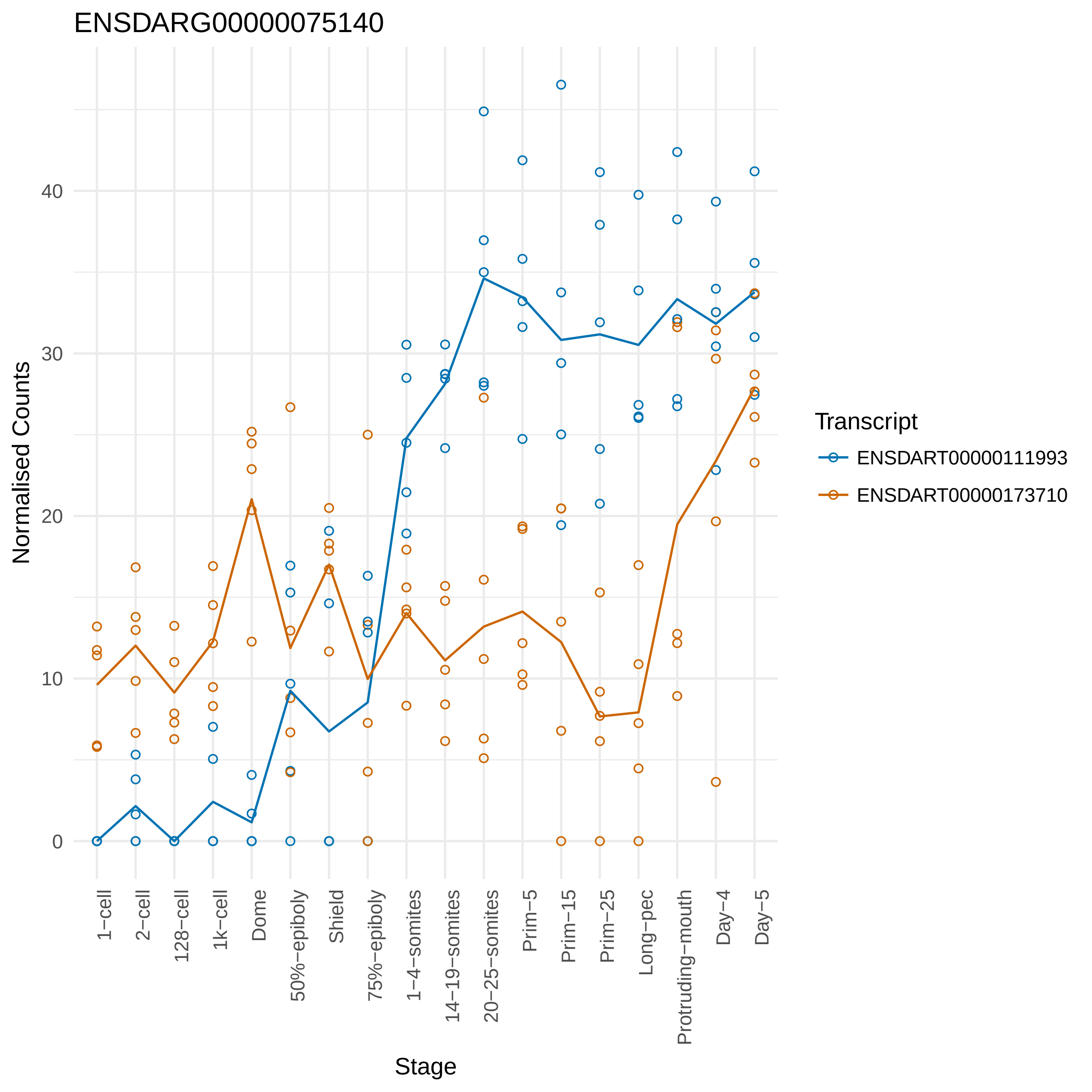

ENSDARG00000075140
Normalised Counts
0
10
20
30
40
Stage
1−cell
2−cell
128−cell
1k−cell
Dome
50%−epiboly
Shield
75%−epiboly
1−4−somites
14−19−somites
20−25−somites
Prim−5
Prim−15
Prim−25
Long−pec
Protruding−mouth
Day−4
Day−5
Transcript
ENSDART00000111993
ENSDART00000173710

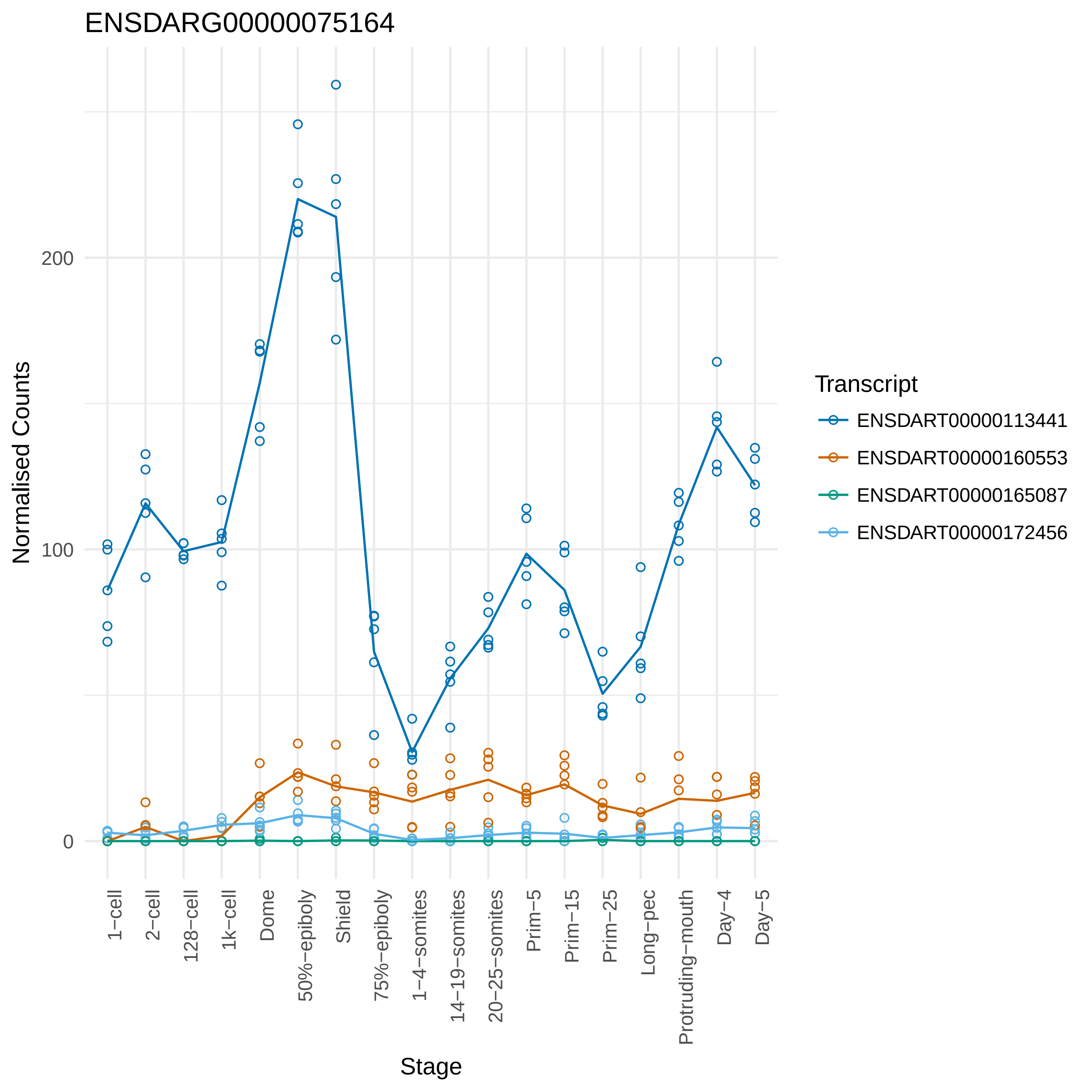

ENSDARG00000075164
Normalised Counts
0
100
200
Stage
1−cell
2−cell
128−cell
1k−cell
Dome
50%−epiboly
Shield
75%−epiboly
1−4−somites
14−19−somites
20−25−somites
Prim−5
Prim−15
Prim−25
Long−pec
Protruding−mouth
Day−4
Day−5
Transcript
ENSDART00000113441
ENSDART00000160553
ENSDART00000165087
ENSDART00000172456

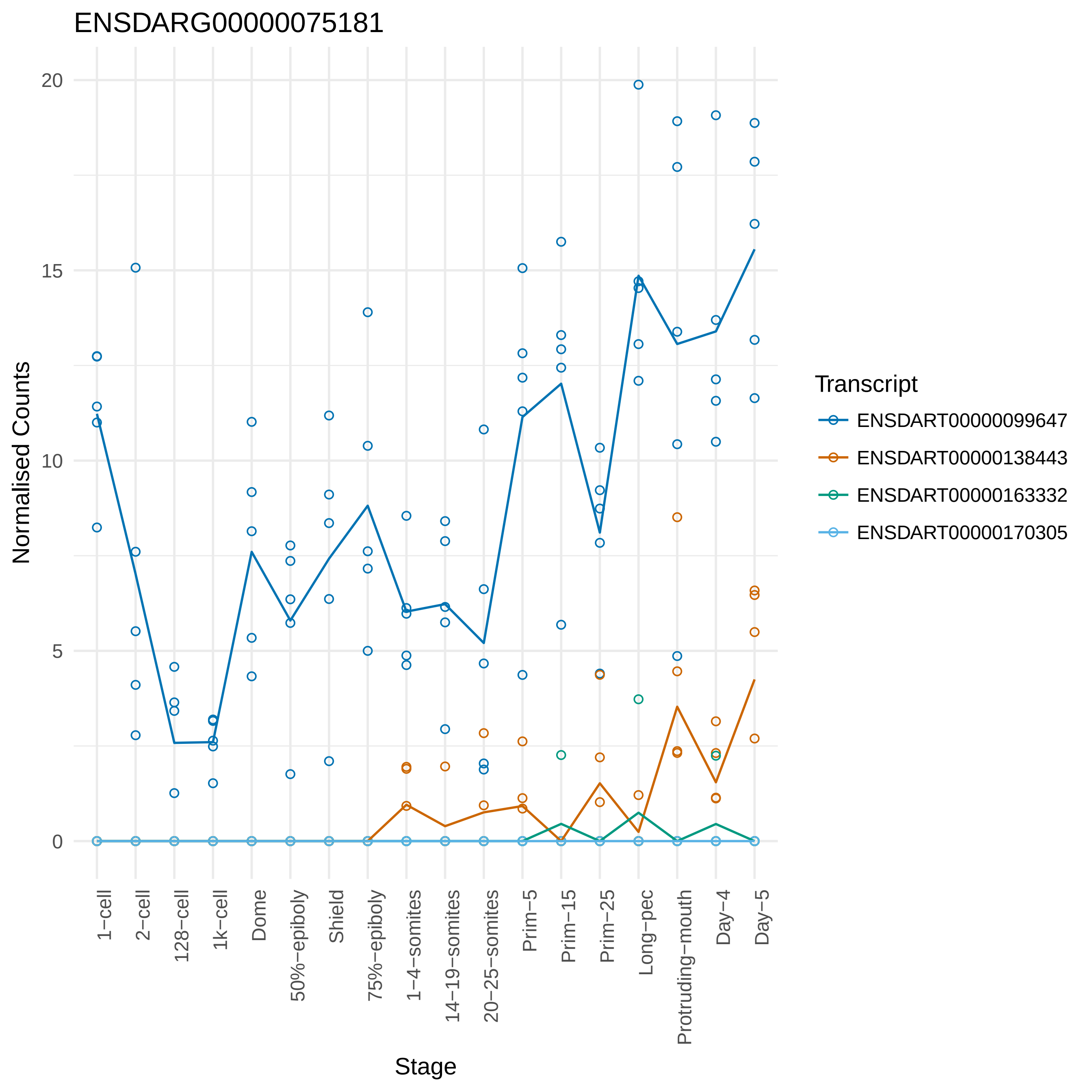

ENSDARG00000075181
Normalised Counts
20
15
10
5
0
1−cell
2−cell
128−cell
1k−cell
Dome
50%−epiboly
Shield
75%−epiboly
1−4−somites
14−19−somites
20−25−somites
Prim−5
Prim−15
Prim−25
Long−pec
Protruding−mouth
Day−4
Day−5
Stage
Transcript
ENSDART00000099647
ENSDART00000138443
ENSDART00000163332
ENSDART00000170305

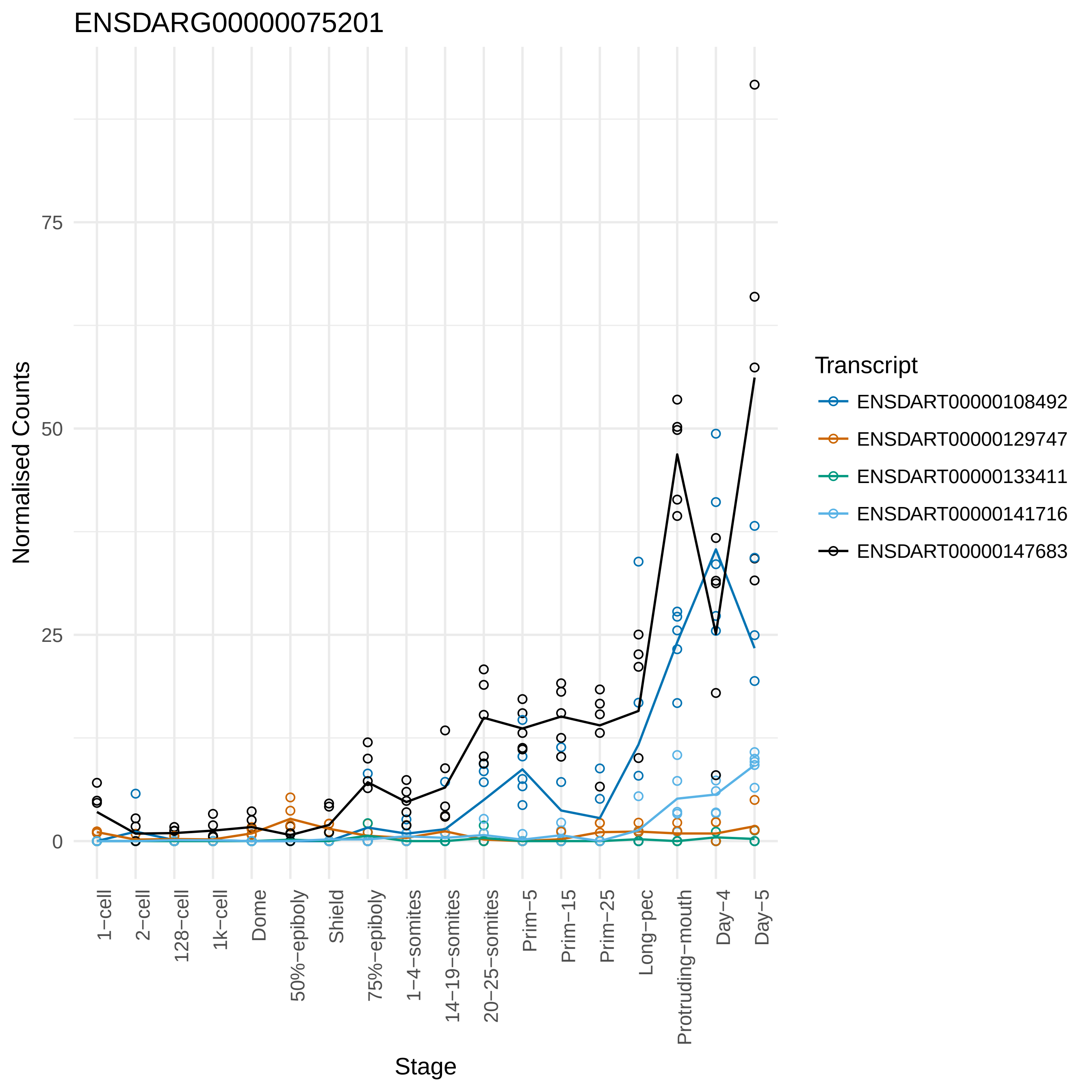
ENSDARG00000075201
Normalised Counts
0
25
50
75
Stage
1−cell
2−cell
128−cell
1k−cell
Dome
50%−epiboly
Shield
75%−epiboly
1−4−somites
14−19−somites
20−25−somites
Prim−5
Prim−15
Prim−25
Long−pec
Protruding−mouth
Day−4
Day−5
Transcript
ENSDART00000108492
ENSDART00000129747
ENSDART00000133411
ENSDART00000141716
ENSDART00000147683

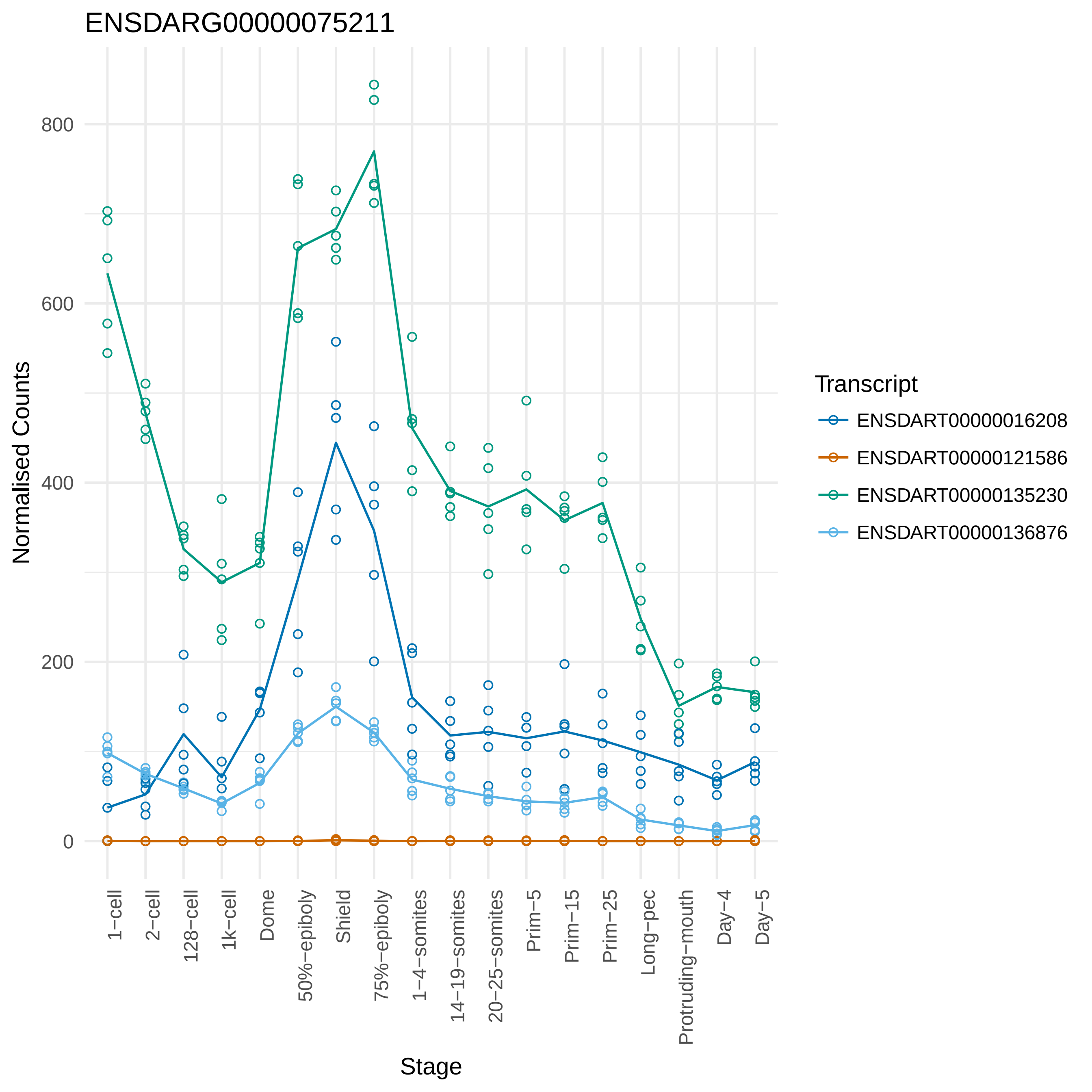

ENSDARG00000075211
Normalised Counts
0
200
400
600
800
Stage
1−cell
2−cell
128−cell
1k−cell
Dome
50%−epiboly
Shield
75%−epiboly
1−4−somites
14−19−somites
20−25−somites
Prim−5
Prim−15
Prim−25
Long−pec
Protruding−mouth
Day−4
Day−5
Transcript
ENSDART00000016208
ENSDART00000121586
ENSDART00000135230
ENSDART00000136876

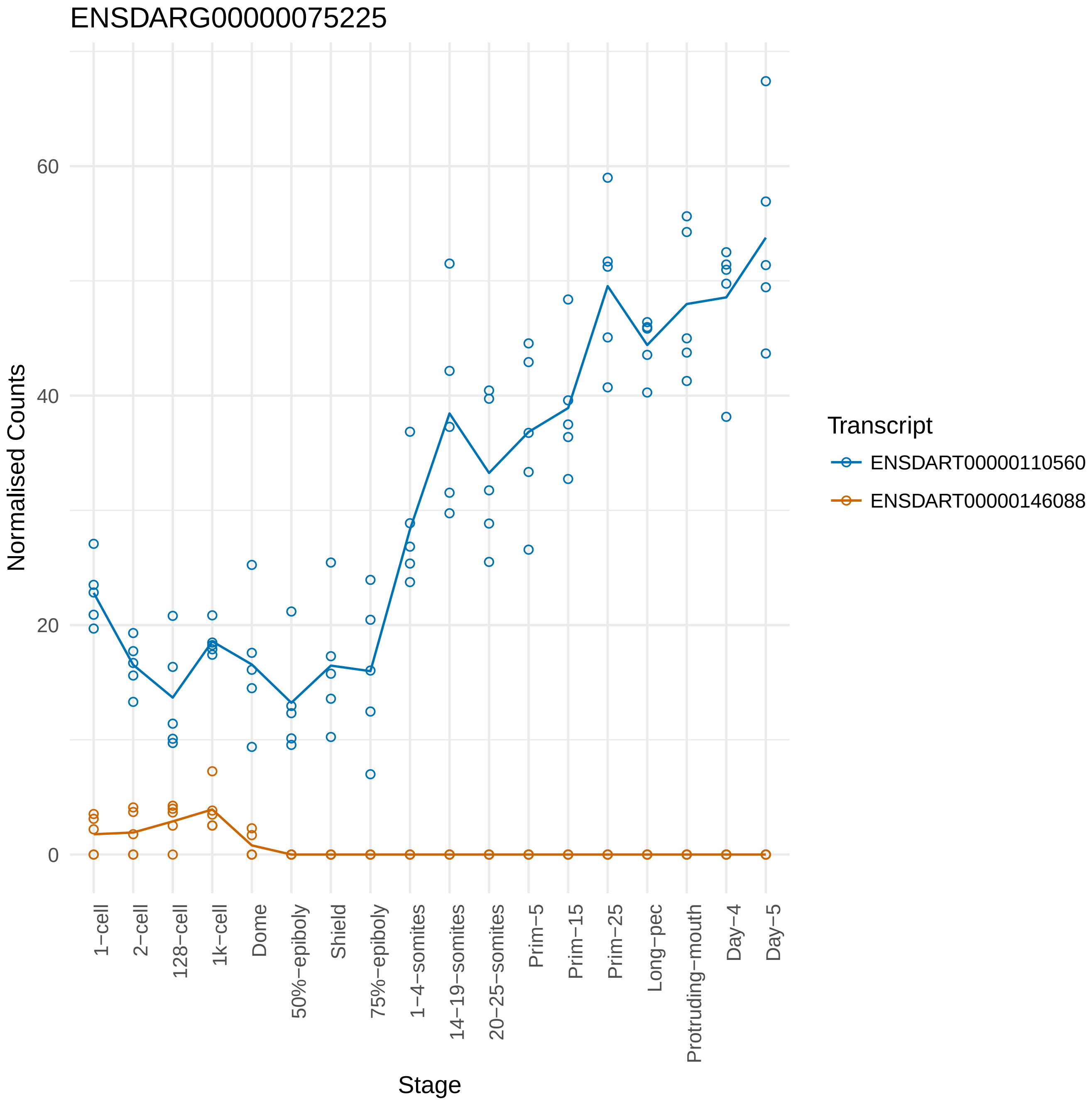

ENSDARG00000075225
Normalised Counts
0
20
40
60
Stage
1−cell
2−cell
128−cell
1k−cell
Dome
50%−epiboly
Shield
75%−epiboly
1−4−somites
14−19−somites
20−25−somites
Prim−5
Prim−15
Prim−25
Long−pec
Protruding−mouth
Day−4
Day−5
Transcript
ENSDART00000110560
ENSDART00000146088

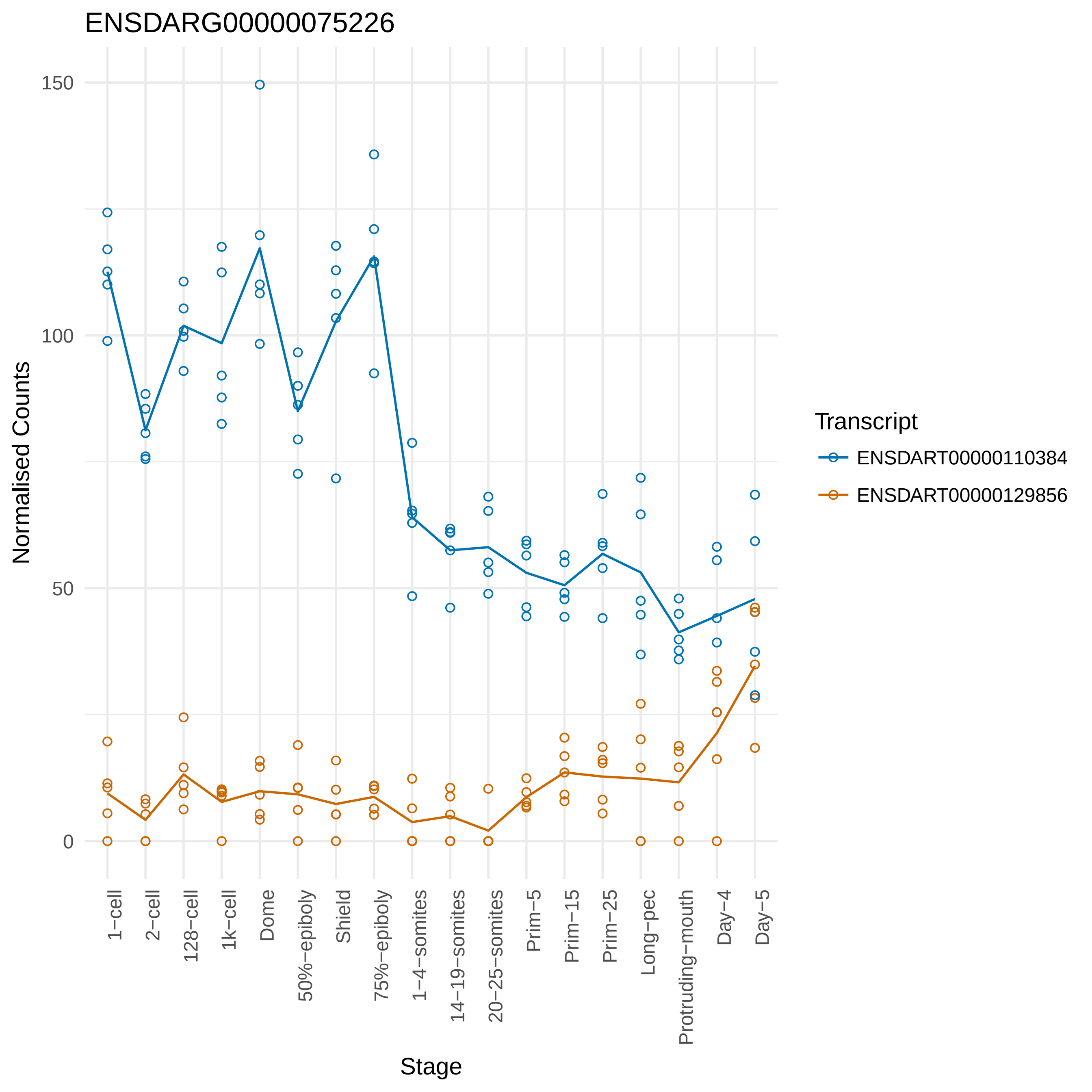
ENSDARG00000075226
Normalised Counts
150
100
50
0
1−cell
2−cell
128−cell
1k−cell
Dome
50%−epiboly
Shield
75%−epiboly
1−4−somites
14−19−somites
20−25−somites
Prim−5
Prim−15
Prim−25
Long−pec
Protruding−mouth
Day−4
Day−5
Stage
Transcript
ENSDART00000110384
ENSDART00000129856

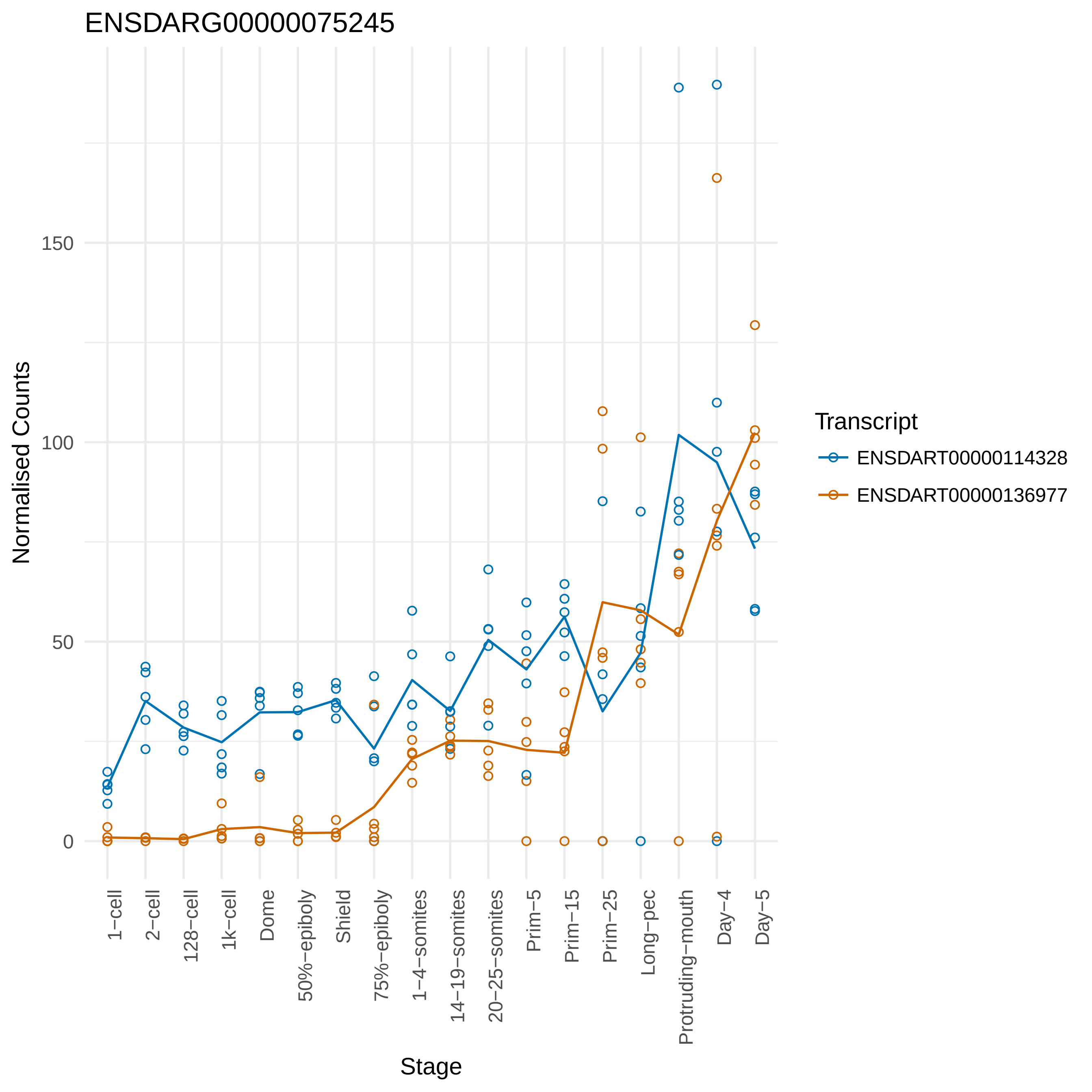

ENSDARG00000075245
Normalised Counts
0
50
100
150
1−cell
2−cell
128−cell
1k−cell
Dome
50%−epiboly
Shield
75%−epiboly
1−4−somites
14−19−somites
20−25−somites
Prim−5
Prim−15
Prim−25
Long−pec
Protruding−mouth
Day−4
Day−5
Stage
Transcript
ENSDART00000114328
ENSDART00000136977

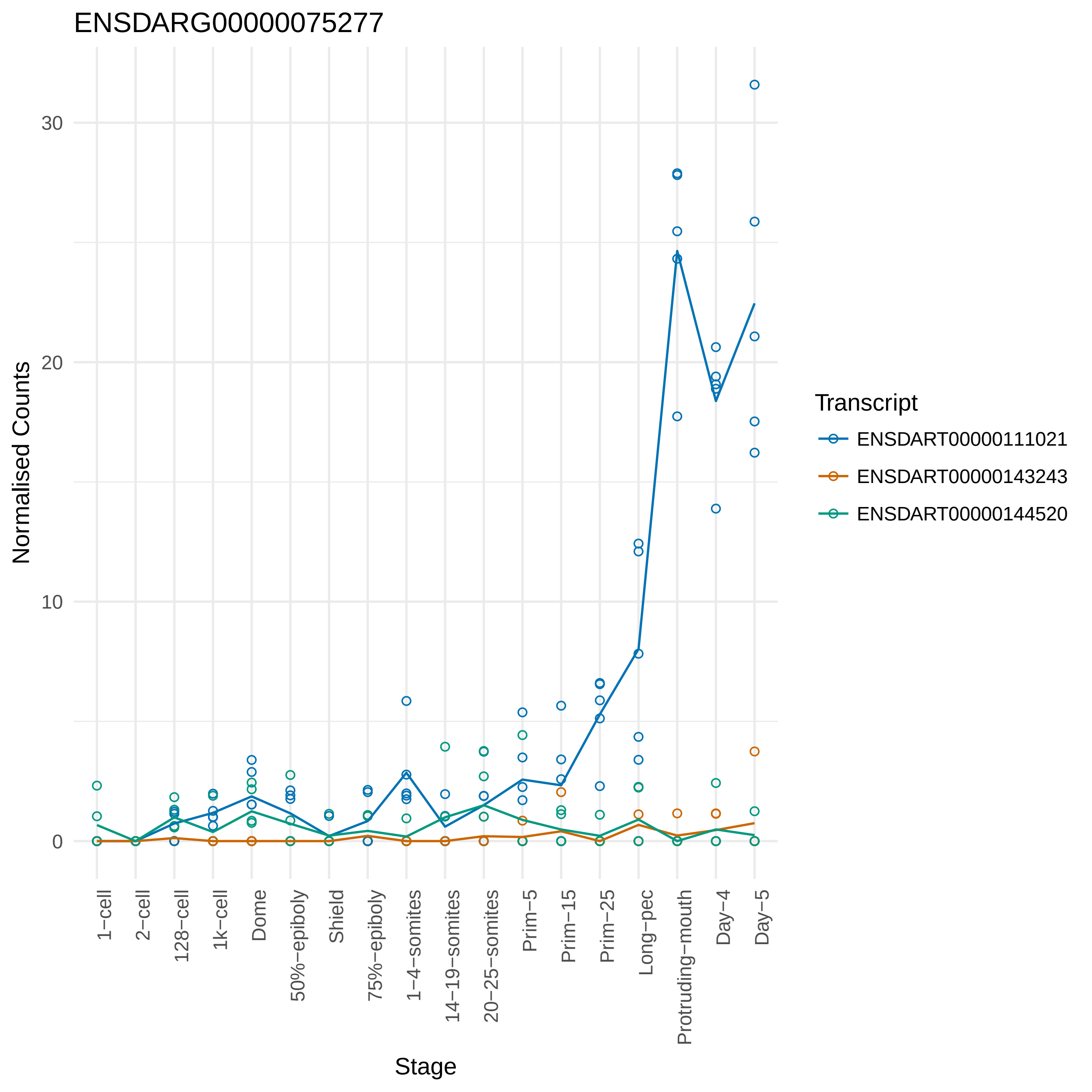
ENSDARG00000075277
Normalised Counts
30
20
10
0
1−cell
2−cell
128−cell
1k−cell
Dome
50%−epiboly
Shield
75%−epiboly
1−4−somites
14−19−somites
20−25−somites
Prim−5
Prim−15
Prim−25
Long−pec
Protruding−mouth
Day−4
Day−5
Stage
Transcript
ENSDART00000111021
ENSDART00000143243
ENSDART00000144520

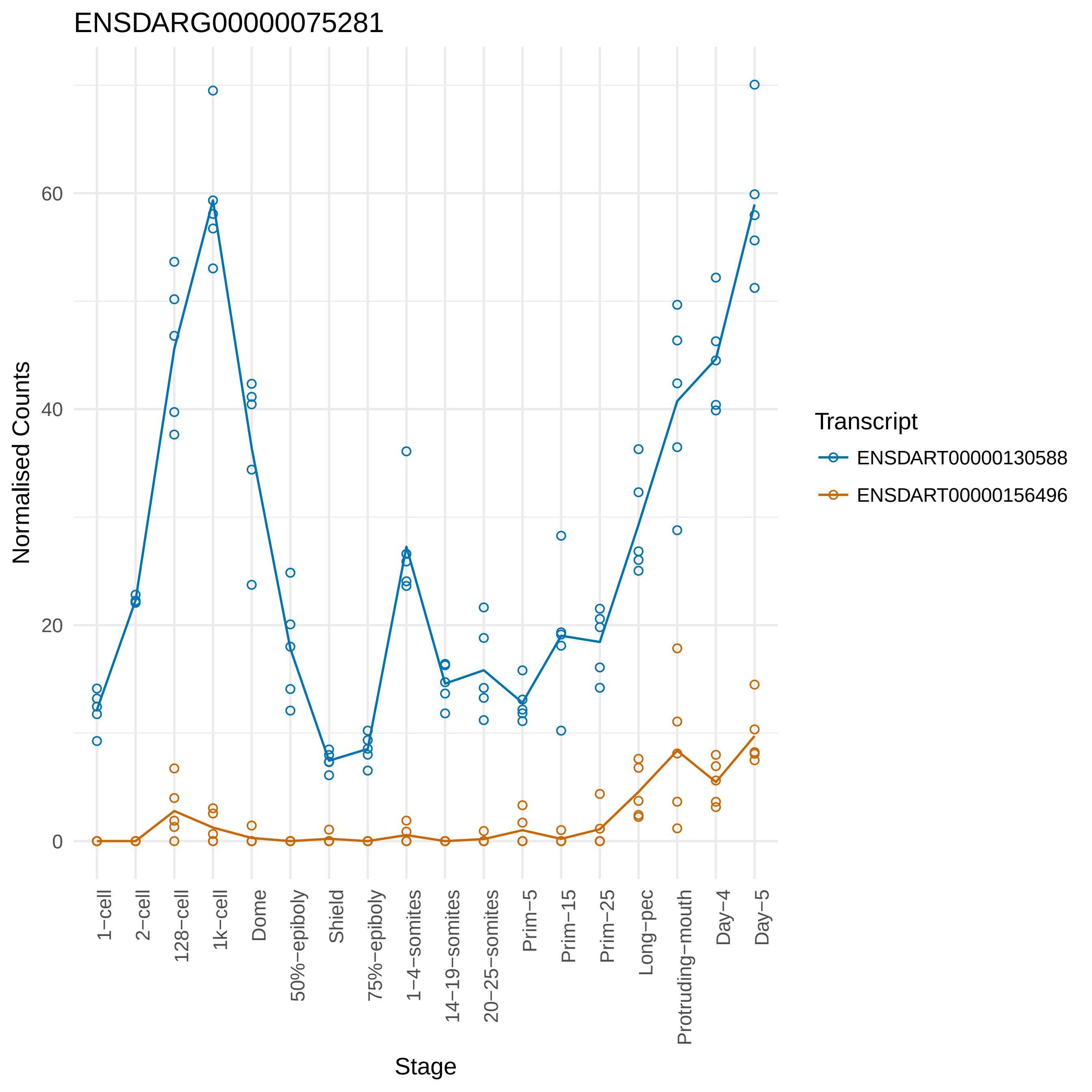

ENSDARG00000075281
Normalised Counts
0
20
40
60
Stage
1−cell
2−cell
128−cell
1k−cell
Dome
50%−epiboly
Shield
75%−epiboly
1−4−somites
14−19−somites
20−25−somites
Prim−5
Prim−15
Prim−25
Long−pec
Protruding−mouth
Day−4
Day−5
Transcript
ENSDART00000130588
ENSDART00000156496

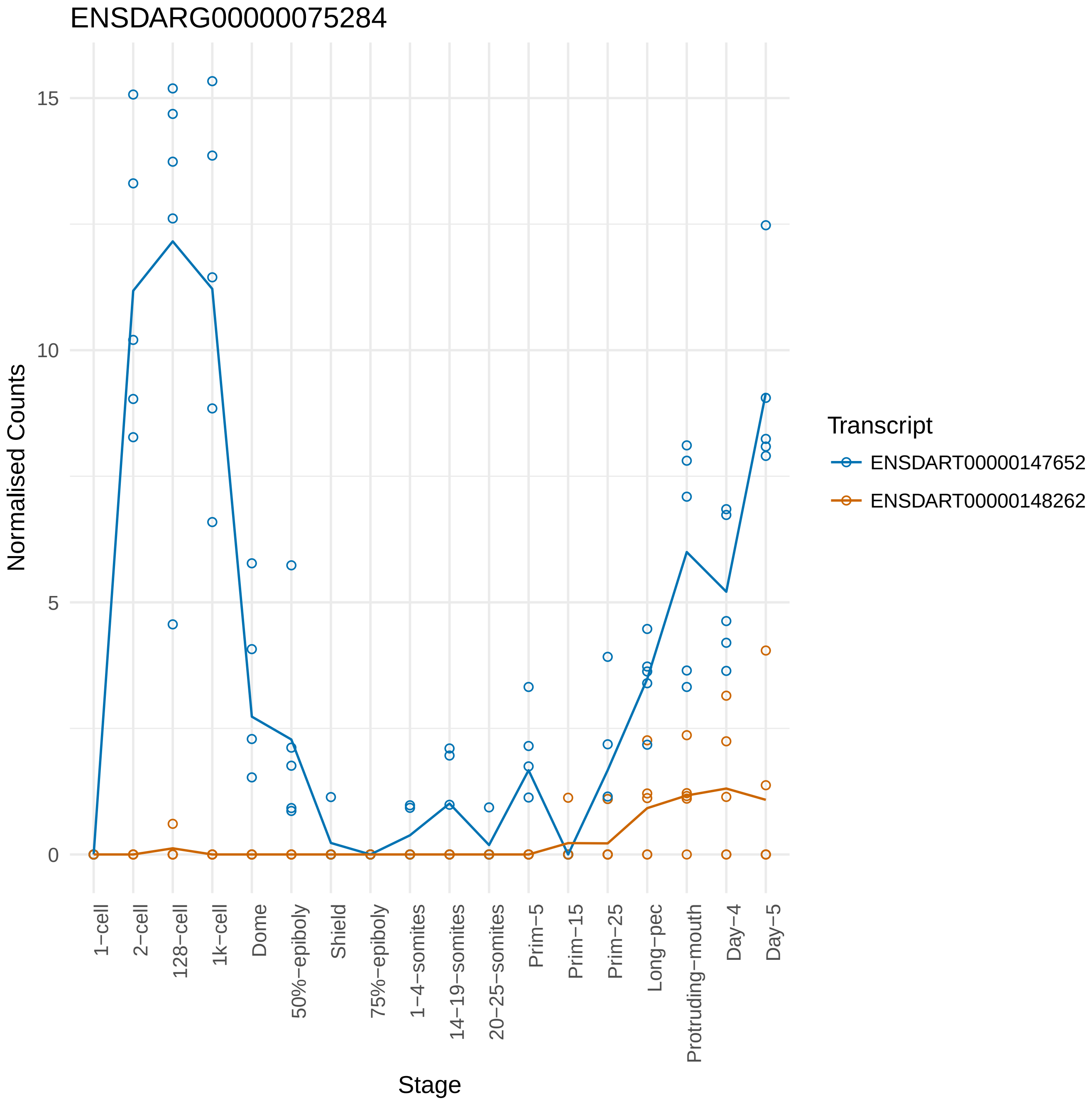

ENSDARG00000075284
Normalised Counts
0
5
10
15
1−cell
2−cell
128−cell
1k−cell
Dome
50%−epiboly
Shield
75%−epiboly
1−4−somites
14−19−somites
20−25−somites
Prim−5
Prim−15
Prim−25
Long−pec
Protruding−mouth
Day−4
Day−5
Stage
Transcript
ENSDART00000147652
ENSDART00000148262

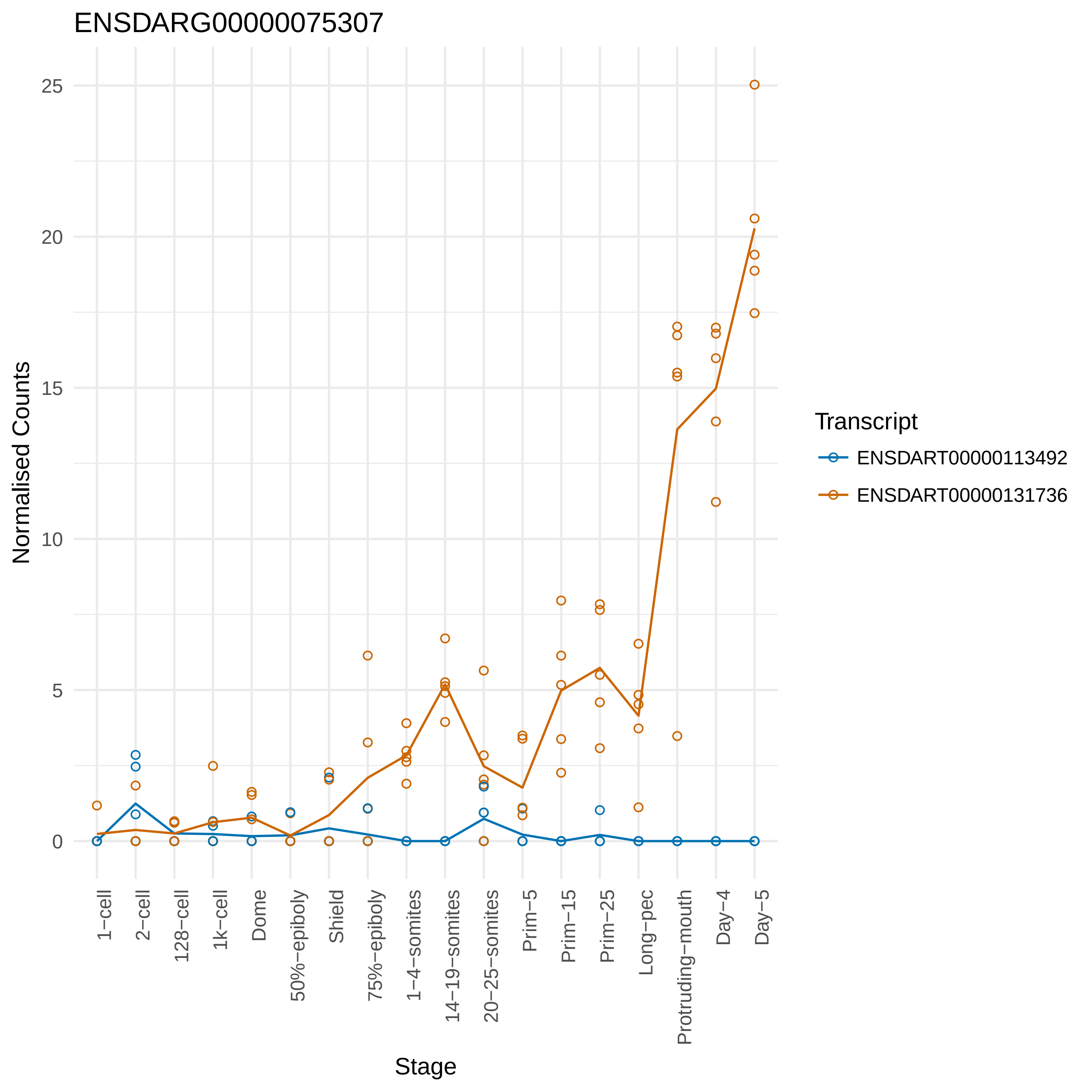
ENSDARG00000075307
Normalised Counts
25
20
15
10
5
0
1−cell
2−cell
128−cell
1k−cell
Dome
50%−epiboly
Shield
75%−epiboly
1−4−somites
14−19−somites
20−25−somites
Prim−5
Prim−15
Prim−25
Long−pec
Protruding−mouth
Day−4
Day−5
Stage
Transcript
ENSDART00000113492
ENSDART00000131736

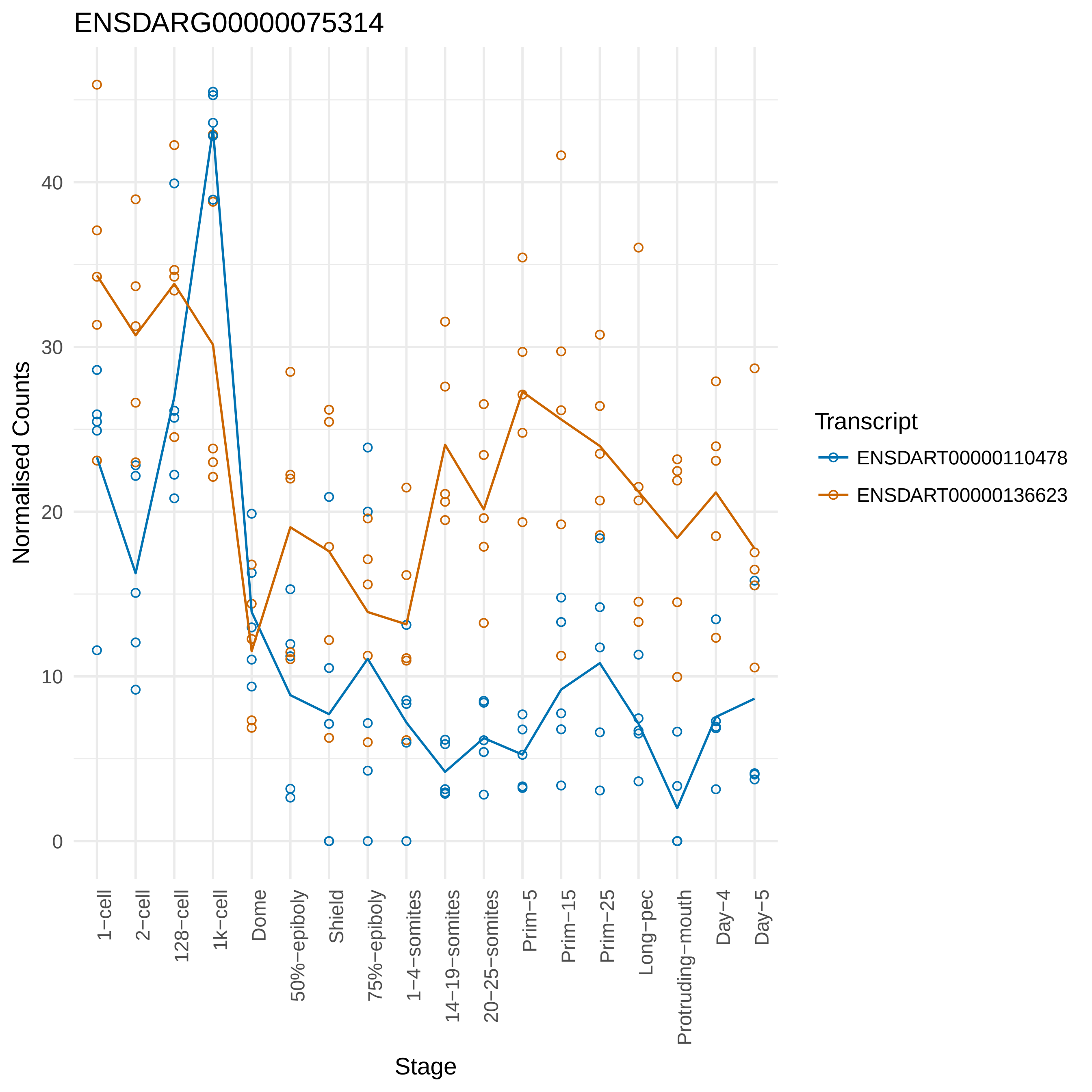

ENSDARG00000075314
Normalised Counts
0
10
20
30
40
Stage
1−cell
2−cell
128−cell
1k−cell
Dome
50%−epiboly
Shield
75%−epiboly
1−4−somites
14−19−somites
20−25−somites
Prim−5
Prim−15
Prim−25
Long−pec
Protruding−mouth
Day−4
Day−5
Transcript
ENSDART00000110478
ENSDART00000136623

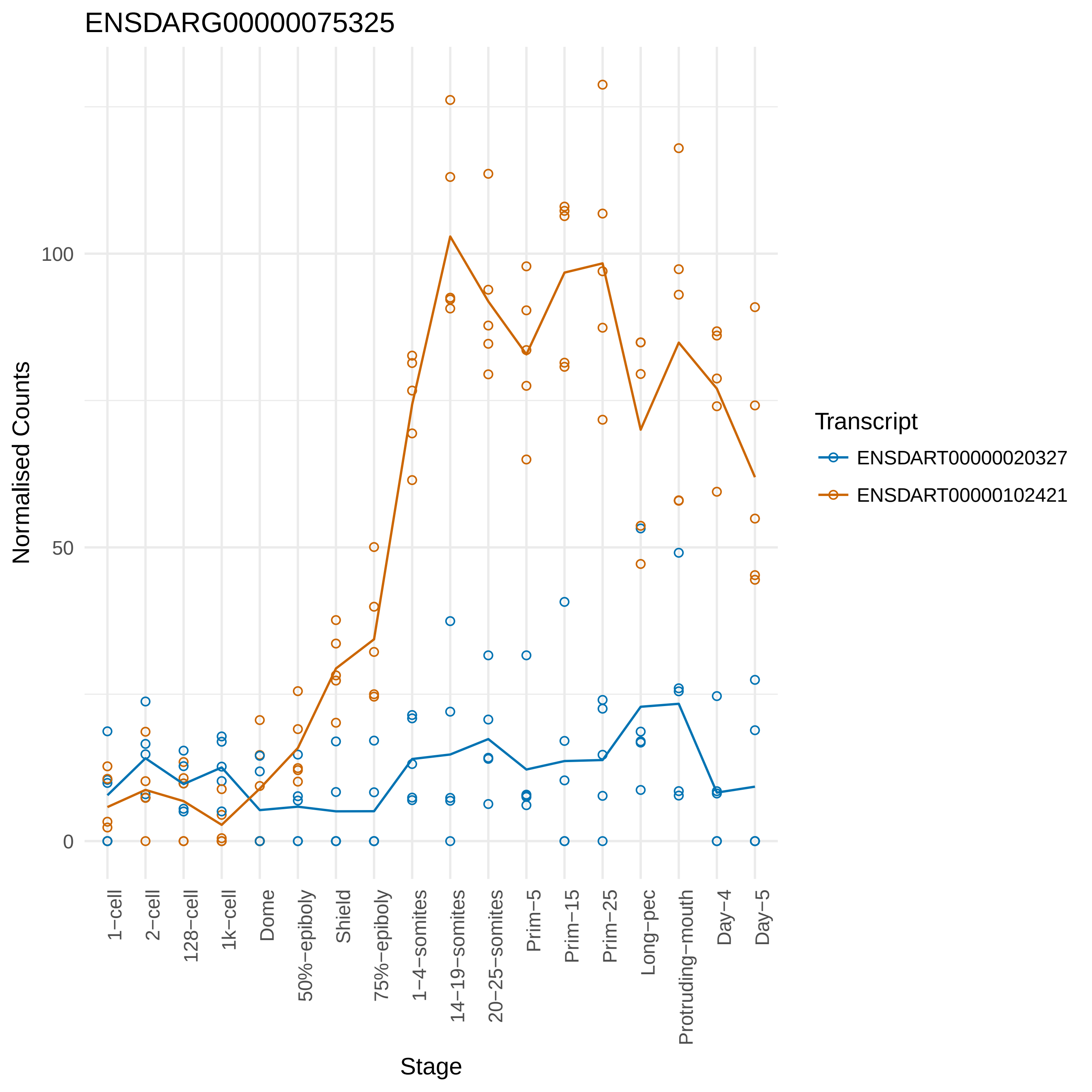

ENSDARG00000075325
Normalised Counts
100
50
0
1−cell
2−cell
128−cell
1k−cell
Dome
50%−epiboly
Shield
75%−epiboly
1−4−somites
14−19−somites
20−25−somites
Prim−5
Prim−15
Prim−25
Long−pec
Protruding−mouth
Day−4
Day−5
Stage
Transcript
ENSDART00000020327
ENSDART00000102421

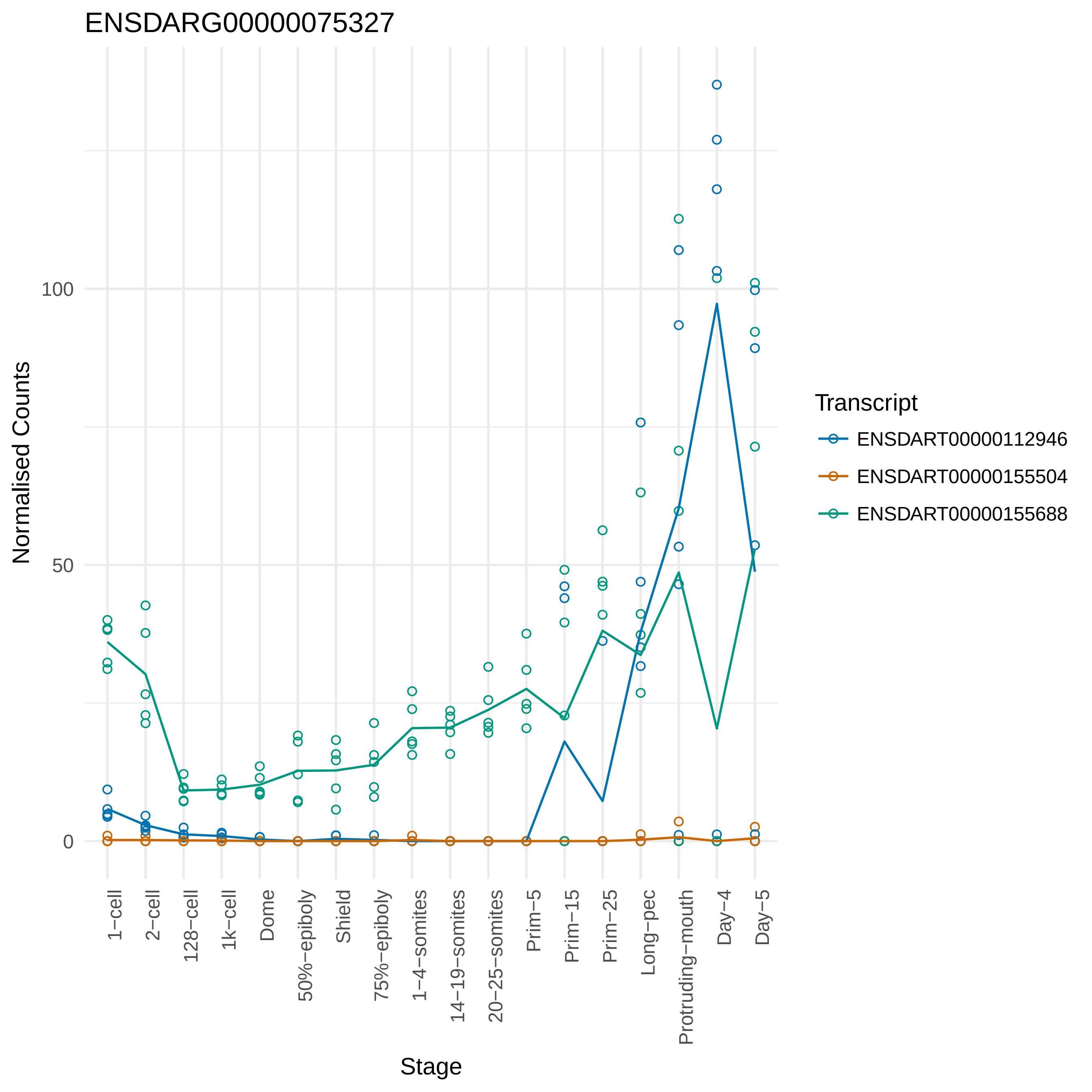

ENSDARG00000075327
Normalised Counts
0
50
100
Stage
1−cell
2−cell
128−cell
1k−cell
Dome
50%−epiboly
Shield
75%−epiboly
1−4−somites
14−19−somites
20−25−somites
Prim−5
Prim−15
Prim−25
Long−pec
Protruding−mouth
Day−4
Day−5
Transcript
ENSDART00000112946
ENSDART00000155504
ENSDART00000155688

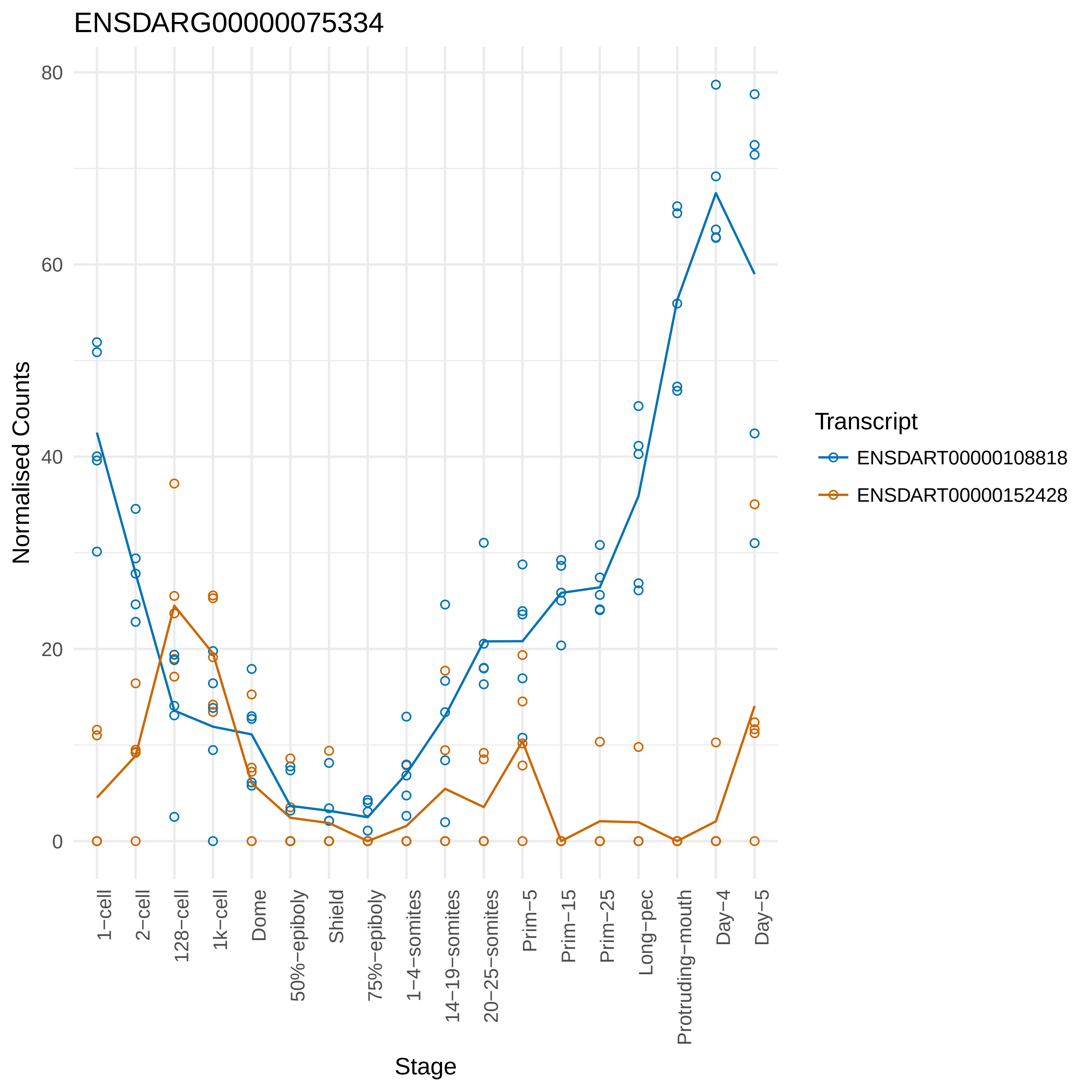

ENSDARG00000075334
Normalised Counts
0
20
40
60
80
Stage
1−cell
2−cell
128−cell
1k−cell
Dome
50%−epiboly
Shield
75%−epiboly
1−4−somites
14−19−somites
20−25−somites
Prim−5
Prim−15
Prim−25
Long−pec
Protruding−mouth
Day−4
Day−5
Transcript
ENSDART00000108818
ENSDART00000152428

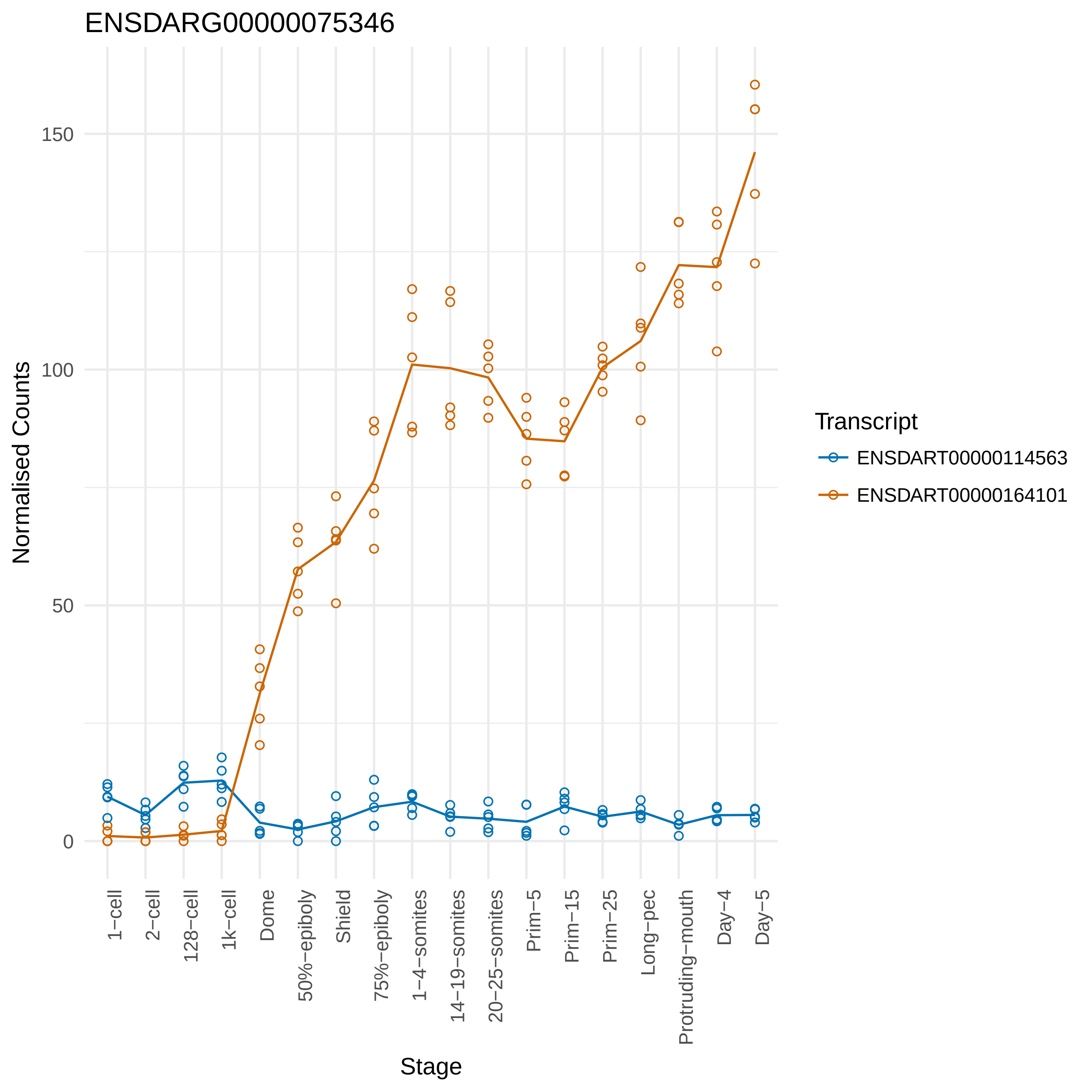

ENSDARG00000075346
Normalised Counts
150
100
50
0
1−cell
2−cell
128−cell
1k−cell
Dome
50%−epiboly
Shield
75%−epiboly
1−4−somites
14−19−somites
20−25−somites
Prim−5
Prim−15
Prim−25
Long−pec
Protruding−mouth
Day−4
Day−5
Stage
Transcript
ENSDART00000114563
ENSDART00000164101

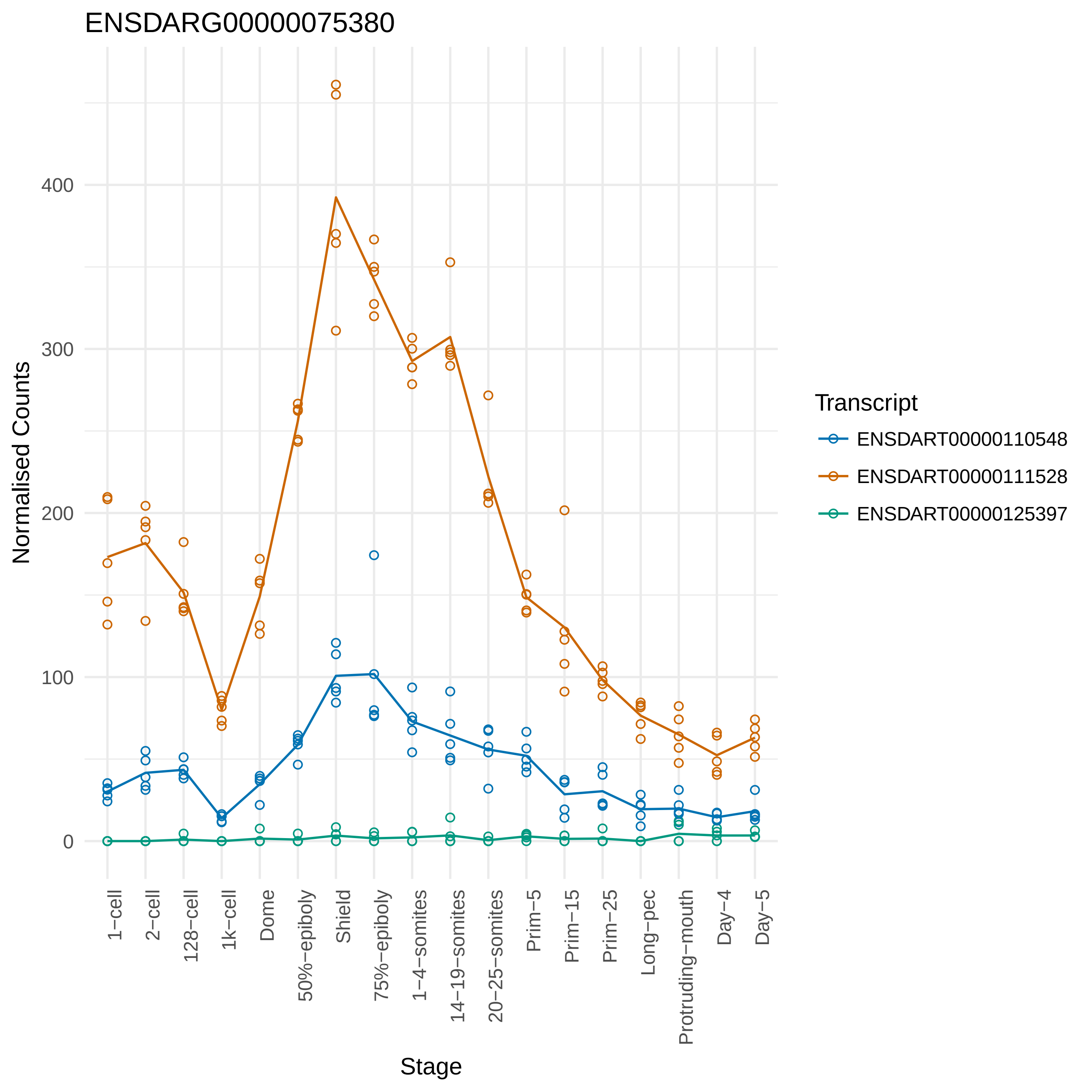

ENSDARG00000075380
Normalised Counts
400
300
200
100
0
Stage
1−cell
2−cell
128−cell
1k−cell
Dome
50%−epiboly
Shield
75%−epiboly
1−4−somites
14−19−somites
20−25−somites
Prim−5
Prim−15
Prim−25
Long−pec
Protruding−mouth
Day−4
Day−5
Transcript
ENSDART00000110548
ENSDART00000111528
ENSDART00000125397

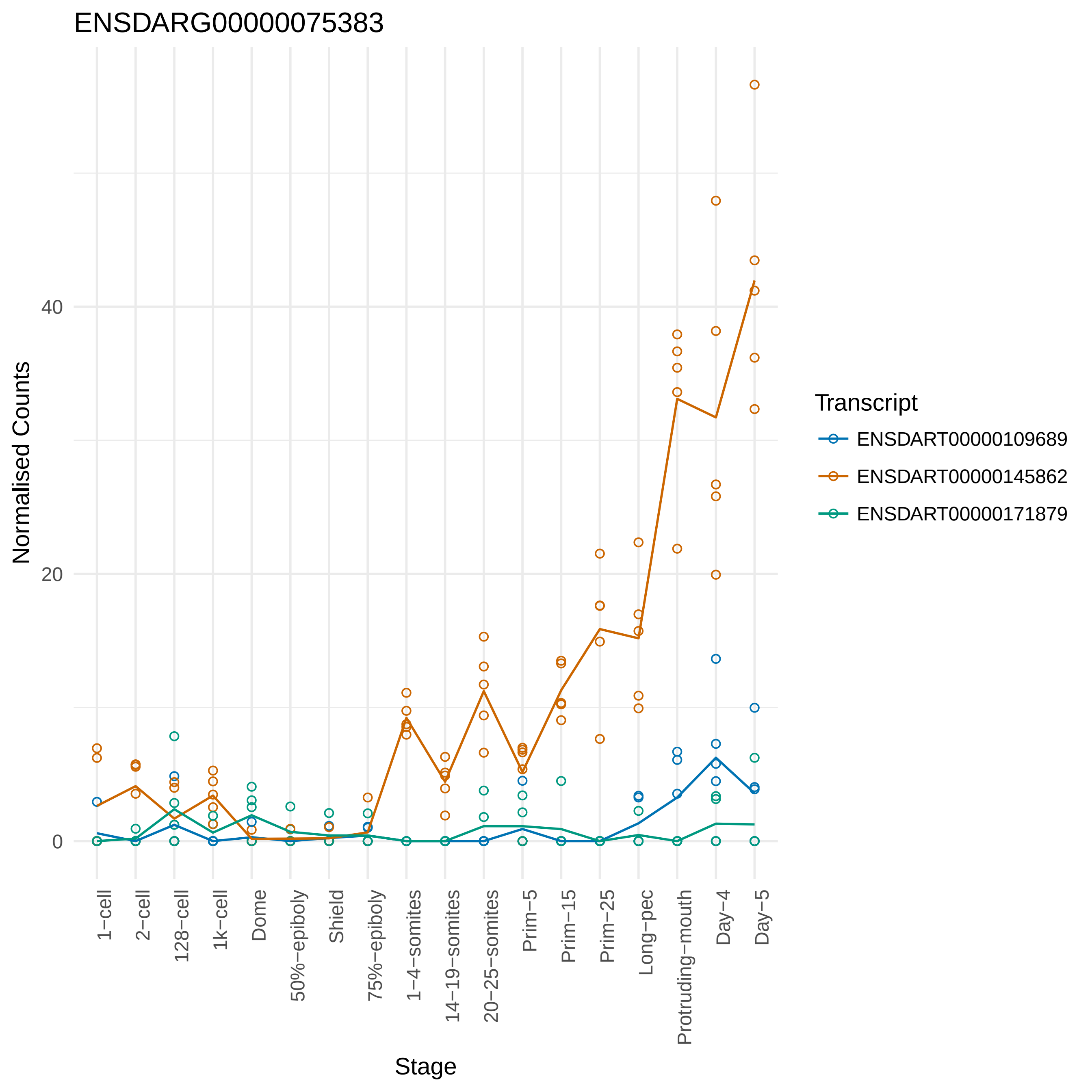

ENSDARG00000075383
Normalised Counts
0
20
40
1−cell
2−cell
128−cell
1k−cell
Dome
50%−epiboly
Shield
75%−epiboly
1−4−somites
14−19−somites
20−25−somites
Prim−5
Prim−15
Prim−25
Long−pec
Protruding−mouth
Day−4
Day−5
Stage
Transcript
ENSDART00000109689
ENSDART00000145862
ENSDART00000171879

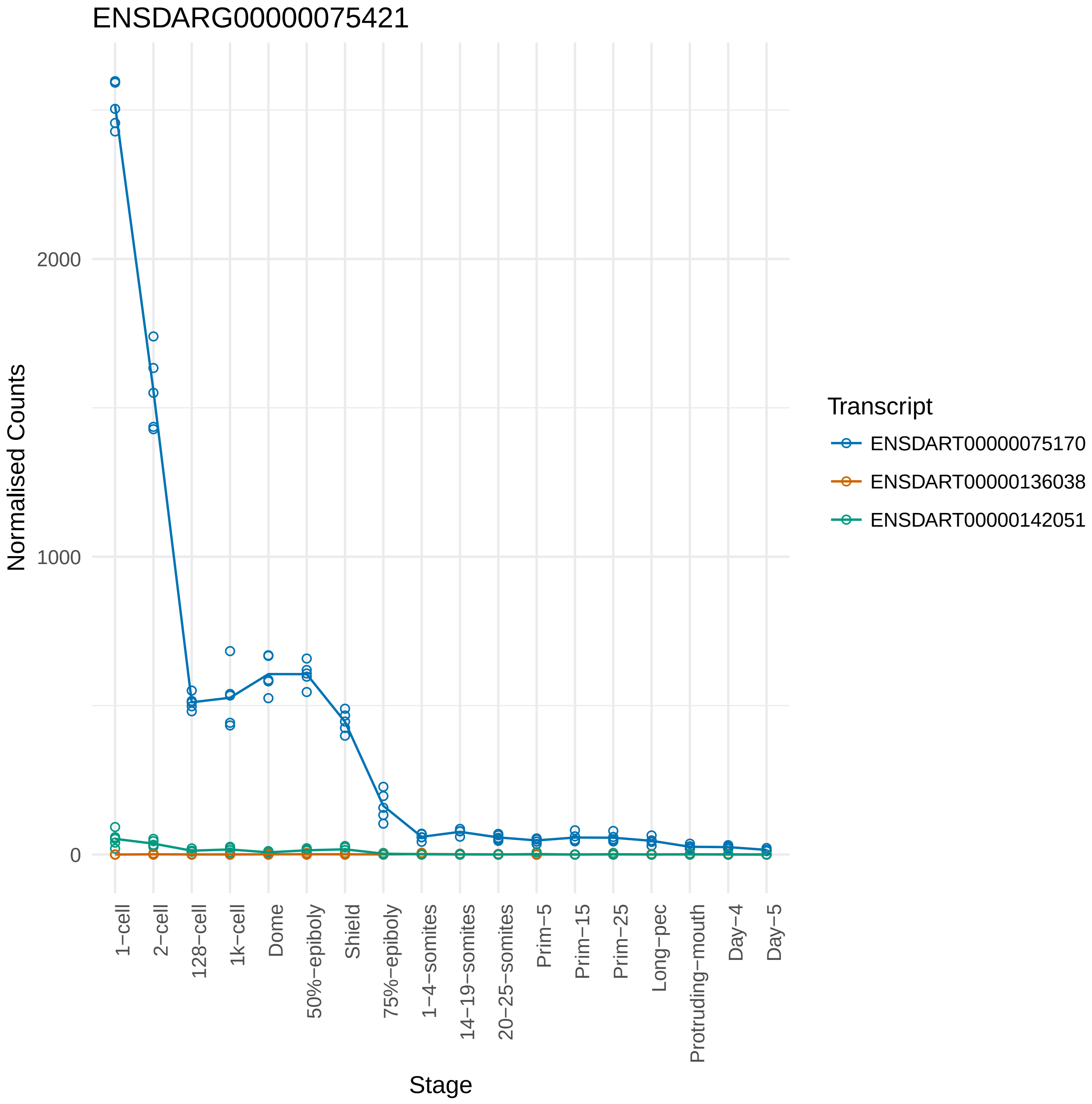

ENSDARG00000075421
Normalised Counts
2000
1000
0
Stage
1−cell
2−cell
128−cell
1k−cell
Dome
50%−epiboly
Shield
75%−epiboly
1−4−somites
14−19−somites
20−25−somites
Prim−5
Prim−15
Prim−25
Long−pec
Protruding−mouth
Day−4
Day−5
Transcript
ENSDART00000075170
ENSDART00000136038
ENSDART00000142051

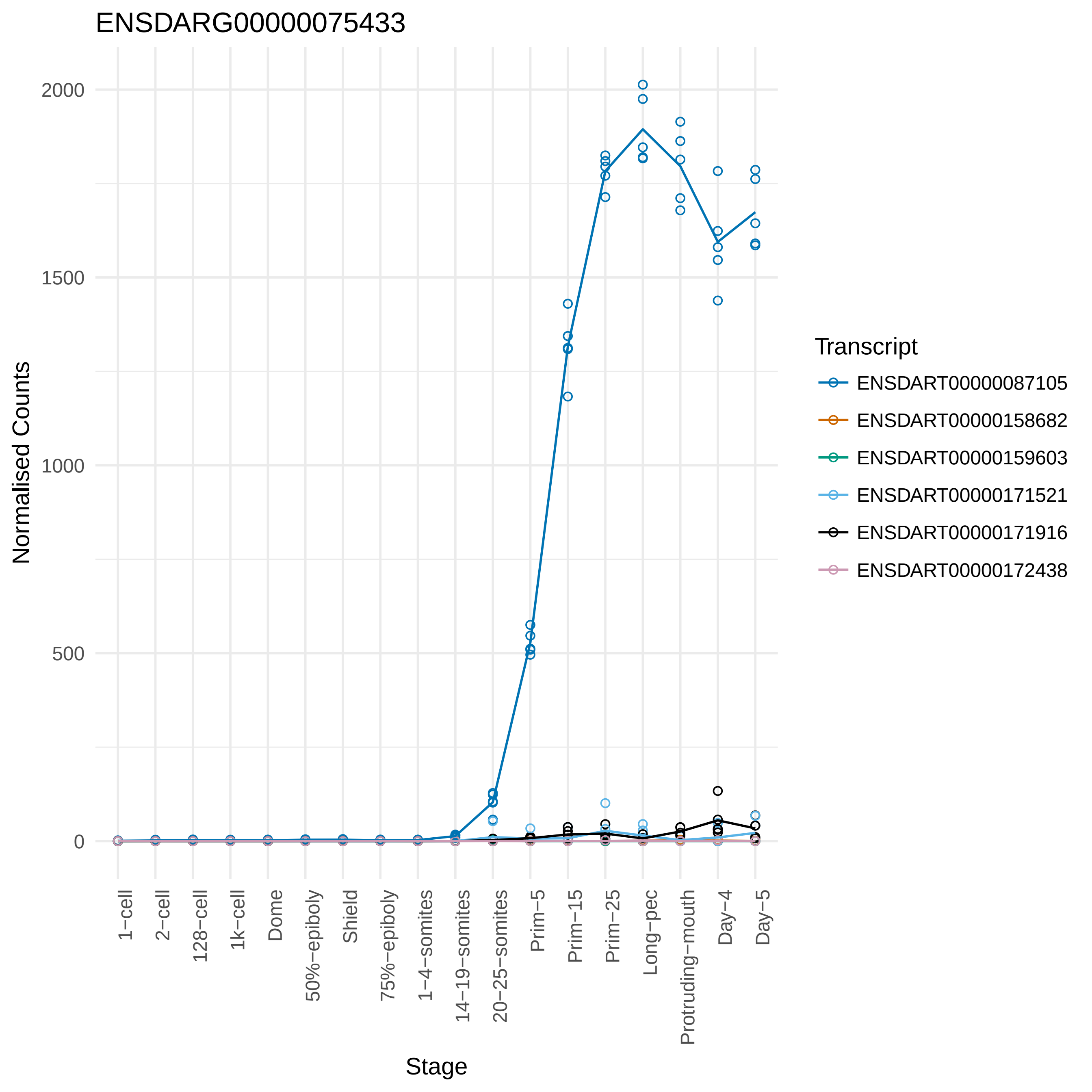

ENSDARG00000075433
Normalised Counts
2000
1500
1000
500
0
1−cell
2−cell
128−cell
1k−cell
Dome
50%−epiboly
Shield
75%−epiboly
1−4−somites
14−19−somites
20−25−somites
Prim−5
Prim−15
Prim−25
Long−pec
Protruding−mouth
Day−4
Day−5
Stage
Transcript
ENSDART00000087105
ENSDART00000158682
ENSDART00000159603
ENSDART00000171521
ENSDART00000171916
ENSDART00000172438

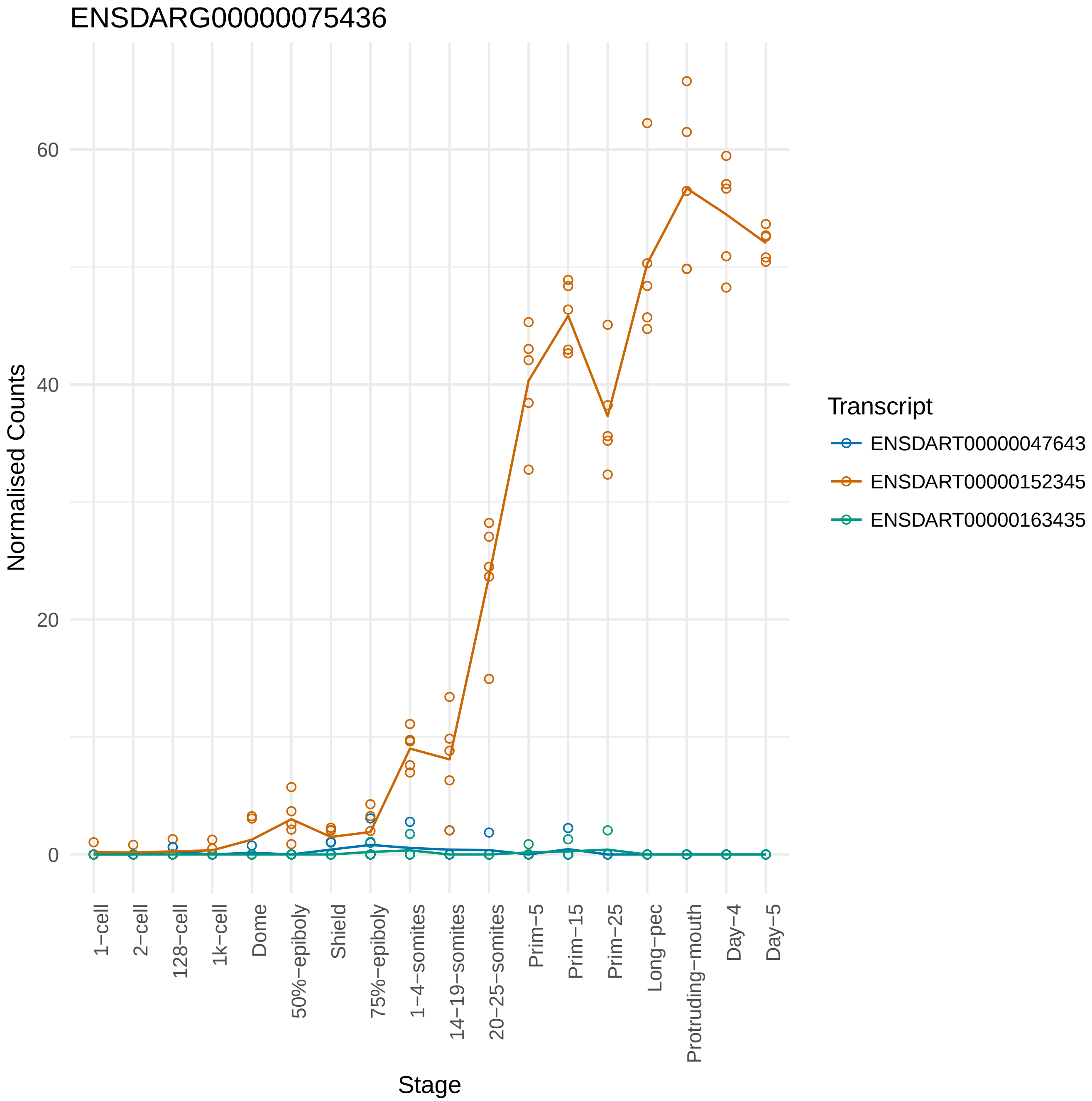

ENSDARG00000075436
Normalised Counts
0
20
40
60
1−cell
2−cell
128−cell
1k−cell
Dome
50%−epiboly
Shield
75%−epiboly
1−4−somites
14−19−somites
20−25−somites
Prim−5
Prim−15
Prim−25
Long−pec
Protruding−mouth
Day−4
Day−5
Stage
Transcript
ENSDART00000047643
ENSDART00000152345
ENSDART00000163435

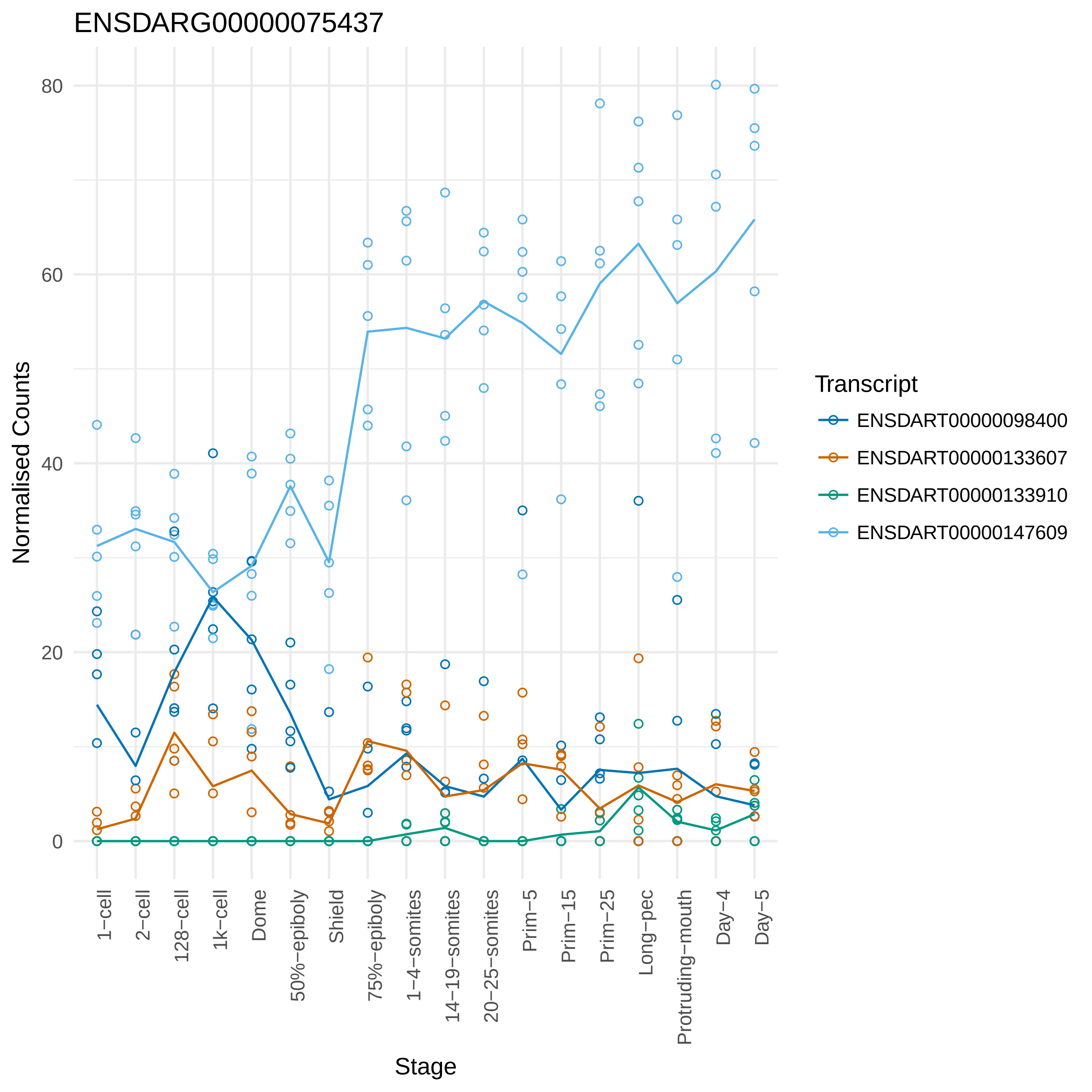

ENSDARG00000075437
Normalised Counts
80
60
40
20
0
1−cell
2−cell
128−cell
1k−cell
Dome
50%−epiboly
Shield
75%−epiboly
1−4−somites
14−19−somites
20−25−somites
Prim−5
Prim−15
Prim−25
Long−pec
Protruding−mouth
Day−4
Day−5
Stage
Transcript
ENSDART00000098400
ENSDART00000133607
ENSDART00000133910
ENSDART00000147609

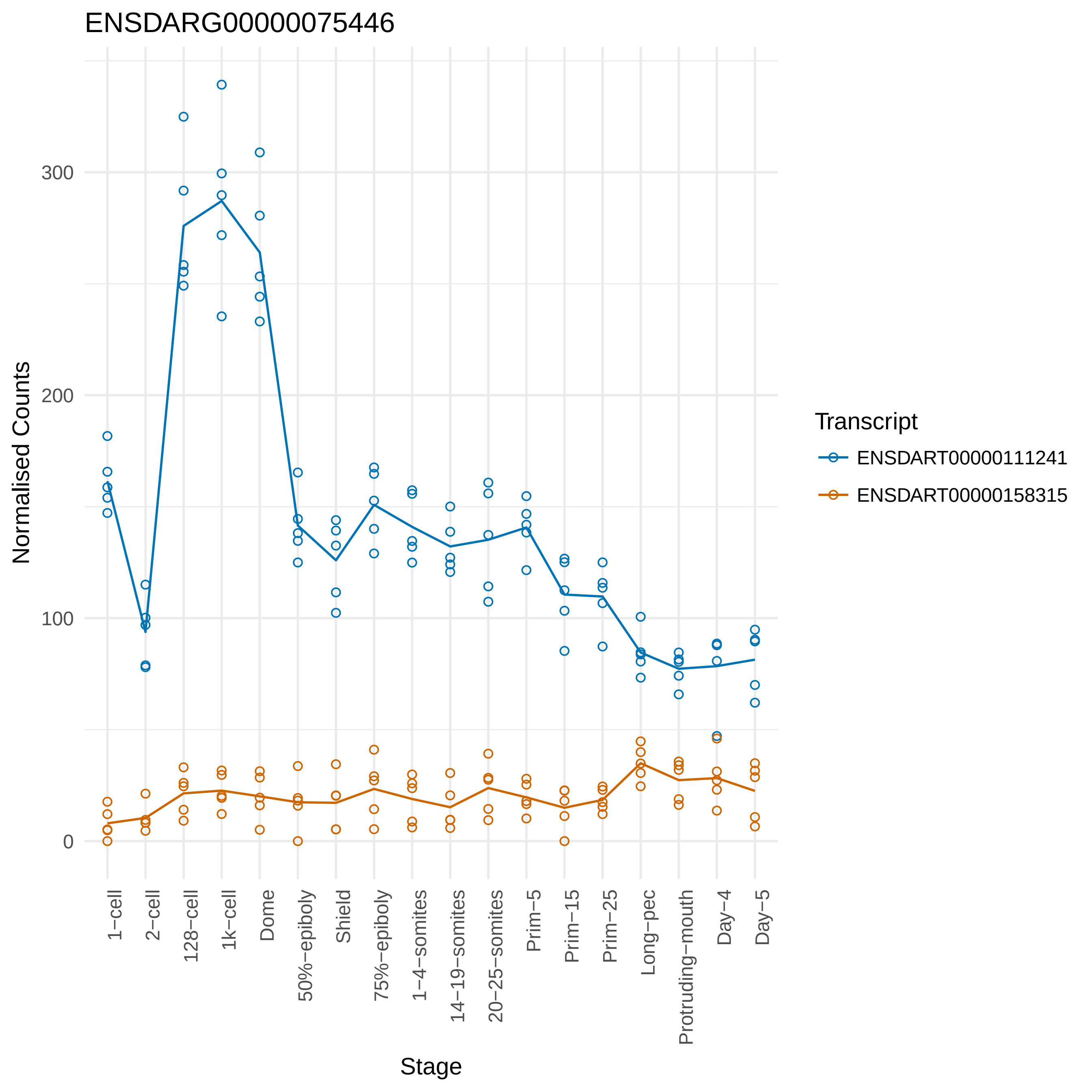

ENSDARG00000075446
Normalised Counts
300
200
100
0
1−cell
2−cell
128−cell
1k−cell
Dome
50%−epiboly
Shield
75%−epiboly
1−4−somites
14−19−somites
20−25−somites
Prim−5
Prim−15
Prim−25
Long−pec
Protruding−mouth
Day−4
Day−5
Stage
Transcript
ENSDART00000111241
ENSDART00000158315

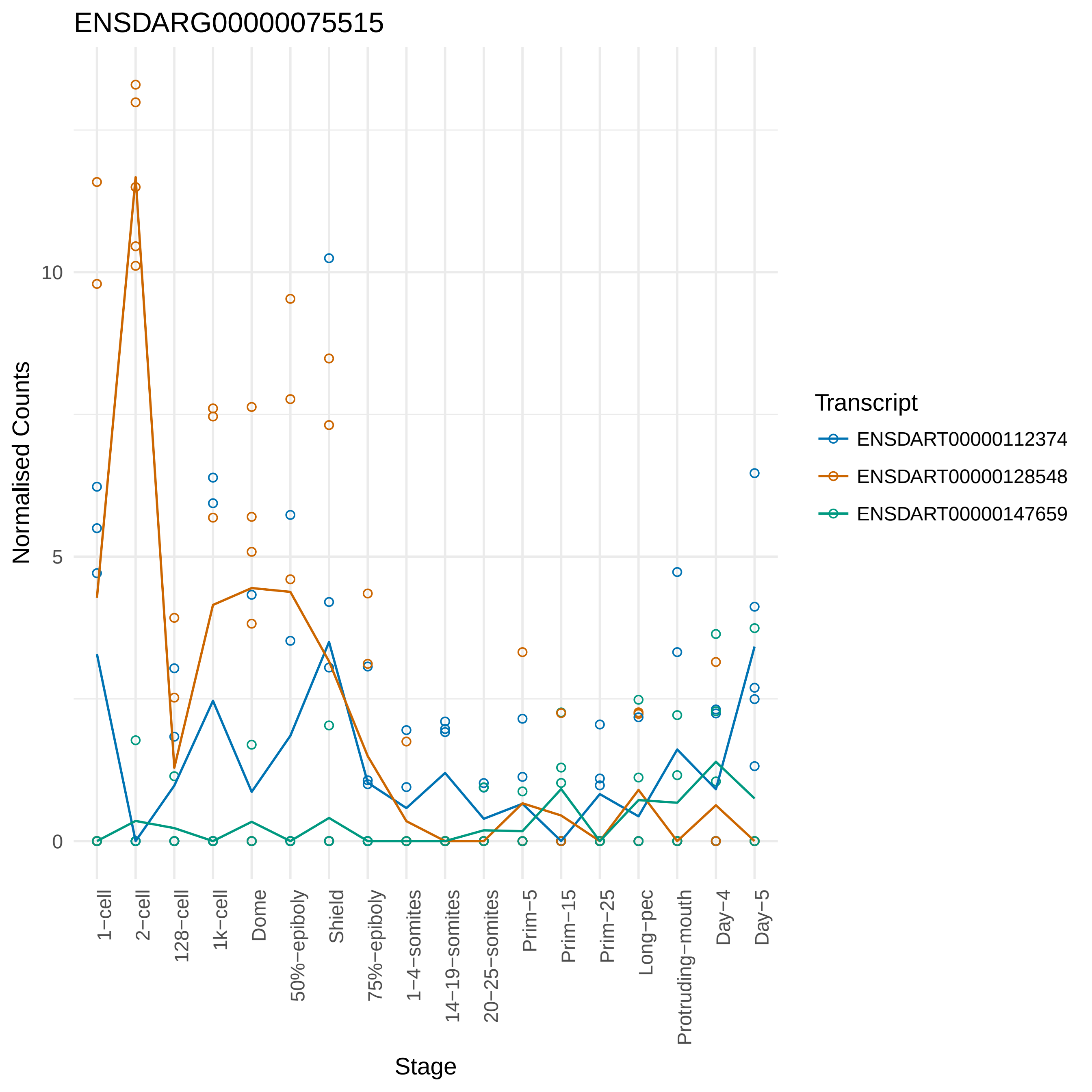

ENSDARG00000075515
Normalised Counts
10
5
0
1−cell
2−cell
128−cell
1k−cell
Dome
50%−epiboly
Shield
75%−epiboly
1−4−somites
14−19−somites
20−25−somites
Prim−5
Prim−15
Prim−25
Long−pec
Protruding−mouth
Day−4
Day−5
Stage
Transcript
ENSDART00000112374
ENSDART00000128548
ENSDART00000147659

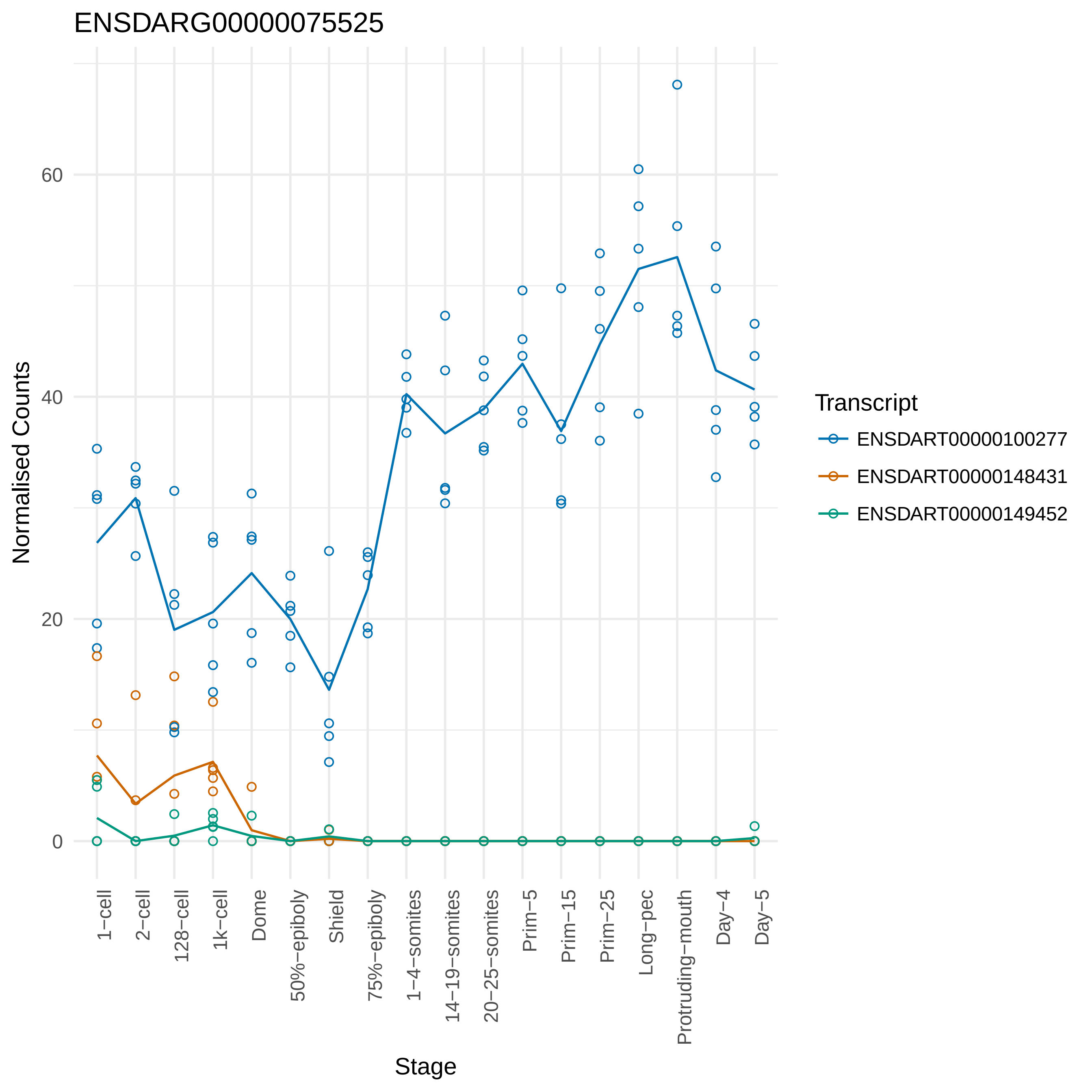

ENSDARG00000075525
Normalised Counts
60
40
20
0
1−cell
2−cell
128−cell
1k−cell
Dome
50%−epiboly
Shield
75%−epiboly
1−4−somites
14−19−somites
20−25−somites
Prim−5
Prim−15
Prim−25
Long−pec
Protruding−mouth
Day−4
Day−5
Stage
Transcript
ENSDART00000100277
ENSDART00000148431
ENSDART00000149452

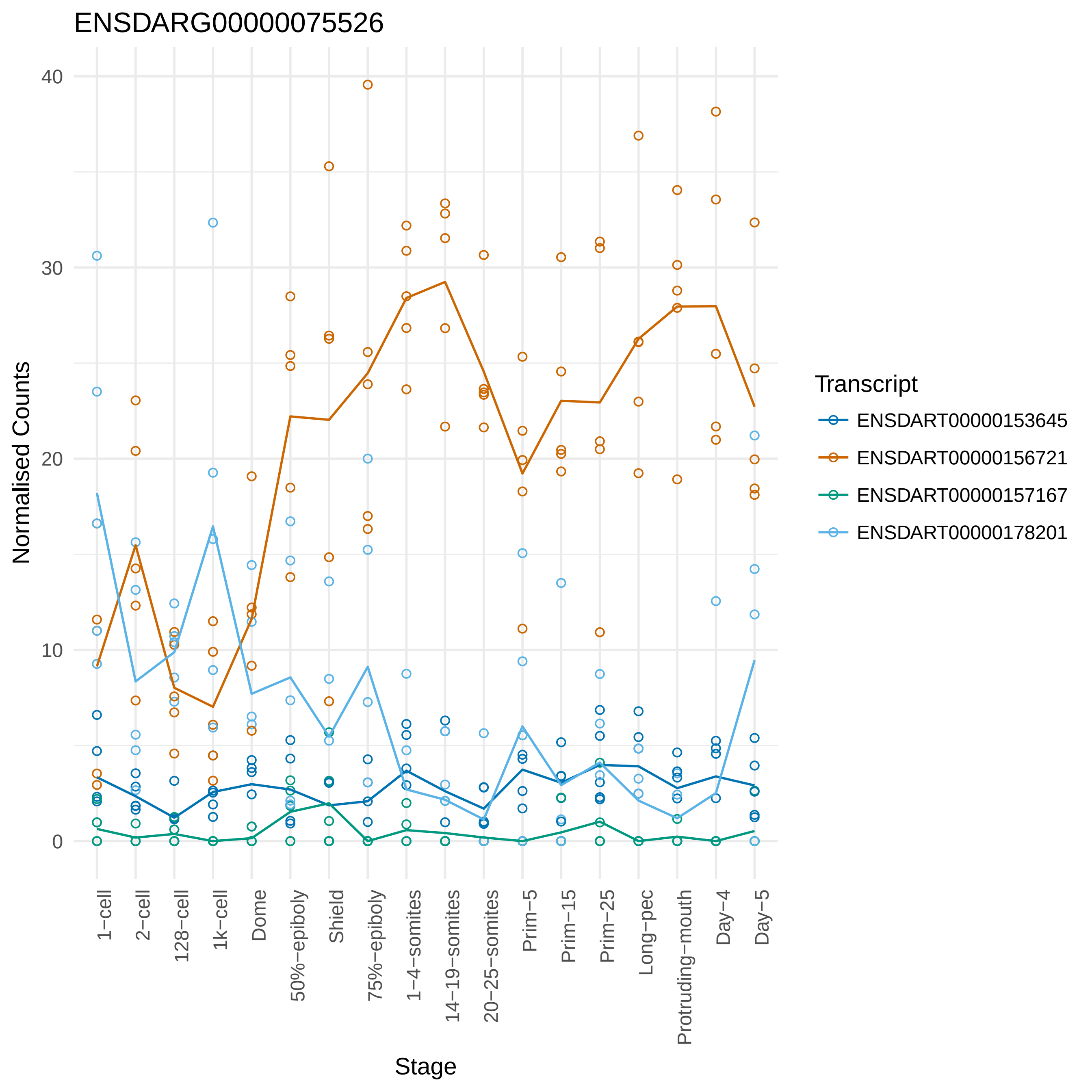

ENSDARG00000075526
Normalised Counts
0
10
20
30
40
1−cell
2−cell
128−cell
1k−cell
Dome
50%−epiboly
Shield
75%−epiboly
1−4−somites
14−19−somites
20−25−somites
Prim−5
Prim−15
Prim−25
Long−pec
Protruding−mouth
Day−4
Day−5
Stage
Transcript
ENSDART00000153645
ENSDART00000156721
ENSDART00000157167
ENSDART00000178201

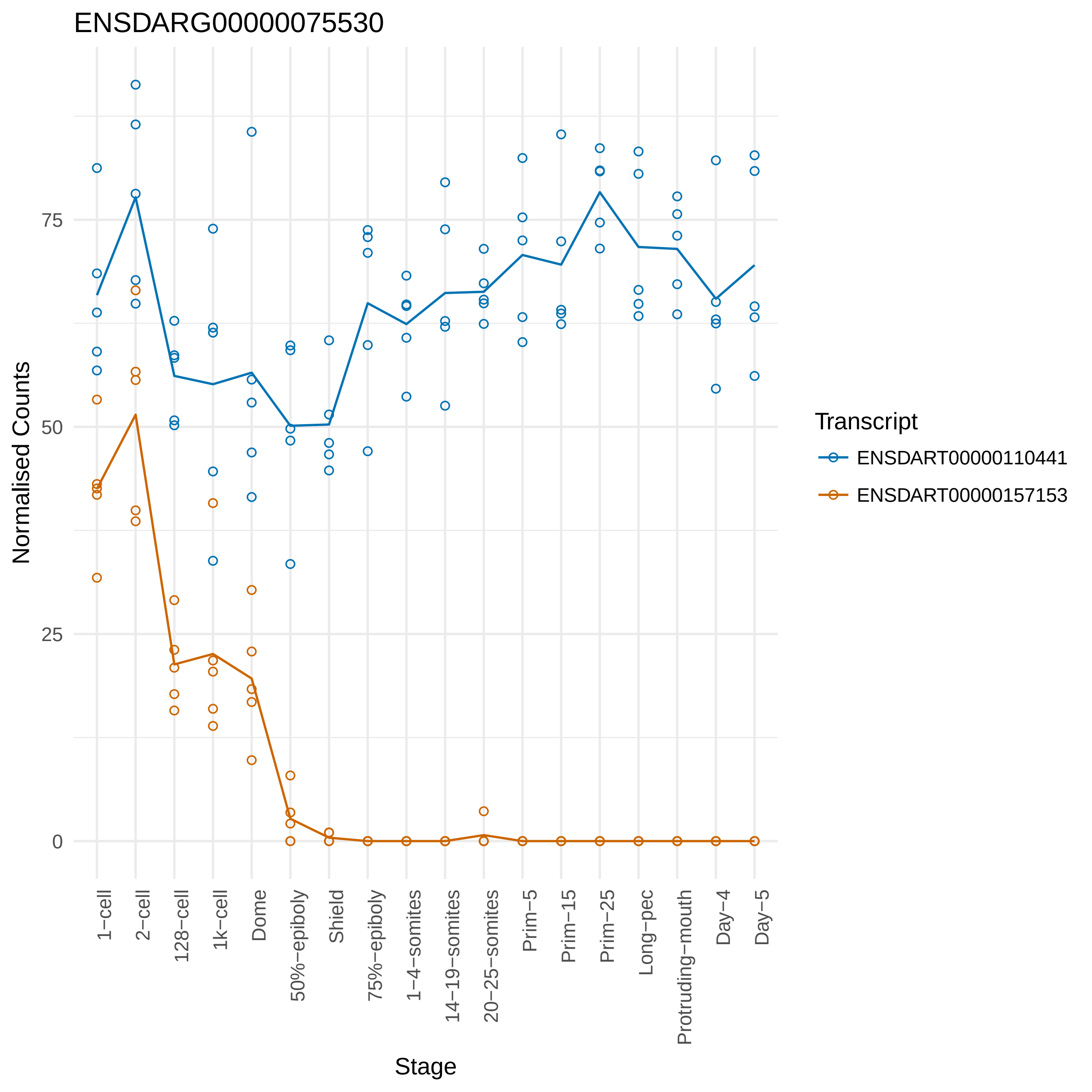
ENSDARG00000075530
Normalised Counts
0
25
50
75
Stage
1−cell
2−cell
128−cell
1k−cell
Dome
50%−epiboly
Shield
75%−epiboly
1−4−somites
14−19−somites
20−25−somites
Prim−5
Prim−15
Prim−25
Long−pec
Protruding−mouth
Day−4
Day−5
Transcript
ENSDART00000110441
ENSDART00000157153

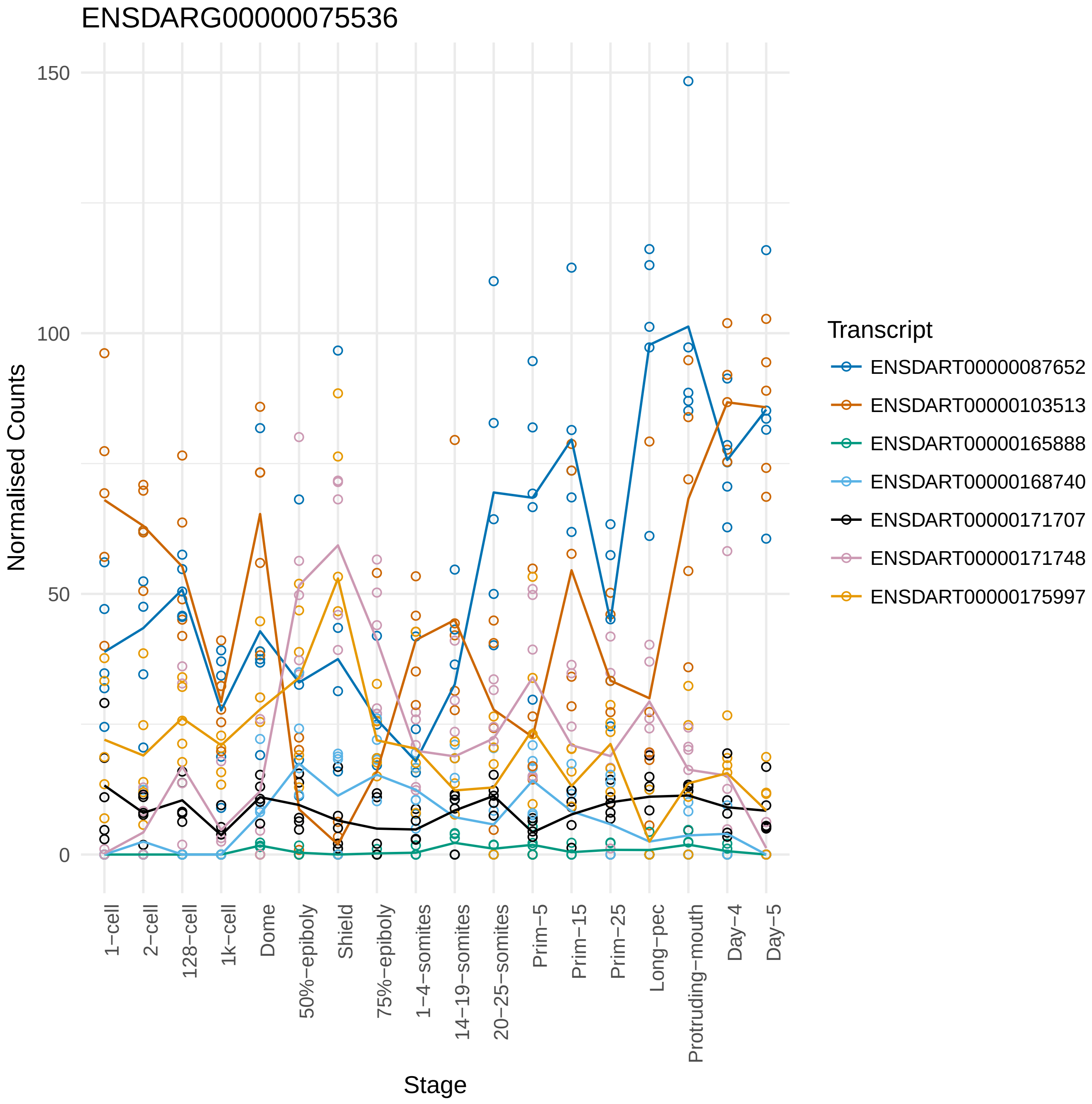
ENSDARG00000075536
Normalised Counts
150
100
50
0
1−cell
2−cell
128−cell
1k−cell
Dome
50%−epiboly
Shield
75%−epiboly
1−4−somites
14−19−somites
20−25−somites
Prim−5
Prim−15
Prim−25
Long−pec
Protruding−mouth
Day−4
Day−5
Stage
Transcript
ENSDART00000087652
ENSDART00000103513
ENSDART00000165888
ENSDART00000168740
ENSDART00000171707
ENSDART00000171748
ENSDART00000175997

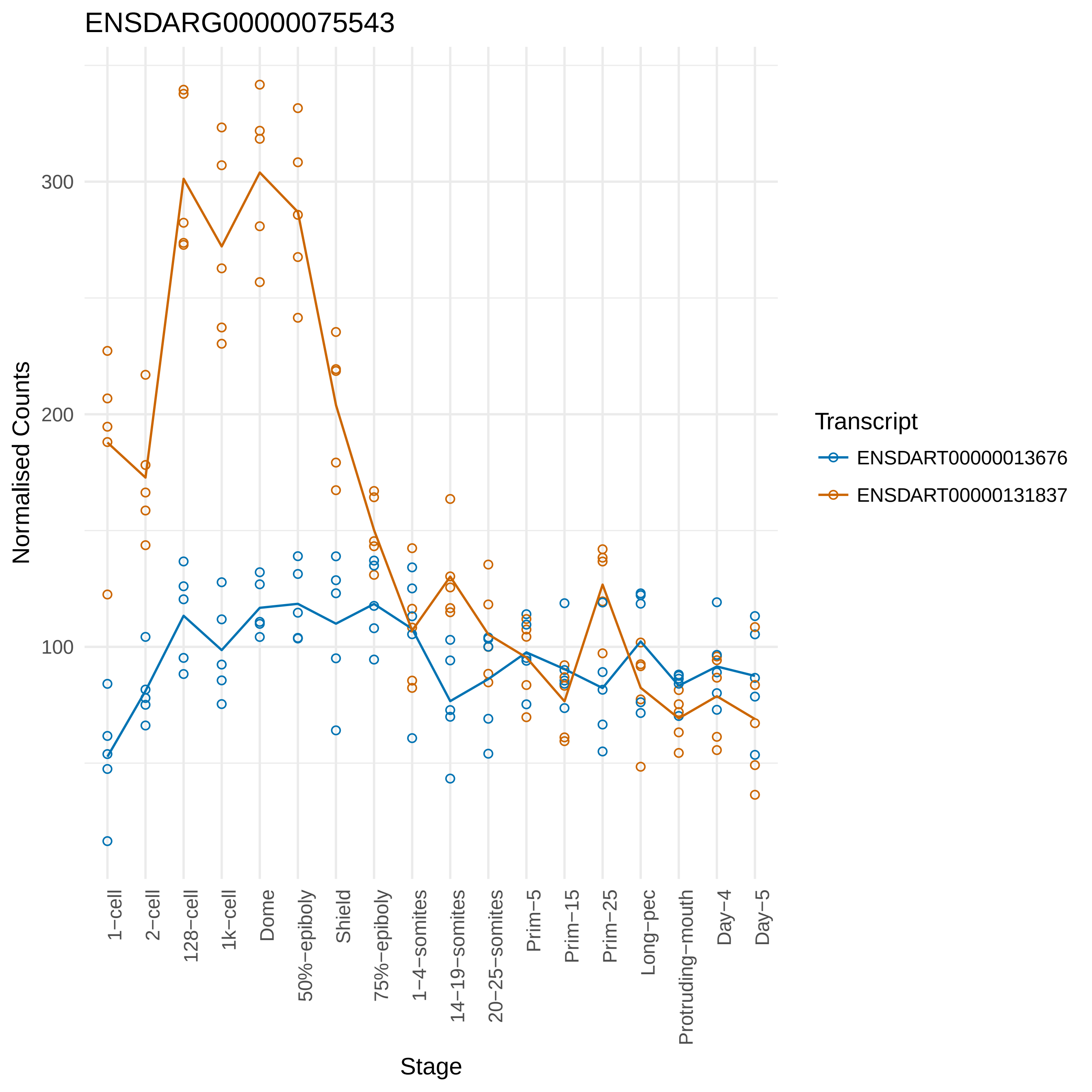

ENSDARG00000075543
Normalised Counts
300
200
100
1−cell
2−cell
128−cell
1k−cell
Dome
50%−epiboly
Shield
75%−epiboly
1−4−somites
14−19−somites
20−25−somites
Prim−5
Prim−15
Prim−25
Long−pec
Protruding−mouth
Day−4
Day−5
Stage
Transcript
ENSDART00000013676
ENSDART00000131837

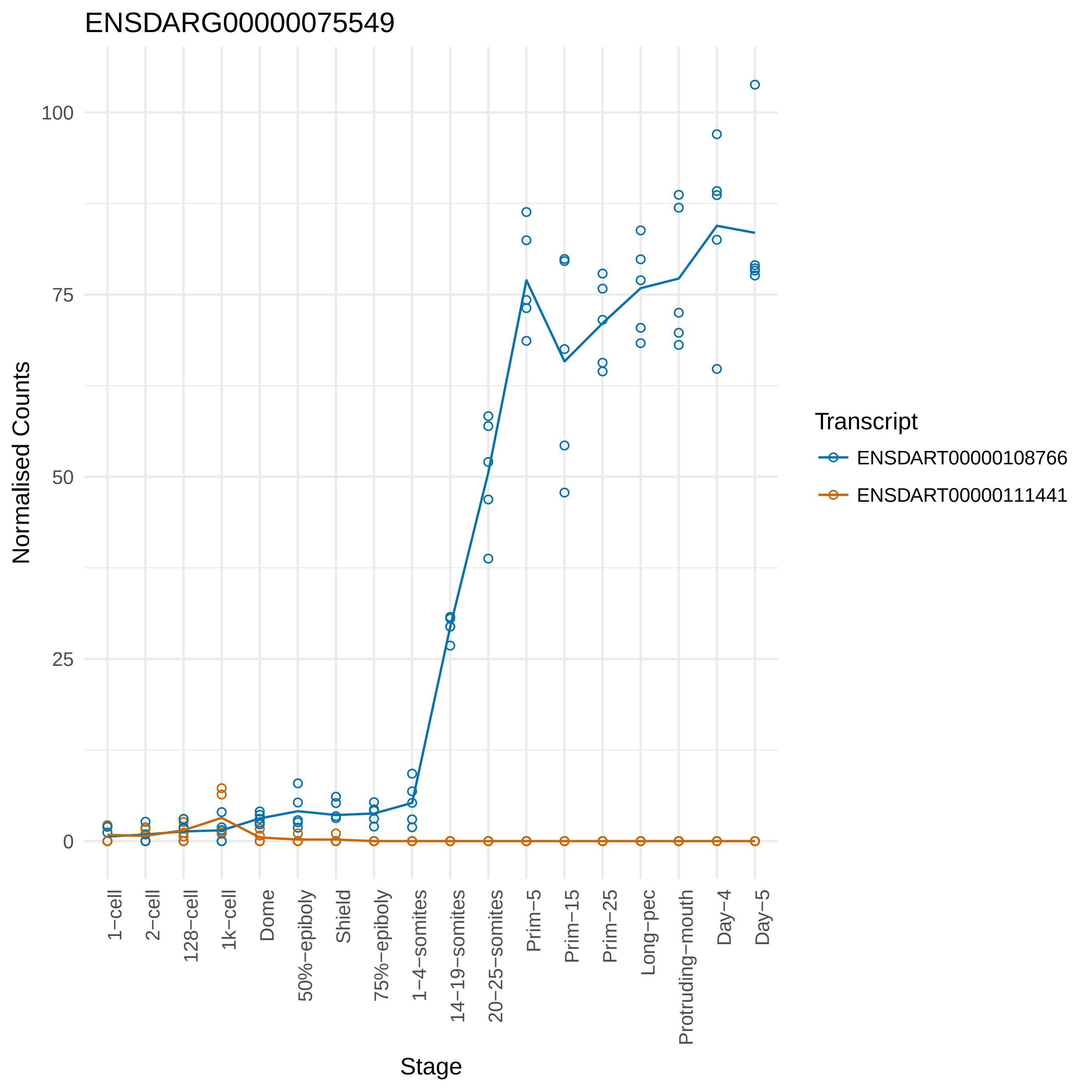

ENSDARG00000075549
Normalised Counts
100
75
50
25
0
1−cell
2−cell
128−cell
1k−cell
Dome
50%−epiboly
Shield
75%−epiboly
1−4−somites
14−19−somites
20−25−somites
Prim−5
Prim−15
Prim−25
Long−pec
Protruding−mouth
Day−4
Day−5
Stage
Transcript
ENSDART00000108766
ENSDART00000111441

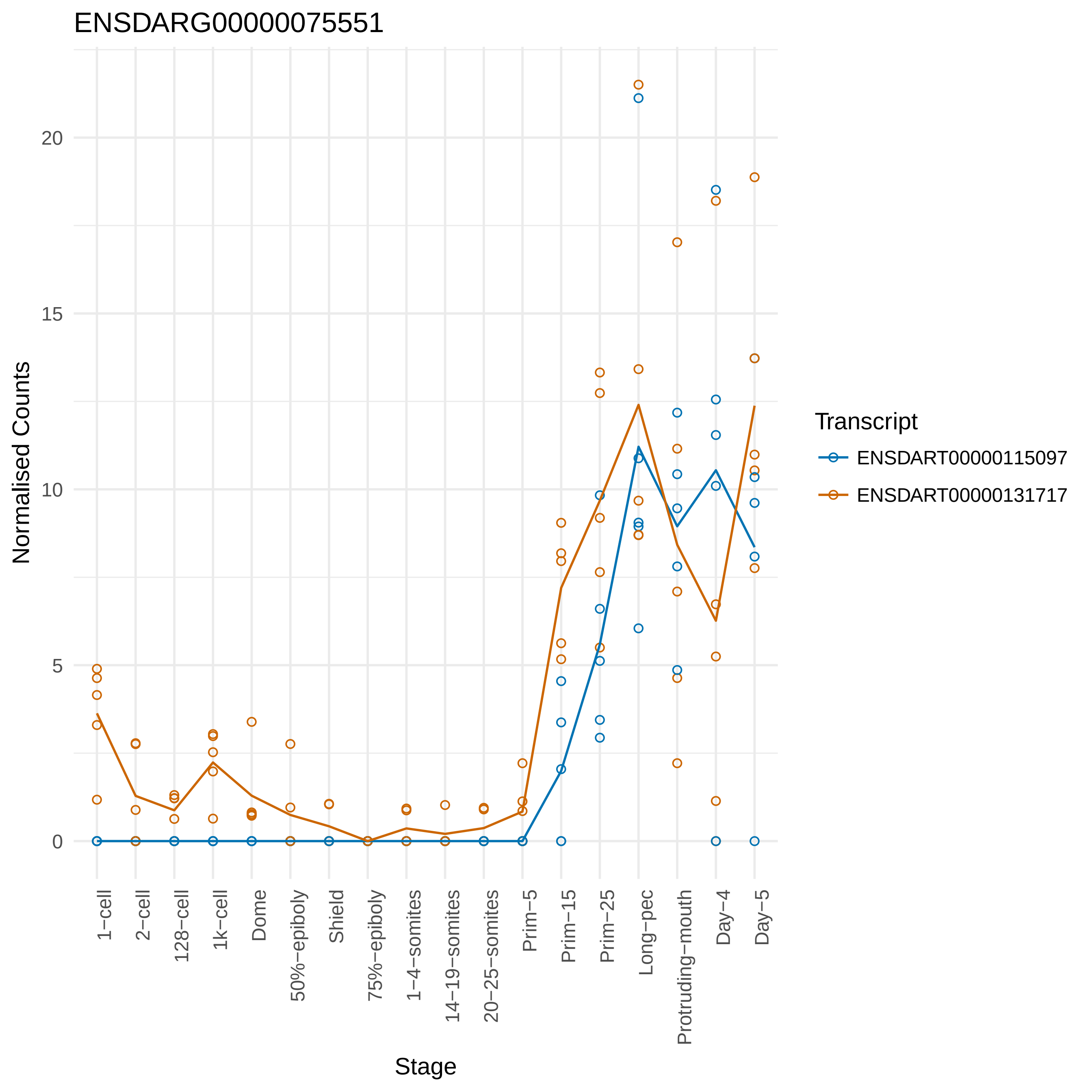

ENSDARG00000075551
Normalised Counts
0
5
10
15
20
1−cell
2−cell
128−cell
1k−cell
Dome
50%−epiboly
Shield
75%−epiboly
1−4−somites
14−19−somites
20−25−somites
Prim−5
Prim−15
Prim−25
Long−pec
Protruding−mouth
Day−4
Day−5
Stage
Transcript
ENSDART00000115097
ENSDART00000131717

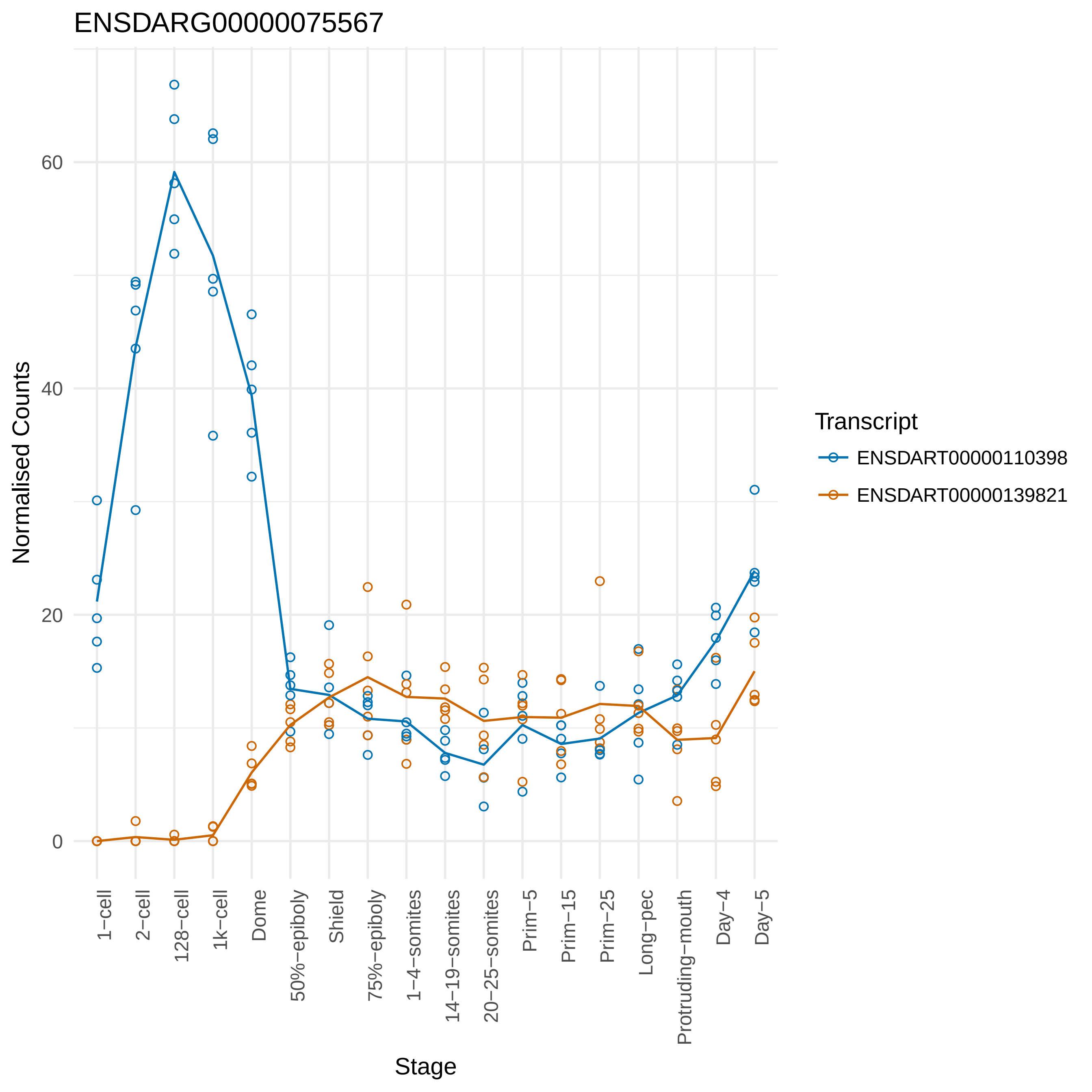

ENSDARG00000075567
Normalised Counts
60
40
20
0
1−cell
2−cell
128−cell
1k−cell
Dome
50%−epiboly
Shield
75%−epiboly
1−4−somites
14−19−somites
20−25−somites
Prim−5
Prim−15
Prim−25
Long−pec
Protruding−mouth
Day−4
Day−5
Stage
Transcript
ENSDART00000110398
ENSDART00000139821

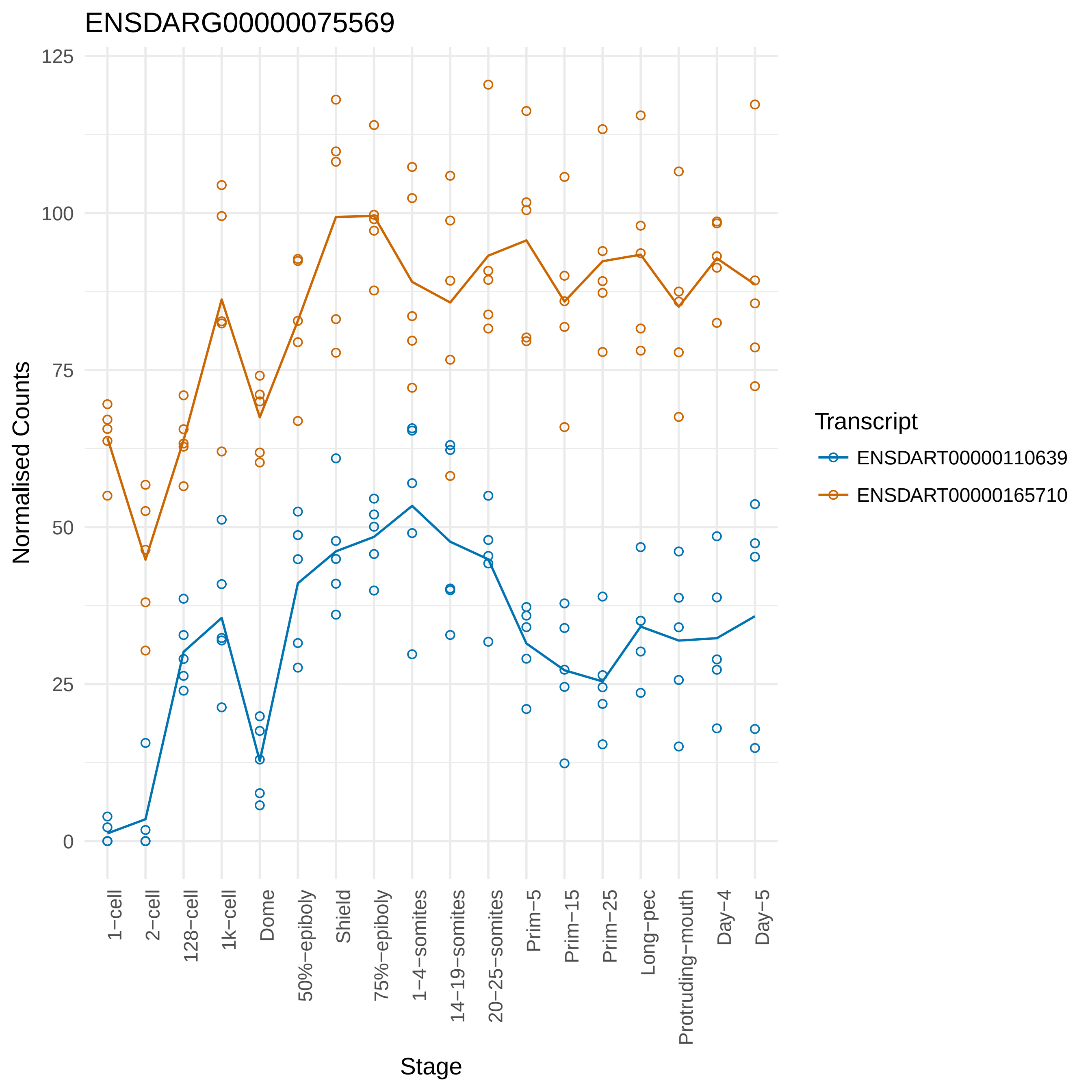

ENSDARG00000075569
Normalised Counts
125
100
75
50
25
0
1−cell
2−cell
128−cell
1k−cell
Dome
50%−epiboly
Shield
75%−epiboly
1−4−somites
14−19−somites
20−25−somites
Prim−5
Prim−15
Prim−25
Long−pec
Protruding−mouth
Day−4
Day−5
Stage
Transcript
ENSDART00000110639
ENSDART00000165710

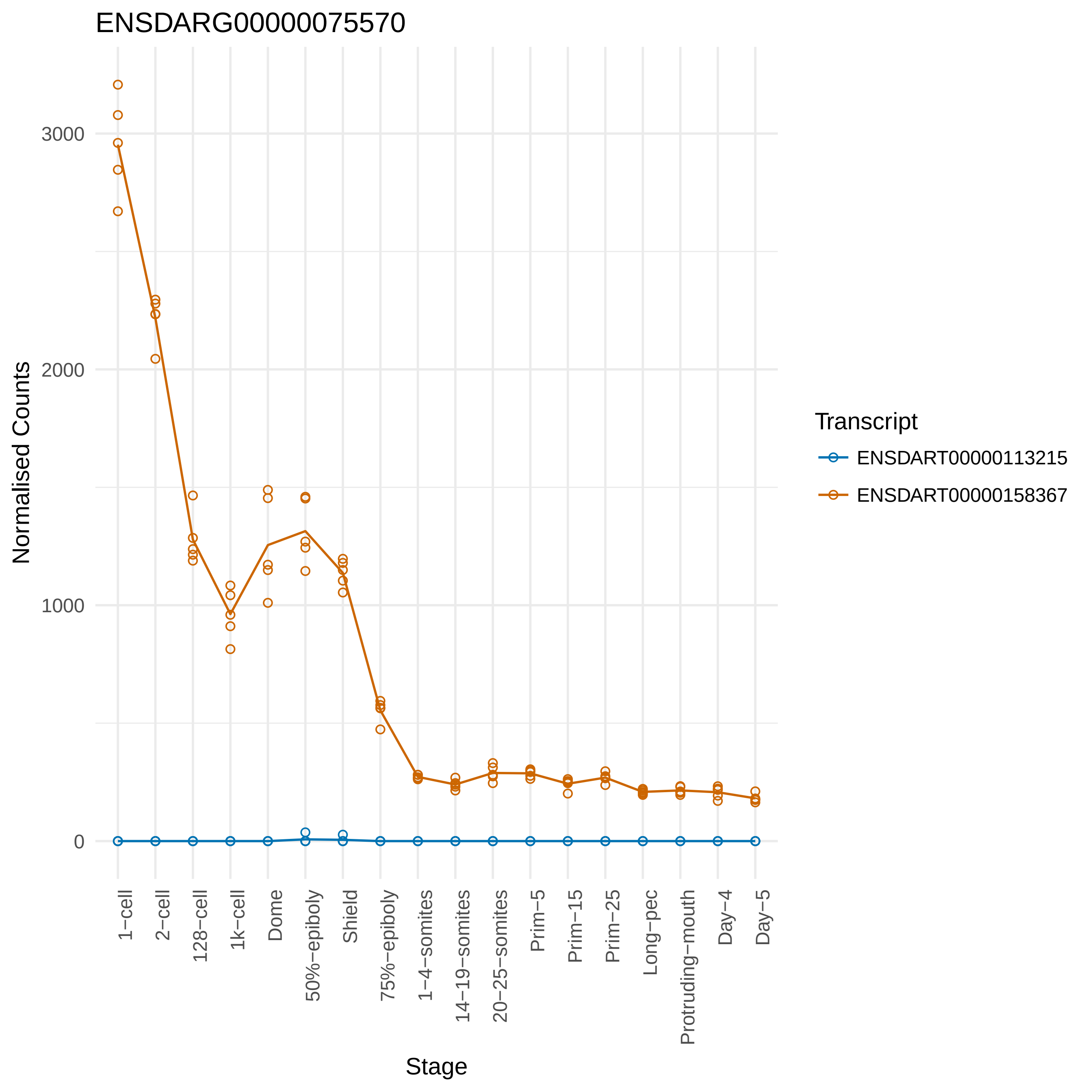

ENSDARG00000075570
Normalised Counts
3000
2000
1000
0
1−cell
2−cell
128−cell
1k−cell
Dome
50%−epiboly
Shield
75%−epiboly
1−4−somites
14−19−somites
20−25−somites
Prim−5
Prim−15
Prim−25
Long−pec
Protruding−mouth
Day−4
Day−5
Stage
Transcript
ENSDART00000113215
ENSDART00000158367

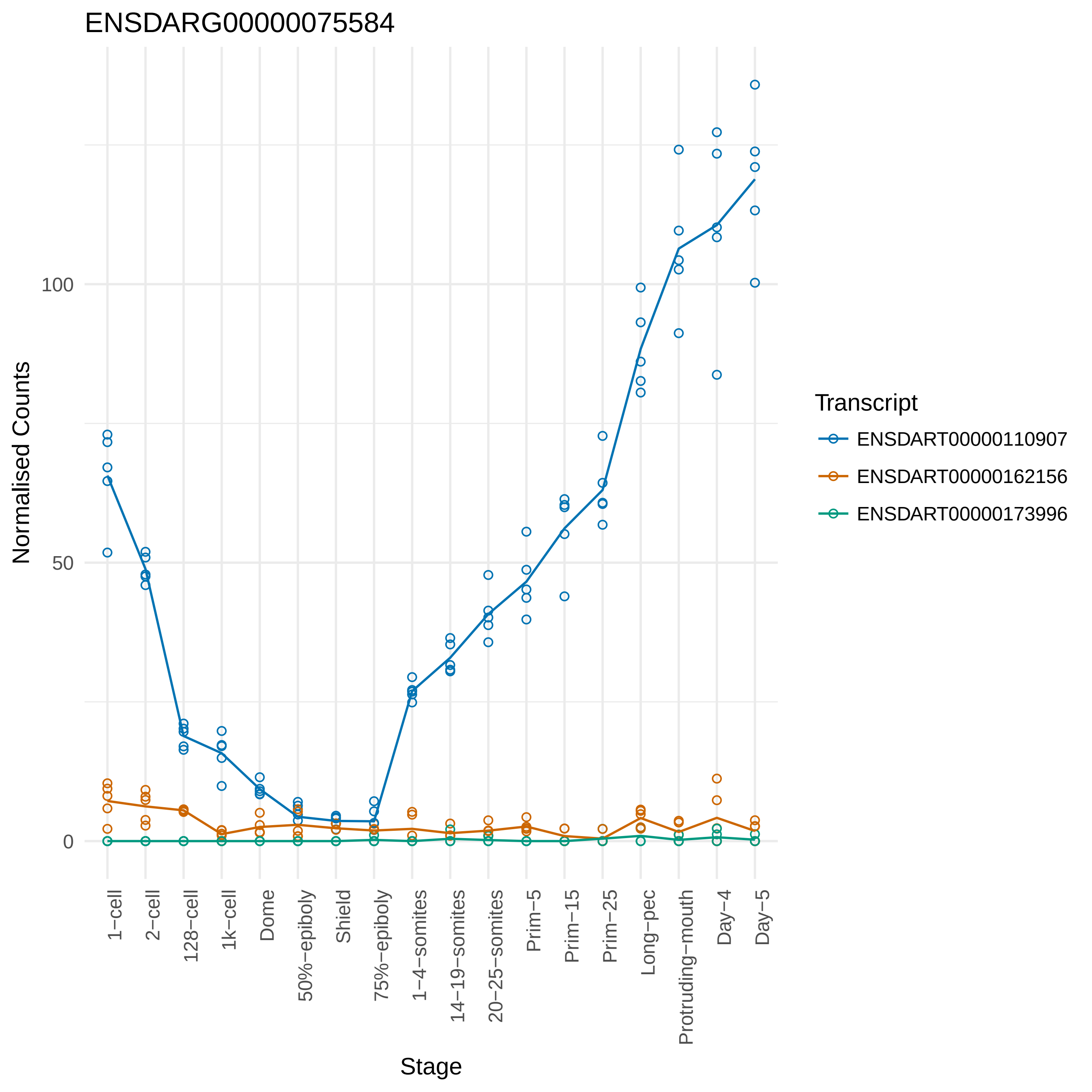

ENSDARG00000075584
Normalised Counts
0
50
100
Stage
1−cell
2−cell
128−cell
1k−cell
Dome
50%−epiboly
Shield
75%−epiboly
1−4−somites
14−19−somites
20−25−somites
Prim−5
Prim−15
Prim−25
Long−pec
Protruding−mouth
Day−4
Day−5
Transcript
ENSDART00000110907
ENSDART00000162156
ENSDART00000173996

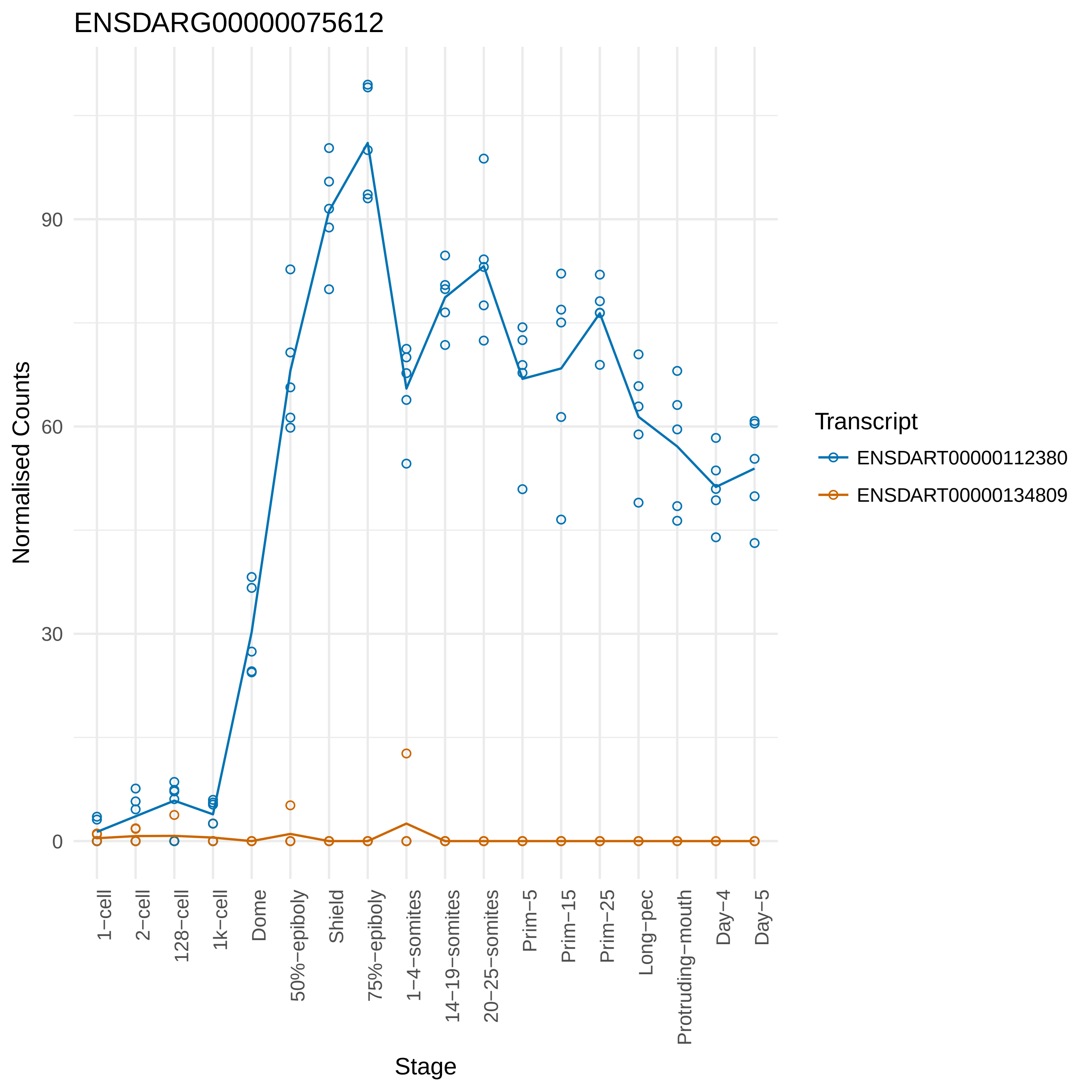
ENSDARG00000075612
Normalised Counts
0
30
60
90
1−cell
2−cell
128−cell
1k−cell
Dome
50%−epiboly
Shield
75%−epiboly
1−4−somites
14−19−somites
20−25−somites
Prim−5
Prim−15
Prim−25
Long−pec
Protruding−mouth
Day−4
Day−5
Stage
Transcript
ENSDART00000112380
ENSDART00000134809

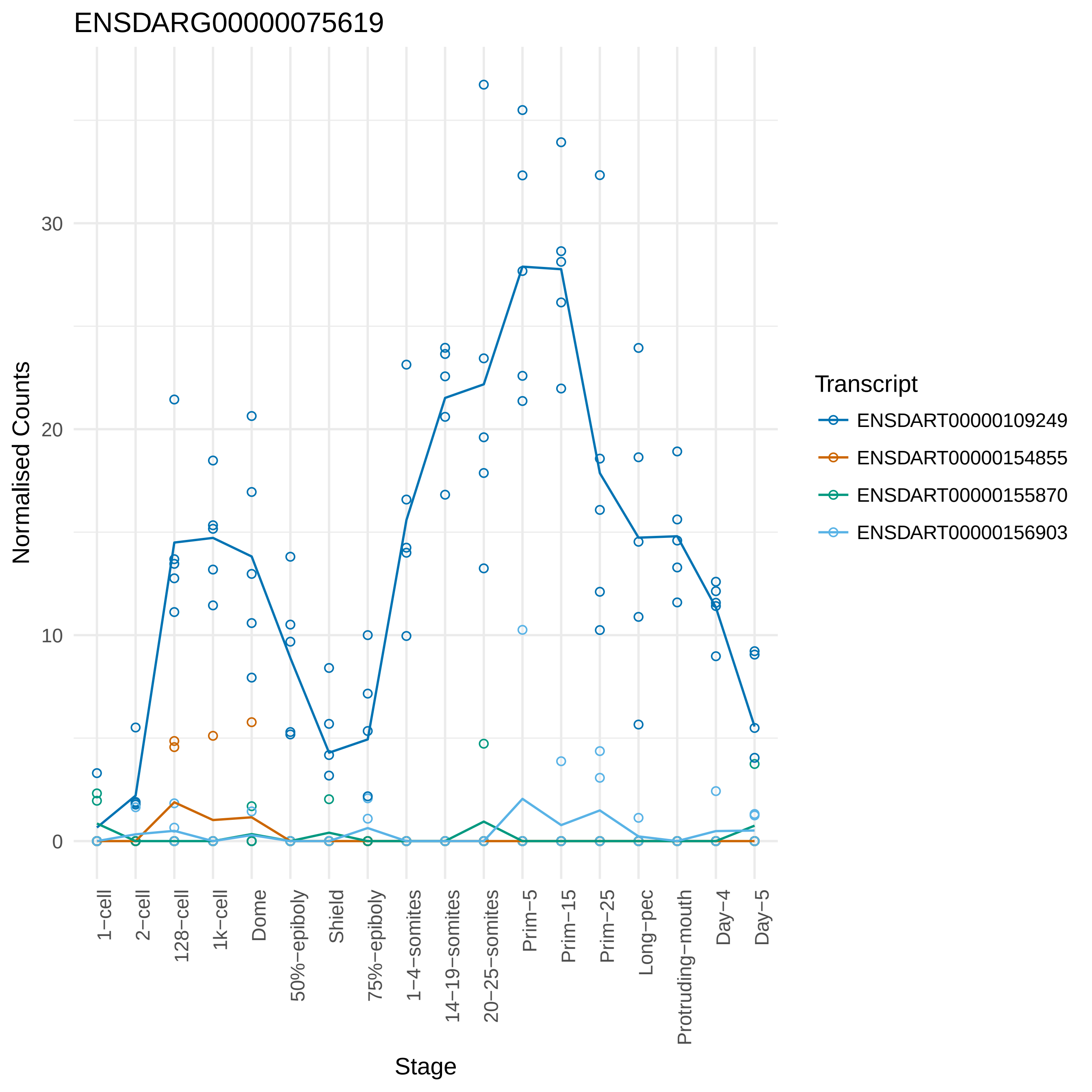
ENSDARG00000075619
Normalised Counts
0
10
20
30
Stage
1−cell
2−cell
128−cell
1k−cell
Dome
50%−epiboly
Shield
75%−epiboly
1−4−somites
14−19−somites
20−25−somites
Prim−5
Prim−15
Prim−25
Long−pec
Protruding−mouth
Day−4
Day−5
Transcript
ENSDART00000109249
ENSDART00000154855
ENSDART00000155870
ENSDART00000156903

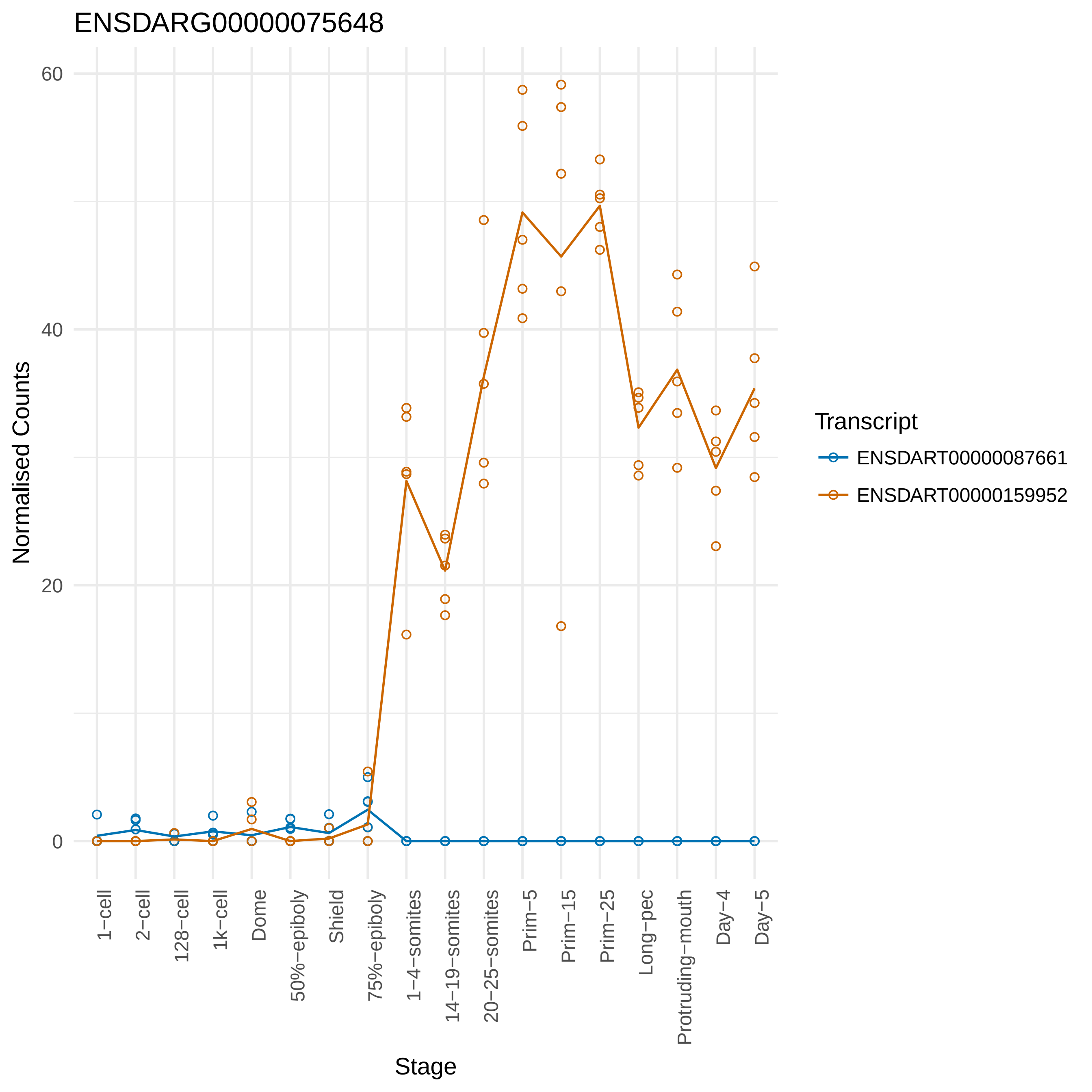
ENSDARG00000075648
Normalised Counts
60
40
20
0
1−cell
2−cell
128−cell
1k−cell
Dome
50%−epiboly
Shield
75%−epiboly
1−4−somites
14−19−somites
20−25−somites
Prim−5
Prim−15
Prim−25
Long−pec
Protruding−mouth
Day−4
Day−5
Stage
Transcript
ENSDART00000087661
ENSDART00000159952

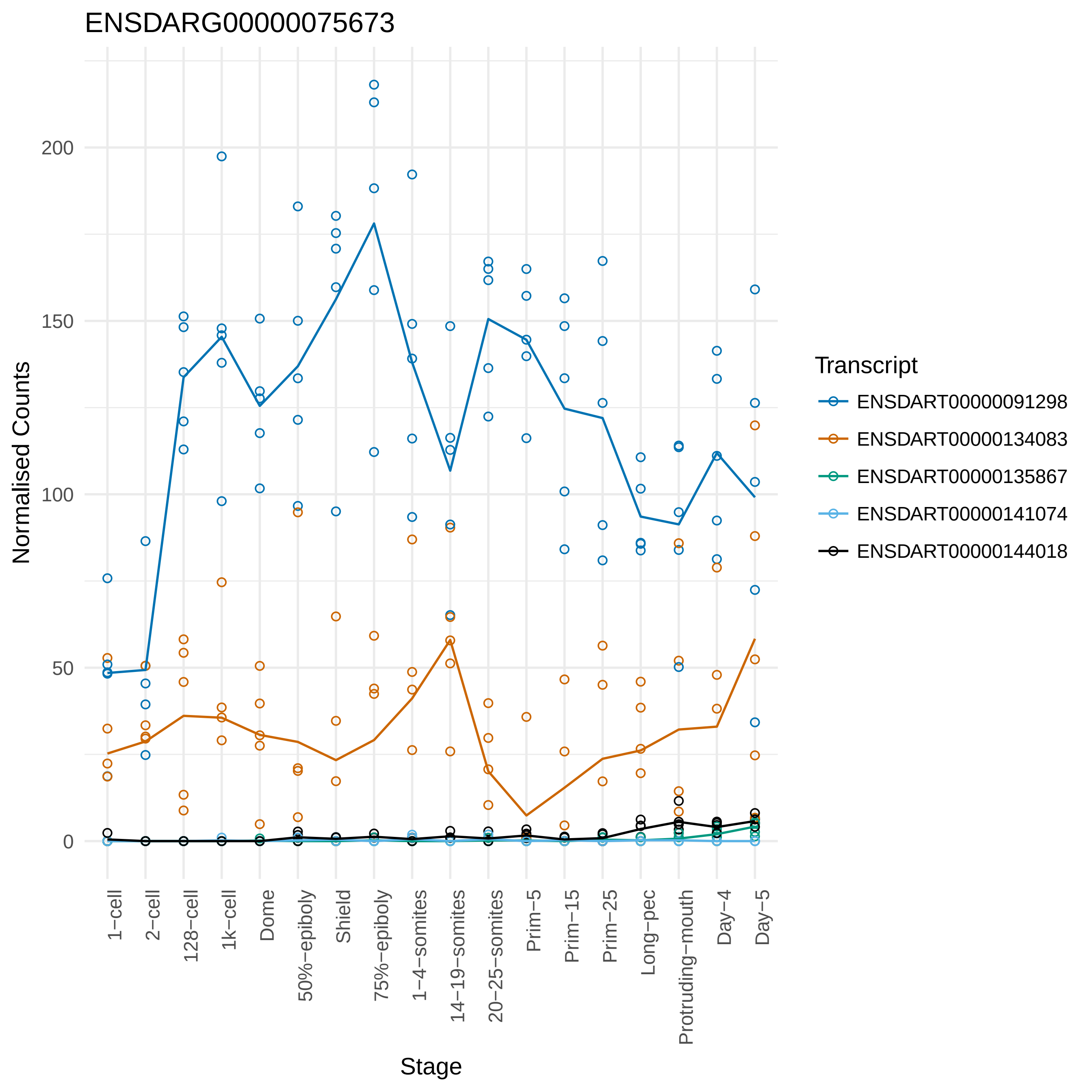

ENSDARG00000075673
Normalised Counts
0
50
100
150
200
1−cell
2−cell
128−cell
1k−cell
Dome
50%−epiboly
Shield
75%−epiboly
1−4−somites
14−19−somites
20−25−somites
Prim−5
Prim−15
Prim−25
Long−pec
Protruding−mouth
Day−4
Day−5
Stage
Transcript
ENSDART00000091298
ENSDART00000134083
ENSDART00000135867
ENSDART00000141074
ENSDART00000144018

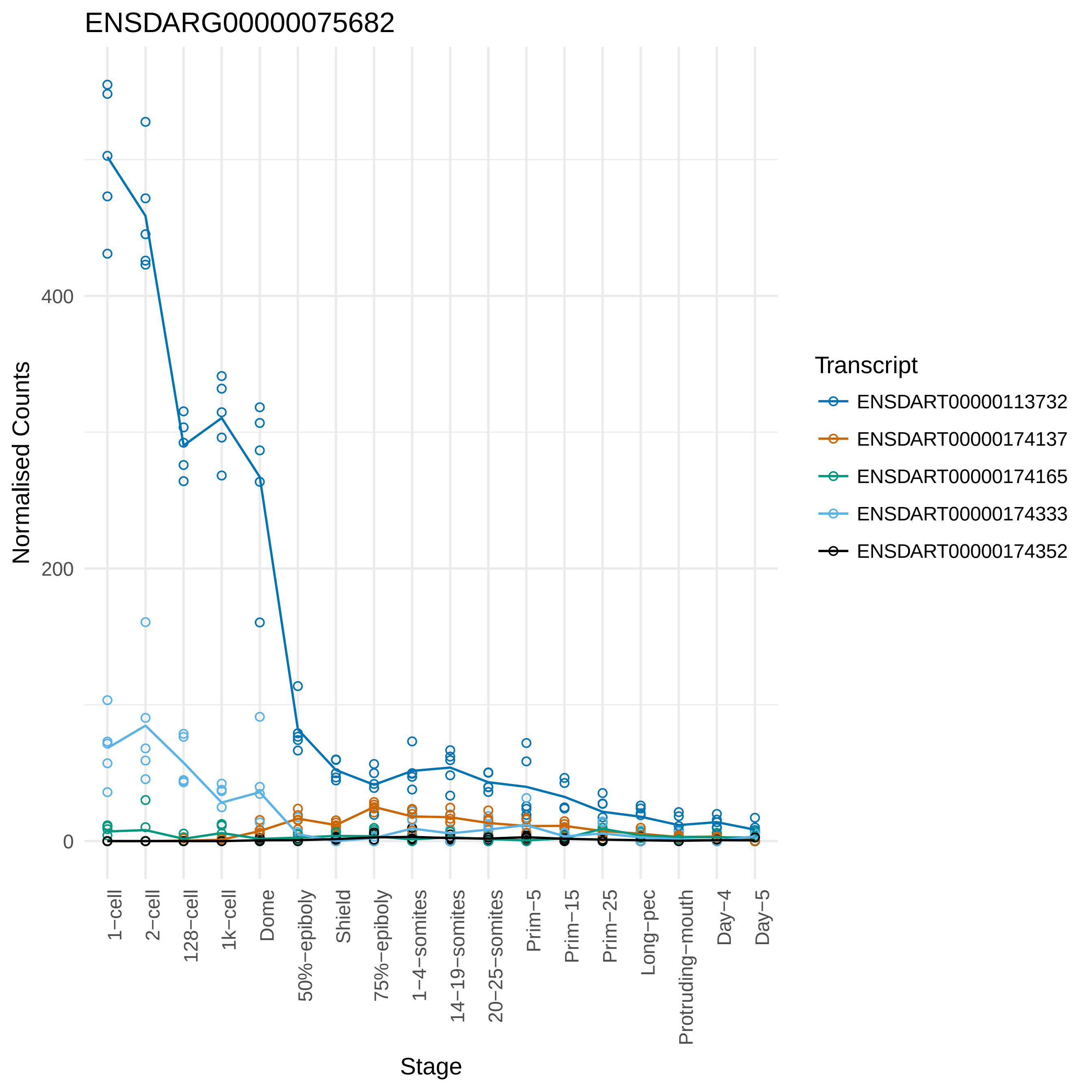
ENSDARG00000075682
Normalised Counts
0
200
400
1−cell
2−cell
128−cell
1k−cell
Dome
50%−epiboly
Shield
75%−epiboly
1−4−somites
14−19−somites
20−25−somites
Prim−5
Prim−15
Prim−25
Long−pec
Protruding−mouth
Day−4
Day−5
Stage
Transcript
ENSDART00000113732
ENSDART00000174137
ENSDART00000174165
ENSDART00000174333
ENSDART00000174352

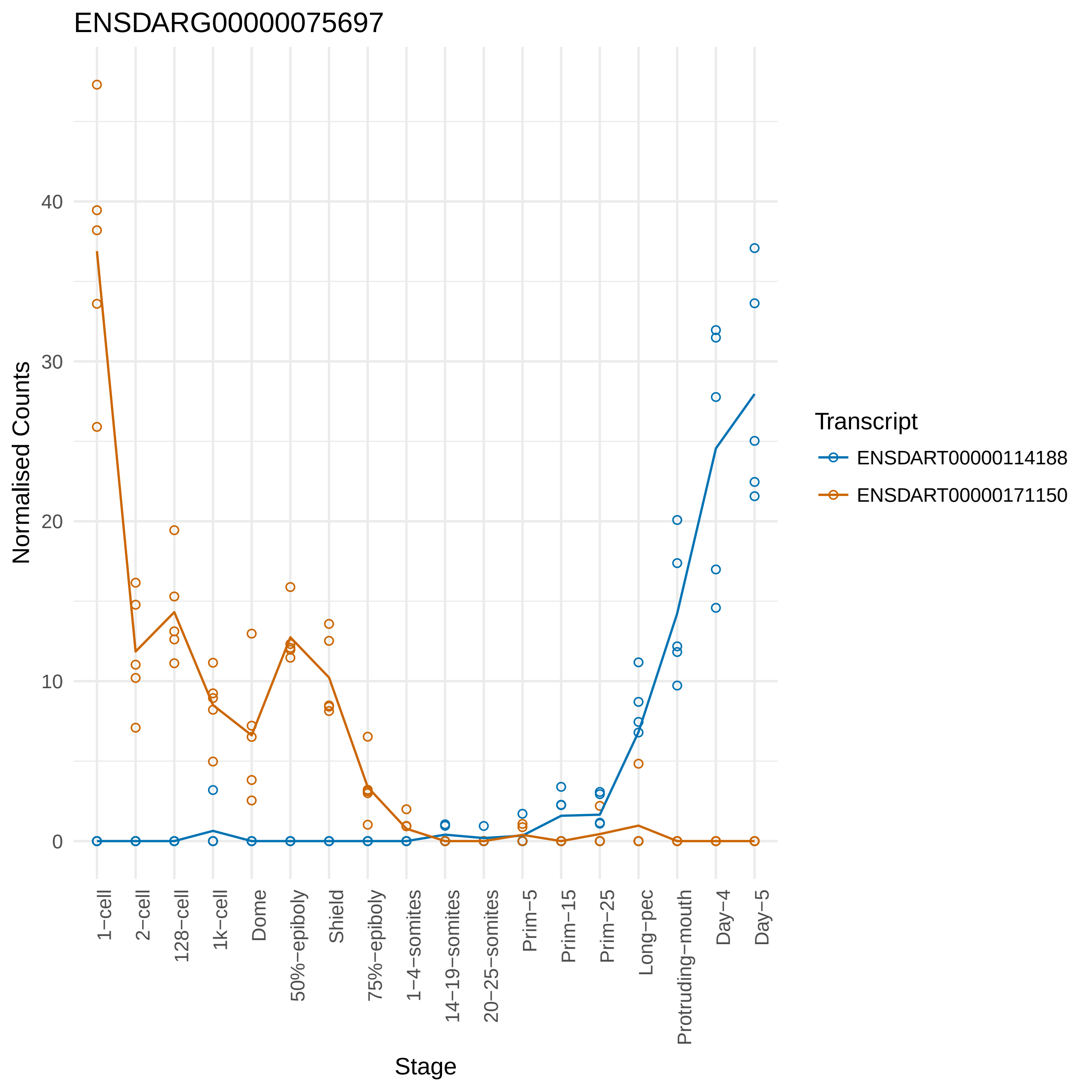
ENSDARG00000075697
Normalised Counts
0
10
20
30
40
Stage
1−cell
2−cell
128−cell
1k−cell
Dome
50%−epiboly
Shield
75%−epiboly
1−4−somites
14−19−somites
20−25−somites
Prim−5
Prim−15
Prim−25
Long−pec
Protruding−mouth
Day−4
Day−5
Transcript
ENSDART00000114188
ENSDART00000171150

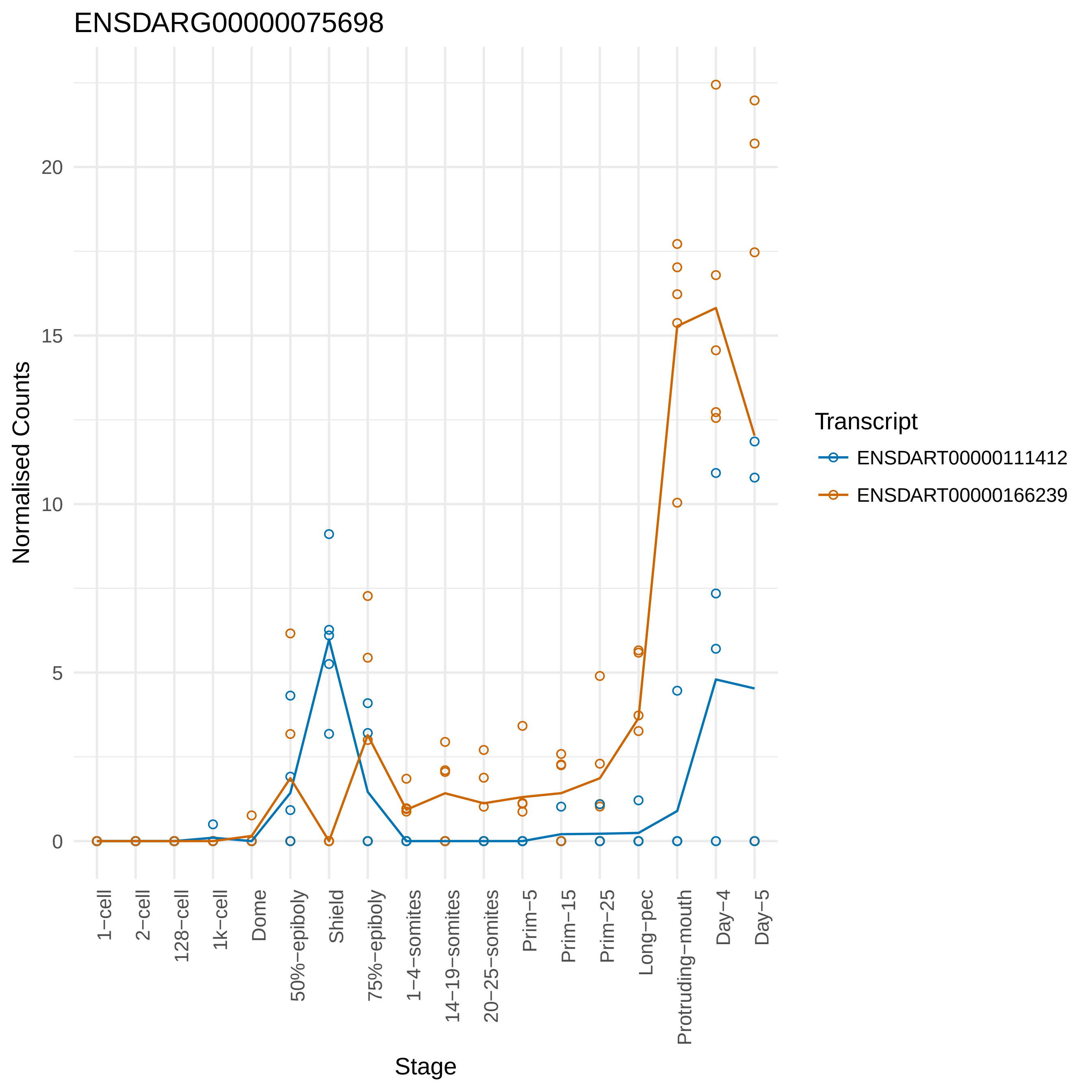

ENSDARG00000075698
Normalised Counts
0
5
10
15
20
Stage
1−cell
2−cell
128−cell
1k−cell
Dome
50%−epiboly
Shield
75%−epiboly
1−4−somites
14−19−somites
20−25−somites
Prim−5
Prim−15
Prim−25
Long−pec
Protruding−mouth
Day−4
Day−5
Transcript
ENSDART00000111412
ENSDART00000166239

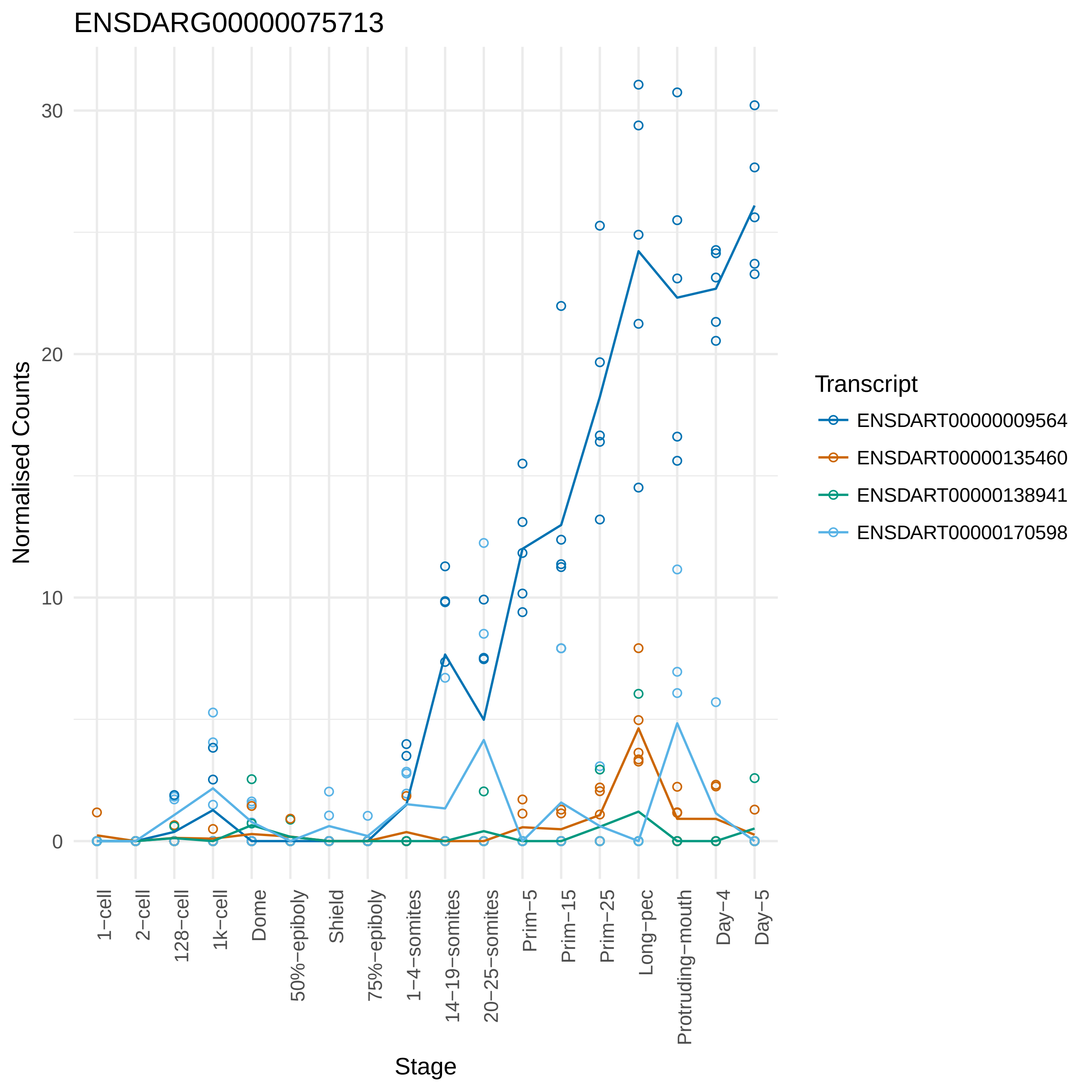
ENSDARG00000075713
Normalised Counts
0
10
20
30
1−cell
2−cell
128−cell
1k−cell
Dome
50%−epiboly
Shield
75%−epiboly
1−4−somites
14−19−somites
20−25−somites
Prim−5
Prim−15
Prim−25
Long−pec
Protruding−mouth
Day−4
Day−5
Stage
Transcript
ENSDART00000009564
ENSDART00000135460
ENSDART00000138941
ENSDART00000170598

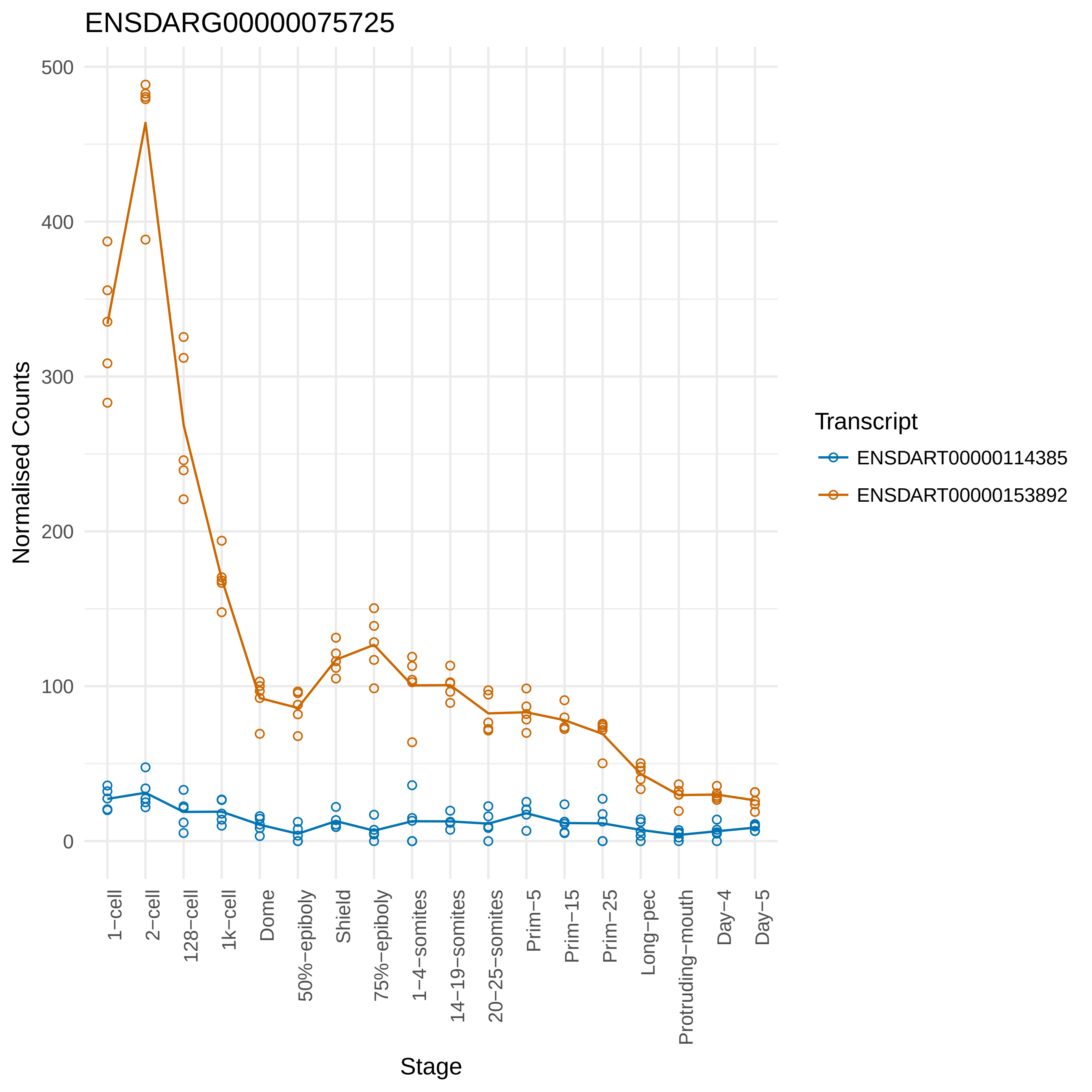
ENSDARG00000075725
Normalised Counts
500
400
300
200
100
0
1−cell
2−cell
128−cell
1k−cell
Dome
50%−epiboly
Shield
75%−epiboly
1−4−somites
14−19−somites
20−25−somites
Prim−5
Prim−15
Prim−25
Long−pec
Protruding−mouth
Day−4
Day−5
Stage
Transcript
ENSDART00000114385
ENSDART00000153892

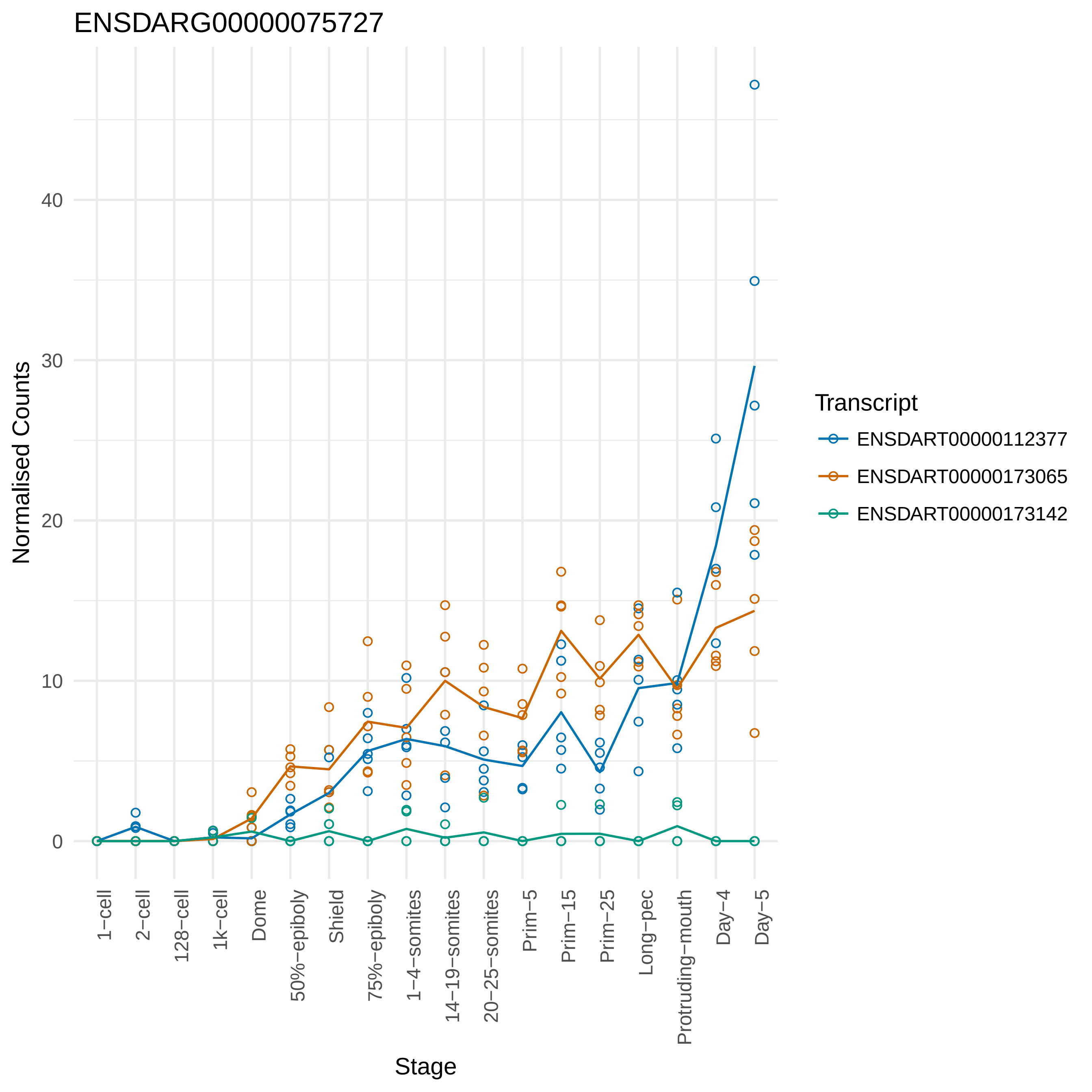
ENSDARG00000075727
Normalised Counts
0
10
20
30
40
Stage
1−cell
2−cell
128−cell
1k−cell
Dome
50%−epiboly
Shield
75%−epiboly
1−4−somites
14−19−somites
20−25−somites
Prim−5
Prim−15
Prim−25
Long−pec
Protruding−mouth
Day−4
Day−5
Transcript
ENSDART00000112377
ENSDART00000173065
ENSDART00000173142

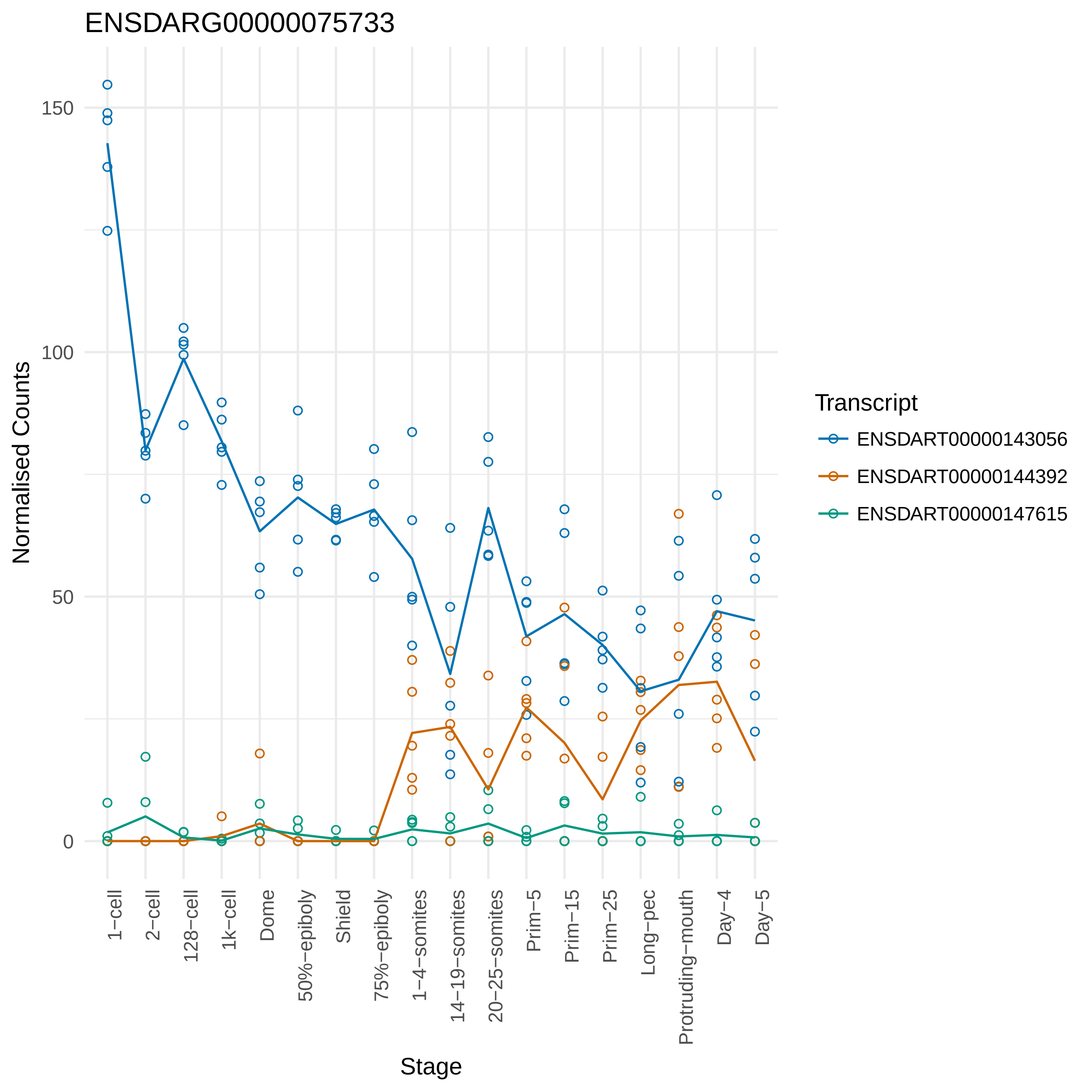
ENSDARG00000075733
Normalised Counts
150
100
50
0
1−cell
2−cell
128−cell
1k−cell
Dome
50%−epiboly
Shield
75%−epiboly
1−4−somites
14−19−somites
20−25−somites
Prim−5
Prim−15
Prim−25
Long−pec
Protruding−mouth
Day−4
Day−5
Stage
Transcript
ENSDART00000143056
ENSDART00000144392
ENSDART00000147615

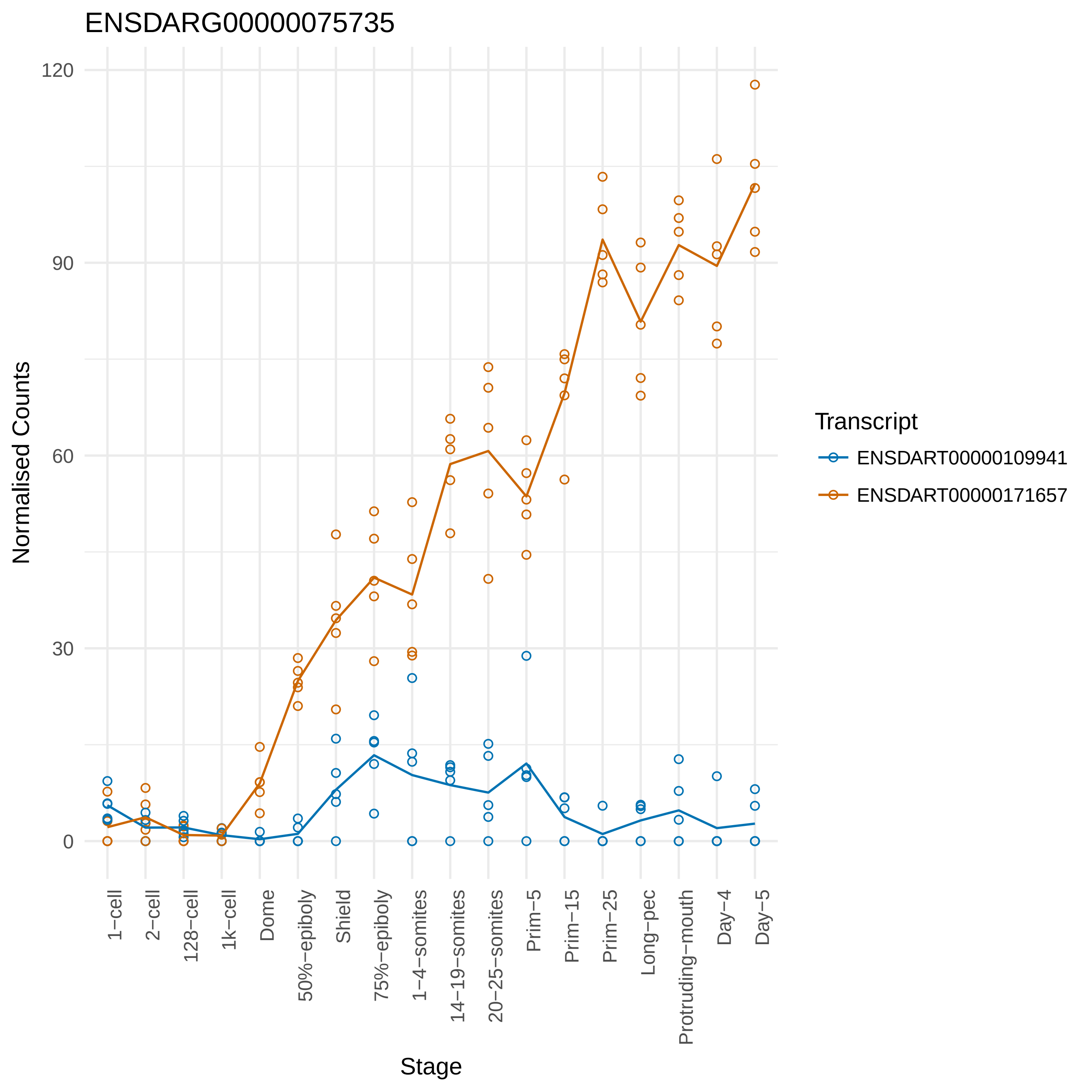

ENSDARG00000075735
Normalised Counts
0
30
60
90
120
1−cell
2−cell
128−cell
1k−cell
Dome
50%−epiboly
Shield
75%−epiboly
1−4−somites
14−19−somites
20−25−somites
Prim−5
Prim−15
Prim−25
Long−pec
Protruding−mouth
Day−4
Day−5
Stage
Transcript
ENSDART00000109941
ENSDART00000171657

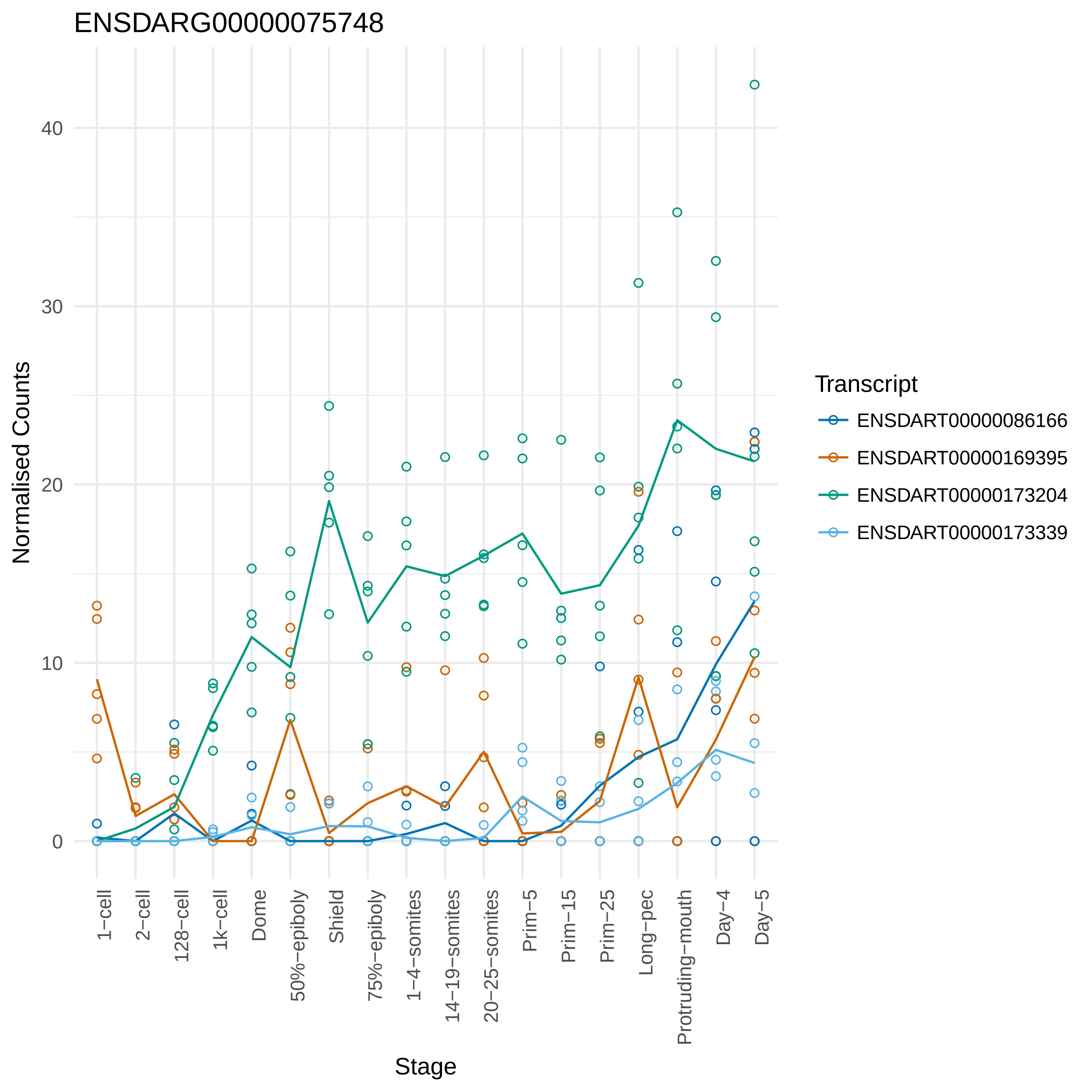

ENSDARG00000075748
Normalised Counts
0
10
20
30
40
Stage
1−cell
2−cell
128−cell
1k−cell
Dome
50%−epiboly
Shield
75%−epiboly
1−4−somites
14−19−somites
20−25−somites
Prim−5
Prim−15
Prim−25
Long−pec
Protruding−mouth
Day−4
Day−5
Transcript
ENSDART00000086166
ENSDART00000169395
ENSDART00000173204
ENSDART00000173339

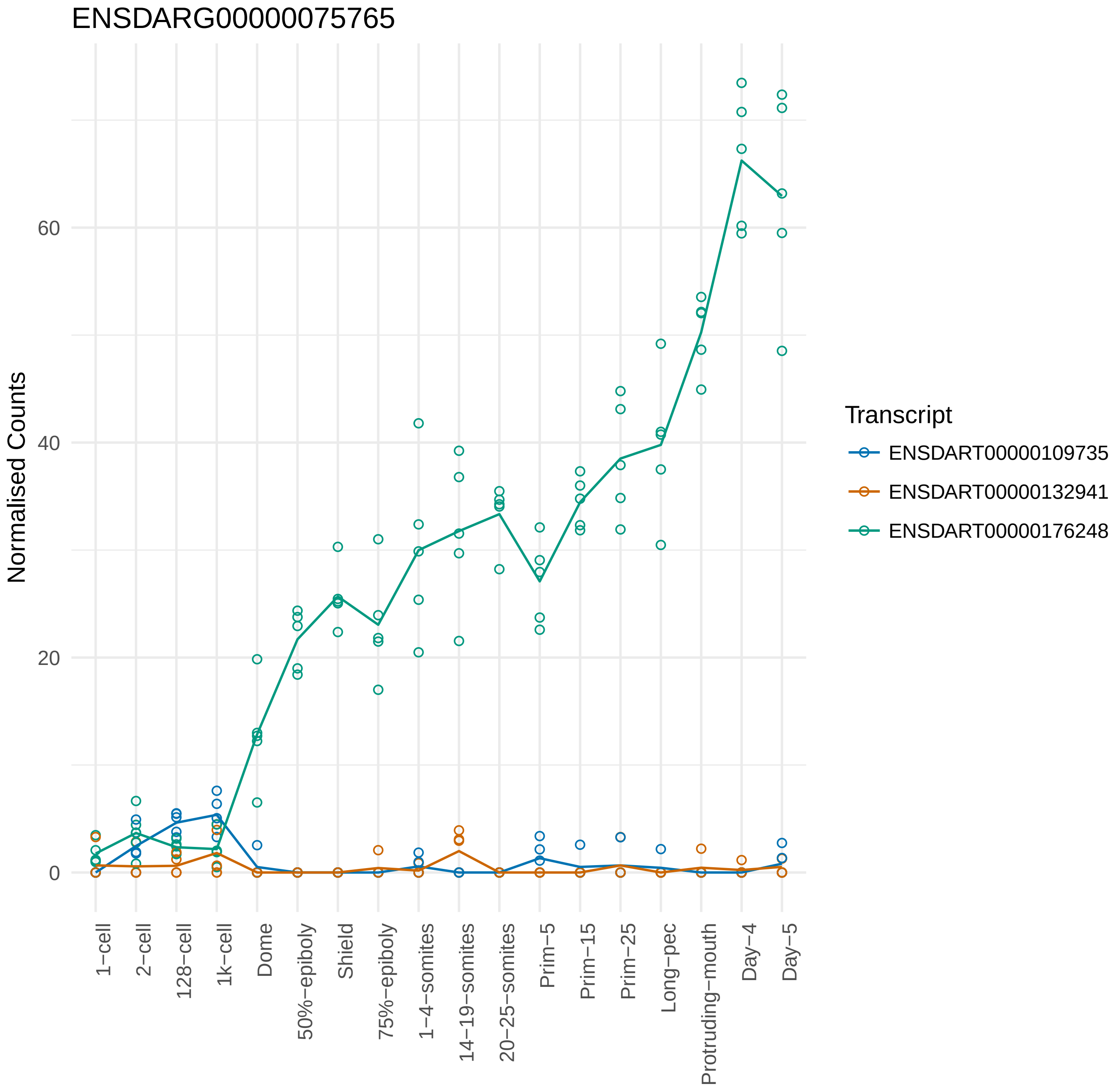

ENSDARG00000075765
Normalised Counts
0
20
40
60
Stage
1−cell
2−cell
128−cell
1k−cell
Dome
50%−epiboly
Shield
75%−epiboly
1−4−somites
14−19−somites
20−25−somites
Prim−5
Prim−15
Prim−25
Long−pec
Protruding−mouth
Day−4
Day−5
Transcript
ENSDART00000109735
ENSDART00000132941
ENSDART00000176248

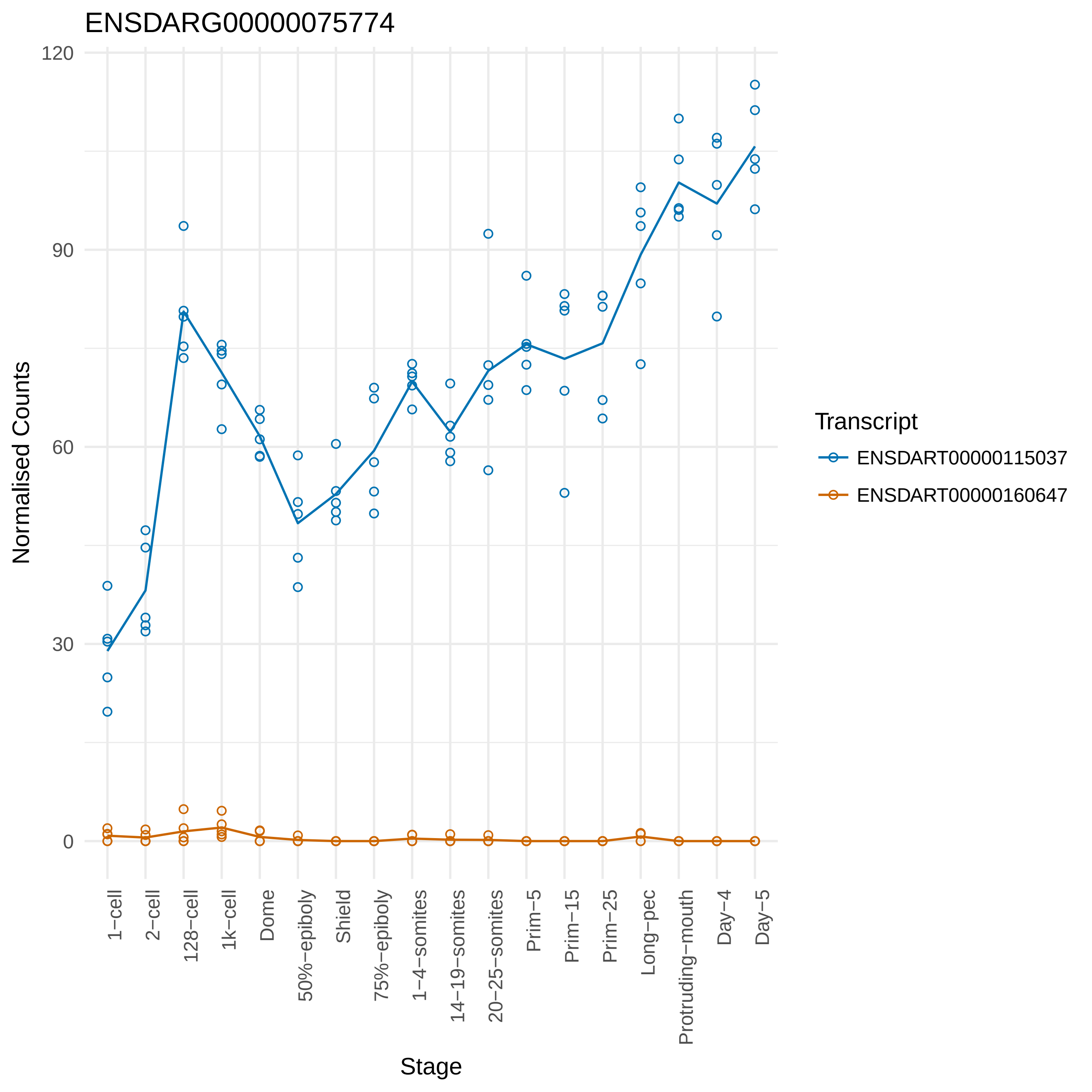

ENSDARG00000075774
Normalised Counts
120
90
60
30
0
Stage
1−cell
2−cell
128−cell
1k−cell
Dome
50%−epiboly
Shield
75%−epiboly
1−4−somites
14−19−somites
20−25−somites
Prim−5
Prim−15
Prim−25
Long−pec
Protruding−mouth
Day−4
Day−5
Transcript
ENSDART00000115037
ENSDART00000160647

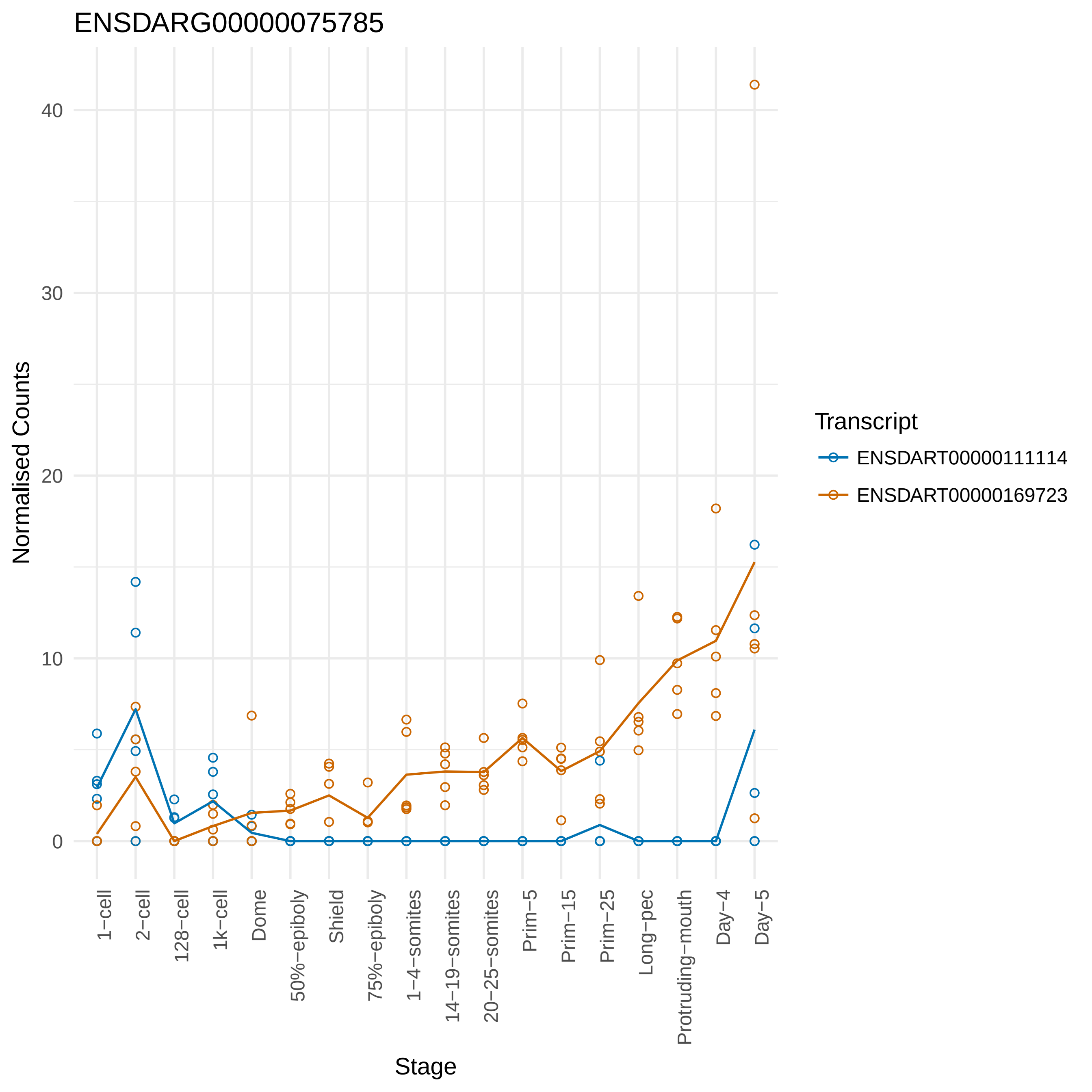

ENSDARG00000075785
Normalised Counts
40
30
20
10
0
1−cell
2−cell
128−cell
1k−cell
Dome
50%−epiboly
Shield
75%−epiboly
1−4−somites
14−19−somites
20−25−somites
Prim−5
Prim−15
Prim−25
Long−pec
Protruding−mouth
Day−4
Day−5
Stage
Transcript
ENSDART00000111114
ENSDART00000169723

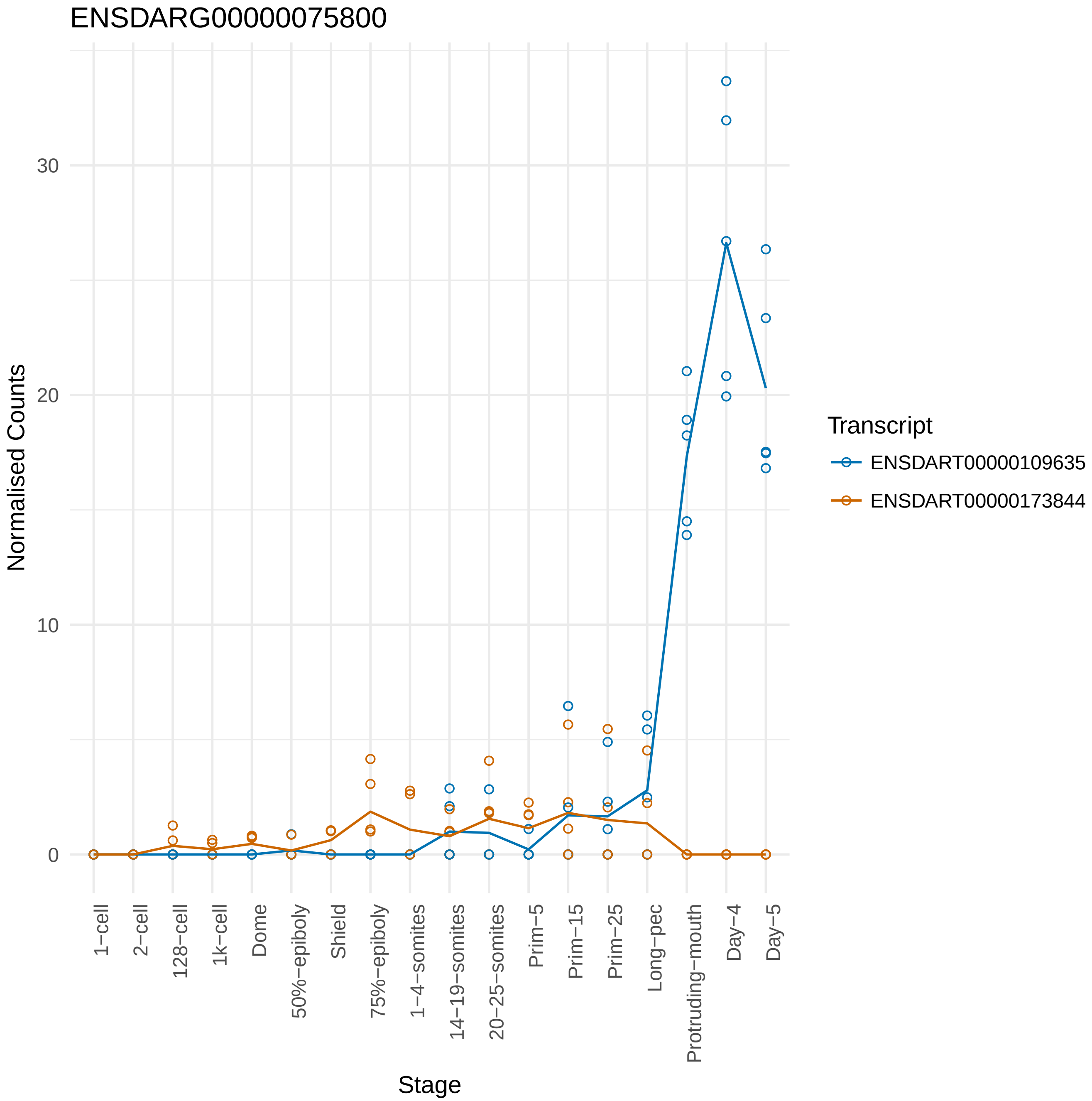

ENSDARG00000075800
Normalised Counts
0
10
20
30
1−cell
2−cell
128−cell
1k−cell
Dome
50%−epiboly
Shield
75%−epiboly
1−4−somites
14−19−somites
20−25−somites
Prim−5
Prim−15
Prim−25
Long−pec
Protruding−mouth
Day−4
Day−5
Stage
Transcript
ENSDART00000109635
ENSDART00000173844

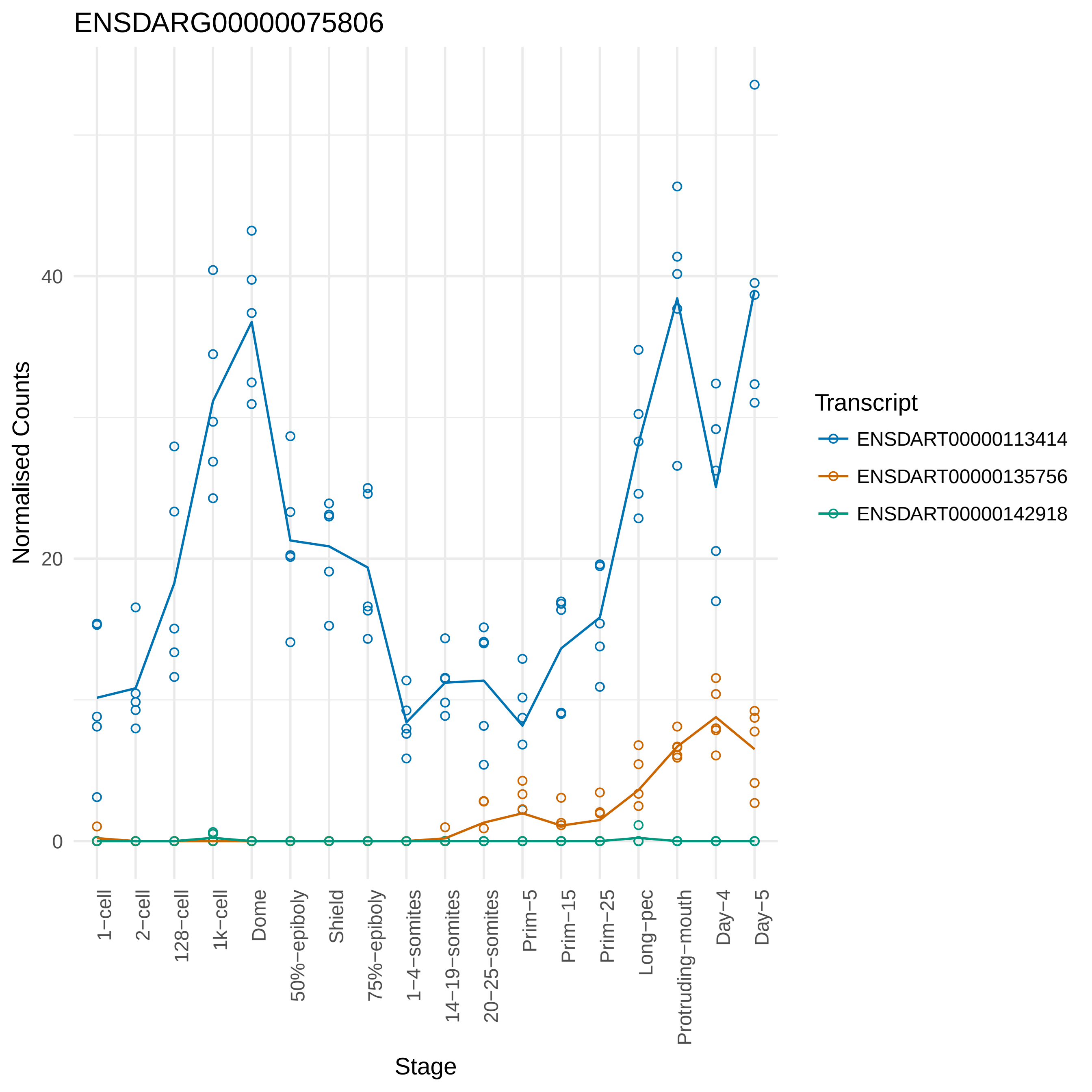

ENSDARG00000075806
Normalised Counts
0
20
40
Stage
1−cell
2−cell
128−cell
1k−cell
Dome
50%−epiboly
Shield
75%−epiboly
1−4−somites
14−19−somites
20−25−somites
Prim−5
Prim−15
Prim−25
Long−pec
Protruding−mouth
Day−4
Day−5
Transcript
ENSDART00000113414
ENSDART00000135756
ENSDART00000142918

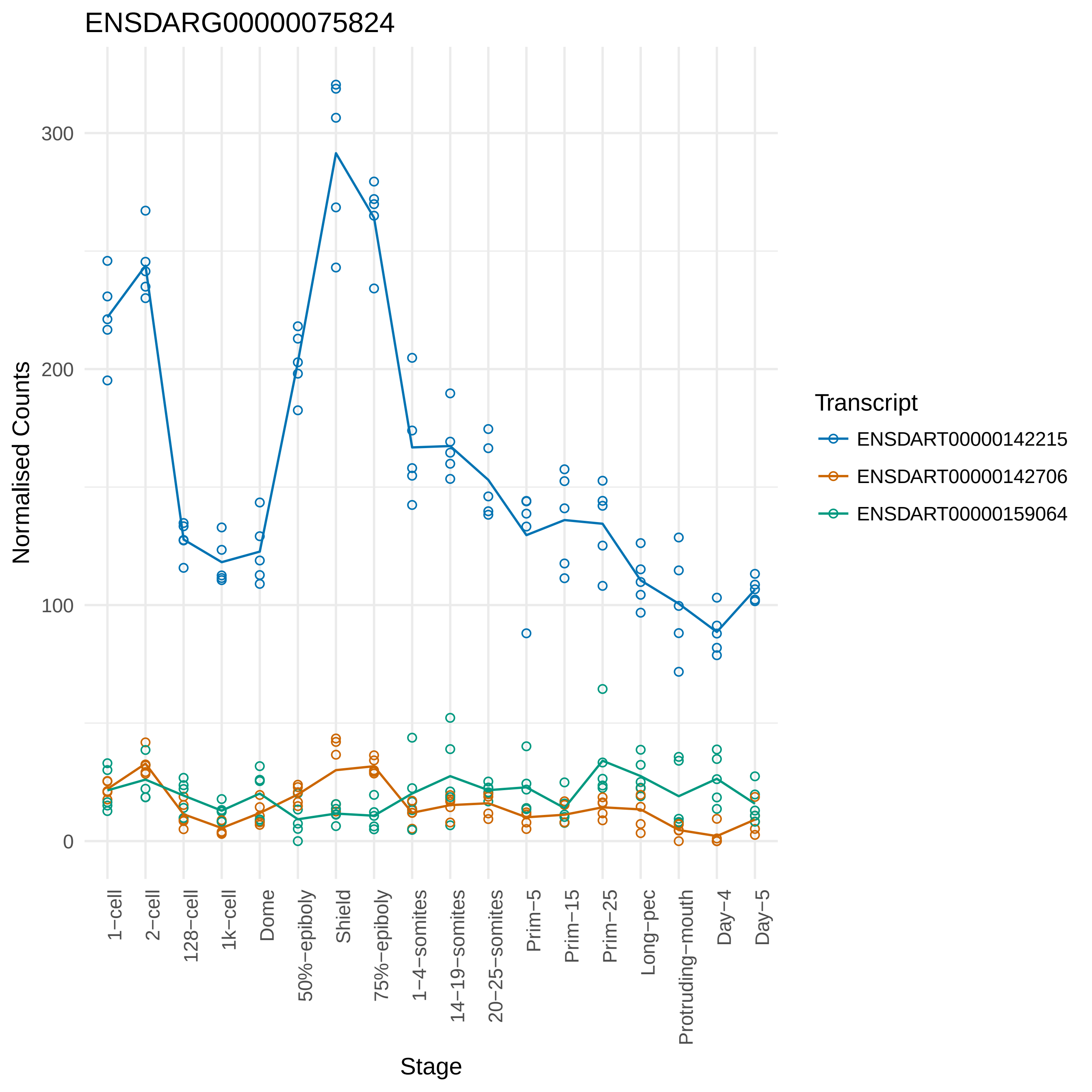

ENSDARG00000075824
Normalised Counts
300
200
100
0
1−cell
2−cell
128−cell
1k−cell
Dome
50%−epiboly
Shield
75%−epiboly
1−4−somites
14−19−somites
20−25−somites
Prim−5
Prim−15
Prim−25
Long−pec
Protruding−mouth
Day−4
Day−5
Stage
Transcript
ENSDART00000142215
ENSDART00000142706
ENSDART00000159064

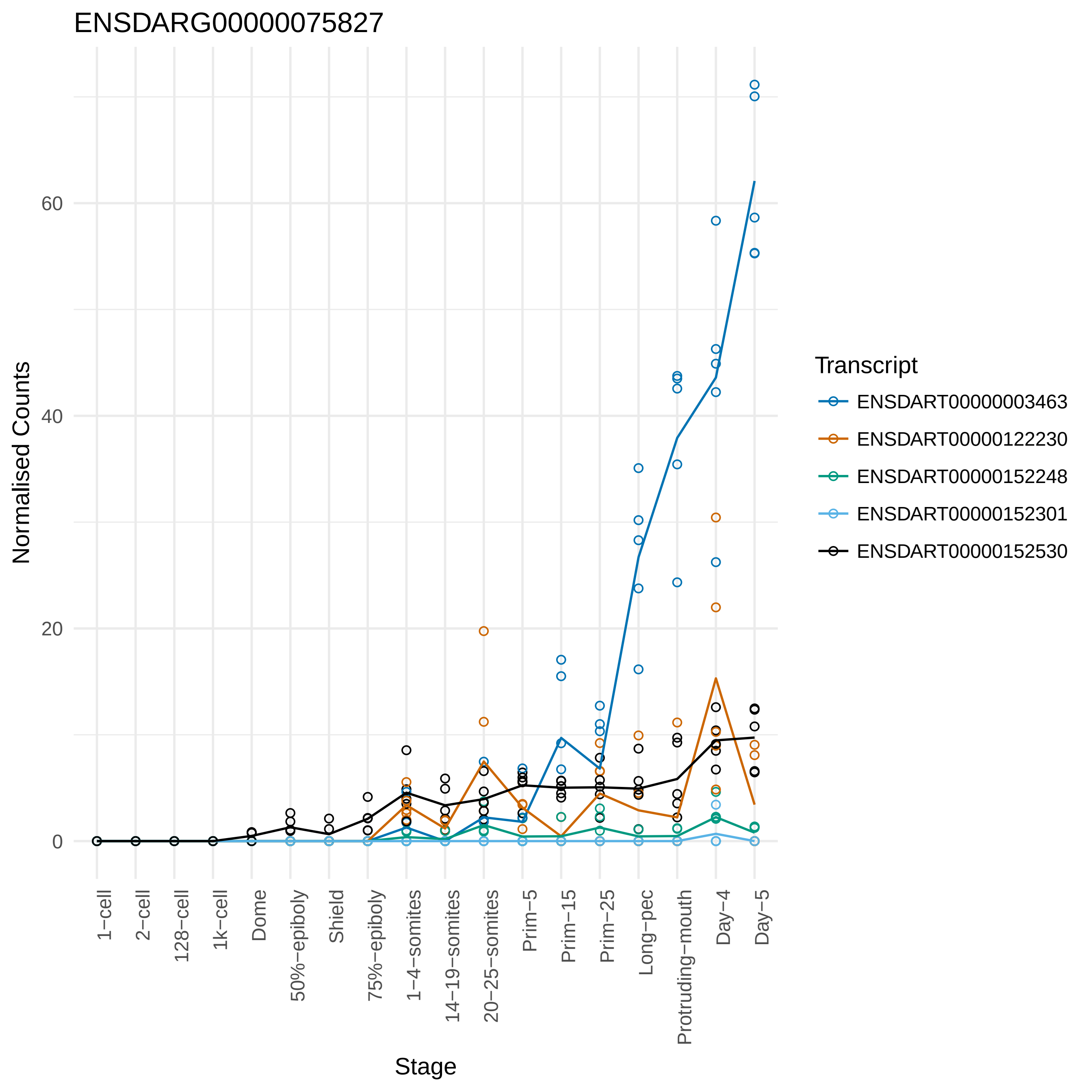

ENSDARG00000075827
Normalised Counts
0
20
40
60
Stage
1−cell
2−cell
128−cell
1k−cell
Dome
50%−epiboly
Shield
75%−epiboly
1−4−somites
14−19−somites
20−25−somites
Prim−5
Prim−15
Prim−25
Long−pec
Protruding−mouth
Day−4
Day−5
Transcript
ENSDART00000003463
ENSDART00000122230
ENSDART00000152248
ENSDART00000152301
ENSDART00000152530

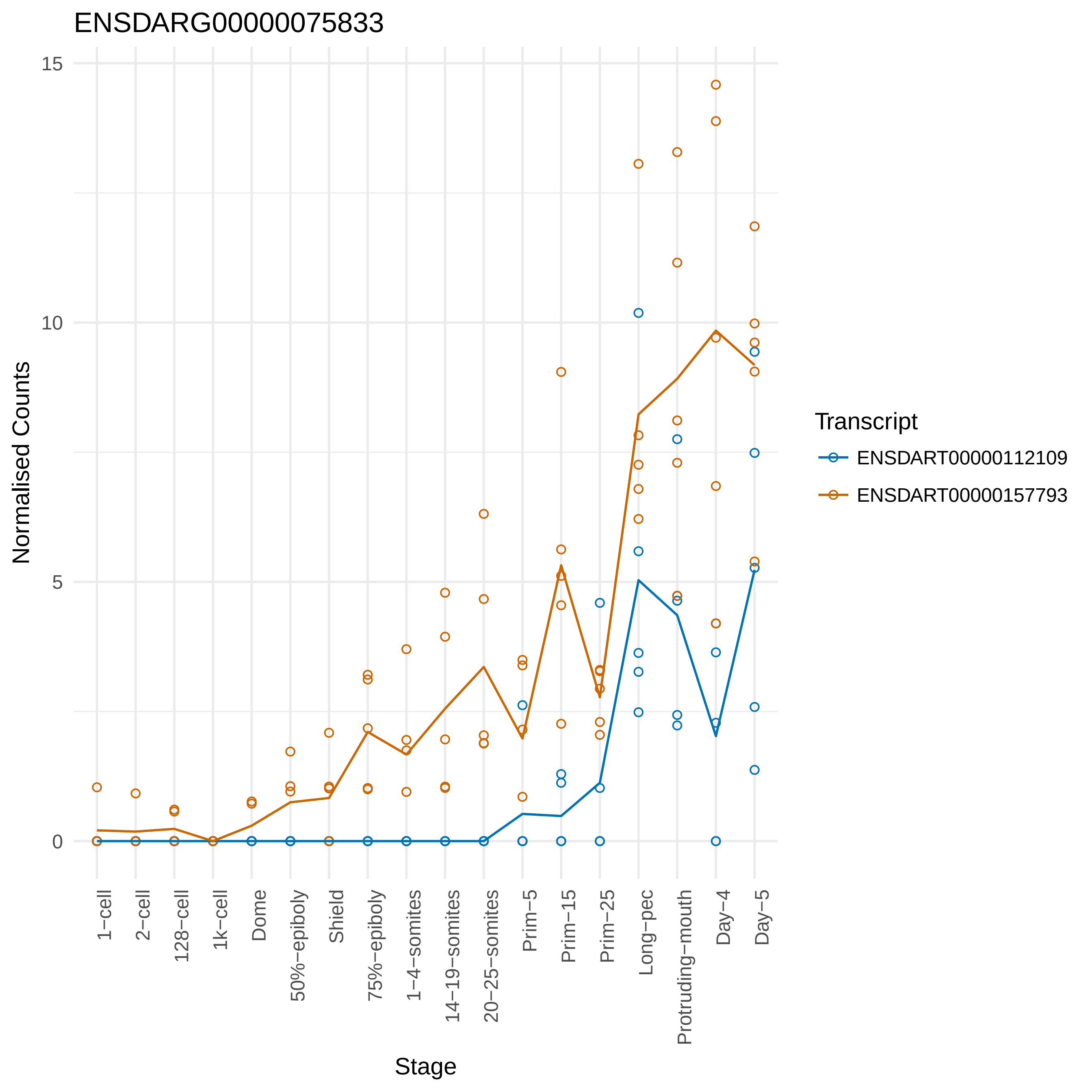

ENSDARG00000075833
Normalised Counts
15
10
5
0
Stage
1−cell
2−cell
128−cell
1k−cell
Dome
50%−epiboly
Shield
75%−epiboly
1−4−somites
14−19−somites
20−25−somites
Prim−5
Prim−15
Prim−25
Long−pec
Protruding−mouth
Day−4
Day−5
Transcript
ENSDART00000112109
ENSDART00000157793

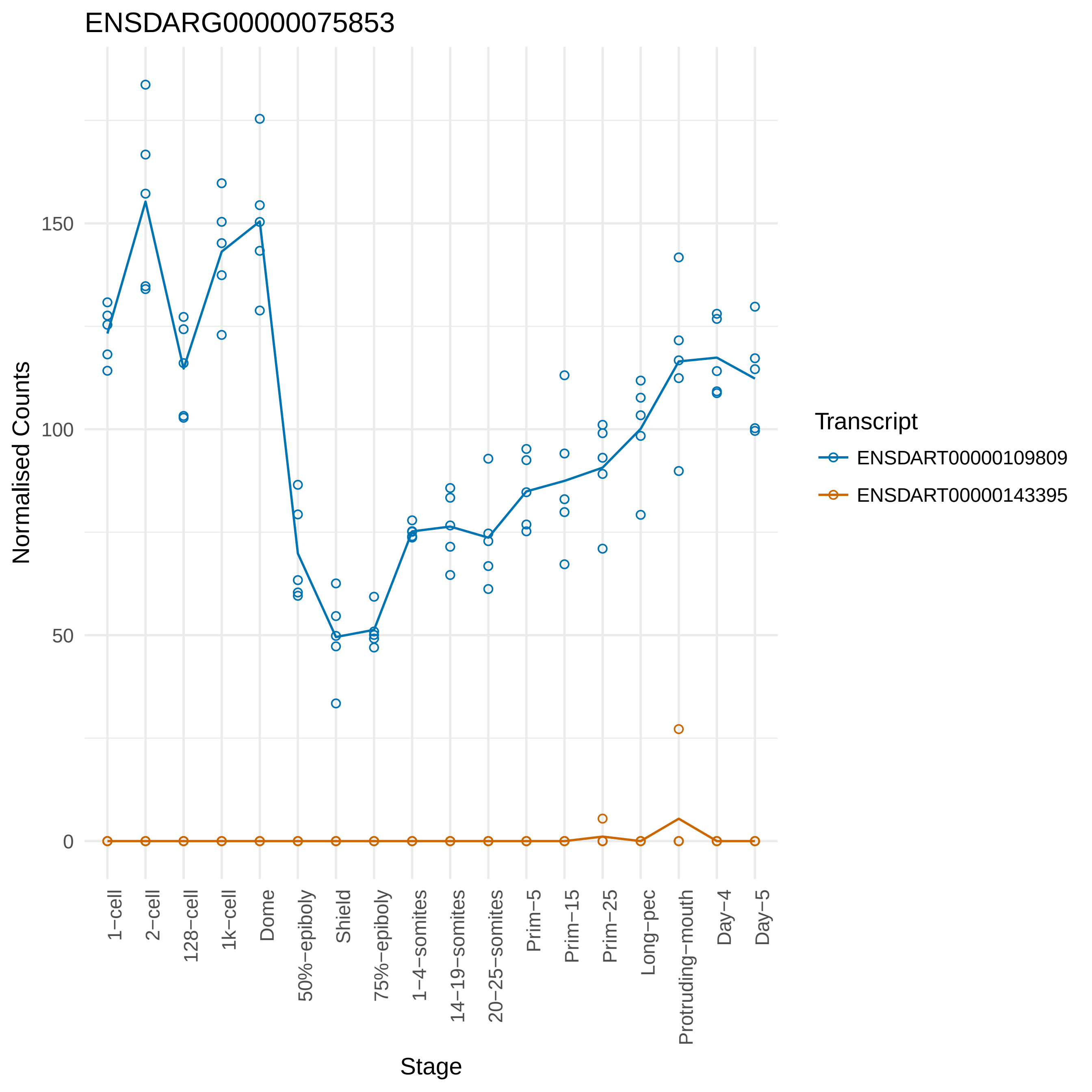
ENSDARG00000075853
Normalised Counts
150
100
50
0
Stage
1−cell
2−cell
128−cell
1k−cell
Dome
50%−epiboly
Shield
75%−epiboly
1−4−somites
14−19−somites
20−25−somites
Prim−5
Prim−15
Prim−25
Long−pec
Protruding−mouth
Day−4
Day−5
Transcript
ENSDART00000109809
ENSDART00000143395

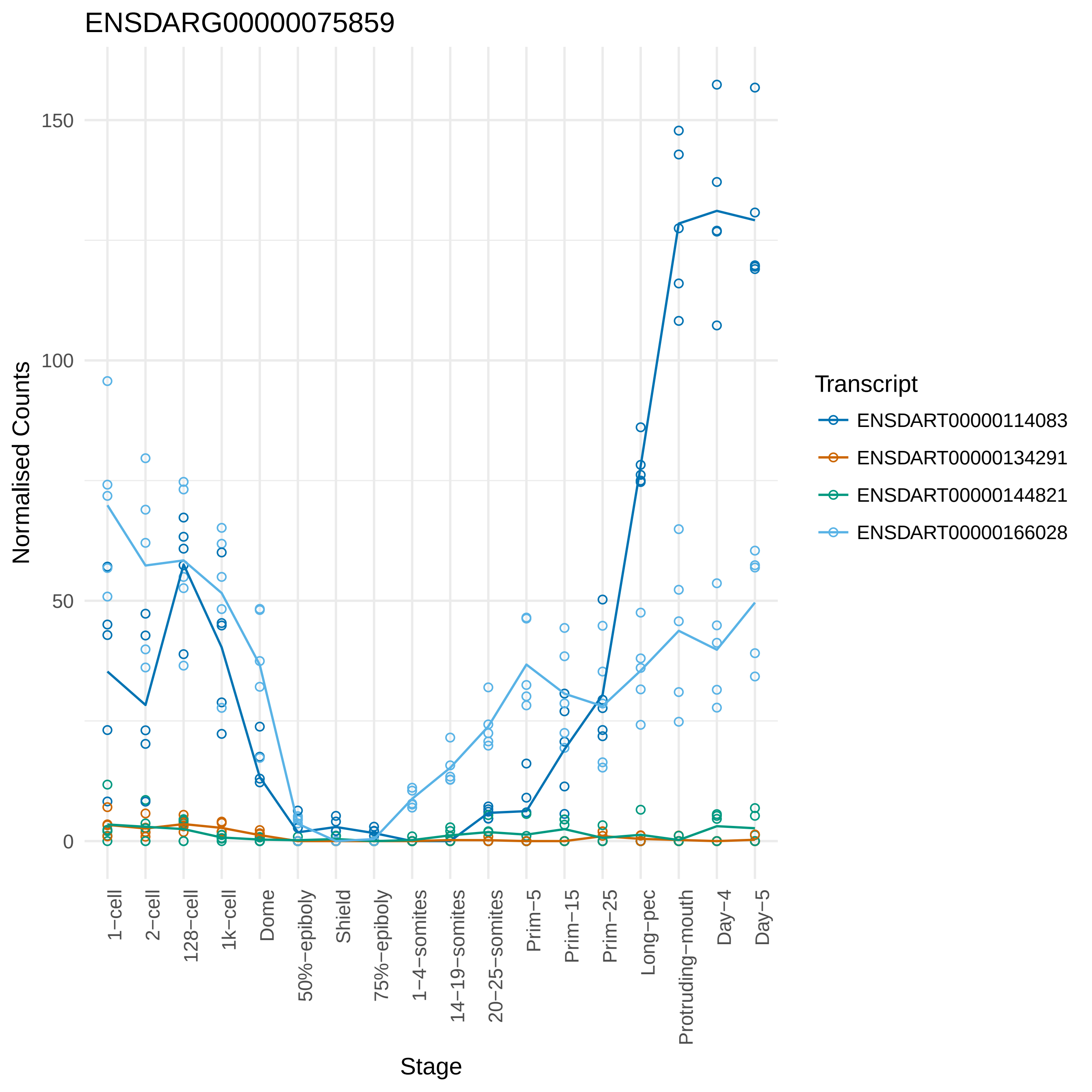

ENSDARG00000075859
Normalised Counts
150
100
50
0
1−cell
2−cell
128−cell
1k−cell
Dome
50%−epiboly
Shield
75%−epiboly
1−4−somites
14−19−somites
20−25−somites
Prim−5
Prim−15
Prim−25
Long−pec
Protruding−mouth
Day−4
Day−5
Stage
Transcript
ENSDART00000114083
ENSDART00000134291
ENSDART00000144821
ENSDART00000166028

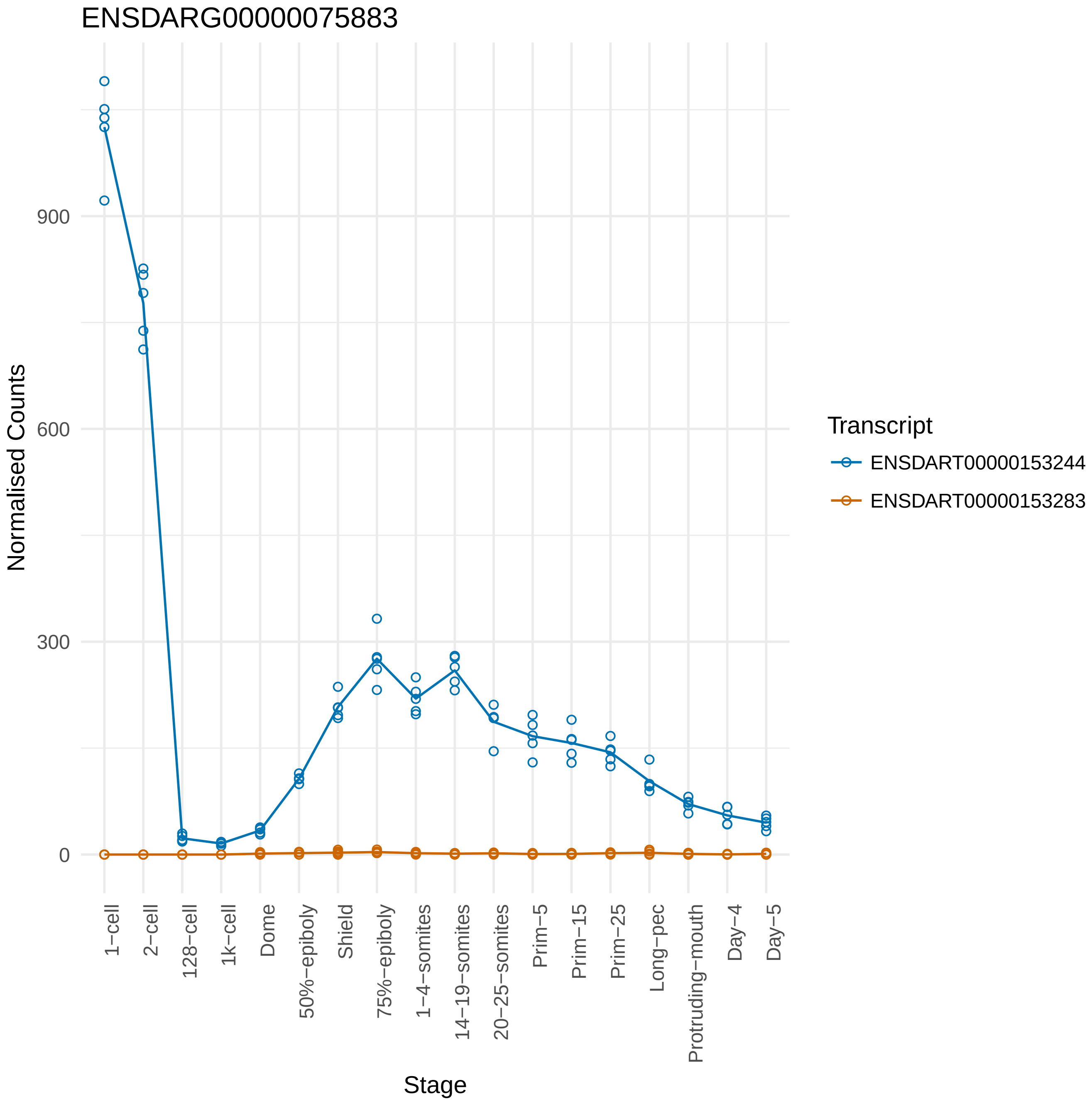

ENSDARG00000075883
Normalised Counts
0
300
600
900
1−cell
2−cell
128−cell
1k−cell
Dome
50%−epiboly
Shield
75%−epiboly
1−4−somites
14−19−somites
20−25−somites
Prim−5
Prim−15
Prim−25
Long−pec
Protruding−mouth
Day−4
Day−5
Stage
Transcript
ENSDART00000153244
ENSDART00000153283

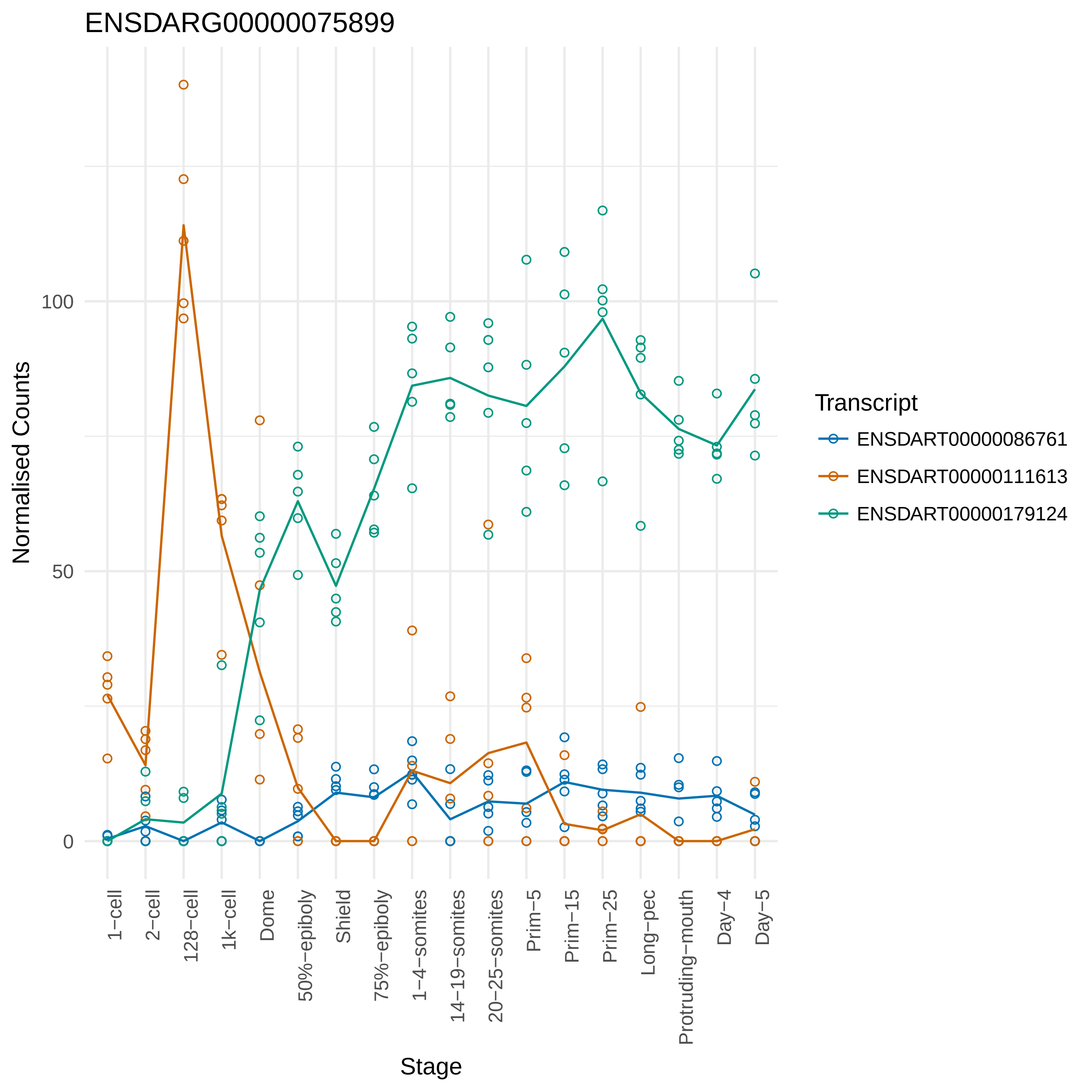
ENSDARG00000075899
Normalised Counts
100
50
0
1−cell
2−cell
128−cell
1k−cell
Dome
50%−epiboly
Shield
75%−epiboly
1−4−somites
14−19−somites
20−25−somites
Prim−5
Prim−15
Prim−25
Long−pec
Protruding−mouth
Day−4
Day−5
Stage
Transcript
ENSDART00000086761
ENSDART00000111613
ENSDART00000179124

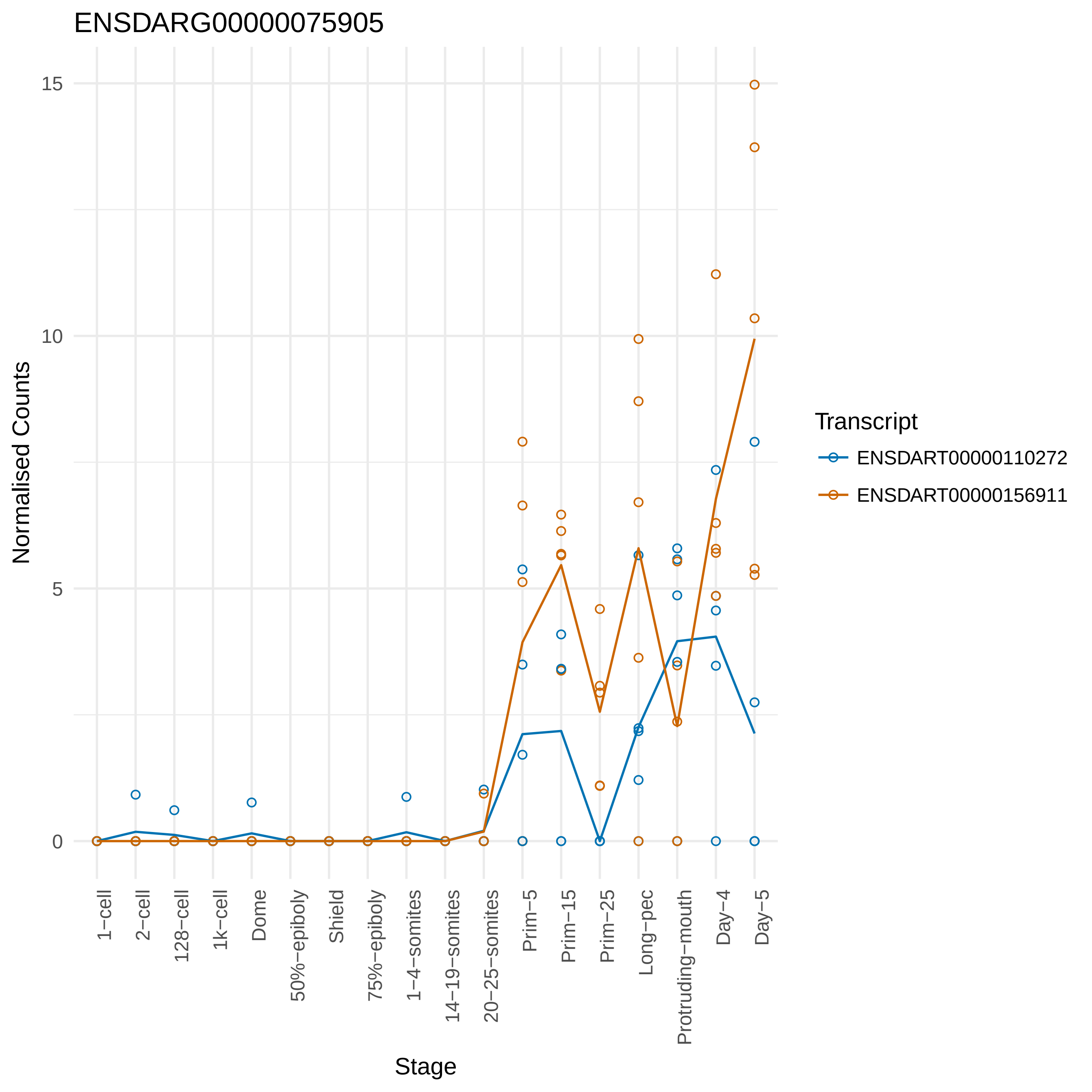

ENSDARG00000075905
Normalised Counts
15
10
5
0
1−cell
2−cell
128−cell
1k−cell
Dome
50%−epiboly
Shield
75%−epiboly
1−4−somites
14−19−somites
20−25−somites
Prim−5
Prim−15
Prim−25
Long−pec
Protruding−mouth
Day−4
Day−5
Stage
Transcript
ENSDART00000110272
ENSDART00000156911

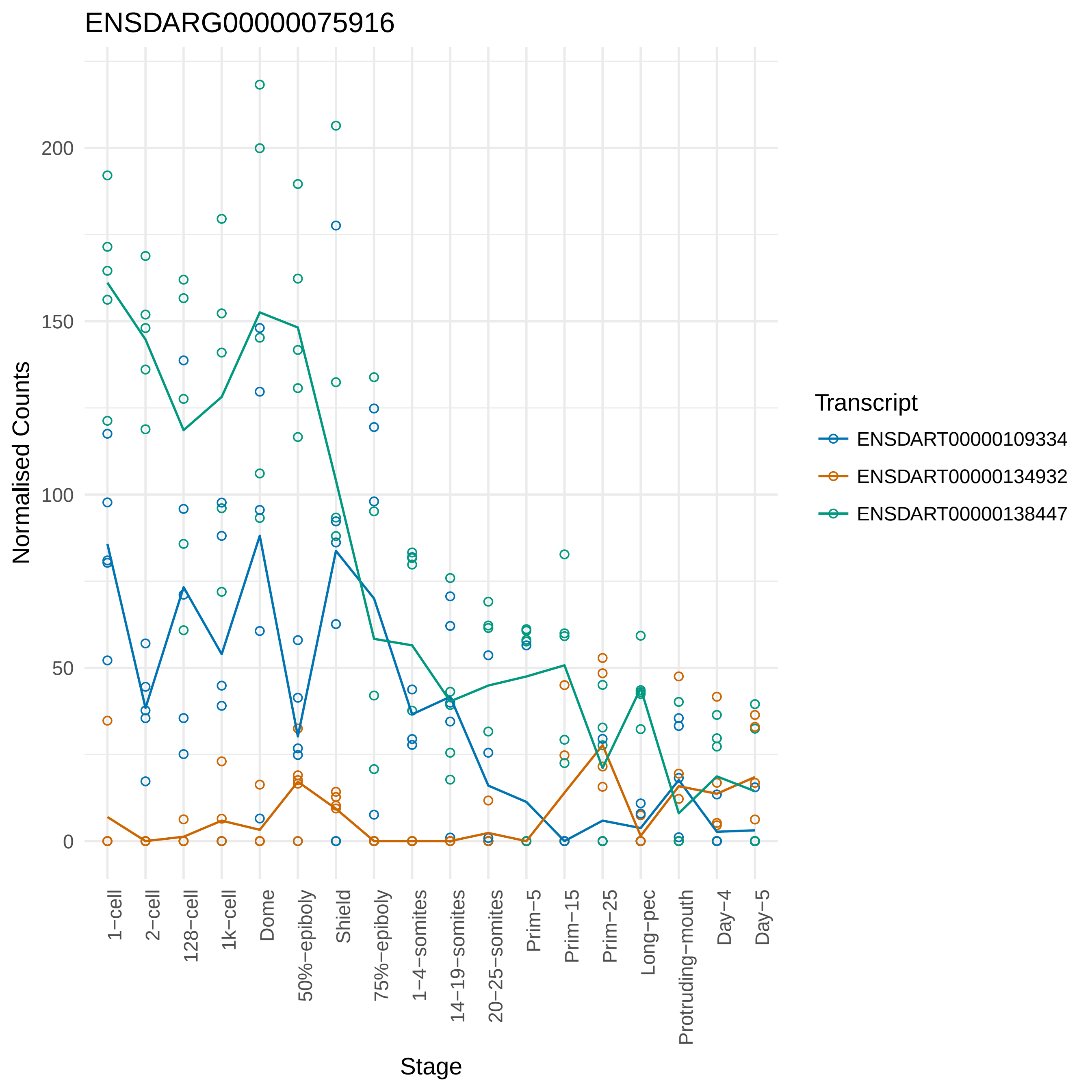
ENSDARG00000075916
Normalised Counts
200
150
100
50
0
1−cell
2−cell
128−cell
1k−cell
Dome
50%−epiboly
Shield
75%−epiboly
1−4−somites
14−19−somites
20−25−somites
Prim−5
Prim−15
Prim−25
Long−pec
Protruding−mouth
Day−4
Day−5
Stage
Transcript
ENSDART00000109334
ENSDART00000134932
ENSDART00000138447

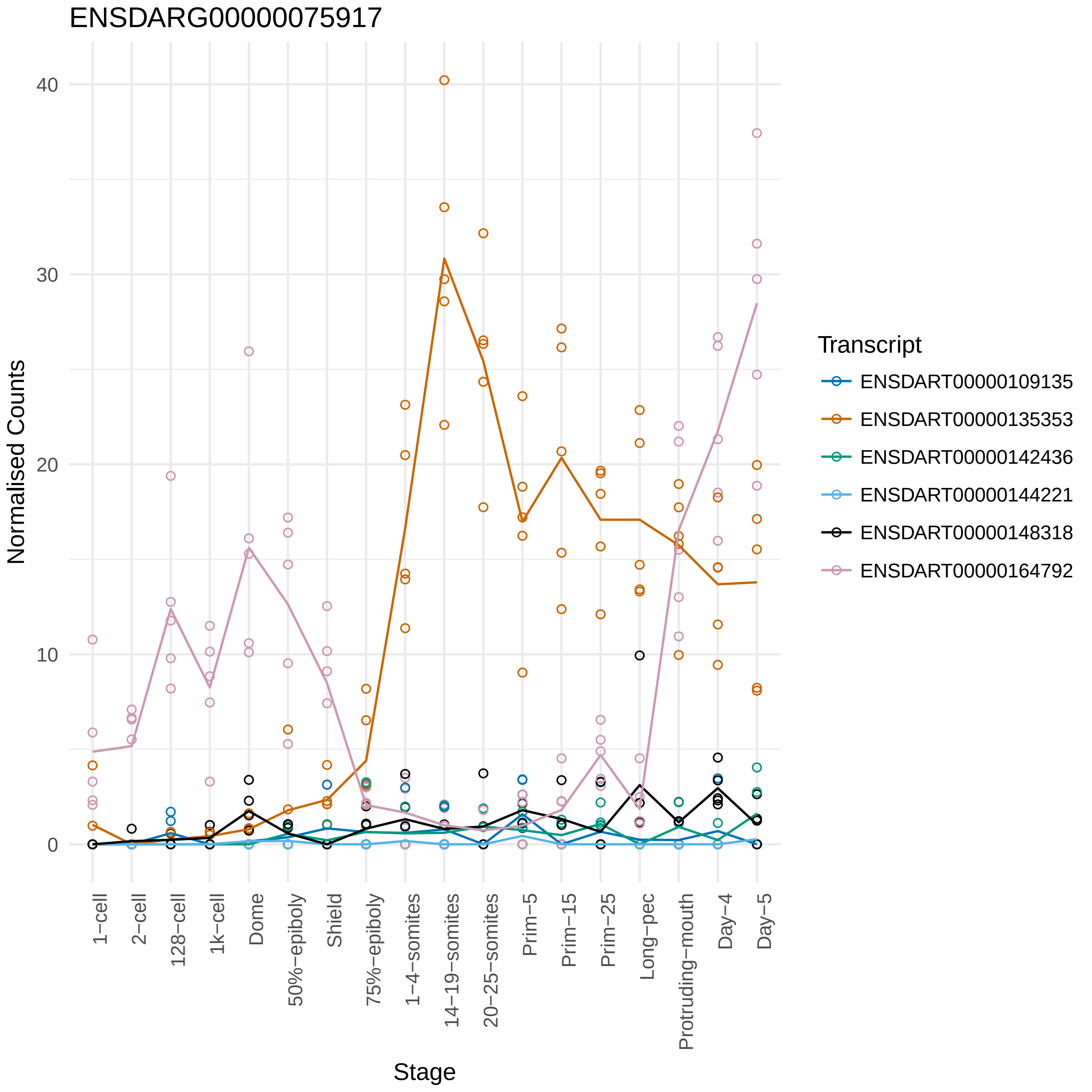

ENSDARG00000075917
Normalised Counts
40
30
20
10
0
1−cell
2−cell
128−cell
1k−cell
Dome
50%−epiboly
Shield
75%−epiboly
1−4−somites
14−19−somites
20−25−somites
Prim−5
Prim−15
Prim−25
Long−pec
Protruding−mouth
Day−4
Day−5
Stage
Transcript
ENSDART00000109135
ENSDART00000135353
ENSDART00000142436
ENSDART00000144221
ENSDART00000148318
ENSDART00000164792

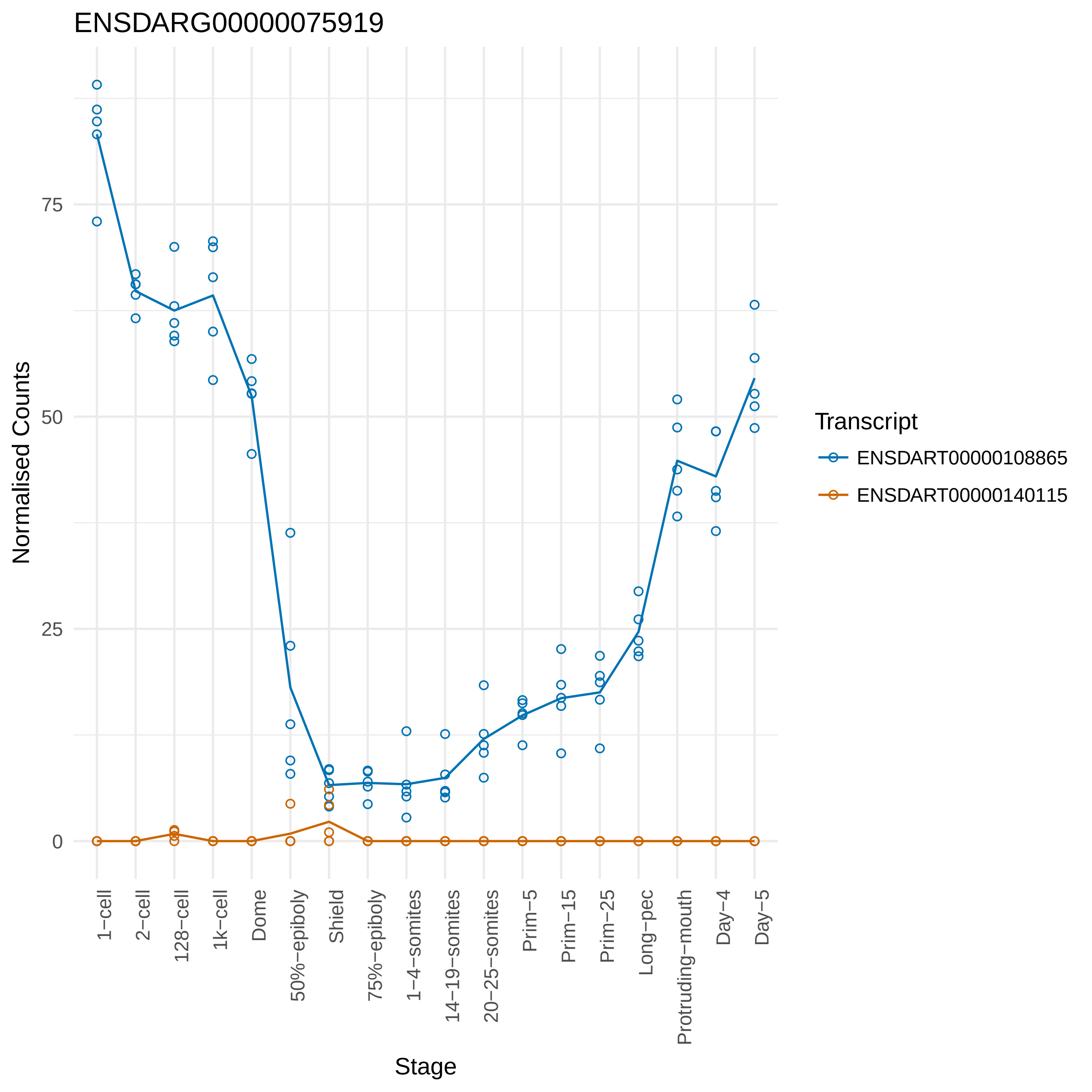

ENSDARG00000075919
Normalised Counts
75
50
25
0
1−cell
2−cell
128−cell
1k−cell
Dome
50%−epiboly
Shield
75%−epiboly
1−4−somites
14−19−somites
20−25−somites
Prim−5
Prim−15
Prim−25
Long−pec
Protruding−mouth
Day−4
Day−5
Stage
Transcript
ENSDART00000108865
ENSDART00000140115

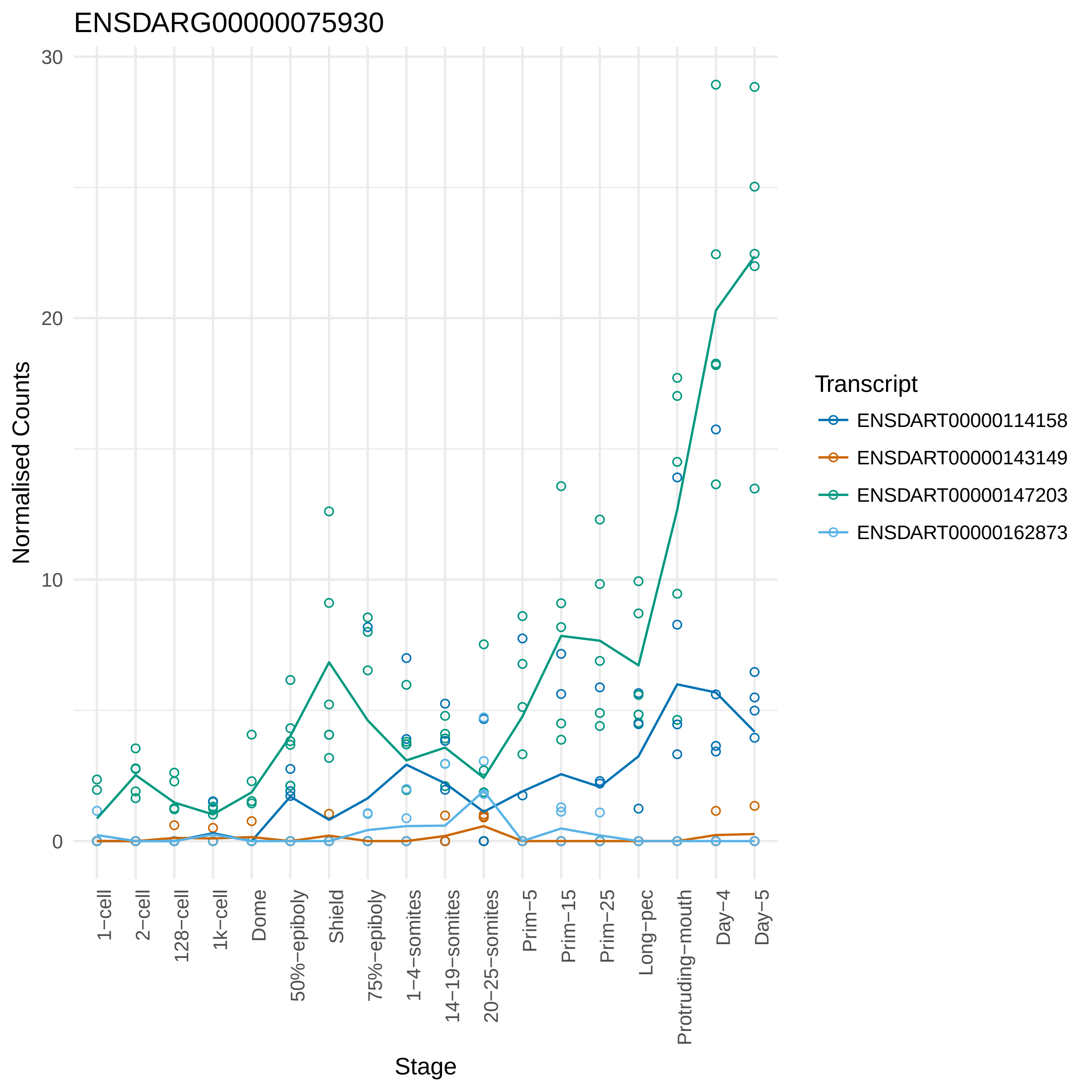

ENSDARG00000075930
Normalised Counts
30
20
10
0
1−cell
2−cell
128−cell
1k−cell
Dome
50%−epiboly
Shield
75%−epiboly
1−4−somites
14−19−somites
20−25−somites
Prim−5
Prim−15
Prim−25
Long−pec
Protruding−mouth
Day−4
Day−5
Stage
Transcript
ENSDART00000114158
ENSDART00000143149
ENSDART00000147203
ENSDART00000162873

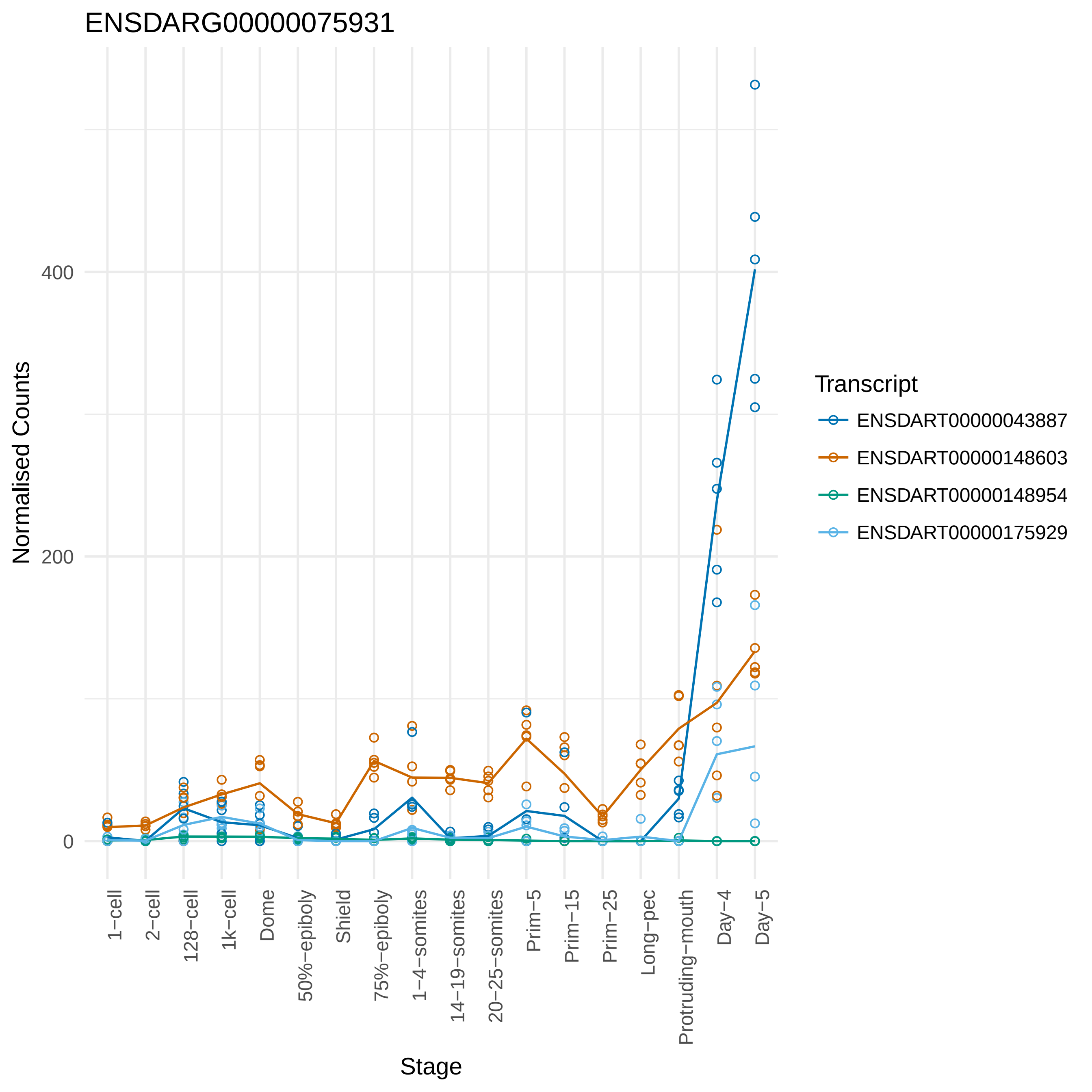
ENSDARG00000075931
Normalised Counts
400
200
0
1−cell
2−cell
128−cell
1k−cell
Dome
50%−epiboly
Shield
75%−epiboly
1−4−somites
14−19−somites
20−25−somites
Prim−5
Prim−15
Prim−25
Long−pec
Protruding−mouth
Day−4
Day−5
Stage
Transcript
ENSDART00000043887
ENSDART00000148603
ENSDART00000148954
ENSDART00000175929

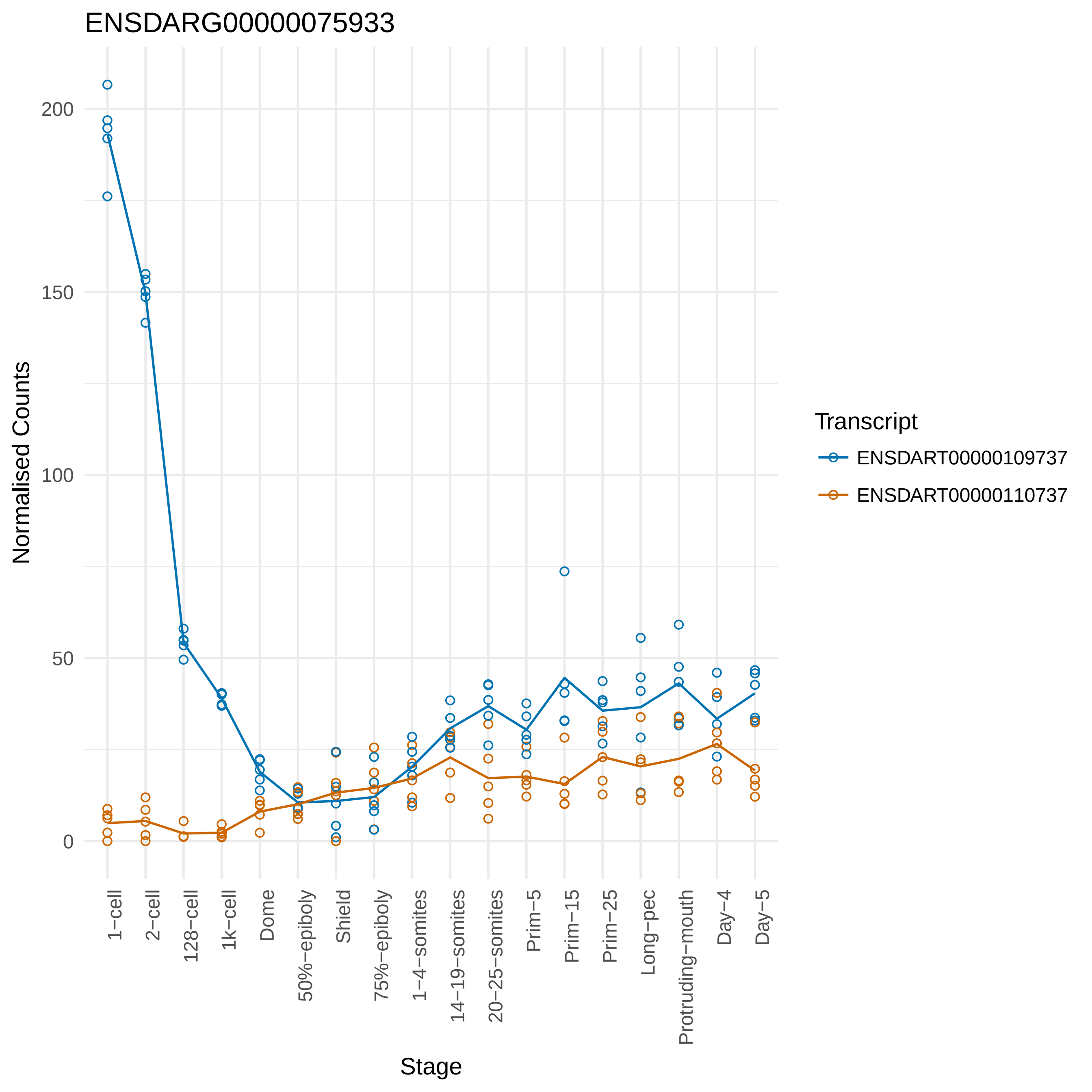
ENSDARG00000075933
Normalised Counts
0
50
100
150
200
1−cell
2−cell
128−cell
1k−cell
Dome
50%−epiboly
Shield
75%−epiboly
1−4−somites
14−19−somites
20−25−somites
Prim−5
Prim−15
Prim−25
Long−pec
Protruding−mouth
Day−4
Day−5
Stage
Transcript
ENSDART00000109737
ENSDART00000110737

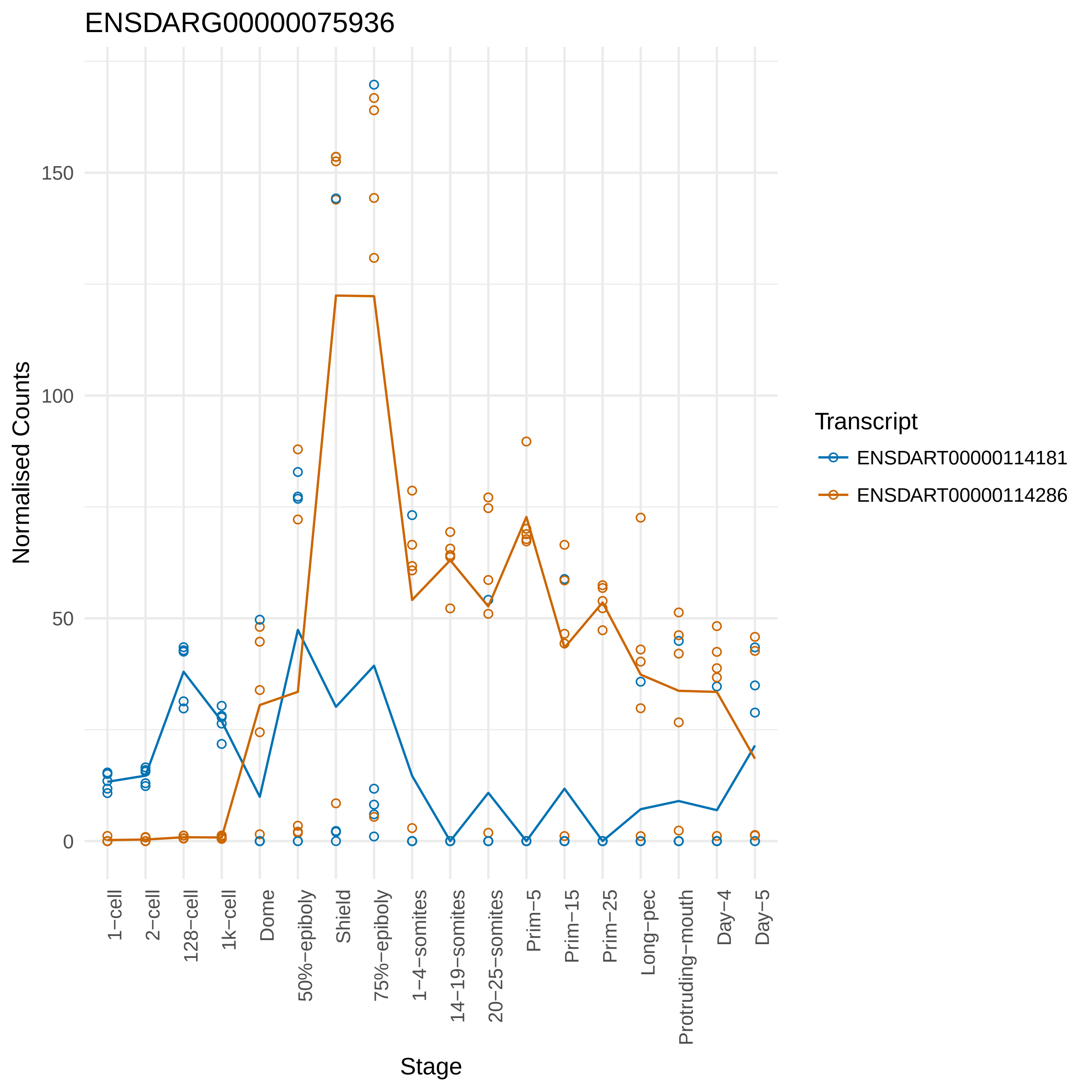
ENSDARG00000075936
Normalised Counts
150
100
50
0
1−cell
2−cell
128−cell
1k−cell
Dome
50%−epiboly
Shield
75%−epiboly
1−4−somites
14−19−somites
20−25−somites
Prim−5
Prim−15
Prim−25
Long−pec
Protruding−mouth
Day−4
Day−5
Stage
Transcript
ENSDART00000114181
ENSDART00000114286

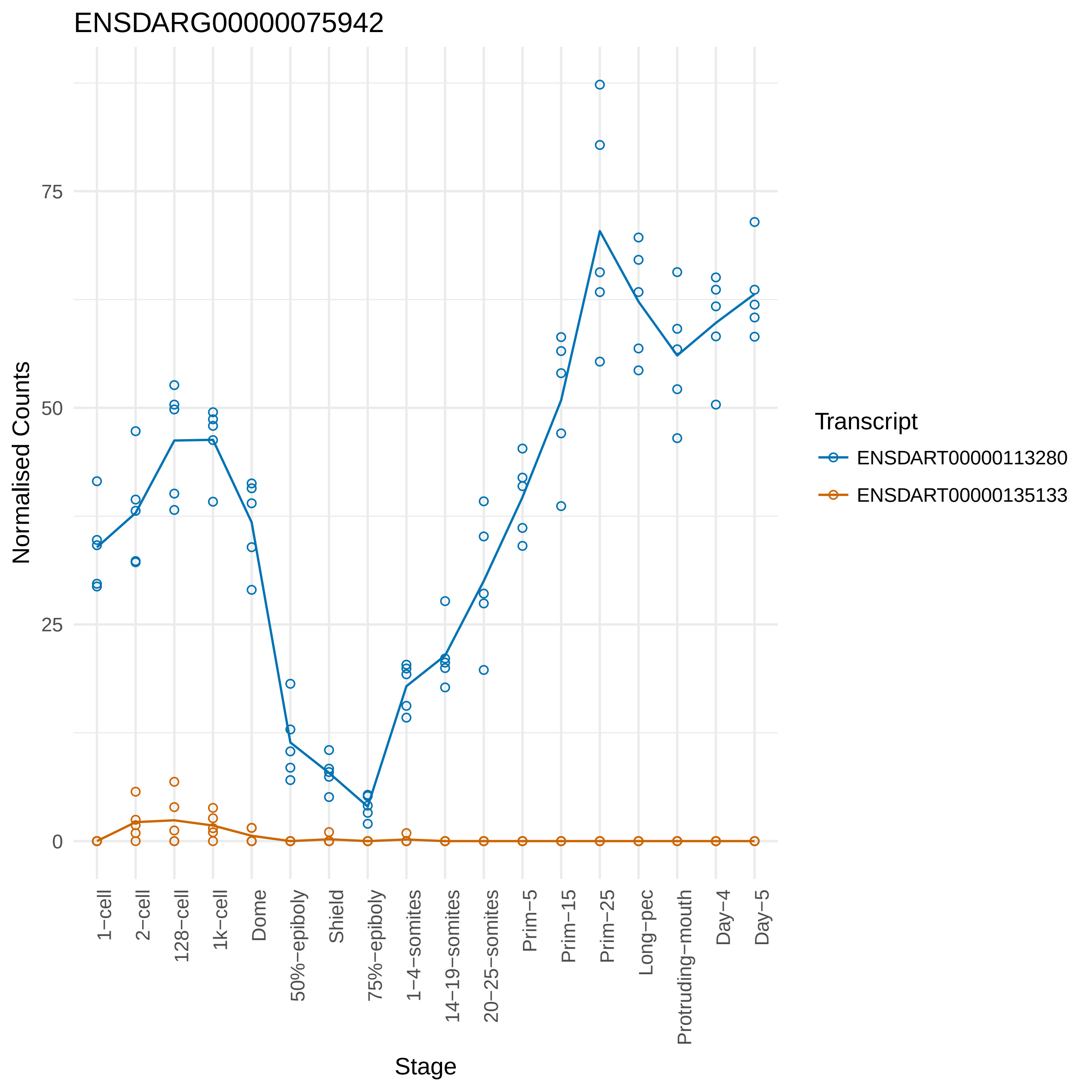

ENSDARG00000075942
Normalised Counts
0
25
50
75
1−cell
2−cell
128−cell
1k−cell
Dome
50%−epiboly
Shield
75%−epiboly
1−4−somites
14−19−somites
20−25−somites
Prim−5
Prim−15
Prim−25
Long−pec
Protruding−mouth
Day−4
Day−5
Stage
Transcript
ENSDART00000113280
ENSDART00000135133

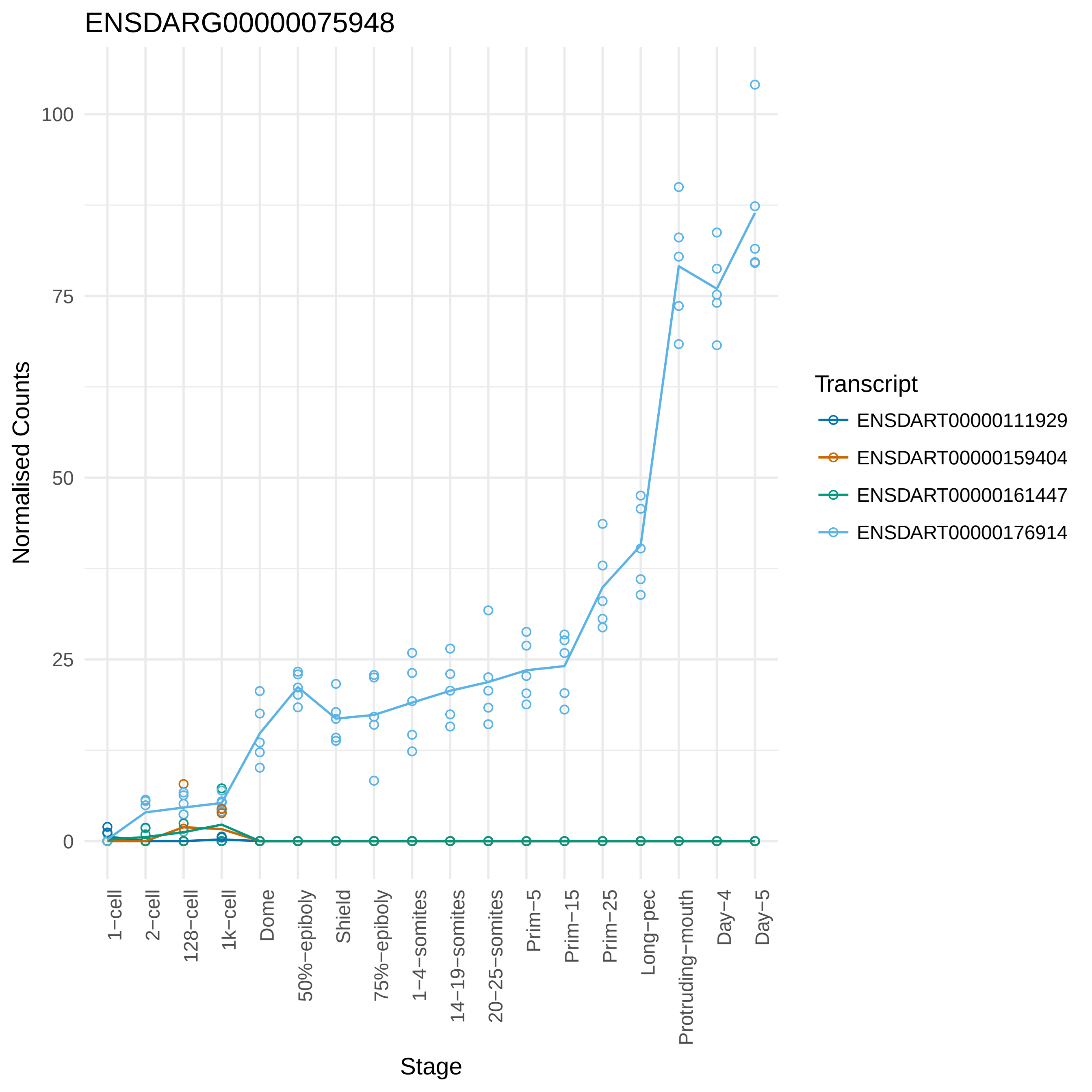

ENSDARG00000075948
Normalised Counts
100
75
50
25
0
1−cell
2−cell
128−cell
1k−cell
Dome
50%−epiboly
Shield
75%−epiboly
1−4−somites
14−19−somites
20−25−somites
Prim−5
Prim−15
Prim−25
Long−pec
Protruding−mouth
Day−4
Day−5
Stage
Transcript
ENSDART00000111929
ENSDART00000159404
ENSDART00000161447
ENSDART00000176914

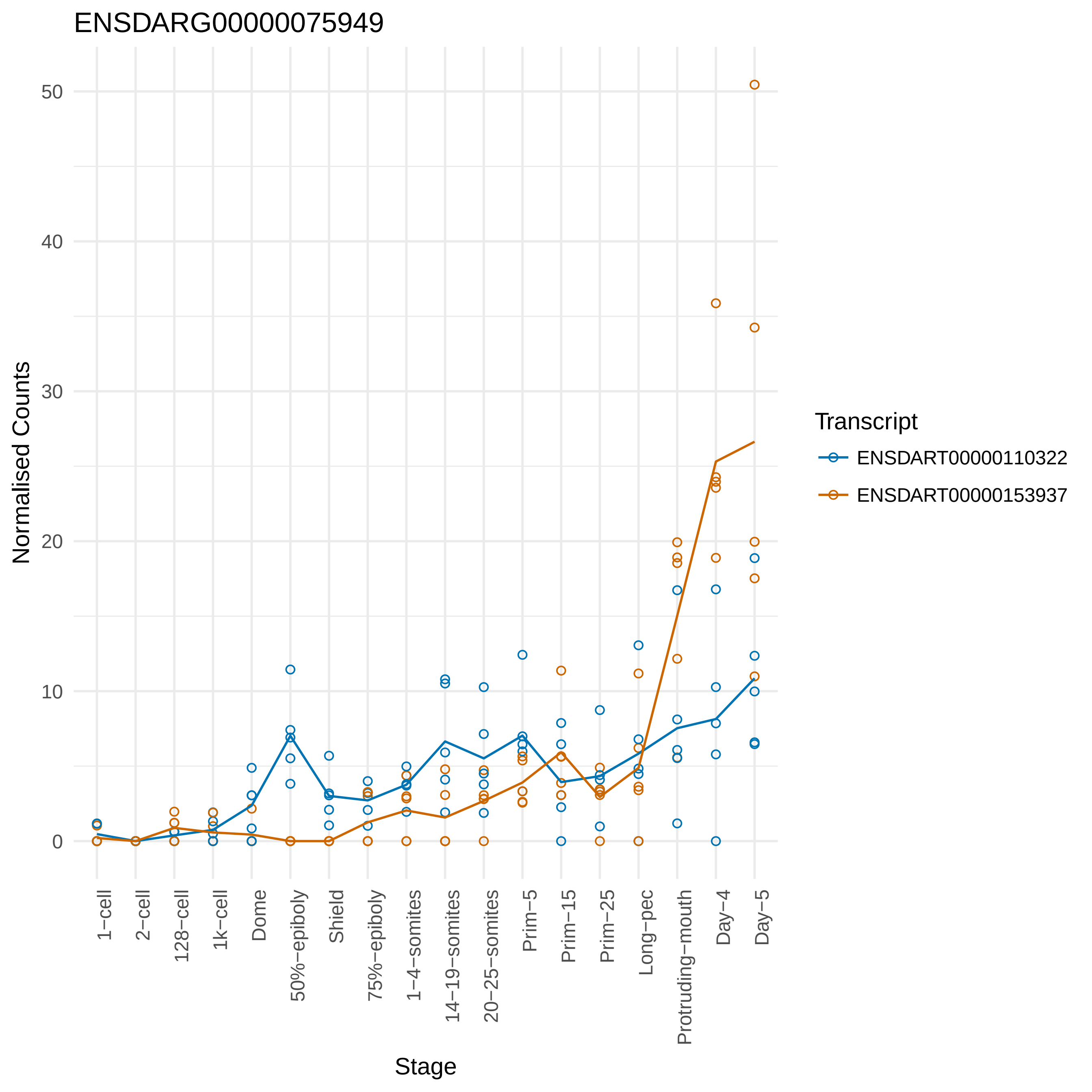

ENSDARG00000075949
Normalised Counts
0
10
20
30
40
50
1−cell
2−cell
128−cell
1k−cell
Dome
50%−epiboly
Shield
75%−epiboly
1−4−somites
14−19−somites
20−25−somites
Prim−5
Prim−15
Prim−25
Long−pec
Protruding−mouth
Day−4
Day−5
Stage
Transcript
ENSDART00000110322
ENSDART00000153937

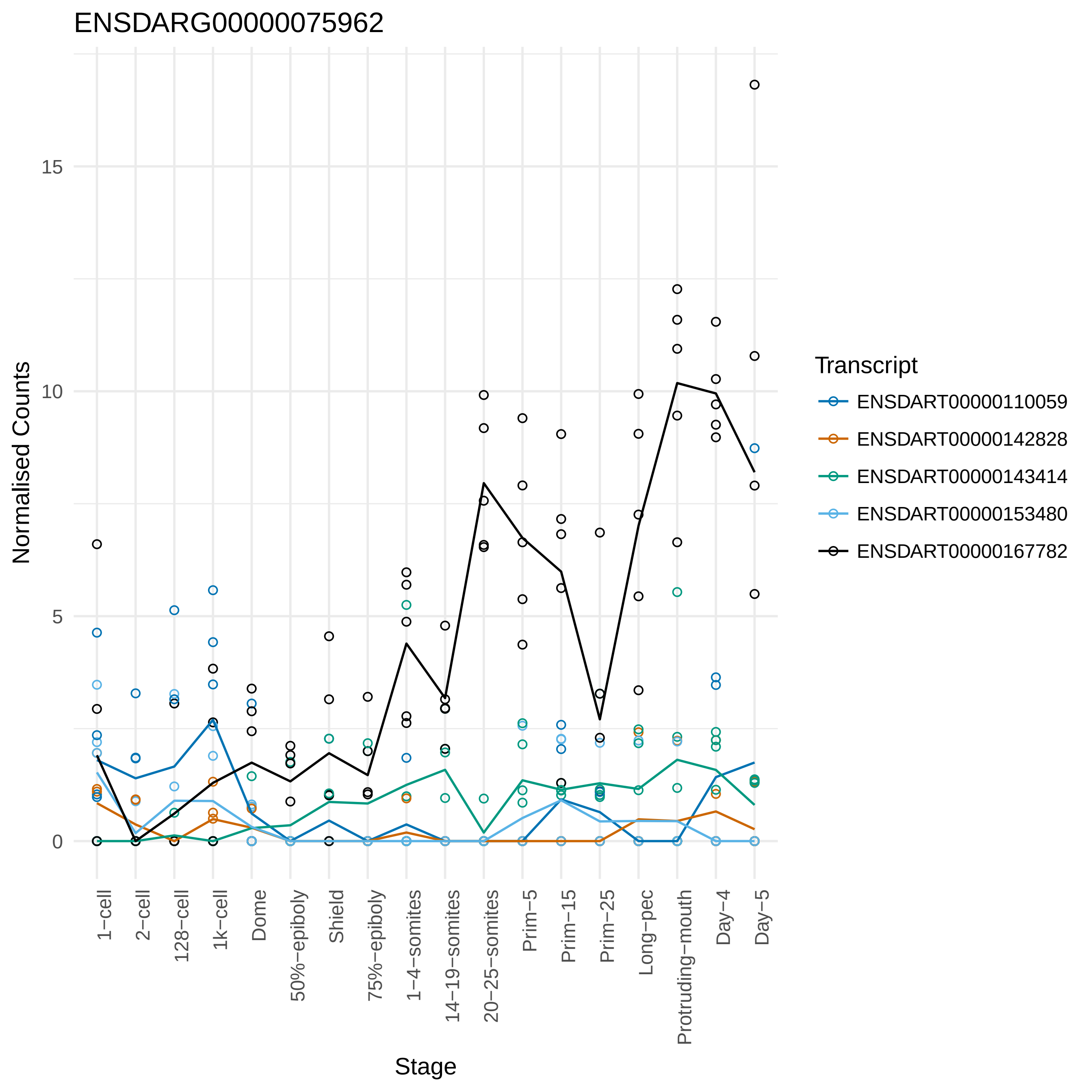
ENSDARG00000075962
Normalised Counts
0
5
10
15
Stage
1−cell
2−cell
128−cell
1k−cell
Dome
50%−epiboly
Shield
75%−epiboly
1−4−somites
14−19−somites
20−25−somites
Prim−5
Prim−15
Prim−25
Long−pec
Protruding−mouth
Day−4
Day−5
Transcript
ENSDART00000110059
ENSDART00000142828
ENSDART00000143414
ENSDART00000153480
ENSDART00000167782

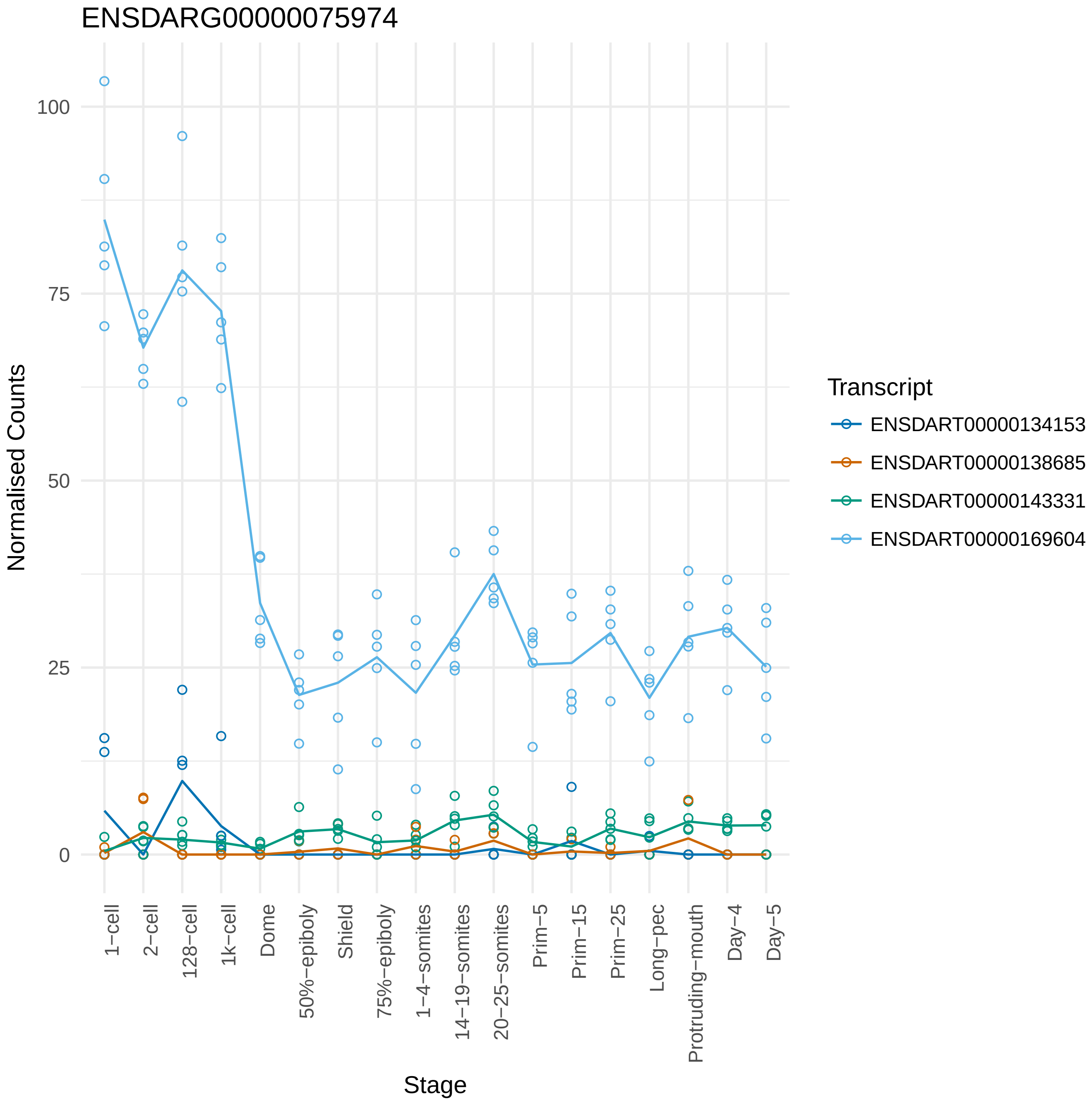
ENSDARG00000075974
Normalised Counts
100
75
50
25
0
Stage
1−cell
2−cell
128−cell
1k−cell
Dome
50%−epiboly
Shield
75%−epiboly
1−4−somites
14−19−somites
20−25−somites
Prim−5
Prim−15
Prim−25
Long−pec
Protruding−mouth
Day−4
Day−5
Transcript
ENSDART00000134153
ENSDART00000138685
ENSDART00000143331
ENSDART00000169604

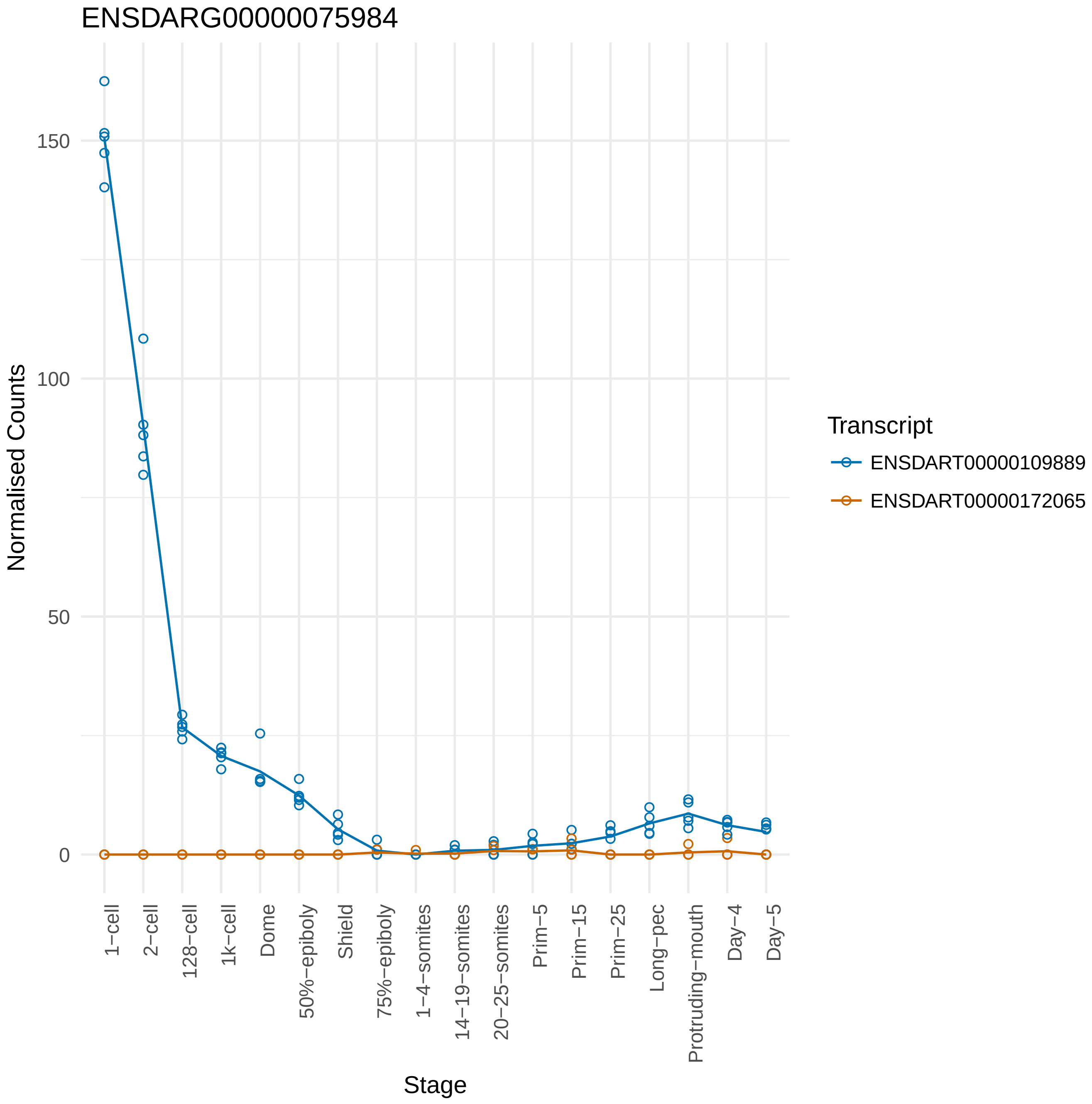

ENSDARG00000075984
Normalised Counts
150
100
50
0
1−cell
2−cell
128−cell
1k−cell
Dome
50%−epiboly
Shield
75%−epiboly
1−4−somites
14−19−somites
20−25−somites
Prim−5
Prim−15
Prim−25
Long−pec
Protruding−mouth
Day−4
Day−5
Stage
Transcript
ENSDART00000109889
ENSDART00000172065

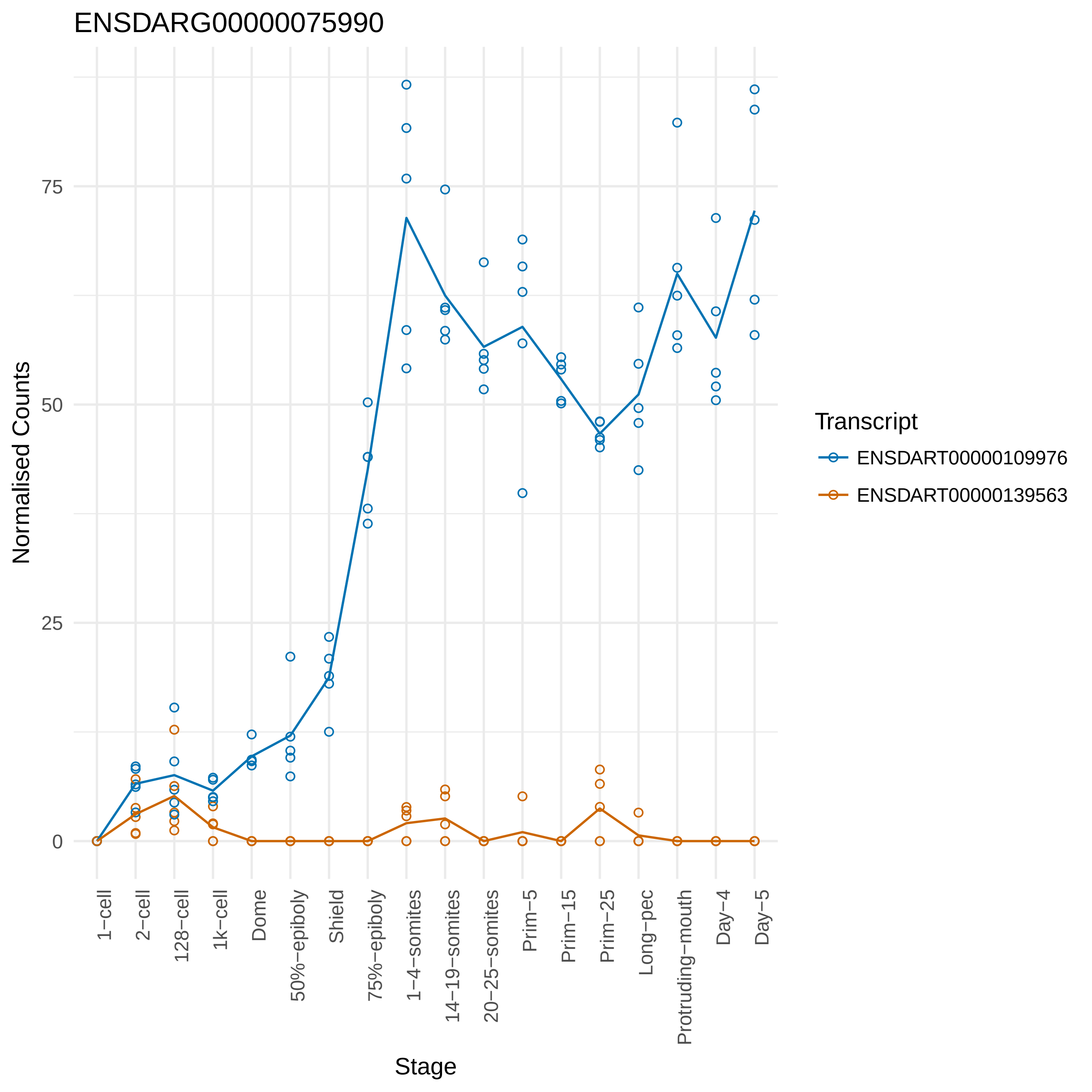
ENSDARG00000075990
Normalised Counts
0
25
50
75
Stage
1−cell
2−cell
128−cell
1k−cell
Dome
50%−epiboly
Shield
75%−epiboly
1−4−somites
14−19−somites
20−25−somites
Prim−5
Prim−15
Prim−25
Long−pec
Protruding−mouth
Day−4
Day−5
Transcript
ENSDART00000109976
ENSDART00000139563

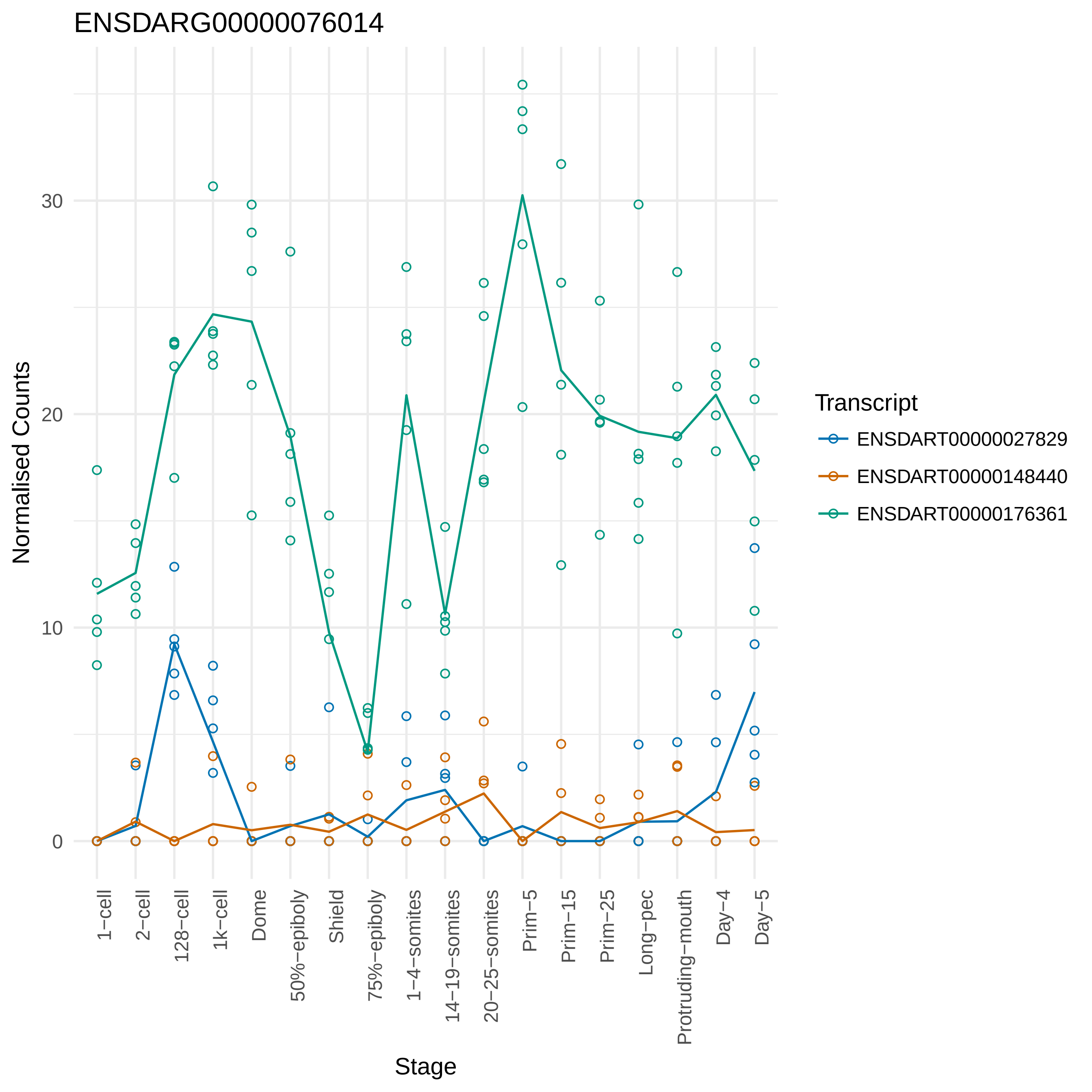

ENSDARG00000076014
Normalised Counts
30
20
10
0
1−cell
2−cell
128−cell
1k−cell
Dome
50%−epiboly
Shield
75%−epiboly
1−4−somites
14−19−somites
20−25−somites
Prim−5
Prim−15
Prim−25
Long−pec
Protruding−mouth
Day−4
Day−5
Stage
Transcript
ENSDART00000027829
ENSDART00000148440
ENSDART00000176361

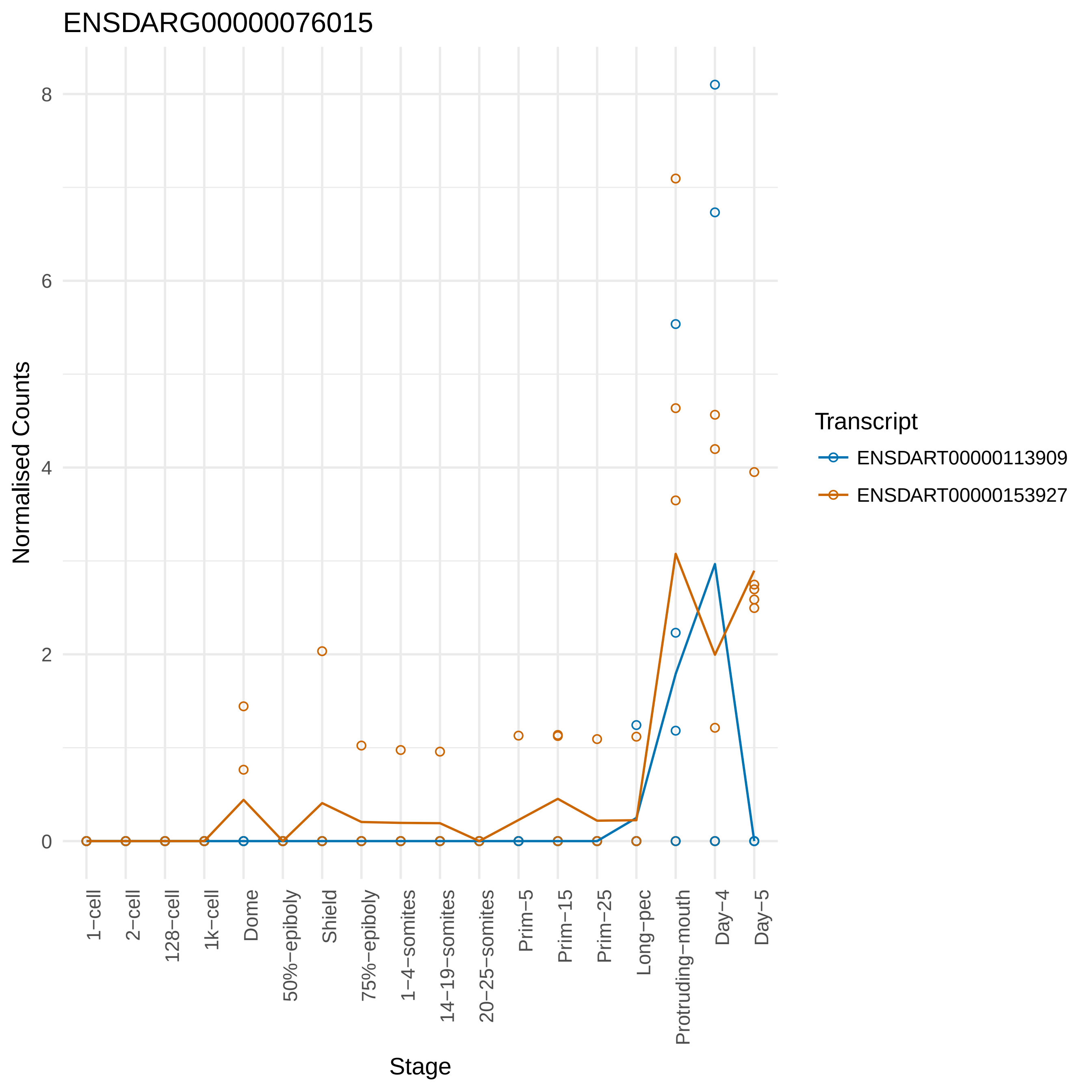
ENSDARG00000076015
Normalised Counts
0
2
4
6
8
1−cell
2−cell
128−cell
1k−cell
Dome
50%−epiboly
Shield
75%−epiboly
1−4−somites
14−19−somites
20−25−somites
Prim−5
Prim−15
Prim−25
Long−pec
Protruding−mouth
Day−4
Day−5
Stage
Transcript
ENSDART00000113909
ENSDART00000153927

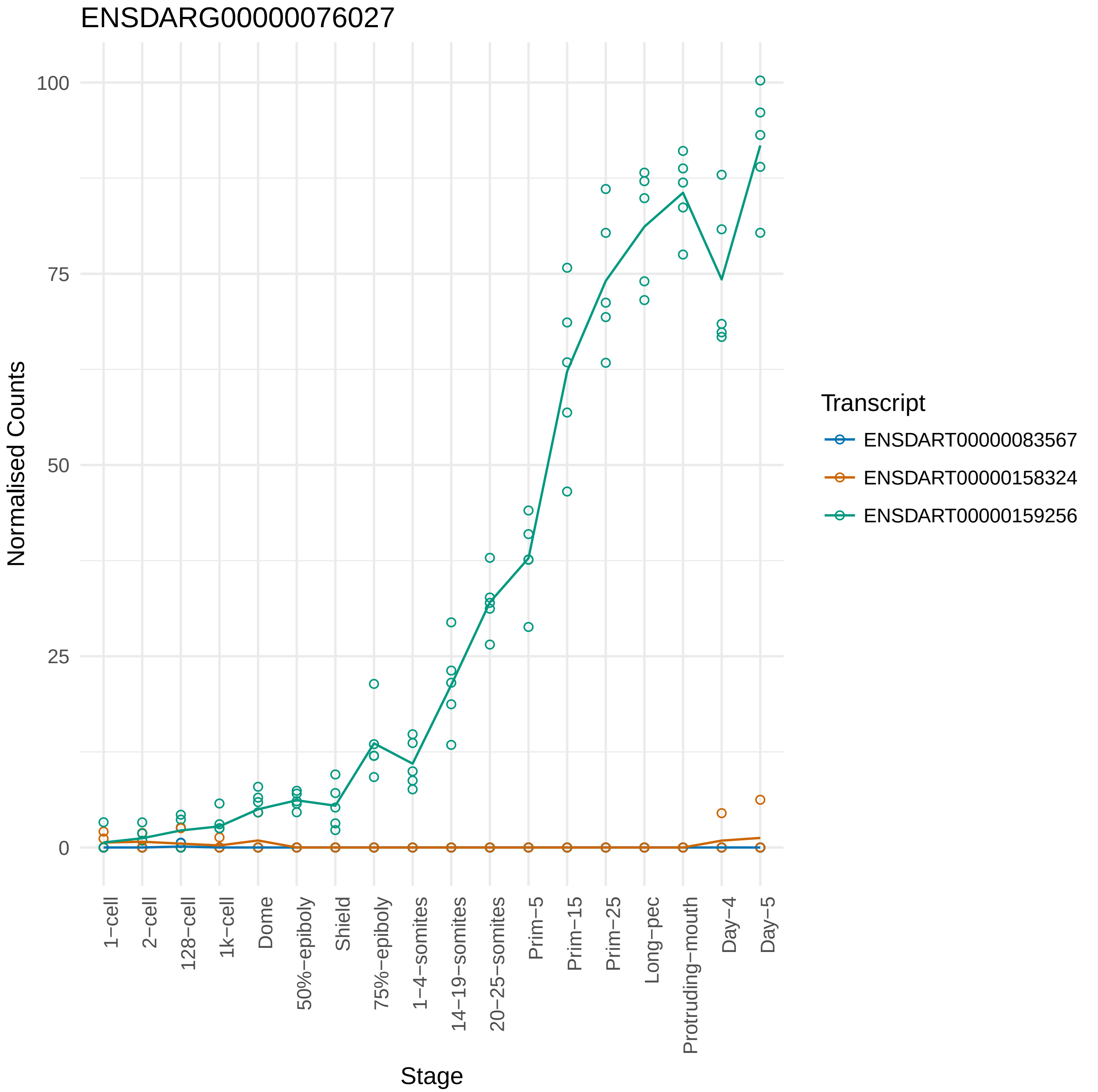

ENSDARG00000076027
Normalised Counts
100
75
50
25
0
1−cell
2−cell
128−cell
1k−cell
Dome
50%−epiboly
Shield
75%−epiboly
1−4−somites
14−19−somites
20−25−somites
Prim−5
Prim−15
Prim−25
Long−pec
Protruding−mouth
Day−4
Day−5
Stage
Transcript
ENSDART00000083567
ENSDART00000158324
ENSDART00000159256

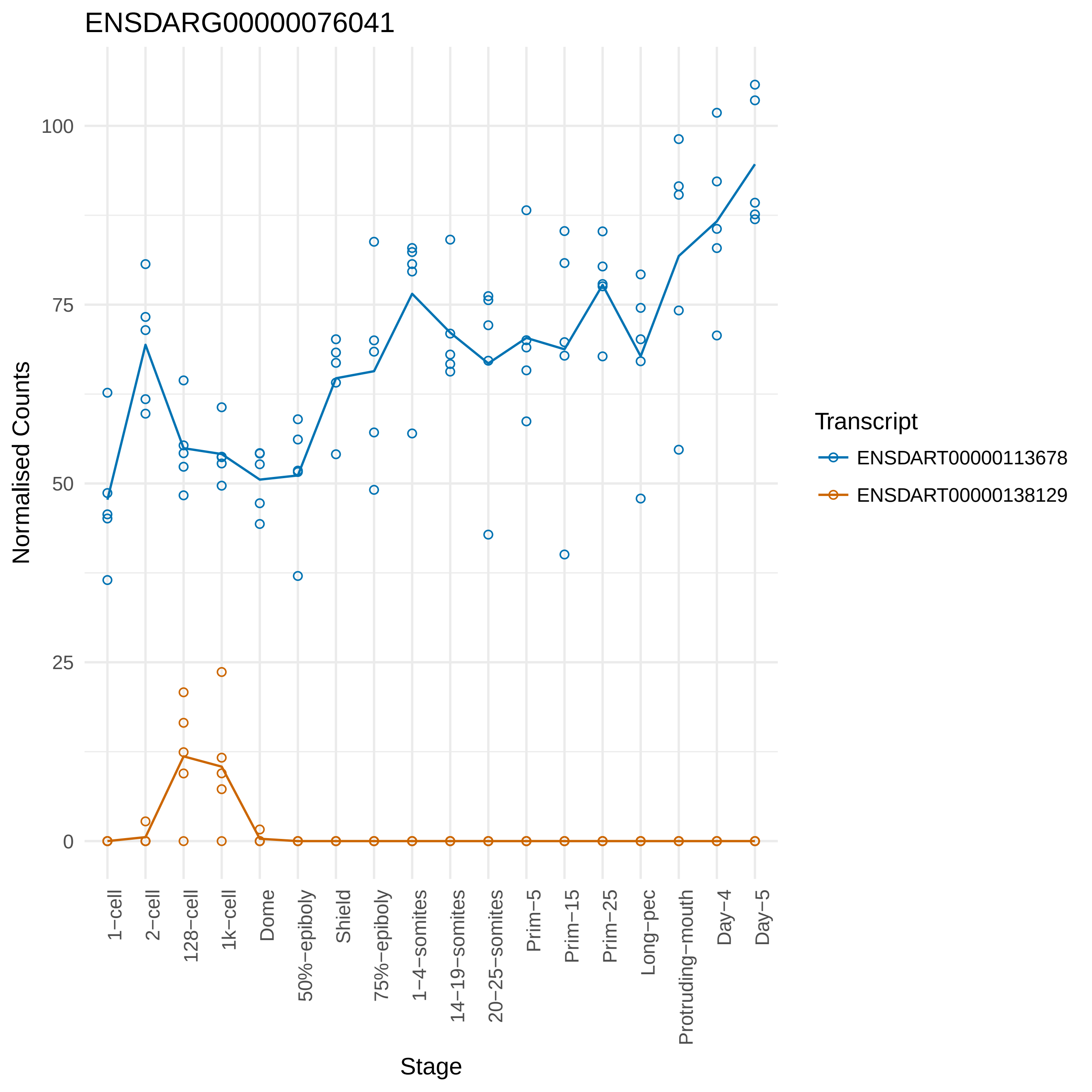
ENSDARG00000076041
Normalised Counts
100
75
50
25
0
1−cell
2−cell
128−cell
1k−cell
Dome
50%−epiboly
Shield
75%−epiboly
1−4−somites
14−19−somites
20−25−somites
Prim−5
Prim−15
Prim−25
Long−pec
Protruding−mouth
Day−4
Day−5
Stage
Transcript
ENSDART00000113678
ENSDART00000138129

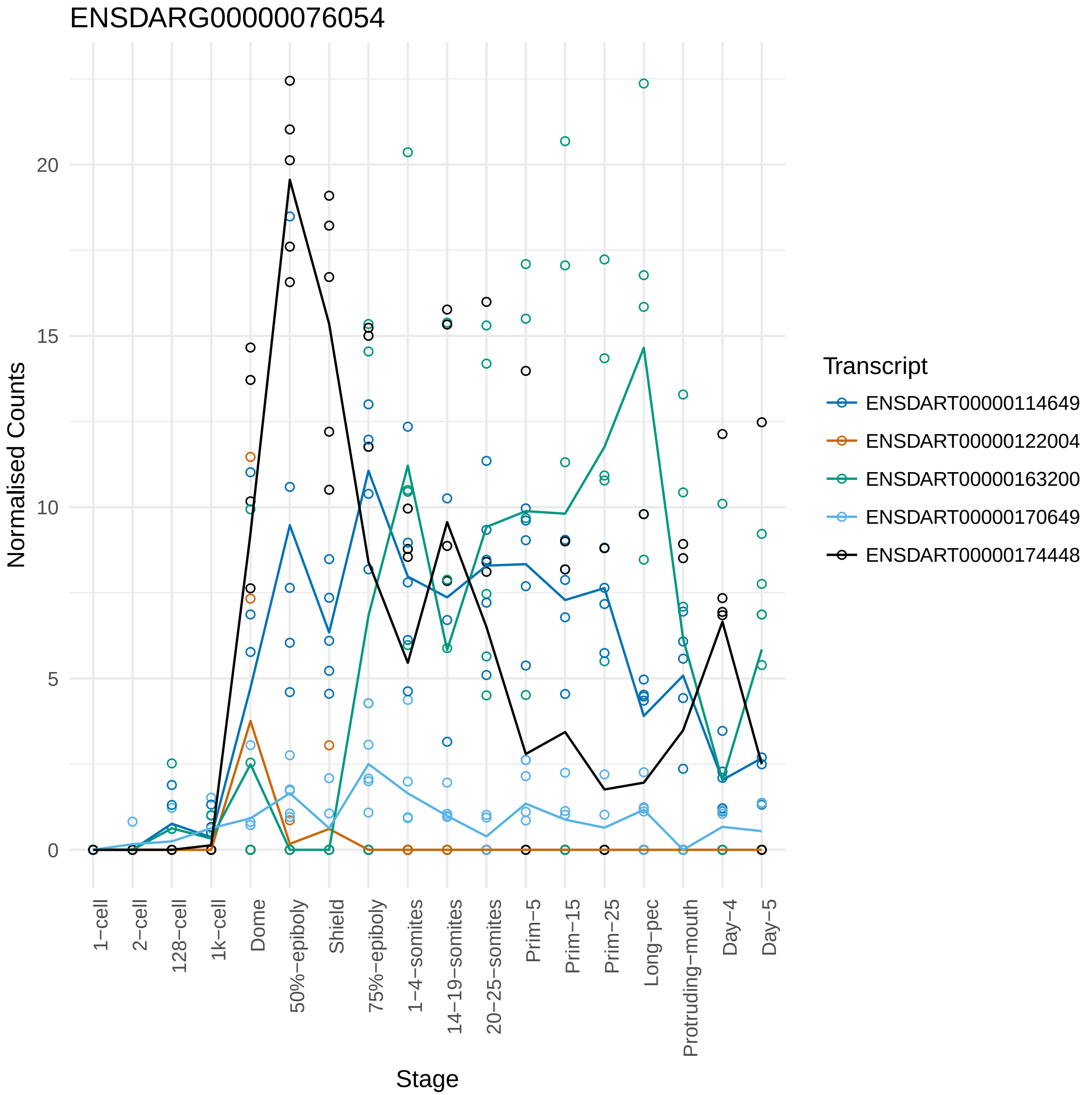
ENSDARG00000076054
Normalised Counts
20
15
10
5
0
Stage
1−cell
2−cell
128−cell
1k−cell
Dome
50%−epiboly
Shield
75%−epiboly
1−4−somites
14−19−somites
20−25−somites
Prim−5
Prim−15
Prim−25
Long−pec
Protruding−mouth
Day−4
Day−5
Transcript
ENSDART00000114649
ENSDART00000122004
ENSDART00000163200
ENSDART00000170649
ENSDART00000174448

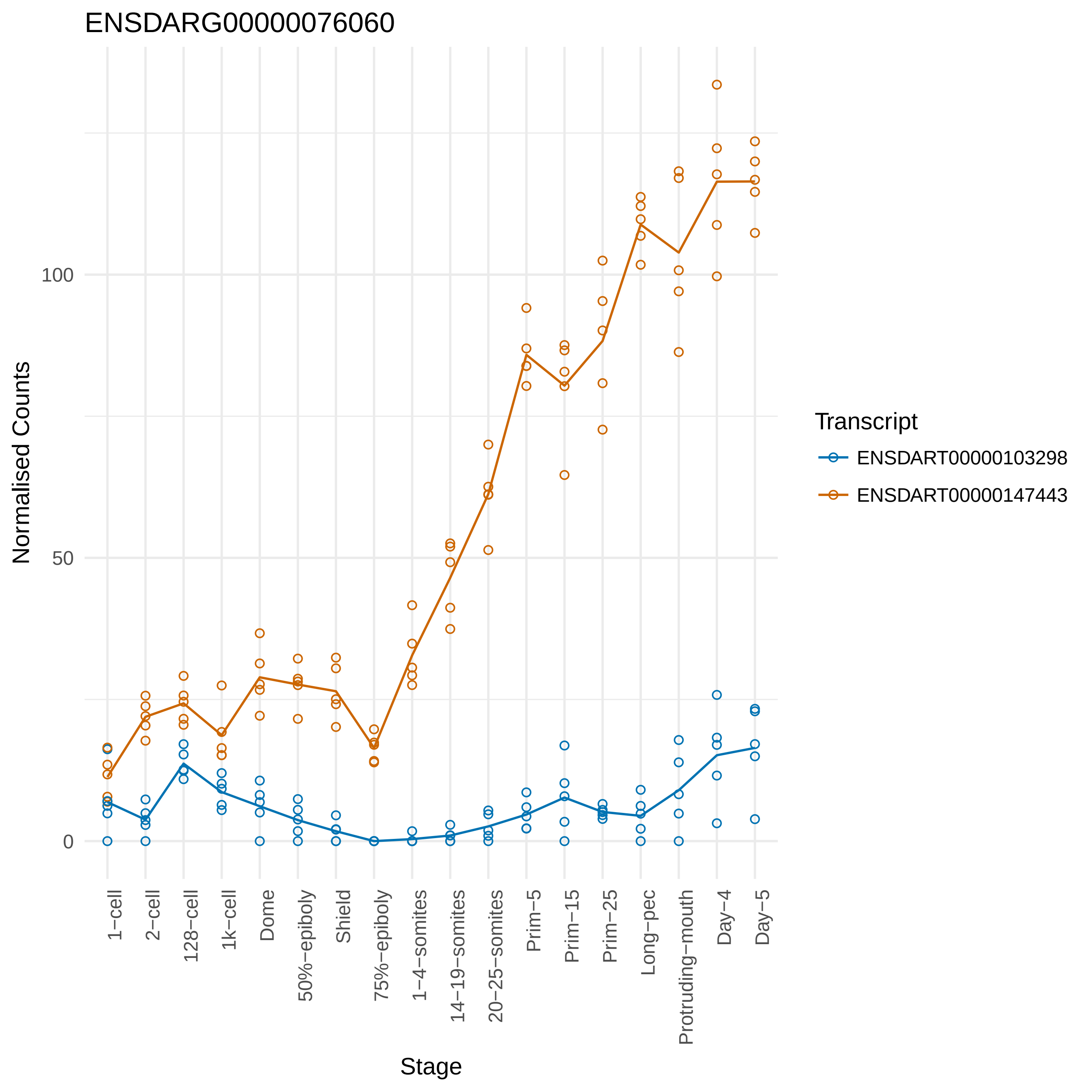

ENSDARG00000076060
Normalised Counts
100
50
0
1−cell
2−cell
128−cell
1k−cell
Dome
50%−epiboly
Shield
75%−epiboly
1−4−somites
14−19−somites
20−25−somites
Prim−5
Prim−15
Prim−25
Long−pec
Protruding−mouth
Day−4
Day−5
Stage
Transcript
ENSDART00000103298
ENSDART00000147443

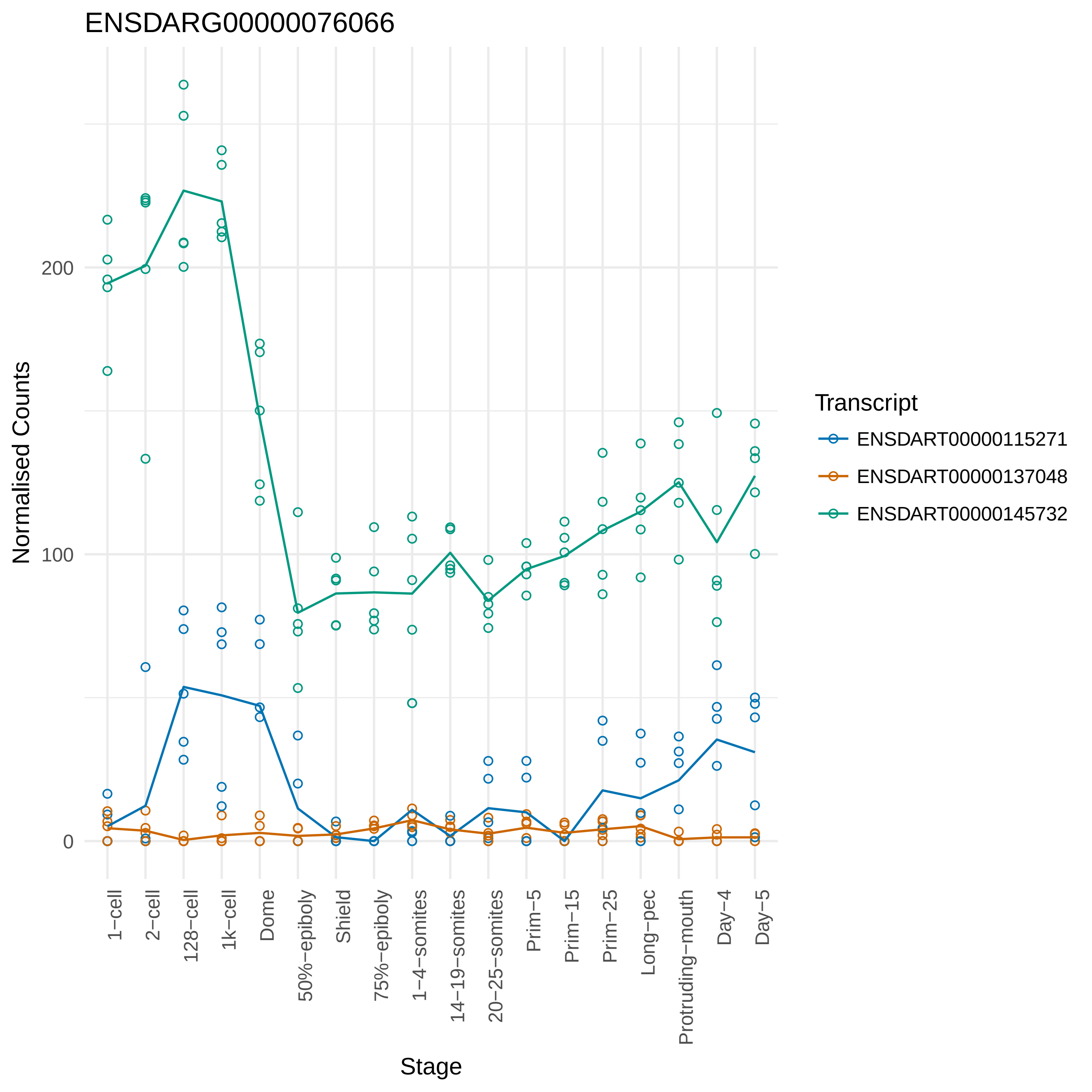

ENSDARG00000076066
Normalised Counts
0
100
200
Stage
1−cell
2−cell
128−cell
1k−cell
Dome
50%−epiboly
Shield
75%−epiboly
1−4−somites
14−19−somites
20−25−somites
Prim−5
Prim−15
Prim−25
Long−pec
Protruding−mouth
Day−4
Day−5
Transcript
ENSDART00000115271
ENSDART00000137048
ENSDART00000145732

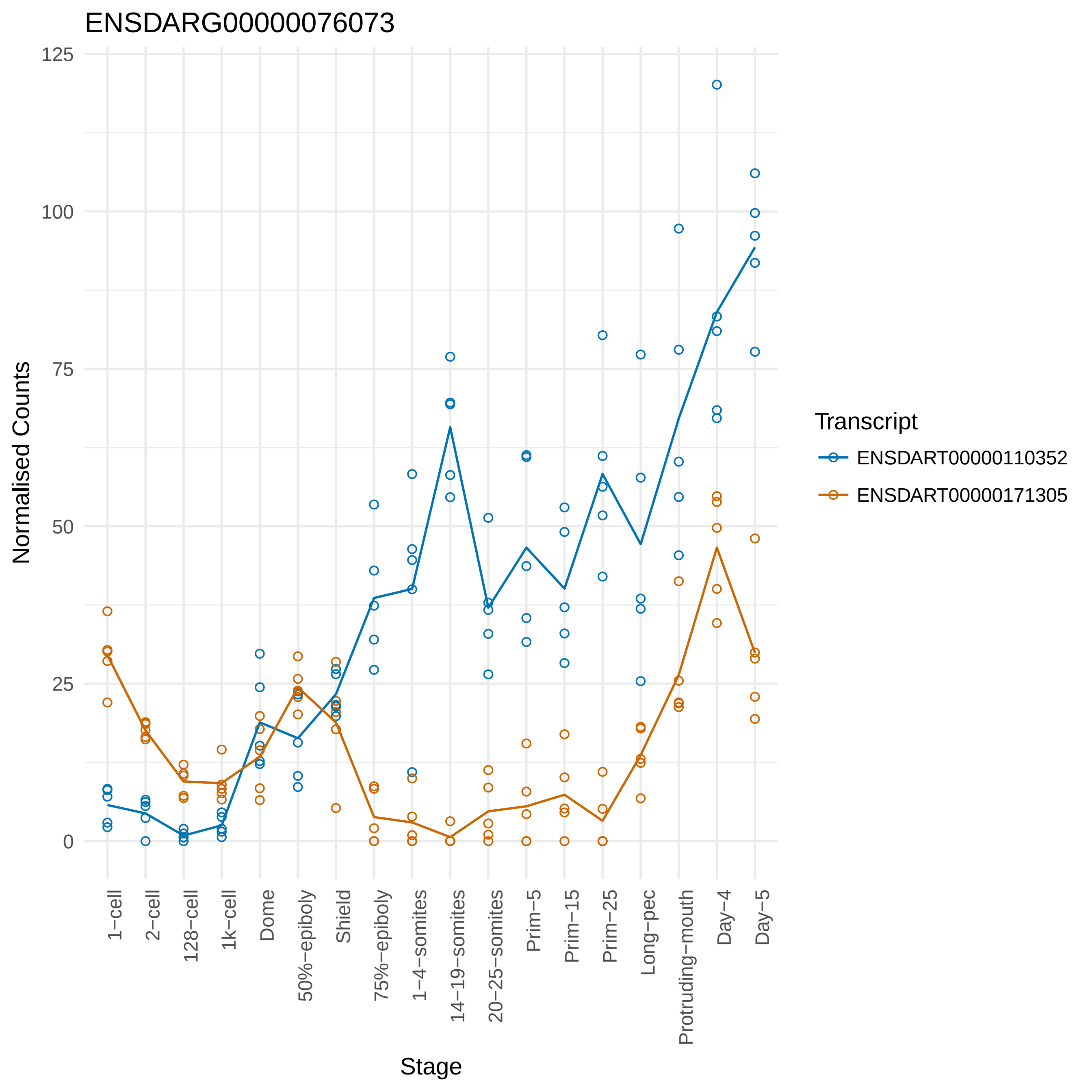
ENSDARG00000076073
Normalised Counts
125
100
75
50
25
0
1−cell
2−cell
128−cell
1k−cell
Dome
50%−epiboly
Shield
75%−epiboly
1−4−somites
14−19−somites
20−25−somites
Prim−5
Prim−15
Prim−25
Long−pec
Protruding−mouth
Day−4
Day−5
Stage
Transcript
ENSDART00000110352
ENSDART00000171305

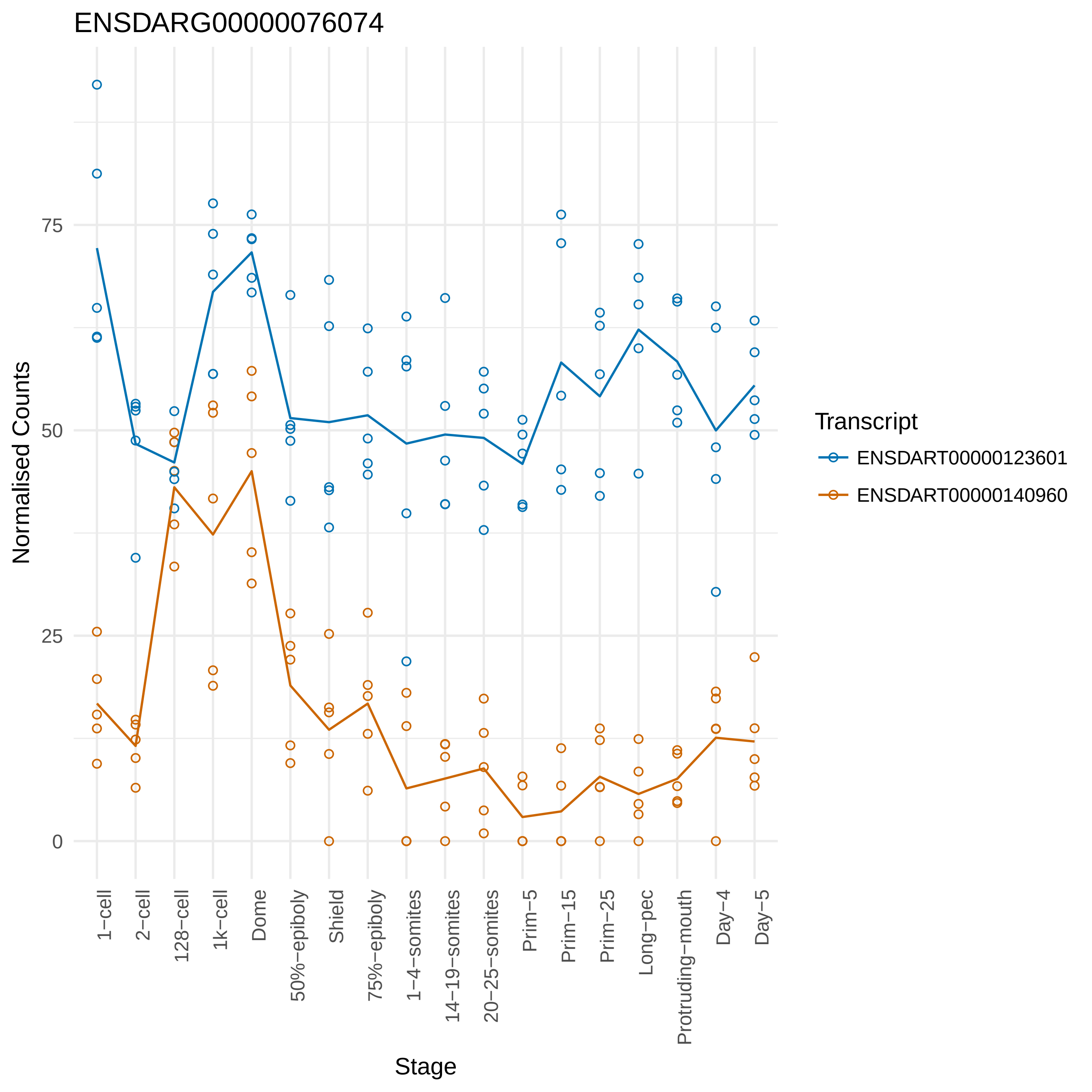
ENSDARG00000076074
Normalised Counts
75
50
25
0
1−cell
2−cell
128−cell
1k−cell
Dome
50%−epiboly
Shield
75%−epiboly
1−4−somites
14−19−somites
20−25−somites
Prim−5
Prim−15
Prim−25
Long−pec
Protruding−mouth
Day−4
Day−5
Stage
Transcript
ENSDART00000123601
ENSDART00000140960

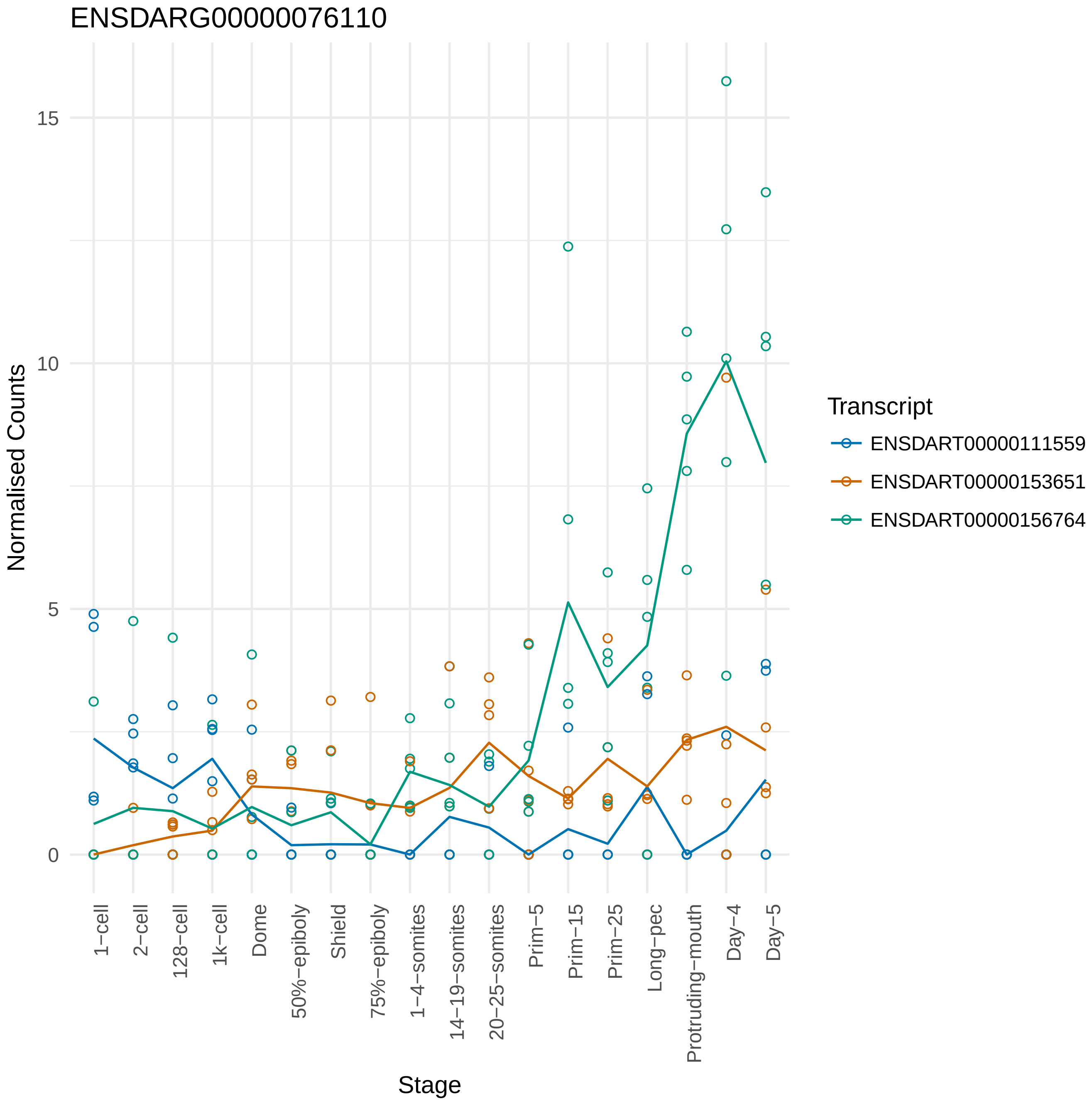
ENSDARG00000076110
Normalised Counts
15
10
5
0
1−cell
2−cell
128−cell
1k−cell
Dome
50%−epiboly
Shield
75%−epiboly
1−4−somites
14−19−somites
20−25−somites
Prim−5
Prim−15
Prim−25
Long−pec
Protruding−mouth
Day−4
Day−5
Stage
Transcript
ENSDART00000111559
ENSDART00000153651
ENSDART00000156764

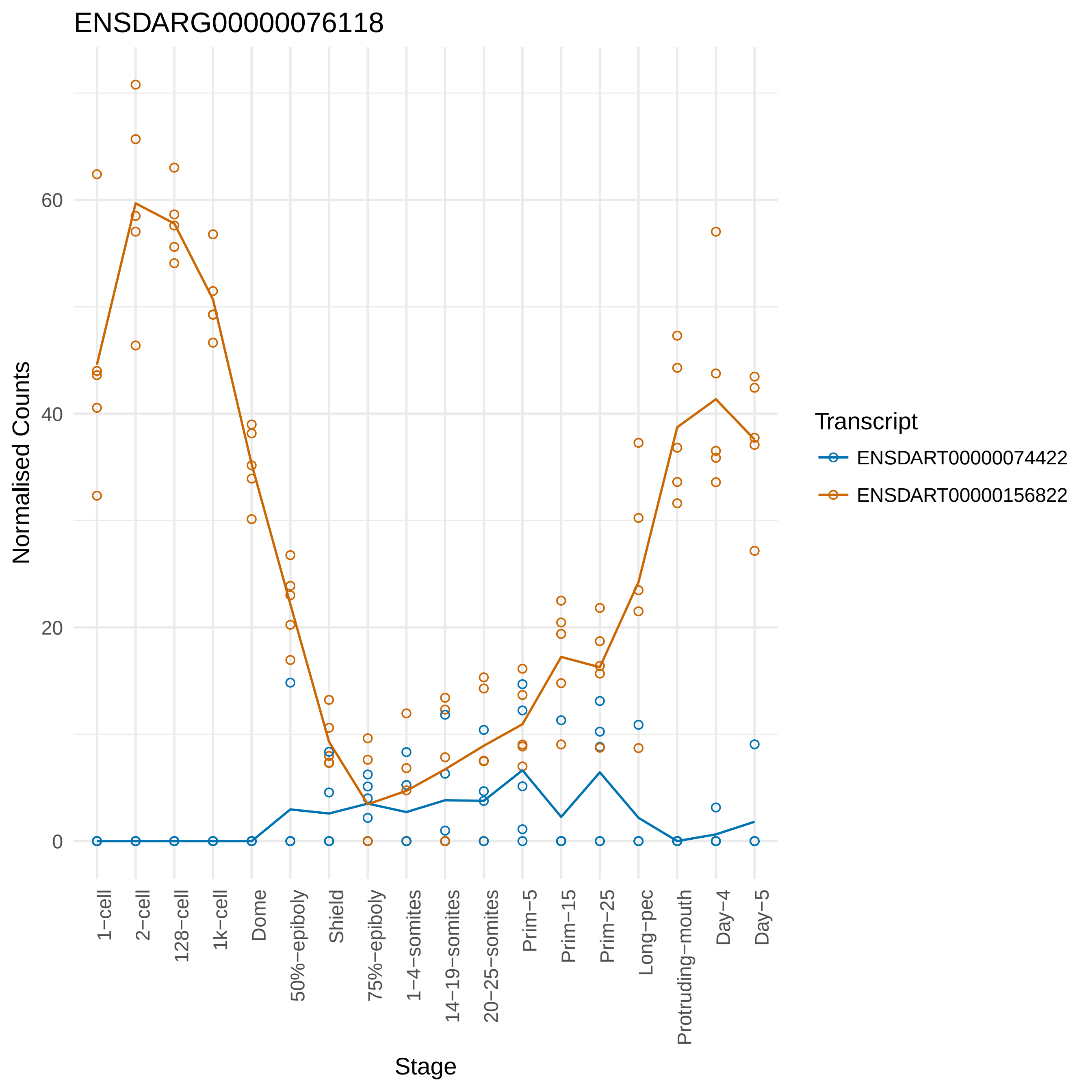
ENSDARG00000076118
Normalised Counts
0
20
40
60
Stage
1−cell
2−cell
128−cell
1k−cell
Dome
50%−epiboly
Shield
75%−epiboly
1−4−somites
14−19−somites
20−25−somites
Prim−5
Prim−15
Prim−25
Long−pec
Protruding−mouth
Day−4
Day−5
Transcript
ENSDART00000074422
ENSDART00000156822

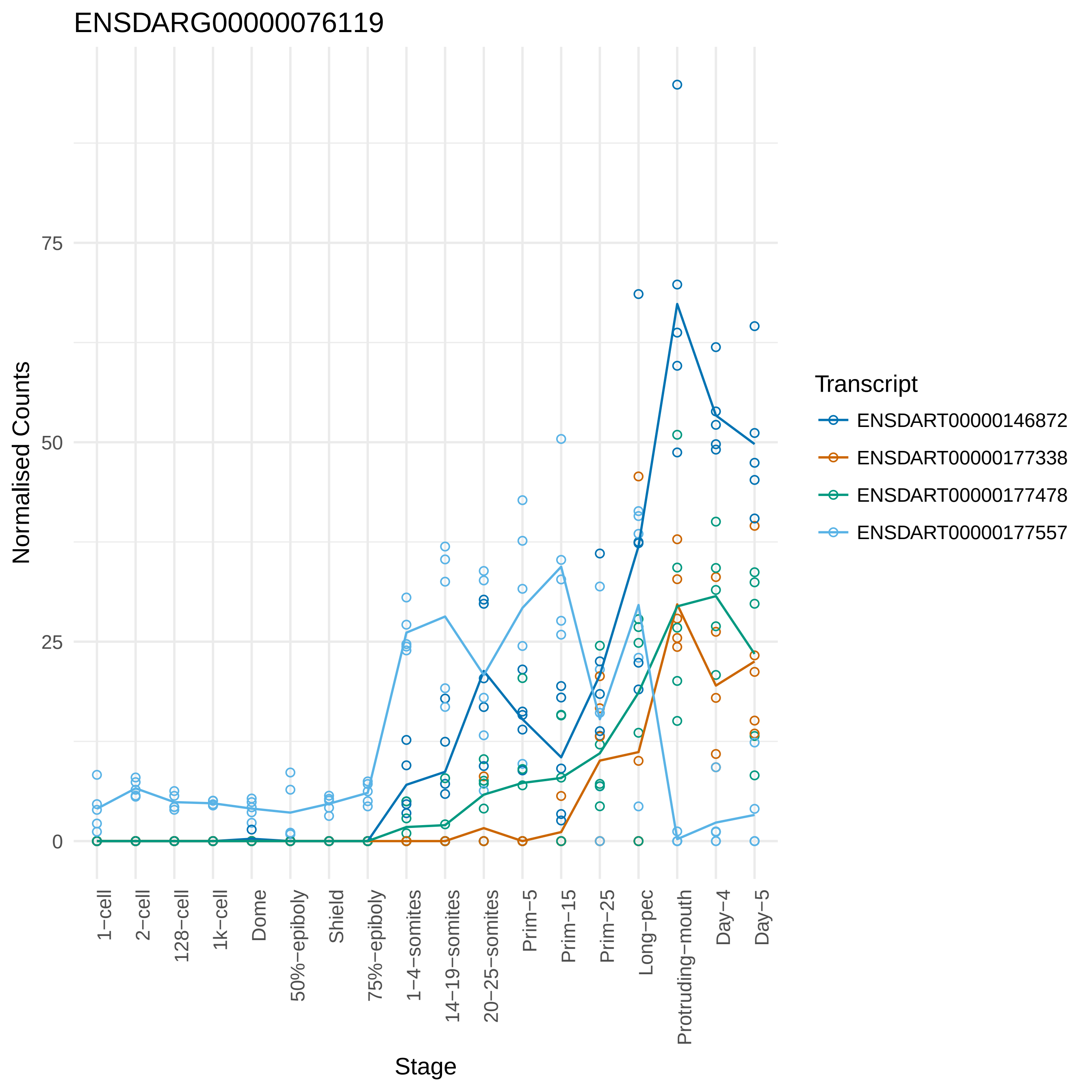

ENSDARG00000076119
Normalised Counts
0
25
50
75
Stage
1−cell
2−cell
128−cell
1k−cell
Dome
50%−epiboly
Shield
75%−epiboly
1−4−somites
14−19−somites
20−25−somites
Prim−5
Prim−15
Prim−25
Long−pec
Protruding−mouth
Day−4
Day−5
Transcript
ENSDART00000146872
ENSDART00000177338
ENSDART00000177478
ENSDART00000177557

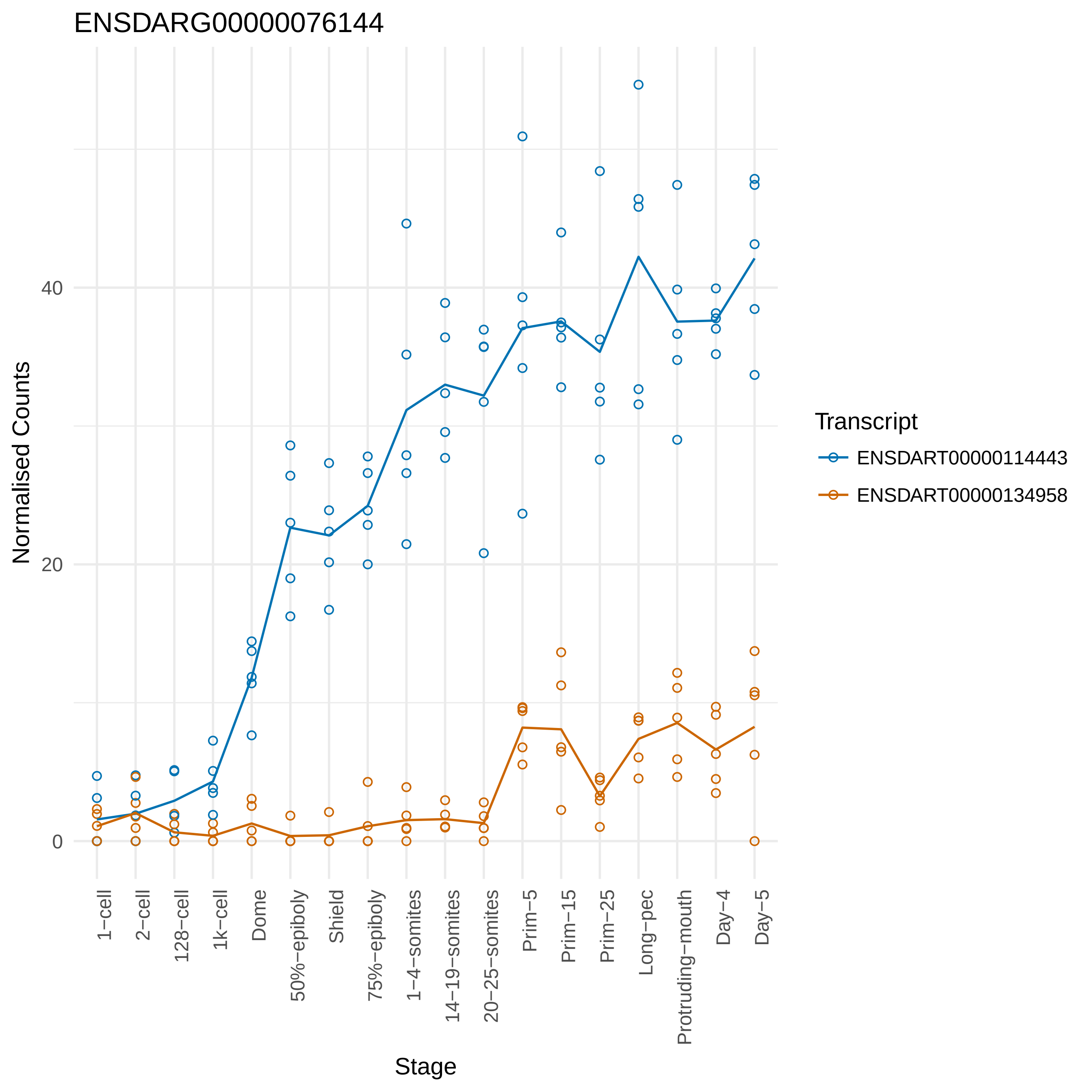

ENSDARG00000076144
Normalised Counts
0
20
40
Stage
1−cell
2−cell
128−cell
1k−cell
Dome
50%−epiboly
Shield
75%−epiboly
1−4−somites
14−19−somites
20−25−somites
Prim−5
Prim−15
Prim−25
Long−pec
Protruding−mouth
Day−4
Day−5
Transcript
ENSDART00000114443
ENSDART00000134958

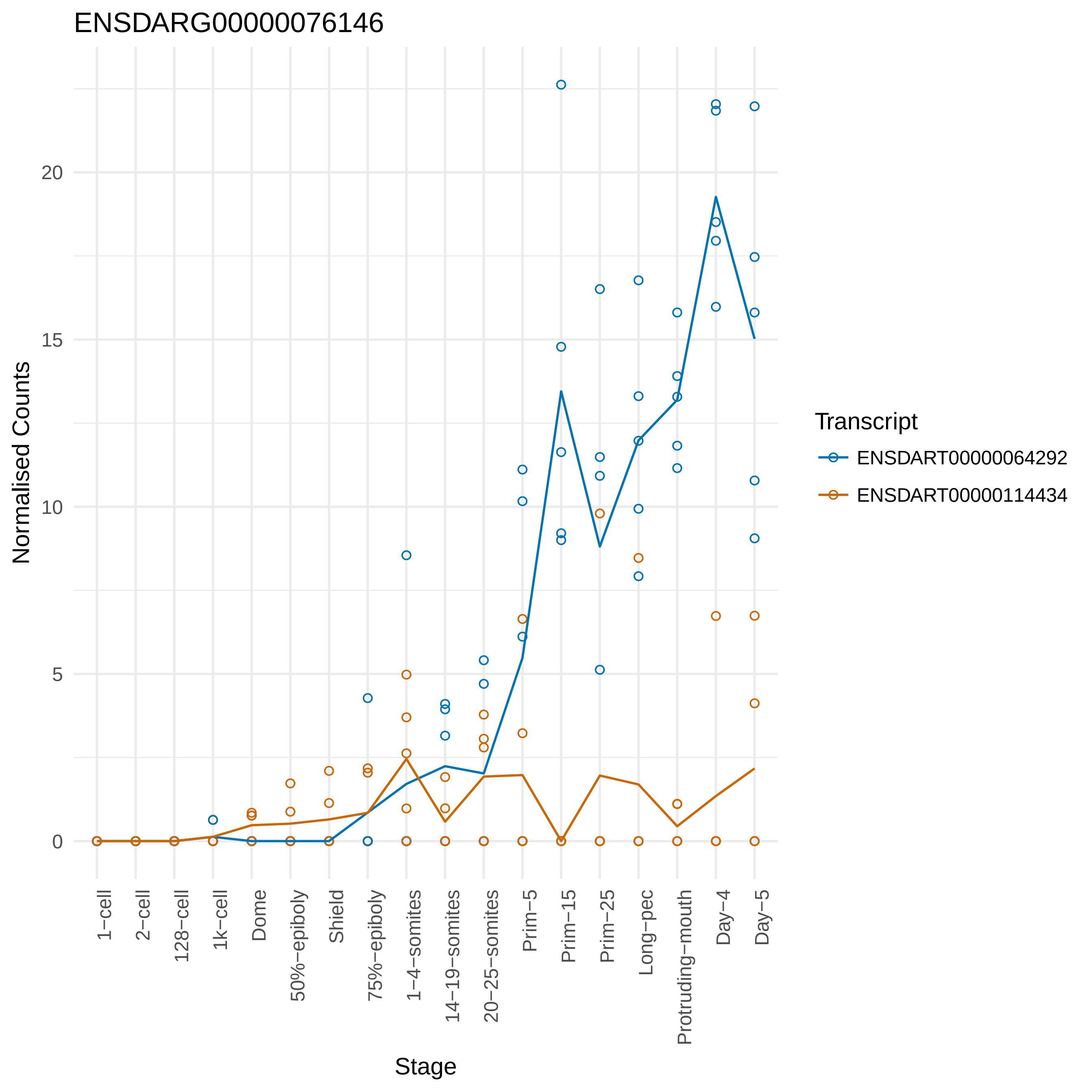

ENSDARG00000076146
Normalised Counts
0
5
10
15
20
Stage
1−cell
2−cell
128−cell
1k−cell
Dome
50%−epiboly
Shield
75%−epiboly
1−4−somites
14−19−somites
20−25−somites
Prim−5
Prim−15
Prim−25
Long−pec
Protruding−mouth
Day−4
Day−5
Transcript
ENSDART00000064292
ENSDART00000114434

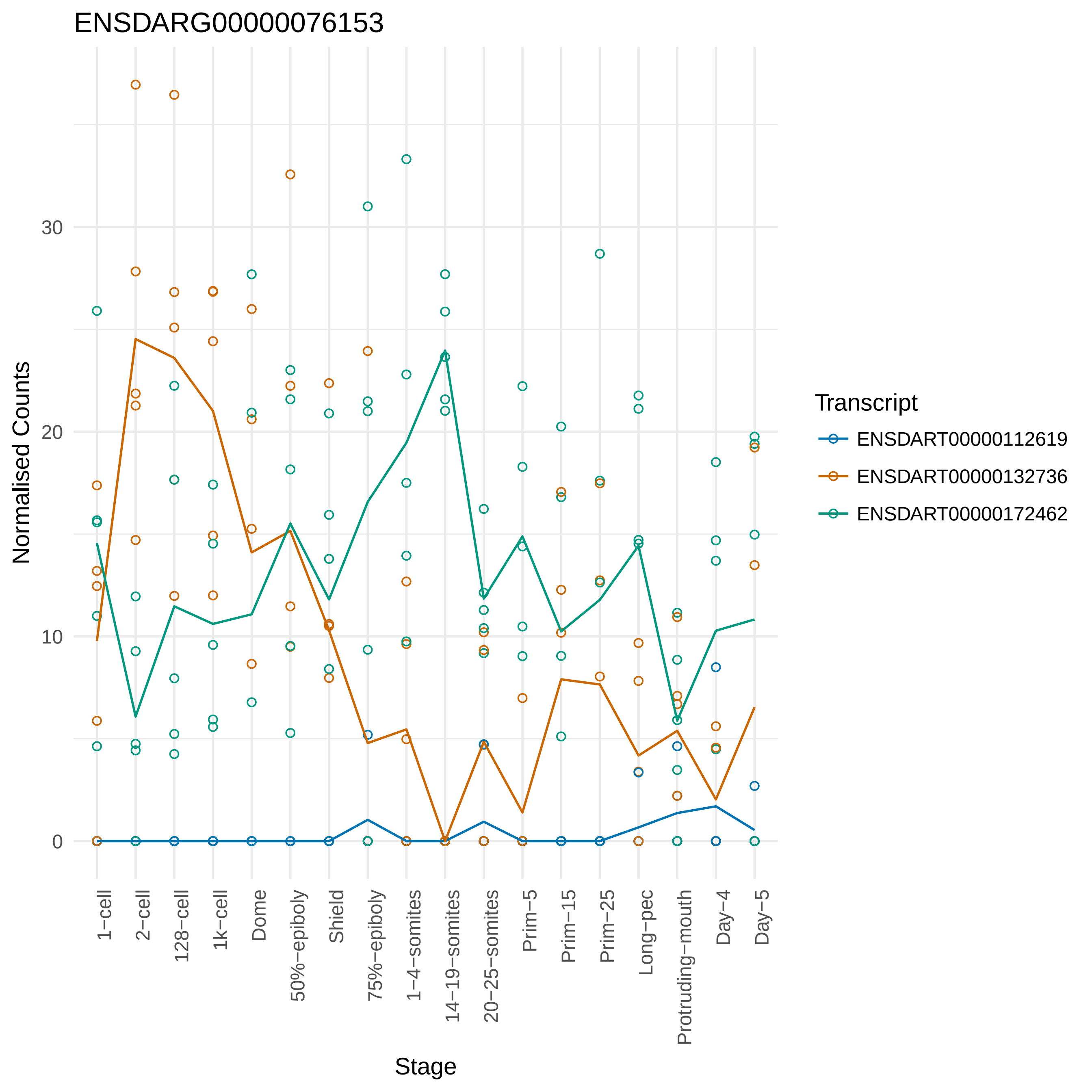

ENSDARG00000076153
Normalised Counts
0
10
20
30
1−cell
2−cell
128−cell
1k−cell
Dome
50%−epiboly
Shield
75%−epiboly
1−4−somites
14−19−somites
20−25−somites
Prim−5
Prim−15
Prim−25
Long−pec
Protruding−mouth
Day−4
Day−5
Stage
Transcript
ENSDART00000112619
ENSDART00000132736
ENSDART00000172462

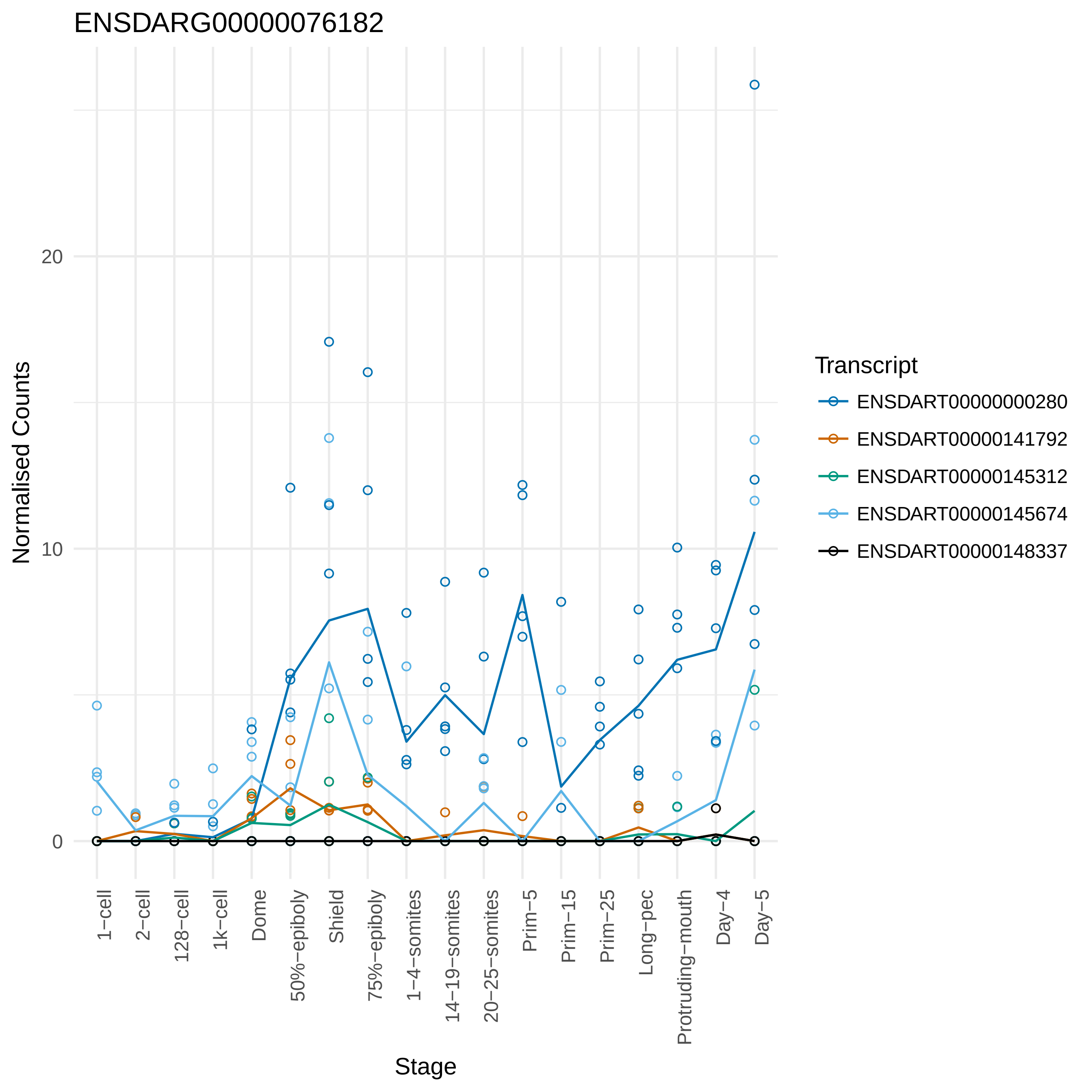
ENSDARG00000076182
Normalised Counts
20
10
0
1−cell
2−cell
128−cell
1k−cell
Dome
50%−epiboly
Shield
75%−epiboly
1−4−somites
14−19−somites
20−25−somites
Prim−5
Prim−15
Prim−25
Long−pec
Protruding−mouth
Day−4
Day−5
Stage
Transcript
ENSDART00000000280
ENSDART00000141792
ENSDART00000145312
ENSDART00000145674
ENSDART00000148337

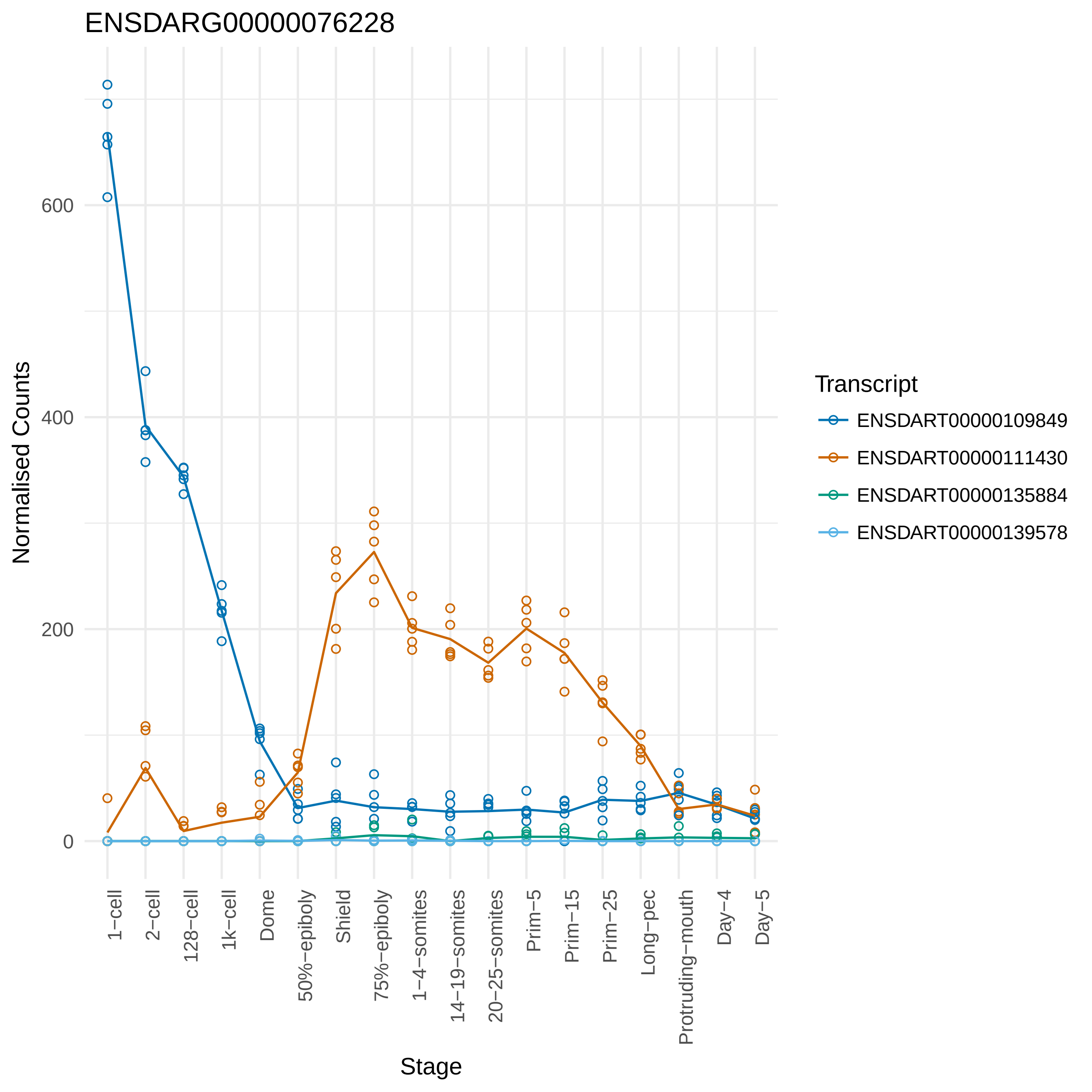
ENSDARG00000076228
Normalised Counts
600
400
200
0
1−cell
2−cell
128−cell
1k−cell
Dome
50%−epiboly
Shield
75%−epiboly
1−4−somites
14−19−somites
20−25−somites
Prim−5
Prim−15
Prim−25
Long−pec
Protruding−mouth
Day−4
Day−5
Stage
Transcript
ENSDART00000109849
ENSDART00000111430
ENSDART00000135884
ENSDART00000139578

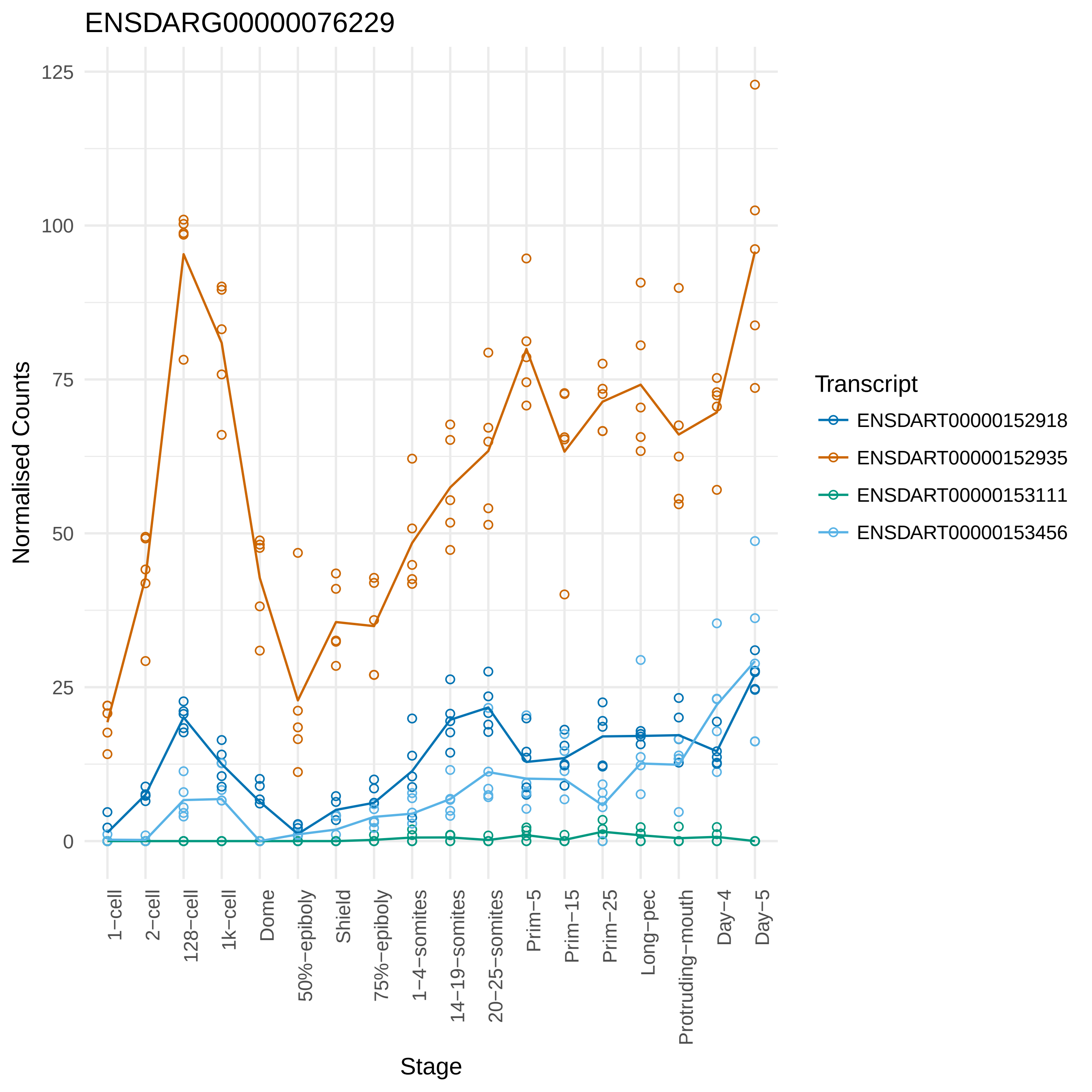
ENSDARG00000076229
Normalised Counts
125
100
75
50
25
0
1−cell
2−cell
128−cell
1k−cell
Dome
50%−epiboly
Shield
75%−epiboly
1−4−somites
14−19−somites
20−25−somites
Prim−5
Prim−15
Prim−25
Long−pec
Protruding−mouth
Day−4
Day−5
Stage
Transcript
ENSDART00000152918
ENSDART00000152935
ENSDART00000153111
ENSDART00000153456

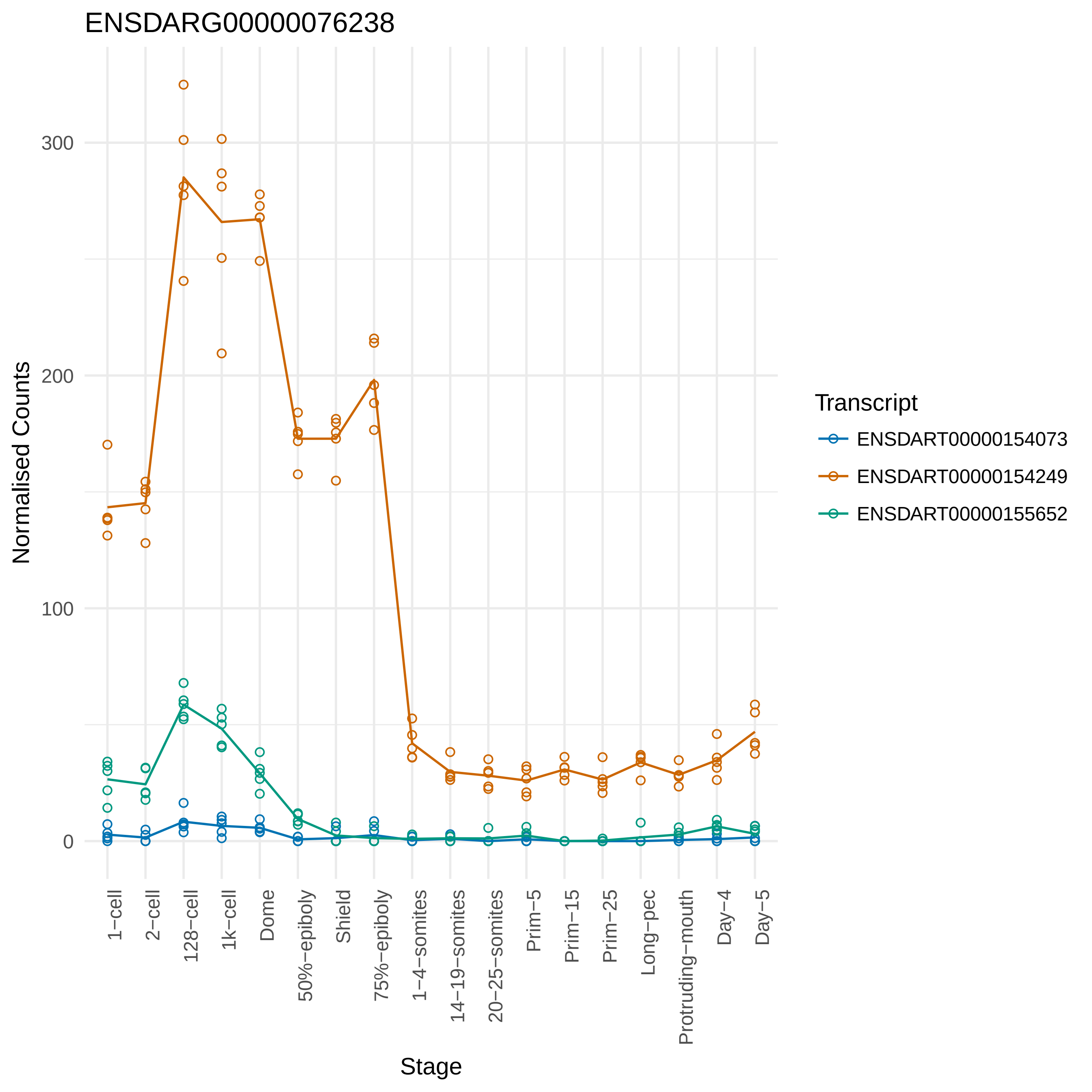

ENSDARG00000076238
Normalised Counts
300
200
100
0
1−cell
2−cell
128−cell
1k−cell
Dome
50%−epiboly
Shield
75%−epiboly
1−4−somites
14−19−somites
20−25−somites
Prim−5
Prim−15
Prim−25
Long−pec
Protruding−mouth
Day−4
Day−5
Stage
Transcript
ENSDART00000154073
ENSDART00000154249
ENSDART00000155652

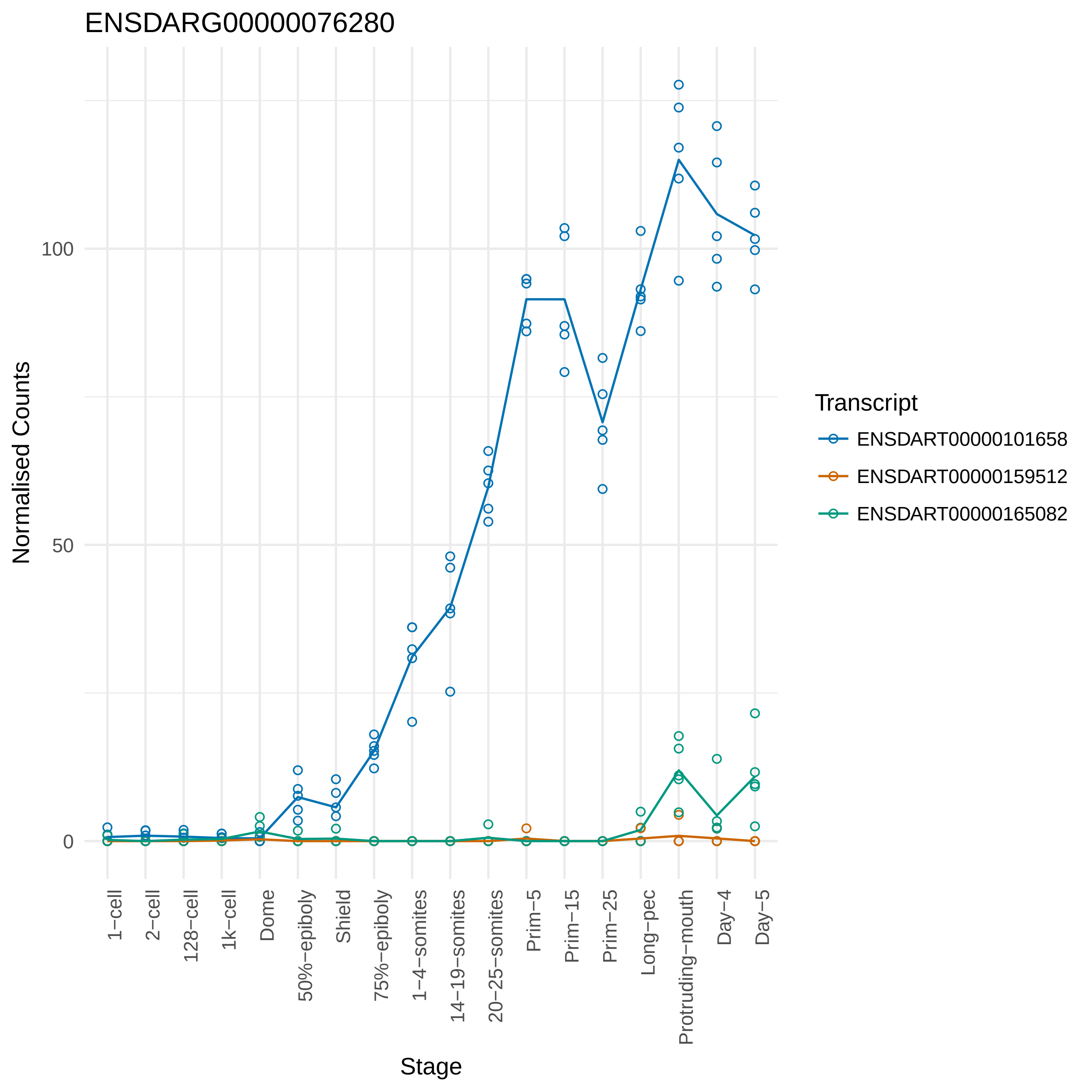

ENSDARG00000076280
Normalised Counts
100
50
0
1−cell
2−cell
128−cell
1k−cell
Dome
50%−epiboly
Shield
75%−epiboly
1−4−somites
14−19−somites
20−25−somites
Prim−5
Prim−15
Prim−25
Long−pec
Protruding−mouth
Day−4
Day−5
Stage
Transcript
ENSDART00000101658
ENSDART00000159512
ENSDART00000165082

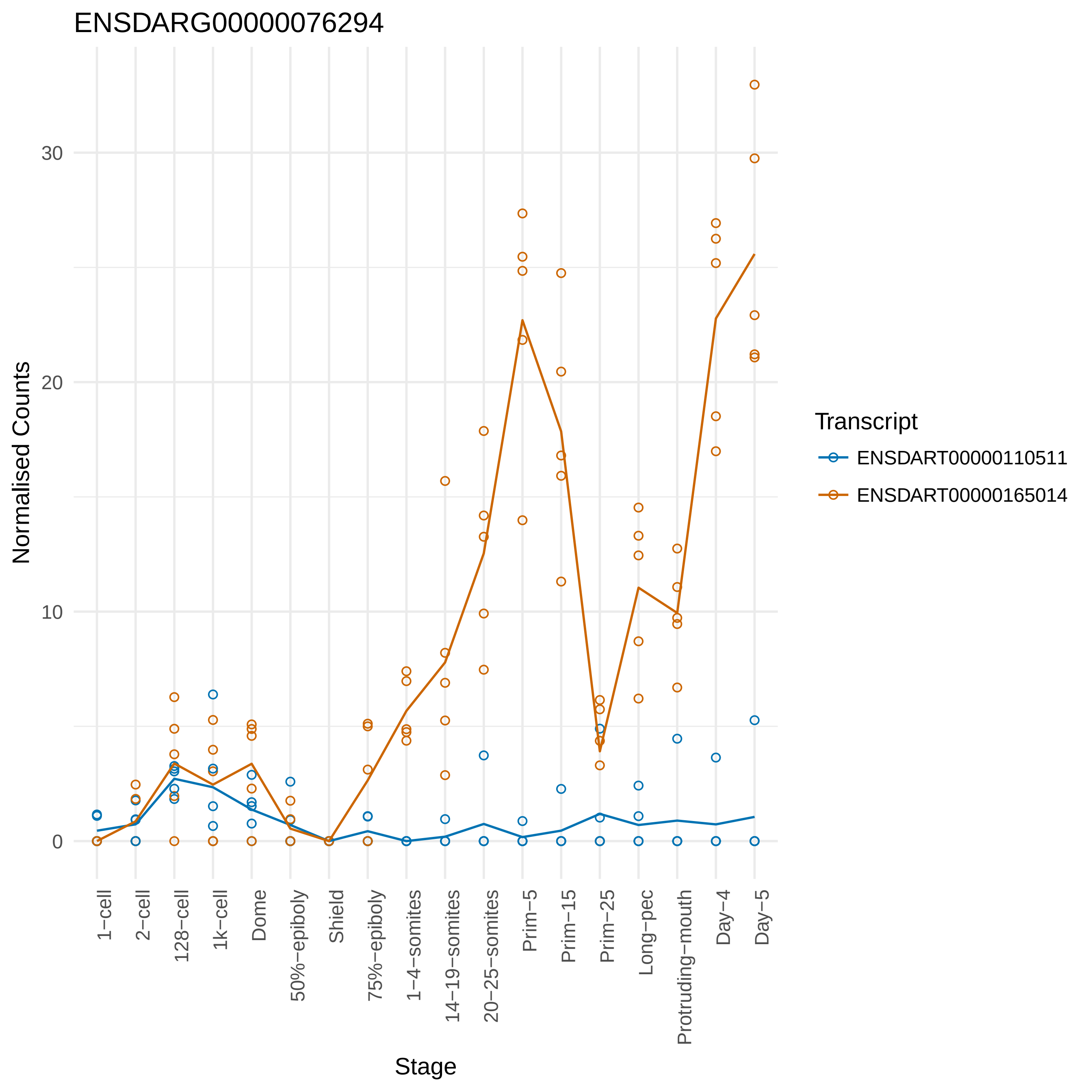
ENSDARG00000076294
Normalised Counts
0
10
20
30
1−cell
2−cell
128−cell
1k−cell
Dome
50%−epiboly
Shield
75%−epiboly
1−4−somites
14−19−somites
20−25−somites
Prim−5
Prim−15
Prim−25
Long−pec
Protruding−mouth
Day−4
Day−5
Stage
Transcript
ENSDART00000110511
ENSDART00000165014

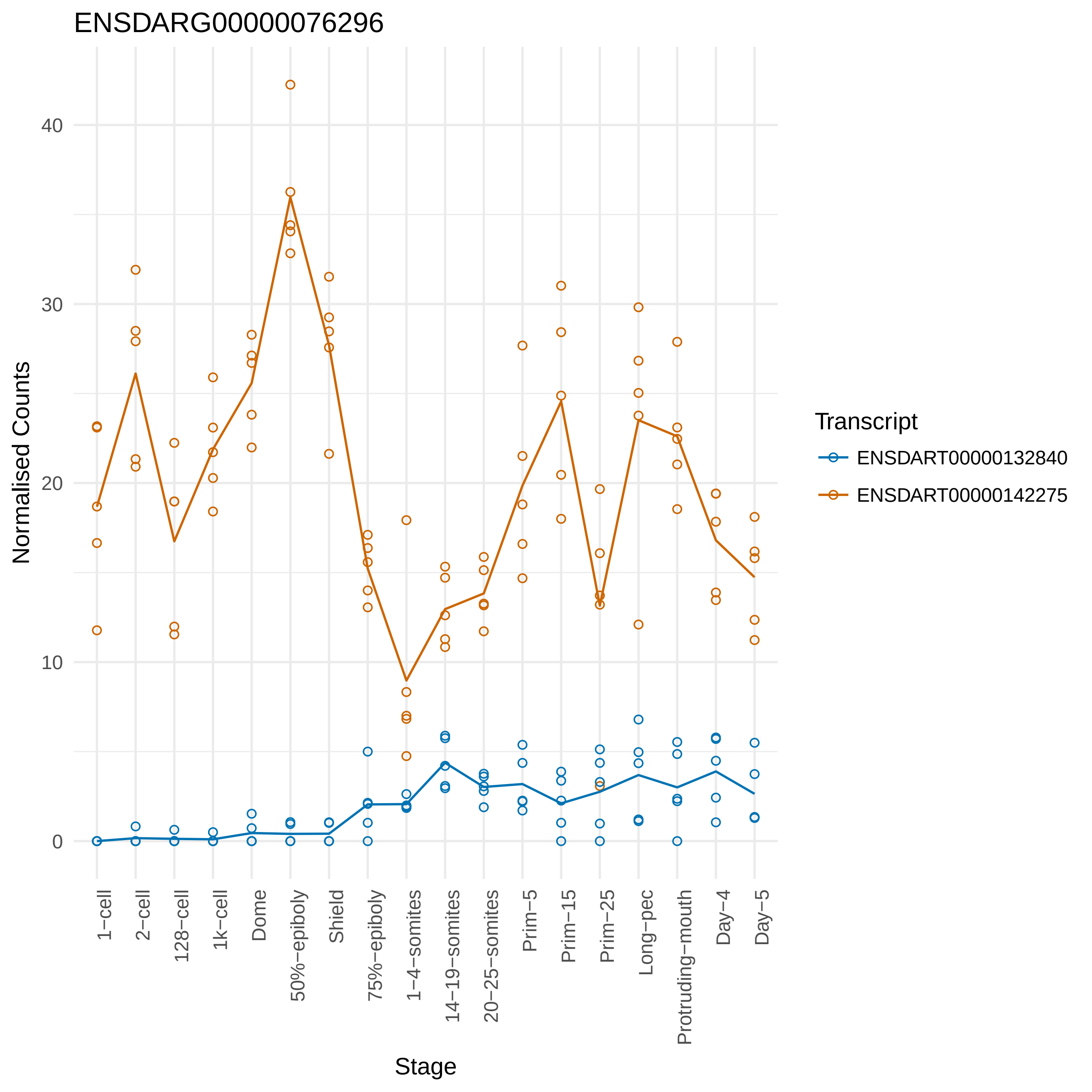

ENSDARG00000076296
Normalised Counts
0
10
20
30
40
Stage
1−cell
2−cell
128−cell
1k−cell
Dome
50%−epiboly
Shield
75%−epiboly
1−4−somites
14−19−somites
20−25−somites
Prim−5
Prim−15
Prim−25
Long−pec
Protruding−mouth
Day−4
Day−5
Transcript
ENSDART00000132840
ENSDART00000142275

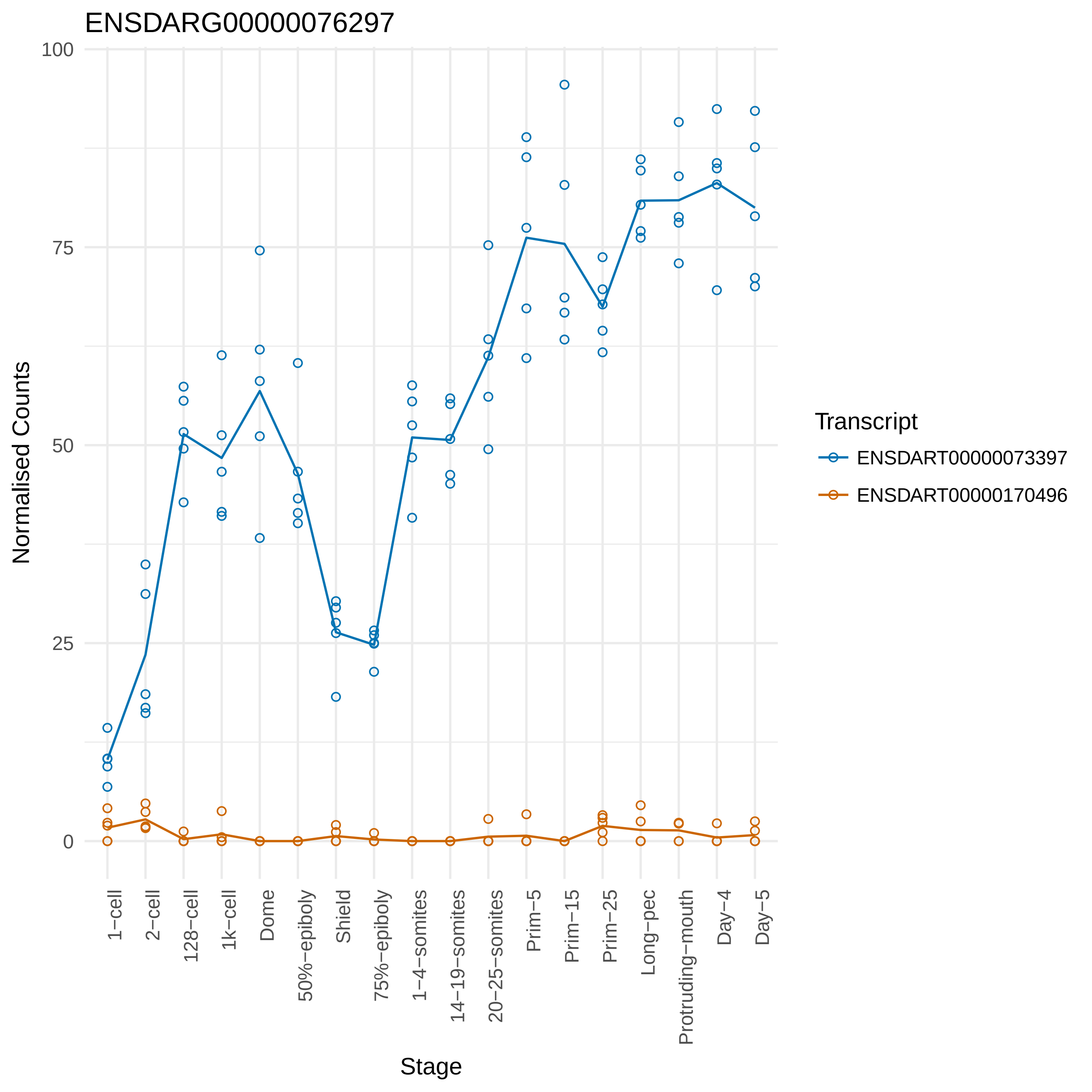

ENSDARG00000076297
100
75
50
25
0
Normalised Counts
1−cell
2−cell
128−cell
1k−cell
Dome
50%−epiboly
Shield
75%−epiboly
1−4−somites
14−19−somites
20−25−somites
Prim−5
Prim−15
Prim−25
Long−pec
Protruding−mouth
Day−4
Day−5
Stage
Transcript
ENSDART00000073397
ENSDART00000170496

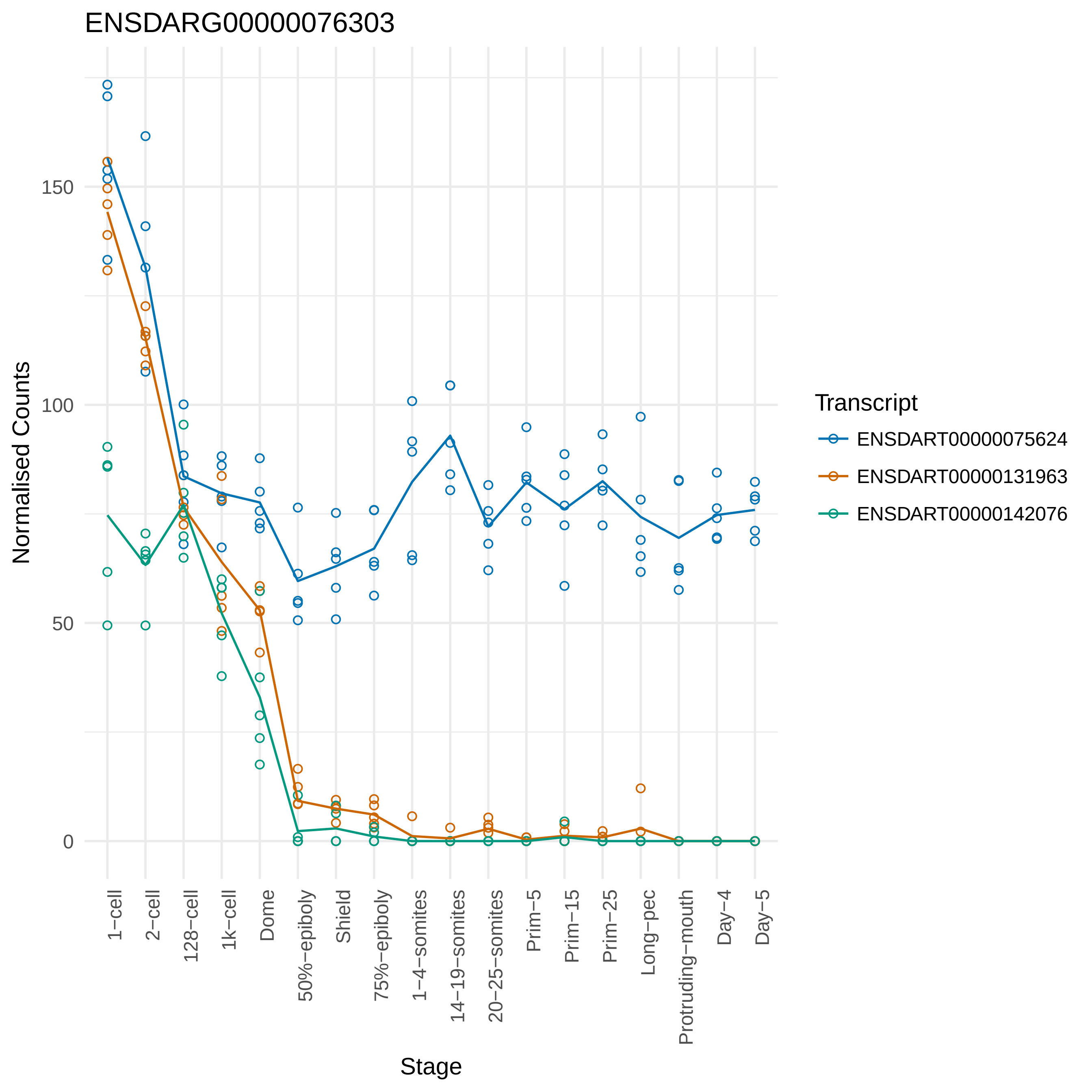
ENSDARG00000076303
Normalised Counts
0
50
100
150
Stage
1−cell
2−cell
128−cell
1k−cell
Dome
50%−epiboly
Shield
75%−epiboly
1−4−somites
14−19−somites
20−25−somites
Prim−5
Prim−15
Prim−25
Long−pec
Protruding−mouth
Day−4
Day−5
Transcript
ENSDART00000075624
ENSDART00000131963
ENSDART00000142076

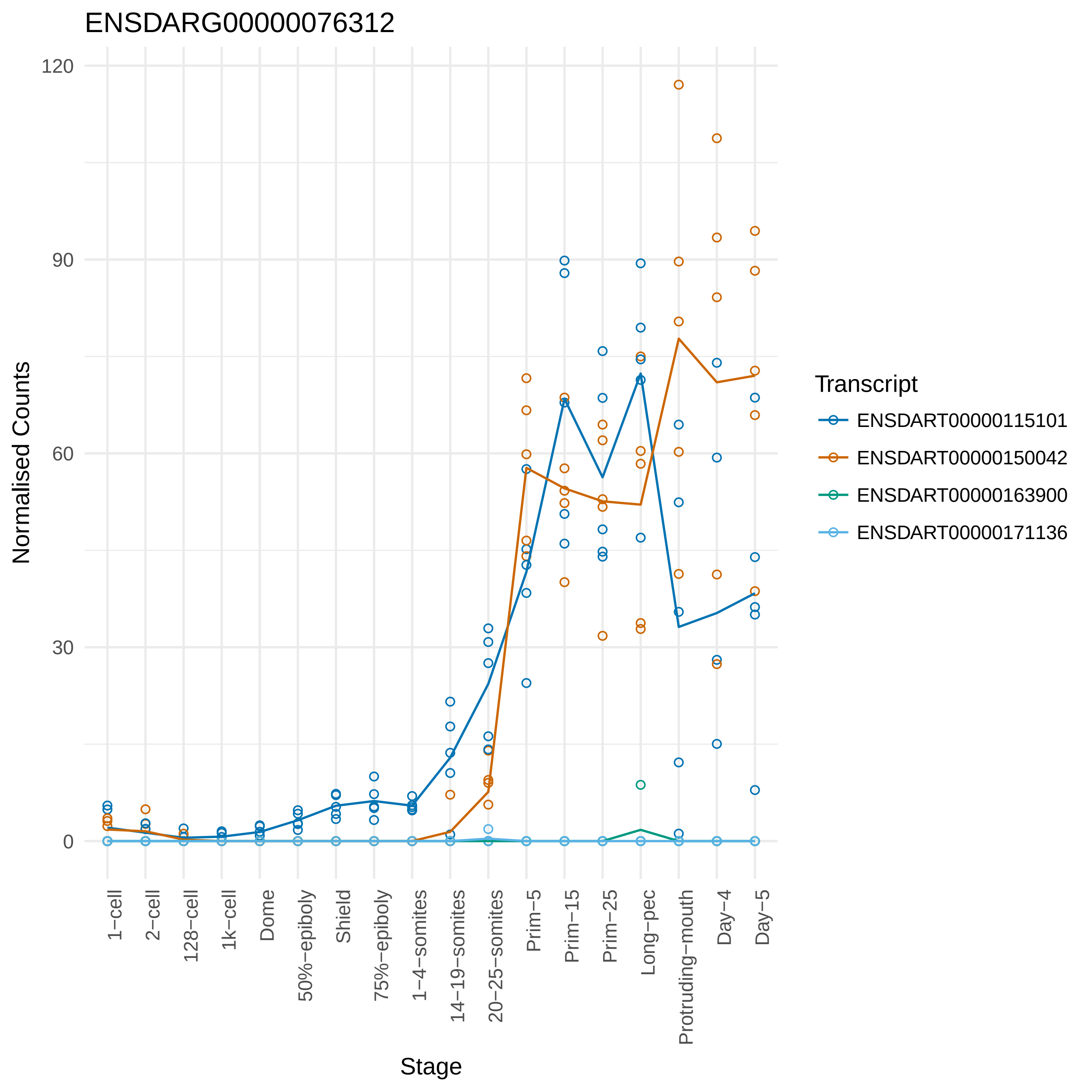

ENSDARG00000076312
Normalised Counts
120
90
60
30
0
1−cell
2−cell
128−cell
1k−cell
Dome
50%−epiboly
Shield
75%−epiboly
1−4−somites
14−19−somites
20−25−somites
Prim−5
Prim−15
Prim−25
Long−pec
Protruding−mouth
Day−4
Day−5
Stage
Transcript
ENSDART00000115101
ENSDART00000150042
ENSDART00000163900
ENSDART00000171136

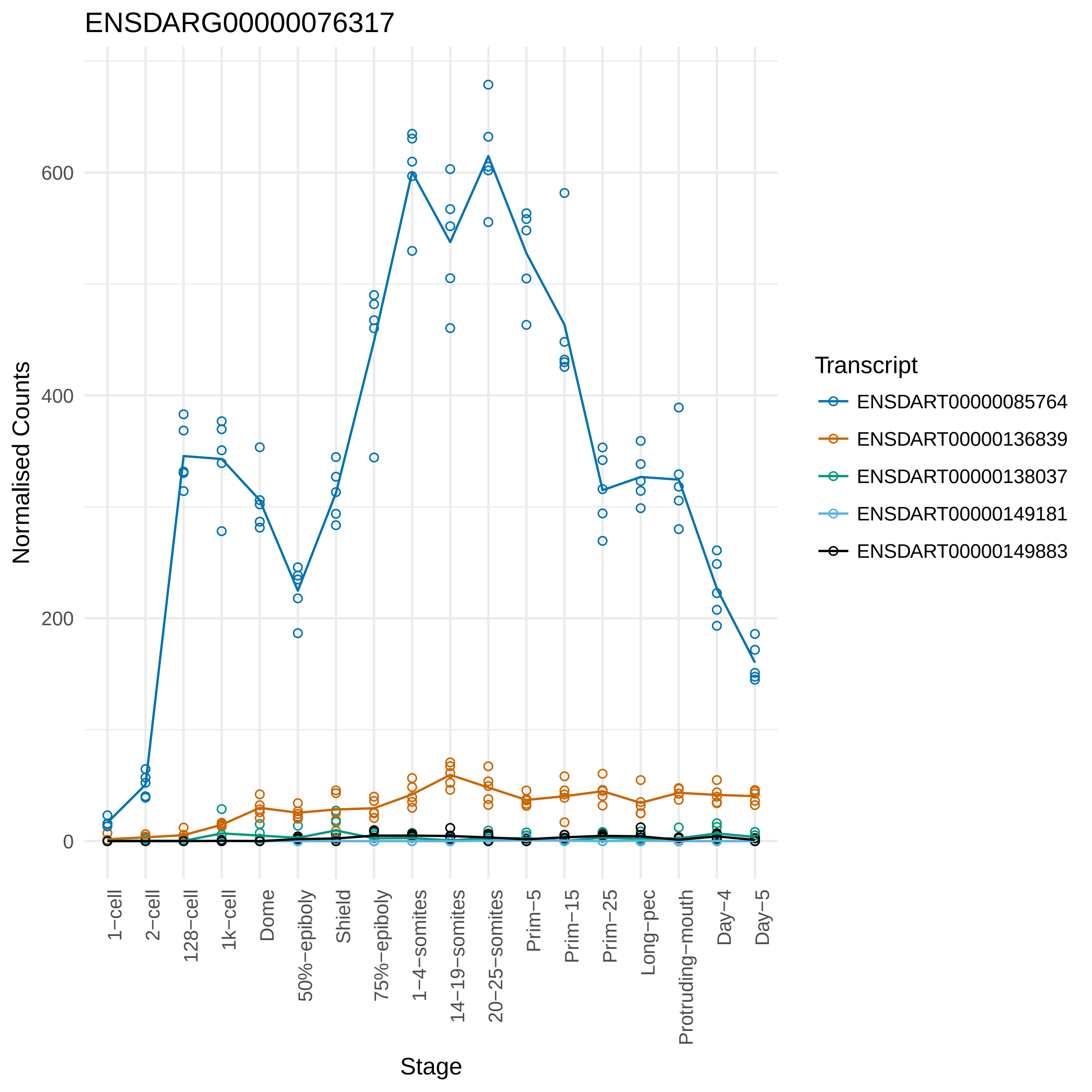
ENSDARG00000076317
Normalised Counts
600
400
200
0
1−cell
2−cell
128−cell
1k−cell
Dome
50%−epiboly
Shield
75%−epiboly
1−4−somites
14−19−somites
20−25−somites
Prim−5
Prim−15
Prim−25
Long−pec
Protruding−mouth
Day−4
Day−5
Stage
Transcript
ENSDART00000085764
ENSDART00000136839
ENSDART00000138037
ENSDART00000149181
ENSDART00000149883

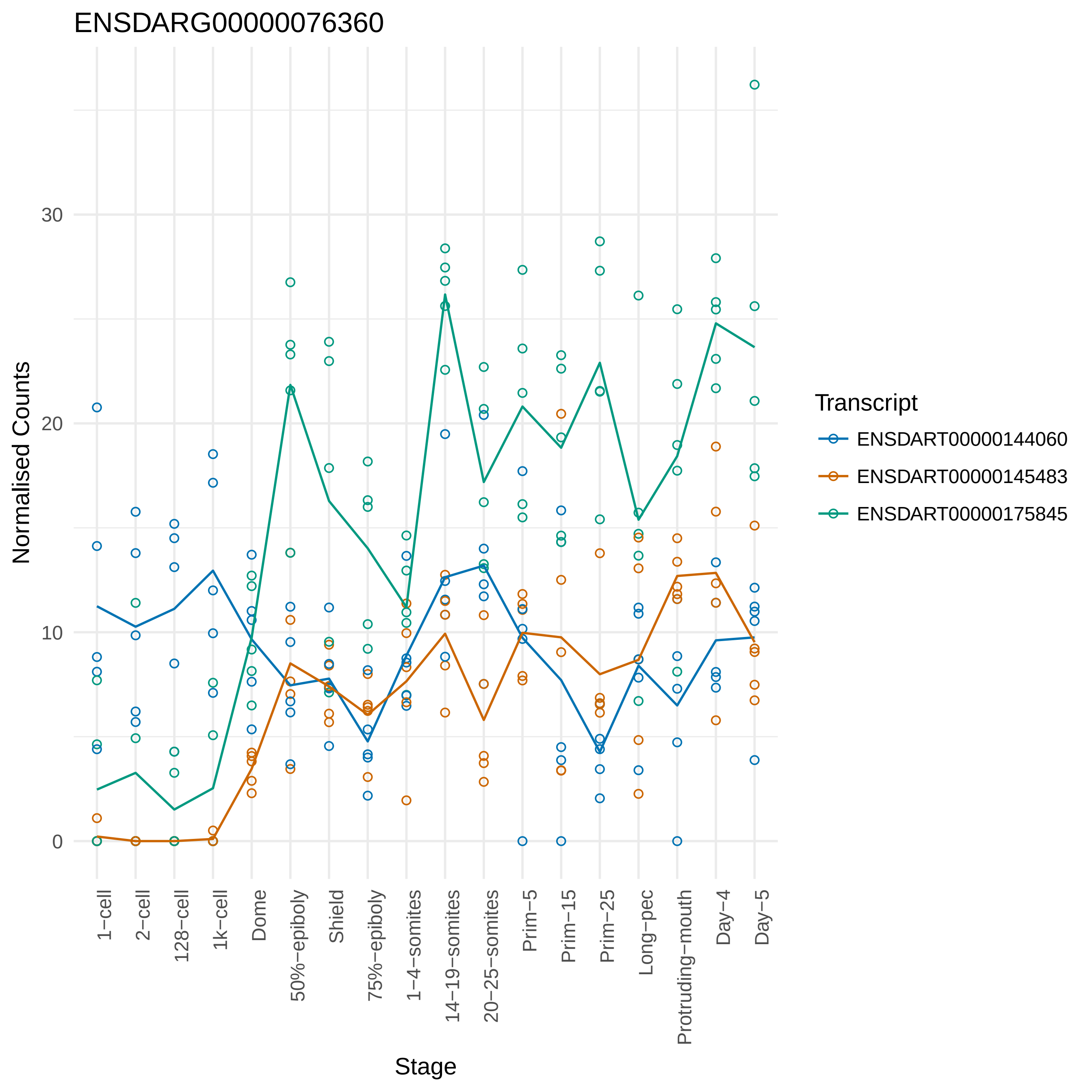

ENSDARG00000076360
Normalised Counts
30
20
10
0
1−cell
2−cell
128−cell
1k−cell
Dome
50%−epiboly
Shield
75%−epiboly
1−4−somites
14−19−somites
20−25−somites
Prim−5
Prim−15
Prim−25
Long−pec
Protruding−mouth
Day−4
Day−5
Stage
Transcript
ENSDART00000144060
ENSDART00000145483
ENSDART00000175845

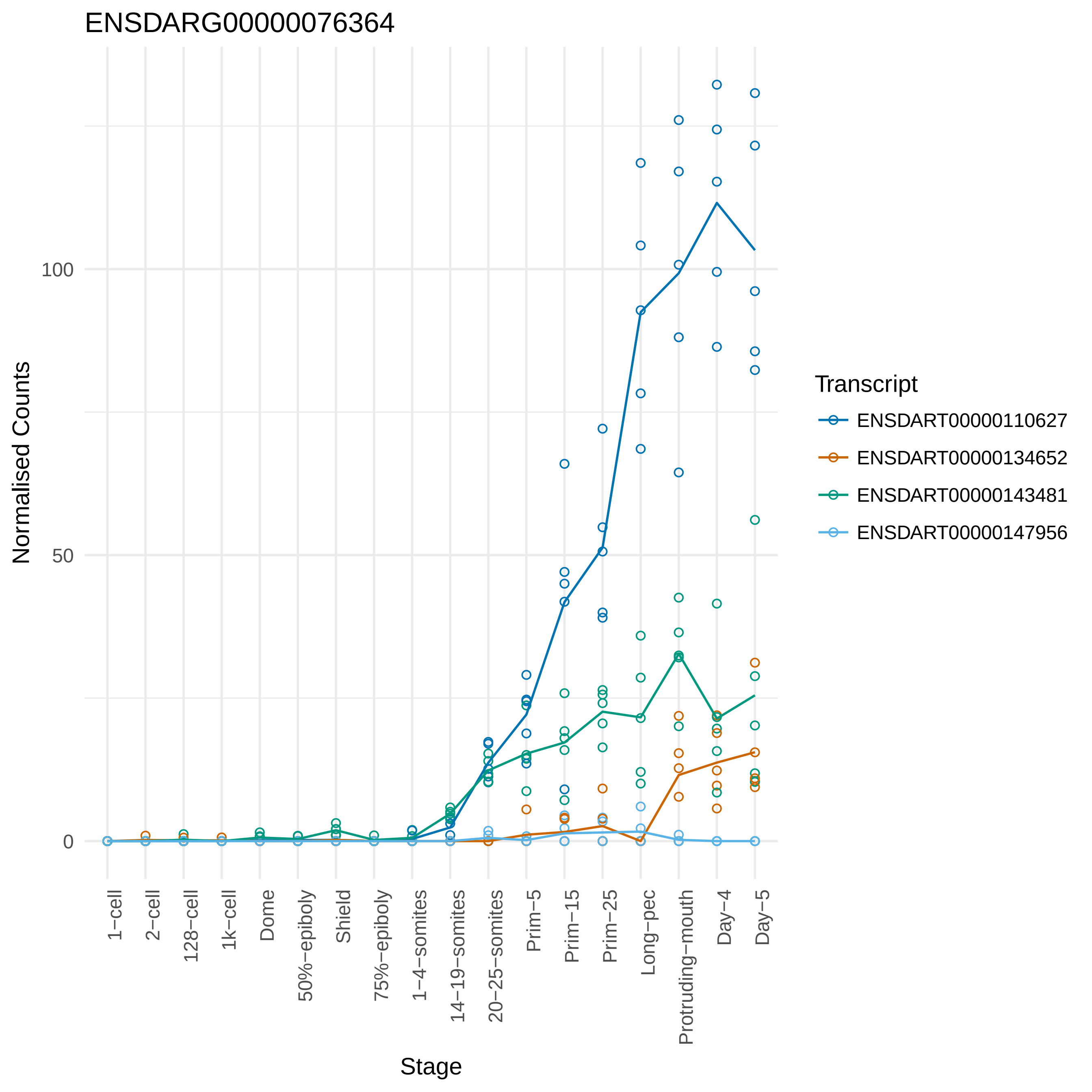
ENSDARG00000076364
Normalised Counts
100
50
0
Stage
1−cell
2−cell
128−cell
1k−cell
Dome
50%−epiboly
Shield
75%−epiboly
1−4−somites
14−19−somites
20−25−somites
Prim−5
Prim−15
Prim−25
Long−pec
Protruding−mouth
Day−4
Day−5
Transcript
ENSDART00000110627
ENSDART00000134652
ENSDART00000143481
ENSDART00000147956

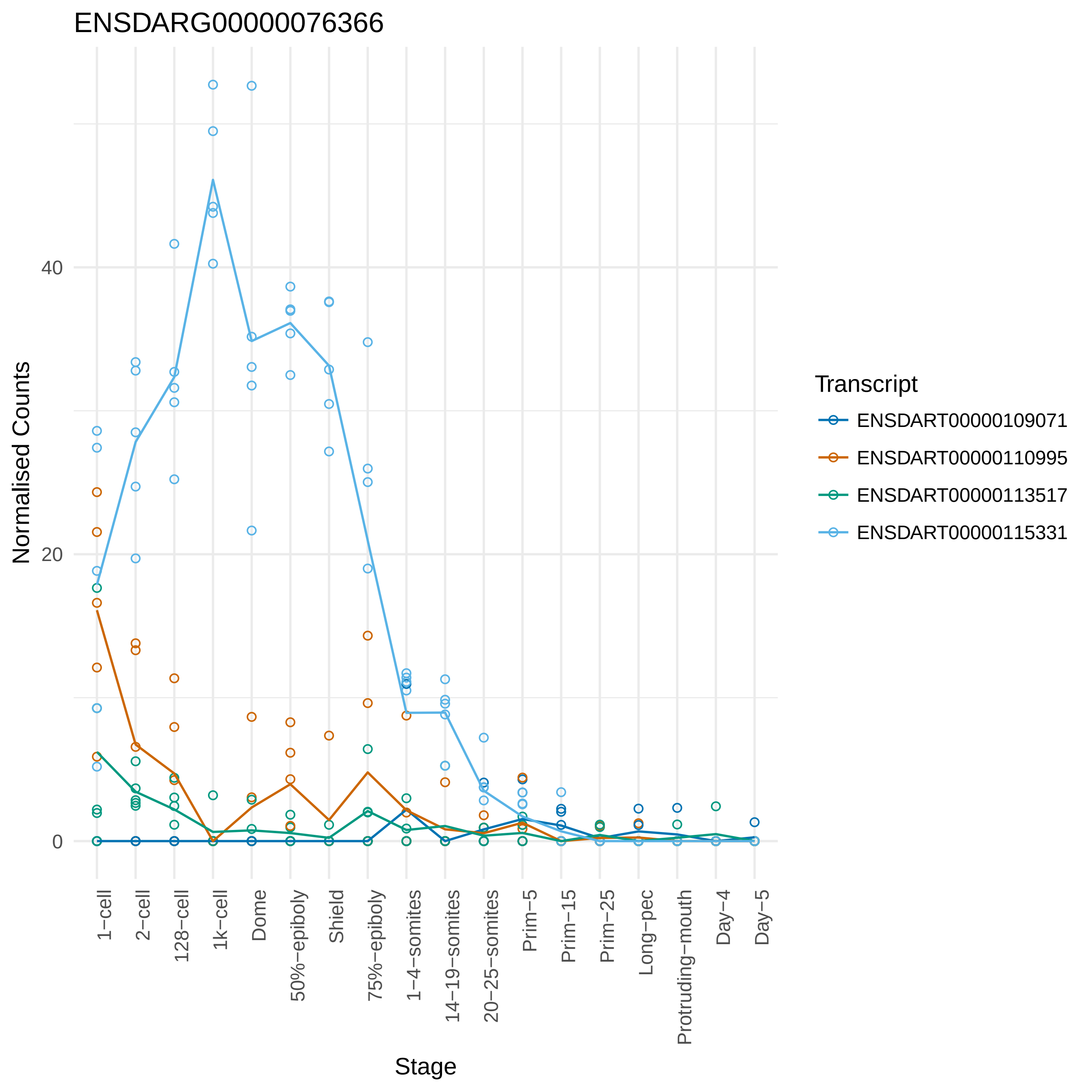

ENSDARG00000076366
Normalised Counts
0
20
40
1−cell
2−cell
128−cell
1k−cell
Dome
50%−epiboly
Shield
75%−epiboly
1−4−somites
14−19−somites
20−25−somites
Prim−5
Prim−15
Prim−25
Long−pec
Protruding−mouth
Day−4
Day−5
Stage
Transcript
ENSDART00000109071
ENSDART00000110995
ENSDART00000113517
ENSDART00000115331

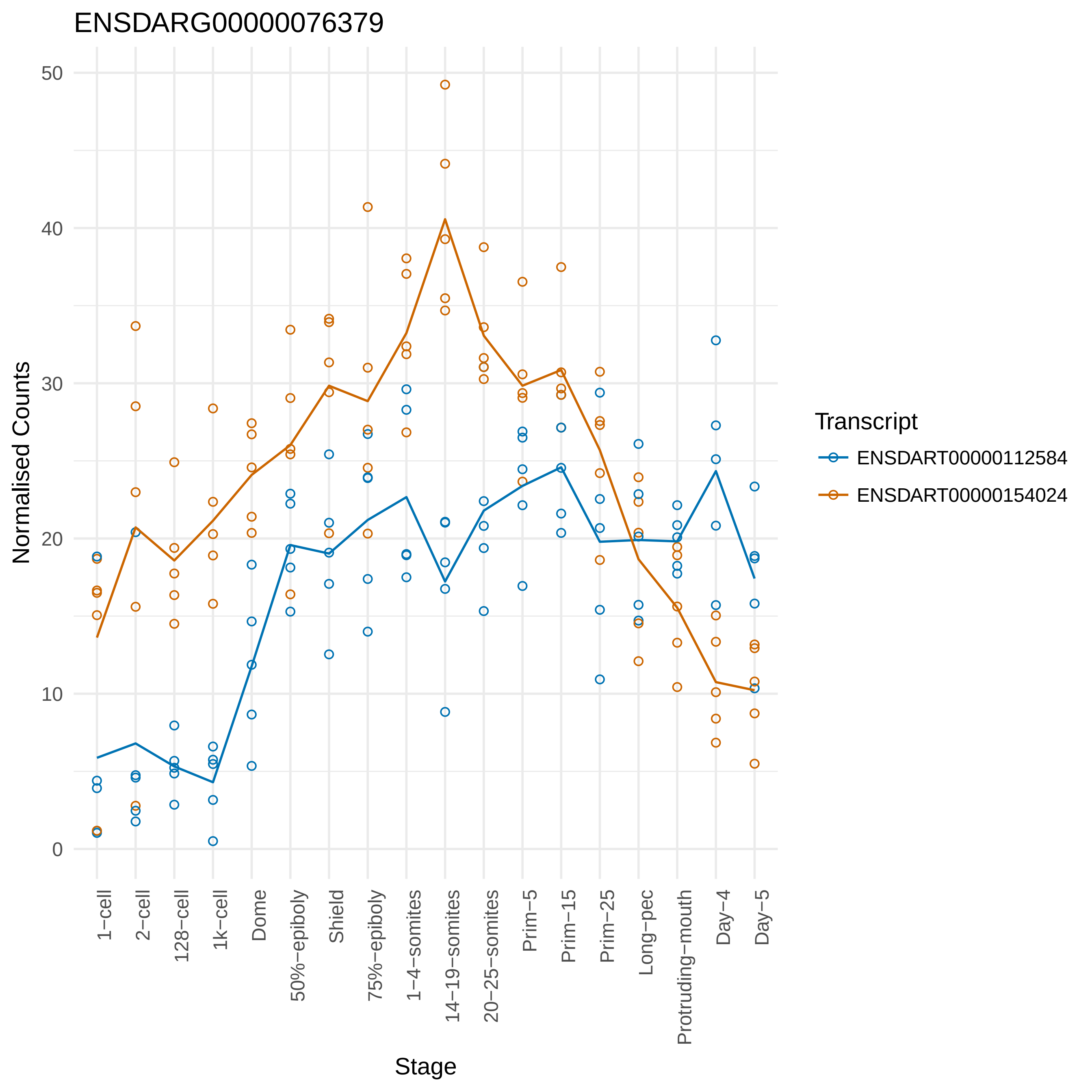

ENSDARG00000076379
Normalised Counts
0
10
20
30
40
50
1−cell
2−cell
128−cell
1k−cell
Dome
50%−epiboly
Shield
75%−epiboly
1−4−somites
14−19−somites
20−25−somites
Prim−5
Prim−15
Prim−25
Long−pec
Protruding−mouth
Day−4
Day−5
Stage
Transcript
ENSDART00000112584
ENSDART00000154024

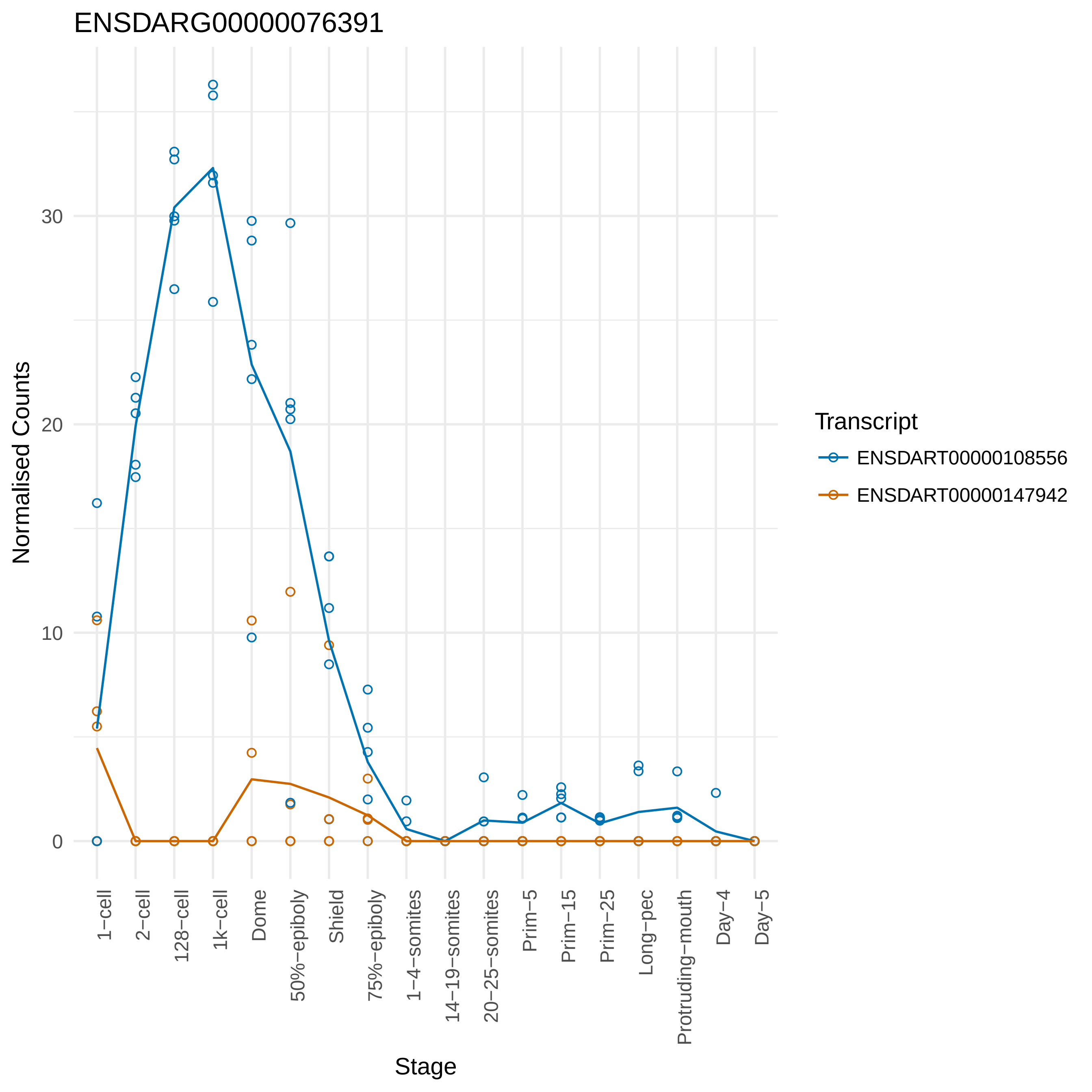

ENSDARG00000076391
Normalised Counts
30
20
10
0
1−cell
2−cell
128−cell
1k−cell
Dome
50%−epiboly
Shield
75%−epiboly
1−4−somites
14−19−somites
20−25−somites
Prim−5
Prim−15
Prim−25
Long−pec
Protruding−mouth
Day−4
Day−5
Stage
Transcript
ENSDART00000108556
ENSDART00000147942

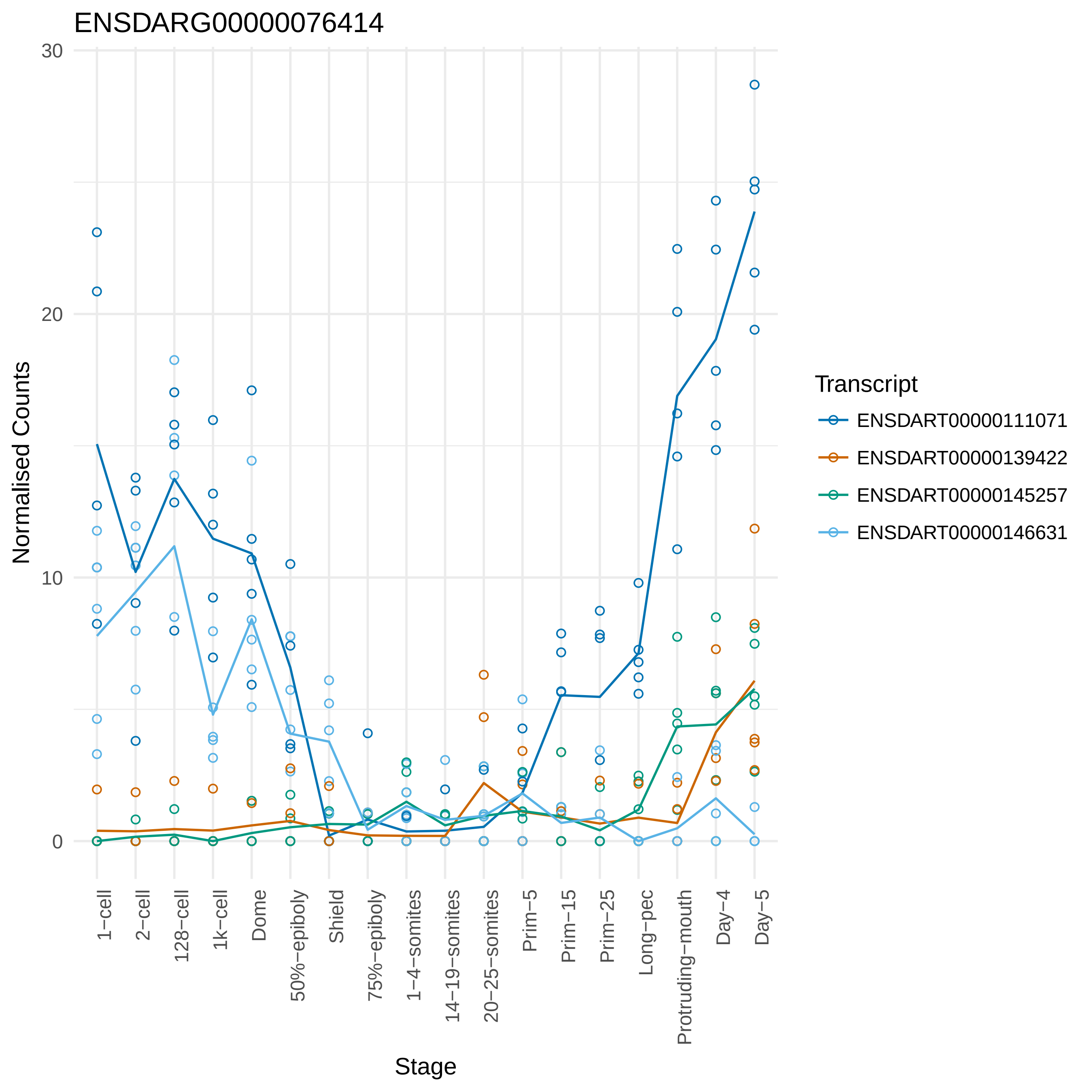

ENSDARG00000076414
Normalised Counts
30
20
10
0
1−cell
2−cell
128−cell
1k−cell
Dome
50%−epiboly
Shield
75%−epiboly
1−4−somites
14−19−somites
20−25−somites
Prim−5
Prim−15
Prim−25
Long−pec
Protruding−mouth
Day−4
Day−5
Stage
Transcript
ENSDART00000111071
ENSDART00000139422
ENSDART00000145257
ENSDART00000146631

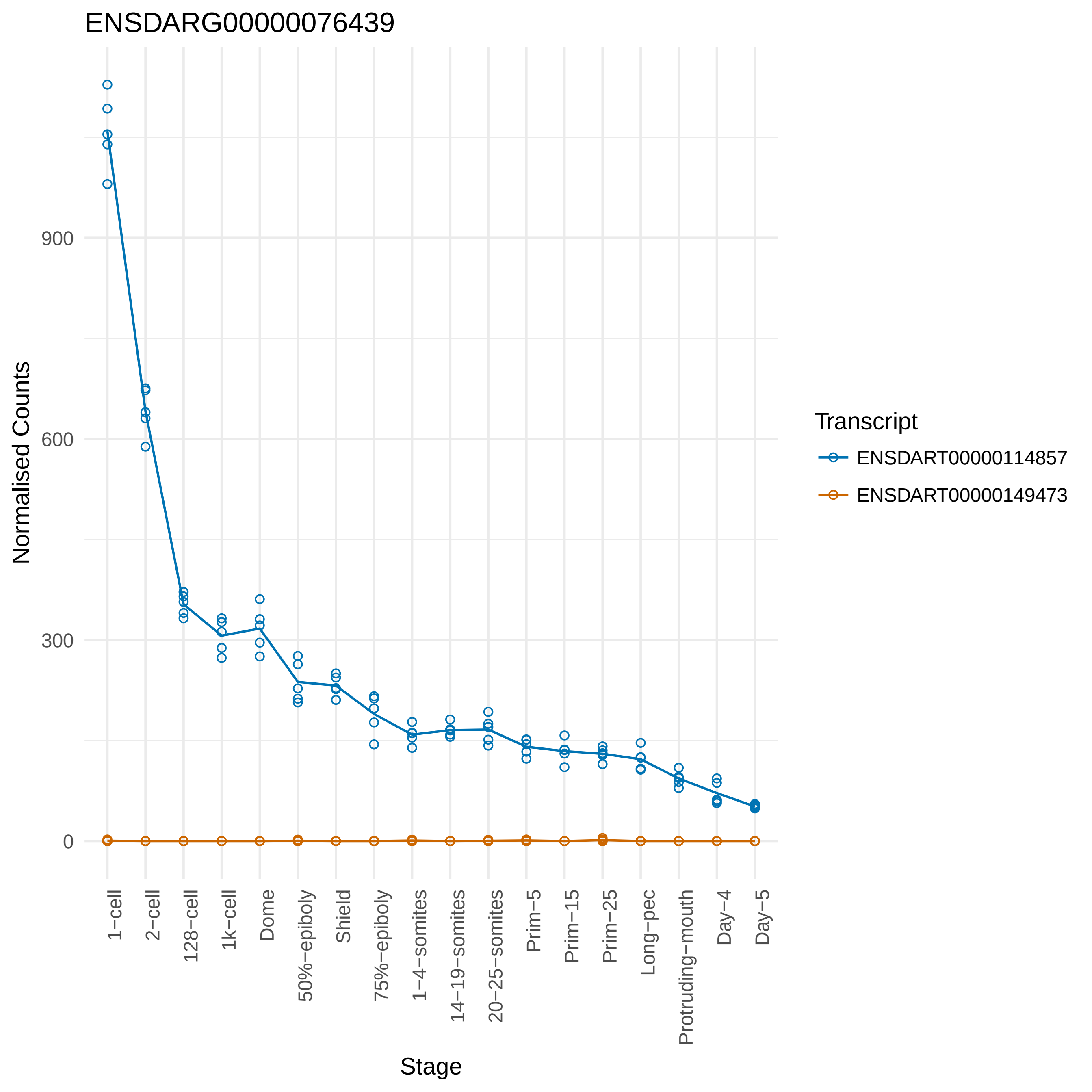

ENSDARG00000076439
Normalised Counts
900
600
300
0
1−cell
2−cell
128−cell
1k−cell
Dome
50%−epiboly
Shield
75%−epiboly
1−4−somites
14−19−somites
20−25−somites
Prim−5
Prim−15
Prim−25
Long−pec
Protruding−mouth
Day−4
Day−5
Stage
Transcript
ENSDART00000114857
ENSDART00000149473

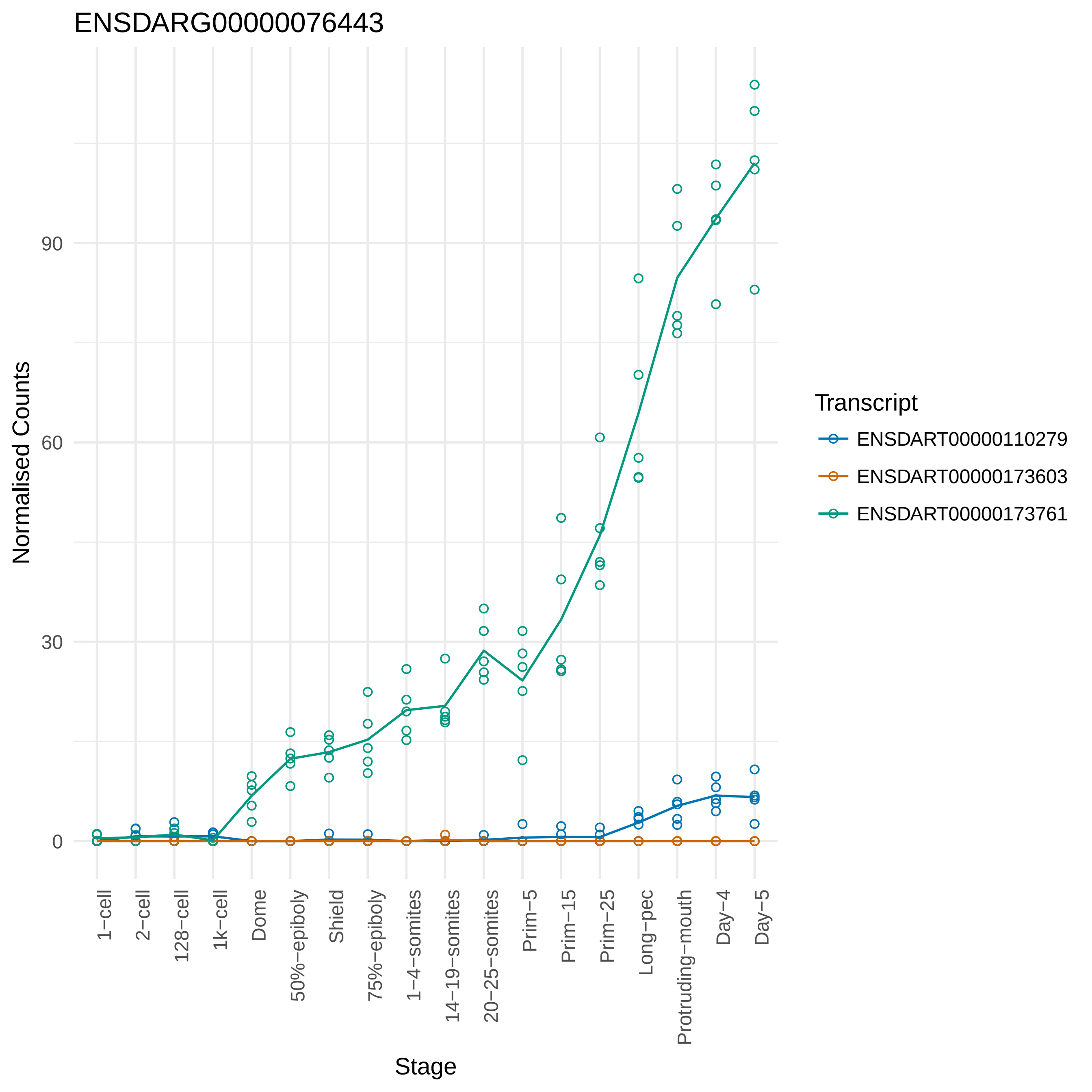
ENSDARG00000076443
Normalised Counts
0
30
60
90
Stage
1−cell
2−cell
128−cell
1k−cell
Dome
50%−epiboly
Shield
75%−epiboly
1−4−somites
14−19−somites
20−25−somites
Prim−5
Prim−15
Prim−25
Long−pec
Protruding−mouth
Day−4
Day−5
Transcript
ENSDART00000110279
ENSDART00000173603
ENSDART00000173761

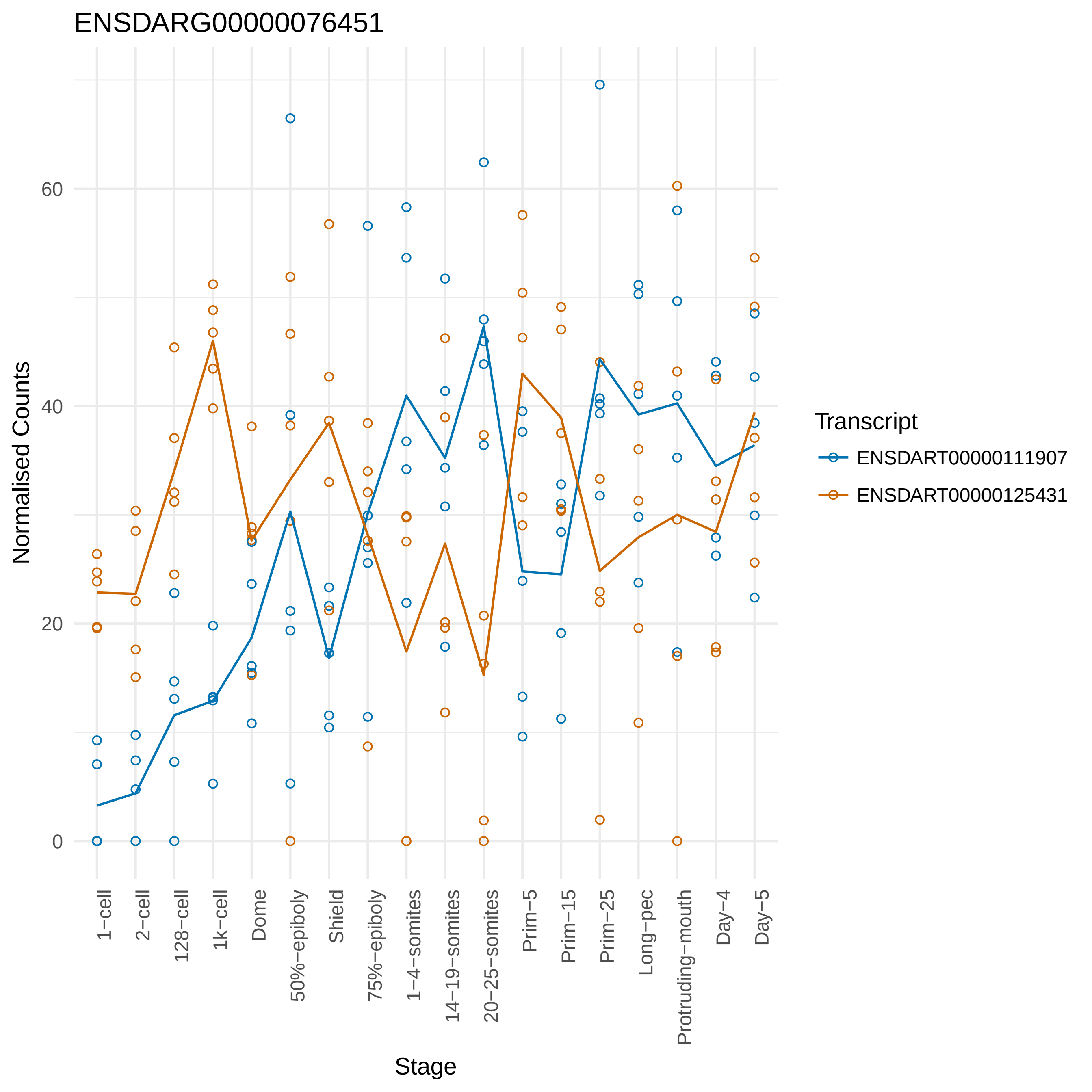

ENSDARG00000076451
Normalised Counts
0
20
40
60
1−cell
2−cell
128−cell
1k−cell
Dome
50%−epiboly
Shield
75%−epiboly
1−4−somites
14−19−somites
20−25−somites
Prim−5
Prim−15
Prim−25
Long−pec
Protruding−mouth
Day−4
Day−5
Stage
Transcript
ENSDART00000111907
ENSDART00000125431

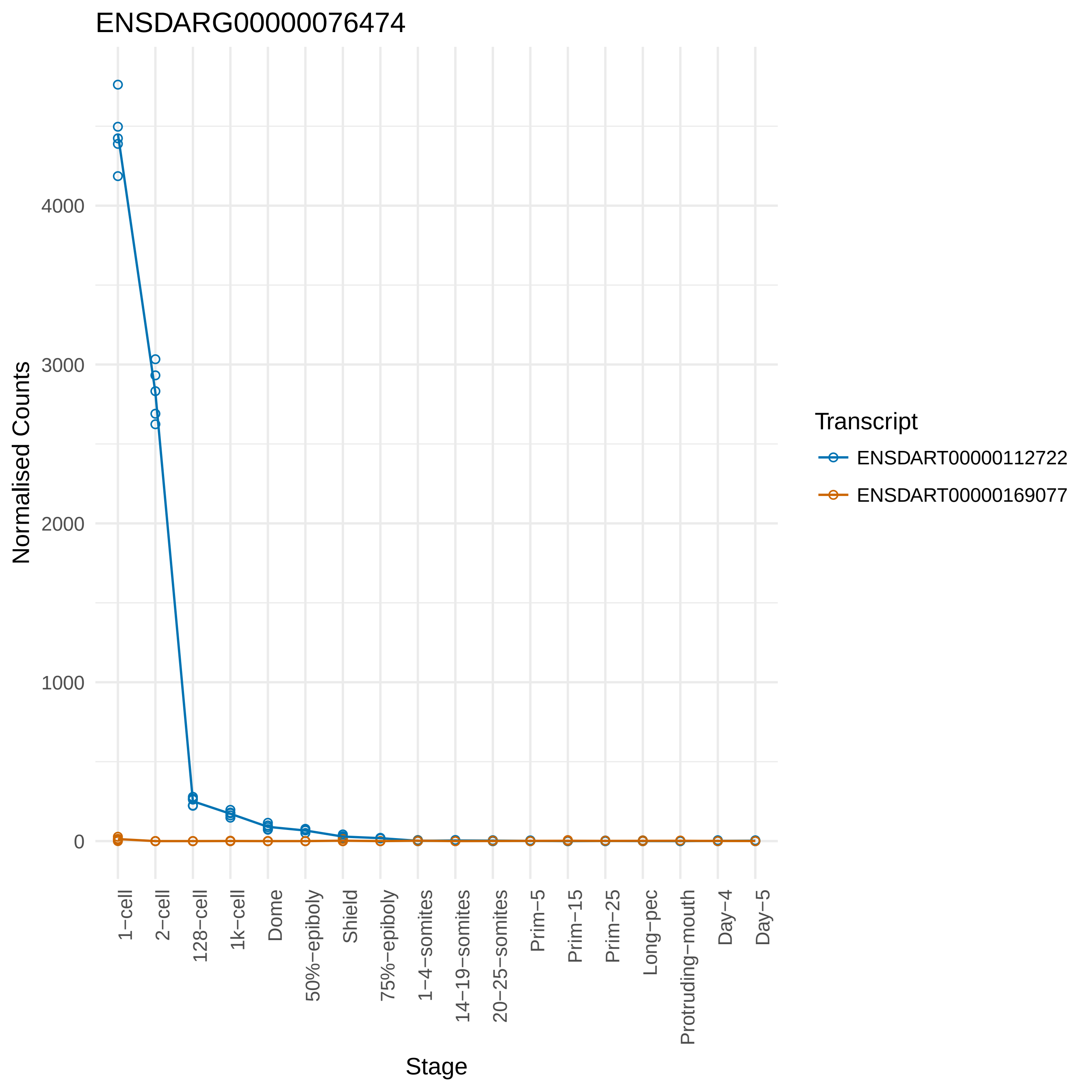
ENSDARG00000076474
Normalised Counts
4000
3000
2000
1000
0
1−cell
2−cell
128−cell
1k−cell
Dome
50%−epiboly
Shield
75%−epiboly
1−4−somites
14−19−somites
20−25−somites
Prim−5
Prim−15
Prim−25
Long−pec
Protruding−mouth
Day−4
Day−5
Stage
Transcript
ENSDART00000112722
ENSDART00000169077

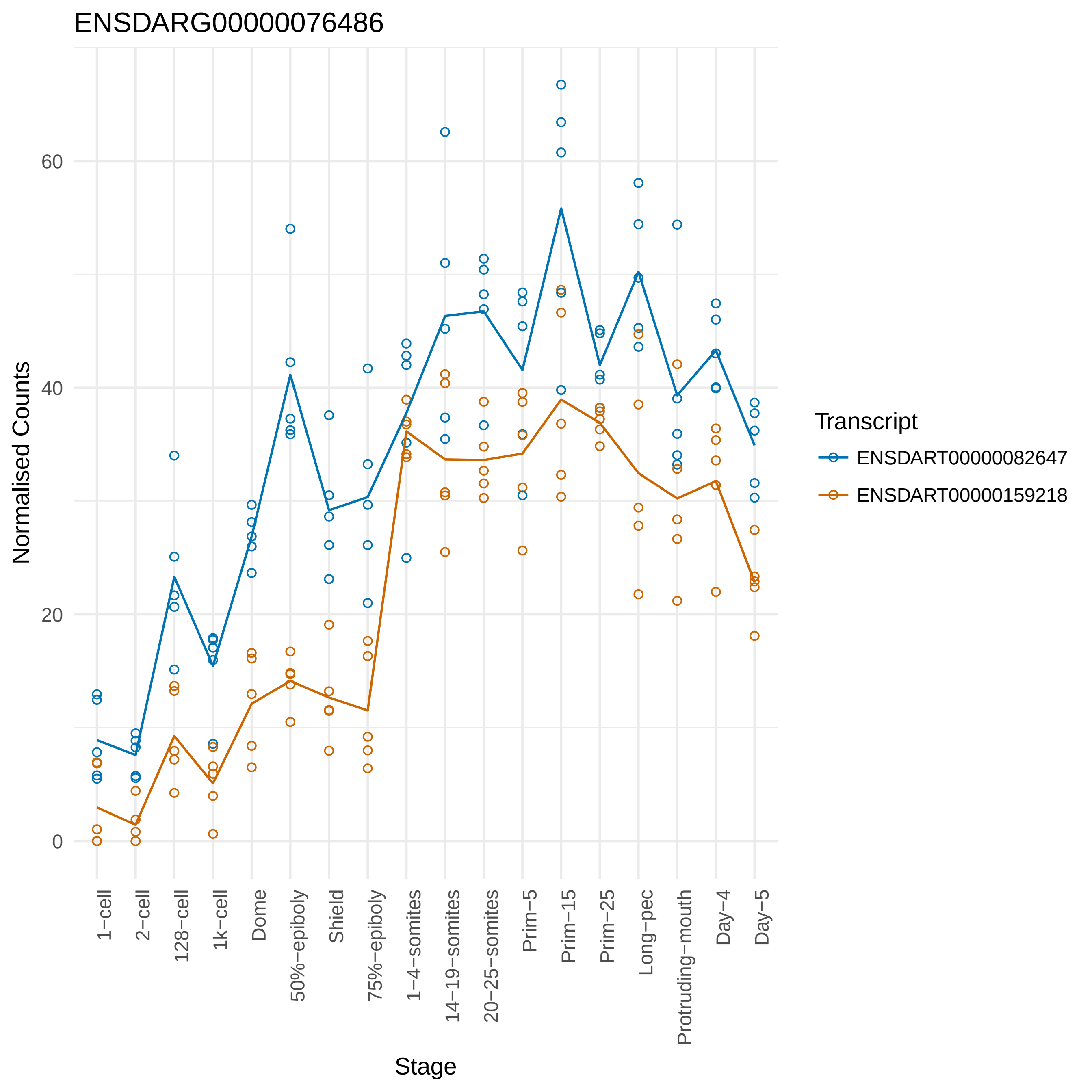

ENSDARG00000076486
Normalised Counts
0
20
40
60
Stage
1−cell
2−cell
128−cell
1k−cell
Dome
50%−epiboly
Shield
75%−epiboly
1−4−somites
14−19−somites
20−25−somites
Prim−5
Prim−15
Prim−25
Long−pec
Protruding−mouth
Day−4
Day−5
Transcript
ENSDART00000082647
ENSDART00000159218

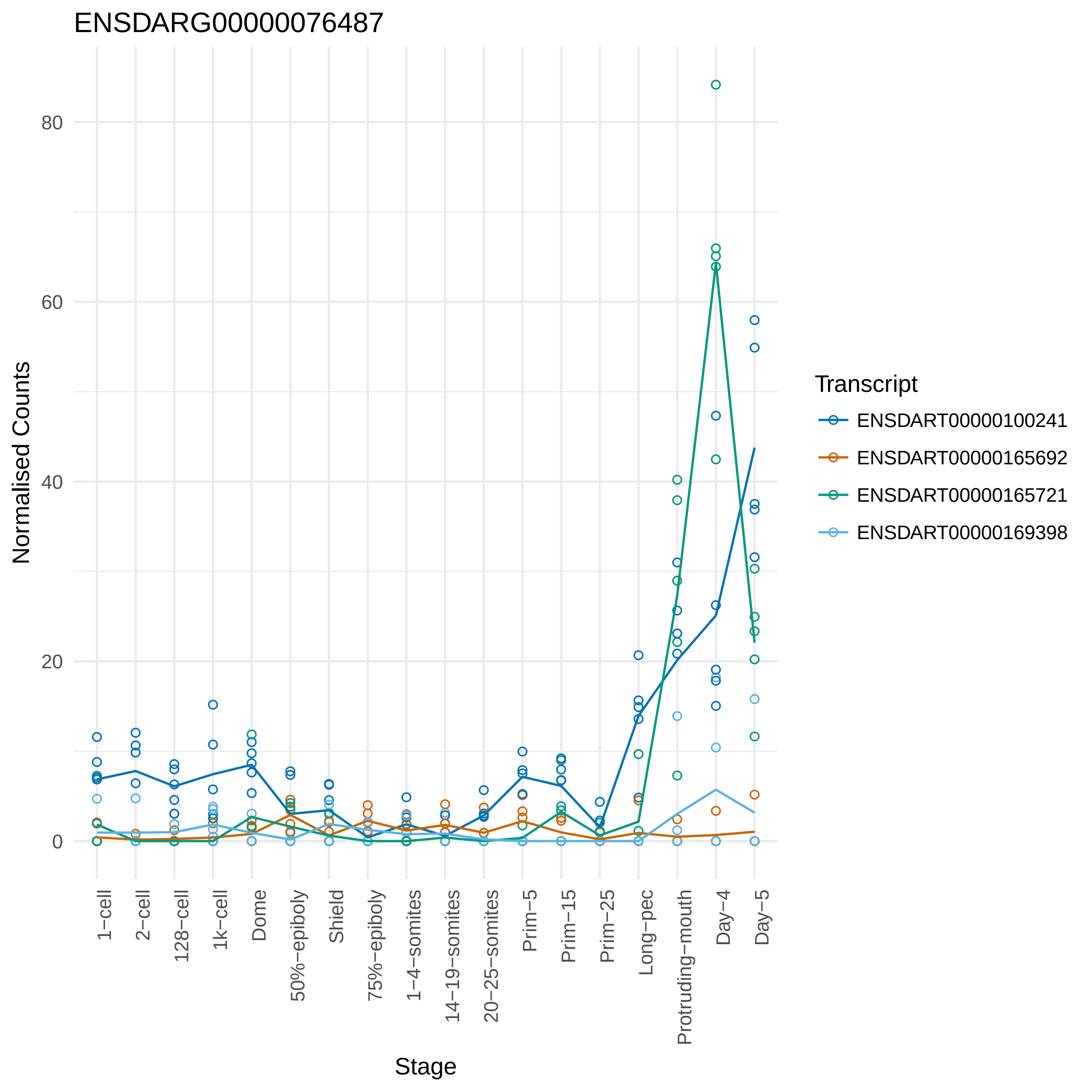

ENSDARG00000076487
Normalised Counts
0
20
40
60
80
1−cell
2−cell
128−cell
1k−cell
Dome
50%−epiboly
Shield
75%−epiboly
1−4−somites
14−19−somites
20−25−somites
Prim−5
Prim−15
Prim−25
Long−pec
Protruding−mouth
Day−4
Day−5
Stage
Transcript
ENSDART00000100241
ENSDART00000165692
ENSDART00000165721
ENSDART00000169398

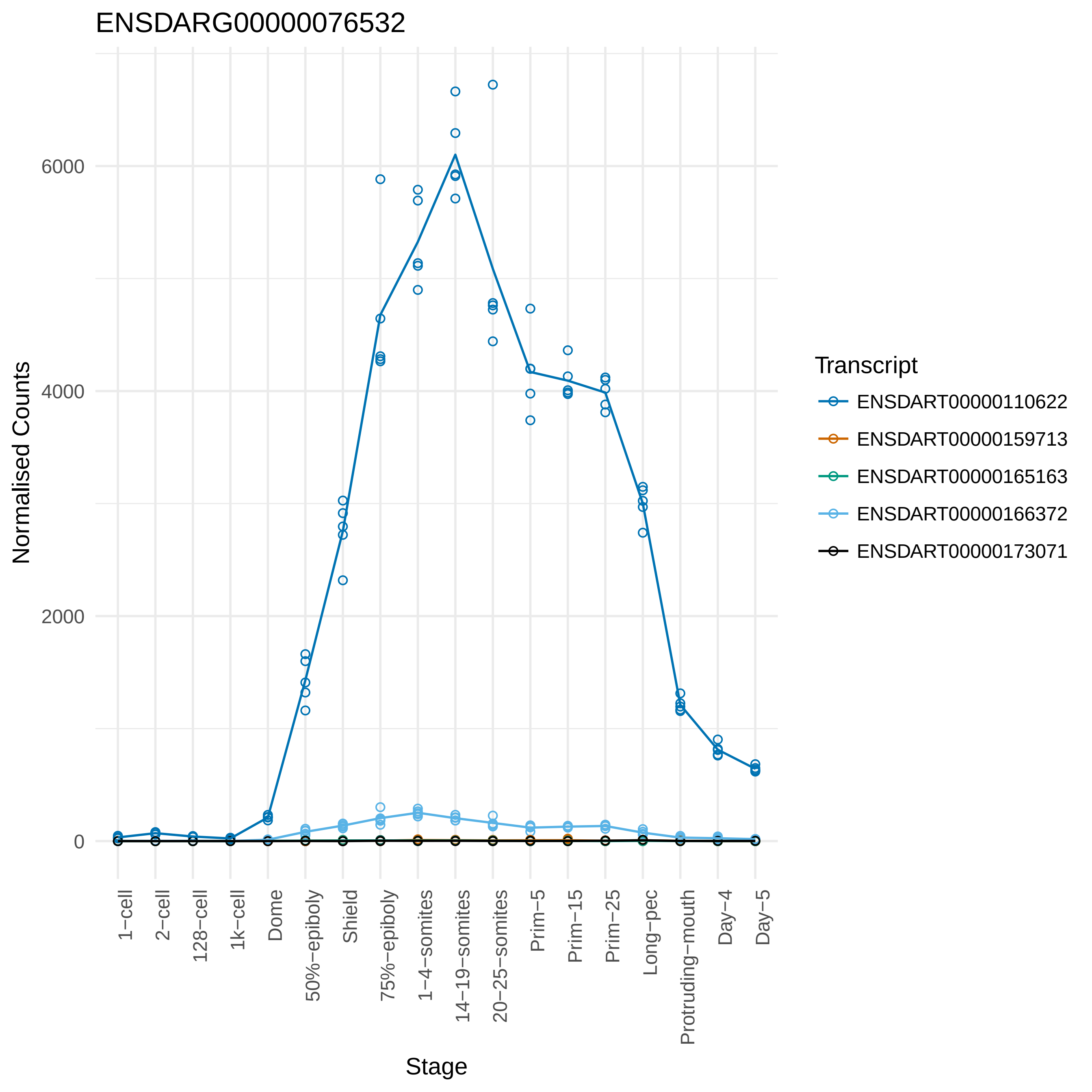
ENSDARG00000076532
Normalised Counts
6000
4000
2000
0
1−cell
2−cell
128−cell
1k−cell
Dome
50%−epiboly
Shield
75%−epiboly
1−4−somites
14−19−somites
20−25−somites
Prim−5
Prim−15
Prim−25
Long−pec
Protruding−mouth
Day−4
Day−5
Stage
Transcript
ENSDART00000110622
ENSDART00000159713
ENSDART00000165163
ENSDART00000166372
ENSDART00000173071

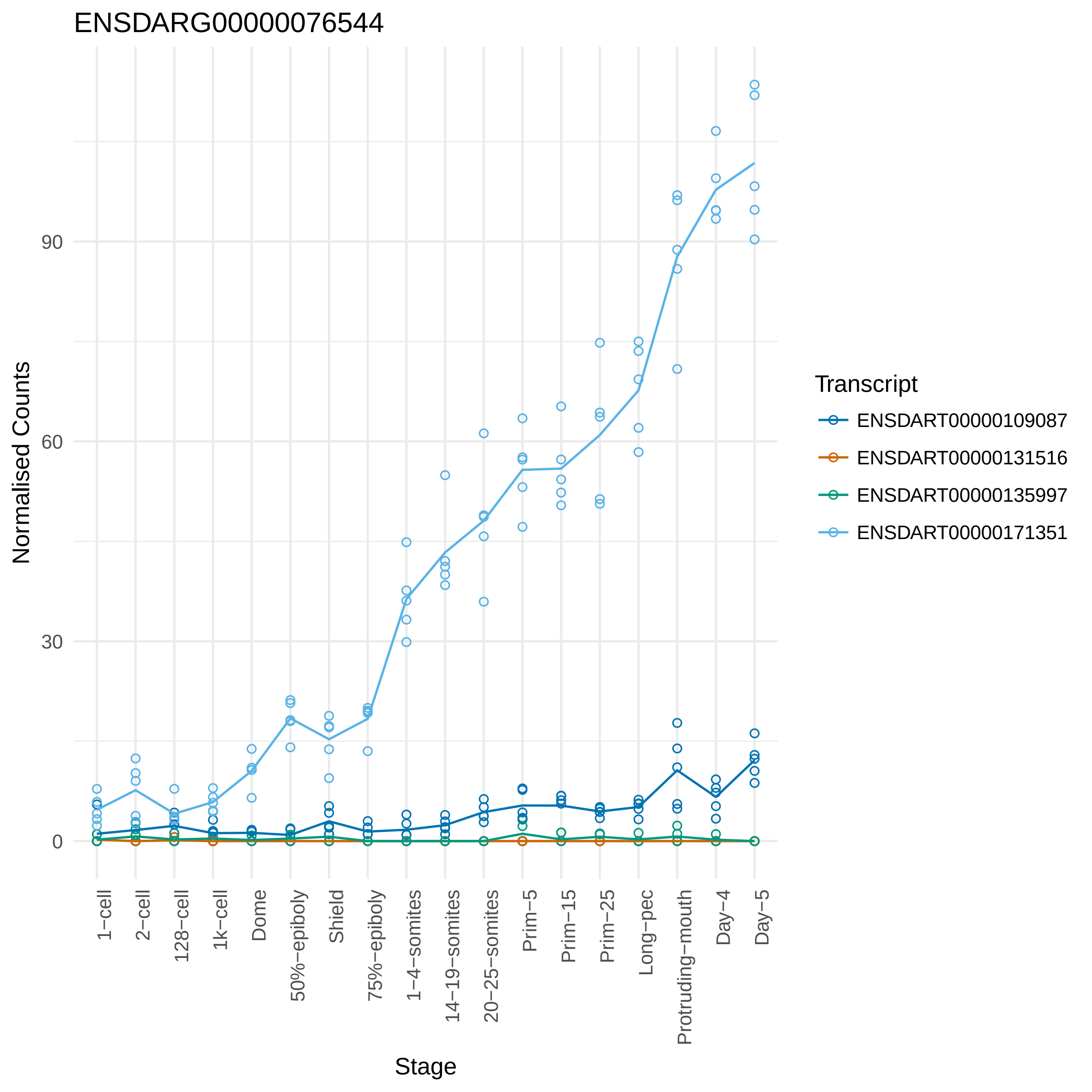

ENSDARG00000076544
Normalised Counts
0
30
60
90
Stage
1−cell
2−cell
128−cell
1k−cell
Dome
50%−epiboly
Shield
75%−epiboly
1−4−somites
14−19−somites
20−25−somites
Prim−5
Prim−15
Prim−25
Long−pec
Protruding−mouth
Day−4
Day−5
Transcript
ENSDART00000109087
ENSDART00000131516
ENSDART00000135997
ENSDART00000171351

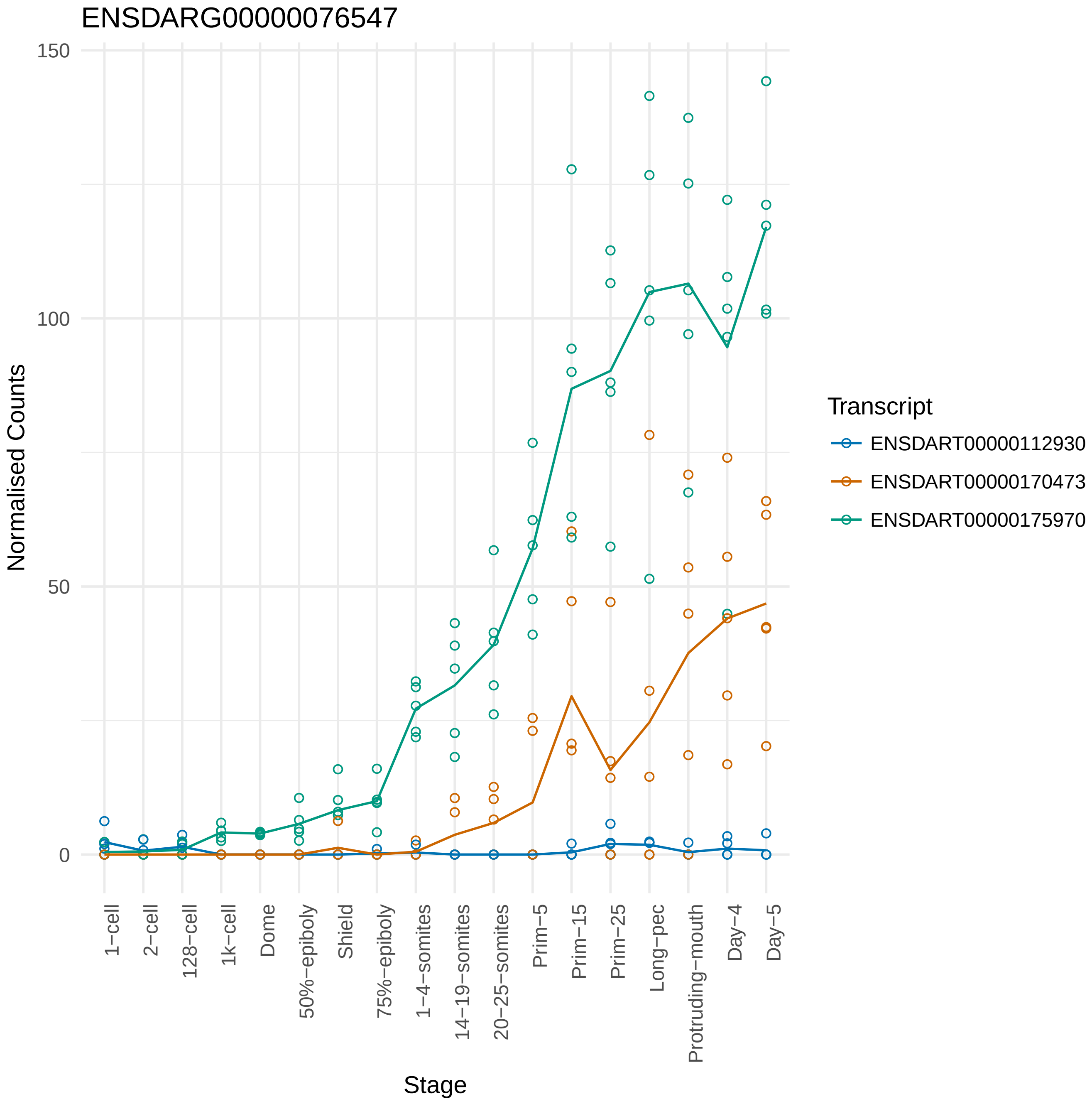

ENSDARG00000076547
150
100
50
0
Normalised Counts
1−cell
2−cell
128−cell
1k−cell
Dome
50%−epiboly
Shield
75%−epiboly
1−4−somites
14−19−somites
20−25−somites
Prim−5
Prim−15
Prim−25
Long−pec
Protruding−mouth
Day−4
Day−5
Stage
Transcript
ENSDART00000112930
ENSDART00000170473
ENSDART00000175970

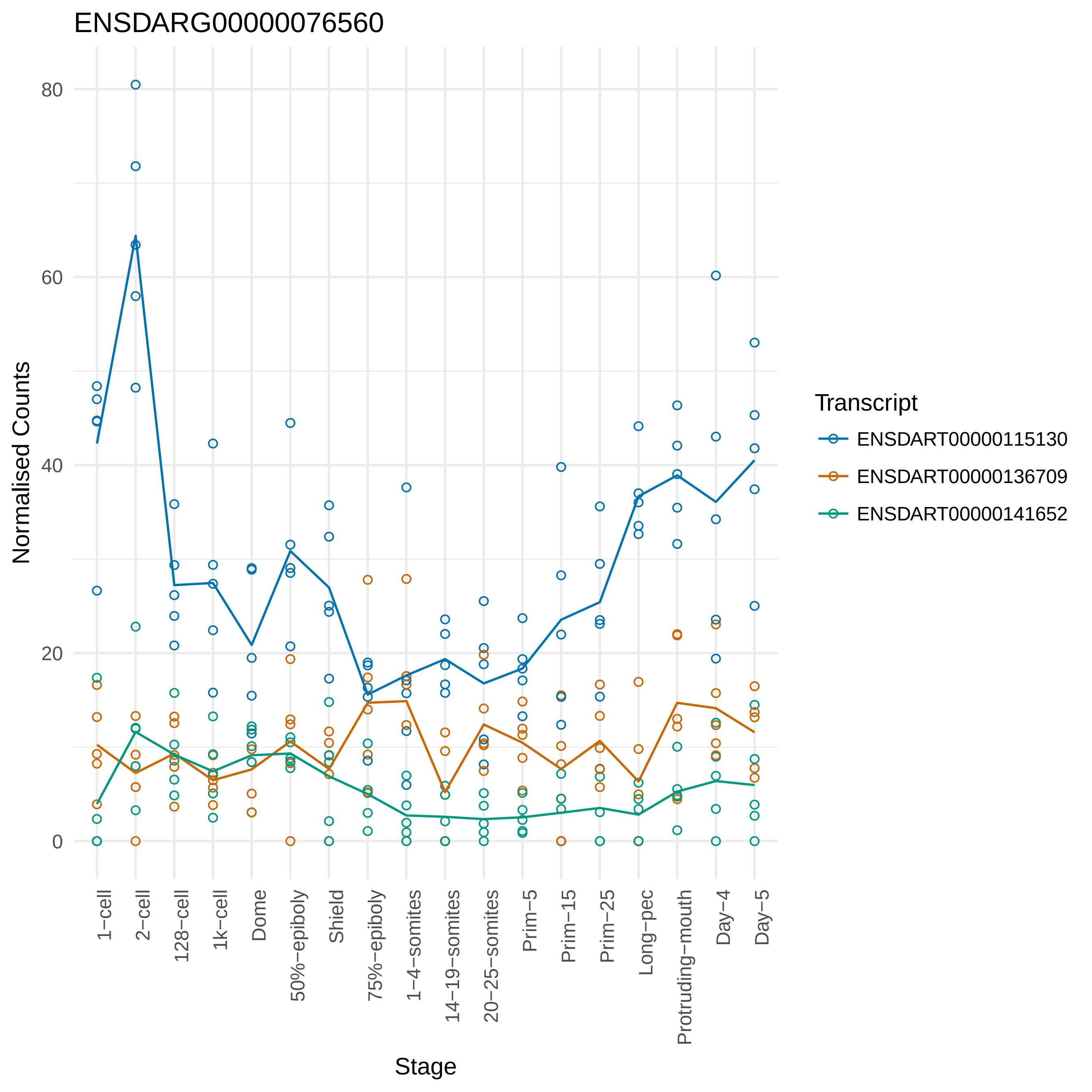

ENSDARG00000076560
Normalised Counts
0
20
40
60
80
1−cell
2−cell
128−cell
1k−cell
Dome
50%−epiboly
Shield
75%−epiboly
1−4−somites
14−19−somites
20−25−somites
Prim−5
Prim−15
Prim−25
Long−pec
Protruding−mouth
Day−4
Day−5
Stage
Transcript
ENSDART00000115130
ENSDART00000136709
ENSDART00000141652

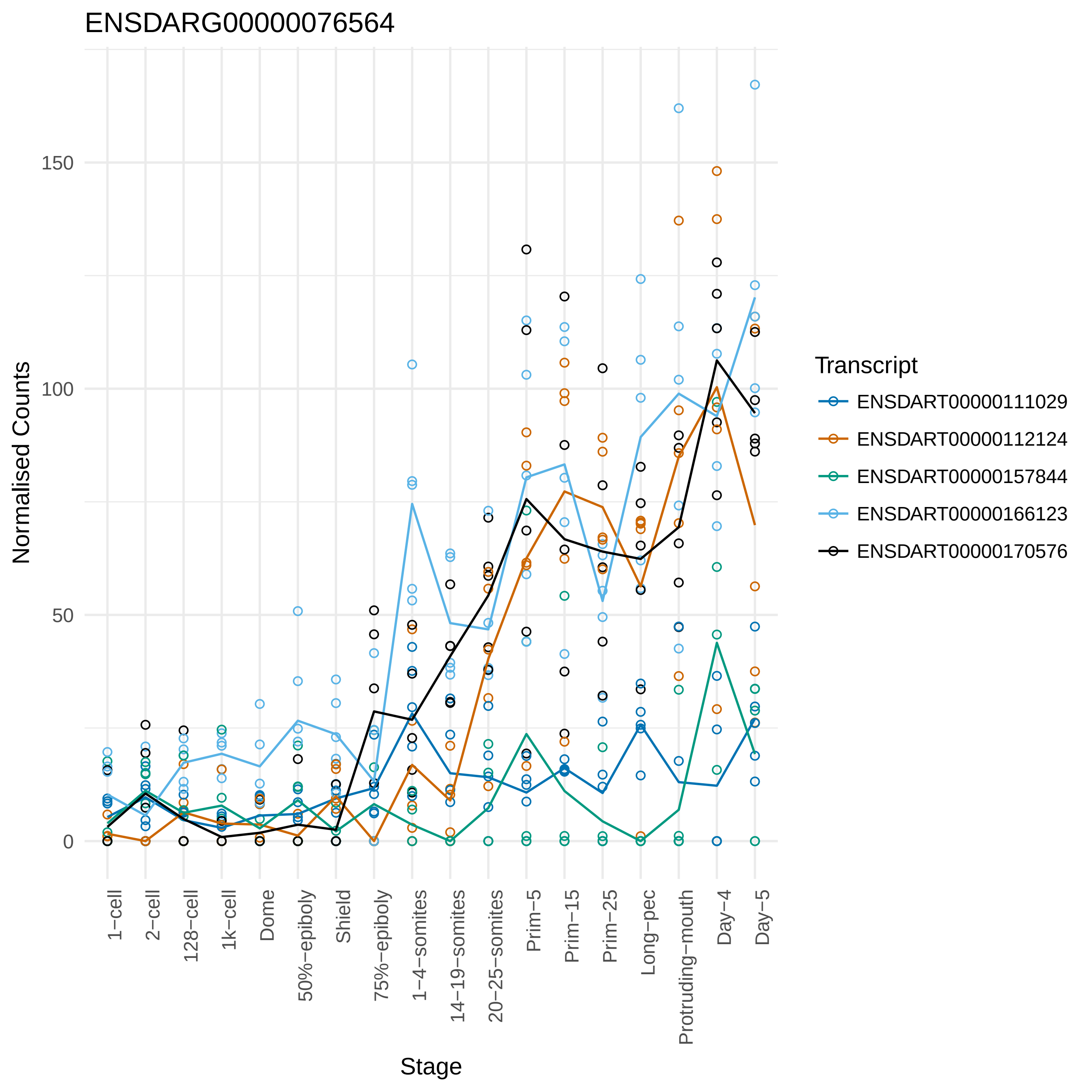
ENSDARG00000076564
Normalised Counts
150
100
50
0
1−cell
2−cell
128−cell
1k−cell
Dome
50%−epiboly
Shield
75%−epiboly
1−4−somites
14−19−somites
20−25−somites
Prim−5
Prim−15
Prim−25
Long−pec
Protruding−mouth
Day−4
Day−5
Stage
Transcript
ENSDART00000111029
ENSDART00000112124
ENSDART00000157844
ENSDART00000166123
ENSDART00000170576

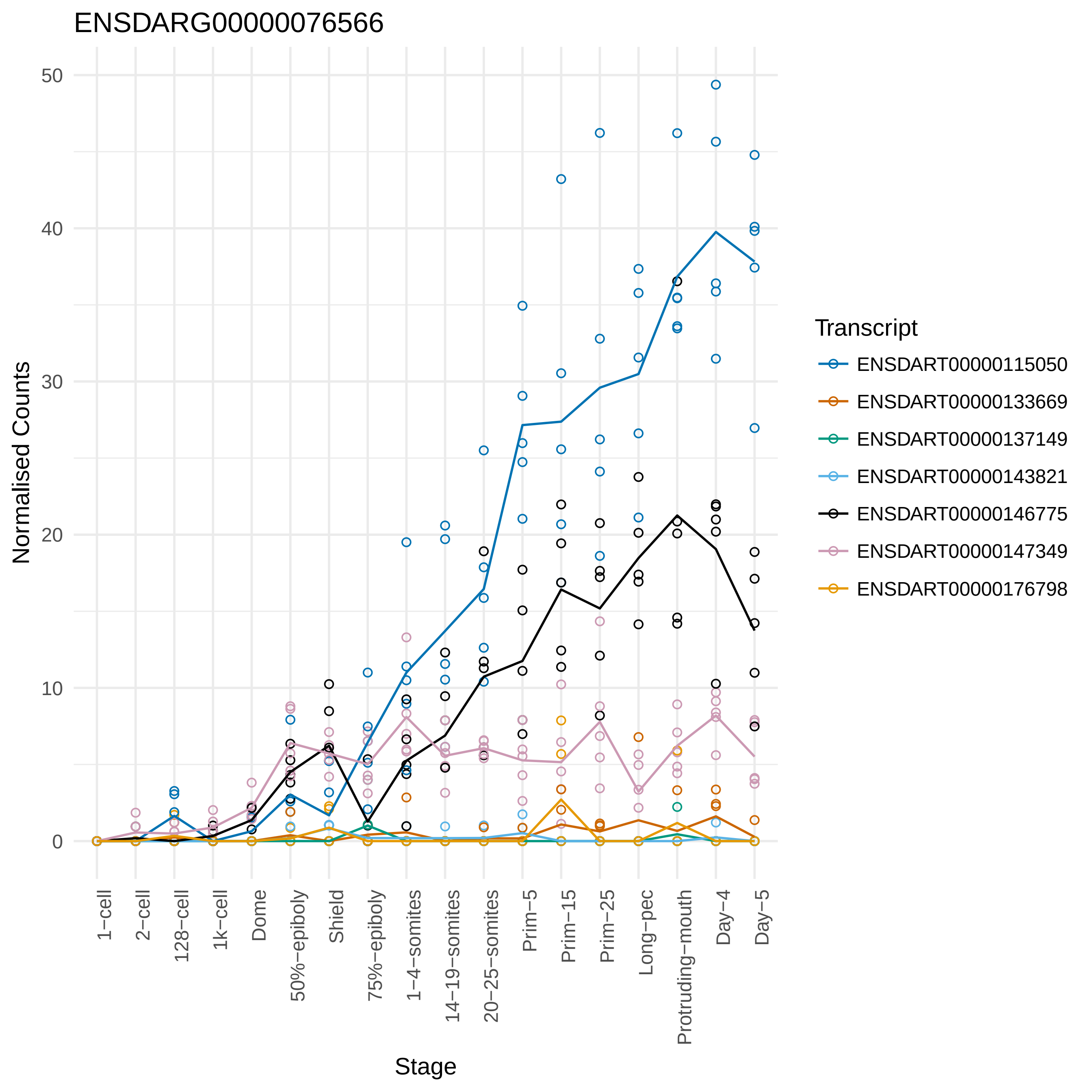
ENSDARG00000076566
Normalised Counts
0
10
20
30
40
50
Stage
1−cell
2−cell
128−cell
1k−cell
Dome
50%−epiboly
Shield
75%−epiboly
1−4−somites
14−19−somites
20−25−somites
Prim−5
Prim−15
Prim−25
Long−pec
Protruding−mouth
Day−4
Day−5
Transcript
ENSDART00000115050
ENSDART00000133669
ENSDART00000137149
ENSDART00000143821
ENSDART00000146775
ENSDART00000147349
ENSDART00000176798

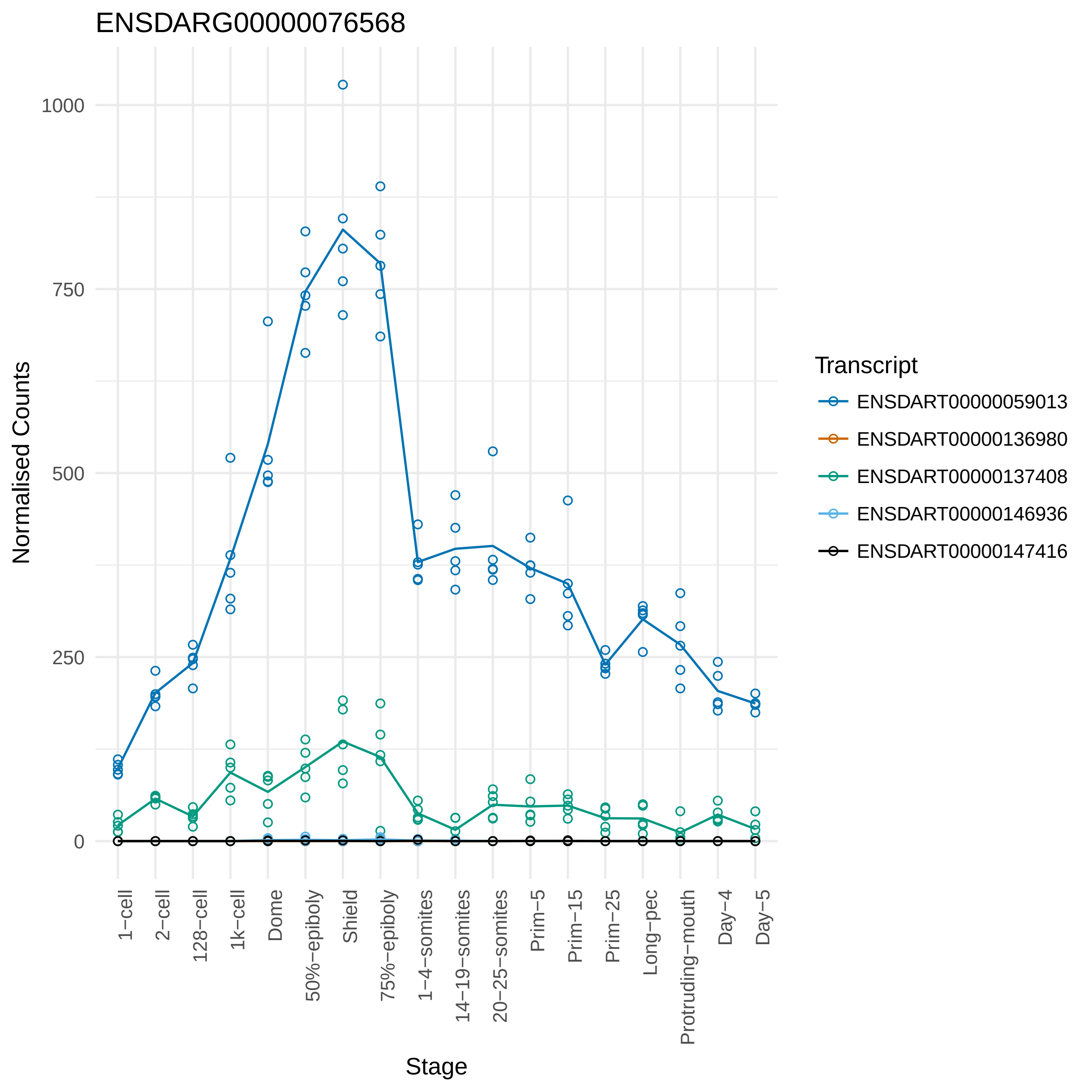

ENSDARG00000076568
Normalised Counts
1000
750
500
250
0
1−cell
2−cell
128−cell
1k−cell
Dome
50%−epiboly
Shield
75%−epiboly
1−4−somites
14−19−somites
20−25−somites
Prim−5
Prim−15
Prim−25
Long−pec
Protruding−mouth
Day−4
Day−5
Stage
Transcript
ENSDART00000059013
ENSDART00000136980
ENSDART00000137408
ENSDART00000146936
ENSDART00000147416

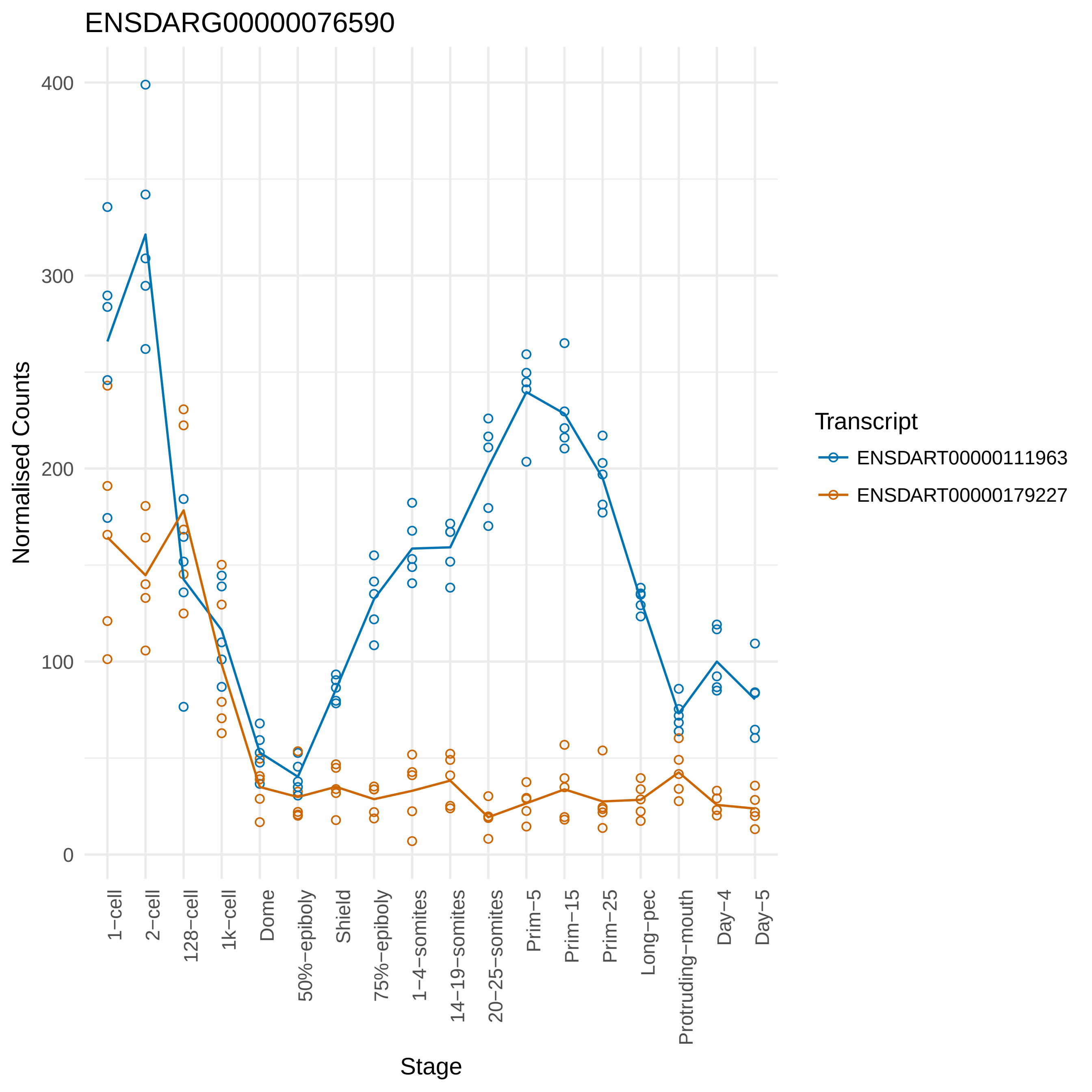

ENSDARG00000076590
Normalised Counts
400
300
200
100
0
1−cell
2−cell
128−cell
1k−cell
Dome
50%−epiboly
Shield
75%−epiboly
1−4−somites
14−19−somites
20−25−somites
Prim−5
Prim−15
Prim−25
Long−pec
Protruding−mouth
Day−4
Day−5
Stage
Transcript
ENSDART00000111963
ENSDART00000179227

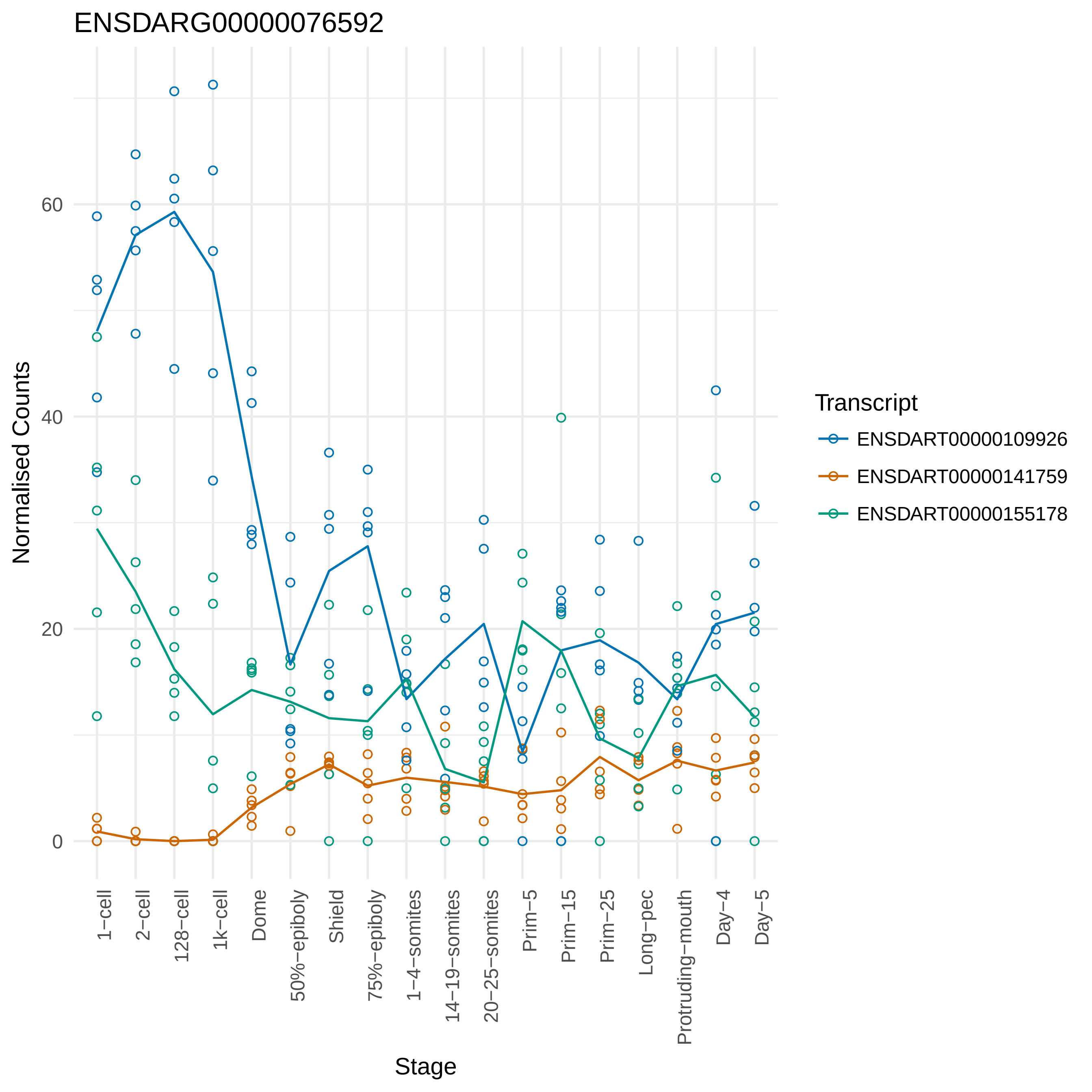
ENSDARG00000076592
Normalised Counts
0
20
40
60
Stage
1−cell
2−cell
128−cell
1k−cell
Dome
50%−epiboly
Shield
75%−epiboly
1−4−somites
14−19−somites
20−25−somites
Prim−5
Prim−15
Prim−25
Long−pec
Protruding−mouth
Day−4
Day−5
Transcript
ENSDART00000109926
ENSDART00000141759
ENSDART00000155178

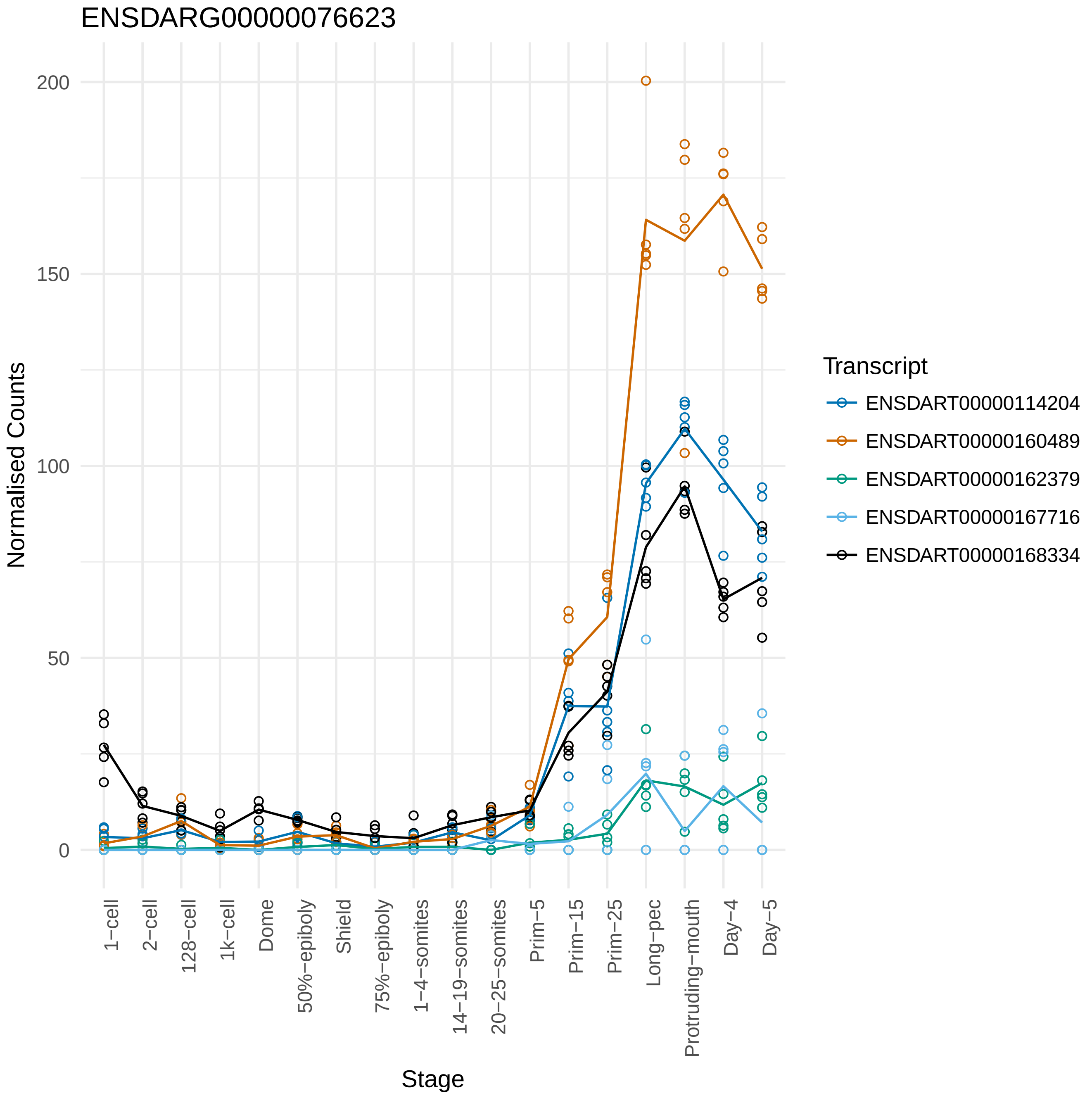

ENSDARG00000076623
Normalised Counts
0
50
100
150
200
1−cell
2−cell
128−cell
1k−cell
Dome
50%−epiboly
Shield
75%−epiboly
1−4−somites
14−19−somites
20−25−somites
Prim−5
Prim−15
Prim−25
Long−pec
Protruding−mouth
Day−4
Day−5
Stage
Transcript
ENSDART00000114204
ENSDART00000160489
ENSDART00000162379
ENSDART00000167716
ENSDART00000168334

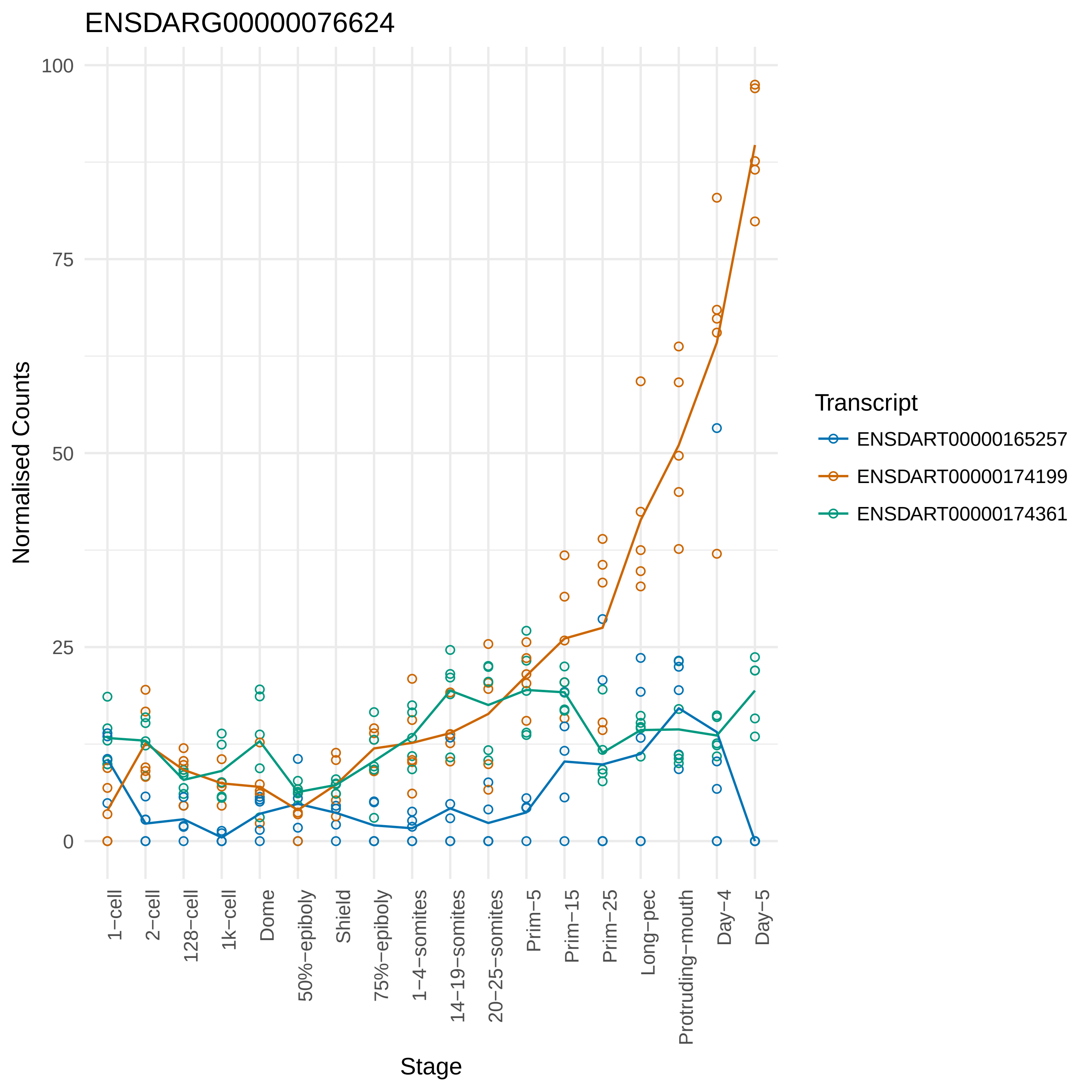

ENSDARG00000076624
Normalised Counts
100
75
50
25
0
Stage
1−cell
2−cell
128−cell
1k−cell
Dome
50%−epiboly
Shield
75%−epiboly
1−4−somites
14−19−somites
20−25−somites
Prim−5
Prim−15
Prim−25
Long−pec
Protruding−mouth
Day−4
Day−5
Transcript
ENSDART00000165257
ENSDART00000174199
ENSDART00000174361

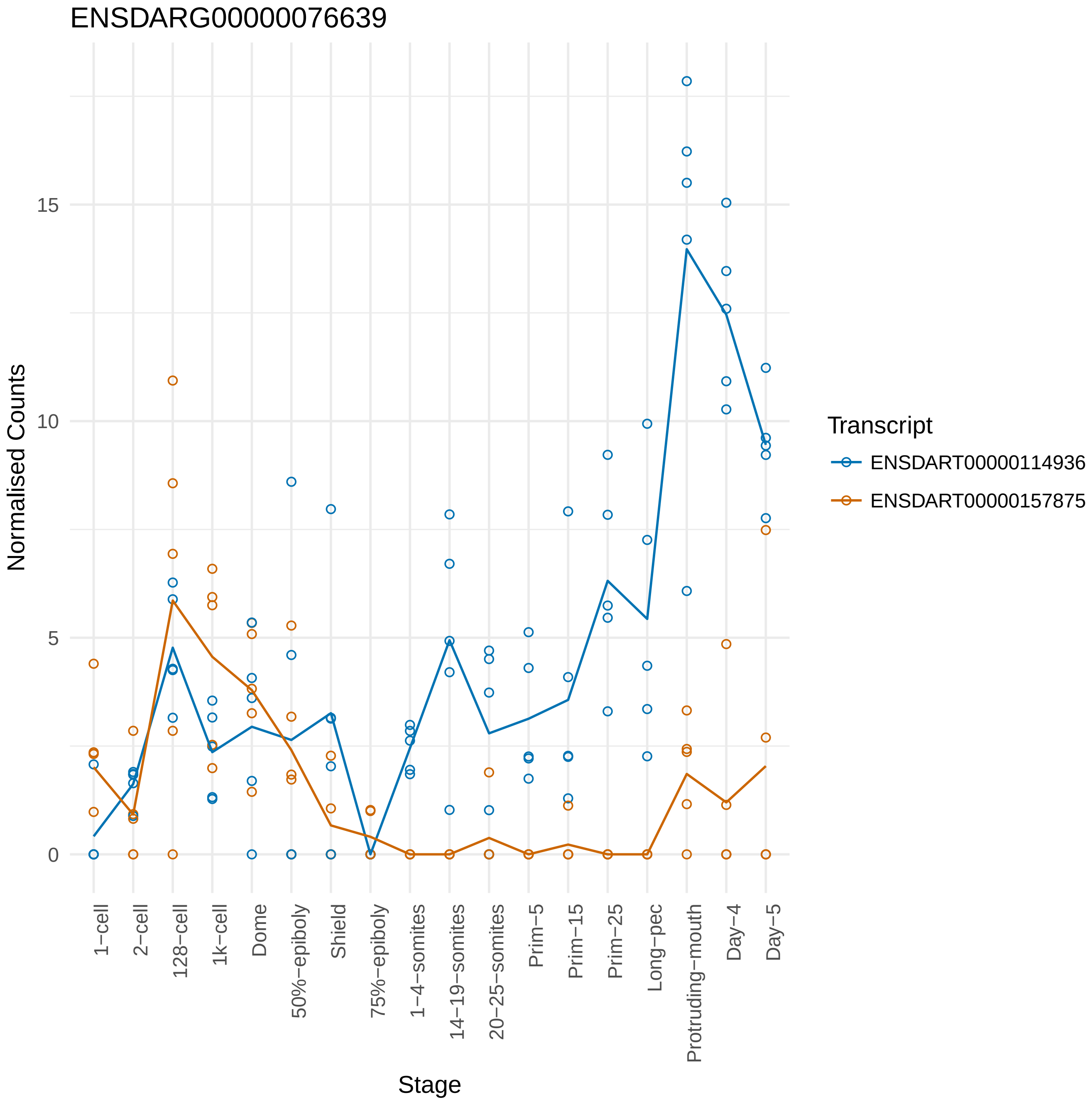

ENSDARG00000076639
Normalised Counts
0
5
10
15
Stage
1−cell
2−cell
128−cell
1k−cell
Dome
50%−epiboly
Shield
75%−epiboly
1−4−somites
14−19−somites
20−25−somites
Prim−5
Prim−15
Prim−25
Long−pec
Protruding−mouth
Day−4
Day−5
Transcript
ENSDART00000114936
ENSDART00000157875

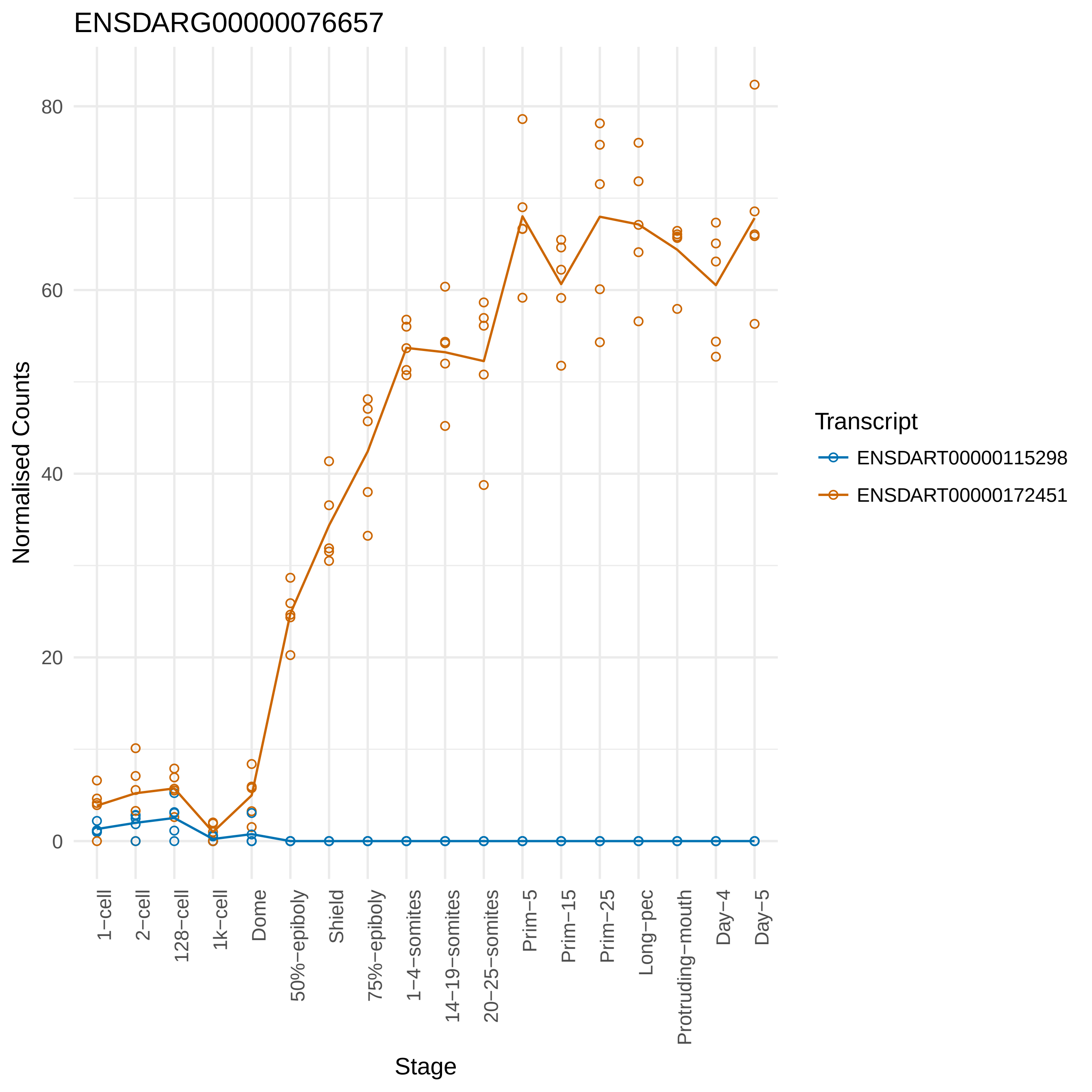
ENSDARG00000076657
Normalised Counts
80
60
40
20
0
1−cell
2−cell
128−cell
1k−cell
Dome
50%−epiboly
Shield
75%−epiboly
1−4−somites
14−19−somites
20−25−somites
Prim−5
Prim−15
Prim−25
Long−pec
Protruding−mouth
Day−4
Day−5
Stage
Transcript
ENSDART00000115298
ENSDART00000172451

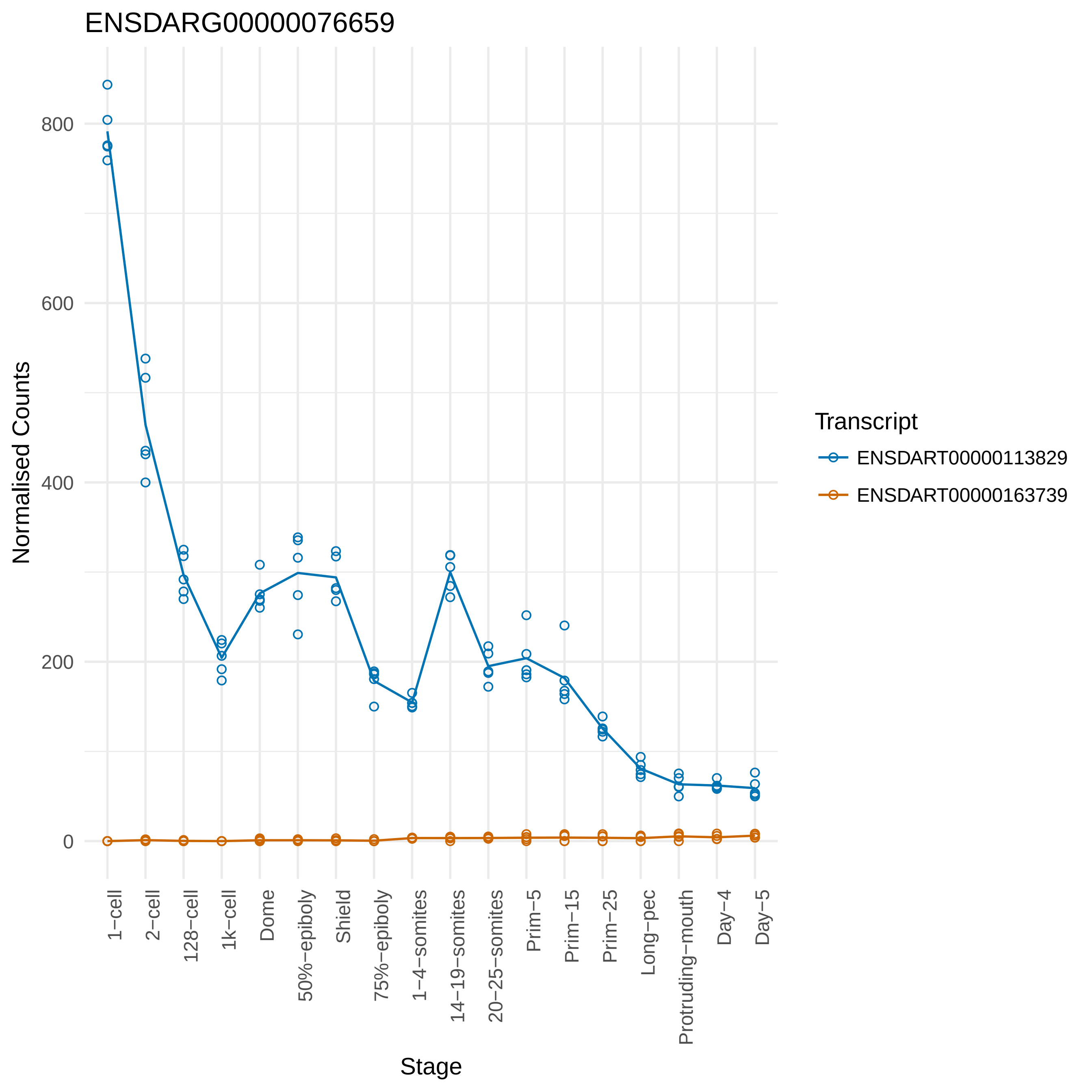
ENSDARG00000076659
Normalised Counts
800
600
400
200
0
1−cell
2−cell
128−cell
1k−cell
Dome
50%−epiboly
Shield
75%−epiboly
1−4−somites
14−19−somites
20−25−somites
Prim−5
Prim−15
Prim−25
Long−pec
Protruding−mouth
Day−4
Day−5
Stage
Transcript
ENSDART00000113829
ENSDART00000163739

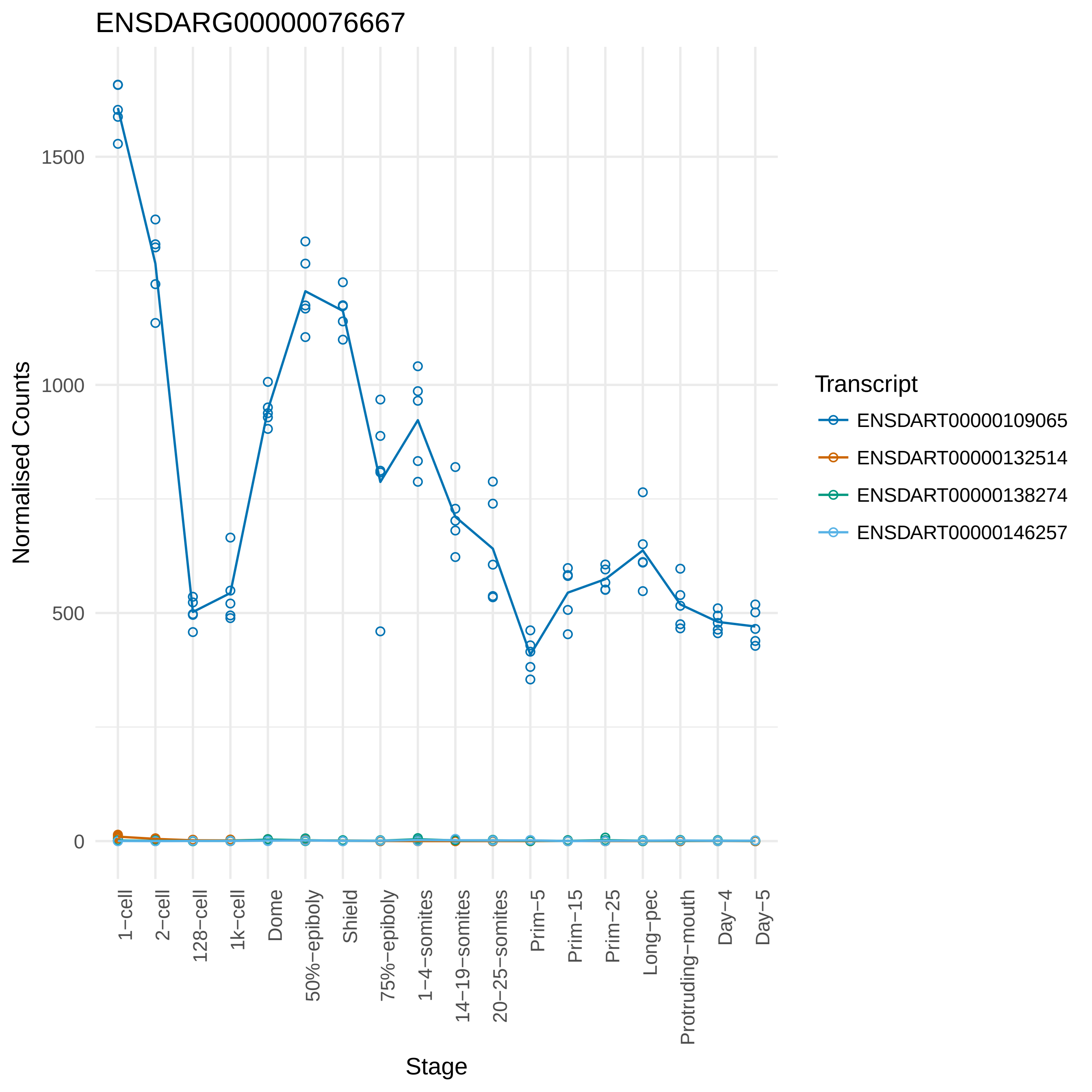

ENSDARG00000076667
Normalised Counts
1500
1000
500
0
1−cell
2−cell
128−cell
1k−cell
Dome
50%−epiboly
Shield
75%−epiboly
1−4−somites
14−19−somites
20−25−somites
Prim−5
Prim−15
Prim−25
Long−pec
Protruding−mouth
Day−4
Day−5
Stage
Transcript
ENSDART00000109065
ENSDART00000132514
ENSDART00000138274
ENSDART00000146257

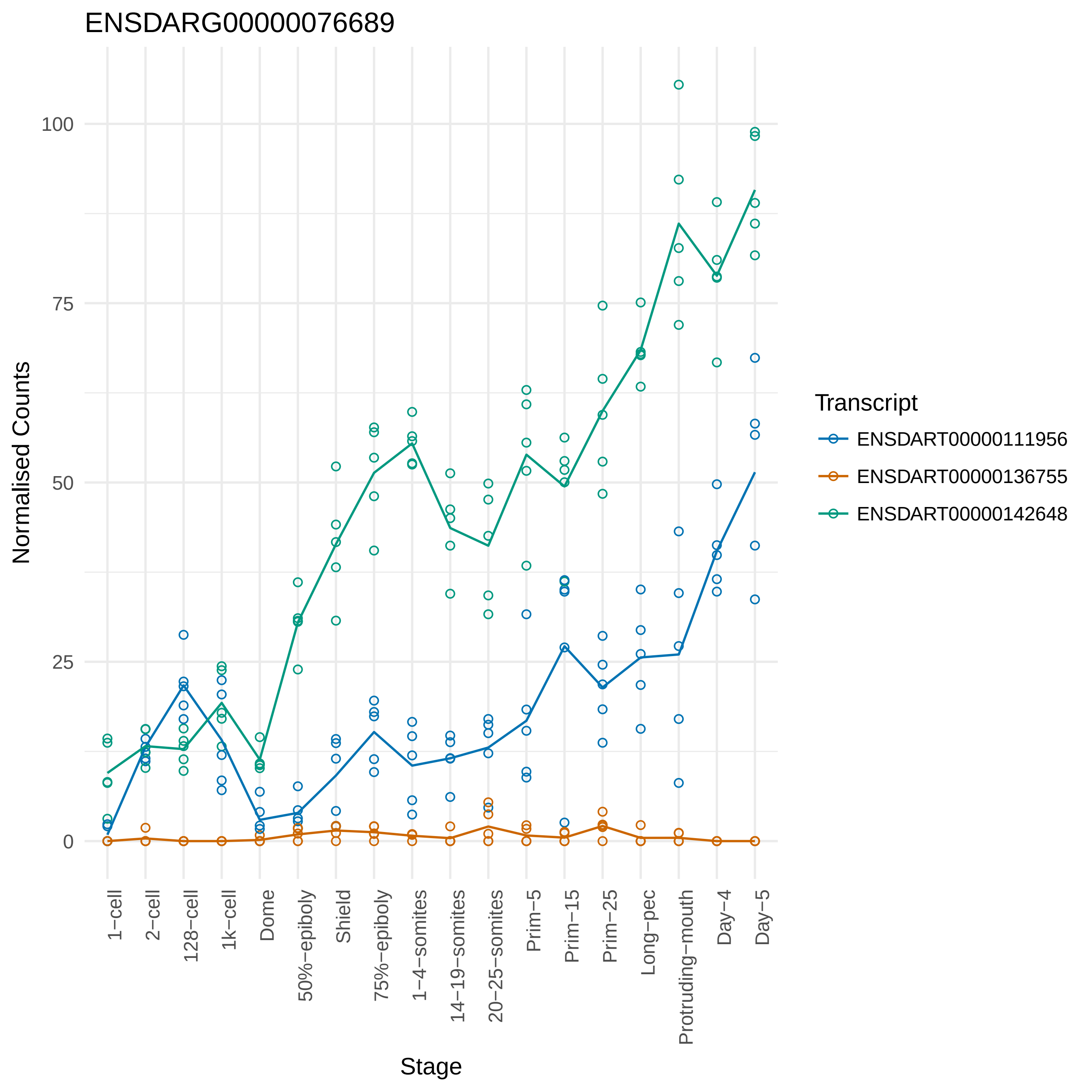
ENSDARG00000076689
Normalised Counts
100
75
50
25
0
1−cell
2−cell
128−cell
1k−cell
Dome
50%−epiboly
Shield
75%−epiboly
1−4−somites
14−19−somites
20−25−somites
Prim−5
Prim−15
Prim−25
Long−pec
Protruding−mouth
Day−4
Day−5
Stage
Transcript
ENSDART00000111956
ENSDART00000136755
ENSDART00000142648

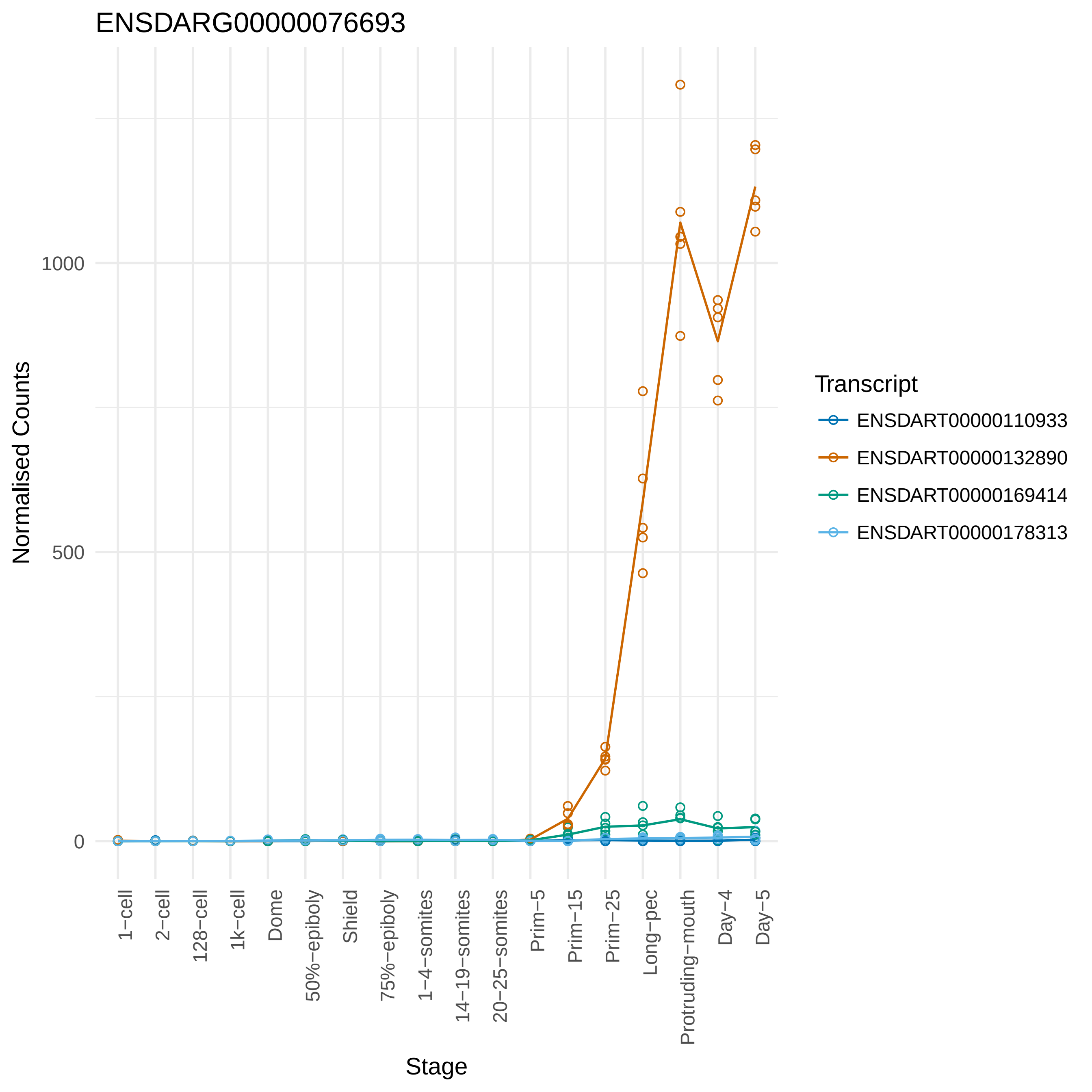

ENSDARG00000076693
Normalised Counts
1000
500
0
1−cell
2−cell
128−cell
1k−cell
Dome
50%−epiboly
Shield
75%−epiboly
1−4−somites
14−19−somites
20−25−somites
Prim−5
Prim−15
Prim−25
Long−pec
Protruding−mouth
Day−4
Day−5
Stage
Transcript
ENSDART00000110933
ENSDART00000132890
ENSDART00000169414
ENSDART00000178313

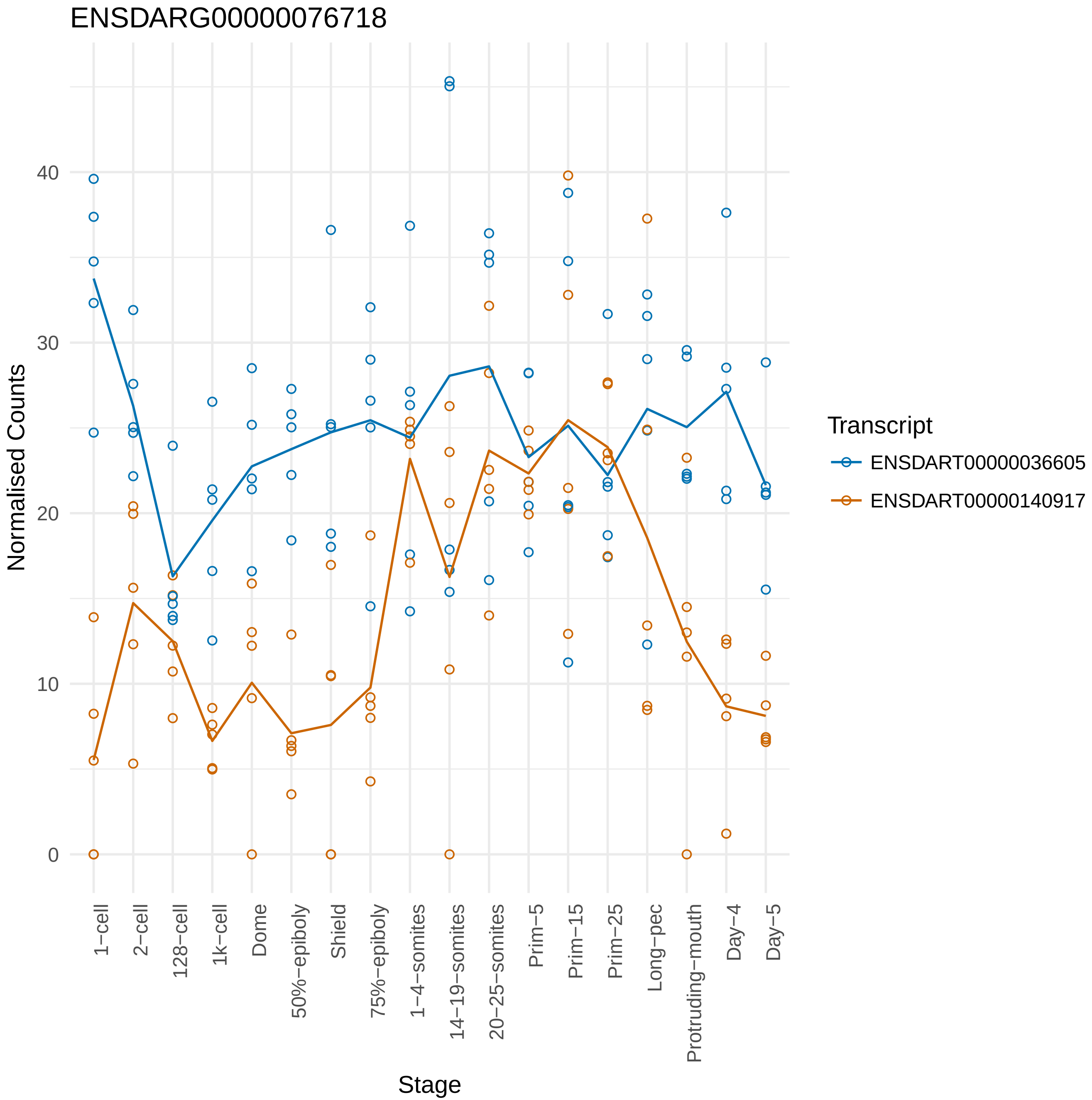

ENSDARG00000076718
Normalised Counts
0
10
20
30
40
Stage
1−cell
2−cell
128−cell
1k−cell
Dome
50%−epiboly
Shield
75%−epiboly
1−4−somites
14−19−somites
20−25−somites
Prim−5
Prim−15
Prim−25
Long−pec
Protruding−mouth
Day−4
Day−5
Transcript
ENSDART00000036605
ENSDART00000140917

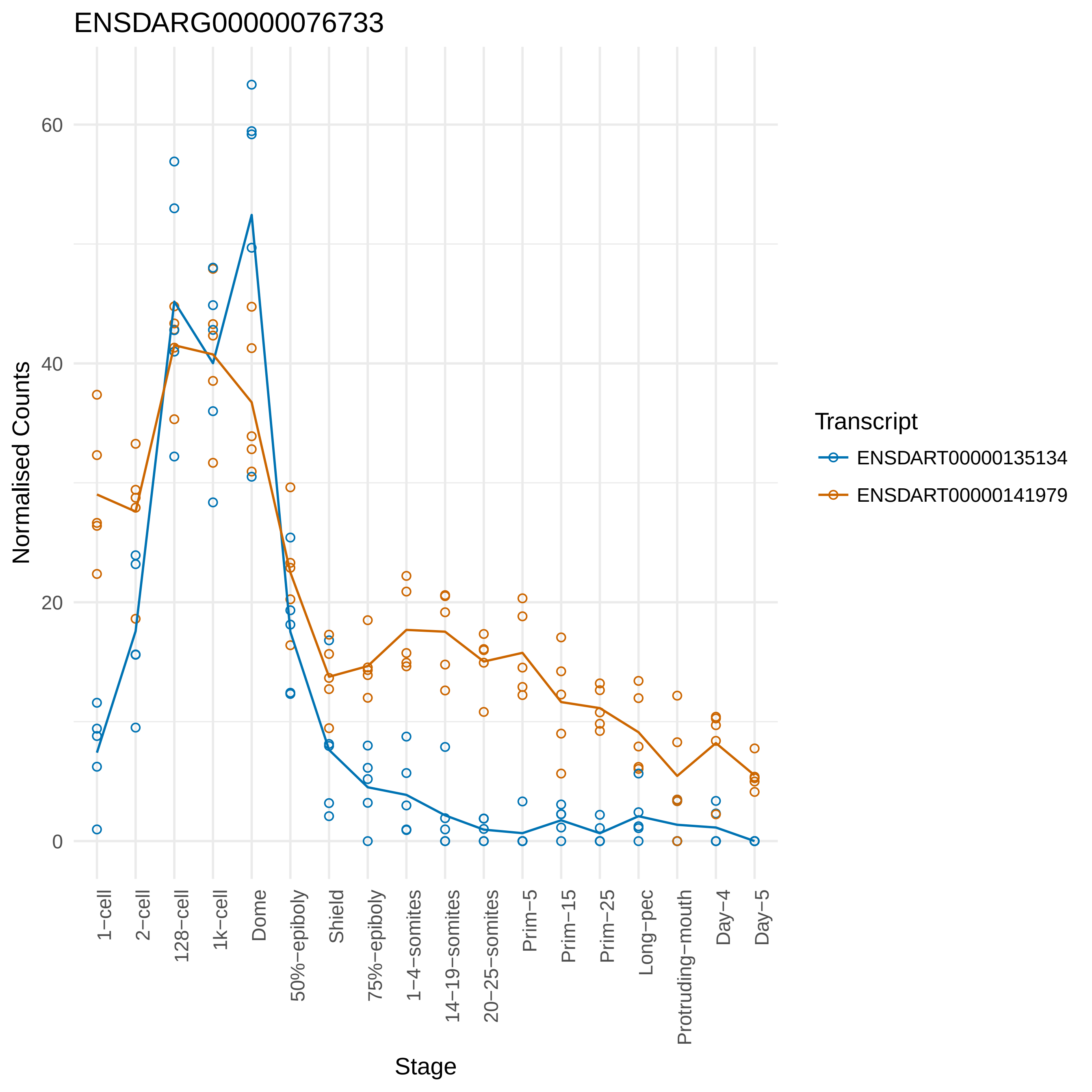

ENSDARG00000076733
Normalised Counts
60
40
20
0
1−cell
2−cell
128−cell
1k−cell
Dome
50%−epiboly
Shield
75%−epiboly
1−4−somites
14−19−somites
20−25−somites
Prim−5
Prim−15
Prim−25
Long−pec
Protruding−mouth
Day−4
Day−5
Stage
Transcript
ENSDART00000135134
ENSDART00000141979

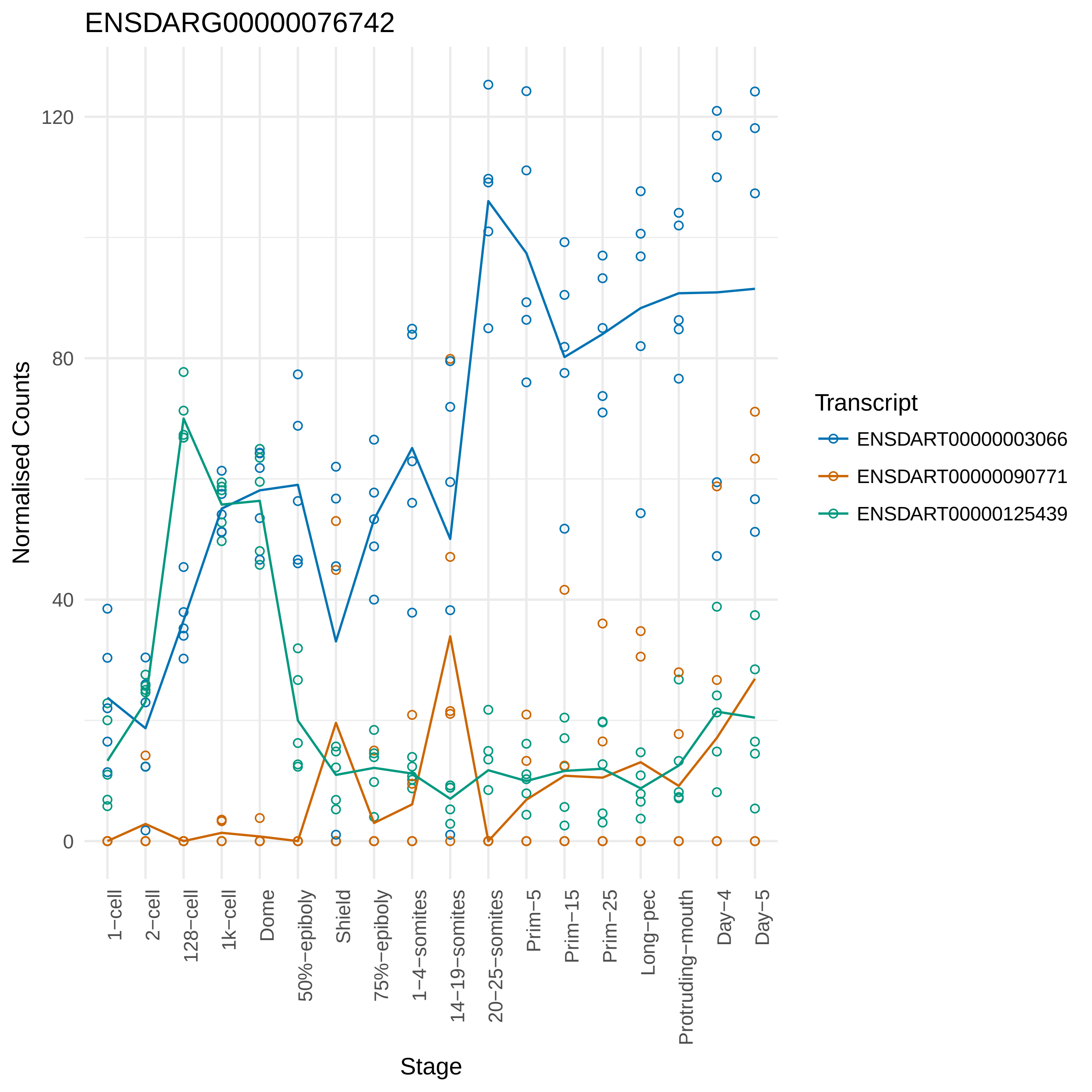

ENSDARG00000076742
Normalised Counts
0
40
80
120
Stage
1−cell
2−cell
128−cell
1k−cell
Dome
50%−epiboly
Shield
75%−epiboly
1−4−somites
14−19−somites
20−25−somites
Prim−5
Prim−15
Prim−25
Long−pec
Protruding−mouth
Day−4
Day−5
Transcript
ENSDART00000003066
ENSDART00000090771
ENSDART00000125439

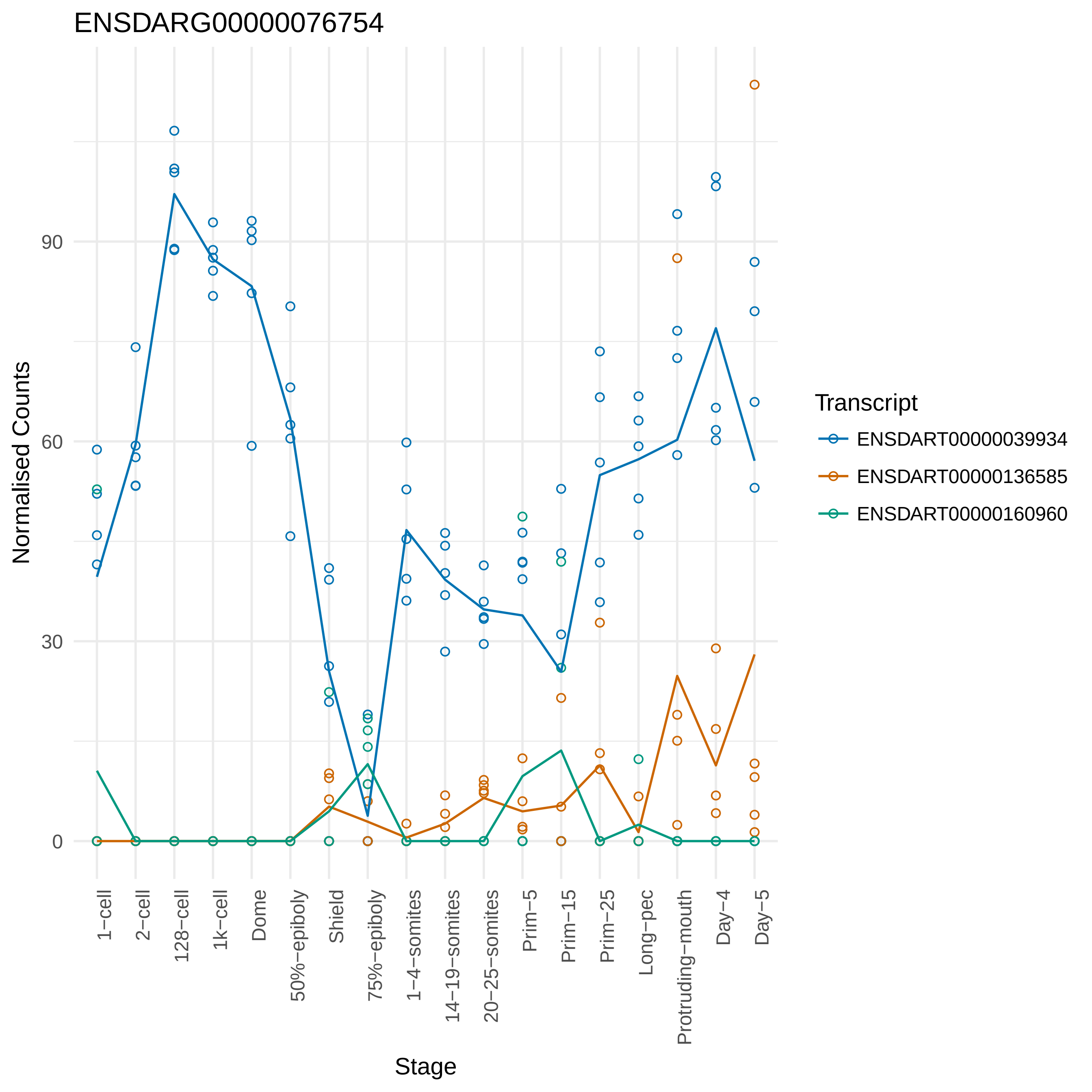
ENSDARG00000076754
Normalised Counts
0
30
60
90
Stage
1−cell
2−cell
128−cell
1k−cell
Dome
50%−epiboly
Shield
75%−epiboly
1−4−somites
14−19−somites
20−25−somites
Prim−5
Prim−15
Prim−25
Long−pec
Protruding−mouth
Day−4
Day−5
Transcript
ENSDART00000039934
ENSDART00000136585
ENSDART00000160960

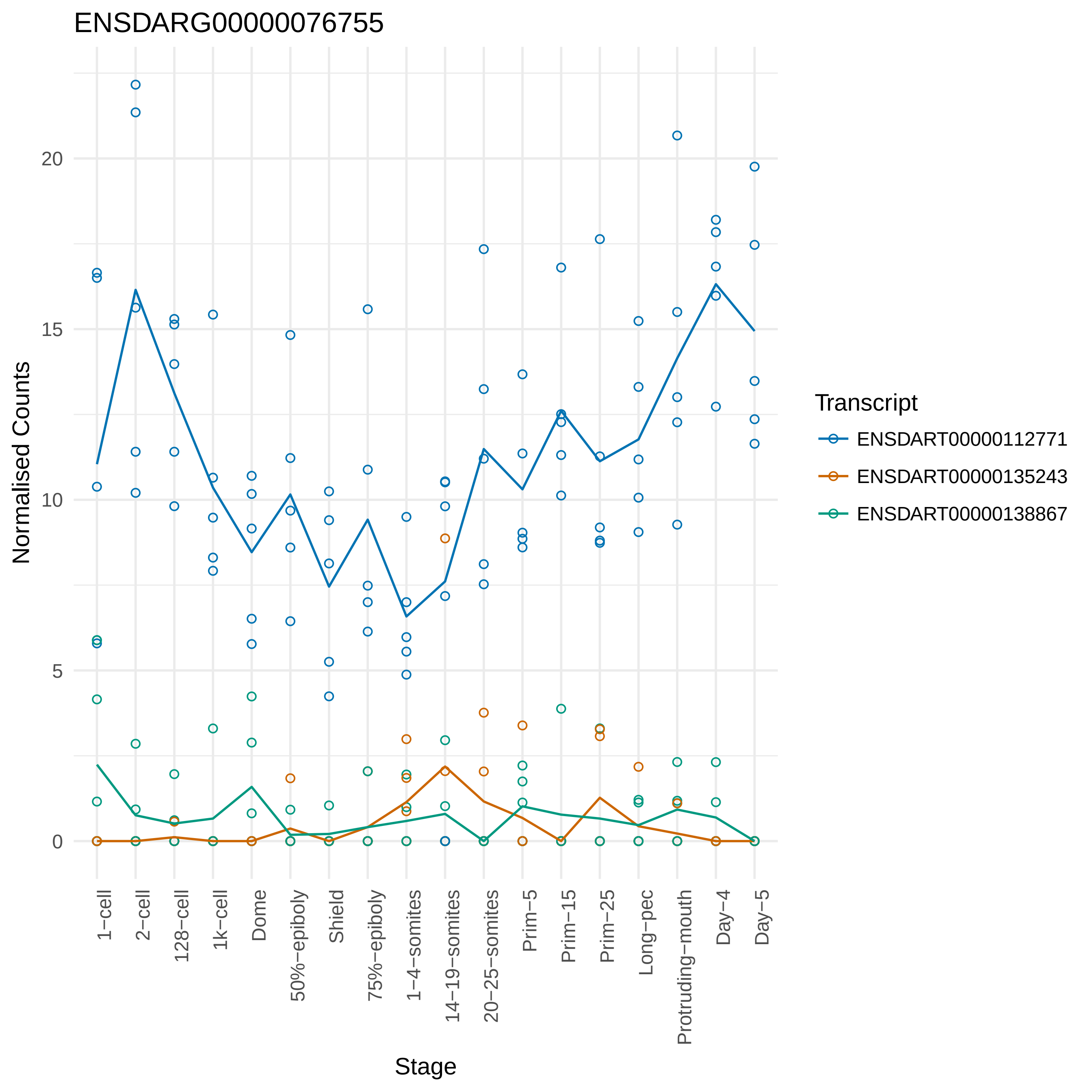

ENSDARG00000076755
Normalised Counts
0
5
10
15
20
1−cell
2−cell
128−cell
1k−cell
Dome
50%−epiboly
Shield
75%−epiboly
1−4−somites
14−19−somites
20−25−somites
Prim−5
Prim−15
Prim−25
Long−pec
Protruding−mouth
Day−4
Day−5
Stage
Transcript
ENSDART00000112771
ENSDART00000135243
ENSDART00000138867

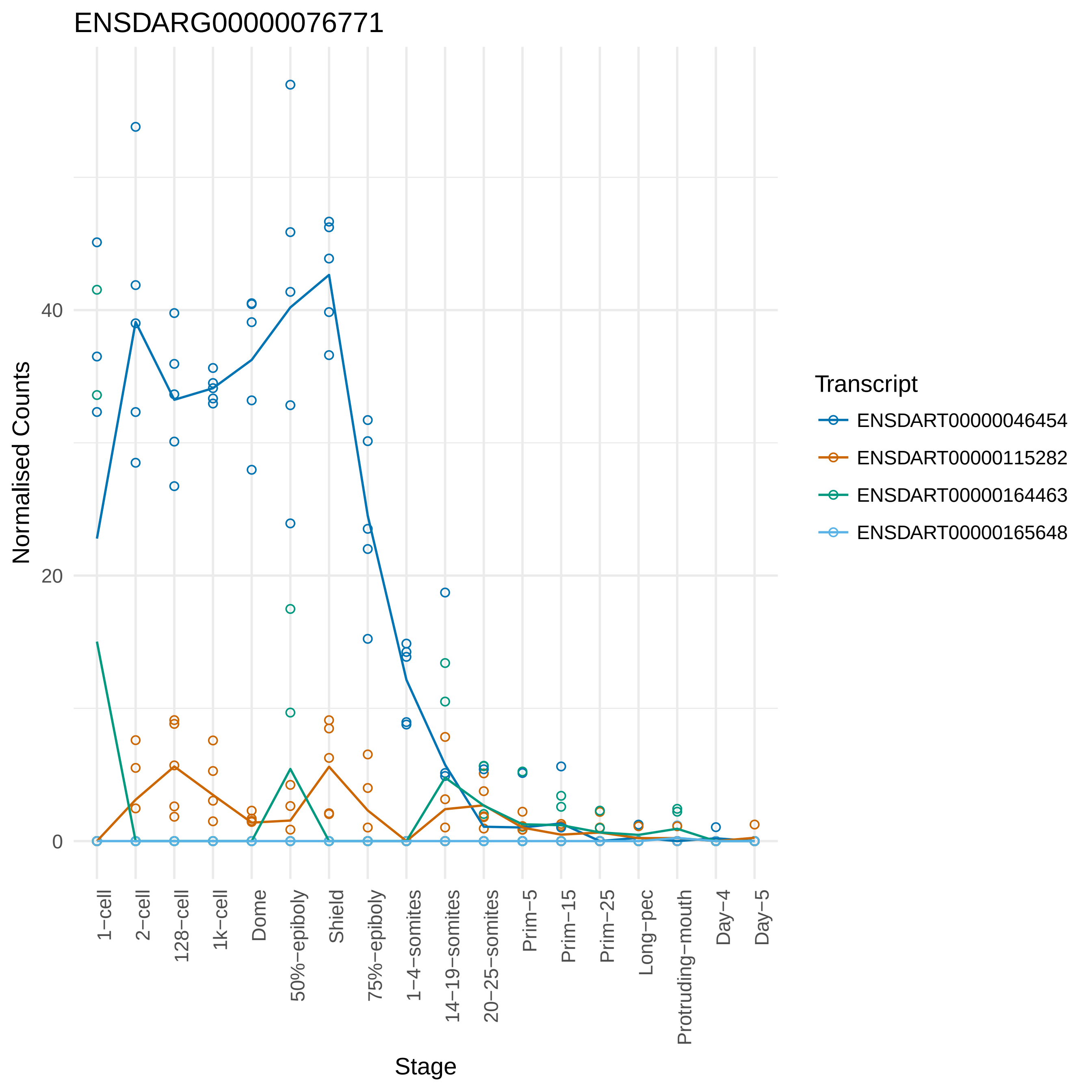

ENSDARG00000076771
Normalised Counts
0
20
40
1−cell
2−cell
128−cell
1k−cell
Dome
50%−epiboly
Shield
75%−epiboly
1−4−somites
14−19−somites
20−25−somites
Prim−5
Prim−15
Prim−25
Long−pec
Protruding−mouth
Day−4
Day−5
Stage
Transcript
ENSDART00000046454
ENSDART00000115282
ENSDART00000164463
ENSDART00000165648

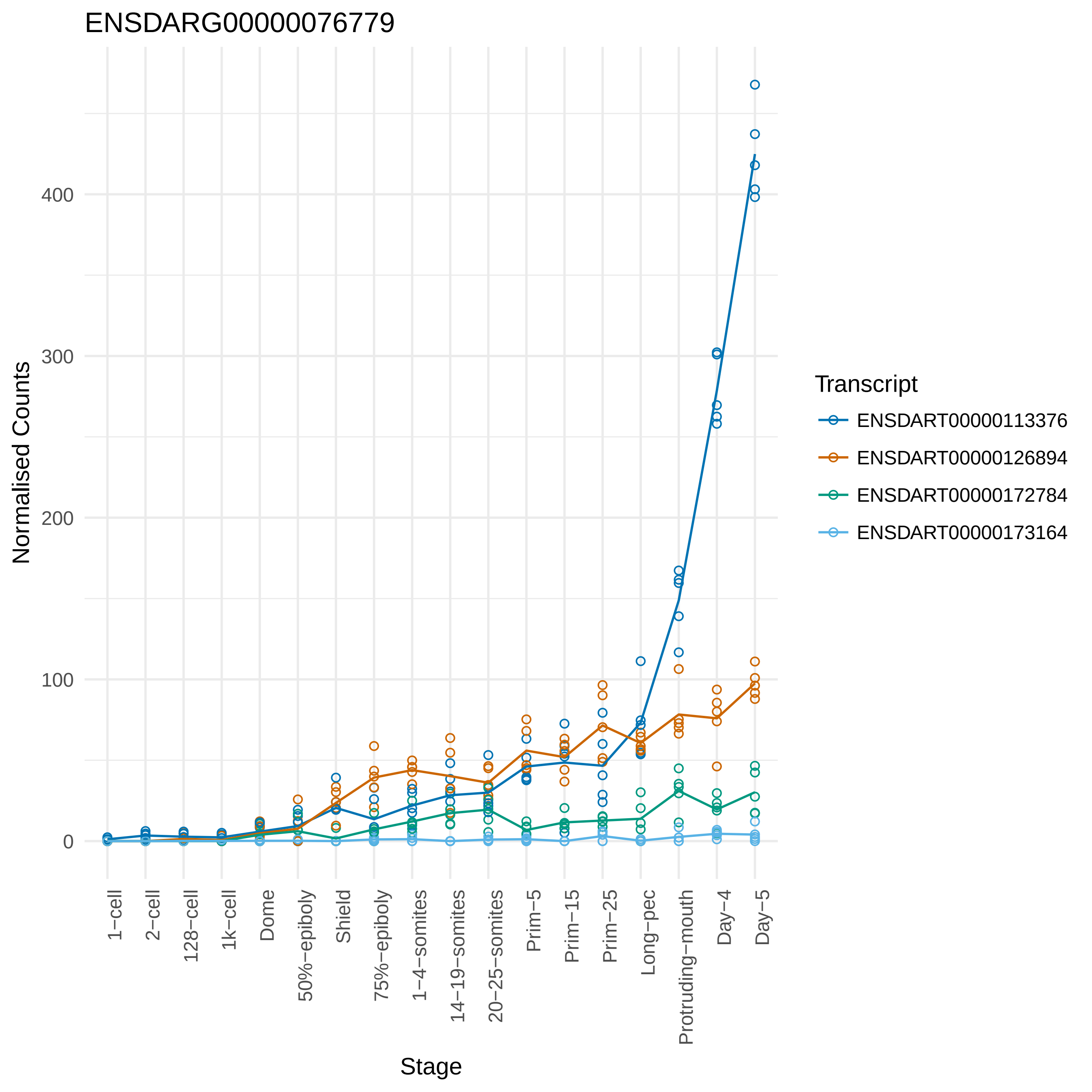

ENSDARG00000076779
Normalised Counts
0
100
200
300
400
Stage
1−cell
2−cell
128−cell
1k−cell
Dome
50%−epiboly
Shield
75%−epiboly
1−4−somites
14−19−somites
20−25−somites
Prim−5
Prim−15
Prim−25
Long−pec
Protruding−mouth
Day−4
Day−5
Transcript
ENSDART00000113376
ENSDART00000126894
ENSDART00000172784
ENSDART00000173164

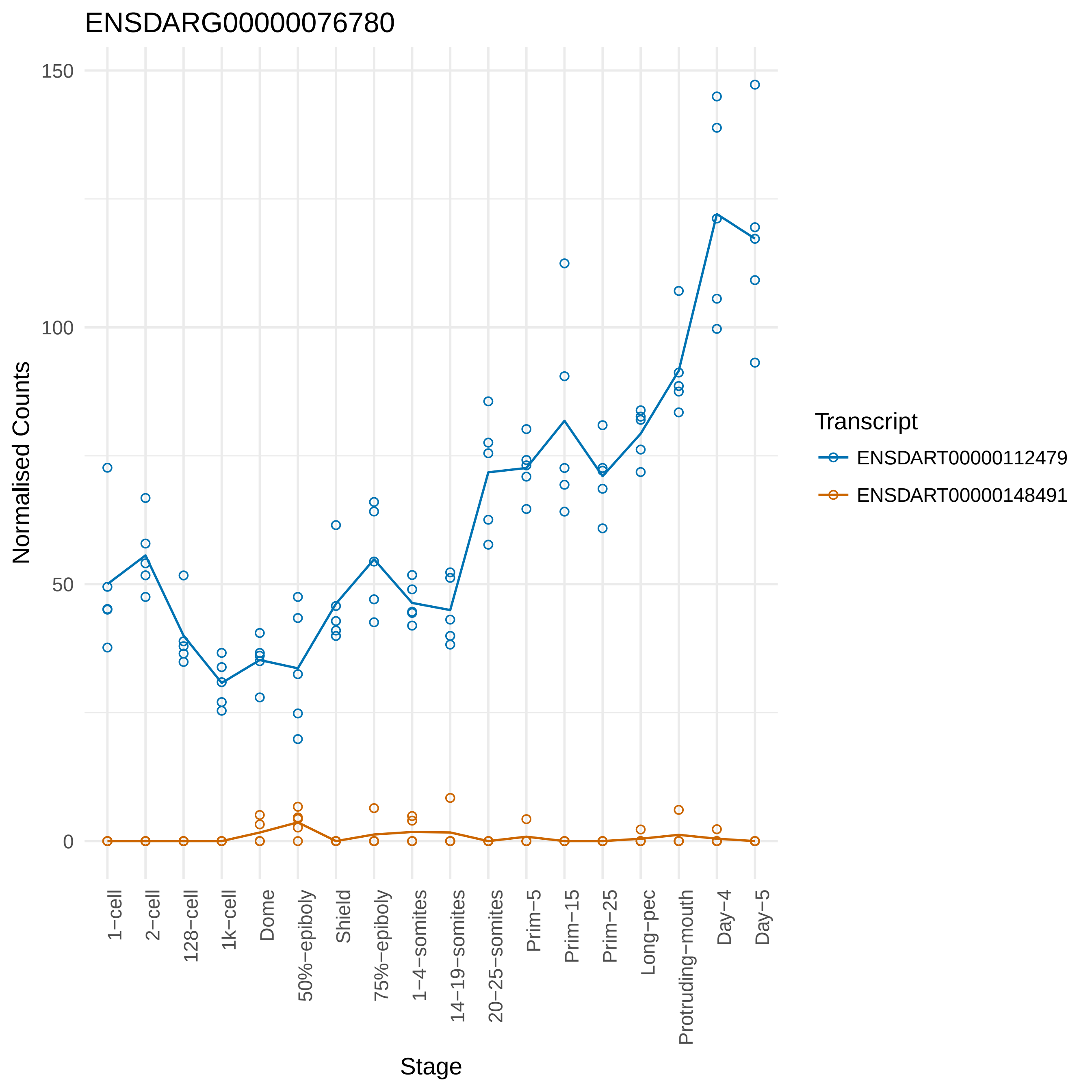

ENSDARG00000076780
Normalised Counts
0
50
100
150
Stage
1−cell
2−cell
128−cell
1k−cell
Dome
50%−epiboly
Shield
75%−epiboly
1−4−somites
14−19−somites
20−25−somites
Prim−5
Prim−15
Prim−25
Long−pec
Protruding−mouth
Day−4
Day−5
Transcript
ENSDART00000112479
ENSDART00000148491

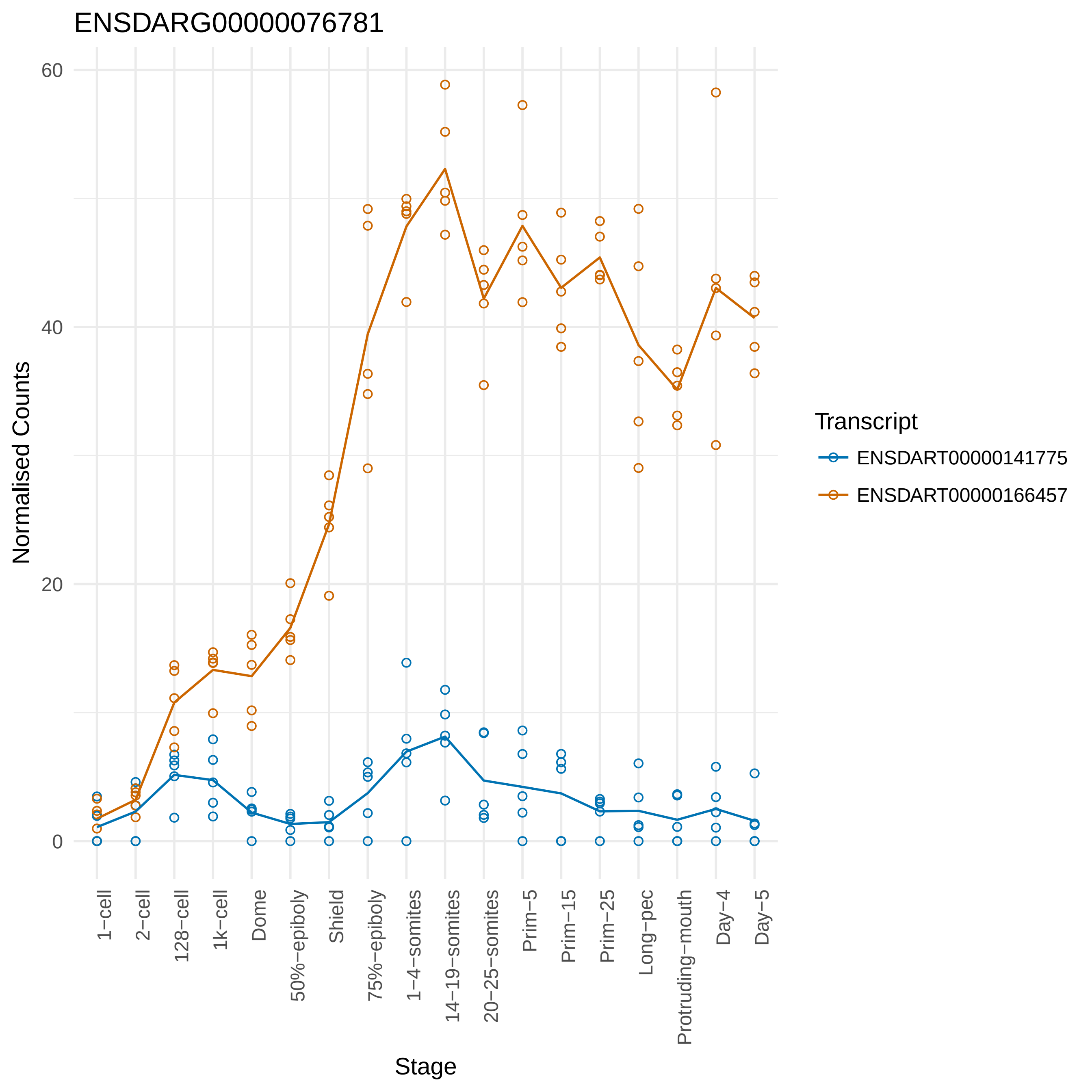

ENSDARG00000076781
Normalised Counts
60
40
20
0
1−cell
2−cell
128−cell
1k−cell
Dome
50%−epiboly
Shield
75%−epiboly
1−4−somites
14−19−somites
20−25−somites
Prim−5
Prim−15
Prim−25
Long−pec
Protruding−mouth
Day−4
Day−5
Stage
Transcript
ENSDART00000141775
ENSDART00000166457

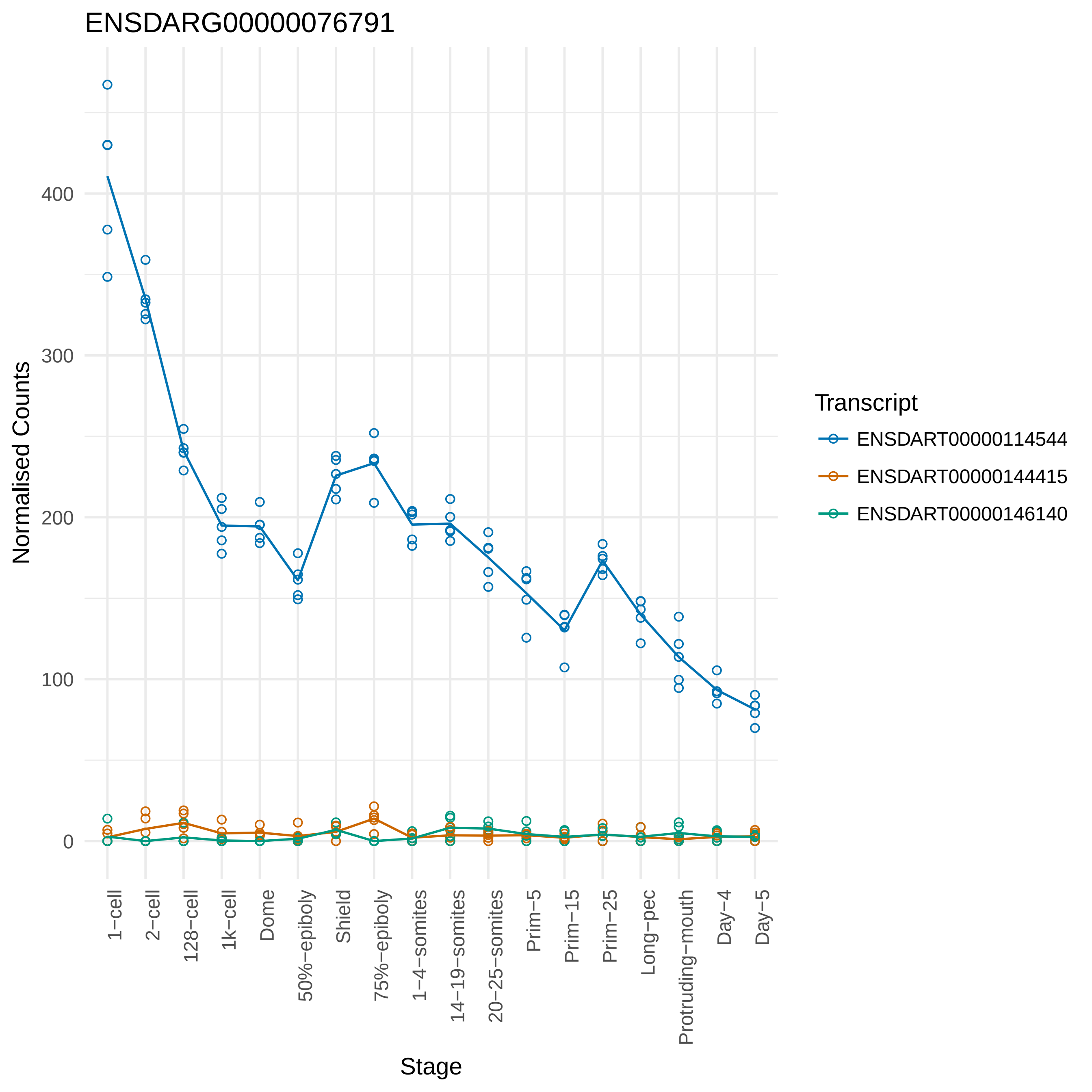
ENSDARG00000076791
Normalised Counts
400
300
200
100
0
1−cell
2−cell
128−cell
1k−cell
Dome
50%−epiboly
Shield
75%−epiboly
1−4−somites
14−19−somites
20−25−somites
Prim−5
Prim−15
Prim−25
Long−pec
Protruding−mouth
Day−4
Day−5
Stage
Transcript
ENSDART00000114544
ENSDART00000144415
ENSDART00000146140

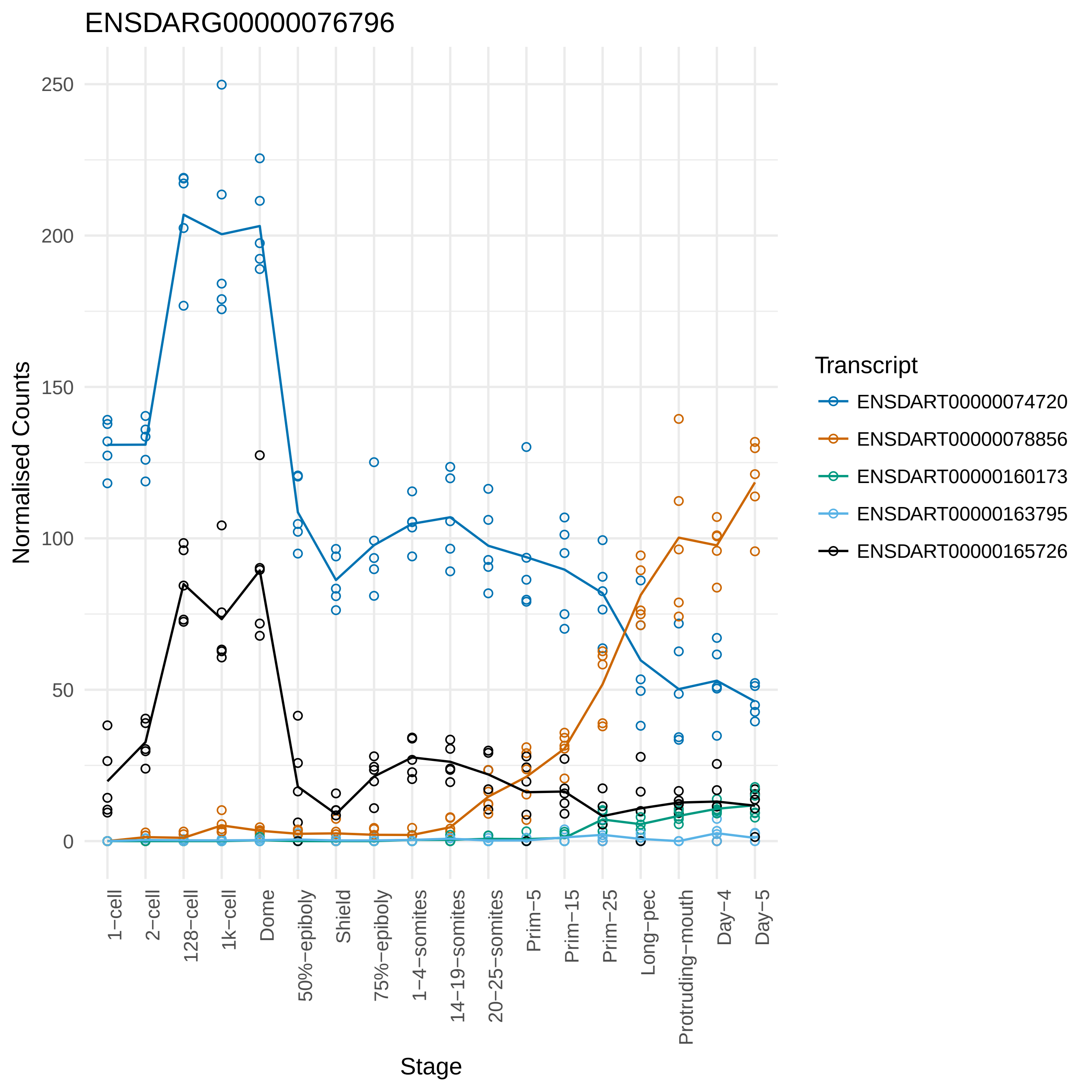
ENSDARG00000076796
Normalised Counts
250
200
150
100
50
0
1−cell
2−cell
128−cell
1k−cell
Dome
50%−epiboly
Shield
75%−epiboly
1−4−somites
14−19−somites
20−25−somites
Prim−5
Prim−15
Prim−25
Long−pec
Protruding−mouth
Day−4
Day−5
Stage
Transcript
ENSDART00000074720
ENSDART00000078856
ENSDART00000160173
ENSDART00000163795
ENSDART00000165726

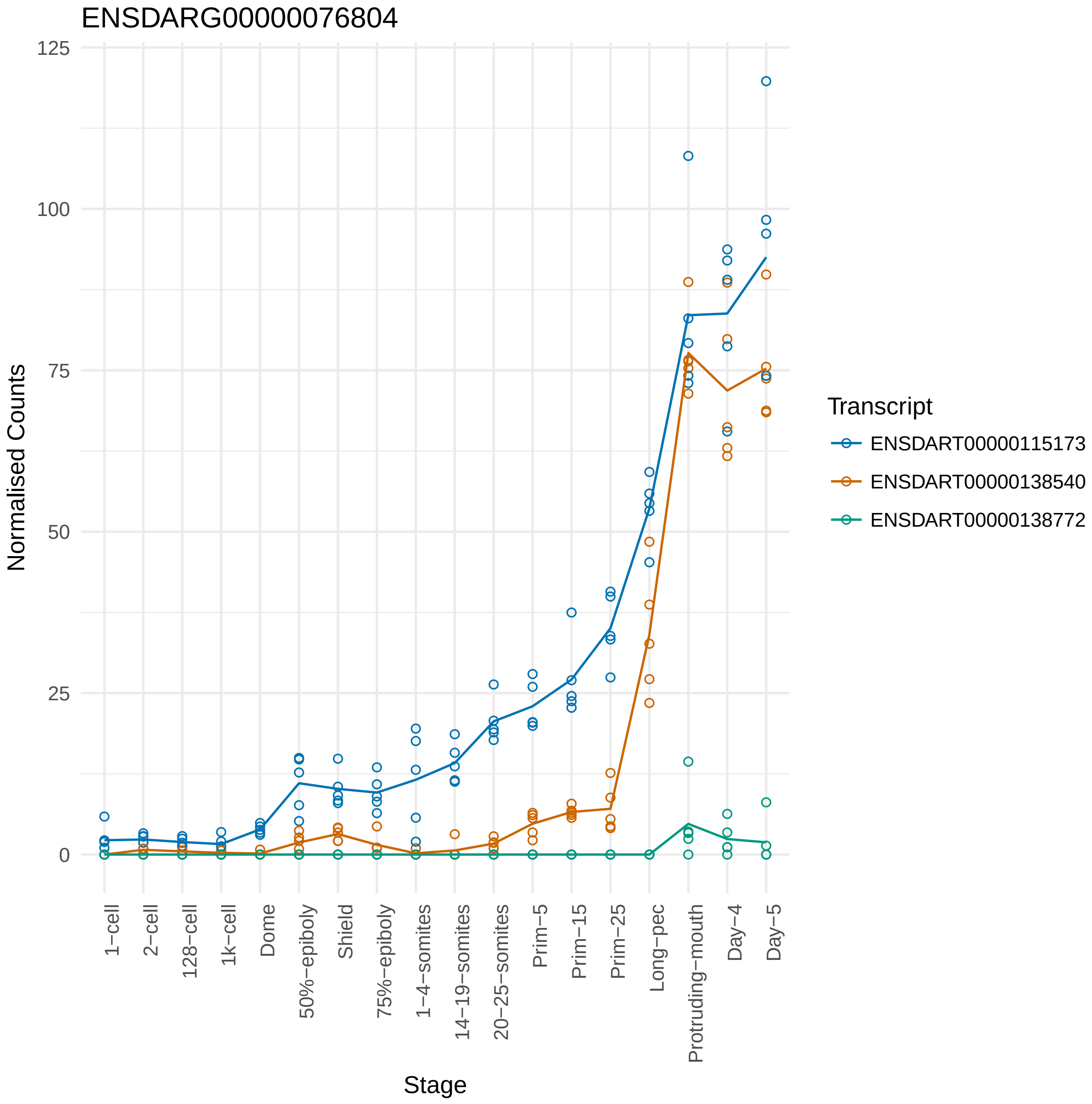
ENSDARG00000076804
Normalised Counts
125
100
75
50
25
0
1−cell
2−cell
128−cell
1k−cell
Dome
50%−epiboly
Shield
75%−epiboly
1−4−somites
14−19−somites
20−25−somites
Prim−5
Prim−15
Prim−25
Long−pec
Protruding−mouth
Day−4
Day−5
Stage
Transcript
ENSDART00000115173
ENSDART00000138540
ENSDART00000138772

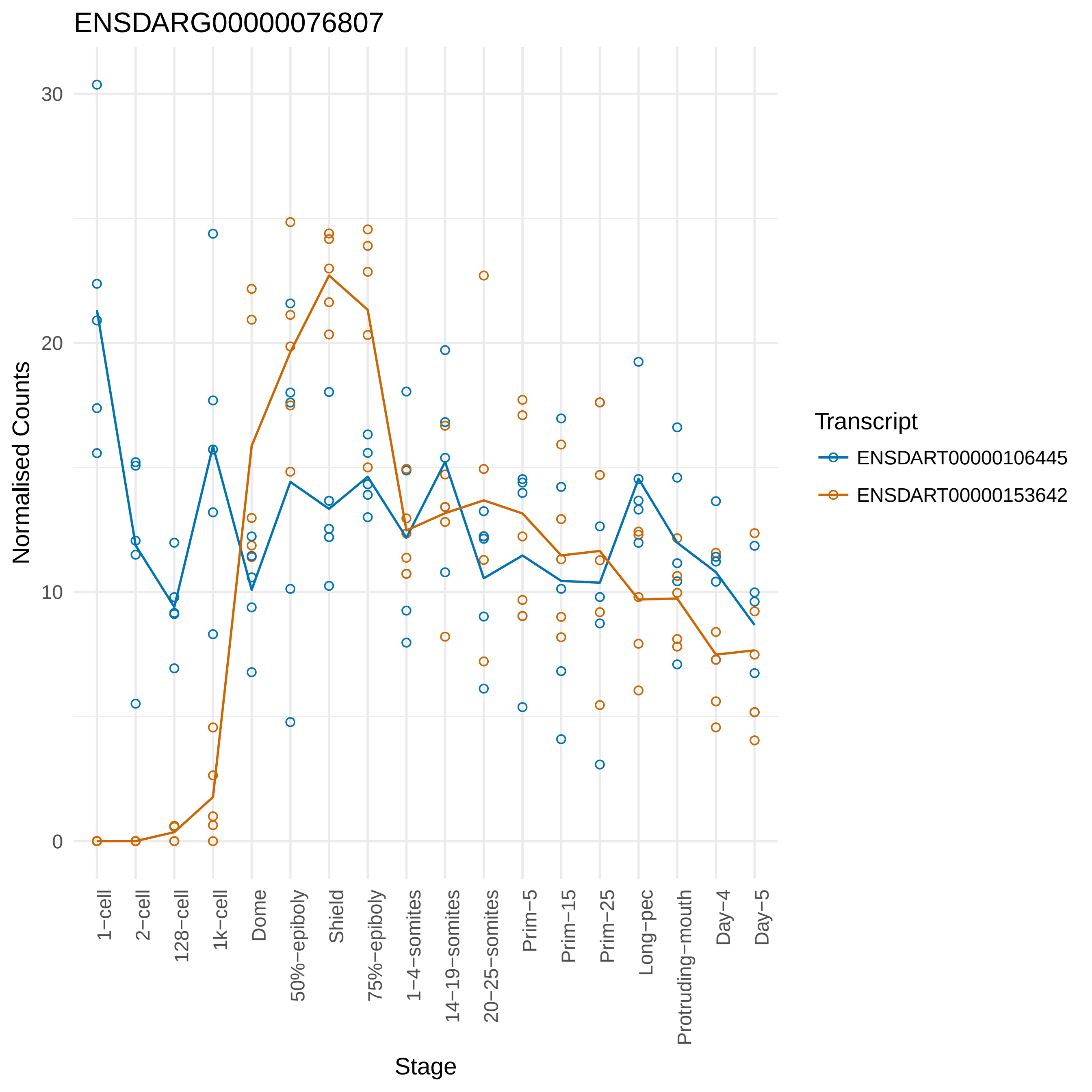

ENSDARG00000076807
Normalised Counts
0
10
20
30
Stage
1−cell
2−cell
128−cell
1k−cell
Dome
50%−epiboly
Shield
75%−epiboly
1−4−somites
14−19−somites
20−25−somites
Prim−5
Prim−15
Prim−25
Long−pec
Protruding−mouth
Day−4
Day−5
Transcript
ENSDART00000106445
ENSDART00000153642

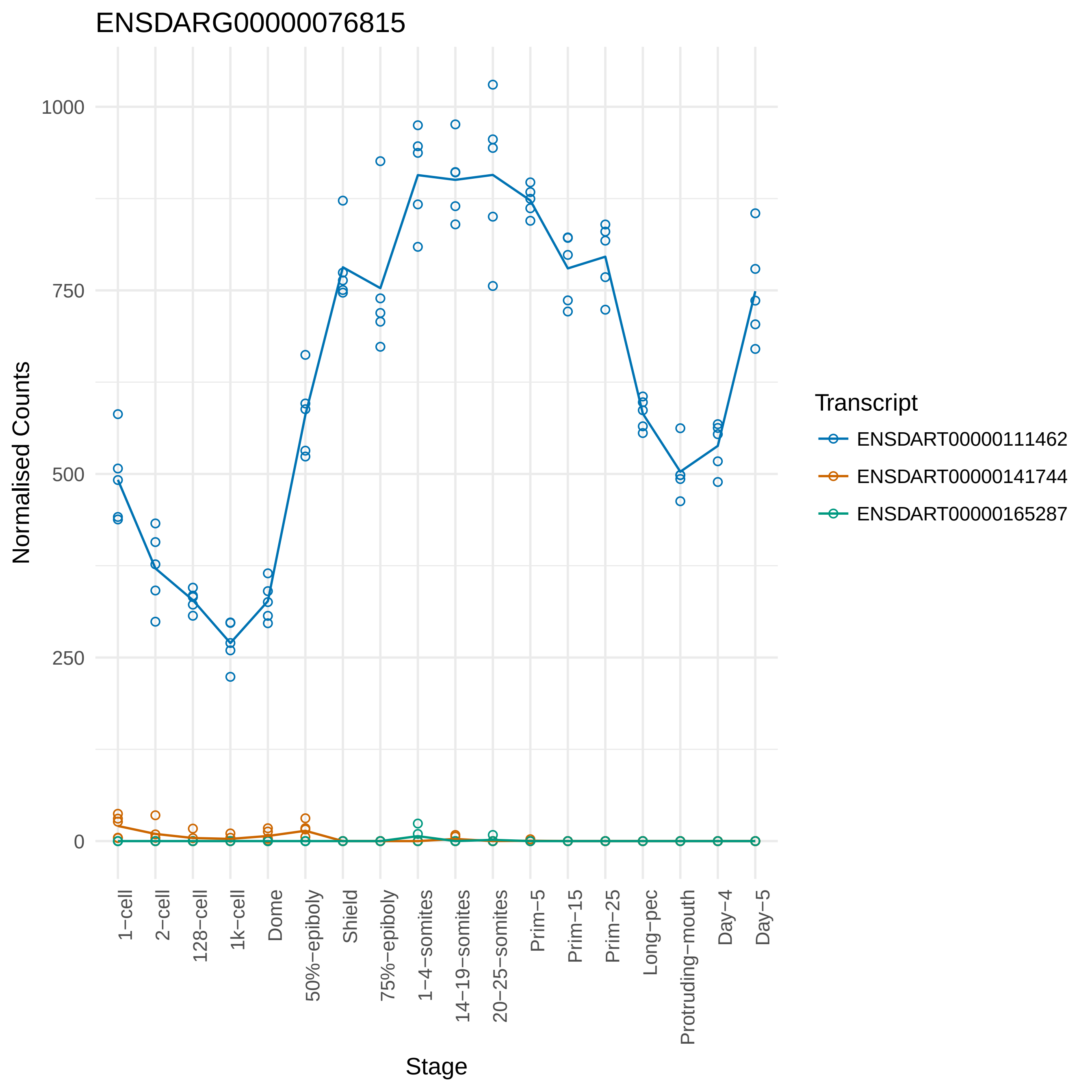

ENSDARG00000076815
Normalised Counts
1000
750
500
250
0
1−cell
2−cell
128−cell
1k−cell
Dome
50%−epiboly
Shield
75%−epiboly
1−4−somites
14−19−somites
20−25−somites
Prim−5
Prim−15
Prim−25
Long−pec
Protruding−mouth
Day−4
Day−5
Stage
Transcript
ENSDART00000111462
ENSDART00000141744
ENSDART00000165287

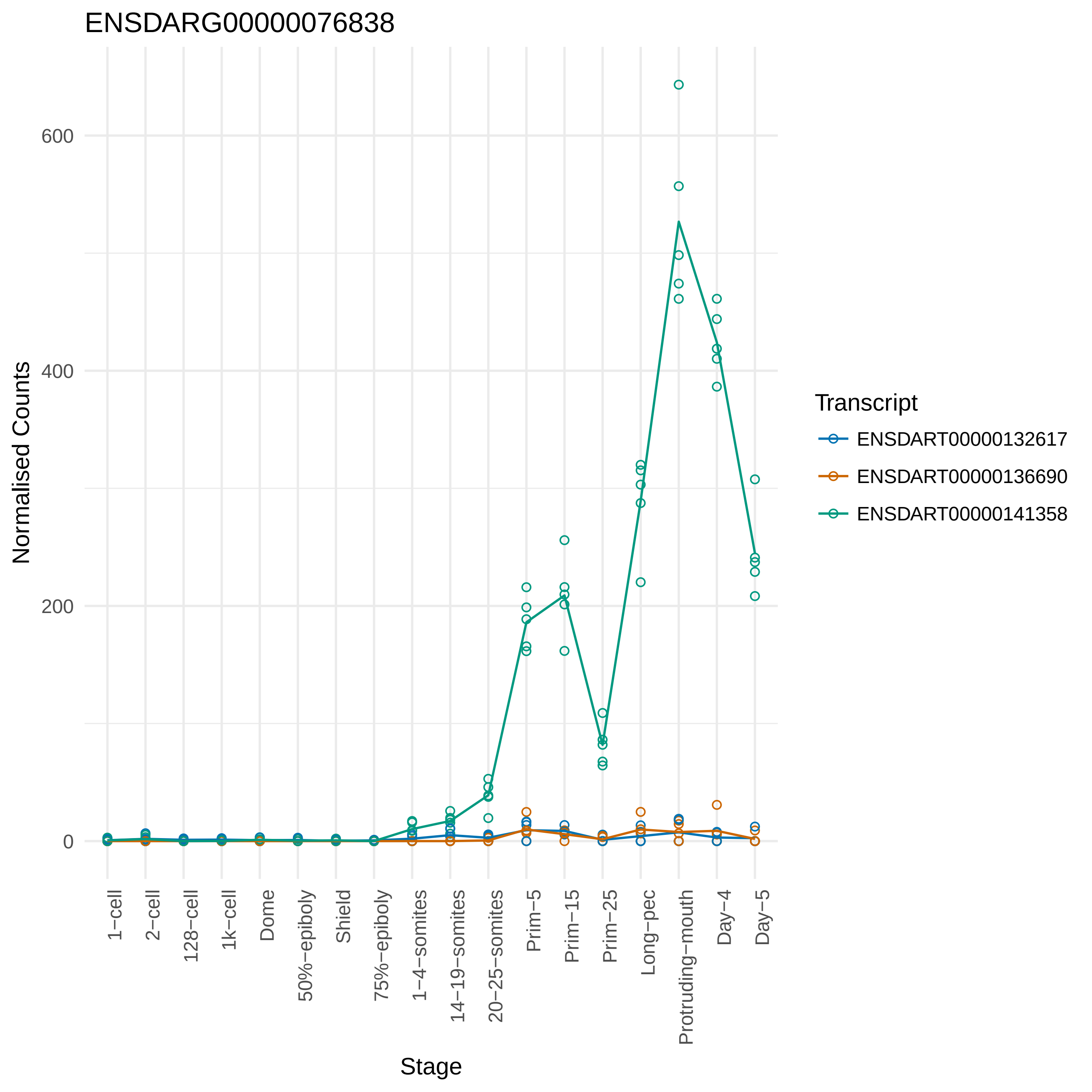

ENSDARG00000076838
Normalised Counts
600
400
200
0
1−cell
2−cell
128−cell
1k−cell
Dome
50%−epiboly
Shield
75%−epiboly
1−4−somites
14−19−somites
20−25−somites
Prim−5
Prim−15
Prim−25
Long−pec
Protruding−mouth
Day−4
Day−5
Stage
Transcript
ENSDART00000132617
ENSDART00000136690
ENSDART00000141358

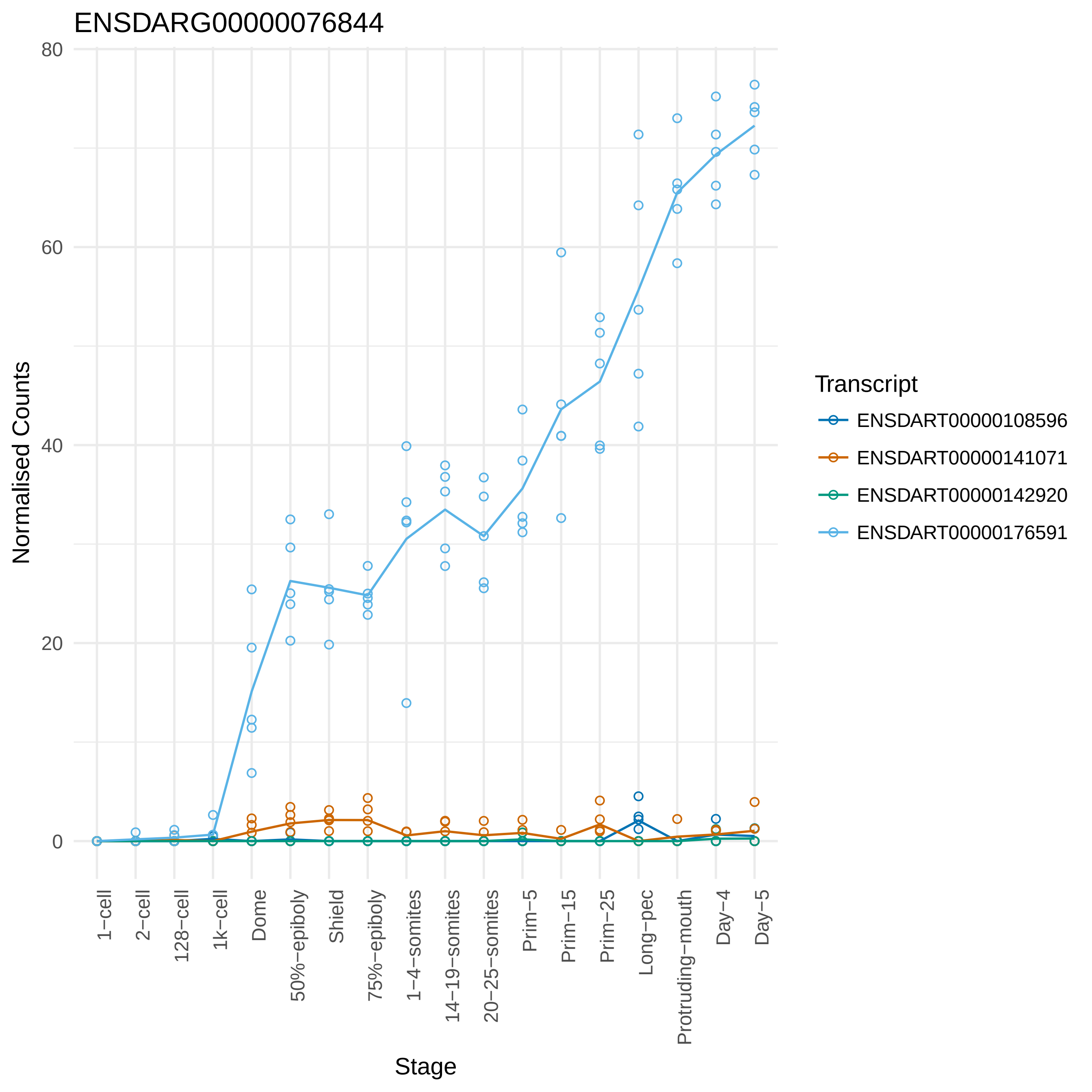
ENSDARG00000076844
Normalised Counts
0
20
40
60
80
Stage
1−cell
2−cell
128−cell
1k−cell
Dome
50%−epiboly
Shield
75%−epiboly
1−4−somites
14−19−somites
20−25−somites
Prim−5
Prim−15
Prim−25
Long−pec
Protruding−mouth
Day−4
Day−5
Transcript
ENSDART00000108596
ENSDART00000141071
ENSDART00000142920
ENSDART00000176591

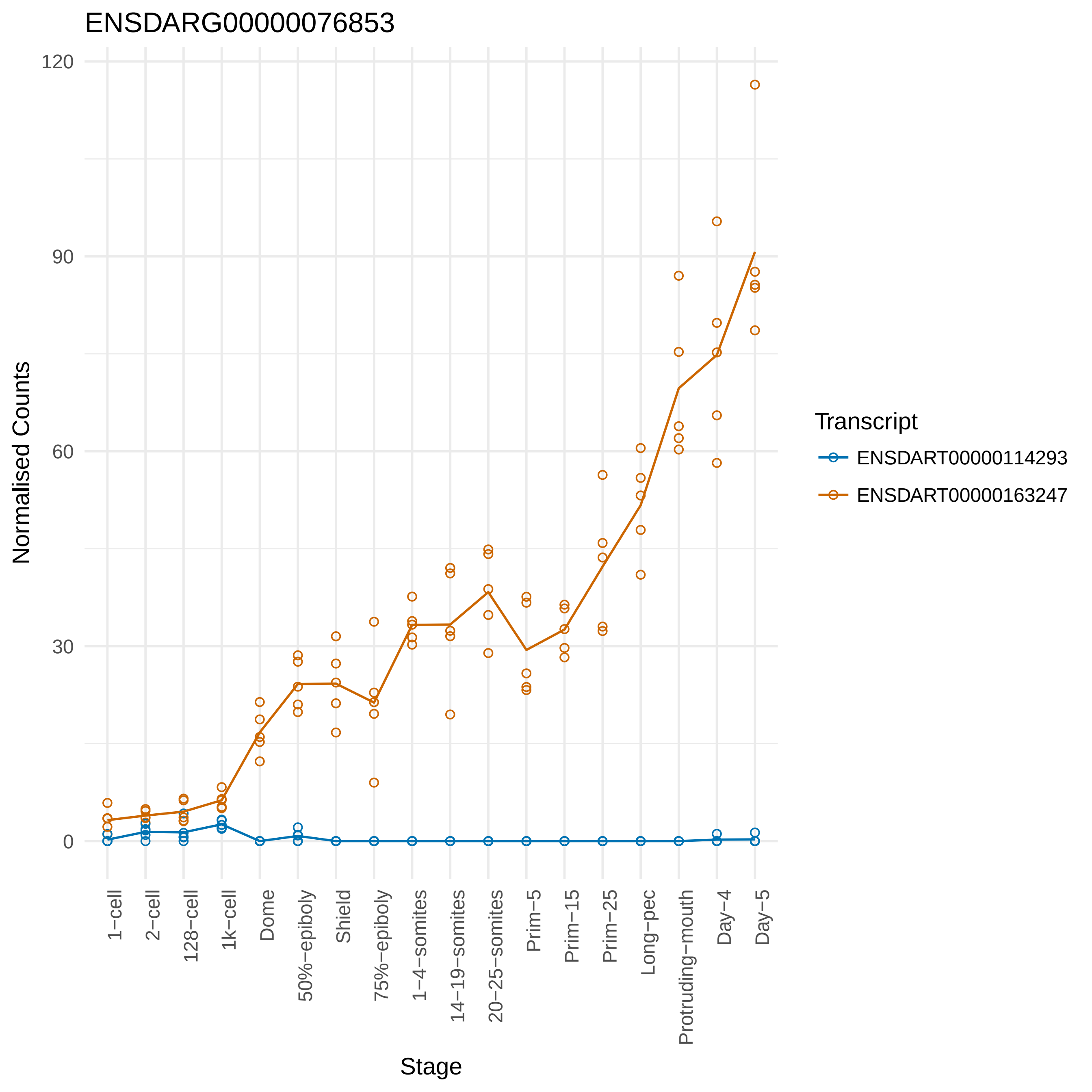

ENSDARG00000076853
Normalised Counts
120
90
60
30
0
1−cell
2−cell
128−cell
1k−cell
Dome
50%−epiboly
Shield
75%−epiboly
1−4−somites
14−19−somites
20−25−somites
Prim−5
Prim−15
Prim−25
Long−pec
Protruding−mouth
Day−4
Day−5
Stage
Transcript
ENSDART00000114293
ENSDART00000163247

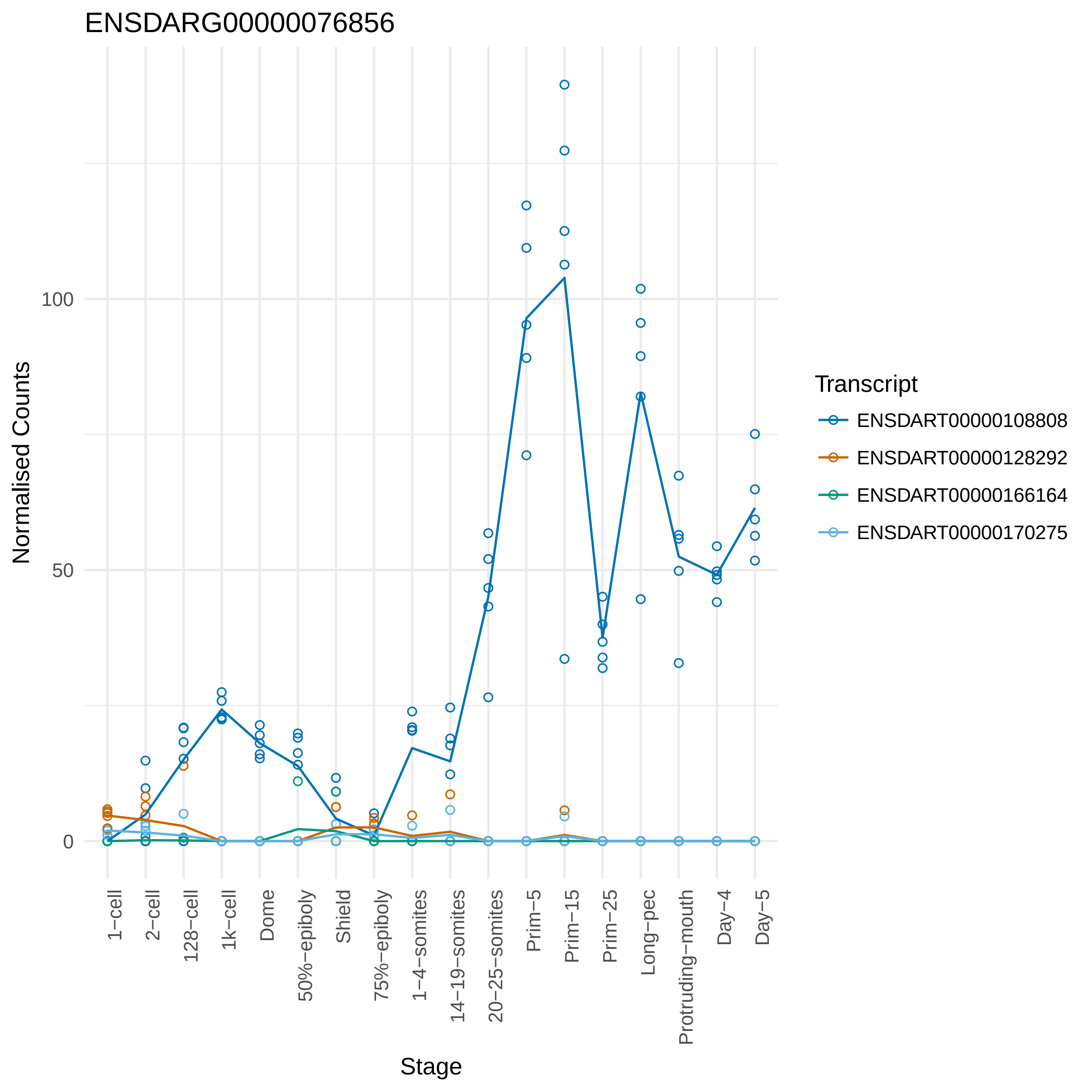

ENSDARG00000076856
Normalised Counts
100
50
0
1−cell
2−cell
128−cell
1k−cell
Dome
50%−epiboly
Shield
75%−epiboly
1−4−somites
14−19−somites
20−25−somites
Prim−5
Prim−15
Prim−25
Long−pec
Protruding−mouth
Day−4
Day−5
Stage
Transcript
ENSDART00000108808
ENSDART00000128292
ENSDART00000166164
ENSDART00000170275

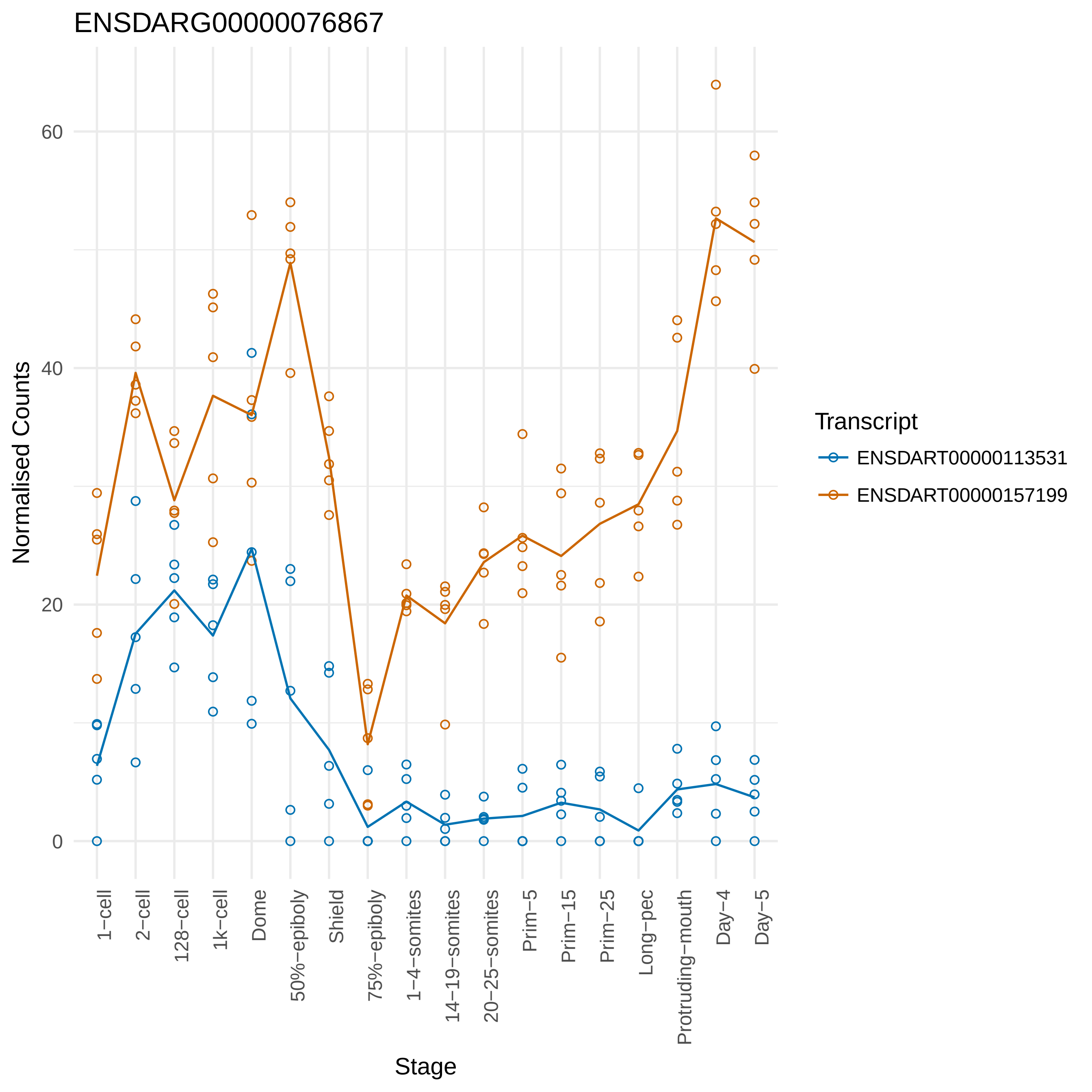
ENSDARG00000076867
Normalised Counts
0
20
40
60
Stage
1−cell
2−cell
128−cell
1k−cell
Dome
50%−epiboly
Shield
75%−epiboly
1−4−somites
14−19−somites
20−25−somites
Prim−5
Prim−15
Prim−25
Long−pec
Protruding−mouth
Day−4
Day−5
Transcript
ENSDART00000113531
ENSDART00000157199

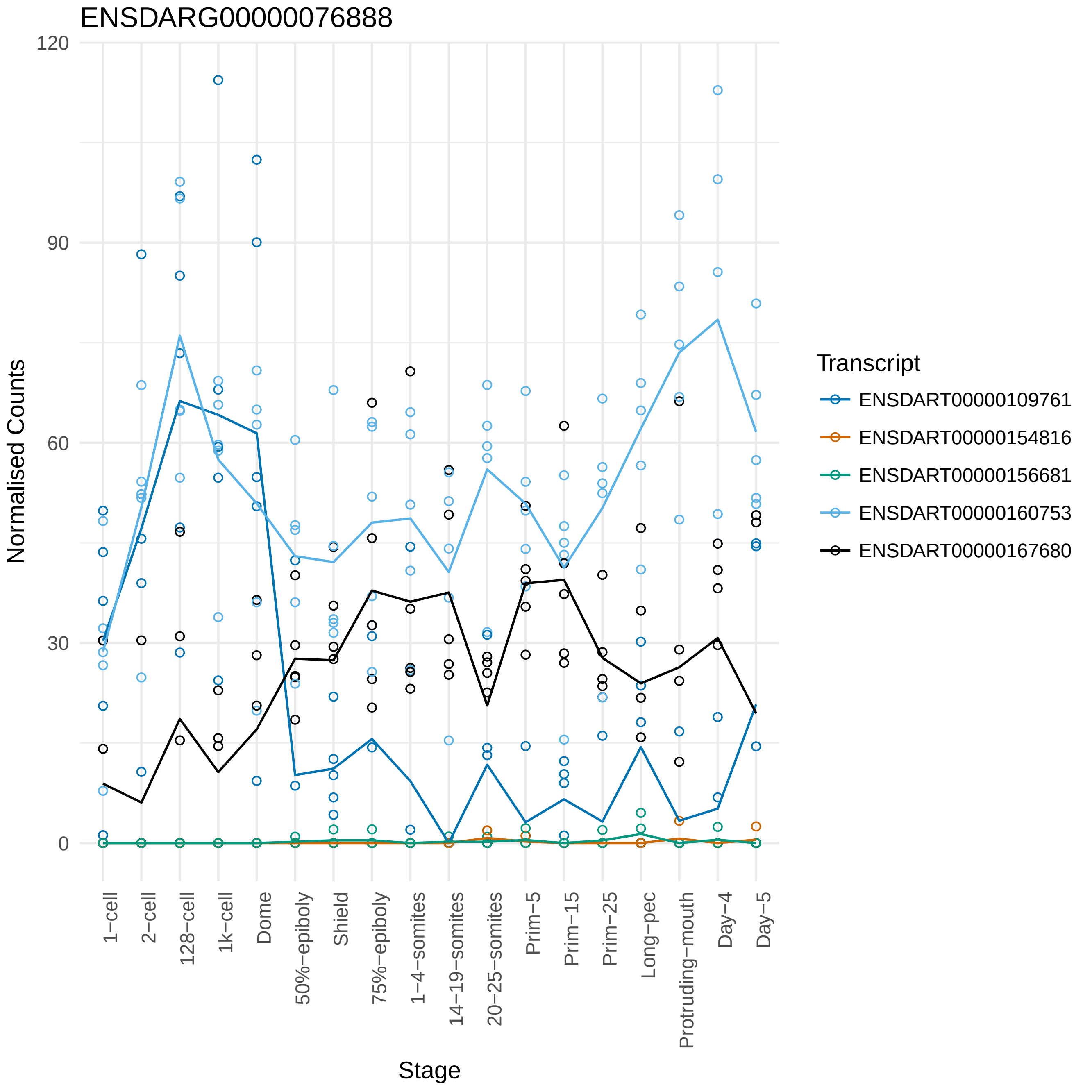
ENSDARG00000076888
Normalised Counts
120
90
60
30
0
1−cell
2−cell
128−cell
1k−cell
Dome
50%−epiboly
Shield
75%−epiboly
1−4−somites
14−19−somites
20−25−somites
Prim−5
Prim−15
Prim−25
Long−pec
Protruding−mouth
Day−4
Day−5
Stage
Transcript
ENSDART00000109761
ENSDART00000154816
ENSDART00000156681
ENSDART00000160753
ENSDART00000167680

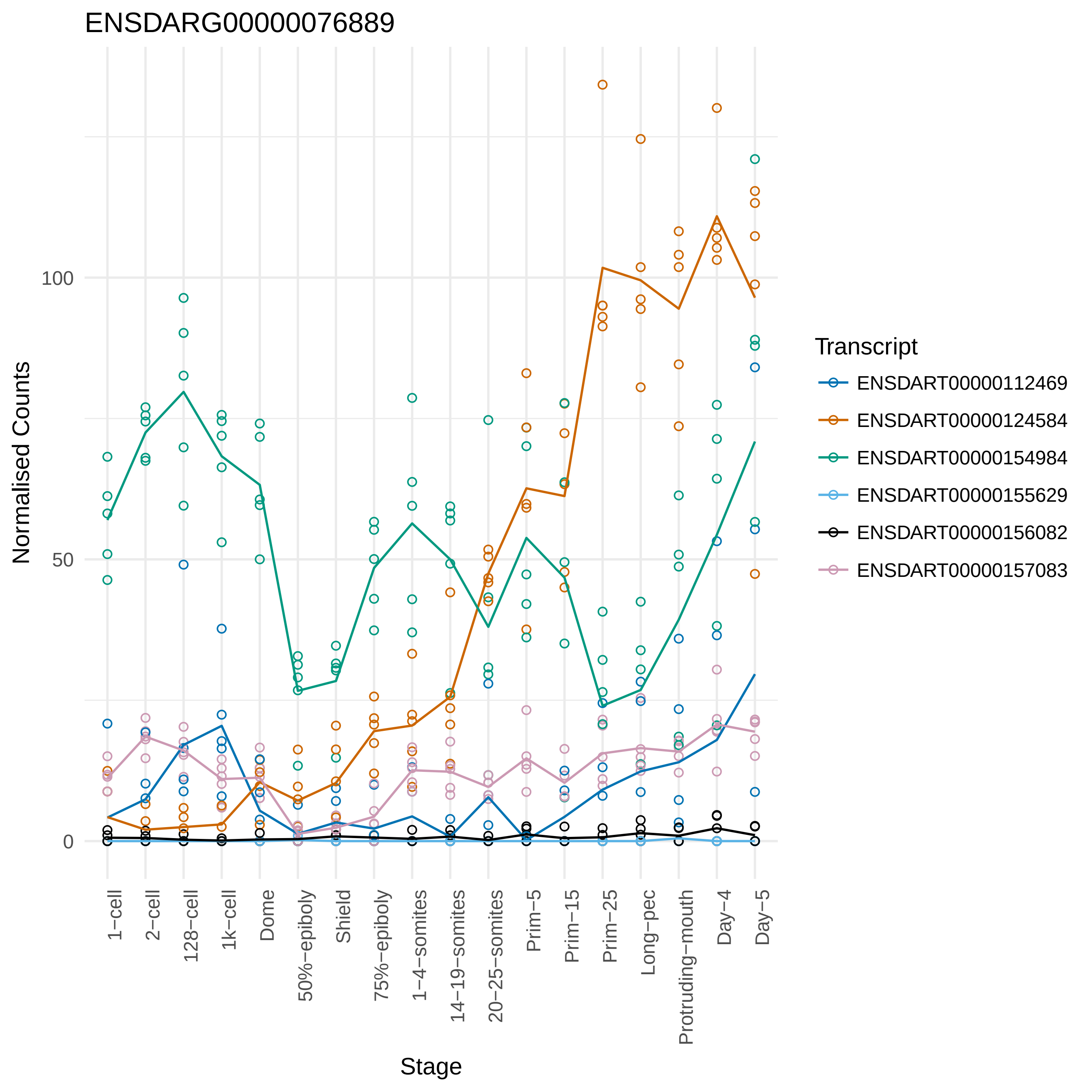

ENSDARG00000076889
Normalised Counts
100
50
0
1−cell
2−cell
128−cell
1k−cell
Dome
50%−epiboly
Shield
75%−epiboly
1−4−somites
14−19−somites
20−25−somites
Prim−5
Prim−15
Prim−25
Long−pec
Protruding−mouth
Day−4
Day−5
Stage
Transcript
ENSDART00000112469
ENSDART00000124584
ENSDART00000154984
ENSDART00000155629
ENSDART00000156082
ENSDART00000157083

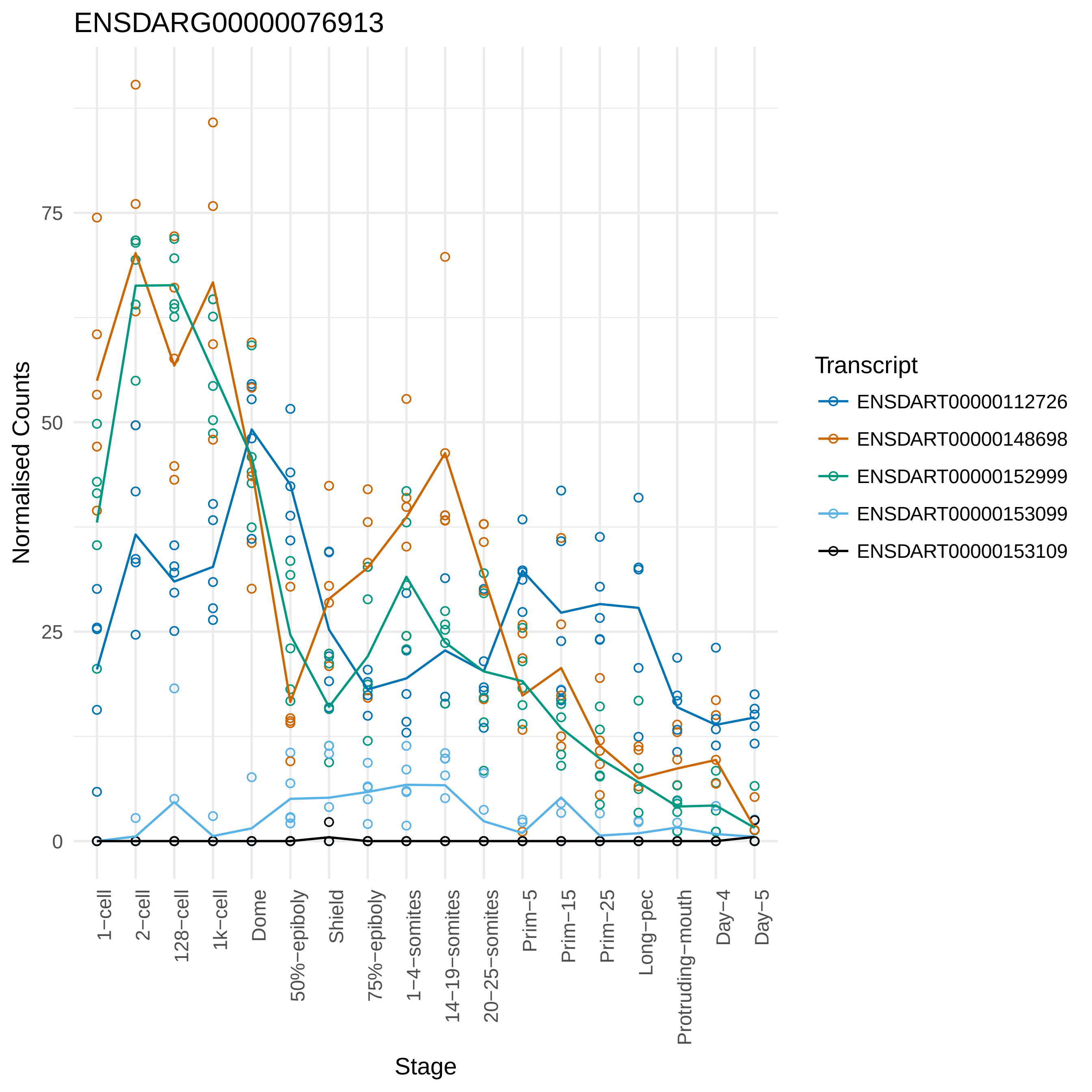
ENSDARG00000076913
Normalised Counts
0
25
50
75
Stage
1−cell
2−cell
128−cell
1k−cell
Dome
50%−epiboly
Shield
75%−epiboly
1−4−somites
14−19−somites
20−25−somites
Prim−5
Prim−15
Prim−25
Long−pec
Protruding−mouth
Day−4
Day−5
Transcript
ENSDART00000112726
ENSDART00000148698
ENSDART00000152999
ENSDART00000153099
ENSDART00000153109

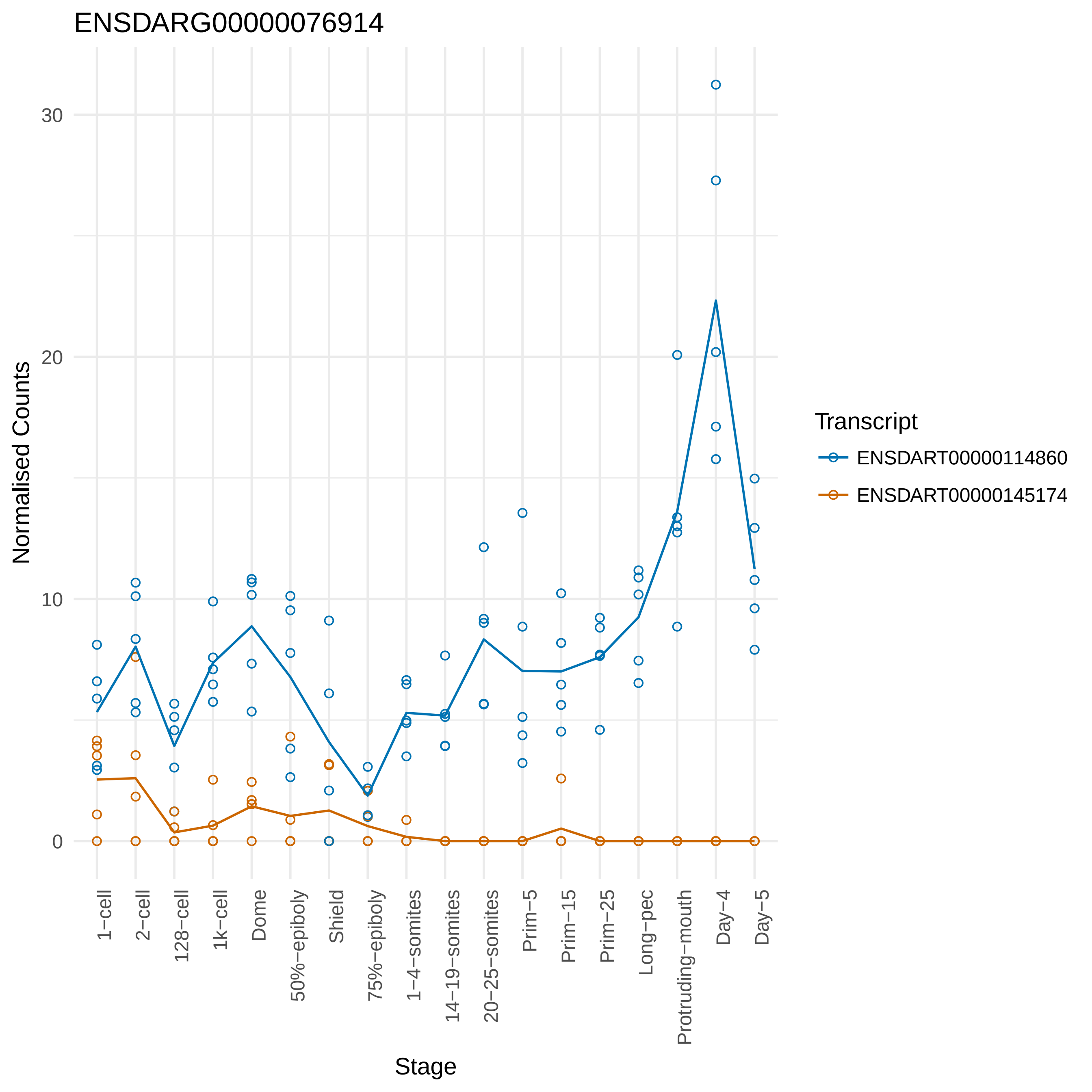
ENSDARG00000076914
Normalised Counts
0
10
20
30
1−cell
2−cell
128−cell
1k−cell
Dome
50%−epiboly
Shield
75%−epiboly
1−4−somites
14−19−somites
20−25−somites
Prim−5
Prim−15
Prim−25
Long−pec
Protruding−mouth
Day−4
Day−5
Stage
Transcript
ENSDART00000114860
ENSDART00000145174

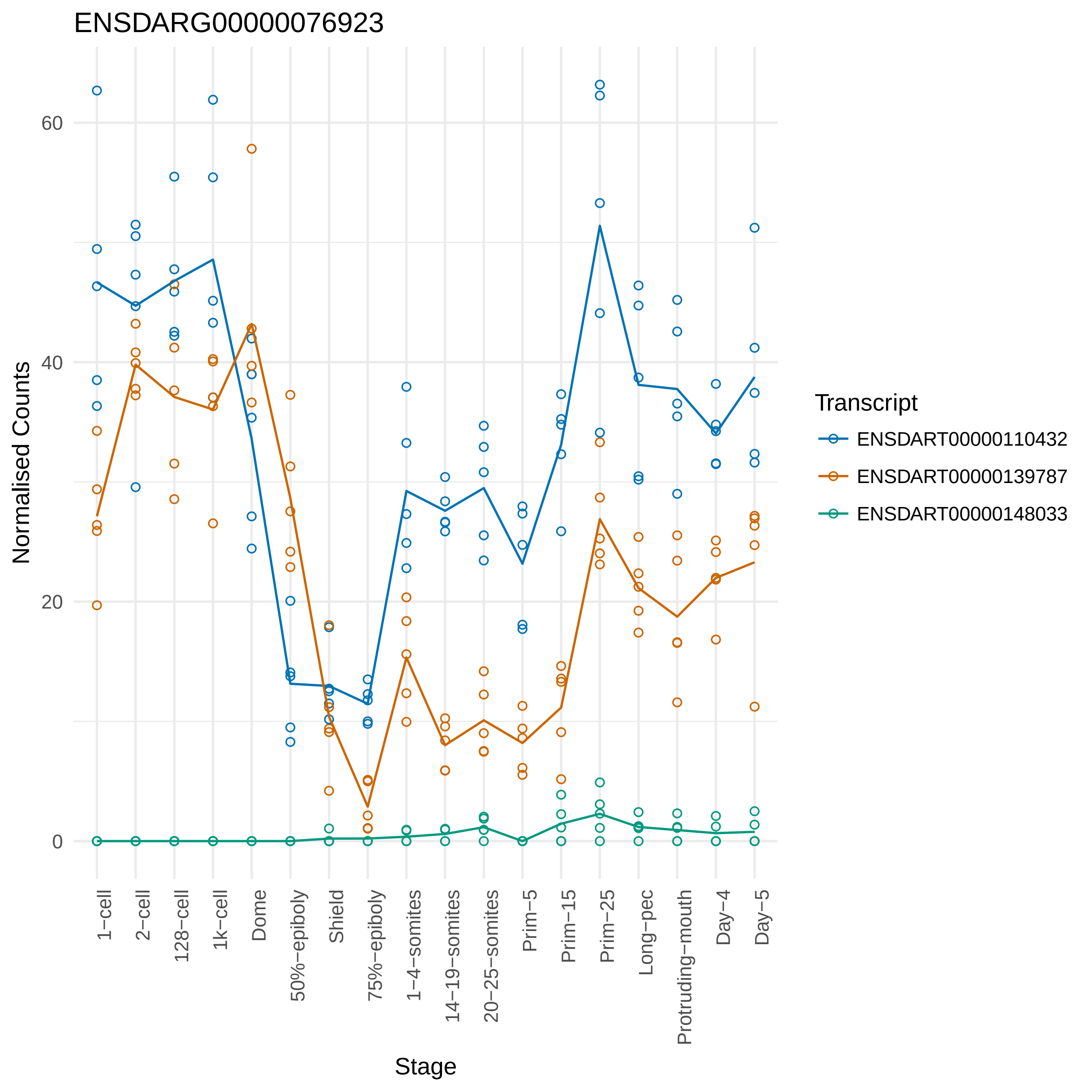
ENSDARG00000076923
Normalised Counts
60
40
20
0
1−cell
2−cell
128−cell
1k−cell
Dome
50%−epiboly
Shield
75%−epiboly
1−4−somites
14−19−somites
20−25−somites
Prim−5
Prim−15
Prim−25
Long−pec
Protruding−mouth
Day−4
Day−5
Stage
Transcript
ENSDART00000110432
ENSDART00000139787
ENSDART00000148033

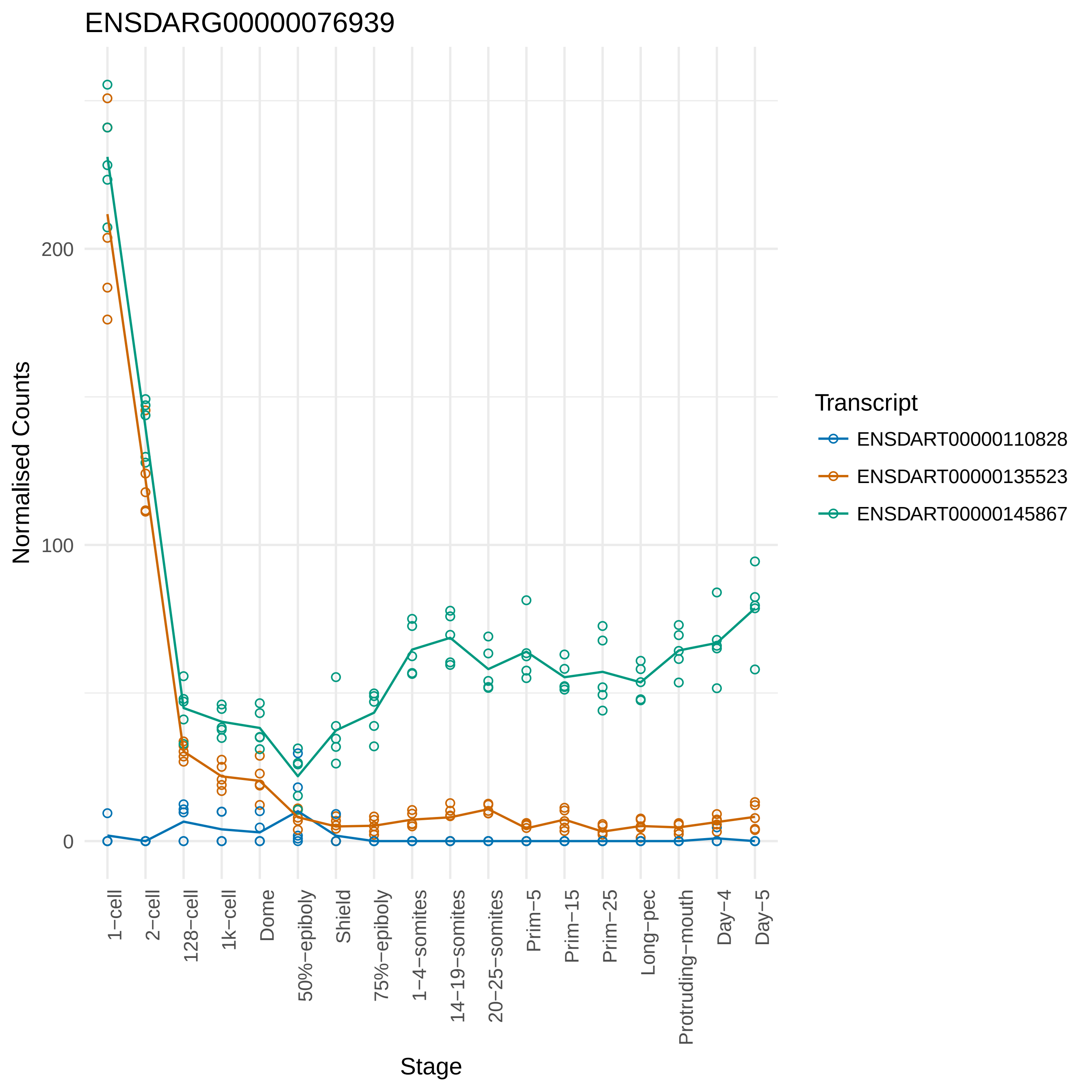
ENSDARG00000076939
Normalised Counts
0
100
200
Stage
1−cell
2−cell
128−cell
1k−cell
Dome
50%−epiboly
Shield
75%−epiboly
1−4−somites
14−19−somites
20−25−somites
Prim−5
Prim−15
Prim−25
Long−pec
Protruding−mouth
Day−4
Day−5
Transcript
ENSDART00000110828
ENSDART00000135523
ENSDART00000145867

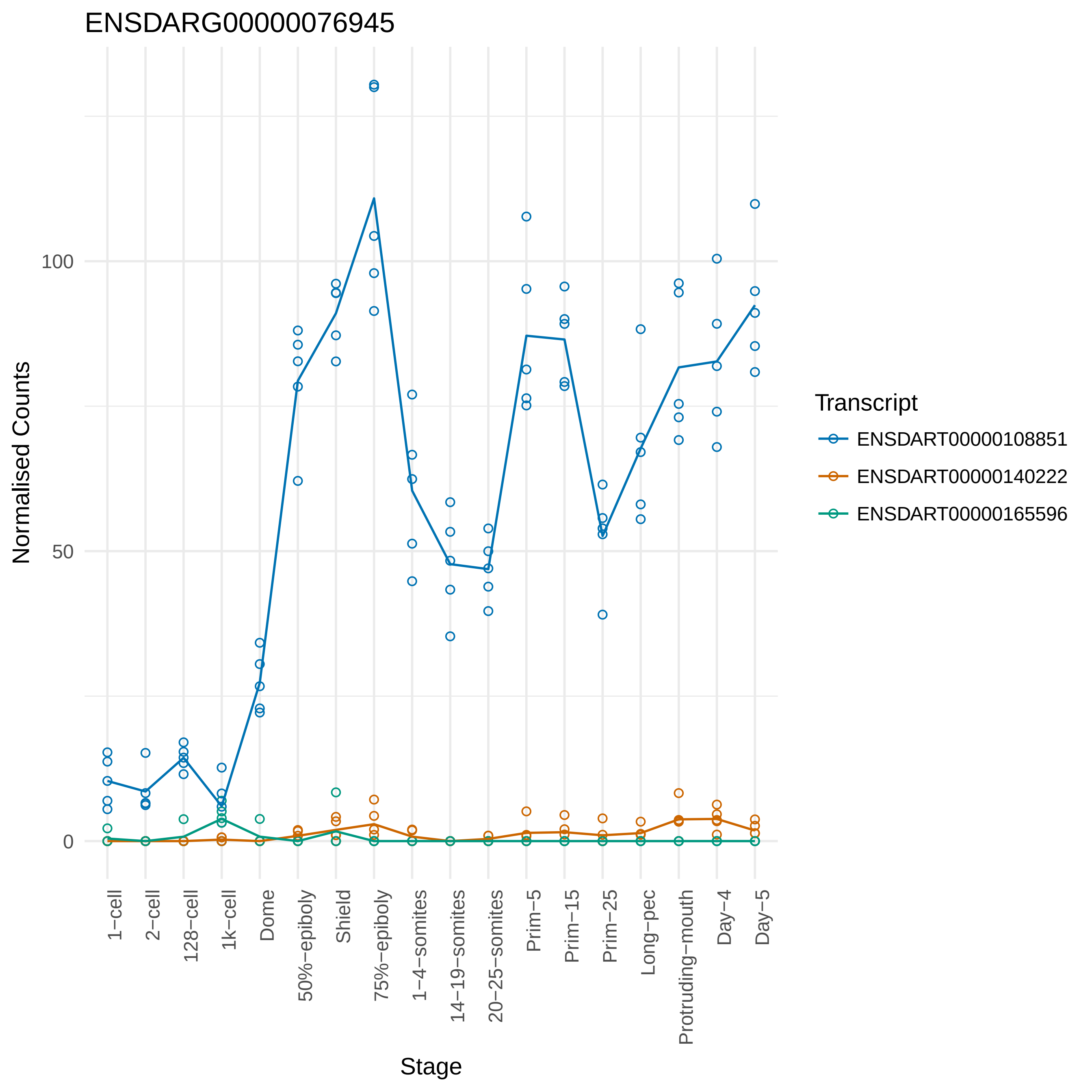

ENSDARG00000076945
Normalised Counts
100
50
0
1−cell
2−cell
128−cell
1k−cell
Dome
50%−epiboly
Shield
75%−epiboly
1−4−somites
14−19−somites
20−25−somites
Prim−5
Prim−15
Prim−25
Long−pec
Protruding−mouth
Day−4
Day−5
Stage
Transcript
ENSDART00000108851
ENSDART00000140222
ENSDART00000165596

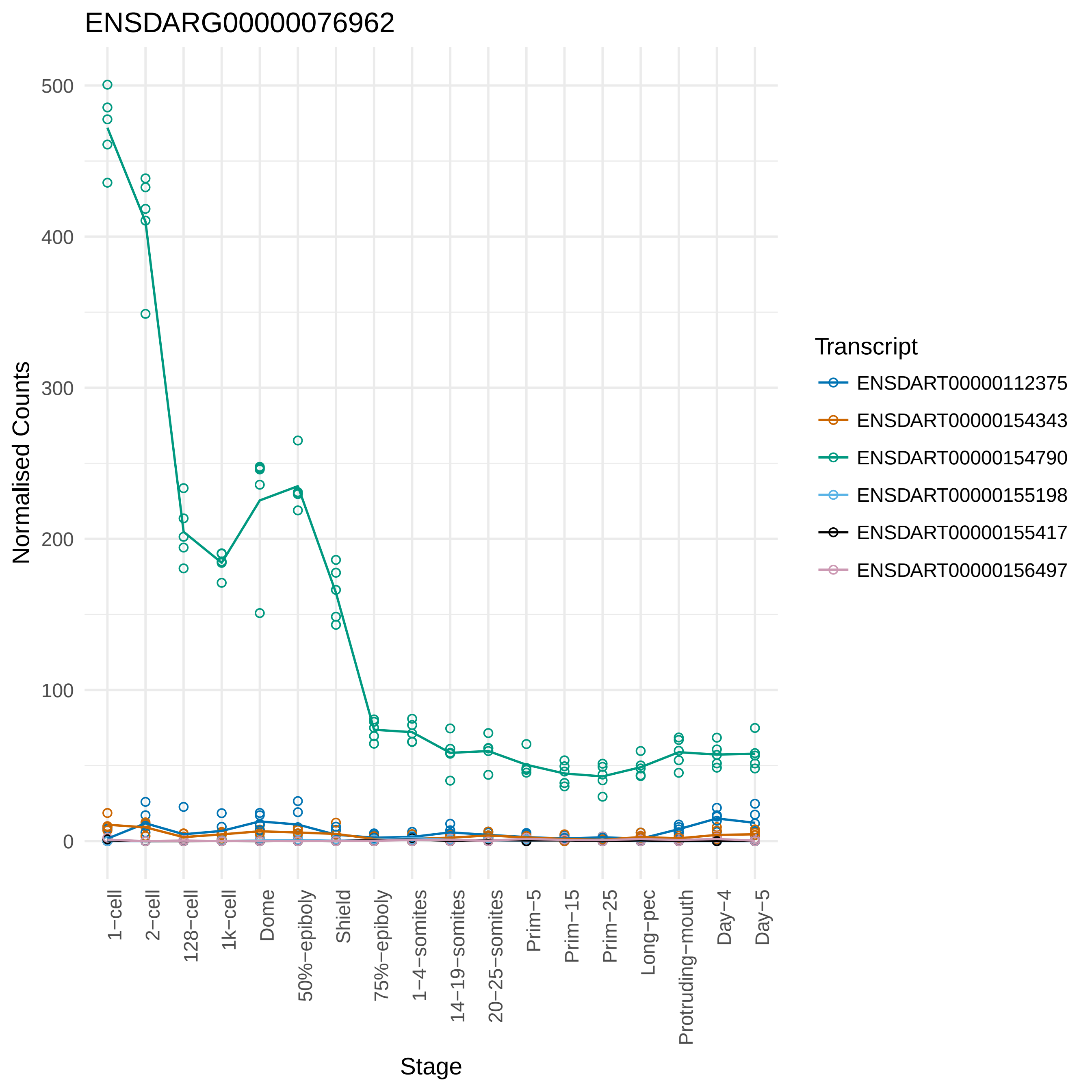

ENSDARG00000076962
Normalised Counts
0
100
200
300
400
500
1−cell
2−cell
128−cell
1k−cell
Dome
50%−epiboly
Shield
75%−epiboly
1−4−somites
14−19−somites
20−25−somites
Prim−5
Prim−15
Prim−25
Long−pec
Protruding−mouth
Day−4
Day−5
Stage
Transcript
ENSDART00000112375
ENSDART00000154343
ENSDART00000154790
ENSDART00000155198
ENSDART00000155417
ENSDART00000156497

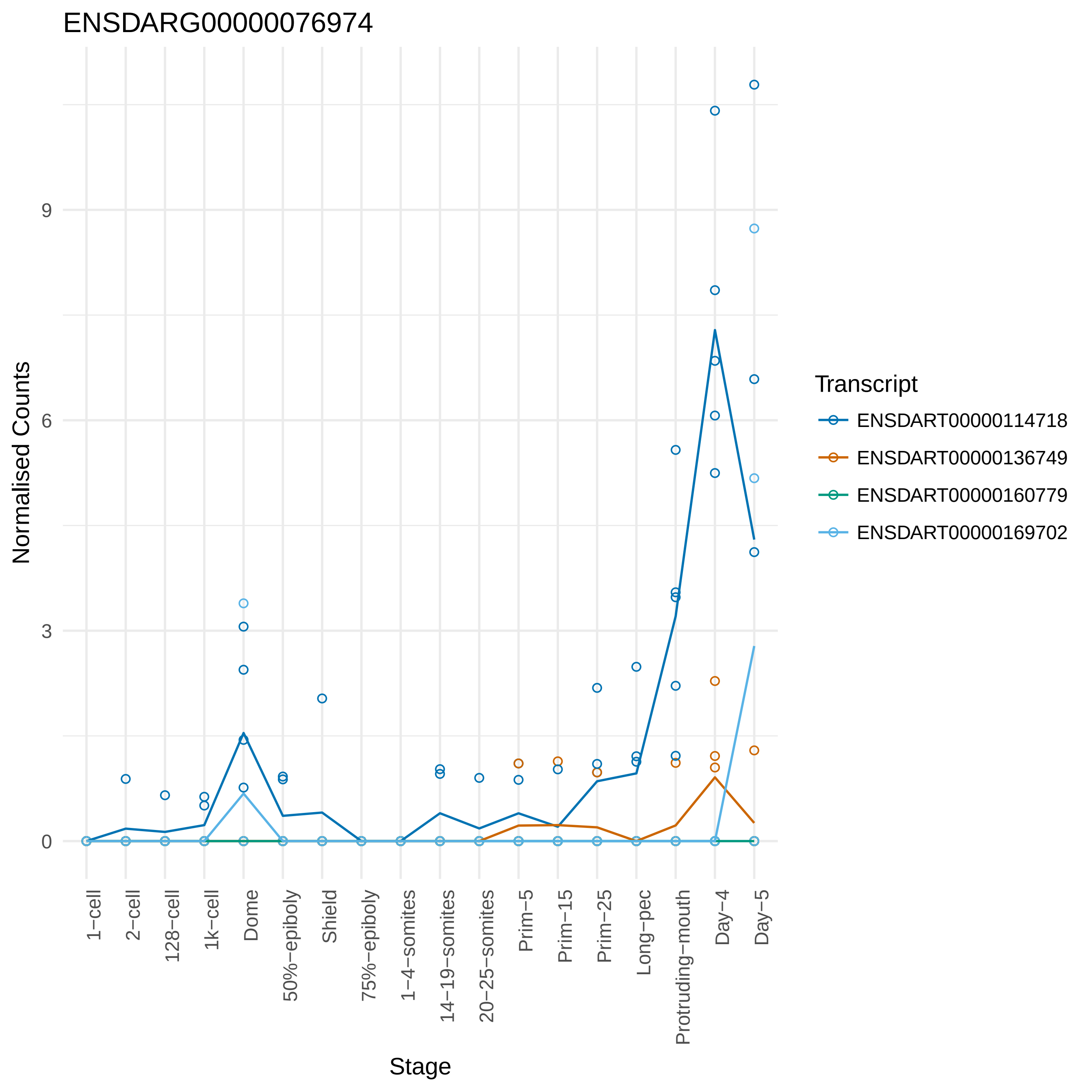

ENSDARG00000076974
Normalised Counts
0
3
6
9
Stage
1−cell
2−cell
128−cell
1k−cell
Dome
50%−epiboly
Shield
75%−epiboly
1−4−somites
14−19−somites
20−25−somites
Prim−5
Prim−15
Prim−25
Long−pec
Protruding−mouth
Day−4
Day−5
Transcript
ENSDART00000114718
ENSDART00000136749
ENSDART00000160779
ENSDART00000169702

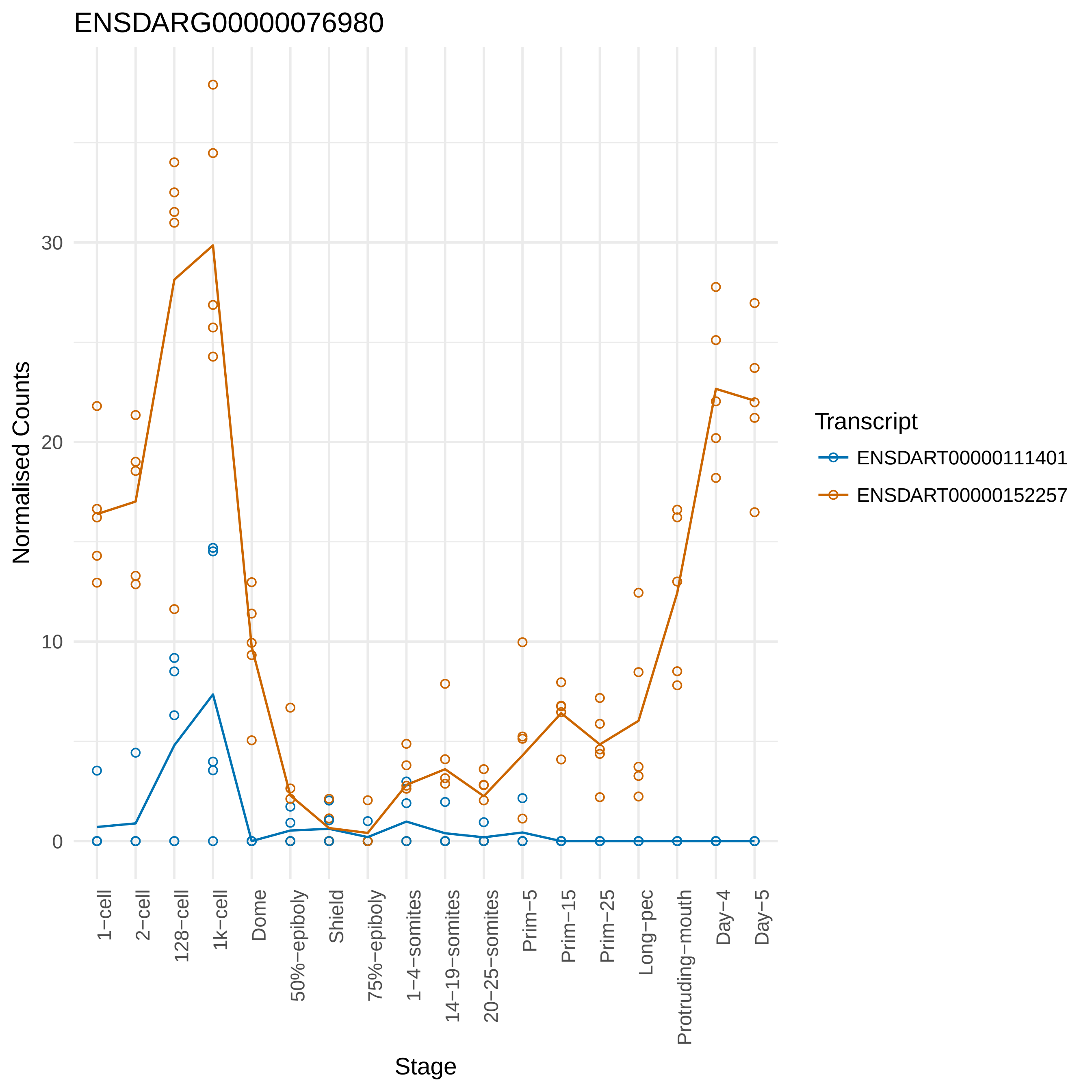
ENSDARG00000076980
Normalised Counts
0
10
20
30
Stage
1−cell
2−cell
128−cell
1k−cell
Dome
50%−epiboly
Shield
75%−epiboly
1−4−somites
14−19−somites
20−25−somites
Prim−5
Prim−15
Prim−25
Long−pec
Protruding−mouth
Day−4
Day−5
Transcript
ENSDART00000111401
ENSDART00000152257

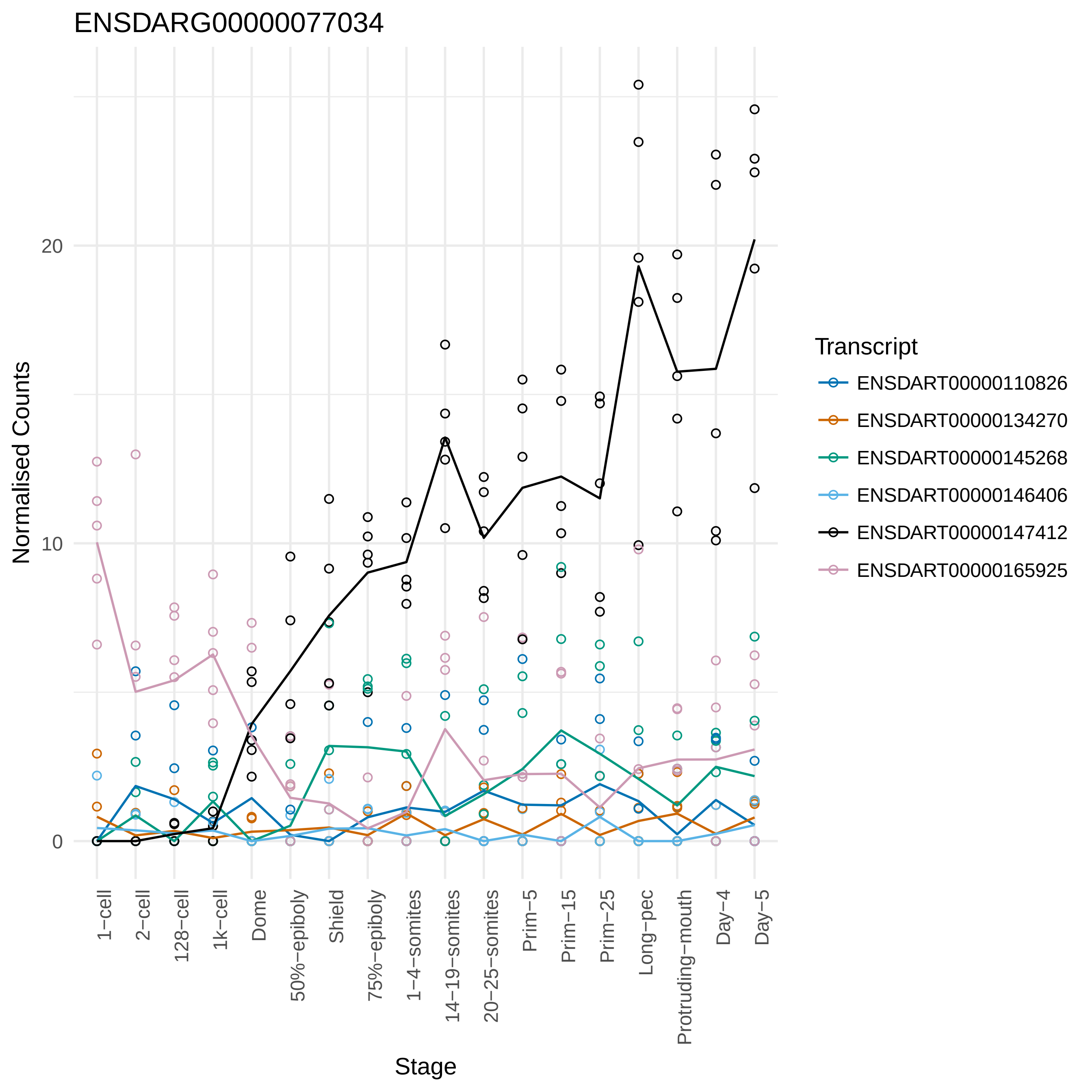

ENSDARG00000077034
Normalised Counts
0
10
20
1−cell
2−cell
128−cell
1k−cell
Dome
50%−epiboly
Shield
75%−epiboly
1−4−somites
14−19−somites
20−25−somites
Prim−5
Prim−15
Prim−25
Long−pec
Protruding−mouth
Day−4
Day−5
Stage
Transcript
ENSDART00000110826
ENSDART00000134270
ENSDART00000145268
ENSDART00000146406
ENSDART00000147412
ENSDART00000165925

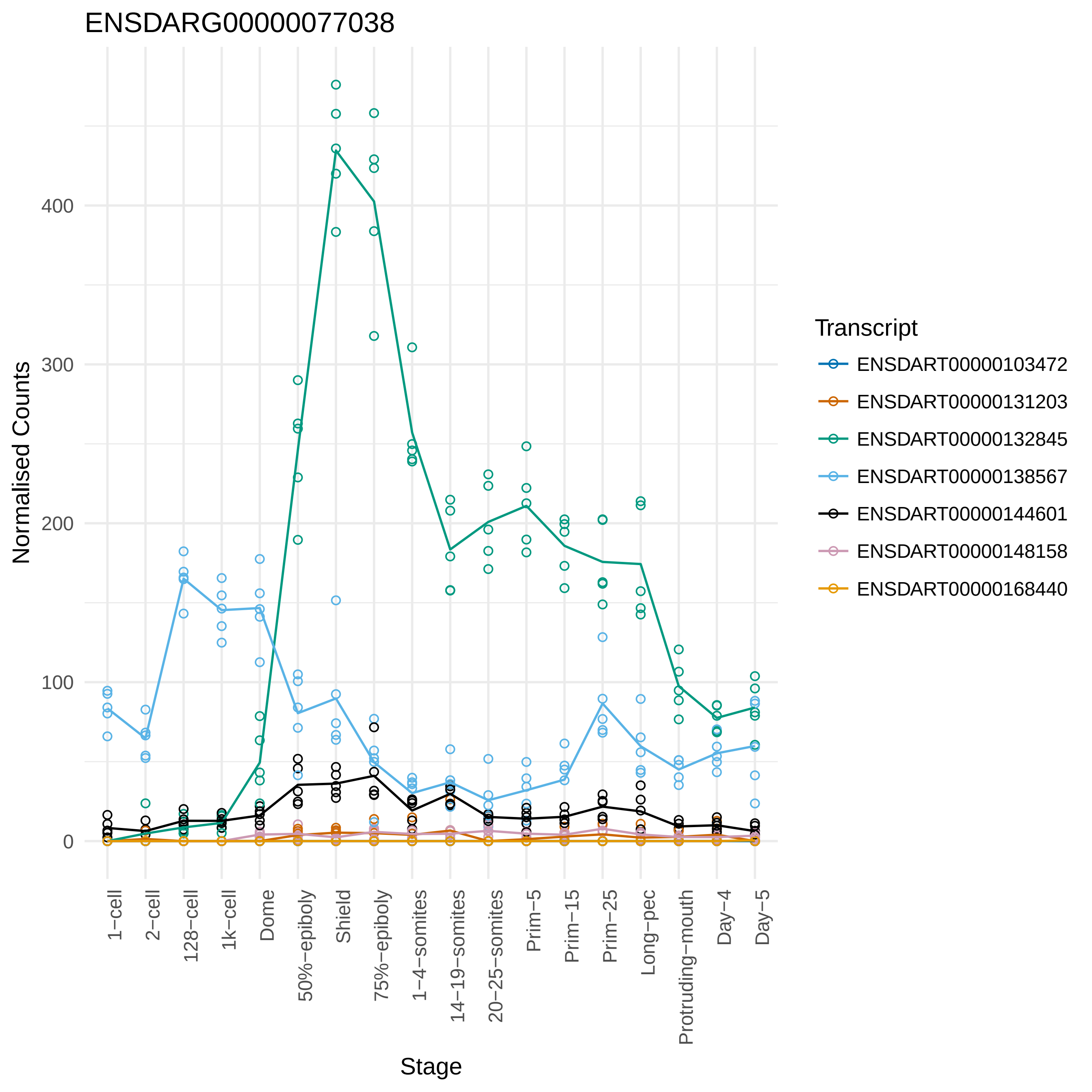

ENSDARG00000077038
Normalised Counts
0
100
200
300
400
Stage
1−cell
2−cell
128−cell
1k−cell
Dome
50%−epiboly
Shield
75%−epiboly
1−4−somites
14−19−somites
20−25−somites
Prim−5
Prim−15
Prim−25
Long−pec
Protruding−mouth
Day−4
Day−5
Transcript
ENSDART00000103472
ENSDART00000131203
ENSDART00000132845
ENSDART00000138567
ENSDART00000144601
ENSDART00000148158
ENSDART00000168440

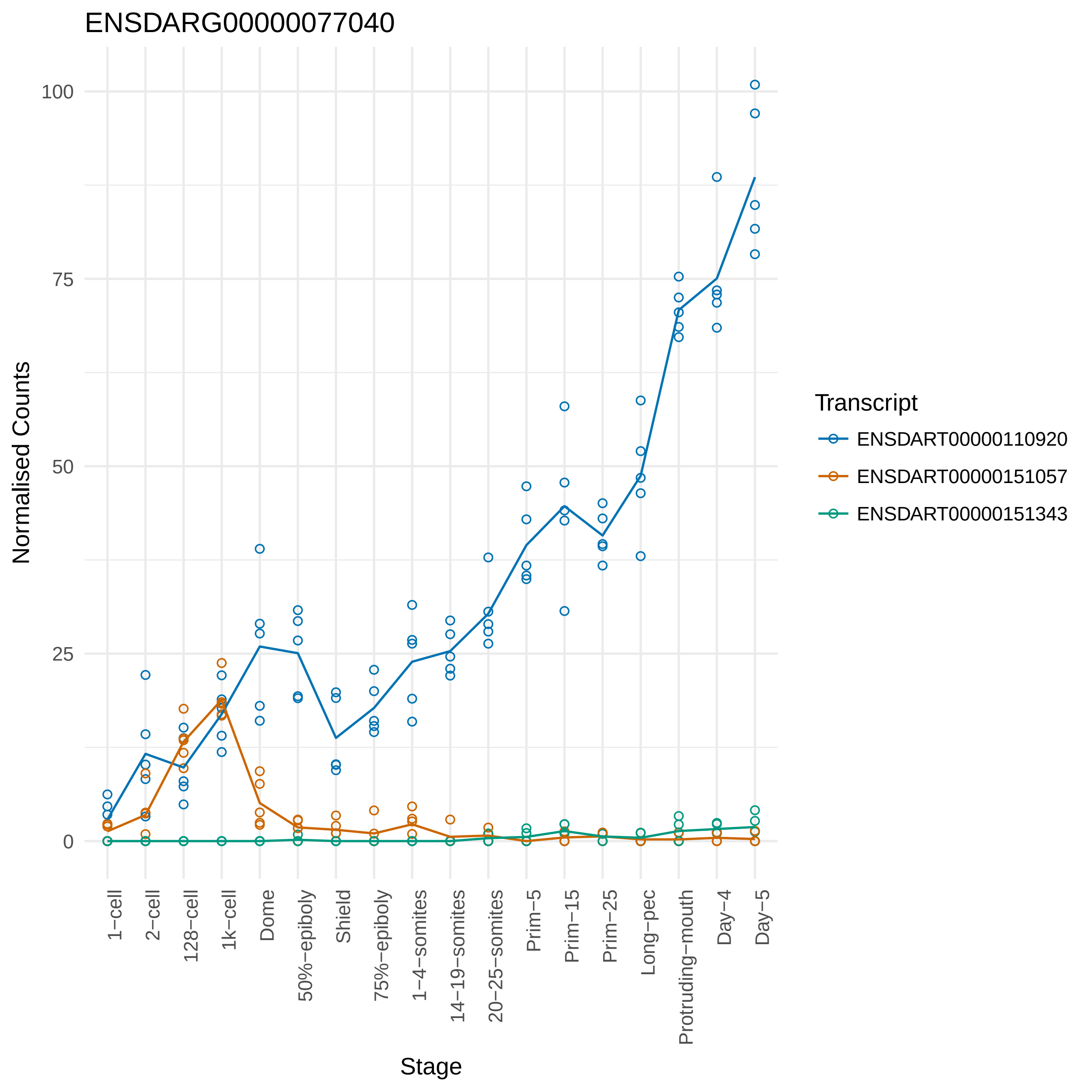
ENSDARG00000077040
Normalised Counts
100
75
50
25
0
1−cell
2−cell
128−cell
1k−cell
Dome
50%−epiboly
Shield
75%−epiboly
1−4−somites
14−19−somites
20−25−somites
Prim−5
Prim−15
Prim−25
Long−pec
Protruding−mouth
Day−4
Day−5
Stage
Transcript
ENSDART00000110920
ENSDART00000151057
ENSDART00000151343

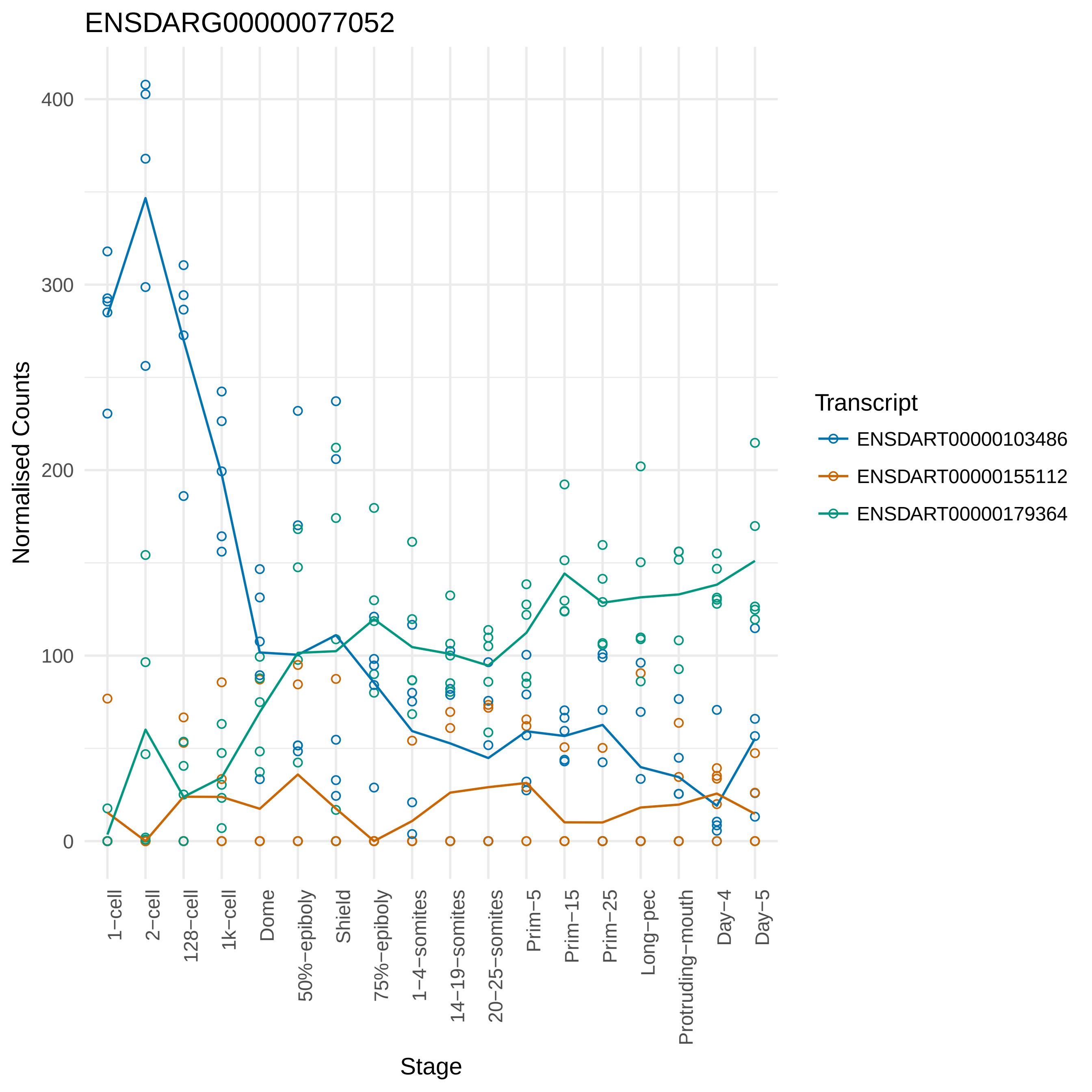

ENSDARG00000077052
Normalised Counts
400
300
200
100
0
1−cell
2−cell
128−cell
1k−cell
Dome
50%−epiboly
Shield
75%−epiboly
1−4−somites
14−19−somites
20−25−somites
Prim−5
Prim−15
Prim−25
Long−pec
Protruding−mouth
Day−4
Day−5
Stage
Transcript
ENSDART00000103486
ENSDART00000155112
ENSDART00000179364

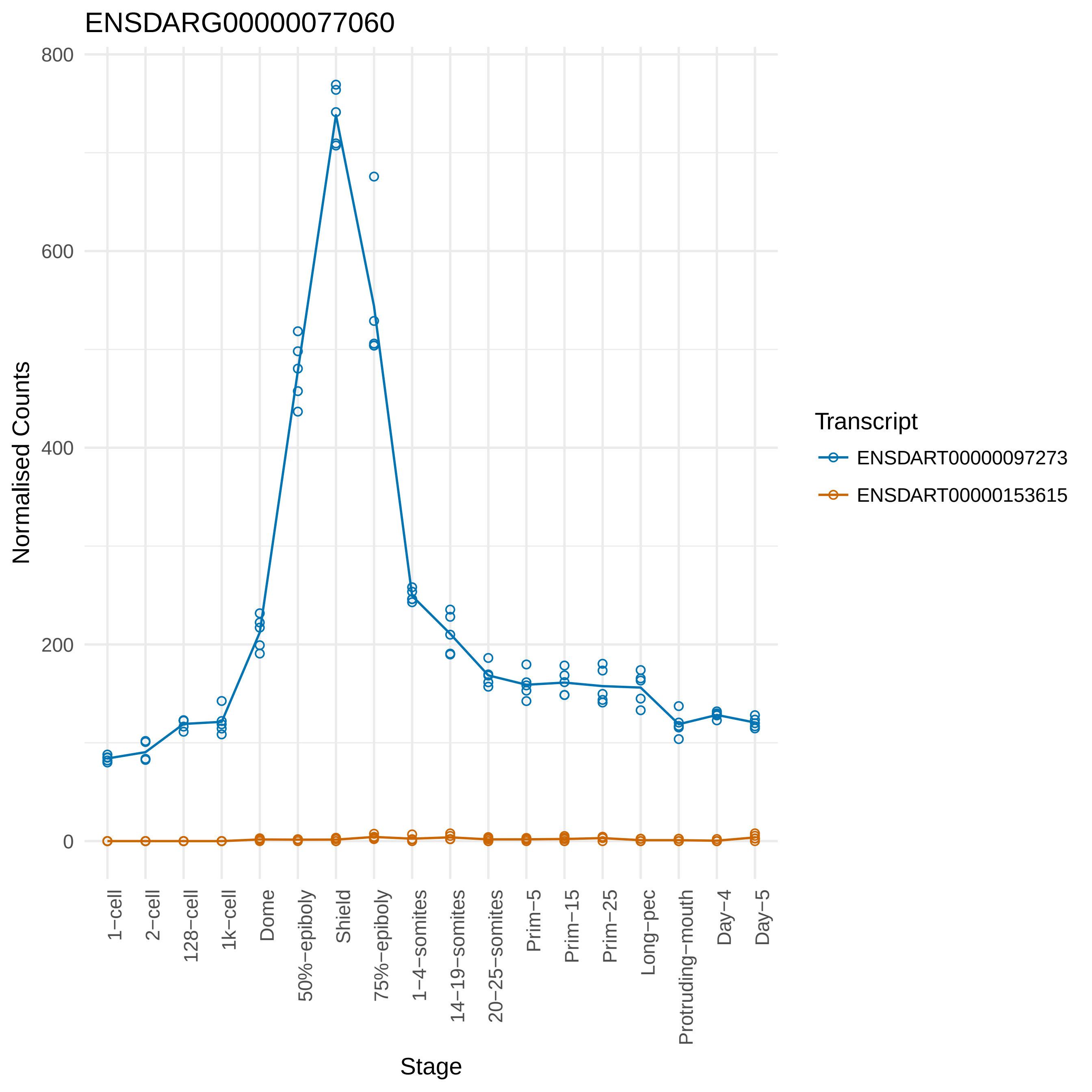

ENSDARG00000077060
Normalised Counts
0
200
400
600
800
Stage
1−cell
2−cell
128−cell
1k−cell
Dome
50%−epiboly
Shield
75%−epiboly
1−4−somites
14−19−somites
20−25−somites
Prim−5
Prim−15
Prim−25
Long−pec
Protruding−mouth
Day−4
Day−5
Transcript
ENSDART00000097273
ENSDART00000153615

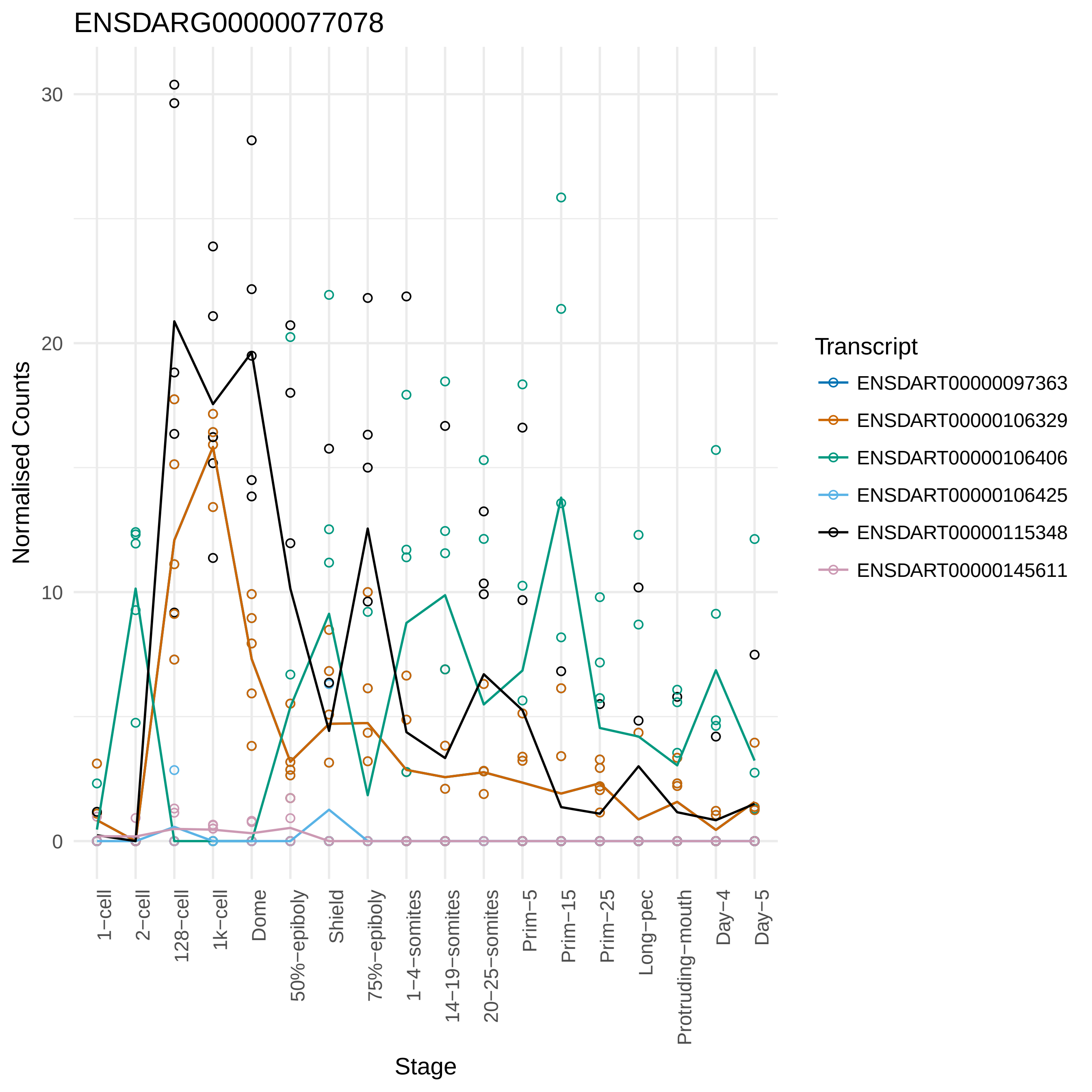
ENSDARG00000077078
Normalised Counts
0
10
20
30
1−cell
2−cell
128−cell
1k−cell
Dome
50%−epiboly
Shield
75%−epiboly
1−4−somites
14−19−somites
20−25−somites
Prim−5
Prim−15
Prim−25
Long−pec
Protruding−mouth
Day−4
Day−5
Stage
Transcript
ENSDART00000097363
ENSDART00000106329
ENSDART00000106406
ENSDART00000106425
ENSDART00000115348
ENSDART00000145611

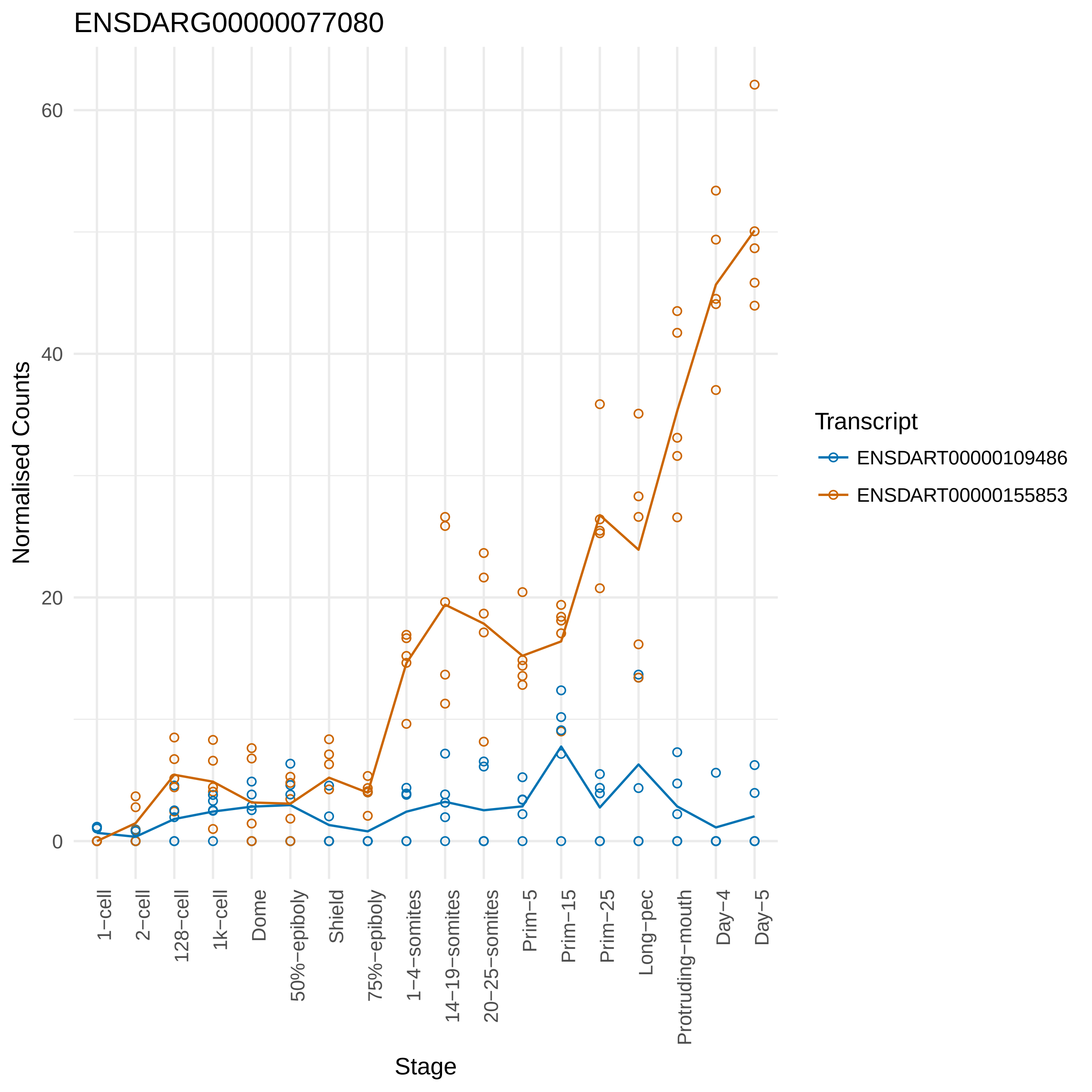
ENSDARG00000077080
Normalised Counts
60
40
20
0
1−cell
2−cell
128−cell
1k−cell
Dome
50%−epiboly
Shield
75%−epiboly
1−4−somites
14−19−somites
20−25−somites
Prim−5
Prim−15
Prim−25
Long−pec
Protruding−mouth
Day−4
Day−5
Stage
Transcript
ENSDART00000109486
ENSDART00000155853

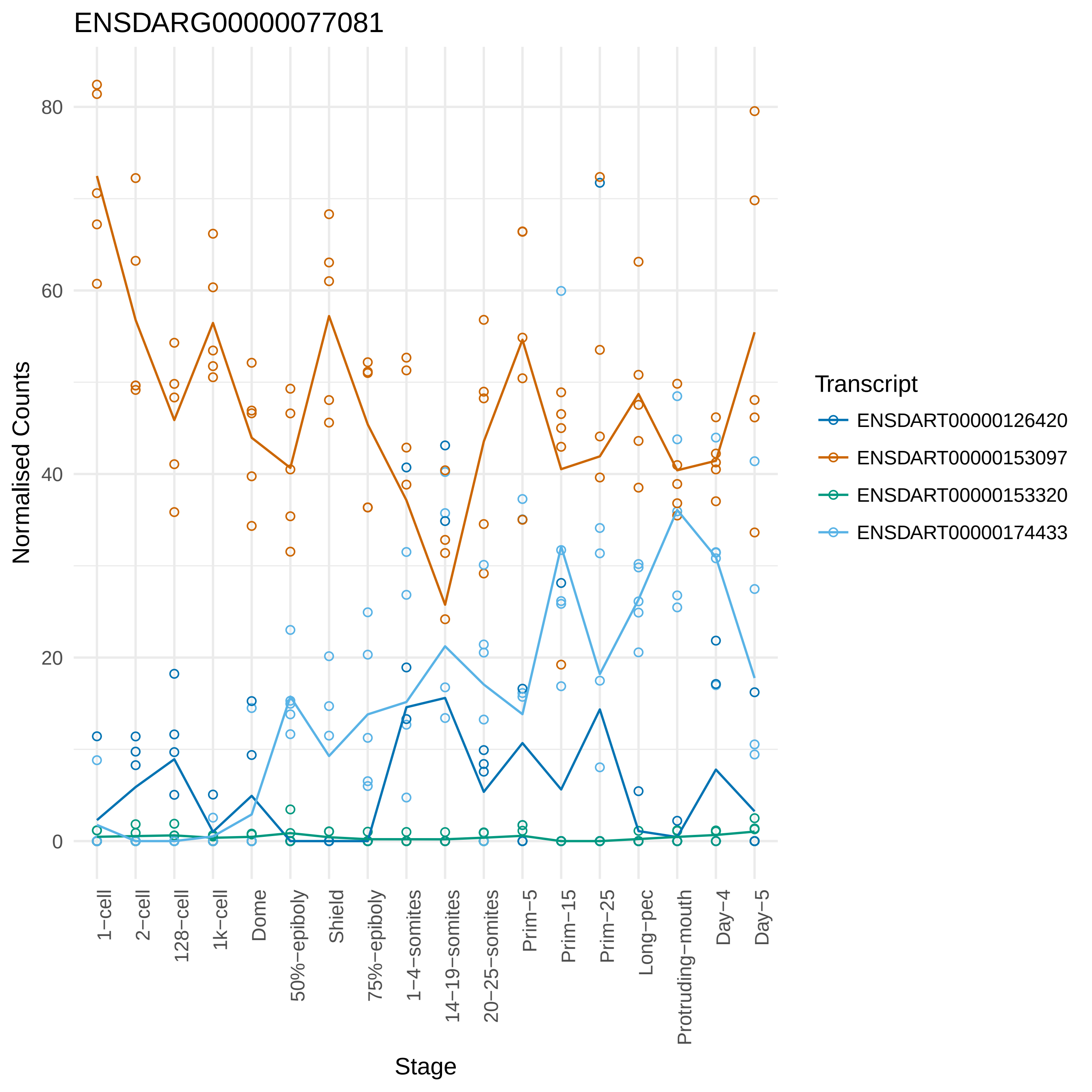
ENSDARG00000077081
Normalised Counts
80
60
40
20
0
1−cell
2−cell
128−cell
1k−cell
Dome
50%−epiboly
Shield
75%−epiboly
1−4−somites
14−19−somites
20−25−somites
Prim−5
Prim−15
Prim−25
Long−pec
Protruding−mouth
Day−4
Day−5
Stage
Transcript
ENSDART00000126420
ENSDART00000153097
ENSDART00000153320
ENSDART00000174433

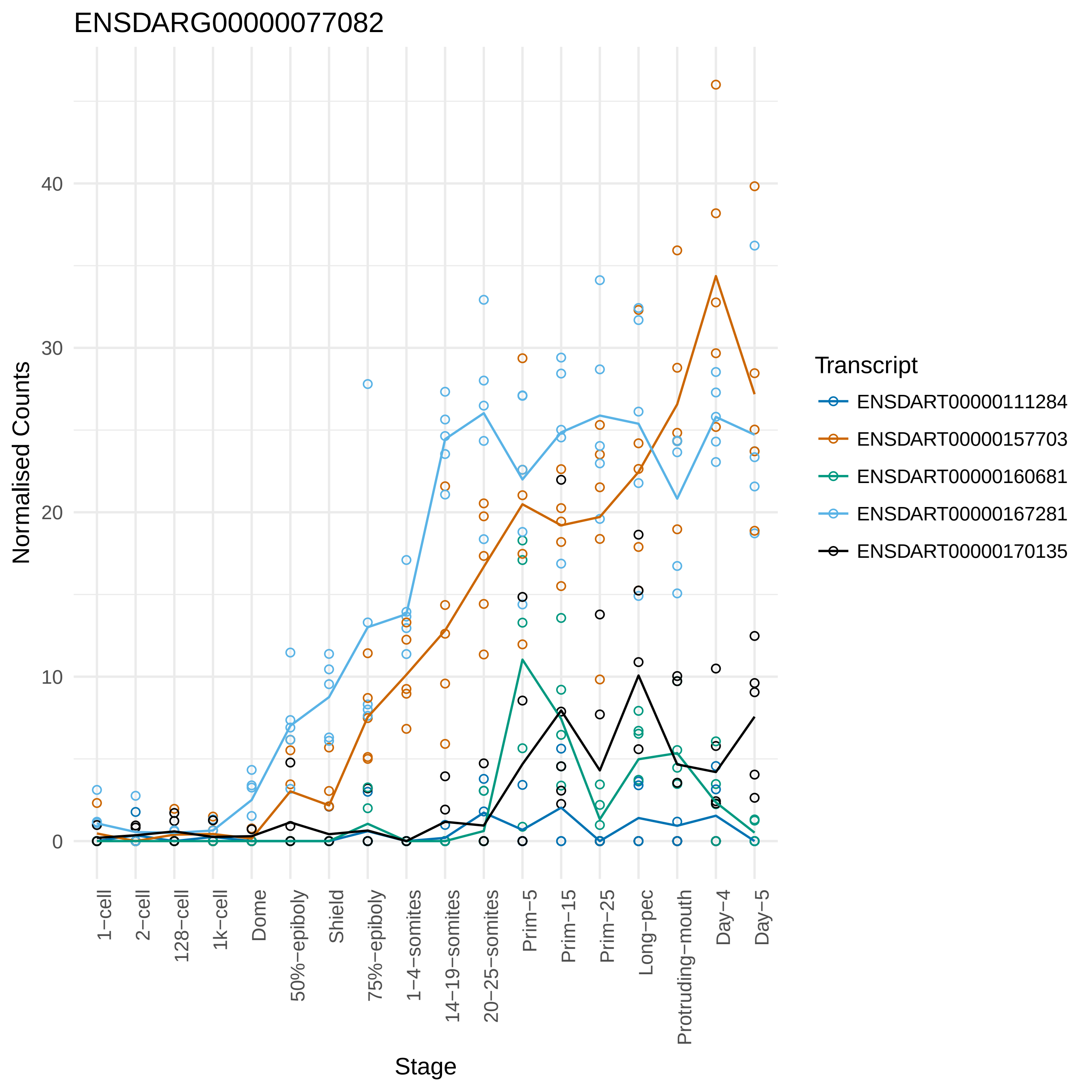
ENSDARG00000077082
Normalised Counts
0
10
20
30
40
1−cell
2−cell
128−cell
1k−cell
Dome
50%−epiboly
Shield
75%−epiboly
1−4−somites
14−19−somites
20−25−somites
Prim−5
Prim−15
Prim−25
Long−pec
Protruding−mouth
Day−4
Day−5
Stage
Transcript
ENSDART00000111284
ENSDART00000157703
ENSDART00000160681
ENSDART00000167281
ENSDART00000170135

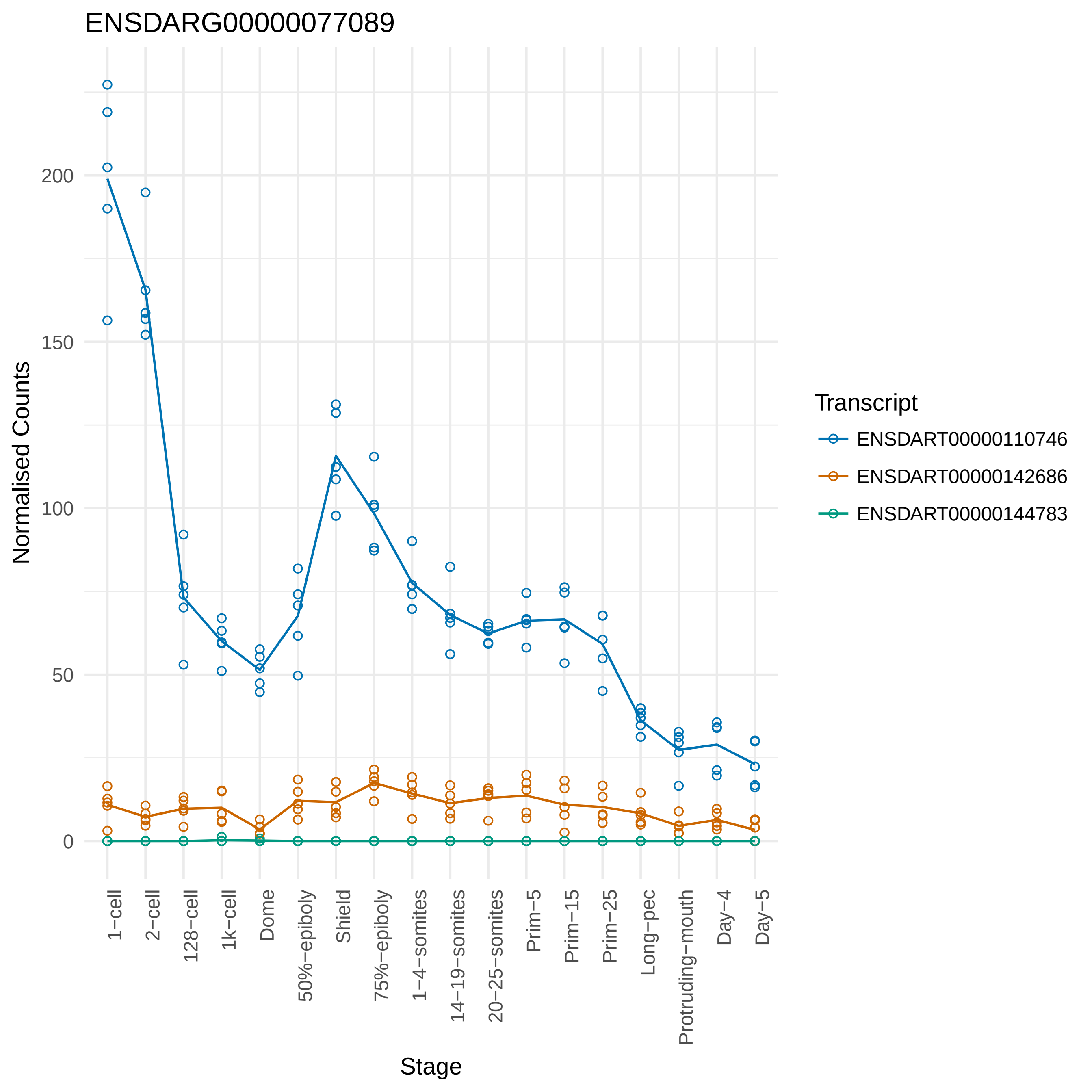
ENSDARG00000077089
Normalised Counts
0
50
100
150
200
1−cell
2−cell
128−cell
1k−cell
Dome
50%−epiboly
Shield
75%−epiboly
1−4−somites
14−19−somites
20−25−somites
Prim−5
Prim−15
Prim−25
Long−pec
Protruding−mouth
Day−4
Day−5
Stage
Transcript
ENSDART00000110746
ENSDART00000142686
ENSDART00000144783

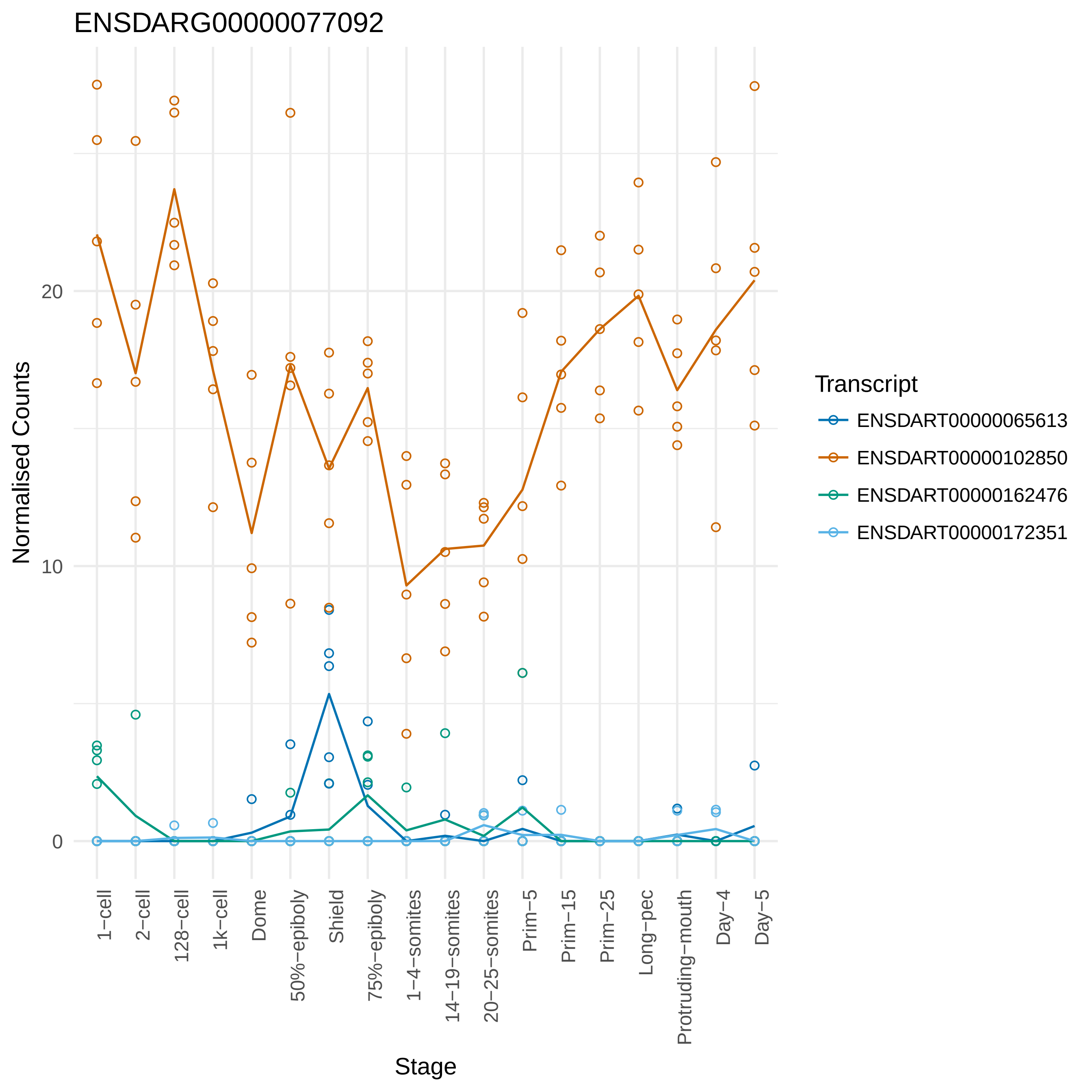
ENSDARG00000077092
Normalised Counts
20
10
0
1−cell
2−cell
128−cell
1k−cell
Dome
50%−epiboly
Shield
75%−epiboly
1−4−somites
14−19−somites
20−25−somites
Prim−5
Prim−15
Prim−25
Long−pec
Protruding−mouth
Day−4
Day−5
Stage
Transcript
ENSDART00000065613
ENSDART00000102850
ENSDART00000162476
ENSDART00000172351

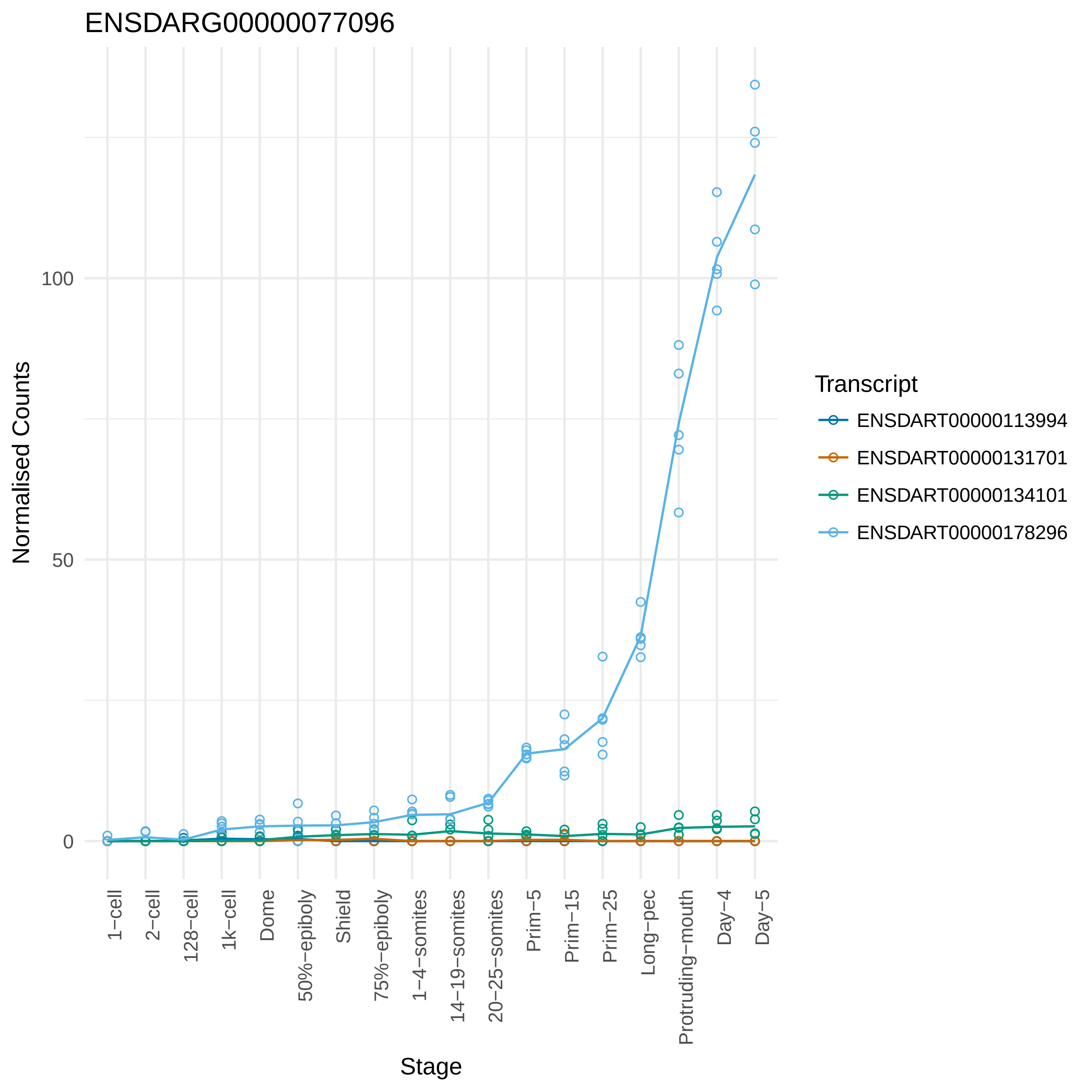

ENSDARG00000077096
Normalised Counts
100
50
0
1−cell
2−cell
128−cell
1k−cell
Dome
50%−epiboly
Shield
75%−epiboly
1−4−somites
14−19−somites
20−25−somites
Prim−5
Prim−15
Prim−25
Long−pec
Protruding−mouth
Day−4
Day−5
Stage
Transcript
ENSDART00000113994
ENSDART00000131701
ENSDART00000134101
ENSDART00000178296

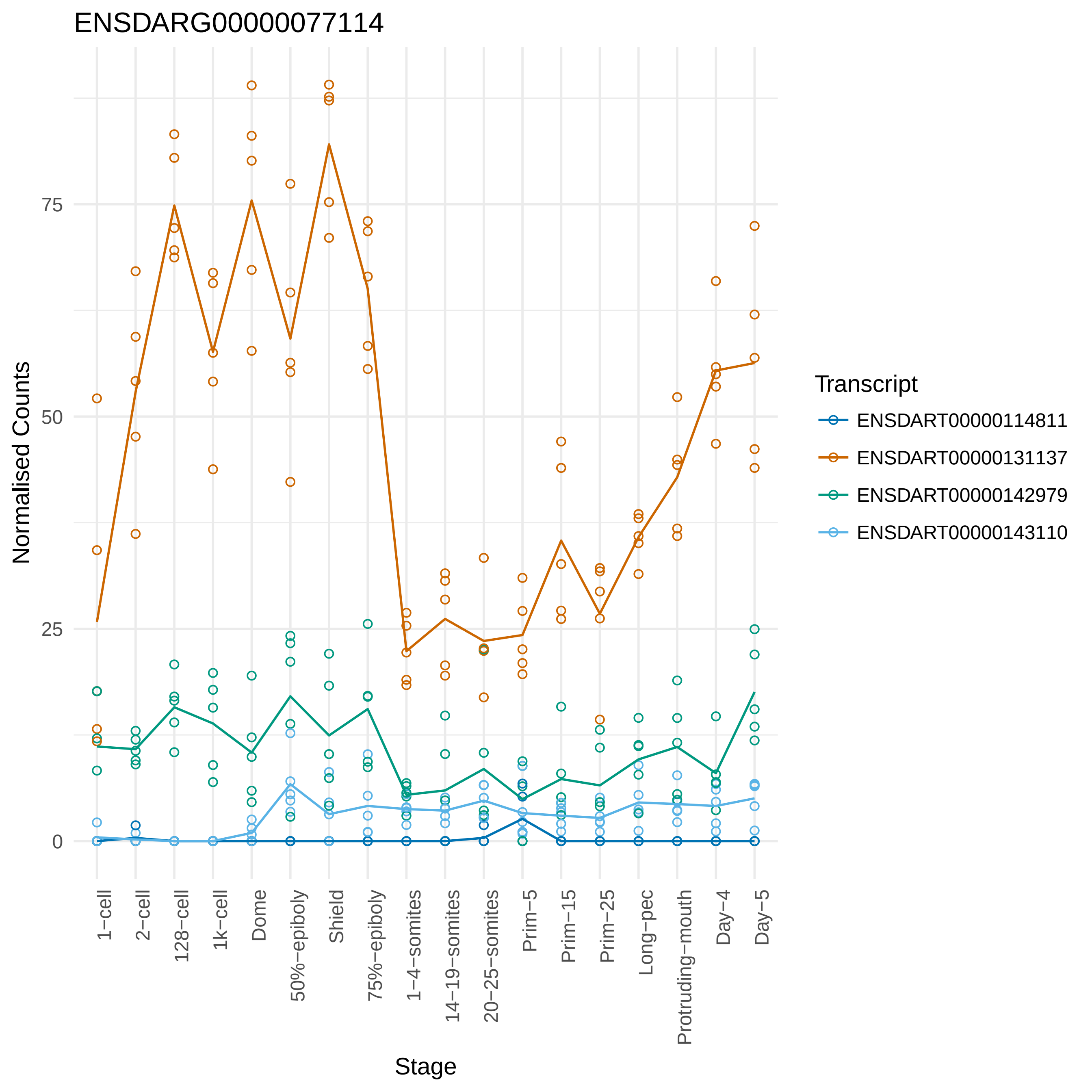
ENSDARG00000077114
Normalised Counts
0
25
50
75
Stage
1−cell
2−cell
128−cell
1k−cell
Dome
50%−epiboly
Shield
75%−epiboly
1−4−somites
14−19−somites
20−25−somites
Prim−5
Prim−15
Prim−25
Long−pec
Protruding−mouth
Day−4
Day−5
Transcript
ENSDART00000114811
ENSDART00000131137
ENSDART00000142979
ENSDART00000143110

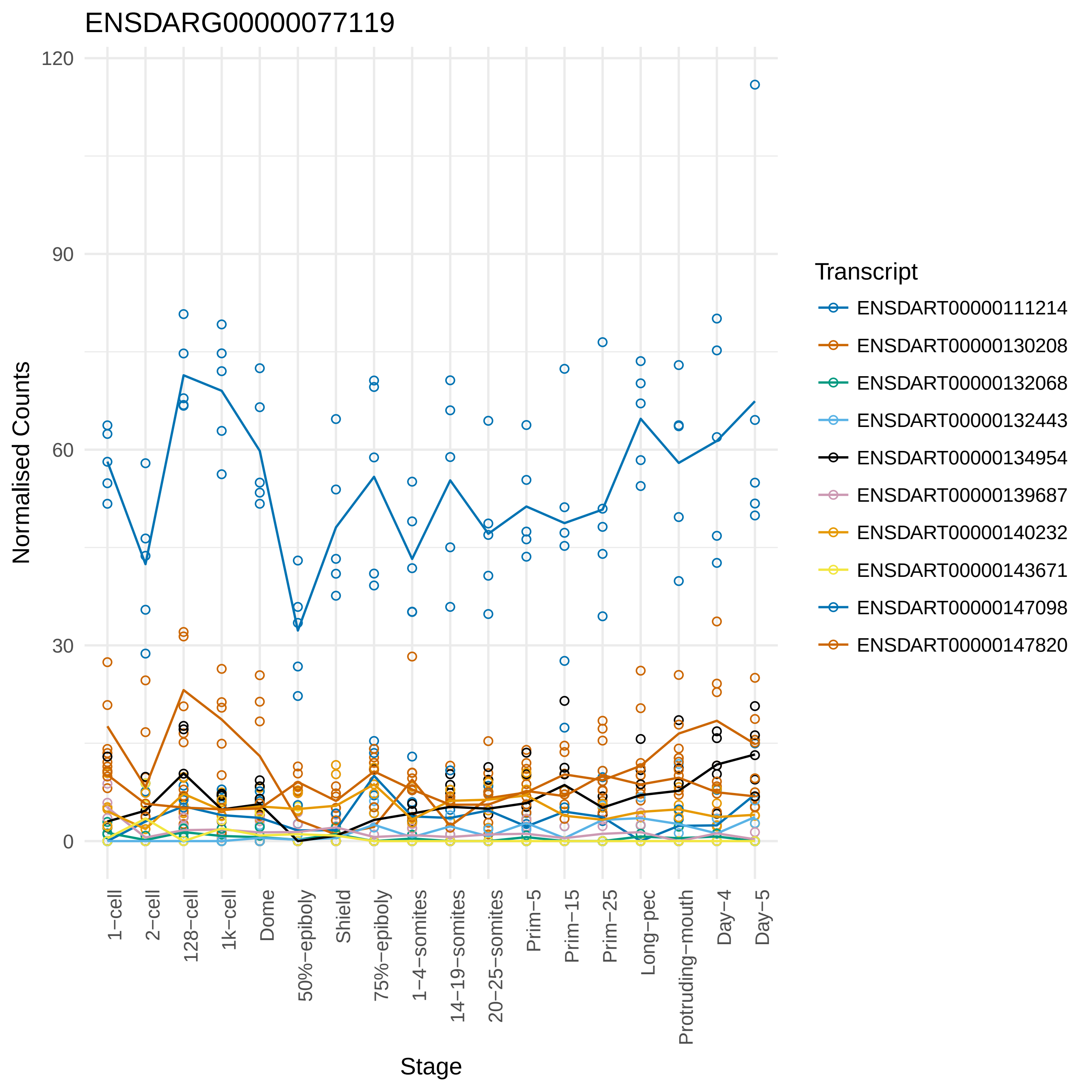

ENSDARG00000077119
Normalised Counts
120
90
60
30
0
1−cell
2−cell
128−cell
1k−cell
Dome
50%−epiboly
Shield
75%−epiboly
1−4−somites
14−19−somites
20−25−somites
Prim−5
Prim−15
Prim−25
Long−pec
Protruding−mouth
Day−4
Day−5
Stage
Transcript
ENSDART00000111214
ENSDART00000130208
ENSDART00000132068
ENSDART00000132443
ENSDART00000134954
ENSDART00000139687
ENSDART00000140232
ENSDART00000143671
ENSDART00000147098
ENSDART00000147820

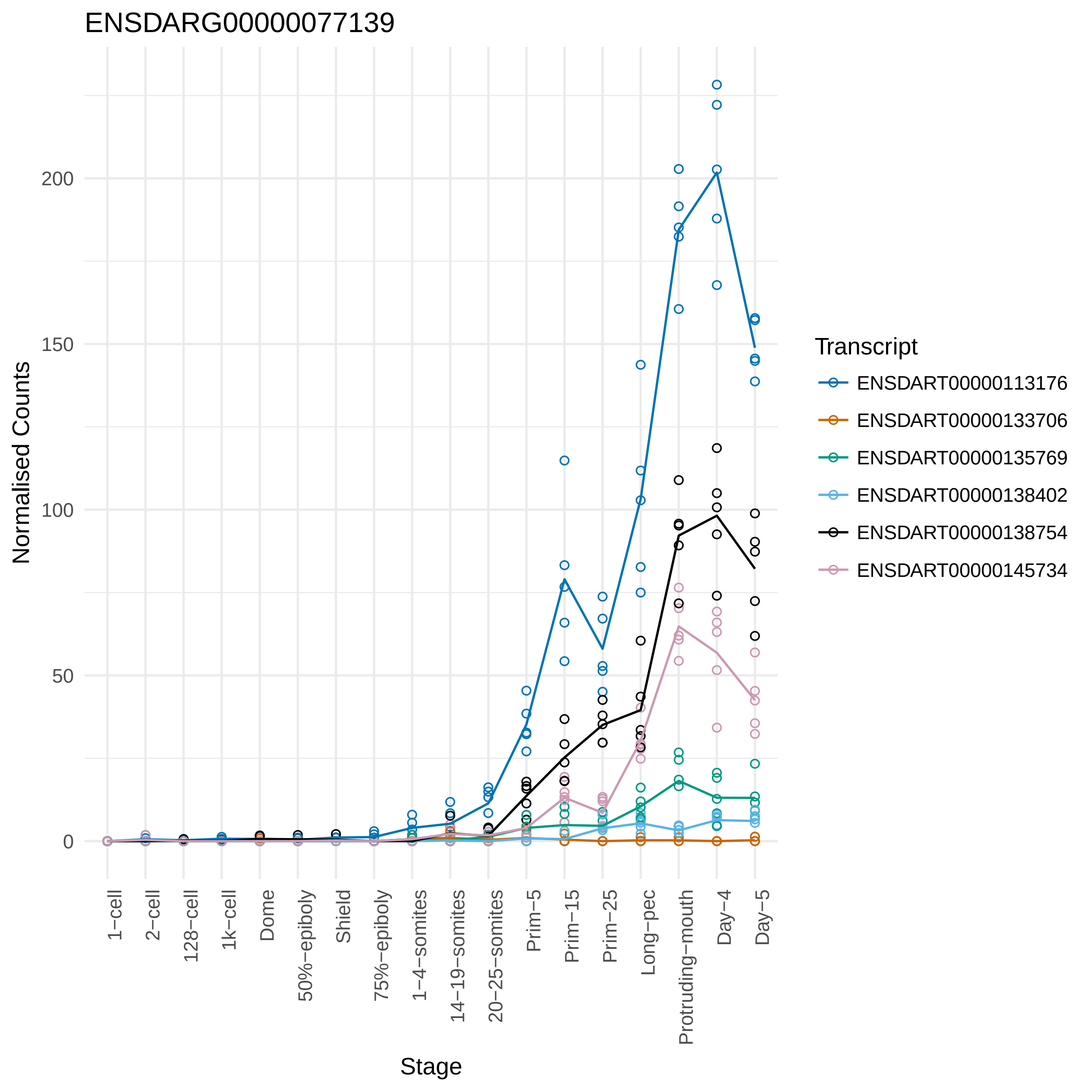
ENSDARG00000077139
Normalised Counts
200
150
100
50
0
1−cell
2−cell
128−cell
1k−cell
Dome
50%−epiboly
Shield
75%−epiboly
1−4−somites
14−19−somites
20−25−somites
Prim−5
Prim−15
Prim−25
Long−pec
Protruding−mouth
Day−4
Day−5
Stage
Transcript
ENSDART00000113176
ENSDART00000133706
ENSDART00000135769
ENSDART00000138402
ENSDART00000138754
ENSDART00000145734

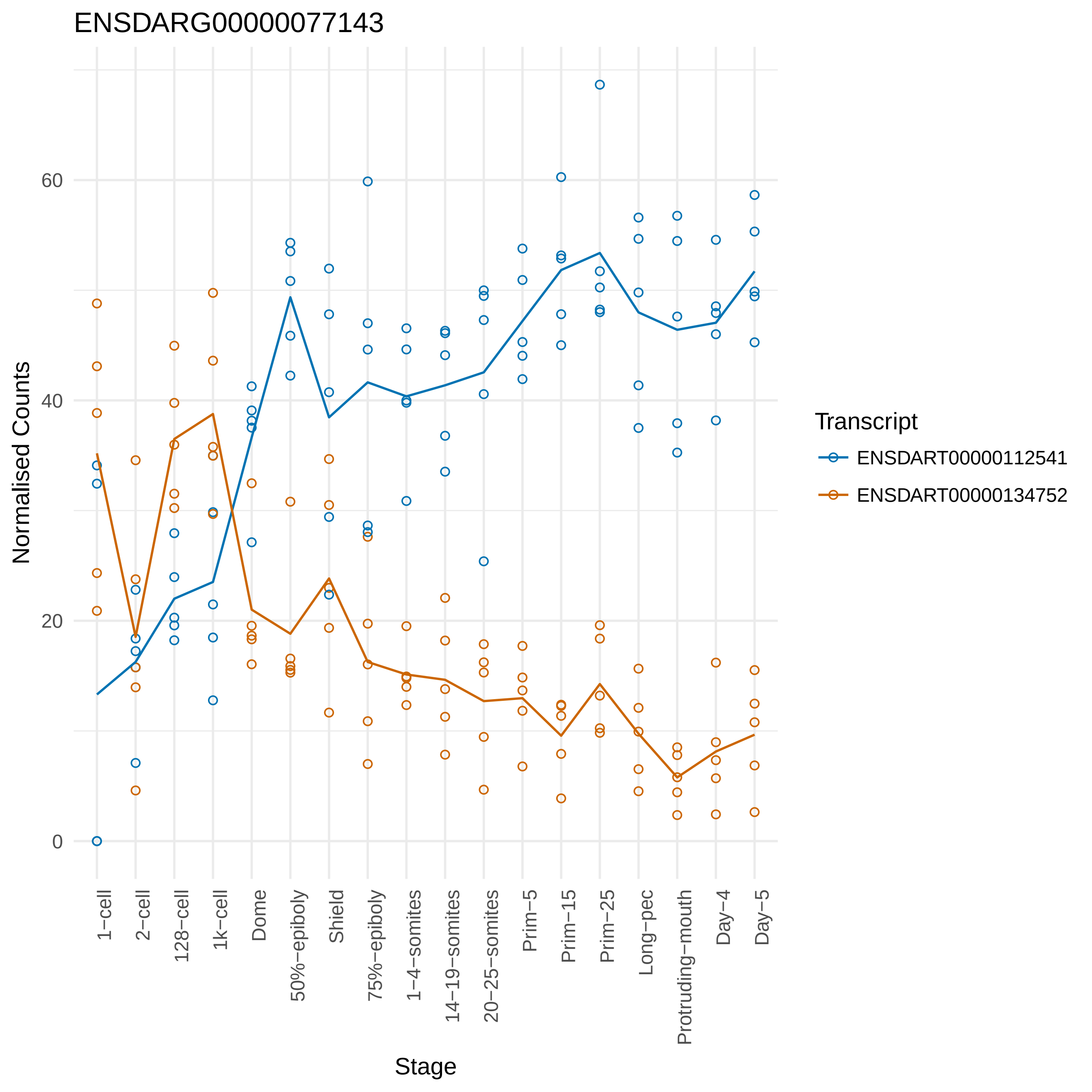

ENSDARG00000077143
Normalised Counts
60
40
20
0
1−cell
2−cell
128−cell
1k−cell
Dome
50%−epiboly
Shield
75%−epiboly
1−4−somites
14−19−somites
20−25−somites
Prim−5
Prim−15
Prim−25
Long−pec
Protruding−mouth
Day−4
Day−5
Stage
Transcript
ENSDART00000112541
ENSDART00000134752

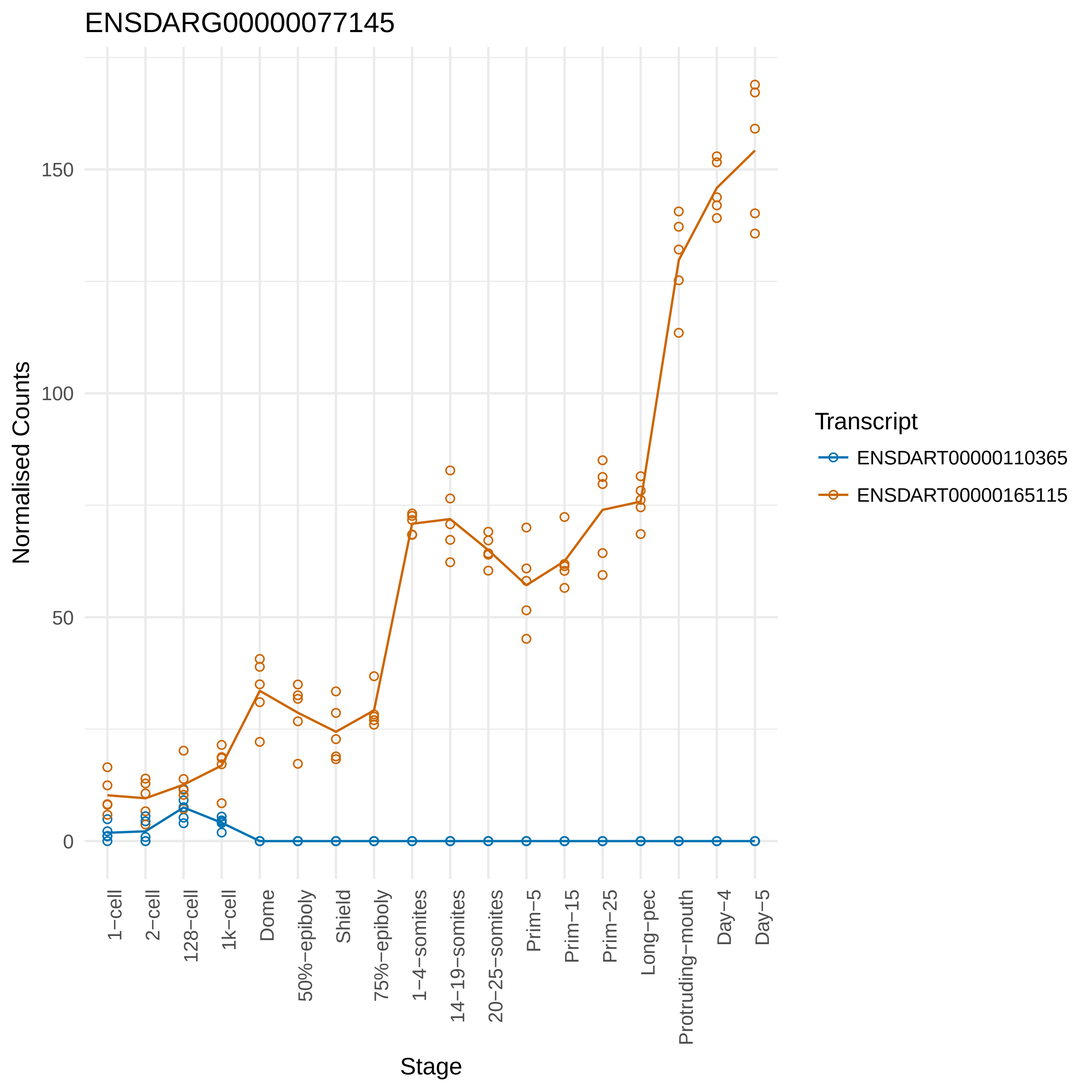

ENSDARG00000077145
Normalised Counts
0
50
100
150
1−cell
2−cell
128−cell
1k−cell
Dome
50%−epiboly
Shield
75%−epiboly
1−4−somites
14−19−somites
20−25−somites
Prim−5
Prim−15
Prim−25
Long−pec
Protruding−mouth
Day−4
Day−5
Stage
Transcript
ENSDART00000110365
ENSDART00000165115

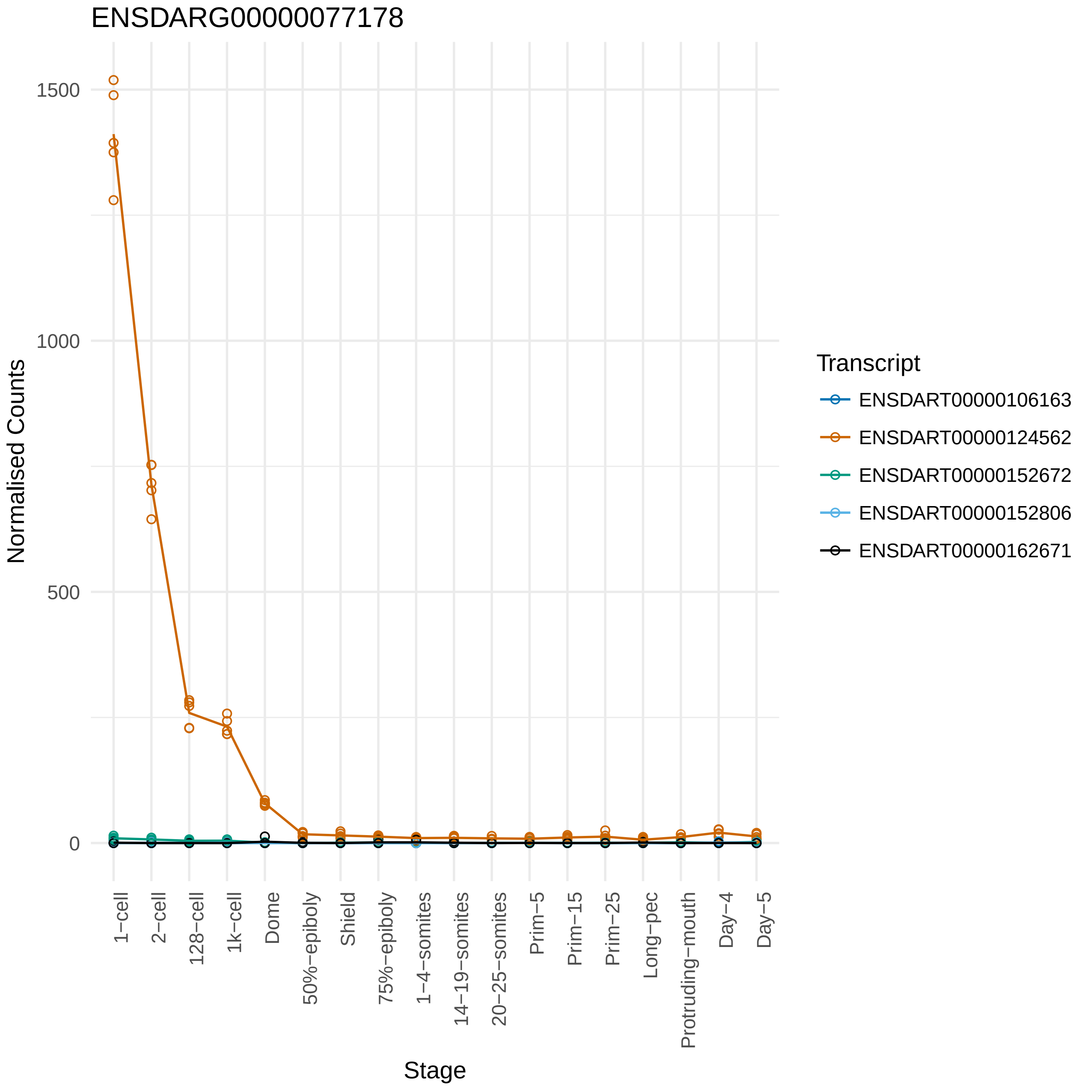

ENSDARG00000077178
Normalised Counts
1500
1000
500
0
Stage
1−cell
2−cell
128−cell
1k−cell
Dome
50%−epiboly
Shield
75%−epiboly
1−4−somites
14−19−somites
20−25−somites
Prim−5
Prim−15
Prim−25
Long−pec
Protruding−mouth
Day−4
Day−5
Transcript
ENSDART00000106163
ENSDART00000124562
ENSDART00000152672
ENSDART00000152806
ENSDART00000162671

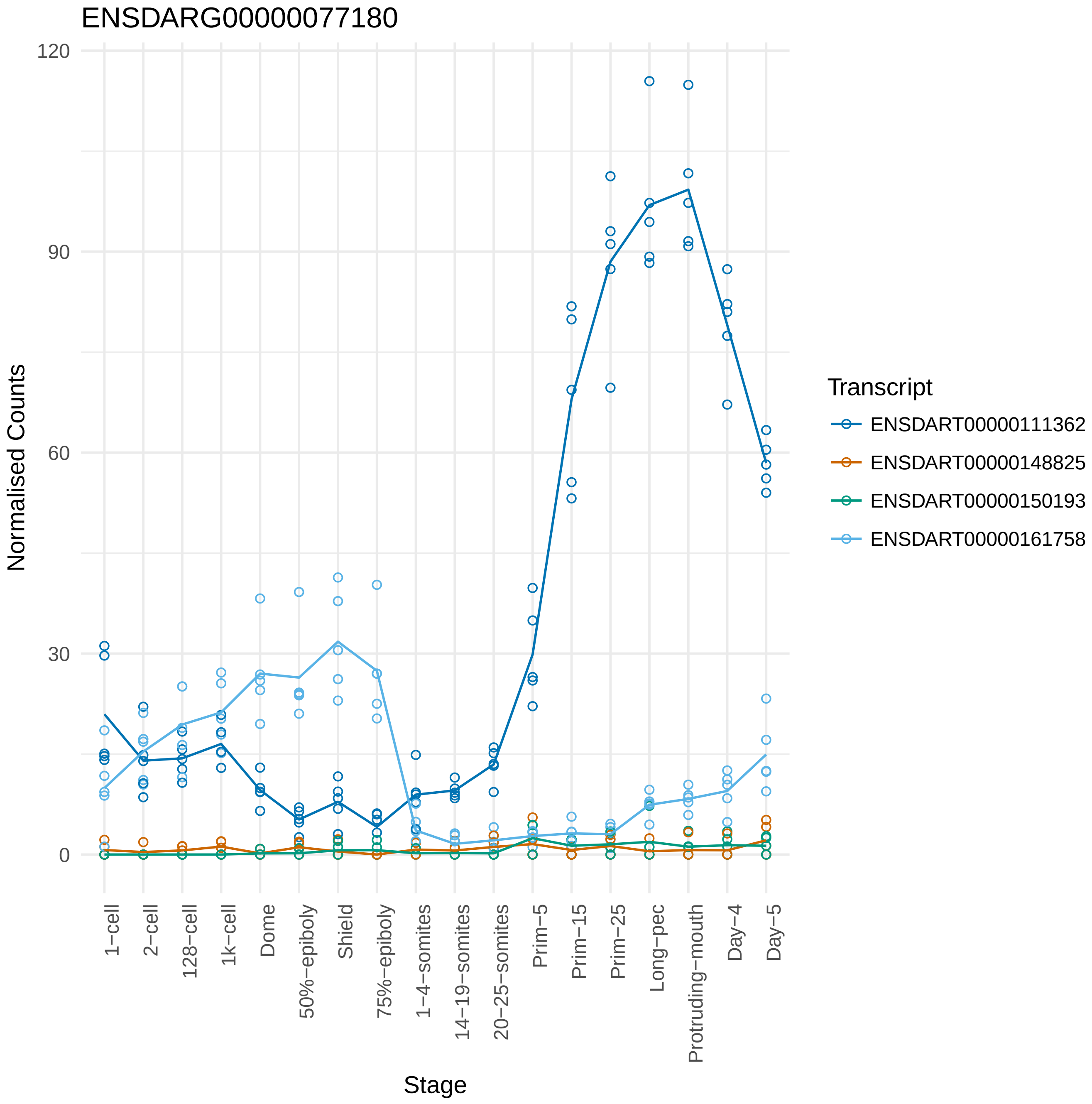

ENSDARG00000077180
Normalised Counts
0
30
60
90
120
1−cell
2−cell
128−cell
1k−cell
Dome
50%−epiboly
Shield
75%−epiboly
1−4−somites
14−19−somites
20−25−somites
Prim−5
Prim−15
Prim−25
Long−pec
Protruding−mouth
Day−4
Day−5
Stage
Transcript
ENSDART00000111362
ENSDART00000148825
ENSDART00000150193
ENSDART00000161758

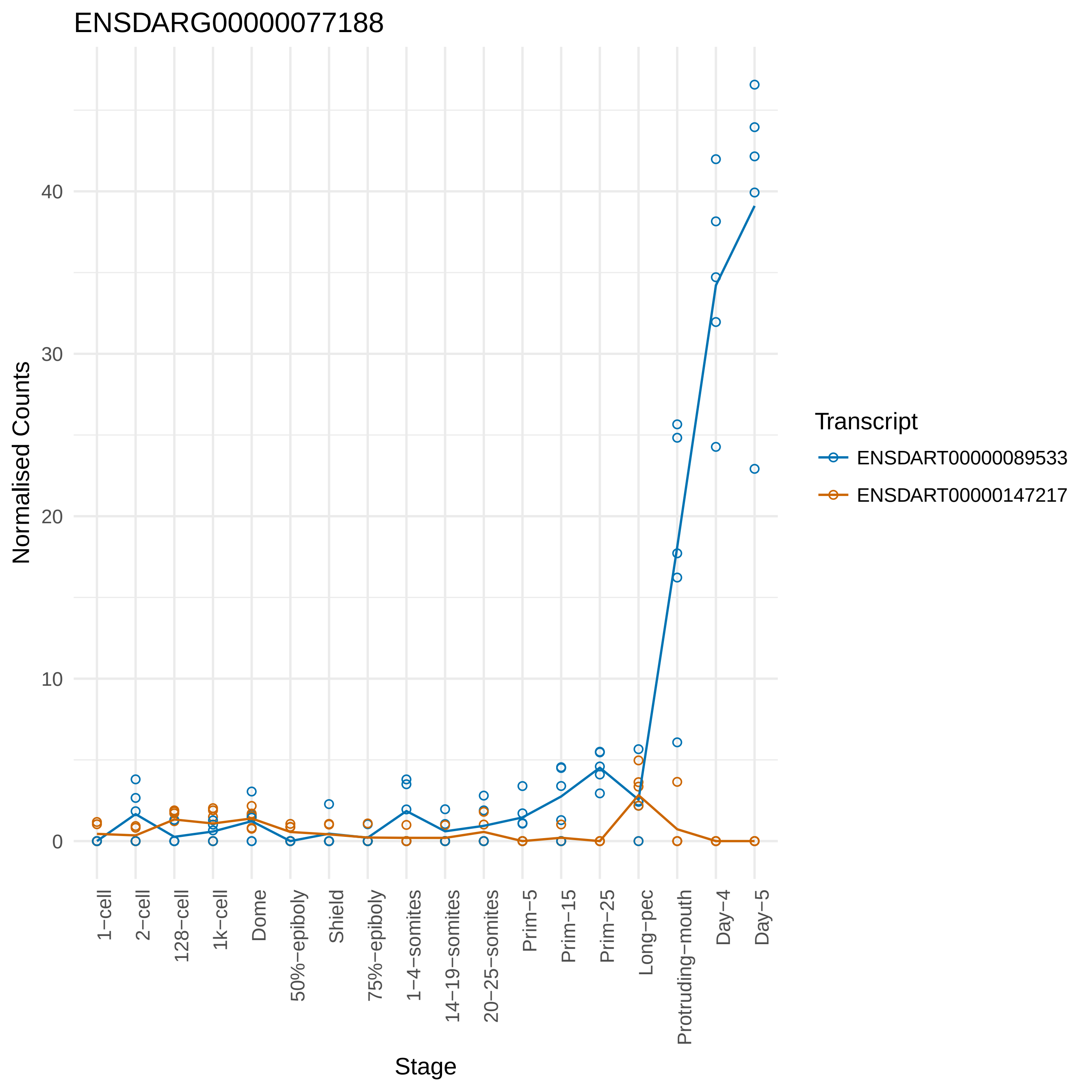

ENSDARG00000077188
Normalised Counts
0
10
20
30
40
1−cell
2−cell
128−cell
1k−cell
Dome
50%−epiboly
Shield
75%−epiboly
1−4−somites
14−19−somites
20−25−somites
Prim−5
Prim−15
Prim−25
Long−pec
Protruding−mouth
Day−4
Day−5
Stage
Transcript
ENSDART00000089533
ENSDART00000147217

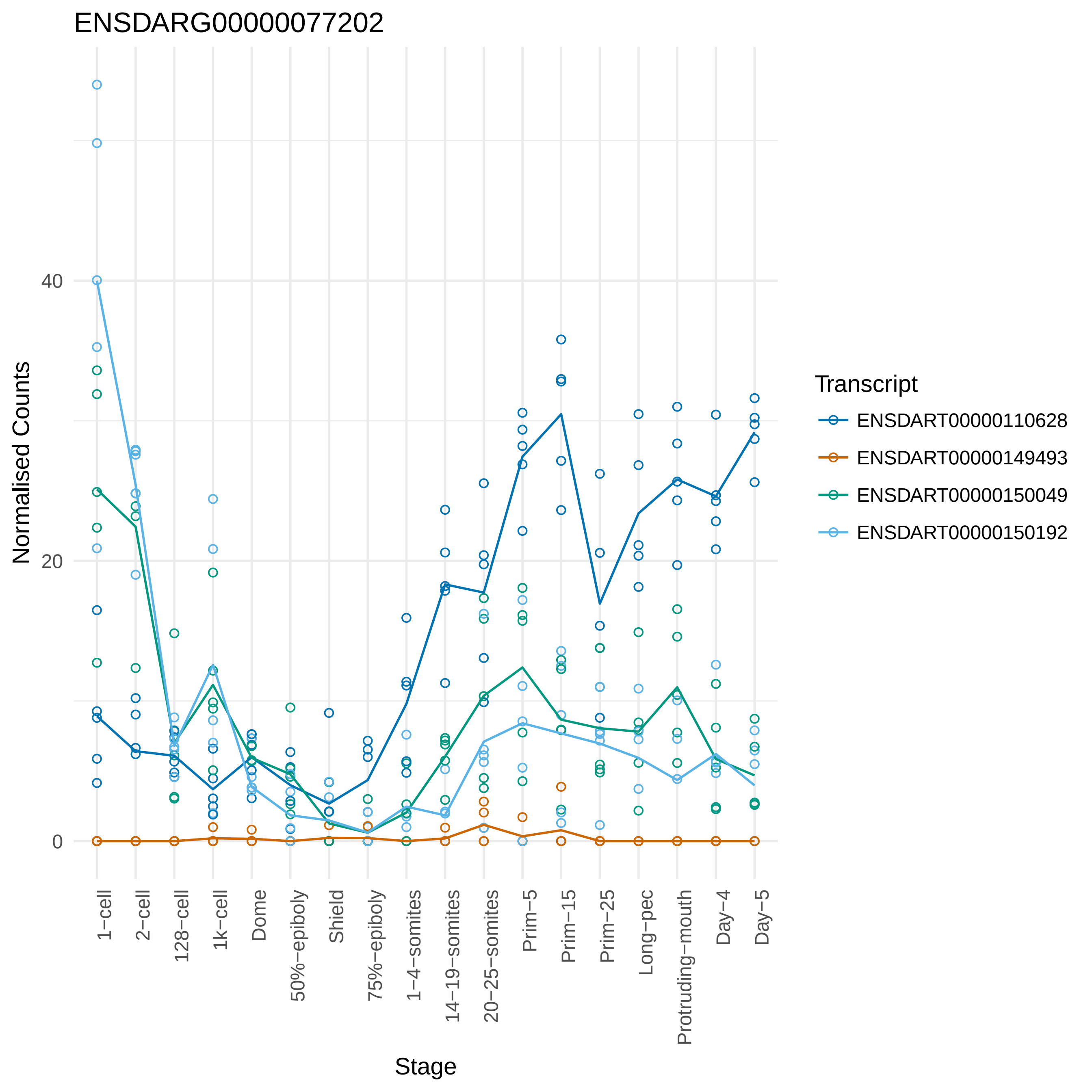

ENSDARG00000077202
Normalised Counts
0
20
40
Stage
1−cell
2−cell
128−cell
1k−cell
Dome
50%−epiboly
Shield
75%−epiboly
1−4−somites
14−19−somites
20−25−somites
Prim−5
Prim−15
Prim−25
Long−pec
Protruding−mouth
Day−4
Day−5
Transcript
ENSDART00000110628
ENSDART00000149493
ENSDART00000150049
ENSDART00000150192

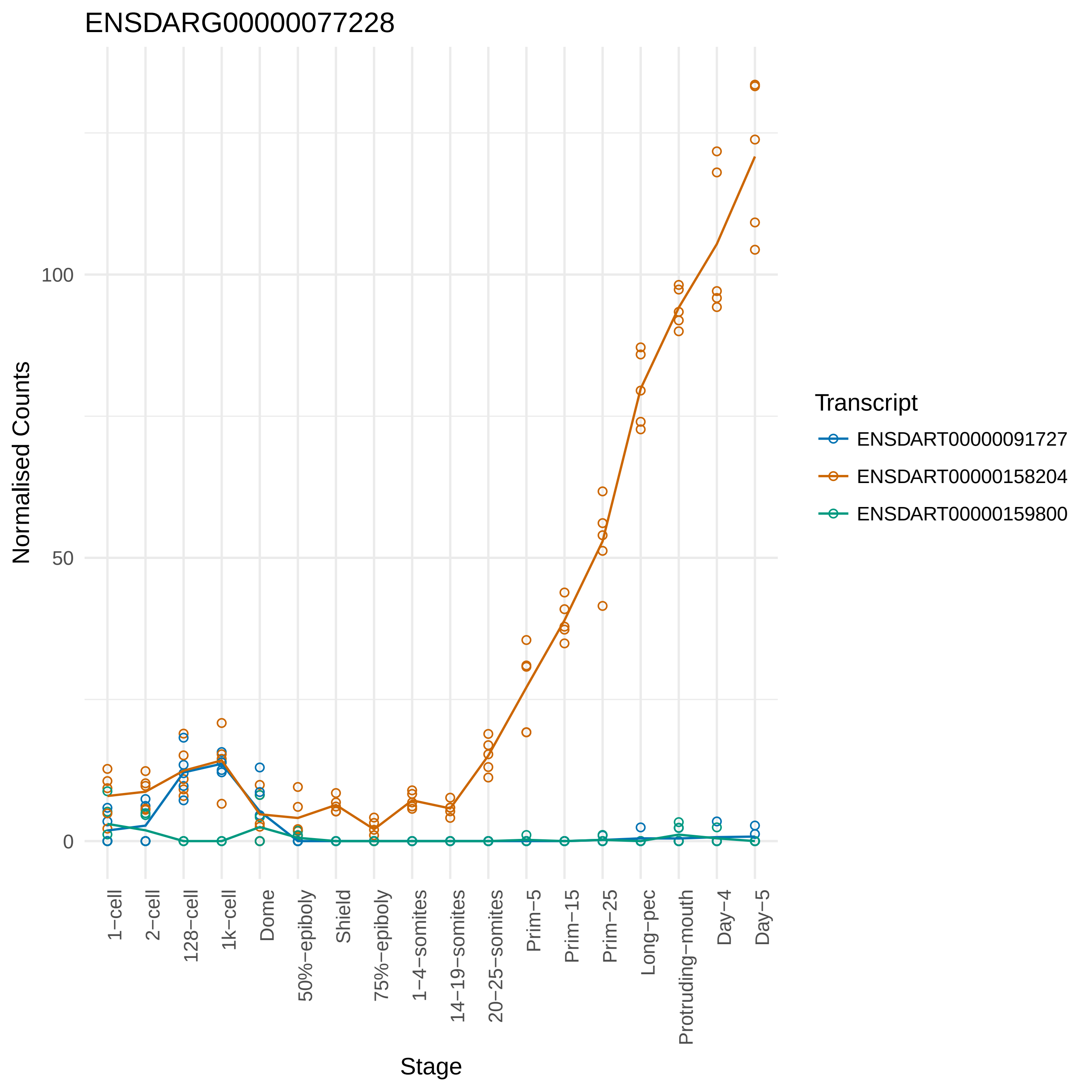

ENSDARG00000077228
Normalised Counts
100
50
0
1−cell
2−cell
128−cell
1k−cell
Dome
50%−epiboly
Shield
75%−epiboly
1−4−somites
14−19−somites
20−25−somites
Prim−5
Prim−15
Prim−25
Long−pec
Protruding−mouth
Day−4
Day−5
Stage
Transcript
ENSDART00000091727
ENSDART00000158204
ENSDART00000159800

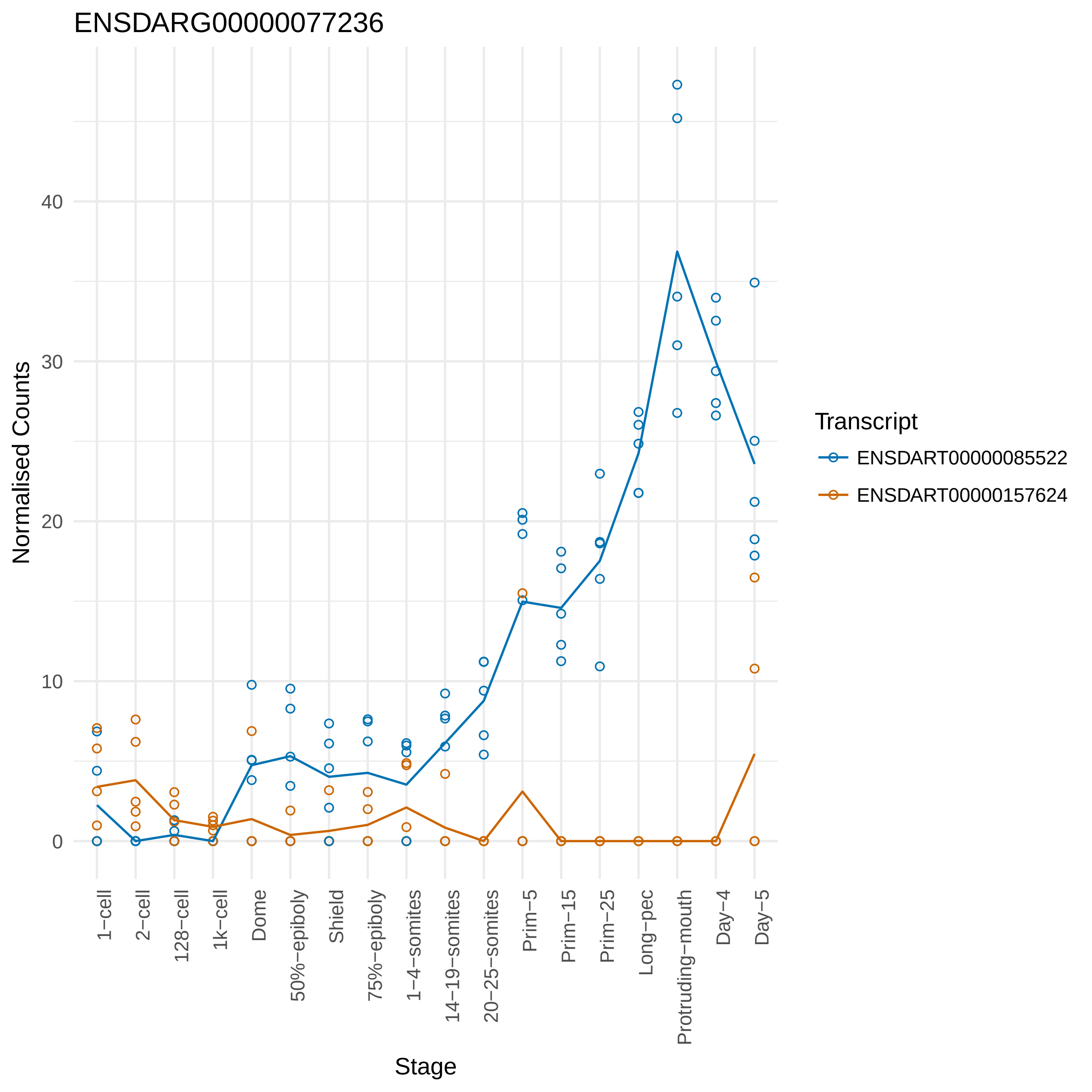
ENSDARG00000077236
Normalised Counts
0
10
20
30
40
1−cell
2−cell
128−cell
1k−cell
Dome
50%−epiboly
Shield
75%−epiboly
1−4−somites
14−19−somites
20−25−somites
Prim−5
Prim−15
Prim−25
Long−pec
Protruding−mouth
Day−4
Day−5
Stage
Transcript
ENSDART00000085522
ENSDART00000157624

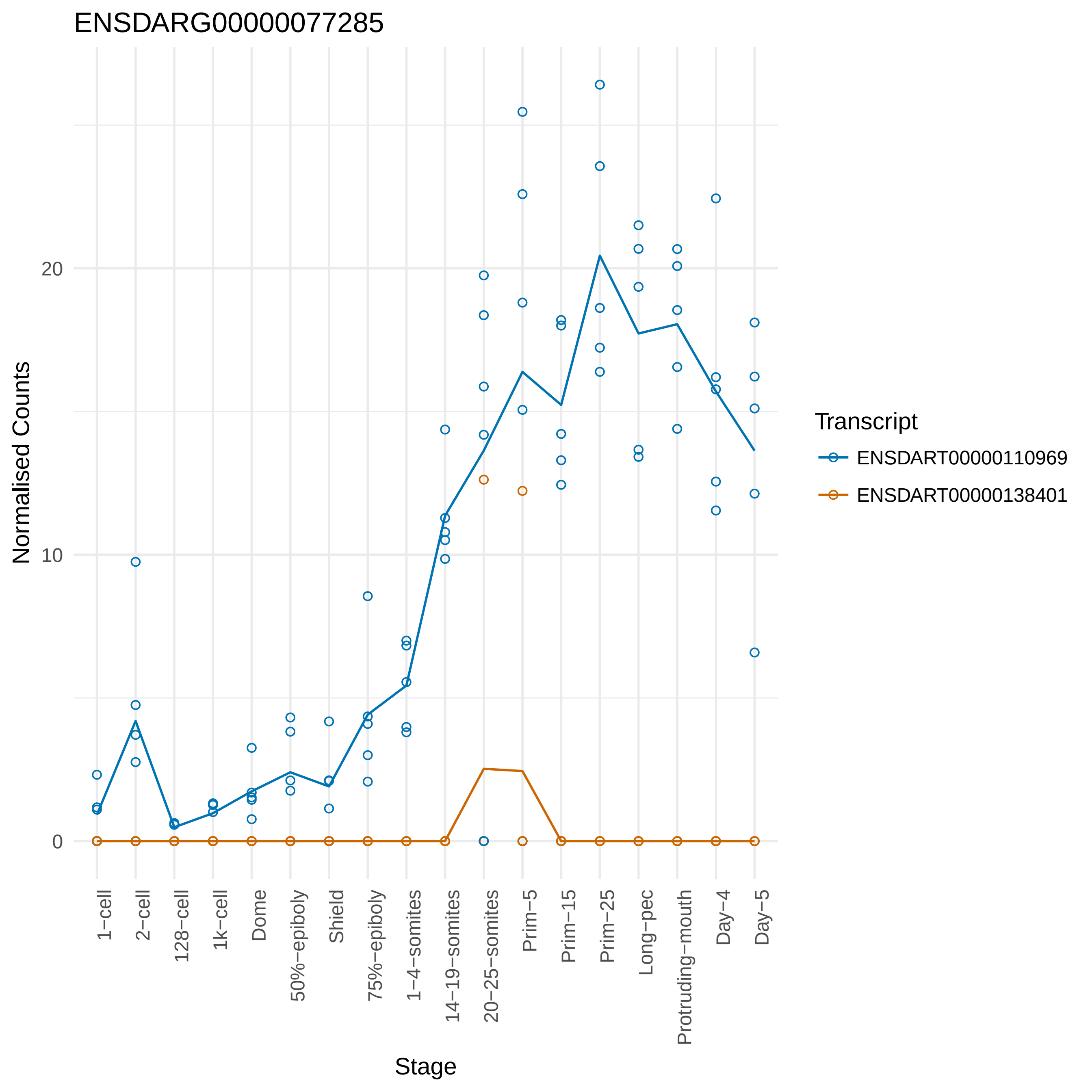

ENSDARG00000077285
Normalised Counts
0
10
20
Stage
1−cell
2−cell
128−cell
1k−cell
Dome
50%−epiboly
Shield
75%−epiboly
1−4−somites
14−19−somites
20−25−somites
Prim−5
Prim−15
Prim−25
Long−pec
Protruding−mouth
Day−4
Day−5
Transcript
ENSDART00000110969
ENSDART00000138401

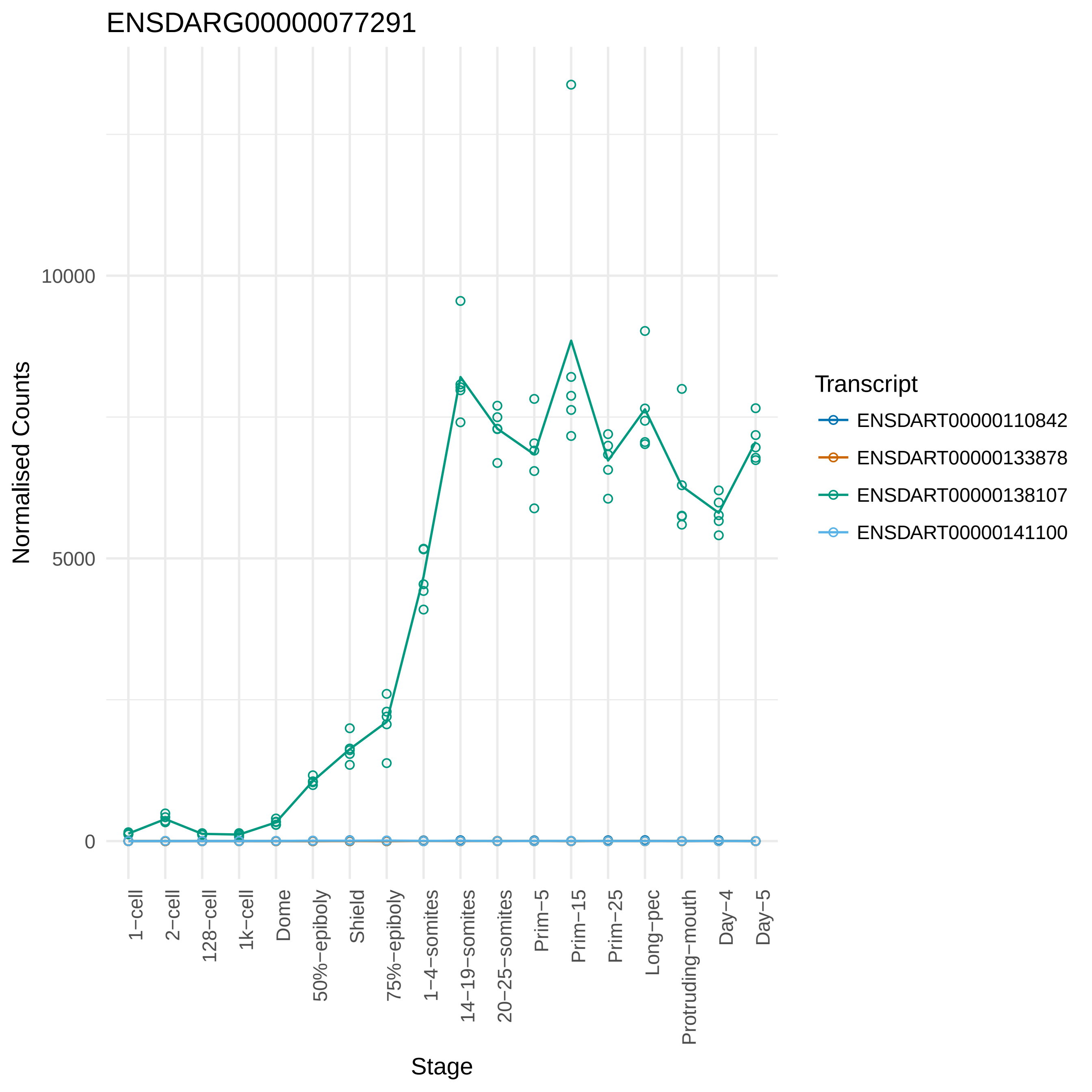

ENSDARG00000077291
Normalised Counts
10000
5000
0
1−cell
2−cell
128−cell
1k−cell
Dome
50%−epiboly
Shield
75%−epiboly
1−4−somites
14−19−somites
20−25−somites
Prim−5
Prim−15
Prim−25
Long−pec
Protruding−mouth
Day−4
Day−5
Stage
Transcript
ENSDART00000110842
ENSDART00000133878
ENSDART00000138107
ENSDART00000141100

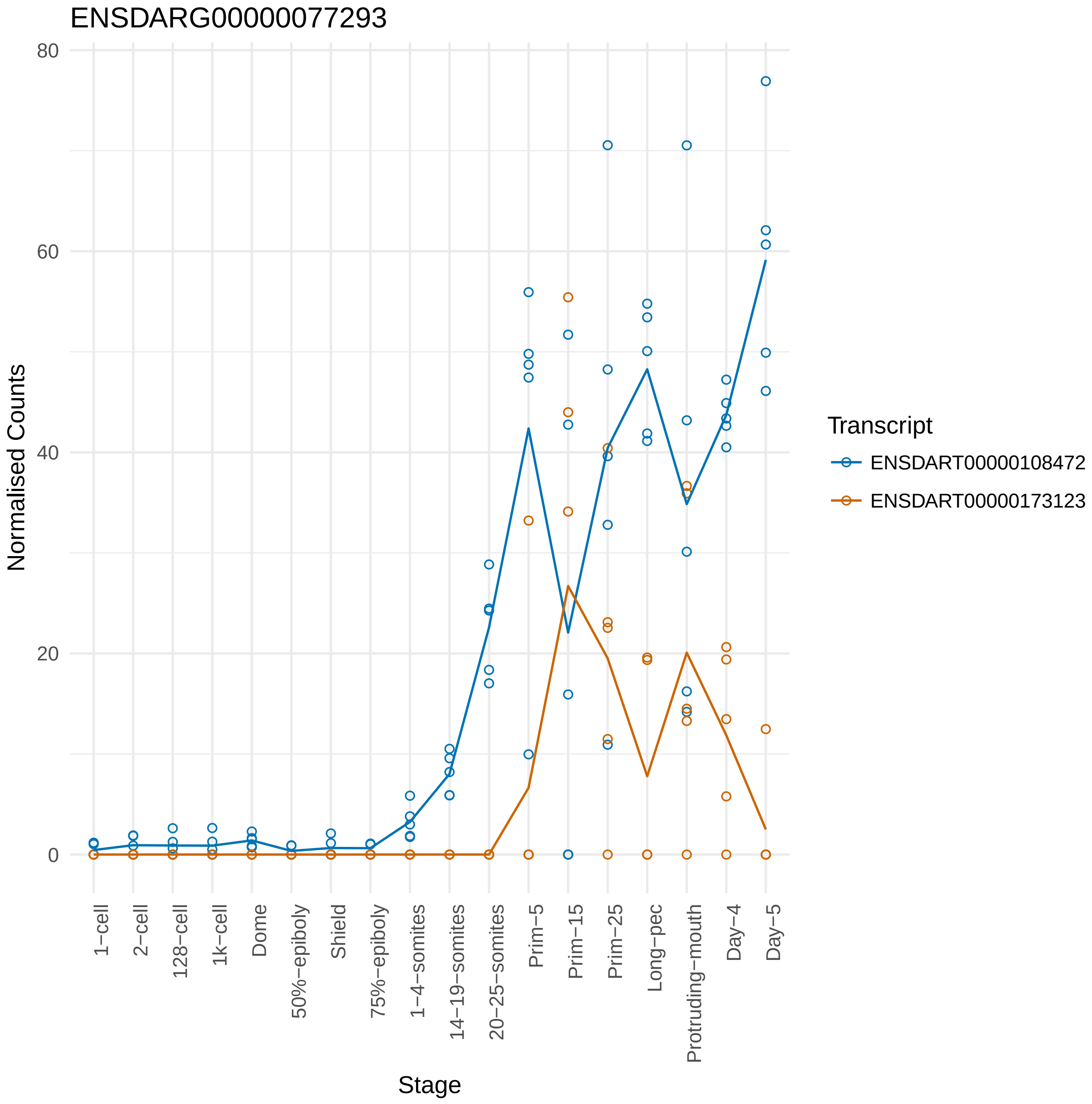

ENSDARG00000077293
Normalised Counts
0
20
40
60
80
1−cell
2−cell
128−cell
1k−cell
Dome
50%−epiboly
Shield
75%−epiboly
1−4−somites
14−19−somites
20−25−somites
Prim−5
Prim−15
Prim−25
Long−pec
Protruding−mouth
Day−4
Day−5
Stage
Transcript
ENSDART00000108472
ENSDART00000173123

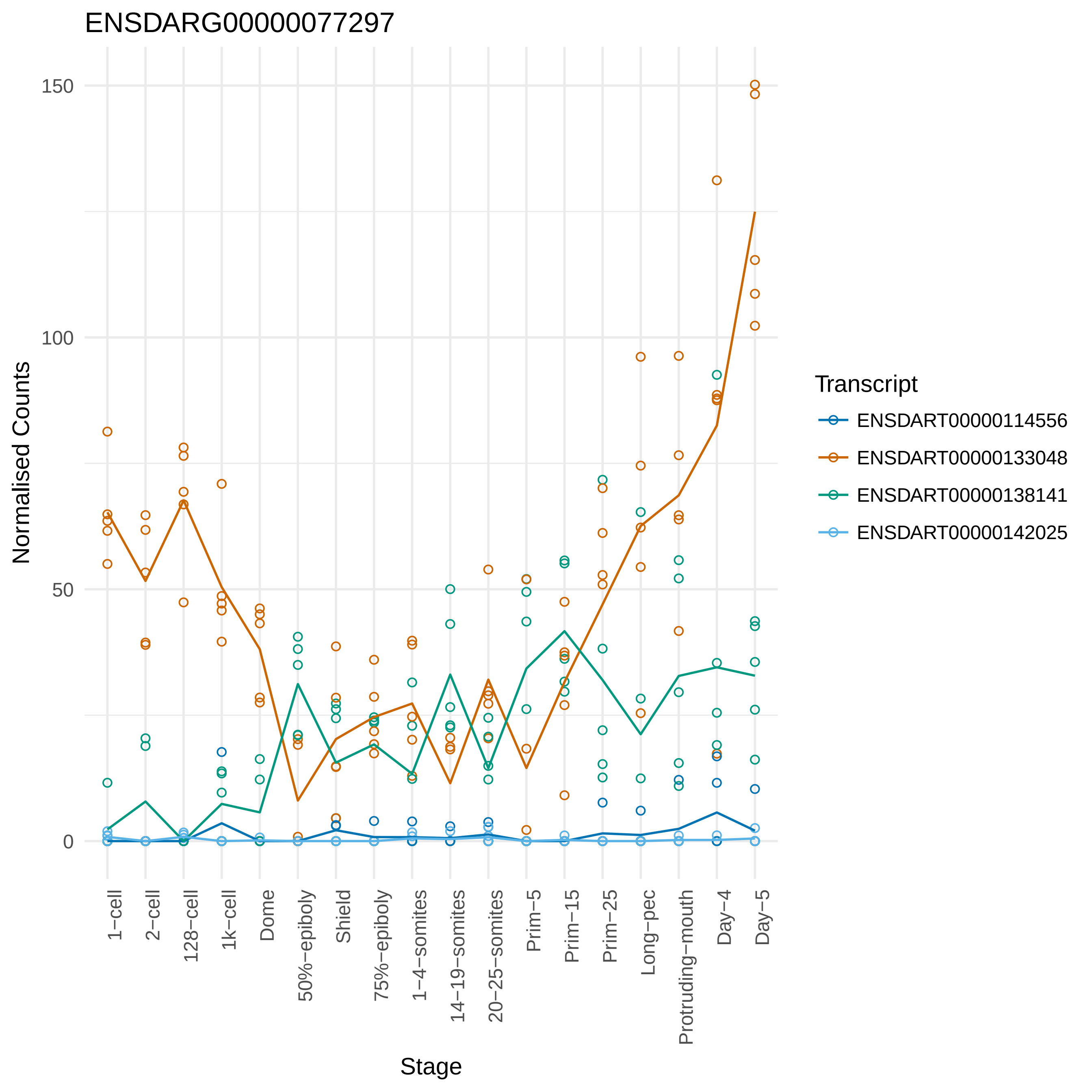

ENSDARG00000077297
Normalised Counts
150
100
50
0
1−cell
2−cell
128−cell
1k−cell
Dome
50%−epiboly
Shield
75%−epiboly
1−4−somites
14−19−somites
20−25−somites
Prim−5
Prim−15
Prim−25
Long−pec
Protruding−mouth
Day−4
Day−5
Stage
Transcript
ENSDART00000114556
ENSDART00000133048
ENSDART00000138141
ENSDART00000142025

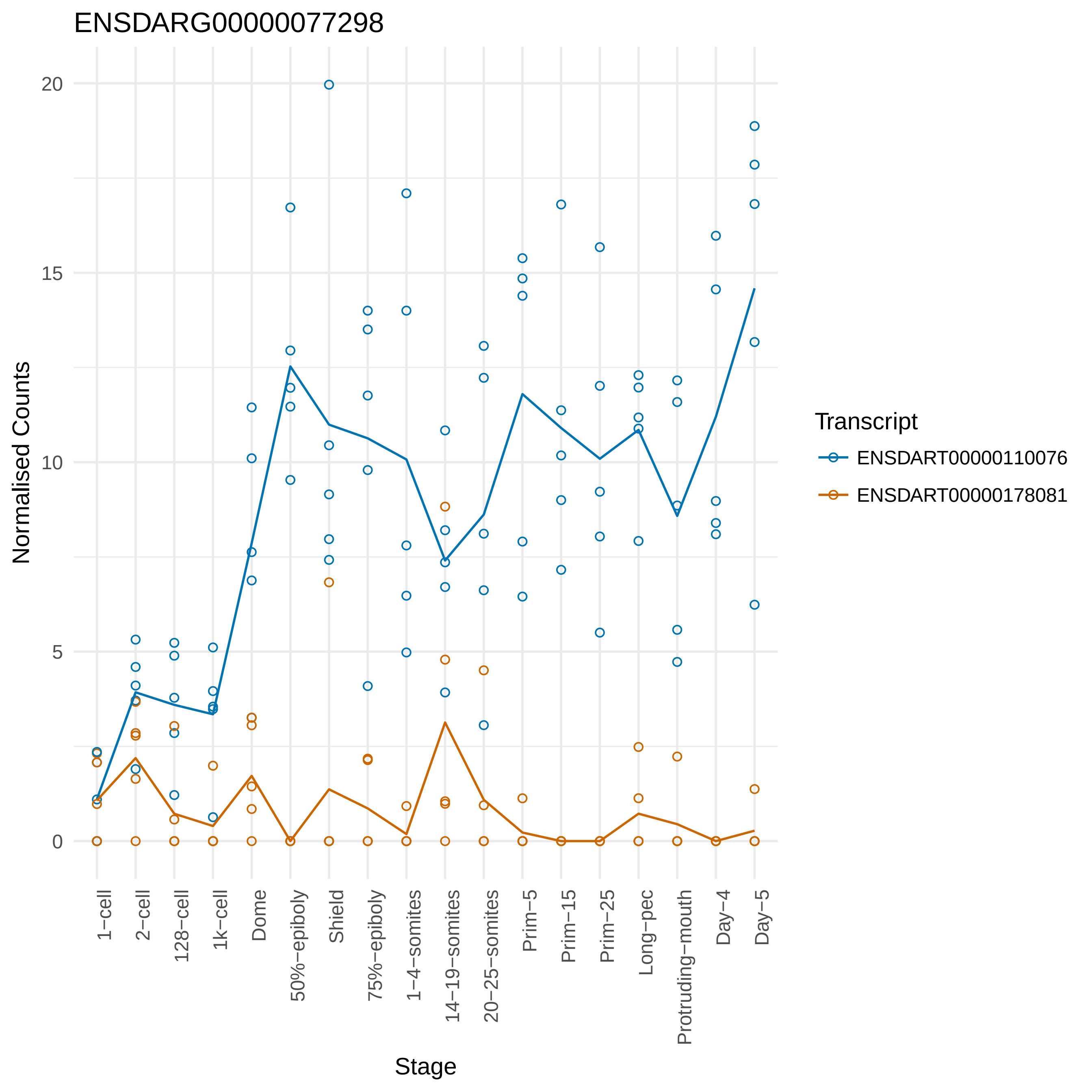

ENSDARG00000077298
Normalised Counts
0
5
10
15
20
1−cell
2−cell
128−cell
1k−cell
Dome
50%−epiboly
Shield
75%−epiboly
1−4−somites
14−19−somites
20−25−somites
Prim−5
Prim−15
Prim−25
Long−pec
Protruding−mouth
Day−4
Day−5
Stage
Transcript
ENSDART00000110076
ENSDART00000178081

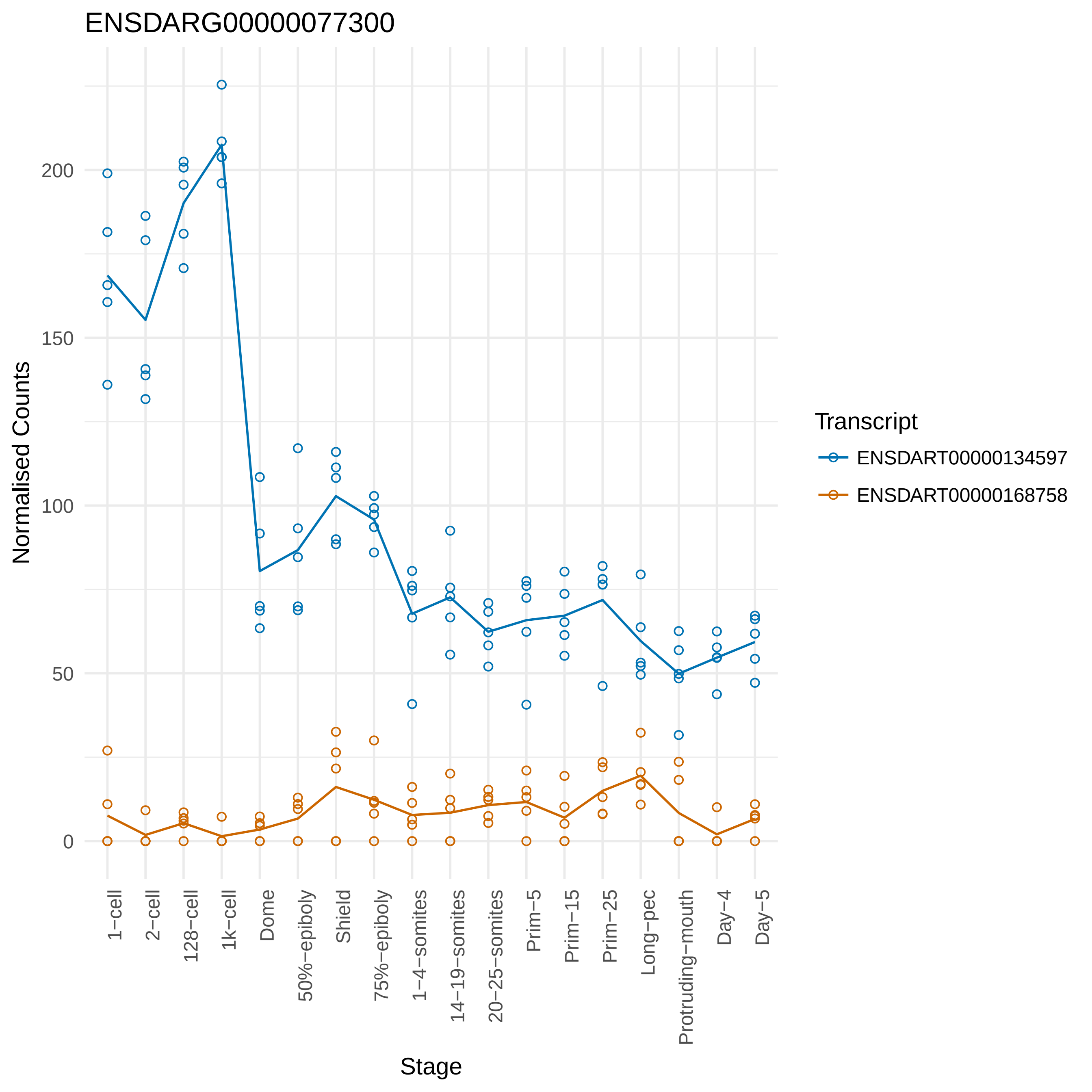

ENSDARG00000077300
Normalised Counts
200
150
100
50
0
1−cell
2−cell
128−cell
1k−cell
Dome
50%−epiboly
Shield
75%−epiboly
1−4−somites
14−19−somites
20−25−somites
Prim−5
Prim−15
Prim−25
Long−pec
Protruding−mouth
Day−4
Day−5
Stage
Transcript
ENSDART00000134597
ENSDART00000168758

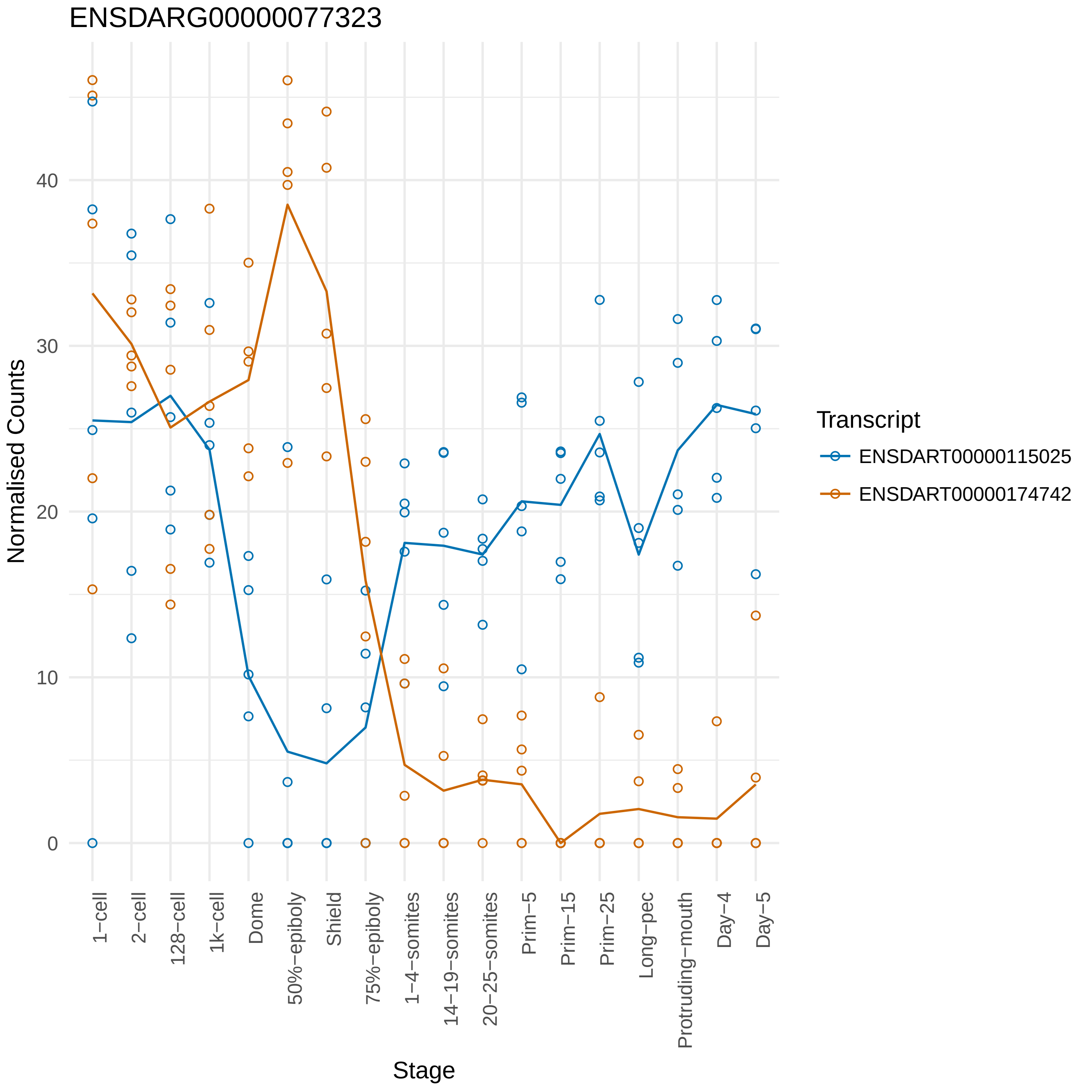

ENSDARG00000077323
Normalised Counts
0
10
20
30
40
Stage
1−cell
2−cell
128−cell
1k−cell
Dome
50%−epiboly
Shield
75%−epiboly
1−4−somites
14−19−somites
20−25−somites
Prim−5
Prim−15
Prim−25
Long−pec
Protruding−mouth
Day−4
Day−5
Transcript
ENSDART00000115025
ENSDART00000174742

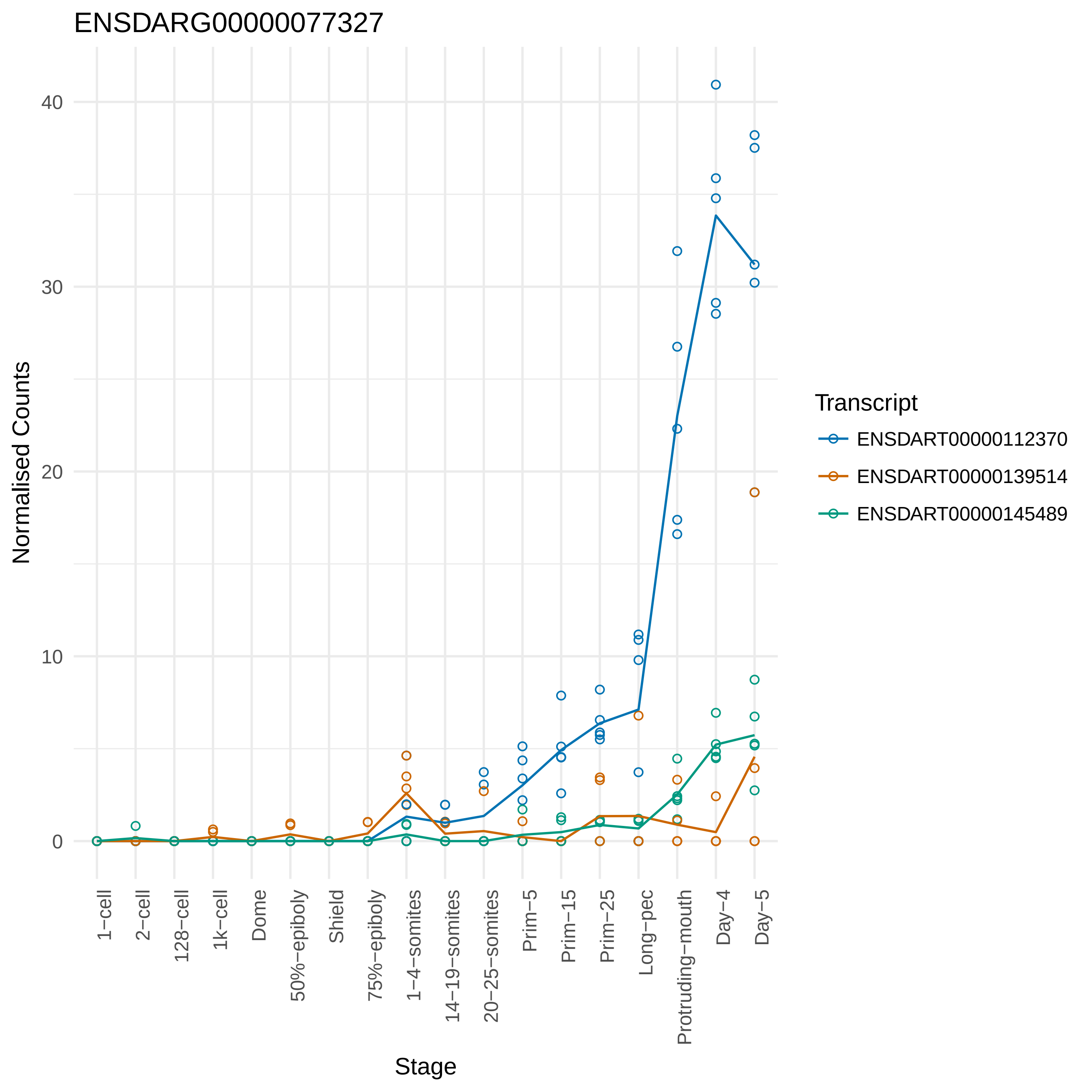
ENSDARG00000077327
Normalised Counts
0
10
20
30
40
1−cell
2−cell
128−cell
1k−cell
Dome
50%−epiboly
Shield
75%−epiboly
1−4−somites
14−19−somites
20−25−somites
Prim−5
Prim−15
Prim−25
Long−pec
Protruding−mouth
Day−4
Day−5
Stage
Transcript
ENSDART00000112370
ENSDART00000139514
ENSDART00000145489

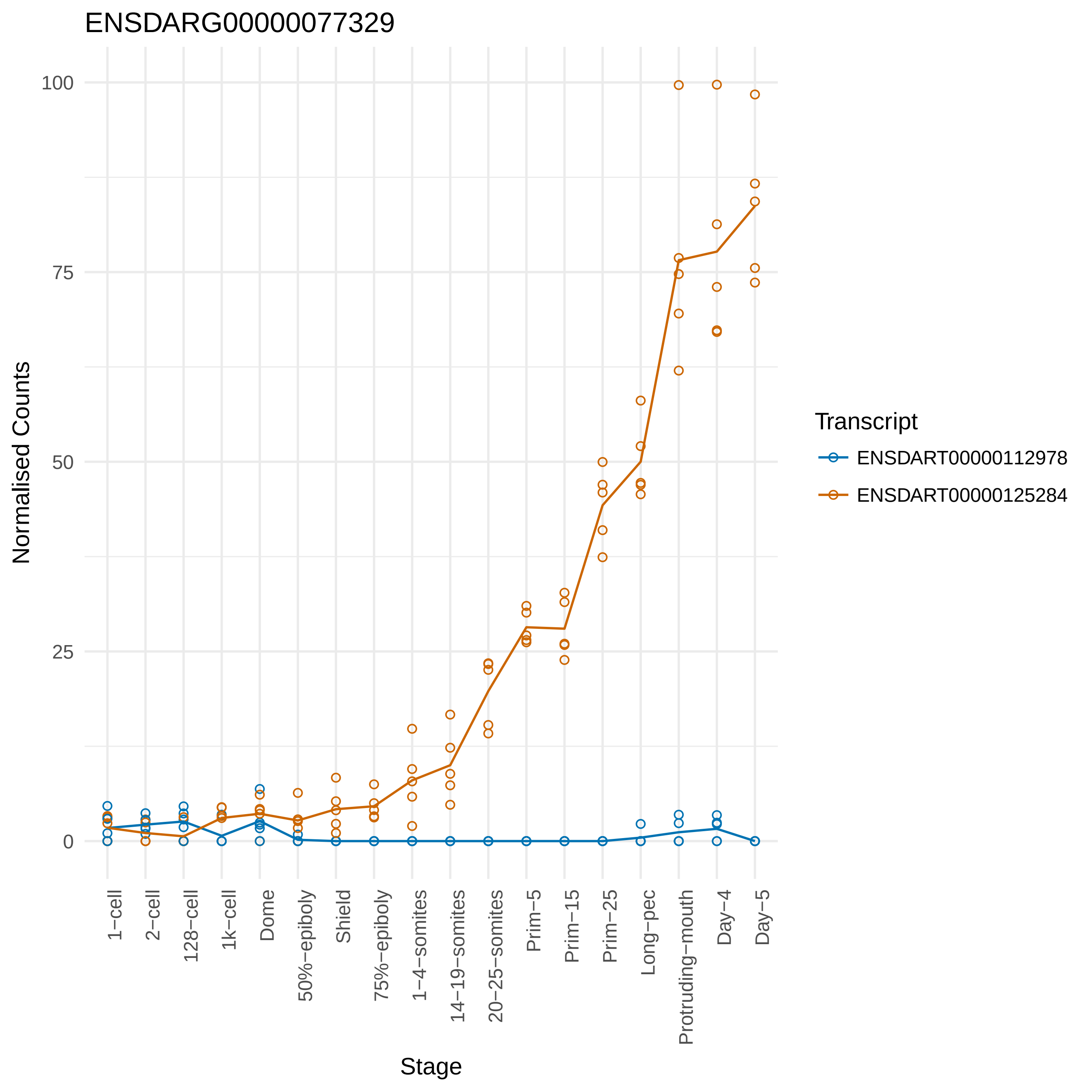
ENSDARG00000077329
Normalised Counts
100
75
50
25
0
1−cell
2−cell
128−cell
1k−cell
Dome
50%−epiboly
Shield
75%−epiboly
1−4−somites
14−19−somites
20−25−somites
Prim−5
Prim−15
Prim−25
Long−pec
Protruding−mouth
Day−4
Day−5
Stage
Transcript
ENSDART00000112978
ENSDART00000125284

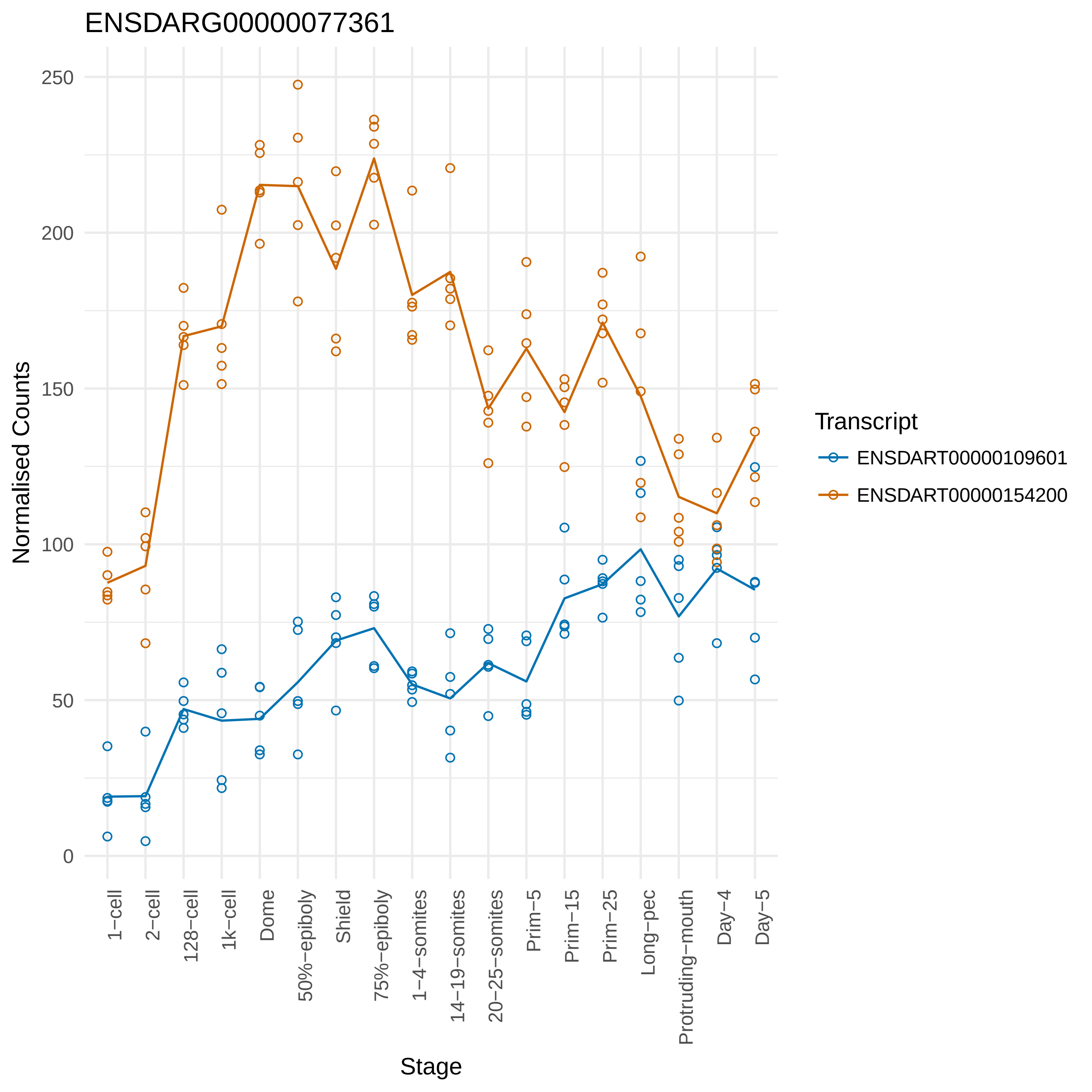

ENSDARG00000077361
Normalised Counts
250
200
150
100
50
0
1−cell
2−cell
128−cell
1k−cell
Dome
50%−epiboly
Shield
75%−epiboly
1−4−somites
14−19−somites
20−25−somites
Prim−5
Prim−15
Prim−25
Long−pec
Protruding−mouth
Day−4
Day−5
Stage
Transcript
ENSDART00000109601
ENSDART00000154200

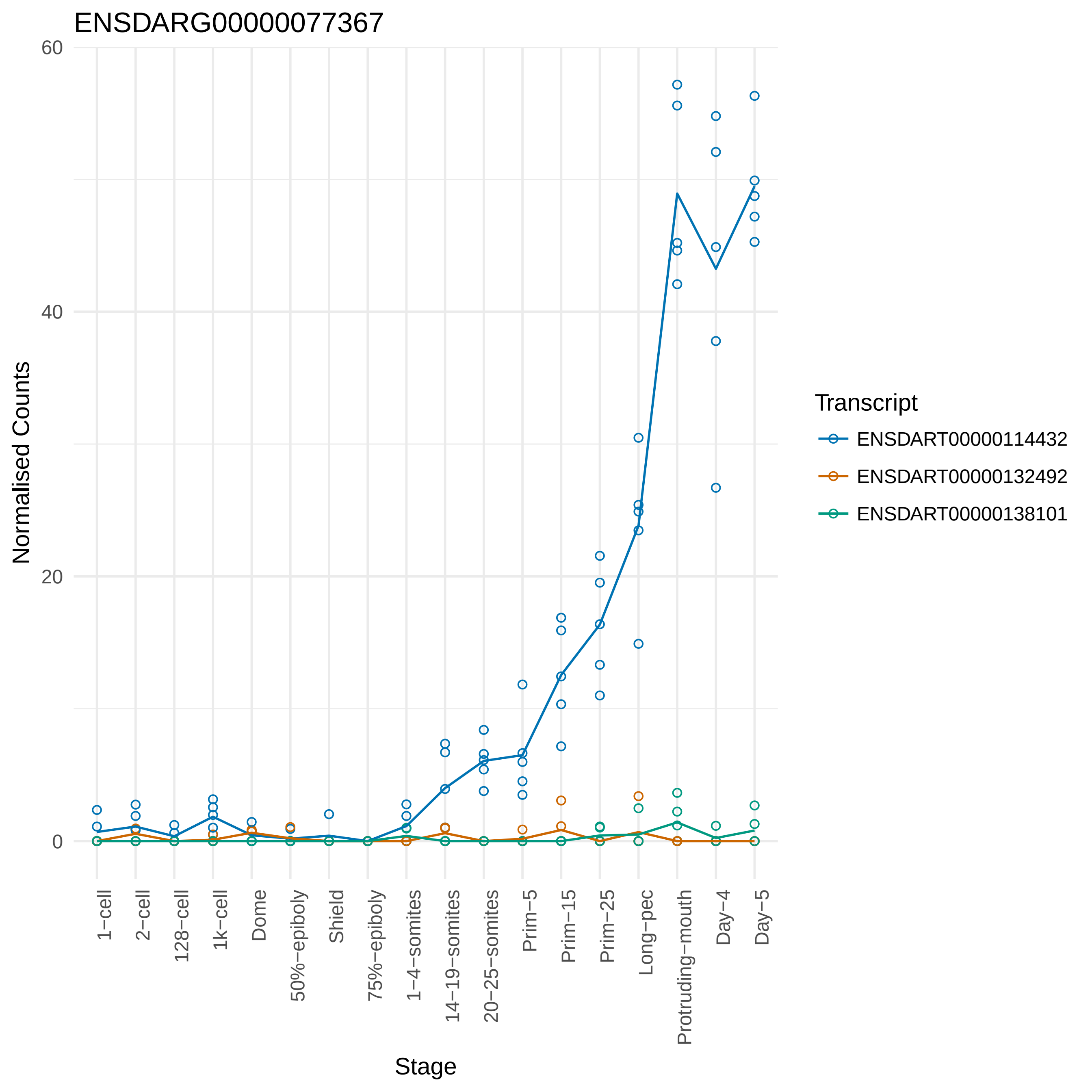

ENSDARG00000077367
Normalised Counts
0
20
40
60
Stage
1−cell
2−cell
128−cell
1k−cell
Dome
50%−epiboly
Shield
75%−epiboly
1−4−somites
14−19−somites
20−25−somites
Prim−5
Prim−15
Prim−25
Long−pec
Protruding−mouth
Day−4
Day−5
Transcript
ENSDART00000114432
ENSDART00000132492
ENSDART00000138101

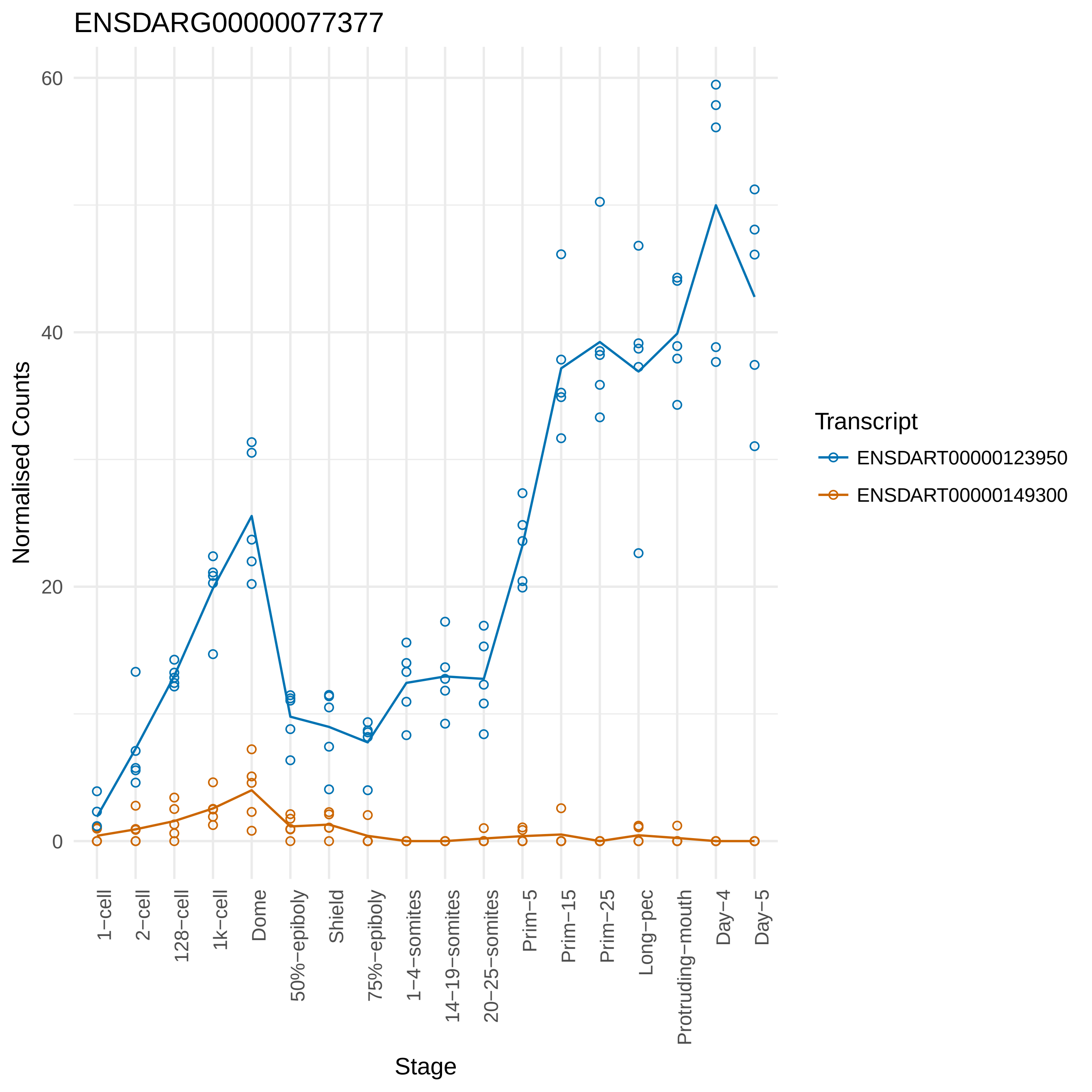

ENSDARG00000077377
Normalised Counts
60
40
20
0
Stage
1−cell
2−cell
128−cell
1k−cell
Dome
50%−epiboly
Shield
75%−epiboly
1−4−somites
14−19−somites
20−25−somites
Prim−5
Prim−15
Prim−25
Long−pec
Protruding−mouth
Day−4
Day−5
Transcript
ENSDART00000123950
ENSDART00000149300

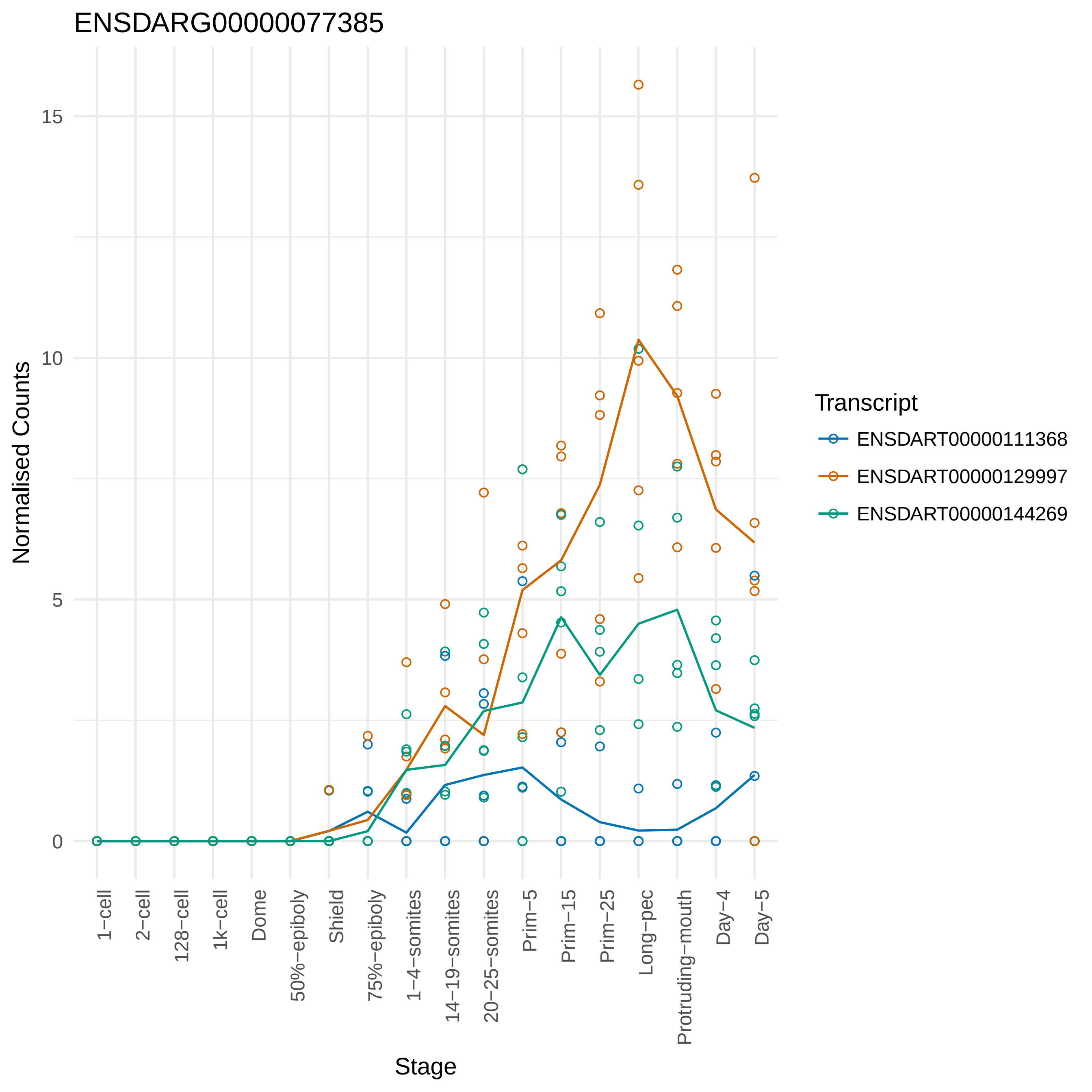

ENSDARG00000077385
Normalised Counts
0
5
10
15
1−cell
2−cell
128−cell
1k−cell
Dome
50%−epiboly
Shield
75%−epiboly
1−4−somites
14−19−somites
20−25−somites
Prim−5
Prim−15
Prim−25
Long−pec
Protruding−mouth
Day−4
Day−5
Stage
Transcript
ENSDART00000111368
ENSDART00000129997
ENSDART00000144269

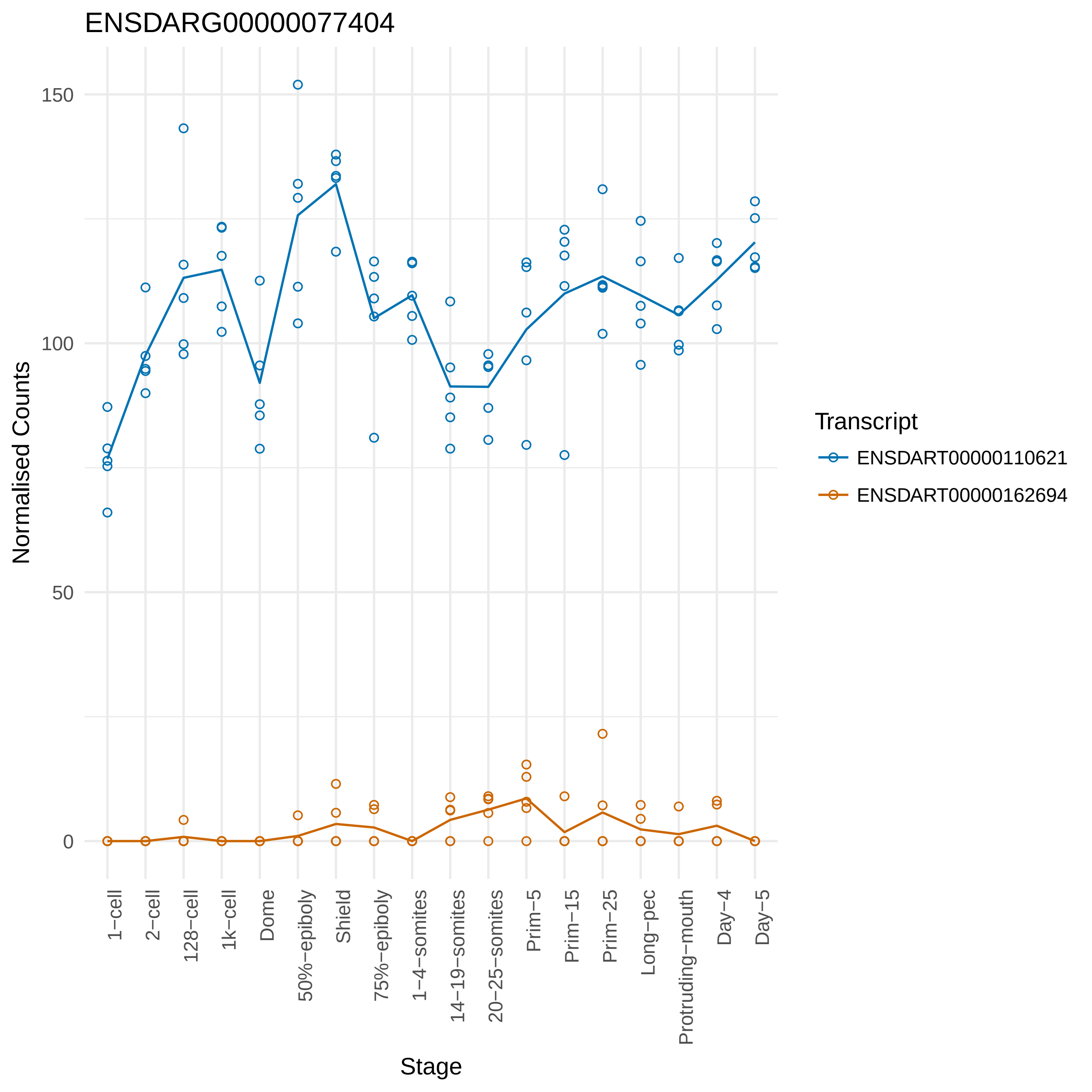

ENSDARG00000077404
Normalised Counts
150
100
50
0
1−cell
2−cell
128−cell
1k−cell
Dome
50%−epiboly
Shield
75%−epiboly
1−4−somites
14−19−somites
20−25−somites
Prim−5
Prim−15
Prim−25
Long−pec
Protruding−mouth
Day−4
Day−5
Stage
Transcript
ENSDART00000110621
ENSDART00000162694

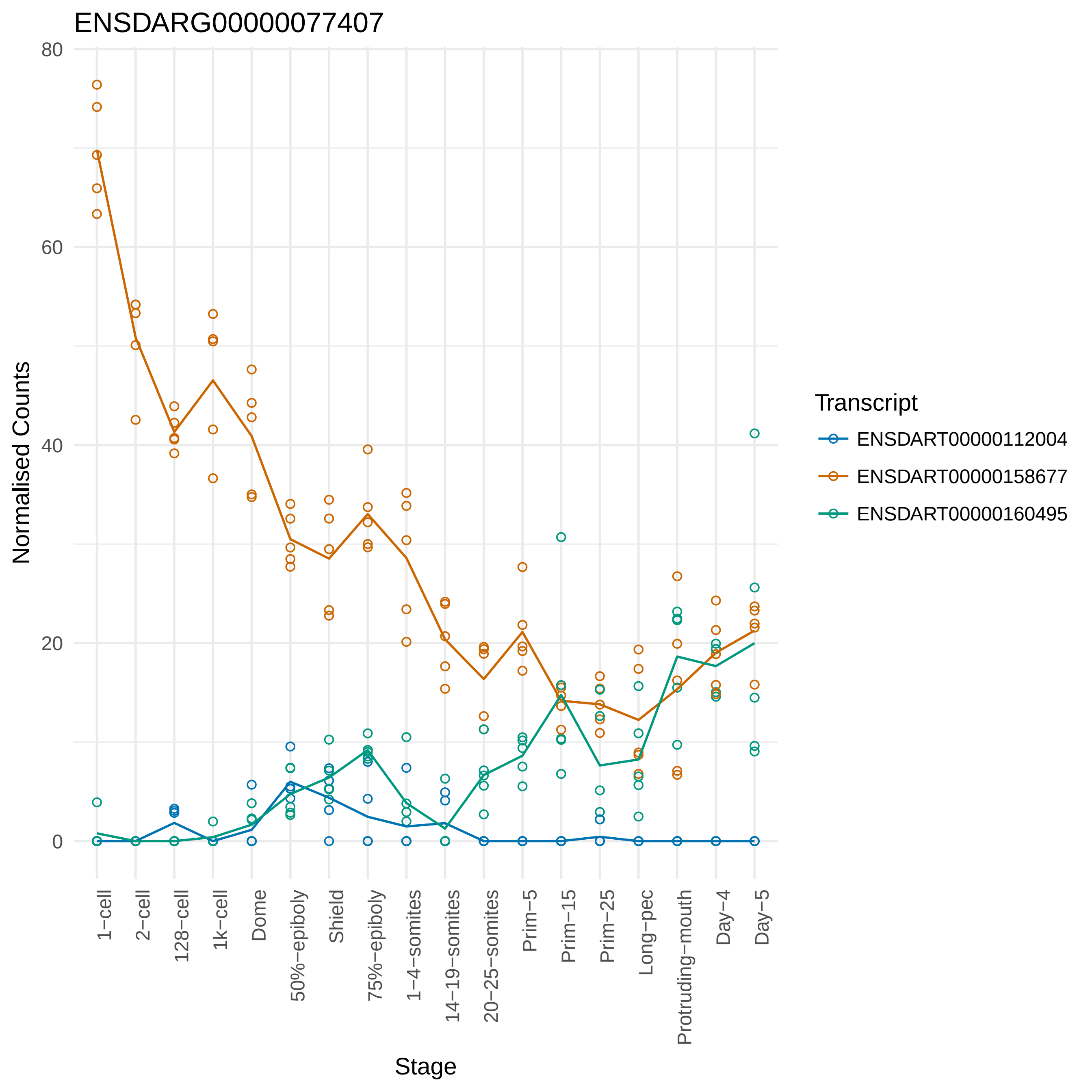
ENSDARG00000077407
Normalised Counts
0
20
40
60
80
Stage
1−cell
2−cell
128−cell
1k−cell
Dome
50%−epiboly
Shield
75%−epiboly
1−4−somites
14−19−somites
20−25−somites
Prim−5
Prim−15
Prim−25
Long−pec
Protruding−mouth
Day−4
Day−5
Transcript
ENSDART00000112004
ENSDART00000158677
ENSDART00000160495

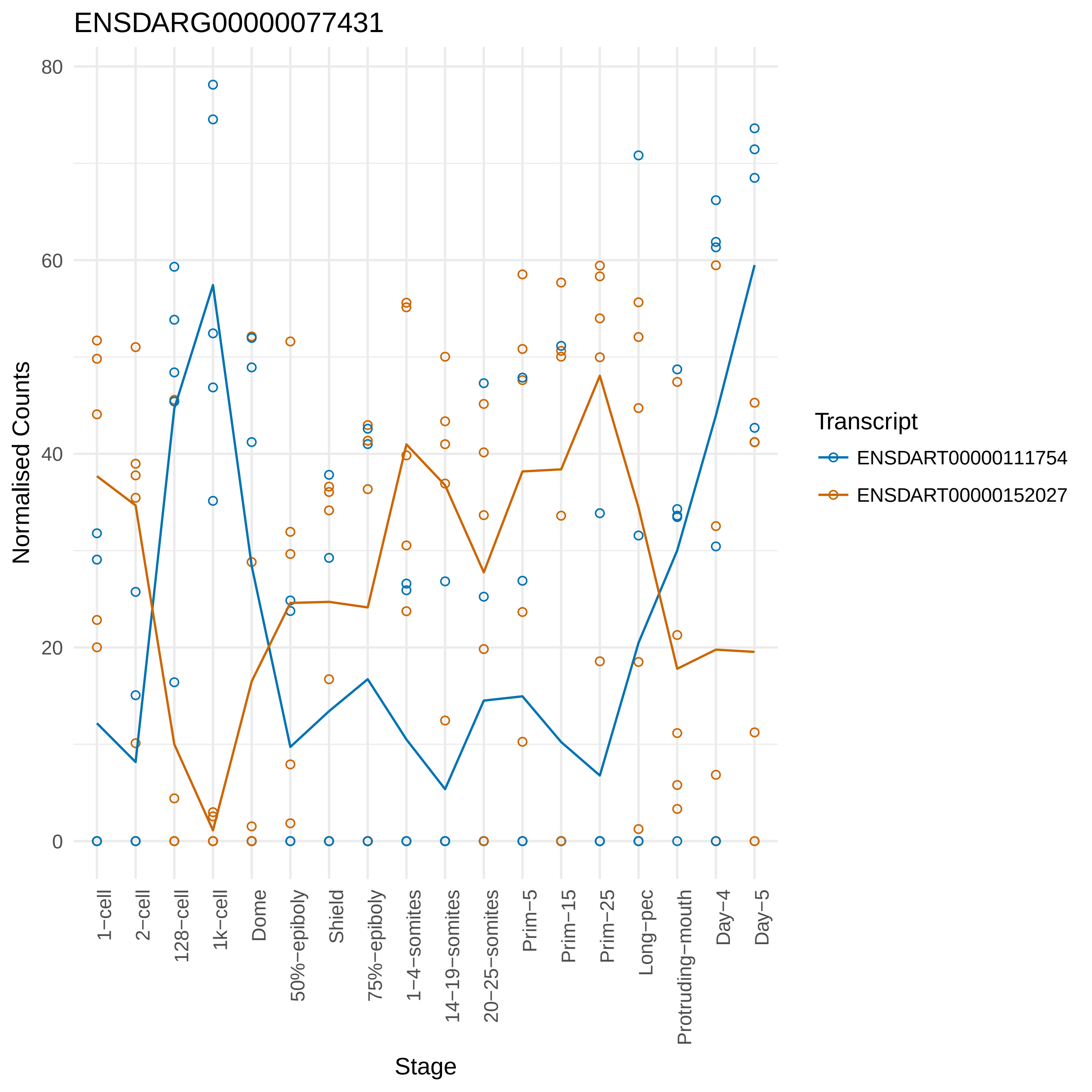

ENSDARG00000077431
Normalised Counts
0
20
40
60
80
1−cell
2−cell
128−cell
1k−cell
Dome
50%−epiboly
Shield
75%−epiboly
1−4−somites
14−19−somites
20−25−somites
Prim−5
Prim−15
Prim−25
Long−pec
Protruding−mouth
Day−4
Day−5
Stage
Transcript
ENSDART00000111754
ENSDART00000152027

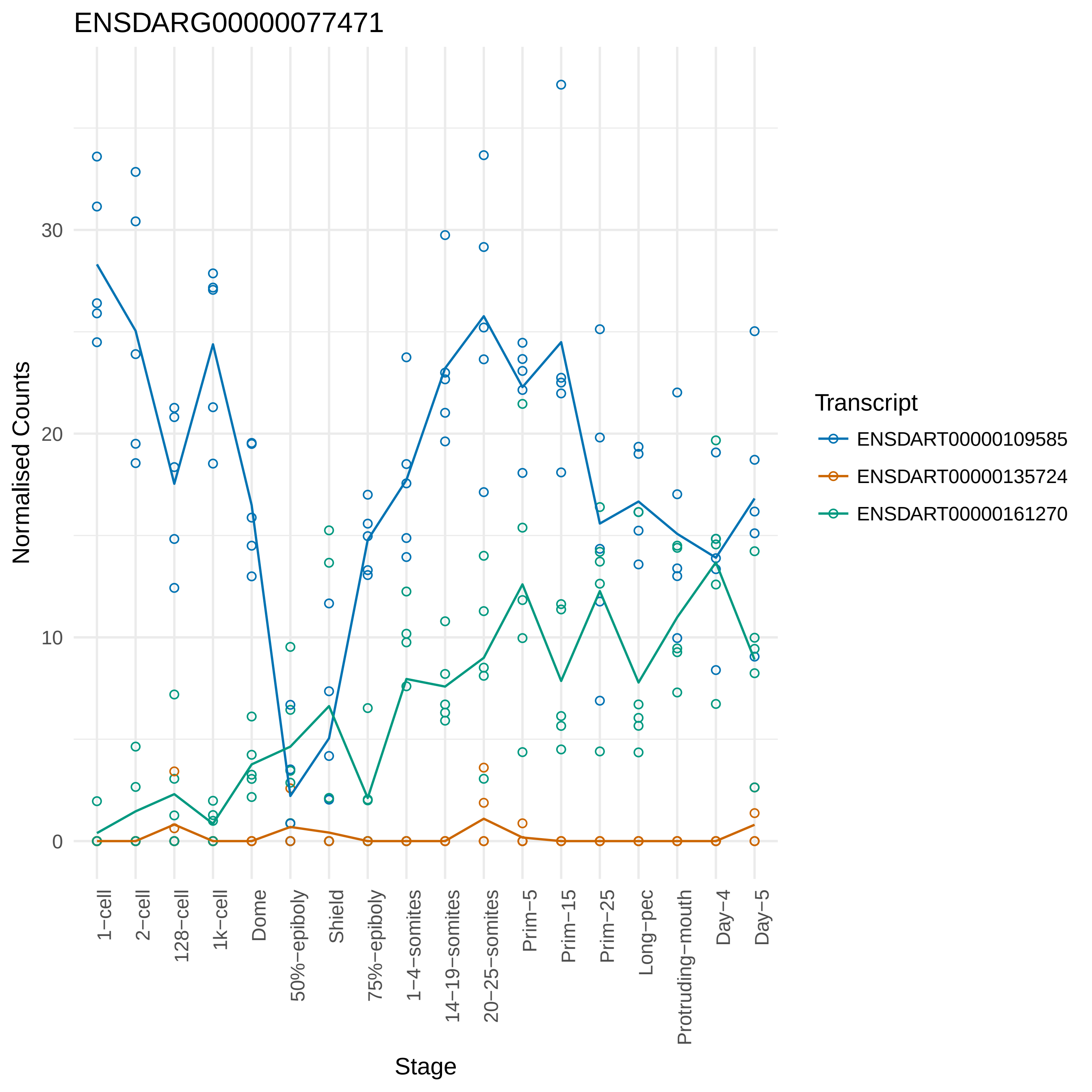
ENSDARG00000077471
Normalised Counts
30
20
10
0
1−cell
2−cell
128−cell
1k−cell
Dome
50%−epiboly
Shield
75%−epiboly
1−4−somites
14−19−somites
20−25−somites
Prim−5
Prim−15
Prim−25
Long−pec
Protruding−mouth
Day−4
Day−5
Stage
Transcript
ENSDART00000109585
ENSDART00000135724
ENSDART00000161270

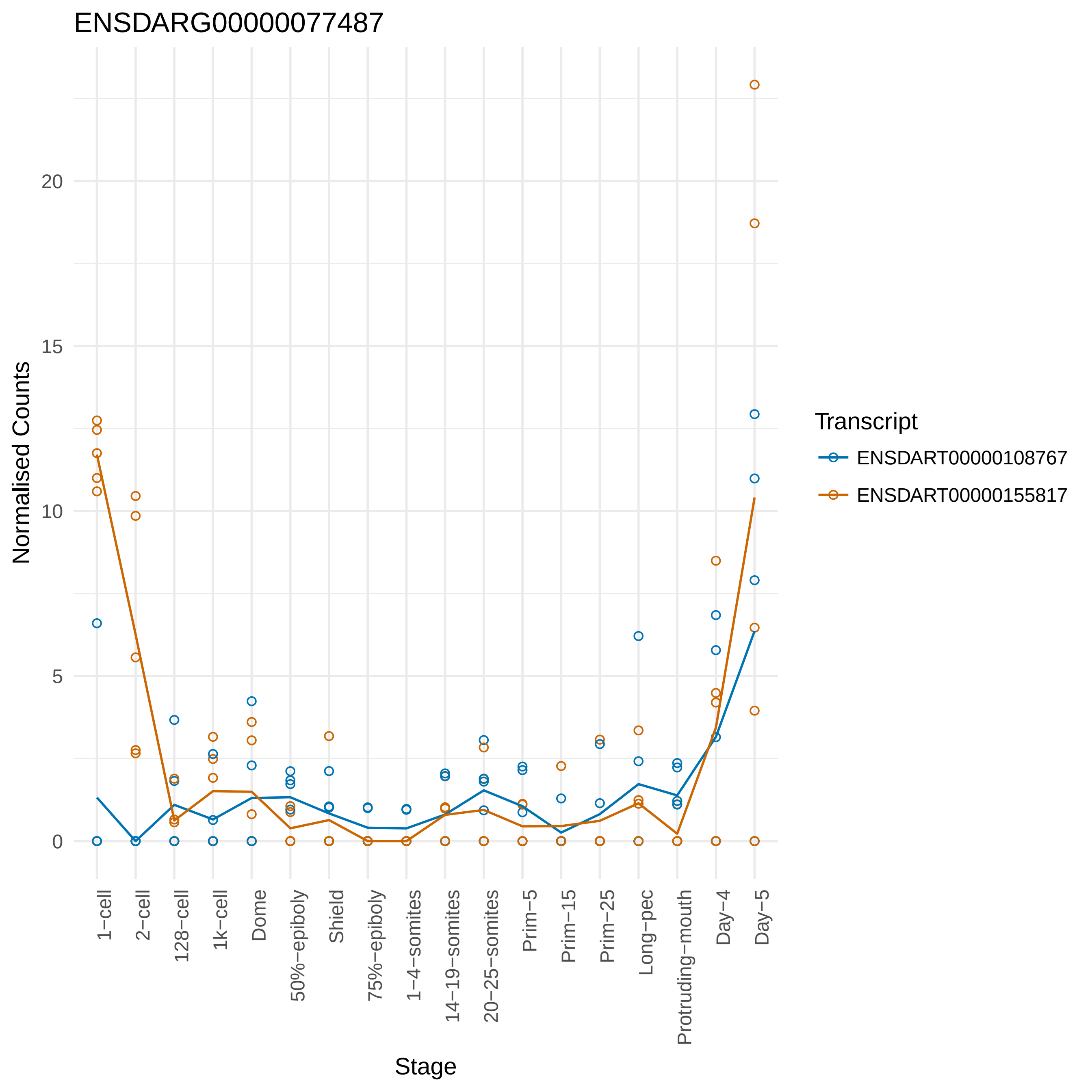

ENSDARG00000077487
Normalised Counts
20
15
10
5
0
1−cell
2−cell
128−cell
1k−cell
Dome
50%−epiboly
Shield
75%−epiboly
1−4−somites
14−19−somites
20−25−somites
Prim−5
Prim−15
Prim−25
Long−pec
Protruding−mouth
Day−4
Day−5
Stage
Transcript
ENSDART00000108767
ENSDART00000155817

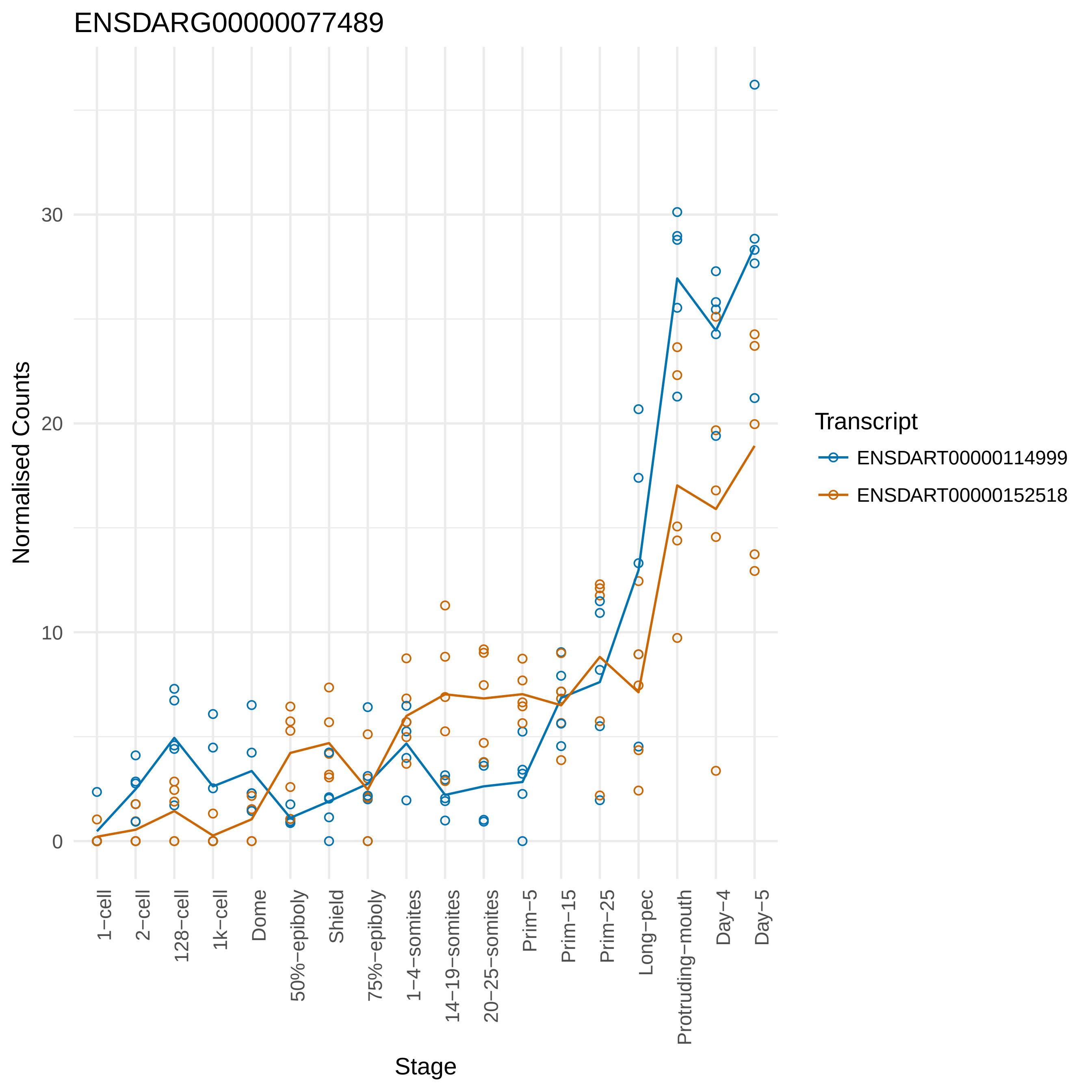

ENSDARG00000077489
Normalised Counts
30
20
10
0
1−cell
2−cell
128−cell
1k−cell
Dome
50%−epiboly
Shield
75%−epiboly
1−4−somites
14−19−somites
20−25−somites
Prim−5
Prim−15
Prim−25
Long−pec
Protruding−mouth
Day−4
Day−5
Stage
Transcript
ENSDART00000114999
ENSDART00000152518

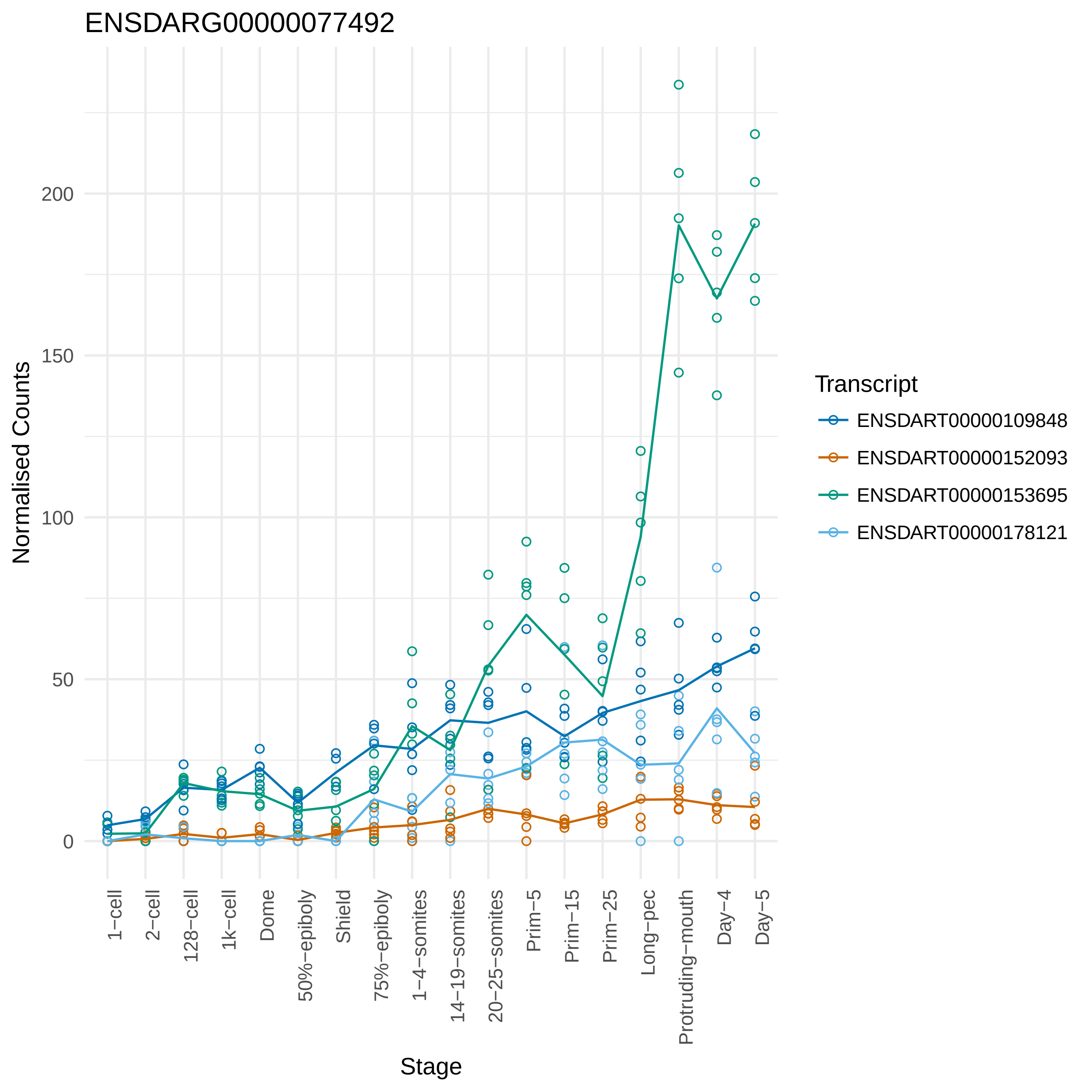
ENSDARG00000077492
Normalised Counts
200
150
100
50
0
1−cell
2−cell
128−cell
1k−cell
Dome
50%−epiboly
Shield
75%−epiboly
1−4−somites
14−19−somites
20−25−somites
Prim−5
Prim−15
Prim−25
Long−pec
Protruding−mouth
Day−4
Day−5
Stage
Transcript
ENSDART00000109848
ENSDART00000152093
ENSDART00000153695
ENSDART00000178121

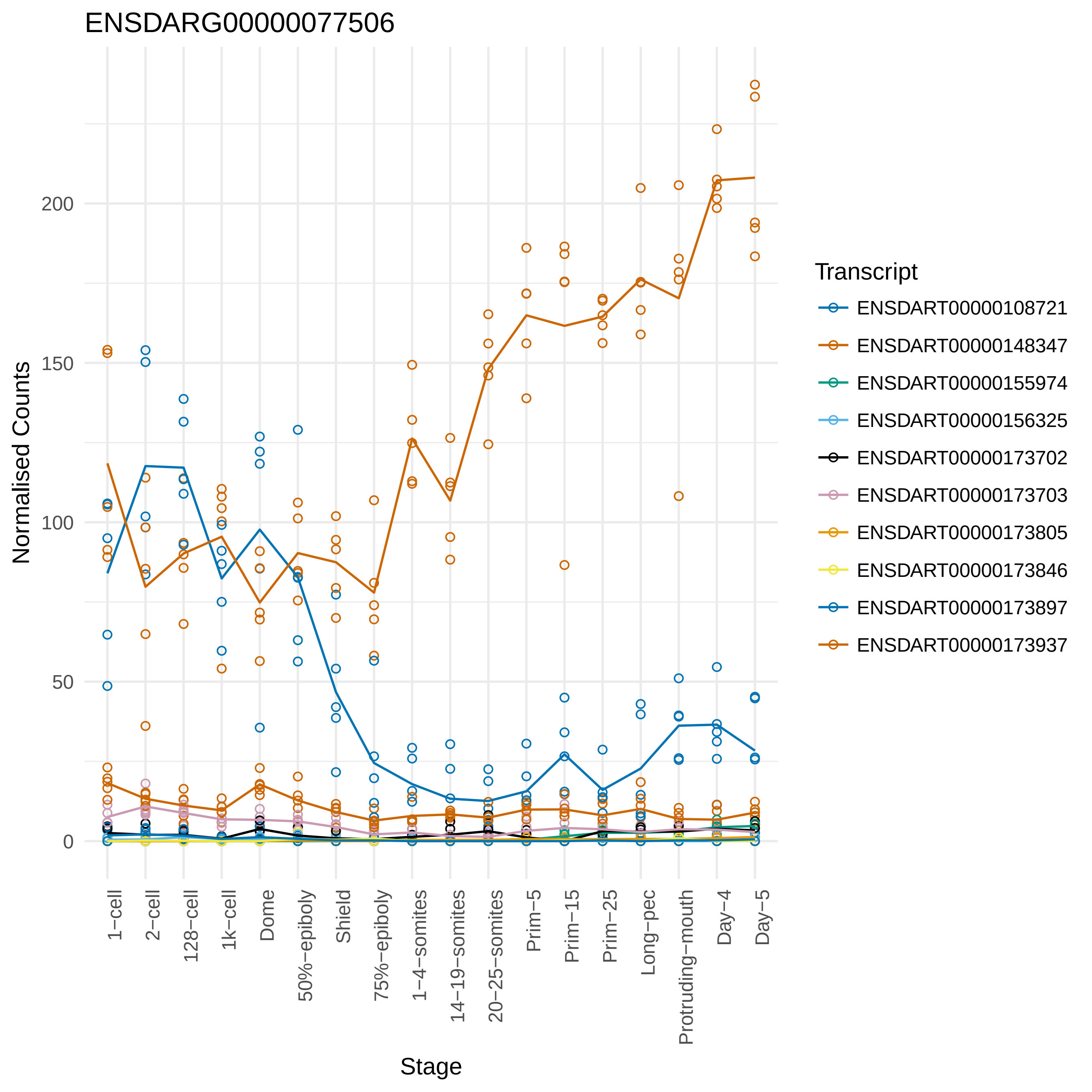

ENSDARG00000077506
Normalised Counts
0
50
100
150
200
Stage
1−cell
2−cell
128−cell
1k−cell
Dome
50%−epiboly
Shield
75%−epiboly
1−4−somites
14−19−somites
20−25−somites
Prim−5
Prim−15
Prim−25
Long−pec
Protruding−mouth
Day−4
Day−5
Transcript
ENSDART00000108721
ENSDART00000148347
ENSDART00000155974
ENSDART00000156325
ENSDART00000173702
ENSDART00000173703
ENSDART00000173805
ENSDART00000173846
ENSDART00000173897
ENSDART00000173937

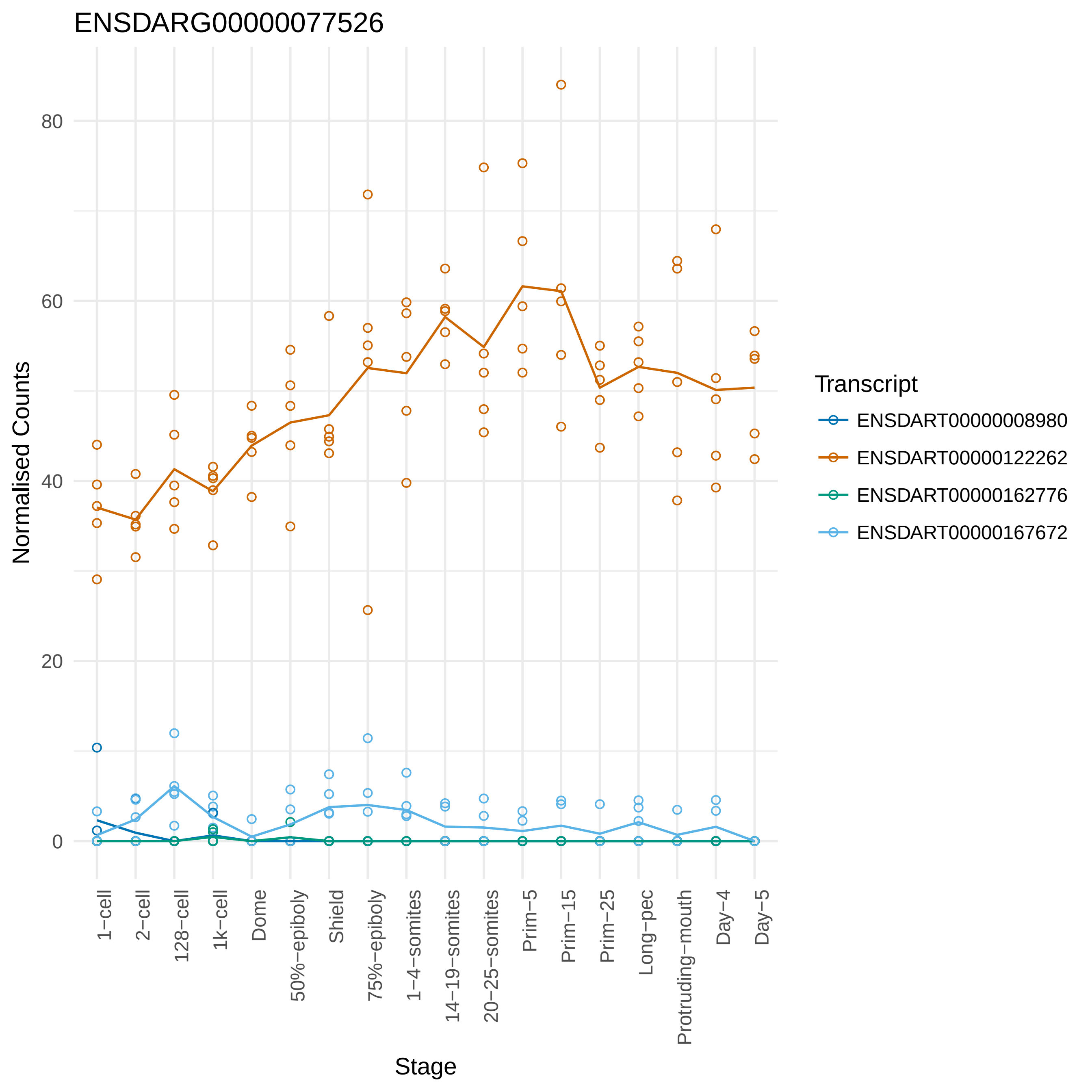

ENSDARG00000077526
Normalised Counts
0
20
40
60
80
Stage
1−cell
2−cell
128−cell
1k−cell
Dome
50%−epiboly
Shield
75%−epiboly
1−4−somites
14−19−somites
20−25−somites
Prim−5
Prim−15
Prim−25
Long−pec
Protruding−mouth
Day−4
Day−5
Transcript
ENSDART00000008980
ENSDART00000122262
ENSDART00000162776
ENSDART00000167672

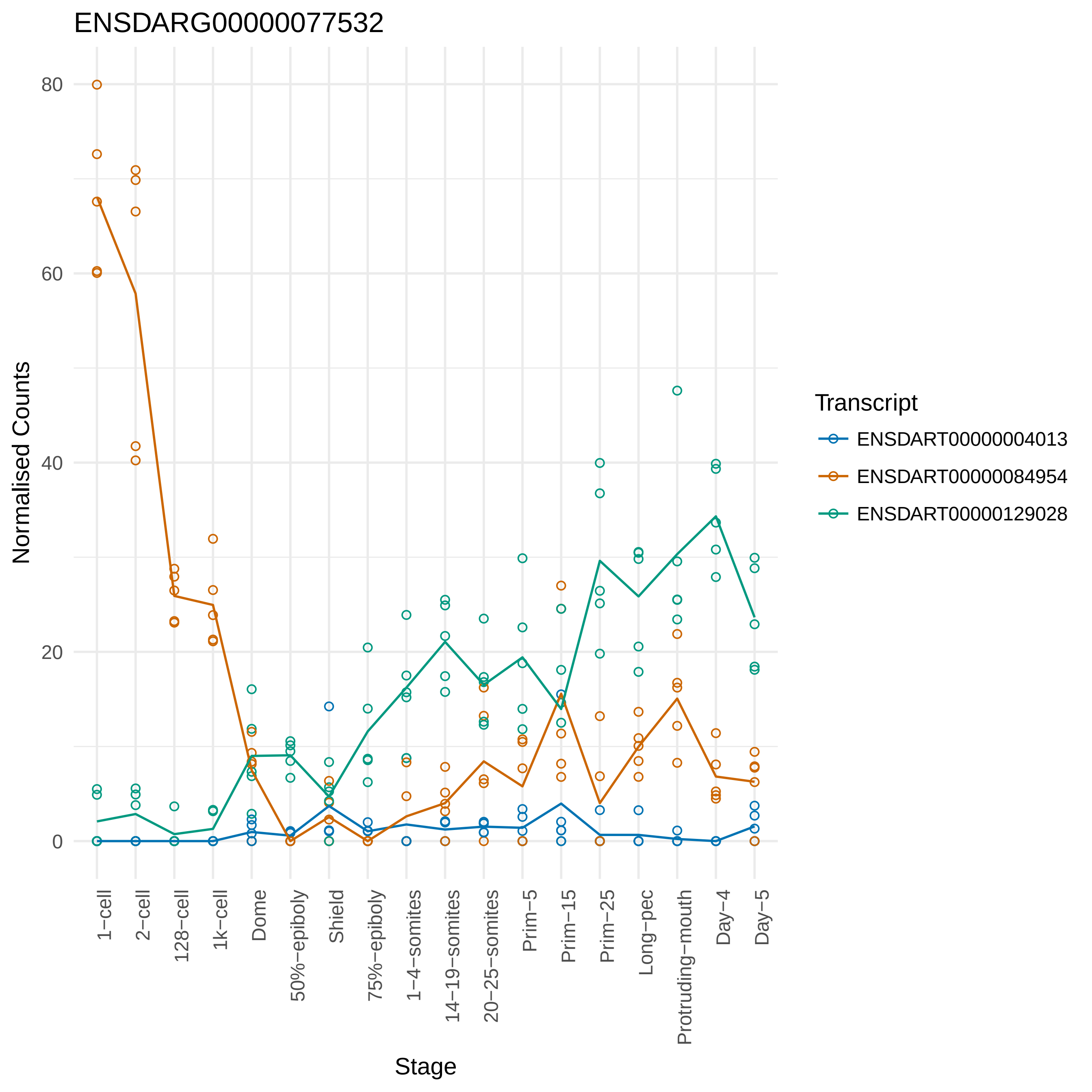
ENSDARG00000077532
Normalised Counts
0
20
40
60
80
1−cell
2−cell
128−cell
1k−cell
Dome
50%−epiboly
Shield
75%−epiboly
1−4−somites
14−19−somites
20−25−somites
Prim−5
Prim−15
Prim−25
Long−pec
Protruding−mouth
Day−4
Day−5
Stage
Transcript
ENSDART00000004013
ENSDART00000084954
ENSDART00000129028

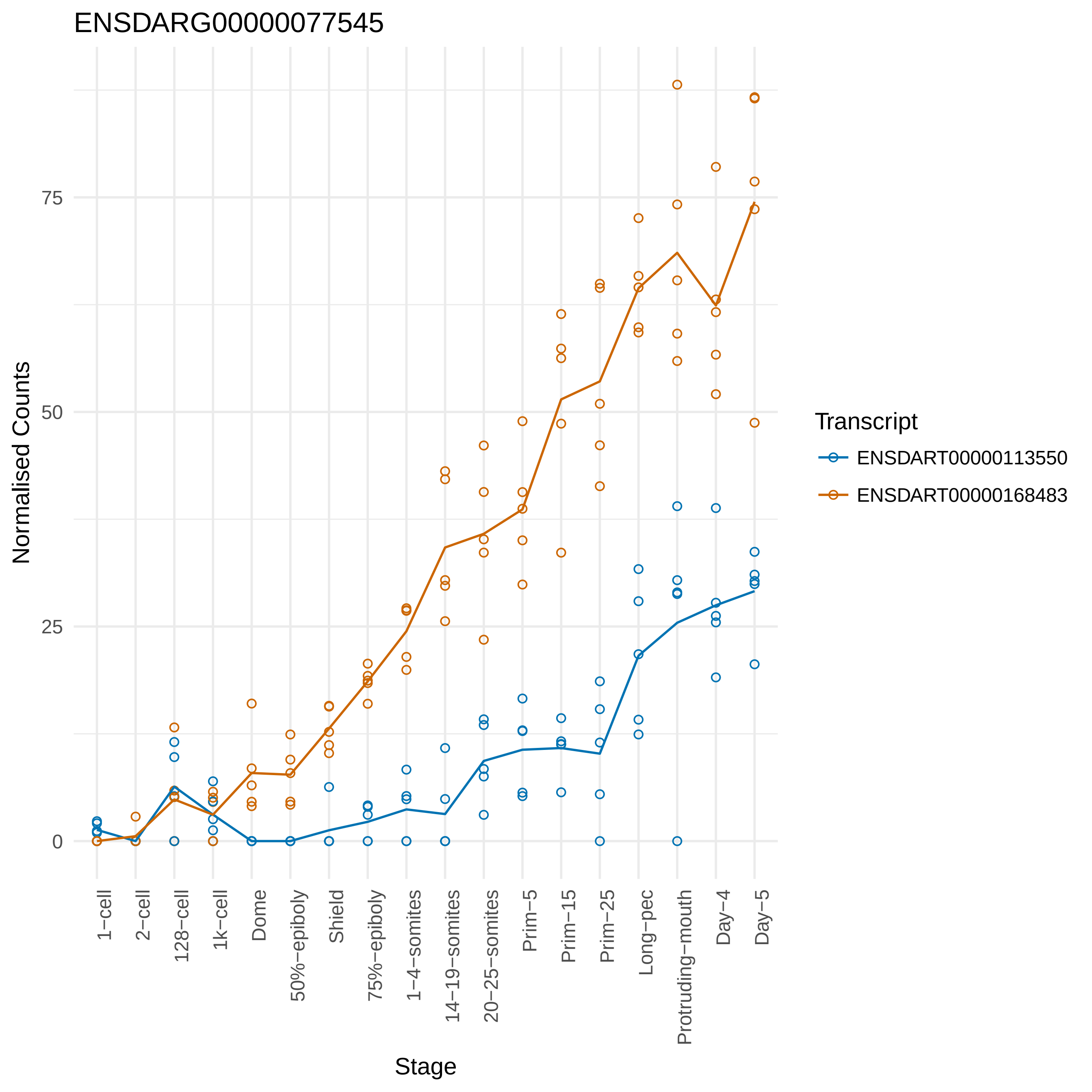
ENSDARG00000077545
Normalised Counts
0
25
50
75
Stage
1−cell
2−cell
128−cell
1k−cell
Dome
50%−epiboly
Shield
75%−epiboly
1−4−somites
14−19−somites
20−25−somites
Prim−5
Prim−15
Prim−25
Long−pec
Protruding−mouth
Day−4
Day−5
Transcript
ENSDART00000113550
ENSDART00000168483

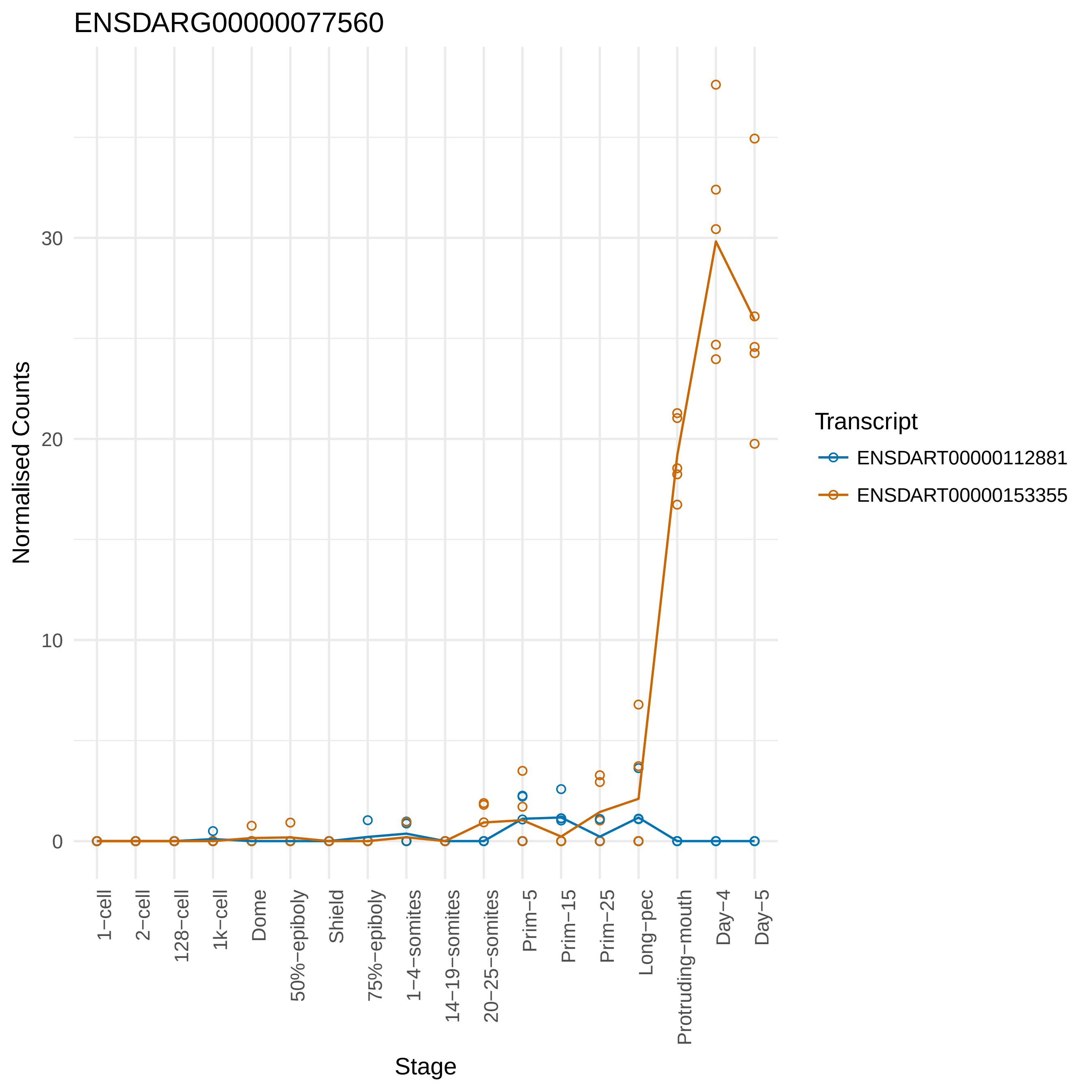

ENSDARG00000077560
Normalised Counts
0
10
20
30
Stage
1−cell
2−cell
128−cell
1k−cell
Dome
50%−epiboly
Shield
75%−epiboly
1−4−somites
14−19−somites
20−25−somites
Prim−5
Prim−15
Prim−25
Long−pec
Protruding−mouth
Day−4
Day−5
Transcript
ENSDART00000112881
ENSDART00000153355

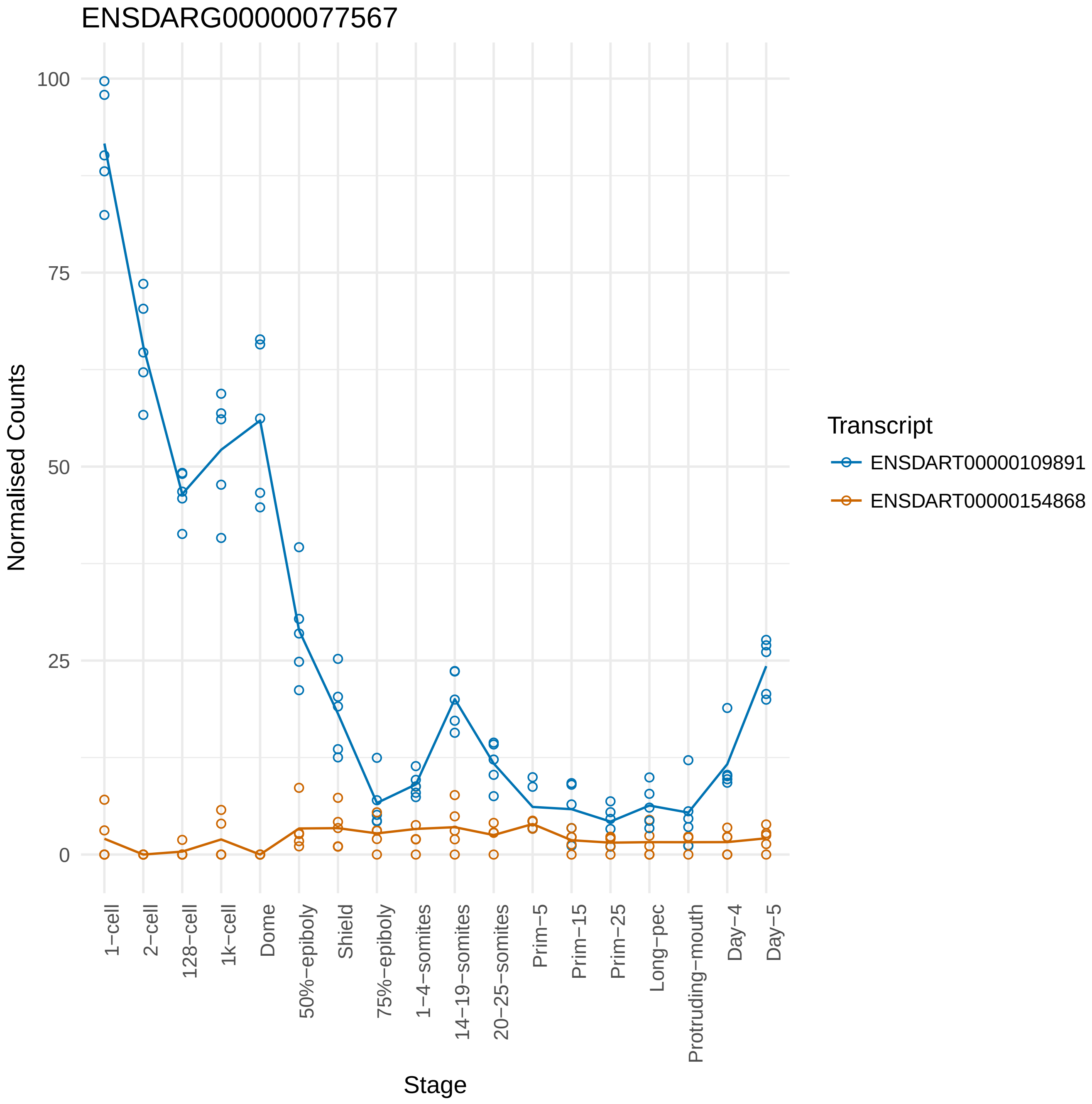

ENSDARG00000077567
Normalised Counts
100
75
50
25
0
1−cell
2−cell
128−cell
1k−cell
Dome
50%−epiboly
Shield
75%−epiboly
1−4−somites
14−19−somites
20−25−somites
Prim−5
Prim−15
Prim−25
Long−pec
Protruding−mouth
Day−4
Day−5
Stage
Transcript
ENSDART00000109891
ENSDART00000154868

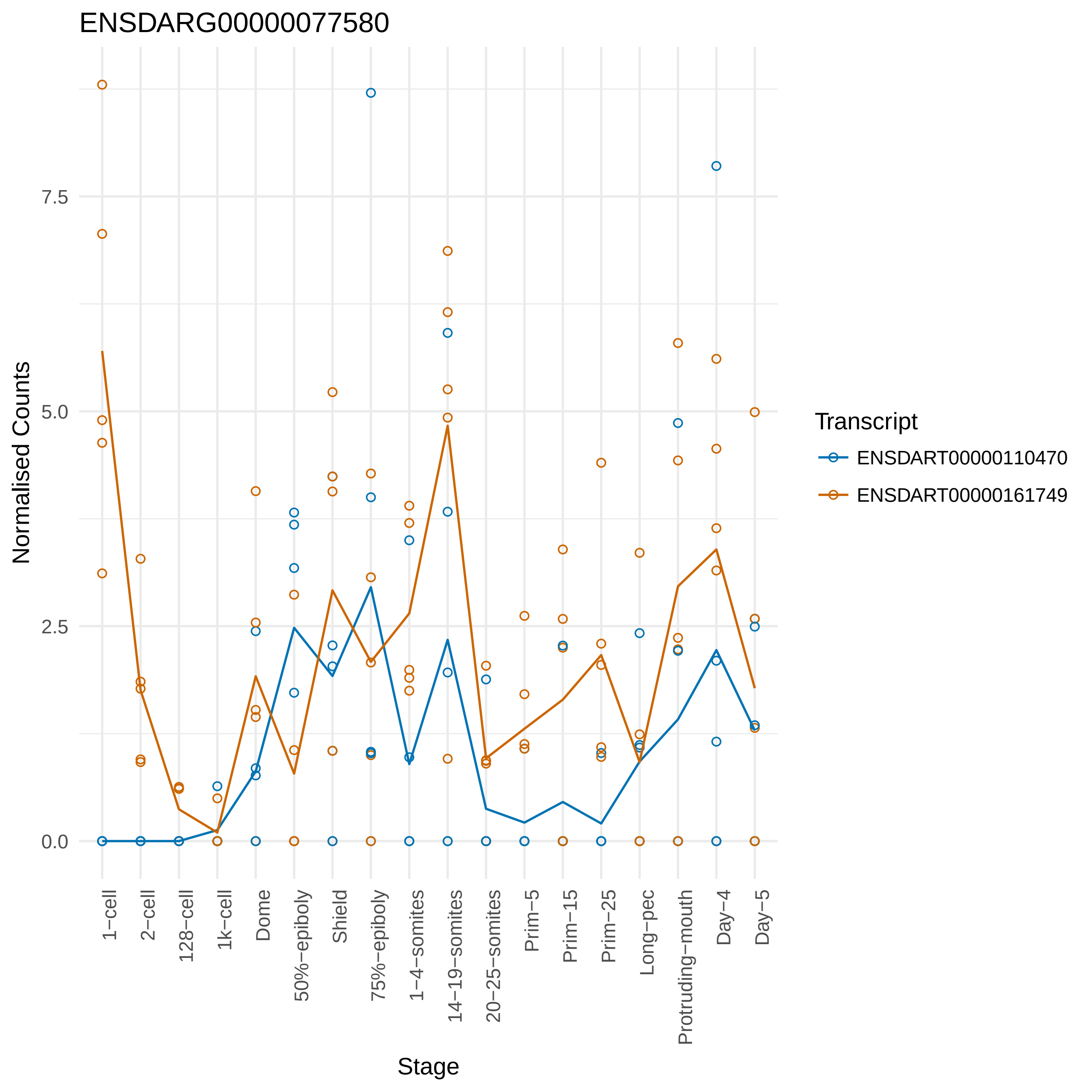

ENSDARG00000077580
Normalised Counts
0.0
2.5
5.0
7.5
Stage
1−cell
2−cell
128−cell
1k−cell
Dome
50%−epiboly
Shield
75%−epiboly
1−4−somites
14−19−somites
20−25−somites
Prim−5
Prim−15
Prim−25
Long−pec
Protruding−mouth
Day−4
Day−5
Transcript
ENSDART00000110470
ENSDART00000161749

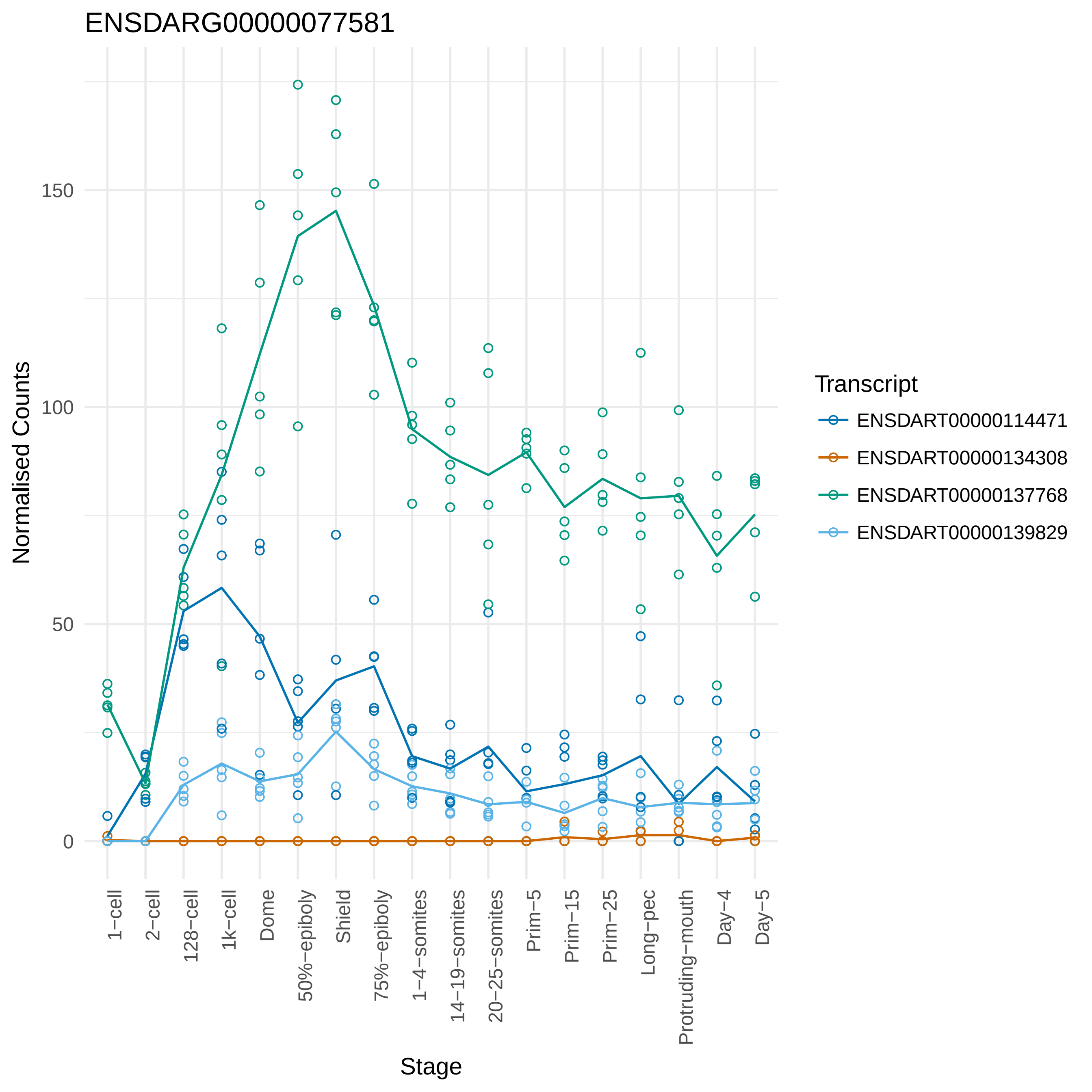

ENSDARG00000077581
Normalised Counts
150
100
50
0
1−cell
2−cell
128−cell
1k−cell
Dome
50%−epiboly
Shield
75%−epiboly
1−4−somites
14−19−somites
20−25−somites
Prim−5
Prim−15
Prim−25
Long−pec
Protruding−mouth
Day−4
Day−5
Stage
Transcript
ENSDART00000114471
ENSDART00000134308
ENSDART00000137768
ENSDART00000139829

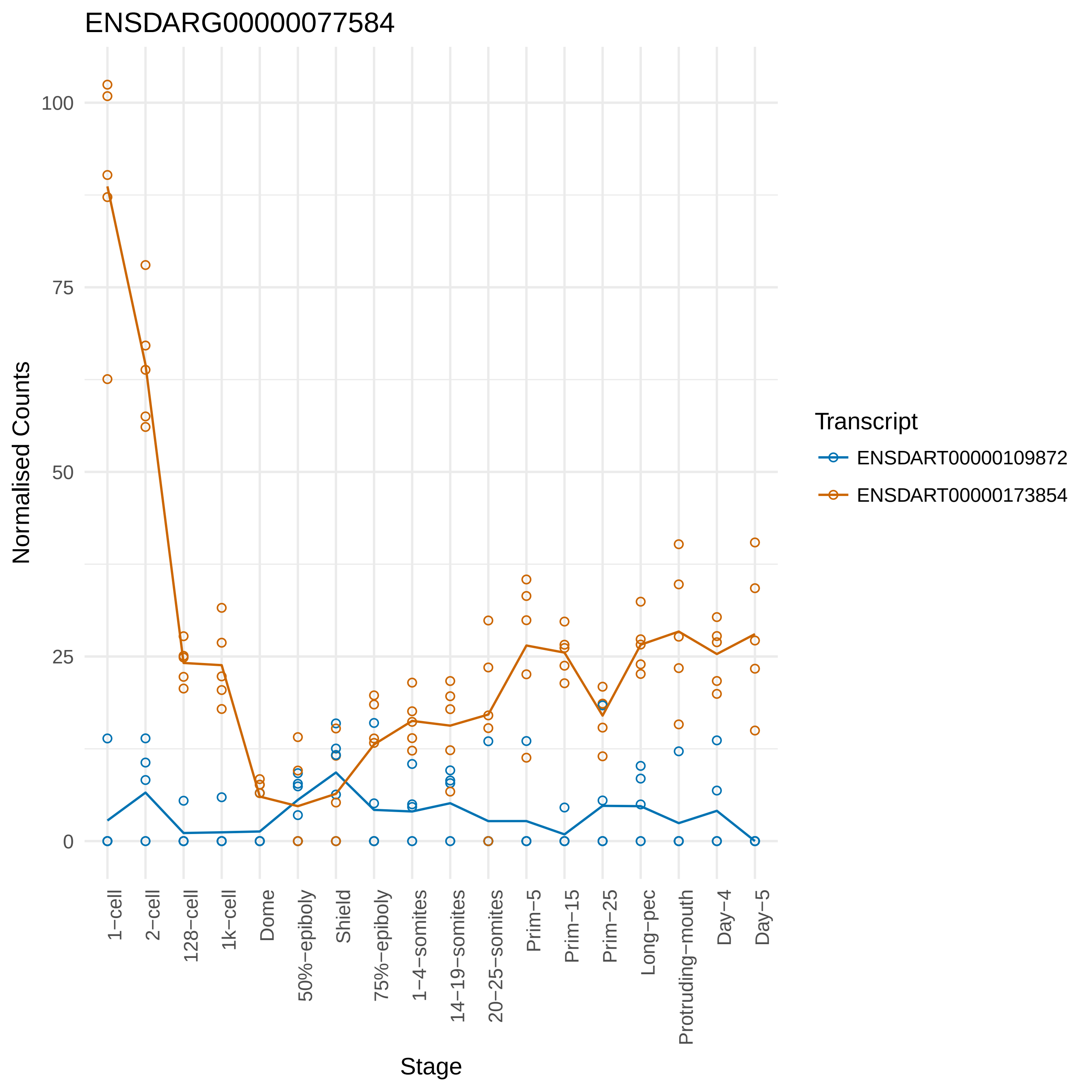

ENSDARG00000077584
Normalised Counts
100
75
50
25
0
1−cell
2−cell
128−cell
1k−cell
Dome
50%−epiboly
Shield
75%−epiboly
1−4−somites
14−19−somites
20−25−somites
Prim−5
Prim−15
Prim−25
Long−pec
Protruding−mouth
Day−4
Day−5
Stage
Transcript
ENSDART00000109872
ENSDART00000173854

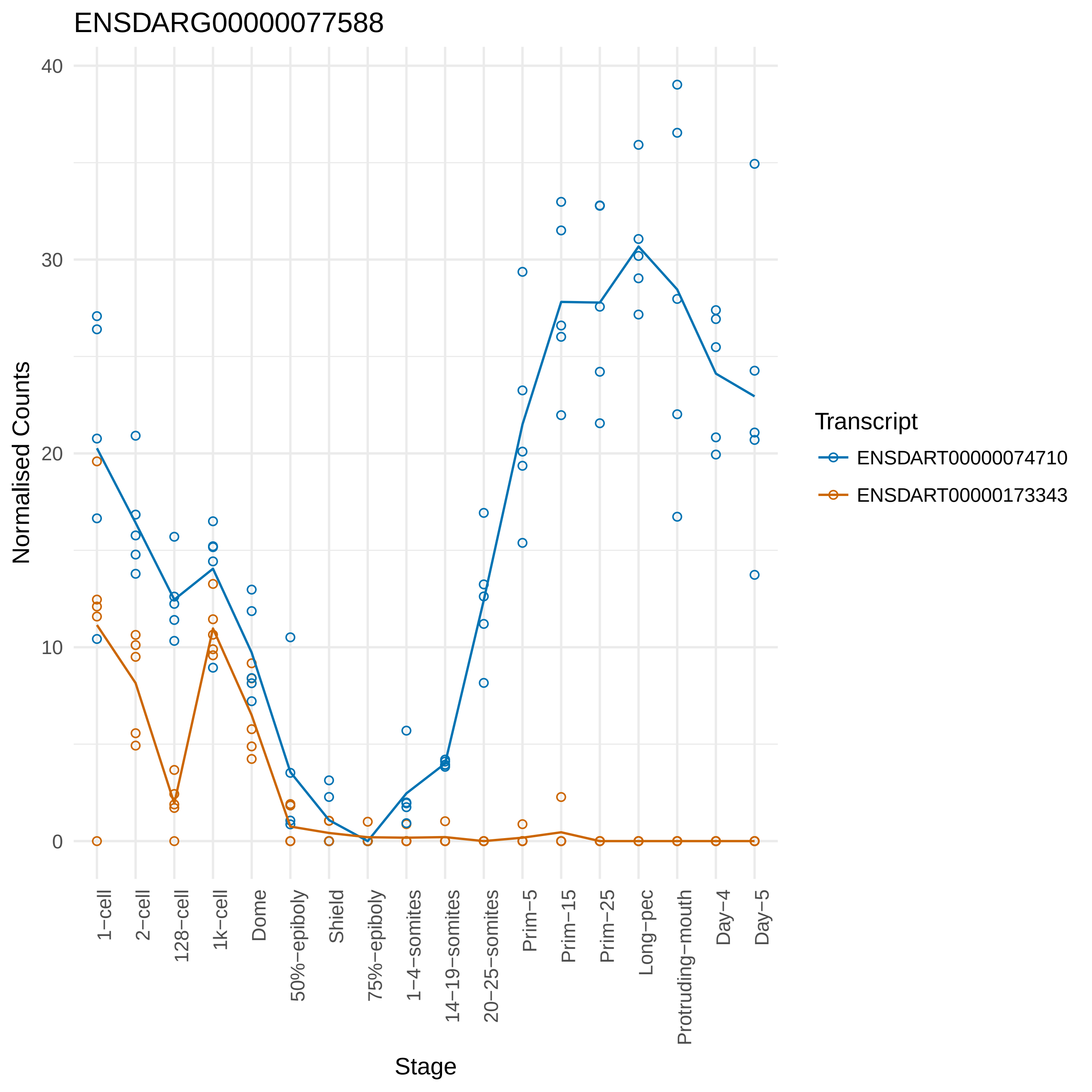
ENSDARG00000077588
Normalised Counts
0
10
20
30
40
Stage
1−cell
2−cell
128−cell
1k−cell
Dome
50%−epiboly
Shield
75%−epiboly
1−4−somites
14−19−somites
20−25−somites
Prim−5
Prim−15
Prim−25
Long−pec
Protruding−mouth
Day−4
Day−5
Transcript
ENSDART00000074710
ENSDART00000173343

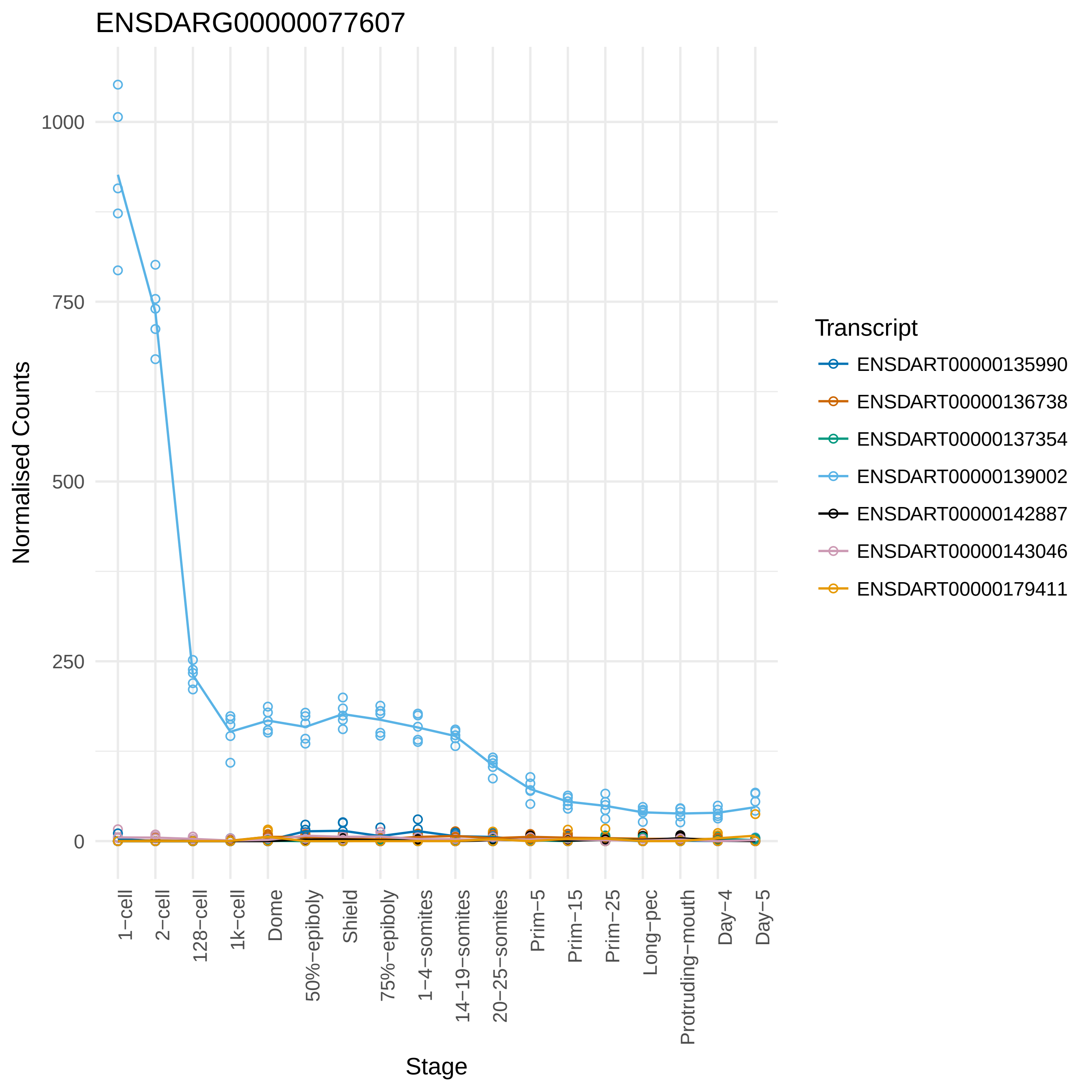

ENSDARG00000077607
Normalised Counts
1000
750
500
250
0
1−cell
2−cell
128−cell
1k−cell
Dome
50%−epiboly
Shield
75%−epiboly
1−4−somites
14−19−somites
20−25−somites
Prim−5
Prim−15
Prim−25
Long−pec
Protruding−mouth
Day−4
Day−5
Stage
Transcript
ENSDART00000135990
ENSDART00000136738
ENSDART00000137354
ENSDART00000139002
ENSDART00000142887
ENSDART00000143046
ENSDART00000179411

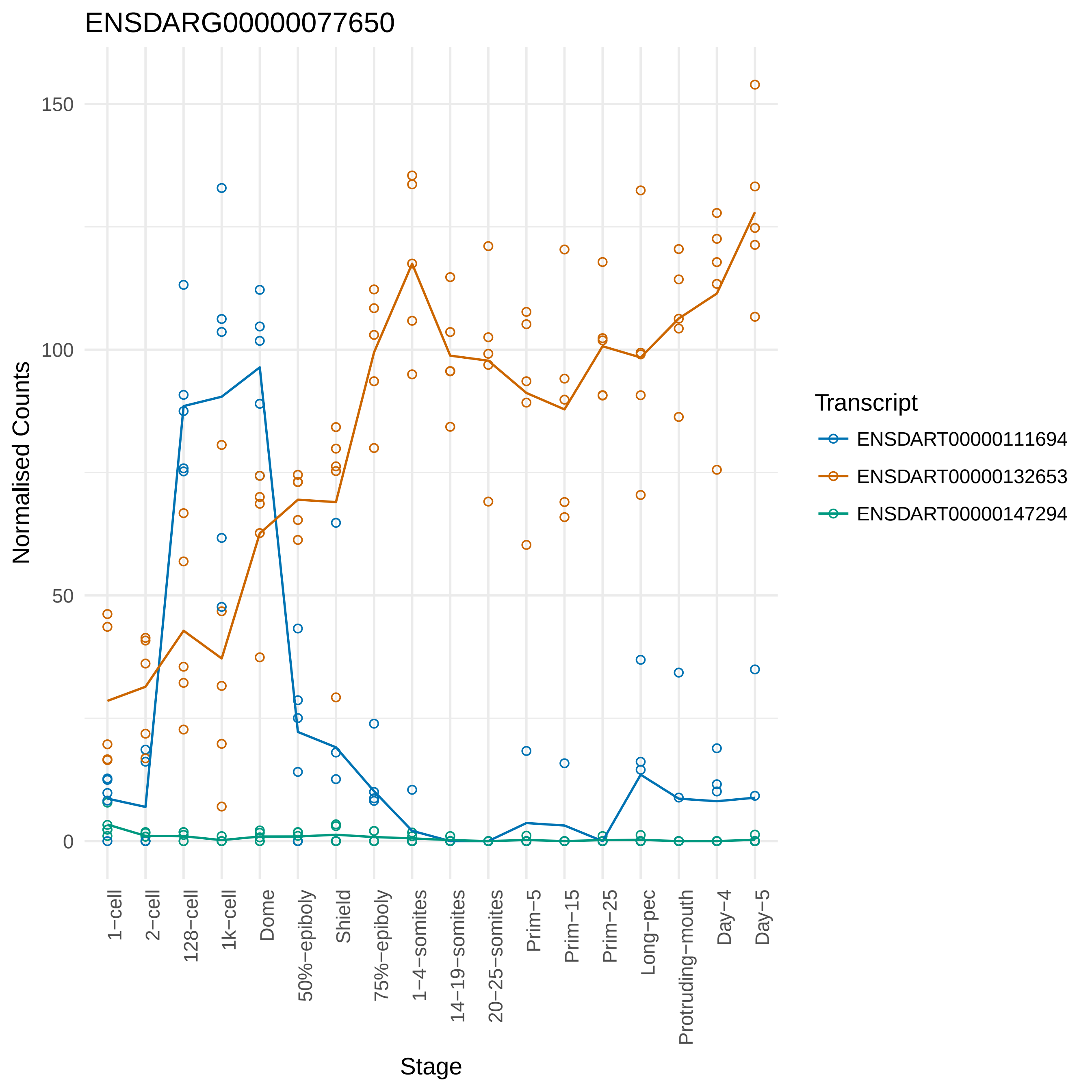

ENSDARG00000077650
Normalised Counts
150
100
50
0
1−cell
2−cell
128−cell
1k−cell
Dome
50%−epiboly
Shield
75%−epiboly
1−4−somites
14−19−somites
20−25−somites
Prim−5
Prim−15
Prim−25
Long−pec
Protruding−mouth
Day−4
Day−5
Stage
Transcript
ENSDART00000111694
ENSDART00000132653
ENSDART00000147294

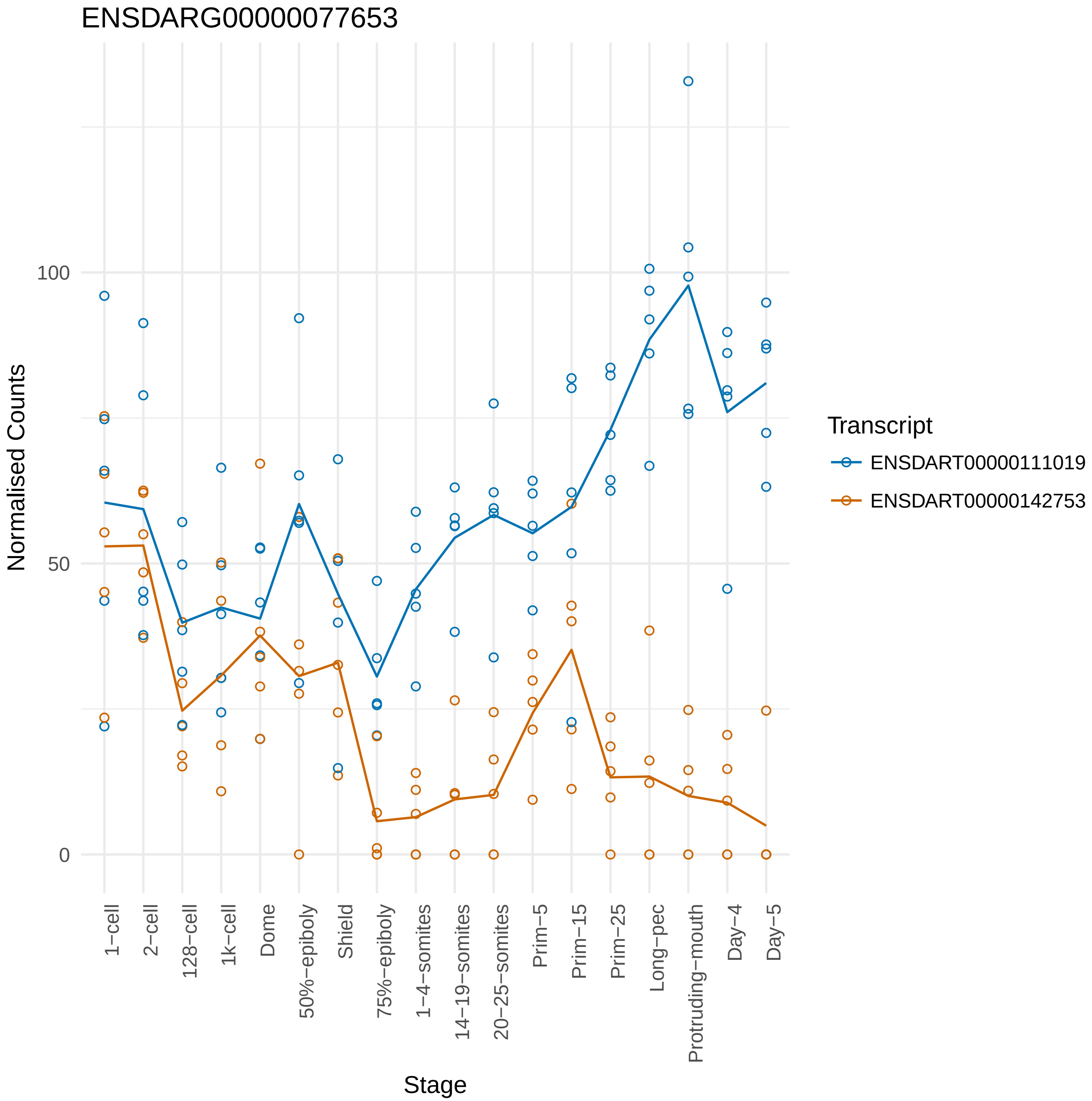
ENSDARG00000077653
Normalised Counts
100
50
0
1−cell
2−cell
128−cell
1k−cell
Dome
50%−epiboly
Shield
75%−epiboly
1−4−somites
14−19−somites
20−25−somites
Prim−5
Prim−15
Prim−25
Long−pec
Protruding−mouth
Day−4
Day−5
Stage
Transcript
ENSDART00000111019
ENSDART00000142753

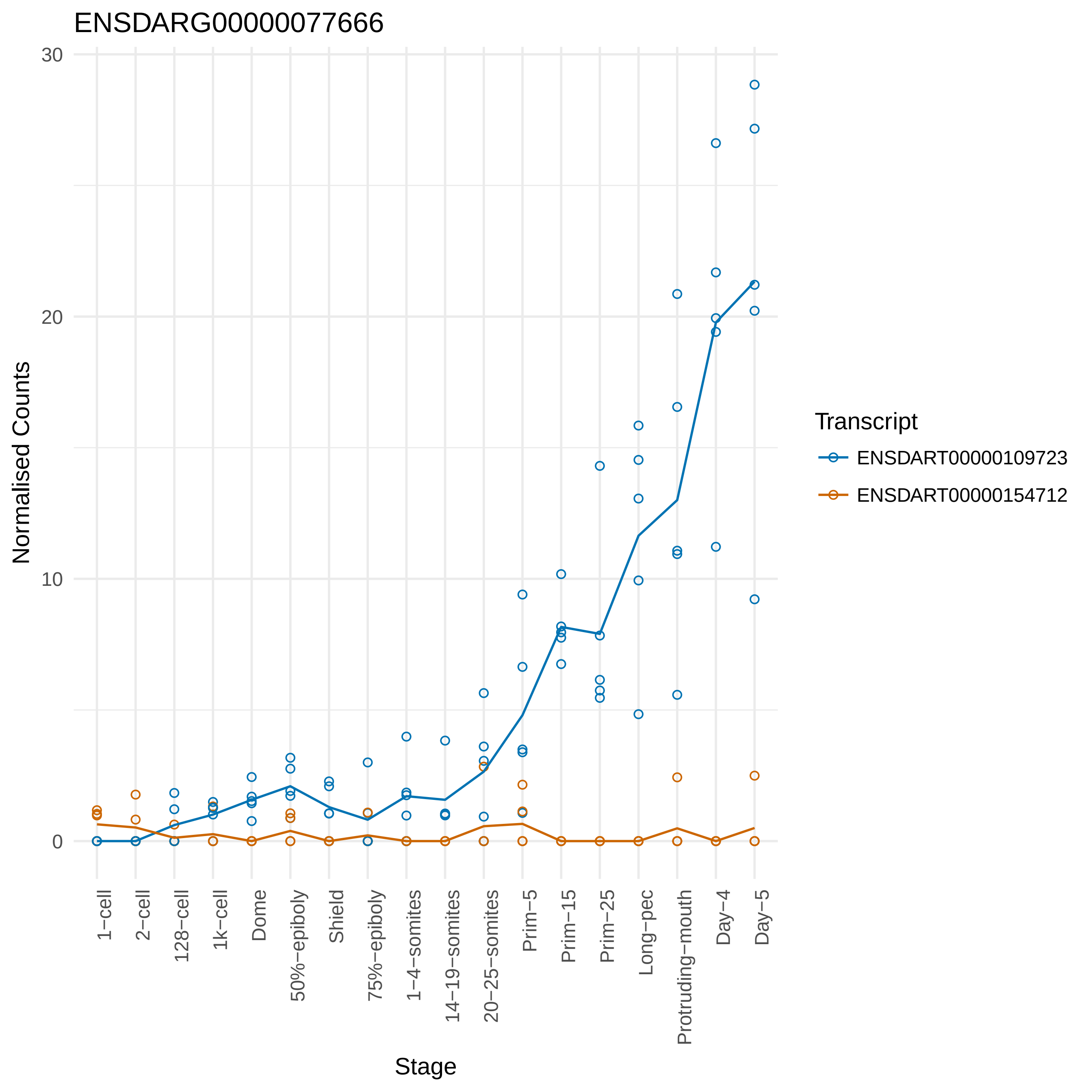
ENSDARG00000077666
Normalised Counts
30
20
10
0
Stage
1−cell
2−cell
128−cell
1k−cell
Dome
50%−epiboly
Shield
75%−epiboly
1−4−somites
14−19−somites
20−25−somites
Prim−5
Prim−15
Prim−25
Long−pec
Protruding−mouth
Day−4
Day−5
Transcript
ENSDART00000109723
ENSDART00000154712

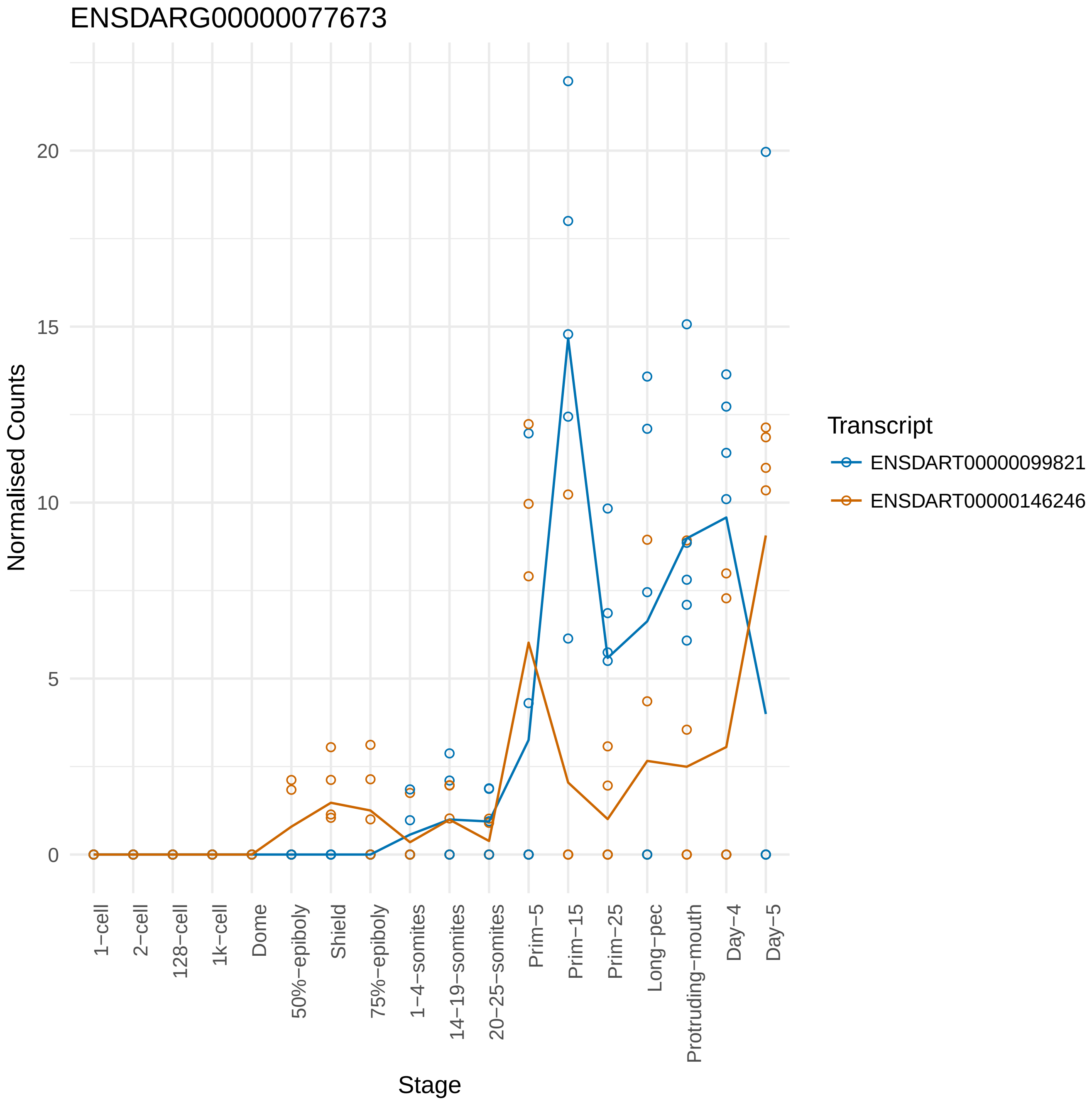

ENSDARG00000077673
Normalised Counts
0
5
10
15
20
1−cell
2−cell
128−cell
1k−cell
Dome
50%−epiboly
Shield
75%−epiboly
1−4−somites
14−19−somites
20−25−somites
Prim−5
Prim−15
Prim−25
Long−pec
Protruding−mouth
Day−4
Day−5
Stage
Transcript
ENSDART00000099821
ENSDART00000146246

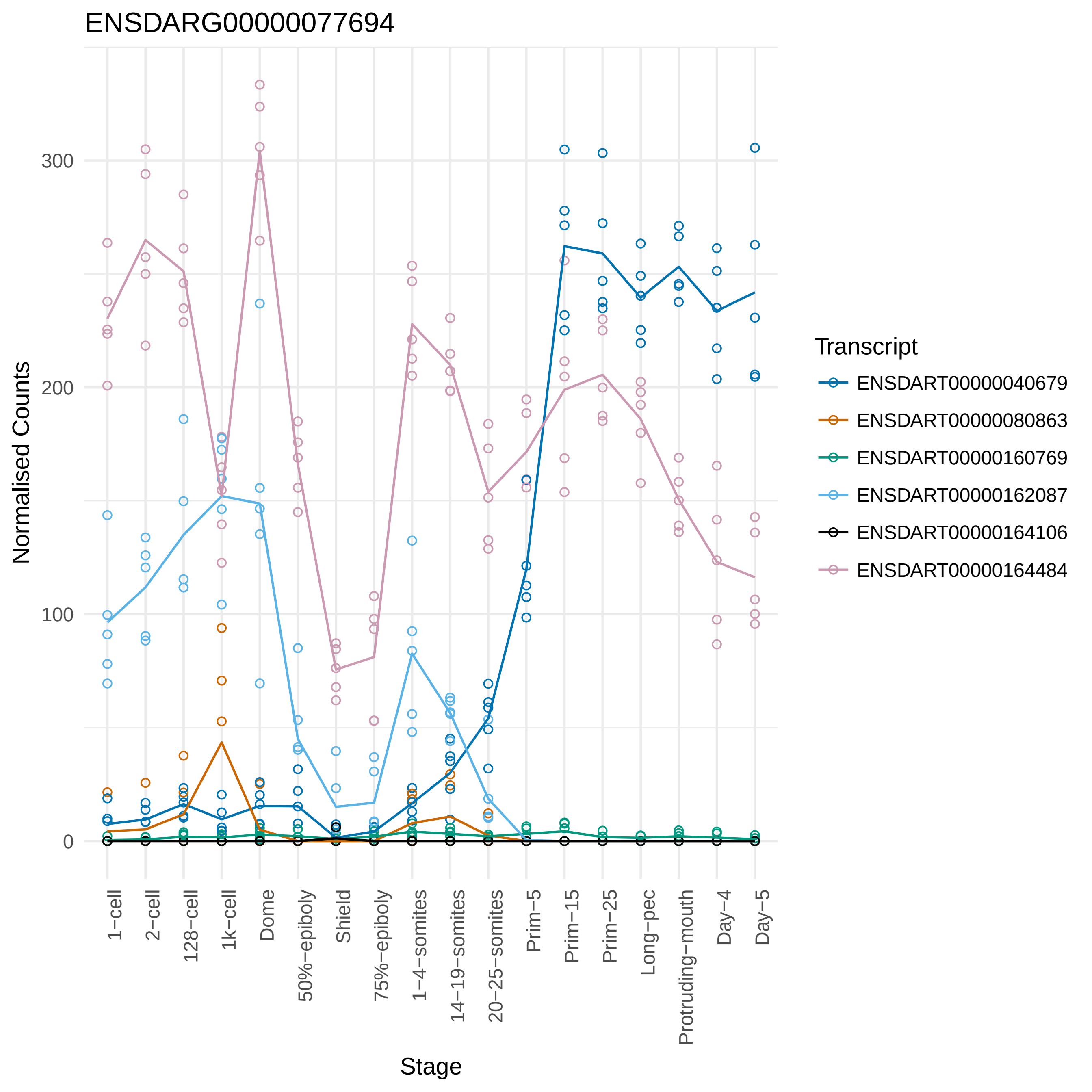

ENSDARG00000077694
Normalised Counts
300
200
100
0
1−cell
2−cell
128−cell
1k−cell
Dome
50%−epiboly
Shield
75%−epiboly
1−4−somites
14−19−somites
20−25−somites
Prim−5
Prim−15
Prim−25
Long−pec
Protruding−mouth
Day−4
Day−5
Stage
Transcript
ENSDART00000040679
ENSDART00000080863
ENSDART00000160769
ENSDART00000162087
ENSDART00000164106
ENSDART00000164484

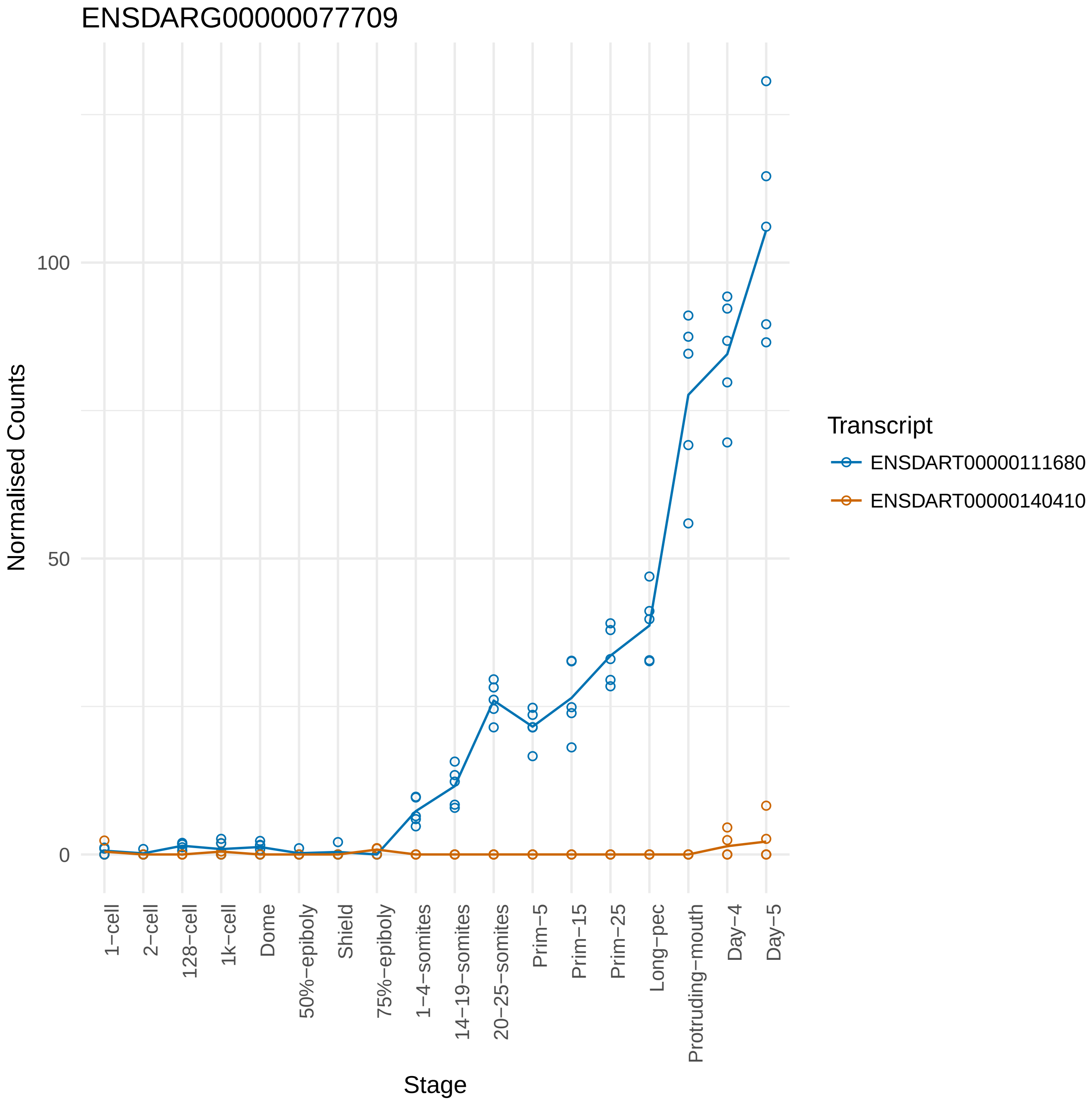

ENSDARG00000077709
Normalised Counts
0
50
100
Stage
1−cell
2−cell
128−cell
1k−cell
Dome
50%−epiboly
Shield
75%−epiboly
1−4−somites
14−19−somites
20−25−somites
Prim−5
Prim−15
Prim−25
Long−pec
Protruding−mouth
Day−4
Day−5
Transcript
ENSDART00000111680
ENSDART00000140410

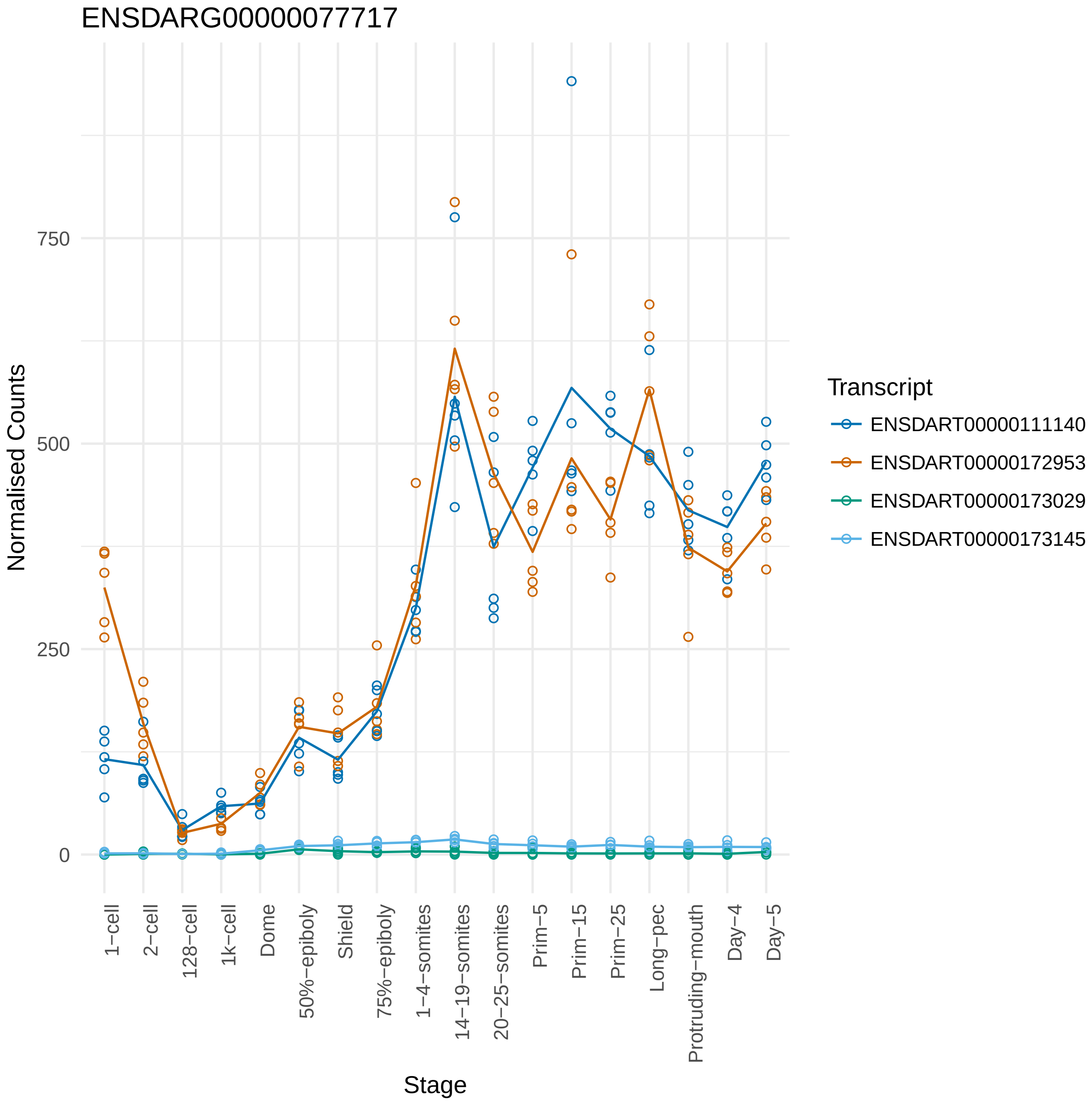

ENSDARG00000077717
Normalised Counts
750
500
250
0
1−cell
2−cell
128−cell
1k−cell
Dome
50%−epiboly
Shield
75%−epiboly
1−4−somites
14−19−somites
20−25−somites
Prim−5
Prim−15
Prim−25
Long−pec
Protruding−mouth
Day−4
Day−5
Stage
Transcript
ENSDART00000111140
ENSDART00000172953
ENSDART00000173029
ENSDART00000173145

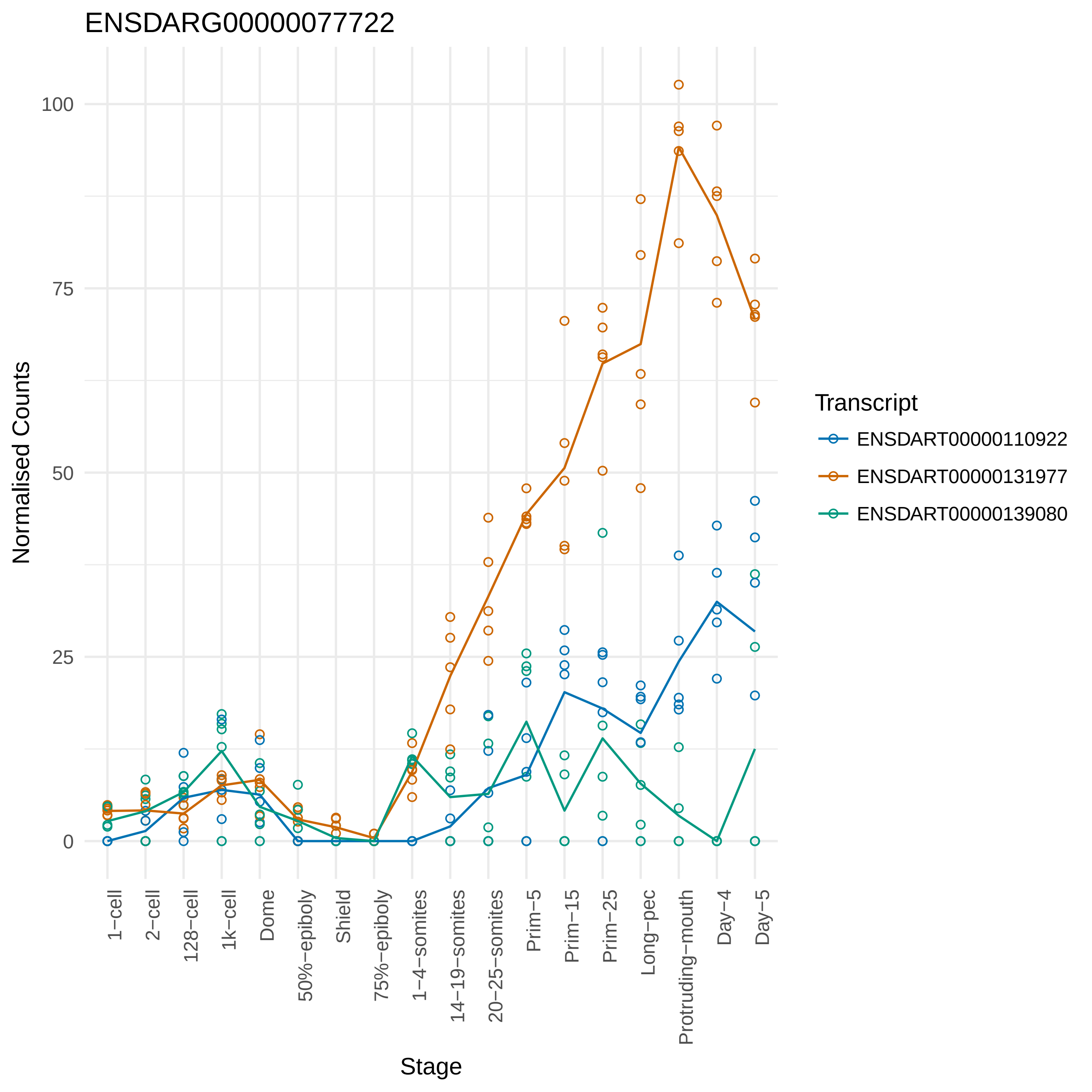

ENSDARG00000077722
Normalised Counts
100
75
50
25
0
1−cell
2−cell
128−cell
1k−cell
Dome
50%−epiboly
Shield
75%−epiboly
1−4−somites
14−19−somites
20−25−somites
Prim−5
Prim−15
Prim−25
Long−pec
Protruding−mouth
Day−4
Day−5
Stage
Transcript
ENSDART00000110922
ENSDART00000131977
ENSDART00000139080

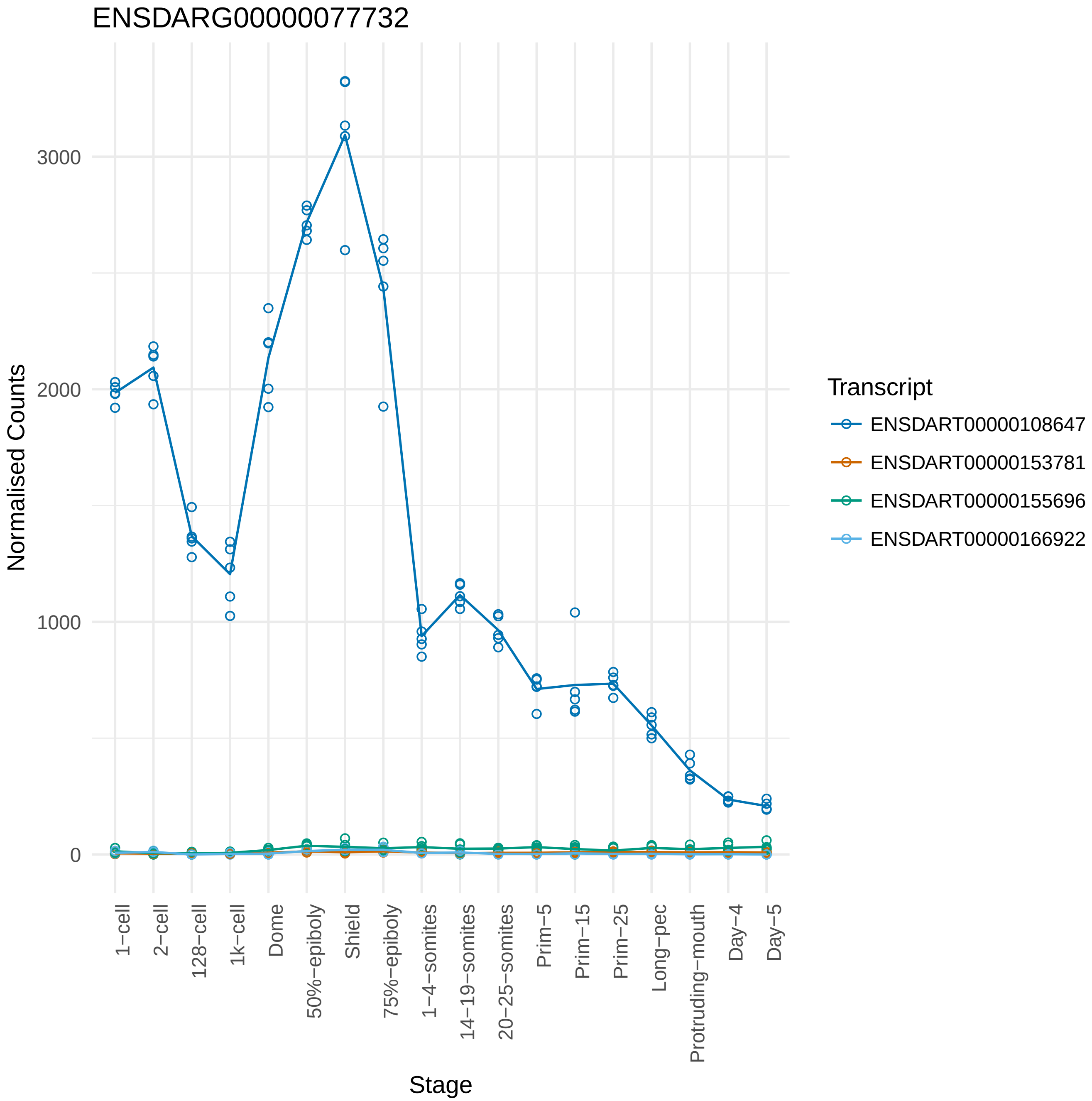

ENSDARG00000077732
Normalised Counts
3000
2000
1000
0
1−cell
2−cell
128−cell
1k−cell
Dome
50%−epiboly
Shield
75%−epiboly
1−4−somites
14−19−somites
20−25−somites
Prim−5
Prim−15
Prim−25
Long−pec
Protruding−mouth
Day−4
Day−5
Stage
Transcript
ENSDART00000108647
ENSDART00000153781
ENSDART00000155696
ENSDART00000166922

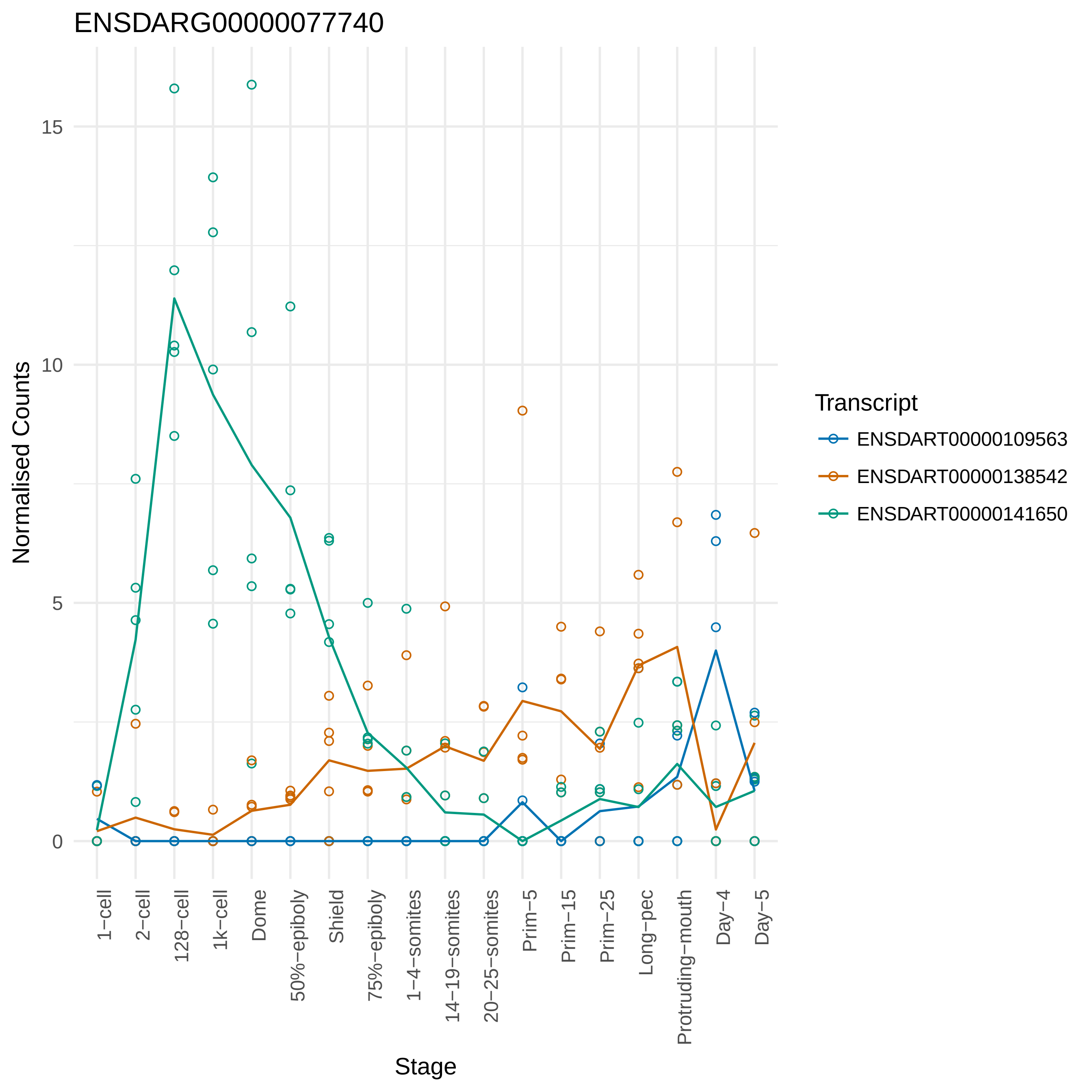
ENSDARG00000077740
Normalised Counts
15
10
5
0
1−cell
2−cell
128−cell
1k−cell
Dome
50%−epiboly
Shield
75%−epiboly
1−4−somites
14−19−somites
20−25−somites
Prim−5
Prim−15
Prim−25
Long−pec
Protruding−mouth
Day−4
Day−5
Stage
Transcript
ENSDART00000109563
ENSDART00000138542
ENSDART00000141650

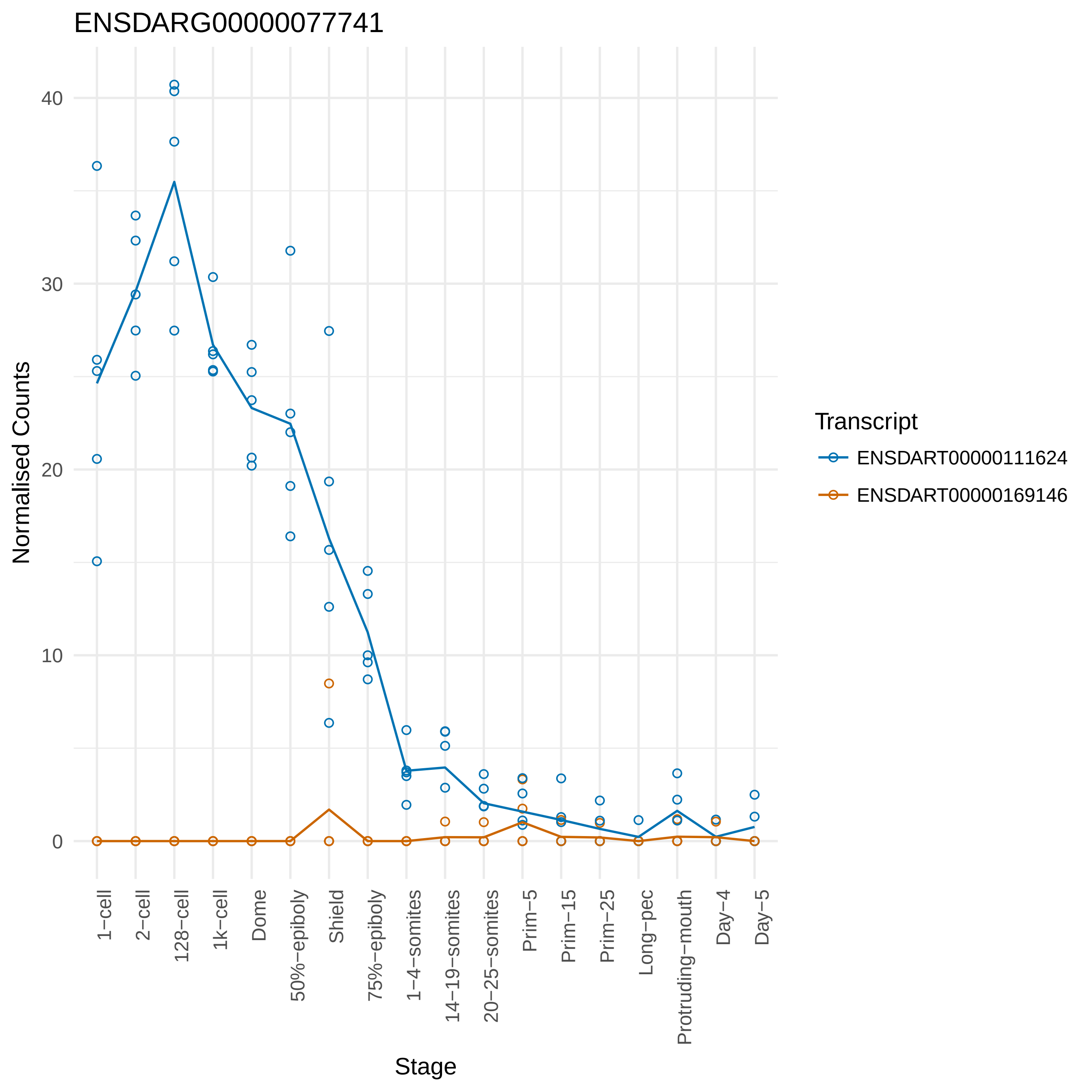

ENSDARG00000077741
Normalised Counts
40
30
20
10
0
1−cell
2−cell
128−cell
1k−cell
Dome
50%−epiboly
Shield
75%−epiboly
1−4−somites
14−19−somites
20−25−somites
Prim−5
Prim−15
Prim−25
Long−pec
Protruding−mouth
Day−4
Day−5
Stage
Transcript
ENSDART00000111624
ENSDART00000169146

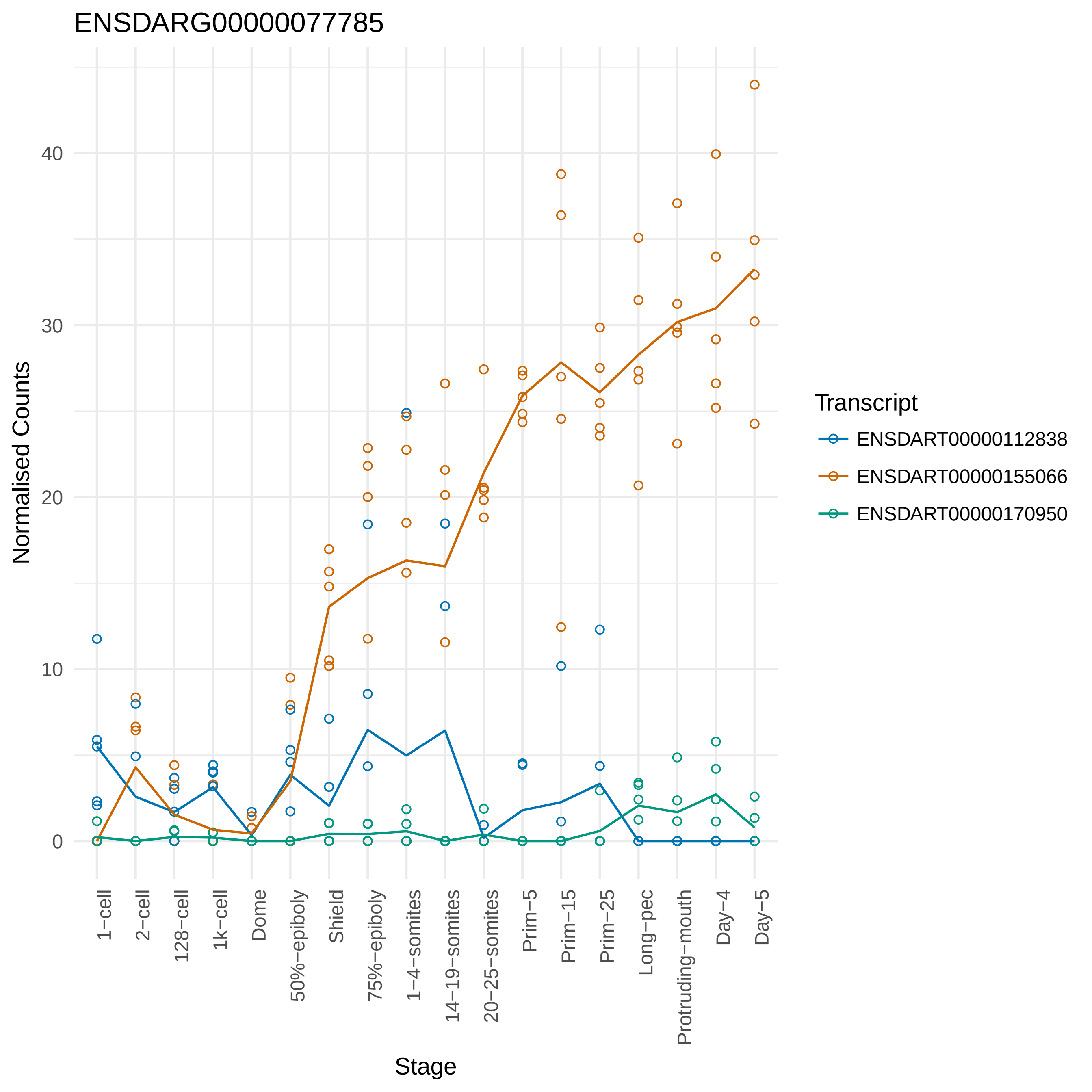

ENSDARG00000077785
Normalised Counts
0
10
20
30
40
Stage
1−cell
2−cell
128−cell
1k−cell
Dome
50%−epiboly
Shield
75%−epiboly
1−4−somites
14−19−somites
20−25−somites
Prim−5
Prim−15
Prim−25
Long−pec
Protruding−mouth
Day−4
Day−5
Transcript
ENSDART00000112838
ENSDART00000155066
ENSDART00000170950

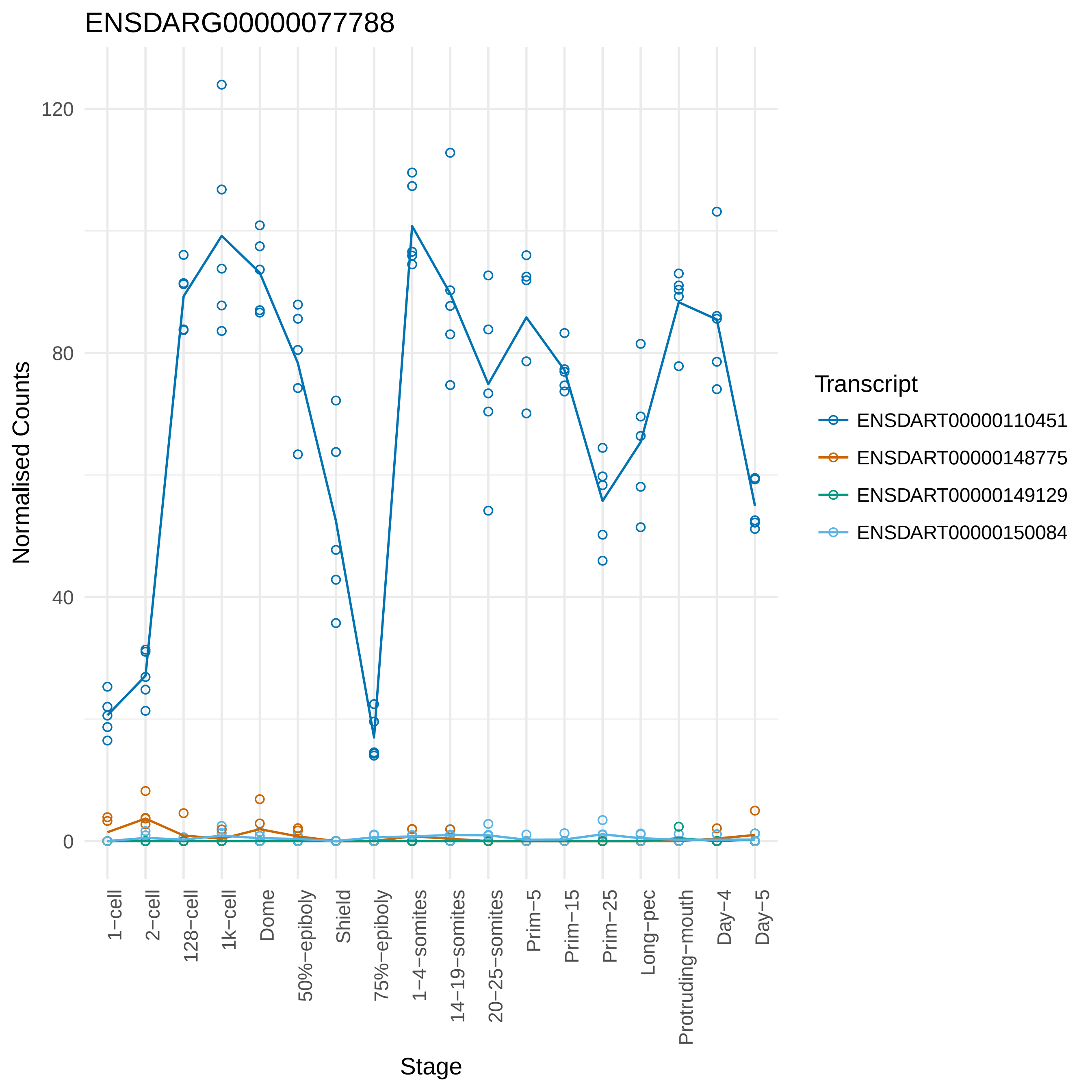
ENSDARG00000077788
Normalised Counts
0
40
80
120
Stage
1−cell
2−cell
128−cell
1k−cell
Dome
50%−epiboly
Shield
75%−epiboly
1−4−somites
14−19−somites
20−25−somites
Prim−5
Prim−15
Prim−25
Long−pec
Protruding−mouth
Day−4
Day−5
Transcript
ENSDART00000110451
ENSDART00000148775
ENSDART00000149129
ENSDART00000150084

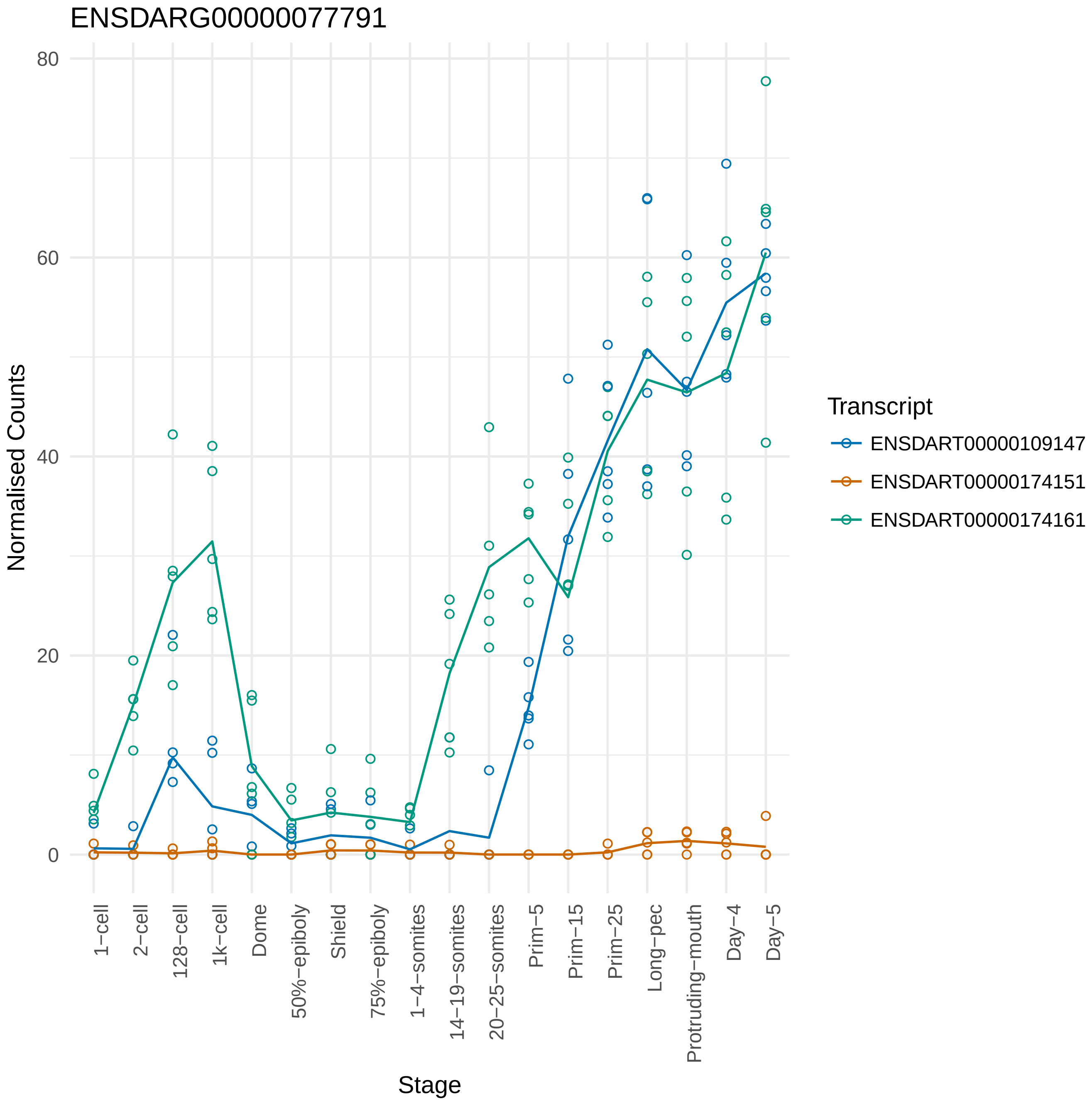
ENSDARG00000077791
Normalised Counts
80
60
40
20
0
1−cell
2−cell
128−cell
1k−cell
Dome
50%−epiboly
Shield
75%−epiboly
1−4−somites
14−19−somites
20−25−somites
Prim−5
Prim−15
Prim−25
Long−pec
Protruding−mouth
Day−4
Day−5
Stage
Transcript
ENSDART00000109147
ENSDART00000174151
ENSDART00000174161

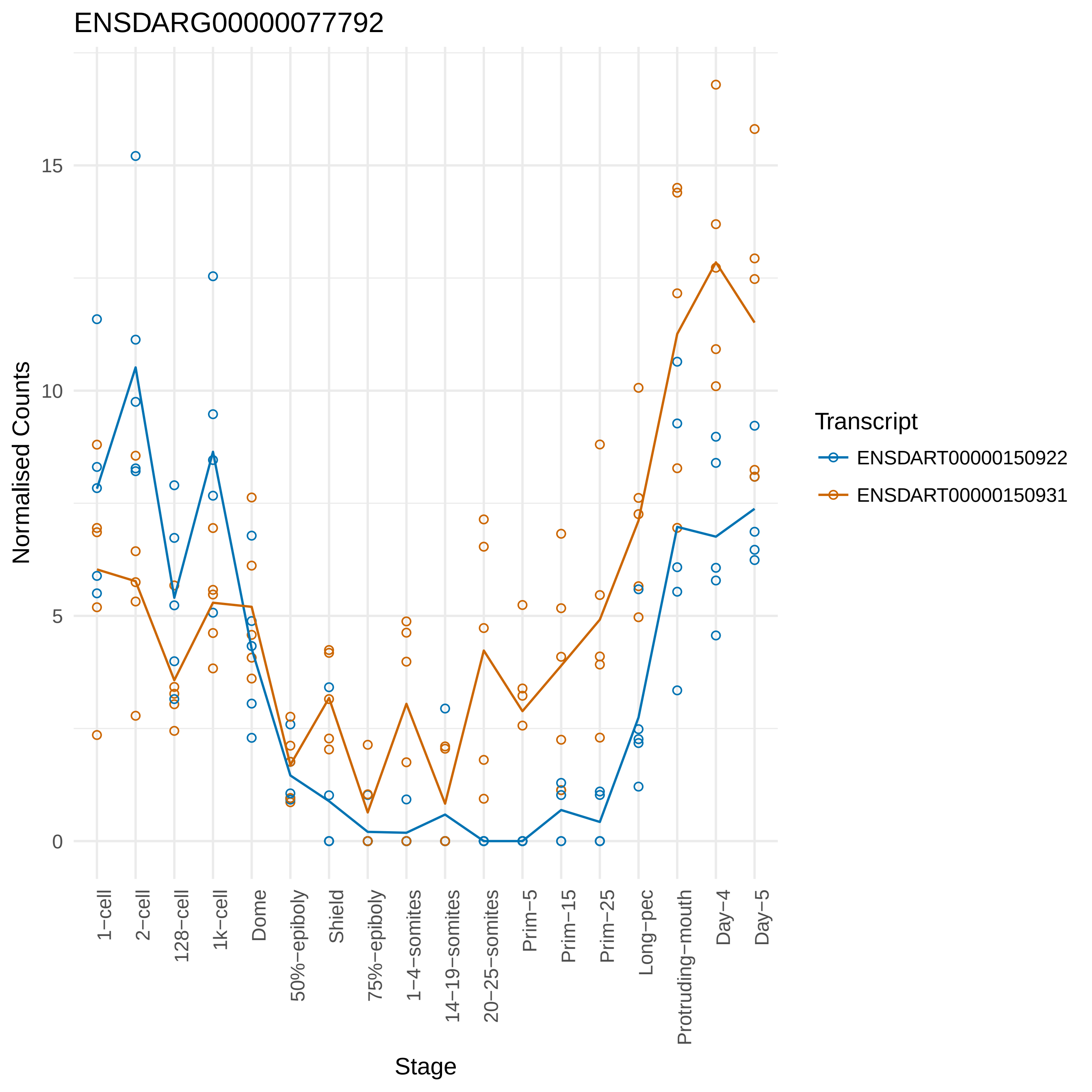
ENSDARG00000077792
Normalised Counts
15
10
5
0
1−cell
2−cell
128−cell
1k−cell
Dome
50%−epiboly
Shield
75%−epiboly
1−4−somites
14−19−somites
20−25−somites
Prim−5
Prim−15
Prim−25
Long−pec
Protruding−mouth
Day−4
Day−5
Stage
Transcript
ENSDART00000150922
ENSDART00000150931

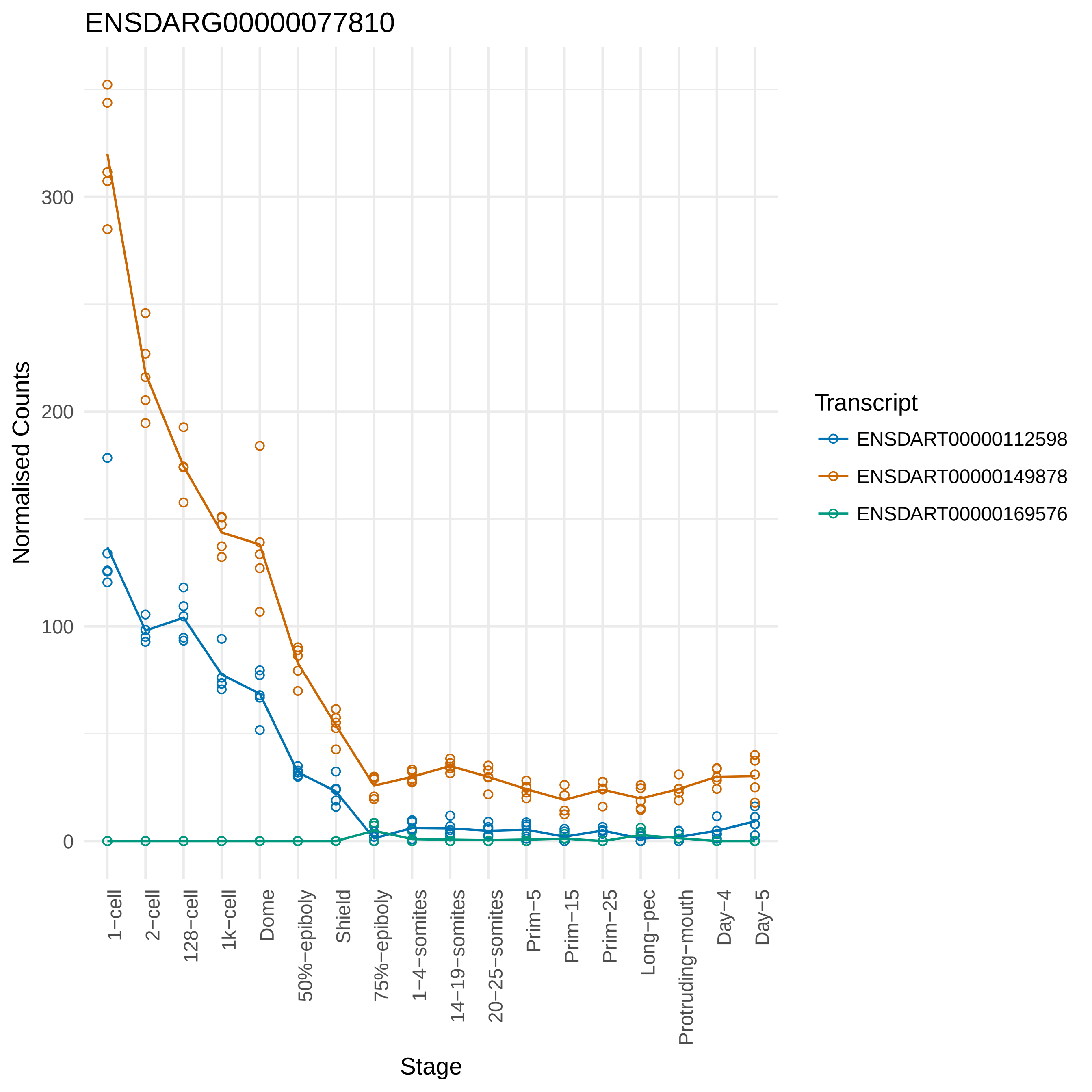
ENSDARG00000077810
Normalised Counts
300
200
100
0
1−cell
2−cell
128−cell
1k−cell
Dome
50%−epiboly
Shield
75%−epiboly
1−4−somites
14−19−somites
20−25−somites
Prim−5
Prim−15
Prim−25
Long−pec
Protruding−mouth
Day−4
Day−5
Stage
Transcript
ENSDART00000112598
ENSDART00000149878
ENSDART00000169576

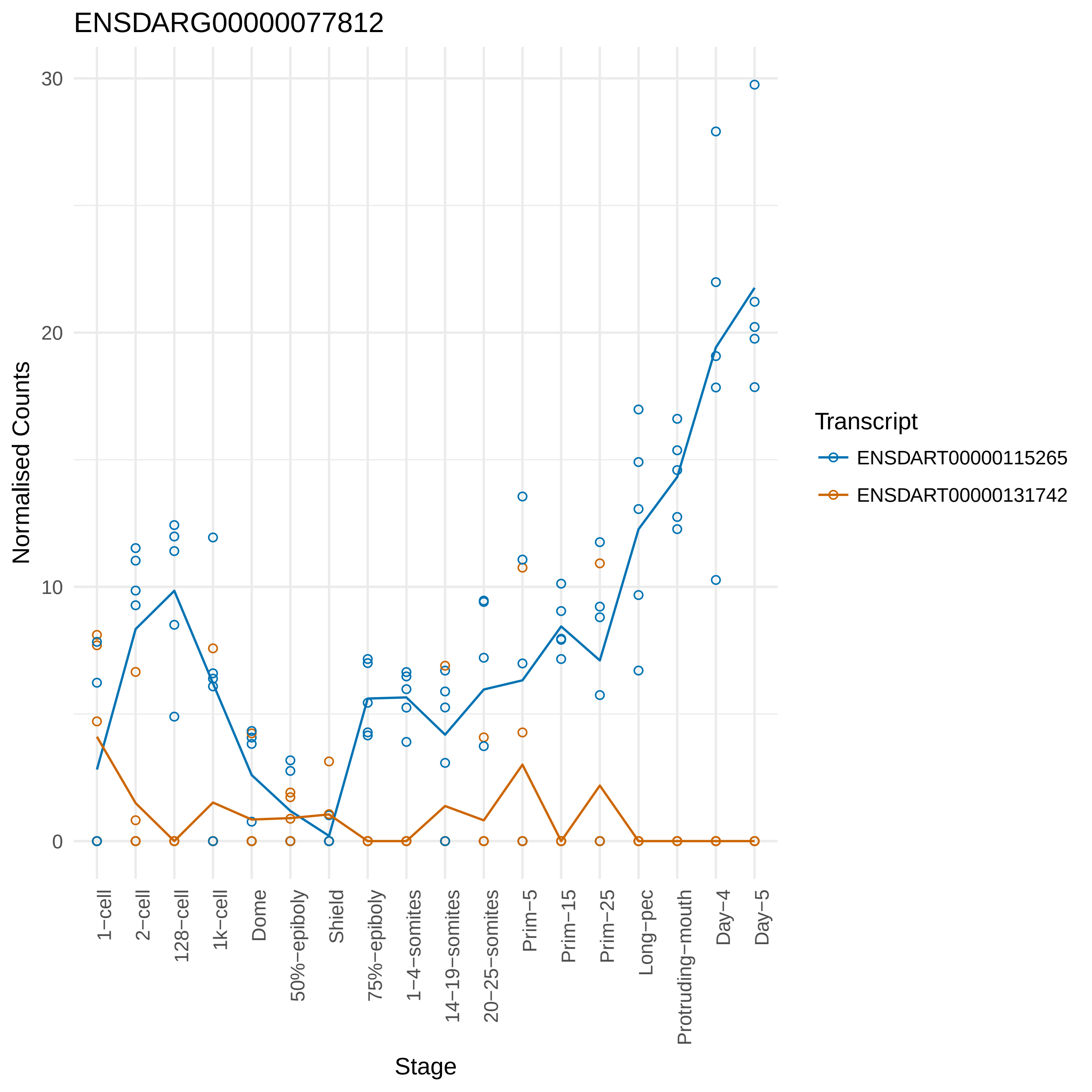

ENSDARG00000077812
Normalised Counts
30
20
10
0
1−cell
2−cell
128−cell
1k−cell
Dome
50%−epiboly
Shield
75%−epiboly
1−4−somites
14−19−somites
20−25−somites
Prim−5
Prim−15
Prim−25
Long−pec
Protruding−mouth
Day−4
Day−5
Stage
Transcript
ENSDART00000115265
ENSDART00000131742

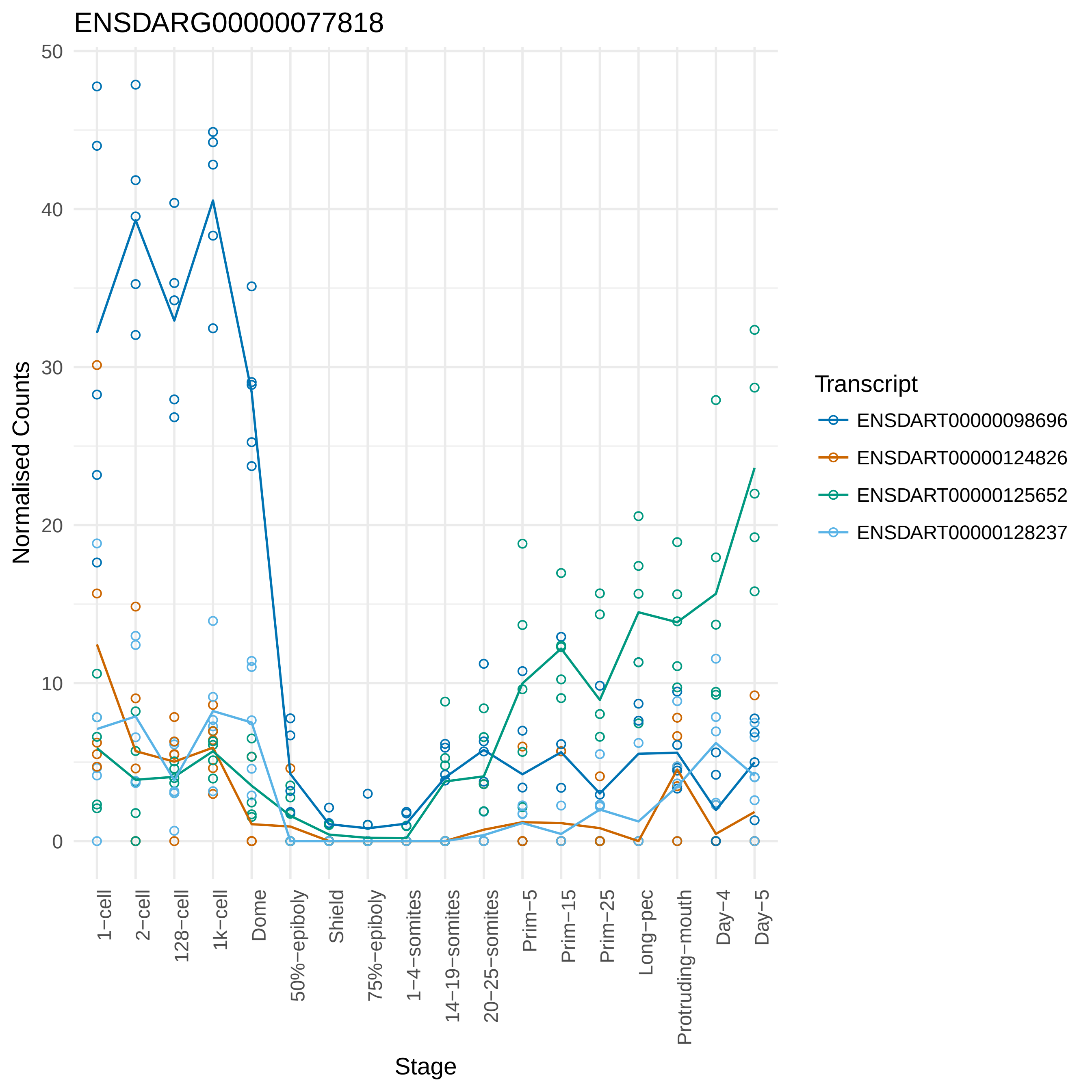
ENSDARG00000077818
Normalised Counts
50
40
30
20
10
0
1−cell
2−cell
128−cell
1k−cell
Dome
50%−epiboly
Shield
75%−epiboly
1−4−somites
14−19−somites
20−25−somites
Prim−5
Prim−15
Prim−25
Long−pec
Protruding−mouth
Day−4
Day−5
Stage
Transcript
ENSDART00000098696
ENSDART00000124826
ENSDART00000125652
ENSDART00000128237

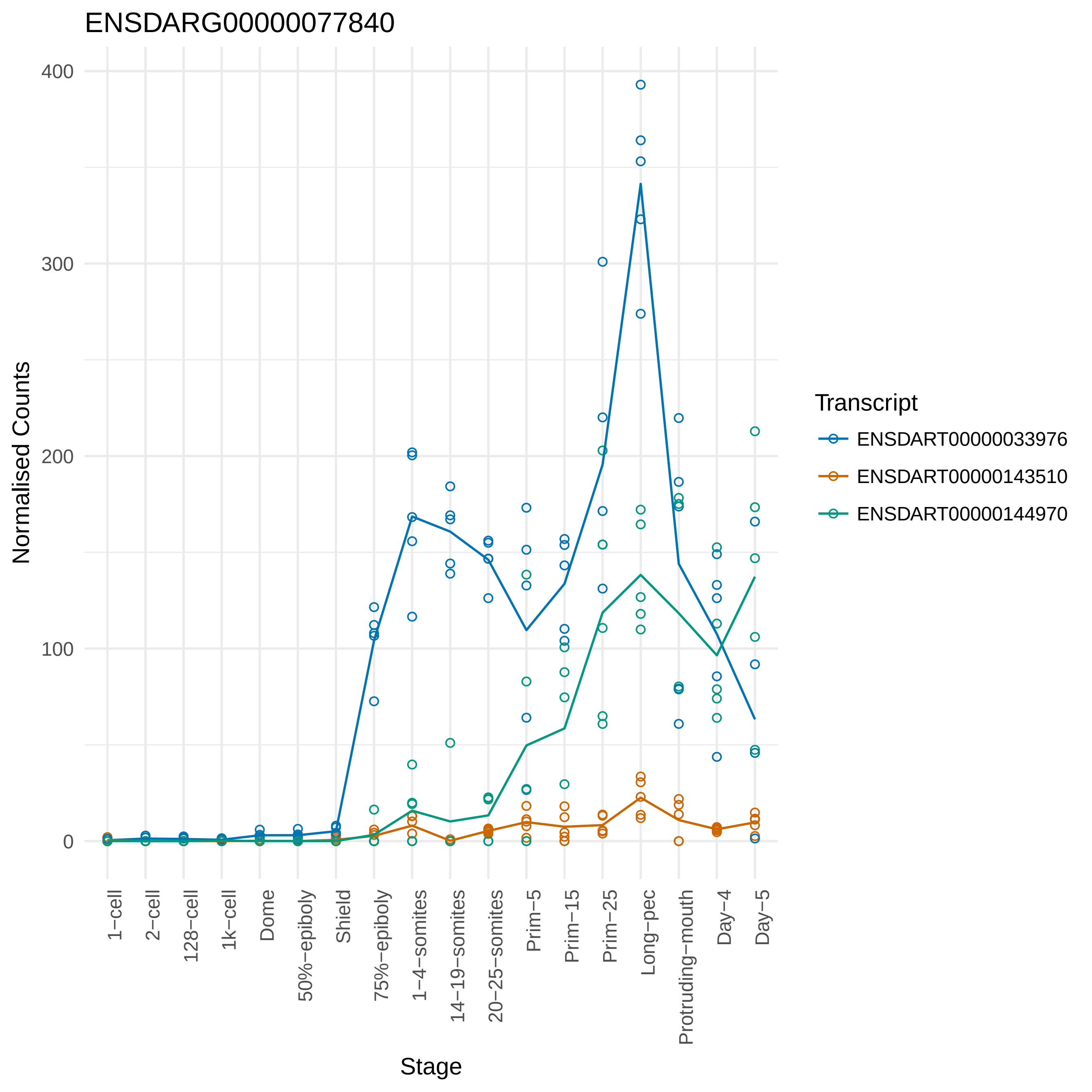

ENSDARG00000077840
Normalised Counts
400
300
200
100
0
1−cell
2−cell
128−cell
1k−cell
Dome
50%−epiboly
Shield
75%−epiboly
1−4−somites
14−19−somites
20−25−somites
Prim−5
Prim−15
Prim−25
Long−pec
Protruding−mouth
Day−4
Day−5
Stage
Transcript
ENSDART00000033976
ENSDART00000143510
ENSDART00000144970

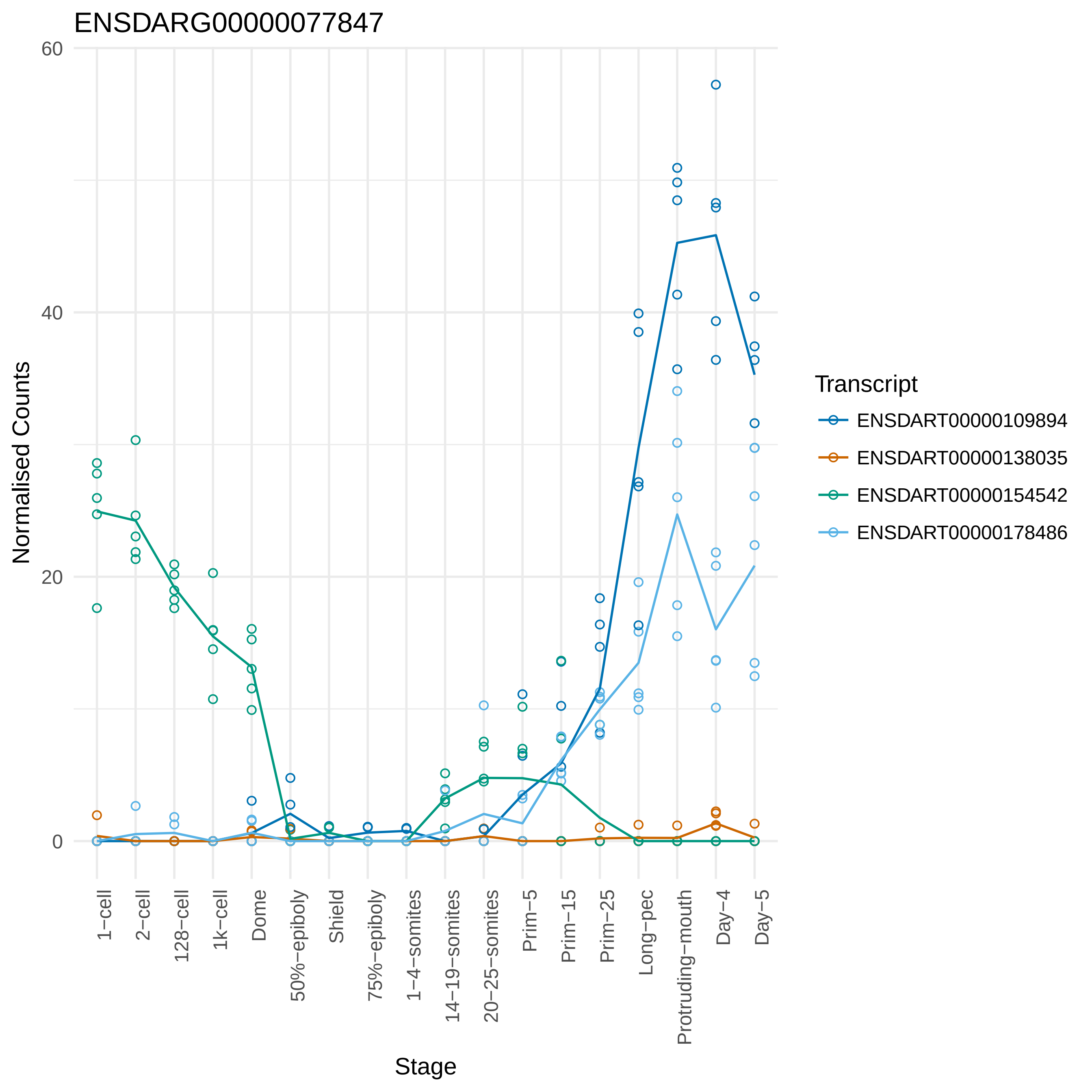
ENSDARG00000077847
Normalised Counts
0
20
40
60
1−cell
2−cell
128−cell
1k−cell
Dome
50%−epiboly
Shield
75%−epiboly
1−4−somites
14−19−somites
20−25−somites
Prim−5
Prim−15
Prim−25
Long−pec
Protruding−mouth
Day−4
Day−5
Stage
Transcript
ENSDART00000109894
ENSDART00000138035
ENSDART00000154542
ENSDART00000178486

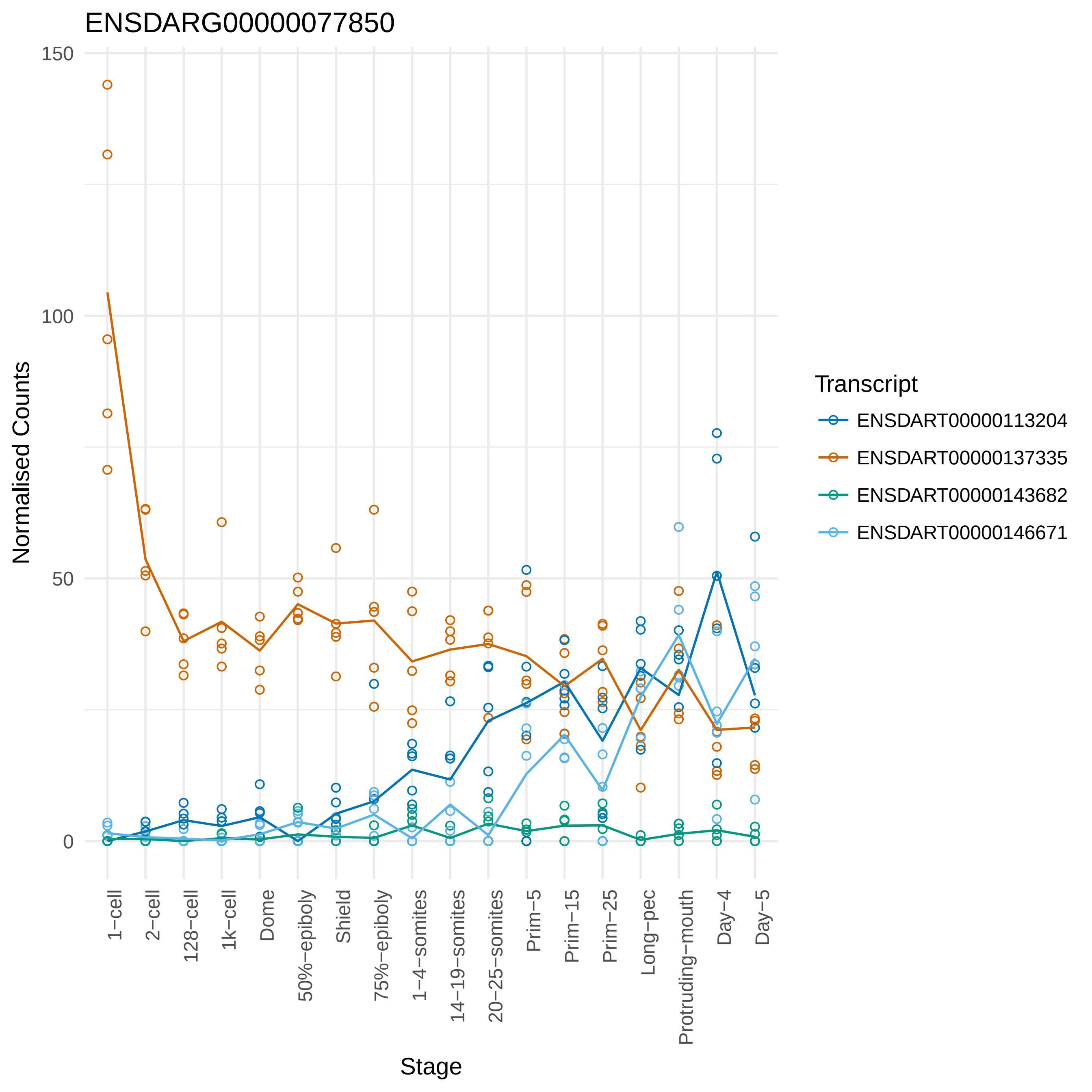
ENSDARG00000077850
Normalised Counts
150
100
50
0
1−cell
2−cell
128−cell
1k−cell
Dome
50%−epiboly
Shield
75%−epiboly
1−4−somites
14−19−somites
20−25−somites
Prim−5
Prim−15
Prim−25
Long−pec
Protruding−mouth
Day−4
Day−5
Stage
Transcript
ENSDART00000113204
ENSDART00000137335
ENSDART00000143682
ENSDART00000146671

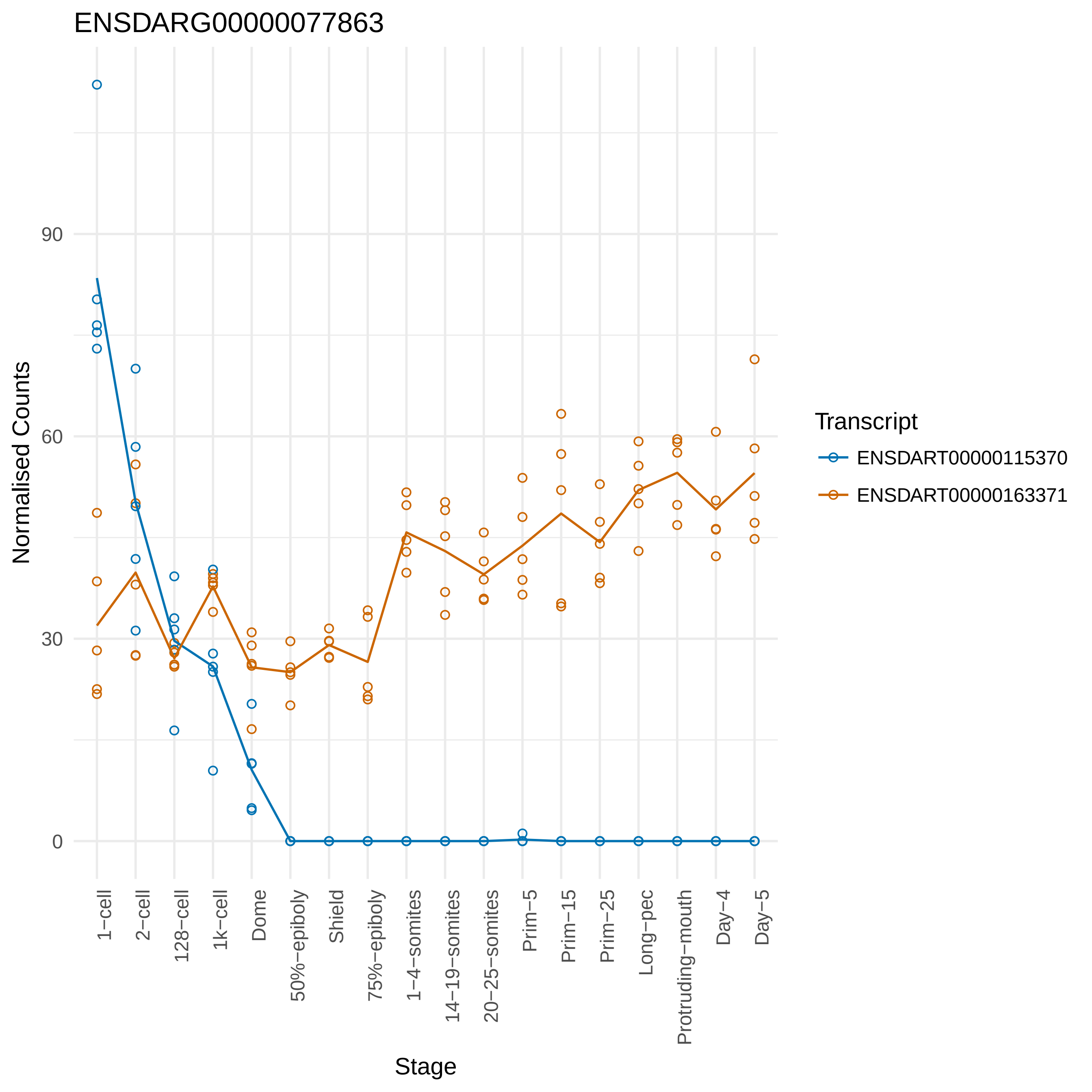

ENSDARG00000077863
Normalised Counts
0
30
60
90
1−cell
2−cell
128−cell
1k−cell
Dome
50%−epiboly
Shield
75%−epiboly
1−4−somites
14−19−somites
20−25−somites
Prim−5
Prim−15
Prim−25
Long−pec
Protruding−mouth
Day−4
Day−5
Stage
Transcript
ENSDART00000115370
ENSDART00000163371

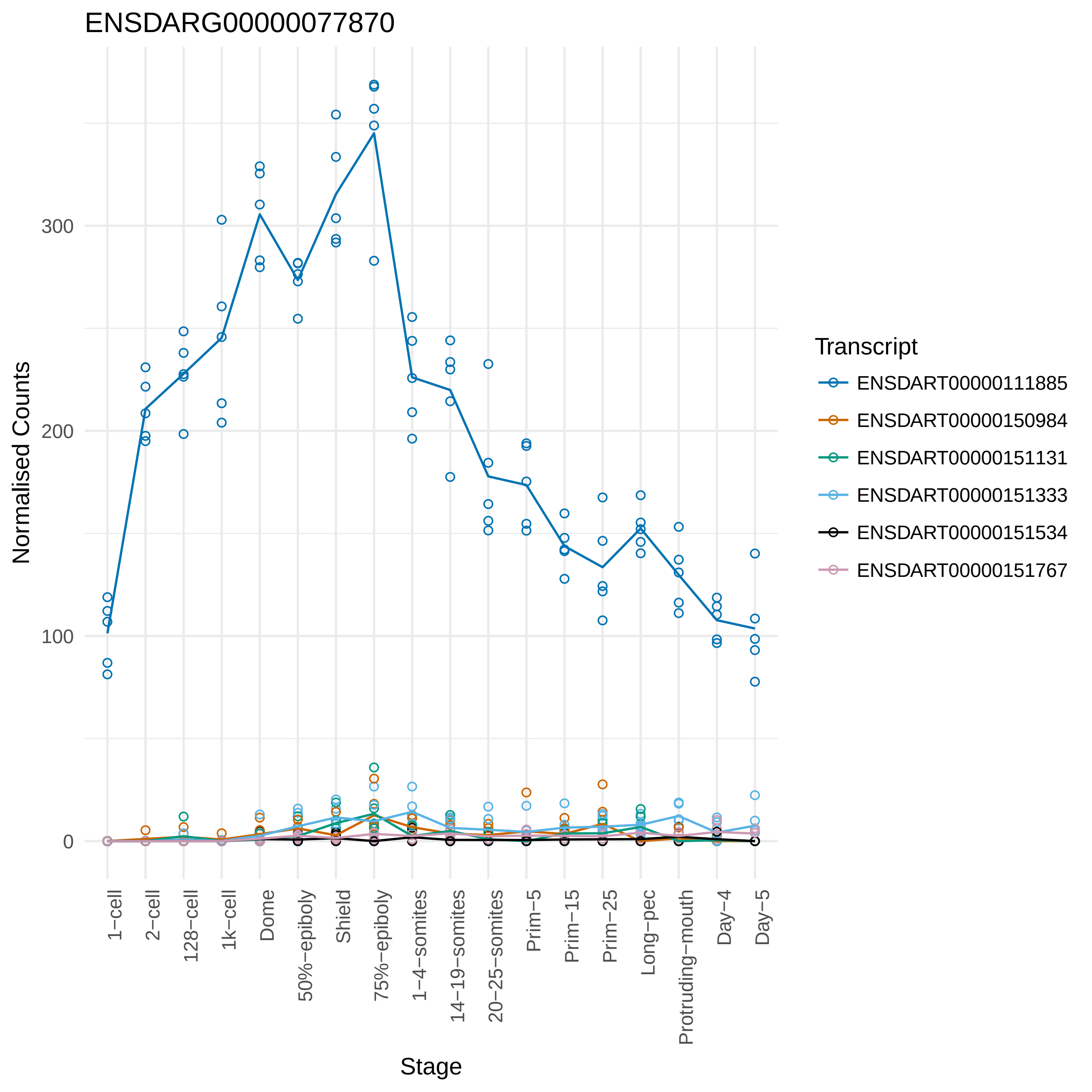
ENSDARG00000077870
Normalised Counts
300
200
100
0
1−cell
2−cell
128−cell
1k−cell
Dome
50%−epiboly
Shield
75%−epiboly
1−4−somites
14−19−somites
20−25−somites
Prim−5
Prim−15
Prim−25
Long−pec
Protruding−mouth
Day−4
Day−5
Stage
Transcript
ENSDART00000111885
ENSDART00000150984
ENSDART00000151131
ENSDART00000151333
ENSDART00000151534
ENSDART00000151767

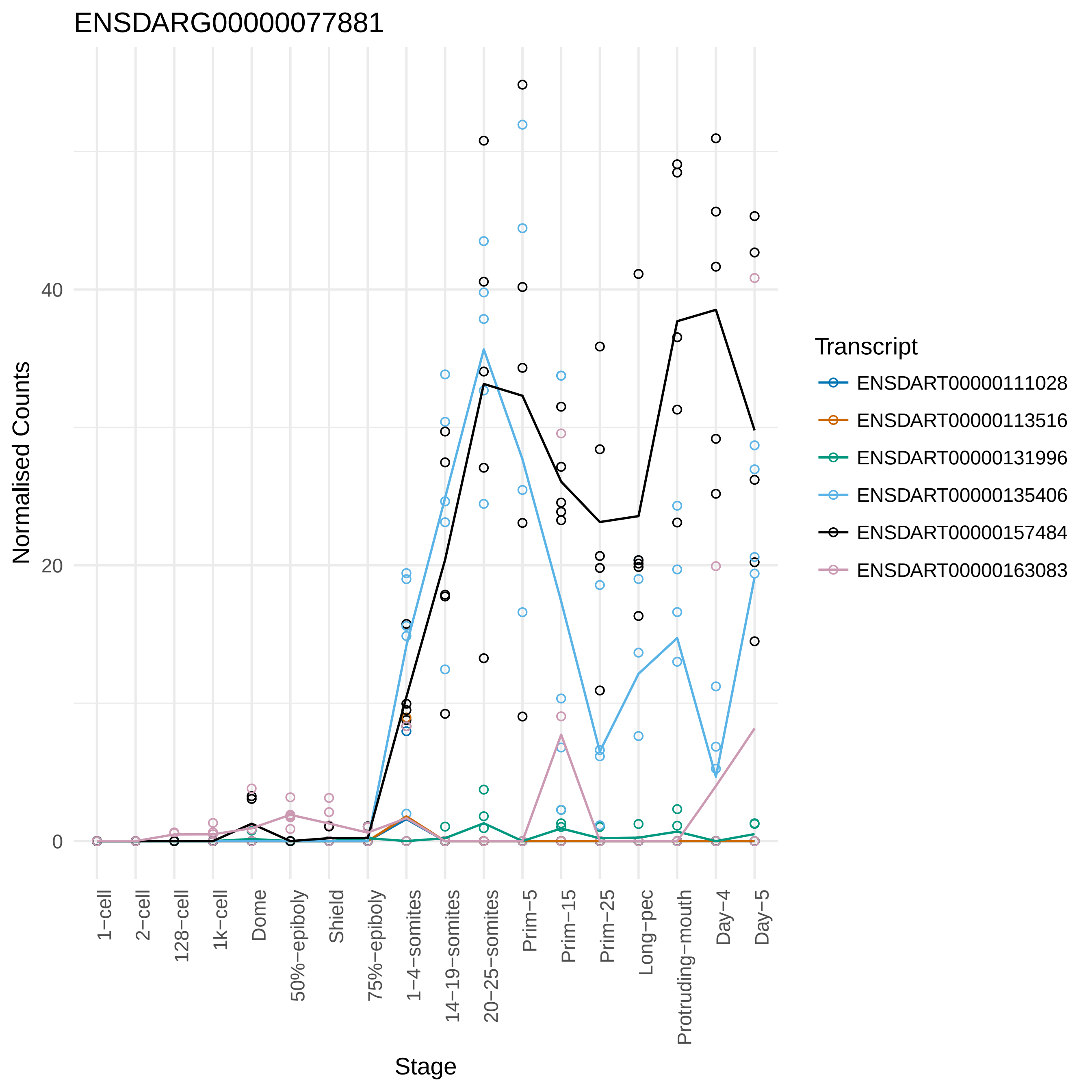

ENSDARG00000077881
Normalised Counts
0
20
40
Stage
1−cell
2−cell
128−cell
1k−cell
Dome
50%−epiboly
Shield
75%−epiboly
1−4−somites
14−19−somites
20−25−somites
Prim−5
Prim−15
Prim−25
Long−pec
Protruding−mouth
Day−4
Day−5
Transcript
ENSDART00000111028
ENSDART00000113516
ENSDART00000131996
ENSDART00000135406
ENSDART00000157484
ENSDART00000163083

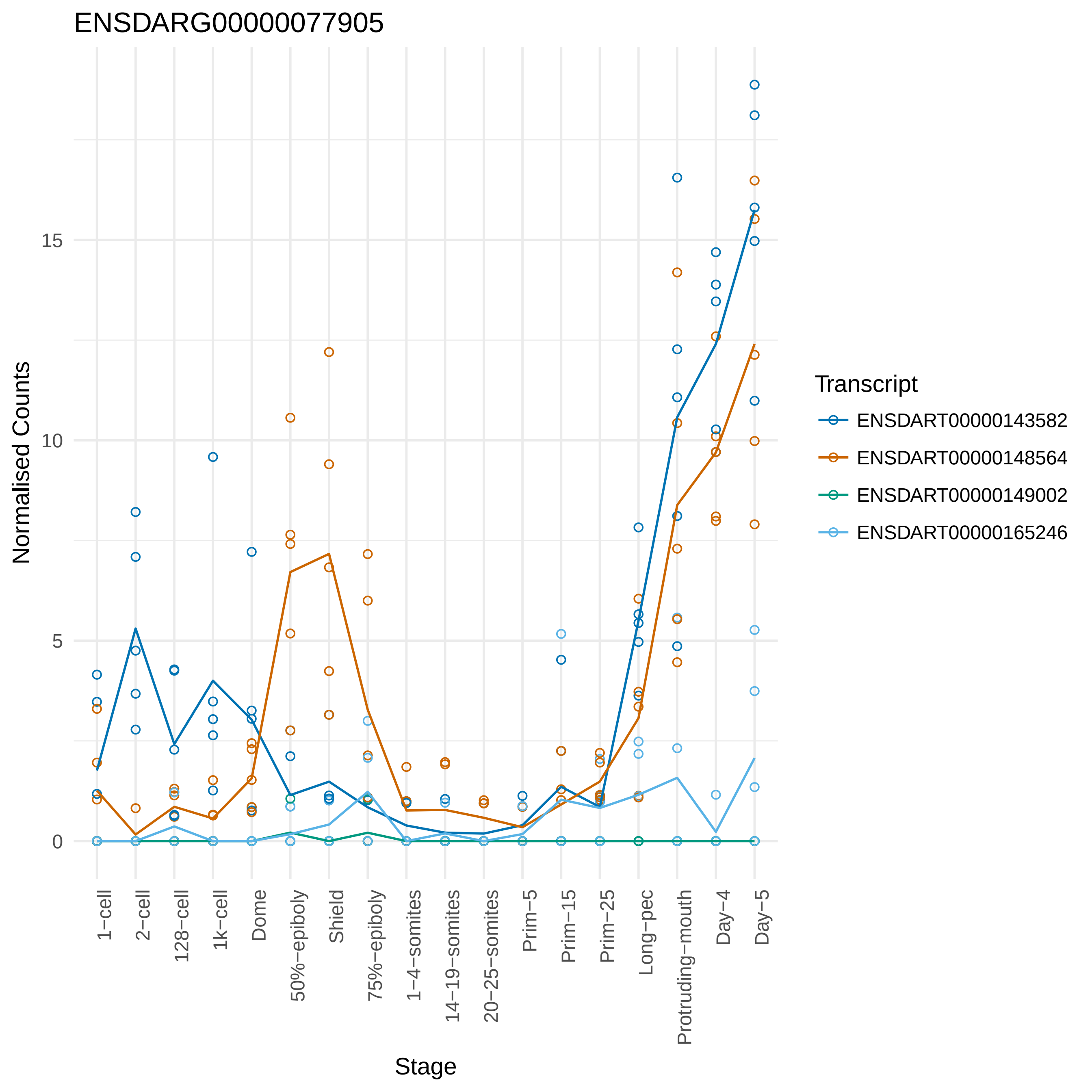

ENSDARG00000077905
Normalised Counts
15
10
5
0
1−cell
2−cell
128−cell
1k−cell
Dome
50%−epiboly
Shield
75%−epiboly
1−4−somites
14−19−somites
20−25−somites
Prim−5
Prim−15
Prim−25
Long−pec
Protruding−mouth
Day−4
Day−5
Stage
Transcript
ENSDART00000143582
ENSDART00000148564
ENSDART00000149002
ENSDART00000165246

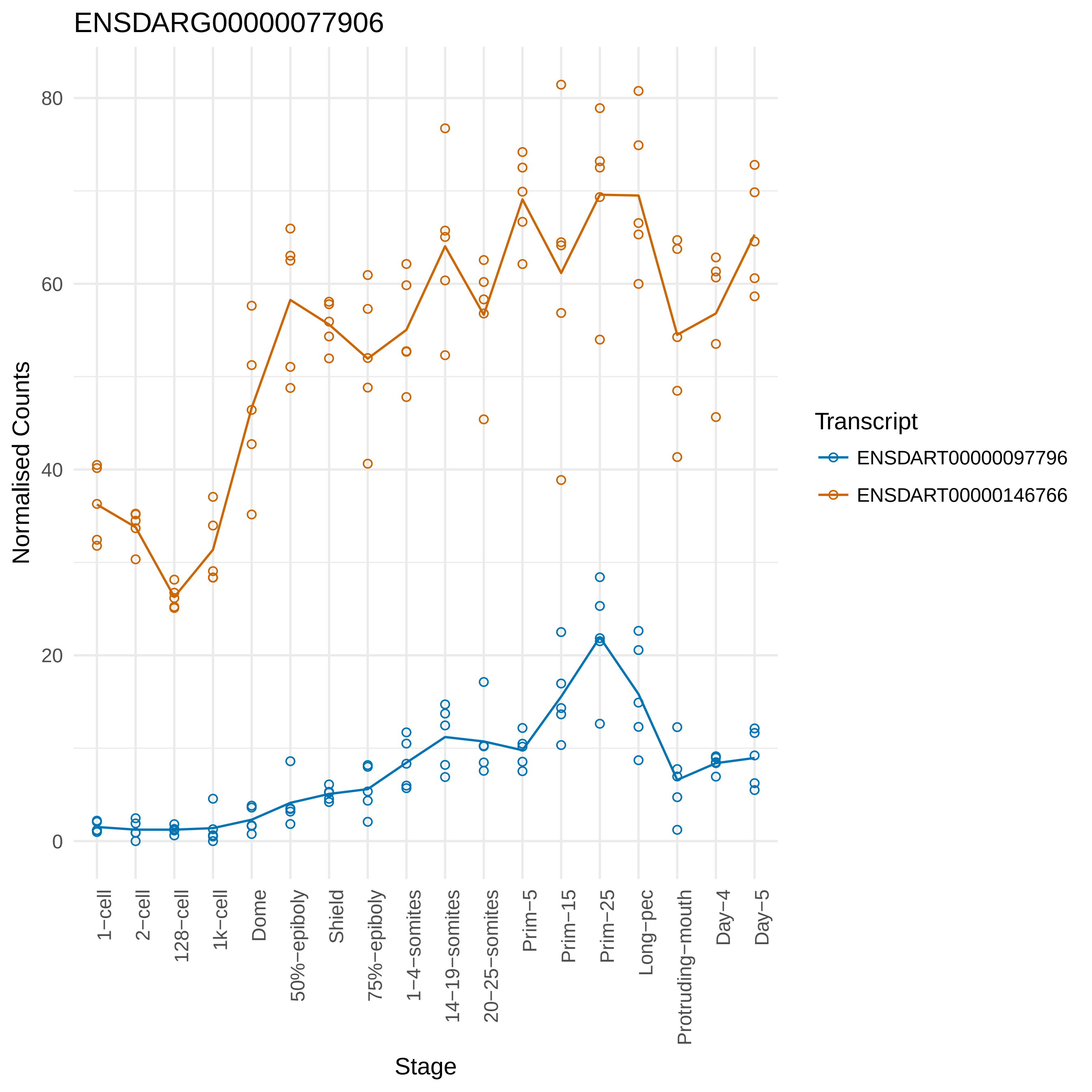

ENSDARG00000077906
Normalised Counts
80
60
40
20
0
1−cell
2−cell
128−cell
1k−cell
Dome
50%−epiboly
Shield
75%−epiboly
1−4−somites
14−19−somites
20−25−somites
Prim−5
Prim−15
Prim−25
Long−pec
Protruding−mouth
Day−4
Day−5
Stage
Transcript
ENSDART00000097796
ENSDART00000146766

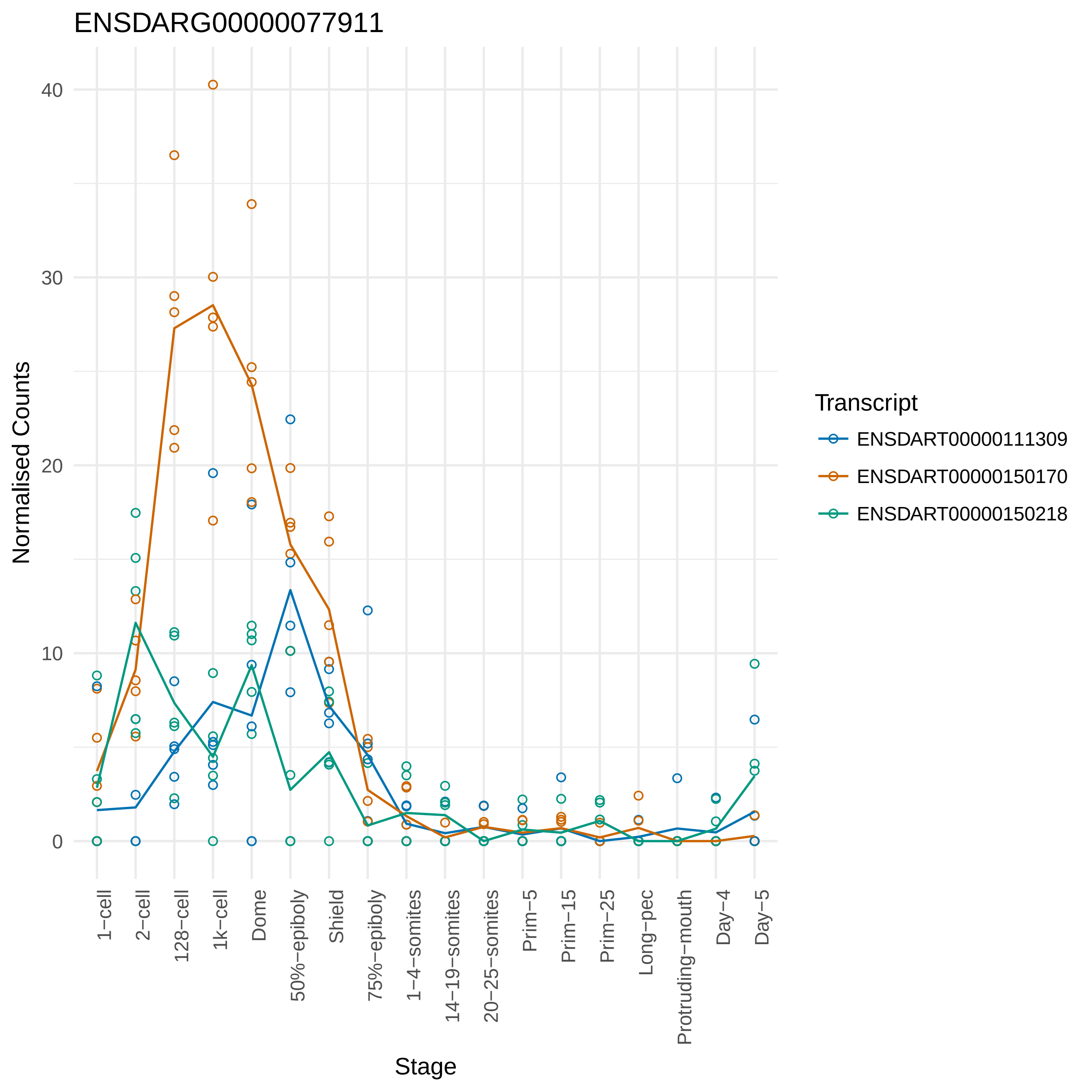
ENSDARG00000077911
Normalised Counts
0
10
20
30
40
1−cell
2−cell
128−cell
1k−cell
Dome
50%−epiboly
Shield
75%−epiboly
1−4−somites
14−19−somites
20−25−somites
Prim−5
Prim−15
Prim−25
Long−pec
Protruding−mouth
Day−4
Day−5
Stage
Transcript
ENSDART00000111309
ENSDART00000150170
ENSDART00000150218

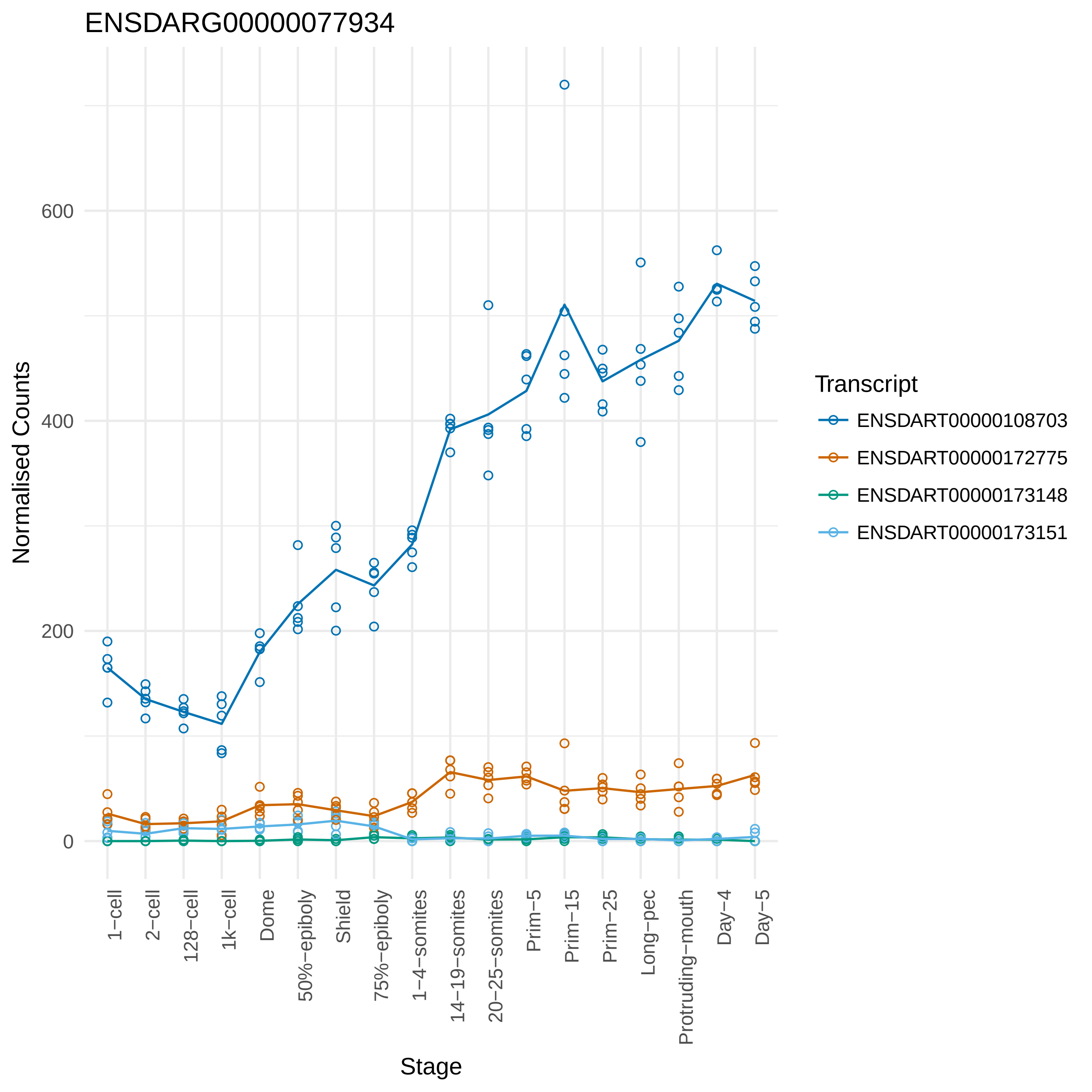

ENSDARG00000077934
Normalised Counts
600
400
200
0
1−cell
2−cell
128−cell
1k−cell
Dome
50%−epiboly
Shield
75%−epiboly
1−4−somites
14−19−somites
20−25−somites
Prim−5
Prim−15
Prim−25
Long−pec
Protruding−mouth
Day−4
Day−5
Stage
Transcript
ENSDART00000108703
ENSDART00000172775
ENSDART00000173148
ENSDART00000173151

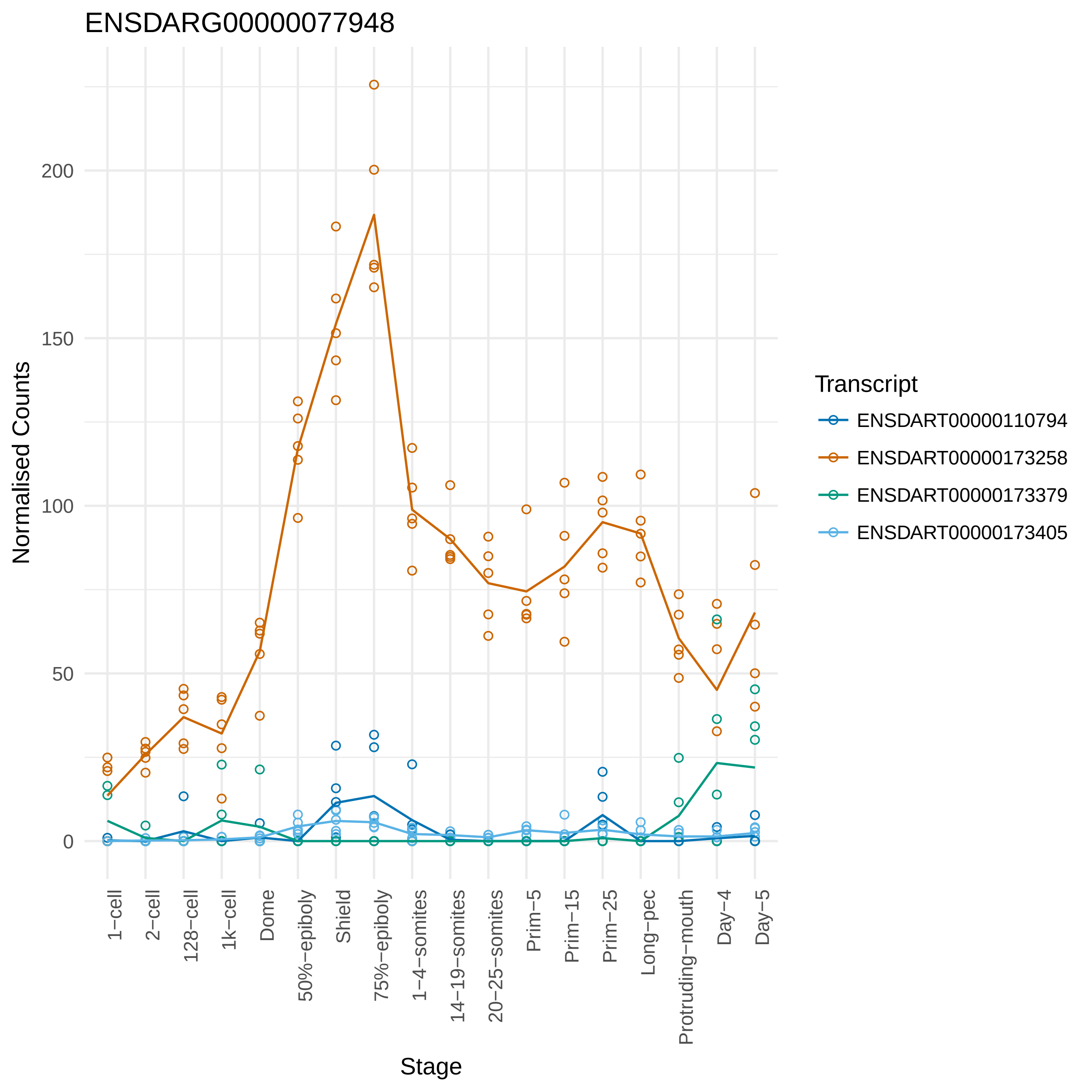

ENSDARG00000077948
Normalised Counts
200
150
100
50
0
1−cell
2−cell
128−cell
1k−cell
Dome
50%−epiboly
Shield
75%−epiboly
1−4−somites
14−19−somites
20−25−somites
Prim−5
Prim−15
Prim−25
Long−pec
Protruding−mouth
Day−4
Day−5
Stage
Transcript
ENSDART00000110794
ENSDART00000173258
ENSDART00000173379
ENSDART00000173405

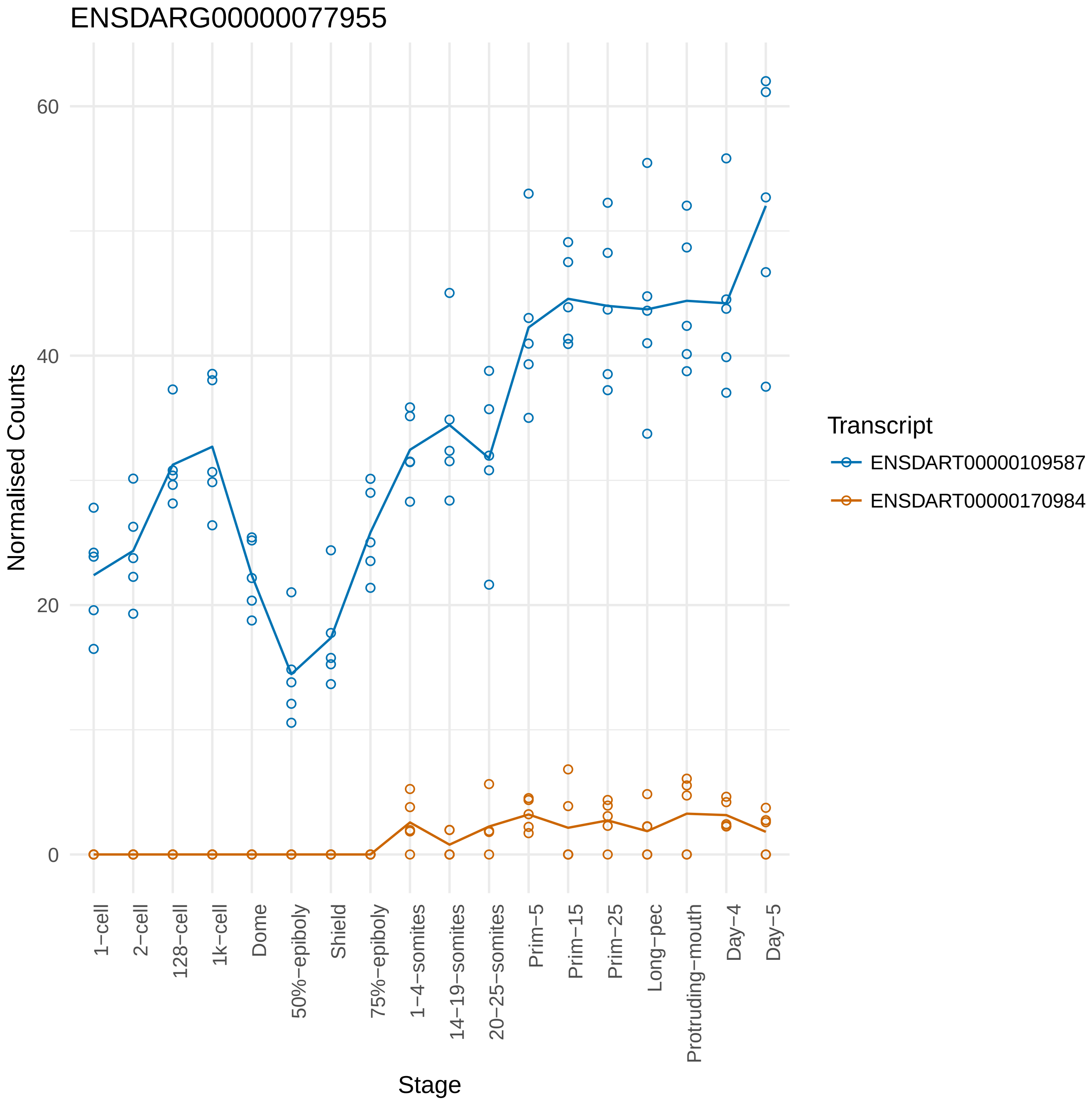

ENSDARG00000077955
Normalised Counts
60
40
20
0
1−cell
2−cell
128−cell
1k−cell
Dome
50%−epiboly
Shield
75%−epiboly
1−4−somites
14−19−somites
20−25−somites
Prim−5
Prim−15
Prim−25
Long−pec
Protruding−mouth
Day−4
Day−5
Stage
Transcript
ENSDART00000109587
ENSDART00000170984

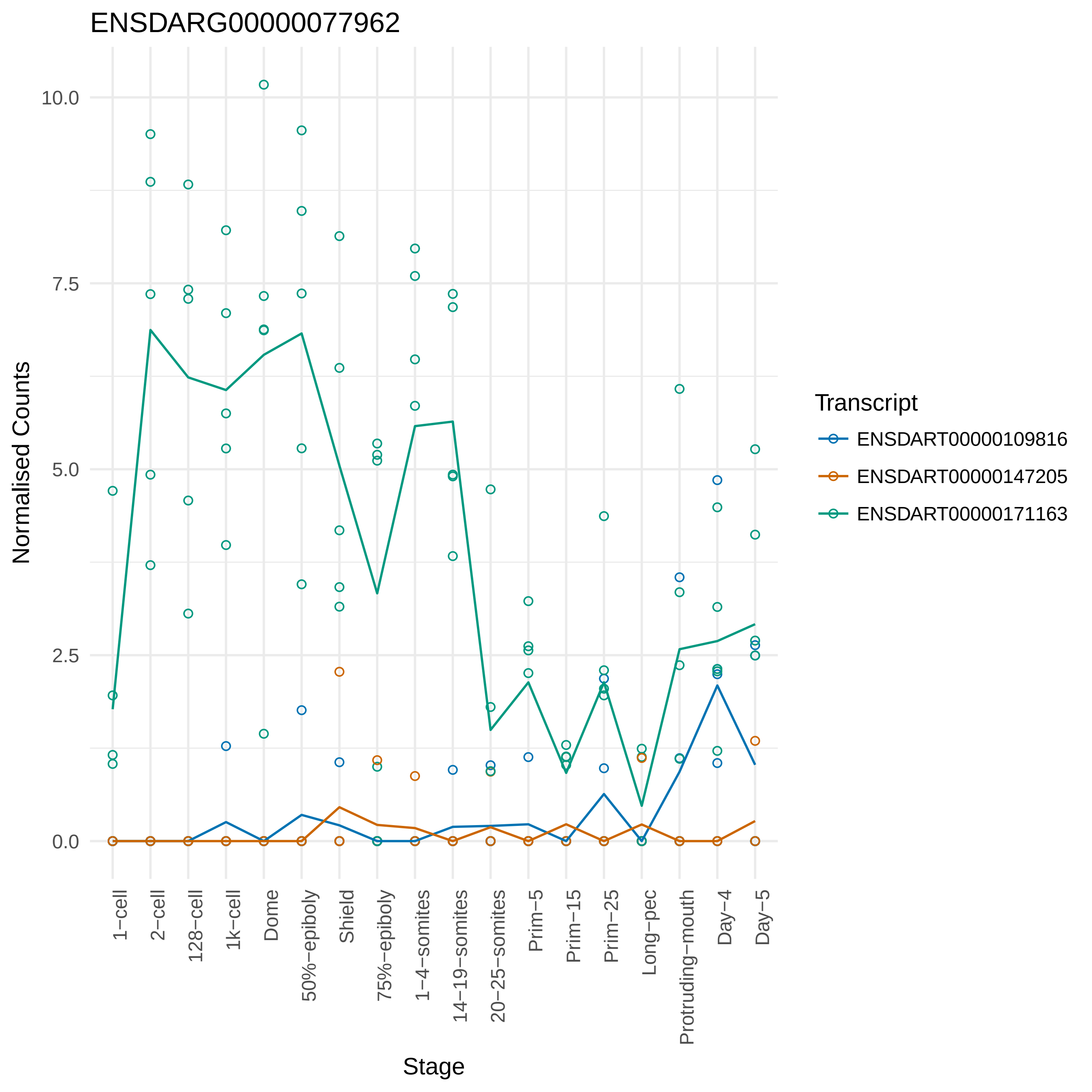

ENSDARG00000077962
Normalised Counts
10.0
7.5
5.0
2.5
0.0
1−cell
2−cell
128−cell
1k−cell
Dome
50%−epiboly
Shield
75%−epiboly
1−4−somites
14−19−somites
20−25−somites
Prim−5
Prim−15
Prim−25
Long−pec
Protruding−mouth
Day−4
Day−5
Stage
Transcript
ENSDART00000109816
ENSDART00000147205
ENSDART00000171163

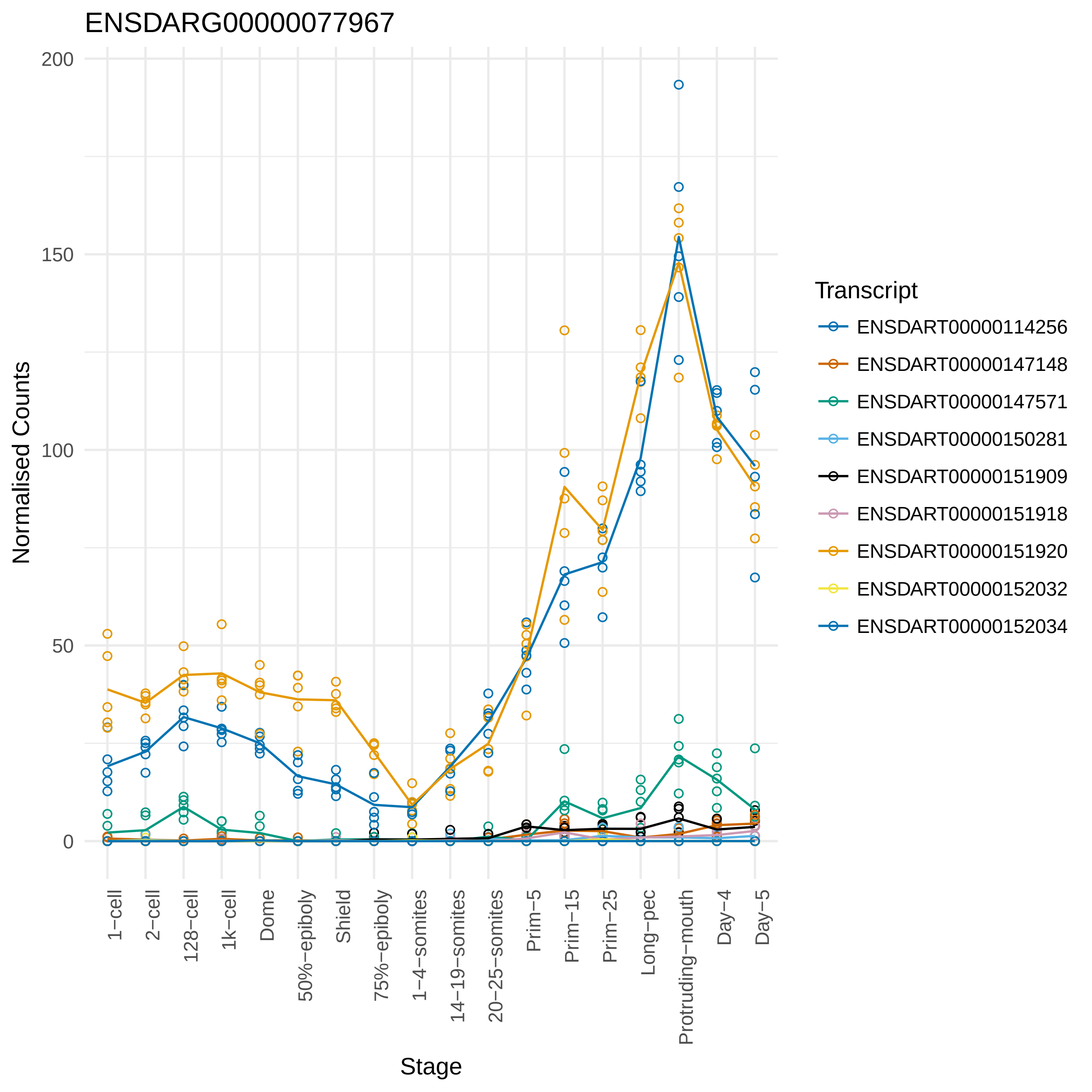

ENSDARG00000077967
Normalised Counts
200
150
100
50
0
1−cell
2−cell
128−cell
1k−cell
Dome
50%−epiboly
Shield
75%−epiboly
1−4−somites
14−19−somites
20−25−somites
Prim−5
Prim−15
Prim−25
Long−pec
Protruding−mouth
Day−4
Day−5
Stage
Transcript
ENSDART00000114256
ENSDART00000147148
ENSDART00000147571
ENSDART00000150281
ENSDART00000151909
ENSDART00000151918
ENSDART00000151920
ENSDART00000152032
ENSDART00000152034

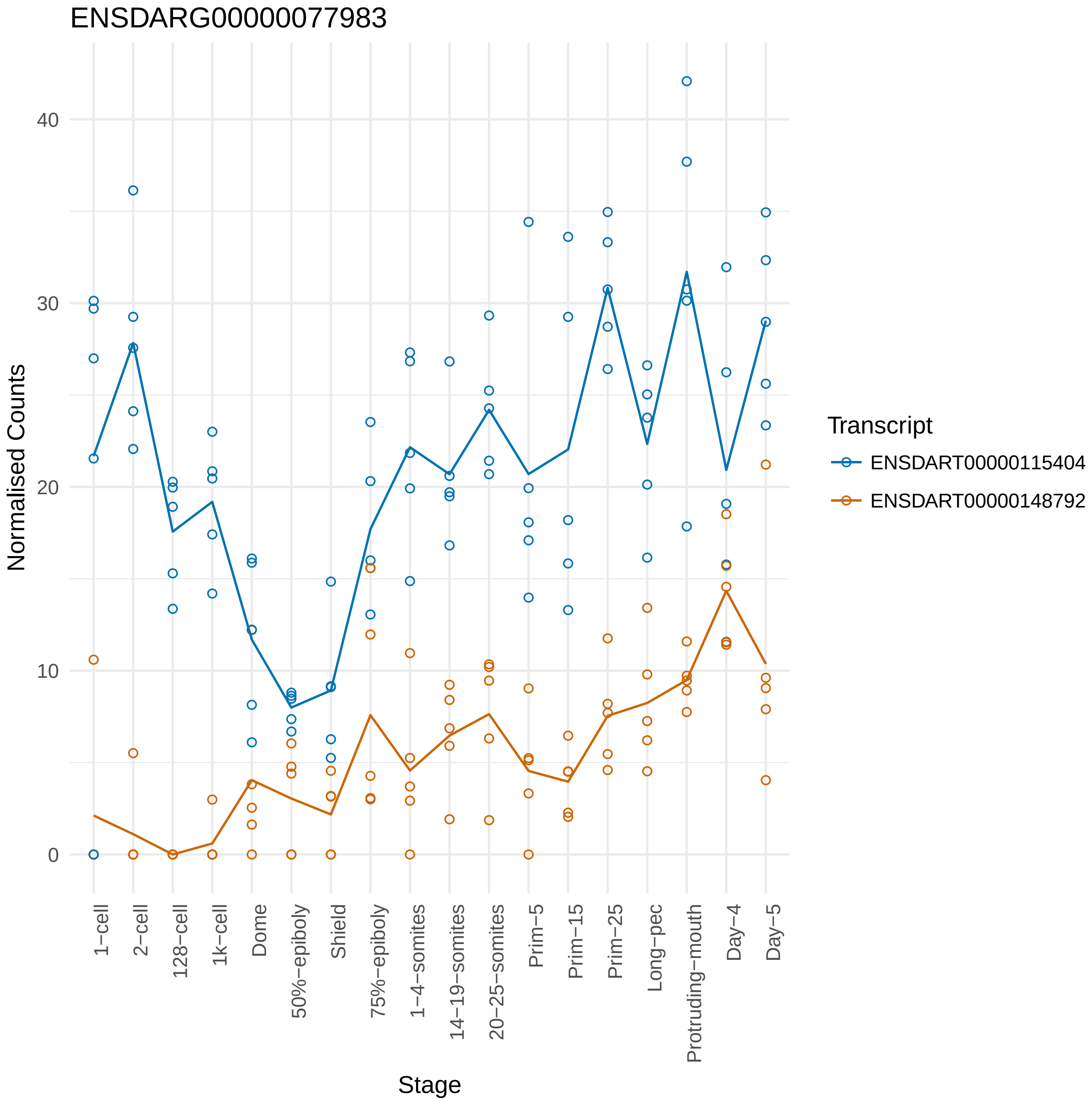

ENSDARG00000077983
Normalised Counts
40
30
20
10
0
1−cell
2−cell
128−cell
1k−cell
Dome
50%−epiboly
Shield
75%−epiboly
1−4−somites
14−19−somites
20−25−somites
Prim−5
Prim−15
Prim−25
Long−pec
Protruding−mouth
Day−4
Day−5
Stage
Transcript
ENSDART00000115404
ENSDART00000148792

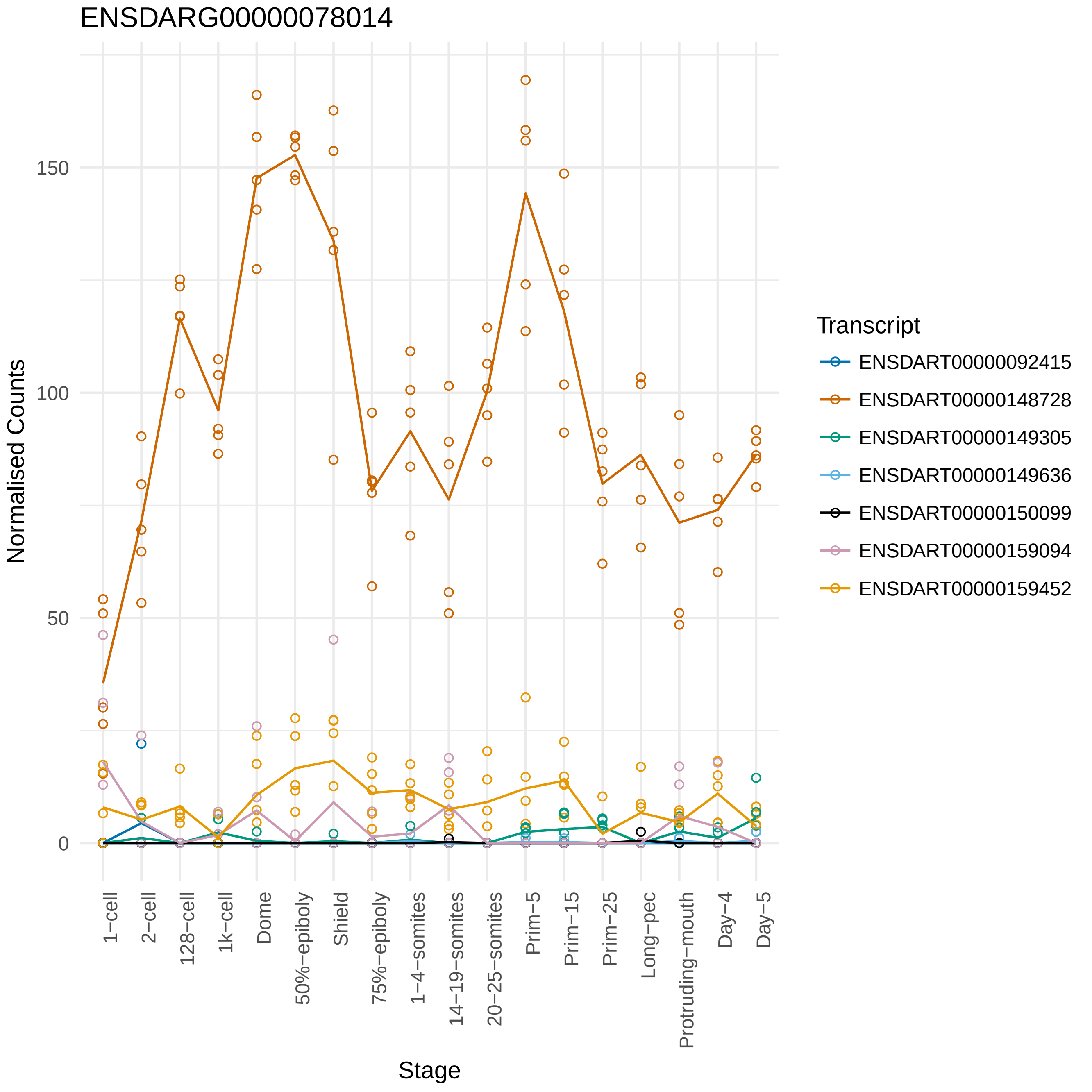
ENSDARG00000078014
Normalised Counts
0
50
100
150
1−cell
2−cell
128−cell
1k−cell
Dome
50%−epiboly
Shield
75%−epiboly
1−4−somites
14−19−somites
20−25−somites
Prim−5
Prim−15
Prim−25
Long−pec
Protruding−mouth
Day−4
Day−5
Stage
Transcript
ENSDART00000092415
ENSDART00000148728
ENSDART00000149305
ENSDART00000149636
ENSDART00000150099
ENSDART00000159094
ENSDART00000159452

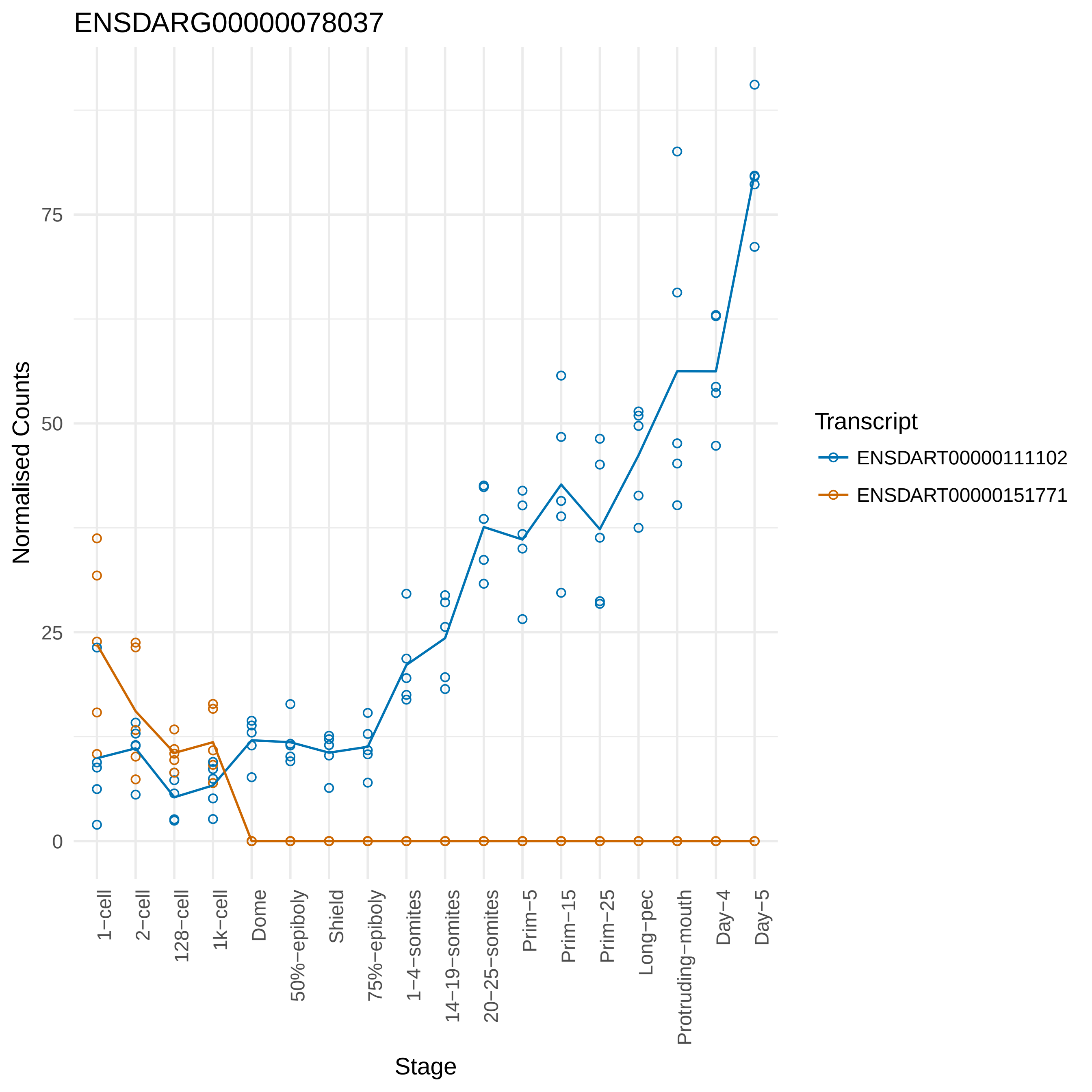

ENSDARG00000078037
Normalised Counts
0
25
50
75
1−cell
2−cell
128−cell
1k−cell
Dome
50%−epiboly
Shield
75%−epiboly
1−4−somites
14−19−somites
20−25−somites
Prim−5
Prim−15
Prim−25
Long−pec
Protruding−mouth
Day−4
Day−5
Stage
Transcript
ENSDART00000111102
ENSDART00000151771

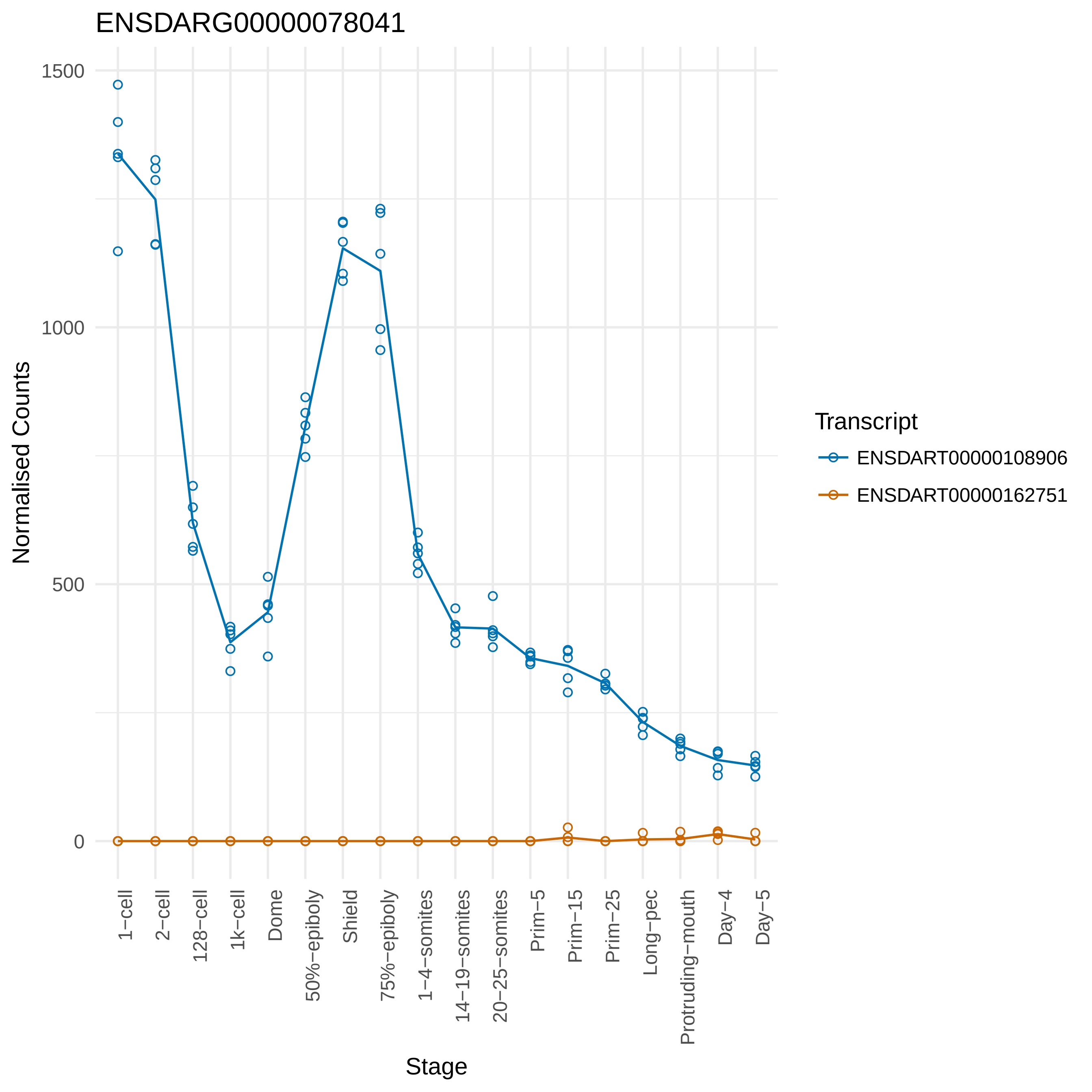
ENSDARG00000078041
Normalised Counts
1500
1000
500
0
1−cell
2−cell
128−cell
1k−cell
Dome
50%−epiboly
Shield
75%−epiboly
1−4−somites
14−19−somites
20−25−somites
Prim−5
Prim−15
Prim−25
Long−pec
Protruding−mouth
Day−4
Day−5
Stage
Transcript
ENSDART00000108906
ENSDART00000162751

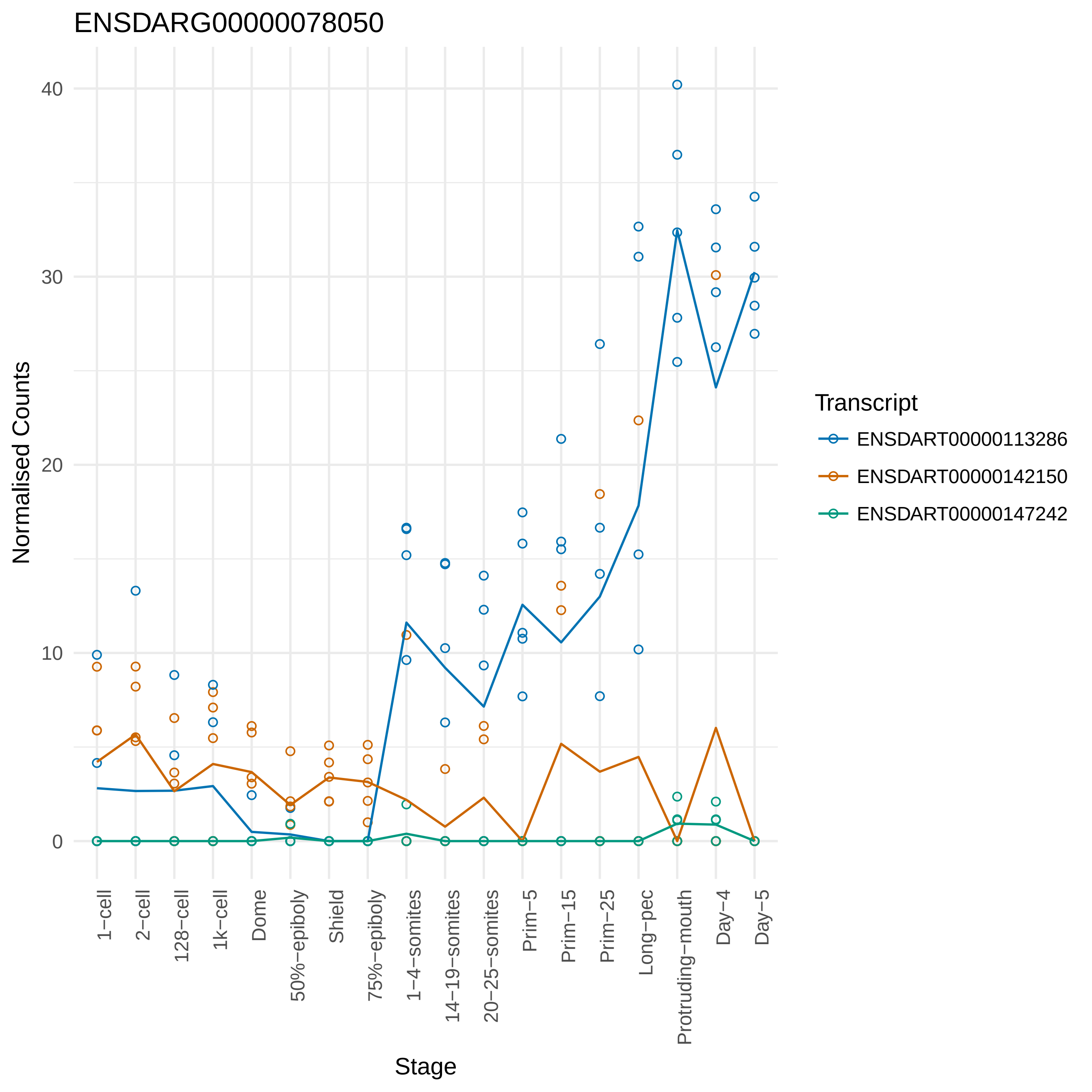
ENSDARG00000078050
Normalised Counts
40
30
20
10
0
1−cell
2−cell
128−cell
1k−cell
Dome
50%−epiboly
Shield
75%−epiboly
1−4−somites
14−19−somites
20−25−somites
Prim−5
Prim−15
Prim−25
Long−pec
Protruding−mouth
Day−4
Day−5
Stage
Transcript
ENSDART00000113286
ENSDART00000142150
ENSDART00000147242

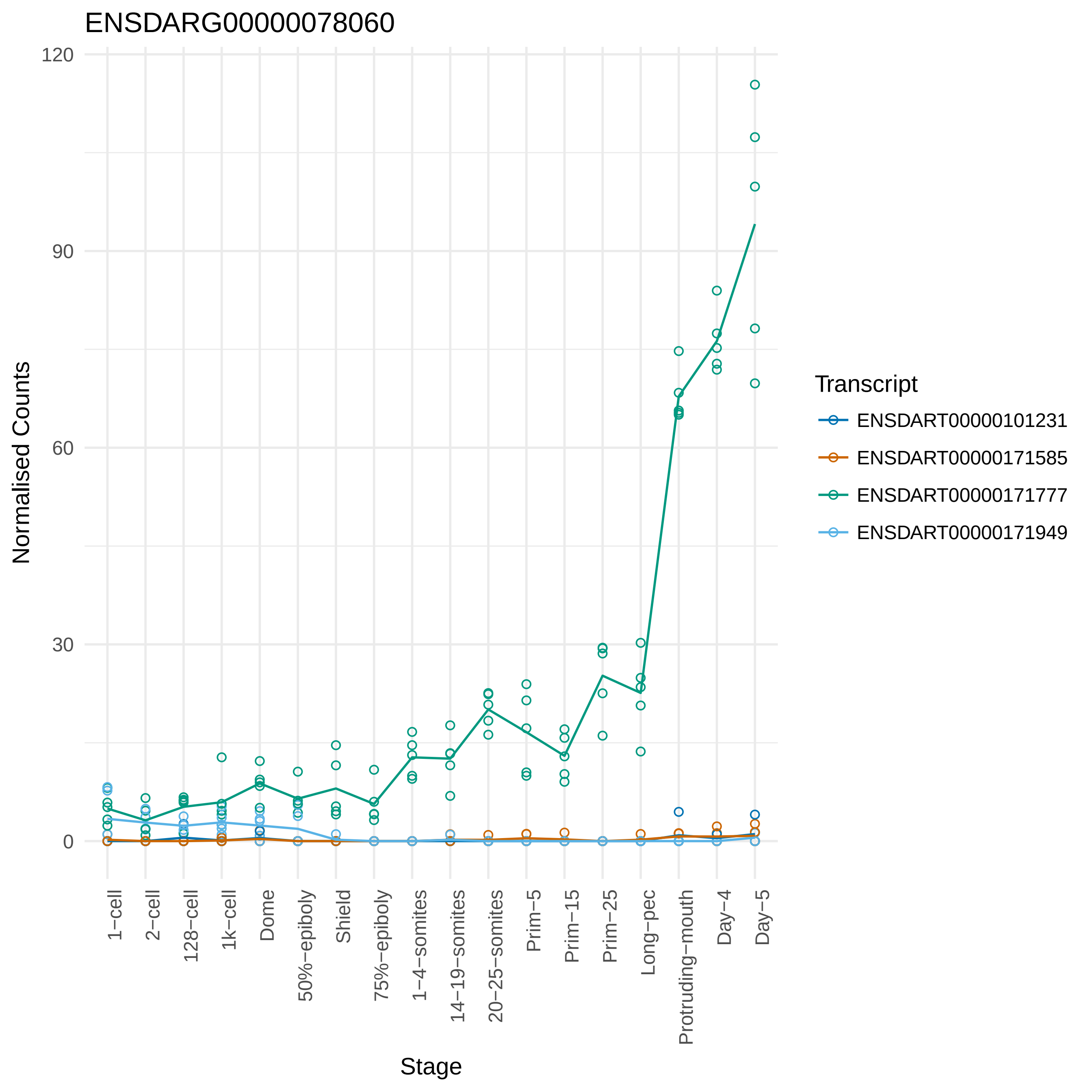
ENSDARG00000078060
Normalised Counts
120
90
60
30
0
1−cell
2−cell
128−cell
1k−cell
Dome
50%−epiboly
Shield
75%−epiboly
1−4−somites
14−19−somites
20−25−somites
Prim−5
Prim−15
Prim−25
Long−pec
Protruding−mouth
Day−4
Day−5
Stage
Transcript
ENSDART00000101231
ENSDART00000171585
ENSDART00000171777
ENSDART00000171949

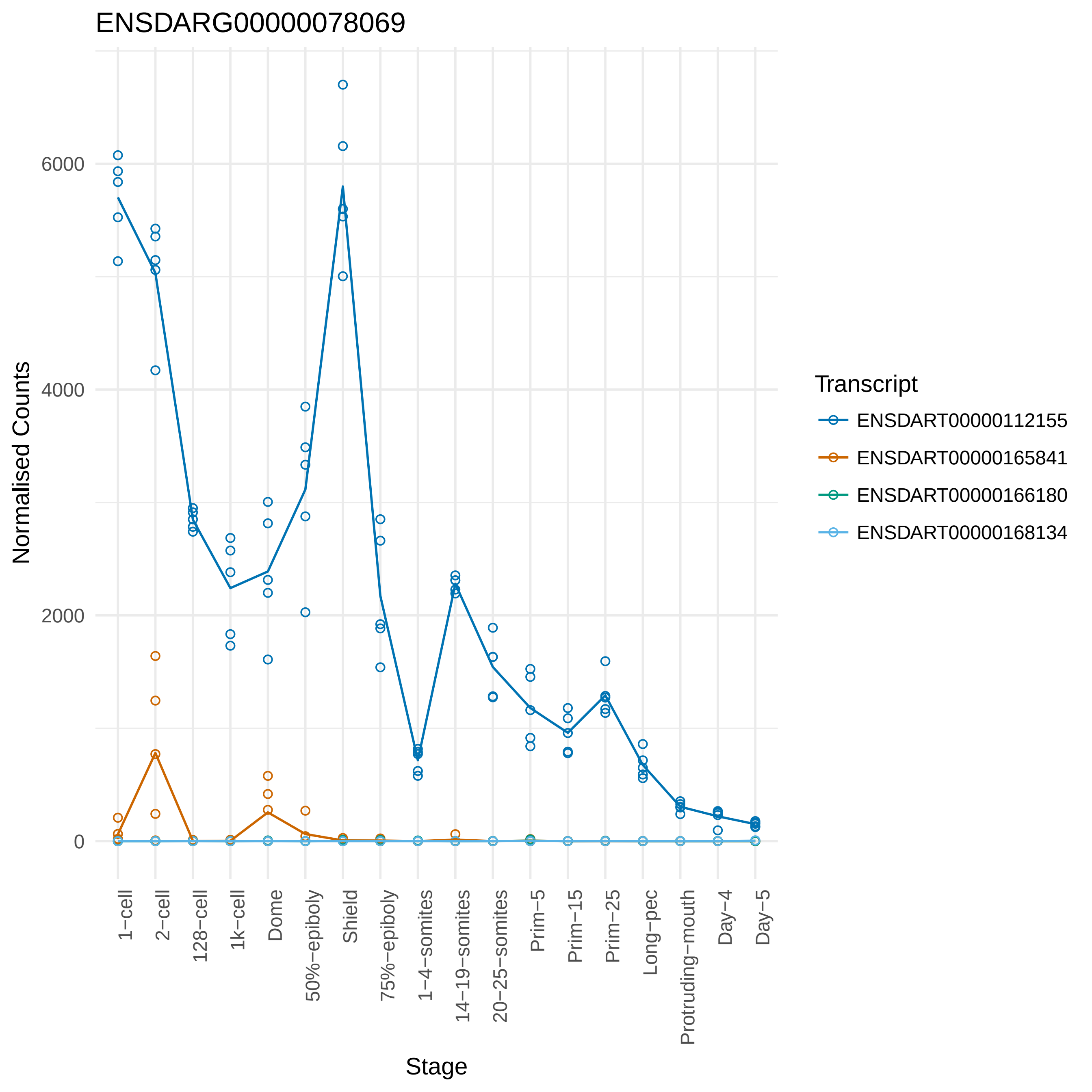
ENSDARG00000078069
Normalised Counts
6000
4000
2000
0
1−cell
2−cell
128−cell
1k−cell
Dome
50%−epiboly
Shield
75%−epiboly
1−4−somites
14−19−somites
20−25−somites
Prim−5
Prim−15
Prim−25
Long−pec
Protruding−mouth
Day−4
Day−5
Stage
Transcript
ENSDART00000112155
ENSDART00000165841
ENSDART00000166180
ENSDART00000168134

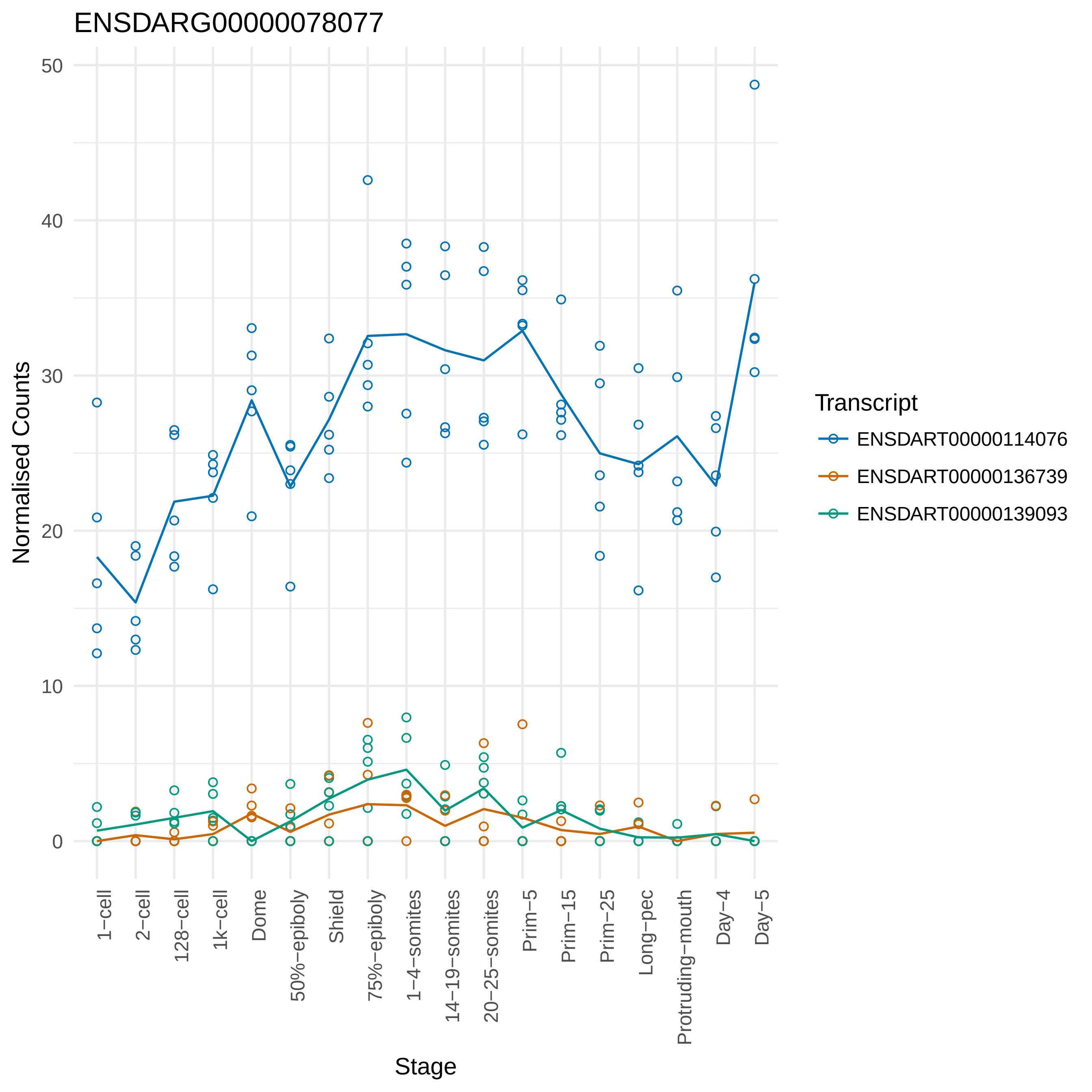

ENSDARG00000078077
Normalised Counts
50
40
30
20
10
0
1−cell
2−cell
128−cell
1k−cell
Dome
50%−epiboly
Shield
75%−epiboly
1−4−somites
14−19−somites
20−25−somites
Prim−5
Prim−15
Prim−25
Long−pec
Protruding−mouth
Day−4
Day−5
Stage
Transcript
ENSDART00000114076
ENSDART00000136739
ENSDART00000139093

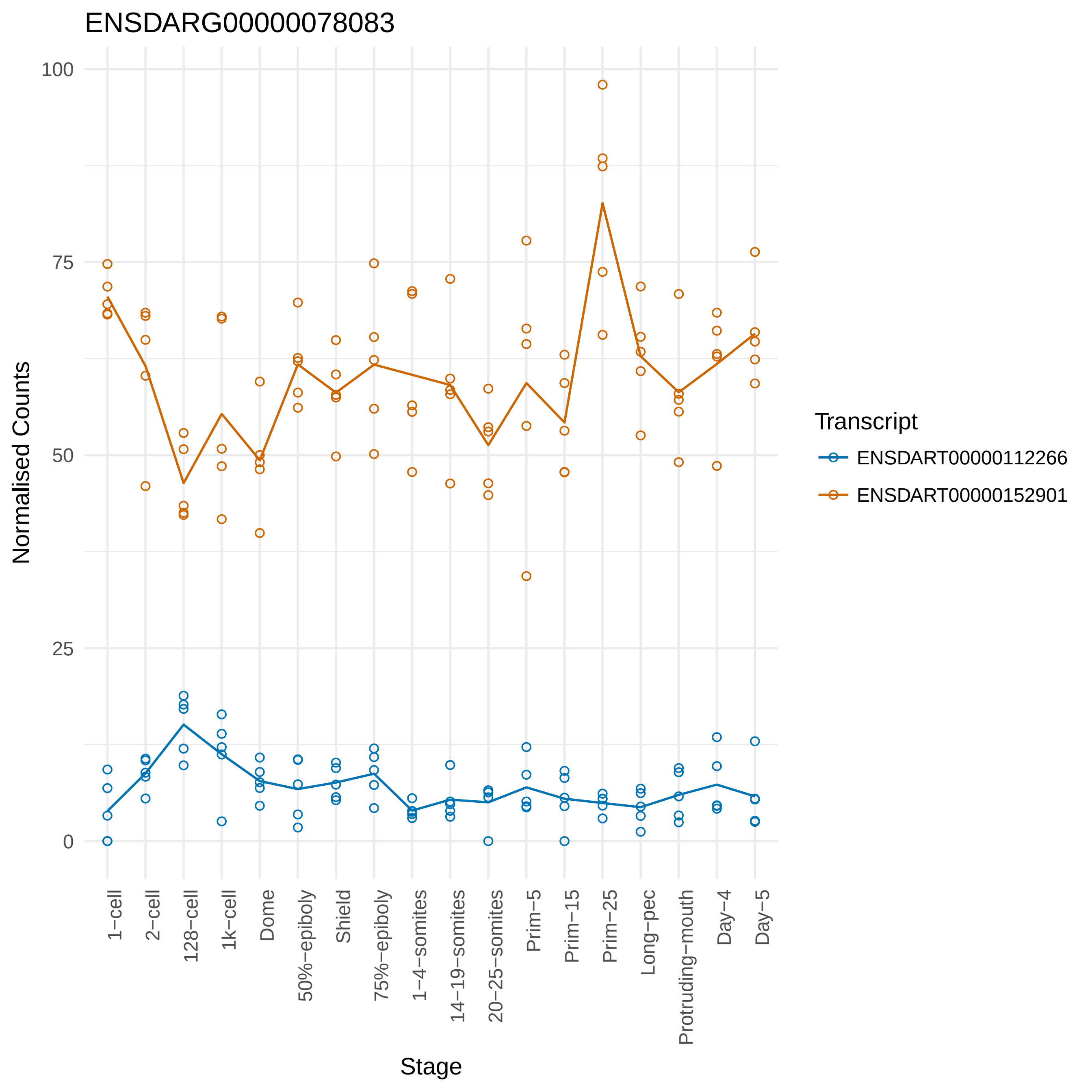

ENSDARG00000078083
Normalised Counts
100
75
50
25
0
1−cell
2−cell
128−cell
1k−cell
Dome
50%−epiboly
Shield
75%−epiboly
1−4−somites
14−19−somites
20−25−somites
Prim−5
Prim−15
Prim−25
Long−pec
Protruding−mouth
Day−4
Day−5
Stage
Transcript
ENSDART00000112266
ENSDART00000152901

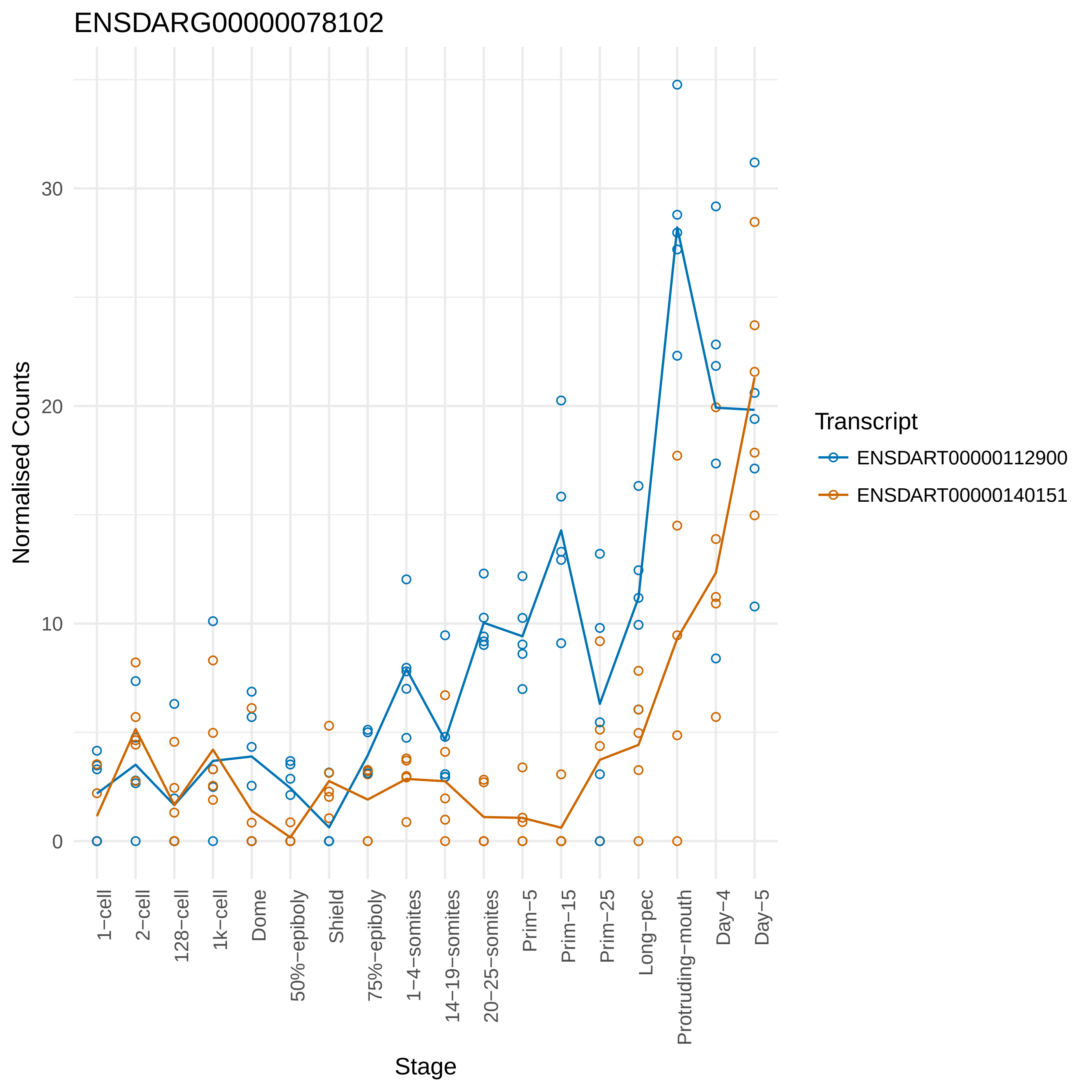

ENSDARG00000078102
Normalised Counts
0
10
20
30
1−cell
2−cell
128−cell
1k−cell
Dome
50%−epiboly
Shield
75%−epiboly
1−4−somites
14−19−somites
20−25−somites
Prim−5
Prim−15
Prim−25
Long−pec
Protruding−mouth
Day−4
Day−5
Stage
Transcript
ENSDART00000112900
ENSDART00000140151

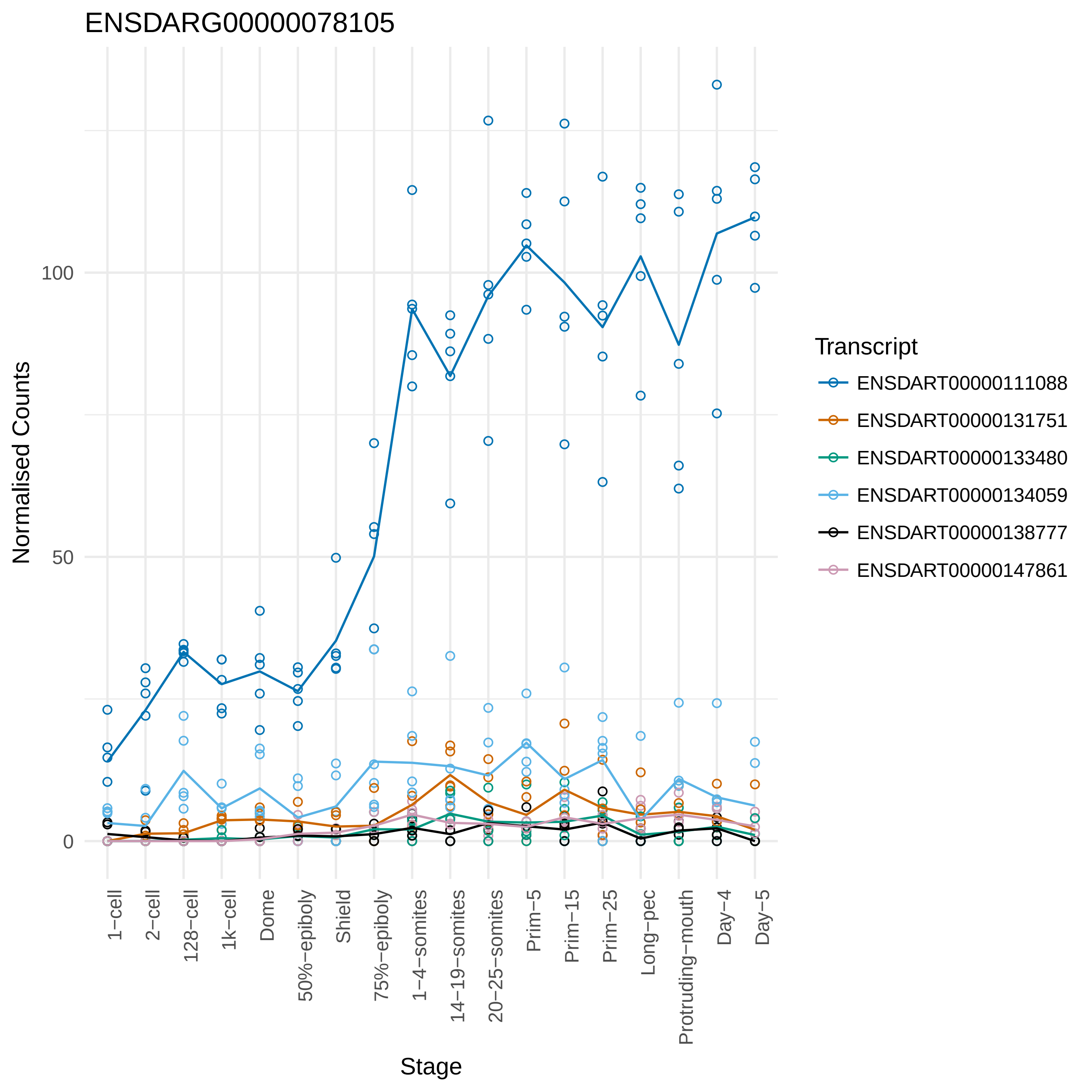

ENSDARG00000078105
Normalised Counts
0
50
100
1−cell
2−cell
128−cell
1k−cell
Dome
50%−epiboly
Shield
75%−epiboly
1−4−somites
14−19−somites
20−25−somites
Prim−5
Prim−15
Prim−25
Long−pec
Protruding−mouth
Day−4
Day−5
Stage
Transcript
ENSDART00000111088
ENSDART00000131751
ENSDART00000133480
ENSDART00000134059
ENSDART00000138777
ENSDART00000147861

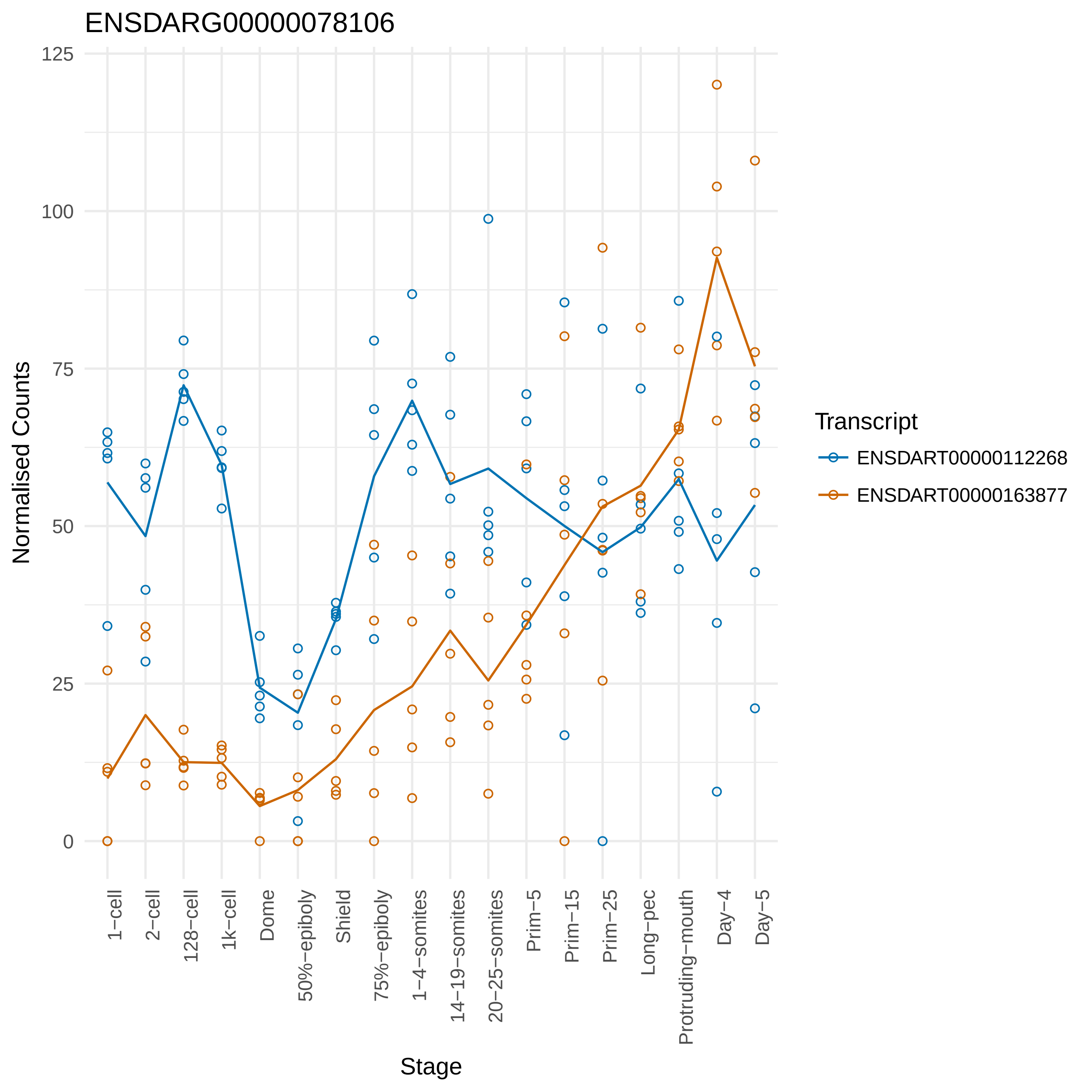
ENSDARG00000078106
Normalised Counts
0
25
50
75
100
125
1−cell
2−cell
128−cell
1k−cell
Dome
50%−epiboly
Shield
75%−epiboly
1−4−somites
14−19−somites
20−25−somites
Prim−5
Prim−15
Prim−25
Long−pec
Protruding−mouth
Day−4
Day−5
Stage
Transcript
ENSDART00000112268
ENSDART00000163877

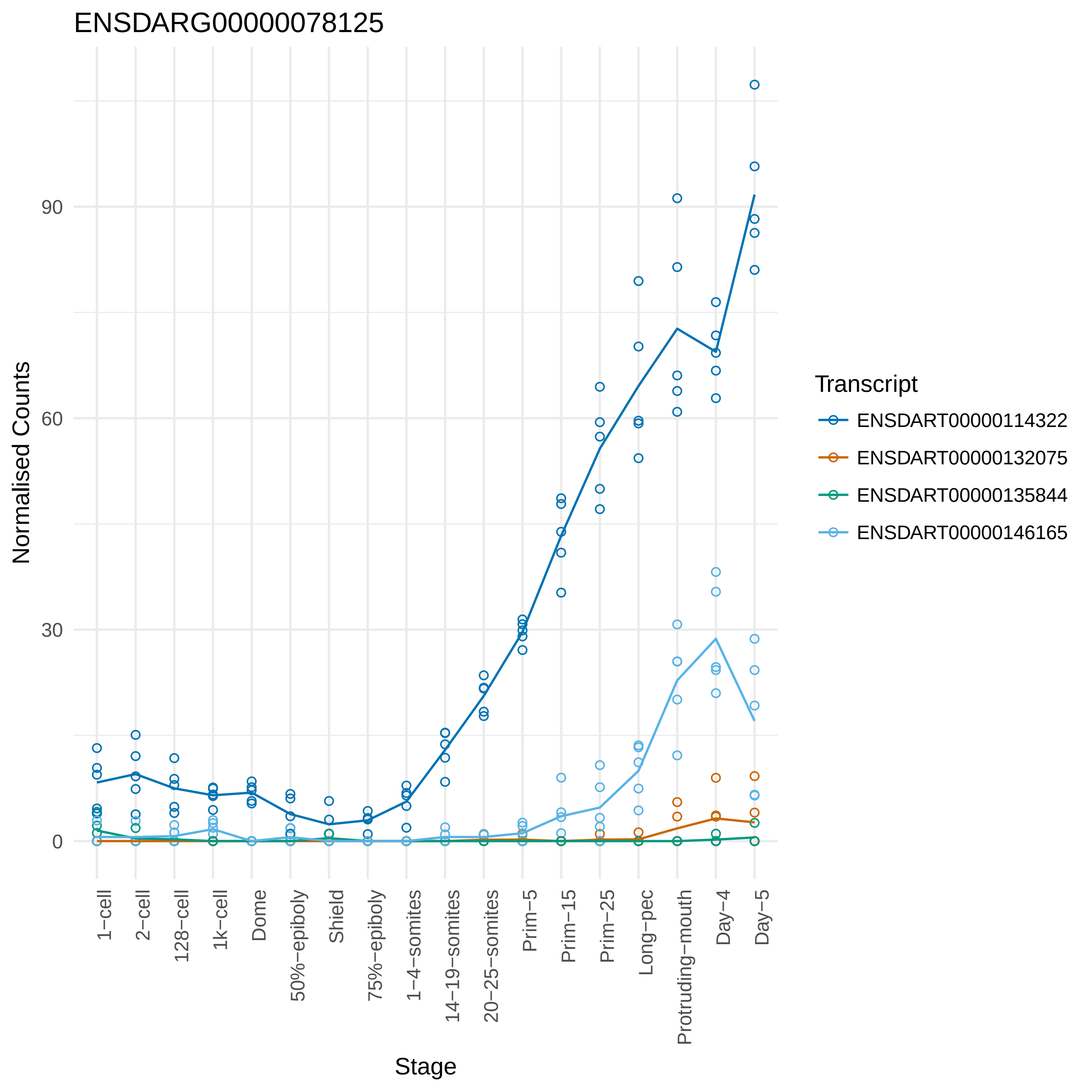

ENSDARG00000078125
Normalised Counts
0
30
60
90
1−cell
2−cell
128−cell
1k−cell
Dome
50%−epiboly
Shield
75%−epiboly
1−4−somites
14−19−somites
20−25−somites
Prim−5
Prim−15
Prim−25
Long−pec
Protruding−mouth
Day−4
Day−5
Stage
Transcript
ENSDART00000114322
ENSDART00000132075
ENSDART00000135844
ENSDART00000146165

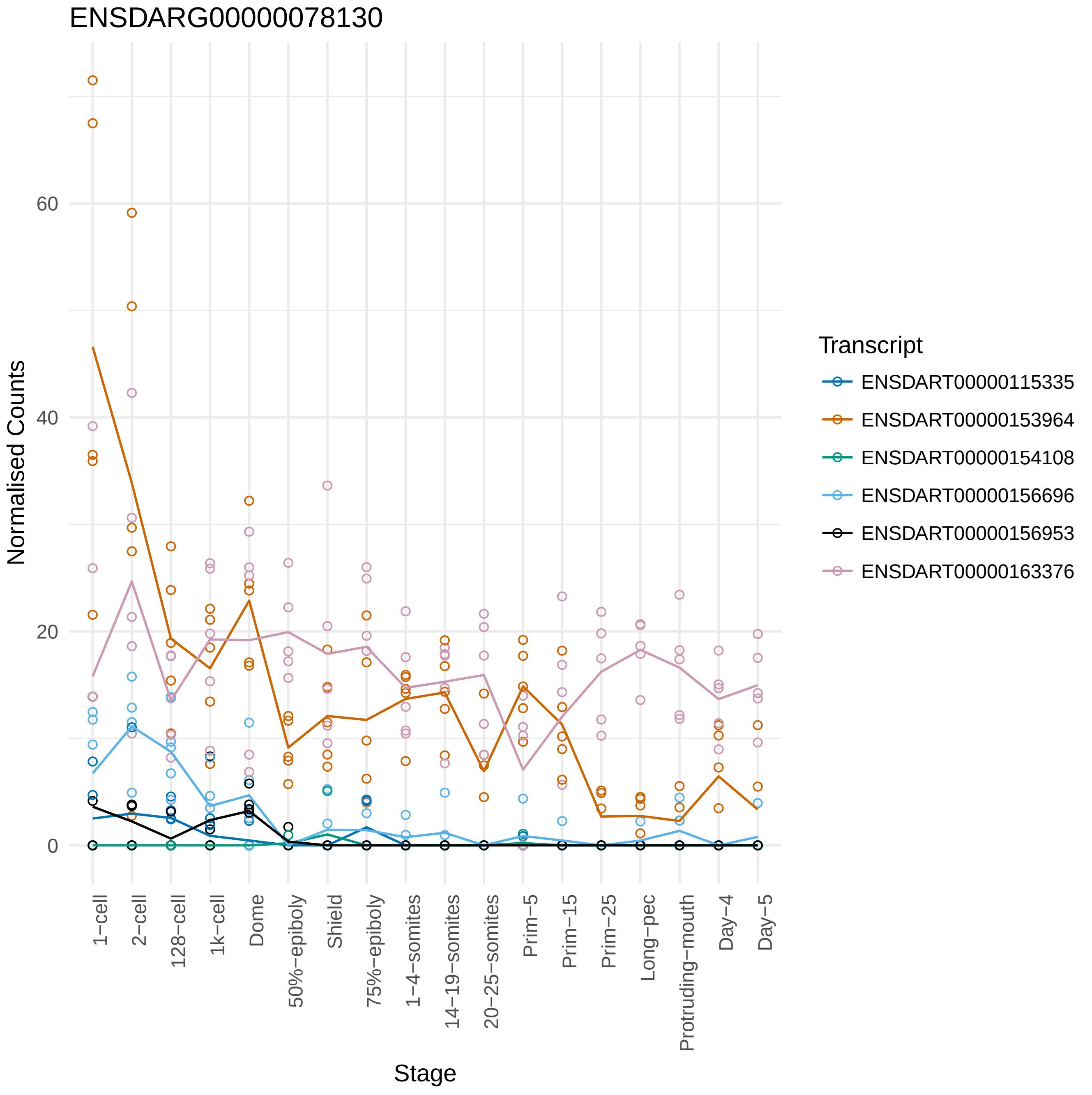
ENSDARG00000078130
Normalised Counts
0
20
40
60
Stage
1−cell
2−cell
128−cell
1k−cell
Dome
50%−epiboly
Shield
75%−epiboly
1−4−somites
14−19−somites
20−25−somites
Prim−5
Prim−15
Prim−25
Long−pec
Protruding−mouth
Day−4
Day−5
Transcript
ENSDART00000115335
ENSDART00000153964
ENSDART00000154108
ENSDART00000156696
ENSDART00000156953
ENSDART00000163376

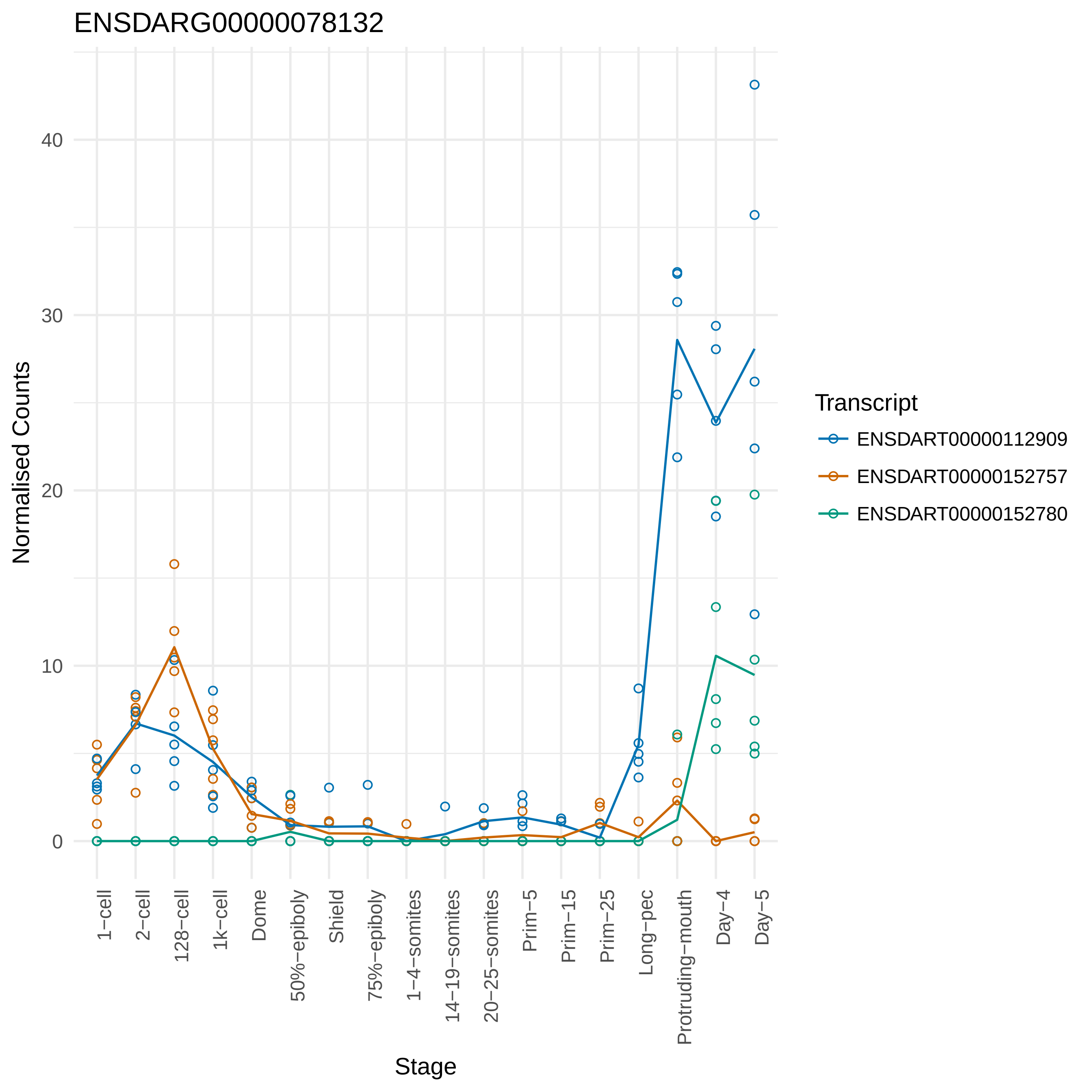
ENSDARG00000078132
Normalised Counts
0
10
20
30
40
1−cell
2−cell
128−cell
1k−cell
Dome
50%−epiboly
Shield
75%−epiboly
1−4−somites
14−19−somites
20−25−somites
Prim−5
Prim−15
Prim−25
Long−pec
Protruding−mouth
Day−4
Day−5
Stage
Transcript
ENSDART00000112909
ENSDART00000152757
ENSDART00000152780

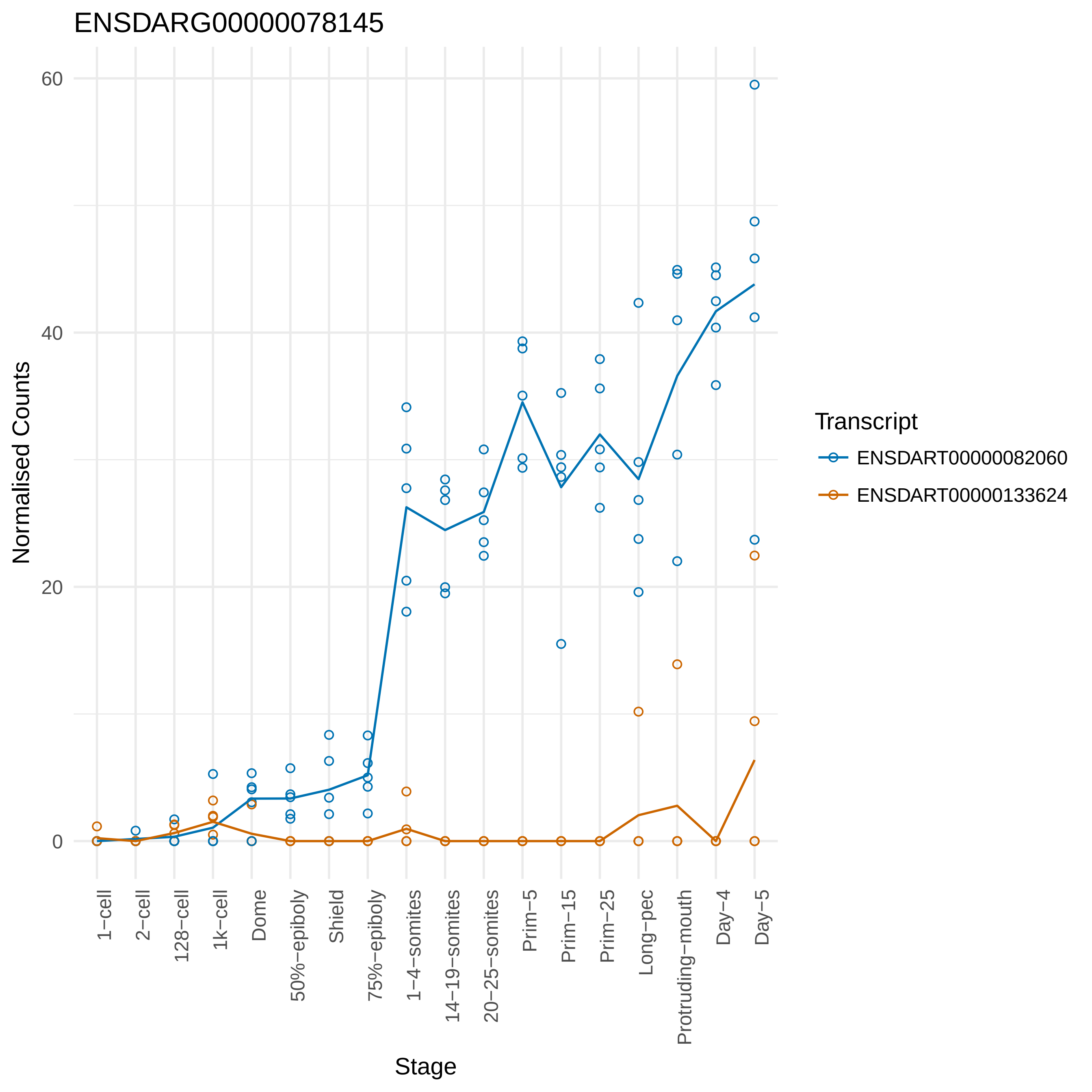

ENSDARG00000078145
Normalised Counts
0
20
40
60
1−cell
2−cell
128−cell
1k−cell
Dome
50%−epiboly
Shield
75%−epiboly
1−4−somites
14−19−somites
20−25−somites
Prim−5
Prim−15
Prim−25
Long−pec
Protruding−mouth
Day−4
Day−5
Stage
Transcript
ENSDART00000082060
ENSDART00000133624

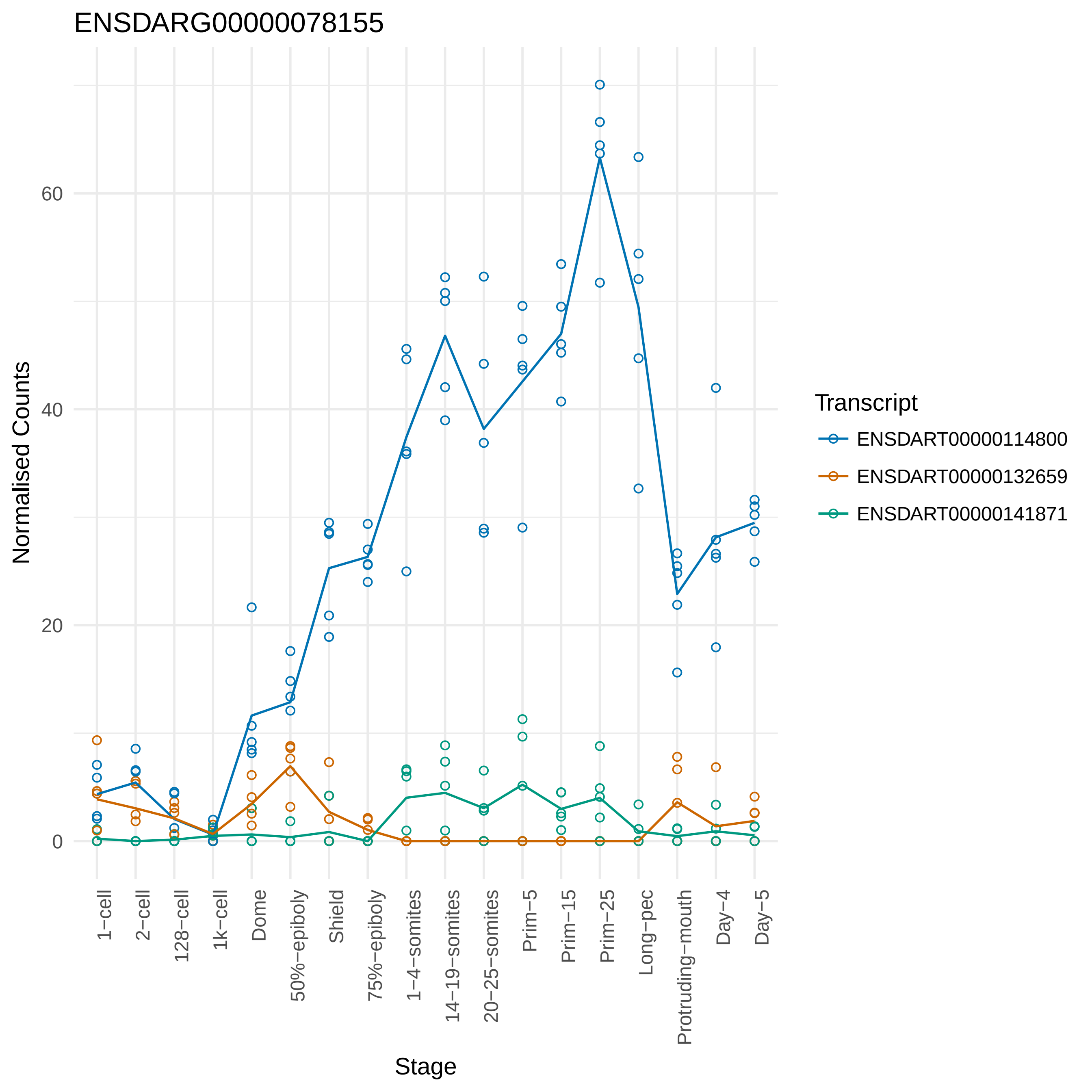
ENSDARG00000078155
Normalised Counts
60
40
20
0
1−cell
2−cell
128−cell
1k−cell
Dome
50%−epiboly
Shield
75%−epiboly
1−4−somites
14−19−somites
20−25−somites
Prim−5
Prim−15
Prim−25
Long−pec
Protruding−mouth
Day−4
Day−5
Stage
Transcript
ENSDART00000114800
ENSDART00000132659
ENSDART00000141871

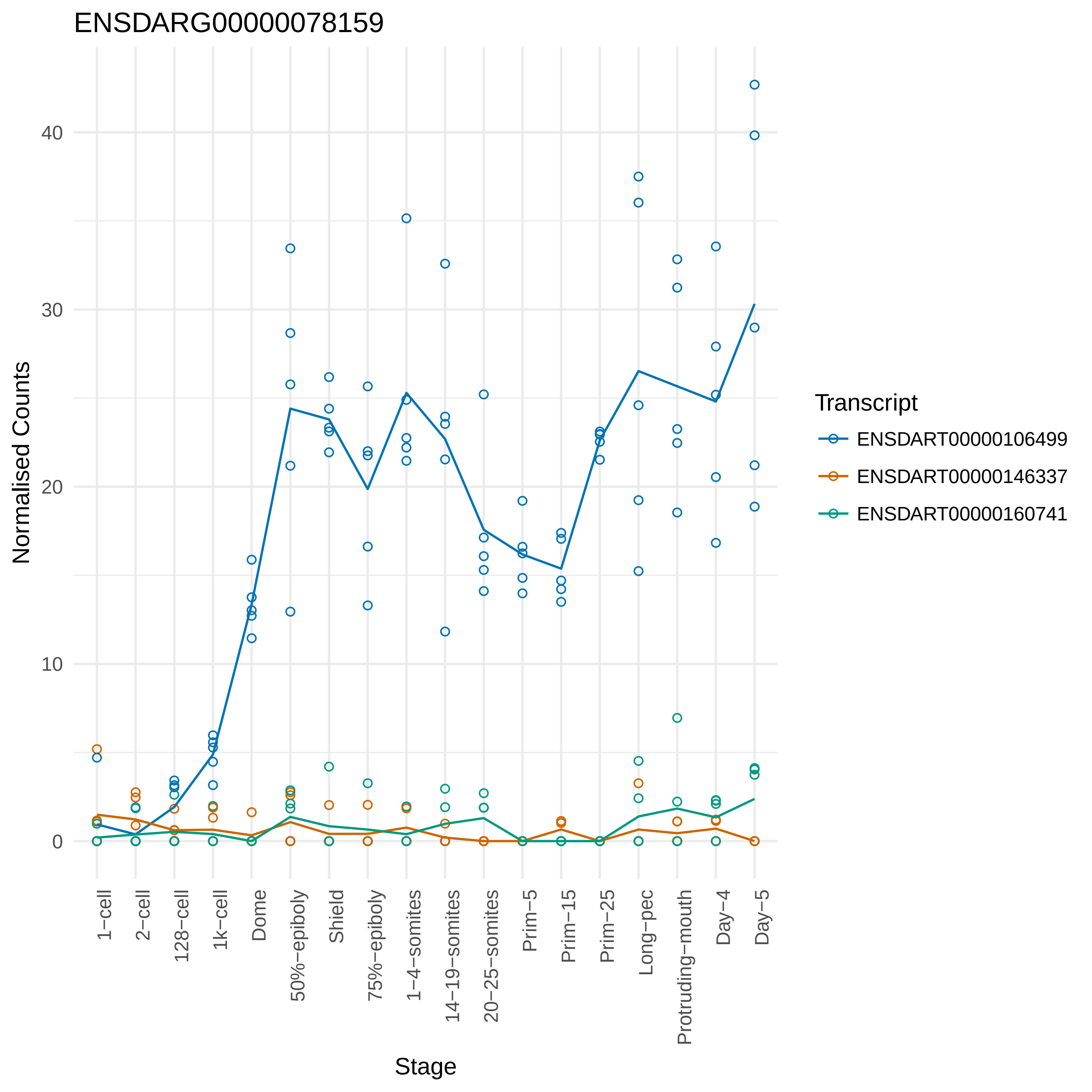
ENSDARG00000078159
Normalised Counts
0
10
20
30
40
Stage
1−cell
2−cell
128−cell
1k−cell
Dome
50%−epiboly
Shield
75%−epiboly
1−4−somites
14−19−somites
20−25−somites
Prim−5
Prim−15
Prim−25
Long−pec
Protruding−mouth
Day−4
Day−5
Transcript
ENSDART00000106499
ENSDART00000146337
ENSDART00000160741

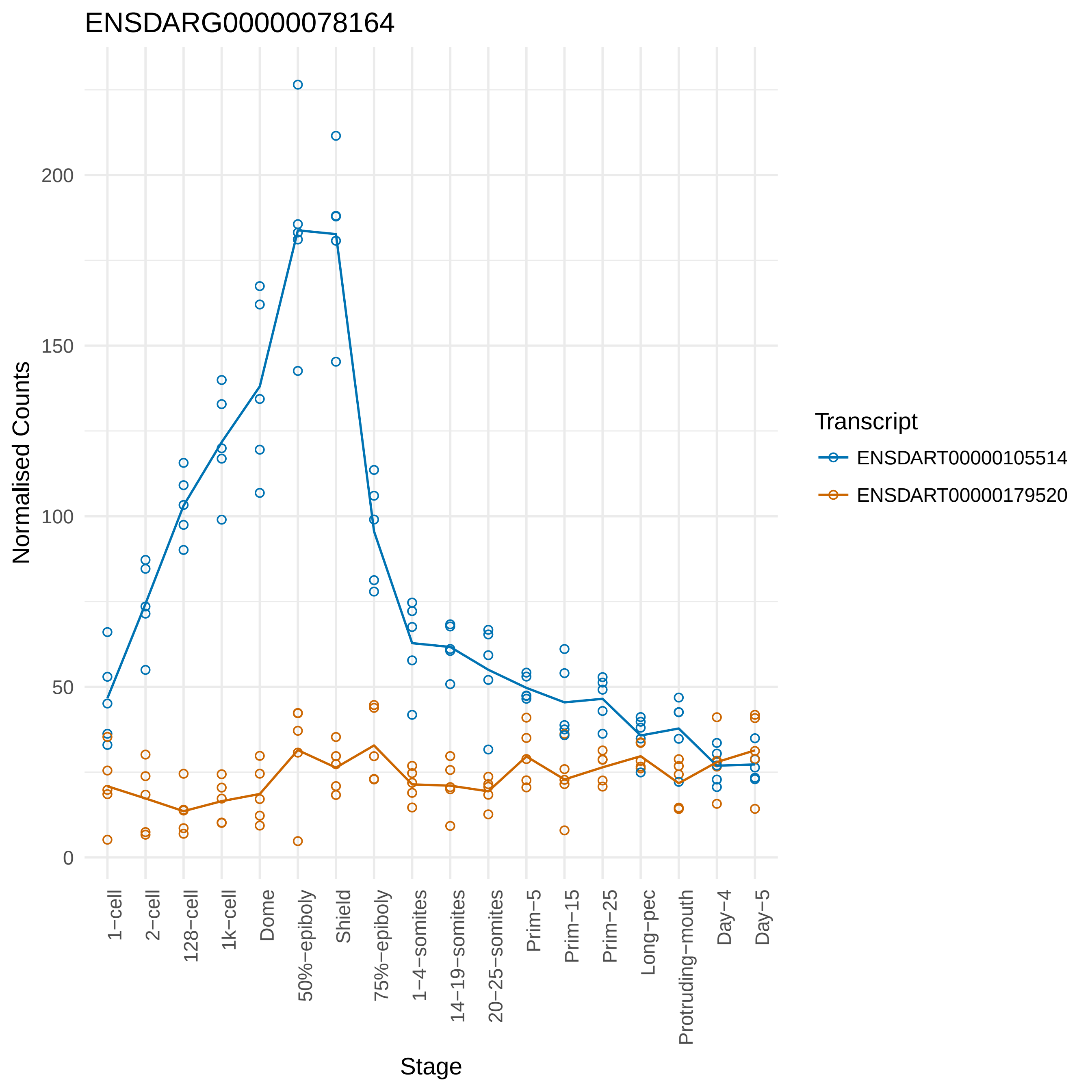

ENSDARG00000078164
Normalised Counts
0
50
100
150
200
Stage
1−cell
2−cell
128−cell
1k−cell
Dome
50%−epiboly
Shield
75%−epiboly
1−4−somites
14−19−somites
20−25−somites
Prim−5
Prim−15
Prim−25
Long−pec
Protruding−mouth
Day−4
Day−5
Transcript
ENSDART00000105514
ENSDART00000179520

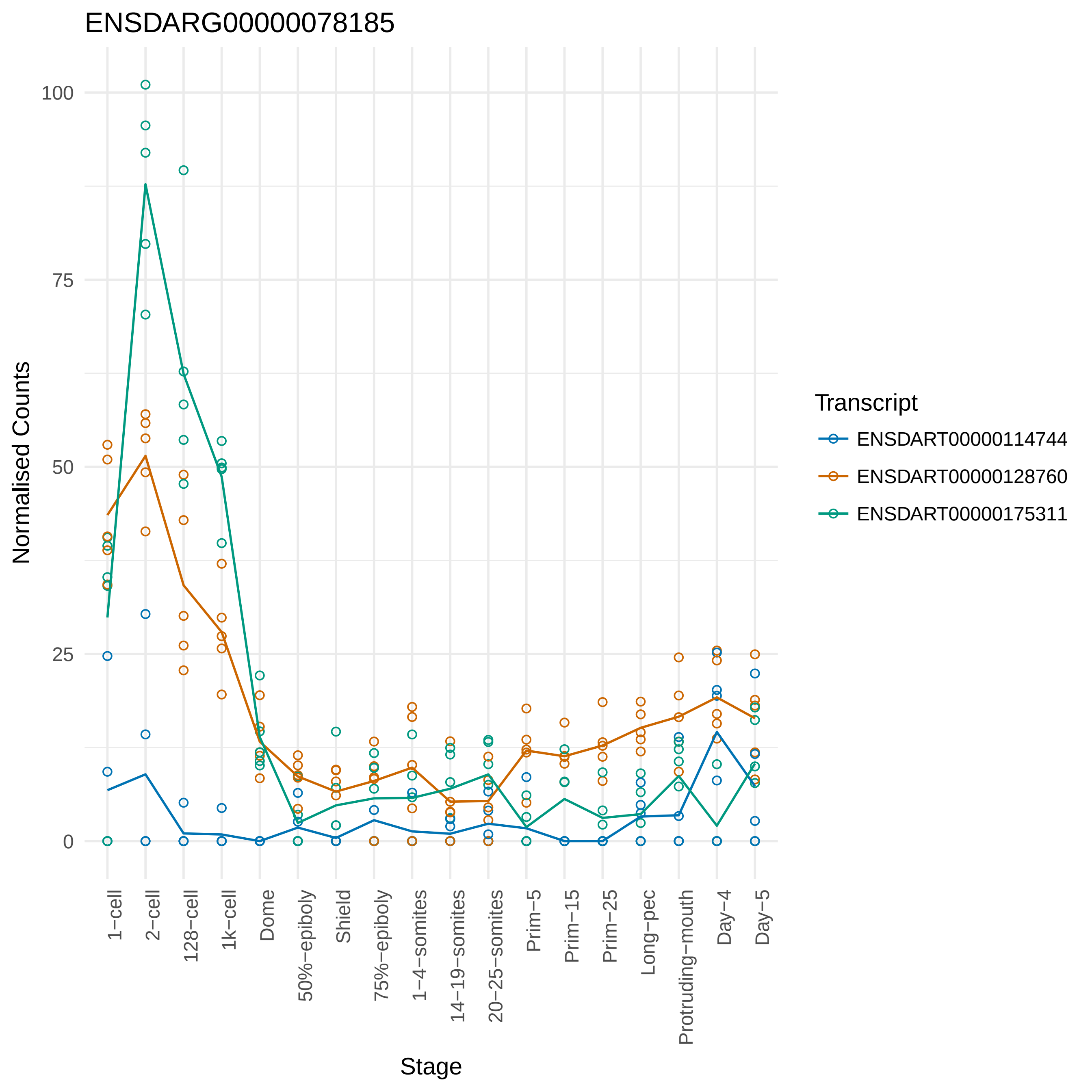

ENSDARG00000078185
Normalised Counts
Stage
Transcript
ENSDART00000114744
ENSDART00000128760
ENSDART00000175311
0
25
50
75
100
1−cell
2−cell
128−cell
1k−cell
Dome
50%−epiboly
Shield
75%−epiboly
1−4−somites
14−19−somites
20−25−somites
Prim−5
Prim−15
Prim−25
Long−pec
Protruding−mouth
Day−4
Day−5

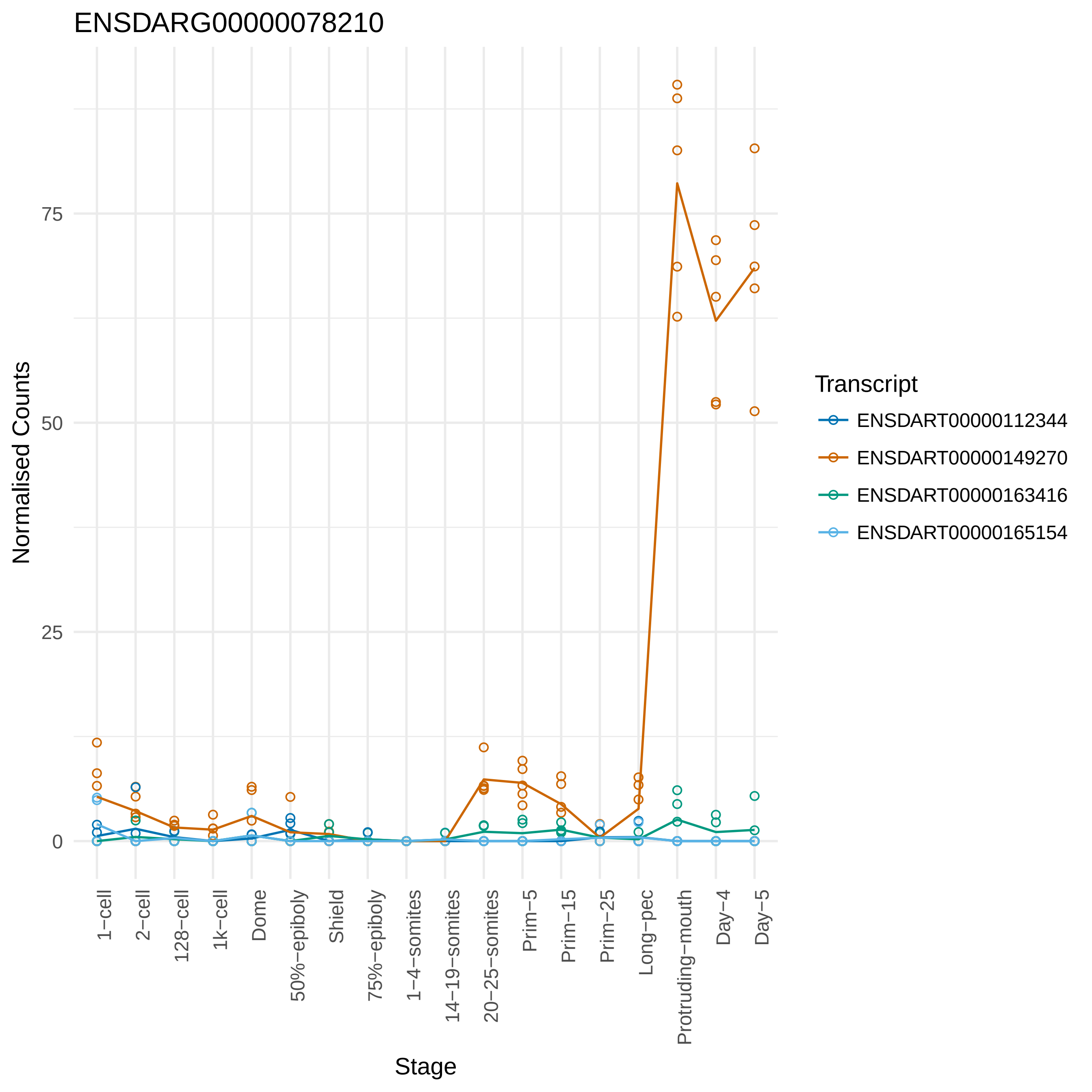

ENSDARG00000078210
Normalised Counts
0
25
50
75
1−cell
2−cell
128−cell
1k−cell
Dome
50%−epiboly
Shield
75%−epiboly
1−4−somites
14−19−somites
20−25−somites
Prim−5
Prim−15
Prim−25
Long−pec
Protruding−mouth
Day−4
Day−5
Stage
Transcript
ENSDART00000112344
ENSDART00000149270
ENSDART00000163416
ENSDART00000165154

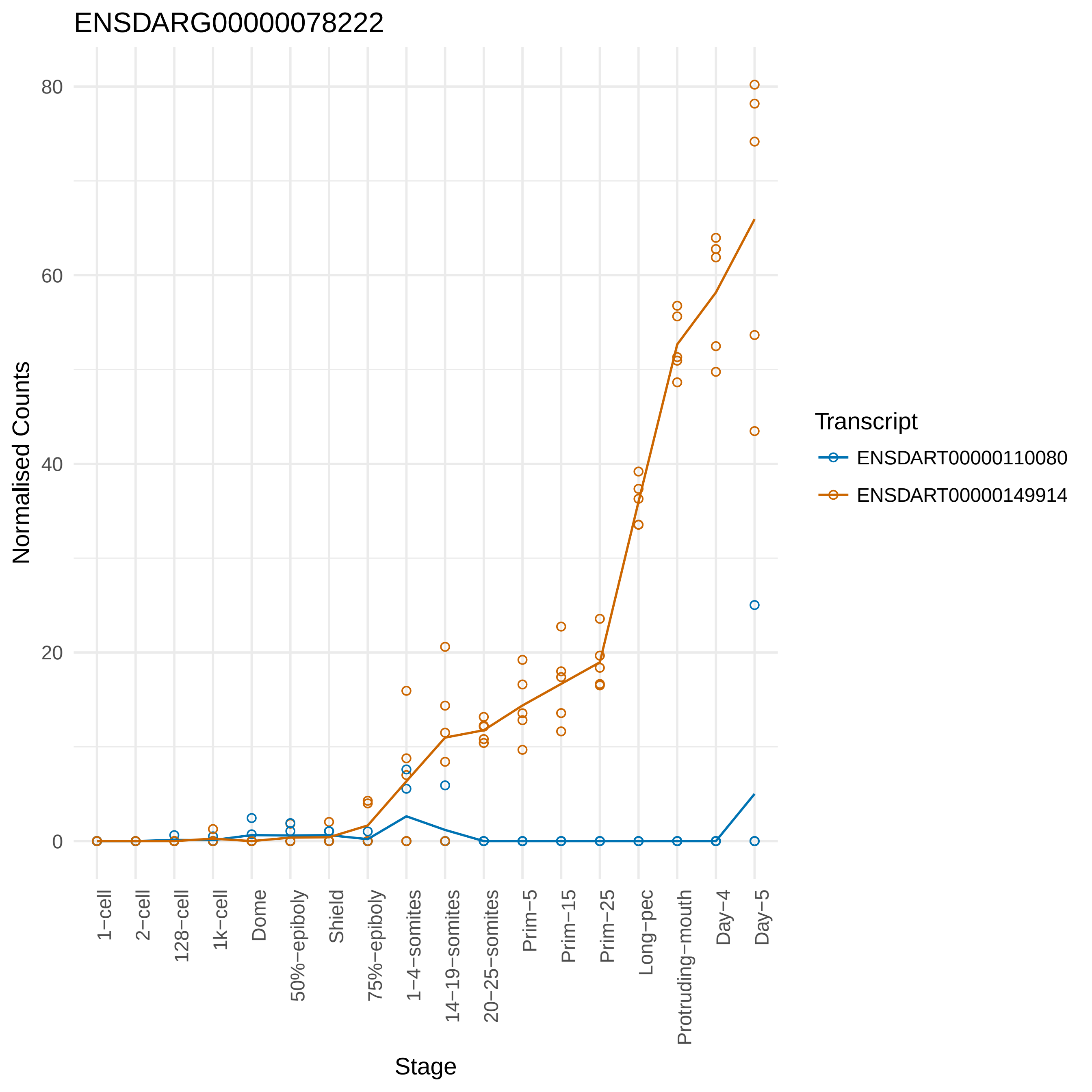

ENSDARG00000078222
Normalised Counts
0
20
40
60
80
1−cell
2−cell
128−cell
1k−cell
Dome
50%−epiboly
Shield
75%−epiboly
1−4−somites
14−19−somites
20−25−somites
Prim−5
Prim−15
Prim−25
Long−pec
Protruding−mouth
Day−4
Day−5
Stage
Transcript
ENSDART00000110080
ENSDART00000149914

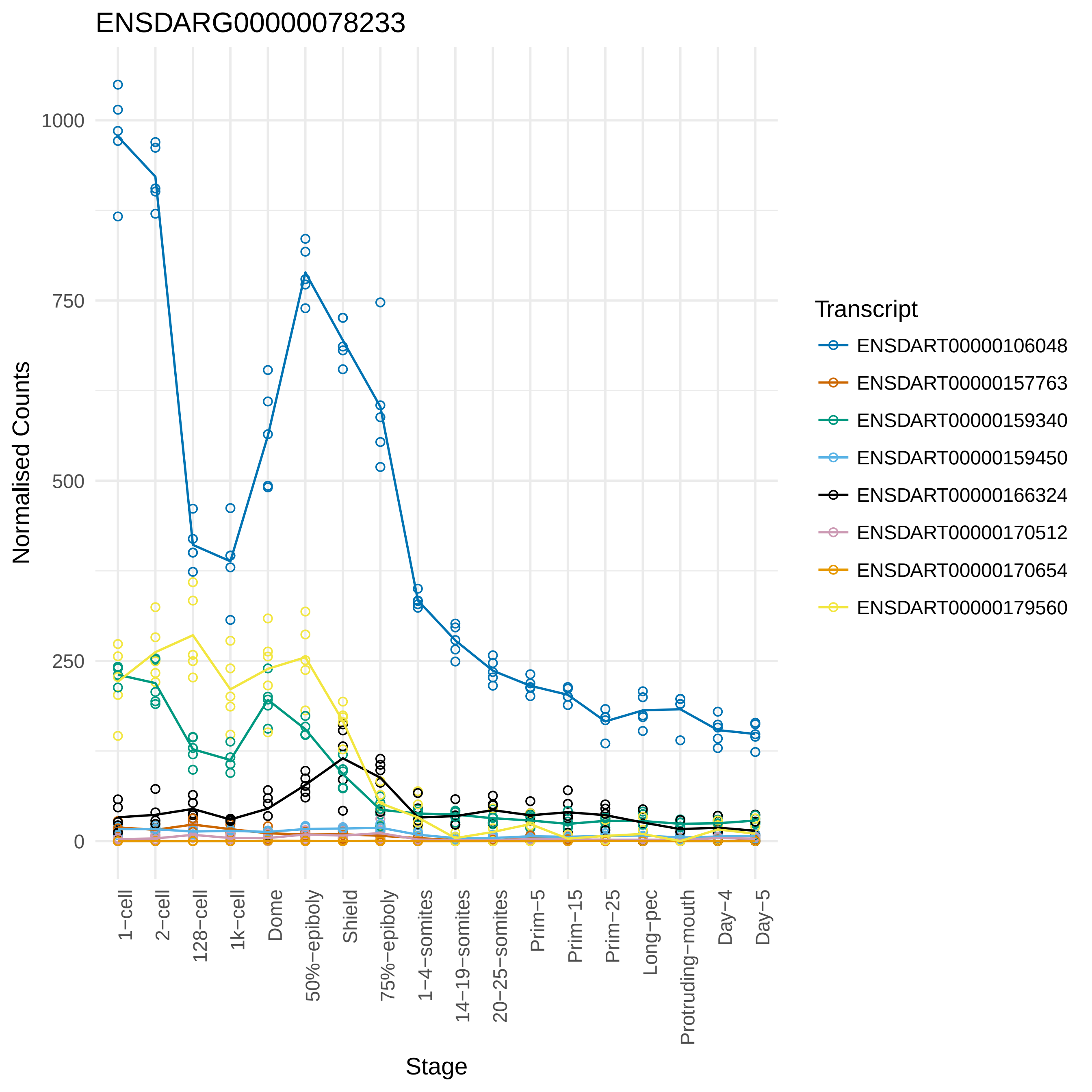

ENSDARG00000078233
Normalised Counts
0
250
500
750
1000
Stage
1−cell
2−cell
128−cell
1k−cell
Dome
50%−epiboly
Shield
75%−epiboly
1−4−somites
14−19−somites
20−25−somites
Prim−5
Prim−15
Prim−25
Long−pec
Protruding−mouth
Day−4
Day−5
Transcript
ENSDART00000106048
ENSDART00000157763
ENSDART00000159340
ENSDART00000159450
ENSDART00000166324
ENSDART00000170512
ENSDART00000170654
ENSDART00000179560

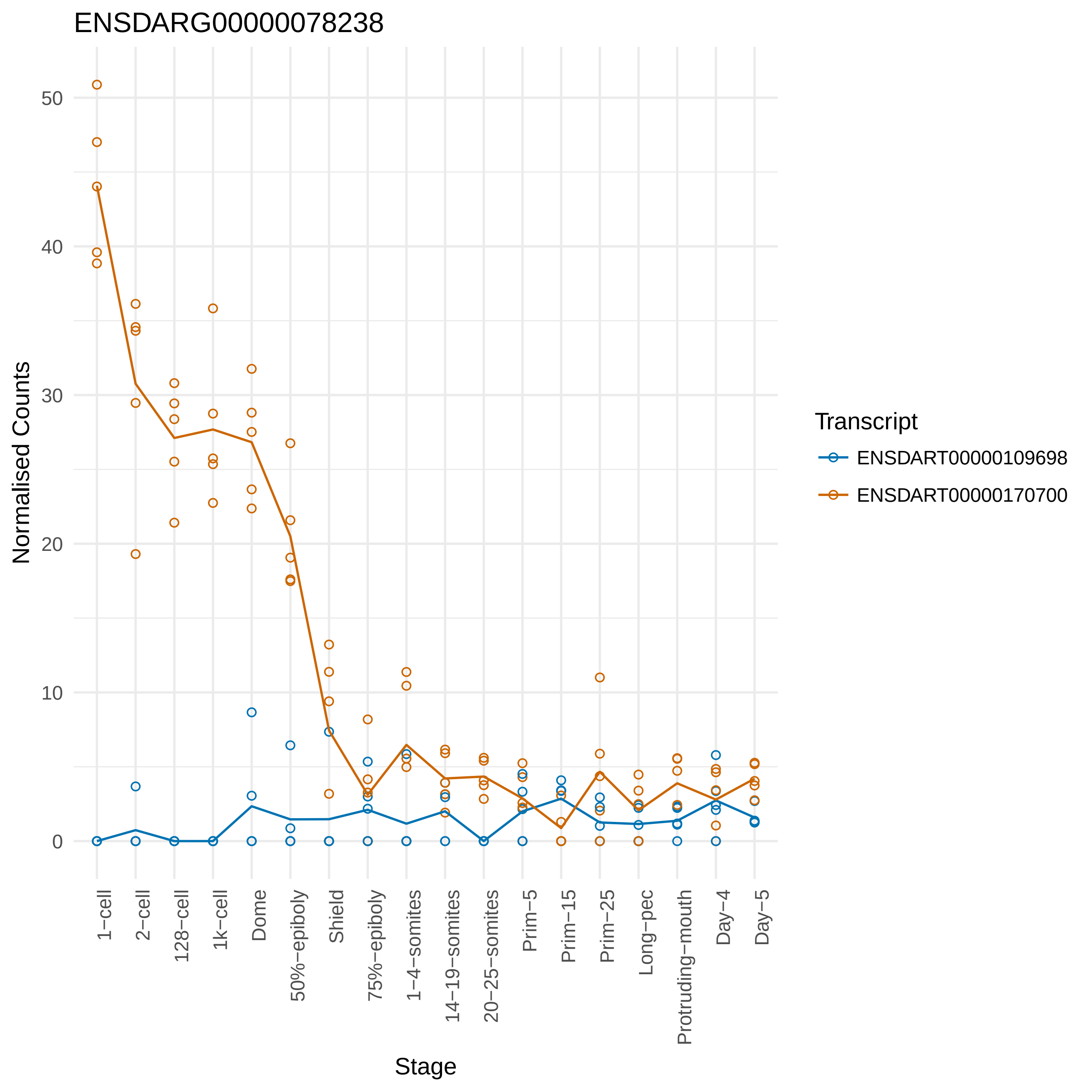

ENSDARG00000078238
Normalised Counts
50
40
30
20
10
0
1−cell
2−cell
128−cell
1k−cell
Dome
50%−epiboly
Shield
75%−epiboly
1−4−somites
14−19−somites
20−25−somites
Prim−5
Prim−15
Prim−25
Long−pec
Protruding−mouth
Day−4
Day−5
Stage
Transcript
ENSDART00000109698
ENSDART00000170700

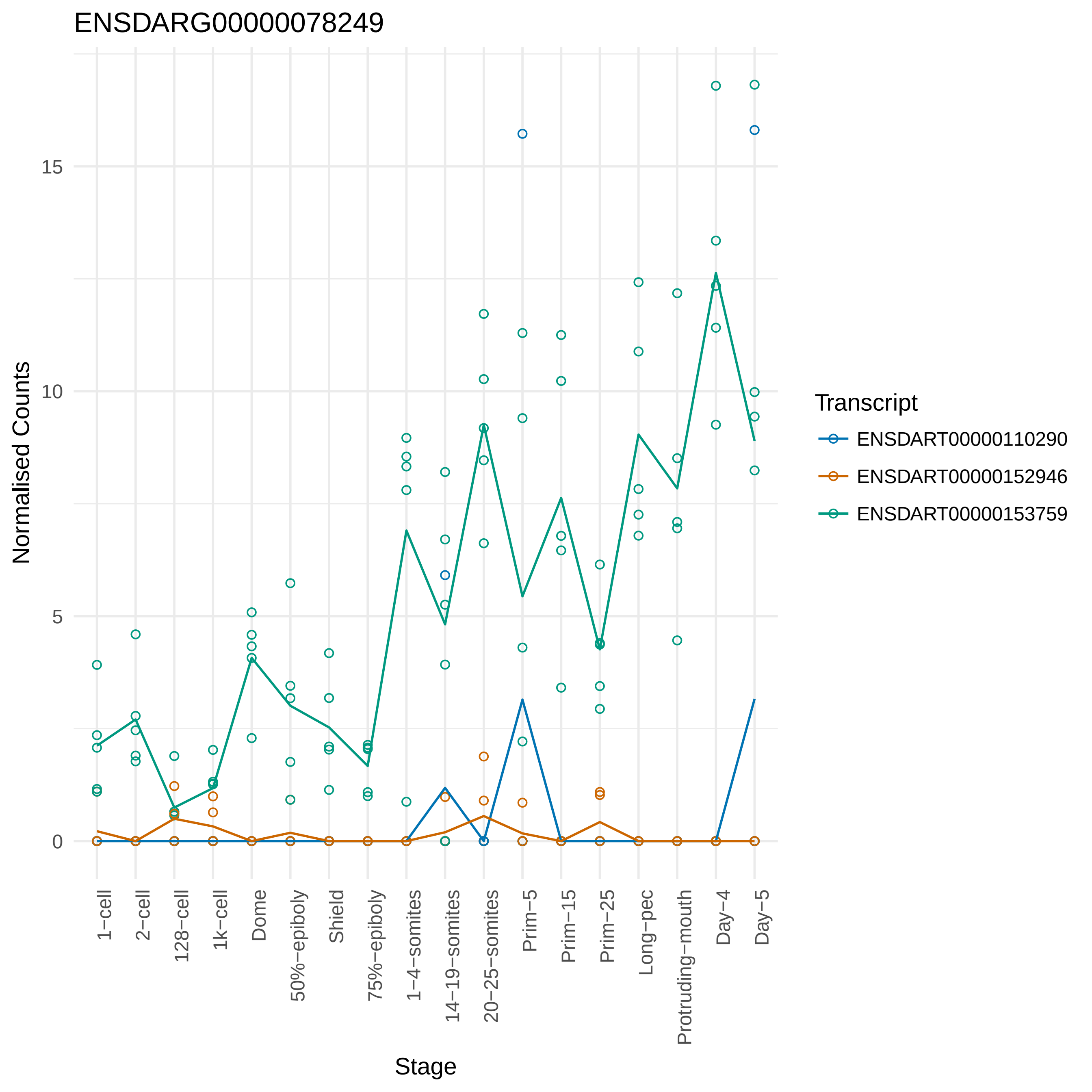

ENSDARG00000078249
Normalised Counts
0
5
10
15
1−cell
2−cell
128−cell
1k−cell
Dome
50%−epiboly
Shield
75%−epiboly
1−4−somites
14−19−somites
20−25−somites
Prim−5
Prim−15
Prim−25
Long−pec
Protruding−mouth
Day−4
Day−5
Stage
Transcript
ENSDART00000110290
ENSDART00000152946
ENSDART00000153759

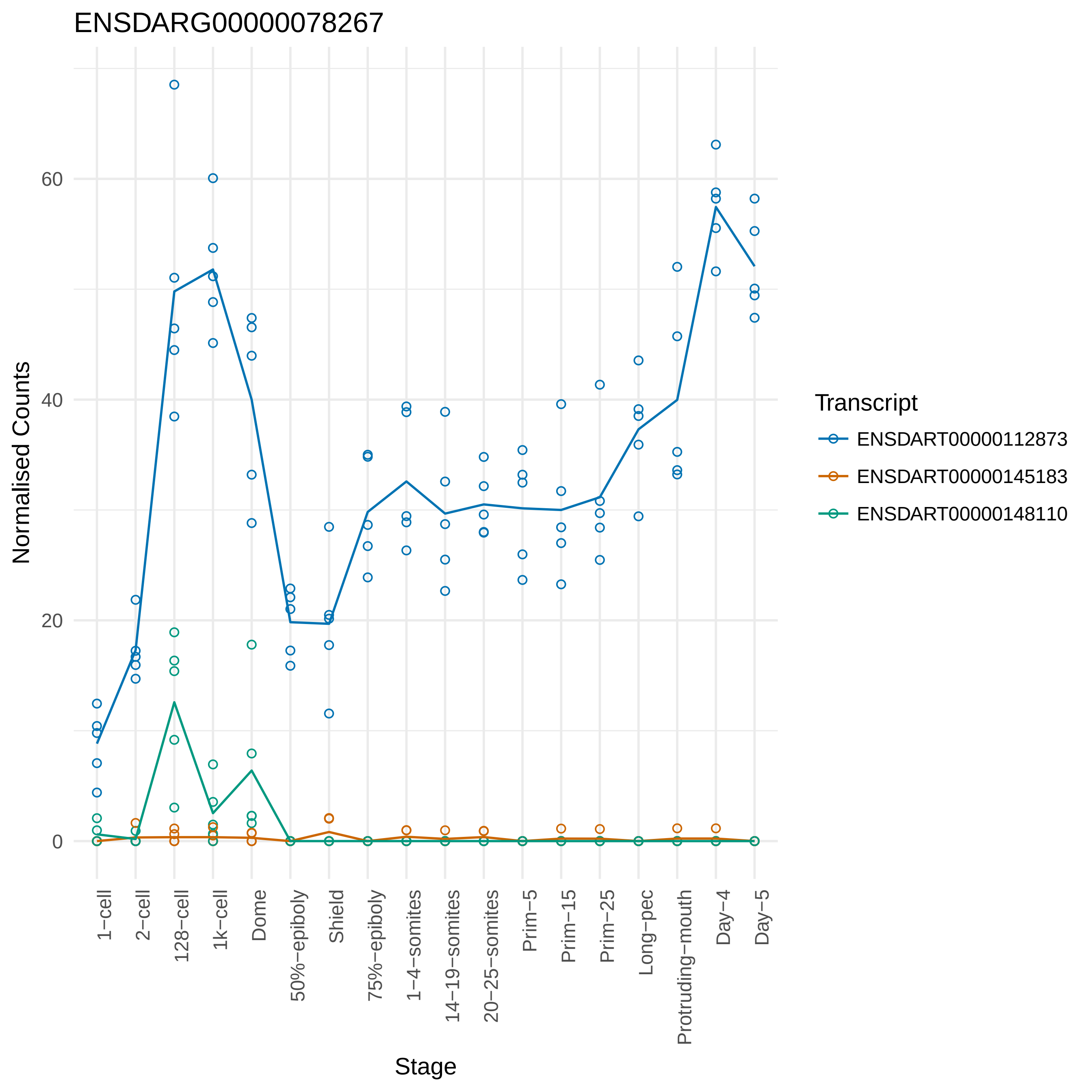
ENSDARG00000078267
Normalised Counts
60
40
20
0
1−cell
2−cell
128−cell
1k−cell
Dome
50%−epiboly
Shield
75%−epiboly
1−4−somites
14−19−somites
20−25−somites
Prim−5
Prim−15
Prim−25
Long−pec
Protruding−mouth
Day−4
Day−5
Stage
Transcript
ENSDART00000112873
ENSDART00000145183
ENSDART00000148110

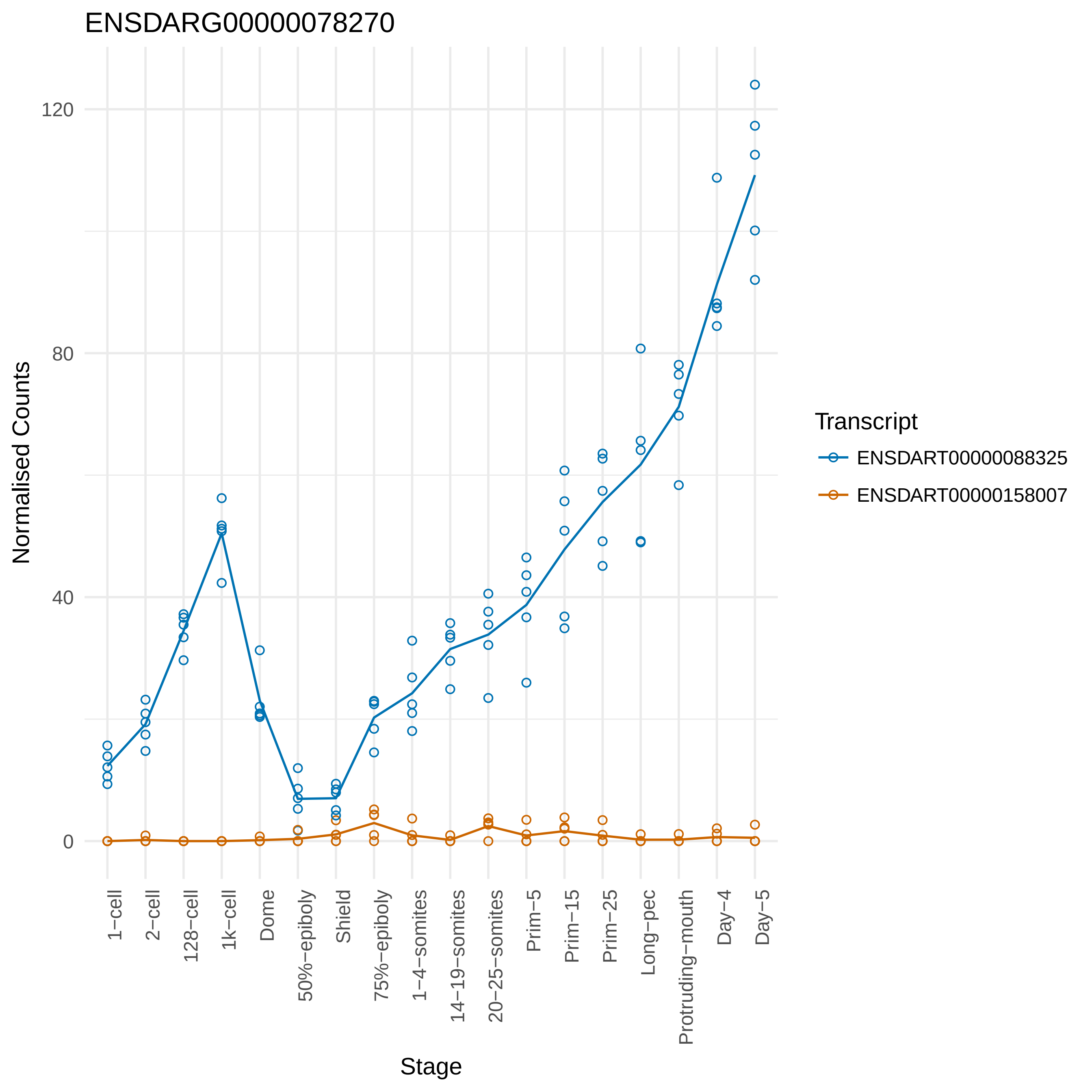
ENSDARG00000078270
Normalised Counts
120
80
40
0
1−cell
2−cell
128−cell
1k−cell
Dome
50%−epiboly
Shield
75%−epiboly
1−4−somites
14−19−somites
20−25−somites
Prim−5
Prim−15
Prim−25
Long−pec
Protruding−mouth
Day−4
Day−5
Stage
Transcript
ENSDART00000088325
ENSDART00000158007

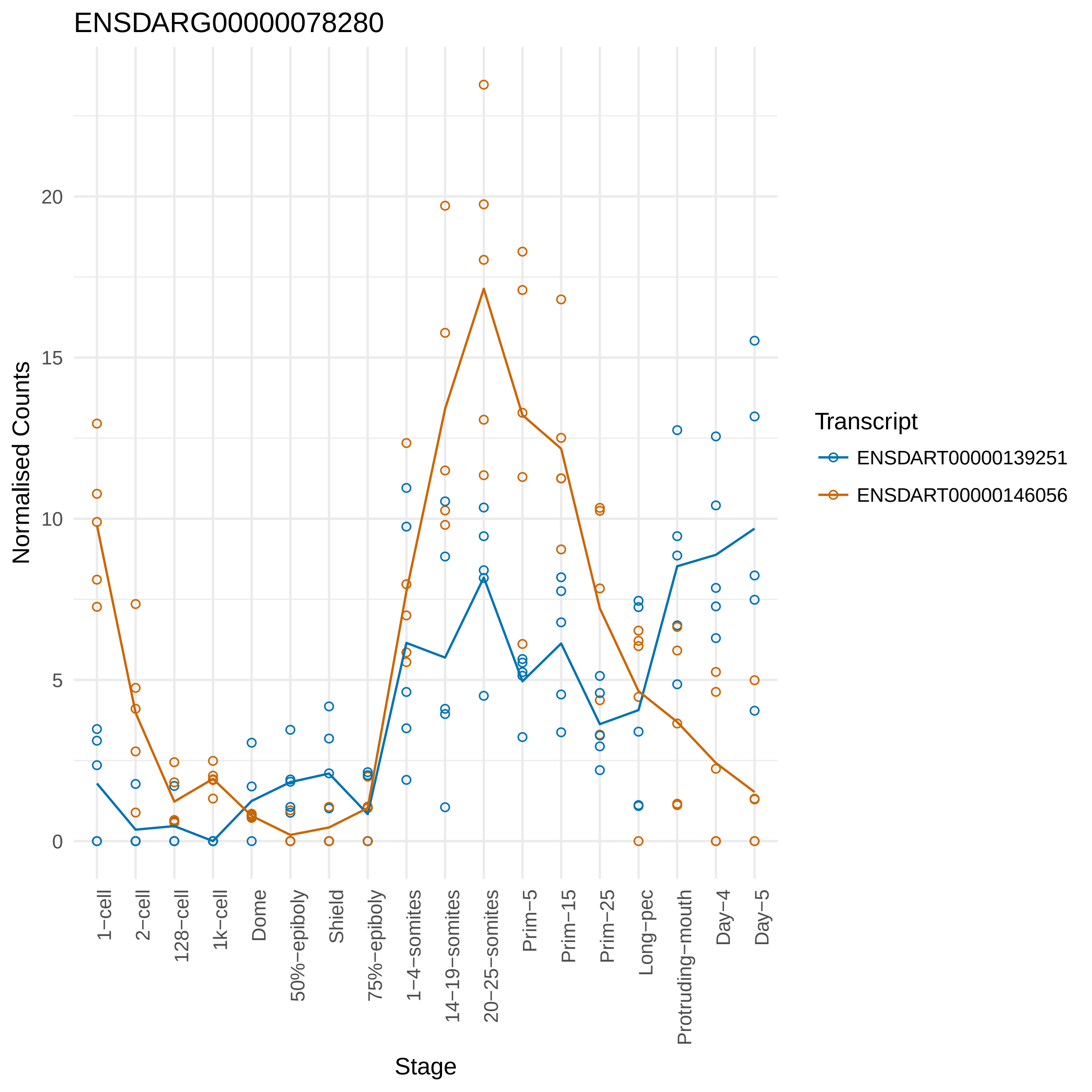

ENSDARG00000078280
Normalised Counts
0
5
10
15
20
Stage
1−cell
2−cell
128−cell
1k−cell
Dome
50%−epiboly
Shield
75%−epiboly
1−4−somites
14−19−somites
20−25−somites
Prim−5
Prim−15
Prim−25
Long−pec
Protruding−mouth
Day−4
Day−5
Transcript
ENSDART00000139251
ENSDART00000146056

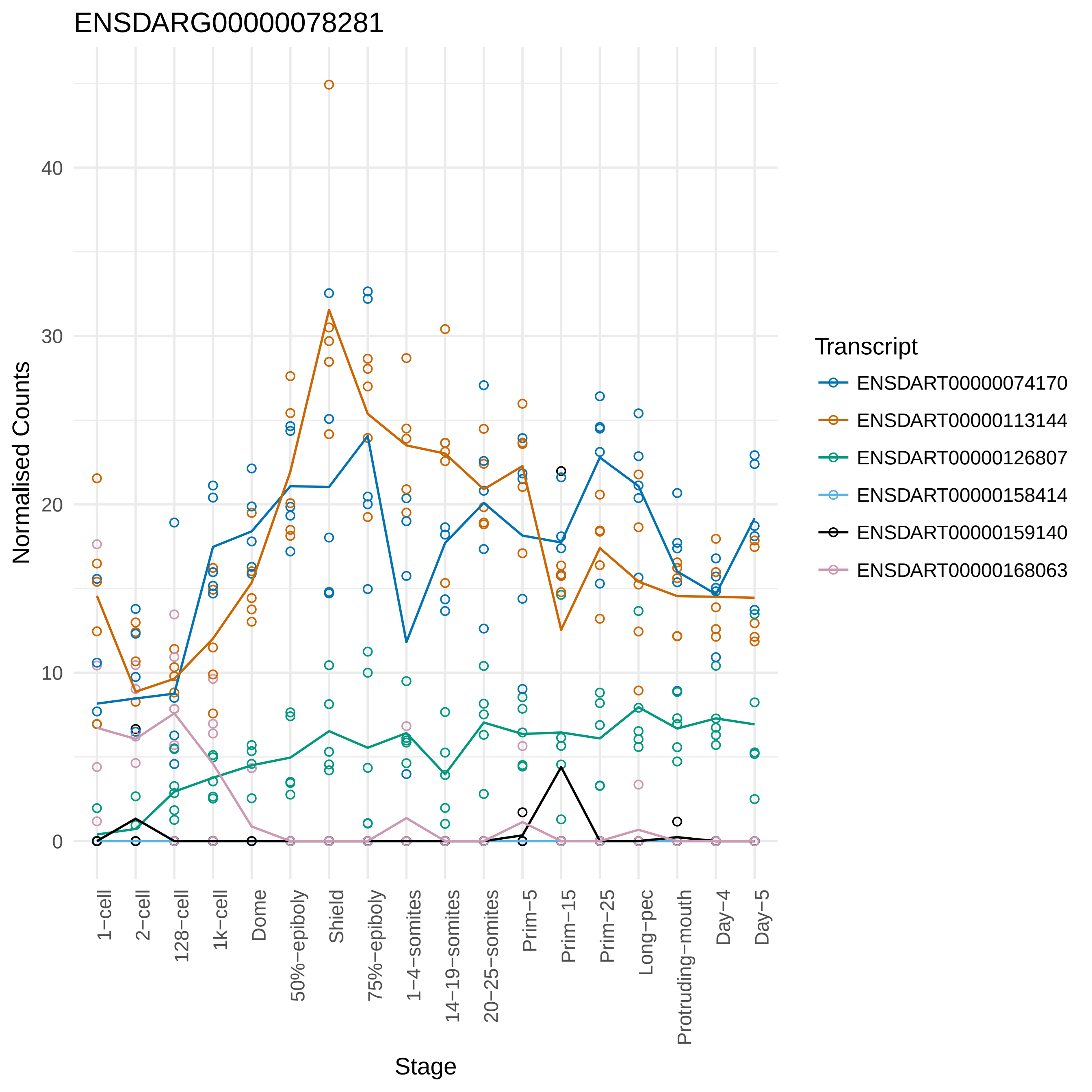

ENSDARG00000078281
Normalised Counts
0
10
20
30
40
1−cell
2−cell
128−cell
1k−cell
Dome
50%−epiboly
Shield
75%−epiboly
1−4−somites
14−19−somites
20−25−somites
Prim−5
Prim−15
Prim−25
Long−pec
Protruding−mouth
Day−4
Day−5
Stage
Transcript
ENSDART00000074170
ENSDART00000113144
ENSDART00000126807
ENSDART00000158414
ENSDART00000159140
ENSDART00000168063

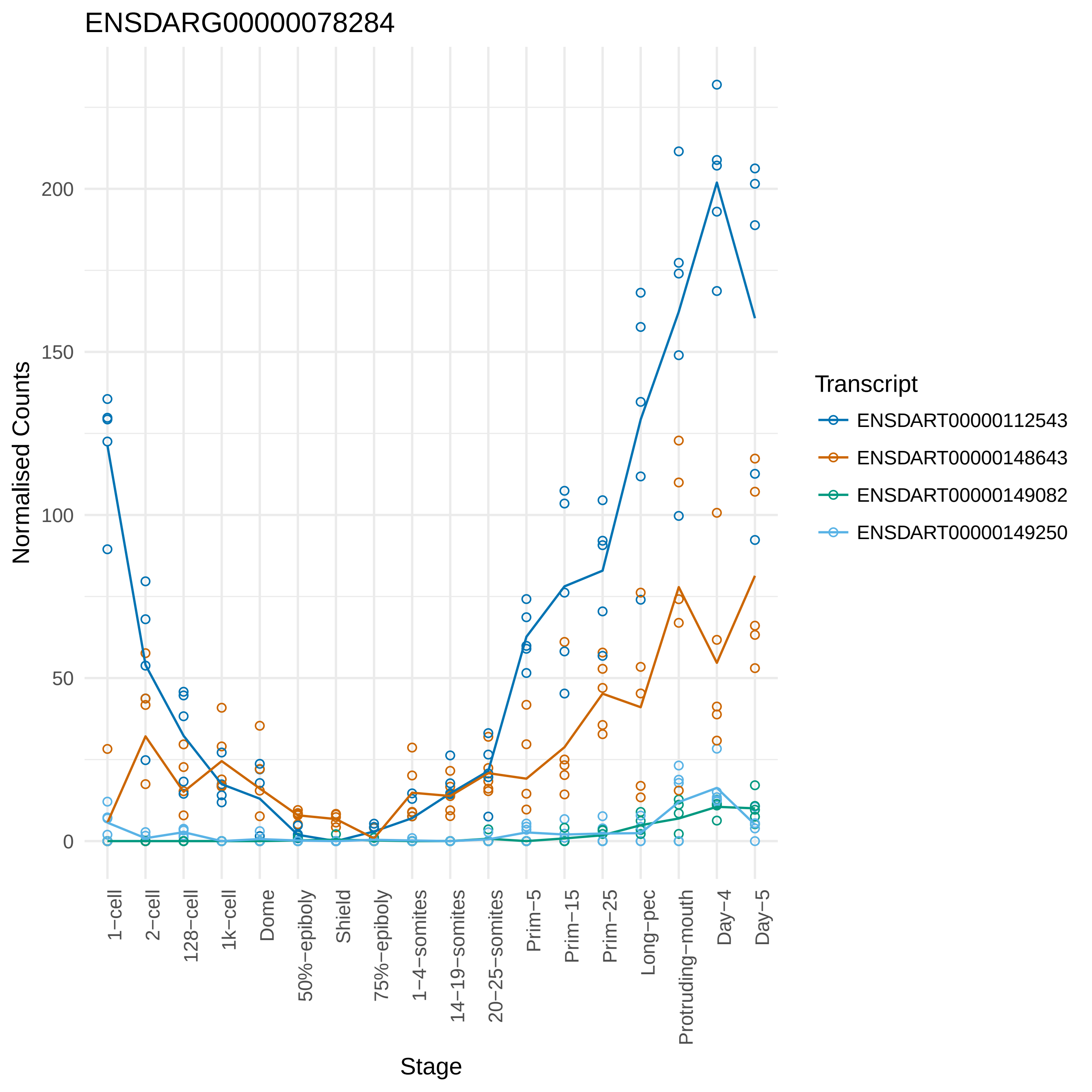
ENSDARG00000078284
Normalised Counts
0
50
100
150
200
Stage
1−cell
2−cell
128−cell
1k−cell
Dome
50%−epiboly
Shield
75%−epiboly
1−4−somites
14−19−somites
20−25−somites
Prim−5
Prim−15
Prim−25
Long−pec
Protruding−mouth
Day−4
Day−5
Transcript
ENSDART00000112543
ENSDART00000148643
ENSDART00000149082
ENSDART00000149250

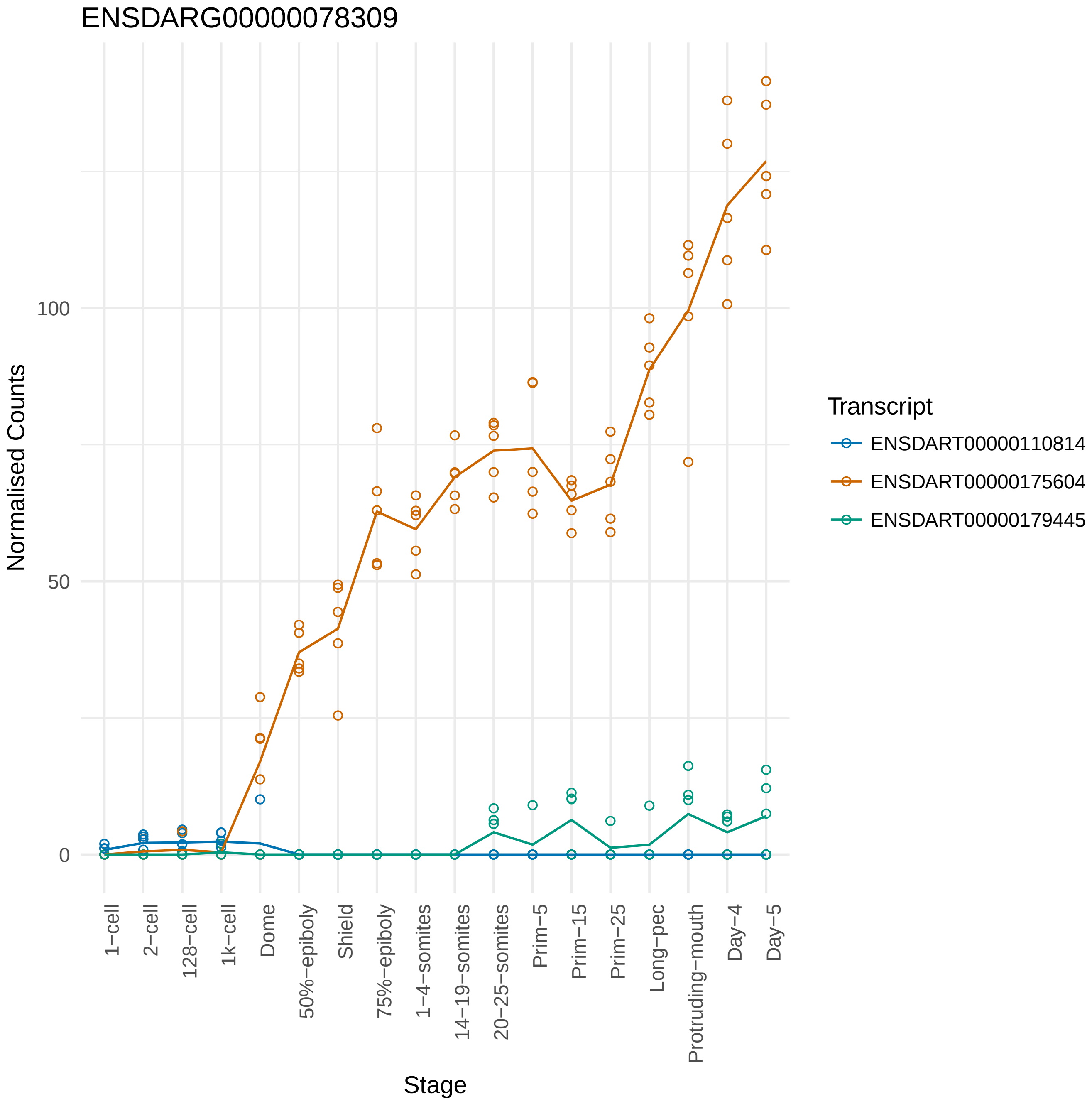

ENSDARG00000078309
Normalised Counts
100
50
0
1−cell
2−cell
128−cell
1k−cell
Dome
50%−epiboly
Shield
75%−epiboly
1−4−somites
14−19−somites
20−25−somites
Prim−5
Prim−15
Prim−25
Long−pec
Protruding−mouth
Day−4
Day−5
Stage
Transcript
ENSDART00000110814
ENSDART00000175604
ENSDART00000179445

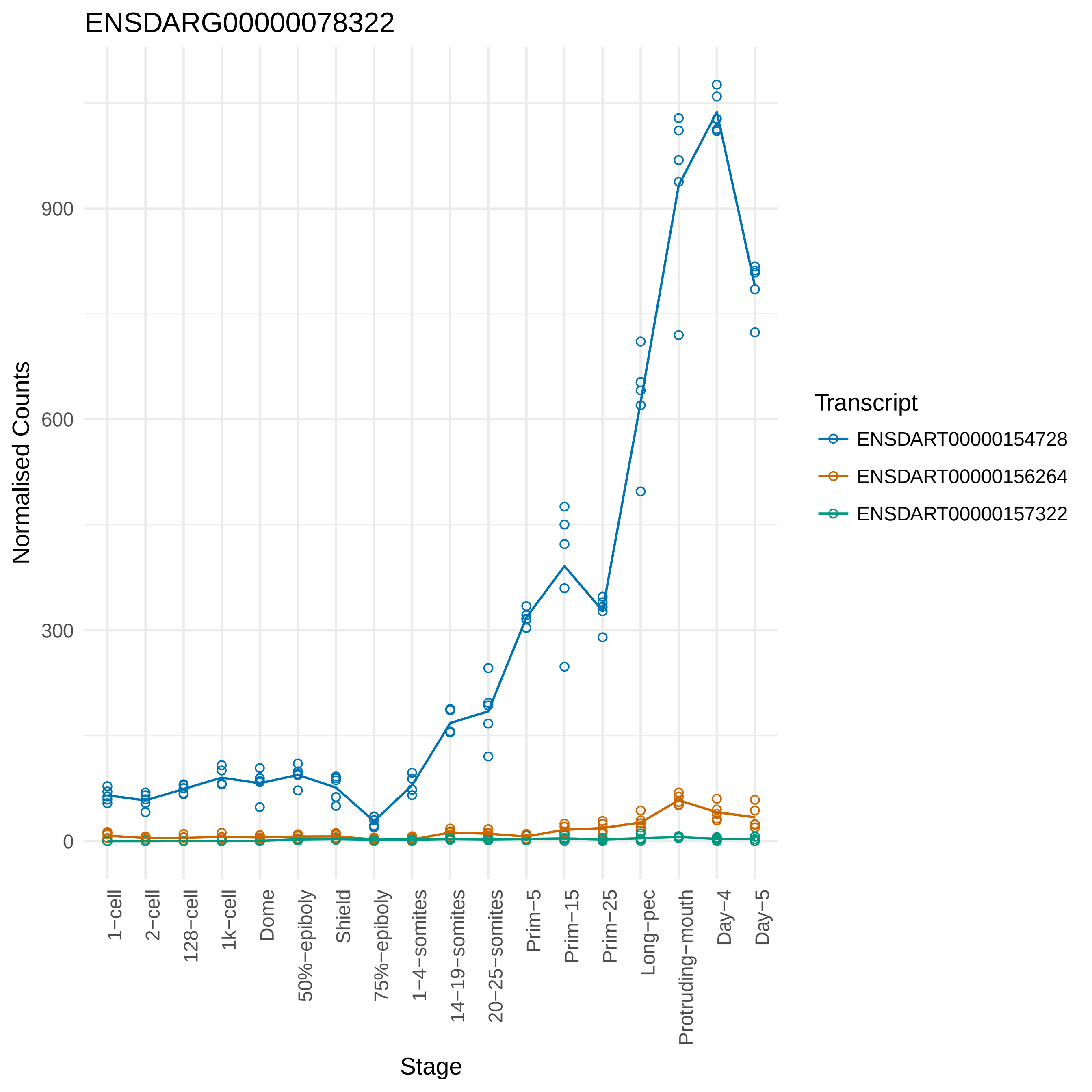
ENSDARG00000078322
Normalised Counts
0
300
600
900
Stage
1−cell
2−cell
128−cell
1k−cell
Dome
50%−epiboly
Shield
75%−epiboly
1−4−somites
14−19−somites
20−25−somites
Prim−5
Prim−15
Prim−25
Long−pec
Protruding−mouth
Day−4
Day−5
Transcript
ENSDART00000154728
ENSDART00000156264
ENSDART00000157322

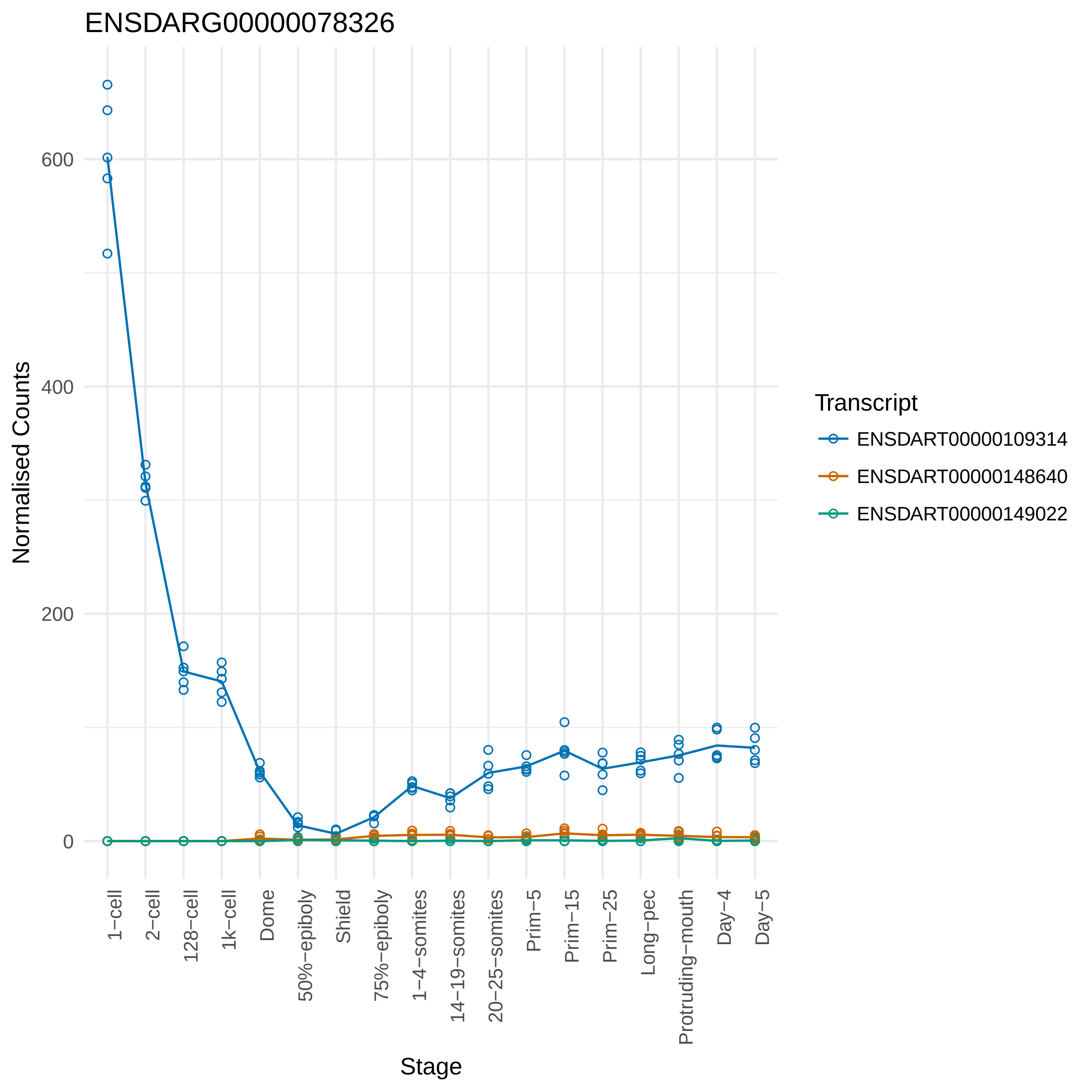

ENSDARG00000078326
Normalised Counts
600
400
200
0
1−cell
2−cell
128−cell
1k−cell
Dome
50%−epiboly
Shield
75%−epiboly
1−4−somites
14−19−somites
20−25−somites
Prim−5
Prim−15
Prim−25
Long−pec
Protruding−mouth
Day−4
Day−5
Stage
Transcript
ENSDART00000109314
ENSDART00000148640
ENSDART00000149022

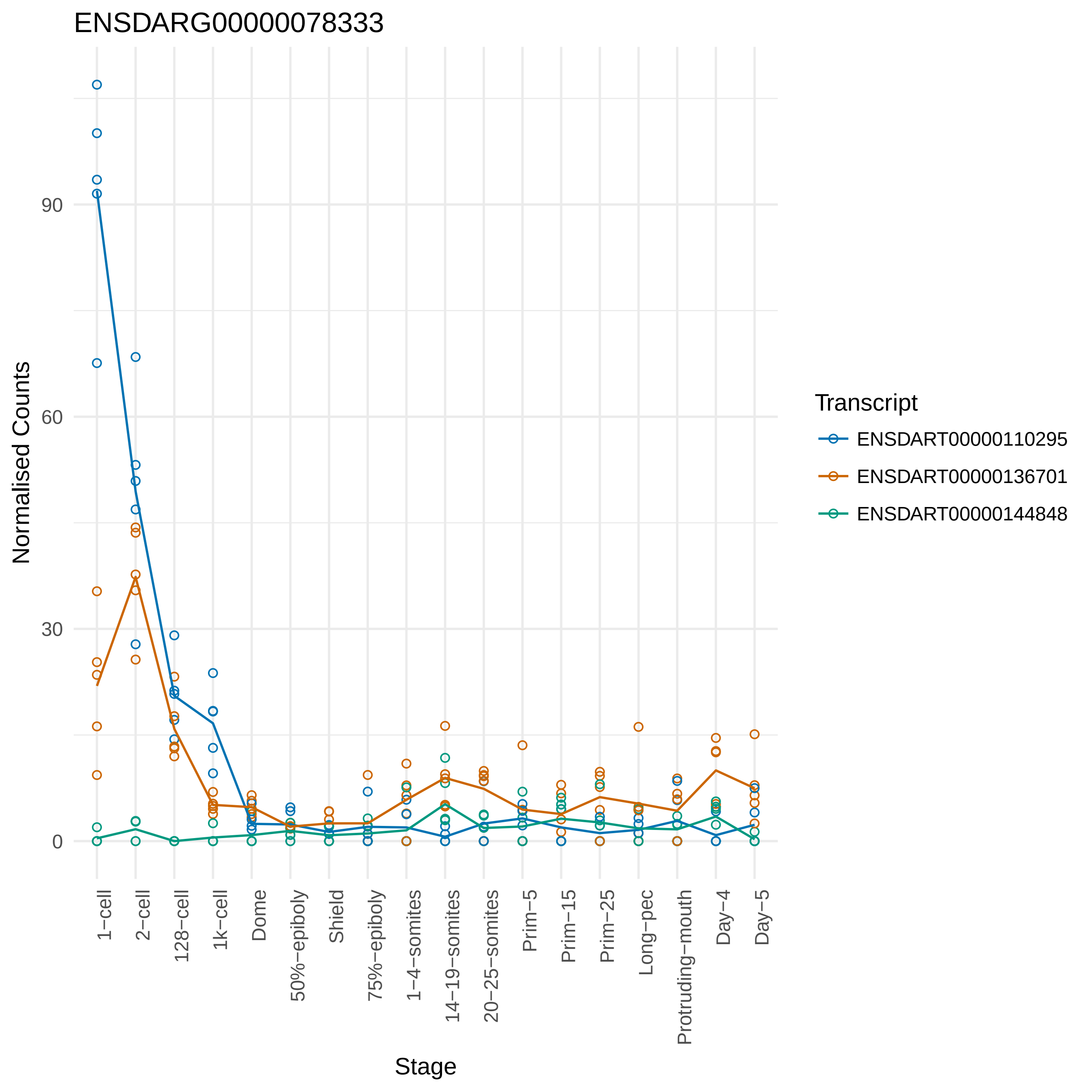

ENSDARG00000078333
Normalised Counts
0
30
60
90
Stage
1−cell
2−cell
128−cell
1k−cell
Dome
50%−epiboly
Shield
75%−epiboly
1−4−somites
14−19−somites
20−25−somites
Prim−5
Prim−15
Prim−25
Long−pec
Protruding−mouth
Day−4
Day−5
Transcript
ENSDART00000110295
ENSDART00000136701
ENSDART00000144848

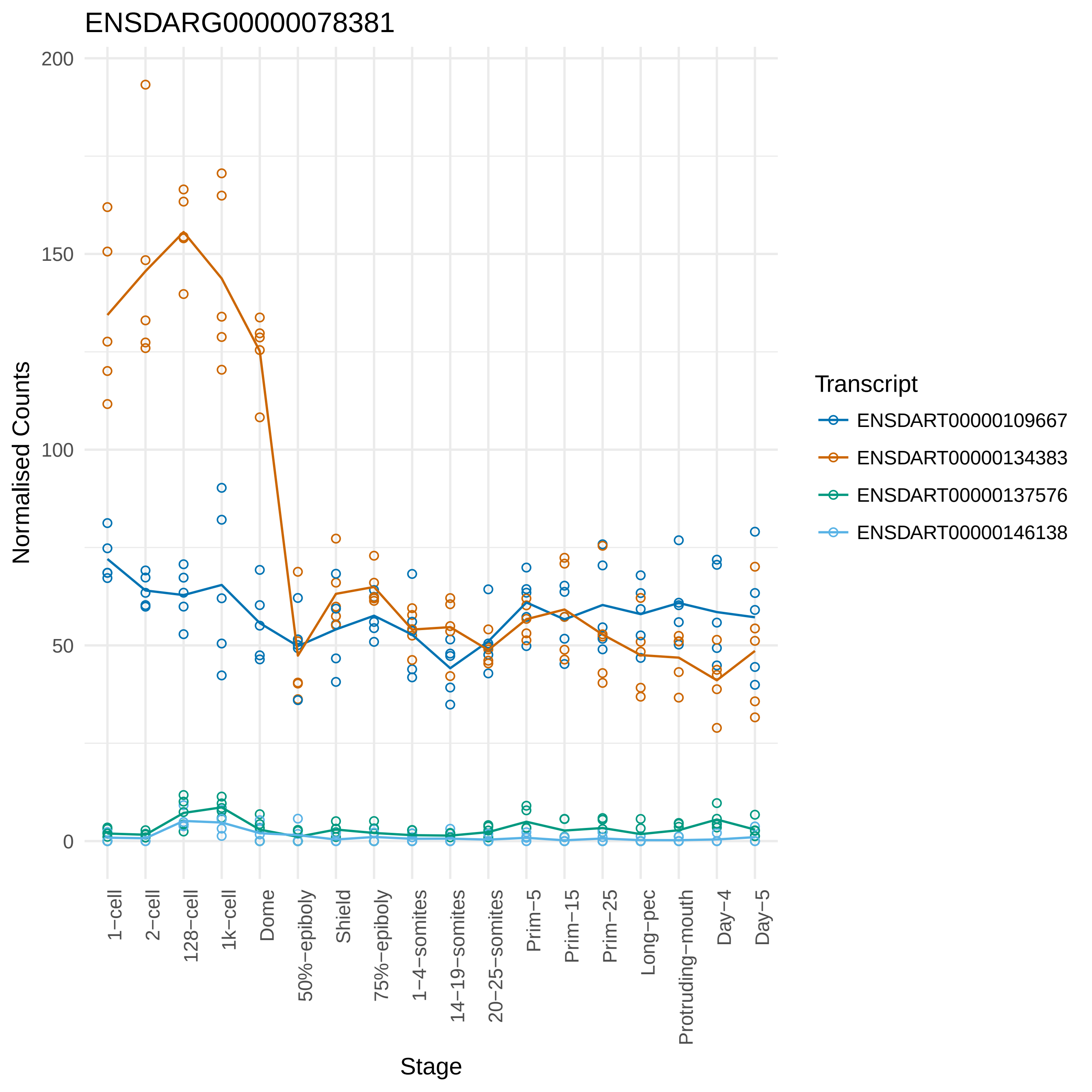

ENSDARG00000078381
Normalised Counts
200
150
100
50
0
1−cell
2−cell
128−cell
1k−cell
Dome
50%−epiboly
Shield
75%−epiboly
1−4−somites
14−19−somites
20−25−somites
Prim−5
Prim−15
Prim−25
Long−pec
Protruding−mouth
Day−4
Day−5
Stage
Transcript
ENSDART00000109667
ENSDART00000134383
ENSDART00000137576
ENSDART00000146138

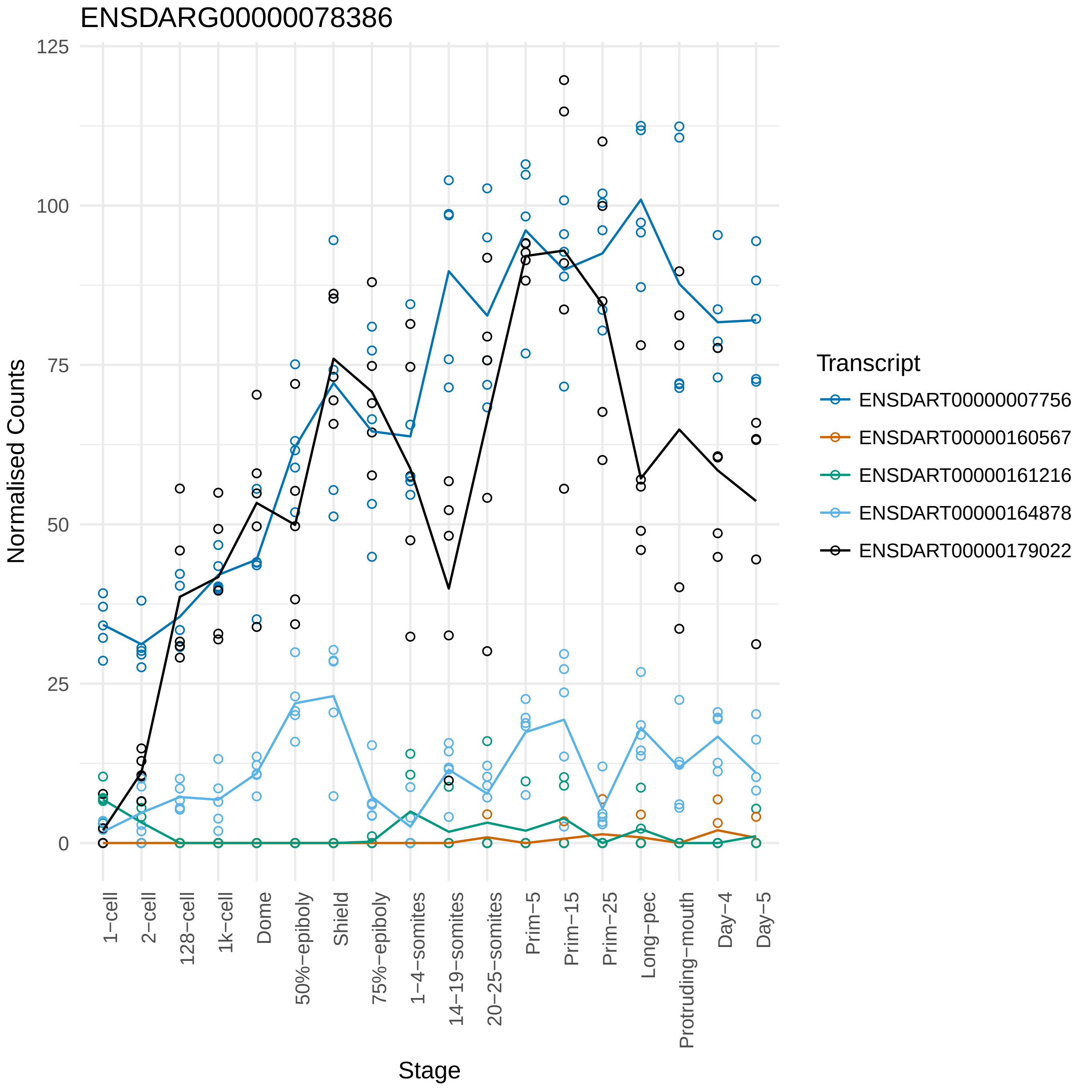
ENSDARG00000078386
Normalised Counts
125
100
75
50
25
0
1−cell
2−cell
128−cell
1k−cell
Dome
50%−epiboly
Shield
75%−epiboly
1−4−somites
14−19−somites
20−25−somites
Prim−5
Prim−15
Prim−25
Long−pec
Protruding−mouth
Day−4
Day−5
Stage
Transcript
ENSDART00000007756
ENSDART00000160567
ENSDART00000161216
ENSDART00000164878
ENSDART00000179022

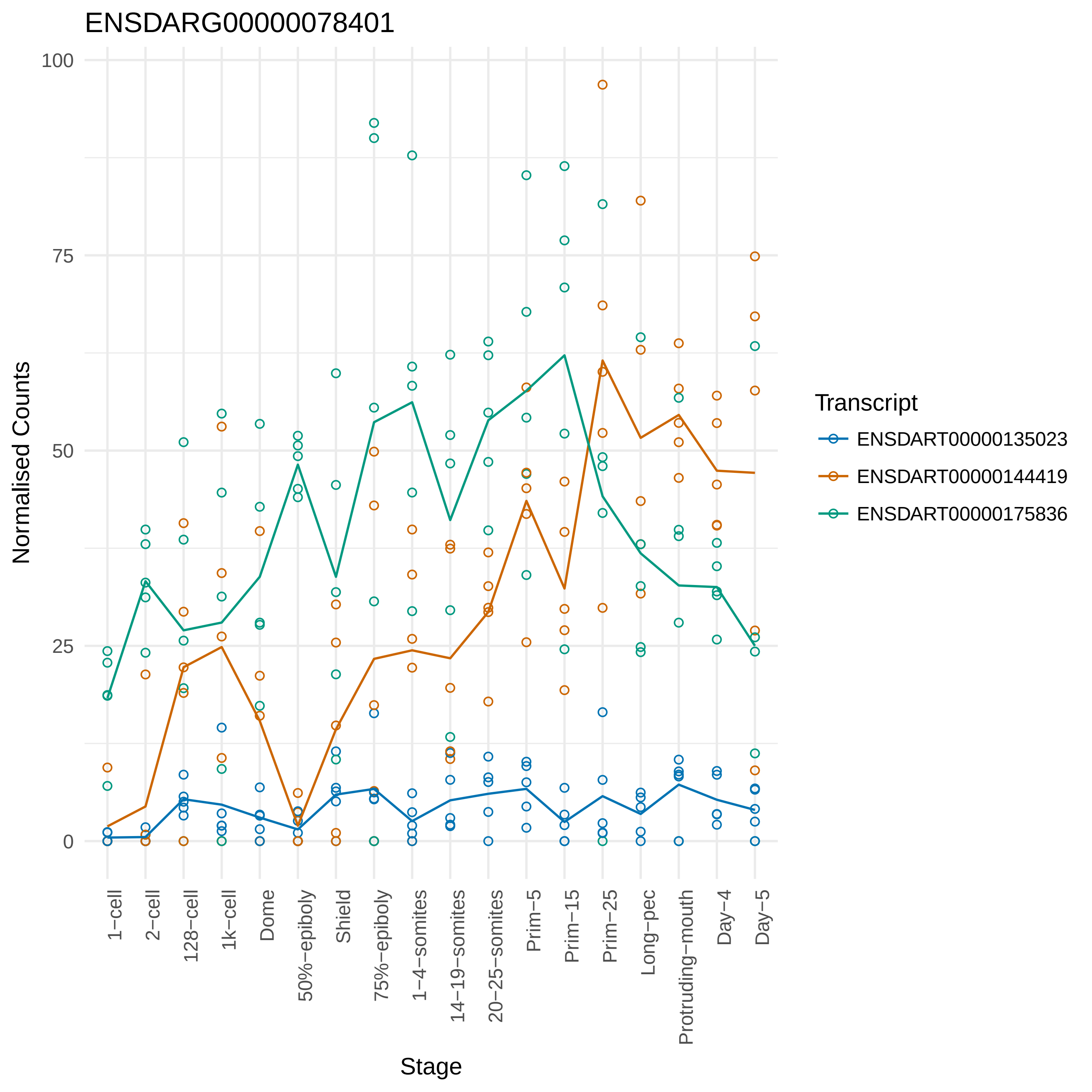
ENSDARG00000078401
Normalised Counts
100
75
50
25
0
1−cell
2−cell
128−cell
1k−cell
Dome
50%−epiboly
Shield
75%−epiboly
1−4−somites
14−19−somites
20−25−somites
Prim−5
Prim−15
Prim−25
Long−pec
Protruding−mouth
Day−4
Day−5
Stage
Transcript
ENSDART00000135023
ENSDART00000144419
ENSDART00000175836

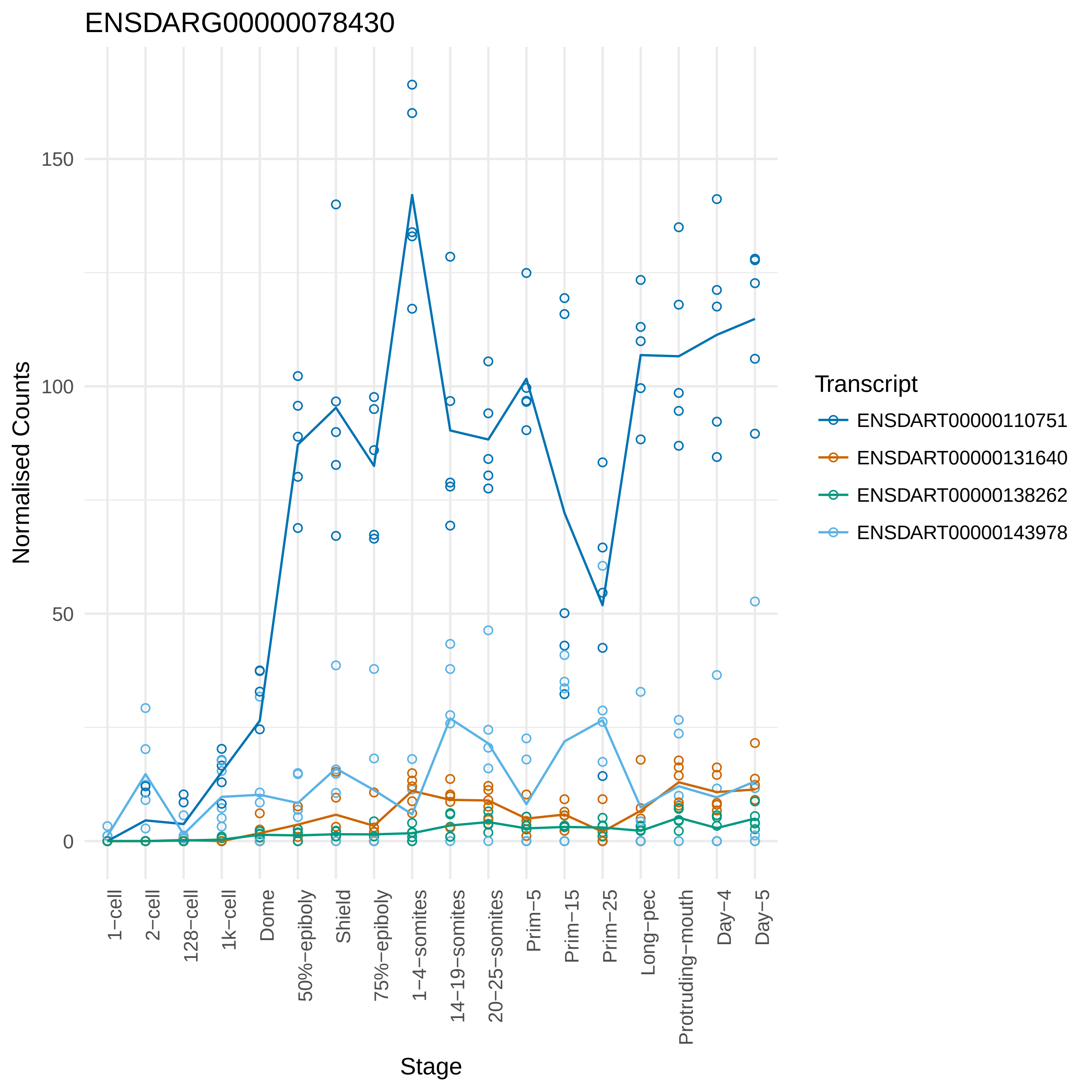

ENSDARG00000078430
Normalised Counts
150
100
50
0
1−cell
2−cell
128−cell
1k−cell
Dome
50%−epiboly
Shield
75%−epiboly
1−4−somites
14−19−somites
20−25−somites
Prim−5
Prim−15
Prim−25
Long−pec
Protruding−mouth
Day−4
Day−5
Stage
Transcript
ENSDART00000110751
ENSDART00000131640
ENSDART00000138262
ENSDART00000143978

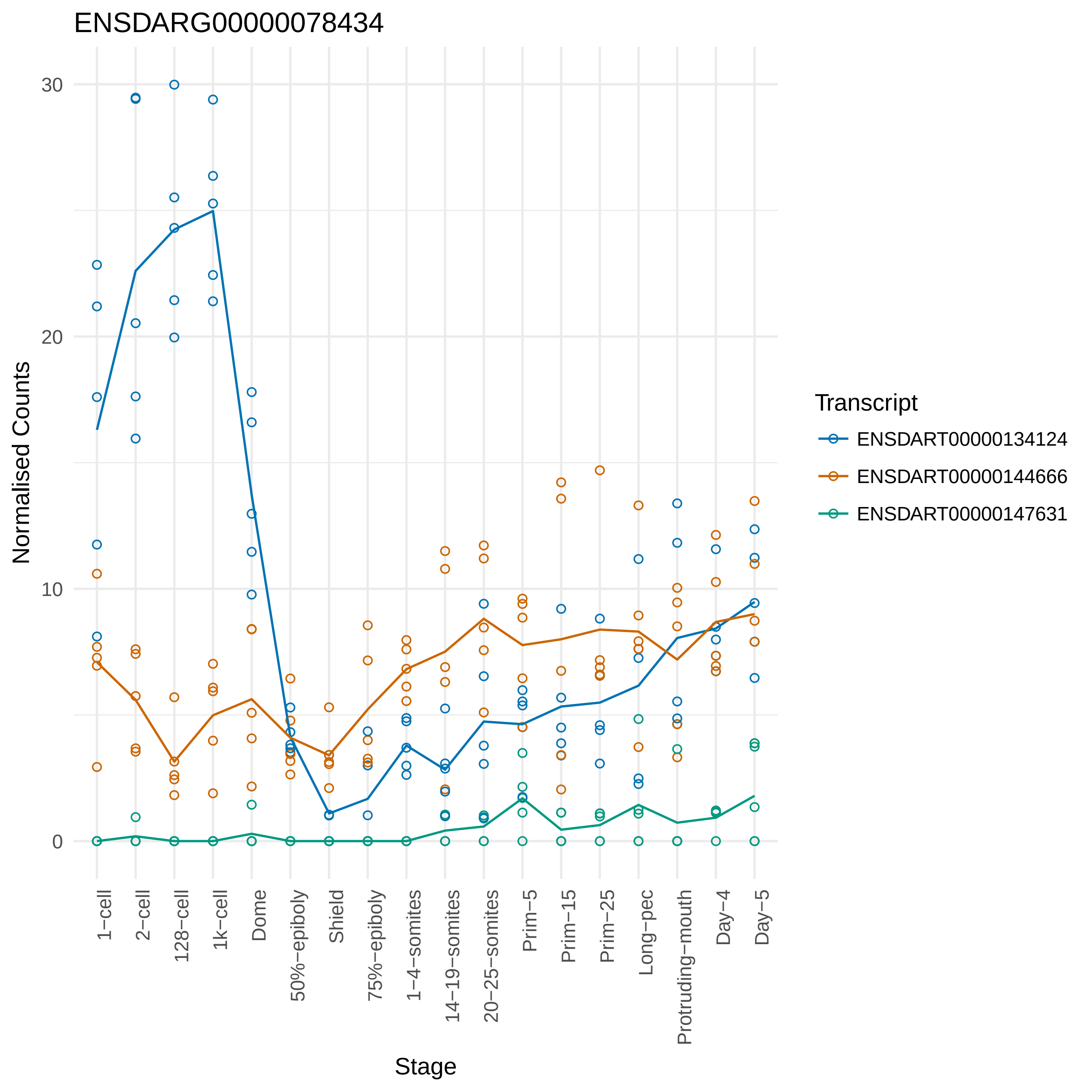

ENSDARG00000078434
Normalised Counts
0
10
20
30
1−cell
2−cell
128−cell
1k−cell
Dome
50%−epiboly
Shield
75%−epiboly
1−4−somites
14−19−somites
20−25−somites
Prim−5
Prim−15
Prim−25
Long−pec
Protruding−mouth
Day−4
Day−5
Stage
Transcript
ENSDART00000134124
ENSDART00000144666
ENSDART00000147631

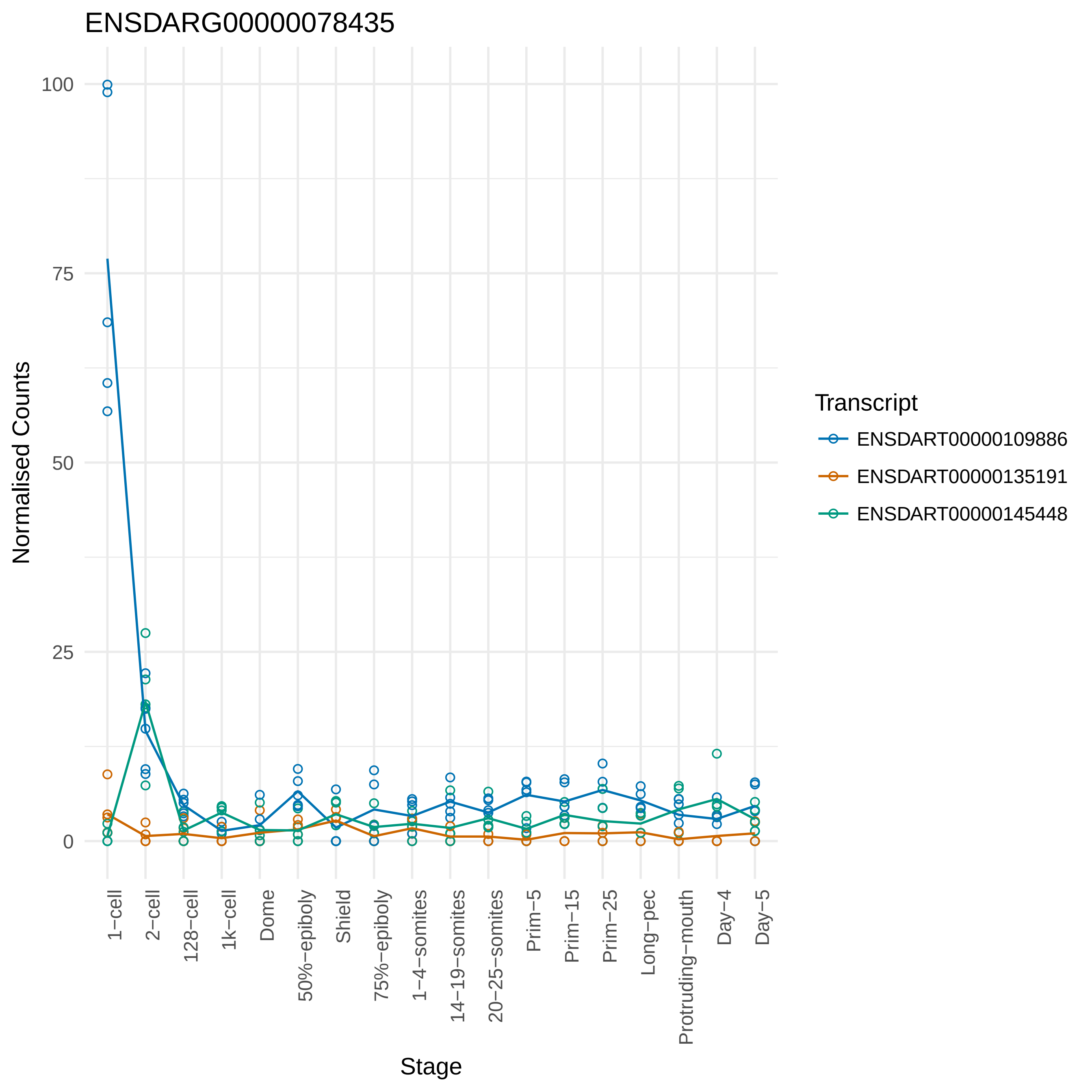
ENSDARG00000078435
Normalised Counts
100
75
50
25
0
1−cell
2−cell
128−cell
1k−cell
Dome
50%−epiboly
Shield
75%−epiboly
1−4−somites
14−19−somites
20−25−somites
Prim−5
Prim−15
Prim−25
Long−pec
Protruding−mouth
Day−4
Day−5
Stage
Transcript
ENSDART00000109886
ENSDART00000135191
ENSDART00000145448

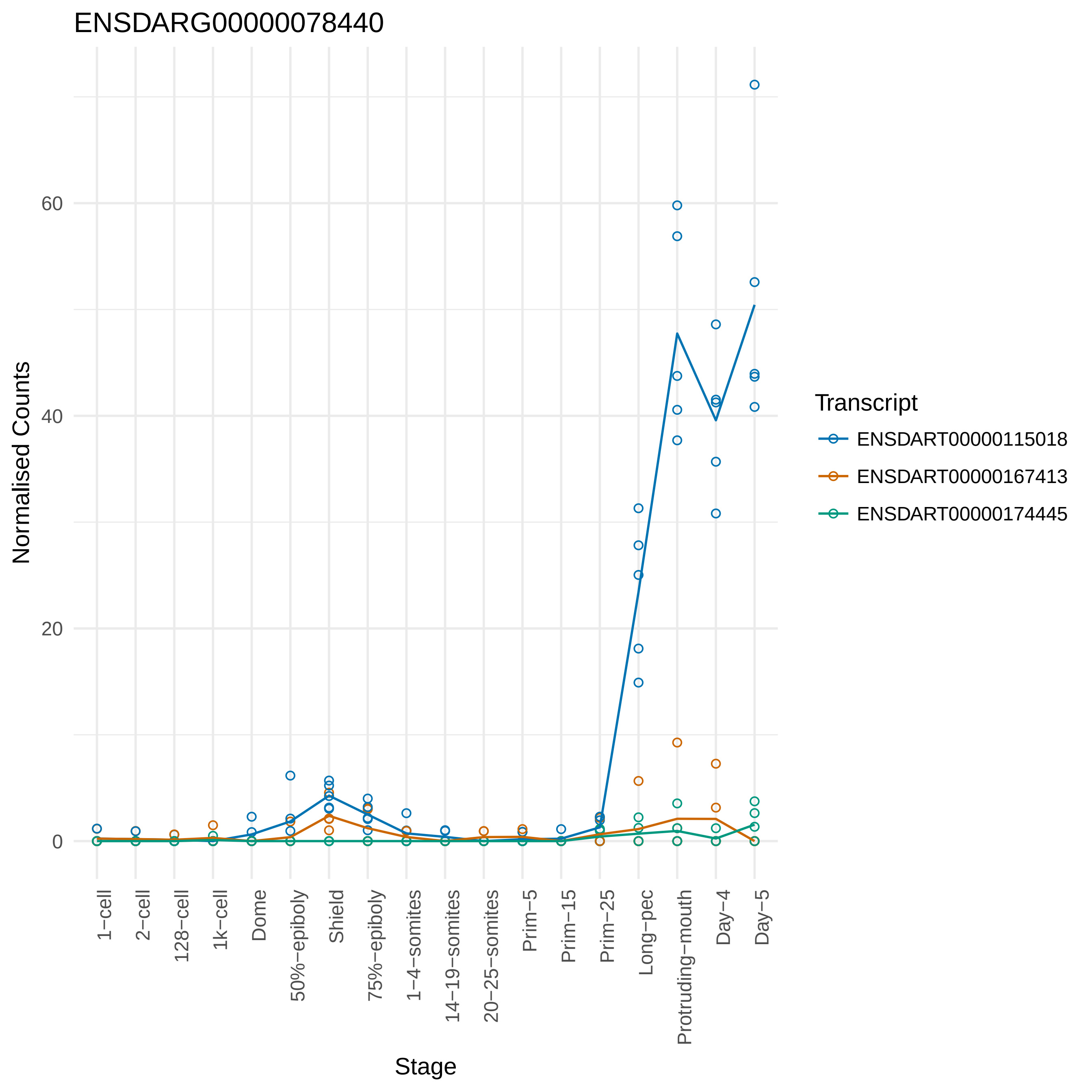

ENSDARG00000078440
Normalised Counts
60
40
20
0
1−cell
2−cell
128−cell
1k−cell
Dome
50%−epiboly
Shield
75%−epiboly
1−4−somites
14−19−somites
20−25−somites
Prim−5
Prim−15
Prim−25
Long−pec
Protruding−mouth
Day−4
Day−5
Stage
Transcript
ENSDART00000115018
ENSDART00000167413
ENSDART00000174445

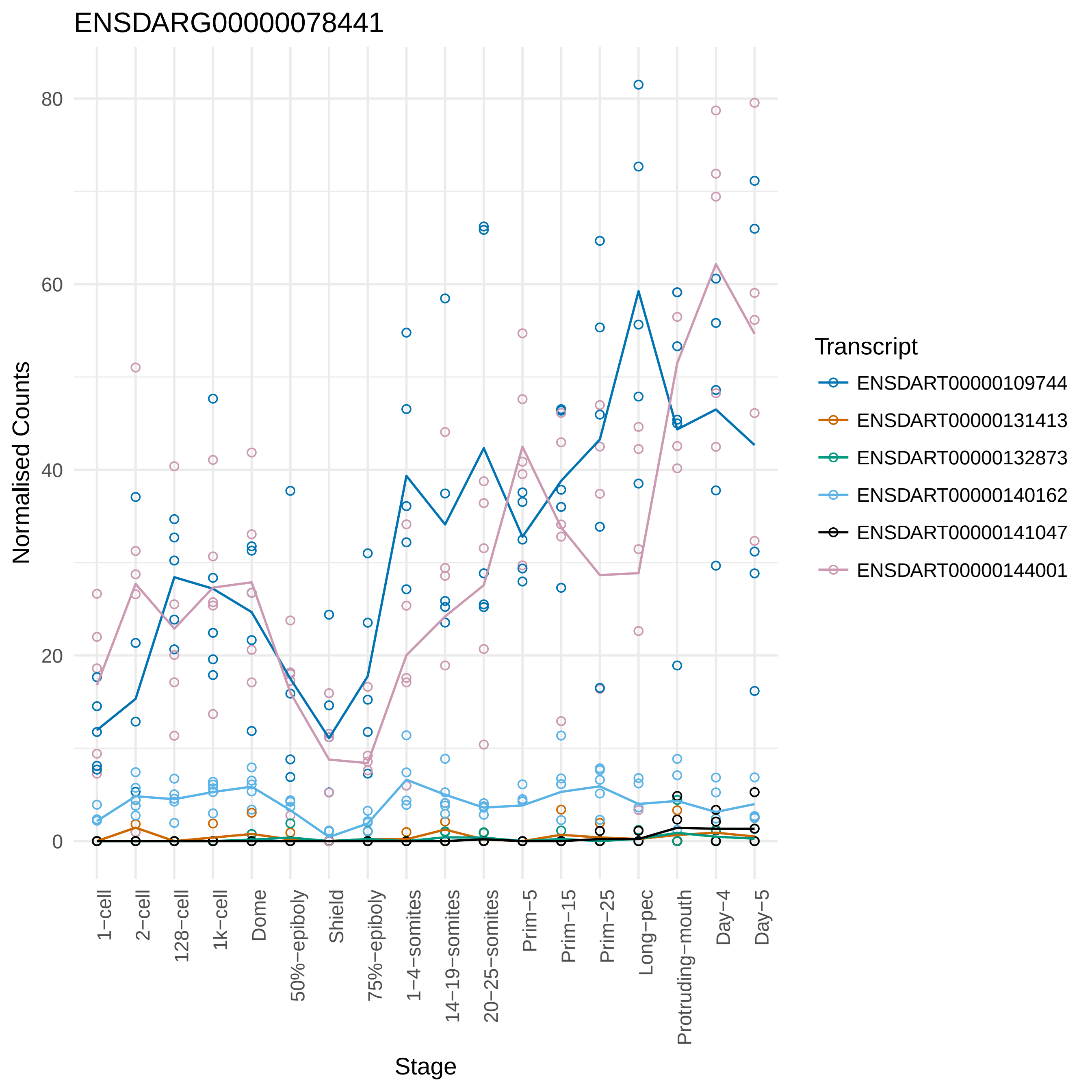
ENSDARG00000078441
Normalised Counts
0
20
40
60
80
Stage
1−cell
2−cell
128−cell
1k−cell
Dome
50%−epiboly
Shield
75%−epiboly
1−4−somites
14−19−somites
20−25−somites
Prim−5
Prim−15
Prim−25
Long−pec
Protruding−mouth
Day−4
Day−5
Transcript
ENSDART00000109744
ENSDART00000131413
ENSDART00000132873
ENSDART00000140162
ENSDART00000141047
ENSDART00000144001

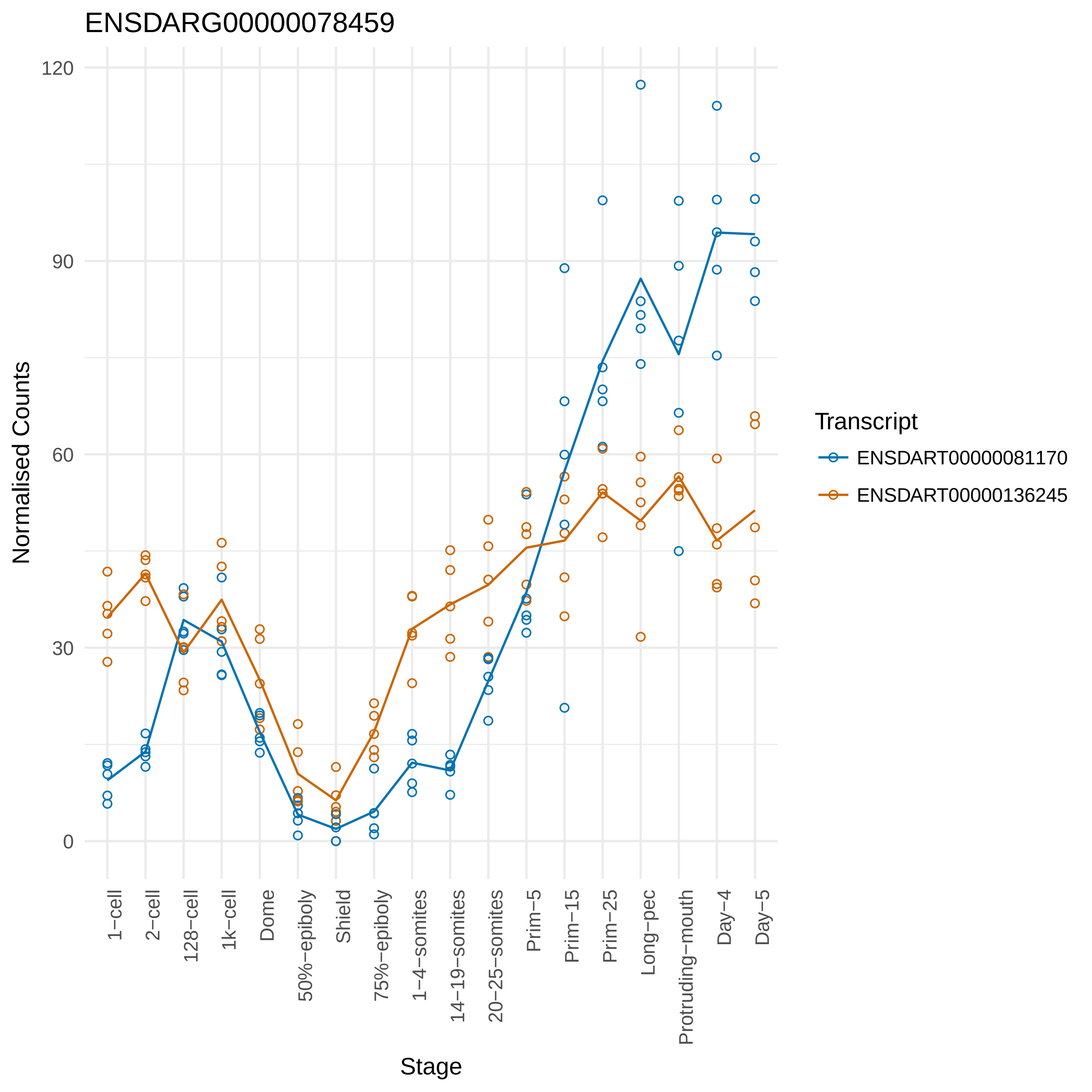

ENSDARG00000078459
Normalised Counts
120
90
60
30
0
1−cell
2−cell
128−cell
1k−cell
Dome
50%−epiboly
Shield
75%−epiboly
1−4−somites
14−19−somites
20−25−somites
Prim−5
Prim−15
Prim−25
Long−pec
Protruding−mouth
Day−4
Day−5
Stage
Transcript
ENSDART00000081170
ENSDART00000136245

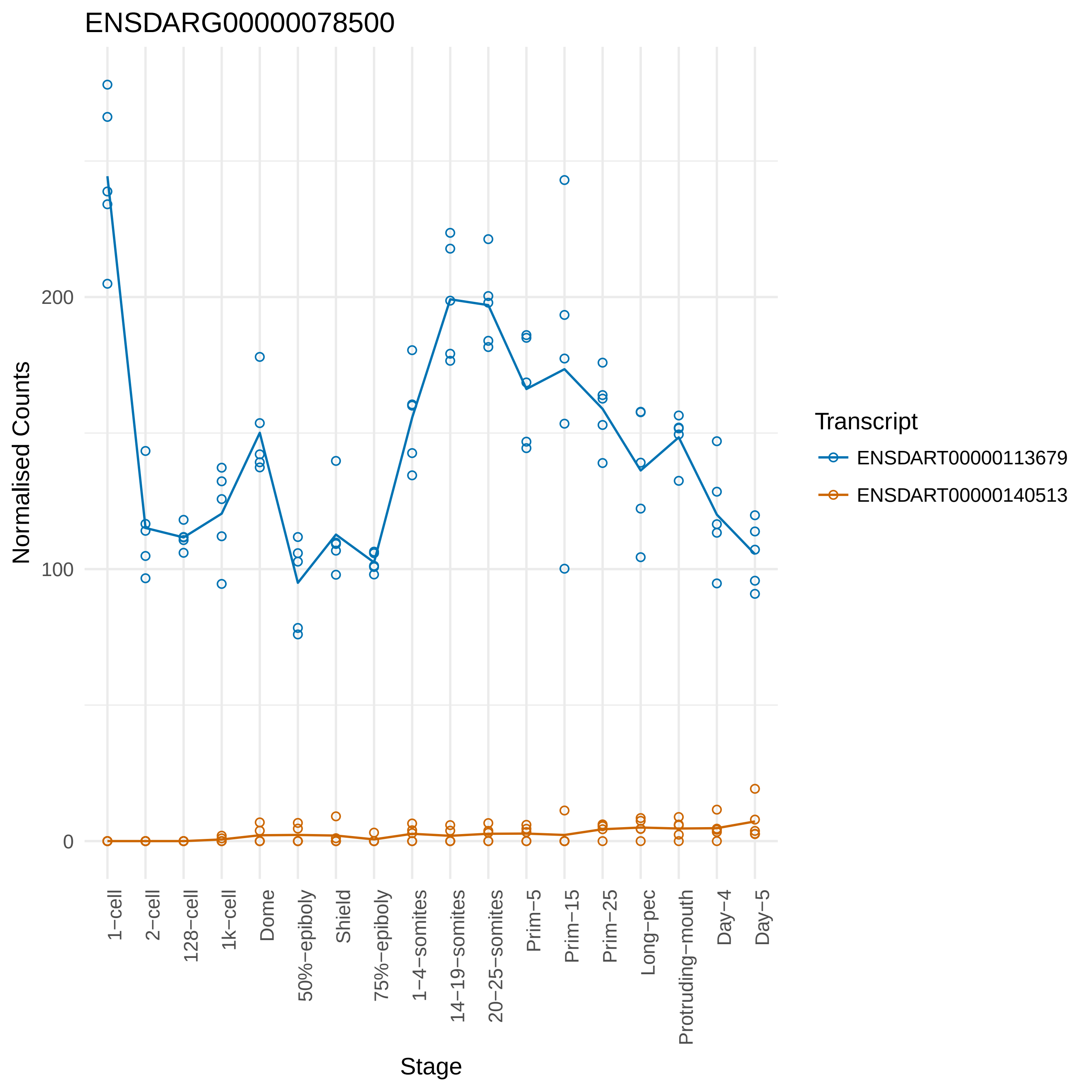
ENSDARG00000078500
Normalised Counts
200
100
0
1−cell
2−cell
128−cell
1k−cell
Dome
50%−epiboly
Shield
75%−epiboly
1−4−somites
14−19−somites
20−25−somites
Prim−5
Prim−15
Prim−25
Long−pec
Protruding−mouth
Day−4
Day−5
Stage
Transcript
ENSDART00000113679
ENSDART00000140513

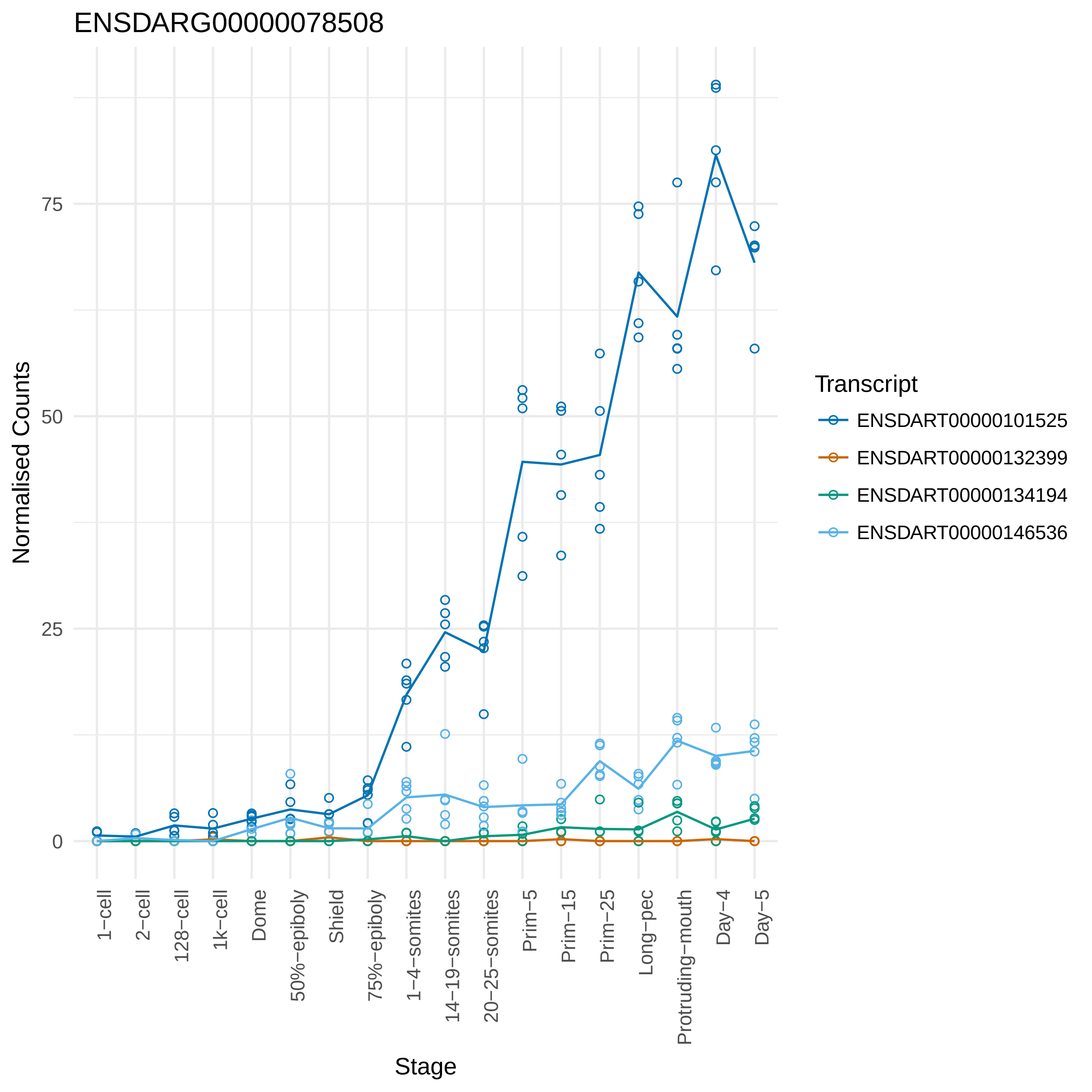
ENSDARG00000078508
Normalised Counts
75
50
25
0
1−cell
2−cell
128−cell
1k−cell
Dome
50%−epiboly
Shield
75%−epiboly
1−4−somites
14−19−somites
20−25−somites
Prim−5
Prim−15
Prim−25
Long−pec
Protruding−mouth
Day−4
Day−5
Stage
Transcript
ENSDART00000101525
ENSDART00000132399
ENSDART00000134194
ENSDART00000146536

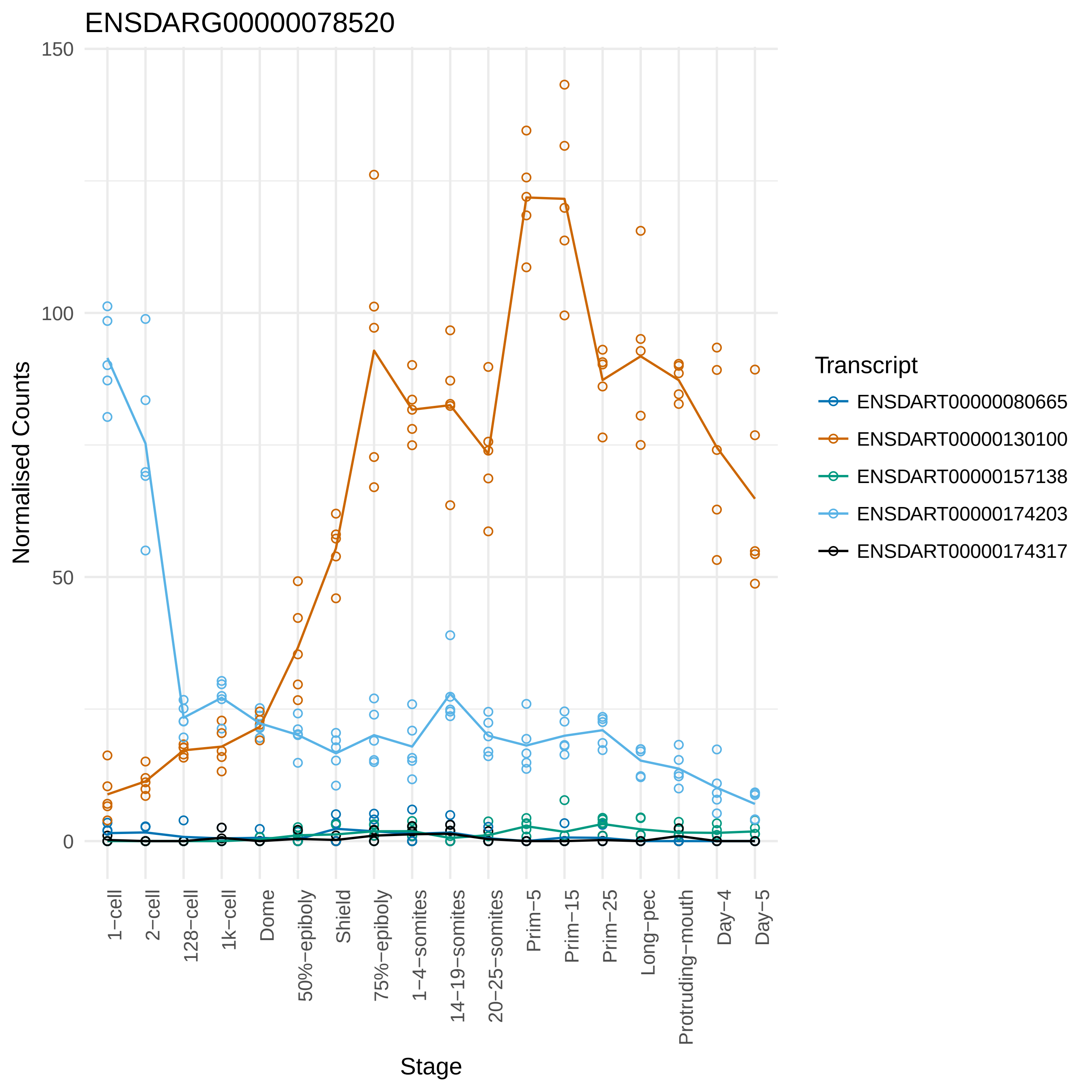

ENSDARG00000078520
Normalised Counts
150
100
50
0
1−cell
2−cell
128−cell
1k−cell
Dome
50%−epiboly
Shield
75%−epiboly
1−4−somites
14−19−somites
20−25−somites
Prim−5
Prim−15
Prim−25
Long−pec
Protruding−mouth
Day−4
Day−5
Stage
Transcript
ENSDART00000080665
ENSDART00000130100
ENSDART00000157138
ENSDART00000174203
ENSDART00000174317

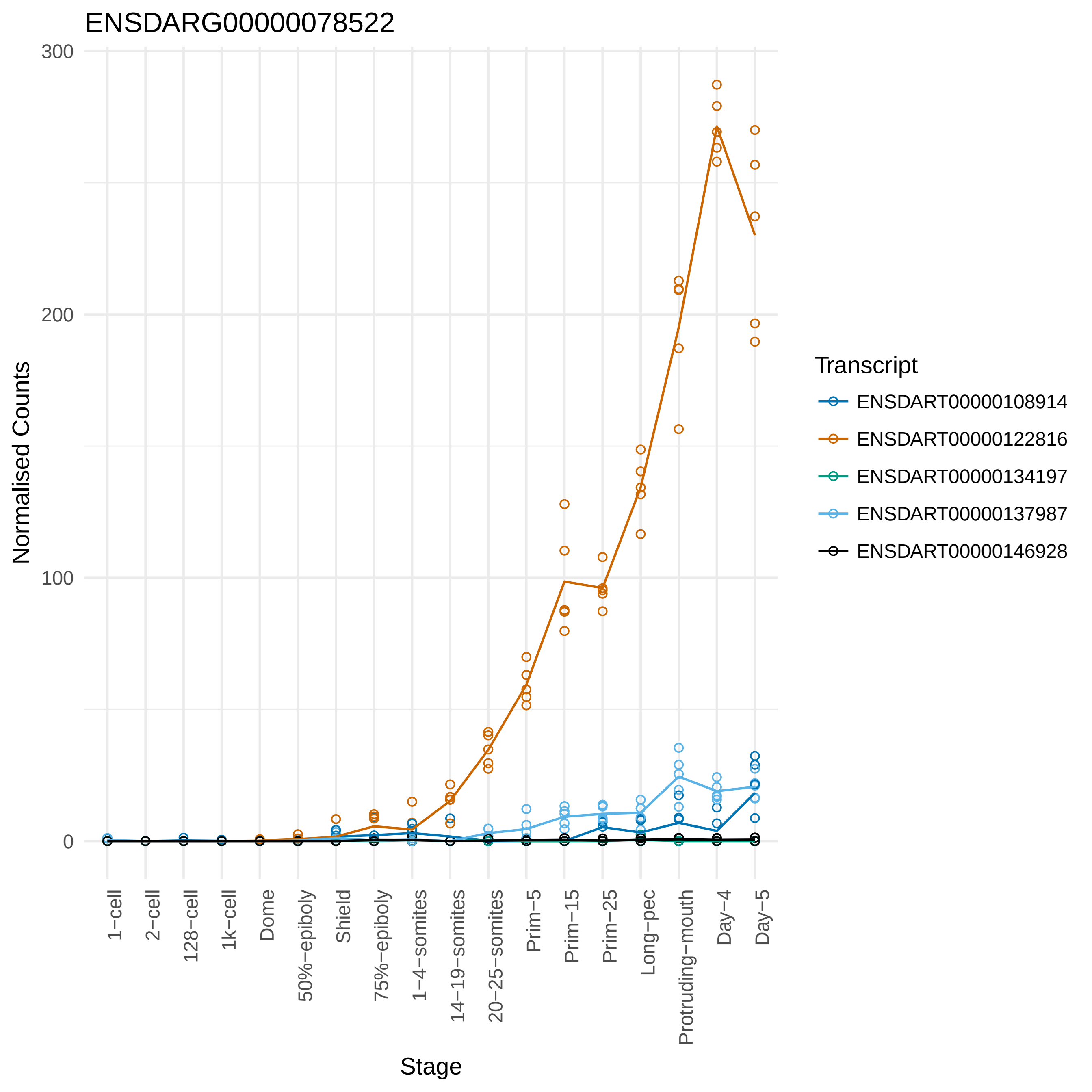

ENSDARG00000078522
Normalised Counts
300
200
100
0
1−cell
2−cell
128−cell
1k−cell
Dome
50%−epiboly
Shield
75%−epiboly
1−4−somites
14−19−somites
20−25−somites
Prim−5
Prim−15
Prim−25
Long−pec
Protruding−mouth
Day−4
Day−5
Stage
Transcript
ENSDART00000108914
ENSDART00000122816
ENSDART00000134197
ENSDART00000137987
ENSDART00000146928

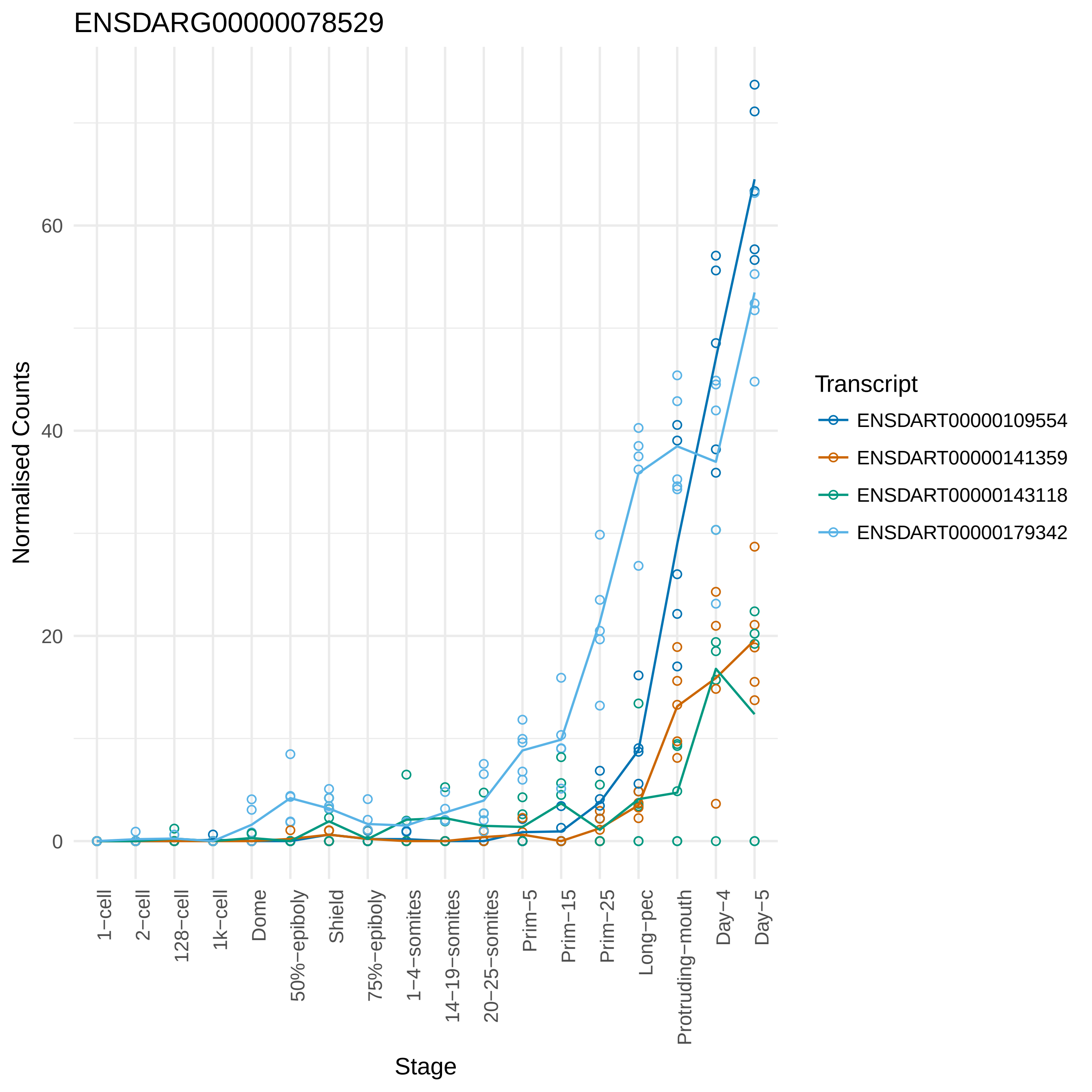

ENSDARG00000078529
Normalised Counts
0
20
40
60
1−cell
2−cell
128−cell
1k−cell
Dome
50%−epiboly
Shield
75%−epiboly
1−4−somites
14−19−somites
20−25−somites
Prim−5
Prim−15
Prim−25
Long−pec
Protruding−mouth
Day−4
Day−5
Stage
Transcript
ENSDART00000109554
ENSDART00000141359
ENSDART00000143118
ENSDART00000179342

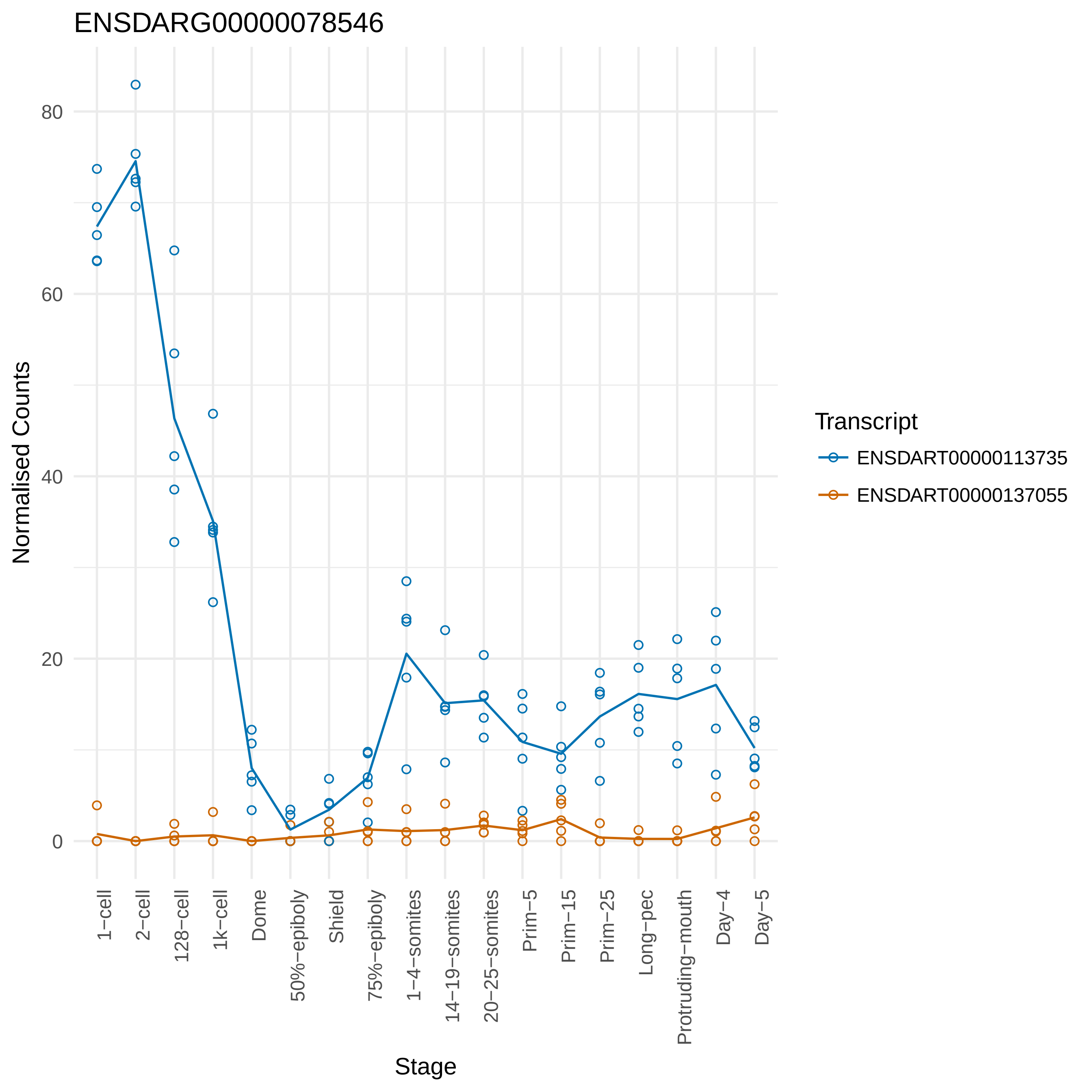

ENSDARG00000078546
Normalised Counts
80
60
40
20
0
1−cell
2−cell
128−cell
1k−cell
Dome
50%−epiboly
Shield
75%−epiboly
1−4−somites
14−19−somites
20−25−somites
Prim−5
Prim−15
Prim−25
Long−pec
Protruding−mouth
Day−4
Day−5
Stage
Transcript
ENSDART00000113735
ENSDART00000137055

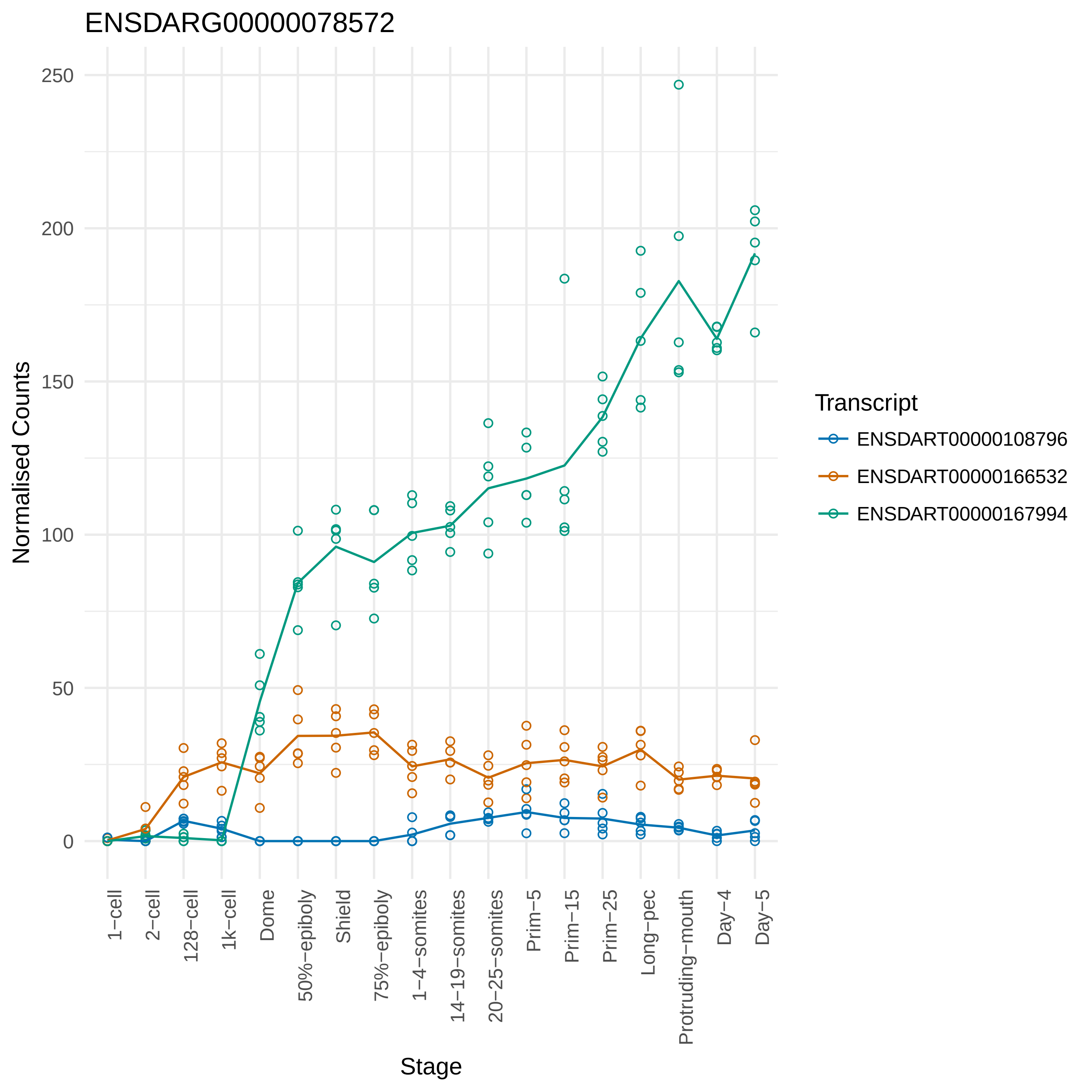
ENSDARG00000078572
Normalised Counts
0
50
100
150
200
250
Stage
1−cell
2−cell
128−cell
1k−cell
Dome
50%−epiboly
Shield
75%−epiboly
1−4−somites
14−19−somites
20−25−somites
Prim−5
Prim−15
Prim−25
Long−pec
Protruding−mouth
Day−4
Day−5
Transcript
ENSDART00000108796
ENSDART00000166532
ENSDART00000167994

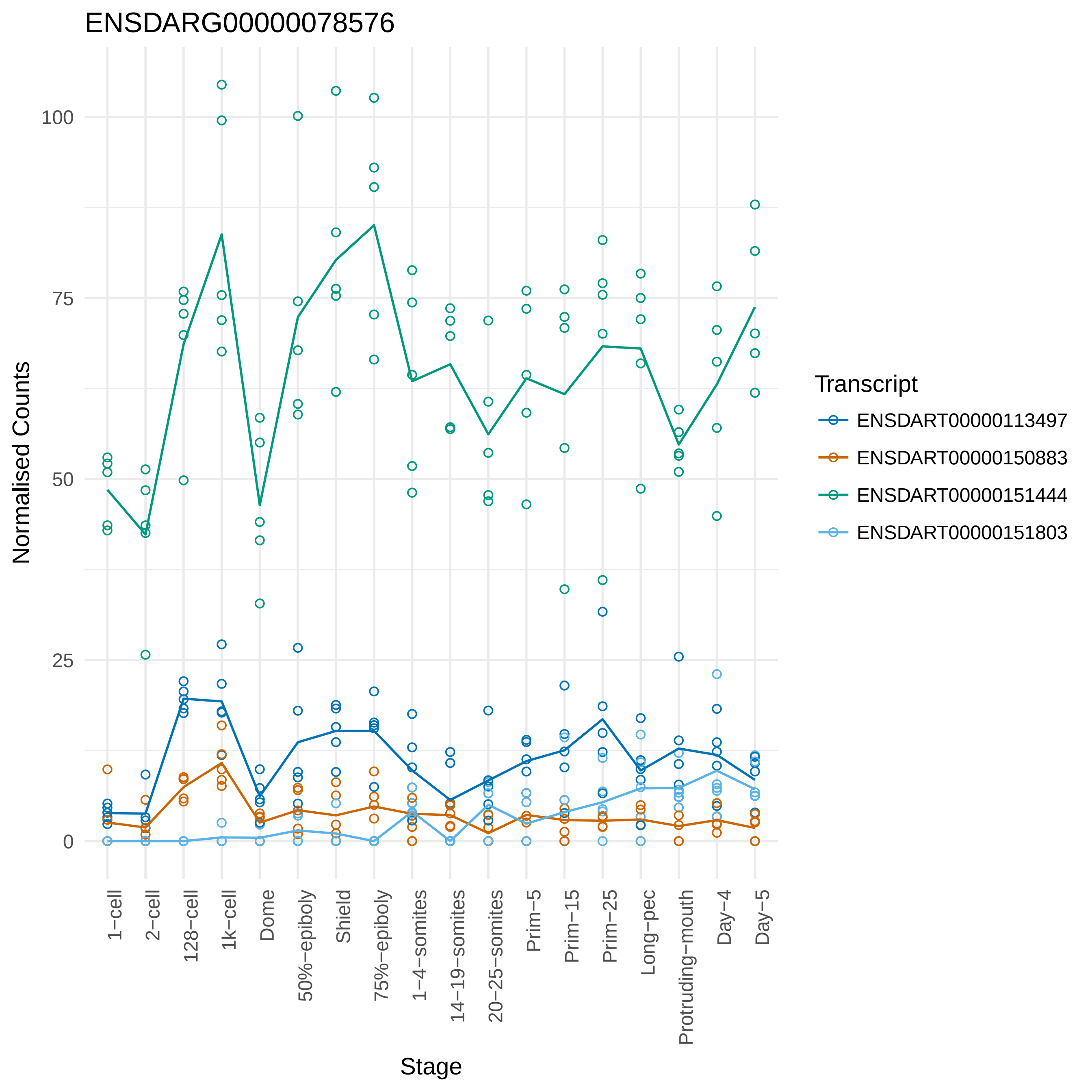
ENSDARG00000078576
Normalised Counts
100
75
50
25
0
1−cell
2−cell
128−cell
1k−cell
Dome
50%−epiboly
Shield
75%−epiboly
1−4−somites
14−19−somites
20−25−somites
Prim−5
Prim−15
Prim−25
Long−pec
Protruding−mouth
Day−4
Day−5
Stage
Transcript
ENSDART00000113497
ENSDART00000150883
ENSDART00000151444
ENSDART00000151803

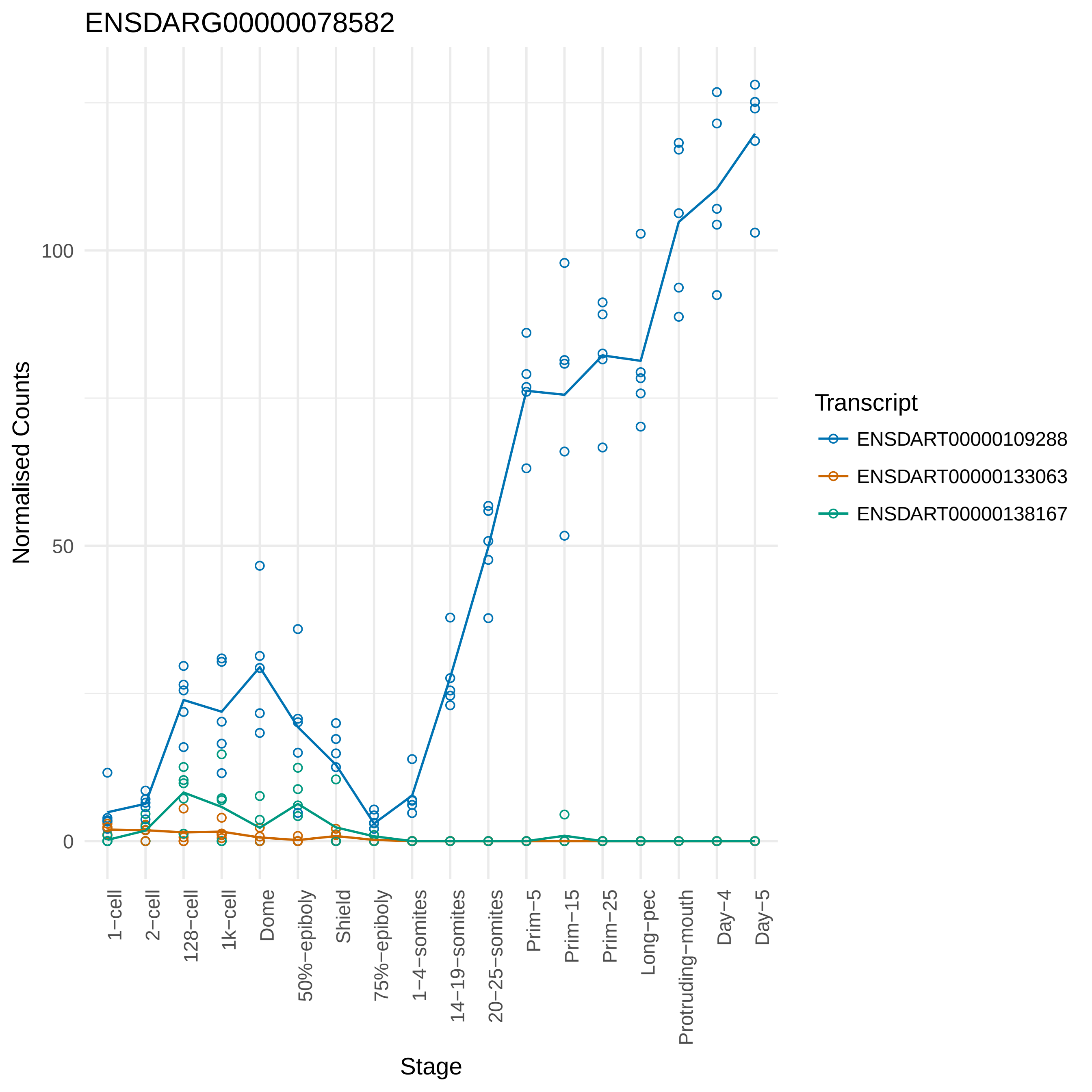

ENSDARG00000078582
Normalised Counts
100
50
0
Stage
1−cell
2−cell
128−cell
1k−cell
Dome
50%−epiboly
Shield
75%−epiboly
1−4−somites
14−19−somites
20−25−somites
Prim−5
Prim−15
Prim−25
Long−pec
Protruding−mouth
Day−4
Day−5
Transcript
ENSDART00000109288
ENSDART00000133063
ENSDART00000138167

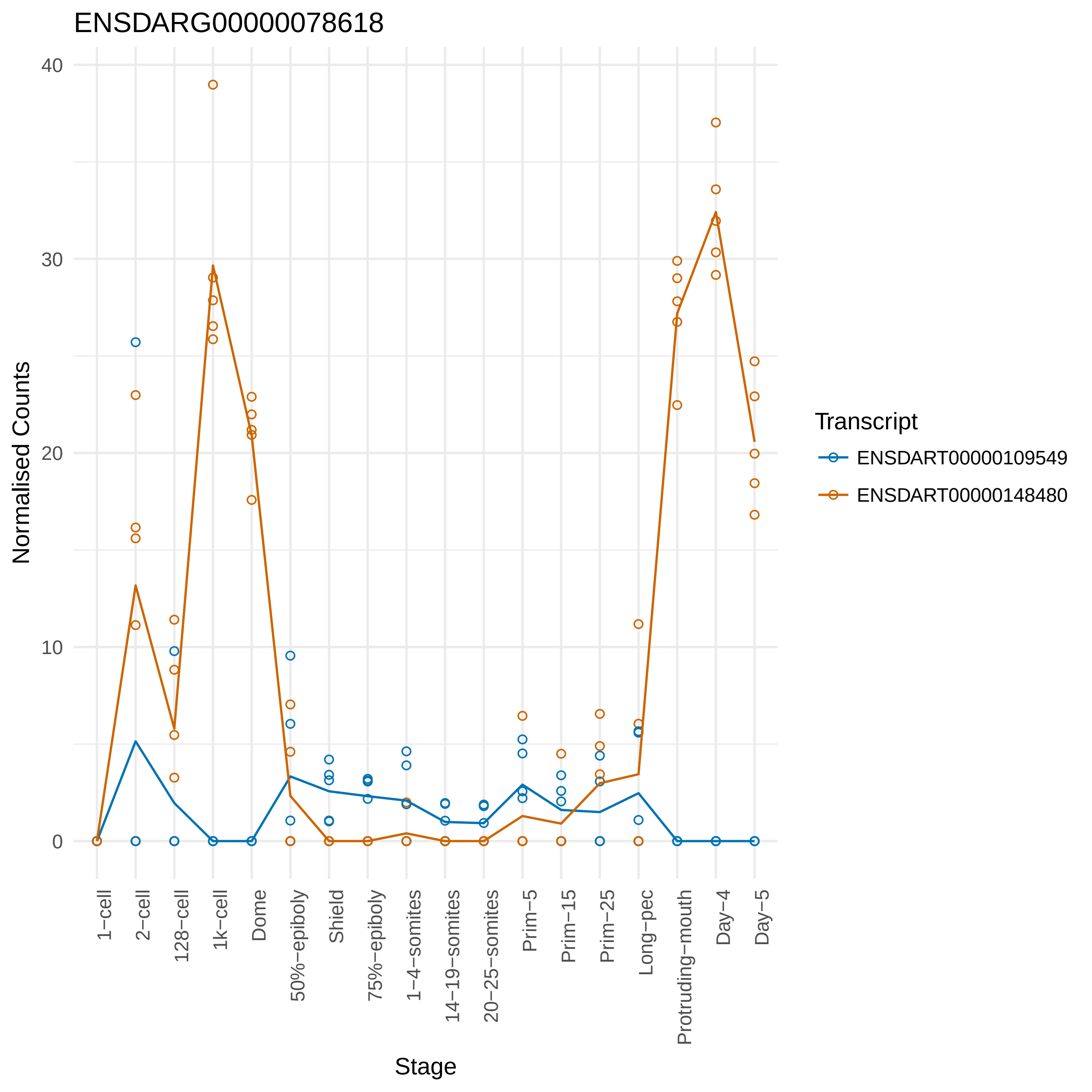
ENSDARG00000078618
Normalised Counts
0
10
20
30
40
Stage
1−cell
2−cell
128−cell
1k−cell
Dome
50%−epiboly
Shield
75%−epiboly
1−4−somites
14−19−somites
20−25−somites
Prim−5
Prim−15
Prim−25
Long−pec
Protruding−mouth
Day−4
Day−5
Transcript
ENSDART00000109549
ENSDART00000148480

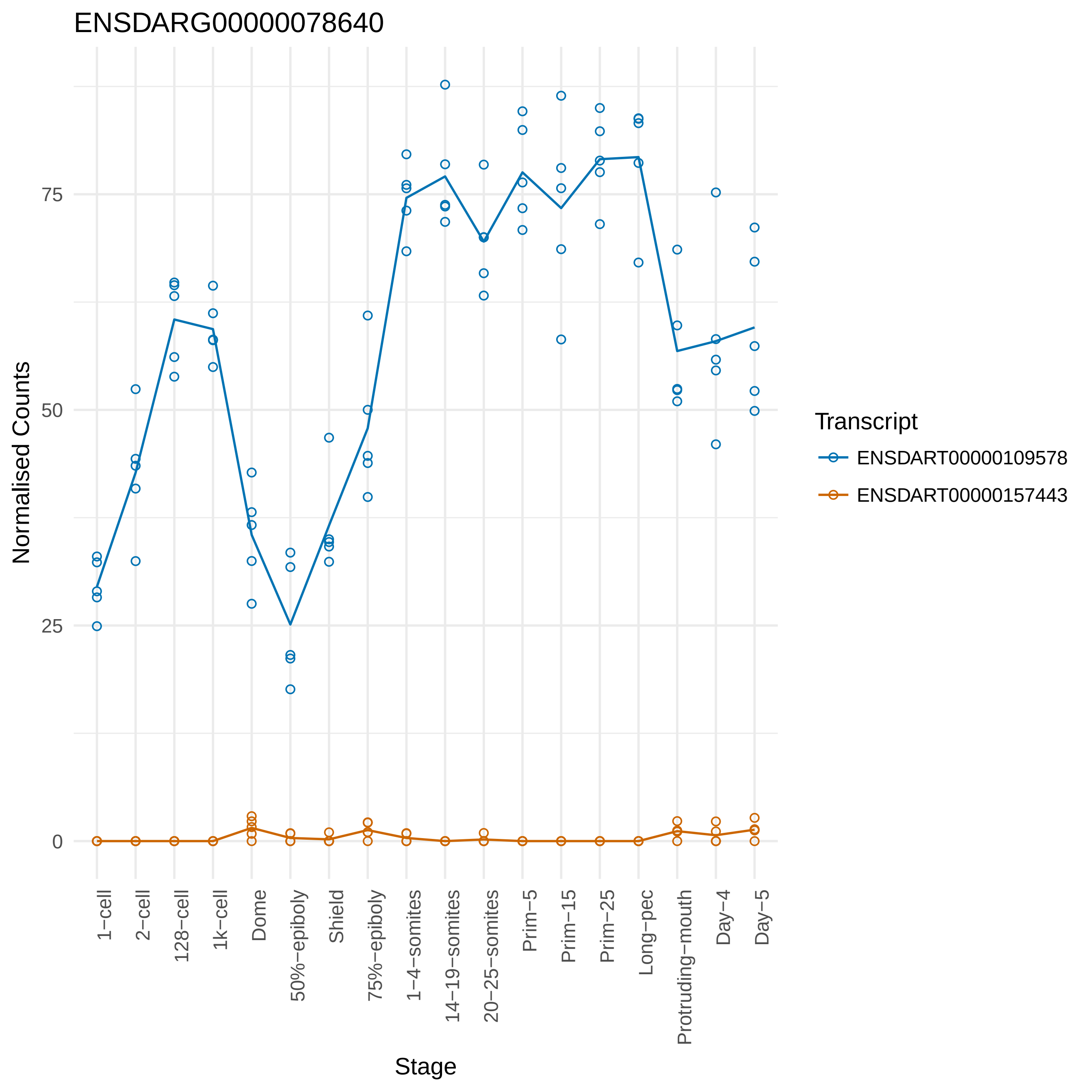

ENSDARG00000078640
Normalised Counts
75
50
25
0
1−cell
2−cell
128−cell
1k−cell
Dome
50%−epiboly
Shield
75%−epiboly
1−4−somites
14−19−somites
20−25−somites
Prim−5
Prim−15
Prim−25
Long−pec
Protruding−mouth
Day−4
Day−5
Stage
Transcript
ENSDART00000109578
ENSDART00000157443

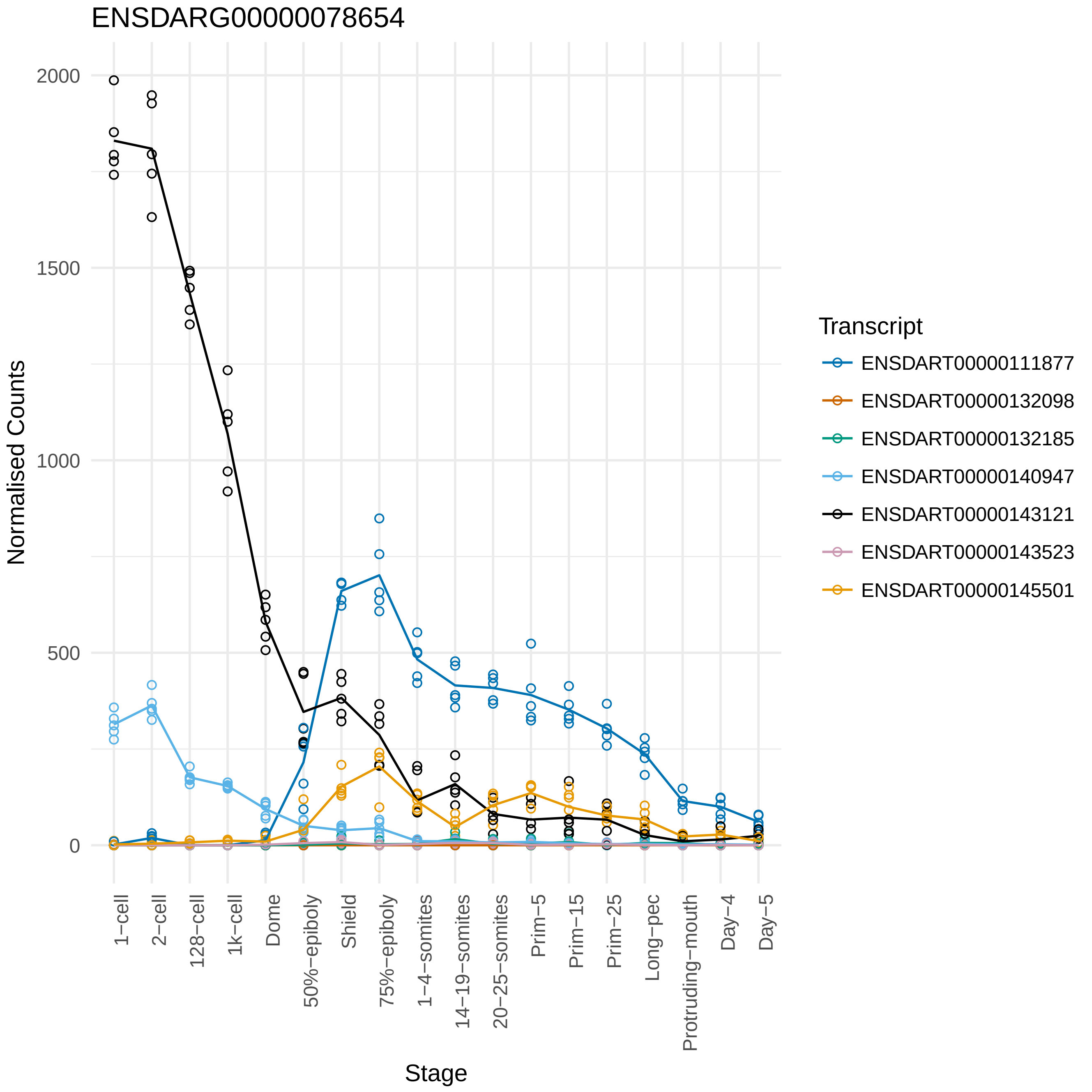

ENSDARG00000078654
Normalised Counts
2000
1500
1000
500
0
Stage
1−cell
2−cell
128−cell
1k−cell
Dome
50%−epiboly
Shield
75%−epiboly
1−4−somites
14−19−somites
20−25−somites
Prim−5
Prim−15
Prim−25
Long−pec
Protruding−mouth
Day−4
Day−5
Transcript
ENSDART00000111877
ENSDART00000132098
ENSDART00000132185
ENSDART00000140947
ENSDART00000143121
ENSDART00000143523
ENSDART00000145501

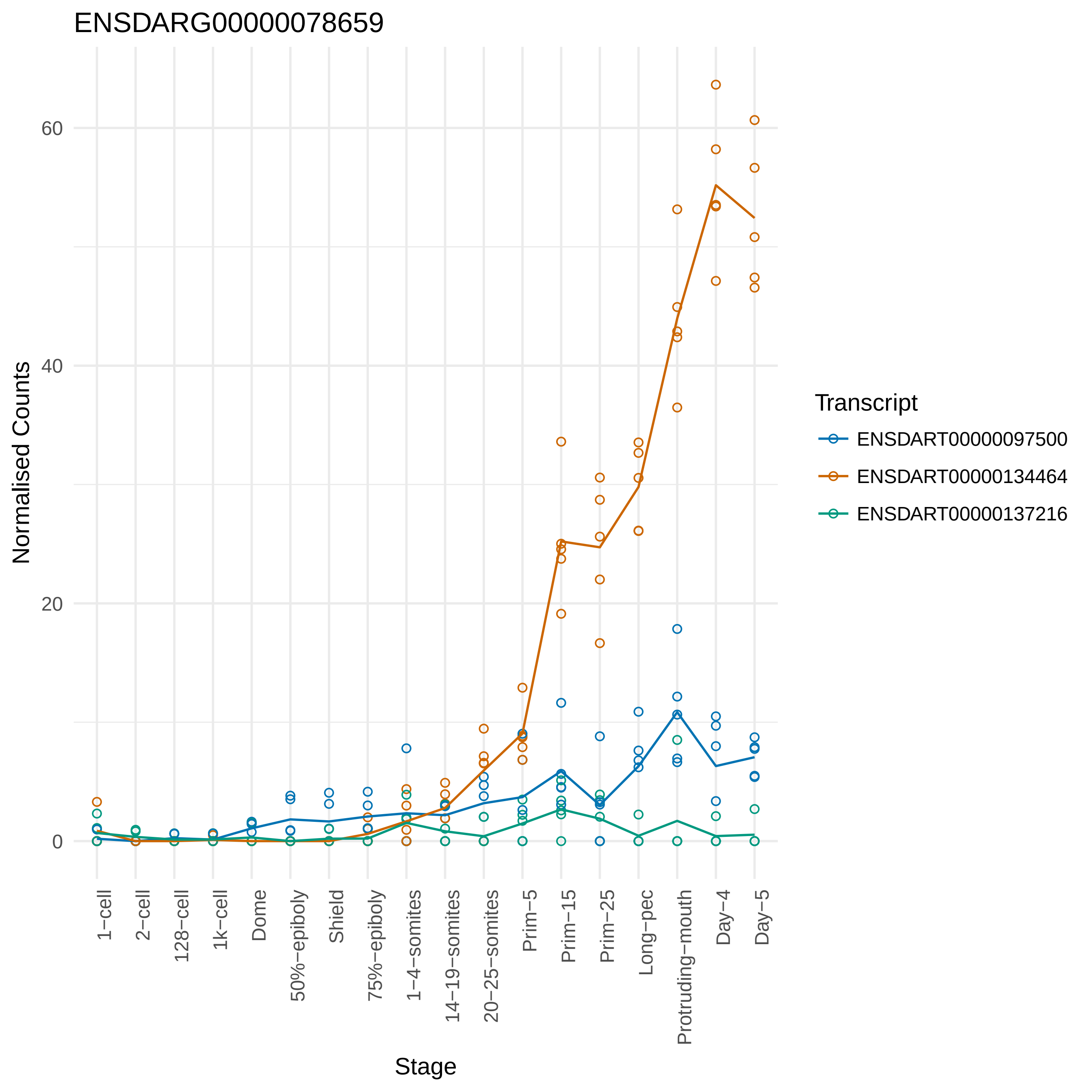
ENSDARG00000078659
Normalised Counts
0
20
40
60
Stage
1−cell
2−cell
128−cell
1k−cell
Dome
50%−epiboly
Shield
75%−epiboly
1−4−somites
14−19−somites
20−25−somites
Prim−5
Prim−15
Prim−25
Long−pec
Protruding−mouth
Day−4
Day−5
Transcript
ENSDART00000097500
ENSDART00000134464
ENSDART00000137216

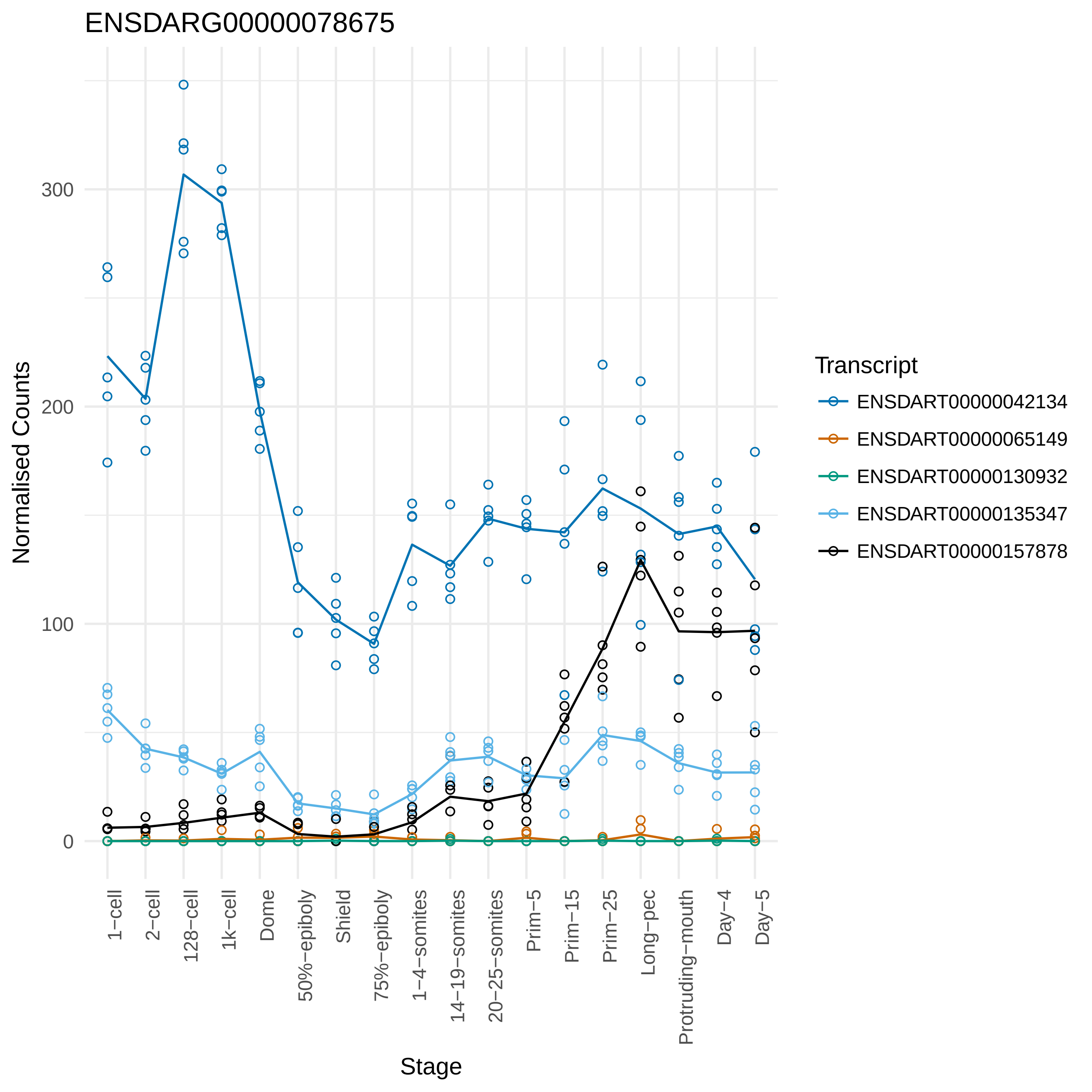
ENSDARG00000078675
Normalised Counts
300
200
100
0
1−cell
2−cell
128−cell
1k−cell
Dome
50%−epiboly
Shield
75%−epiboly
1−4−somites
14−19−somites
20−25−somites
Prim−5
Prim−15
Prim−25
Long−pec
Protruding−mouth
Day−4
Day−5
Stage
Transcript
ENSDART00000042134
ENSDART00000065149
ENSDART00000130932
ENSDART00000135347
ENSDART00000157878

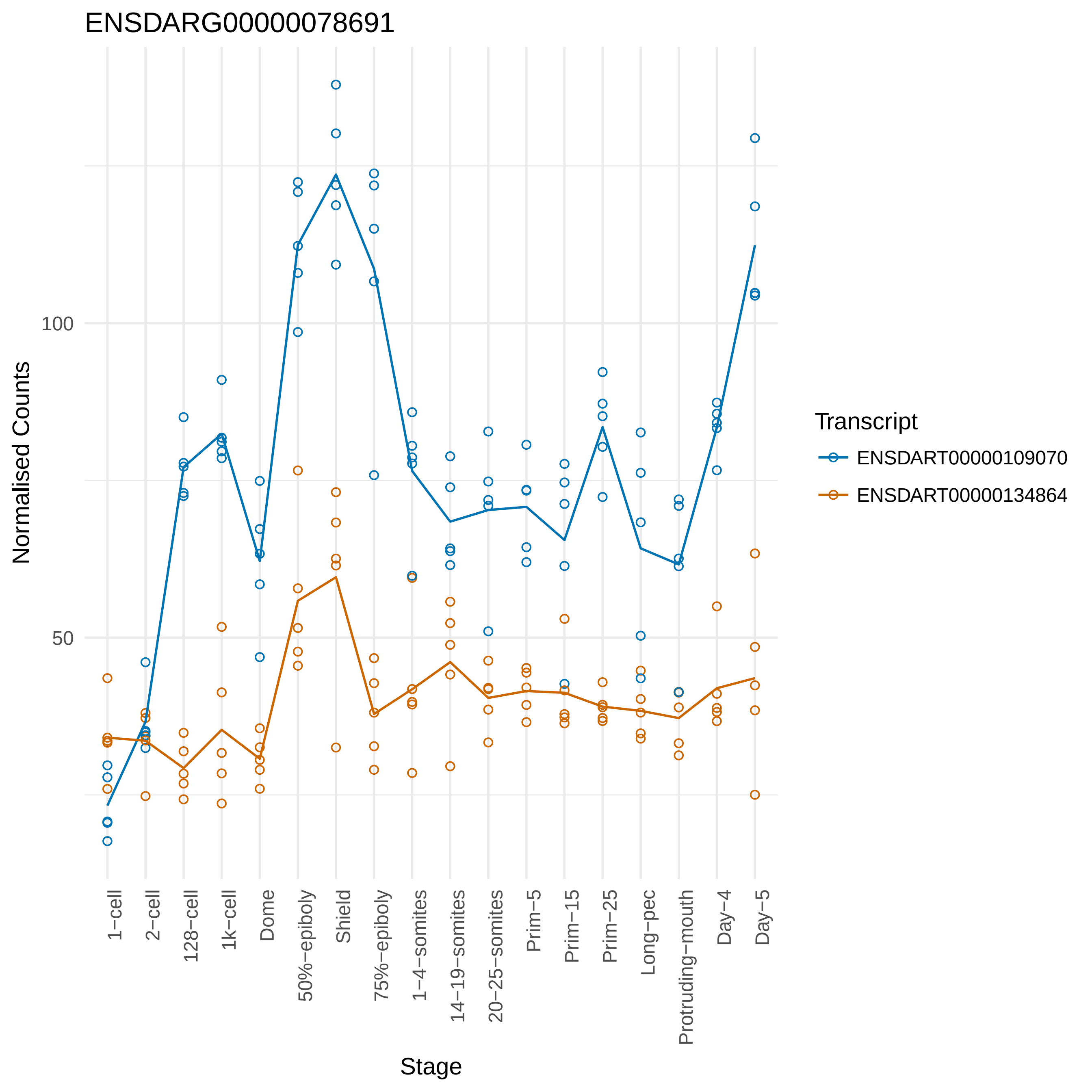

ENSDARG00000078691
Normalised Counts
100
50
Stage
1−cell
2−cell
128−cell
1k−cell
Dome
50%−epiboly
Shield
75%−epiboly
1−4−somites
14−19−somites
20−25−somites
Prim−5
Prim−15
Prim−25
Long−pec
Protruding−mouth
Day−4
Day−5
Transcript
ENSDART00000109070
ENSDART00000134864

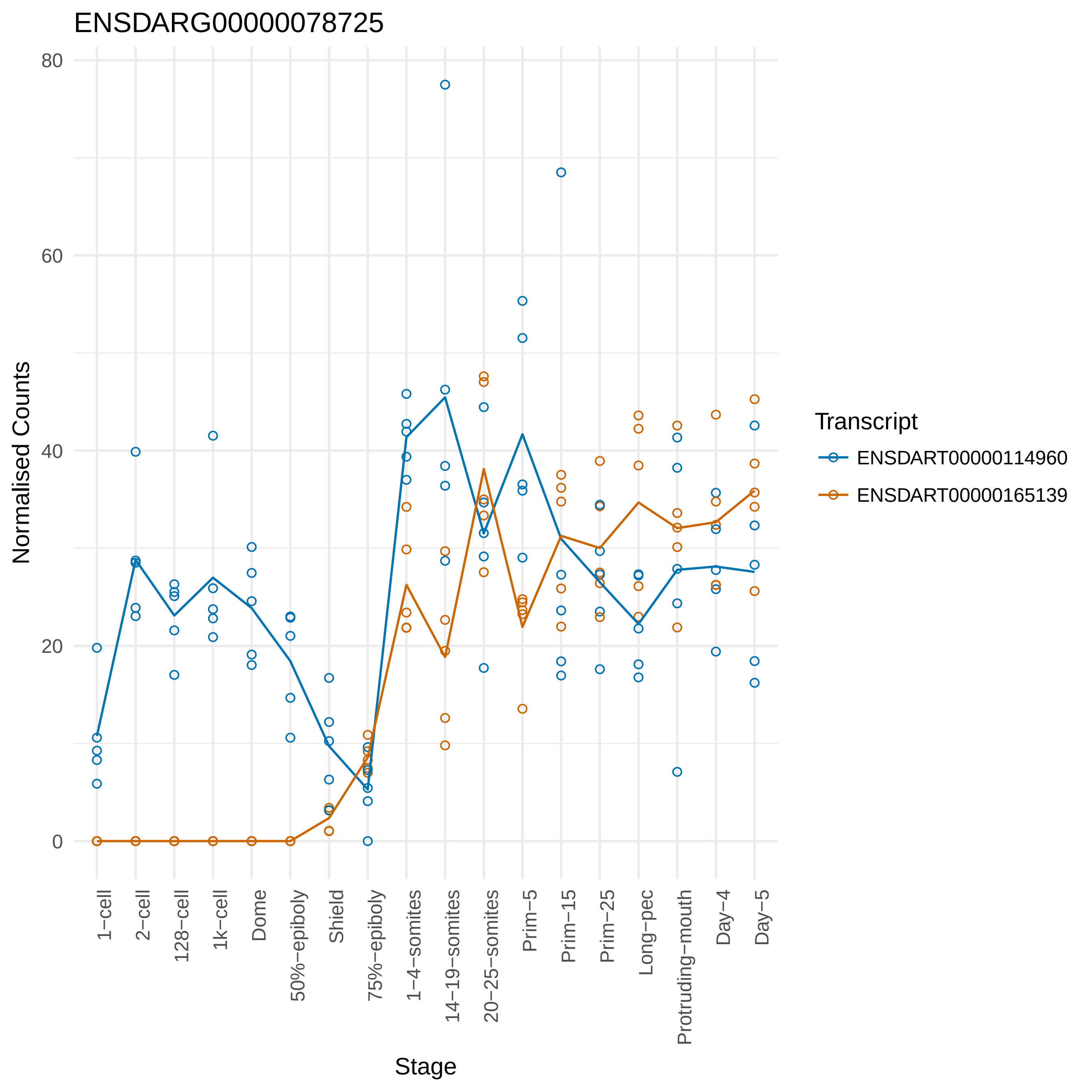

ENSDARG00000078725
Normalised Counts
0
20
40
60
80
1−cell
2−cell
128−cell
1k−cell
Dome
50%−epiboly
Shield
75%−epiboly
1−4−somites
14−19−somites
20−25−somites
Prim−5
Prim−15
Prim−25
Long−pec
Protruding−mouth
Day−4
Day−5
Stage
Transcript
ENSDART00000114960
ENSDART00000165139

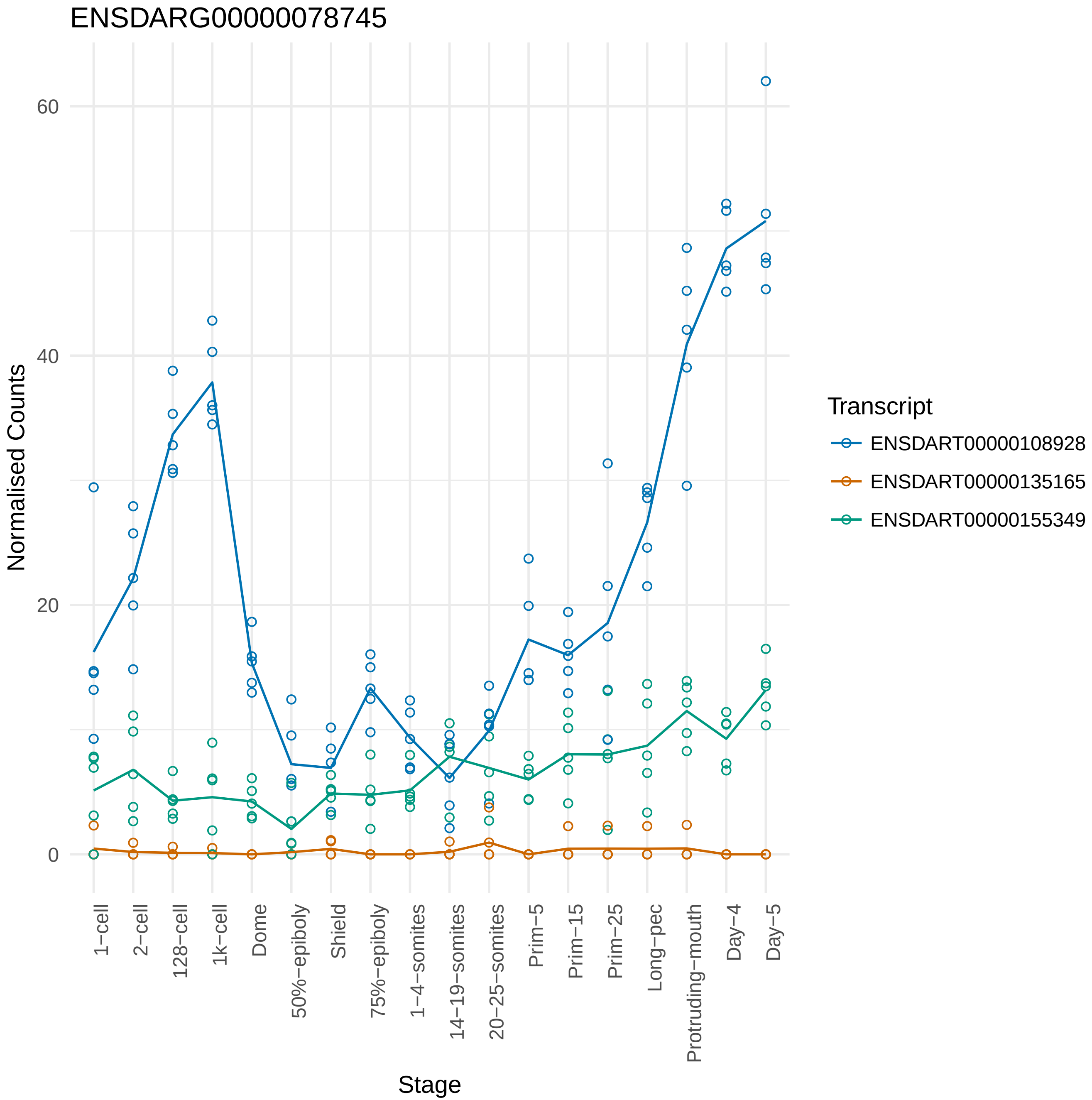
ENSDARG00000078745
Normalised Counts
0
20
40
60
Stage
1−cell
2−cell
128−cell
1k−cell
Dome
50%−epiboly
Shield
75%−epiboly
1−4−somites
14−19−somites
20−25−somites
Prim−5
Prim−15
Prim−25
Long−pec
Protruding−mouth
Day−4
Day−5
Transcript
ENSDART00000108928
ENSDART00000135165
ENSDART00000155349

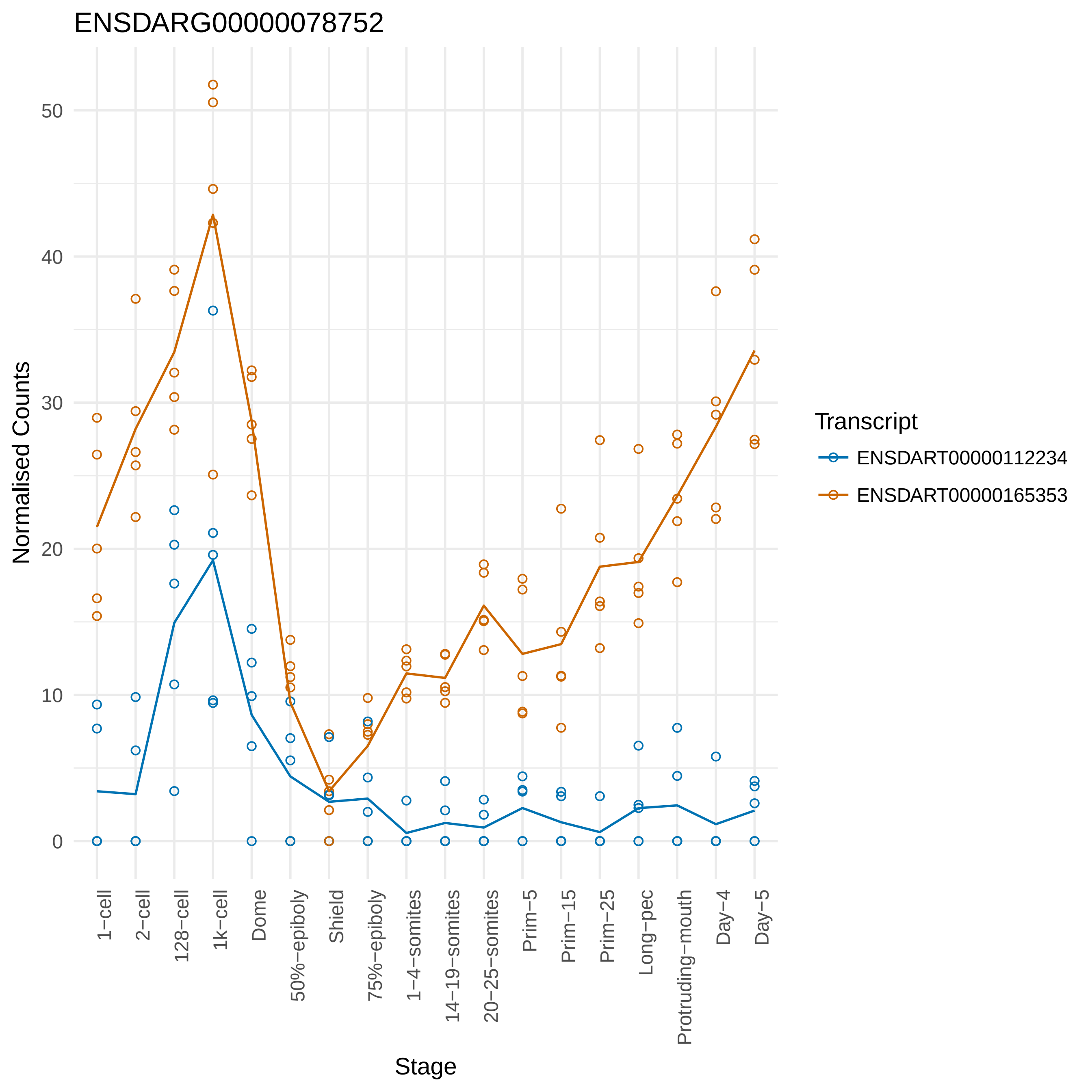

ENSDARG00000078752
Normalised Counts
0
10
20
30
40
50
Stage
1−cell
2−cell
128−cell
1k−cell
Dome
50%−epiboly
Shield
75%−epiboly
1−4−somites
14−19−somites
20−25−somites
Prim−5
Prim−15
Prim−25
Long−pec
Protruding−mouth
Day−4
Day−5
Transcript
ENSDART00000112234
ENSDART00000165353

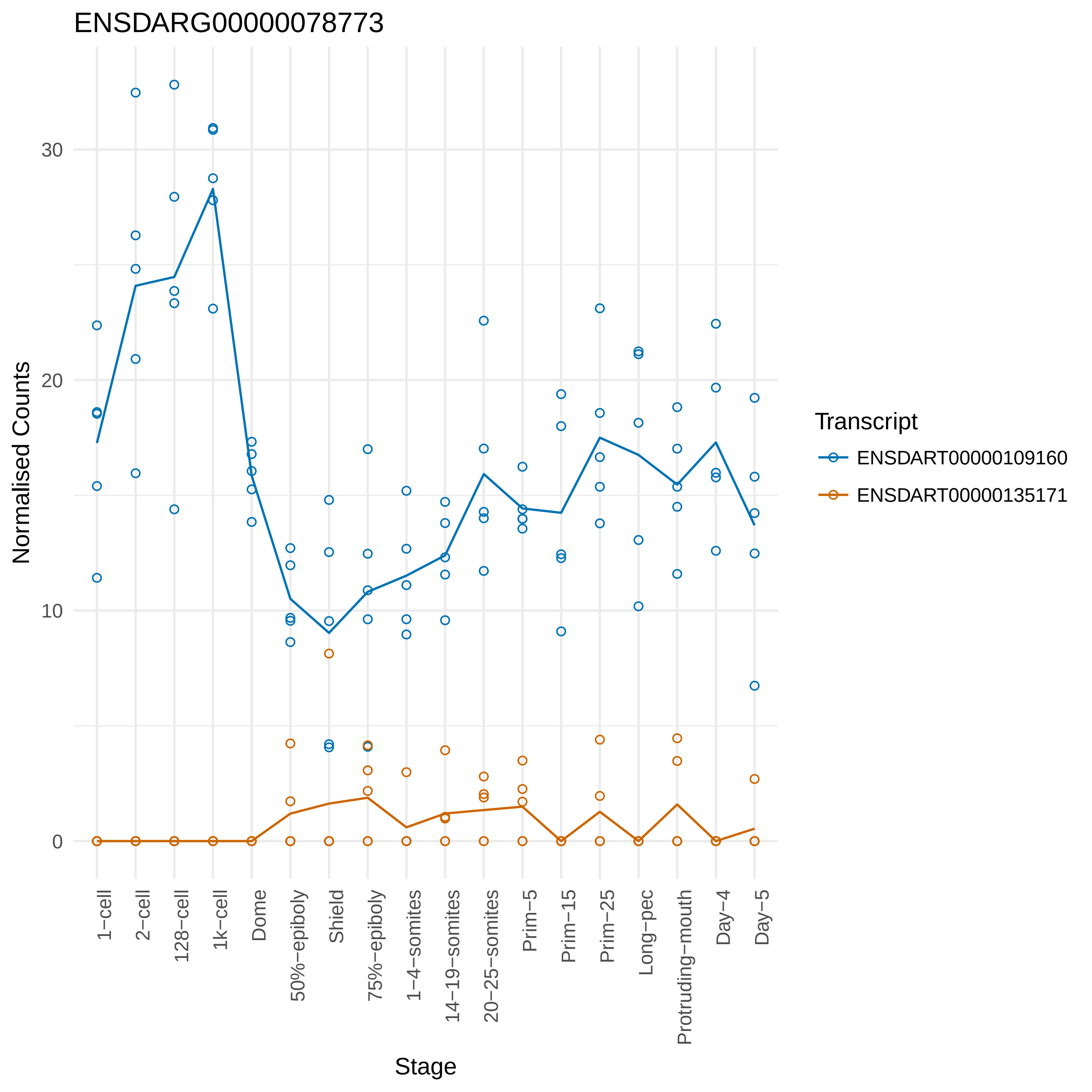
ENSDARG00000078773
Normalised Counts
30
20
10
0
1−cell
2−cell
128−cell
1k−cell
Dome
50%−epiboly
Shield
75%−epiboly
1−4−somites
14−19−somites
20−25−somites
Prim−5
Prim−15
Prim−25
Long−pec
Protruding−mouth
Day−4
Day−5
Stage
Transcript
ENSDART00000109160
ENSDART00000135171

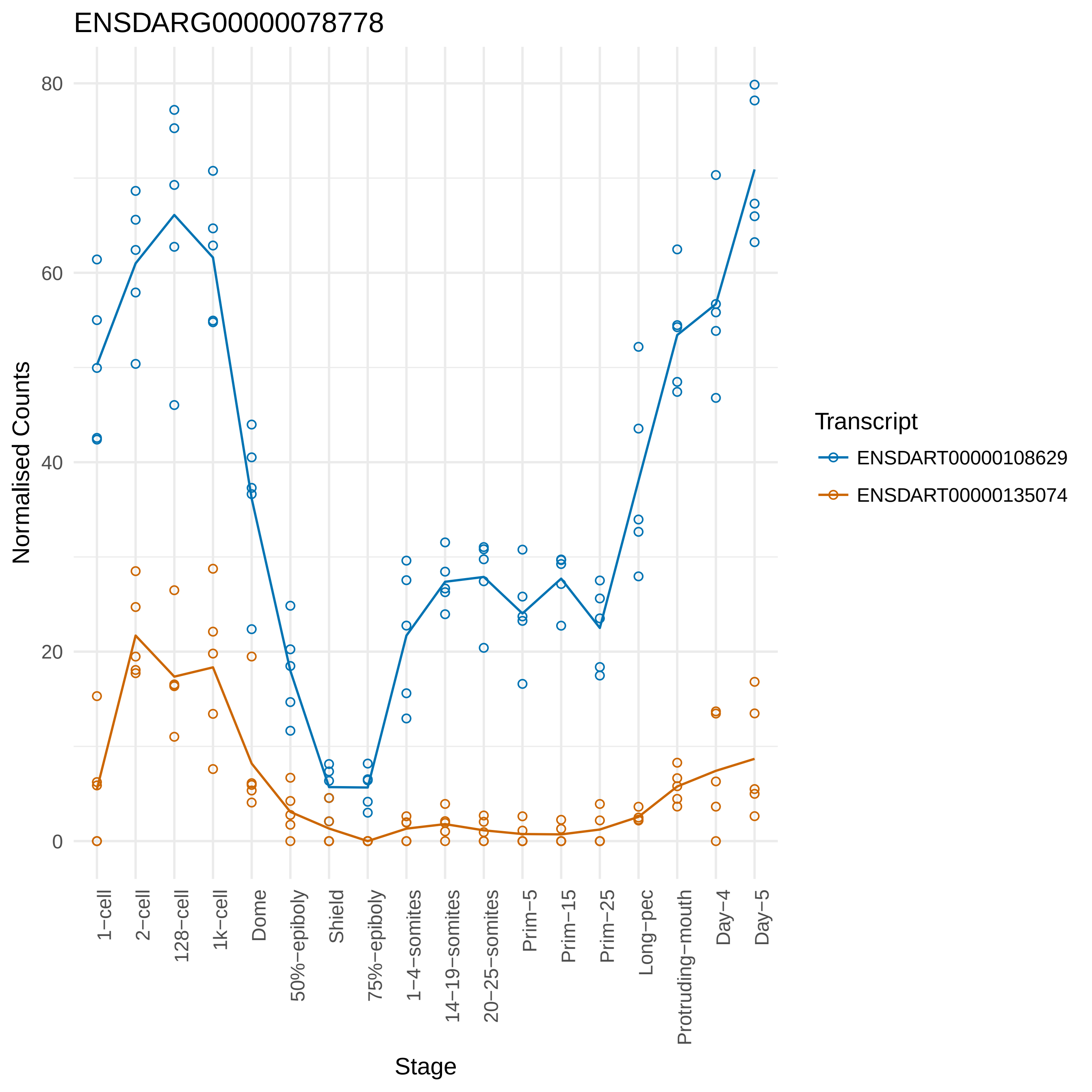
ENSDARG00000078778
Normalised Counts
80
60
40
20
0
1−cell
2−cell
128−cell
1k−cell
Dome
50%−epiboly
Shield
75%−epiboly
1−4−somites
14−19−somites
20−25−somites
Prim−5
Prim−15
Prim−25
Long−pec
Protruding−mouth
Day−4
Day−5
Stage
Transcript
ENSDART00000108629
ENSDART00000135074

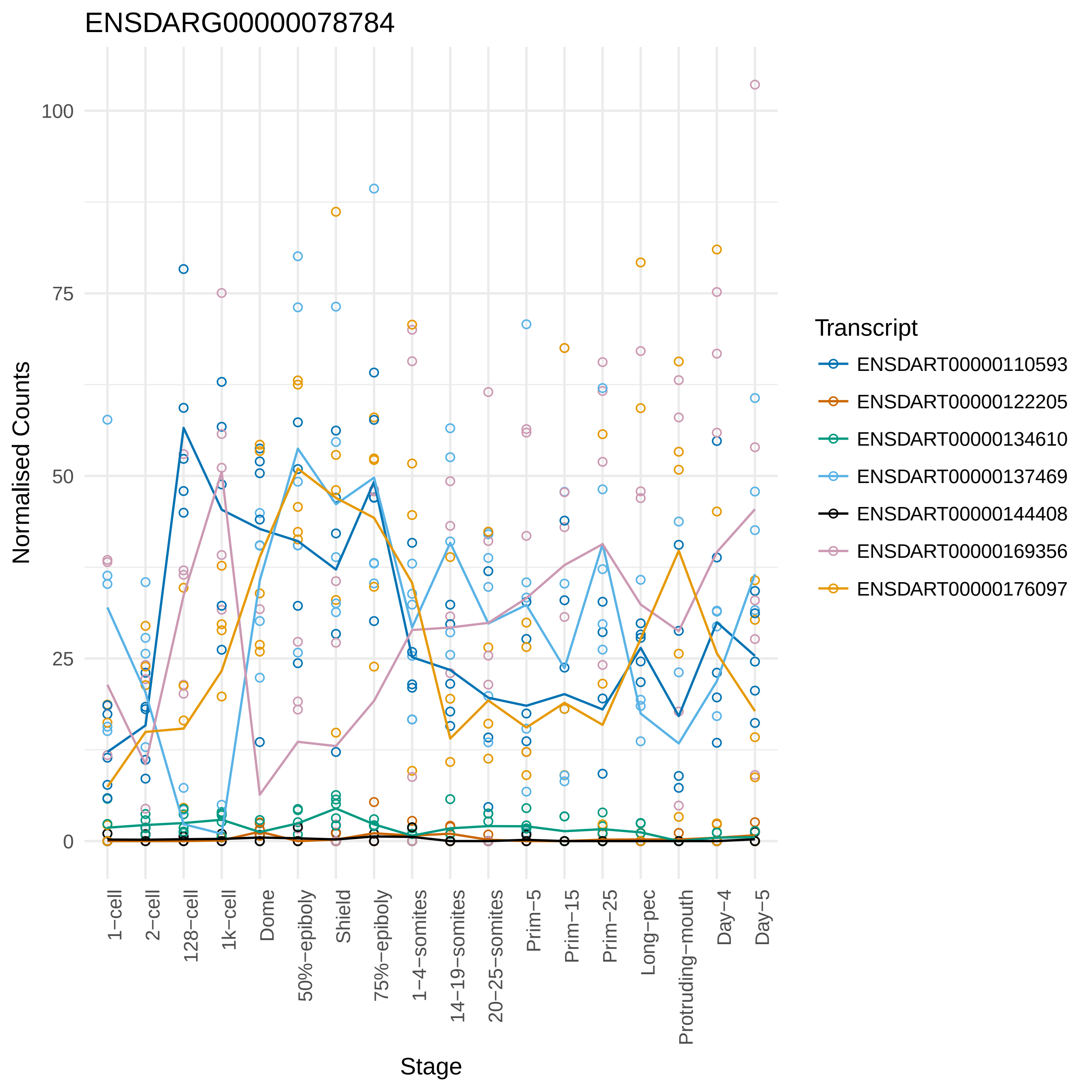

ENSDARG00000078784
Normalised Counts
100
75
50
25
0
1−cell
2−cell
128−cell
1k−cell
Dome
50%−epiboly
Shield
75%−epiboly
1−4−somites
14−19−somites
20−25−somites
Prim−5
Prim−15
Prim−25
Long−pec
Protruding−mouth
Day−4
Day−5
Stage
Transcript
ENSDART00000110593
ENSDART00000122205
ENSDART00000134610
ENSDART00000137469
ENSDART00000144408
ENSDART00000169356
ENSDART00000176097

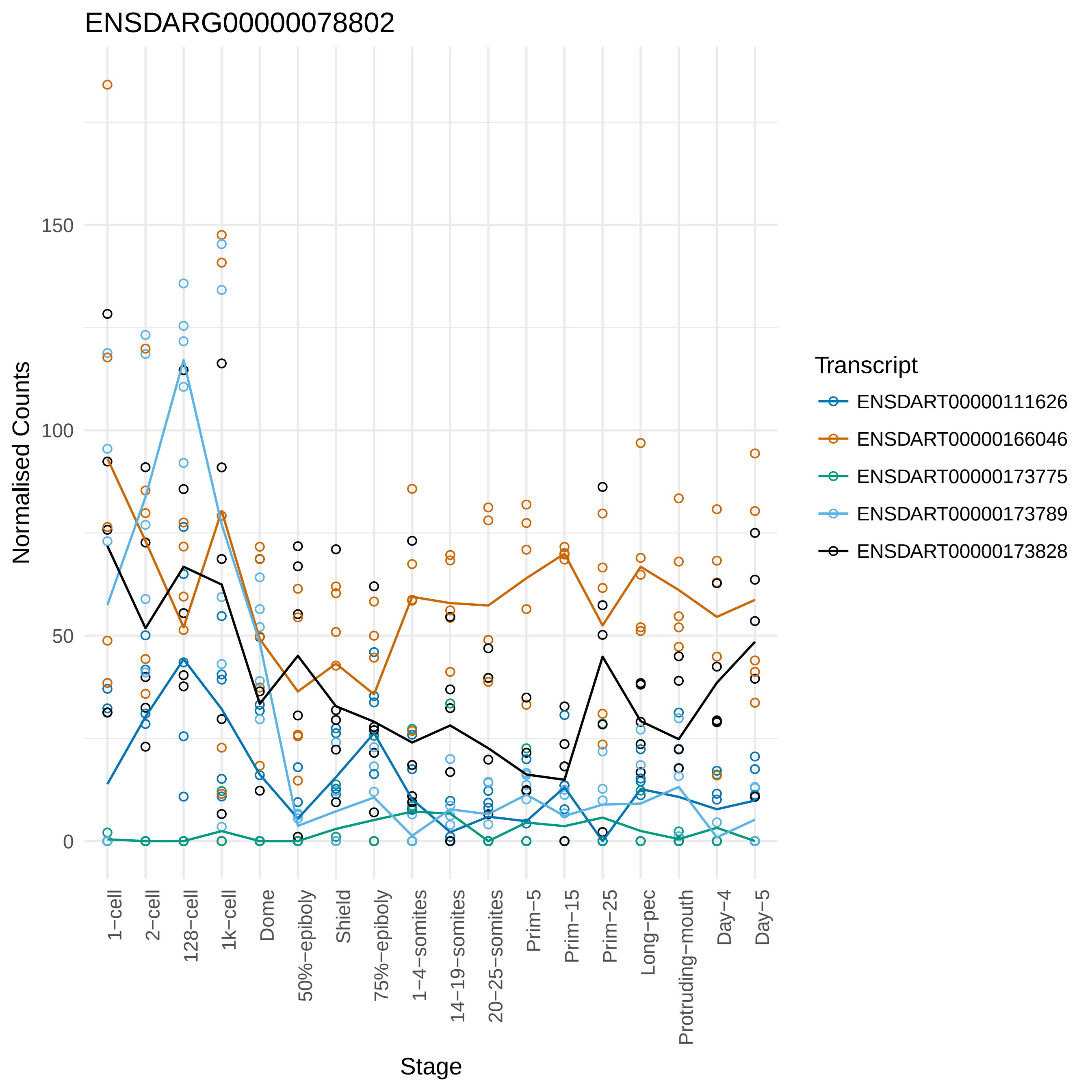
ENSDARG00000078802
Normalised Counts
150
100
50
0
1−cell
2−cell
128−cell
1k−cell
Dome
50%−epiboly
Shield
75%−epiboly
1−4−somites
14−19−somites
20−25−somites
Prim−5
Prim−15
Prim−25
Long−pec
Protruding−mouth
Day−4
Day−5
Stage
Transcript
ENSDART00000111626
ENSDART00000166046
ENSDART00000173775
ENSDART00000173789
ENSDART00000173828

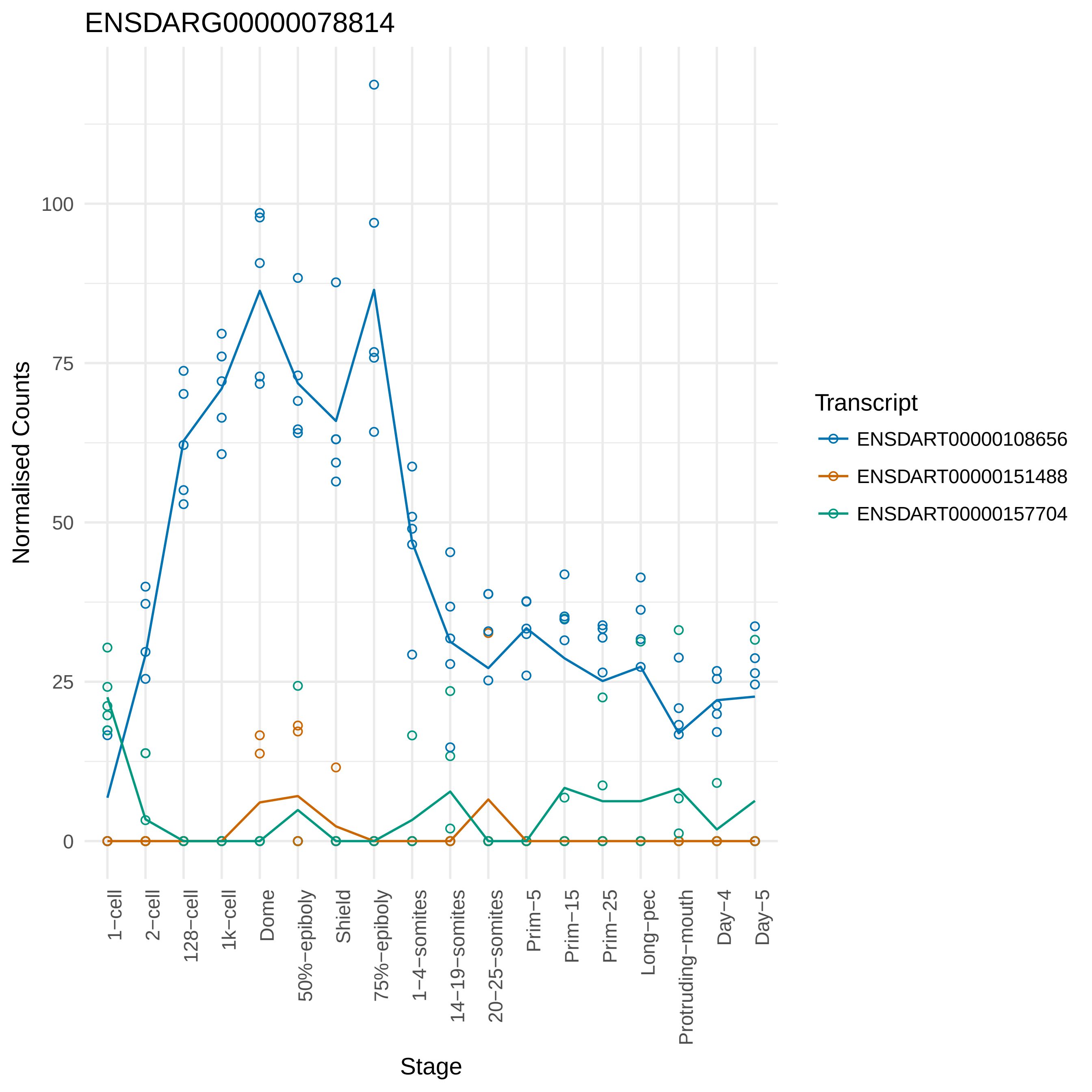
ENSDARG00000078814
Normalised Counts
100
75
50
25
0
1−cell
2−cell
128−cell
1k−cell
Dome
50%−epiboly
Shield
75%−epiboly
1−4−somites
14−19−somites
20−25−somites
Prim−5
Prim−15
Prim−25
Long−pec
Protruding−mouth
Day−4
Day−5
Stage
Transcript
ENSDART00000108656
ENSDART00000151488
ENSDART00000157704

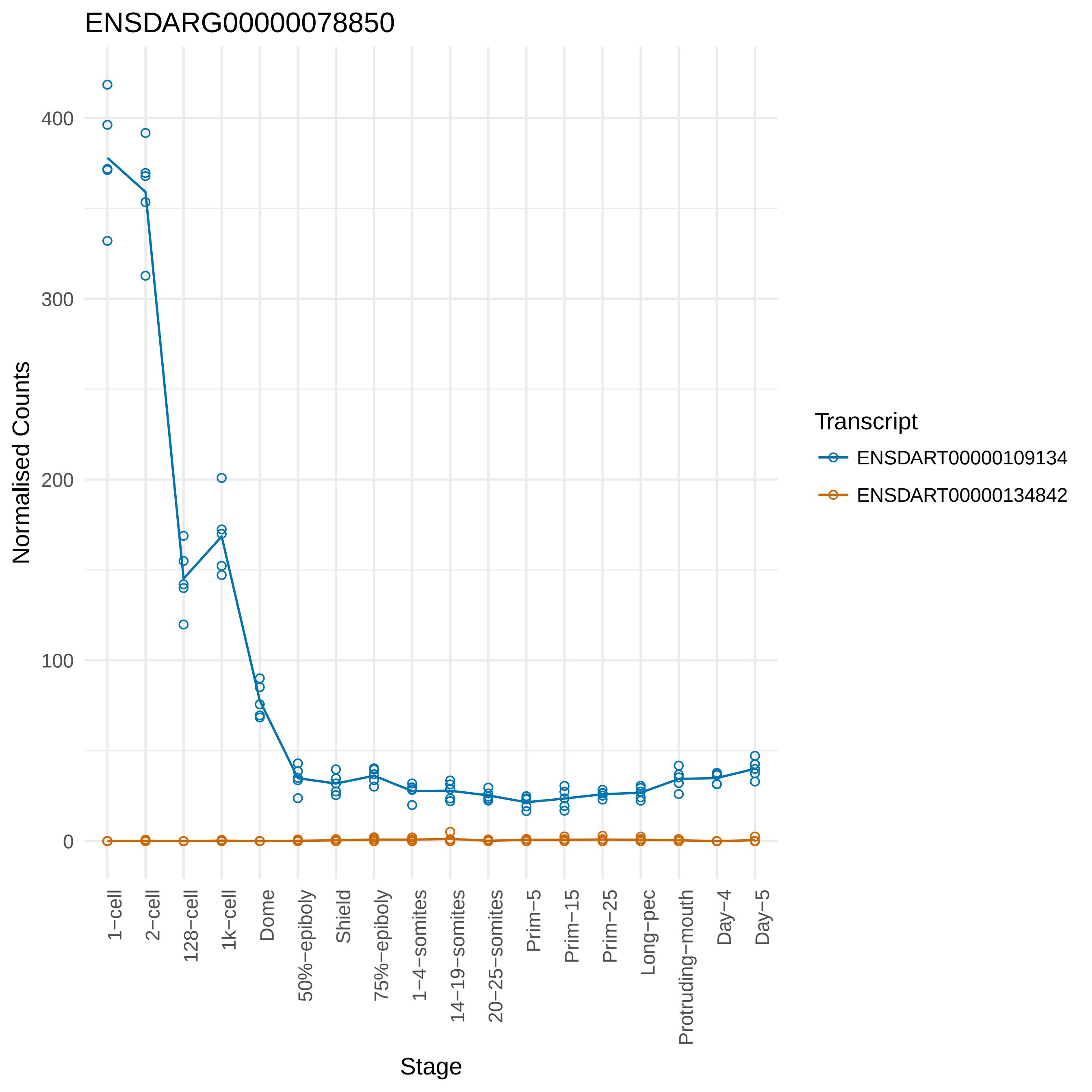

ENSDARG00000078850
Normalised Counts
0
100
200
300
400
Stage
1−cell
2−cell
128−cell
1k−cell
Dome
50%−epiboly
Shield
75%−epiboly
1−4−somites
14−19−somites
20−25−somites
Prim−5
Prim−15
Prim−25
Long−pec
Protruding−mouth
Day−4
Day−5
Transcript
ENSDART00000109134
ENSDART00000134842

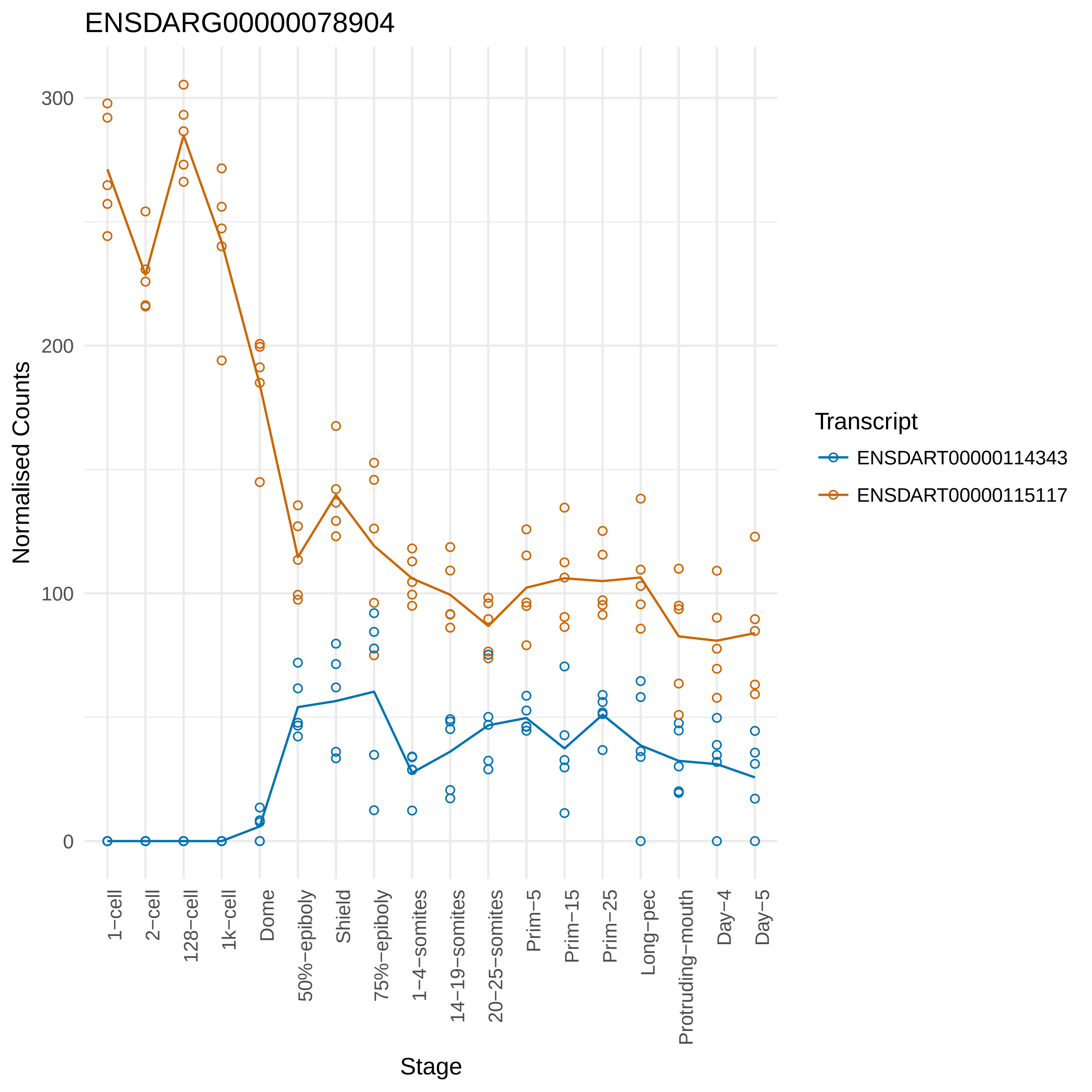

ENSDARG00000078904
Normalised Counts
0
100
200
300
Stage
1−cell
2−cell
128−cell
1k−cell
Dome
50%−epiboly
Shield
75%−epiboly
1−4−somites
14−19−somites
20−25−somites
Prim−5
Prim−15
Prim−25
Long−pec
Protruding−mouth
Day−4
Day−5
Transcript
ENSDART00000114343
ENSDART00000115117

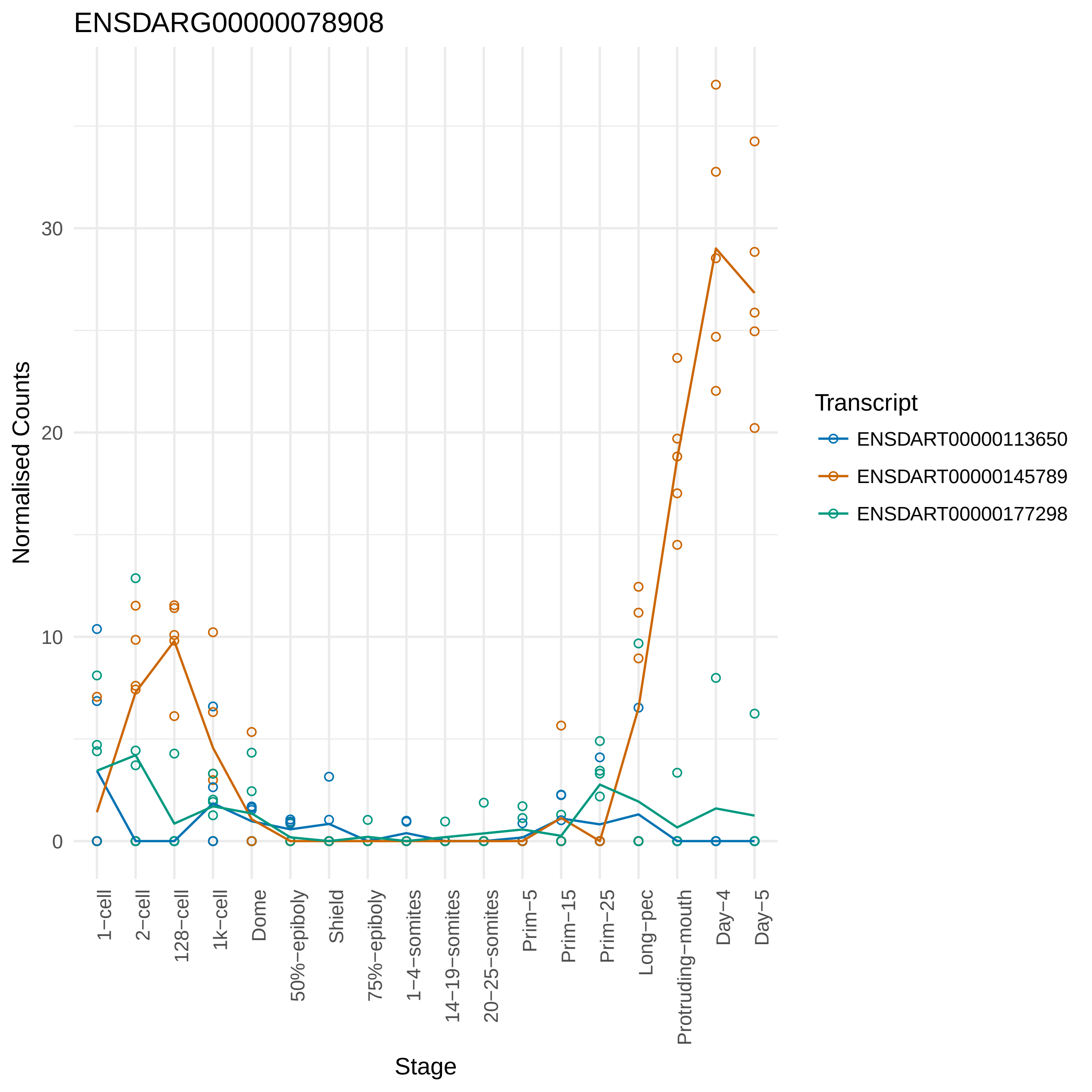
ENSDARG00000078908
Normalised Counts
0
10
20
30
1−cell
2−cell
128−cell
1k−cell
Dome
50%−epiboly
Shield
75%−epiboly
1−4−somites
14−19−somites
20−25−somites
Prim−5
Prim−15
Prim−25
Long−pec
Protruding−mouth
Day−4
Day−5
Stage
Transcript
ENSDART00000113650
ENSDART00000145789
ENSDART00000177298

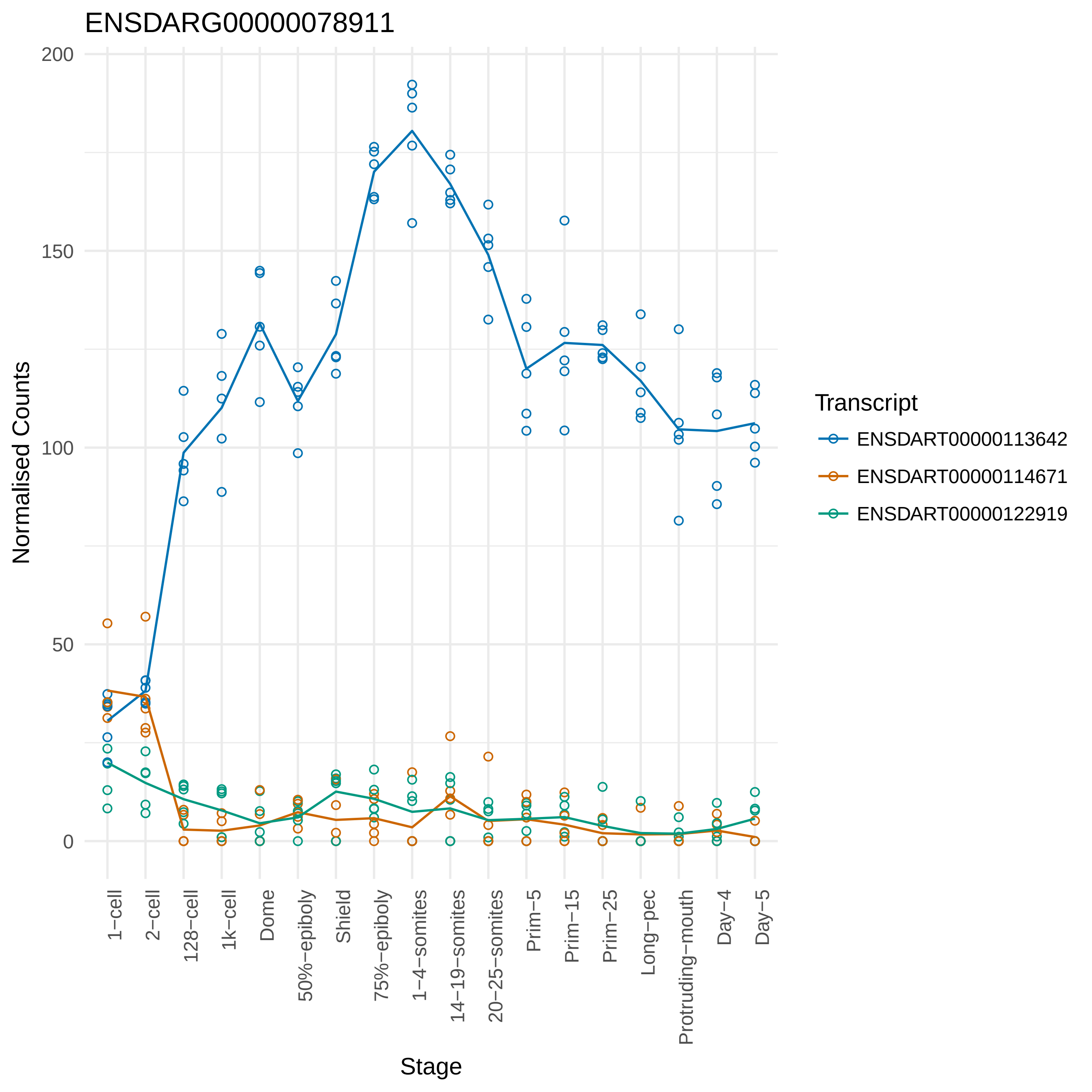

ENSDARG00000078911
Normalised Counts
0
50
100
150
200
Stage
1−cell
2−cell
128−cell
1k−cell
Dome
50%−epiboly
Shield
75%−epiboly
1−4−somites
14−19−somites
20−25−somites
Prim−5
Prim−15
Prim−25
Long−pec
Protruding−mouth
Day−4
Day−5
Transcript
ENSDART00000113642
ENSDART00000114671
ENSDART00000122919

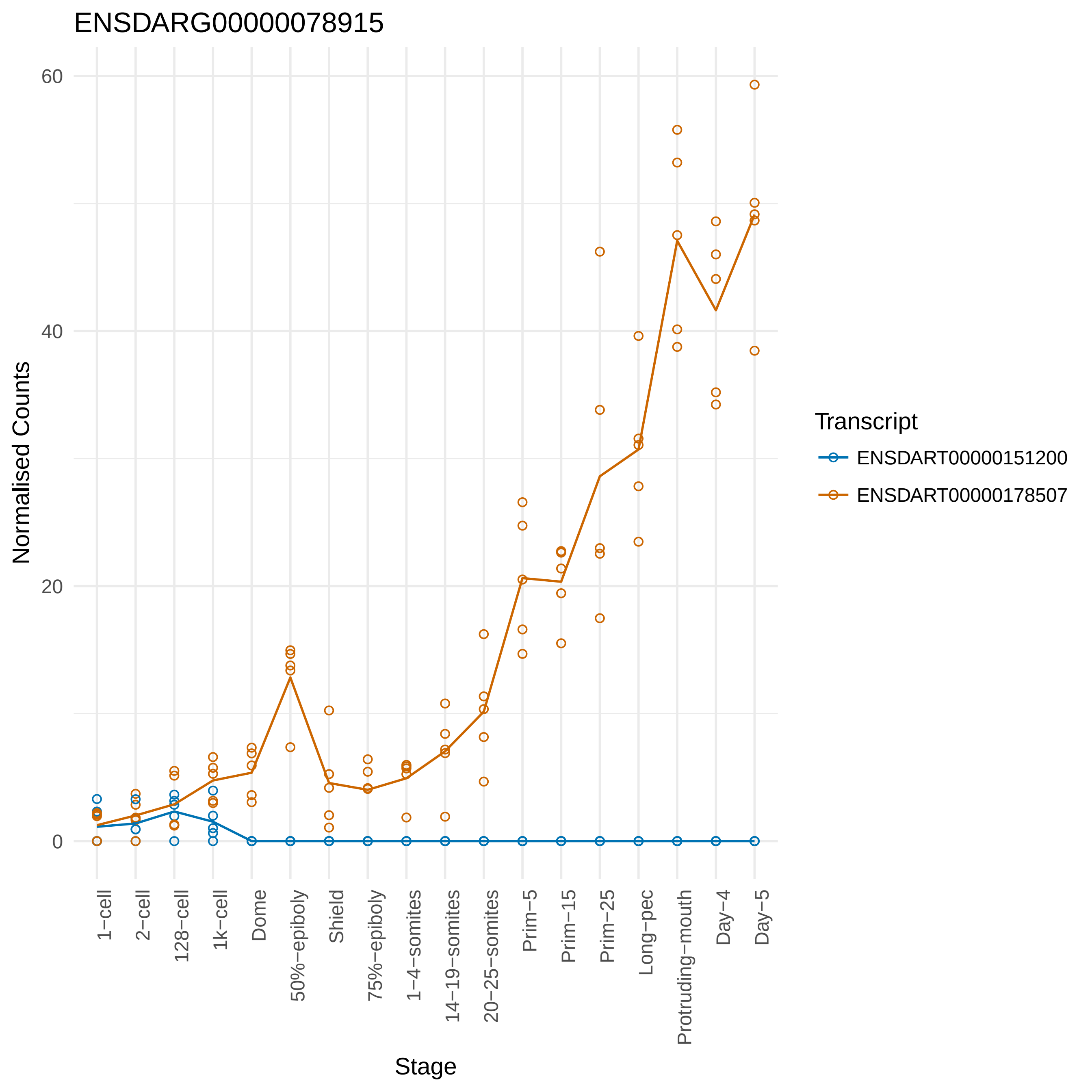
ENSDARG00000078915
Normalised Counts
60
40
20
0
Stage
1−cell
2−cell
128−cell
1k−cell
Dome
50%−epiboly
Shield
75%−epiboly
1−4−somites
14−19−somites
20−25−somites
Prim−5
Prim−15
Prim−25
Long−pec
Protruding−mouth
Day−4
Day−5
Transcript
ENSDART00000151200
ENSDART00000178507

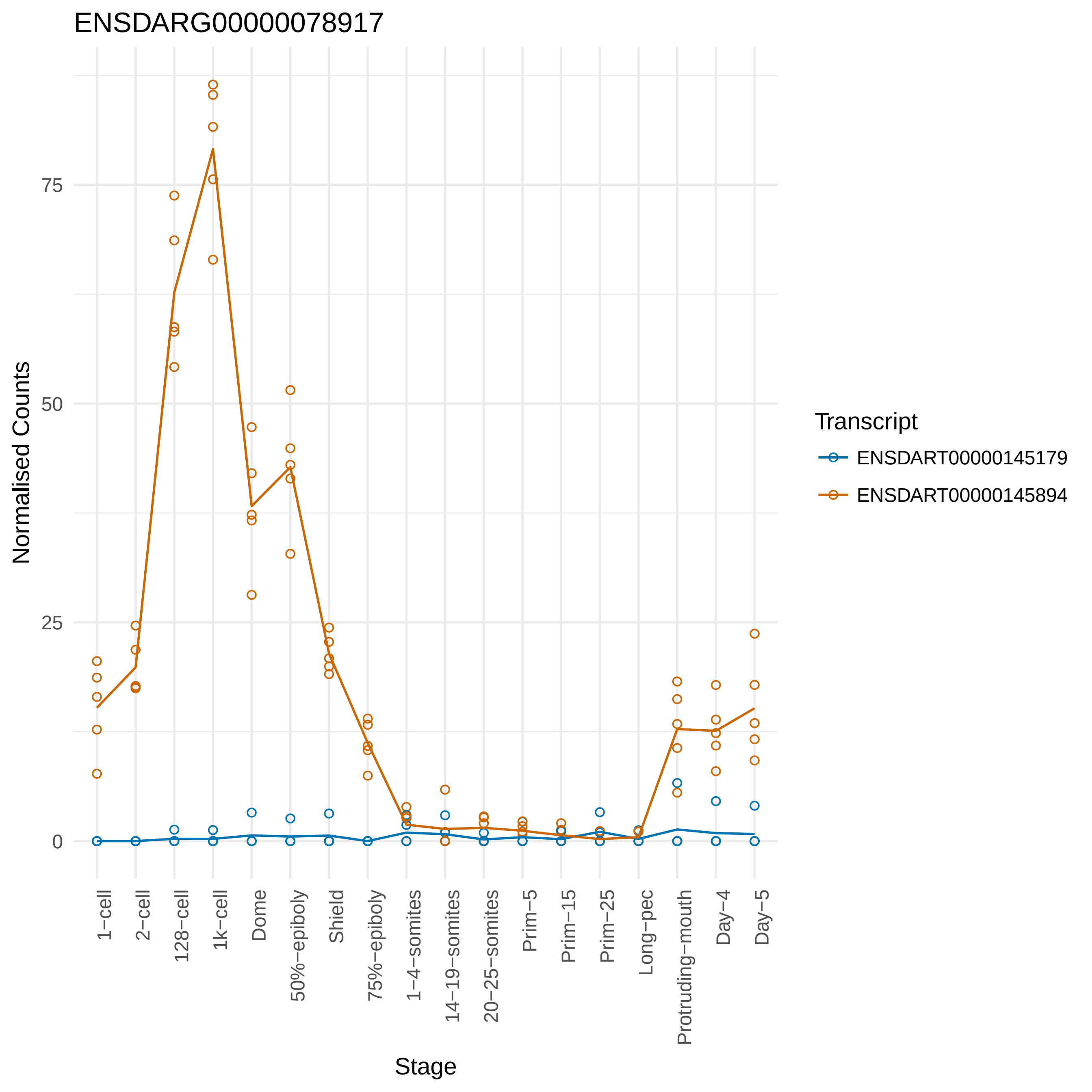

ENSDARG00000078917
Normalised Counts
75
50
25
0
1−cell
2−cell
128−cell
1k−cell
Dome
50%−epiboly
Shield
75%−epiboly
1−4−somites
14−19−somites
20−25−somites
Prim−5
Prim−15
Prim−25
Long−pec
Protruding−mouth
Day−4
Day−5
Stage
Transcript
ENSDART00000145179
ENSDART00000145894

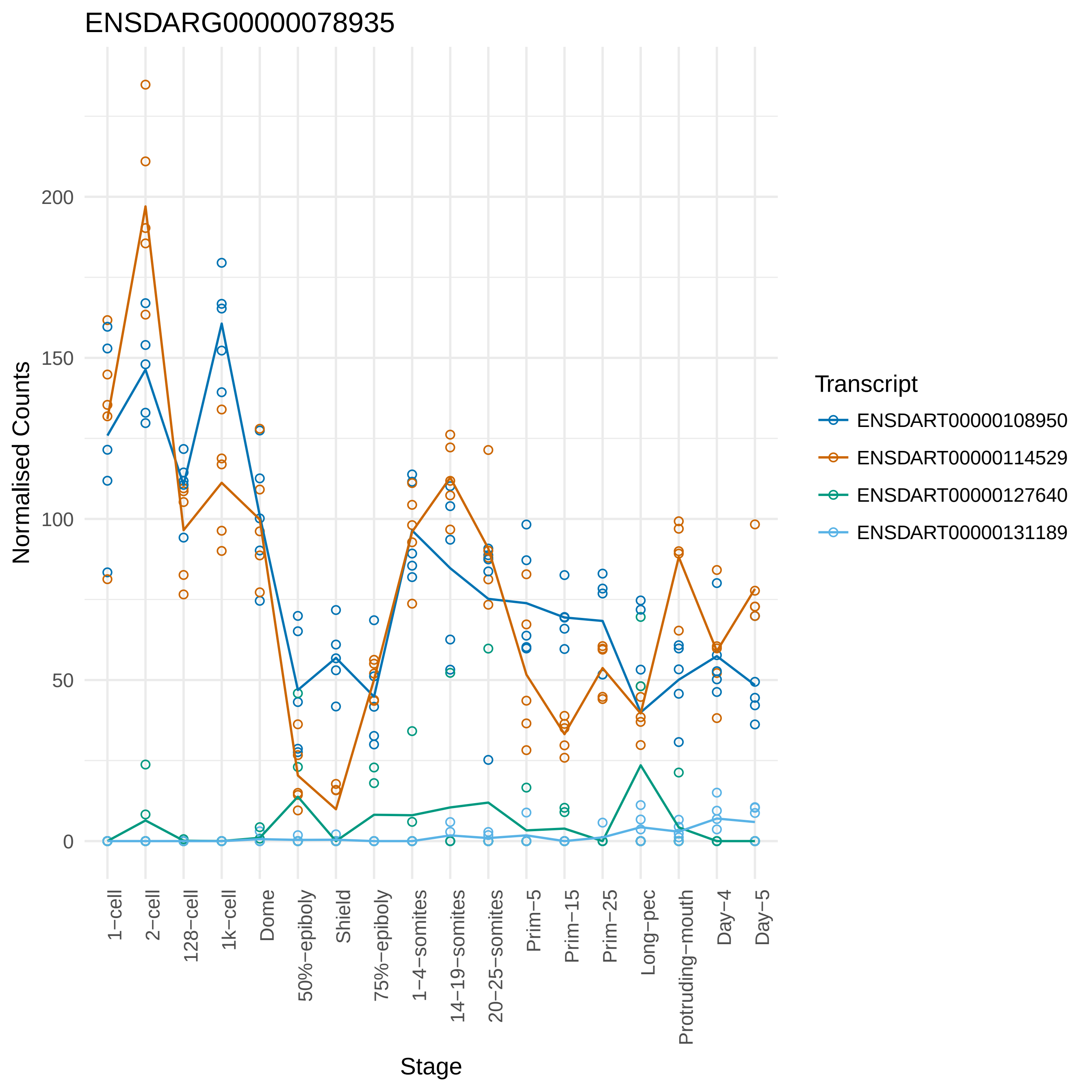

ENSDARG00000078935
Normalised Counts
0
50
100
150
200
1−cell
2−cell
128−cell
1k−cell
Dome
50%−epiboly
Shield
75%−epiboly
1−4−somites
14−19−somites
20−25−somites
Prim−5
Prim−15
Prim−25
Long−pec
Protruding−mouth
Day−4
Day−5
Stage
Transcript
ENSDART00000108950
ENSDART00000114529
ENSDART00000127640
ENSDART00000131189

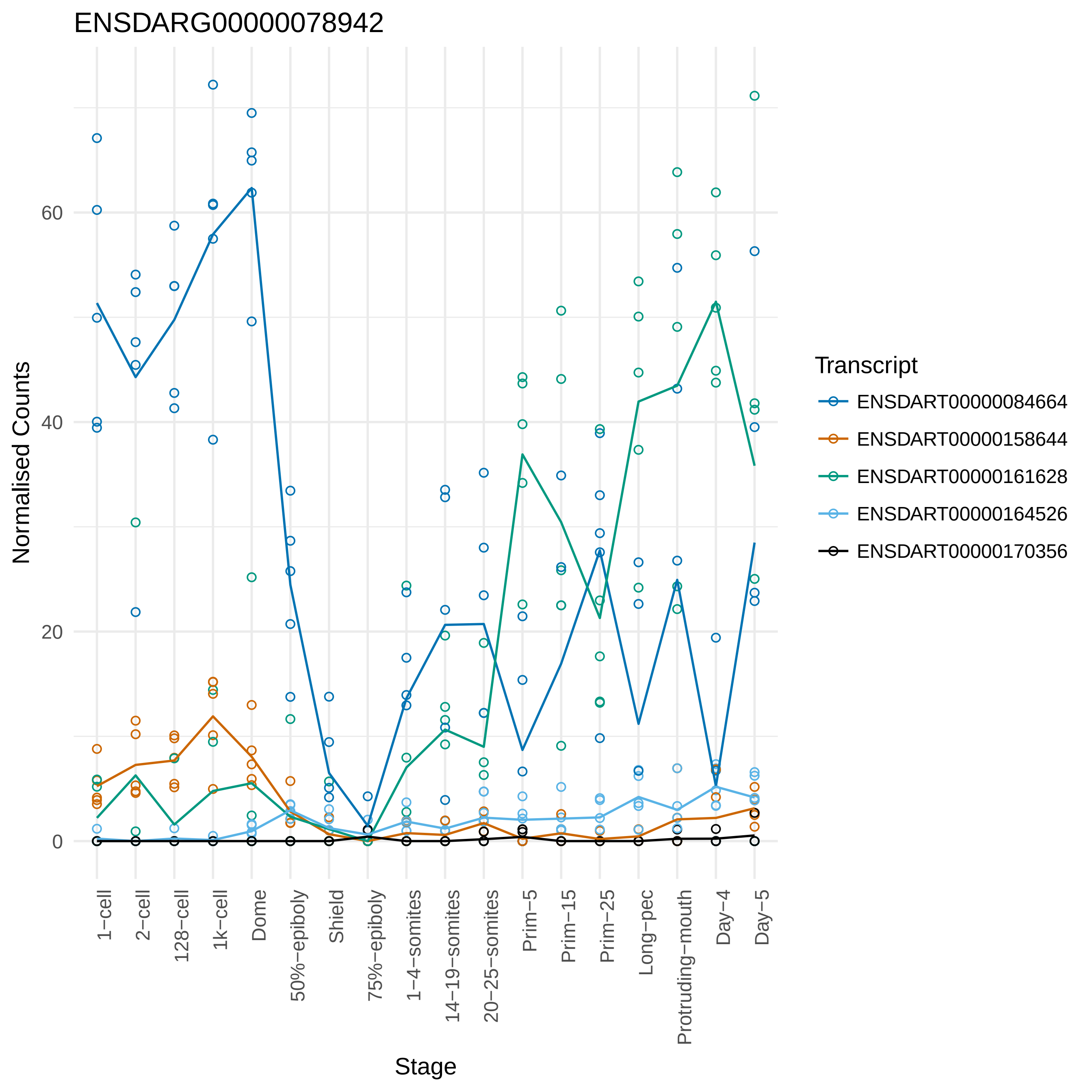
ENSDARG00000078942
Normalised Counts
60
40
20
0
1−cell
2−cell
128−cell
1k−cell
Dome
50%−epiboly
Shield
75%−epiboly
1−4−somites
14−19−somites
20−25−somites
Prim−5
Prim−15
Prim−25
Long−pec
Protruding−mouth
Day−4
Day−5
Stage
Transcript
ENSDART00000084664
ENSDART00000158644
ENSDART00000161628
ENSDART00000164526
ENSDART00000170356

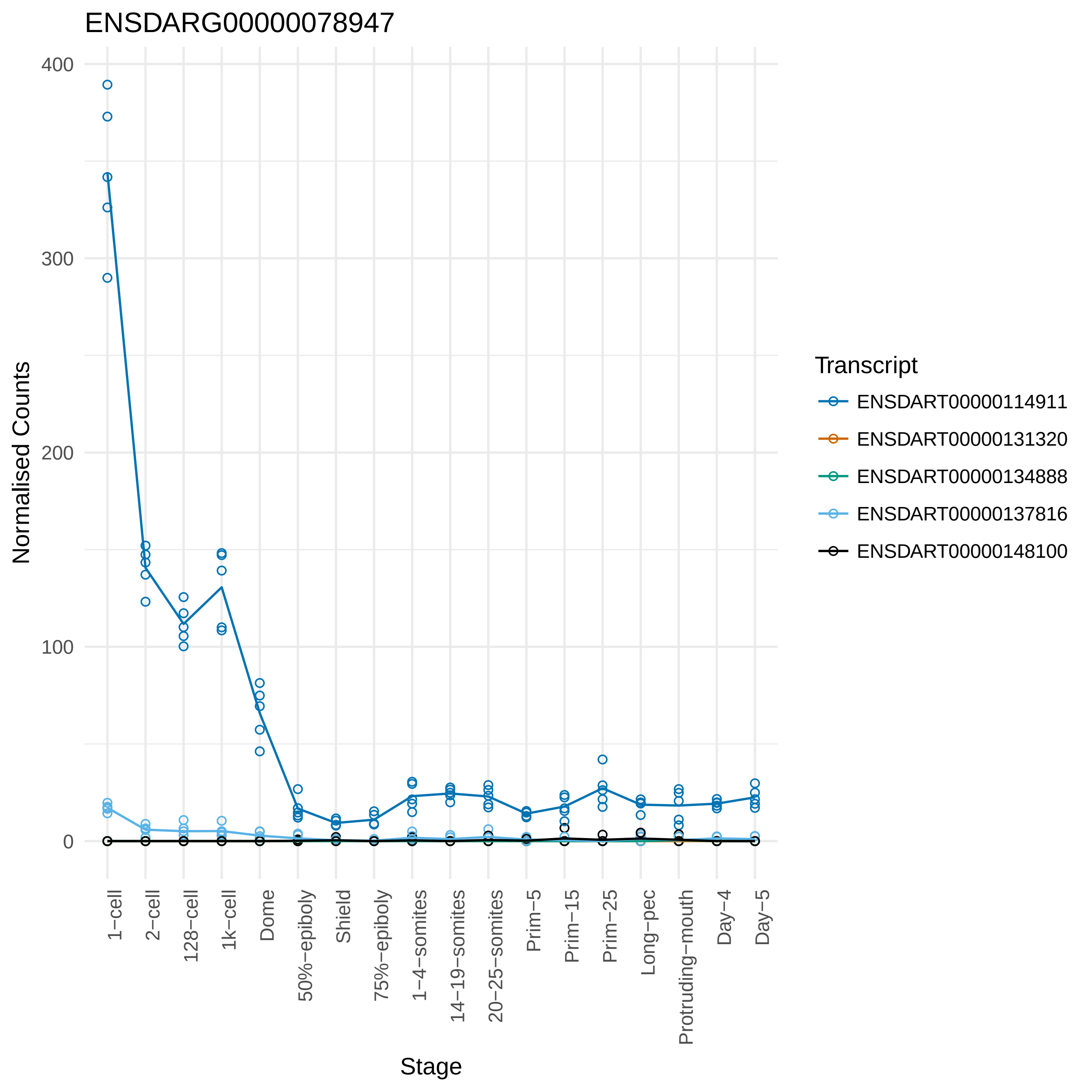

ENSDARG00000078947
Normalised Counts
400
300
200
100
0
1−cell
2−cell
128−cell
1k−cell
Dome
50%−epiboly
Shield
75%−epiboly
1−4−somites
14−19−somites
20−25−somites
Prim−5
Prim−15
Prim−25
Long−pec
Protruding−mouth
Day−4
Day−5
Stage
Transcript
ENSDART00000114911
ENSDART00000131320
ENSDART00000134888
ENSDART00000137816
ENSDART00000148100

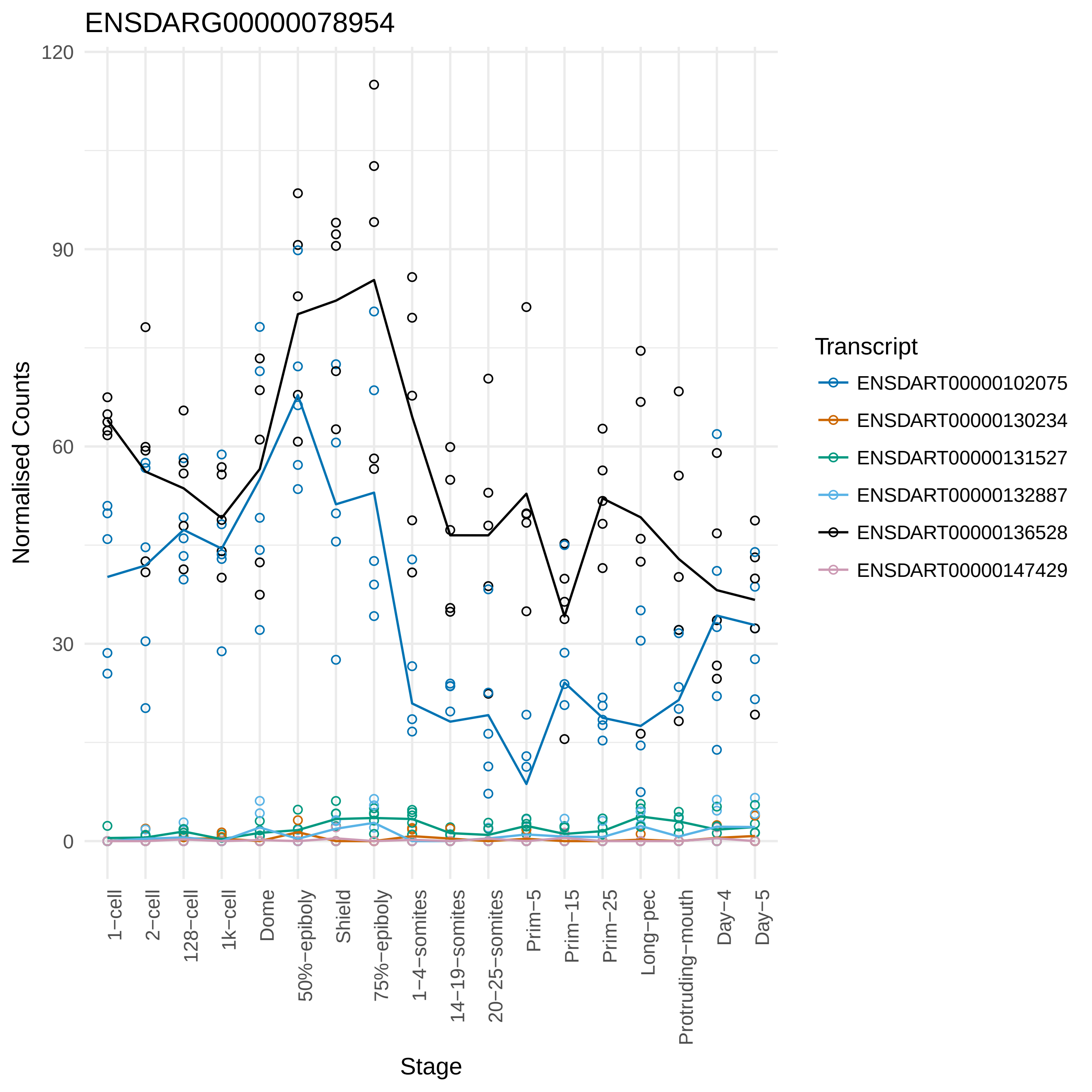
ENSDARG00000078954
Normalised Counts
120
90
60
30
0
1−cell
2−cell
128−cell
1k−cell
Dome
50%−epiboly
Shield
75%−epiboly
1−4−somites
14−19−somites
20−25−somites
Prim−5
Prim−15
Prim−25
Long−pec
Protruding−mouth
Day−4
Day−5
Stage
Transcript
ENSDART00000102075
ENSDART00000130234
ENSDART00000131527
ENSDART00000132887
ENSDART00000136528
ENSDART00000147429

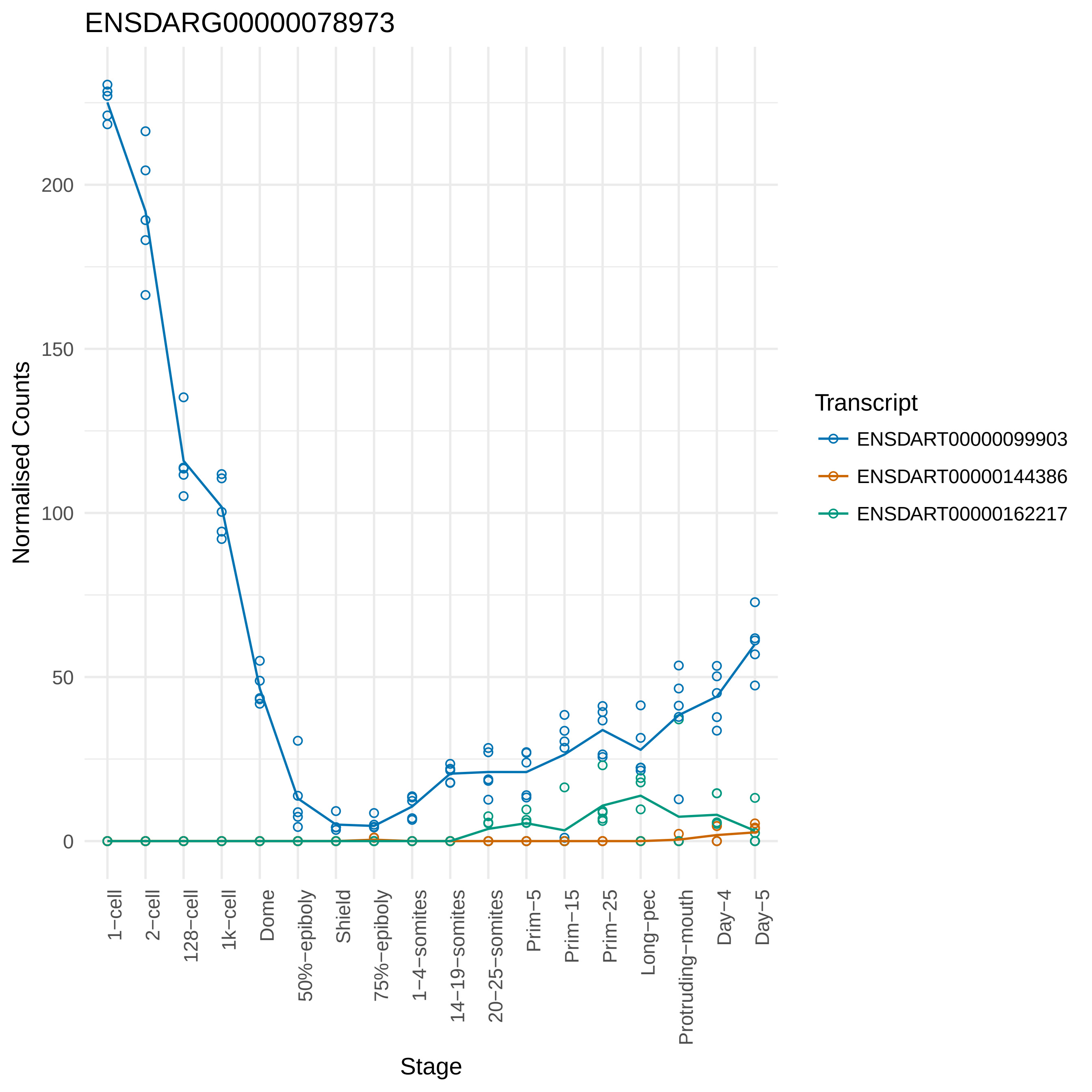

ENSDARG00000078973
Normalised Counts
0
50
100
150
200
Stage
1−cell
2−cell
128−cell
1k−cell
Dome
50%−epiboly
Shield
75%−epiboly
1−4−somites
14−19−somites
20−25−somites
Prim−5
Prim−15
Prim−25
Long−pec
Protruding−mouth
Day−4
Day−5
Transcript
ENSDART00000099903
ENSDART00000144386
ENSDART00000162217

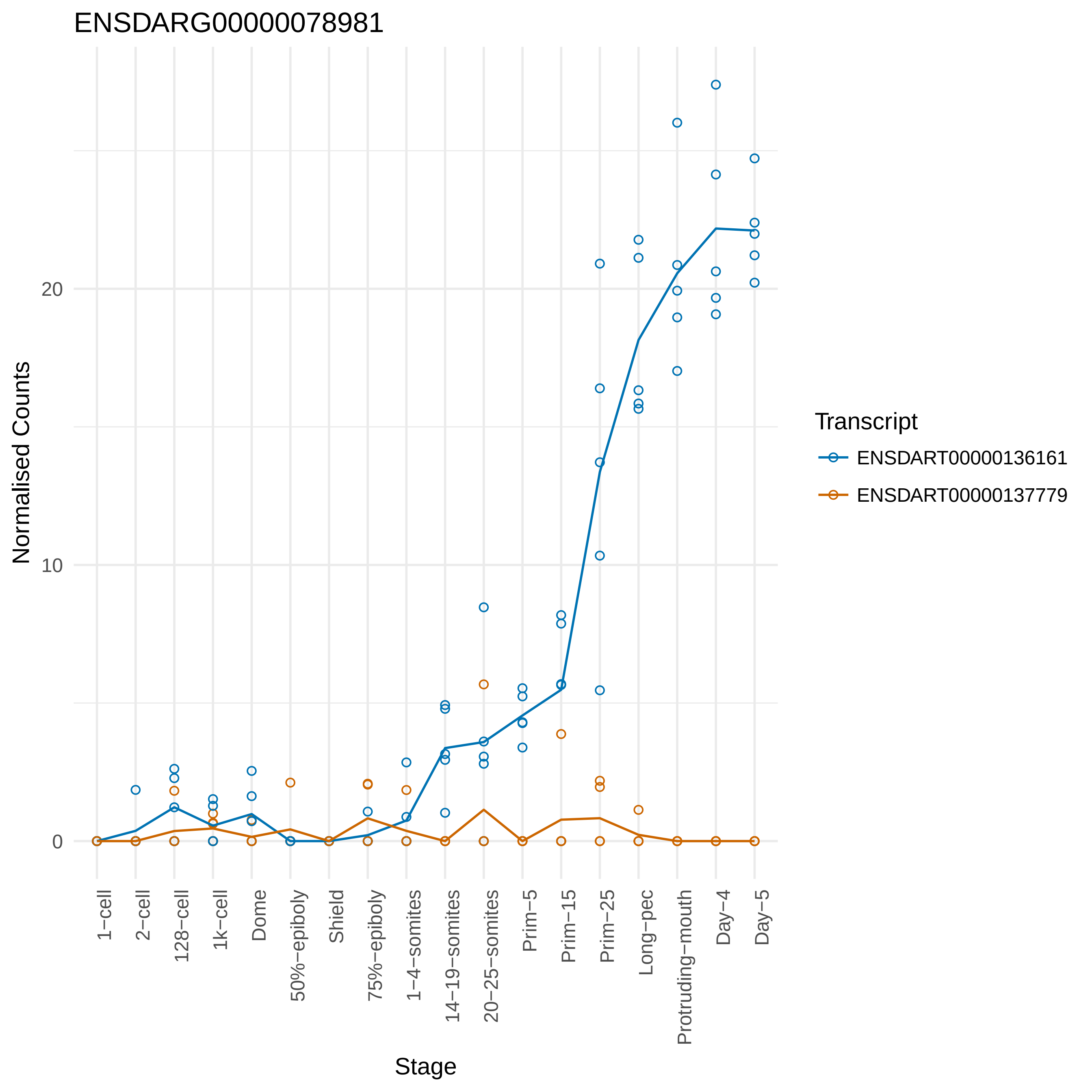

ENSDARG00000078981
Normalised Counts
20
10
0
1−cell
2−cell
128−cell
1k−cell
Dome
50%−epiboly
Shield
75%−epiboly
1−4−somites
14−19−somites
20−25−somites
Prim−5
Prim−15
Prim−25
Long−pec
Protruding−mouth
Day−4
Day−5
Stage
Transcript
ENSDART00000136161
ENSDART00000137779

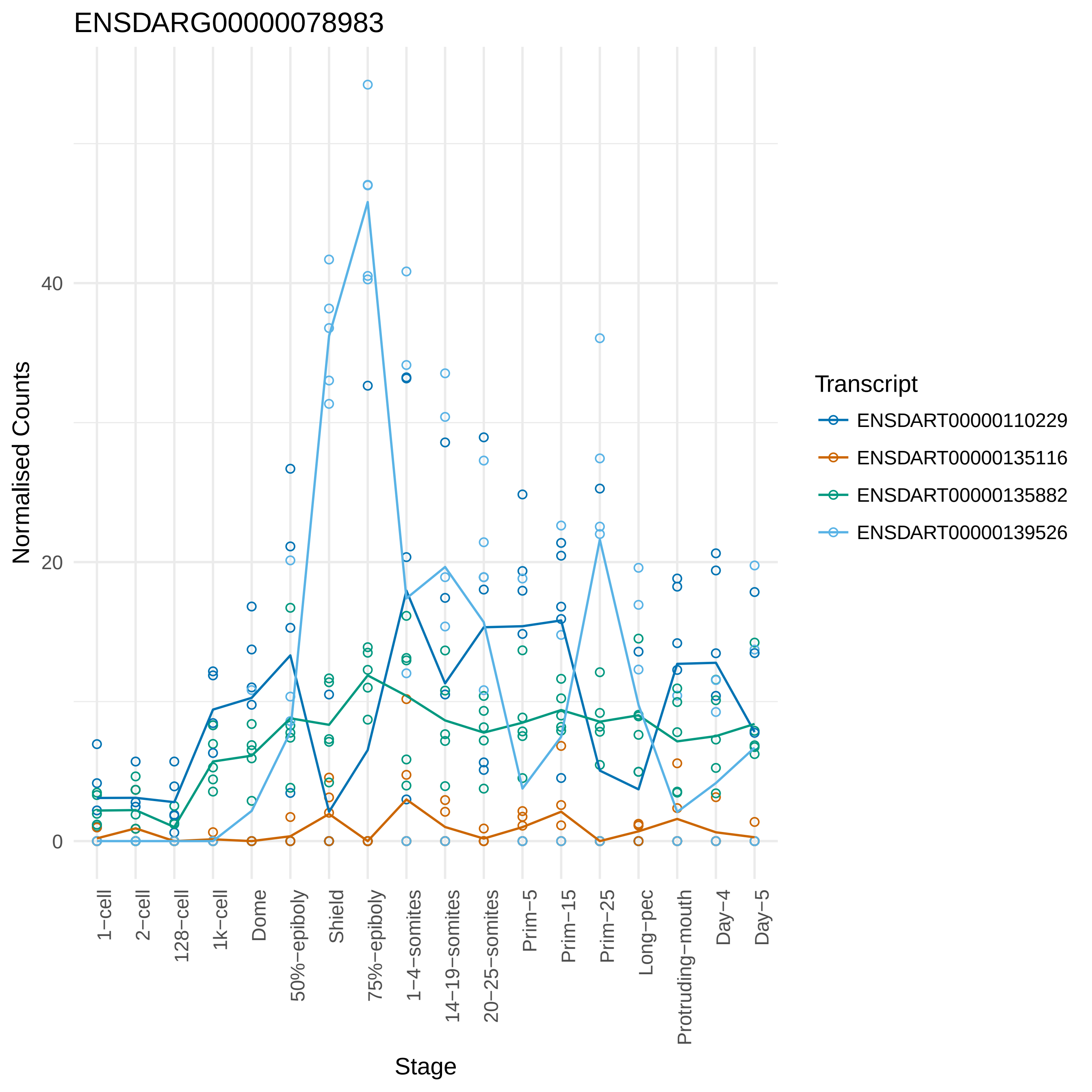

ENSDARG00000078983
Normalised Counts
0
20
40
Stage
1−cell
2−cell
128−cell
1k−cell
Dome
50%−epiboly
Shield
75%−epiboly
1−4−somites
14−19−somites
20−25−somites
Prim−5
Prim−15
Prim−25
Long−pec
Protruding−mouth
Day−4
Day−5
Transcript
ENSDART00000110229
ENSDART00000135116
ENSDART00000135882
ENSDART00000139526

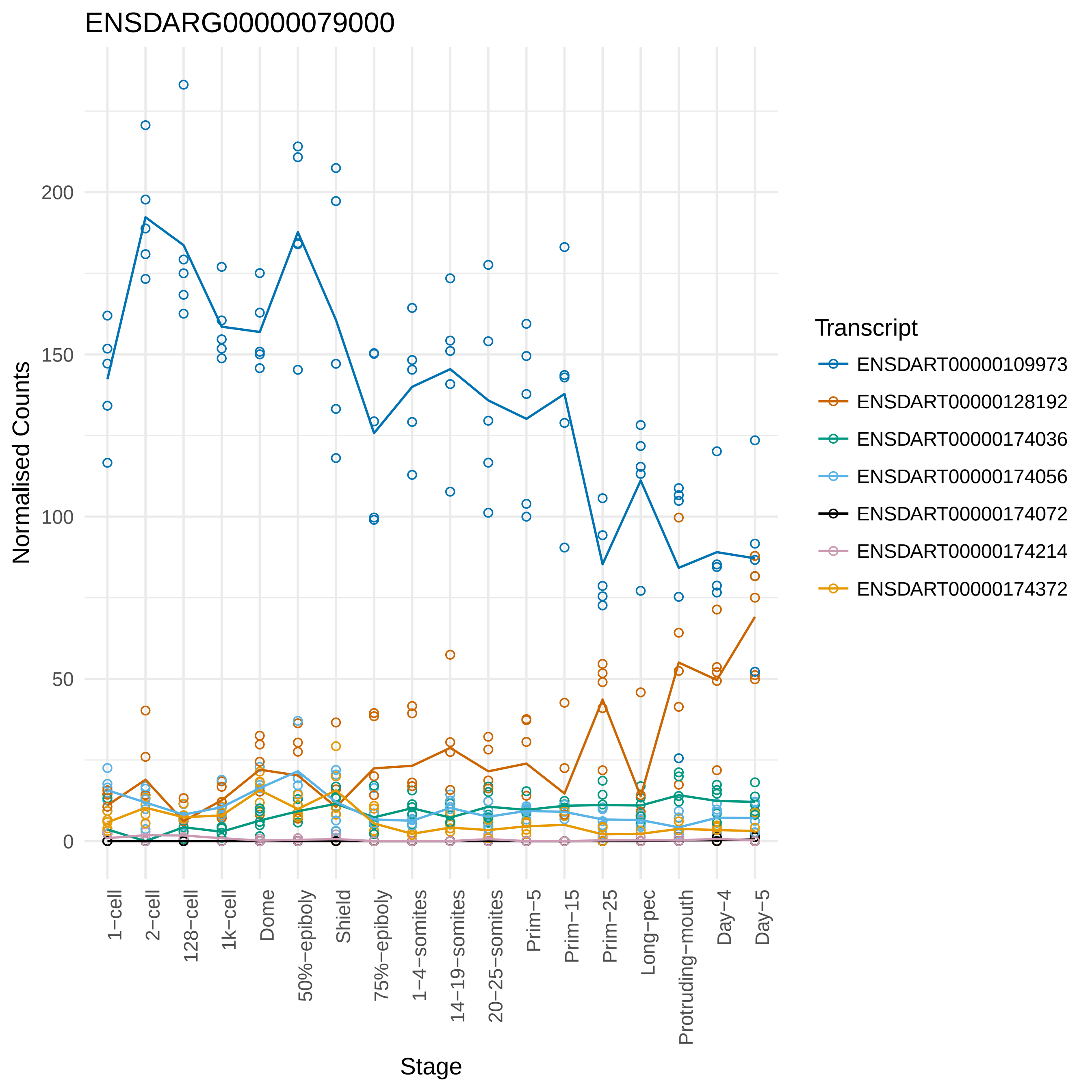
ENSDARG00000079000
Normalised Counts
0
50
100
150
200
Stage
1−cell
2−cell
128−cell
1k−cell
Dome
50%−epiboly
Shield
75%−epiboly
1−4−somites
14−19−somites
20−25−somites
Prim−5
Prim−15
Prim−25
Long−pec
Protruding−mouth
Day−4
Day−5
Transcript
ENSDART00000109973
ENSDART00000128192
ENSDART00000174036
ENSDART00000174056
ENSDART00000174072
ENSDART00000174214
ENSDART00000174372

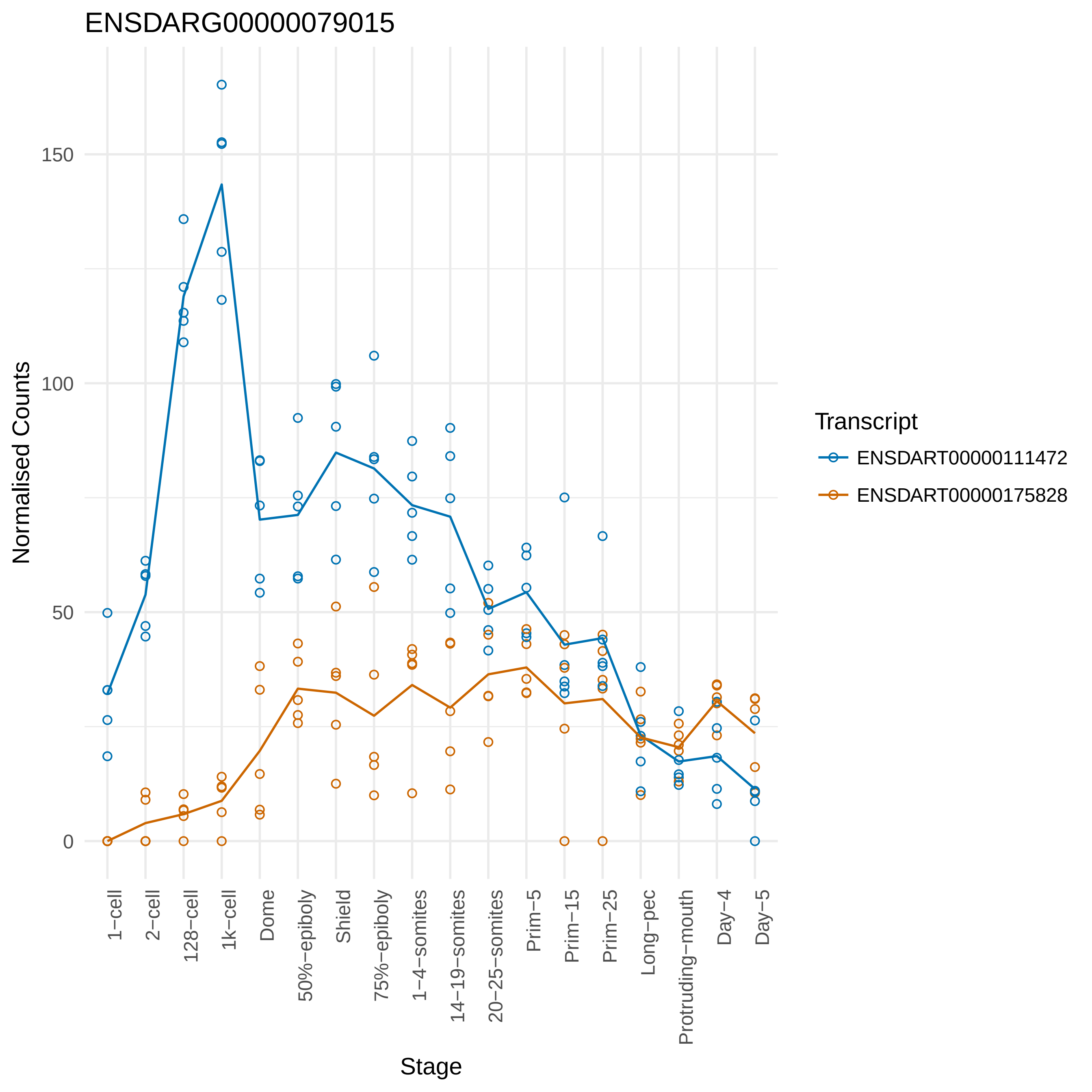
ENSDARG00000079015
Normalised Counts
150
100
50
0
1−cell
2−cell
128−cell
1k−cell
Dome
50%−epiboly
Shield
75%−epiboly
1−4−somites
14−19−somites
20−25−somites
Prim−5
Prim−15
Prim−25
Long−pec
Protruding−mouth
Day−4
Day−5
Stage
Transcript
ENSDART00000111472
ENSDART00000175828

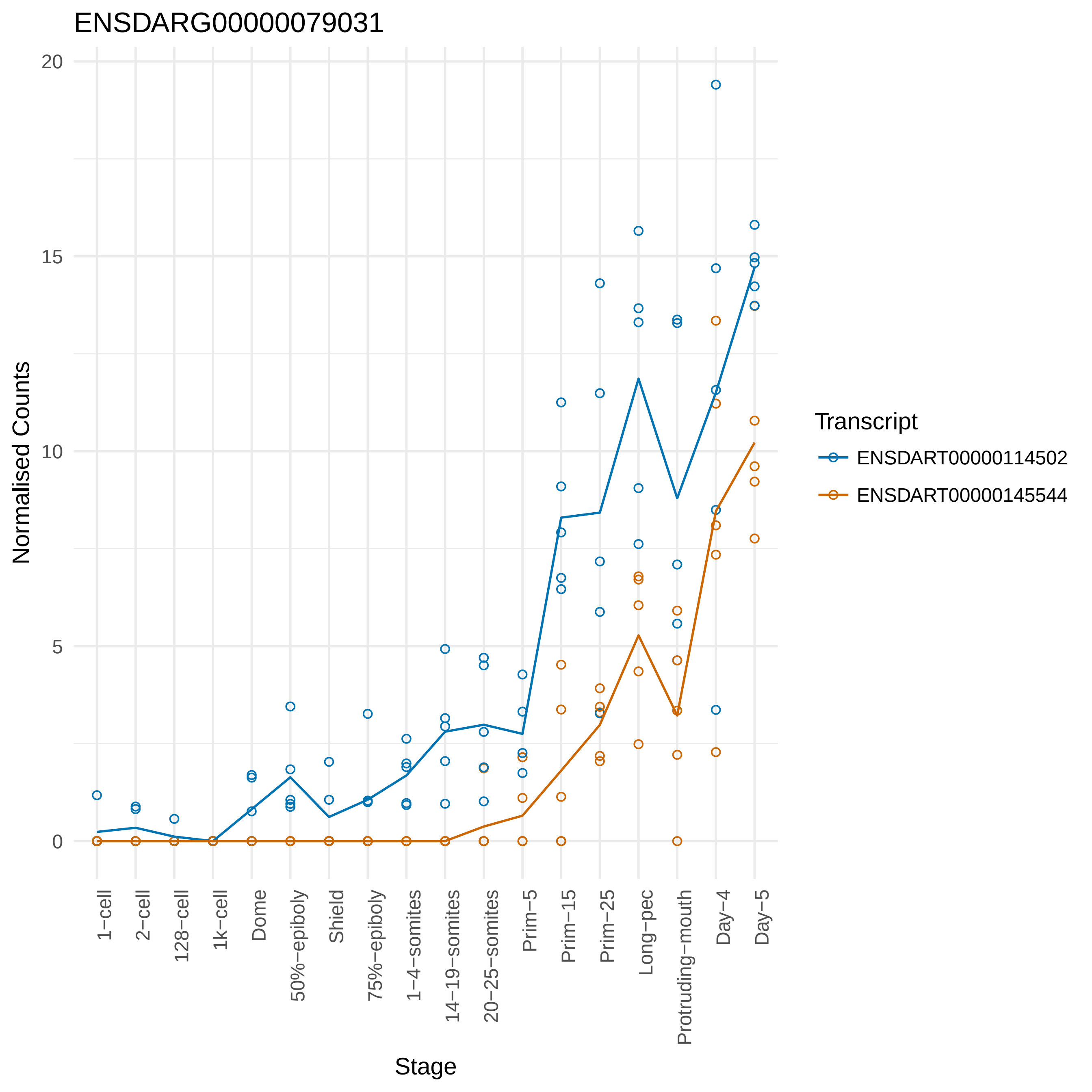

ENSDARG00000079031
Normalised Counts
20
15
10
5
0
1−cell
2−cell
128−cell
1k−cell
Dome
50%−epiboly
Shield
75%−epiboly
1−4−somites
14−19−somites
20−25−somites
Prim−5
Prim−15
Prim−25
Long−pec
Protruding−mouth
Day−4
Day−5
Stage
Transcript
ENSDART00000114502
ENSDART00000145544

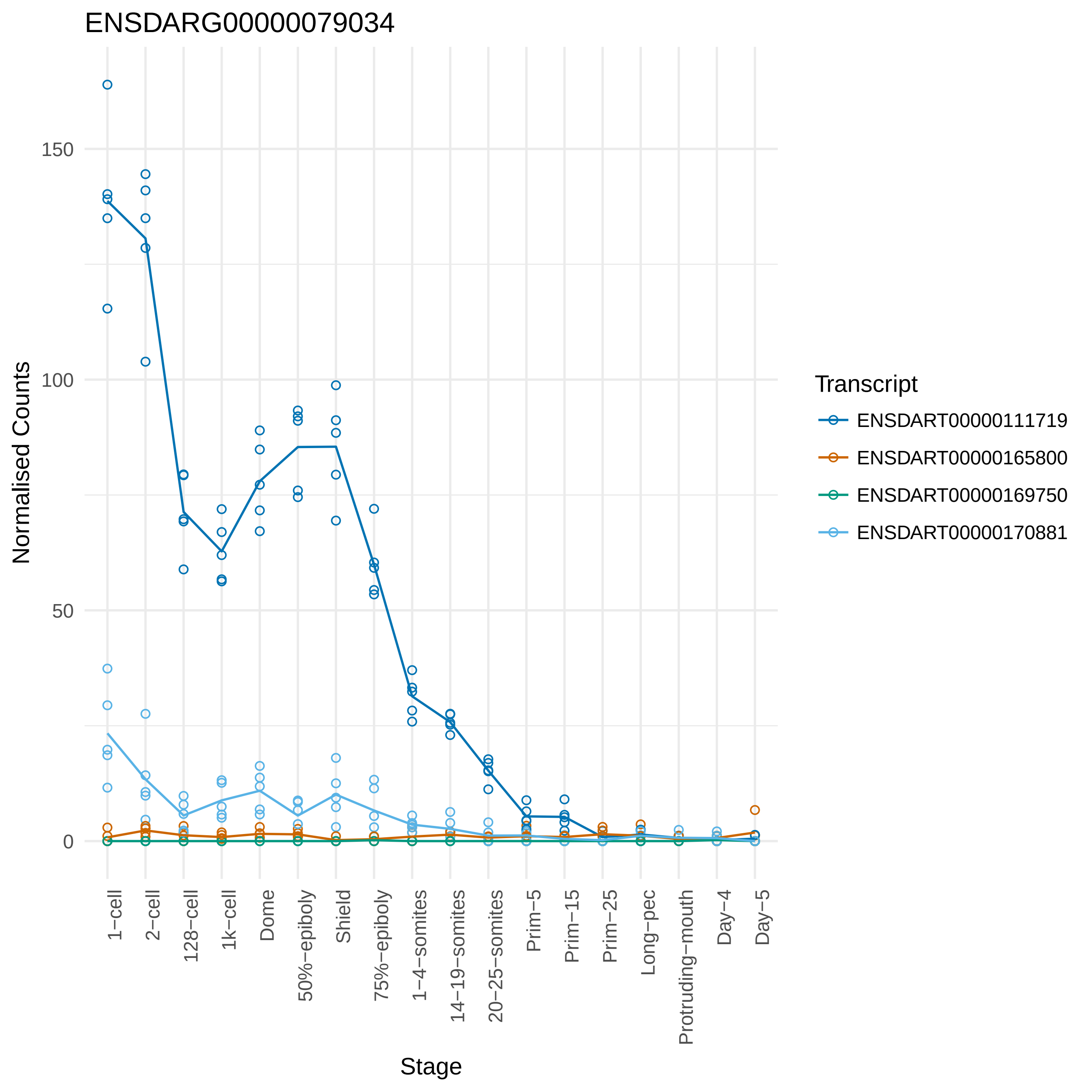
ENSDARG00000079034
Normalised Counts
150
100
50
0
1−cell
2−cell
128−cell
1k−cell
Dome
50%−epiboly
Shield
75%−epiboly
1−4−somites
14−19−somites
20−25−somites
Prim−5
Prim−15
Prim−25
Long−pec
Protruding−mouth
Day−4
Day−5
Stage
Transcript
ENSDART00000111719
ENSDART00000165800
ENSDART00000169750
ENSDART00000170881

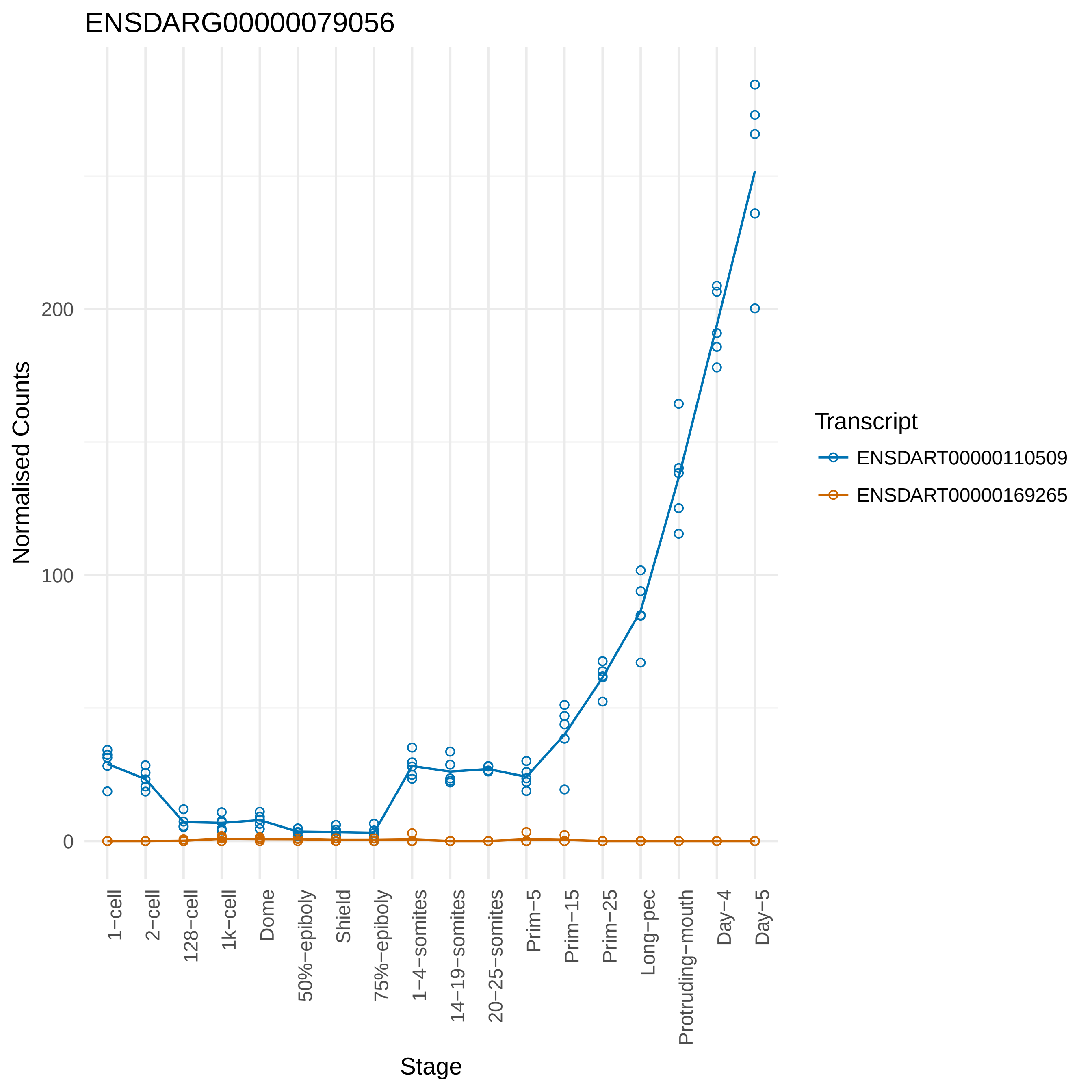
ENSDARG00000079056
Normalised Counts
200
100
0
1−cell
2−cell
128−cell
1k−cell
Dome
50%−epiboly
Shield
75%−epiboly
1−4−somites
14−19−somites
20−25−somites
Prim−5
Prim−15
Prim−25
Long−pec
Protruding−mouth
Day−4
Day−5
Stage
Transcript
ENSDART00000110509
ENSDART00000169265

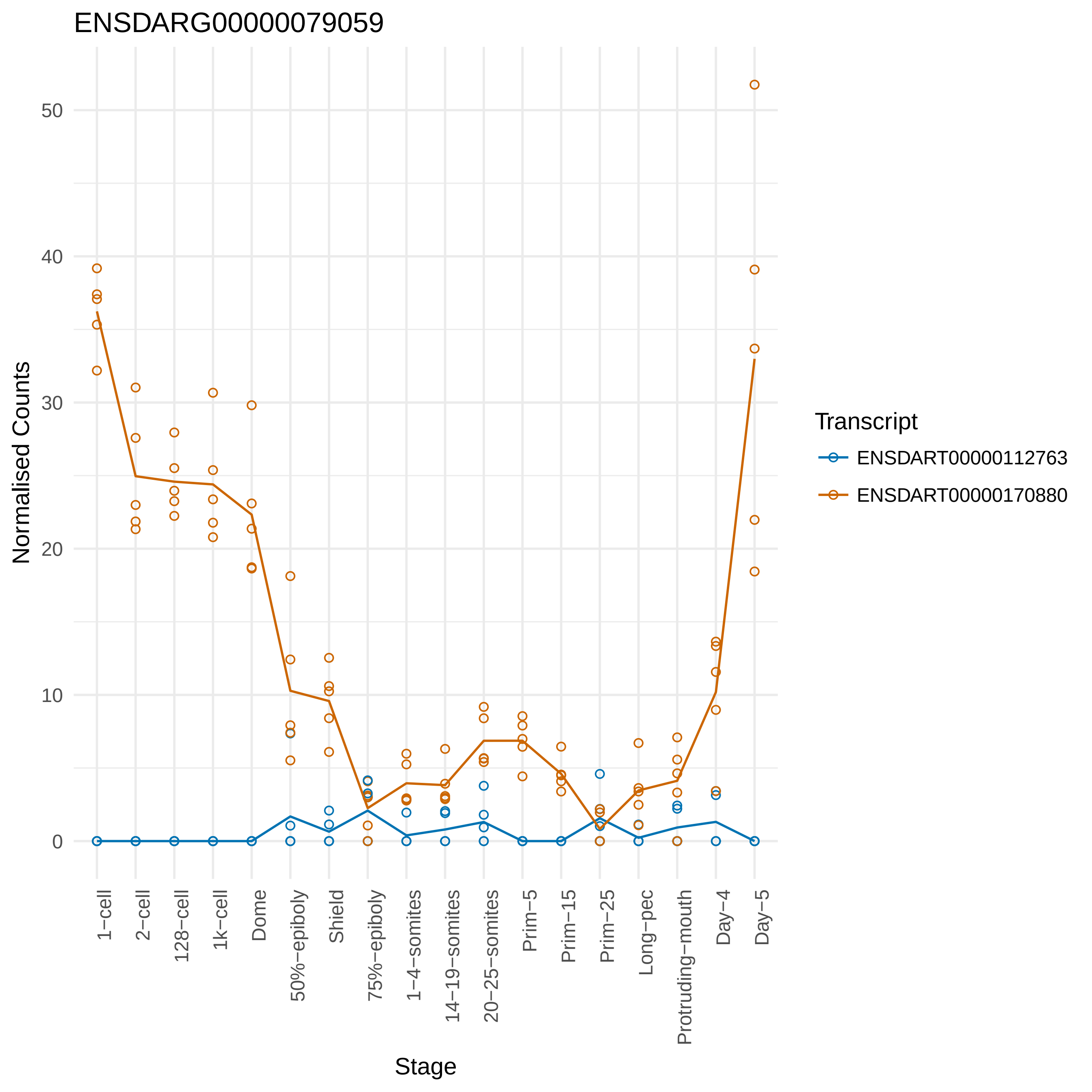

ENSDARG00000079059
Normalised Counts
0
10
20
30
40
50
1−cell
2−cell
128−cell
1k−cell
Dome
50%−epiboly
Shield
75%−epiboly
1−4−somites
14−19−somites
20−25−somites
Prim−5
Prim−15
Prim−25
Long−pec
Protruding−mouth
Day−4
Day−5
Stage
Transcript
ENSDART00000112763
ENSDART00000170880

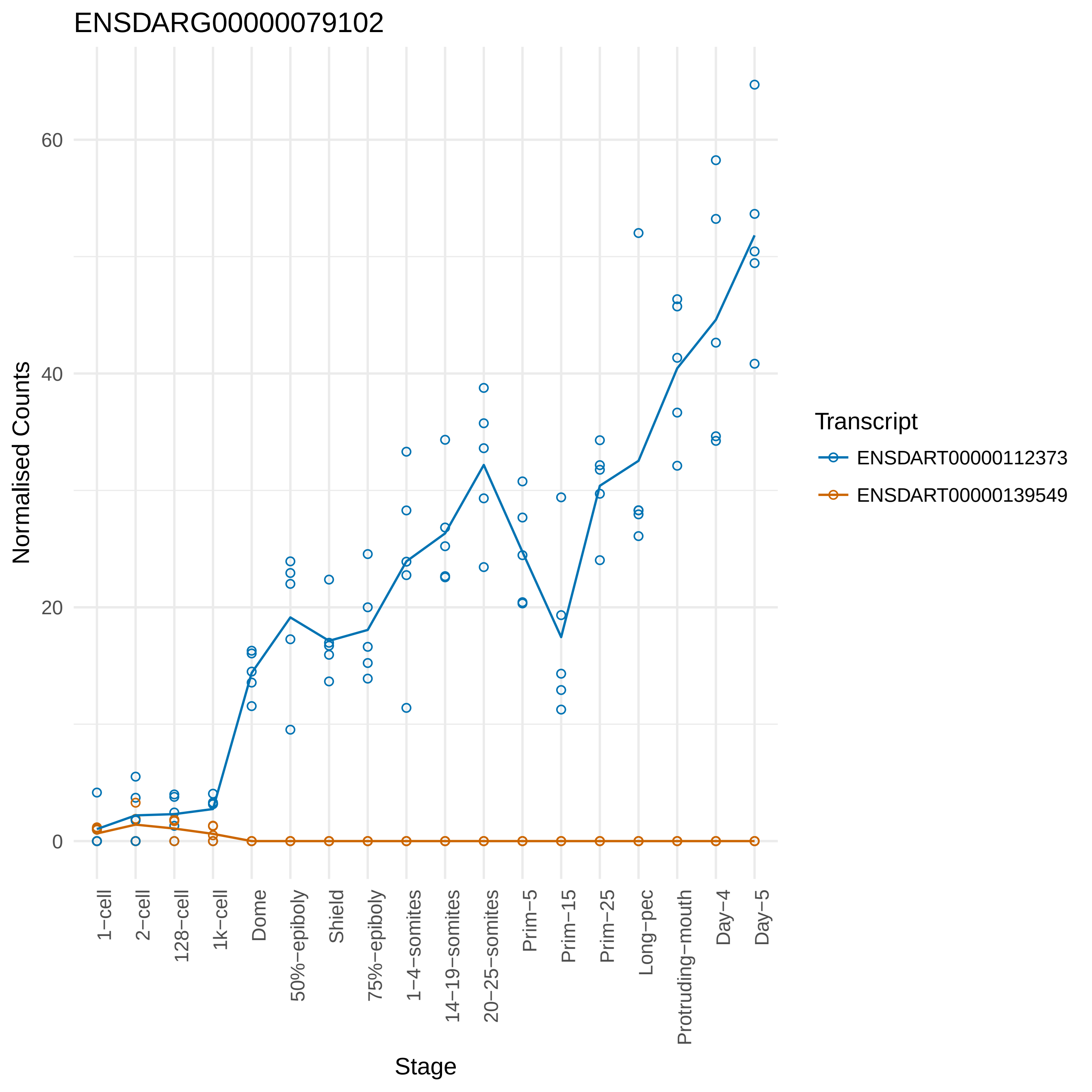
ENSDARG00000079102
Normalised Counts
0
20
40
60
Stage
1−cell
2−cell
128−cell
1k−cell
Dome
50%−epiboly
Shield
75%−epiboly
1−4−somites
14−19−somites
20−25−somites
Prim−5
Prim−15
Prim−25
Long−pec
Protruding−mouth
Day−4
Day−5
Transcript
ENSDART00000112373
ENSDART00000139549

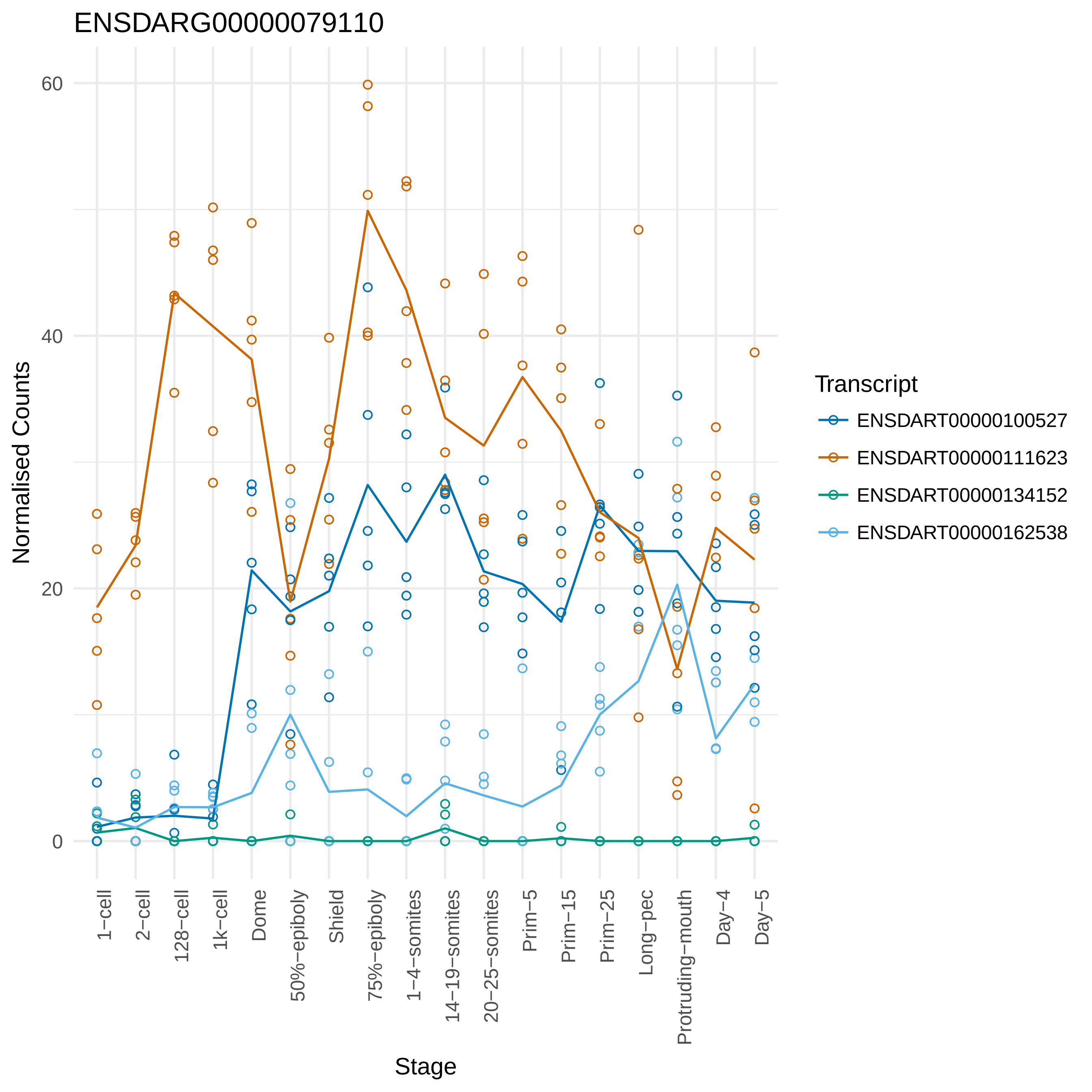

ENSDARG00000079110
Normalised Counts
0
20
40
60
1−cell
2−cell
128−cell
1k−cell
Dome
50%−epiboly
Shield
75%−epiboly
1−4−somites
14−19−somites
20−25−somites
Prim−5
Prim−15
Prim−25
Long−pec
Protruding−mouth
Day−4
Day−5
Stage
Transcript
ENSDART00000100527
ENSDART00000111623
ENSDART00000134152
ENSDART00000162538

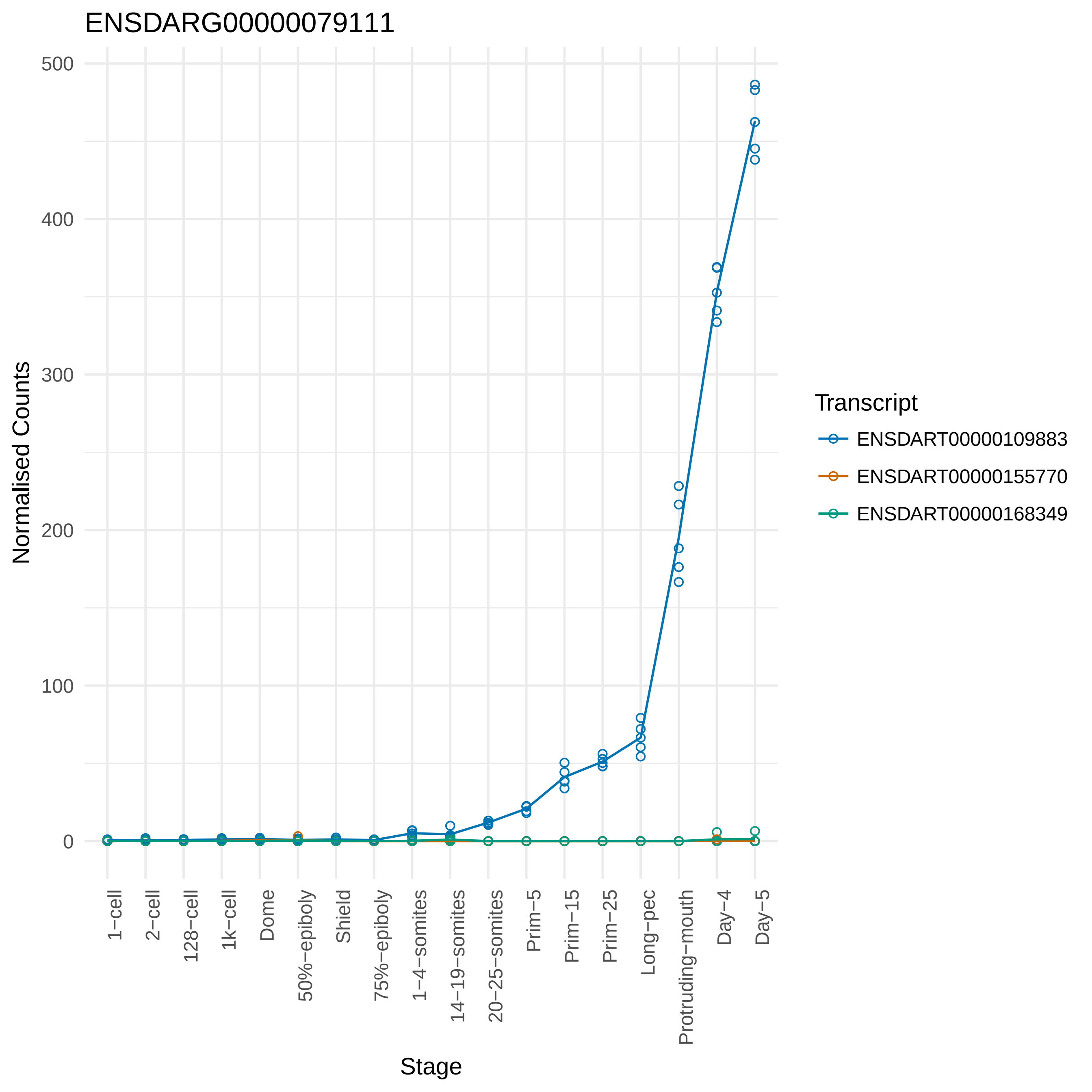

ENSDARG00000079111
Normalised Counts
0
100
200
300
400
500
1−cell
2−cell
128−cell
1k−cell
Dome
50%−epiboly
Shield
75%−epiboly
1−4−somites
14−19−somites
20−25−somites
Prim−5
Prim−15
Prim−25
Long−pec
Protruding−mouth
Day−4
Day−5
Stage
Transcript
ENSDART00000109883
ENSDART00000155770
ENSDART00000168349

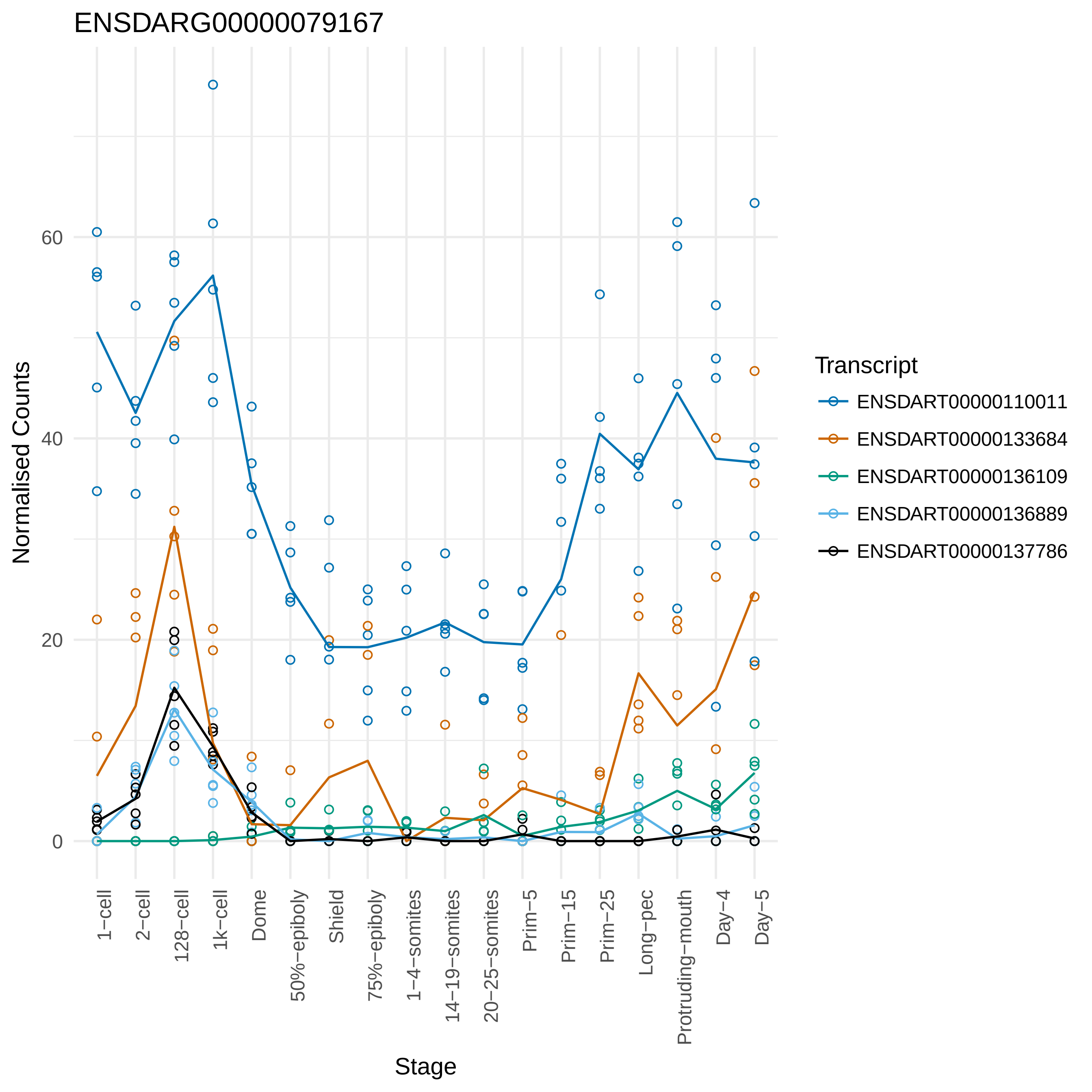
ENSDARG00000079167
Normalised Counts
60
40
20
0
1−cell
2−cell
128−cell
1k−cell
Dome
50%−epiboly
Shield
75%−epiboly
1−4−somites
14−19−somites
20−25−somites
Prim−5
Prim−15
Prim−25
Long−pec
Protruding−mouth
Day−4
Day−5
Stage
Transcript
ENSDART00000110011
ENSDART00000133684
ENSDART00000136109
ENSDART00000136889
ENSDART00000137786

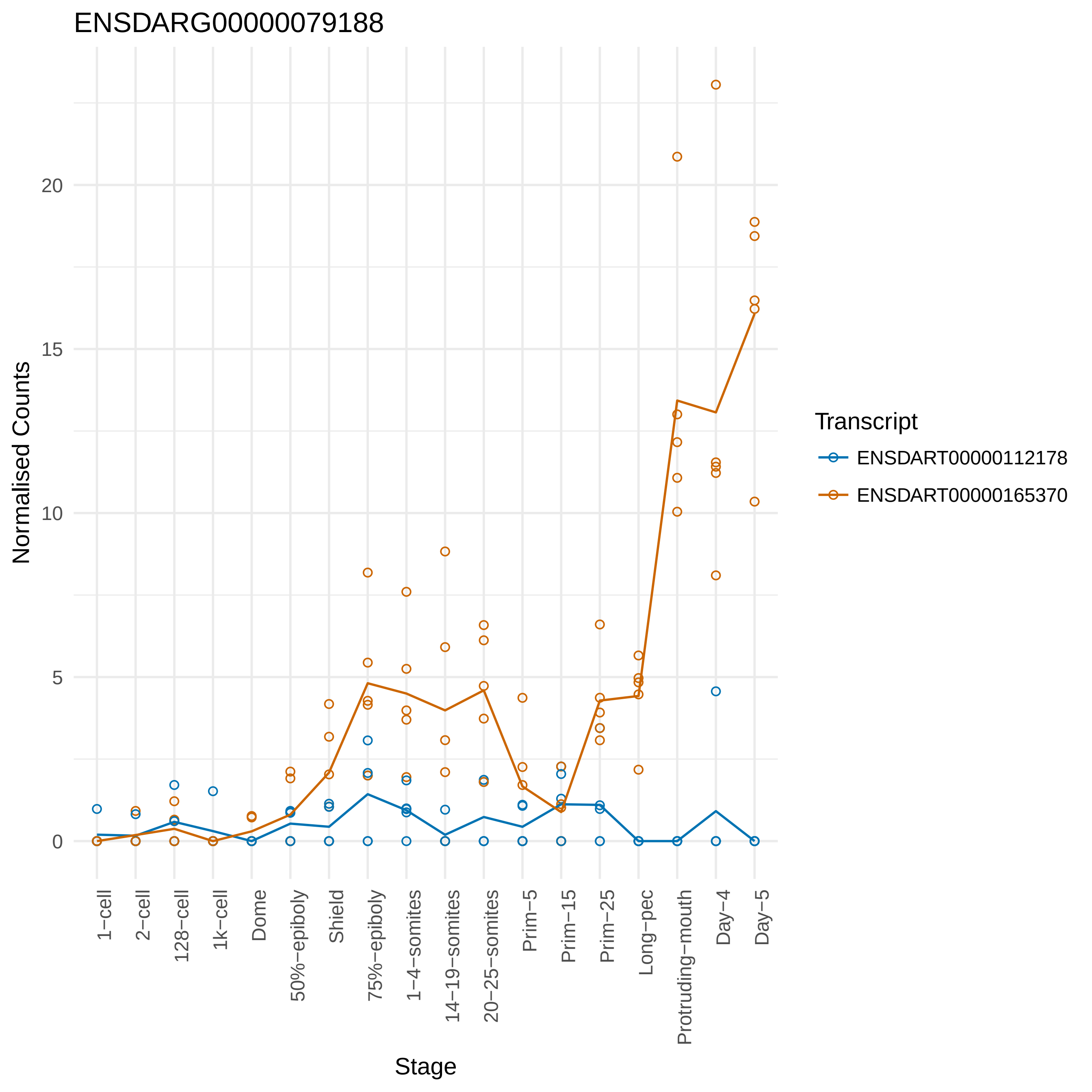

ENSDARG00000079188
Normalised Counts
0
5
10
15
20
1−cell
2−cell
128−cell
1k−cell
Dome
50%−epiboly
Shield
75%−epiboly
1−4−somites
14−19−somites
20−25−somites
Prim−5
Prim−15
Prim−25
Long−pec
Protruding−mouth
Day−4
Day−5
Stage
Transcript
ENSDART00000112178
ENSDART00000165370

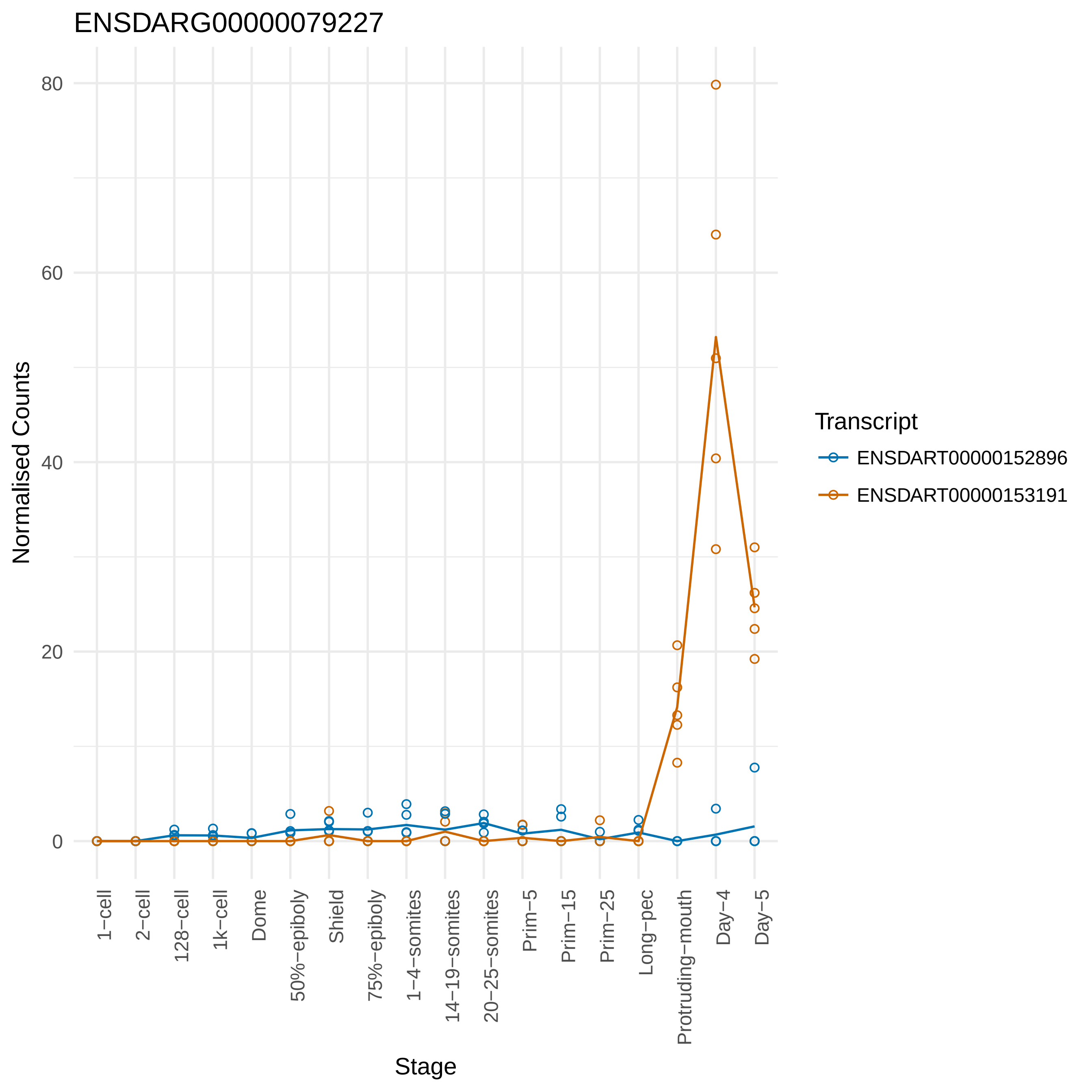

ENSDARG00000079227
Normalised Counts
0
20
40
60
80
1−cell
2−cell
128−cell
1k−cell
Dome
50%−epiboly
Shield
75%−epiboly
1−4−somites
14−19−somites
20−25−somites
Prim−5
Prim−15
Prim−25
Long−pec
Protruding−mouth
Day−4
Day−5
Stage
Transcript
ENSDART00000152896
ENSDART00000153191

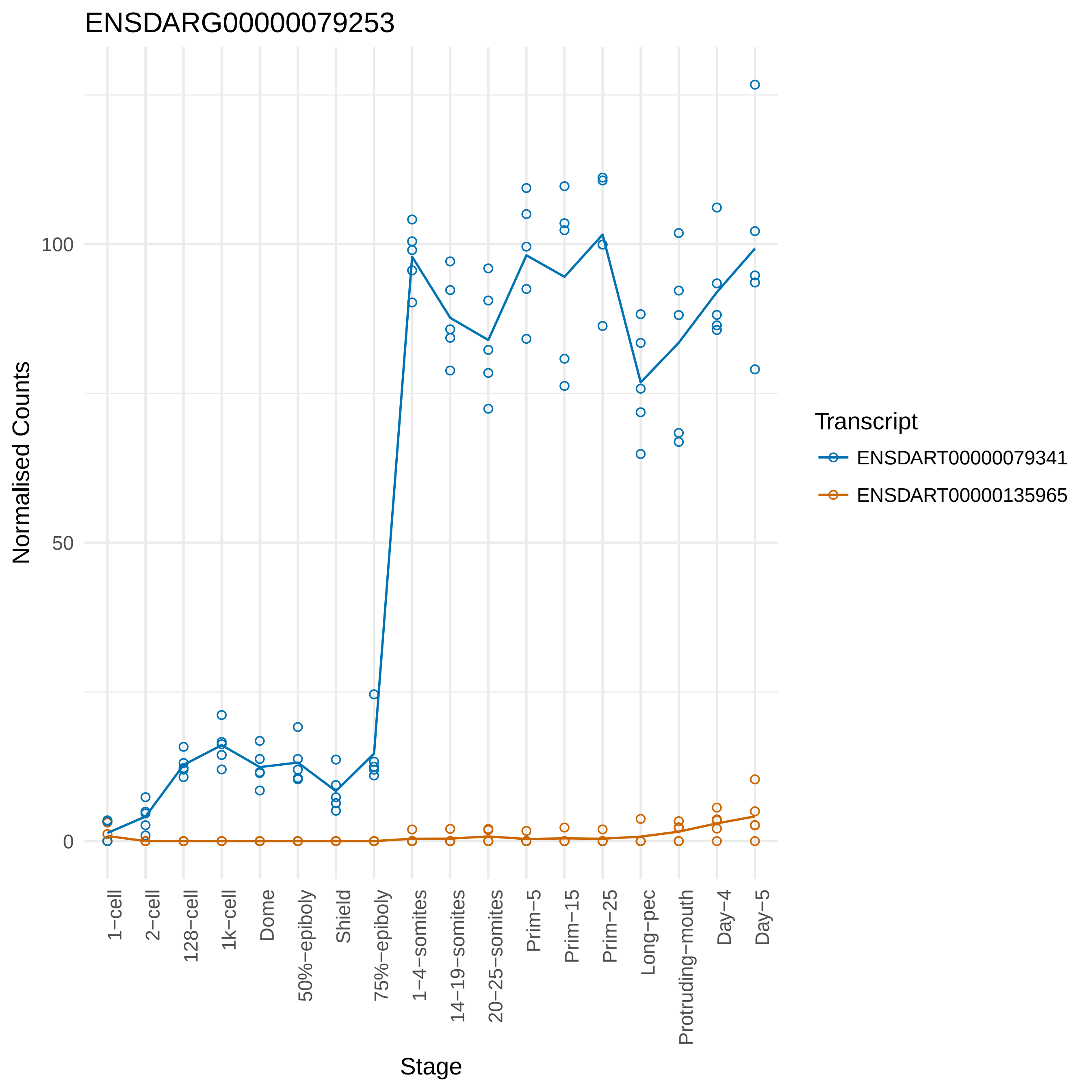
ENSDARG00000079253
Normalised Counts
100
50
0
1−cell
2−cell
128−cell
1k−cell
Dome
50%−epiboly
Shield
75%−epiboly
1−4−somites
14−19−somites
20−25−somites
Prim−5
Prim−15
Prim−25
Long−pec
Protruding−mouth
Day−4
Day−5
Stage
Transcript
ENSDART00000079341
ENSDART00000135965

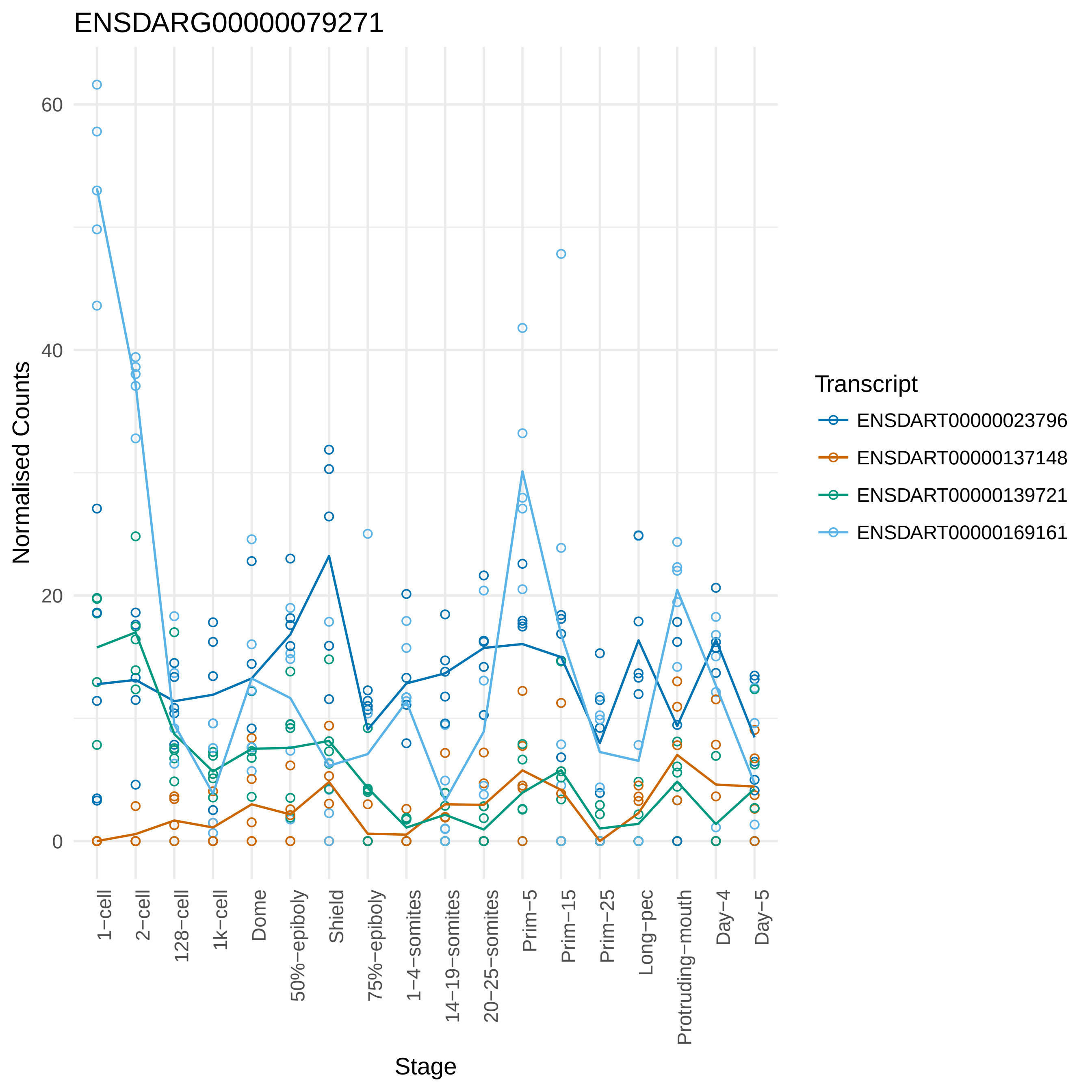
ENSDARG00000079271
Normalised Counts
60
40
20
0
1−cell
2−cell
128−cell
1k−cell
Dome
50%−epiboly
Shield
75%−epiboly
1−4−somites
14−19−somites
20−25−somites
Prim−5
Prim−15
Prim−25
Long−pec
Protruding−mouth
Day−4
Day−5
Stage
Transcript
ENSDART00000023796
ENSDART00000137148
ENSDART00000139721
ENSDART00000169161

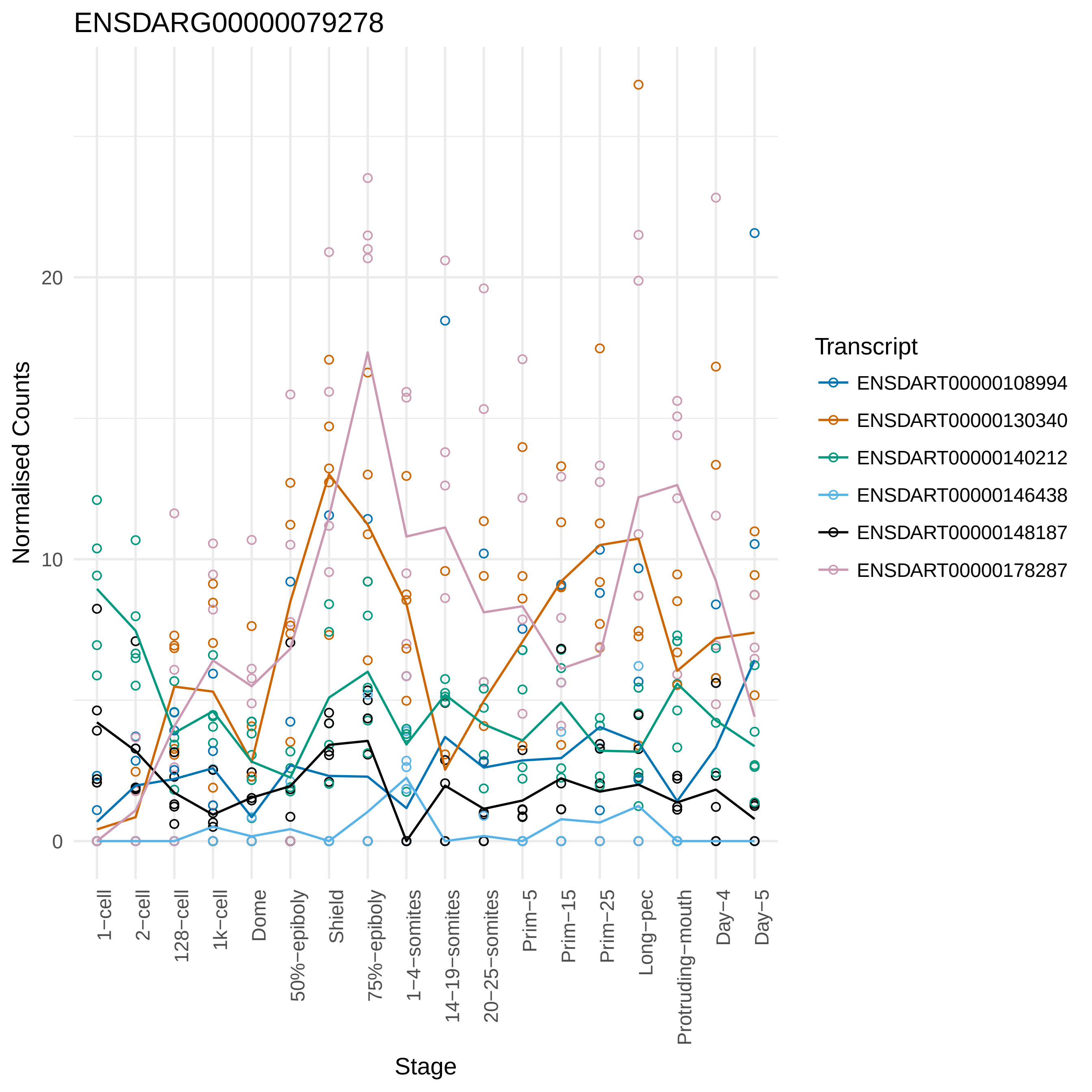

ENSDARG00000079278
Normalised Counts
0
10
20
1−cell
2−cell
128−cell
1k−cell
Dome
50%−epiboly
Shield
75%−epiboly
1−4−somites
14−19−somites
20−25−somites
Prim−5
Prim−15
Prim−25
Long−pec
Protruding−mouth
Day−4
Day−5
Stage
Transcript
ENSDART00000108994
ENSDART00000130340
ENSDART00000140212
ENSDART00000146438
ENSDART00000148187
ENSDART00000178287

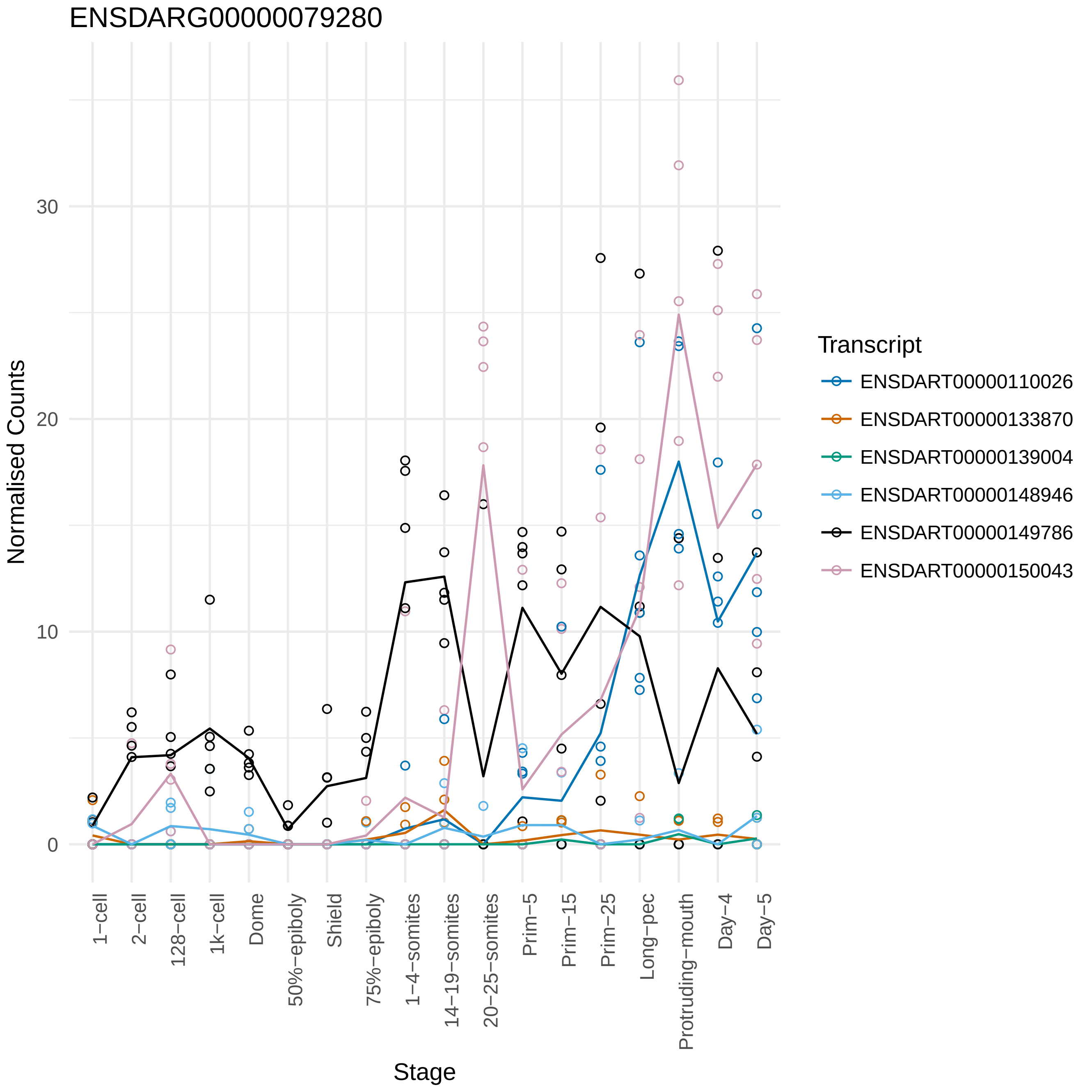

ENSDARG00000079280
Normalised Counts
0
10
20
30
1−cell
2−cell
128−cell
1k−cell
Dome
50%−epiboly
Shield
75%−epiboly
1−4−somites
14−19−somites
20−25−somites
Prim−5
Prim−15
Prim−25
Long−pec
Protruding−mouth
Day−4
Day−5
Stage
Transcript
ENSDART00000110026
ENSDART00000133870
ENSDART00000139004
ENSDART00000148946
ENSDART00000149786
ENSDART00000150043

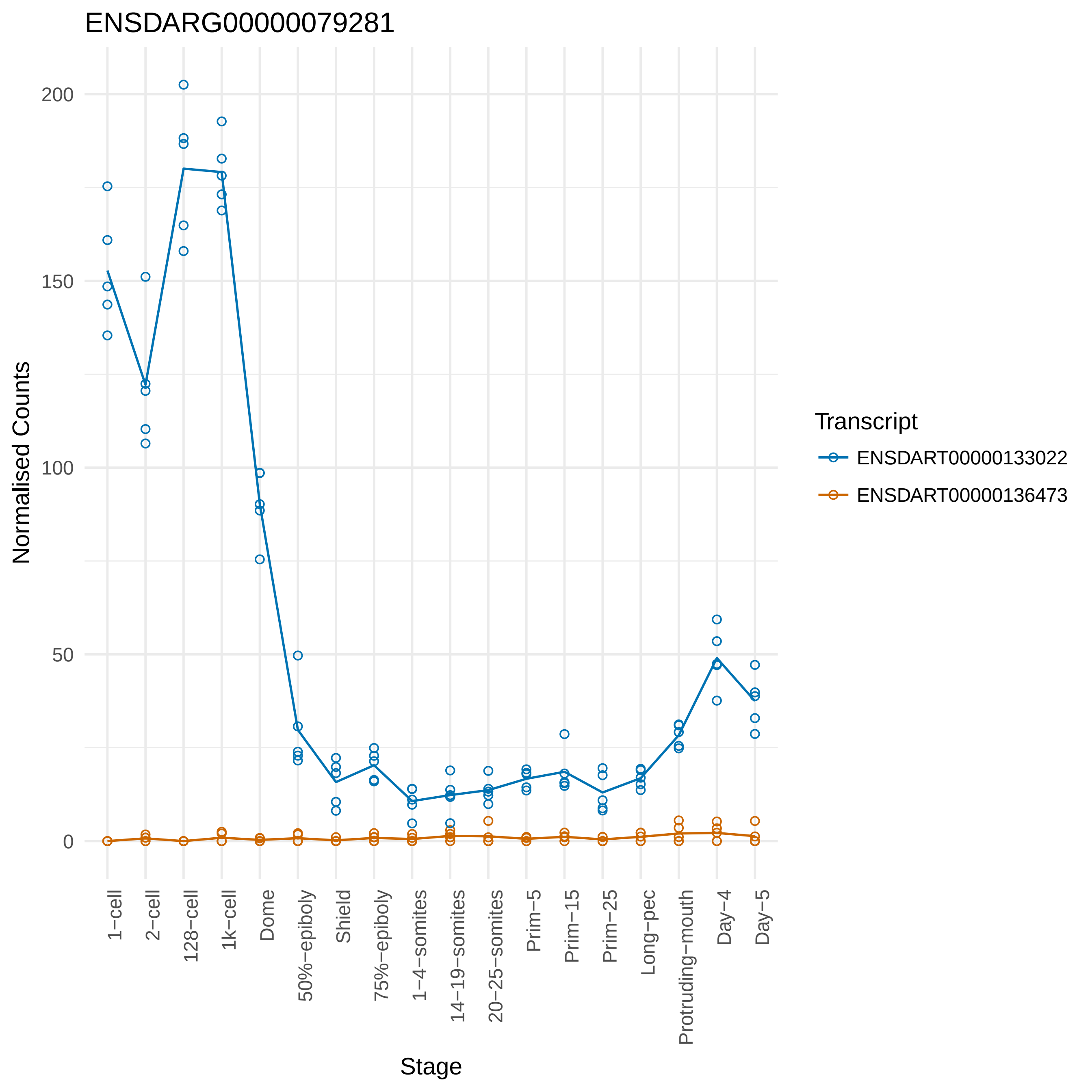

ENSDARG00000079281
Normalised Counts
200
150
100
50
0
1−cell
2−cell
128−cell
1k−cell
Dome
50%−epiboly
Shield
75%−epiboly
1−4−somites
14−19−somites
20−25−somites
Prim−5
Prim−15
Prim−25
Long−pec
Protruding−mouth
Day−4
Day−5
Stage
Transcript
ENSDART00000133022
ENSDART00000136473

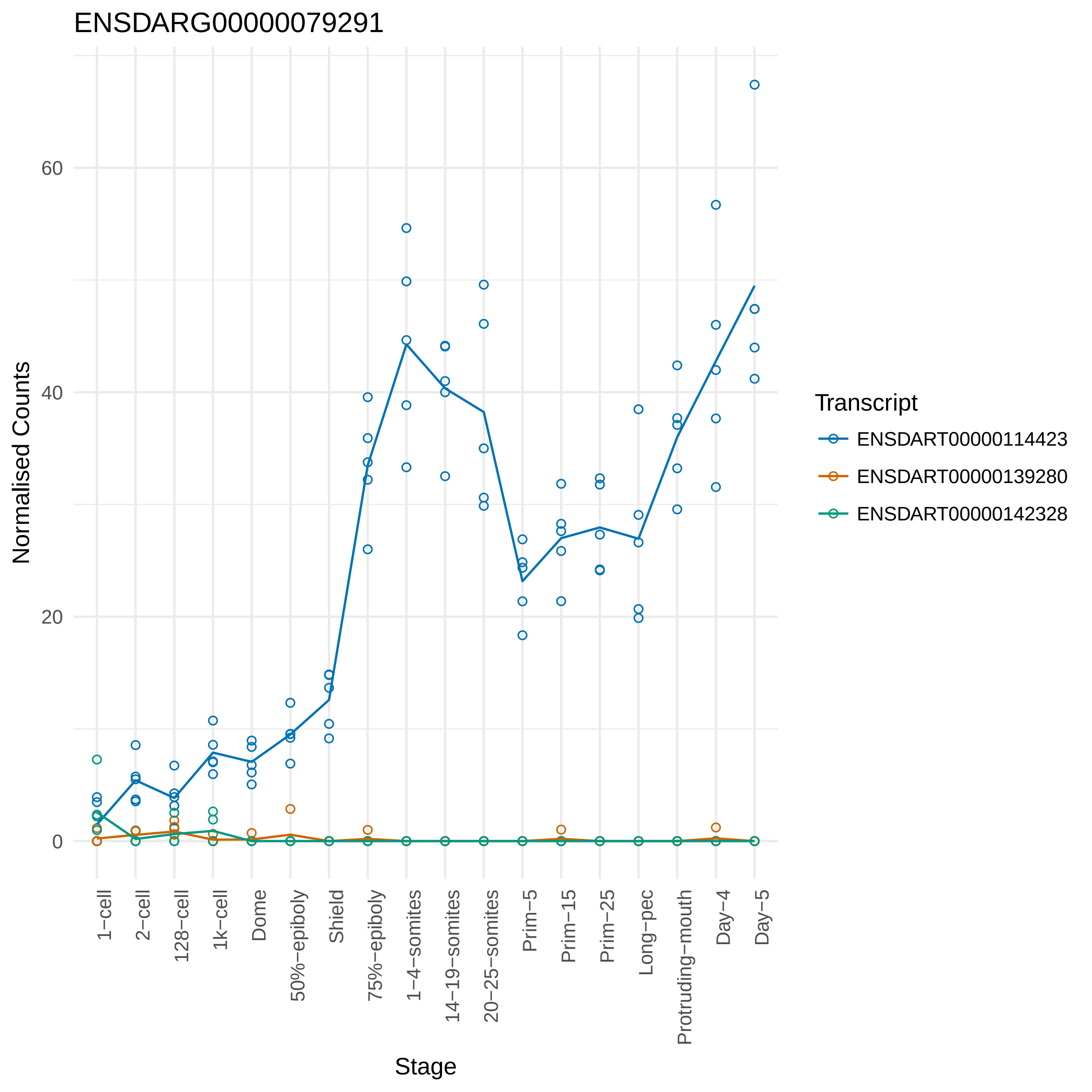

ENSDARG00000079291
Normalised Counts
0
20
40
60
Stage
1−cell
2−cell
128−cell
1k−cell
Dome
50%−epiboly
Shield
75%−epiboly
1−4−somites
14−19−somites
20−25−somites
Prim−5
Prim−15
Prim−25
Long−pec
Protruding−mouth
Day−4
Day−5
Transcript
ENSDART00000114423
ENSDART00000139280
ENSDART00000142328

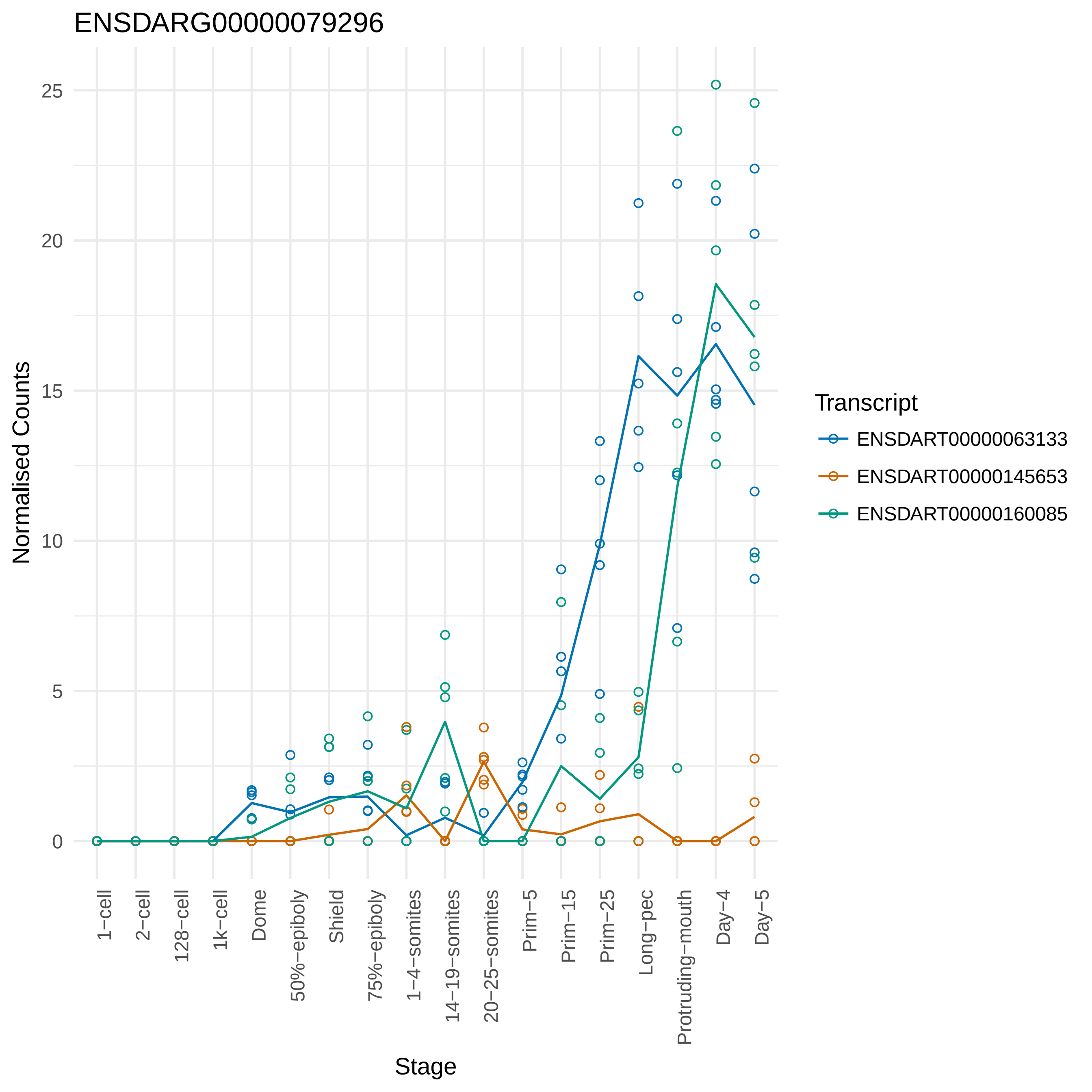
ENSDARG00000079296
Normalised Counts
0
5
10
15
20
25
1−cell
2−cell
128−cell
1k−cell
Dome
50%−epiboly
Shield
75%−epiboly
1−4−somites
14−19−somites
20−25−somites
Prim−5
Prim−15
Prim−25
Long−pec
Protruding−mouth
Day−4
Day−5
Stage
Transcript
ENSDART00000063133
ENSDART00000145653
ENSDART00000160085

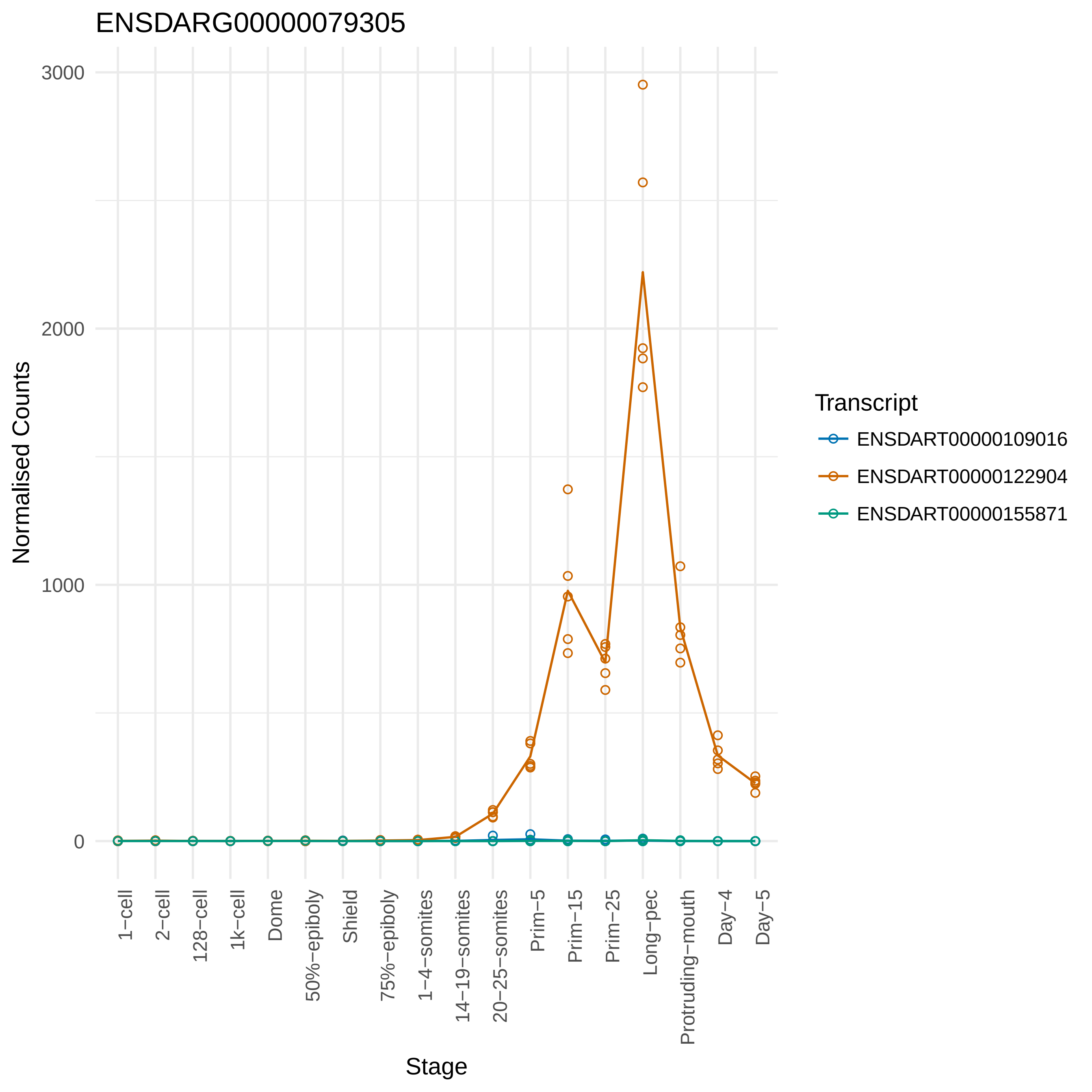

ENSDARG00000079305
Normalised Counts
3000
2000
1000
0
1−cell
2−cell
128−cell
1k−cell
Dome
50%−epiboly
Shield
75%−epiboly
1−4−somites
14−19−somites
20−25−somites
Prim−5
Prim−15
Prim−25
Long−pec
Protruding−mouth
Day−4
Day−5
Stage
Transcript
ENSDART00000109016
ENSDART00000122904
ENSDART00000155871

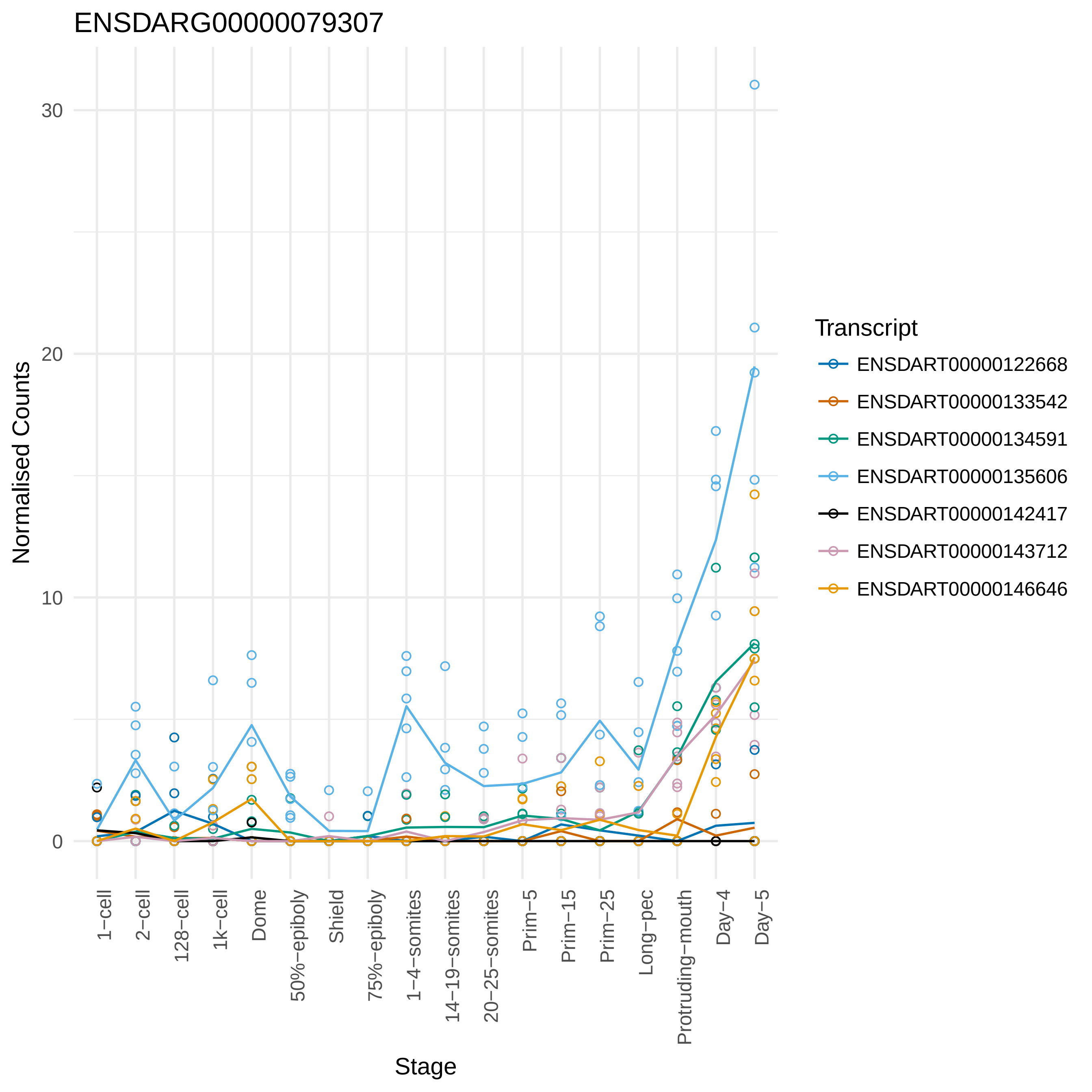

ENSDARG00000079307
Normalised Counts
0
10
20
30
1−cell
2−cell
128−cell
1k−cell
Dome
50%−epiboly
Shield
75%−epiboly
1−4−somites
14−19−somites
20−25−somites
Prim−5
Prim−15
Prim−25
Long−pec
Protruding−mouth
Day−4
Day−5
Stage
Transcript
ENSDART00000122668
ENSDART00000133542
ENSDART00000134591
ENSDART00000135606
ENSDART00000142417
ENSDART00000143712
ENSDART00000146646

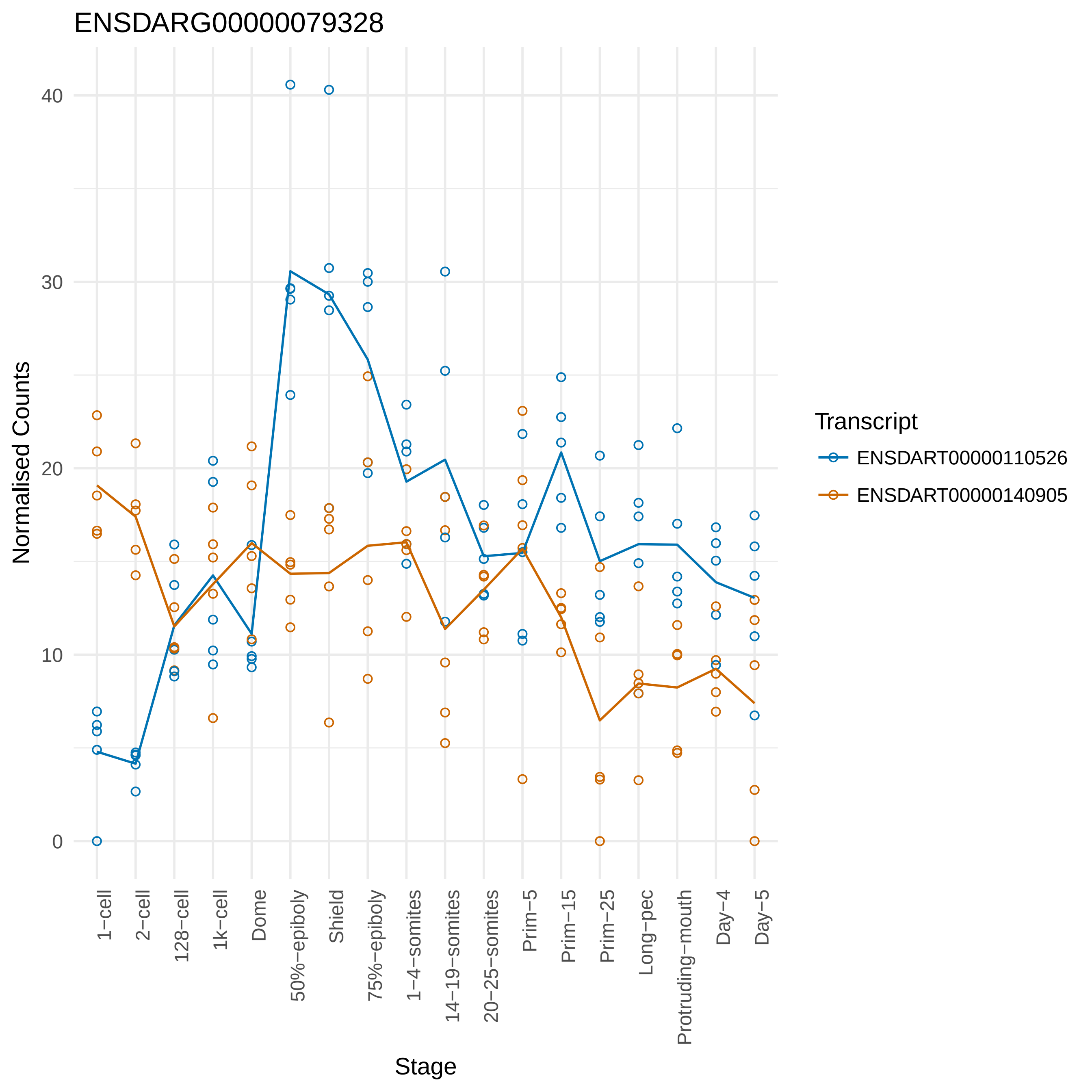
ENSDARG00000079328
Normalised Counts
0
10
20
30
40
1−cell
2−cell
128−cell
1k−cell
Dome
50%−epiboly
Shield
75%−epiboly
1−4−somites
14−19−somites
20−25−somites
Prim−5
Prim−15
Prim−25
Long−pec
Protruding−mouth
Day−4
Day−5
Stage
Transcript
ENSDART00000110526
ENSDART00000140905

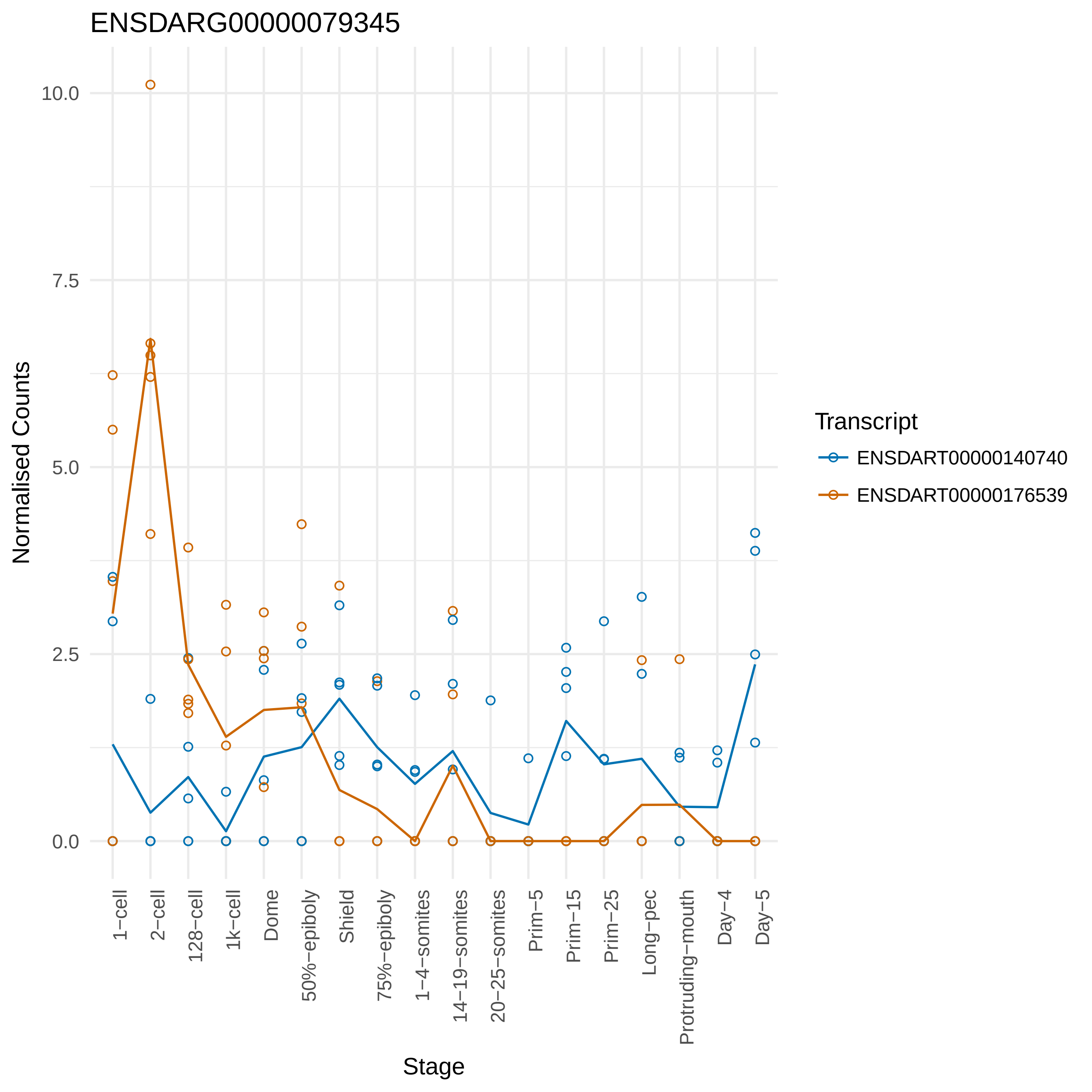
ENSDARG00000079345
Normalised Counts
10.0
7.5
5.0
2.5
0.0
1−cell
2−cell
128−cell
1k−cell
Dome
50%−epiboly
Shield
75%−epiboly
1−4−somites
14−19−somites
20−25−somites
Prim−5
Prim−15
Prim−25
Long−pec
Protruding−mouth
Day−4
Day−5
Stage
Transcript
ENSDART00000140740
ENSDART00000176539

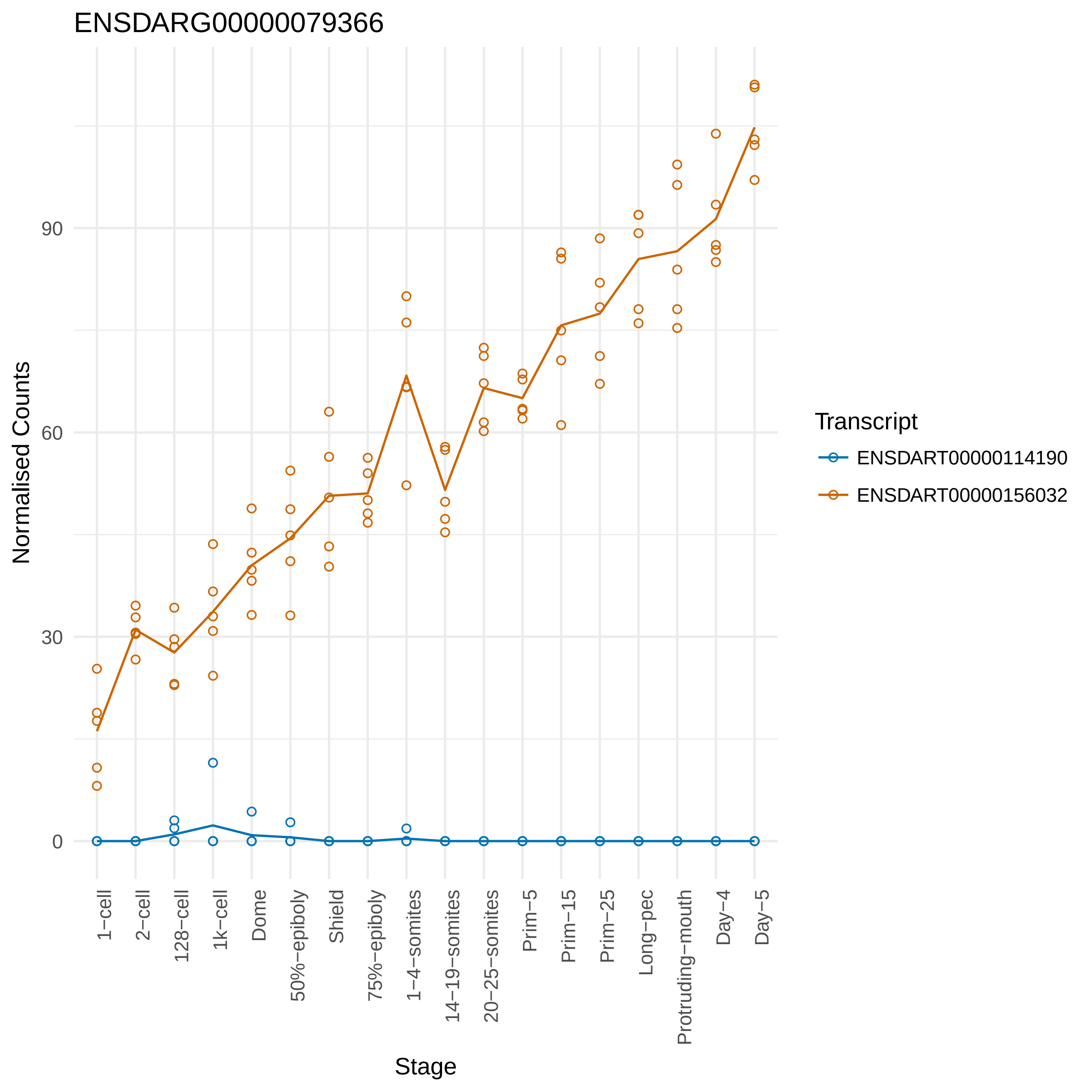

ENSDARG00000079366
Normalised Counts
0
30
60
90
1−cell
2−cell
128−cell
1k−cell
Dome
50%−epiboly
Shield
75%−epiboly
1−4−somites
14−19−somites
20−25−somites
Prim−5
Prim−15
Prim−25
Long−pec
Protruding−mouth
Day−4
Day−5
Stage
Transcript
ENSDART00000114190
ENSDART00000156032

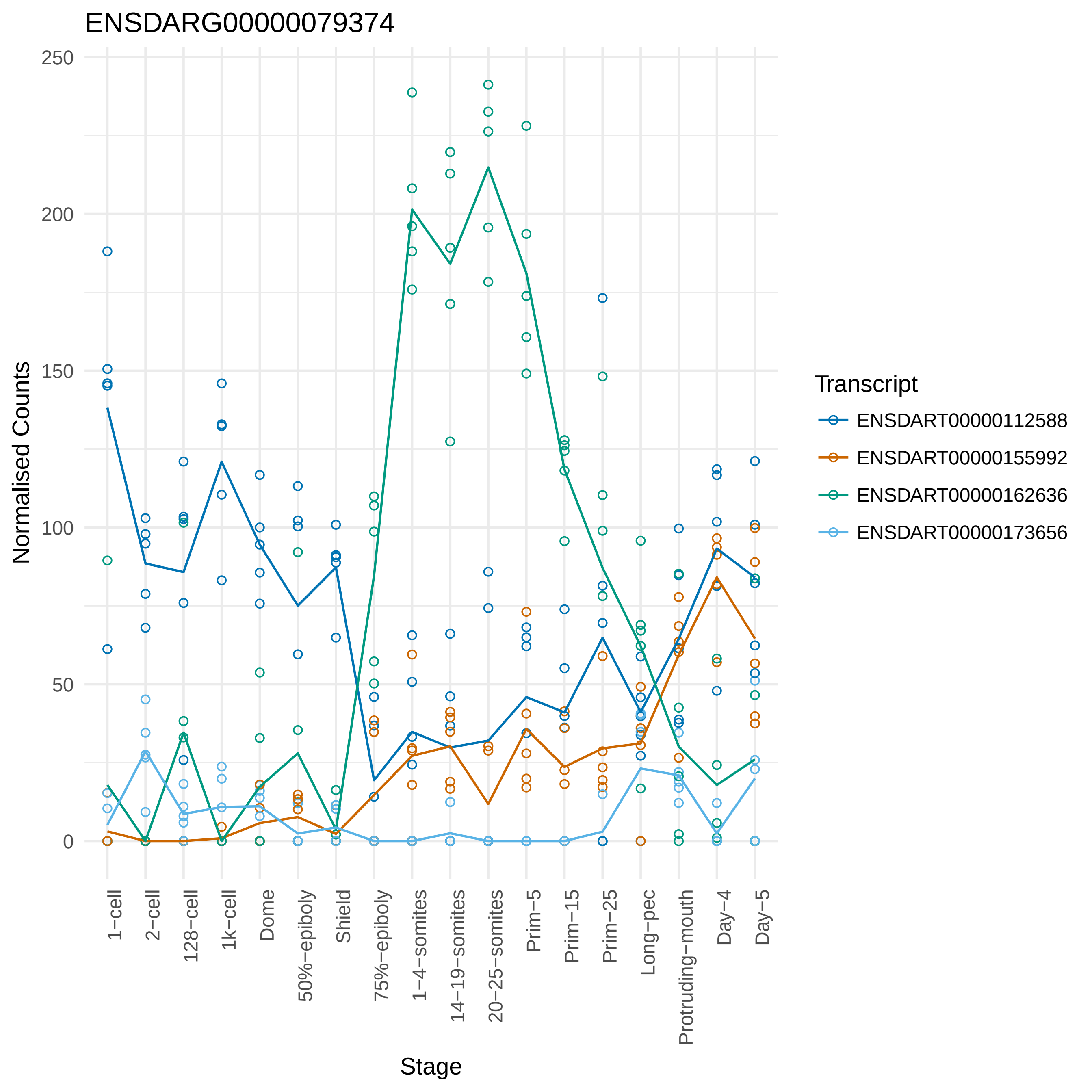

ENSDARG00000079374
Normalised Counts
250
200
150
100
50
0
1−cell
2−cell
128−cell
1k−cell
Dome
50%−epiboly
Shield
75%−epiboly
1−4−somites
14−19−somites
20−25−somites
Prim−5
Prim−15
Prim−25
Long−pec
Protruding−mouth
Day−4
Day−5
Stage
Transcript
ENSDART00000112588
ENSDART00000155992
ENSDART00000162636
ENSDART00000173656

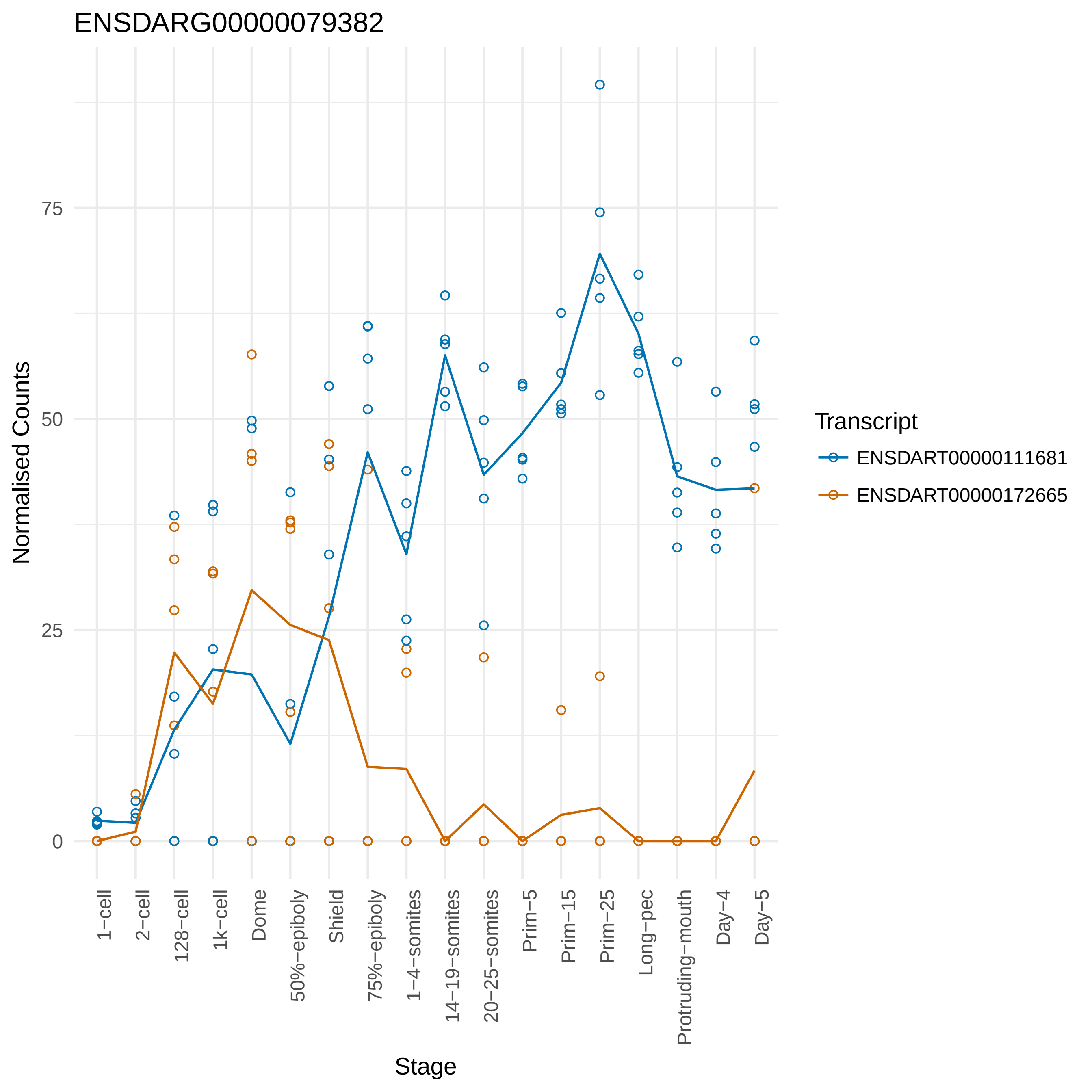

ENSDARG00000079382
Normalised Counts
0
25
50
75
1−cell
2−cell
128−cell
1k−cell
Dome
50%−epiboly
Shield
75%−epiboly
1−4−somites
14−19−somites
20−25−somites
Prim−5
Prim−15
Prim−25
Long−pec
Protruding−mouth
Day−4
Day−5
Stage
Transcript
ENSDART00000111681
ENSDART00000172665

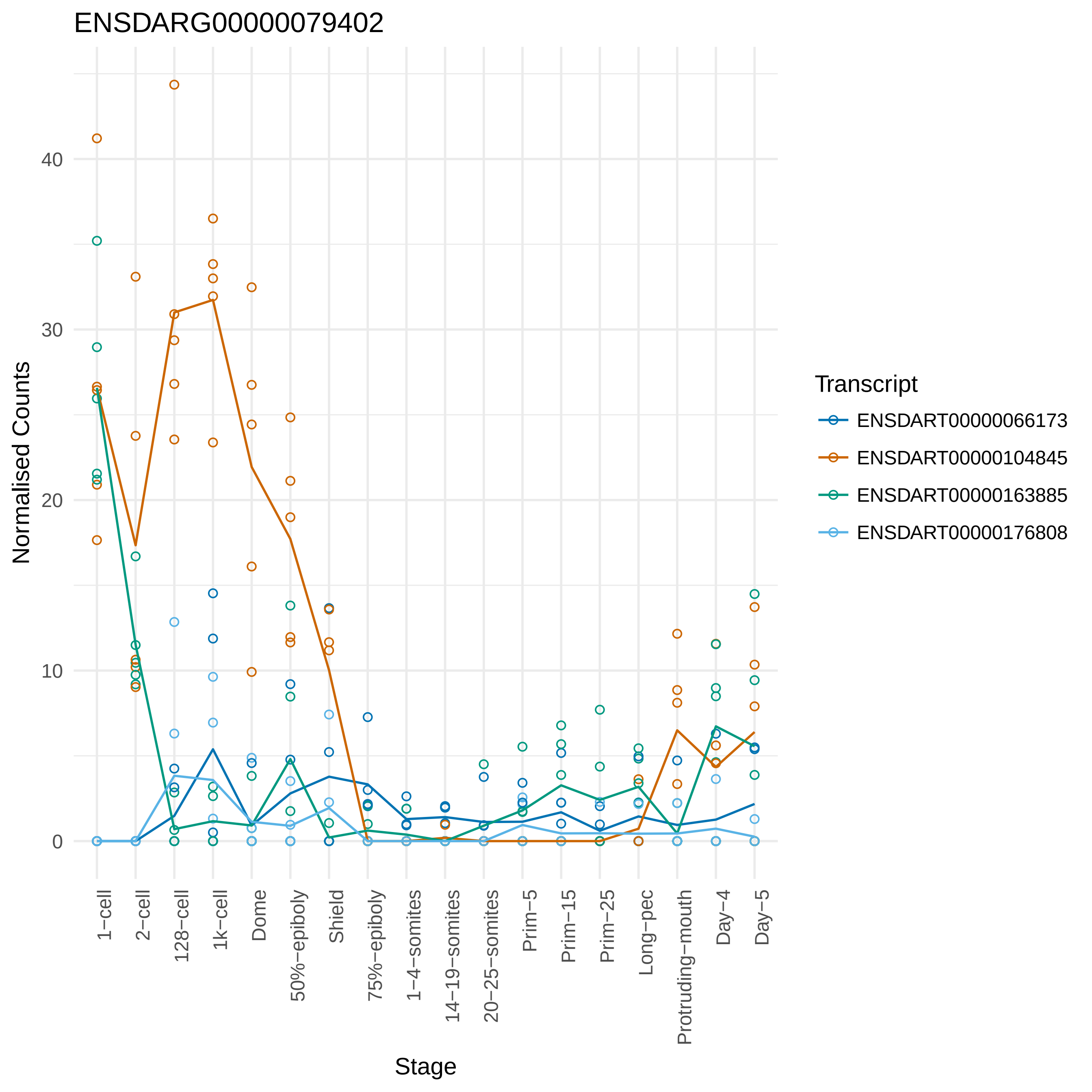

ENSDARG00000079402
Normalised Counts
0
10
20
30
40
Stage
1−cell
2−cell
128−cell
1k−cell
Dome
50%−epiboly
Shield
75%−epiboly
1−4−somites
14−19−somites
20−25−somites
Prim−5
Prim−15
Prim−25
Long−pec
Protruding−mouth
Day−4
Day−5
Transcript
ENSDART00000066173
ENSDART00000104845
ENSDART00000163885
ENSDART00000176808

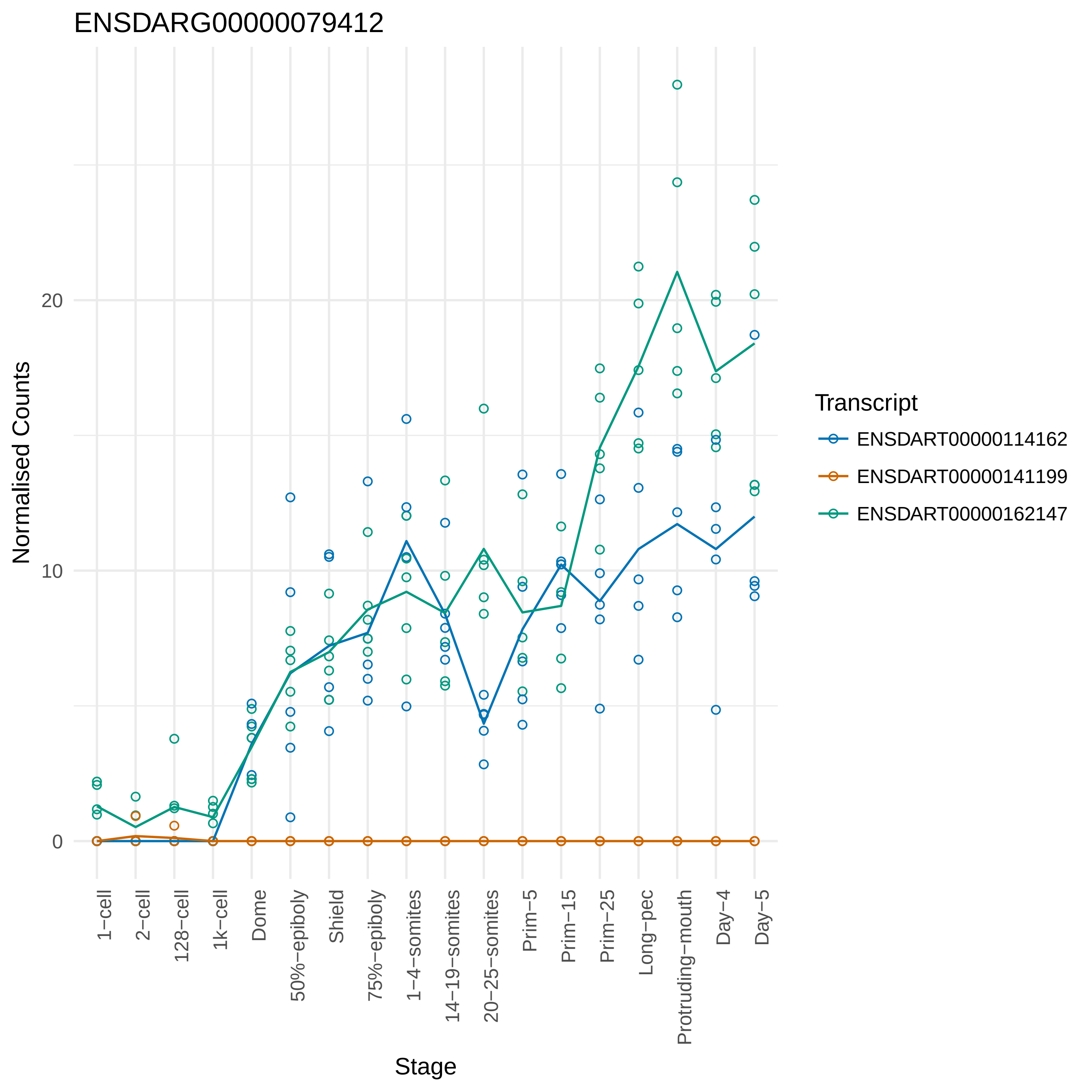
ENSDARG00000079412
Normalised Counts
0
10
20
Stage
1−cell
2−cell
128−cell
1k−cell
Dome
50%−epiboly
Shield
75%−epiboly
1−4−somites
14−19−somites
20−25−somites
Prim−5
Prim−15
Prim−25
Long−pec
Protruding−mouth
Day−4
Day−5
Transcript
ENSDART00000114162
ENSDART00000141199
ENSDART00000162147

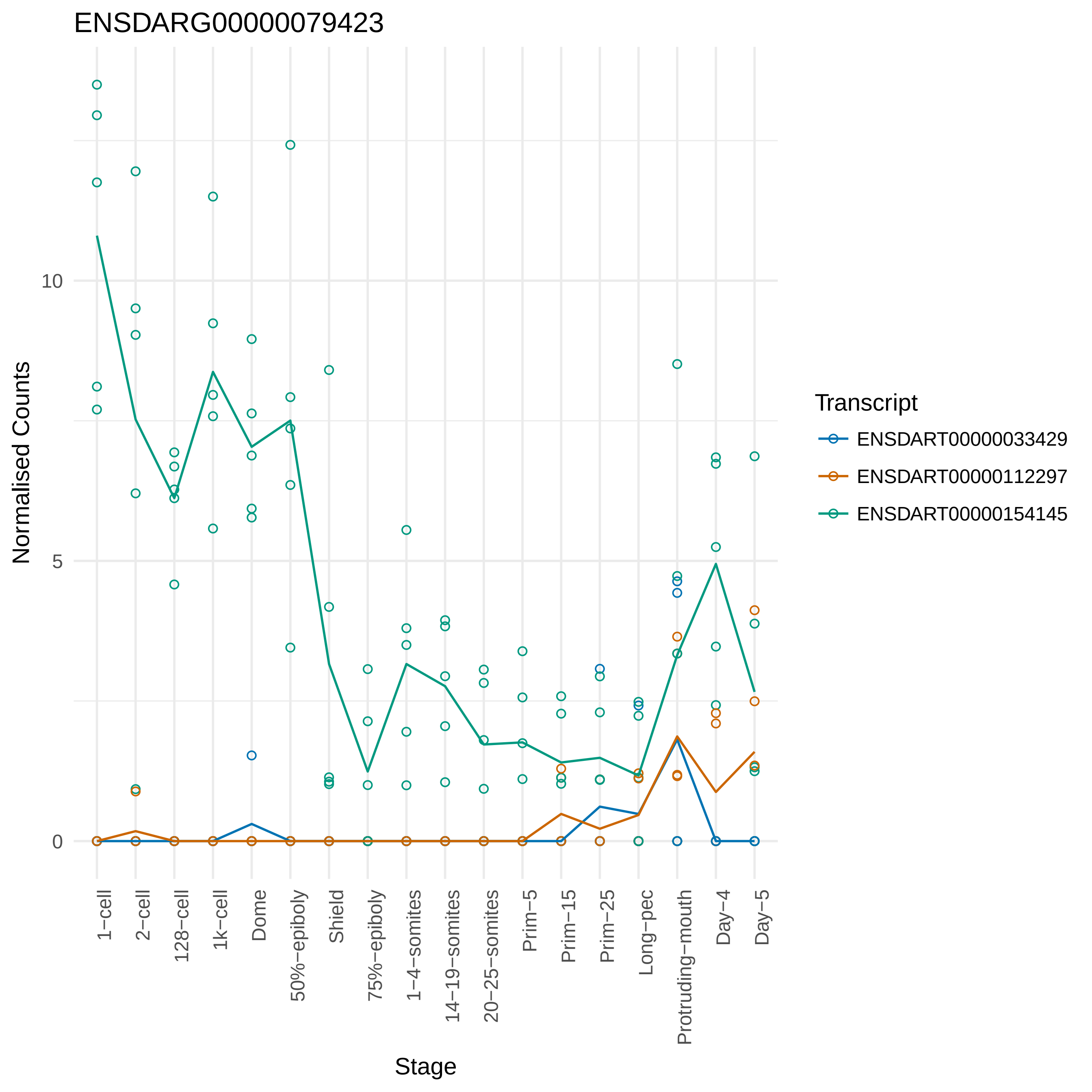

ENSDARG00000079423
Normalised Counts
10
5
0
1−cell
2−cell
128−cell
1k−cell
Dome
50%−epiboly
Shield
75%−epiboly
1−4−somites
14−19−somites
20−25−somites
Prim−5
Prim−15
Prim−25
Long−pec
Protruding−mouth
Day−4
Day−5
Stage
Transcript
ENSDART00000033429
ENSDART00000112297
ENSDART00000154145

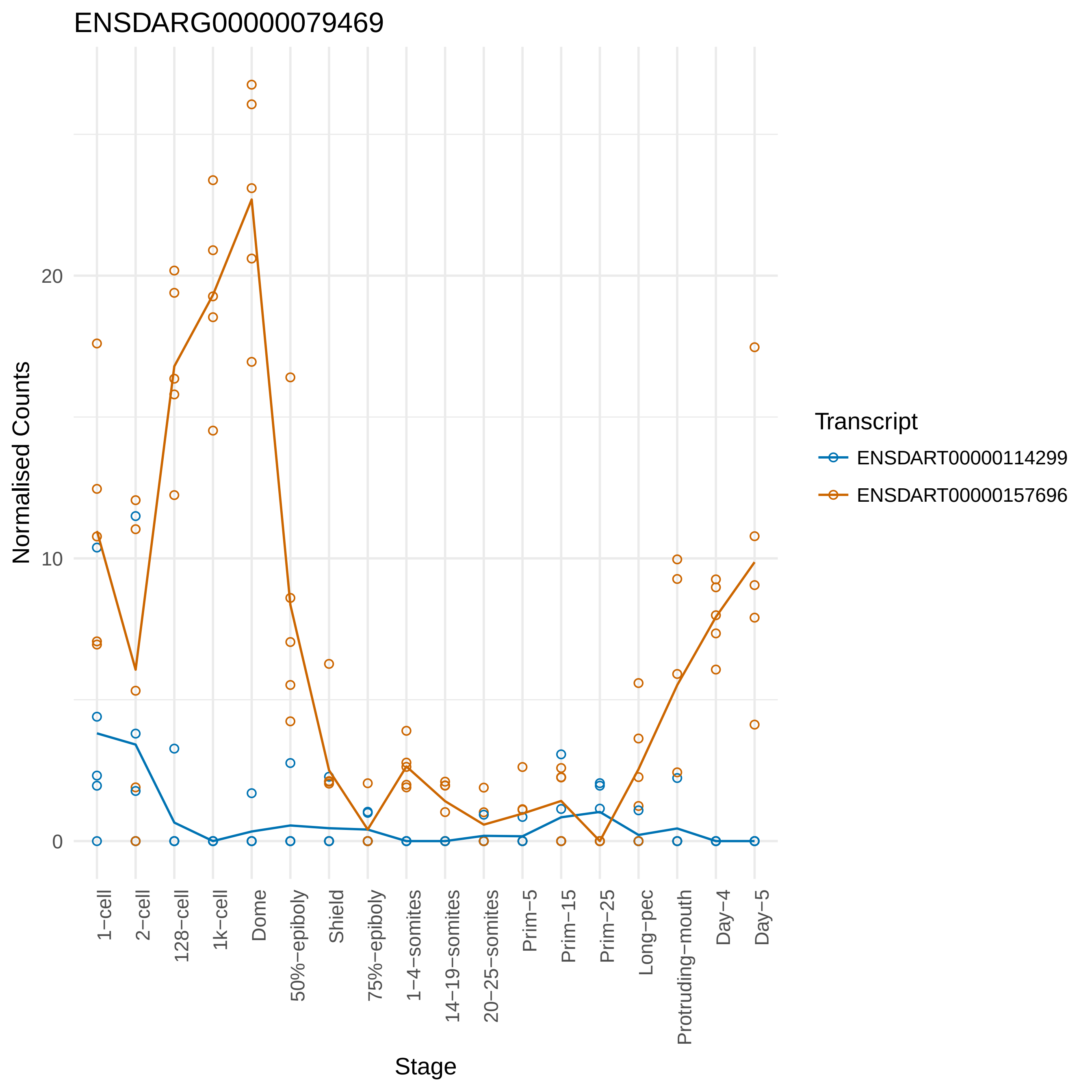

ENSDARG00000079469
Normalised Counts
20
10
0
1−cell
2−cell
128−cell
1k−cell
Dome
50%−epiboly
Shield
75%−epiboly
1−4−somites
14−19−somites
20−25−somites
Prim−5
Prim−15
Prim−25
Long−pec
Protruding−mouth
Day−4
Day−5
Stage
Transcript
ENSDART00000114299
ENSDART00000157696

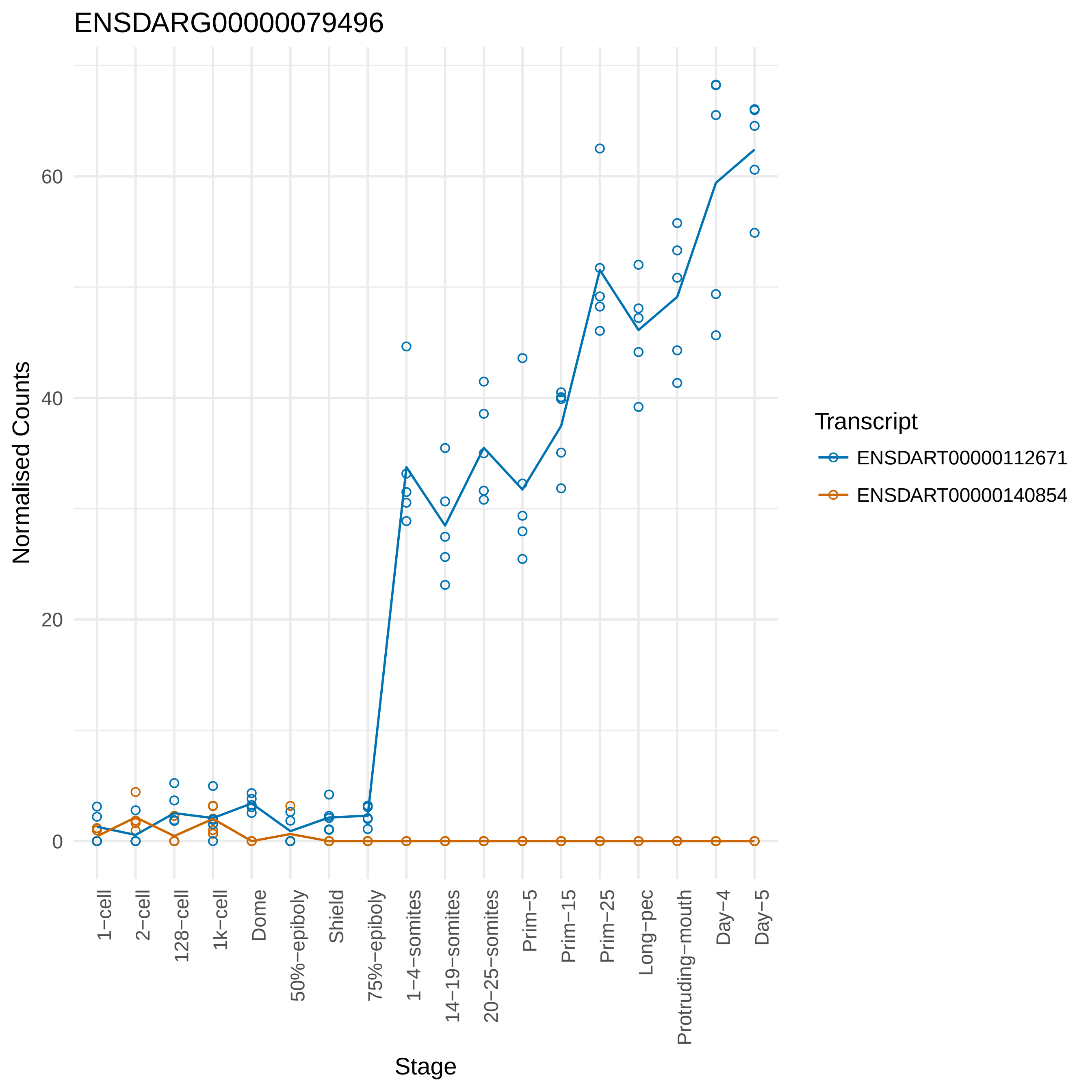

ENSDARG00000079496
Normalised Counts
0
20
40
60
Stage
1−cell
2−cell
128−cell
1k−cell
Dome
50%−epiboly
Shield
75%−epiboly
1−4−somites
14−19−somites
20−25−somites
Prim−5
Prim−15
Prim−25
Long−pec
Protruding−mouth
Day−4
Day−5
Transcript
ENSDART00000112671
ENSDART00000140854

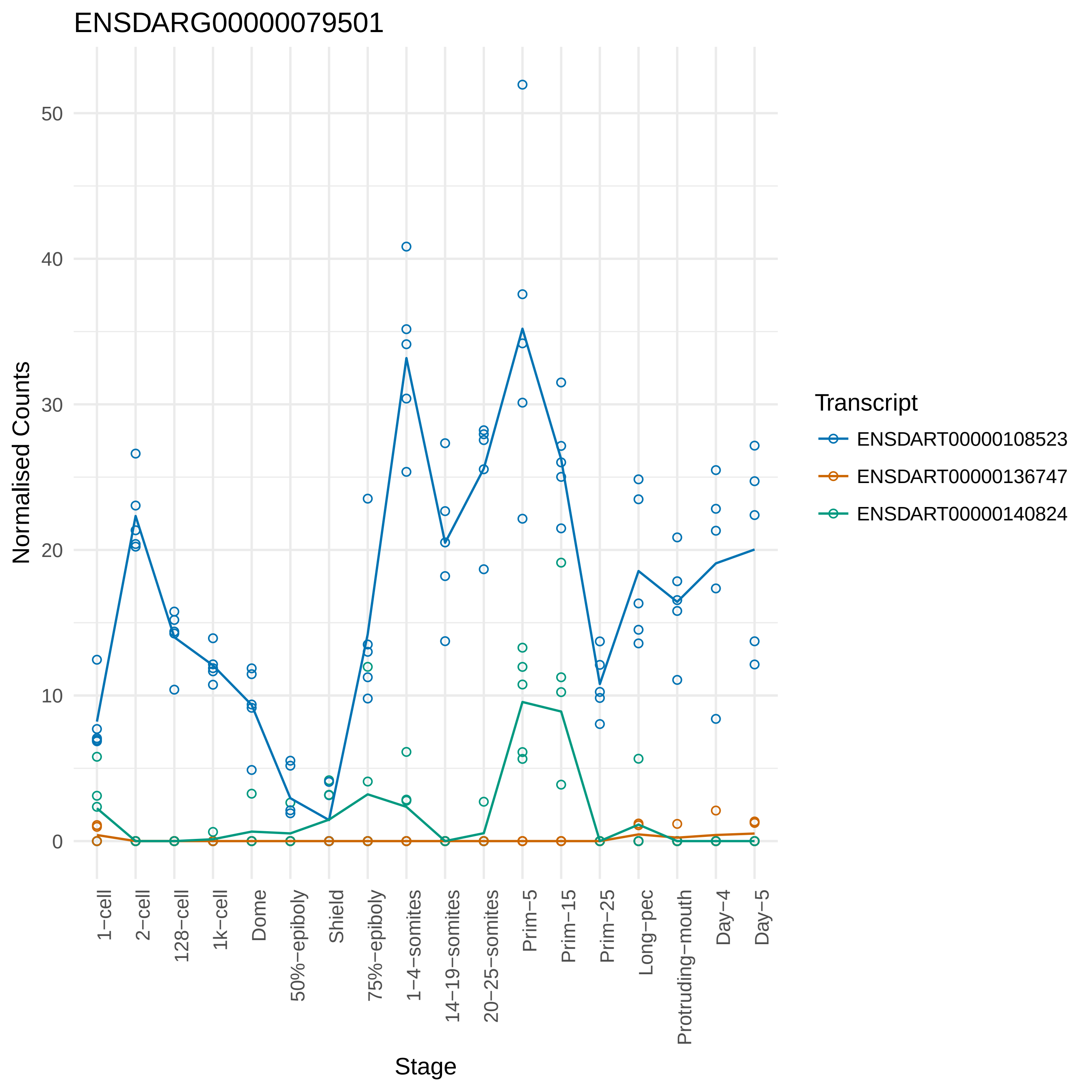
ENSDARG00000079501
Normalised Counts
50
40
30
20
10
0
1−cell
2−cell
128−cell
1k−cell
Dome
50%−epiboly
Shield
75%−epiboly
1−4−somites
14−19−somites
20−25−somites
Prim−5
Prim−15
Prim−25
Long−pec
Protruding−mouth
Day−4
Day−5
Stage
Transcript
ENSDART00000108523
ENSDART00000136747
ENSDART00000140824

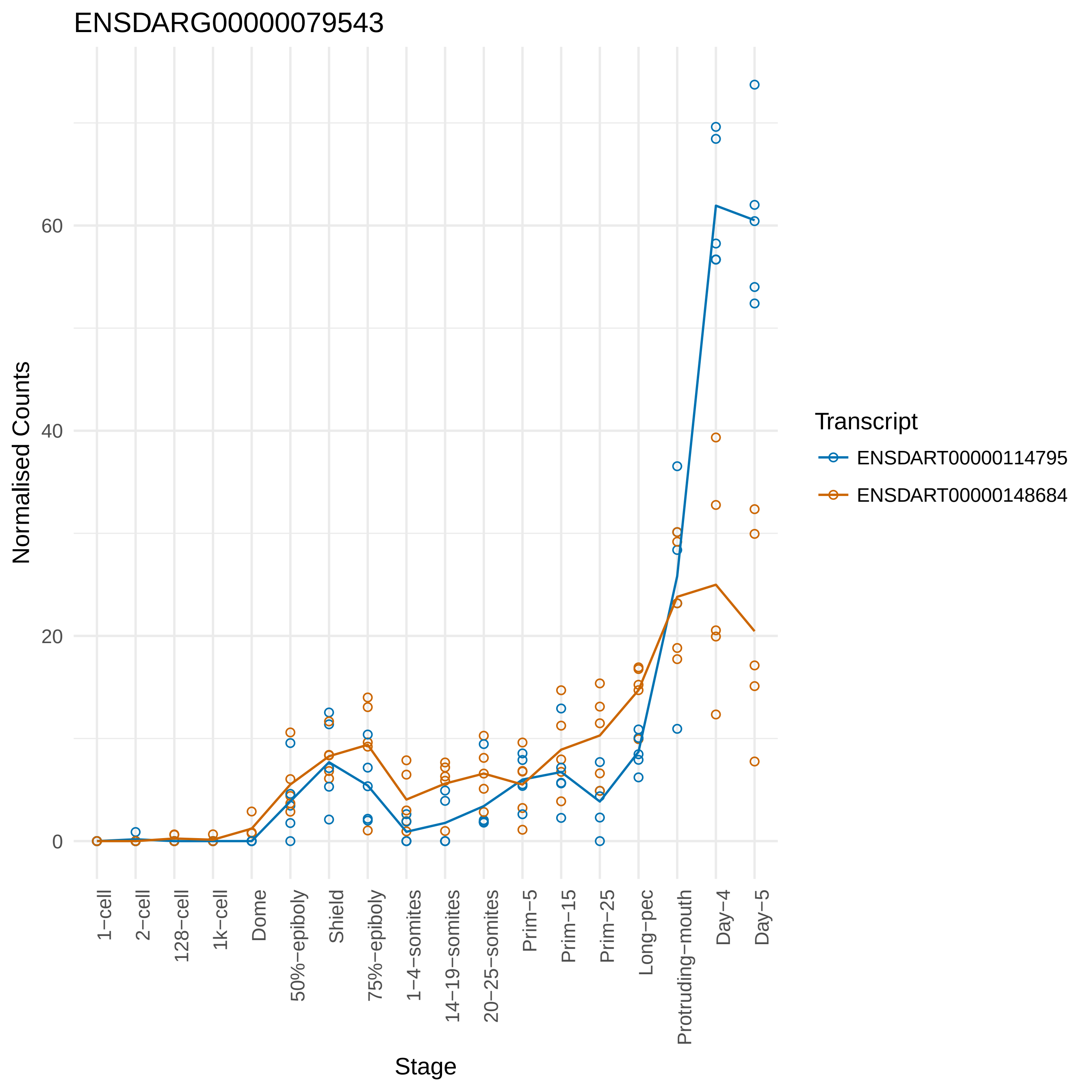

ENSDARG00000079543
Normalised Counts
0
20
40
60
1−cell
2−cell
128−cell
1k−cell
Dome
50%−epiboly
Shield
75%−epiboly
1−4−somites
14−19−somites
20−25−somites
Prim−5
Prim−15
Prim−25
Long−pec
Protruding−mouth
Day−4
Day−5
Stage
Transcript
ENSDART00000114795
ENSDART00000148684

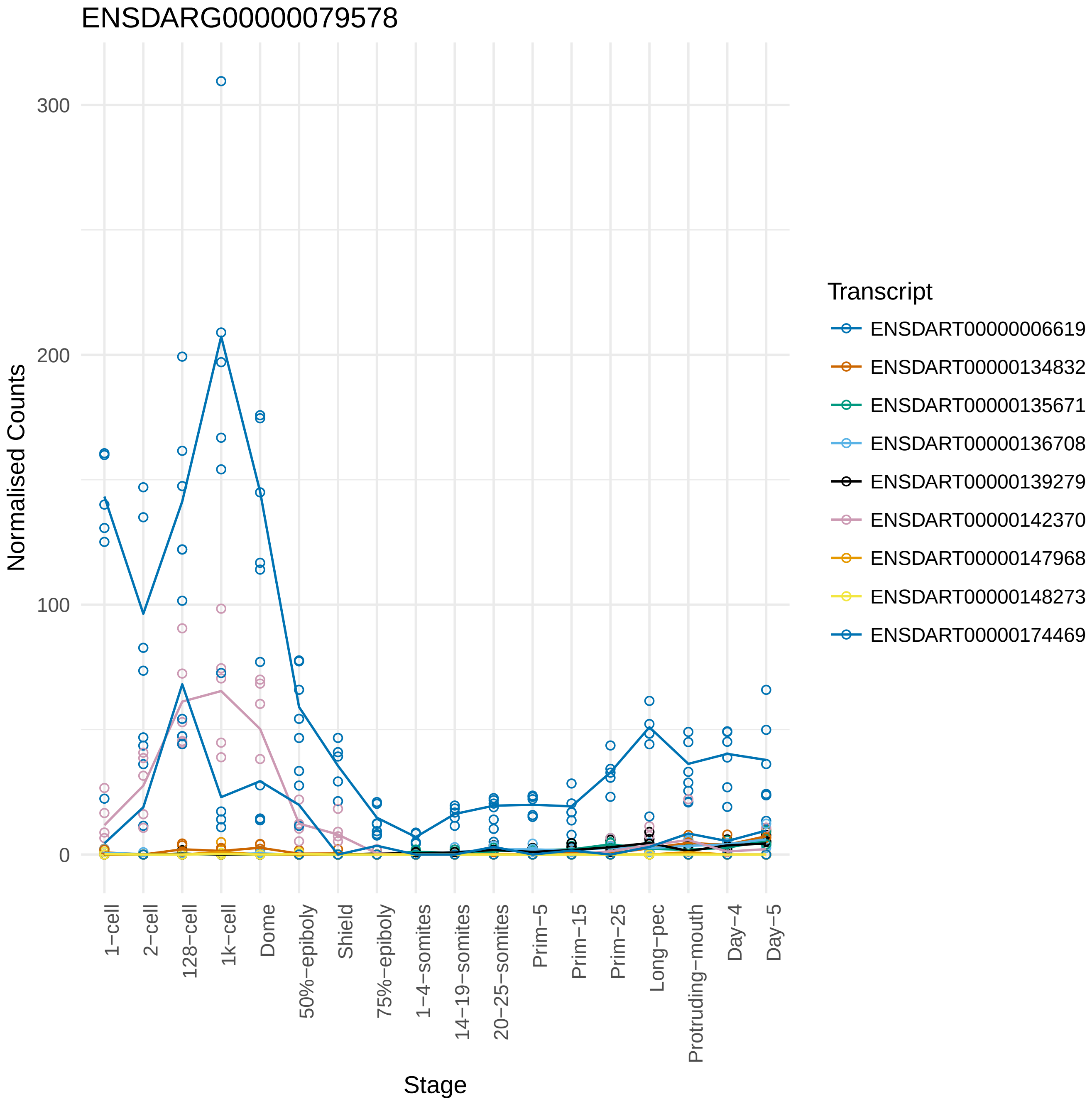

ENSDARG00000079578
Normalised Counts
300
200
100
0
1−cell
2−cell
128−cell
1k−cell
Dome
50%−epiboly
Shield
75%−epiboly
1−4−somites
14−19−somites
20−25−somites
Prim−5
Prim−15
Prim−25
Long−pec
Protruding−mouth
Day−4
Day−5
Stage
Transcript
ENSDART00000006619
ENSDART00000134832
ENSDART00000135671
ENSDART00000136708
ENSDART00000139279
ENSDART00000142370
ENSDART00000147968
ENSDART00000148273
ENSDART00000174469

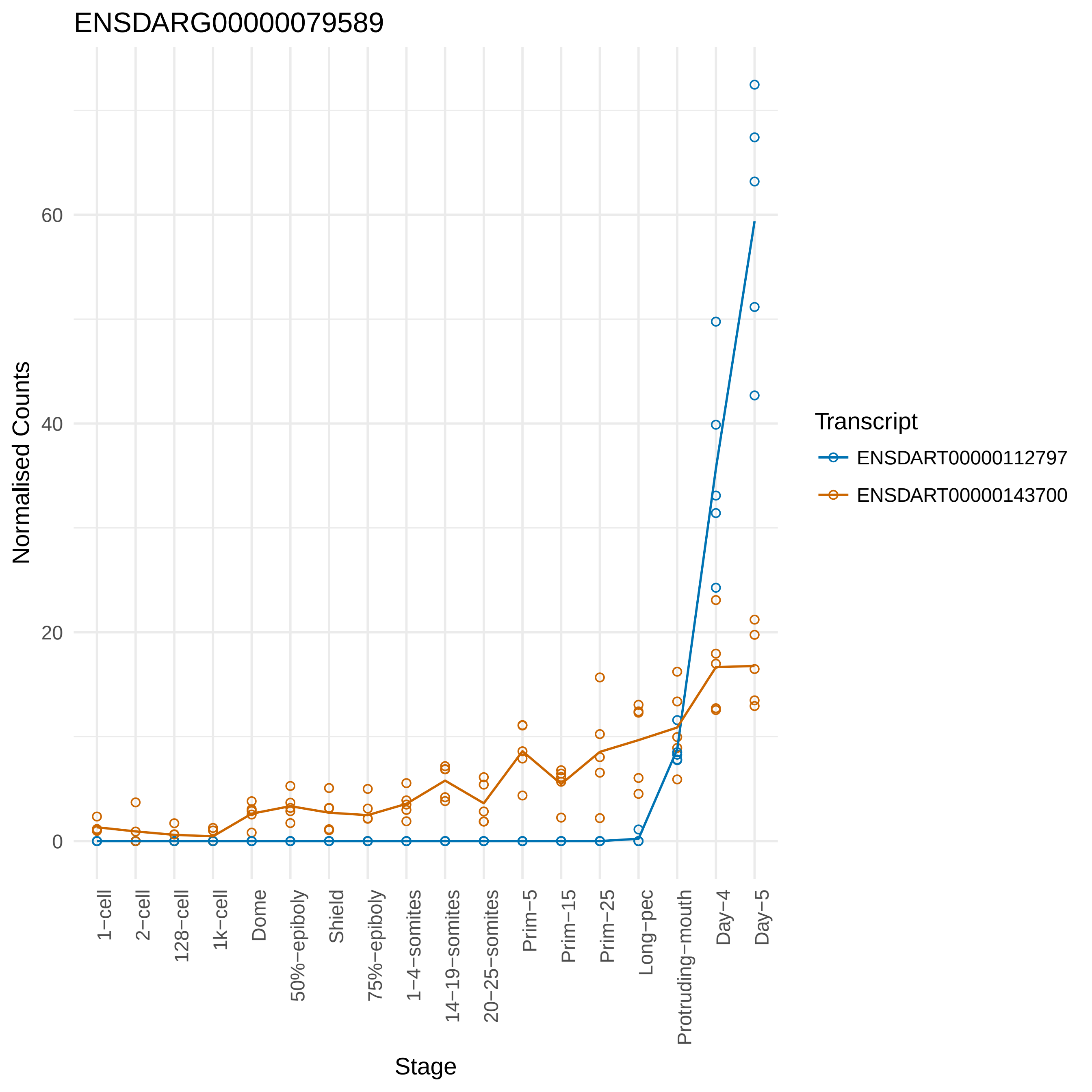

ENSDARG00000079589
Normalised Counts
60
40
20
0
1−cell
2−cell
128−cell
1k−cell
Dome
50%−epiboly
Shield
75%−epiboly
1−4−somites
14−19−somites
20−25−somites
Prim−5
Prim−15
Prim−25
Long−pec
Protruding−mouth
Day−4
Day−5
Stage
Transcript
ENSDART00000112797
ENSDART00000143700

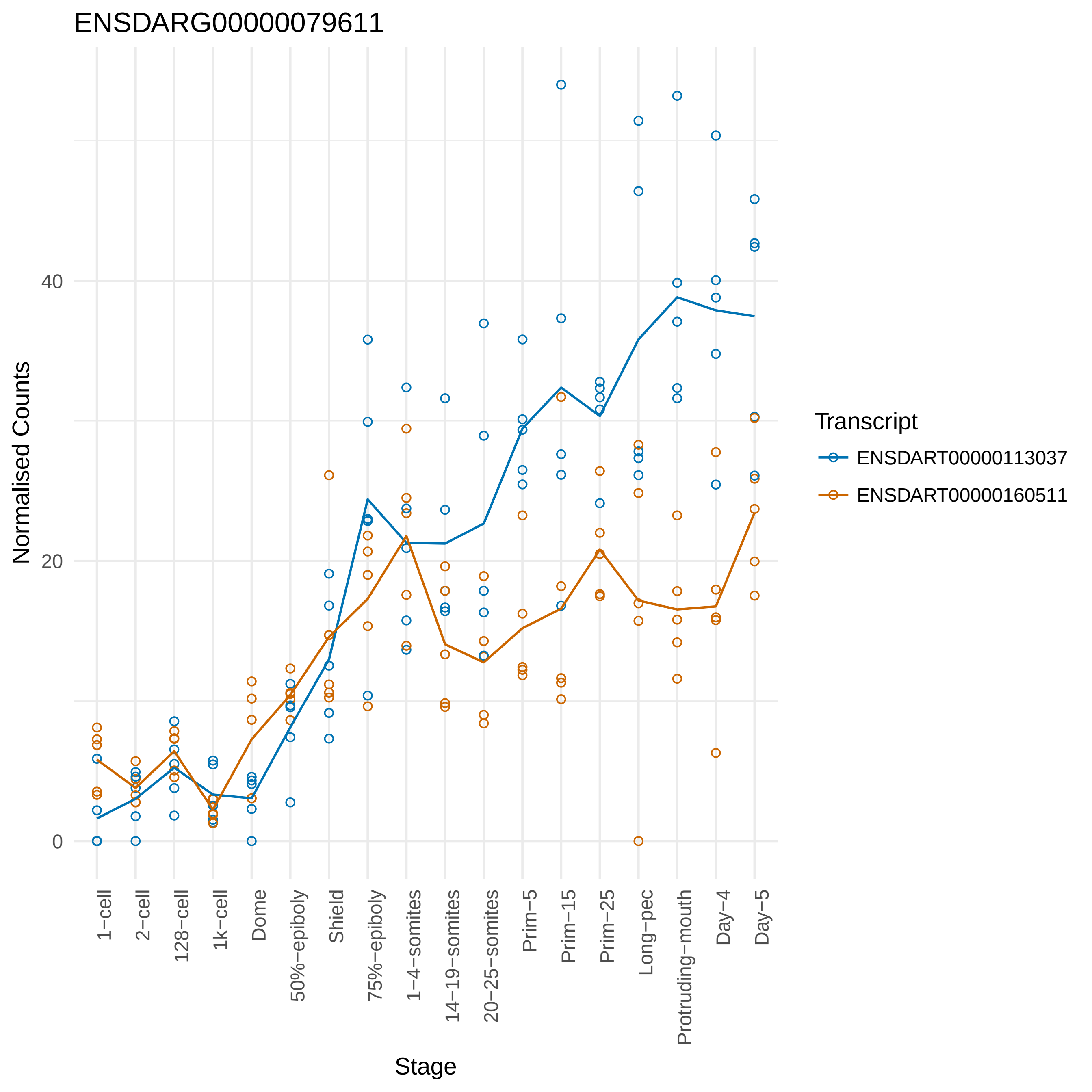

ENSDARG00000079611
Normalised Counts
0
20
40
1−cell
2−cell
128−cell
1k−cell
Dome
50%−epiboly
Shield
75%−epiboly
1−4−somites
14−19−somites
20−25−somites
Prim−5
Prim−15
Prim−25
Long−pec
Protruding−mouth
Day−4
Day−5
Stage
Transcript
ENSDART00000113037
ENSDART00000160511

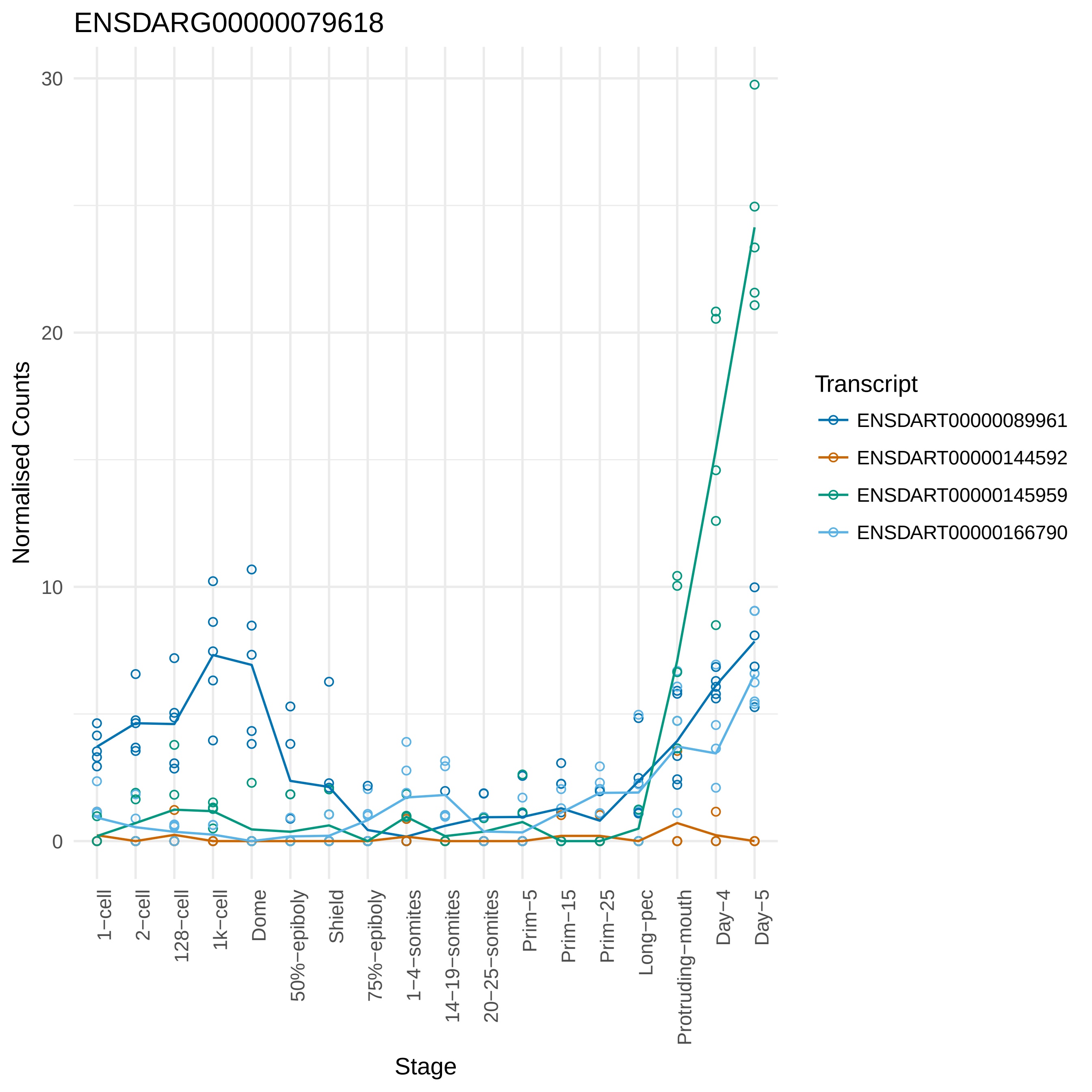
ENSDARG00000079618
Normalised Counts
30
20
10
0
Stage
1−cell
2−cell
128−cell
1k−cell
Dome
50%−epiboly
Shield
75%−epiboly
1−4−somites
14−19−somites
20−25−somites
Prim−5
Prim−15
Prim−25
Long−pec
Protruding−mouth
Day−4
Day−5
Transcript
ENSDART00000089961
ENSDART00000144592
ENSDART00000145959
ENSDART00000166790

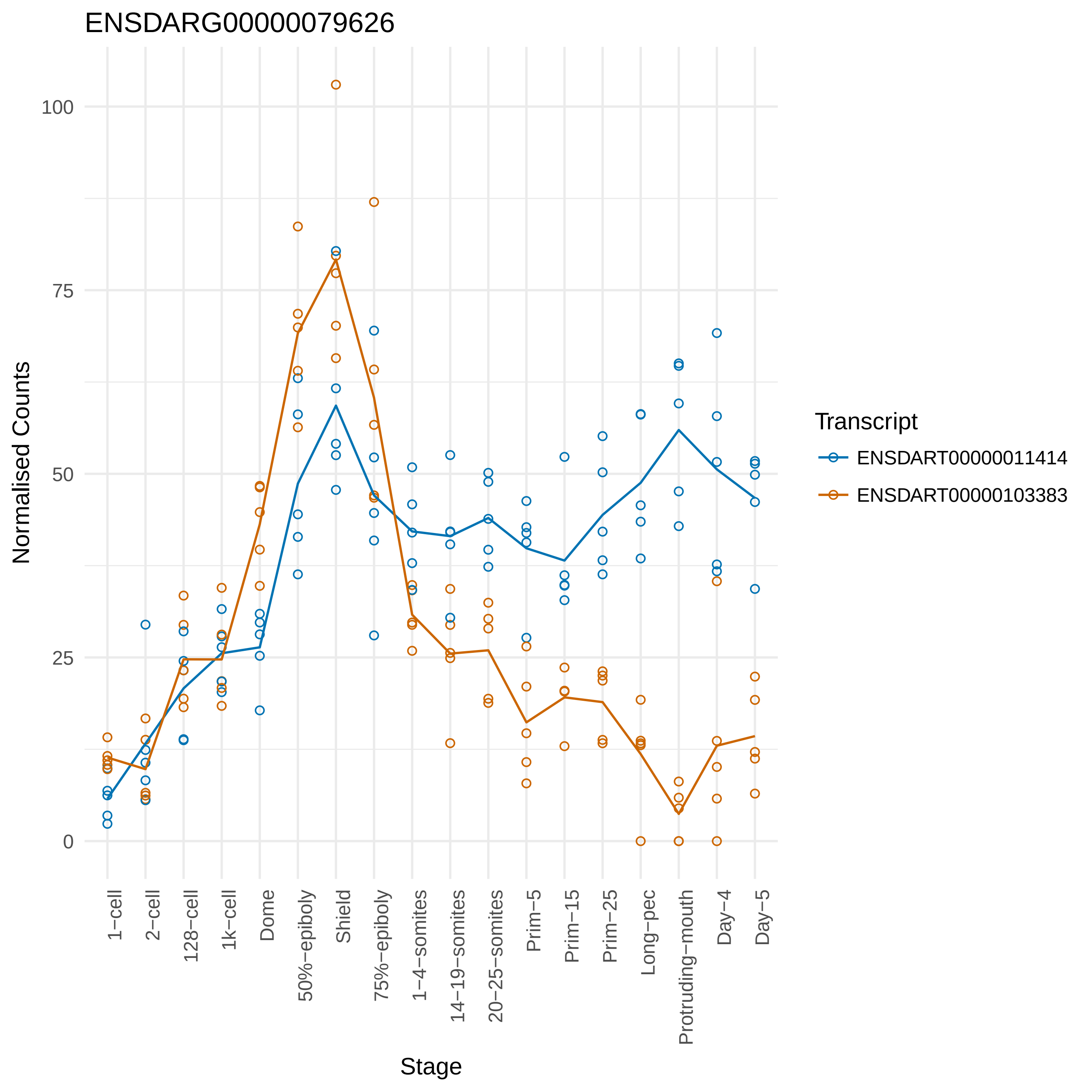
ENSDARG00000079626
Normalised Counts
100
75
50
25
0
Stage
1−cell
2−cell
128−cell
1k−cell
Dome
50%−epiboly
Shield
75%−epiboly
1−4−somites
14−19−somites
20−25−somites
Prim−5
Prim−15
Prim−25
Long−pec
Protruding−mouth
Day−4
Day−5
Transcript
ENSDART00000011414
ENSDART00000103383

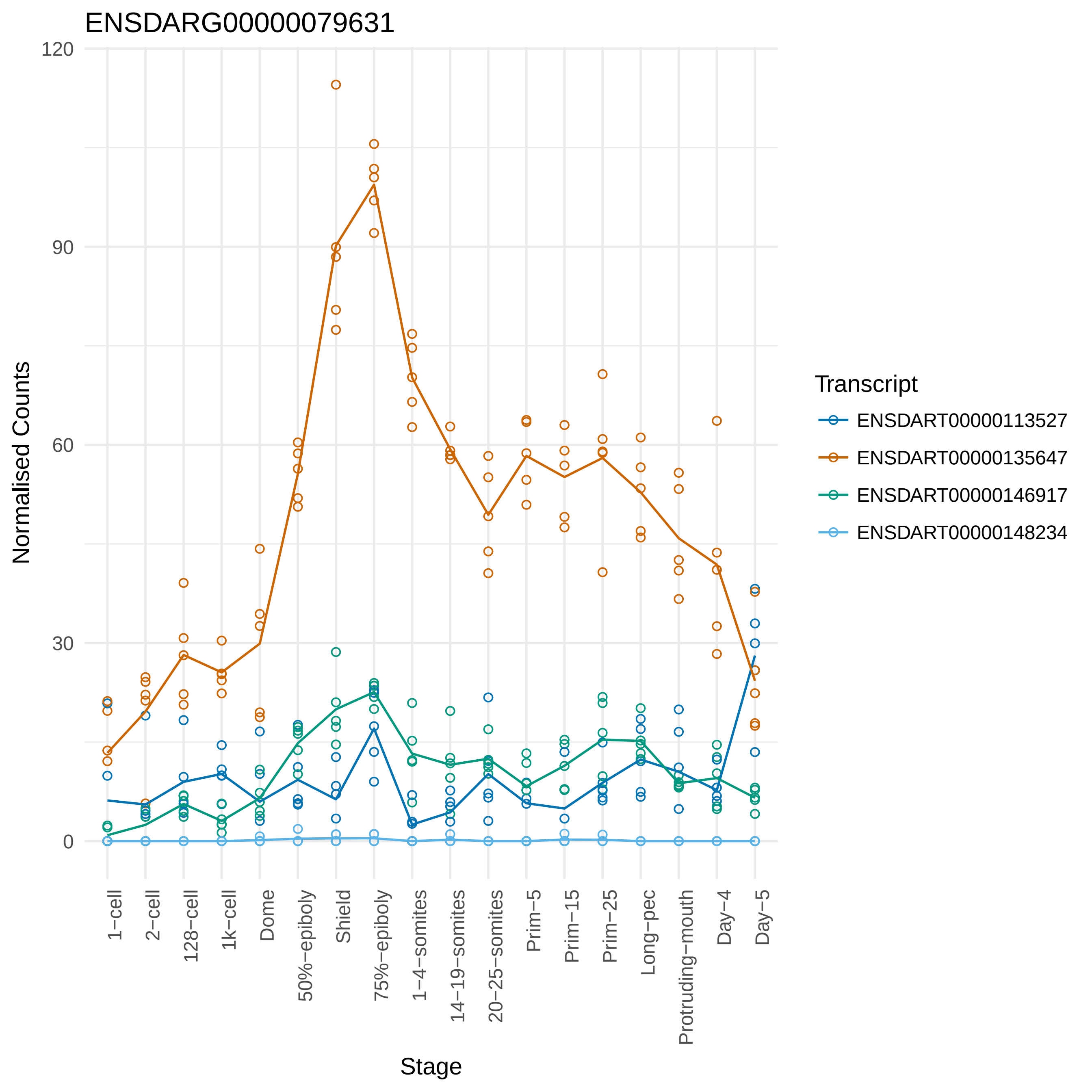
ENSDARG00000079631
Normalised Counts
120
90
60
30
0
1−cell
2−cell
128−cell
1k−cell
Dome
50%−epiboly
Shield
75%−epiboly
1−4−somites
14−19−somites
20−25−somites
Prim−5
Prim−15
Prim−25
Long−pec
Protruding−mouth
Day−4
Day−5
Stage
Transcript
ENSDART00000113527
ENSDART00000135647
ENSDART00000146917
ENSDART00000148234

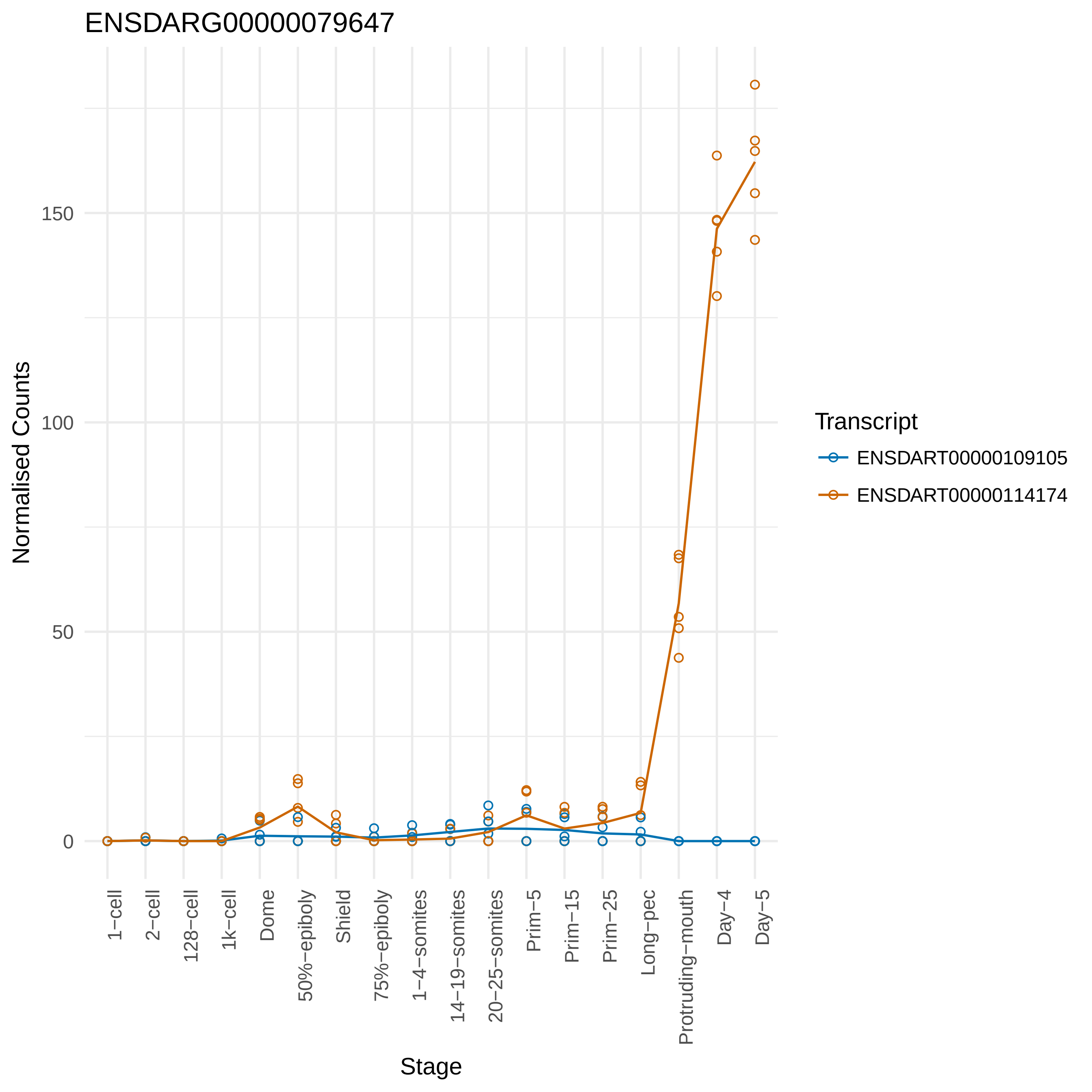

ENSDARG00000079647
Normalised Counts
0
50
100
150
Stage
1−cell
2−cell
128−cell
1k−cell
Dome
50%−epiboly
Shield
75%−epiboly
1−4−somites
14−19−somites
20−25−somites
Prim−5
Prim−15
Prim−25
Long−pec
Protruding−mouth
Day−4
Day−5
Transcript
ENSDART00000109105
ENSDART00000114174

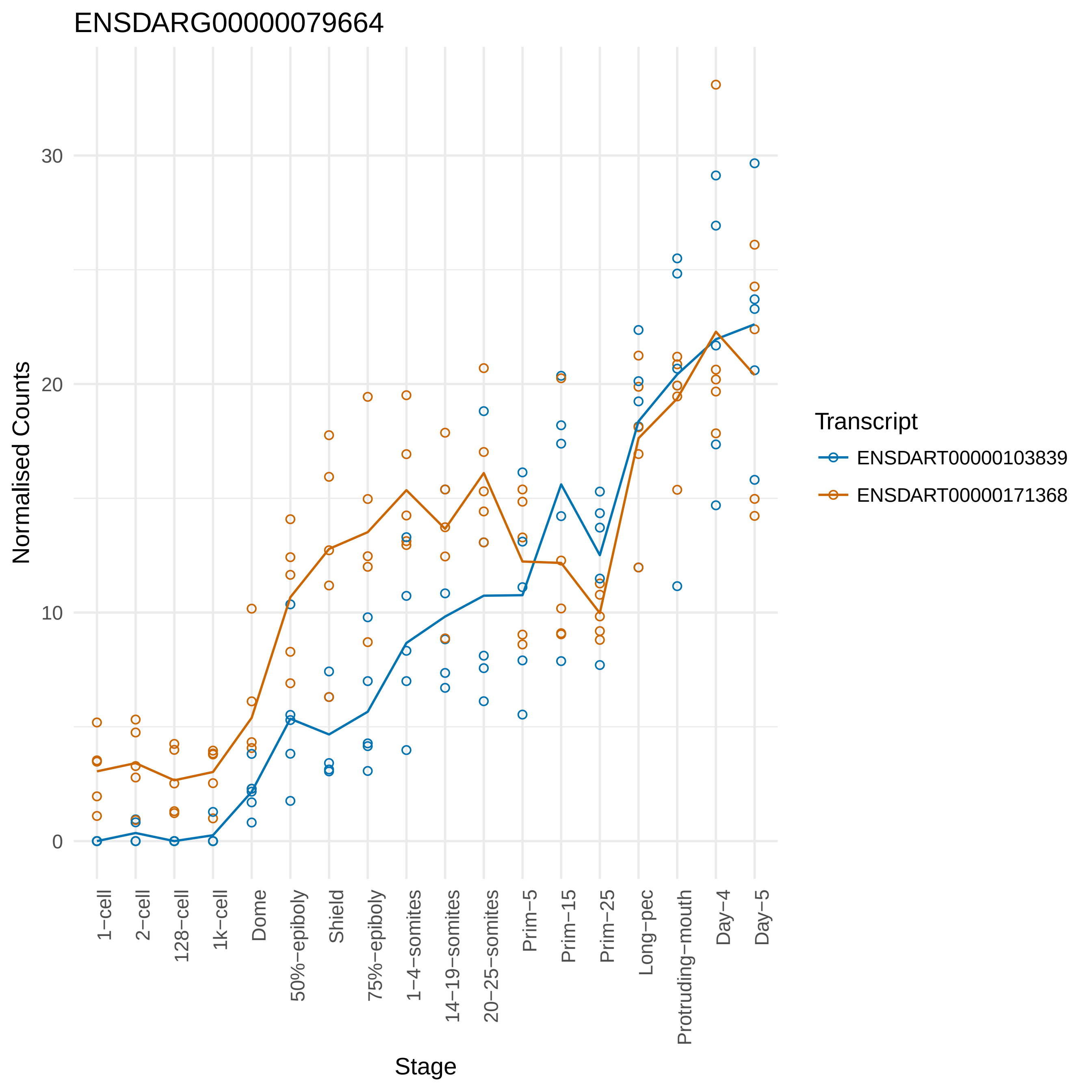

ENSDARG00000079664
Normalised Counts
0
10
20
30
1−cell
2−cell
128−cell
1k−cell
Dome
50%−epiboly
Shield
75%−epiboly
1−4−somites
14−19−somites
20−25−somites
Prim−5
Prim−15
Prim−25
Long−pec
Protruding−mouth
Day−4
Day−5
Stage
Transcript
ENSDART00000103839
ENSDART00000171368

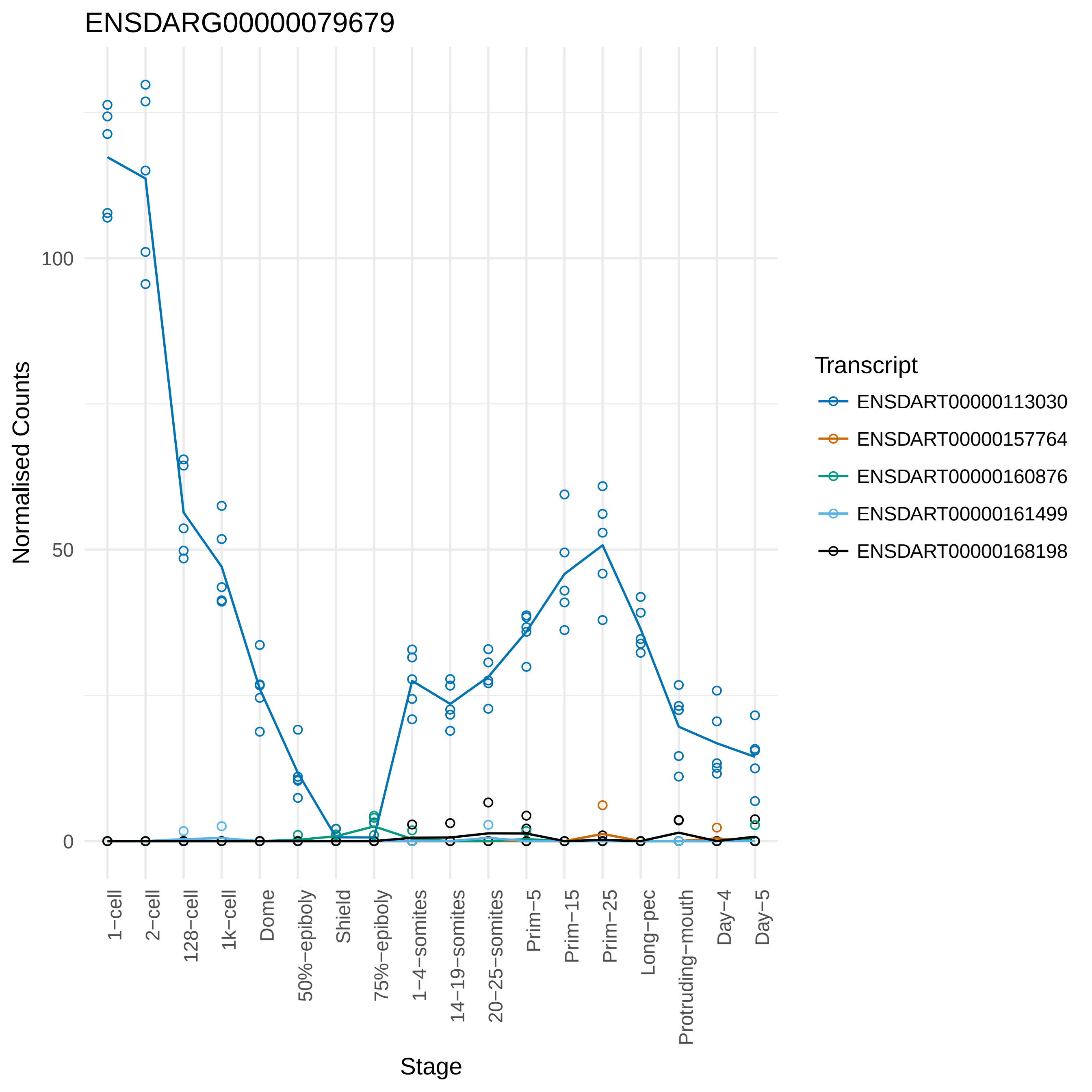

ENSDARG00000079679
Normalised Counts
100
50
0
1−cell
2−cell
128−cell
1k−cell
Dome
50%−epiboly
Shield
75%−epiboly
1−4−somites
14−19−somites
20−25−somites
Prim−5
Prim−15
Prim−25
Long−pec
Protruding−mouth
Day−4
Day−5
Stage
Transcript
ENSDART00000113030
ENSDART00000157764
ENSDART00000160876
ENSDART00000161499
ENSDART00000168198

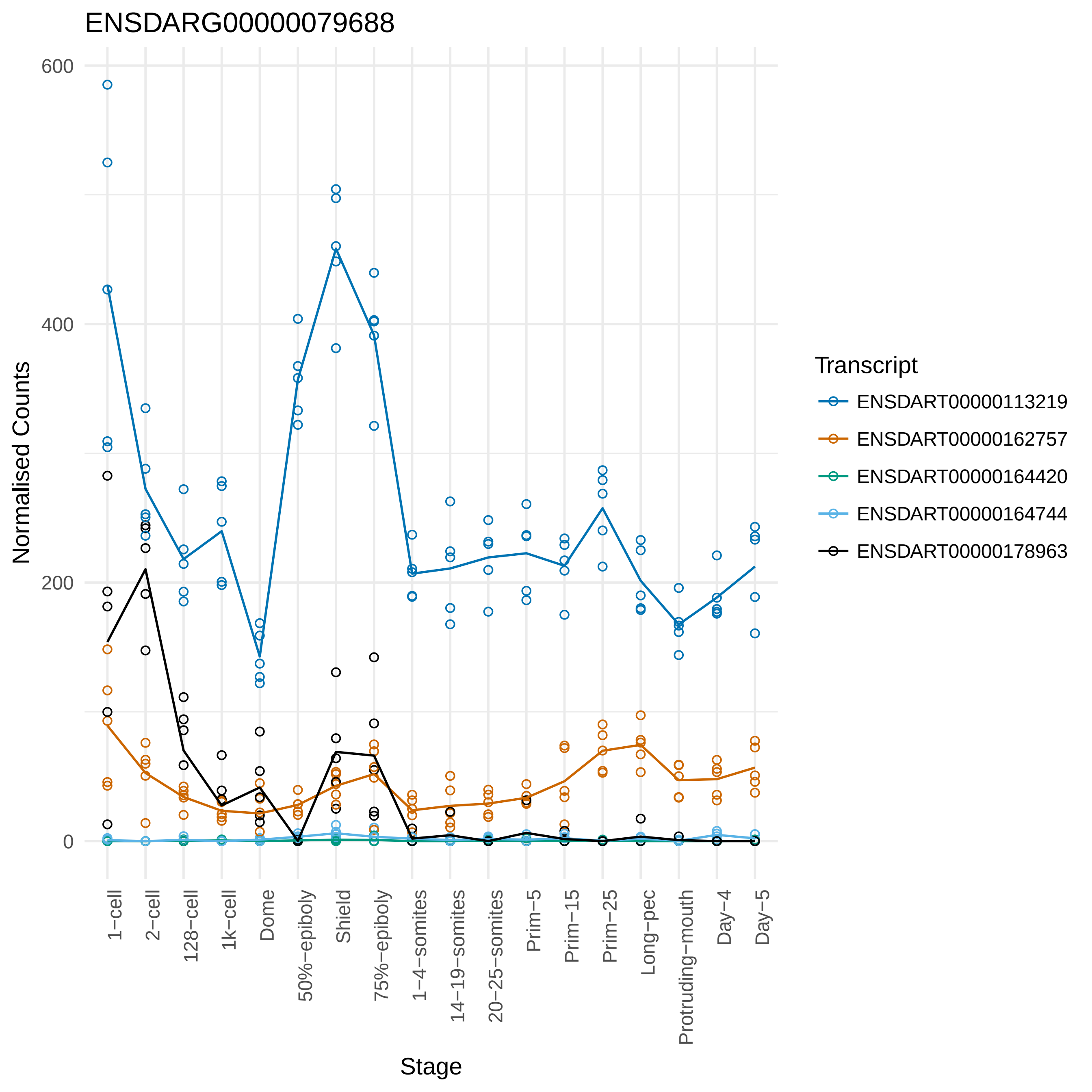

ENSDARG00000079688
Normalised Counts
600
400
200
0
1−cell
2−cell
128−cell
1k−cell
Dome
50%−epiboly
Shield
75%−epiboly
1−4−somites
14−19−somites
20−25−somites
Prim−5
Prim−15
Prim−25
Long−pec
Protruding−mouth
Day−4
Day−5
Stage
Transcript
ENSDART00000113219
ENSDART00000162757
ENSDART00000164420
ENSDART00000164744
ENSDART00000178963

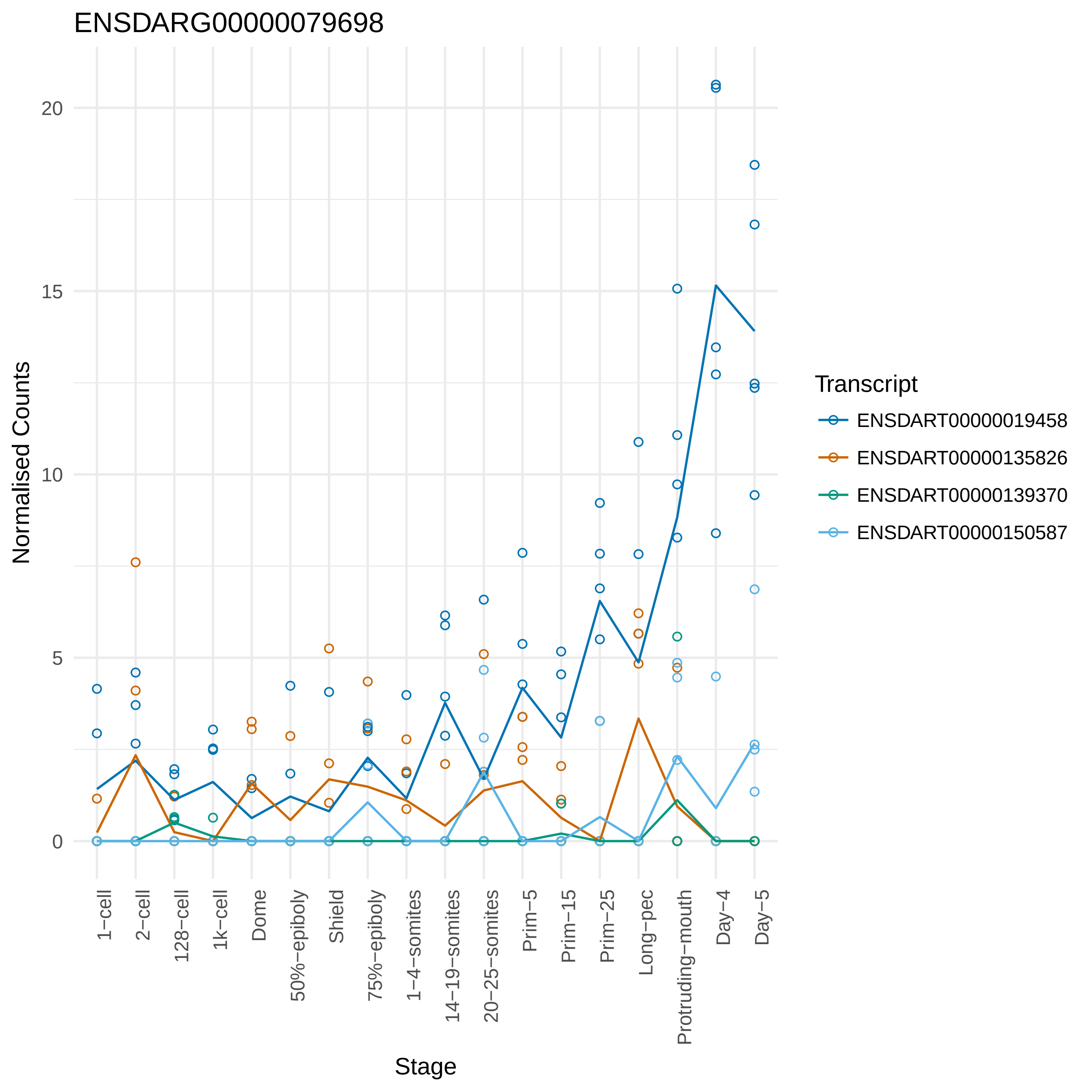
ENSDARG00000079698
Normalised Counts
0
5
10
15
20
1−cell
2−cell
128−cell
1k−cell
Dome
50%−epiboly
Shield
75%−epiboly
1−4−somites
14−19−somites
20−25−somites
Prim−5
Prim−15
Prim−25
Long−pec
Protruding−mouth
Day−4
Day−5
Stage
Transcript
ENSDART00000019458
ENSDART00000135826
ENSDART00000139370
ENSDART00000150587

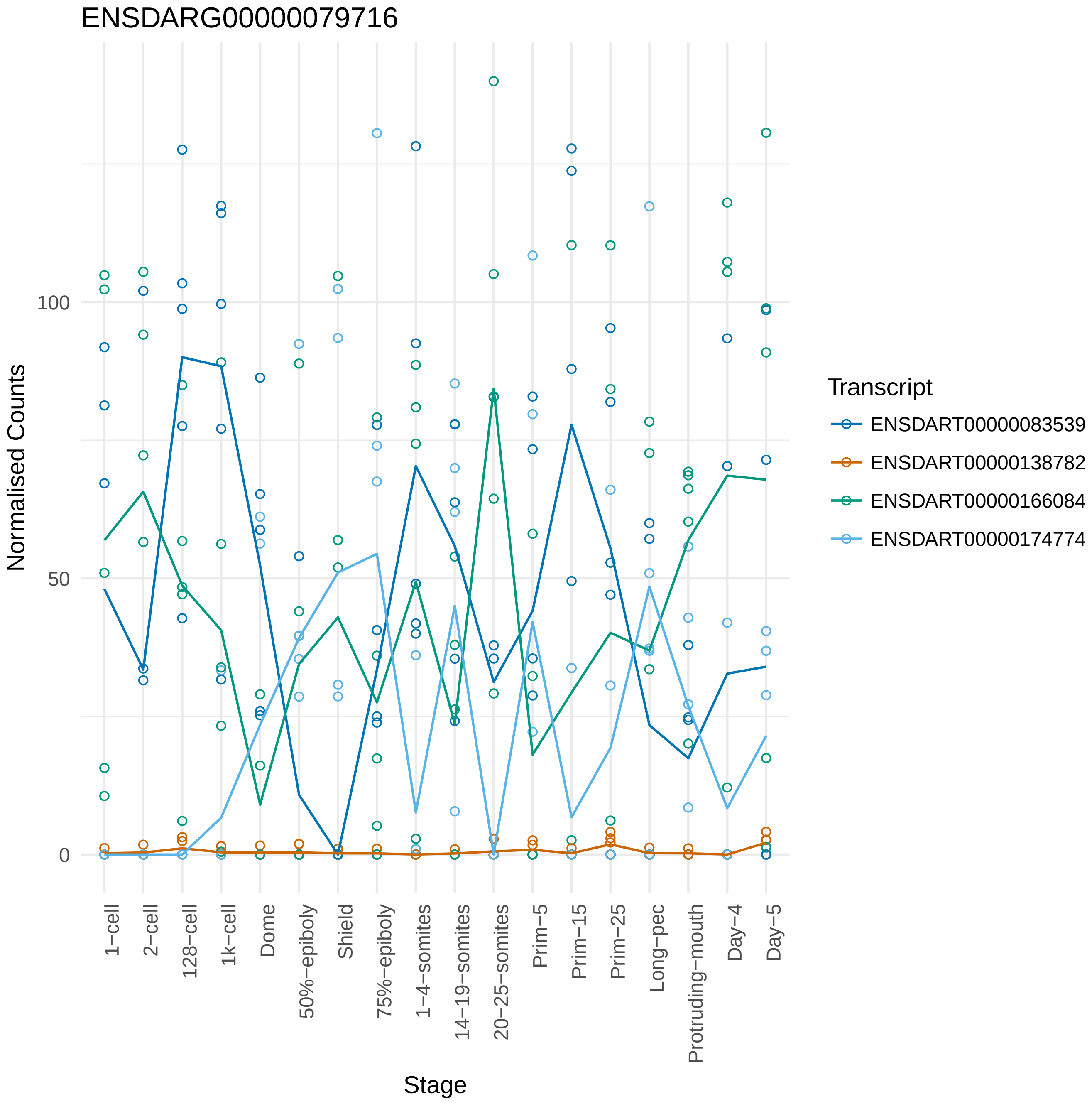

ENSDARG00000079716
Normalised Counts
100
50
0
1−cell
2−cell
128−cell
1k−cell
Dome
50%−epiboly
Shield
75%−epiboly
1−4−somites
14−19−somites
20−25−somites
Prim−5
Prim−15
Prim−25
Long−pec
Protruding−mouth
Day−4
Day−5
Stage
Transcript
ENSDART00000083539
ENSDART00000138782
ENSDART00000166084
ENSDART00000174774

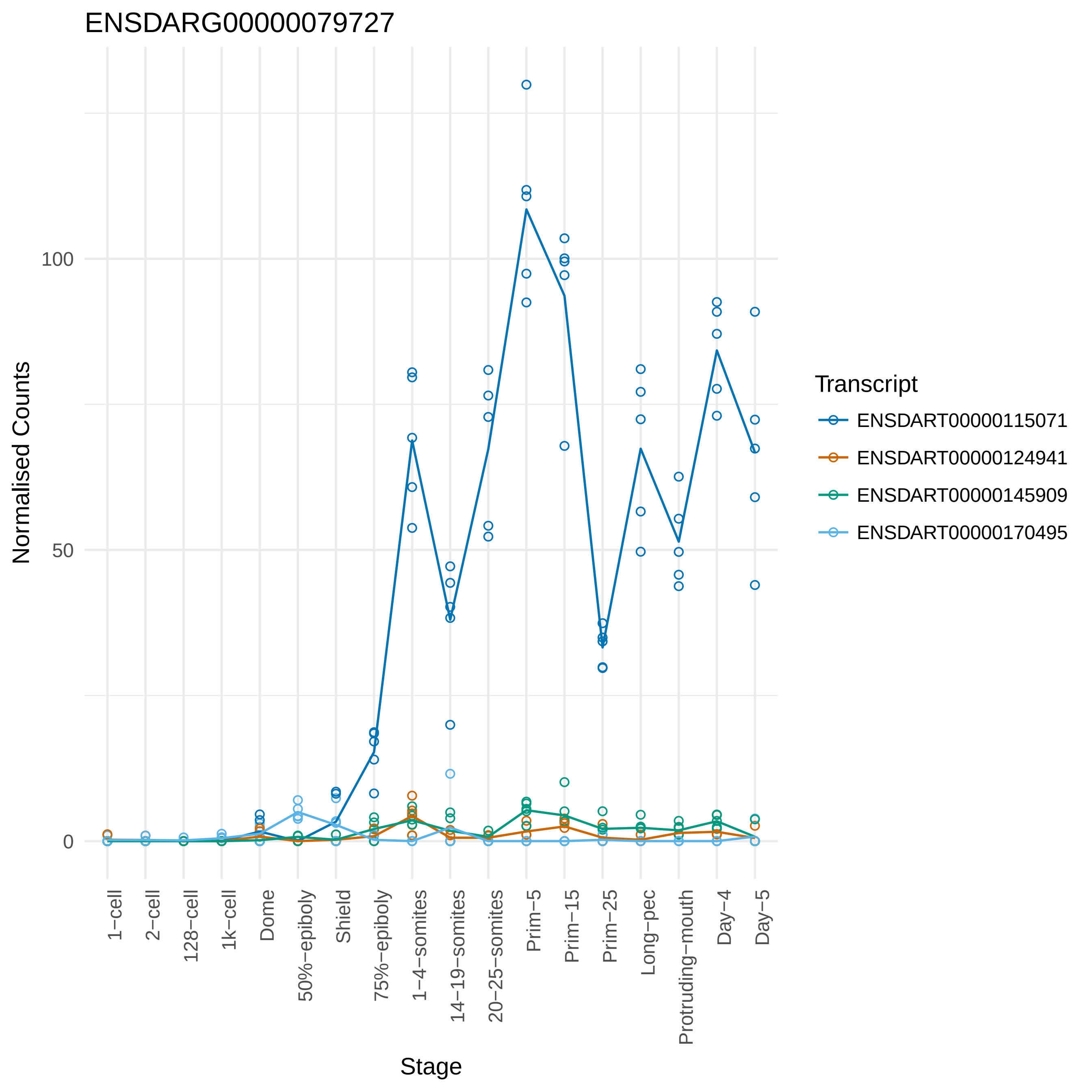

ENSDARG00000079727
Normalised Counts
100
50
0
1−cell
2−cell
128−cell
1k−cell
Dome
50%−epiboly
Shield
75%−epiboly
1−4−somites
14−19−somites
20−25−somites
Prim−5
Prim−15
Prim−25
Long−pec
Protruding−mouth
Day−4
Day−5
Stage
Transcript
ENSDART00000115071
ENSDART00000124941
ENSDART00000145909
ENSDART00000170495

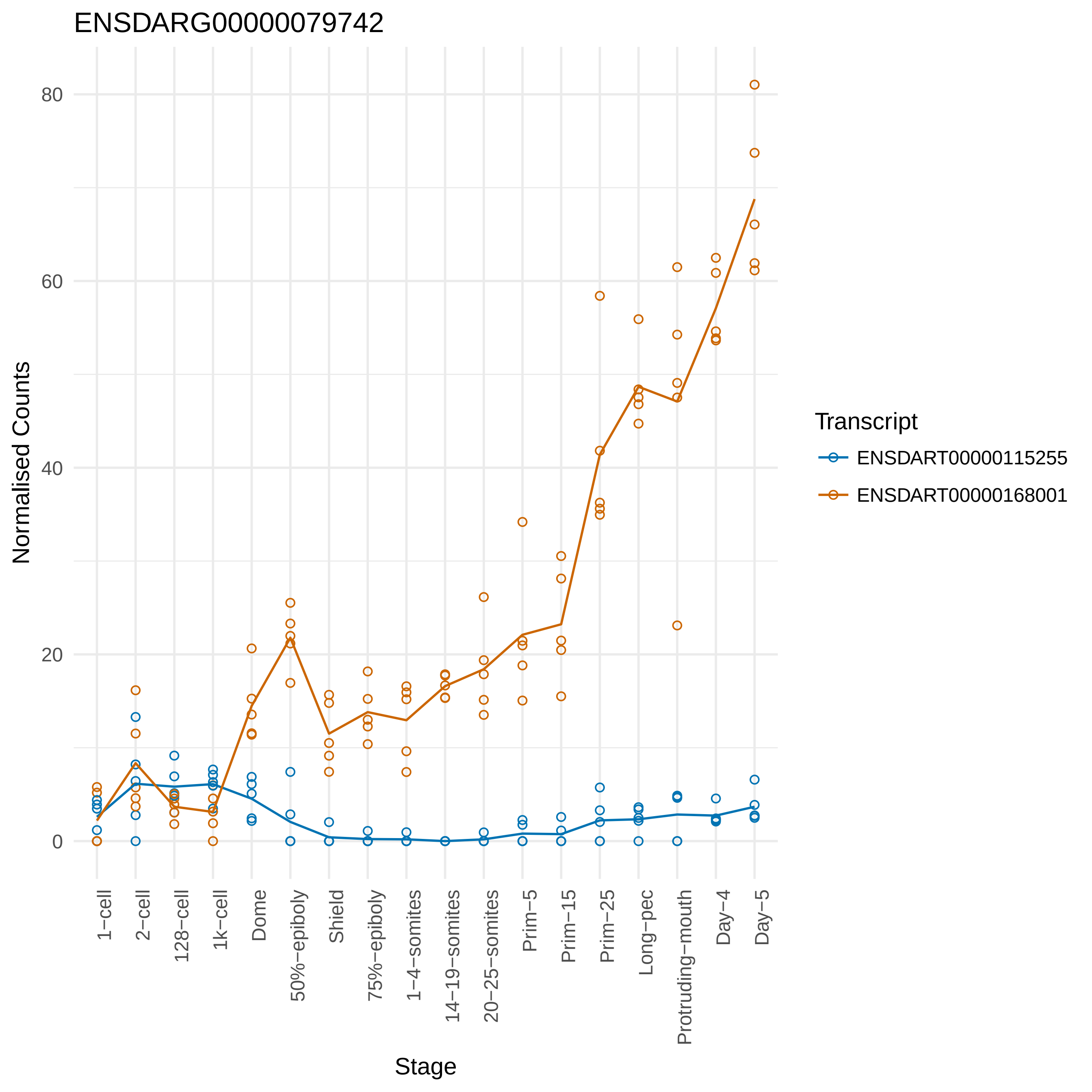

ENSDARG00000079742
Normalised Counts
80
60
40
20
0
1−cell
2−cell
128−cell
1k−cell
Dome
50%−epiboly
Shield
75%−epiboly
1−4−somites
14−19−somites
20−25−somites
Prim−5
Prim−15
Prim−25
Long−pec
Protruding−mouth
Day−4
Day−5
Stage
Transcript
ENSDART00000115255
ENSDART00000168001

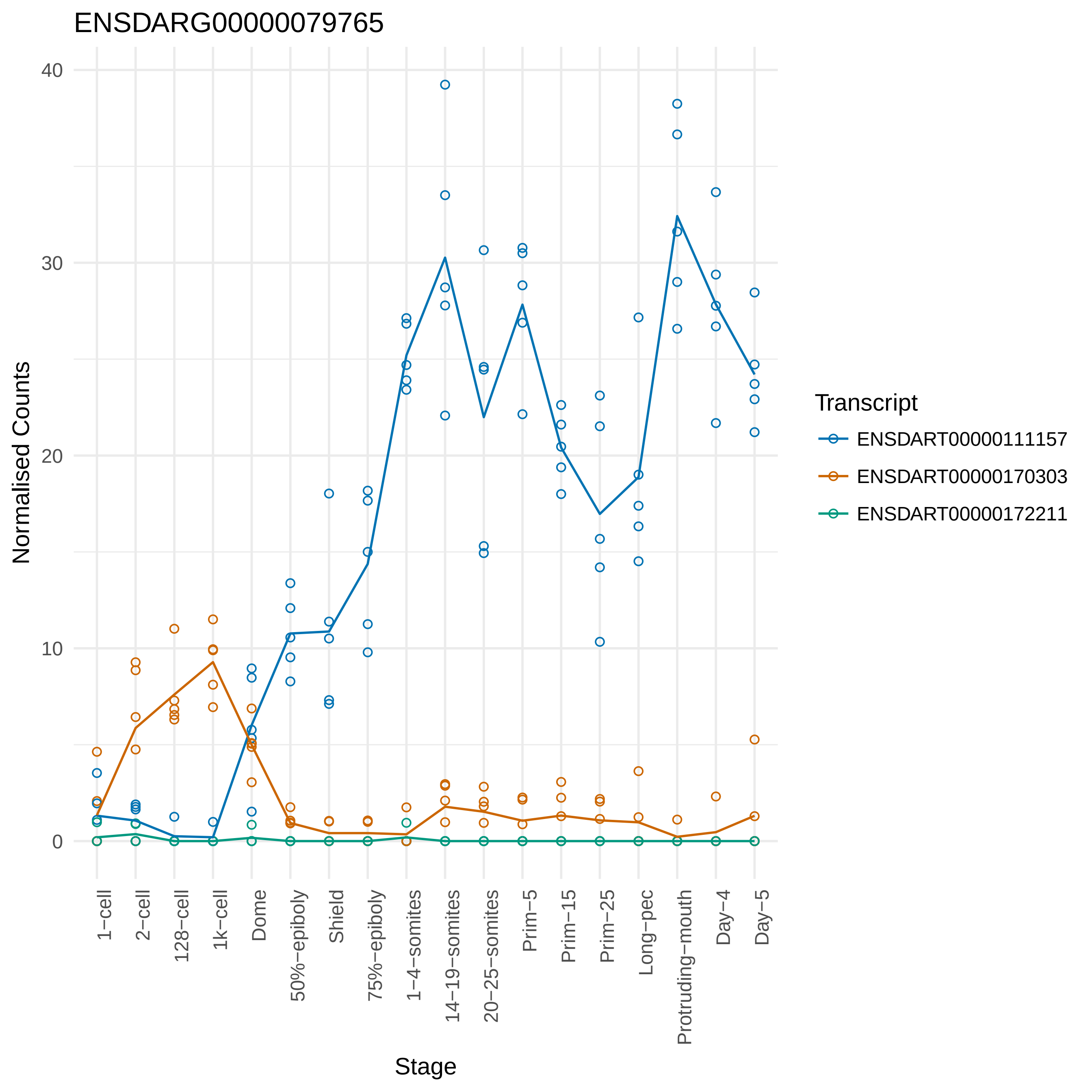

ENSDARG00000079765
Normalised Counts
40
30
20
10
0
1−cell
2−cell
128−cell
1k−cell
Dome
50%−epiboly
Shield
75%−epiboly
1−4−somites
14−19−somites
20−25−somites
Prim−5
Prim−15
Prim−25
Long−pec
Protruding−mouth
Day−4
Day−5
Stage
Transcript
ENSDART00000111157
ENSDART00000170303
ENSDART00000172211

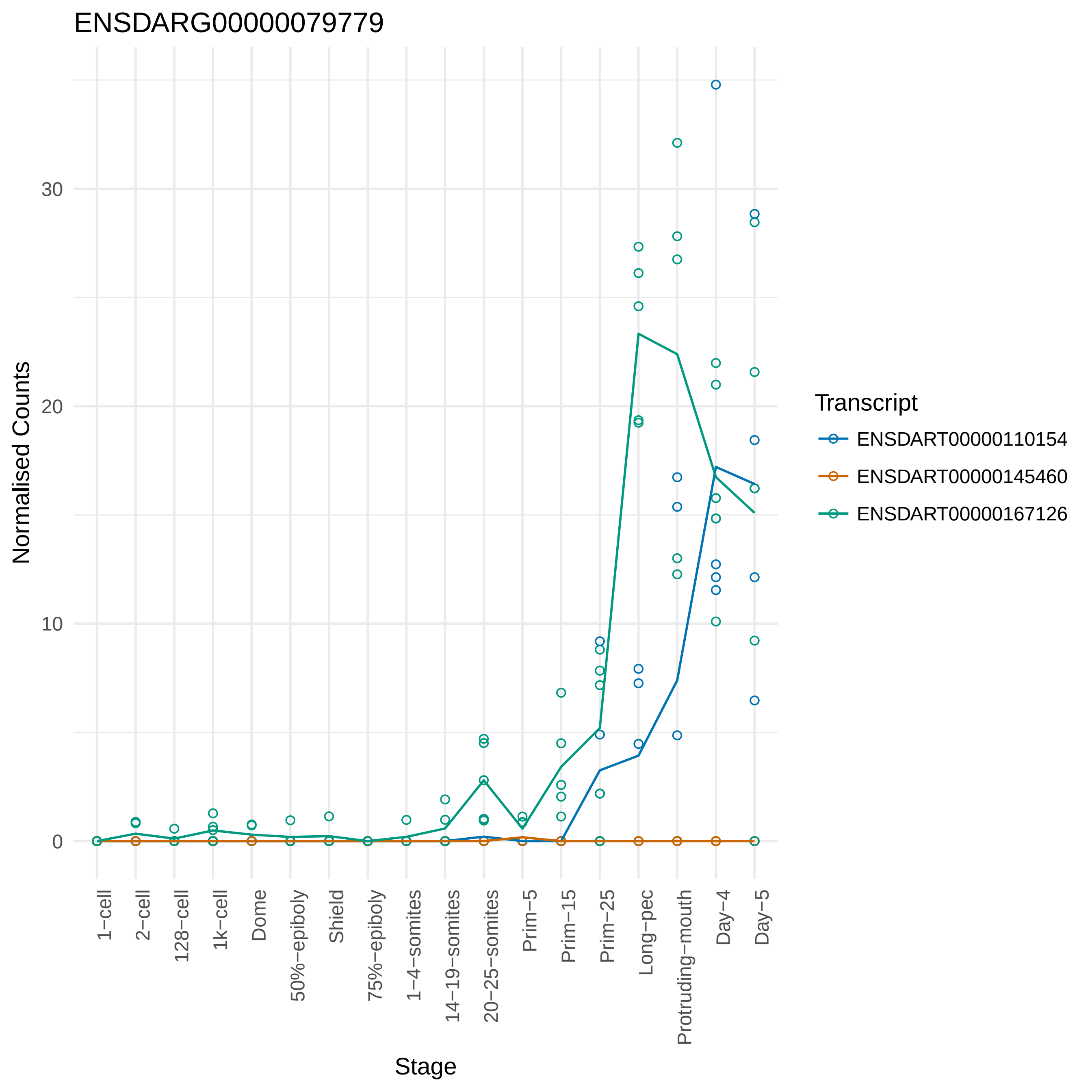
ENSDARG00000079779
Normalised Counts
0
10
20
30
1−cell
2−cell
128−cell
1k−cell
Dome
50%−epiboly
Shield
75%−epiboly
1−4−somites
14−19−somites
20−25−somites
Prim−5
Prim−15
Prim−25
Long−pec
Protruding−mouth
Day−4
Day−5
Stage
Transcript
ENSDART00000110154
ENSDART00000145460
ENSDART00000167126

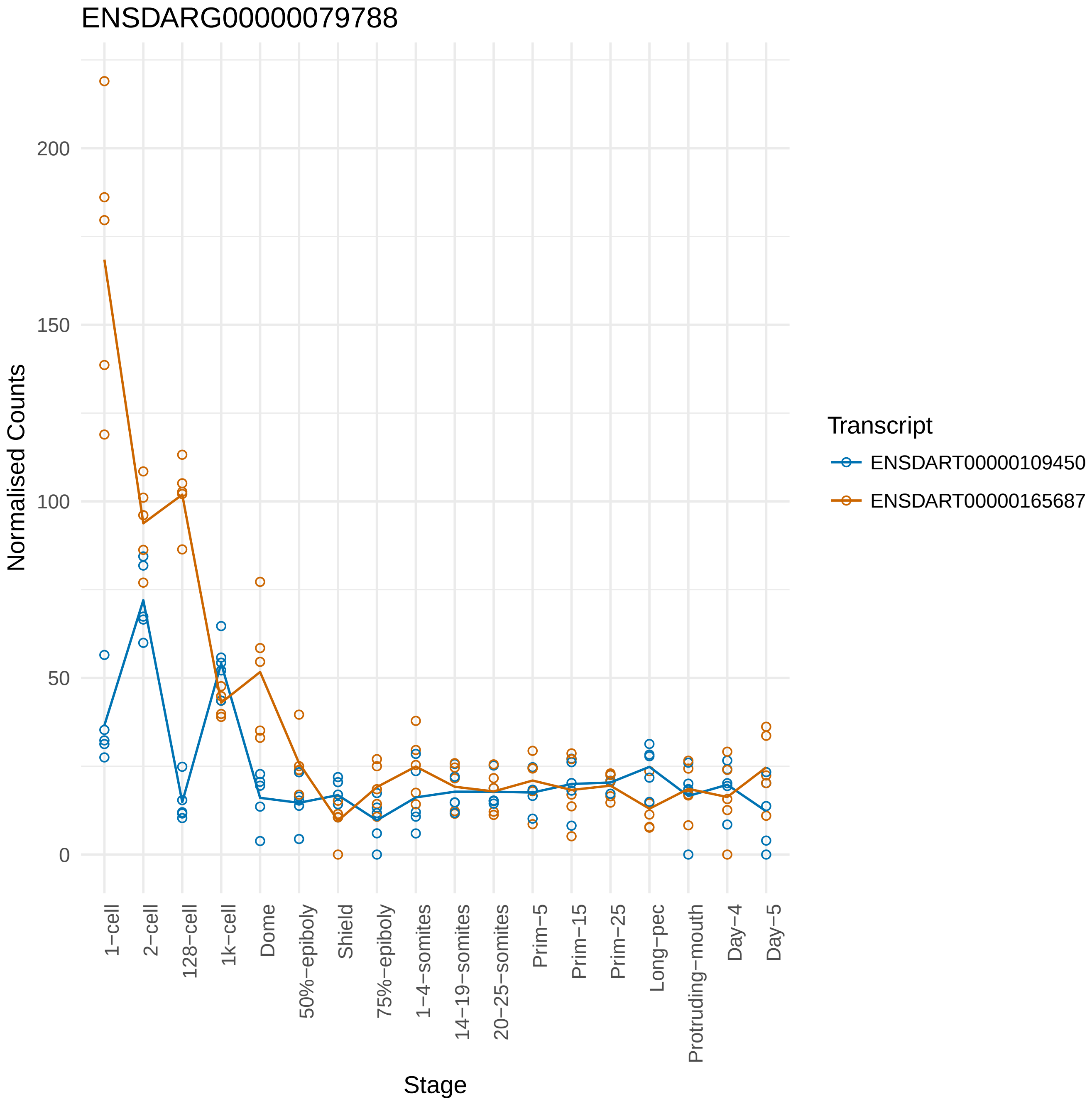

ENSDARG00000079788
Normalised Counts
200
150
100
50
0
1−cell
2−cell
128−cell
1k−cell
Dome
50%−epiboly
Shield
75%−epiboly
1−4−somites
14−19−somites
20−25−somites
Prim−5
Prim−15
Prim−25
Long−pec
Protruding−mouth
Day−4
Day−5
Stage
Transcript
ENSDART00000109450
ENSDART00000165687

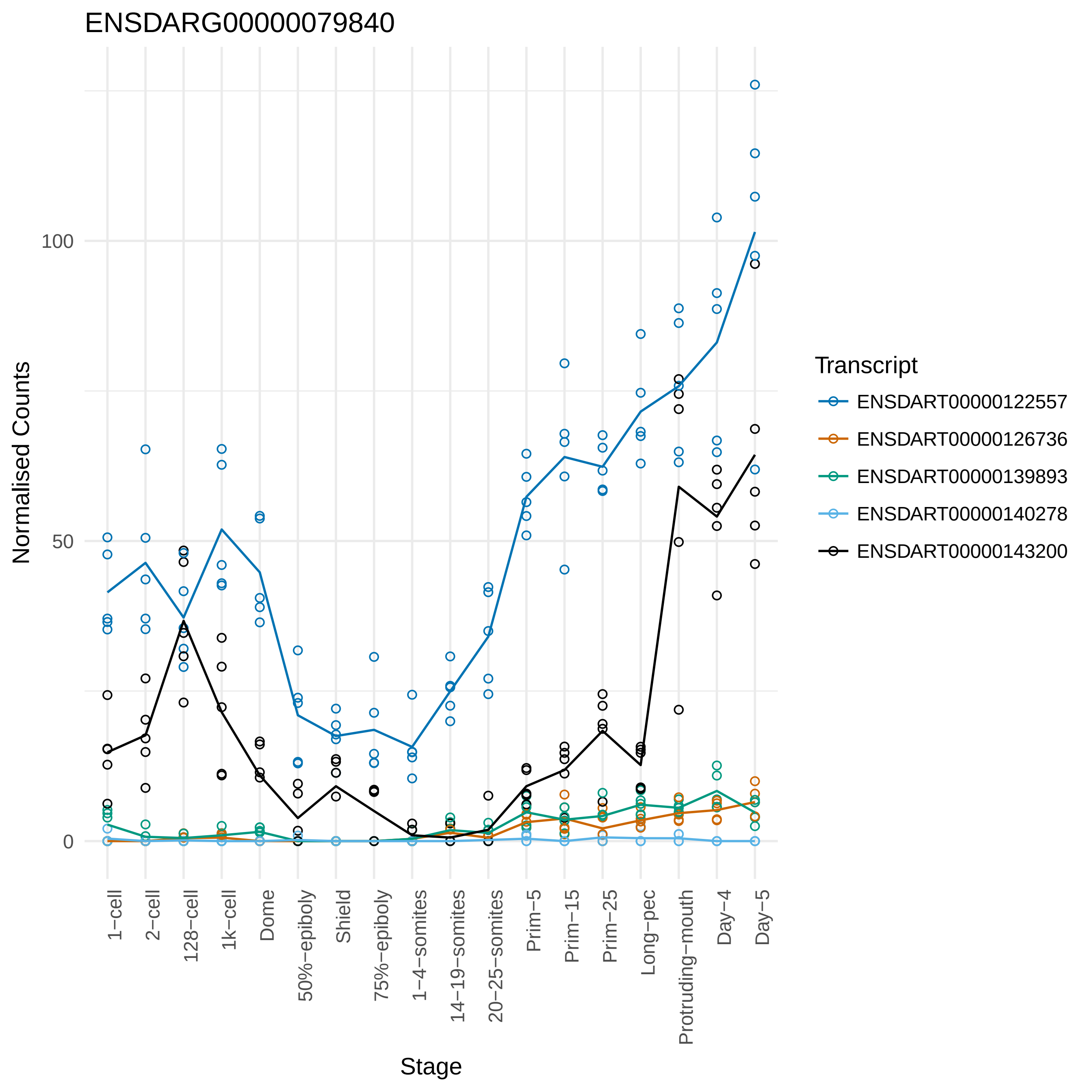

ENSDARG00000079840
Normalised Counts
100
50
0
1−cell
2−cell
128−cell
1k−cell
Dome
50%−epiboly
Shield
75%−epiboly
1−4−somites
14−19−somites
20−25−somites
Prim−5
Prim−15
Prim−25
Long−pec
Protruding−mouth
Day−4
Day−5
Stage
Transcript
ENSDART00000122557
ENSDART00000126736
ENSDART00000139893
ENSDART00000140278
ENSDART00000143200

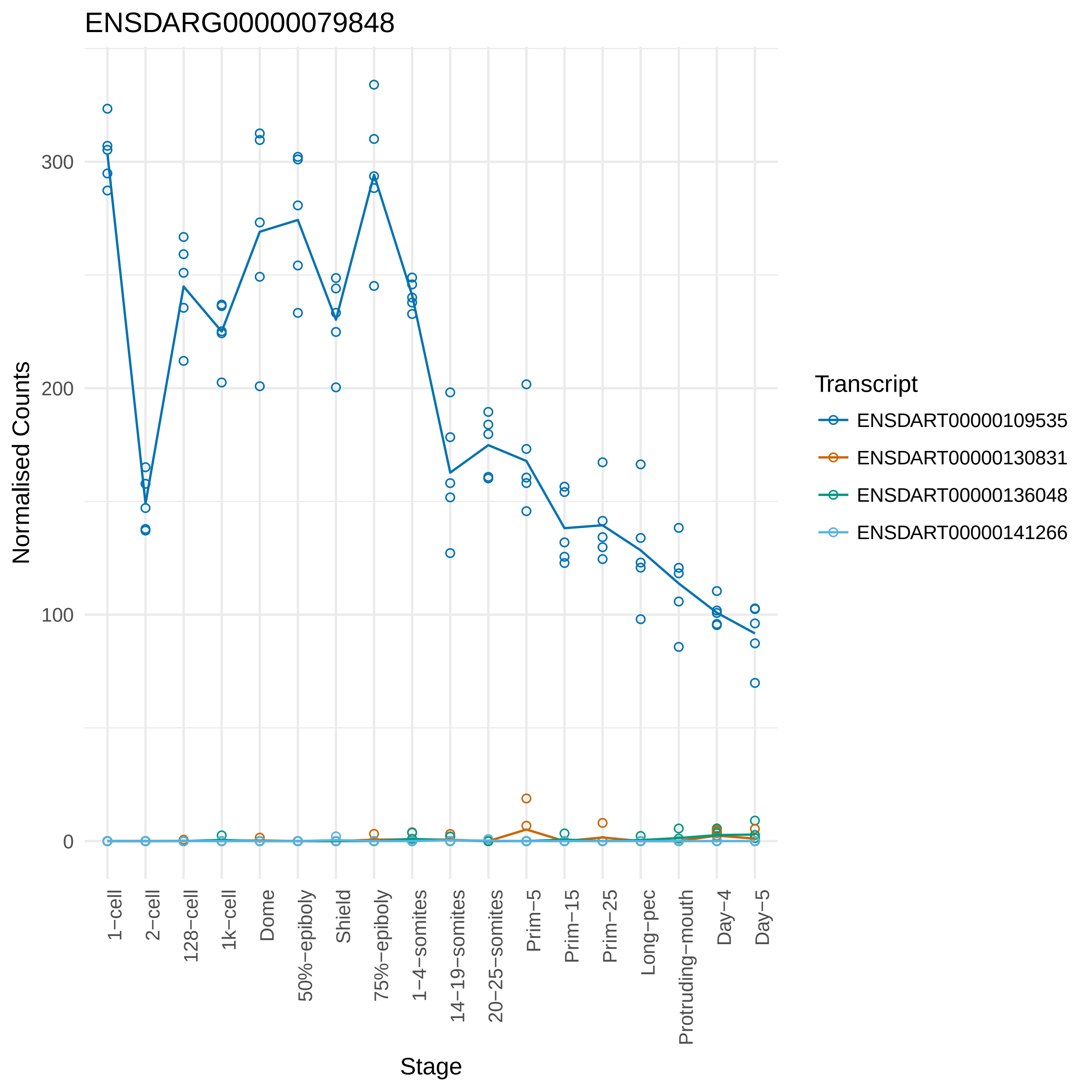

ENSDARG00000079848
Normalised Counts
300
200
100
0
1−cell
2−cell
128−cell
1k−cell
Dome
50%−epiboly
Shield
75%−epiboly
1−4−somites
14−19−somites
20−25−somites
Prim−5
Prim−15
Prim−25
Long−pec
Protruding−mouth
Day−4
Day−5
Stage
Transcript
ENSDART00000109535
ENSDART00000130831
ENSDART00000136048
ENSDART00000141266

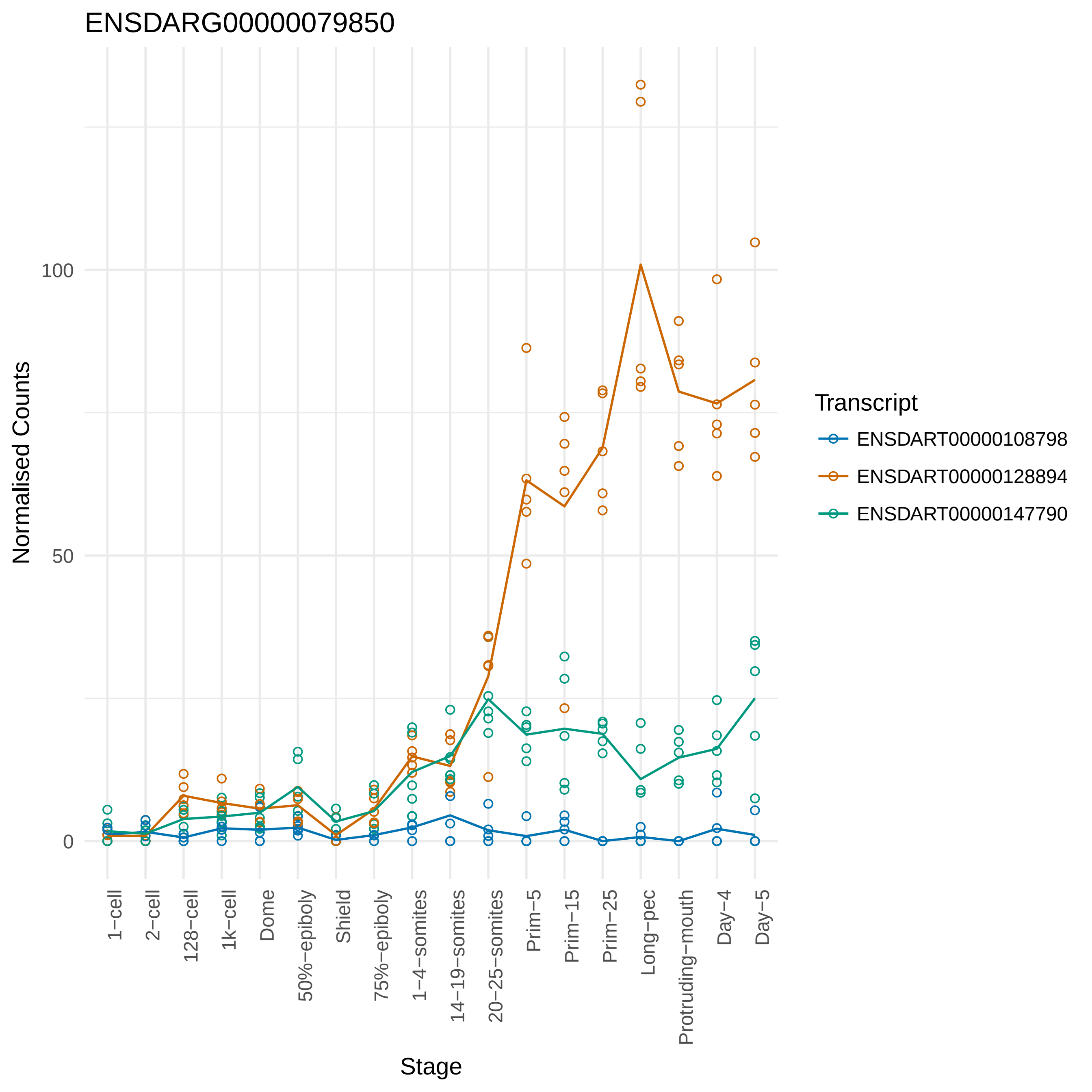

ENSDARG00000079850
Normalised Counts
100
50
0
1−cell
2−cell
128−cell
1k−cell
Dome
50%−epiboly
Shield
75%−epiboly
1−4−somites
14−19−somites
20−25−somites
Prim−5
Prim−15
Prim−25
Long−pec
Protruding−mouth
Day−4
Day−5
Stage
Transcript
ENSDART00000108798
ENSDART00000128894
ENSDART00000147790

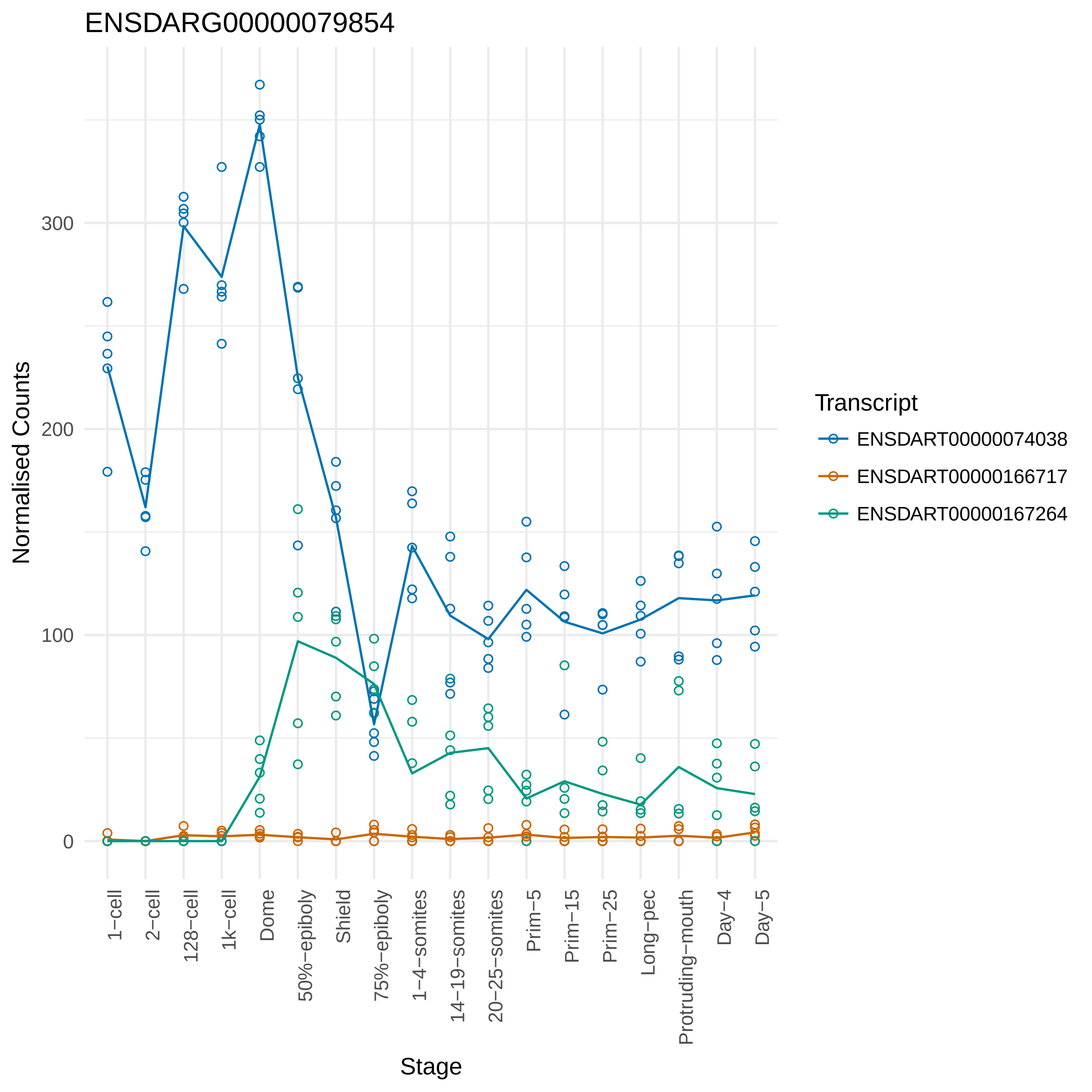

ENSDARG00000079854
Normalised Counts
300
200
100
0
1−cell
2−cell
128−cell
1k−cell
Dome
50%−epiboly
Shield
75%−epiboly
1−4−somites
14−19−somites
20−25−somites
Prim−5
Prim−15
Prim−25
Long−pec
Protruding−mouth
Day−4
Day−5
Stage
Transcript
ENSDART00000074038
ENSDART00000166717
ENSDART00000167264

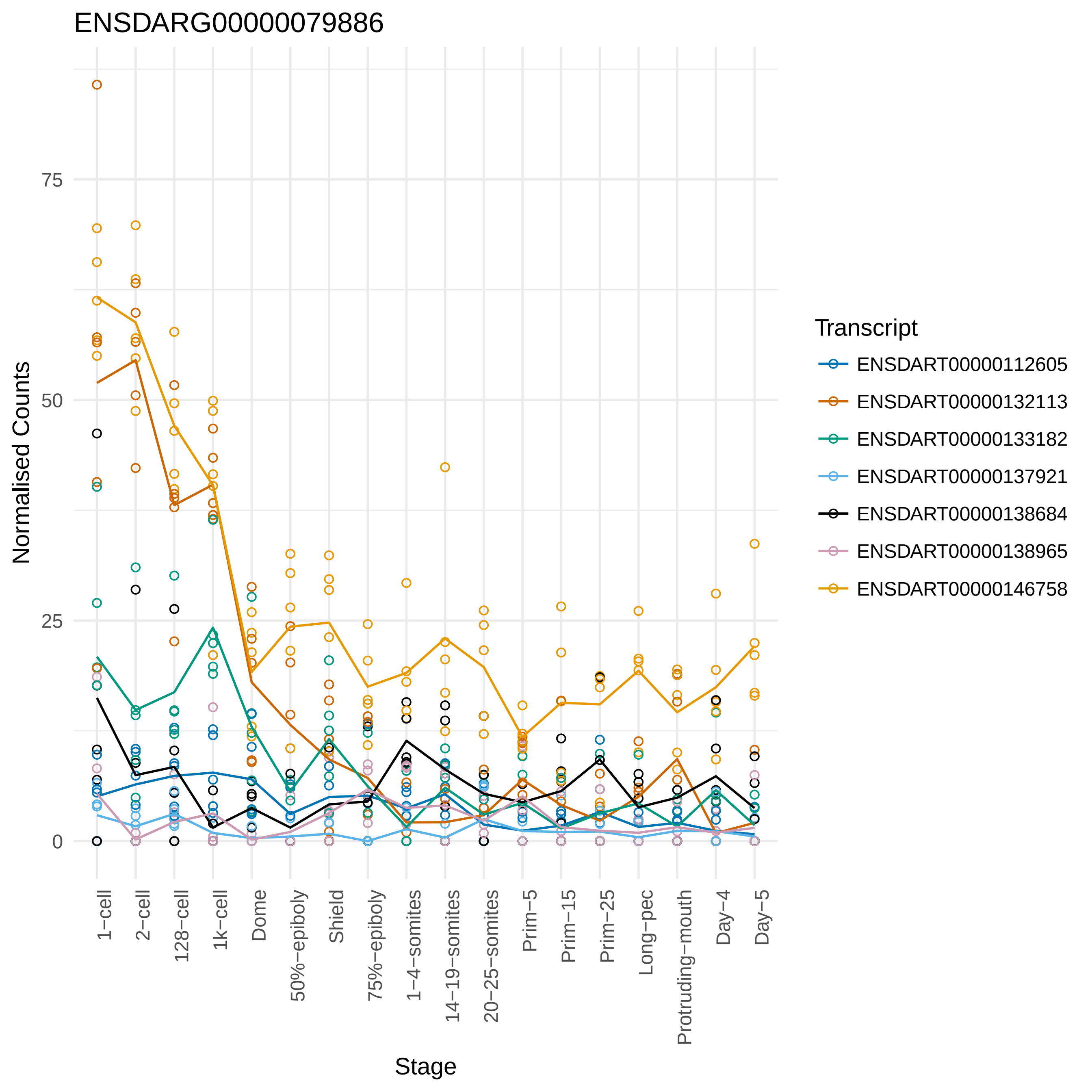

ENSDARG00000079886
Normalised Counts
0
25
50
75
1−cell
2−cell
128−cell
1k−cell
Dome
50%−epiboly
Shield
75%−epiboly
1−4−somites
14−19−somites
20−25−somites
Prim−5
Prim−15
Prim−25
Long−pec
Protruding−mouth
Day−4
Day−5
Stage
Transcript
ENSDART00000112605
ENSDART00000132113
ENSDART00000133182
ENSDART00000137921
ENSDART00000138684
ENSDART00000138965
ENSDART00000146758

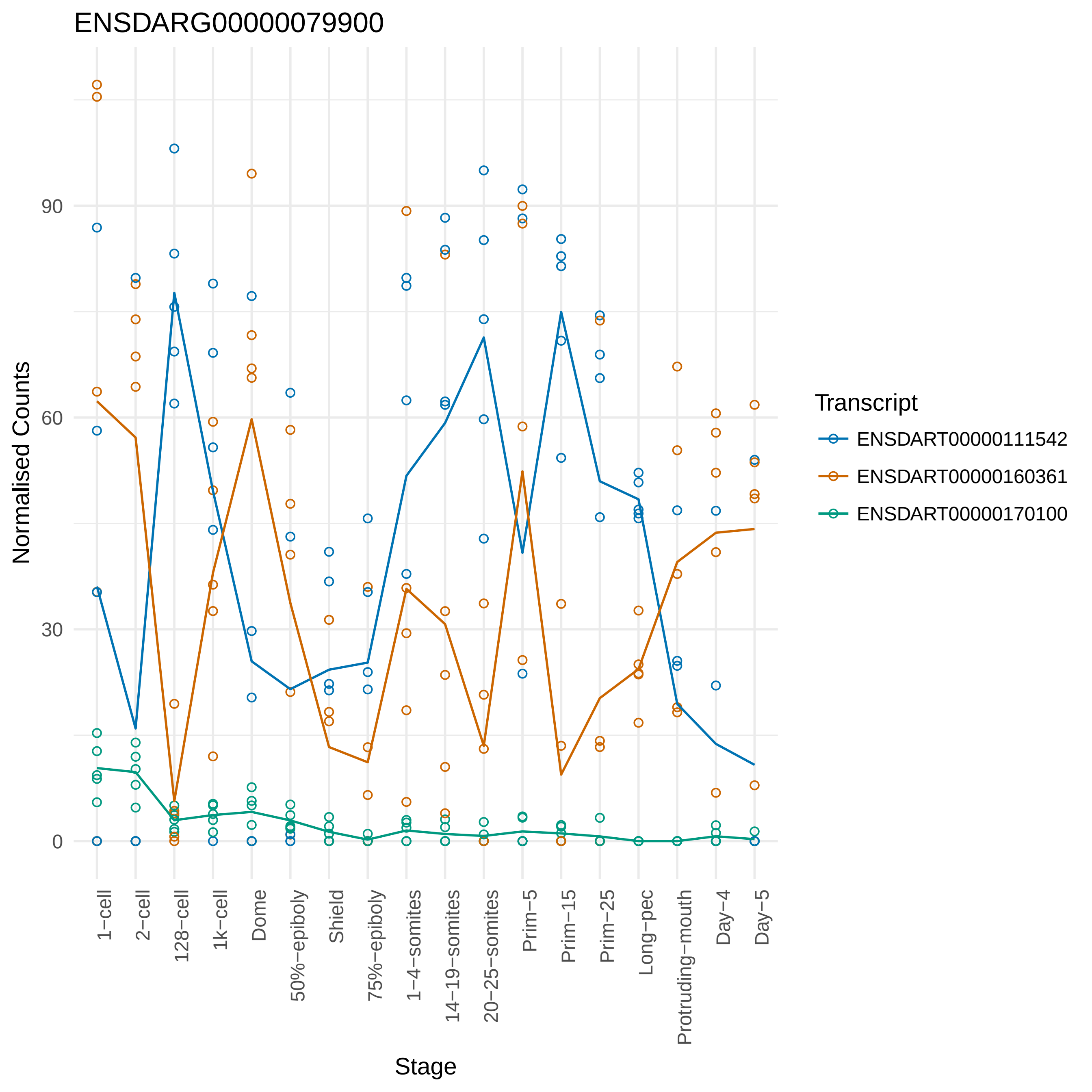

ENSDARG00000079900
Normalised Counts
90
60
30
0
1−cell
2−cell
128−cell
1k−cell
Dome
50%−epiboly
Shield
75%−epiboly
1−4−somites
14−19−somites
20−25−somites
Prim−5
Prim−15
Prim−25
Long−pec
Protruding−mouth
Day−4
Day−5
Stage
Transcript
ENSDART00000111542
ENSDART00000160361
ENSDART00000170100

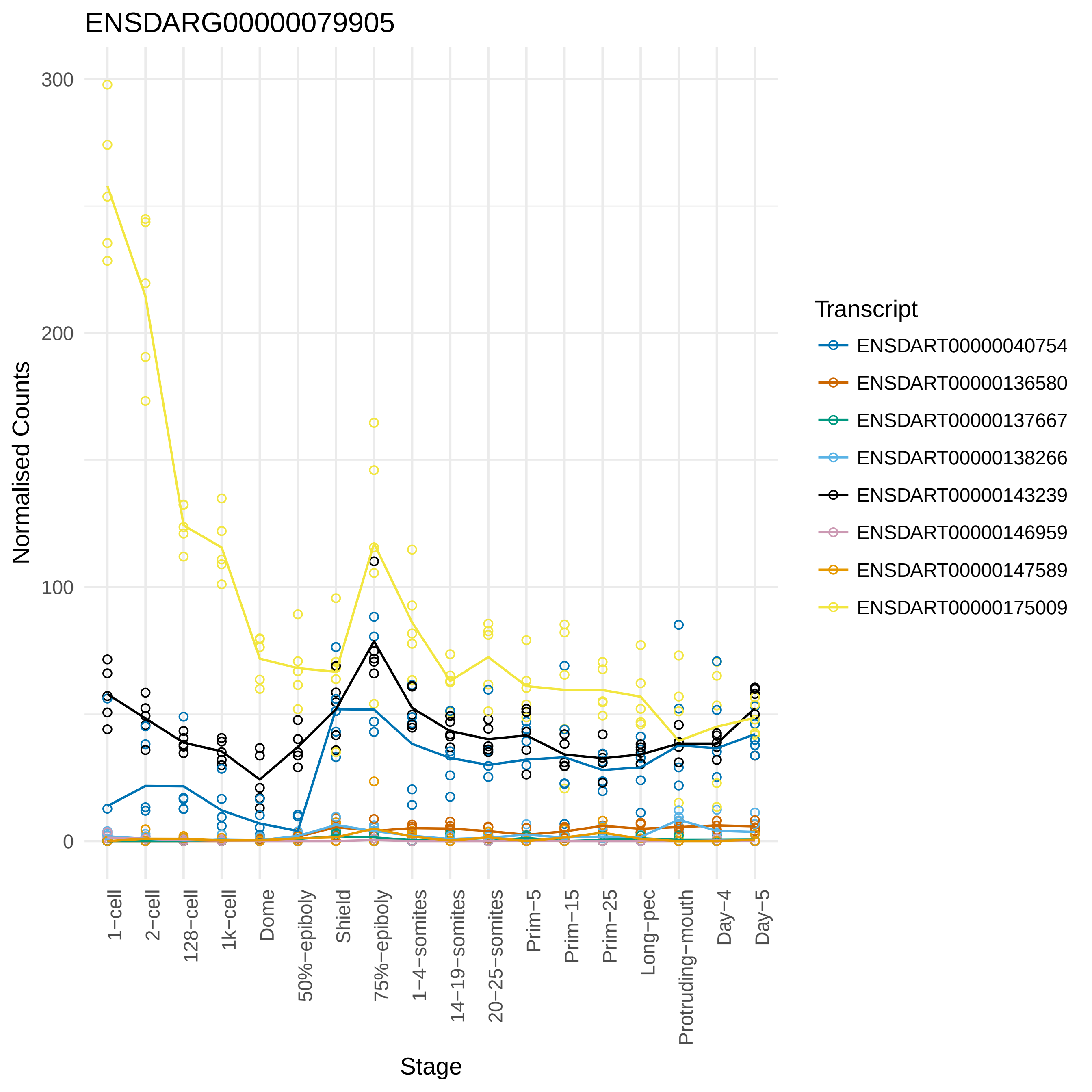

ENSDARG00000079905
Normalised Counts
300
200
100
0
1−cell
2−cell
128−cell
1k−cell
Dome
50%−epiboly
Shield
75%−epiboly
1−4−somites
14−19−somites
20−25−somites
Prim−5
Prim−15
Prim−25
Long−pec
Protruding−mouth
Day−4
Day−5
Stage
Transcript
ENSDART00000040754
ENSDART00000136580
ENSDART00000137667
ENSDART00000138266
ENSDART00000143239
ENSDART00000146959
ENSDART00000147589
ENSDART00000175009

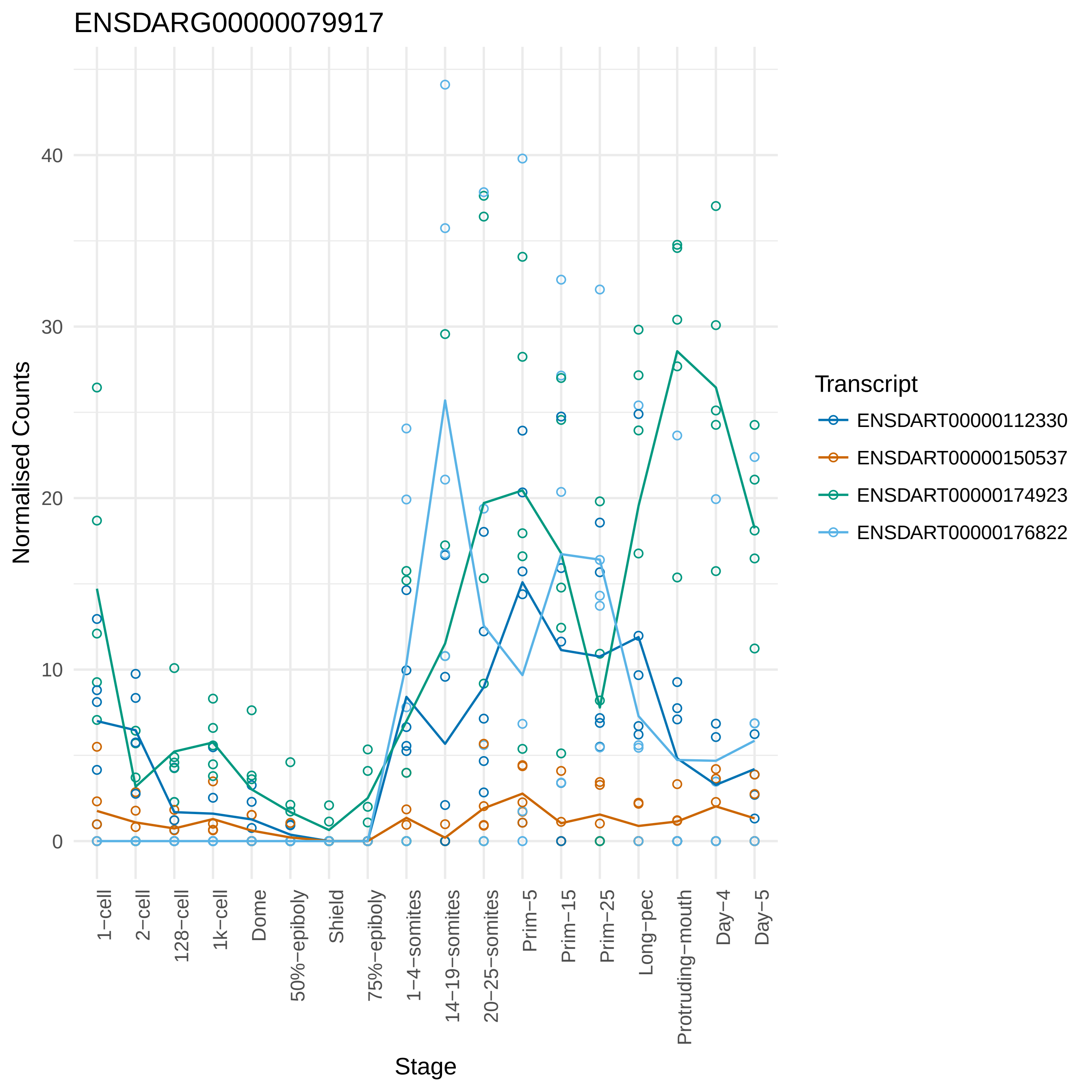

ENSDARG00000079917
Normalised Counts
0
10
20
30
40
Stage
1−cell
2−cell
128−cell
1k−cell
Dome
50%−epiboly
Shield
75%−epiboly
1−4−somites
14−19−somites
20−25−somites
Prim−5
Prim−15
Prim−25
Long−pec
Protruding−mouth
Day−4
Day−5
Transcript
ENSDART00000112330
ENSDART00000150537
ENSDART00000174923
ENSDART00000176822

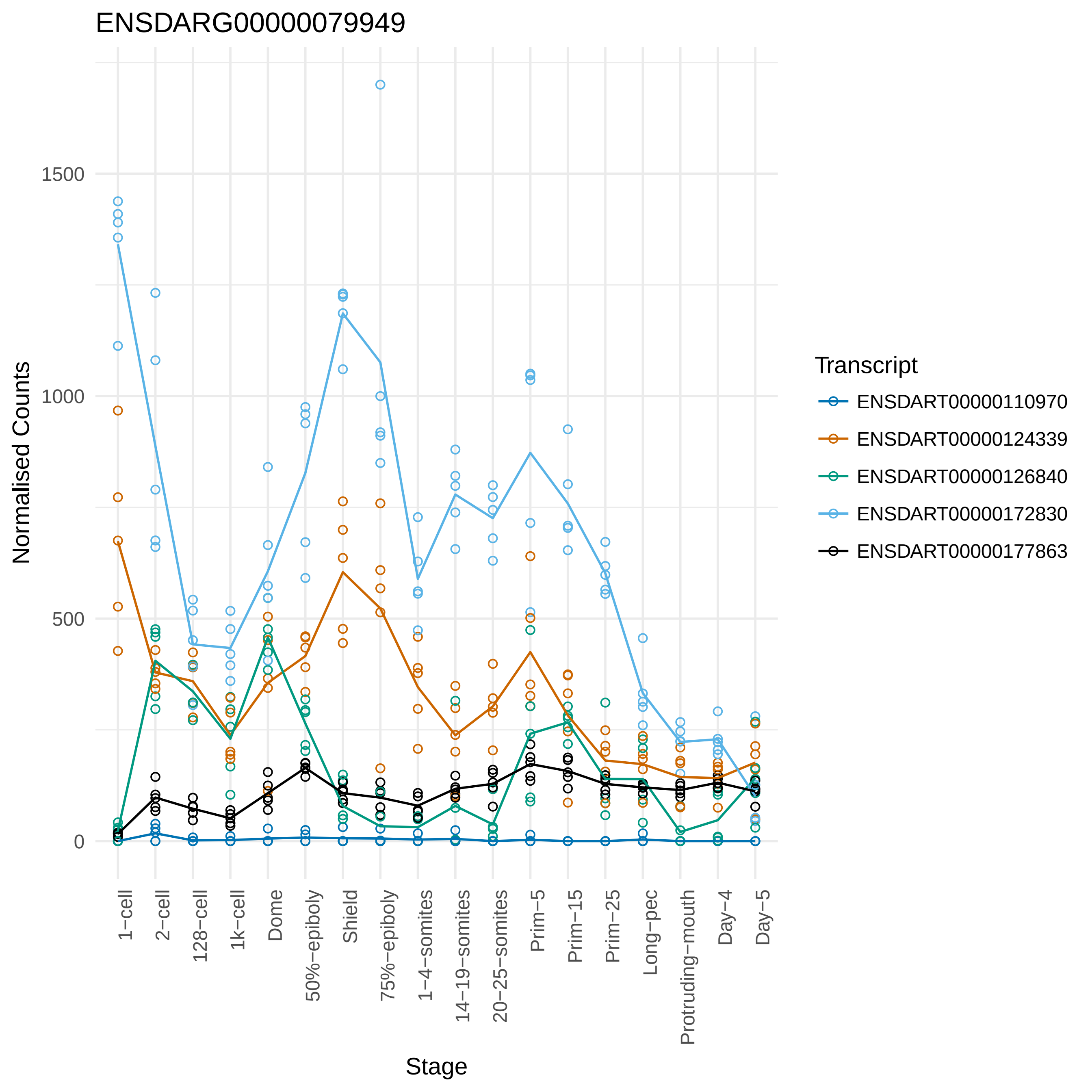
ENSDARG00000079949
Normalised Counts
0
500
1000
1500
1−cell
2−cell
128−cell
1k−cell
Dome
50%−epiboly
Shield
75%−epiboly
1−4−somites
14−19−somites
20−25−somites
Prim−5
Prim−15
Prim−25
Long−pec
Protruding−mouth
Day−4
Day−5
Stage
Transcript
ENSDART00000110970
ENSDART00000124339
ENSDART00000126840
ENSDART00000172830
ENSDART00000177863

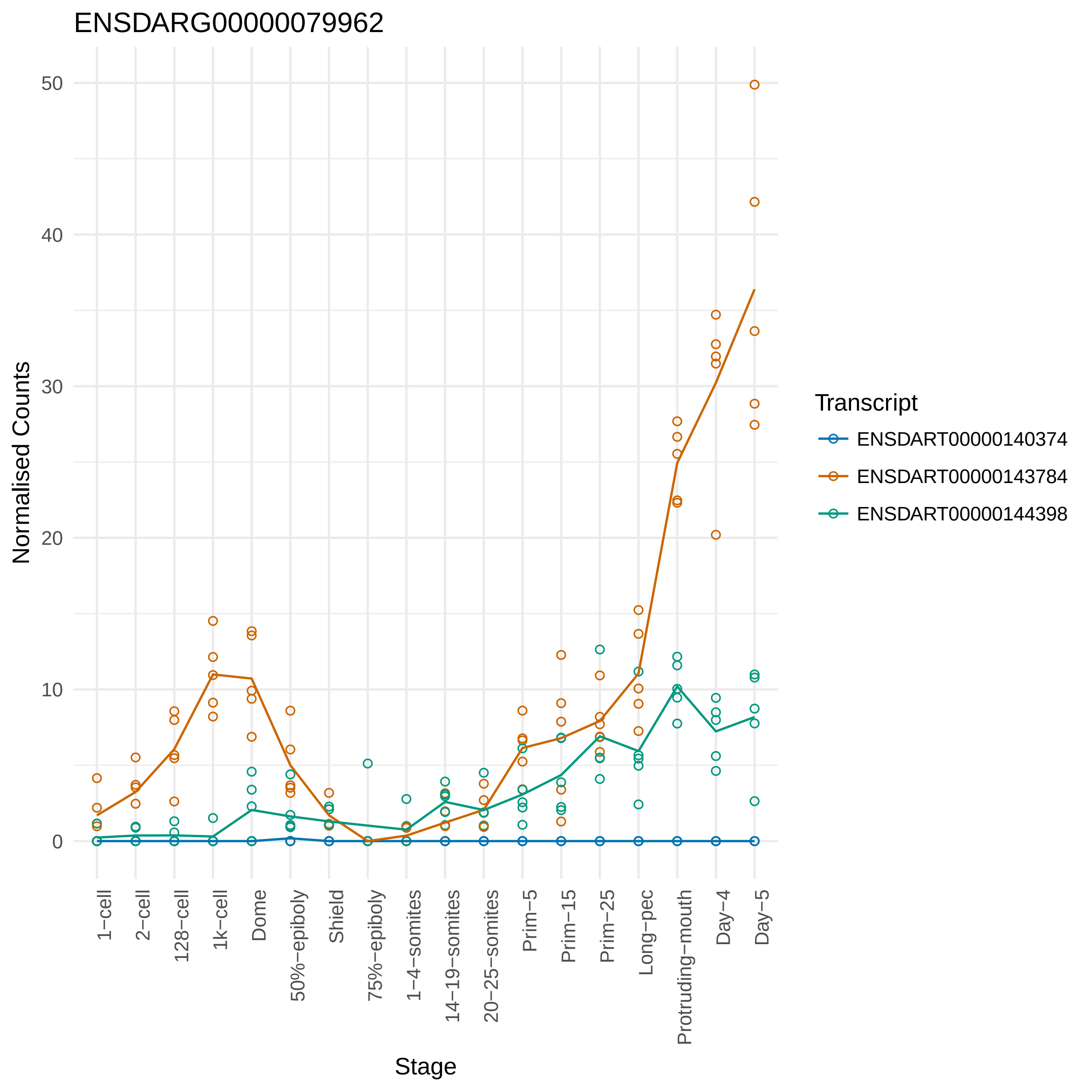

ENSDARG00000079962
Normalised Counts
0
10
20
30
40
50
1−cell
2−cell
128−cell
1k−cell
Dome
50%−epiboly
Shield
75%−epiboly
1−4−somites
14−19−somites
20−25−somites
Prim−5
Prim−15
Prim−25
Long−pec
Protruding−mouth
Day−4
Day−5
Stage
Transcript
ENSDART00000140374
ENSDART00000143784
ENSDART00000144398

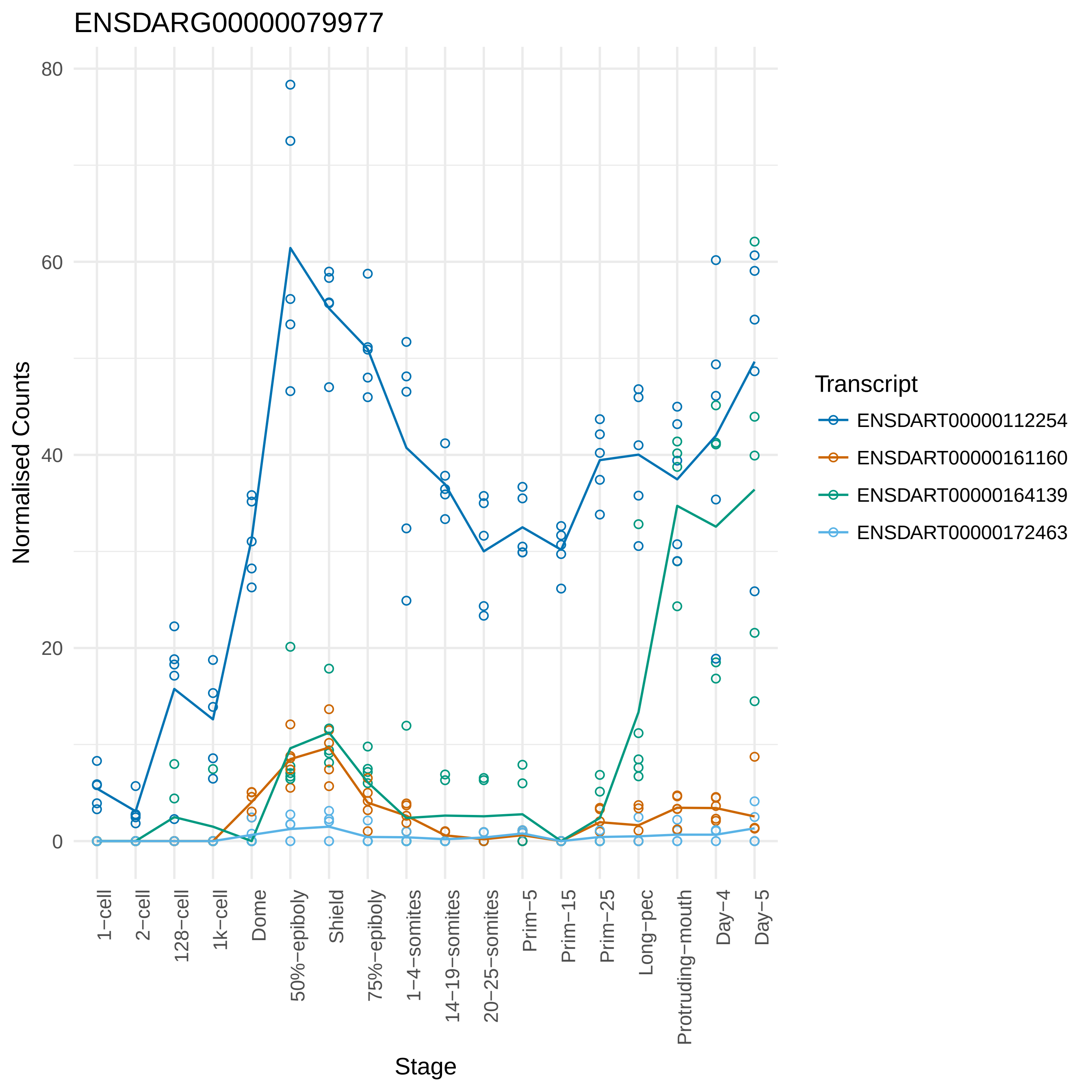
ENSDARG00000079977
Normalised Counts
0
20
40
60
80
Stage
1−cell
2−cell
128−cell
1k−cell
Dome
50%−epiboly
Shield
75%−epiboly
1−4−somites
14−19−somites
20−25−somites
Prim−5
Prim−15
Prim−25
Long−pec
Protruding−mouth
Day−4
Day−5
Transcript
ENSDART00000112254
ENSDART00000161160
ENSDART00000164139
ENSDART00000172463

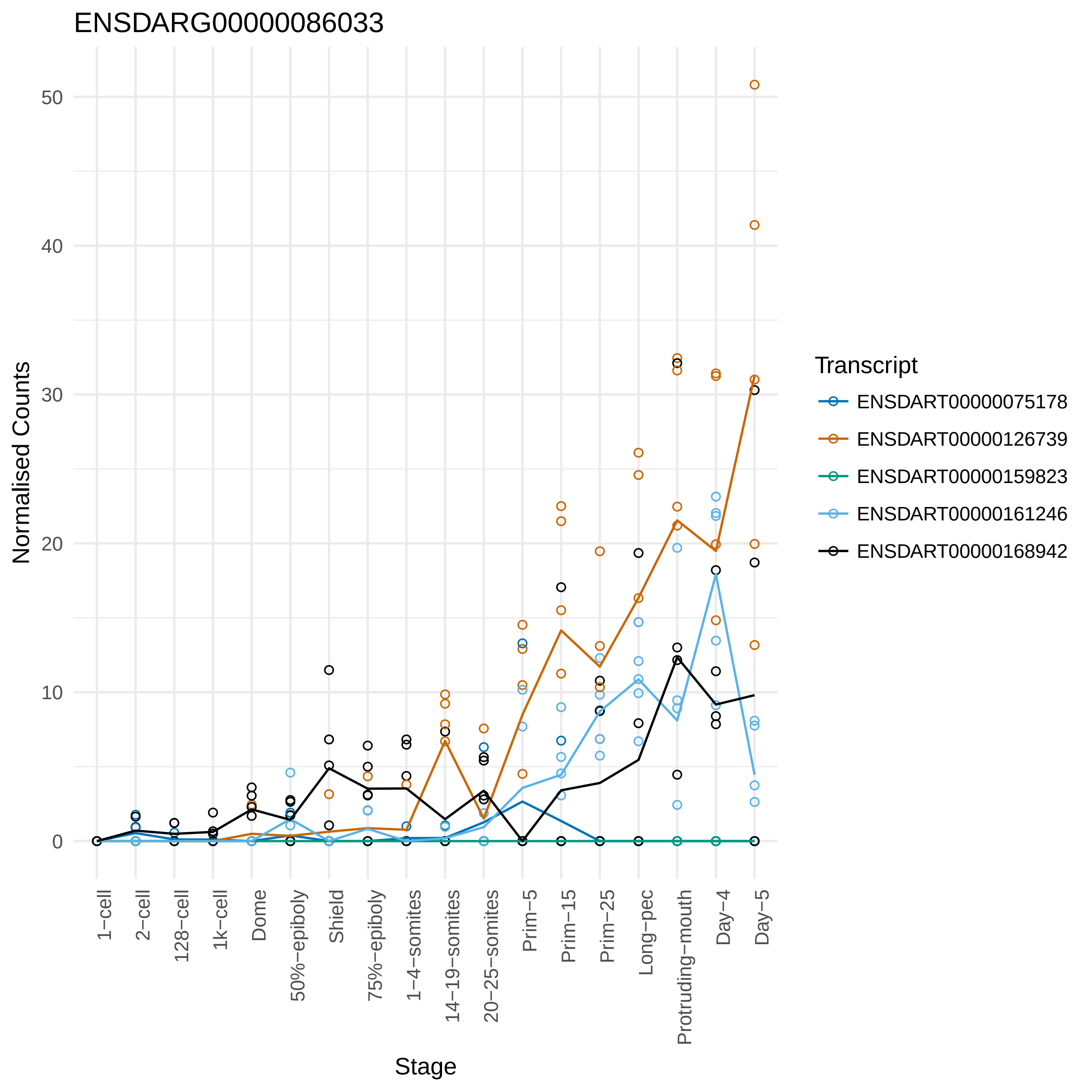

ENSDARG00000086033
Normalised Counts
0
10
20
30
40
50
1−cell
2−cell
128−cell
1k−cell
Dome
50%−epiboly
Shield
75%−epiboly
1−4−somites
14−19−somites
20−25−somites
Prim−5
Prim−15
Prim−25
Long−pec
Protruding−mouth
Day−4
Day−5
Stage
Transcript
ENSDART00000075178
ENSDART00000126739
ENSDART00000159823
ENSDART00000161246
ENSDART00000168942

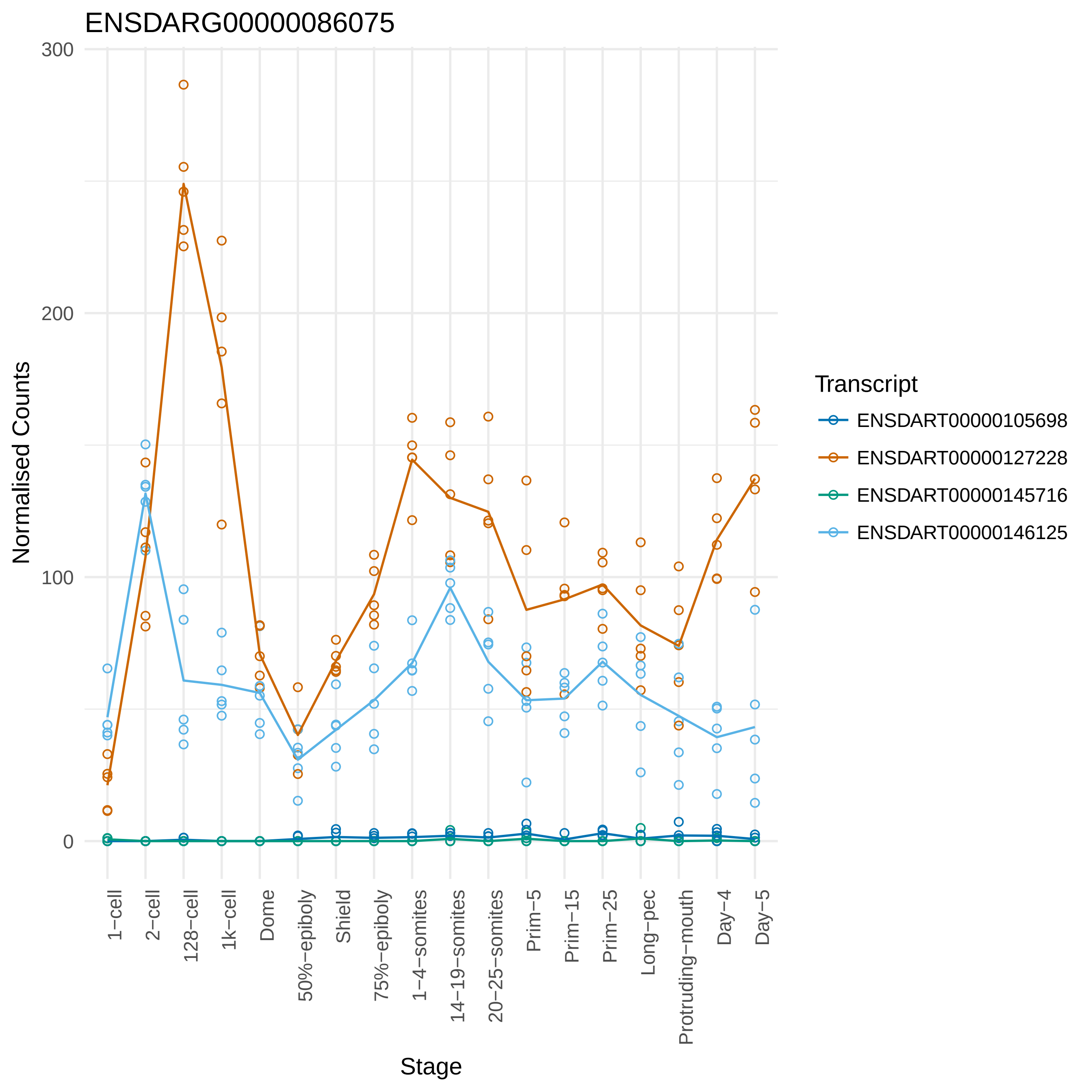

ENSDARG00000086075
Normalised Counts
300
200
100
0
Stage
1−cell
2−cell
128−cell
1k−cell
Dome
50%−epiboly
Shield
75%−epiboly
1−4−somites
14−19−somites
20−25−somites
Prim−5
Prim−15
Prim−25
Long−pec
Protruding−mouth
Day−4
Day−5
Transcript
ENSDART00000105698
ENSDART00000127228
ENSDART00000145716
ENSDART00000146125

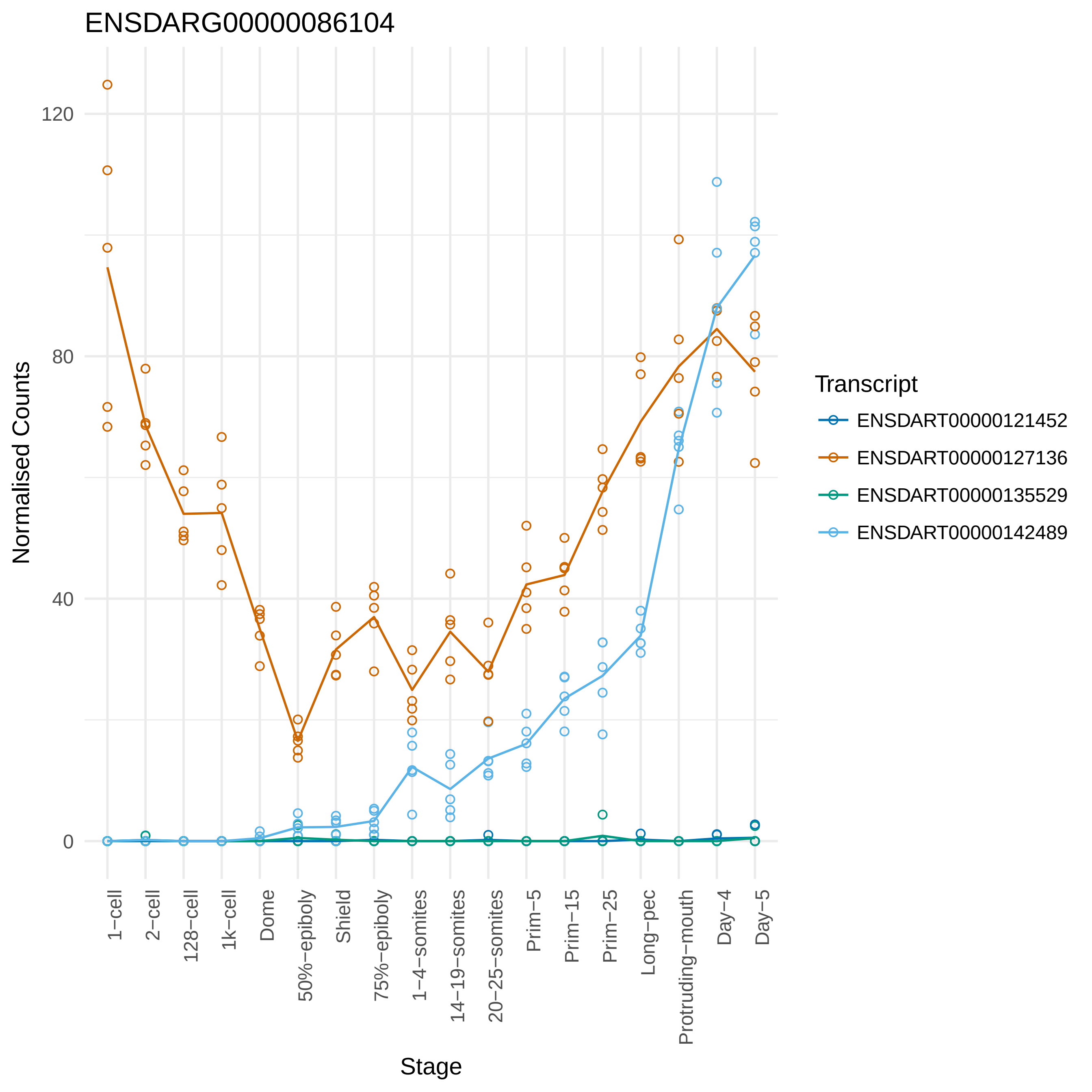
ENSDARG00000086104
Normalised Counts
120
80
40
0
1−cell
2−cell
128−cell
1k−cell
Dome
50%−epiboly
Shield
75%−epiboly
1−4−somites
14−19−somites
20−25−somites
Prim−5
Prim−15
Prim−25
Long−pec
Protruding−mouth
Day−4
Day−5
Stage
Transcript
ENSDART00000121452
ENSDART00000127136
ENSDART00000135529
ENSDART00000142489

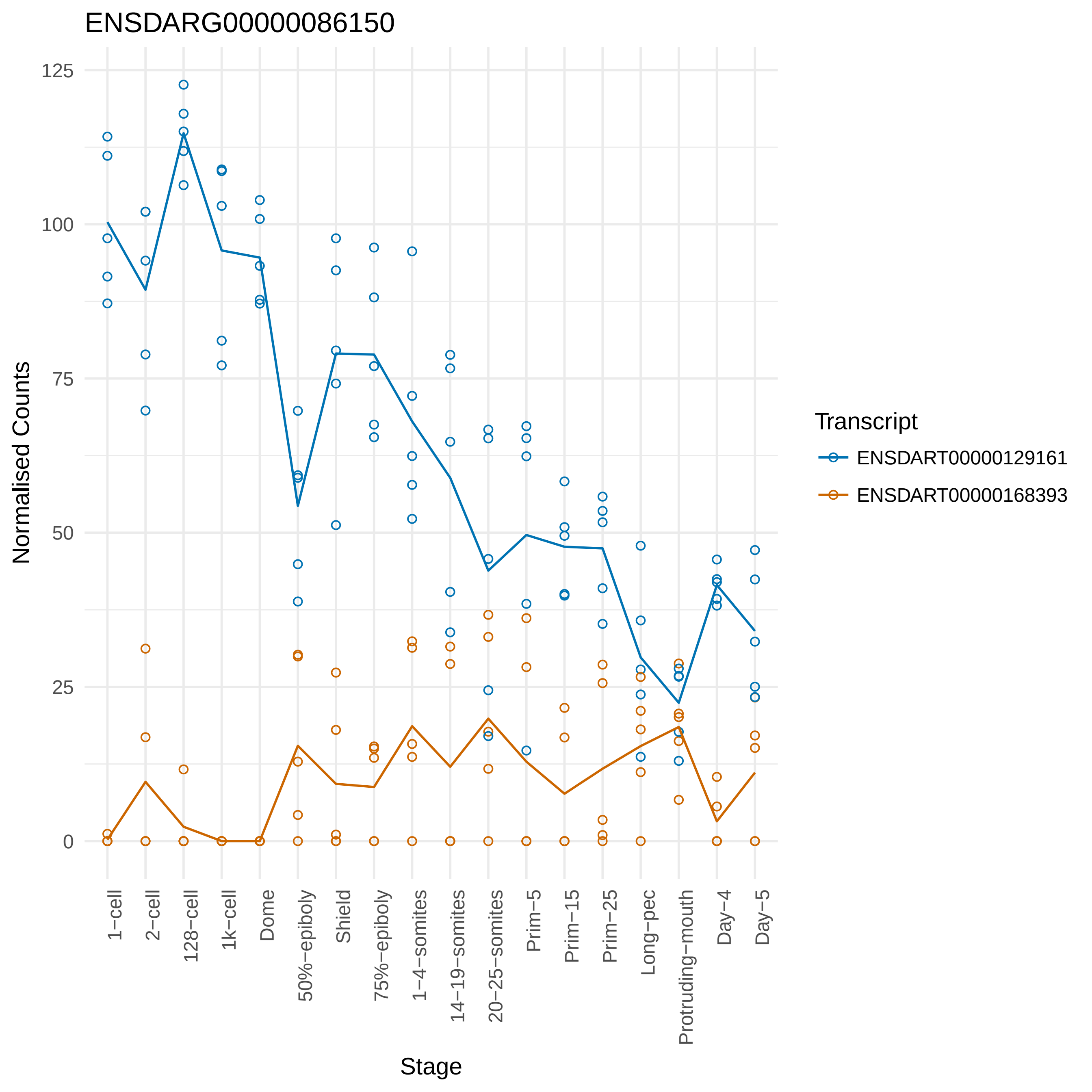
ENSDARG00000086150
Normalised Counts
125
100
75
50
25
0
1−cell
2−cell
128−cell
1k−cell
Dome
50%−epiboly
Shield
75%−epiboly
1−4−somites
14−19−somites
20−25−somites
Prim−5
Prim−15
Prim−25
Long−pec
Protruding−mouth
Day−4
Day−5
Stage
Transcript
ENSDART00000129161
ENSDART00000168393

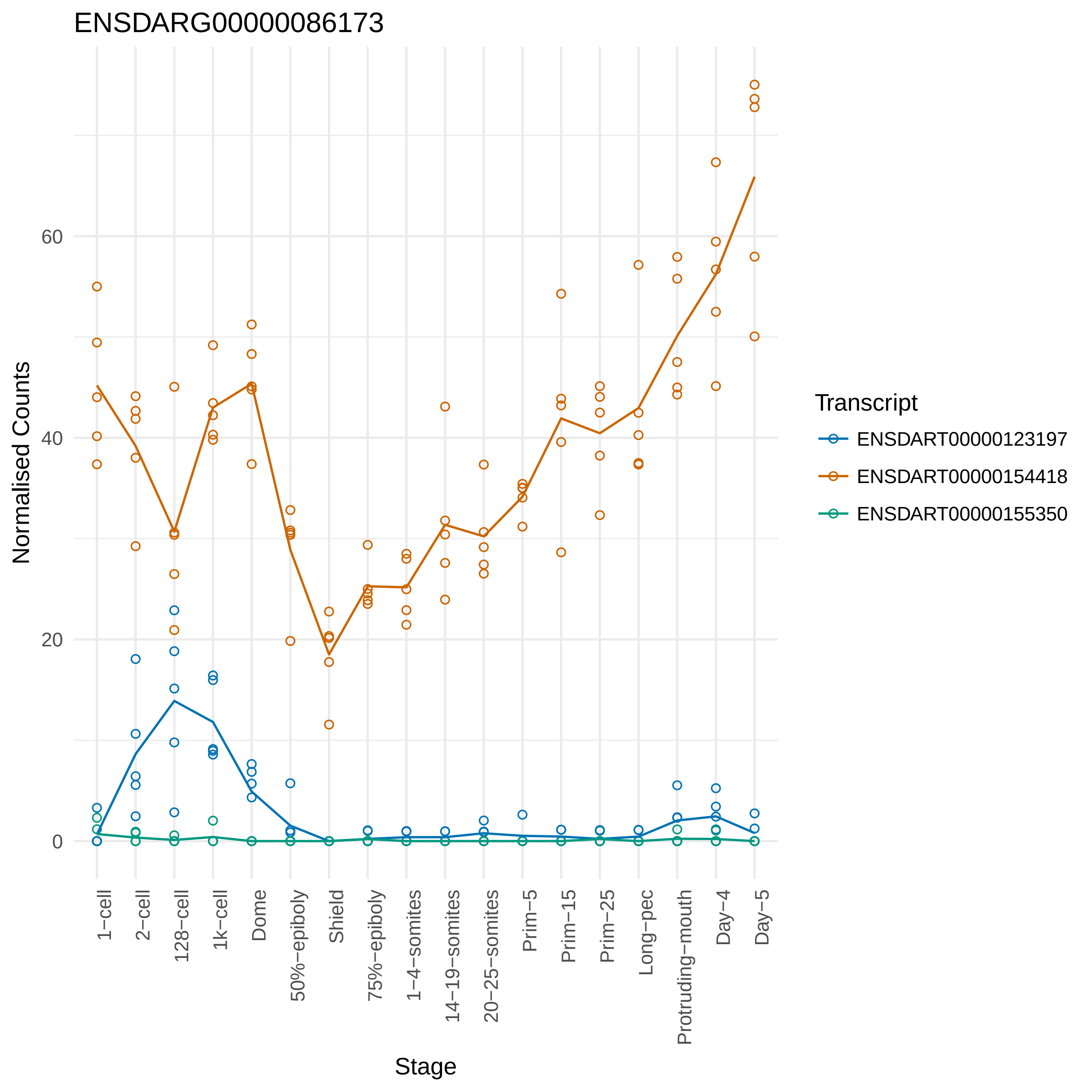
ENSDARG00000086173
Normalised Counts
0
20
40
60
Stage
1−cell
2−cell
128−cell
1k−cell
Dome
50%−epiboly
Shield
75%−epiboly
1−4−somites
14−19−somites
20−25−somites
Prim−5
Prim−15
Prim−25
Long−pec
Protruding−mouth
Day−4
Day−5
Transcript
ENSDART00000123197
ENSDART00000154418
ENSDART00000155350

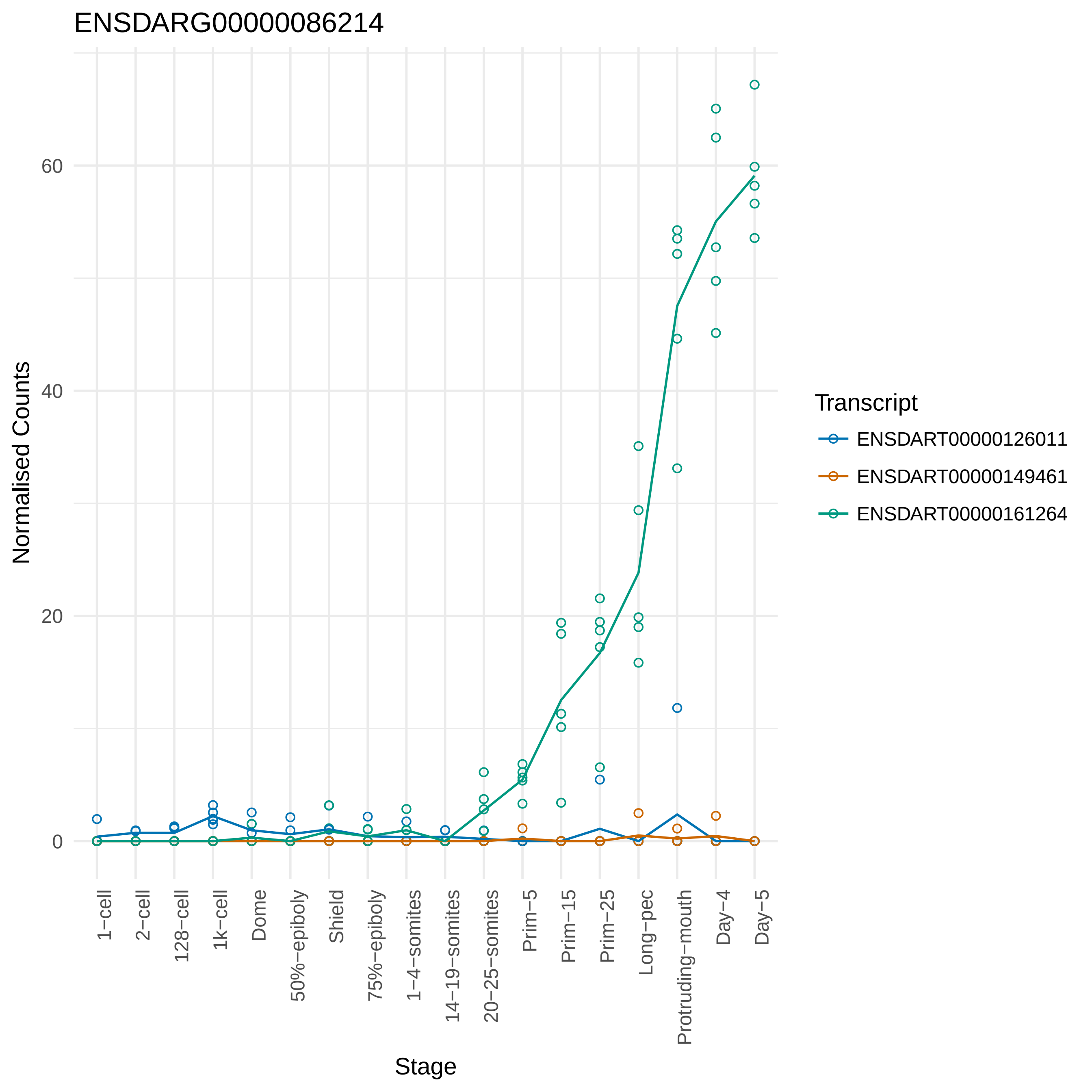

ENSDARG00000086214
Normalised Counts
0
20
40
60
1−cell
2−cell
128−cell
1k−cell
Dome
50%−epiboly
Shield
75%−epiboly
1−4−somites
14−19−somites
20−25−somites
Prim−5
Prim−15
Prim−25
Long−pec
Protruding−mouth
Day−4
Day−5
Stage
Transcript
ENSDART00000126011
ENSDART00000149461
ENSDART00000161264

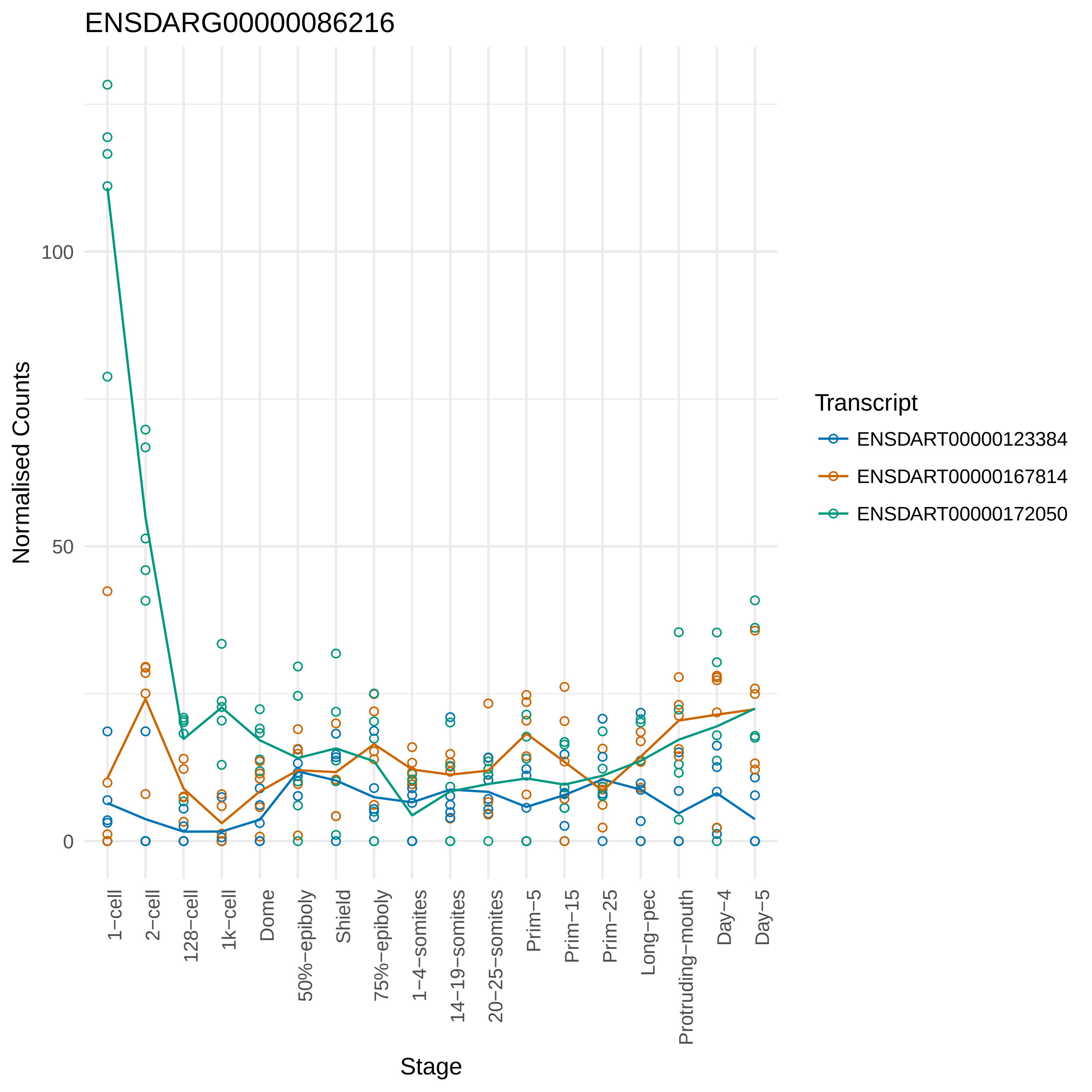
ENSDARG00000086216
Normalised Counts
100
50
0
Stage
1−cell
2−cell
128−cell
1k−cell
Dome
50%−epiboly
Shield
75%−epiboly
1−4−somites
14−19−somites
20−25−somites
Prim−5
Prim−15
Prim−25
Long−pec
Protruding−mouth
Day−4
Day−5
Transcript
ENSDART00000123384
ENSDART00000167814
ENSDART00000172050

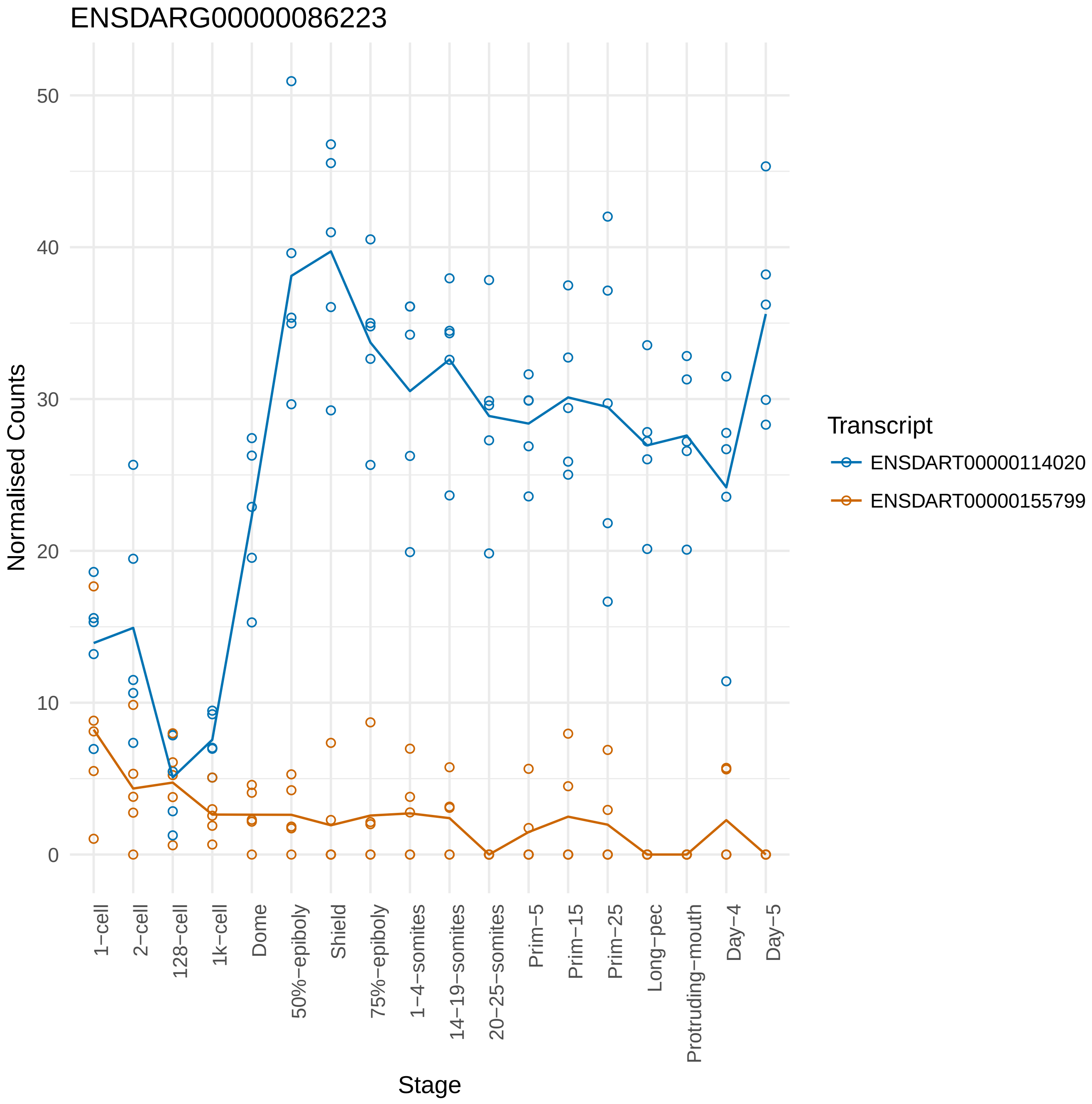

ENSDARG00000086223
Normalised Counts
0
10
20
30
40
50
1−cell
2−cell
128−cell
1k−cell
Dome
50%−epiboly
Shield
75%−epiboly
1−4−somites
14−19−somites
20−25−somites
Prim−5
Prim−15
Prim−25
Long−pec
Protruding−mouth
Day−4
Day−5
Stage
Transcript
ENSDART00000114020
ENSDART00000155799

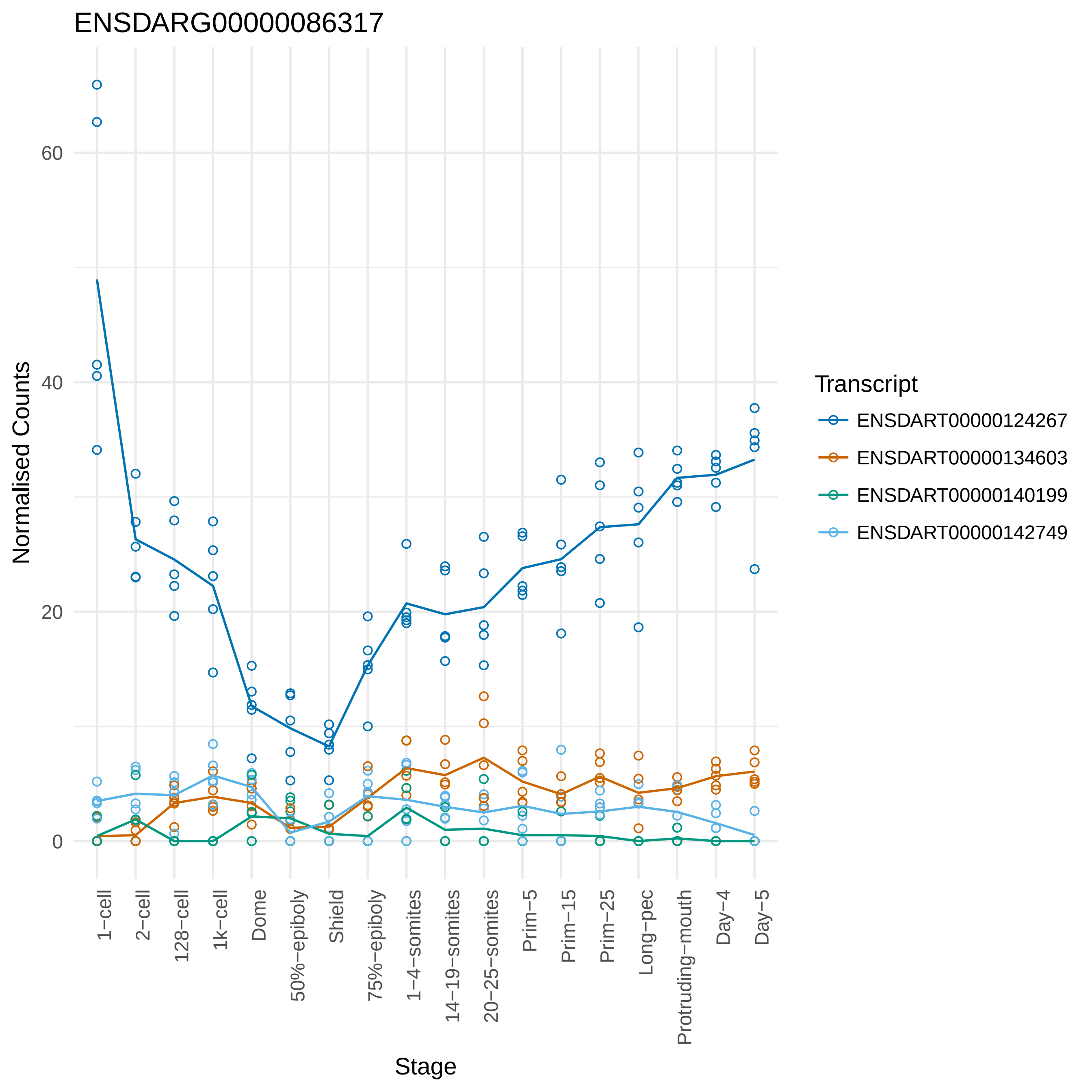
ENSDARG00000086317
Normalised Counts
0
20
40
60
1−cell
2−cell
128−cell
1k−cell
Dome
50%−epiboly
Shield
75%−epiboly
1−4−somites
14−19−somites
20−25−somites
Prim−5
Prim−15
Prim−25
Long−pec
Protruding−mouth
Day−4
Day−5
Stage
Transcript
ENSDART00000124267
ENSDART00000134603
ENSDART00000140199
ENSDART00000142749

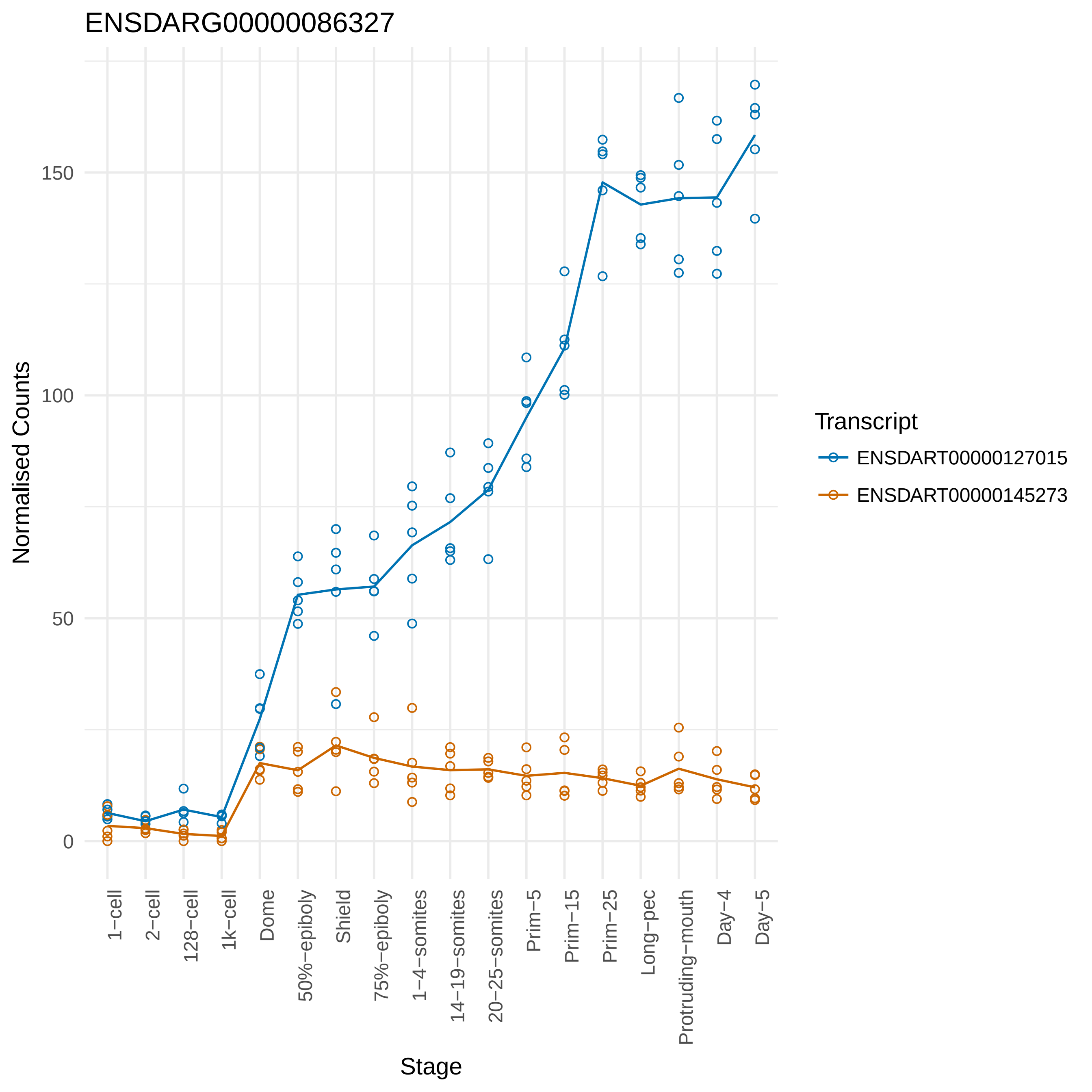

ENSDARG00000086327
Normalised Counts
0
50
100
150
1−cell
2−cell
128−cell
1k−cell
Dome
50%−epiboly
Shield
75%−epiboly
1−4−somites
14−19−somites
20−25−somites
Prim−5
Prim−15
Prim−25
Long−pec
Protruding−mouth
Day−4
Day−5
Stage
Transcript
ENSDART00000127015
ENSDART00000145273

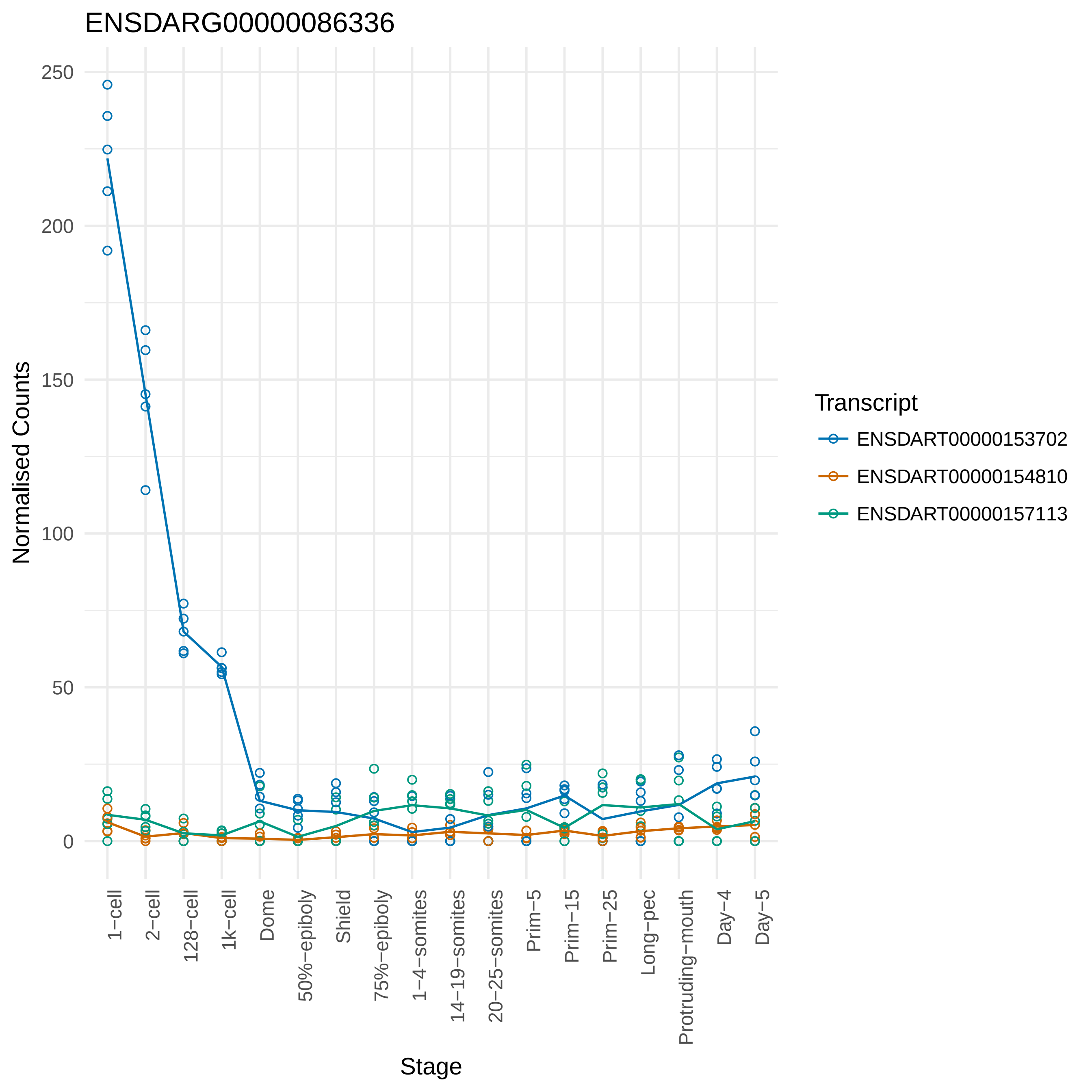
ENSDARG00000086336
Normalised Counts
250
200
150
100
50
0
1−cell
2−cell
128−cell
1k−cell
Dome
50%−epiboly
Shield
75%−epiboly
1−4−somites
14−19−somites
20−25−somites
Prim−5
Prim−15
Prim−25
Long−pec
Protruding−mouth
Day−4
Day−5
Stage
Transcript
ENSDART00000153702
ENSDART00000154810
ENSDART00000157113

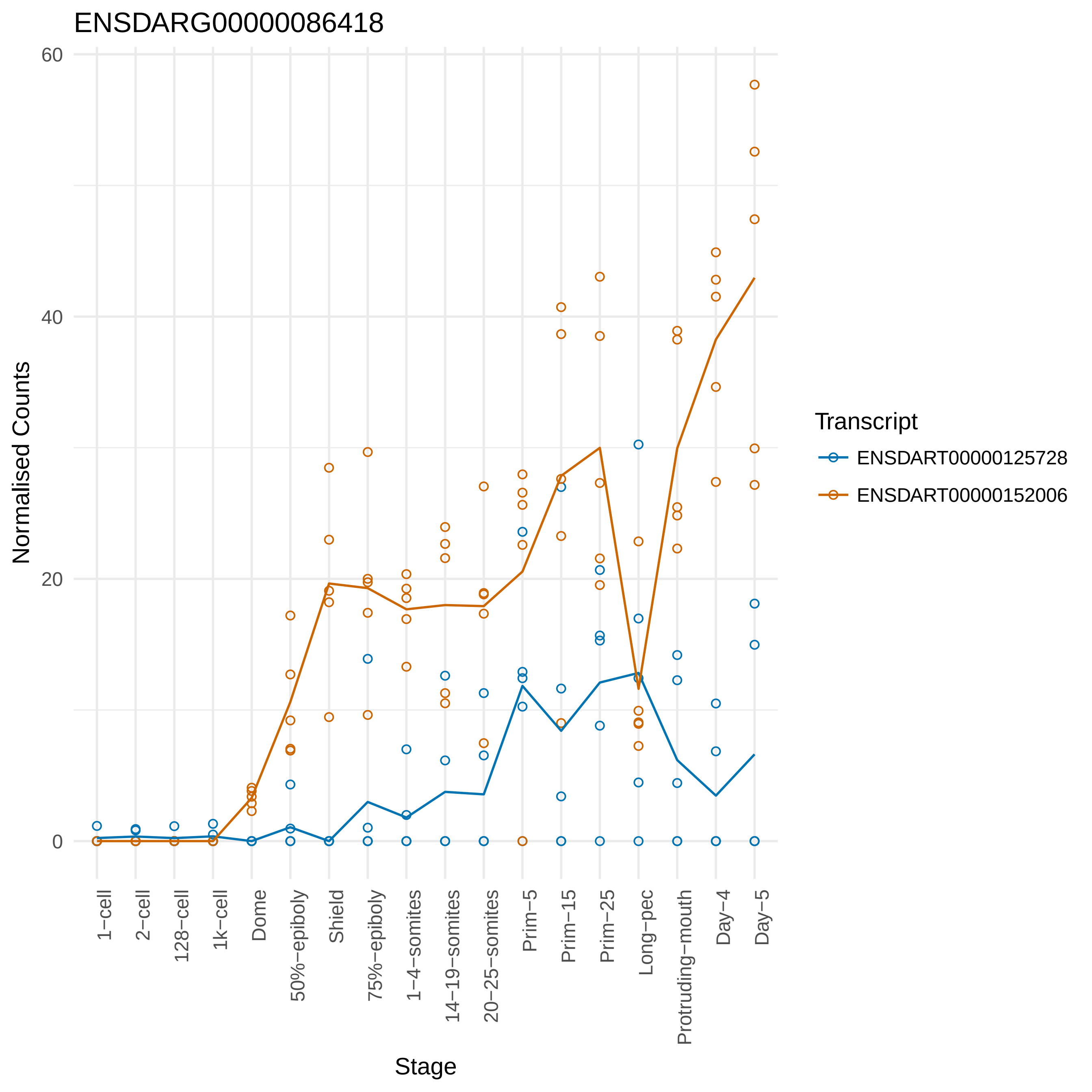
ENSDARG00000086418
Normalised Counts
0
20
40
60
1−cell
2−cell
128−cell
1k−cell
Dome
50%−epiboly
Shield
75%−epiboly
1−4−somites
14−19−somites
20−25−somites
Prim−5
Prim−15
Prim−25
Long−pec
Protruding−mouth
Day−4
Day−5
Stage
Transcript
ENSDART00000125728
ENSDART00000152006

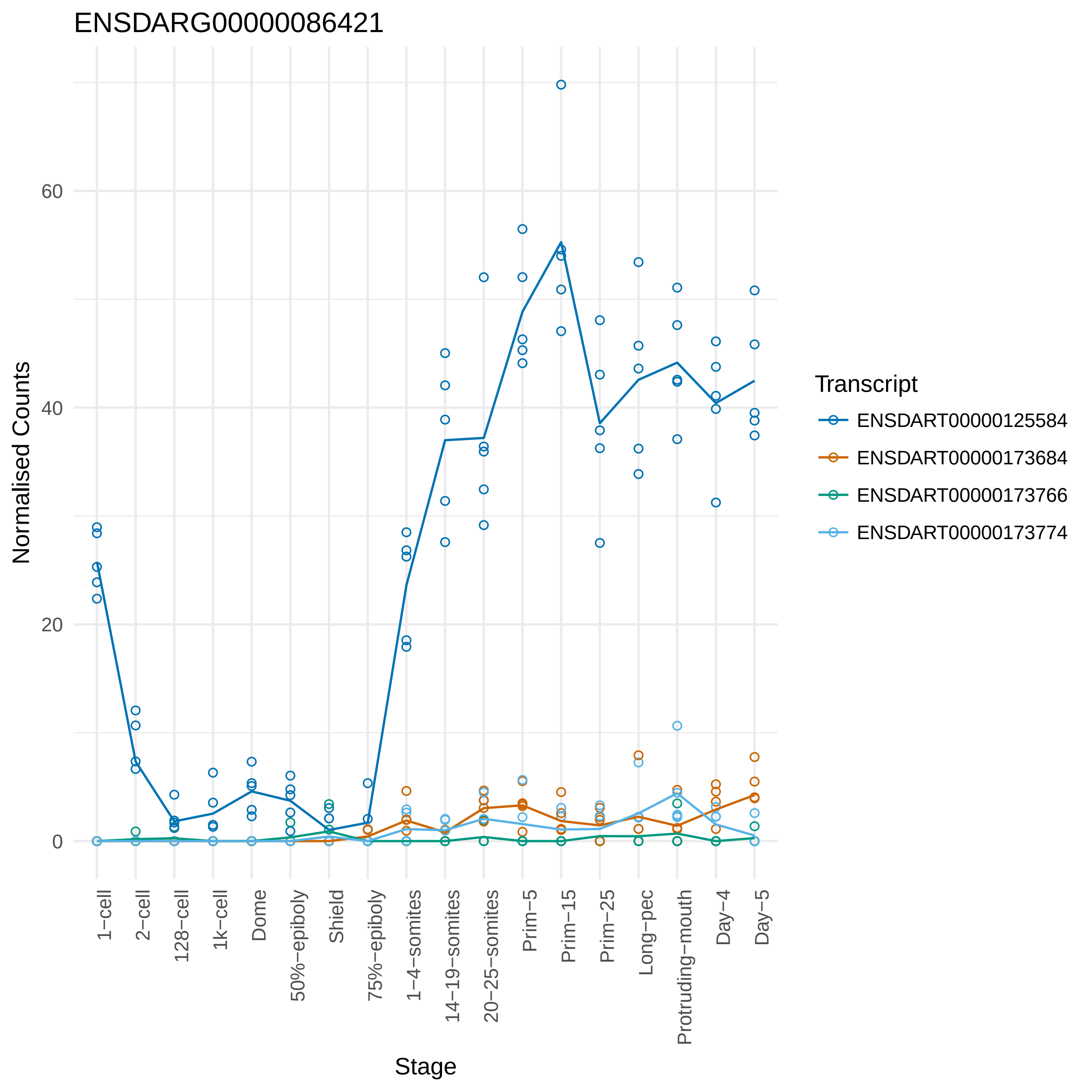
ENSDARG00000086421
Normalised Counts
0
20
40
60
Stage
1−cell
2−cell
128−cell
1k−cell
Dome
50%−epiboly
Shield
75%−epiboly
1−4−somites
14−19−somites
20−25−somites
Prim−5
Prim−15
Prim−25
Long−pec
Protruding−mouth
Day−4
Day−5
Transcript
ENSDART00000125584
ENSDART00000173684
ENSDART00000173766
ENSDART00000173774

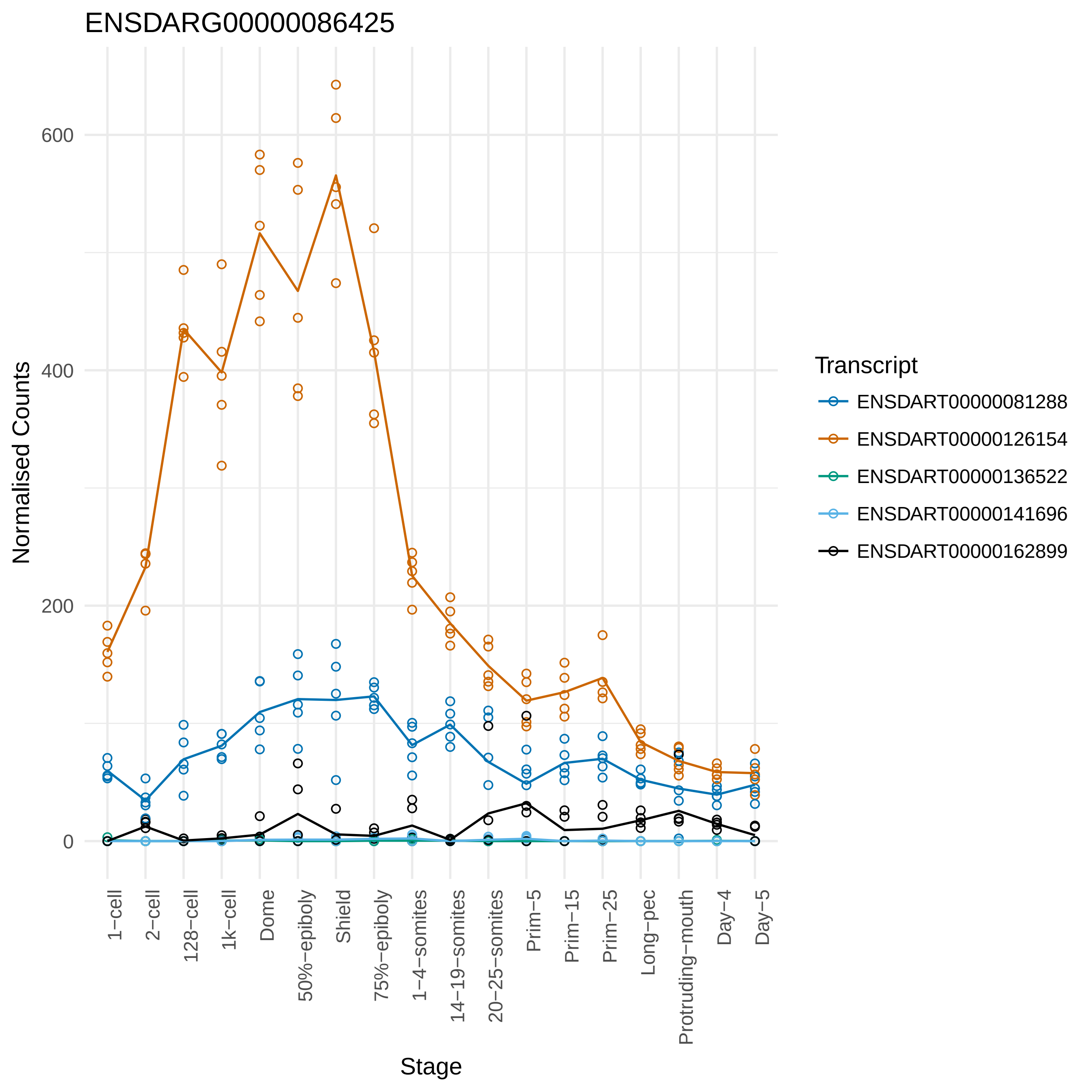
ENSDARG00000086425
Normalised Counts
600
400
200
0
1−cell
2−cell
128−cell
1k−cell
Dome
50%−epiboly
Shield
75%−epiboly
1−4−somites
14−19−somites
20−25−somites
Prim−5
Prim−15
Prim−25
Long−pec
Protruding−mouth
Day−4
Day−5
Stage
Transcript
ENSDART00000081288
ENSDART00000126154
ENSDART00000136522
ENSDART00000141696
ENSDART00000162899

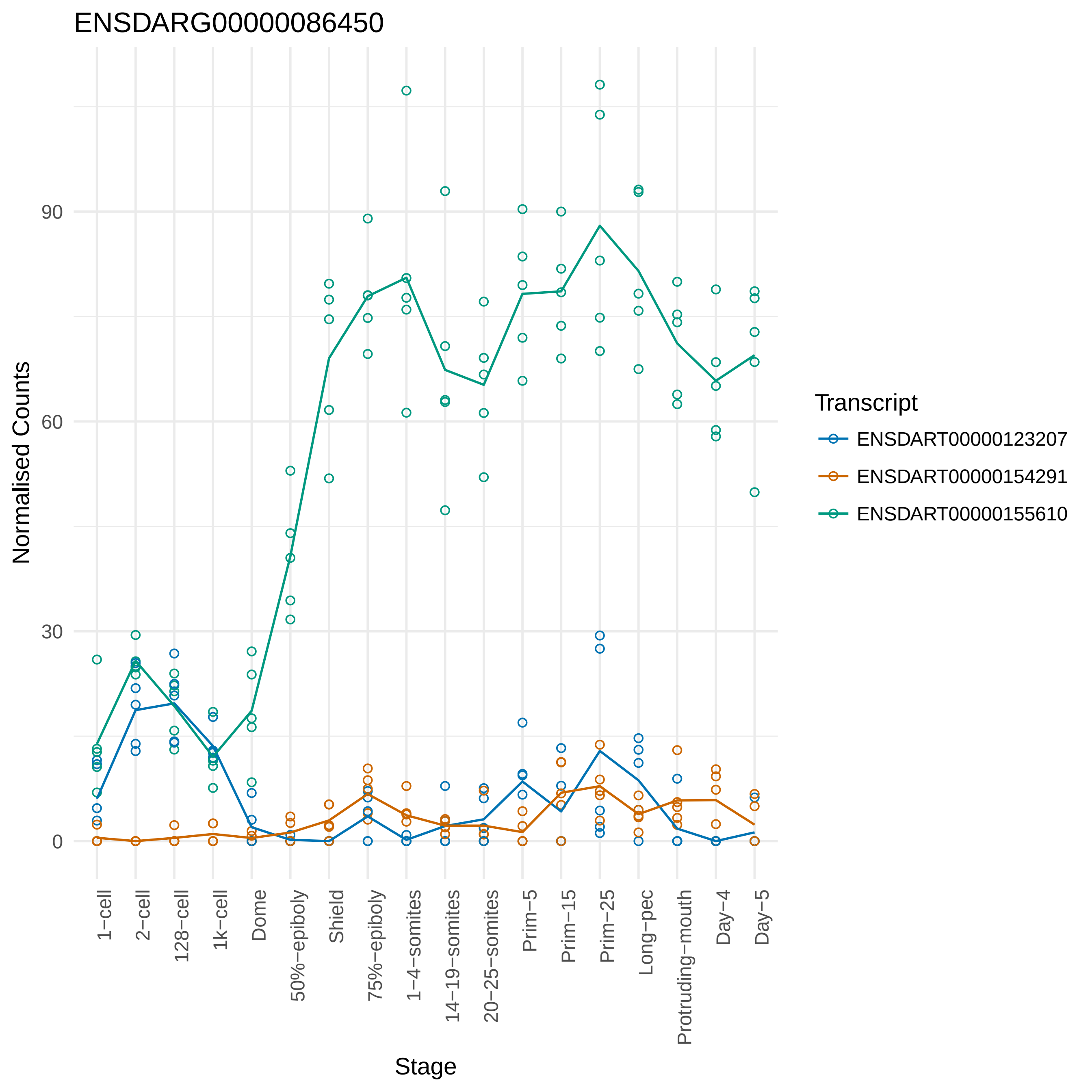

ENSDARG00000086450
Normalised Counts
0
30
60
90
Stage
1−cell
2−cell
128−cell
1k−cell
Dome
50%−epiboly
Shield
75%−epiboly
1−4−somites
14−19−somites
20−25−somites
Prim−5
Prim−15
Prim−25
Long−pec
Protruding−mouth
Day−4
Day−5
Transcript
ENSDART00000123207
ENSDART00000154291
ENSDART00000155610

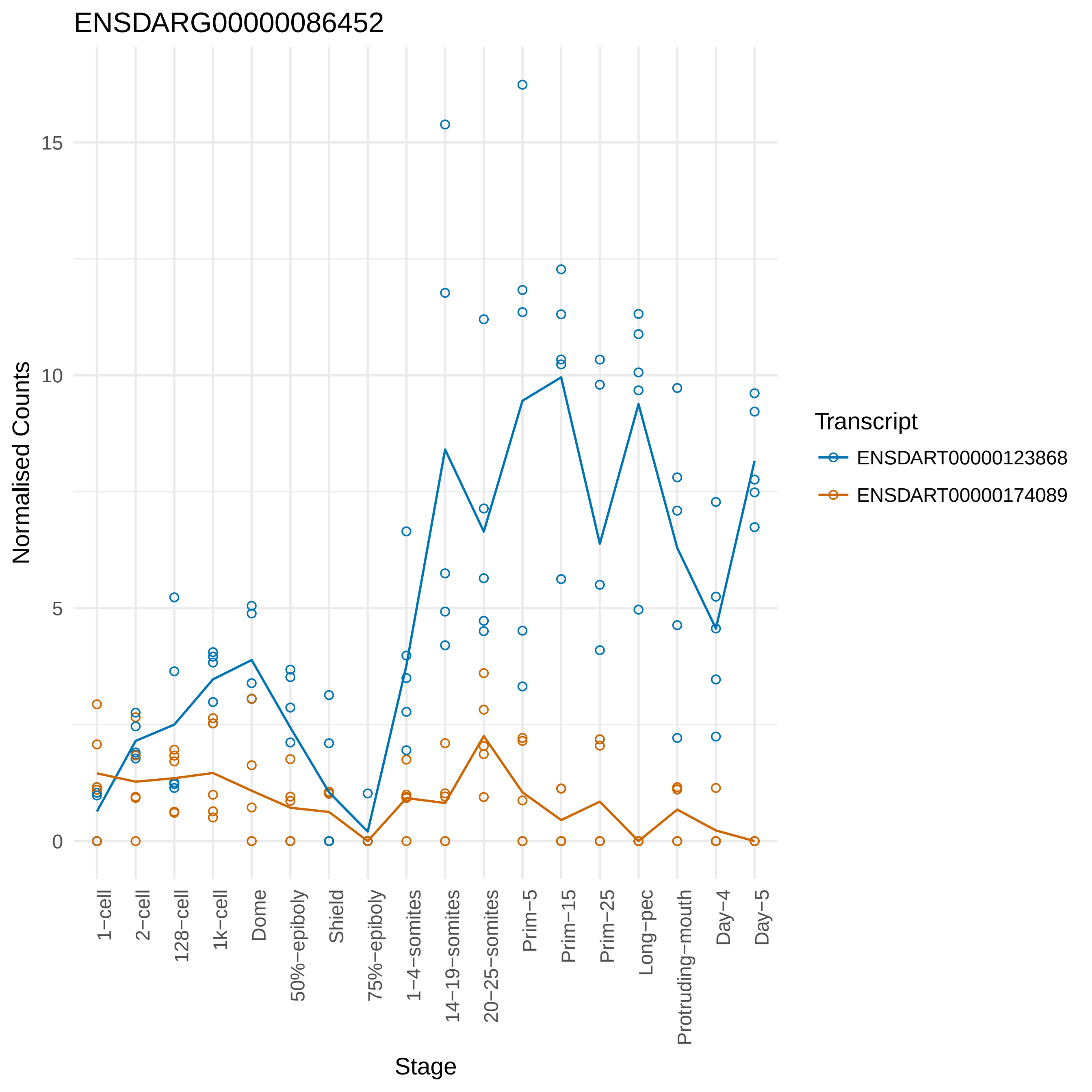

ENSDARG00000086452
Normalised Counts
0
5
10
15
Stage
1−cell
2−cell
128−cell
1k−cell
Dome
50%−epiboly
Shield
75%−epiboly
1−4−somites
14−19−somites
20−25−somites
Prim−5
Prim−15
Prim−25
Long−pec
Protruding−mouth
Day−4
Day−5
Transcript
ENSDART00000123868
ENSDART00000174089

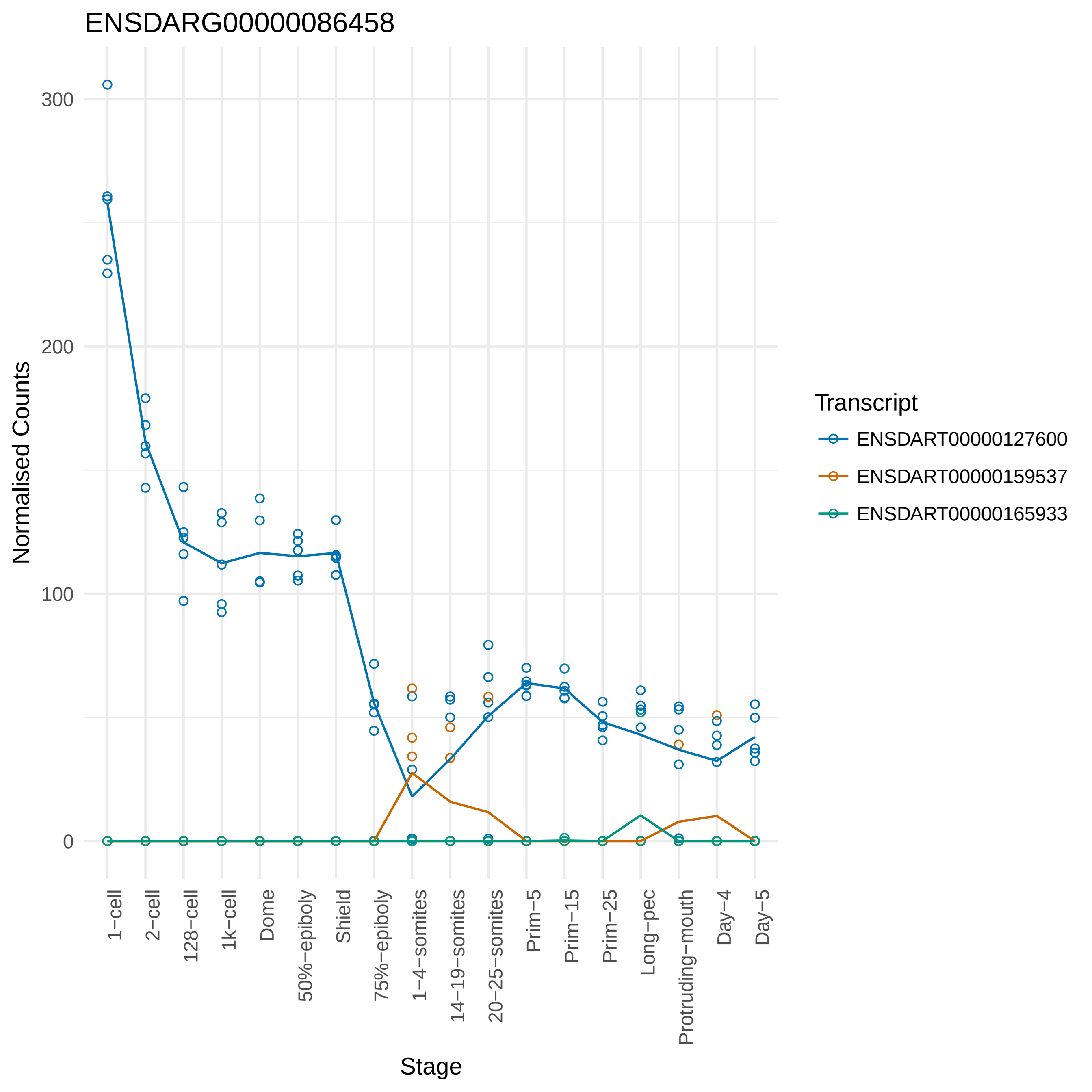
ENSDARG00000086458
Normalised Counts
300
200
100
0
1−cell
2−cell
128−cell
1k−cell
Dome
50%−epiboly
Shield
75%−epiboly
1−4−somites
14−19−somites
20−25−somites
Prim−5
Prim−15
Prim−25
Long−pec
Protruding−mouth
Day−4
Day−5
Stage
Transcript
ENSDART00000127600
ENSDART00000159537
ENSDART00000165933

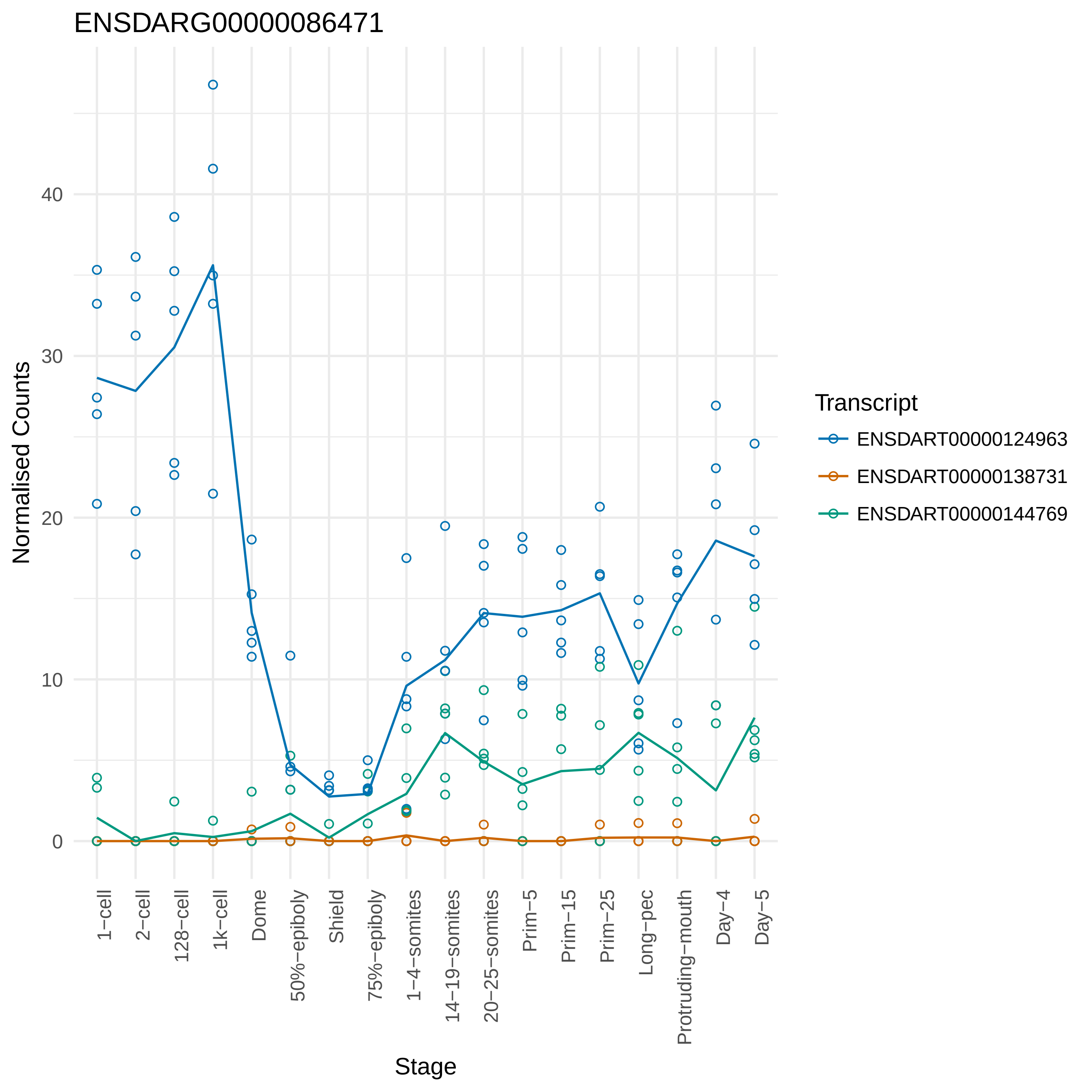
ENSDARG00000086471
Normalised Counts
0
10
20
30
40
1−cell
2−cell
128−cell
1k−cell
Dome
50%−epiboly
Shield
75%−epiboly
1−4−somites
14−19−somites
20−25−somites
Prim−5
Prim−15
Prim−25
Long−pec
Protruding−mouth
Day−4
Day−5
Stage
Transcript
ENSDART00000124963
ENSDART00000138731
ENSDART00000144769

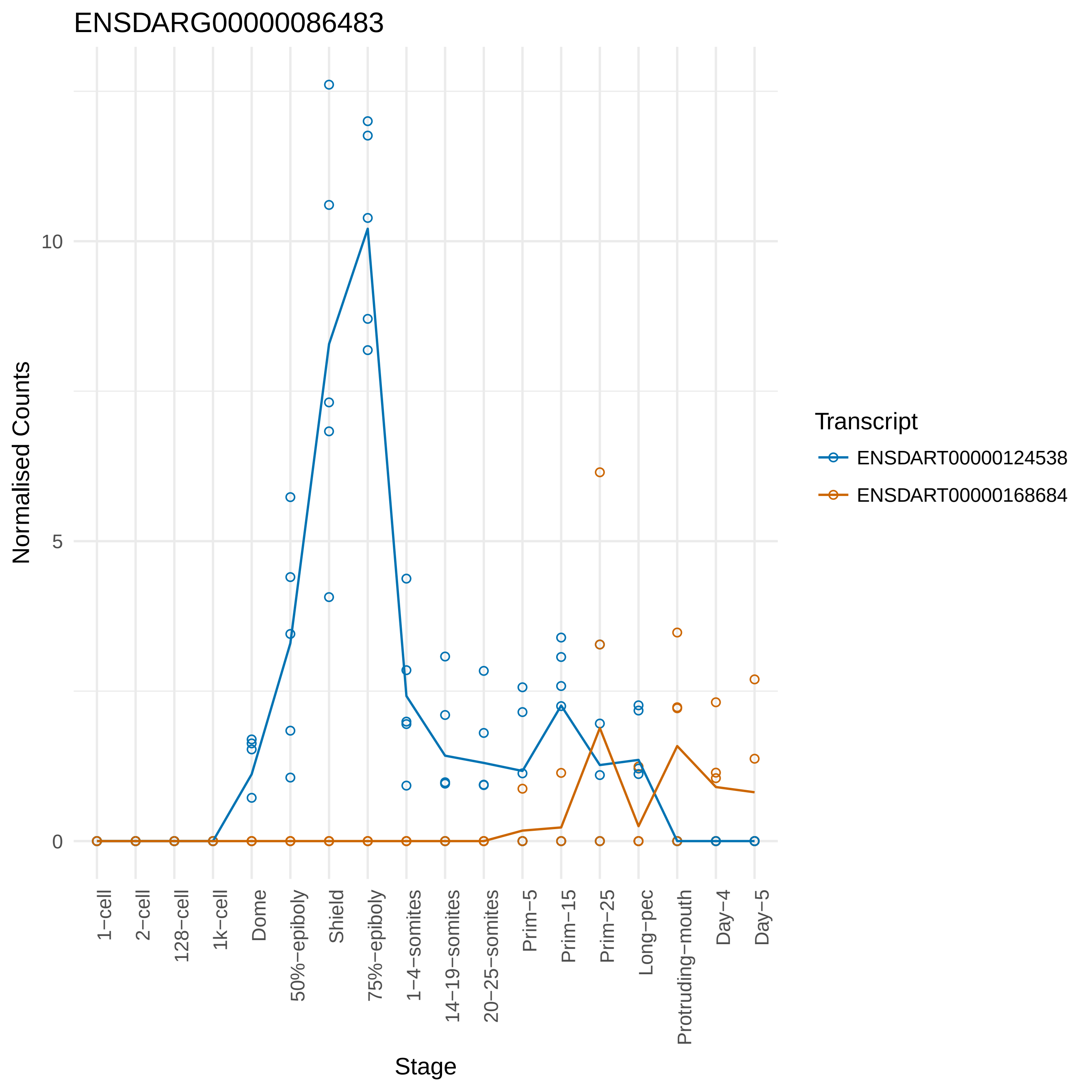

ENSDARG00000086483
Normalised Counts
10
5
0
1−cell
2−cell
128−cell
1k−cell
Dome
50%−epiboly
Shield
75%−epiboly
1−4−somites
14−19−somites
20−25−somites
Prim−5
Prim−15
Prim−25
Long−pec
Protruding−mouth
Day−4
Day−5
Stage
Transcript
ENSDART00000124538
ENSDART00000168684

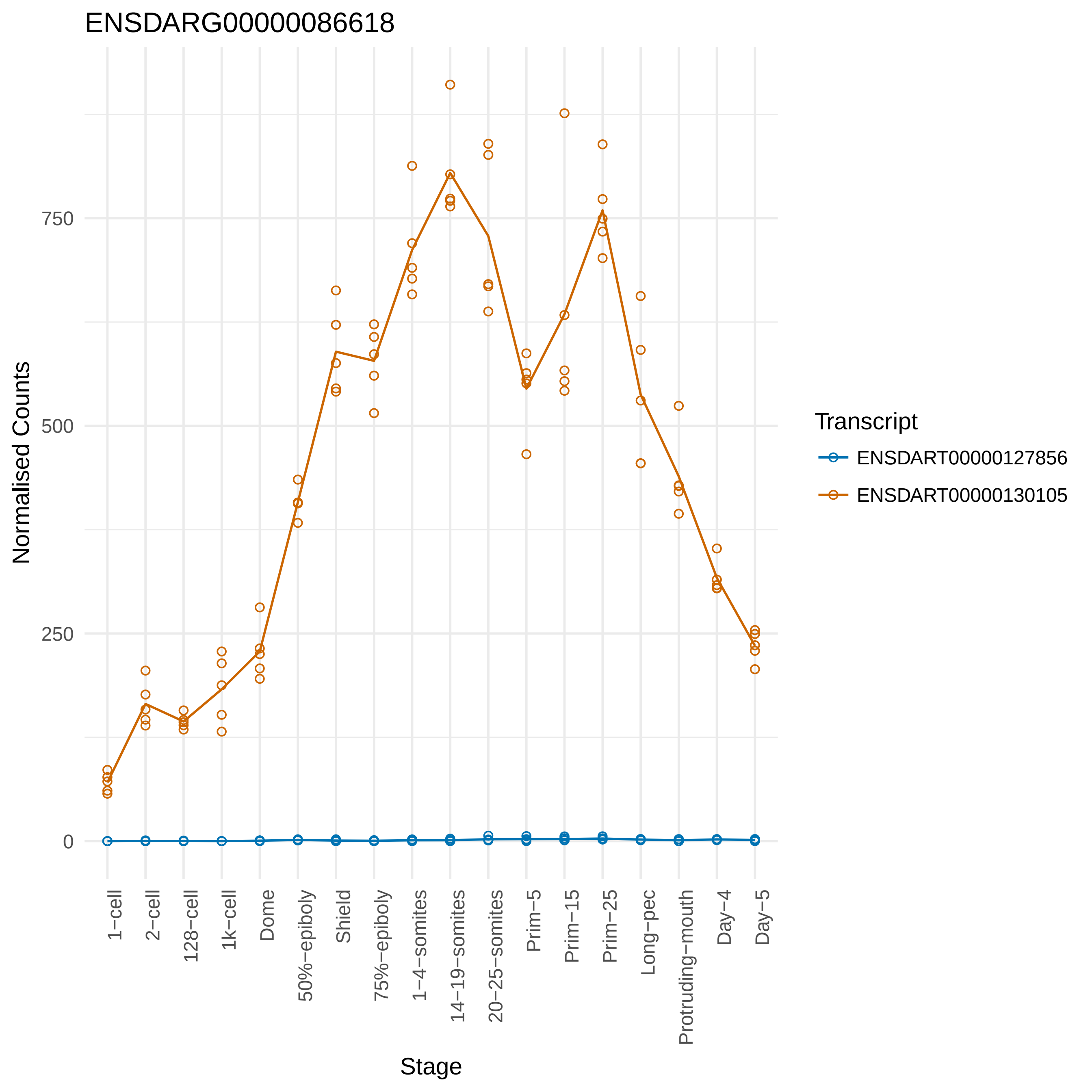

ENSDARG00000086618
Normalised Counts
750
500
250
0
1−cell
2−cell
128−cell
1k−cell
Dome
50%−epiboly
Shield
75%−epiboly
1−4−somites
14−19−somites
20−25−somites
Prim−5
Prim−15
Prim−25
Long−pec
Protruding−mouth
Day−4
Day−5
Stage
Transcript
ENSDART00000127856
ENSDART00000130105

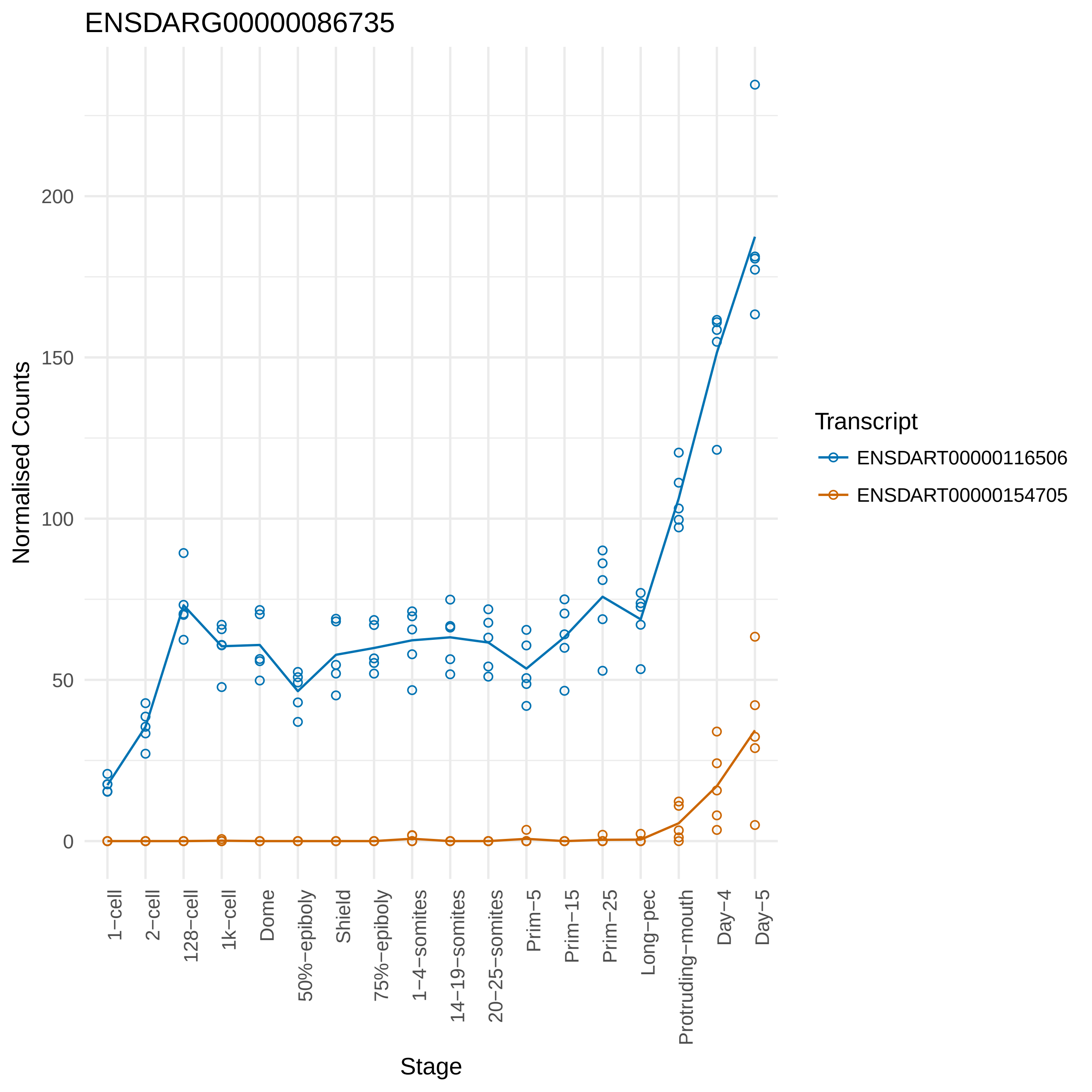

ENSDARG00000086735
Normalised Counts
0
50
100
150
200
1−cell
2−cell
128−cell
1k−cell
Dome
50%−epiboly
Shield
75%−epiboly
1−4−somites
14−19−somites
20−25−somites
Prim−5
Prim−15
Prim−25
Long−pec
Protruding−mouth
Day−4
Day−5
Stage
Transcript
ENSDART00000116506
ENSDART00000154705

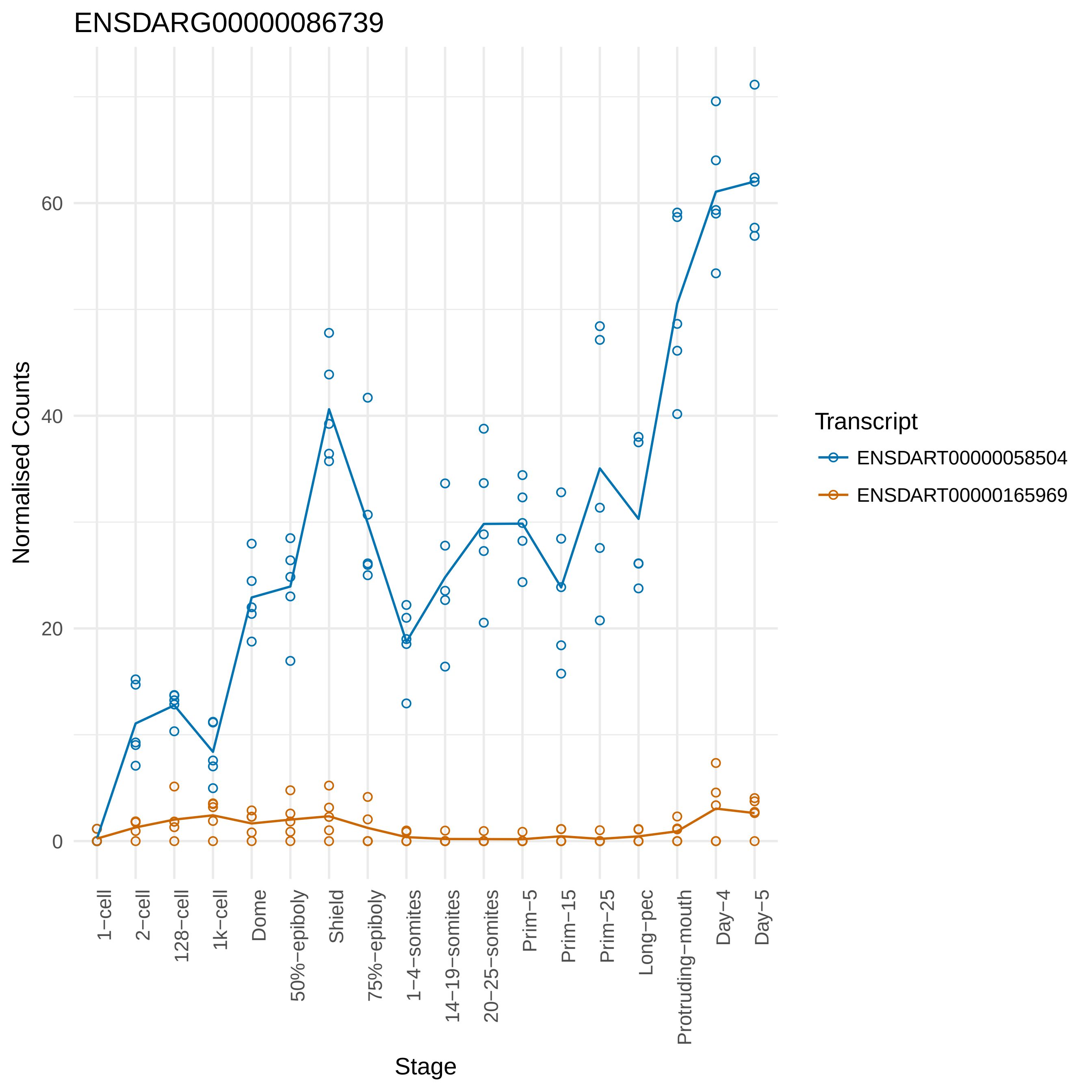
ENSDARG00000086739
Normalised Counts
0
20
40
60
Stage
1−cell
2−cell
128−cell
1k−cell
Dome
50%−epiboly
Shield
75%−epiboly
1−4−somites
14−19−somites
20−25−somites
Prim−5
Prim−15
Prim−25
Long−pec
Protruding−mouth
Day−4
Day−5
Transcript
ENSDART00000058504
ENSDART00000165969

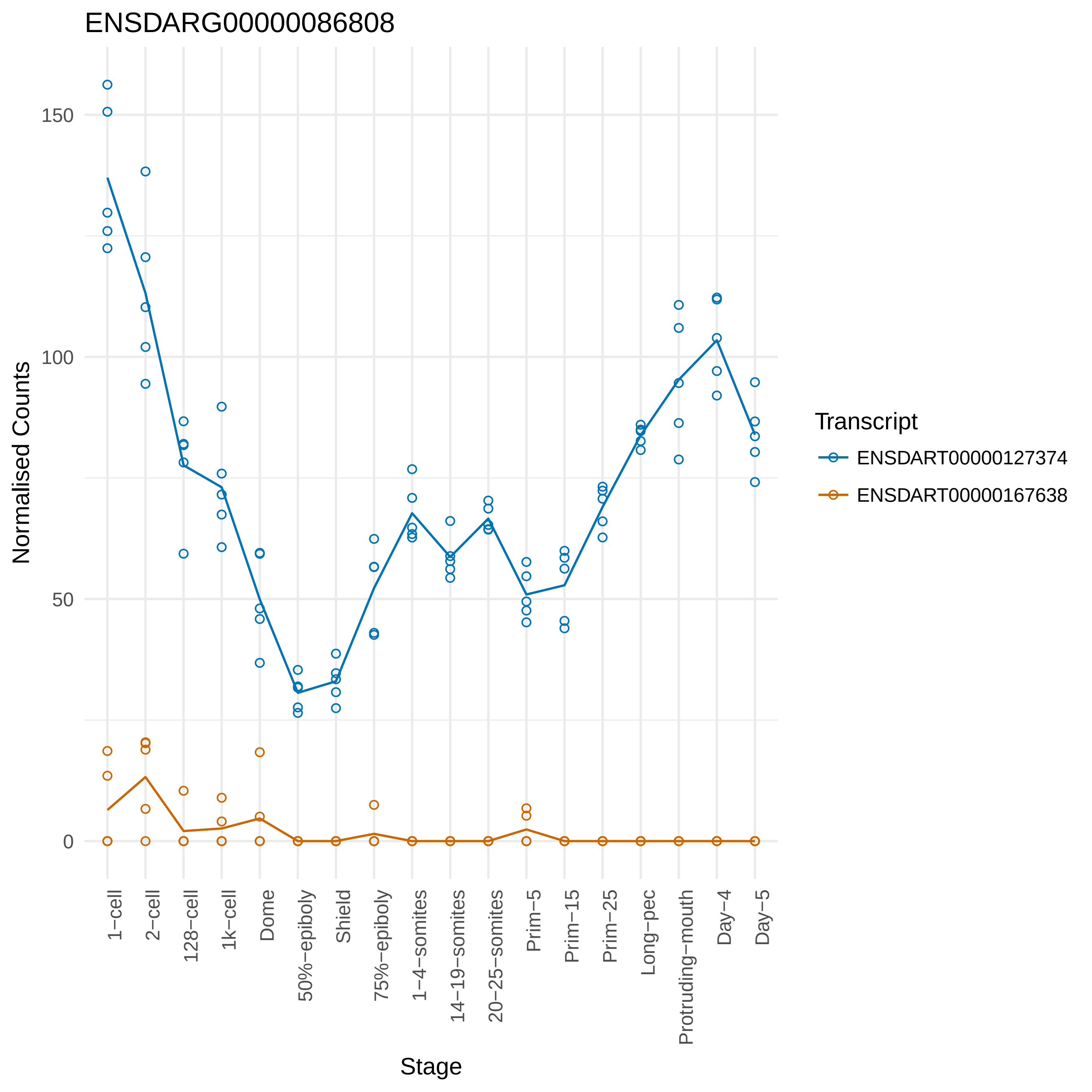

ENSDARG00000086808
Normalised Counts
150
100
50
0
1−cell
2−cell
128−cell
1k−cell
Dome
50%−epiboly
Shield
75%−epiboly
1−4−somites
14−19−somites
20−25−somites
Prim−5
Prim−15
Prim−25
Long−pec
Protruding−mouth
Day−4
Day−5
Stage
Transcript
ENSDART00000127374
ENSDART00000167638

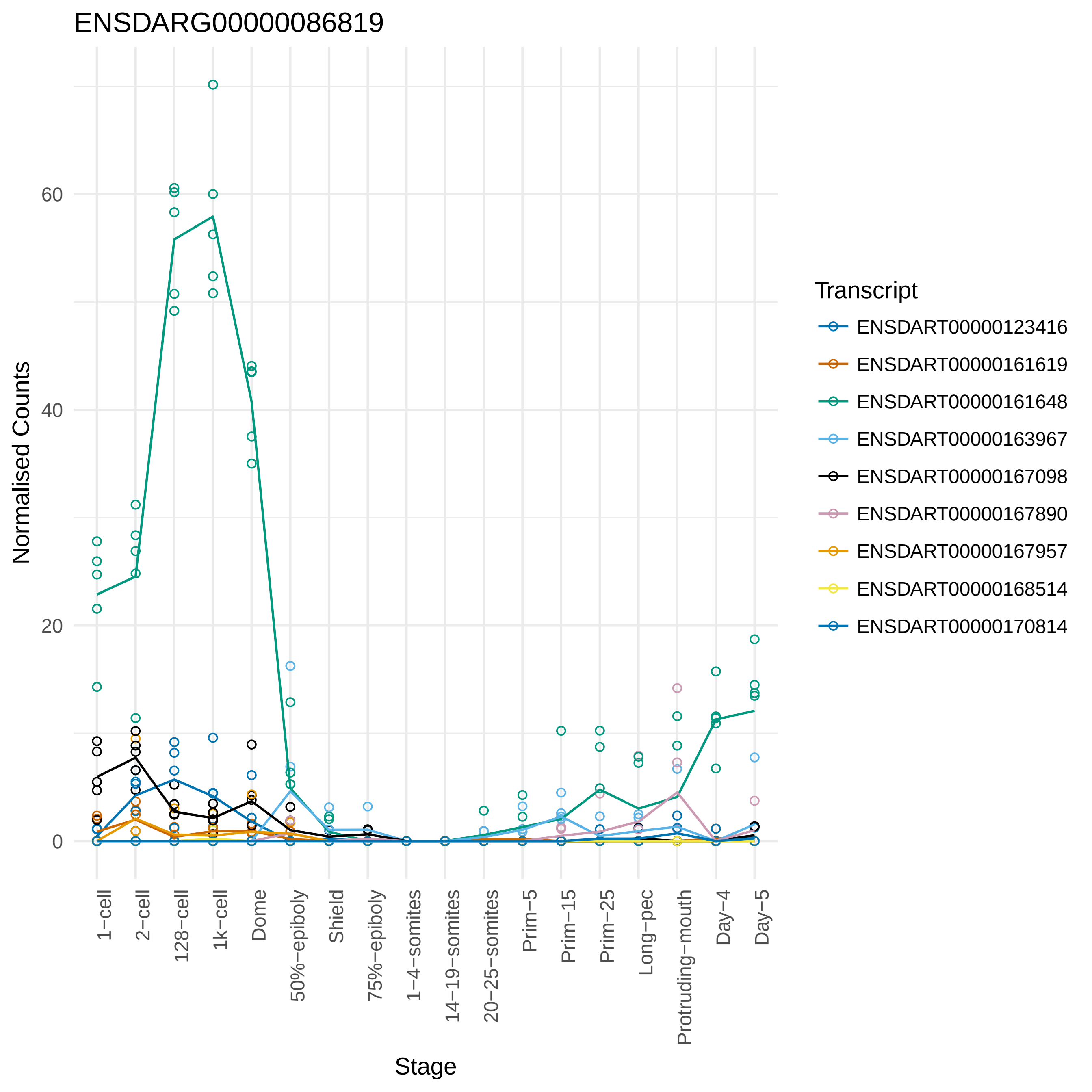

ENSDARG00000086819
Normalised Counts
0
20
40
60
1−cell
2−cell
128−cell
1k−cell
Dome
50%−epiboly
Shield
75%−epiboly
1−4−somites
14−19−somites
20−25−somites
Prim−5
Prim−15
Prim−25
Long−pec
Protruding−mouth
Day−4
Day−5
Stage
Transcript
ENSDART00000123416
ENSDART00000161619
ENSDART00000161648
ENSDART00000163967
ENSDART00000167098
ENSDART00000167890
ENSDART00000167957
ENSDART00000168514
ENSDART00000170814

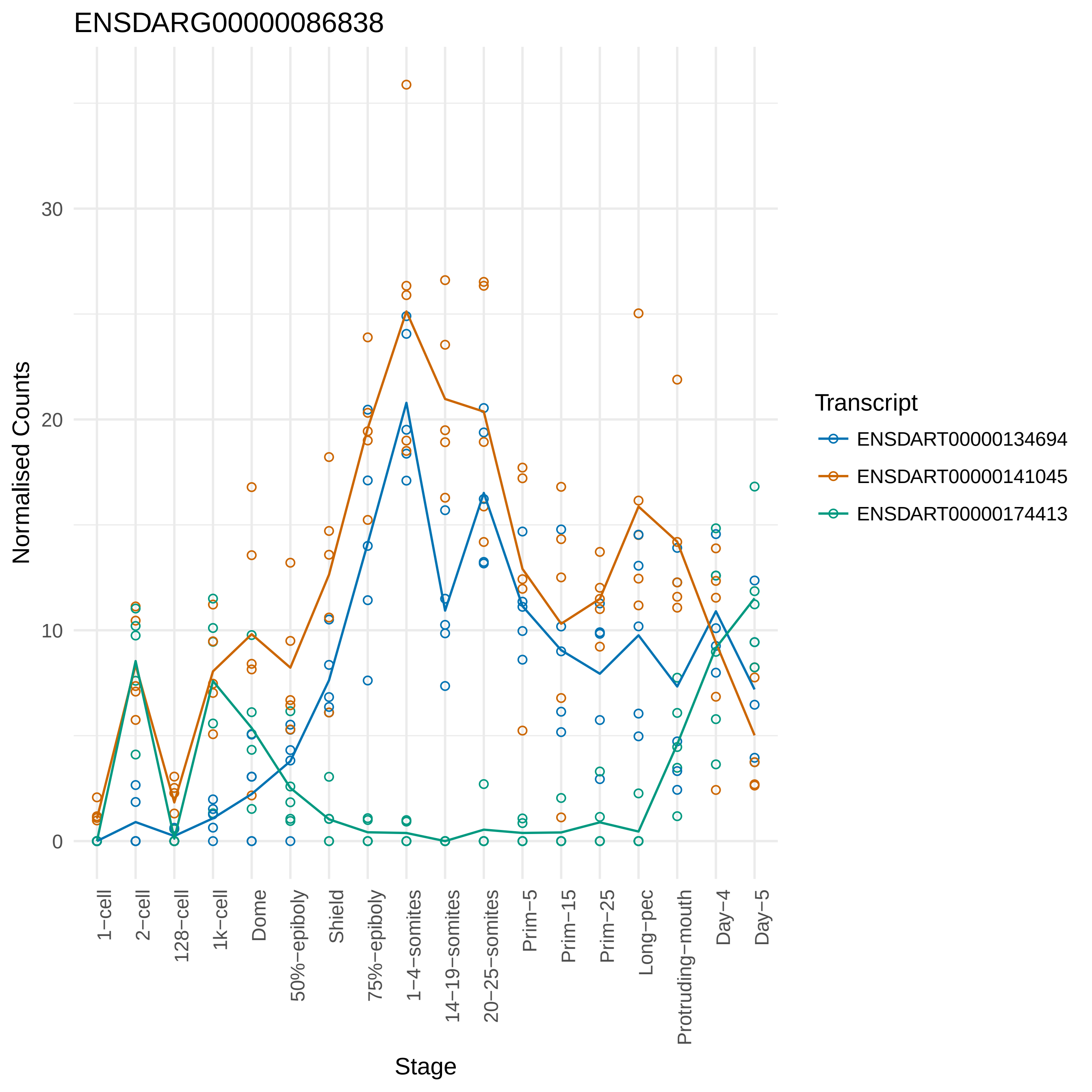

ENSDARG00000086838
Normalised Counts
30
20
10
0
1−cell
2−cell
128−cell
1k−cell
Dome
50%−epiboly
Shield
75%−epiboly
1−4−somites
14−19−somites
20−25−somites
Prim−5
Prim−15
Prim−25
Long−pec
Protruding−mouth
Day−4
Day−5
Stage
Transcript
ENSDART00000134694
ENSDART00000141045
ENSDART00000174413

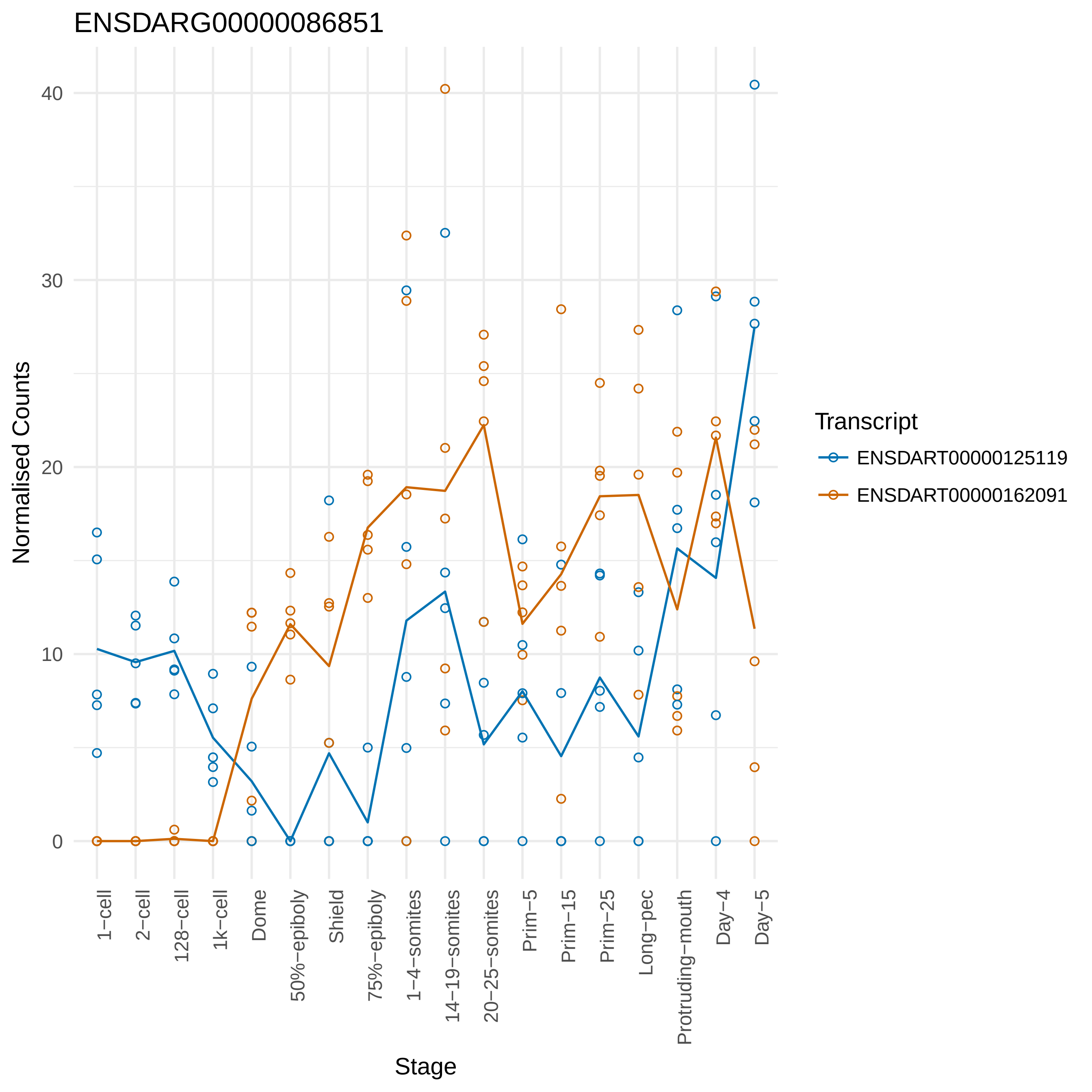

ENSDARG00000086851
Normalised Counts
0
10
20
30
40
1−cell
2−cell
128−cell
1k−cell
Dome
50%−epiboly
Shield
75%−epiboly
1−4−somites
14−19−somites
20−25−somites
Prim−5
Prim−15
Prim−25
Long−pec
Protruding−mouth
Day−4
Day−5
Stage
Transcript
ENSDART00000125119
ENSDART00000162091

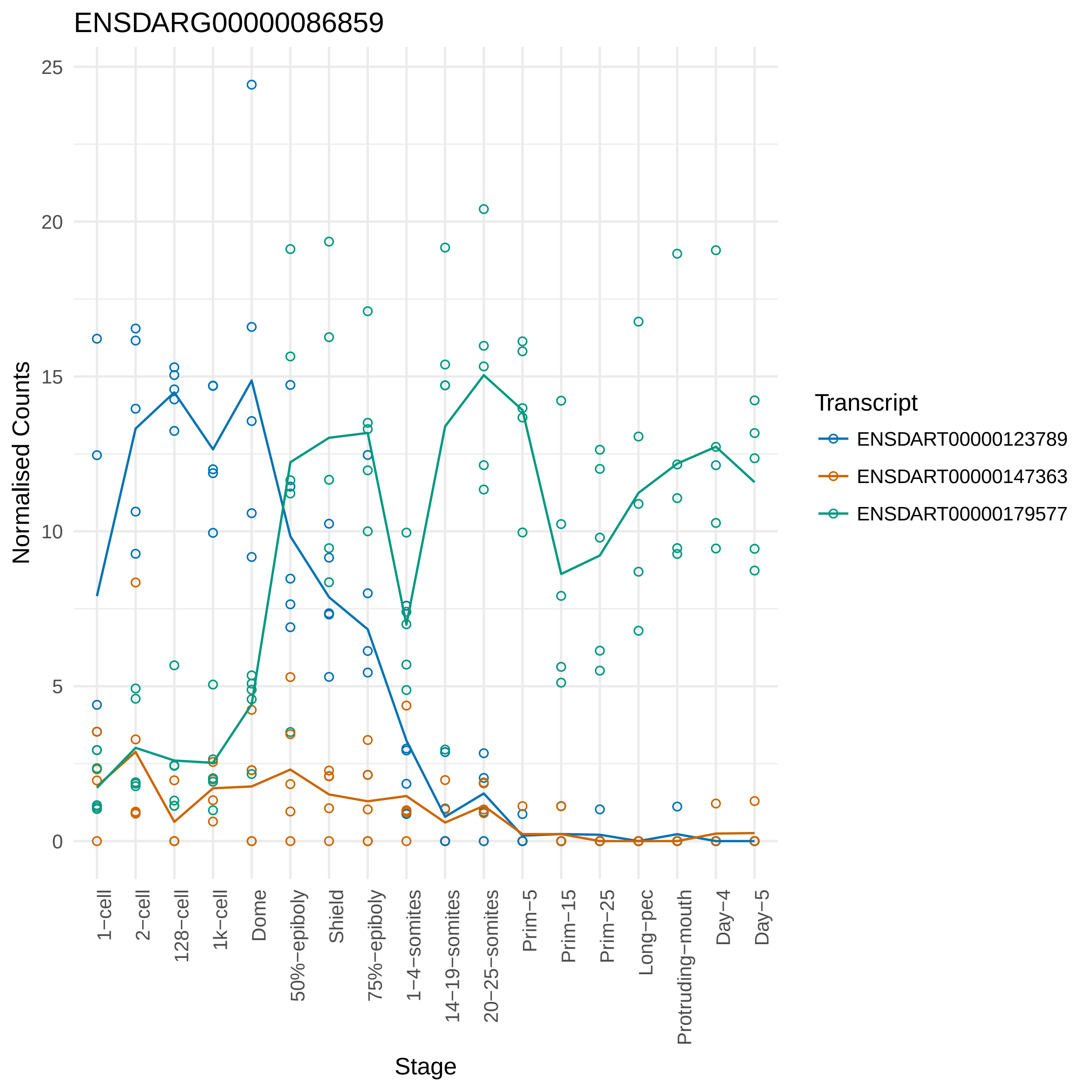
ENSDARG00000086859
Normalised Counts
25
20
15
10
5
0
1−cell
2−cell
128−cell
1k−cell
Dome
50%−epiboly
Shield
75%−epiboly
1−4−somites
14−19−somites
20−25−somites
Prim−5
Prim−15
Prim−25
Long−pec
Protruding−mouth
Day−4
Day−5
Stage
Transcript
ENSDART00000123789
ENSDART00000147363
ENSDART00000179577

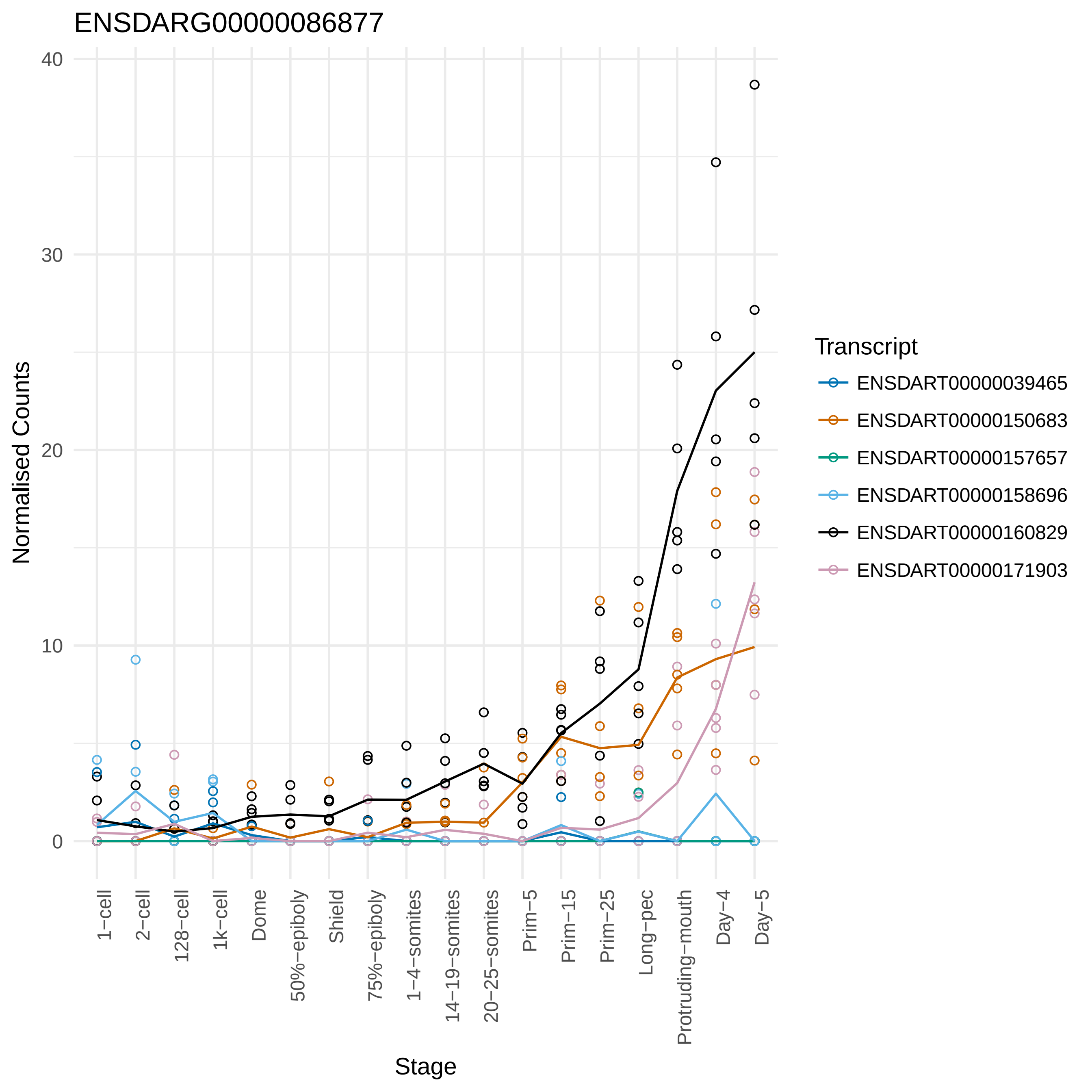

ENSDARG00000086877
Normalised Counts
40
30
20
10
0
1−cell
2−cell
128−cell
1k−cell
Dome
50%−epiboly
Shield
75%−epiboly
1−4−somites
14−19−somites
20−25−somites
Prim−5
Prim−15
Prim−25
Long−pec
Protruding−mouth
Day−4
Day−5
Stage
Transcript
ENSDART00000039465
ENSDART00000150683
ENSDART00000157657
ENSDART00000158696
ENSDART00000160829
ENSDART00000171903

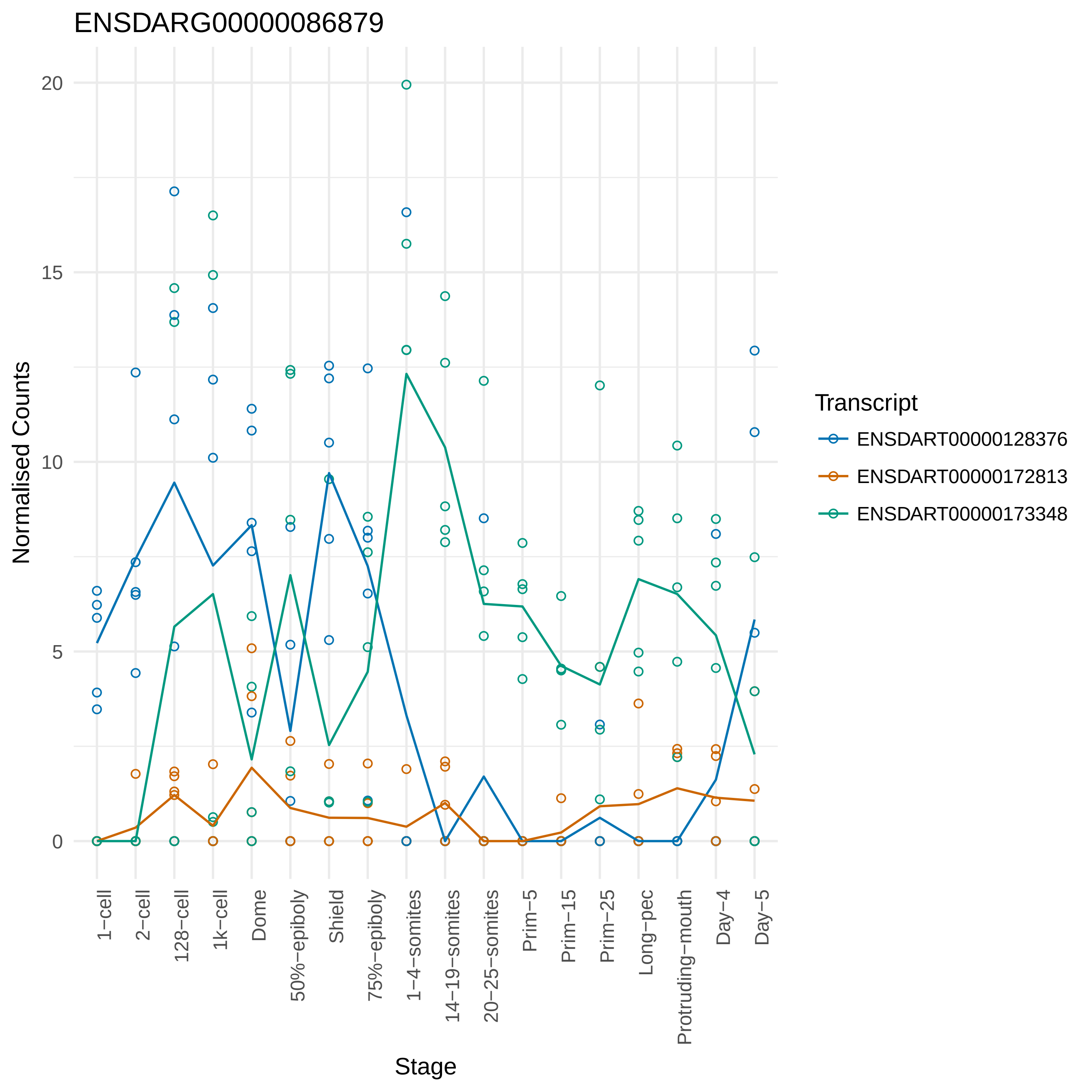
ENSDARG00000086879
Normalised Counts
0
5
10
15
20
Stage
1−cell
2−cell
128−cell
1k−cell
Dome
50%−epiboly
Shield
75%−epiboly
1−4−somites
14−19−somites
20−25−somites
Prim−5
Prim−15
Prim−25
Long−pec
Protruding−mouth
Day−4
Day−5
Transcript
ENSDART00000128376
ENSDART00000172813
ENSDART00000173348

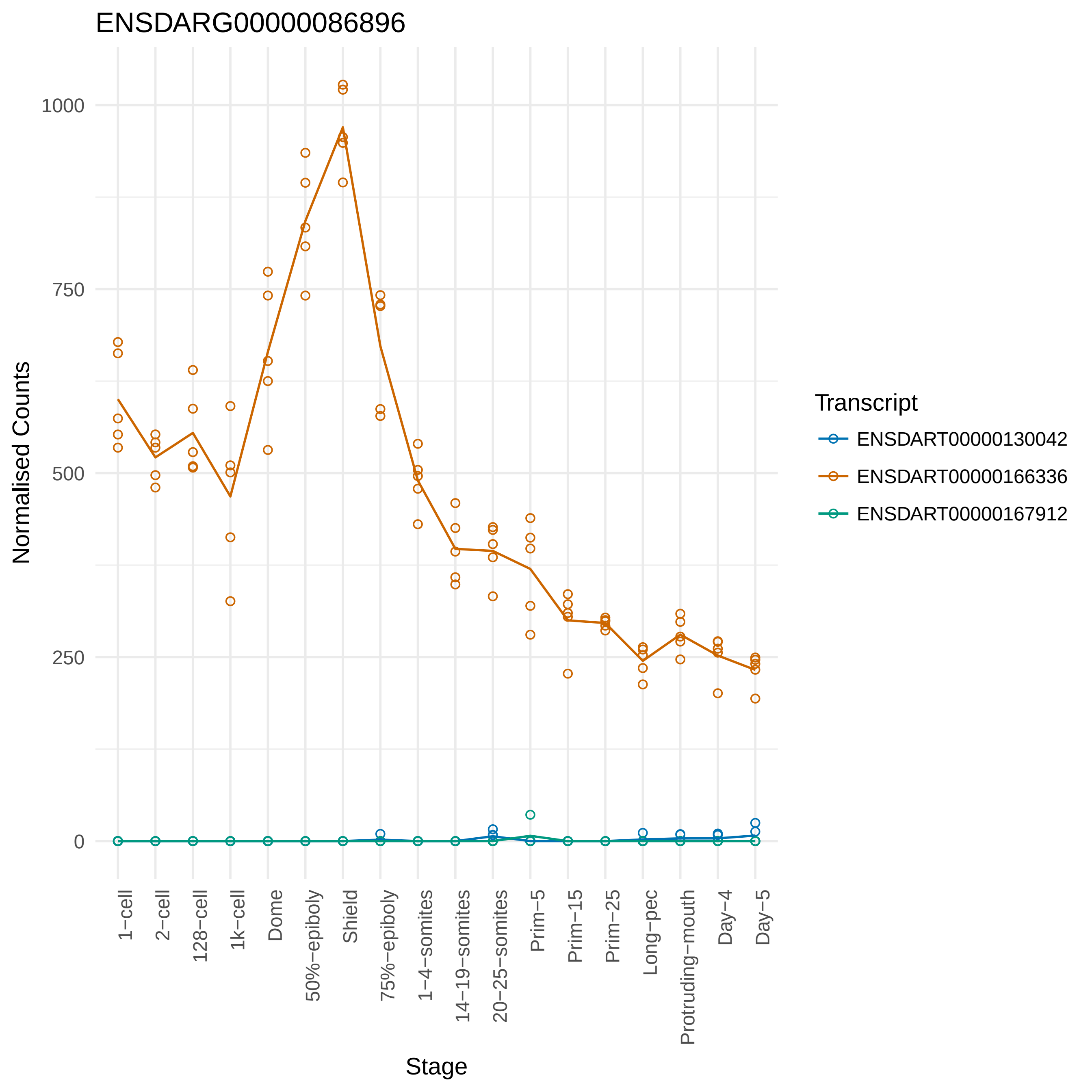

ENSDARG00000086896
Normalised Counts
1000
750
500
250
0
1−cell
2−cell
128−cell
1k−cell
Dome
50%−epiboly
Shield
75%−epiboly
1−4−somites
14−19−somites
20−25−somites
Prim−5
Prim−15
Prim−25
Long−pec
Protruding−mouth
Day−4
Day−5
Stage
Transcript
ENSDART00000130042
ENSDART00000166336
ENSDART00000167912

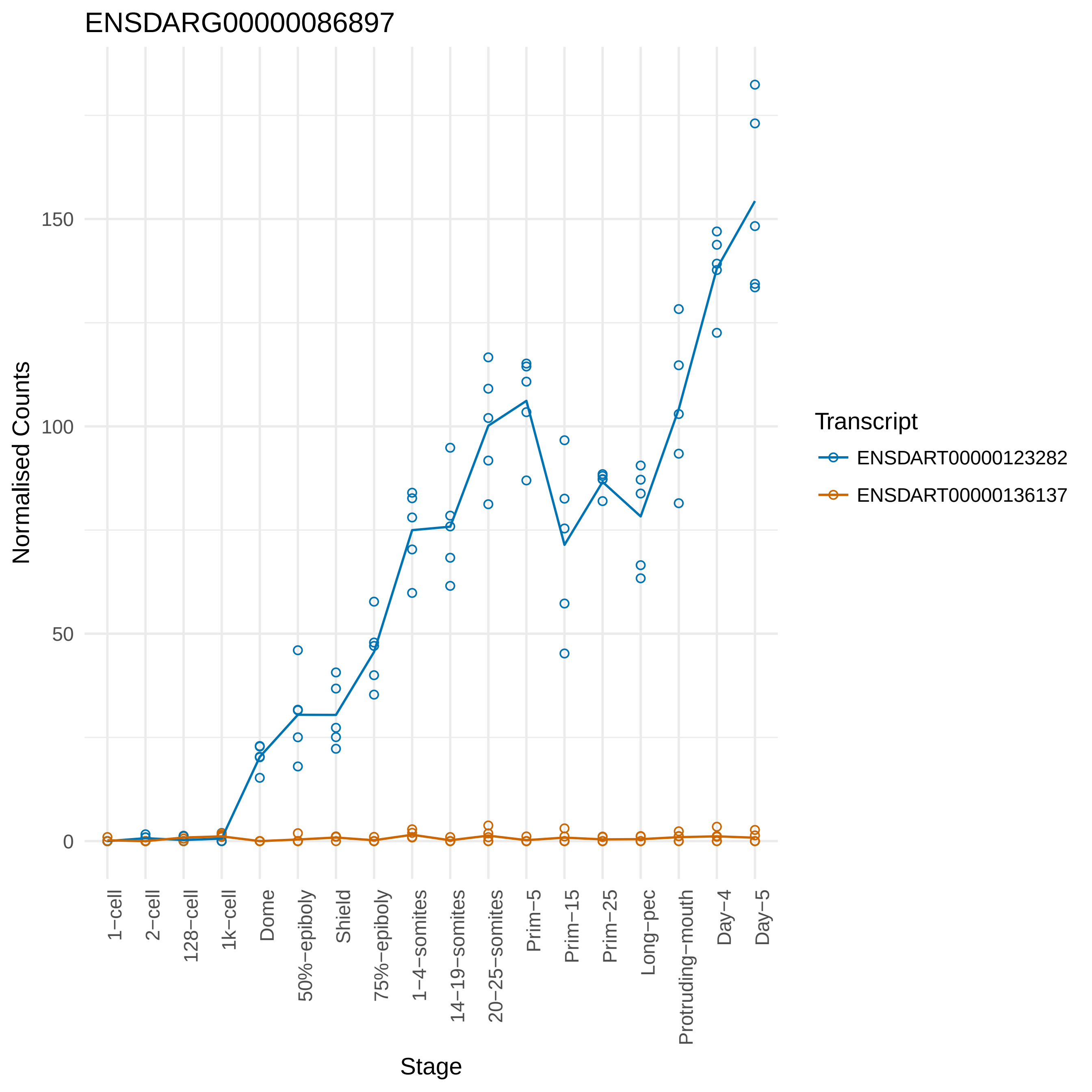
ENSDARG00000086897
Normalised Counts
150
100
50
0
1−cell
2−cell
128−cell
1k−cell
Dome
50%−epiboly
Shield
75%−epiboly
1−4−somites
14−19−somites
20−25−somites
Prim−5
Prim−15
Prim−25
Long−pec
Protruding−mouth
Day−4
Day−5
Stage
Transcript
ENSDART00000123282
ENSDART00000136137

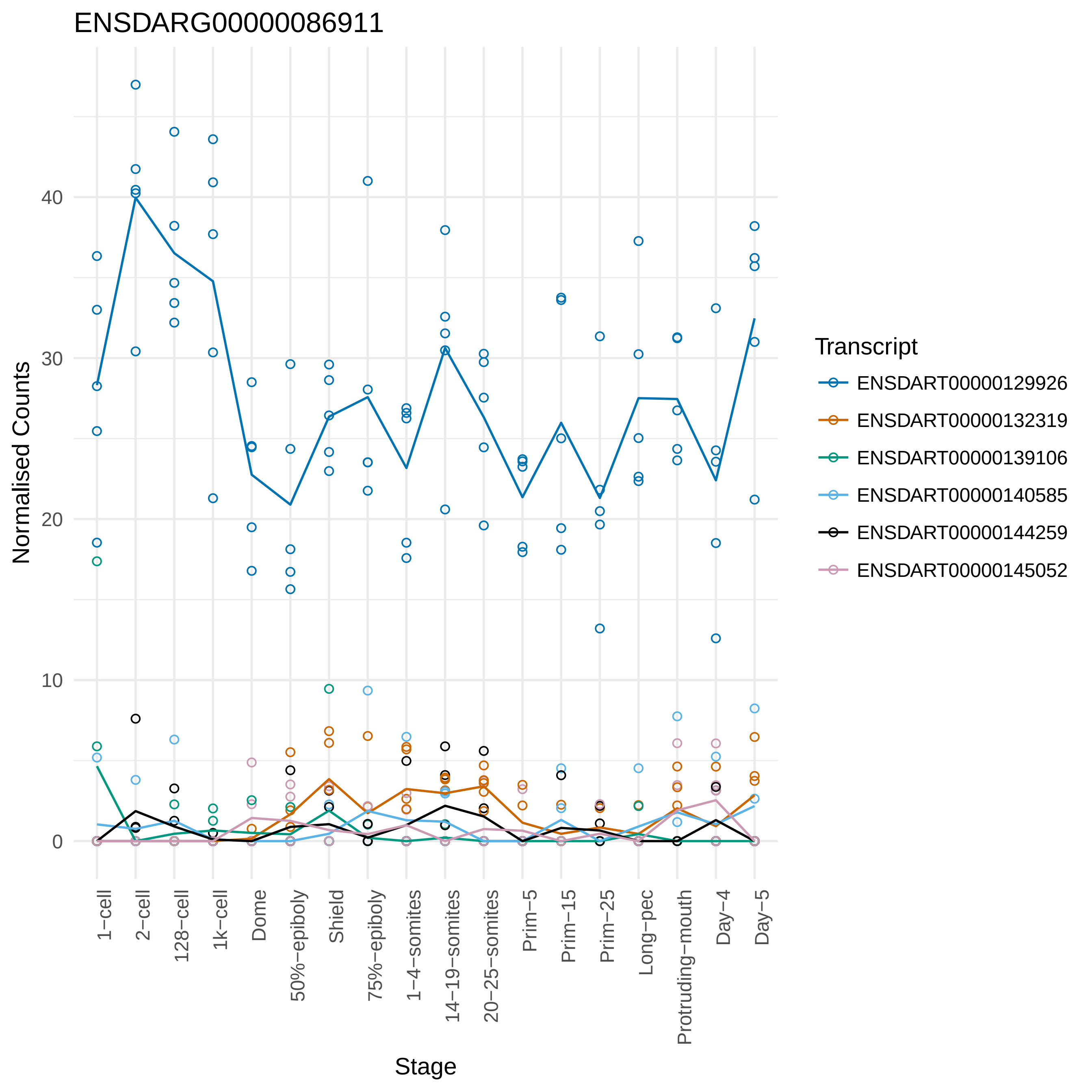
ENSDARG00000086911
Normalised Counts
0
10
20
30
40
Stage
1−cell
2−cell
128−cell
1k−cell
Dome
50%−epiboly
Shield
75%−epiboly
1−4−somites
14−19−somites
20−25−somites
Prim−5
Prim−15
Prim−25
Long−pec
Protruding−mouth
Day−4
Day−5
Transcript
ENSDART00000129926
ENSDART00000132319
ENSDART00000139106
ENSDART00000140585
ENSDART00000144259
ENSDART00000145052

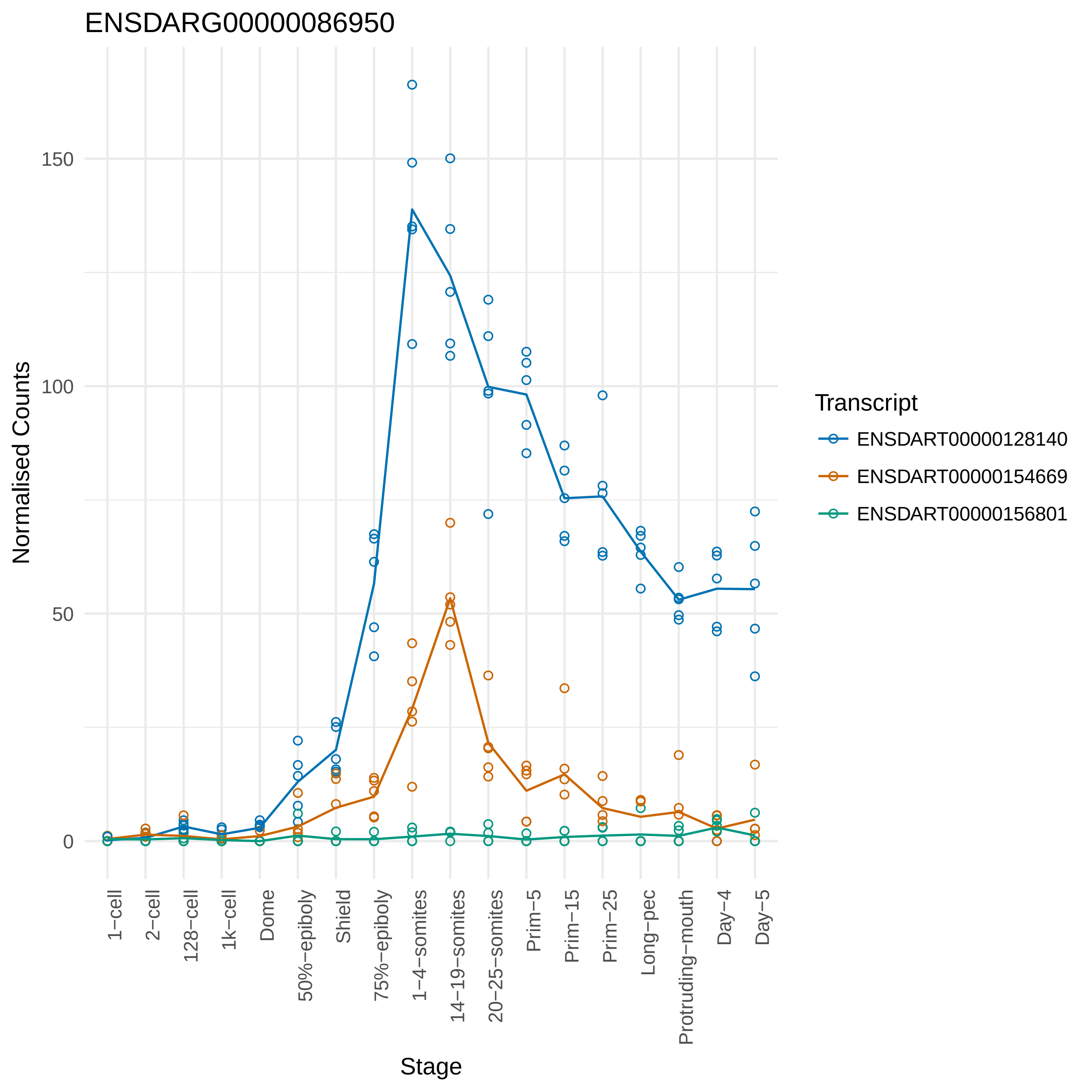

ENSDARG00000086950
Normalised Counts
150
100
50
0
1−cell
2−cell
128−cell
1k−cell
Dome
50%−epiboly
Shield
75%−epiboly
1−4−somites
14−19−somites
20−25−somites
Prim−5
Prim−15
Prim−25
Long−pec
Protruding−mouth
Day−4
Day−5
Stage
Transcript
ENSDART00000128140
ENSDART00000154669
ENSDART00000156801

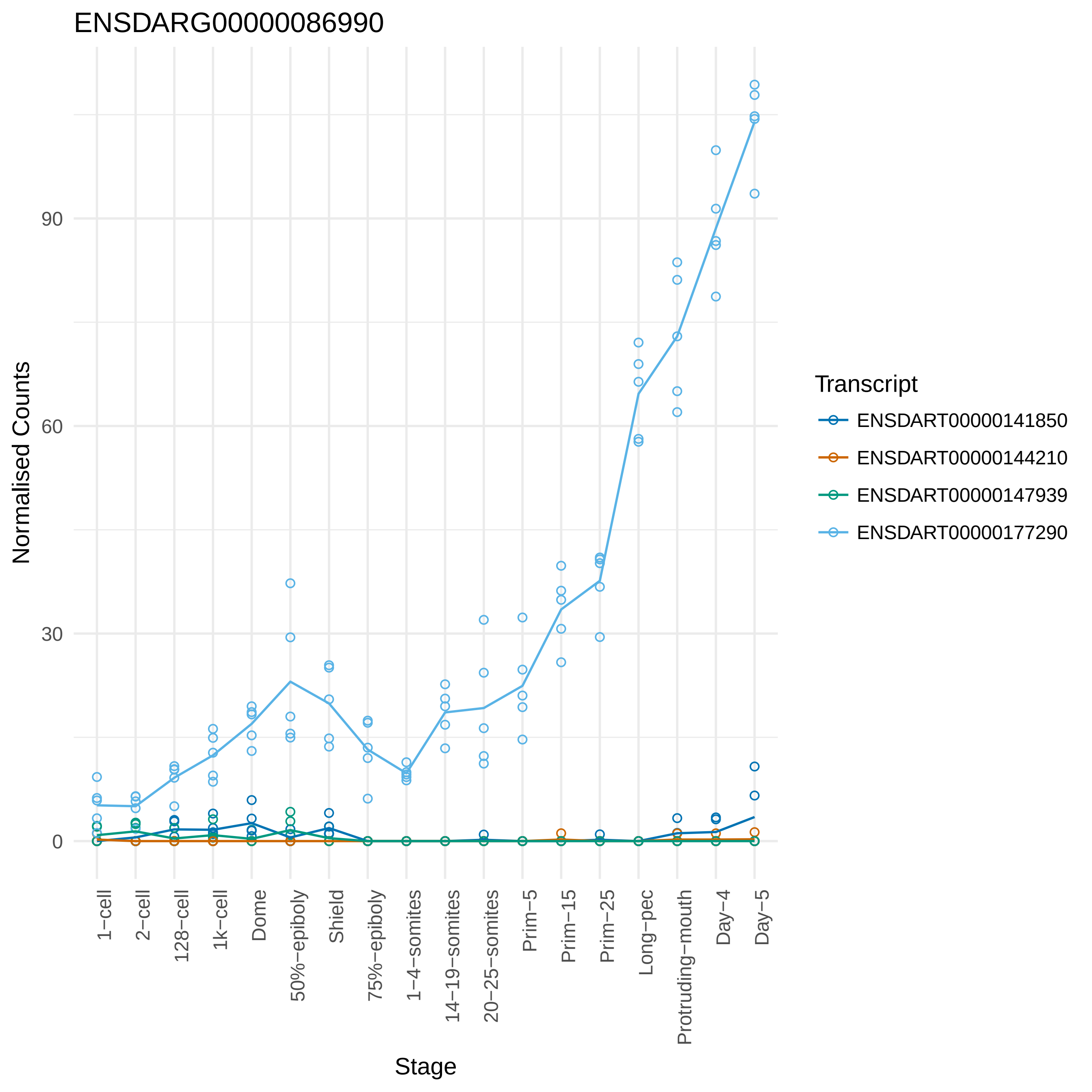

ENSDARG00000086990
Normalised Counts
0
30
60
90
Stage
1−cell
2−cell
128−cell
1k−cell
Dome
50%−epiboly
Shield
75%−epiboly
1−4−somites
14−19−somites
20−25−somites
Prim−5
Prim−15
Prim−25
Long−pec
Protruding−mouth
Day−4
Day−5
Transcript
ENSDART00000141850
ENSDART00000144210
ENSDART00000147939
ENSDART00000177290

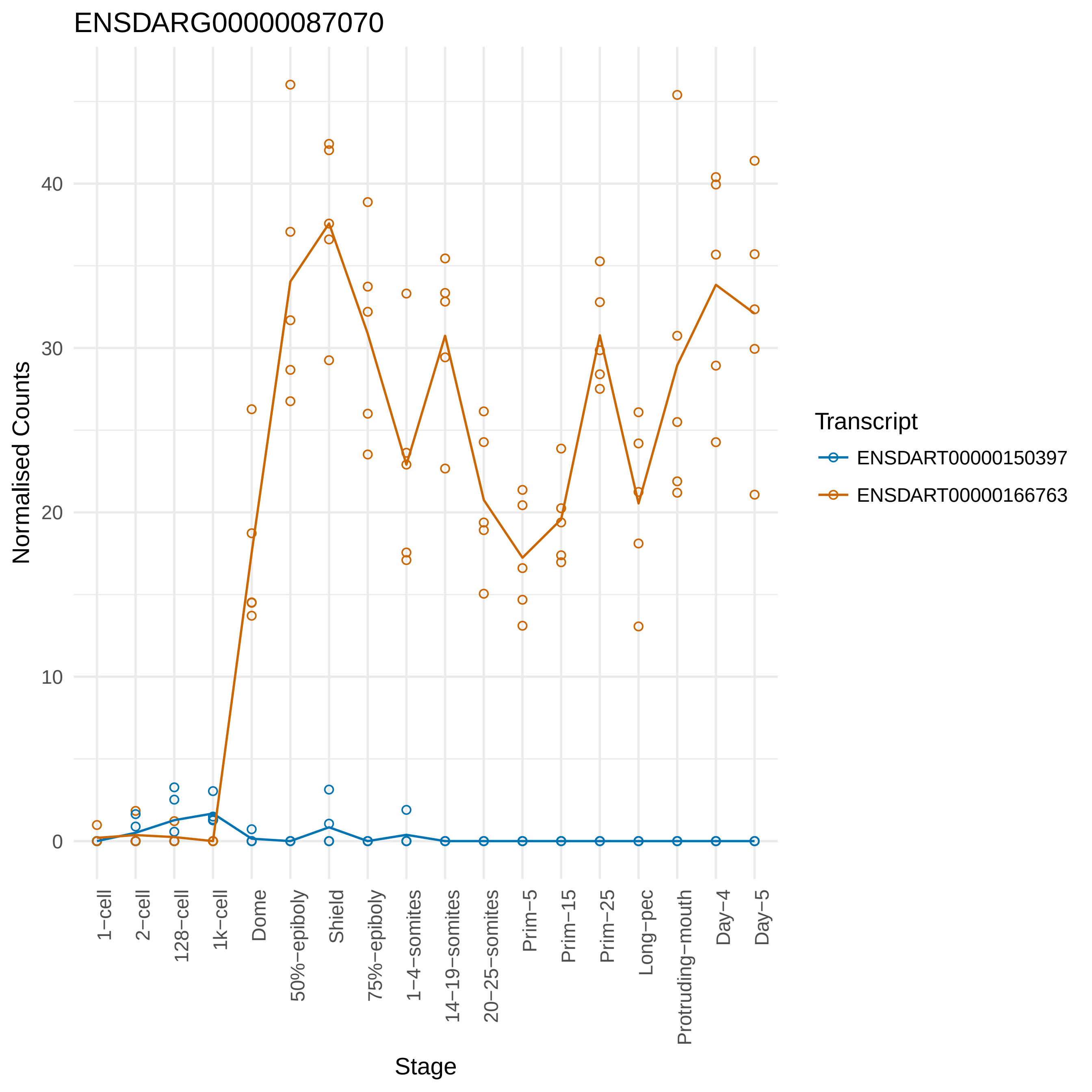
ENSDARG00000087070
Normalised Counts
0
10
20
30
40
Stage
1−cell
2−cell
128−cell
1k−cell
Dome
50%−epiboly
Shield
75%−epiboly
1−4−somites
14−19−somites
20−25−somites
Prim−5
Prim−15
Prim−25
Long−pec
Protruding−mouth
Day−4
Day−5
Transcript
ENSDART00000150397
ENSDART00000166763

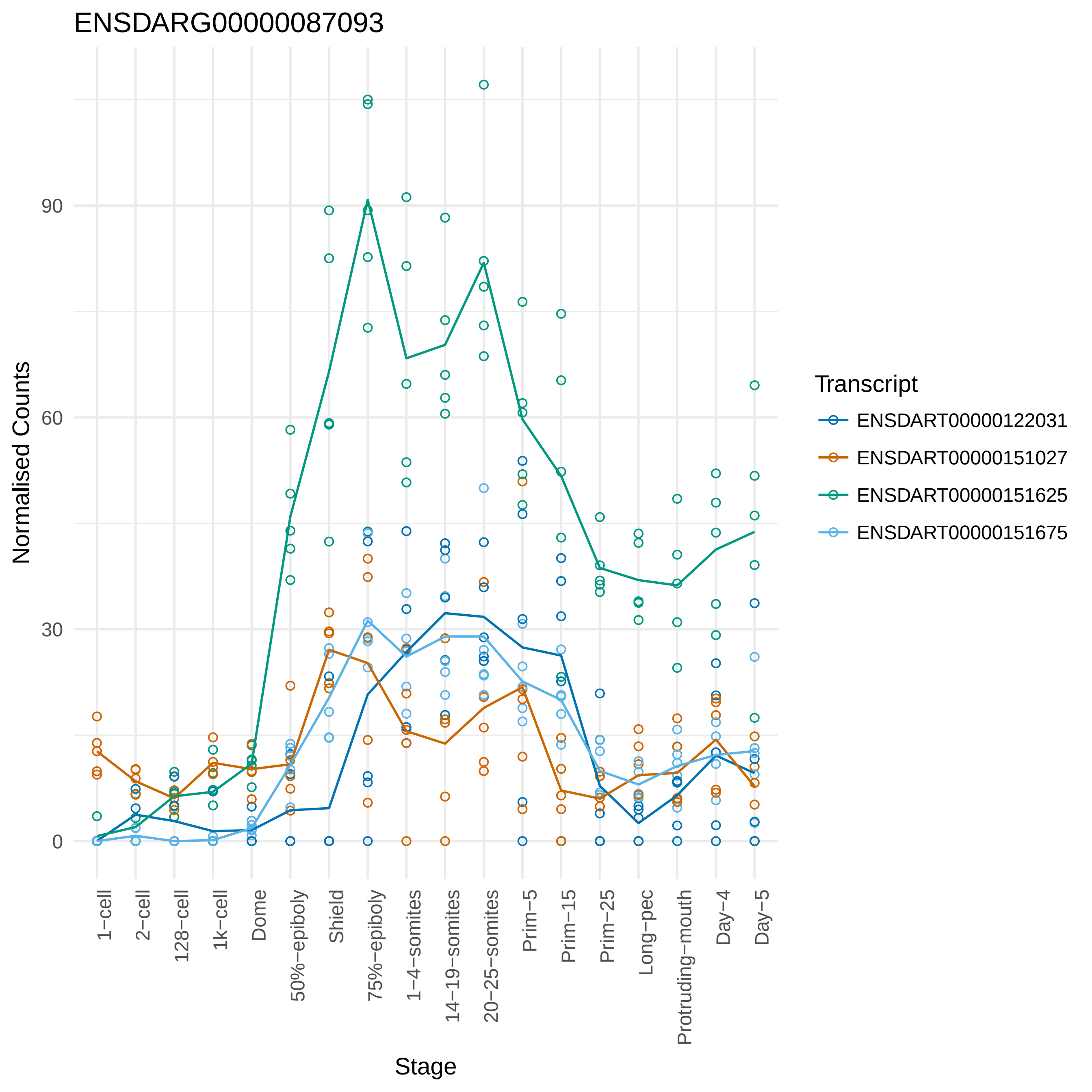

ENSDARG00000087093
Normalised Counts
0
30
60
90
1−cell
2−cell
128−cell
1k−cell
Dome
50%−epiboly
Shield
75%−epiboly
1−4−somites
14−19−somites
20−25−somites
Prim−5
Prim−15
Prim−25
Long−pec
Protruding−mouth
Day−4
Day−5
Stage
Transcript
ENSDART00000122031
ENSDART00000151027
ENSDART00000151625
ENSDART00000151675

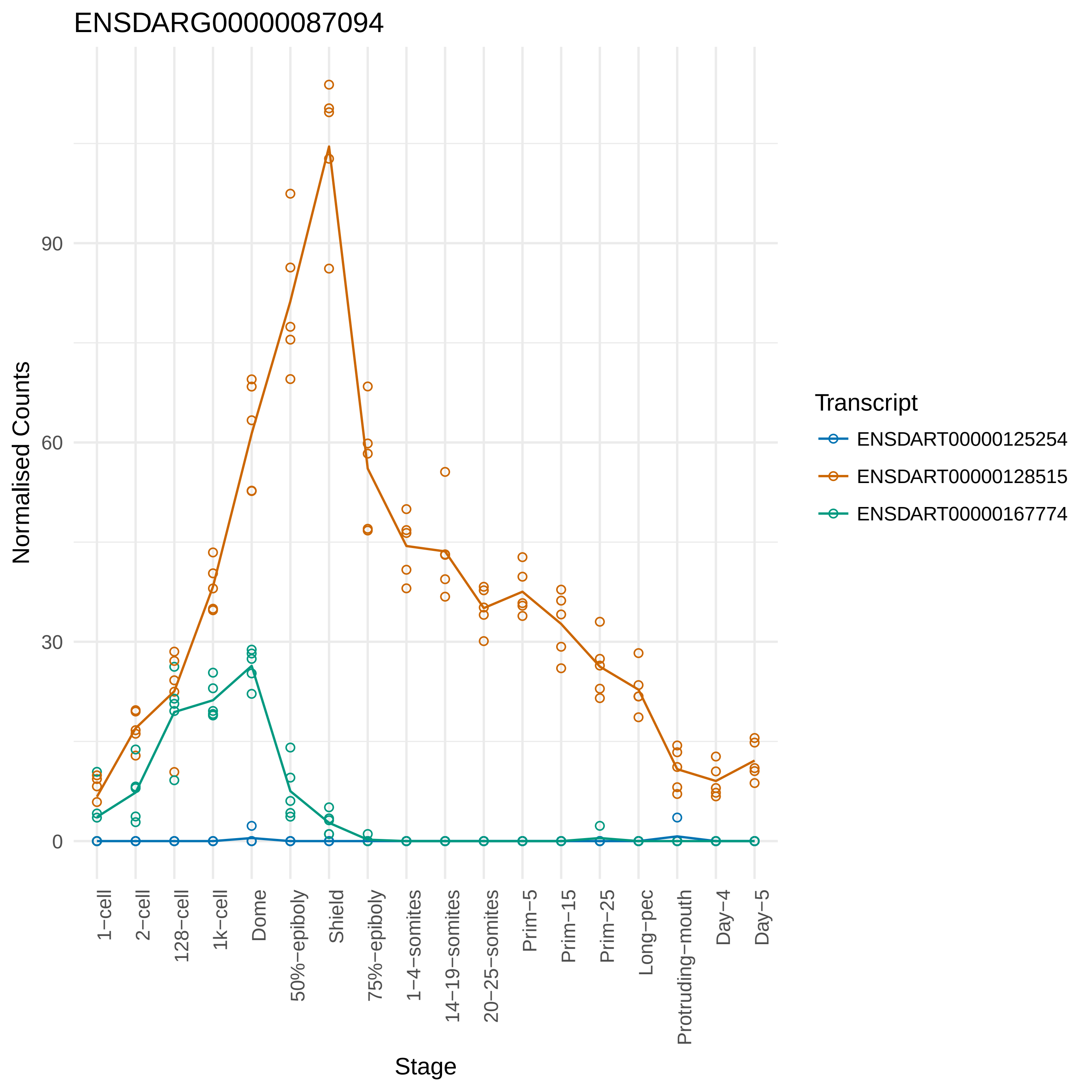

ENSDARG00000087094
Normalised Counts
0
30
60
90
Stage
1−cell
2−cell
128−cell
1k−cell
Dome
50%−epiboly
Shield
75%−epiboly
1−4−somites
14−19−somites
20−25−somites
Prim−5
Prim−15
Prim−25
Long−pec
Protruding−mouth
Day−4
Day−5
Transcript
ENSDART00000125254
ENSDART00000128515
ENSDART00000167774

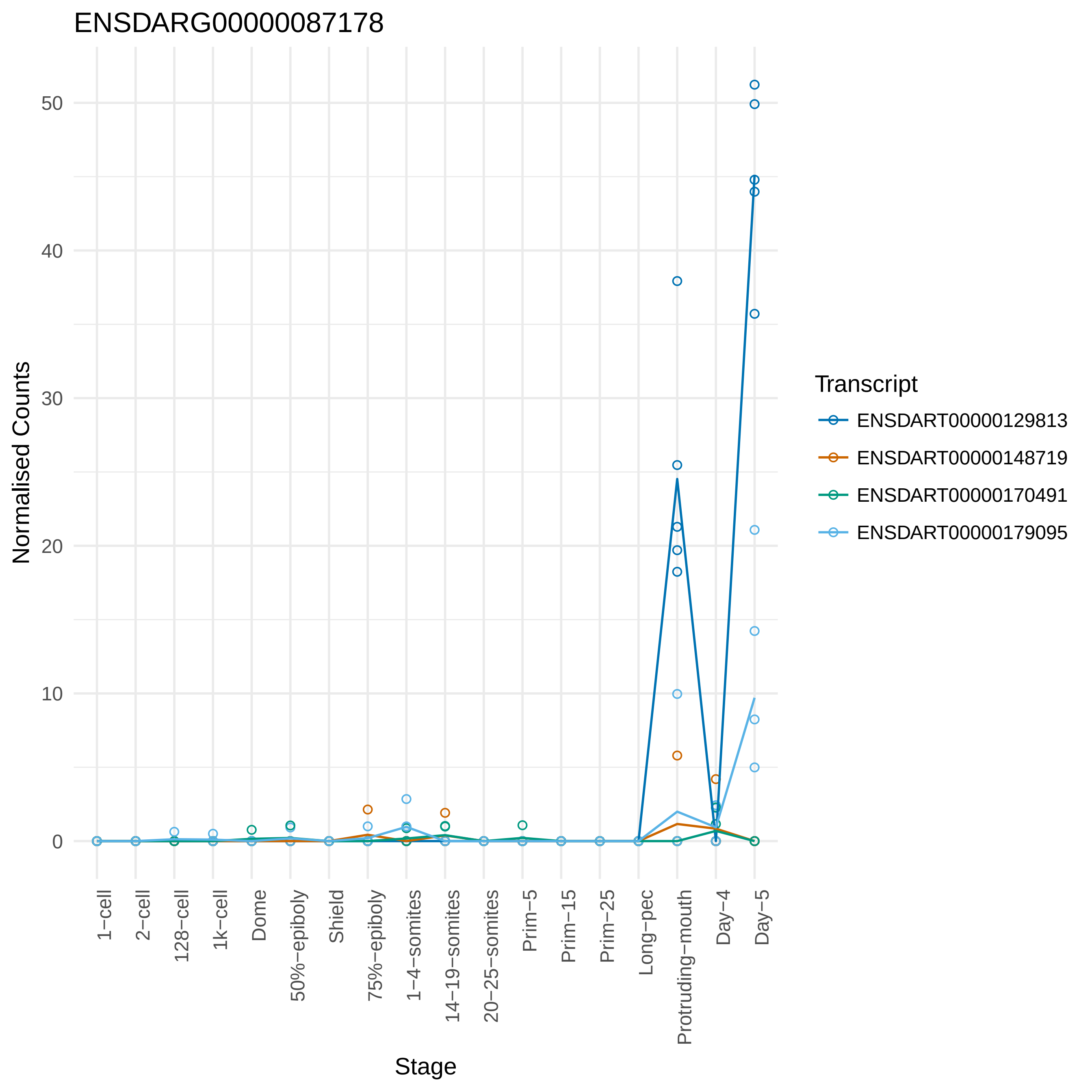

ENSDARG00000087178
Normalised Counts
0
10
20
30
40
50
1−cell
2−cell
128−cell
1k−cell
Dome
50%−epiboly
Shield
75%−epiboly
1−4−somites
14−19−somites
20−25−somites
Prim−5
Prim−15
Prim−25
Long−pec
Protruding−mouth
Day−4
Day−5
Stage
Transcript
ENSDART00000129813
ENSDART00000148719
ENSDART00000170491
ENSDART00000179095

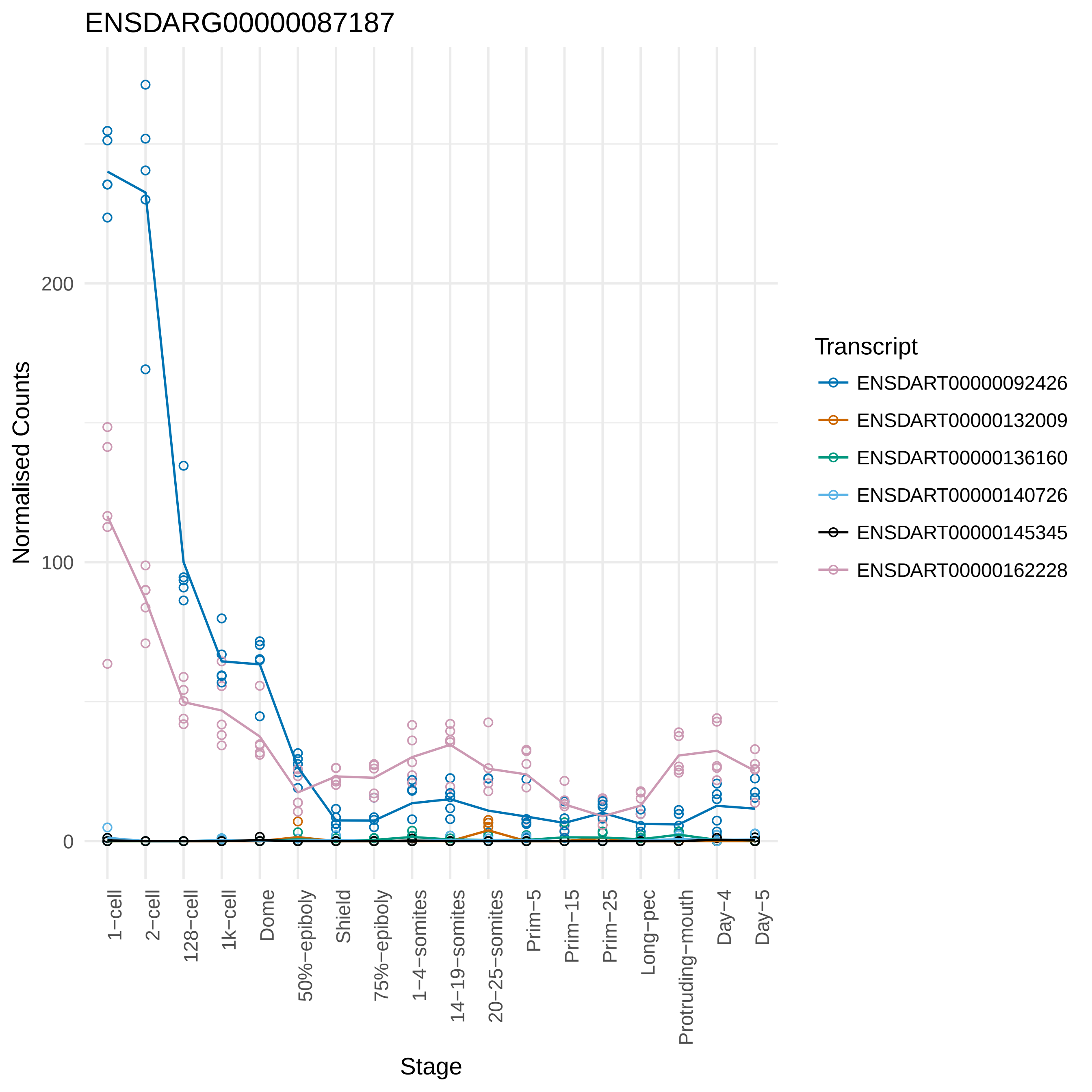

ENSDARG00000087187
Normalised Counts
200
100
0
Stage
1−cell
2−cell
128−cell
1k−cell
Dome
50%−epiboly
Shield
75%−epiboly
1−4−somites
14−19−somites
20−25−somites
Prim−5
Prim−15
Prim−25
Long−pec
Protruding−mouth
Day−4
Day−5
Transcript
ENSDART00000092426
ENSDART00000132009
ENSDART00000136160
ENSDART00000140726
ENSDART00000145345
ENSDART00000162228

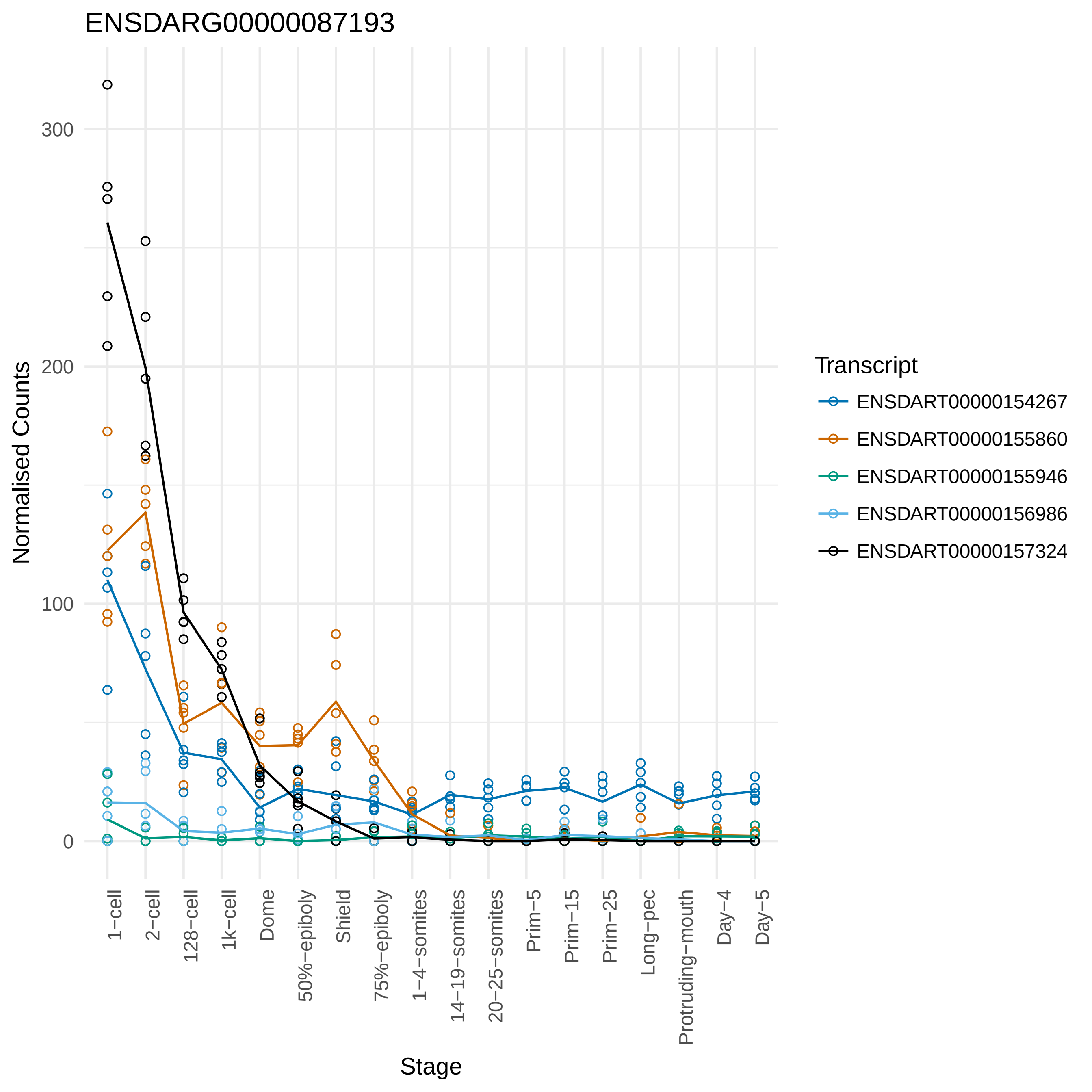

ENSDARG00000087193
Normalised Counts
300
200
100
0
1−cell
2−cell
128−cell
1k−cell
Dome
50%−epiboly
Shield
75%−epiboly
1−4−somites
14−19−somites
20−25−somites
Prim−5
Prim−15
Prim−25
Long−pec
Protruding−mouth
Day−4
Day−5
Stage
Transcript
ENSDART00000154267
ENSDART00000155860
ENSDART00000155946
ENSDART00000156986
ENSDART00000157324

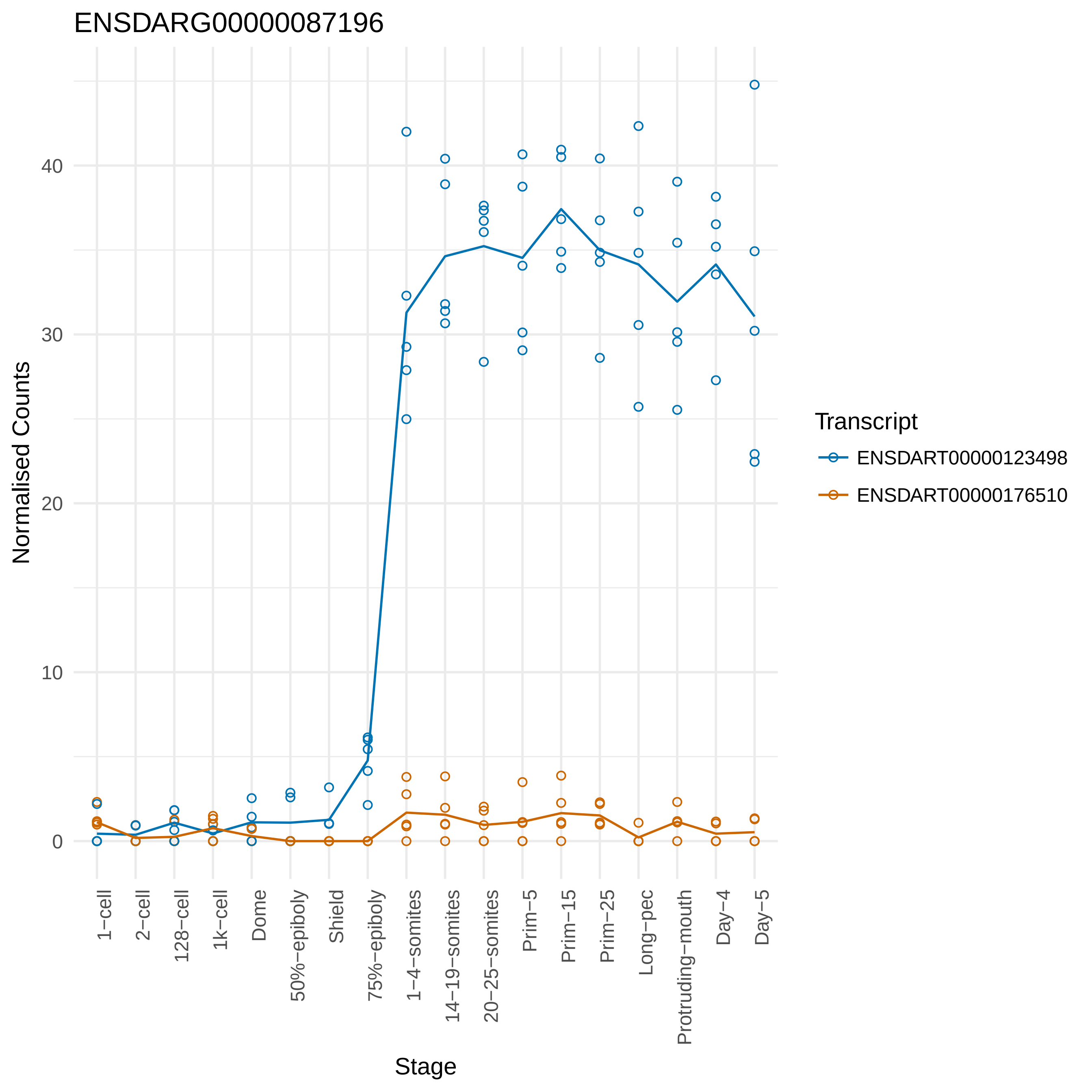

ENSDARG00000087196
Normalised Counts
0
10
20
30
40
Stage
1−cell
2−cell
128−cell
1k−cell
Dome
50%−epiboly
Shield
75%−epiboly
1−4−somites
14−19−somites
20−25−somites
Prim−5
Prim−15
Prim−25
Long−pec
Protruding−mouth
Day−4
Day−5
Transcript
ENSDART00000123498
ENSDART00000176510

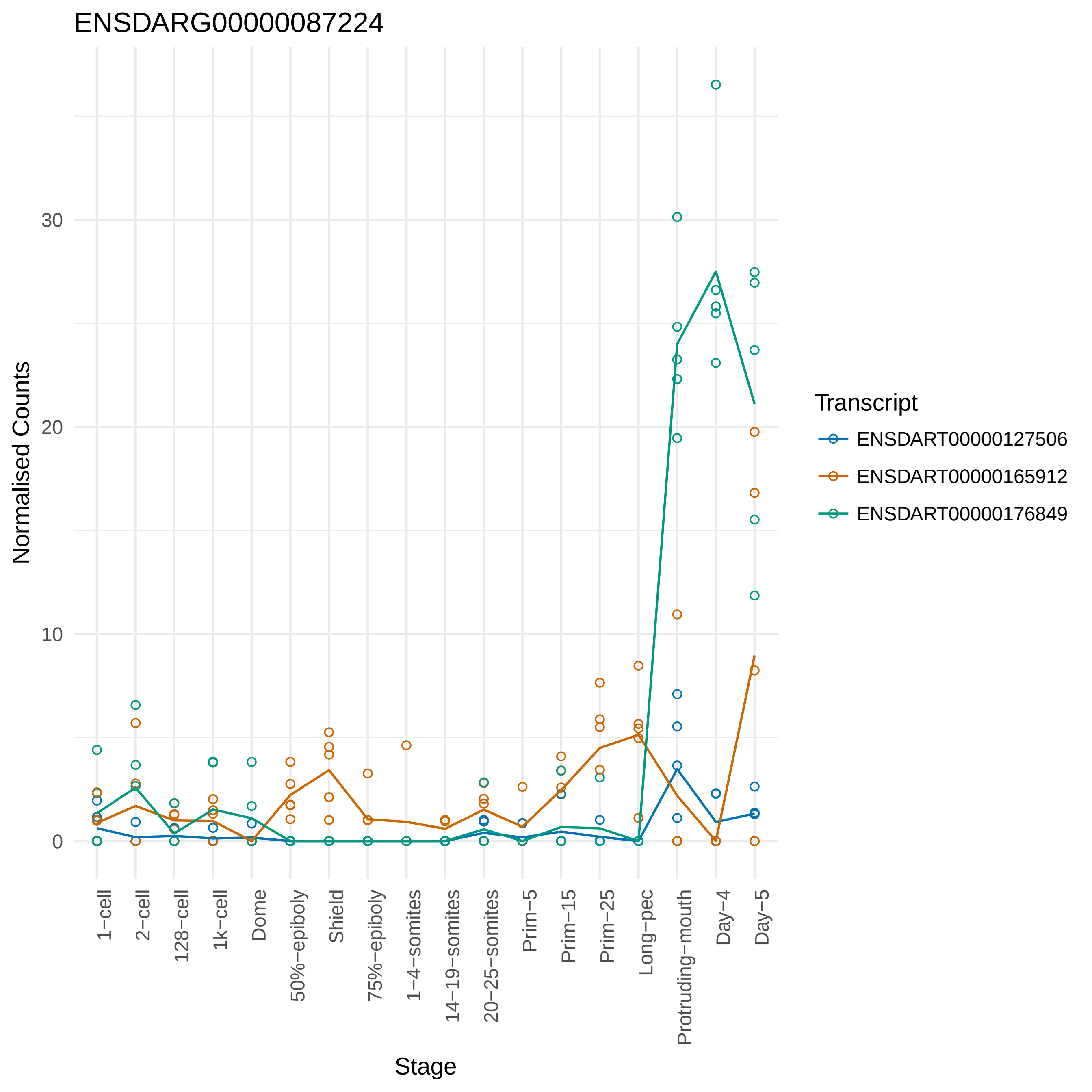

ENSDARG00000087224
Normalised Counts
0
10
20
30
1−cell
2−cell
128−cell
1k−cell
Dome
50%−epiboly
Shield
75%−epiboly
1−4−somites
14−19−somites
20−25−somites
Prim−5
Prim−15
Prim−25
Long−pec
Protruding−mouth
Day−4
Day−5
Stage
Transcript
ENSDART00000127506
ENSDART00000165912
ENSDART00000176849

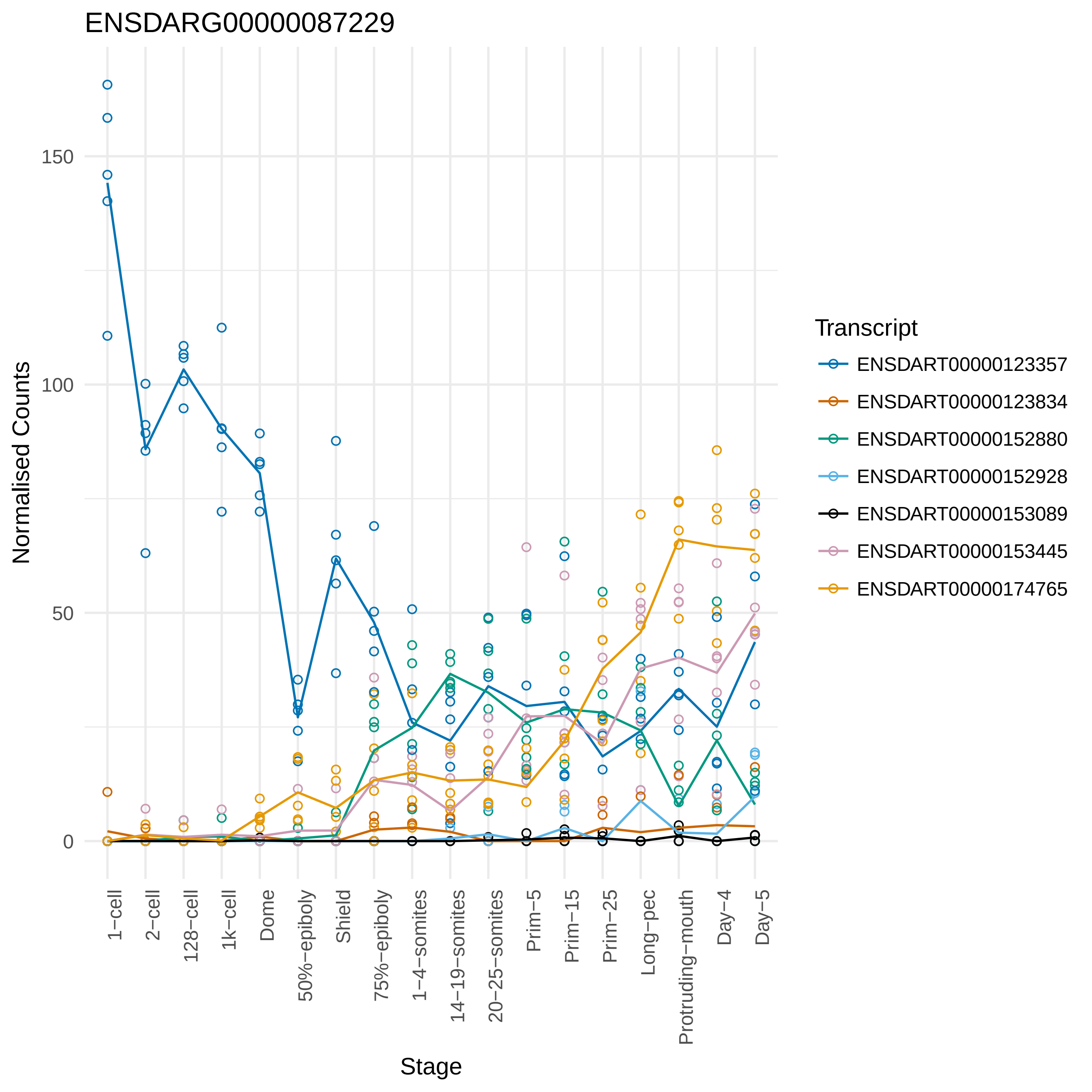

ENSDARG00000087229
Normalised Counts
150
100
50
0
Stage
1−cell
2−cell
128−cell
1k−cell
Dome
50%−epiboly
Shield
75%−epiboly
1−4−somites
14−19−somites
20−25−somites
Prim−5
Prim−15
Prim−25
Long−pec
Protruding−mouth
Day−4
Day−5
Transcript
ENSDART00000123357
ENSDART00000123834
ENSDART00000152880
ENSDART00000152928
ENSDART00000153089
ENSDART00000153445
ENSDART00000174765

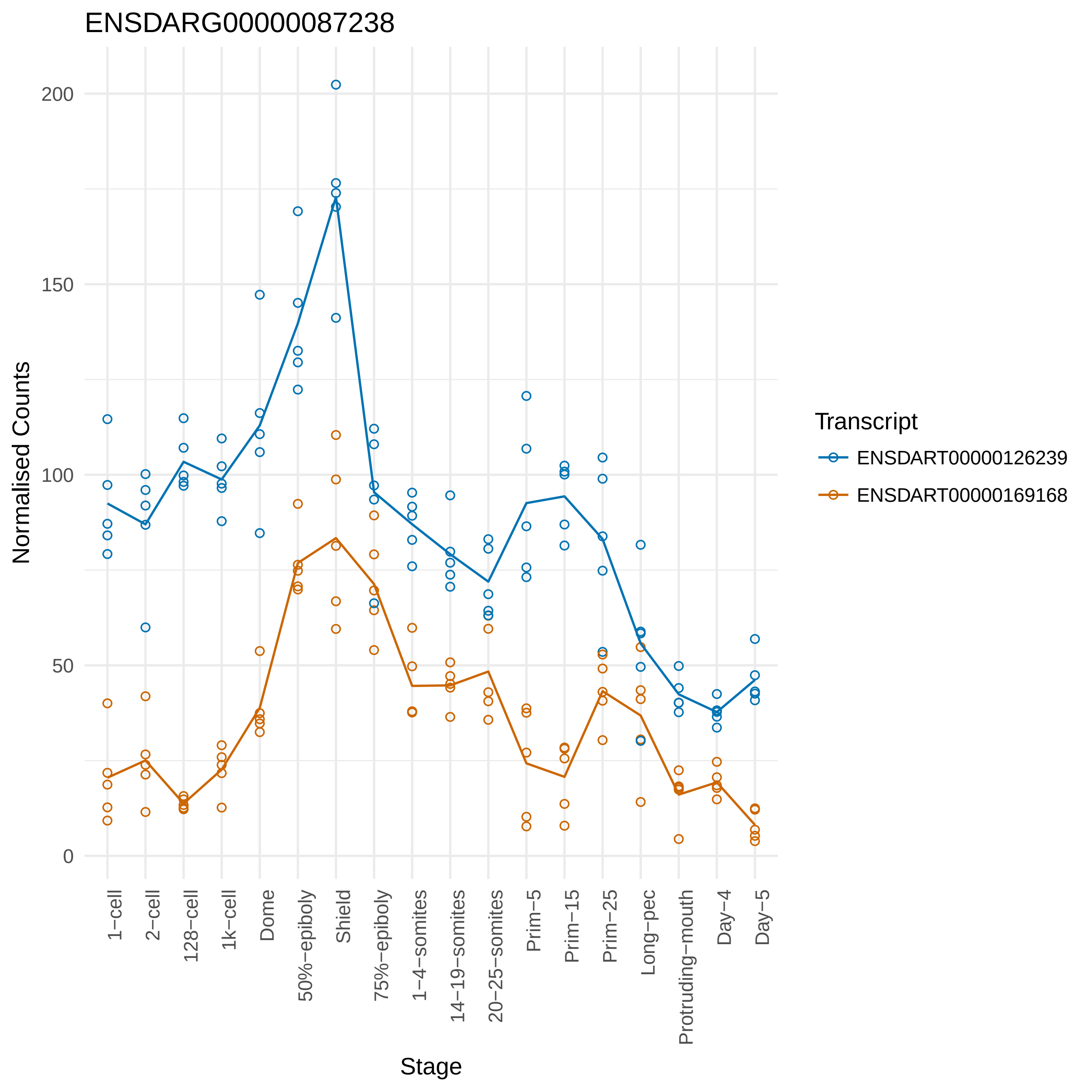
ENSDARG00000087238
Normalised Counts
200
150
100
50
0
1−cell
2−cell
128−cell
1k−cell
Dome
50%−epiboly
Shield
75%−epiboly
1−4−somites
14−19−somites
20−25−somites
Prim−5
Prim−15
Prim−25
Long−pec
Protruding−mouth
Day−4
Day−5
Stage
Transcript
ENSDART00000126239
ENSDART00000169168

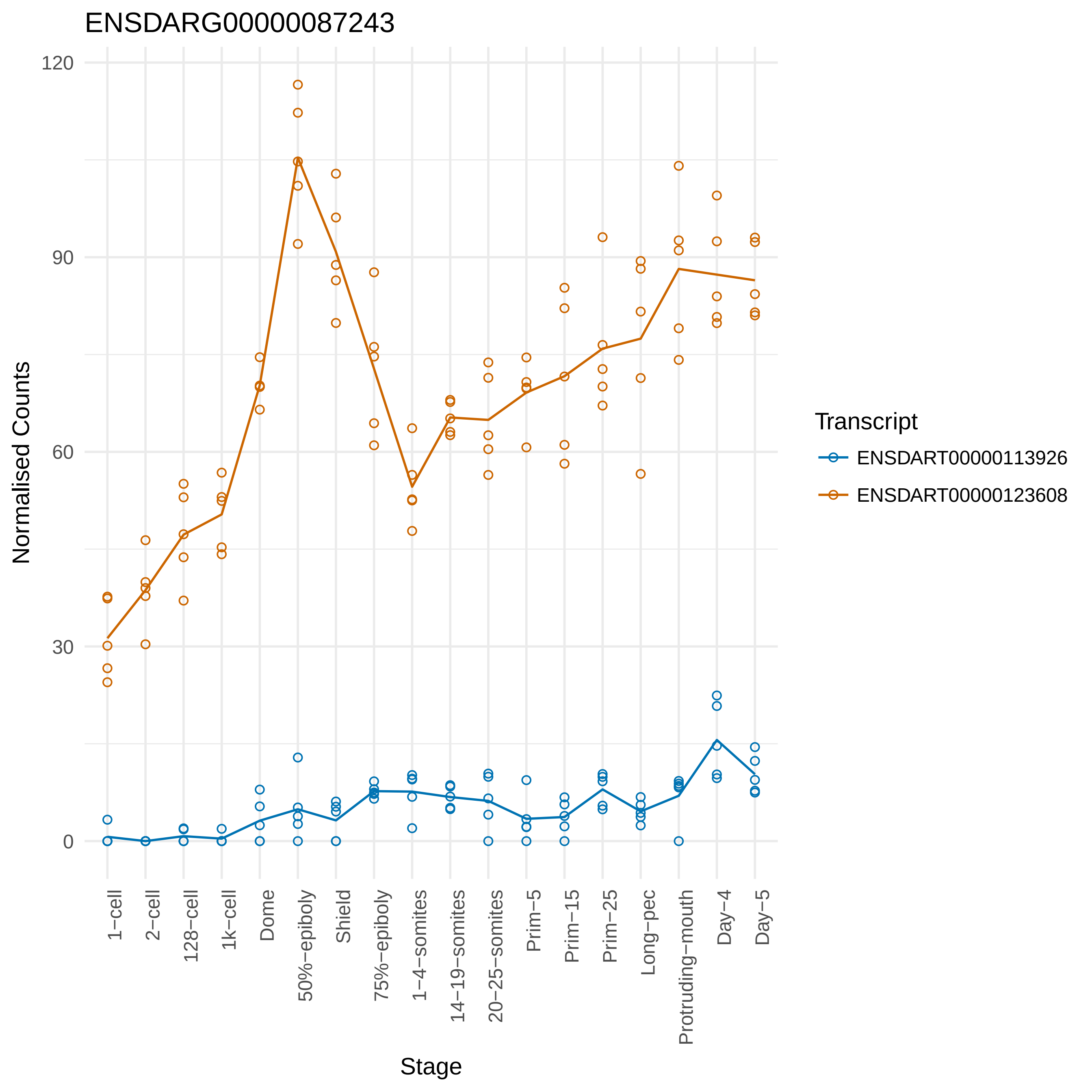
ENSDARG00000087243
Normalised Counts
120
90
60
30
0
1−cell
2−cell
128−cell
1k−cell
Dome
50%−epiboly
Shield
75%−epiboly
1−4−somites
14−19−somites
20−25−somites
Prim−5
Prim−15
Prim−25
Long−pec
Protruding−mouth
Day−4
Day−5
Stage
Transcript
ENSDART00000113926
ENSDART00000123608

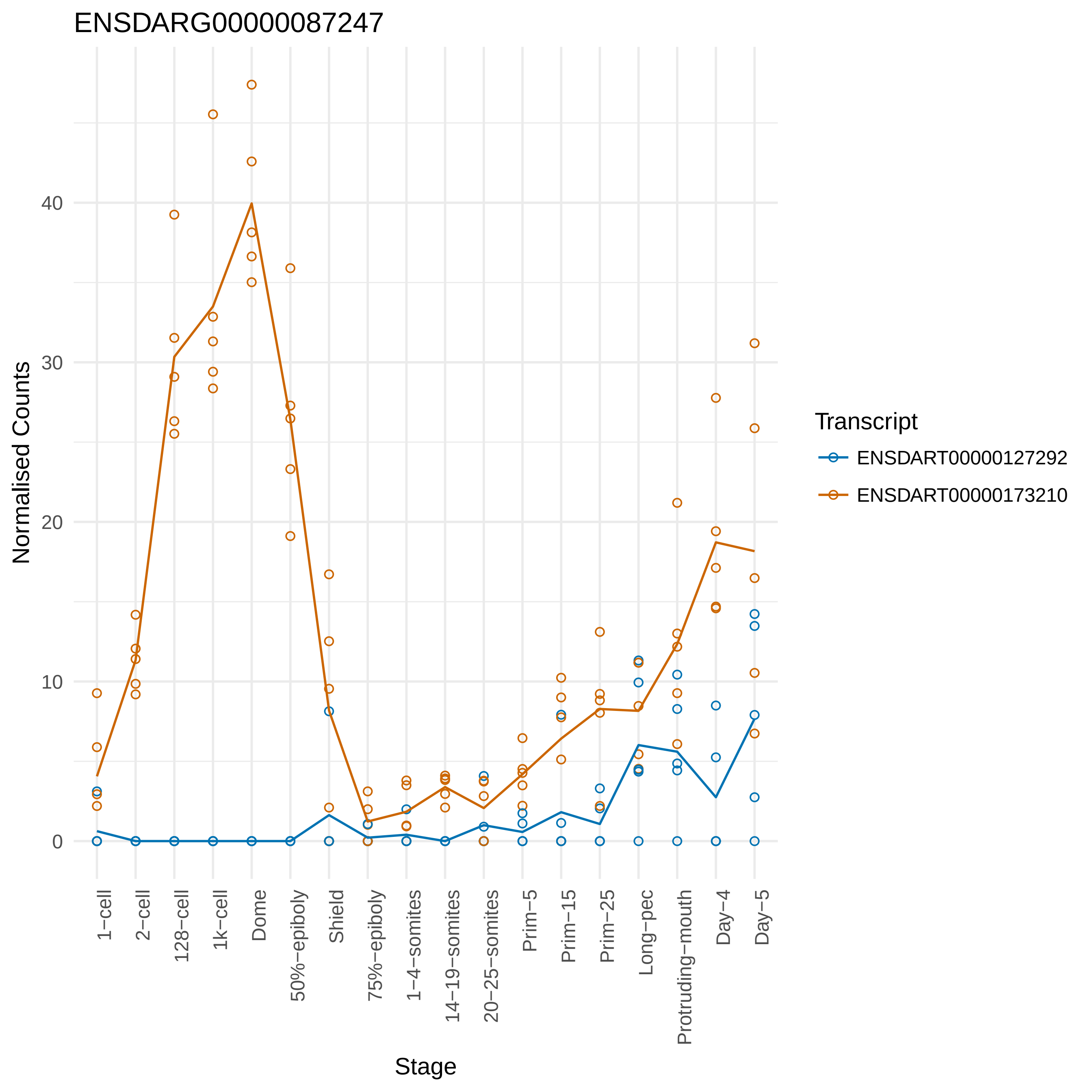
ENSDARG00000087247
Normalised Counts
0
10
20
30
40
Stage
1−cell
2−cell
128−cell
1k−cell
Dome
50%−epiboly
Shield
75%−epiboly
1−4−somites
14−19−somites
20−25−somites
Prim−5
Prim−15
Prim−25
Long−pec
Protruding−mouth
Day−4
Day−5
Transcript
ENSDART00000127292
ENSDART00000173210

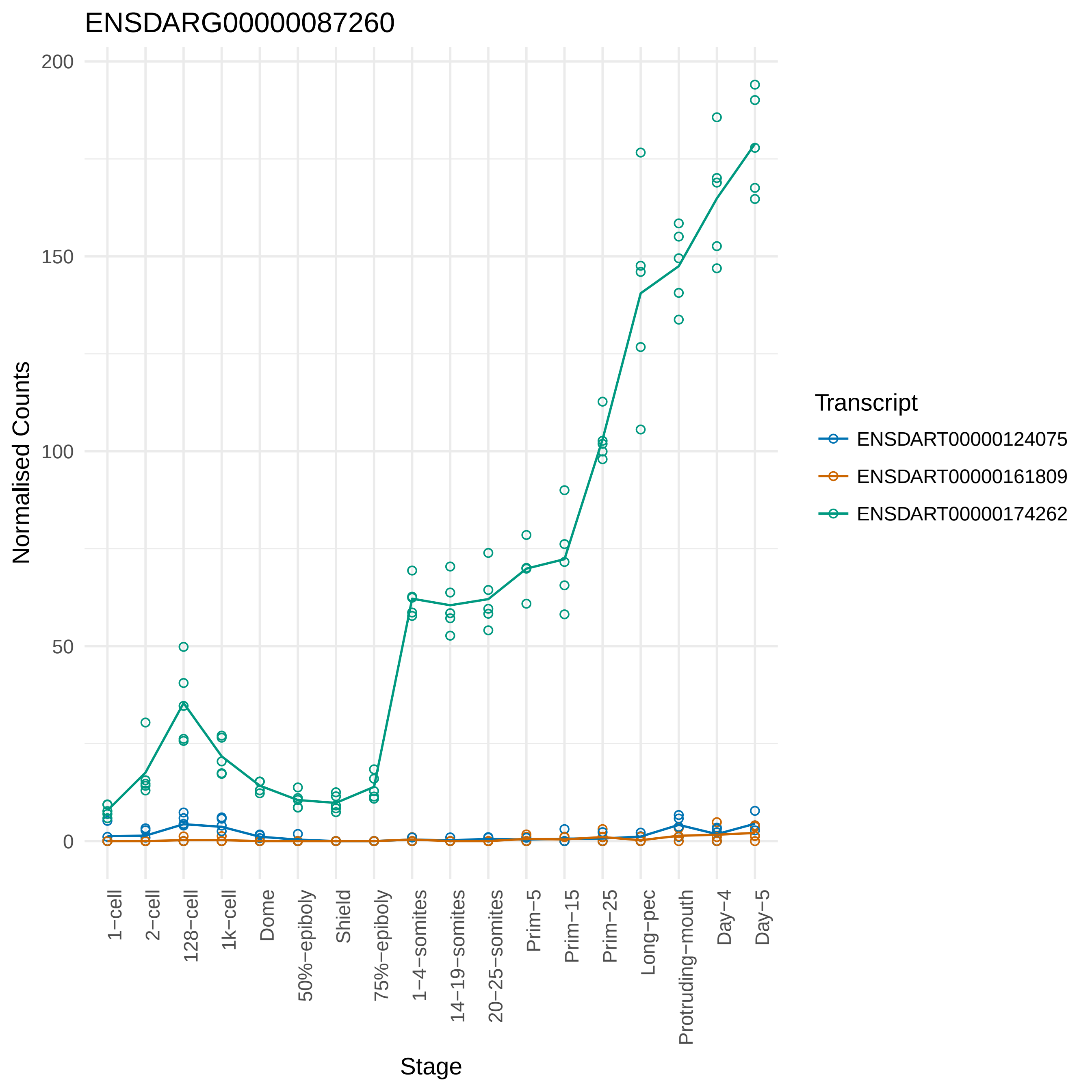

ENSDARG00000087260
200
150
100
50
0
Normalised Counts
1−cell
2−cell
128−cell
1k−cell
Dome
50%−epiboly
Shield
75%−epiboly
1−4−somites
14−19−somites
20−25−somites
Prim−5
Prim−15
Prim−25
Long−pec
Protruding−mouth
Day−4
Day−5
Stage
Transcript
ENSDART00000124075
ENSDART00000161809
ENSDART00000174262

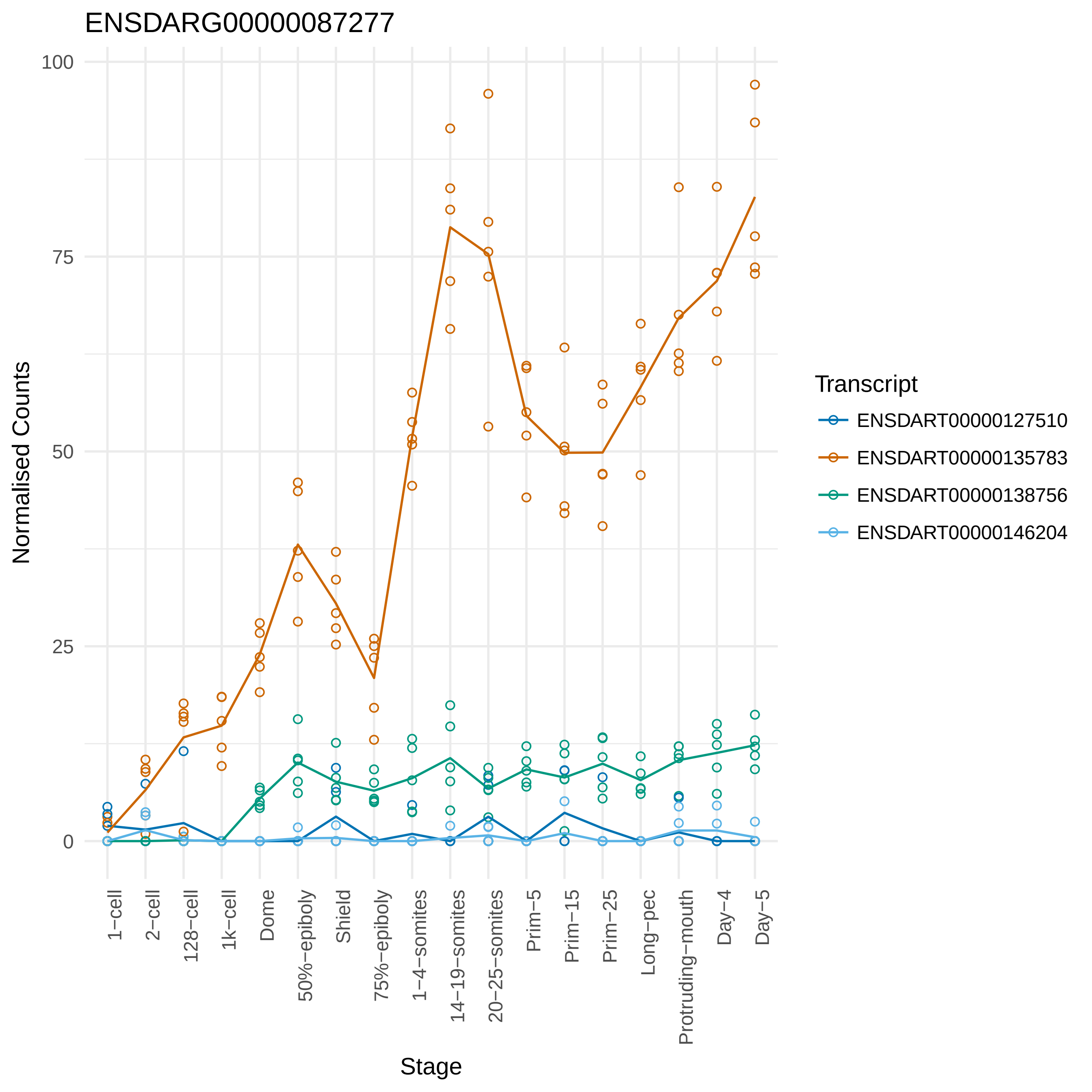

ENSDARG00000087277
Normalised Counts
100
75
50
25
0
1−cell
2−cell
128−cell
1k−cell
Dome
50%−epiboly
Shield
75%−epiboly
1−4−somites
14−19−somites
20−25−somites
Prim−5
Prim−15
Prim−25
Long−pec
Protruding−mouth
Day−4
Day−5
Stage
Transcript
ENSDART00000127510
ENSDART00000135783
ENSDART00000138756
ENSDART00000146204

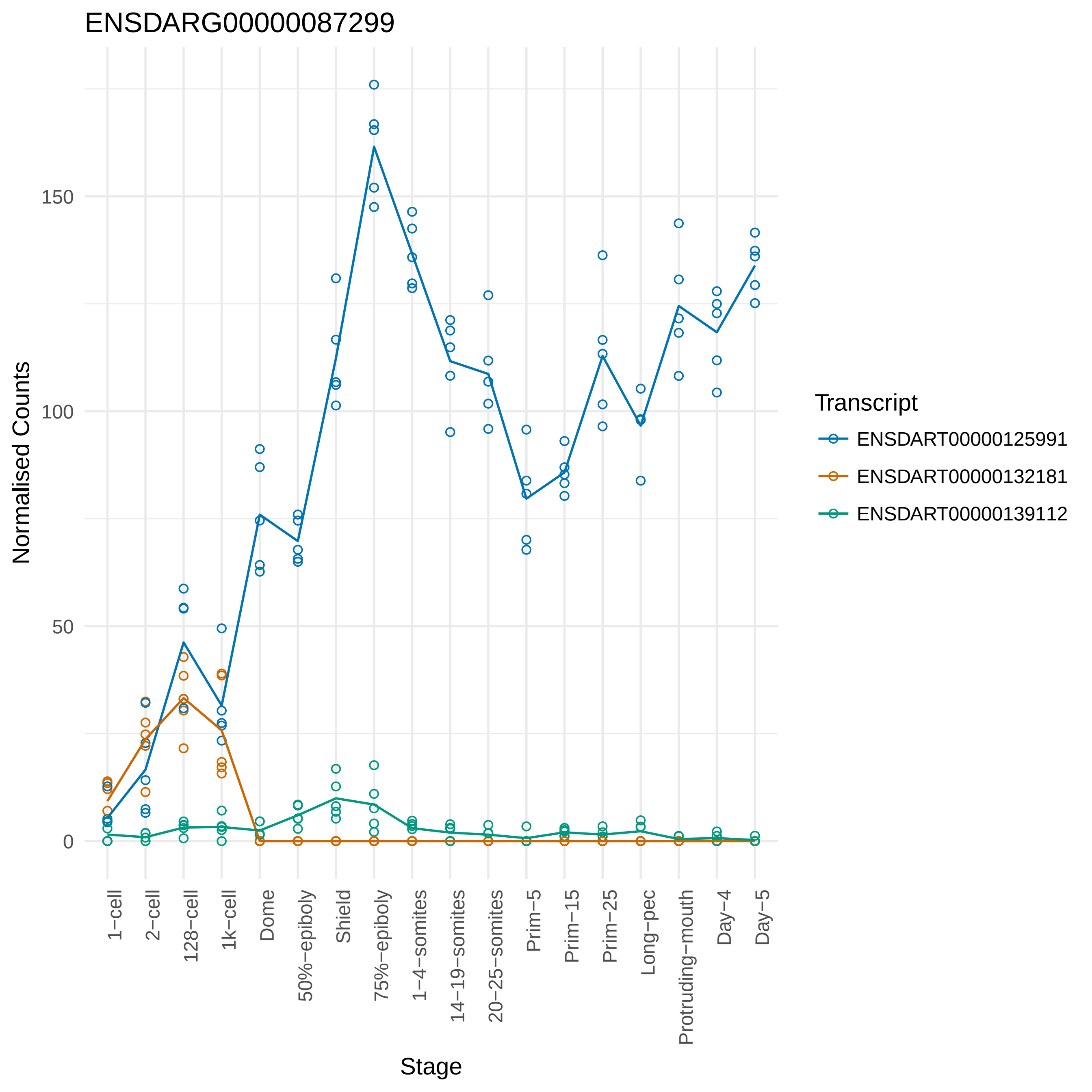

ENSDARG00000087299
Normalised Counts
0
50
100
150
1−cell
2−cell
128−cell
1k−cell
Dome
50%−epiboly
Shield
75%−epiboly
1−4−somites
14−19−somites
20−25−somites
Prim−5
Prim−15
Prim−25
Long−pec
Protruding−mouth
Day−4
Day−5
Stage
Transcript
ENSDART00000125991
ENSDART00000132181
ENSDART00000139112

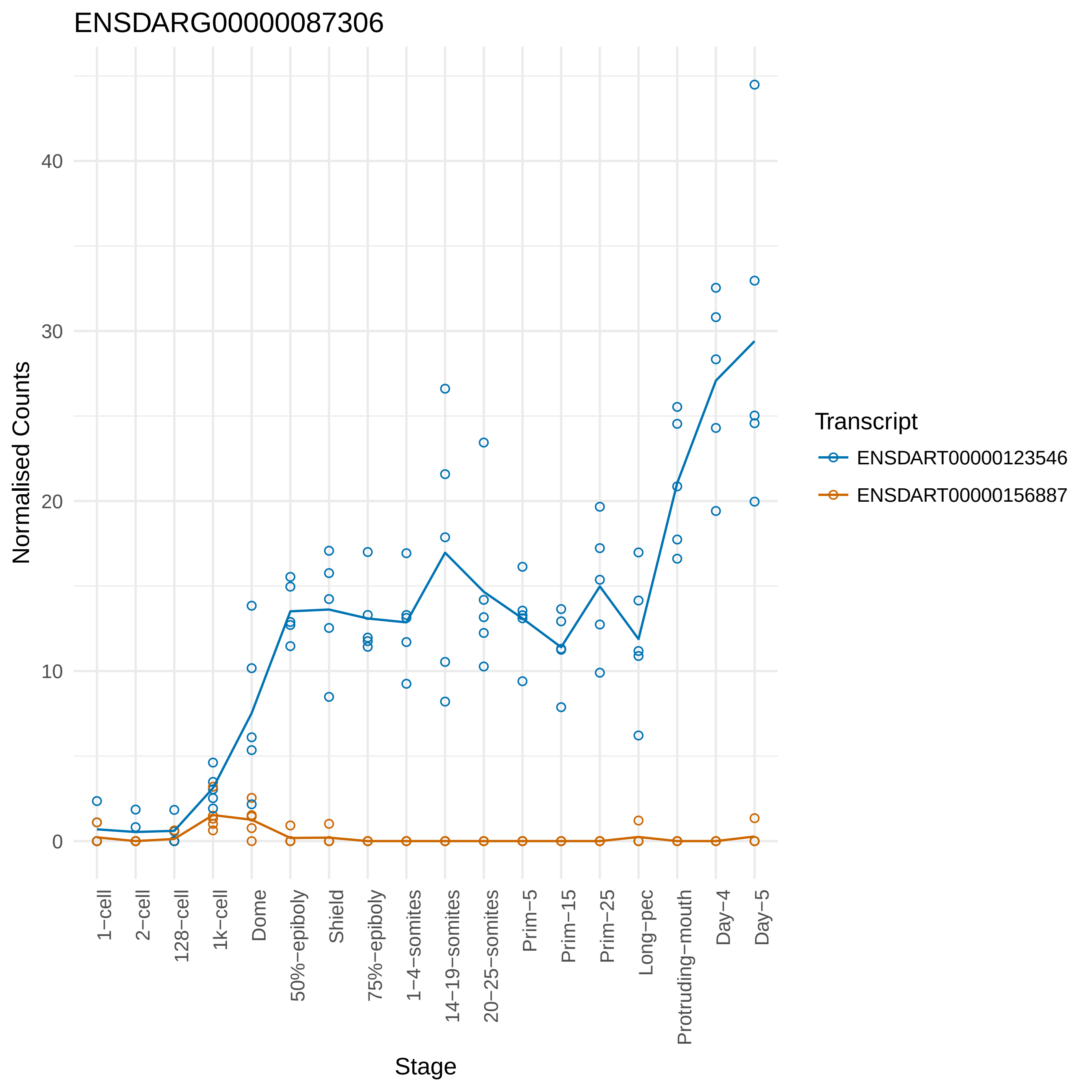
ENSDARG00000087306
Normalised Counts
0
10
20
30
40
Stage
1−cell
2−cell
128−cell
1k−cell
Dome
50%−epiboly
Shield
75%−epiboly
1−4−somites
14−19−somites
20−25−somites
Prim−5
Prim−15
Prim−25
Long−pec
Protruding−mouth
Day−4
Day−5
Transcript
ENSDART00000123546
ENSDART00000156887

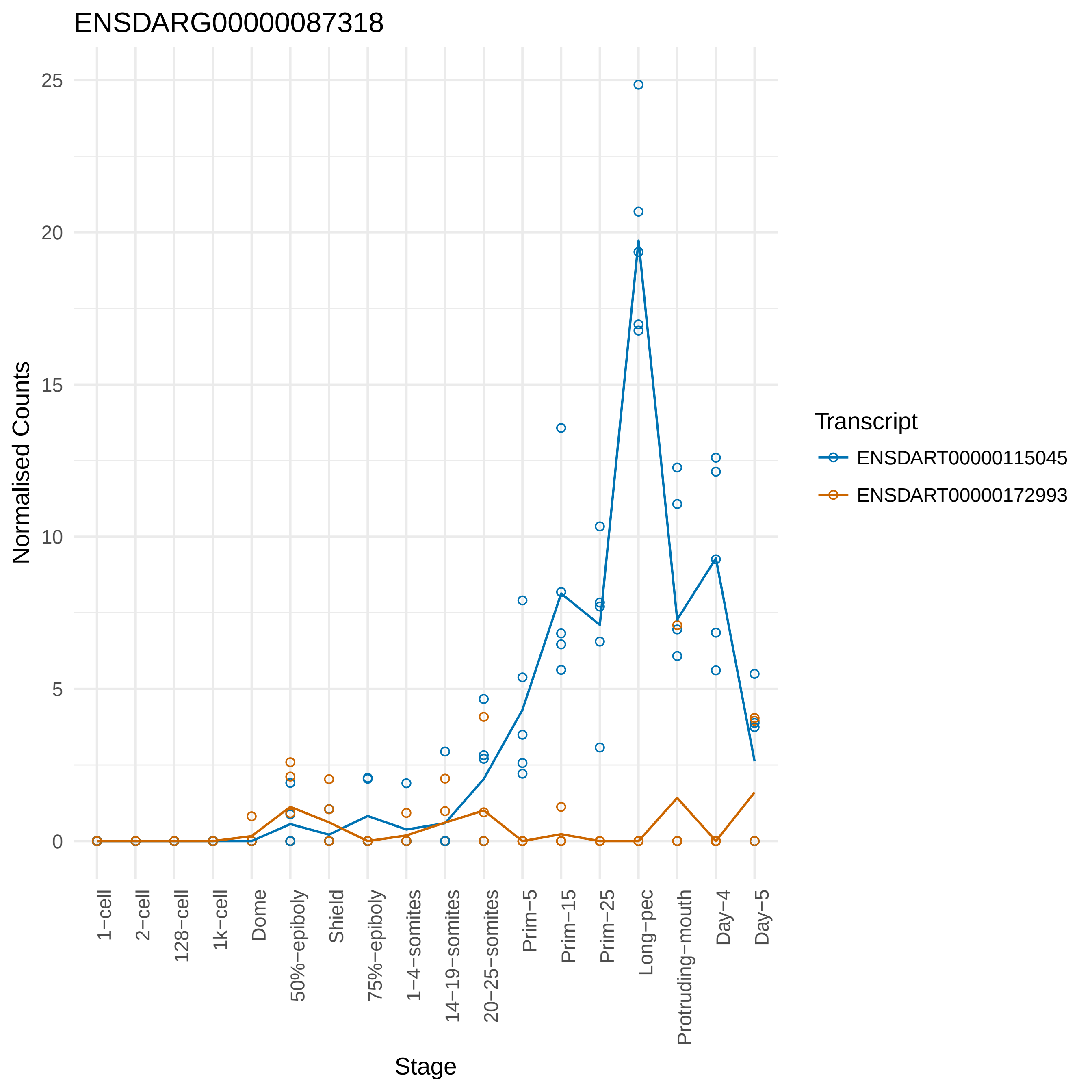
ENSDARG00000087318
Normalised Counts
25
20
15
10
5
0
1−cell
2−cell
128−cell
1k−cell
Dome
50%−epiboly
Shield
75%−epiboly
1−4−somites
14−19−somites
20−25−somites
Prim−5
Prim−15
Prim−25
Long−pec
Protruding−mouth
Day−4
Day−5
Stage
Transcript
ENSDART00000115045
ENSDART00000172993

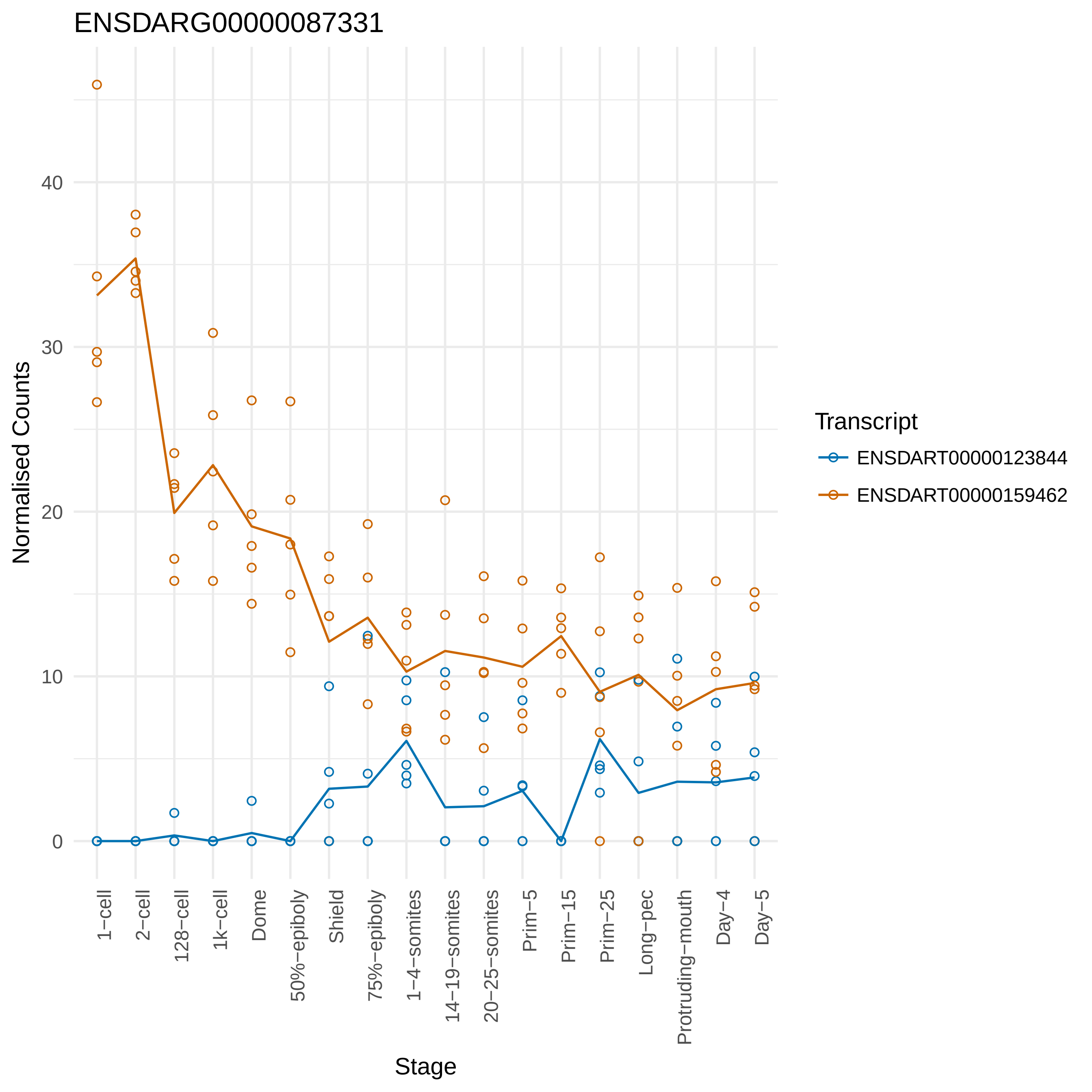

ENSDARG00000087331
Normalised Counts
0
10
20
30
40
Stage
1−cell
2−cell
128−cell
1k−cell
Dome
50%−epiboly
Shield
75%−epiboly
1−4−somites
14−19−somites
20−25−somites
Prim−5
Prim−15
Prim−25
Long−pec
Protruding−mouth
Day−4
Day−5
Transcript
ENSDART00000123844
ENSDART00000159462

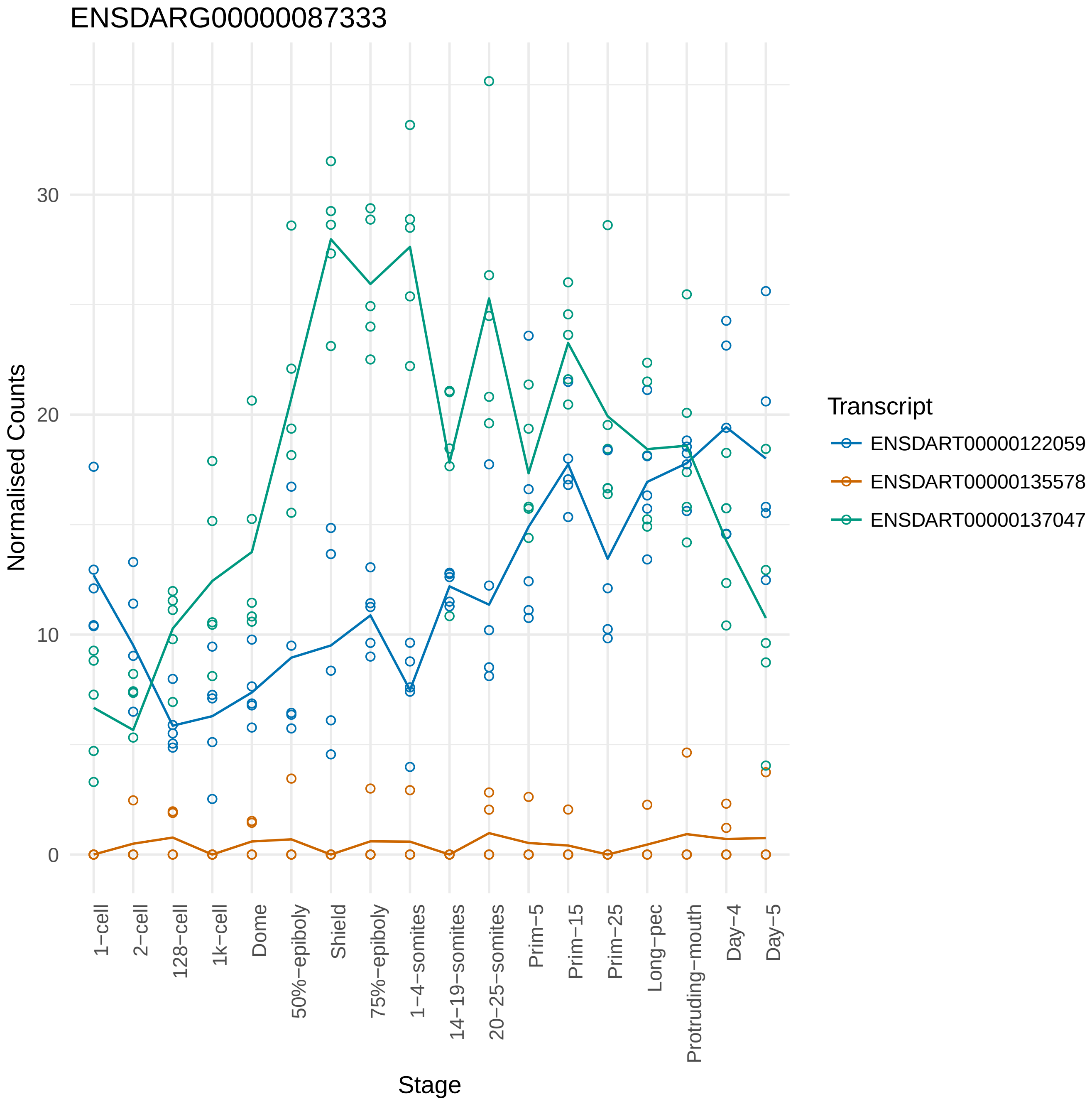

ENSDARG00000087333
Normalised Counts
0
10
20
30
Stage
1−cell
2−cell
128−cell
1k−cell
Dome
50%−epiboly
Shield
75%−epiboly
1−4−somites
14−19−somites
20−25−somites
Prim−5
Prim−15
Prim−25
Long−pec
Protruding−mouth
Day−4
Day−5
Transcript
ENSDART00000122059
ENSDART00000135578
ENSDART00000137047

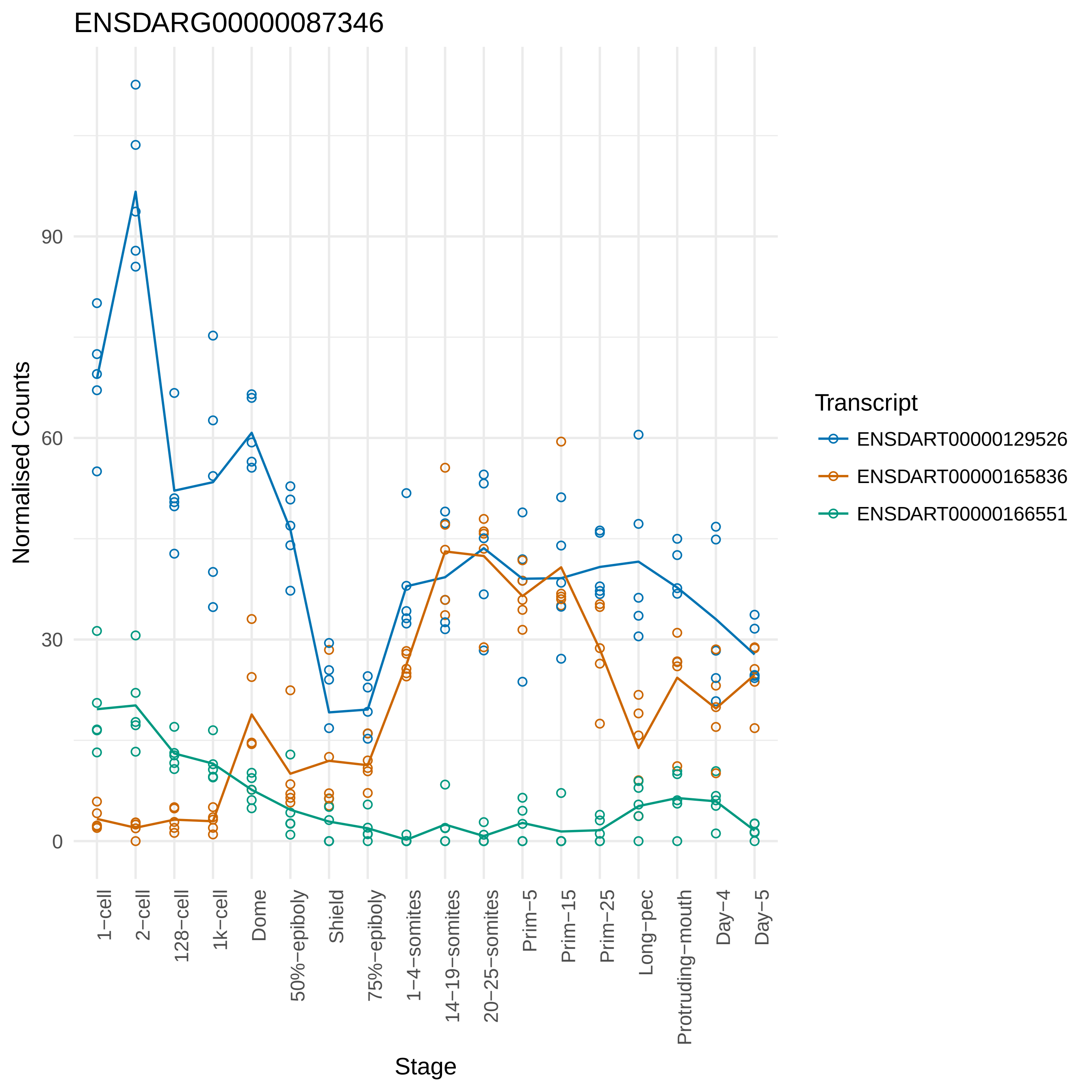
ENSDARG00000087346
Normalised Counts
0
30
60
90
Stage
1−cell
2−cell
128−cell
1k−cell
Dome
50%−epiboly
Shield
75%−epiboly
1−4−somites
14−19−somites
20−25−somites
Prim−5
Prim−15
Prim−25
Long−pec
Protruding−mouth
Day−4
Day−5
Transcript
ENSDART00000129526
ENSDART00000165836
ENSDART00000166551

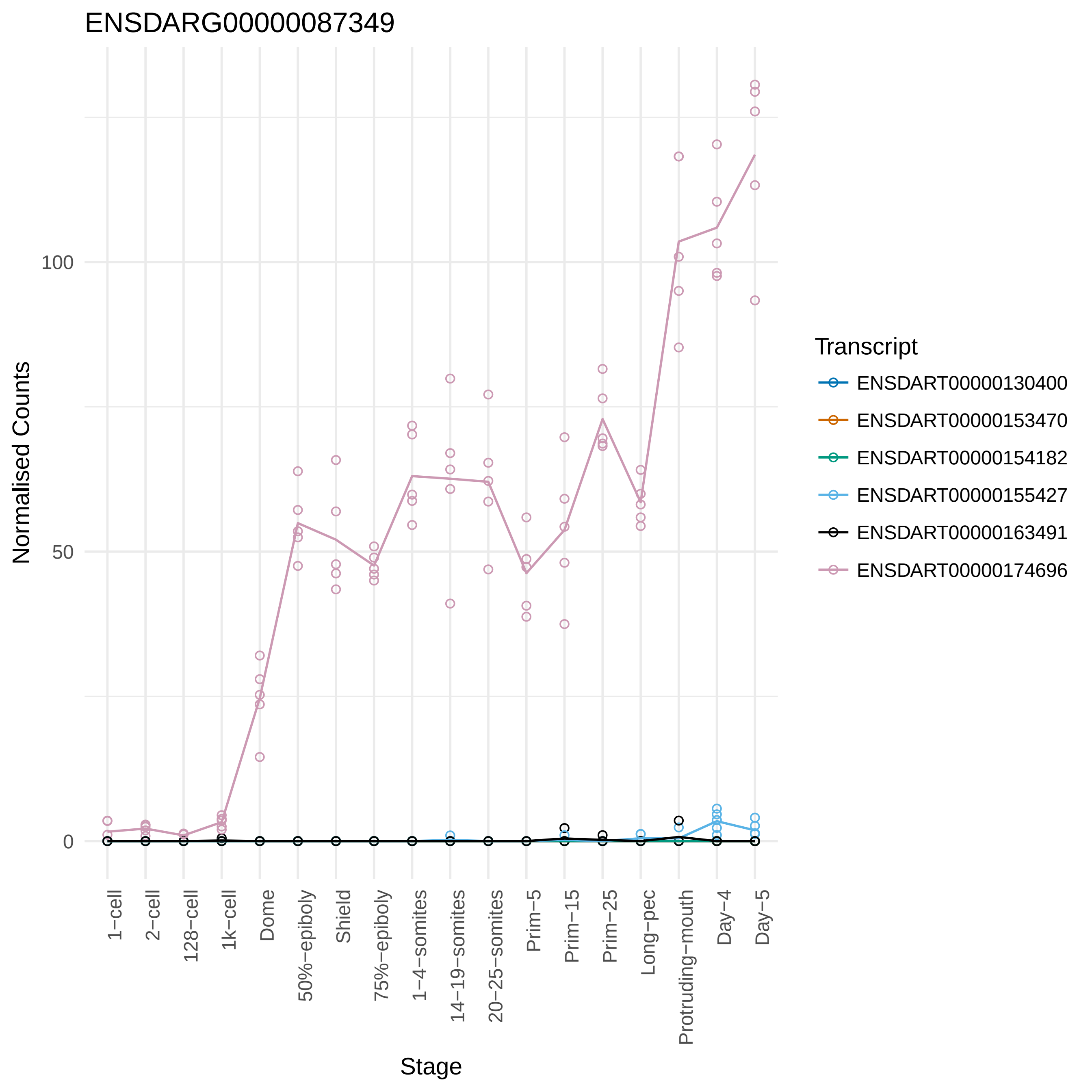

ENSDARG00000087349
Normalised Counts
100
50
0
1−cell
2−cell
128−cell
1k−cell
Dome
50%−epiboly
Shield
75%−epiboly
1−4−somites
14−19−somites
20−25−somites
Prim−5
Prim−15
Prim−25
Long−pec
Protruding−mouth
Day−4
Day−5
Stage
Transcript
ENSDART00000130400
ENSDART00000153470
ENSDART00000154182
ENSDART00000155427
ENSDART00000163491
ENSDART00000174696

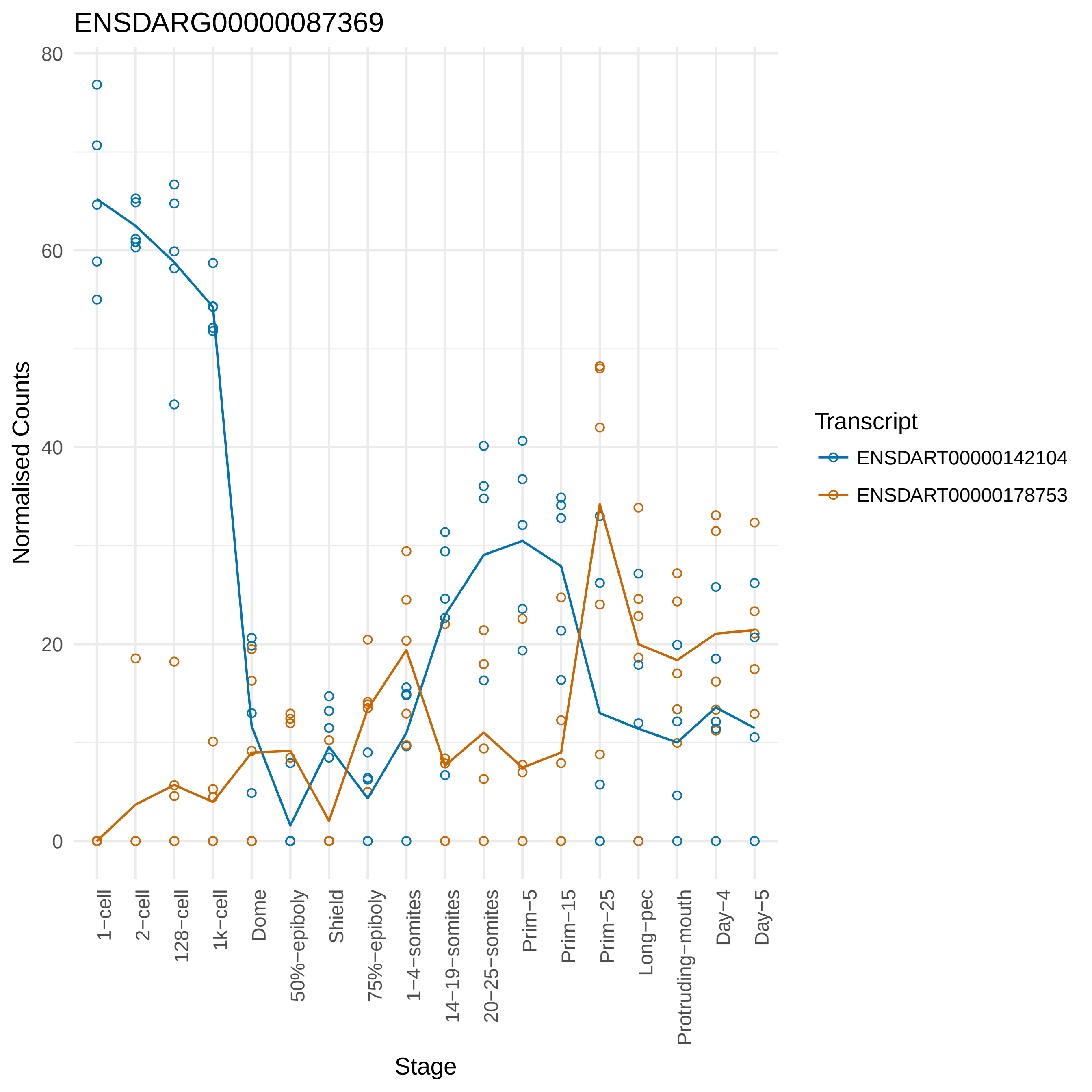

ENSDARG00000087369
Normalised Counts
80
60
40
20
0
1−cell
2−cell
128−cell
1k−cell
Dome
50%−epiboly
Shield
75%−epiboly
1−4−somites
14−19−somites
20−25−somites
Prim−5
Prim−15
Prim−25
Long−pec
Protruding−mouth
Day−4
Day−5
Stage
Transcript
ENSDART00000142104
ENSDART00000178753

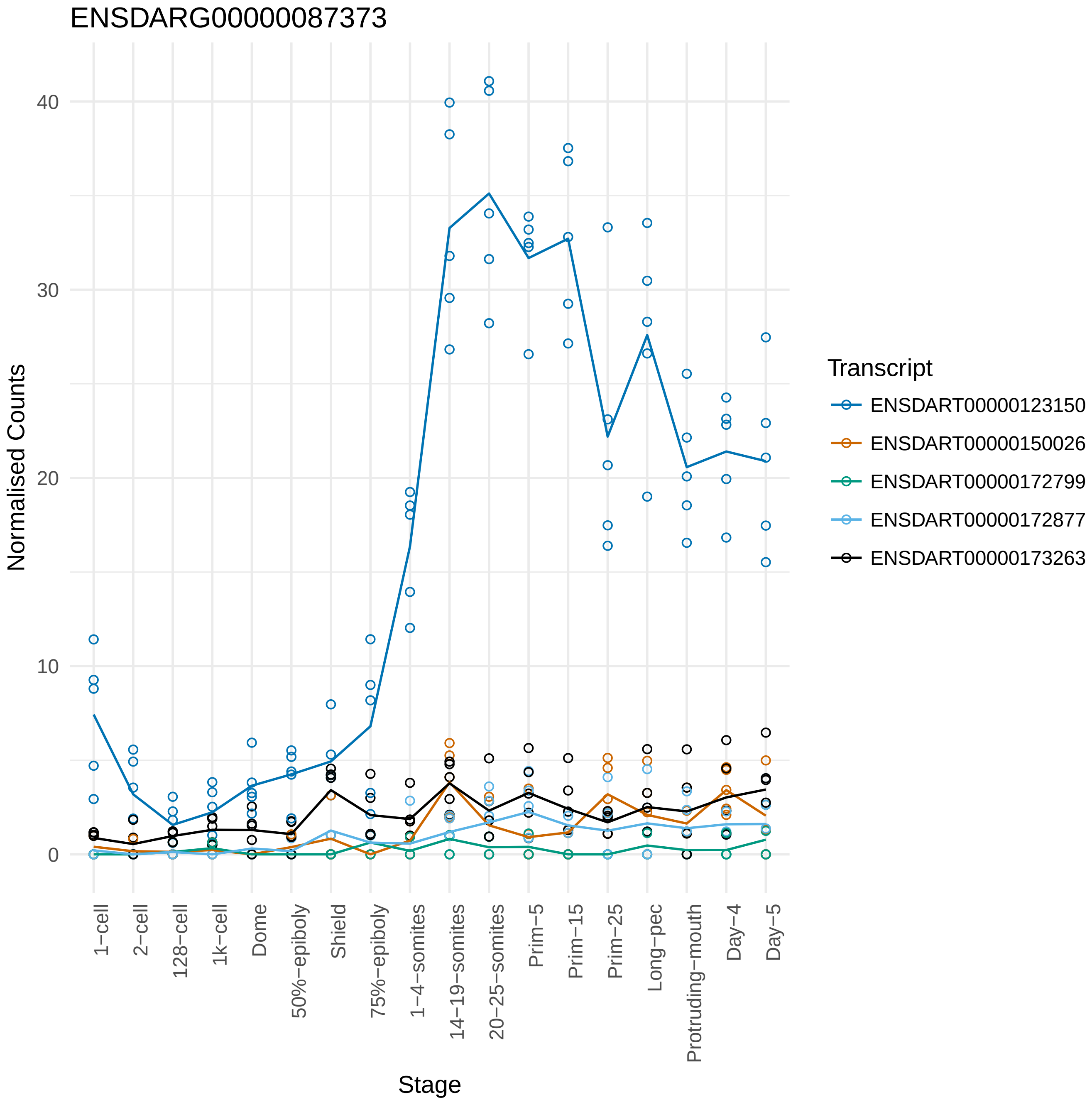
ENSDARG00000087373
Normalised Counts
40
30
20
10
0
1−cell
2−cell
128−cell
1k−cell
Dome
50%−epiboly
Shield
75%−epiboly
1−4−somites
14−19−somites
20−25−somites
Prim−5
Prim−15
Prim−25
Long−pec
Protruding−mouth
Day−4
Day−5
Stage
Transcript
ENSDART00000123150
ENSDART00000150026
ENSDART00000172799
ENSDART00000172877
ENSDART00000173263

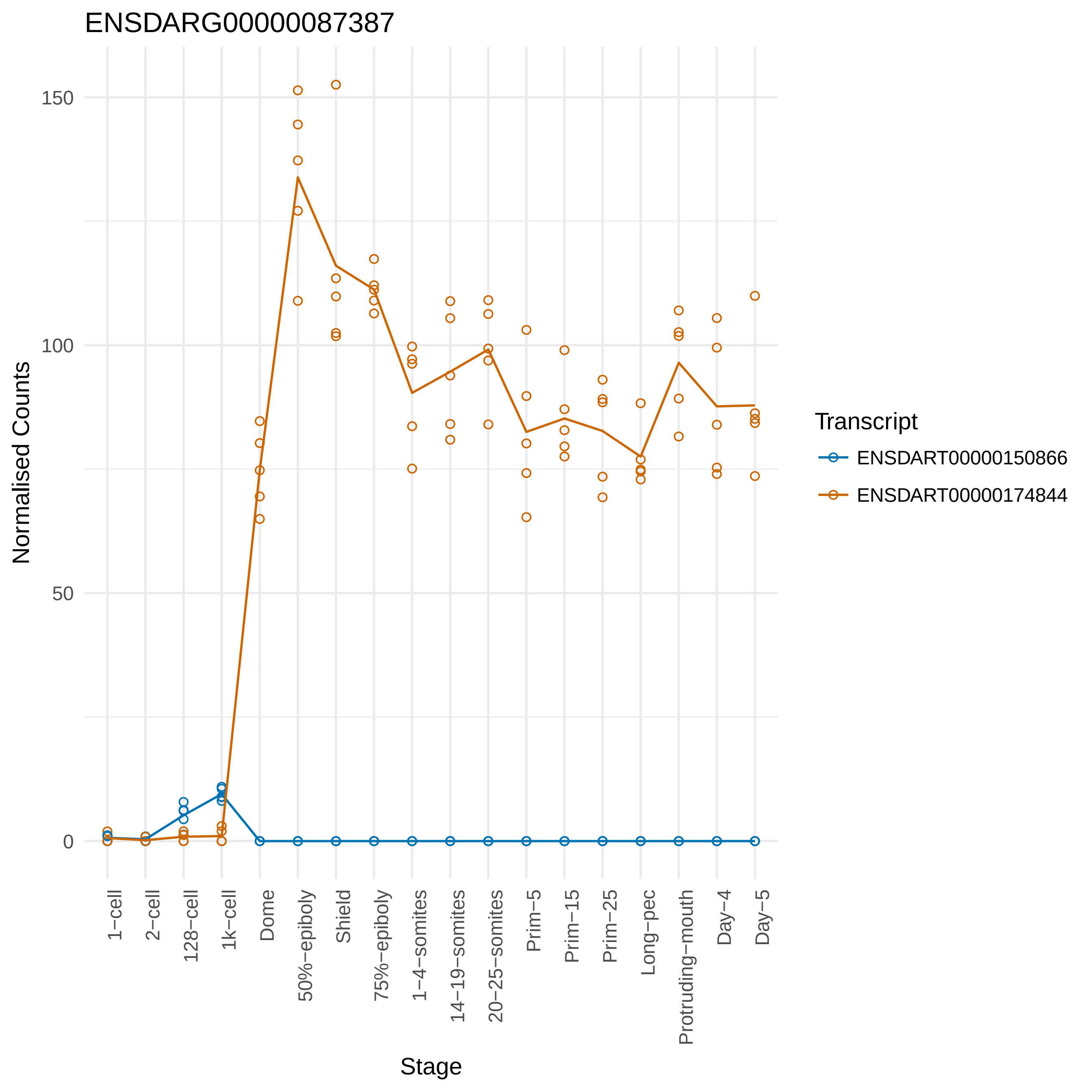

ENSDARG00000087387
Normalised Counts
150
100
50
0
1−cell
2−cell
128−cell
1k−cell
Dome
50%−epiboly
Shield
75%−epiboly
1−4−somites
14−19−somites
20−25−somites
Prim−5
Prim−15
Prim−25
Long−pec
Protruding−mouth
Day−4
Day−5
Stage
Transcript
ENSDART00000150866
ENSDART00000174844

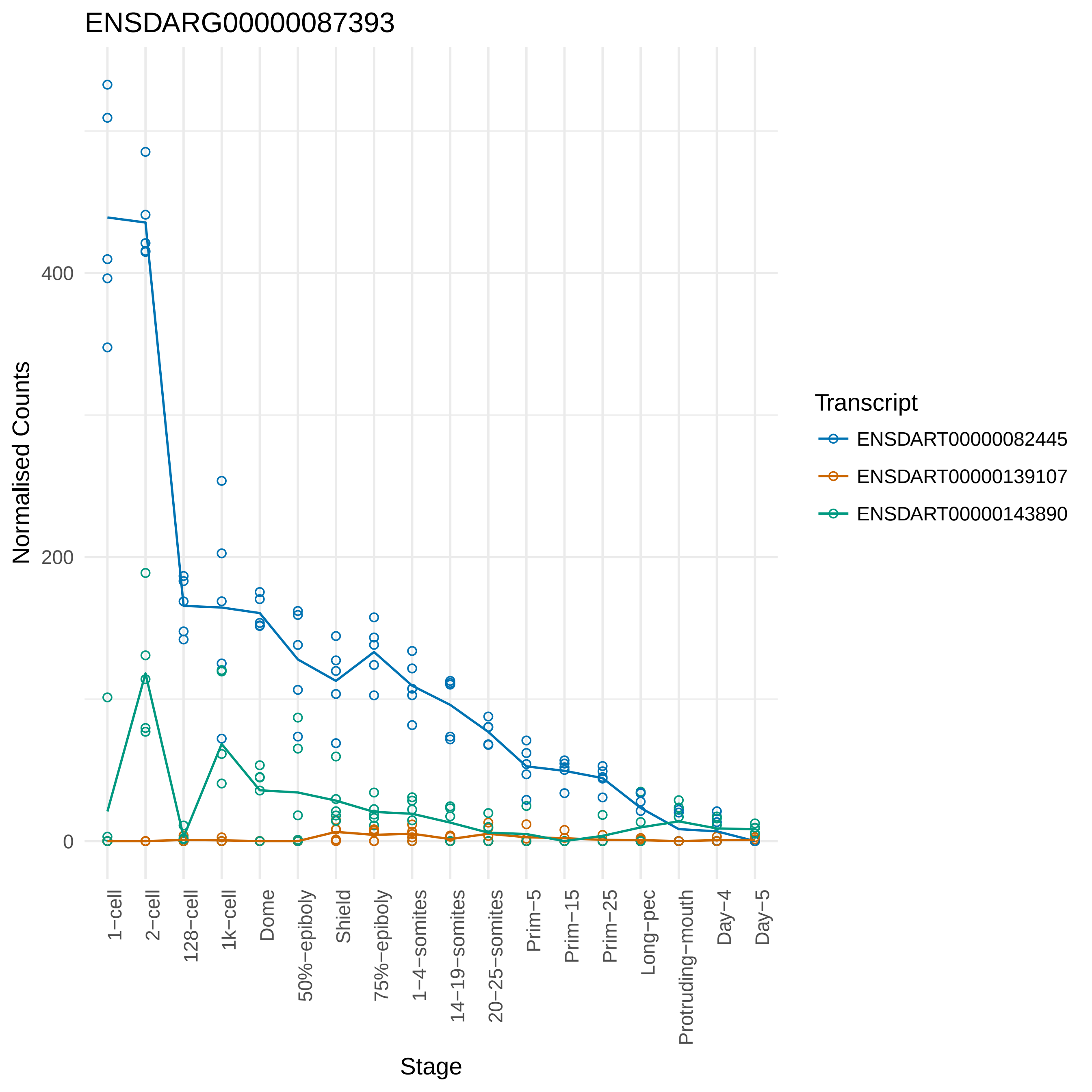
ENSDARG00000087393
Normalised Counts
400
200
0
1−cell
2−cell
128−cell
1k−cell
Dome
50%−epiboly
Shield
75%−epiboly
1−4−somites
14−19−somites
20−25−somites
Prim−5
Prim−15
Prim−25
Long−pec
Protruding−mouth
Day−4
Day−5
Stage
Transcript
ENSDART00000082445
ENSDART00000139107
ENSDART00000143890

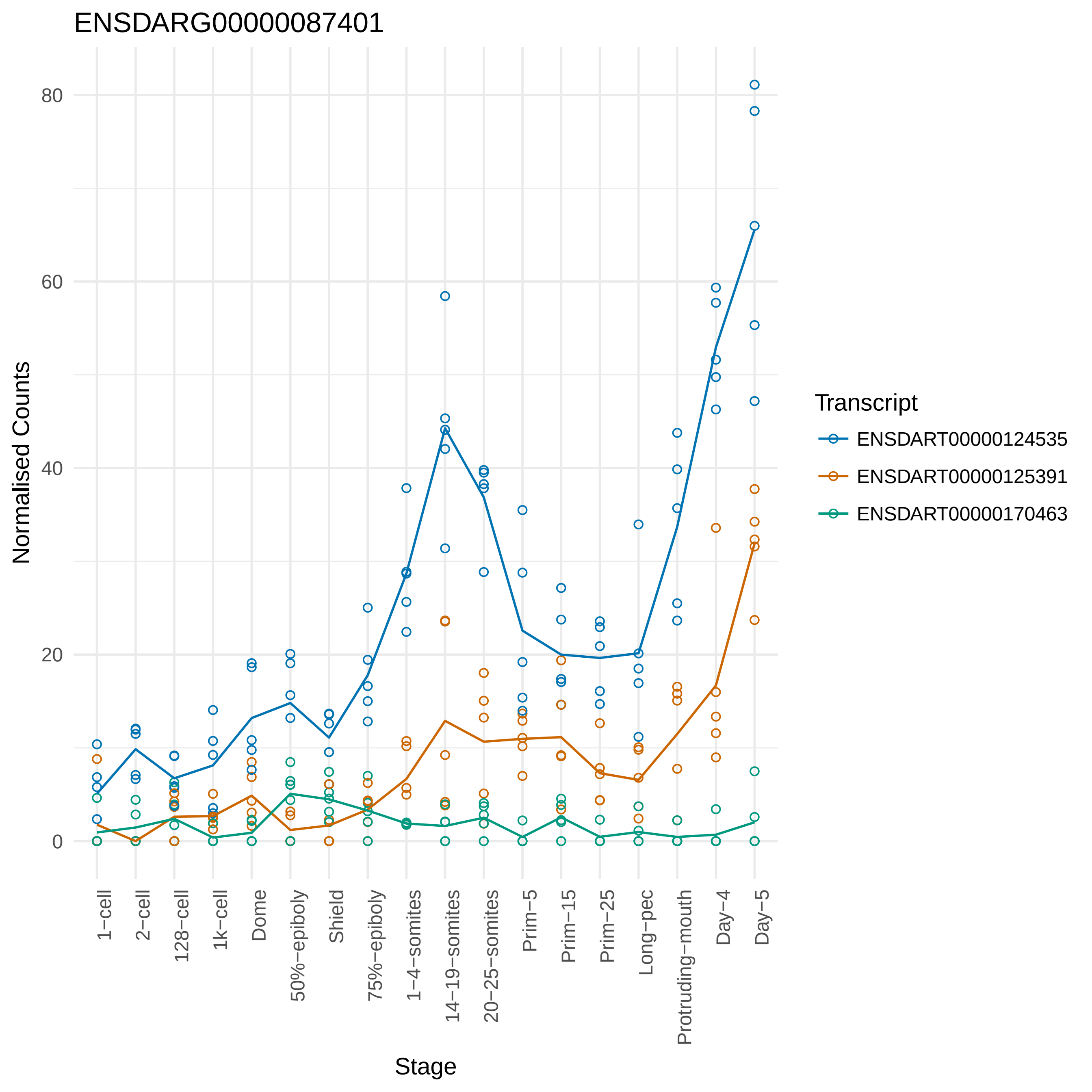
ENSDARG00000087401
Normalised Counts
0
20
40
60
80
Stage
1−cell
2−cell
128−cell
1k−cell
Dome
50%−epiboly
Shield
75%−epiboly
1−4−somites
14−19−somites
20−25−somites
Prim−5
Prim−15
Prim−25
Long−pec
Protruding−mouth
Day−4
Day−5
Transcript
ENSDART00000124535
ENSDART00000125391
ENSDART00000170463

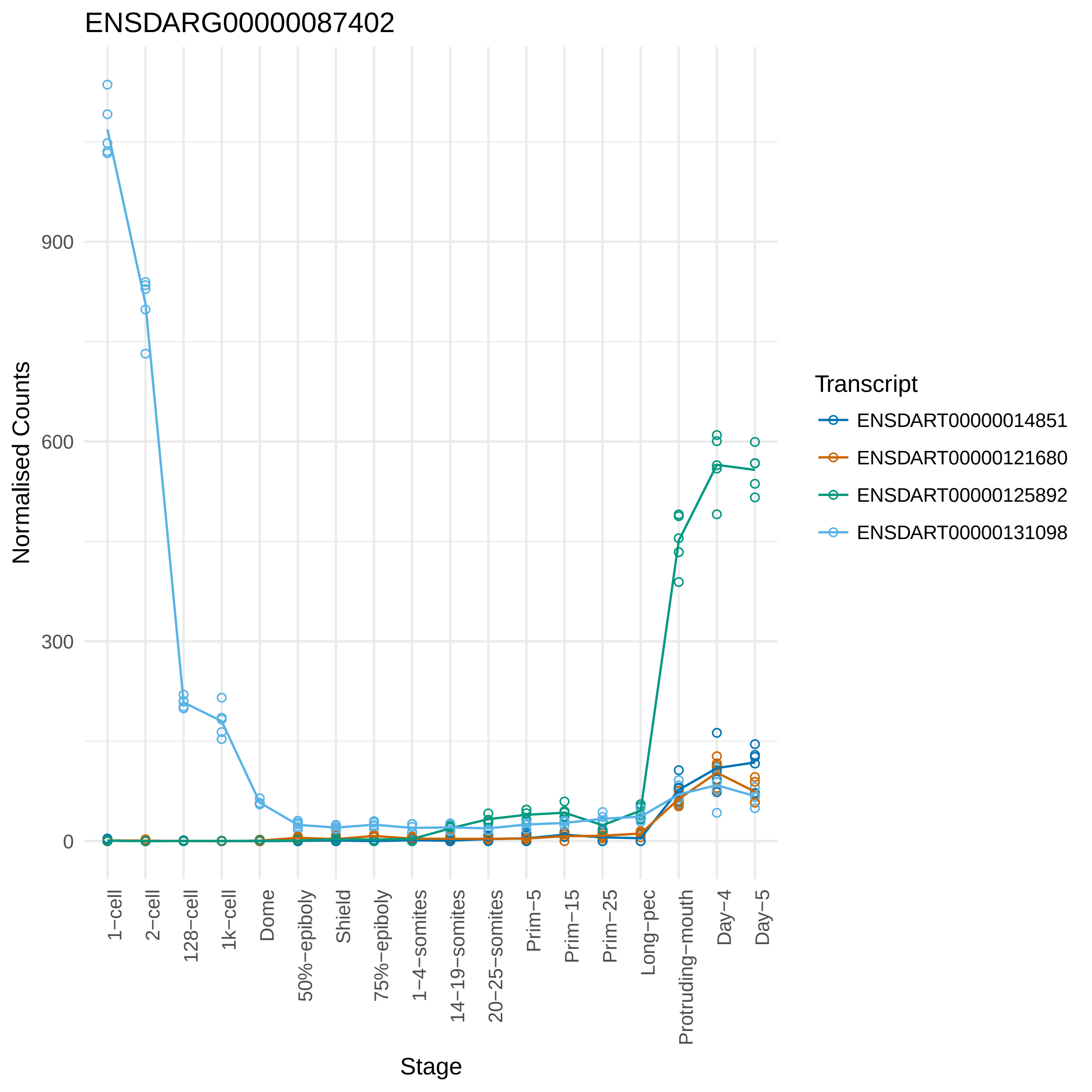

ENSDARG00000087402
Normalised Counts
900
600
300
0
1−cell
2−cell
128−cell
1k−cell
Dome
50%−epiboly
Shield
75%−epiboly
1−4−somites
14−19−somites
20−25−somites
Prim−5
Prim−15
Prim−25
Long−pec
Protruding−mouth
Day−4
Day−5
Stage
Transcript
ENSDART00000014851
ENSDART00000121680
ENSDART00000125892
ENSDART00000131098

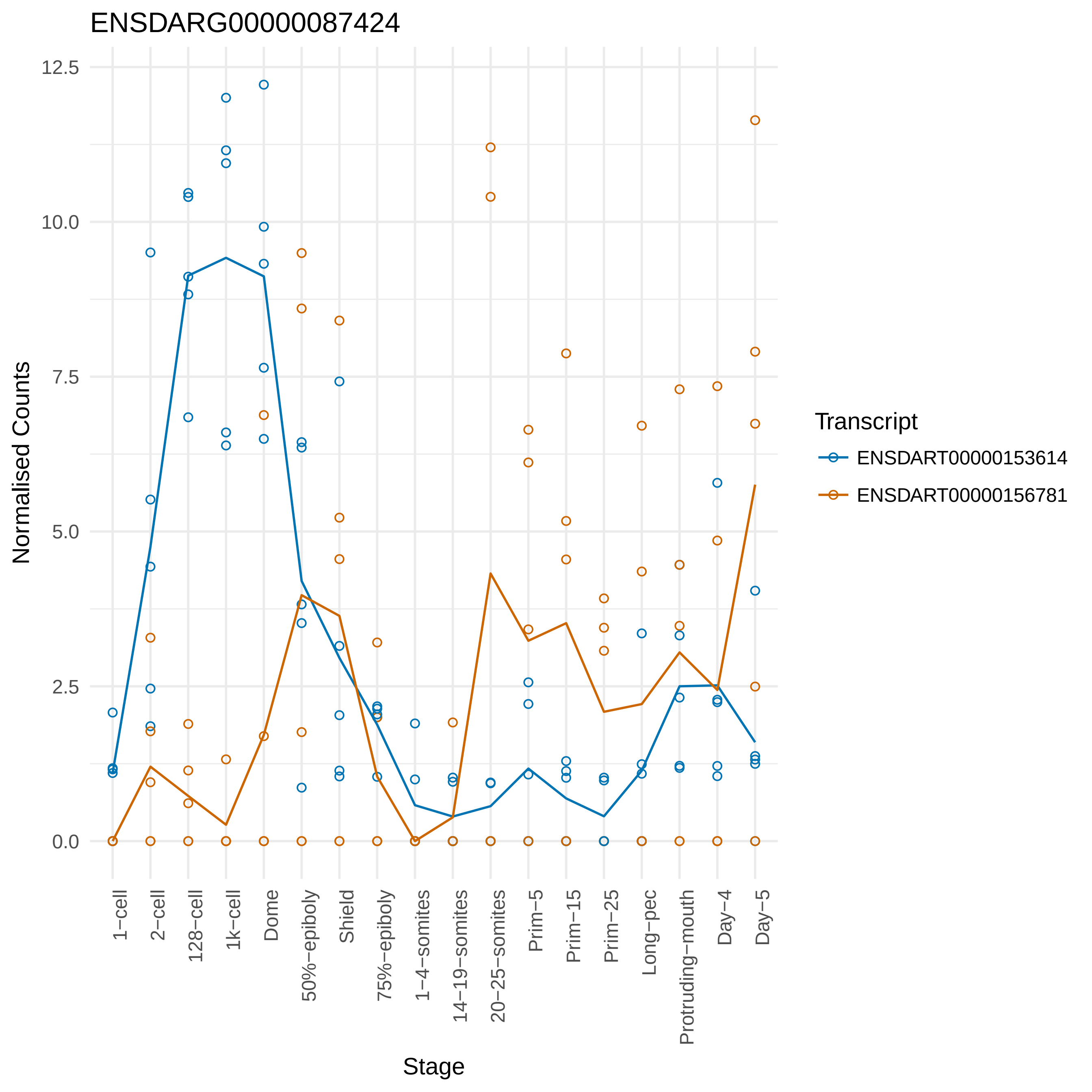

ENSDARG00000087424
12.5
10.0
7.5
5.0
2.5
0.0
Normalised Counts
1−cell
2−cell
128−cell
1k−cell
Dome
50%−epiboly
Shield
75%−epiboly
1−4−somites
14−19−somites
20−25−somites
Prim−5
Prim−15
Prim−25
Long−pec
Protruding−mouth
Day−4
Day−5
Stage
Transcript
ENSDART00000153614
ENSDART00000156781

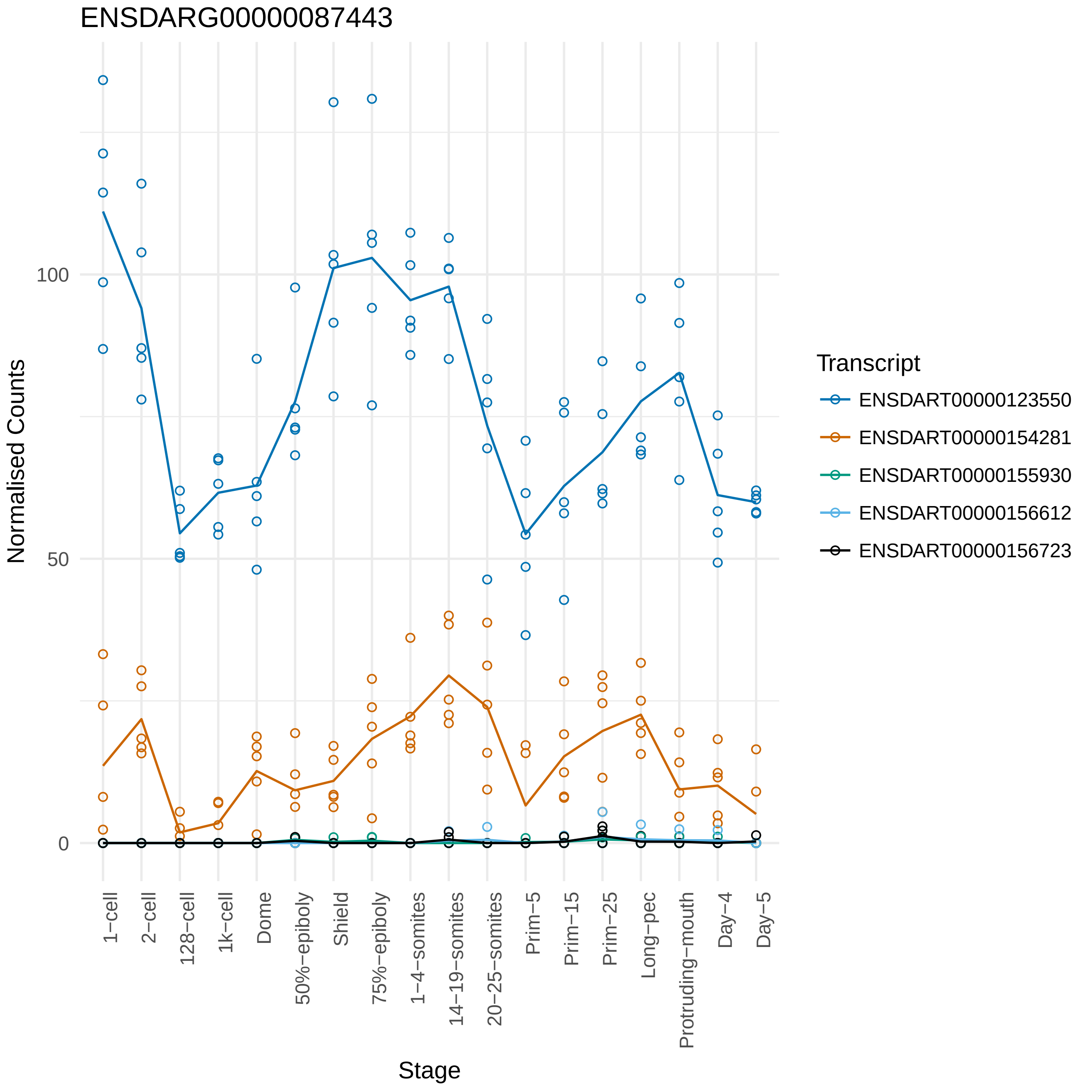
ENSDARG00000087443
Normalised Counts
100
50
0
1−cell
2−cell
128−cell
1k−cell
Dome
50%−epiboly
Shield
75%−epiboly
1−4−somites
14−19−somites
20−25−somites
Prim−5
Prim−15
Prim−25
Long−pec
Protruding−mouth
Day−4
Day−5
Stage
Transcript
ENSDART00000123550
ENSDART00000154281
ENSDART00000155930
ENSDART00000156612
ENSDART00000156723

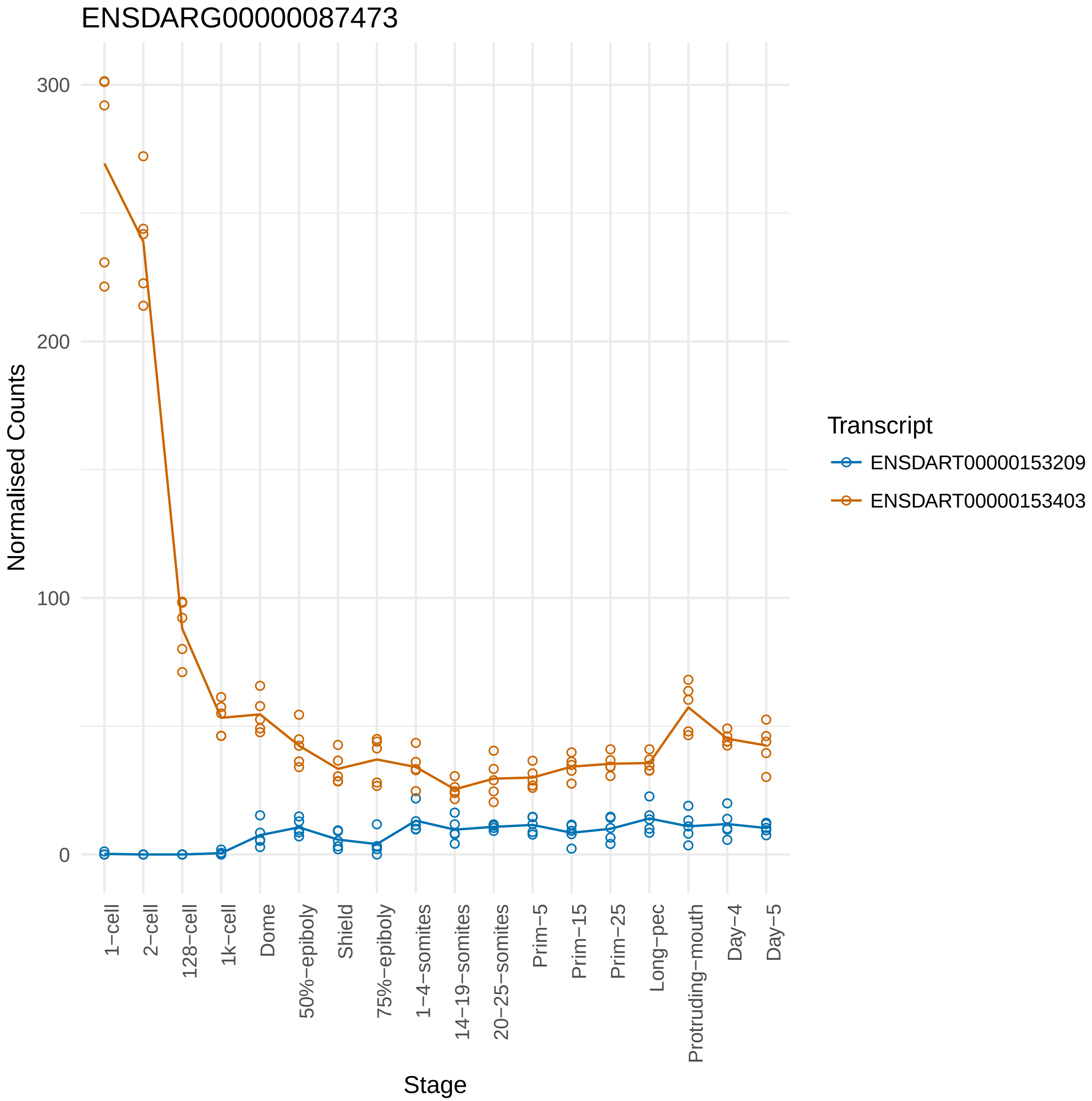

ENSDARG00000087473
Normalised Counts
300
200
100
0
1−cell
2−cell
128−cell
1k−cell
Dome
50%−epiboly
Shield
75%−epiboly
1−4−somites
14−19−somites
20−25−somites
Prim−5
Prim−15
Prim−25
Long−pec
Protruding−mouth
Day−4
Day−5
Stage
Transcript
ENSDART00000153209
ENSDART00000153403

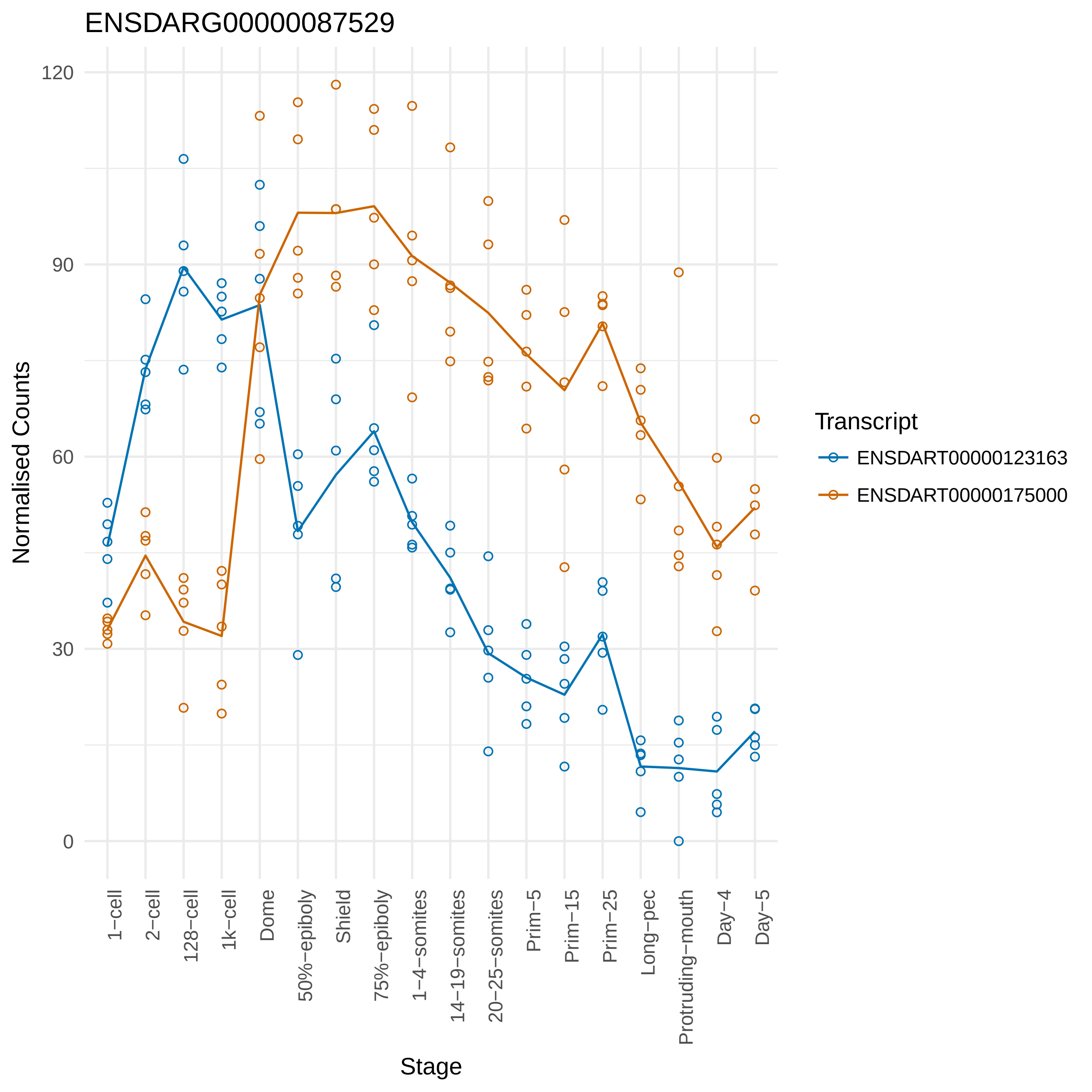

ENSDARG00000087529
Normalised Counts
0
30
60
90
120
Stage
1−cell
2−cell
128−cell
1k−cell
Dome
50%−epiboly
Shield
75%−epiboly
1−4−somites
14−19−somites
20−25−somites
Prim−5
Prim−15
Prim−25
Long−pec
Protruding−mouth
Day−4
Day−5
Transcript
ENSDART00000123163
ENSDART00000175000

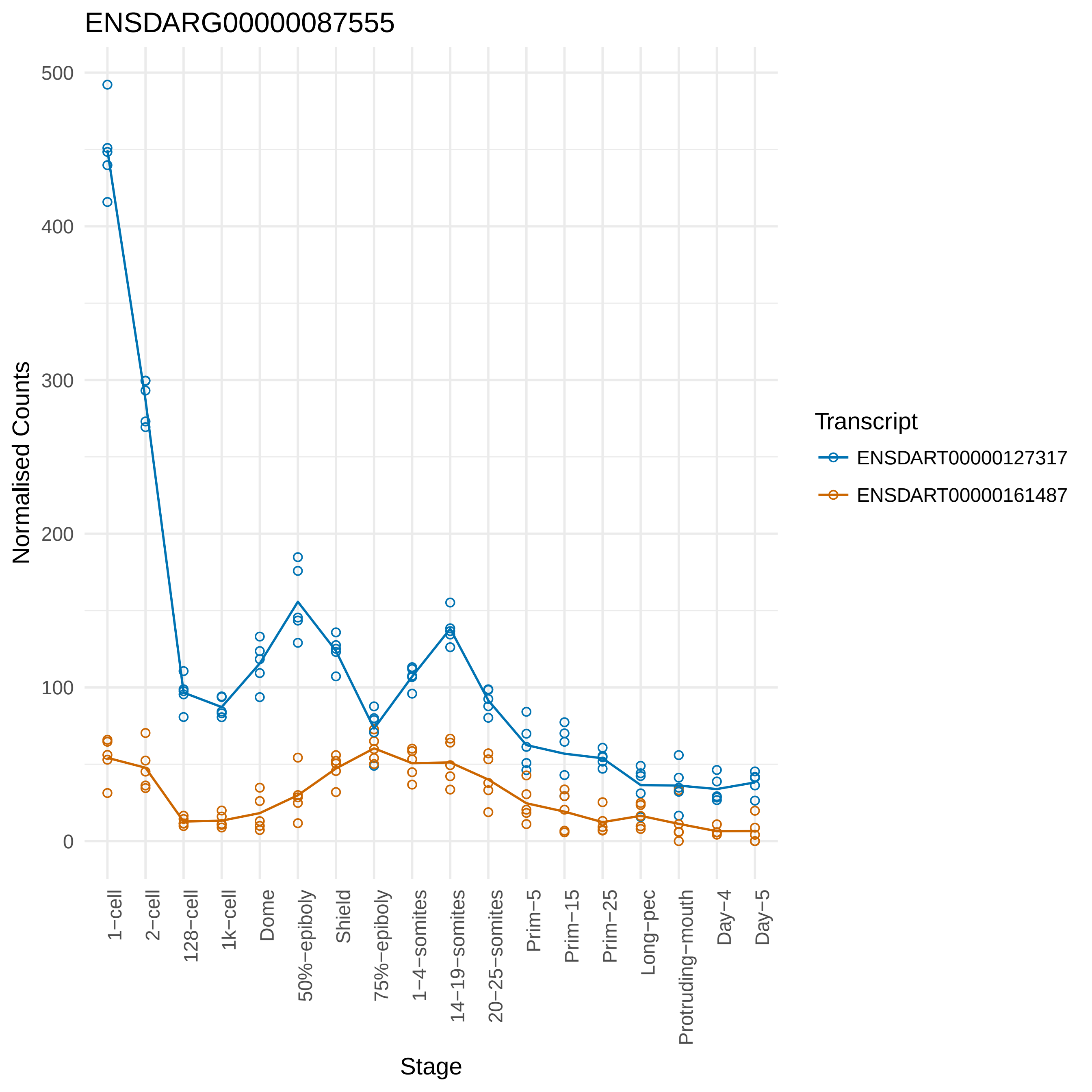
ENSDARG00000087555
Normalised Counts
500
400
300
200
100
0
1−cell
2−cell
128−cell
1k−cell
Dome
50%−epiboly
Shield
75%−epiboly
1−4−somites
14−19−somites
20−25−somites
Prim−5
Prim−15
Prim−25
Long−pec
Protruding−mouth
Day−4
Day−5
Stage
Transcript
ENSDART00000127317
ENSDART00000161487

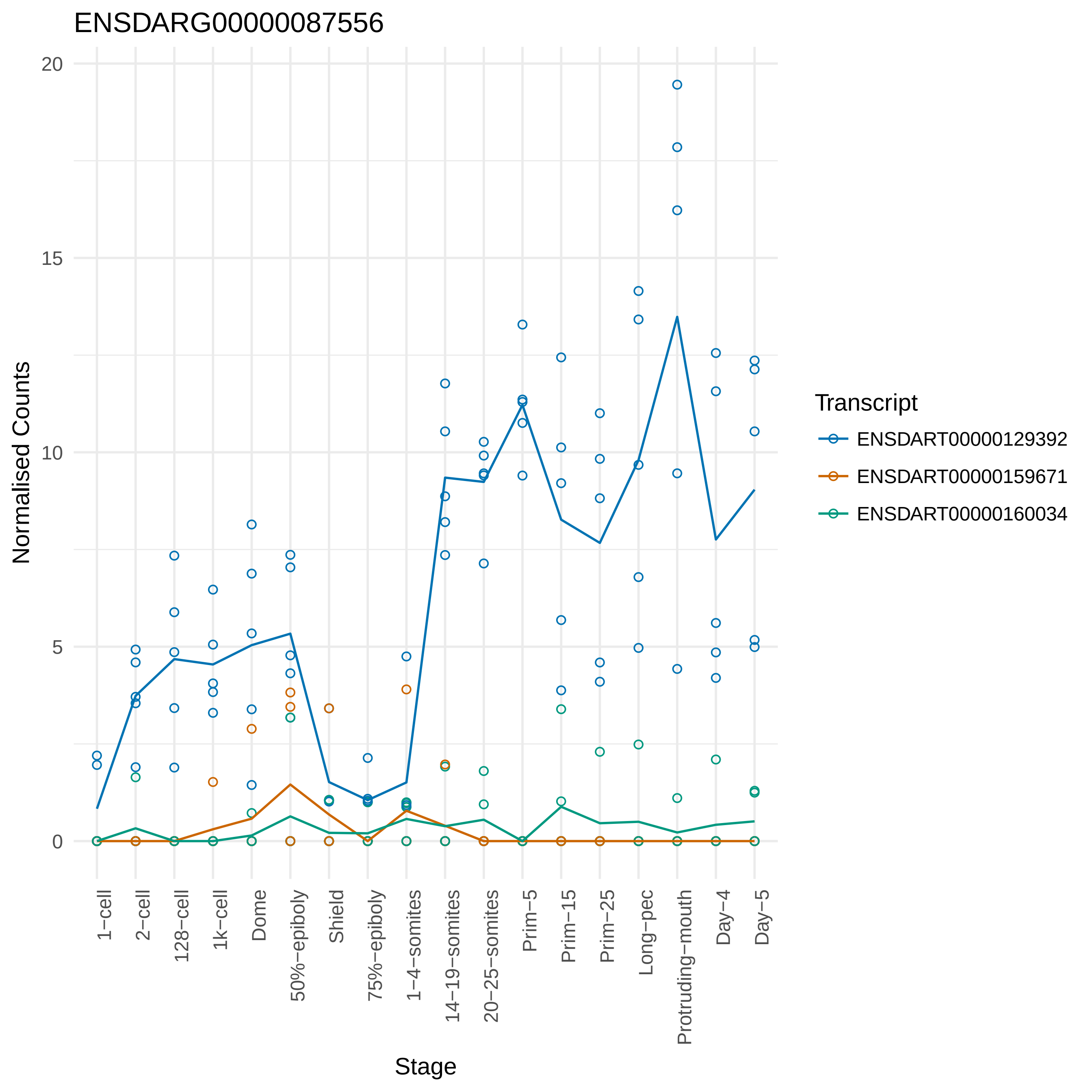

ENSDARG00000087556
Normalised Counts
0
5
10
15
20
1−cell
2−cell
128−cell
1k−cell
Dome
50%−epiboly
Shield
75%−epiboly
1−4−somites
14−19−somites
20−25−somites
Prim−5
Prim−15
Prim−25
Long−pec
Protruding−mouth
Day−4
Day−5
Stage
Transcript
ENSDART00000129392
ENSDART00000159671
ENSDART00000160034

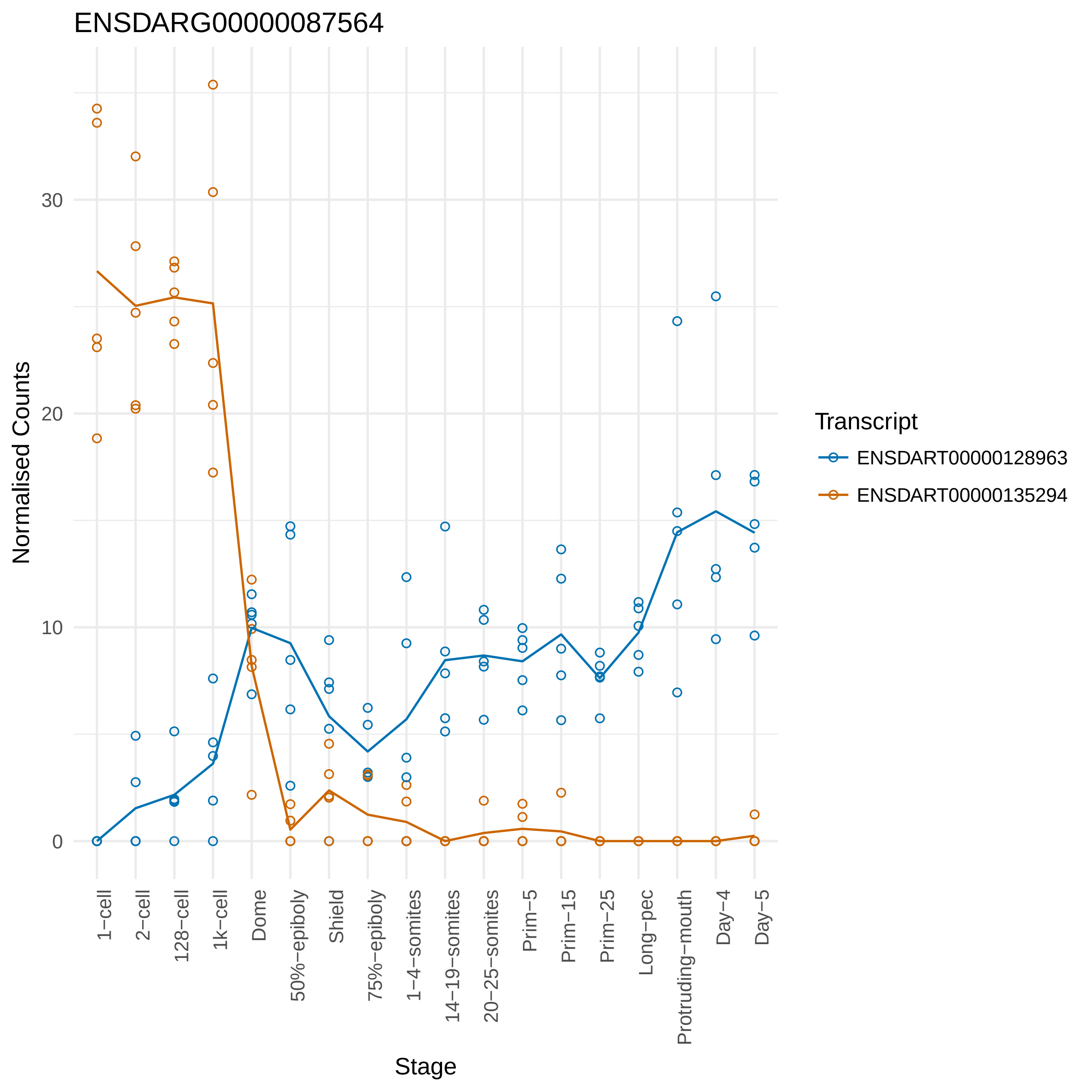

ENSDARG00000087564
Normalised Counts
0
10
20
30
1−cell
2−cell
128−cell
1k−cell
Dome
50%−epiboly
Shield
75%−epiboly
1−4−somites
14−19−somites
20−25−somites
Prim−5
Prim−15
Prim−25
Long−pec
Protruding−mouth
Day−4
Day−5
Stage
Transcript
ENSDART00000128963
ENSDART00000135294

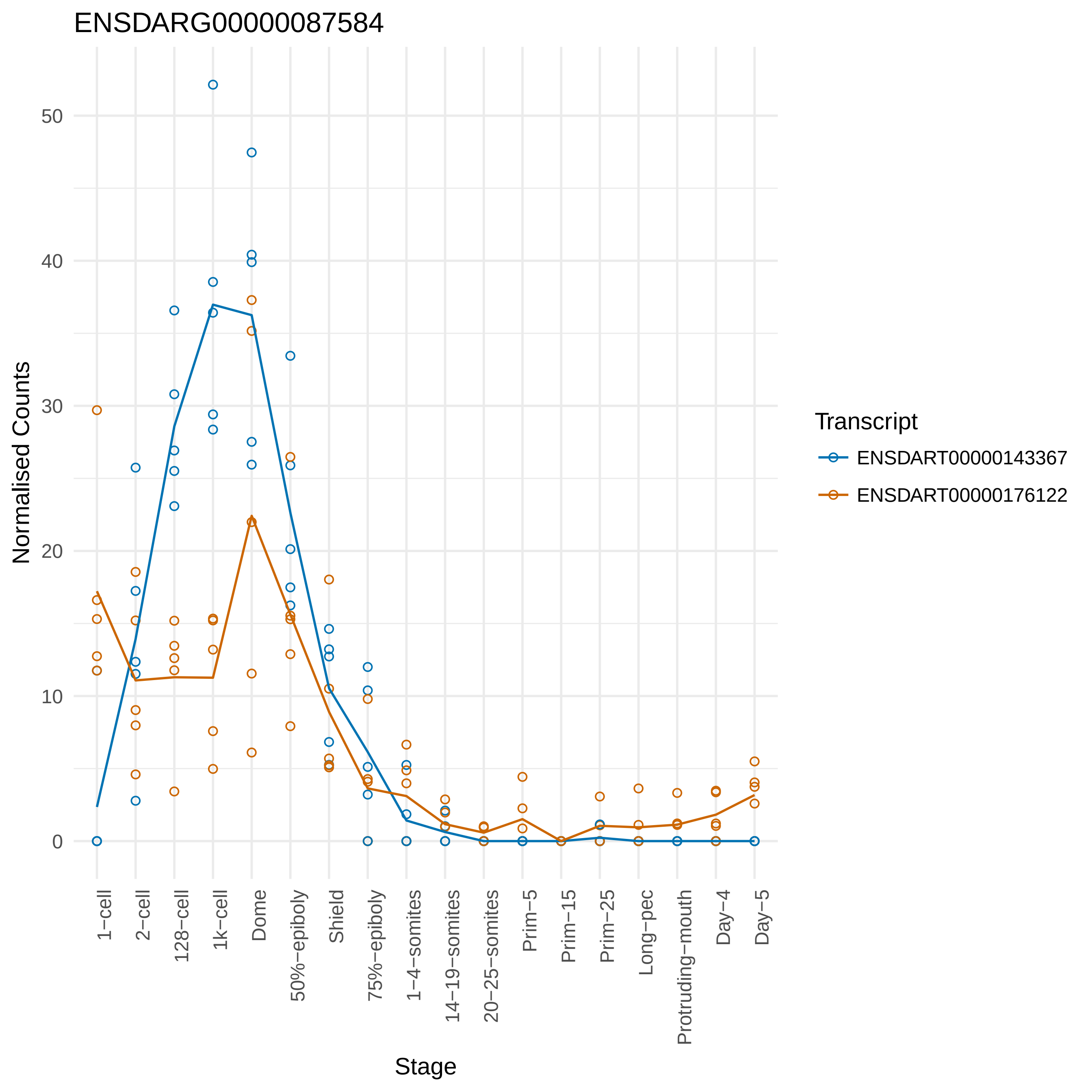
ENSDARG00000087584
Normalised Counts
0
10
20
30
40
50
Stage
1−cell
2−cell
128−cell
1k−cell
Dome
50%−epiboly
Shield
75%−epiboly
1−4−somites
14−19−somites
20−25−somites
Prim−5
Prim−15
Prim−25
Long−pec
Protruding−mouth
Day−4
Day−5
Transcript
ENSDART00000143367
ENSDART00000176122

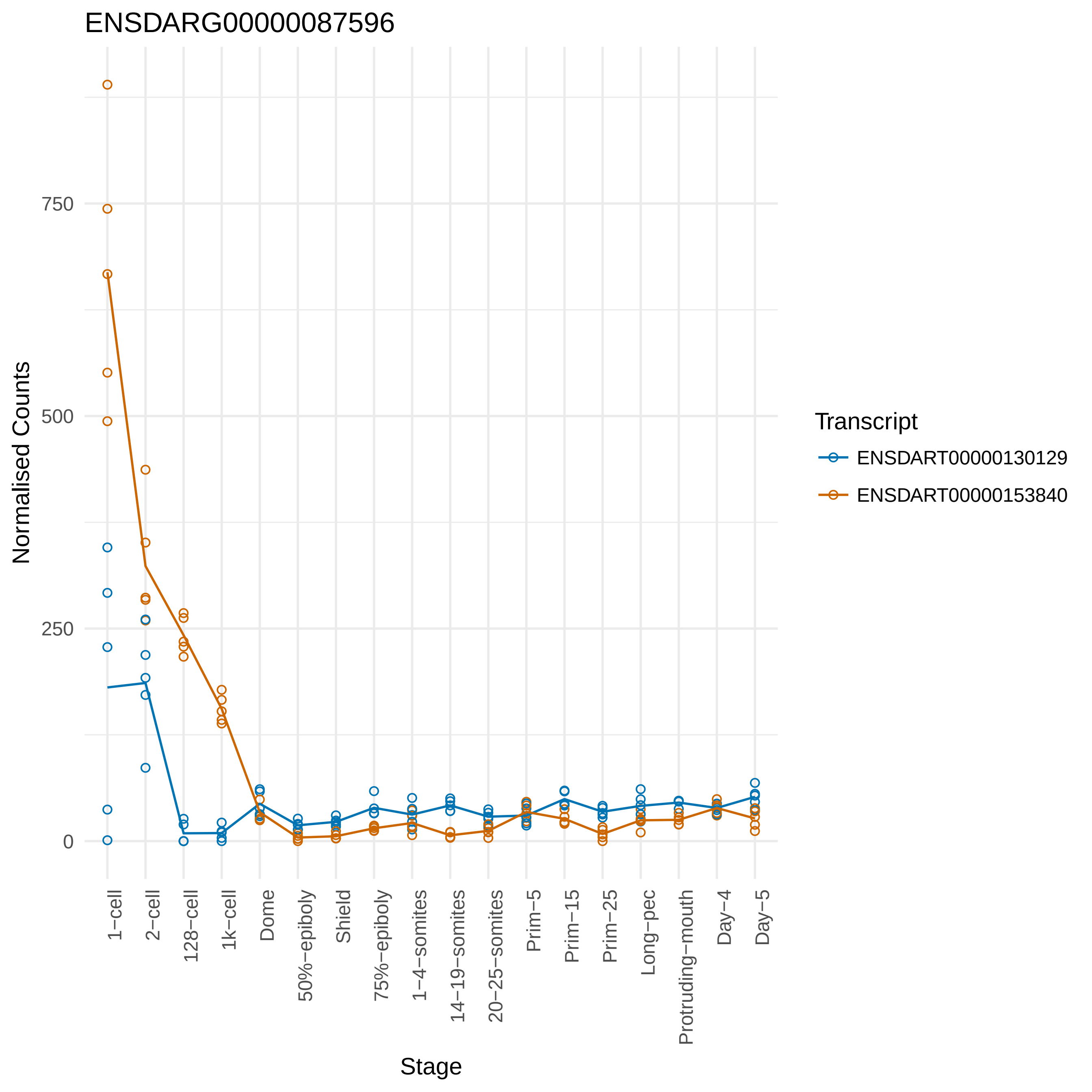
ENSDARG00000087596
Normalised Counts
750
500
250
0
Stage
1−cell
2−cell
128−cell
1k−cell
Dome
50%−epiboly
Shield
75%−epiboly
1−4−somites
14−19−somites
20−25−somites
Prim−5
Prim−15
Prim−25
Long−pec
Protruding−mouth
Day−4
Day−5
Transcript
ENSDART00000130129
ENSDART00000153840

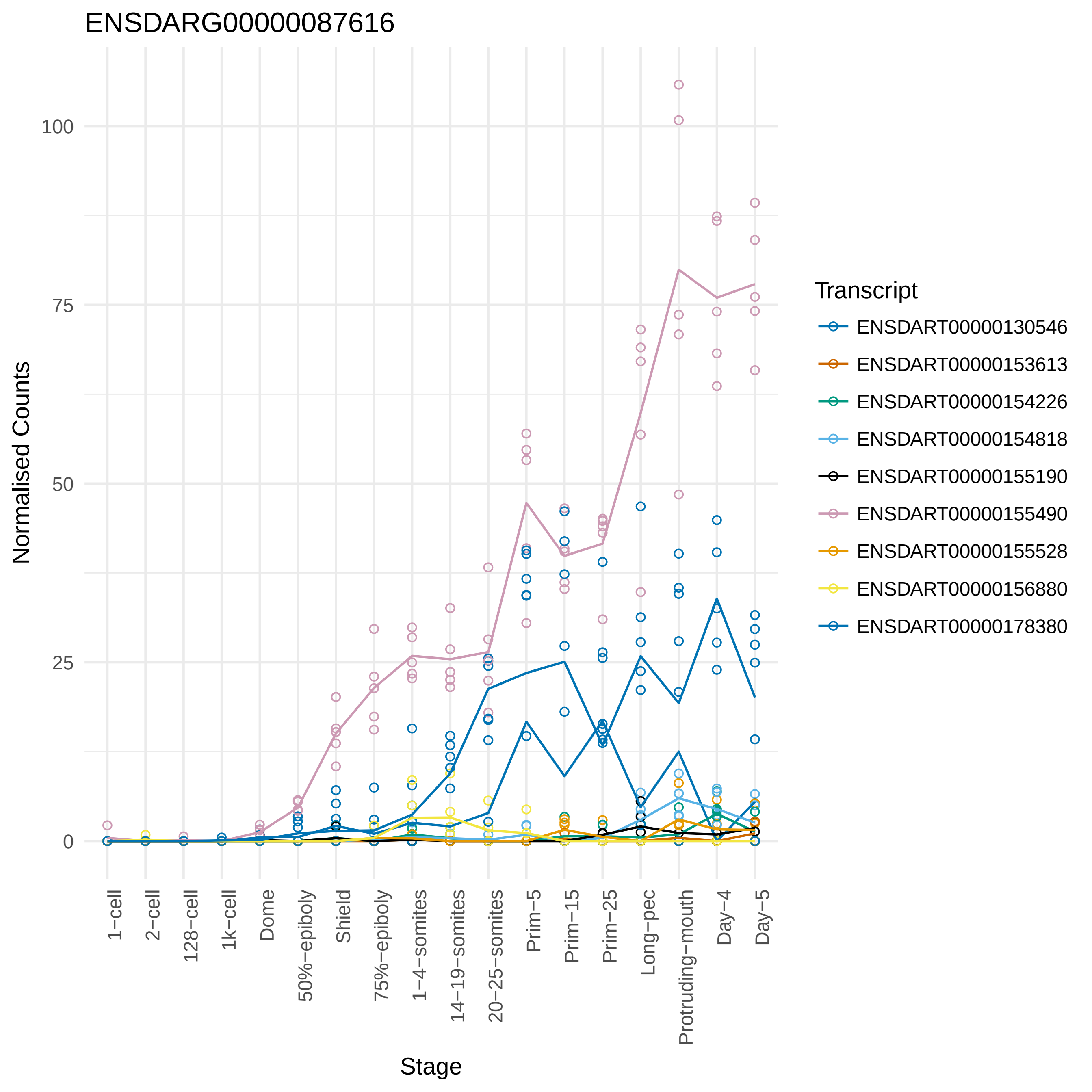

ENSDARG00000087616
Normalised Counts
100
75
50
25
0
1−cell
2−cell
128−cell
1k−cell
Dome
50%−epiboly
Shield
75%−epiboly
1−4−somites
14−19−somites
20−25−somites
Prim−5
Prim−15
Prim−25
Long−pec
Protruding−mouth
Day−4
Day−5
Stage
Transcript
ENSDART00000130546
ENSDART00000153613
ENSDART00000154226
ENSDART00000154818
ENSDART00000155190
ENSDART00000155490
ENSDART00000155528
ENSDART00000156880
ENSDART00000178380

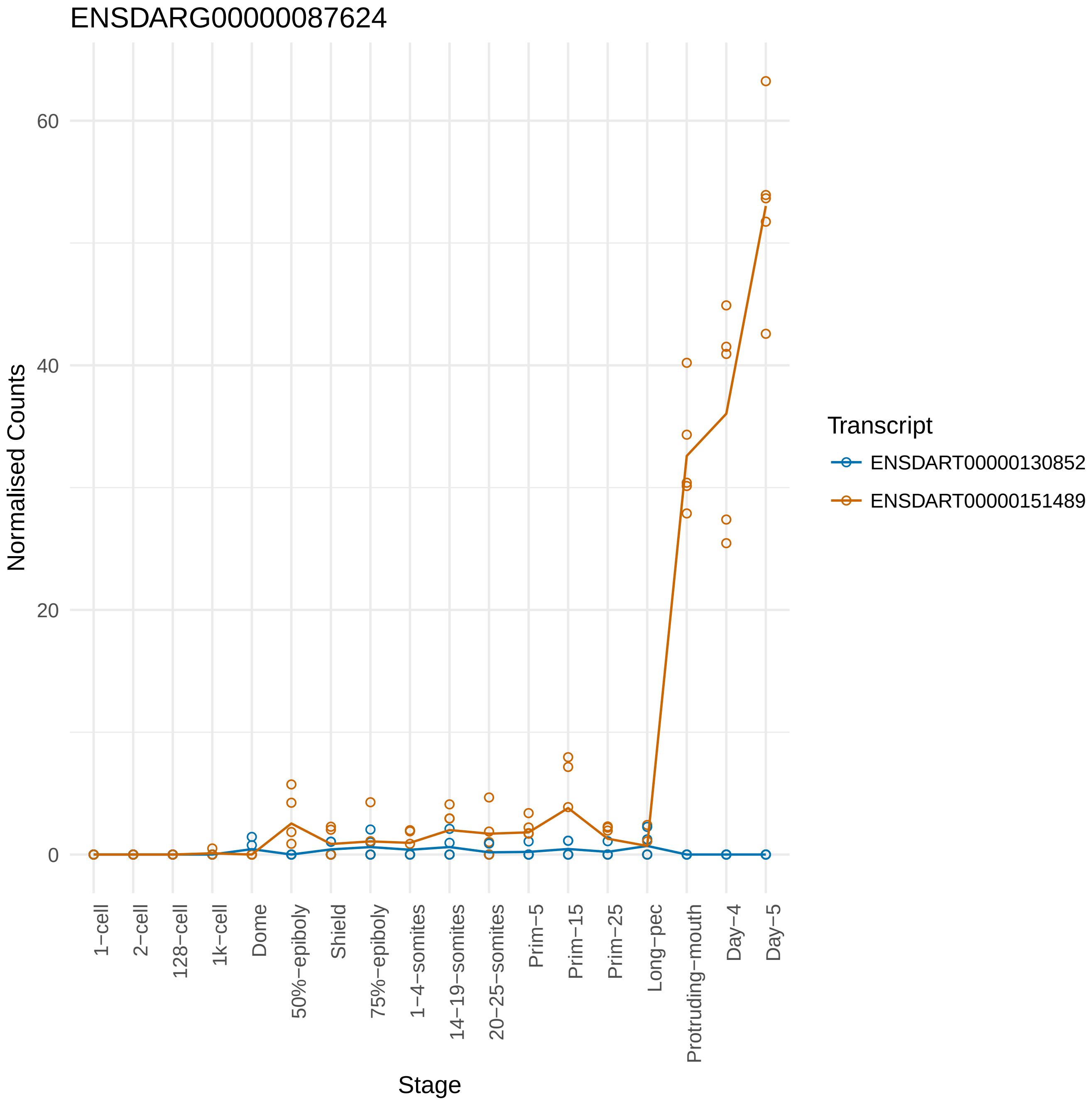

ENSDARG00000087624
Normalised Counts
60
40
20
0
1−cell
2−cell
128−cell
1k−cell
Dome
50%−epiboly
Shield
75%−epiboly
1−4−somites
14−19−somites
20−25−somites
Prim−5
Prim−15
Prim−25
Long−pec
Protruding−mouth
Day−4
Day−5
Stage
Transcript
ENSDART00000130852
ENSDART00000151489

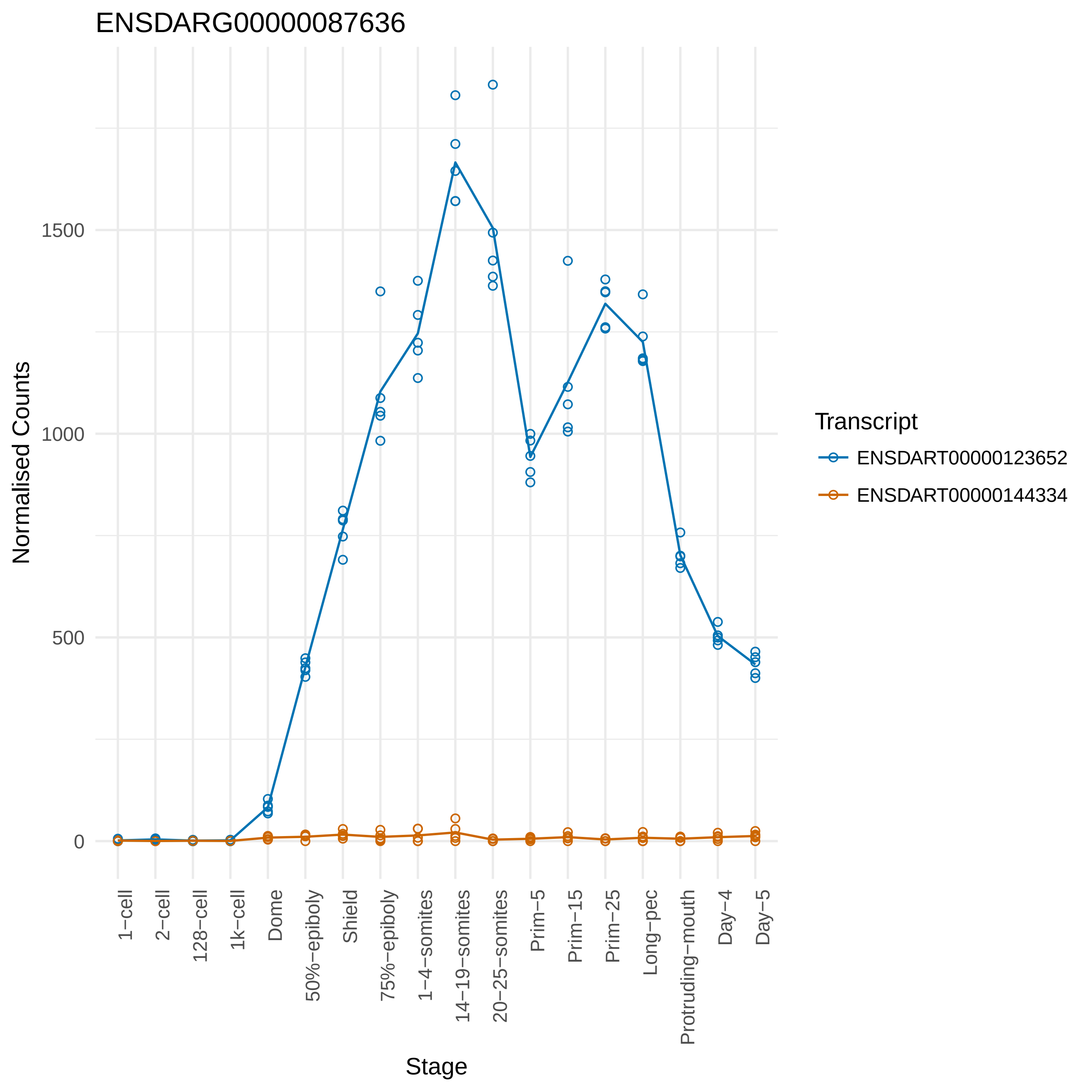
ENSDARG00000087636
Normalised Counts
1500
1000
500
0
1−cell
2−cell
128−cell
1k−cell
Dome
50%−epiboly
Shield
75%−epiboly
1−4−somites
14−19−somites
20−25−somites
Prim−5
Prim−15
Prim−25
Long−pec
Protruding−mouth
Day−4
Day−5
Stage
Transcript
ENSDART00000123652
ENSDART00000144334

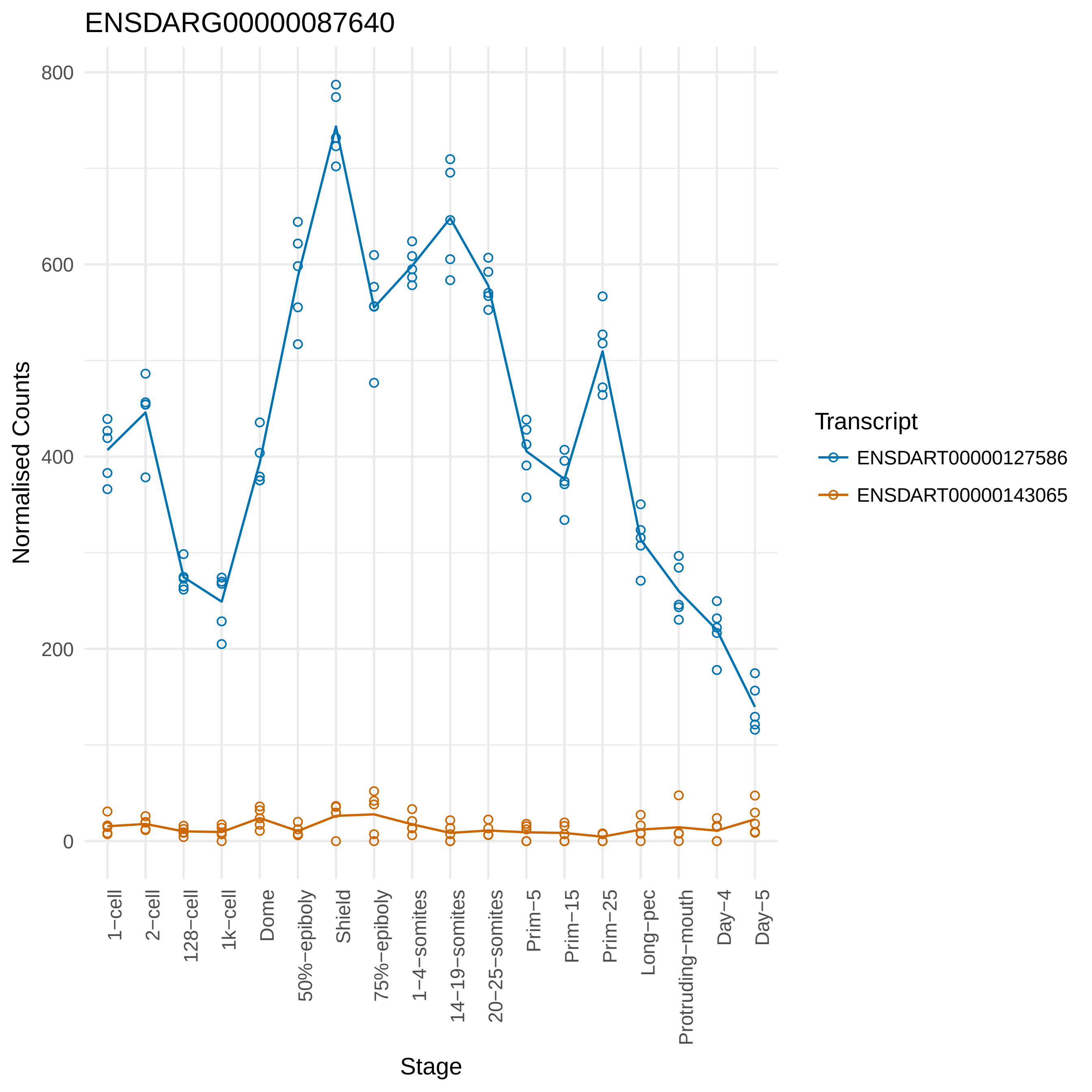

ENSDARG00000087640
Normalised Counts
800
600
400
200
0
1−cell
2−cell
128−cell
1k−cell
Dome
50%−epiboly
Shield
75%−epiboly
1−4−somites
14−19−somites
20−25−somites
Prim−5
Prim−15
Prim−25
Long−pec
Protruding−mouth
Day−4
Day−5
Stage
Transcript
ENSDART00000127586
ENSDART00000143065

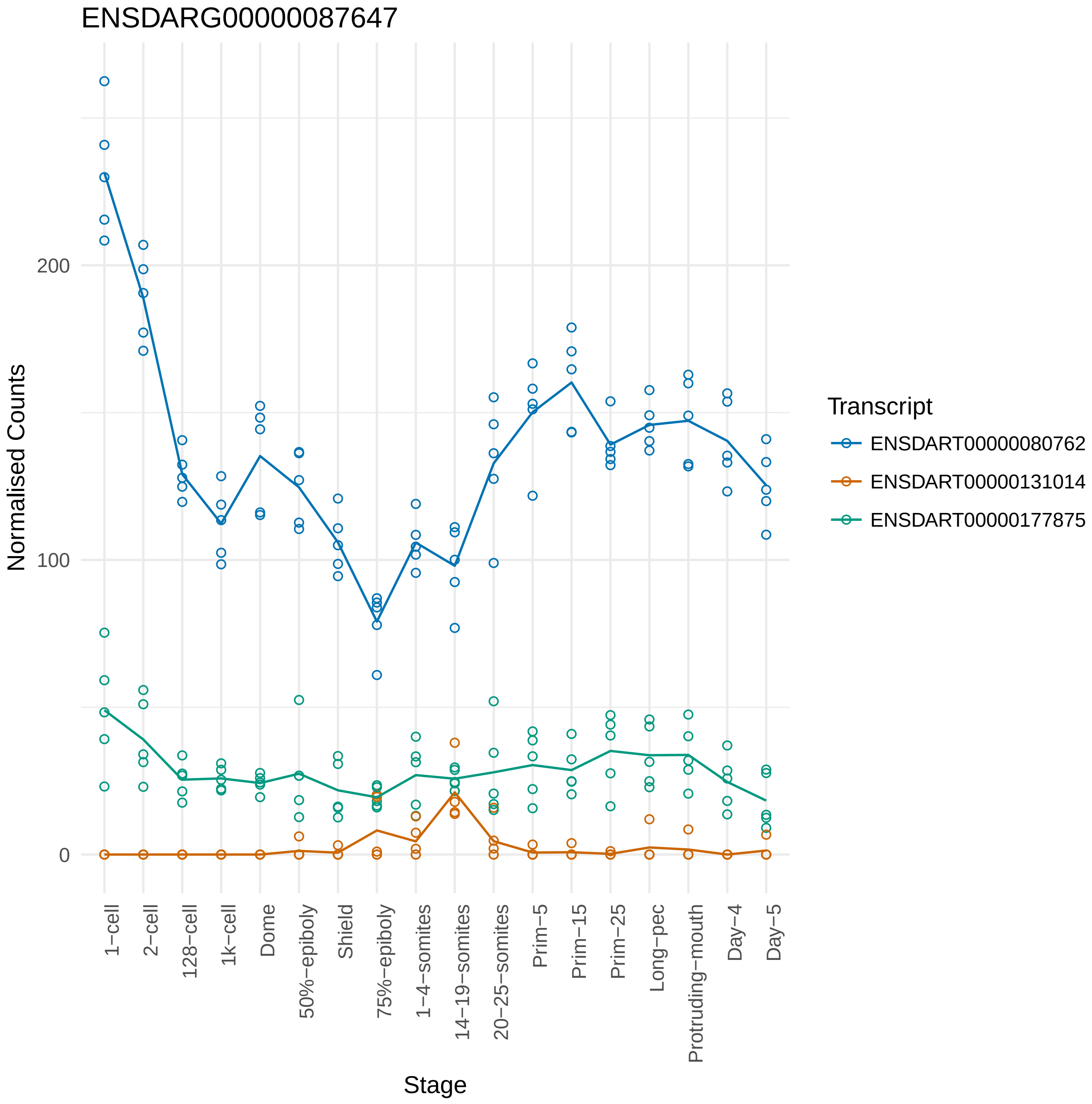

ENSDARG00000087647
Normalised Counts
200
100
0
1−cell
2−cell
128−cell
1k−cell
Dome
50%−epiboly
Shield
75%−epiboly
1−4−somites
14−19−somites
20−25−somites
Prim−5
Prim−15
Prim−25
Long−pec
Protruding−mouth
Day−4
Day−5
Stage
Transcript
ENSDART00000080762
ENSDART00000131014
ENSDART00000177875

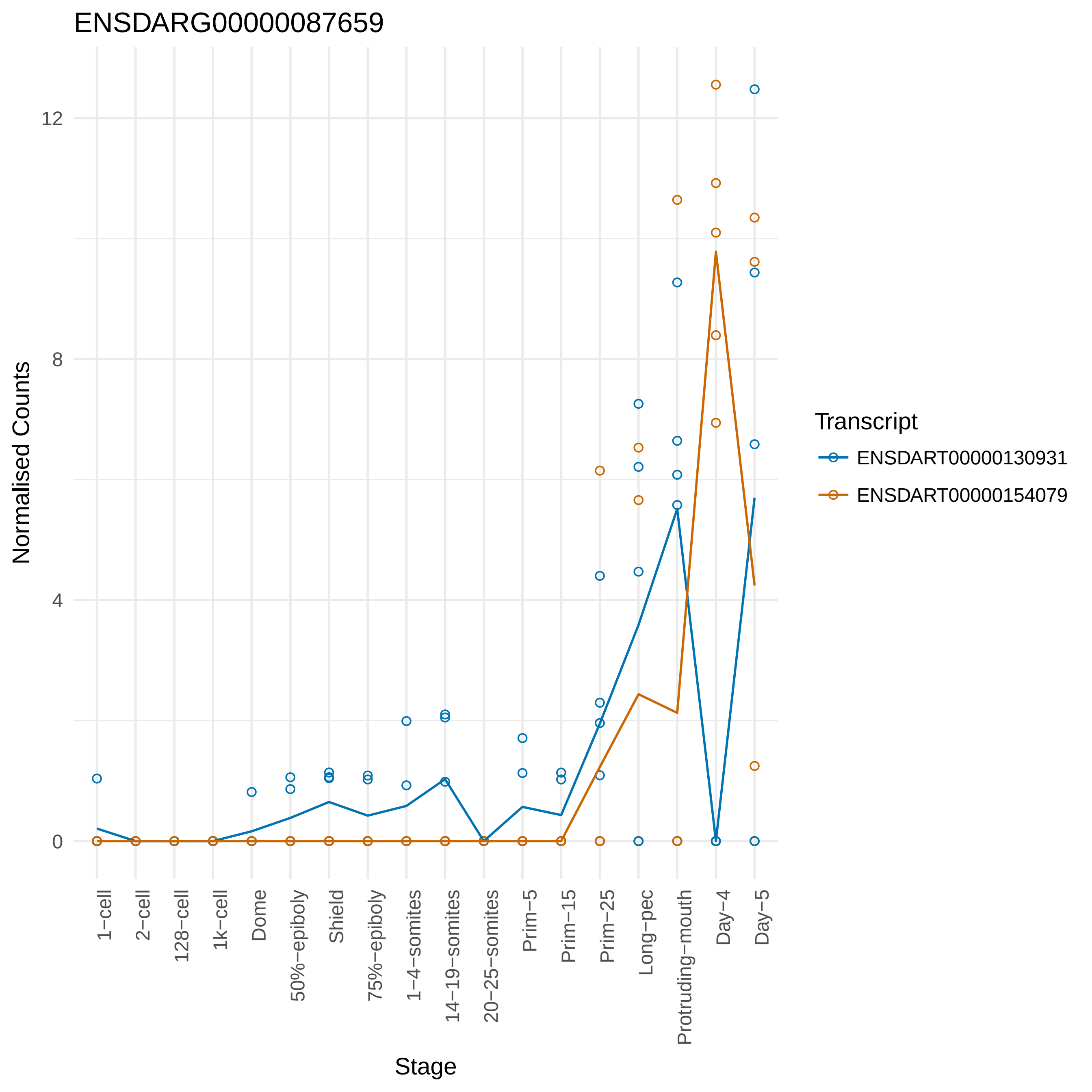
ENSDARG00000087659
Normalised Counts
0
4
8
12
1−cell
2−cell
128−cell
1k−cell
Dome
50%−epiboly
Shield
75%−epiboly
1−4−somites
14−19−somites
20−25−somites
Prim−5
Prim−15
Prim−25
Long−pec
Protruding−mouth
Day−4
Day−5
Stage
Transcript
ENSDART00000130931
ENSDART00000154079

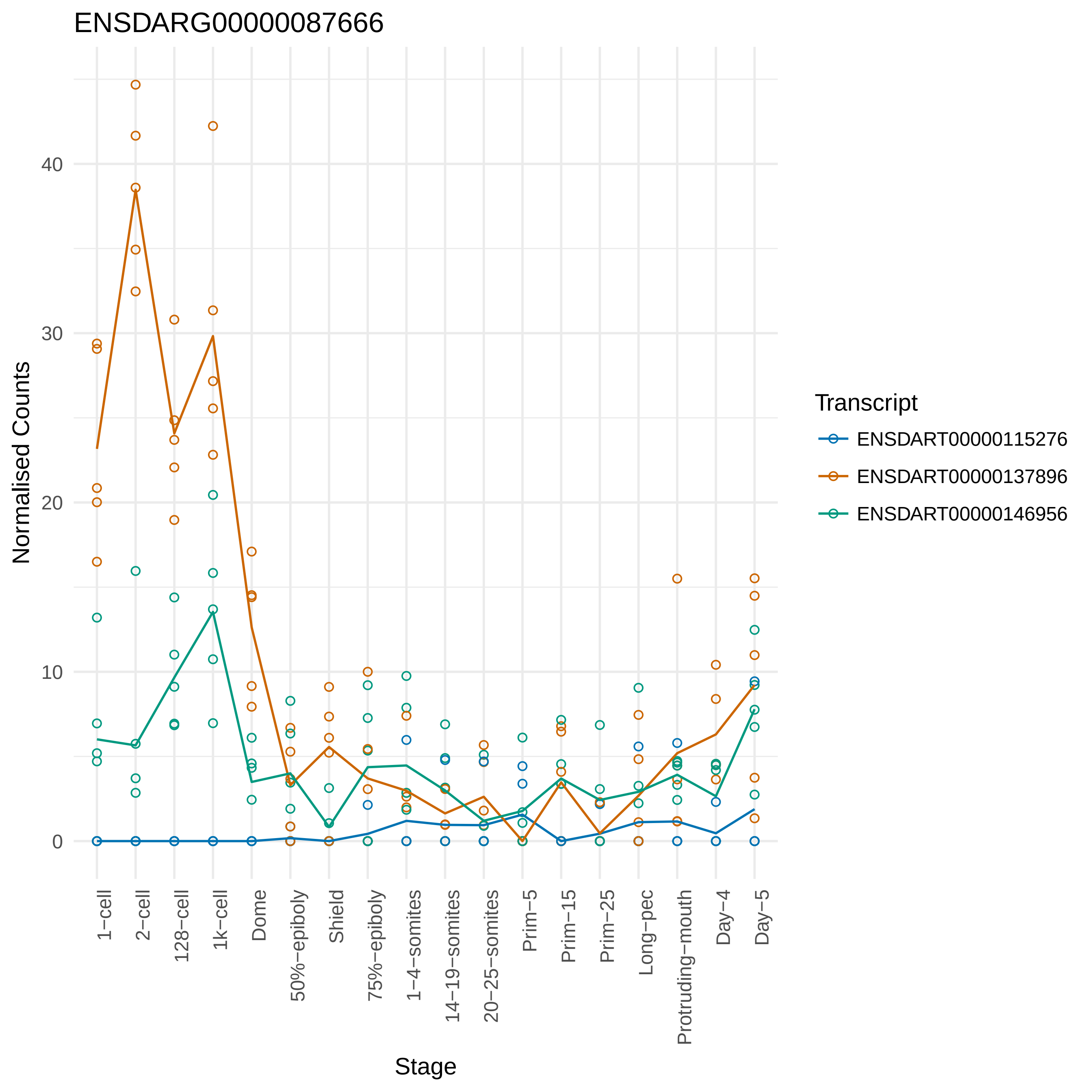

ENSDARG00000087666
Normalised Counts
0
10
20
30
40
Stage
1−cell
2−cell
128−cell
1k−cell
Dome
50%−epiboly
Shield
75%−epiboly
1−4−somites
14−19−somites
20−25−somites
Prim−5
Prim−15
Prim−25
Long−pec
Protruding−mouth
Day−4
Day−5
Transcript
ENSDART00000115276
ENSDART00000137896
ENSDART00000146956

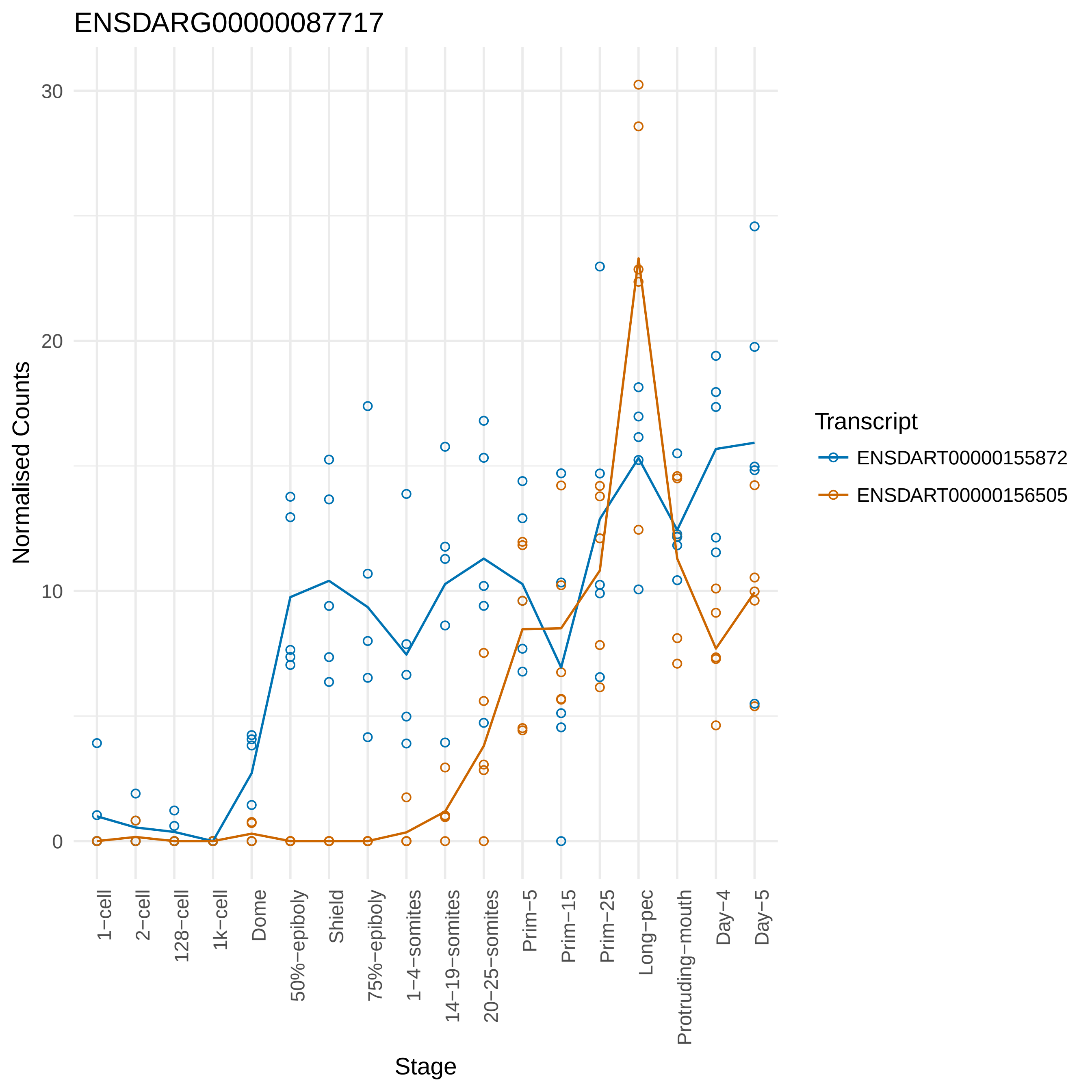
ENSDARG00000087717
Normalised Counts
30
20
10
0
1−cell
2−cell
128−cell
1k−cell
Dome
50%−epiboly
Shield
75%−epiboly
1−4−somites
14−19−somites
20−25−somites
Prim−5
Prim−15
Prim−25
Long−pec
Protruding−mouth
Day−4
Day−5
Stage
Transcript
ENSDART00000155872
ENSDART00000156505

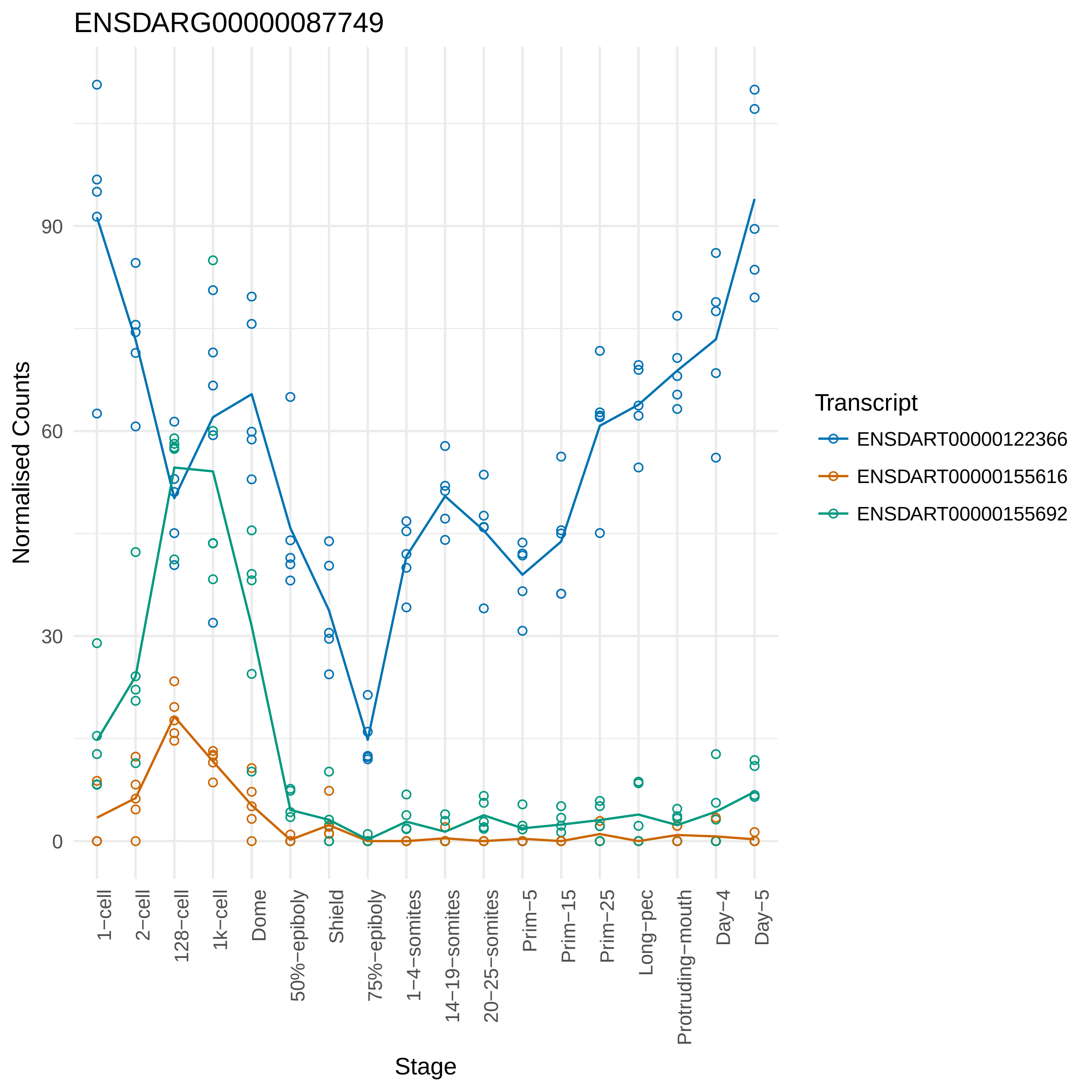

ENSDARG00000087749
Normalised Counts
0
30
60
90
1−cell
2−cell
128−cell
1k−cell
Dome
50%−epiboly
Shield
75%−epiboly
1−4−somites
14−19−somites
20−25−somites
Prim−5
Prim−15
Prim−25
Long−pec
Protruding−mouth
Day−4
Day−5
Stage
Transcript
ENSDART00000122366
ENSDART00000155616
ENSDART00000155692

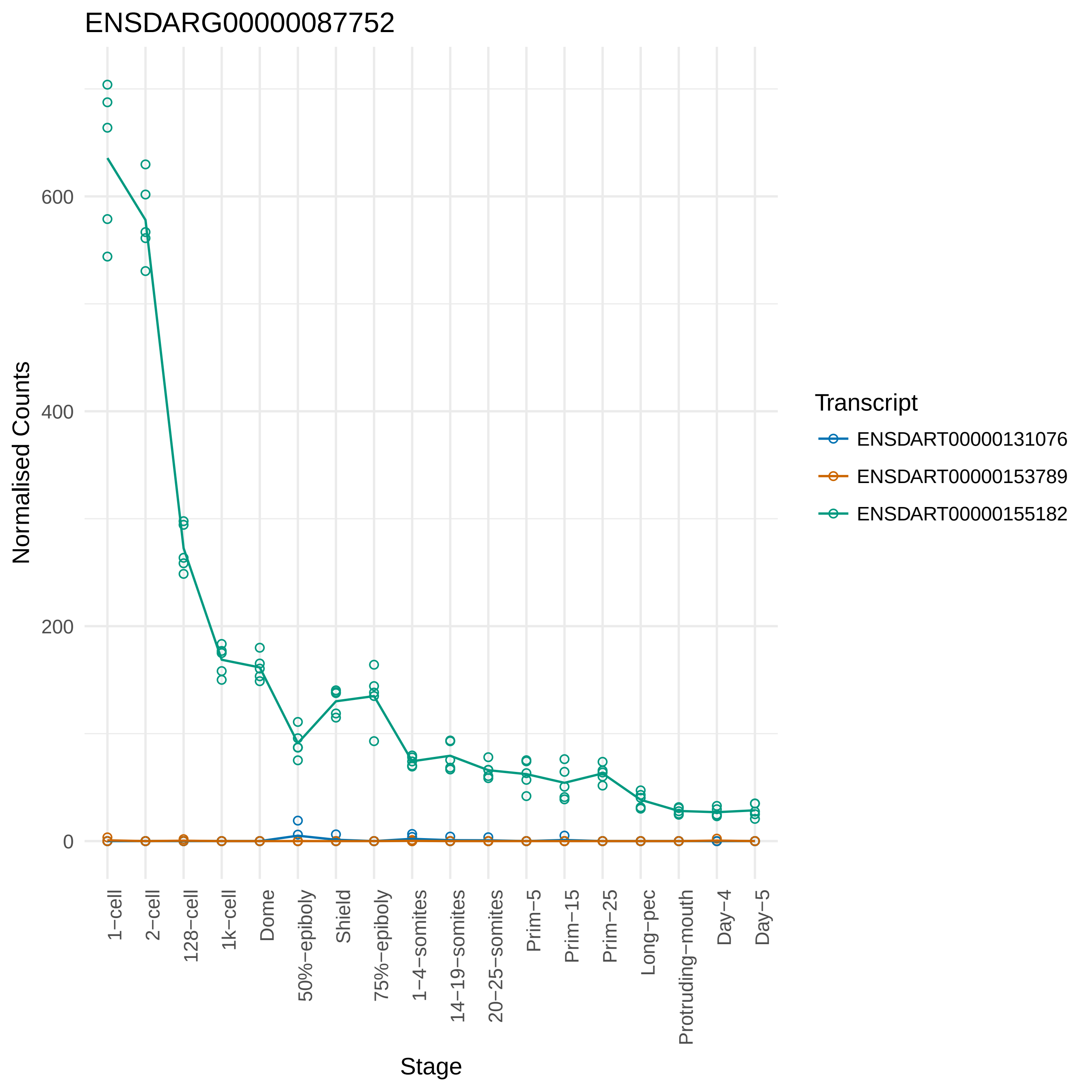

ENSDARG00000087752
Normalised Counts
0
200
400
600
Stage
1−cell
2−cell
128−cell
1k−cell
Dome
50%−epiboly
Shield
75%−epiboly
1−4−somites
14−19−somites
20−25−somites
Prim−5
Prim−15
Prim−25
Long−pec
Protruding−mouth
Day−4
Day−5
Transcript
ENSDART00000131076
ENSDART00000153789
ENSDART00000155182

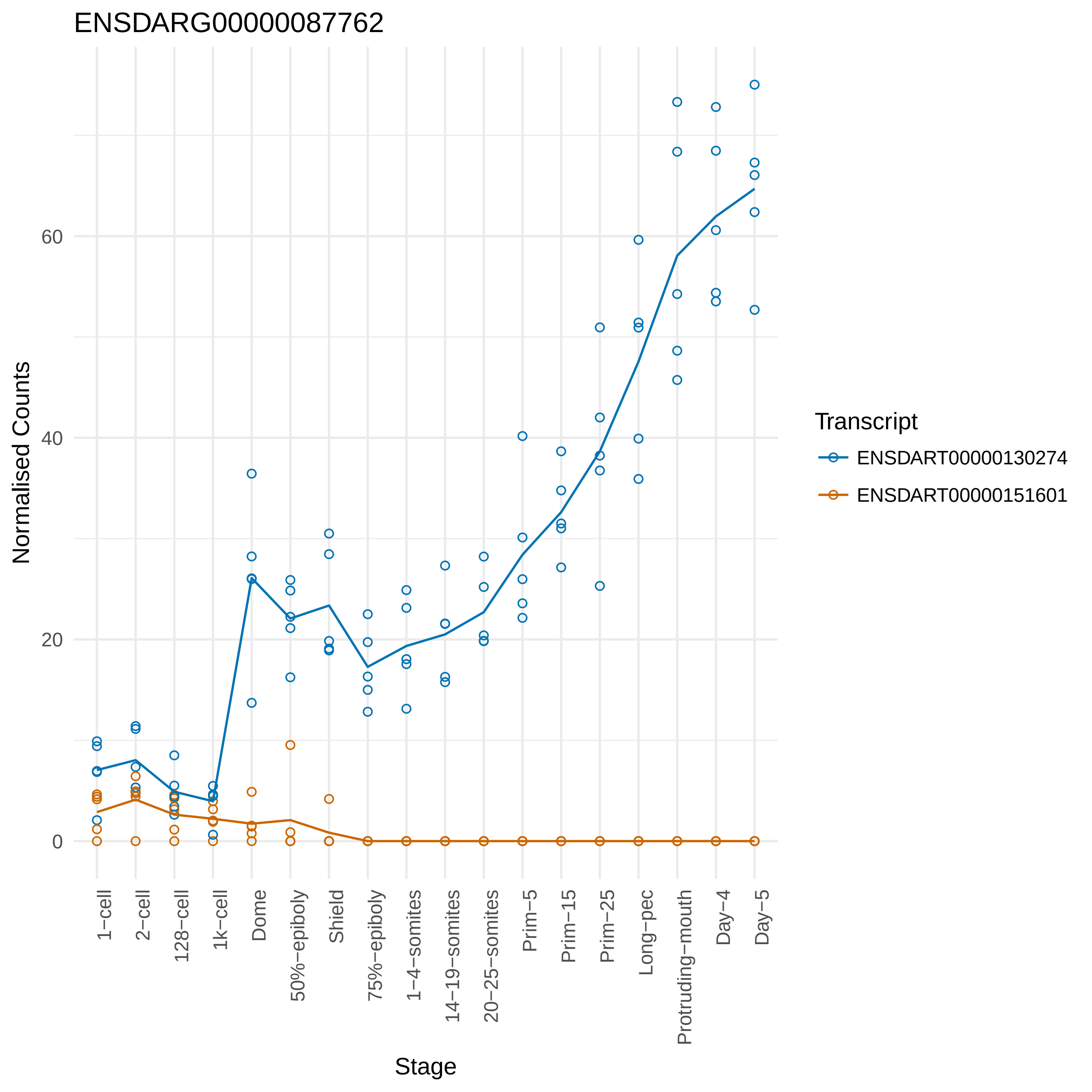
ENSDARG00000087762
Normalised Counts
0
20
40
60
Stage
1−cell
2−cell
128−cell
1k−cell
Dome
50%−epiboly
Shield
75%−epiboly
1−4−somites
14−19−somites
20−25−somites
Prim−5
Prim−15
Prim−25
Long−pec
Protruding−mouth
Day−4
Day−5
Transcript
ENSDART00000130274
ENSDART00000151601

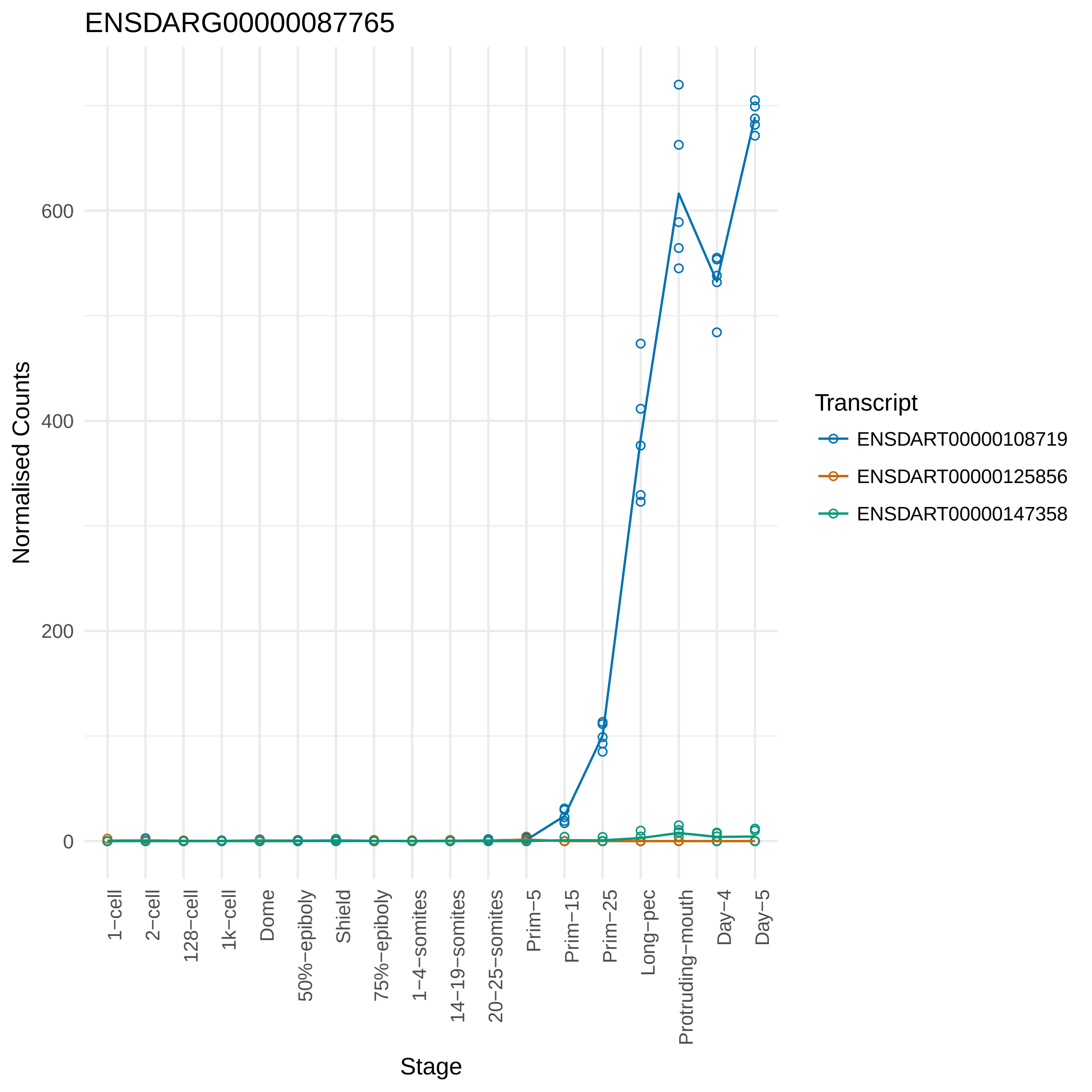
ENSDARG00000087765
Normalised Counts
600
400
200
0
1−cell
2−cell
128−cell
1k−cell
Dome
50%−epiboly
Shield
75%−epiboly
1−4−somites
14−19−somites
20−25−somites
Prim−5
Prim−15
Prim−25
Long−pec
Protruding−mouth
Day−4
Day−5
Stage
Transcript
ENSDART00000108719
ENSDART00000125856
ENSDART00000147358

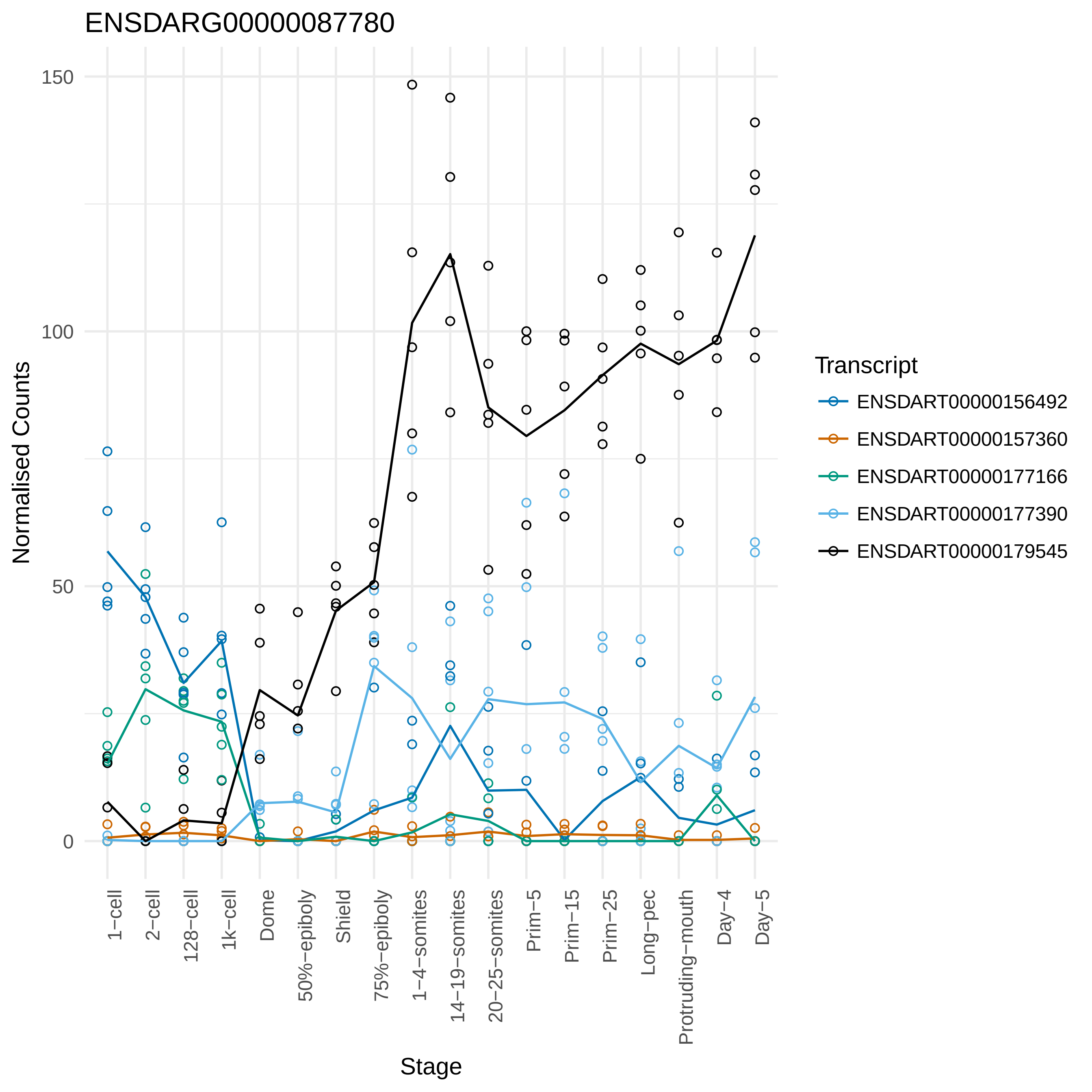

ENSDARG00000087780
Normalised Counts
150
100
50
0
1−cell
2−cell
128−cell
1k−cell
Dome
50%−epiboly
Shield
75%−epiboly
1−4−somites
14−19−somites
20−25−somites
Prim−5
Prim−15
Prim−25
Long−pec
Protruding−mouth
Day−4
Day−5
Stage
Transcript
ENSDART00000156492
ENSDART00000157360
ENSDART00000177166
ENSDART00000177390
ENSDART00000179545

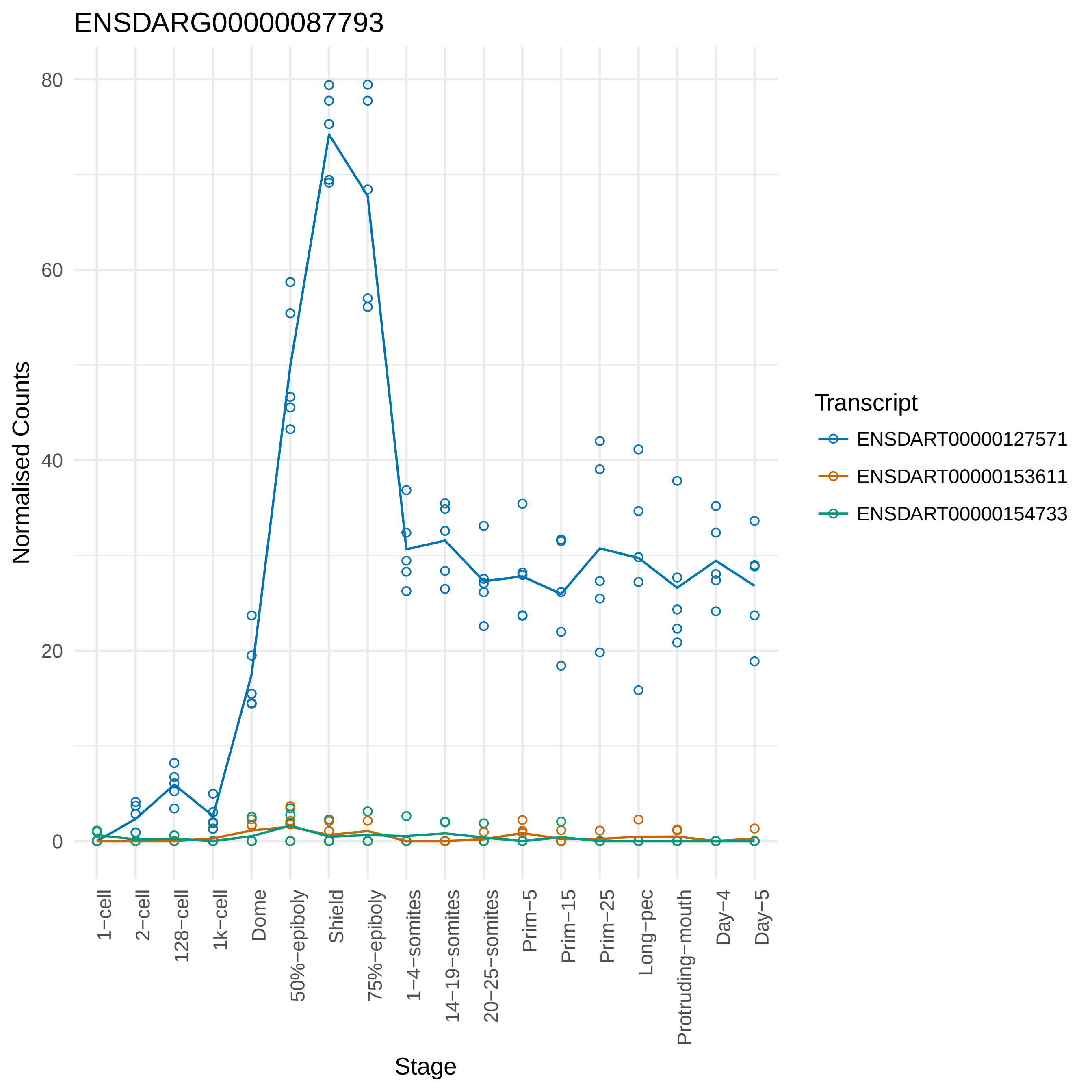

ENSDARG00000087793
Normalised Counts
80
60
40
20
0
1−cell
2−cell
128−cell
1k−cell
Dome
50%−epiboly
Shield
75%−epiboly
1−4−somites
14−19−somites
20−25−somites
Prim−5
Prim−15
Prim−25
Long−pec
Protruding−mouth
Day−4
Day−5
Stage
Transcript
ENSDART00000127571
ENSDART00000153611
ENSDART00000154733

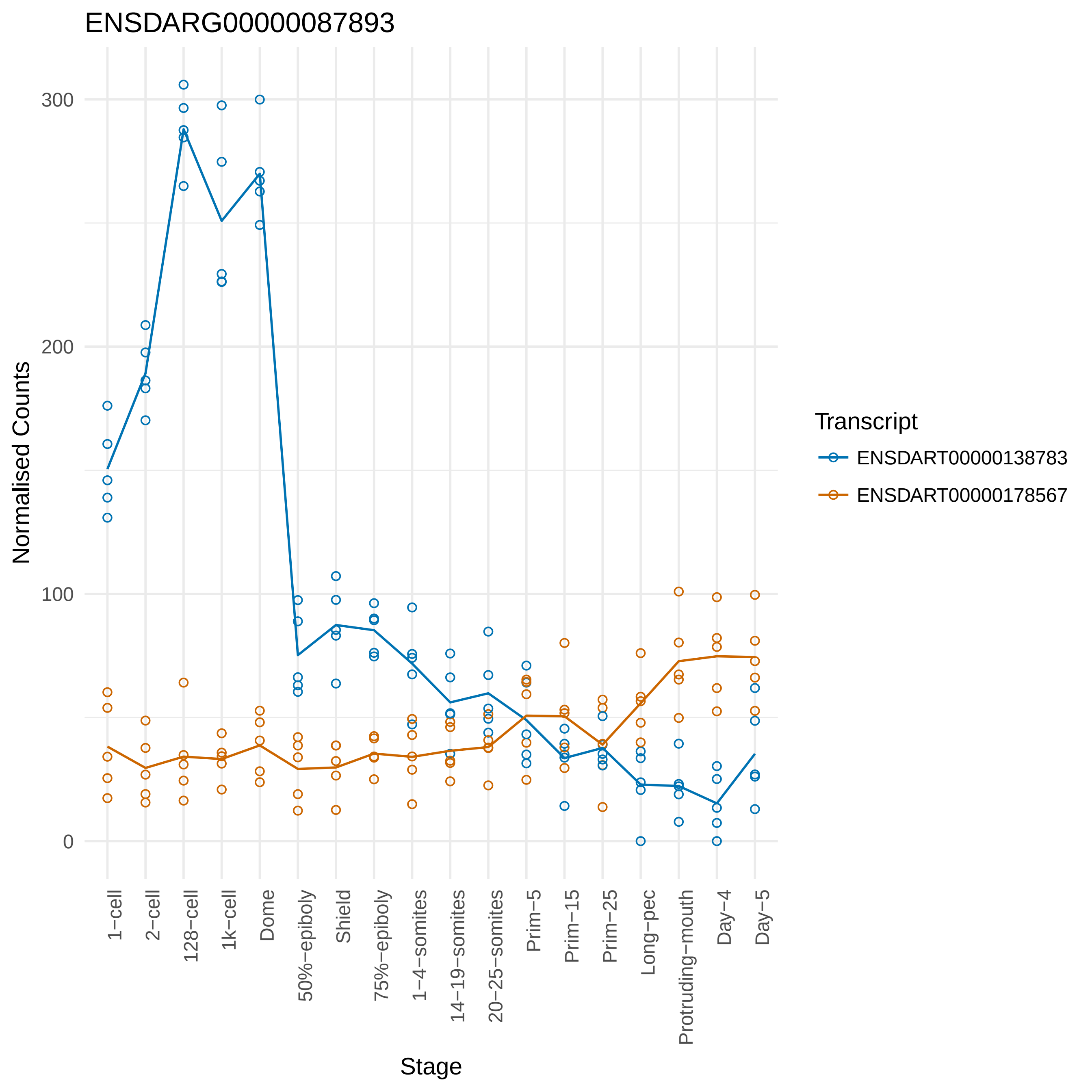

ENSDARG00000087893
Normalised Counts
300
200
100
0
1−cell
2−cell
128−cell
1k−cell
Dome
50%−epiboly
Shield
75%−epiboly
1−4−somites
14−19−somites
20−25−somites
Prim−5
Prim−15
Prim−25
Long−pec
Protruding−mouth
Day−4
Day−5
Stage
Transcript
ENSDART00000138783
ENSDART00000178567

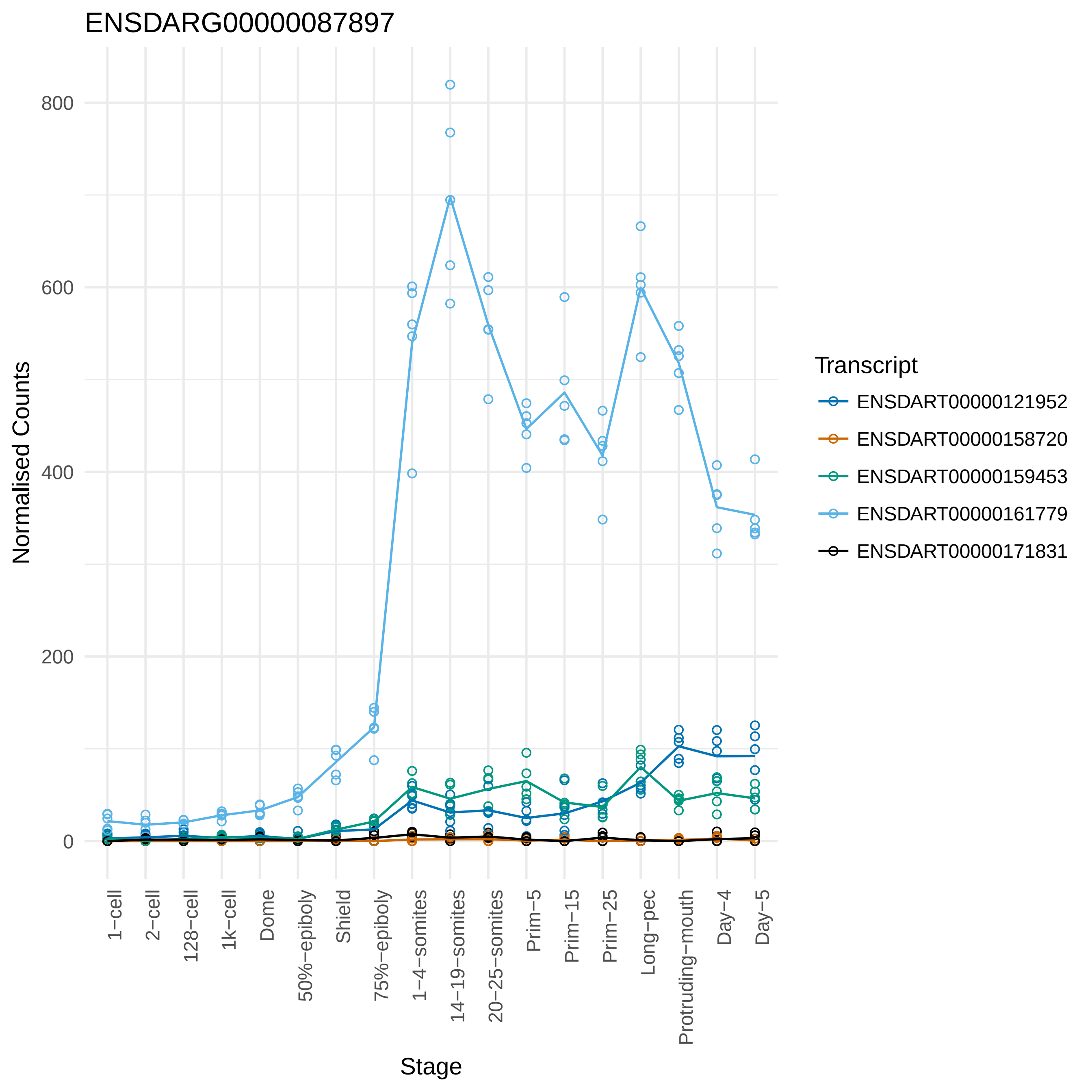
ENSDARG00000087897
Normalised Counts
800
600
400
200
0
1−cell
2−cell
128−cell
1k−cell
Dome
50%−epiboly
Shield
75%−epiboly
1−4−somites
14−19−somites
20−25−somites
Prim−5
Prim−15
Prim−25
Long−pec
Protruding−mouth
Day−4
Day−5
Stage
Transcript
ENSDART00000121952
ENSDART00000158720
ENSDART00000159453
ENSDART00000161779
ENSDART00000171831

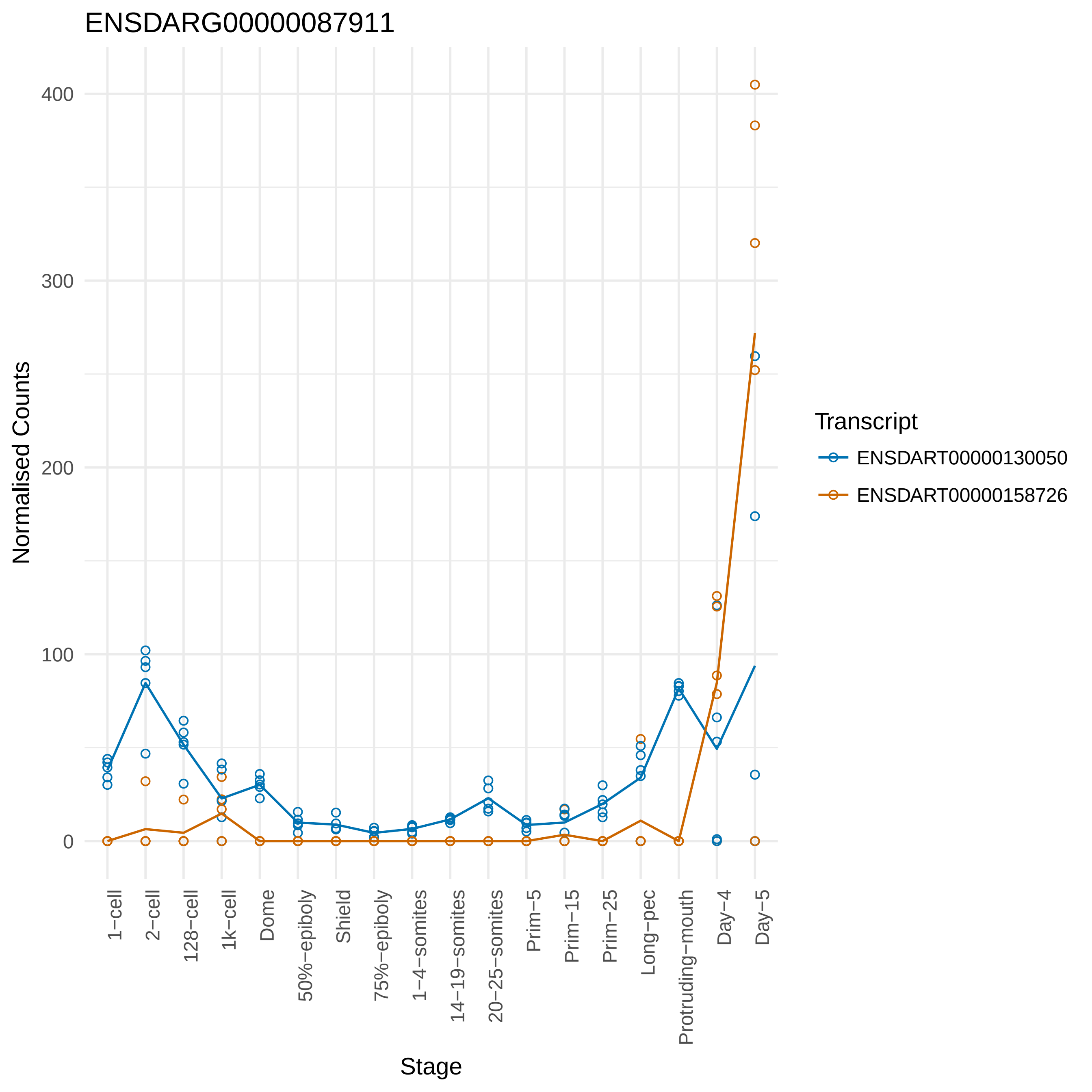

ENSDARG00000087911
Normalised Counts
400
300
200
100
0
1−cell
2−cell
128−cell
1k−cell
Dome
50%−epiboly
Shield
75%−epiboly
1−4−somites
14−19−somites
20−25−somites
Prim−5
Prim−15
Prim−25
Long−pec
Protruding−mouth
Day−4
Day−5
Stage
Transcript
ENSDART00000130050
ENSDART00000158726

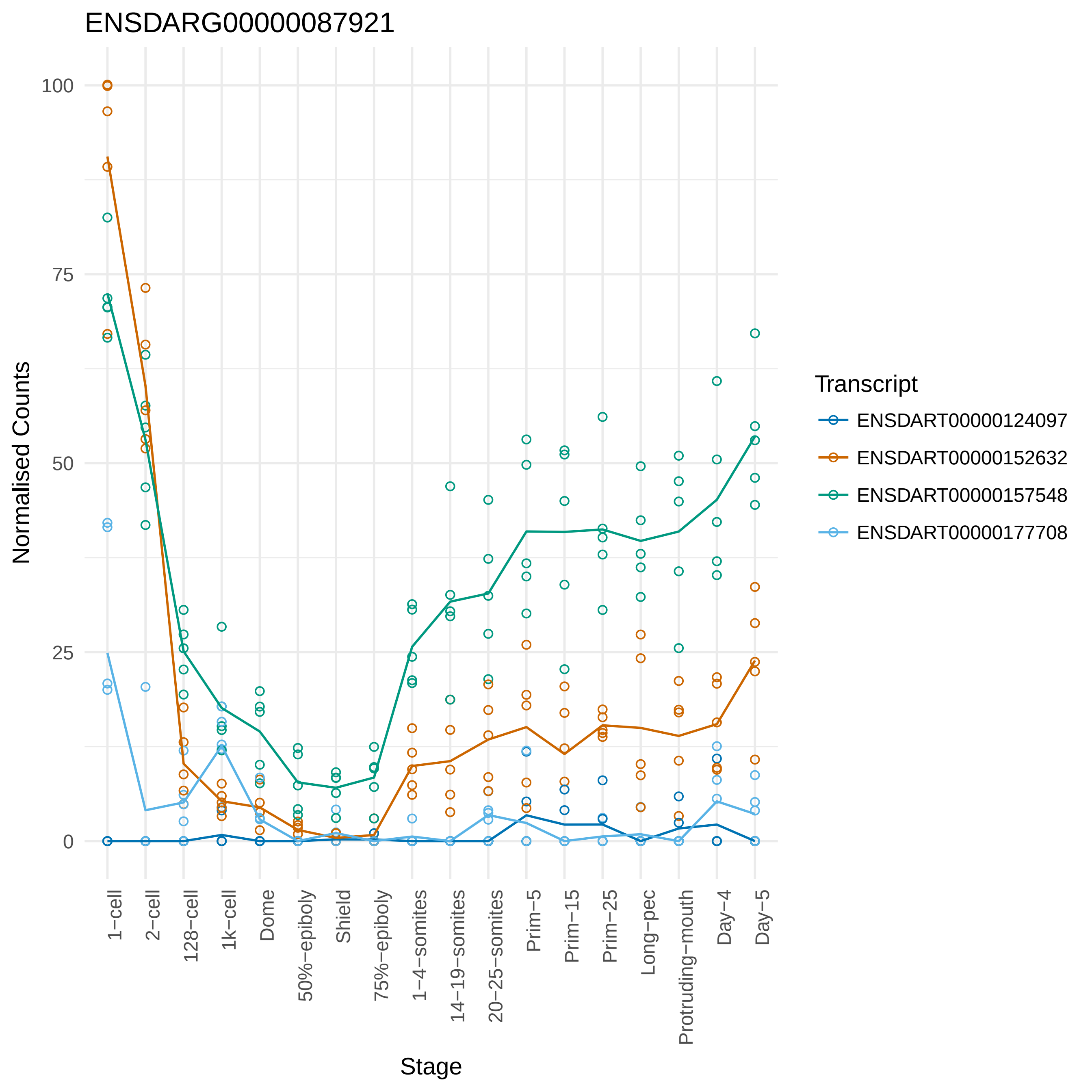

ENSDARG00000087921
Normalised Counts
100
75
50
25
0
1−cell
2−cell
128−cell
1k−cell
Dome
50%−epiboly
Shield
75%−epiboly
1−4−somites
14−19−somites
20−25−somites
Prim−5
Prim−15
Prim−25
Long−pec
Protruding−mouth
Day−4
Day−5
Stage
Transcript
ENSDART00000124097
ENSDART00000152632
ENSDART00000157548
ENSDART00000177708

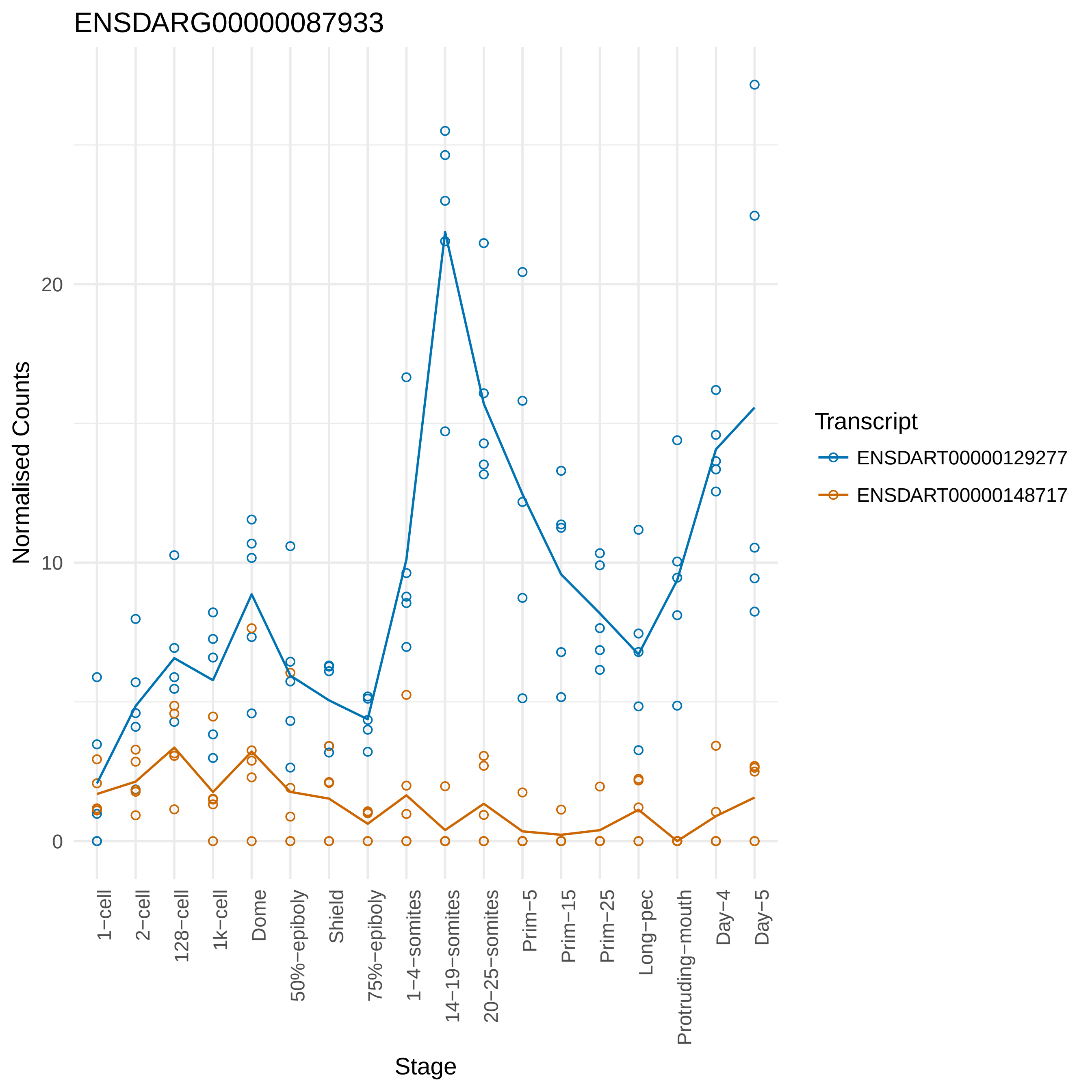

ENSDARG00000087933
Normalised Counts
0
10
20
1−cell
2−cell
128−cell
1k−cell
Dome
50%−epiboly
Shield
75%−epiboly
1−4−somites
14−19−somites
20−25−somites
Prim−5
Prim−15
Prim−25
Long−pec
Protruding−mouth
Day−4
Day−5
Stage
Transcript
ENSDART00000129277
ENSDART00000148717

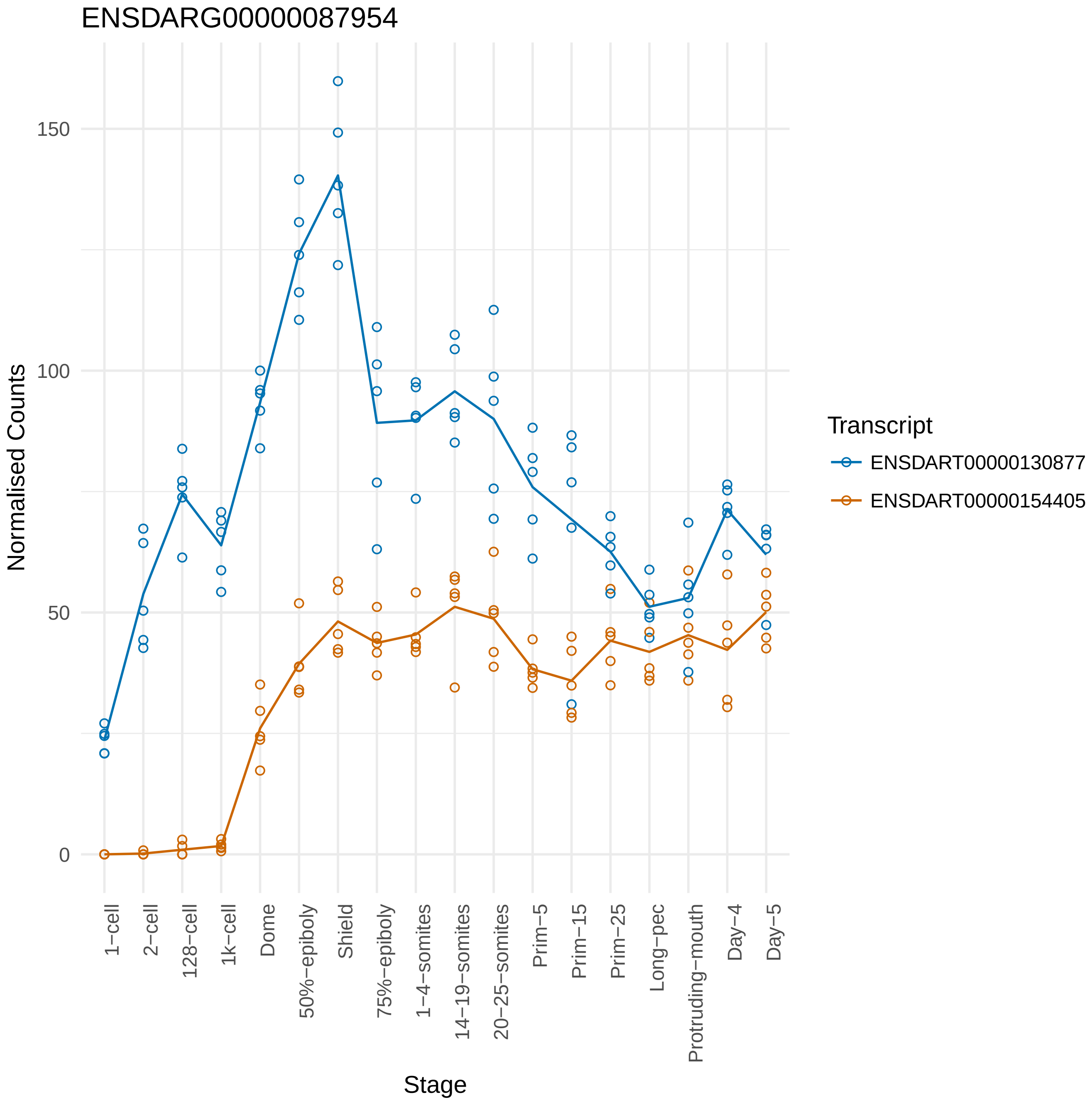

ENSDARG00000087954
Normalised Counts
0
50
100
150
1−cell
2−cell
128−cell
1k−cell
Dome
50%−epiboly
Shield
75%−epiboly
1−4−somites
14−19−somites
20−25−somites
Prim−5
Prim−15
Prim−25
Long−pec
Protruding−mouth
Day−4
Day−5
Stage
Transcript
ENSDART00000130877
ENSDART00000154405

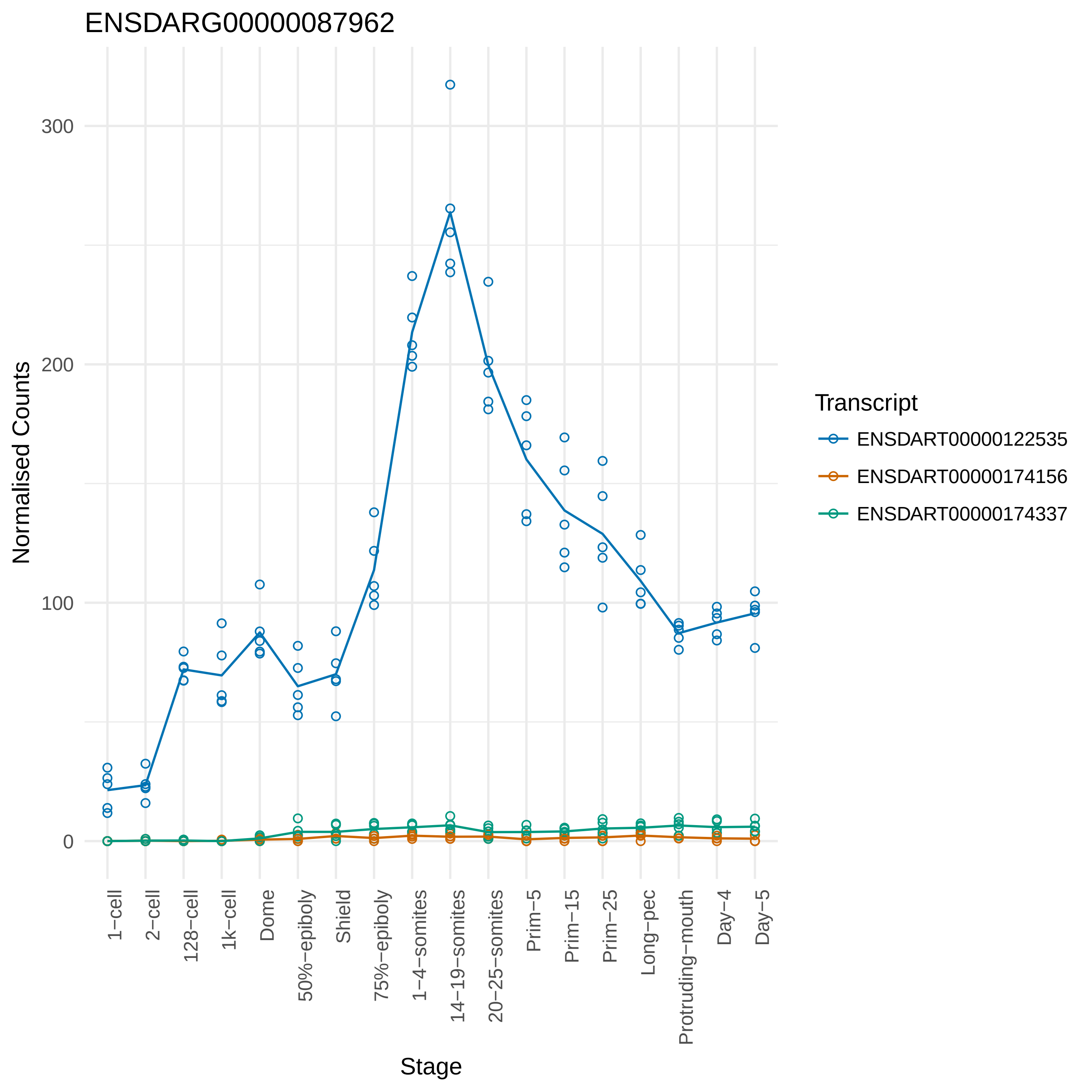
ENSDARG00000087962
Normalised Counts
300
200
100
0
1−cell
2−cell
128−cell
1k−cell
Dome
50%−epiboly
Shield
75%−epiboly
1−4−somites
14−19−somites
20−25−somites
Prim−5
Prim−15
Prim−25
Long−pec
Protruding−mouth
Day−4
Day−5
Stage
Transcript
ENSDART00000122535
ENSDART00000174156
ENSDART00000174337

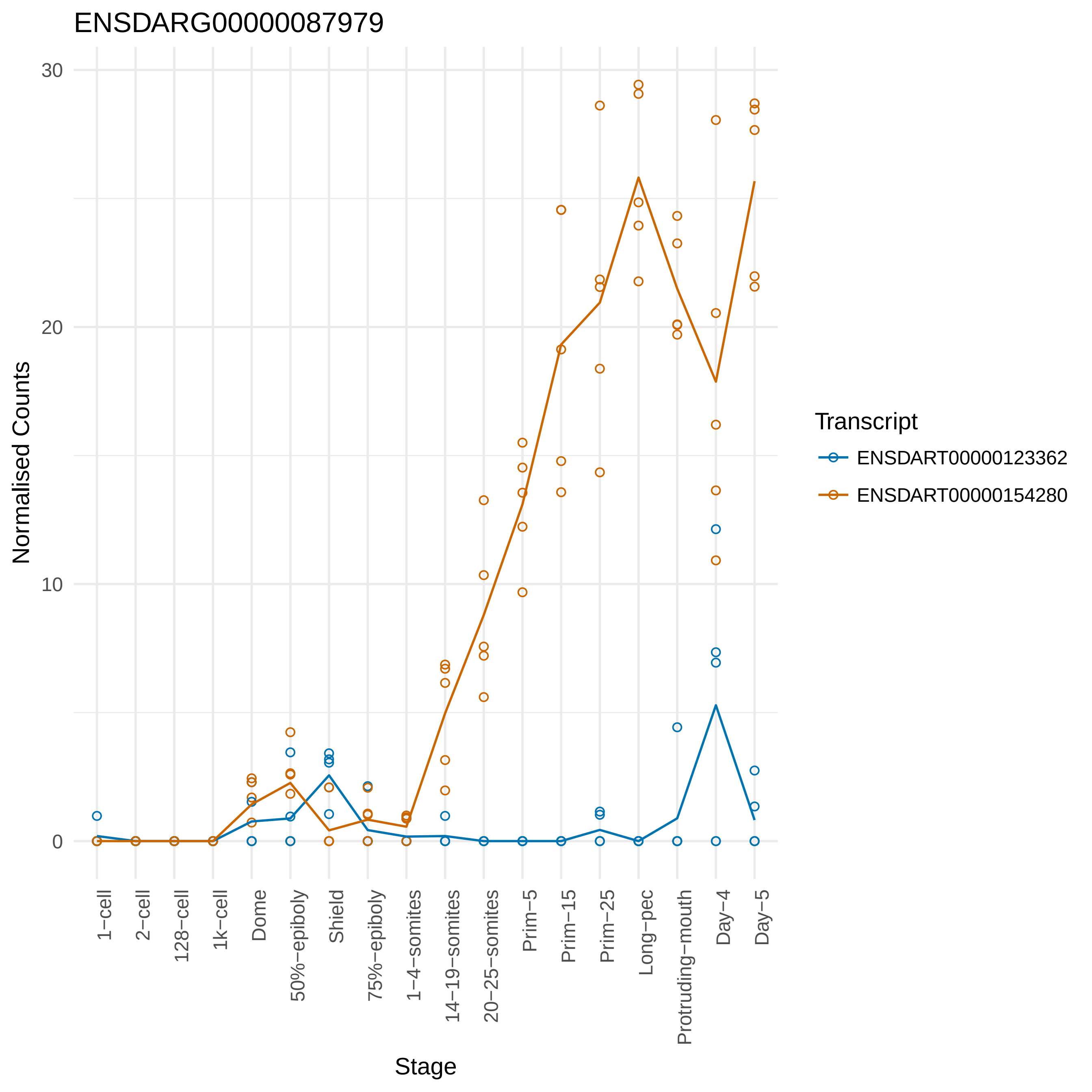

ENSDARG00000087979
Normalised Counts
30
20
10
0
1−cell
2−cell
128−cell
1k−cell
Dome
50%−epiboly
Shield
75%−epiboly
1−4−somites
14−19−somites
20−25−somites
Prim−5
Prim−15
Prim−25
Long−pec
Protruding−mouth
Day−4
Day−5
Stage
Transcript
ENSDART00000123362
ENSDART00000154280

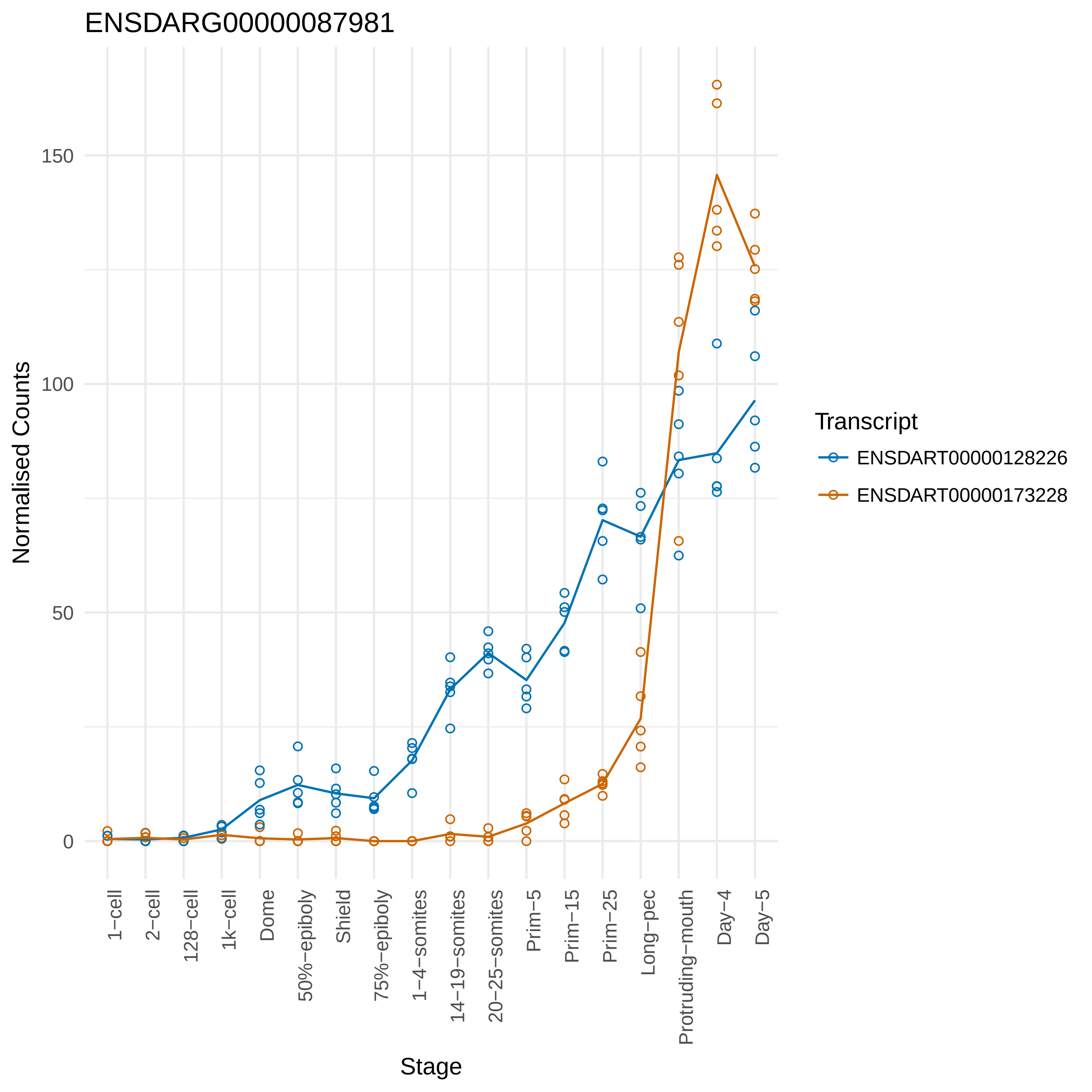

ENSDARG00000087981
Normalised Counts
150
100
50
0
1−cell
2−cell
128−cell
1k−cell
Dome
50%−epiboly
Shield
75%−epiboly
1−4−somites
14−19−somites
20−25−somites
Prim−5
Prim−15
Prim−25
Long−pec
Protruding−mouth
Day−4
Day−5
Stage
Transcript
ENSDART00000128226
ENSDART00000173228

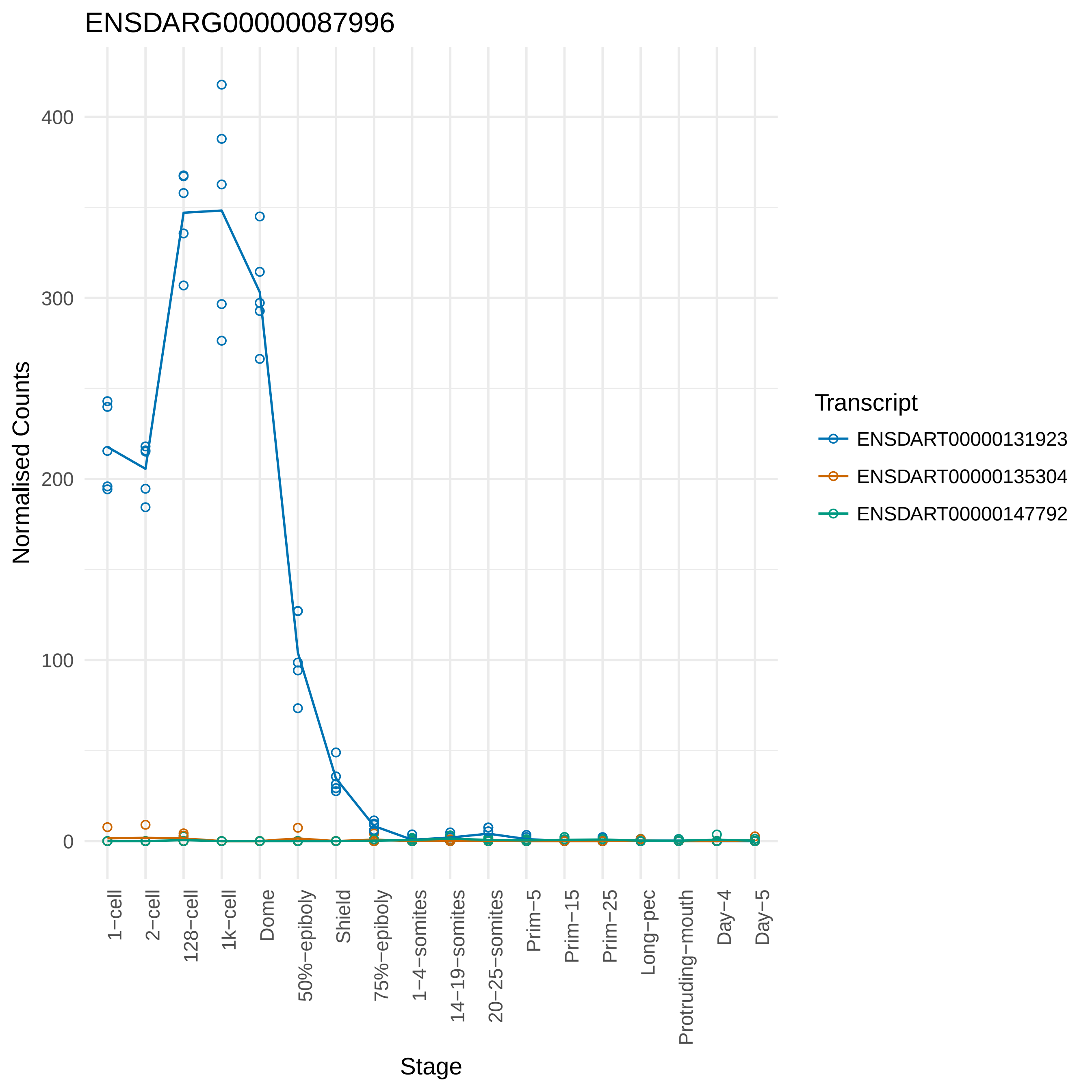

ENSDARG00000087996
Normalised Counts
0
100
200
300
400
Stage
1−cell
2−cell
128−cell
1k−cell
Dome
50%−epiboly
Shield
75%−epiboly
1−4−somites
14−19−somites
20−25−somites
Prim−5
Prim−15
Prim−25
Long−pec
Protruding−mouth
Day−4
Day−5
Transcript
ENSDART00000131923
ENSDART00000135304
ENSDART00000147792

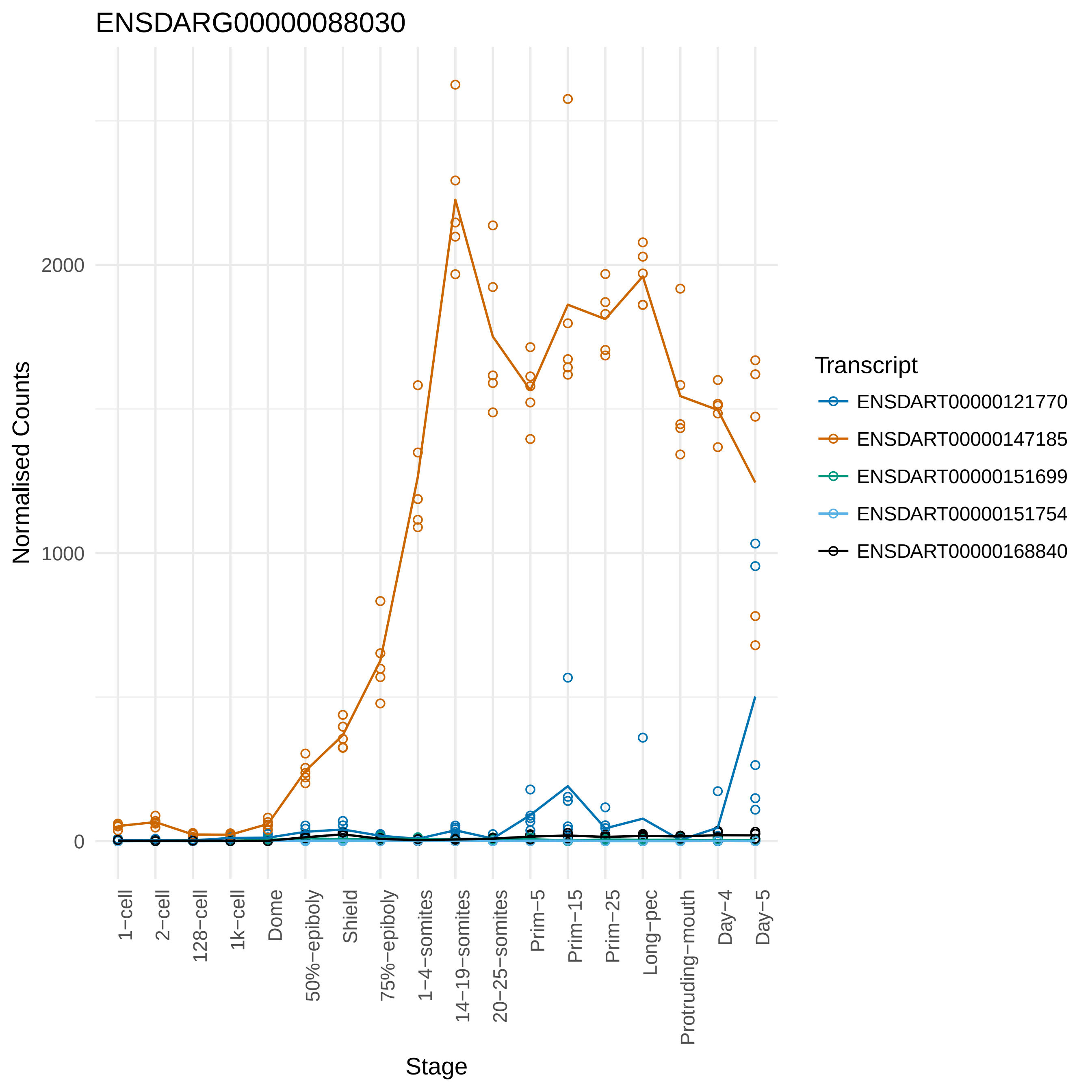

ENSDARG00000088030
Normalised Counts
2000
1000
0
1−cell
2−cell
128−cell
1k−cell
Dome
50%−epiboly
Shield
75%−epiboly
1−4−somites
14−19−somites
20−25−somites
Prim−5
Prim−15
Prim−25
Long−pec
Protruding−mouth
Day−4
Day−5
Stage
Transcript
ENSDART00000121770
ENSDART00000147185
ENSDART00000151699
ENSDART00000151754
ENSDART00000168840

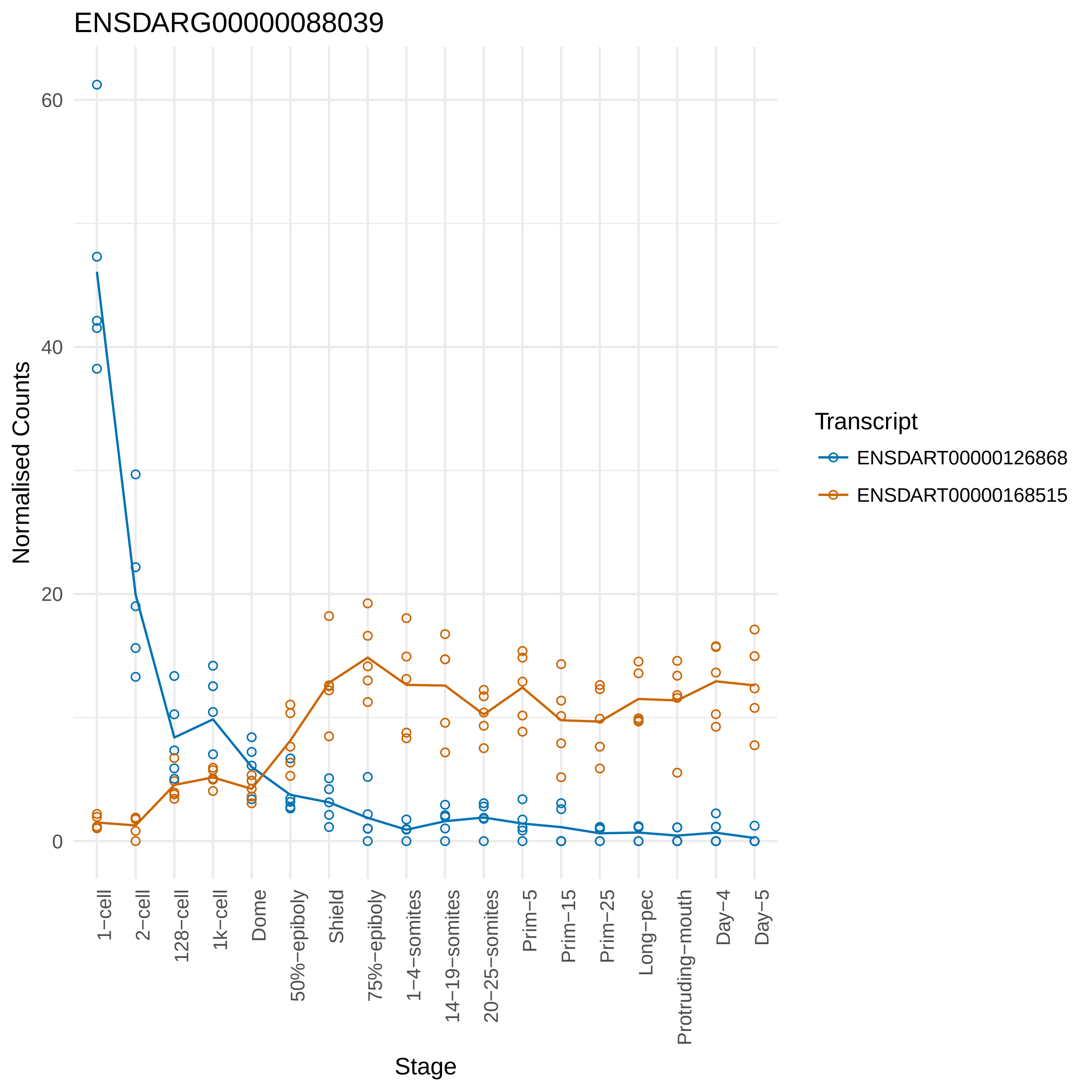

ENSDARG00000088039
Normalised Counts
0
20
40
60
Stage
1−cell
2−cell
128−cell
1k−cell
Dome
50%−epiboly
Shield
75%−epiboly
1−4−somites
14−19−somites
20−25−somites
Prim−5
Prim−15
Prim−25
Long−pec
Protruding−mouth
Day−4
Day−5
Transcript
ENSDART00000126868
ENSDART00000168515

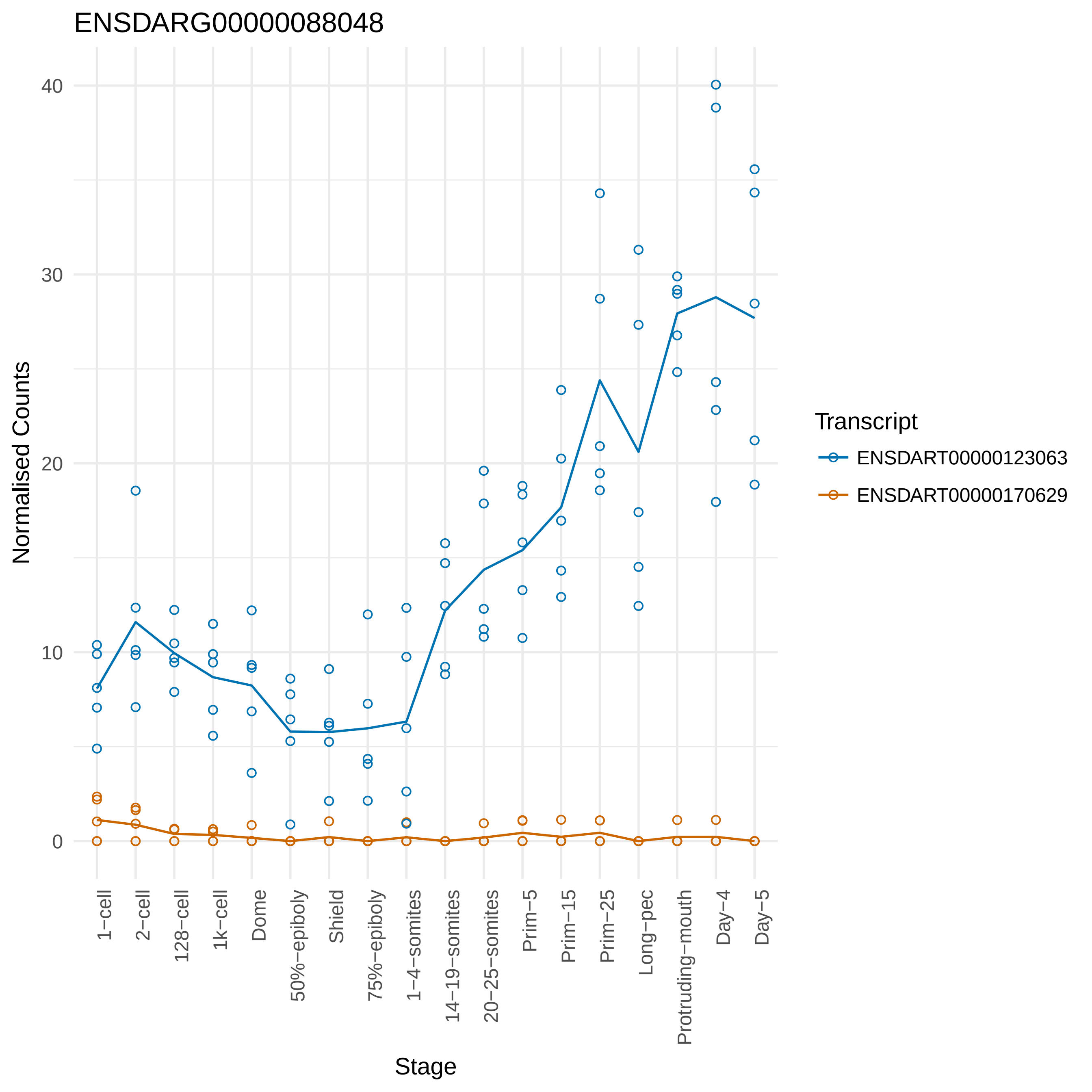
ENSDARG00000088048
Normalised Counts
0
10
20
30
40
Stage
1−cell
2−cell
128−cell
1k−cell
Dome
50%−epiboly
Shield
75%−epiboly
1−4−somites
14−19−somites
20−25−somites
Prim−5
Prim−15
Prim−25
Long−pec
Protruding−mouth
Day−4
Day−5
Transcript
ENSDART00000123063
ENSDART00000170629

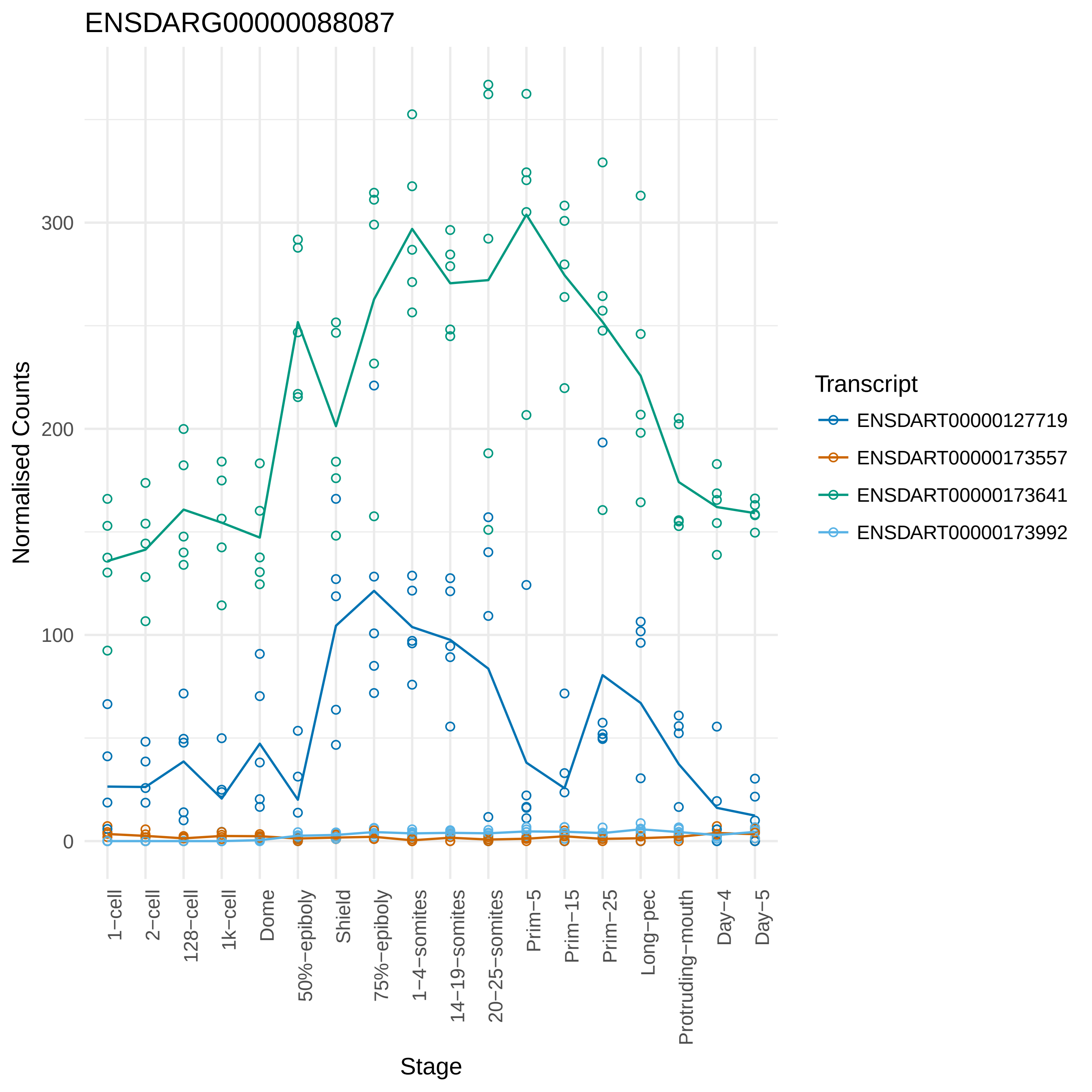

ENSDARG00000088087
Normalised Counts
0
100
200
300
Stage
1−cell
2−cell
128−cell
1k−cell
Dome
50%−epiboly
Shield
75%−epiboly
1−4−somites
14−19−somites
20−25−somites
Prim−5
Prim−15
Prim−25
Long−pec
Protruding−mouth
Day−4
Day−5
Transcript
ENSDART00000127719
ENSDART00000173557
ENSDART00000173641
ENSDART00000173992

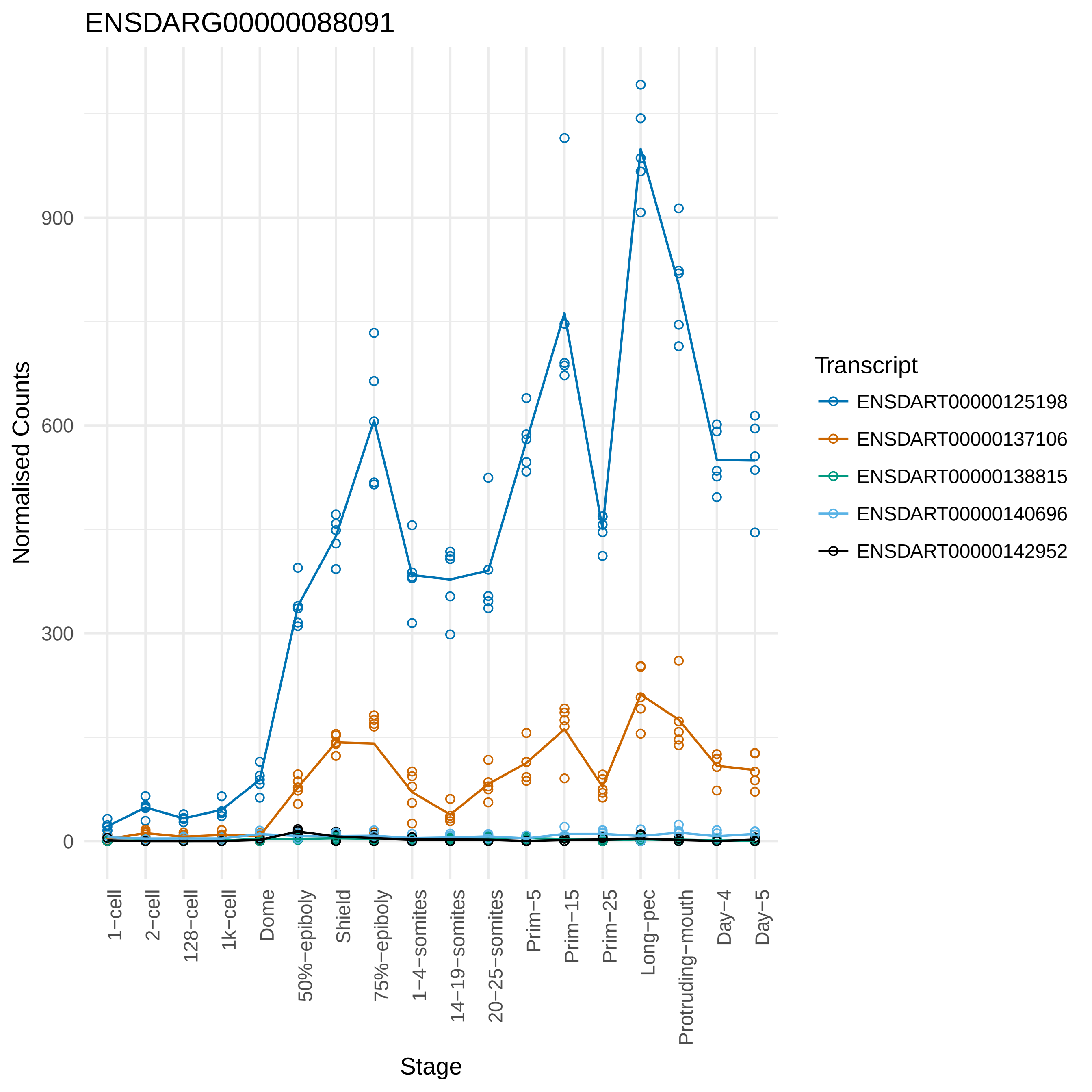

ENSDARG00000088091
Normalised Counts
900
600
300
0
1−cell
2−cell
128−cell
1k−cell
Dome
50%−epiboly
Shield
75%−epiboly
1−4−somites
14−19−somites
20−25−somites
Prim−5
Prim−15
Prim−25
Long−pec
Protruding−mouth
Day−4
Day−5
Stage
Transcript
ENSDART00000125198
ENSDART00000137106
ENSDART00000138815
ENSDART00000140696
ENSDART00000142952

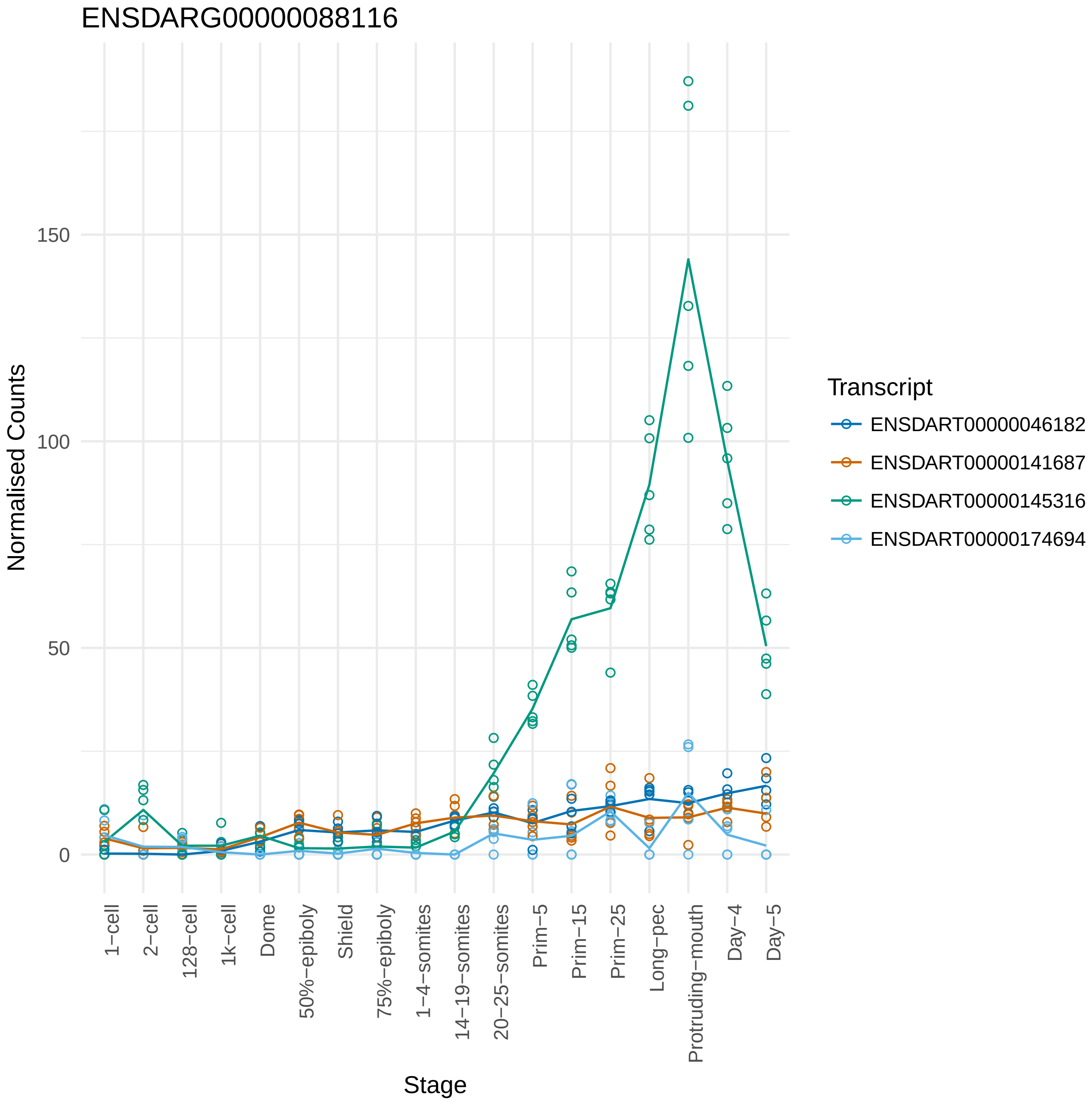

ENSDARG00000088116
Normalised Counts
150
100
50
0
1−cell
2−cell
128−cell
1k−cell
Dome
50%−epiboly
Shield
75%−epiboly
1−4−somites
14−19−somites
20−25−somites
Prim−5
Prim−15
Prim−25
Long−pec
Protruding−mouth
Day−4
Day−5
Stage
Transcript
ENSDART00000046182
ENSDART00000141687
ENSDART00000145316
ENSDART00000174694

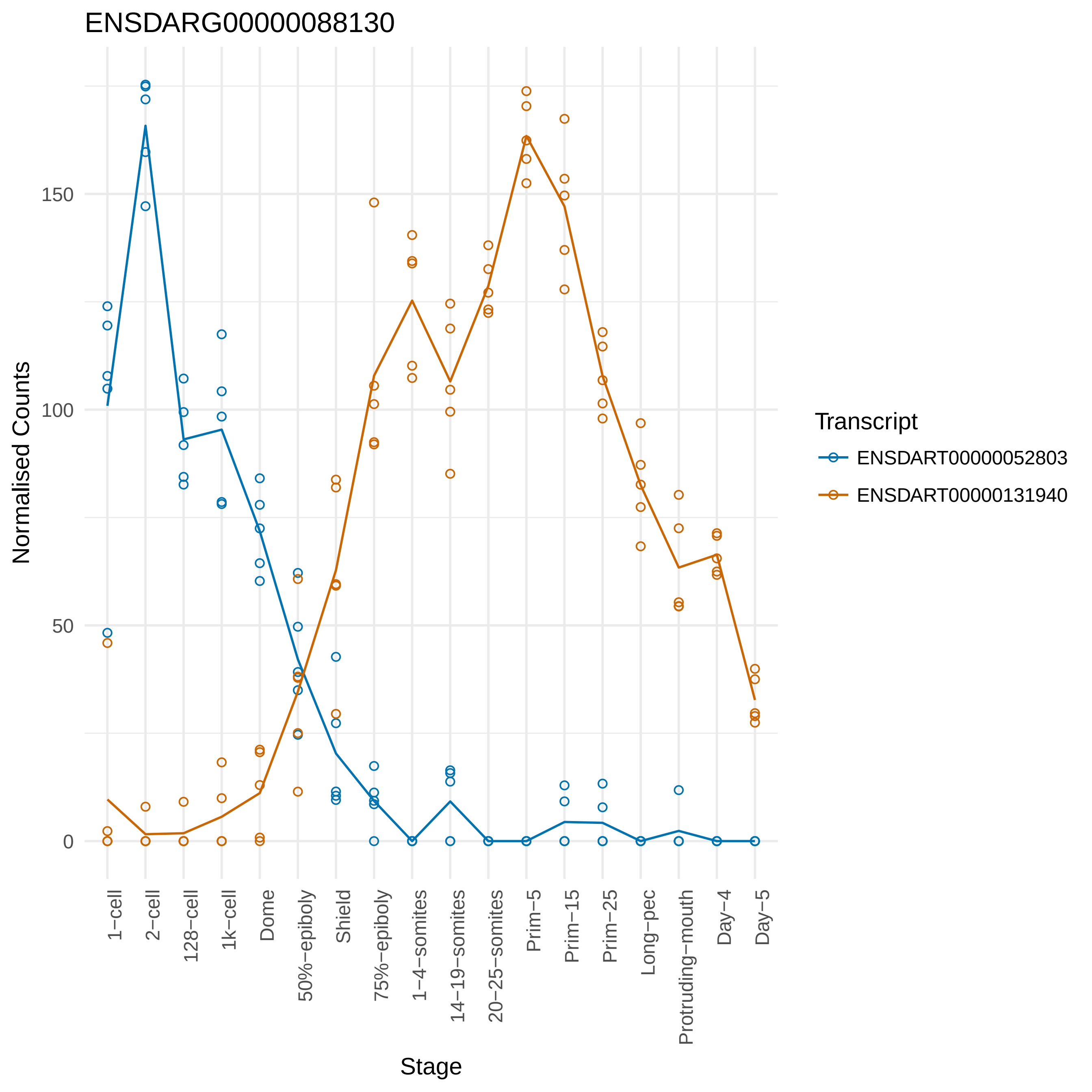
ENSDARG00000088130
Normalised Counts
150
100
50
0
1−cell
2−cell
128−cell
1k−cell
Dome
50%−epiboly
Shield
75%−epiboly
1−4−somites
14−19−somites
20−25−somites
Prim−5
Prim−15
Prim−25
Long−pec
Protruding−mouth
Day−4
Day−5
Stage
Transcript
ENSDART00000052803
ENSDART00000131940

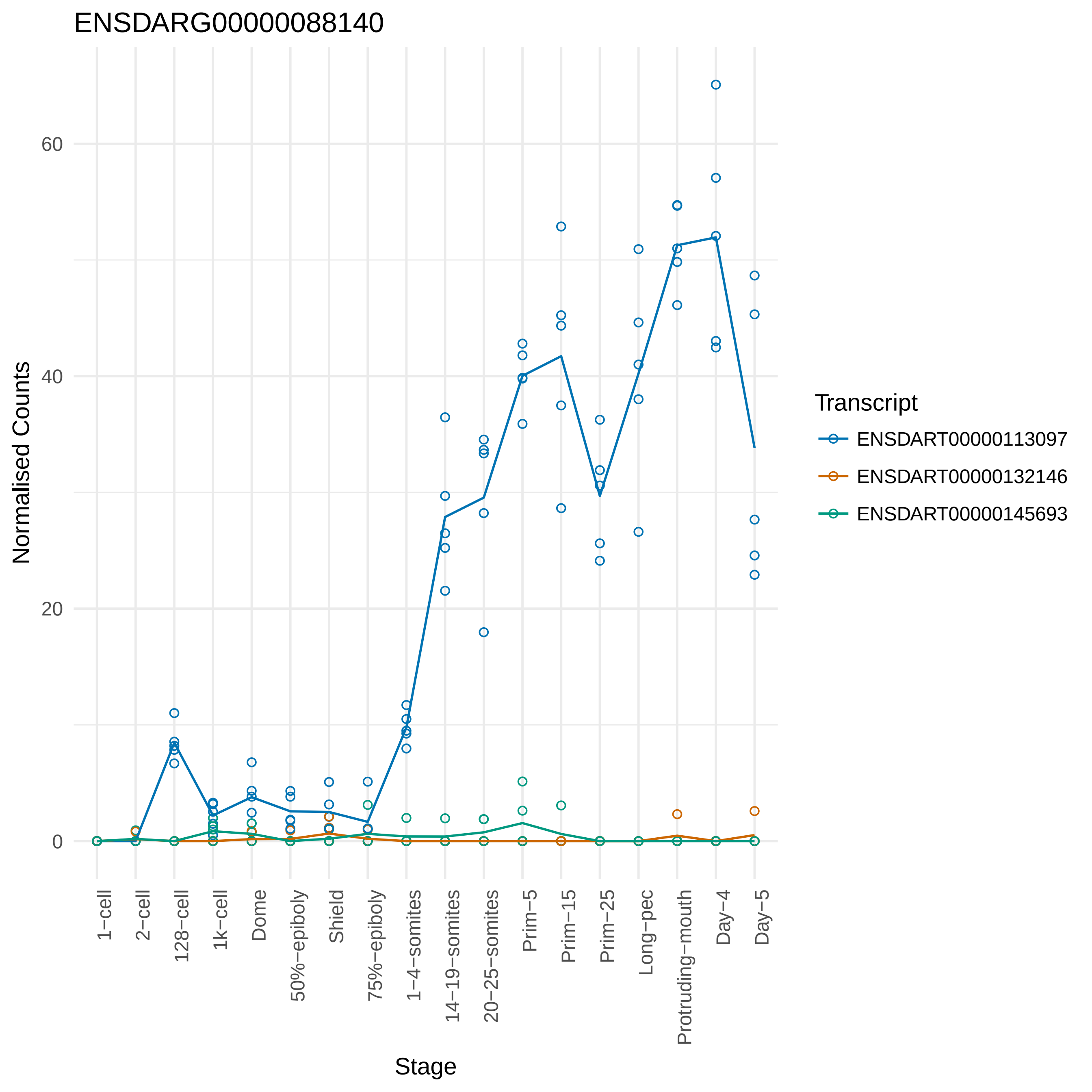

ENSDARG00000088140
Normalised Counts
60
40
20
0
1−cell
2−cell
128−cell
1k−cell
Dome
50%−epiboly
Shield
75%−epiboly
1−4−somites
14−19−somites
20−25−somites
Prim−5
Prim−15
Prim−25
Long−pec
Protruding−mouth
Day−4
Day−5
Stage
Transcript
ENSDART00000113097
ENSDART00000132146
ENSDART00000145693

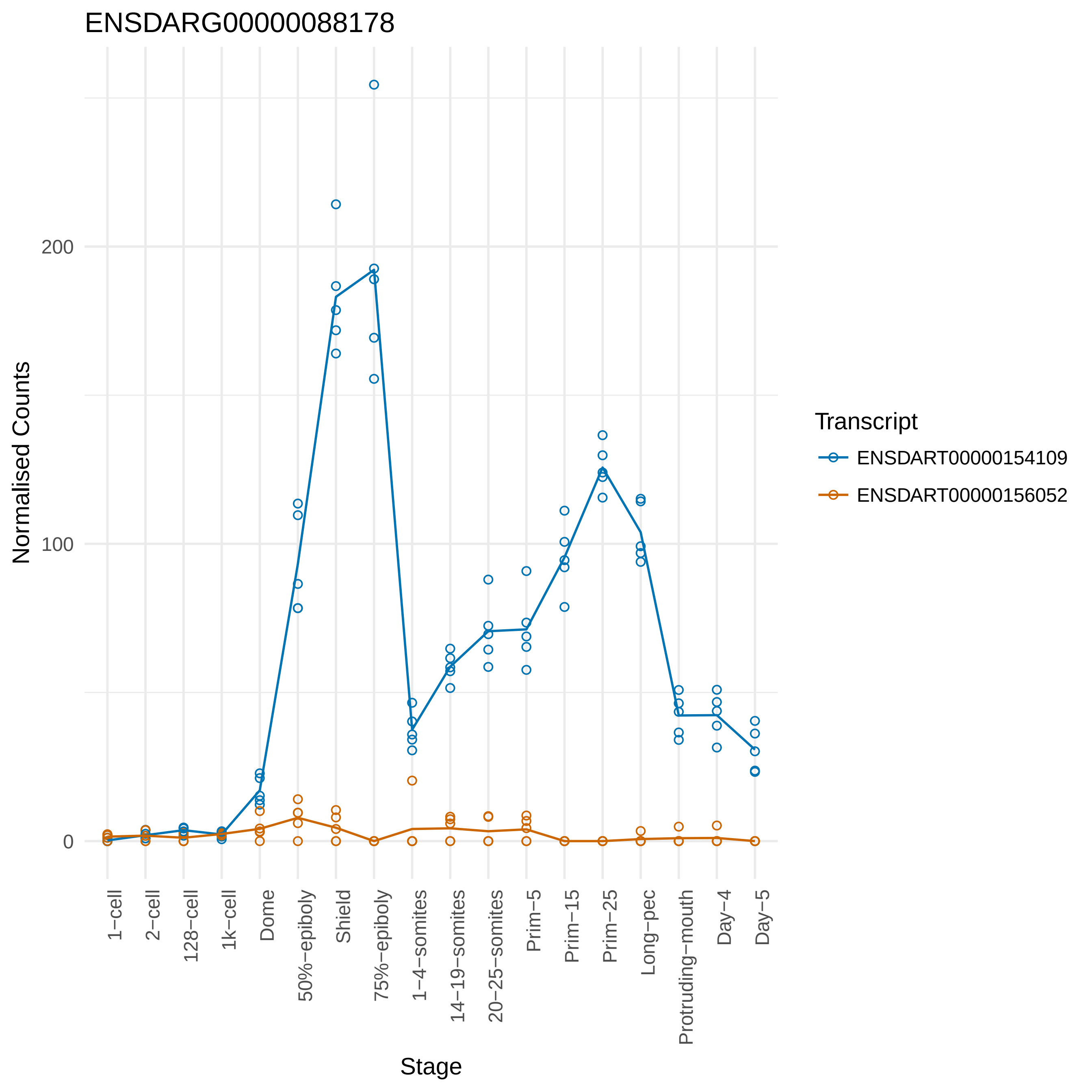
ENSDARG00000088178
Normalised Counts
200
100
0
1−cell
2−cell
128−cell
1k−cell
Dome
50%−epiboly
Shield
75%−epiboly
1−4−somites
14−19−somites
20−25−somites
Prim−5
Prim−15
Prim−25
Long−pec
Protruding−mouth
Day−4
Day−5
Stage
Transcript
ENSDART00000154109
ENSDART00000156052

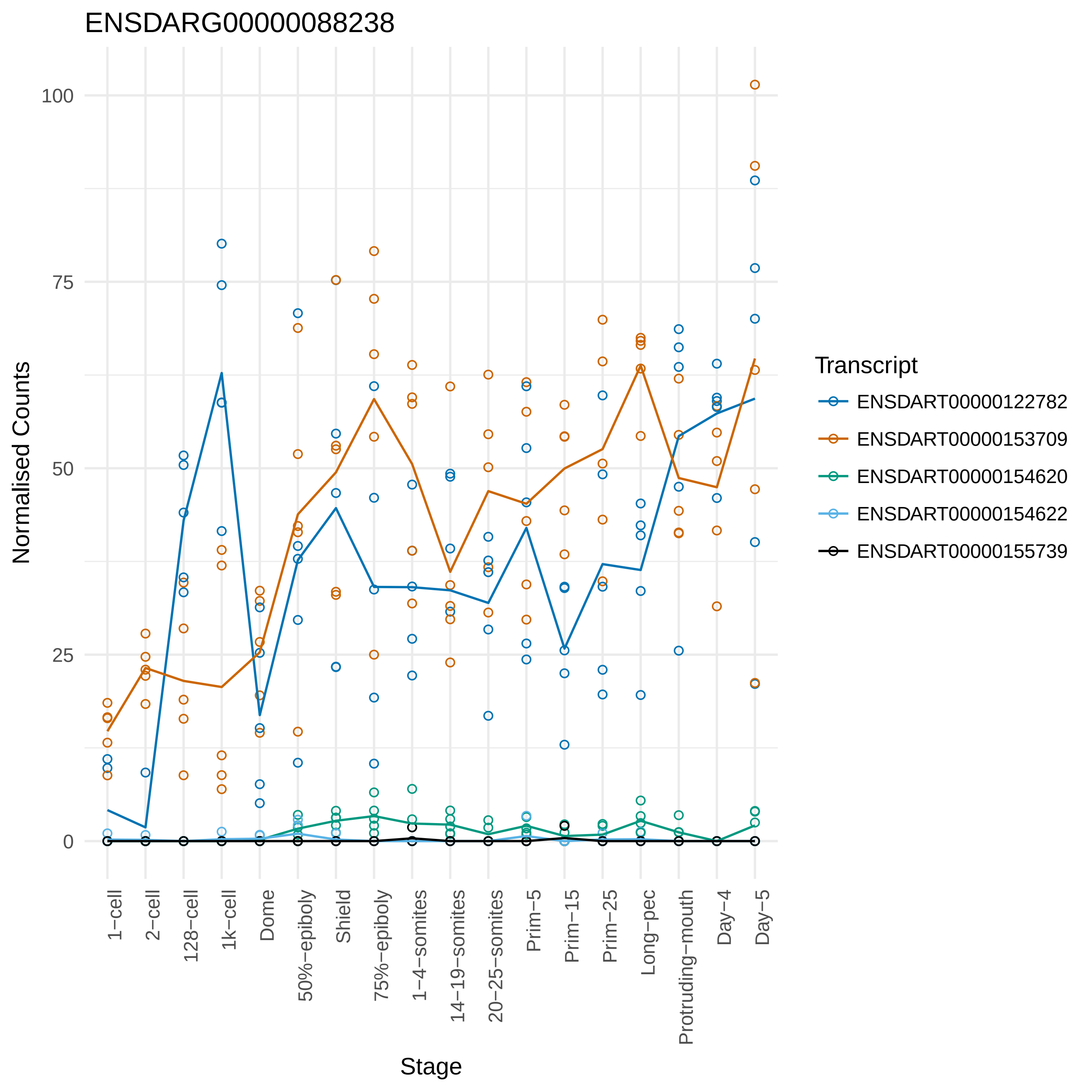

ENSDARG00000088238
Normalised Counts
100
75
50
25
0
1−cell
2−cell
128−cell
1k−cell
Dome
50%−epiboly
Shield
75%−epiboly
1−4−somites
14−19−somites
20−25−somites
Prim−5
Prim−15
Prim−25
Long−pec
Protruding−mouth
Day−4
Day−5
Stage
Transcript
ENSDART00000122782
ENSDART00000153709
ENSDART00000154620
ENSDART00000154622
ENSDART00000155739

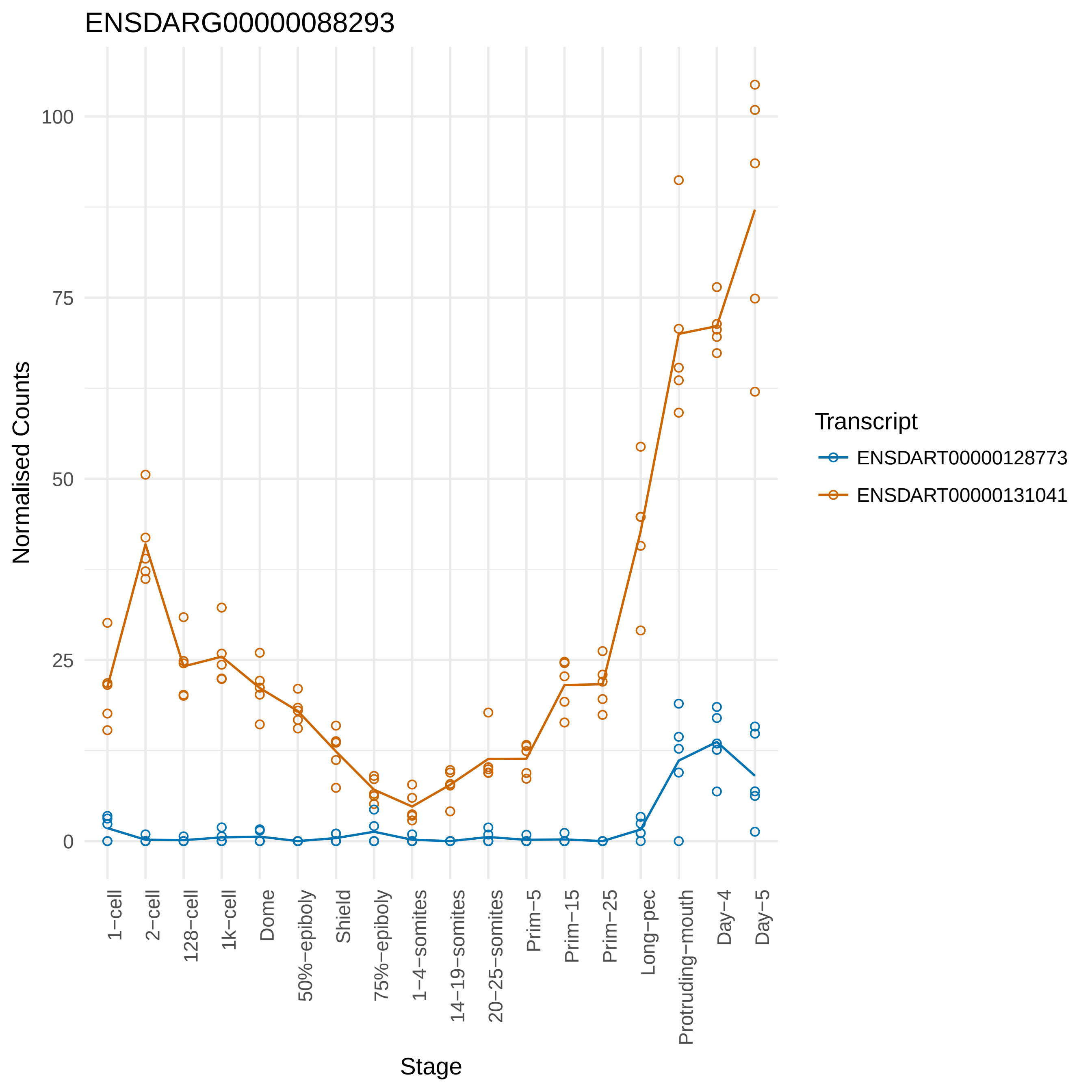

ENSDARG00000088293
Normalised Counts
100
75
50
25
0
1−cell
2−cell
128−cell
1k−cell
Dome
50%−epiboly
Shield
75%−epiboly
1−4−somites
14−19−somites
20−25−somites
Prim−5
Prim−15
Prim−25
Long−pec
Protruding−mouth
Day−4
Day−5
Stage
Transcript
ENSDART00000128773
ENSDART00000131041

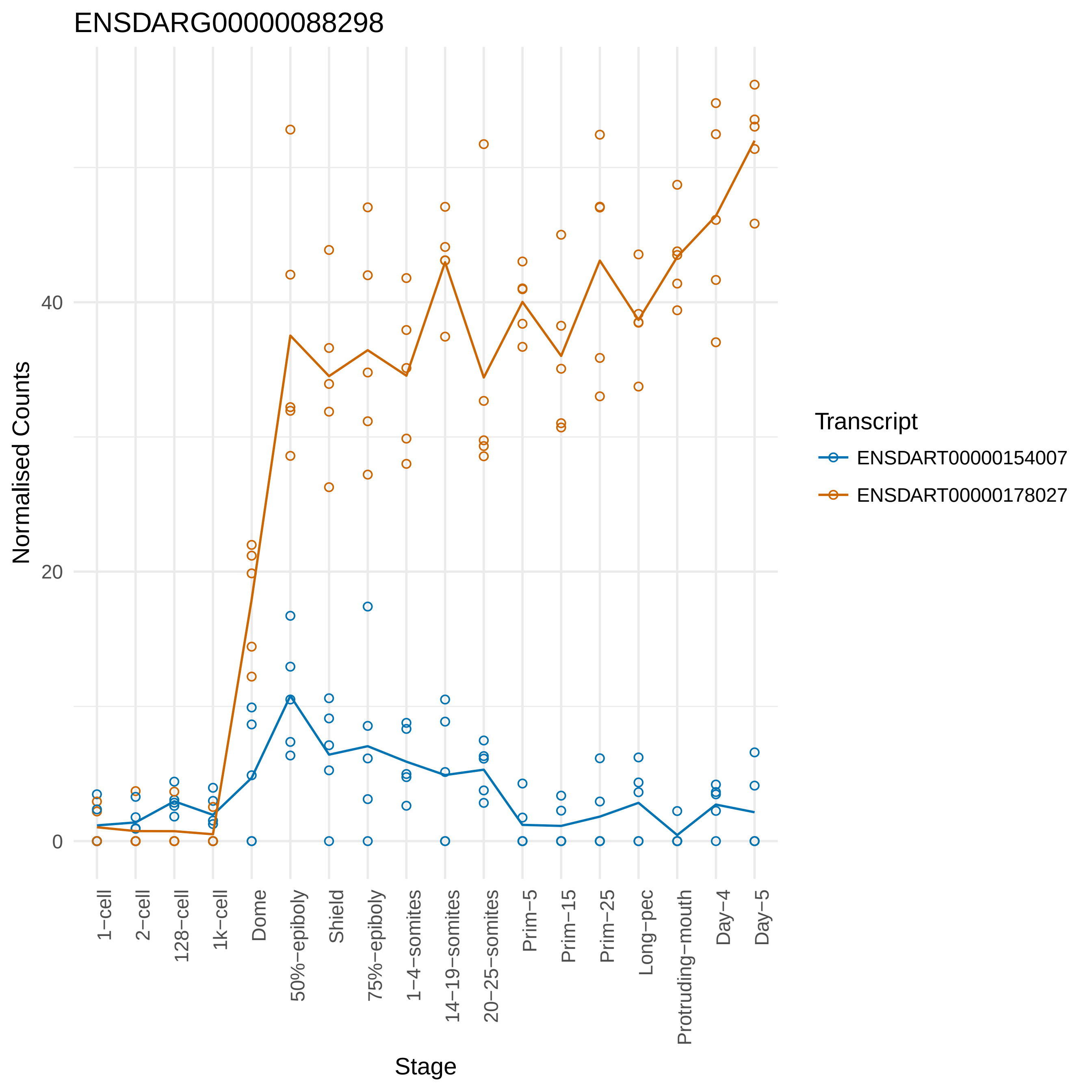
ENSDARG00000088298
Normalised Counts
0
20
40
Stage
1−cell
2−cell
128−cell
1k−cell
Dome
50%−epiboly
Shield
75%−epiboly
1−4−somites
14−19−somites
20−25−somites
Prim−5
Prim−15
Prim−25
Long−pec
Protruding−mouth
Day−4
Day−5
Transcript
ENSDART00000154007
ENSDART00000178027

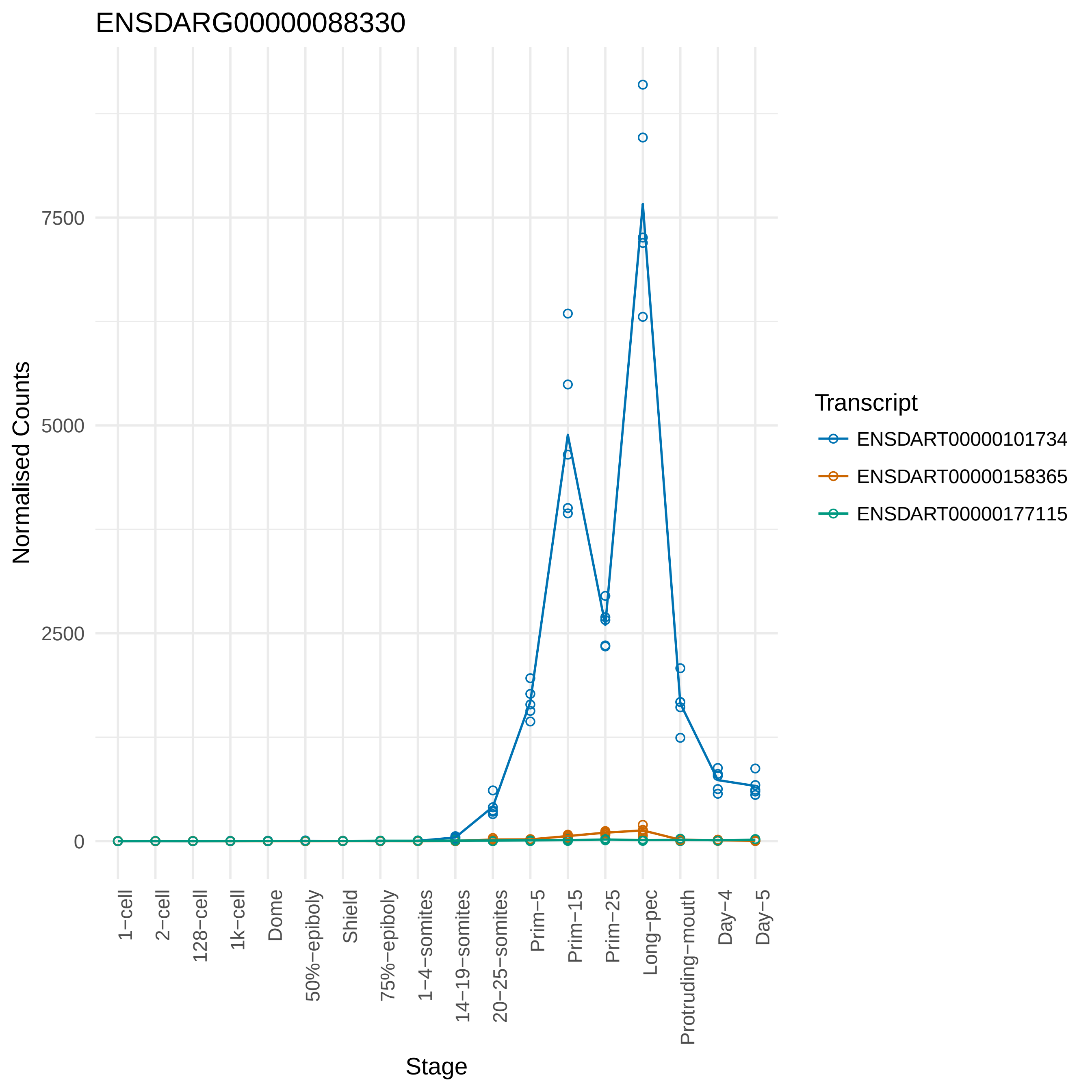

ENSDARG00000088330
Normalised Counts
0
2500
5000
7500
Stage
1−cell
2−cell
128−cell
1k−cell
Dome
50%−epiboly
Shield
75%−epiboly
1−4−somites
14−19−somites
20−25−somites
Prim−5
Prim−15
Prim−25
Long−pec
Protruding−mouth
Day−4
Day−5
Transcript
ENSDART00000101734
ENSDART00000158365
ENSDART00000177115

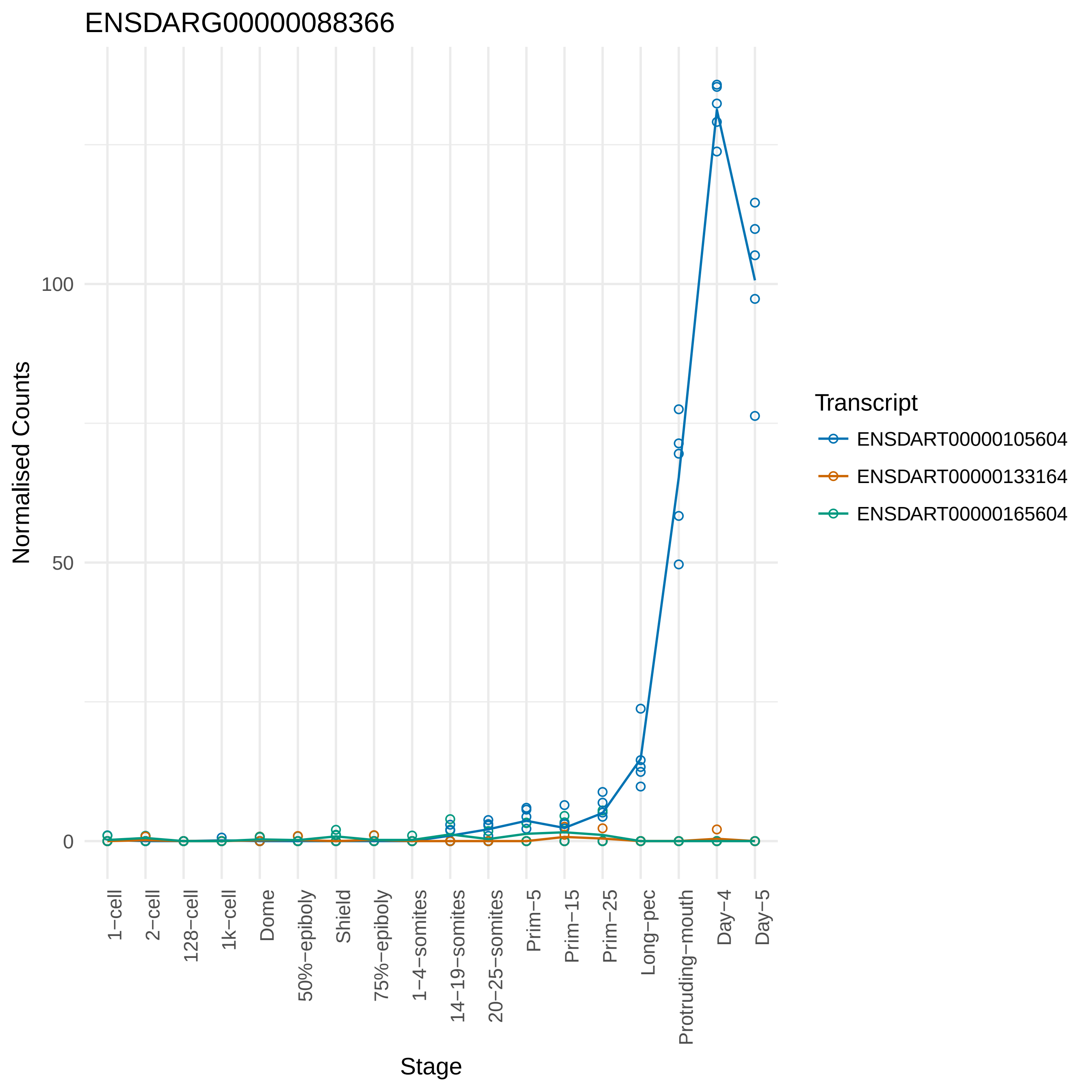
ENSDARG00000088366
Normalised Counts
0
50
100
Stage
1−cell
2−cell
128−cell
1k−cell
Dome
50%−epiboly
Shield
75%−epiboly
1−4−somites
14−19−somites
20−25−somites
Prim−5
Prim−15
Prim−25
Long−pec
Protruding−mouth
Day−4
Day−5
Transcript
ENSDART00000105604
ENSDART00000133164
ENSDART00000165604

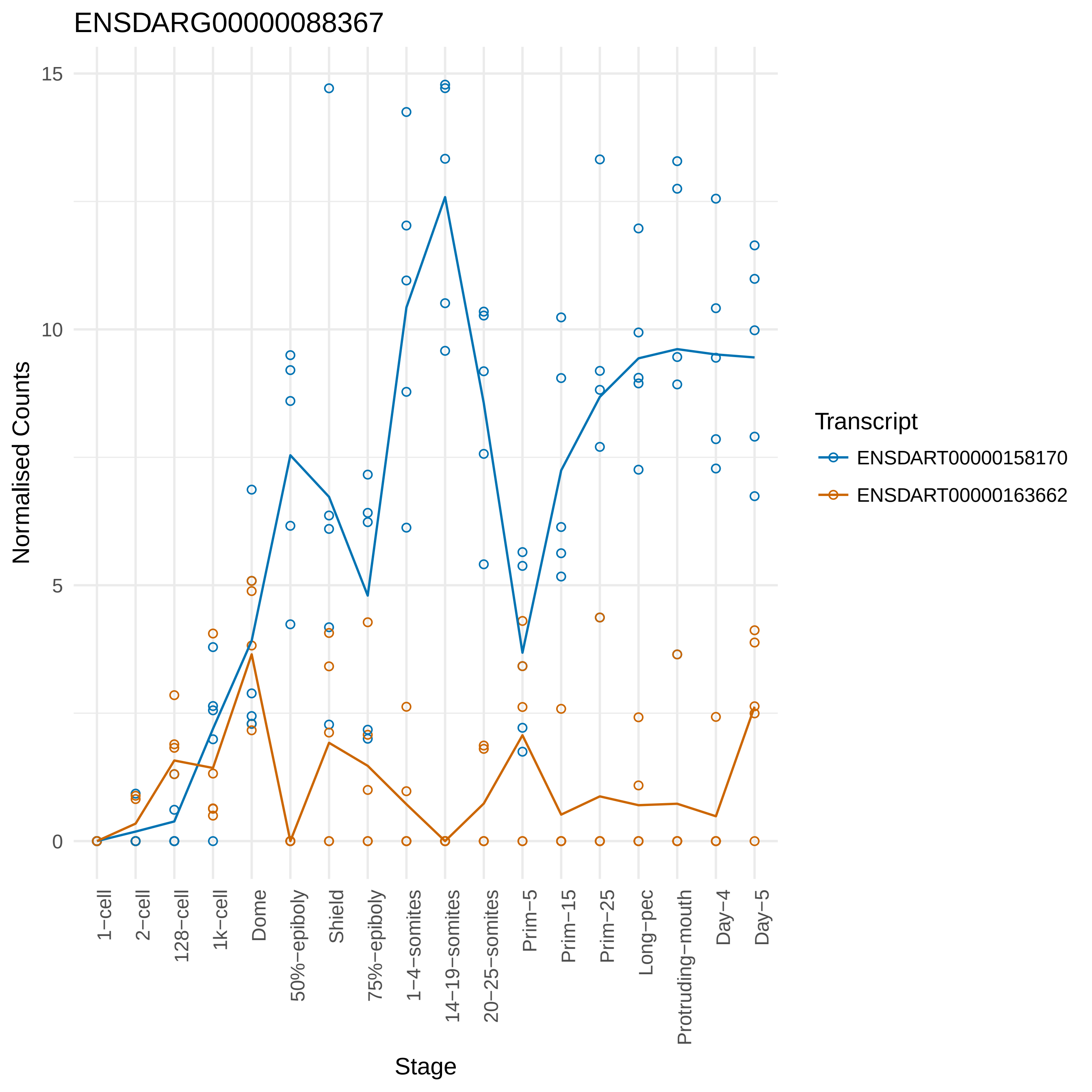

ENSDARG00000088367
Normalised Counts
15
10
5
0
1−cell
2−cell
128−cell
1k−cell
Dome
50%−epiboly
Shield
75%−epiboly
1−4−somites
14−19−somites
20−25−somites
Prim−5
Prim−15
Prim−25
Long−pec
Protruding−mouth
Day−4
Day−5
Stage
Transcript
ENSDART00000158170
ENSDART00000163662

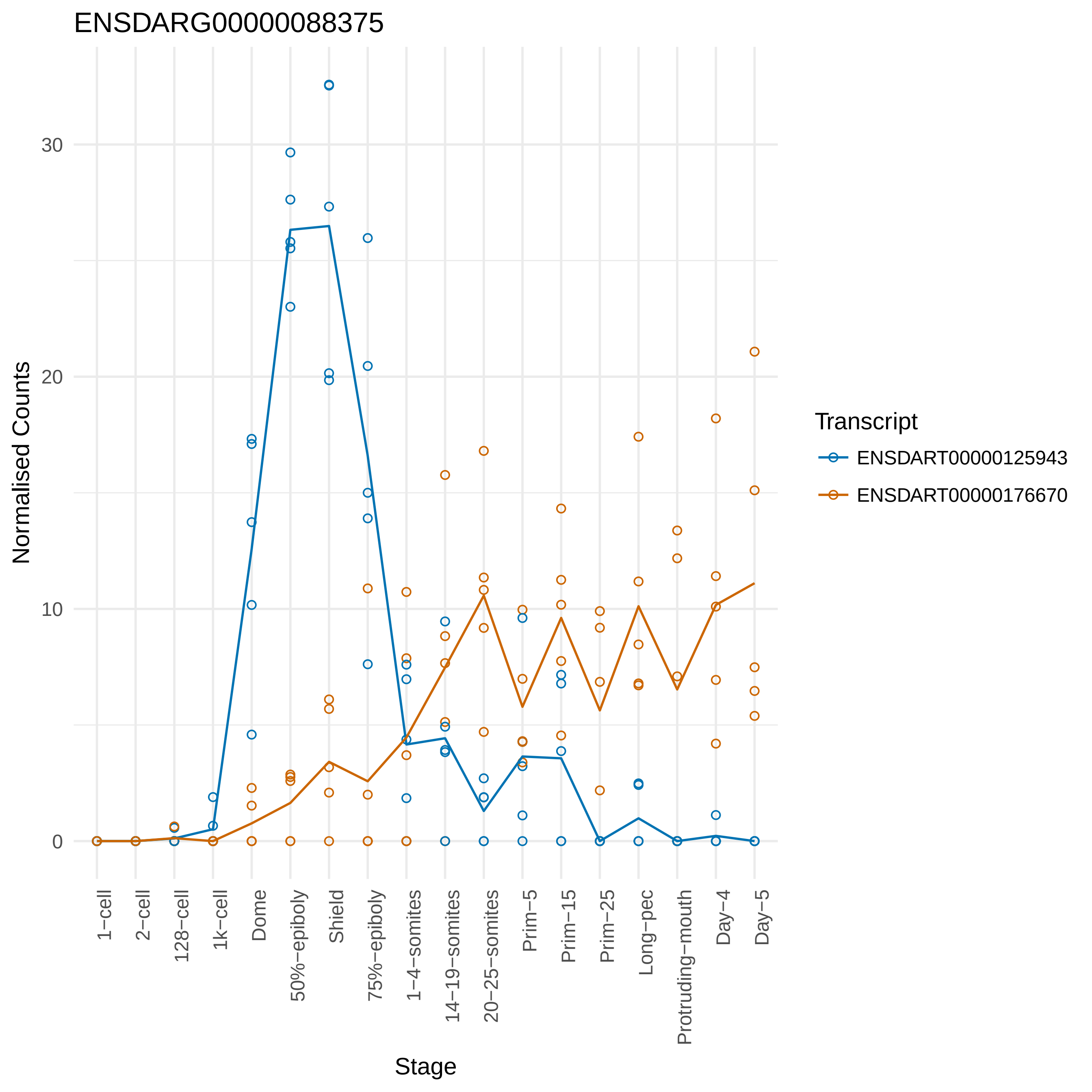

ENSDARG00000088375
Normalised Counts
30
20
10
0
1−cell
2−cell
128−cell
1k−cell
Dome
50%−epiboly
Shield
75%−epiboly
1−4−somites
14−19−somites
20−25−somites
Prim−5
Prim−15
Prim−25
Long−pec
Protruding−mouth
Day−4
Day−5
Stage
Transcript
ENSDART00000125943
ENSDART00000176670

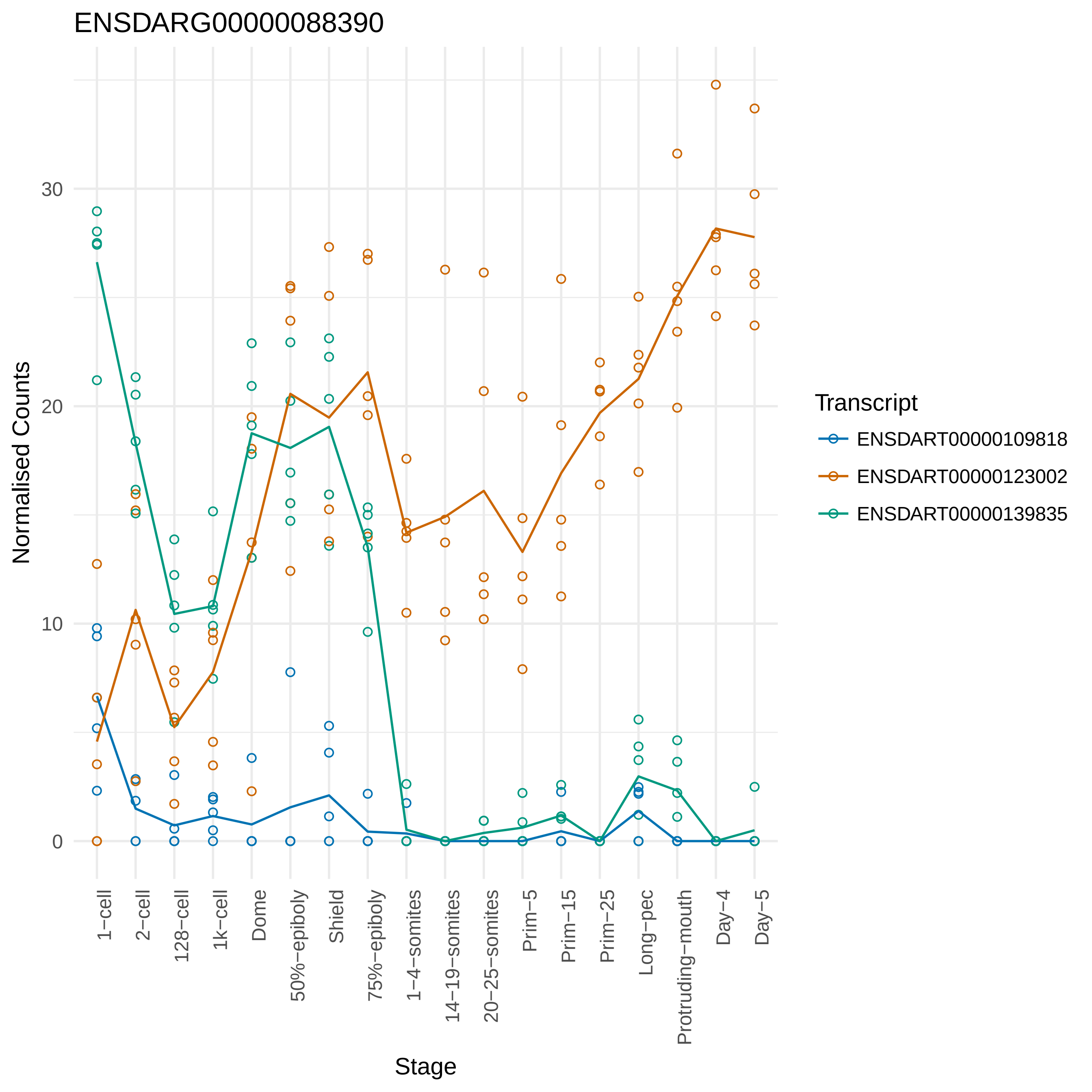

ENSDARG00000088390
Normalised Counts
30
20
10
0
1−cell
2−cell
128−cell
1k−cell
Dome
50%−epiboly
Shield
75%−epiboly
1−4−somites
14−19−somites
20−25−somites
Prim−5
Prim−15
Prim−25
Long−pec
Protruding−mouth
Day−4
Day−5
Stage
Transcript
ENSDART00000109818
ENSDART00000123002
ENSDART00000139835

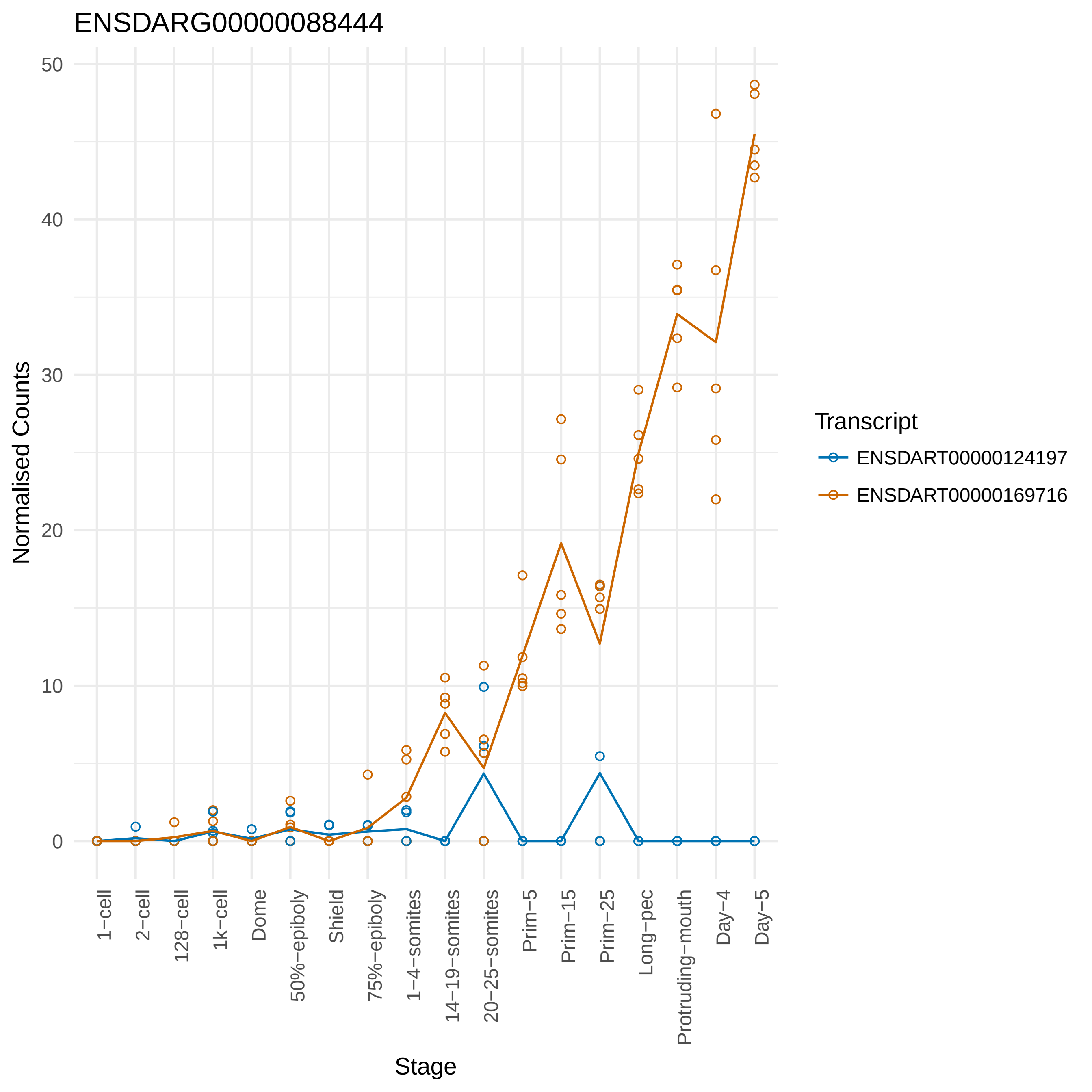
ENSDARG00000088444
Normalised Counts
50
40
30
20
10
0
1−cell
2−cell
128−cell
1k−cell
Dome
50%−epiboly
Shield
75%−epiboly
1−4−somites
14−19−somites
20−25−somites
Prim−5
Prim−15
Prim−25
Long−pec
Protruding−mouth
Day−4
Day−5
Stage
Transcript
ENSDART00000124197
ENSDART00000169716

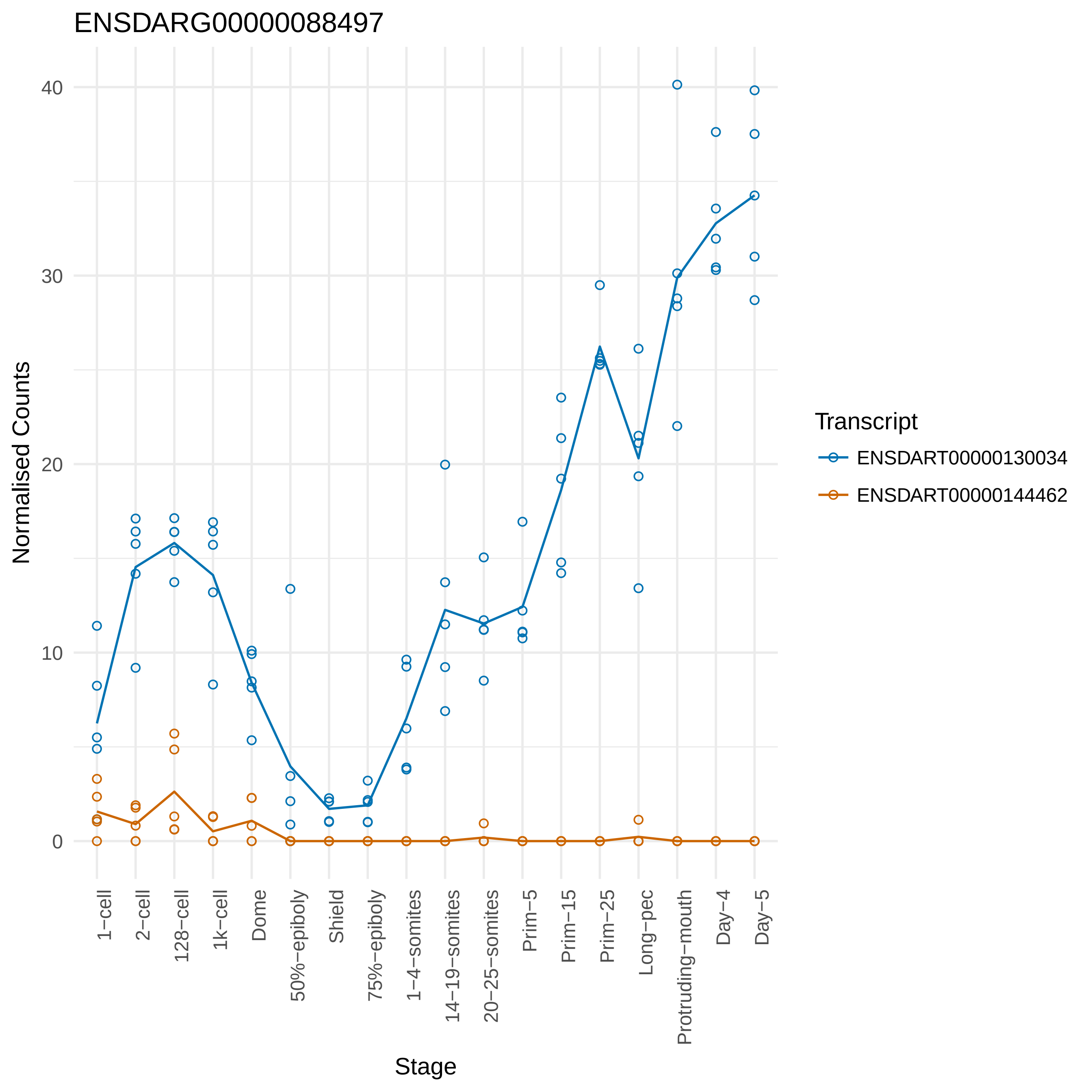

ENSDARG00000088497
Normalised Counts
Stage
0
10
20
30
40
1−cell
2−cell
128−cell
1k−cell
Dome
50%−epiboly
Shield
75%−epiboly
1−4−somites
14−19−somites
20−25−somites
Prim−5
Prim−15
Prim−25
Long−pec
Protruding−mouth
Day−4
Day−5
Transcript
ENSDART00000130034
ENSDART00000144462

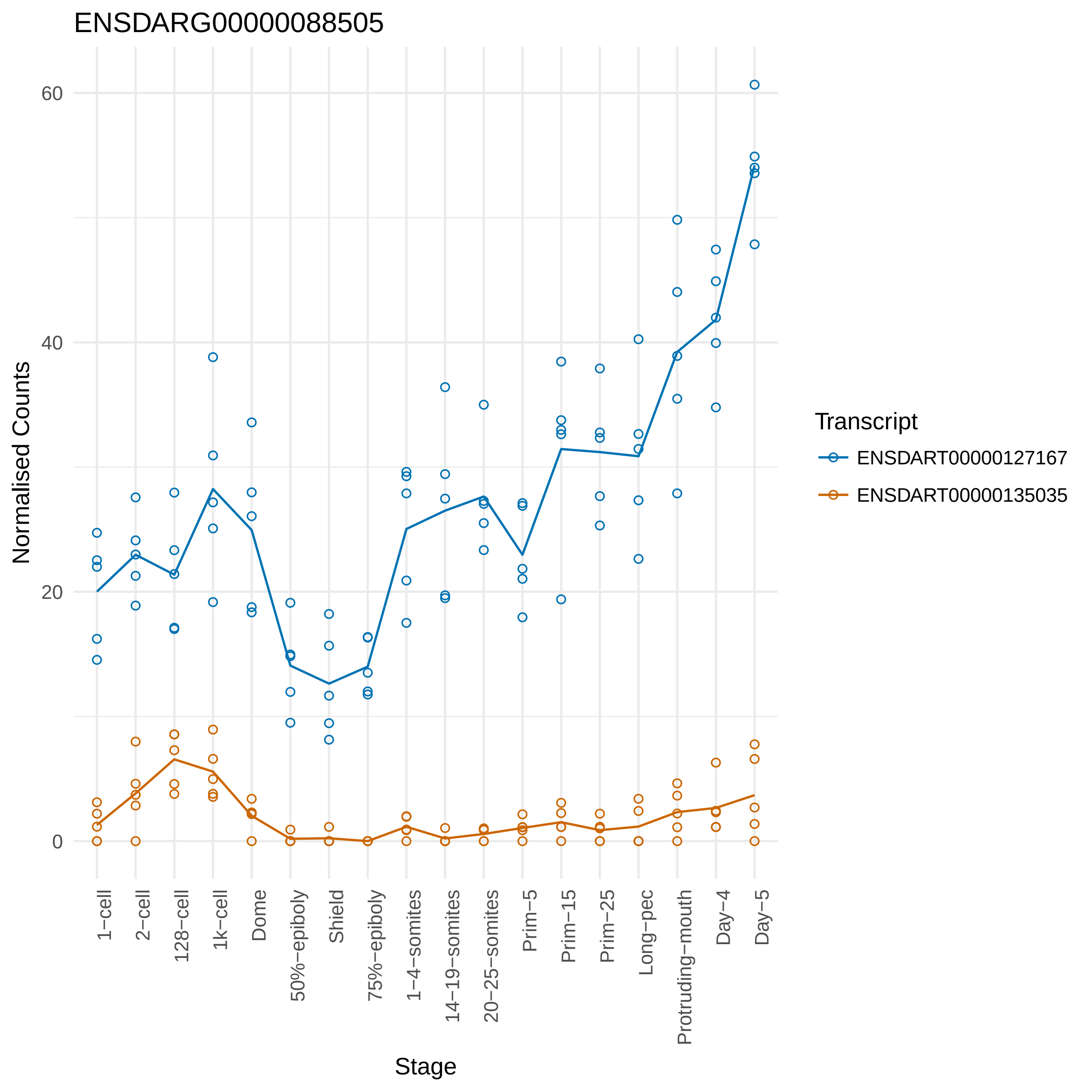
ENSDARG00000088505
Normalised Counts
60
40
20
0
1−cell
2−cell
128−cell
1k−cell
Dome
50%−epiboly
Shield
75%−epiboly
1−4−somites
14−19−somites
20−25−somites
Prim−5
Prim−15
Prim−25
Long−pec
Protruding−mouth
Day−4
Day−5
Stage
Transcript
ENSDART00000127167
ENSDART00000135035

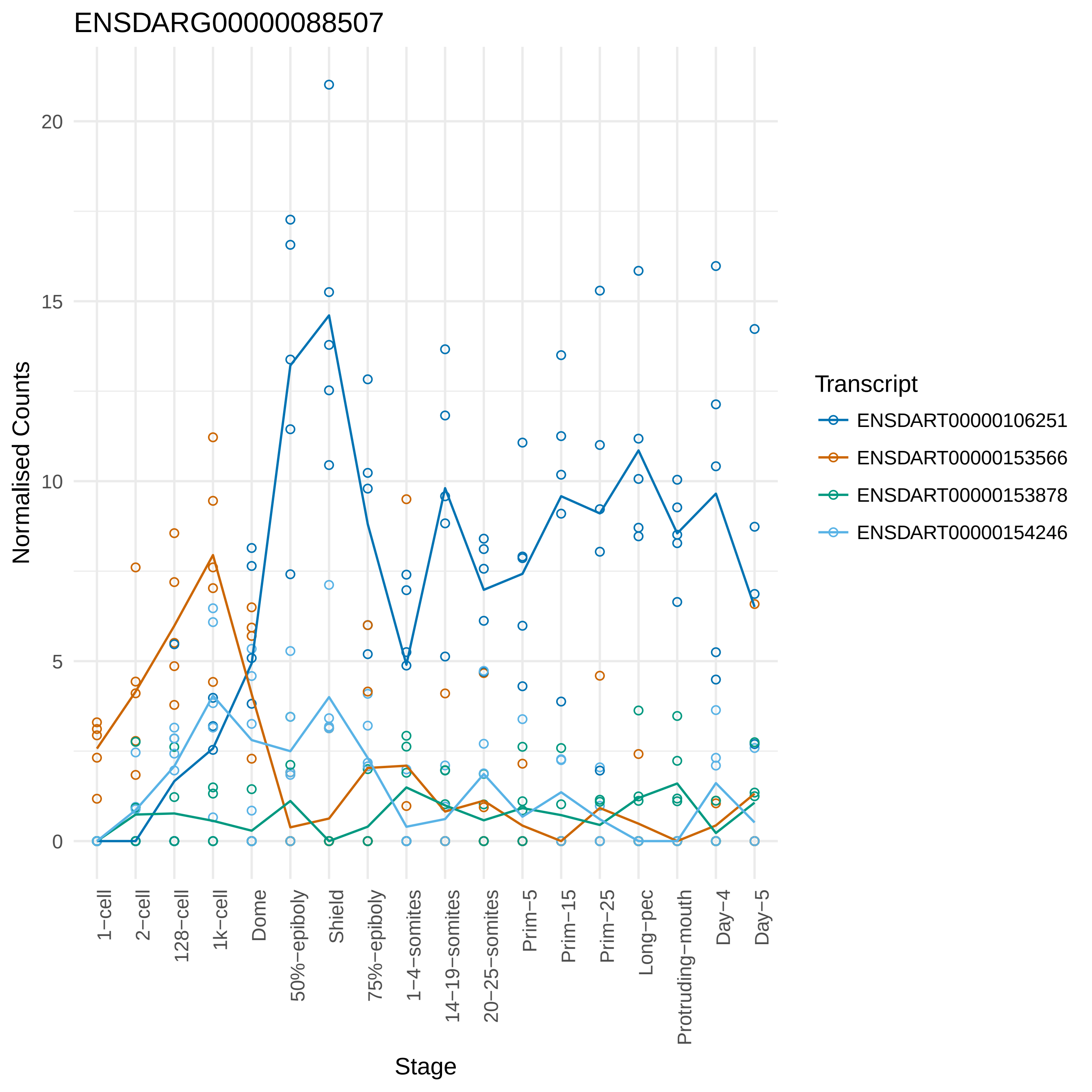

ENSDARG00000088507
Normalised Counts
0
5
10
15
20
1−cell
2−cell
128−cell
1k−cell
Dome
50%−epiboly
Shield
75%−epiboly
1−4−somites
14−19−somites
20−25−somites
Prim−5
Prim−15
Prim−25
Long−pec
Protruding−mouth
Day−4
Day−5
Stage
Transcript
ENSDART00000106251
ENSDART00000153566
ENSDART00000153878
ENSDART00000154246

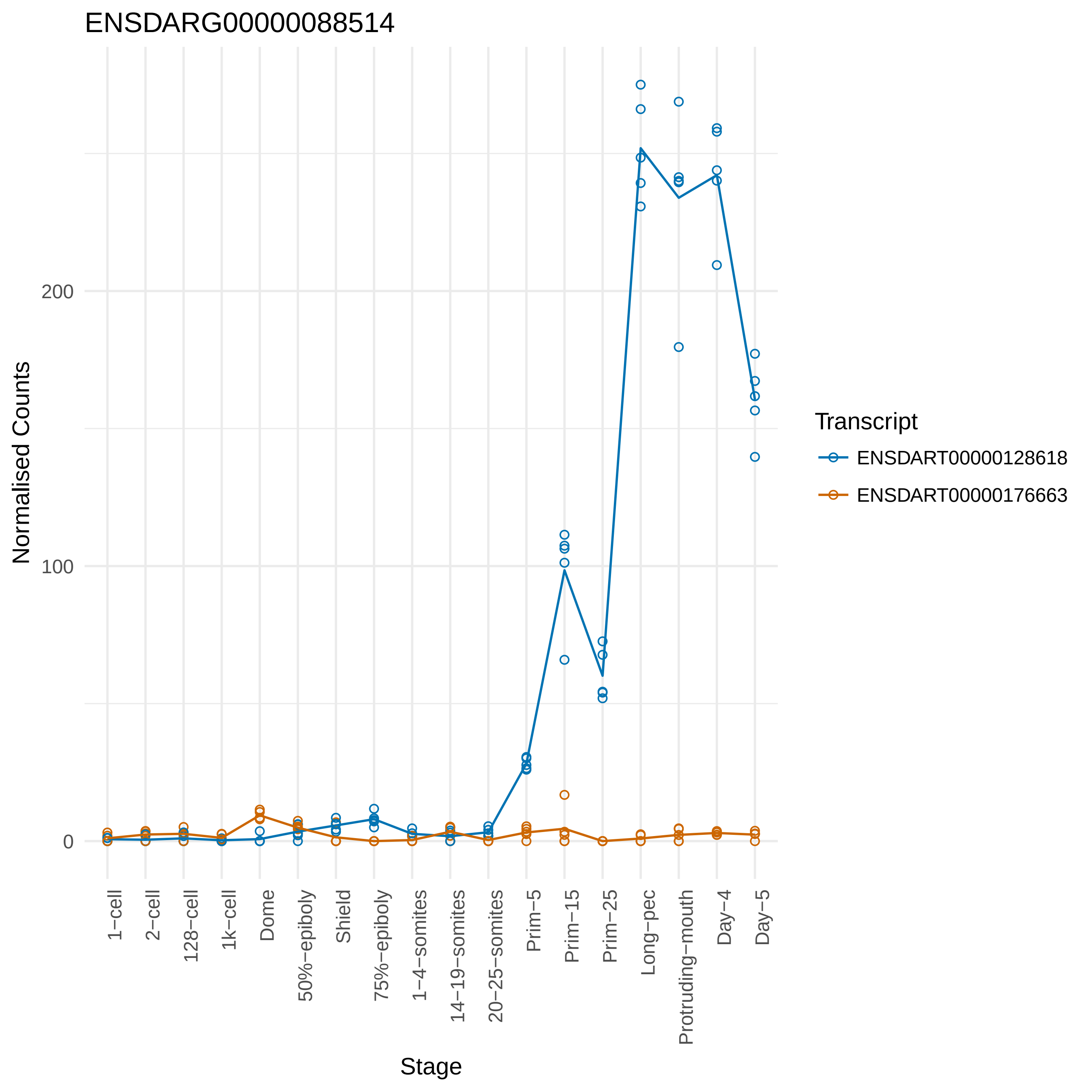

ENSDARG00000088514
Normalised Counts
0
100
200
Stage
1−cell
2−cell
128−cell
1k−cell
Dome
50%−epiboly
Shield
75%−epiboly
1−4−somites
14−19−somites
20−25−somites
Prim−5
Prim−15
Prim−25
Long−pec
Protruding−mouth
Day−4
Day−5
Transcript
ENSDART00000128618
ENSDART00000176663

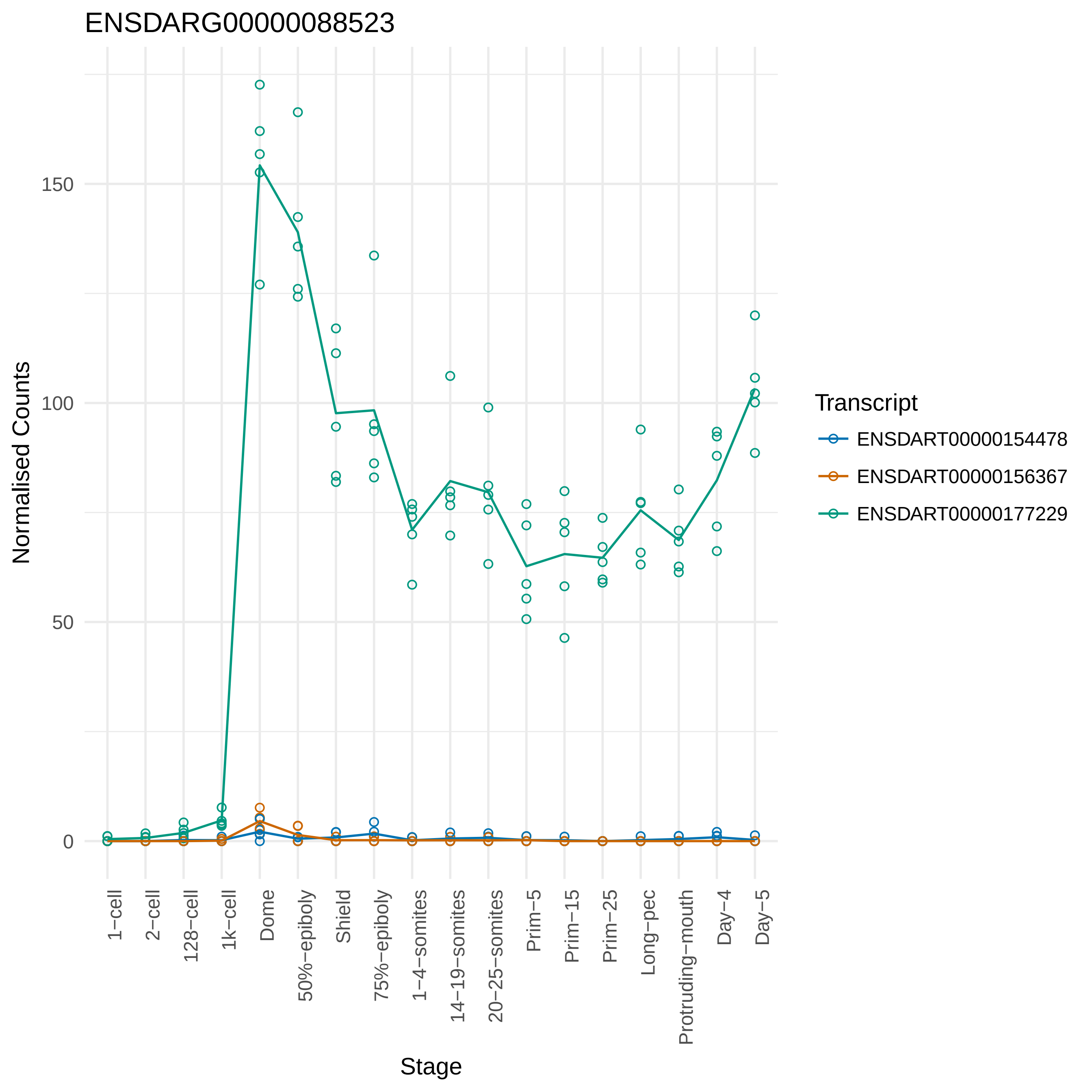

ENSDARG00000088523
Normalised Counts
150
100
50
0
1−cell
2−cell
128−cell
1k−cell
Dome
50%−epiboly
Shield
75%−epiboly
1−4−somites
14−19−somites
20−25−somites
Prim−5
Prim−15
Prim−25
Long−pec
Protruding−mouth
Day−4
Day−5
Stage
Transcript
ENSDART00000154478
ENSDART00000156367
ENSDART00000177229

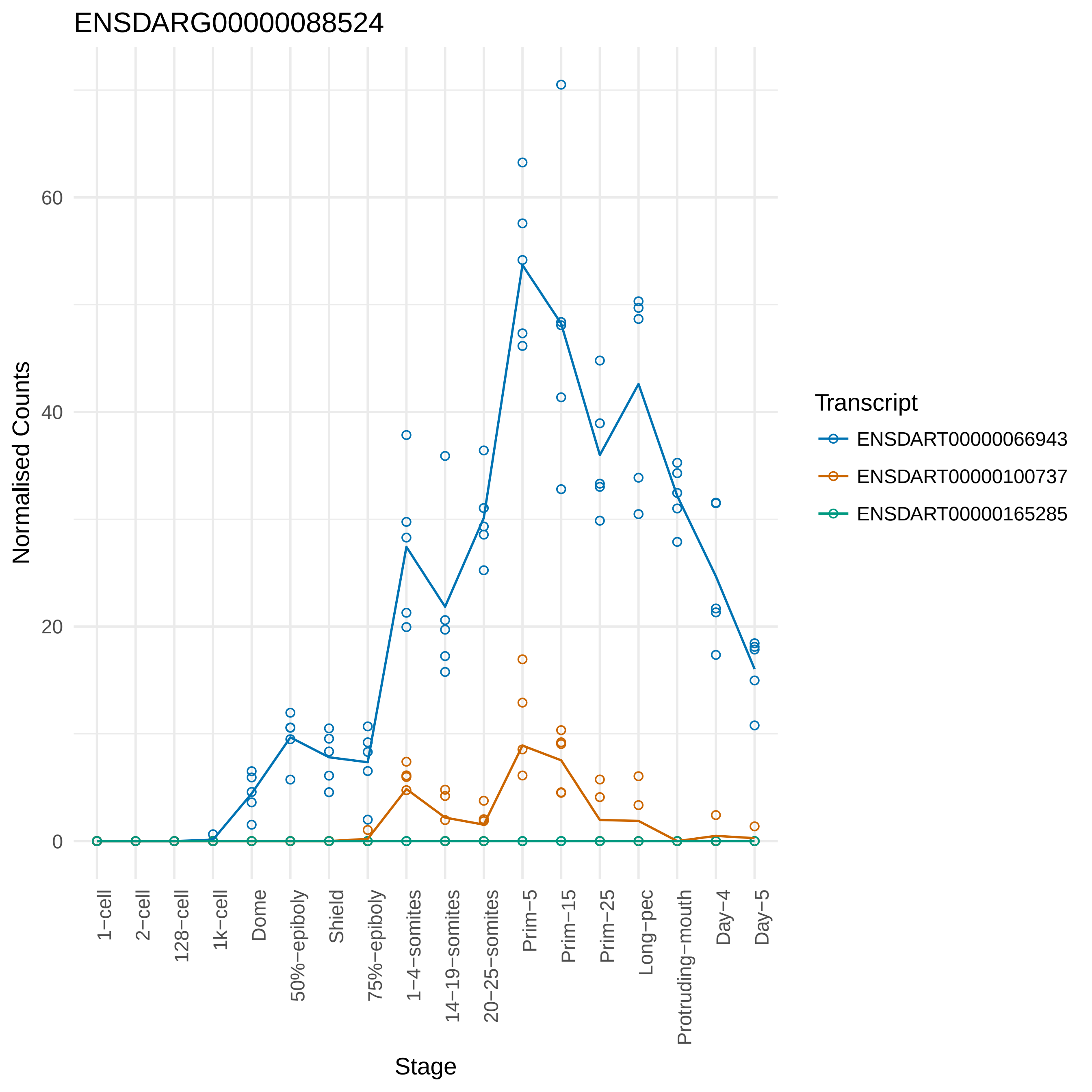
ENSDARG00000088524
Normalised Counts
0
20
40
60
Stage
1−cell
2−cell
128−cell
1k−cell
Dome
50%−epiboly
Shield
75%−epiboly
1−4−somites
14−19−somites
20−25−somites
Prim−5
Prim−15
Prim−25
Long−pec
Protruding−mouth
Day−4
Day−5
Transcript
ENSDART00000066943
ENSDART00000100737
ENSDART00000165285

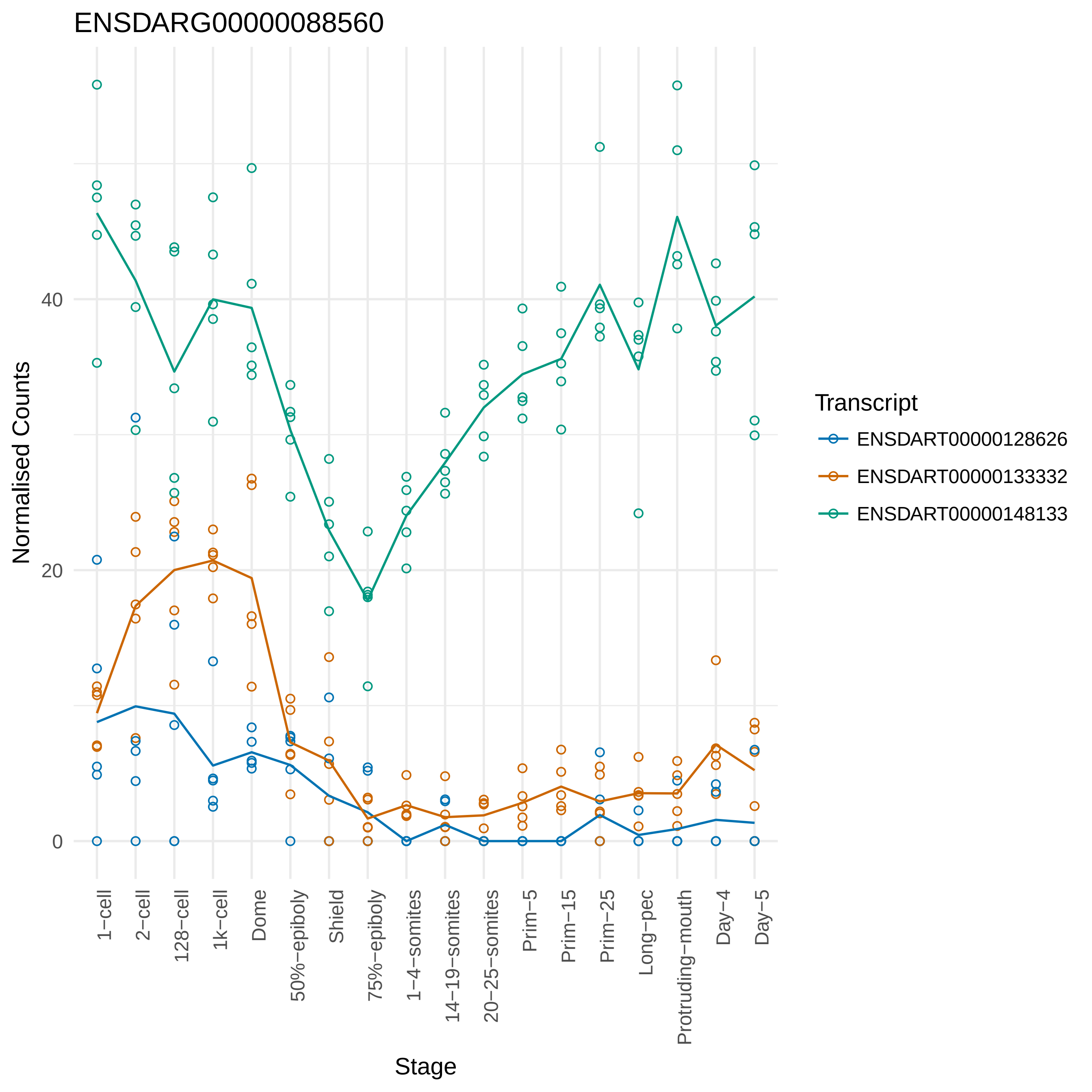
ENSDARG00000088560
Normalised Counts
0
20
40
Stage
1−cell
2−cell
128−cell
1k−cell
Dome
50%−epiboly
Shield
75%−epiboly
1−4−somites
14−19−somites
20−25−somites
Prim−5
Prim−15
Prim−25
Long−pec
Protruding−mouth
Day−4
Day−5
Transcript
ENSDART00000128626
ENSDART00000133332
ENSDART00000148133

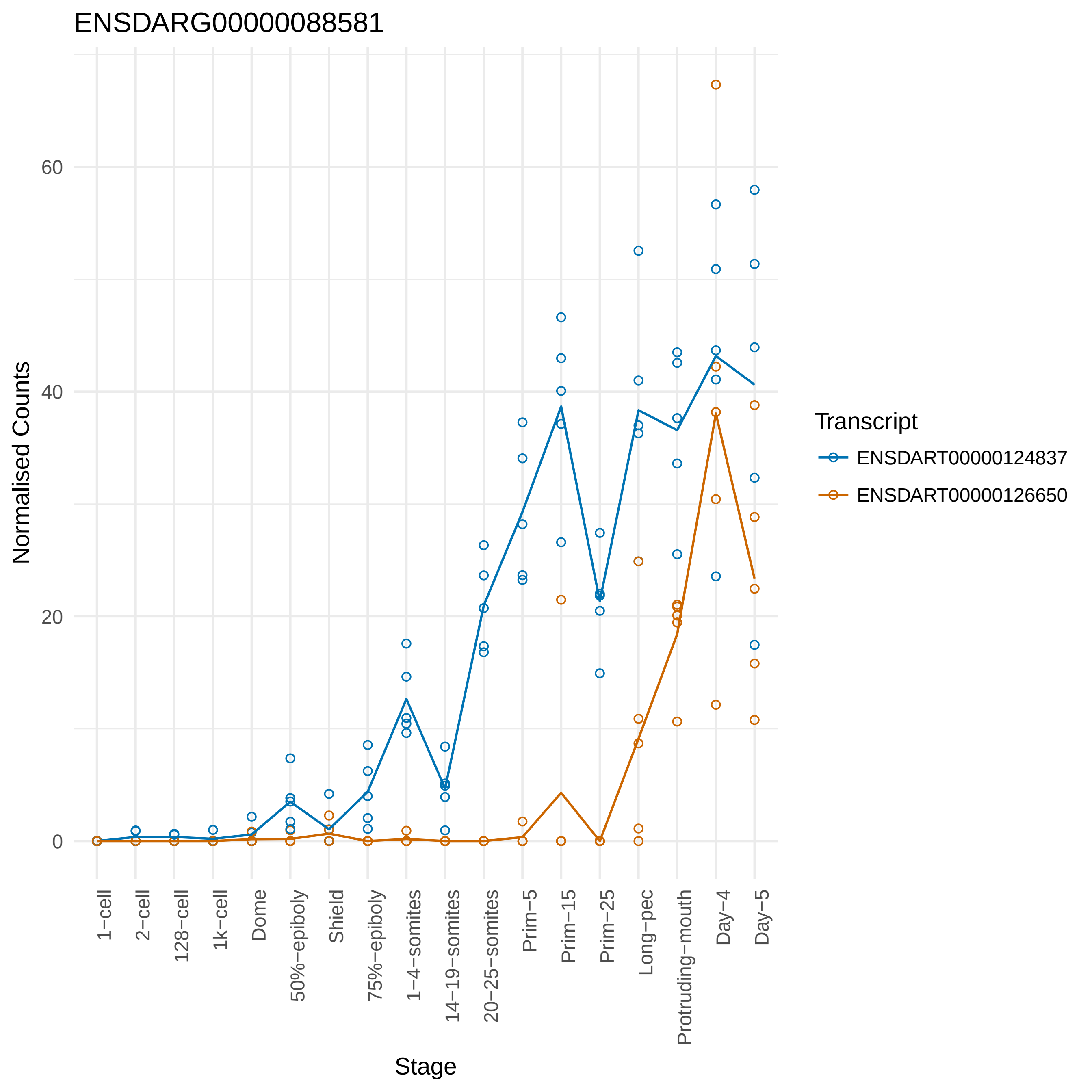
ENSDARG00000088581
Normalised Counts
0
20
40
60
1−cell
2−cell
128−cell
1k−cell
Dome
50%−epiboly
Shield
75%−epiboly
1−4−somites
14−19−somites
20−25−somites
Prim−5
Prim−15
Prim−25
Long−pec
Protruding−mouth
Day−4
Day−5
Stage
Transcript
ENSDART00000124837
ENSDART00000126650

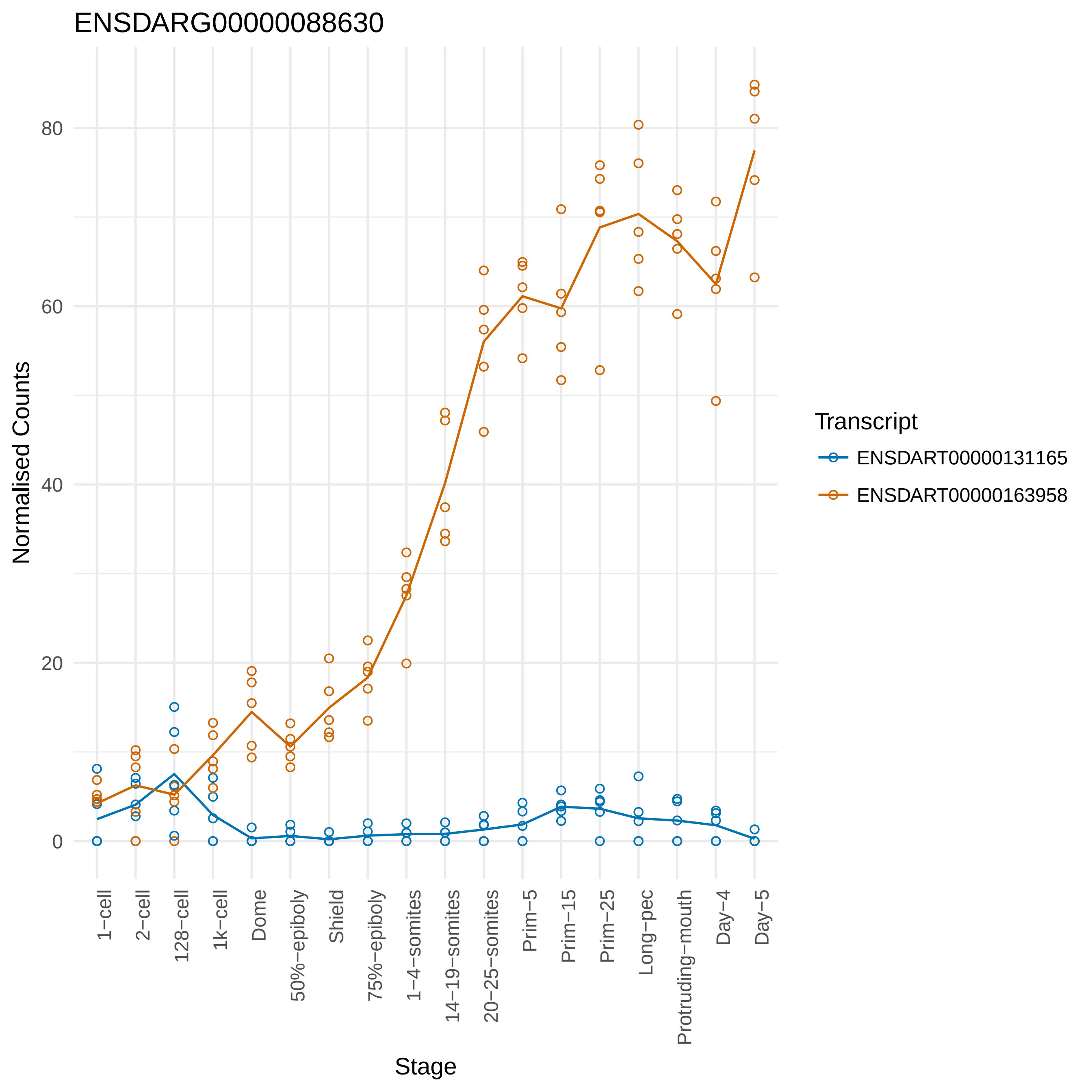

ENSDARG00000088630
Normalised Counts
0
20
40
60
80
Stage
1−cell
2−cell
128−cell
1k−cell
Dome
50%−epiboly
Shield
75%−epiboly
1−4−somites
14−19−somites
20−25−somites
Prim−5
Prim−15
Prim−25
Long−pec
Protruding−mouth
Day−4
Day−5
Transcript
ENSDART00000131165
ENSDART00000163958

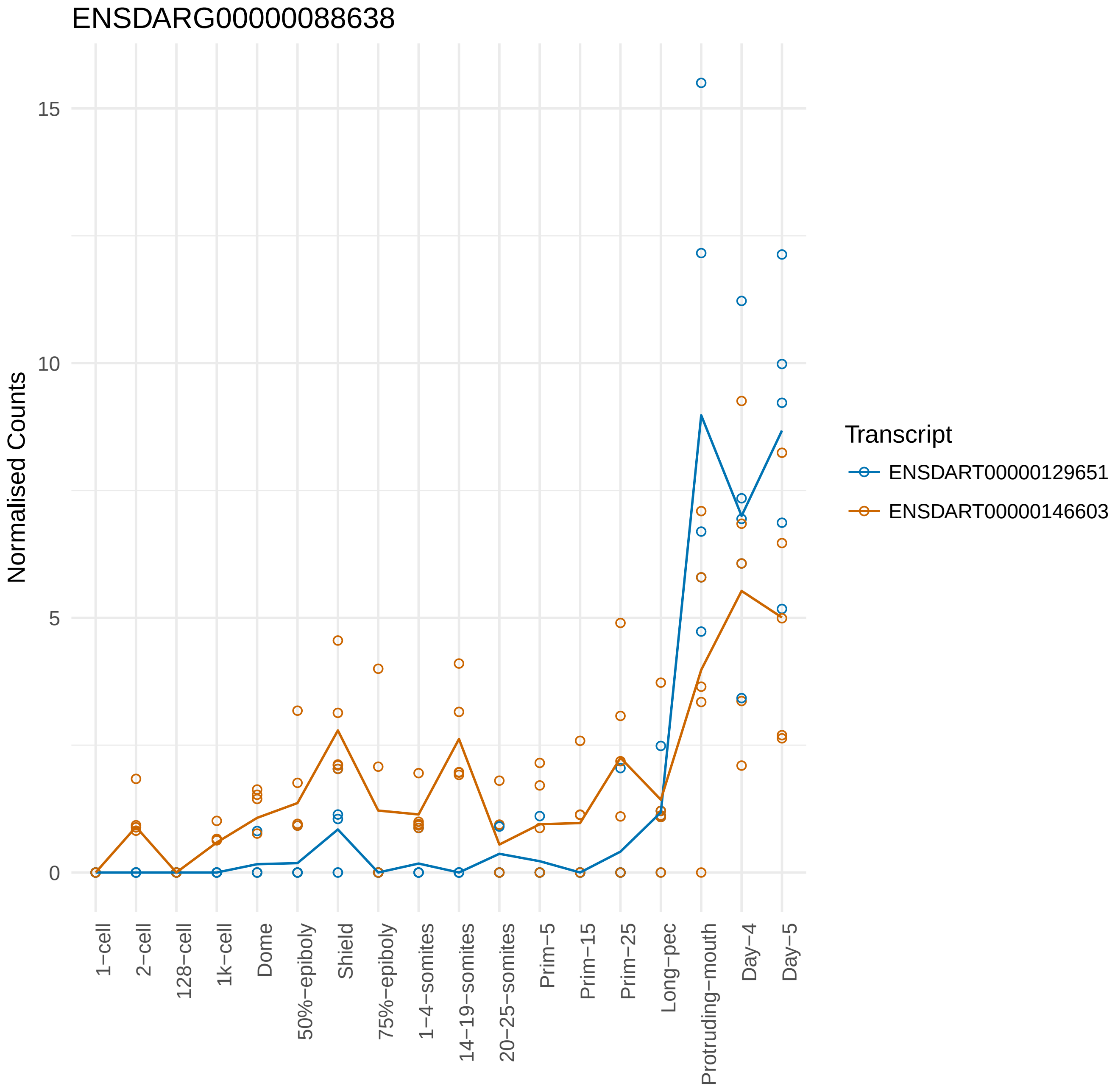

ENSDARG00000088638
Normalised Counts
15
10
5
0
1−cell
2−cell
128−cell
1k−cell
Dome
50%−epiboly
Shield
75%−epiboly
1−4−somites
14−19−somites
20−25−somites
Prim−5
Prim−15
Prim−25
Long−pec
Protruding−mouth
Day−4
Day−5
Stage
Transcript
ENSDART00000129651
ENSDART00000146603

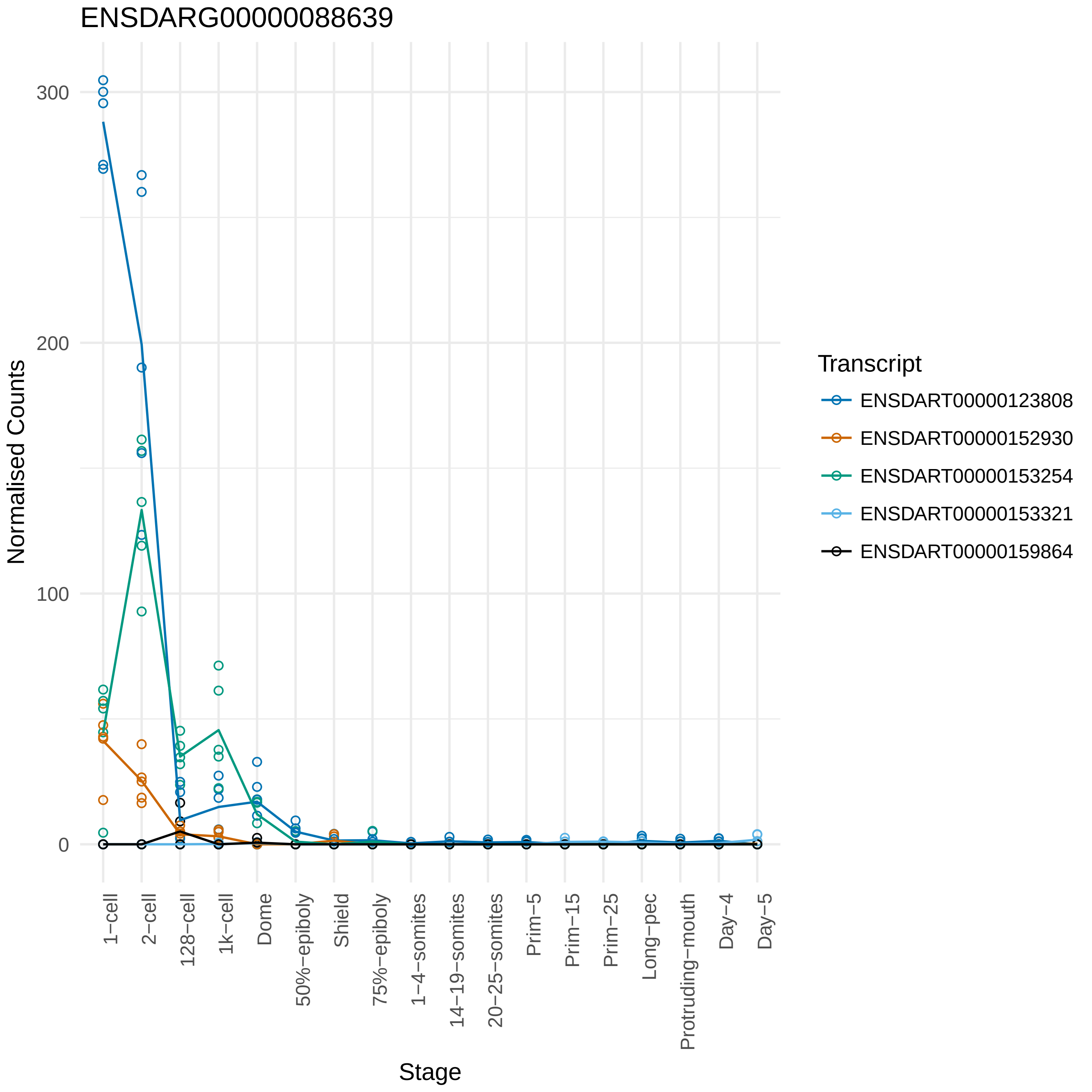

ENSDARG00000088639
Normalised Counts
300
200
100
0
1−cell
2−cell
128−cell
1k−cell
Dome
50%−epiboly
Shield
75%−epiboly
1−4−somites
14−19−somites
20−25−somites
Prim−5
Prim−15
Prim−25
Long−pec
Protruding−mouth
Day−4
Day−5
Stage
Transcript
ENSDART00000123808
ENSDART00000152930
ENSDART00000153254
ENSDART00000153321
ENSDART00000159864

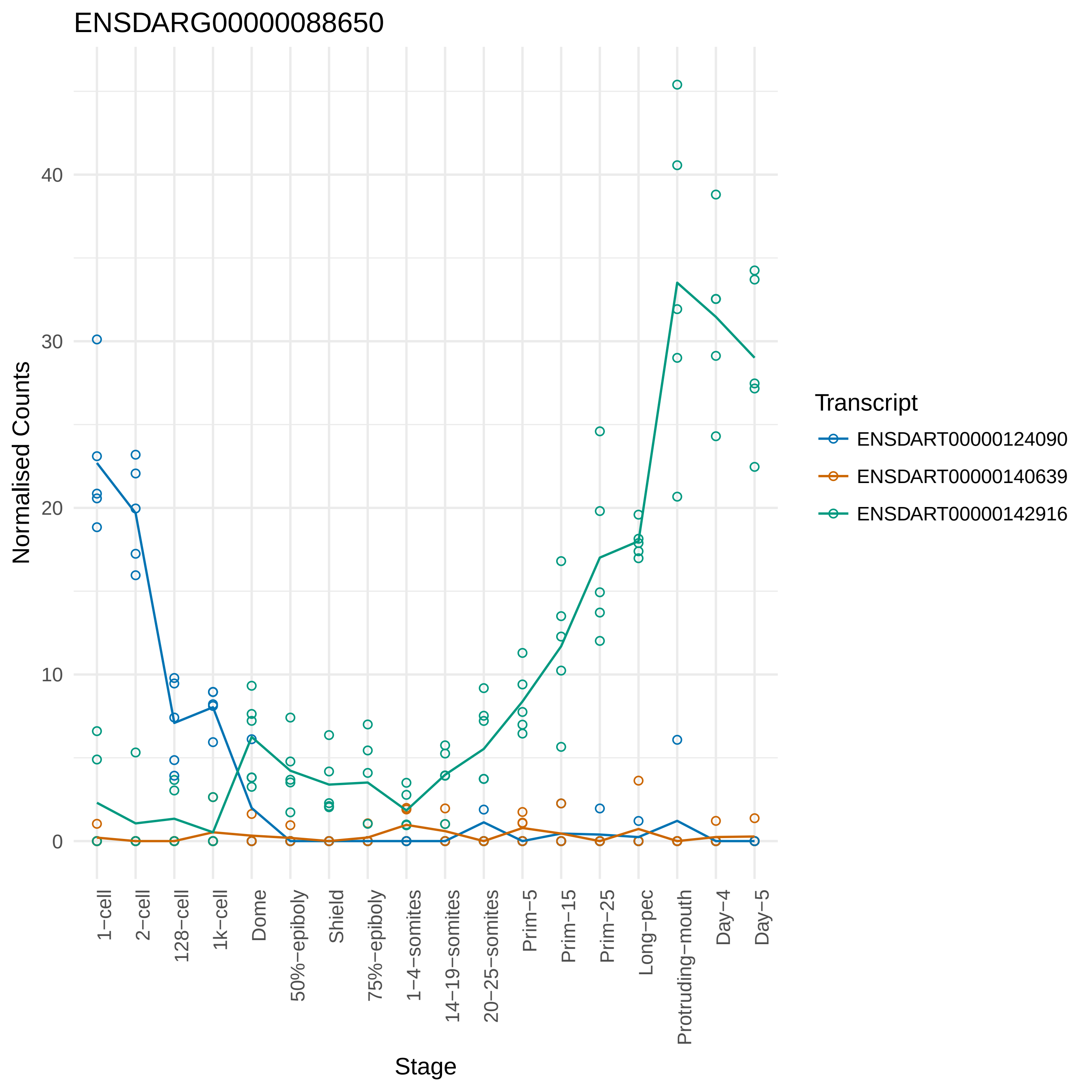

ENSDARG00000088650
Normalised Counts
0
10
20
30
40
1−cell
2−cell
128−cell
1k−cell
Dome
50%−epiboly
Shield
75%−epiboly
1−4−somites
14−19−somites
20−25−somites
Prim−5
Prim−15
Prim−25
Long−pec
Protruding−mouth
Day−4
Day−5
Stage
Transcript
ENSDART00000124090
ENSDART00000140639
ENSDART00000142916

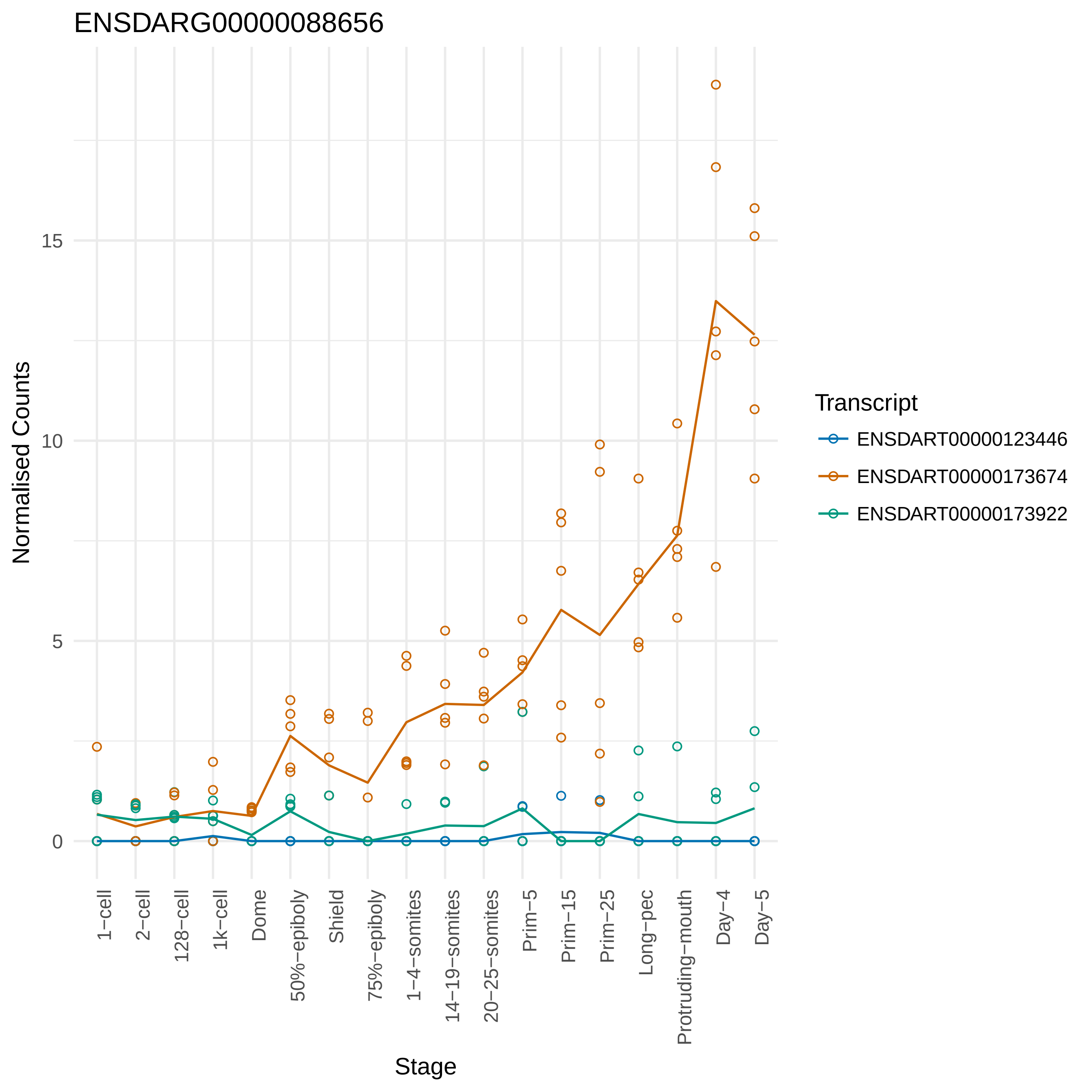

ENSDARG00000088656
Normalised Counts
15
10
5
0
1−cell
2−cell
128−cell
1k−cell
Dome
50%−epiboly
Shield
75%−epiboly
1−4−somites
14−19−somites
20−25−somites
Prim−5
Prim−15
Prim−25
Long−pec
Protruding−mouth
Day−4
Day−5
Stage
Transcript
ENSDART00000123446
ENSDART00000173674
ENSDART00000173922

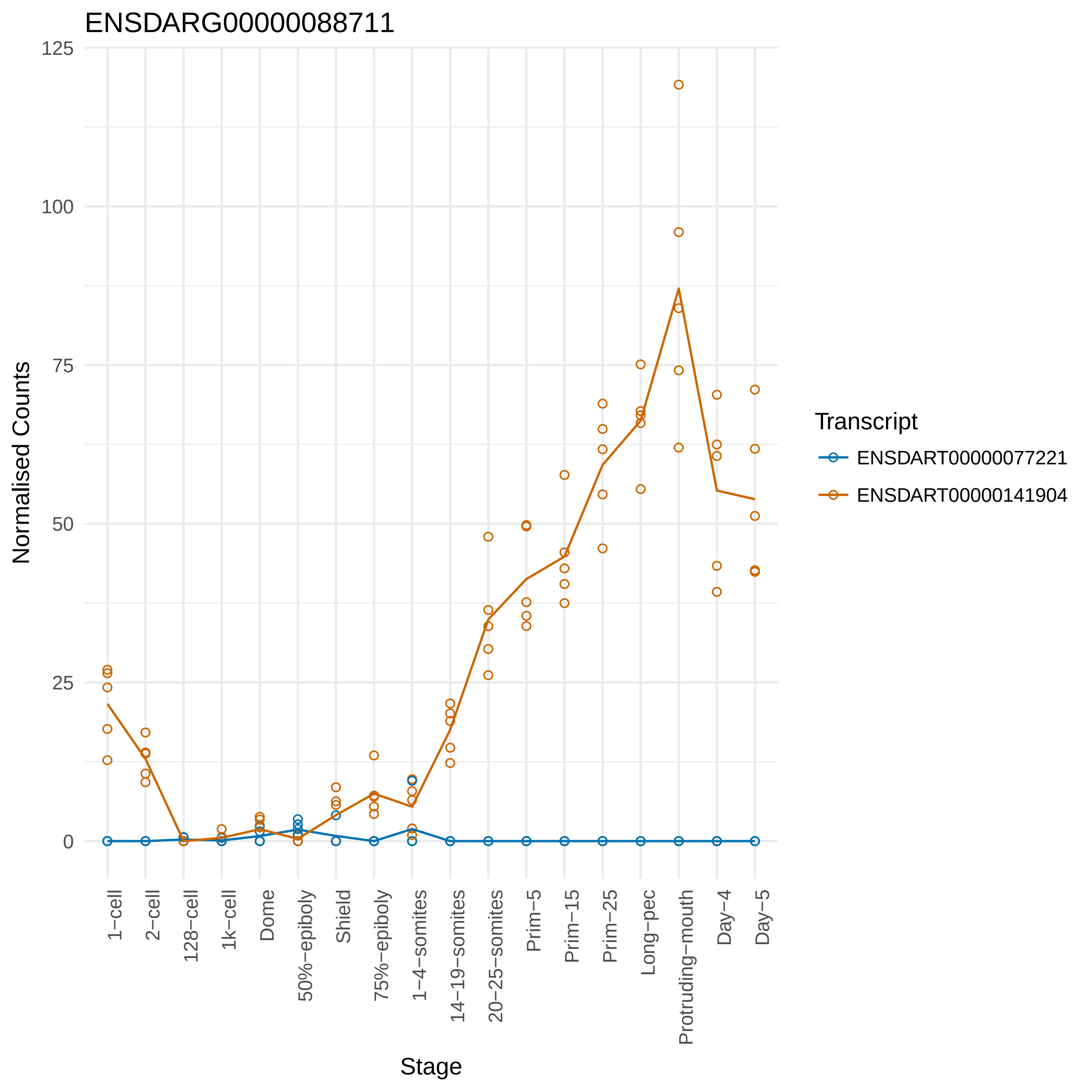

ENSDARG00000088711
Normalised Counts
125
100
75
50
25
0
1−cell
2−cell
128−cell
1k−cell
Dome
50%−epiboly
Shield
75%−epiboly
1−4−somites
14−19−somites
20−25−somites
Prim−5
Prim−15
Prim−25
Long−pec
Protruding−mouth
Day−4
Day−5
Stage
Transcript
ENSDART00000077221
ENSDART00000141904

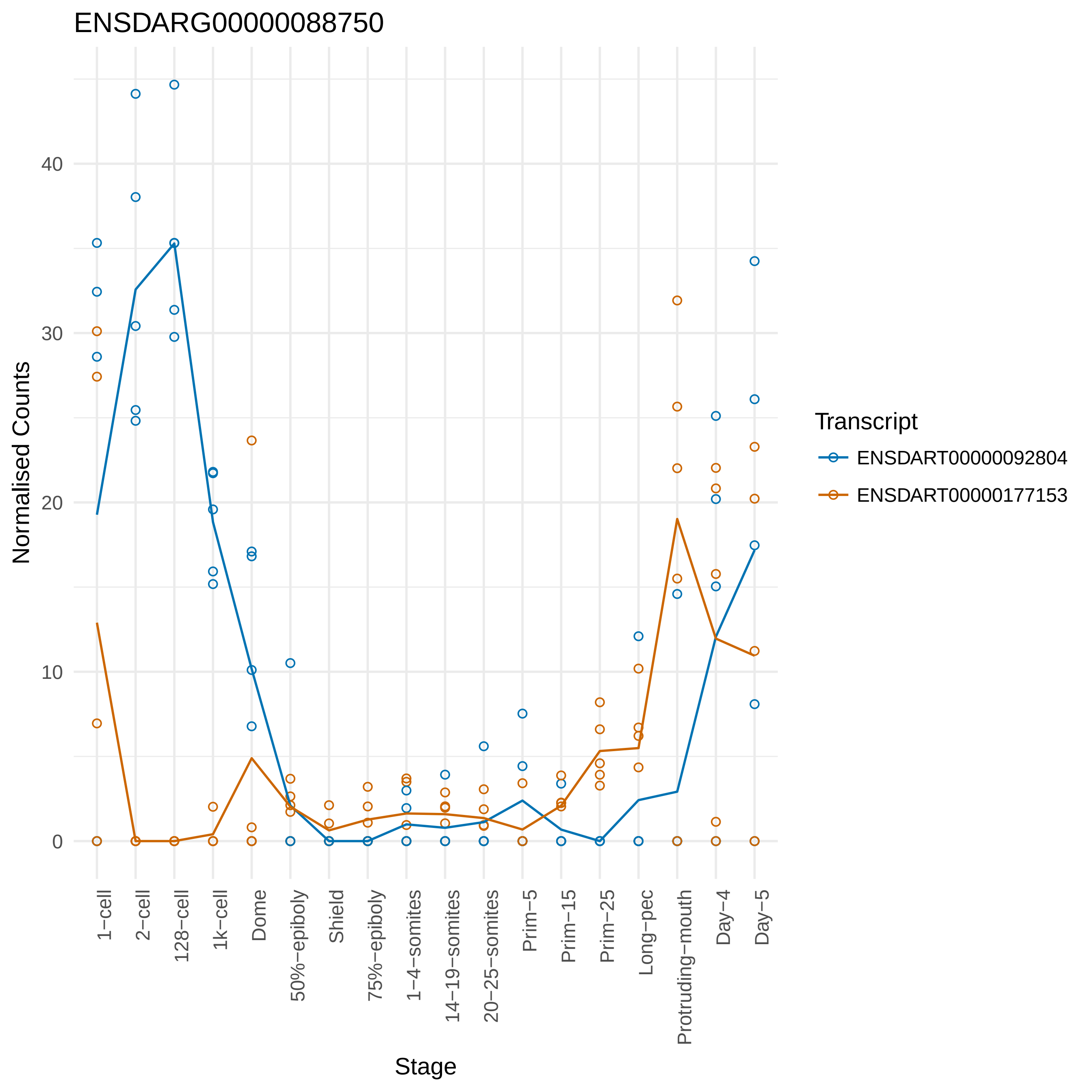

ENSDARG00000088750
Normalised Counts
0
10
20
30
40
Stage
1−cell
2−cell
128−cell
1k−cell
Dome
50%−epiboly
Shield
75%−epiboly
1−4−somites
14−19−somites
20−25−somites
Prim−5
Prim−15
Prim−25
Long−pec
Protruding−mouth
Day−4
Day−5
Transcript
ENSDART00000092804
ENSDART00000177153

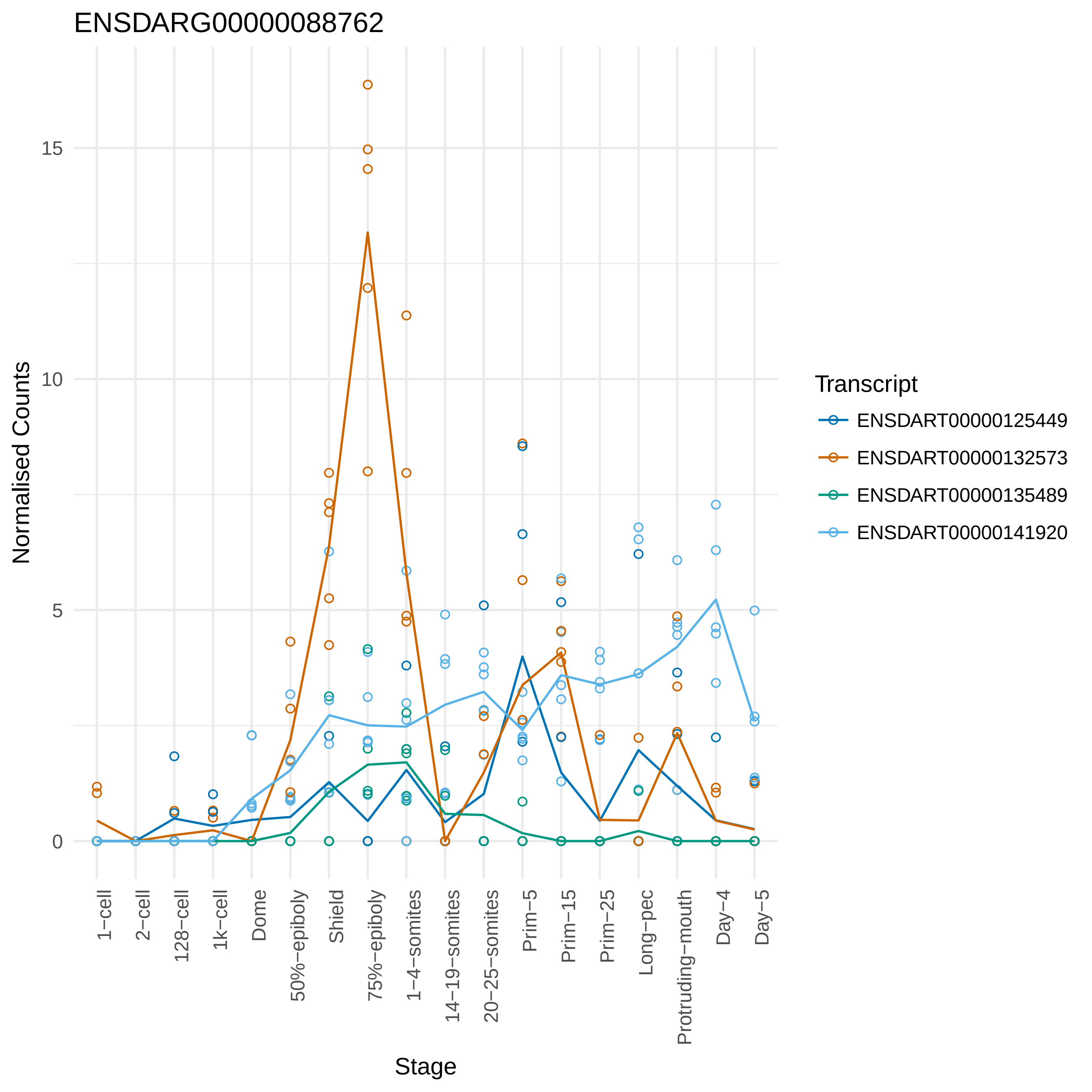

ENSDARG00000088762
Normalised Counts
0
5
10
15
Stage
1−cell
2−cell
128−cell
1k−cell
Dome
50%−epiboly
Shield
75%−epiboly
1−4−somites
14−19−somites
20−25−somites
Prim−5
Prim−15
Prim−25
Long−pec
Protruding−mouth
Day−4
Day−5
Transcript
ENSDART00000125449
ENSDART00000132573
ENSDART00000135489
ENSDART00000141920

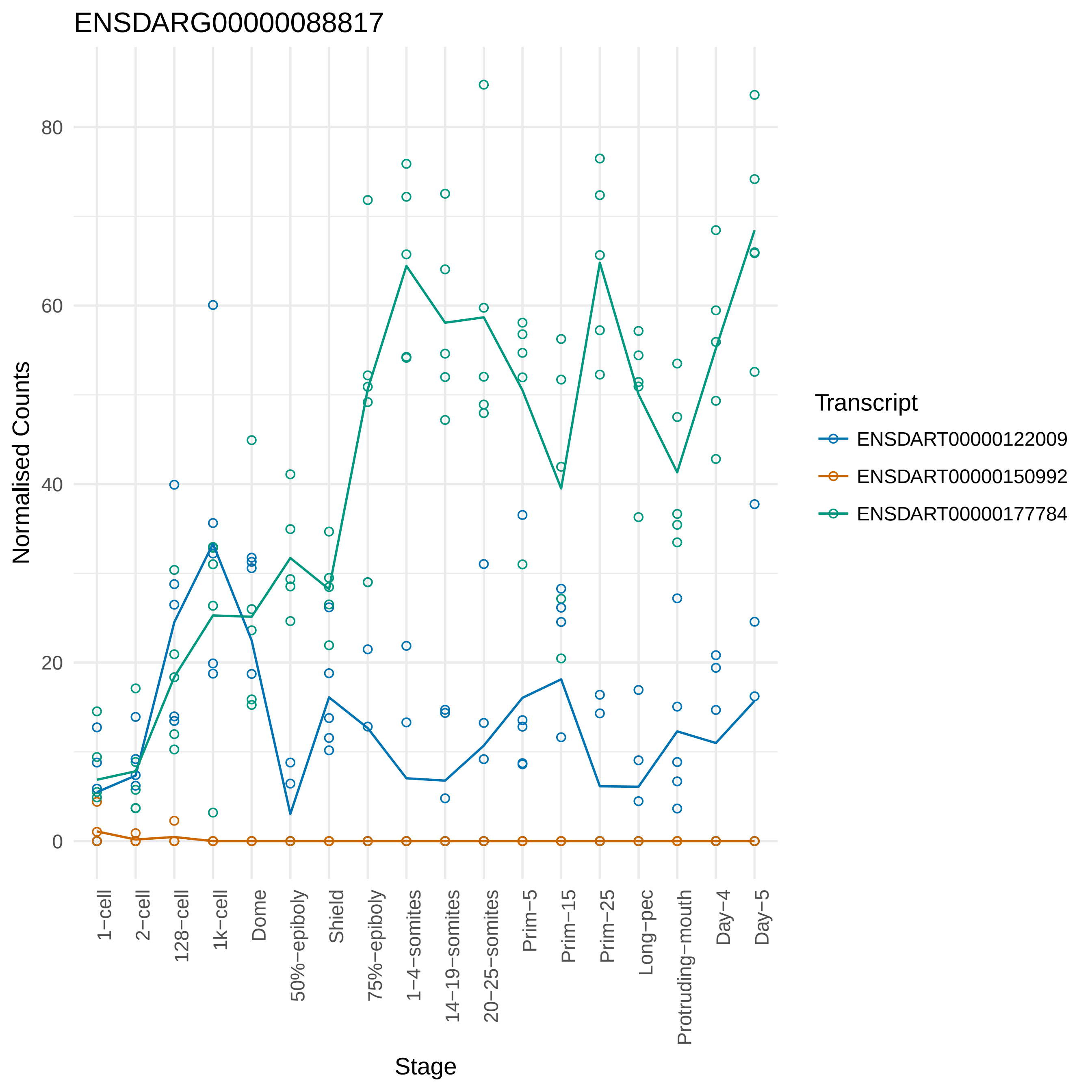

ENSDARG00000088817
Normalised Counts
0
20
40
60
80
1−cell
2−cell
128−cell
1k−cell
Dome
50%−epiboly
Shield
75%−epiboly
1−4−somites
14−19−somites
20−25−somites
Prim−5
Prim−15
Prim−25
Long−pec
Protruding−mouth
Day−4
Day−5
Stage
Transcript
ENSDART00000122009
ENSDART00000150992
ENSDART00000177784

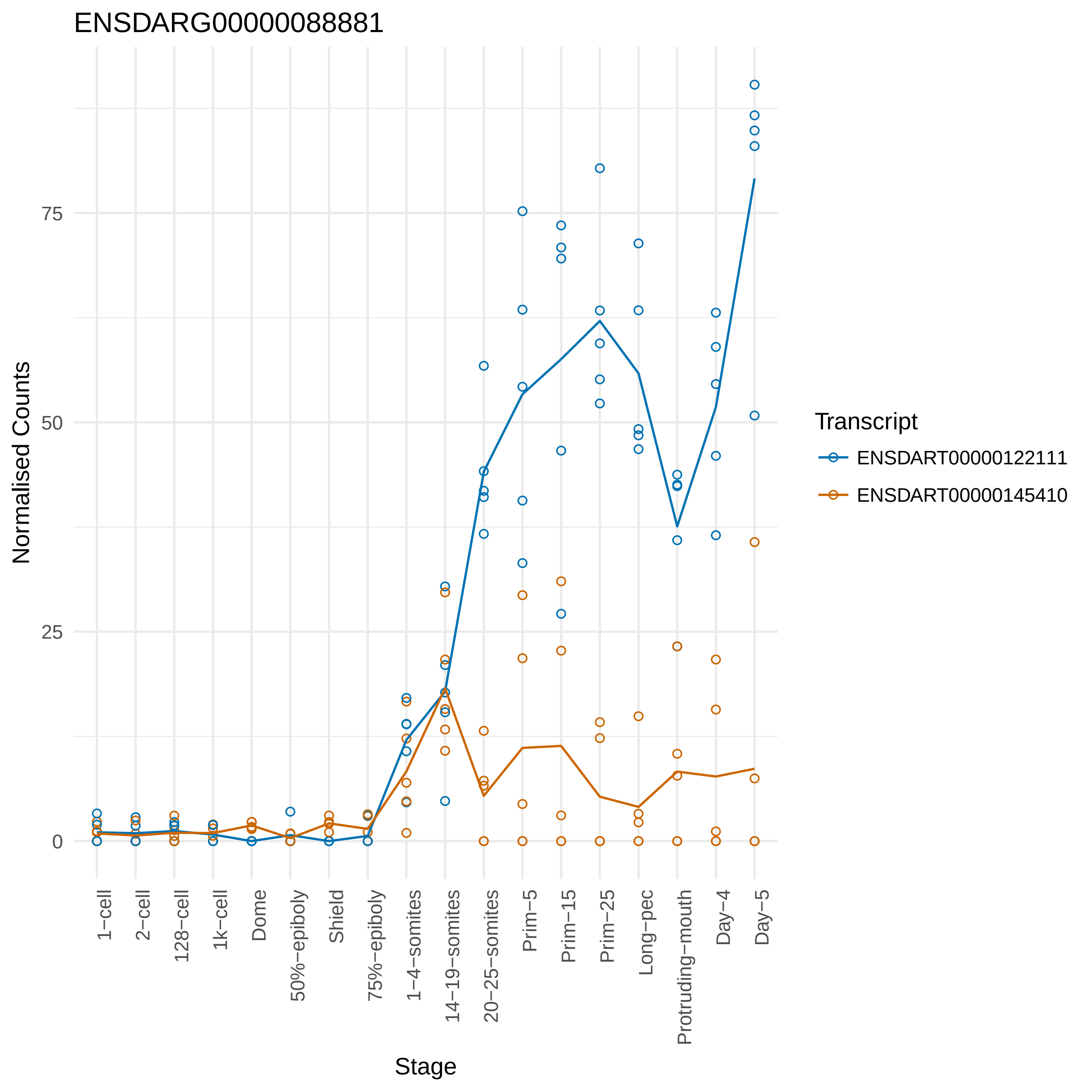

ENSDARG00000088881
Normalised Counts
75
50
25
0
1−cell
2−cell
128−cell
1k−cell
Dome
50%−epiboly
Shield
75%−epiboly
1−4−somites
14−19−somites
20−25−somites
Prim−5
Prim−15
Prim−25
Long−pec
Protruding−mouth
Day−4
Day−5
Stage
Transcript
ENSDART00000122111
ENSDART00000145410

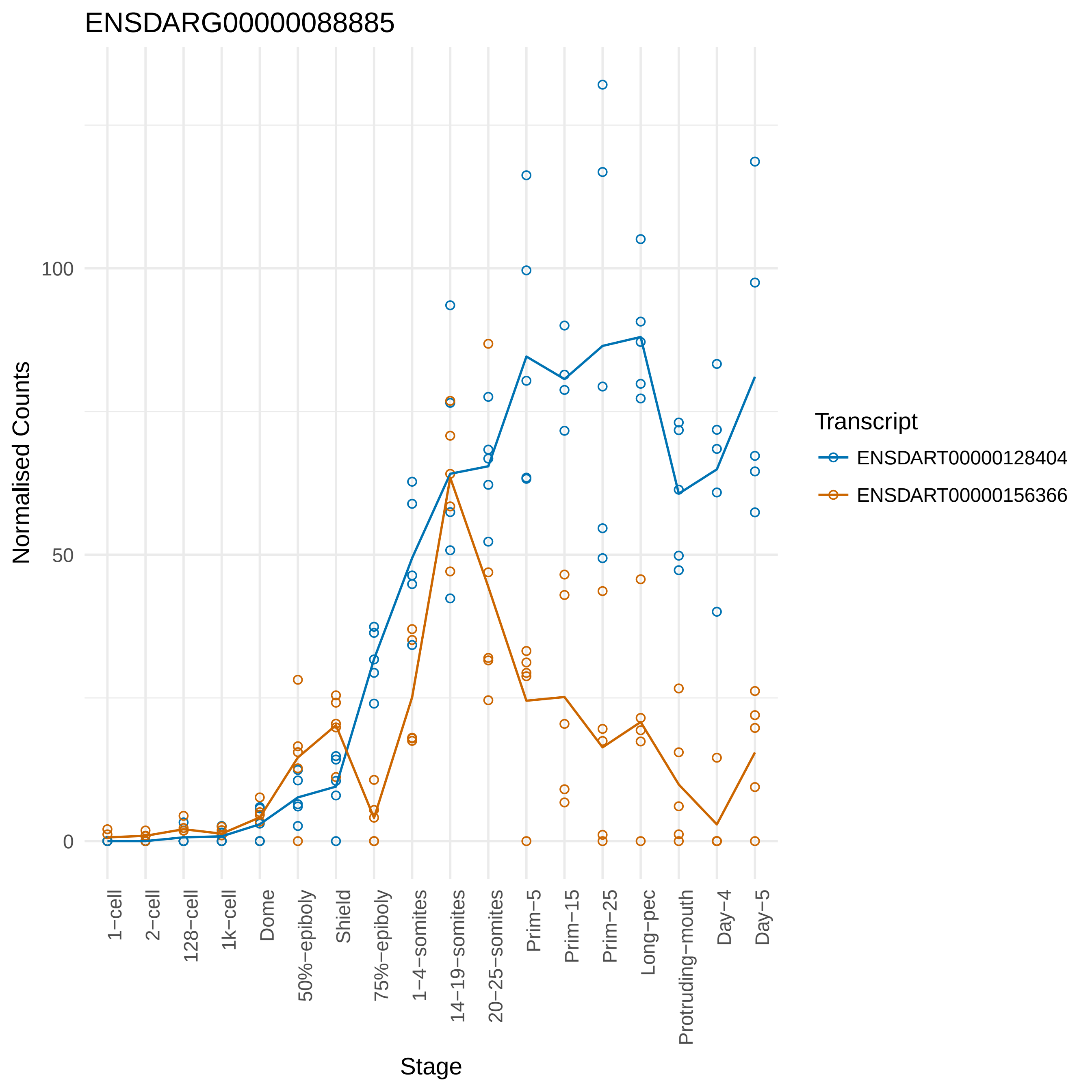
ENSDARG00000088885
Normalised Counts
100
50
0
Stage
1−cell
2−cell
128−cell
1k−cell
Dome
50%−epiboly
Shield
75%−epiboly
1−4−somites
14−19−somites
20−25−somites
Prim−5
Prim−15
Prim−25
Long−pec
Protruding−mouth
Day−4
Day−5
Transcript
ENSDART00000128404
ENSDART00000156366

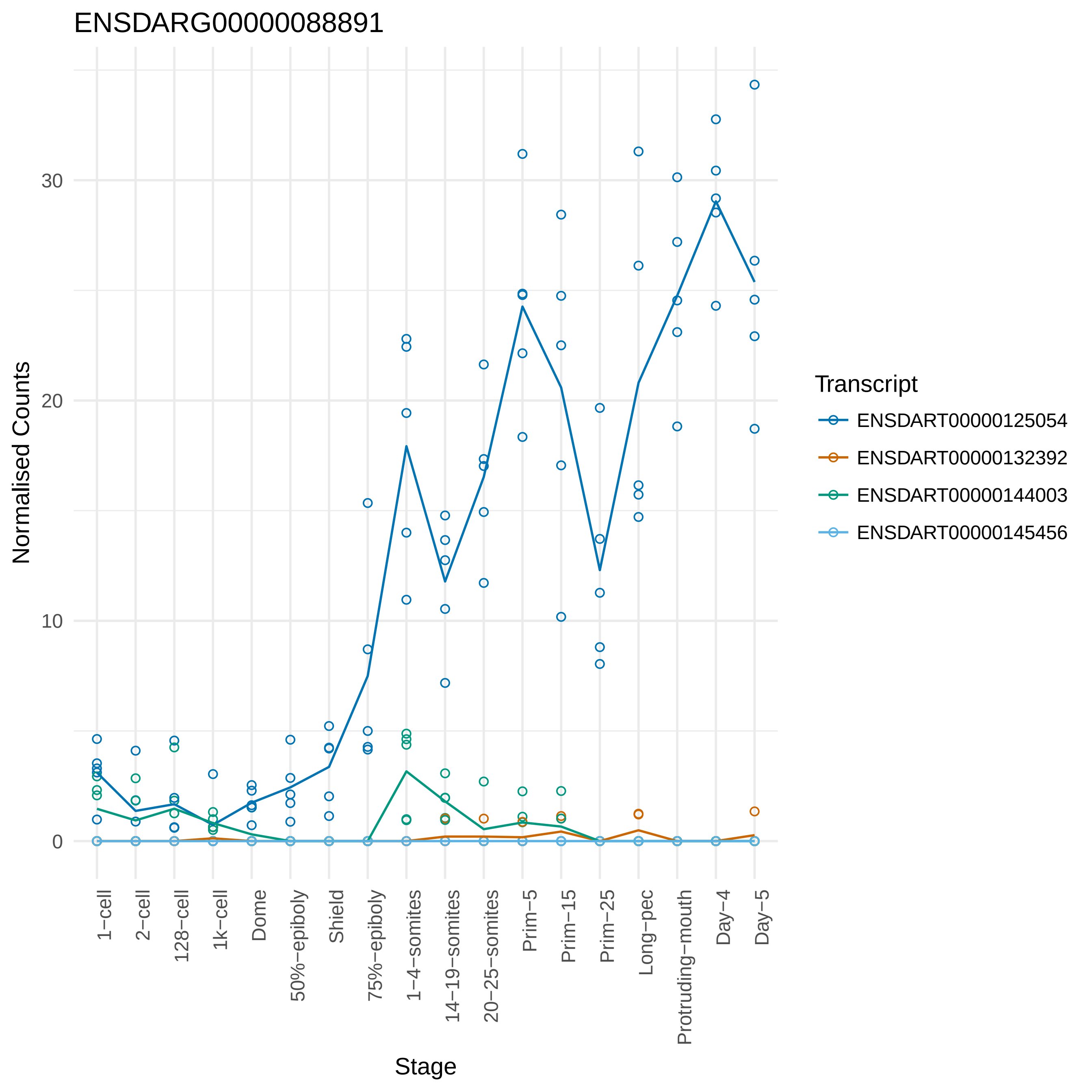

ENSDARG00000088891
Normalised Counts
30
20
10
0
1−cell
2−cell
128−cell
1k−cell
Dome
50%−epiboly
Shield
75%−epiboly
1−4−somites
14−19−somites
20−25−somites
Prim−5
Prim−15
Prim−25
Long−pec
Protruding−mouth
Day−4
Day−5
Stage
Transcript
ENSDART00000125054
ENSDART00000132392
ENSDART00000144003
ENSDART00000145456

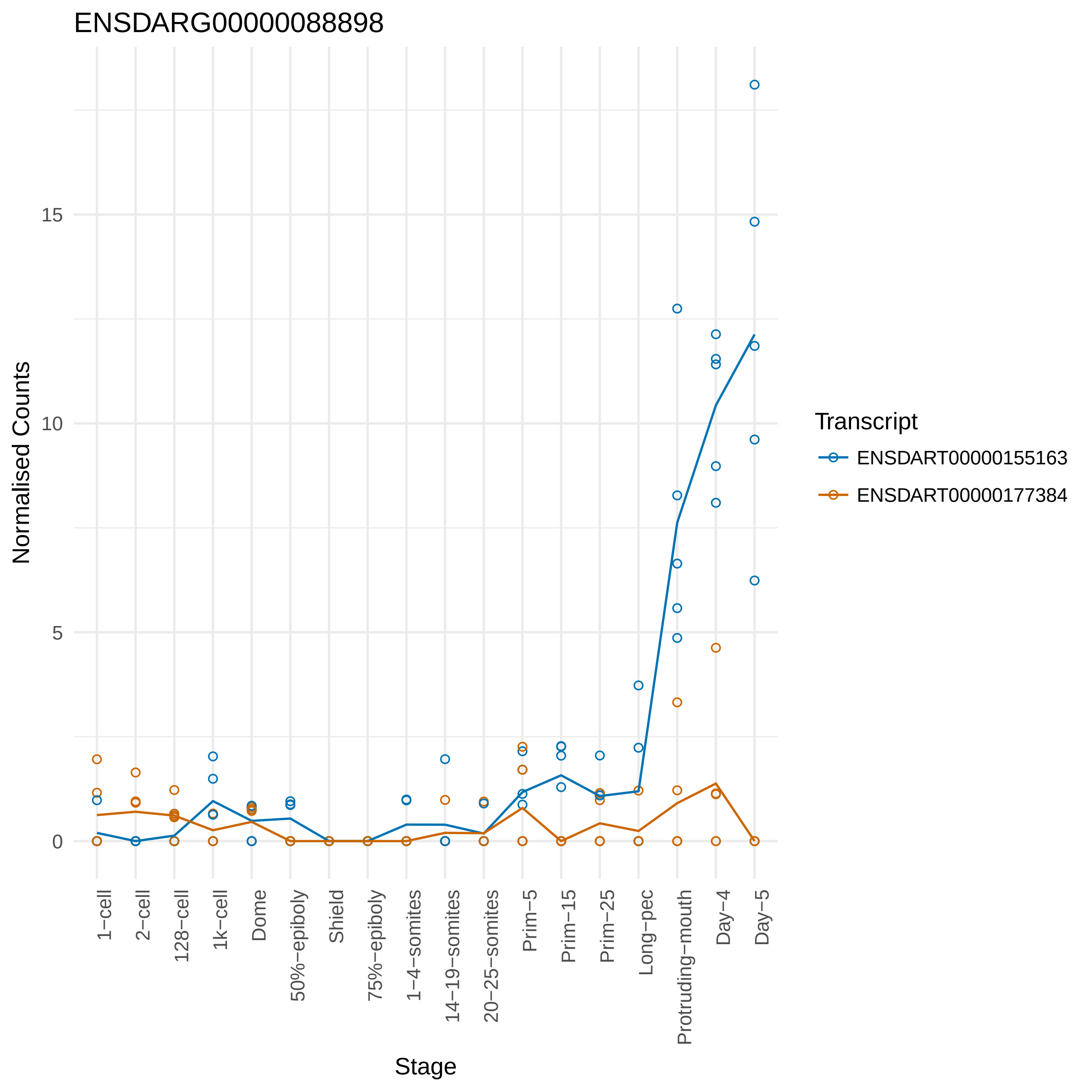

ENSDARG00000088898
Normalised Counts
0
5
10
15
1−cell
2−cell
128−cell
1k−cell
Dome
50%−epiboly
Shield
75%−epiboly
1−4−somites
14−19−somites
20−25−somites
Prim−5
Prim−15
Prim−25
Long−pec
Protruding−mouth
Day−4
Day−5
Stage
Transcript
ENSDART00000155163
ENSDART00000177384

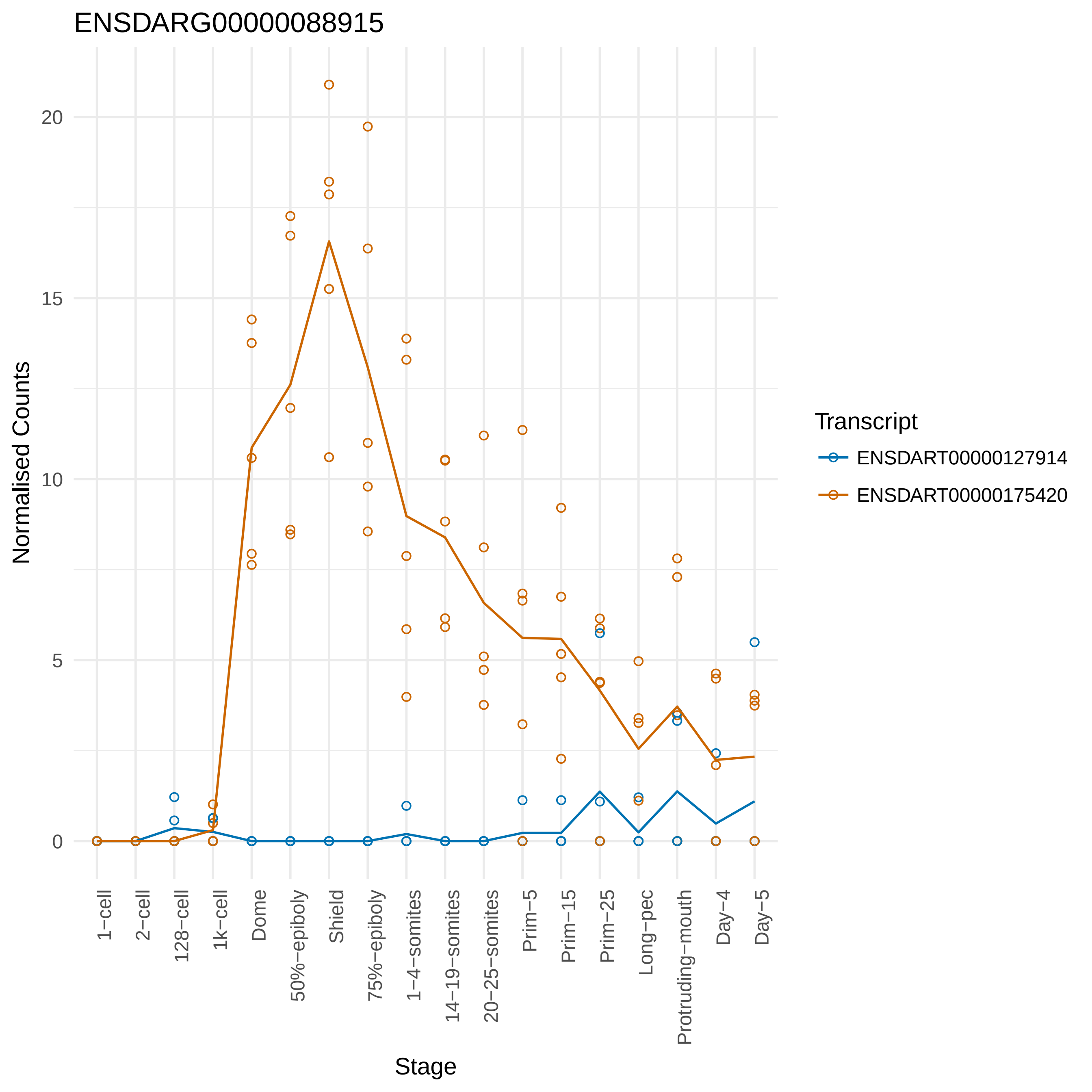

ENSDARG00000088915
Normalised Counts
20
15
10
5
0
1−cell
2−cell
128−cell
1k−cell
Dome
50%−epiboly
Shield
75%−epiboly
1−4−somites
14−19−somites
20−25−somites
Prim−5
Prim−15
Prim−25
Long−pec
Protruding−mouth
Day−4
Day−5
Stage
Transcript
ENSDART00000127914
ENSDART00000175420

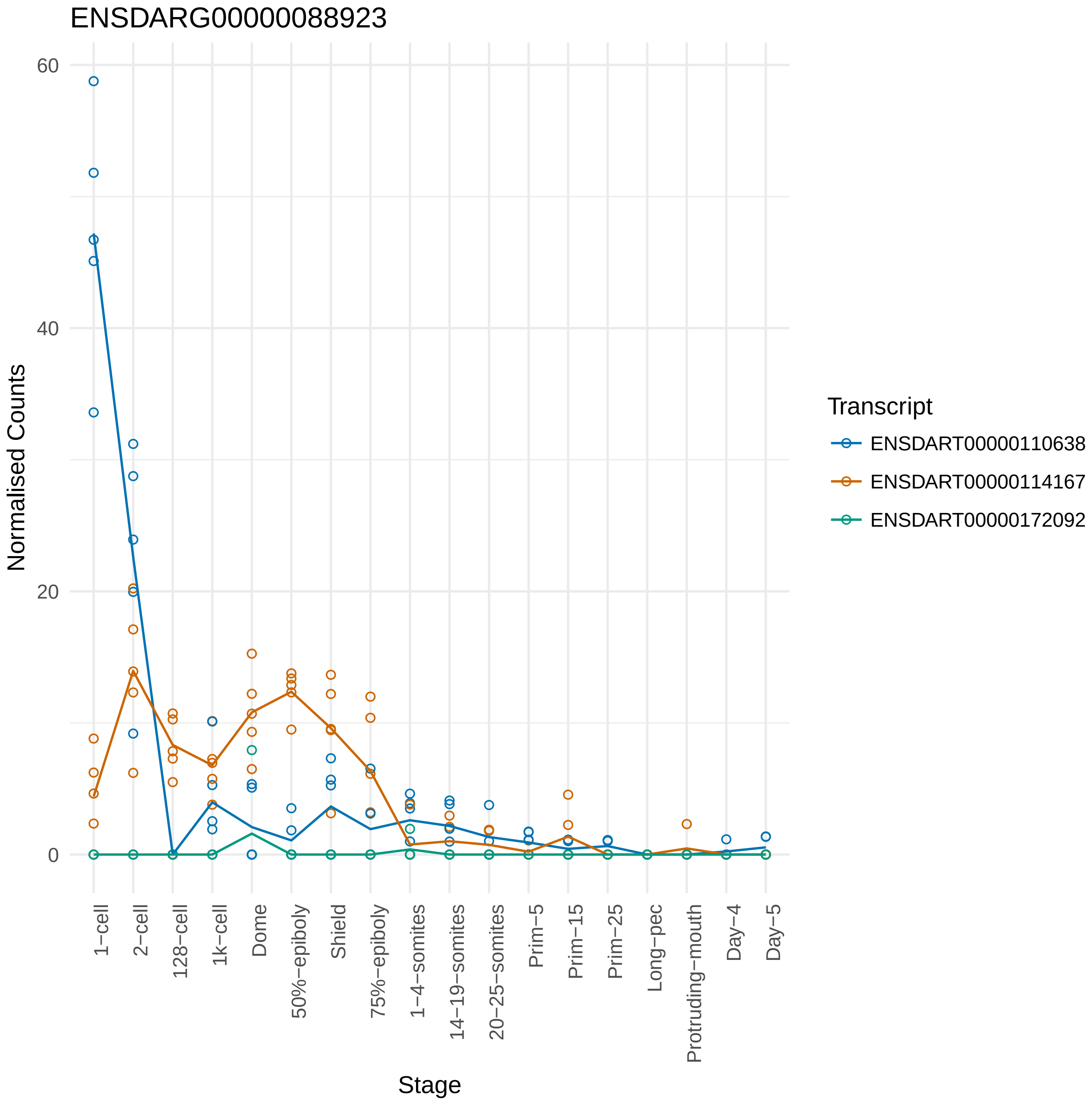

ENSDARG00000088923
Normalised Counts
60
40
20
0
1−cell
2−cell
128−cell
1k−cell
Dome
50%−epiboly
Shield
75%−epiboly
1−4−somites
14−19−somites
20−25−somites
Prim−5
Prim−15
Prim−25
Long−pec
Protruding−mouth
Day−4
Day−5
Stage
Transcript
ENSDART00000110638
ENSDART00000114167
ENSDART00000172092

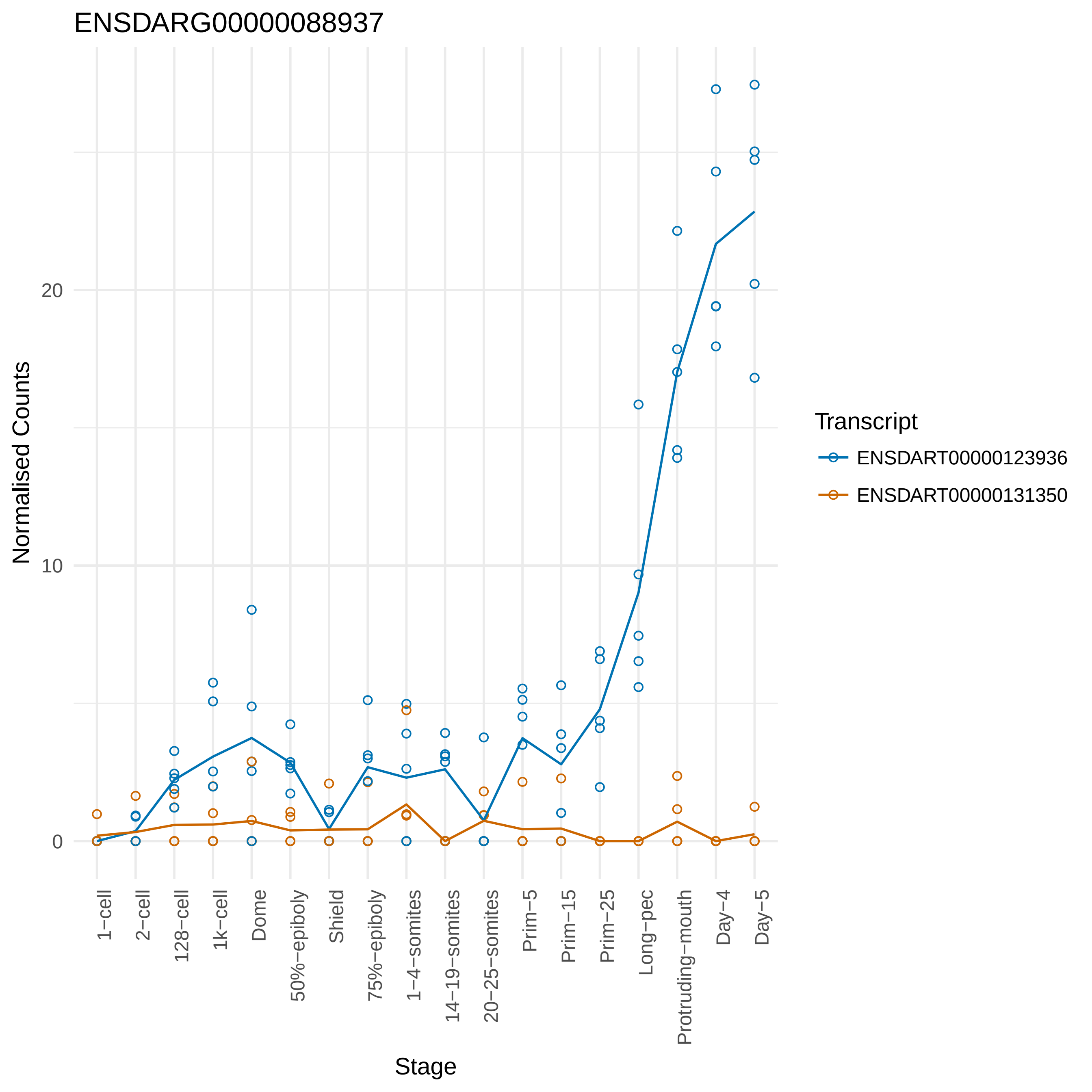

ENSDARG00000088937
Normalised Counts
0
10
20
Stage
1−cell
2−cell
128−cell
1k−cell
Dome
50%−epiboly
Shield
75%−epiboly
1−4−somites
14−19−somites
20−25−somites
Prim−5
Prim−15
Prim−25
Long−pec
Protruding−mouth
Day−4
Day−5
Transcript
ENSDART00000123936
ENSDART00000131350

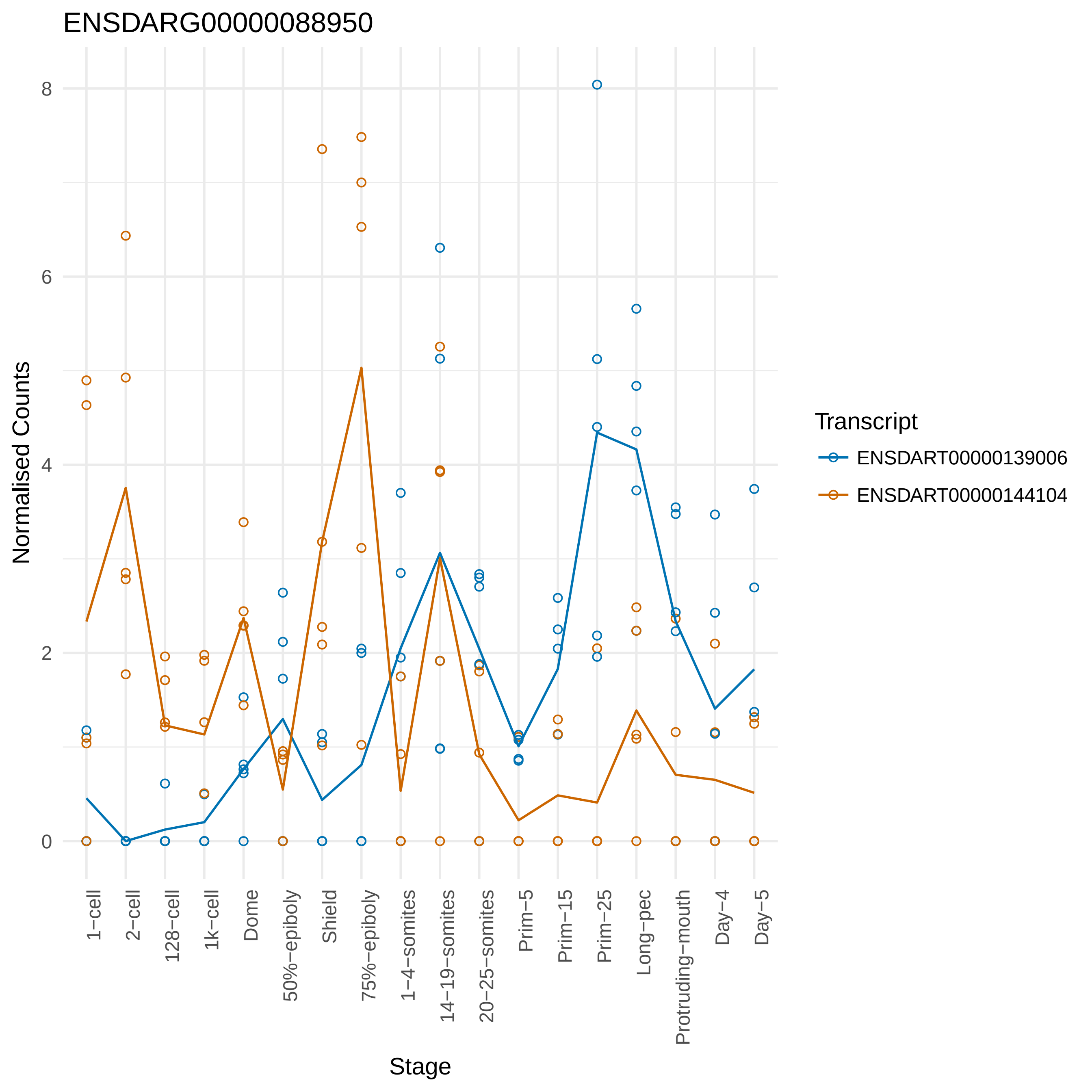

ENSDARG00000088950
Normalised Counts
8
6
4
2
0
1−cell
2−cell
128−cell
1k−cell
Dome
50%−epiboly
Shield
75%−epiboly
1−4−somites
14−19−somites
20−25−somites
Prim−5
Prim−15
Prim−25
Long−pec
Protruding−mouth
Day−4
Day−5
Stage
Transcript
ENSDART00000139006
ENSDART00000144104

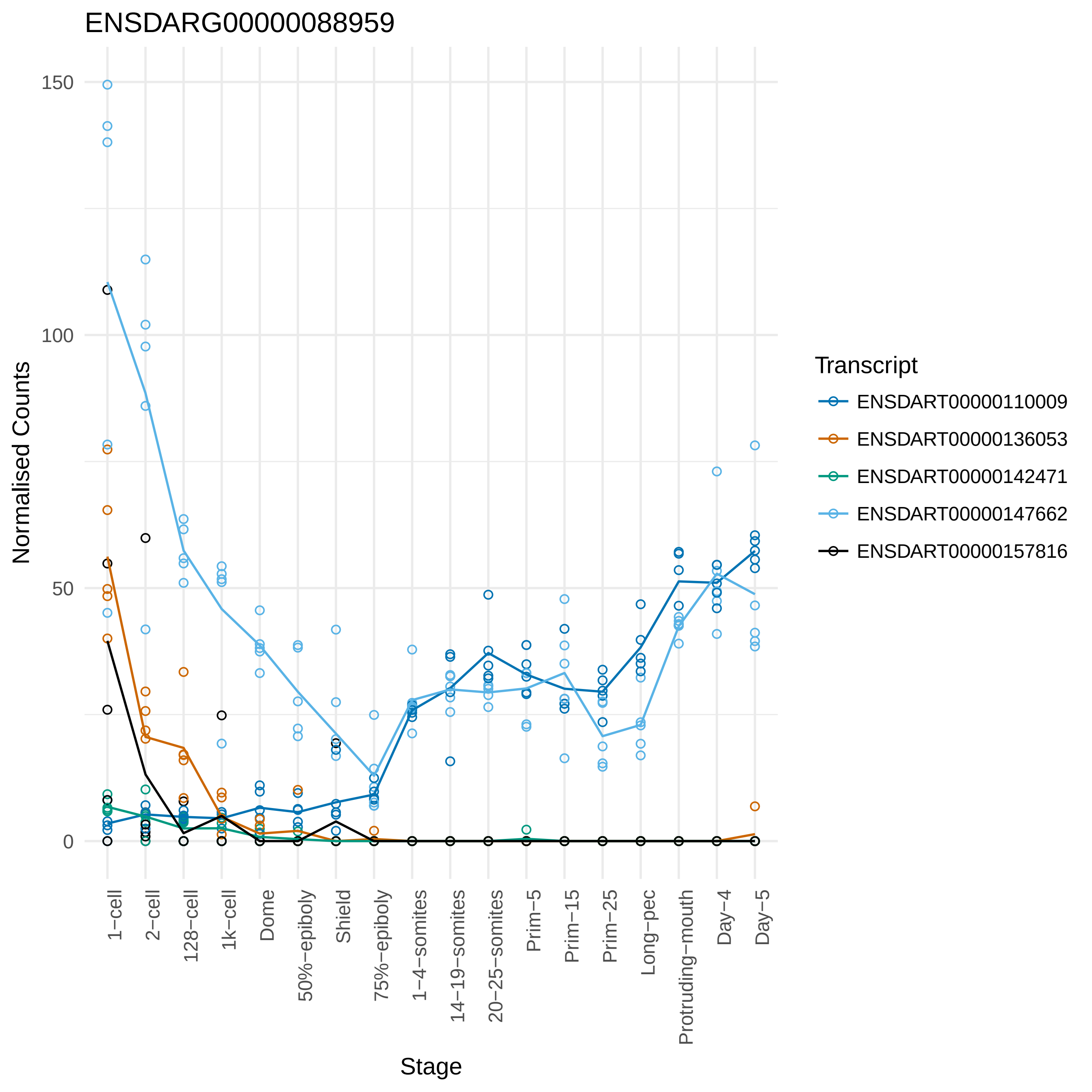
ENSDARG00000088959
Normalised Counts
150
100
50
0
Stage
1−cell
2−cell
128−cell
1k−cell
Dome
50%−epiboly
Shield
75%−epiboly
1−4−somites
14−19−somites
20−25−somites
Prim−5
Prim−15
Prim−25
Long−pec
Protruding−mouth
Day−4
Day−5
Transcript
ENSDART00000110009
ENSDART00000136053
ENSDART00000142471
ENSDART00000147662
ENSDART00000157816

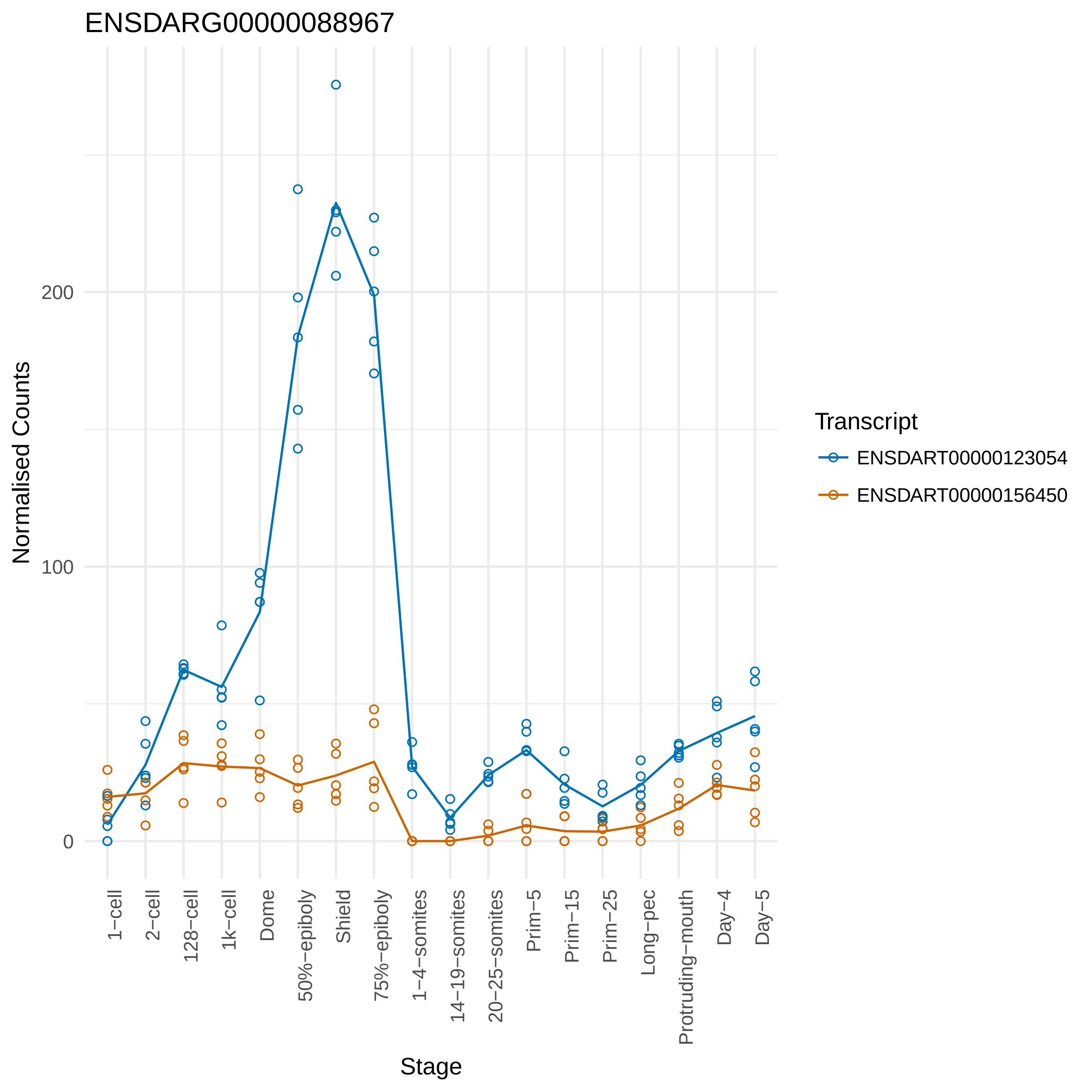
ENSDARG00000088967
Normalised Counts
0
100
200
Stage
1−cell
2−cell
128−cell
1k−cell
Dome
50%−epiboly
Shield
75%−epiboly
1−4−somites
14−19−somites
20−25−somites
Prim−5
Prim−15
Prim−25
Long−pec
Protruding−mouth
Day−4
Day−5
Transcript
ENSDART00000123054
ENSDART00000156450

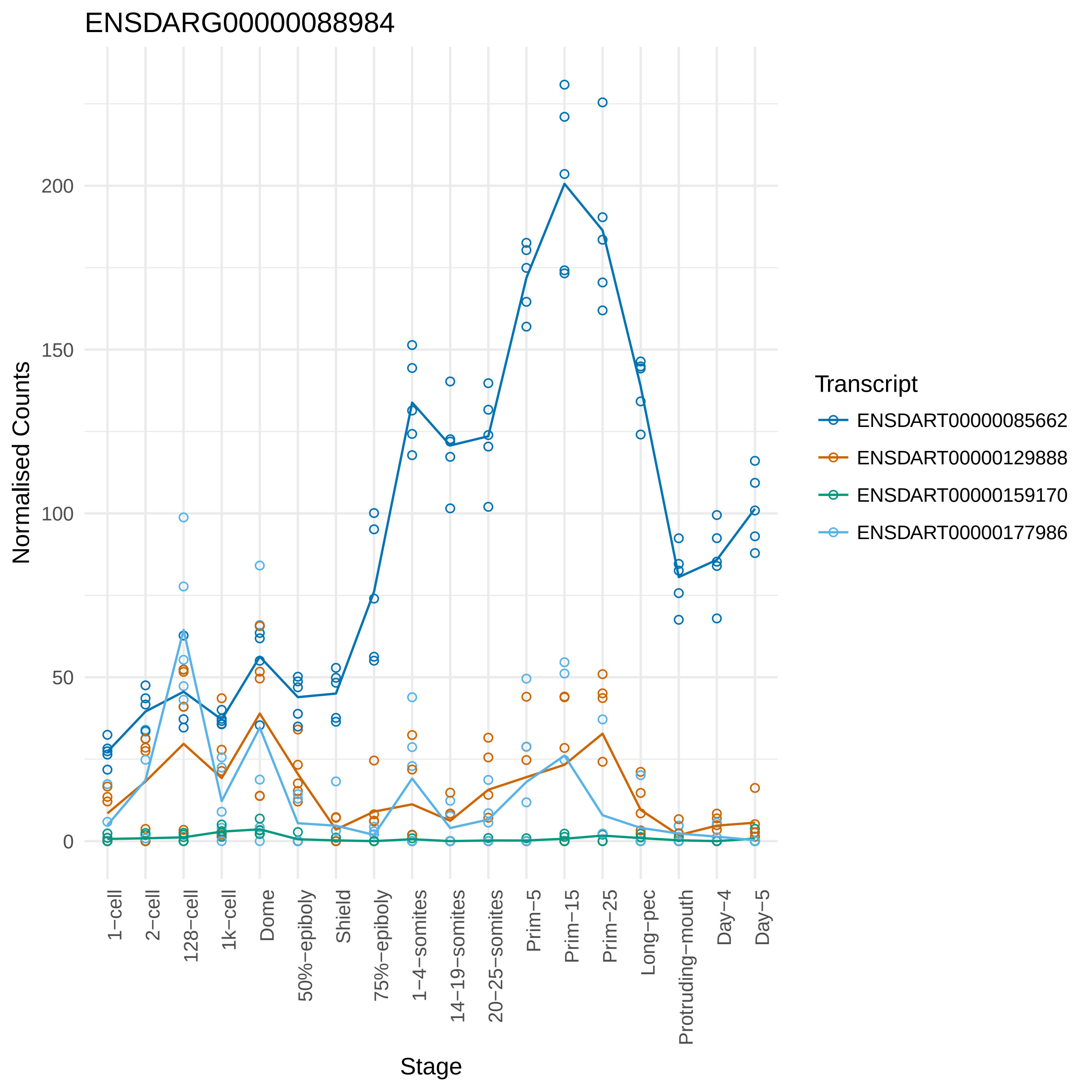

ENSDARG00000088984
Normalised Counts
0
50
100
150
200
Stage
1−cell
2−cell
128−cell
1k−cell
Dome
50%−epiboly
Shield
75%−epiboly
1−4−somites
14−19−somites
20−25−somites
Prim−5
Prim−15
Prim−25
Long−pec
Protruding−mouth
Day−4
Day−5
Transcript
ENSDART00000085662
ENSDART00000129888
ENSDART00000159170
ENSDART00000177986

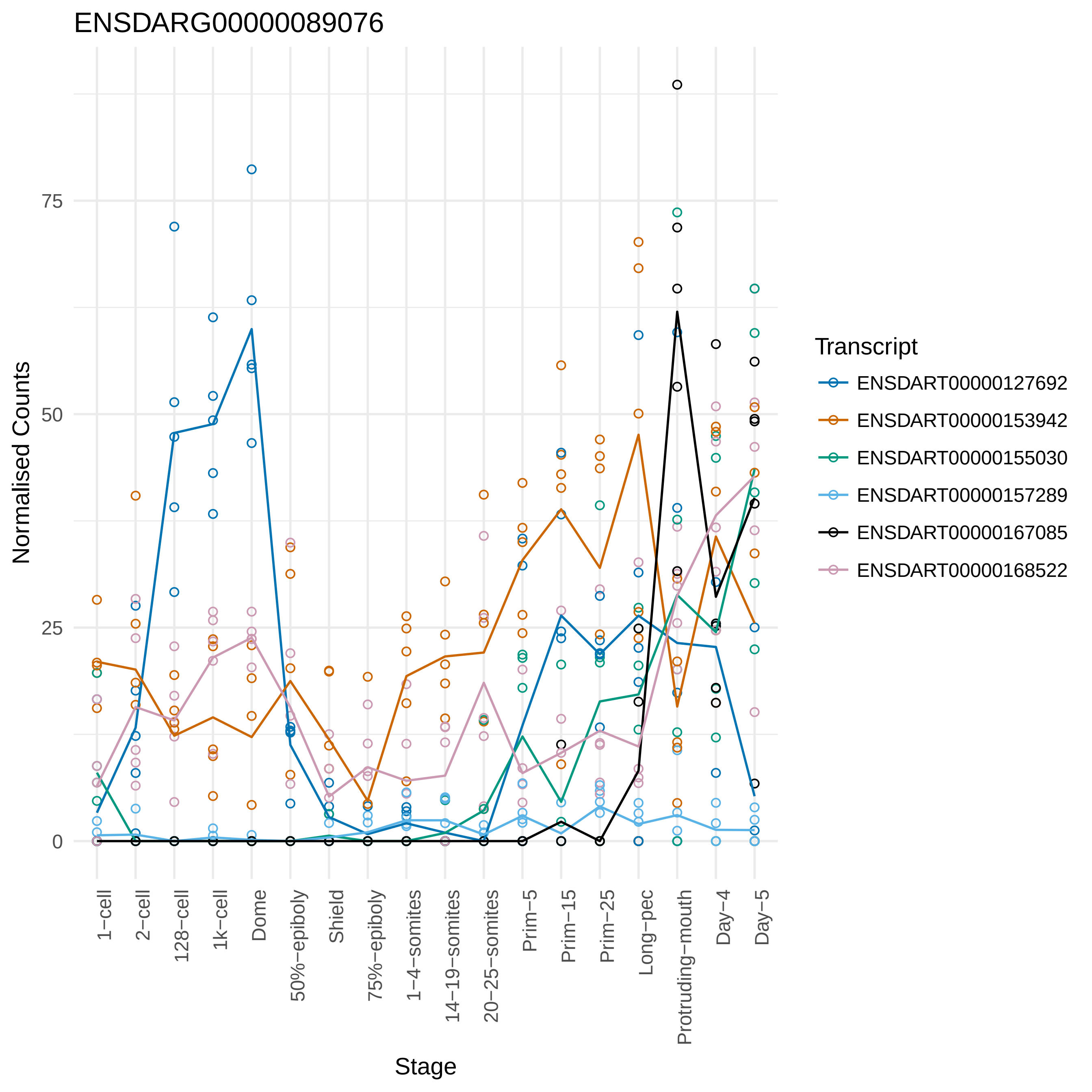
ENSDARG00000089076
Normalised Counts
75
50
25
0
1−cell
2−cell
128−cell
1k−cell
Dome
50%−epiboly
Shield
75%−epiboly
1−4−somites
14−19−somites
20−25−somites
Prim−5
Prim−15
Prim−25
Long−pec
Protruding−mouth
Day−4
Day−5
Stage
Transcript
ENSDART00000127692
ENSDART00000153942
ENSDART00000155030
ENSDART00000157289
ENSDART00000167085
ENSDART00000168522

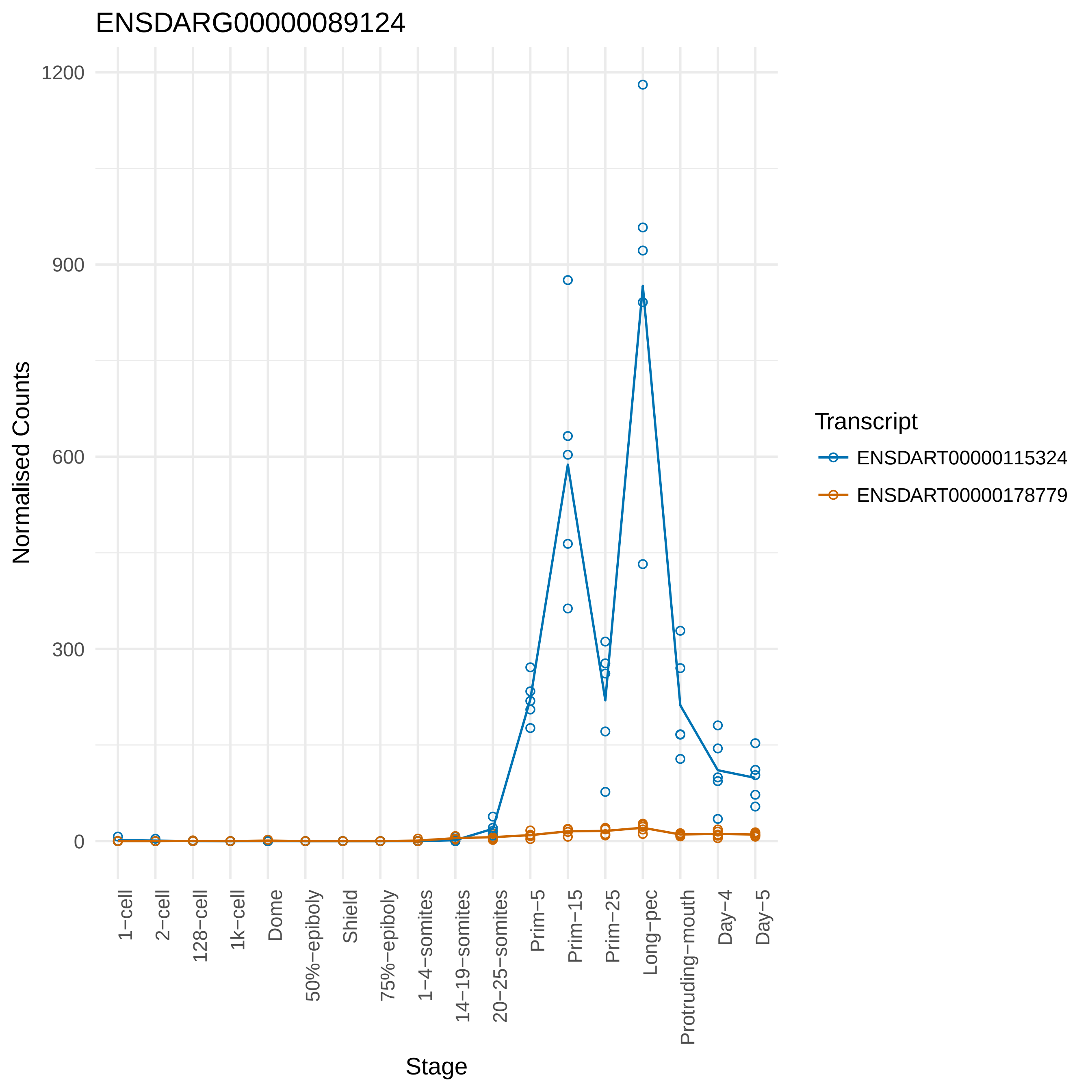

ENSDARG00000089124
Normalised Counts
1200
900
600
300
0
1−cell
2−cell
128−cell
1k−cell
Dome
50%−epiboly
Shield
75%−epiboly
1−4−somites
14−19−somites
20−25−somites
Prim−5
Prim−15
Prim−25
Long−pec
Protruding−mouth
Day−4
Day−5
Stage
Transcript
ENSDART00000115324
ENSDART00000178779

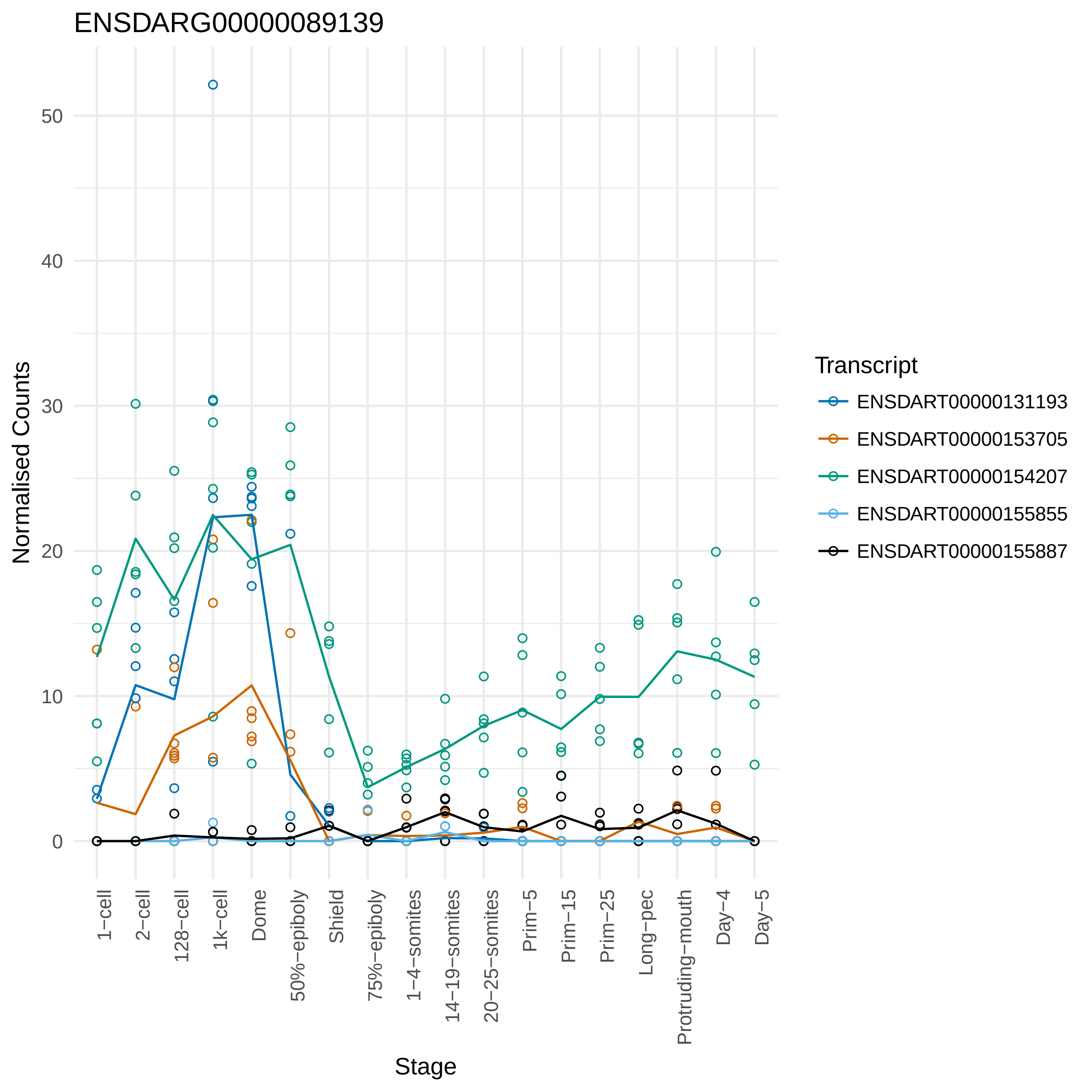

ENSDARG00000089139
Normalised Counts
0
10
20
30
40
50
Stage
1−cell
2−cell
128−cell
1k−cell
Dome
50%−epiboly
Shield
75%−epiboly
1−4−somites
14−19−somites
20−25−somites
Prim−5
Prim−15
Prim−25
Long−pec
Protruding−mouth
Day−4
Day−5
Transcript
ENSDART00000131193
ENSDART00000153705
ENSDART00000154207
ENSDART00000155855
ENSDART00000155887

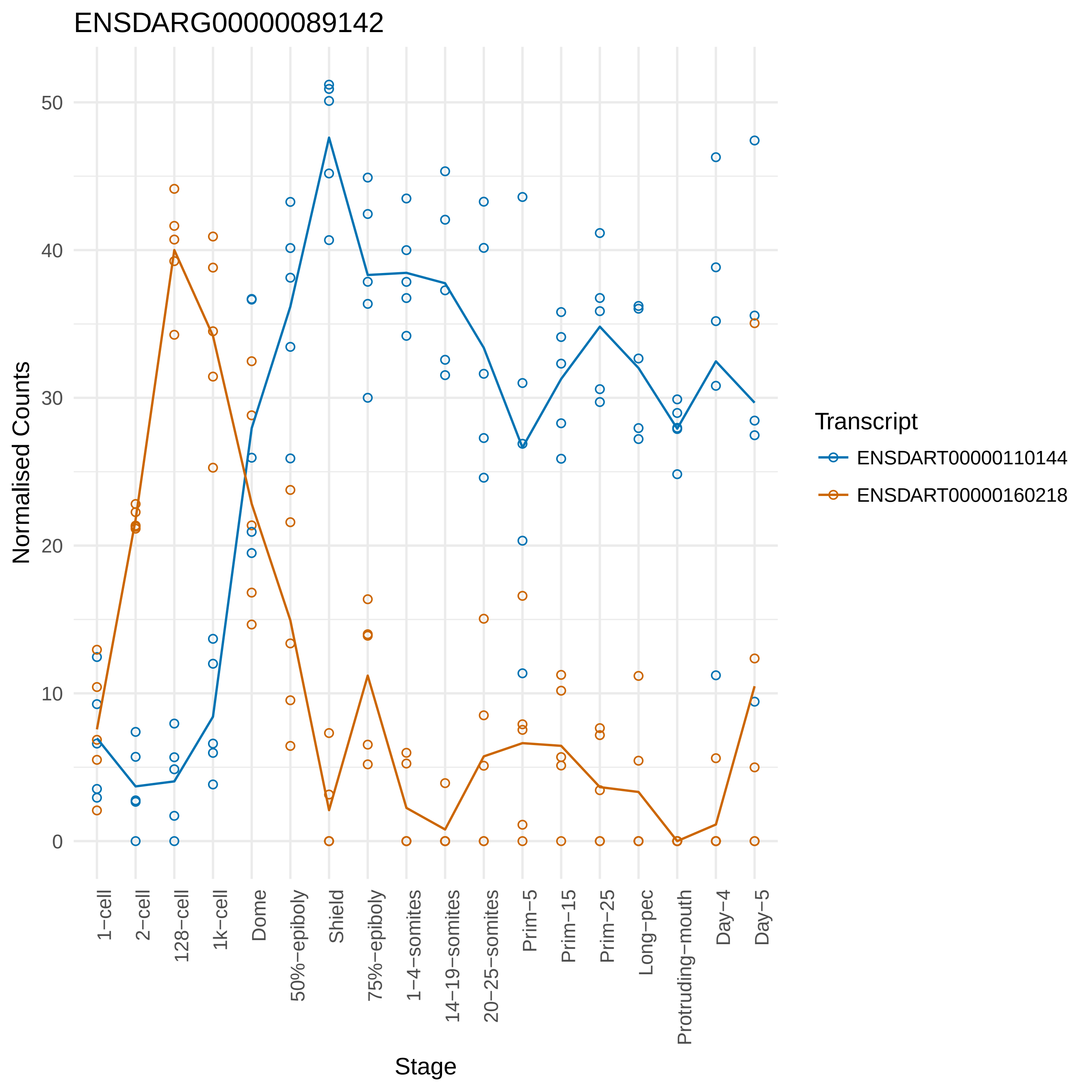

ENSDARG00000089142
Normalised Counts
0
10
20
30
40
50
Stage
1−cell
2−cell
128−cell
1k−cell
Dome
50%−epiboly
Shield
75%−epiboly
1−4−somites
14−19−somites
20−25−somites
Prim−5
Prim−15
Prim−25
Long−pec
Protruding−mouth
Day−4
Day−5
Transcript
ENSDART00000110144
ENSDART00000160218

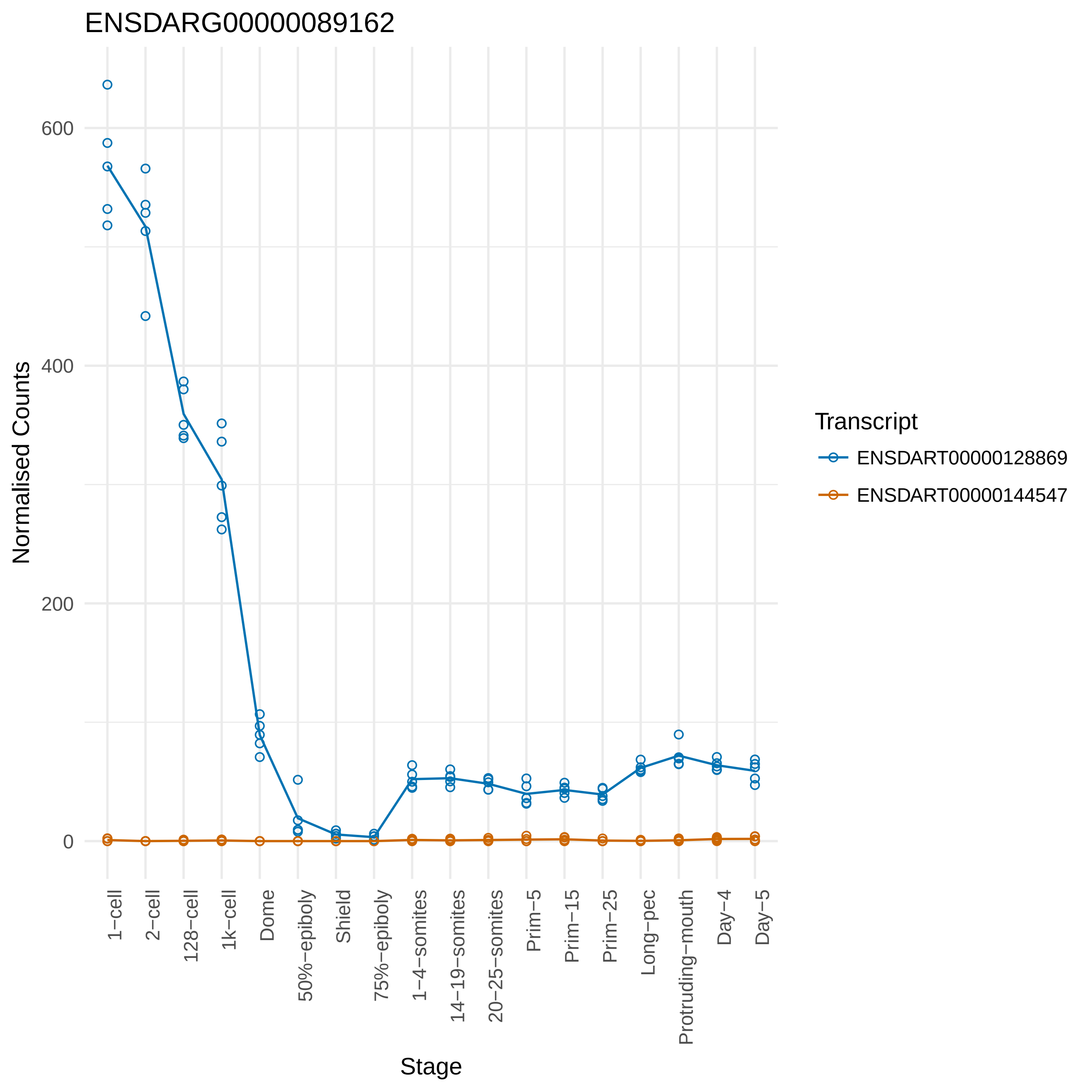

ENSDARG00000089162
Normalised Counts
600
400
200
0
1−cell
2−cell
128−cell
1k−cell
Dome
50%−epiboly
Shield
75%−epiboly
1−4−somites
14−19−somites
20−25−somites
Prim−5
Prim−15
Prim−25
Long−pec
Protruding−mouth
Day−4
Day−5
Stage
Transcript
ENSDART00000128869
ENSDART00000144547

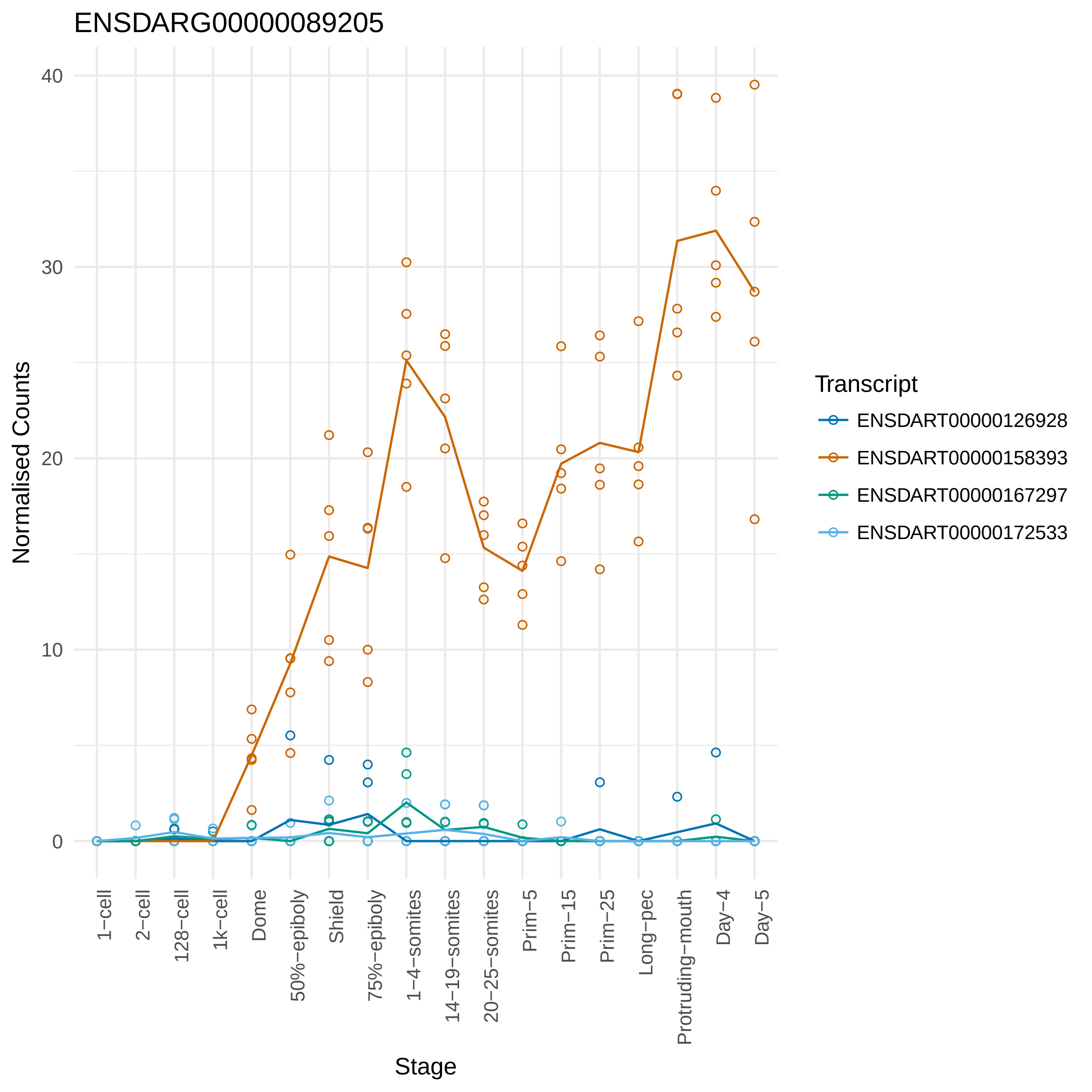
ENSDARG00000089205
Normalised Counts
0
10
20
30
40
Stage
1−cell
2−cell
128−cell
1k−cell
Dome
50%−epiboly
Shield
75%−epiboly
1−4−somites
14−19−somites
20−25−somites
Prim−5
Prim−15
Prim−25
Long−pec
Protruding−mouth
Day−4
Day−5
Transcript
ENSDART00000126928
ENSDART00000158393
ENSDART00000167297
ENSDART00000172533

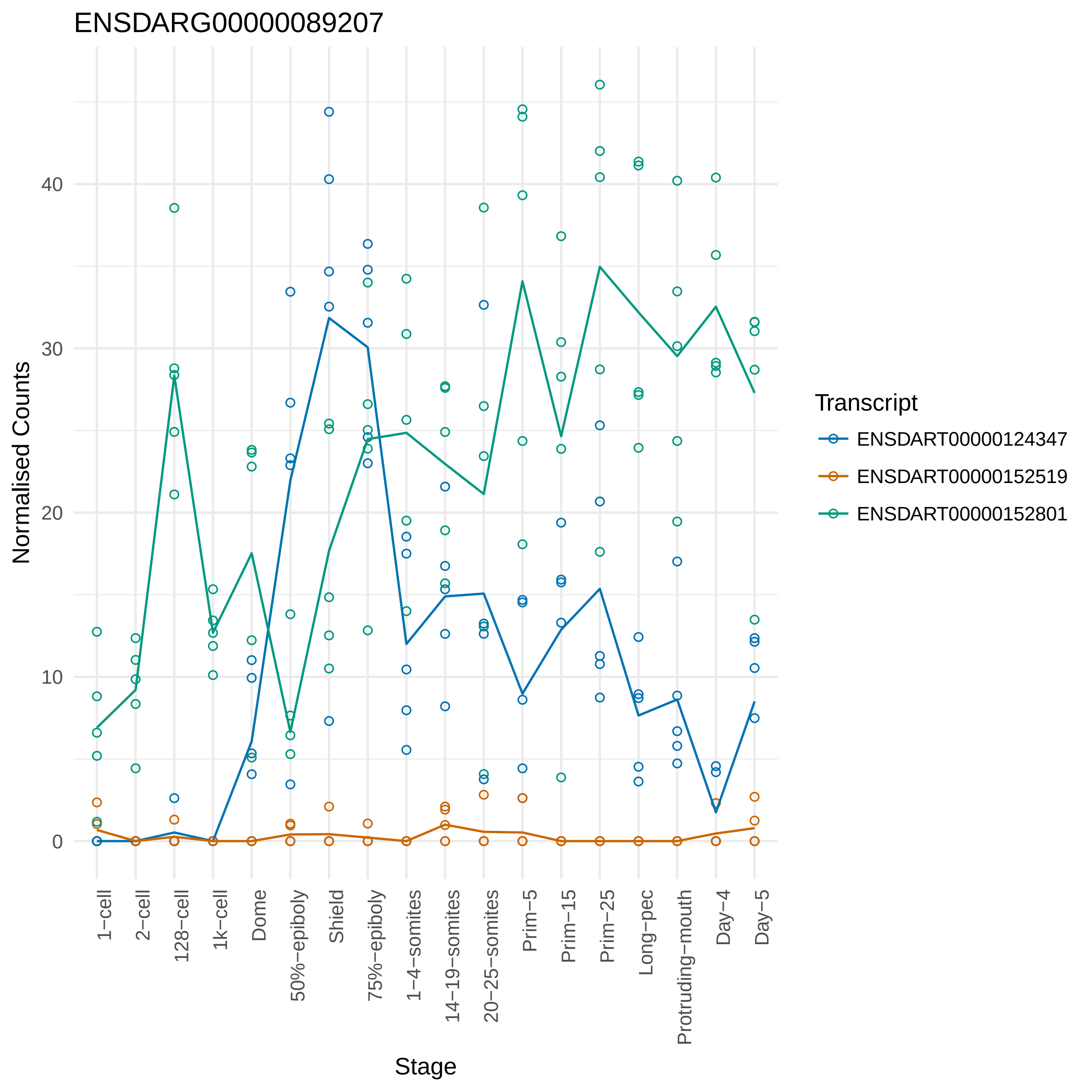
ENSDARG00000089207
Normalised Counts
0
10
20
30
40
Stage
1−cell
2−cell
128−cell
1k−cell
Dome
50%−epiboly
Shield
75%−epiboly
1−4−somites
14−19−somites
20−25−somites
Prim−5
Prim−15
Prim−25
Long−pec
Protruding−mouth
Day−4
Day−5
Transcript
ENSDART00000124347
ENSDART00000152519
ENSDART00000152801

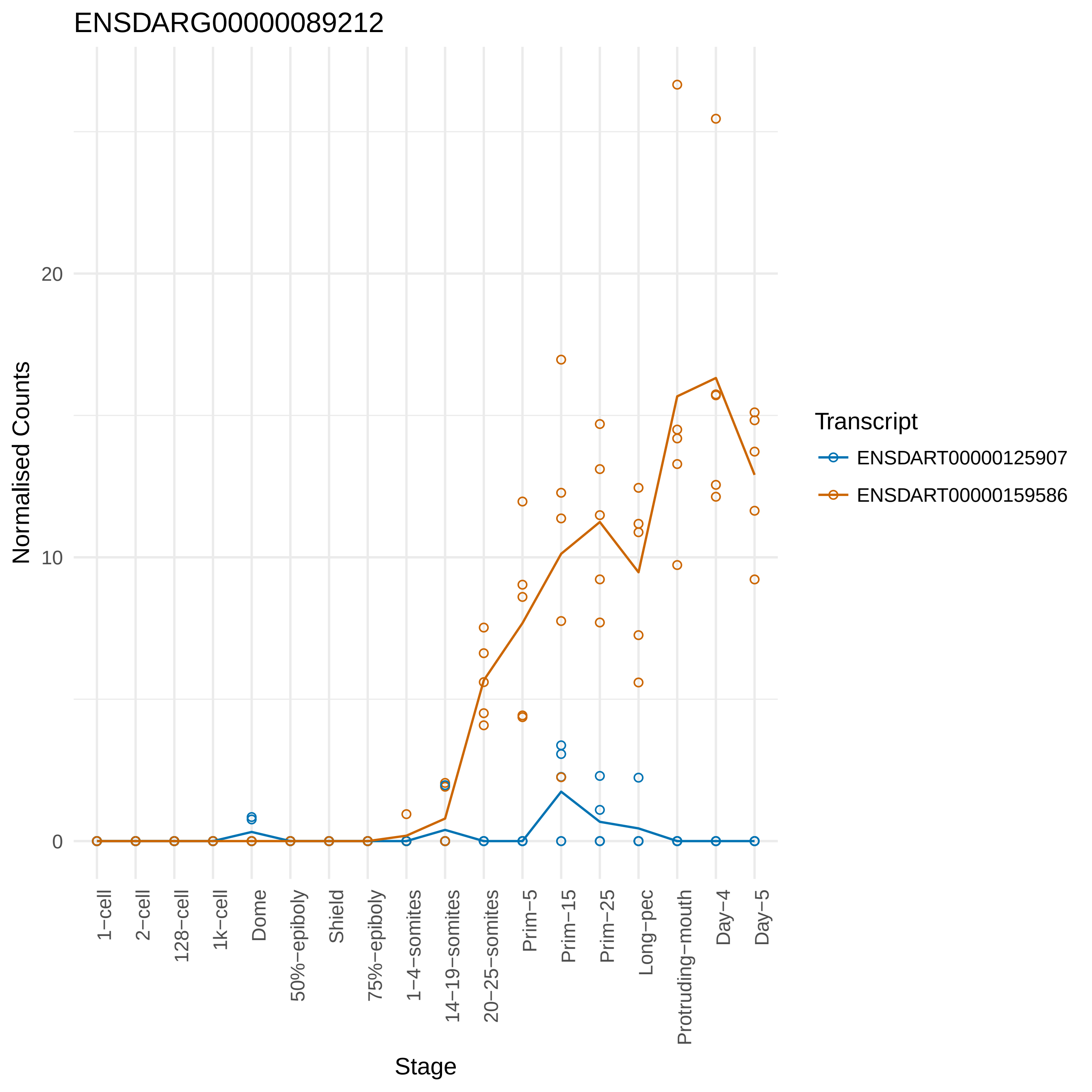

ENSDARG00000089212
Normalised Counts
0
10
20
1−cell
2−cell
128−cell
1k−cell
Dome
50%−epiboly
Shield
75%−epiboly
1−4−somites
14−19−somites
20−25−somites
Prim−5
Prim−15
Prim−25
Long−pec
Protruding−mouth
Day−4
Day−5
Stage
Transcript
ENSDART00000125907
ENSDART00000159586

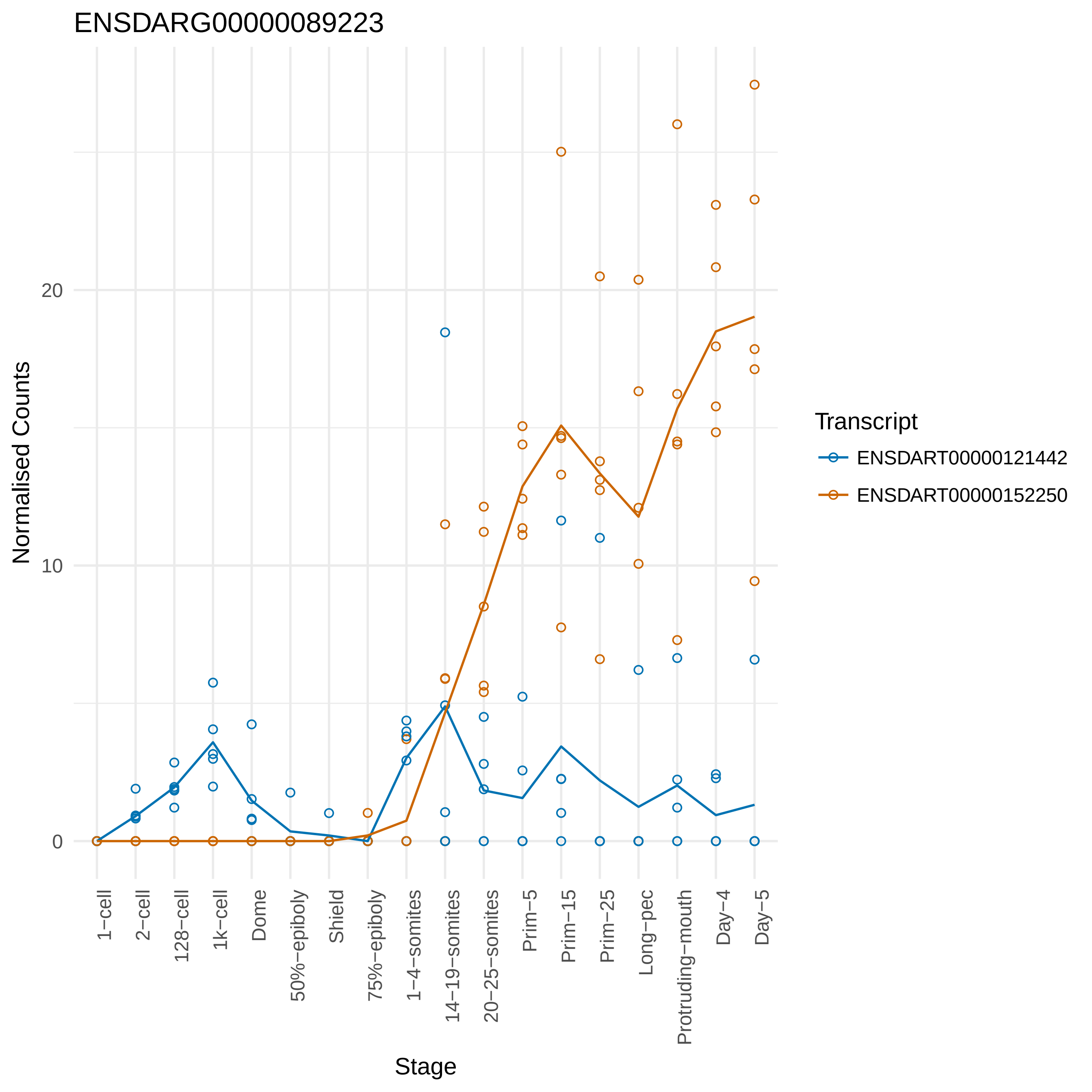
ENSDARG00000089223
Normalised Counts
0
10
20
1−cell
2−cell
128−cell
1k−cell
Dome
50%−epiboly
Shield
75%−epiboly
1−4−somites
14−19−somites
20−25−somites
Prim−5
Prim−15
Prim−25
Long−pec
Protruding−mouth
Day−4
Day−5
Stage
Transcript
ENSDART00000121442
ENSDART00000152250

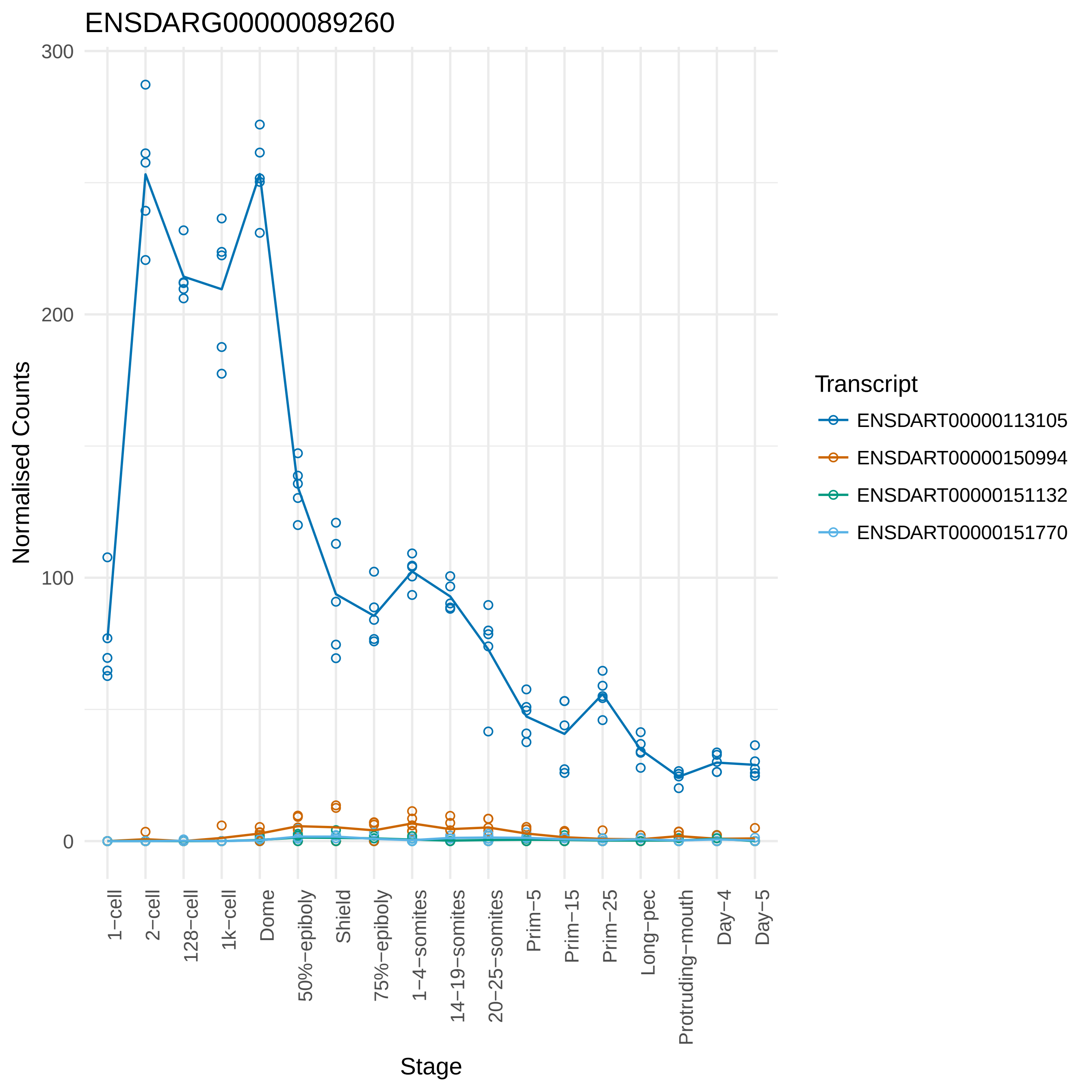
ENSDARG00000089260
Normalised Counts
300
200
100
0
1−cell
2−cell
128−cell
1k−cell
Dome
50%−epiboly
Shield
75%−epiboly
1−4−somites
14−19−somites
20−25−somites
Prim−5
Prim−15
Prim−25
Long−pec
Protruding−mouth
Day−4
Day−5
Stage
Transcript
ENSDART00000113105
ENSDART00000150994
ENSDART00000151132
ENSDART00000151770

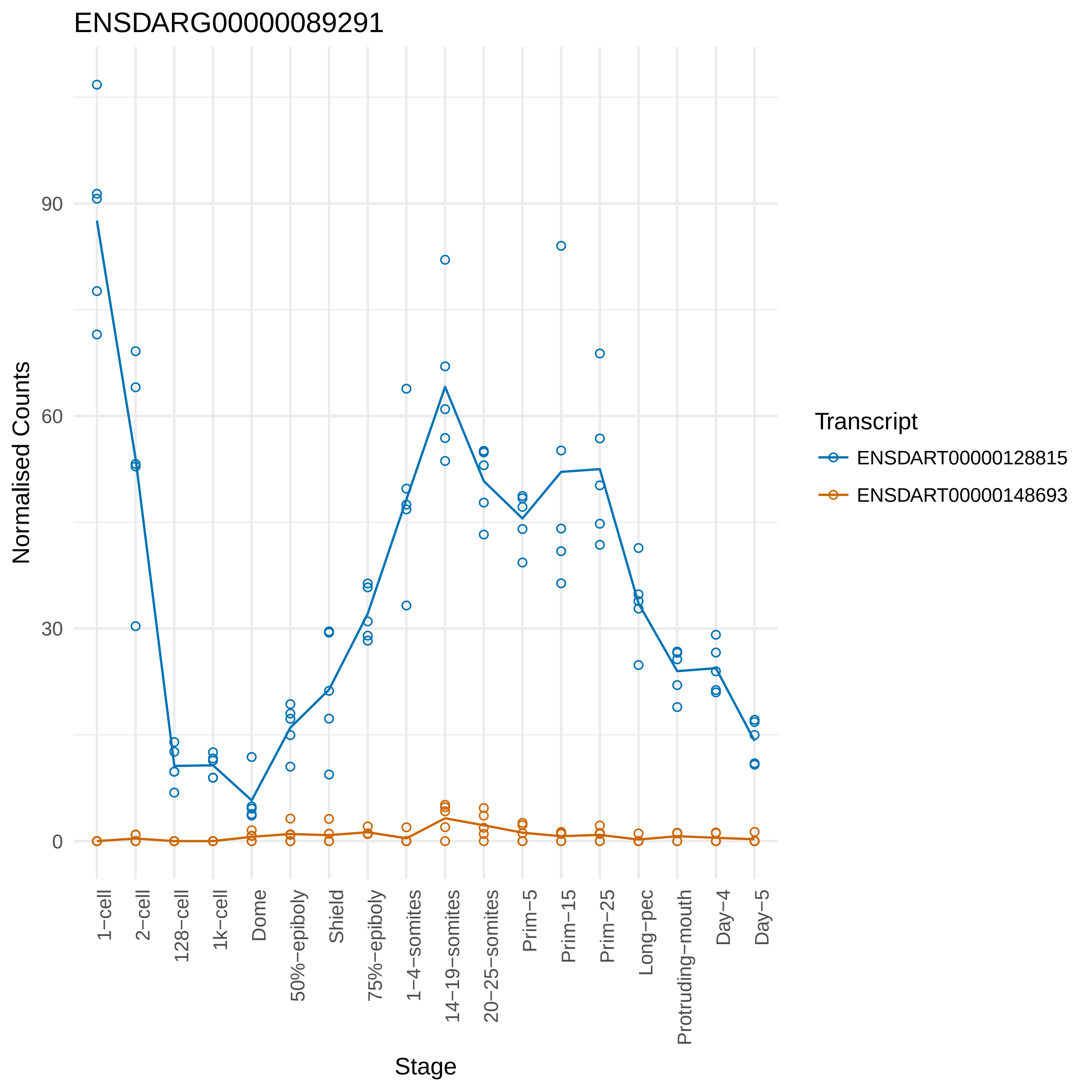

ENSDARG00000089291
Normalised Counts
90
60
30
0
1−cell
2−cell
128−cell
1k−cell
Dome
50%−epiboly
Shield
75%−epiboly
1−4−somites
14−19−somites
20−25−somites
Prim−5
Prim−15
Prim−25
Long−pec
Protruding−mouth
Day−4
Day−5
Stage
Transcript
ENSDART00000128815
ENSDART00000148693

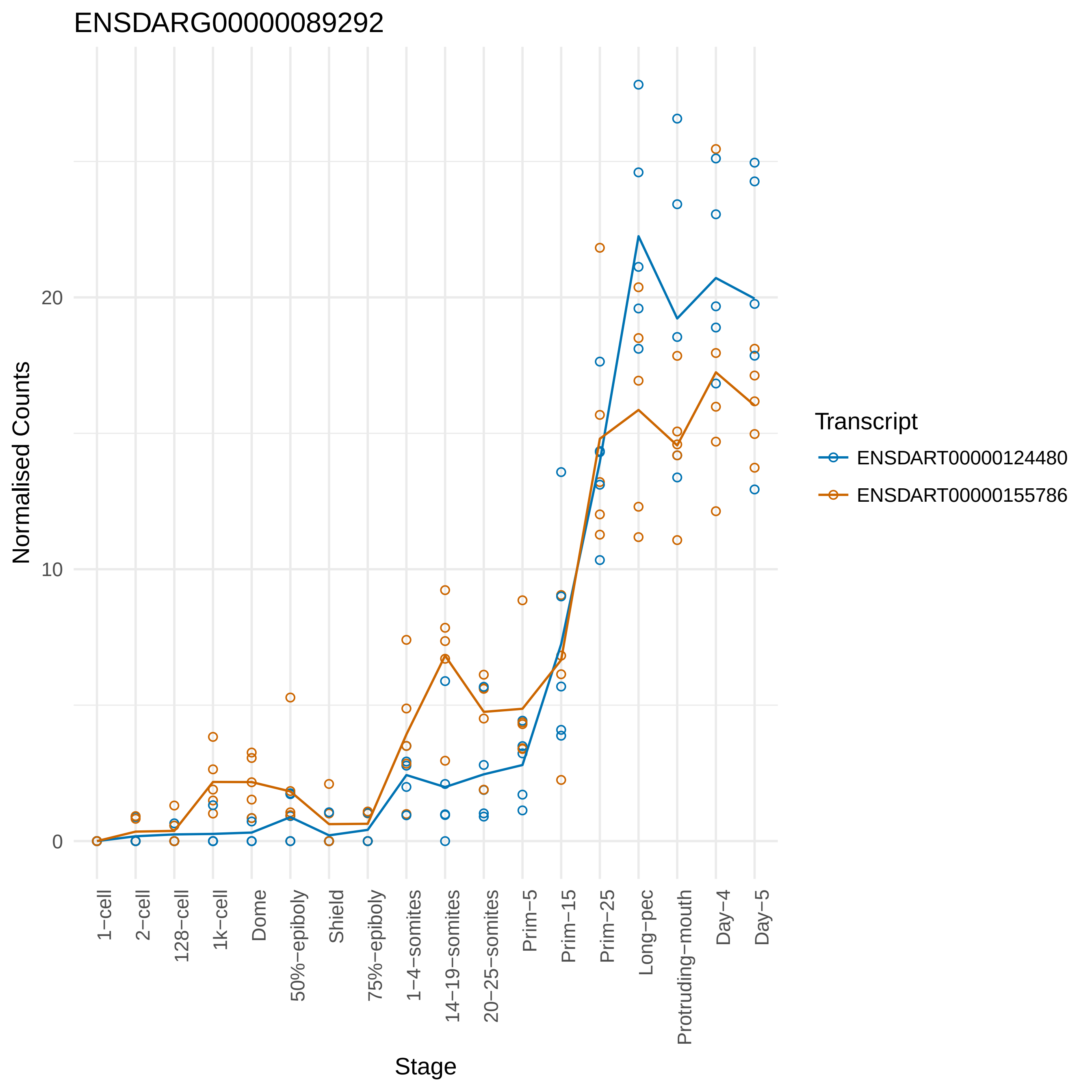
ENSDARG00000089292
Normalised Counts
0
10
20
Stage
1−cell
2−cell
128−cell
1k−cell
Dome
50%−epiboly
Shield
75%−epiboly
1−4−somites
14−19−somites
20−25−somites
Prim−5
Prim−15
Prim−25
Long−pec
Protruding−mouth
Day−4
Day−5
Transcript
ENSDART00000124480
ENSDART00000155786

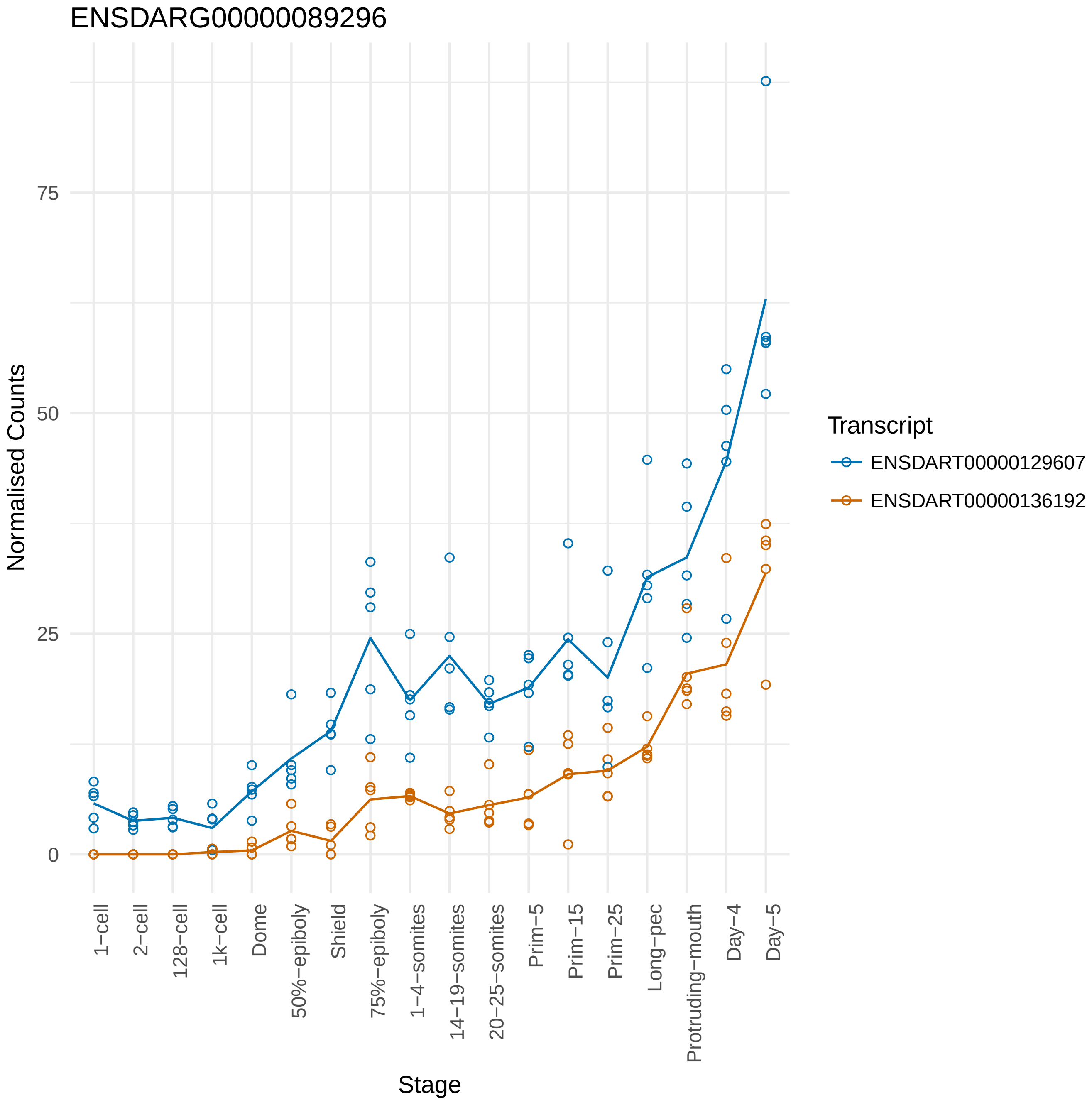
ENSDARG00000089296
Normalised Counts
0
25
50
75
Stage
1−cell
2−cell
128−cell
1k−cell
Dome
50%−epiboly
Shield
75%−epiboly
1−4−somites
14−19−somites
20−25−somites
Prim−5
Prim−15
Prim−25
Long−pec
Protruding−mouth
Day−4
Day−5
Transcript
ENSDART00000129607
ENSDART00000136192

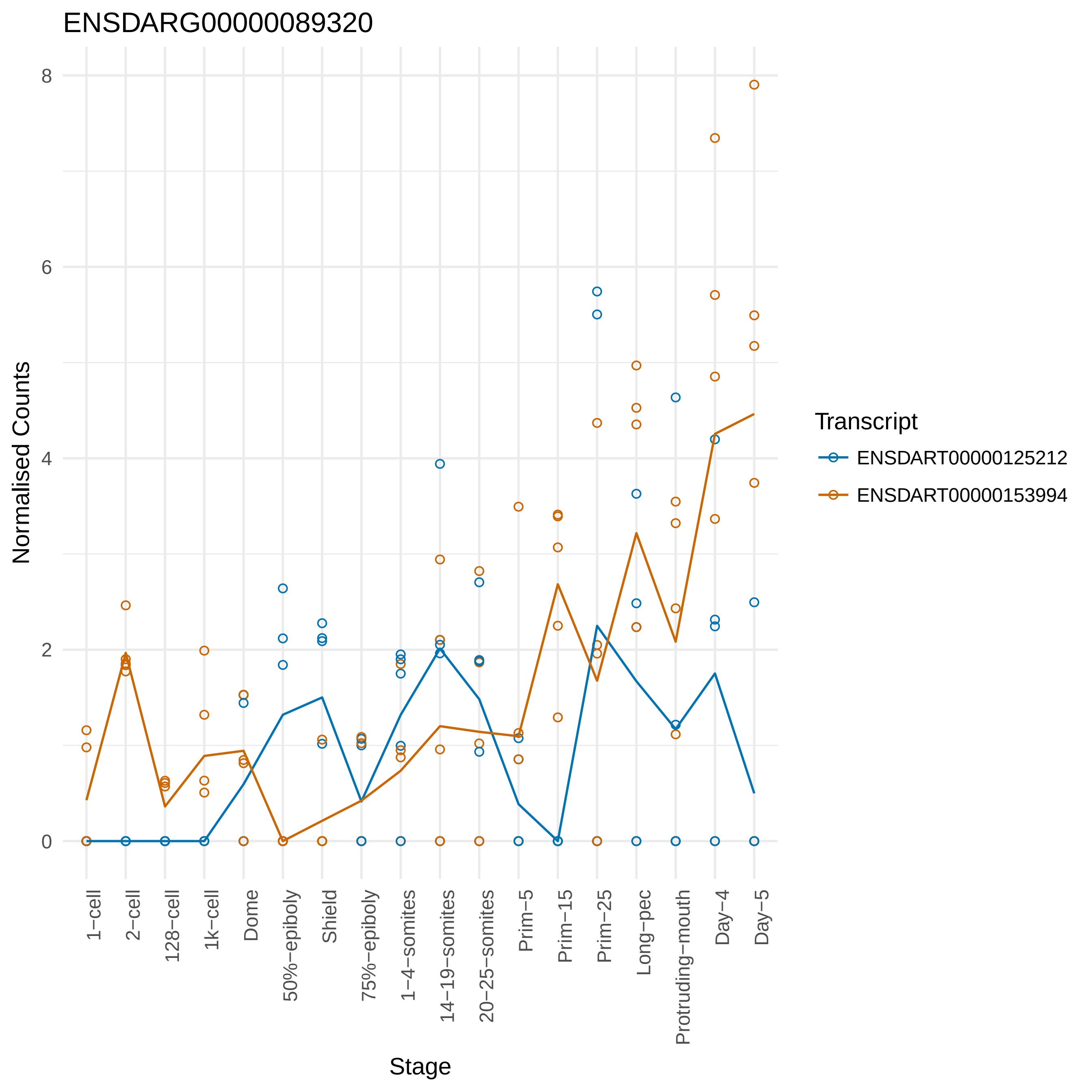
ENSDARG00000089320
Normalised Counts
0
2
4
6
8
Stage
1−cell
2−cell
128−cell
1k−cell
Dome
50%−epiboly
Shield
75%−epiboly
1−4−somites
14−19−somites
20−25−somites
Prim−5
Prim−15
Prim−25
Long−pec
Protruding−mouth
Day−4
Day−5
Transcript
ENSDART00000125212
ENSDART00000153994

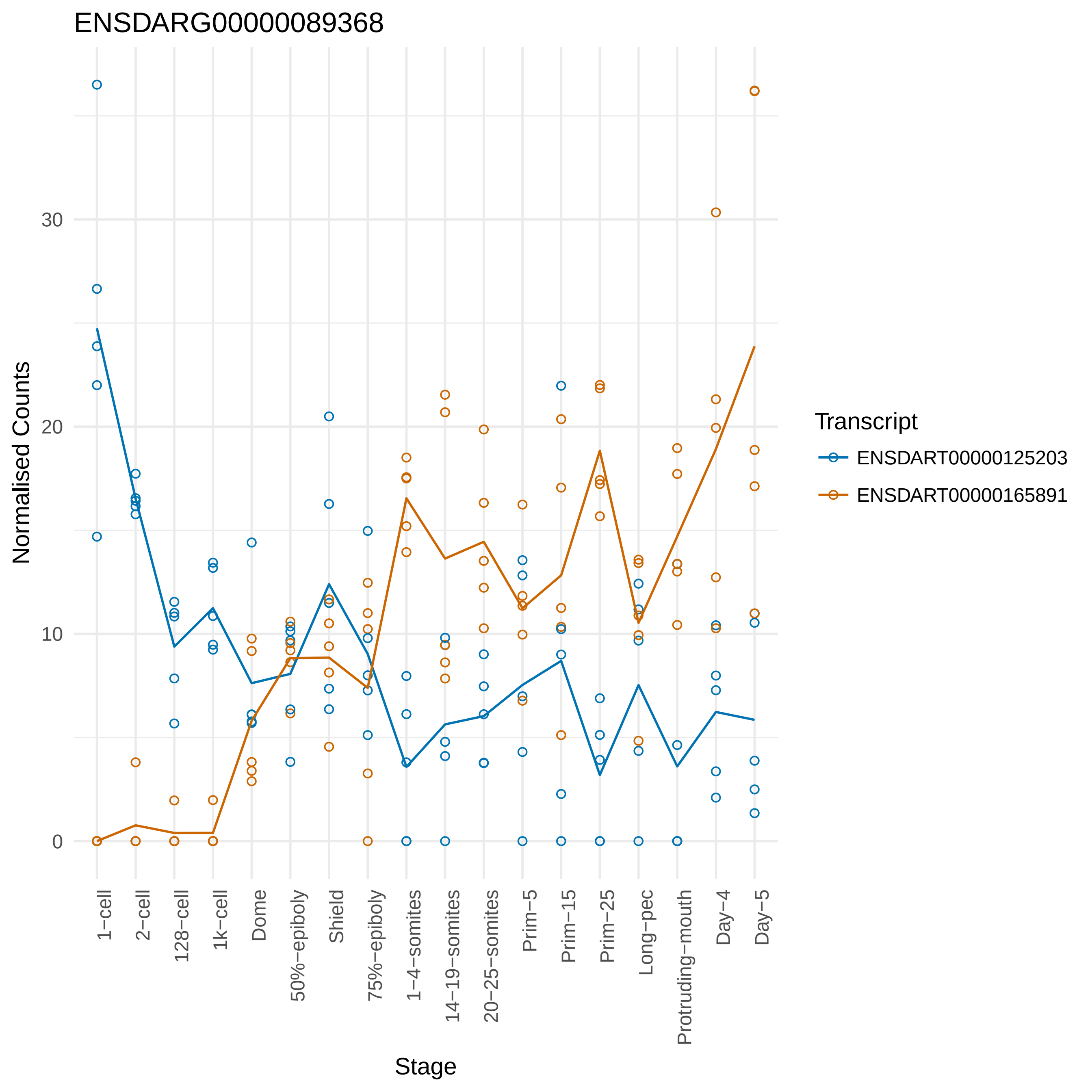
ENSDARG00000089368
Normalised Counts
0
10
20
30
Stage
1−cell
2−cell
128−cell
1k−cell
Dome
50%−epiboly
Shield
75%−epiboly
1−4−somites
14−19−somites
20−25−somites
Prim−5
Prim−15
Prim−25
Long−pec
Protruding−mouth
Day−4
Day−5
Transcript
ENSDART00000125203
ENSDART00000165891

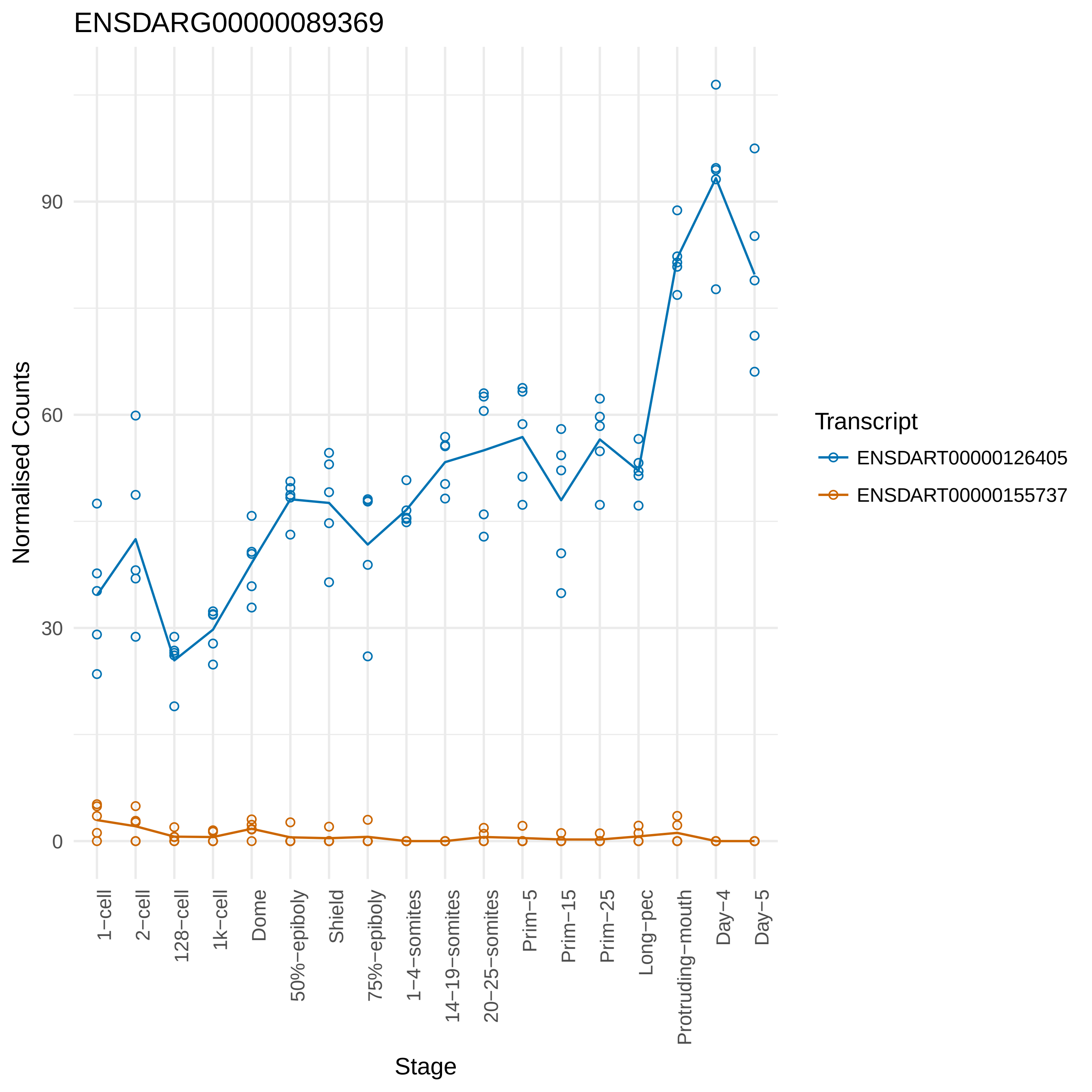
ENSDARG00000089369
Normalised Counts
0
30
60
90
Stage
1−cell
2−cell
128−cell
1k−cell
Dome
50%−epiboly
Shield
75%−epiboly
1−4−somites
14−19−somites
20−25−somites
Prim−5
Prim−15
Prim−25
Long−pec
Protruding−mouth
Day−4
Day−5
Transcript
ENSDART00000126405
ENSDART00000155737

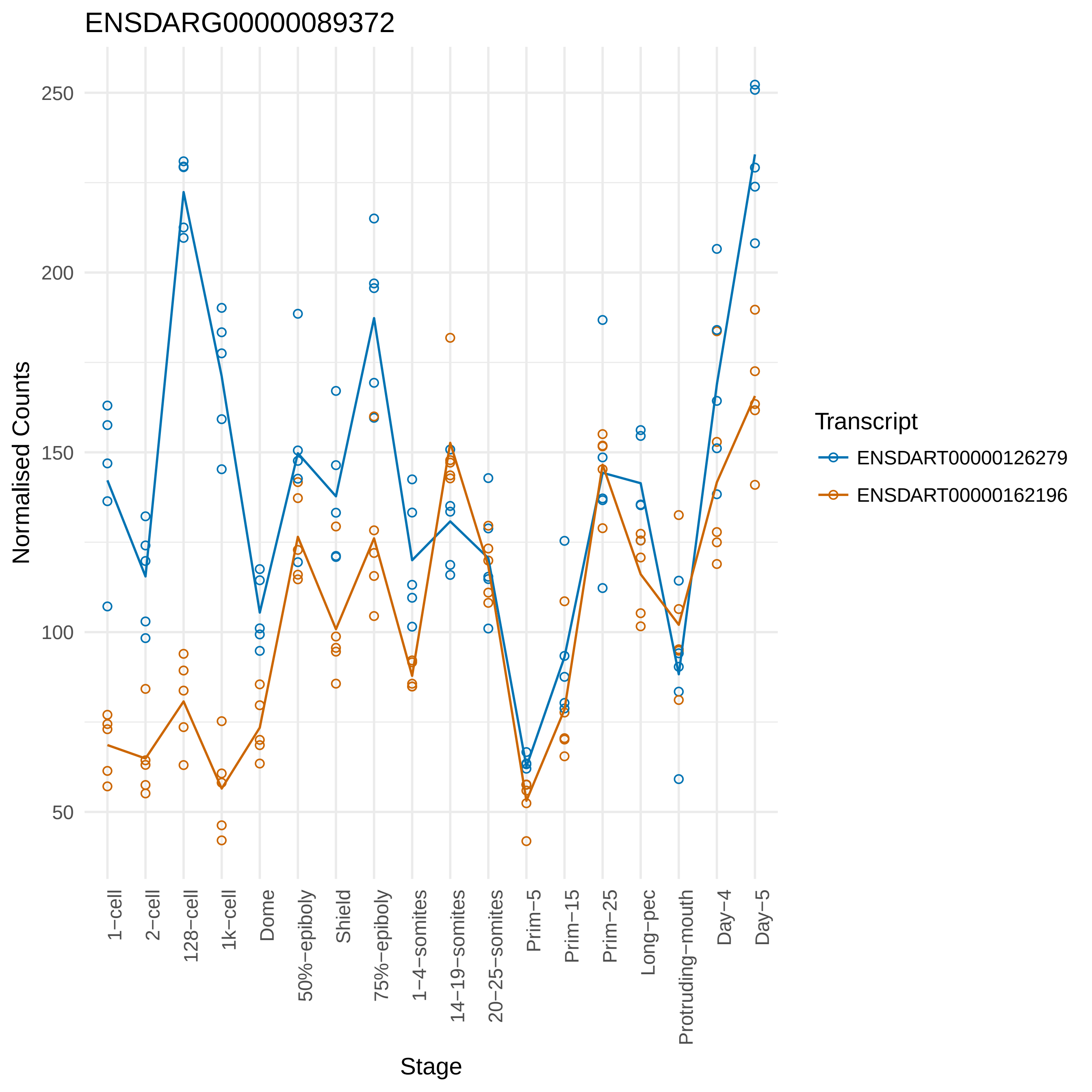
ENSDARG00000089372
Normalised Counts
250
200
150
100
50
1−cell
2−cell
128−cell
1k−cell
Dome
50%−epiboly
Shield
75%−epiboly
1−4−somites
14−19−somites
20−25−somites
Prim−5
Prim−15
Prim−25
Long−pec
Protruding−mouth
Day−4
Day−5
Stage
Transcript
ENSDART00000126279
ENSDART00000162196

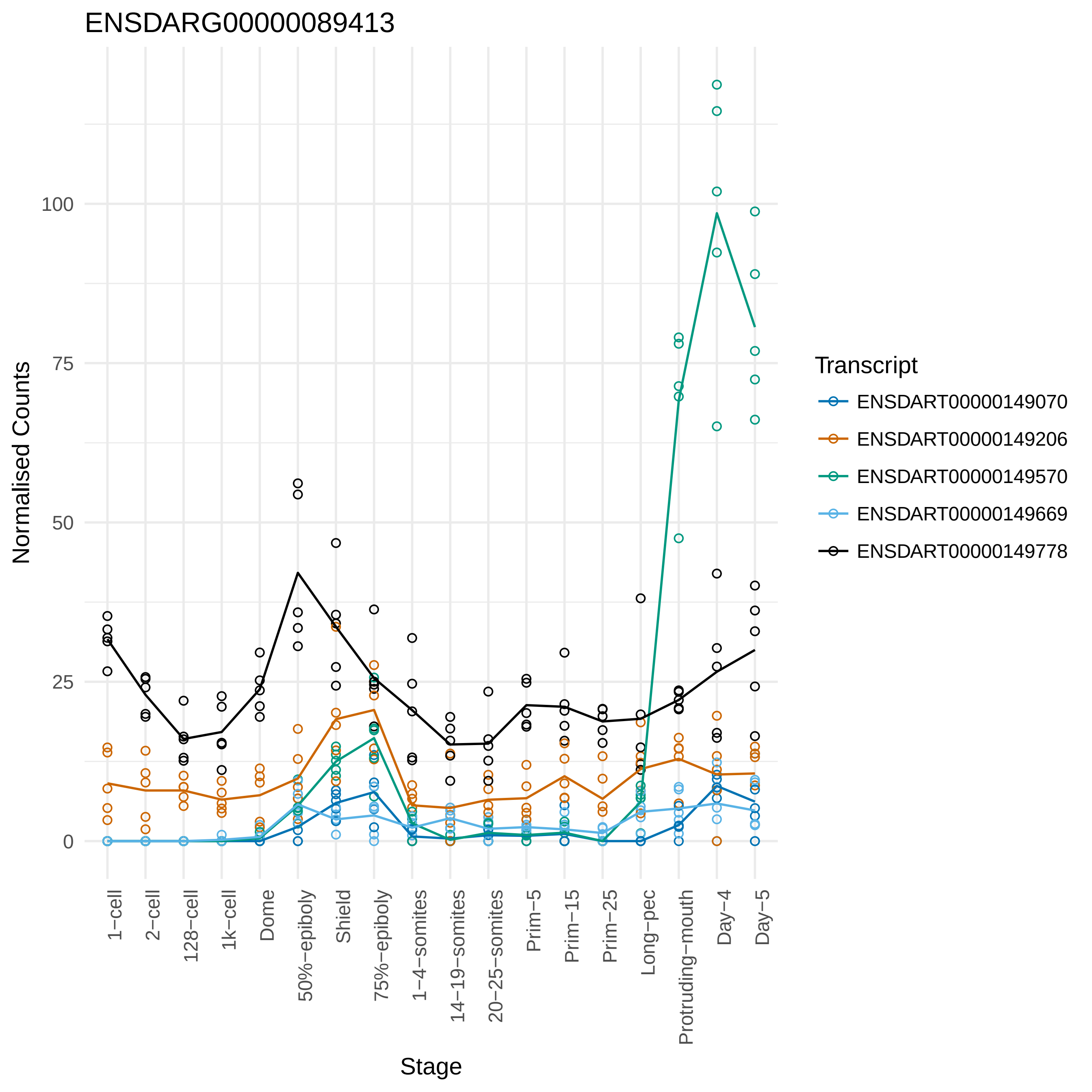

ENSDARG00000089413
Normalised Counts
100
75
50
25
0
1−cell
2−cell
128−cell
1k−cell
Dome
50%−epiboly
Shield
75%−epiboly
1−4−somites
14−19−somites
20−25−somites
Prim−5
Prim−15
Prim−25
Long−pec
Protruding−mouth
Day−4
Day−5
Stage
Transcript
ENSDART00000149070
ENSDART00000149206
ENSDART00000149570
ENSDART00000149669
ENSDART00000149778

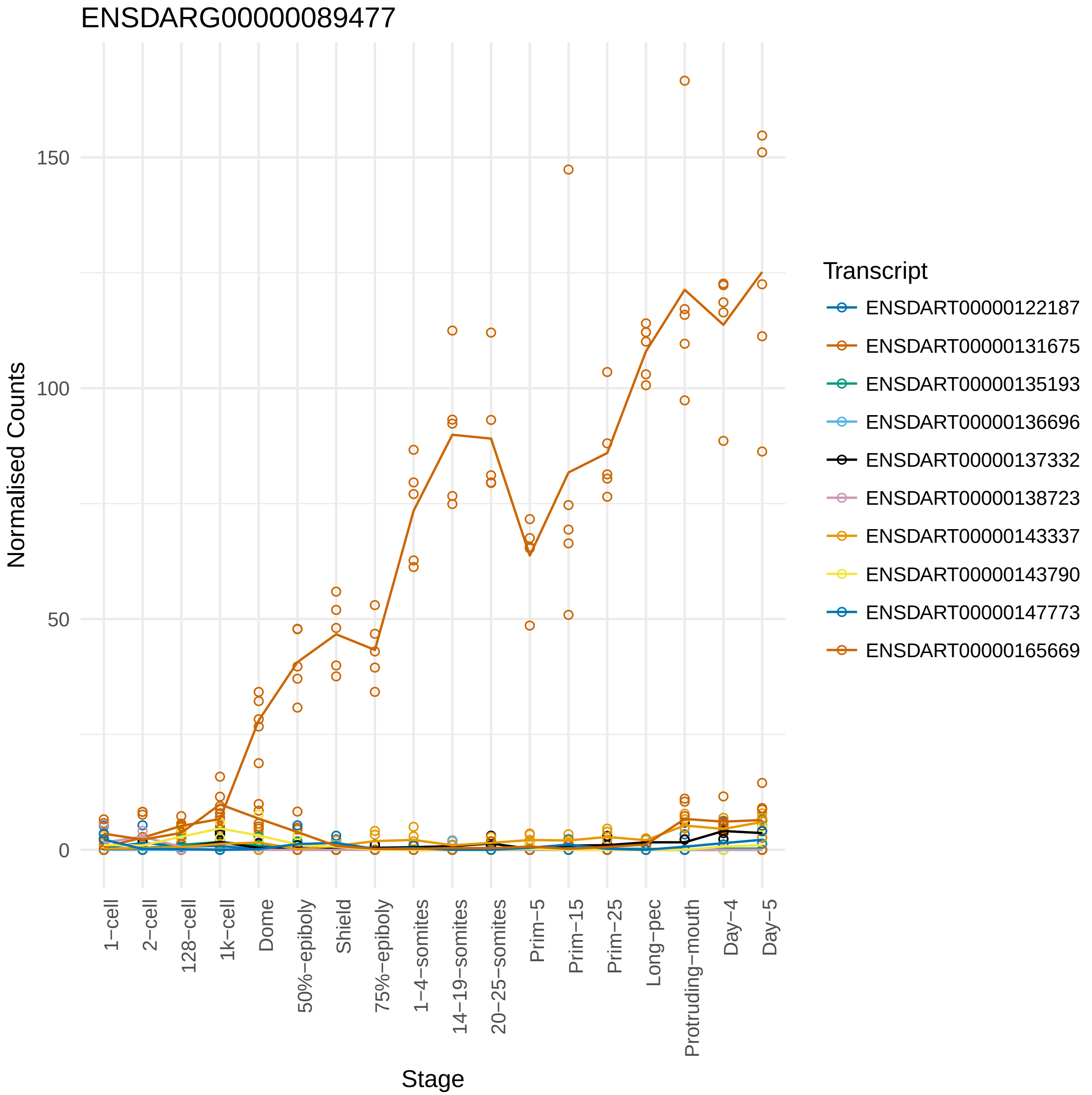
ENSDARG00000089477
Normalised Counts
150
100
50
0
1−cell
2−cell
128−cell
1k−cell
Dome
50%−epiboly
Shield
75%−epiboly
1−4−somites
14−19−somites
20−25−somites
Prim−5
Prim−15
Prim−25
Long−pec
Protruding−mouth
Day−4
Day−5
Stage
Transcript
ENSDART00000122187
ENSDART00000131675
ENSDART00000135193
ENSDART00000136696
ENSDART00000137332
ENSDART00000138723
ENSDART00000143337
ENSDART00000143790
ENSDART00000147773
ENSDART00000165669

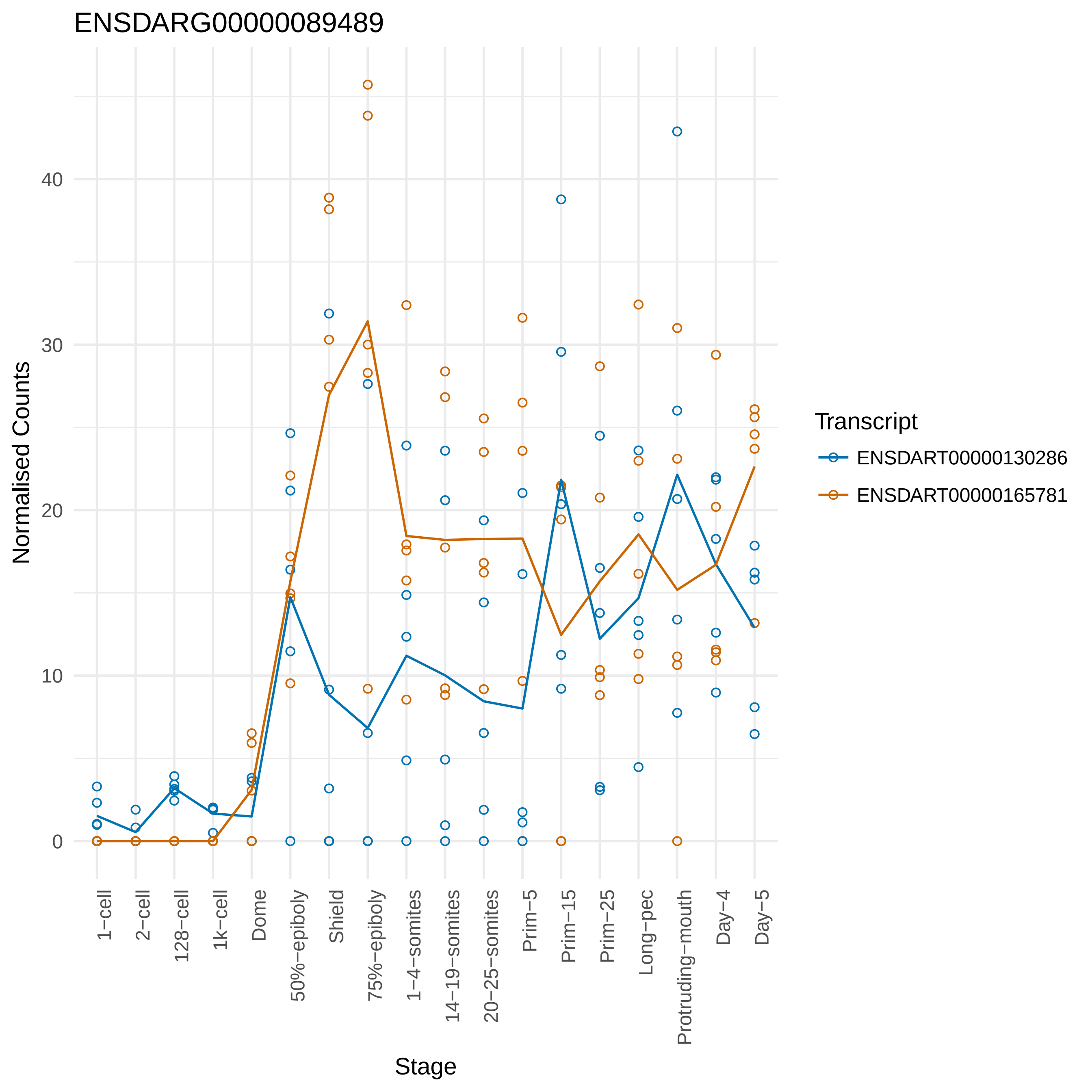
ENSDARG00000089489
Normalised Counts
0
10
20
30
40
Stage
1−cell
2−cell
128−cell
1k−cell
Dome
50%−epiboly
Shield
75%−epiboly
1−4−somites
14−19−somites
20−25−somites
Prim−5
Prim−15
Prim−25
Long−pec
Protruding−mouth
Day−4
Day−5
Transcript
ENSDART00000130286
ENSDART00000165781

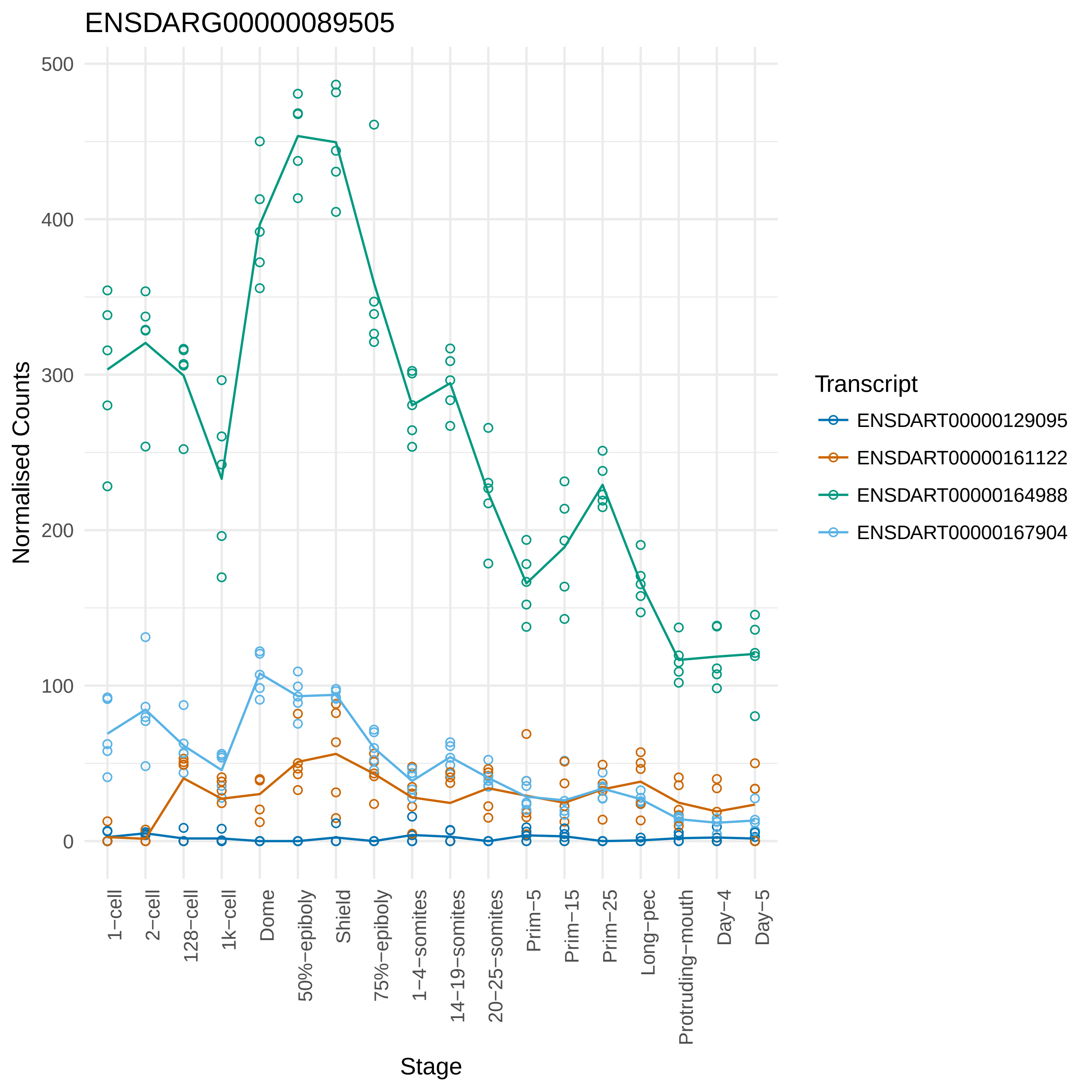

ENSDARG00000089505
Normalised Counts
500
400
300
200
100
0
1−cell
2−cell
128−cell
1k−cell
Dome
50%−epiboly
Shield
75%−epiboly
1−4−somites
14−19−somites
20−25−somites
Prim−5
Prim−15
Prim−25
Long−pec
Protruding−mouth
Day−4
Day−5
Stage
Transcript
ENSDART00000129095
ENSDART00000161122
ENSDART00000164988
ENSDART00000167904

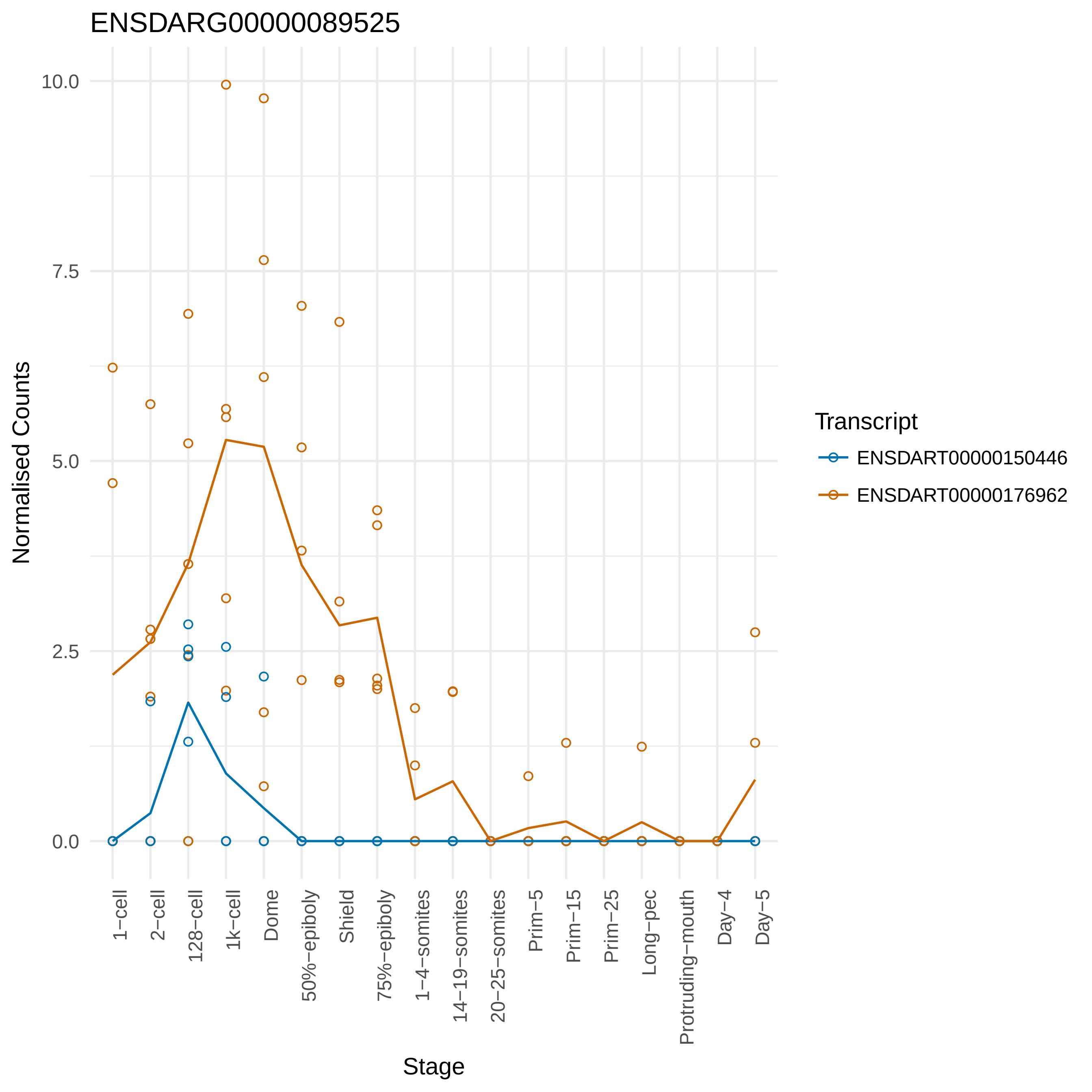
ENSDARG00000089525
Normalised Counts
10.0
7.5
5.0
2.5
0.0
Stage
1−cell
2−cell
128−cell
1k−cell
Dome
50%−epiboly
Shield
75%−epiboly
1−4−somites
14−19−somites
20−25−somites
Prim−5
Prim−15
Prim−25
Long−pec
Protruding−mouth
Day−4
Day−5
Transcript
ENSDART00000150446
ENSDART00000176962

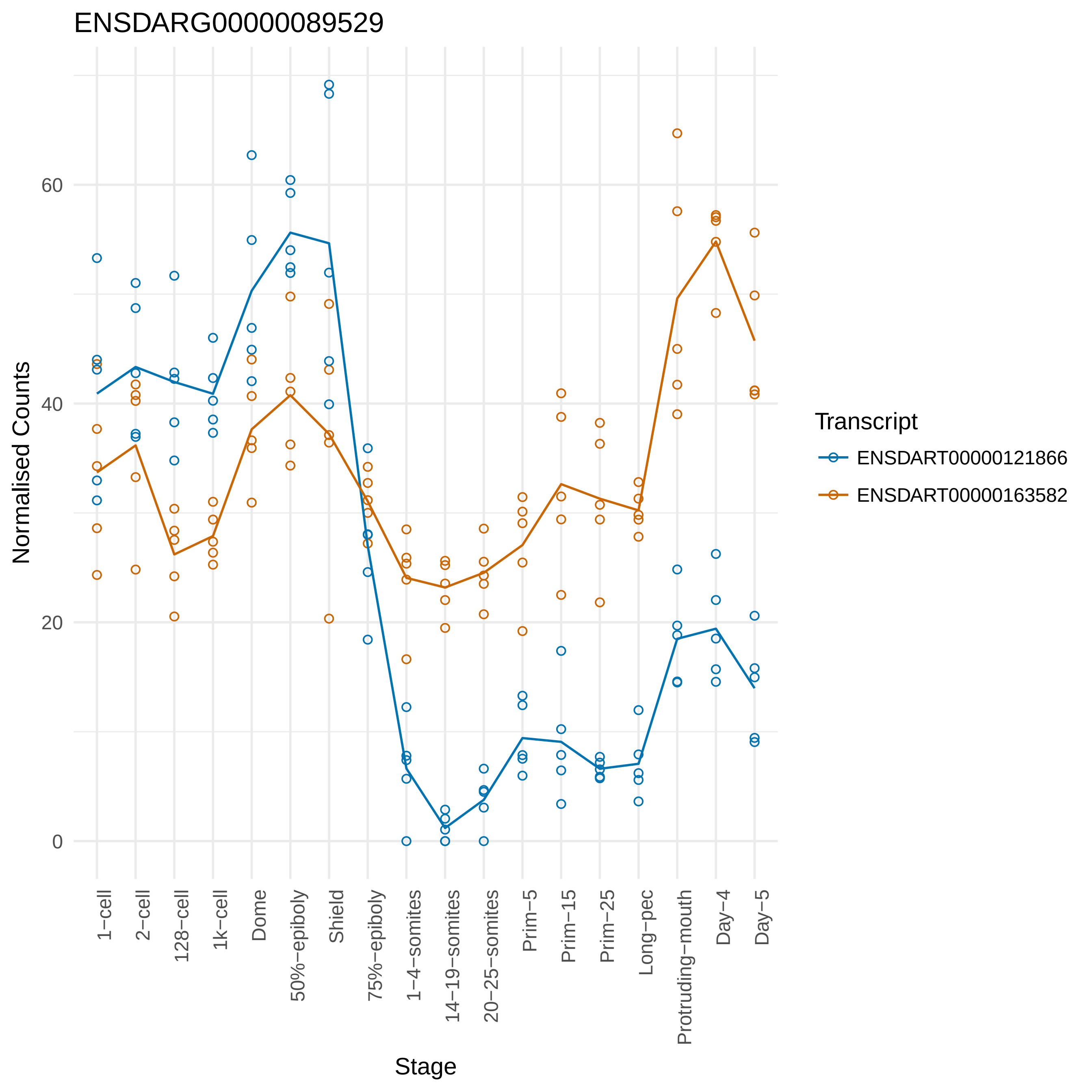

ENSDARG00000089529
Normalised Counts
0
20
40
60
1−cell
2−cell
128−cell
1k−cell
Dome
50%−epiboly
Shield
75%−epiboly
1−4−somites
14−19−somites
20−25−somites
Prim−5
Prim−15
Prim−25
Long−pec
Protruding−mouth
Day−4
Day−5
Stage
Transcript
ENSDART00000121866
ENSDART00000163582

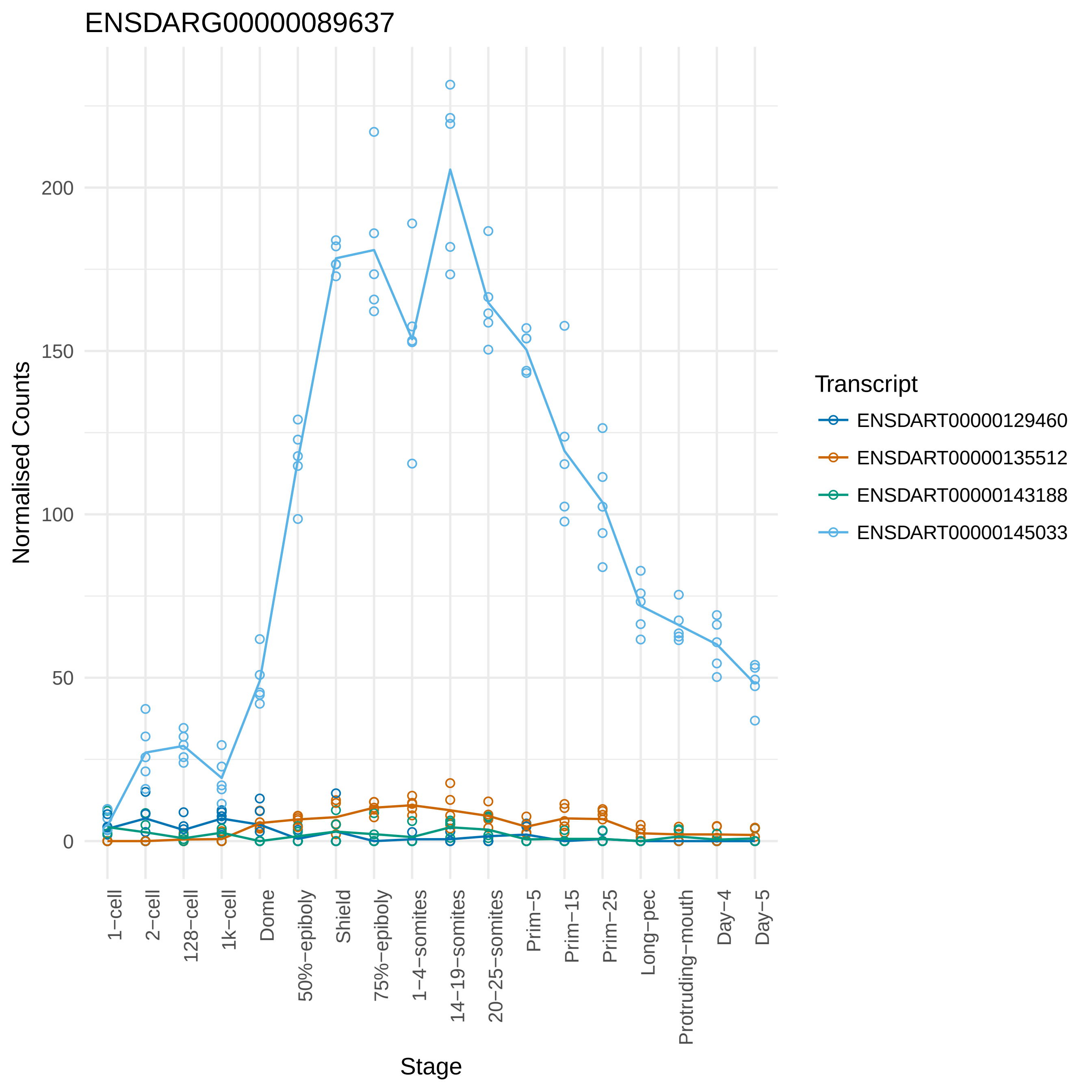

ENSDARG00000089637
Normalised Counts
200
150
100
50
0
1−cell
2−cell
128−cell
1k−cell
Dome
50%−epiboly
Shield
75%−epiboly
1−4−somites
14−19−somites
20−25−somites
Prim−5
Prim−15
Prim−25
Long−pec
Protruding−mouth
Day−4
Day−5
Stage
Transcript
ENSDART00000129460
ENSDART00000135512
ENSDART00000143188
ENSDART00000145033

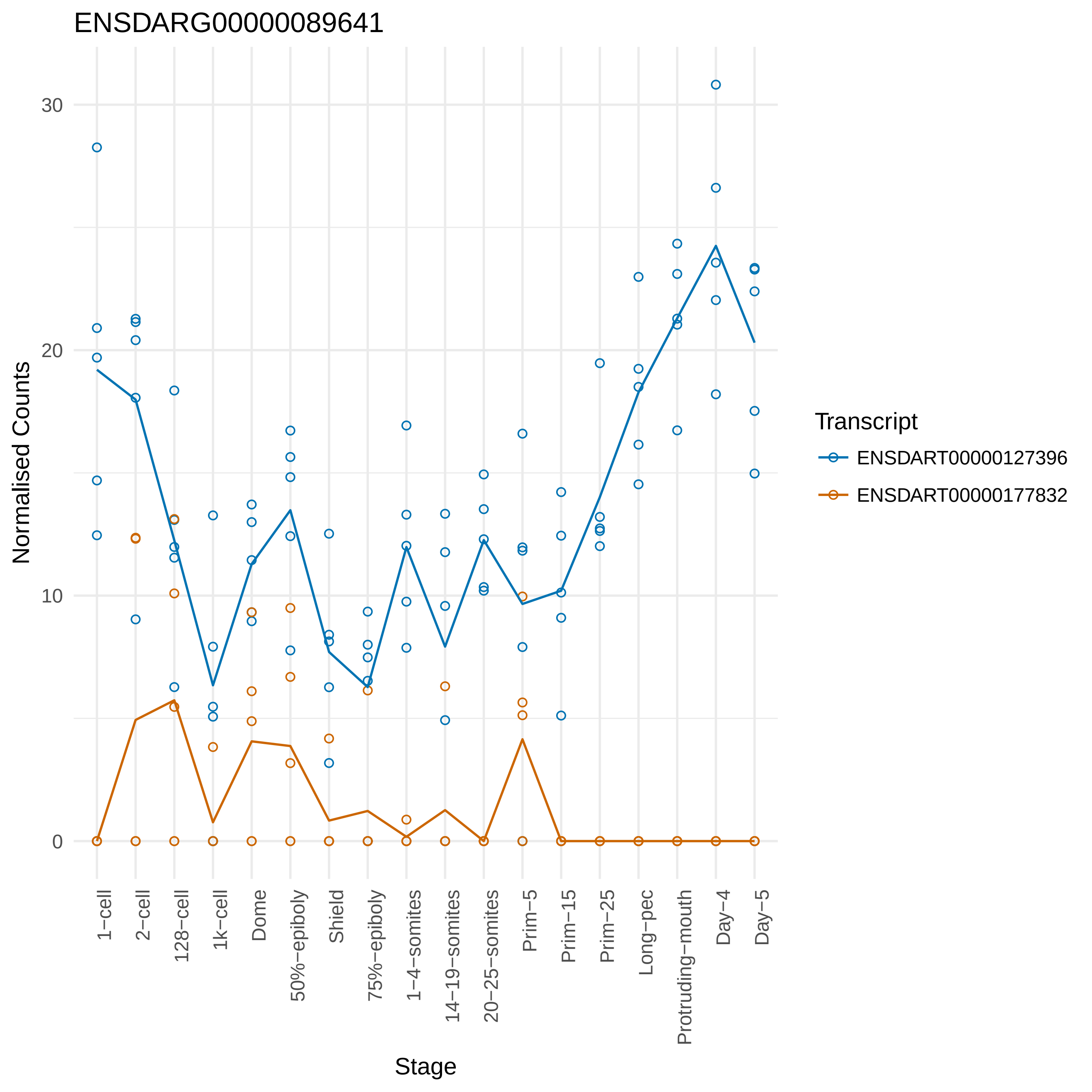
ENSDARG00000089641
Normalised Counts
30
20
10
0
1−cell
2−cell
128−cell
1k−cell
Dome
50%−epiboly
Shield
75%−epiboly
1−4−somites
14−19−somites
20−25−somites
Prim−5
Prim−15
Prim−25
Long−pec
Protruding−mouth
Day−4
Day−5
Stage
Transcript
ENSDART00000127396
ENSDART00000177832

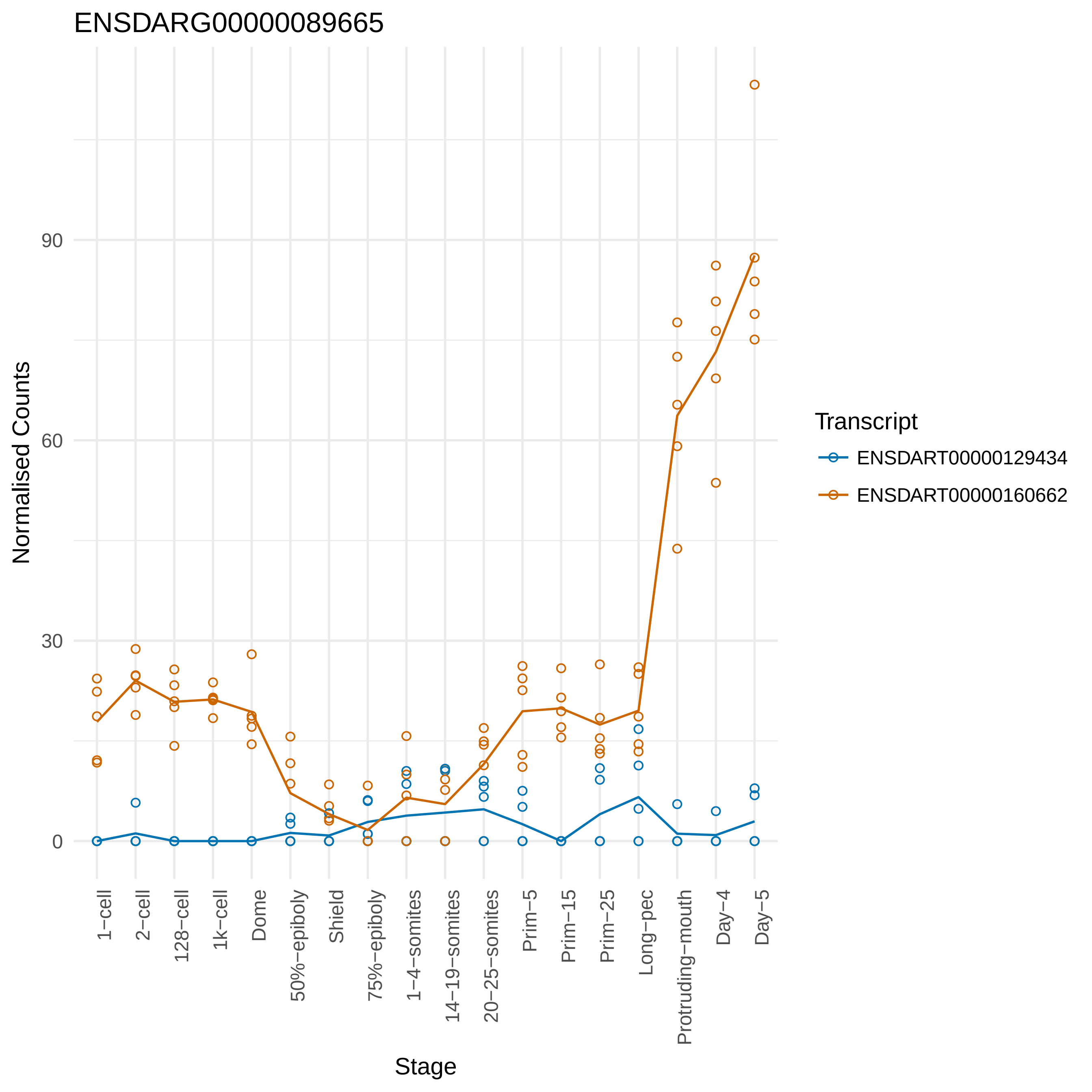

ENSDARG00000089665
Normalised Counts
0
30
60
90
Stage
1−cell
2−cell
128−cell
1k−cell
Dome
50%−epiboly
Shield
75%−epiboly
1−4−somites
14−19−somites
20−25−somites
Prim−5
Prim−15
Prim−25
Long−pec
Protruding−mouth
Day−4
Day−5
Transcript
ENSDART00000129434
ENSDART00000160662

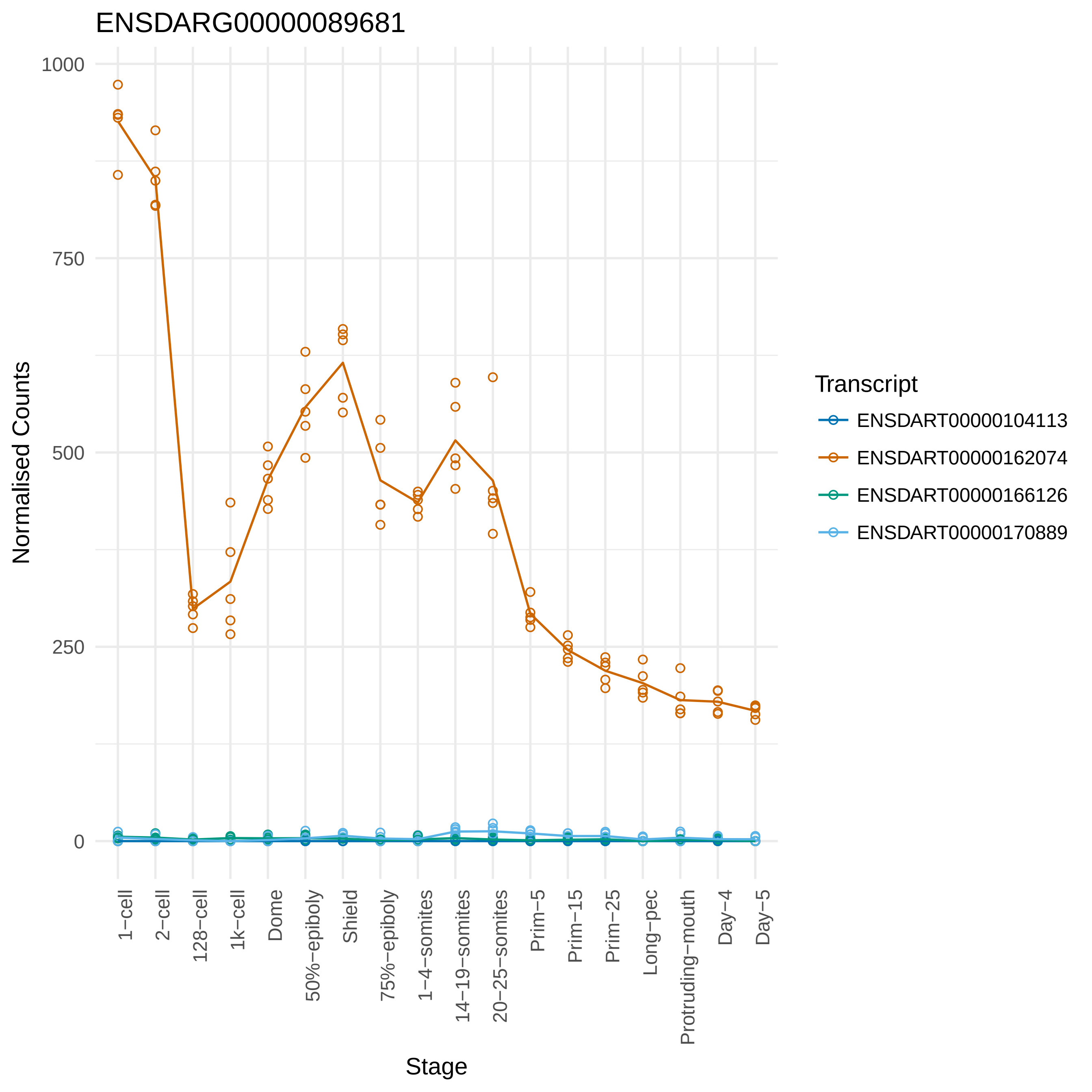

ENSDARG00000089681
Normalised Counts
1000
750
500
250
0
1−cell
2−cell
128−cell
1k−cell
Dome
50%−epiboly
Shield
75%−epiboly
1−4−somites
14−19−somites
20−25−somites
Prim−5
Prim−15
Prim−25
Long−pec
Protruding−mouth
Day−4
Day−5
Stage
Transcript
ENSDART00000104113
ENSDART00000162074
ENSDART00000166126
ENSDART00000170889

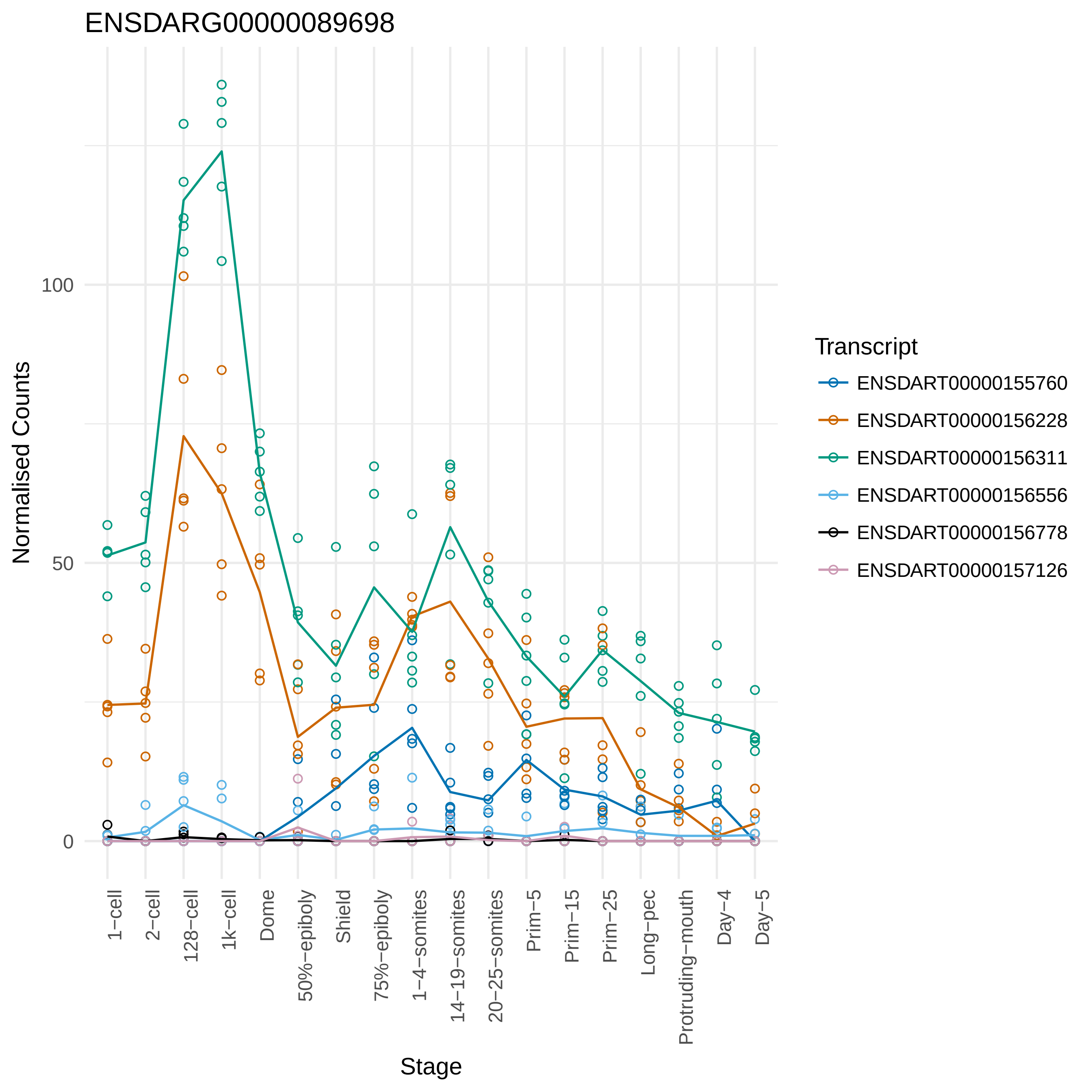
ENSDARG00000089698
Normalised Counts
100
50
0
Stage
1−cell
2−cell
128−cell
1k−cell
Dome
50%−epiboly
Shield
75%−epiboly
1−4−somites
14−19−somites
20−25−somites
Prim−5
Prim−15
Prim−25
Long−pec
Protruding−mouth
Day−4
Day−5
Transcript
ENSDART00000155760
ENSDART00000156228
ENSDART00000156311
ENSDART00000156556
ENSDART00000156778
ENSDART00000157126

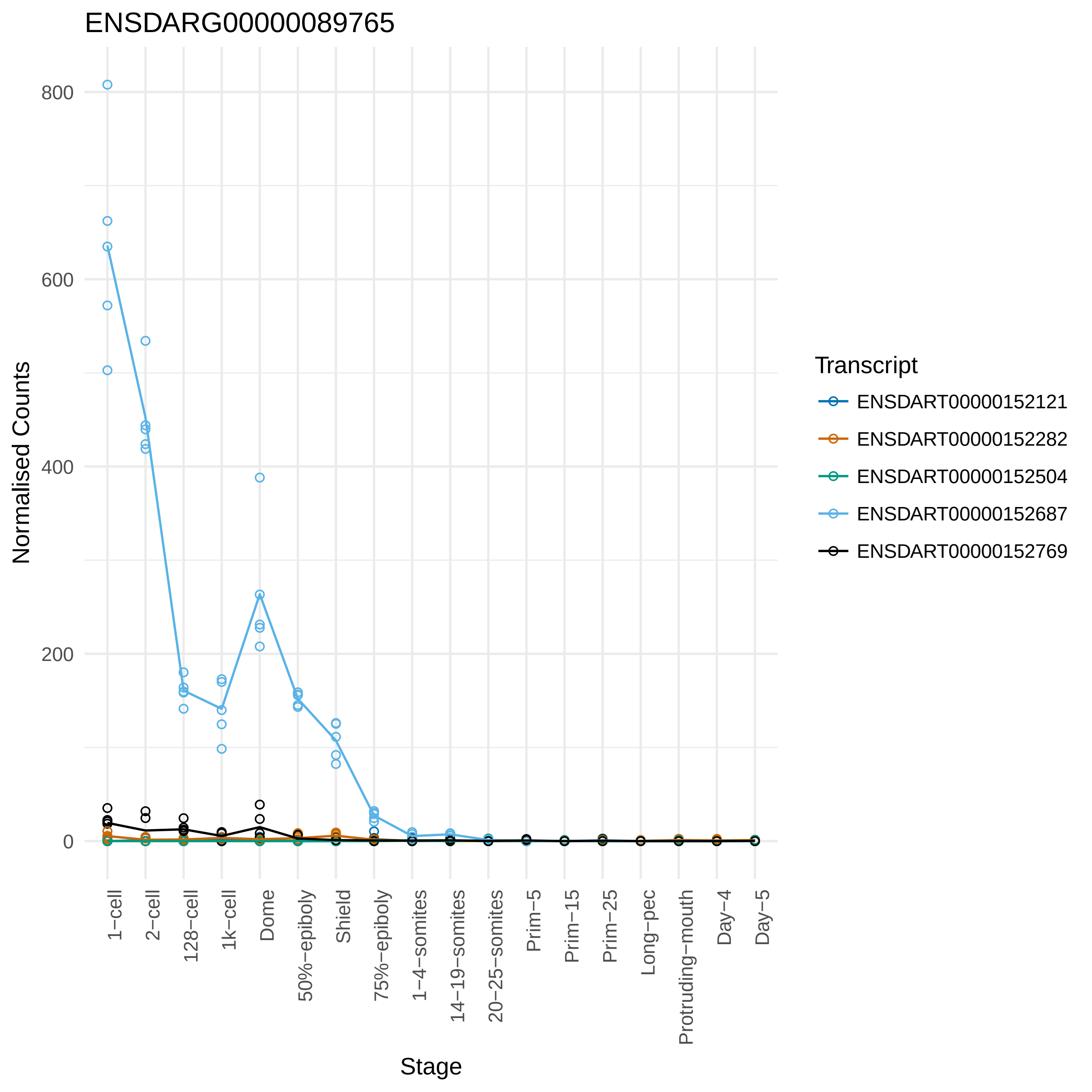

ENSDARG00000089765
Normalised Counts
0
200
400
600
800
Stage
1−cell
2−cell
128−cell
1k−cell
Dome
50%−epiboly
Shield
75%−epiboly
1−4−somites
14−19−somites
20−25−somites
Prim−5
Prim−15
Prim−25
Long−pec
Protruding−mouth
Day−4
Day−5
Transcript
ENSDART00000152121
ENSDART00000152282
ENSDART00000152504
ENSDART00000152687
ENSDART00000152769

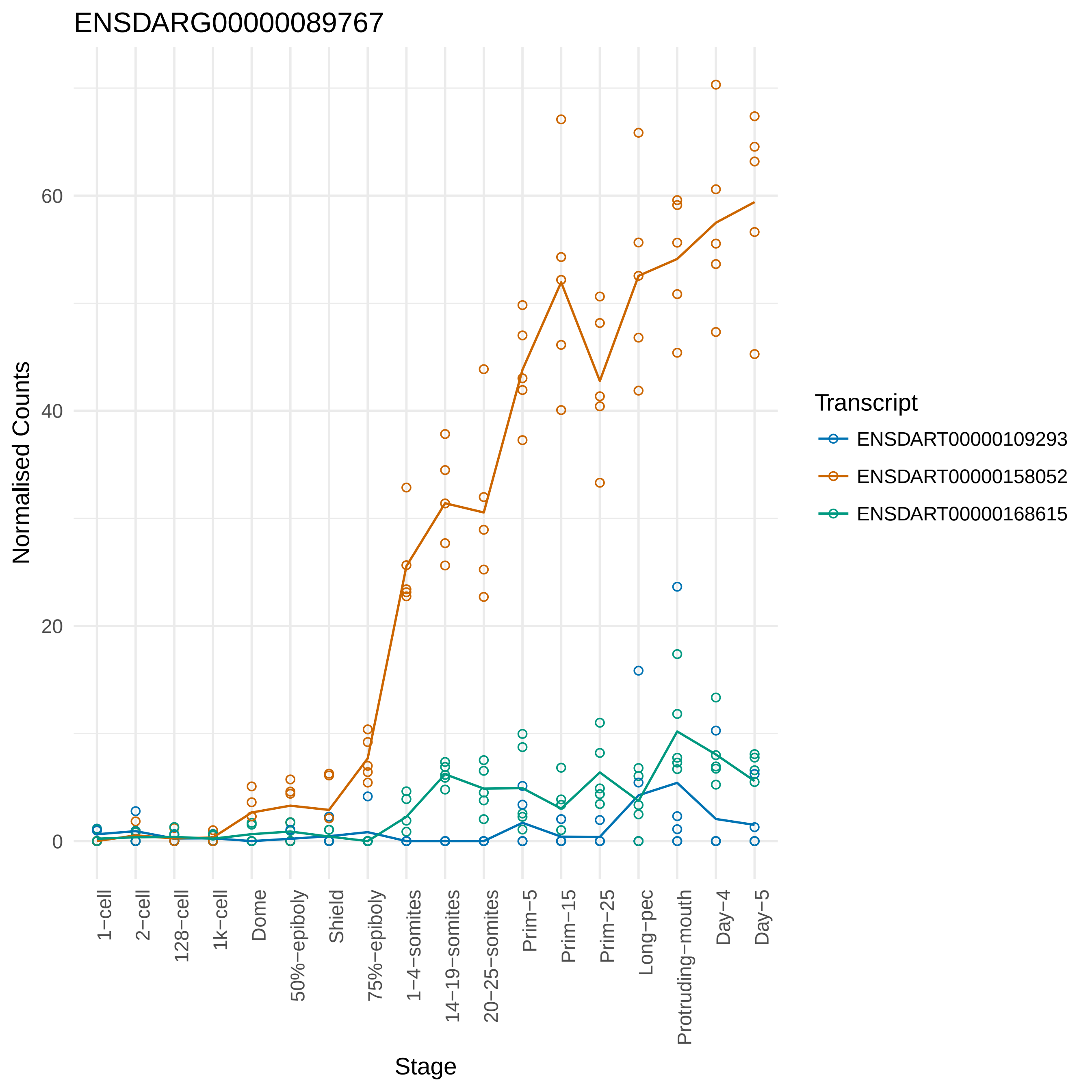

ENSDARG00000089767
Normalised Counts
0
20
40
60
Stage
1−cell
2−cell
128−cell
1k−cell
Dome
50%−epiboly
Shield
75%−epiboly
1−4−somites
14−19−somites
20−25−somites
Prim−5
Prim−15
Prim−25
Long−pec
Protruding−mouth
Day−4
Day−5
Transcript
ENSDART00000109293
ENSDART00000158052
ENSDART00000168615

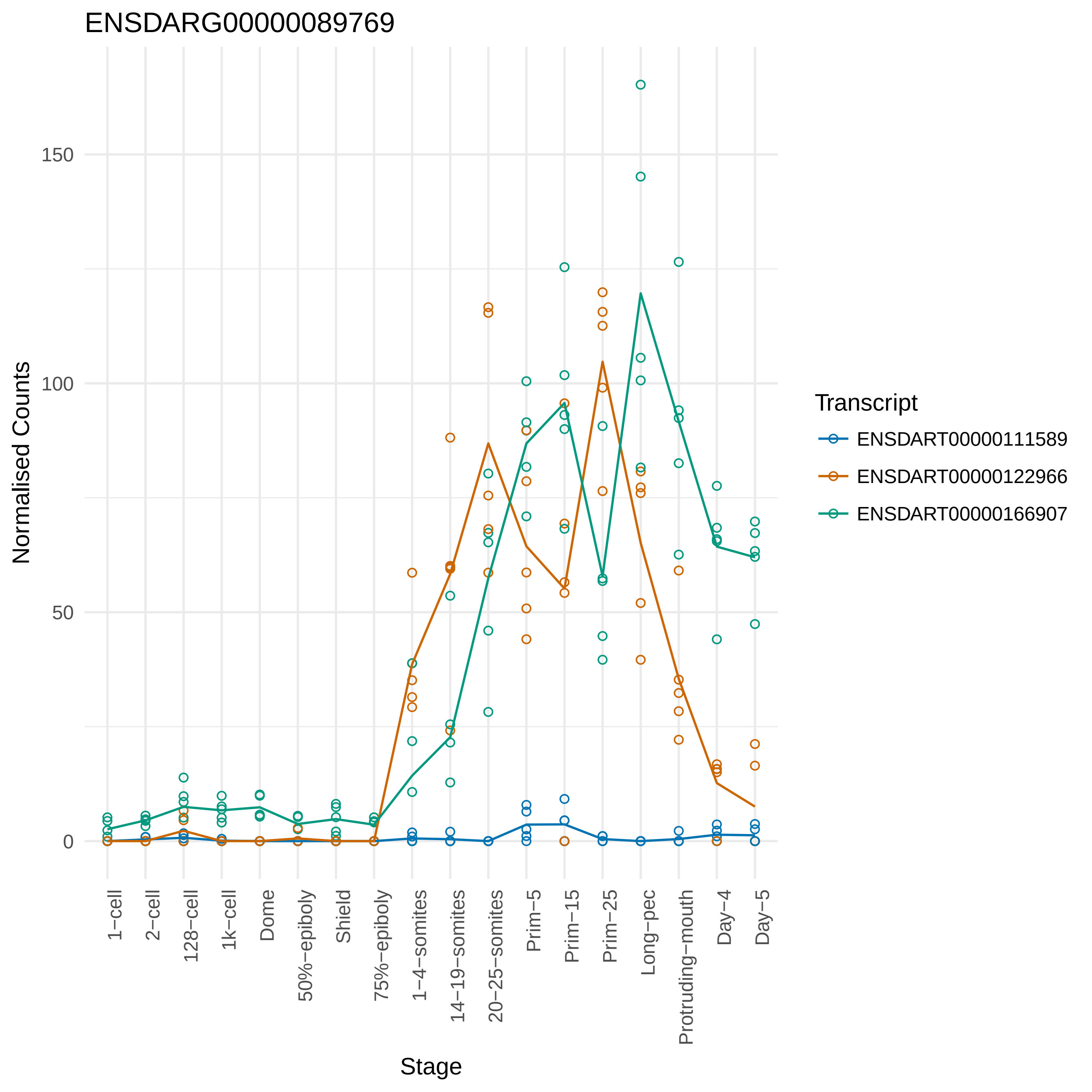
ENSDARG00000089769
Normalised Counts
0
50
100
150
Stage
1−cell
2−cell
128−cell
1k−cell
Dome
50%−epiboly
Shield
75%−epiboly
1−4−somites
14−19−somites
20−25−somites
Prim−5
Prim−15
Prim−25
Long−pec
Protruding−mouth
Day−4
Day−5
Transcript
ENSDART00000111589
ENSDART00000122966
ENSDART00000166907

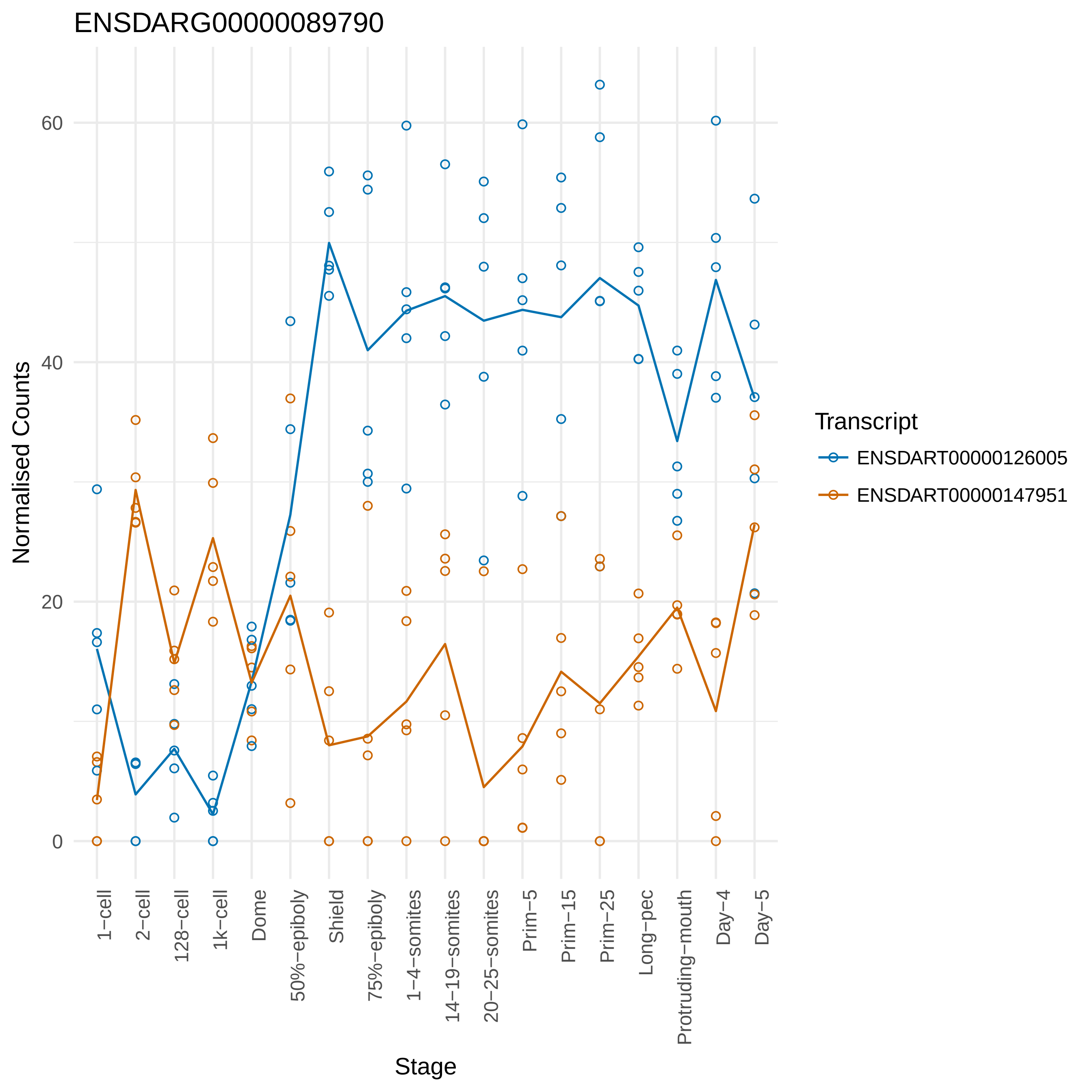
ENSDARG00000089790
Normalised Counts
0
20
40
60
1−cell
2−cell
128−cell
1k−cell
Dome
50%−epiboly
Shield
75%−epiboly
1−4−somites
14−19−somites
20−25−somites
Prim−5
Prim−15
Prim−25
Long−pec
Protruding−mouth
Day−4
Day−5
Stage
Transcript
ENSDART00000126005
ENSDART00000147951

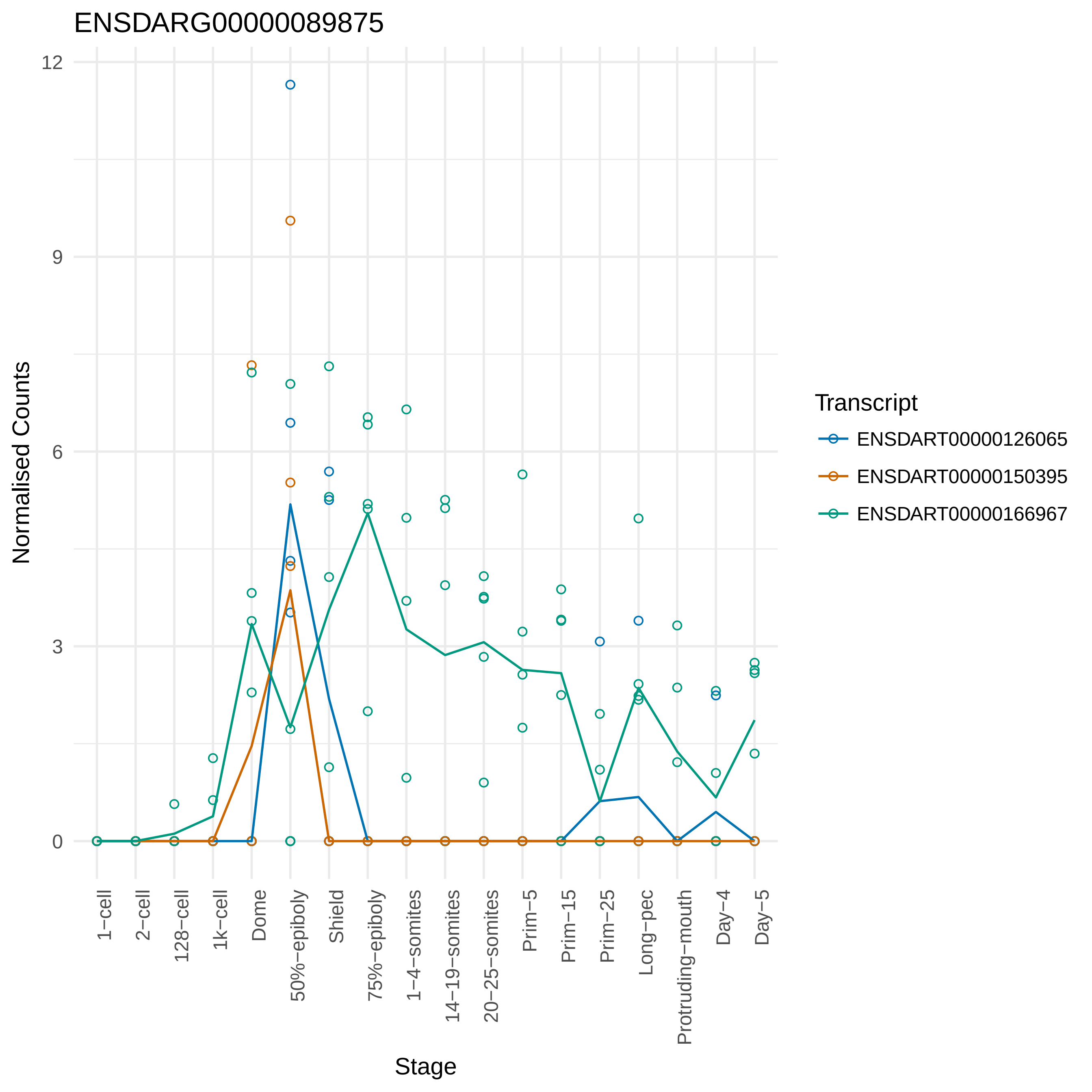
ENSDARG00000089875
Normalised Counts
12
9
6
3
0
1−cell
2−cell
128−cell
1k−cell
Dome
50%−epiboly
Shield
75%−epiboly
1−4−somites
14−19−somites
20−25−somites
Prim−5
Prim−15
Prim−25
Long−pec
Protruding−mouth
Day−4
Day−5
Stage
Transcript
ENSDART00000126065
ENSDART00000150395
ENSDART00000166967

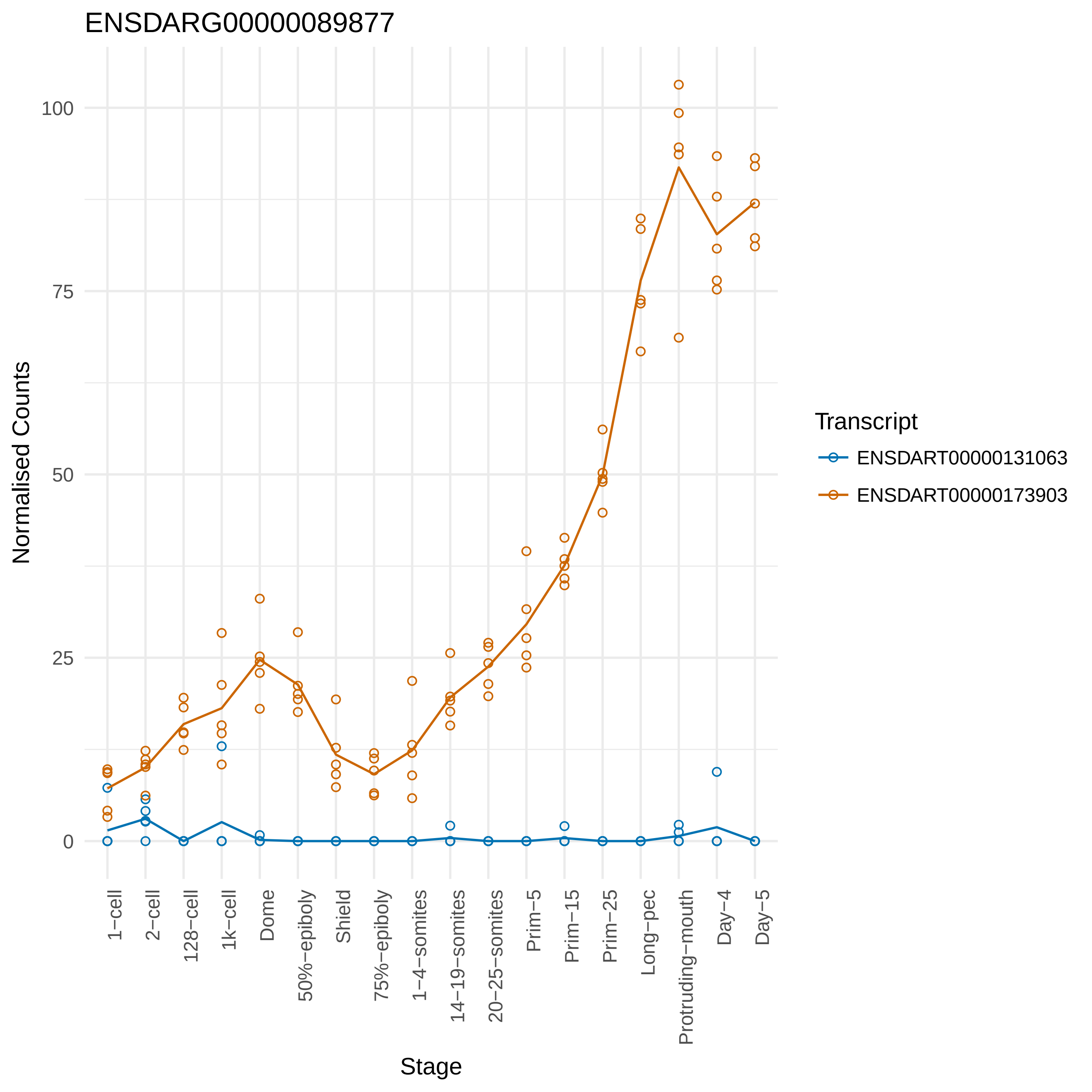

ENSDARG00000089877
Normalised Counts
100
75
50
25
0
1−cell
2−cell
128−cell
1k−cell
Dome
50%−epiboly
Shield
75%−epiboly
1−4−somites
14−19−somites
20−25−somites
Prim−5
Prim−15
Prim−25
Long−pec
Protruding−mouth
Day−4
Day−5
Stage
Transcript
ENSDART00000131063
ENSDART00000173903

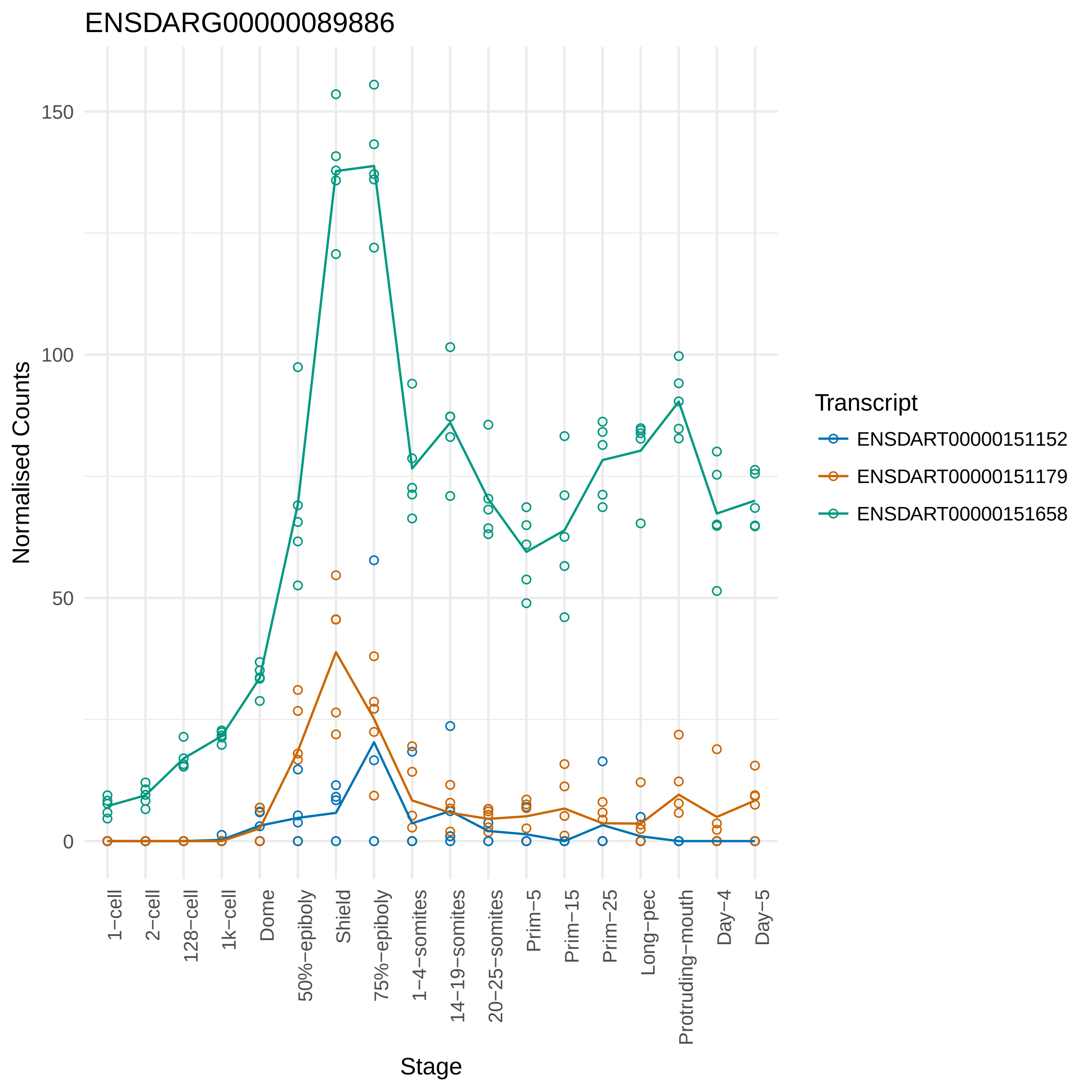
ENSDARG00000089886
Normalised Counts
150
100
50
0
1−cell
2−cell
128−cell
1k−cell
Dome
50%−epiboly
Shield
75%−epiboly
1−4−somites
14−19−somites
20−25−somites
Prim−5
Prim−15
Prim−25
Long−pec
Protruding−mouth
Day−4
Day−5
Stage
Transcript
ENSDART00000151152
ENSDART00000151179
ENSDART00000151658

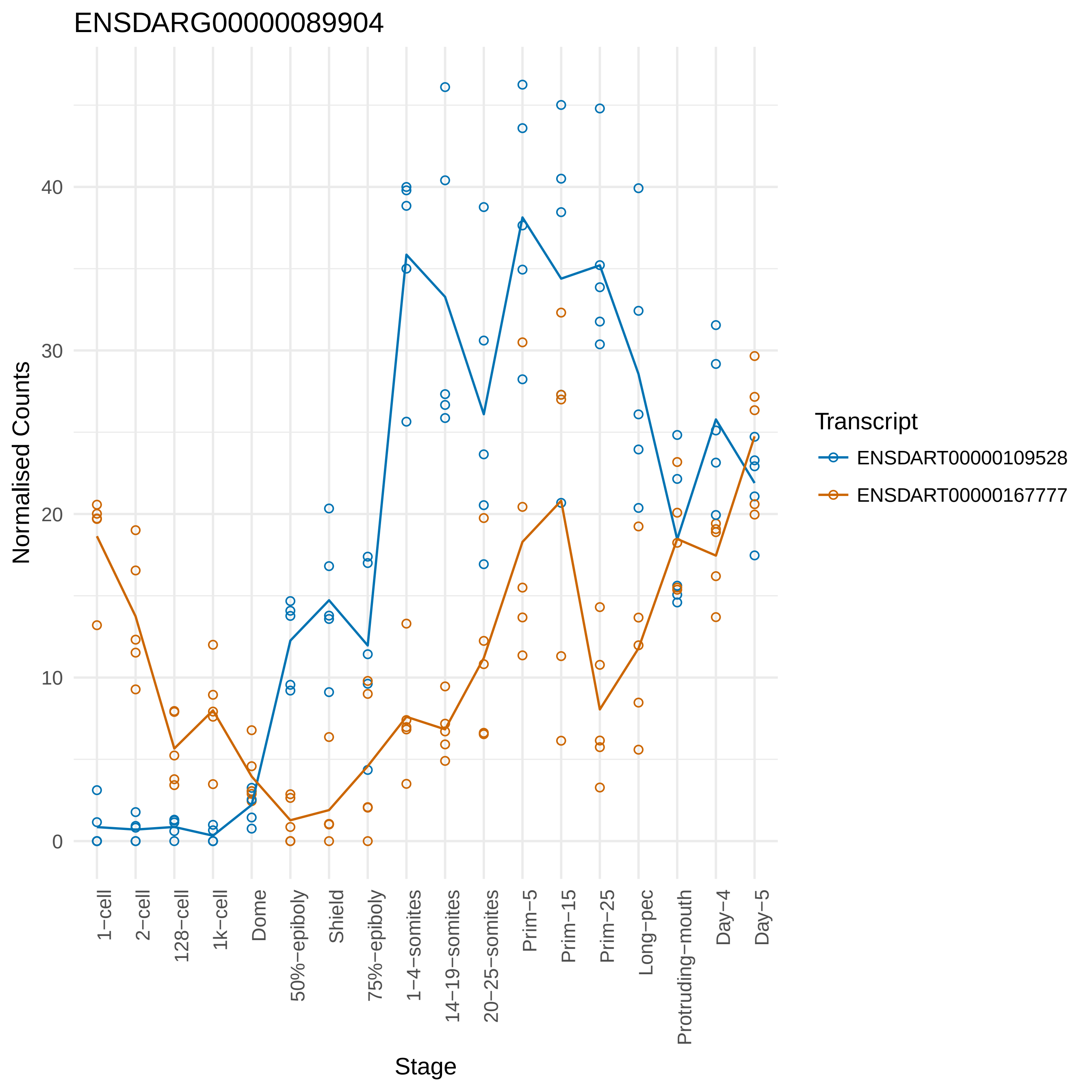
ENSDARG00000089904
Normalised Counts
0
10
20
30
40
Stage
1−cell
2−cell
128−cell
1k−cell
Dome
50%−epiboly
Shield
75%−epiboly
1−4−somites
14−19−somites
20−25−somites
Prim−5
Prim−15
Prim−25
Long−pec
Protruding−mouth
Day−4
Day−5
Transcript
ENSDART00000109528
ENSDART00000167777

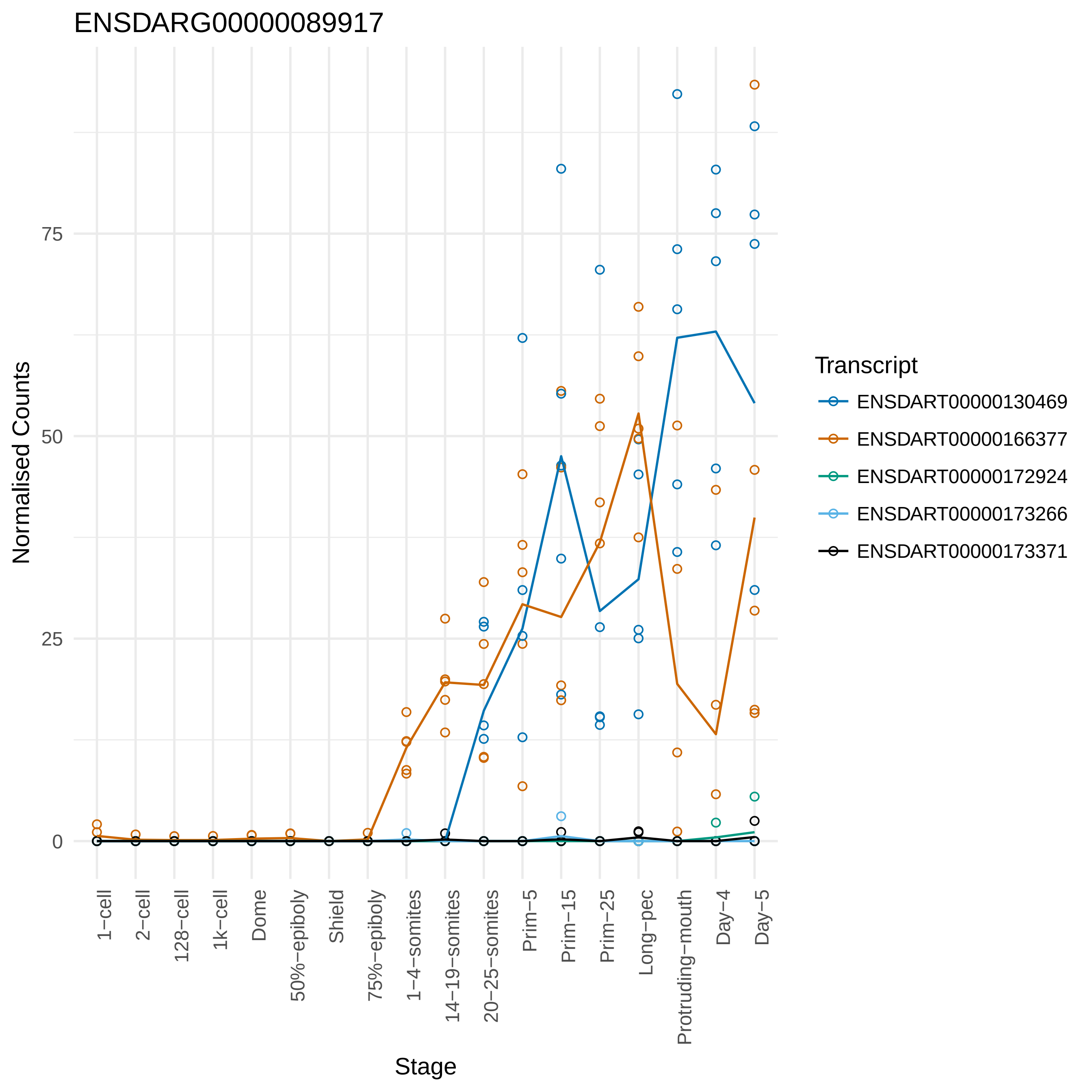

ENSDARG00000089917
Normalised Counts
75
50
25
0
1−cell
2−cell
128−cell
1k−cell
Dome
50%−epiboly
Shield
75%−epiboly
1−4−somites
14−19−somites
20−25−somites
Prim−5
Prim−15
Prim−25
Long−pec
Protruding−mouth
Day−4
Day−5
Stage
Transcript
ENSDART00000130469
ENSDART00000166377
ENSDART00000172924
ENSDART00000173266
ENSDART00000173371

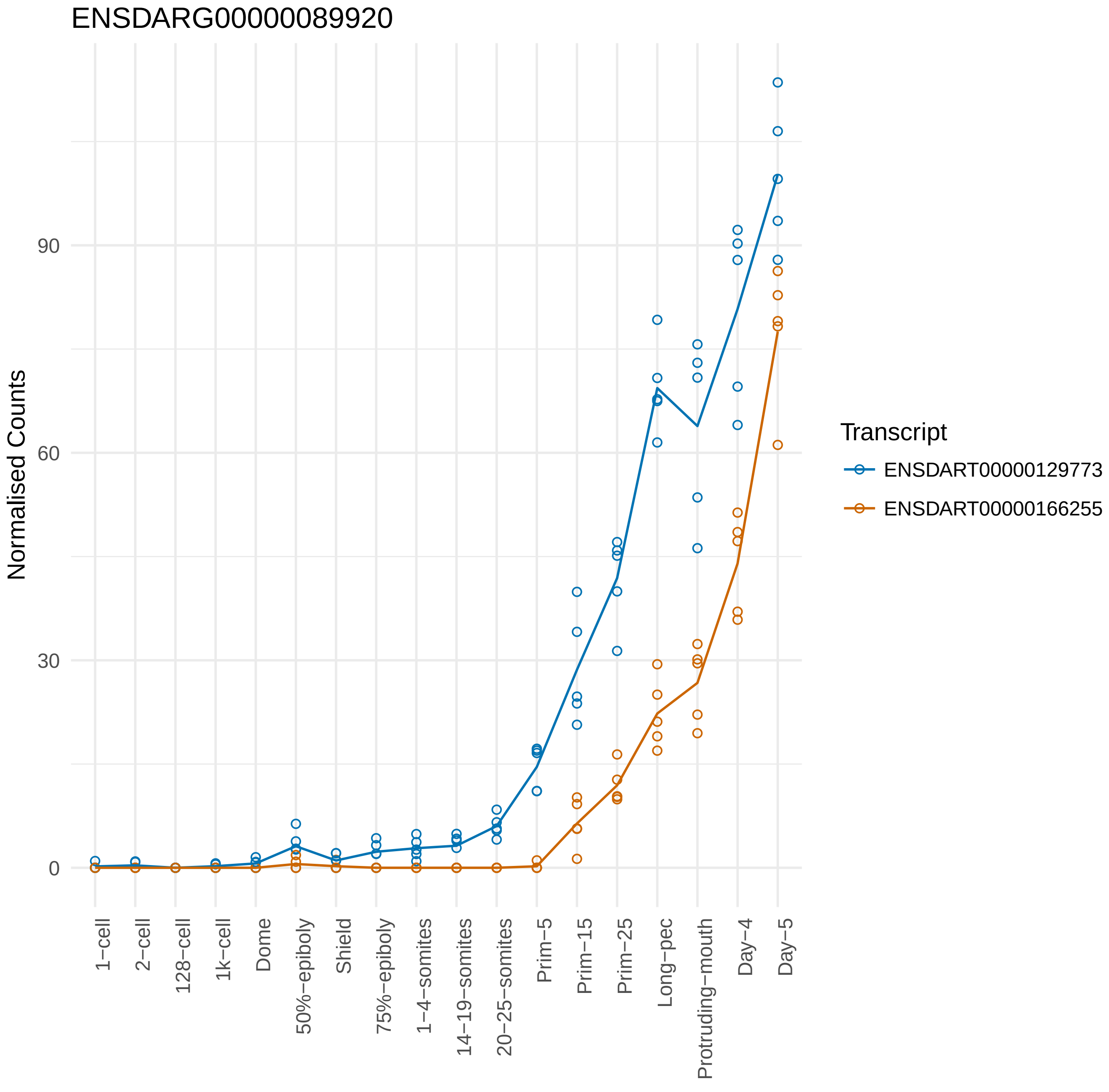
ENSDARG00000089920
Normalised Counts
0
30
60
90
Stage
1−cell
2−cell
128−cell
1k−cell
Dome
50%−epiboly
Shield
75%−epiboly
1−4−somites
14−19−somites
20−25−somites
Prim−5
Prim−15
Prim−25
Long−pec
Protruding−mouth
Day−4
Day−5
Transcript
ENSDART00000129773
ENSDART00000166255

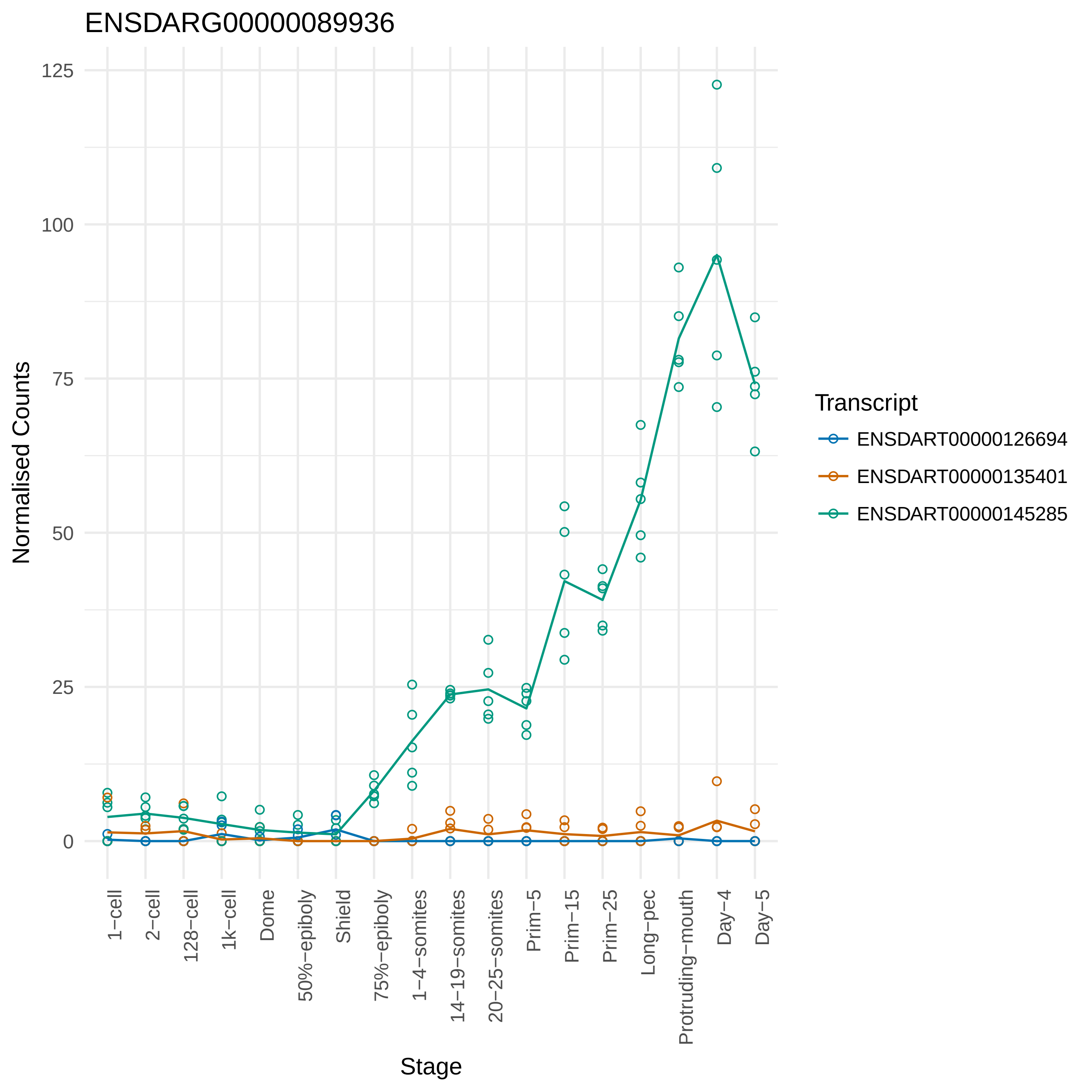

ENSDARG00000089936
Normalised Counts
125
100
75
50
25
0
1−cell
2−cell
128−cell
1k−cell
Dome
50%−epiboly
Shield
75%−epiboly
1−4−somites
14−19−somites
20−25−somites
Prim−5
Prim−15
Prim−25
Long−pec
Protruding−mouth
Day−4
Day−5
Stage
Transcript
ENSDART00000126694
ENSDART00000135401
ENSDART00000145285

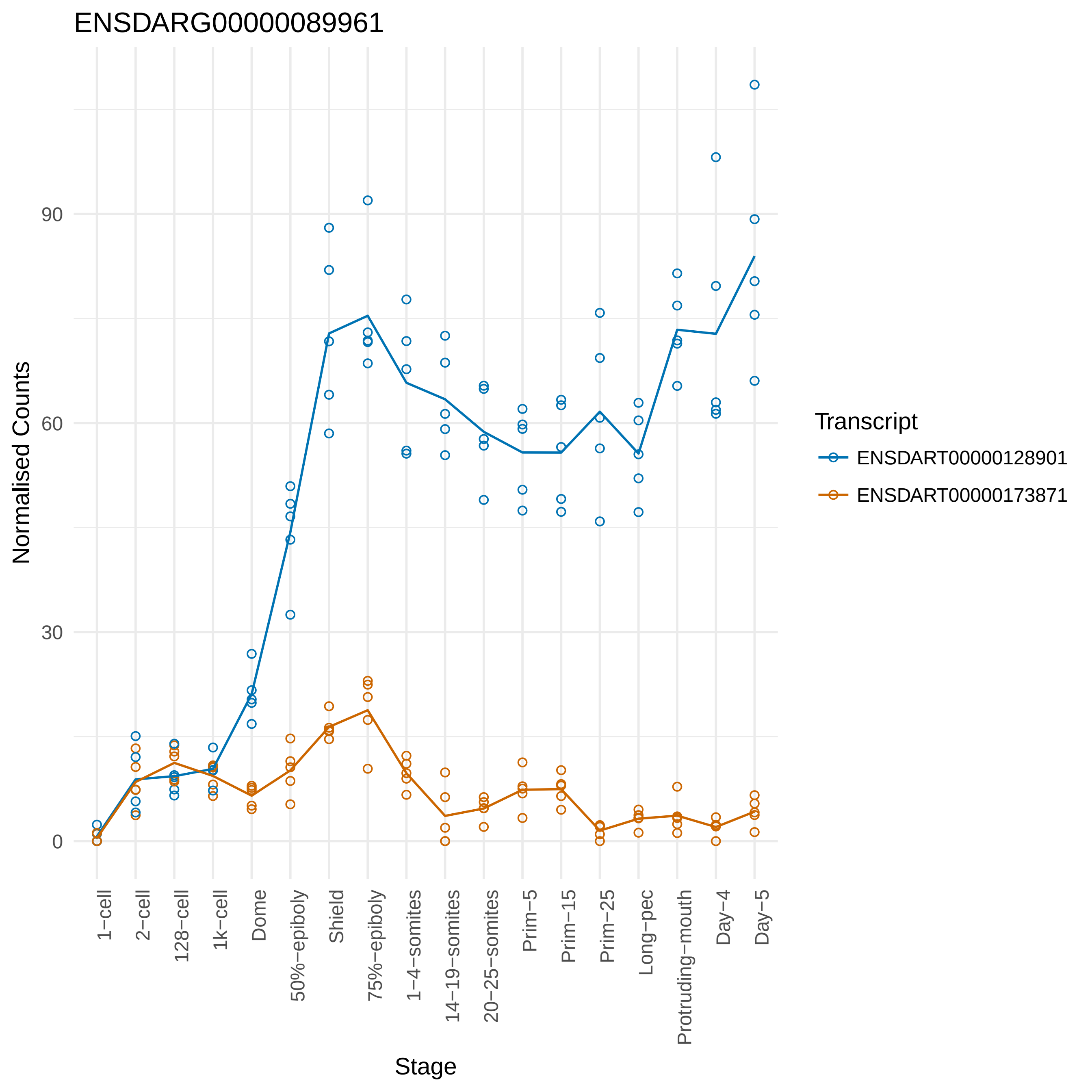
ENSDARG00000089961
Normalised Counts
0
30
60
90
Stage
1−cell
2−cell
128−cell
1k−cell
Dome
50%−epiboly
Shield
75%−epiboly
1−4−somites
14−19−somites
20−25−somites
Prim−5
Prim−15
Prim−25
Long−pec
Protruding−mouth
Day−4
Day−5
Transcript
ENSDART00000128901
ENSDART00000173871

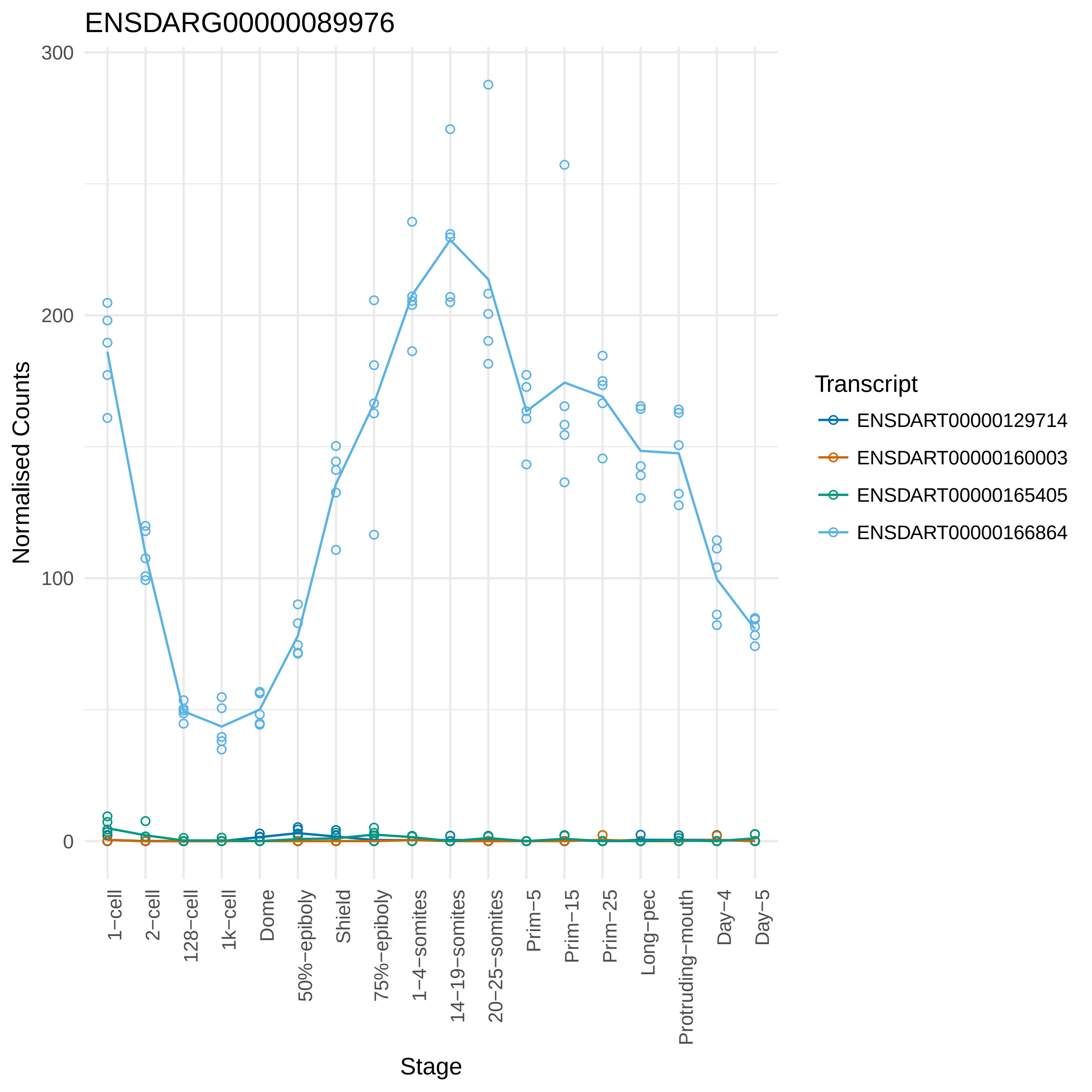

ENSDARG00000089976
300
200
100
0
Normalised Counts
1−cell
2−cell
128−cell
1k−cell
Dome
50%−epiboly
Shield
75%−epiboly
1−4−somites
14−19−somites
20−25−somites
Prim−5
Prim−15
Prim−25
Long−pec
Protruding−mouth
Day−4
Day−5
Stage
Transcript
ENSDART00000129714
ENSDART00000160003
ENSDART00000165405
ENSDART00000166864

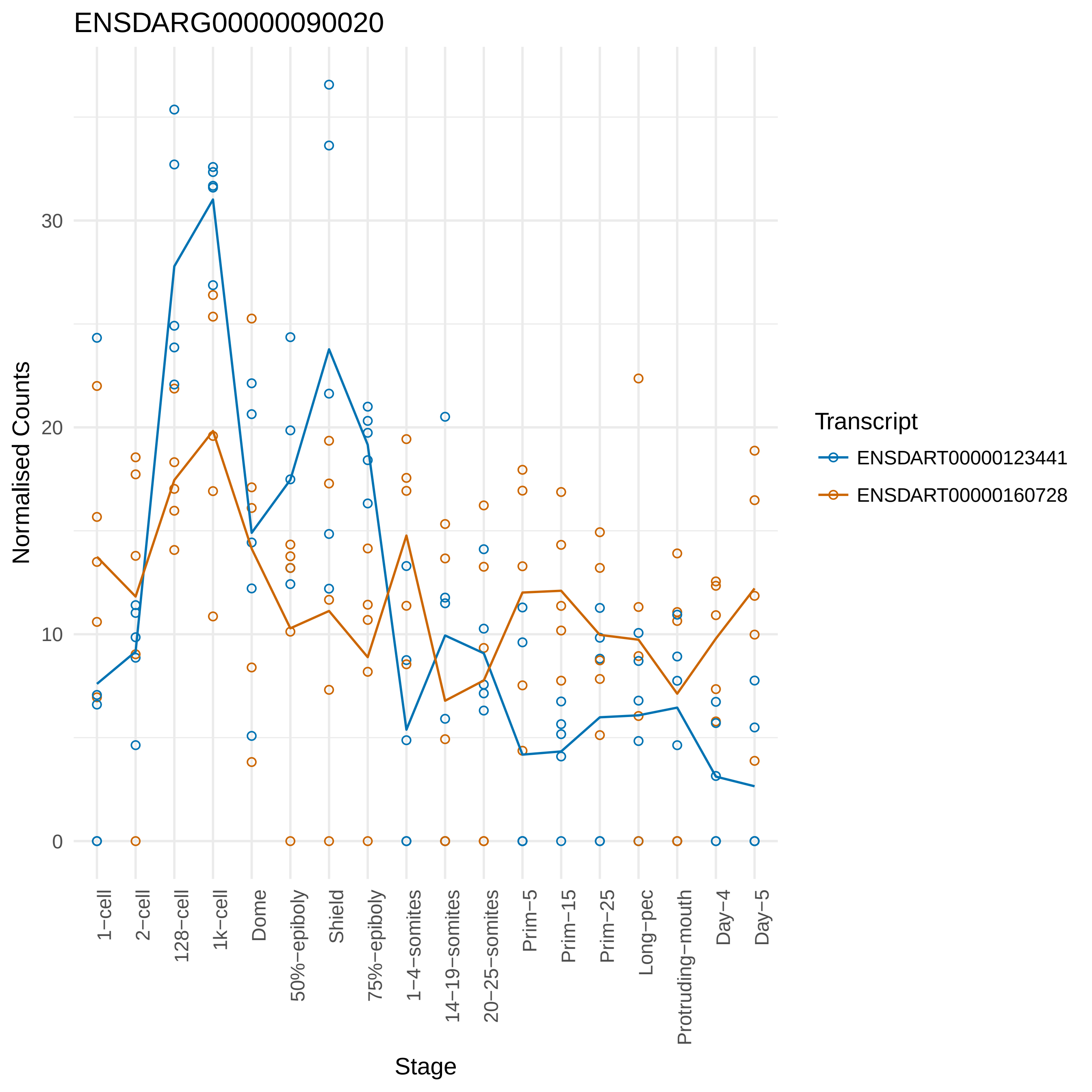
ENSDARG00000090020
Normalised Counts
0
10
20
30
Stage
1−cell
2−cell
128−cell
1k−cell
Dome
50%−epiboly
Shield
75%−epiboly
1−4−somites
14−19−somites
20−25−somites
Prim−5
Prim−15
Prim−25
Long−pec
Protruding−mouth
Day−4
Day−5
Transcript
ENSDART00000123441
ENSDART00000160728

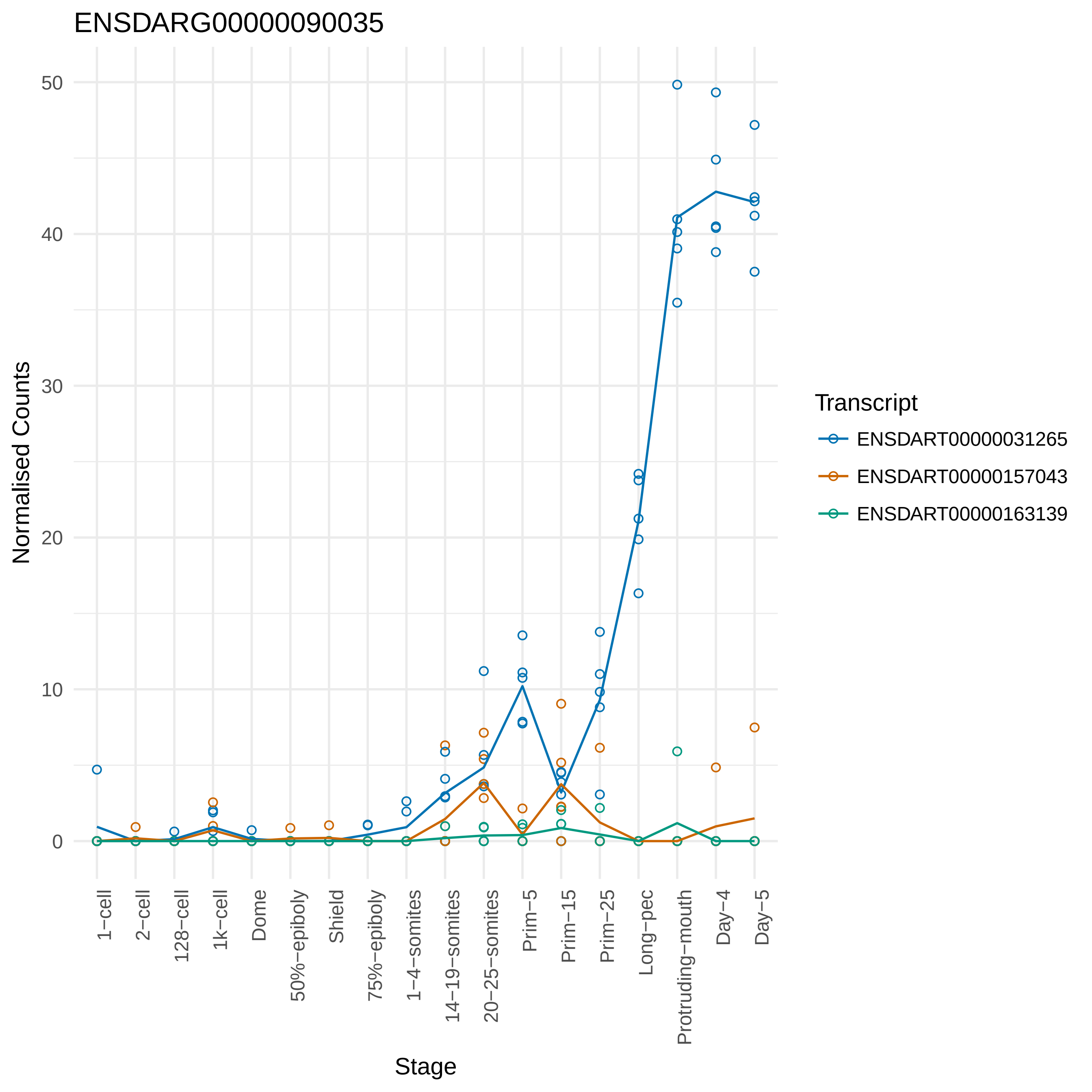

ENSDARG00000090035
Normalised Counts
0
10
20
30
40
50
1−cell
2−cell
128−cell
1k−cell
Dome
50%−epiboly
Shield
75%−epiboly
1−4−somites
14−19−somites
20−25−somites
Prim−5
Prim−15
Prim−25
Long−pec
Protruding−mouth
Day−4
Day−5
Stage
Transcript
ENSDART00000031265
ENSDART00000157043
ENSDART00000163139

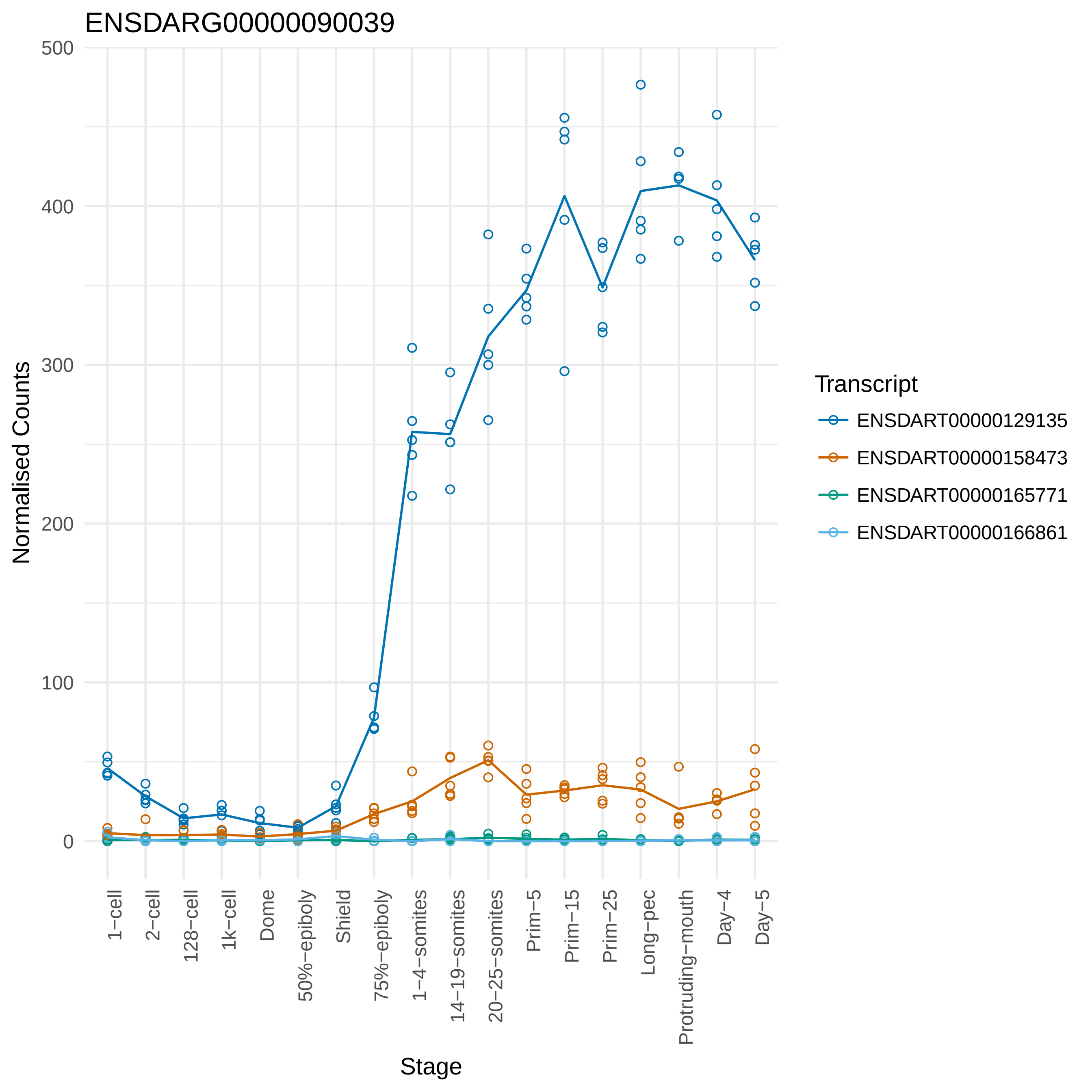
ENSDARG00000090039
Normalised Counts
0
100
200
300
400
500
Stage
1−cell
2−cell
128−cell
1k−cell
Dome
50%−epiboly
Shield
75%−epiboly
1−4−somites
14−19−somites
20−25−somites
Prim−5
Prim−15
Prim−25
Long−pec
Protruding−mouth
Day−4
Day−5
Transcript
ENSDART00000129135
ENSDART00000158473
ENSDART00000165771
ENSDART00000166861

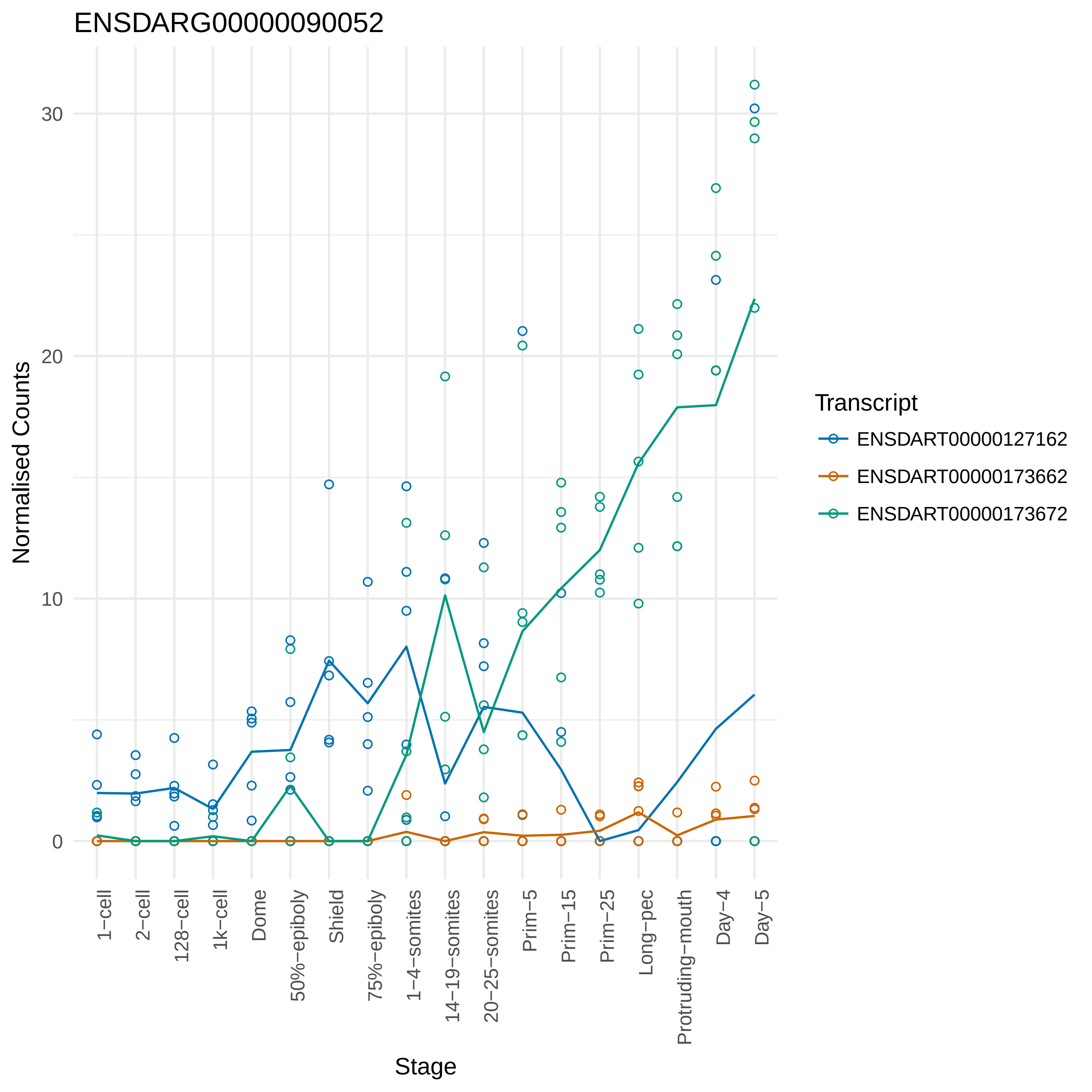
ENSDARG00000090052
Normalised Counts
0
10
20
30
Stage
1−cell
2−cell
128−cell
1k−cell
Dome
50%−epiboly
Shield
75%−epiboly
1−4−somites
14−19−somites
20−25−somites
Prim−5
Prim−15
Prim−25
Long−pec
Protruding−mouth
Day−4
Day−5
Transcript
ENSDART00000127162
ENSDART00000173662
ENSDART00000173672

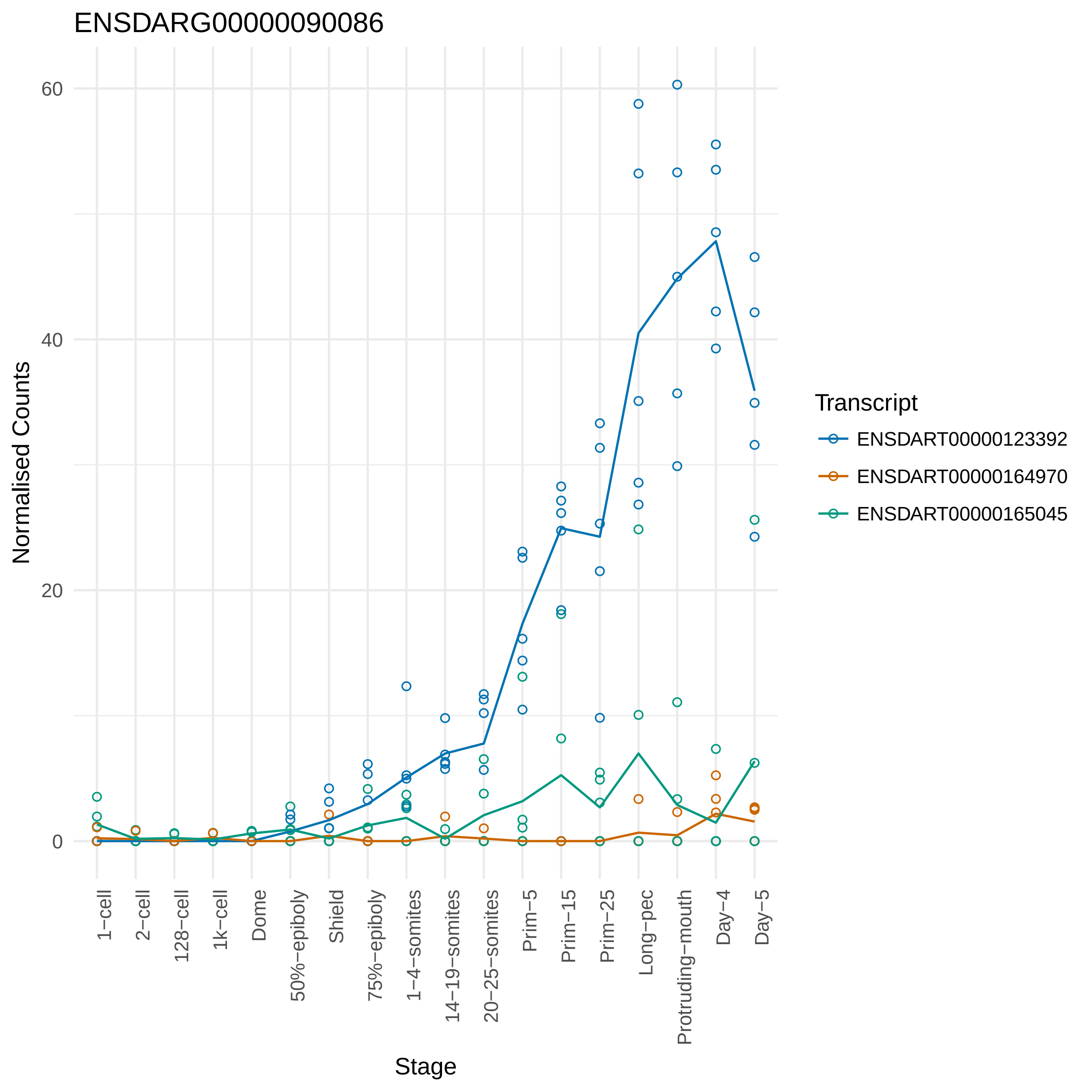

ENSDARG00000090086
Normalised Counts
60
40
20
0
1−cell
2−cell
128−cell
1k−cell
Dome
50%−epiboly
Shield
75%−epiboly
1−4−somites
14−19−somites
20−25−somites
Prim−5
Prim−15
Prim−25
Long−pec
Protruding−mouth
Day−4
Day−5
Stage
Transcript
ENSDART00000123392
ENSDART00000164970
ENSDART00000165045

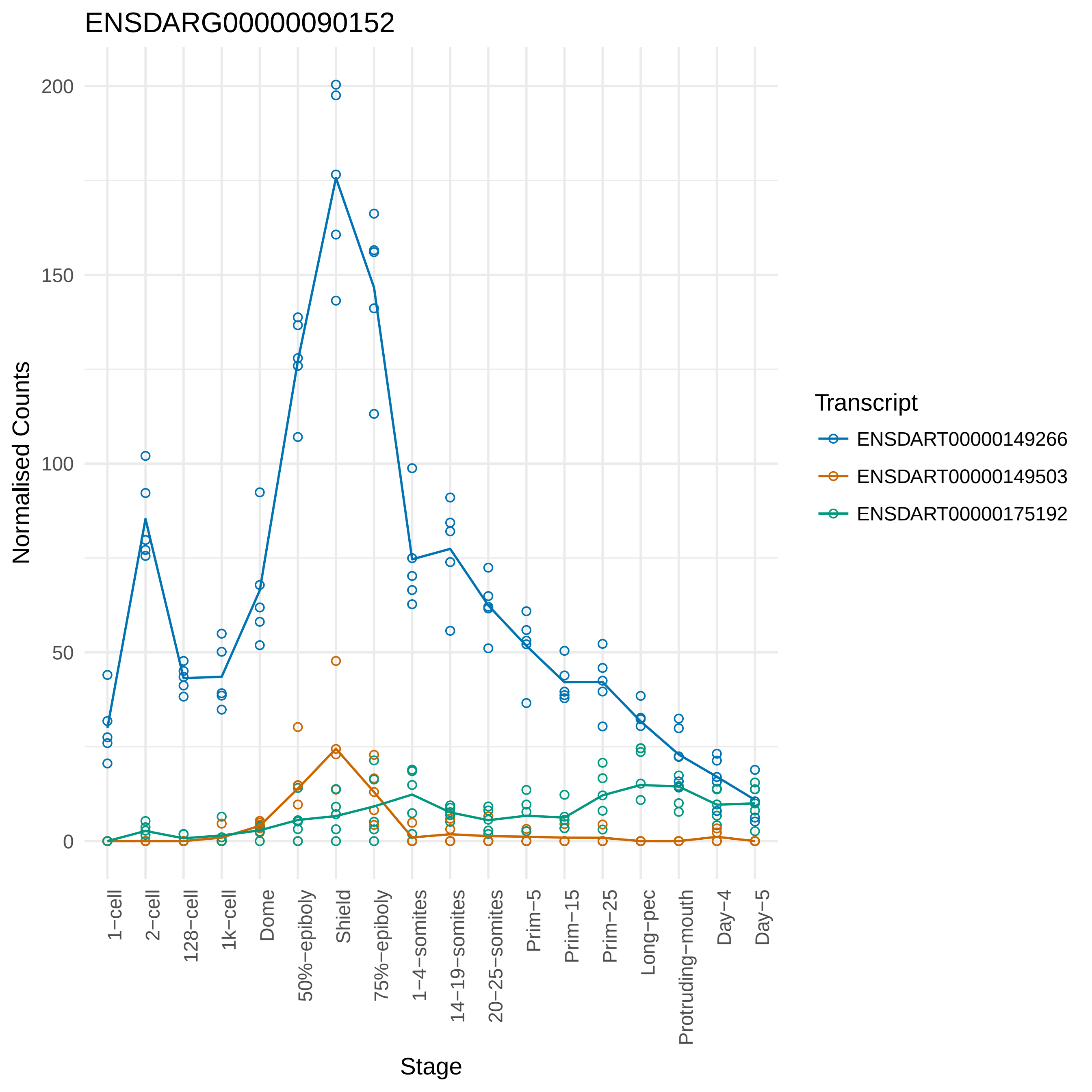

ENSDARG00000090152
Normalised Counts
200
150
100
50
0
1−cell
2−cell
128−cell
1k−cell
Dome
50%−epiboly
Shield
75%−epiboly
1−4−somites
14−19−somites
20−25−somites
Prim−5
Prim−15
Prim−25
Long−pec
Protruding−mouth
Day−4
Day−5
Stage
Transcript
ENSDART00000149266
ENSDART00000149503
ENSDART00000175192

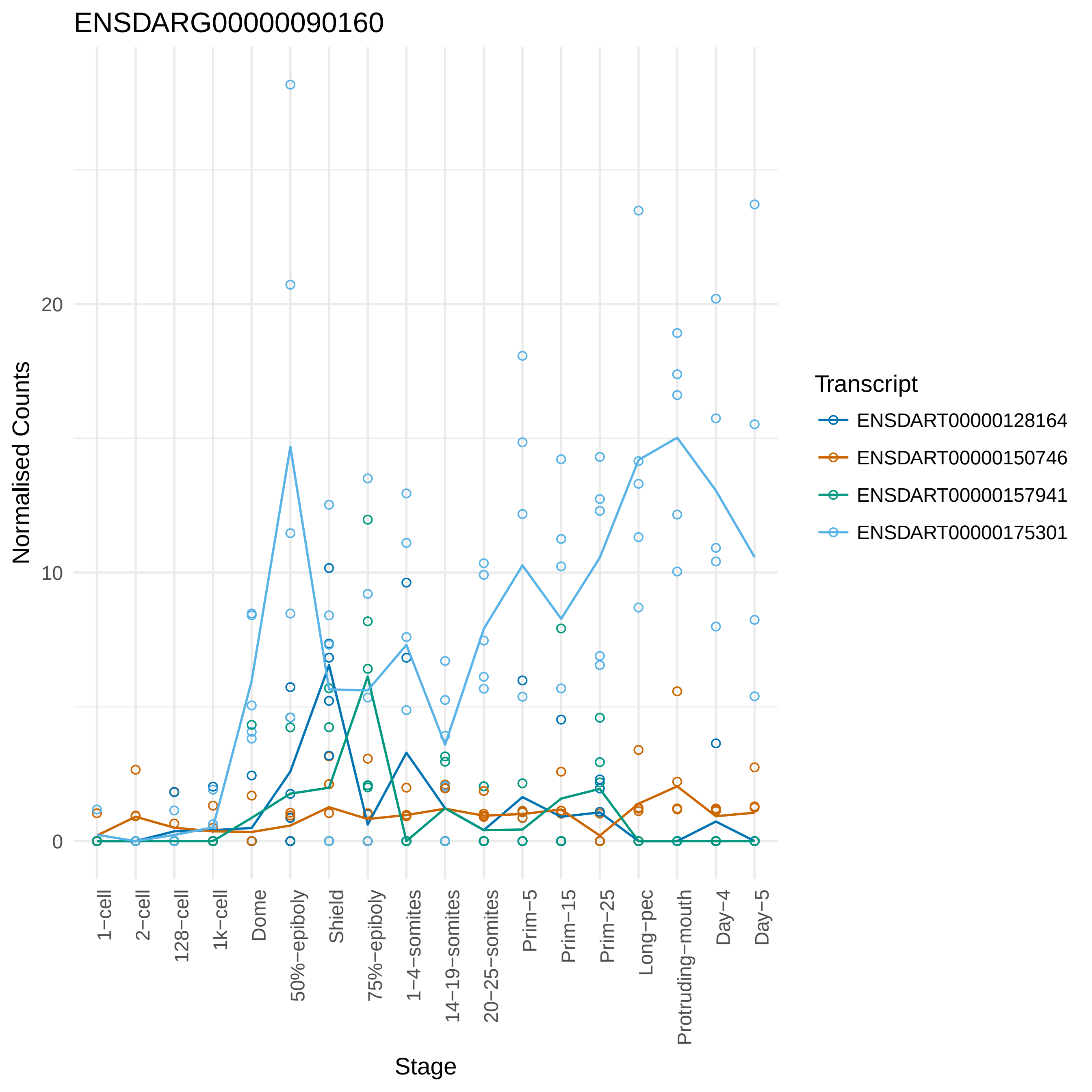
ENSDARG00000090160
Normalised Counts
0
10
20
Stage
1−cell
2−cell
128−cell
1k−cell
Dome
50%−epiboly
Shield
75%−epiboly
1−4−somites
14−19−somites
20−25−somites
Prim−5
Prim−15
Prim−25
Long−pec
Protruding−mouth
Day−4
Day−5
Transcript
ENSDART00000128164
ENSDART00000150746
ENSDART00000157941
ENSDART00000175301

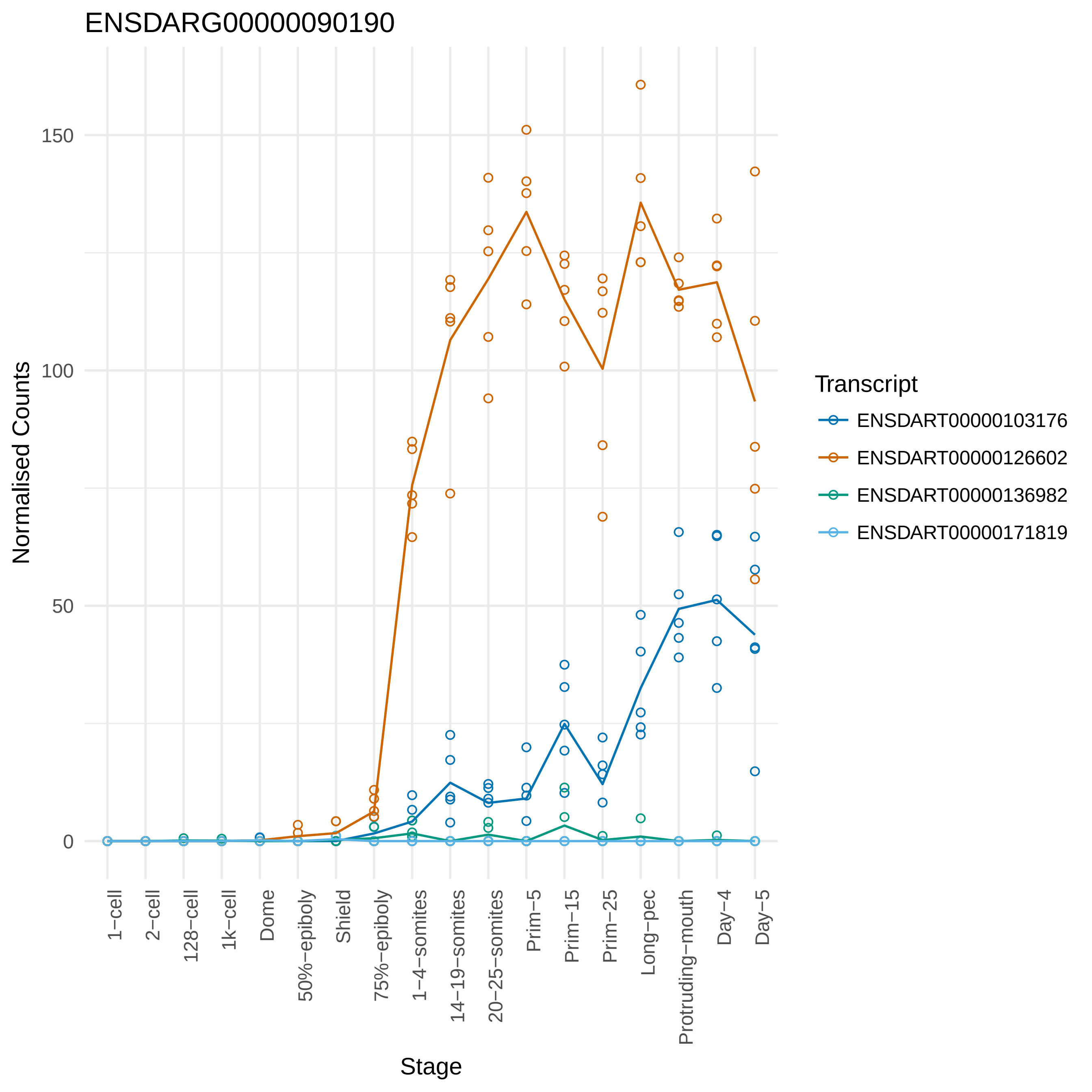
ENSDARG00000090190
Normalised Counts
150
100
50
0
1−cell
2−cell
128−cell
1k−cell
Dome
50%−epiboly
Shield
75%−epiboly
1−4−somites
14−19−somites
20−25−somites
Prim−5
Prim−15
Prim−25
Long−pec
Protruding−mouth
Day−4
Day−5
Stage
Transcript
ENSDART00000103176
ENSDART00000126602
ENSDART00000136982
ENSDART00000171819

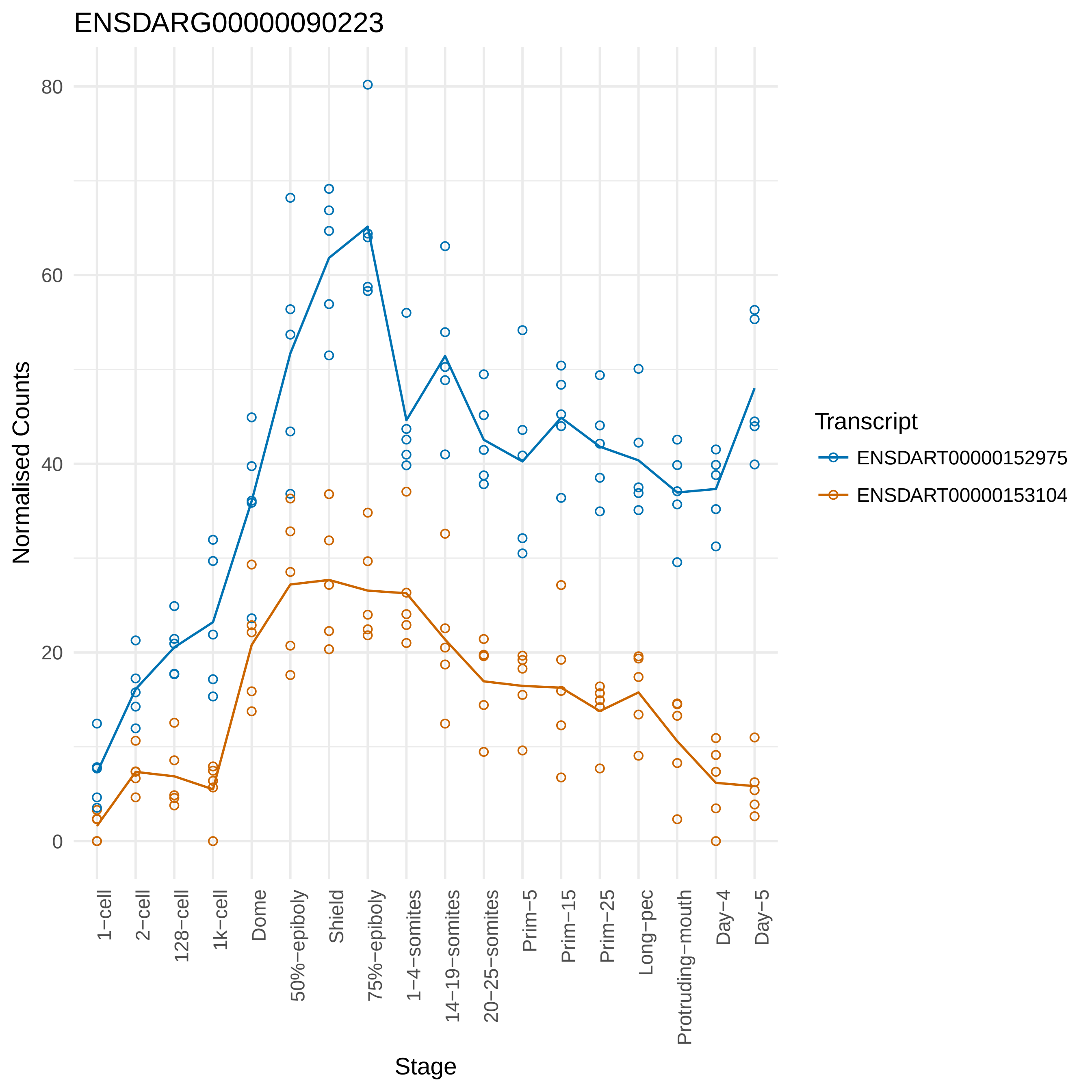
ENSDARG00000090223
Normalised Counts
80
60
40
20
0
1−cell
2−cell
128−cell
1k−cell
Dome
50%−epiboly
Shield
75%−epiboly
1−4−somites
14−19−somites
20−25−somites
Prim−5
Prim−15
Prim−25
Long−pec
Protruding−mouth
Day−4
Day−5
Stage
Transcript
ENSDART00000152975
ENSDART00000153104

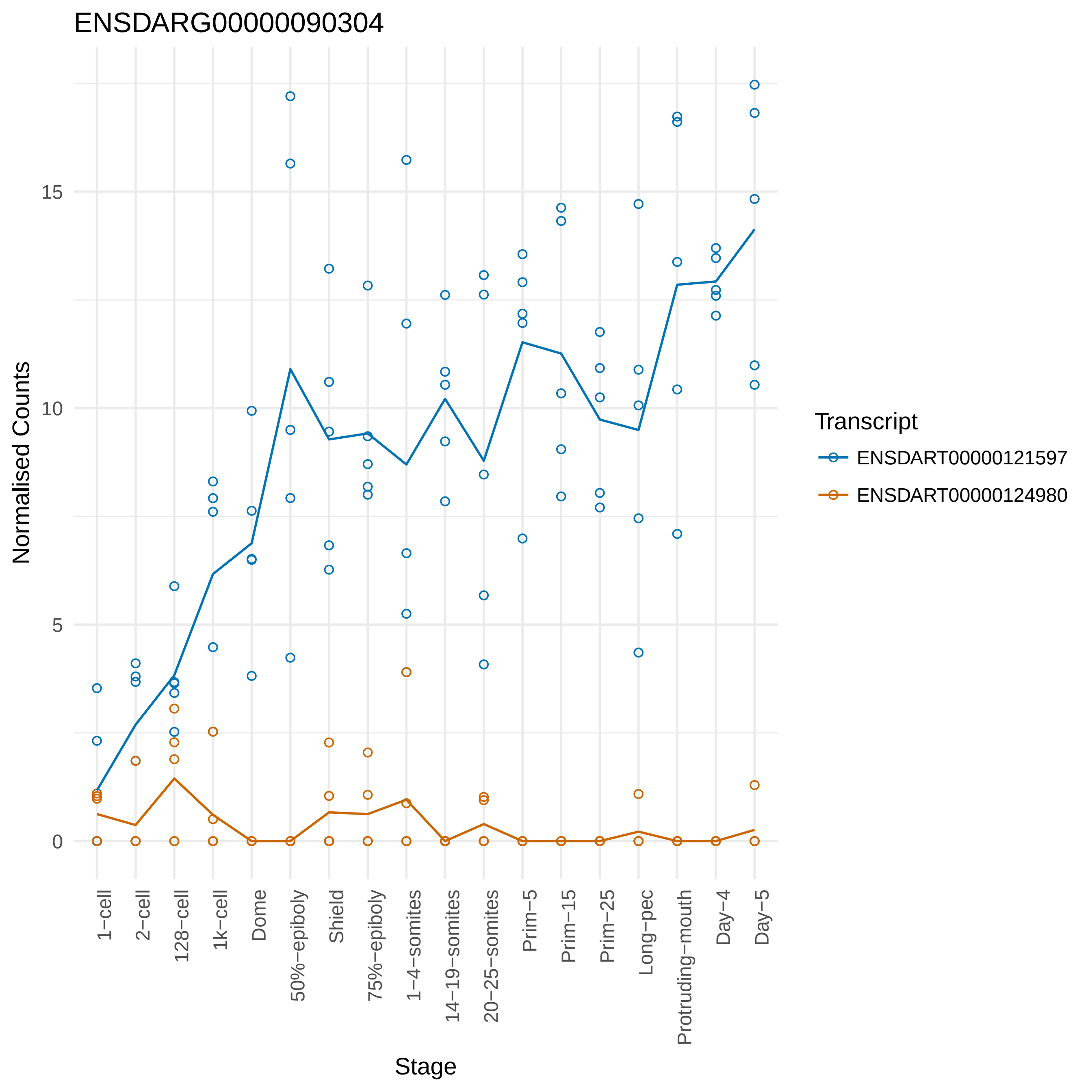

ENSDARG00000090304
Normalised Counts
15
10
5
0
1−cell
2−cell
128−cell
1k−cell
Dome
50%−epiboly
Shield
75%−epiboly
1−4−somites
14−19−somites
20−25−somites
Prim−5
Prim−15
Prim−25
Long−pec
Protruding−mouth
Day−4
Day−5
Stage
Transcript
ENSDART00000121597
ENSDART00000124980

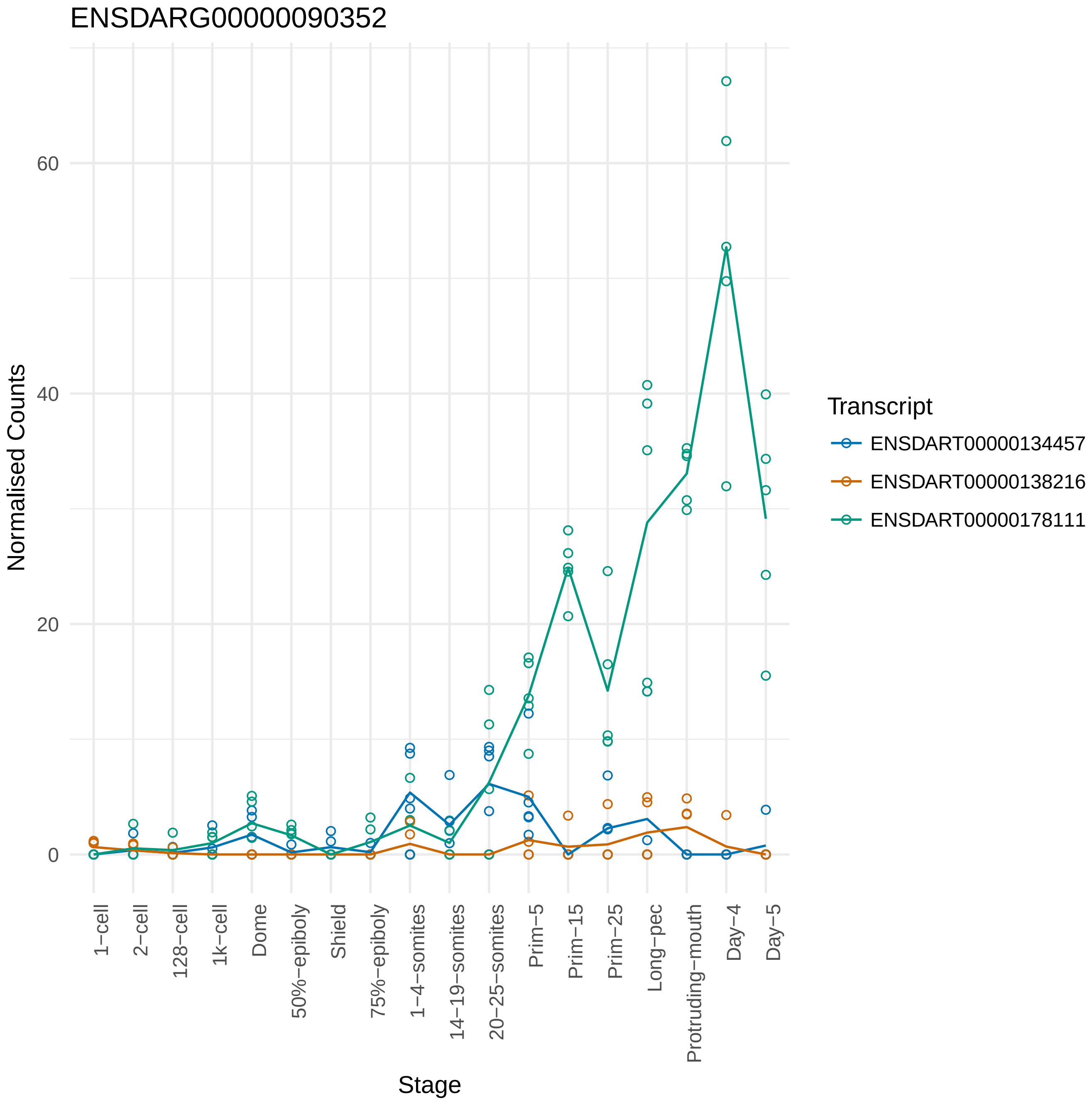
ENSDARG00000090352
Normalised Counts
0
20
40
60
Stage
1−cell
2−cell
128−cell
1k−cell
Dome
50%−epiboly
Shield
75%−epiboly
1−4−somites
14−19−somites
20−25−somites
Prim−5
Prim−15
Prim−25
Long−pec
Protruding−mouth
Day−4
Day−5
Transcript
ENSDART00000134457
ENSDART00000138216
ENSDART00000178111

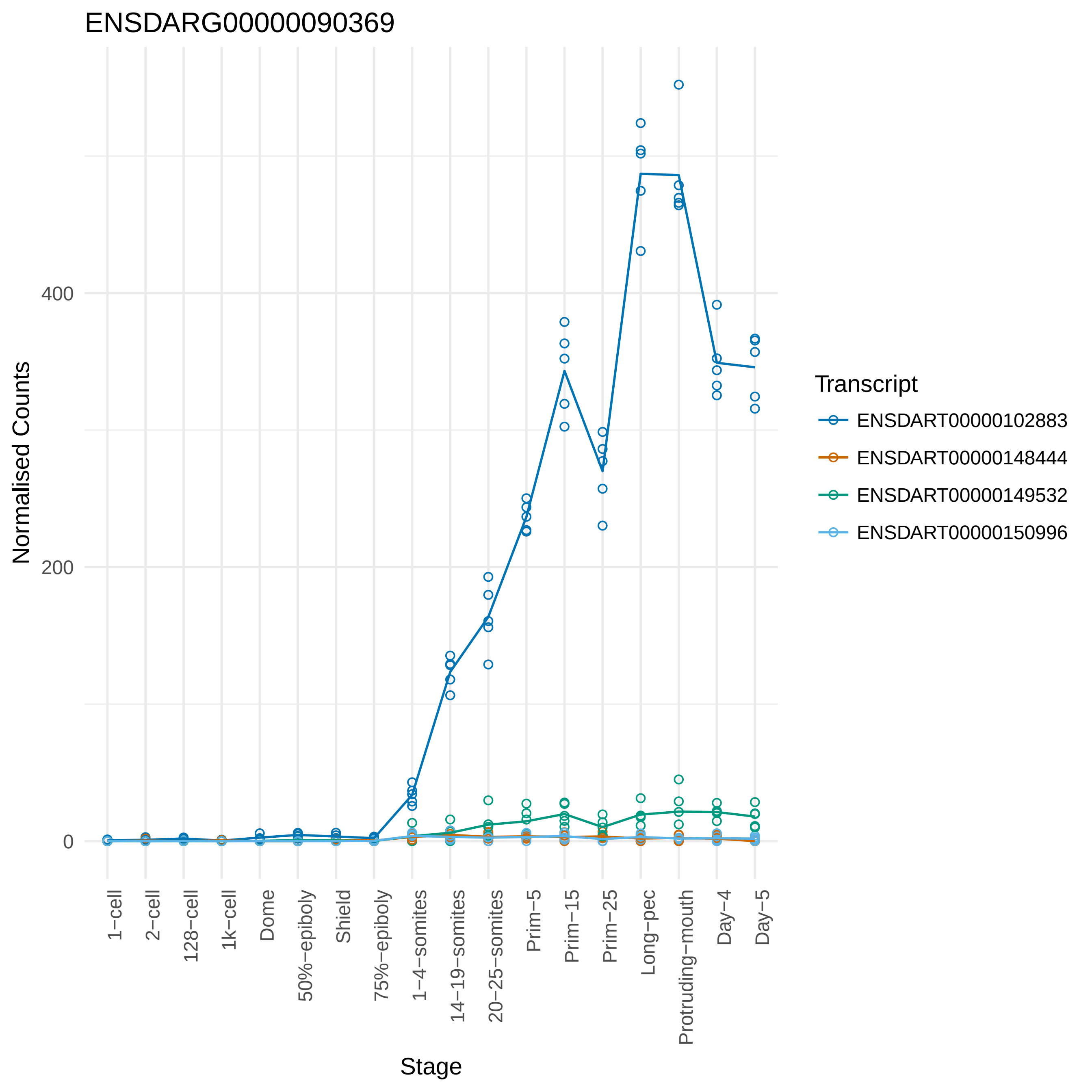

ENSDARG00000090369
Normalised Counts
400
200
0
1−cell
2−cell
128−cell
1k−cell
Dome
50%−epiboly
Shield
75%−epiboly
1−4−somites
14−19−somites
20−25−somites
Prim−5
Prim−15
Prim−25
Long−pec
Protruding−mouth
Day−4
Day−5
Stage
Transcript
ENSDART00000102883
ENSDART00000148444
ENSDART00000149532
ENSDART00000150996

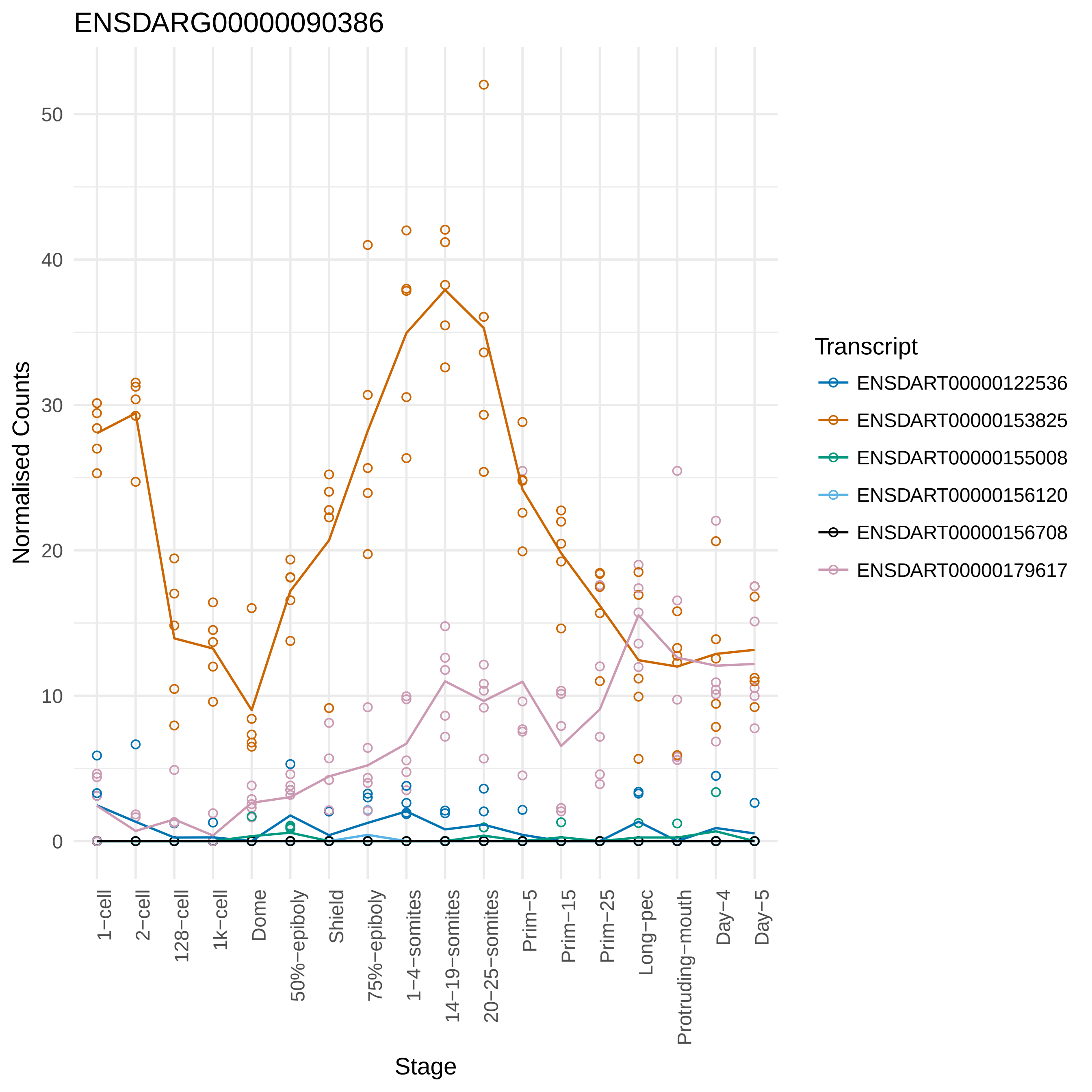

ENSDARG00000090386
Normalised Counts
0
10
20
30
40
50
Stage
1−cell
2−cell
128−cell
1k−cell
Dome
50%−epiboly
Shield
75%−epiboly
1−4−somites
14−19−somites
20−25−somites
Prim−5
Prim−15
Prim−25
Long−pec
Protruding−mouth
Day−4
Day−5
Transcript
ENSDART00000122536
ENSDART00000153825
ENSDART00000155008
ENSDART00000156120
ENSDART00000156708
ENSDART00000179617

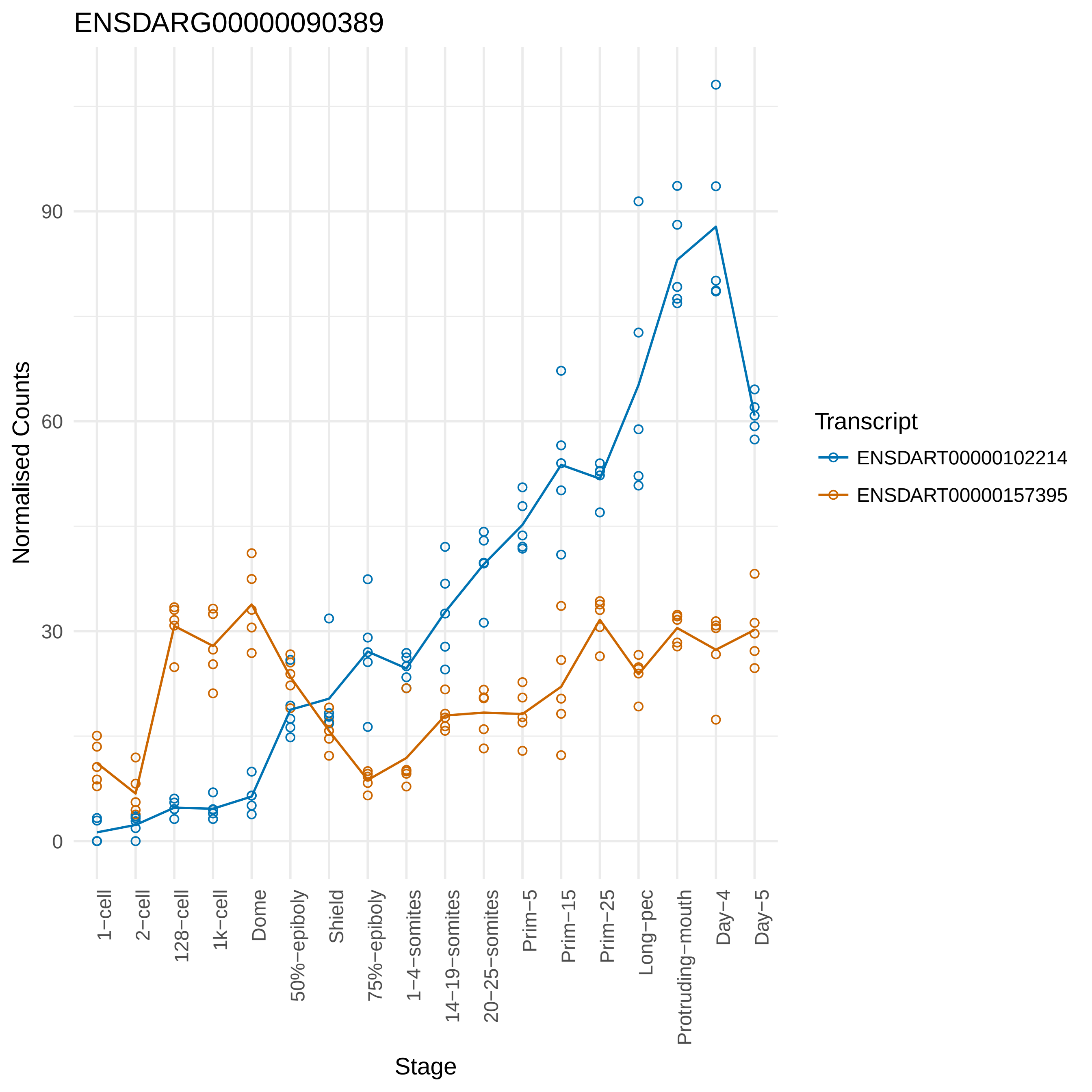

ENSDARG00000090389
Normalised Counts
0
30
60
90
Stage
1−cell
2−cell
128−cell
1k−cell
Dome
50%−epiboly
Shield
75%−epiboly
1−4−somites
14−19−somites
20−25−somites
Prim−5
Prim−15
Prim−25
Long−pec
Protruding−mouth
Day−4
Day−5
Transcript
ENSDART00000102214
ENSDART00000157395

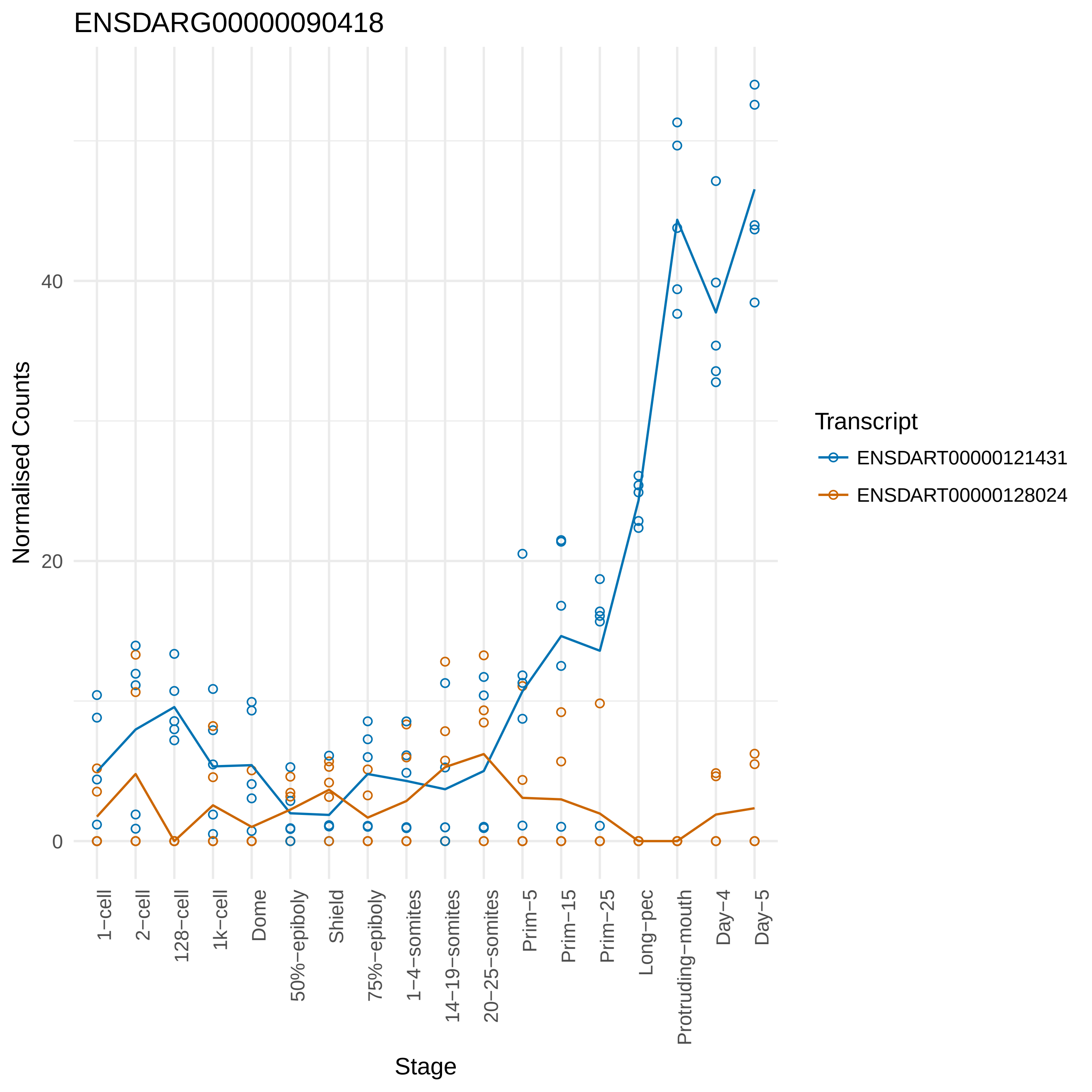

ENSDARG00000090418
Normalised Counts
0
20
40
Stage
1−cell
2−cell
128−cell
1k−cell
Dome
50%−epiboly
Shield
75%−epiboly
1−4−somites
14−19−somites
20−25−somites
Prim−5
Prim−15
Prim−25
Long−pec
Protruding−mouth
Day−4
Day−5
Transcript
ENSDART00000121431
ENSDART00000128024

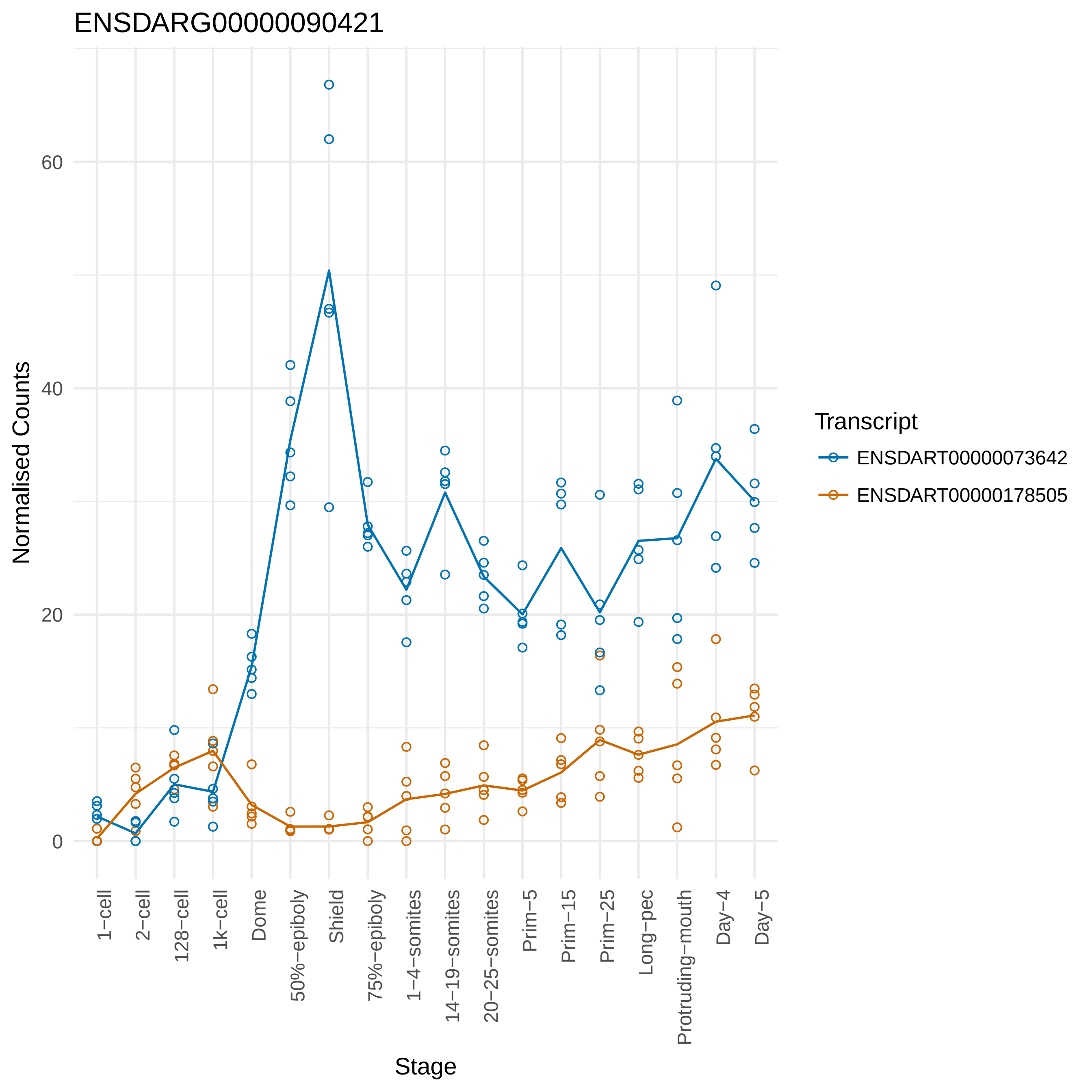

ENSDARG00000090421
Normalised Counts
0
20
40
60
1−cell
2−cell
128−cell
1k−cell
Dome
50%−epiboly
Shield
75%−epiboly
1−4−somites
14−19−somites
20−25−somites
Prim−5
Prim−15
Prim−25
Long−pec
Protruding−mouth
Day−4
Day−5
Stage
Transcript
ENSDART00000073642
ENSDART00000178505

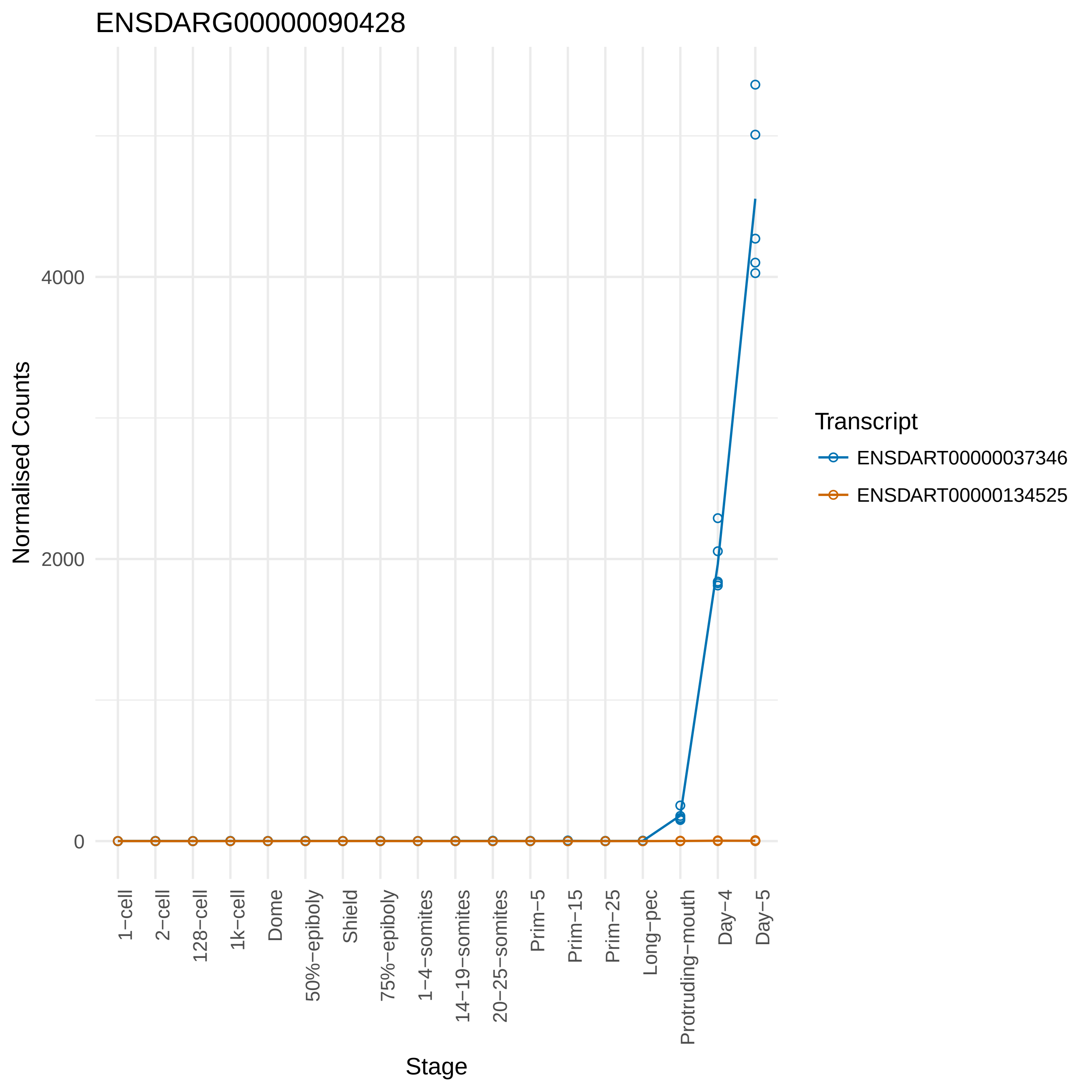
ENSDARG00000090428
Normalised Counts
4000
2000
0
1−cell
2−cell
128−cell
1k−cell
Dome
50%−epiboly
Shield
75%−epiboly
1−4−somites
14−19−somites
20−25−somites
Prim−5
Prim−15
Prim−25
Long−pec
Protruding−mouth
Day−4
Day−5
Stage
Transcript
ENSDART00000037346
ENSDART00000134525

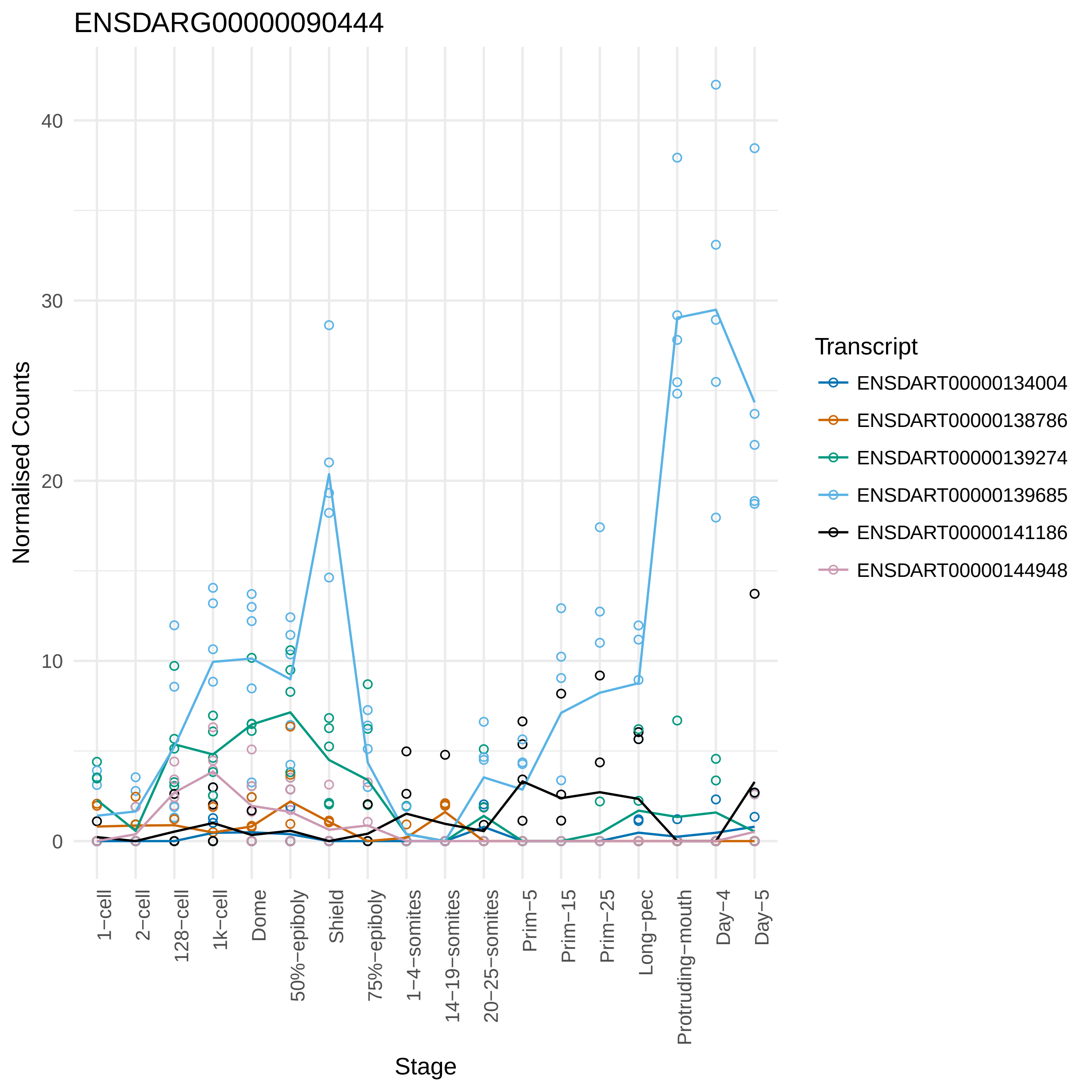

ENSDARG00000090444
Normalised Counts
0
10
20
30
40
1−cell
2−cell
128−cell
1k−cell
Dome
50%−epiboly
Shield
75%−epiboly
1−4−somites
14−19−somites
20−25−somites
Prim−5
Prim−15
Prim−25
Long−pec
Protruding−mouth
Day−4
Day−5
Stage
Transcript
ENSDART00000134004
ENSDART00000138786
ENSDART00000139274
ENSDART00000139685
ENSDART00000141186
ENSDART00000144948

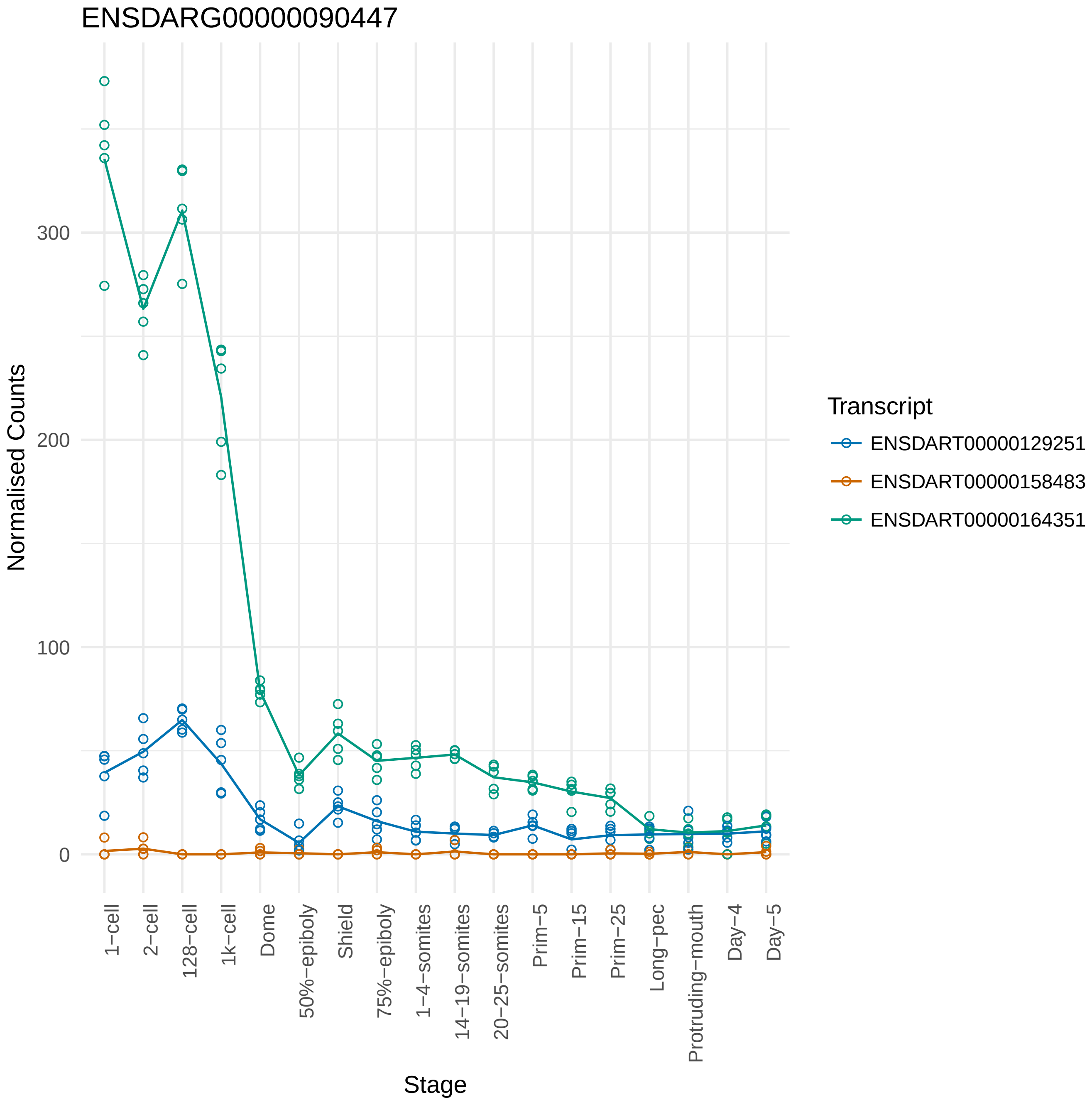
ENSDARG00000090447
Normalised Counts
300
200
100
0
1−cell
2−cell
128−cell
1k−cell
Dome
50%−epiboly
Shield
75%−epiboly
1−4−somites
14−19−somites
20−25−somites
Prim−5
Prim−15
Prim−25
Long−pec
Protruding−mouth
Day−4
Day−5
Stage
Transcript
ENSDART00000129251
ENSDART00000158483
ENSDART00000164351

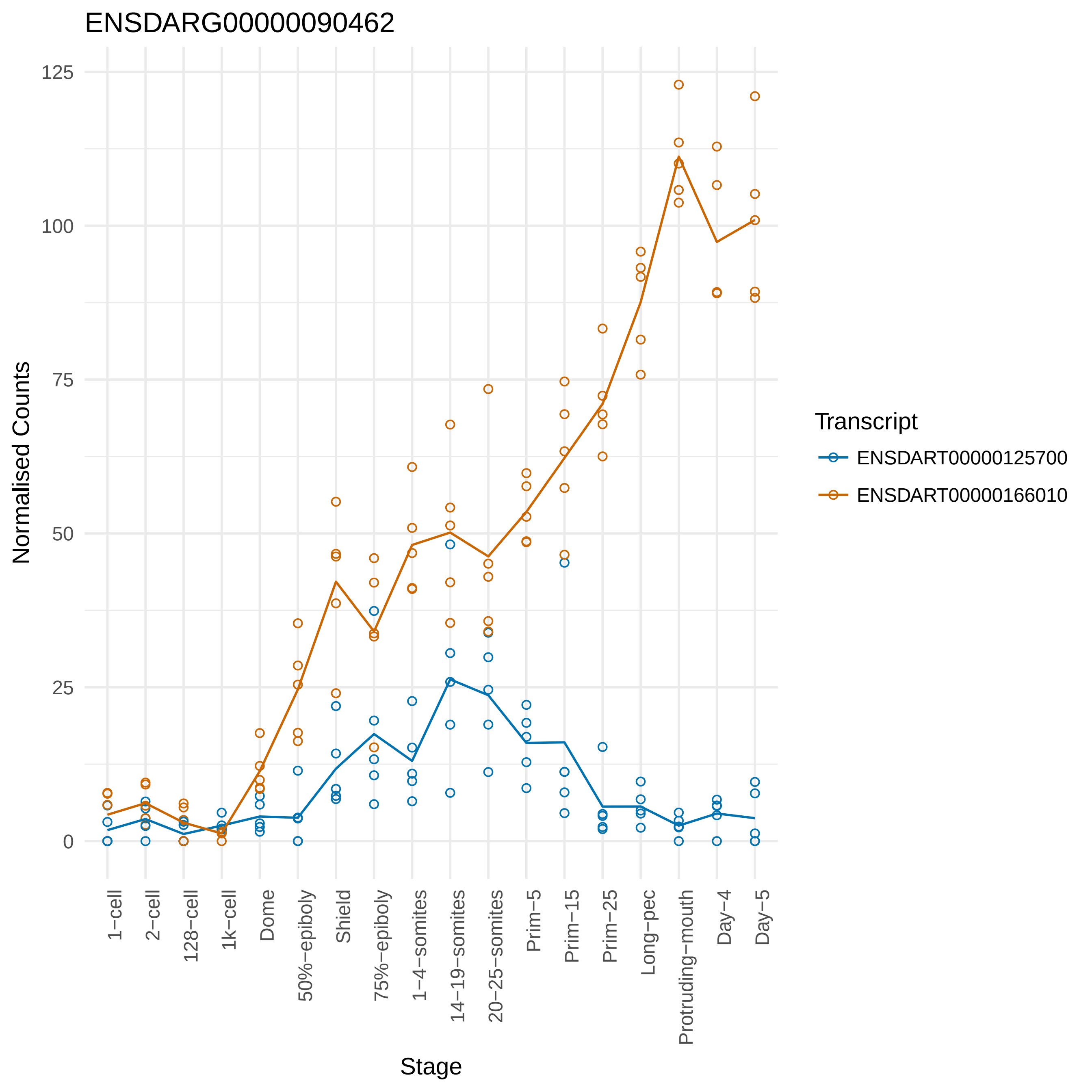
ENSDARG00000090462
Normalised Counts
125
100
75
50
25
0
1−cell
2−cell
128−cell
1k−cell
Dome
50%−epiboly
Shield
75%−epiboly
1−4−somites
14−19−somites
20−25−somites
Prim−5
Prim−15
Prim−25
Long−pec
Protruding−mouth
Day−4
Day−5
Stage
Transcript
ENSDART00000125700
ENSDART00000166010

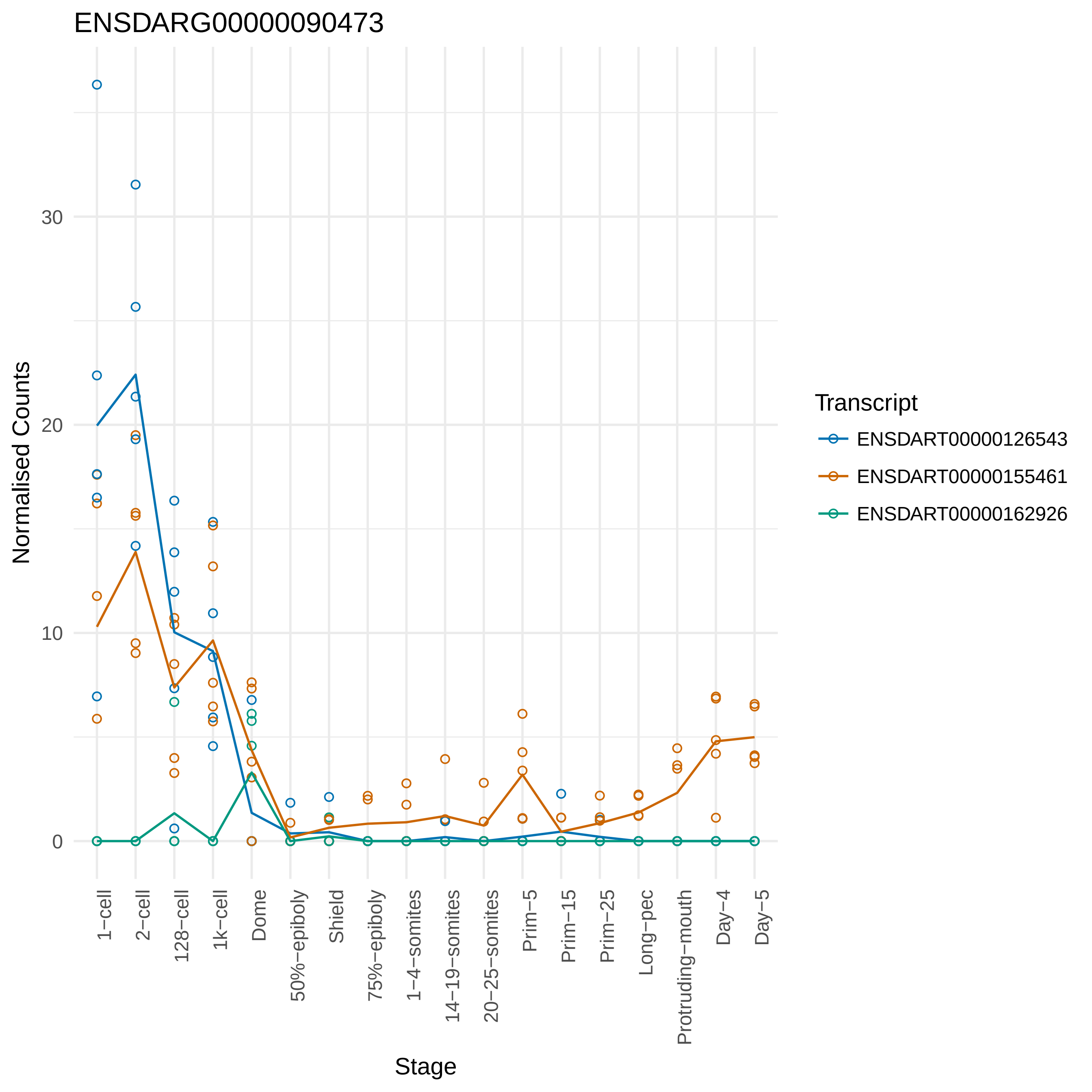

ENSDARG00000090473
Normalised Counts
0
10
20
30
1−cell
2−cell
128−cell
1k−cell
Dome
50%−epiboly
Shield
75%−epiboly
1−4−somites
14−19−somites
20−25−somites
Prim−5
Prim−15
Prim−25
Long−pec
Protruding−mouth
Day−4
Day−5
Stage
Transcript
ENSDART00000126543
ENSDART00000155461
ENSDART00000162926

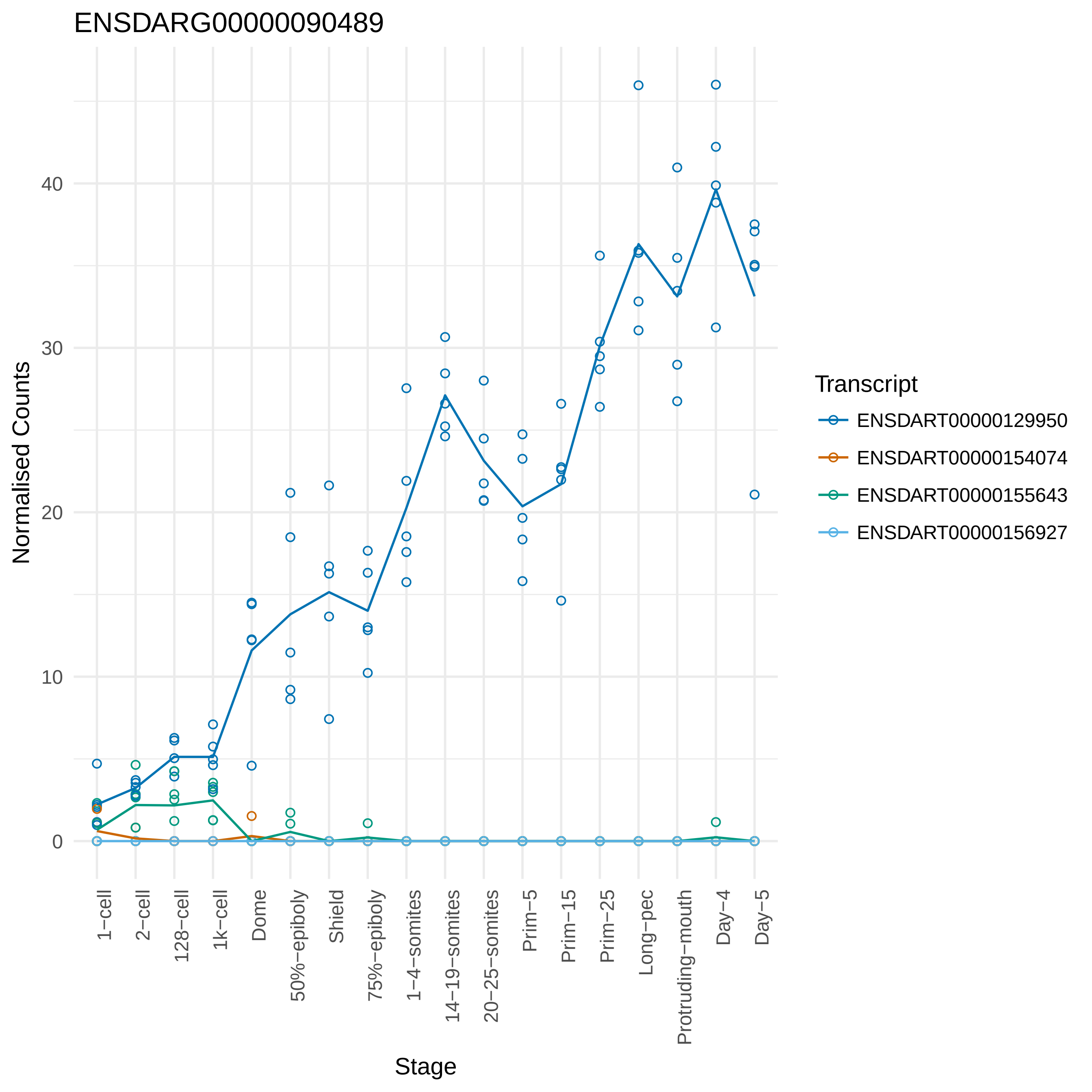

ENSDARG00000090489
Normalised Counts
0
10
20
30
40
Stage
1−cell
2−cell
128−cell
1k−cell
Dome
50%−epiboly
Shield
75%−epiboly
1−4−somites
14−19−somites
20−25−somites
Prim−5
Prim−15
Prim−25
Long−pec
Protruding−mouth
Day−4
Day−5
Transcript
ENSDART00000129950
ENSDART00000154074
ENSDART00000155643
ENSDART00000156927

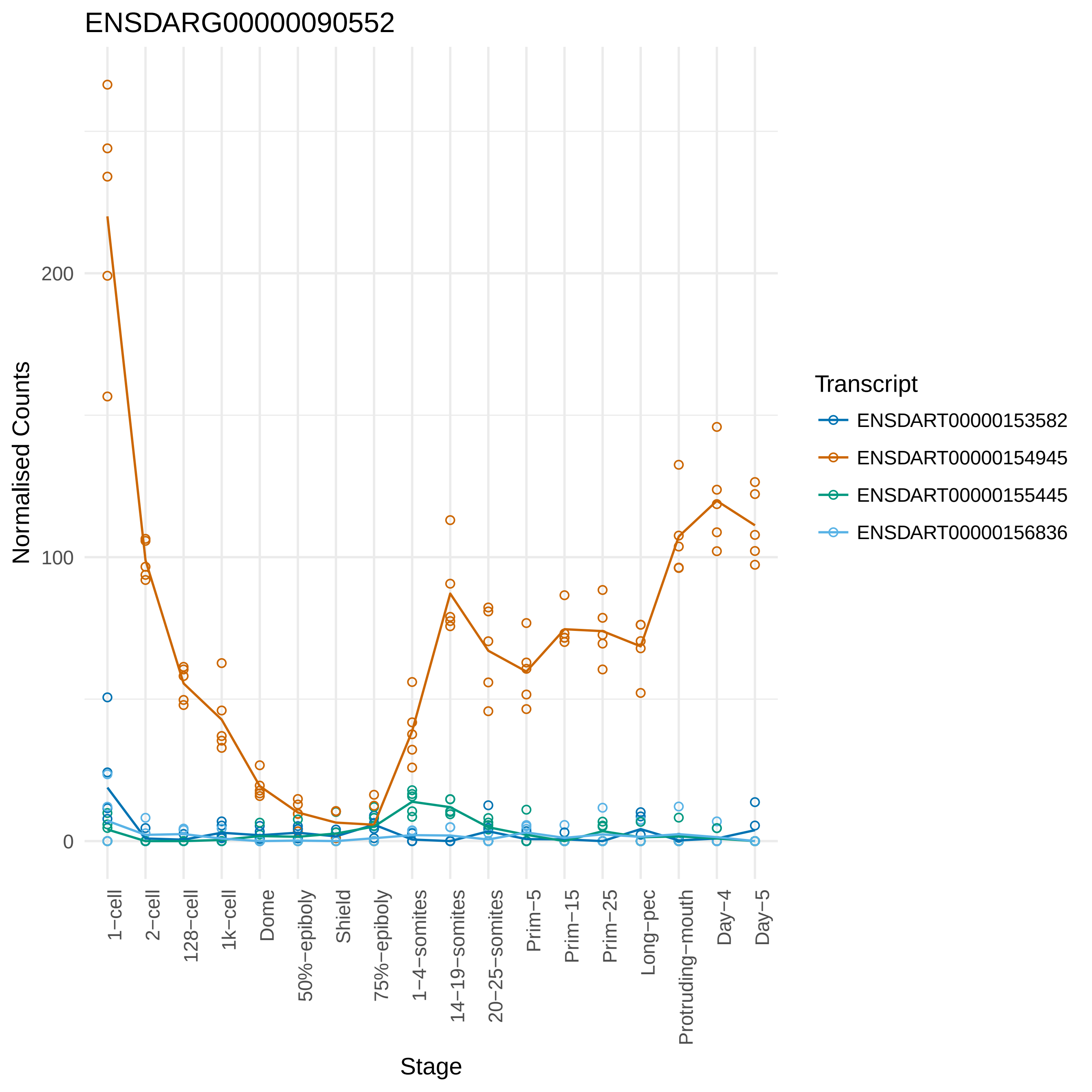

ENSDARG00000090552
Normalised Counts
200
100
0
1−cell
2−cell
128−cell
1k−cell
Dome
50%−epiboly
Shield
75%−epiboly
1−4−somites
14−19−somites
20−25−somites
Prim−5
Prim−15
Prim−25
Long−pec
Protruding−mouth
Day−4
Day−5
Stage
Transcript
ENSDART00000153582
ENSDART00000154945
ENSDART00000155445
ENSDART00000156836

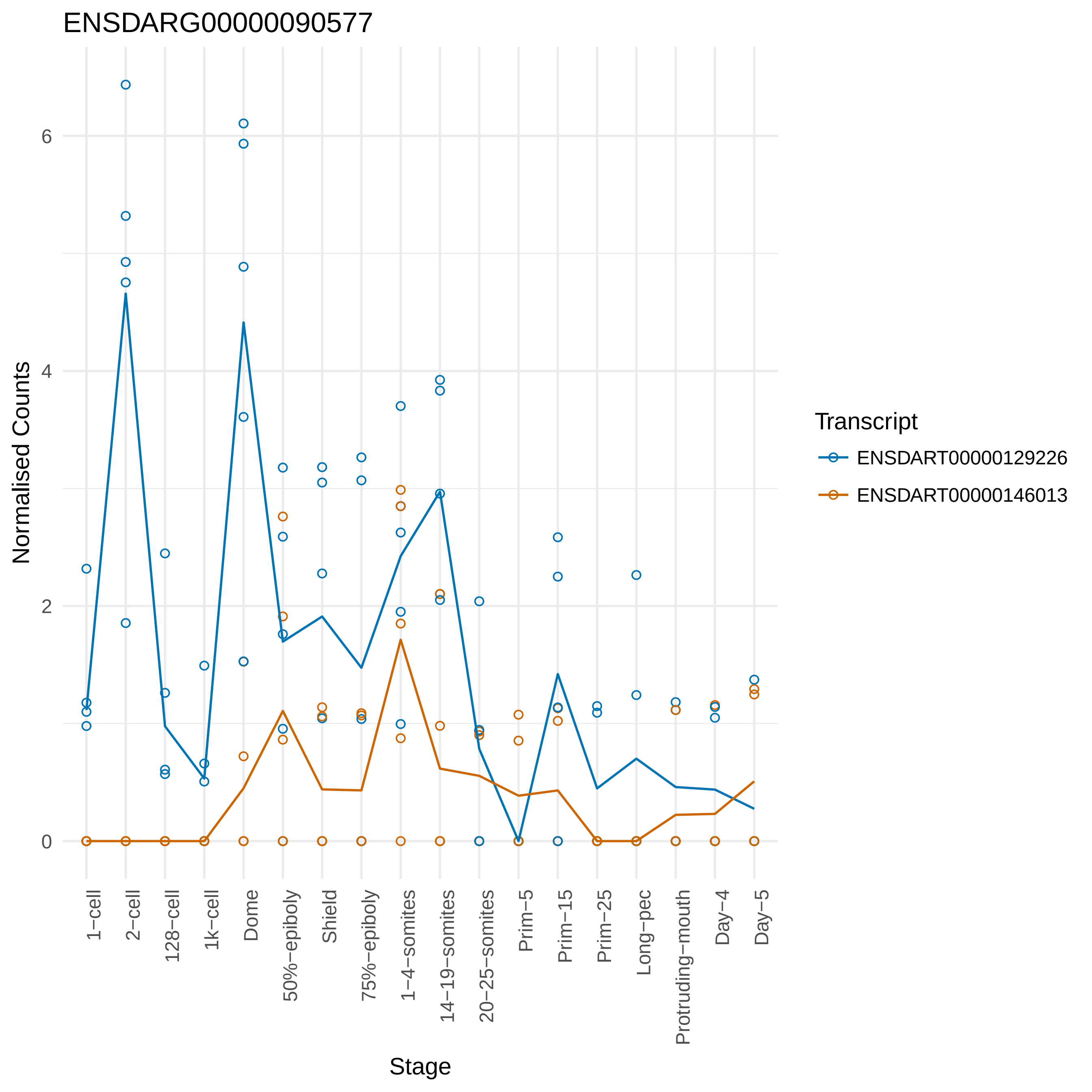

ENSDARG00000090577
Normalised Counts
0
2
4
6
1−cell
2−cell
128−cell
1k−cell
Dome
50%−epiboly
Shield
75%−epiboly
1−4−somites
14−19−somites
20−25−somites
Prim−5
Prim−15
Prim−25
Long−pec
Protruding−mouth
Day−4
Day−5
Stage
Transcript
ENSDART00000129226
ENSDART00000146013

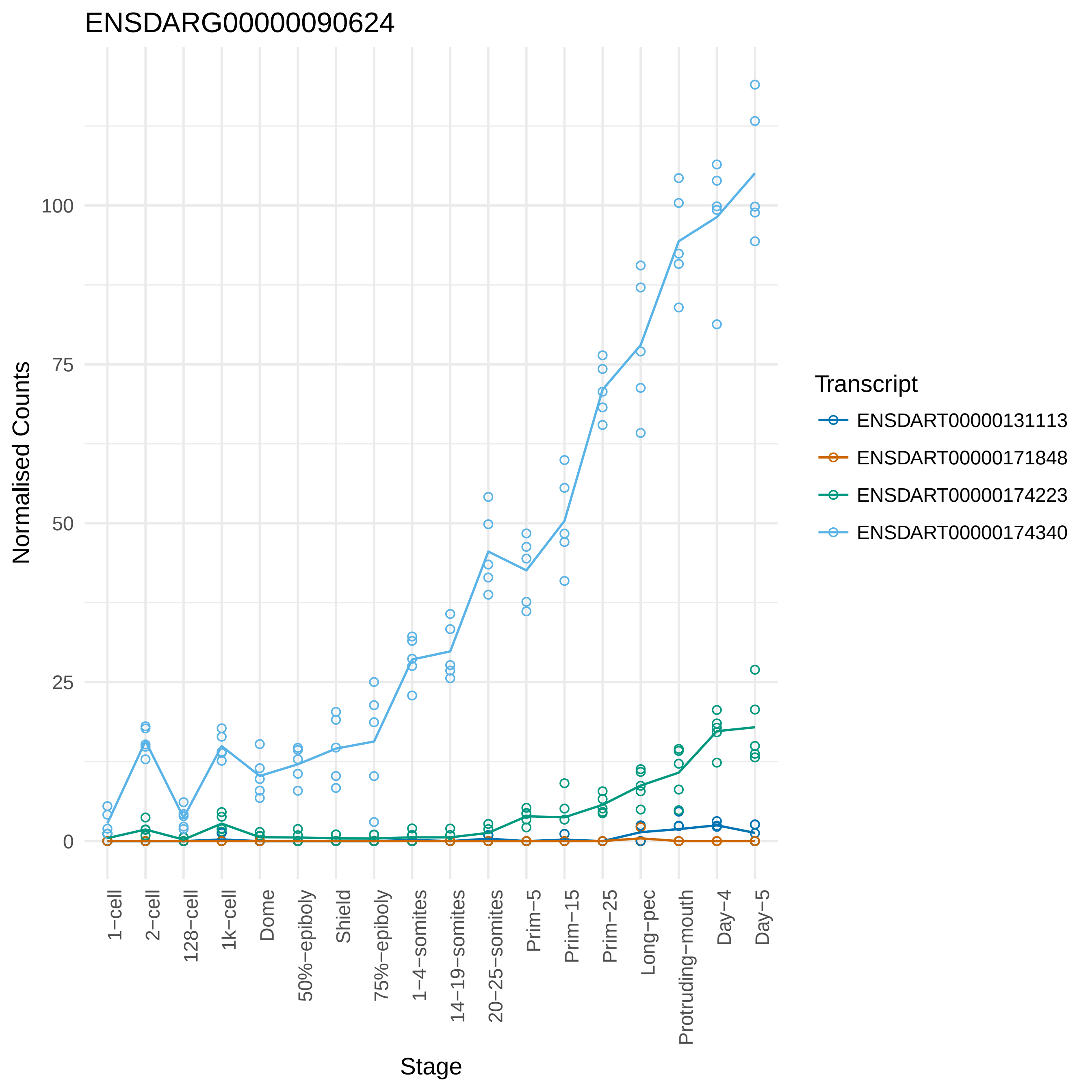
ENSDARG00000090624
Normalised Counts
0
25
50
75
100
1−cell
2−cell
128−cell
1k−cell
Dome
50%−epiboly
Shield
75%−epiboly
1−4−somites
14−19−somites
20−25−somites
Prim−5
Prim−15
Prim−25
Long−pec
Protruding−mouth
Day−4
Day−5
Stage
Transcript
ENSDART00000131113
ENSDART00000171848
ENSDART00000174223
ENSDART00000174340

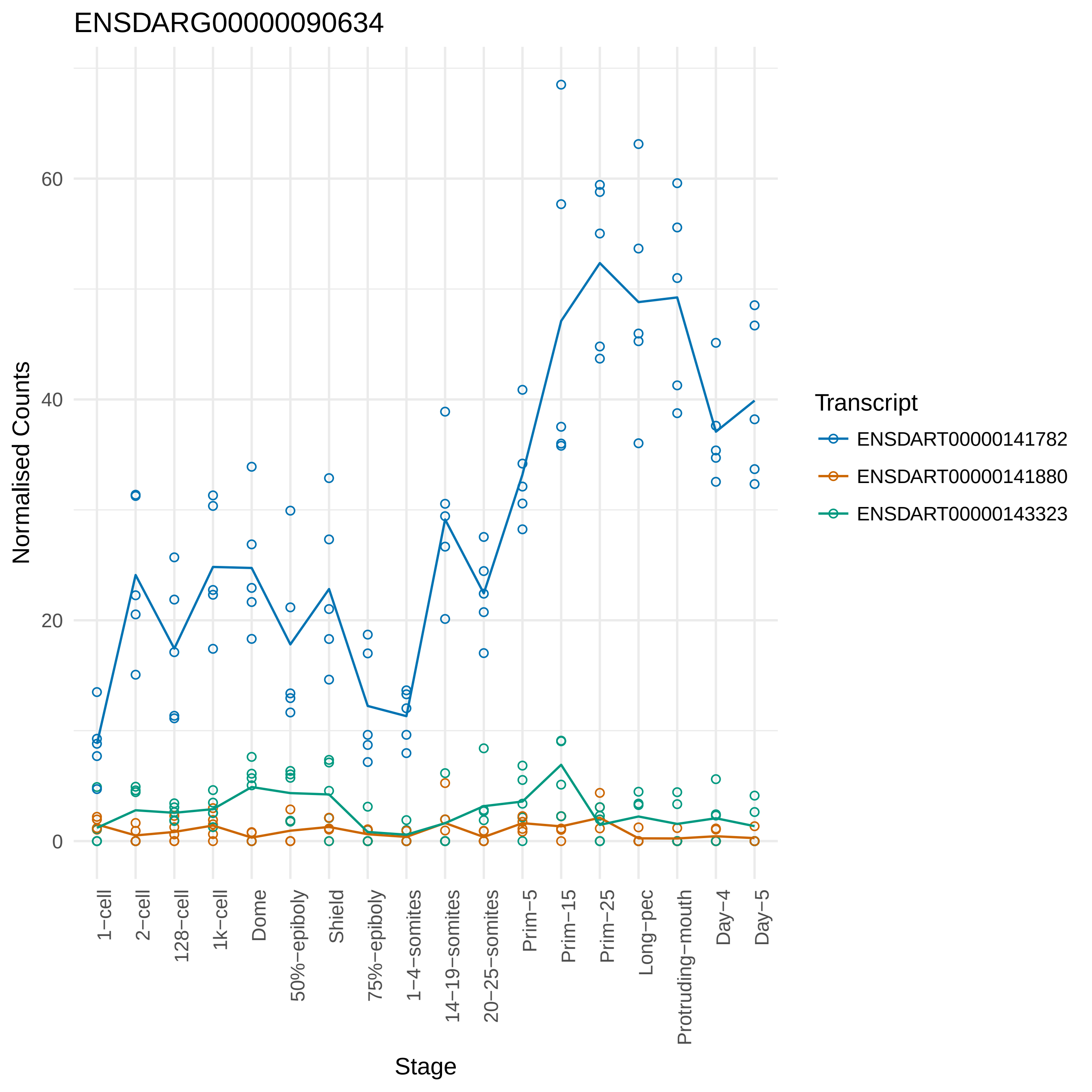

ENSDARG00000090634
Normalised Counts
0
20
40
60
Stage
1−cell
2−cell
128−cell
1k−cell
Dome
50%−epiboly
Shield
75%−epiboly
1−4−somites
14−19−somites
20−25−somites
Prim−5
Prim−15
Prim−25
Long−pec
Protruding−mouth
Day−4
Day−5
Transcript
ENSDART00000141782
ENSDART00000141880
ENSDART00000143323

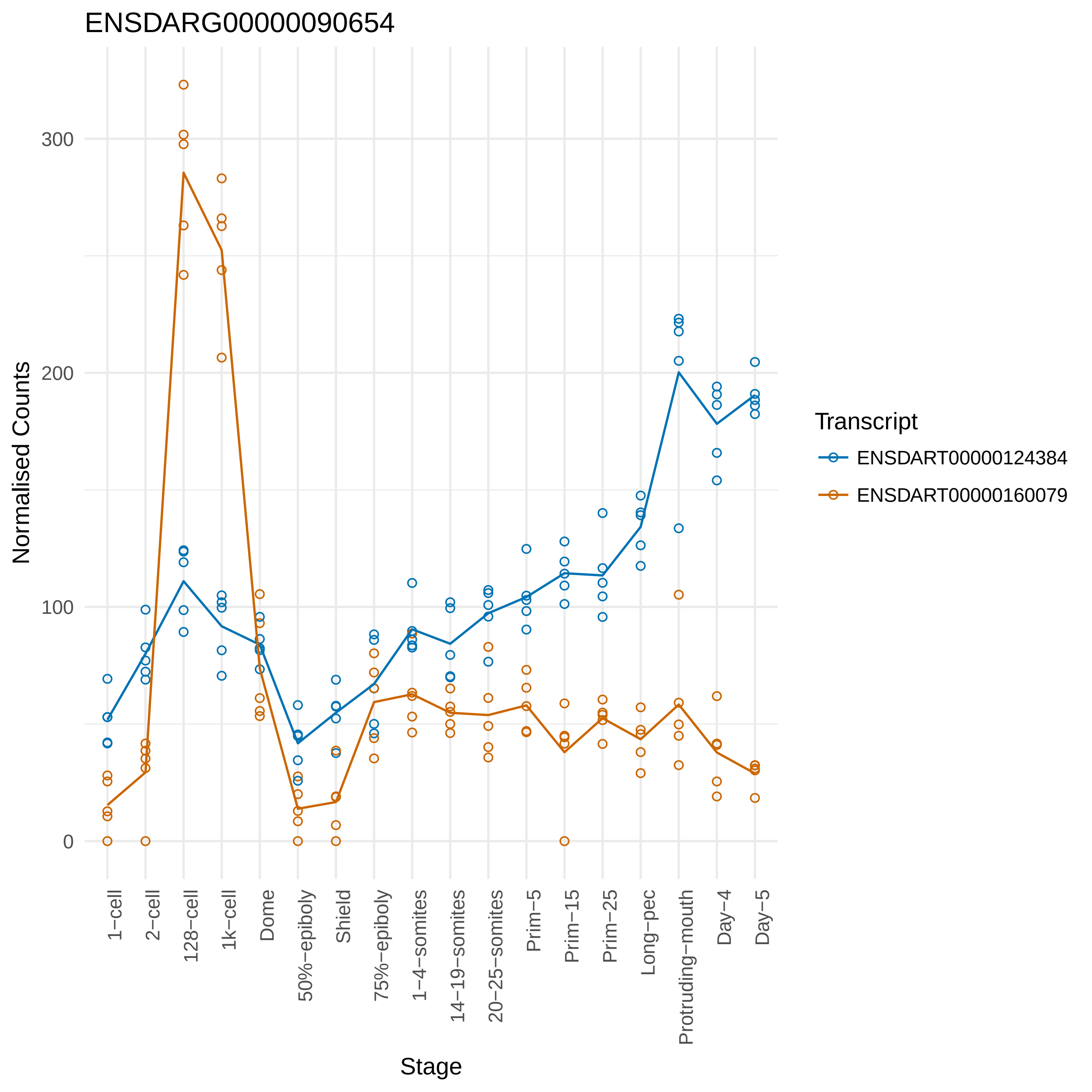

ENSDARG00000090654
Normalised Counts
300
200
100
0
1−cell
2−cell
128−cell
1k−cell
Dome
50%−epiboly
Shield
75%−epiboly
1−4−somites
14−19−somites
20−25−somites
Prim−5
Prim−15
Prim−25
Long−pec
Protruding−mouth
Day−4
Day−5
Stage
Transcript
ENSDART00000124384
ENSDART00000160079

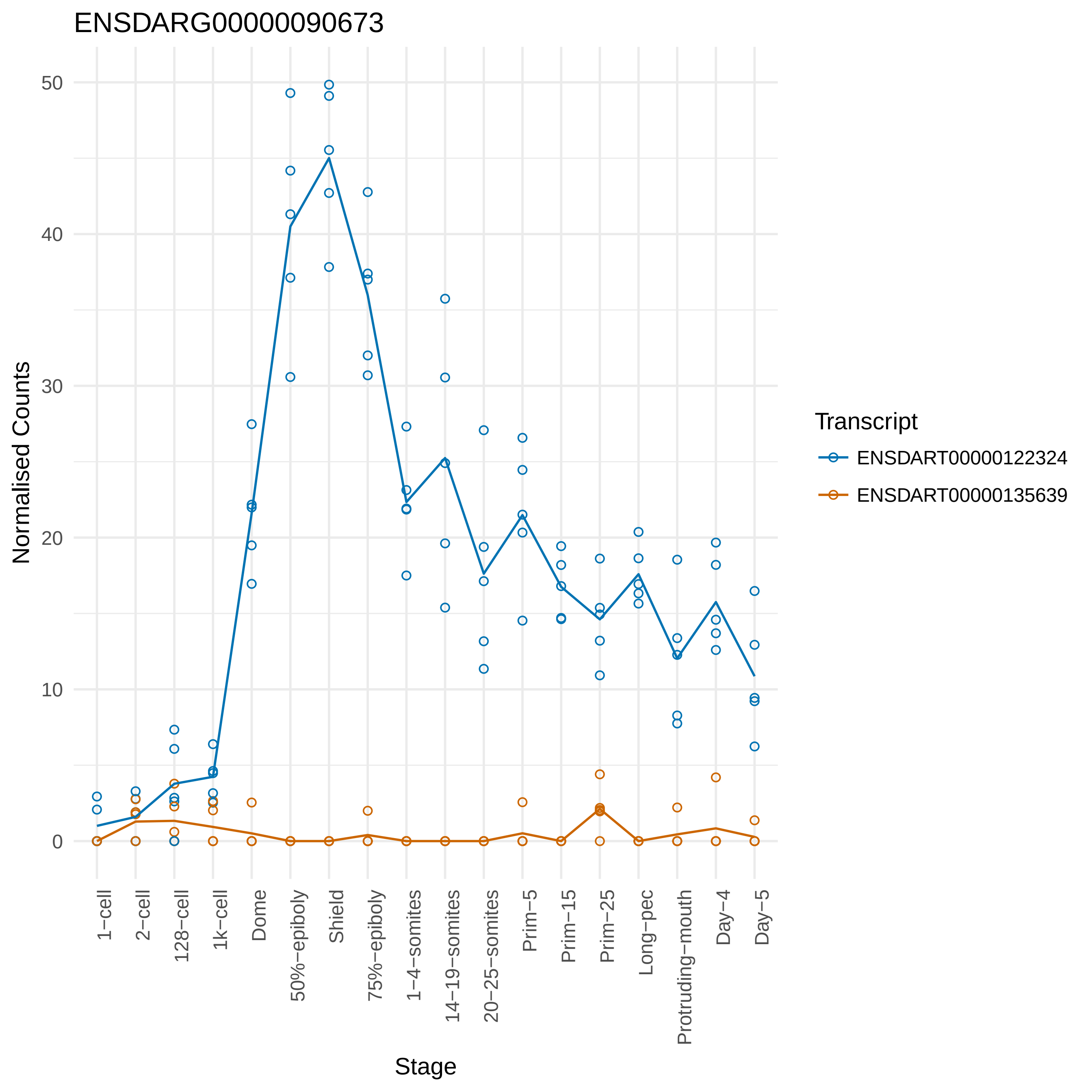

ENSDARG00000090673
Normalised Counts
0
10
20
30
40
50
1−cell
2−cell
128−cell
1k−cell
Dome
50%−epiboly
Shield
75%−epiboly
1−4−somites
14−19−somites
20−25−somites
Prim−5
Prim−15
Prim−25
Long−pec
Protruding−mouth
Day−4
Day−5
Stage
Transcript
ENSDART00000122324
ENSDART00000135639

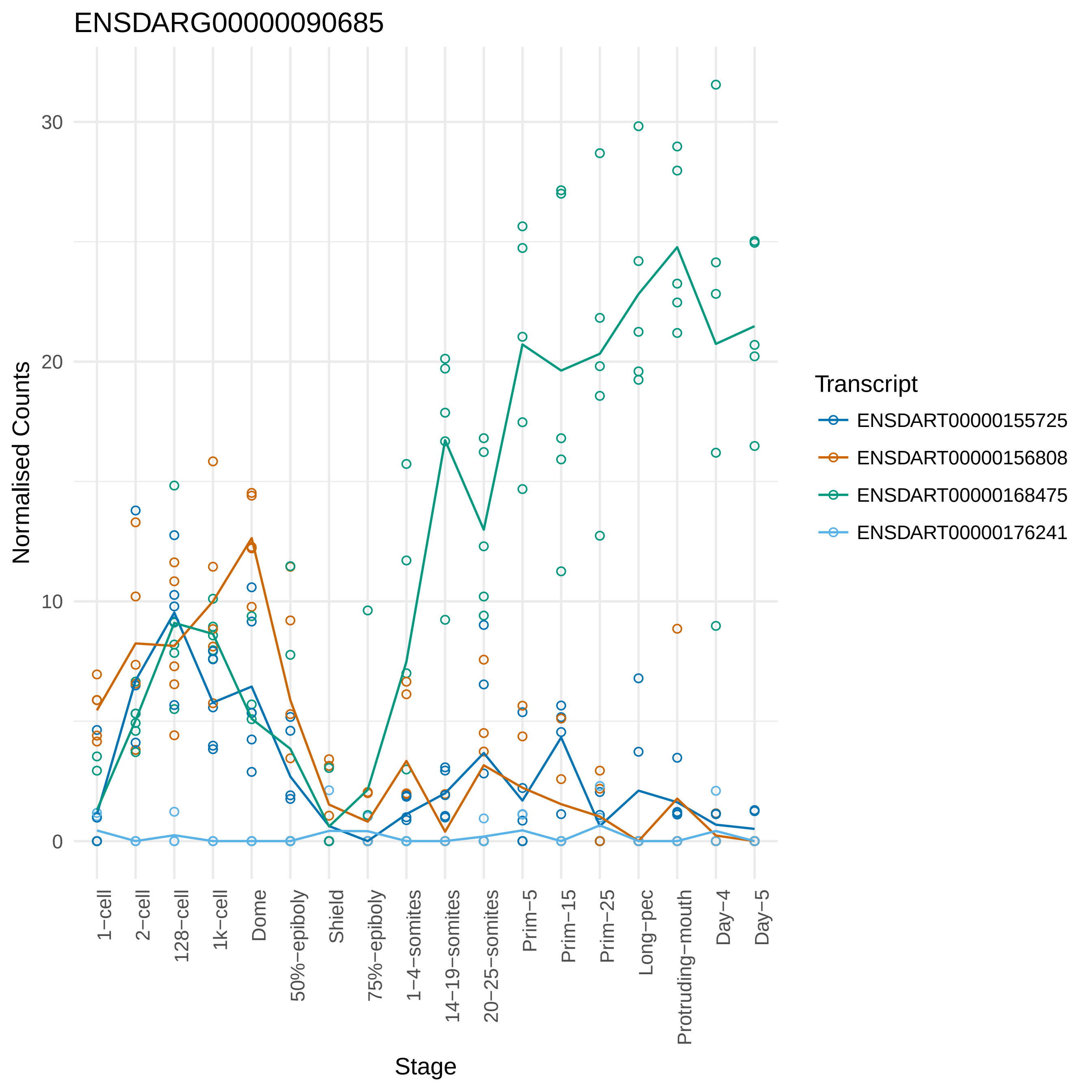

ENSDARG00000090685
Normalised Counts
30
20
10
0
1−cell
2−cell
128−cell
1k−cell
Dome
50%−epiboly
Shield
75%−epiboly
1−4−somites
14−19−somites
20−25−somites
Prim−5
Prim−15
Prim−25
Long−pec
Protruding−mouth
Day−4
Day−5
Stage
Transcript
ENSDART00000155725
ENSDART00000156808
ENSDART00000168475
ENSDART00000176241

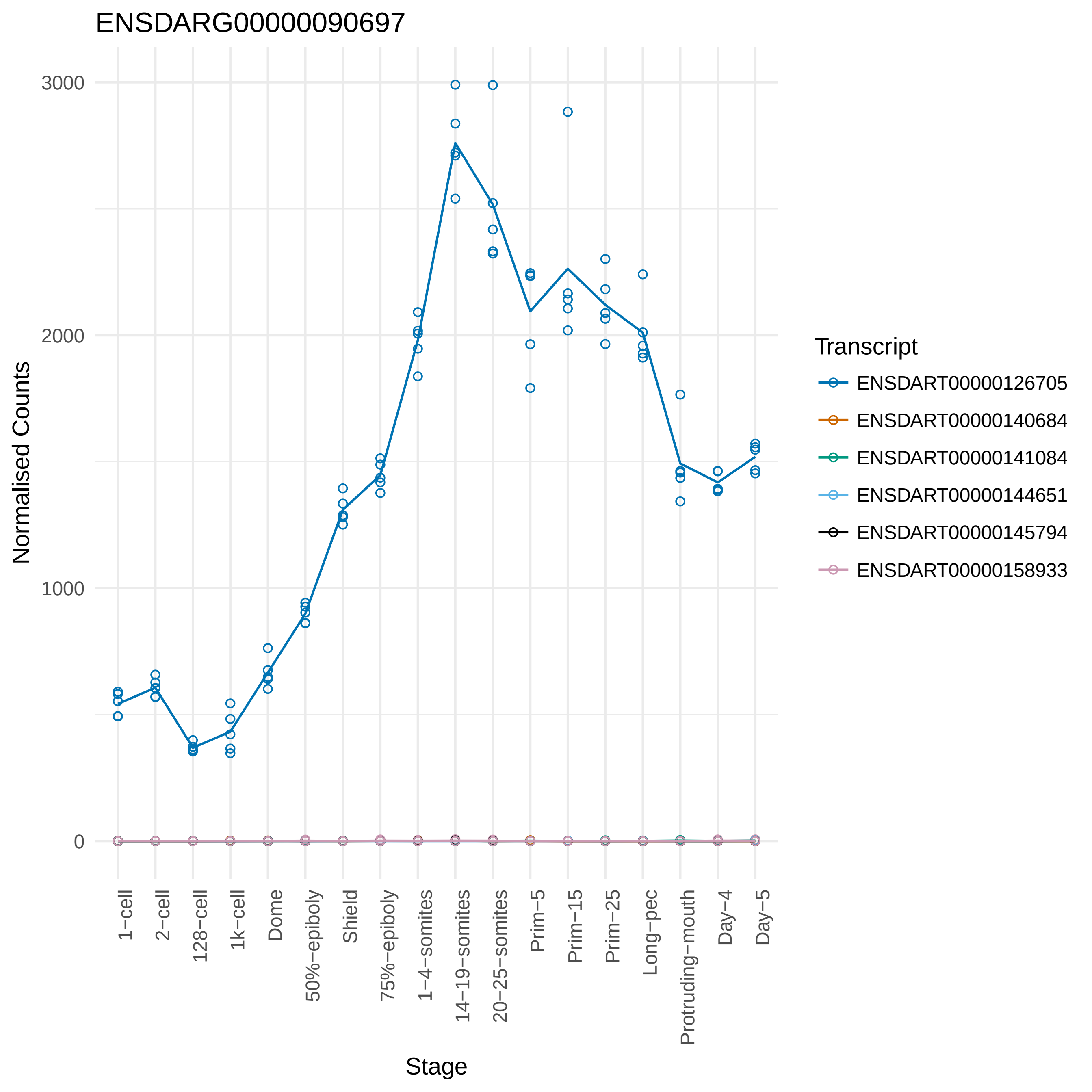
ENSDARG00000090697
Normalised Counts
3000
2000
1000
0
1−cell
2−cell
128−cell
1k−cell
Dome
50%−epiboly
Shield
75%−epiboly
1−4−somites
14−19−somites
20−25−somites
Prim−5
Prim−15
Prim−25
Long−pec
Protruding−mouth
Day−4
Day−5
Stage
Transcript
ENSDART00000126705
ENSDART00000140684
ENSDART00000141084
ENSDART00000144651
ENSDART00000145794
ENSDART00000158933

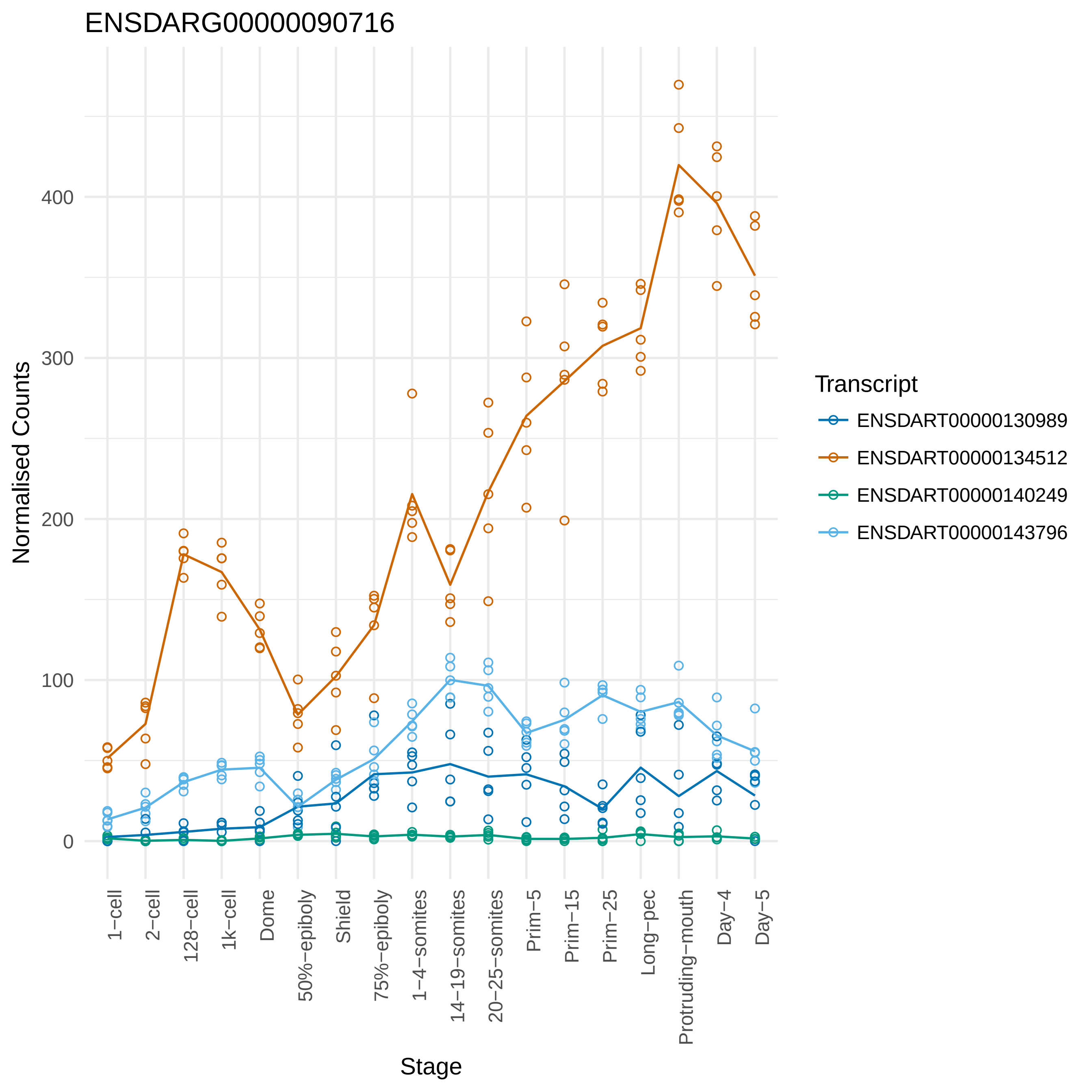

ENSDARG00000090716
Normalised Counts
400
300
200
100
0
1−cell
2−cell
128−cell
1k−cell
Dome
50%−epiboly
Shield
75%−epiboly
1−4−somites
14−19−somites
20−25−somites
Prim−5
Prim−15
Prim−25
Long−pec
Protruding−mouth
Day−4
Day−5
Stage
Transcript
ENSDART00000130989
ENSDART00000134512
ENSDART00000140249
ENSDART00000143796

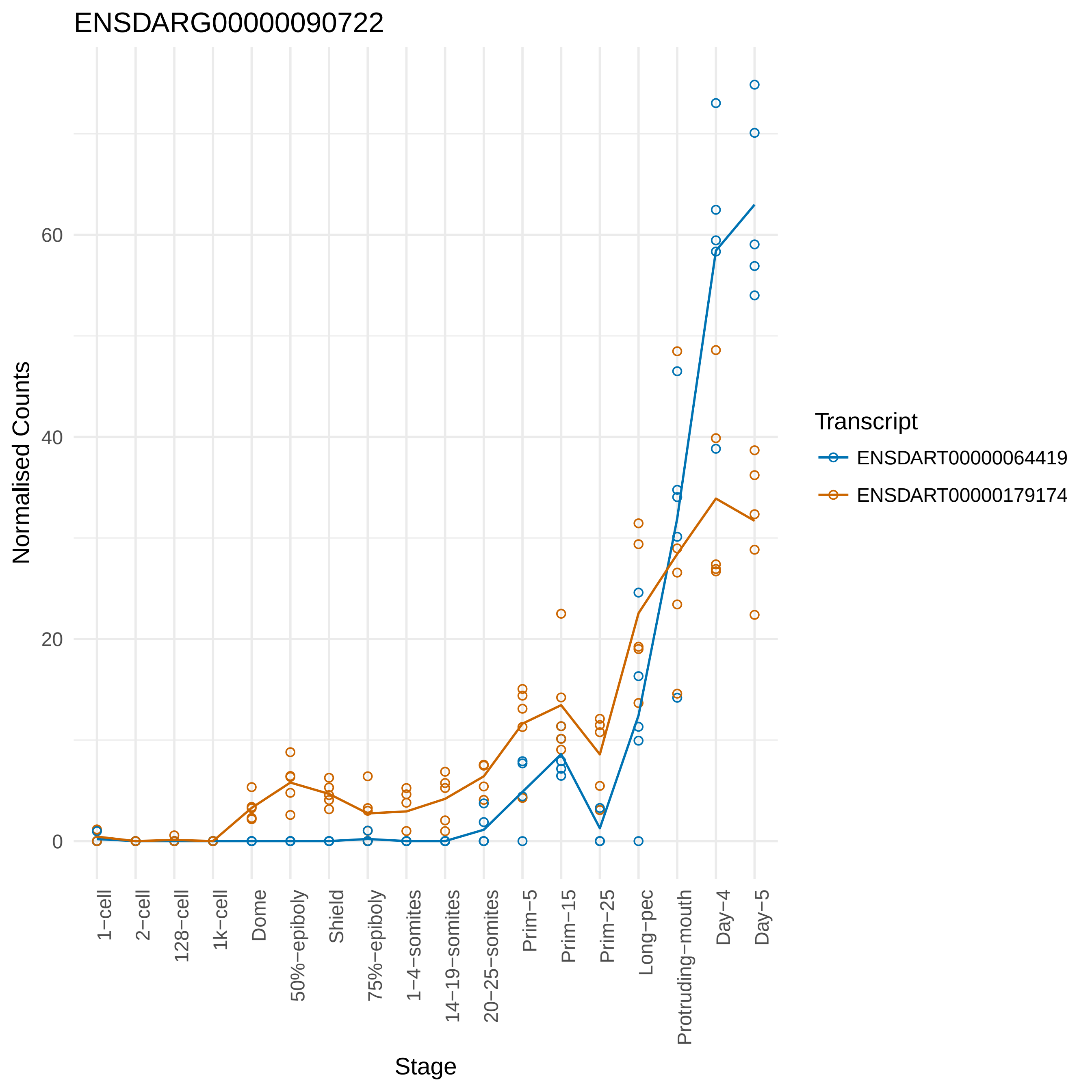

ENSDARG00000090722
Normalised Counts
0
20
40
60
1−cell
2−cell
128−cell
1k−cell
Dome
50%−epiboly
Shield
75%−epiboly
1−4−somites
14−19−somites
20−25−somites
Prim−5
Prim−15
Prim−25
Long−pec
Protruding−mouth
Day−4
Day−5
Stage
Transcript
ENSDART00000064419
ENSDART00000179174

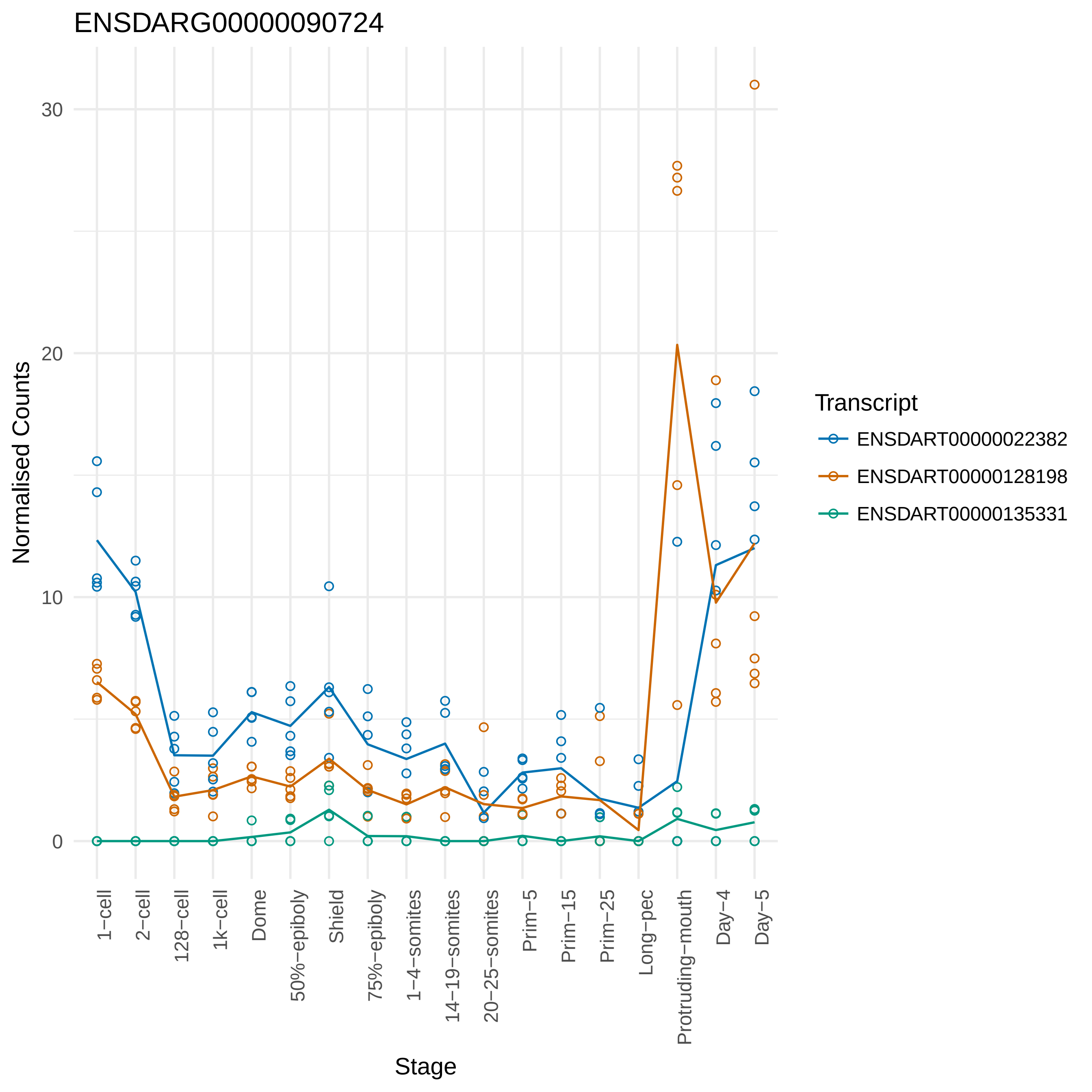
ENSDARG00000090724
Normalised Counts
0
10
20
30
Stage
1−cell
2−cell
128−cell
1k−cell
Dome
50%−epiboly
Shield
75%−epiboly
1−4−somites
14−19−somites
20−25−somites
Prim−5
Prim−15
Prim−25
Long−pec
Protruding−mouth
Day−4
Day−5
Transcript
ENSDART00000022382
ENSDART00000128198
ENSDART00000135331

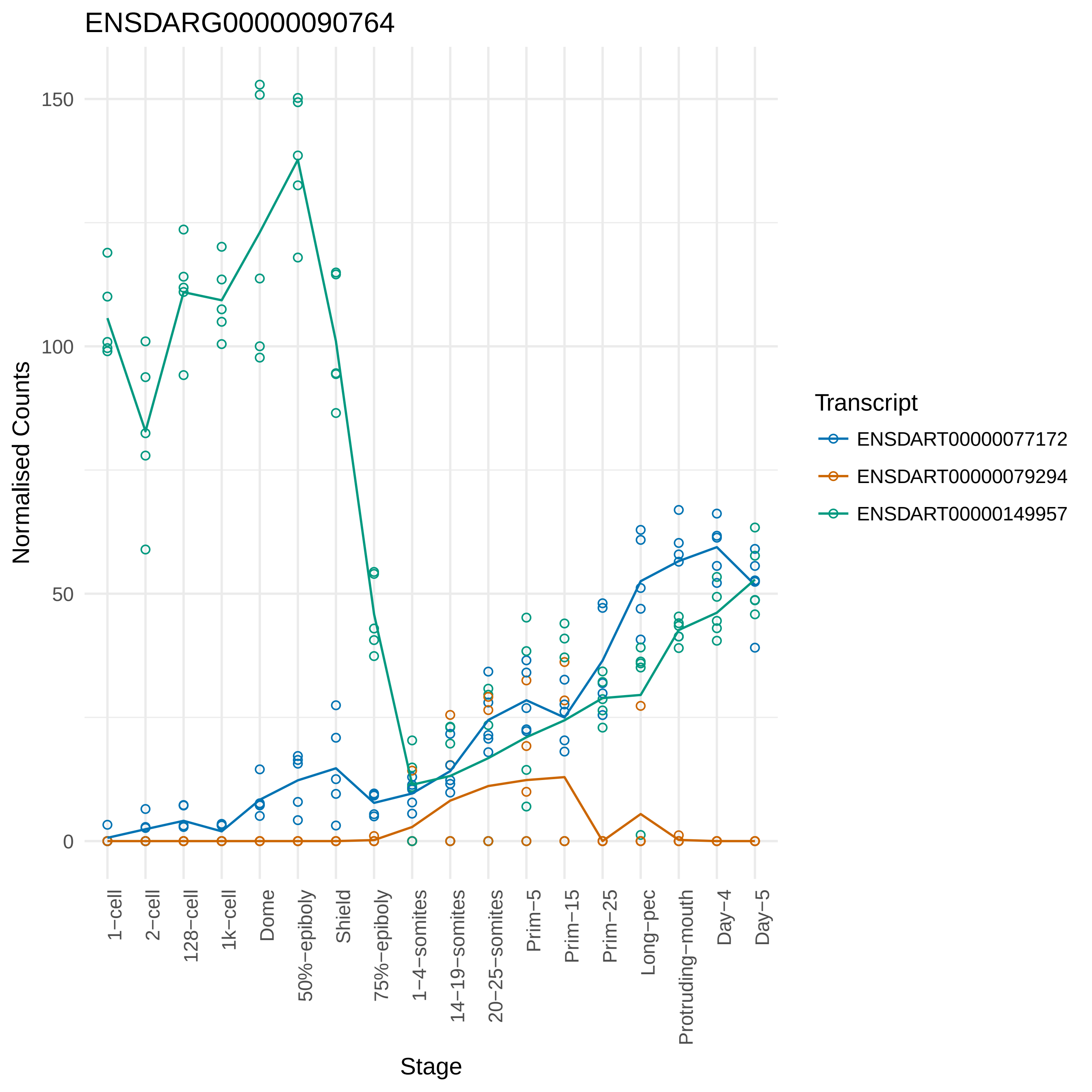

ENSDARG00000090764
Normalised Counts
150
100
50
0
1−cell
2−cell
128−cell
1k−cell
Dome
50%−epiboly
Shield
75%−epiboly
1−4−somites
14−19−somites
20−25−somites
Prim−5
Prim−15
Prim−25
Long−pec
Protruding−mouth
Day−4
Day−5
Stage
Transcript
ENSDART00000077172
ENSDART00000079294
ENSDART00000149957

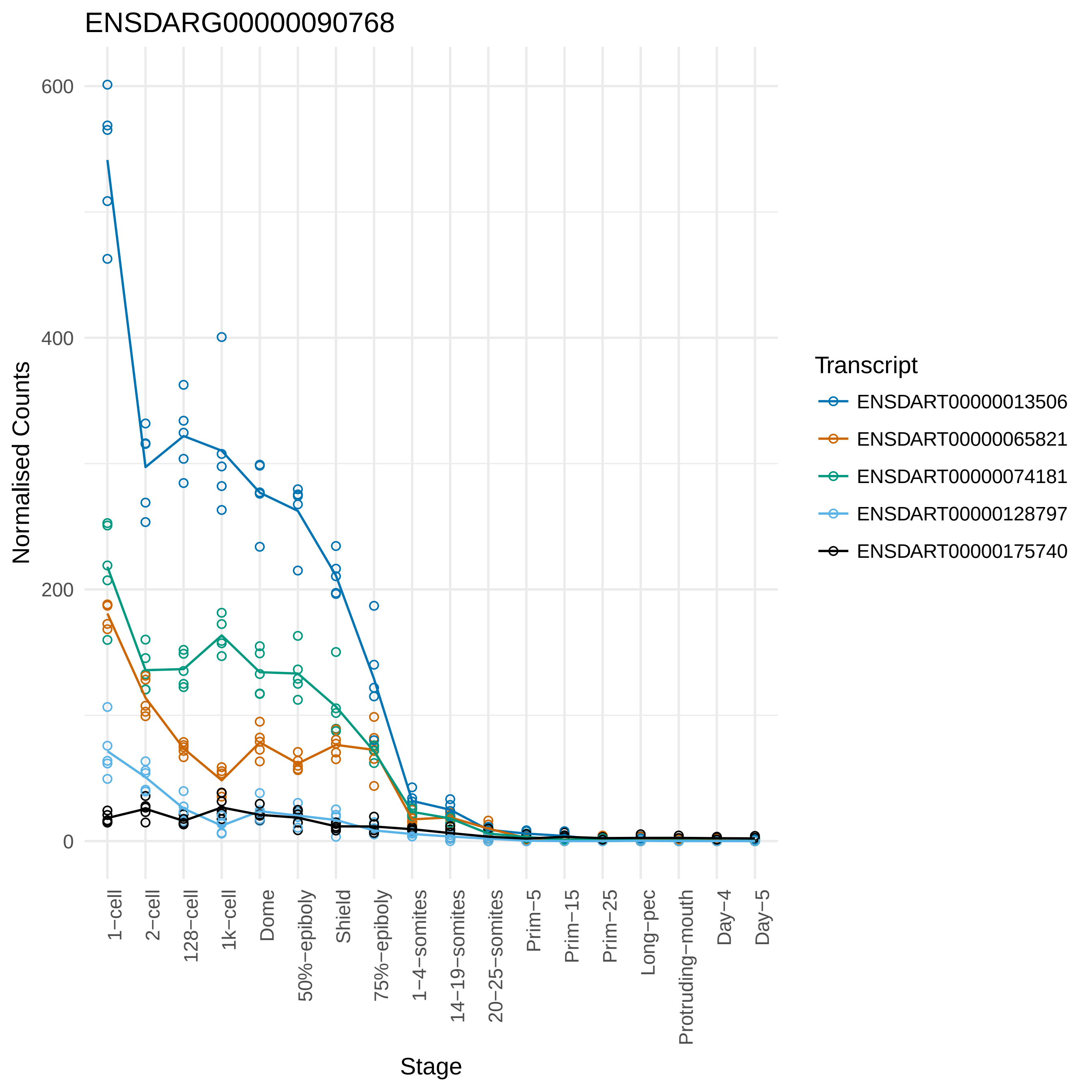

ENSDARG00000090768
Normalised Counts
600
400
200
0
1−cell
2−cell
128−cell
1k−cell
Dome
50%−epiboly
Shield
75%−epiboly
1−4−somites
14−19−somites
20−25−somites
Prim−5
Prim−15
Prim−25
Long−pec
Protruding−mouth
Day−4
Day−5
Stage
Transcript
ENSDART00000013506
ENSDART00000065821
ENSDART00000074181
ENSDART00000128797
ENSDART00000175740

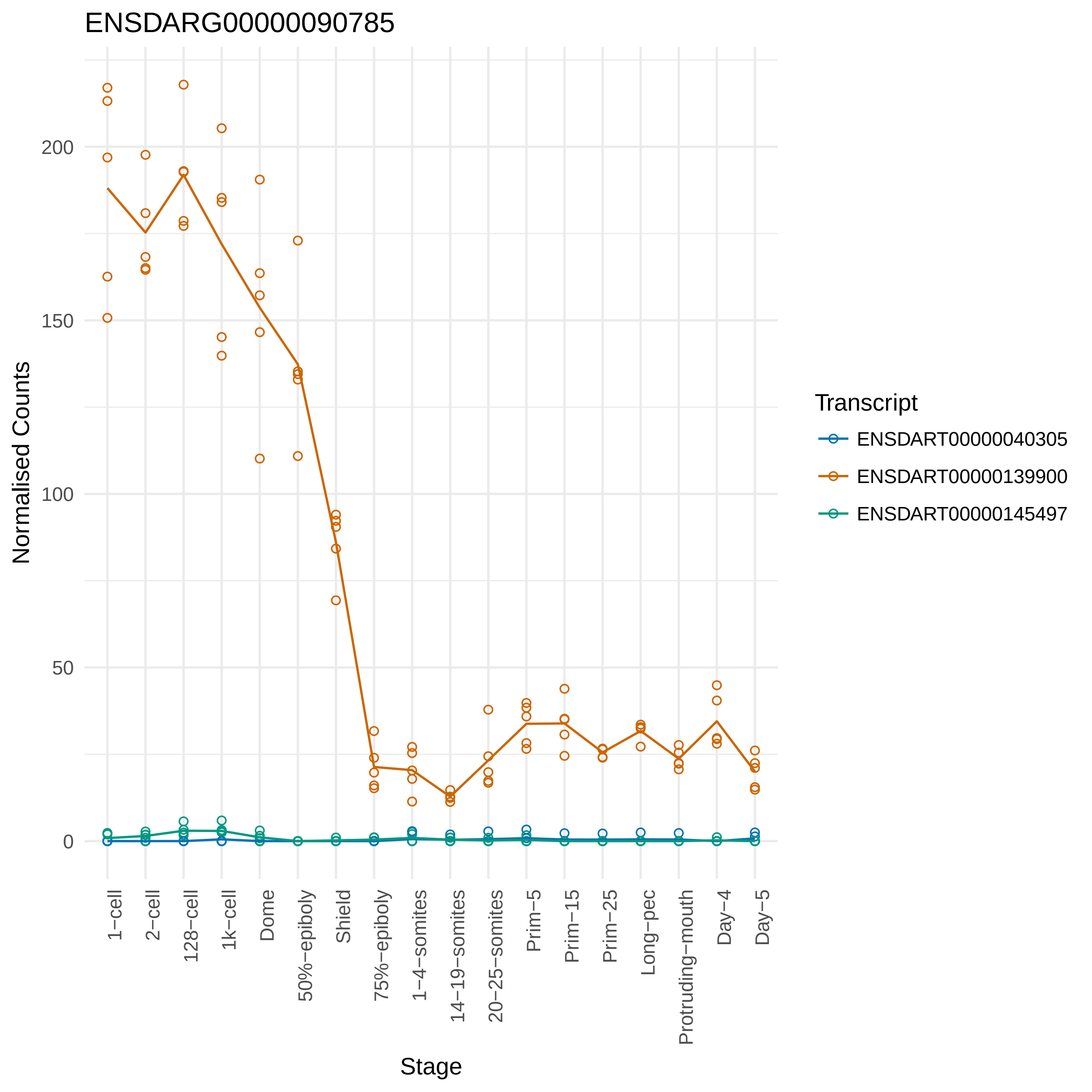

ENSDARG00000090785
Normalised Counts
0
50
100
150
200
1−cell
2−cell
128−cell
1k−cell
Dome
50%−epiboly
Shield
75%−epiboly
1−4−somites
14−19−somites
20−25−somites
Prim−5
Prim−15
Prim−25
Long−pec
Protruding−mouth
Day−4
Day−5
Stage
Transcript
ENSDART00000040305
ENSDART00000139900
ENSDART00000145497

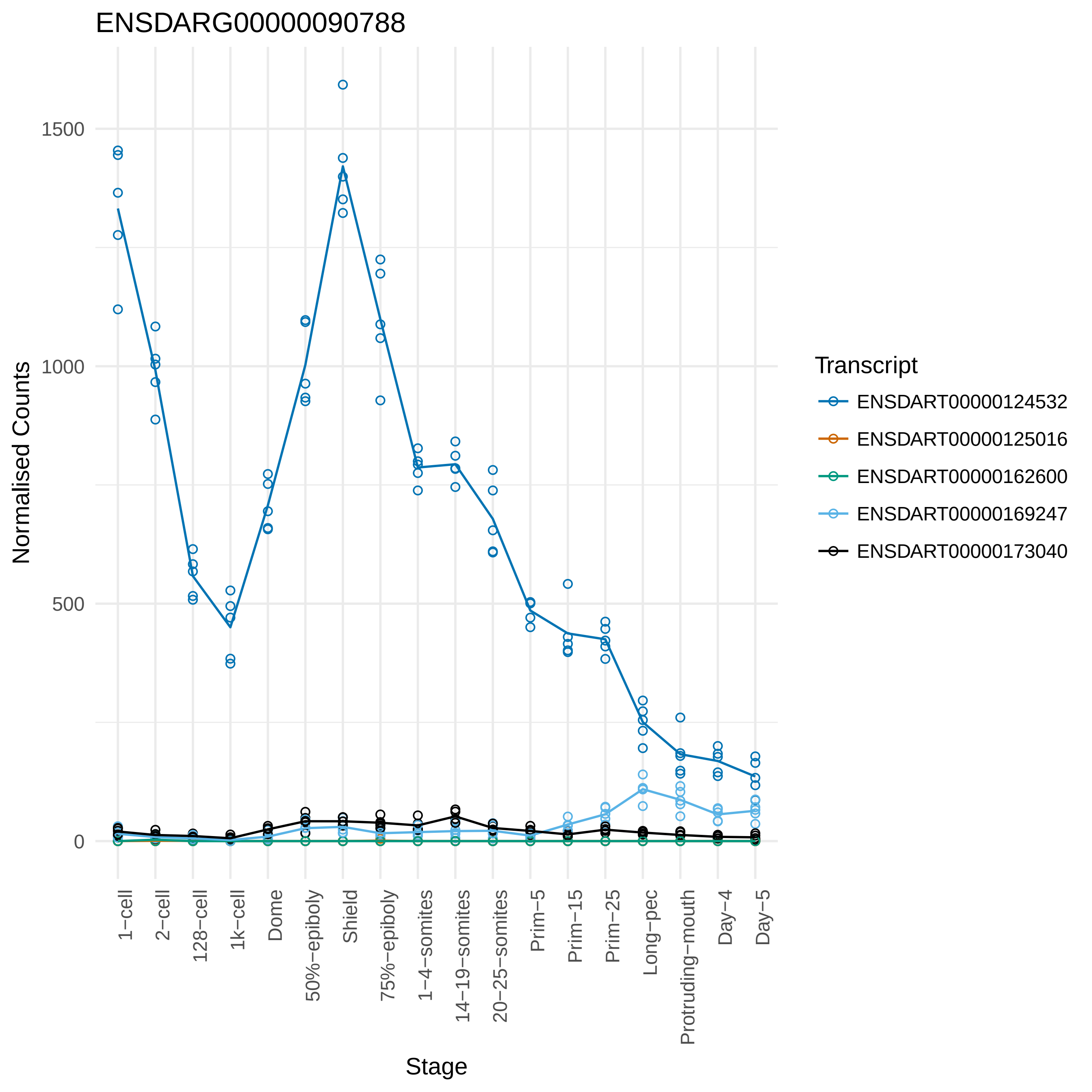

ENSDARG00000090788
Normalised Counts
1500
1000
500
0
1−cell
2−cell
128−cell
1k−cell
Dome
50%−epiboly
Shield
75%−epiboly
1−4−somites
14−19−somites
20−25−somites
Prim−5
Prim−15
Prim−25
Long−pec
Protruding−mouth
Day−4
Day−5
Stage
Transcript
ENSDART00000124532
ENSDART00000125016
ENSDART00000162600
ENSDART00000169247
ENSDART00000173040

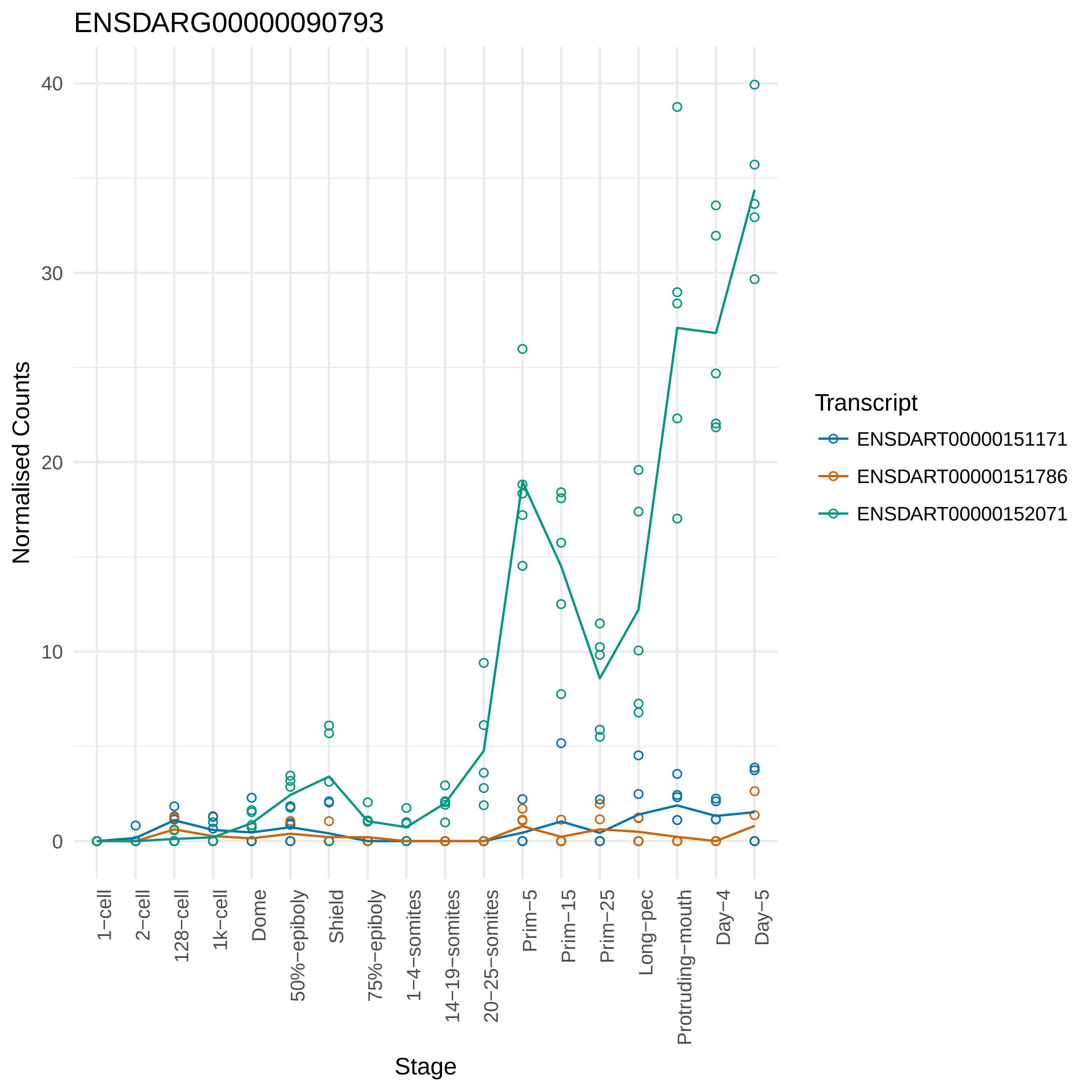

ENSDARG00000090793
Normalised Counts
0
10
20
30
40
Stage
1−cell
2−cell
128−cell
1k−cell
Dome
50%−epiboly
Shield
75%−epiboly
1−4−somites
14−19−somites
20−25−somites
Prim−5
Prim−15
Prim−25
Long−pec
Protruding−mouth
Day−4
Day−5
Transcript
ENSDART00000151171
ENSDART00000151786
ENSDART00000152071

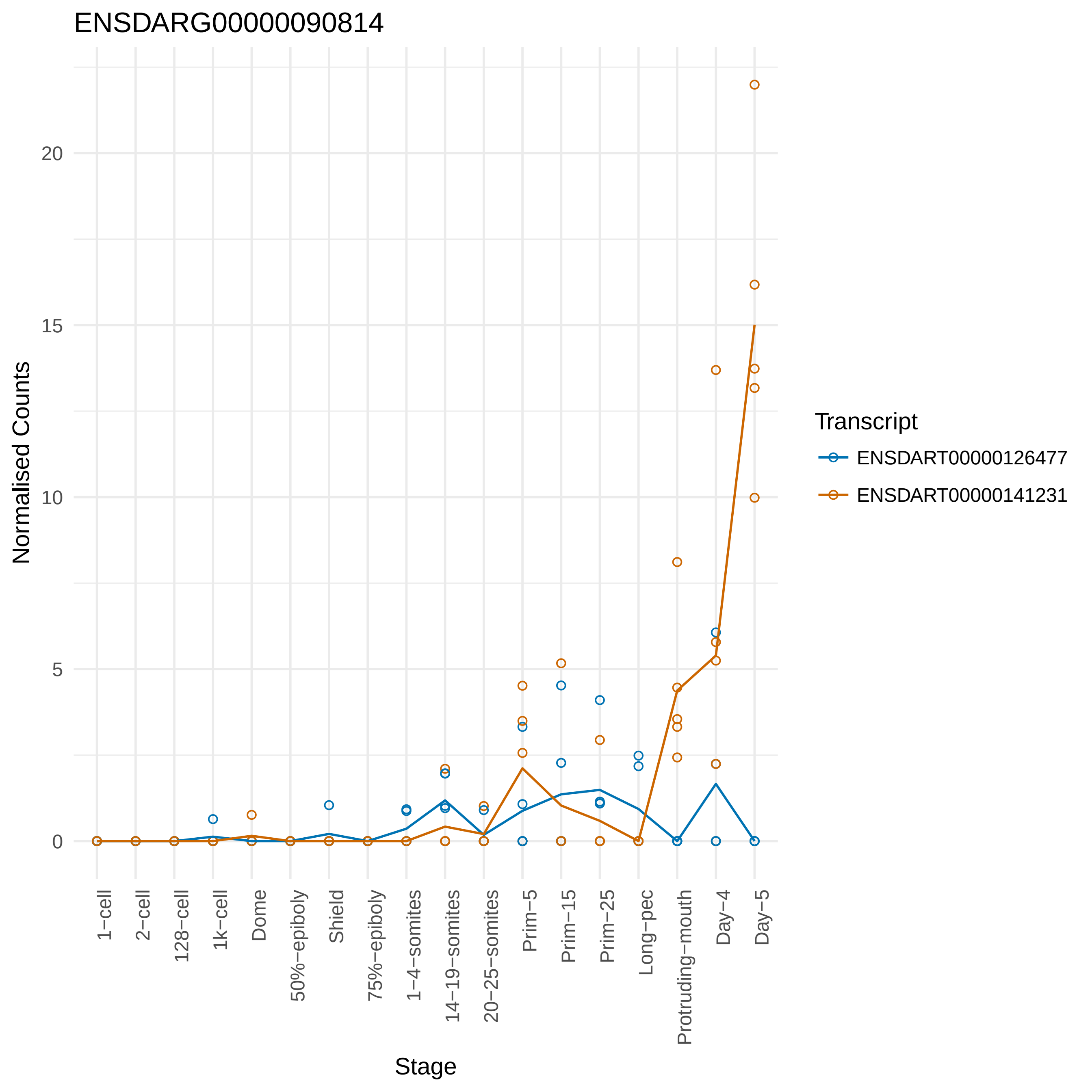
ENSDARG00000090814
Normalised Counts
0
5
10
15
20
Stage
1−cell
2−cell
128−cell
1k−cell
Dome
50%−epiboly
Shield
75%−epiboly
1−4−somites
14−19−somites
20−25−somites
Prim−5
Prim−15
Prim−25
Long−pec
Protruding−mouth
Day−4
Day−5
Transcript
ENSDART00000126477
ENSDART00000141231

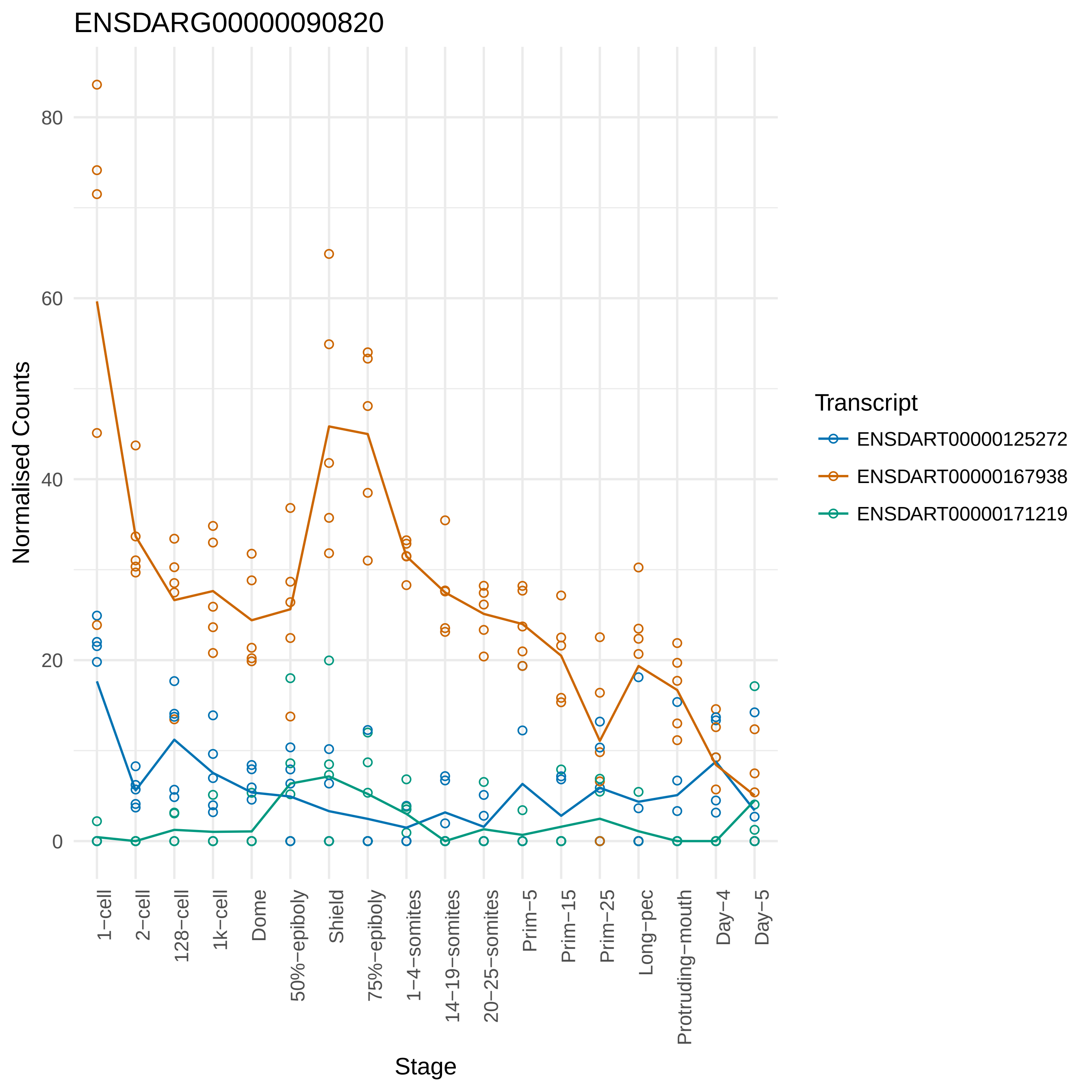

ENSDARG00000090820
Normalised Counts
0
20
40
60
80
1−cell
2−cell
128−cell
1k−cell
Dome
50%−epiboly
Shield
75%−epiboly
1−4−somites
14−19−somites
20−25−somites
Prim−5
Prim−15
Prim−25
Long−pec
Protruding−mouth
Day−4
Day−5
Stage
Transcript
ENSDART00000125272
ENSDART00000167938
ENSDART00000171219

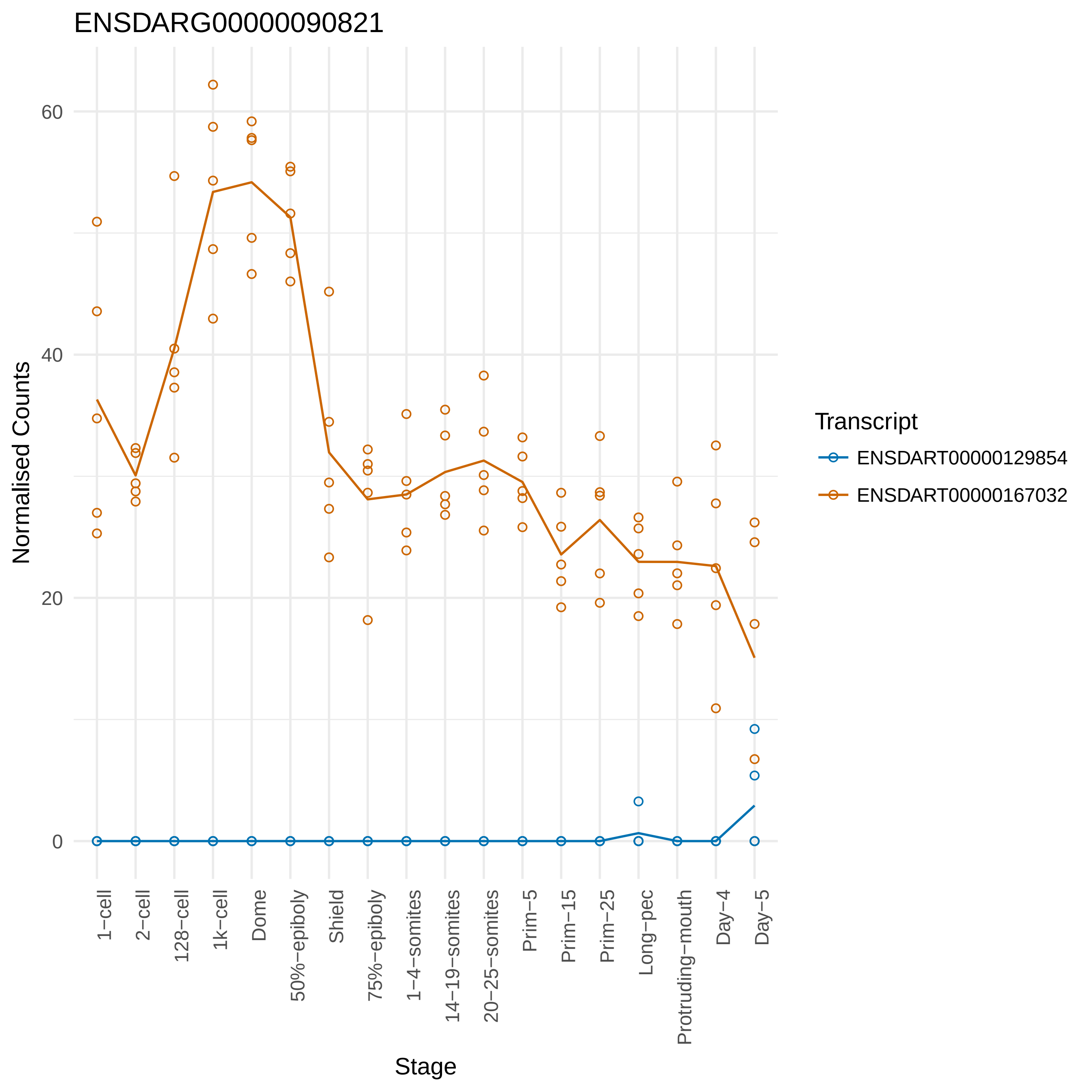
ENSDARG00000090821
Normalised Counts
60
40
20
0
1−cell
2−cell
128−cell
1k−cell
Dome
50%−epiboly
Shield
75%−epiboly
1−4−somites
14−19−somites
20−25−somites
Prim−5
Prim−15
Prim−25
Long−pec
Protruding−mouth
Day−4
Day−5
Stage
Transcript
ENSDART00000129854
ENSDART00000167032

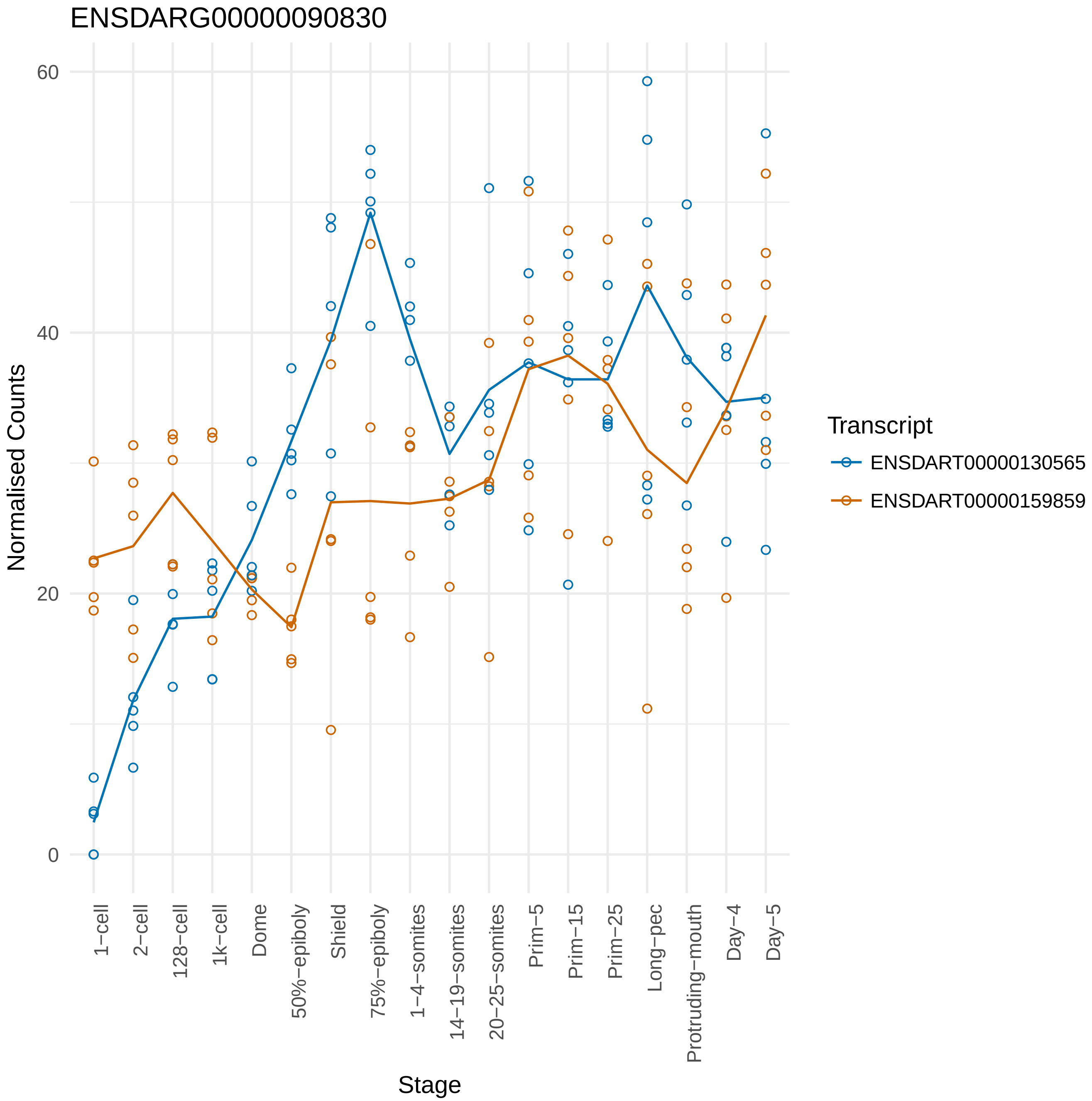

ENSDARG00000090830
Normalised Counts
60
40
20
0
1−cell
2−cell
128−cell
1k−cell
Dome
50%−epiboly
Shield
75%−epiboly
1−4−somites
14−19−somites
20−25−somites
Prim−5
Prim−15
Prim−25
Long−pec
Protruding−mouth
Day−4
Day−5
Stage
Transcript
ENSDART00000130565
ENSDART00000159859

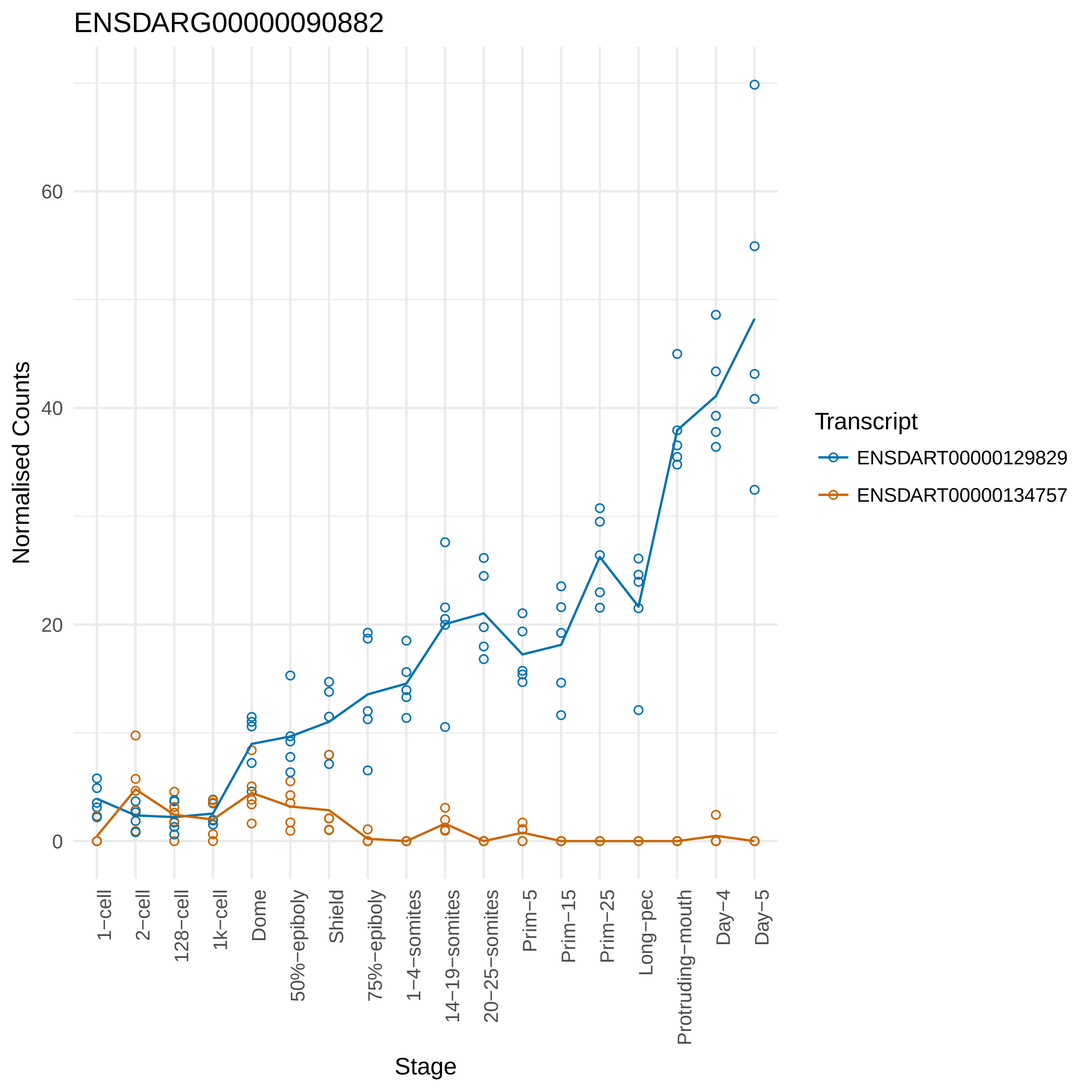

ENSDARG00000090882
Normalised Counts
60
40
20
0
1−cell
2−cell
128−cell
1k−cell
Dome
50%−epiboly
Shield
75%−epiboly
1−4−somites
14−19−somites
20−25−somites
Prim−5
Prim−15
Prim−25
Long−pec
Protruding−mouth
Day−4
Day−5
Stage
Transcript
ENSDART00000129829
ENSDART00000134757

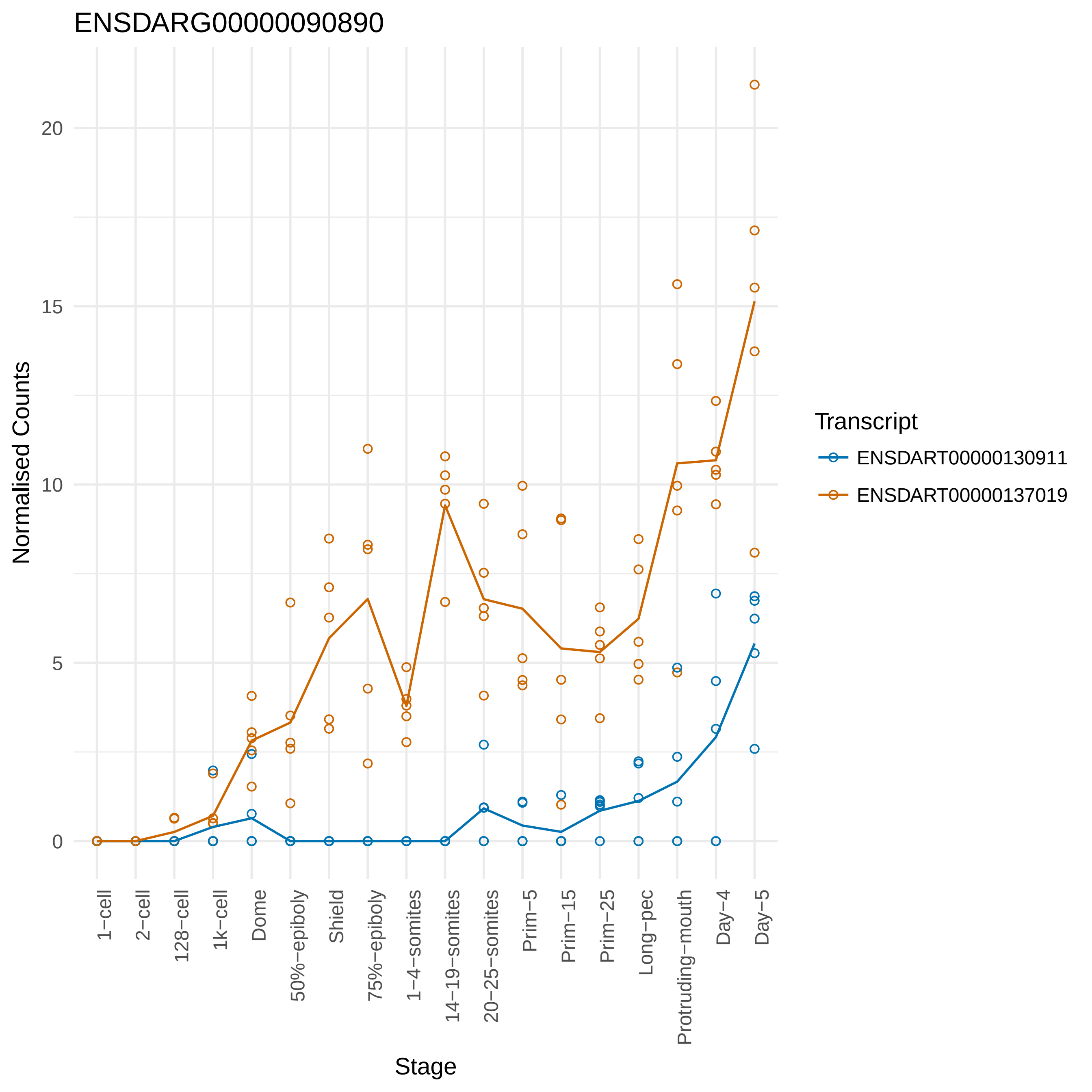

ENSDARG00000090890
Normalised Counts
0
5
10
15
20
1−cell
2−cell
128−cell
1k−cell
Dome
50%−epiboly
Shield
75%−epiboly
1−4−somites
14−19−somites
20−25−somites
Prim−5
Prim−15
Prim−25
Long−pec
Protruding−mouth
Day−4
Day−5
Stage
Transcript
ENSDART00000130911
ENSDART00000137019

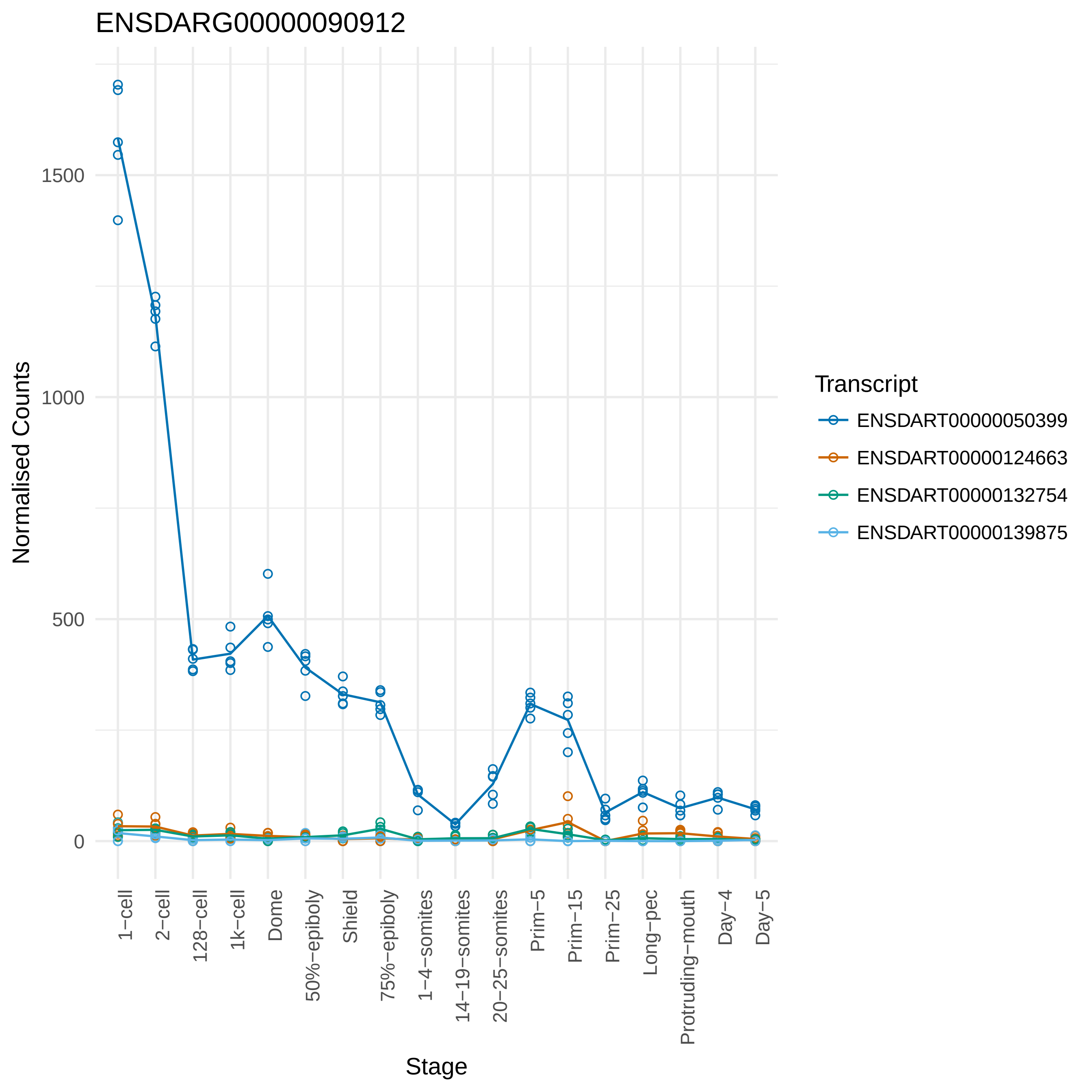
ENSDARG00000090912
Normalised Counts
0
500
1000
1500
1−cell
2−cell
128−cell
1k−cell
Dome
50%−epiboly
Shield
75%−epiboly
1−4−somites
14−19−somites
20−25−somites
Prim−5
Prim−15
Prim−25
Long−pec
Protruding−mouth
Day−4
Day−5
Stage
Transcript
ENSDART00000050399
ENSDART00000124663
ENSDART00000132754
ENSDART00000139875

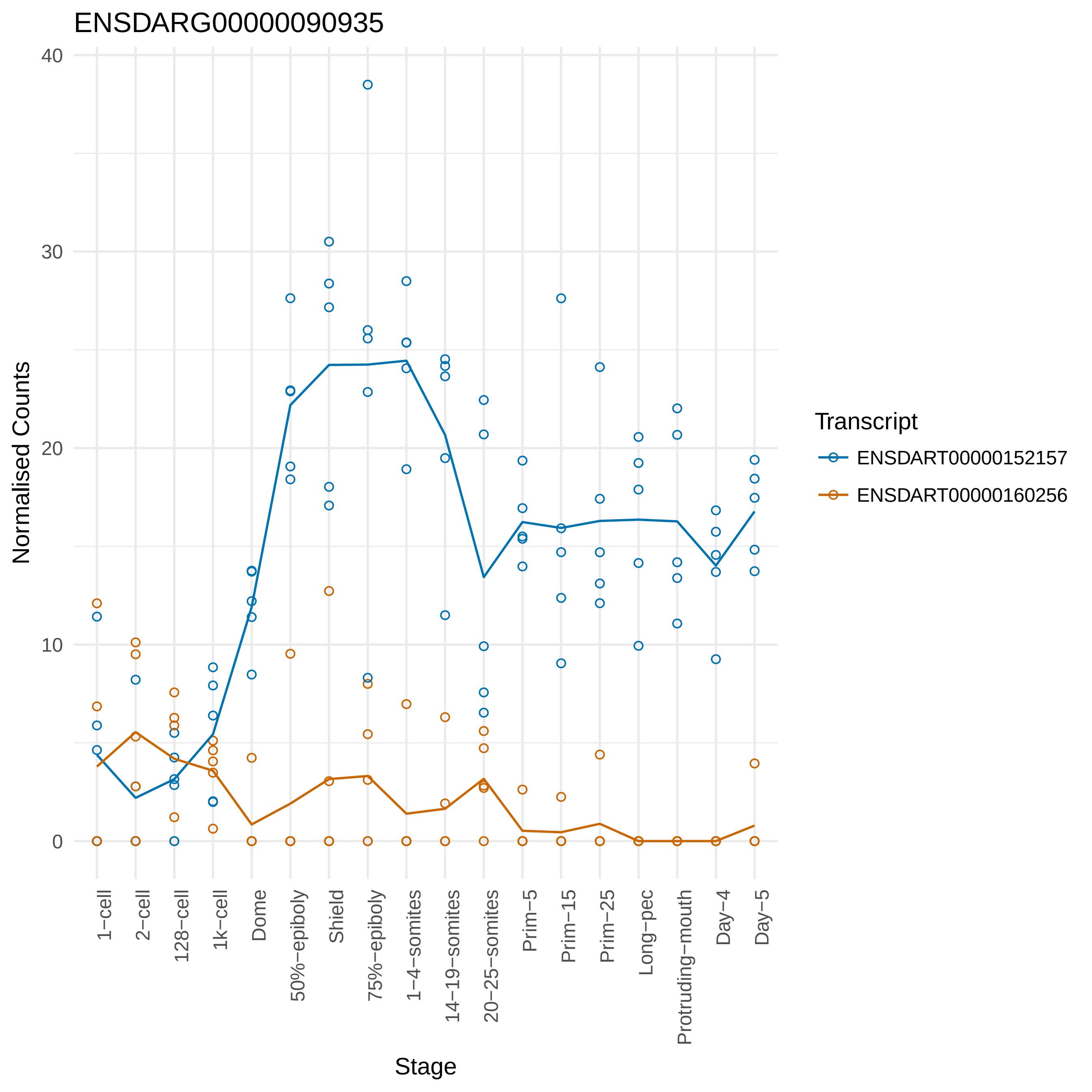

ENSDARG00000090935
Normalised Counts
40
30
20
10
0
Stage
1−cell
2−cell
128−cell
1k−cell
Dome
50%−epiboly
Shield
75%−epiboly
1−4−somites
14−19−somites
20−25−somites
Prim−5
Prim−15
Prim−25
Long−pec
Protruding−mouth
Day−4
Day−5
Transcript
ENSDART00000152157
ENSDART00000160256

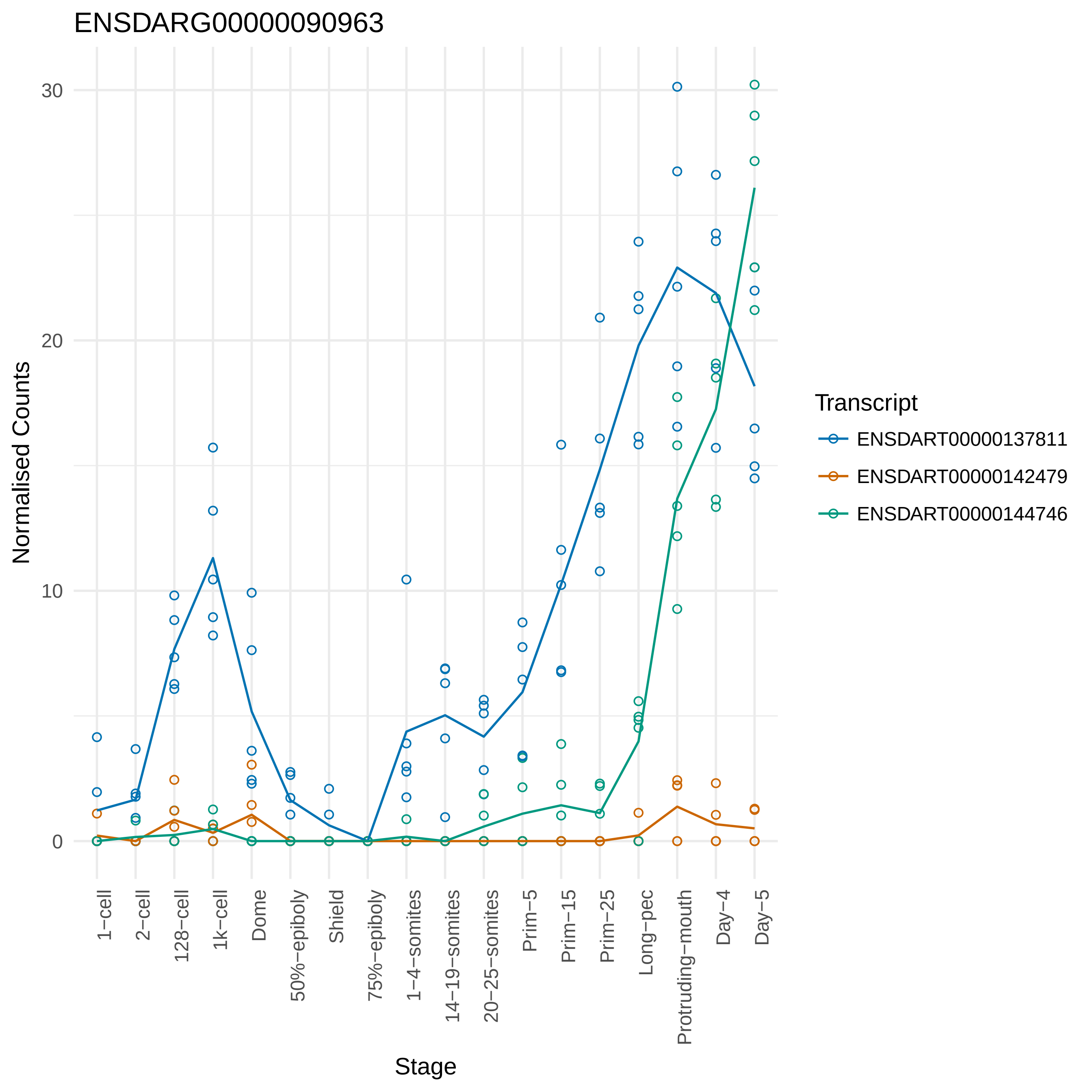

ENSDARG00000090963
Normalised Counts
30
20
10
0
1−cell
2−cell
128−cell
1k−cell
Dome
50%−epiboly
Shield
75%−epiboly
1−4−somites
14−19−somites
20−25−somites
Prim−5
Prim−15
Prim−25
Long−pec
Protruding−mouth
Day−4
Day−5
Stage
Transcript
ENSDART00000137811
ENSDART00000142479
ENSDART00000144746

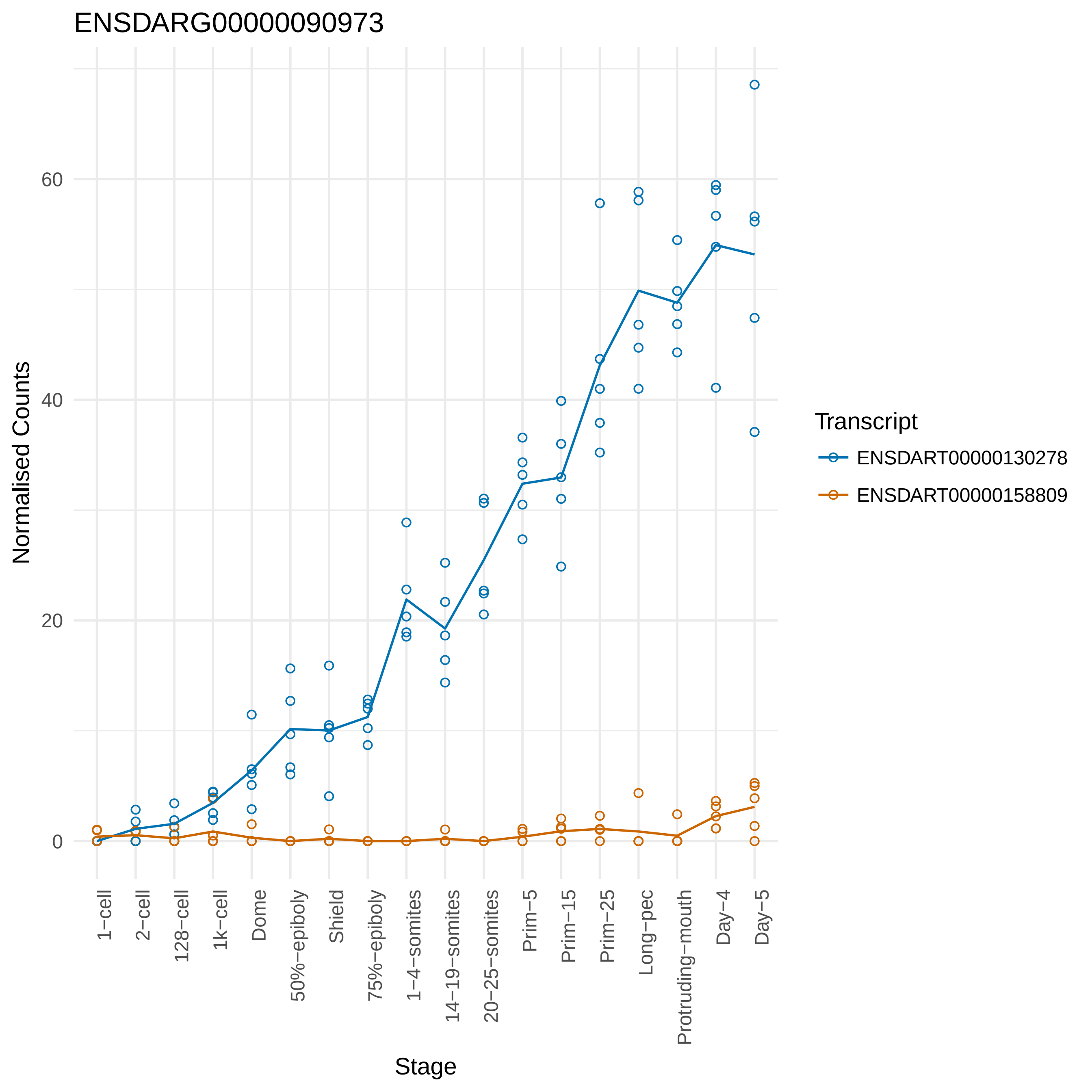

ENSDARG00000090973
Normalised Counts
60
40
20
0
1−cell
2−cell
128−cell
1k−cell
Dome
50%−epiboly
Shield
75%−epiboly
1−4−somites
14−19−somites
20−25−somites
Prim−5
Prim−15
Prim−25
Long−pec
Protruding−mouth
Day−4
Day−5
Stage
Transcript
ENSDART00000130278
ENSDART00000158809

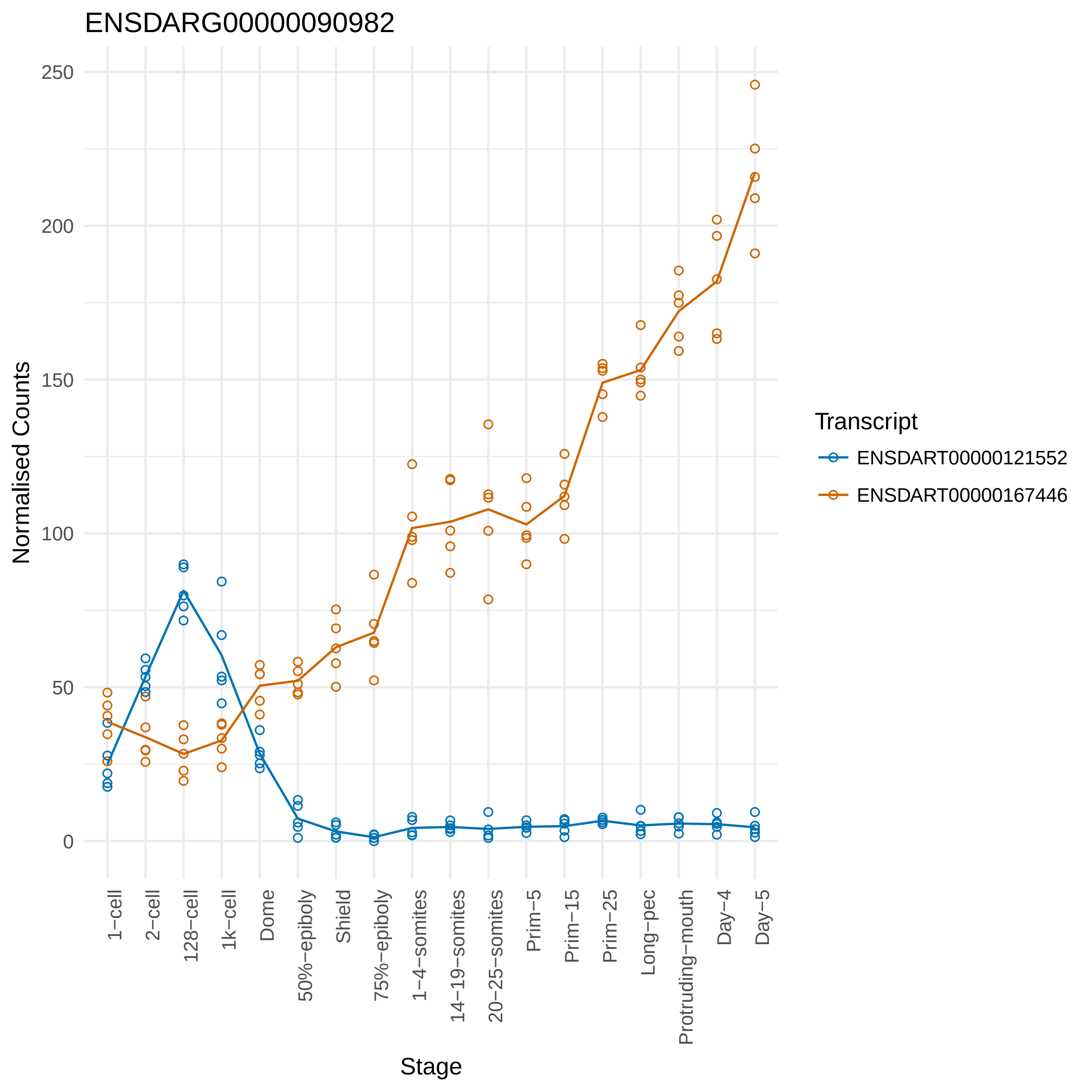
ENSDARG00000090982
Normalised Counts
0
50
100
150
200
250
1−cell
2−cell
128−cell
1k−cell
Dome
50%−epiboly
Shield
75%−epiboly
1−4−somites
14−19−somites
20−25−somites
Prim−5
Prim−15
Prim−25
Long−pec
Protruding−mouth
Day−4
Day−5
Stage
Transcript
ENSDART00000121552
ENSDART00000167446

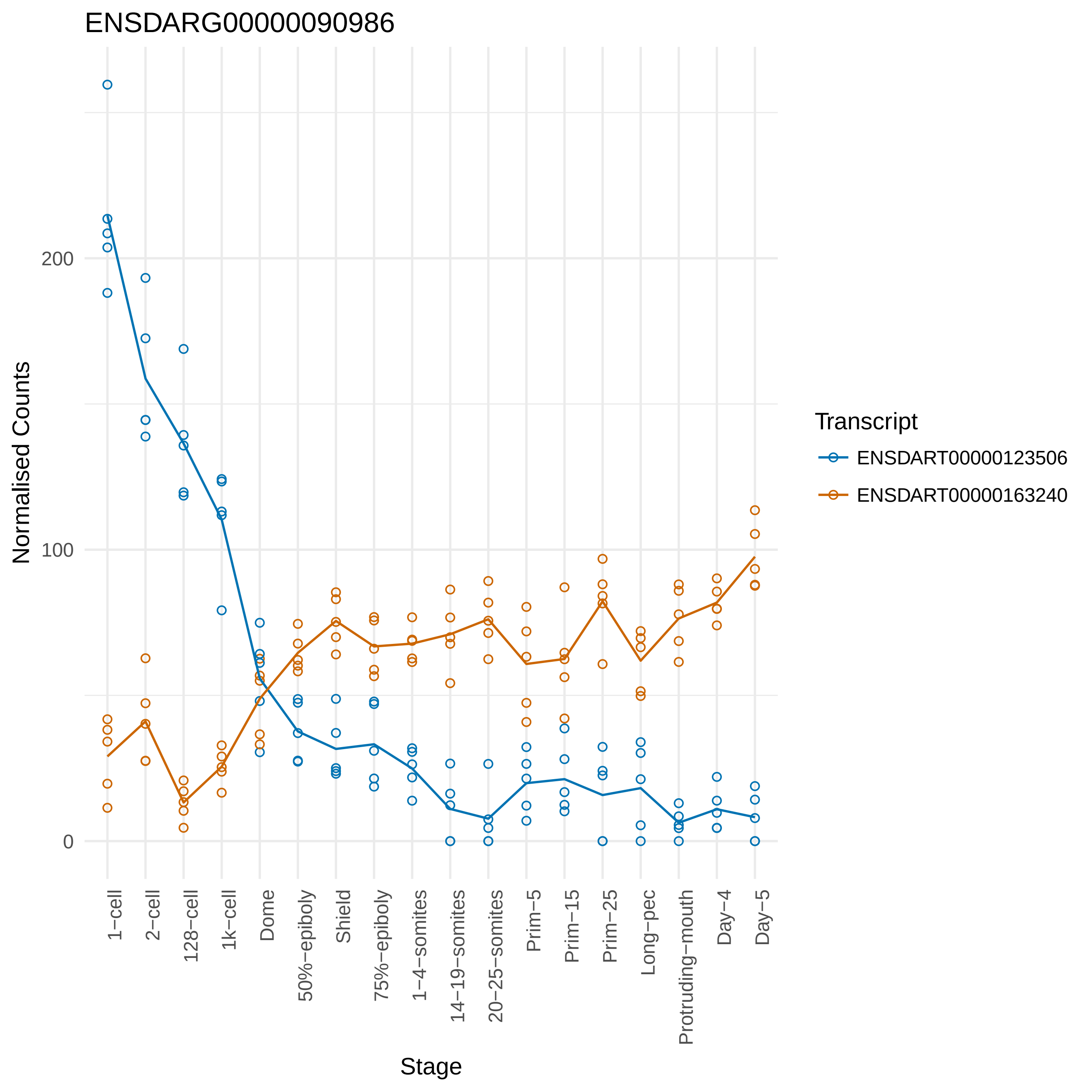

ENSDARG00000090986
Normalised Counts
200
100
0
1−cell
2−cell
128−cell
1k−cell
Dome
50%−epiboly
Shield
75%−epiboly
1−4−somites
14−19−somites
20−25−somites
Prim−5
Prim−15
Prim−25
Long−pec
Protruding−mouth
Day−4
Day−5
Stage
Transcript
ENSDART00000123506
ENSDART00000163240

ENSDARG00000090993

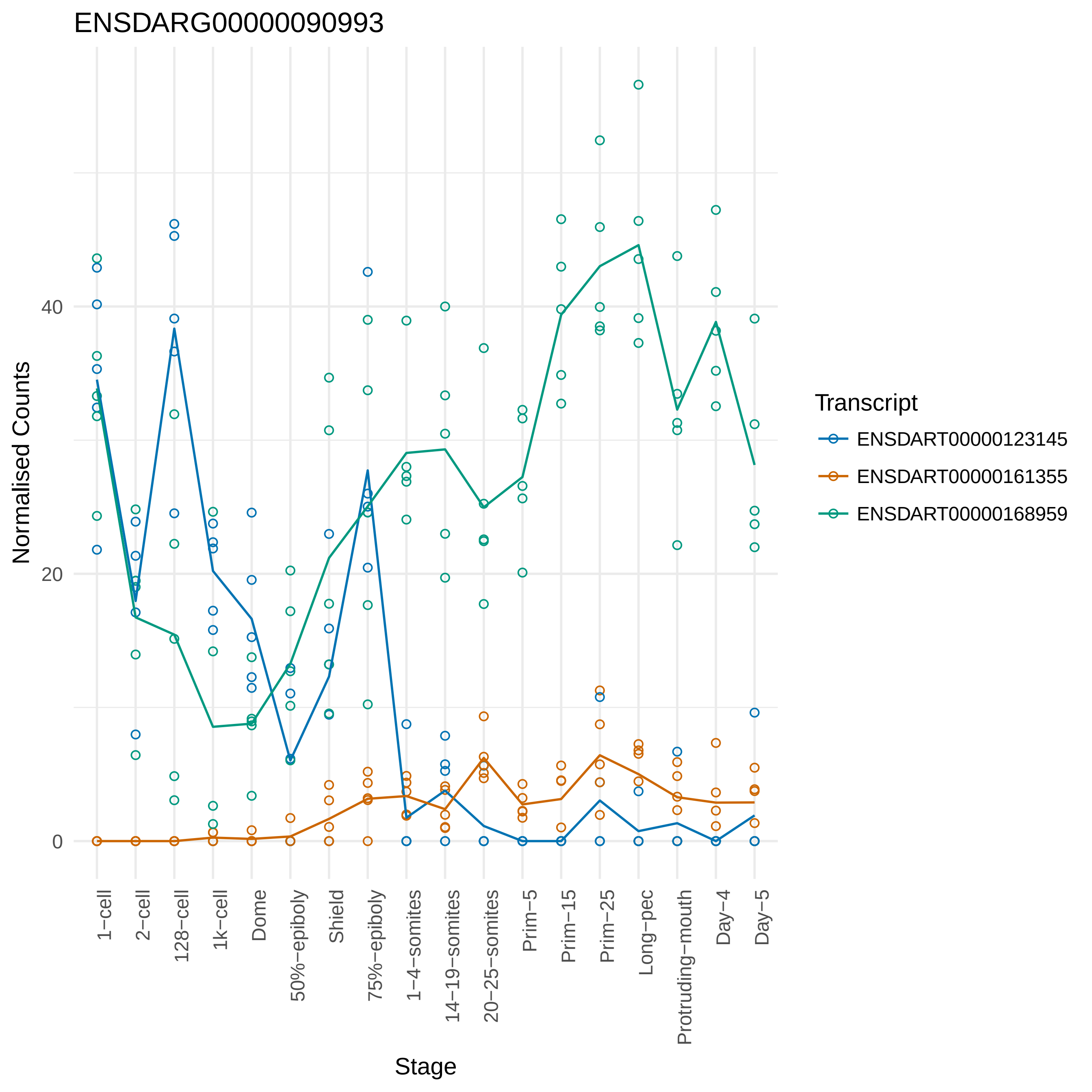

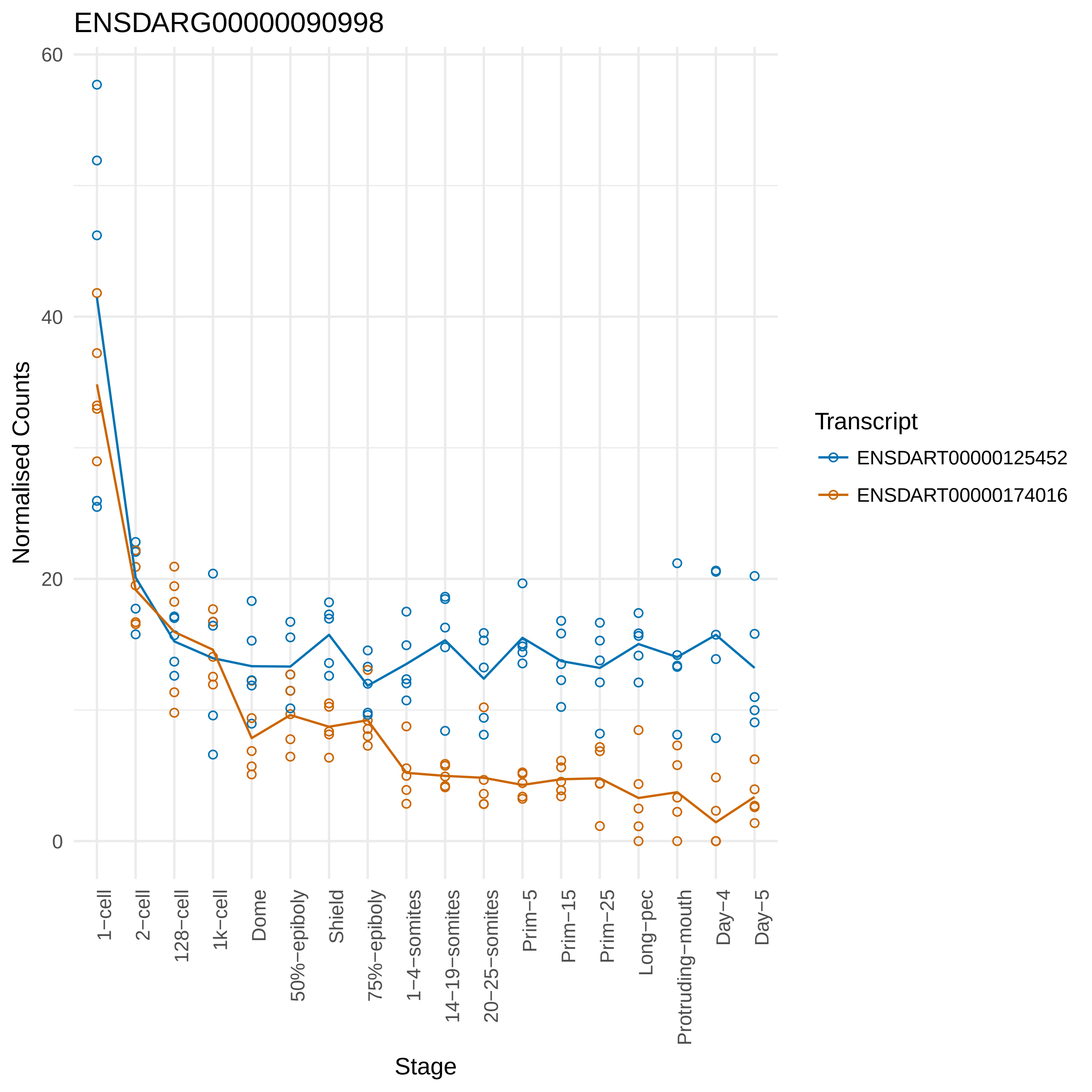
ENSDARG00000090998
Normalised Counts
60
40
20
0
Stage
1−cell
2−cell
128−cell
1k−cell
Dome
50%−epiboly
Shield
75%−epiboly
1−4−somites
14−19−somites
20−25−somites
Prim−5
Prim−15
Prim−25
Long−pec
Protruding−mouth
Day−4
Day−5
Transcript
ENSDART00000125452
ENSDART00000174016

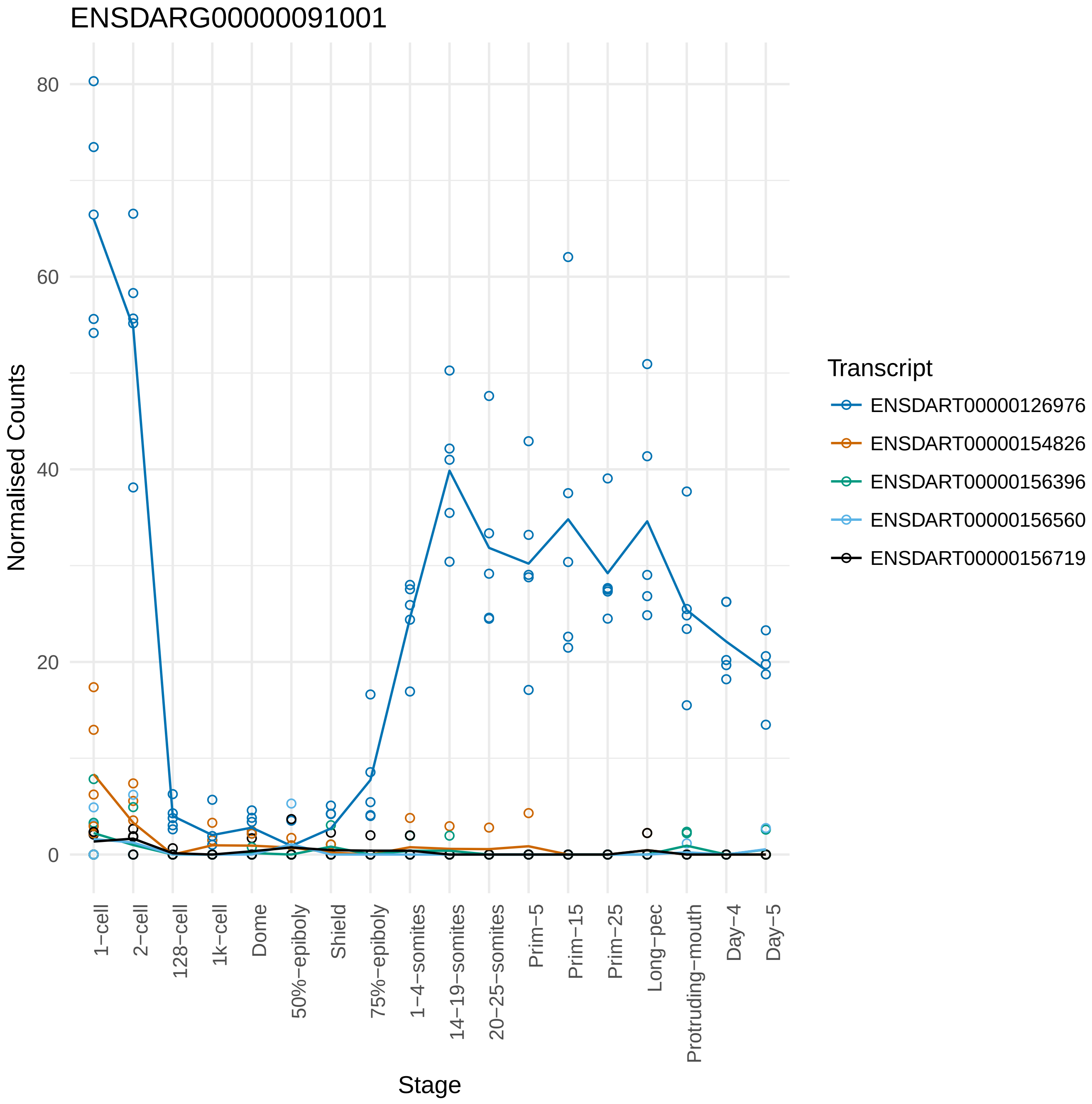

ENSDARG00000091001
Normalised Counts
80
60
40
20
0
1−cell
2−cell
128−cell
1k−cell
Dome
50%−epiboly
Shield
75%−epiboly
1−4−somites
14−19−somites
20−25−somites
Prim−5
Prim−15
Prim−25
Long−pec
Protruding−mouth
Day−4
Day−5
Stage
Transcript
ENSDART00000126976
ENSDART00000154826
ENSDART00000156396
ENSDART00000156560
ENSDART00000156719

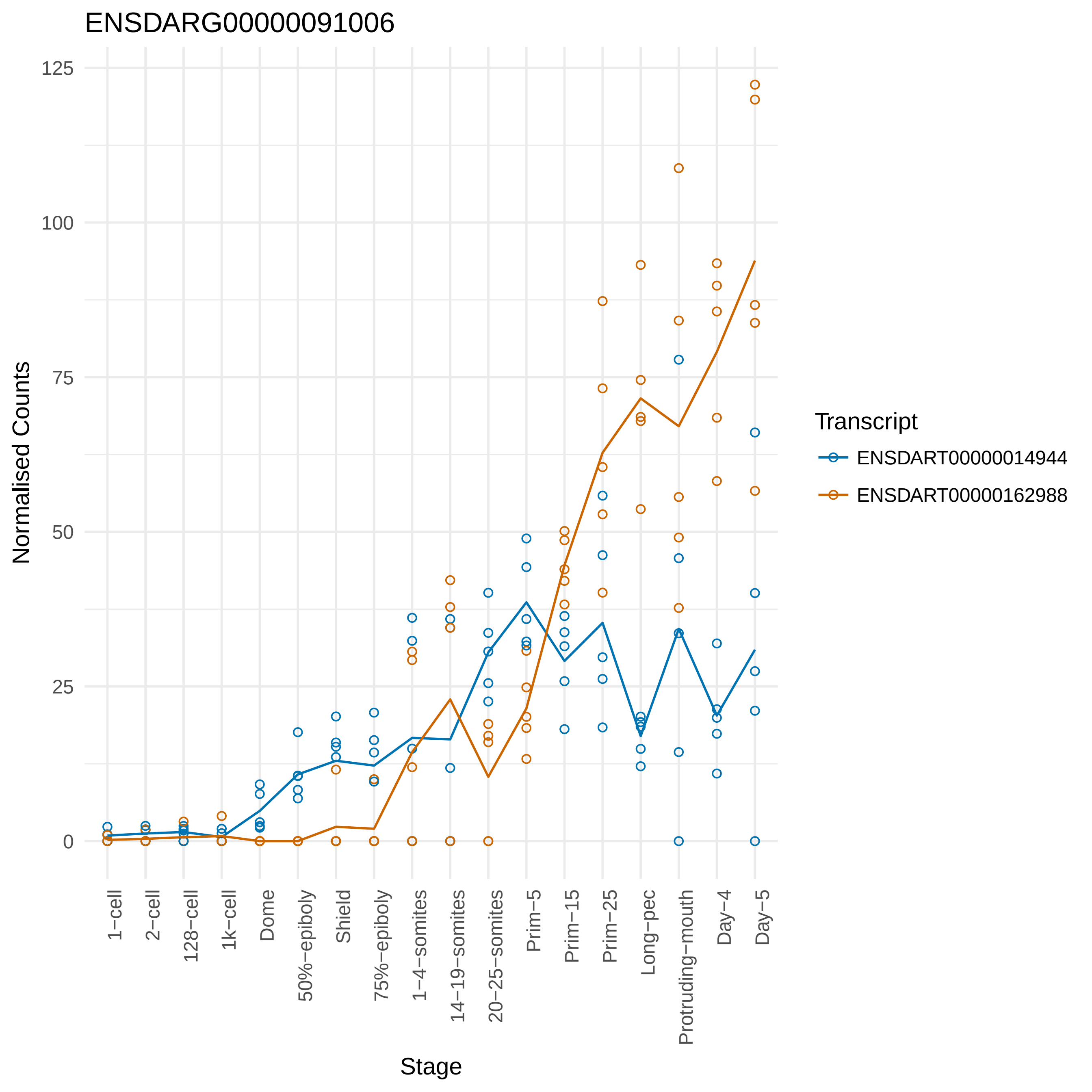

ENSDARG00000091006
Normalised Counts
125
100
75
50
25
0
1−cell
2−cell
128−cell
1k−cell
Dome
50%−epiboly
Shield
75%−epiboly
1−4−somites
14−19−somites
20−25−somites
Prim−5
Prim−15
Prim−25
Long−pec
Protruding−mouth
Day−4
Day−5
Stage
Transcript
ENSDART00000014944
ENSDART00000162988

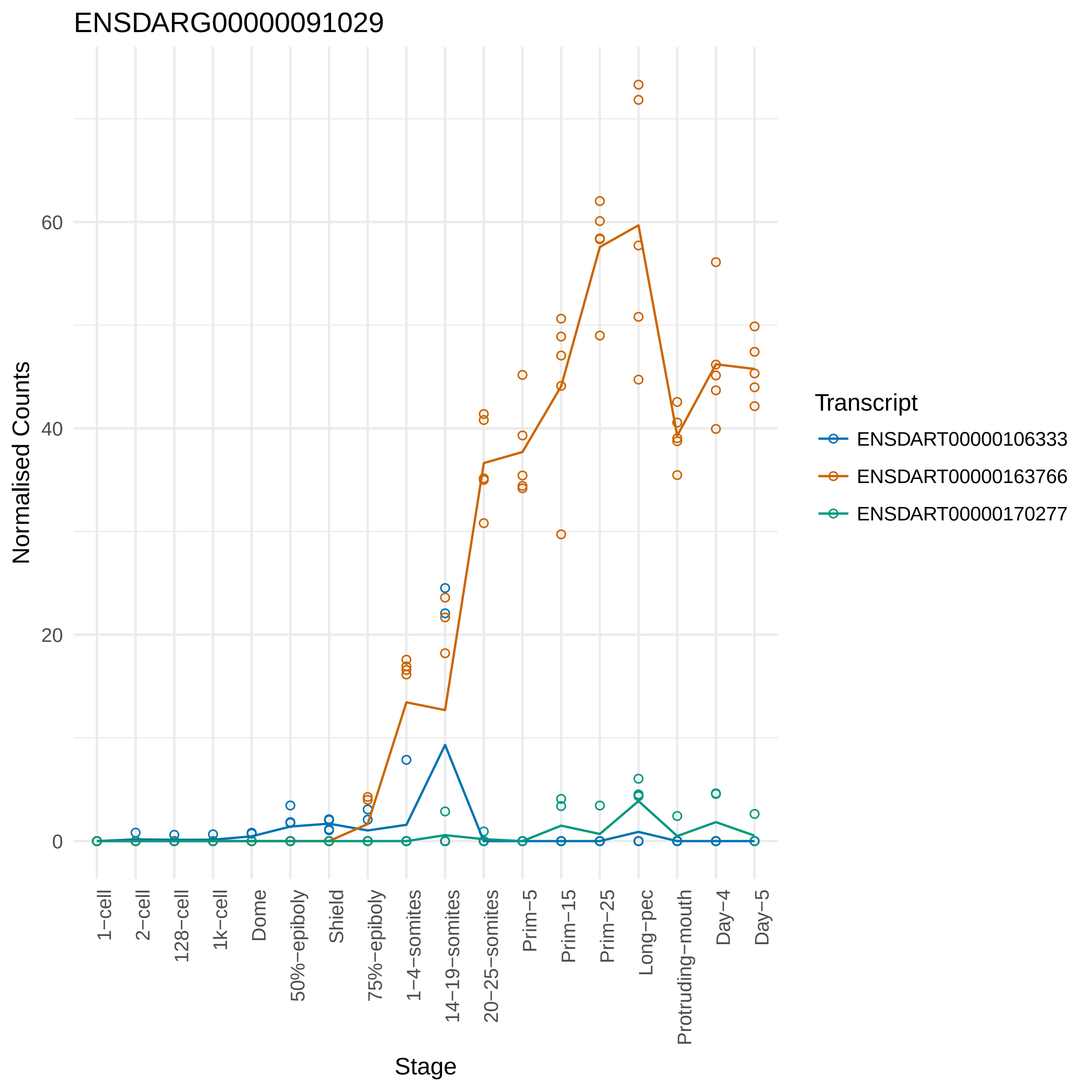

ENSDARG00000091029
Normalised Counts
0
20
40
60
1−cell
2−cell
128−cell
1k−cell
Dome
50%−epiboly
Shield
75%−epiboly
1−4−somites
14−19−somites
20−25−somites
Prim−5
Prim−15
Prim−25
Long−pec
Protruding−mouth
Day−4
Day−5
Stage
Transcript
ENSDART00000106333
ENSDART00000163766
ENSDART00000170277

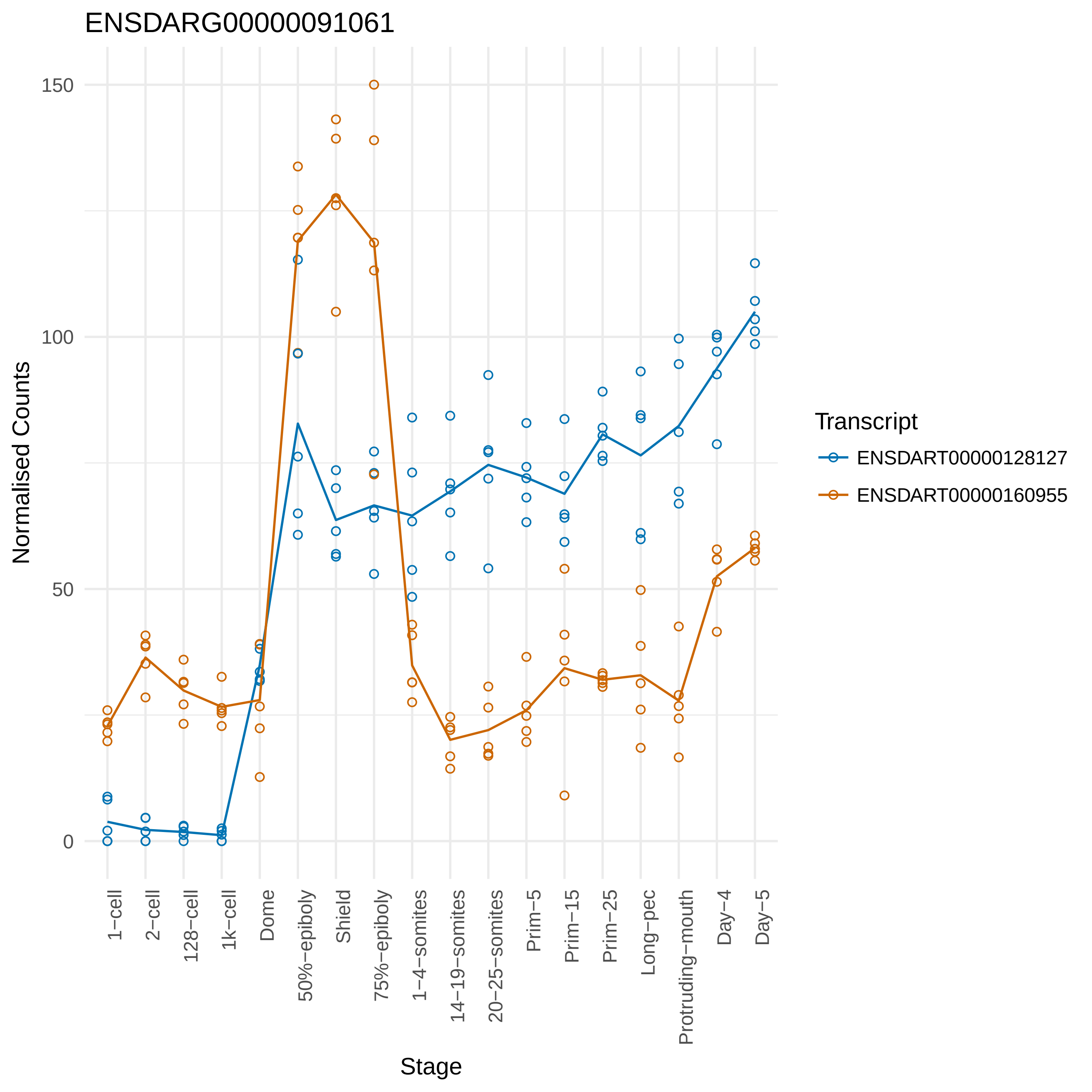
ENSDARG00000091061
Normalised Counts
150
100
50
0
1−cell
2−cell
128−cell
1k−cell
Dome
50%−epiboly
Shield
75%−epiboly
1−4−somites
14−19−somites
20−25−somites
Prim−5
Prim−15
Prim−25
Long−pec
Protruding−mouth
Day−4
Day−5
Stage
Transcript
ENSDART00000128127
ENSDART00000160955

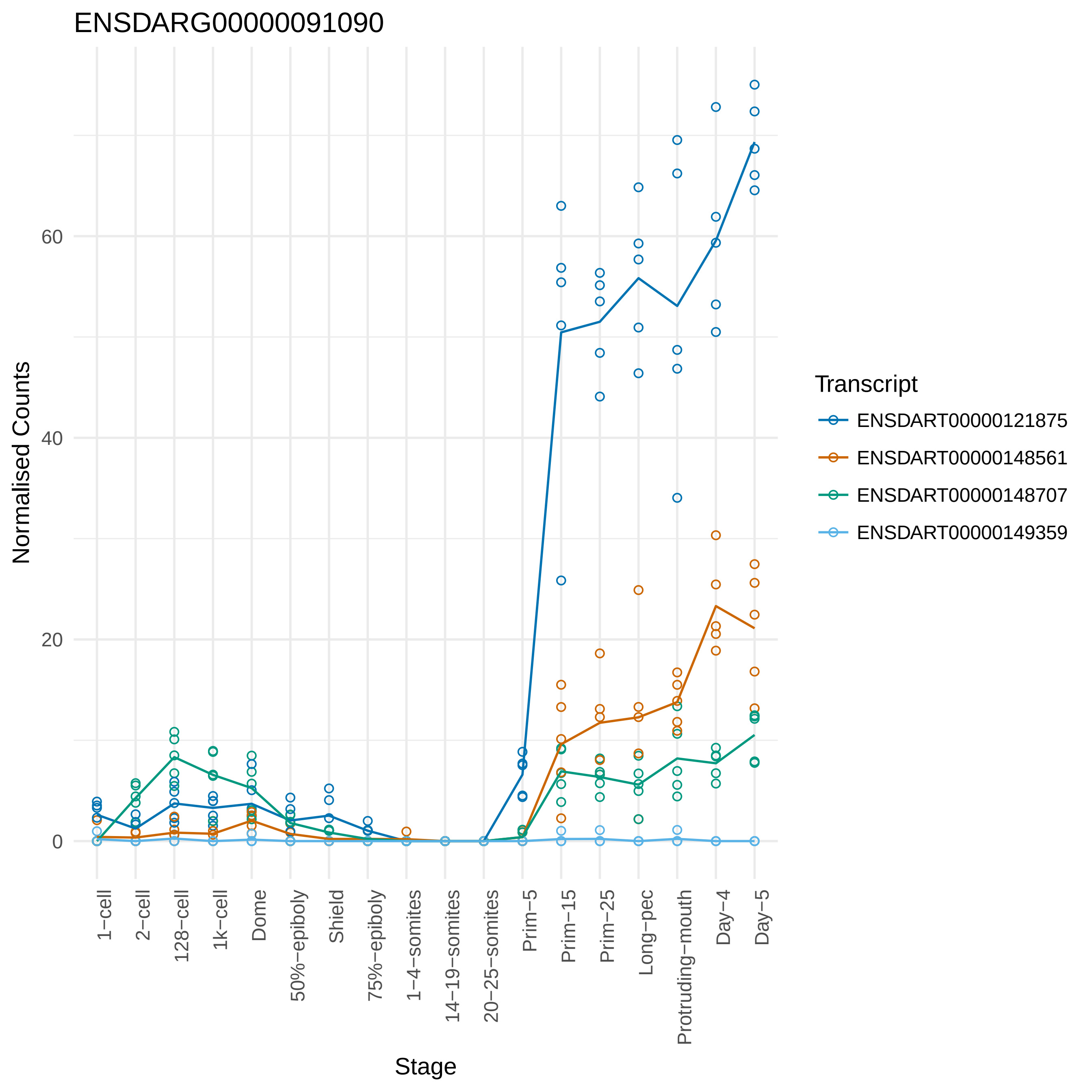
ENSDARG00000091090
Normalised Counts
60
40
20
0
1−cell
2−cell
128−cell
1k−cell
Dome
50%−epiboly
Shield
75%−epiboly
1−4−somites
14−19−somites
20−25−somites
Prim−5
Prim−15
Prim−25
Long−pec
Protruding−mouth
Day−4
Day−5
Stage
Transcript
ENSDART00000121875
ENSDART00000148561
ENSDART00000148707
ENSDART00000149359

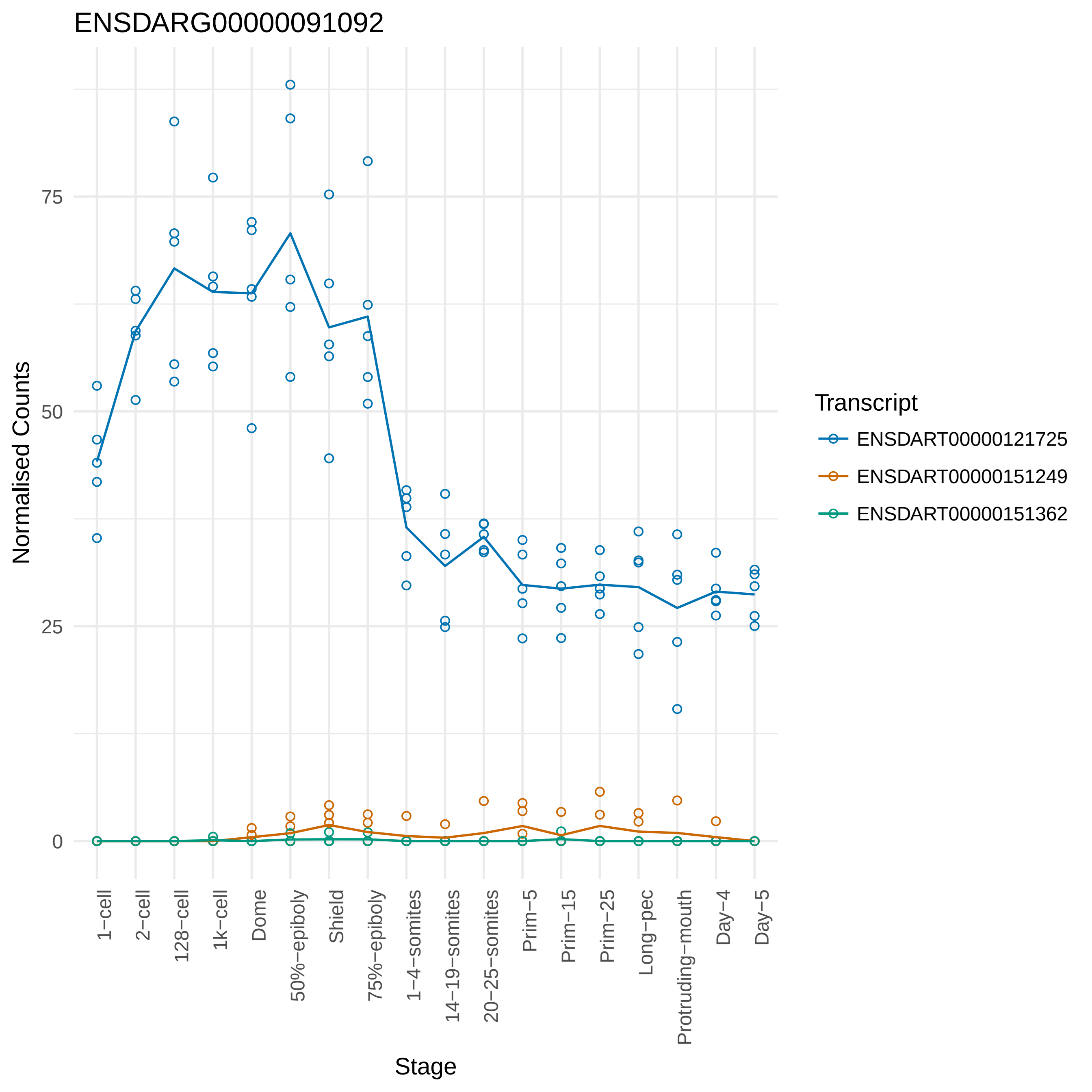
ENSDARG00000091092
Normalised Counts
75
50
25
0
Stage
1−cell
2−cell
128−cell
1k−cell
Dome
50%−epiboly
Shield
75%−epiboly
1−4−somites
14−19−somites
20−25−somites
Prim−5
Prim−15
Prim−25
Long−pec
Protruding−mouth
Day−4
Day−5
Transcript
ENSDART00000121725
ENSDART00000151249
ENSDART00000151362

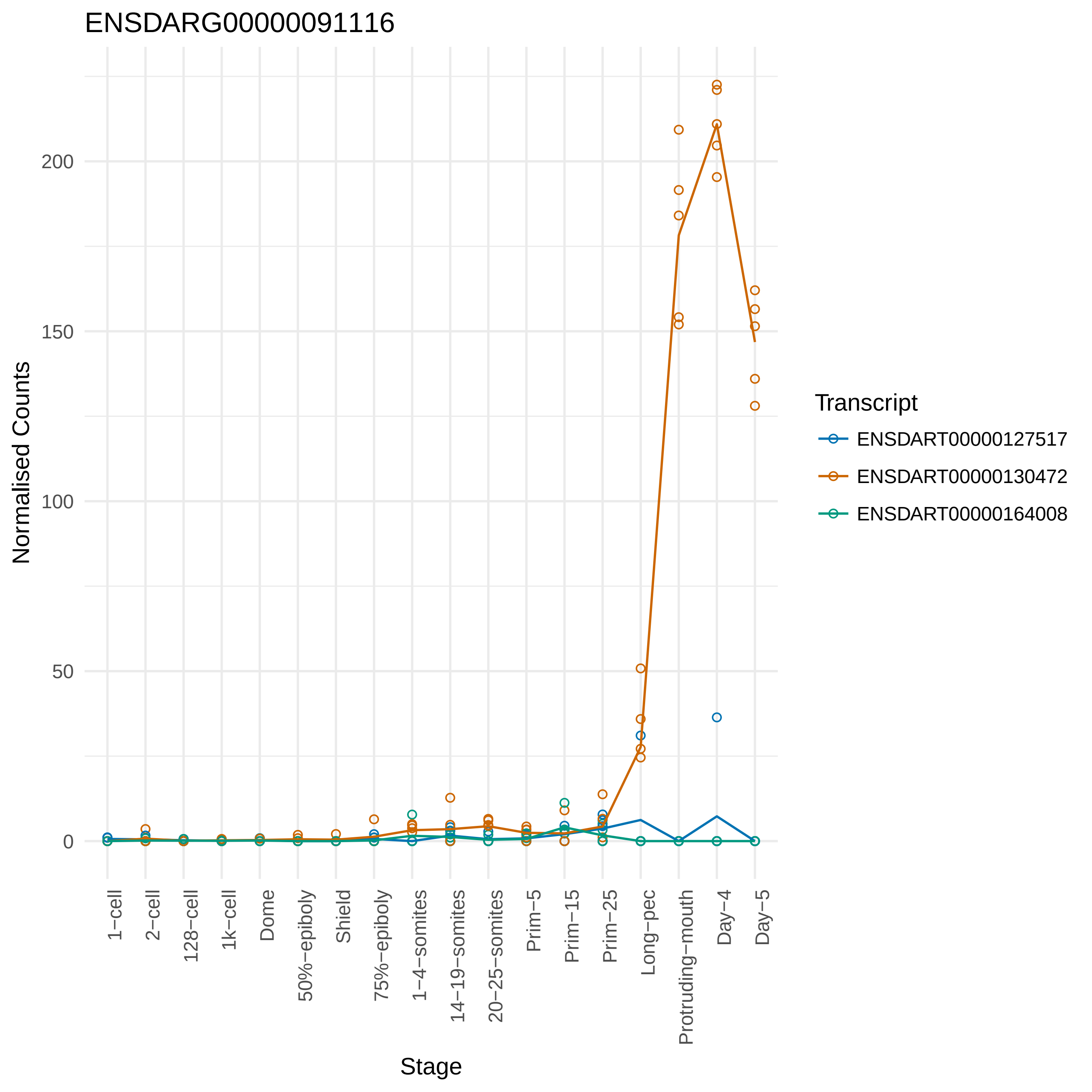

ENSDARG00000091116
Normalised Counts
0
50
100
150
200
Stage
1−cell
2−cell
128−cell
1k−cell
Dome
50%−epiboly
Shield
75%−epiboly
1−4−somites
14−19−somites
20−25−somites
Prim−5
Prim−15
Prim−25
Long−pec
Protruding−mouth
Day−4
Day−5
Transcript
ENSDART00000127517
ENSDART00000130472
ENSDART00000164008

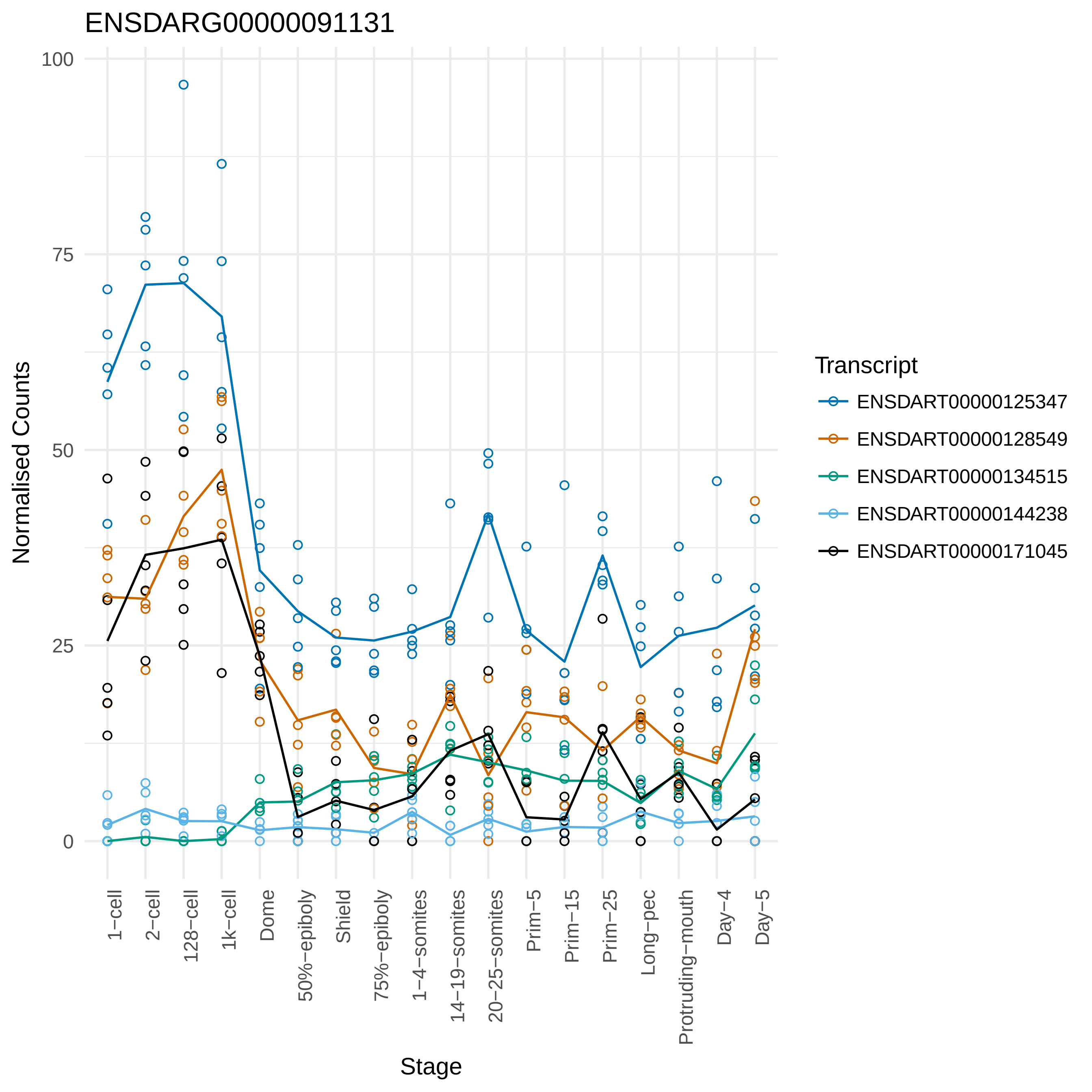

ENSDARG00000091131
Normalised Counts
100
75
50
25
0
1−cell
2−cell
128−cell
1k−cell
Dome
50%−epiboly
Shield
75%−epiboly
1−4−somites
14−19−somites
20−25−somites
Prim−5
Prim−15
Prim−25
Long−pec
Protruding−mouth
Day−4
Day−5
Stage
Transcript
ENSDART00000125347
ENSDART00000128549
ENSDART00000134515
ENSDART00000144238
ENSDART00000171045

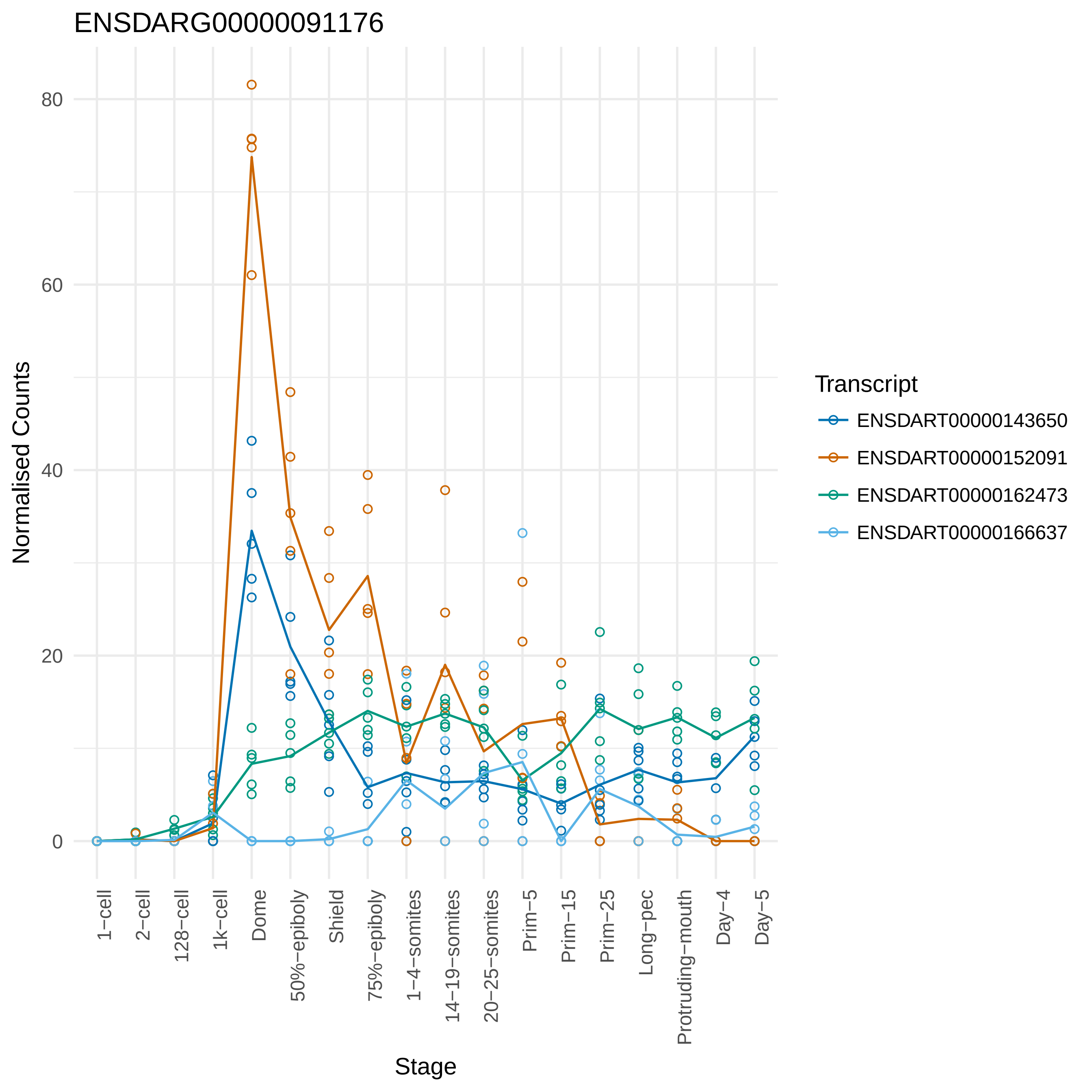

ENSDARG00000091176
Normalised Counts
0
20
40
60
80
Stage
1−cell
2−cell
128−cell
1k−cell
Dome
50%−epiboly
Shield
75%−epiboly
1−4−somites
14−19−somites
20−25−somites
Prim−5
Prim−15
Prim−25
Long−pec
Protruding−mouth
Day−4
Day−5
Transcript
ENSDART00000143650
ENSDART00000152091
ENSDART00000162473
ENSDART00000166637

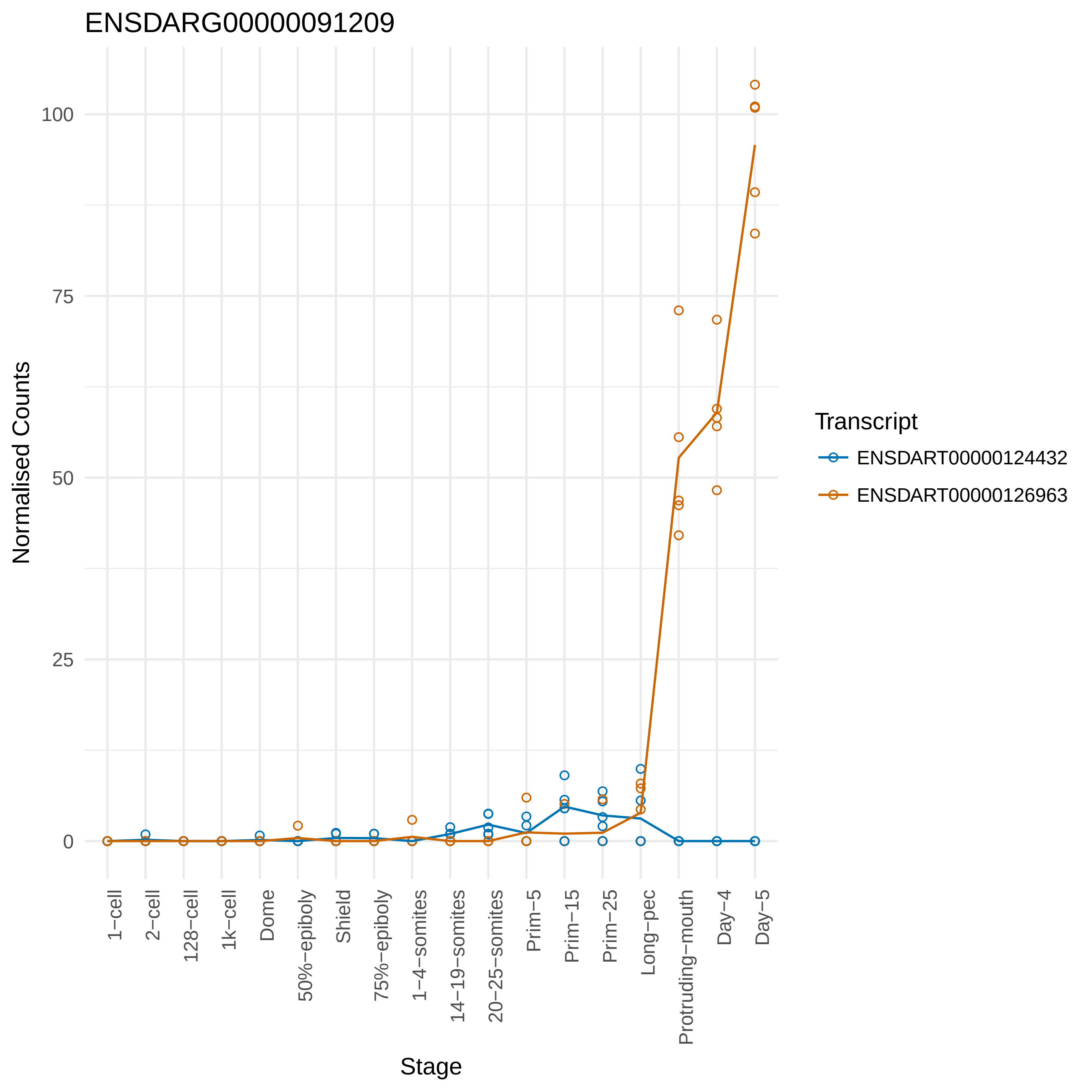
ENSDARG00000091209
Normalised Counts
100
75
50
25
0
1−cell
2−cell
128−cell
1k−cell
Dome
50%−epiboly
Shield
75%−epiboly
1−4−somites
14−19−somites
20−25−somites
Prim−5
Prim−15
Prim−25
Long−pec
Protruding−mouth
Day−4
Day−5
Stage
Transcript
ENSDART00000124432
ENSDART00000126963

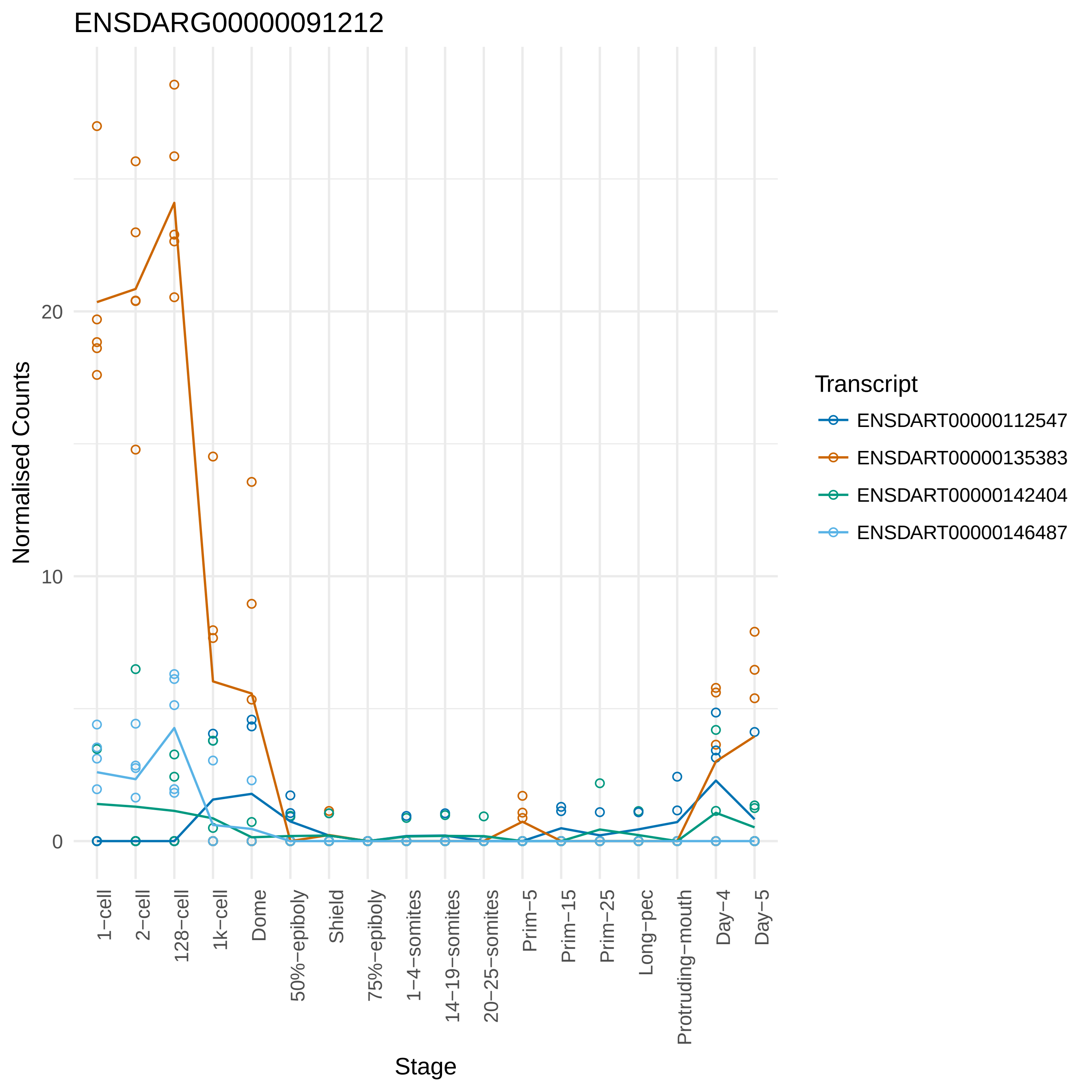

ENSDARG00000091212
Normalised Counts
0
10
20
1−cell
2−cell
128−cell
1k−cell
Dome
50%−epiboly
Shield
75%−epiboly
1−4−somites
14−19−somites
20−25−somites
Prim−5
Prim−15
Prim−25
Long−pec
Protruding−mouth
Day−4
Day−5
Stage
Transcript
ENSDART00000112547
ENSDART00000135383
ENSDART00000142404
ENSDART00000146487

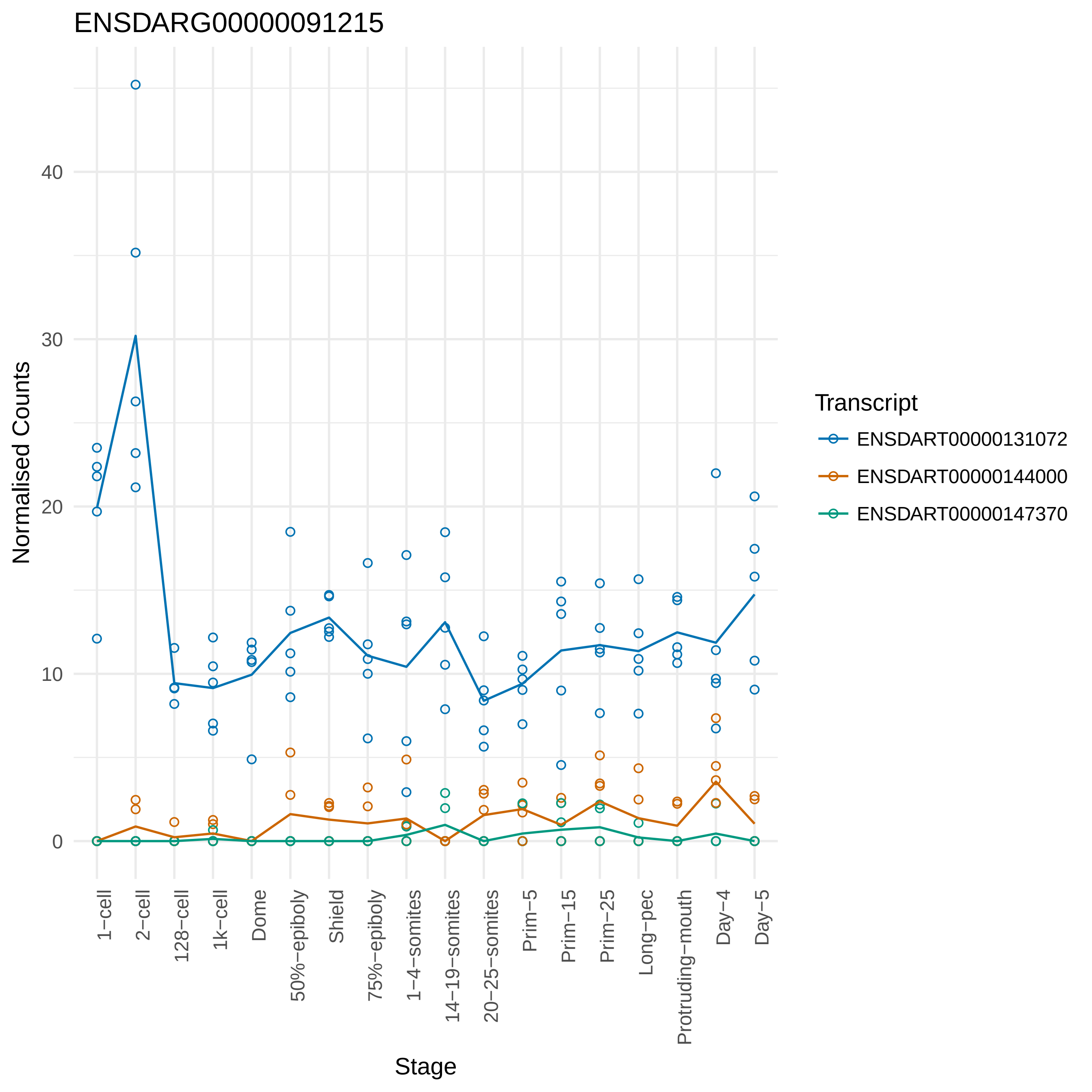

ENSDARG00000091215
Normalised Counts
0
10
20
30
40
Stage
1−cell
2−cell
128−cell
1k−cell
Dome
50%−epiboly
Shield
75%−epiboly
1−4−somites
14−19−somites
20−25−somites
Prim−5
Prim−15
Prim−25
Long−pec
Protruding−mouth
Day−4
Day−5
Transcript
ENSDART00000131072
ENSDART00000144000
ENSDART00000147370

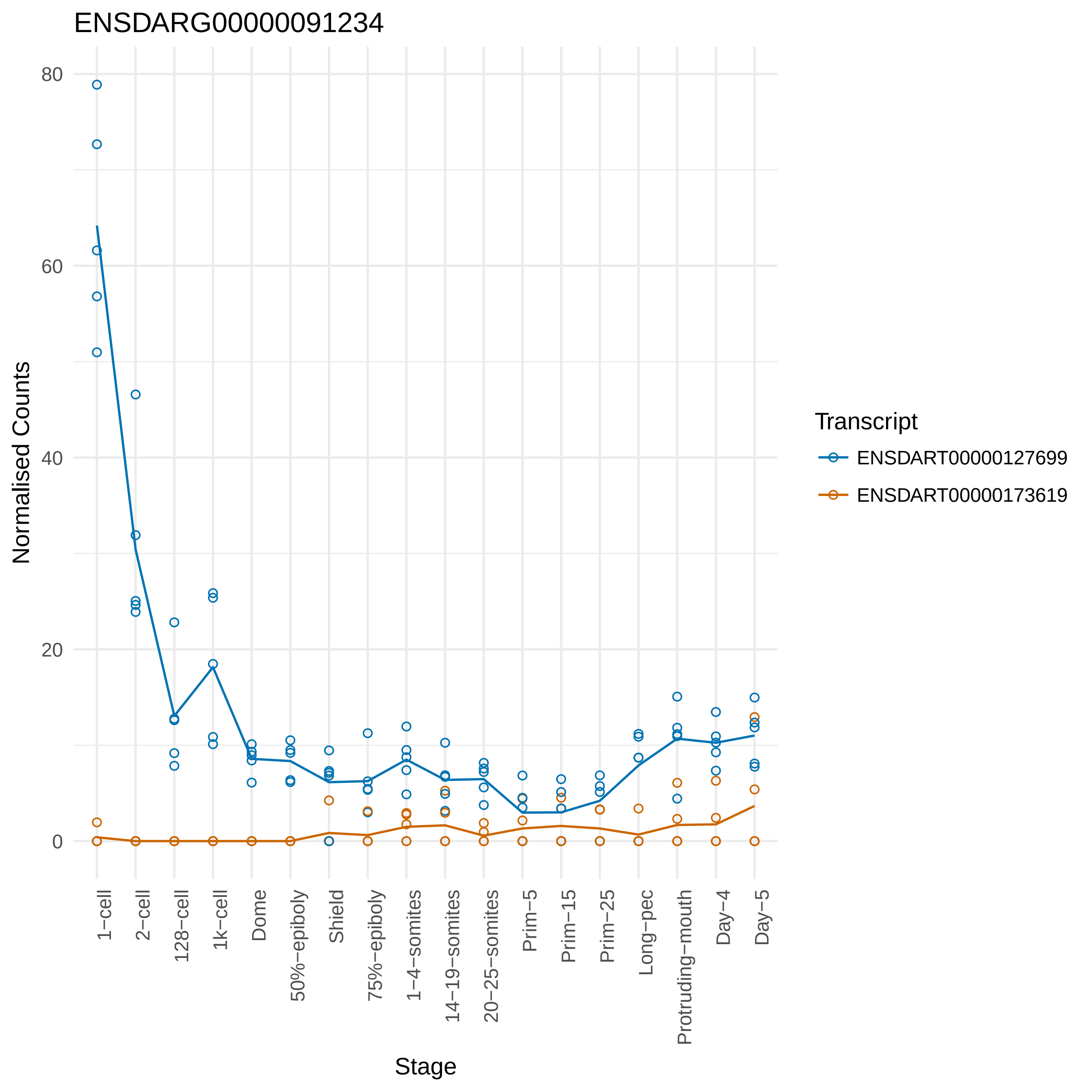
ENSDARG00000091234
Normalised Counts
80
60
40
20
0
1−cell
2−cell
128−cell
1k−cell
Dome
50%−epiboly
Shield
75%−epiboly
1−4−somites
14−19−somites
20−25−somites
Prim−5
Prim−15
Prim−25
Long−pec
Protruding−mouth
Day−4
Day−5
Stage
Transcript
ENSDART00000127699
ENSDART00000173619

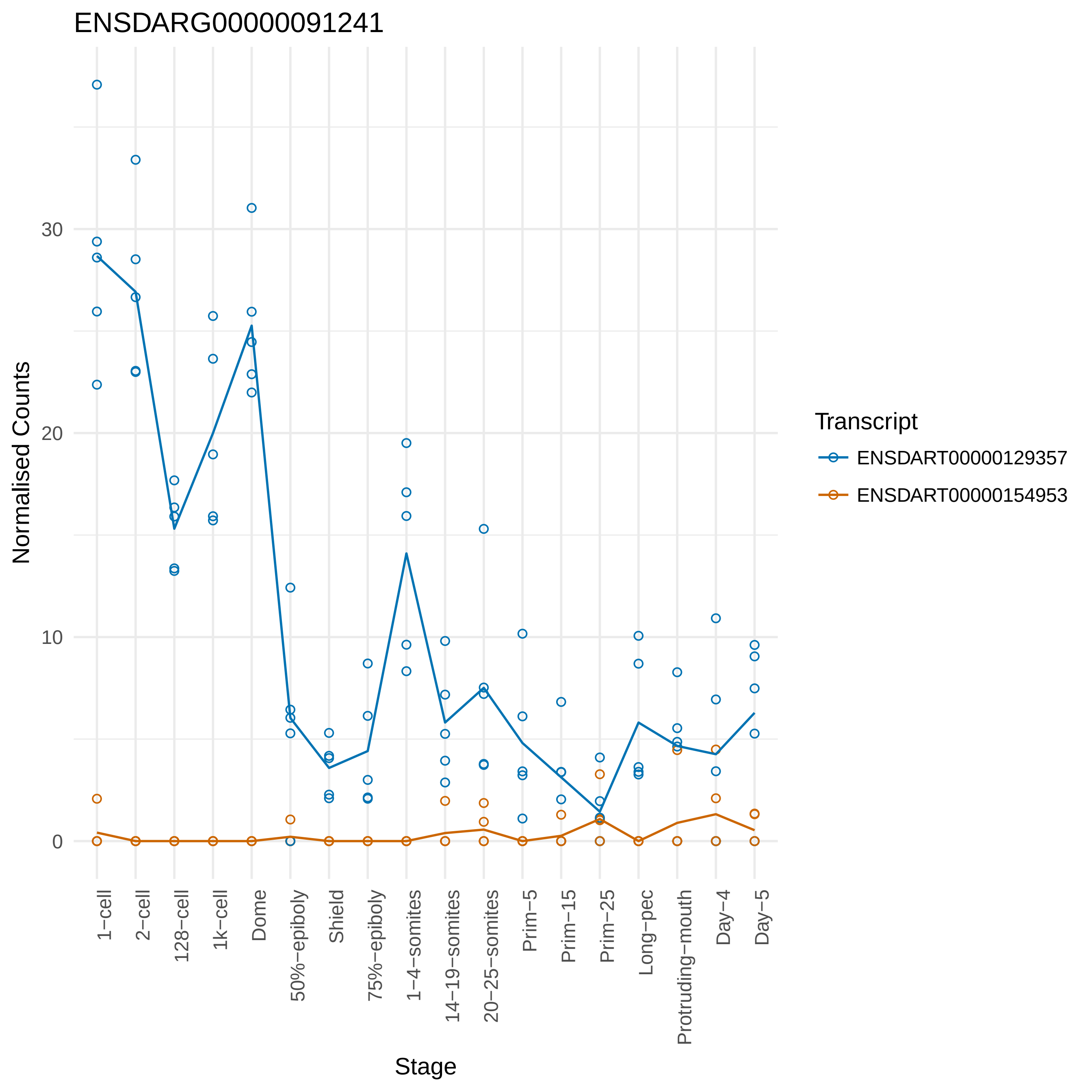
ENSDARG00000091241
Normalised Counts
0
10
20
30
Stage
1−cell
2−cell
128−cell
1k−cell
Dome
50%−epiboly
Shield
75%−epiboly
1−4−somites
14−19−somites
20−25−somites
Prim−5
Prim−15
Prim−25
Long−pec
Protruding−mouth
Day−4
Day−5
Transcript
ENSDART00000129357
ENSDART00000154953

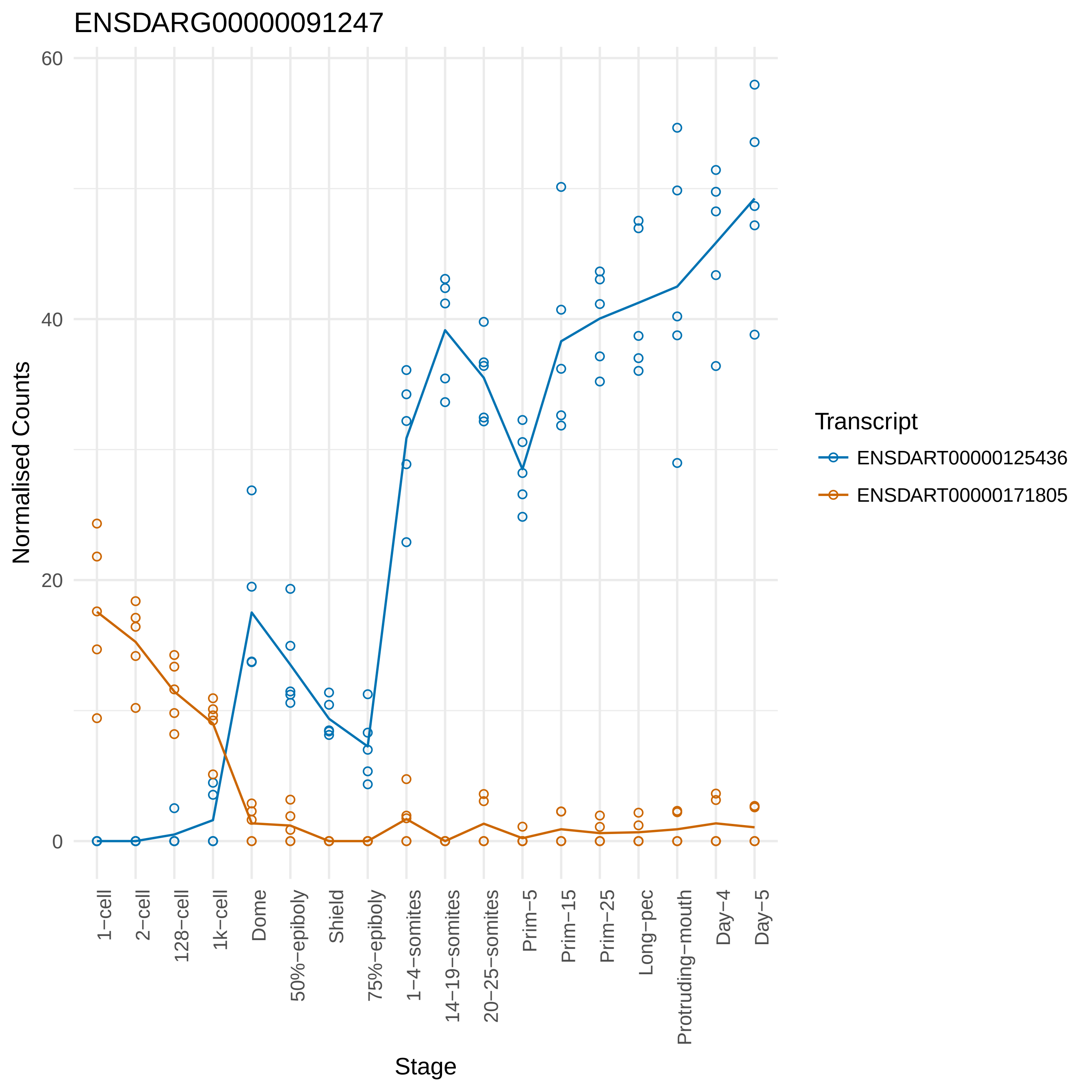
ENSDARG00000091247
Normalised Counts
0
20
40
60
1−cell
2−cell
128−cell
1k−cell
Dome
50%−epiboly
Shield
75%−epiboly
1−4−somites
14−19−somites
20−25−somites
Prim−5
Prim−15
Prim−25
Long−pec
Protruding−mouth
Day−4
Day−5
Stage
Transcript
ENSDART00000125436
ENSDART00000171805

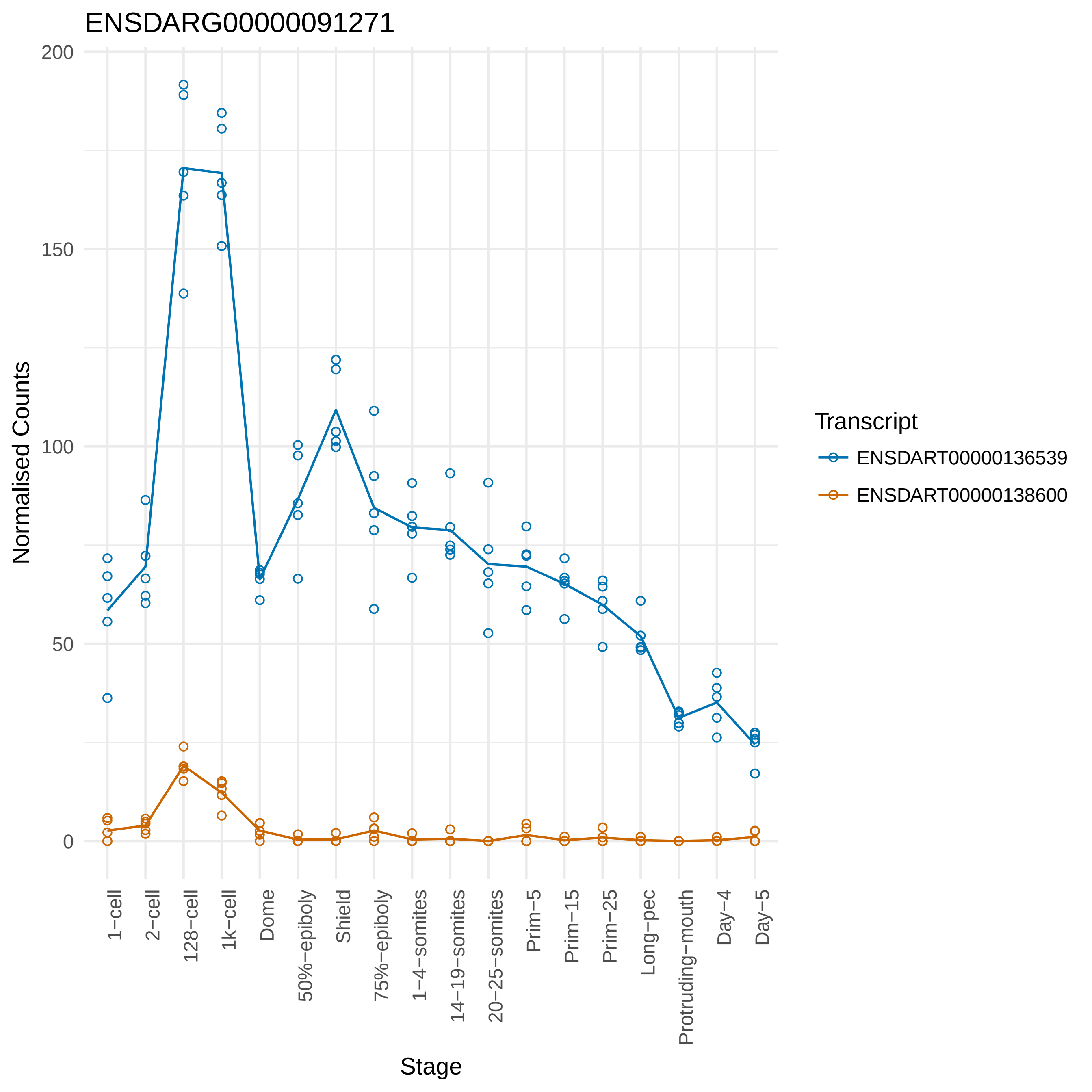

ENSDARG00000091271
Normalised Counts
0
50
100
150
200
1−cell
2−cell
128−cell
1k−cell
Dome
50%−epiboly
Shield
75%−epiboly
1−4−somites
14−19−somites
20−25−somites
Prim−5
Prim−15
Prim−25
Long−pec
Protruding−mouth
Day−4
Day−5
Stage
Transcript
ENSDART00000136539
ENSDART00000138600

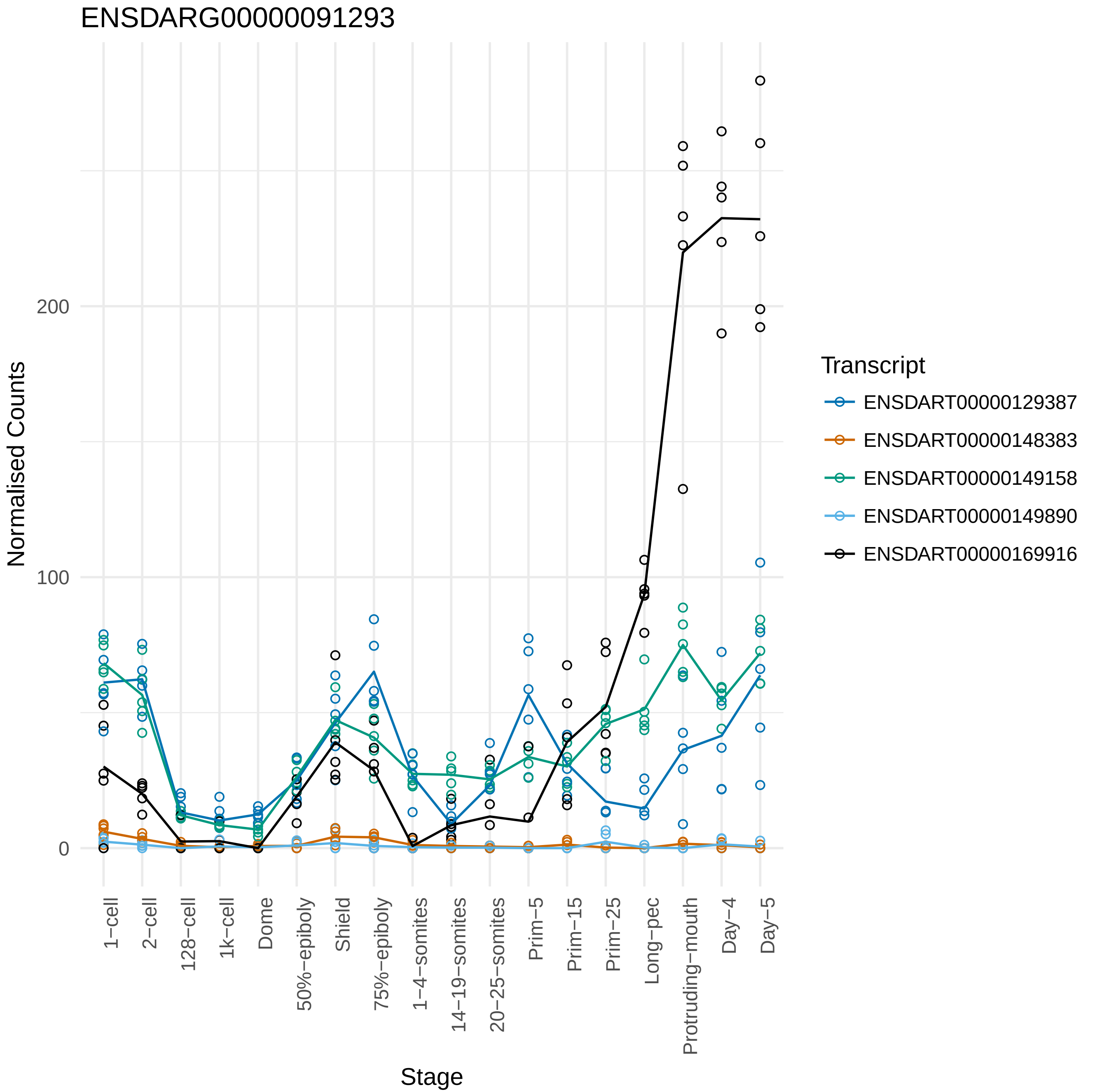

ENSDARG00000091293
Normalised Counts
0
100
200
1−cell
2−cell
128−cell
1k−cell
Dome
50%−epiboly
Shield
75%−epiboly
1−4−somites
14−19−somites
20−25−somites
Prim−5
Prim−15
Prim−25
Long−pec
Protruding−mouth
Day−4
Day−5
Stage
Transcript
ENSDART00000129387
ENSDART00000148383
ENSDART00000149158
ENSDART00000149890
ENSDART00000169916

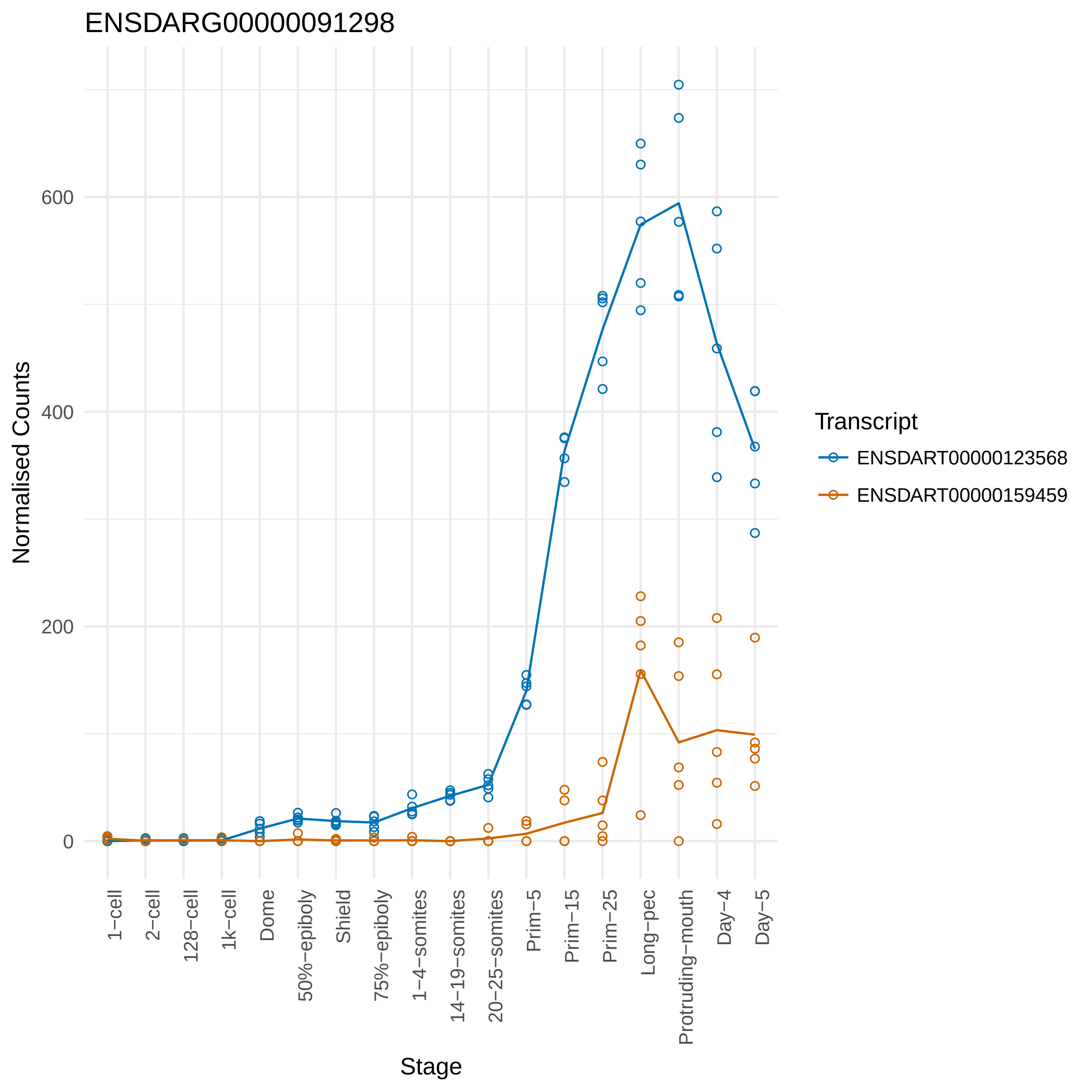

ENSDARG00000091298
Normalised Counts
0
200
400
600
Stage
1−cell
2−cell
128−cell
1k−cell
Dome
50%−epiboly
Shield
75%−epiboly
1−4−somites
14−19−somites
20−25−somites
Prim−5
Prim−15
Prim−25
Long−pec
Protruding−mouth
Day−4
Day−5
Transcript
ENSDART00000123568
ENSDART00000159459

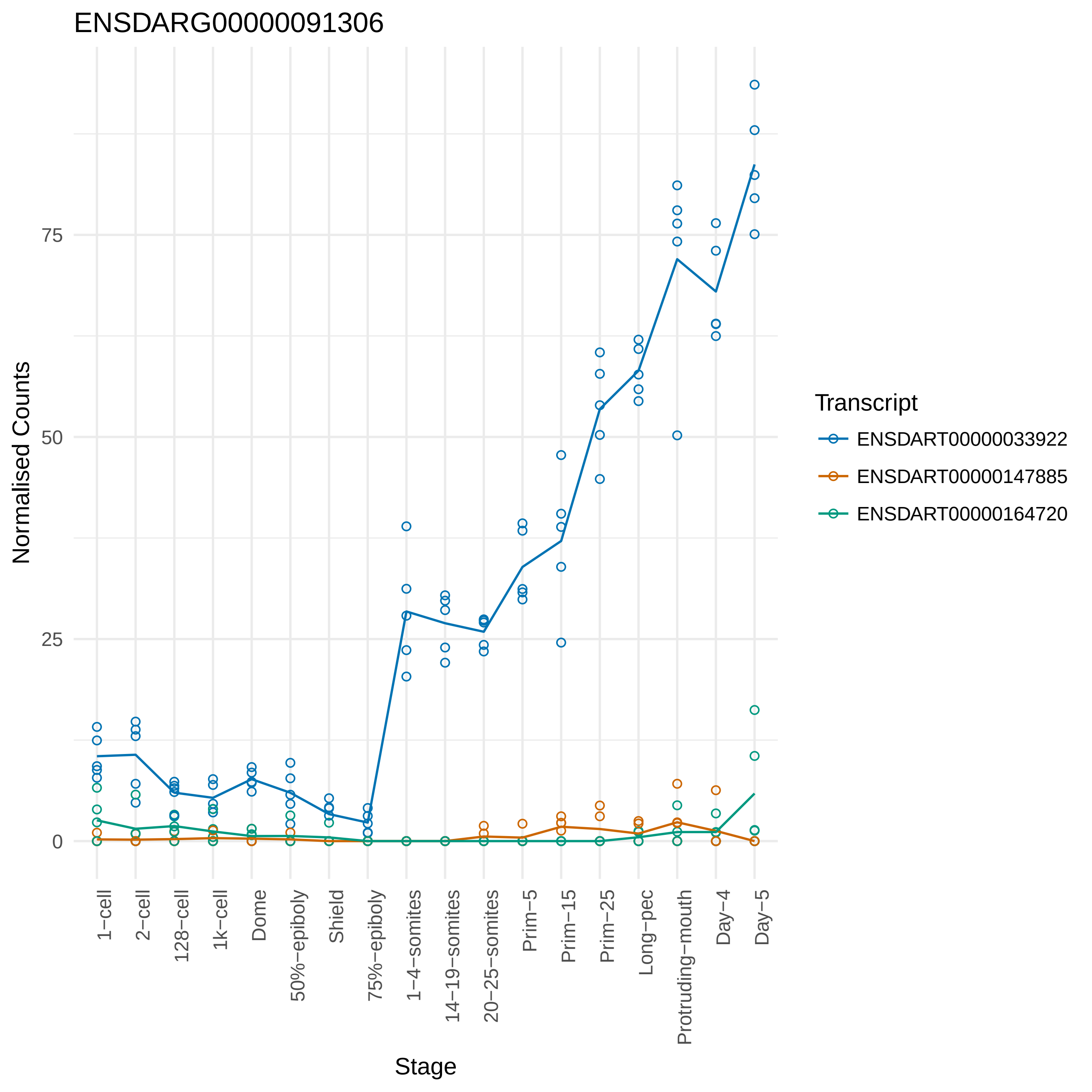
ENSDARG00000091306
Normalised Counts
0
25
50
75
1−cell
2−cell
128−cell
1k−cell
Dome
50%−epiboly
Shield
75%−epiboly
1−4−somites
14−19−somites
20−25−somites
Prim−5
Prim−15
Prim−25
Long−pec
Protruding−mouth
Day−4
Day−5
Stage
Transcript
ENSDART00000033922
ENSDART00000147885
ENSDART00000164720

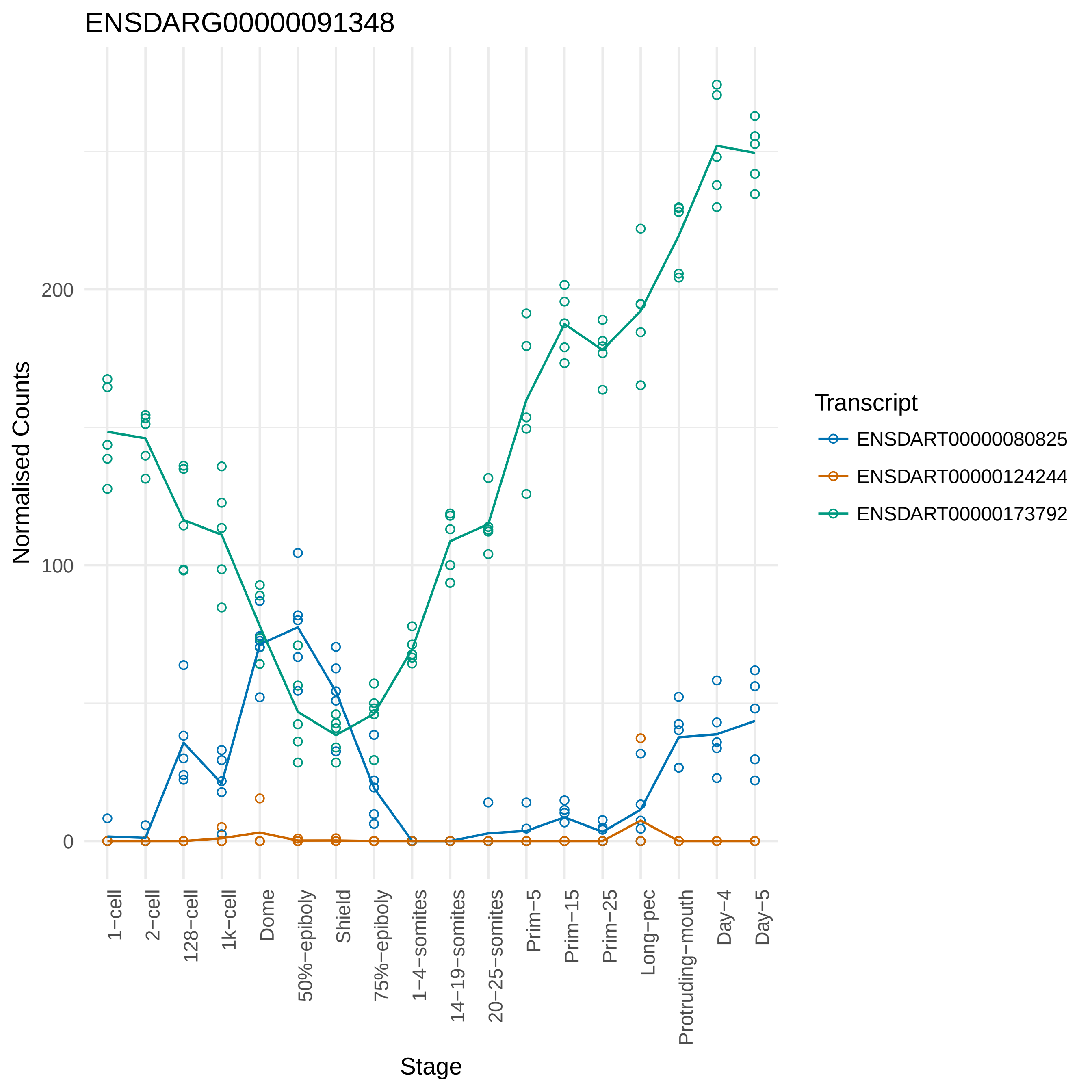
ENSDARG00000091348
Normalised Counts
0
100
200
Stage
1−cell
2−cell
128−cell
1k−cell
Dome
50%−epiboly
Shield
75%−epiboly
1−4−somites
14−19−somites
20−25−somites
Prim−5
Prim−15
Prim−25
Long−pec
Protruding−mouth
Day−4
Day−5
Transcript
ENSDART00000080825
ENSDART00000124244
ENSDART00000173792

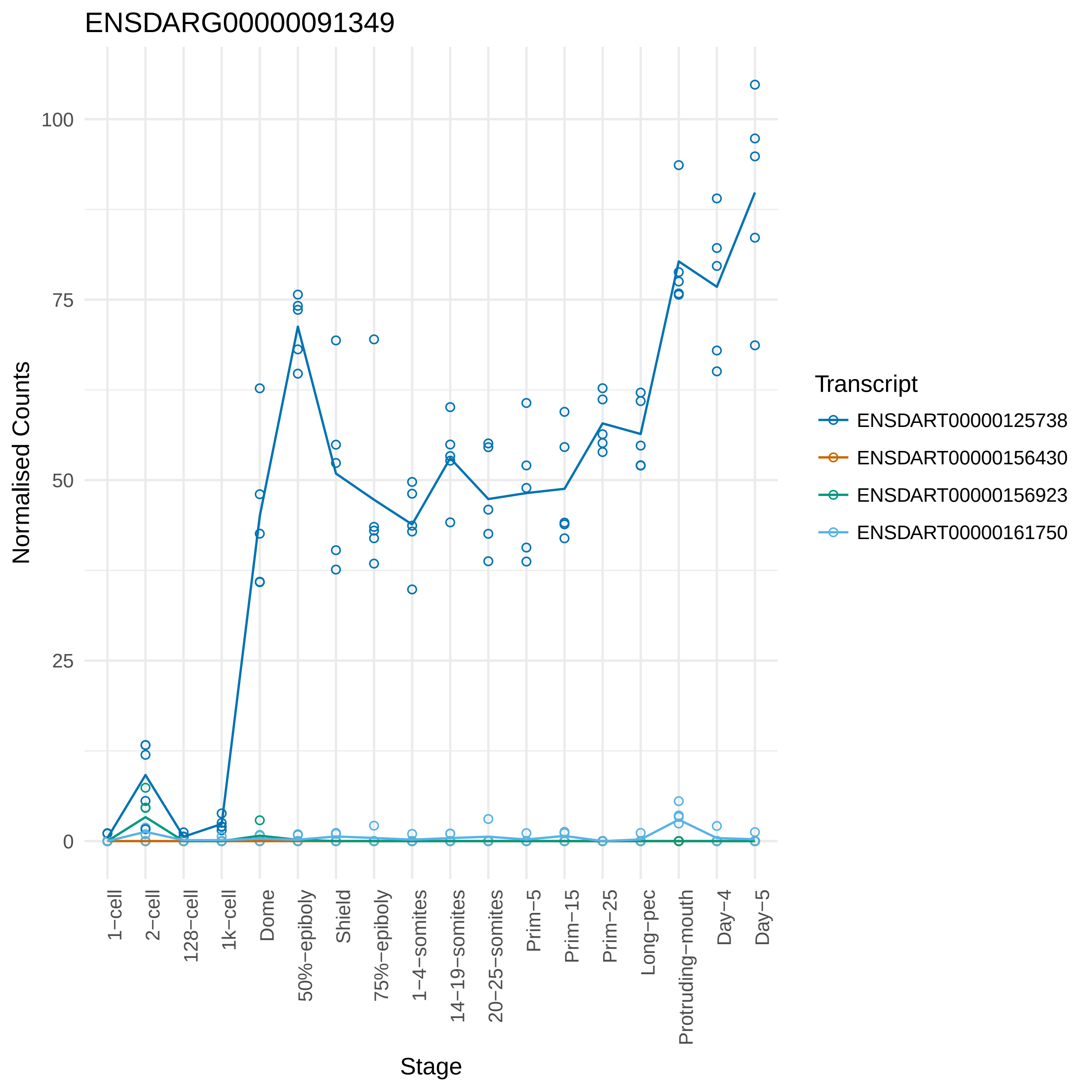

ENSDARG00000091349
Normalised Counts
100
75
50
25
0
1−cell
2−cell
128−cell
1k−cell
Dome
50%−epiboly
Shield
75%−epiboly
1−4−somites
14−19−somites
20−25−somites
Prim−5
Prim−15
Prim−25
Long−pec
Protruding−mouth
Day−4
Day−5
Stage
Transcript
ENSDART00000125738
ENSDART00000156430
ENSDART00000156923
ENSDART00000161750

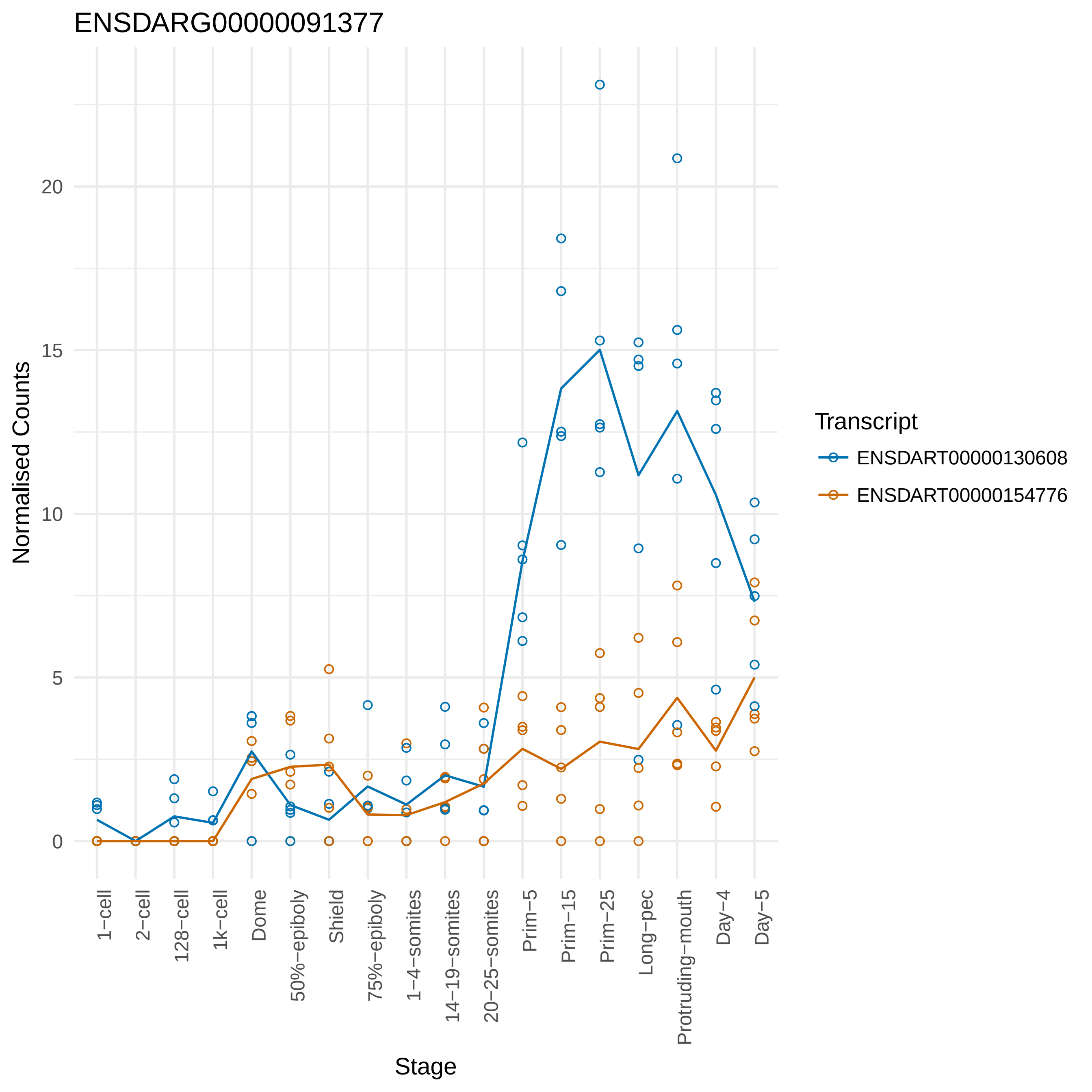
ENSDARG00000091377
Normalised Counts
0
5
10
15
20
1−cell
2−cell
128−cell
1k−cell
Dome
50%−epiboly
Shield
75%−epiboly
1−4−somites
14−19−somites
20−25−somites
Prim−5
Prim−15
Prim−25
Long−pec
Protruding−mouth
Day−4
Day−5
Stage
Transcript
ENSDART00000130608
ENSDART00000154776

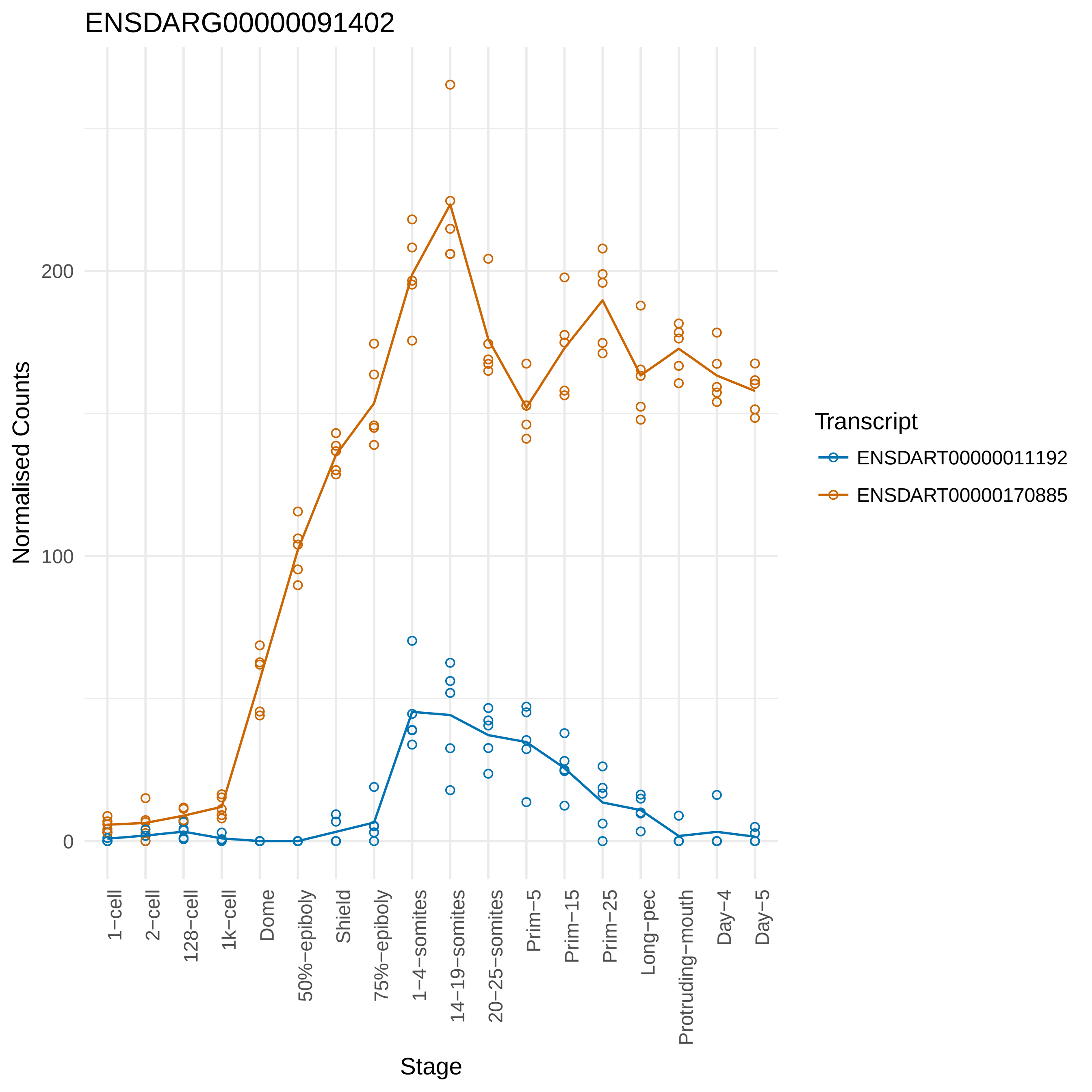
ENSDARG00000091402
Normalised Counts
0
100
200
1−cell
2−cell
128−cell
1k−cell
Dome
50%−epiboly
Shield
75%−epiboly
1−4−somites
14−19−somites
20−25−somites
Prim−5
Prim−15
Prim−25
Long−pec
Protruding−mouth
Day−4
Day−5
Stage
Transcript
ENSDART00000011192
ENSDART00000170885

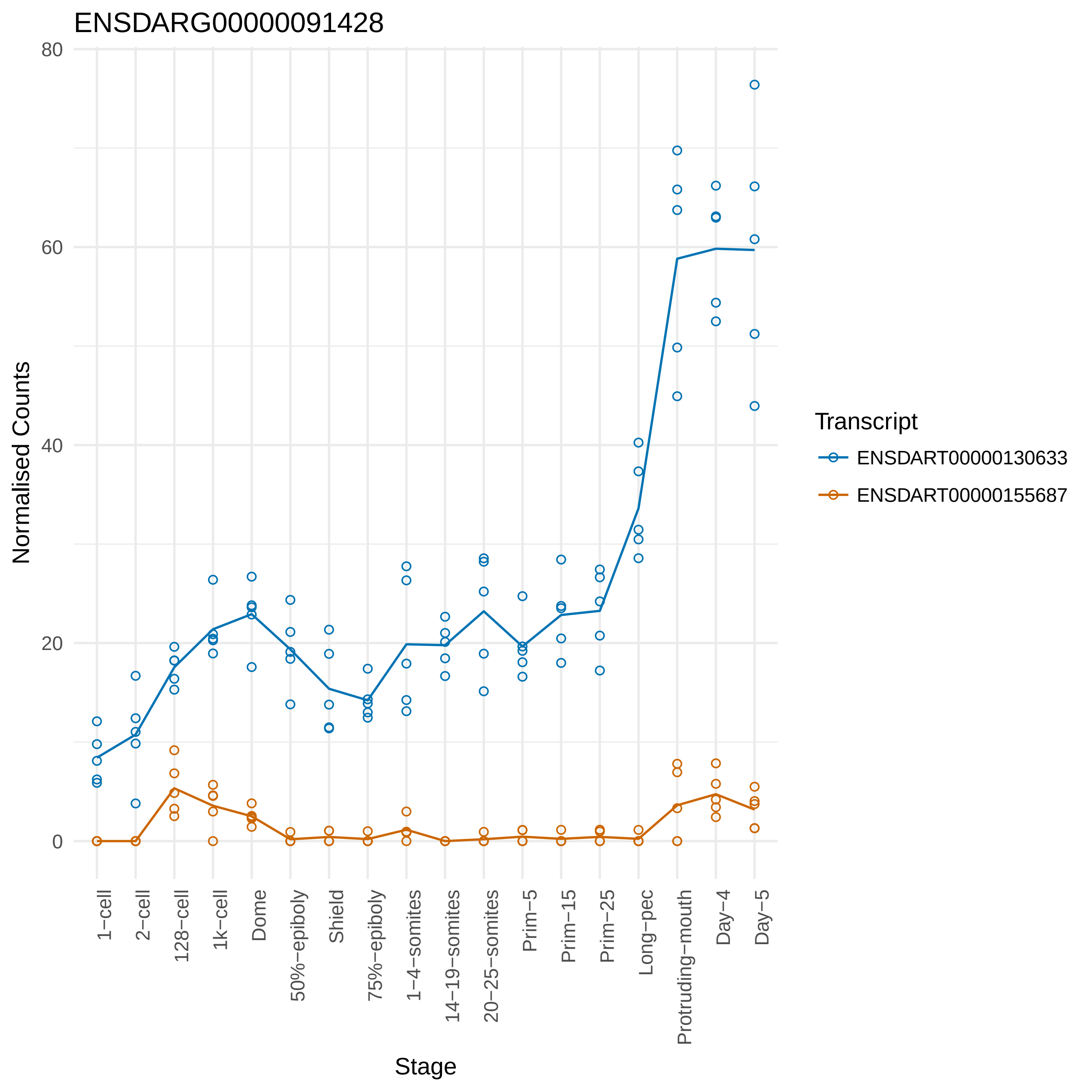
ENSDARG00000091428
Normalised Counts
0
20
40
60
80
Stage
1−cell
2−cell
128−cell
1k−cell
Dome
50%−epiboly
Shield
75%−epiboly
1−4−somites
14−19−somites
20−25−somites
Prim−5
Prim−15
Prim−25
Long−pec
Protruding−mouth
Day−4
Day−5
Transcript
ENSDART00000130633
ENSDART00000155687

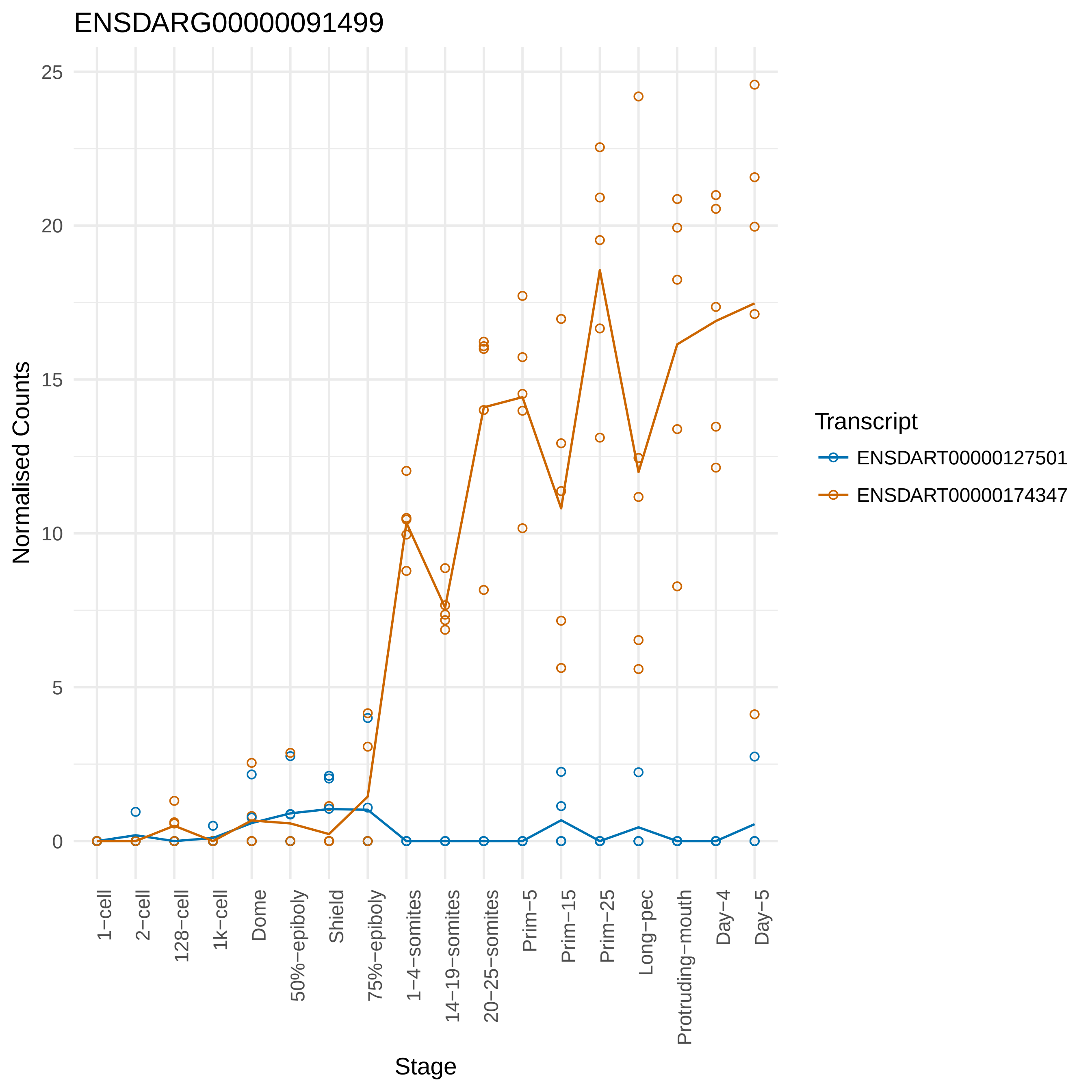
ENSDARG00000091499
Normalised Counts
25
20
15
10
5
0
1−cell
2−cell
128−cell
1k−cell
Dome
50%−epiboly
Shield
75%−epiboly
1−4−somites
14−19−somites
20−25−somites
Prim−5
Prim−15
Prim−25
Long−pec
Protruding−mouth
Day−4
Day−5
Stage
Transcript
ENSDART00000127501
ENSDART00000174347

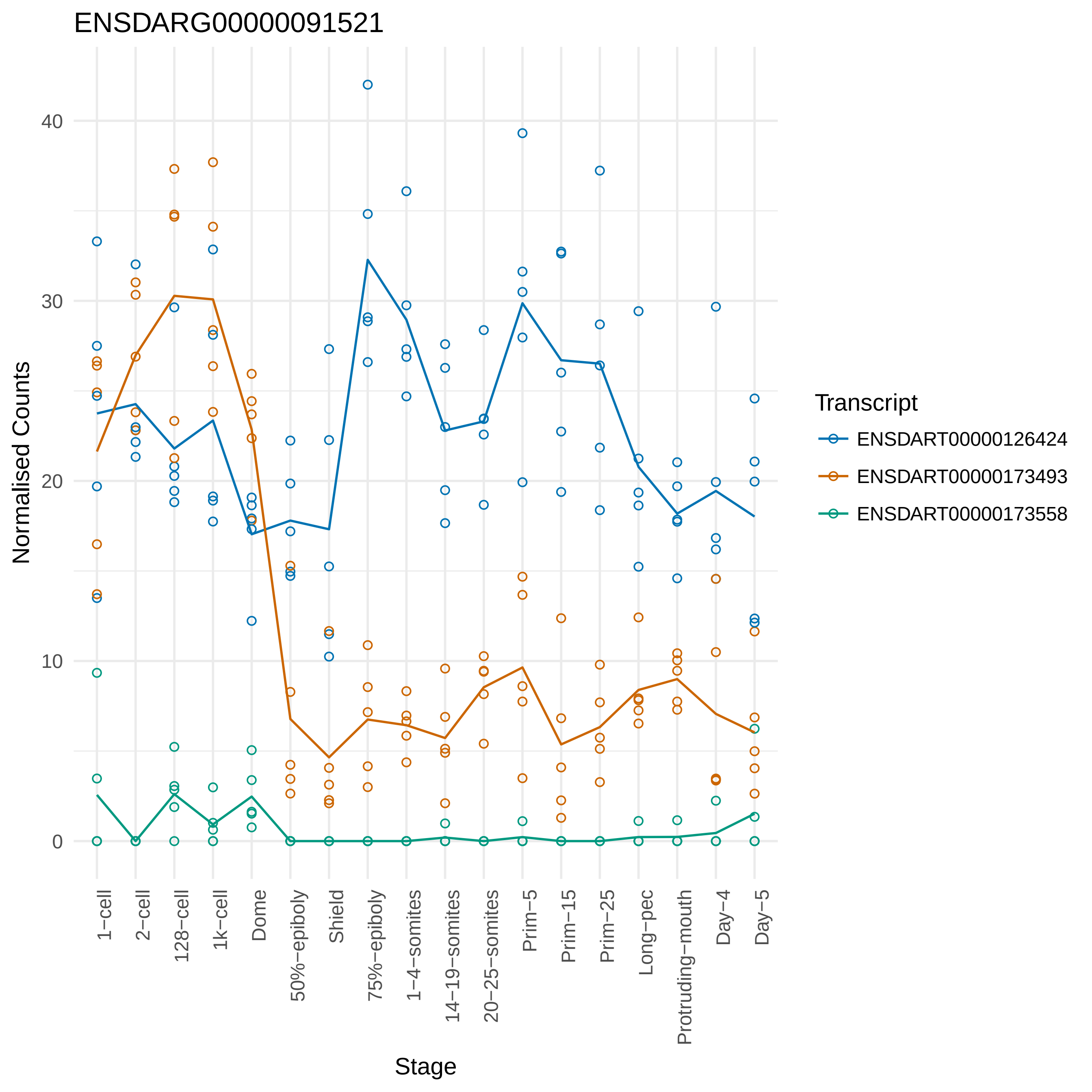
ENSDARG00000091521
Normalised Counts
0
10
20
30
40
Stage
1−cell
2−cell
128−cell
1k−cell
Dome
50%−epiboly
Shield
75%−epiboly
1−4−somites
14−19−somites
20−25−somites
Prim−5
Prim−15
Prim−25
Long−pec
Protruding−mouth
Day−4
Day−5
Transcript
ENSDART00000126424
ENSDART00000173493
ENSDART00000173558

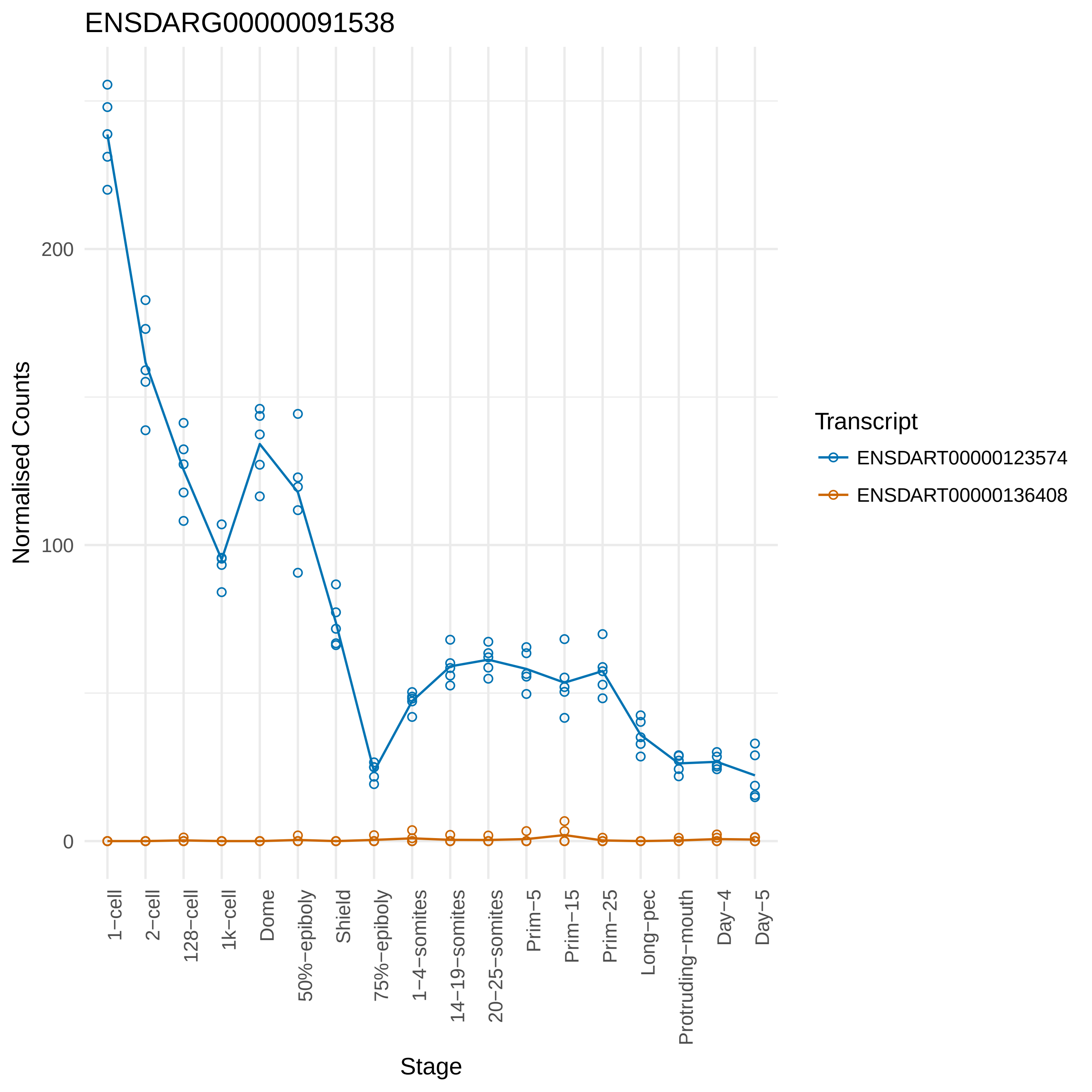

ENSDARG00000091538
Normalised Counts
0
100
200
Stage
1−cell
2−cell
128−cell
1k−cell
Dome
50%−epiboly
Shield
75%−epiboly
1−4−somites
14−19−somites
20−25−somites
Prim−5
Prim−15
Prim−25
Long−pec
Protruding−mouth
Day−4
Day−5
Transcript
ENSDART00000123574
ENSDART00000136408

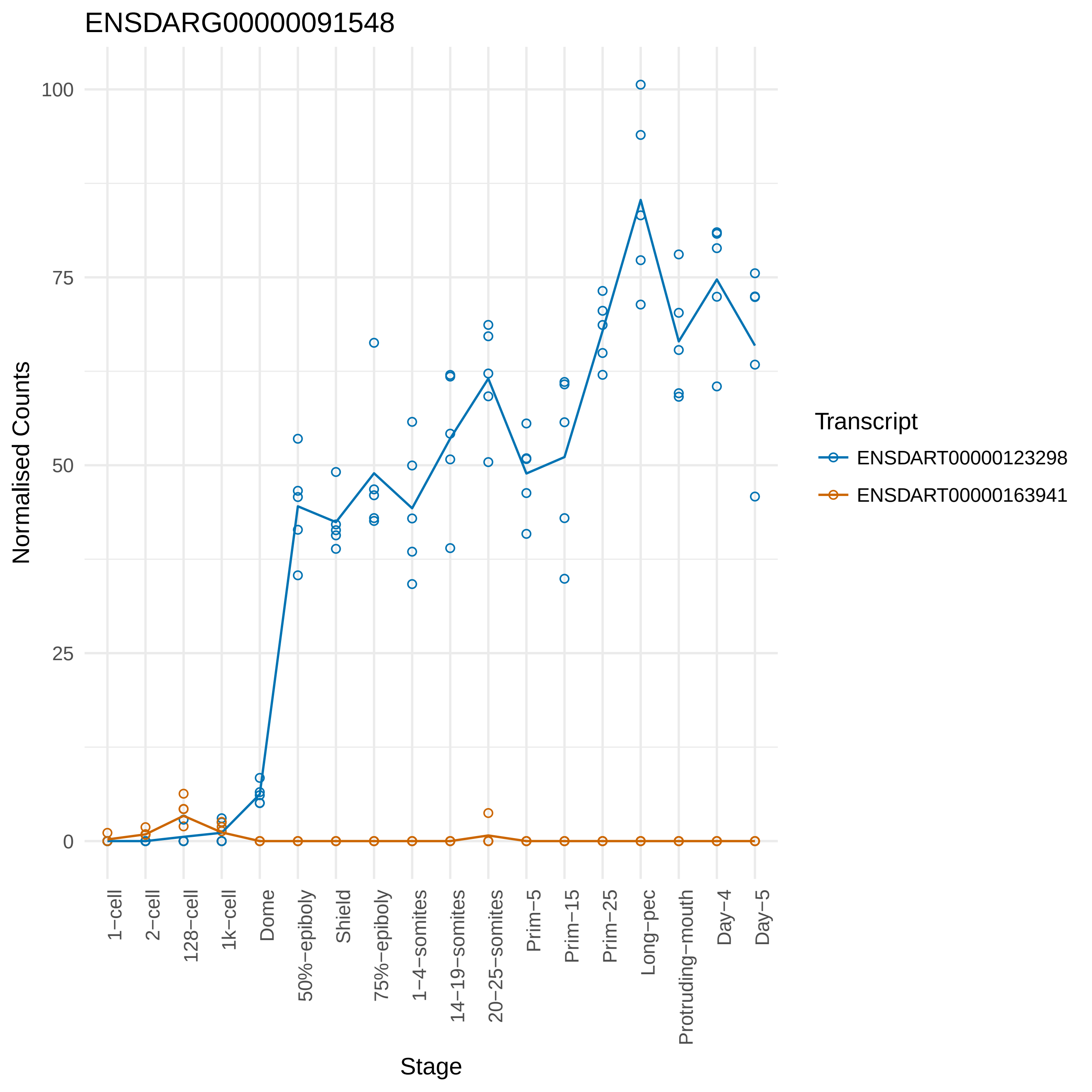

ENSDARG00000091548
Normalised Counts
100
75
50
25
0
1−cell
2−cell
128−cell
1k−cell
Dome
50%−epiboly
Shield
75%−epiboly
1−4−somites
14−19−somites
20−25−somites
Prim−5
Prim−15
Prim−25
Long−pec
Protruding−mouth
Day−4
Day−5
Stage
Transcript
ENSDART00000123298
ENSDART00000163941

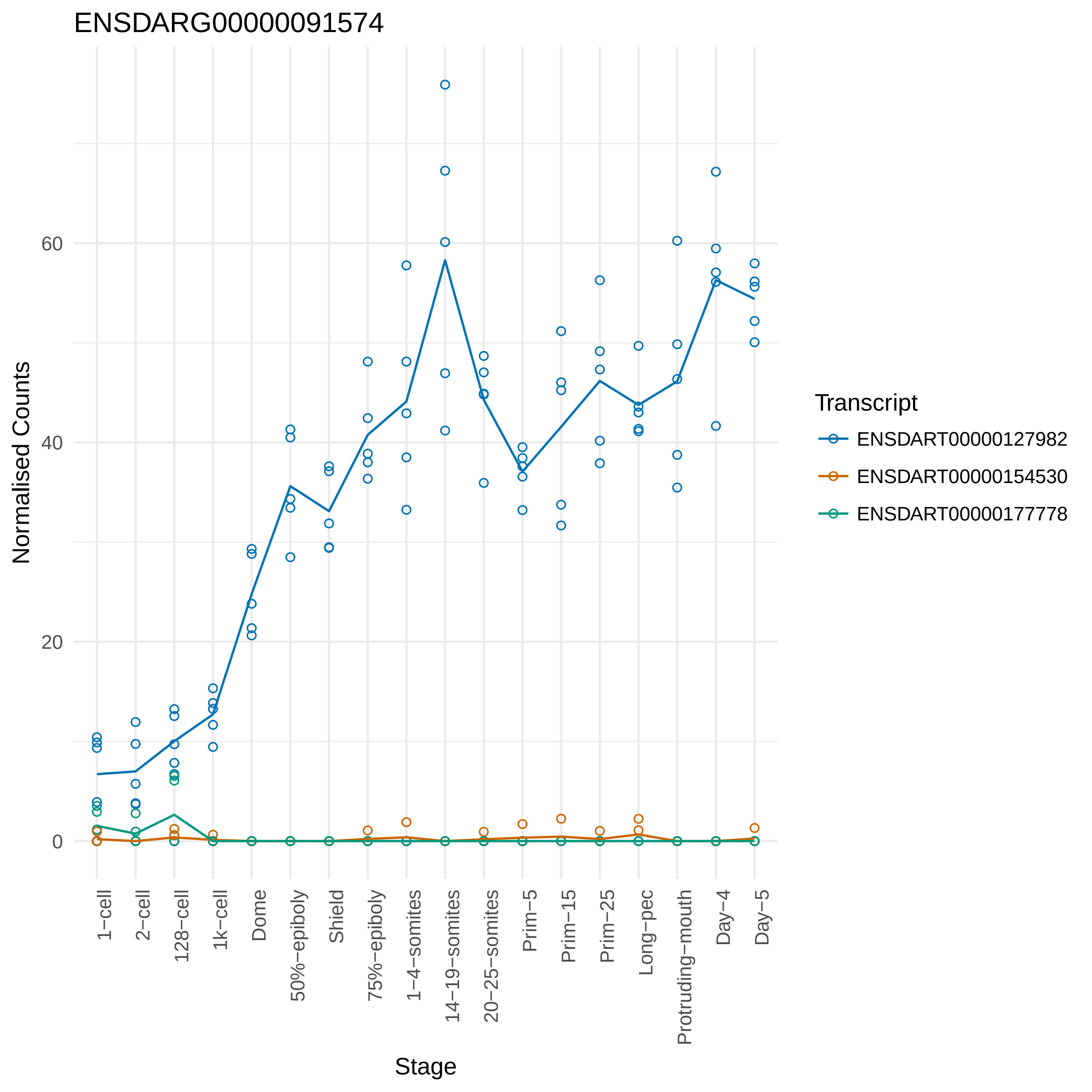
ENSDARG00000091574
Normalised Counts
0
20
40
60
Stage
1−cell
2−cell
128−cell
1k−cell
Dome
50%−epiboly
Shield
75%−epiboly
1−4−somites
14−19−somites
20−25−somites
Prim−5
Prim−15
Prim−25
Long−pec
Protruding−mouth
Day−4
Day−5
Transcript
ENSDART00000127982
ENSDART00000154530
ENSDART00000177778

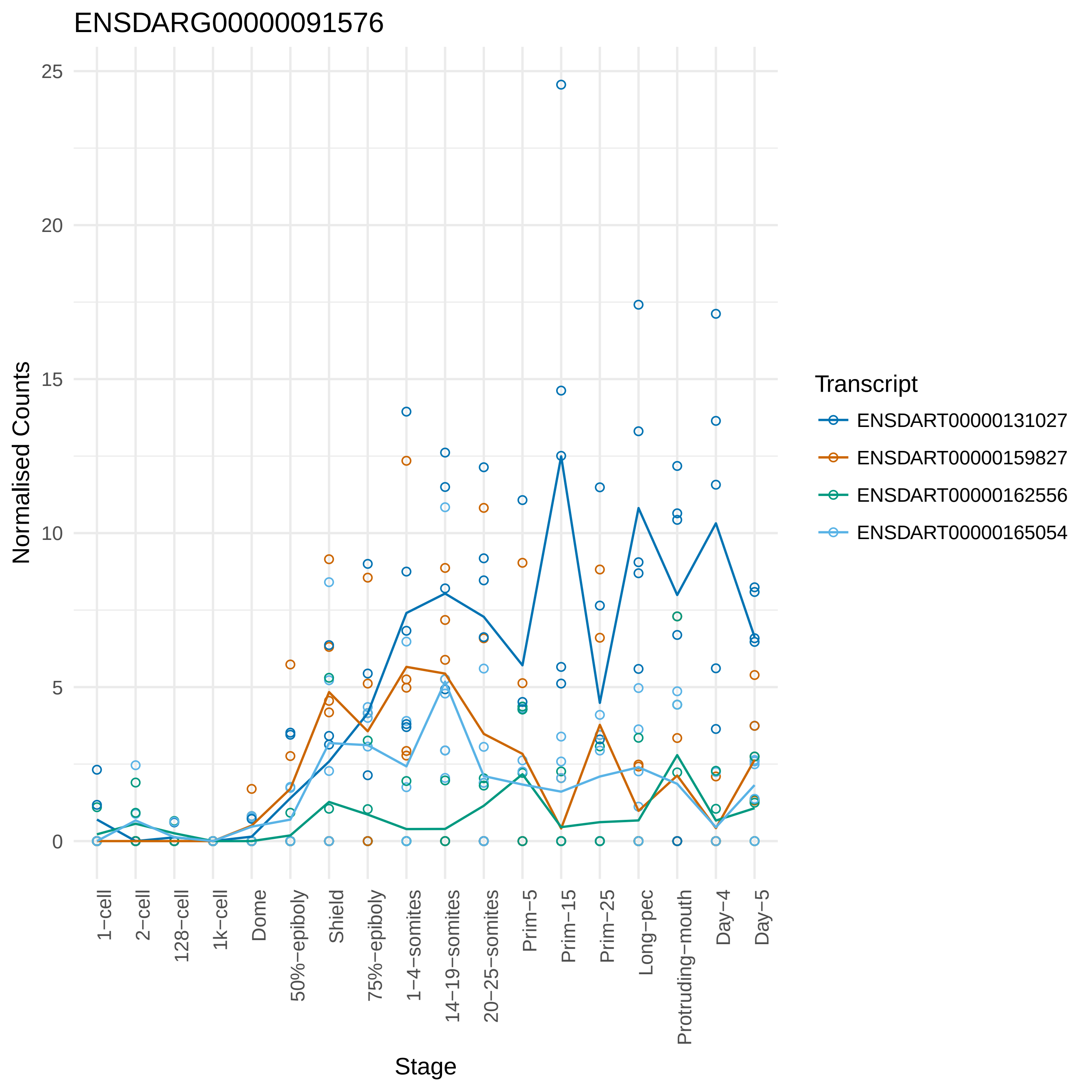
ENSDARG00000091576
Normalised Counts
0
5
10
15
20
25
1−cell
2−cell
128−cell
1k−cell
Dome
50%−epiboly
Shield
75%−epiboly
1−4−somites
14−19−somites
20−25−somites
Prim−5
Prim−15
Prim−25
Long−pec
Protruding−mouth
Day−4
Day−5
Stage
Transcript
ENSDART00000131027
ENSDART00000159827
ENSDART00000162556
ENSDART00000165054

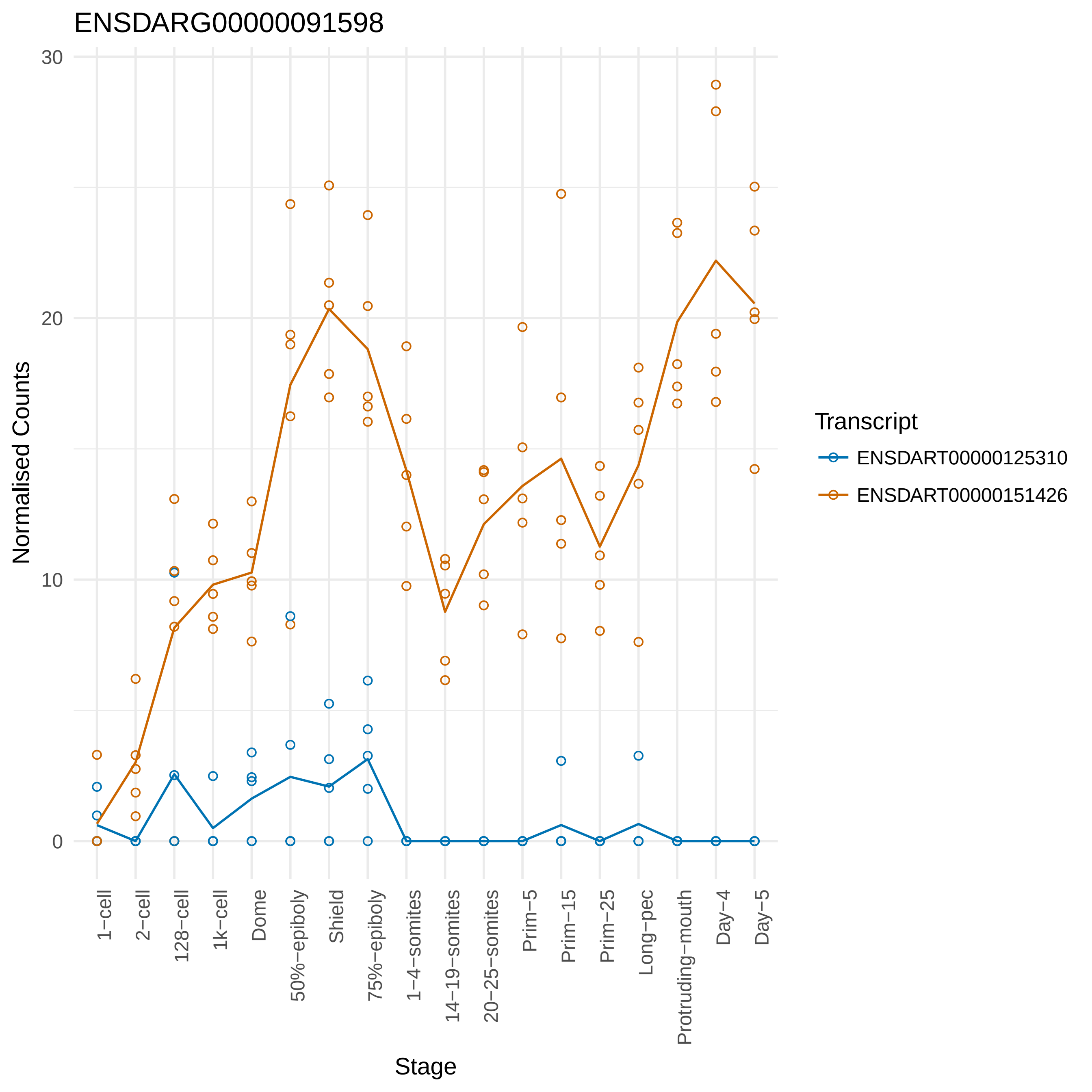

ENSDARG00000091598
Normalised Counts
30
20
10
0
1−cell
2−cell
128−cell
1k−cell
Dome
50%−epiboly
Shield
75%−epiboly
1−4−somites
14−19−somites
20−25−somites
Prim−5
Prim−15
Prim−25
Long−pec
Protruding−mouth
Day−4
Day−5
Stage
Transcript
ENSDART00000125310
ENSDART00000151426

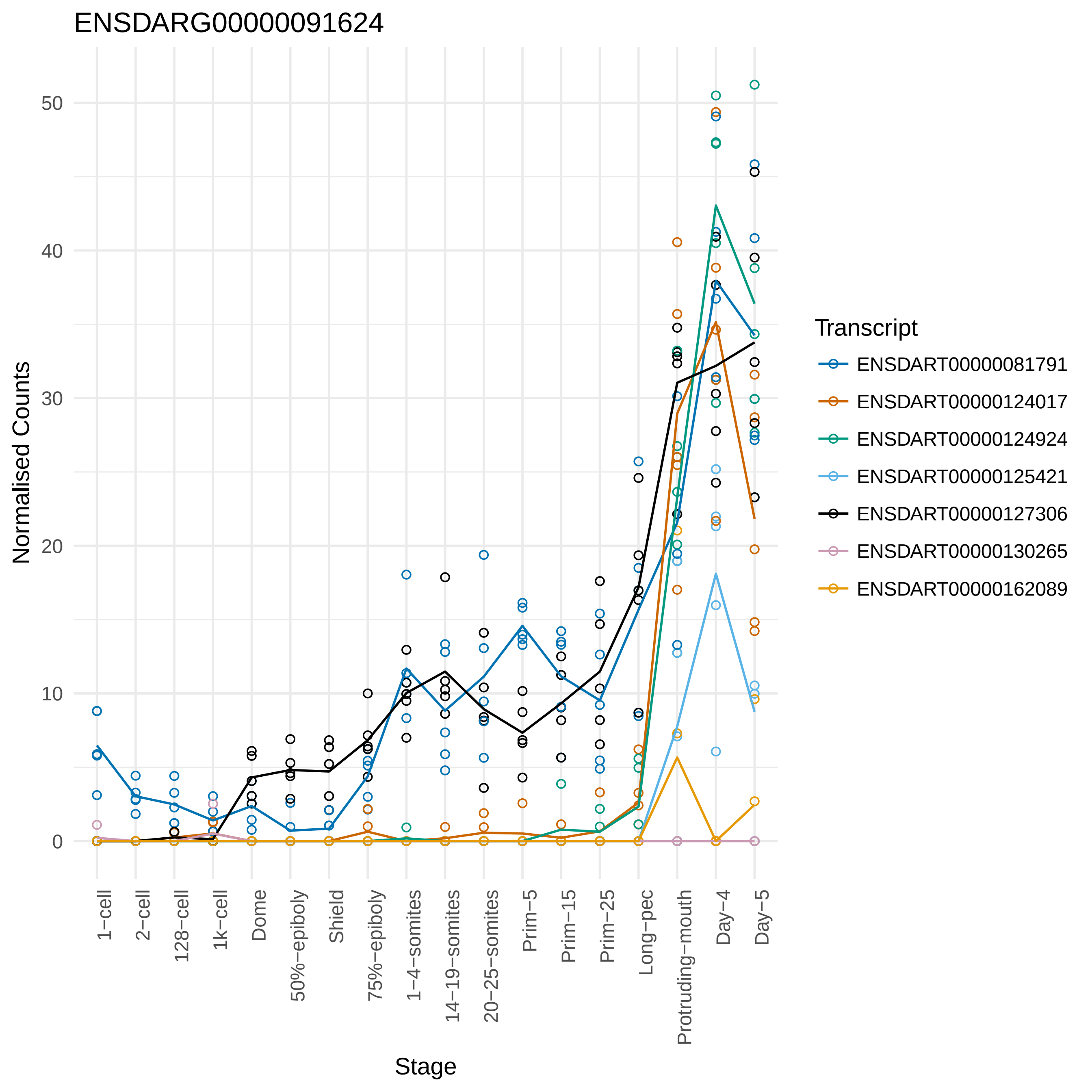
ENSDARG00000091624
Normalised Counts
0
10
20
30
40
50
1−cell
2−cell
128−cell
1k−cell
Dome
50%−epiboly
Shield
75%−epiboly
1−4−somites
14−19−somites
20−25−somites
Prim−5
Prim−15
Prim−25
Long−pec
Protruding−mouth
Day−4
Day−5
Stage
Transcript
ENSDART00000081791
ENSDART00000124017
ENSDART00000124924
ENSDART00000125421
ENSDART00000127306
ENSDART00000130265
ENSDART00000162089

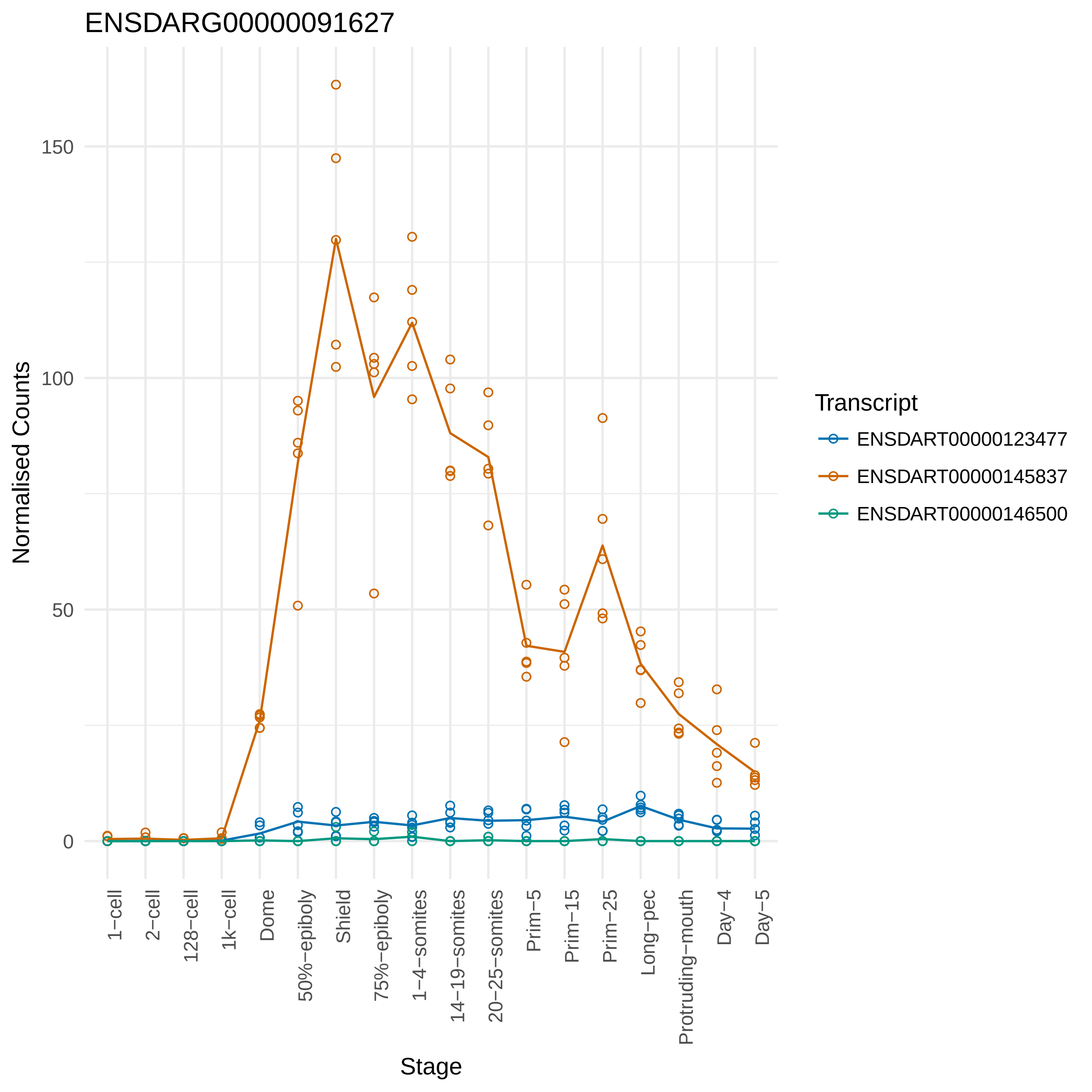

ENSDARG00000091627
Normalised Counts
150
100
50
0
1−cell
2−cell
128−cell
1k−cell
Dome
50%−epiboly
Shield
75%−epiboly
1−4−somites
14−19−somites
20−25−somites
Prim−5
Prim−15
Prim−25
Long−pec
Protruding−mouth
Day−4
Day−5
Stage
Transcript
ENSDART00000123477
ENSDART00000145837
ENSDART00000146500

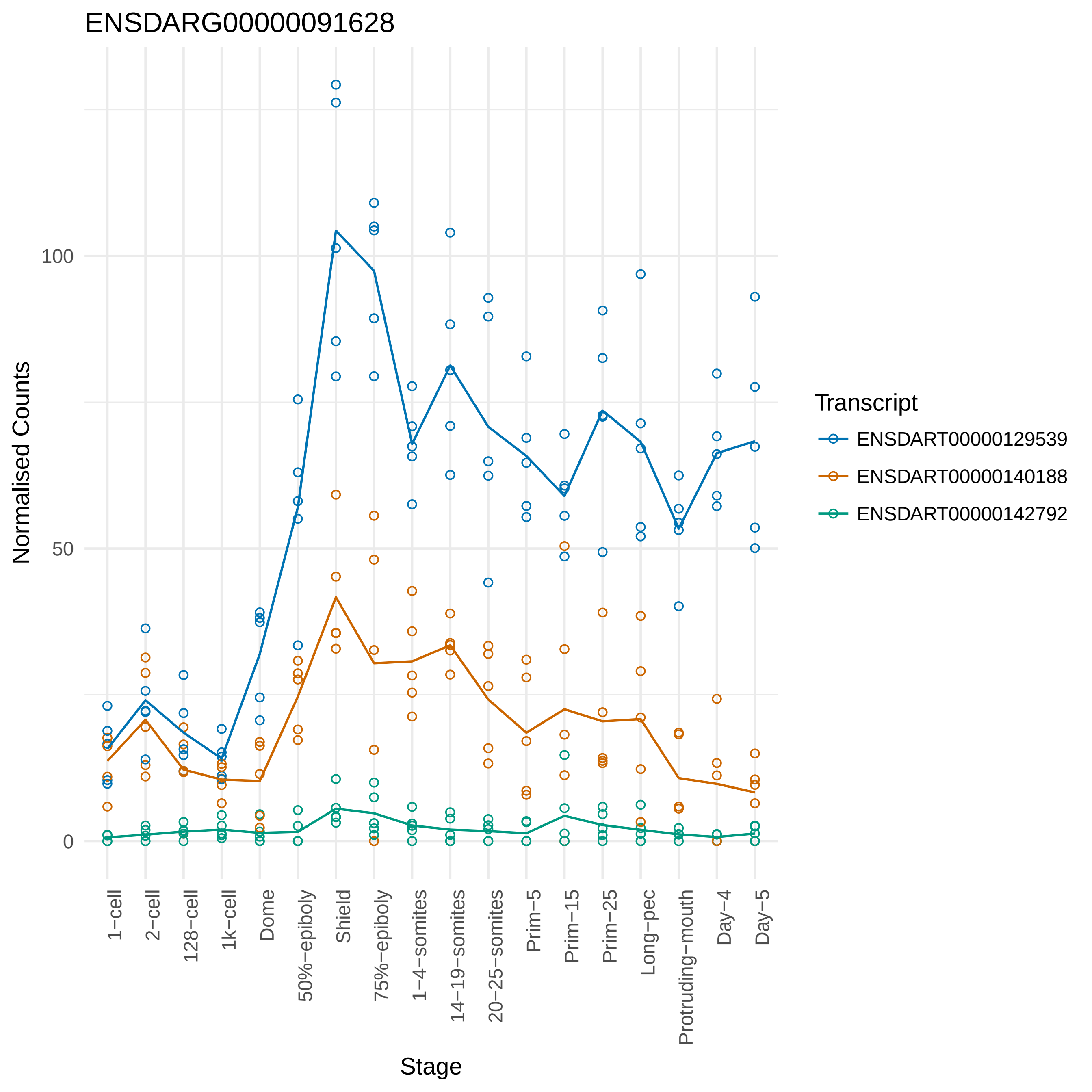

ENSDARG00000091628
Normalised Counts
100
50
0
1−cell
2−cell
128−cell
1k−cell
Dome
50%−epiboly
Shield
75%−epiboly
1−4−somites
14−19−somites
20−25−somites
Prim−5
Prim−15
Prim−25
Long−pec
Protruding−mouth
Day−4
Day−5
Stage
Transcript
ENSDART00000129539
ENSDART00000140188
ENSDART00000142792

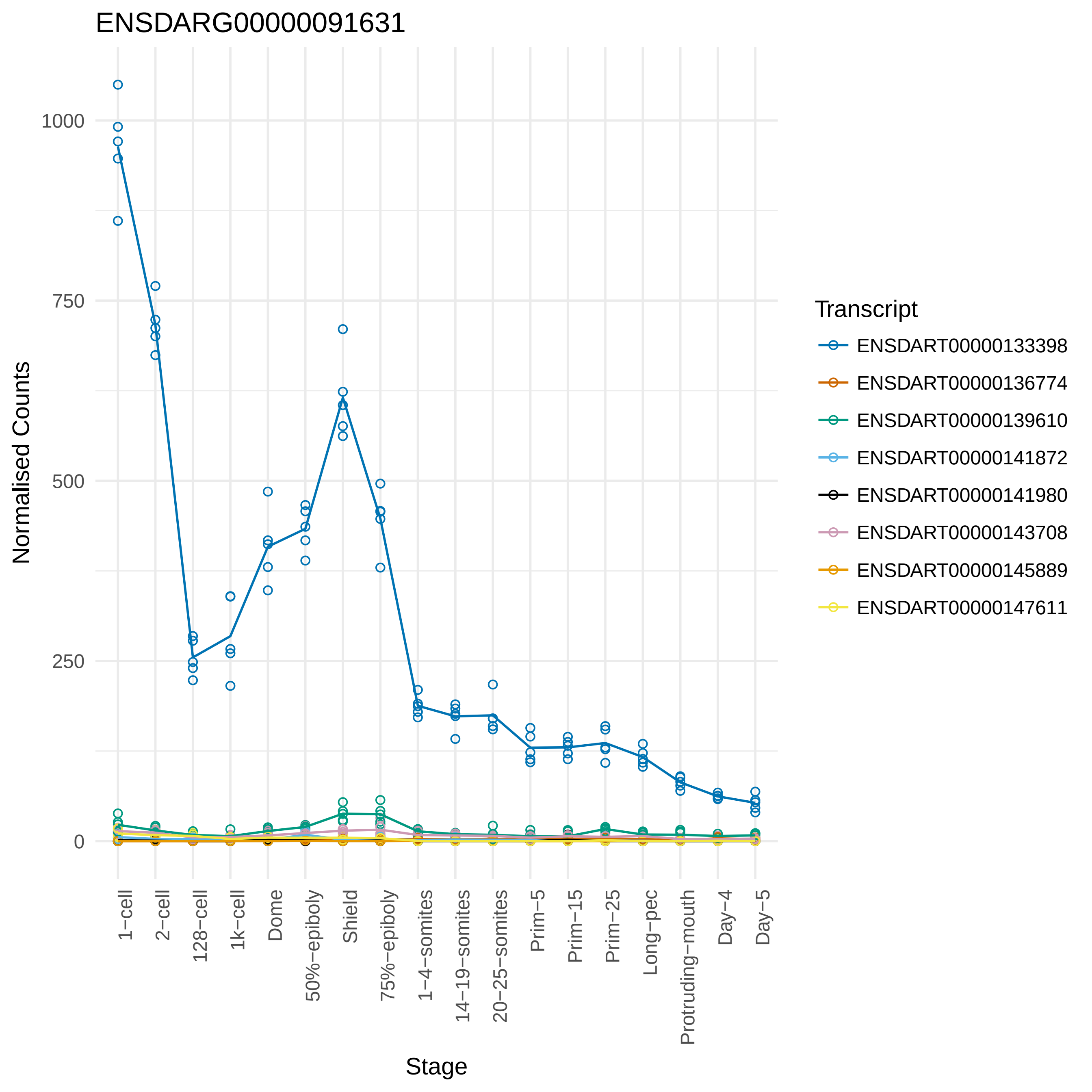

ENSDARG00000091631
Normalised Counts
1000
750
500
250
0
1−cell
2−cell
128−cell
1k−cell
Dome
50%−epiboly
Shield
75%−epiboly
1−4−somites
14−19−somites
20−25−somites
Prim−5
Prim−15
Prim−25
Long−pec
Protruding−mouth
Day−4
Day−5
Stage
Transcript
ENSDART00000133398
ENSDART00000136774
ENSDART00000139610
ENSDART00000141872
ENSDART00000141980
ENSDART00000143708
ENSDART00000145889
ENSDART00000147611

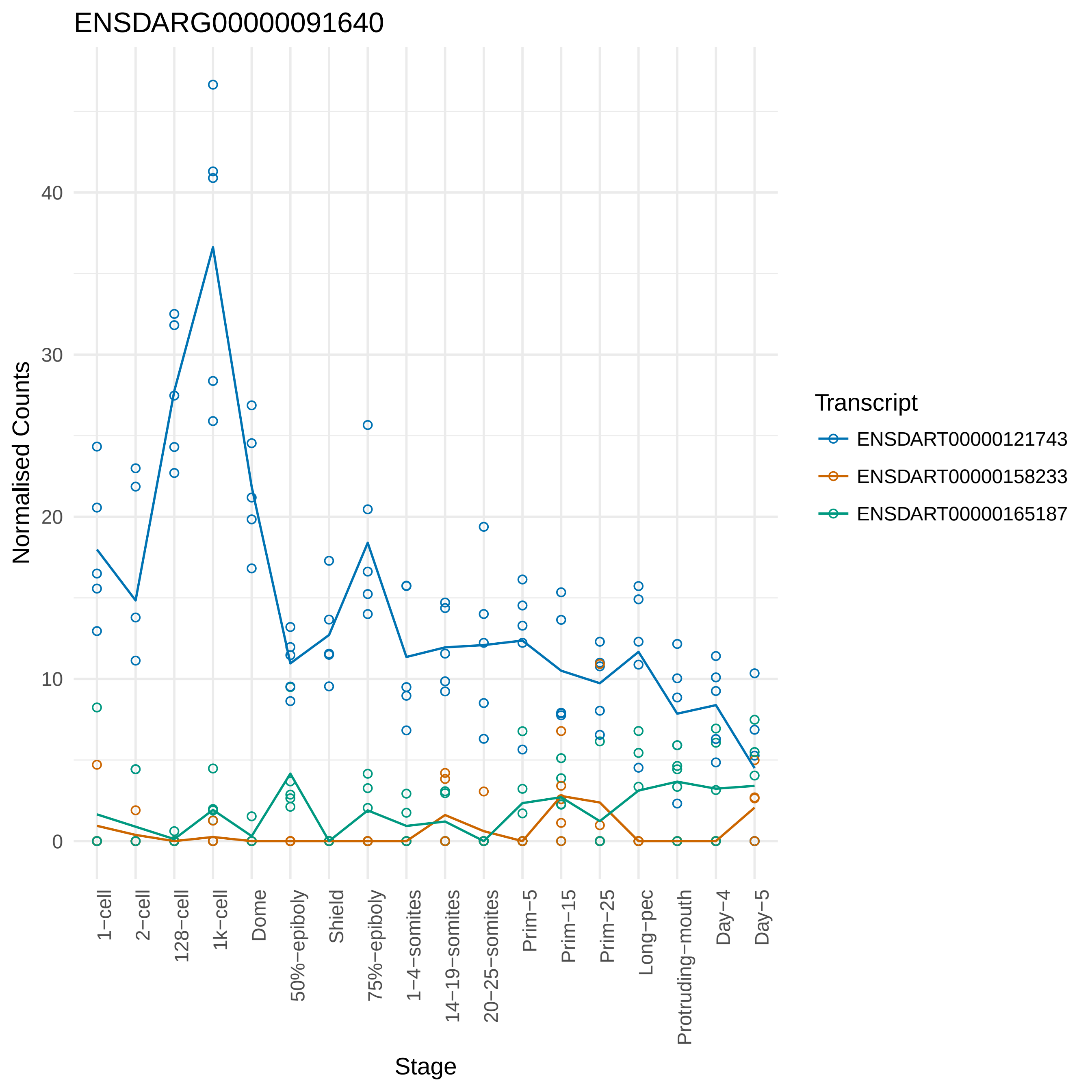
ENSDARG00000091640
Normalised Counts
0
10
20
30
40
Stage
1−cell
2−cell
128−cell
1k−cell
Dome
50%−epiboly
Shield
75%−epiboly
1−4−somites
14−19−somites
20−25−somites
Prim−5
Prim−15
Prim−25
Long−pec
Protruding−mouth
Day−4
Day−5
Transcript
ENSDART00000121743
ENSDART00000158233
ENSDART00000165187

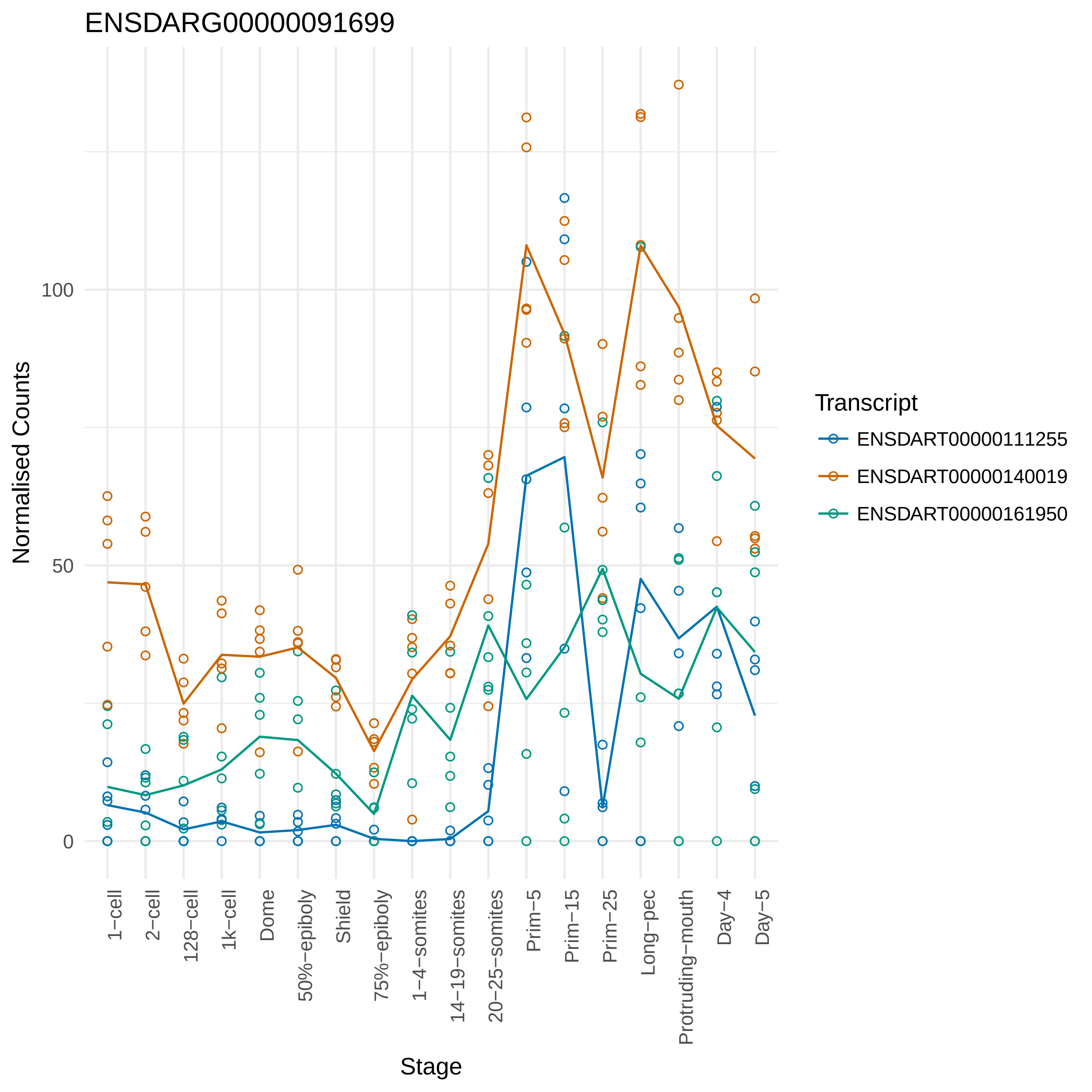

ENSDARG00000091699
Normalised Counts
100
50
0
1−cell
2−cell
128−cell
1k−cell
Dome
50%−epiboly
Shield
75%−epiboly
1−4−somites
14−19−somites
20−25−somites
Prim−5
Prim−15
Prim−25
Long−pec
Protruding−mouth
Day−4
Day−5
Stage
Transcript
ENSDART00000111255
ENSDART00000140019
ENSDART00000161950

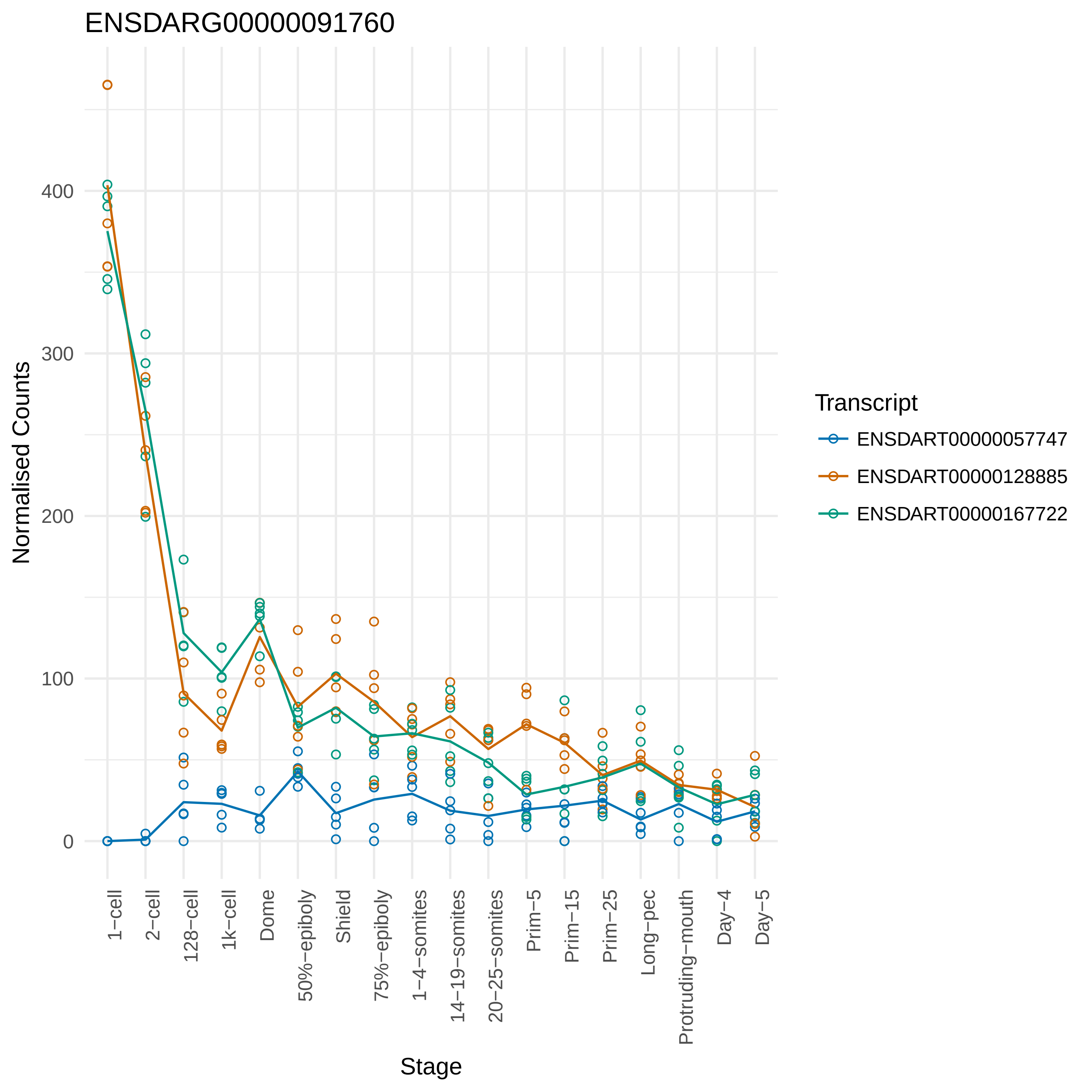
ENSDARG00000091760
Normalised Counts
0
100
200
300
400
1−cell
2−cell
128−cell
1k−cell
Dome
50%−epiboly
Shield
75%−epiboly
1−4−somites
14−19−somites
20−25−somites
Prim−5
Prim−15
Prim−25
Long−pec
Protruding−mouth
Day−4
Day−5
Stage
Transcript
ENSDART00000057747
ENSDART00000128885
ENSDART00000167722

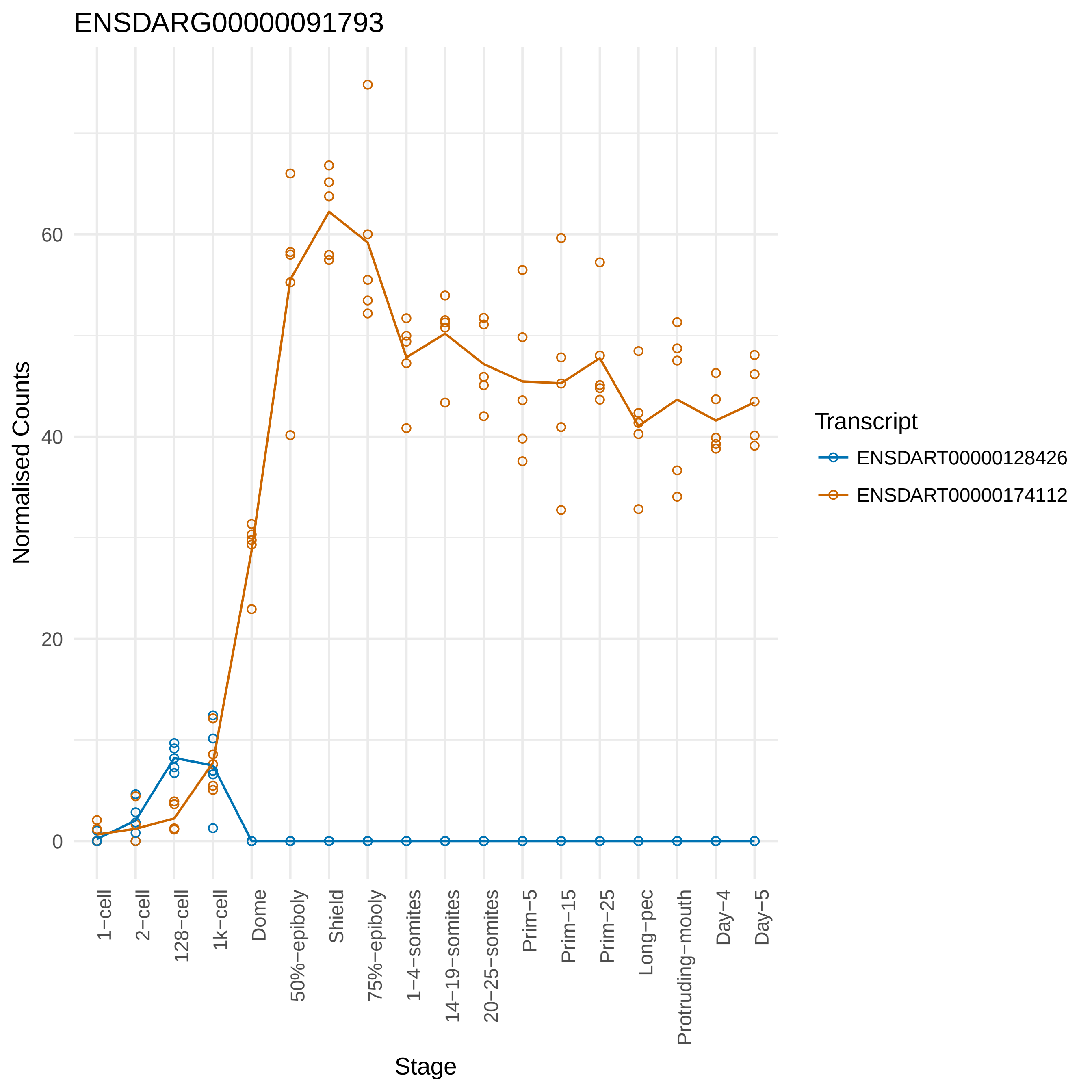
ENSDARG00000091793
Normalised Counts
60
40
20
0
1−cell
2−cell
128−cell
1k−cell
Dome
50%−epiboly
Shield
75%−epiboly
1−4−somites
14−19−somites
20−25−somites
Prim−5
Prim−15
Prim−25
Long−pec
Protruding−mouth
Day−4
Day−5
Stage
Transcript
ENSDART00000128426
ENSDART00000174112

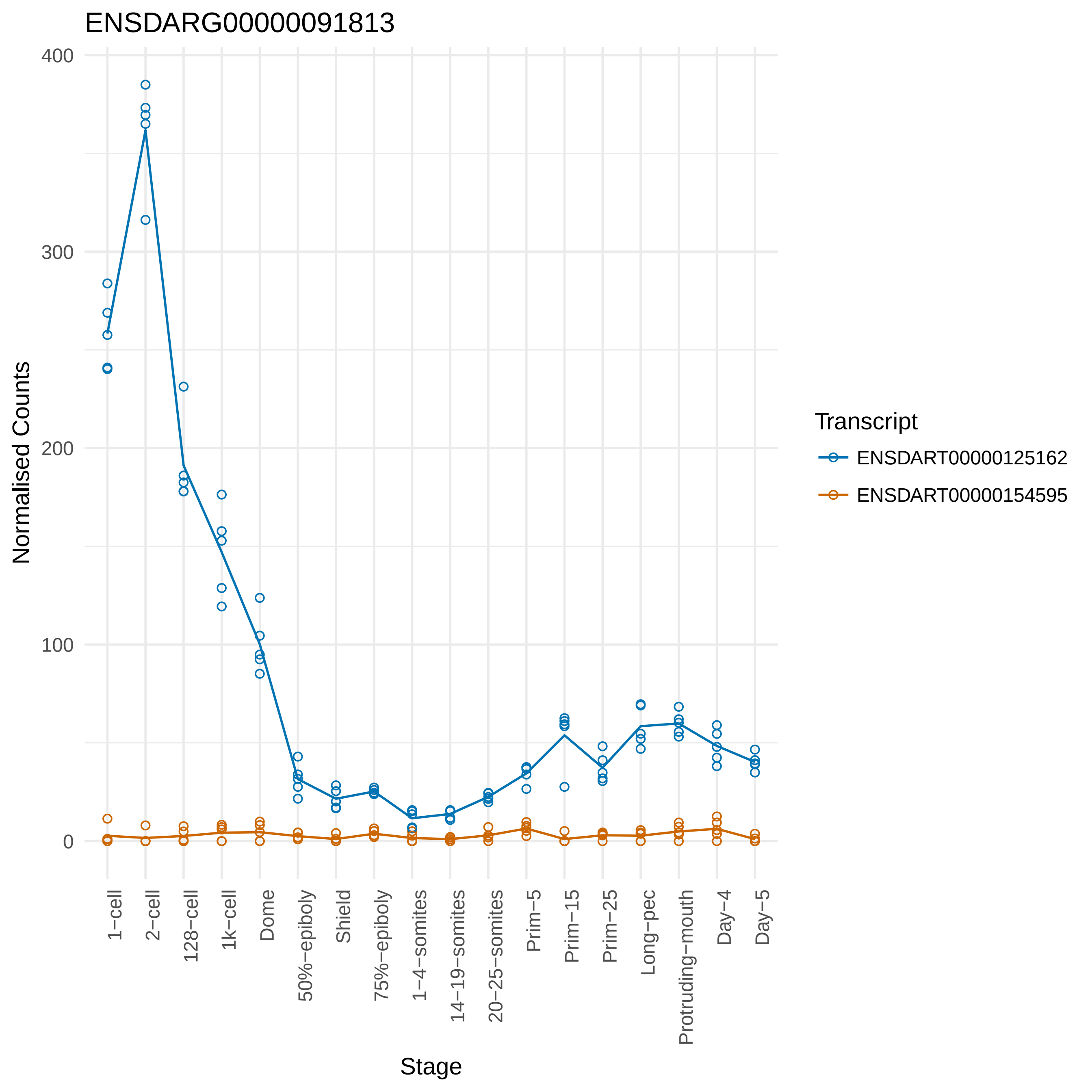

ENSDARG00000091813
Normalised Counts
400
300
200
100
0
1−cell
2−cell
128−cell
1k−cell
Dome
50%−epiboly
Shield
75%−epiboly
1−4−somites
14−19−somites
20−25−somites
Prim−5
Prim−15
Prim−25
Long−pec
Protruding−mouth
Day−4
Day−5
Stage
Transcript
ENSDART00000125162
ENSDART00000154595

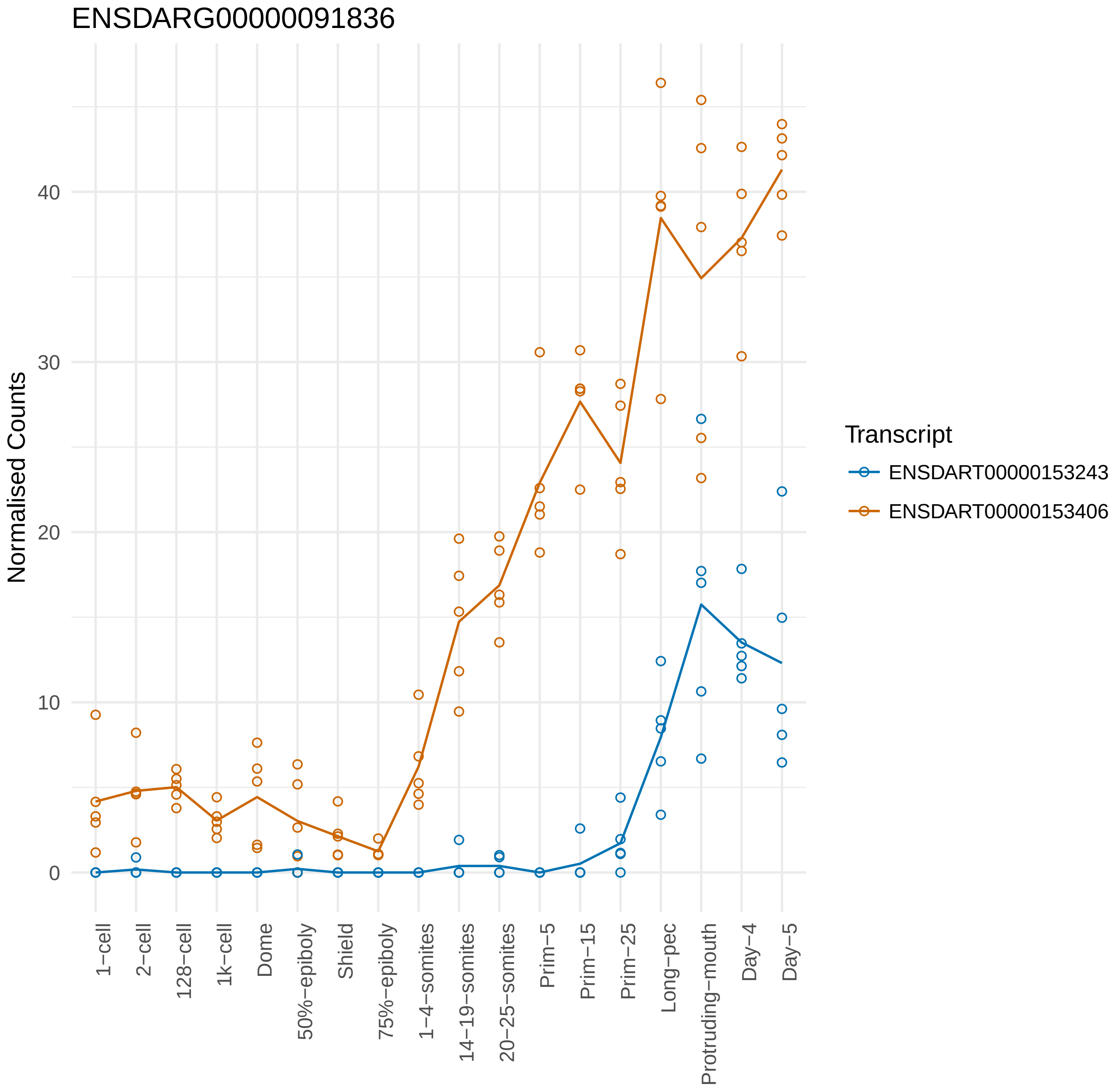
ENSDARG00000091836
Normalised Counts
0
10
20
30
40
Stage
1−cell
2−cell
128−cell
1k−cell
Dome
50%−epiboly
Shield
75%−epiboly
1−4−somites
14−19−somites
20−25−somites
Prim−5
Prim−15
Prim−25
Long−pec
Protruding−mouth
Day−4
Day−5
Transcript
ENSDART00000153243
ENSDART00000153406

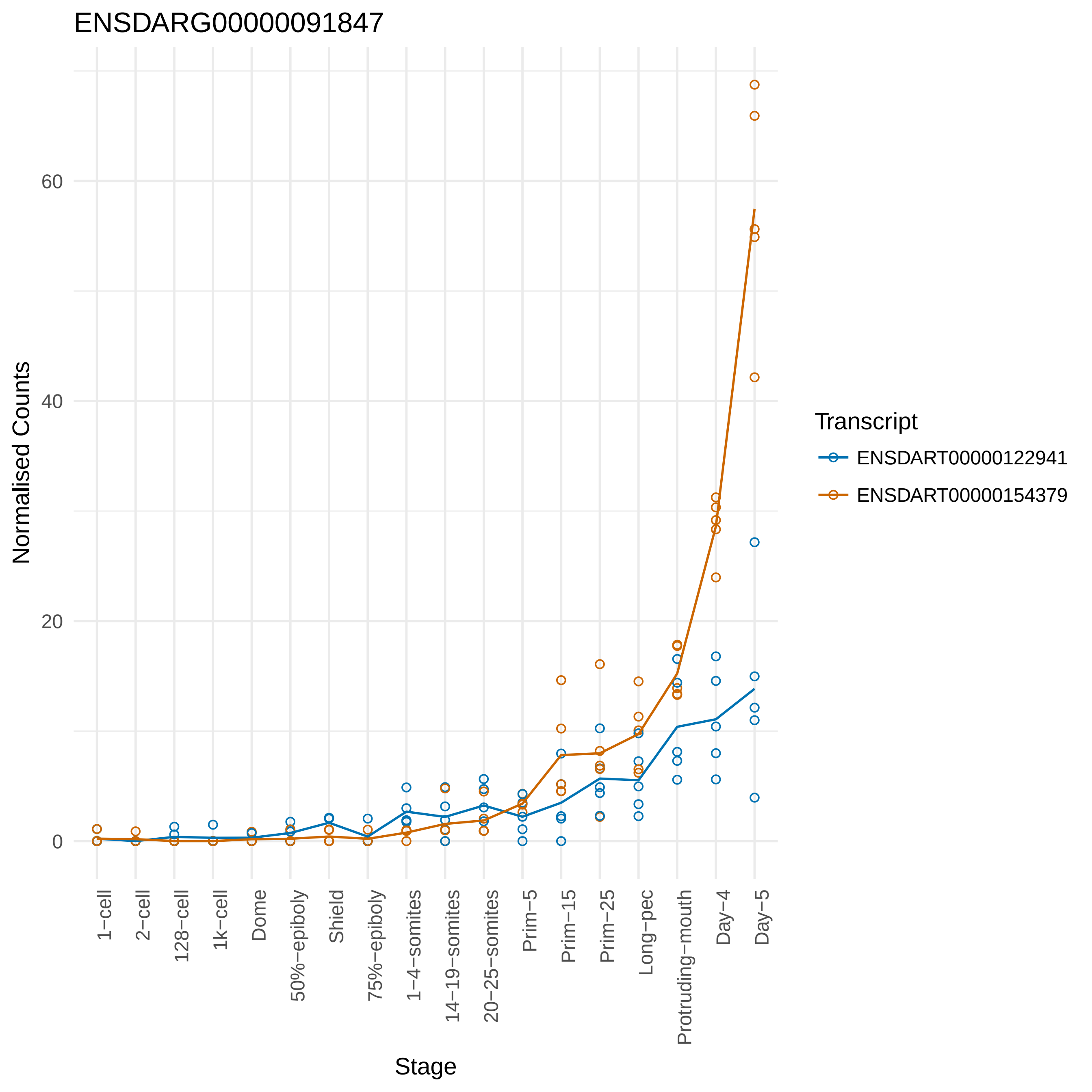

ENSDARG00000091847
Normalised Counts
0
20
40
60
1−cell
2−cell
128−cell
1k−cell
Dome
50%−epiboly
Shield
75%−epiboly
1−4−somites
14−19−somites
20−25−somites
Prim−5
Prim−15
Prim−25
Long−pec
Protruding−mouth
Day−4
Day−5
Stage
Transcript
ENSDART00000122941
ENSDART00000154379

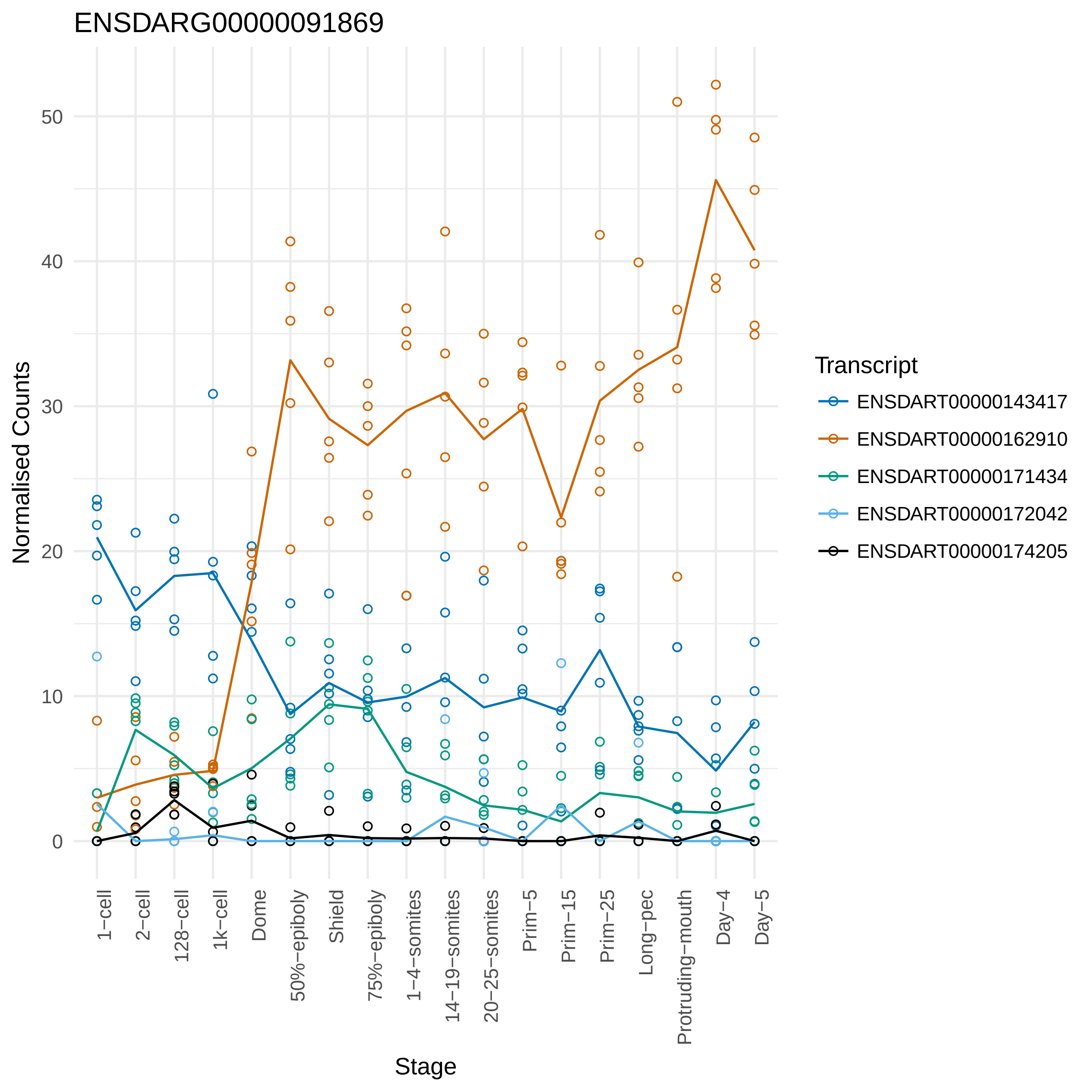

ENSDARG00000091869
Normalised Counts
Stage
Transcript
ENSDART00000143417
ENSDART00000162910
ENSDART00000171434
ENSDART00000172042
ENSDART00000174205
0
10
20
30
40
50
1−cell
2−cell
128−cell
1k−cell
Dome
50%−epiboly
Shield
75%−epiboly
1−4−somites
14−19−somites
20−25−somites
Prim−5
Prim−15
Prim−25
Long−pec
Protruding−mouth
Day−4
Day−5

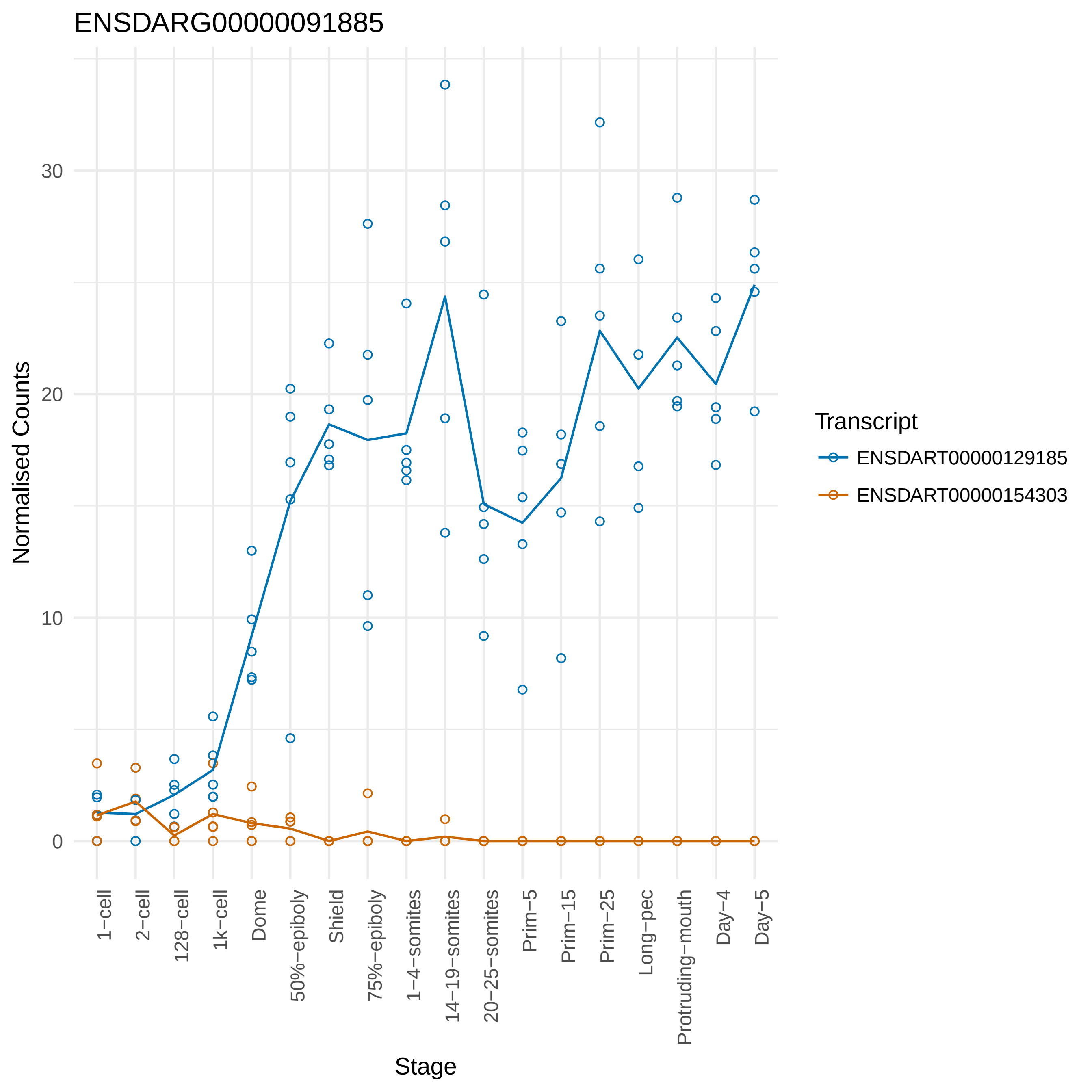
ENSDARG00000091885
Normalised Counts
0
10
20
30
1−cell
2−cell
128−cell
1k−cell
Dome
50%−epiboly
Shield
75%−epiboly
1−4−somites
14−19−somites
20−25−somites
Prim−5
Prim−15
Prim−25
Long−pec
Protruding−mouth
Day−4
Day−5
Stage
Transcript
ENSDART00000129185
ENSDART00000154303

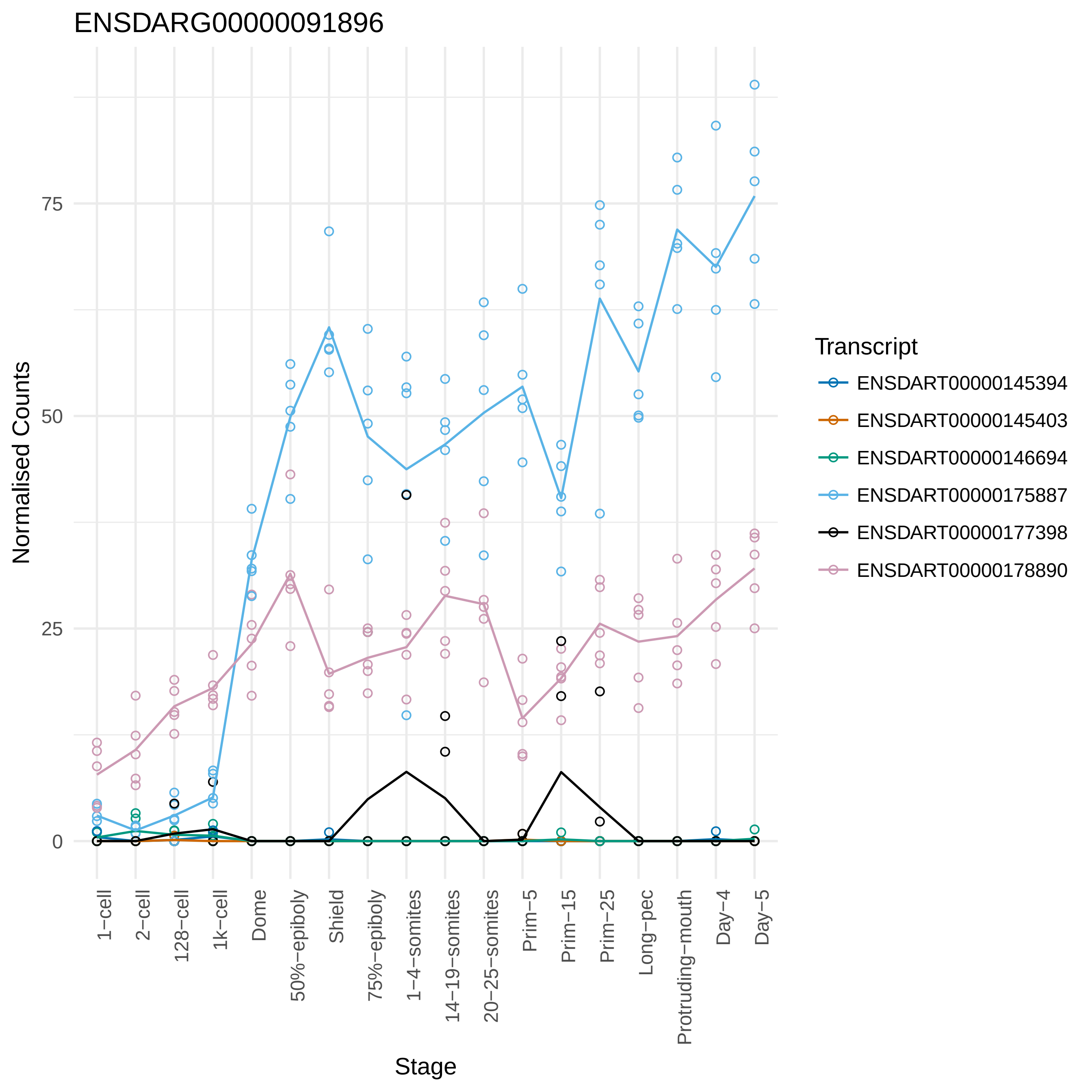
ENSDARG00000091896
Normalised Counts
0
25
50
75
1−cell
2−cell
128−cell
1k−cell
Dome
50%−epiboly
Shield
75%−epiboly
1−4−somites
14−19−somites
20−25−somites
Prim−5
Prim−15
Prim−25
Long−pec
Protruding−mouth
Day−4
Day−5
Stage
Transcript
ENSDART00000145394
ENSDART00000145403
ENSDART00000146694
ENSDART00000175887
ENSDART00000177398
ENSDART00000178890

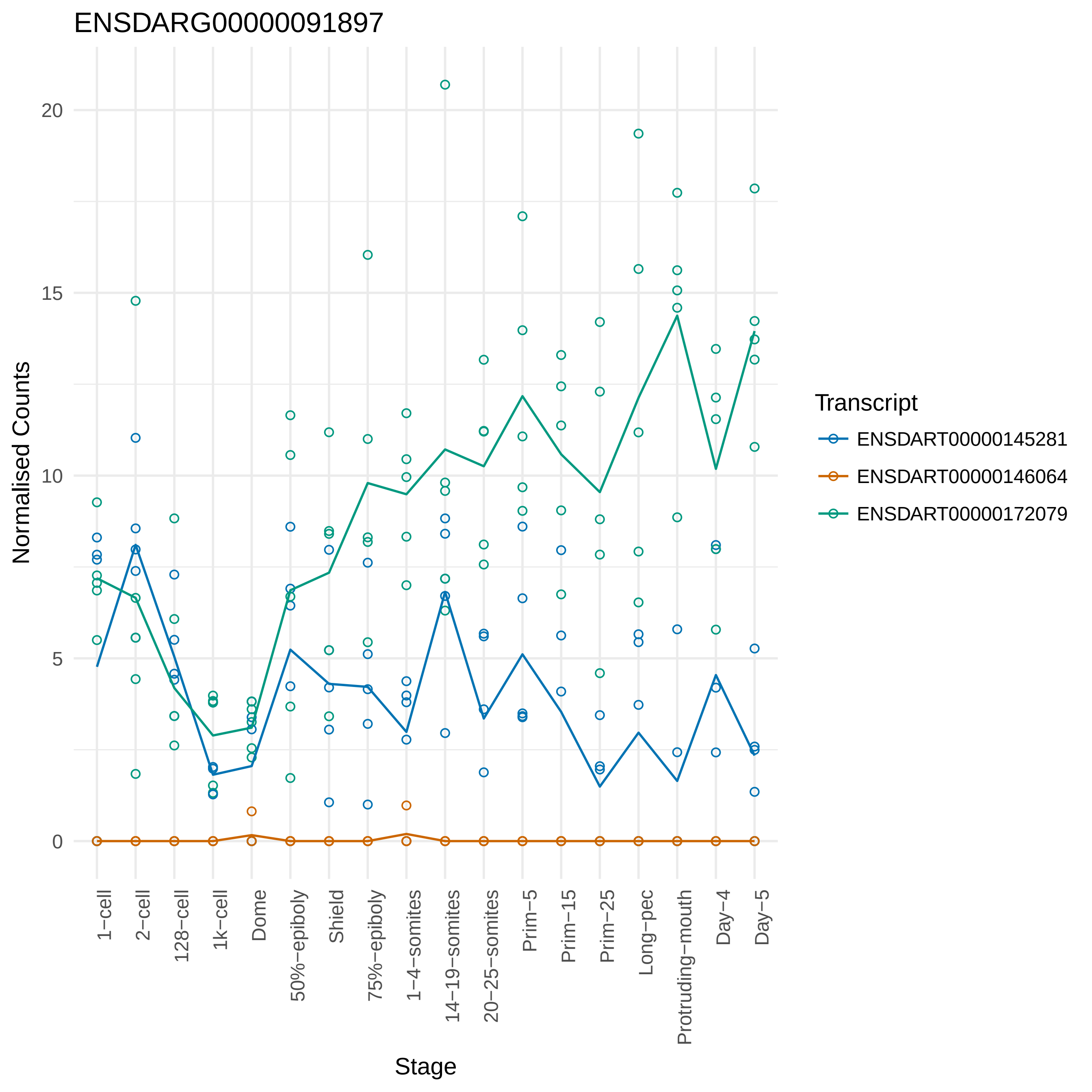
ENSDARG00000091897
Normalised Counts
0
5
10
15
20
Stage
1−cell
2−cell
128−cell
1k−cell
Dome
50%−epiboly
Shield
75%−epiboly
1−4−somites
14−19−somites
20−25−somites
Prim−5
Prim−15
Prim−25
Long−pec
Protruding−mouth
Day−4
Day−5
Transcript
ENSDART00000145281
ENSDART00000146064
ENSDART00000172079

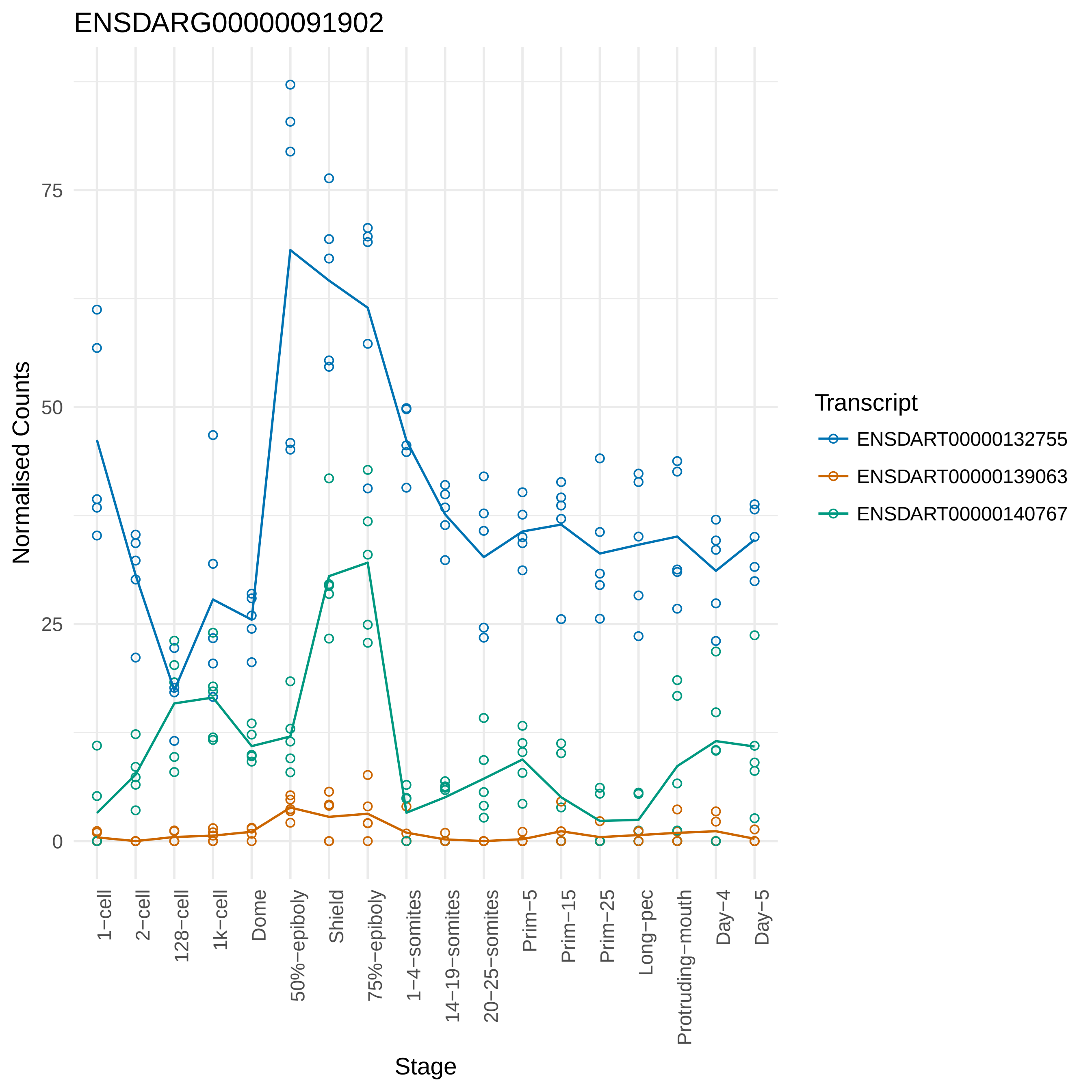
ENSDARG00000091902
Normalised Counts
0
25
50
75
Stage
1−cell
2−cell
128−cell
1k−cell
Dome
50%−epiboly
Shield
75%−epiboly
1−4−somites
14−19−somites
20−25−somites
Prim−5
Prim−15
Prim−25
Long−pec
Protruding−mouth
Day−4
Day−5
Transcript
ENSDART00000132755
ENSDART00000139063
ENSDART00000140767

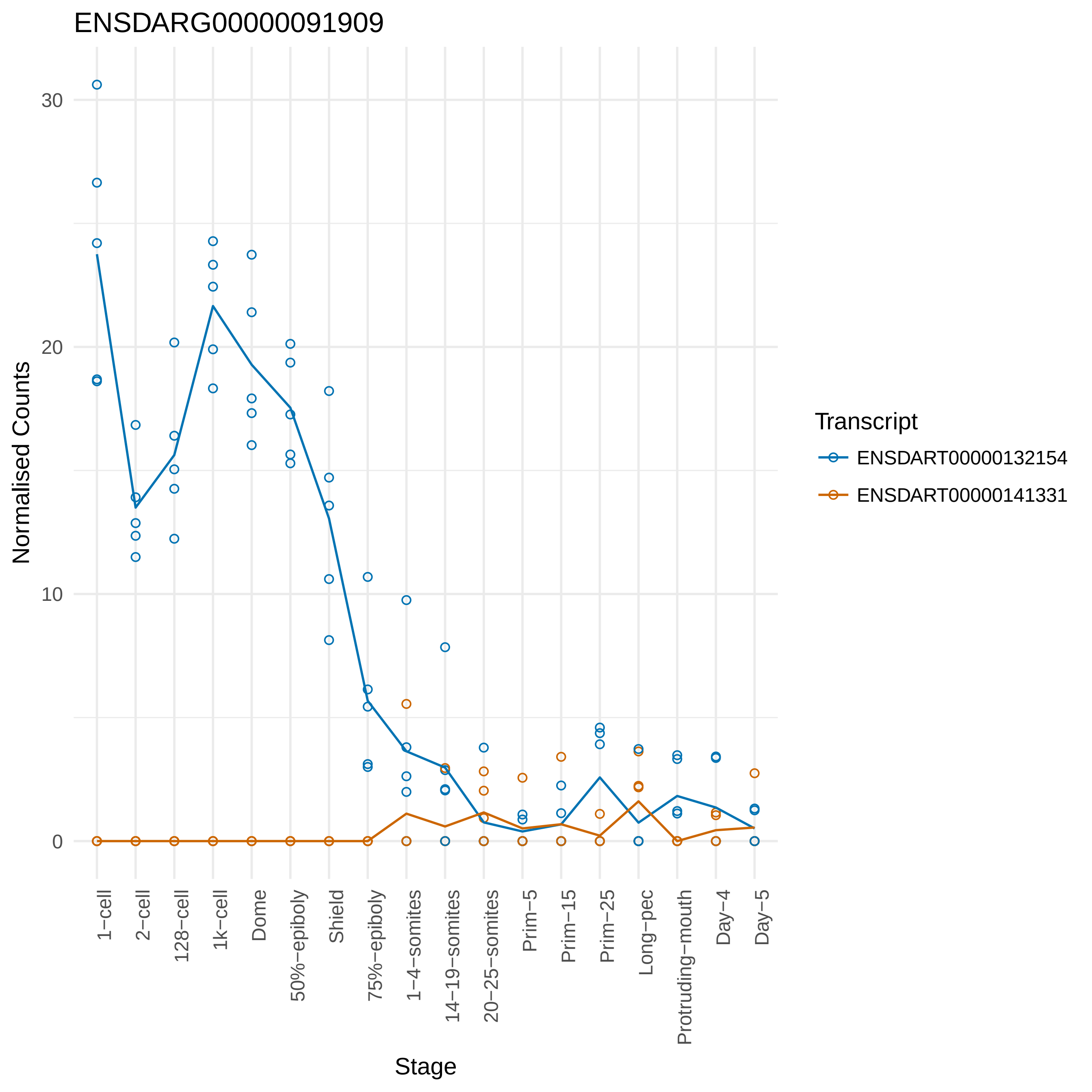

ENSDARG00000091909
Normalised Counts
30
20
10
0
1−cell
2−cell
128−cell
1k−cell
Dome
50%−epiboly
Shield
75%−epiboly
1−4−somites
14−19−somites
20−25−somites
Prim−5
Prim−15
Prim−25
Long−pec
Protruding−mouth
Day−4
Day−5
Stage
Transcript
ENSDART00000132154
ENSDART00000141331

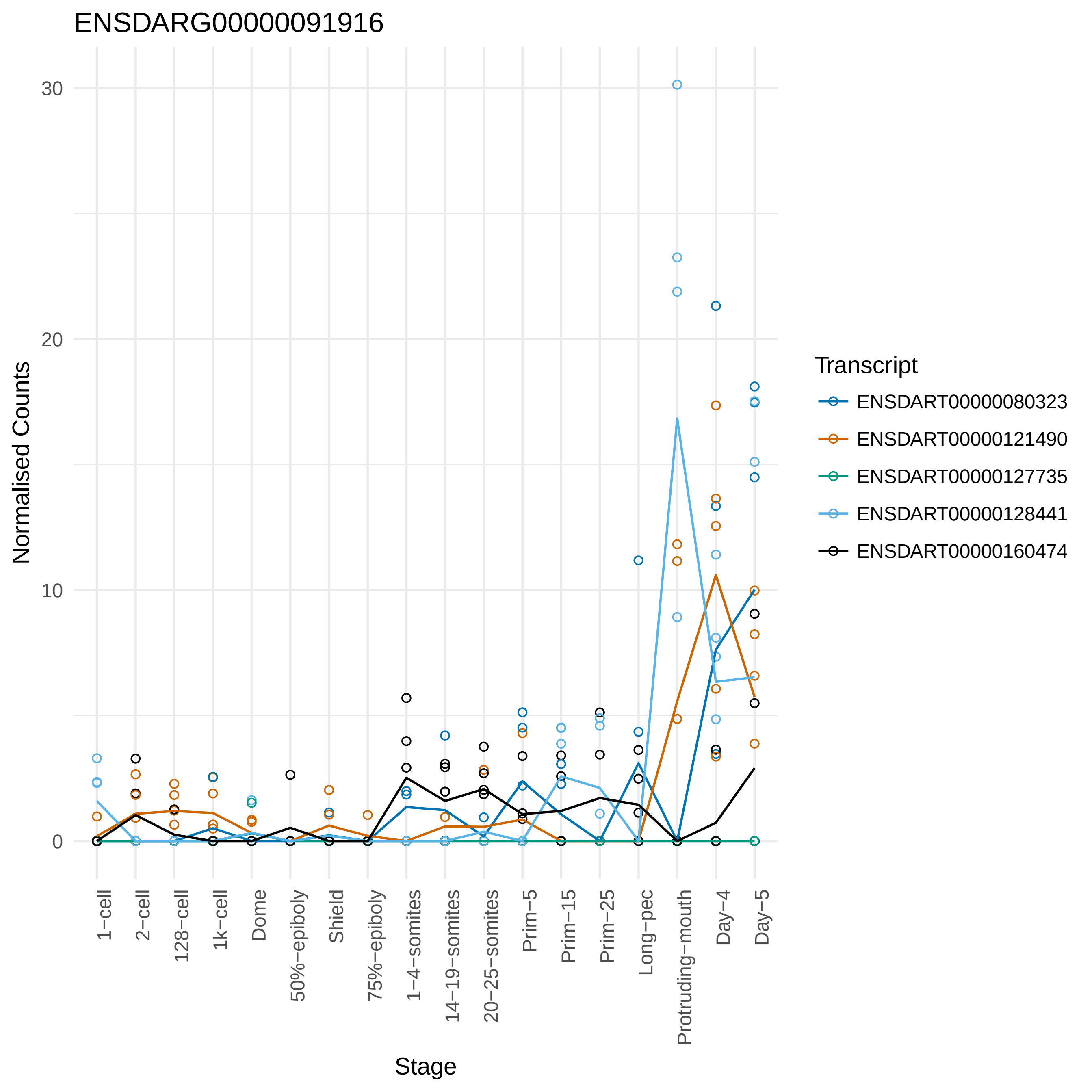

ENSDARG00000091916
Normalised Counts
30
20
10
0
1−cell
2−cell
128−cell
1k−cell
Dome
50%−epiboly
Shield
75%−epiboly
1−4−somites
14−19−somites
20−25−somites
Prim−5
Prim−15
Prim−25
Long−pec
Protruding−mouth
Day−4
Day−5
Stage
Transcript
ENSDART00000080323
ENSDART00000121490
ENSDART00000127735
ENSDART00000128441
ENSDART00000160474

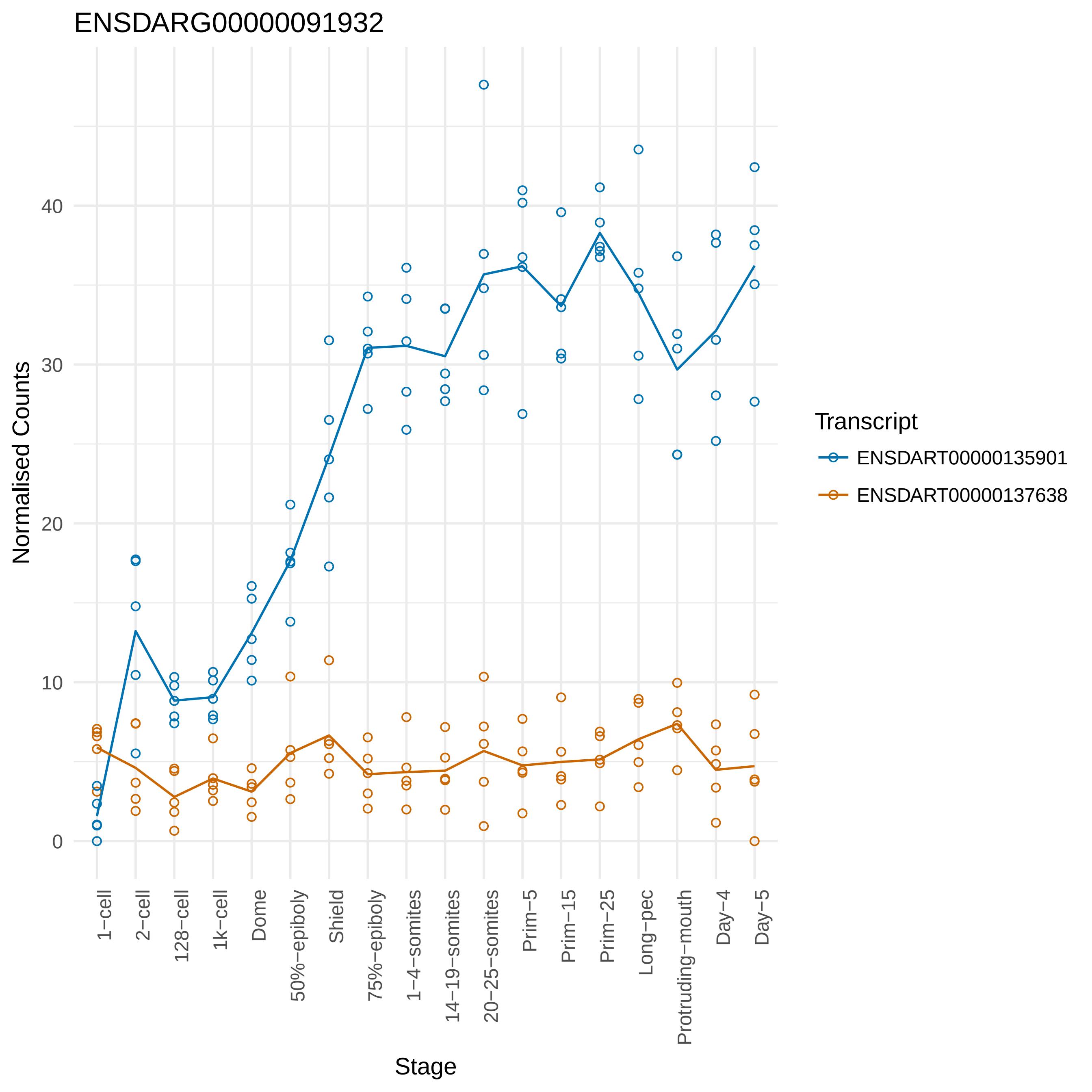
ENSDARG00000091932
Normalised Counts
0
10
20
30
40
Stage
1−cell
2−cell
128−cell
1k−cell
Dome
50%−epiboly
Shield
75%−epiboly
1−4−somites
14−19−somites
20−25−somites
Prim−5
Prim−15
Prim−25
Long−pec
Protruding−mouth
Day−4
Day−5
Transcript
ENSDART00000135901
ENSDART00000137638

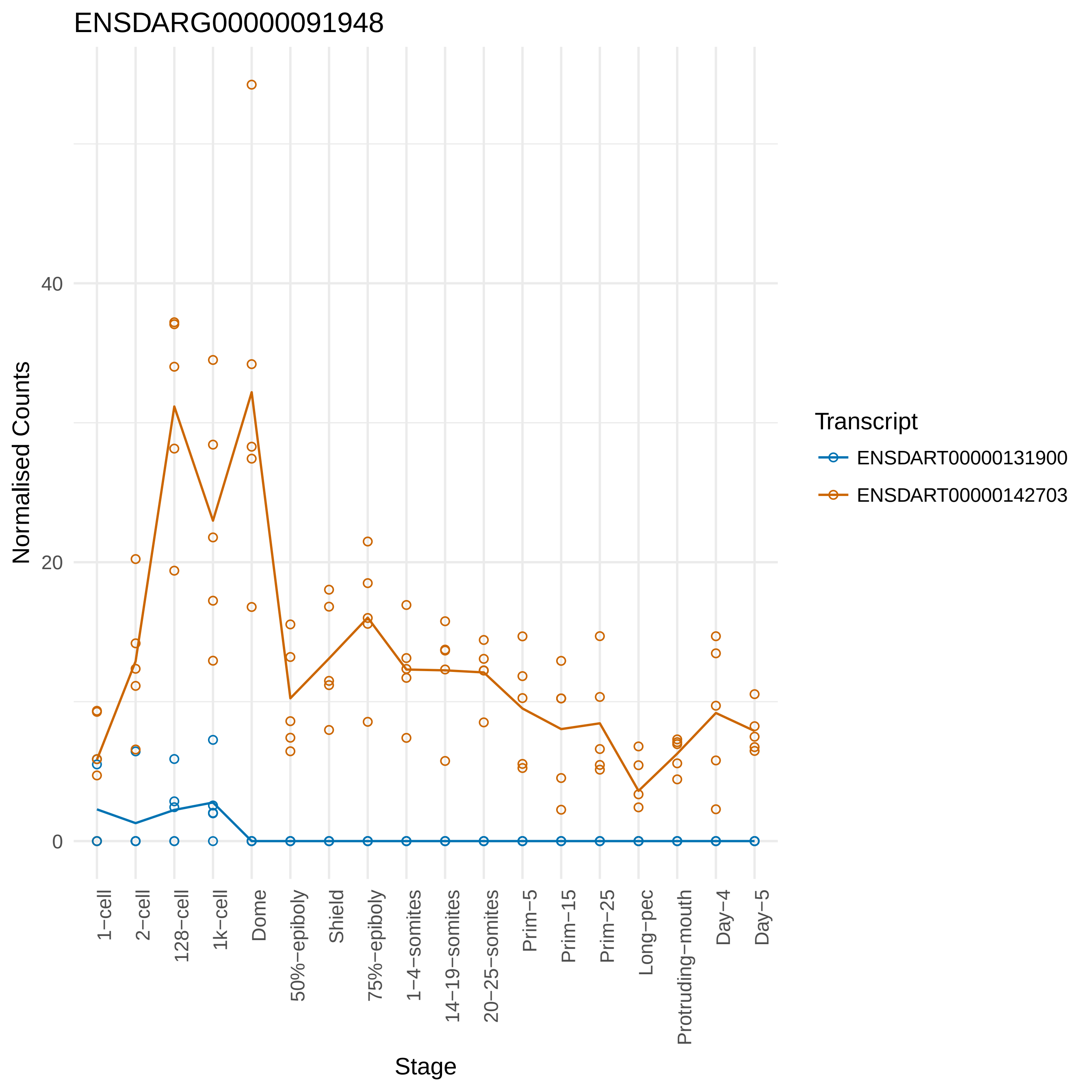

ENSDARG00000091948
Normalised Counts
0
20
40
1−cell
2−cell
128−cell
1k−cell
Dome
50%−epiboly
Shield
75%−epiboly
1−4−somites
14−19−somites
20−25−somites
Prim−5
Prim−15
Prim−25
Long−pec
Protruding−mouth
Day−4
Day−5
Stage
Transcript
ENSDART00000131900
ENSDART00000142703

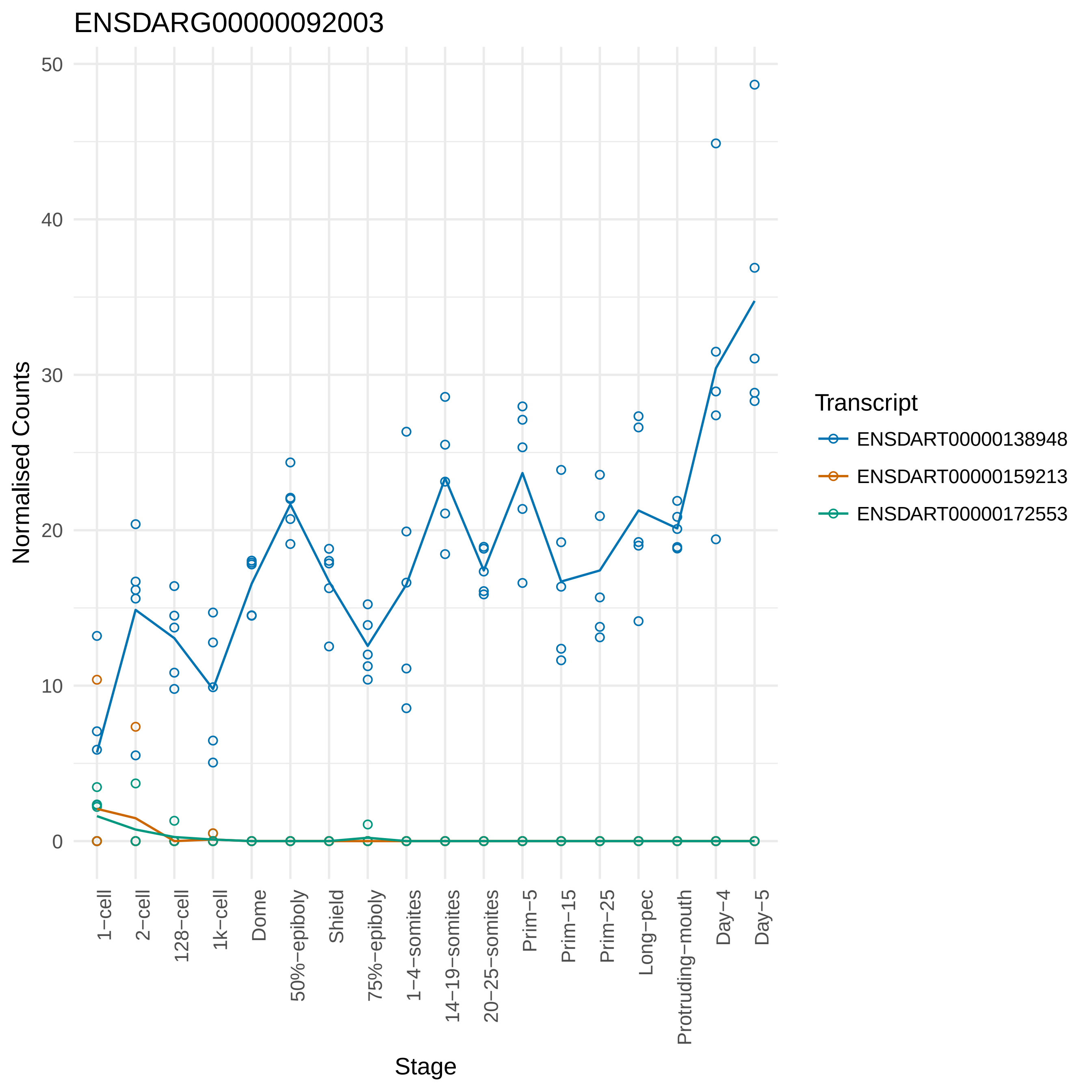
ENSDARG00000092003
Normalised Counts
50
40
30
20
10
0
1−cell
2−cell
128−cell
1k−cell
Dome
50%−epiboly
Shield
75%−epiboly
1−4−somites
14−19−somites
20−25−somites
Prim−5
Prim−15
Prim−25
Long−pec
Protruding−mouth
Day−4
Day−5
Stage
Transcript
ENSDART00000138948
ENSDART00000159213
ENSDART00000172553

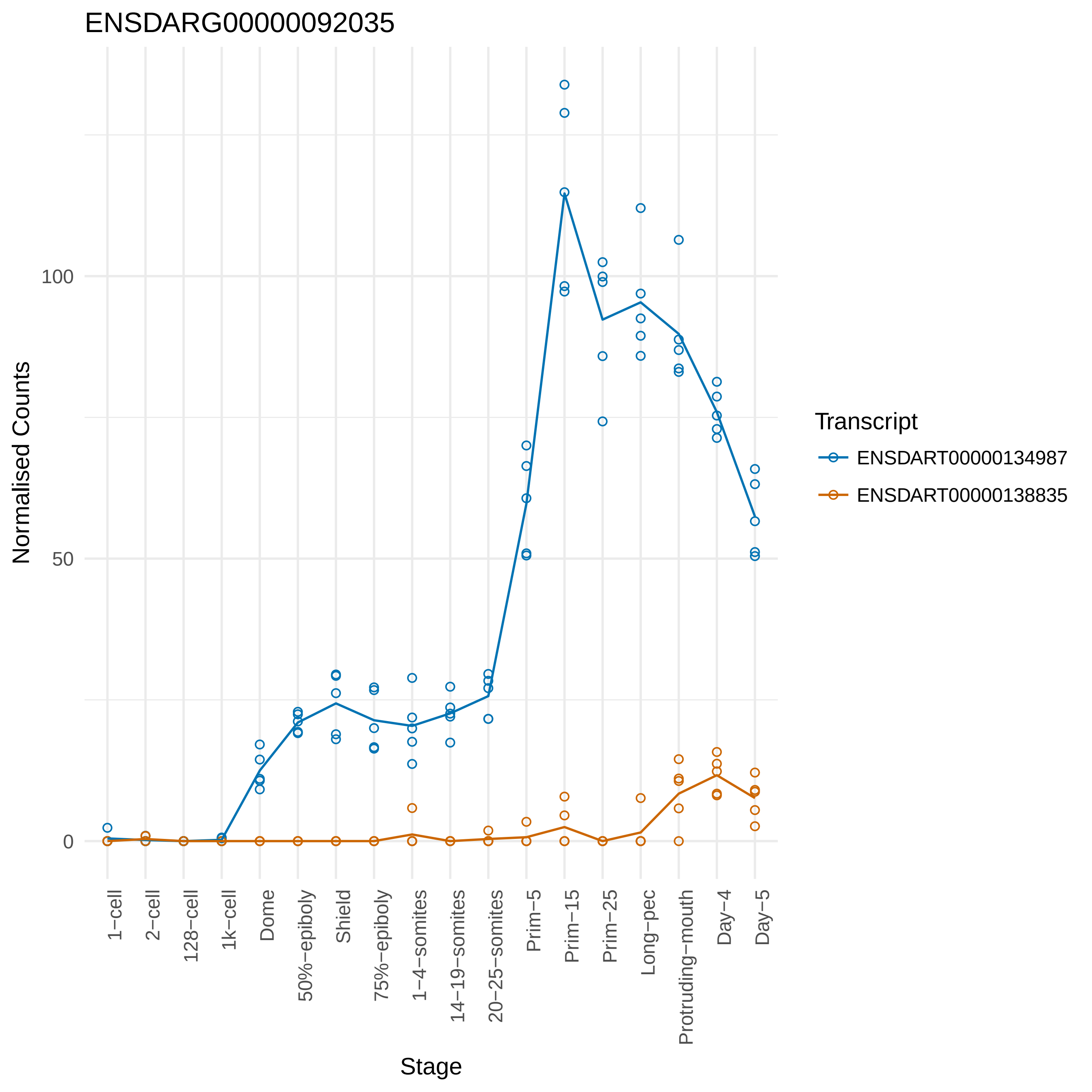

ENSDARG00000092035
Normalised Counts
100
50
0
1−cell
2−cell
128−cell
1k−cell
Dome
50%−epiboly
Shield
75%−epiboly
1−4−somites
14−19−somites
20−25−somites
Prim−5
Prim−15
Prim−25
Long−pec
Protruding−mouth
Day−4
Day−5
Stage
Transcript
ENSDART00000134987
ENSDART00000138835

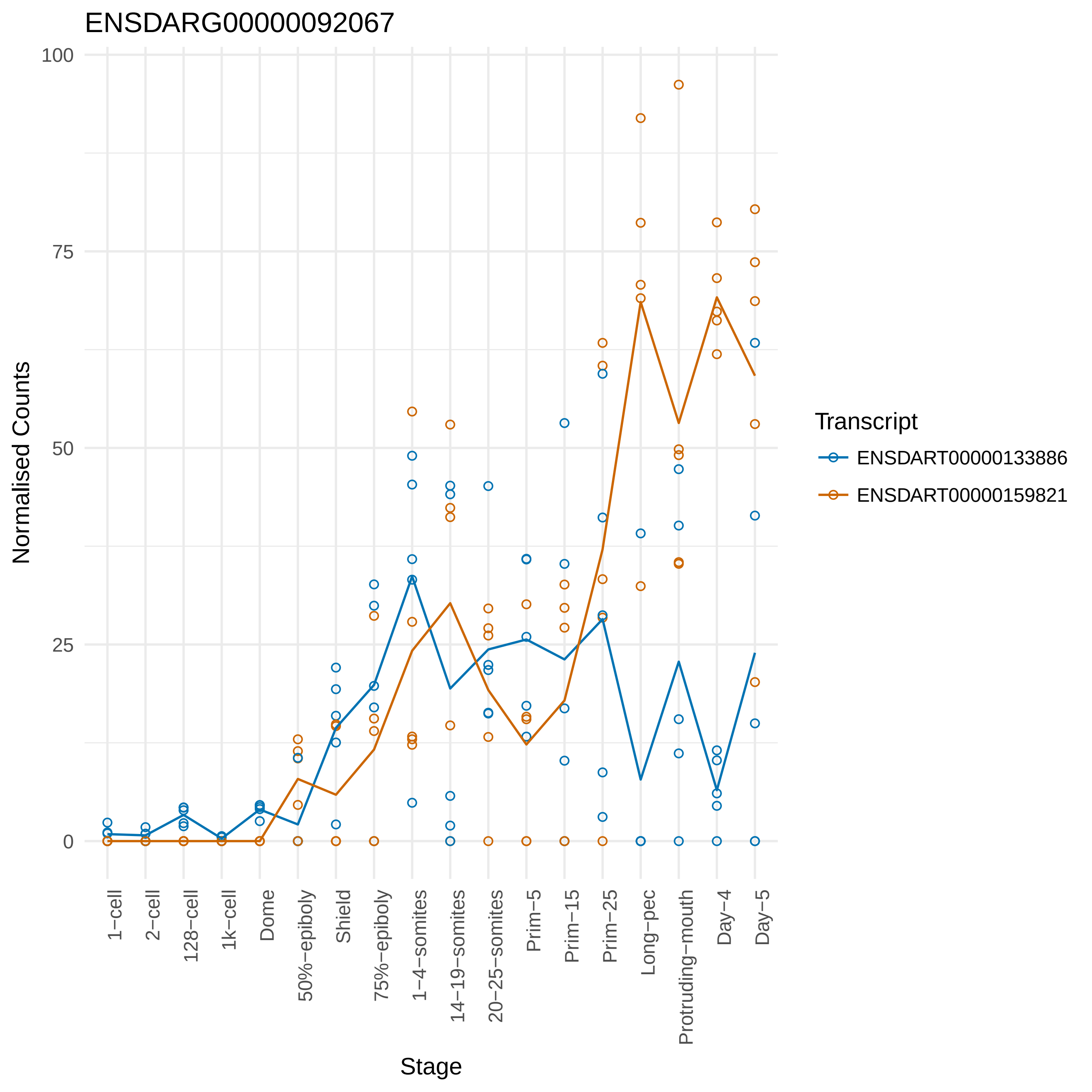
ENSDARG00000092067
Normalised Counts
100
75
50
25
0
1−cell
2−cell
128−cell
1k−cell
Dome
50%−epiboly
Shield
75%−epiboly
1−4−somites
14−19−somites
20−25−somites
Prim−5
Prim−15
Prim−25
Long−pec
Protruding−mouth
Day−4
Day−5
Stage
Transcript
ENSDART00000133886
ENSDART00000159821

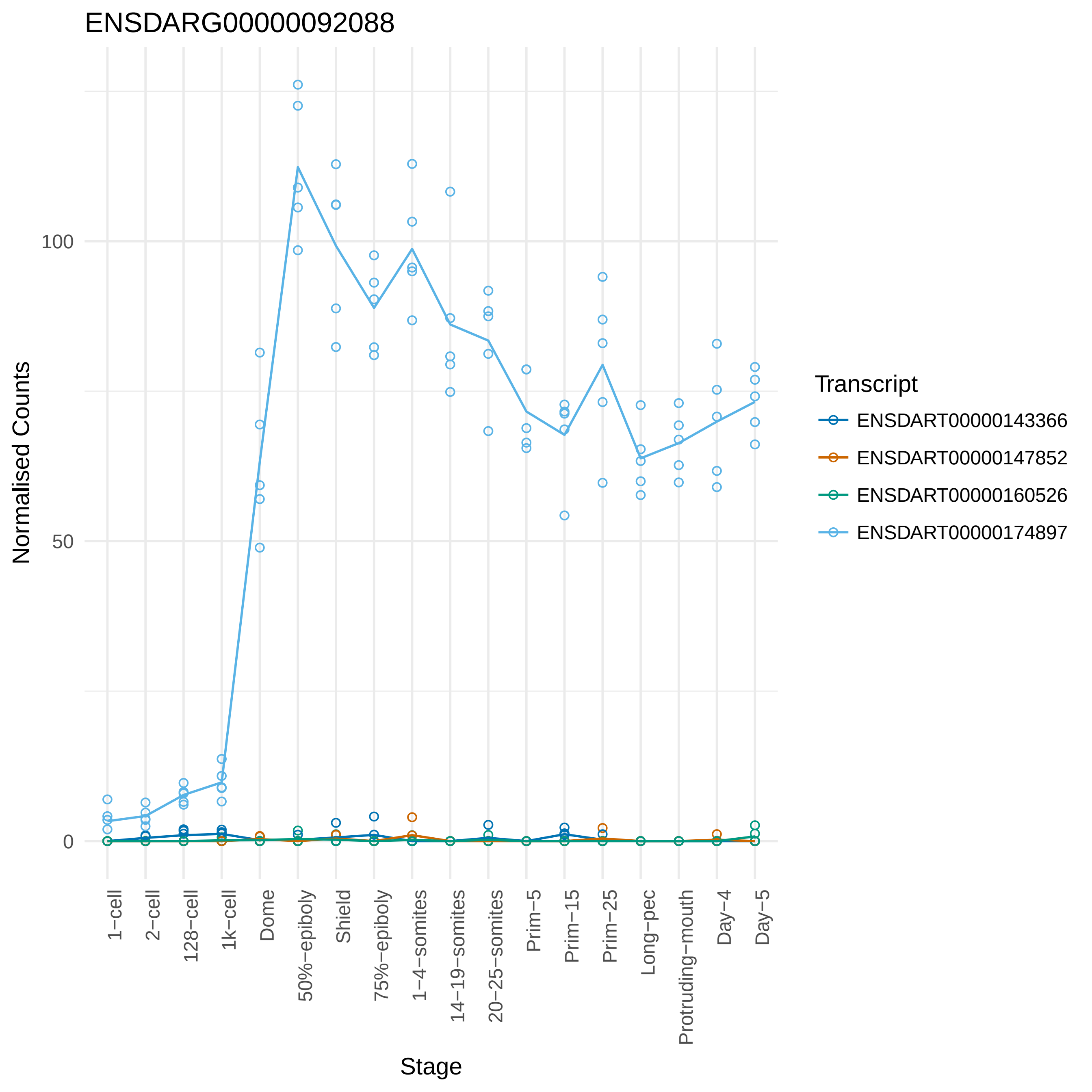

ENSDARG00000092088
Normalised Counts
100
50
0
Stage
1−cell
2−cell
128−cell
1k−cell
Dome
50%−epiboly
Shield
75%−epiboly
1−4−somites
14−19−somites
20−25−somites
Prim−5
Prim−15
Prim−25
Long−pec
Protruding−mouth
Day−4
Day−5
Transcript
ENSDART00000143366
ENSDART00000147852
ENSDART00000160526
ENSDART00000174897

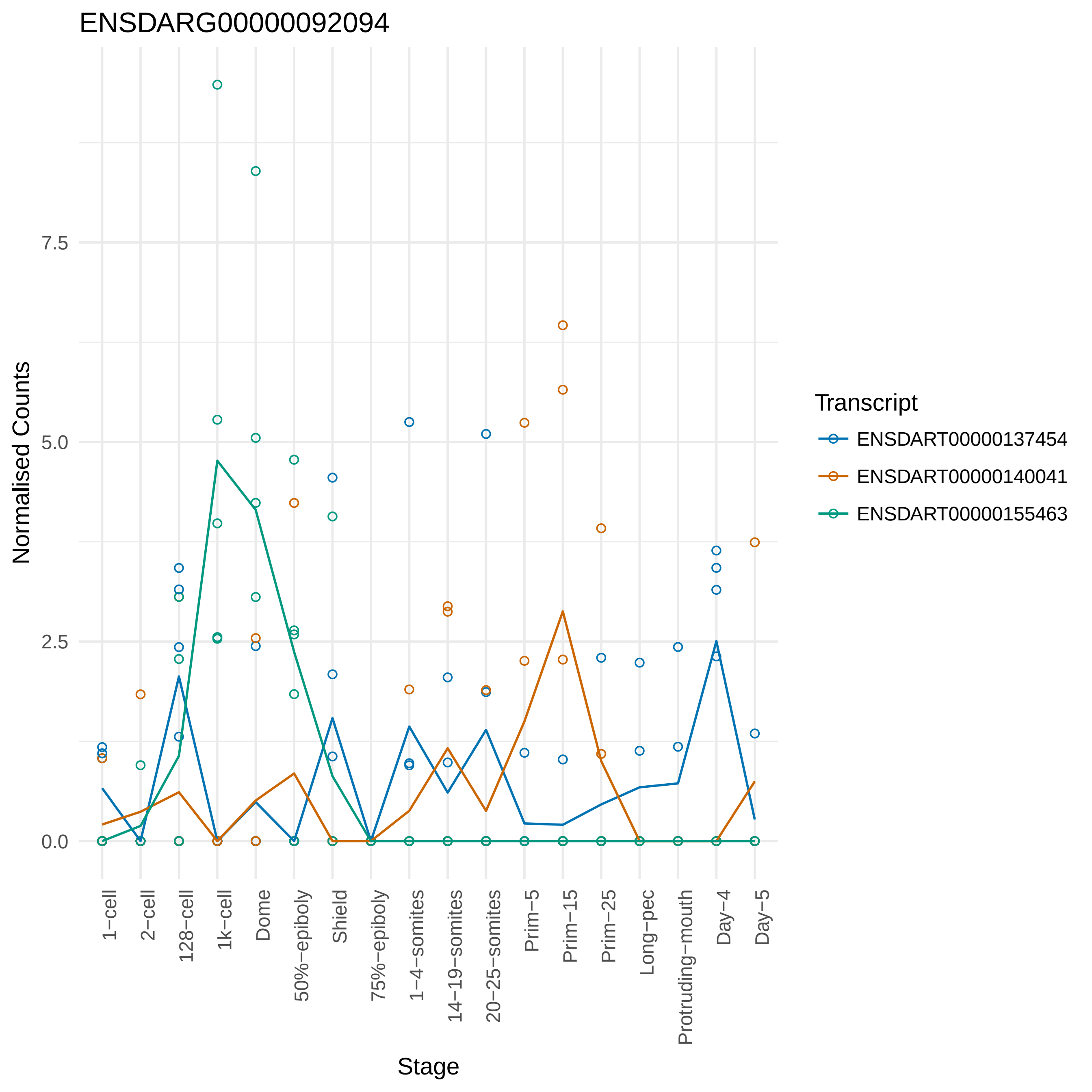

ENSDARG00000092094
Normalised Counts
0.0
2.5
5.0
7.5
Stage
1−cell
2−cell
128−cell
1k−cell
Dome
50%−epiboly
Shield
75%−epiboly
1−4−somites
14−19−somites
20−25−somites
Prim−5
Prim−15
Prim−25
Long−pec
Protruding−mouth
Day−4
Day−5
Transcript
ENSDART00000137454
ENSDART00000140041
ENSDART00000155463

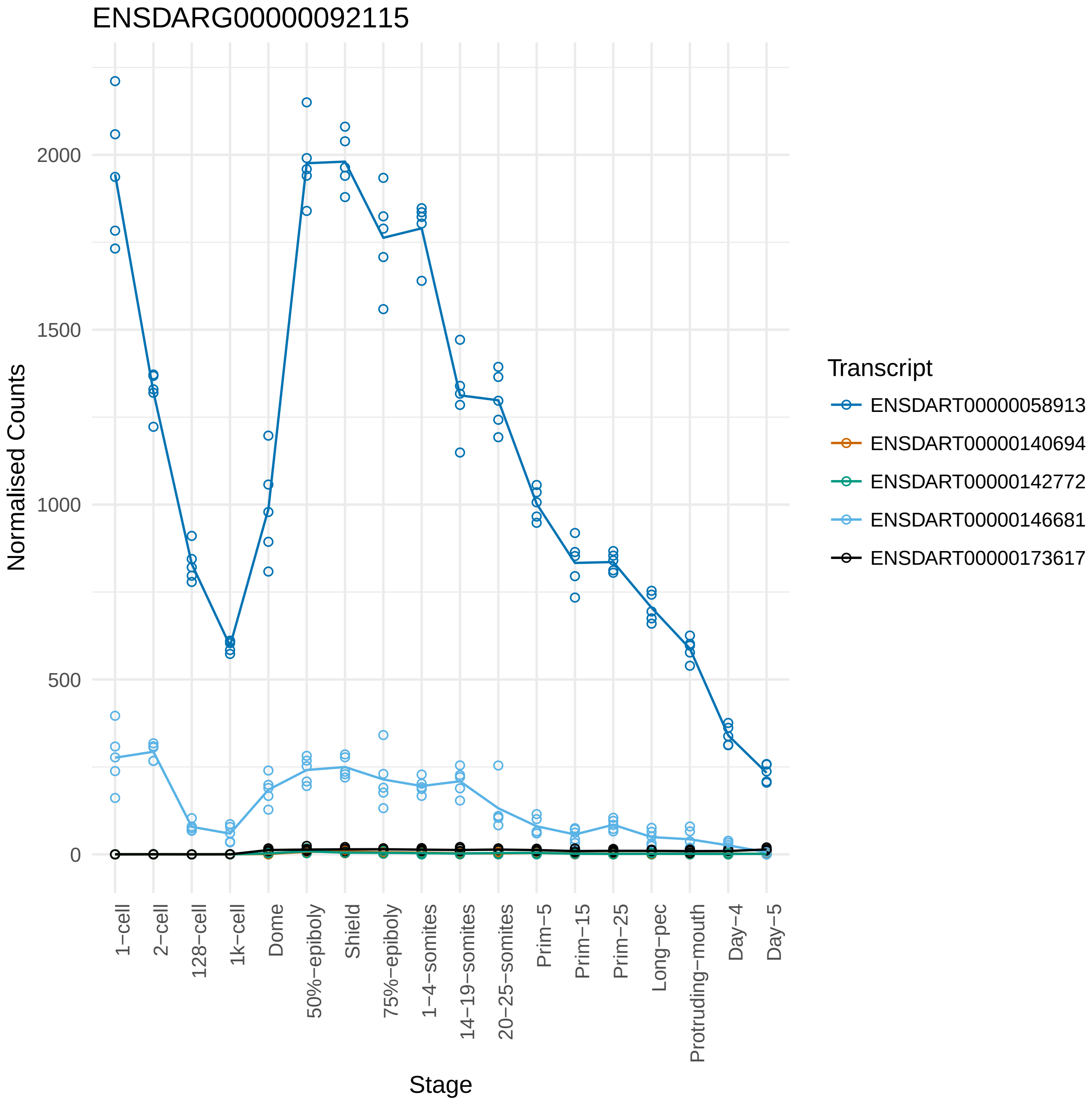

ENSDARG00000092115
Normalised Counts
2000
1500
1000
500
0
1−cell
2−cell
128−cell
1k−cell
Dome
50%−epiboly
Shield
75%−epiboly
1−4−somites
14−19−somites
20−25−somites
Prim−5
Prim−15
Prim−25
Long−pec
Protruding−mouth
Day−4
Day−5
Stage
Transcript
ENSDART00000058913
ENSDART00000140694
ENSDART00000142772
ENSDART00000146681
ENSDART00000173617

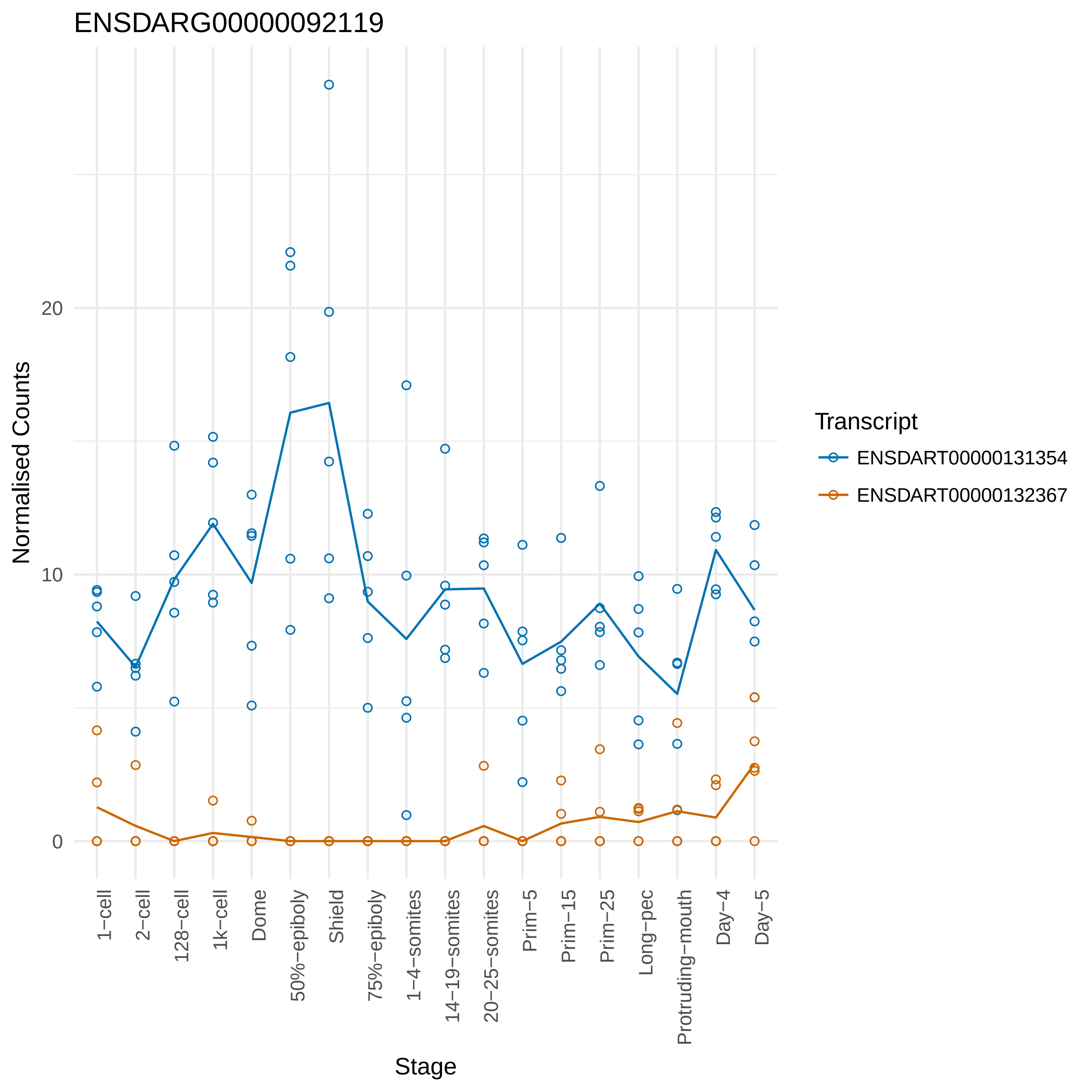

ENSDARG00000092119
Normalised Counts
0
10
20
Stage
1−cell
2−cell
128−cell
1k−cell
Dome
50%−epiboly
Shield
75%−epiboly
1−4−somites
14−19−somites
20−25−somites
Prim−5
Prim−15
Prim−25
Long−pec
Protruding−mouth
Day−4
Day−5
Transcript
ENSDART00000131354
ENSDART00000132367

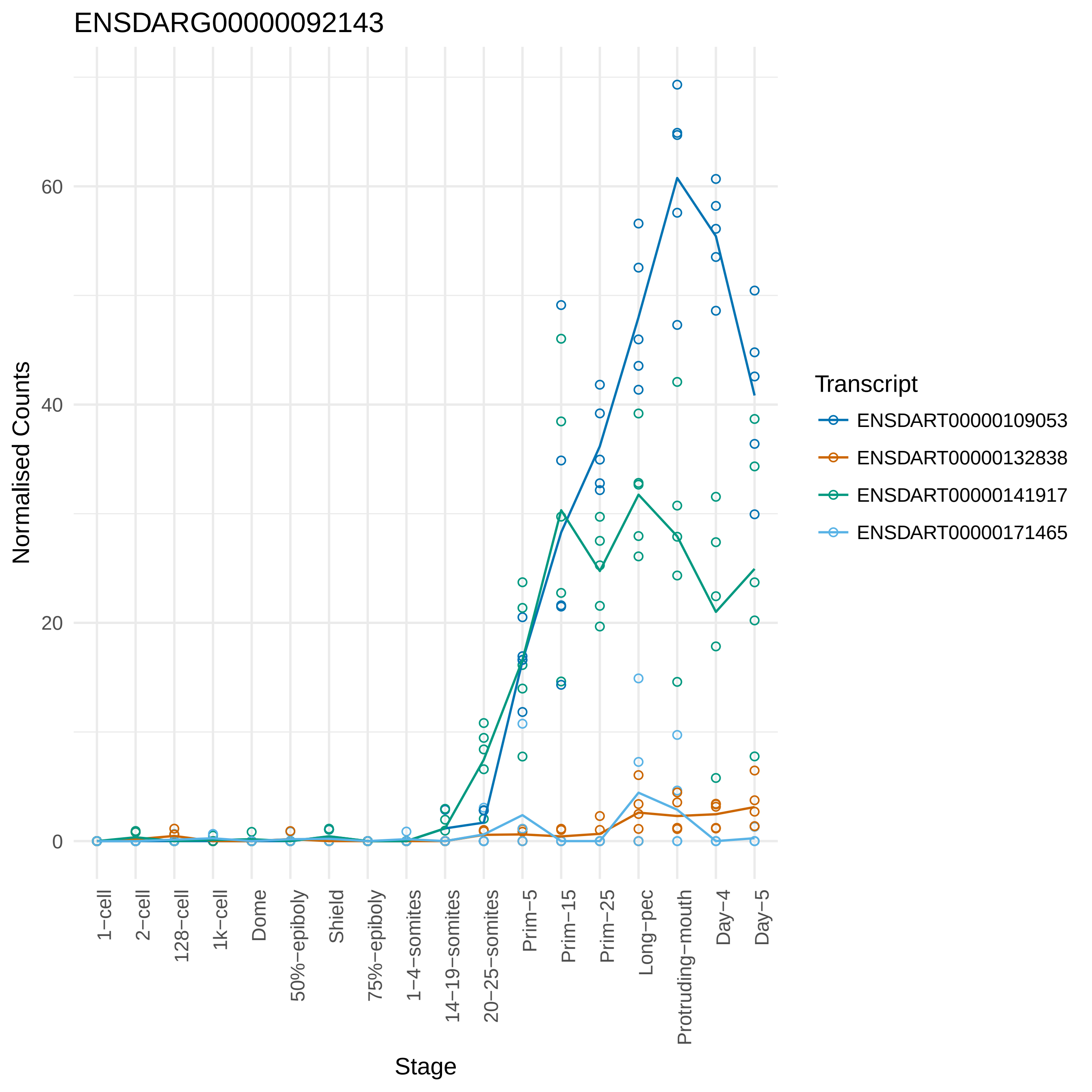

ENSDARG00000092143
Normalised Counts
0
20
40
60
1−cell
2−cell
128−cell
1k−cell
Dome
50%−epiboly
Shield
75%−epiboly
1−4−somites
14−19−somites
20−25−somites
Prim−5
Prim−15
Prim−25
Long−pec
Protruding−mouth
Day−4
Day−5
Stage
Transcript
ENSDART00000109053
ENSDART00000132838
ENSDART00000141917
ENSDART00000171465

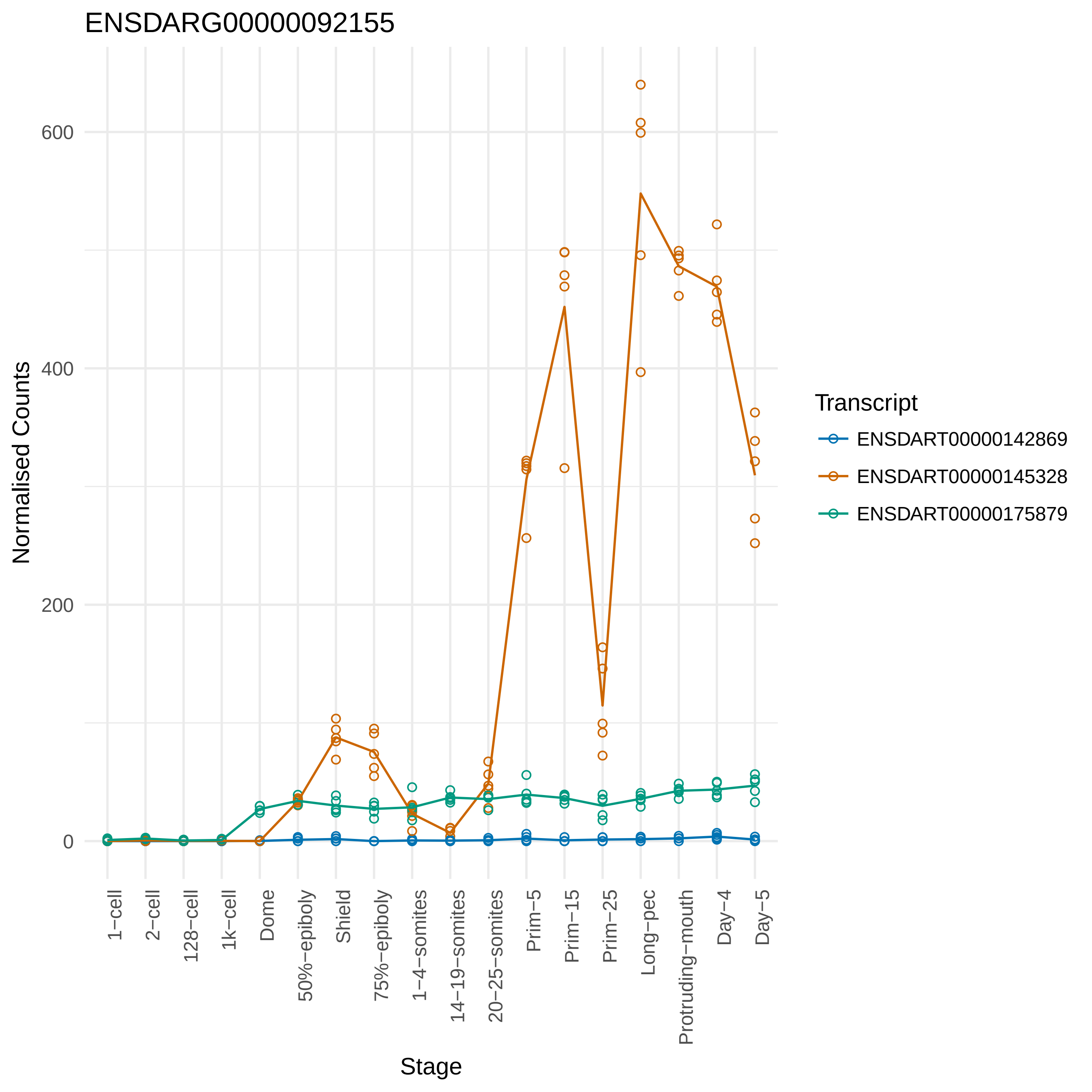

ENSDARG00000092155
Normalised Counts
600
400
200
0
1−cell
2−cell
128−cell
1k−cell
Dome
50%−epiboly
Shield
75%−epiboly
1−4−somites
14−19−somites
20−25−somites
Prim−5
Prim−15
Prim−25
Long−pec
Protruding−mouth
Day−4
Day−5
Stage
Transcript
ENSDART00000142869
ENSDART00000145328
ENSDART00000175879

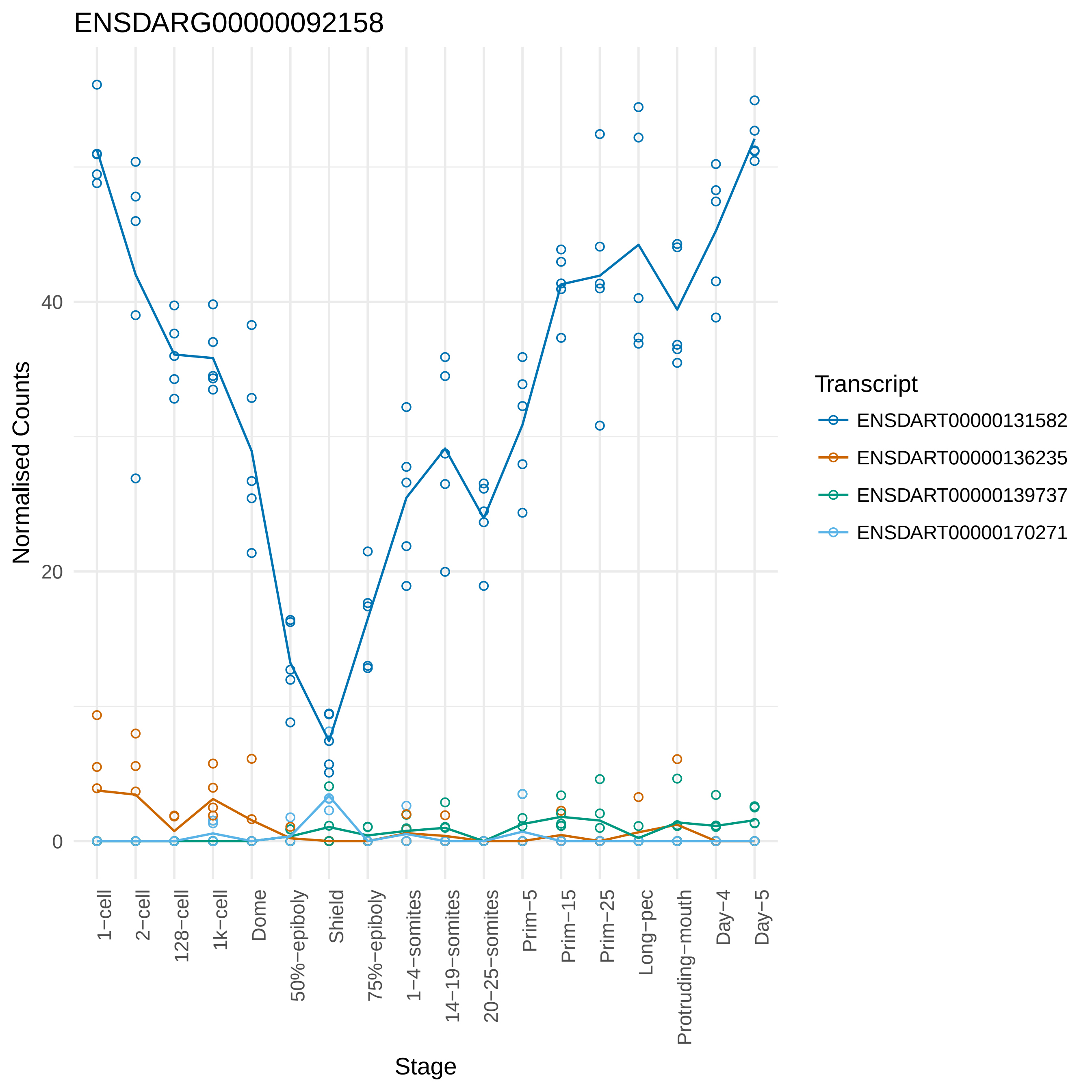

ENSDARG00000092158
Normalised Counts
0
20
40
1−cell
2−cell
128−cell
1k−cell
Dome
50%−epiboly
Shield
75%−epiboly
1−4−somites
14−19−somites
20−25−somites
Prim−5
Prim−15
Prim−25
Long−pec
Protruding−mouth
Day−4
Day−5
Stage
Transcript
ENSDART00000131582
ENSDART00000136235
ENSDART00000139737
ENSDART00000170271

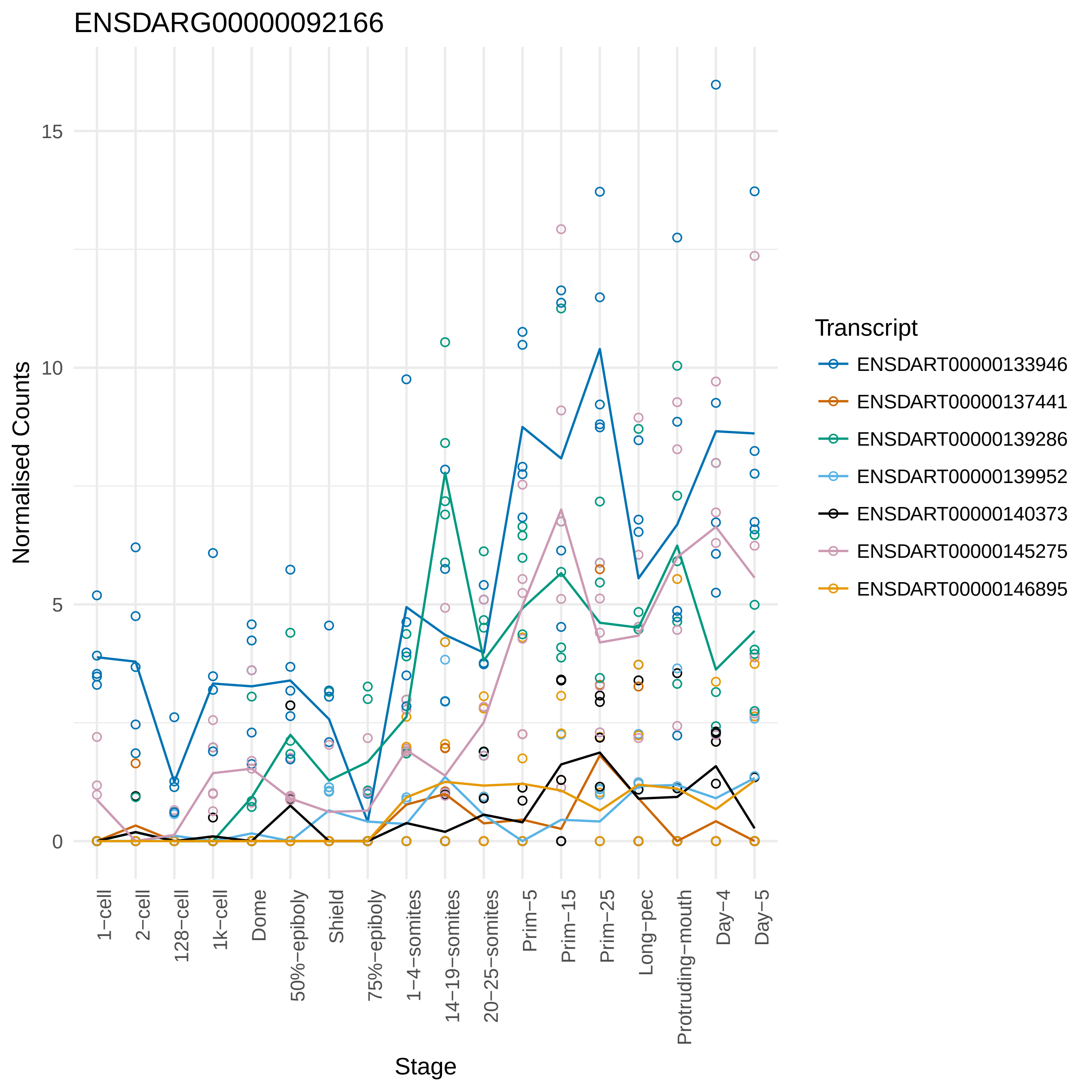

ENSDARG00000092166
Normalised Counts
0
5
10
15
Stage
1−cell
2−cell
128−cell
1k−cell
Dome
50%−epiboly
Shield
75%−epiboly
1−4−somites
14−19−somites
20−25−somites
Prim−5
Prim−15
Prim−25
Long−pec
Protruding−mouth
Day−4
Day−5
Transcript
ENSDART00000133946
ENSDART00000137441
ENSDART00000139286
ENSDART00000139952
ENSDART00000140373
ENSDART00000145275
ENSDART00000146895

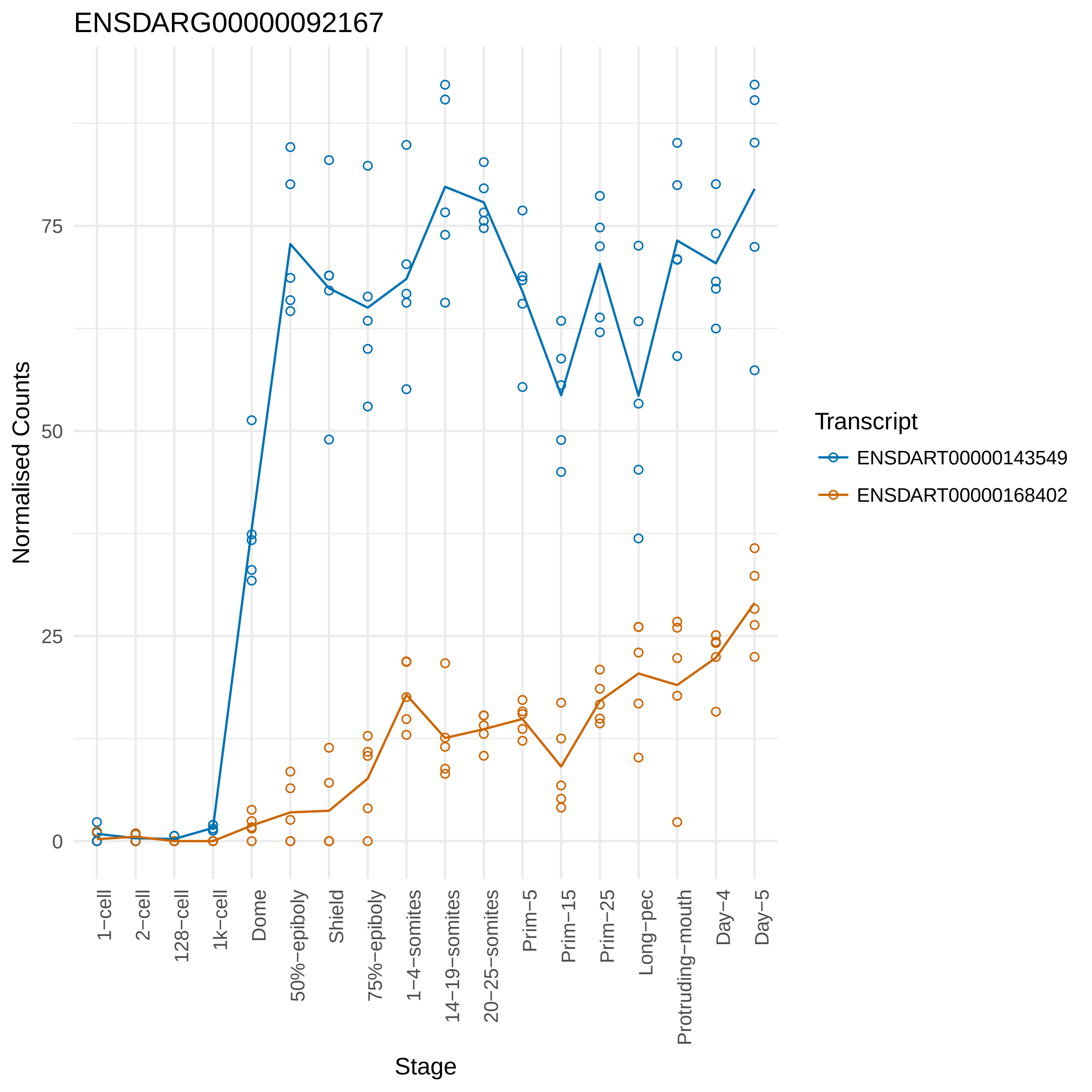

ENSDARG00000092167
Normalised Counts
0
25
50
75
Stage
1−cell
2−cell
128−cell
1k−cell
Dome
50%−epiboly
Shield
75%−epiboly
1−4−somites
14−19−somites
20−25−somites
Prim−5
Prim−15
Prim−25
Long−pec
Protruding−mouth
Day−4
Day−5
Transcript
ENSDART00000143549
ENSDART00000168402

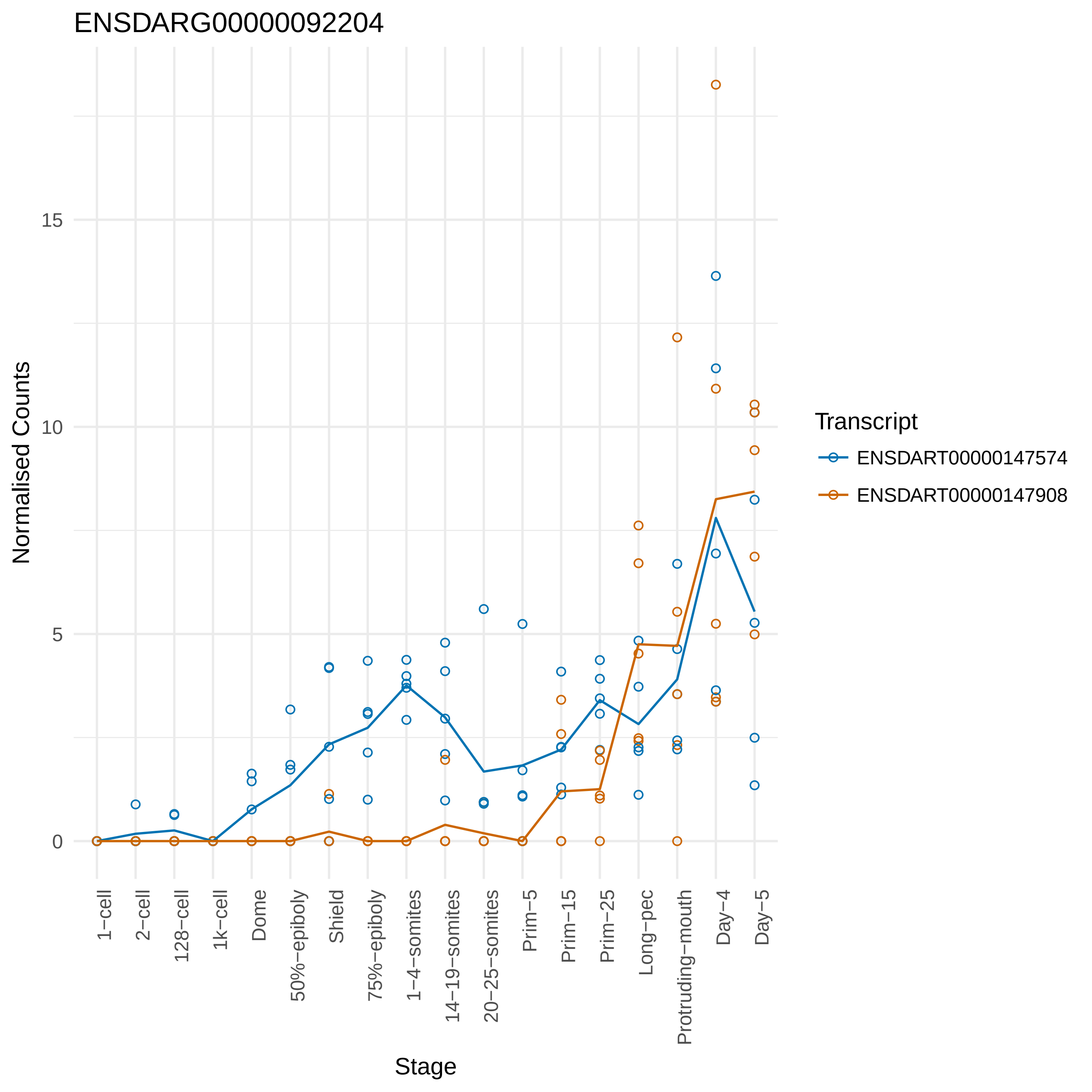
ENSDARG00000092204
Normalised Counts
0
5
10
15
Stage
1−cell
2−cell
128−cell
1k−cell
Dome
50%−epiboly
Shield
75%−epiboly
1−4−somites
14−19−somites
20−25−somites
Prim−5
Prim−15
Prim−25
Long−pec
Protruding−mouth
Day−4
Day−5
Transcript
ENSDART00000147574
ENSDART00000147908

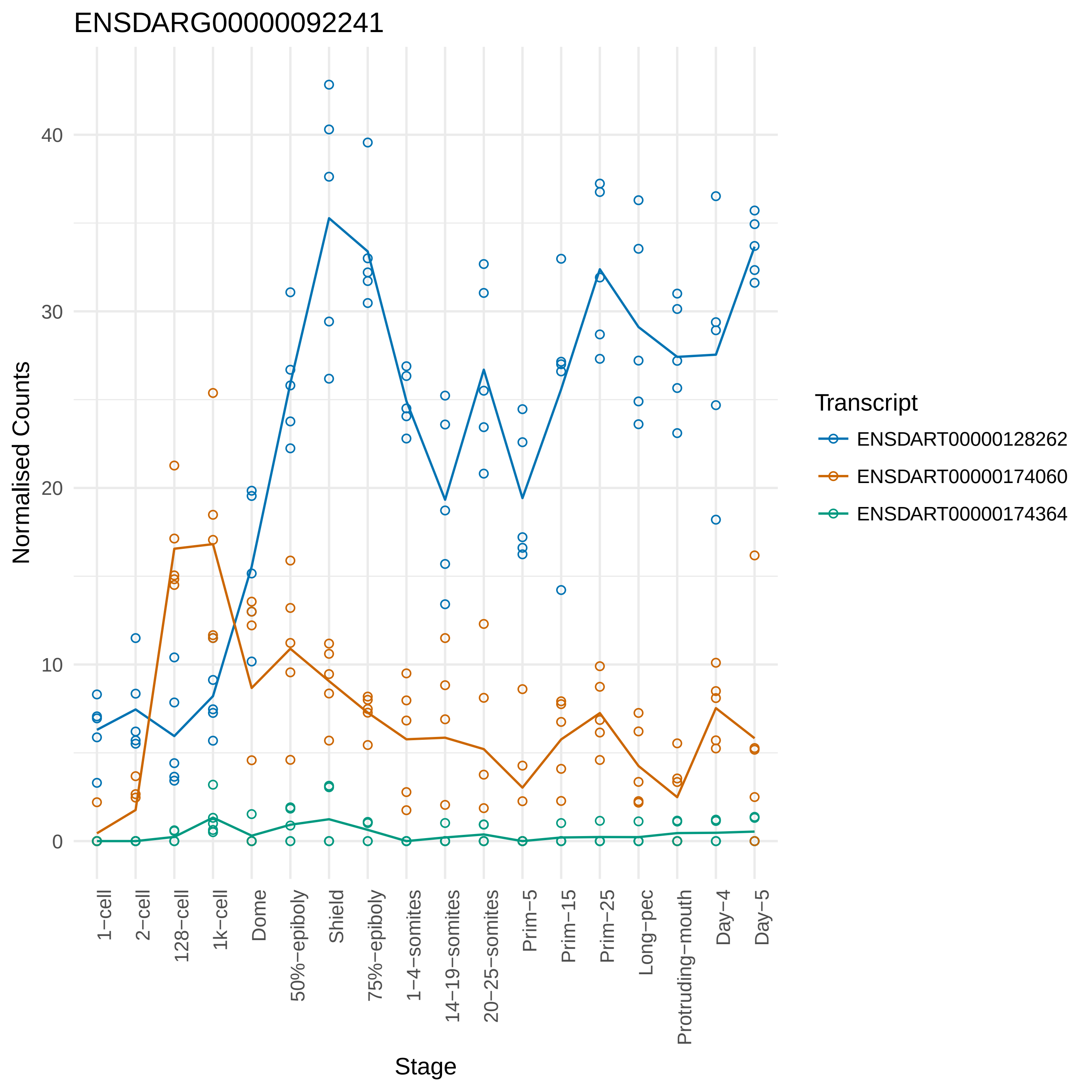
ENSDARG00000092241
Normalised Counts
0
10
20
30
40
Stage
1−cell
2−cell
128−cell
1k−cell
Dome
50%−epiboly
Shield
75%−epiboly
1−4−somites
14−19−somites
20−25−somites
Prim−5
Prim−15
Prim−25
Long−pec
Protruding−mouth
Day−4
Day−5
Transcript
ENSDART00000128262
ENSDART00000174060
ENSDART00000174364

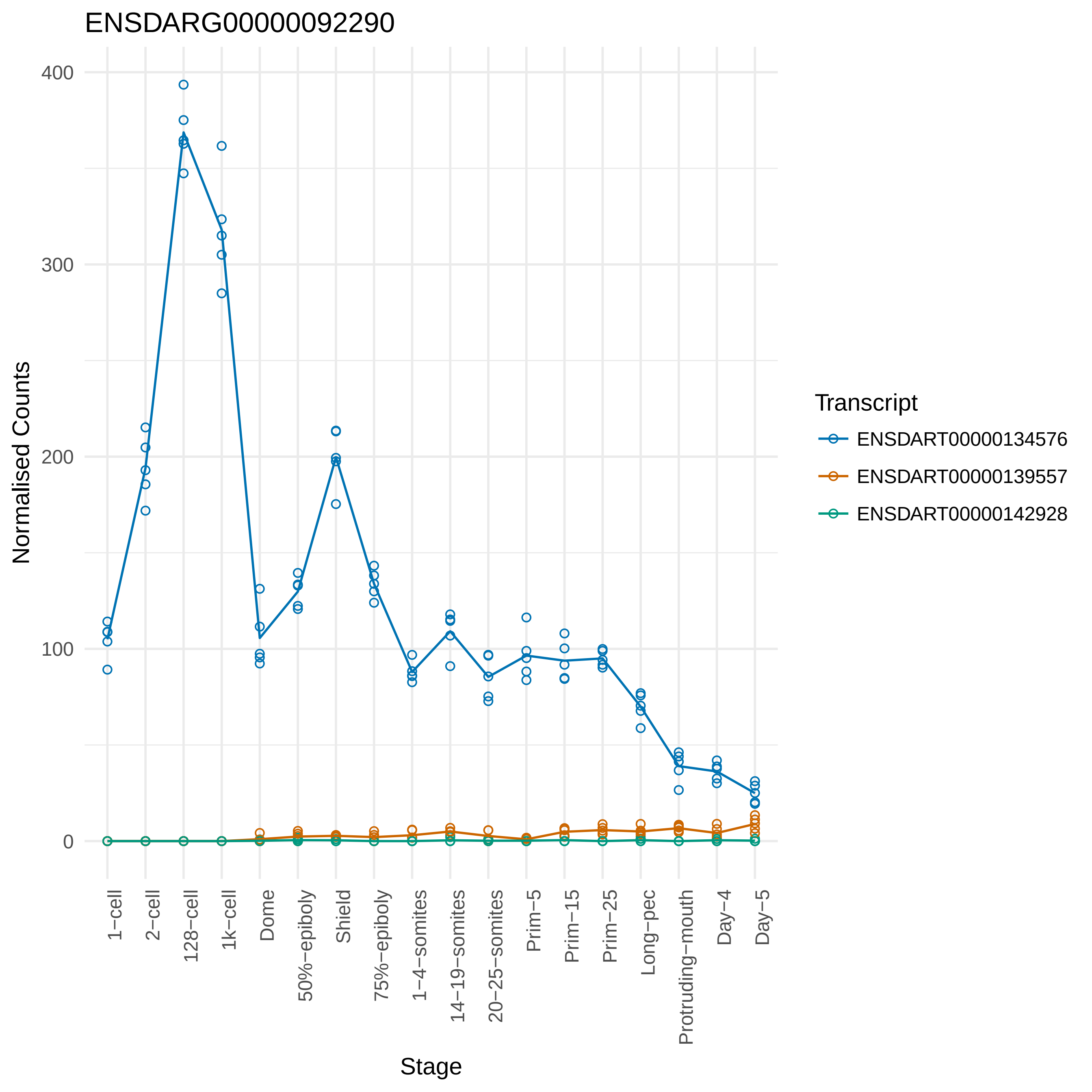
ENSDARG00000092290
Normalised Counts
400
300
200
100
0
1−cell
2−cell
128−cell
1k−cell
Dome
50%−epiboly
Shield
75%−epiboly
1−4−somites
14−19−somites
20−25−somites
Prim−5
Prim−15
Prim−25
Long−pec
Protruding−mouth
Day−4
Day−5
Stage
Transcript
ENSDART00000134576
ENSDART00000139557
ENSDART00000142928

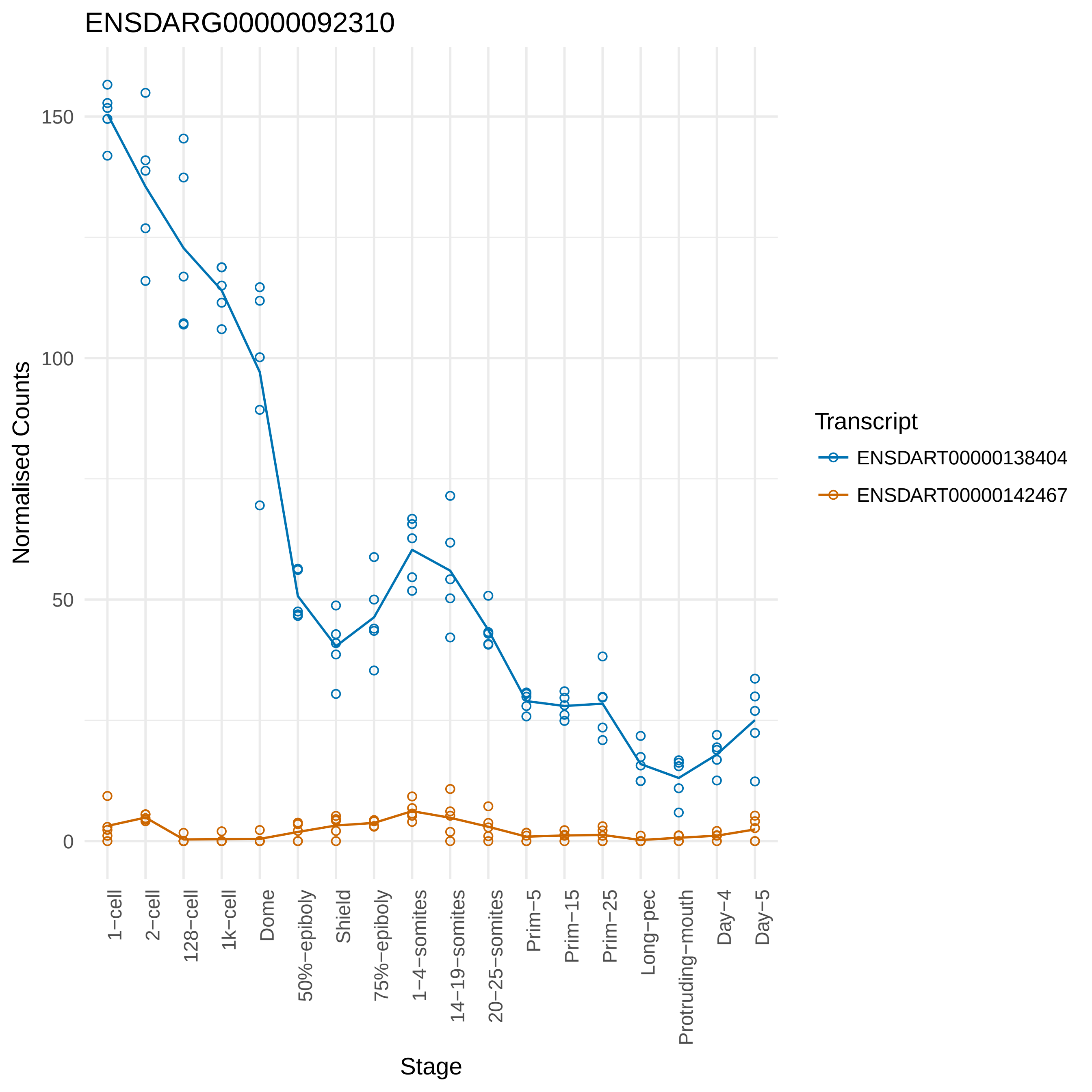

ENSDARG00000092310
Normalised Counts
150
100
50
0
1−cell
2−cell
128−cell
1k−cell
Dome
50%−epiboly
Shield
75%−epiboly
1−4−somites
14−19−somites
20−25−somites
Prim−5
Prim−15
Prim−25
Long−pec
Protruding−mouth
Day−4
Day−5
Stage
Transcript
ENSDART00000138404
ENSDART00000142467

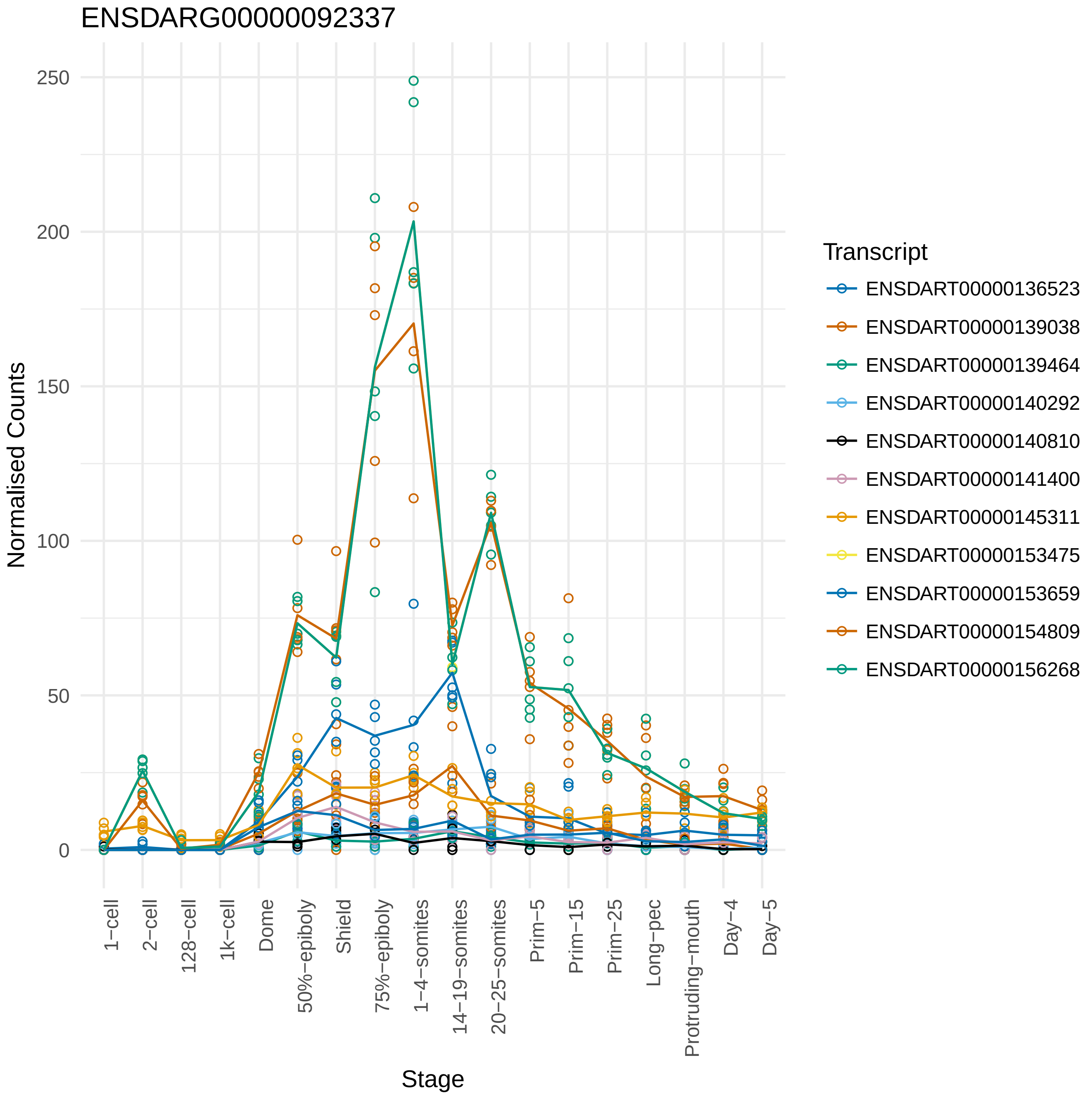

ENSDARG00000092337
Normalised Counts
250
200
150
100
50
0
1−cell
2−cell
128−cell
1k−cell
Dome
50%−epiboly
Shield
75%−epiboly
1−4−somites
14−19−somites
20−25−somites
Prim−5
Prim−15
Prim−25
Long−pec
Protruding−mouth
Day−4
Day−5
Stage
Transcript
ENSDART00000136523
ENSDART00000139038
ENSDART00000139464
ENSDART00000140292
ENSDART00000140810
ENSDART00000141400
ENSDART00000145311
ENSDART00000153475
ENSDART00000153659
ENSDART00000154809
ENSDART00000156268

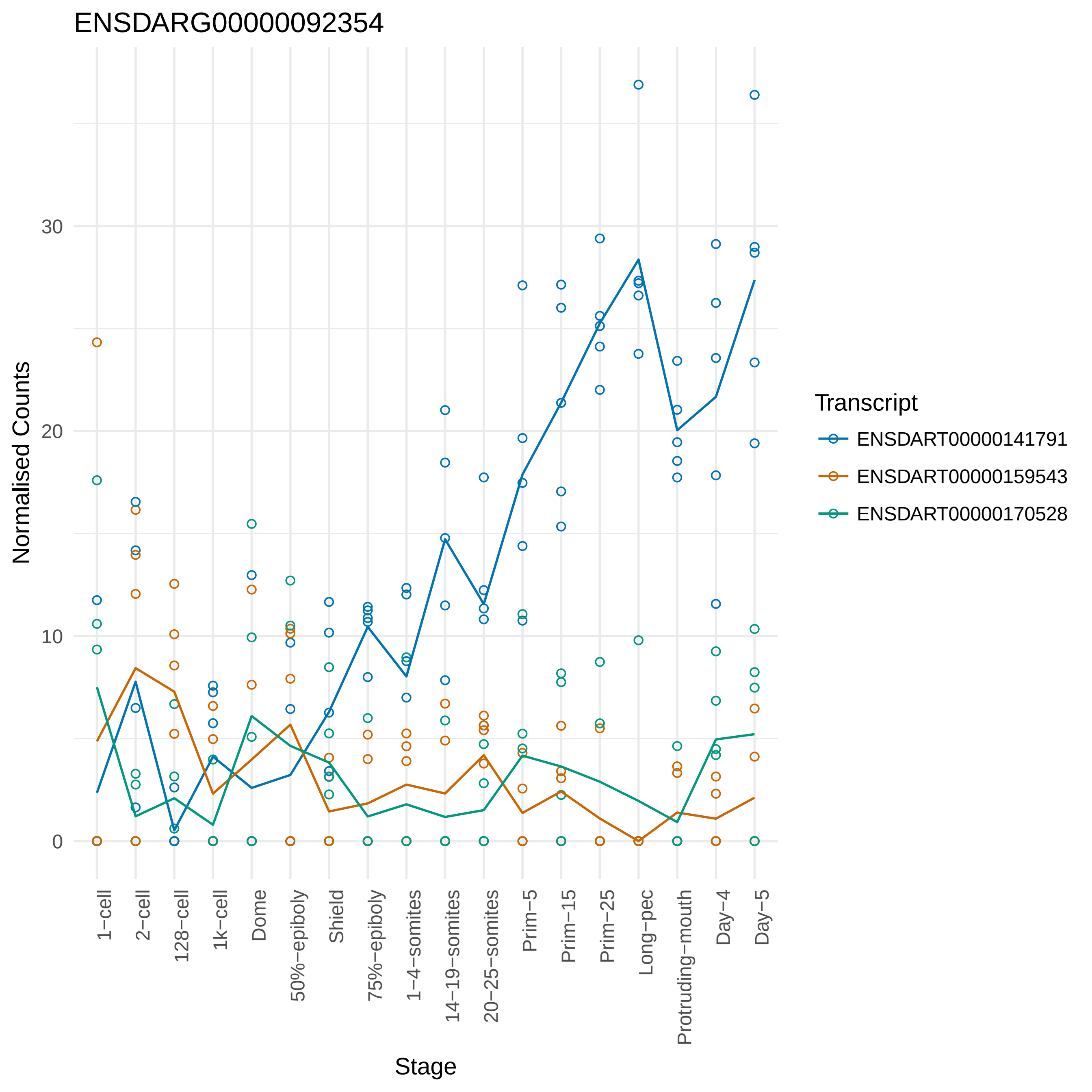

ENSDARG00000092354
Normalised Counts
0
10
20
30
Stage
1−cell
2−cell
128−cell
1k−cell
Dome
50%−epiboly
Shield
75%−epiboly
1−4−somites
14−19−somites
20−25−somites
Prim−5
Prim−15
Prim−25
Long−pec
Protruding−mouth
Day−4
Day−5
Transcript
ENSDART00000141791
ENSDART00000159543
ENSDART00000170528

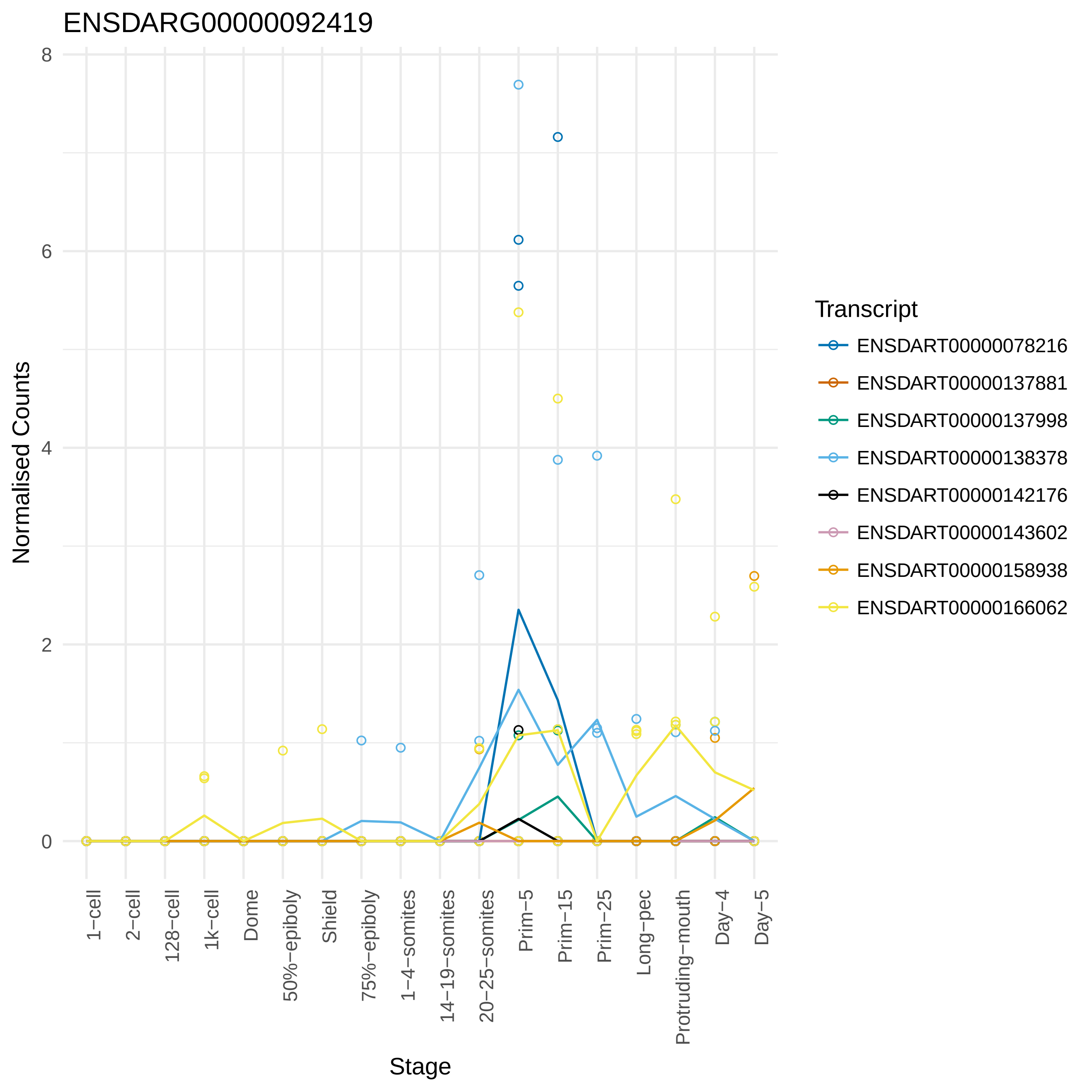

ENSDARG00000092419
Normalised Counts
0
2
4
6
8
1−cell
2−cell
128−cell
1k−cell
Dome
50%−epiboly
Shield
75%−epiboly
1−4−somites
14−19−somites
20−25−somites
Prim−5
Prim−15
Prim−25
Long−pec
Protruding−mouth
Day−4
Day−5
Stage
Transcript
ENSDART00000078216
ENSDART00000137881
ENSDART00000137998
ENSDART00000138378
ENSDART00000142176
ENSDART00000143602
ENSDART00000158938
ENSDART00000166062

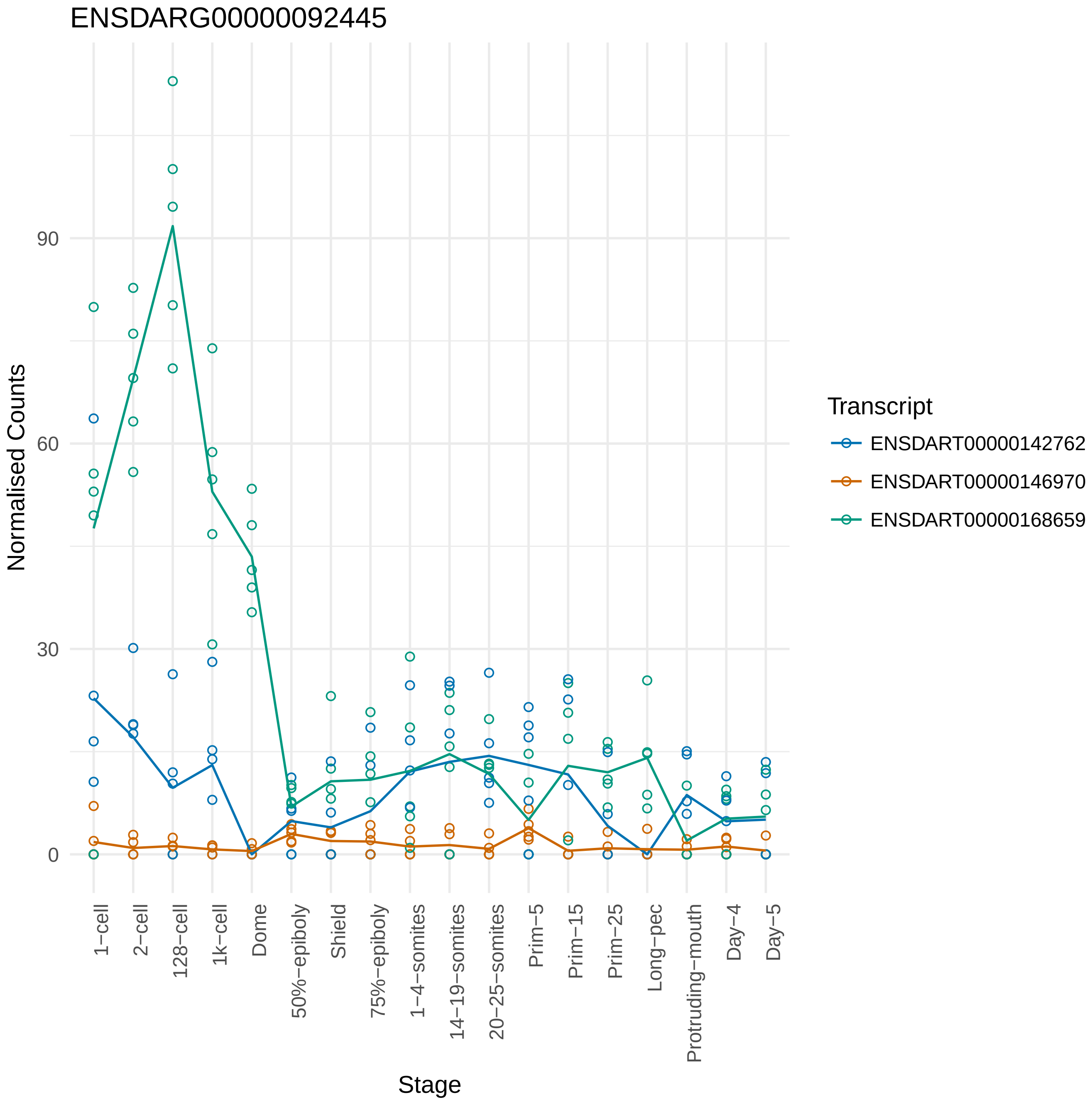
ENSDARG00000092445
Normalised Counts
0
30
60
90
1−cell
2−cell
128−cell
1k−cell
Dome
50%−epiboly
Shield
75%−epiboly
1−4−somites
14−19−somites
20−25−somites
Prim−5
Prim−15
Prim−25
Long−pec
Protruding−mouth
Day−4
Day−5
Stage
Transcript
ENSDART00000142762
ENSDART00000146970
ENSDART00000168659

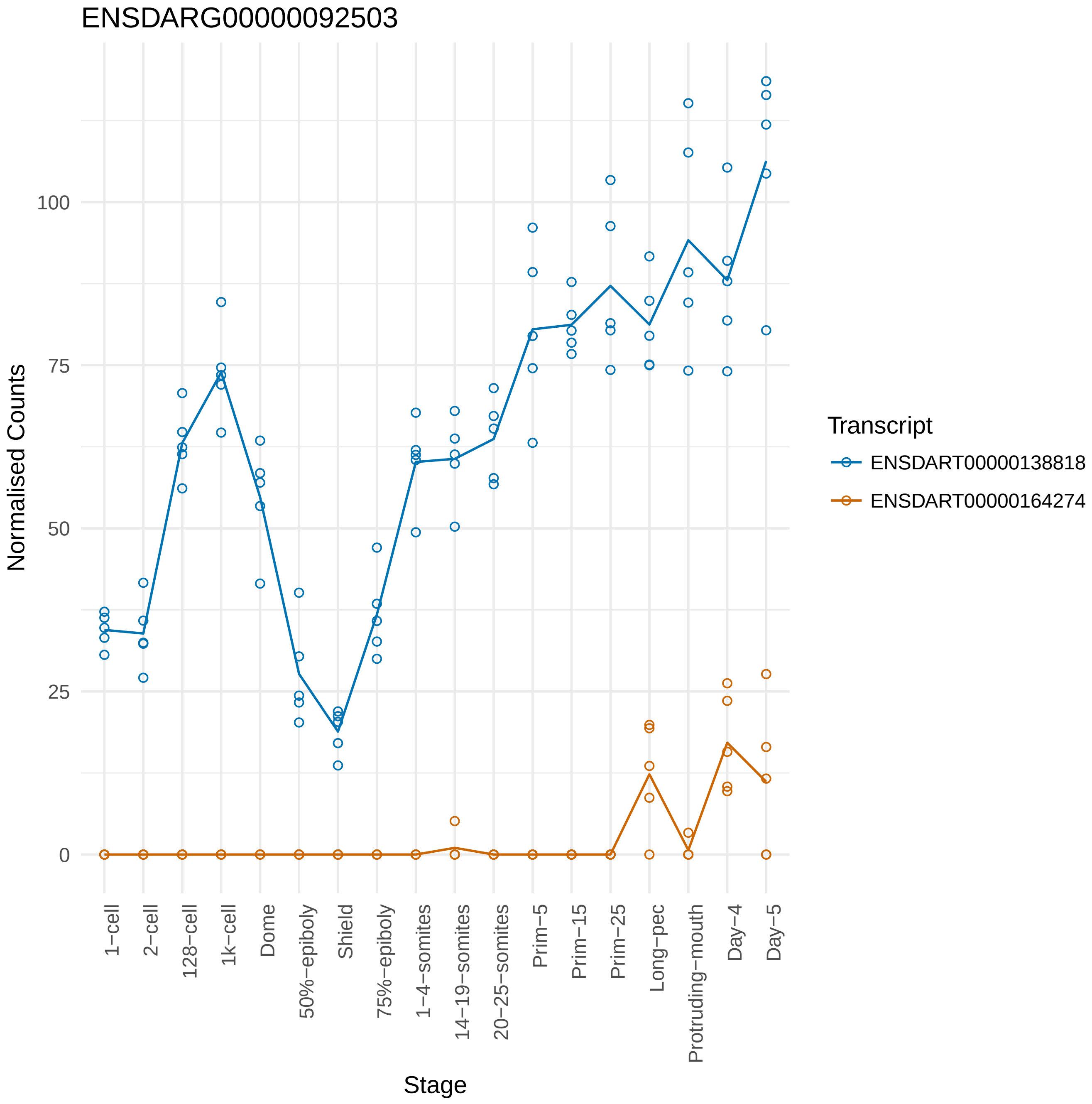
ENSDARG00000092503
Normalised Counts
100
75
50
25
0
Stage
1−cell
2−cell
128−cell
1k−cell
Dome
50%−epiboly
Shield
75%−epiboly
1−4−somites
14−19−somites
20−25−somites
Prim−5
Prim−15
Prim−25
Long−pec
Protruding−mouth
Day−4
Day−5
Transcript
ENSDART00000138818
ENSDART00000164274

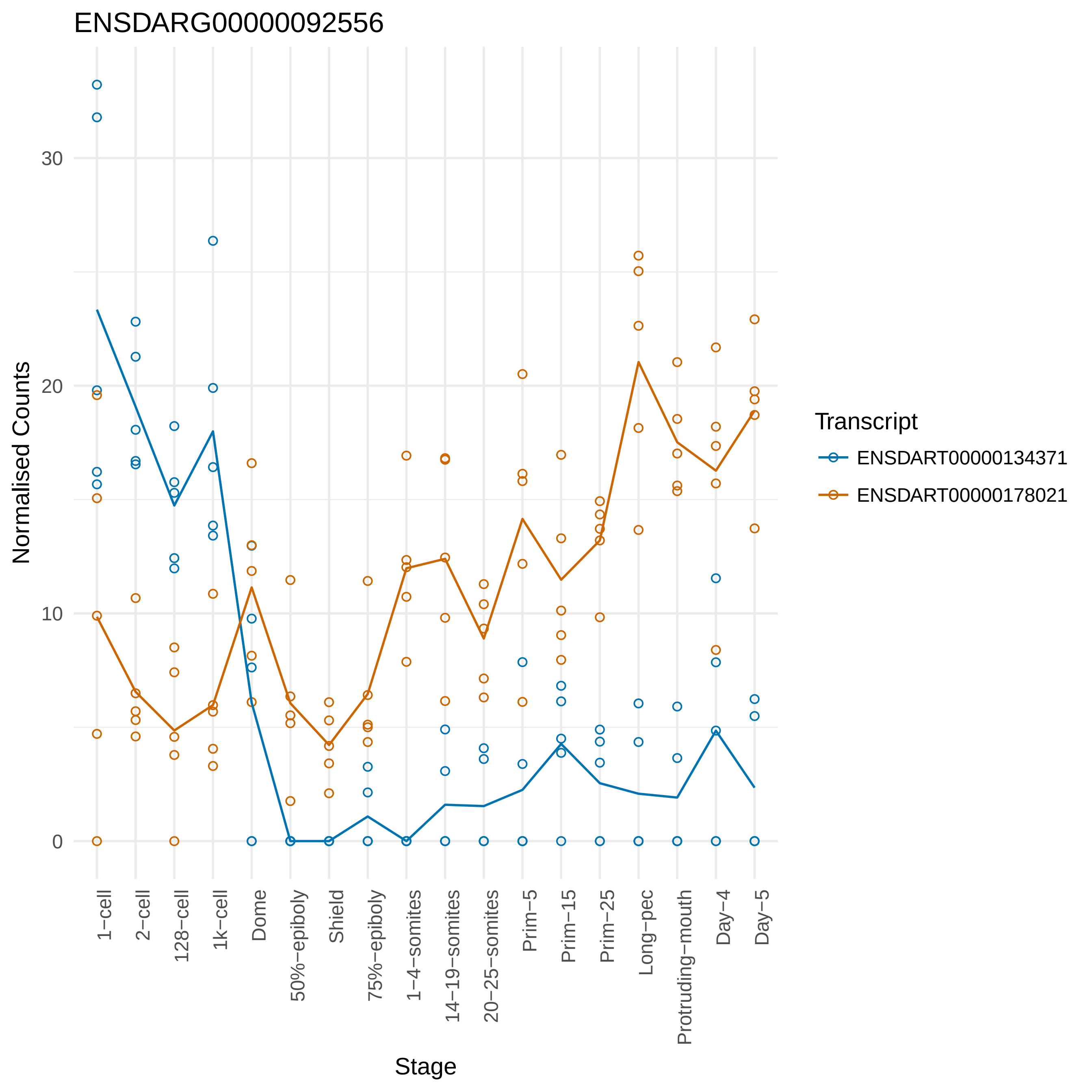
ENSDARG00000092556
Normalised Counts
30
20
10
0
1−cell
2−cell
128−cell
1k−cell
Dome
50%−epiboly
Shield
75%−epiboly
1−4−somites
14−19−somites
20−25−somites
Prim−5
Prim−15
Prim−25
Long−pec
Protruding−mouth
Day−4
Day−5
Stage
Transcript
ENSDART00000134371
ENSDART00000178021

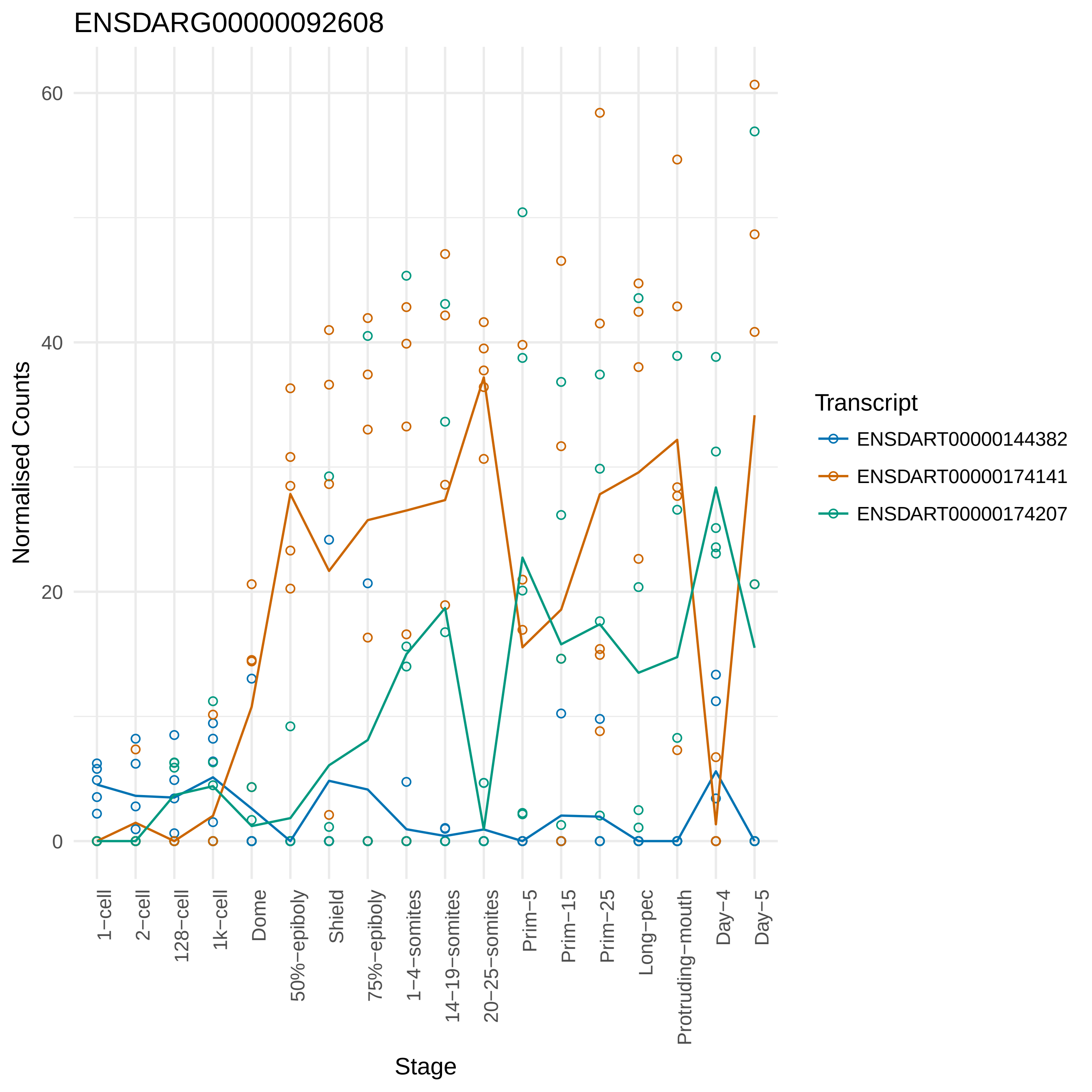
ENSDARG00000092608
Normalised Counts
0
20
40
60
1−cell
2−cell
128−cell
1k−cell
Dome
50%−epiboly
Shield
75%−epiboly
1−4−somites
14−19−somites
20−25−somites
Prim−5
Prim−15
Prim−25
Long−pec
Protruding−mouth
Day−4
Day−5
Stage
Transcript
ENSDART00000144382
ENSDART00000174141
ENSDART00000174207

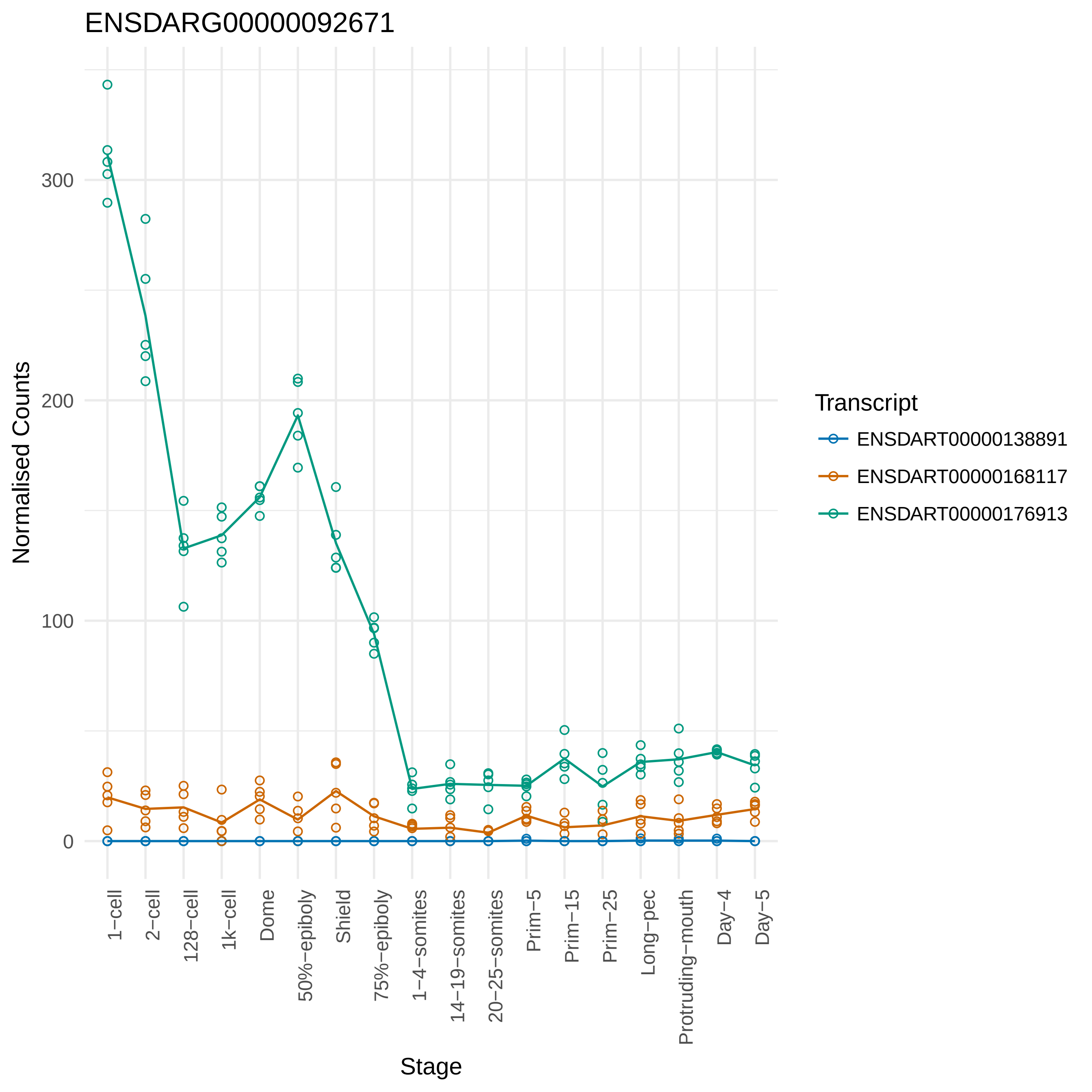

ENSDARG00000092671
Normalised Counts
300
200
100
0
1−cell
2−cell
128−cell
1k−cell
Dome
50%−epiboly
Shield
75%−epiboly
1−4−somites
14−19−somites
20−25−somites
Prim−5
Prim−15
Prim−25
Long−pec
Protruding−mouth
Day−4
Day−5
Stage
Transcript
ENSDART00000138891
ENSDART00000168117
ENSDART00000176913

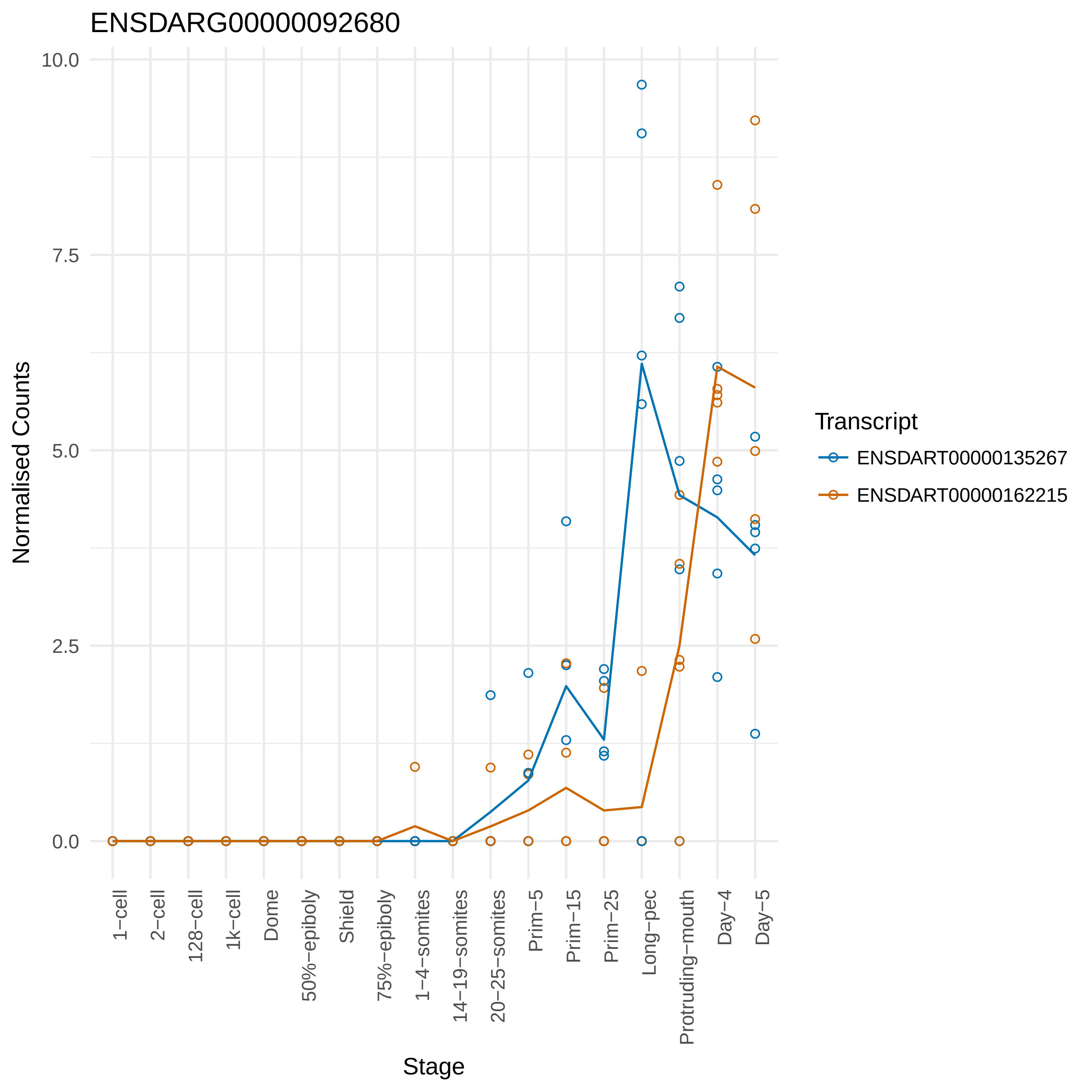
ENSDARG00000092680
Normalised Counts
10.0
7.5
5.0
2.5
0.0
1−cell
2−cell
128−cell
1k−cell
Dome
50%−epiboly
Shield
75%−epiboly
1−4−somites
14−19−somites
20−25−somites
Prim−5
Prim−15
Prim−25
Long−pec
Protruding−mouth
Day−4
Day−5
Stage
Transcript
ENSDART00000135267
ENSDART00000162215

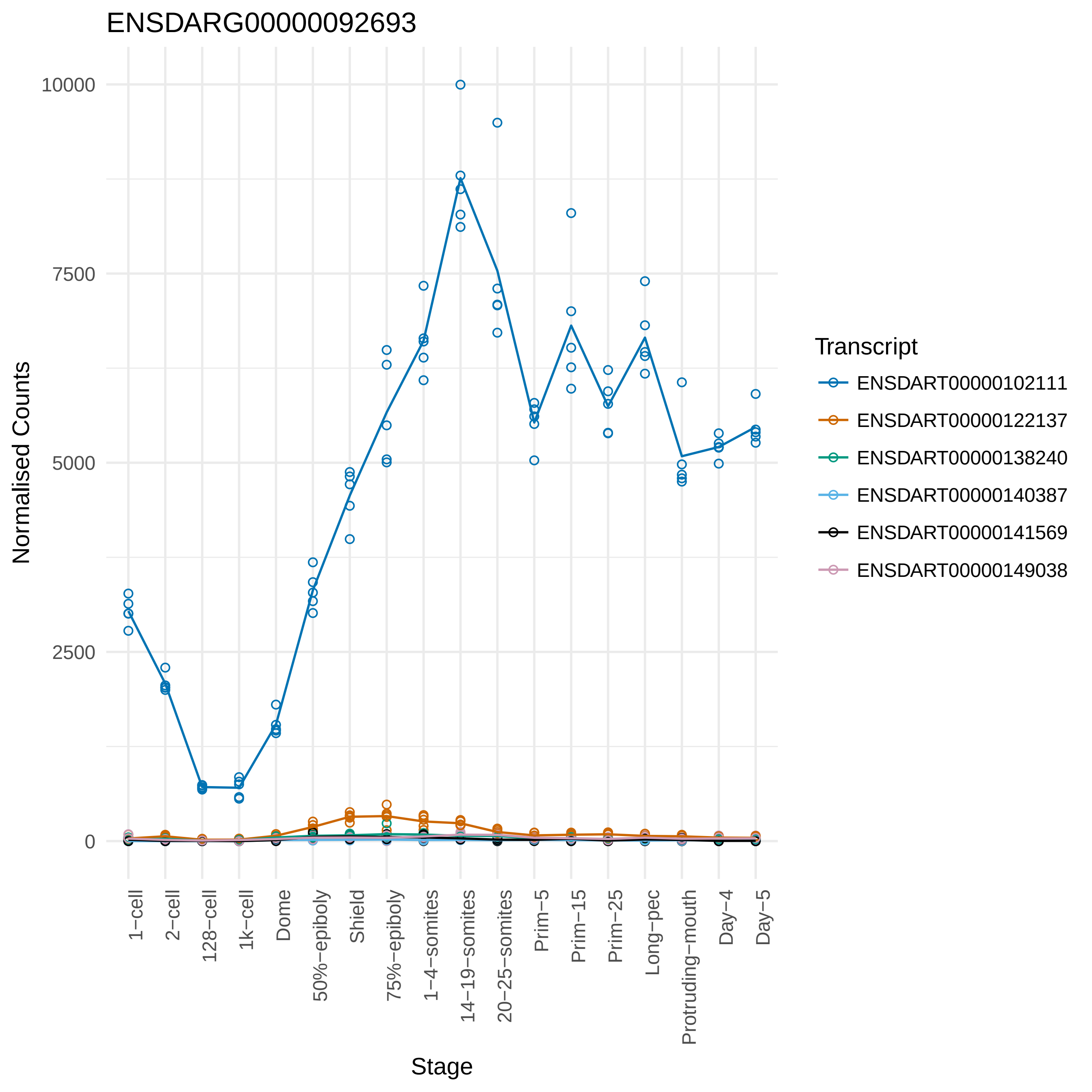
ENSDARG00000092693
Normalised Counts
10000
7500
5000
2500
0
1−cell
2−cell
128−cell
1k−cell
Dome
50%−epiboly
Shield
75%−epiboly
1−4−somites
14−19−somites
20−25−somites
Prim−5
Prim−15
Prim−25
Long−pec
Protruding−mouth
Day−4
Day−5
Stage
Transcript
ENSDART00000102111
ENSDART00000122137
ENSDART00000138240
ENSDART00000140387
ENSDART00000141569
ENSDART00000149038

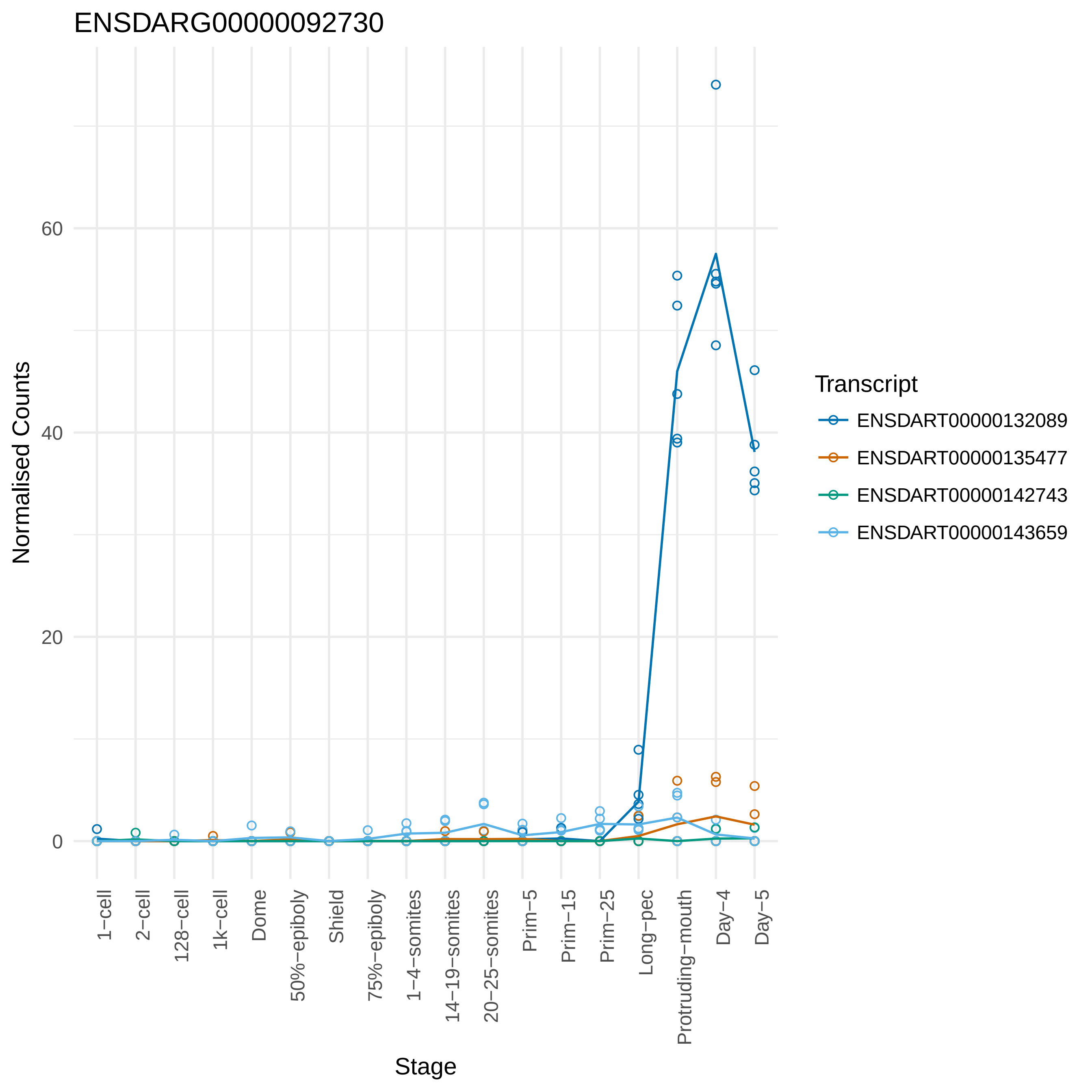

ENSDARG00000092730
Normalised Counts
0
20
40
60
Stage
1−cell
2−cell
128−cell
1k−cell
Dome
50%−epiboly
Shield
75%−epiboly
1−4−somites
14−19−somites
20−25−somites
Prim−5
Prim−15
Prim−25
Long−pec
Protruding−mouth
Day−4
Day−5
Transcript
ENSDART00000132089
ENSDART00000135477
ENSDART00000142743
ENSDART00000143659

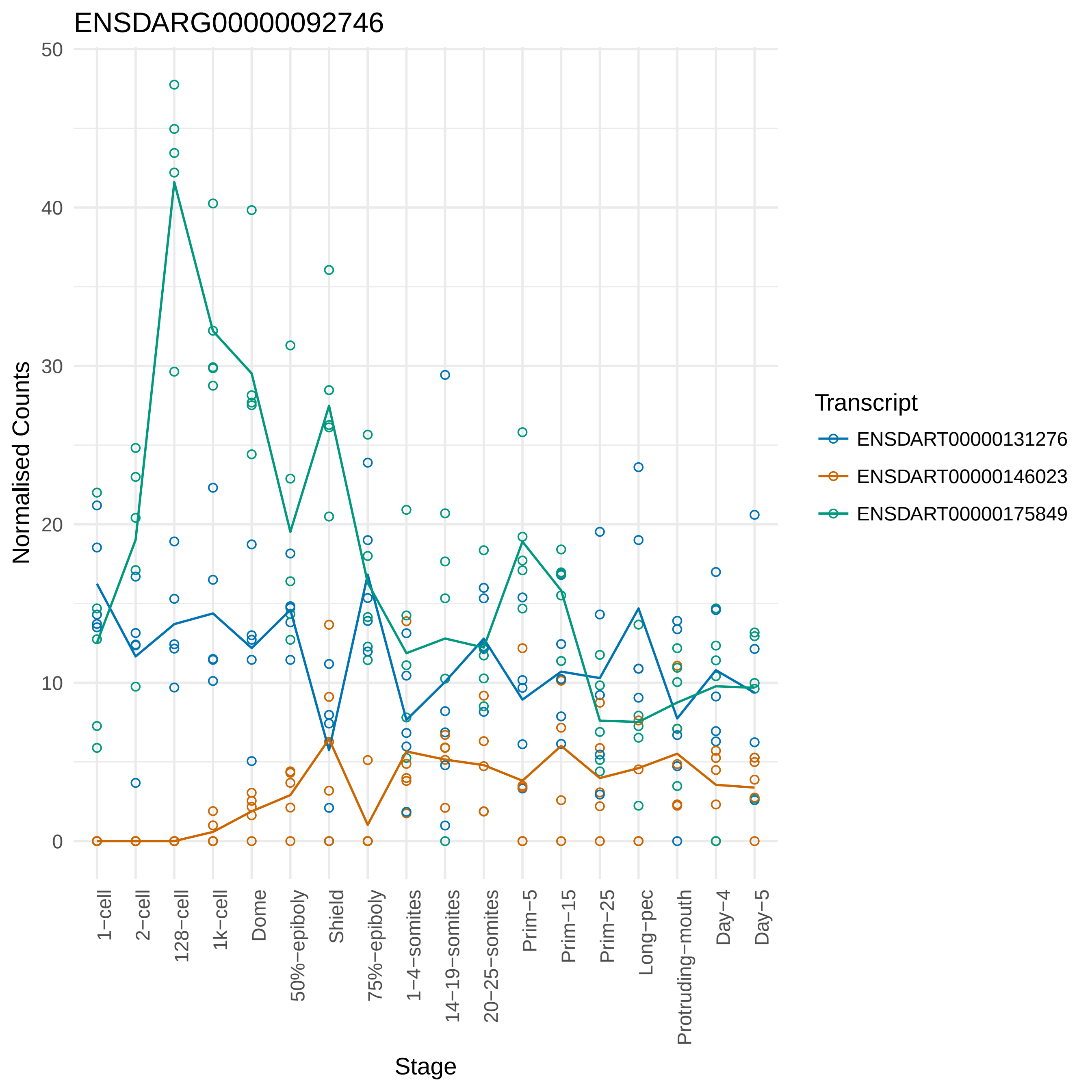
ENSDARG00000092746
Normalised Counts
0
10
20
30
40
50
Stage
1−cell
2−cell
128−cell
1k−cell
Dome
50%−epiboly
Shield
75%−epiboly
1−4−somites
14−19−somites
20−25−somites
Prim−5
Prim−15
Prim−25
Long−pec
Protruding−mouth
Day−4
Day−5
Transcript
ENSDART00000131276
ENSDART00000146023
ENSDART00000175849

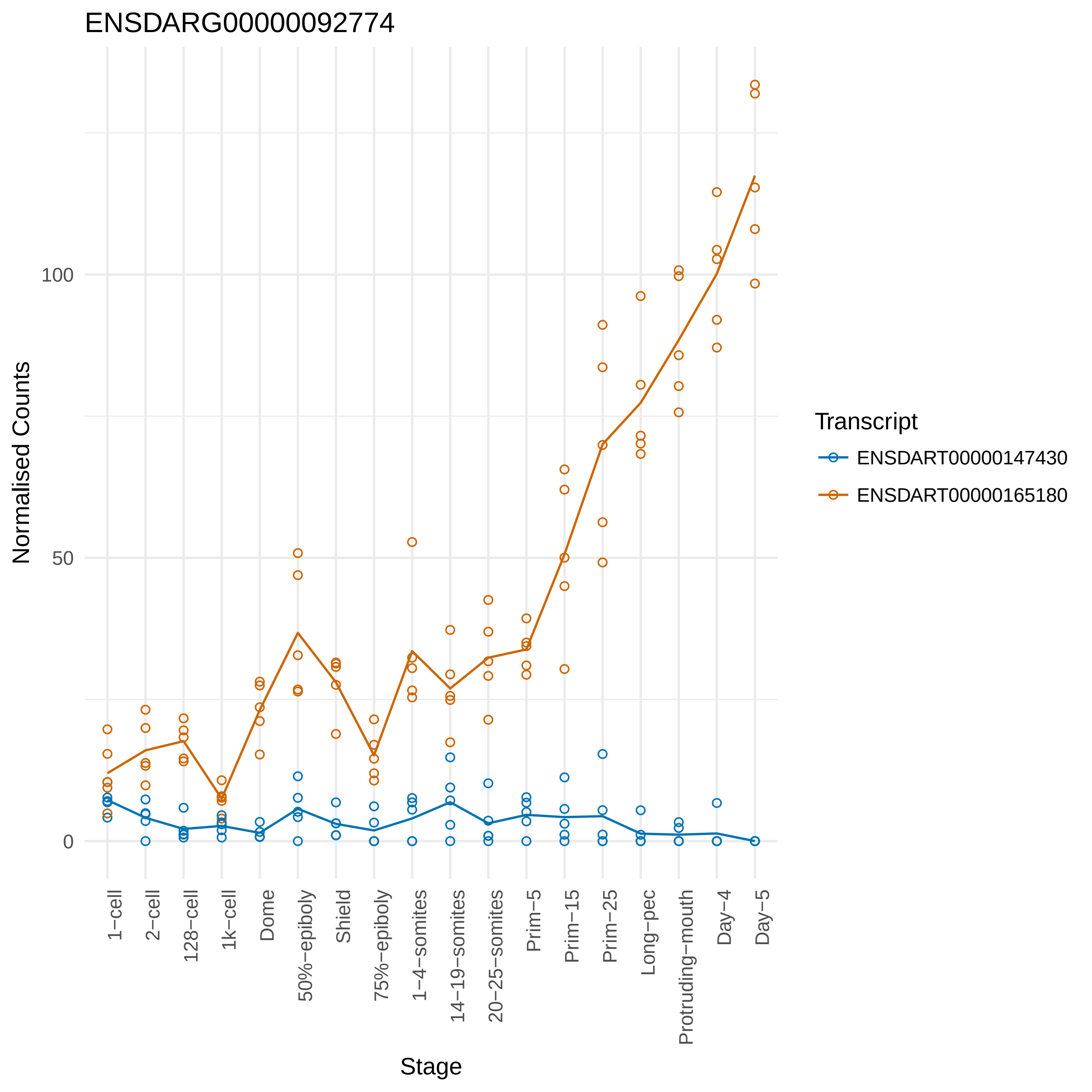

ENSDARG00000092774
Normalised Counts
0
50
100
Stage
1−cell
2−cell
128−cell
1k−cell
Dome
50%−epiboly
Shield
75%−epiboly
1−4−somites
14−19−somites
20−25−somites
Prim−5
Prim−15
Prim−25
Long−pec
Protruding−mouth
Day−4
Day−5
Transcript
ENSDART00000147430
ENSDART00000165180

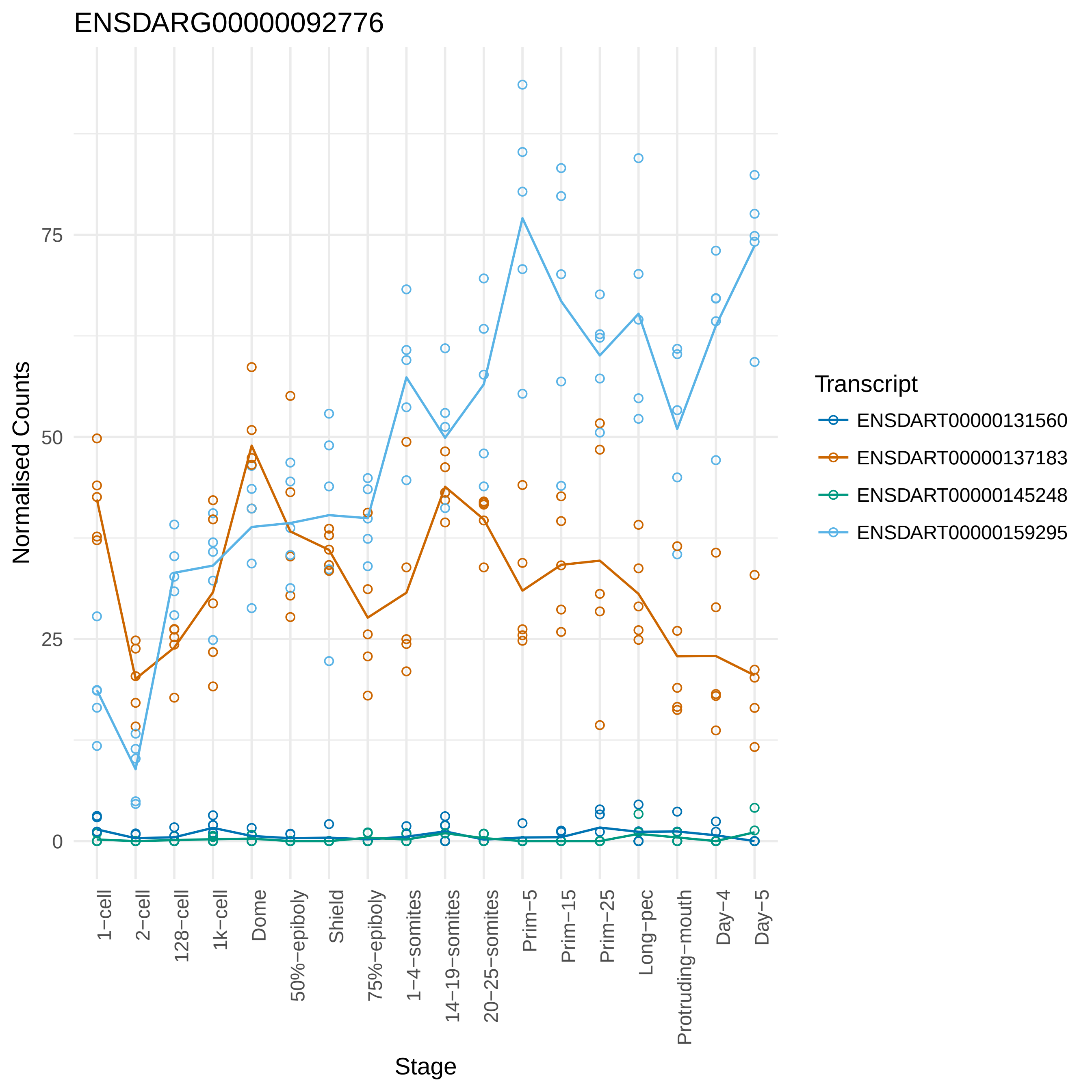

ENSDARG00000092776
Normalised Counts
0
25
50
75
Stage
1−cell
2−cell
128−cell
1k−cell
Dome
50%−epiboly
Shield
75%−epiboly
1−4−somites
14−19−somites
20−25−somites
Prim−5
Prim−15
Prim−25
Long−pec
Protruding−mouth
Day−4
Day−5
Transcript
ENSDART00000131560
ENSDART00000137183
ENSDART00000145248
ENSDART00000159295

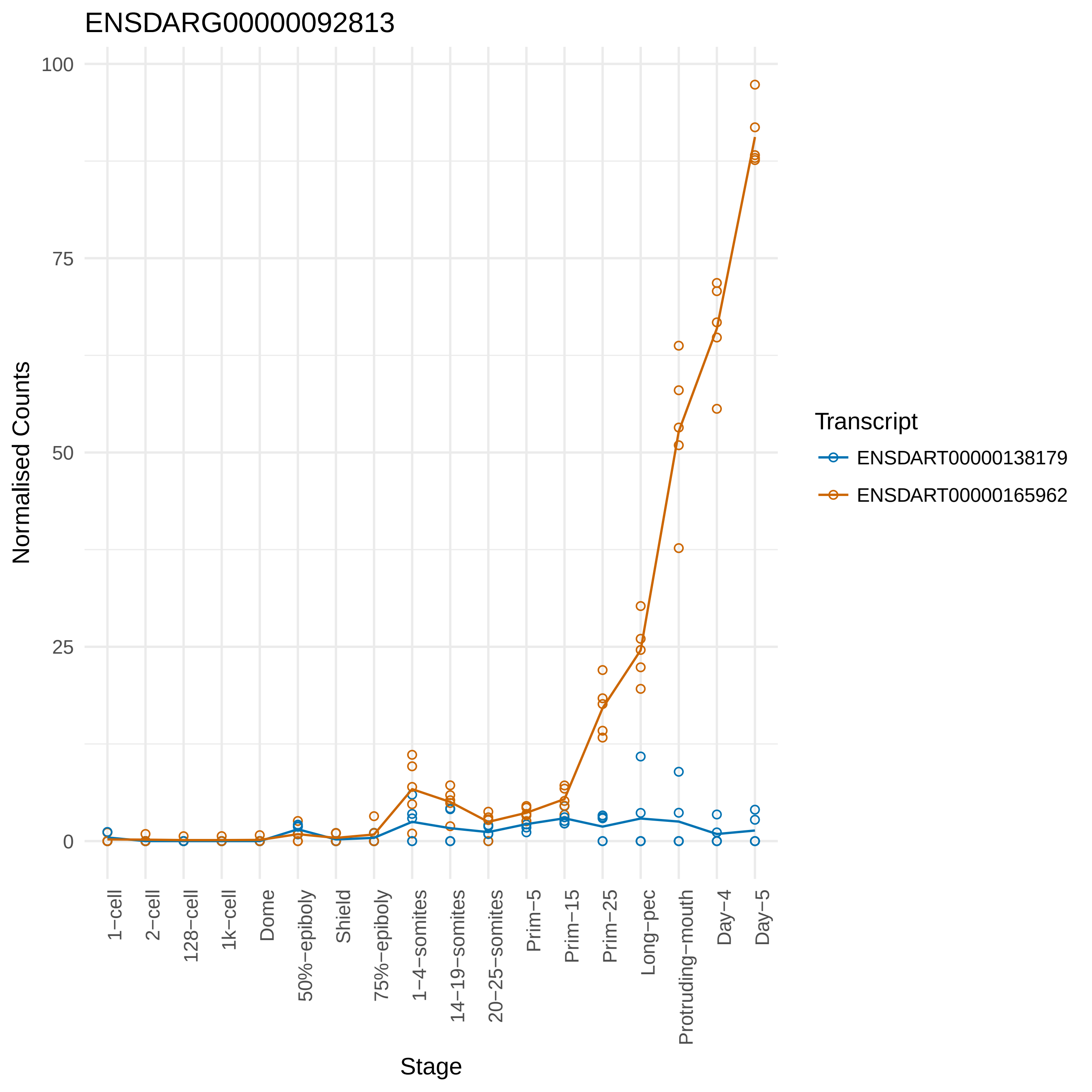

ENSDARG00000092813
Normalised Counts
100
75
50
25
0
1−cell
2−cell
128−cell
1k−cell
Dome
50%−epiboly
Shield
75%−epiboly
1−4−somites
14−19−somites
20−25−somites
Prim−5
Prim−15
Prim−25
Long−pec
Protruding−mouth
Day−4
Day−5
Stage
Transcript
ENSDART00000138179
ENSDART00000165962

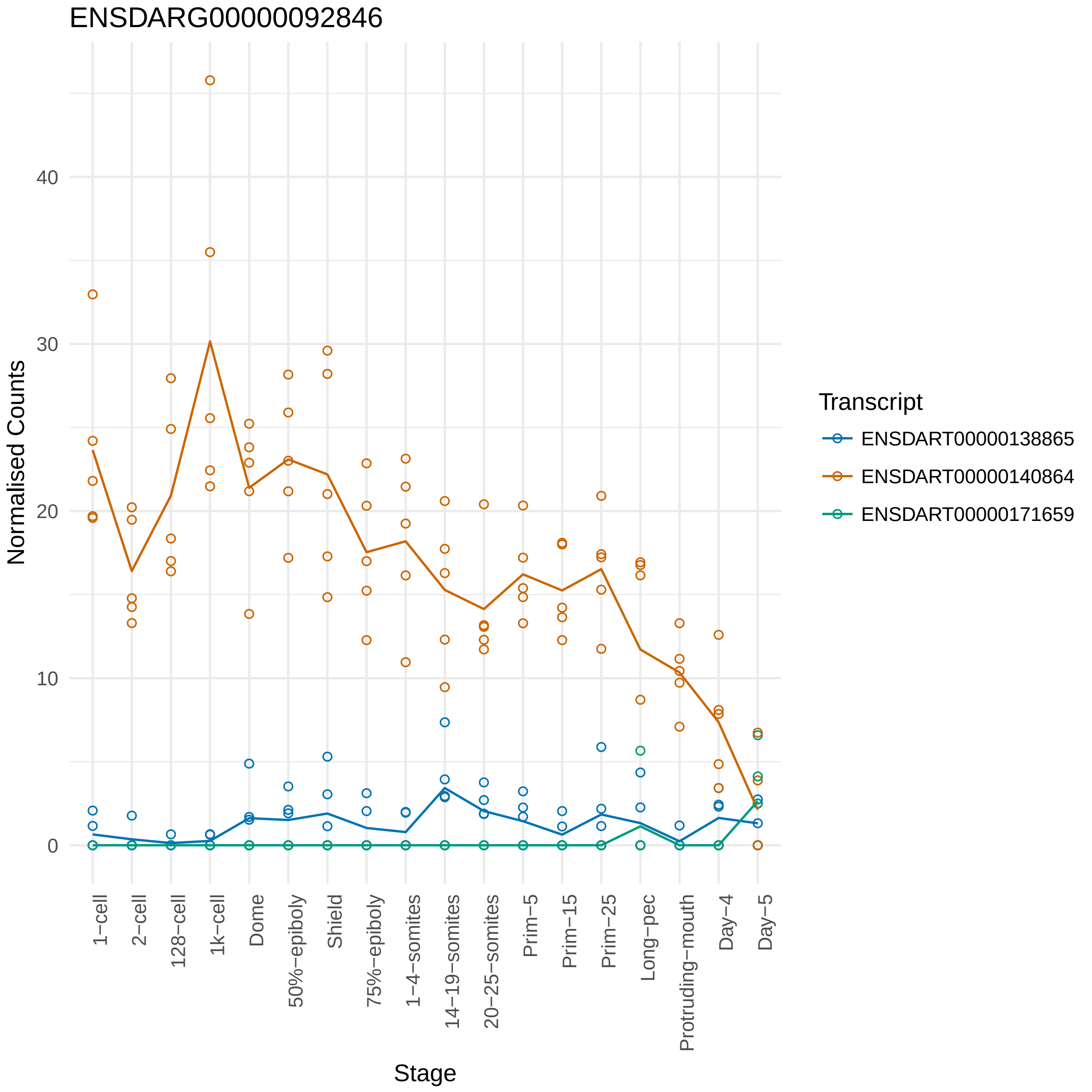
ENSDARG00000092846
Normalised Counts
0
10
20
30
40
Stage
1−cell
2−cell
128−cell
1k−cell
Dome
50%−epiboly
Shield
75%−epiboly
1−4−somites
14−19−somites
20−25−somites
Prim−5
Prim−15
Prim−25
Long−pec
Protruding−mouth
Day−4
Day−5
Transcript
ENSDART00000138865
ENSDART00000140864
ENSDART00000171659

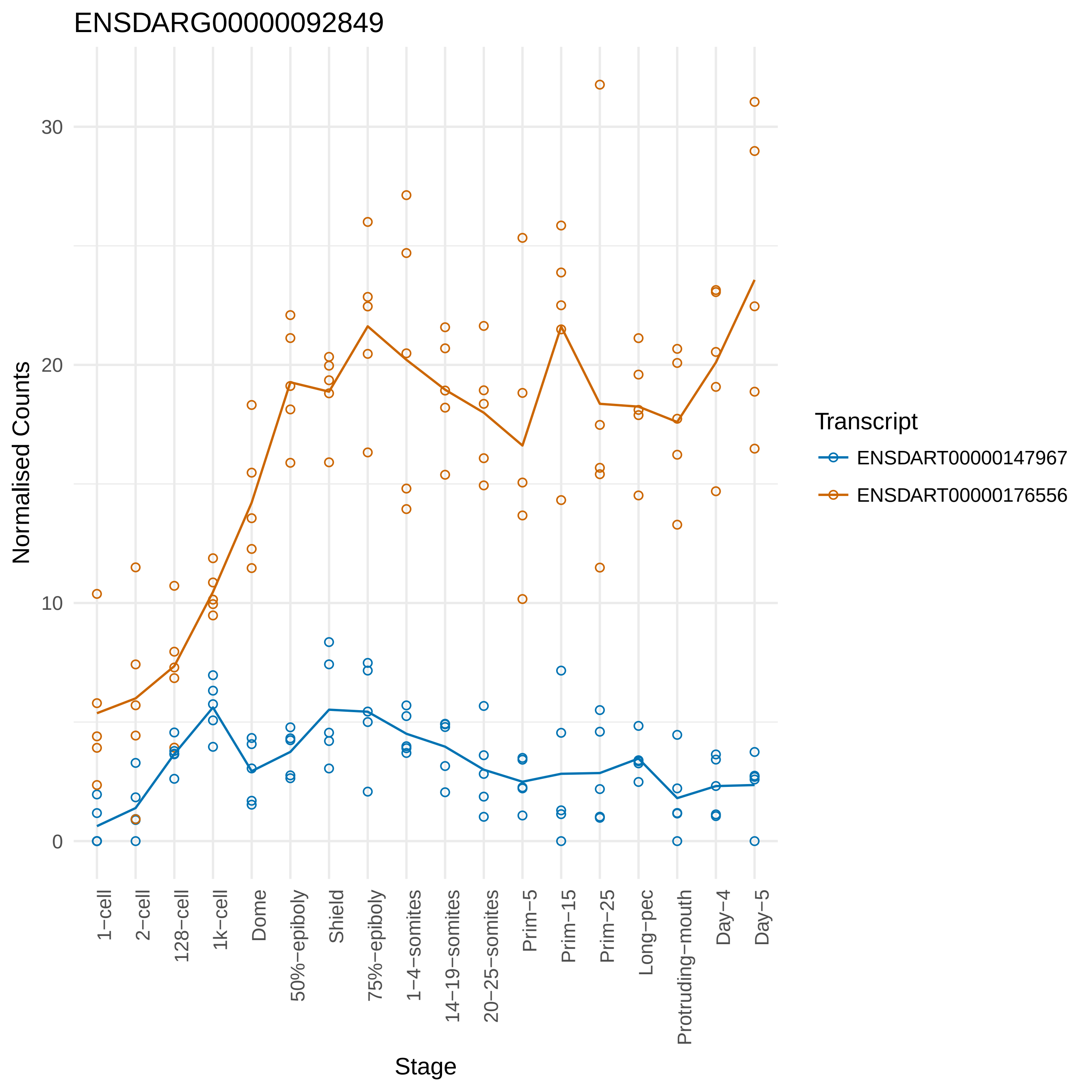
ENSDARG00000092849
Normalised Counts
30
20
10
0
1−cell
2−cell
128−cell
1k−cell
Dome
50%−epiboly
Shield
75%−epiboly
1−4−somites
14−19−somites
20−25−somites
Prim−5
Prim−15
Prim−25
Long−pec
Protruding−mouth
Day−4
Day−5
Stage
Transcript
ENSDART00000147967
ENSDART00000176556

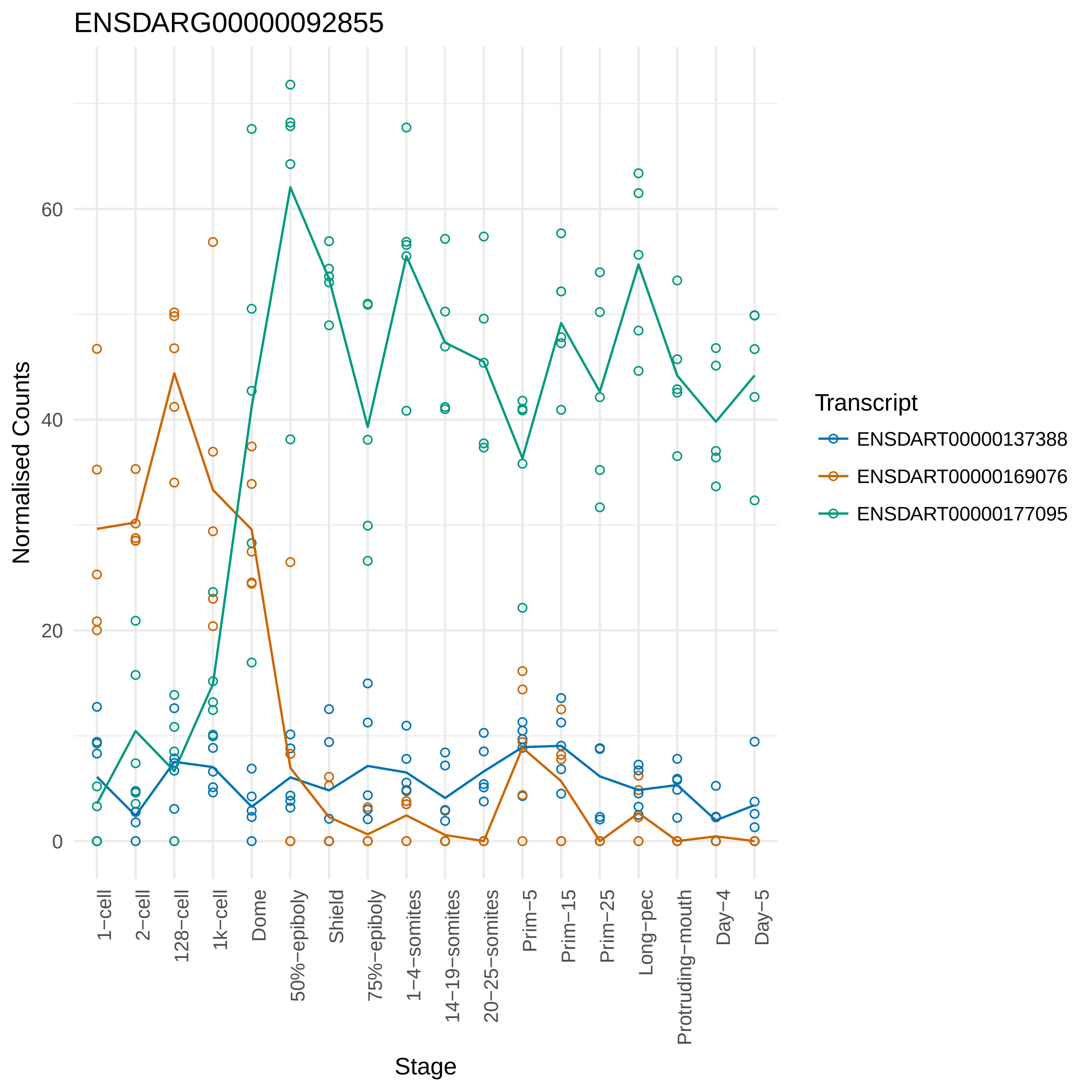
ENSDARG00000092855
Normalised Counts
0
20
40
60
1−cell
2−cell
128−cell
1k−cell
Dome
50%−epiboly
Shield
75%−epiboly
1−4−somites
14−19−somites
20−25−somites
Prim−5
Prim−15
Prim−25
Long−pec
Protruding−mouth
Day−4
Day−5
Stage
Transcript
ENSDART00000137388
ENSDART00000169076
ENSDART00000177095

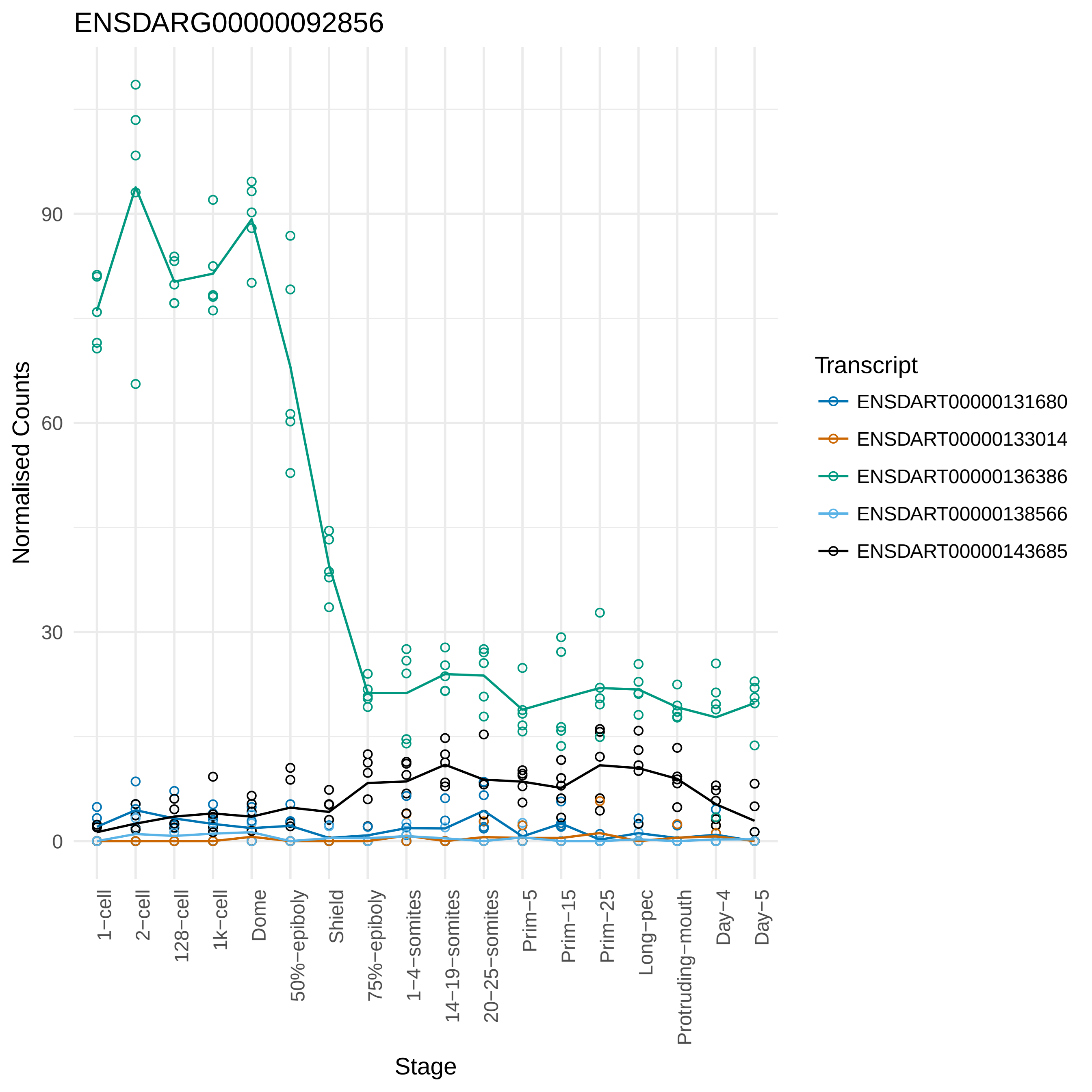

ENSDARG00000092856
Normalised Counts
90
60
30
0
1−cell
2−cell
128−cell
1k−cell
Dome
50%−epiboly
Shield
75%−epiboly
1−4−somites
14−19−somites
20−25−somites
Prim−5
Prim−15
Prim−25
Long−pec
Protruding−mouth
Day−4
Day−5
Stage
Transcript
ENSDART00000131680
ENSDART00000133014
ENSDART00000136386
ENSDART00000138566
ENSDART00000143685

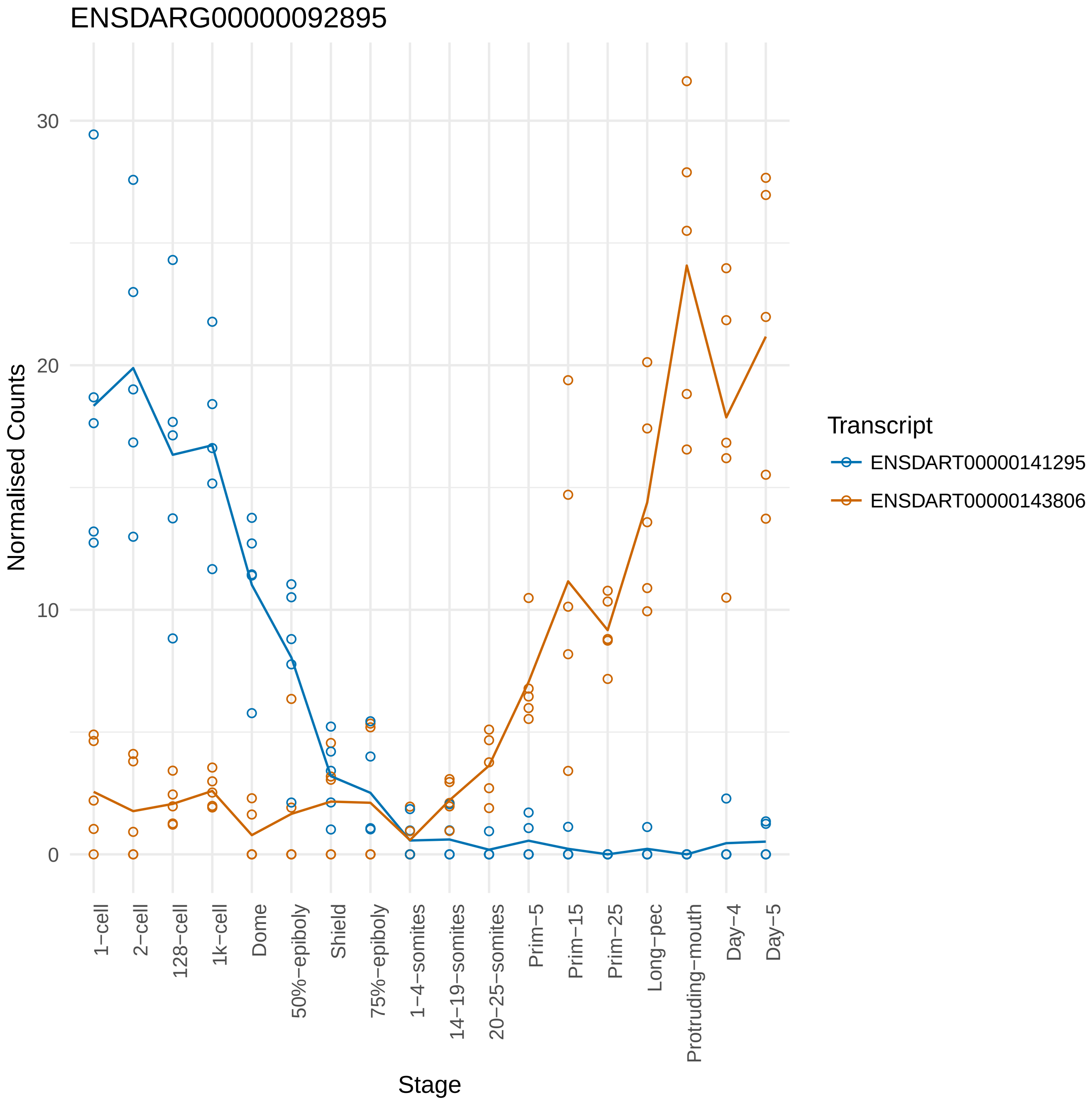

ENSDARG00000092895
Normalised Counts
0
10
20
30
1−cell
2−cell
128−cell
1k−cell
Dome
50%−epiboly
Shield
75%−epiboly
1−4−somites
14−19−somites
20−25−somites
Prim−5
Prim−15
Prim−25
Long−pec
Protruding−mouth
Day−4
Day−5
Stage
Transcript
ENSDART00000141295
ENSDART00000143806

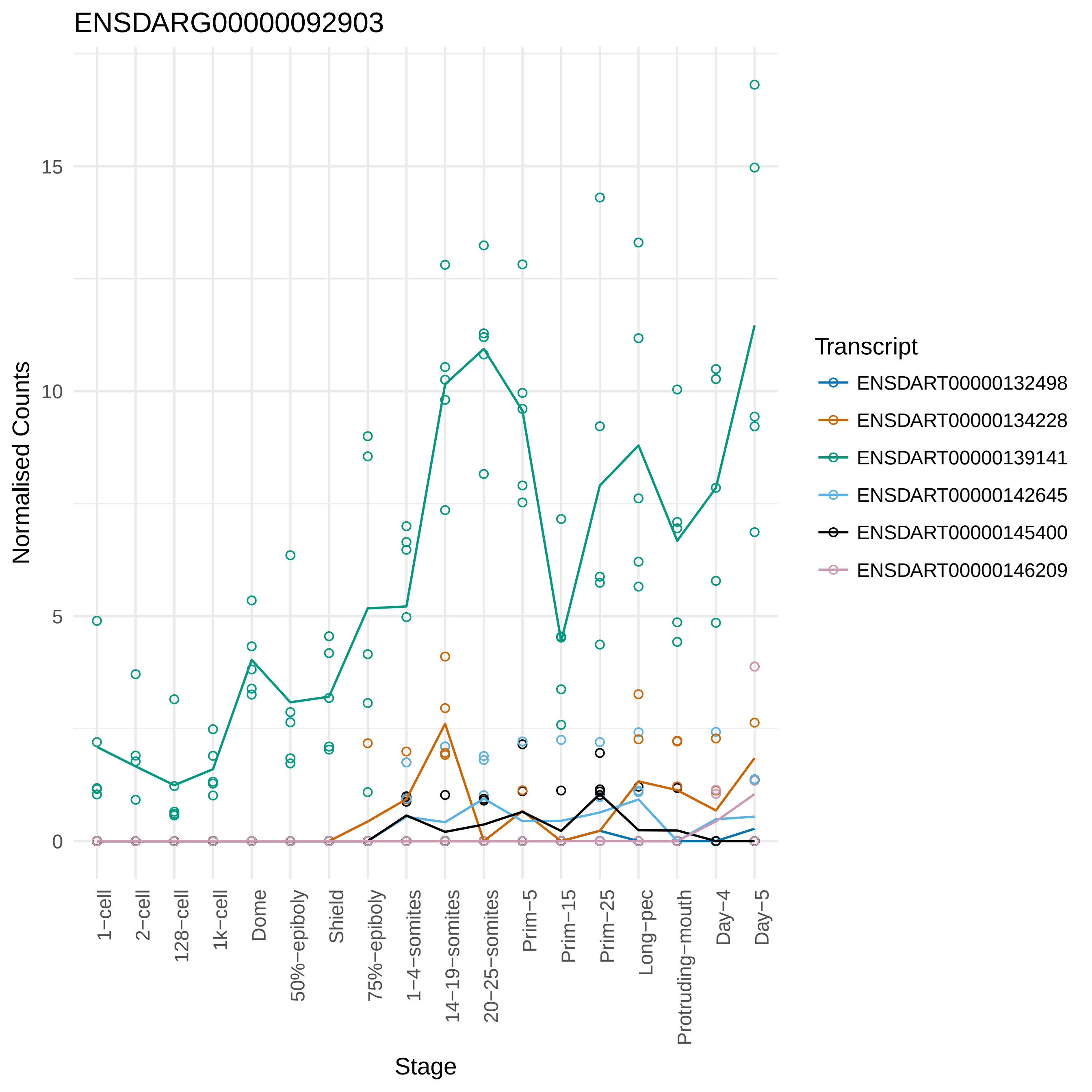

ENSDARG00000092903
Normalised Counts
0
5
10
15
1−cell
2−cell
128−cell
1k−cell
Dome
50%−epiboly
Shield
75%−epiboly
1−4−somites
14−19−somites
20−25−somites
Prim−5
Prim−15
Prim−25
Long−pec
Protruding−mouth
Day−4
Day−5
Stage
Transcript
ENSDART00000132498
ENSDART00000134228
ENSDART00000139141
ENSDART00000142645
ENSDART00000145400
ENSDART00000146209

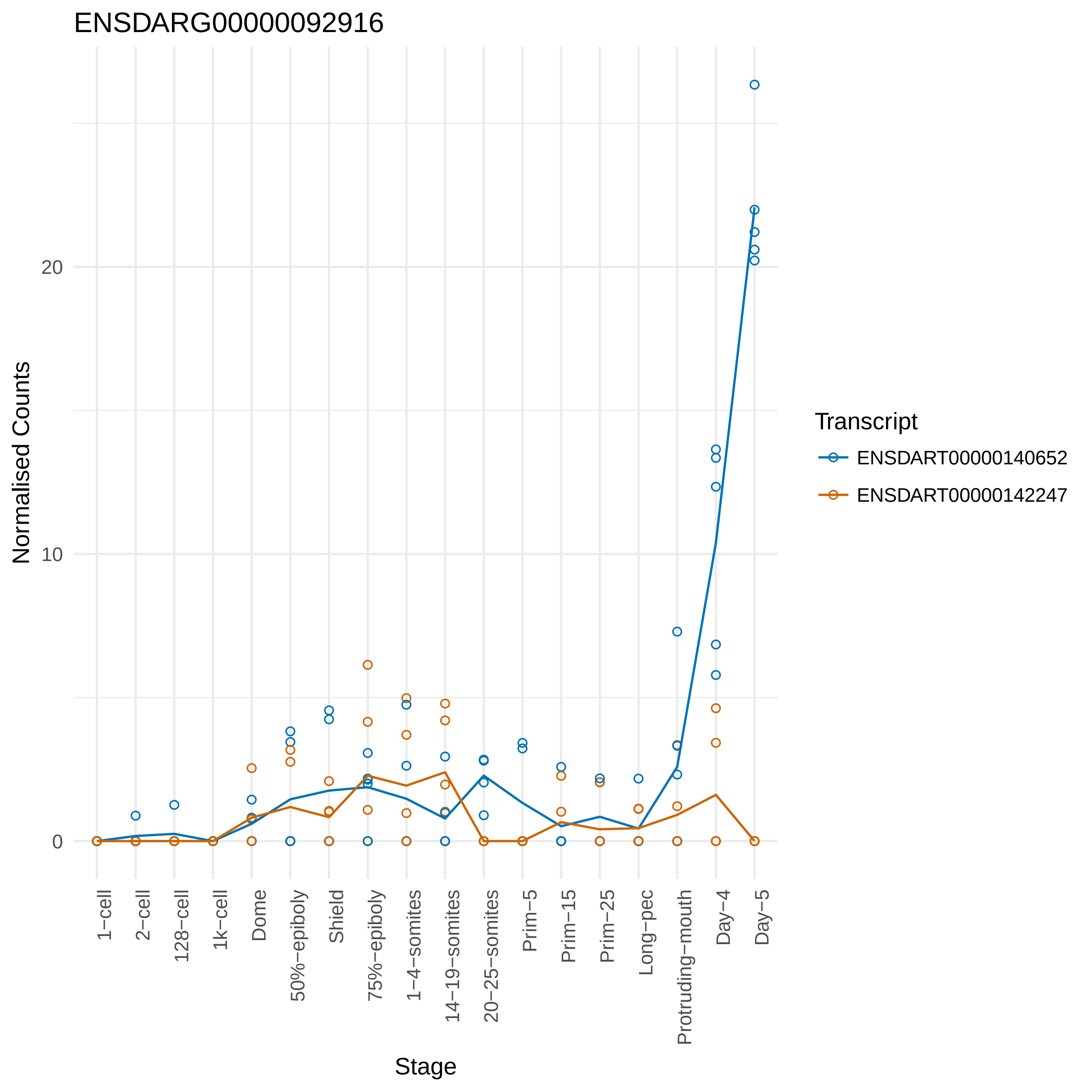
ENSDARG00000092916
Normalised Counts
0
10
20
Stage
1−cell
2−cell
128−cell
1k−cell
Dome
50%−epiboly
Shield
75%−epiboly
1−4−somites
14−19−somites
20−25−somites
Prim−5
Prim−15
Prim−25
Long−pec
Protruding−mouth
Day−4
Day−5
Transcript
ENSDART00000140652
ENSDART00000142247

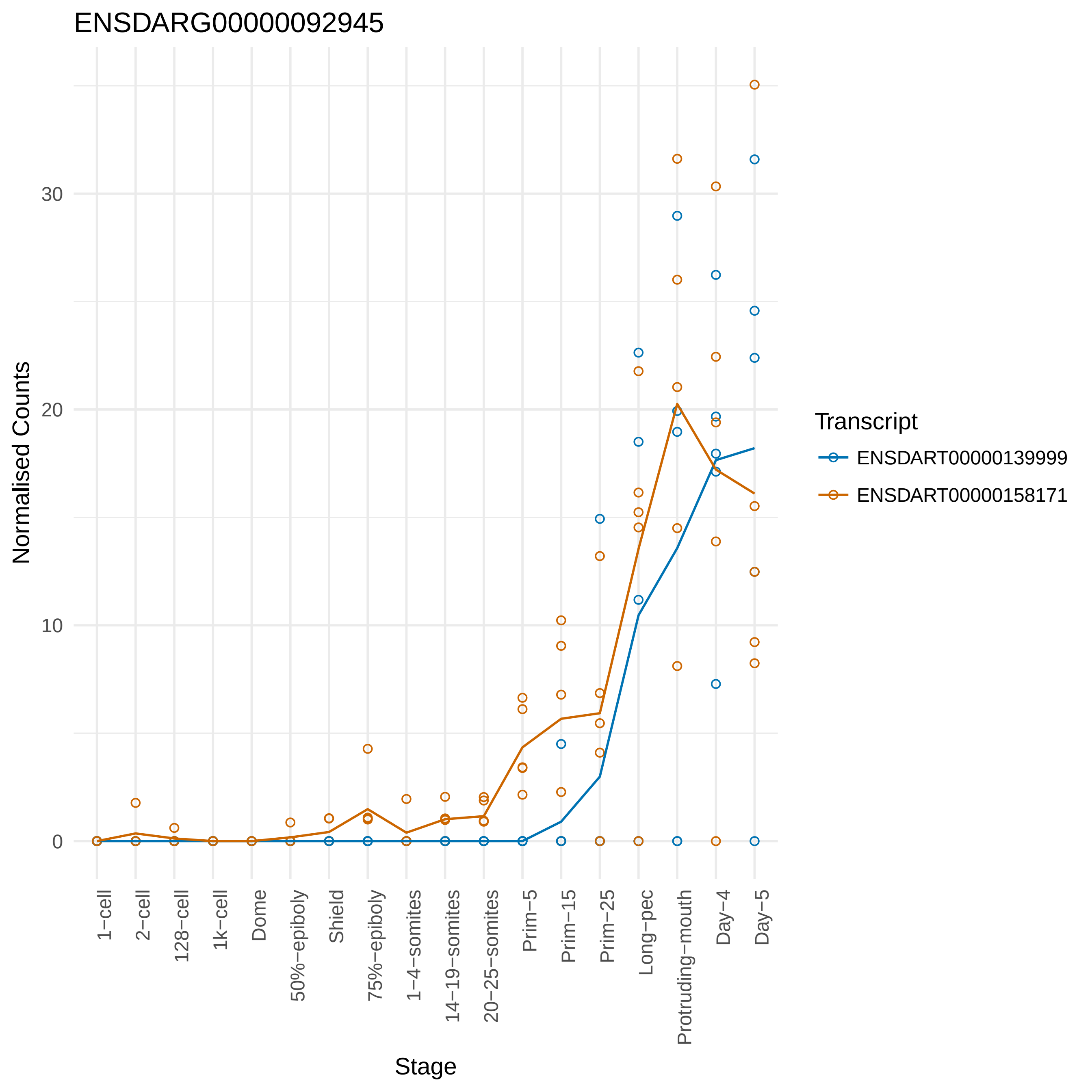
ENSDARG00000092945
Normalised Counts
30
20
10
0
1−cell
2−cell
128−cell
1k−cell
Dome
50%−epiboly
Shield
75%−epiboly
1−4−somites
14−19−somites
20−25−somites
Prim−5
Prim−15
Prim−25
Long−pec
Protruding−mouth
Day−4
Day−5
Stage
Transcript
ENSDART00000139999
ENSDART00000158171

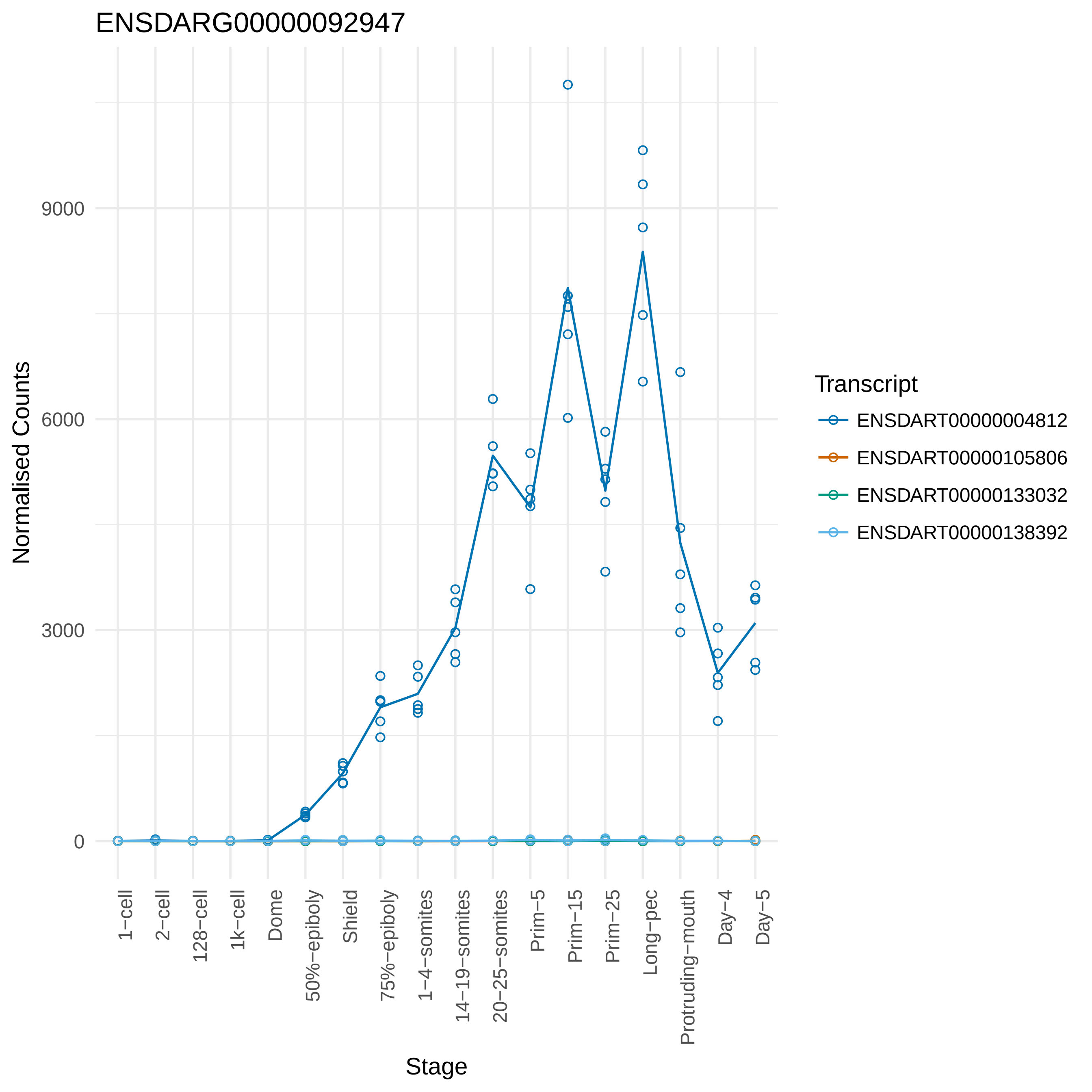

ENSDARG00000092947
Normalised Counts
9000
6000
3000
0
1−cell
2−cell
128−cell
1k−cell
Dome
50%−epiboly
Shield
75%−epiboly
1−4−somites
14−19−somites
20−25−somites
Prim−5
Prim−15
Prim−25
Long−pec
Protruding−mouth
Day−4
Day−5
Stage
Transcript
ENSDART00000004812
ENSDART00000105806
ENSDART00000133032
ENSDART00000138392

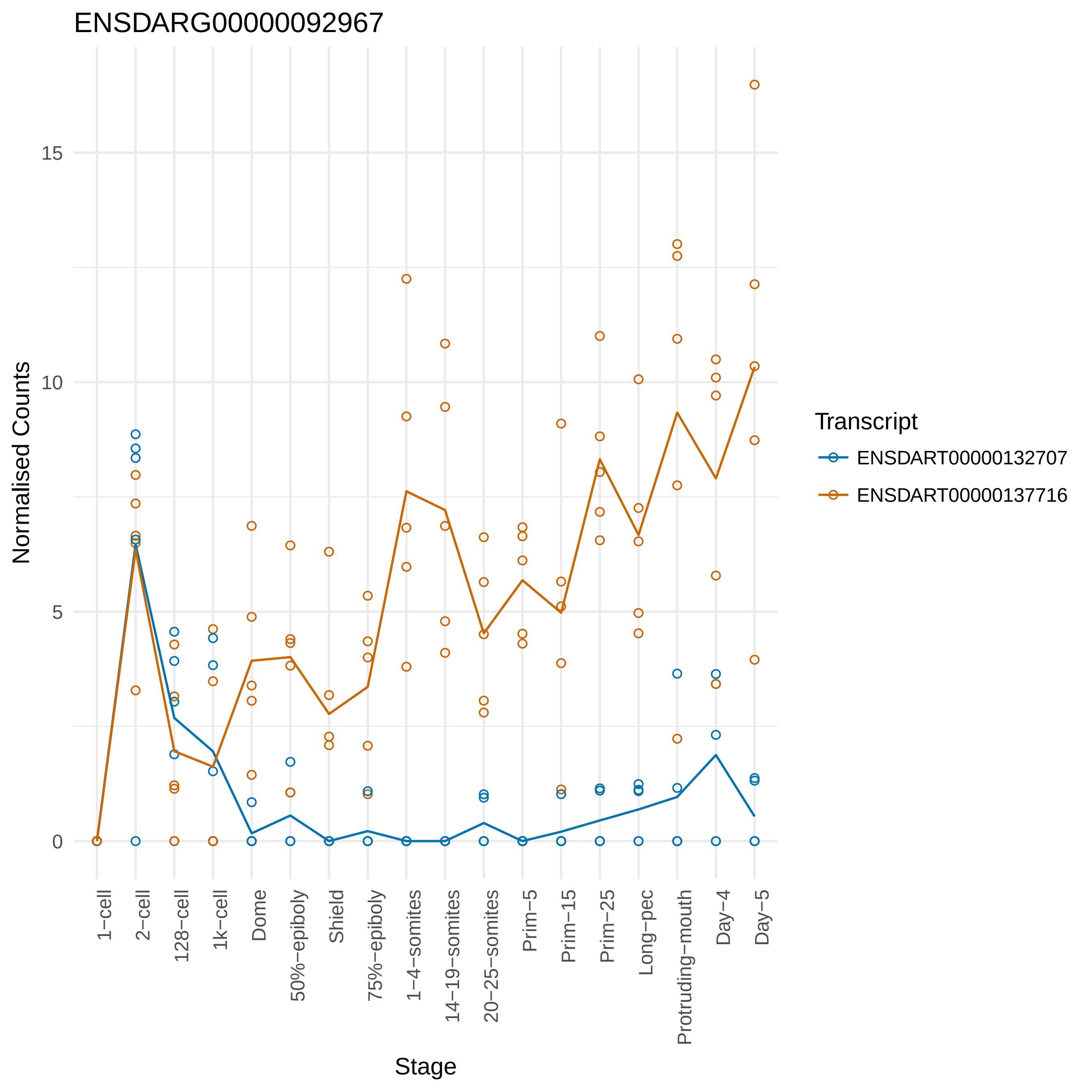
ENSDARG00000092967
Normalised Counts
0
5
10
15
Stage
1−cell
2−cell
128−cell
1k−cell
Dome
50%−epiboly
Shield
75%−epiboly
1−4−somites
14−19−somites
20−25−somites
Prim−5
Prim−15
Prim−25
Long−pec
Protruding−mouth
Day−4
Day−5
Transcript
ENSDART00000132707
ENSDART00000137716

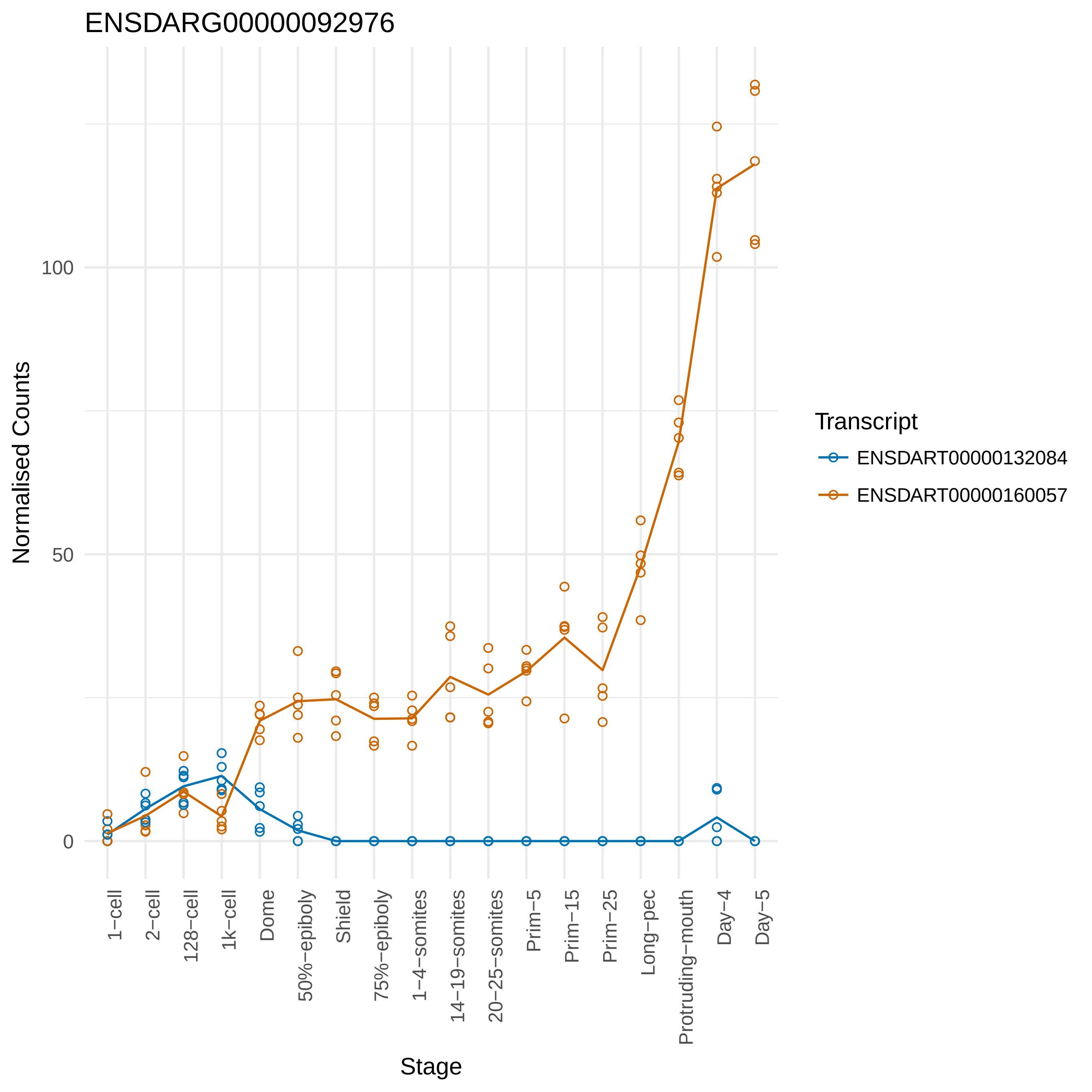
ENSDARG00000092976
Normalised Counts
100
50
0
1−cell
2−cell
128−cell
1k−cell
Dome
50%−epiboly
Shield
75%−epiboly
1−4−somites
14−19−somites
20−25−somites
Prim−5
Prim−15
Prim−25
Long−pec
Protruding−mouth
Day−4
Day−5
Stage
Transcript
ENSDART00000132084
ENSDART00000160057

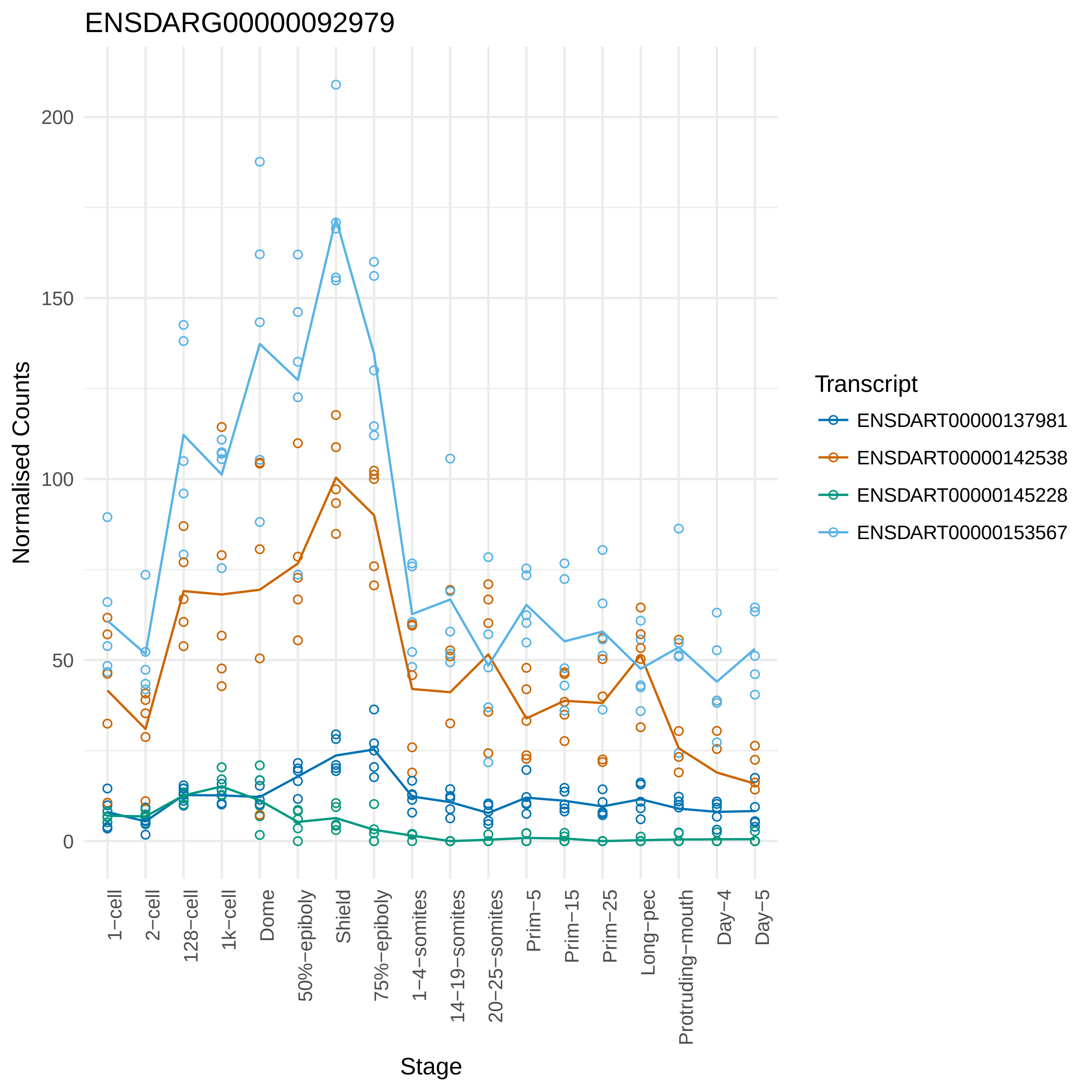

ENSDARG00000092979
Normalised Counts
200
150
100
50
0
1−cell
2−cell
128−cell
1k−cell
Dome
50%−epiboly
Shield
75%−epiboly
1−4−somites
14−19−somites
20−25−somites
Prim−5
Prim−15
Prim−25
Long−pec
Protruding−mouth
Day−4
Day−5
Stage
Transcript
ENSDART00000137981
ENSDART00000142538
ENSDART00000145228
ENSDART00000153567

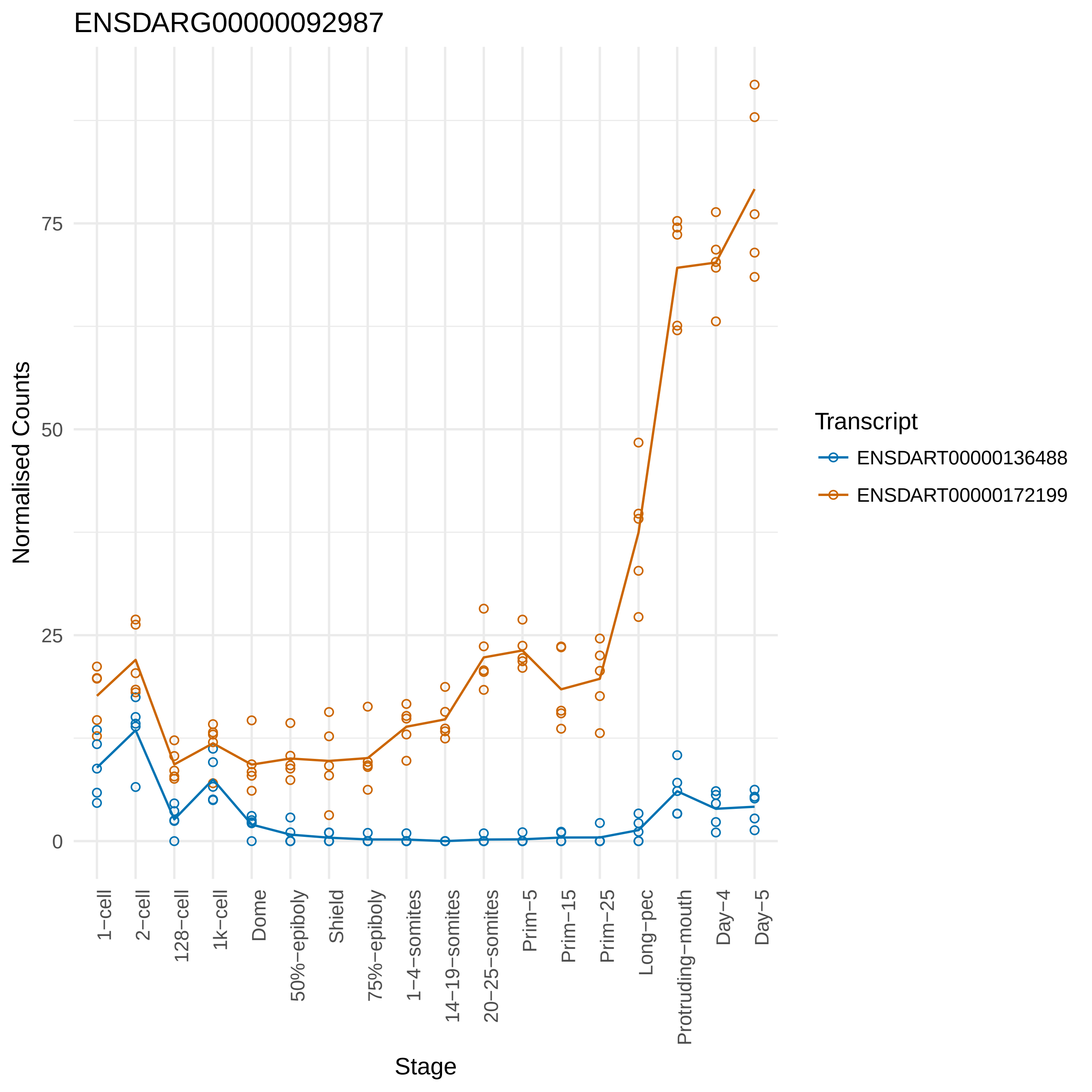

ENSDARG00000092987
Normalised Counts
0
25
50
75
1−cell
2−cell
128−cell
1k−cell
Dome
50%−epiboly
Shield
75%−epiboly
1−4−somites
14−19−somites
20−25−somites
Prim−5
Prim−15
Prim−25
Long−pec
Protruding−mouth
Day−4
Day−5
Stage
Transcript
ENSDART00000136488
ENSDART00000172199

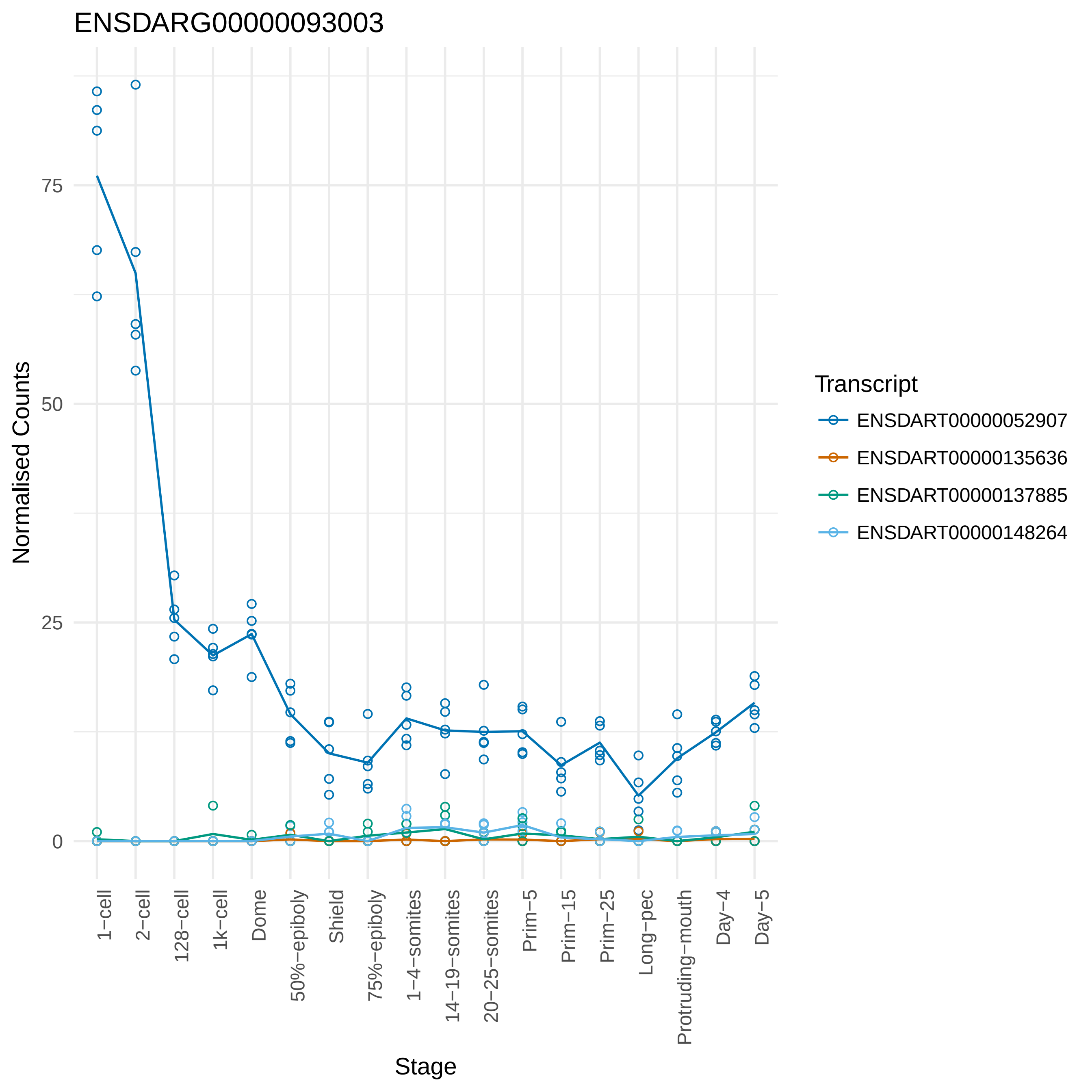

ENSDARG00000093003
Normalised Counts
75
50
25
0
1−cell
2−cell
128−cell
1k−cell
Dome
50%−epiboly
Shield
75%−epiboly
1−4−somites
14−19−somites
20−25−somites
Prim−5
Prim−15
Prim−25
Long−pec
Protruding−mouth
Day−4
Day−5
Stage
Transcript
ENSDART00000052907
ENSDART00000135636
ENSDART00000137885
ENSDART00000148264

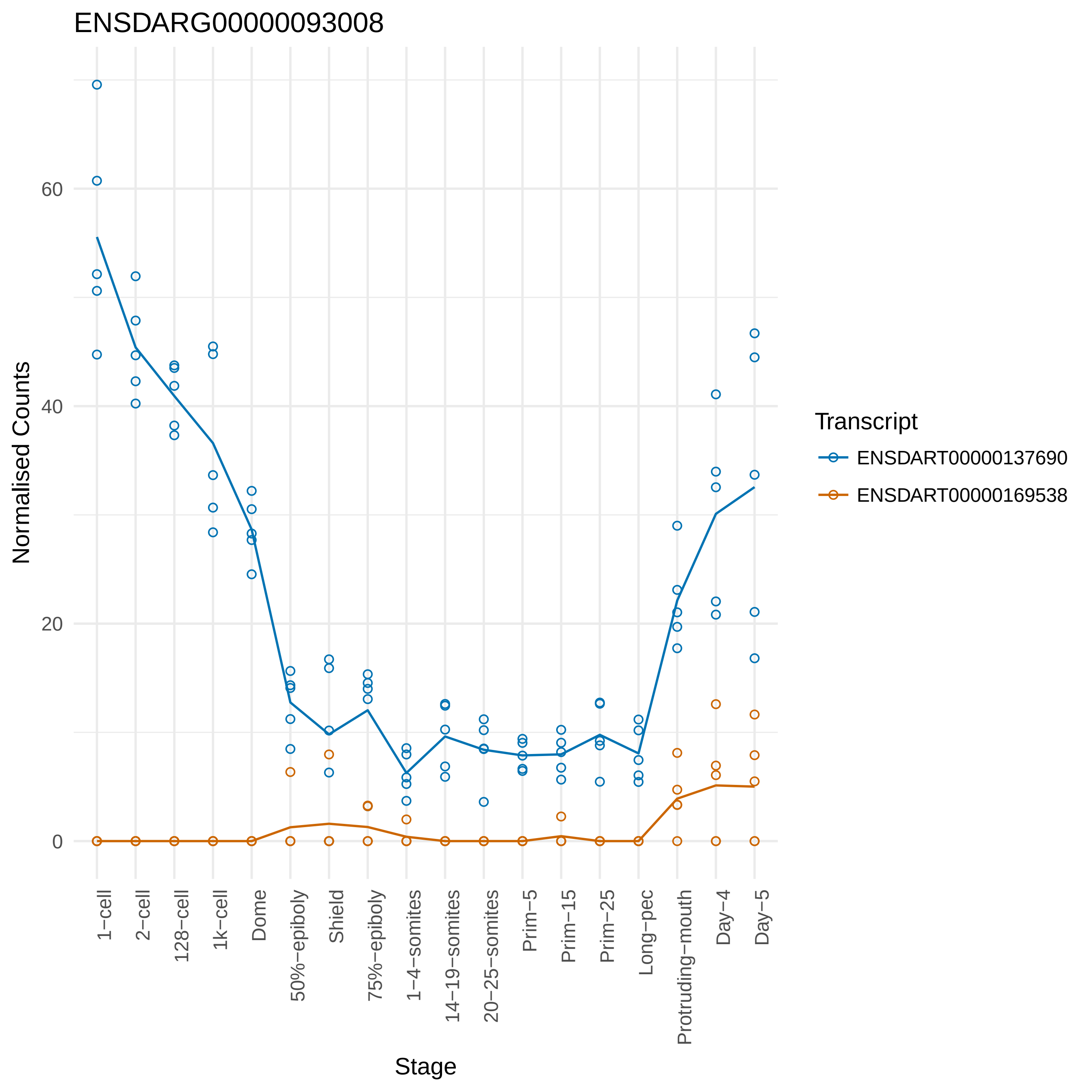

ENSDARG00000093008
Normalised Counts
60
40
20
0
1−cell
2−cell
128−cell
1k−cell
Dome
50%−epiboly
Shield
75%−epiboly
1−4−somites
14−19−somites
20−25−somites
Prim−5
Prim−15
Prim−25
Long−pec
Protruding−mouth
Day−4
Day−5
Stage
Transcript
ENSDART00000137690
ENSDART00000169538

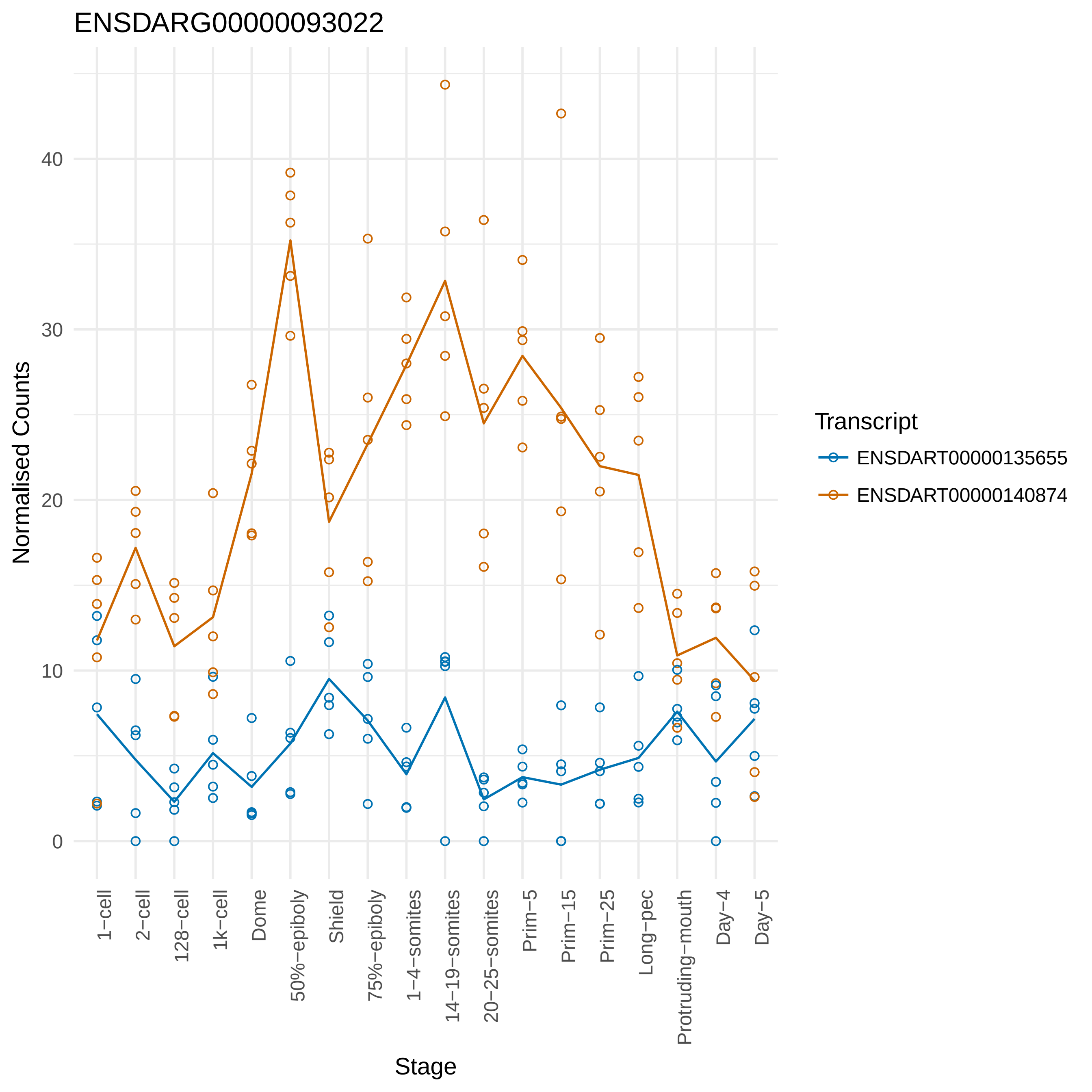

ENSDARG00000093022
Normalised Counts
0
10
20
30
40
Stage
1−cell
2−cell
128−cell
1k−cell
Dome
50%−epiboly
Shield
75%−epiboly
1−4−somites
14−19−somites
20−25−somites
Prim−5
Prim−15
Prim−25
Long−pec
Protruding−mouth
Day−4
Day−5
Transcript
ENSDART00000135655
ENSDART00000140874

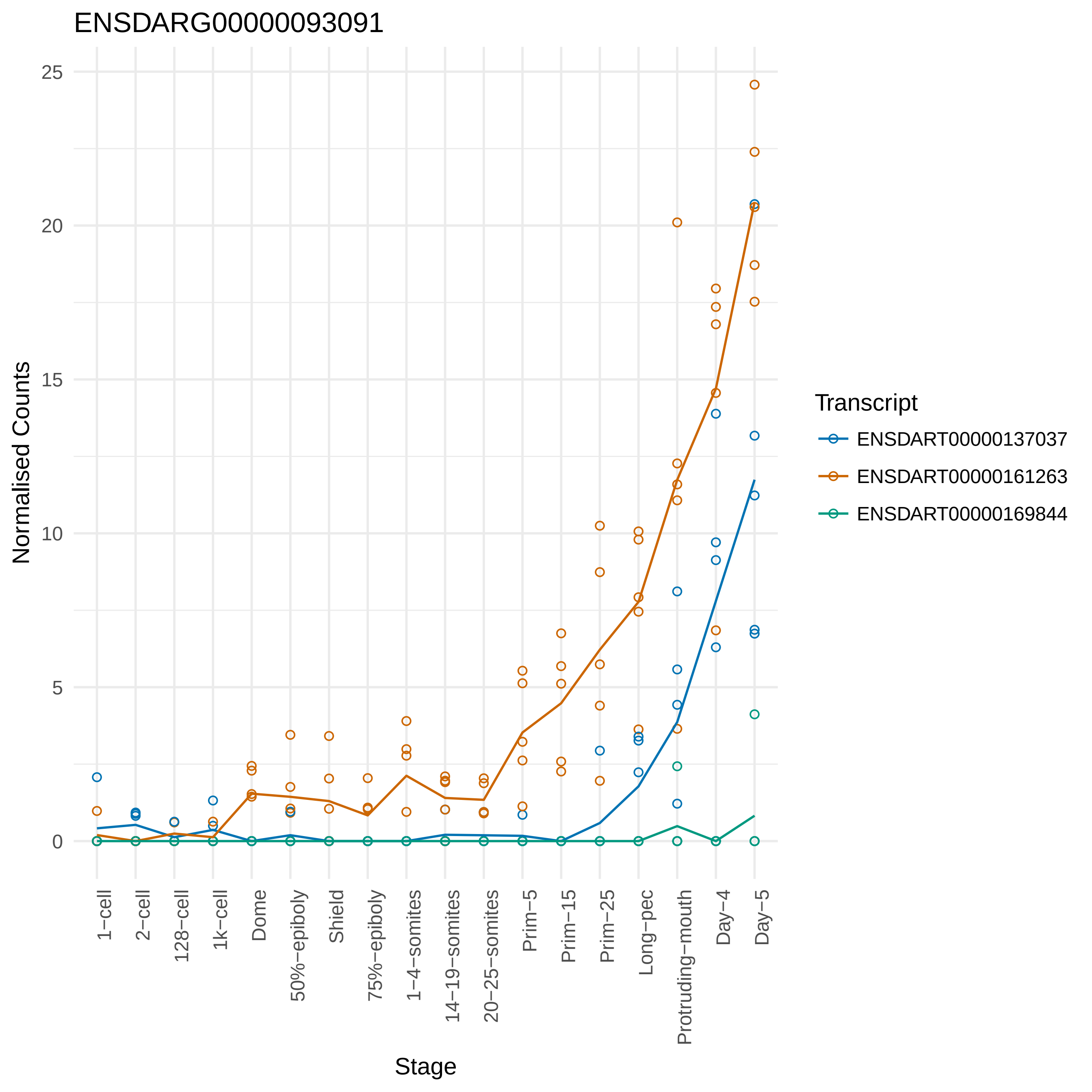
ENSDARG00000093091
Normalised Counts
0
5
10
15
20
25
1−cell
2−cell
128−cell
1k−cell
Dome
50%−epiboly
Shield
75%−epiboly
1−4−somites
14−19−somites
20−25−somites
Prim−5
Prim−15
Prim−25
Long−pec
Protruding−mouth
Day−4
Day−5
Stage
Transcript
ENSDART00000137037
ENSDART00000161263
ENSDART00000169844

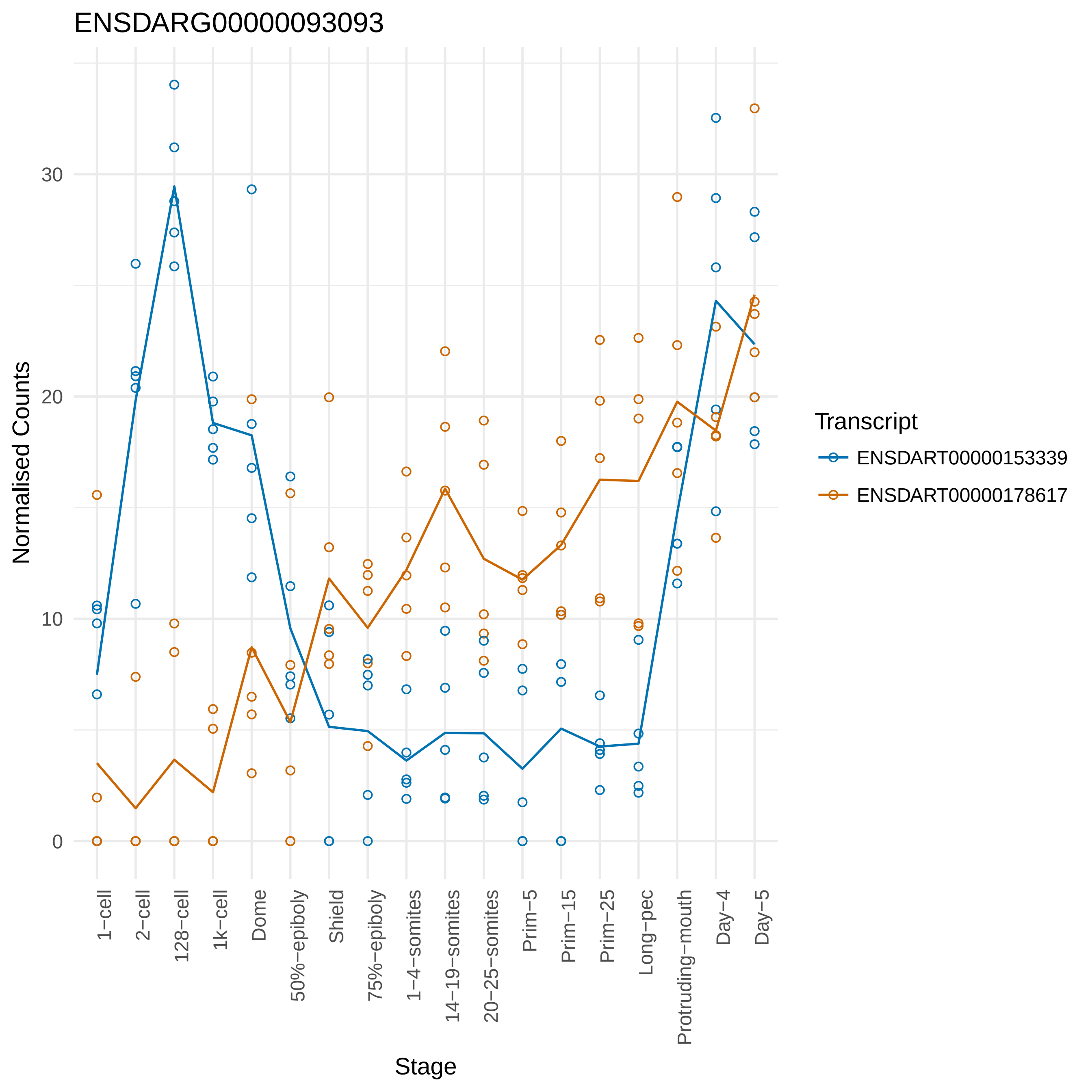

ENSDARG00000093093
Normalised Counts
0
10
20
30
1−cell
2−cell
128−cell
1k−cell
Dome
50%−epiboly
Shield
75%−epiboly
1−4−somites
14−19−somites
20−25−somites
Prim−5
Prim−15
Prim−25
Long−pec
Protruding−mouth
Day−4
Day−5
Stage
Transcript
ENSDART00000153339
ENSDART00000178617

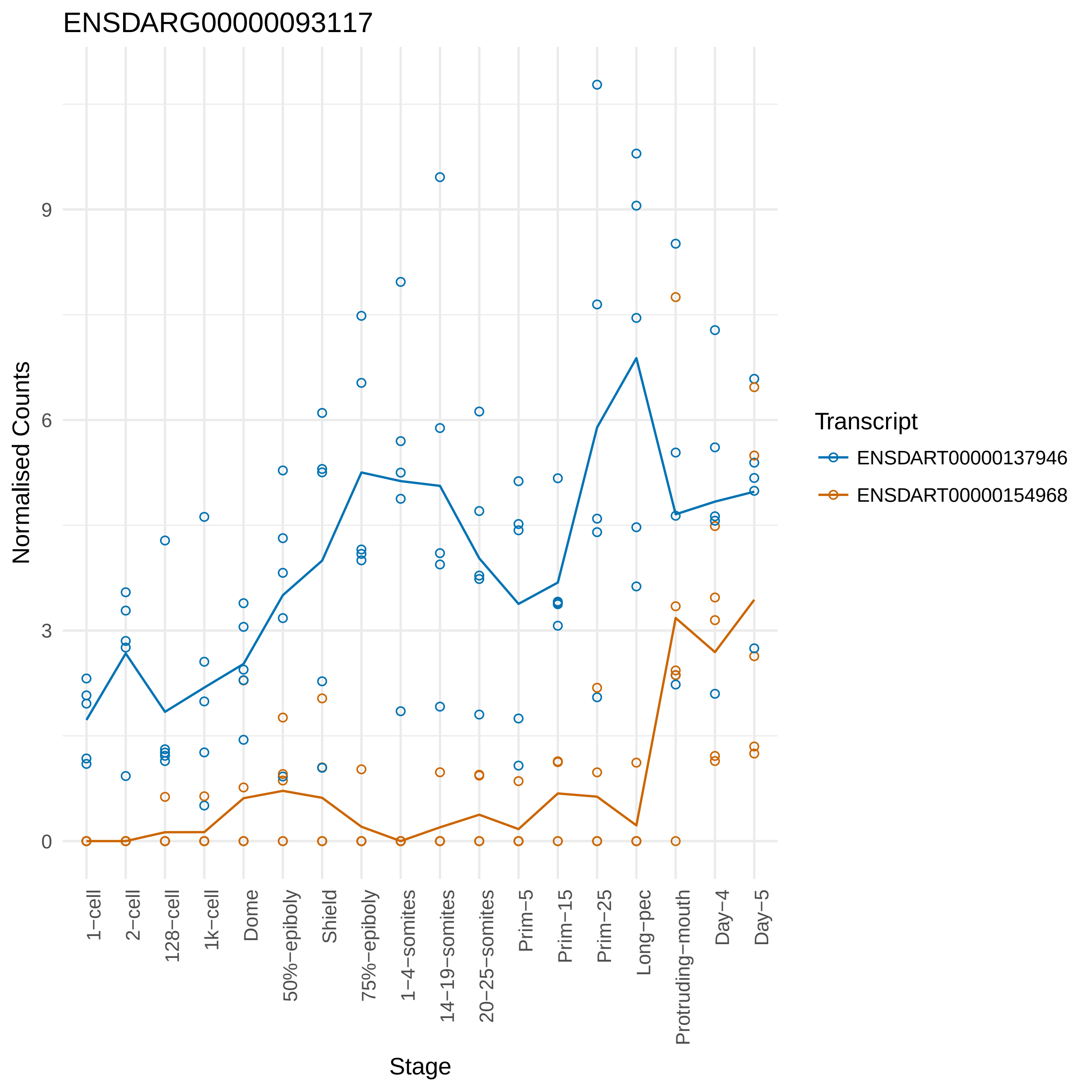

ENSDARG00000093117
Normalised Counts
0
3
6
9
1−cell
2−cell
128−cell
1k−cell
Dome
50%−epiboly
Shield
75%−epiboly
1−4−somites
14−19−somites
20−25−somites
Prim−5
Prim−15
Prim−25
Long−pec
Protruding−mouth
Day−4
Day−5
Stage
Transcript
ENSDART00000137946
ENSDART00000154968

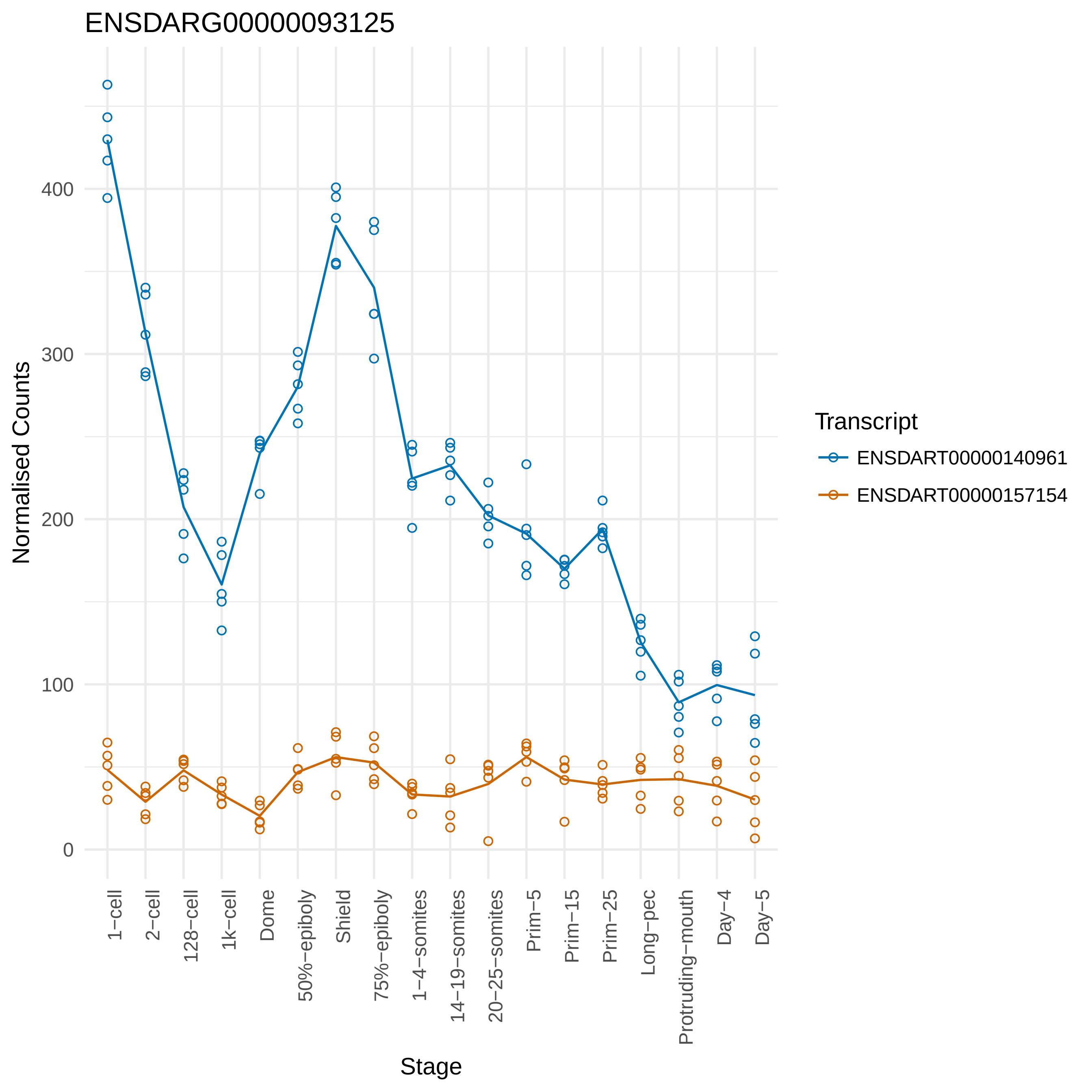

ENSDARG00000093125
Normalised Counts
0
100
200
300
400
Stage
1−cell
2−cell
128−cell
1k−cell
Dome
50%−epiboly
Shield
75%−epiboly
1−4−somites
14−19−somites
20−25−somites
Prim−5
Prim−15
Prim−25
Long−pec
Protruding−mouth
Day−4
Day−5
Transcript
ENSDART00000140961
ENSDART00000157154

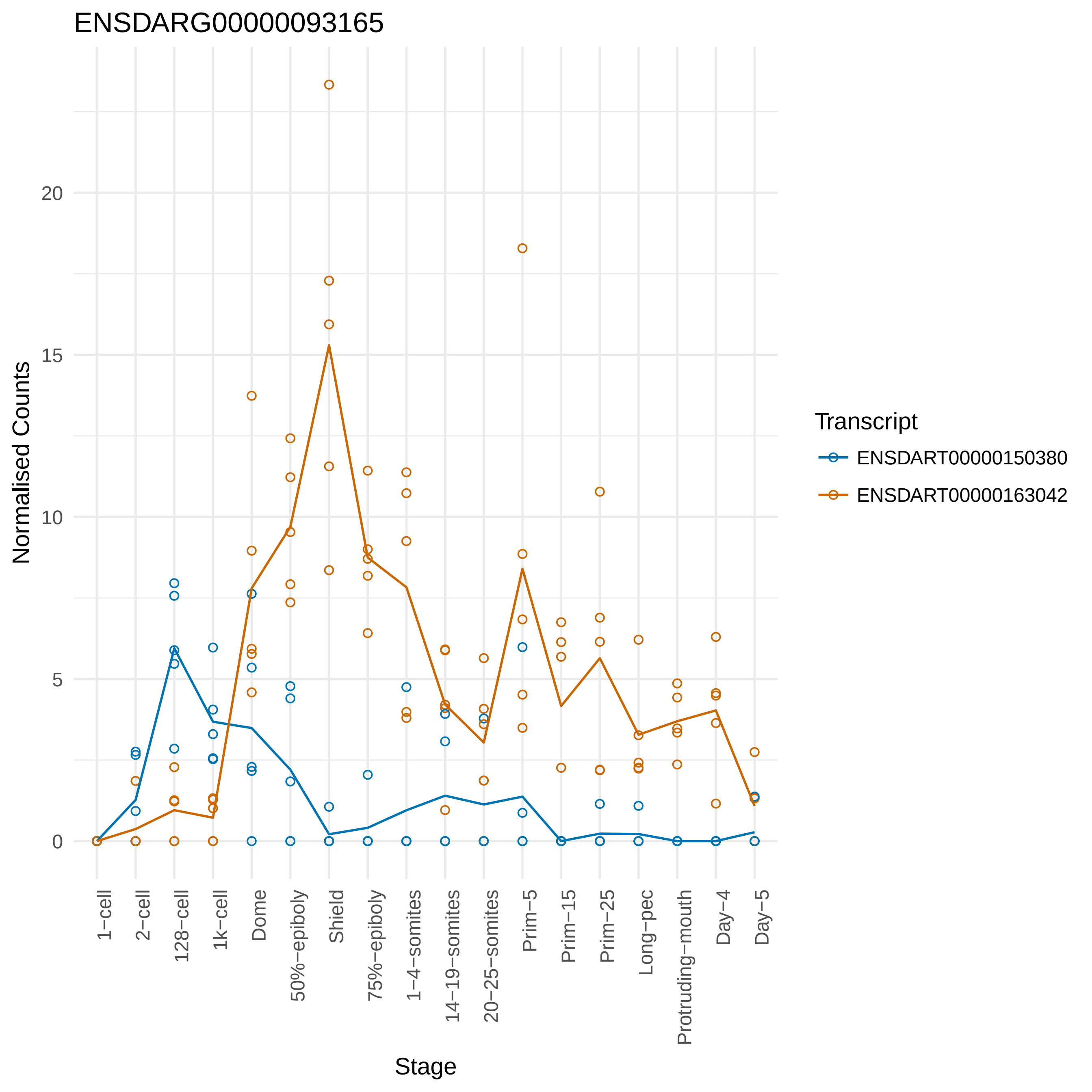
ENSDARG00000093165
Normalised Counts
0
5
10
15
20
Stage
1−cell
2−cell
128−cell
1k−cell
Dome
50%−epiboly
Shield
75%−epiboly
1−4−somites
14−19−somites
20−25−somites
Prim−5
Prim−15
Prim−25
Long−pec
Protruding−mouth
Day−4
Day−5
Transcript
ENSDART00000150380
ENSDART00000163042

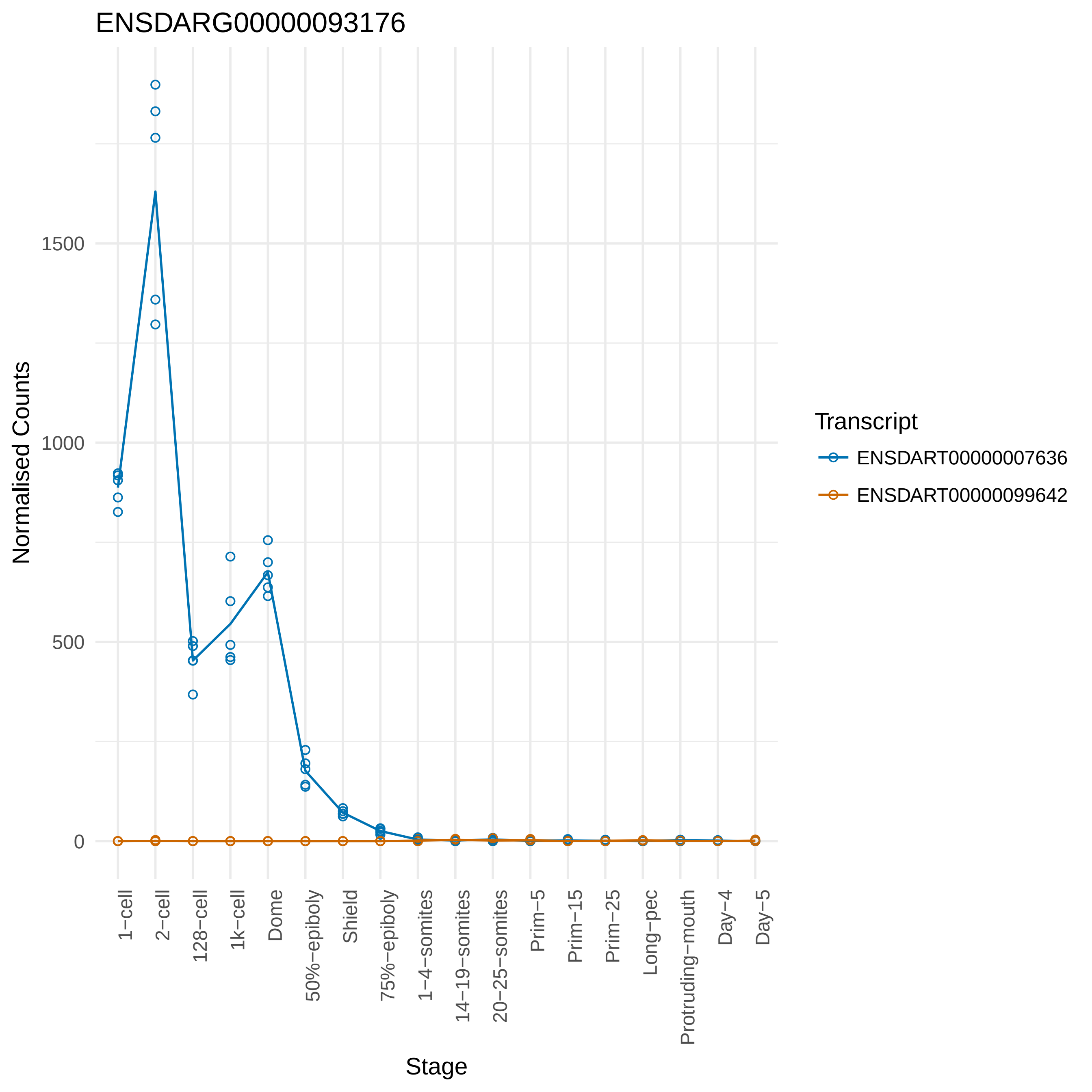
ENSDARG00000093176
Normalised Counts
1500
1000
500
0
1−cell
2−cell
128−cell
1k−cell
Dome
50%−epiboly
Shield
75%−epiboly
1−4−somites
14−19−somites
20−25−somites
Prim−5
Prim−15
Prim−25
Long−pec
Protruding−mouth
Day−4
Day−5
Stage
Transcript
ENSDART00000007636
ENSDART00000099642

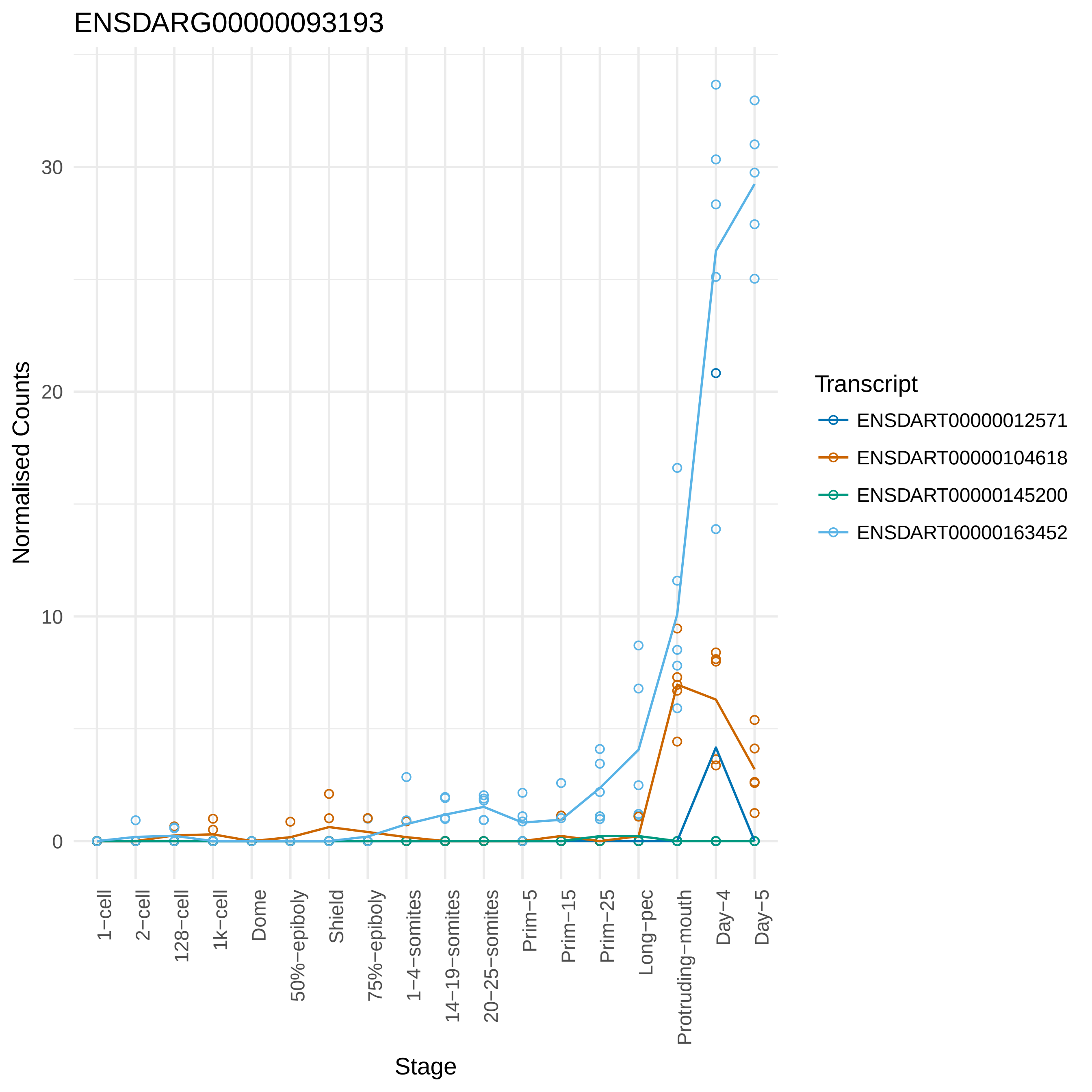

ENSDARG00000093193
Normalised Counts
0
10
20
30
1−cell
2−cell
128−cell
1k−cell
Dome
50%−epiboly
Shield
75%−epiboly
1−4−somites
14−19−somites
20−25−somites
Prim−5
Prim−15
Prim−25
Long−pec
Protruding−mouth
Day−4
Day−5
Stage
Transcript
ENSDART00000012571
ENSDART00000104618
ENSDART00000145200
ENSDART00000163452

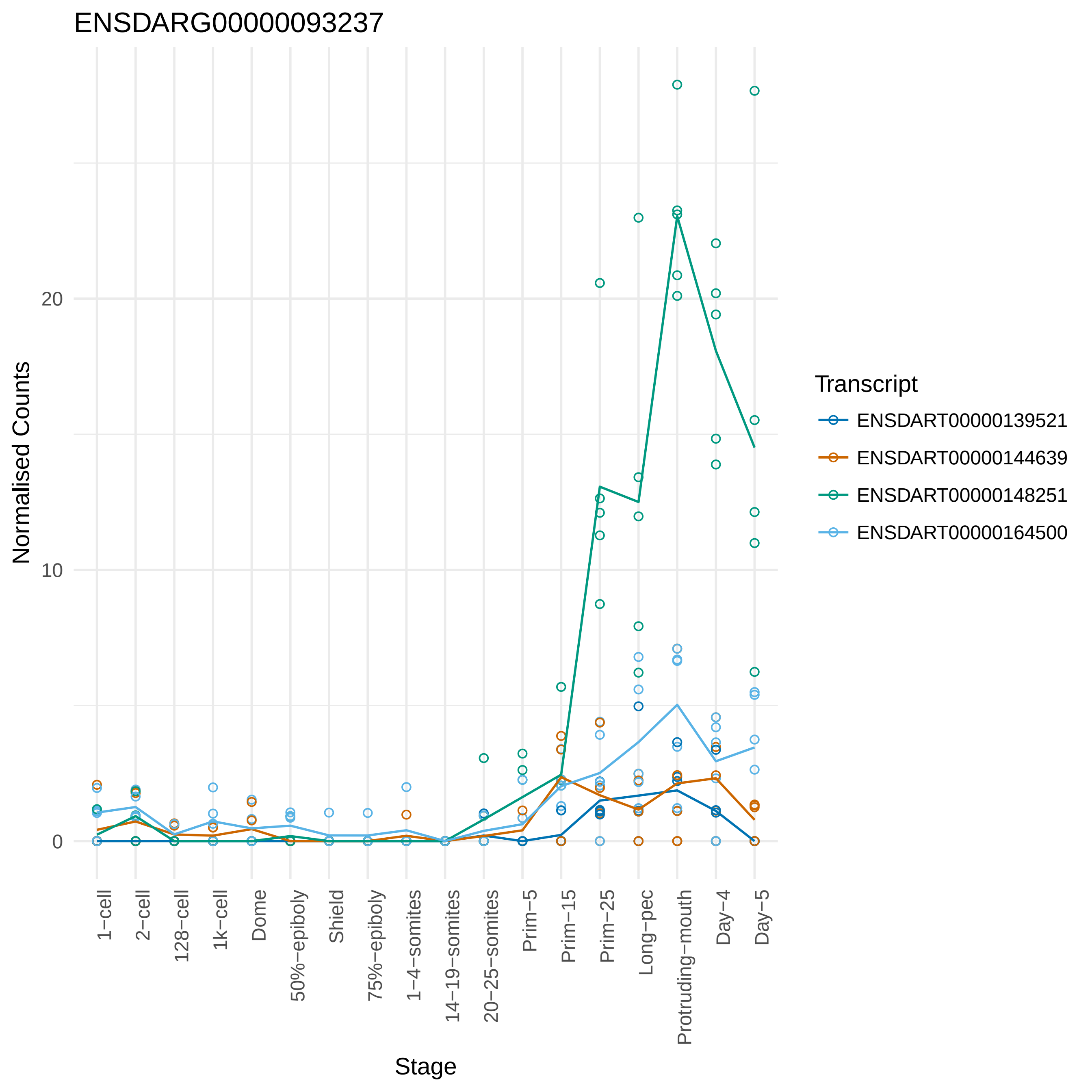
ENSDARG00000093237
Normalised Counts
0
10
20
1−cell
2−cell
128−cell
1k−cell
Dome
50%−epiboly
Shield
75%−epiboly
1−4−somites
14−19−somites
20−25−somites
Prim−5
Prim−15
Prim−25
Long−pec
Protruding−mouth
Day−4
Day−5
Stage
Transcript
ENSDART00000139521
ENSDART00000144639
ENSDART00000148251
ENSDART00000164500

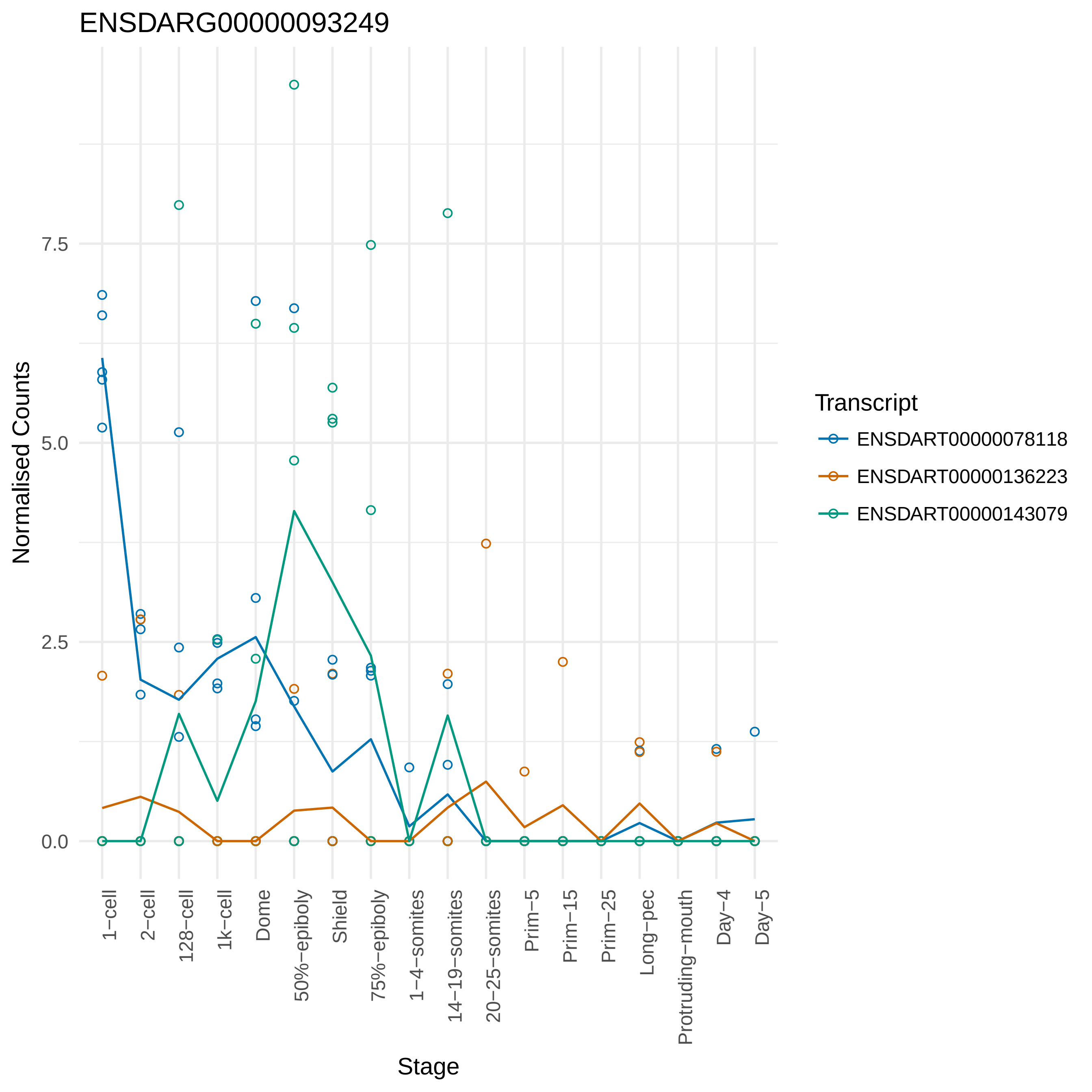

ENSDARG00000093249
Normalised Counts
7.5
5.0
2.5
0.0
1−cell
2−cell
128−cell
1k−cell
Dome
50%−epiboly
Shield
75%−epiboly
1−4−somites
14−19−somites
20−25−somites
Prim−5
Prim−15
Prim−25
Long−pec
Protruding−mouth
Day−4
Day−5
Stage
Transcript
ENSDART00000078118
ENSDART00000136223
ENSDART00000143079

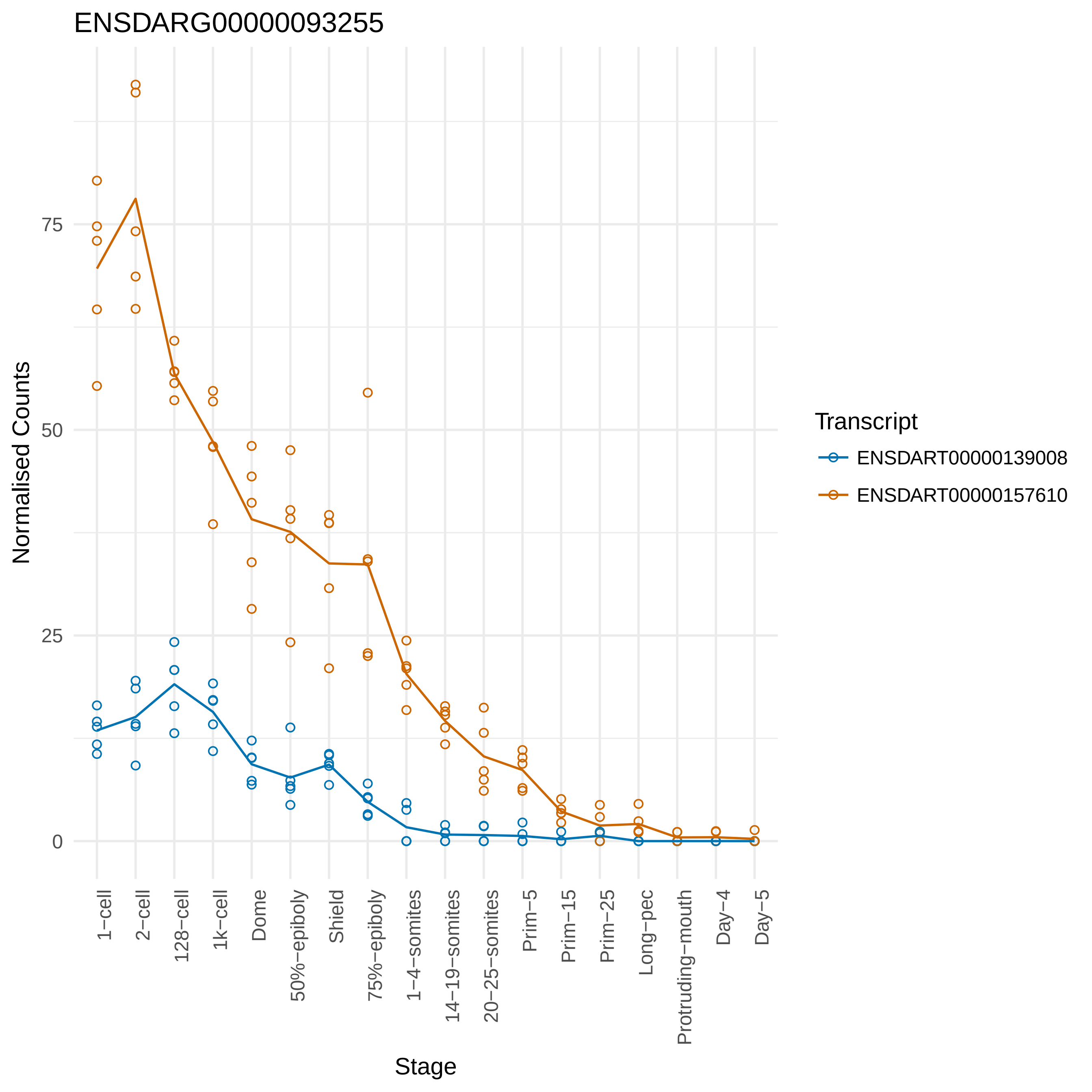
ENSDARG00000093255
Normalised Counts
75
50
25
0
1−cell
2−cell
128−cell
1k−cell
Dome
50%−epiboly
Shield
75%−epiboly
1−4−somites
14−19−somites
20−25−somites
Prim−5
Prim−15
Prim−25
Long−pec
Protruding−mouth
Day−4
Day−5
Stage
Transcript
ENSDART00000139008
ENSDART00000157610

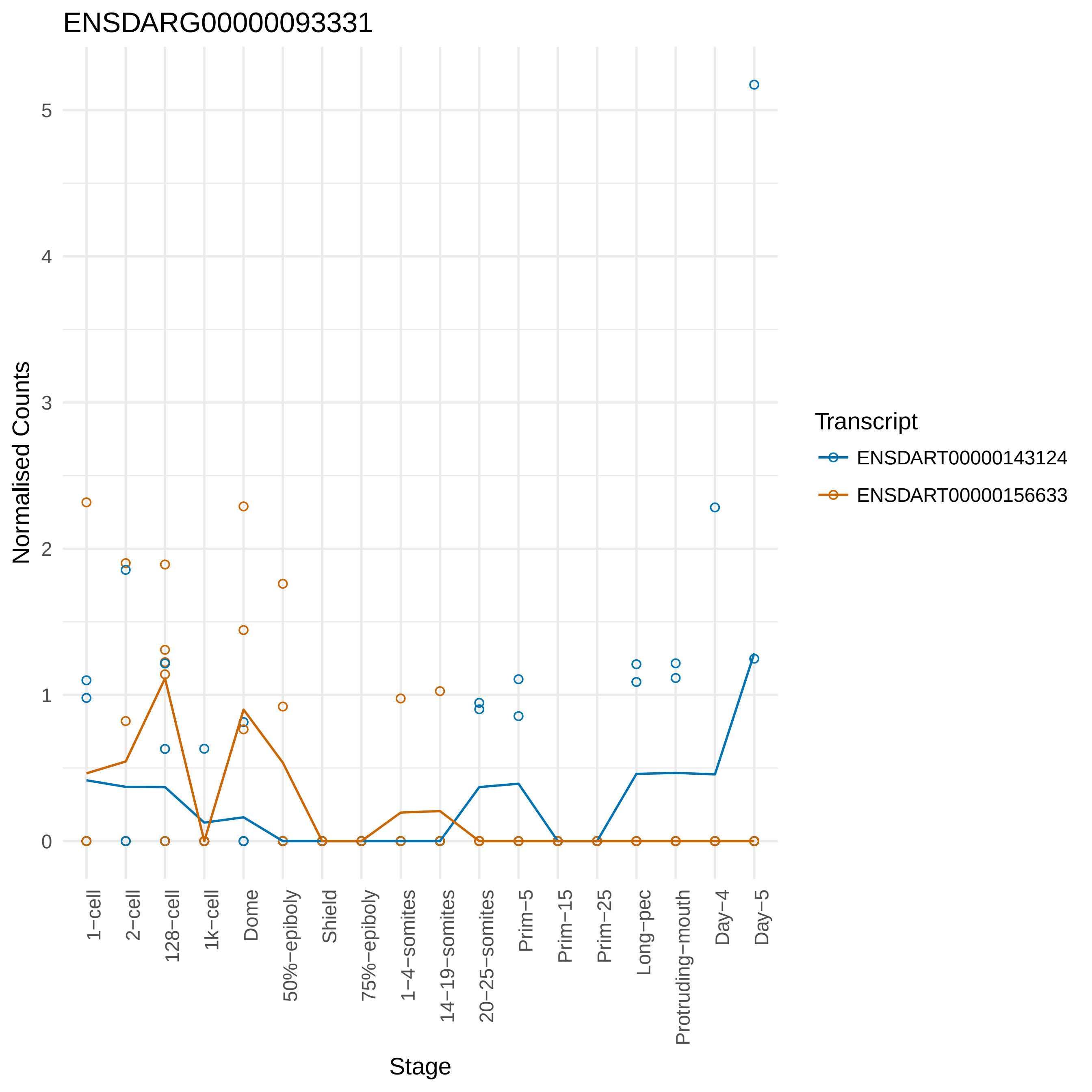
ENSDARG00000093331
Normalised Counts
0
1
2
3
4
5
Stage
1−cell
2−cell
128−cell
1k−cell
Dome
50%−epiboly
Shield
75%−epiboly
1−4−somites
14−19−somites
20−25−somites
Prim−5
Prim−15
Prim−25
Long−pec
Protruding−mouth
Day−4
Day−5
Transcript
ENSDART00000143124
ENSDART00000156633

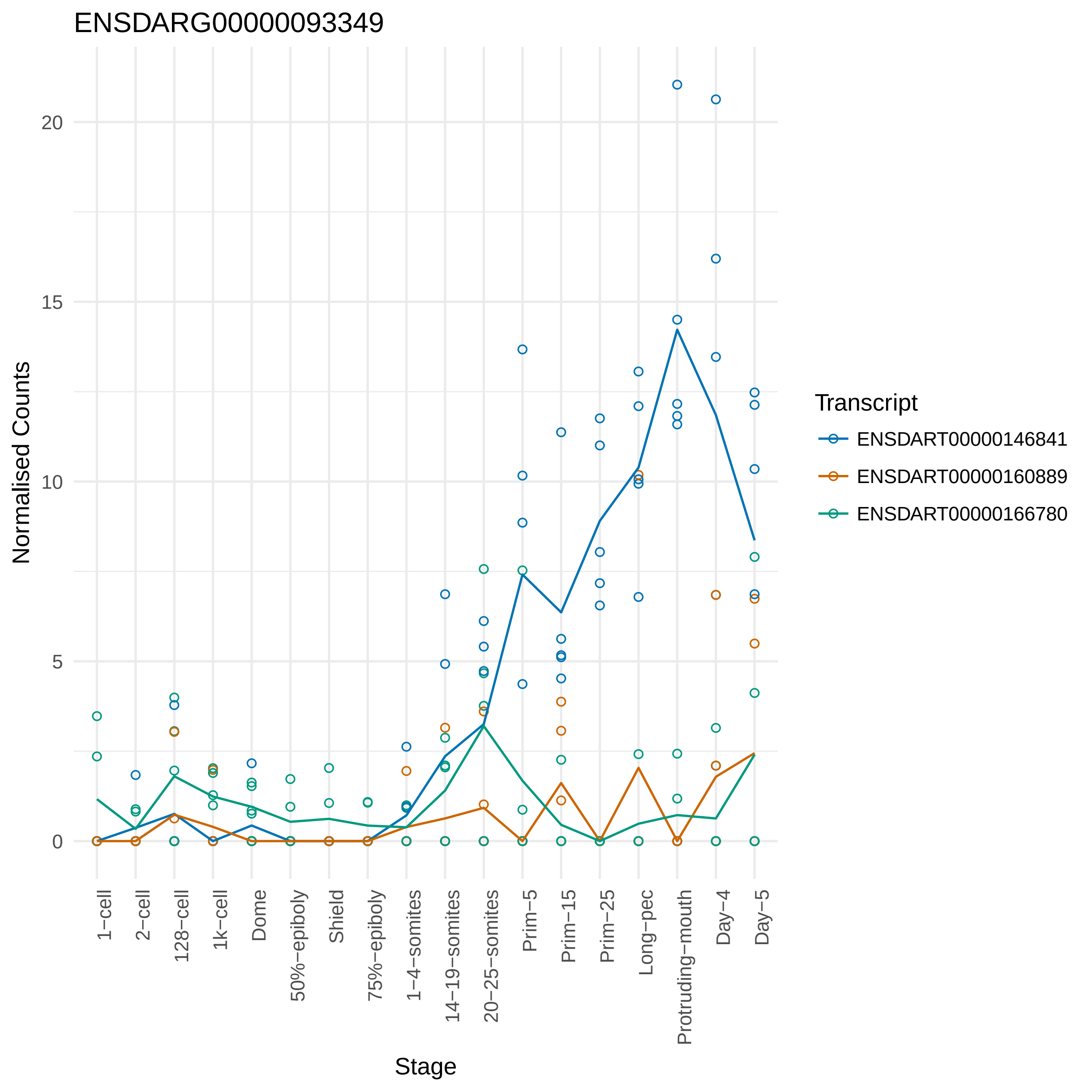

ENSDARG00000093349
Normalised Counts
20
15
10
5
0
1−cell
2−cell
128−cell
1k−cell
Dome
50%−epiboly
Shield
75%−epiboly
1−4−somites
14−19−somites
20−25−somites
Prim−5
Prim−15
Prim−25
Long−pec
Protruding−mouth
Day−4
Day−5
Stage
Transcript
ENSDART00000146841
ENSDART00000160889
ENSDART00000166780

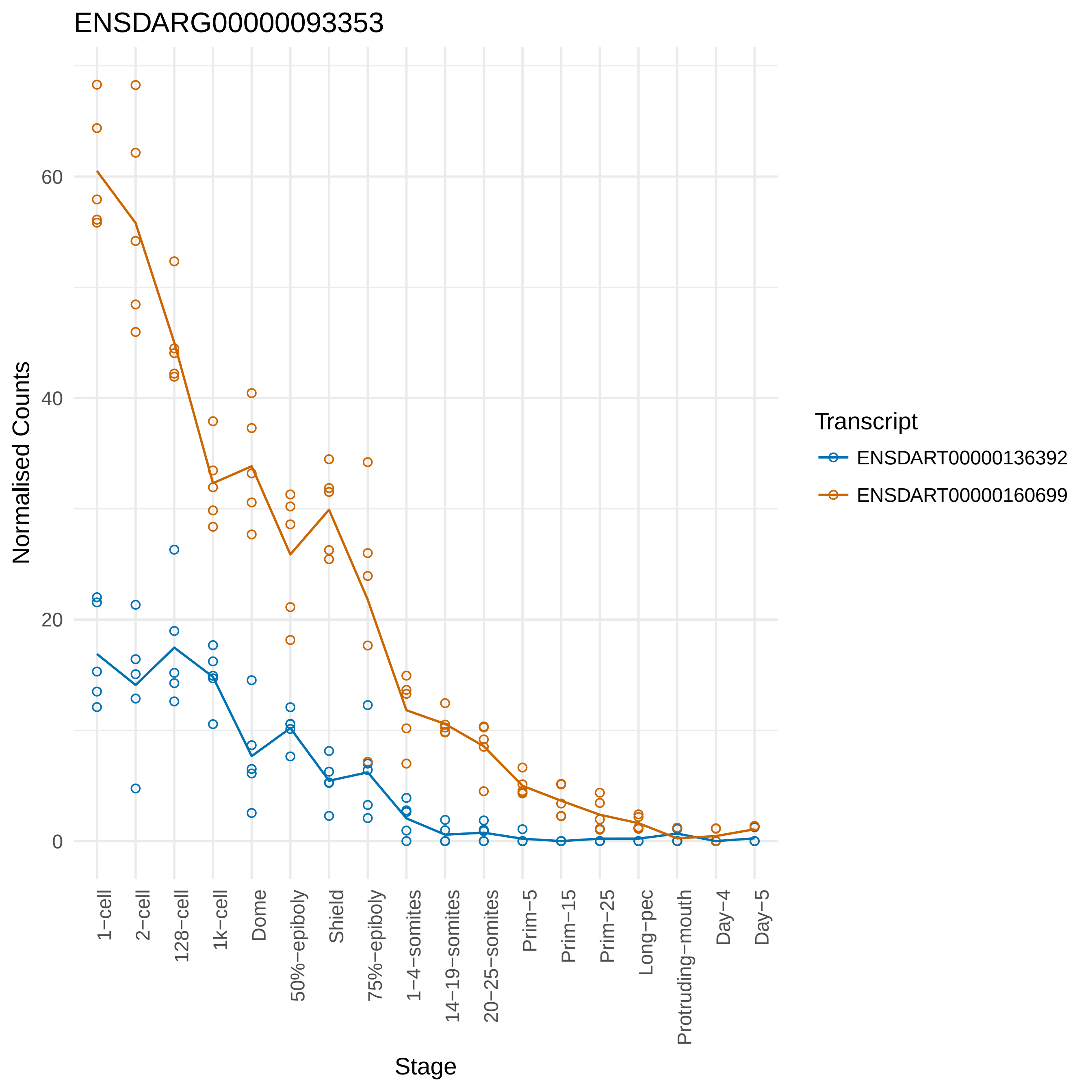

ENSDARG00000093353
Normalised Counts
0
20
40
60
Stage
1−cell
2−cell
128−cell
1k−cell
Dome
50%−epiboly
Shield
75%−epiboly
1−4−somites
14−19−somites
20−25−somites
Prim−5
Prim−15
Prim−25
Long−pec
Protruding−mouth
Day−4
Day−5
Transcript
ENSDART00000136392
ENSDART00000160699

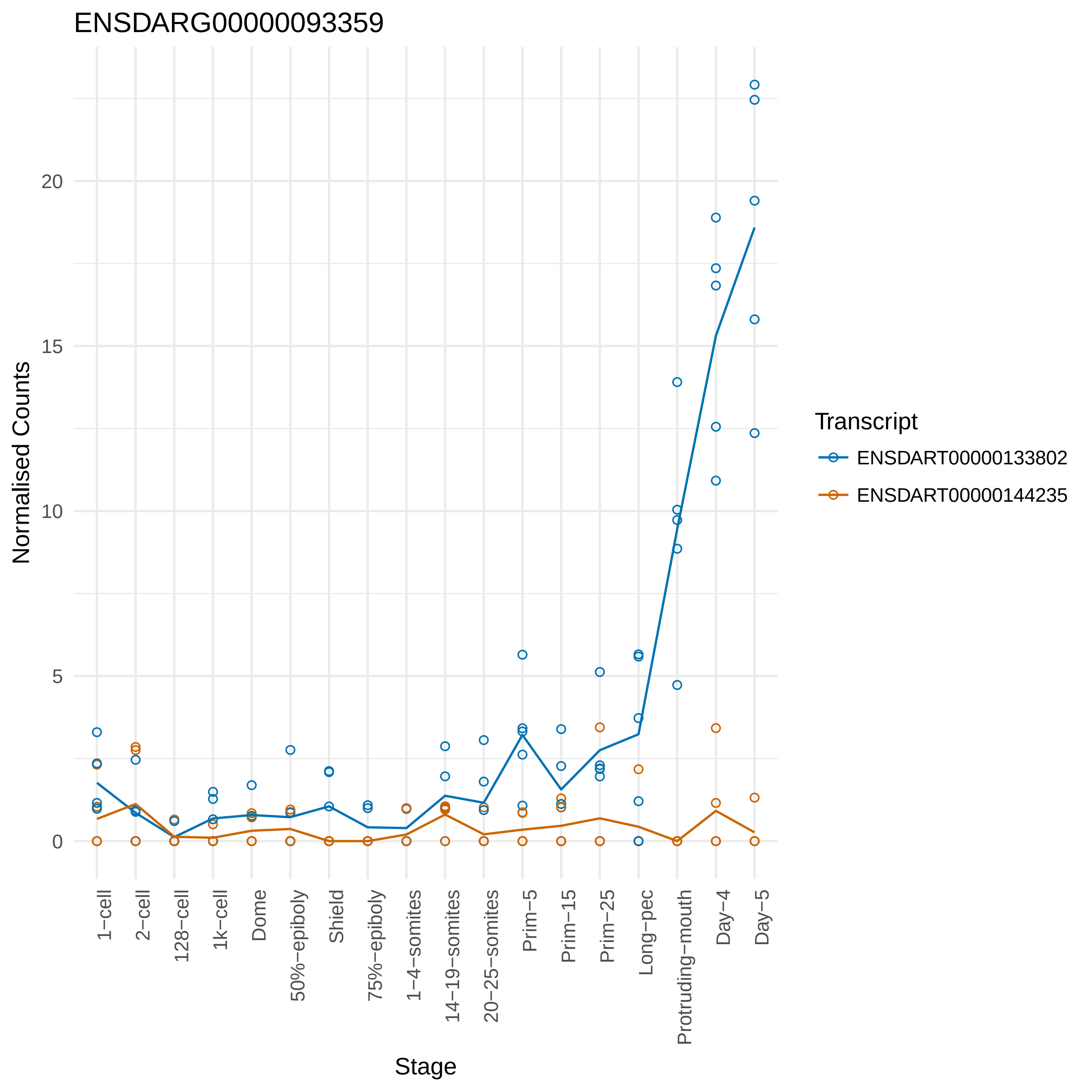

ENSDARG00000093359
Normalised Counts
0
5
10
15
20
Stage
1−cell
2−cell
128−cell
1k−cell
Dome
50%−epiboly
Shield
75%−epiboly
1−4−somites
14−19−somites
20−25−somites
Prim−5
Prim−15
Prim−25
Long−pec
Protruding−mouth
Day−4
Day−5
Transcript
ENSDART00000133802
ENSDART00000144235

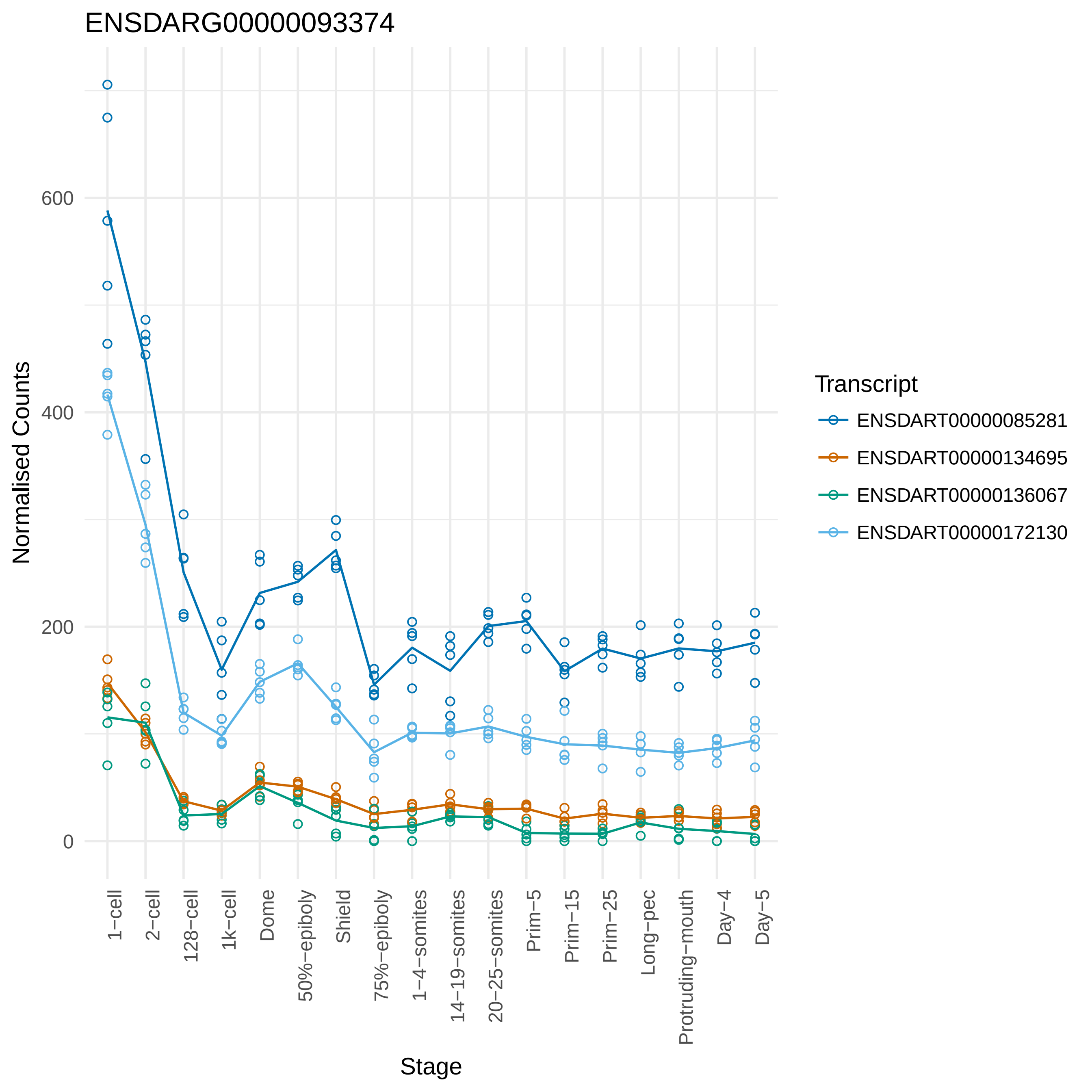

ENSDARG00000093374
Normalised Counts
600
400
200
0
1−cell
2−cell
128−cell
1k−cell
Dome
50%−epiboly
Shield
75%−epiboly
1−4−somites
14−19−somites
20−25−somites
Prim−5
Prim−15
Prim−25
Long−pec
Protruding−mouth
Day−4
Day−5
Stage
Transcript
ENSDART00000085281
ENSDART00000134695
ENSDART00000136067
ENSDART00000172130

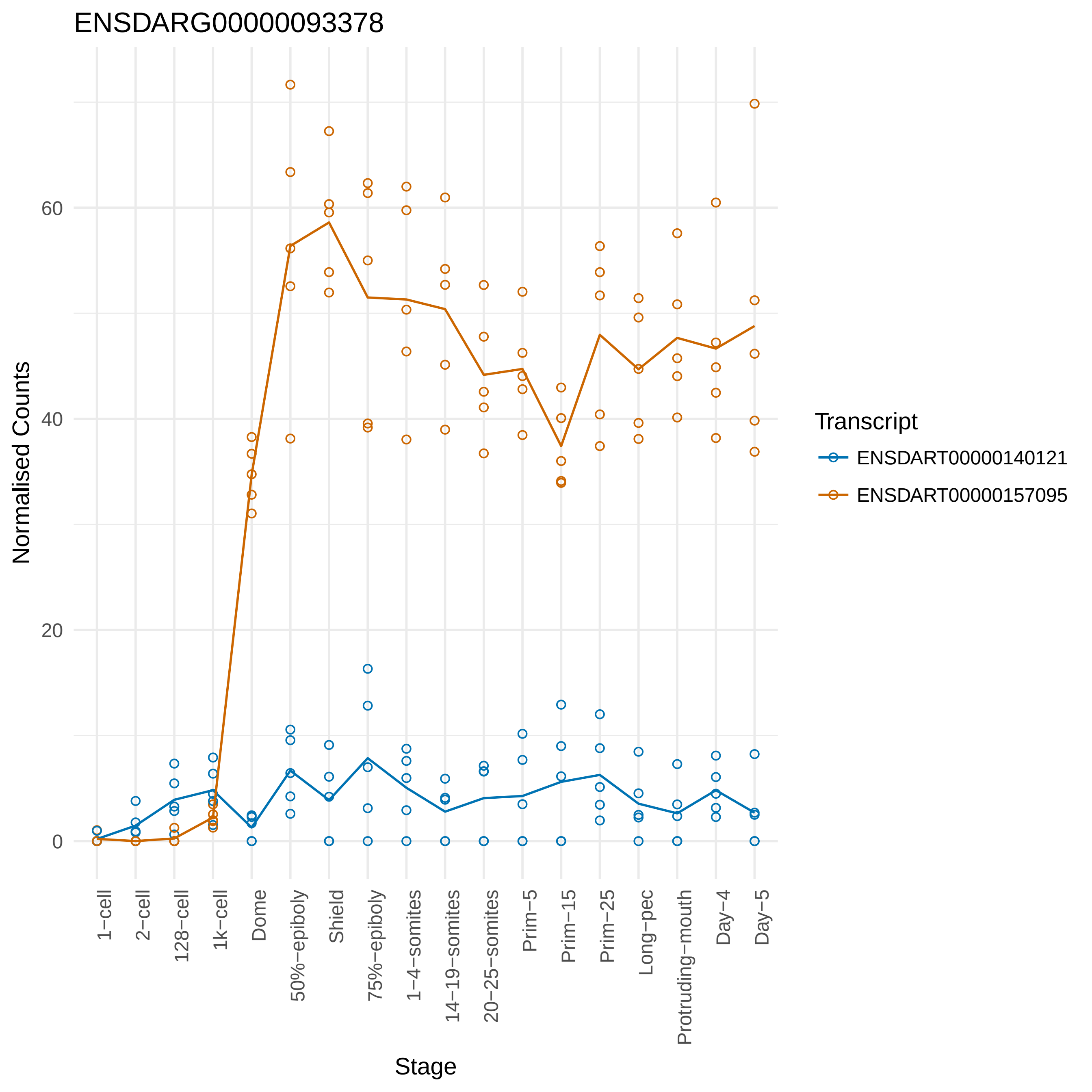
ENSDARG00000093378
Normalised Counts
0
20
40
60
Stage
1−cell
2−cell
128−cell
1k−cell
Dome
50%−epiboly
Shield
75%−epiboly
1−4−somites
14−19−somites
20−25−somites
Prim−5
Prim−15
Prim−25
Long−pec
Protruding−mouth
Day−4
Day−5
Transcript
ENSDART00000140121
ENSDART00000157095

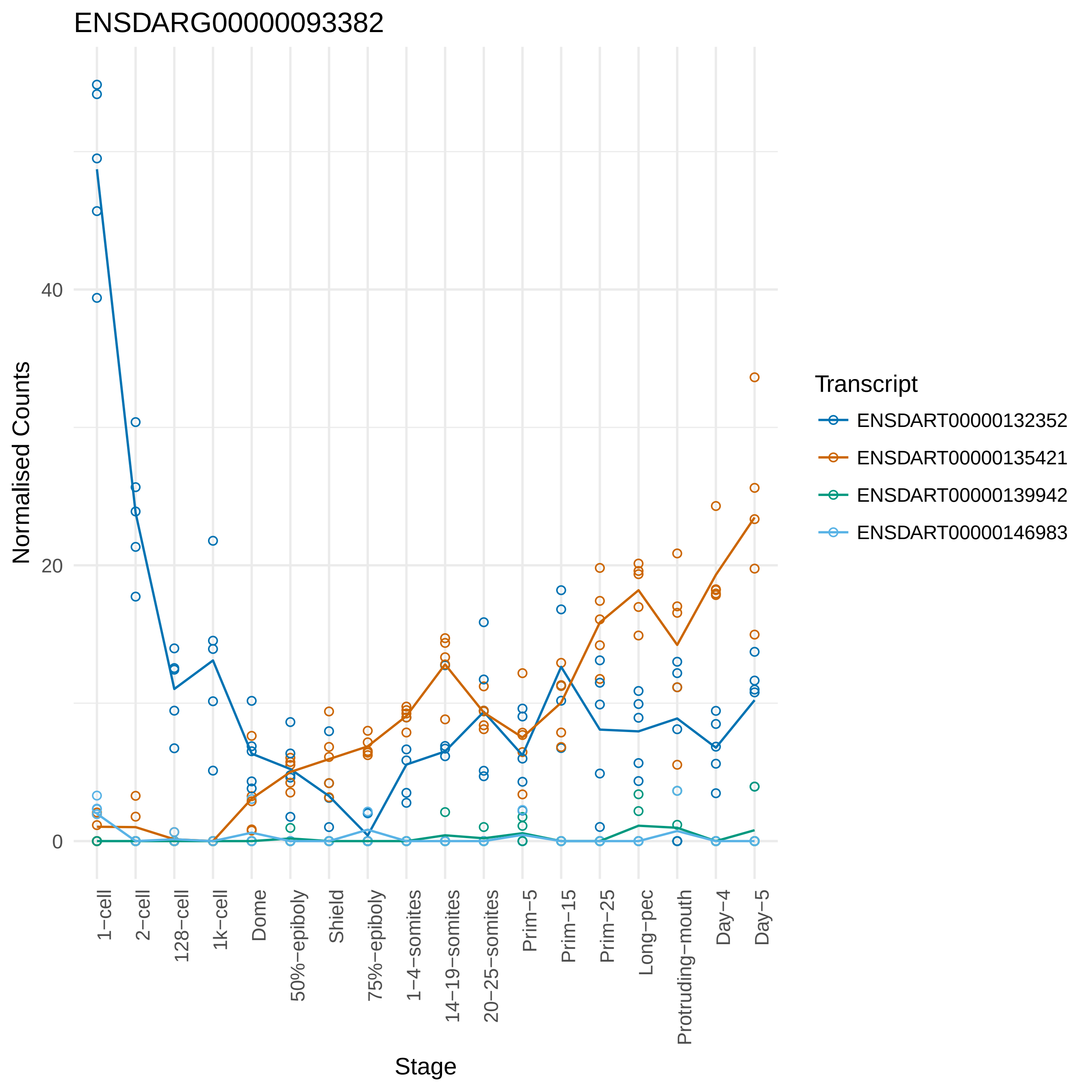

ENSDARG00000093382
Normalised Counts
0
20
40
1−cell
2−cell
128−cell
1k−cell
Dome
50%−epiboly
Shield
75%−epiboly
1−4−somites
14−19−somites
20−25−somites
Prim−5
Prim−15
Prim−25
Long−pec
Protruding−mouth
Day−4
Day−5
Stage
Transcript
ENSDART00000132352
ENSDART00000135421
ENSDART00000139942
ENSDART00000146983

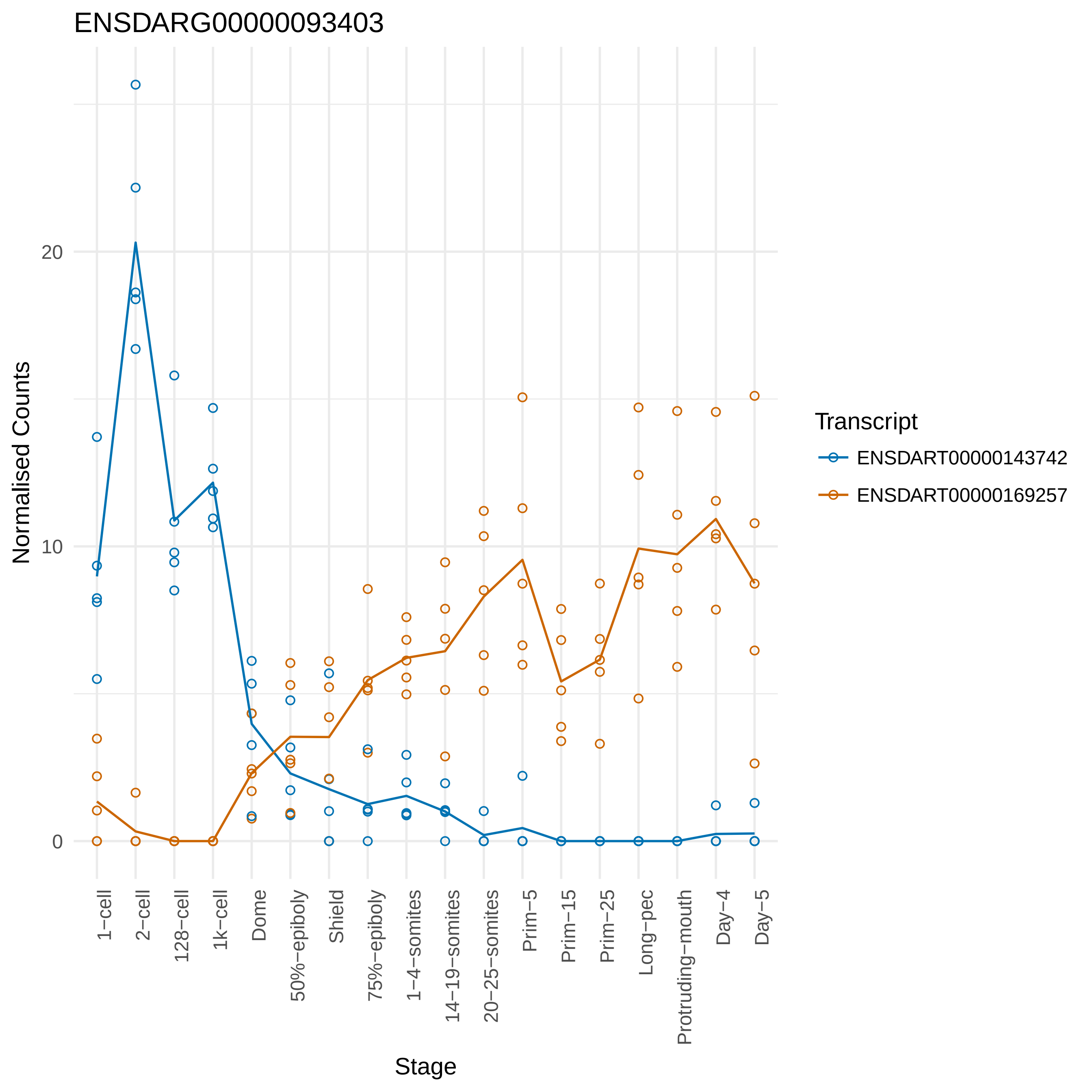

ENSDARG00000093403
Normalised Counts
0
10
20
1−cell
2−cell
128−cell
1k−cell
Dome
50%−epiboly
Shield
75%−epiboly
1−4−somites
14−19−somites
20−25−somites
Prim−5
Prim−15
Prim−25
Long−pec
Protruding−mouth
Day−4
Day−5
Stage
Transcript
ENSDART00000143742
ENSDART00000169257

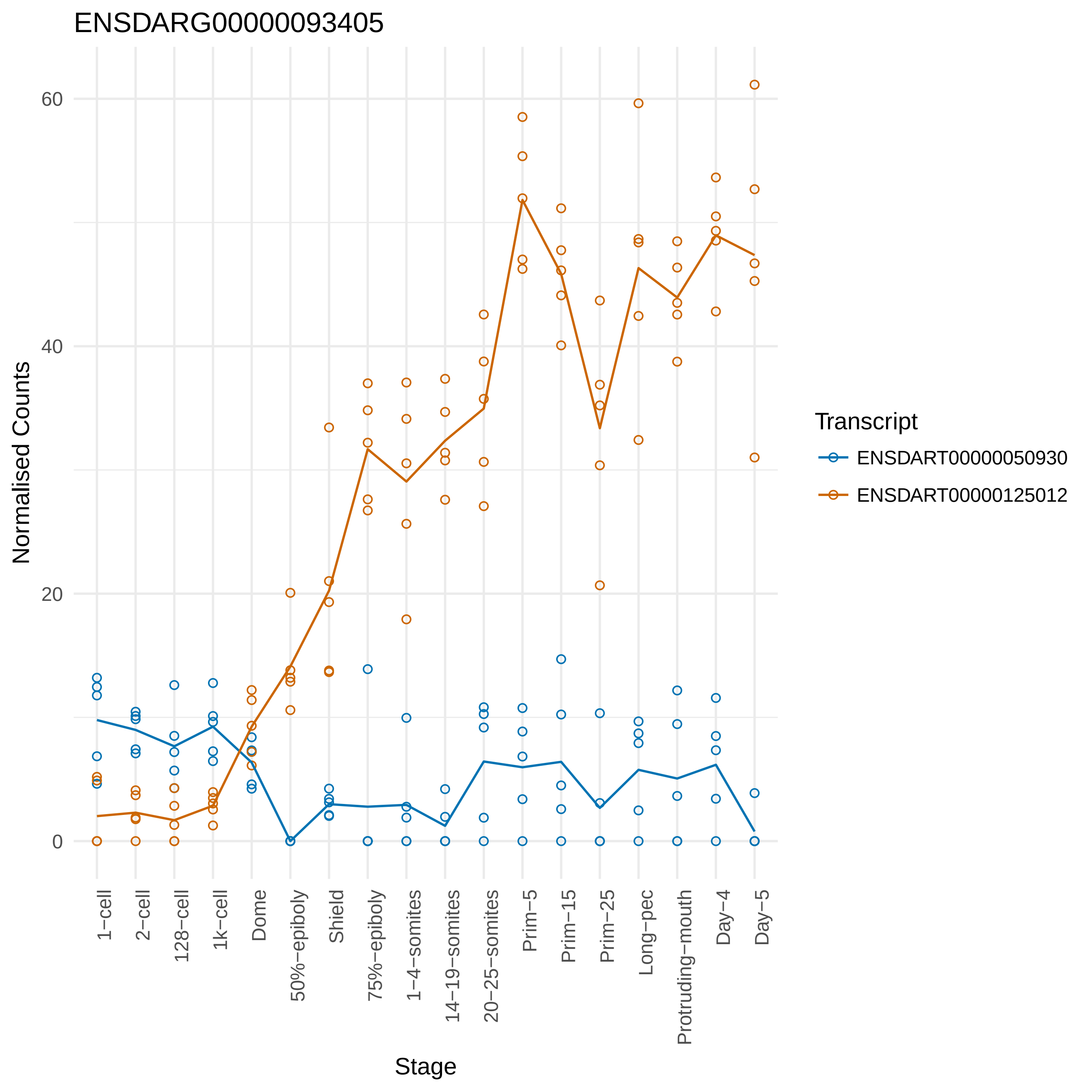
ENSDARG00000093405
Normalised Counts
0
20
40
60
1−cell
2−cell
128−cell
1k−cell
Dome
50%−epiboly
Shield
75%−epiboly
1−4−somites
14−19−somites
20−25−somites
Prim−5
Prim−15
Prim−25
Long−pec
Protruding−mouth
Day−4
Day−5
Stage
Transcript
ENSDART00000050930
ENSDART00000125012

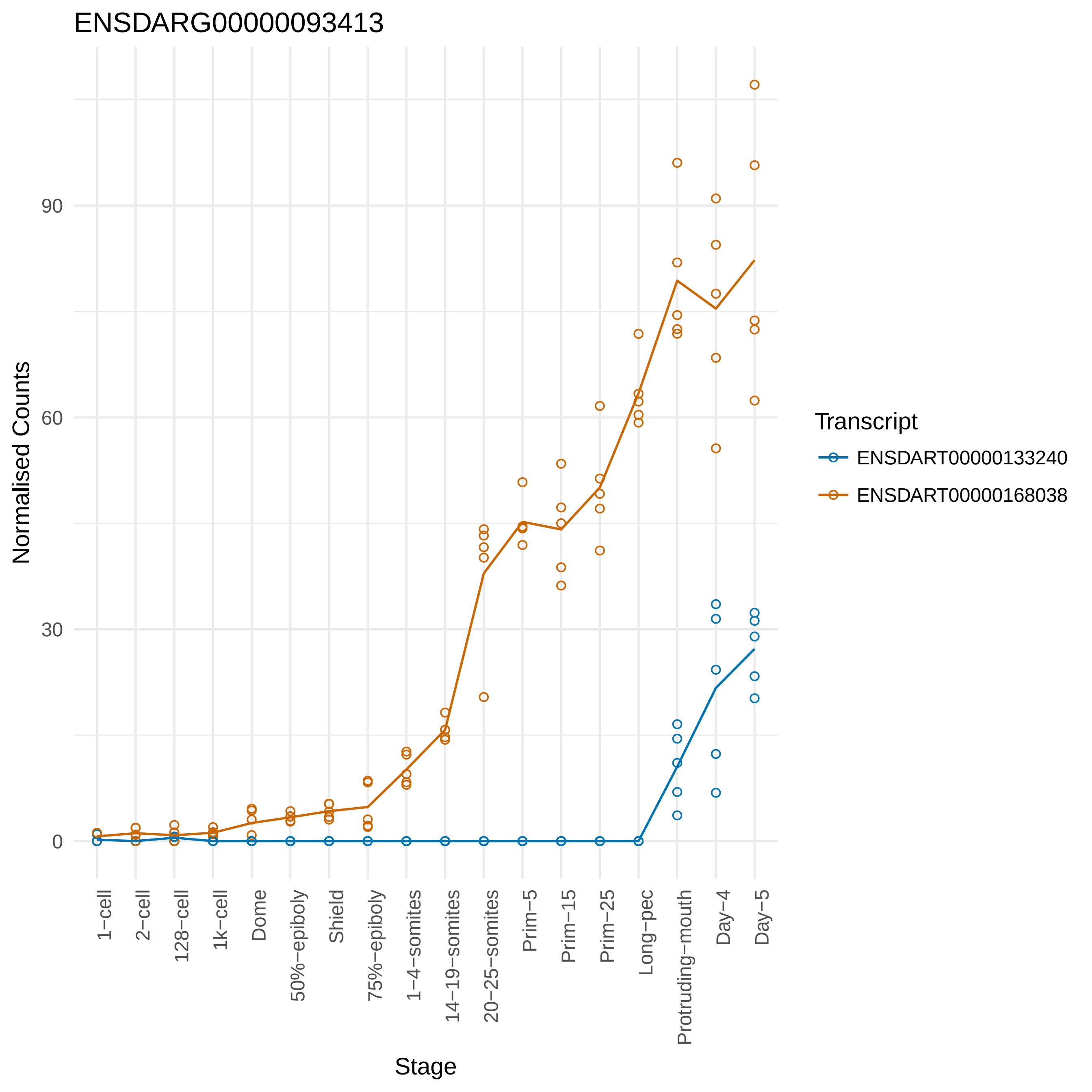

ENSDARG00000093413
Normalised Counts
90
60
30
0
1−cell
2−cell
128−cell
1k−cell
Dome
50%−epiboly
Shield
75%−epiboly
1−4−somites
14−19−somites
20−25−somites
Prim−5
Prim−15
Prim−25
Long−pec
Protruding−mouth
Day−4
Day−5
Stage
Transcript
ENSDART00000133240
ENSDART00000168038

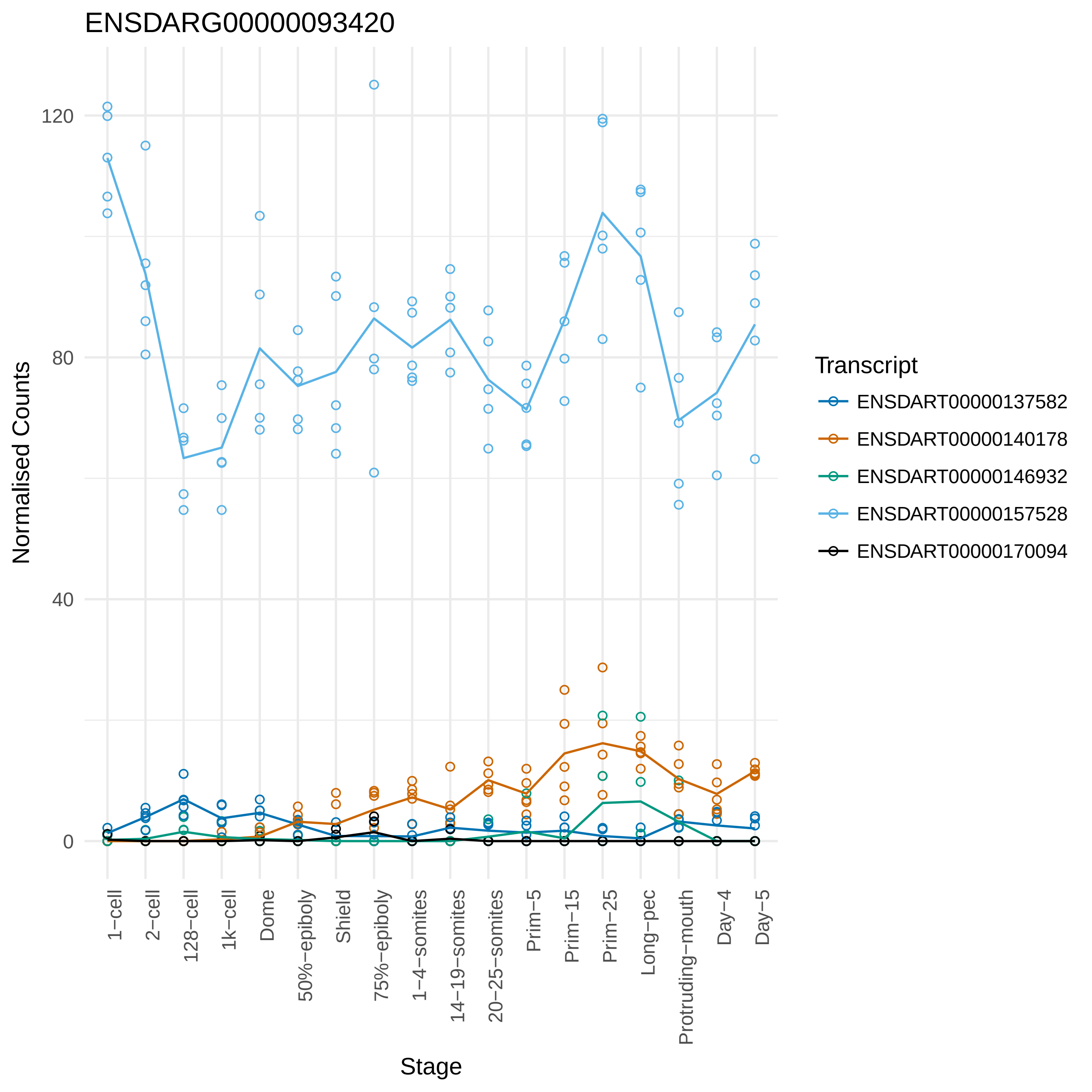

ENSDARG00000093420
Normalised Counts
120
80
40
0
1−cell
2−cell
128−cell
1k−cell
Dome
50%−epiboly
Shield
75%−epiboly
1−4−somites
14−19−somites
20−25−somites
Prim−5
Prim−15
Prim−25
Long−pec
Protruding−mouth
Day−4
Day−5
Stage
Transcript
ENSDART00000137582
ENSDART00000140178
ENSDART00000146932
ENSDART00000157528
ENSDART00000170094

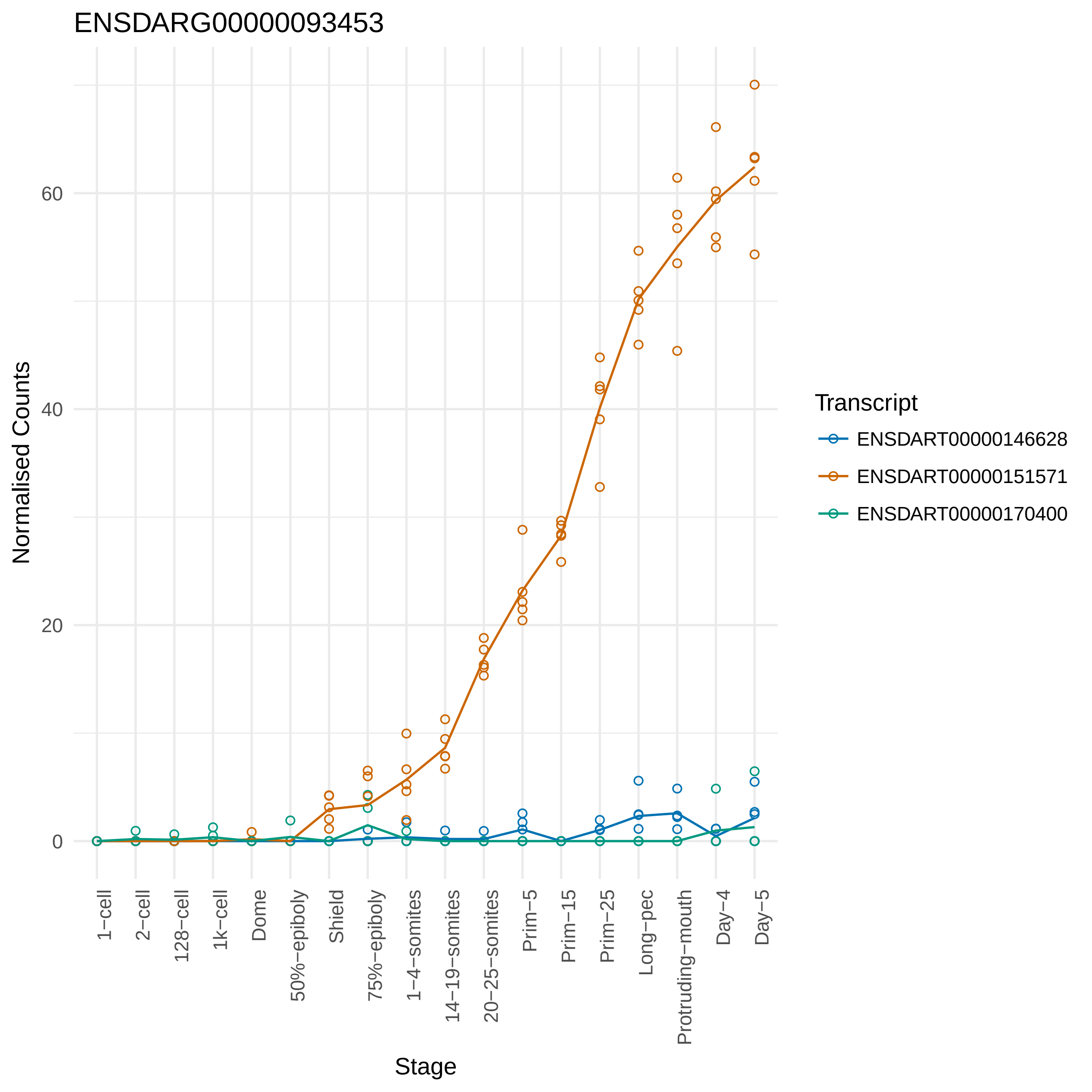

ENSDARG00000093453
Normalised Counts
0
20
40
60
Stage
1−cell
2−cell
128−cell
1k−cell
Dome
50%−epiboly
Shield
75%−epiboly
1−4−somites
14−19−somites
20−25−somites
Prim−5
Prim−15
Prim−25
Long−pec
Protruding−mouth
Day−4
Day−5
Transcript
ENSDART00000146628
ENSDART00000151571
ENSDART00000170400

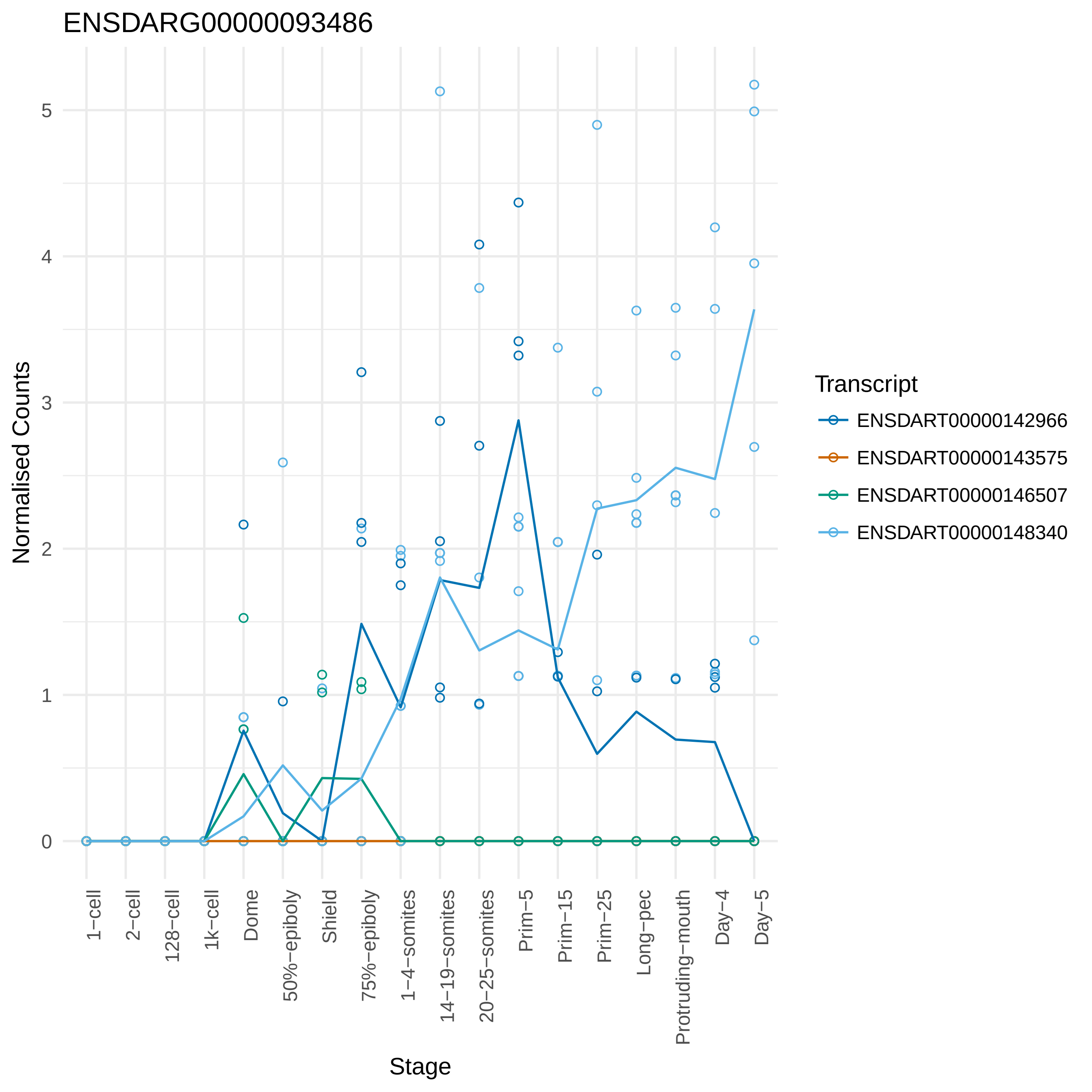
ENSDARG00000093486
Normalised Counts
0
1
2
3
4
5
1−cell
2−cell
128−cell
1k−cell
Dome
50%−epiboly
Shield
75%−epiboly
1−4−somites
14−19−somites
20−25−somites
Prim−5
Prim−15
Prim−25
Long−pec
Protruding−mouth
Day−4
Day−5
Stage
Transcript
ENSDART00000142966
ENSDART00000143575
ENSDART00000146507
ENSDART00000148340

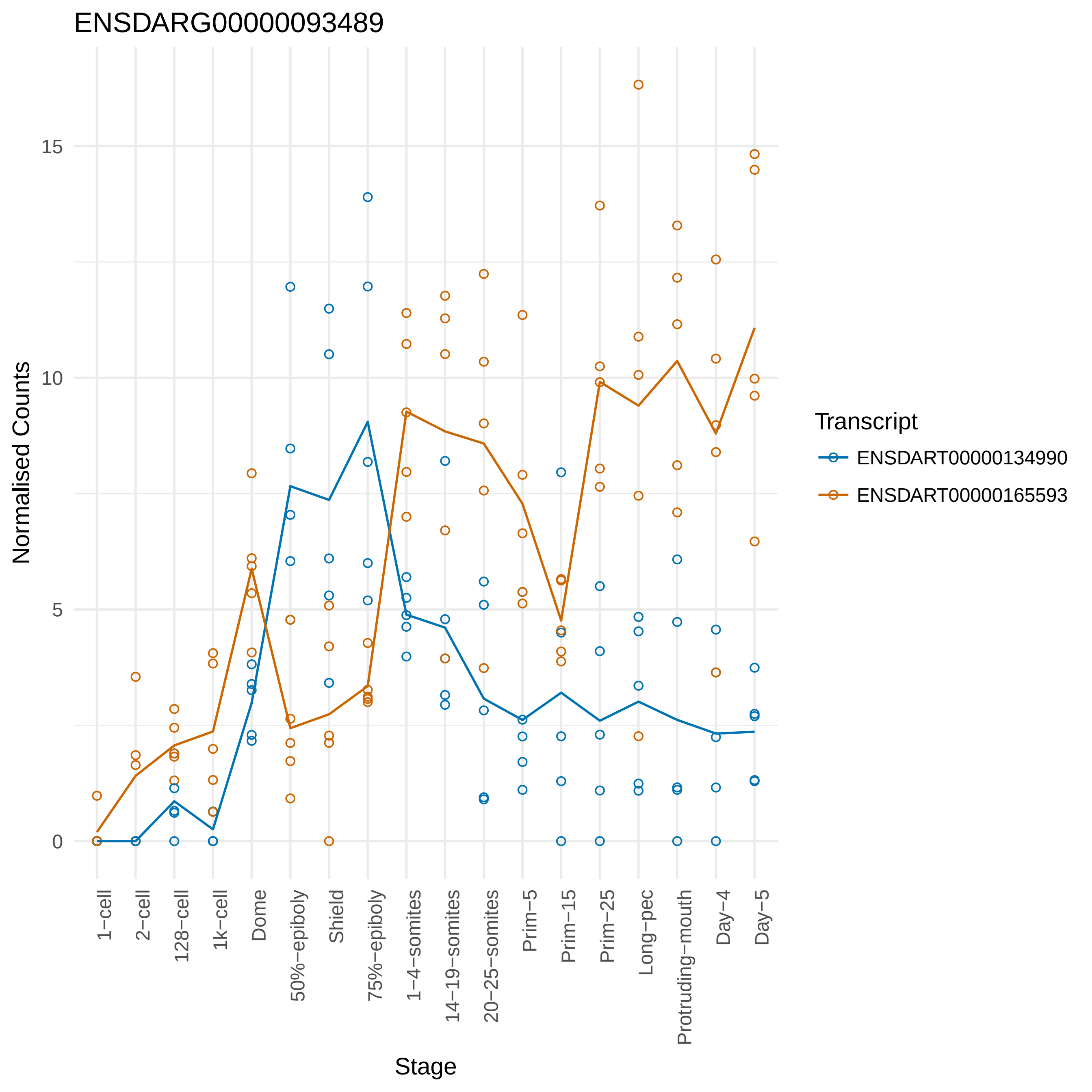
ENSDARG00000093489
Normalised Counts
15
10
5
0
1−cell
2−cell
128−cell
1k−cell
Dome
50%−epiboly
Shield
75%−epiboly
1−4−somites
14−19−somites
20−25−somites
Prim−5
Prim−15
Prim−25
Long−pec
Protruding−mouth
Day−4
Day−5
Stage
Transcript
ENSDART00000134990
ENSDART00000165593

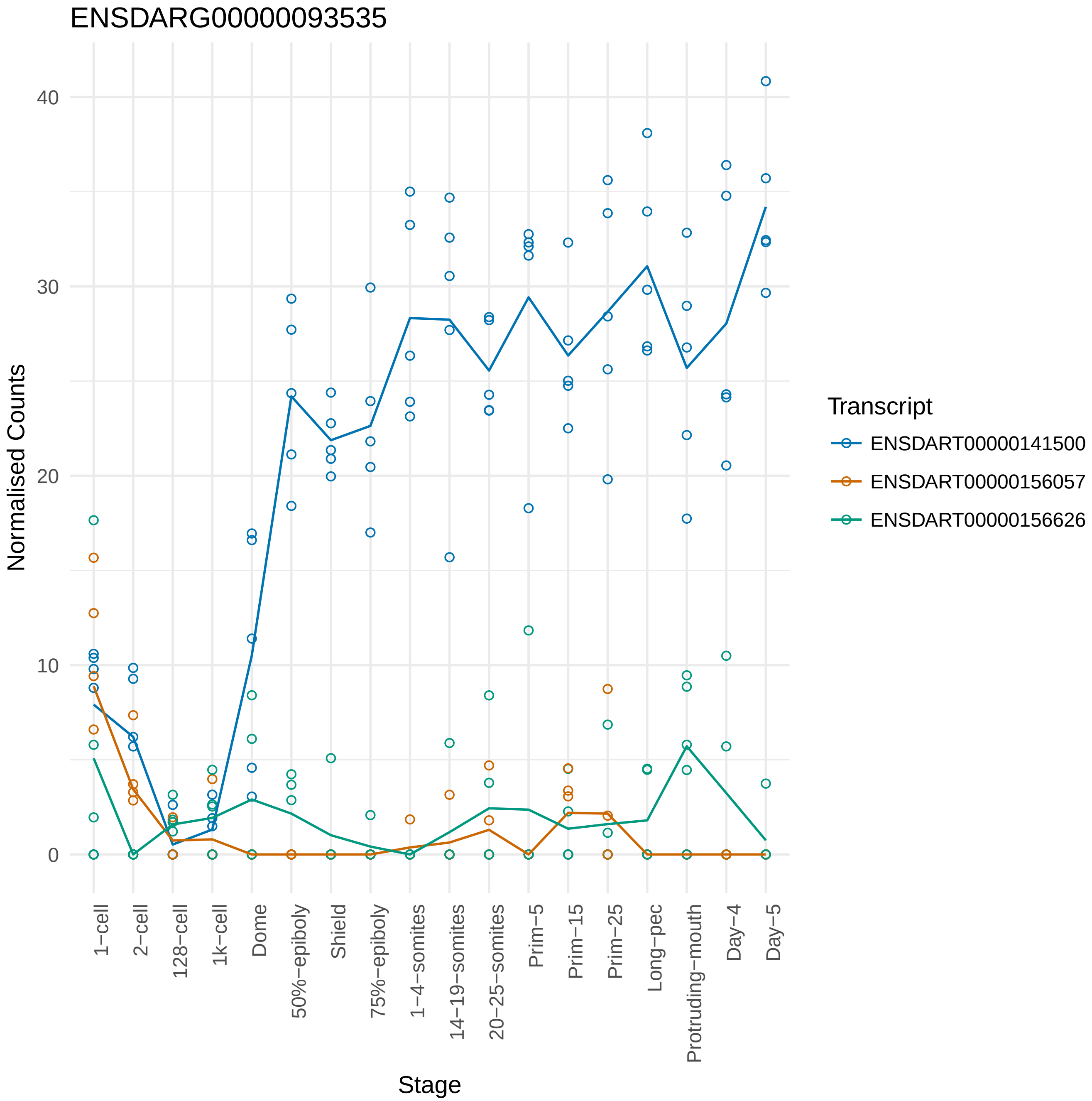

ENSDARG00000093535
Normalised Counts
40
30
20
10
0
1−cell
2−cell
128−cell
1k−cell
Dome
50%−epiboly
Shield
75%−epiboly
1−4−somites
14−19−somites
20−25−somites
Prim−5
Prim−15
Prim−25
Long−pec
Protruding−mouth
Day−4
Day−5
Stage
Transcript
ENSDART00000141500
ENSDART00000156057
ENSDART00000156626

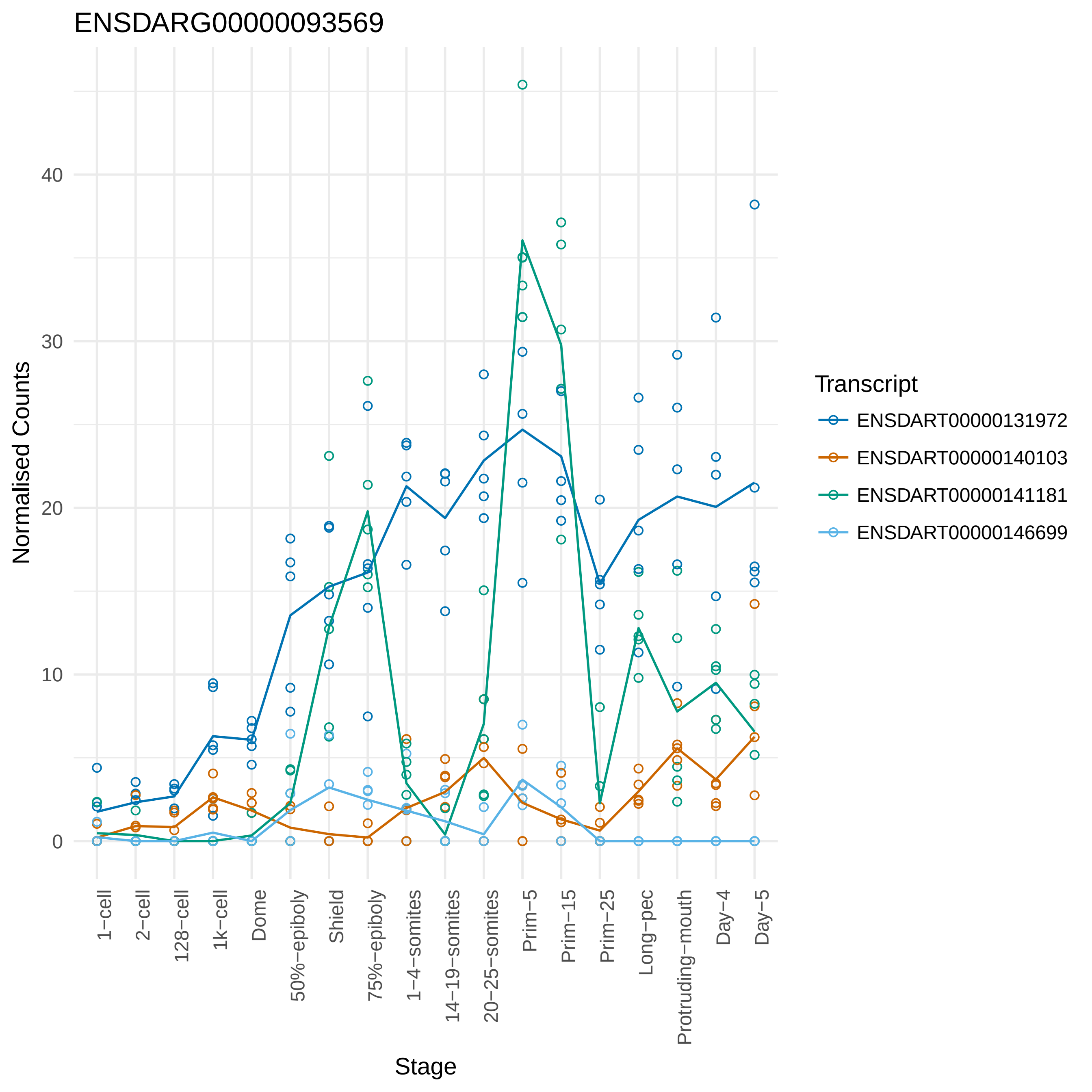

ENSDARG00000093569
Normalised Counts
0
10
20
30
40
1−cell
2−cell
128−cell
1k−cell
Dome
50%−epiboly
Shield
75%−epiboly
1−4−somites
14−19−somites
20−25−somites
Prim−5
Prim−15
Prim−25
Long−pec
Protruding−mouth
Day−4
Day−5
Stage
Transcript
ENSDART00000131972
ENSDART00000140103
ENSDART00000141181
ENSDART00000146699

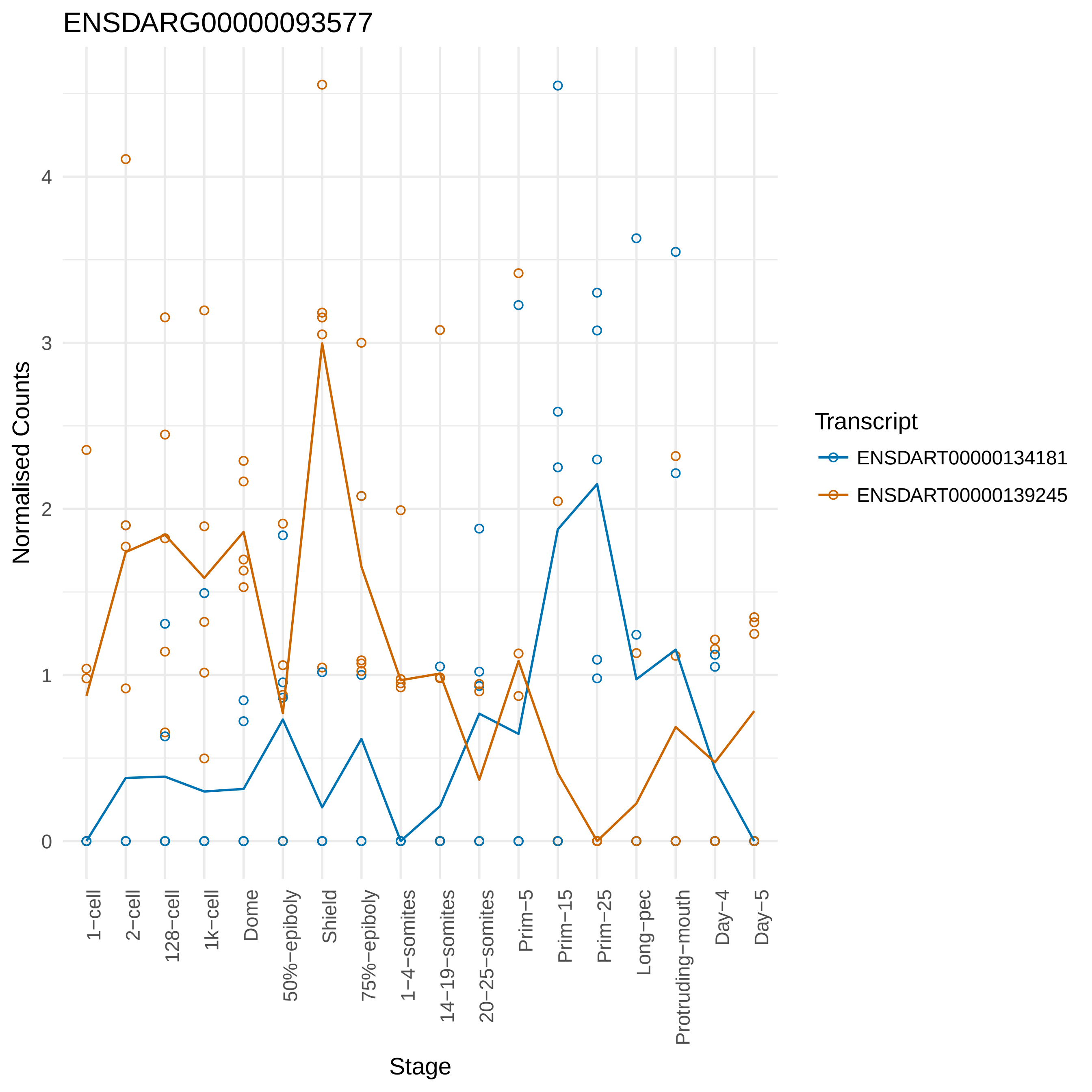
ENSDARG00000093577
Normalised Counts
0
1
2
3
4
1−cell
2−cell
128−cell
1k−cell
Dome
50%−epiboly
Shield
75%−epiboly
1−4−somites
14−19−somites
20−25−somites
Prim−5
Prim−15
Prim−25
Long−pec
Protruding−mouth
Day−4
Day−5
Stage
Transcript
ENSDART00000134181
ENSDART00000139245

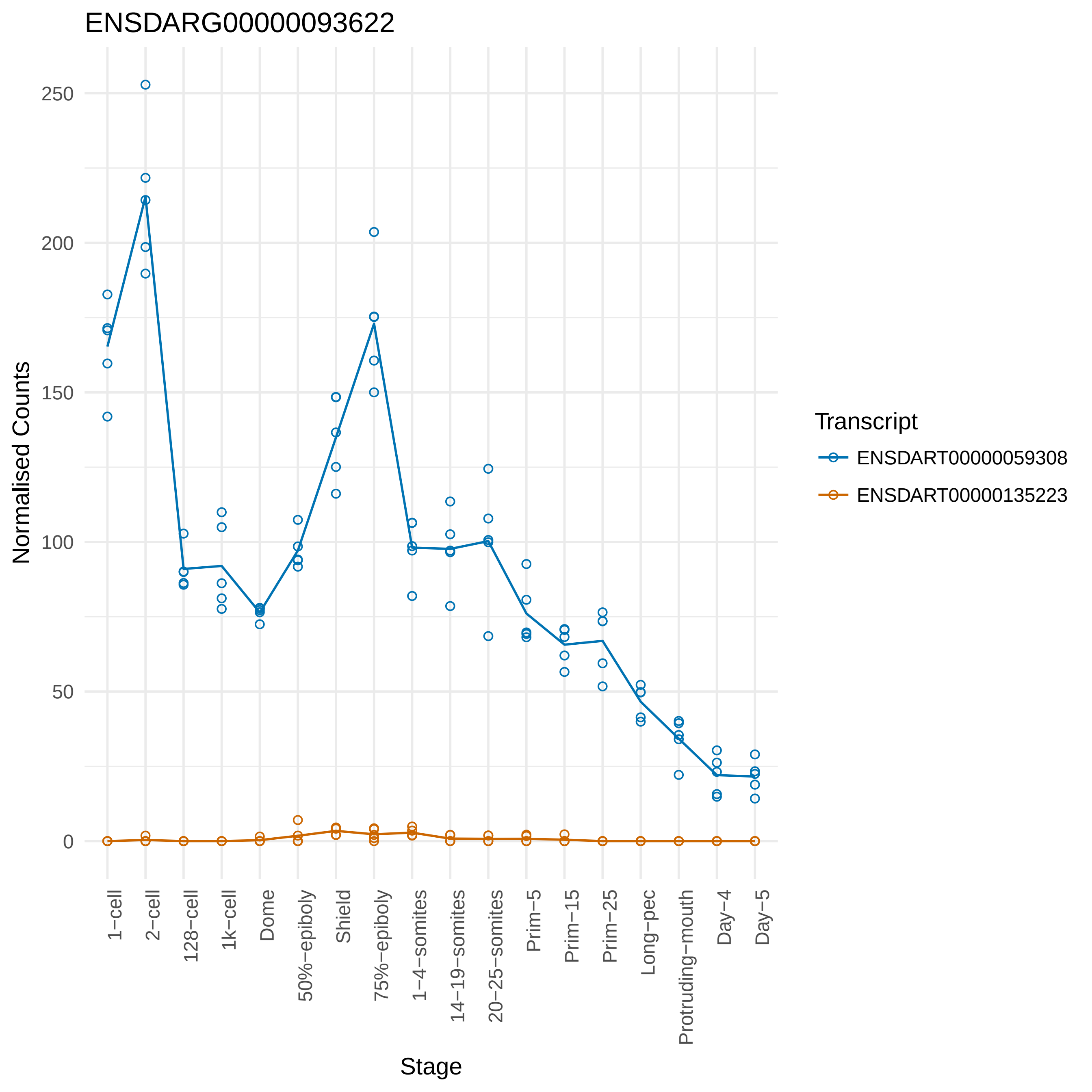

ENSDARG00000093622
Normalised Counts
250
200
150
100
50
0
1−cell
2−cell
128−cell
1k−cell
Dome
50%−epiboly
Shield
75%−epiboly
1−4−somites
14−19−somites
20−25−somites
Prim−5
Prim−15
Prim−25
Long−pec
Protruding−mouth
Day−4
Day−5
Stage
Transcript
ENSDART00000059308
ENSDART00000135223

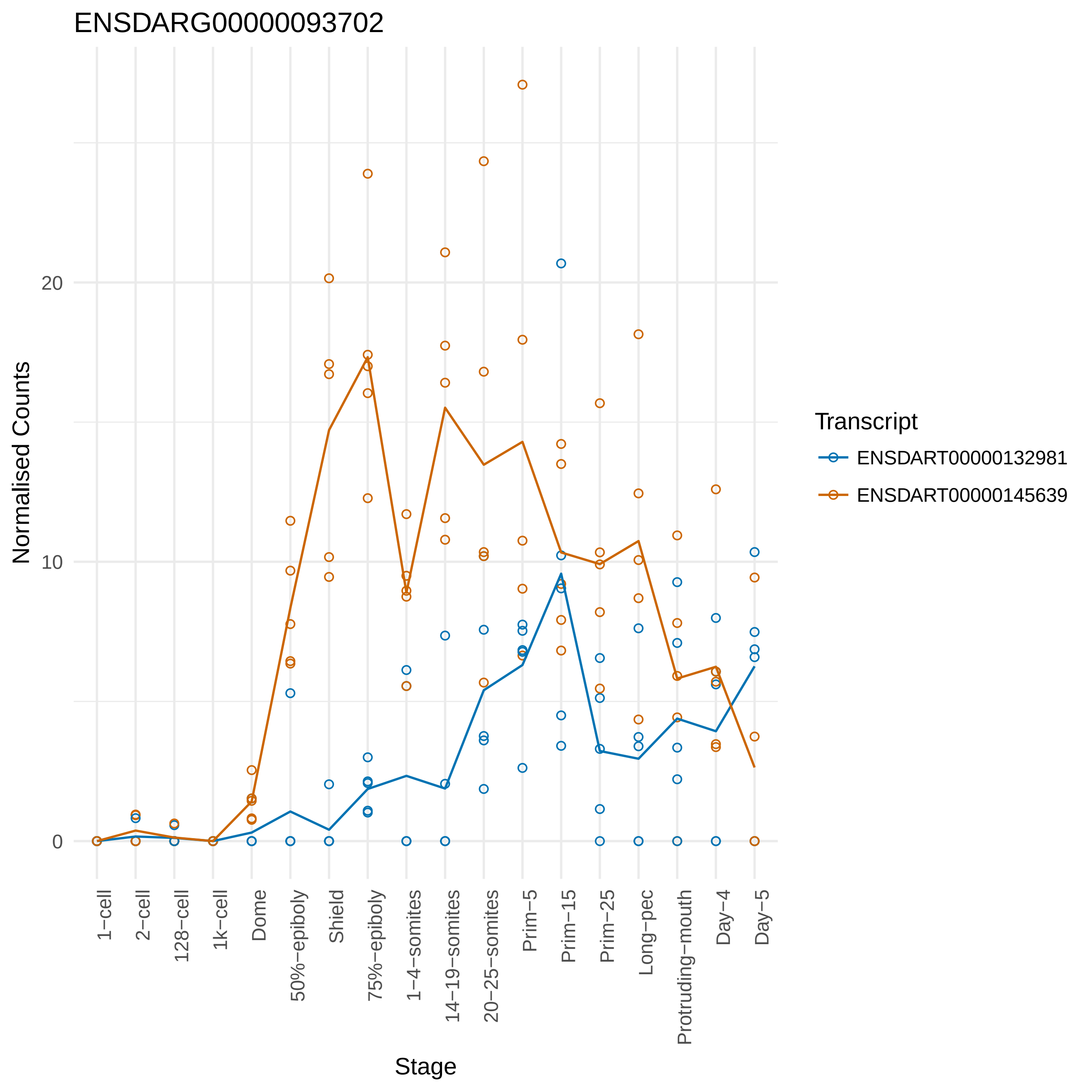

ENSDARG00000093702
Normalised Counts
20
10
0
Stage
1−cell
2−cell
128−cell
1k−cell
Dome
50%−epiboly
Shield
75%−epiboly
1−4−somites
14−19−somites
20−25−somites
Prim−5
Prim−15
Prim−25
Long−pec
Protruding−mouth
Day−4
Day−5
Transcript
ENSDART00000132981
ENSDART00000145639

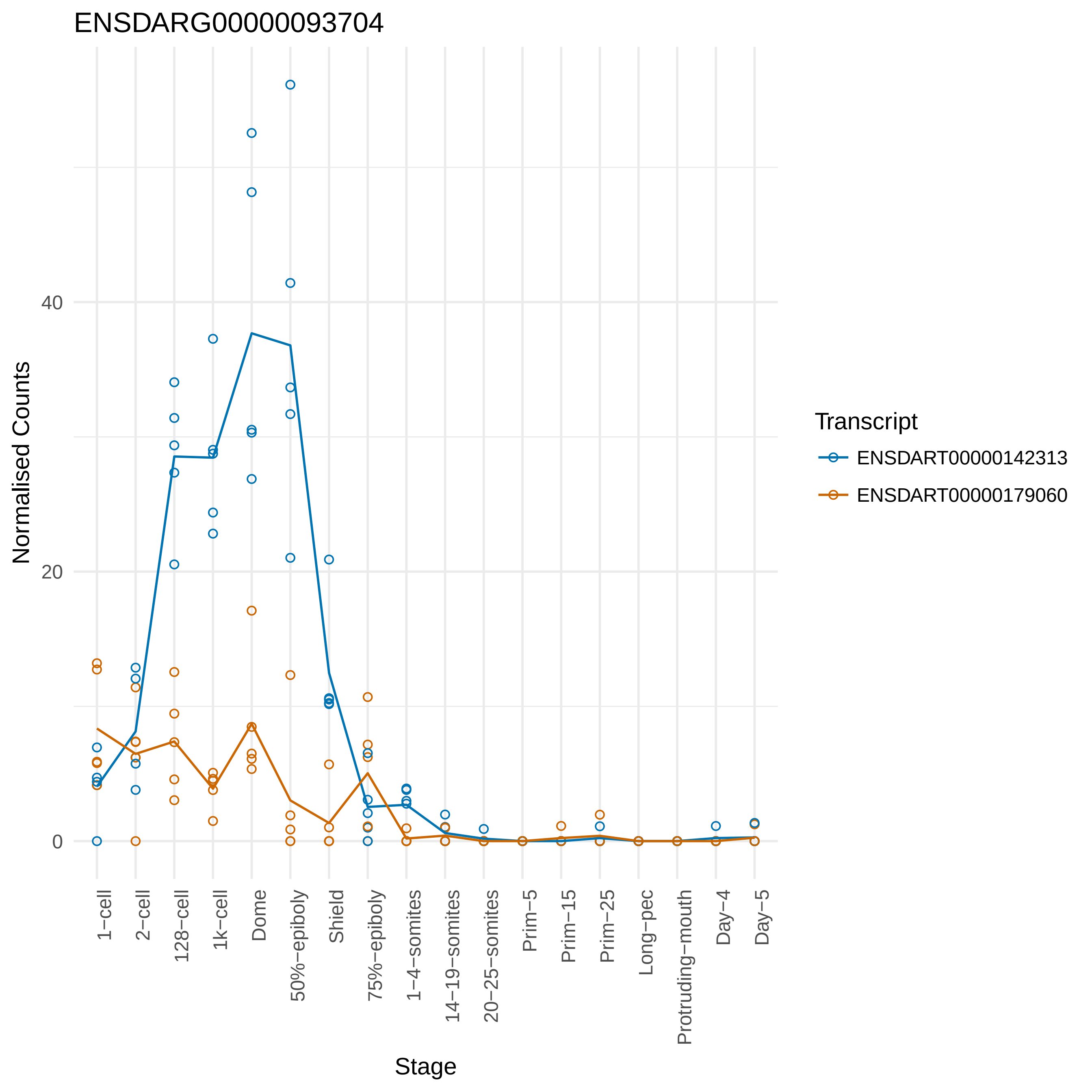

ENSDARG00000093704
Normalised Counts
40
20
0
1−cell
2−cell
128−cell
1k−cell
Dome
50%−epiboly
Shield
75%−epiboly
1−4−somites
14−19−somites
20−25−somites
Prim−5
Prim−15
Prim−25
Long−pec
Protruding−mouth
Day−4
Day−5
Stage
Transcript
ENSDART00000142313
ENSDART00000179060

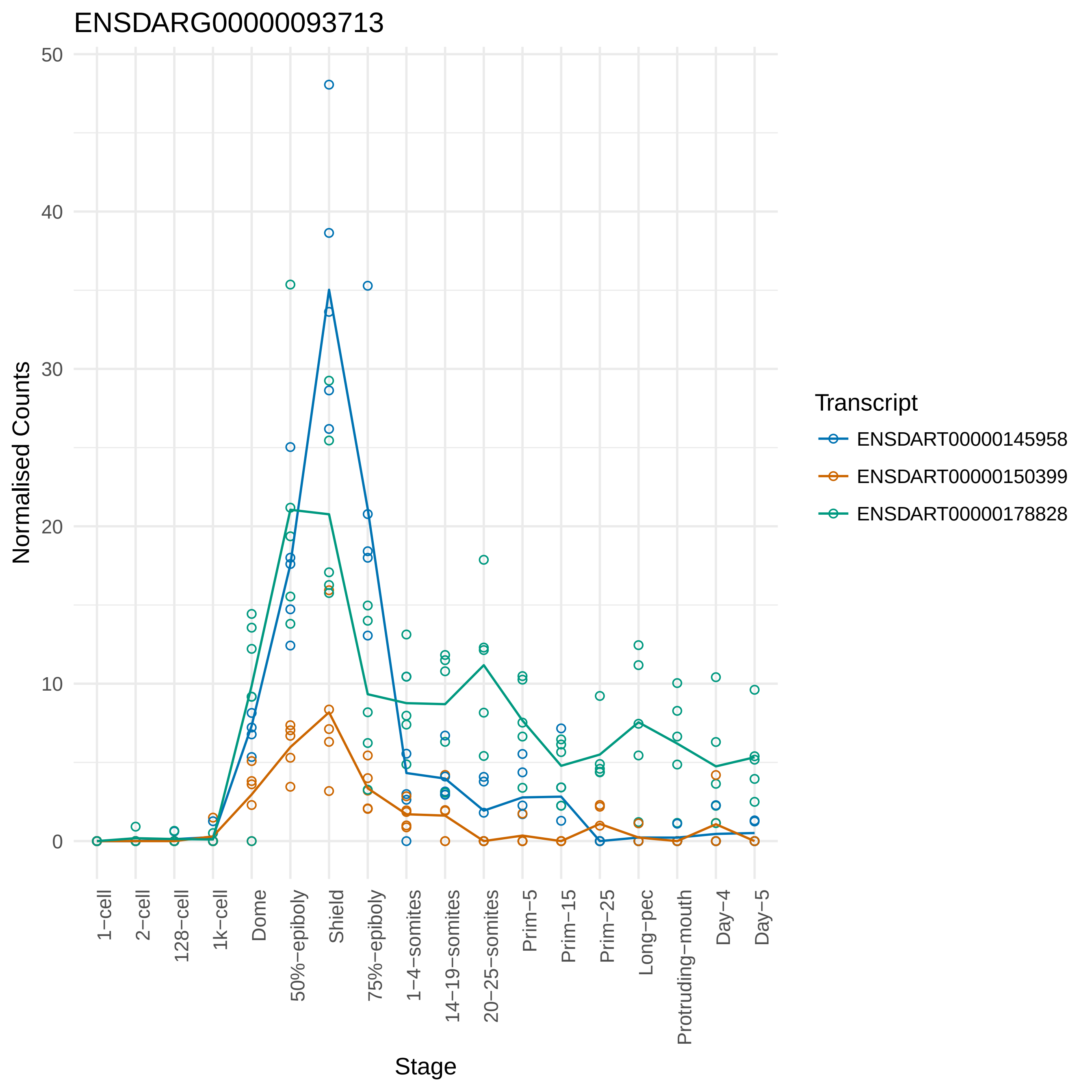
ENSDARG00000093713
Normalised Counts
50
40
30
20
10
0
1−cell
2−cell
128−cell
1k−cell
Dome
50%−epiboly
Shield
75%−epiboly
1−4−somites
14−19−somites
20−25−somites
Prim−5
Prim−15
Prim−25
Long−pec
Protruding−mouth
Day−4
Day−5
Stage
Transcript
ENSDART00000145958
ENSDART00000150399
ENSDART00000178828

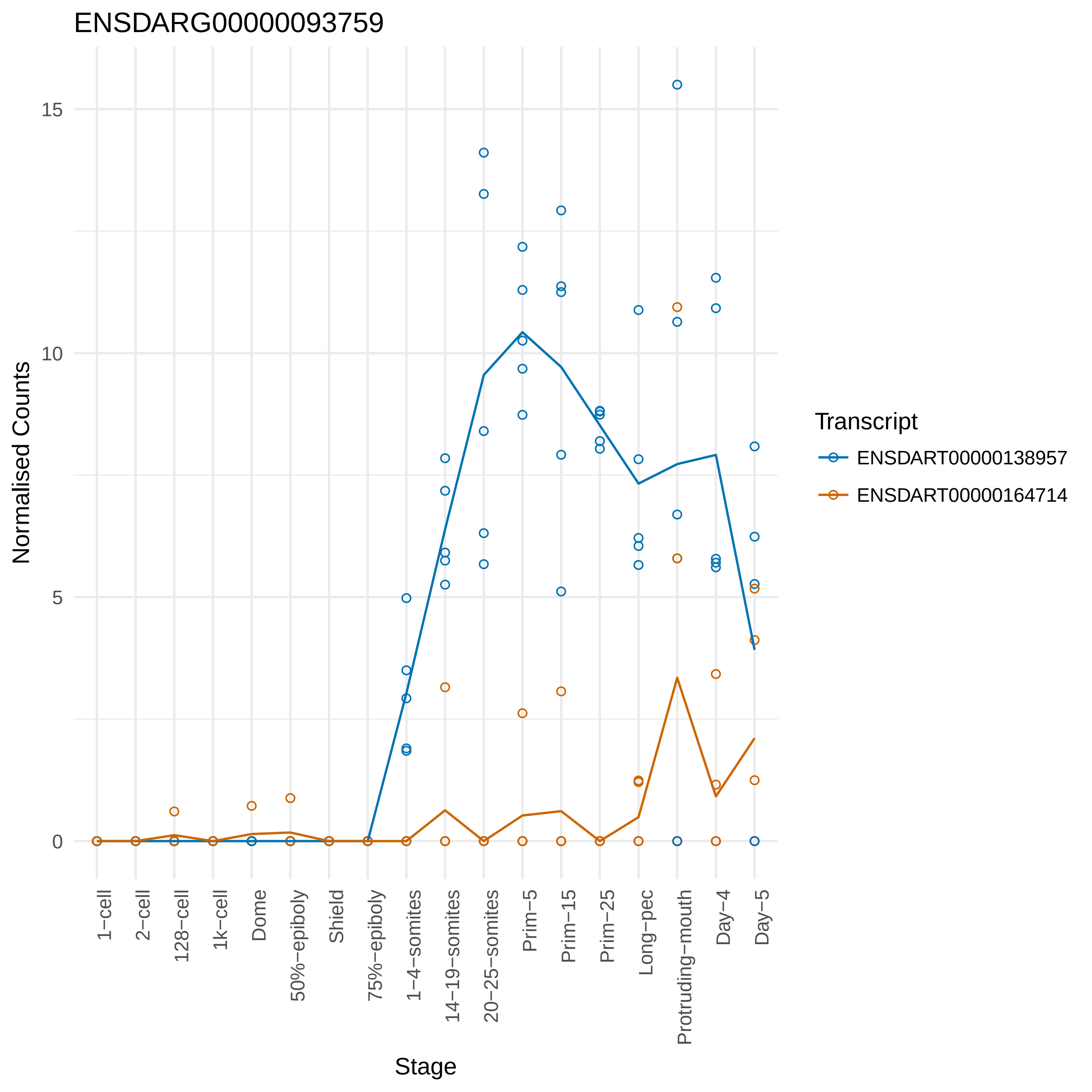
ENSDARG00000093759
Normalised Counts
15
10
5
0
1−cell
2−cell
128−cell
1k−cell
Dome
50%−epiboly
Shield
75%−epiboly
1−4−somites
14−19−somites
20−25−somites
Prim−5
Prim−15
Prim−25
Long−pec
Protruding−mouth
Day−4
Day−5
Stage
Transcript
ENSDART00000138957
ENSDART00000164714

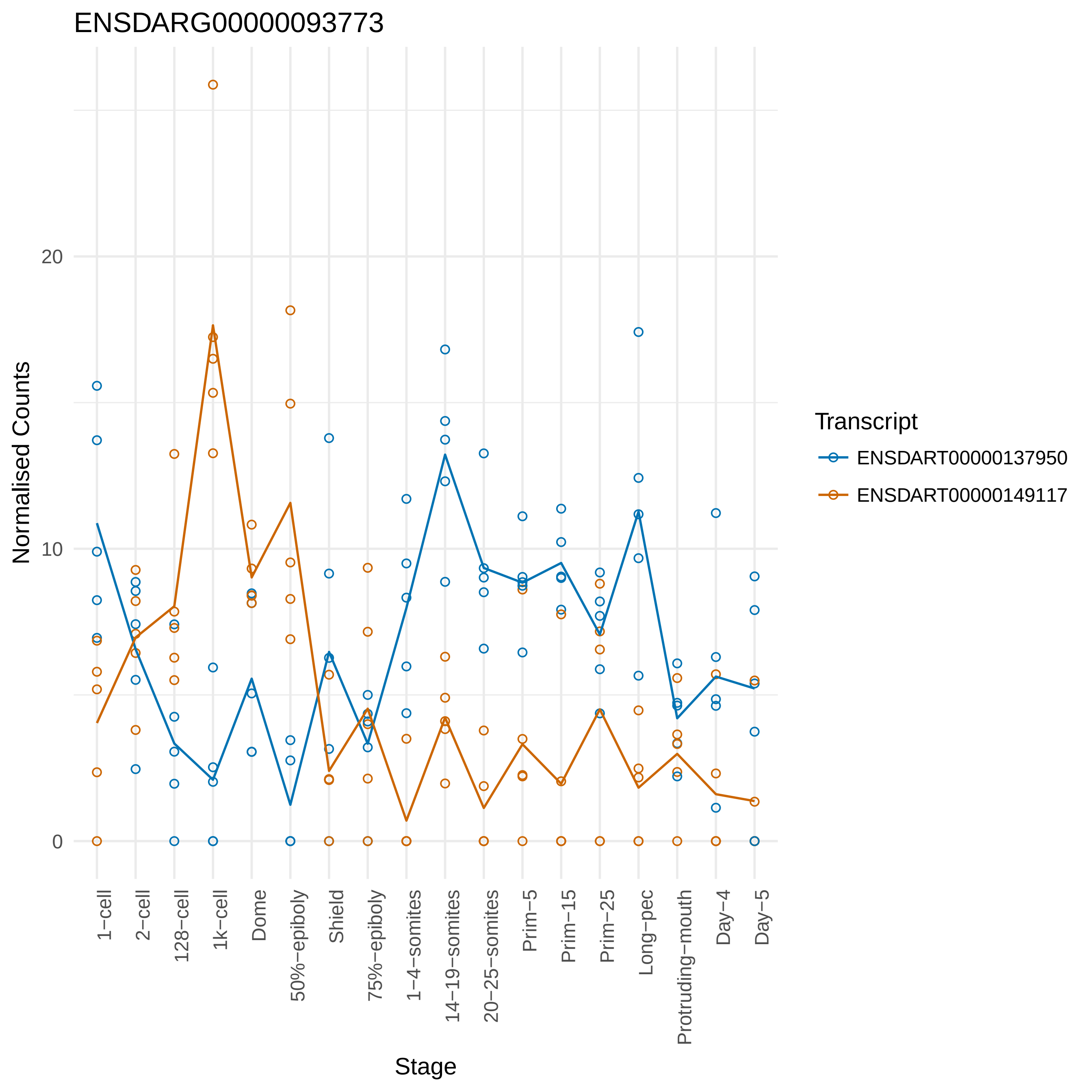

ENSDARG00000093773
Normalised Counts
0
10
20
1−cell
2−cell
128−cell
1k−cell
Dome
50%−epiboly
Shield
75%−epiboly
1−4−somites
14−19−somites
20−25−somites
Prim−5
Prim−15
Prim−25
Long−pec
Protruding−mouth
Day−4
Day−5
Stage
Transcript
ENSDART00000137950
ENSDART00000149117

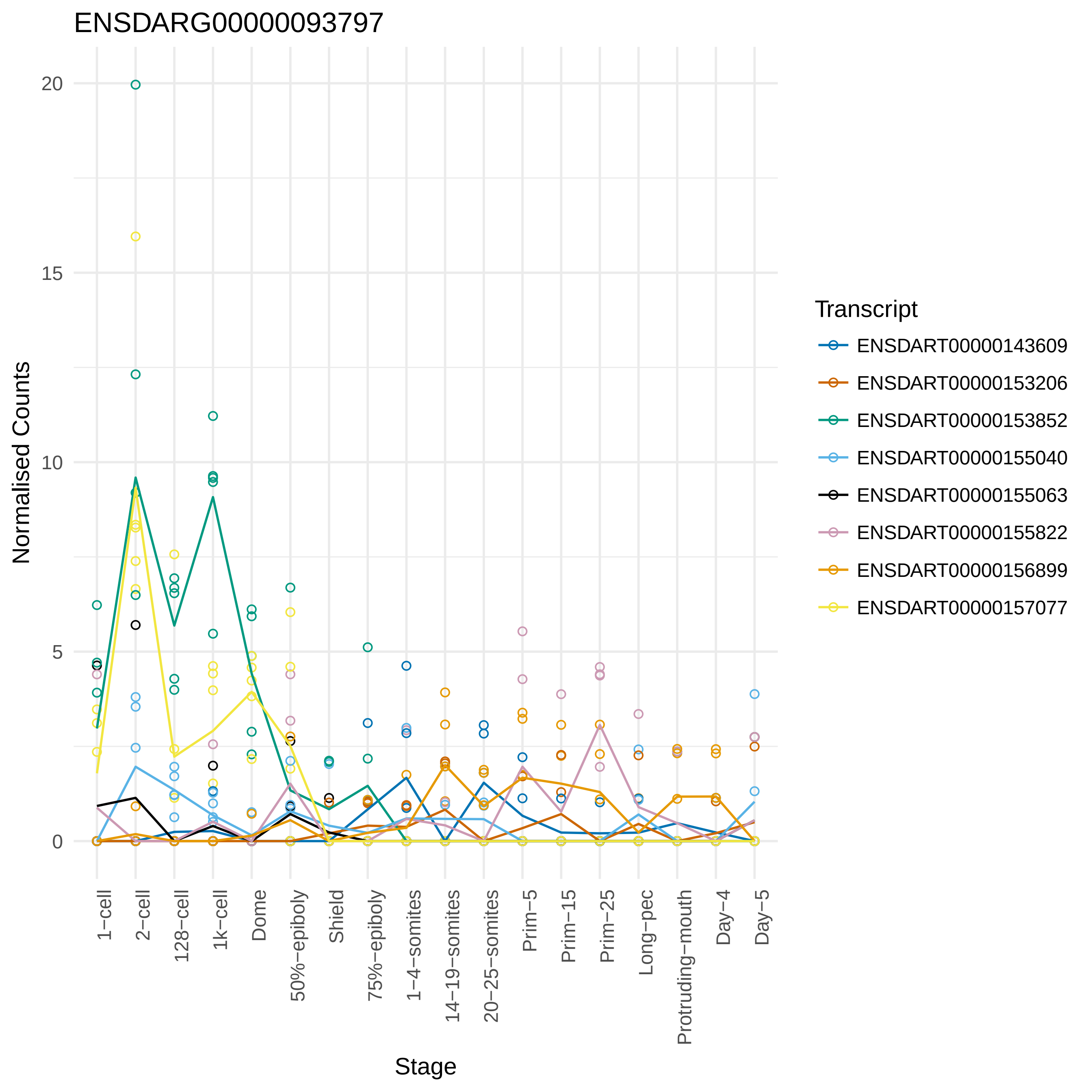

ENSDARG00000093797
Normalised Counts
0
5
10
15
20
1−cell
2−cell
128−cell
1k−cell
Dome
50%−epiboly
Shield
75%−epiboly
1−4−somites
14−19−somites
20−25−somites
Prim−5
Prim−15
Prim−25
Long−pec
Protruding−mouth
Day−4
Day−5
Stage
Transcript
ENSDART00000143609
ENSDART00000153206
ENSDART00000153852
ENSDART00000155040
ENSDART00000155063
ENSDART00000155822
ENSDART00000156899
ENSDART00000157077

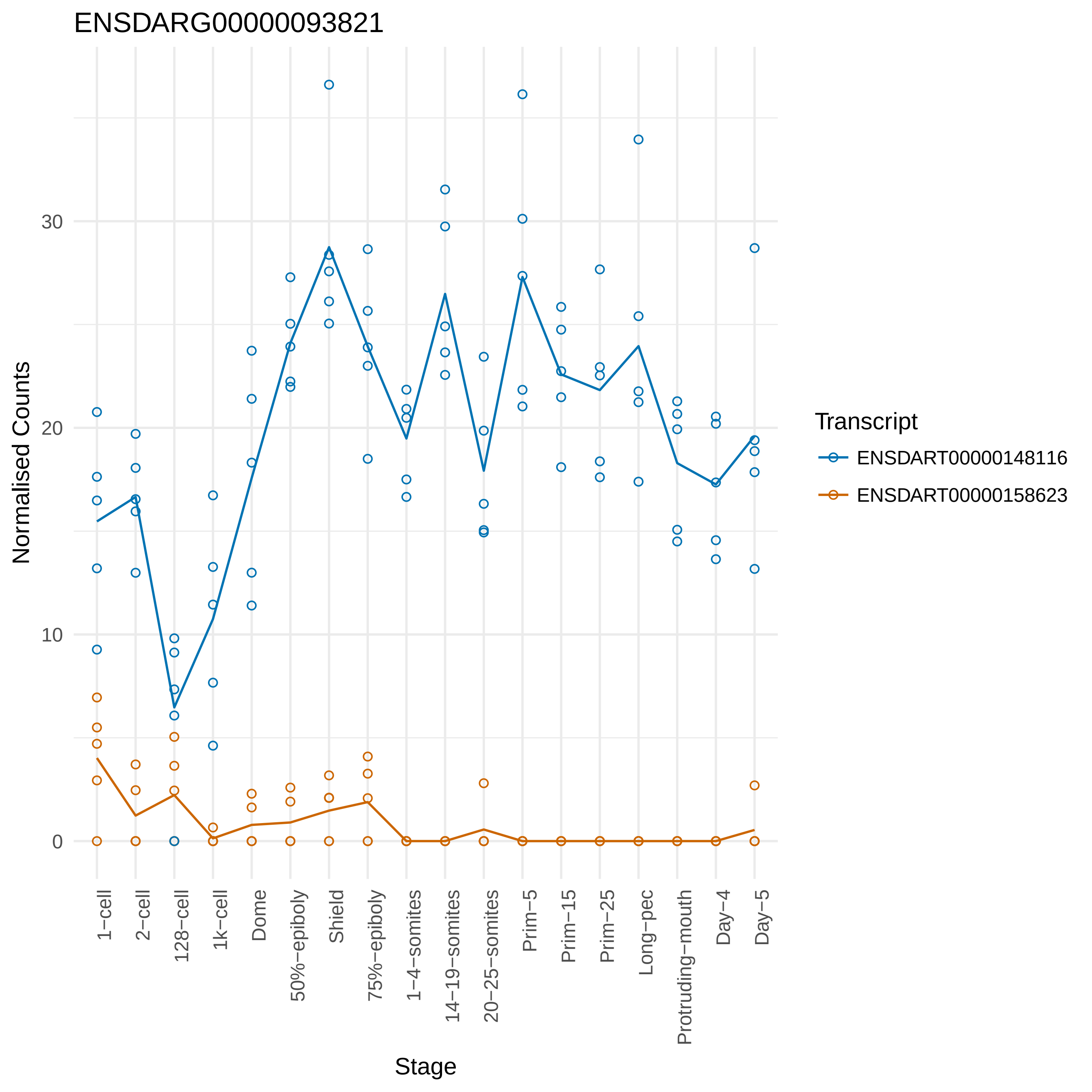
ENSDARG00000093821
Normalised Counts
0
10
20
30
Stage
1−cell
2−cell
128−cell
1k−cell
Dome
50%−epiboly
Shield
75%−epiboly
1−4−somites
14−19−somites
20−25−somites
Prim−5
Prim−15
Prim−25
Long−pec
Protruding−mouth
Day−4
Day−5
Transcript
ENSDART00000148116
ENSDART00000158623

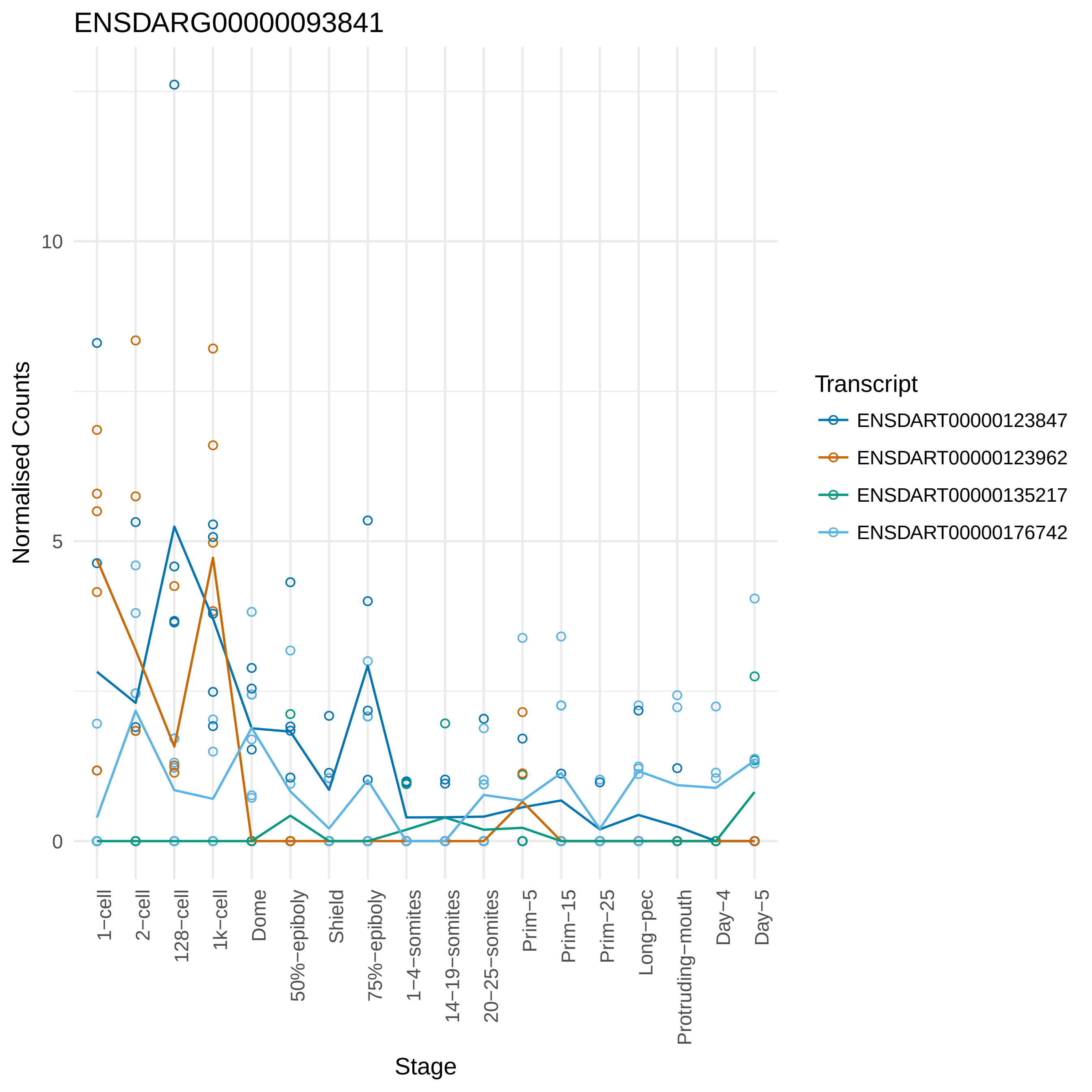

ENSDARG00000093841
Normalised Counts
0
5
10
Stage
1−cell
2−cell
128−cell
1k−cell
Dome
50%−epiboly
Shield
75%−epiboly
1−4−somites
14−19−somites
20−25−somites
Prim−5
Prim−15
Prim−25
Long−pec
Protruding−mouth
Day−4
Day−5
Transcript
ENSDART00000123847
ENSDART00000123962
ENSDART00000135217
ENSDART00000176742

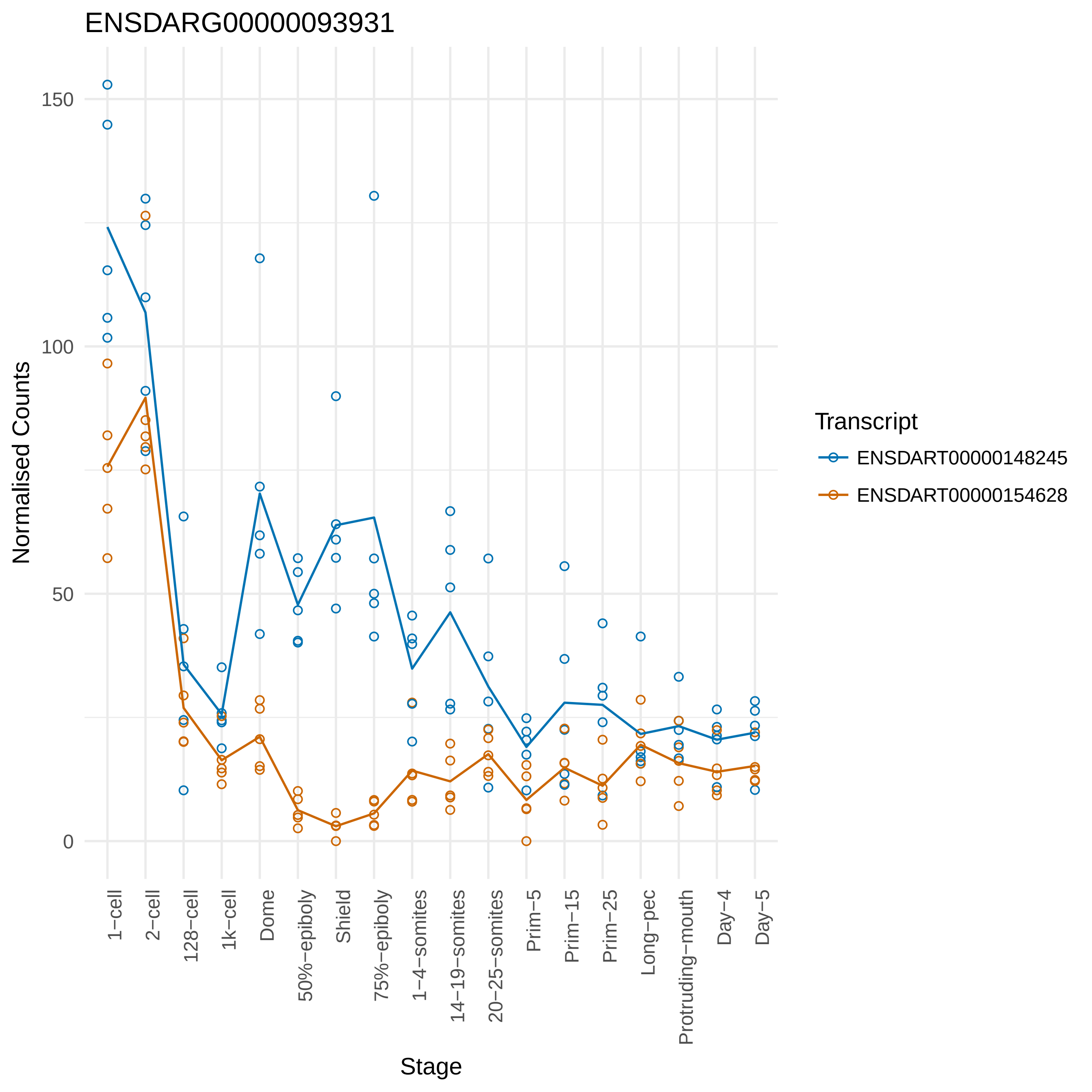
ENSDARG00000093931
Normalised Counts
150
100
50
0
1−cell
2−cell
128−cell
1k−cell
Dome
50%−epiboly
Shield
75%−epiboly
1−4−somites
14−19−somites
20−25−somites
Prim−5
Prim−15
Prim−25
Long−pec
Protruding−mouth
Day−4
Day−5
Stage
Transcript
ENSDART00000148245
ENSDART00000154628

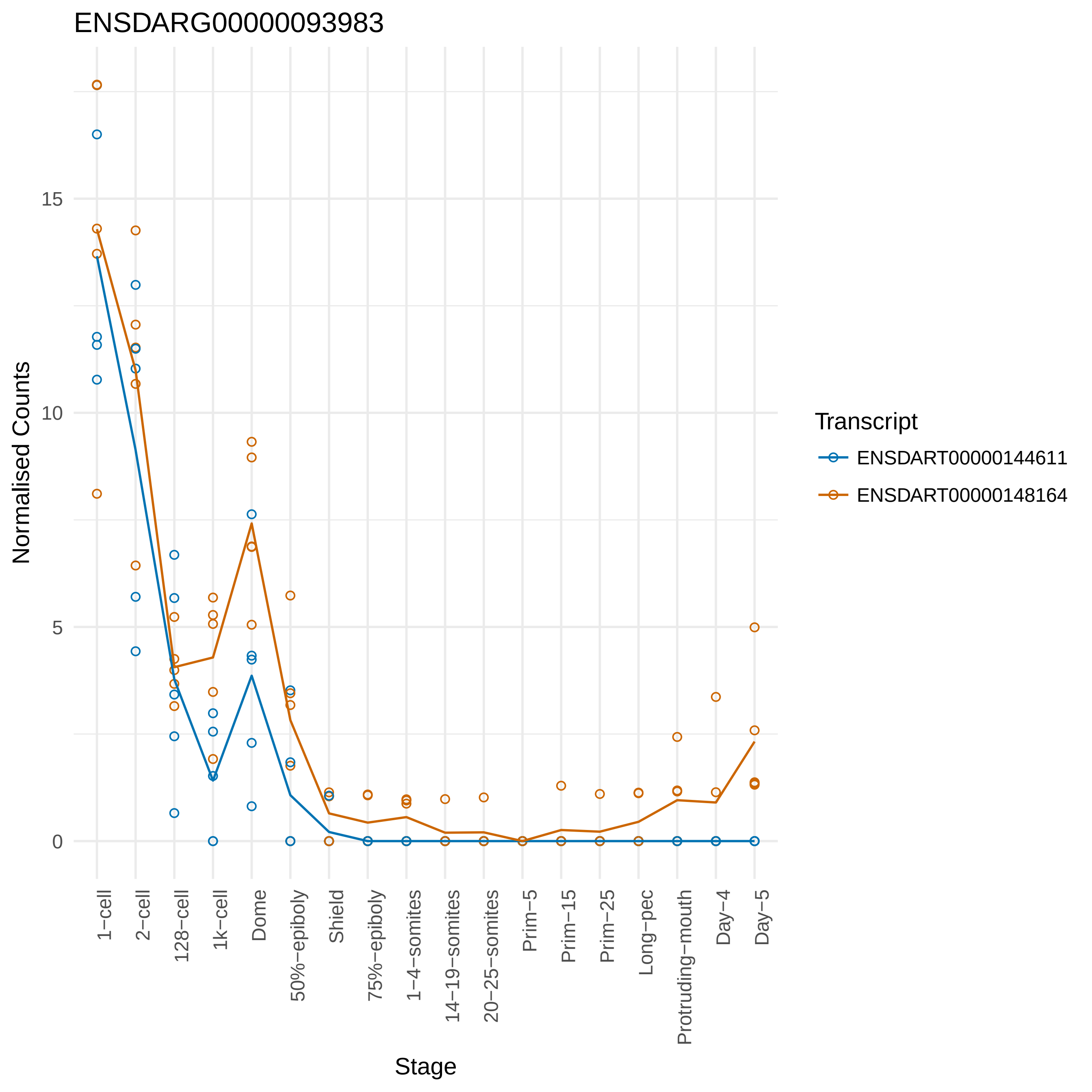
ENSDARG00000093983
Normalised Counts
0
5
10
15
Stage
1−cell
2−cell
128−cell
1k−cell
Dome
50%−epiboly
Shield
75%−epiboly
1−4−somites
14−19−somites
20−25−somites
Prim−5
Prim−15
Prim−25
Long−pec
Protruding−mouth
Day−4
Day−5
Transcript
ENSDART00000144611
ENSDART00000148164

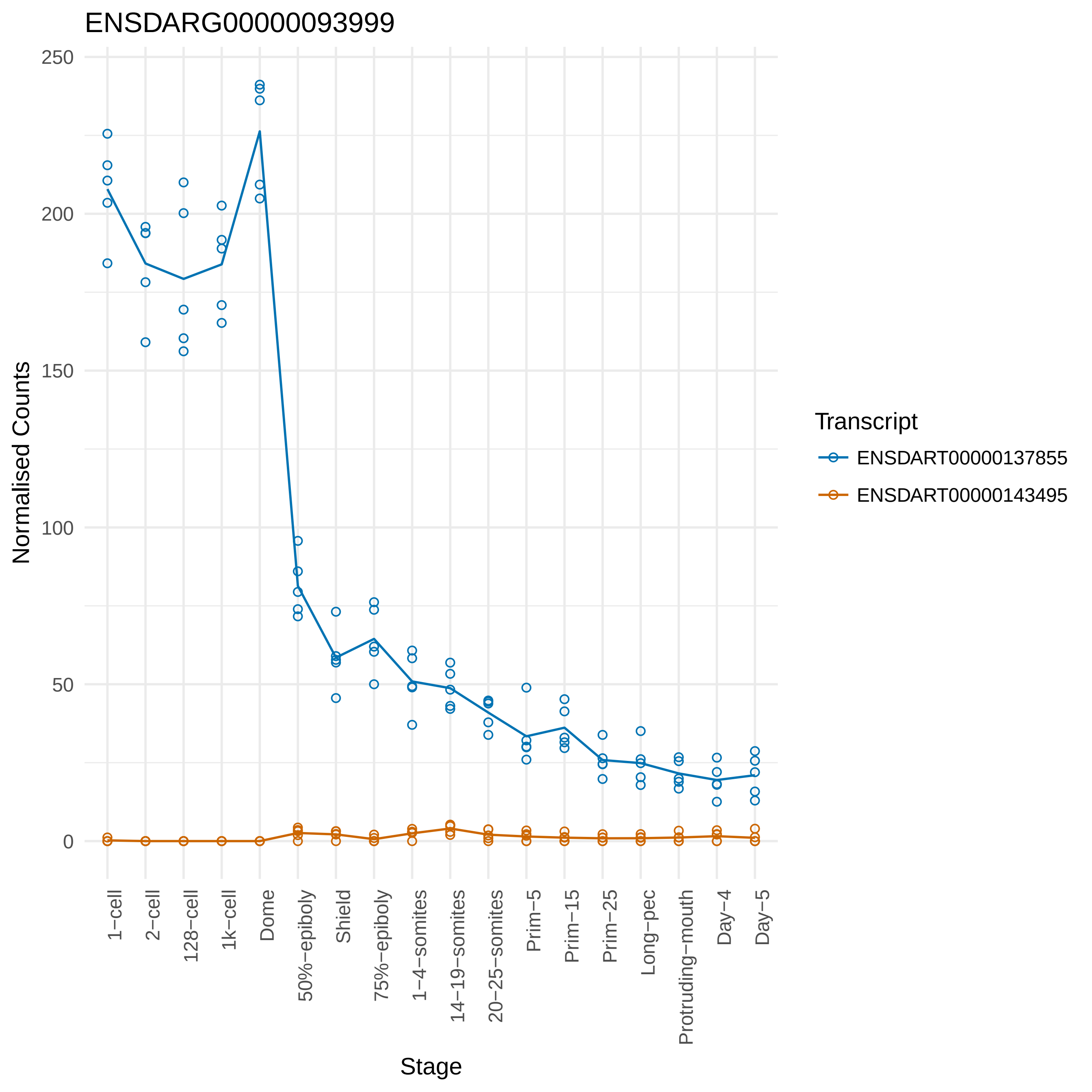

ENSDARG00000093999
Normalised Counts
250
200
150
100
50
0
1−cell
2−cell
128−cell
1k−cell
Dome
50%−epiboly
Shield
75%−epiboly
1−4−somites
14−19−somites
20−25−somites
Prim−5
Prim−15
Prim−25
Long−pec
Protruding−mouth
Day−4
Day−5
Stage
Transcript
ENSDART00000137855
ENSDART00000143495

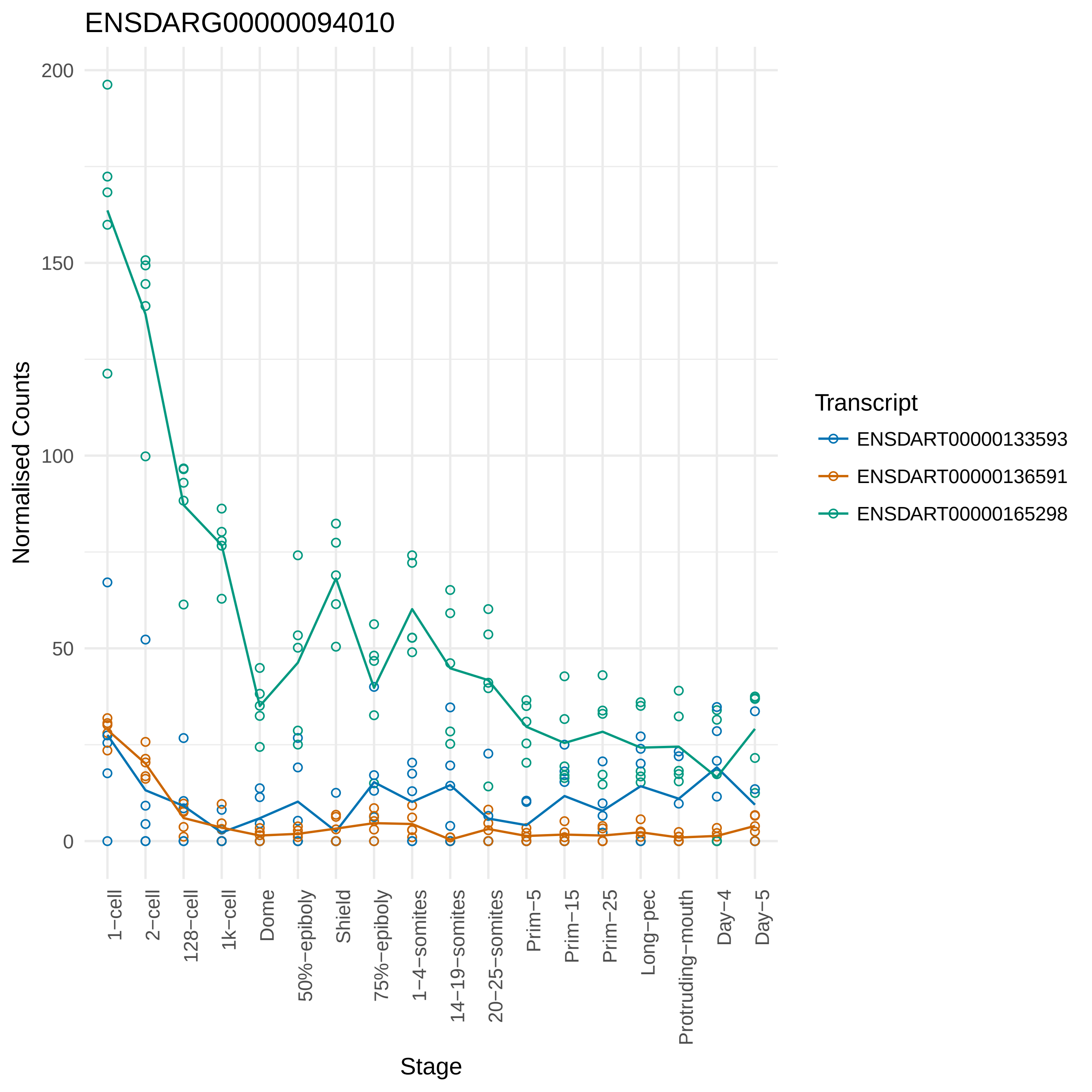

ENSDARG00000094010
Normalised Counts
200
150
100
50
0
1−cell
2−cell
128−cell
1k−cell
Dome
50%−epiboly
Shield
75%−epiboly
1−4−somites
14−19−somites
20−25−somites
Prim−5
Prim−15
Prim−25
Long−pec
Protruding−mouth
Day−4
Day−5
Stage
Transcript
ENSDART00000133593
ENSDART00000136591
ENSDART00000165298

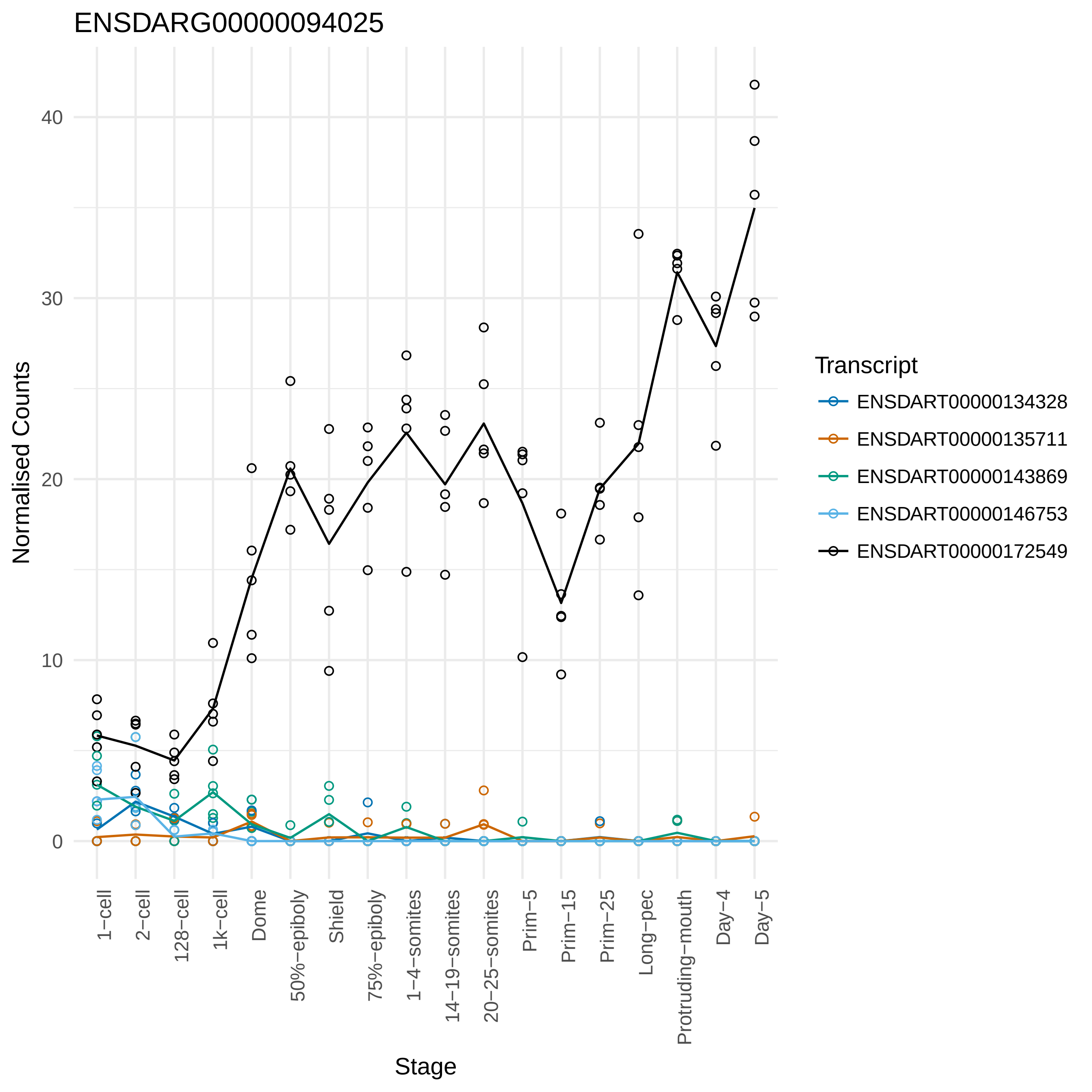

ENSDARG00000094025
Normalised Counts
0
10
20
30
40
Stage
1−cell
2−cell
128−cell
1k−cell
Dome
50%−epiboly
Shield
75%−epiboly
1−4−somites
14−19−somites
20−25−somites
Prim−5
Prim−15
Prim−25
Long−pec
Protruding−mouth
Day−4
Day−5
Transcript
ENSDART00000134328
ENSDART00000135711
ENSDART00000143869
ENSDART00000146753
ENSDART00000172549

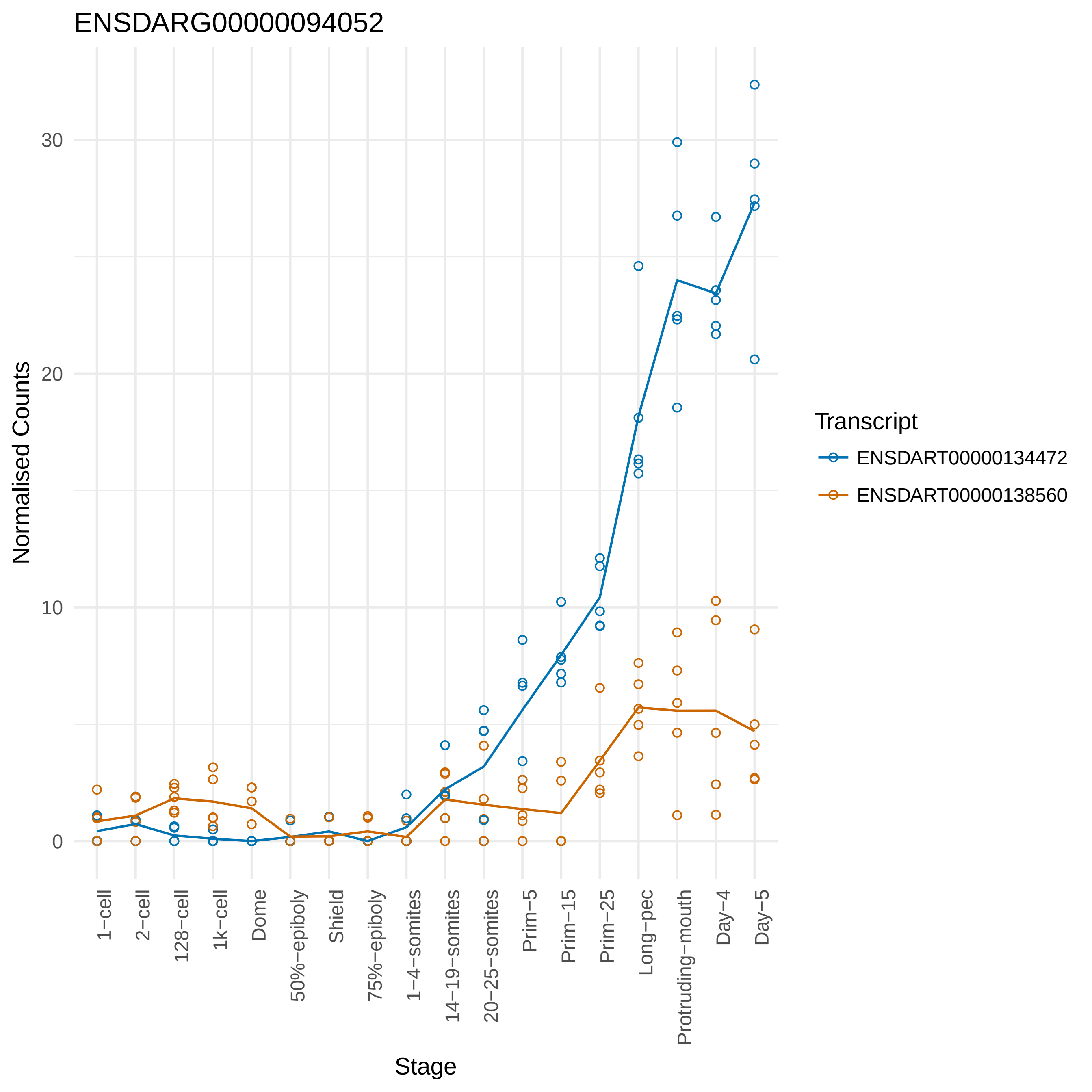

ENSDARG00000094052
Normalised Counts
0
10
20
30
1−cell
2−cell
128−cell
1k−cell
Dome
50%−epiboly
Shield
75%−epiboly
1−4−somites
14−19−somites
20−25−somites
Prim−5
Prim−15
Prim−25
Long−pec
Protruding−mouth
Day−4
Day−5
Stage
Transcript
ENSDART00000134472
ENSDART00000138560

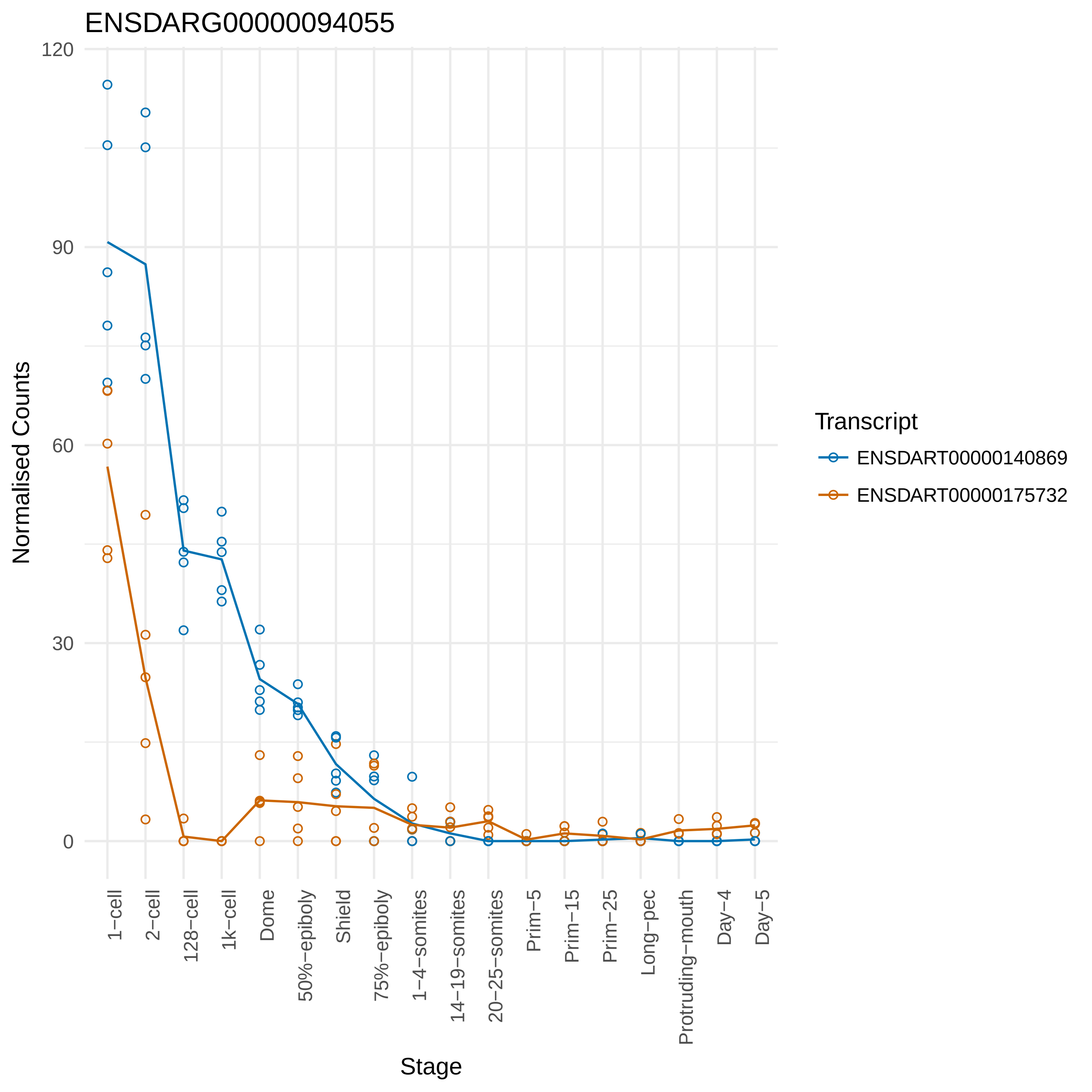

ENSDARG00000094055
Normalised Counts
120
90
60
30
0
1−cell
2−cell
128−cell
1k−cell
Dome
50%−epiboly
Shield
75%−epiboly
1−4−somites
14−19−somites
20−25−somites
Prim−5
Prim−15
Prim−25
Long−pec
Protruding−mouth
Day−4
Day−5
Stage
Transcript
ENSDART00000140869
ENSDART00000175732

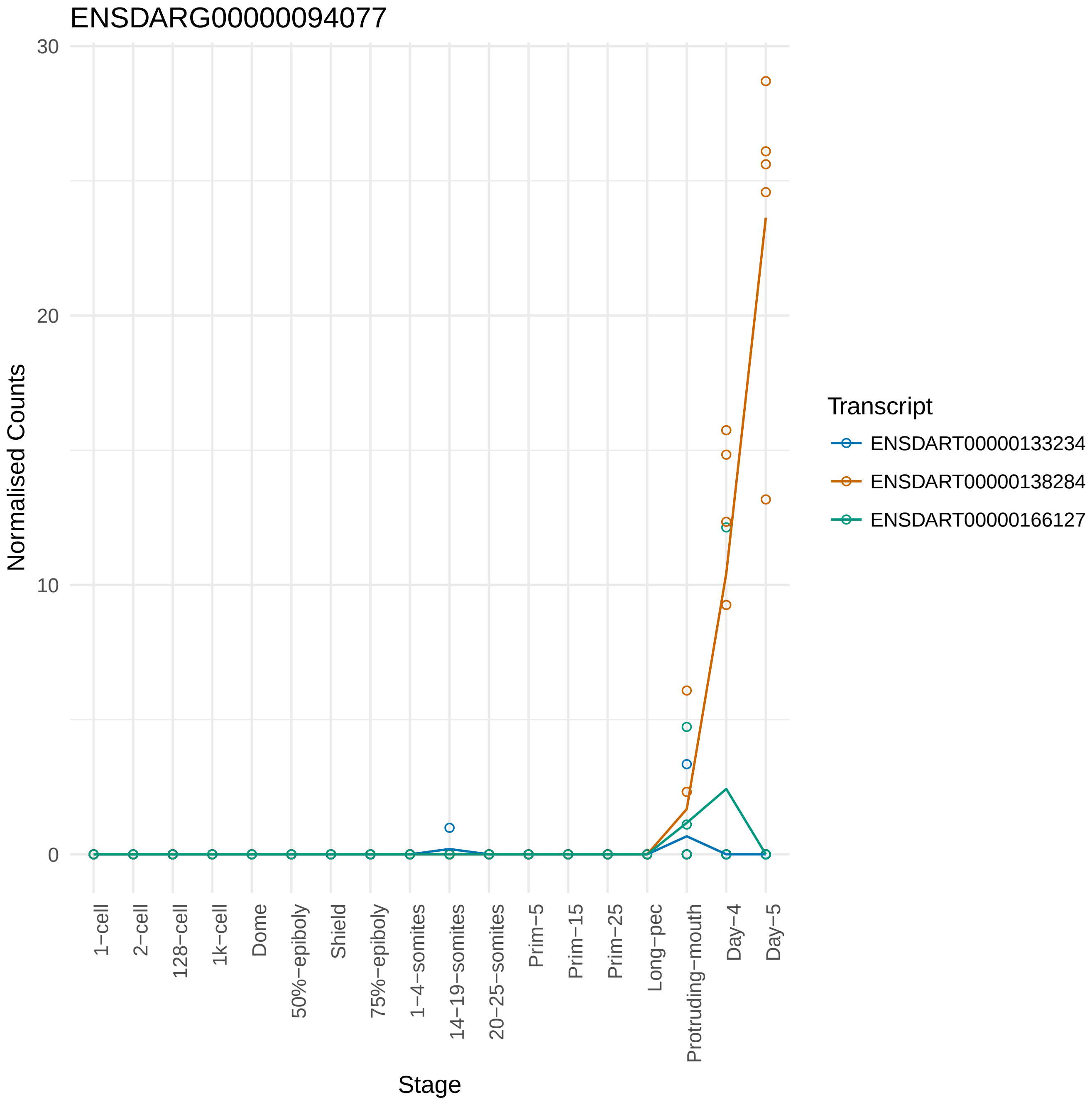
ENSDARG00000094077
Normalised Counts
30
20
10
0
1−cell
2−cell
128−cell
1k−cell
Dome
50%−epiboly
Shield
75%−epiboly
1−4−somites
14−19−somites
20−25−somites
Prim−5
Prim−15
Prim−25
Long−pec
Protruding−mouth
Day−4
Day−5
Stage
Transcript
ENSDART00000133234
ENSDART00000138284
ENSDART00000166127

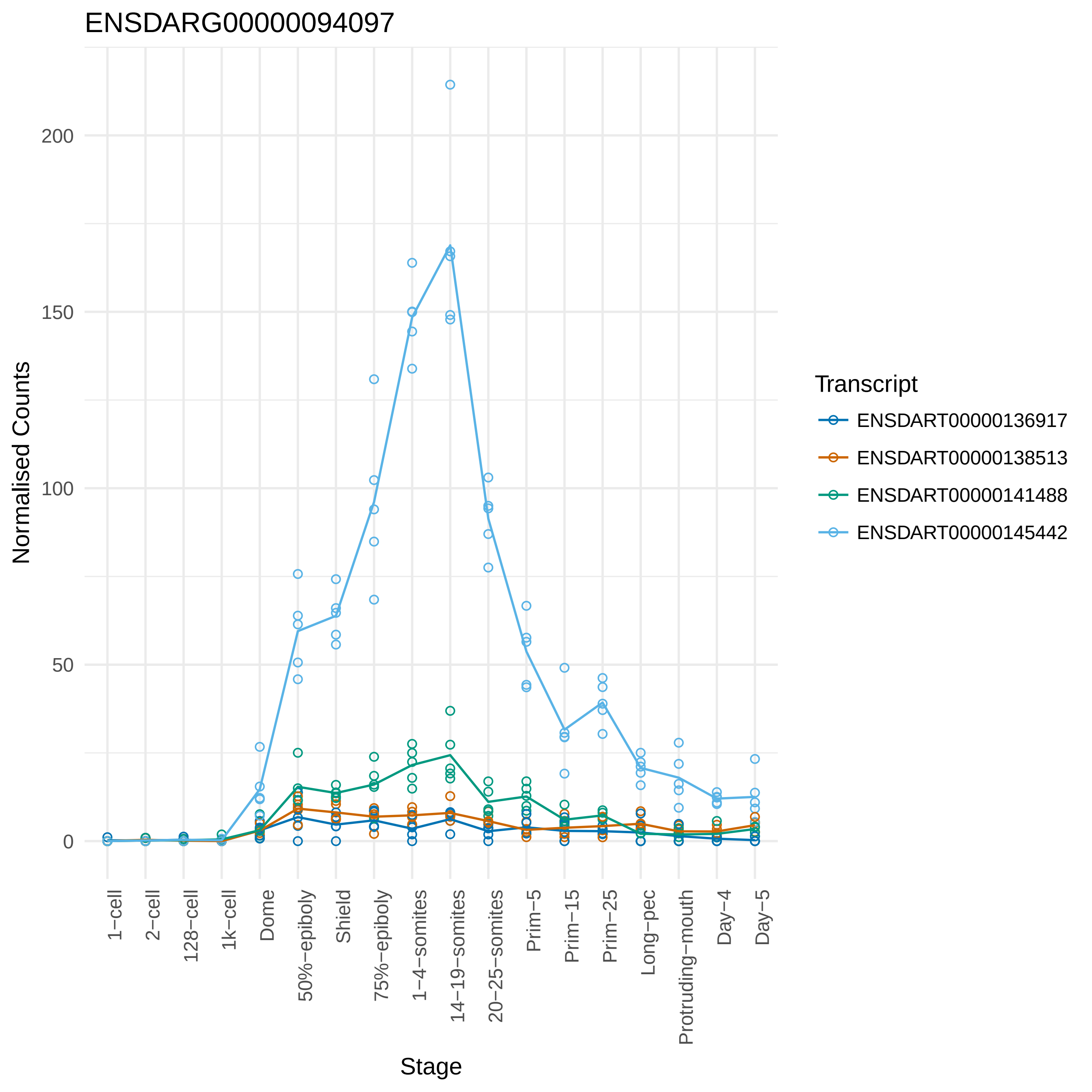

ENSDARG00000094097
Normalised Counts
0
50
100
150
200
1−cell
2−cell
128−cell
1k−cell
Dome
50%−epiboly
Shield
75%−epiboly
1−4−somites
14−19−somites
20−25−somites
Prim−5
Prim−15
Prim−25
Long−pec
Protruding−mouth
Day−4
Day−5
Stage
Transcript
ENSDART00000136917
ENSDART00000138513
ENSDART00000141488
ENSDART00000145442

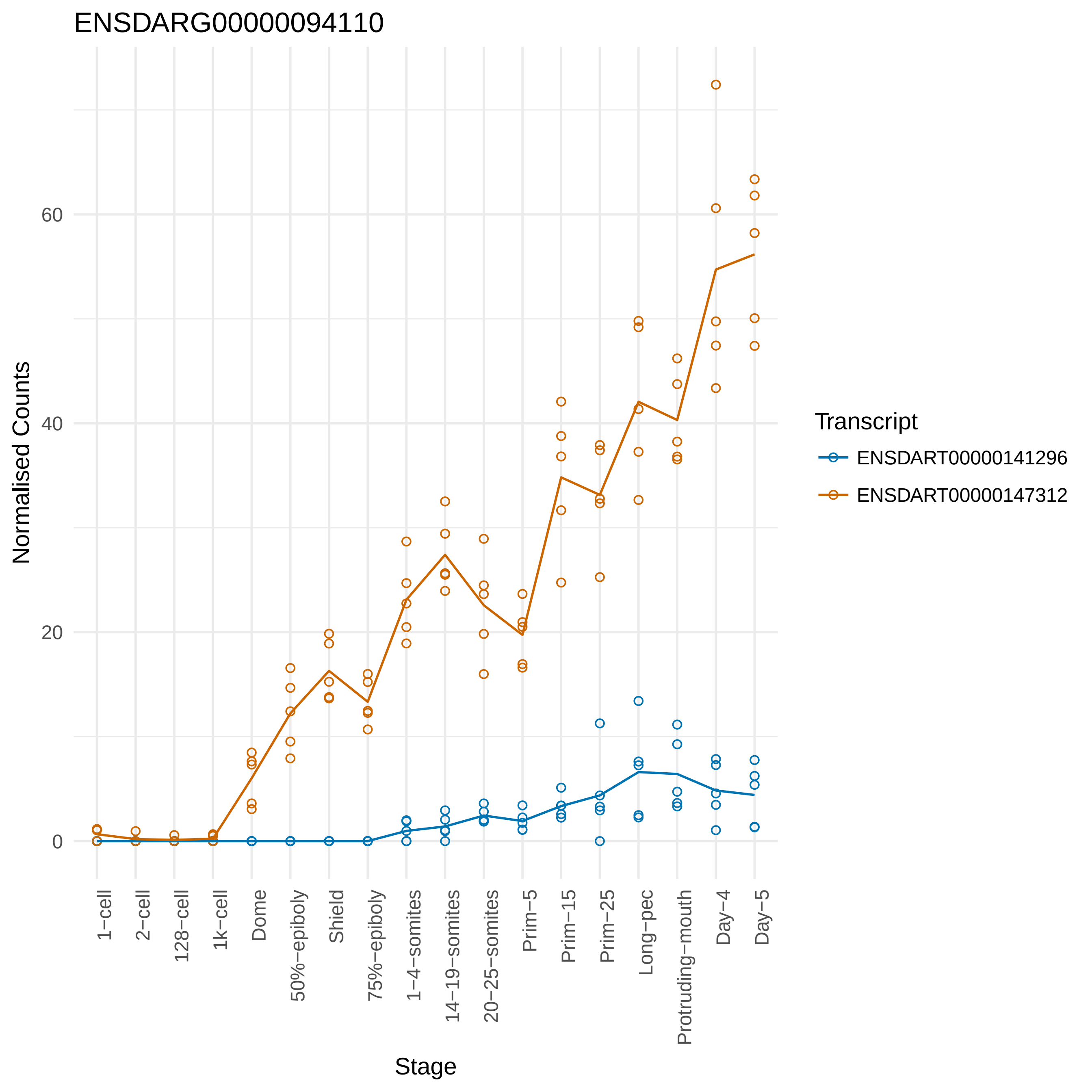

ENSDARG00000094110
Normalised Counts
60
40
20
0
1−cell
2−cell
128−cell
1k−cell
Dome
50%−epiboly
Shield
75%−epiboly
1−4−somites
14−19−somites
20−25−somites
Prim−5
Prim−15
Prim−25
Long−pec
Protruding−mouth
Day−4
Day−5
Stage
Transcript
ENSDART00000141296
ENSDART00000147312

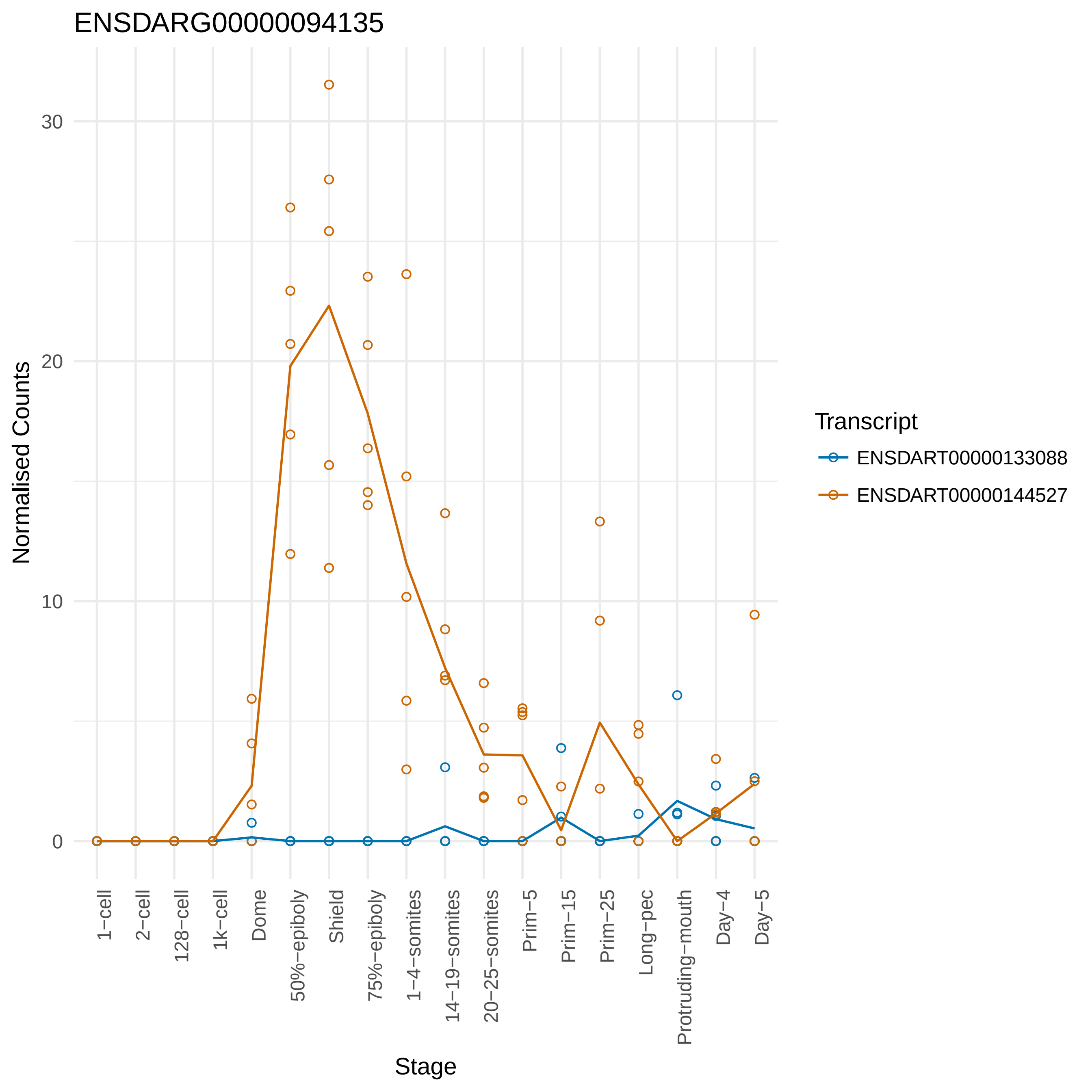

ENSDARG00000094135
Normalised Counts
0
10
20
30
1−cell
2−cell
128−cell
1k−cell
Dome
50%−epiboly
Shield
75%−epiboly
1−4−somites
14−19−somites
20−25−somites
Prim−5
Prim−15
Prim−25
Long−pec
Protruding−mouth
Day−4
Day−5
Stage
Transcript
ENSDART00000133088
ENSDART00000144527

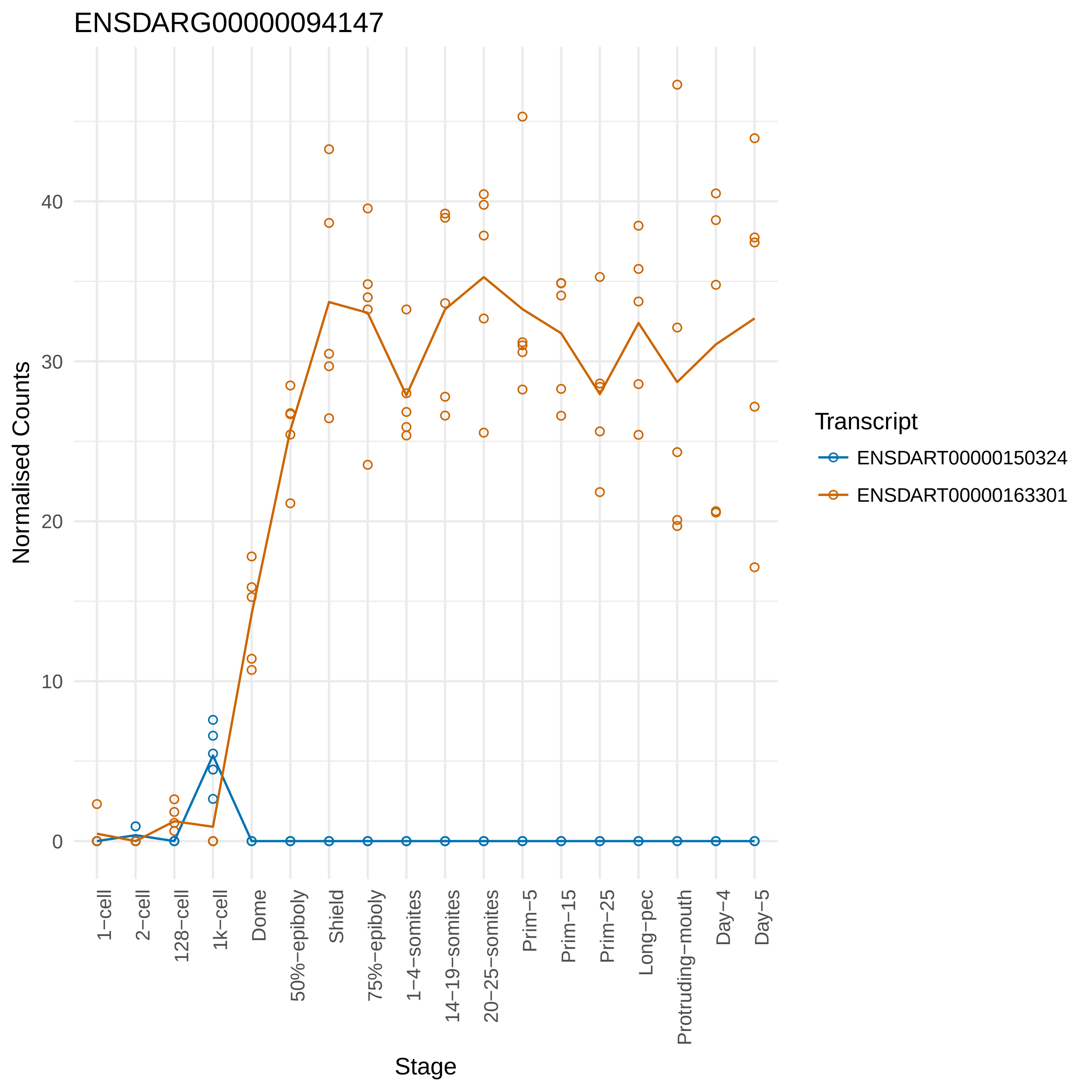
ENSDARG00000094147
Normalised Counts
0
10
20
30
40
Stage
1−cell
2−cell
128−cell
1k−cell
Dome
50%−epiboly
Shield
75%−epiboly
1−4−somites
14−19−somites
20−25−somites
Prim−5
Prim−15
Prim−25
Long−pec
Protruding−mouth
Day−4
Day−5
Transcript
ENSDART00000150324
ENSDART00000163301

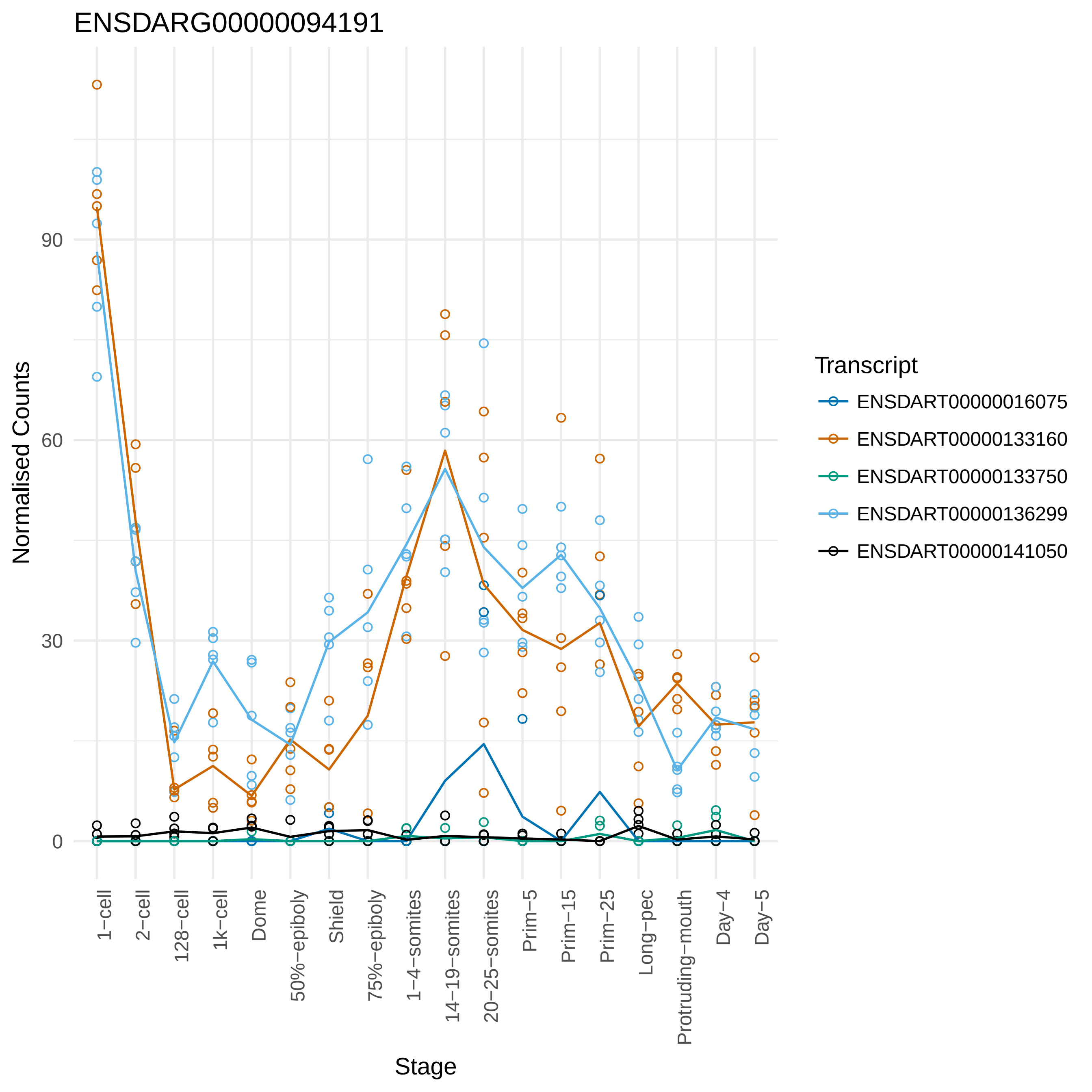

ENSDARG00000094191
Normalised Counts
90
60
30
0
1−cell
2−cell
128−cell
1k−cell
Dome
50%−epiboly
Shield
75%−epiboly
1−4−somites
14−19−somites
20−25−somites
Prim−5
Prim−15
Prim−25
Long−pec
Protruding−mouth
Day−4
Day−5
Stage
Transcript
ENSDART00000016075
ENSDART00000133160
ENSDART00000133750
ENSDART00000136299
ENSDART00000141050

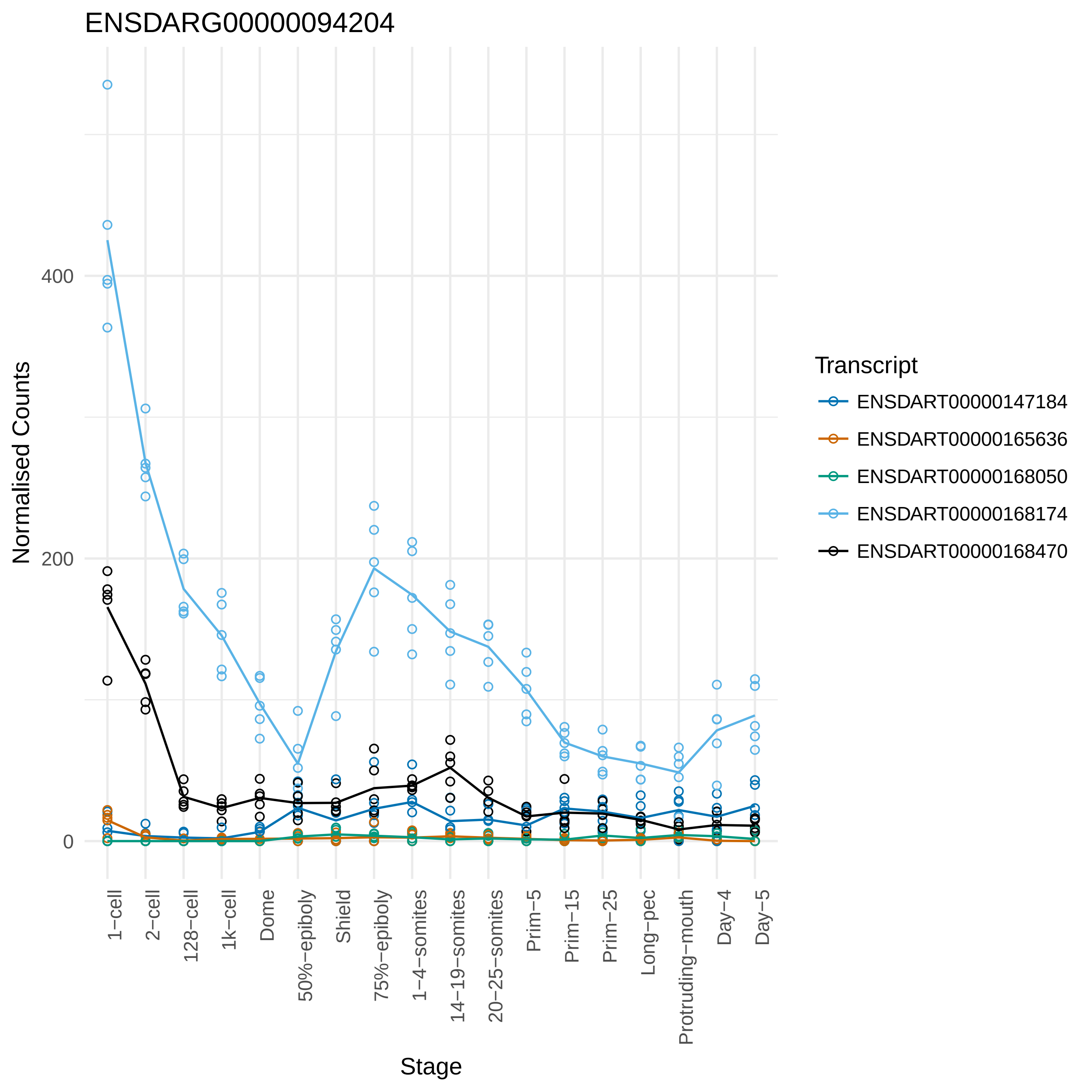

ENSDARG00000094204
Normalised Counts
400
200
0
1−cell
2−cell
128−cell
1k−cell
Dome
50%−epiboly
Shield
75%−epiboly
1−4−somites
14−19−somites
20−25−somites
Prim−5
Prim−15
Prim−25
Long−pec
Protruding−mouth
Day−4
Day−5
Stage
Transcript
ENSDART00000147184
ENSDART00000165636
ENSDART00000168050
ENSDART00000168174
ENSDART00000168470

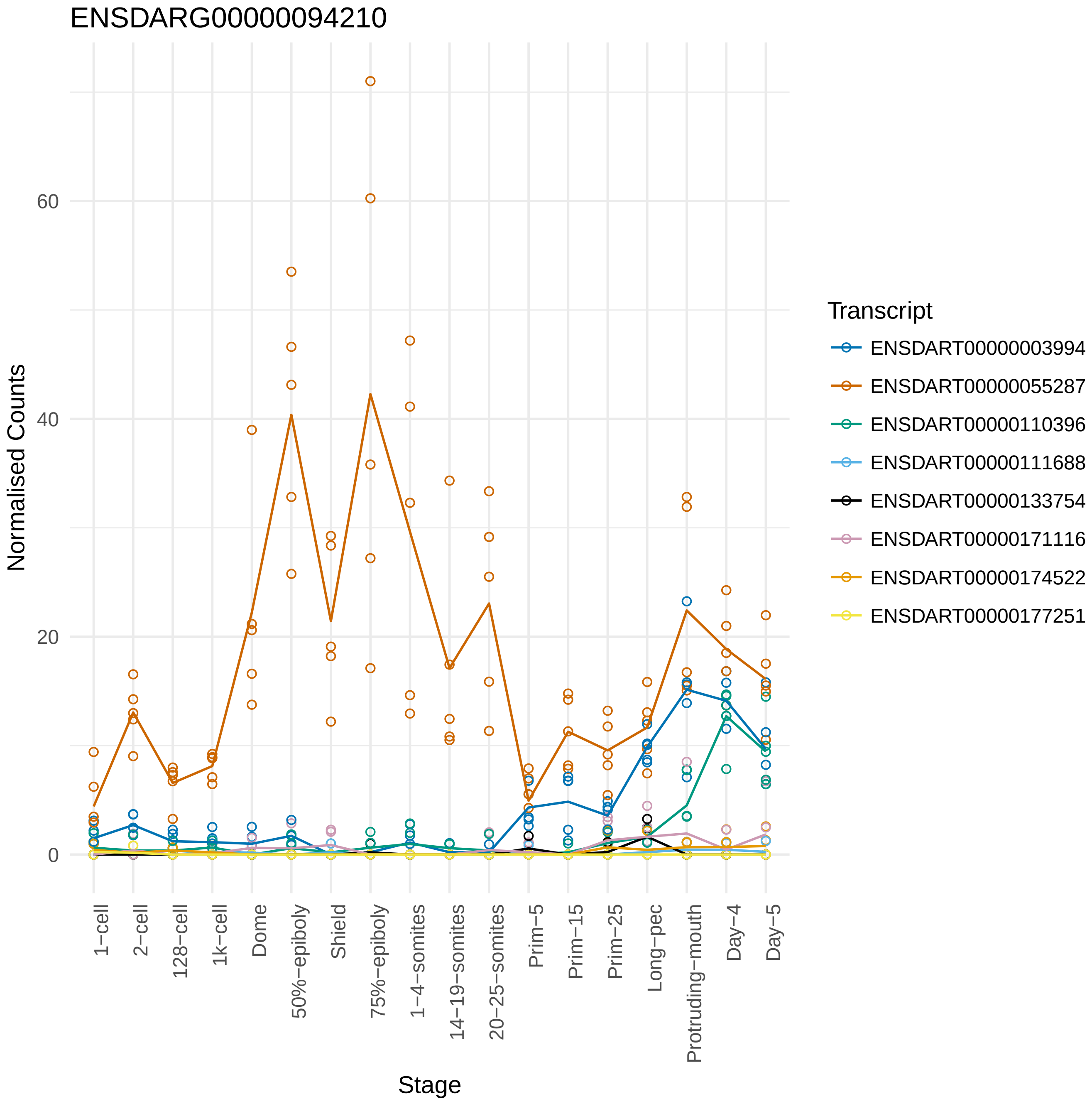

ENSDARG00000094210
Normalised Counts
0
20
40
60
1−cell
2−cell
128−cell
1k−cell
Dome
50%−epiboly
Shield
75%−epiboly
1−4−somites
14−19−somites
20−25−somites
Prim−5
Prim−15
Prim−25
Long−pec
Protruding−mouth
Day−4
Day−5
Stage
Transcript
ENSDART00000003994
ENSDART00000055287
ENSDART00000110396
ENSDART00000111688
ENSDART00000133754
ENSDART00000171116
ENSDART00000174522
ENSDART00000177251

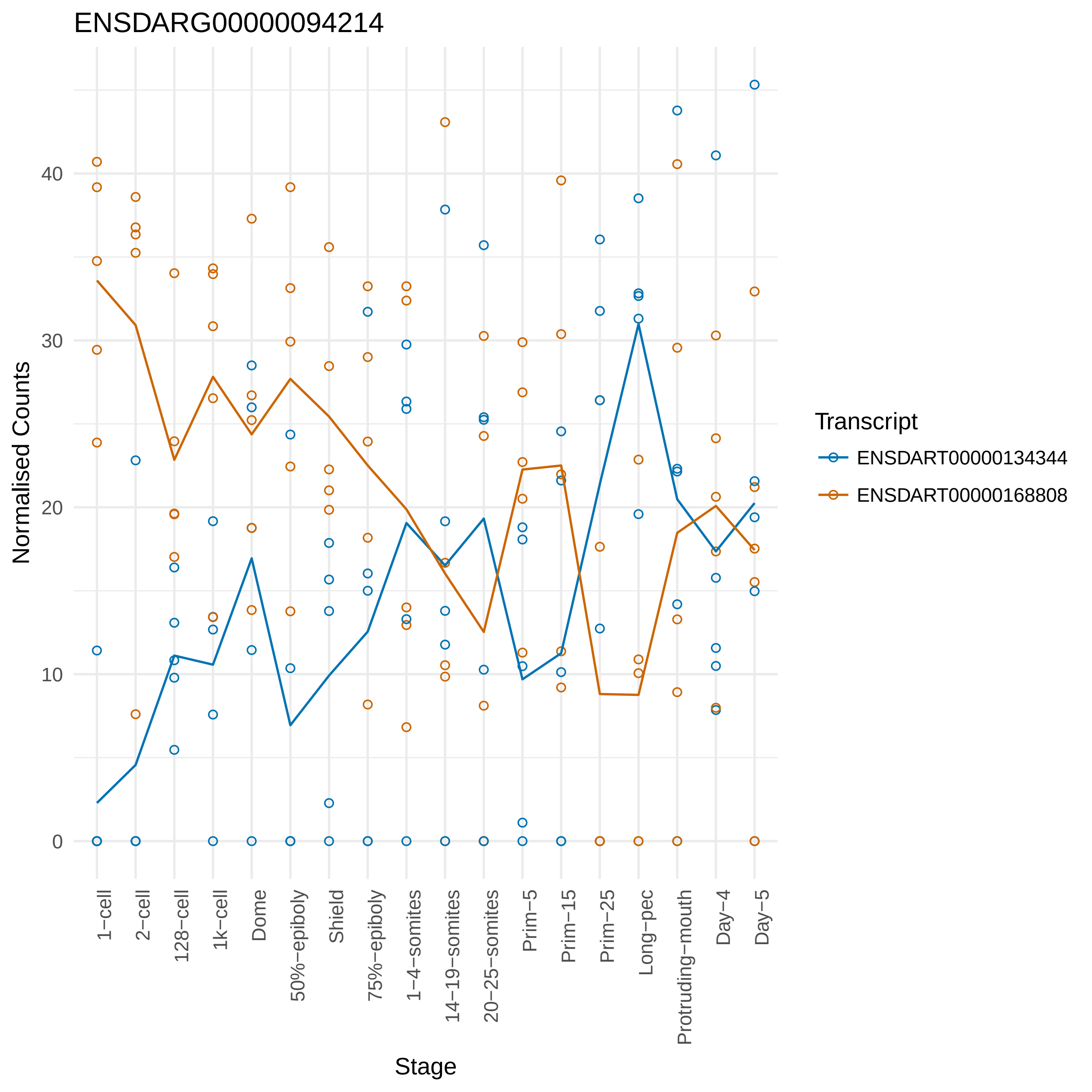
ENSDARG00000094214
Normalised Counts
40
30
20
10
0
1−cell
2−cell
128−cell
1k−cell
Dome
50%−epiboly
Shield
75%−epiboly
1−4−somites
14−19−somites
20−25−somites
Prim−5
Prim−15
Prim−25
Long−pec
Protruding−mouth
Day−4
Day−5
Stage
Transcript
ENSDART00000134344
ENSDART00000168808

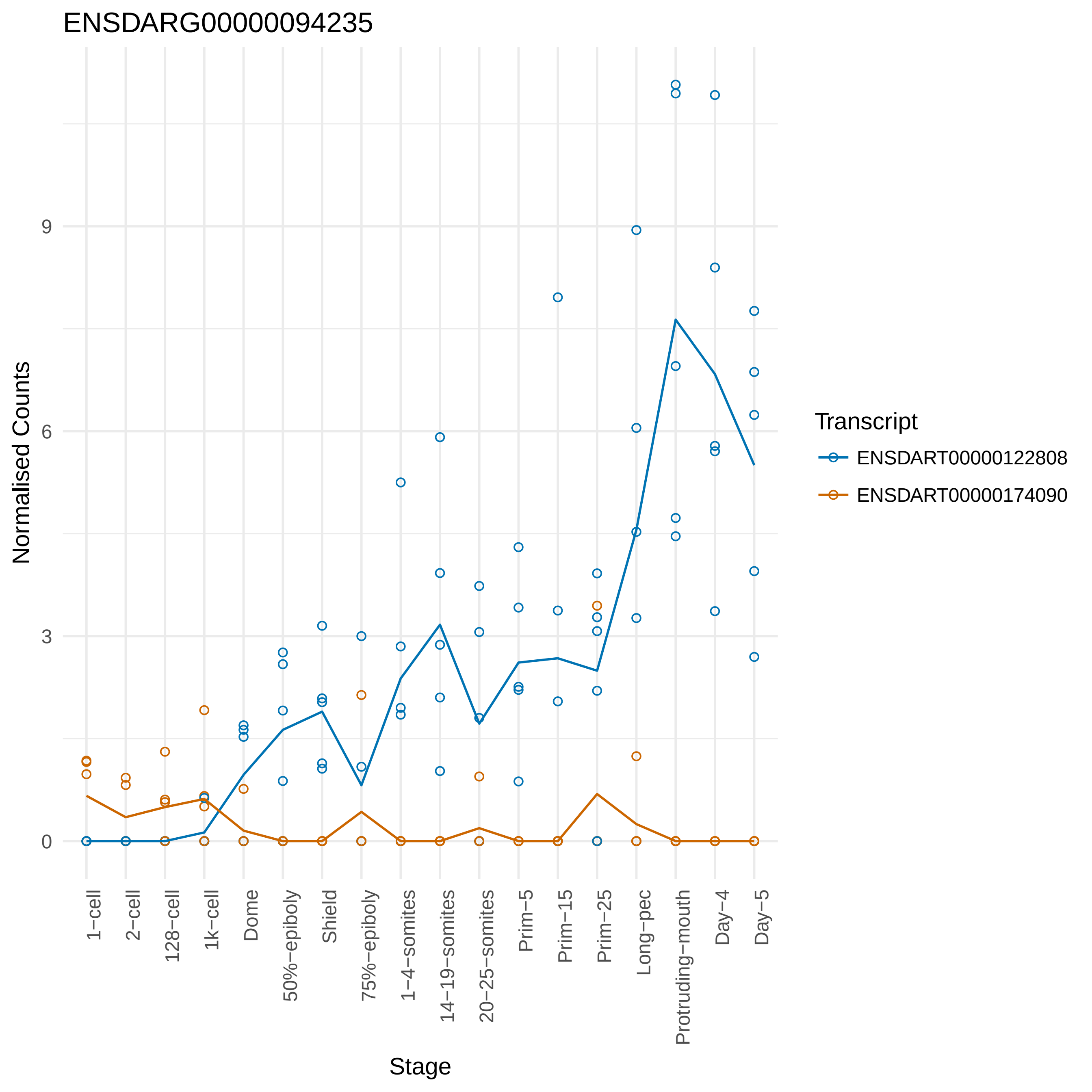

ENSDARG00000094235
Normalised Counts
0
3
6
9
Stage
1−cell
2−cell
128−cell
1k−cell
Dome
50%−epiboly
Shield
75%−epiboly
1−4−somites
14−19−somites
20−25−somites
Prim−5
Prim−15
Prim−25
Long−pec
Protruding−mouth
Day−4
Day−5
Transcript
ENSDART00000122808
ENSDART00000174090

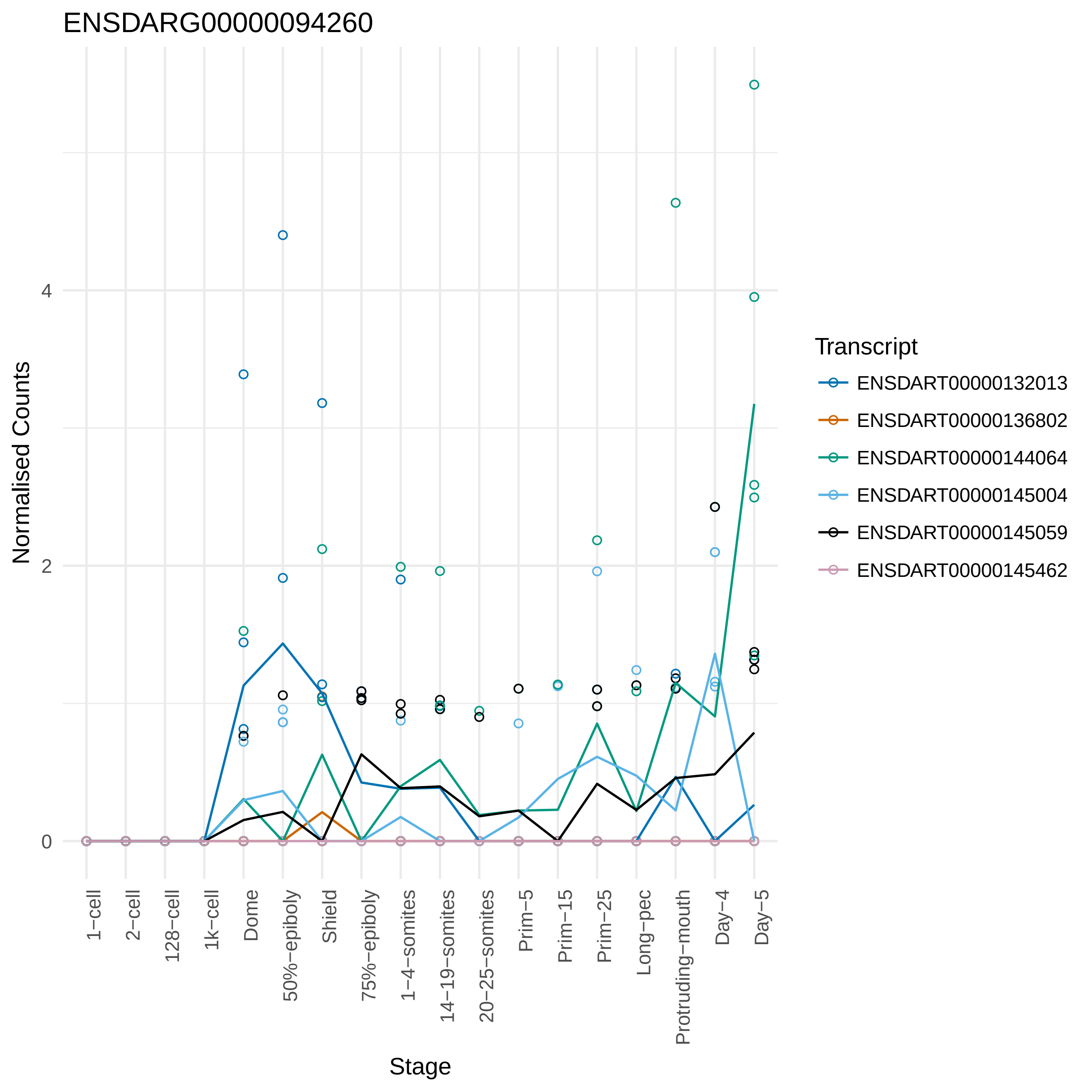
ENSDARG00000094260
Normalised Counts
0
2
4
Stage
1−cell
2−cell
128−cell
1k−cell
Dome
50%−epiboly
Shield
75%−epiboly
1−4−somites
14−19−somites
20−25−somites
Prim−5
Prim−15
Prim−25
Long−pec
Protruding−mouth
Day−4
Day−5
Transcript
ENSDART00000132013
ENSDART00000136802
ENSDART00000144064
ENSDART00000145004
ENSDART00000145059
ENSDART00000145462

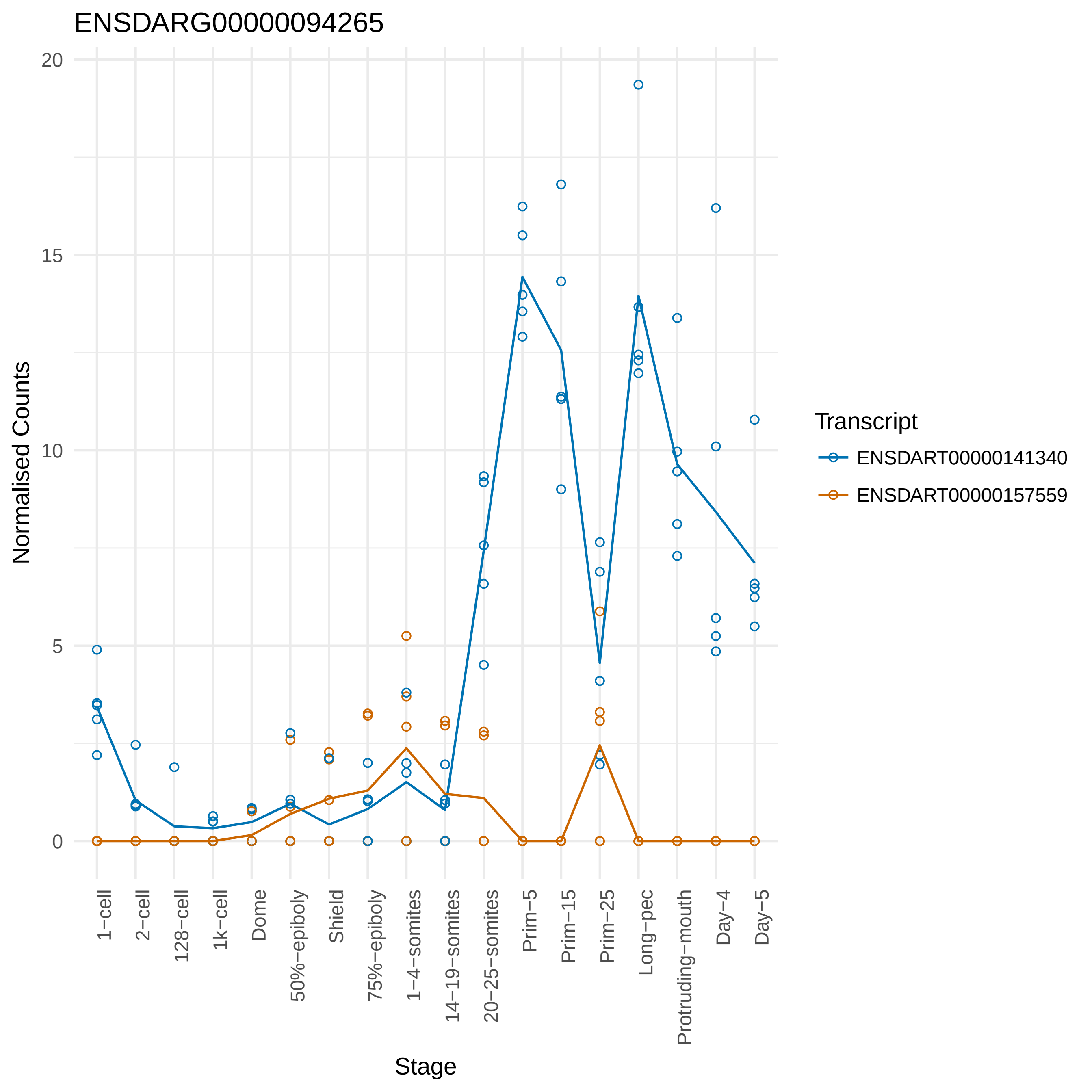

ENSDARG00000094265
Normalised Counts
20
15
10
5
0
1−cell
2−cell
128−cell
1k−cell
Dome
50%−epiboly
Shield
75%−epiboly
1−4−somites
14−19−somites
20−25−somites
Prim−5
Prim−15
Prim−25
Long−pec
Protruding−mouth
Day−4
Day−5
Stage
Transcript
ENSDART00000141340
ENSDART00000157559

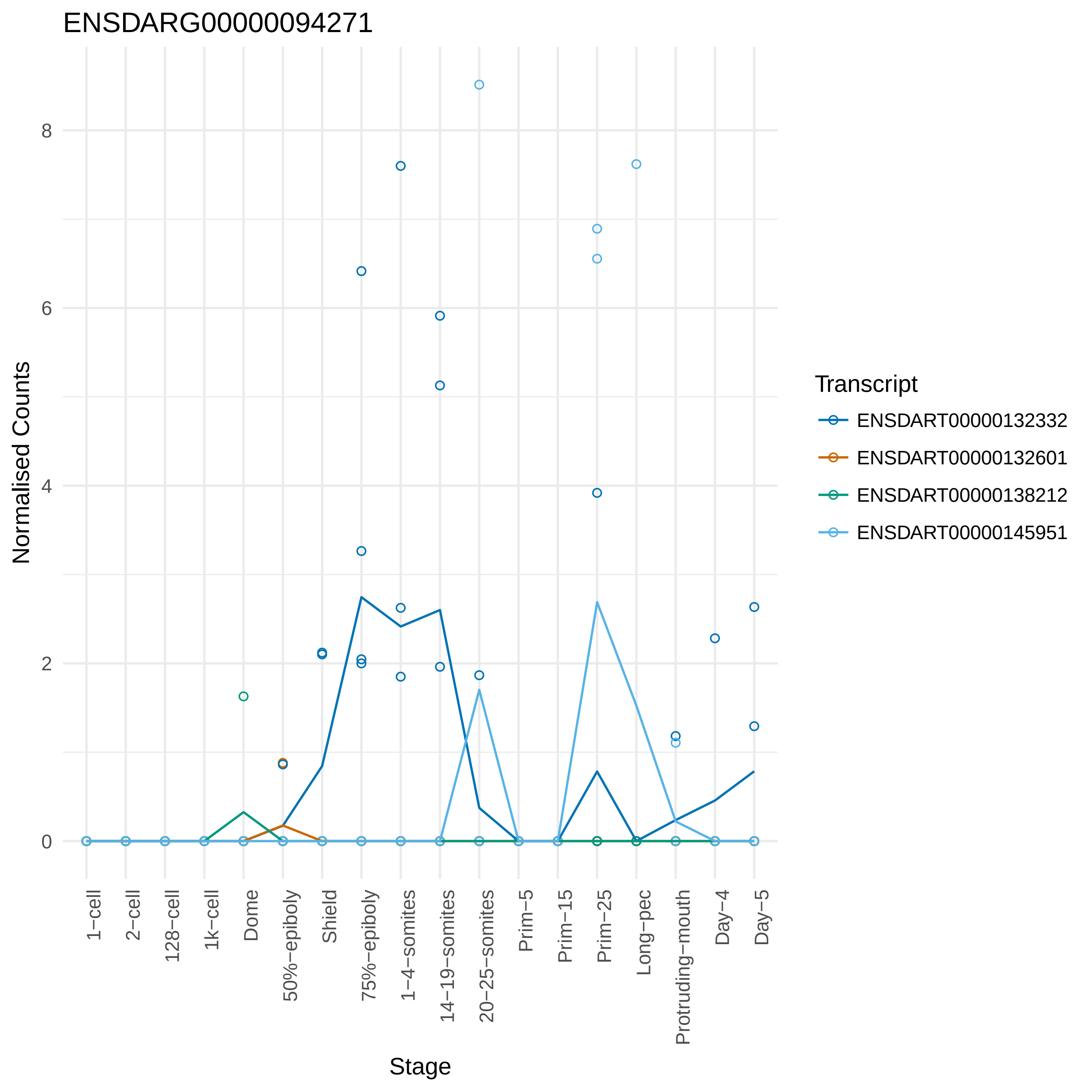

ENSDARG00000094271
Normalised Counts
8
6
4
2
0
1−cell
2−cell
128−cell
1k−cell
Dome
50%−epiboly
Shield
75%−epiboly
1−4−somites
14−19−somites
20−25−somites
Prim−5
Prim−15
Prim−25
Long−pec
Protruding−mouth
Day−4
Day−5
Stage
Transcript
ENSDART00000132332
ENSDART00000132601
ENSDART00000138212
ENSDART00000145951

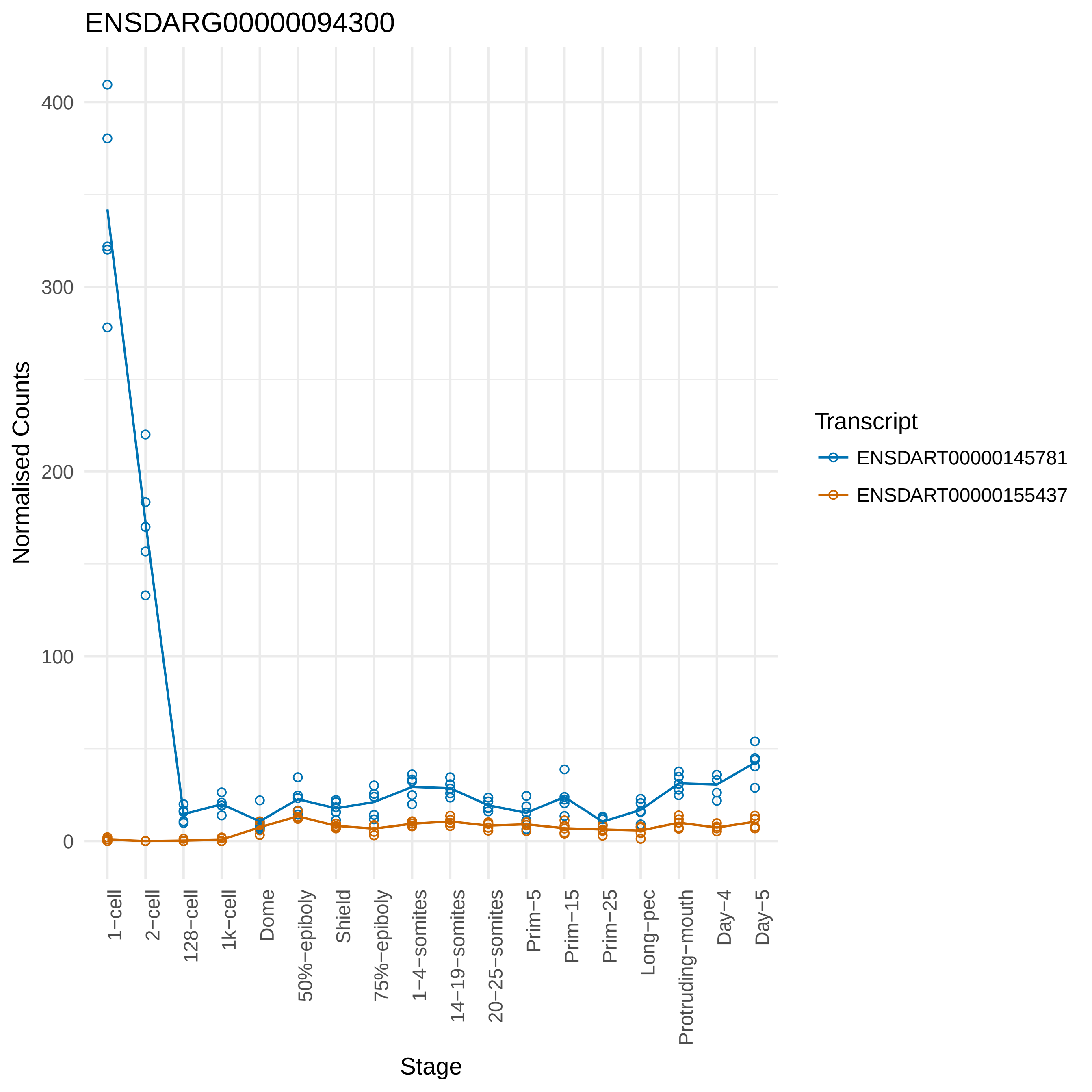

ENSDARG00000094300
Normalised Counts
400
300
200
100
0
1−cell
2−cell
128−cell
1k−cell
Dome
50%−epiboly
Shield
75%−epiboly
1−4−somites
14−19−somites
20−25−somites
Prim−5
Prim−15
Prim−25
Long−pec
Protruding−mouth
Day−4
Day−5
Stage
Transcript
ENSDART00000145781
ENSDART00000155437

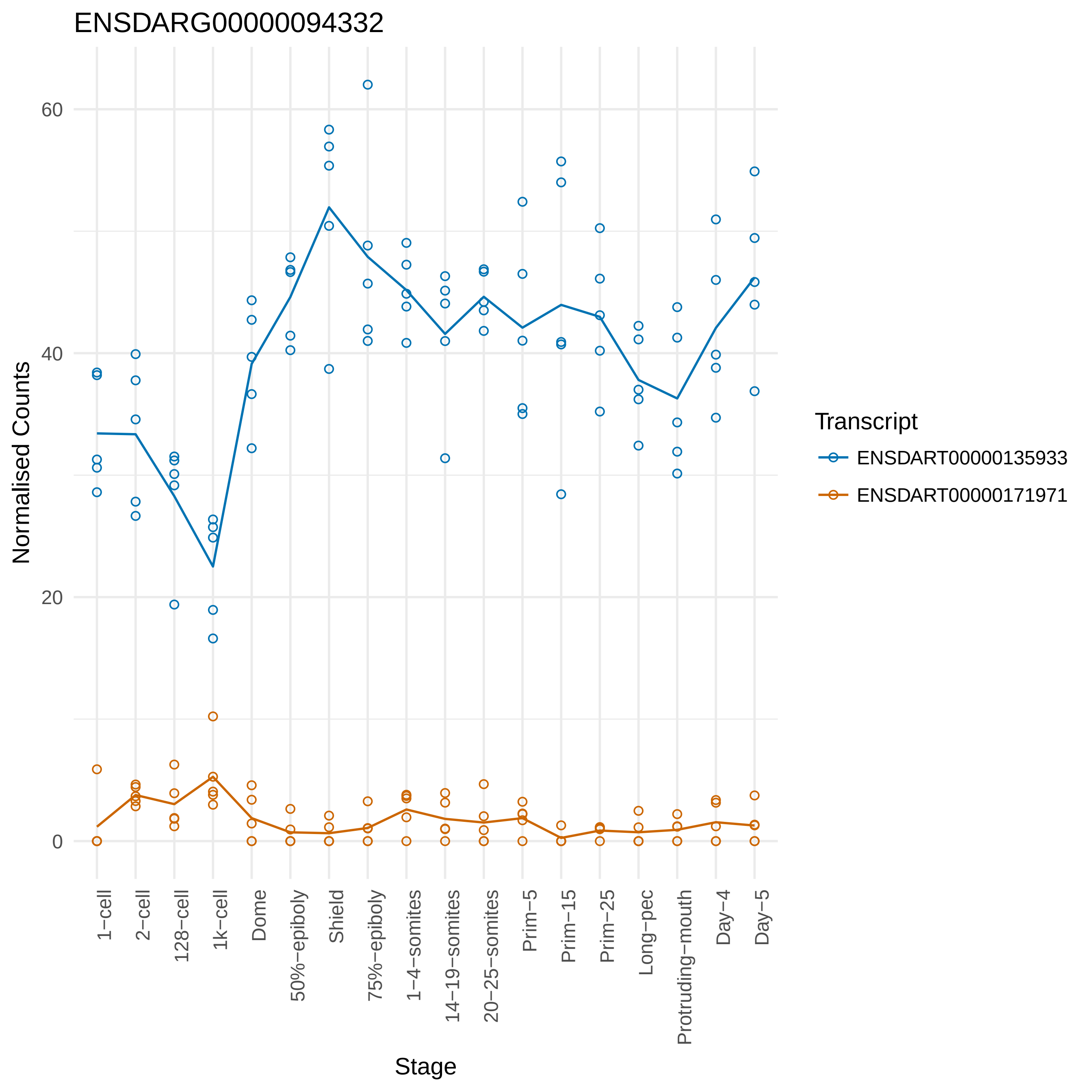

ENSDARG00000094332
Normalised Counts
0
20
40
60
Stage
1−cell
2−cell
128−cell
1k−cell
Dome
50%−epiboly
Shield
75%−epiboly
1−4−somites
14−19−somites
20−25−somites
Prim−5
Prim−15
Prim−25
Long−pec
Protruding−mouth
Day−4
Day−5
Transcript
ENSDART00000135933
ENSDART00000171971

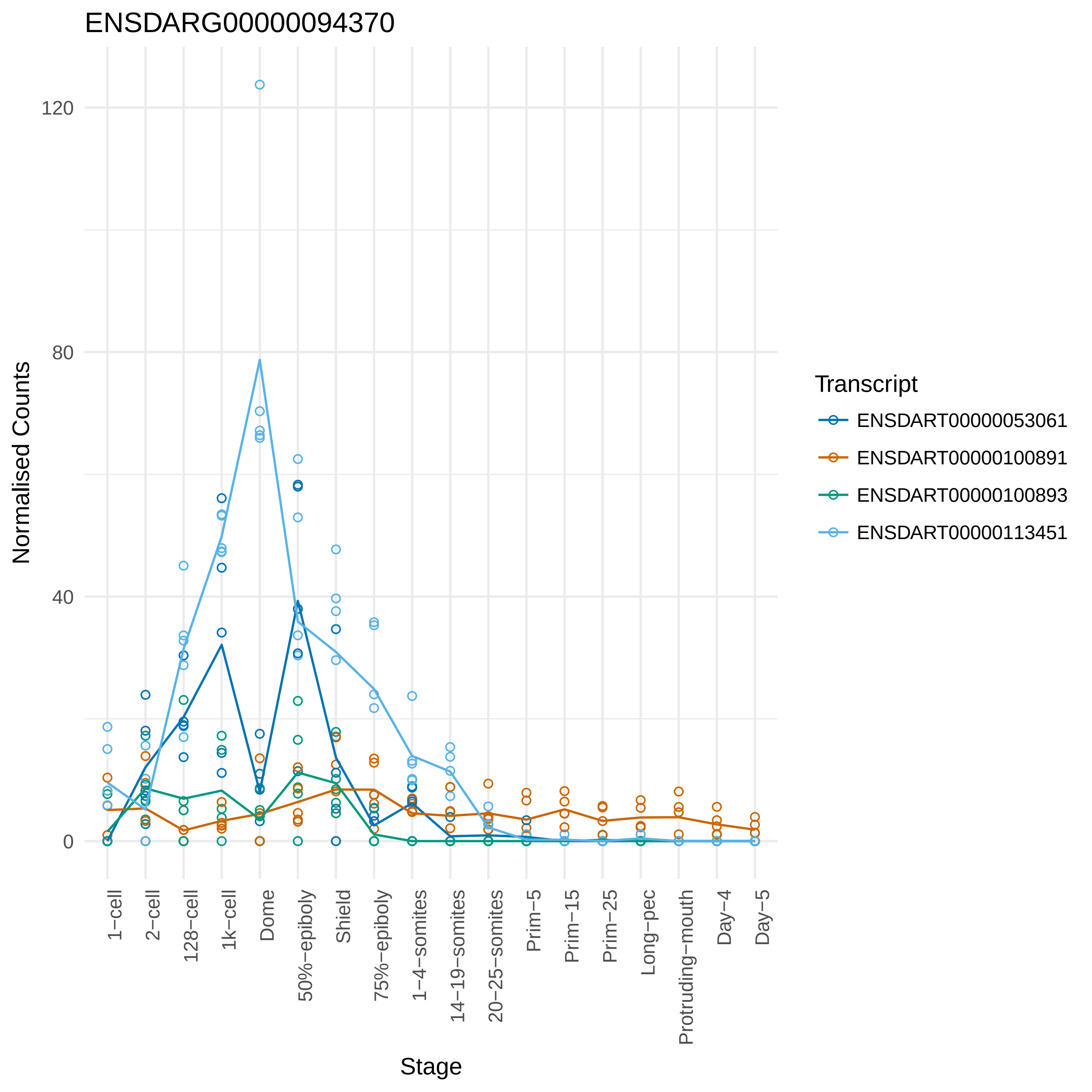

ENSDARG00000094370
Normalised Counts
120
80
40
0
1−cell
2−cell
128−cell
1k−cell
Dome
50%−epiboly
Shield
75%−epiboly
1−4−somites
14−19−somites
20−25−somites
Prim−5
Prim−15
Prim−25
Long−pec
Protruding−mouth
Day−4
Day−5
Stage
Transcript
ENSDART00000053061
ENSDART00000100891
ENSDART00000100893
ENSDART00000113451

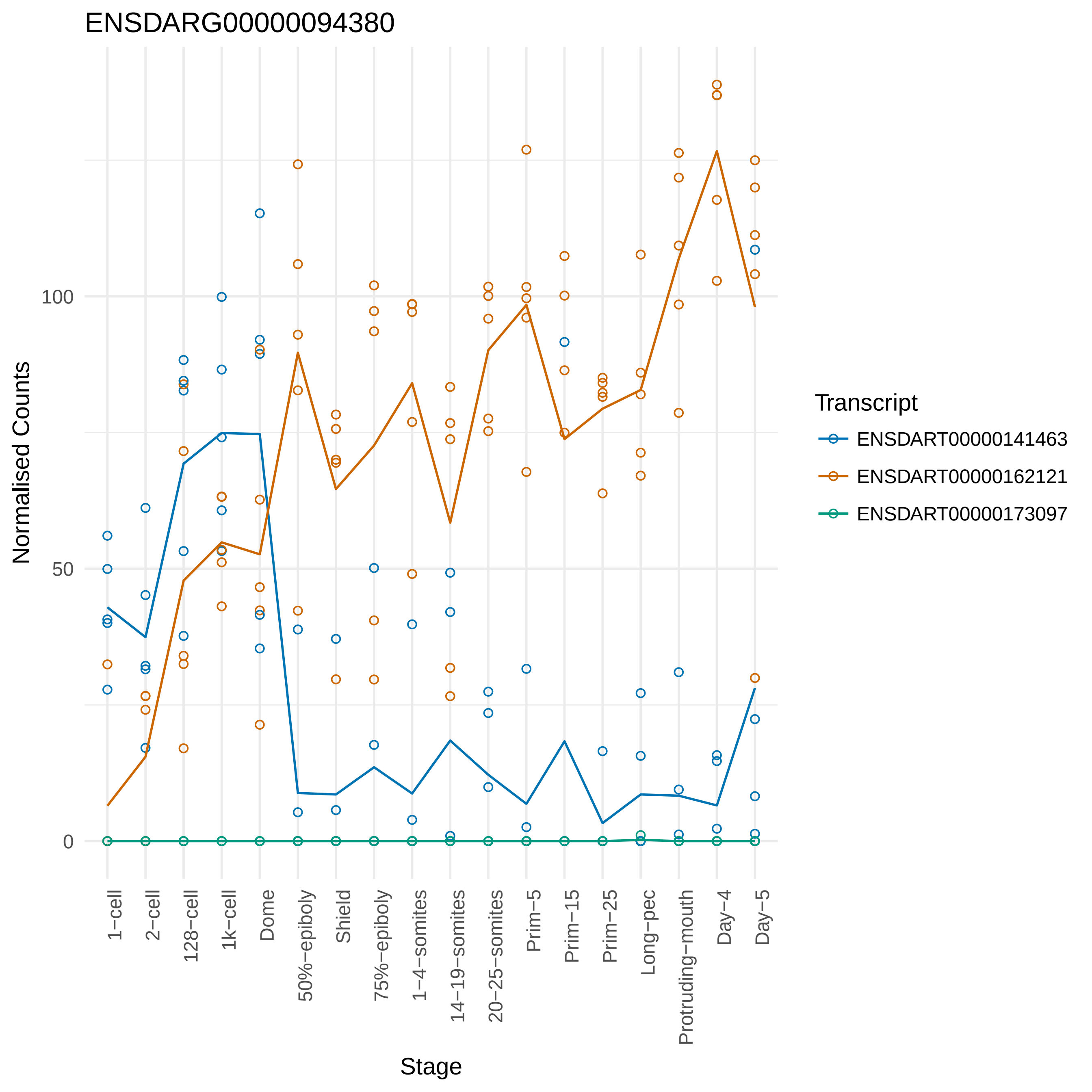

ENSDARG00000094380
Normalised Counts
100
50
0
1−cell
2−cell
128−cell
1k−cell
Dome
50%−epiboly
Shield
75%−epiboly
1−4−somites
14−19−somites
20−25−somites
Prim−5
Prim−15
Prim−25
Long−pec
Protruding−mouth
Day−4
Day−5
Stage
Transcript
ENSDART00000141463
ENSDART00000162121
ENSDART00000173097

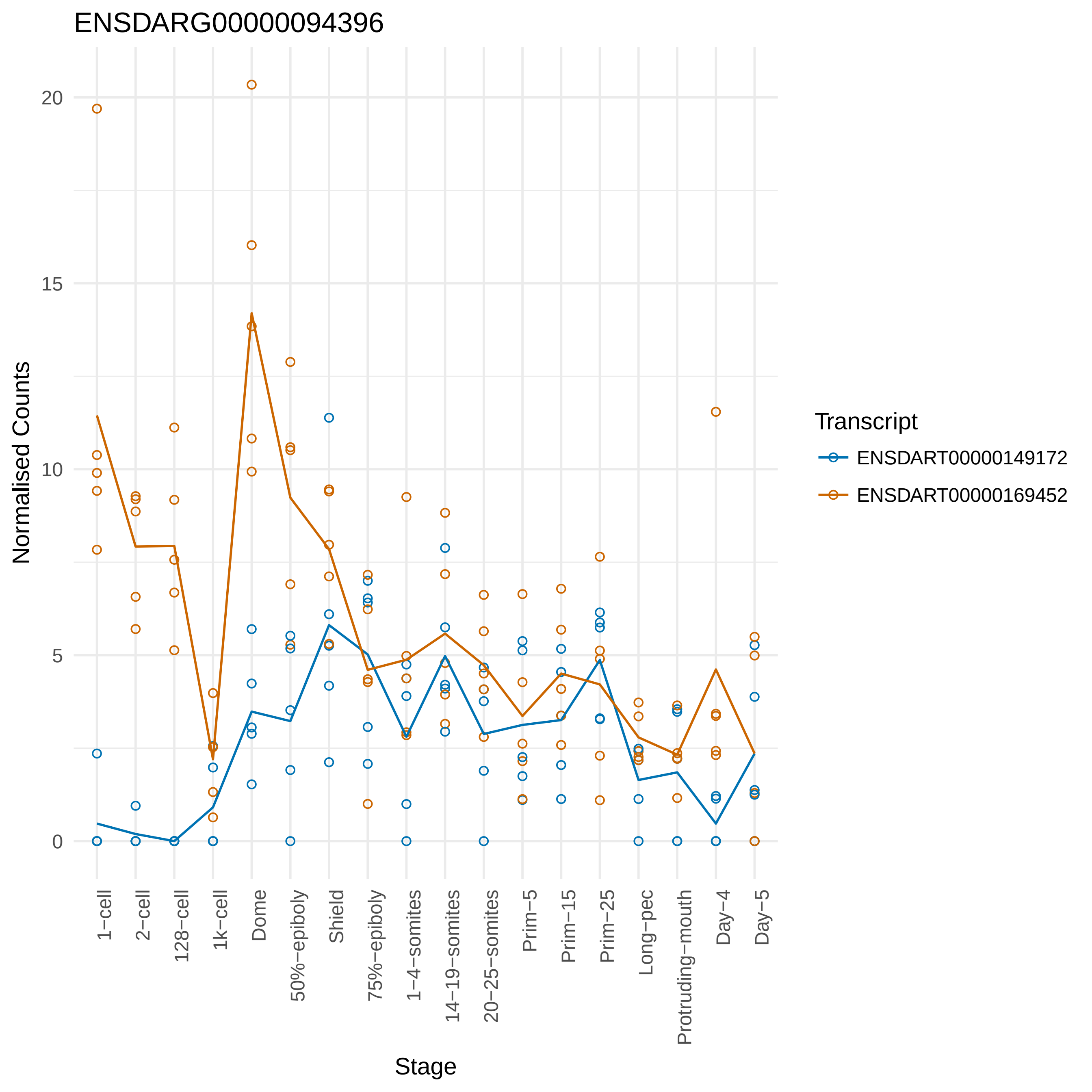
ENSDARG00000094396
Normalised Counts
0
5
10
15
20
Stage
1−cell
2−cell
128−cell
1k−cell
Dome
50%−epiboly
Shield
75%−epiboly
1−4−somites
14−19−somites
20−25−somites
Prim−5
Prim−15
Prim−25
Long−pec
Protruding−mouth
Day−4
Day−5
Transcript
ENSDART00000149172
ENSDART00000169452

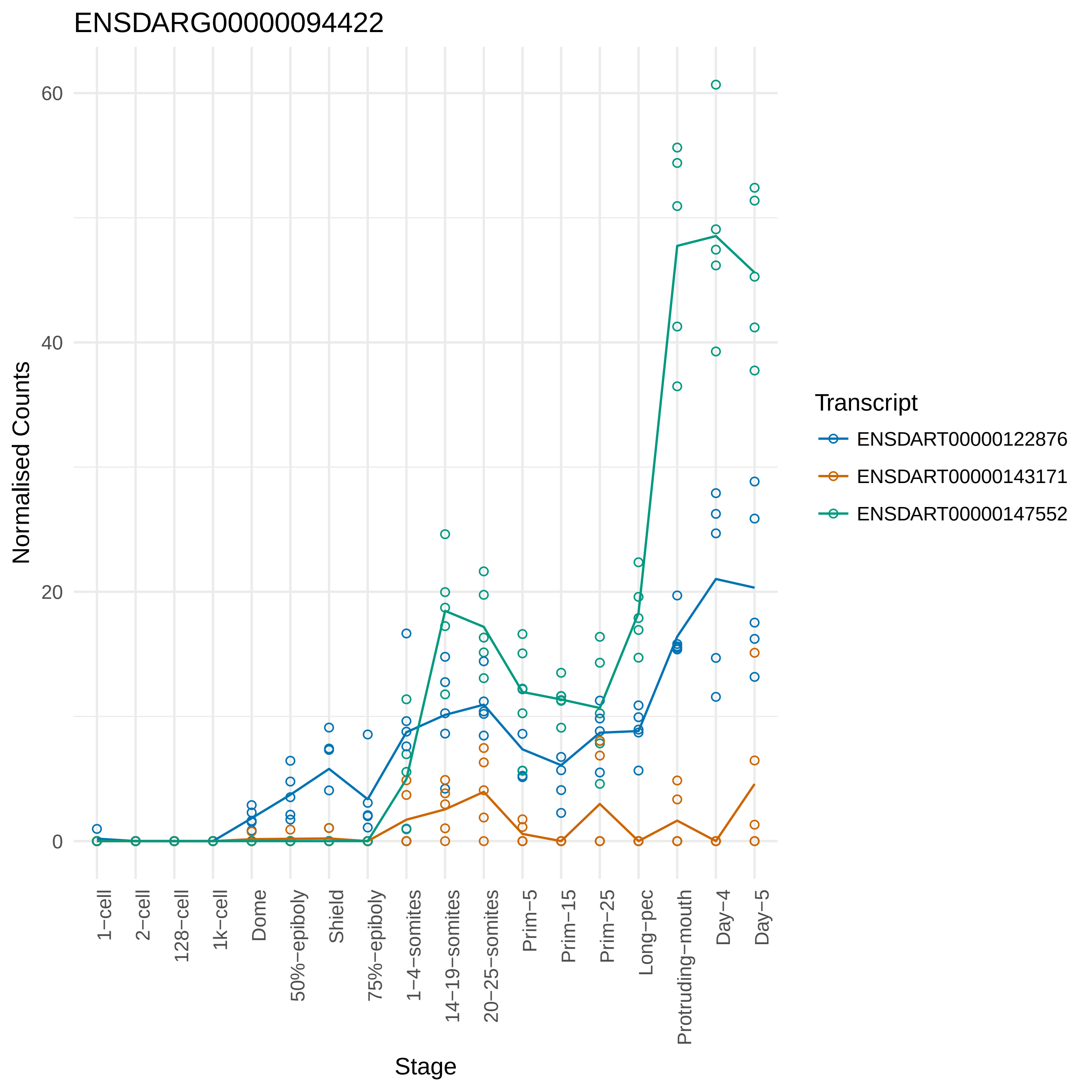
ENSDARG00000094422
Normalised Counts
60
40
20
0
Stage
1−cell
2−cell
128−cell
1k−cell
Dome
50%−epiboly
Shield
75%−epiboly
1−4−somites
14−19−somites
20−25−somites
Prim−5
Prim−15
Prim−25
Long−pec
Protruding−mouth
Day−4
Day−5
Transcript
ENSDART00000122876
ENSDART00000143171
ENSDART00000147552

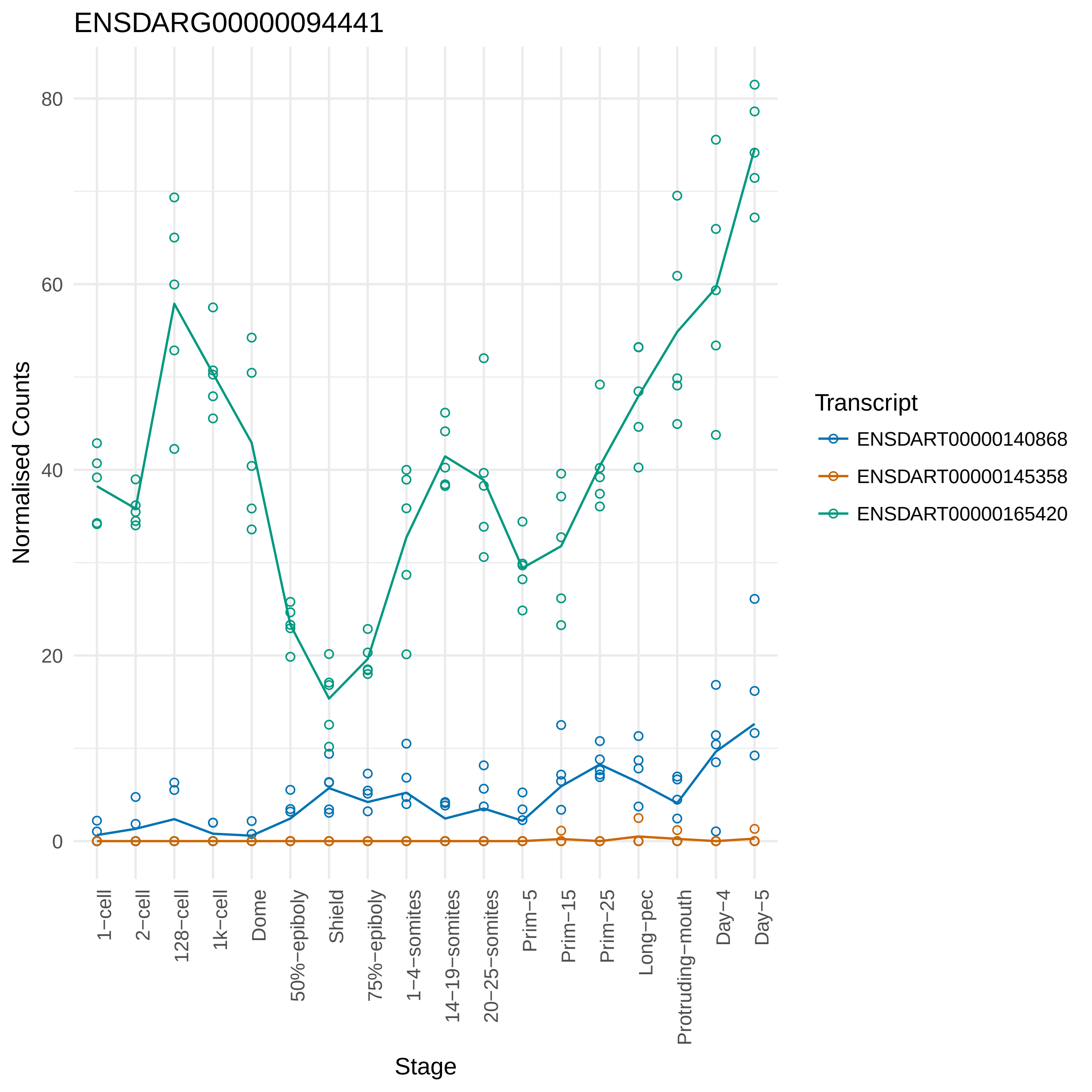
ENSDARG00000094441
Normalised Counts
0
20
40
60
80
1−cell
2−cell
128−cell
1k−cell
Dome
50%−epiboly
Shield
75%−epiboly
1−4−somites
14−19−somites
20−25−somites
Prim−5
Prim−15
Prim−25
Long−pec
Protruding−mouth
Day−4
Day−5
Stage
Transcript
ENSDART00000140868
ENSDART00000145358
ENSDART00000165420

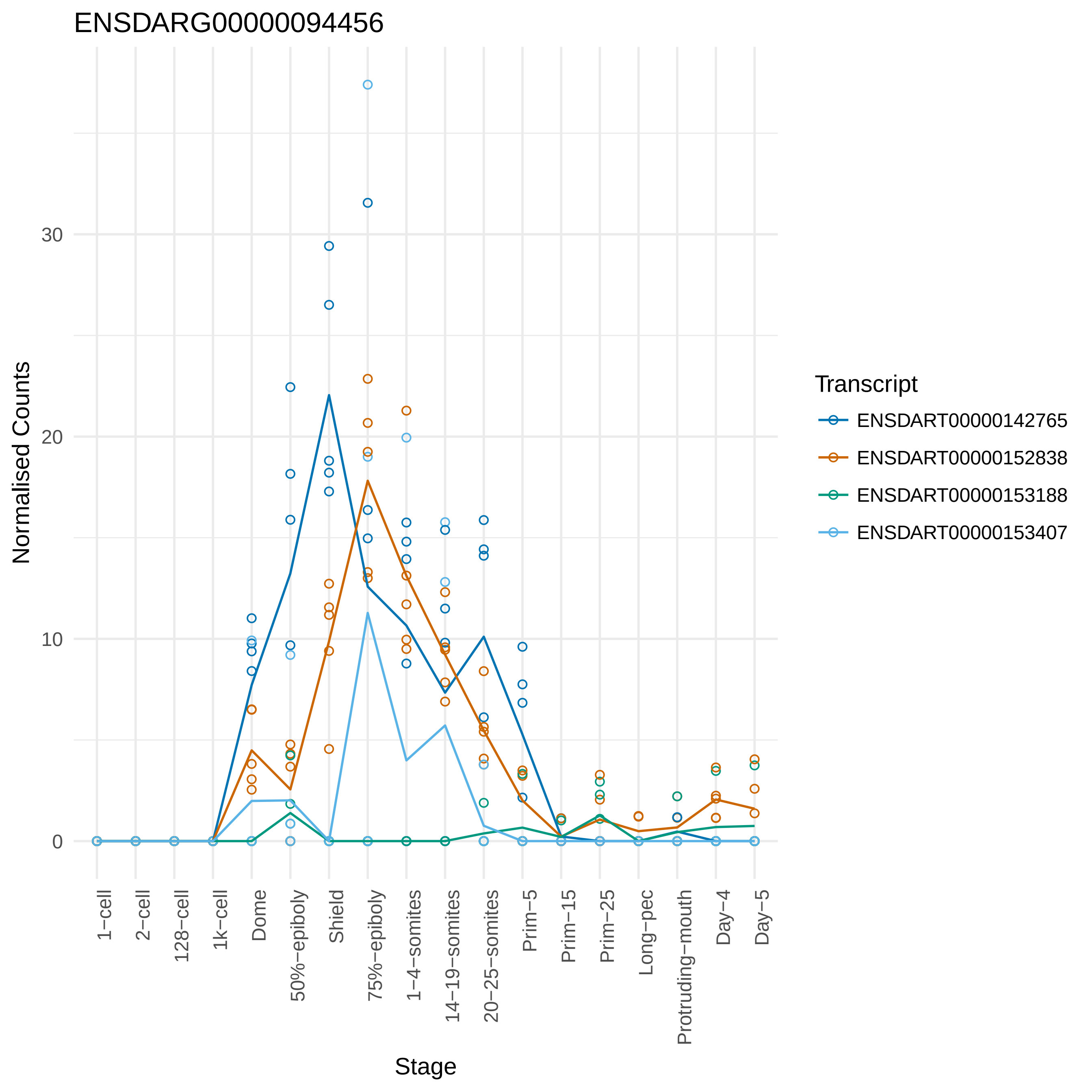
ENSDARG00000094456
Normalised Counts
0
10
20
30
1−cell
2−cell
128−cell
1k−cell
Dome
50%−epiboly
Shield
75%−epiboly
1−4−somites
14−19−somites
20−25−somites
Prim−5
Prim−15
Prim−25
Long−pec
Protruding−mouth
Day−4
Day−5
Stage
Transcript
ENSDART00000142765
ENSDART00000152838
ENSDART00000153188
ENSDART00000153407

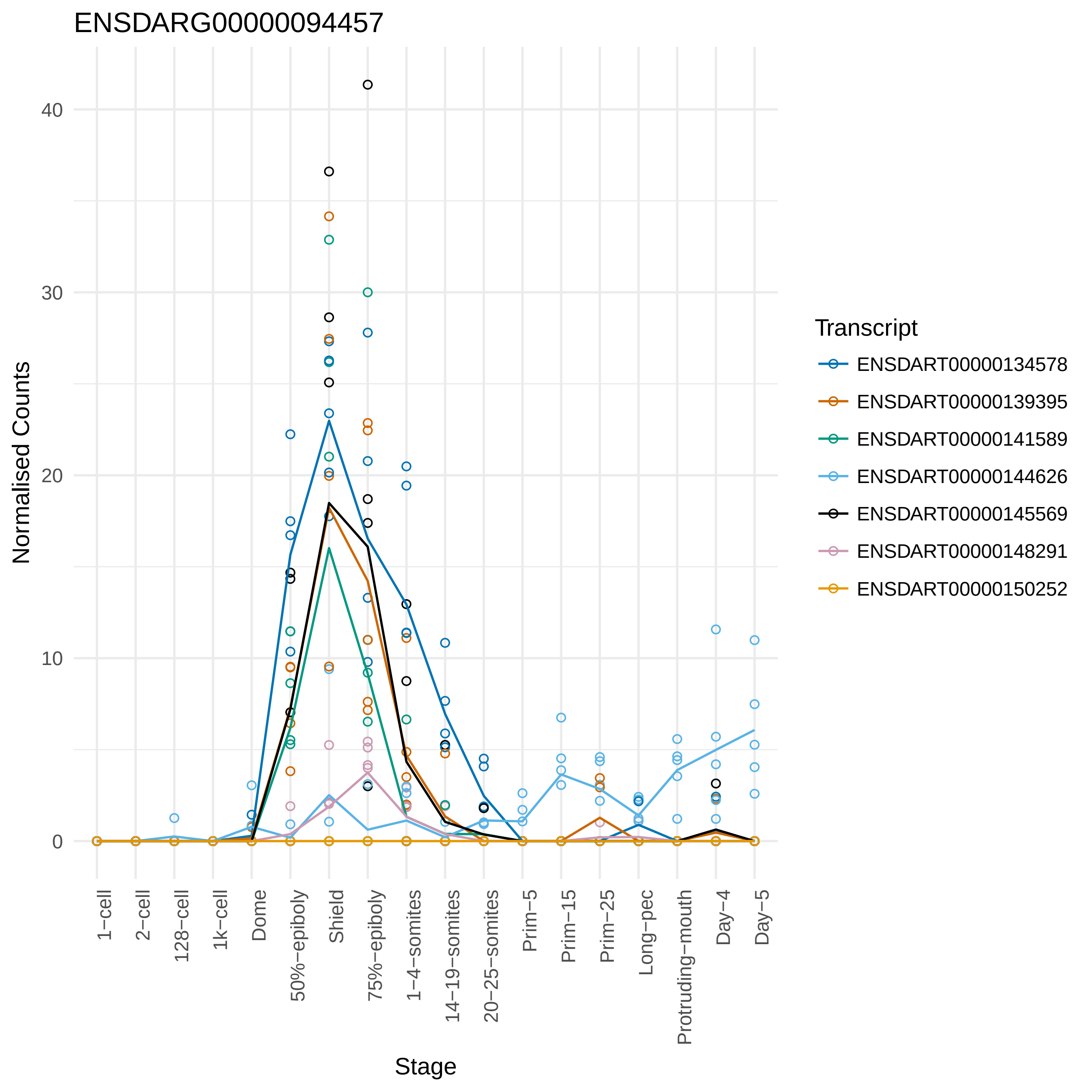

ENSDARG00000094457
Normalised Counts
40
30
20
10
0
1−cell
2−cell
128−cell
1k−cell
Dome
50%−epiboly
Shield
75%−epiboly
1−4−somites
14−19−somites
20−25−somites
Prim−5
Prim−15
Prim−25
Long−pec
Protruding−mouth
Day−4
Day−5
Stage
Transcript
ENSDART00000134578
ENSDART00000139395
ENSDART00000141589
ENSDART00000144626
ENSDART00000145569
ENSDART00000148291
ENSDART00000150252

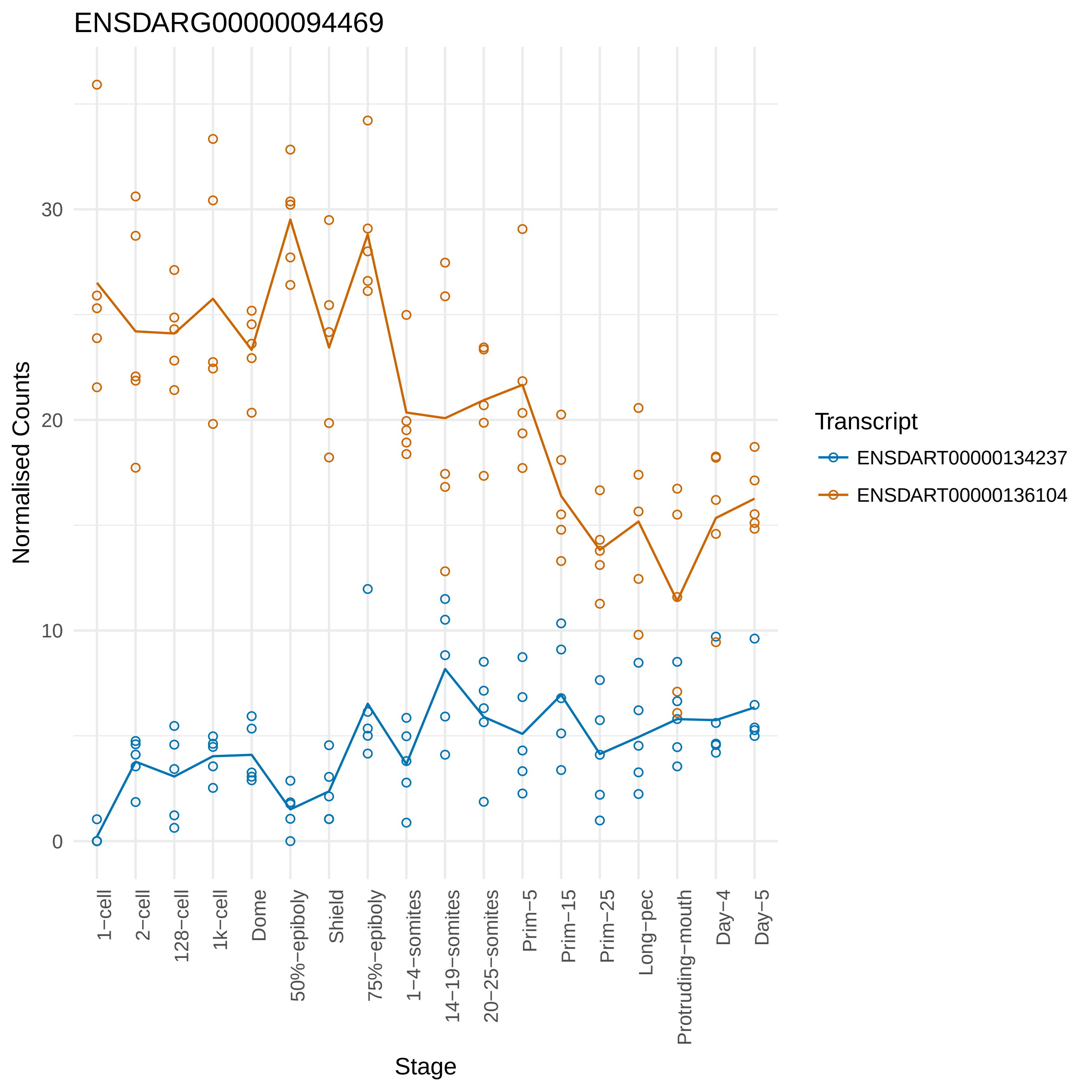
ENSDARG00000094469
Normalised Counts
0
10
20
30
Stage
1−cell
2−cell
128−cell
1k−cell
Dome
50%−epiboly
Shield
75%−epiboly
1−4−somites
14−19−somites
20−25−somites
Prim−5
Prim−15
Prim−25
Long−pec
Protruding−mouth
Day−4
Day−5
Transcript
ENSDART00000134237
ENSDART00000136104

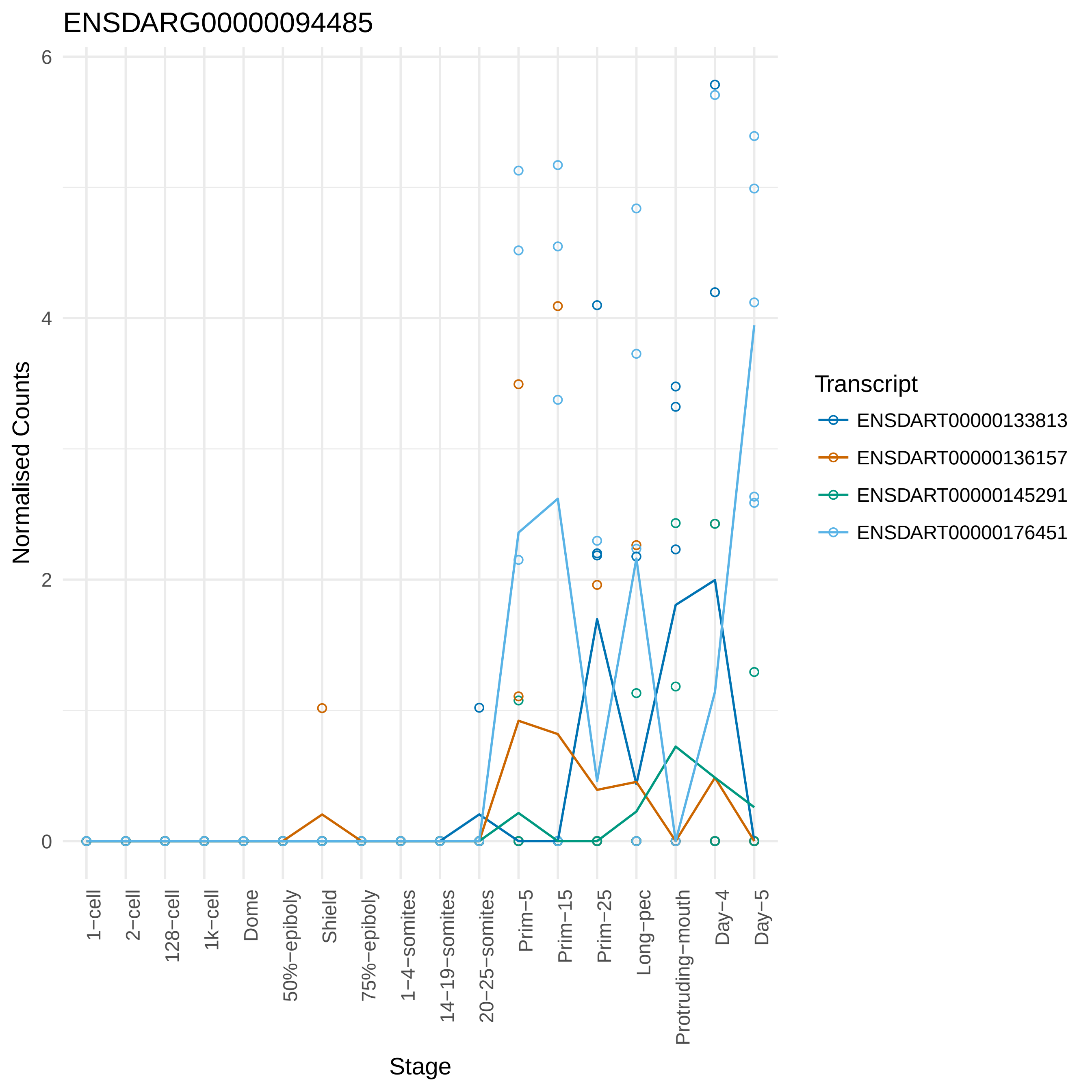
ENSDARG00000094485
Normalised Counts
Stage
0
2
4
6
1−cell
2−cell
128−cell
1k−cell
Dome
50%−epiboly
Shield
75%−epiboly
1−4−somites
14−19−somites
20−25−somites
Prim−5
Prim−15
Prim−25
Long−pec
Protruding−mouth
Day−4
Day−5
Transcript
ENSDART00000133813
ENSDART00000136157
ENSDART00000145291
ENSDART00000176451

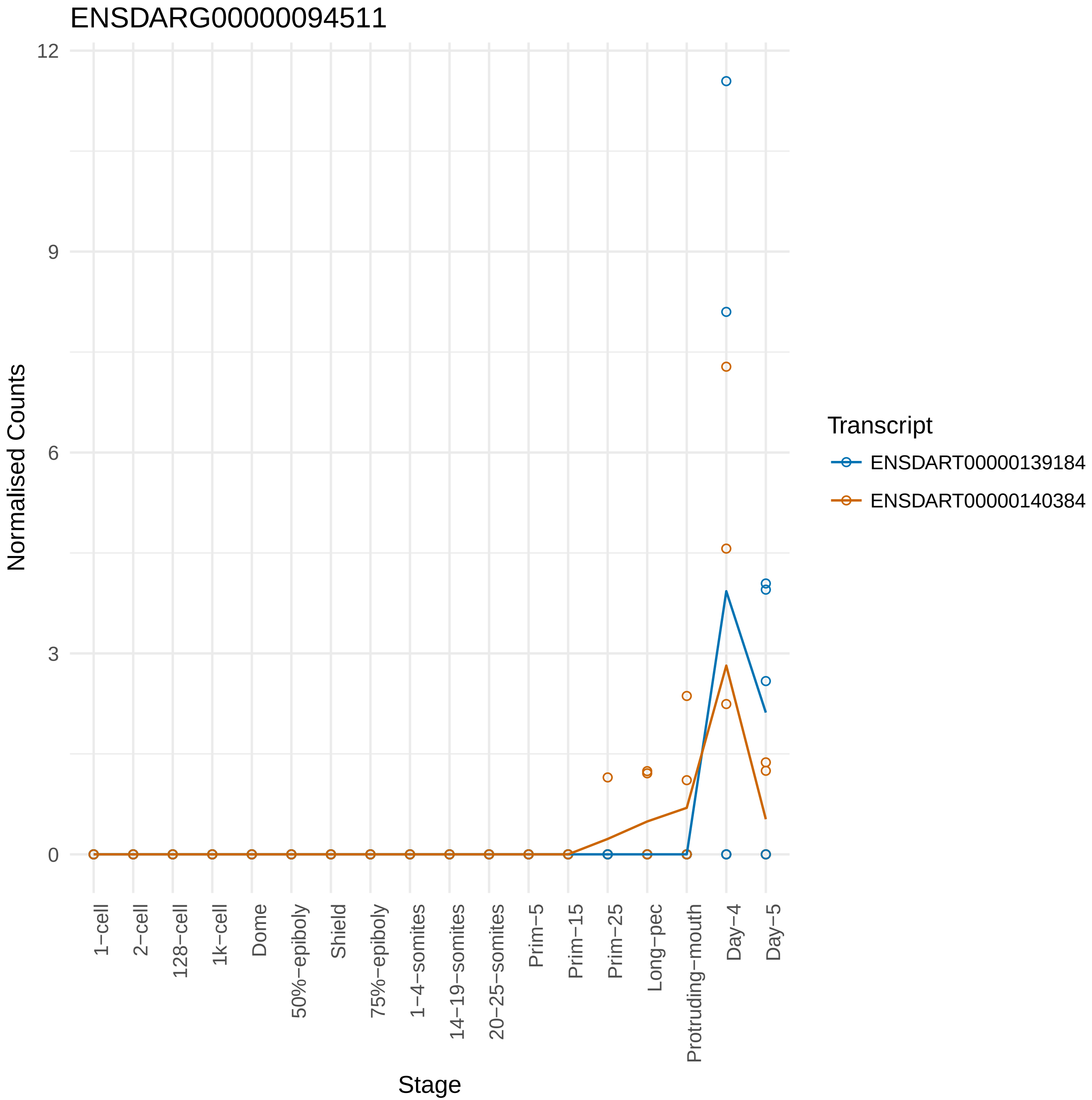
ENSDARG00000094511
Normalised Counts
12
9
6
3
0
1−cell
2−cell
128−cell
1k−cell
Dome
50%−epiboly
Shield
75%−epiboly
1−4−somites
14−19−somites
20−25−somites
Prim−5
Prim−15
Prim−25
Long−pec
Protruding−mouth
Day−4
Day−5
Stage
Transcript
ENSDART00000139184
ENSDART00000140384

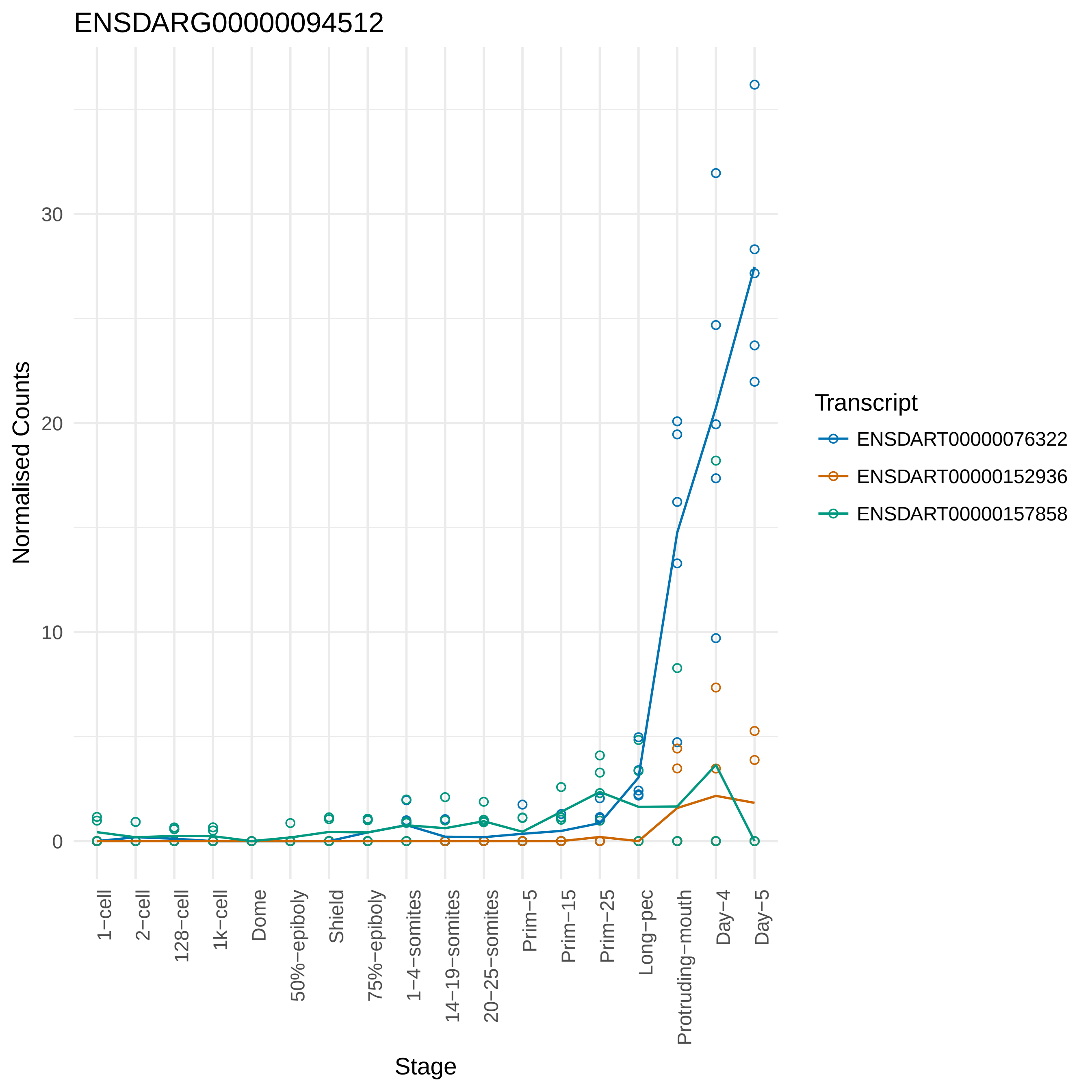
ENSDARG00000094512
Normalised Counts
0
10
20
30
1−cell
2−cell
128−cell
1k−cell
Dome
50%−epiboly
Shield
75%−epiboly
1−4−somites
14−19−somites
20−25−somites
Prim−5
Prim−15
Prim−25
Long−pec
Protruding−mouth
Day−4
Day−5
Stage
Transcript
ENSDART00000076322
ENSDART00000152936
ENSDART00000157858

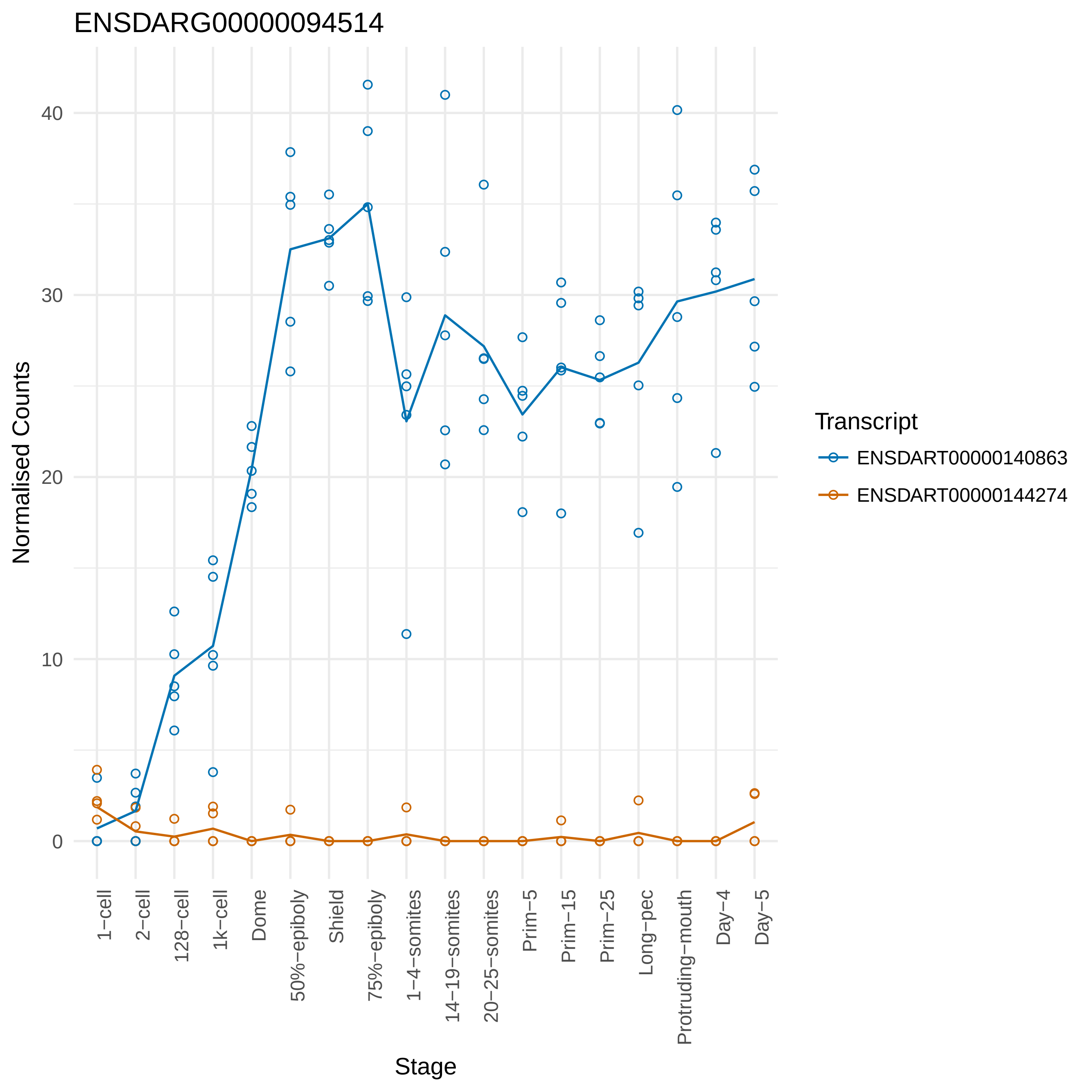

ENSDARG00000094514
Normalised Counts
0
10
20
30
40
1−cell
2−cell
128−cell
1k−cell
Dome
50%−epiboly
Shield
75%−epiboly
1−4−somites
14−19−somites
20−25−somites
Prim−5
Prim−15
Prim−25
Long−pec
Protruding−mouth
Day−4
Day−5
Stage
Transcript
ENSDART00000140863
ENSDART00000144274

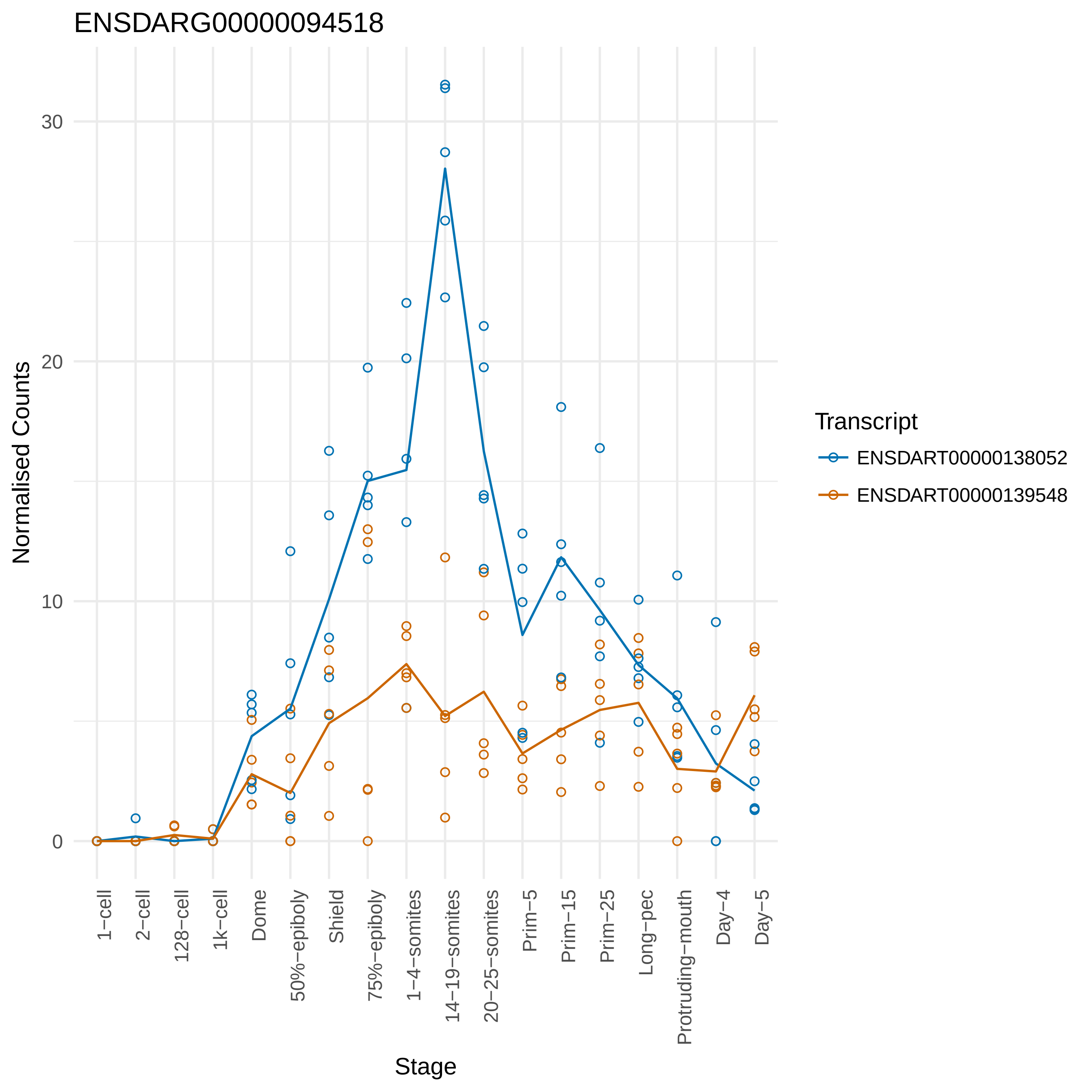

ENSDARG00000094518
Normalised Counts
30
20
10
0
1−cell
2−cell
128−cell
1k−cell
Dome
50%−epiboly
Shield
75%−epiboly
1−4−somites
14−19−somites
20−25−somites
Prim−5
Prim−15
Prim−25
Long−pec
Protruding−mouth
Day−4
Day−5
Stage
Transcript
ENSDART00000138052
ENSDART00000139548

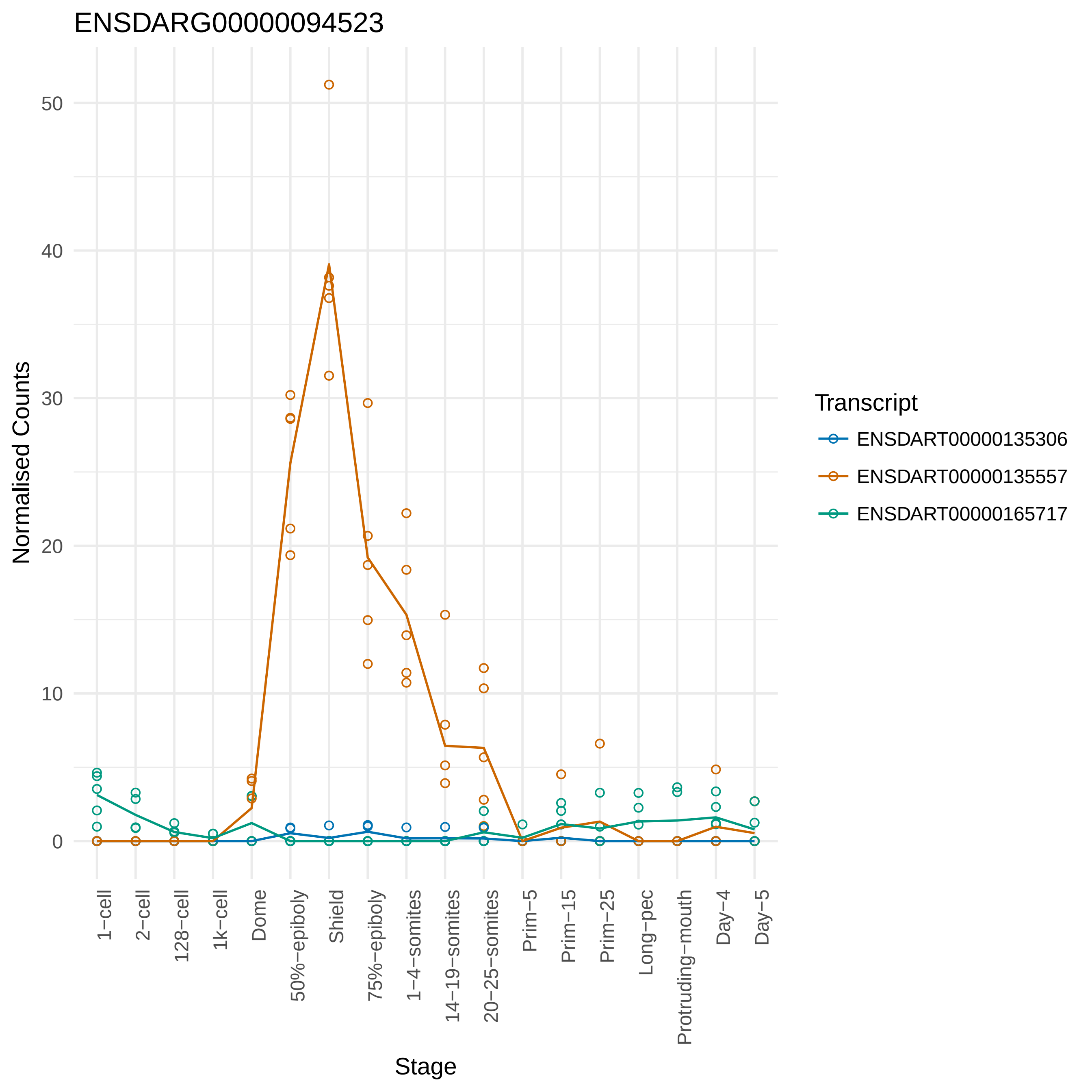

ENSDARG00000094523
Normalised Counts
50
40
30
20
10
0
1−cell
2−cell
128−cell
1k−cell
Dome
50%−epiboly
Shield
75%−epiboly
1−4−somites
14−19−somites
20−25−somites
Prim−5
Prim−15
Prim−25
Long−pec
Protruding−mouth
Day−4
Day−5
Stage
Transcript
ENSDART00000135306
ENSDART00000135557
ENSDART00000165717

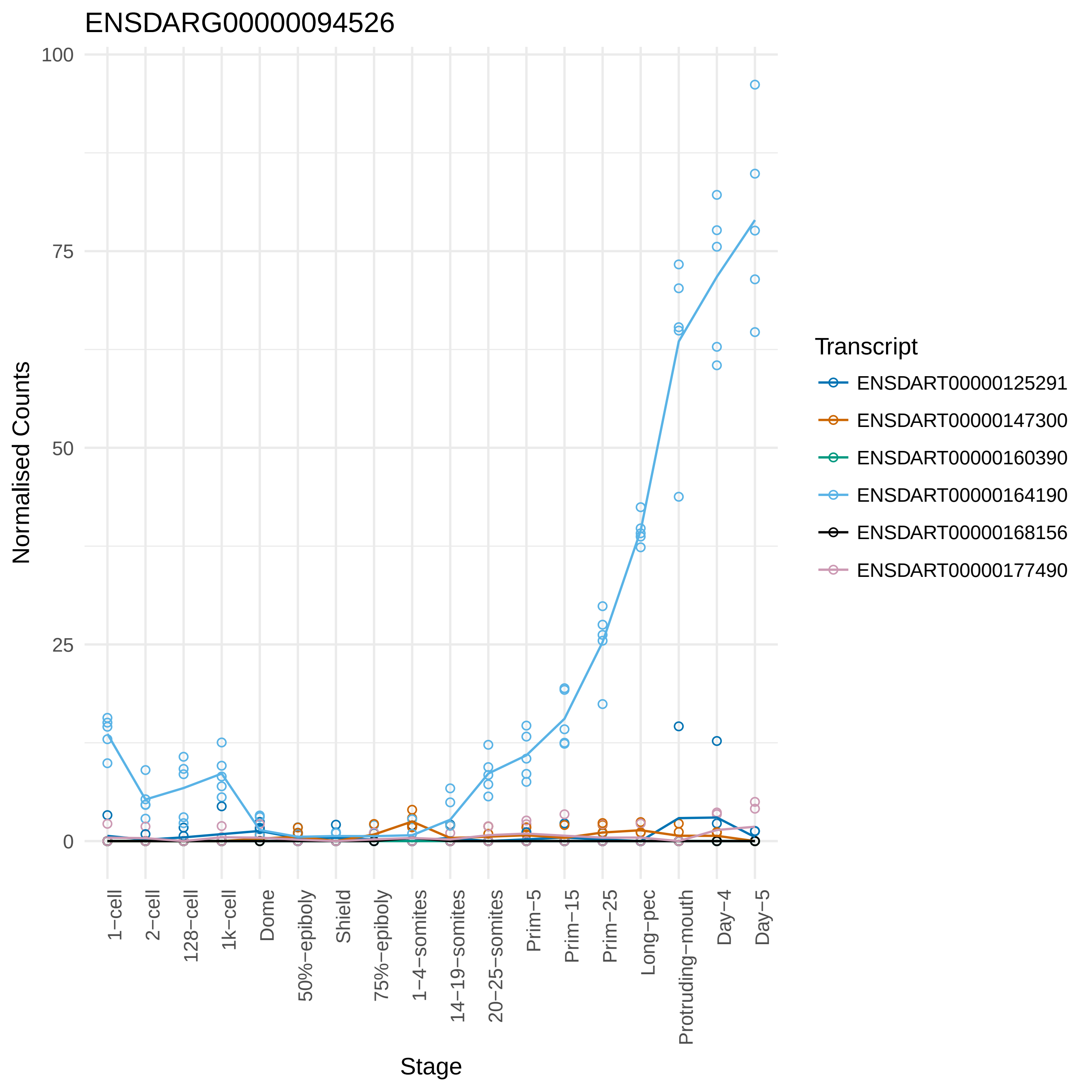

ENSDARG00000094526
Normalised Counts
100
75
50
25
0
1−cell
2−cell
128−cell
1k−cell
Dome
50%−epiboly
Shield
75%−epiboly
1−4−somites
14−19−somites
20−25−somites
Prim−5
Prim−15
Prim−25
Long−pec
Protruding−mouth
Day−4
Day−5
Stage
Transcript
ENSDART00000125291
ENSDART00000147300
ENSDART00000160390
ENSDART00000164190
ENSDART00000168156
ENSDART00000177490

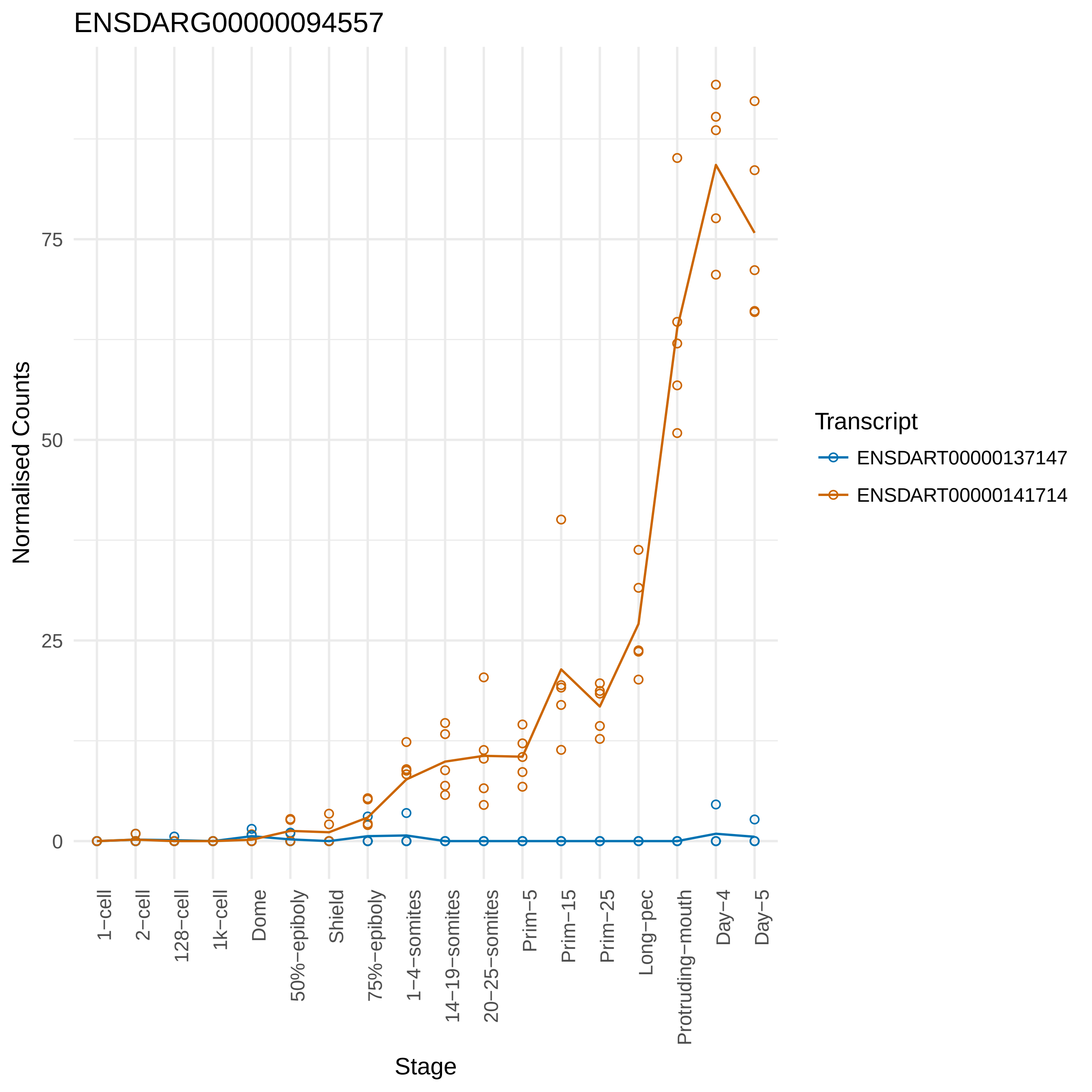
ENSDARG00000094557
Normalised Counts
0
25
50
75
Stage
1−cell
2−cell
128−cell
1k−cell
Dome
50%−epiboly
Shield
75%−epiboly
1−4−somites
14−19−somites
20−25−somites
Prim−5
Prim−15
Prim−25
Long−pec
Protruding−mouth
Day−4
Day−5
Transcript
ENSDART00000137147
ENSDART00000141714

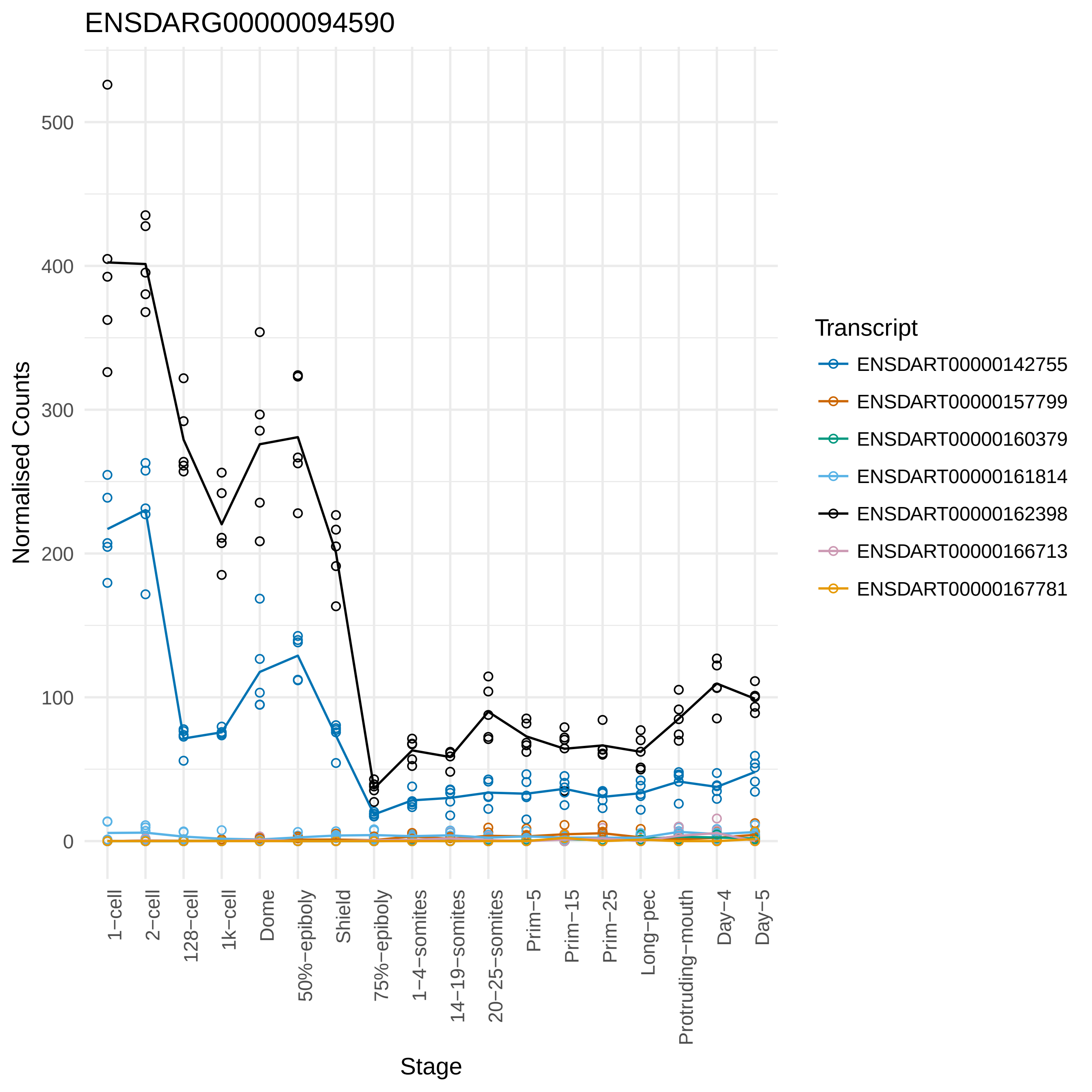
ENSDARG00000094590
Normalised Counts
0
100
200
300
400
500
1−cell
2−cell
128−cell
1k−cell
Dome
50%−epiboly
Shield
75%−epiboly
1−4−somites
14−19−somites
20−25−somites
Prim−5
Prim−15
Prim−25
Long−pec
Protruding−mouth
Day−4
Day−5
Stage
Transcript
ENSDART00000142755
ENSDART00000157799
ENSDART00000160379
ENSDART00000161814
ENSDART00000162398
ENSDART00000166713
ENSDART00000167781

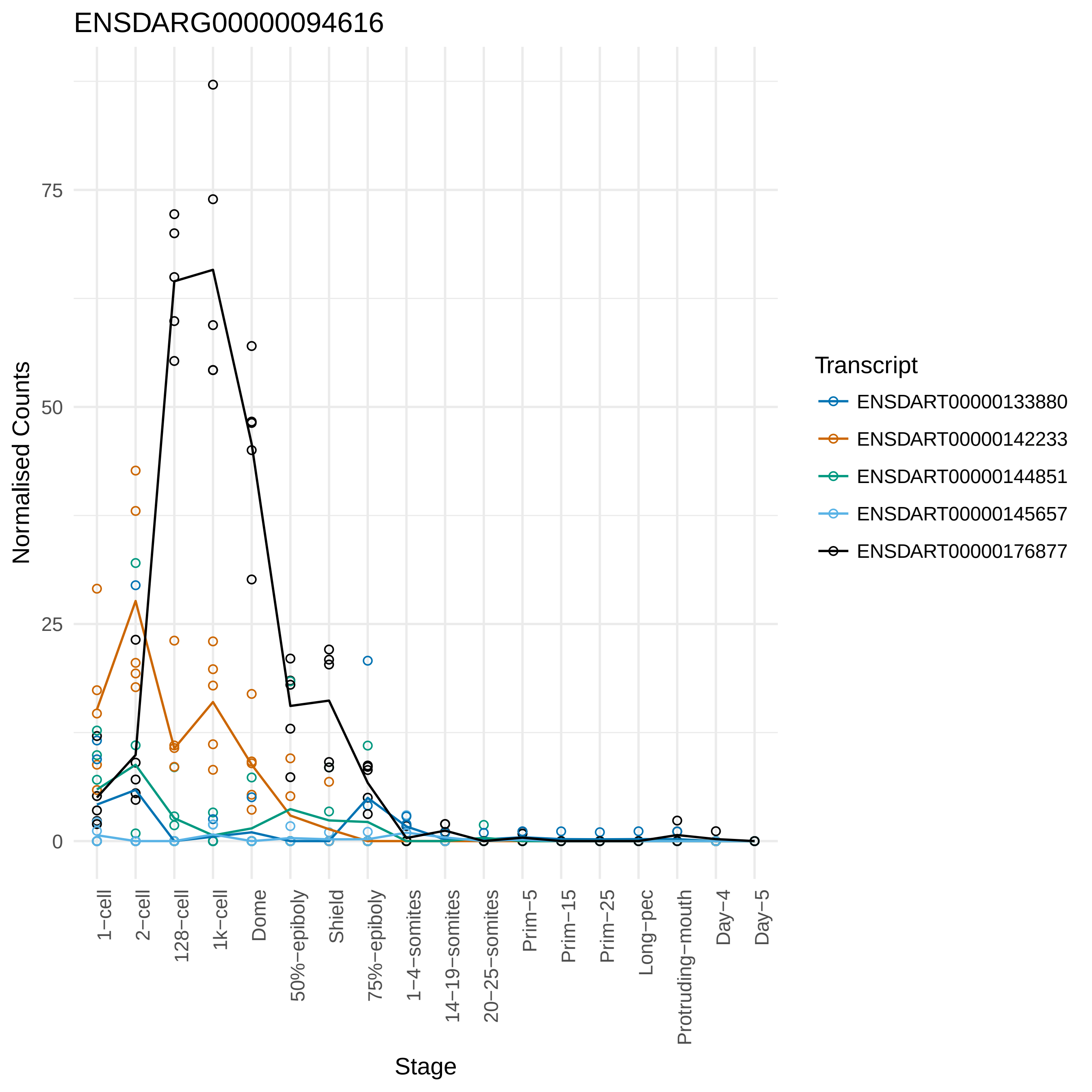

ENSDARG00000094616
Normalised Counts
0
25
50
75
Stage
1−cell
2−cell
128−cell
1k−cell
Dome
50%−epiboly
Shield
75%−epiboly
1−4−somites
14−19−somites
20−25−somites
Prim−5
Prim−15
Prim−25
Long−pec
Protruding−mouth
Day−4
Day−5
Transcript
ENSDART00000133880
ENSDART00000142233
ENSDART00000144851
ENSDART00000145657
ENSDART00000176877

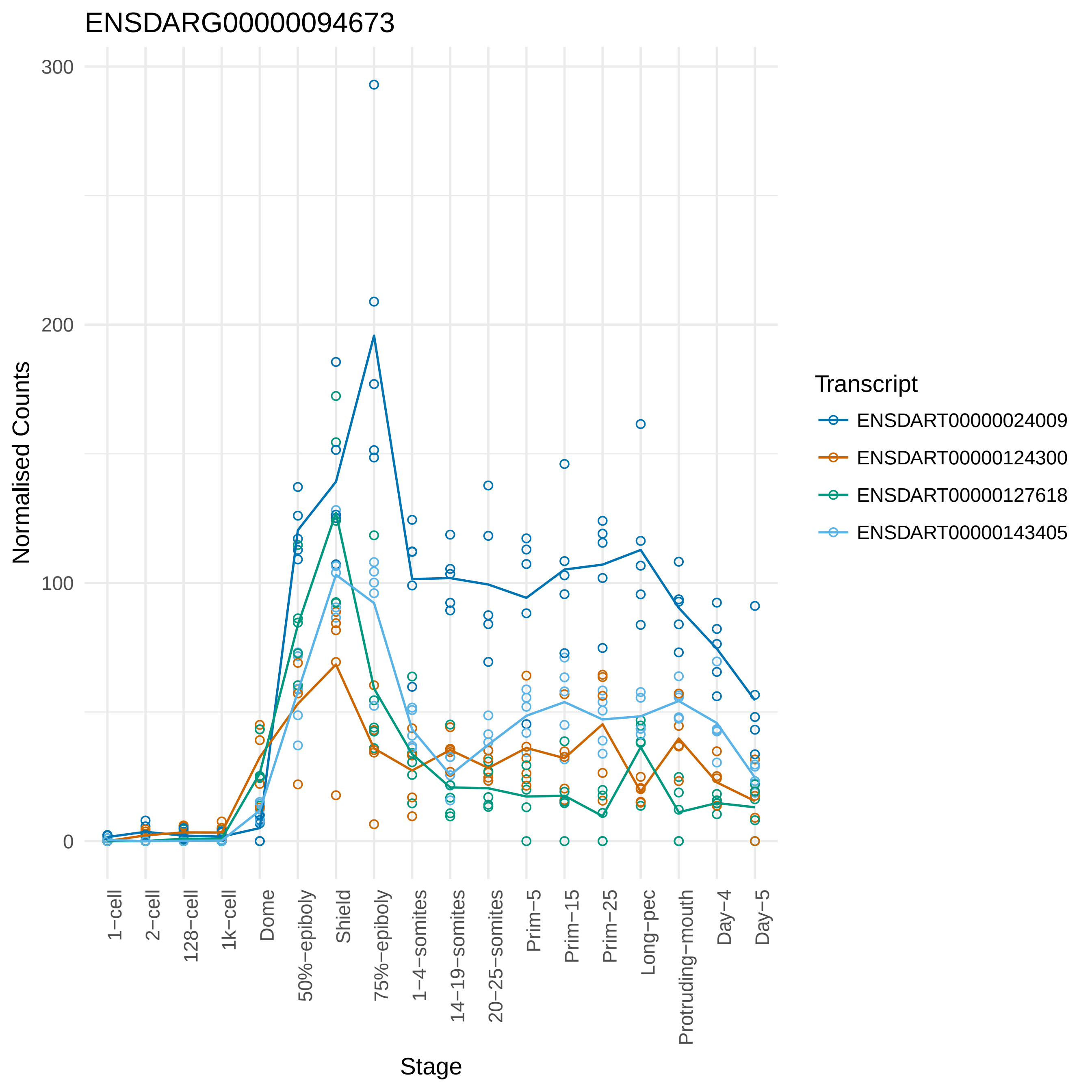
ENSDARG00000094673
Normalised Counts
0
100
200
300
Stage
1−cell
2−cell
128−cell
1k−cell
Dome
50%−epiboly
Shield
75%−epiboly
1−4−somites
14−19−somites
20−25−somites
Prim−5
Prim−15
Prim−25
Long−pec
Protruding−mouth
Day−4
Day−5
Transcript
ENSDART00000024009
ENSDART00000124300
ENSDART00000127618
ENSDART00000143405

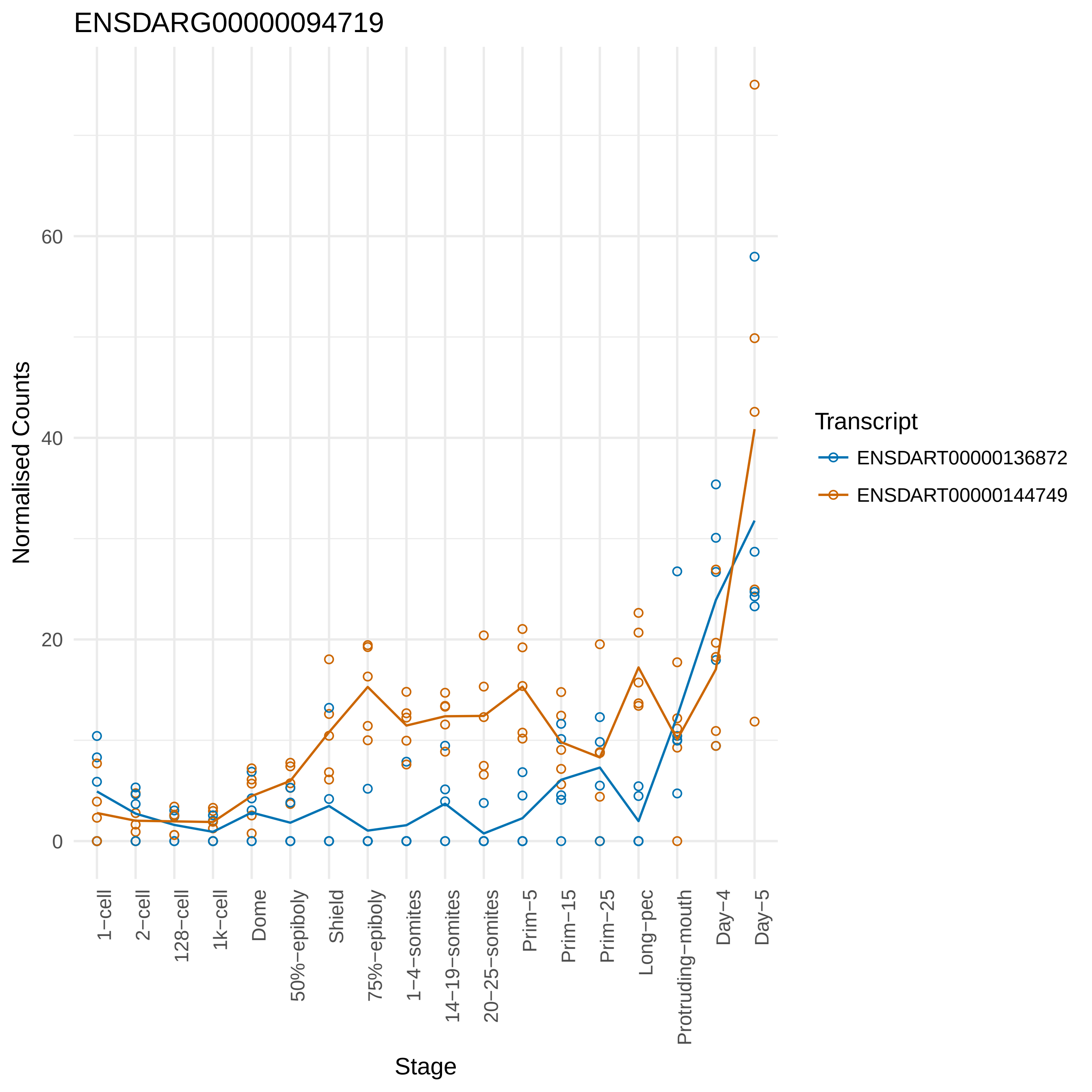

ENSDARG00000094719
Normalised Counts
60
40
20
0
1−cell
2−cell
128−cell
1k−cell
Dome
50%−epiboly
Shield
75%−epiboly
1−4−somites
14−19−somites
20−25−somites
Prim−5
Prim−15
Prim−25
Long−pec
Protruding−mouth
Day−4
Day−5
Stage
Transcript
ENSDART00000136872
ENSDART00000144749

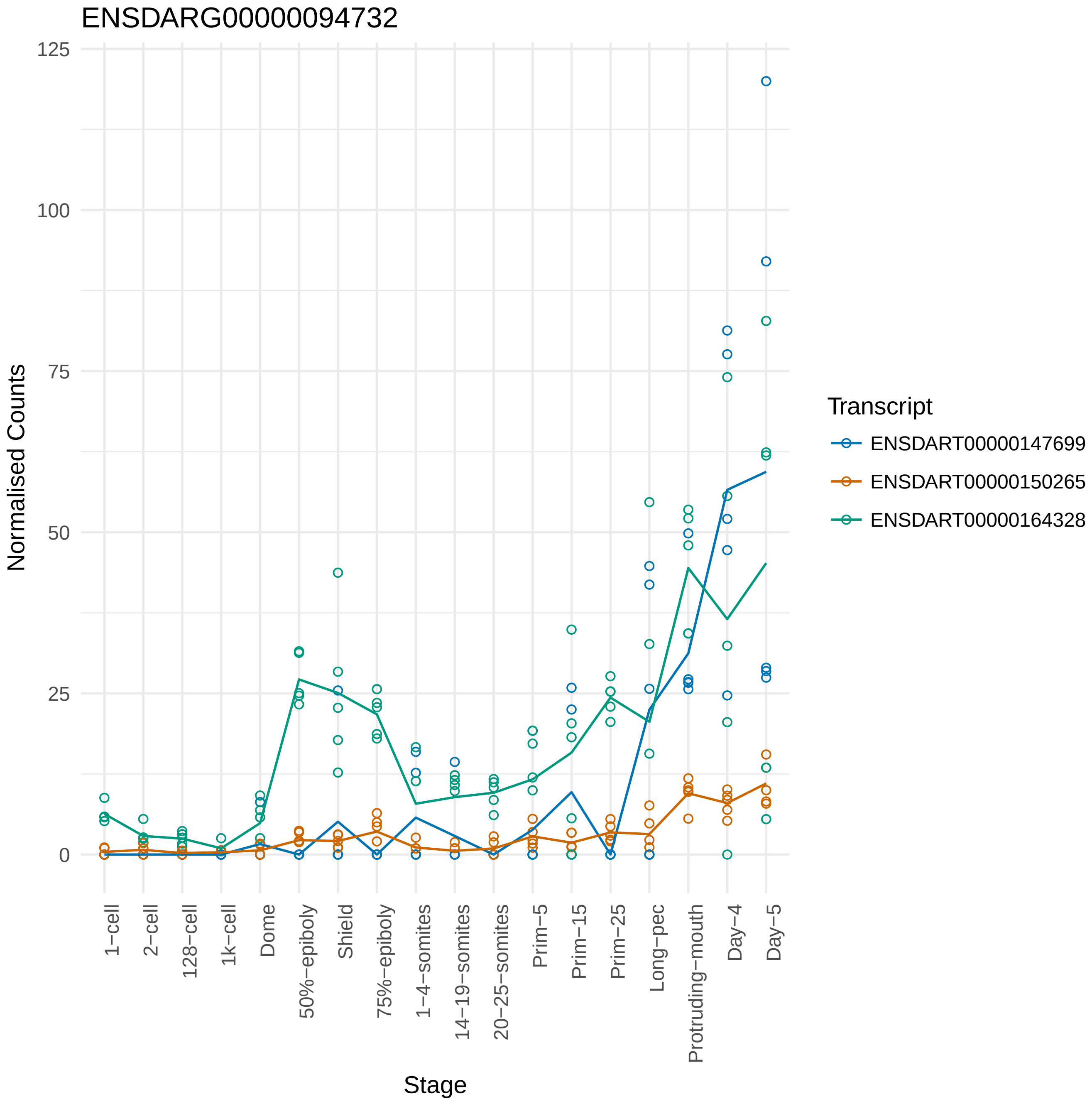

ENSDARG00000094732
Normalised Counts
125
100
75
50
25
0
1−cell
2−cell
128−cell
1k−cell
Dome
50%−epiboly
Shield
75%−epiboly
1−4−somites
14−19−somites
20−25−somites
Prim−5
Prim−15
Prim−25
Long−pec
Protruding−mouth
Day−4
Day−5
Stage
Transcript
ENSDART00000147699
ENSDART00000150265
ENSDART00000164328

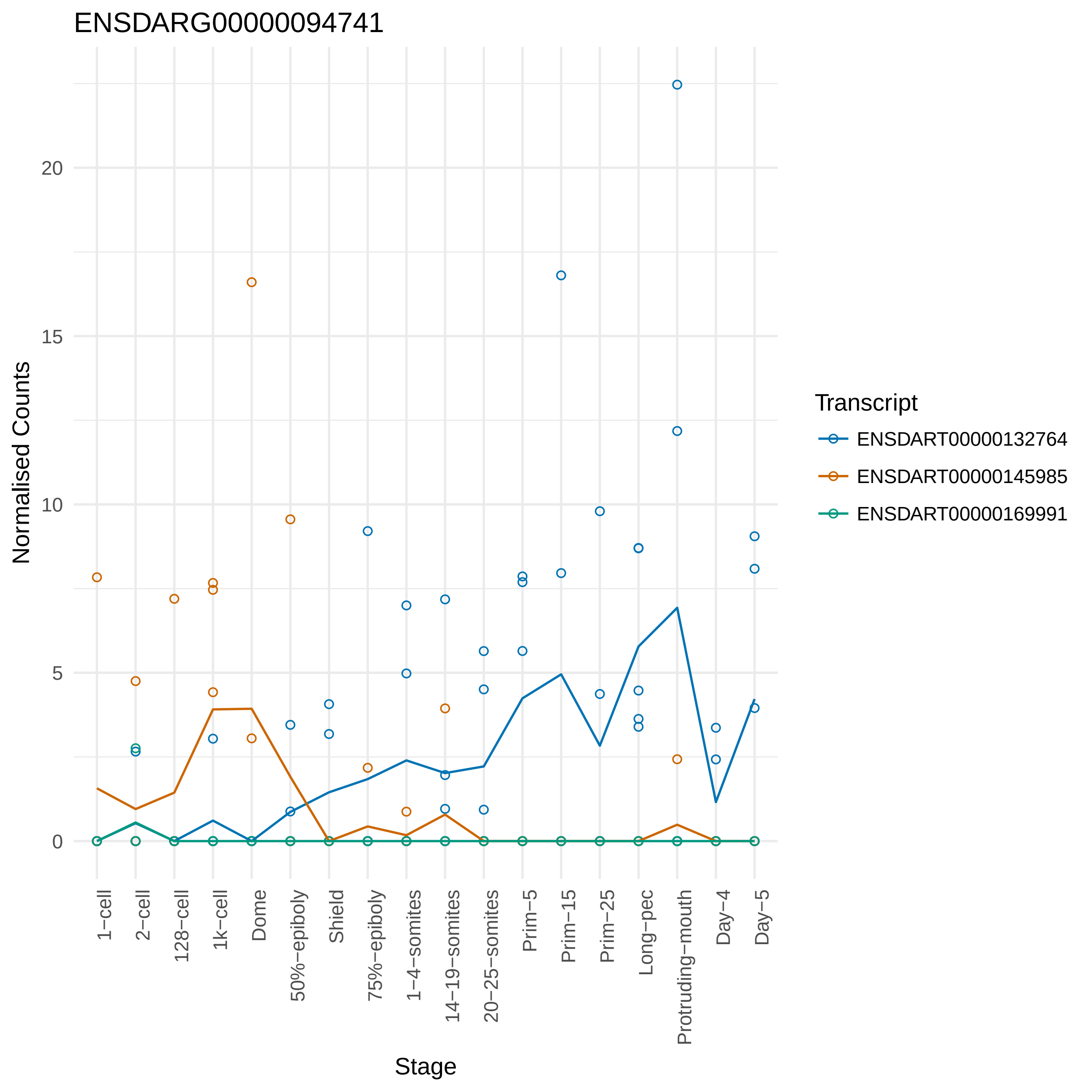
ENSDARG00000094741
Normalised Counts
20
15
10
5
0
1−cell
2−cell
128−cell
1k−cell
Dome
50%−epiboly
Shield
75%−epiboly
1−4−somites
14−19−somites
20−25−somites
Prim−5
Prim−15
Prim−25
Long−pec
Protruding−mouth
Day−4
Day−5
Stage
Transcript
ENSDART00000132764
ENSDART00000145985
ENSDART00000169991

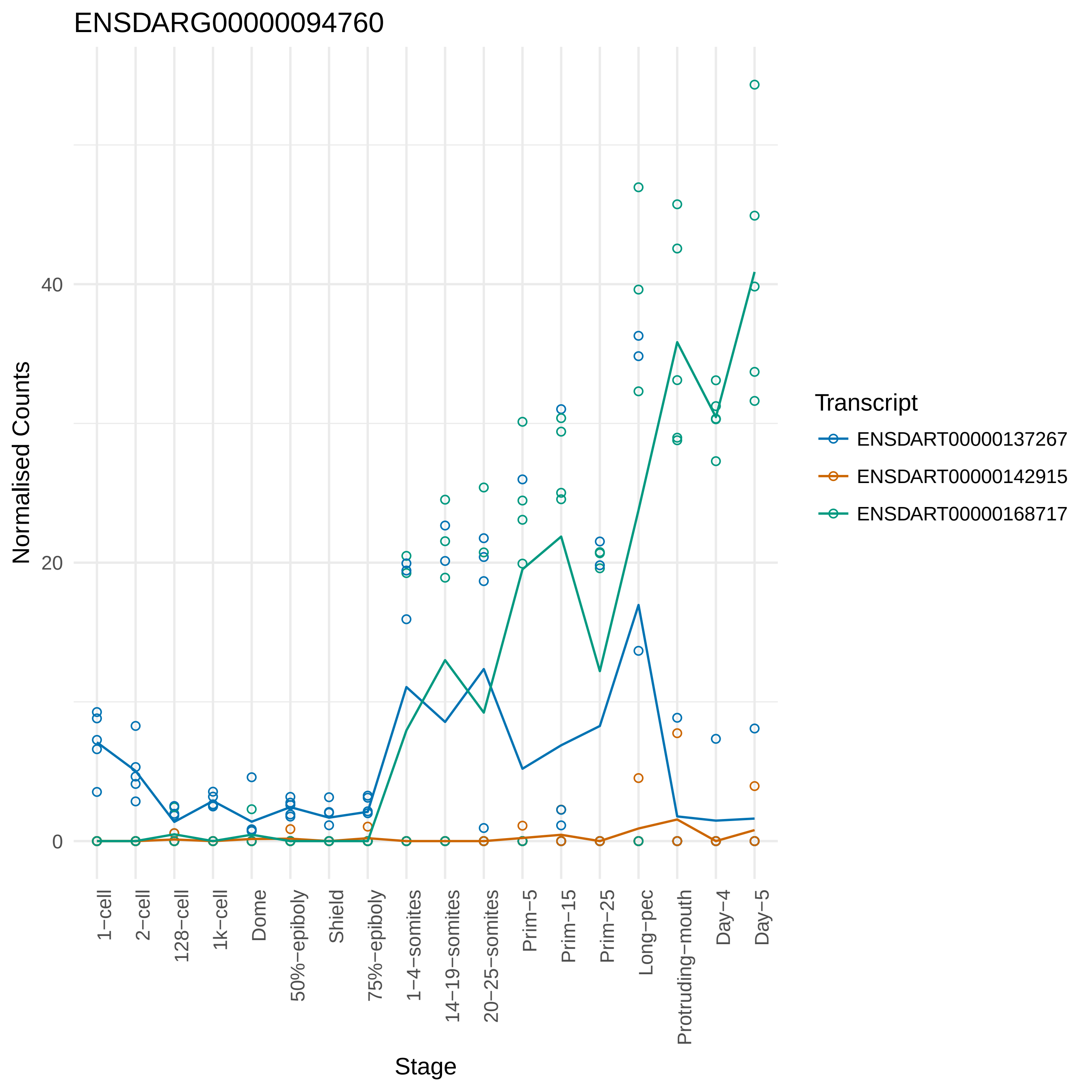
ENSDARG00000094760
Normalised Counts
0
20
40
Stage
1−cell
2−cell
128−cell
1k−cell
Dome
50%−epiboly
Shield
75%−epiboly
1−4−somites
14−19−somites
20−25−somites
Prim−5
Prim−15
Prim−25
Long−pec
Protruding−mouth
Day−4
Day−5
Transcript
ENSDART00000137267
ENSDART00000142915
ENSDART00000168717

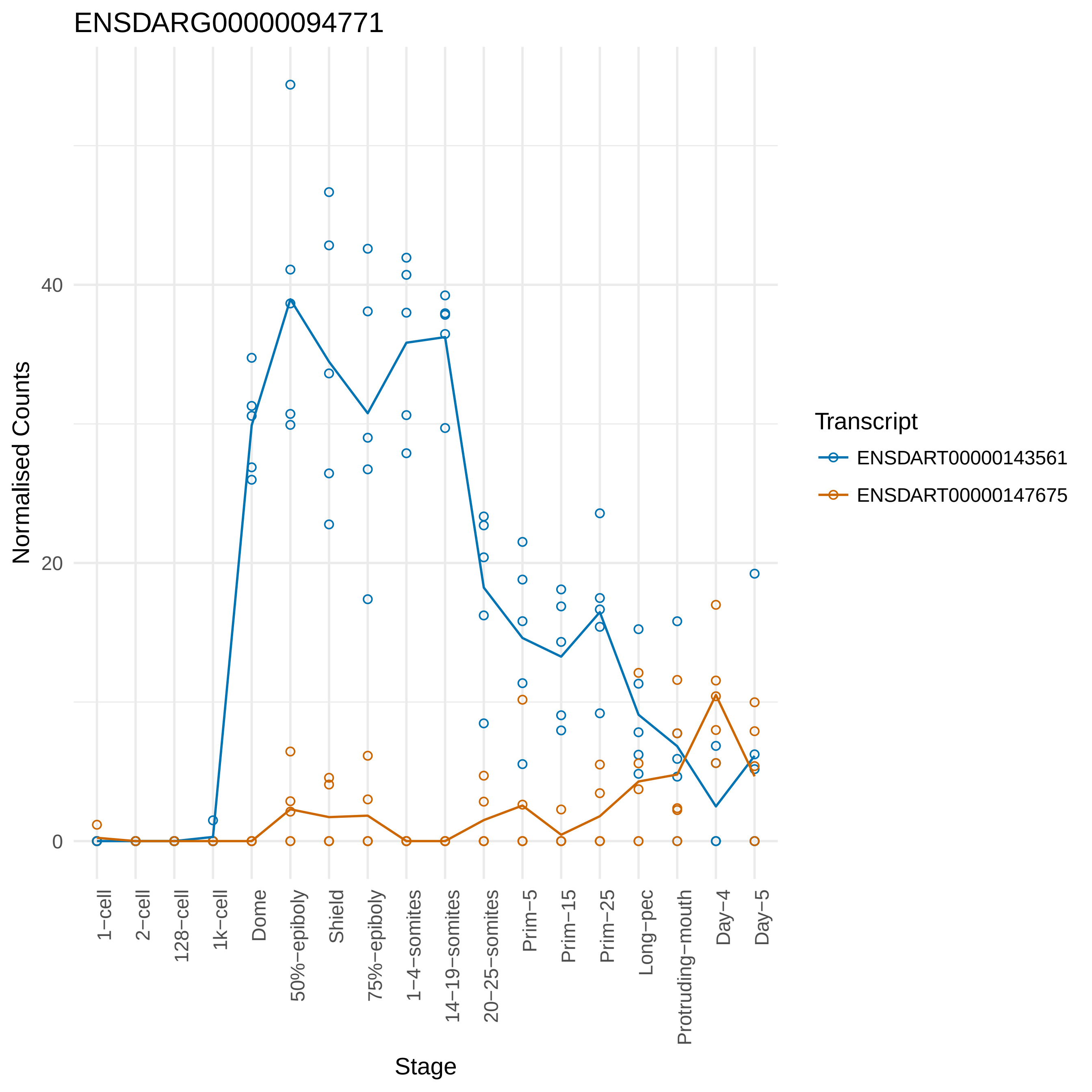

ENSDARG00000094771
Normalised Counts
0
20
40
Stage
1−cell
2−cell
128−cell
1k−cell
Dome
50%−epiboly
Shield
75%−epiboly
1−4−somites
14−19−somites
20−25−somites
Prim−5
Prim−15
Prim−25
Long−pec
Protruding−mouth
Day−4
Day−5
Transcript
ENSDART00000143561
ENSDART00000147675

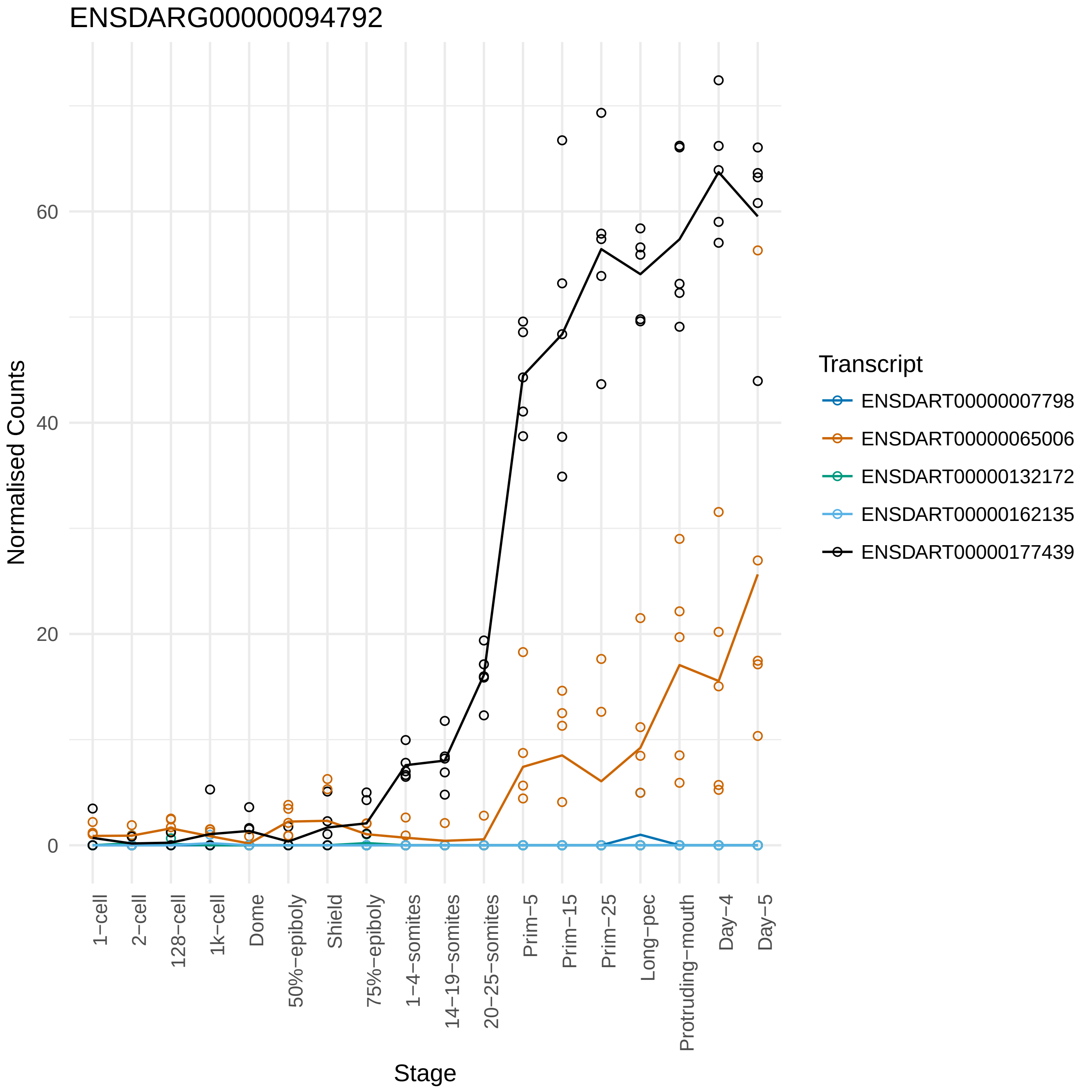

ENSDARG00000094792
Normalised Counts
0
20
40
60
Stage
1−cell
2−cell
128−cell
1k−cell
Dome
50%−epiboly
Shield
75%−epiboly
1−4−somites
14−19−somites
20−25−somites
Prim−5
Prim−15
Prim−25
Long−pec
Protruding−mouth
Day−4
Day−5
Transcript
ENSDART00000007798
ENSDART00000065006
ENSDART00000132172
ENSDART00000162135
ENSDART00000177439

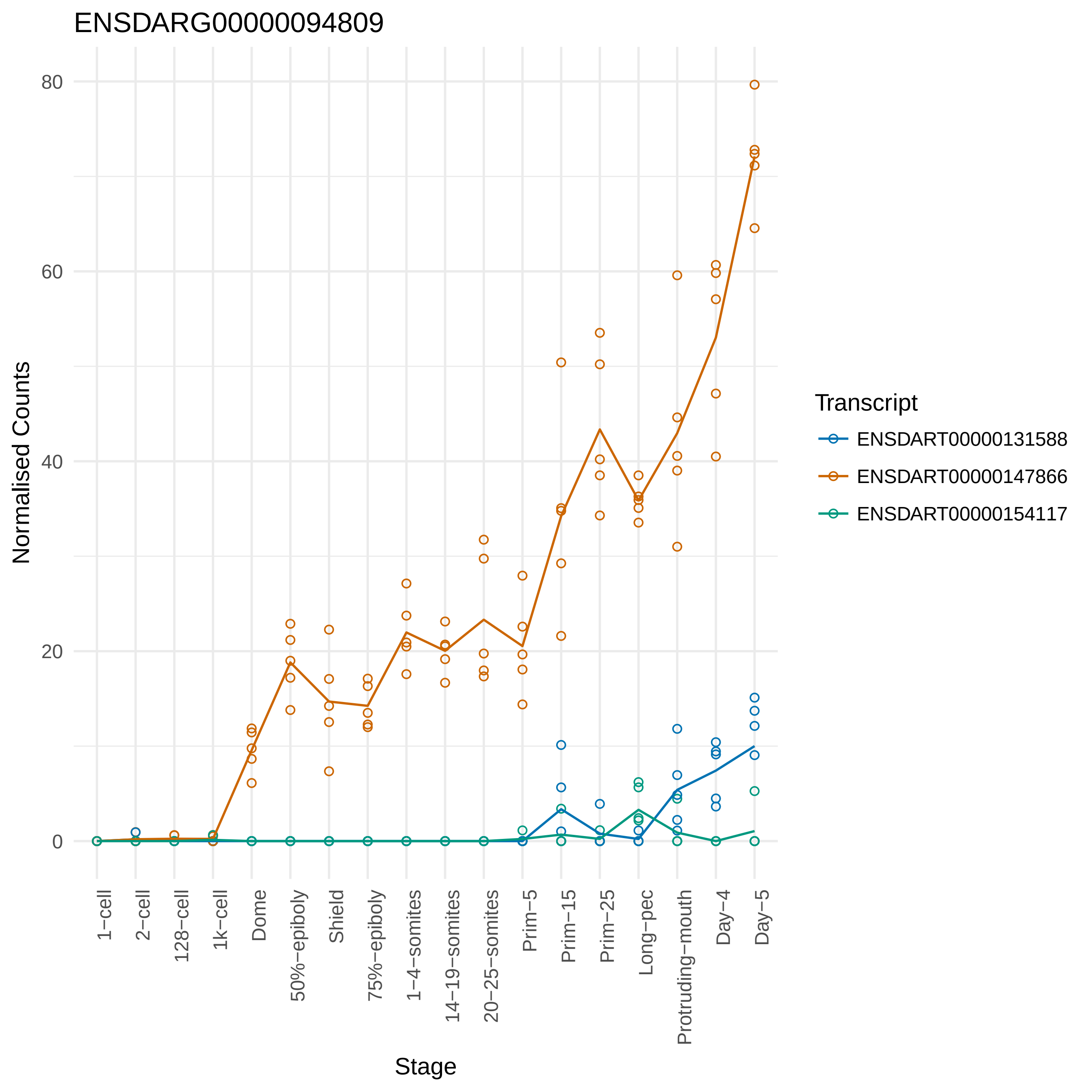
ENSDARG00000094809
Normalised Counts
0
20
40
60
80
Stage
1−cell
2−cell
128−cell
1k−cell
Dome
50%−epiboly
Shield
75%−epiboly
1−4−somites
14−19−somites
20−25−somites
Prim−5
Prim−15
Prim−25
Long−pec
Protruding−mouth
Day−4
Day−5
Transcript
ENSDART00000131588
ENSDART00000147866
ENSDART00000154117

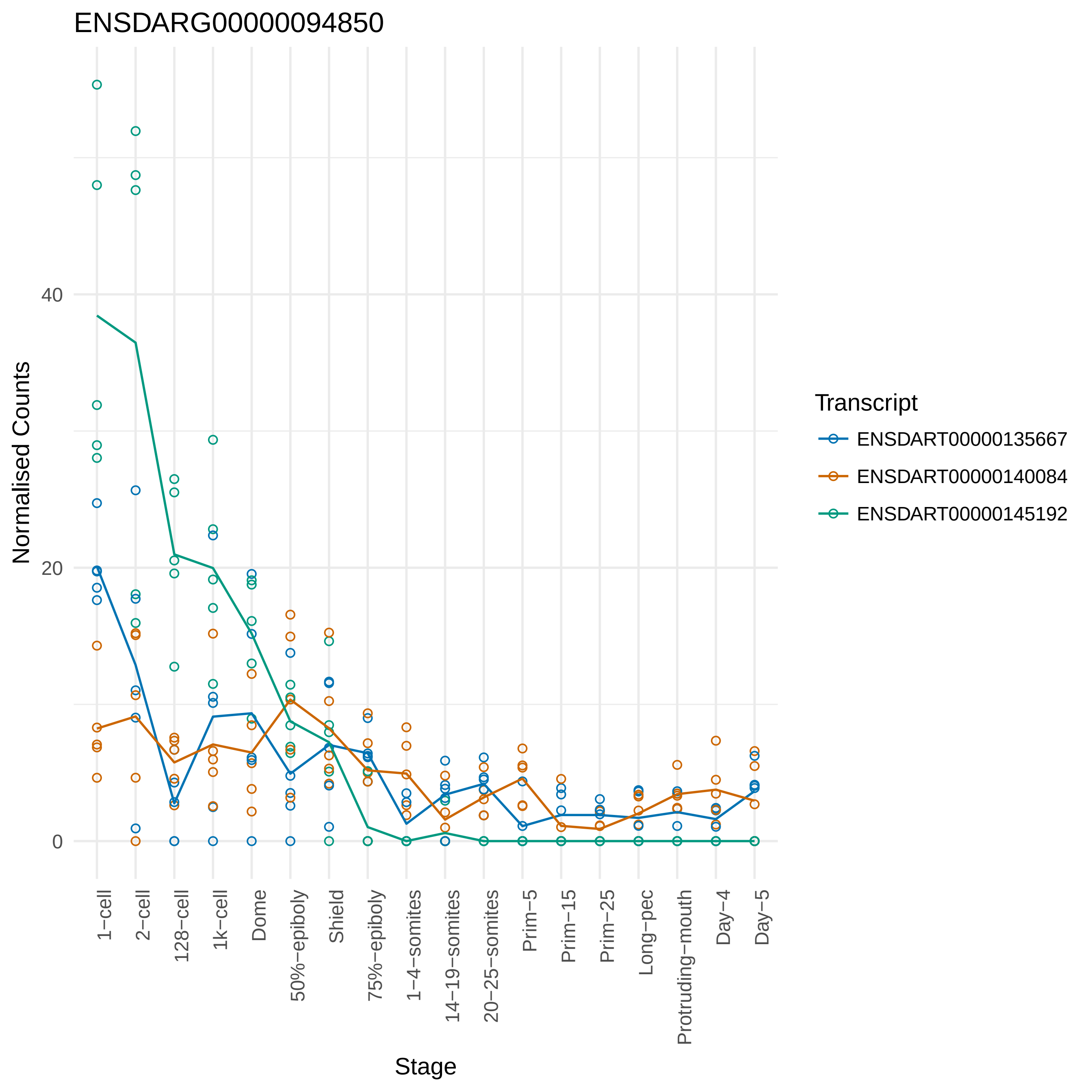
ENSDARG00000094850
Normalised Counts
0
20
40
Stage
1−cell
2−cell
128−cell
1k−cell
Dome
50%−epiboly
Shield
75%−epiboly
1−4−somites
14−19−somites
20−25−somites
Prim−5
Prim−15
Prim−25
Long−pec
Protruding−mouth
Day−4
Day−5
Transcript
ENSDART00000135667
ENSDART00000140084
ENSDART00000145192

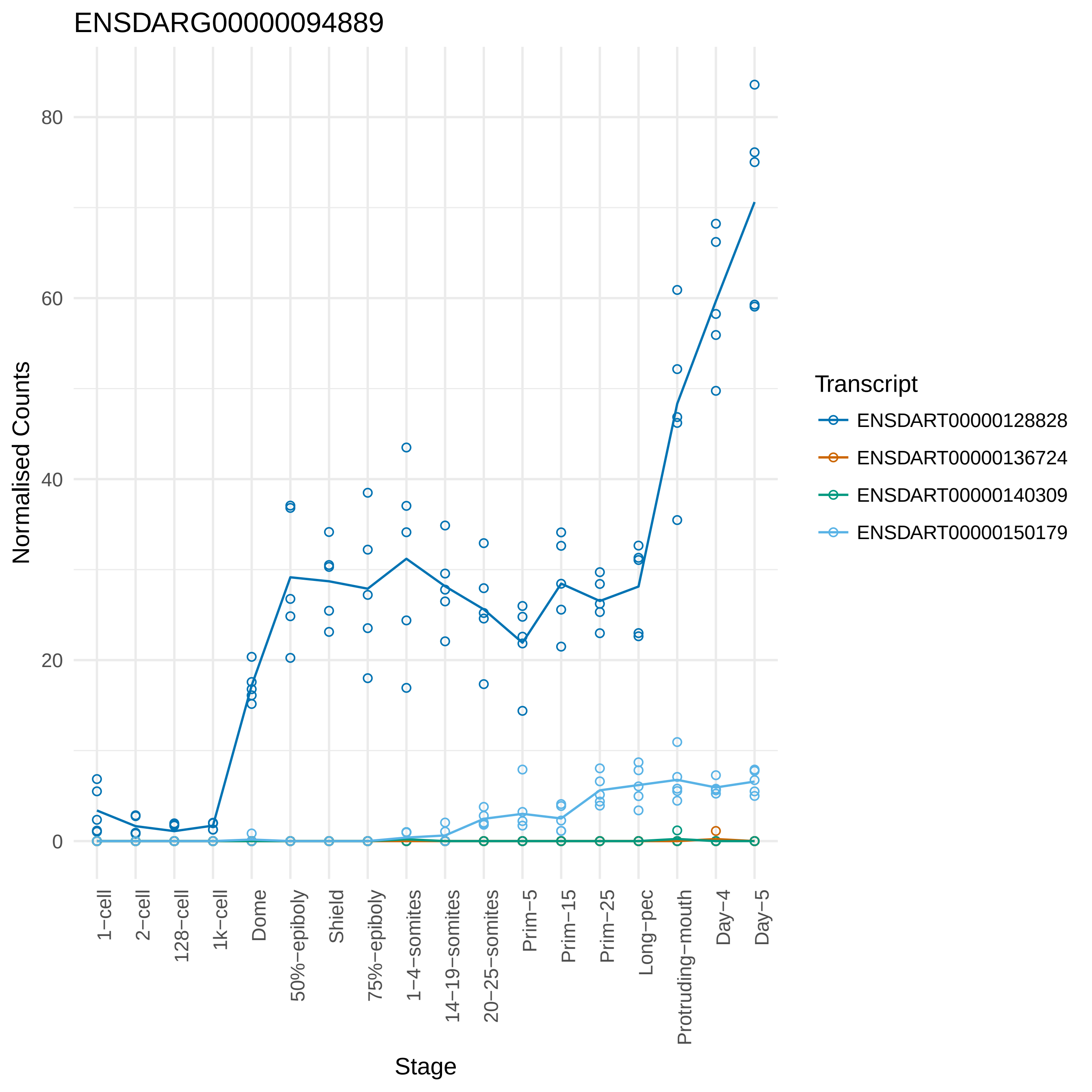
ENSDARG00000094889
Normalised Counts
0
20
40
60
80
Stage
1−cell
2−cell
128−cell
1k−cell
Dome
50%−epiboly
Shield
75%−epiboly
1−4−somites
14−19−somites
20−25−somites
Prim−5
Prim−15
Prim−25
Long−pec
Protruding−mouth
Day−4
Day−5
Transcript
ENSDART00000128828
ENSDART00000136724
ENSDART00000140309
ENSDART00000150179

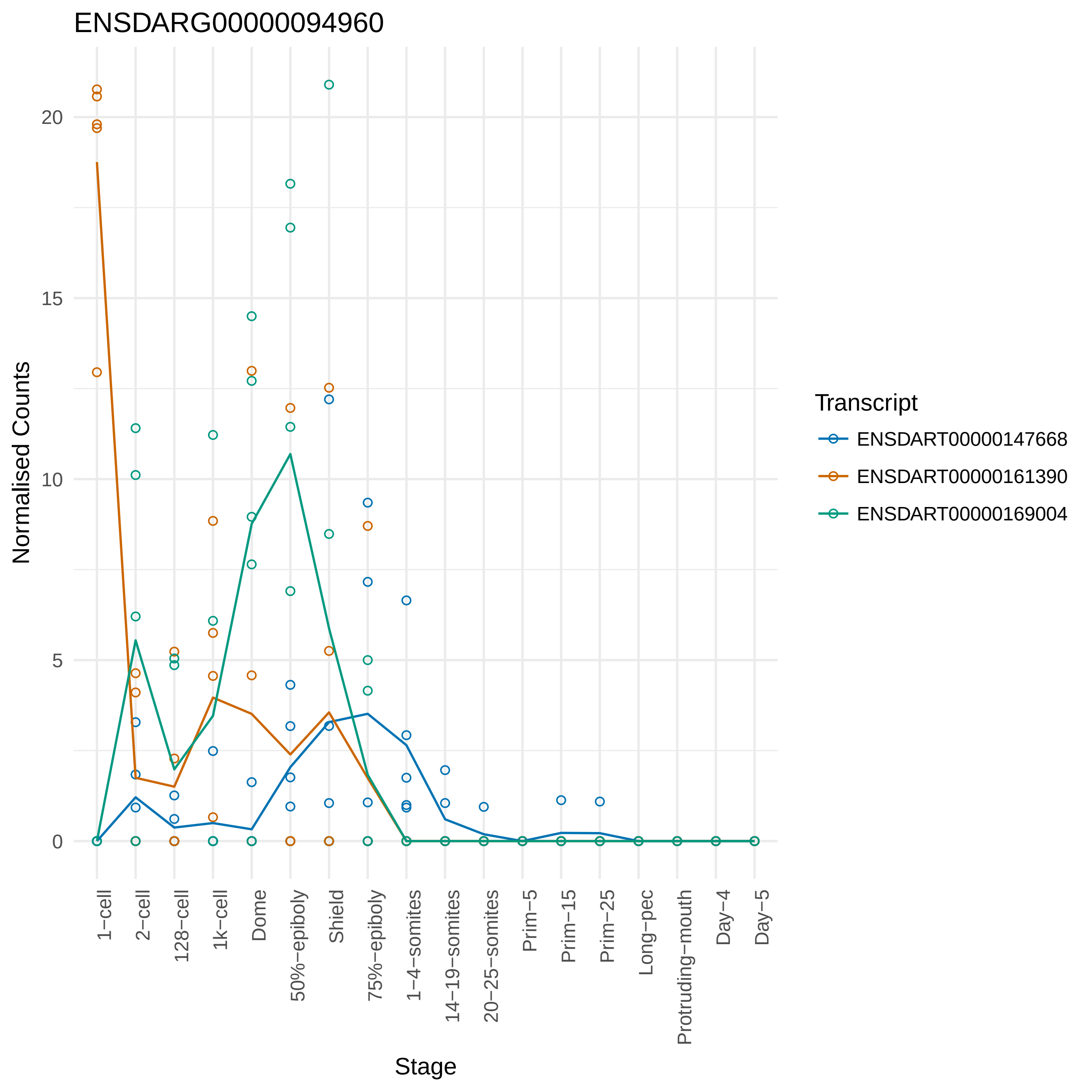
ENSDARG00000094960
Normalised Counts
0
5
10
15
20
Stage
1−cell
2−cell
128−cell
1k−cell
Dome
50%−epiboly
Shield
75%−epiboly
1−4−somites
14−19−somites
20−25−somites
Prim−5
Prim−15
Prim−25
Long−pec
Protruding−mouth
Day−4
Day−5
Transcript
ENSDART00000147668
ENSDART00000161390
ENSDART00000169004

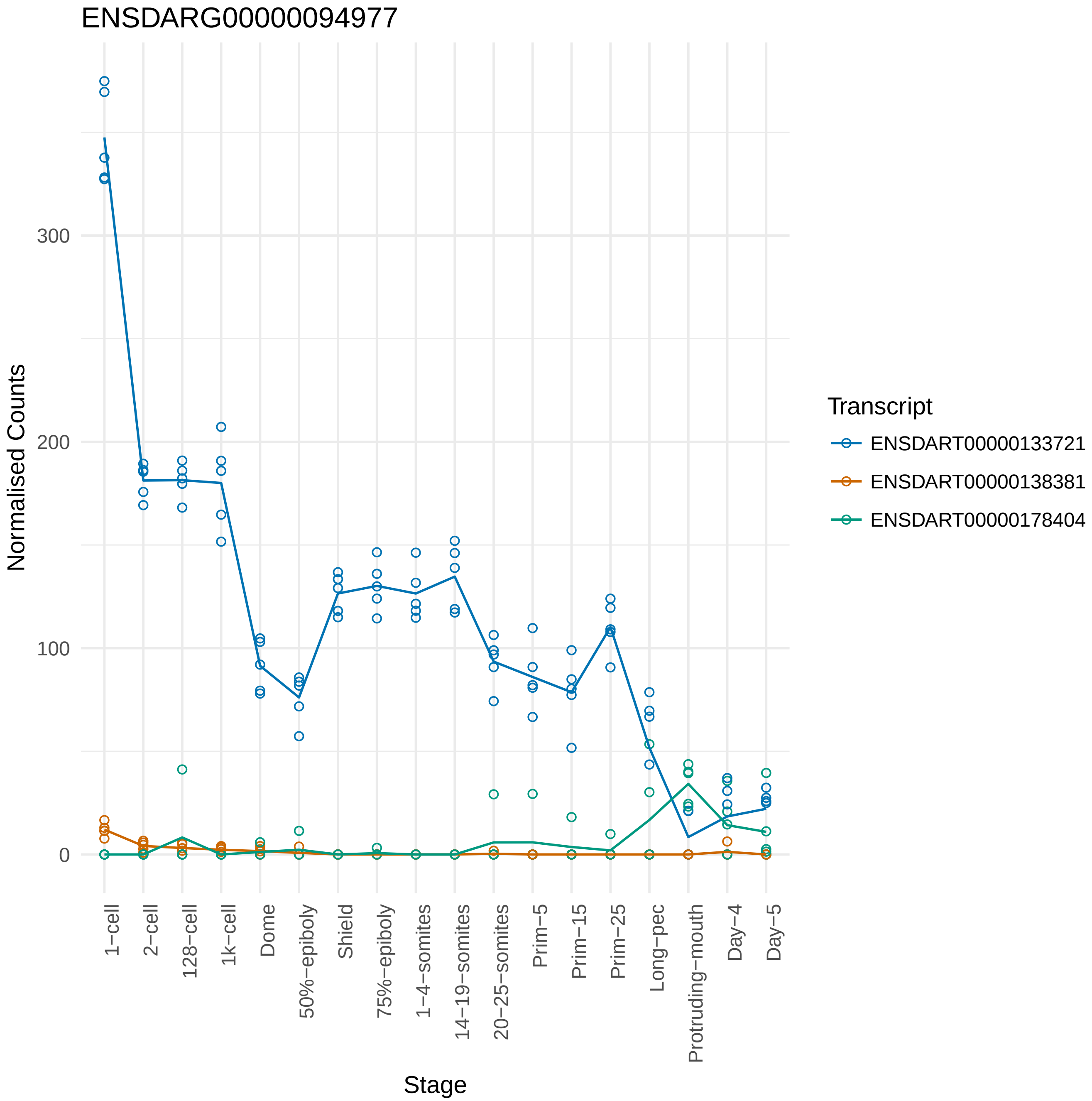

ENSDARG00000094977
Normalised Counts
300
200
100
0
1−cell
2−cell
128−cell
1k−cell
Dome
50%−epiboly
Shield
75%−epiboly
1−4−somites
14−19−somites
20−25−somites
Prim−5
Prim−15
Prim−25
Long−pec
Protruding−mouth
Day−4
Day−5
Stage
Transcript
ENSDART00000133721
ENSDART00000138381
ENSDART00000178404

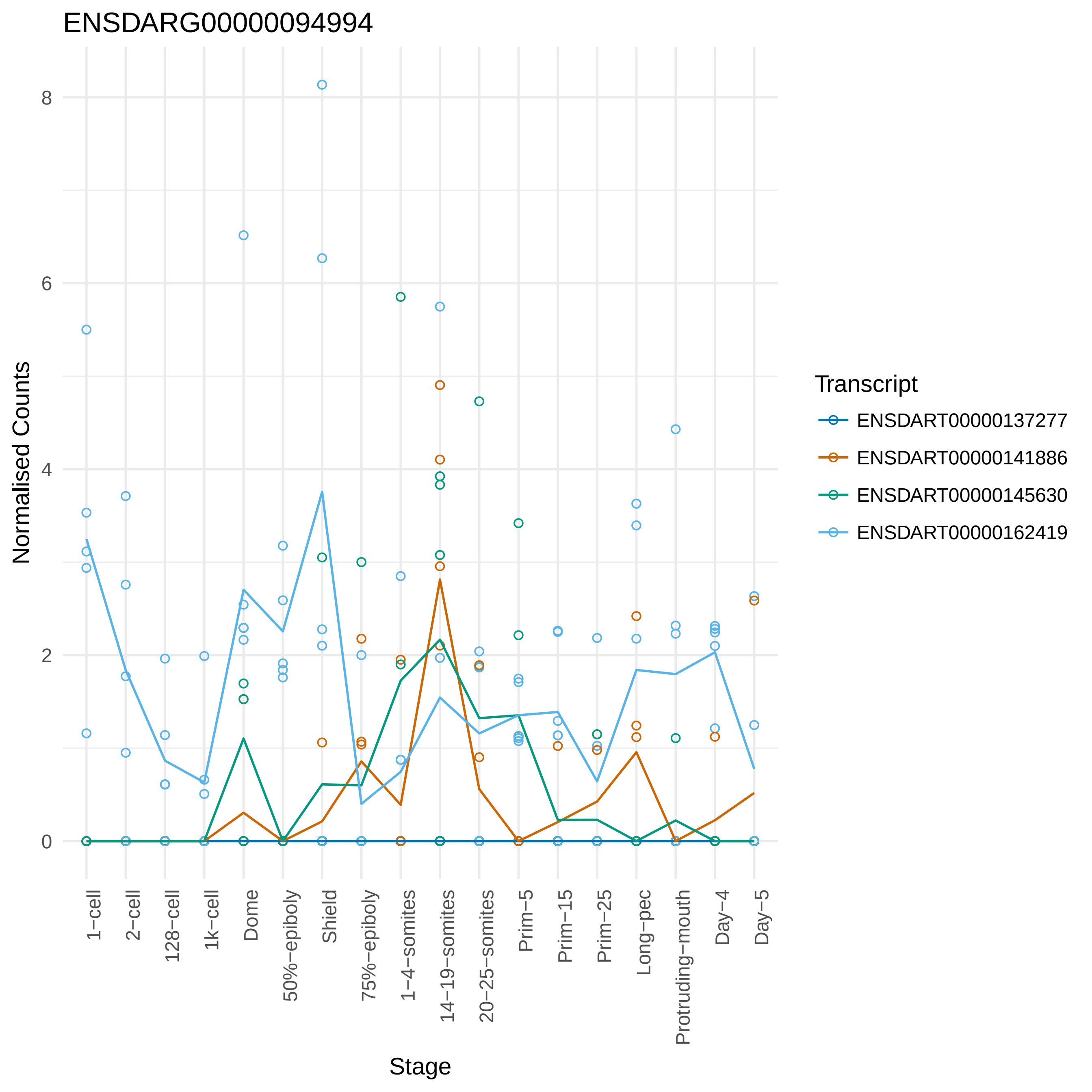
ENSDARG00000094994
Normalised Counts
0
2
4
6
8
1−cell
2−cell
128−cell
1k−cell
Dome
50%−epiboly
Shield
75%−epiboly
1−4−somites
14−19−somites
20−25−somites
Prim−5
Prim−15
Prim−25
Long−pec
Protruding−mouth
Day−4
Day−5
Stage
Transcript
ENSDART00000137277
ENSDART00000141886
ENSDART00000145630
ENSDART00000162419

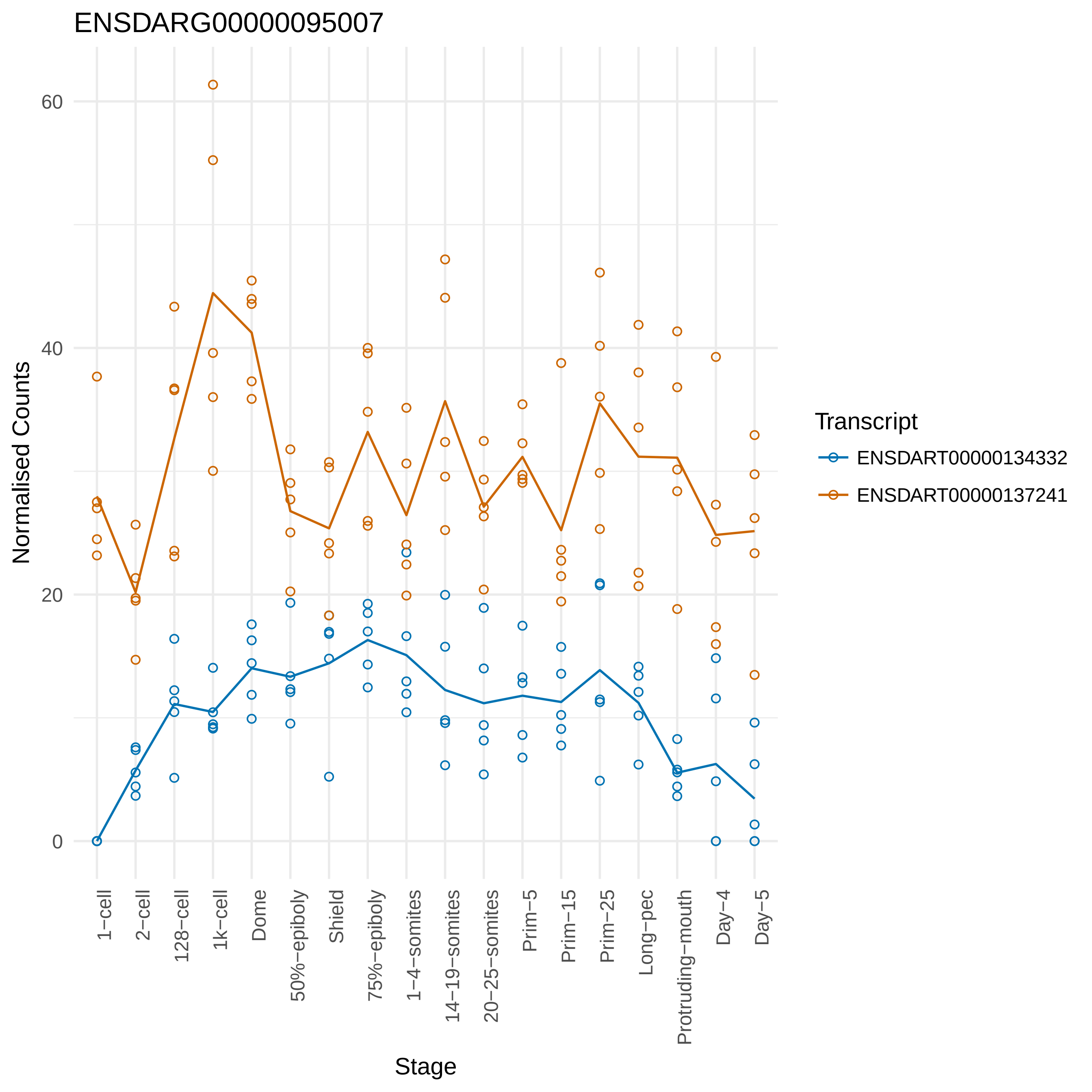

ENSDARG00000095007
Normalised Counts
60
40
20
0
1−cell
2−cell
128−cell
1k−cell
Dome
50%−epiboly
Shield
75%−epiboly
1−4−somites
14−19−somites
20−25−somites
Prim−5
Prim−15
Prim−25
Long−pec
Protruding−mouth
Day−4
Day−5
Stage
Transcript
ENSDART00000134332
ENSDART00000137241

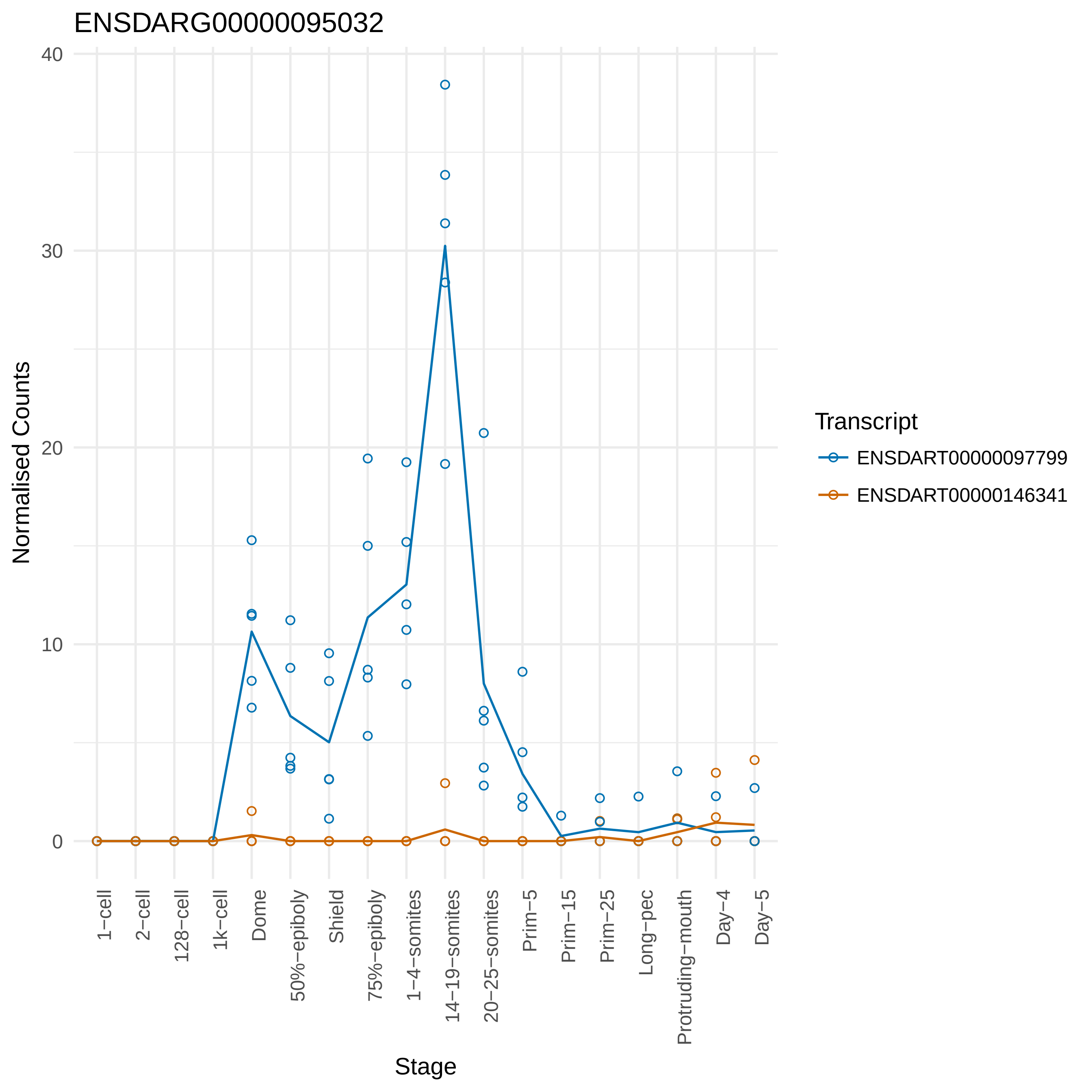

ENSDARG00000095032
Normalised Counts
0
10
20
30
40
1−cell
2−cell
128−cell
1k−cell
Dome
50%−epiboly
Shield
75%−epiboly
1−4−somites
14−19−somites
20−25−somites
Prim−5
Prim−15
Prim−25
Long−pec
Protruding−mouth
Day−4
Day−5
Stage
Transcript
ENSDART00000097799
ENSDART00000146341

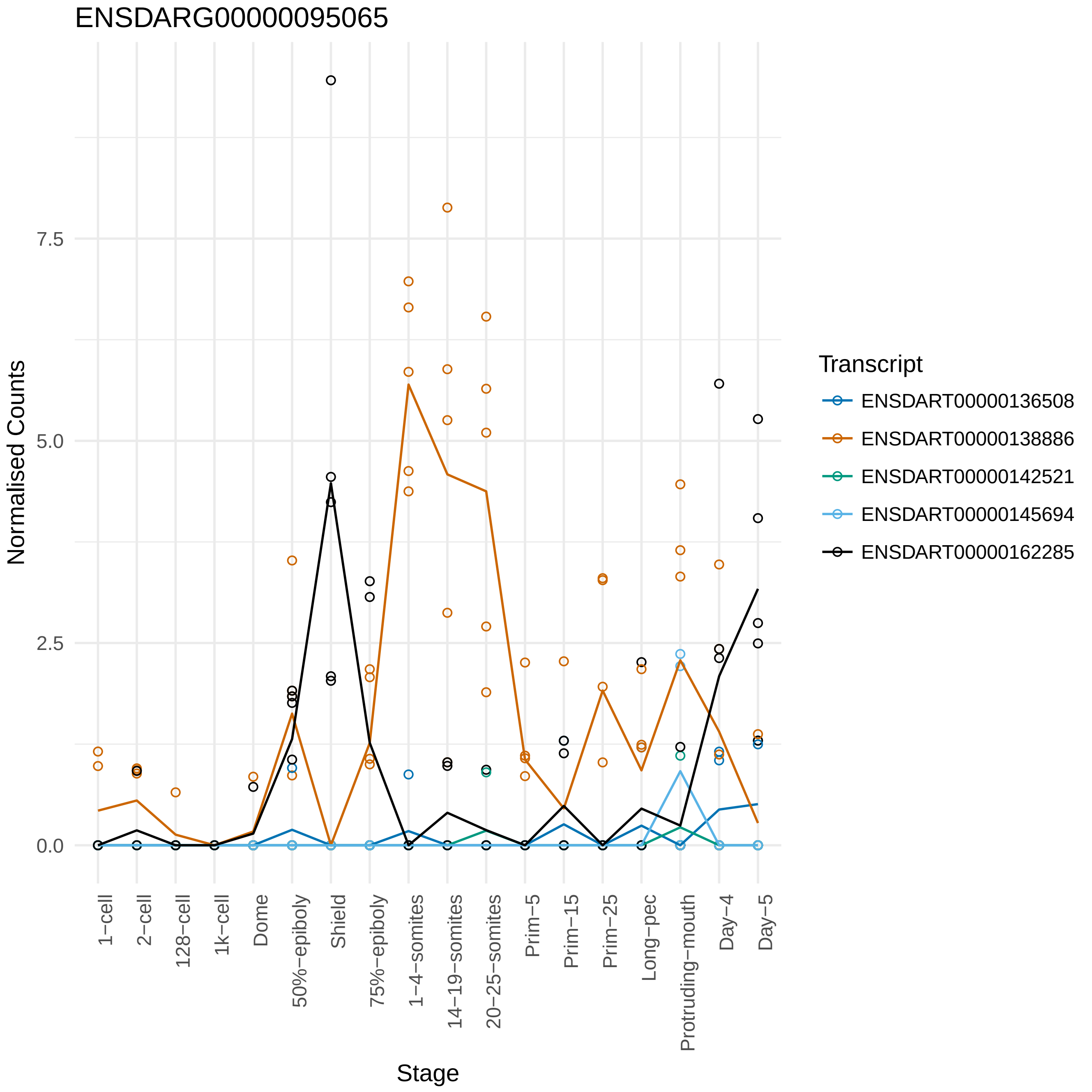
ENSDARG00000095065
Normalised Counts
7.5
5.0
2.5
0.0
Stage
1−cell
2−cell
128−cell
1k−cell
Dome
50%−epiboly
Shield
75%−epiboly
1−4−somites
14−19−somites
20−25−somites
Prim−5
Prim−15
Prim−25
Long−pec
Protruding−mouth
Day−4
Day−5
Transcript
ENSDART00000136508
ENSDART00000138886
ENSDART00000142521
ENSDART00000145694
ENSDART00000162285

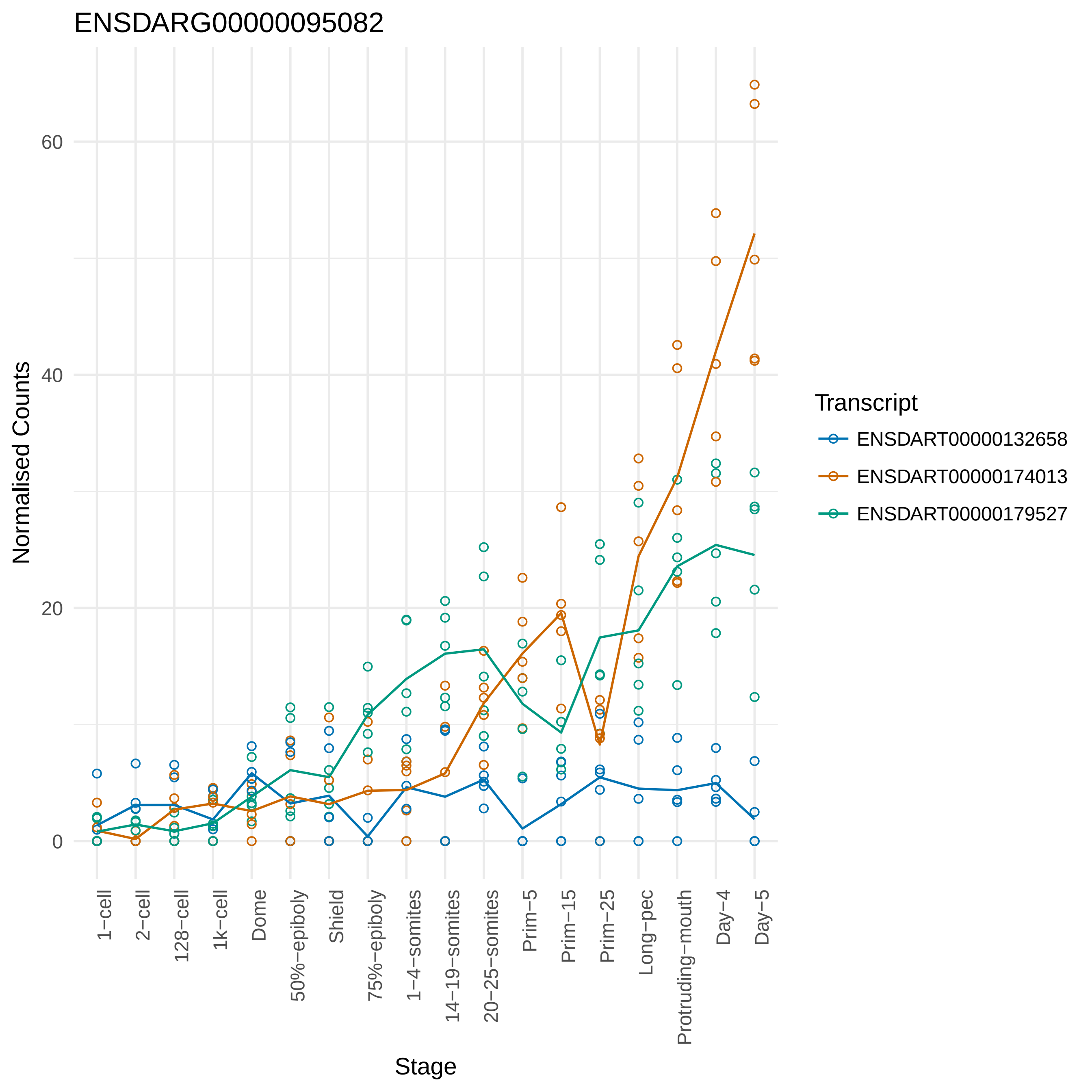
ENSDARG00000095082
Normalised Counts
0
20
40
60
Stage
1−cell
2−cell
128−cell
1k−cell
Dome
50%−epiboly
Shield
75%−epiboly
1−4−somites
14−19−somites
20−25−somites
Prim−5
Prim−15
Prim−25
Long−pec
Protruding−mouth
Day−4
Day−5
Transcript
ENSDART00000132658
ENSDART00000174013
ENSDART00000179527

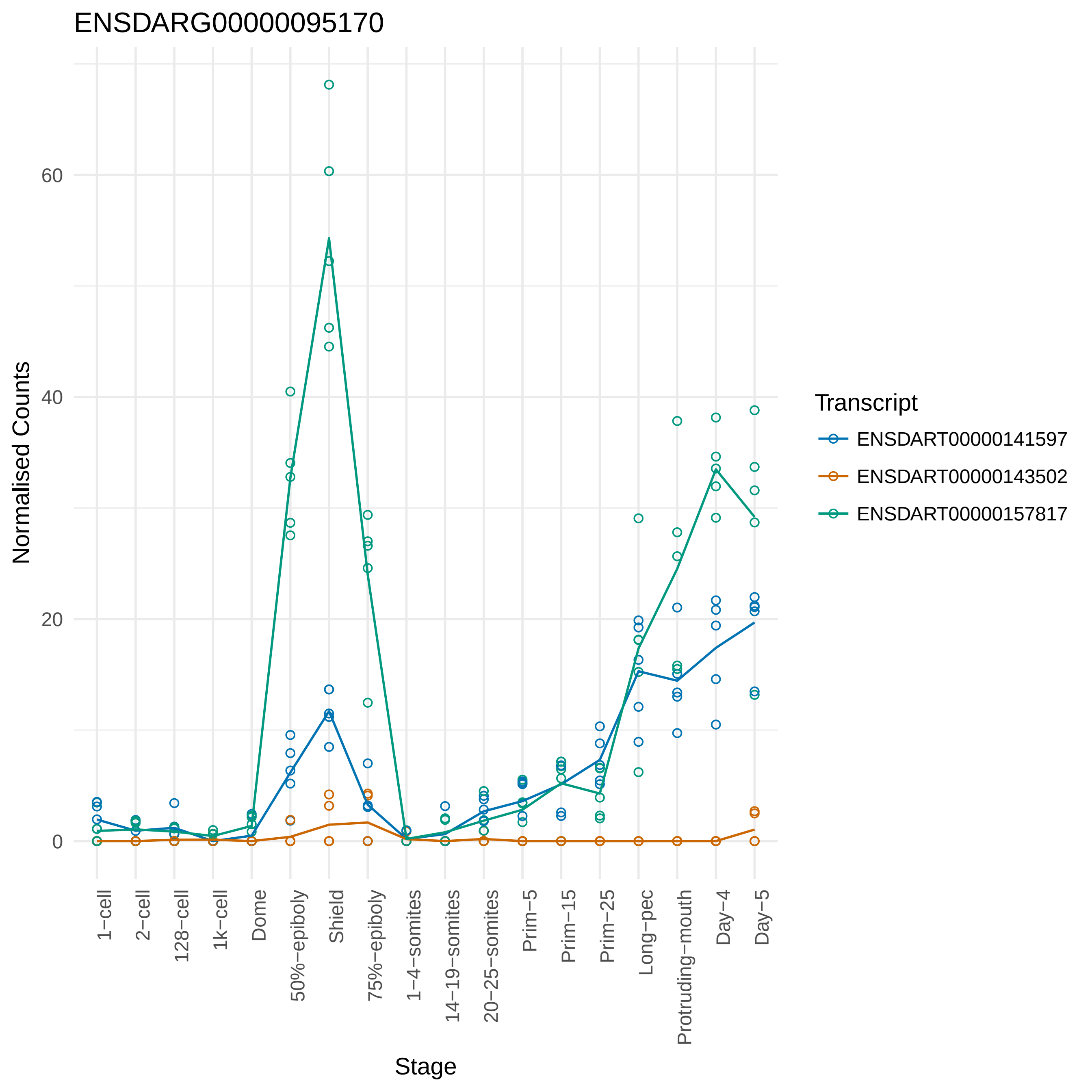
ENSDARG00000095170
Normalised Counts
0
20
40
60
Stage
1−cell
2−cell
128−cell
1k−cell
Dome
50%−epiboly
Shield
75%−epiboly
1−4−somites
14−19−somites
20−25−somites
Prim−5
Prim−15
Prim−25
Long−pec
Protruding−mouth
Day−4
Day−5
Transcript
ENSDART00000141597
ENSDART00000143502
ENSDART00000157817

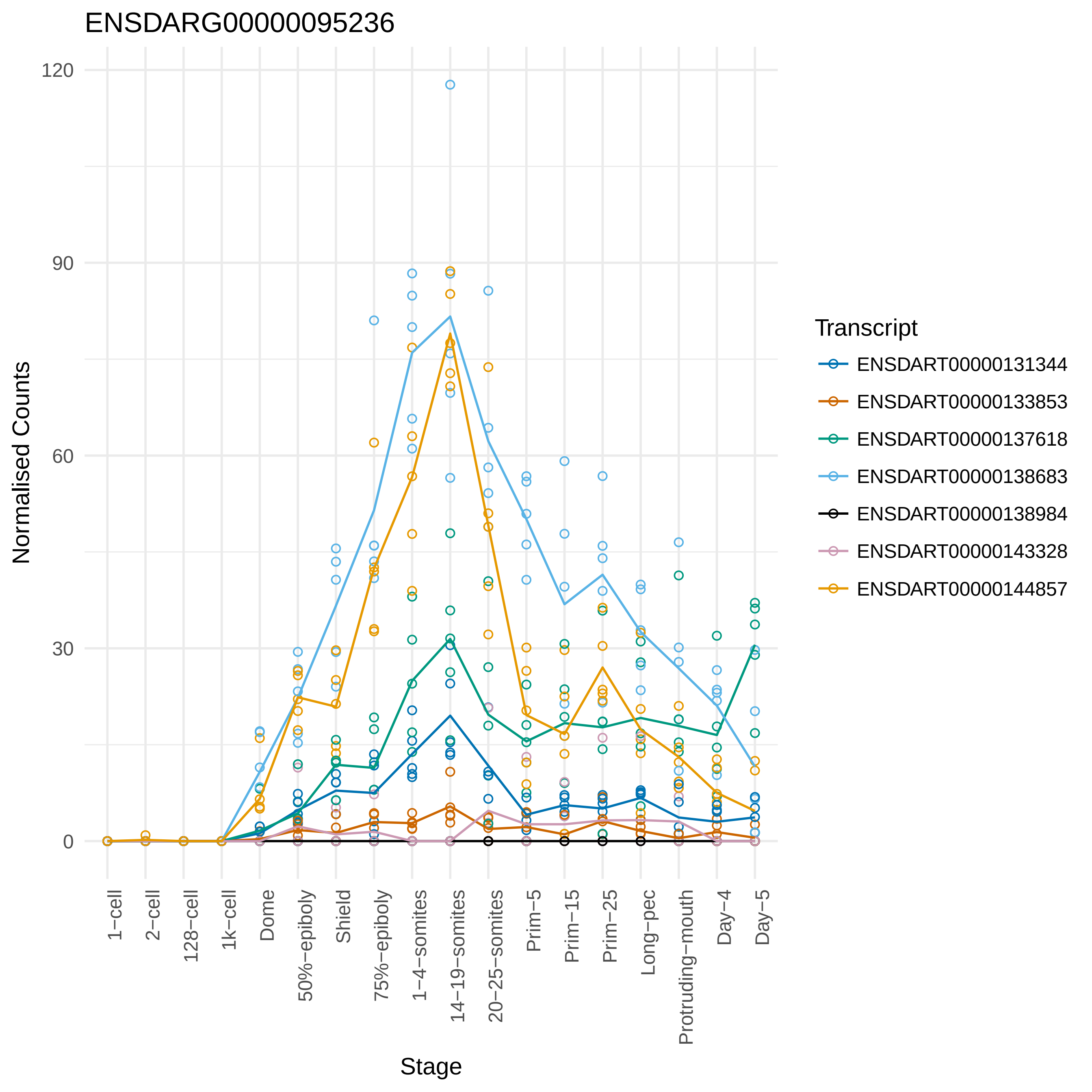
ENSDARG00000095236
Normalised Counts
120
90
60
30
0
1−cell
2−cell
128−cell
1k−cell
Dome
50%−epiboly
Shield
75%−epiboly
1−4−somites
14−19−somites
20−25−somites
Prim−5
Prim−15
Prim−25
Long−pec
Protruding−mouth
Day−4
Day−5
Stage
Transcript
ENSDART00000131344
ENSDART00000133853
ENSDART00000137618
ENSDART00000138683
ENSDART00000138984
ENSDART00000143328
ENSDART00000144857

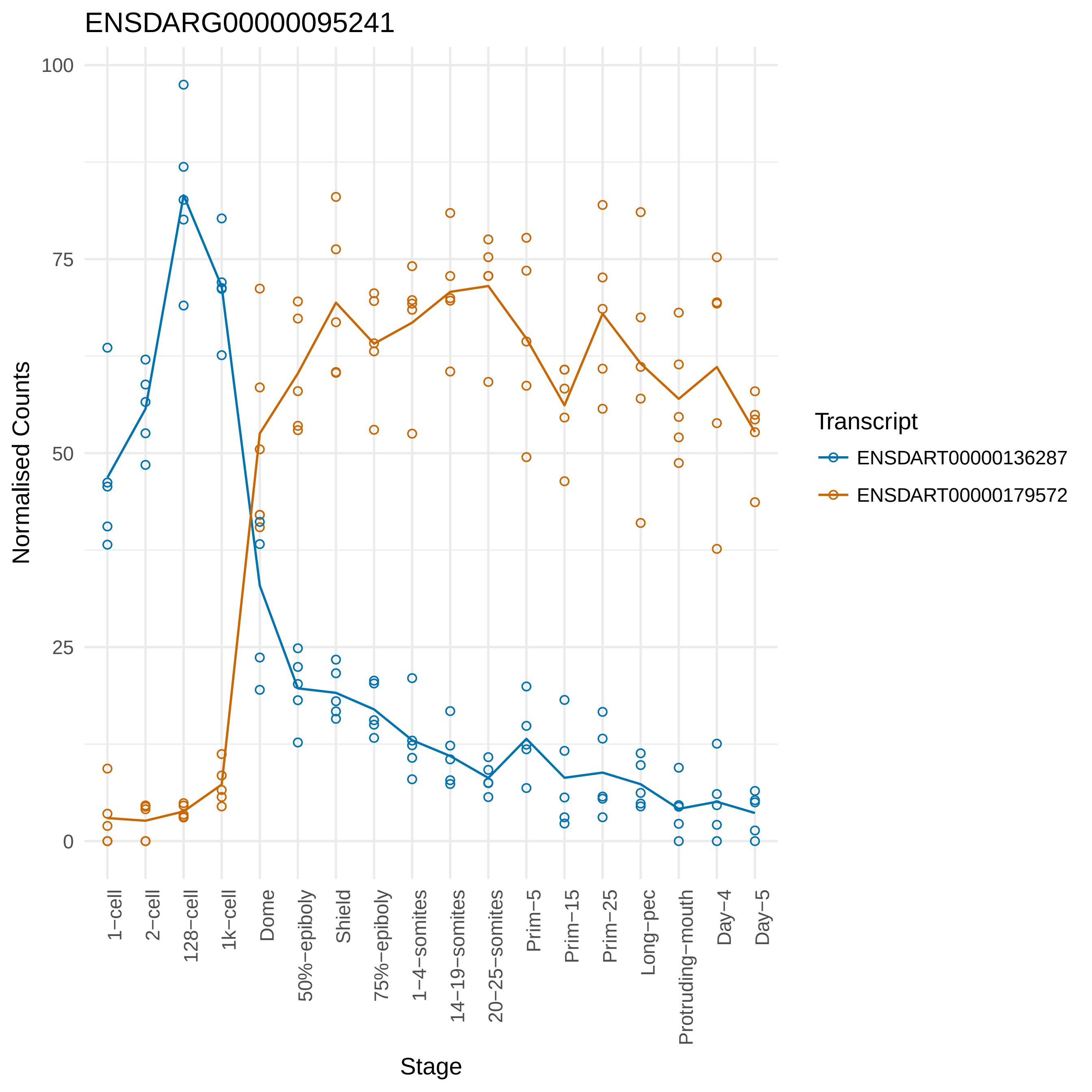

ENSDARG00000095241
Normalised Counts
100
75
50
25
0
1−cell
2−cell
128−cell
1k−cell
Dome
50%−epiboly
Shield
75%−epiboly
1−4−somites
14−19−somites
20−25−somites
Prim−5
Prim−15
Prim−25
Long−pec
Protruding−mouth
Day−4
Day−5
Stage
Transcript
ENSDART00000136287
ENSDART00000179572

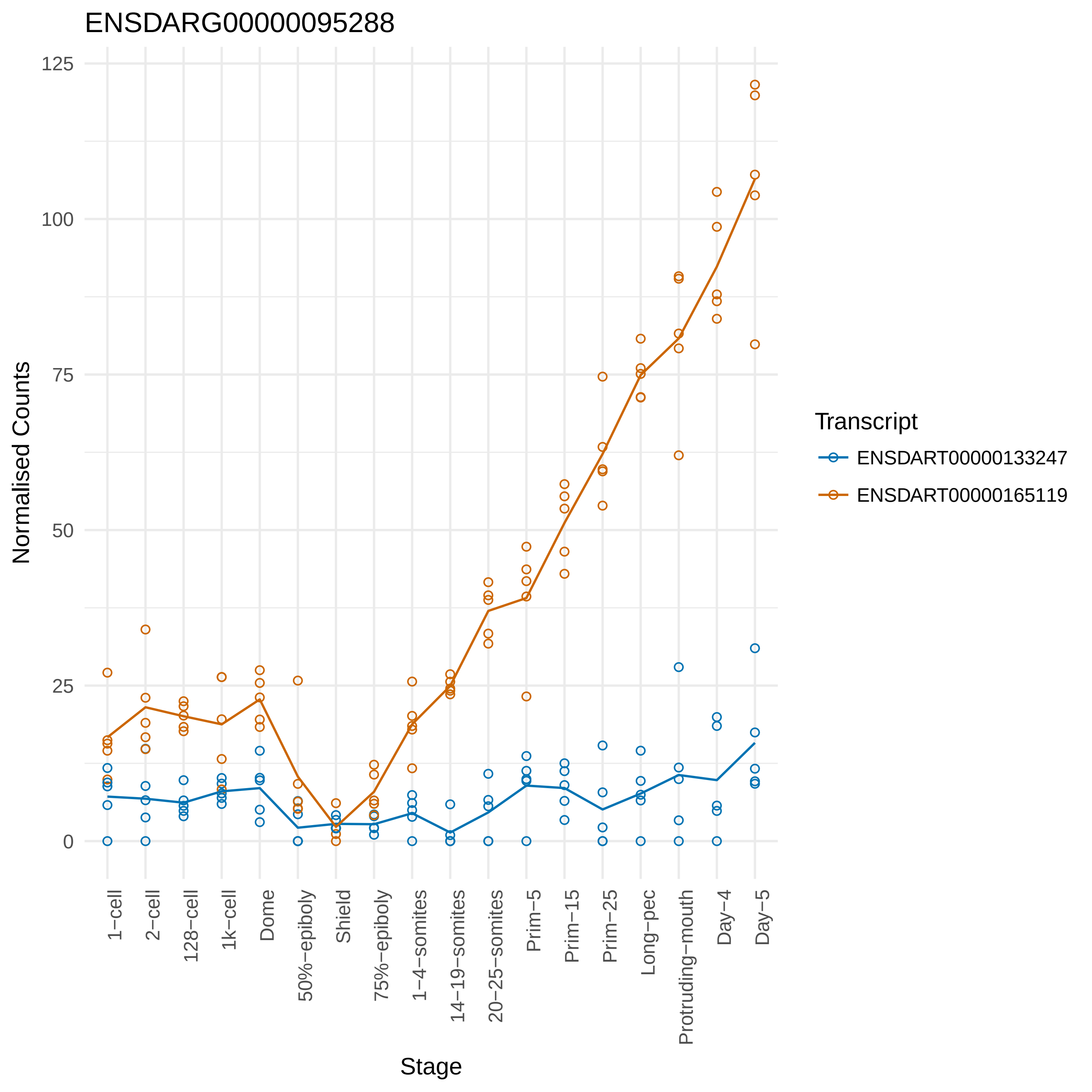
ENSDARG00000095288
Normalised Counts
0
25
50
75
100
125
Stage
1−cell
2−cell
128−cell
1k−cell
Dome
50%−epiboly
Shield
75%−epiboly
1−4−somites
14−19−somites
20−25−somites
Prim−5
Prim−15
Prim−25
Long−pec
Protruding−mouth
Day−4
Day−5
Transcript
ENSDART00000133247
ENSDART00000165119

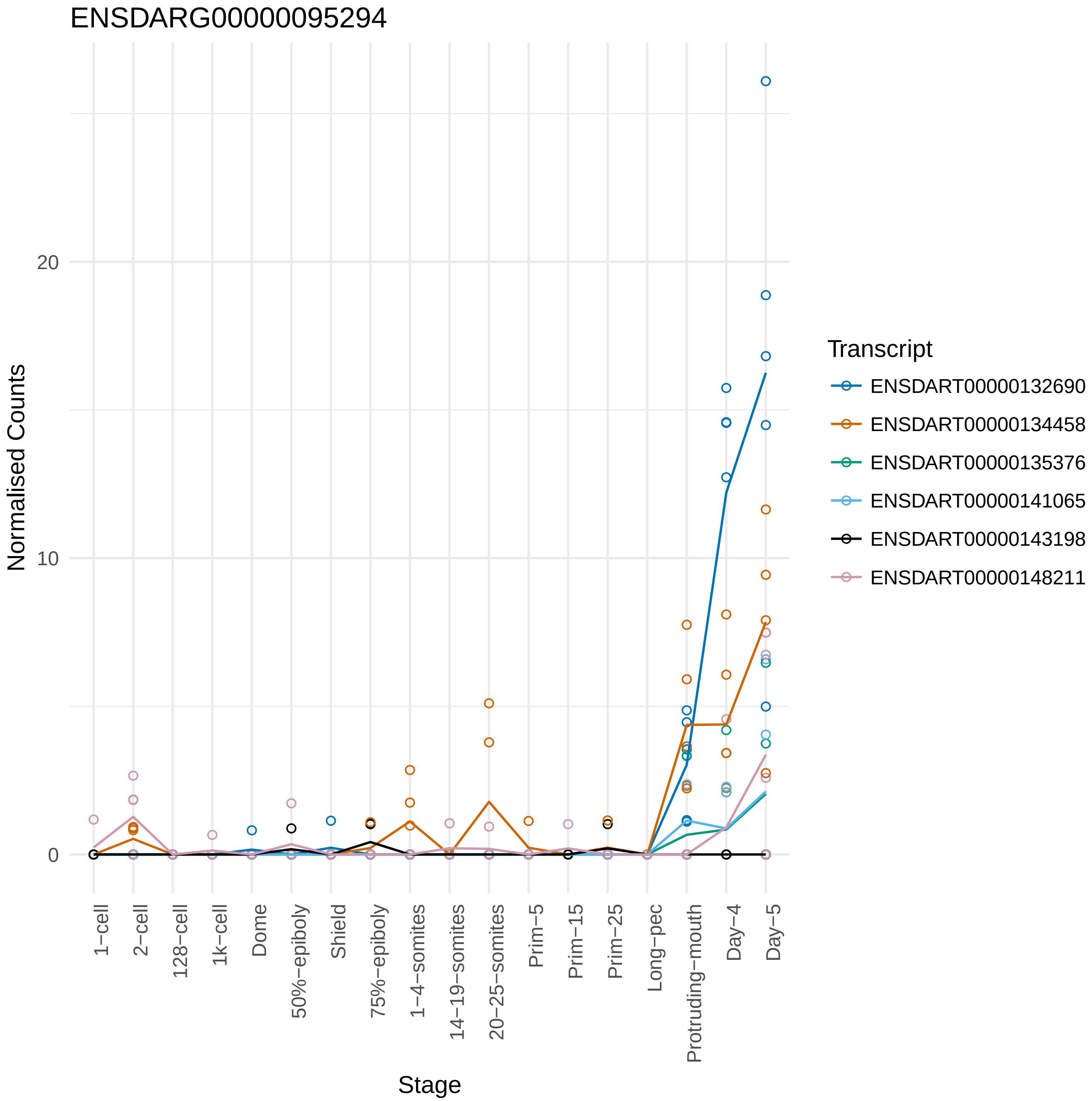

ENSDARG00000095294
Normalised Counts
0
10
20
Stage
1−cell
2−cell
128−cell
1k−cell
Dome
50%−epiboly
Shield
75%−epiboly
1−4−somites
14−19−somites
20−25−somites
Prim−5
Prim−15
Prim−25
Long−pec
Protruding−mouth
Day−4
Day−5
Transcript
ENSDART00000132690
ENSDART00000134458
ENSDART00000135376
ENSDART00000141065
ENSDART00000143198
ENSDART00000148211

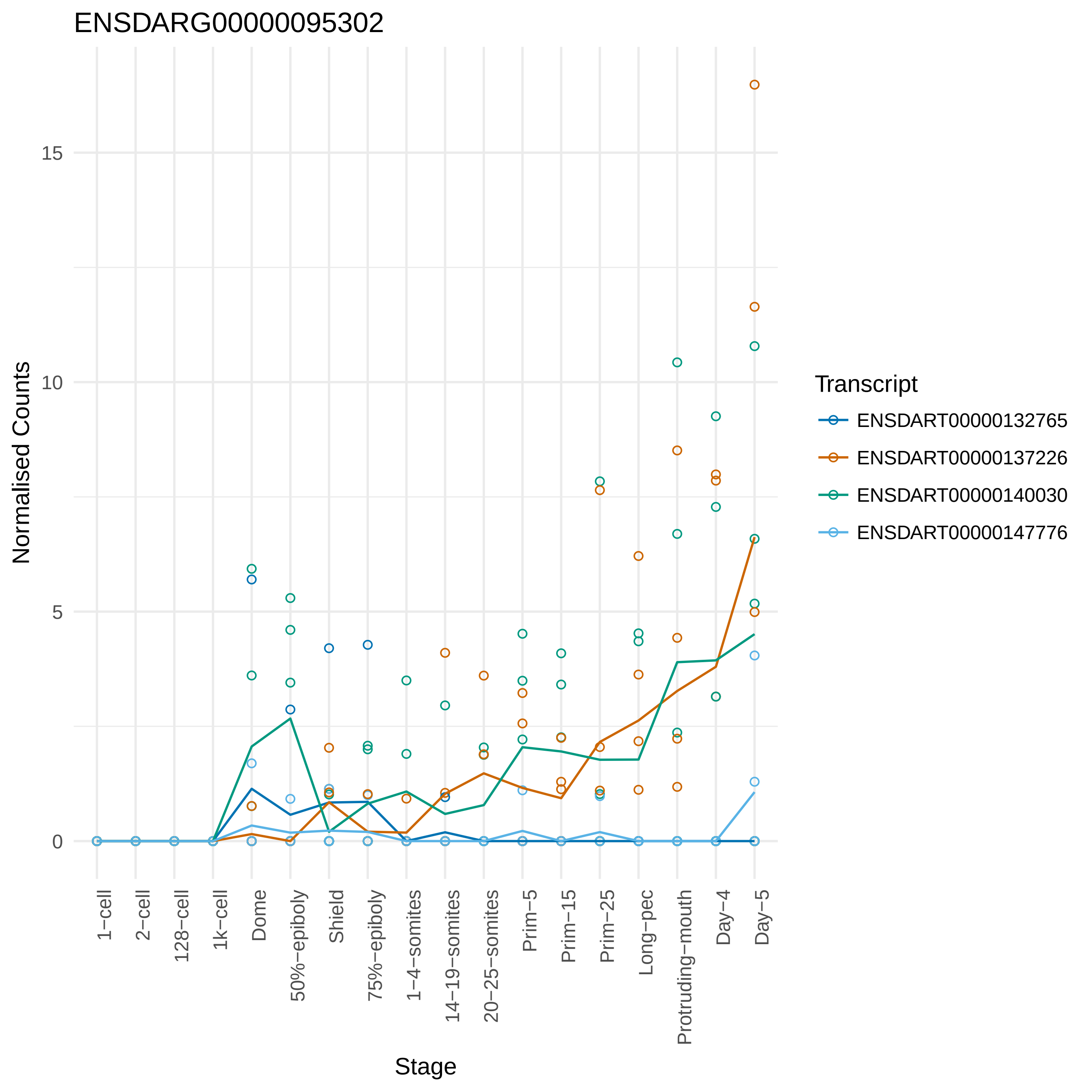
ENSDARG00000095302
Normalised Counts
15
10
5
0
1−cell
2−cell
128−cell
1k−cell
Dome
50%−epiboly
Shield
75%−epiboly
1−4−somites
14−19−somites
20−25−somites
Prim−5
Prim−15
Prim−25
Long−pec
Protruding−mouth
Day−4
Day−5
Stage
Transcript
ENSDART00000132765
ENSDART00000137226
ENSDART00000140030
ENSDART00000147776

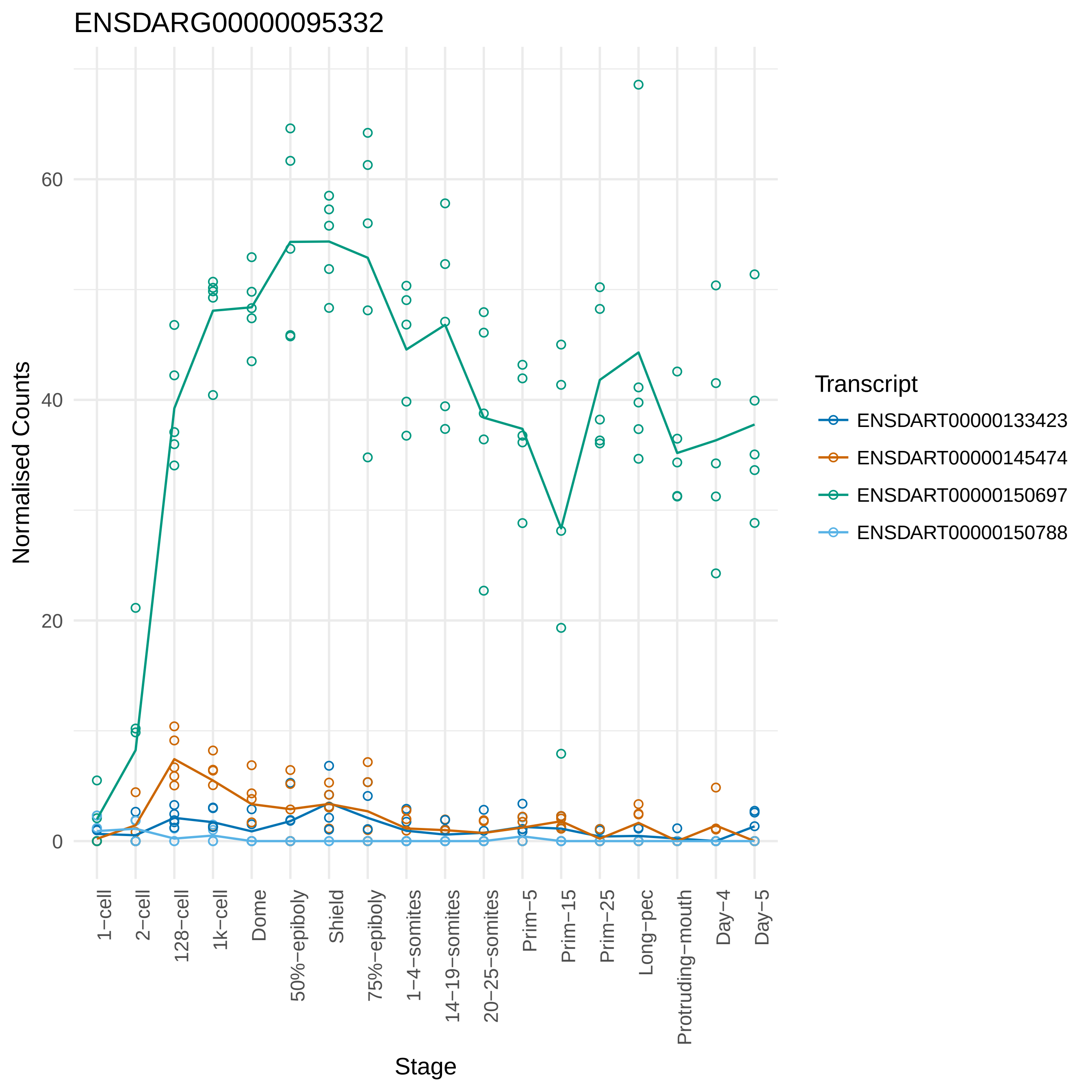

ENSDARG00000095332
Normalised Counts
0
20
40
60
Stage
1−cell
2−cell
128−cell
1k−cell
Dome
50%−epiboly
Shield
75%−epiboly
1−4−somites
14−19−somites
20−25−somites
Prim−5
Prim−15
Prim−25
Long−pec
Protruding−mouth
Day−4
Day−5
Transcript
ENSDART00000133423
ENSDART00000145474
ENSDART00000150697
ENSDART00000150788

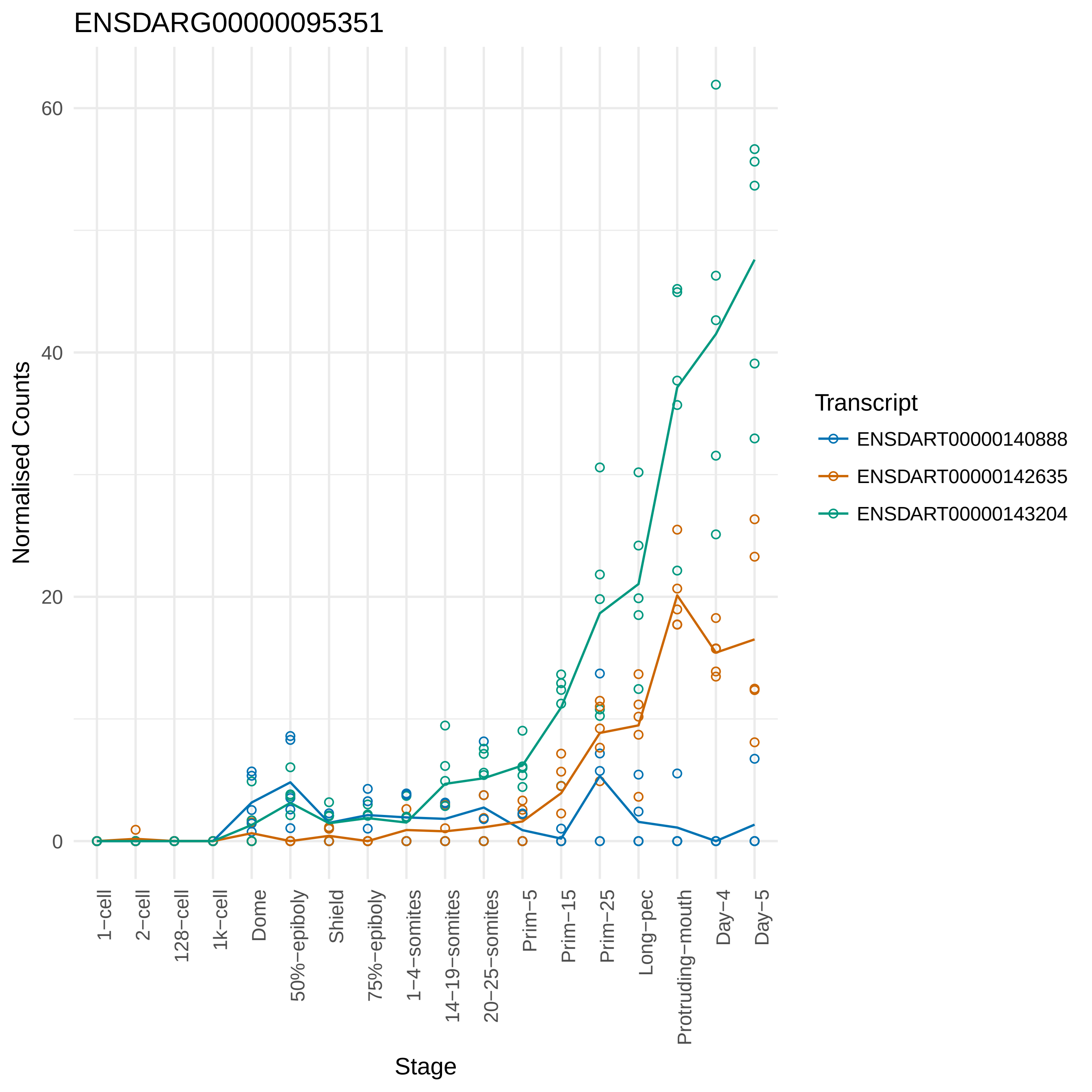
ENSDARG00000095351
Normalised Counts
60
40
20
0
1−cell
2−cell
128−cell
1k−cell
Dome
50%−epiboly
Shield
75%−epiboly
1−4−somites
14−19−somites
20−25−somites
Prim−5
Prim−15
Prim−25
Long−pec
Protruding−mouth
Day−4
Day−5
Stage
Transcript
ENSDART00000140888
ENSDART00000142635
ENSDART00000143204

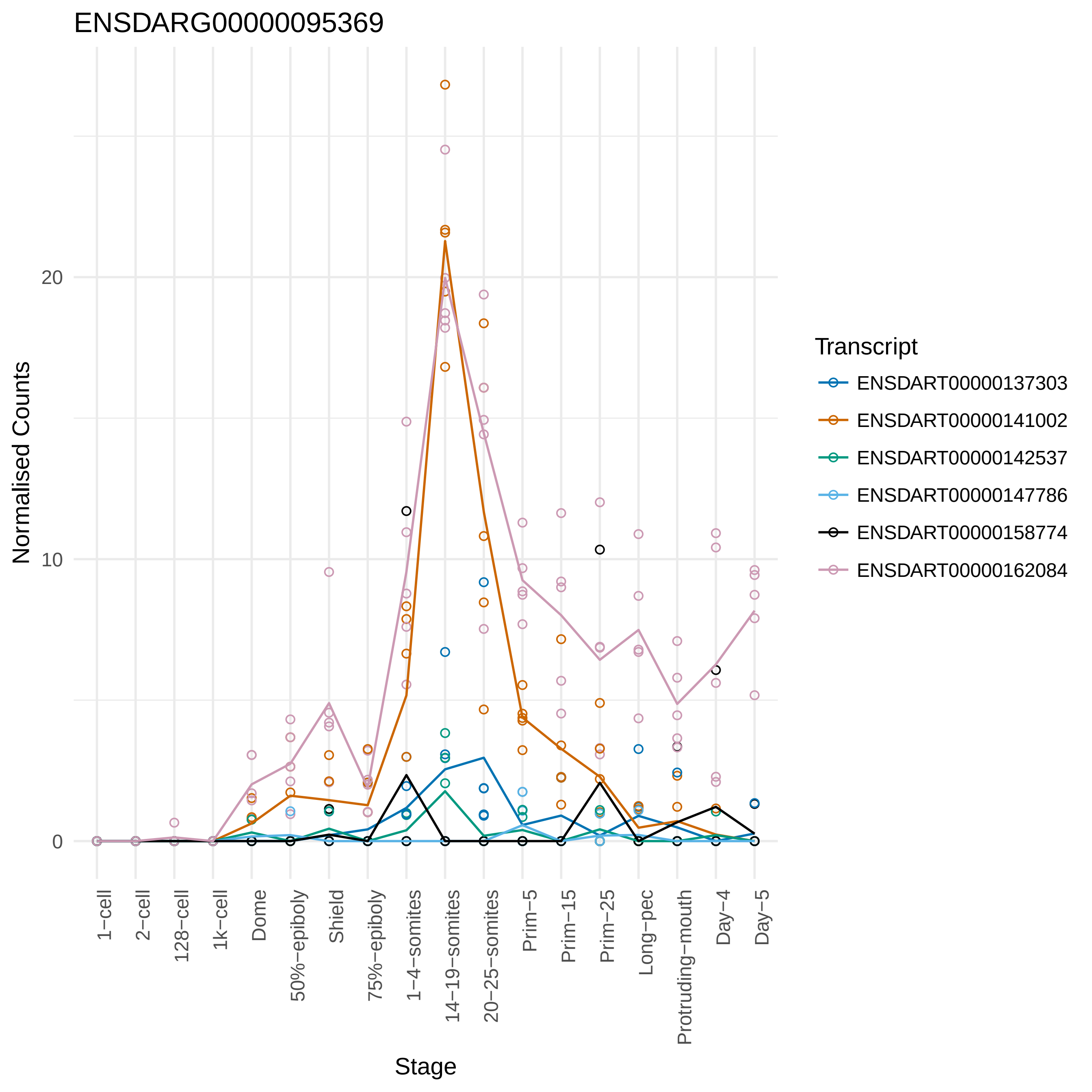

ENSDARG00000095369
Normalised Counts
0
10
20
1−cell
2−cell
128−cell
1k−cell
Dome
50%−epiboly
Shield
75%−epiboly
1−4−somites
14−19−somites
20−25−somites
Prim−5
Prim−15
Prim−25
Long−pec
Protruding−mouth
Day−4
Day−5
Stage
Transcript
ENSDART00000137303
ENSDART00000141002
ENSDART00000142537
ENSDART00000147786
ENSDART00000158774
ENSDART00000162084

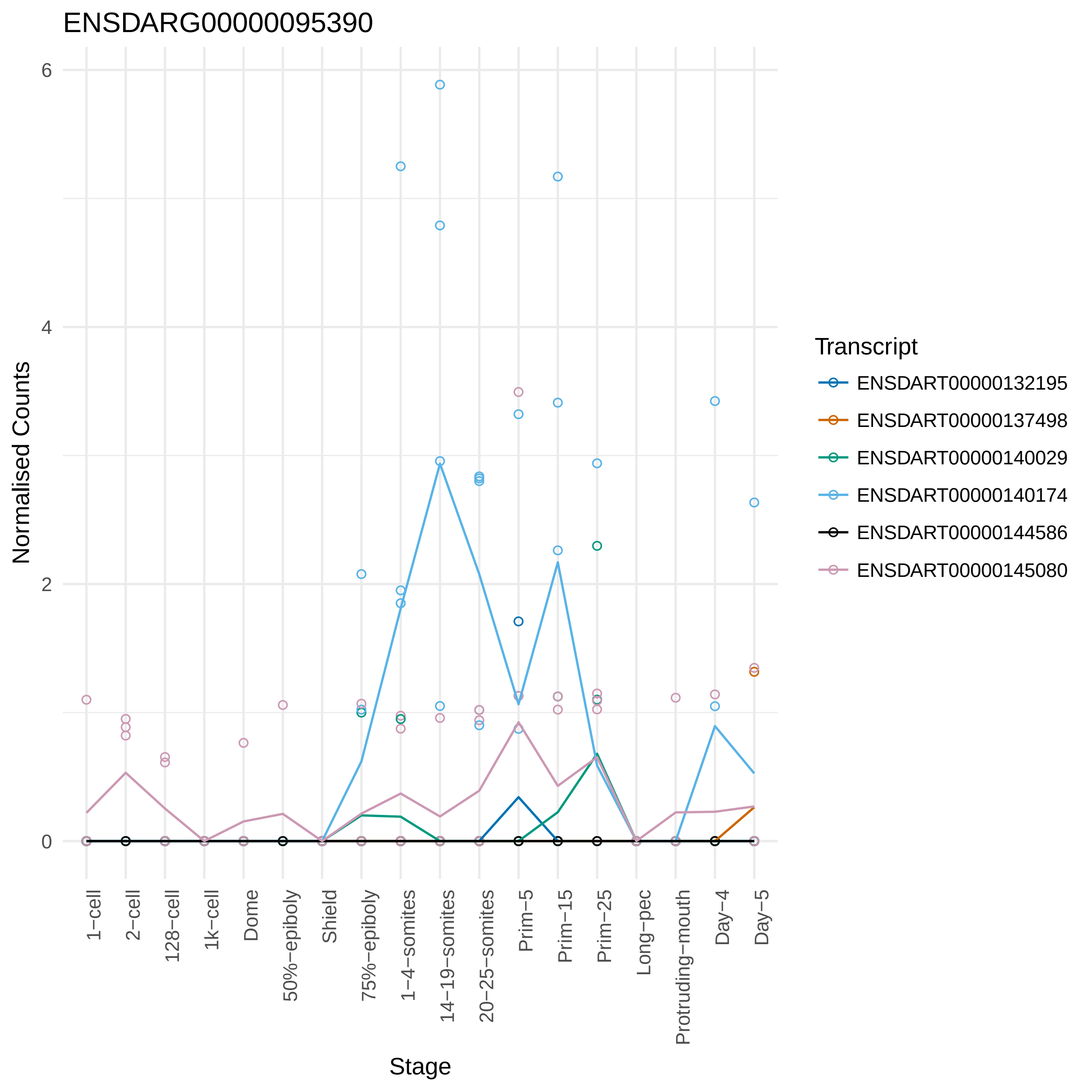
ENSDARG00000095390
Normalised Counts
0
2
4
6
Stage
1−cell
2−cell
128−cell
1k−cell
Dome
50%−epiboly
Shield
75%−epiboly
1−4−somites
14−19−somites
20−25−somites
Prim−5
Prim−15
Prim−25
Long−pec
Protruding−mouth
Day−4
Day−5
Transcript
ENSDART00000132195
ENSDART00000137498
ENSDART00000140029
ENSDART00000140174
ENSDART00000144586
ENSDART00000145080

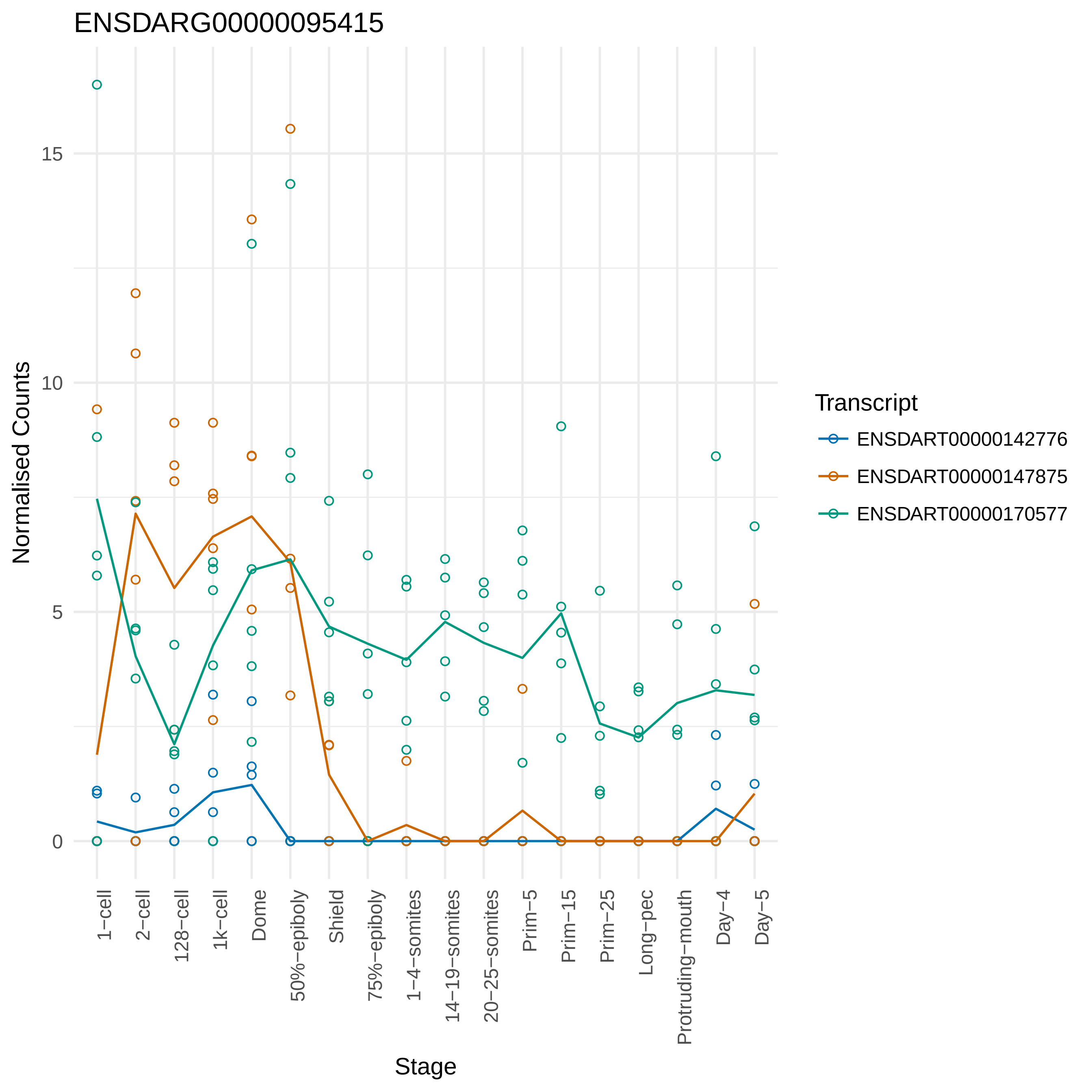

ENSDARG00000095415
Normalised Counts
15
10
5
0
1−cell
2−cell
128−cell
1k−cell
Dome
50%−epiboly
Shield
75%−epiboly
1−4−somites
14−19−somites
20−25−somites
Prim−5
Prim−15
Prim−25
Long−pec
Protruding−mouth
Day−4
Day−5
Stage
Transcript
ENSDART00000142776
ENSDART00000147875
ENSDART00000170577

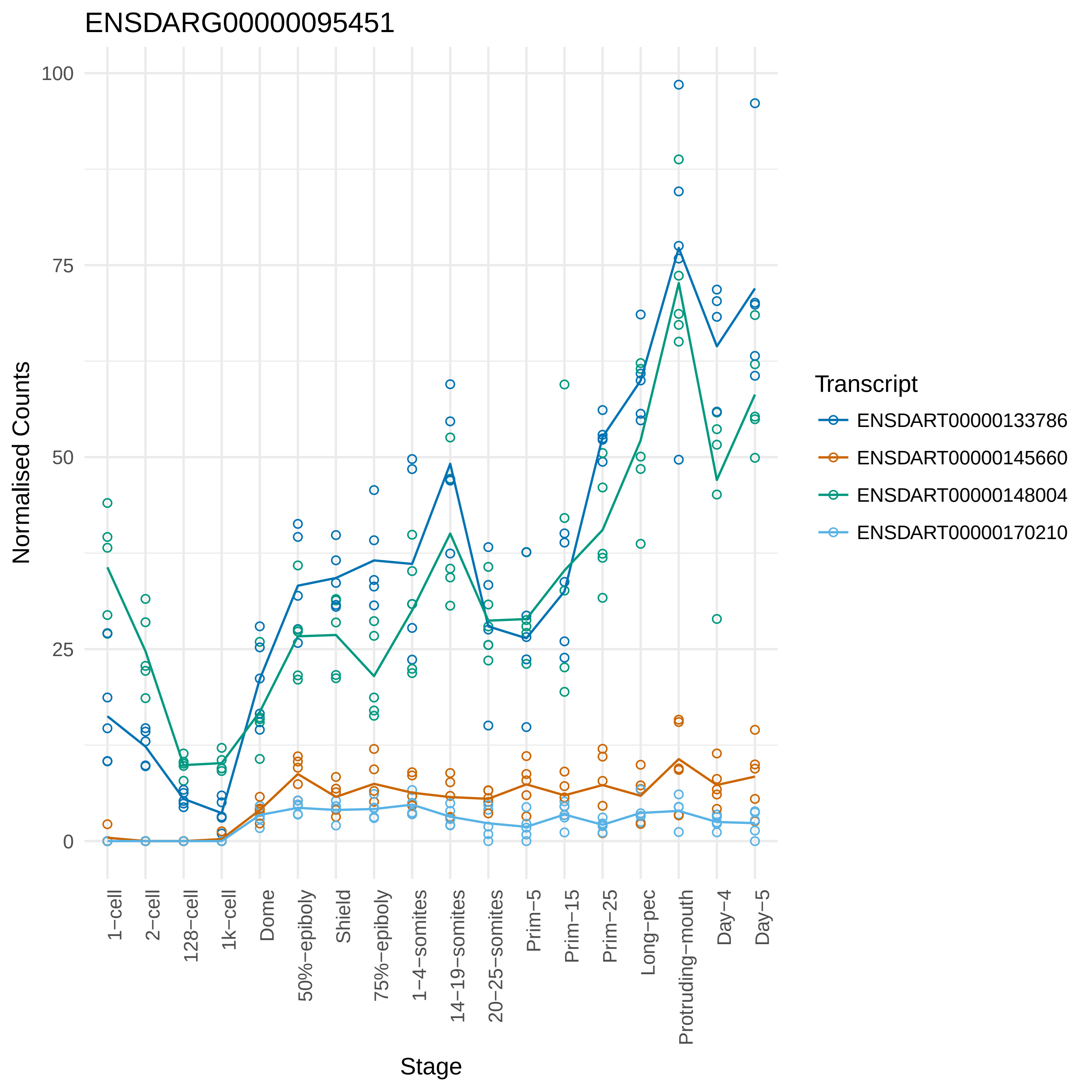

ENSDARG00000095451
Normalised Counts
100
75
50
25
0
1−cell
2−cell
128−cell
1k−cell
Dome
50%−epiboly
Shield
75%−epiboly
1−4−somites
14−19−somites
20−25−somites
Prim−5
Prim−15
Prim−25
Long−pec
Protruding−mouth
Day−4
Day−5
Stage
Transcript
ENSDART00000133786
ENSDART00000145660
ENSDART00000148004
ENSDART00000170210

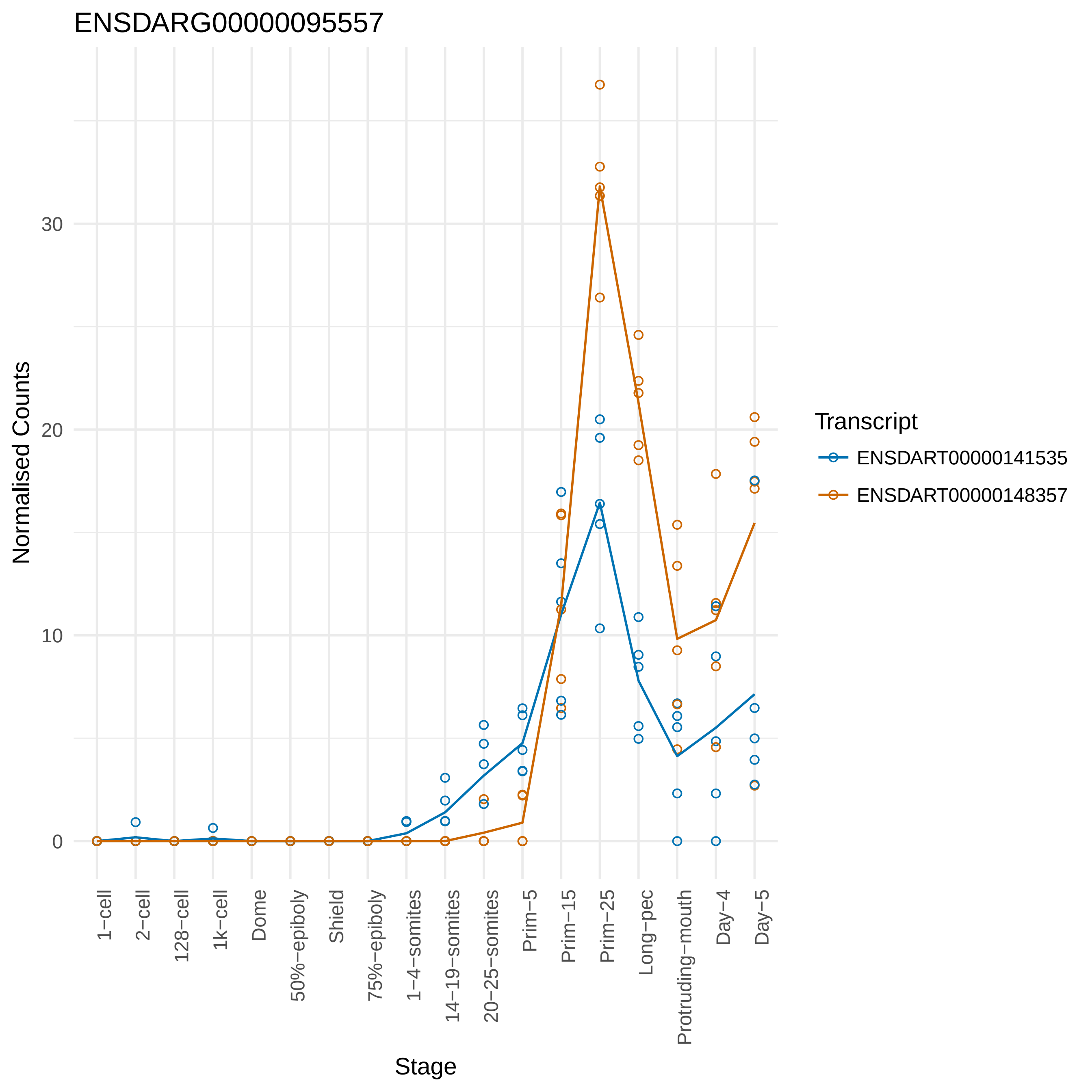

ENSDARG00000095557
Normalised Counts
0
10
20
30
1−cell
2−cell
128−cell
1k−cell
Dome
50%−epiboly
Shield
75%−epiboly
1−4−somites
14−19−somites
20−25−somites
Prim−5
Prim−15
Prim−25
Long−pec
Protruding−mouth
Day−4
Day−5
Stage
Transcript
ENSDART00000141535
ENSDART00000148357

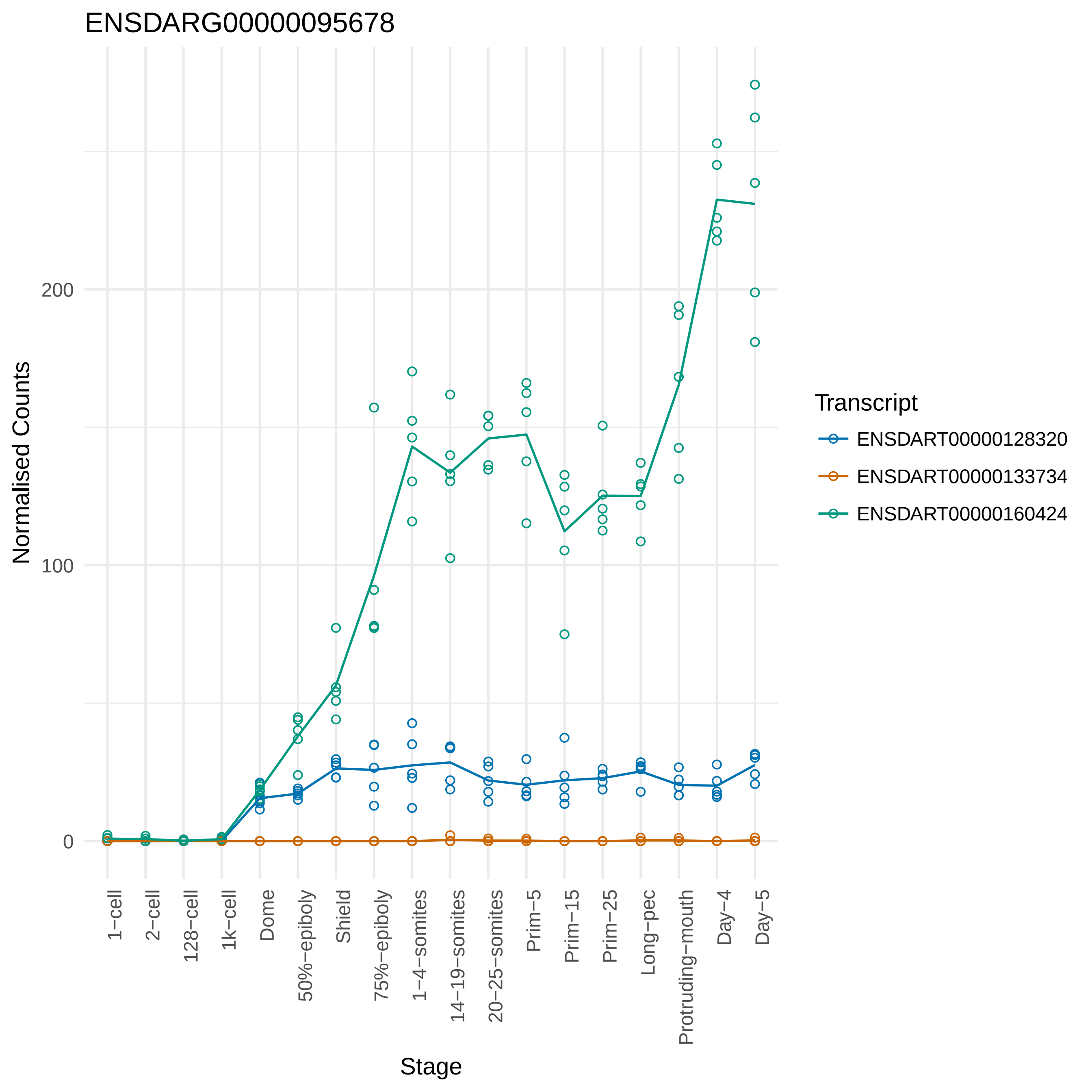
ENSDARG00000095678
Normalised Counts
0
100
200
Stage
1−cell
2−cell
128−cell
1k−cell
Dome
50%−epiboly
Shield
75%−epiboly
1−4−somites
14−19−somites
20−25−somites
Prim−5
Prim−15
Prim−25
Long−pec
Protruding−mouth
Day−4
Day−5
Transcript
ENSDART00000128320
ENSDART00000133734
ENSDART00000160424

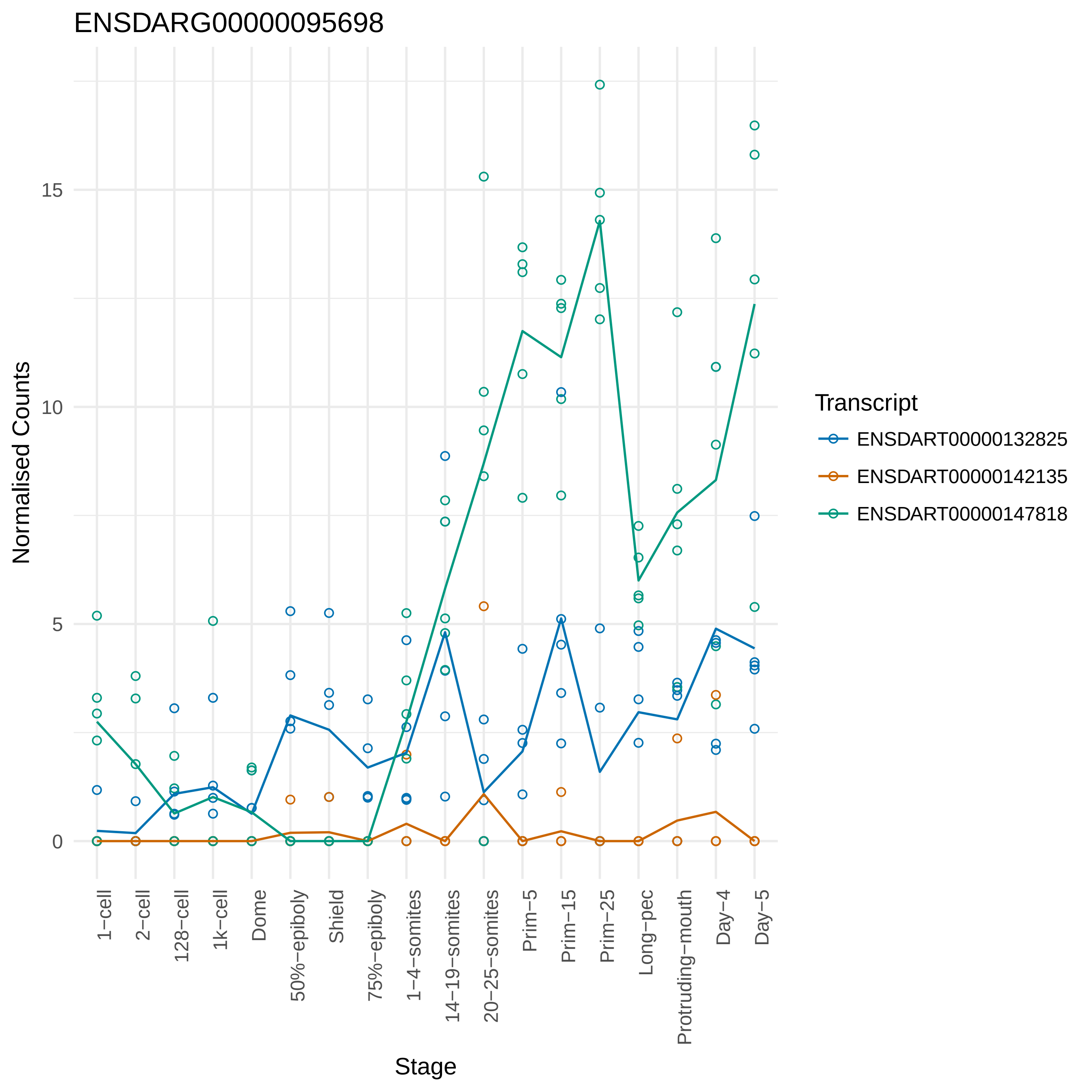

ENSDARG00000095698
Normalised Counts
15
10
5
0
1−cell
2−cell
128−cell
1k−cell
Dome
50%−epiboly
Shield
75%−epiboly
1−4−somites
14−19−somites
20−25−somites
Prim−5
Prim−15
Prim−25
Long−pec
Protruding−mouth
Day−4
Day−5
Stage
Transcript
ENSDART00000132825
ENSDART00000142135
ENSDART00000147818

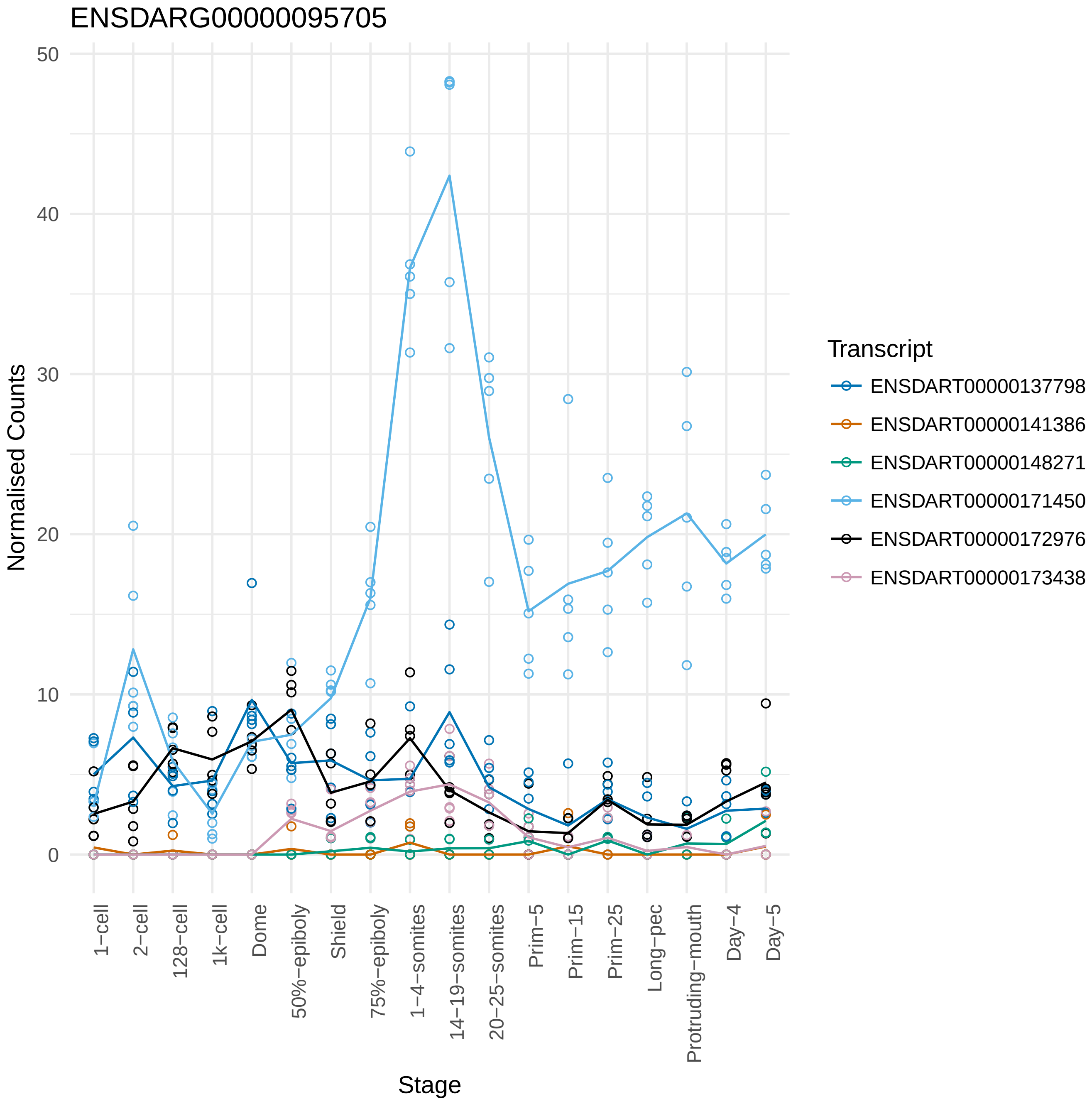

ENSDARG00000095705
Normalised Counts
Stage
50
40
30
20
10
0
1−cell
2−cell
128−cell
1k−cell
Dome
50%−epiboly
Shield
75%−epiboly
1−4−somites
14−19−somites
20−25−somites
Prim−5
Prim−15
Prim−25
Long−pec
Protruding−mouth
Day−4
Day−5
Transcript
ENSDART00000137798
ENSDART00000141386
ENSDART00000148271
ENSDART00000171450
ENSDART00000172976
ENSDART00000173438

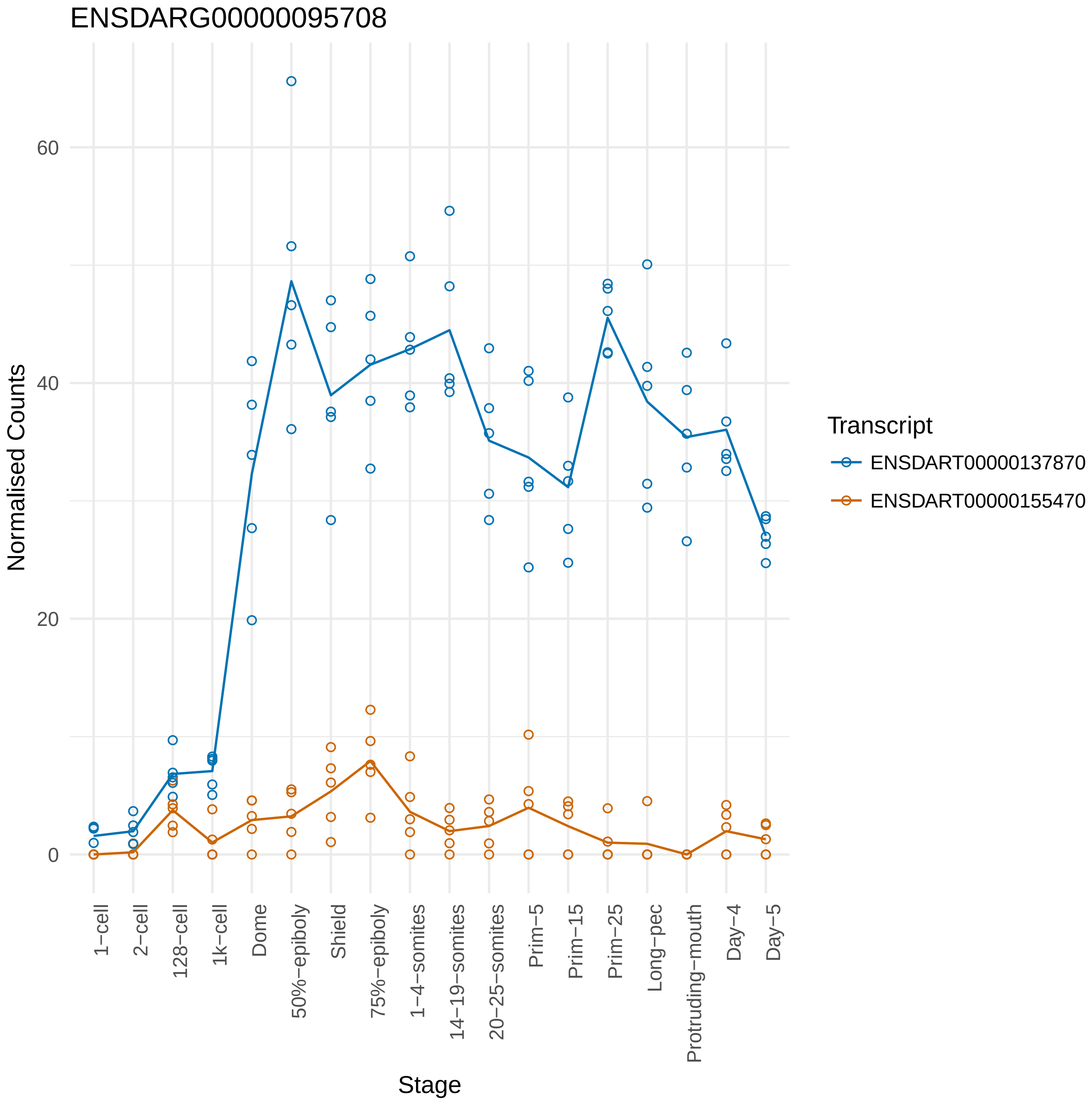

ENSDARG00000095708
Normalised Counts
60
40
20
0
1−cell
2−cell
128−cell
1k−cell
Dome
50%−epiboly
Shield
75%−epiboly
1−4−somites
14−19−somites
20−25−somites
Prim−5
Prim−15
Prim−25
Long−pec
Protruding−mouth
Day−4
Day−5
Stage
Transcript
ENSDART00000137870
ENSDART00000155470

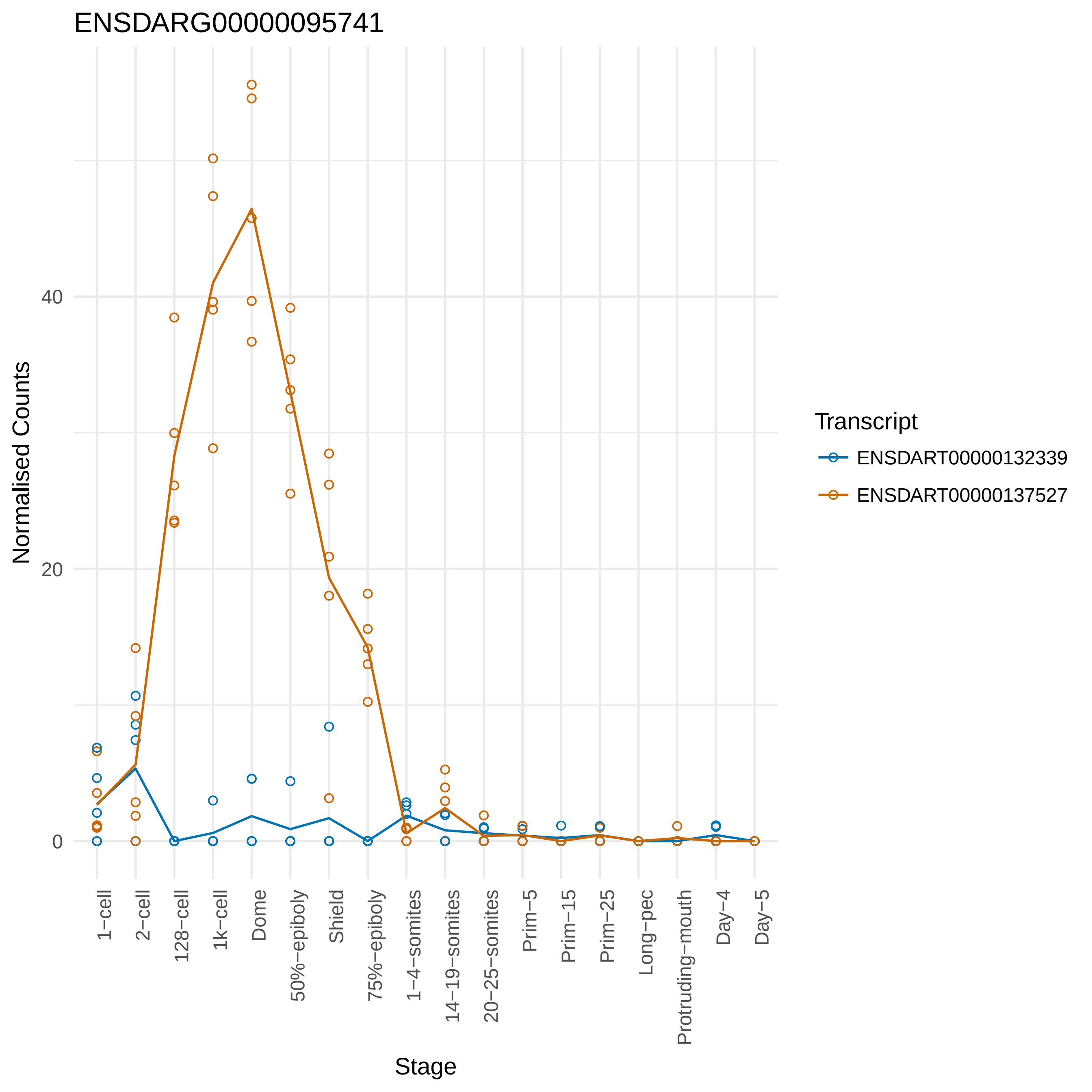

ENSDARG00000095741
Normalised Counts
0
20
40
Stage
1−cell
2−cell
128−cell
1k−cell
Dome
50%−epiboly
Shield
75%−epiboly
1−4−somites
14−19−somites
20−25−somites
Prim−5
Prim−15
Prim−25
Long−pec
Protruding−mouth
Day−4
Day−5
Transcript
ENSDART00000132339
ENSDART00000137527

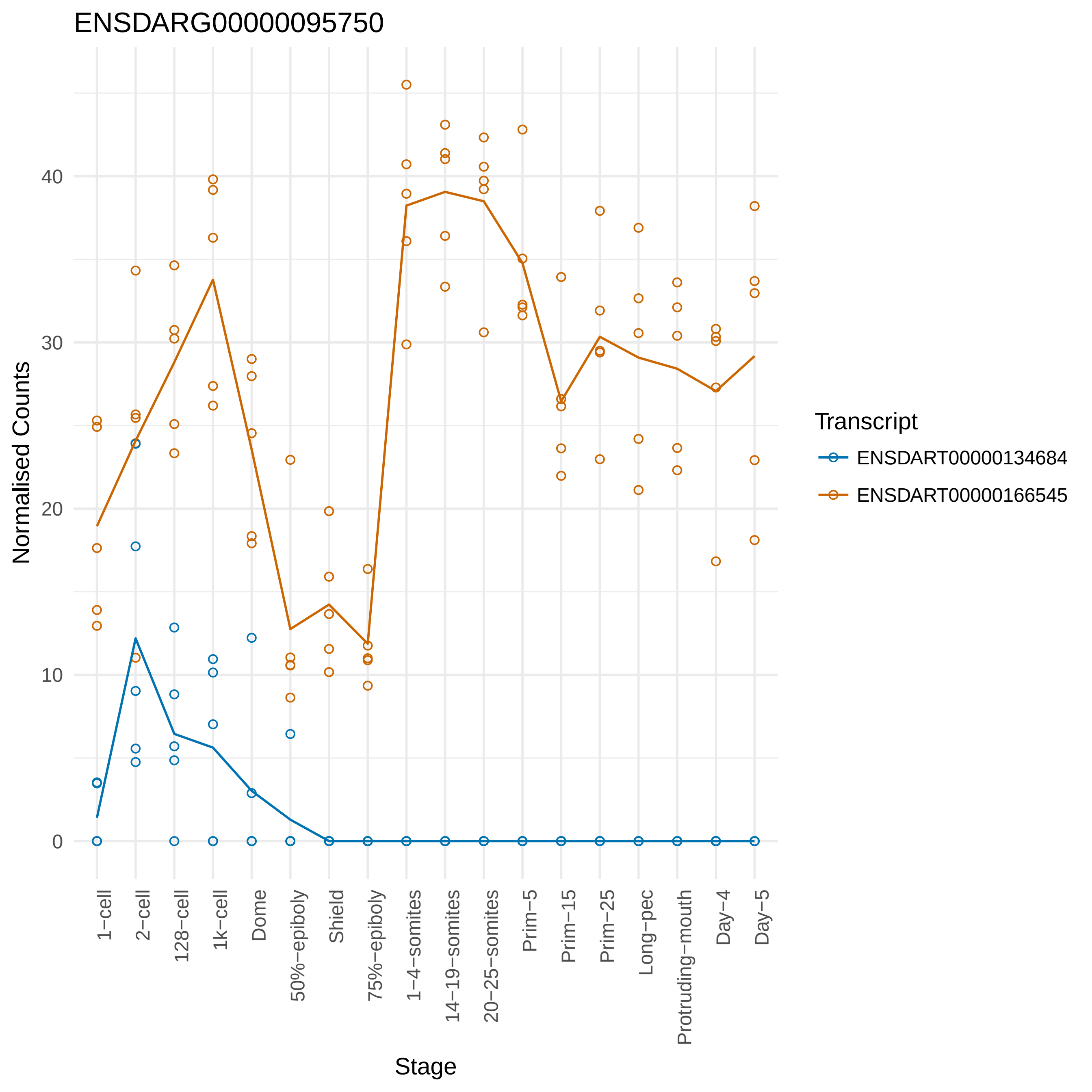

ENSDARG00000095750
Normalised Counts
0
10
20
30
40
1−cell
2−cell
128−cell
1k−cell
Dome
50%−epiboly
Shield
75%−epiboly
1−4−somites
14−19−somites
20−25−somites
Prim−5
Prim−15
Prim−25
Long−pec
Protruding−mouth
Day−4
Day−5
Stage
Transcript
ENSDART00000134684
ENSDART00000166545

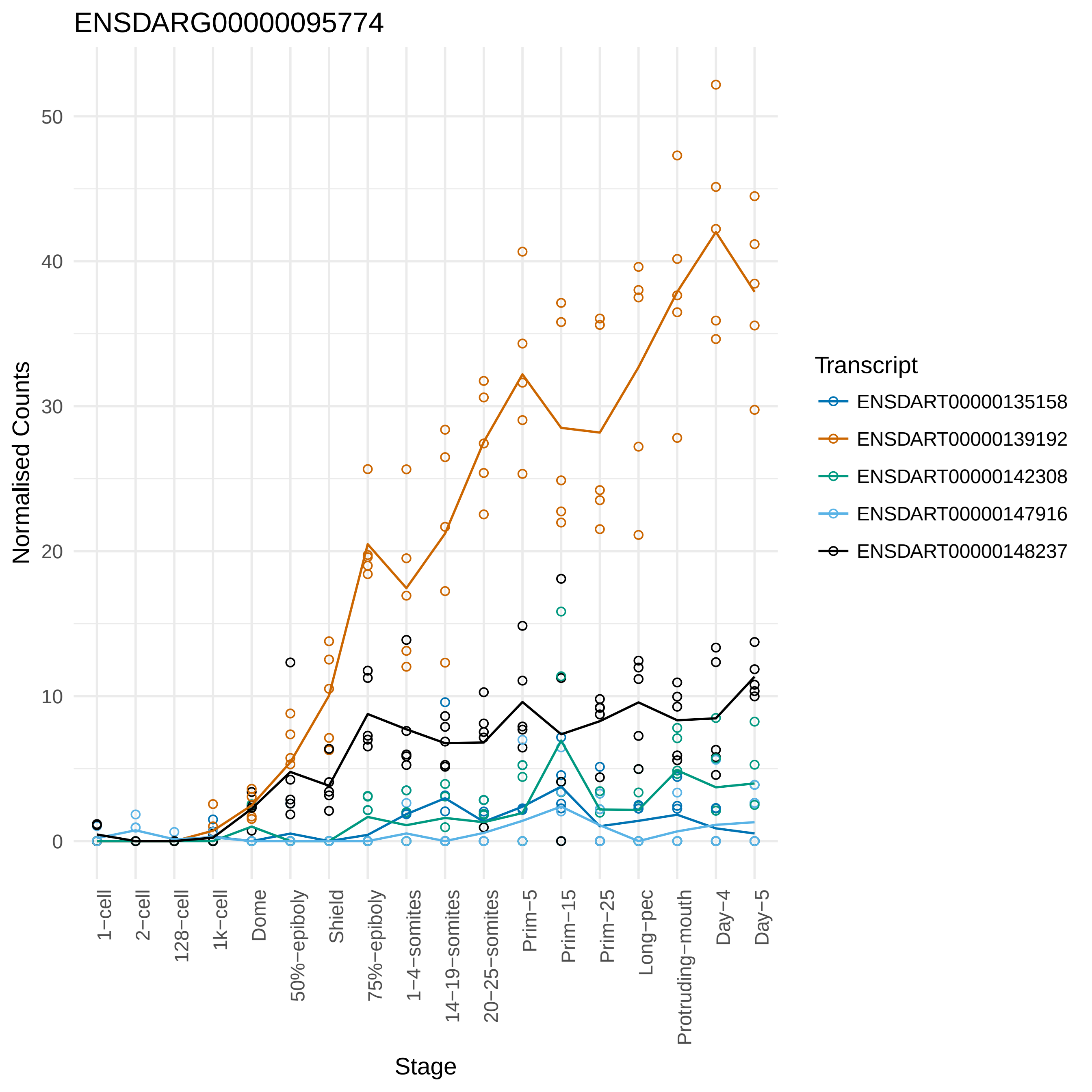
ENSDARG00000095774
Normalised Counts
0
10
20
30
40
50
Stage
1−cell
2−cell
128−cell
1k−cell
Dome
50%−epiboly
Shield
75%−epiboly
1−4−somites
14−19−somites
20−25−somites
Prim−5
Prim−15
Prim−25
Long−pec
Protruding−mouth
Day−4
Day−5
Transcript
ENSDART00000135158
ENSDART00000139192
ENSDART00000142308
ENSDART00000147916
ENSDART00000148237

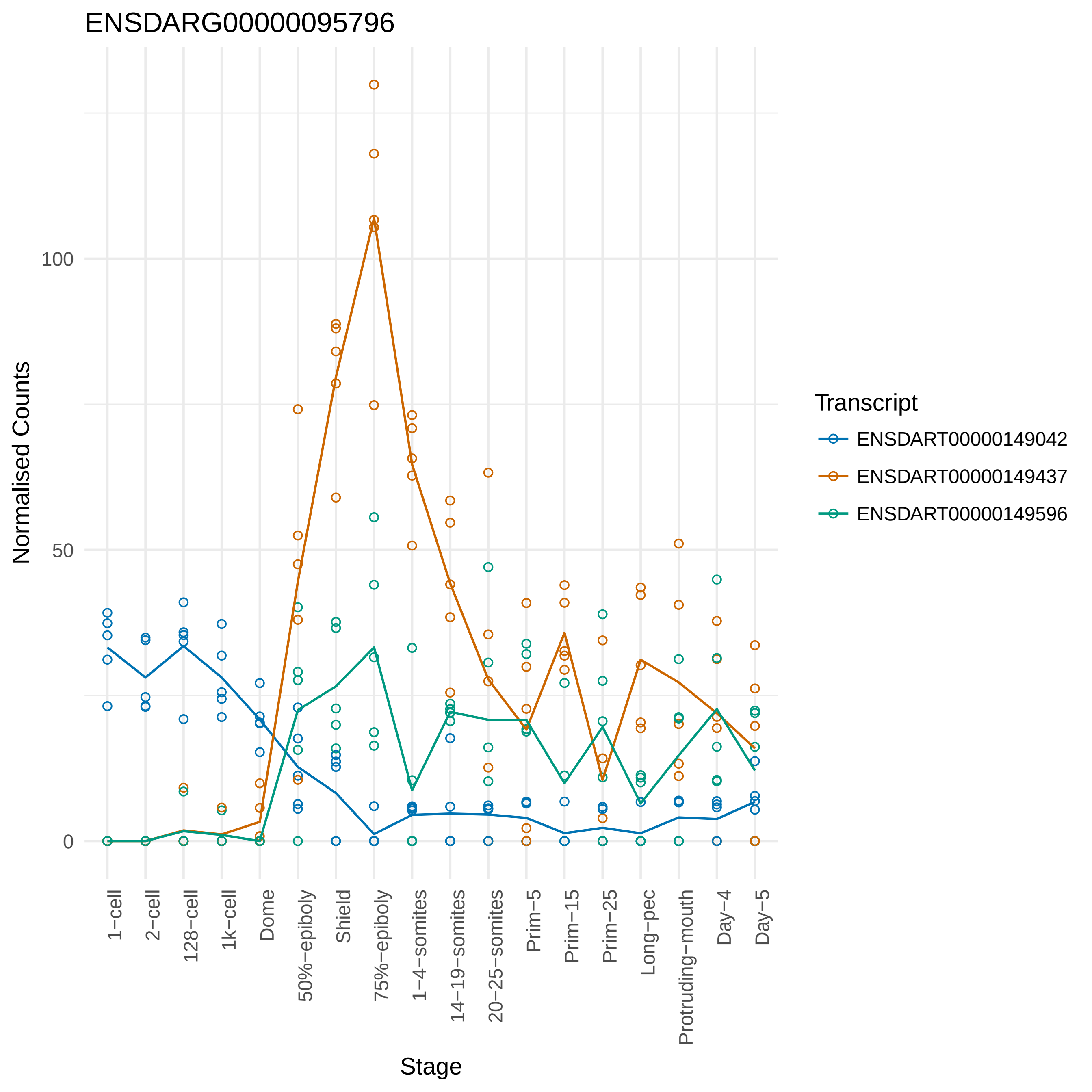

ENSDARG00000095796
Normalised Counts
100
50
0
1−cell
2−cell
128−cell
1k−cell
Dome
50%−epiboly
Shield
75%−epiboly
1−4−somites
14−19−somites
20−25−somites
Prim−5
Prim−15
Prim−25
Long−pec
Protruding−mouth
Day−4
Day−5
Stage
Transcript
ENSDART00000149042
ENSDART00000149437
ENSDART00000149596

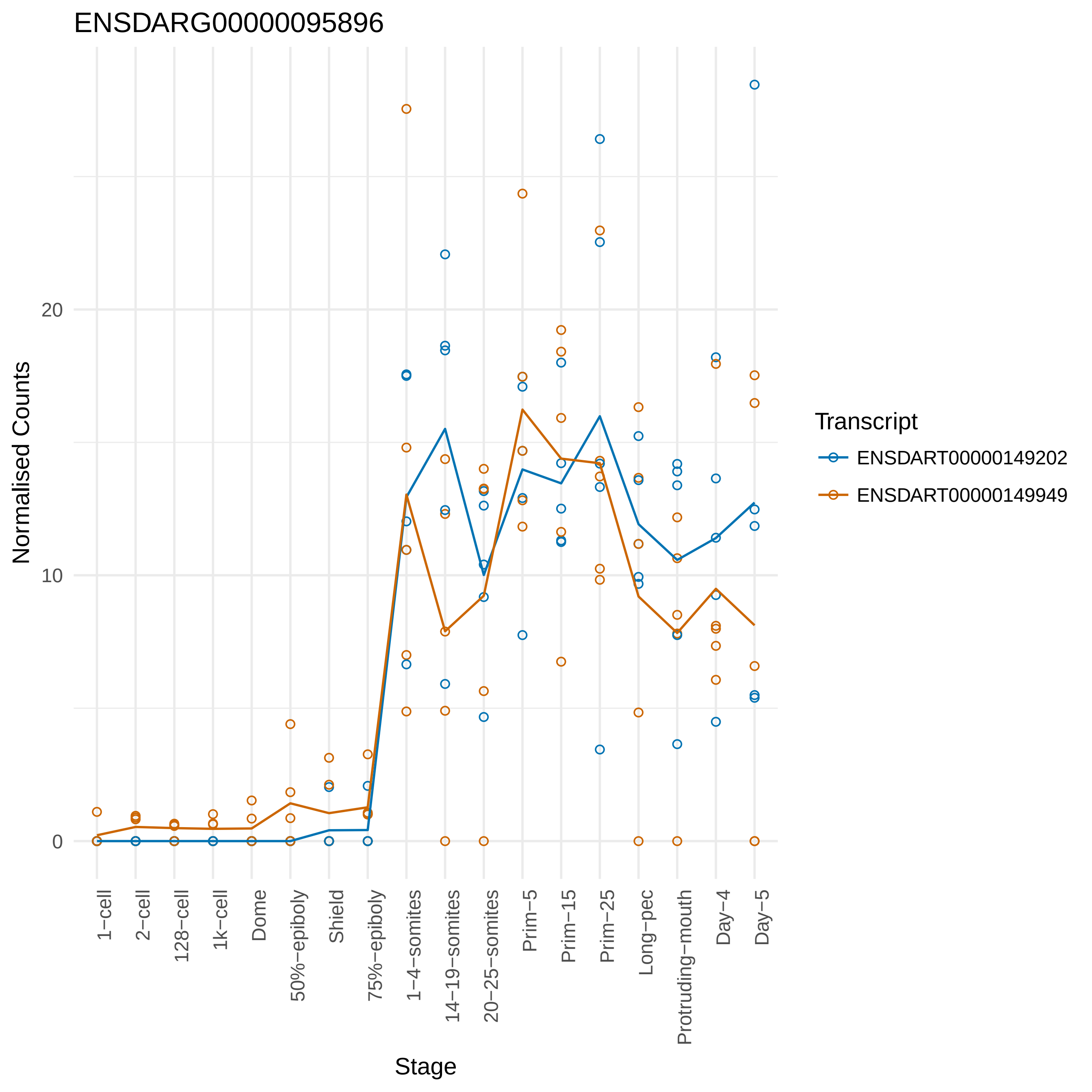

ENSDARG00000095896
Normalised Counts
0
10
20
Stage
1−cell
2−cell
128−cell
1k−cell
Dome
50%−epiboly
Shield
75%−epiboly
1−4−somites
14−19−somites
20−25−somites
Prim−5
Prim−15
Prim−25
Long−pec
Protruding−mouth
Day−4
Day−5
Transcript
ENSDART00000149202
ENSDART00000149949

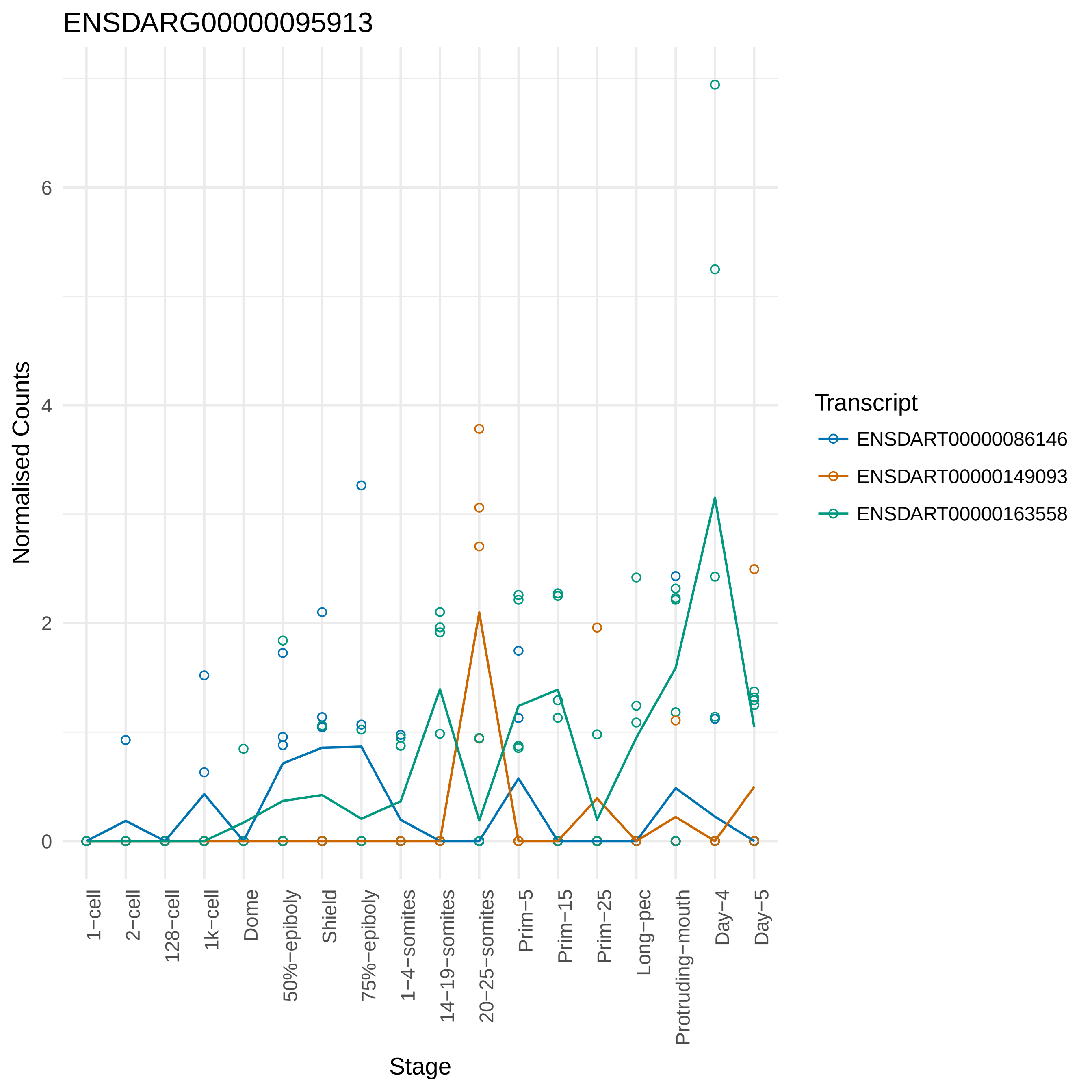

ENSDARG00000095913
Normalised Counts
6
4
2
0
1−cell
2−cell
128−cell
1k−cell
Dome
50%−epiboly
Shield
75%−epiboly
1−4−somites
14−19−somites
20−25−somites
Prim−5
Prim−15
Prim−25
Long−pec
Protruding−mouth
Day−4
Day−5
Stage
Transcript
ENSDART00000086146
ENSDART00000149093
ENSDART00000163558

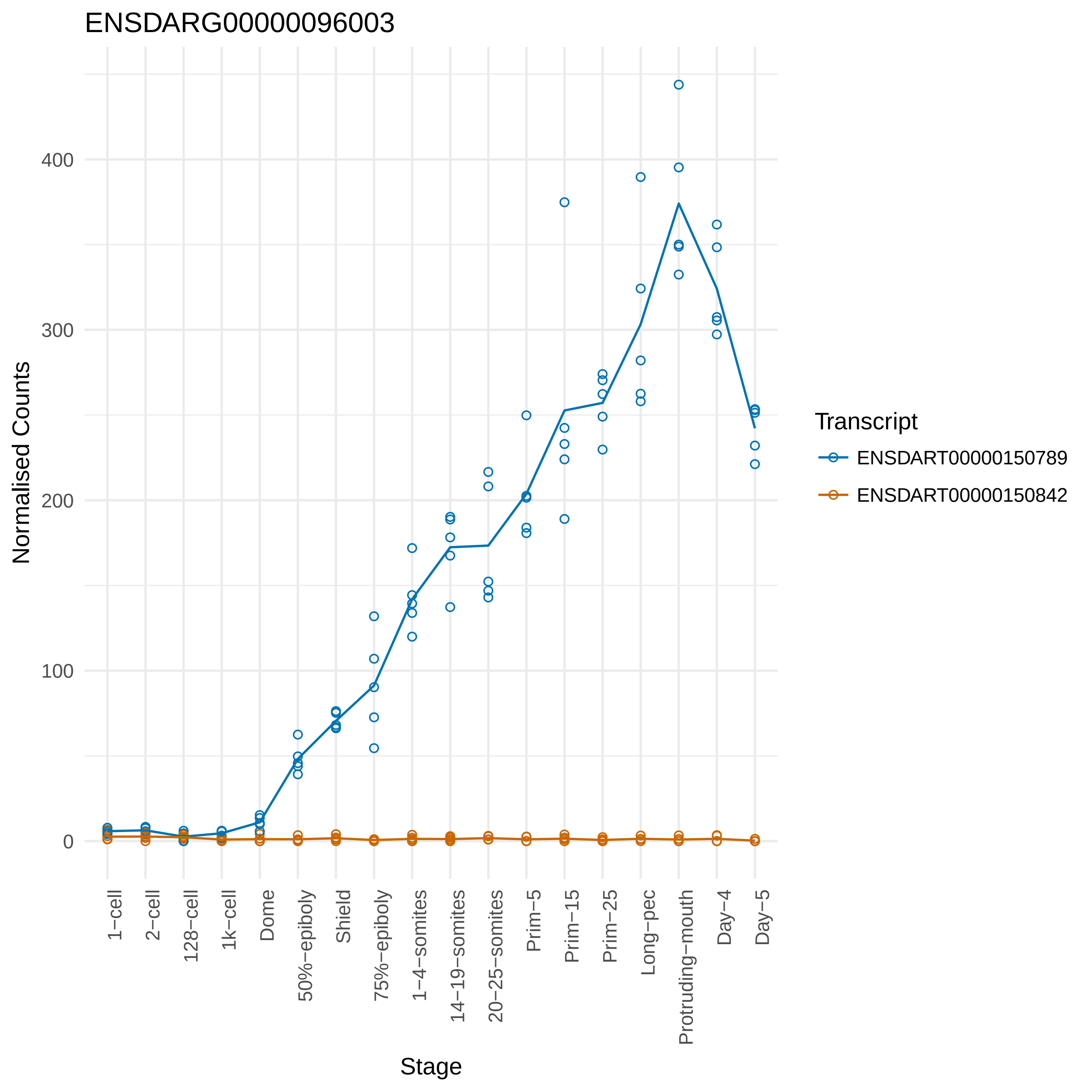
ENSDARG00000096003
Normalised Counts
0
100
200
300
400
Stage
1−cell
2−cell
128−cell
1k−cell
Dome
50%−epiboly
Shield
75%−epiboly
1−4−somites
14−19−somites
20−25−somites
Prim−5
Prim−15
Prim−25
Long−pec
Protruding−mouth
Day−4
Day−5
Transcript
ENSDART00000150789
ENSDART00000150842

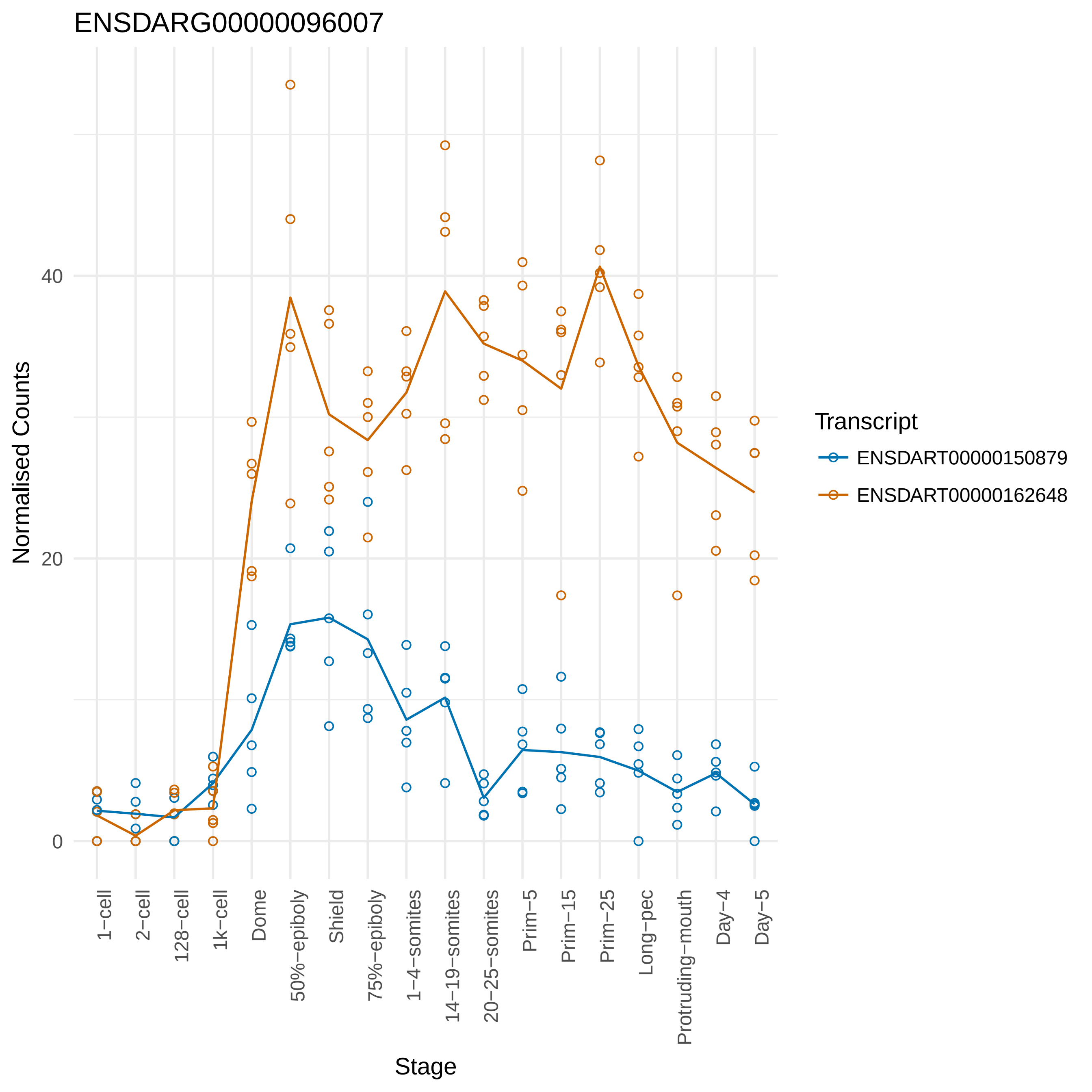
ENSDARG00000096007
Normalised Counts
0
20
40
1−cell
2−cell
128−cell
1k−cell
Dome
50%−epiboly
Shield
75%−epiboly
1−4−somites
14−19−somites
20−25−somites
Prim−5
Prim−15
Prim−25
Long−pec
Protruding−mouth
Day−4
Day−5
Stage
Transcript
ENSDART00000150879
ENSDART00000162648

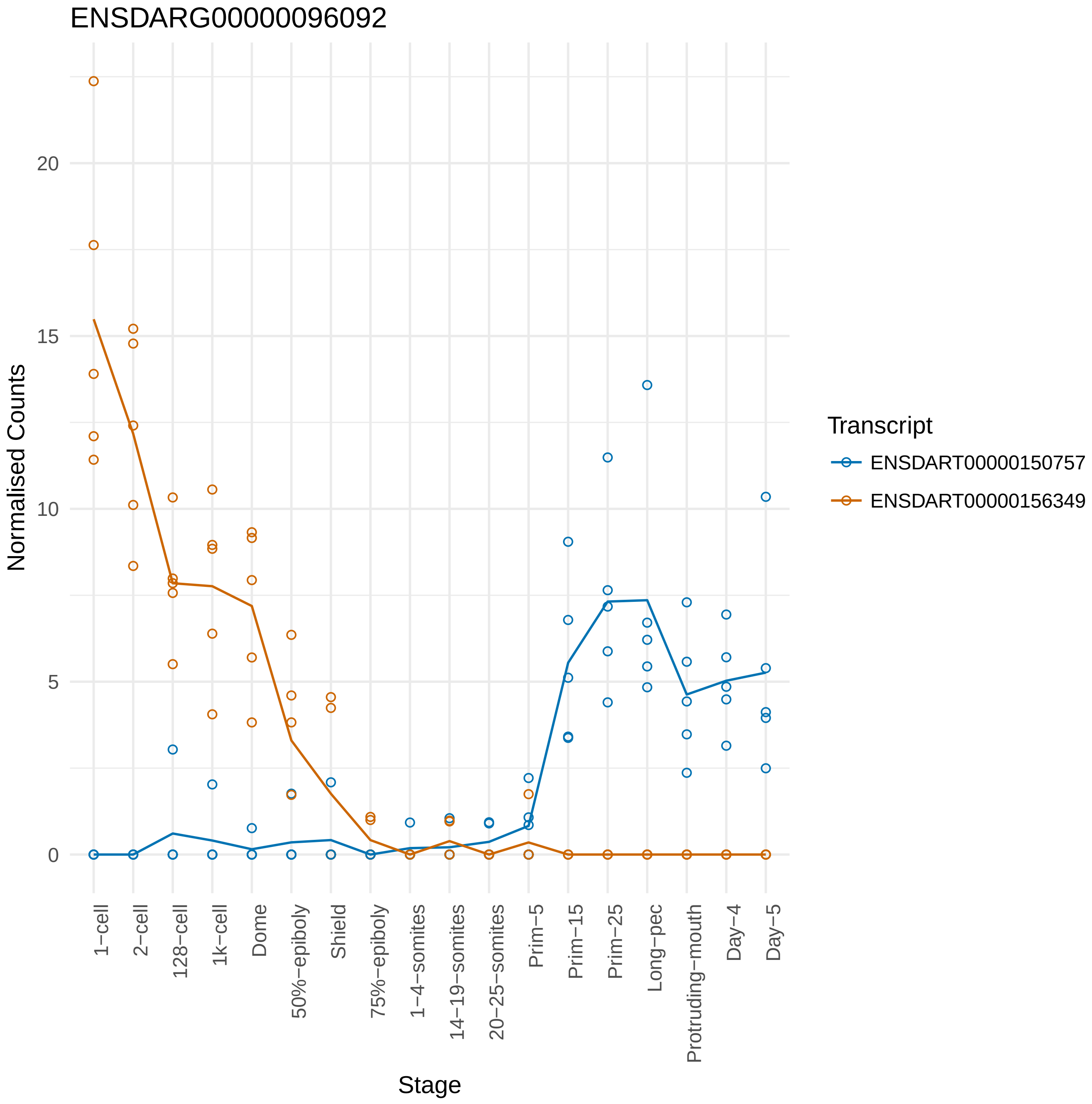
ENSDARG00000096092
Normalised Counts
20
15
10
5
0
1−cell
2−cell
128−cell
1k−cell
Dome
50%−epiboly
Shield
75%−epiboly
1−4−somites
14−19−somites
20−25−somites
Prim−5
Prim−15
Prim−25
Long−pec
Protruding−mouth
Day−4
Day−5
Stage
Transcript
ENSDART00000150757
ENSDART00000156349

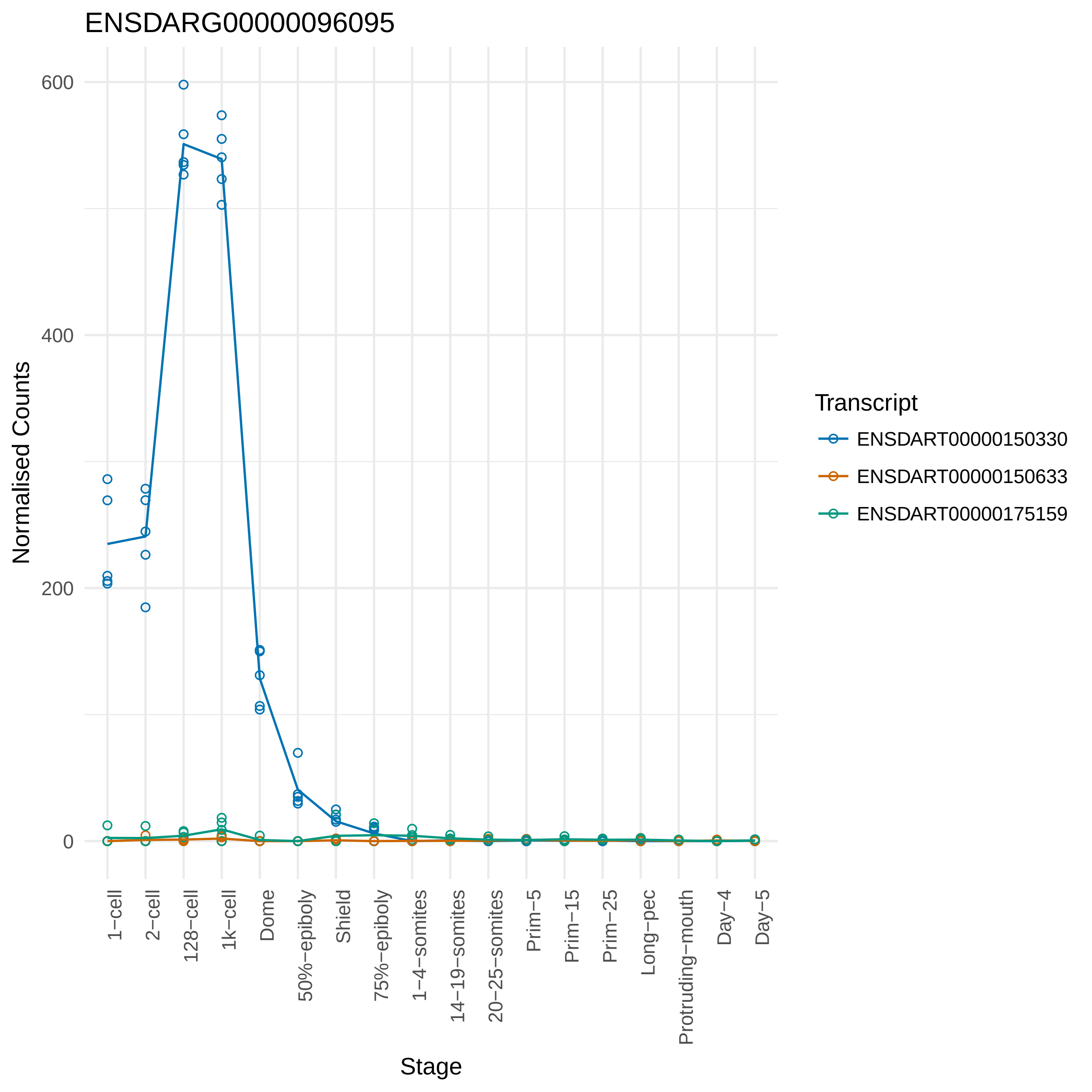

ENSDARG00000096095
Normalised Counts
600
400
200
0
1−cell
2−cell
128−cell
1k−cell
Dome
50%−epiboly
Shield
75%−epiboly
1−4−somites
14−19−somites
20−25−somites
Prim−5
Prim−15
Prim−25
Long−pec
Protruding−mouth
Day−4
Day−5
Stage
Transcript
ENSDART00000150330
ENSDART00000150633
ENSDART00000175159

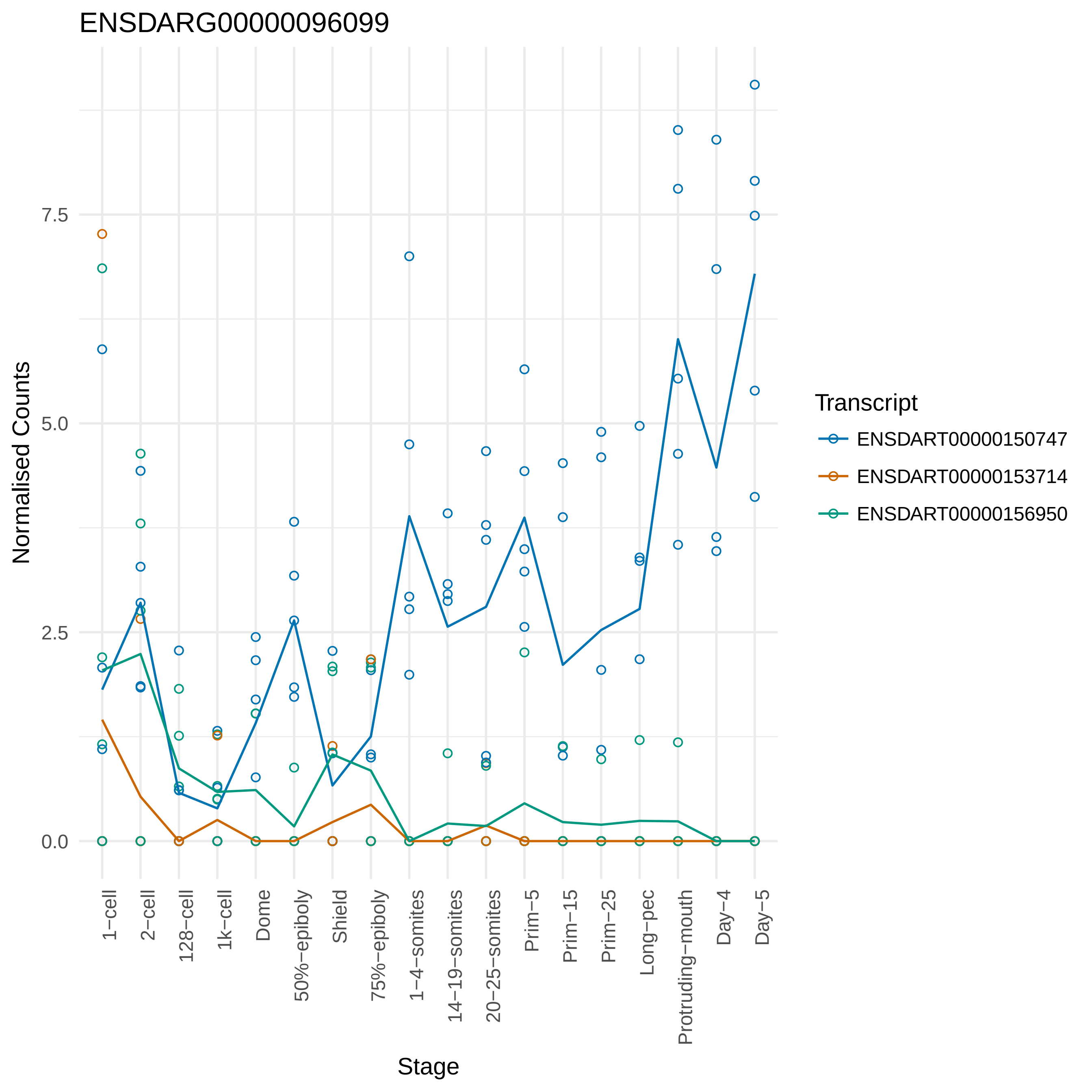
ENSDARG00000096099
Normalised Counts
0.0
2.5
5.0
7.5
Stage
1−cell
2−cell
128−cell
1k−cell
Dome
50%−epiboly
Shield
75%−epiboly
1−4−somites
14−19−somites
20−25−somites
Prim−5
Prim−15
Prim−25
Long−pec
Protruding−mouth
Day−4
Day−5
Transcript
ENSDART00000150747
ENSDART00000153714
ENSDART00000156950

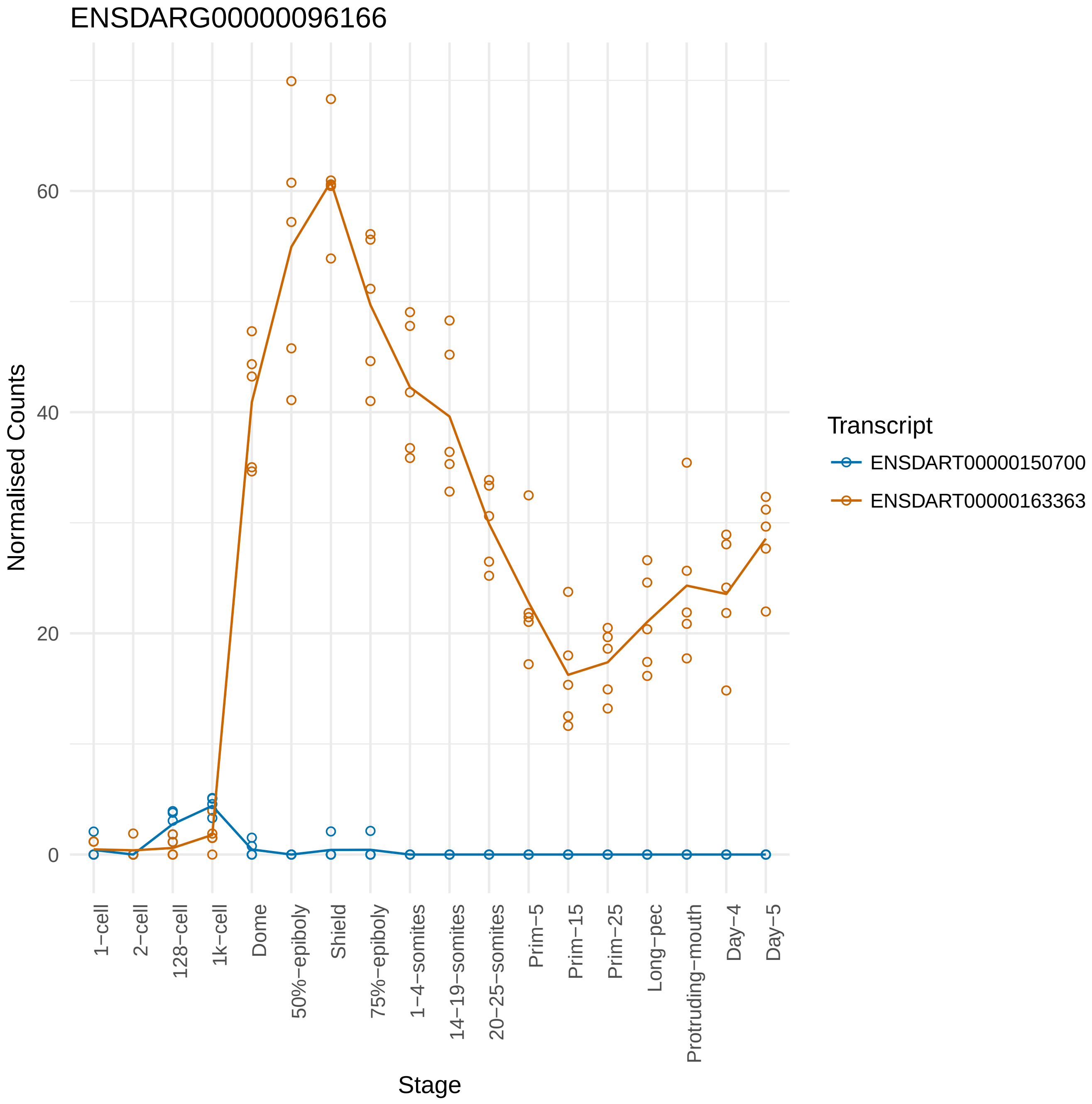
ENSDARG00000096166
Normalised Counts
0
20
40
60
Stage
1−cell
2−cell
128−cell
1k−cell
Dome
50%−epiboly
Shield
75%−epiboly
1−4−somites
14−19−somites
20−25−somites
Prim−5
Prim−15
Prim−25
Long−pec
Protruding−mouth
Day−4
Day−5
Transcript
ENSDART00000150700
ENSDART00000163363

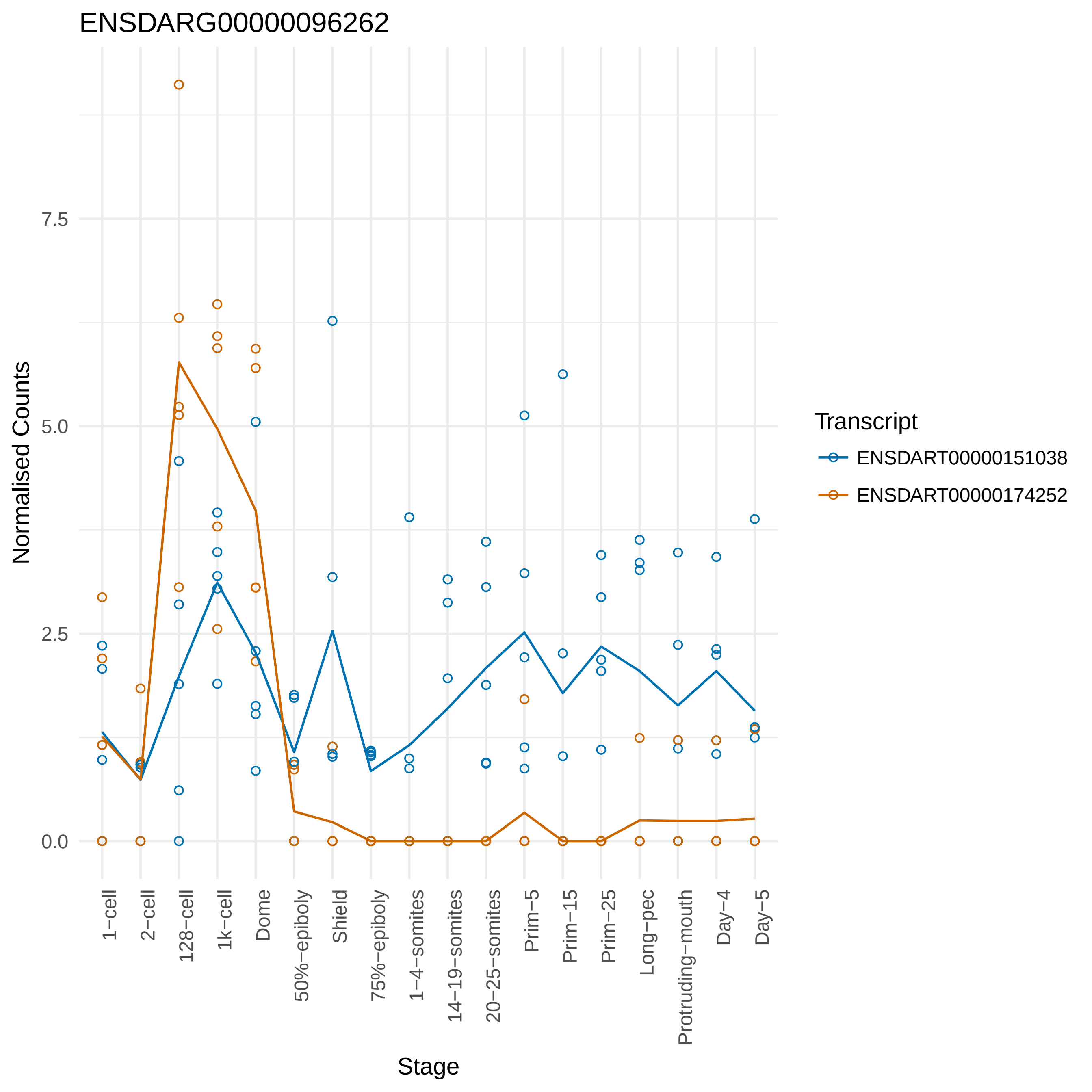

ENSDARG00000096262
Normalised Counts
0.0
2.5
5.0
7.5
1−cell
2−cell
128−cell
1k−cell
Dome
50%−epiboly
Shield
75%−epiboly
1−4−somites
14−19−somites
20−25−somites
Prim−5
Prim−15
Prim−25
Long−pec
Protruding−mouth
Day−4
Day−5
Stage
Transcript
ENSDART00000151038
ENSDART00000174252

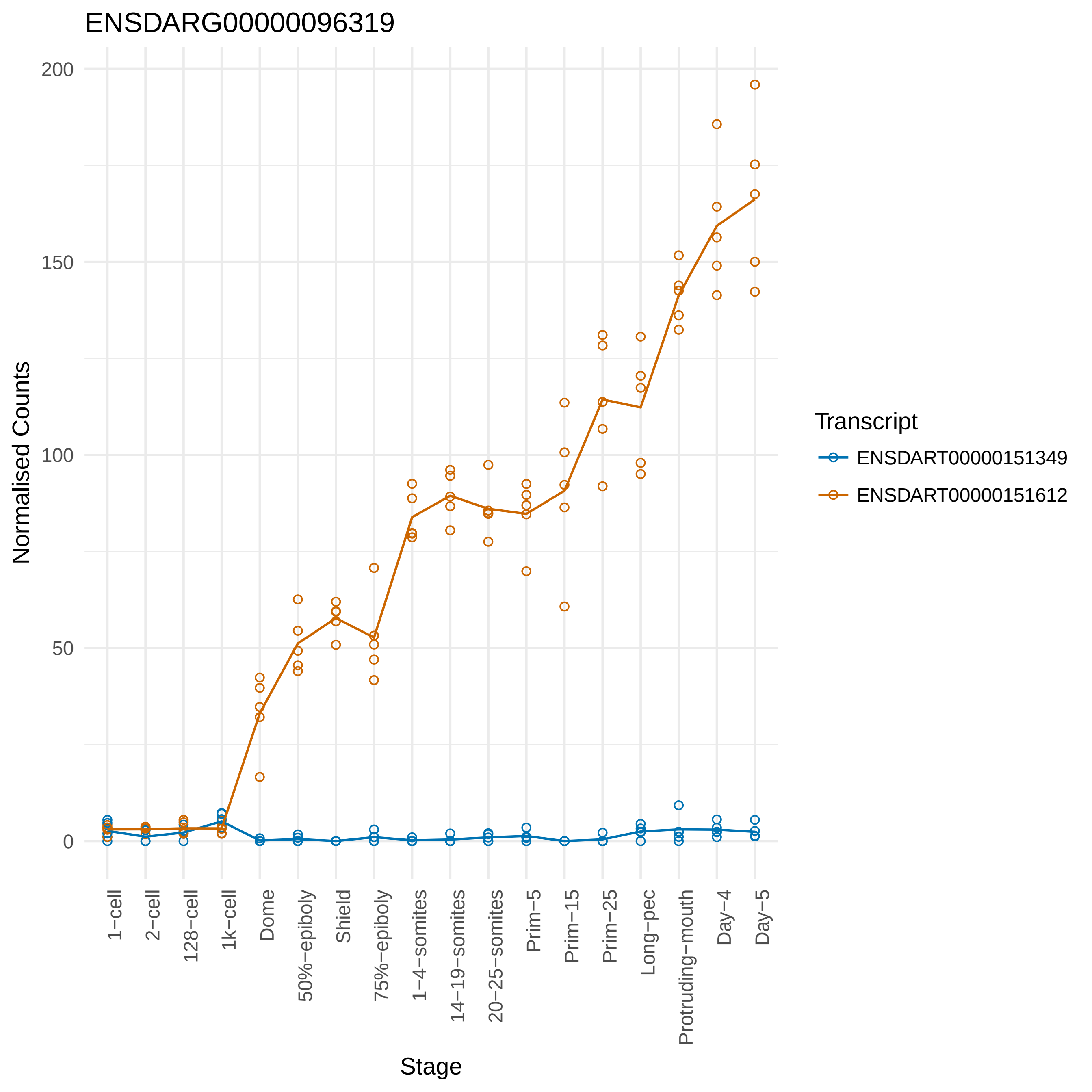
ENSDARG00000096319
Normalised Counts
0
50
100
150
200
Stage
1−cell
2−cell
128−cell
1k−cell
Dome
50%−epiboly
Shield
75%−epiboly
1−4−somites
14−19−somites
20−25−somites
Prim−5
Prim−15
Prim−25
Long−pec
Protruding−mouth
Day−4
Day−5
Transcript
ENSDART00000151349
ENSDART00000151612

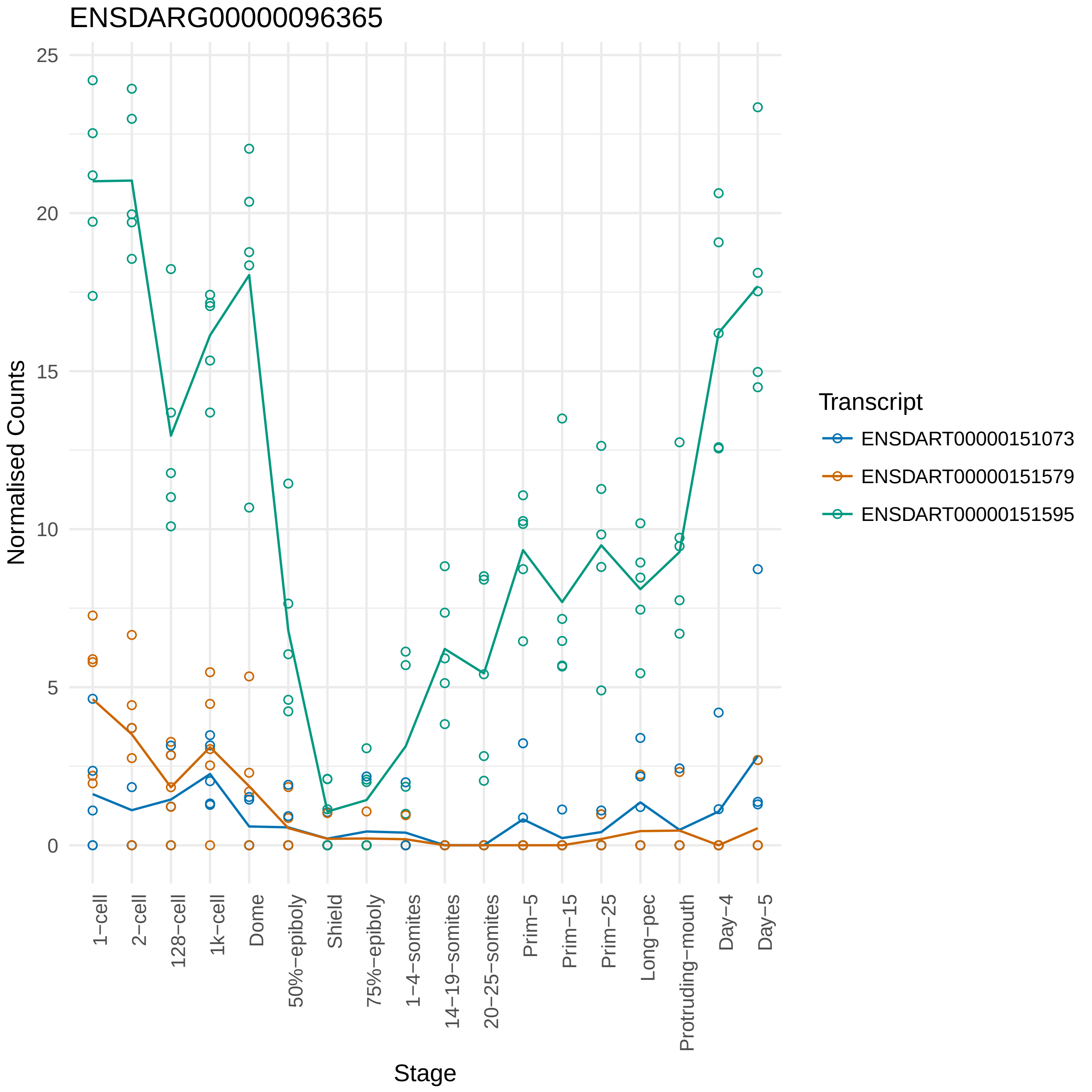

ENSDARG00000096365
Normalised Counts
0
5
10
15
20
25
Stage
1−cell
2−cell
128−cell
1k−cell
Dome
50%−epiboly
Shield
75%−epiboly
1−4−somites
14−19−somites
20−25−somites
Prim−5
Prim−15
Prim−25
Long−pec
Protruding−mouth
Day−4
Day−5
Transcript
ENSDART00000151073
ENSDART00000151579
ENSDART00000151595

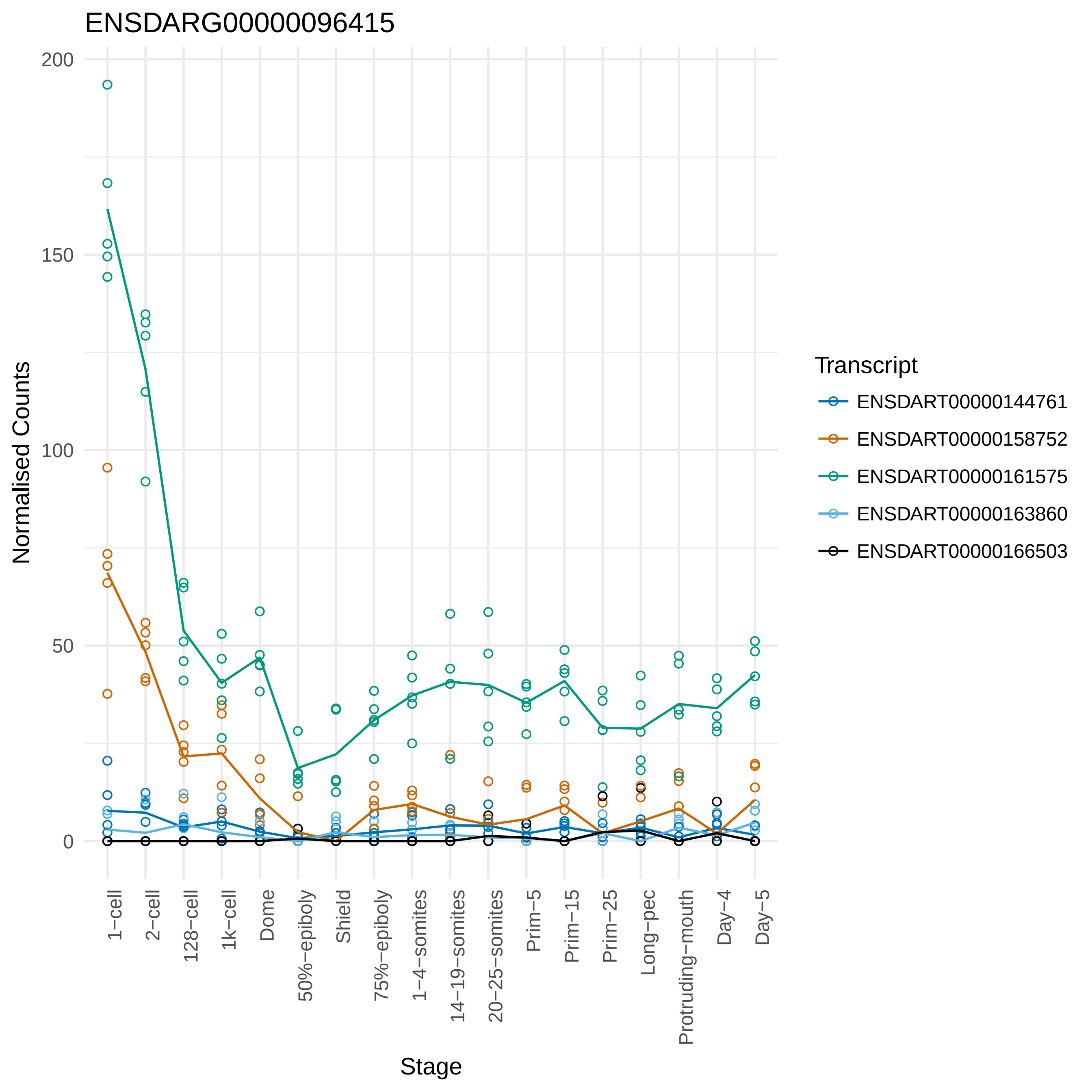
ENSDARG00000096415
Normalised Counts
200
150
100
50
0
1−cell
2−cell
128−cell
1k−cell
Dome
50%−epiboly
Shield
75%−epiboly
1−4−somites
14−19−somites
20−25−somites
Prim−5
Prim−15
Prim−25
Long−pec
Protruding−mouth
Day−4
Day−5
Stage
Transcript
ENSDART00000144761
ENSDART00000158752
ENSDART00000161575
ENSDART00000163860
ENSDART00000166503

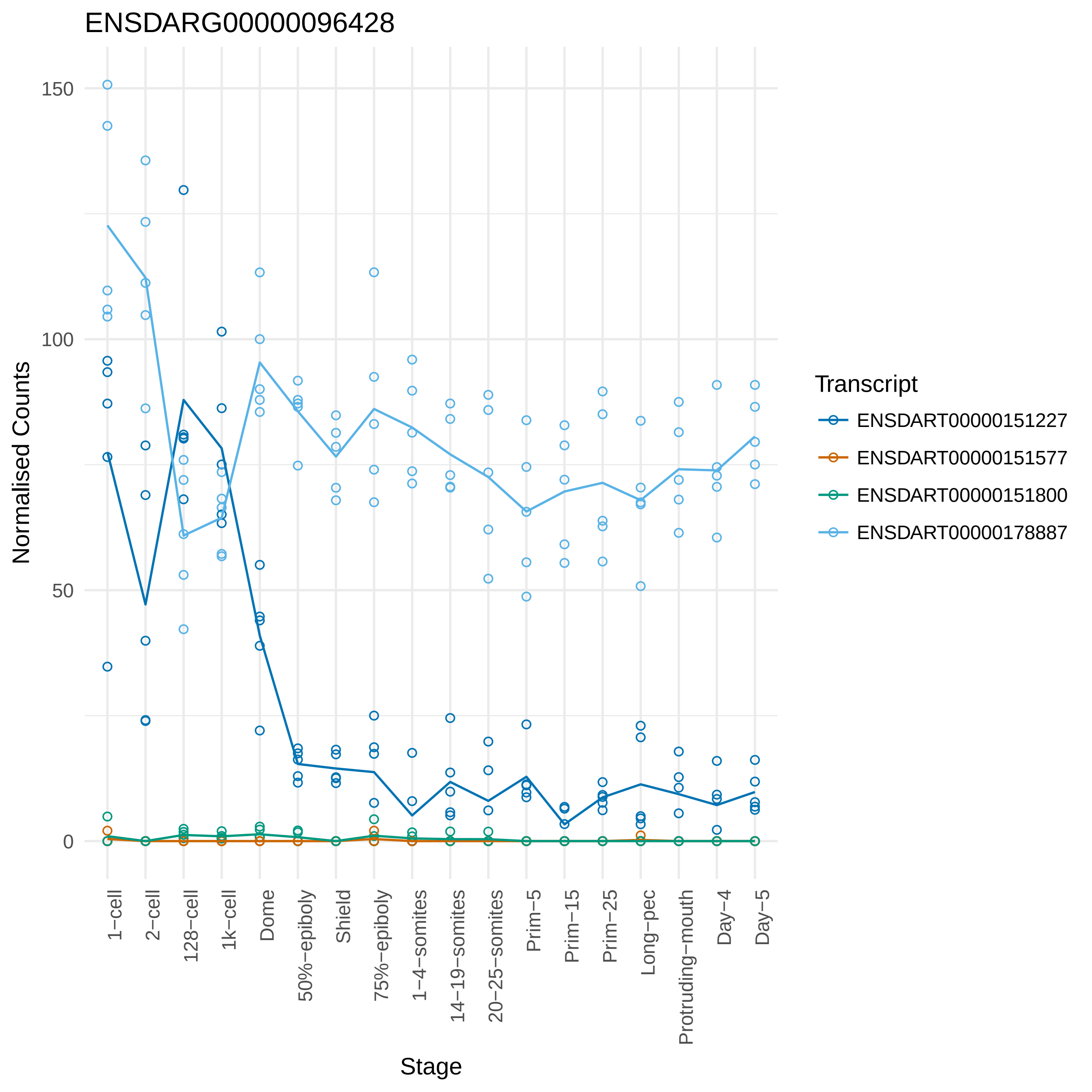

ENSDARG00000096428
Normalised Counts
150
100
50
0
1−cell
2−cell
128−cell
1k−cell
Dome
50%−epiboly
Shield
75%−epiboly
1−4−somites
14−19−somites
20−25−somites
Prim−5
Prim−15
Prim−25
Long−pec
Protruding−mouth
Day−4
Day−5
Stage
Transcript
ENSDART00000151227
ENSDART00000151577
ENSDART00000151800
ENSDART00000178887

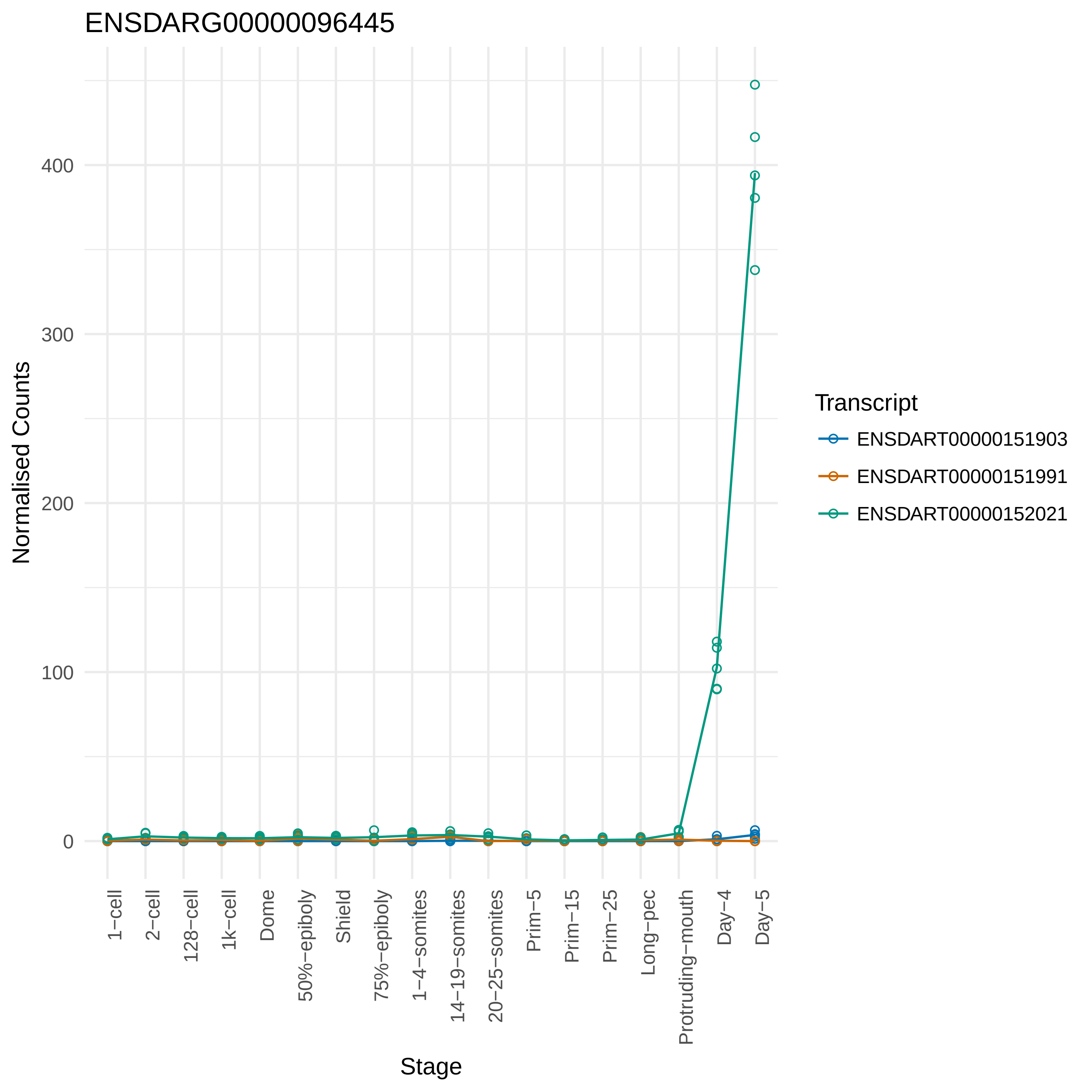
ENSDARG00000096445
Normalised Counts
400
300
200
100
0
1−cell
2−cell
128−cell
1k−cell
Dome
50%−epiboly
Shield
75%−epiboly
1−4−somites
14−19−somites
20−25−somites
Prim−5
Prim−15
Prim−25
Long−pec
Protruding−mouth
Day−4
Day−5
Stage
Transcript
ENSDART00000151903
ENSDART00000151991
ENSDART00000152021

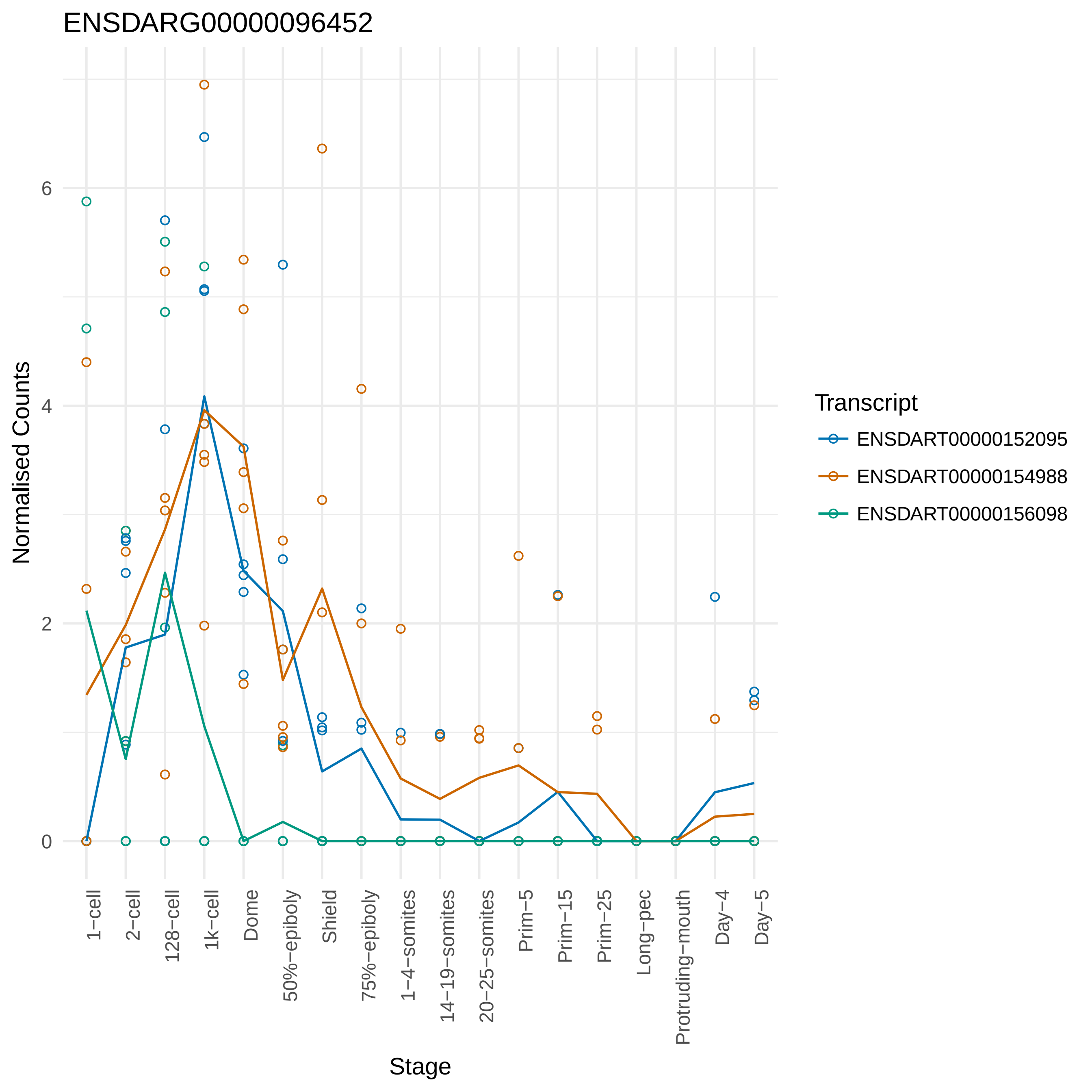

ENSDARG00000096452
Normalised Counts
0
2
4
6
1−cell
2−cell
128−cell
1k−cell
Dome
50%−epiboly
Shield
75%−epiboly
1−4−somites
14−19−somites
20−25−somites
Prim−5
Prim−15
Prim−25
Long−pec
Protruding−mouth
Day−4
Day−5
Stage
Transcript
ENSDART00000152095
ENSDART00000154988
ENSDART00000156098

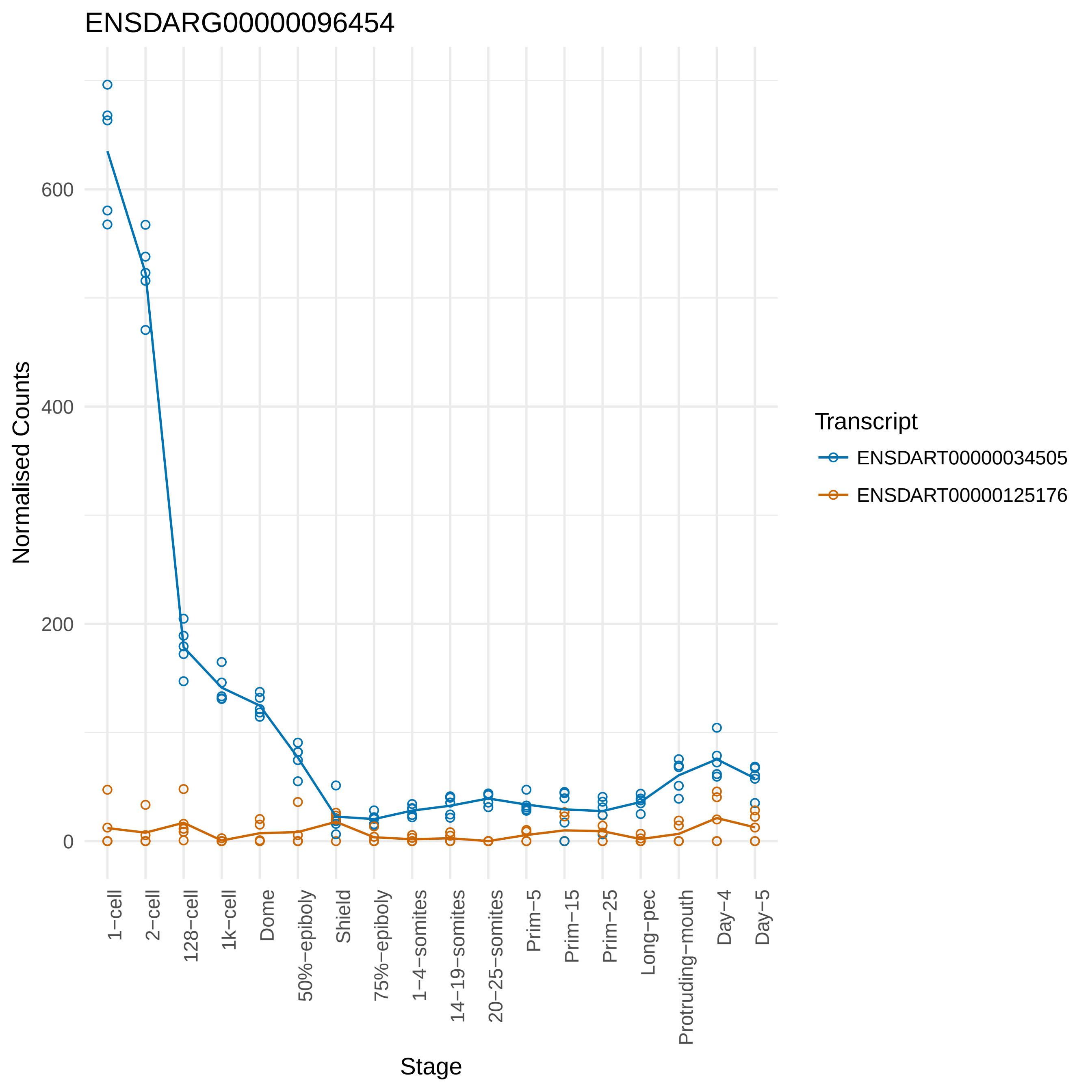

ENSDARG00000096454
Normalised Counts
600
400
200
0
1−cell
2−cell
128−cell
1k−cell
Dome
50%−epiboly
Shield
75%−epiboly
1−4−somites
14−19−somites
20−25−somites
Prim−5
Prim−15
Prim−25
Long−pec
Protruding−mouth
Day−4
Day−5
Stage
Transcript
ENSDART00000034505
ENSDART00000125176

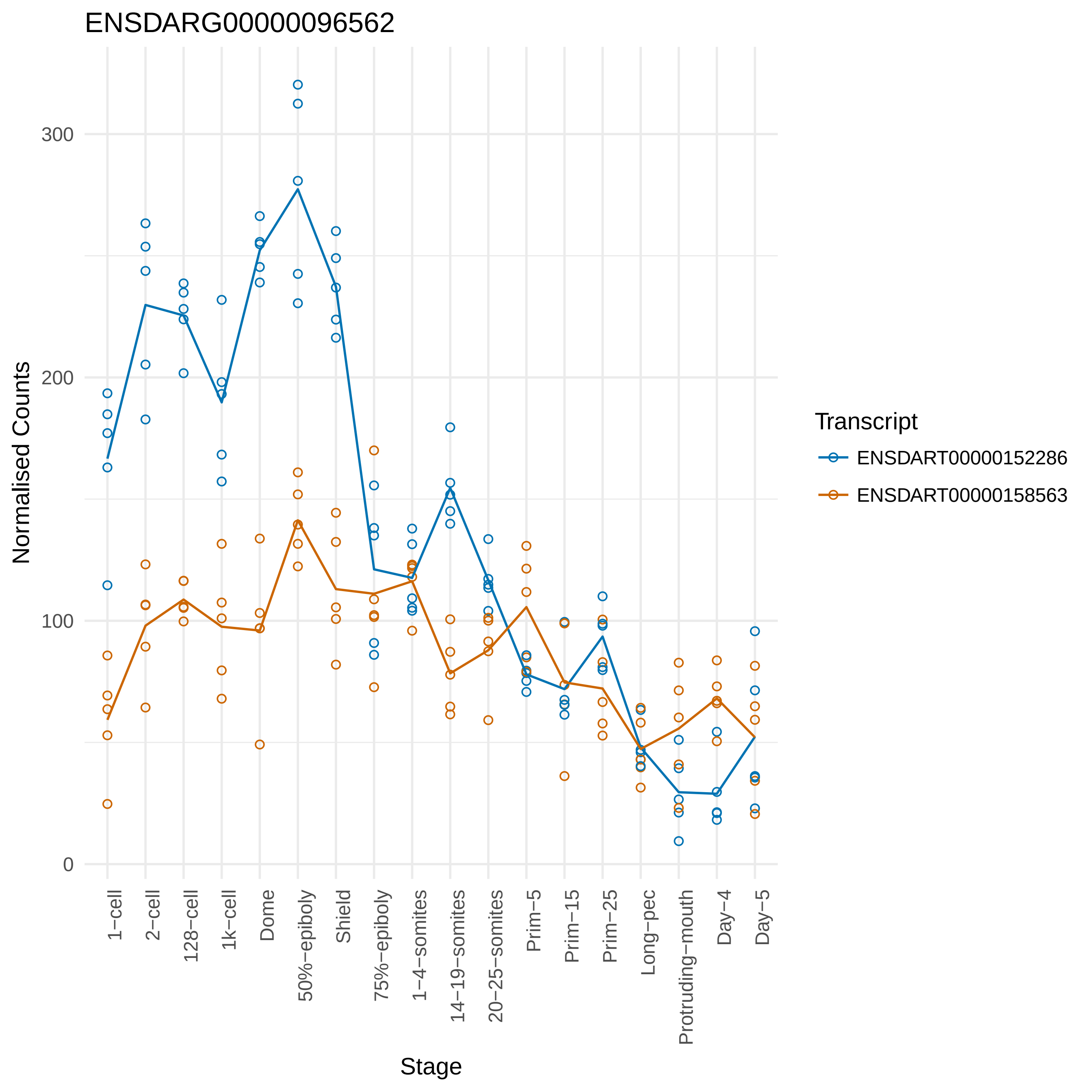
ENSDARG00000096562
Normalised Counts
300
200
100
0
1−cell
2−cell
128−cell
1k−cell
Dome
50%−epiboly
Shield
75%−epiboly
1−4−somites
14−19−somites
20−25−somites
Prim−5
Prim−15
Prim−25
Long−pec
Protruding−mouth
Day−4
Day−5
Stage
Transcript
ENSDART00000152286
ENSDART00000158563

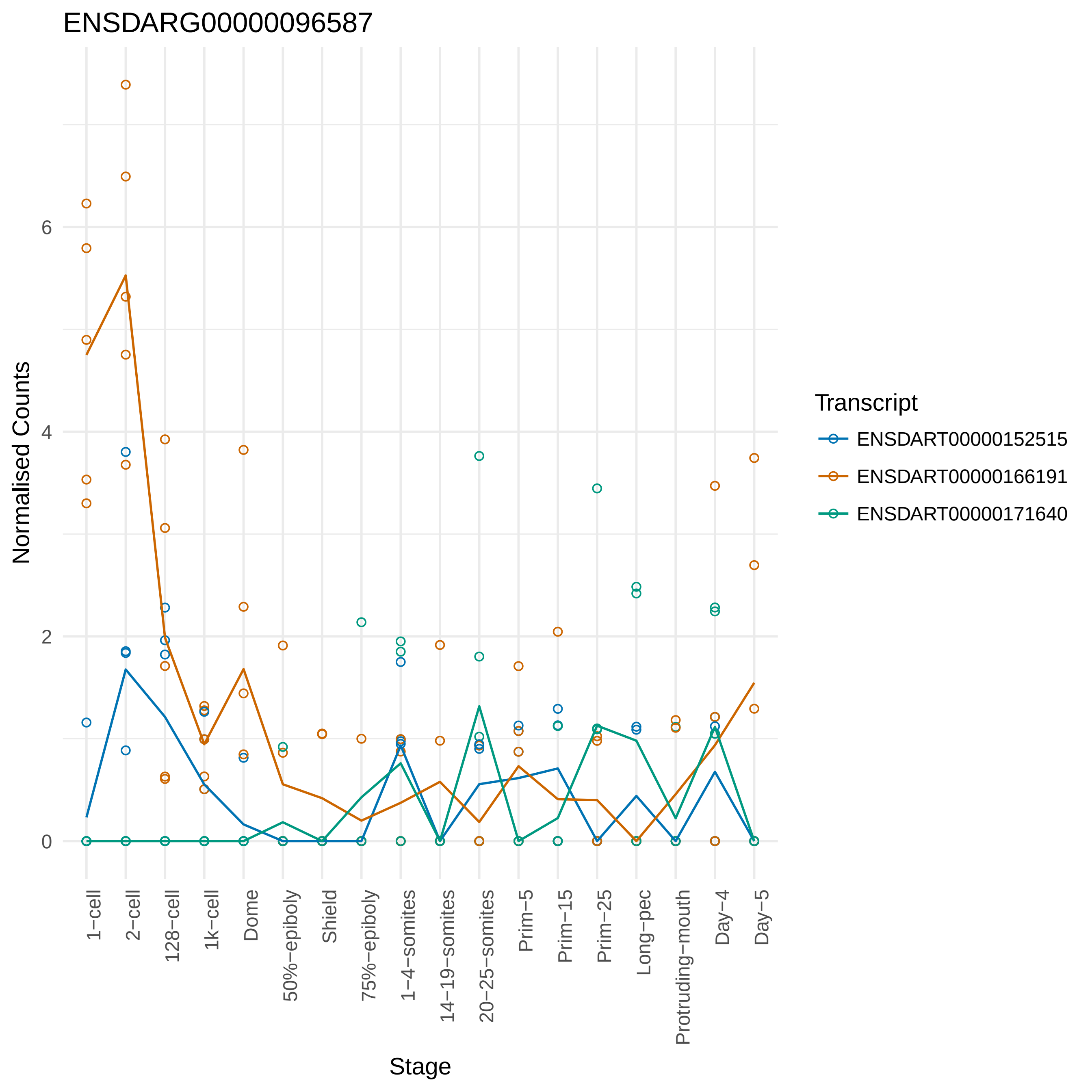
ENSDARG00000096587
Normalised Counts
0
2
4
6
Stage
1−cell
2−cell
128−cell
1k−cell
Dome
50%−epiboly
Shield
75%−epiboly
1−4−somites
14−19−somites
20−25−somites
Prim−5
Prim−15
Prim−25
Long−pec
Protruding−mouth
Day−4
Day−5
Transcript
ENSDART00000152515
ENSDART00000166191
ENSDART00000171640

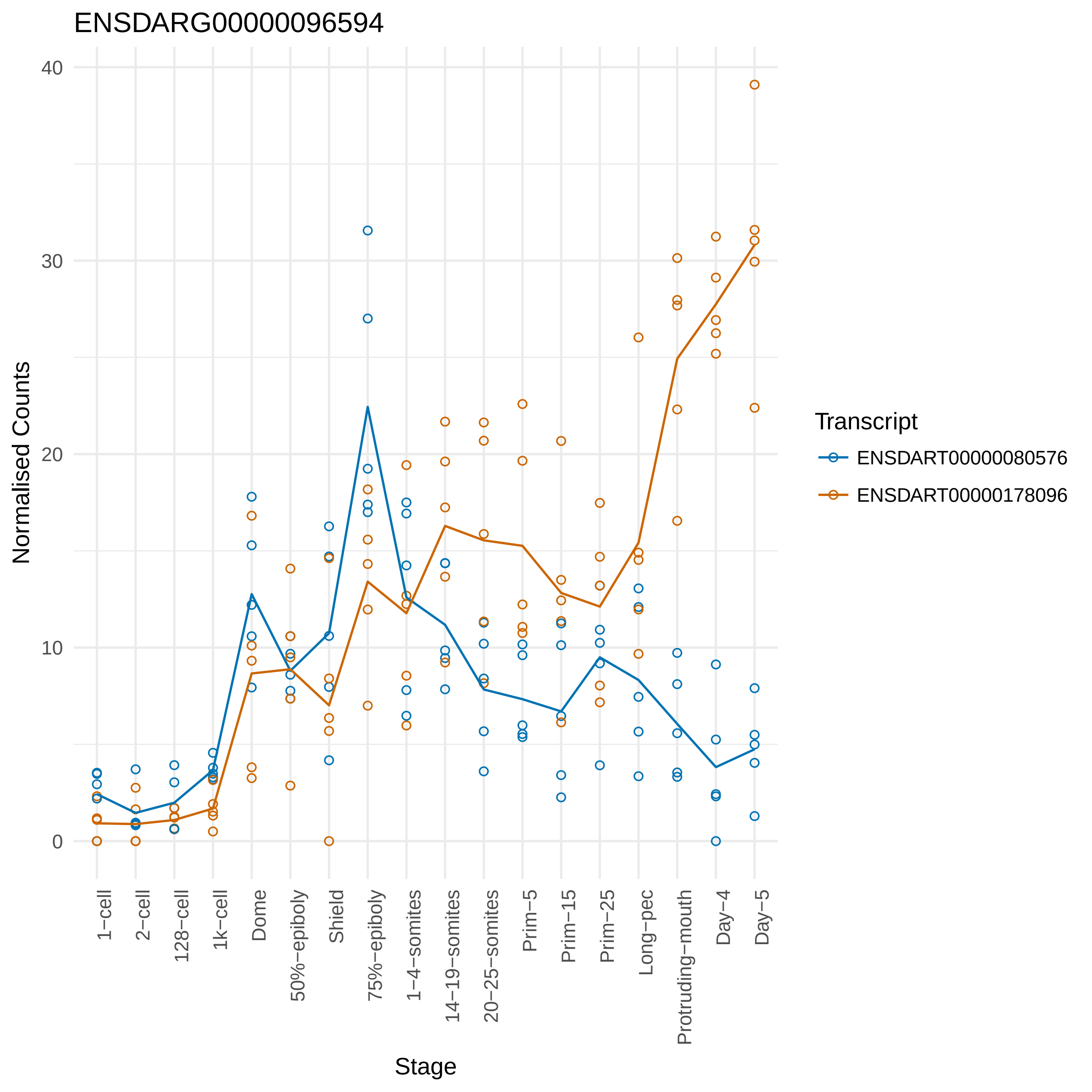

ENSDARG00000096594
Normalised Counts
0
10
20
30
40
Stage
1−cell
2−cell
128−cell
1k−cell
Dome
50%−epiboly
Shield
75%−epiboly
1−4−somites
14−19−somites
20−25−somites
Prim−5
Prim−15
Prim−25
Long−pec
Protruding−mouth
Day−4
Day−5
Transcript
ENSDART00000080576
ENSDART00000178096

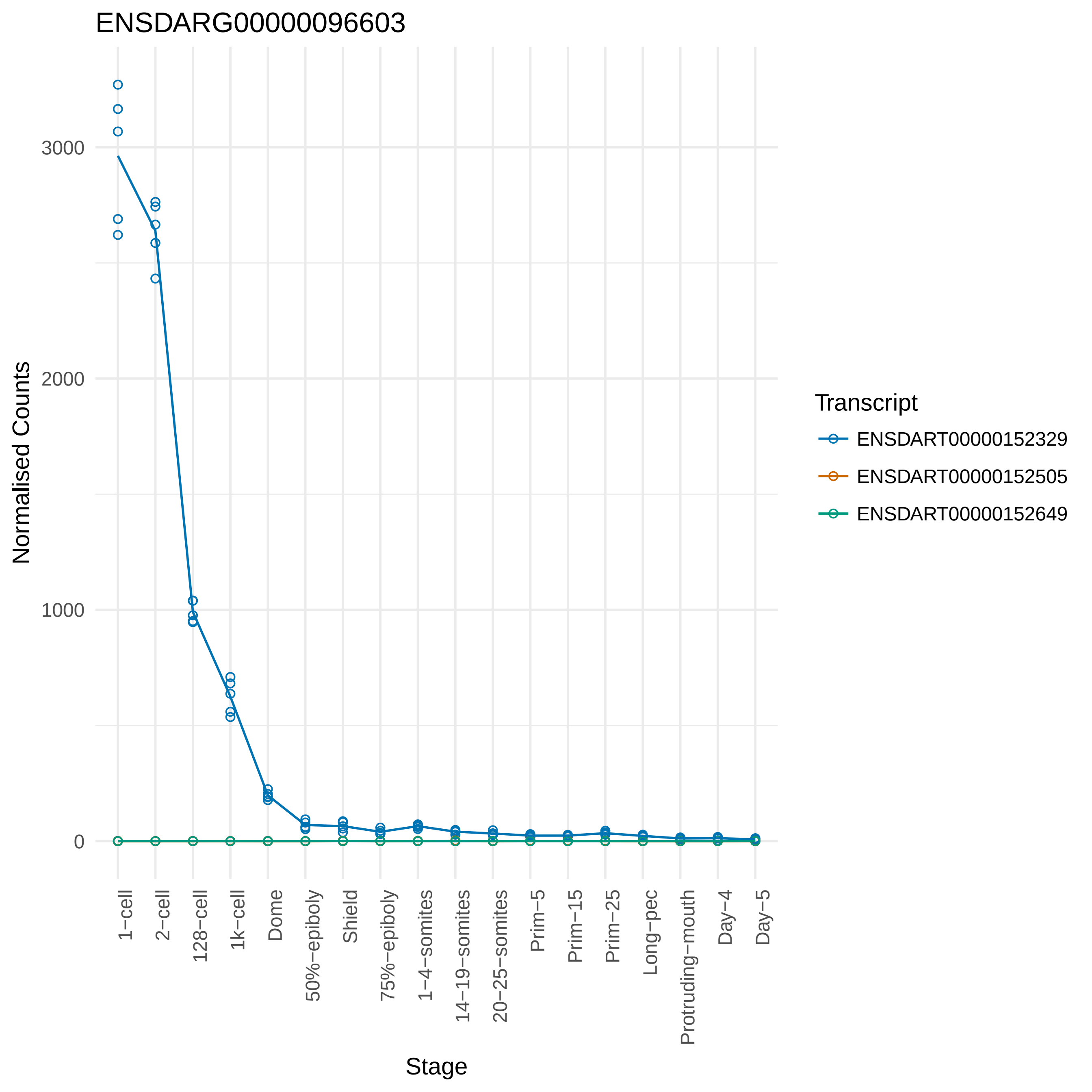
ENSDARG00000096603
Normalised Counts
3000
2000
1000
0
1−cell
2−cell
128−cell
1k−cell
Dome
50%−epiboly
Shield
75%−epiboly
1−4−somites
14−19−somites
20−25−somites
Prim−5
Prim−15
Prim−25
Long−pec
Protruding−mouth
Day−4
Day−5
Stage
Transcript
ENSDART00000152329
ENSDART00000152505
ENSDART00000152649

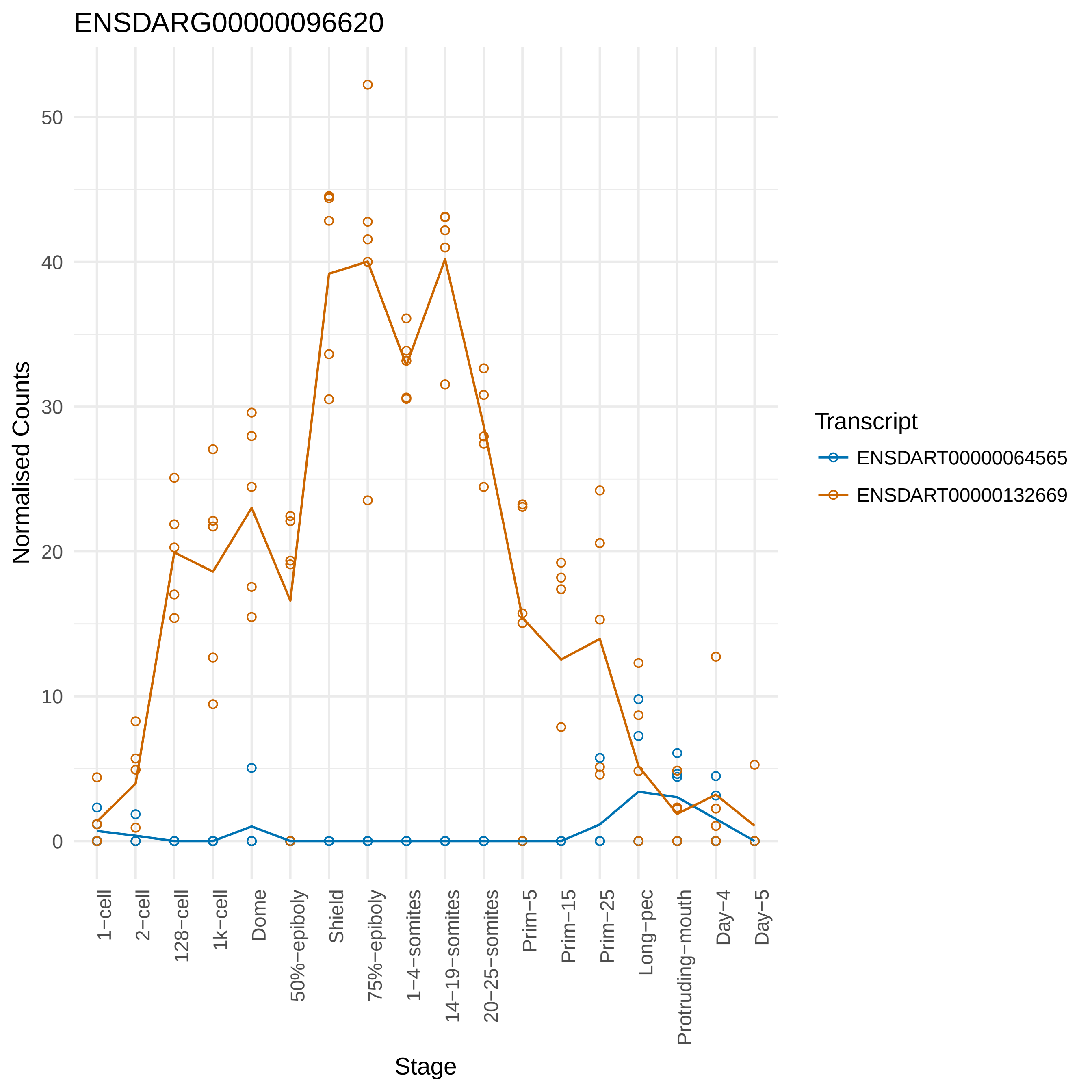

ENSDARG00000096620
Normalised Counts
0
10
20
30
40
50
1−cell
2−cell
128−cell
1k−cell
Dome
50%−epiboly
Shield
75%−epiboly
1−4−somites
14−19−somites
20−25−somites
Prim−5
Prim−15
Prim−25
Long−pec
Protruding−mouth
Day−4
Day−5
Stage
Transcript
ENSDART00000064565
ENSDART00000132669

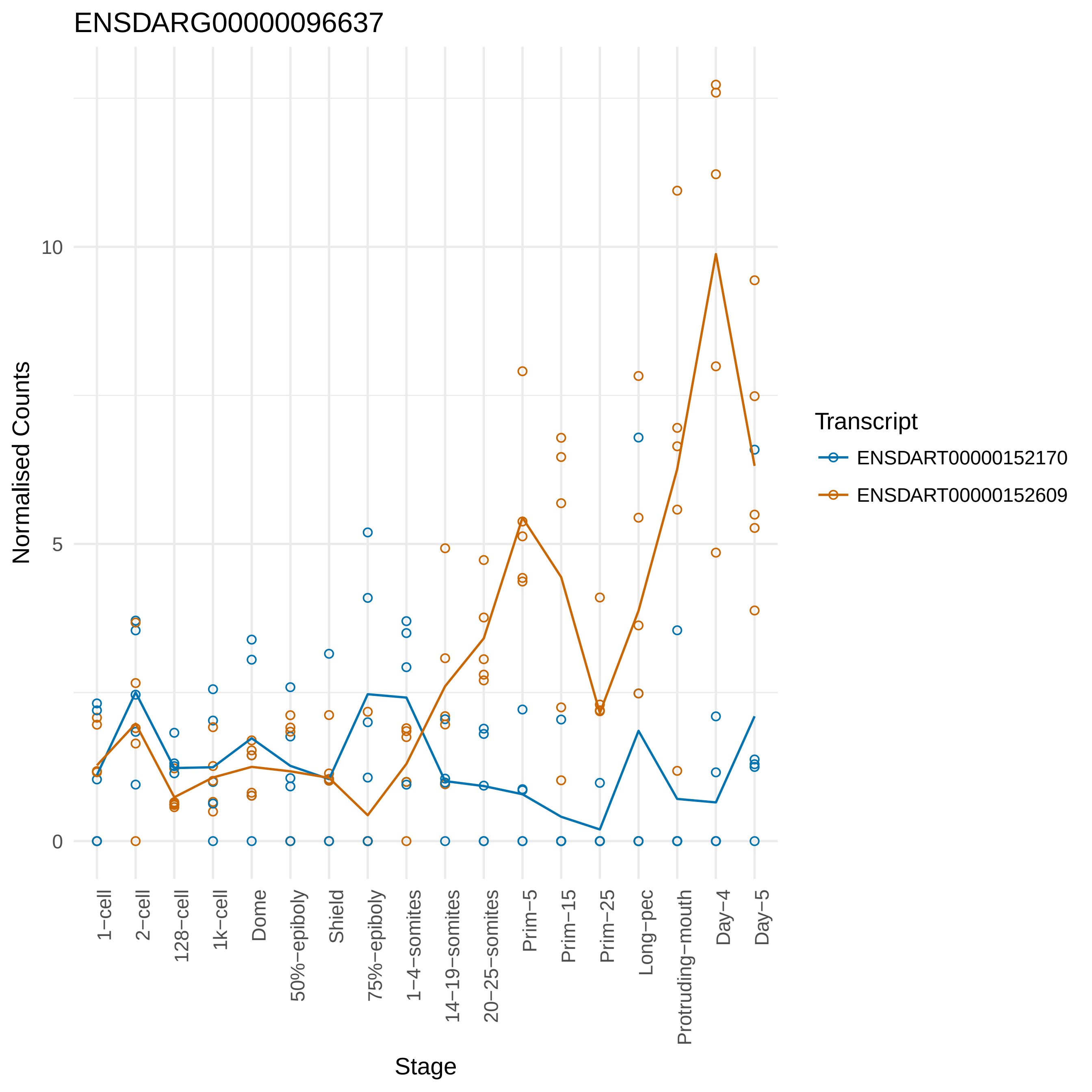

ENSDARG00000096637
Normalised Counts
0
5
10
Stage
1−cell
2−cell
128−cell
1k−cell
Dome
50%−epiboly
Shield
75%−epiboly
1−4−somites
14−19−somites
20−25−somites
Prim−5
Prim−15
Prim−25
Long−pec
Protruding−mouth
Day−4
Day−5
Transcript
ENSDART00000152170
ENSDART00000152609

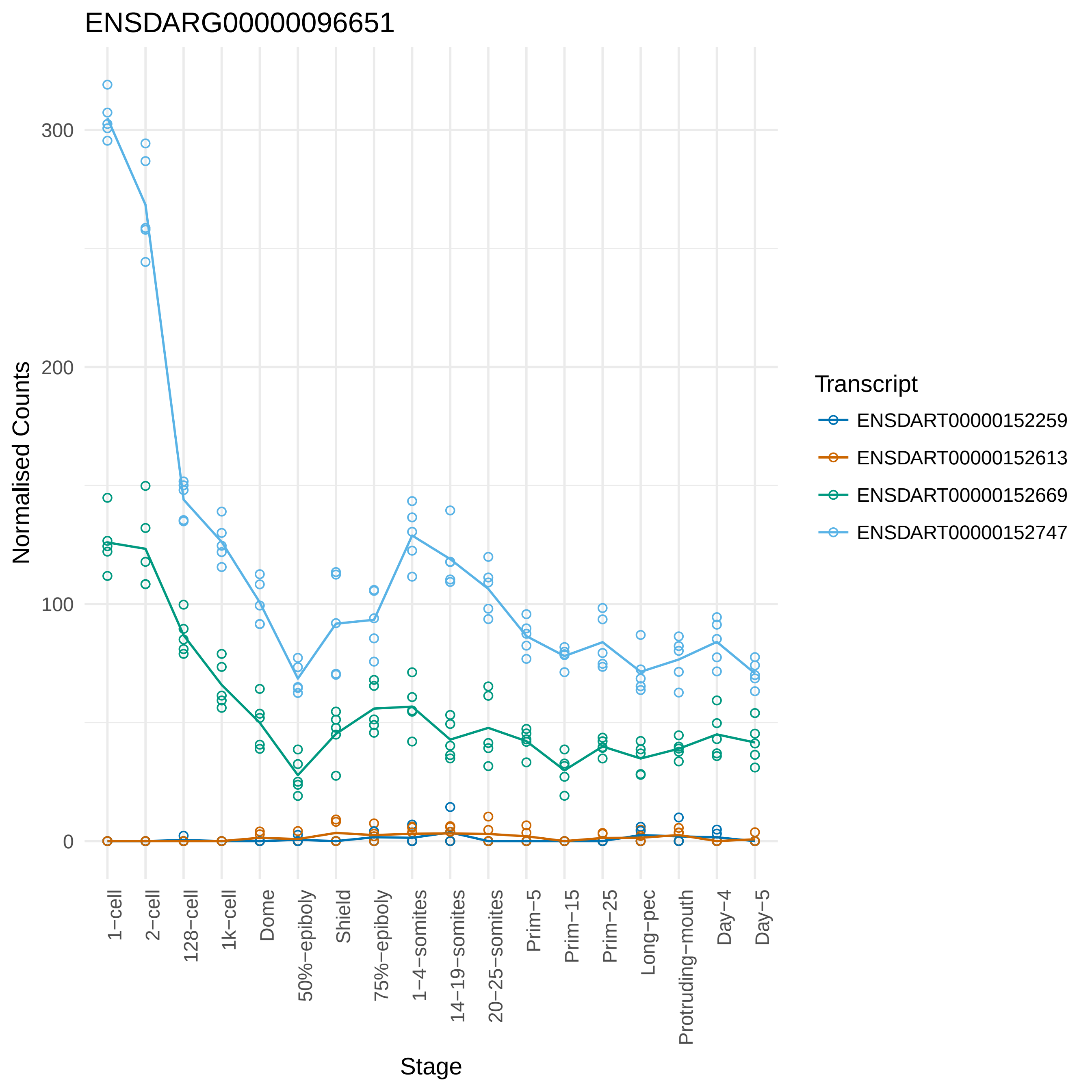

ENSDARG00000096651
Normalised Counts
300
200
100
0
1−cell
2−cell
128−cell
1k−cell
Dome
50%−epiboly
Shield
75%−epiboly
1−4−somites
14−19−somites
20−25−somites
Prim−5
Prim−15
Prim−25
Long−pec
Protruding−mouth
Day−4
Day−5
Stage
Transcript
ENSDART00000152259
ENSDART00000152613
ENSDART00000152669
ENSDART00000152747

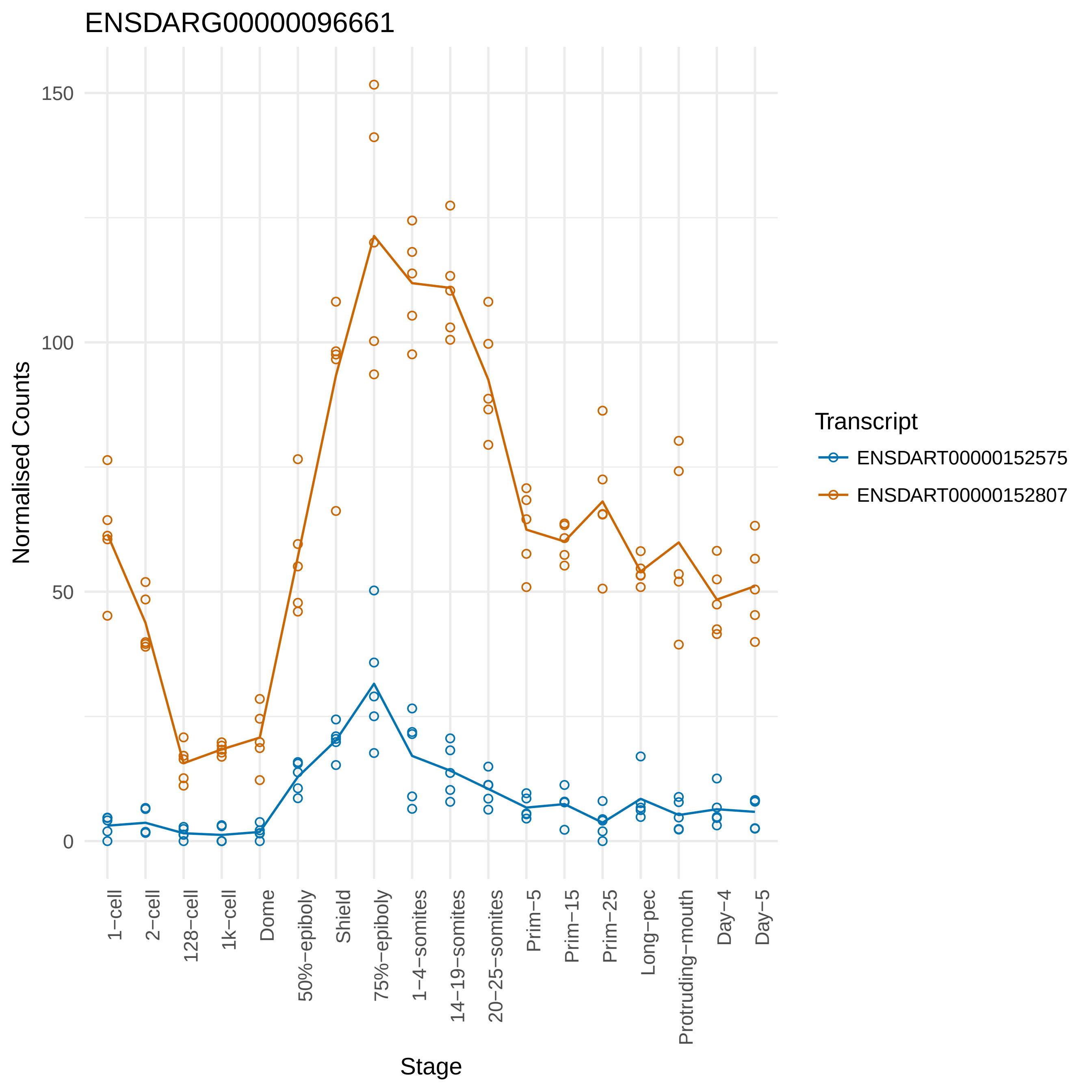
ENSDARG00000096661
Normalised Counts
150
100
50
0
1−cell
2−cell
128−cell
1k−cell
Dome
50%−epiboly
Shield
75%−epiboly
1−4−somites
14−19−somites
20−25−somites
Prim−5
Prim−15
Prim−25
Long−pec
Protruding−mouth
Day−4
Day−5
Stage
Transcript
ENSDART00000152575
ENSDART00000152807

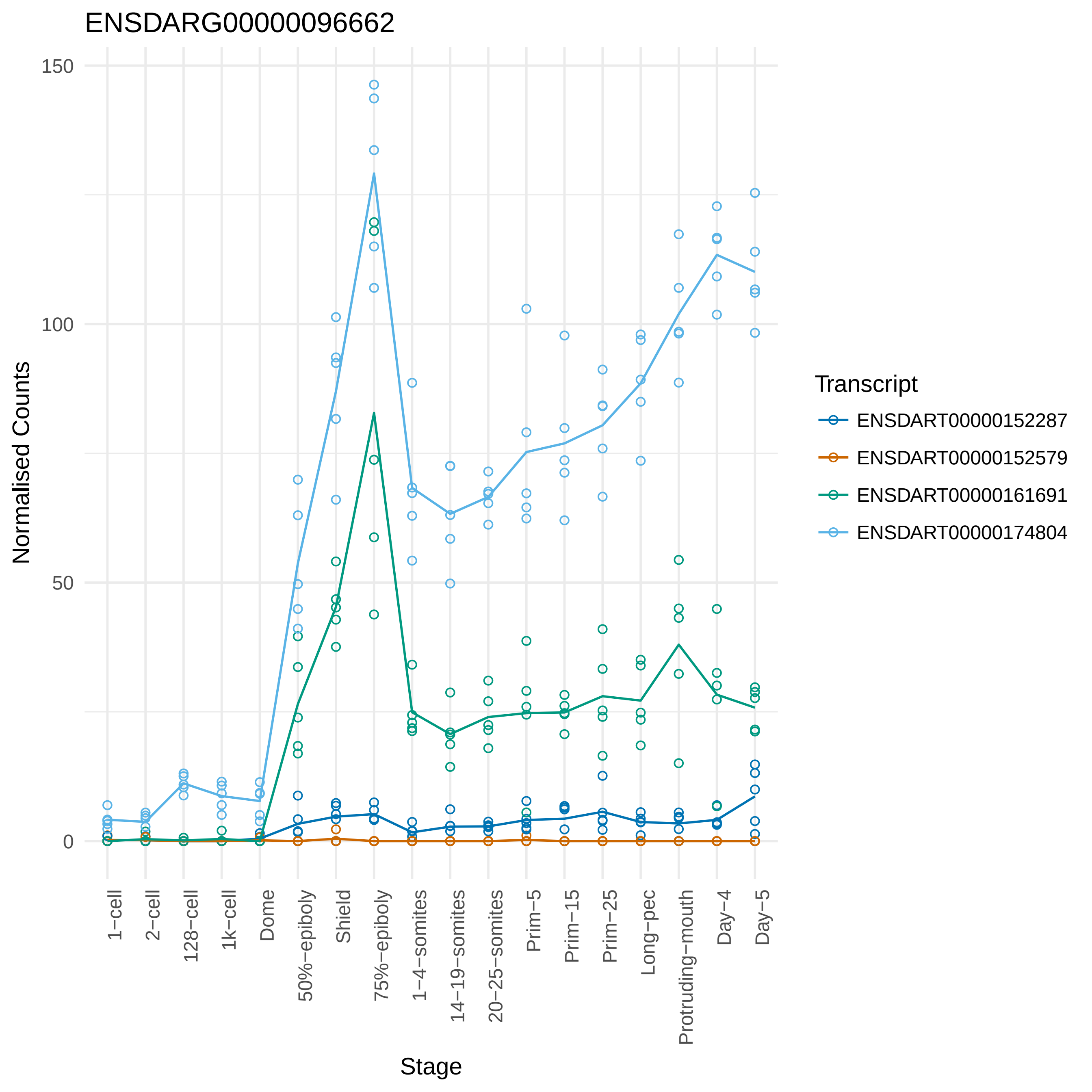

ENSDARG00000096662
Normalised Counts
150
100
50
0
1−cell
2−cell
128−cell
1k−cell
Dome
50%−epiboly
Shield
75%−epiboly
1−4−somites
14−19−somites
20−25−somites
Prim−5
Prim−15
Prim−25
Long−pec
Protruding−mouth
Day−4
Day−5
Stage
Transcript
ENSDART00000152287
ENSDART00000152579
ENSDART00000161691
ENSDART00000174804

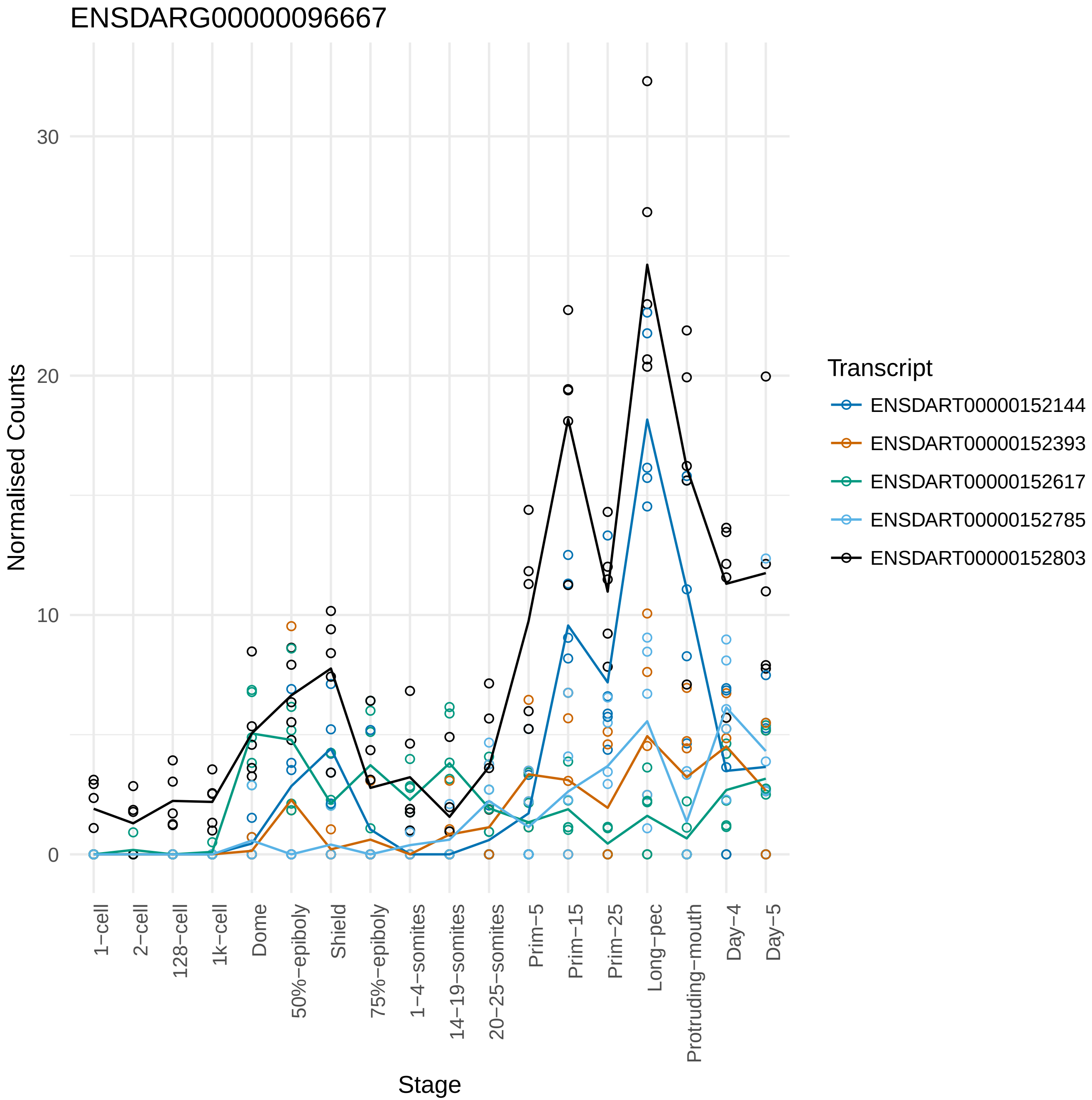
ENSDARG00000096667
Normalised Counts
30
20
10
0
1−cell
2−cell
128−cell
1k−cell
Dome
50%−epiboly
Shield
75%−epiboly
1−4−somites
14−19−somites
20−25−somites
Prim−5
Prim−15
Prim−25
Long−pec
Protruding−mouth
Day−4
Day−5
Stage
Transcript
ENSDART00000152144
ENSDART00000152393
ENSDART00000152617
ENSDART00000152785
ENSDART00000152803

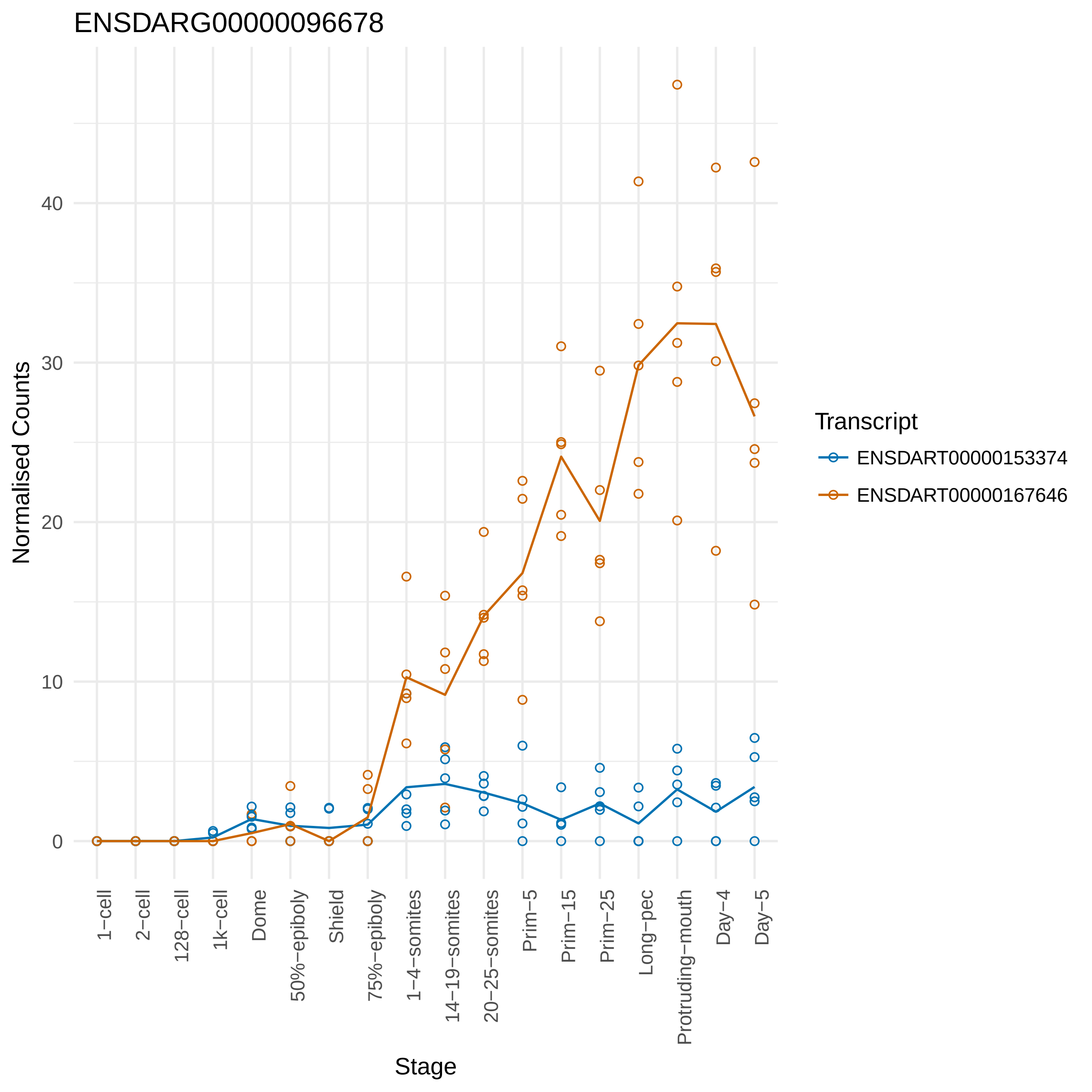

ENSDARG00000096678
Normalised Counts
0
10
20
30
40
Stage
1−cell
2−cell
128−cell
1k−cell
Dome
50%−epiboly
Shield
75%−epiboly
1−4−somites
14−19−somites
20−25−somites
Prim−5
Prim−15
Prim−25
Long−pec
Protruding−mouth
Day−4
Day−5
Transcript
ENSDART00000153374
ENSDART00000167646

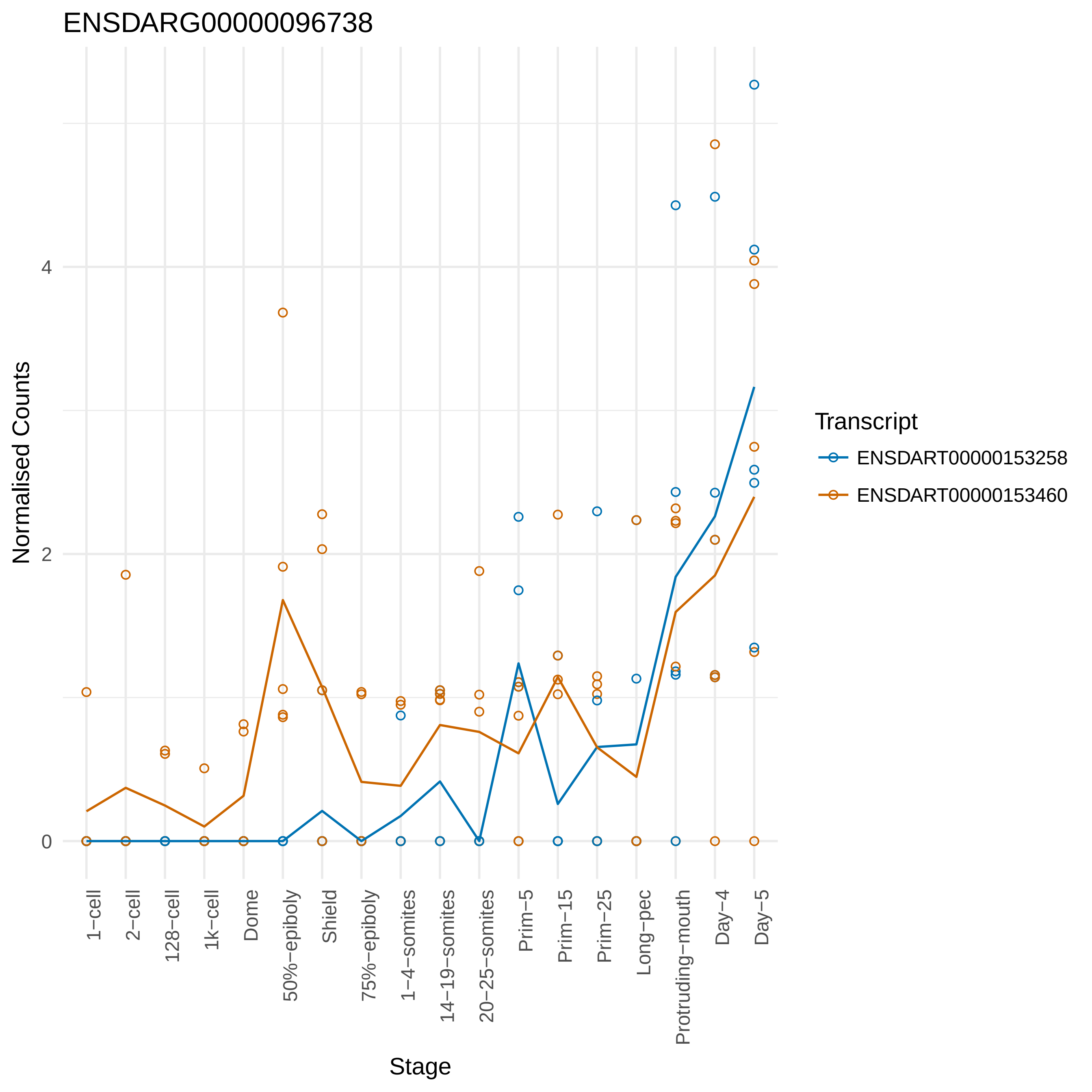
ENSDARG00000096738
Normalised Counts
0
2
4
1−cell
2−cell
128−cell
1k−cell
Dome
50%−epiboly
Shield
75%−epiboly
1−4−somites
14−19−somites
20−25−somites
Prim−5
Prim−15
Prim−25
Long−pec
Protruding−mouth
Day−4
Day−5
Stage
Transcript
ENSDART00000153258
ENSDART00000153460

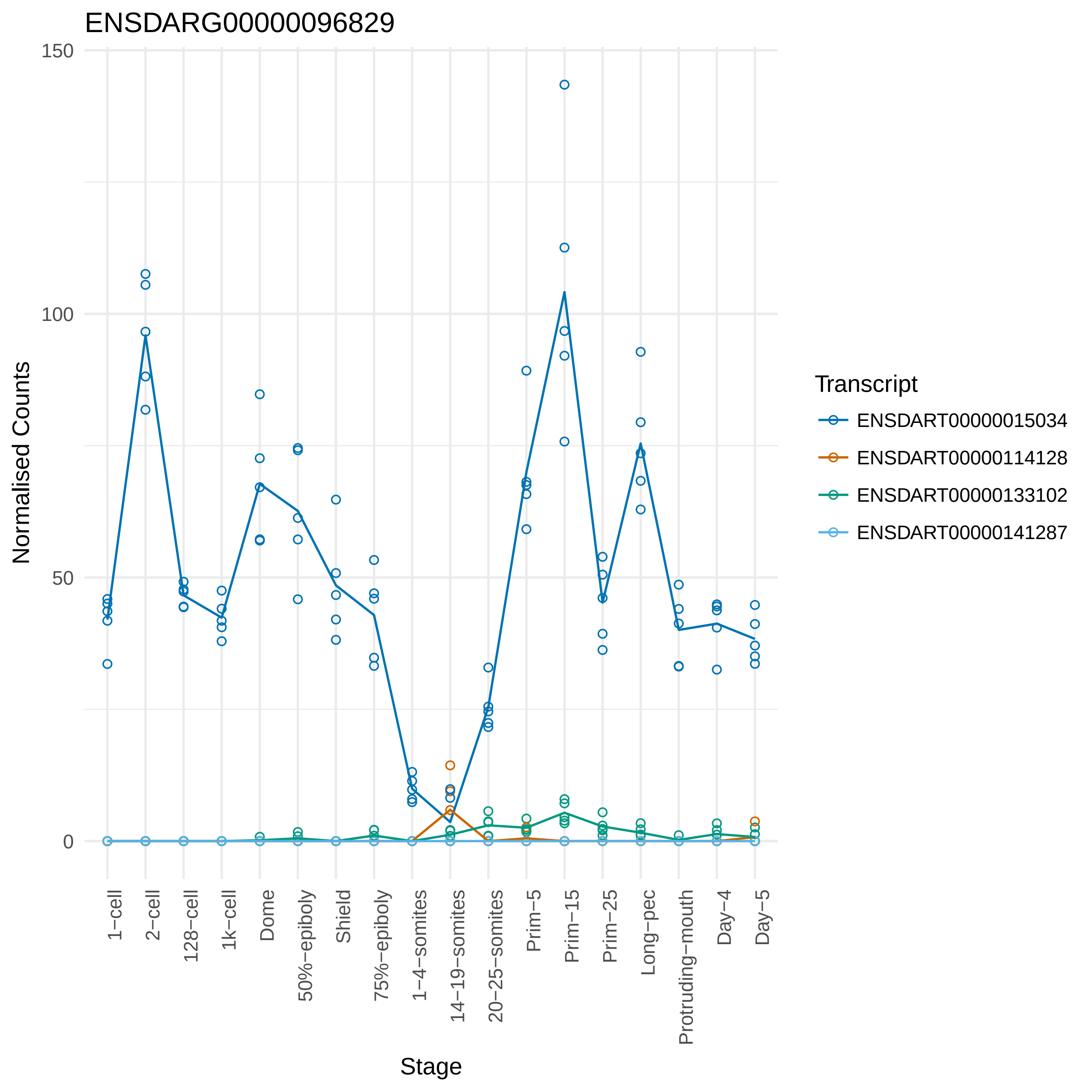
ENSDARG00000096829
Normalised Counts
150
100
50
0
1−cell
2−cell
128−cell
1k−cell
Dome
50%−epiboly
Shield
75%−epiboly
1−4−somites
14−19−somites
20−25−somites
Prim−5
Prim−15
Prim−25
Long−pec
Protruding−mouth
Day−4
Day−5
Stage
Transcript
ENSDART00000015034
ENSDART00000114128
ENSDART00000133102
ENSDART00000141287

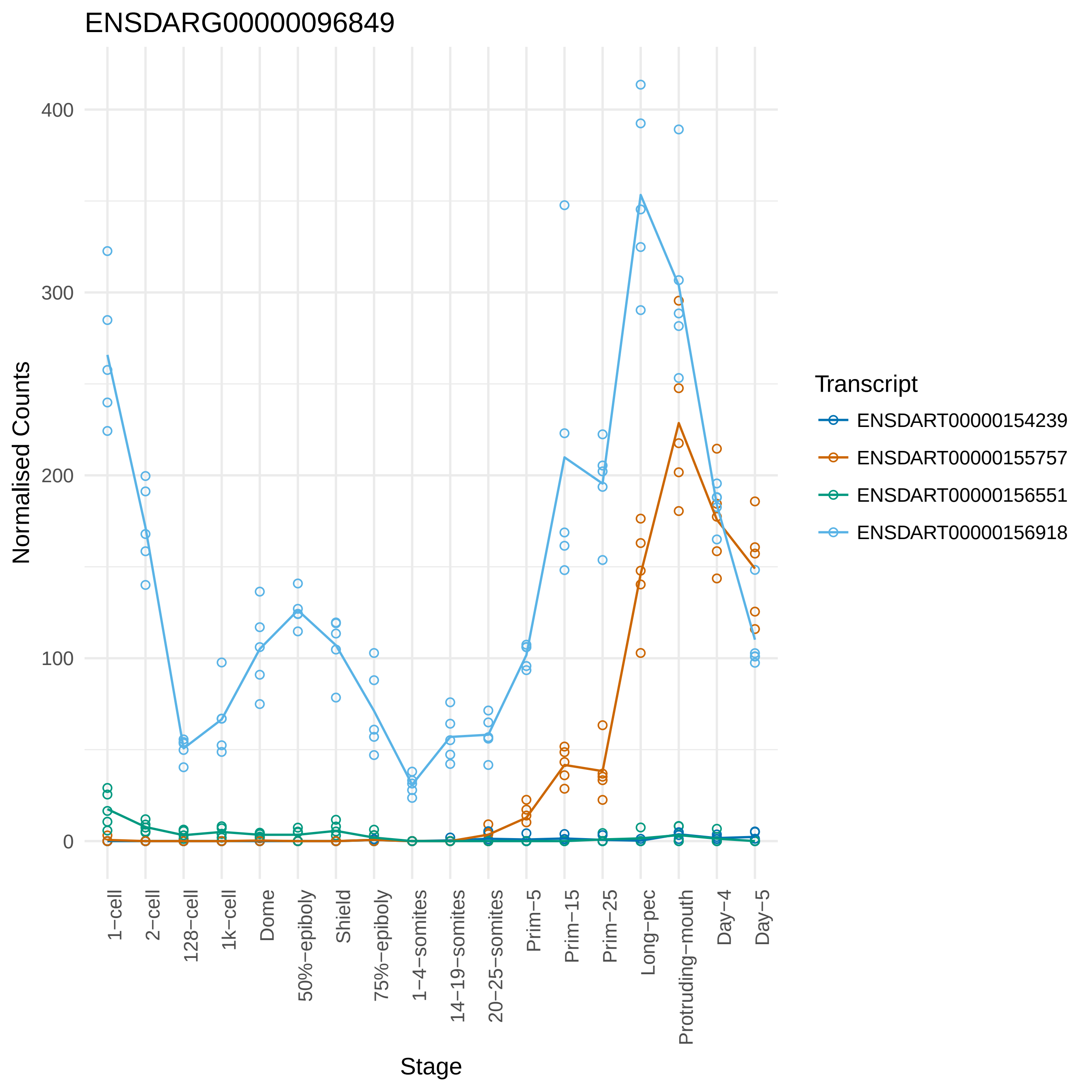

ENSDARG00000096849
Normalised Counts
400
300
200
100
0
1−cell
2−cell
128−cell
1k−cell
Dome
50%−epiboly
Shield
75%−epiboly
1−4−somites
14−19−somites
20−25−somites
Prim−5
Prim−15
Prim−25
Long−pec
Protruding−mouth
Day−4
Day−5
Stage
Transcript
ENSDART00000154239
ENSDART00000155757
ENSDART00000156551
ENSDART00000156918

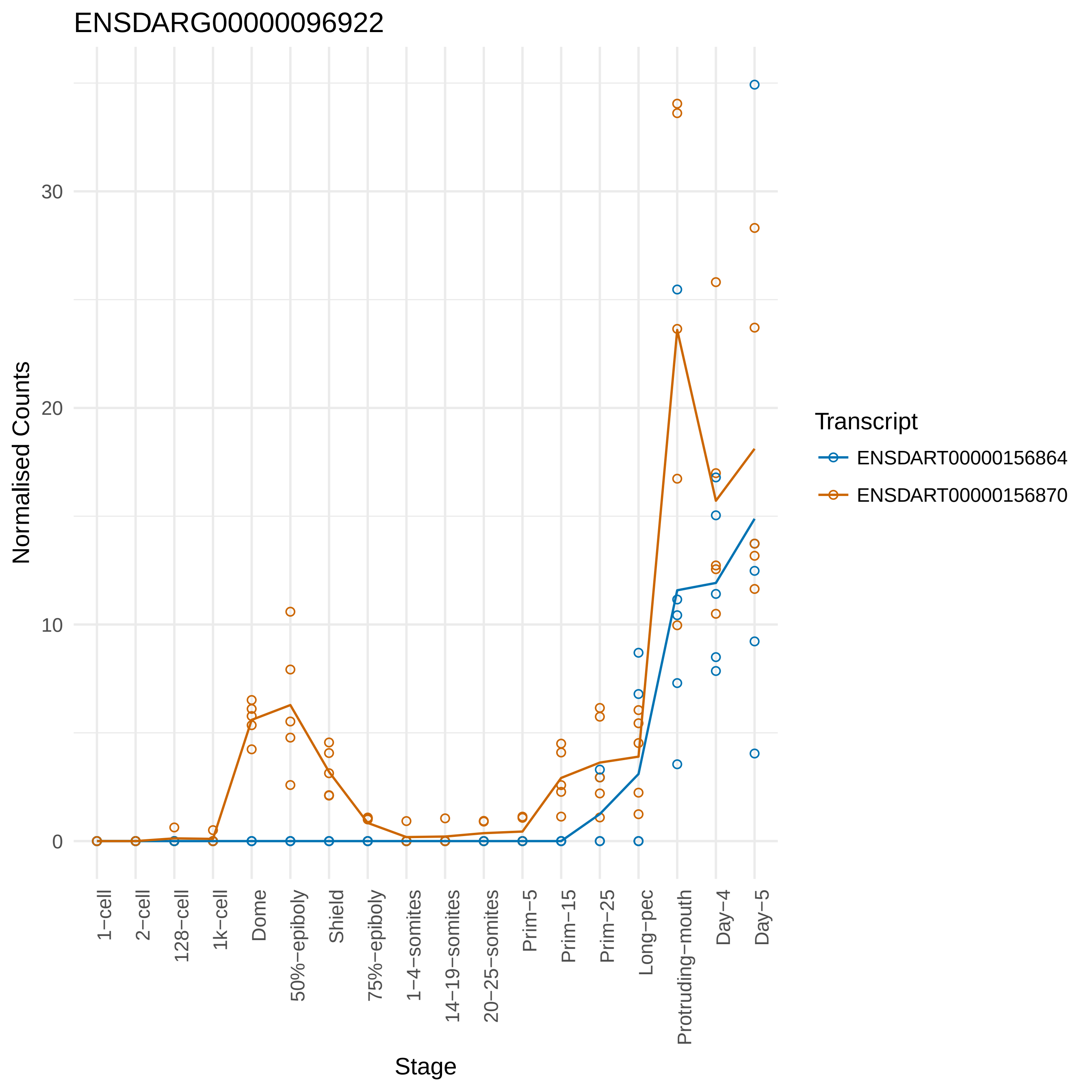

ENSDARG00000096922
Normalised Counts
0
10
20
30
1−cell
2−cell
128−cell
1k−cell
Dome
50%−epiboly
Shield
75%−epiboly
1−4−somites
14−19−somites
20−25−somites
Prim−5
Prim−15
Prim−25
Long−pec
Protruding−mouth
Day−4
Day−5
Stage
Transcript
ENSDART00000156864
ENSDART00000156870

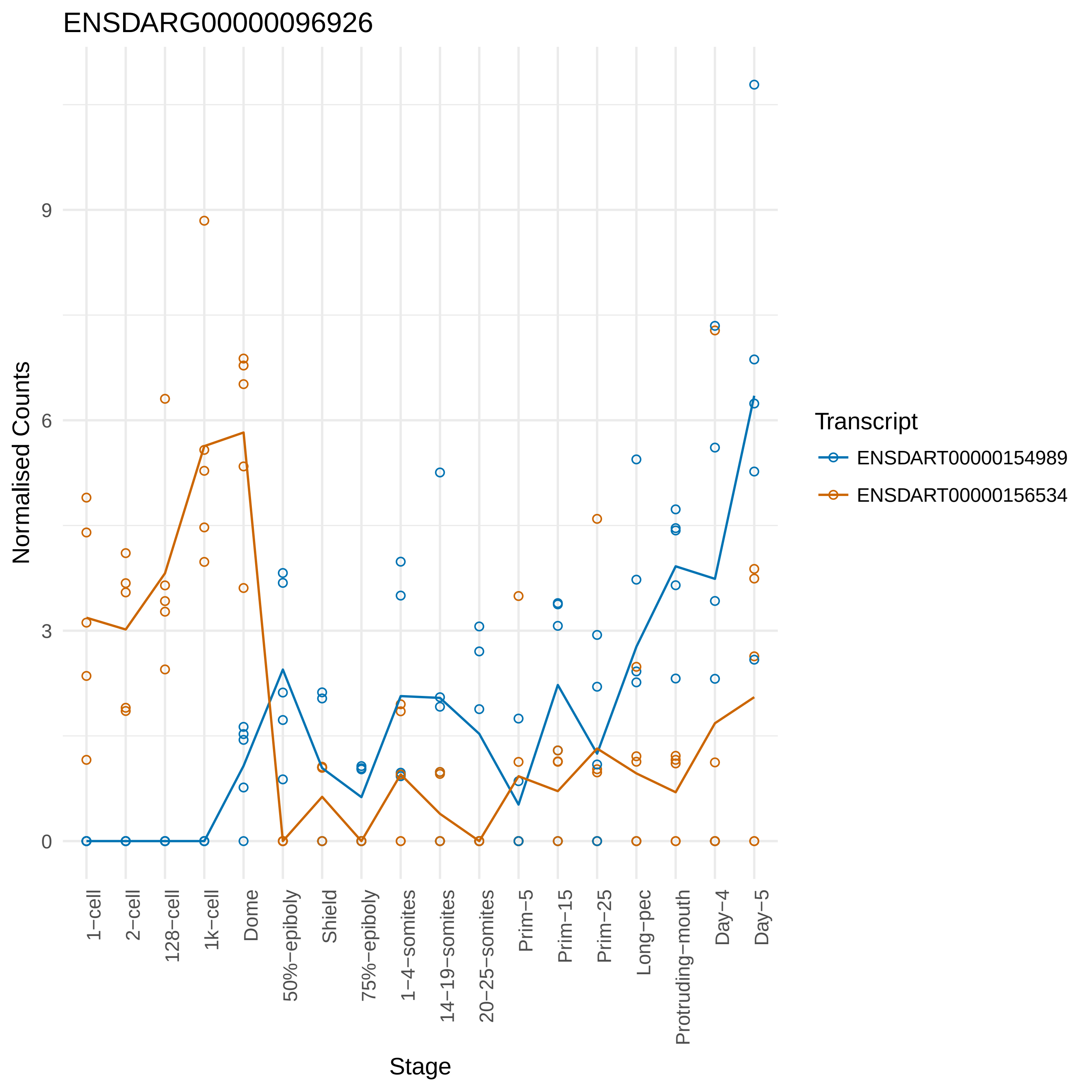
ENSDARG00000096926
Normalised Counts
0
3
6
9
Stage
1−cell
2−cell
128−cell
1k−cell
Dome
50%−epiboly
Shield
75%−epiboly
1−4−somites
14−19−somites
20−25−somites
Prim−5
Prim−15
Prim−25
Long−pec
Protruding−mouth
Day−4
Day−5
Transcript
ENSDART00000154989
ENSDART00000156534

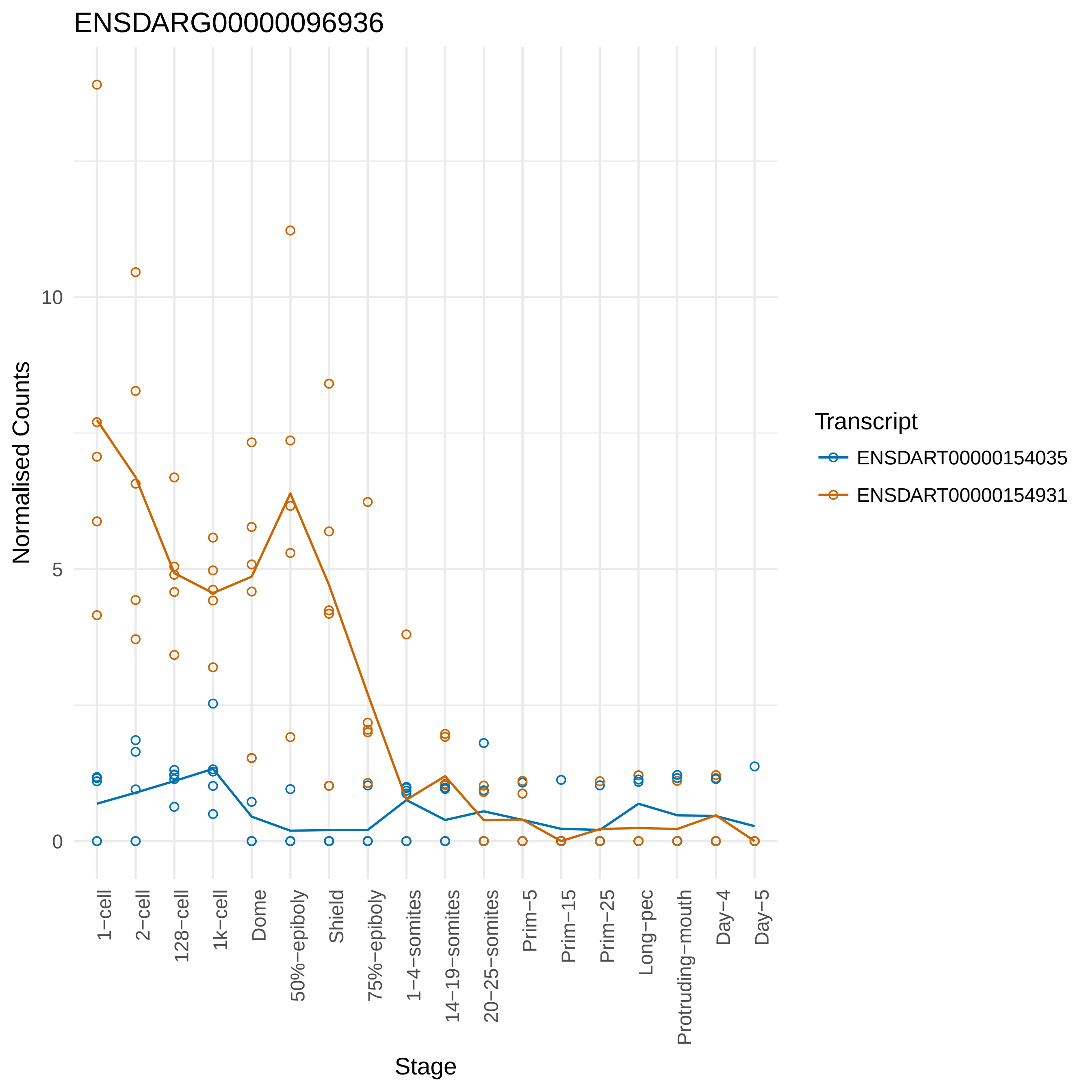
ENSDARG00000096936
Normalised Counts
10
5
0
1−cell
2−cell
128−cell
1k−cell
Dome
50%−epiboly
Shield
75%−epiboly
1−4−somites
14−19−somites
20−25−somites
Prim−5
Prim−15
Prim−25
Long−pec
Protruding−mouth
Day−4
Day−5
Stage
Transcript
ENSDART00000154035
ENSDART00000154931

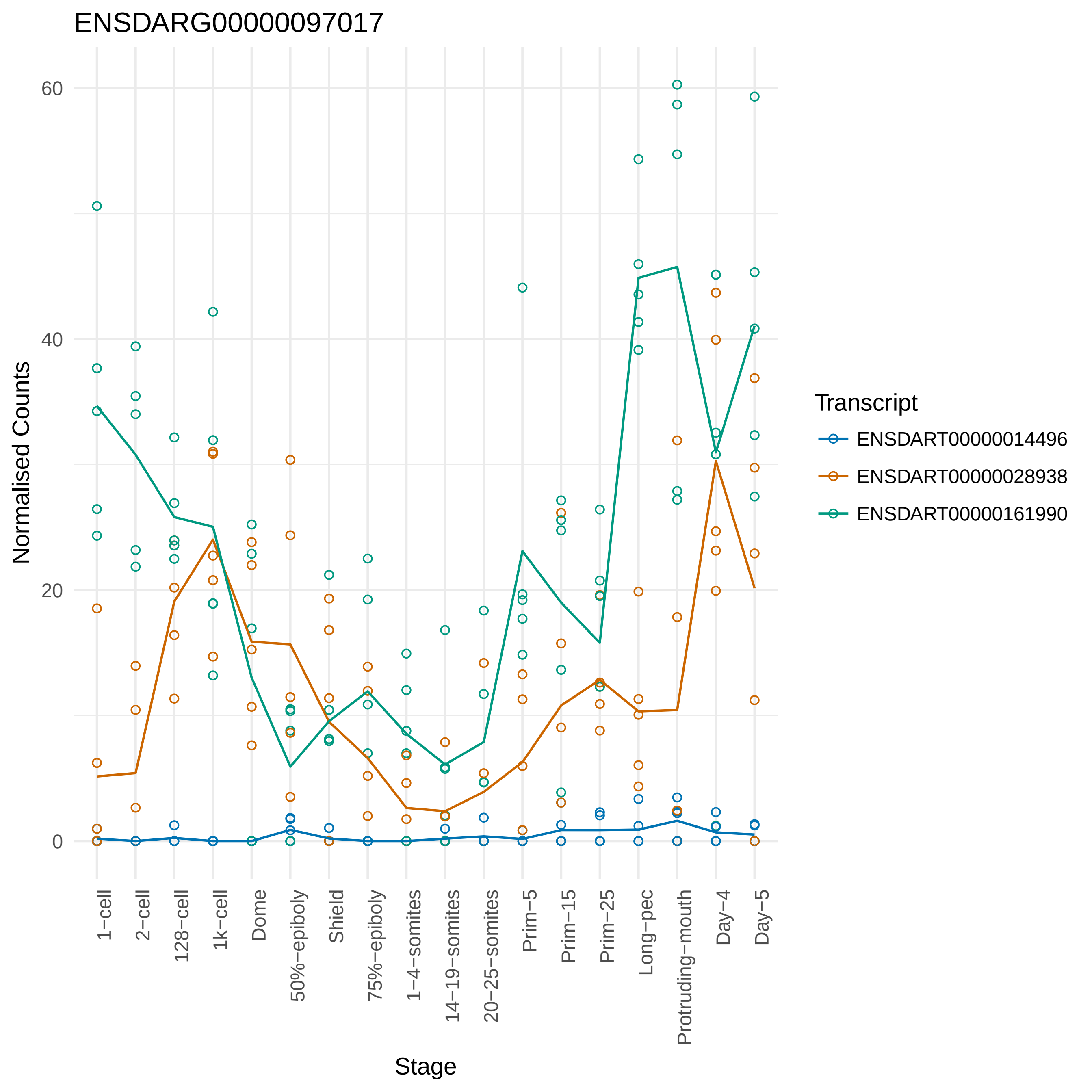

ENSDARG00000097017
Normalised Counts
0
20
40
60
1−cell
2−cell
128−cell
1k−cell
Dome
50%−epiboly
Shield
75%−epiboly
1−4−somites
14−19−somites
20−25−somites
Prim−5
Prim−15
Prim−25
Long−pec
Protruding−mouth
Day−4
Day−5
Stage
Transcript
ENSDART00000014496
ENSDART00000028938
ENSDART00000161990

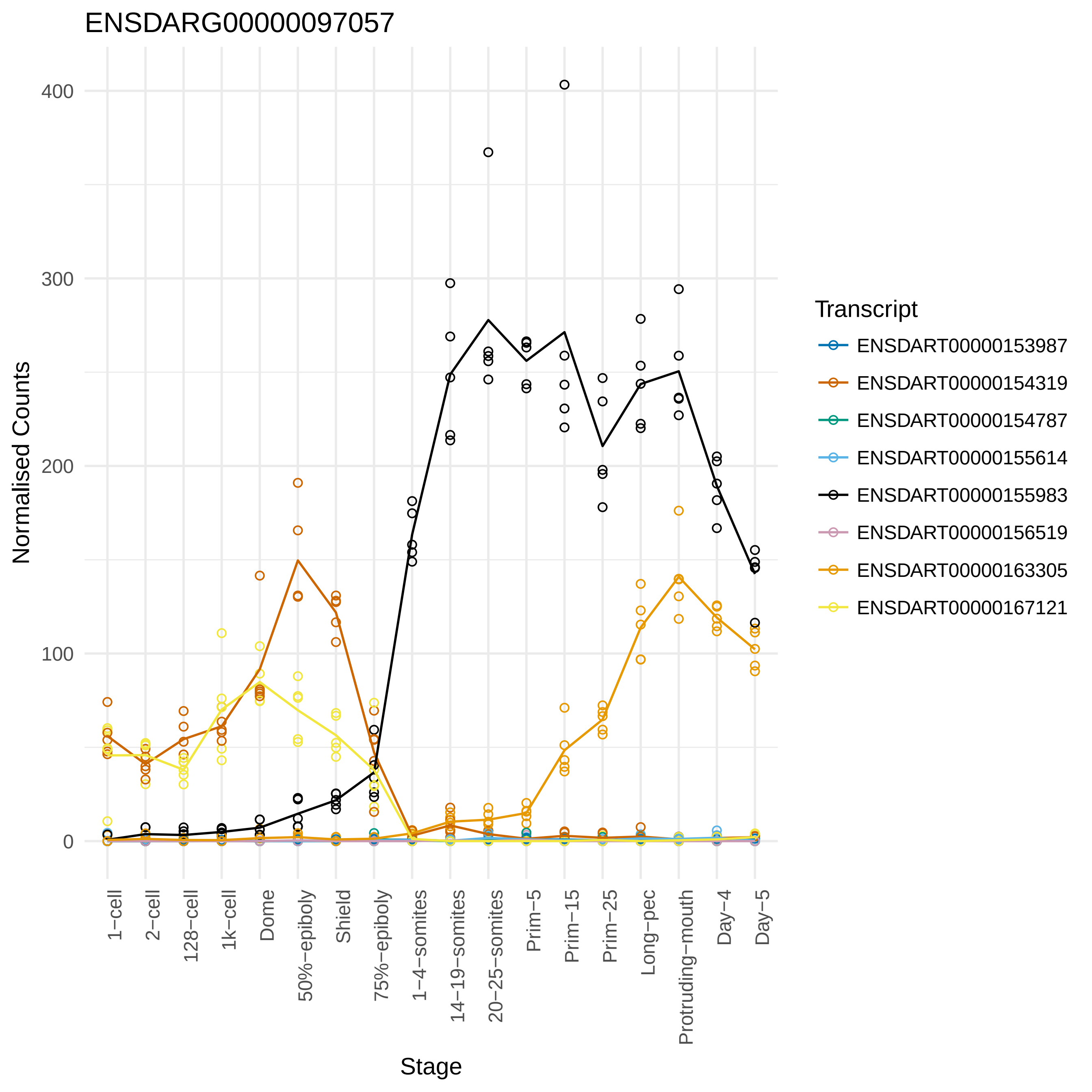

ENSDARG00000097057
Normalised Counts
400
300
200
100
0
1−cell
2−cell
128−cell
1k−cell
Dome
50%−epiboly
Shield
75%−epiboly
1−4−somites
14−19−somites
20−25−somites
Prim−5
Prim−15
Prim−25
Long−pec
Protruding−mouth
Day−4
Day−5
Stage
Transcript
ENSDART00000153987
ENSDART00000154319
ENSDART00000154787
ENSDART00000155614
ENSDART00000155983
ENSDART00000156519
ENSDART00000163305
ENSDART00000167121

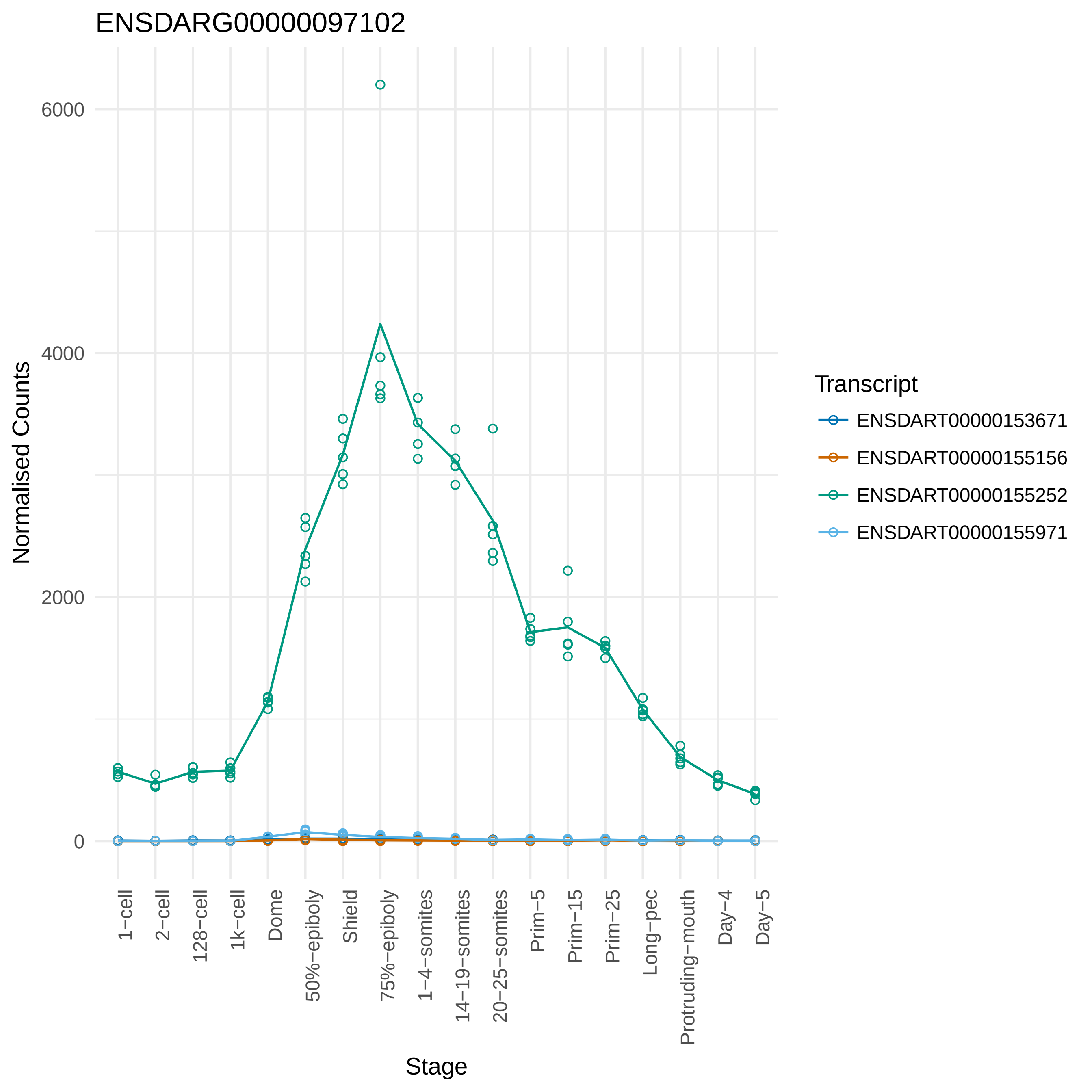
ENSDARG00000097102
Normalised Counts
6000
4000
2000
0
1−cell
2−cell
128−cell
1k−cell
Dome
50%−epiboly
Shield
75%−epiboly
1−4−somites
14−19−somites
20−25−somites
Prim−5
Prim−15
Prim−25
Long−pec
Protruding−mouth
Day−4
Day−5
Stage
Transcript
ENSDART00000153671
ENSDART00000155156
ENSDART00000155252
ENSDART00000155971

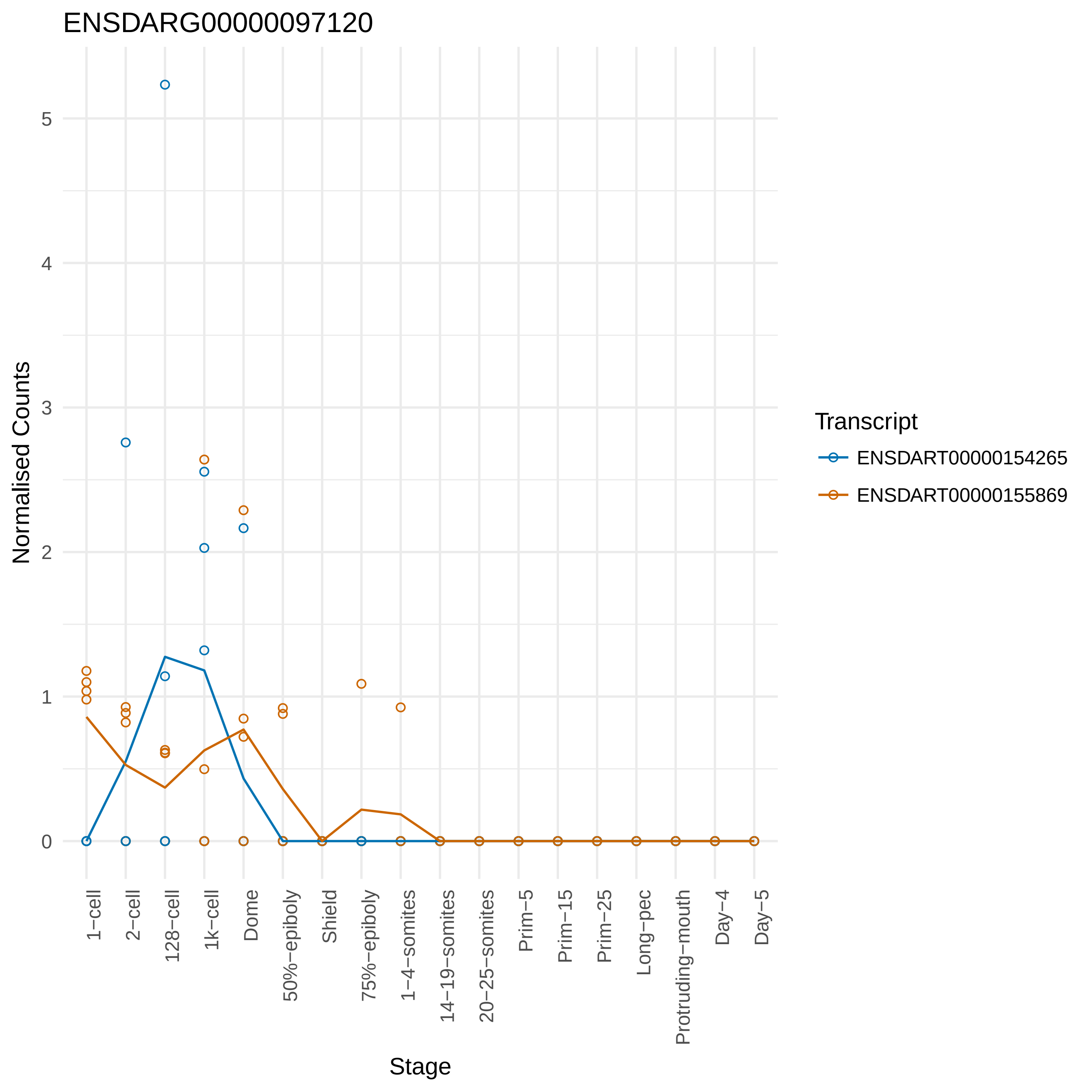

ENSDARG00000097120
Normalised Counts
0
1
2
3
4
5
Stage
1−cell
2−cell
128−cell
1k−cell
Dome
50%−epiboly
Shield
75%−epiboly
1−4−somites
14−19−somites
20−25−somites
Prim−5
Prim−15
Prim−25
Long−pec
Protruding−mouth
Day−4
Day−5
Transcript
ENSDART00000154265
ENSDART00000155869

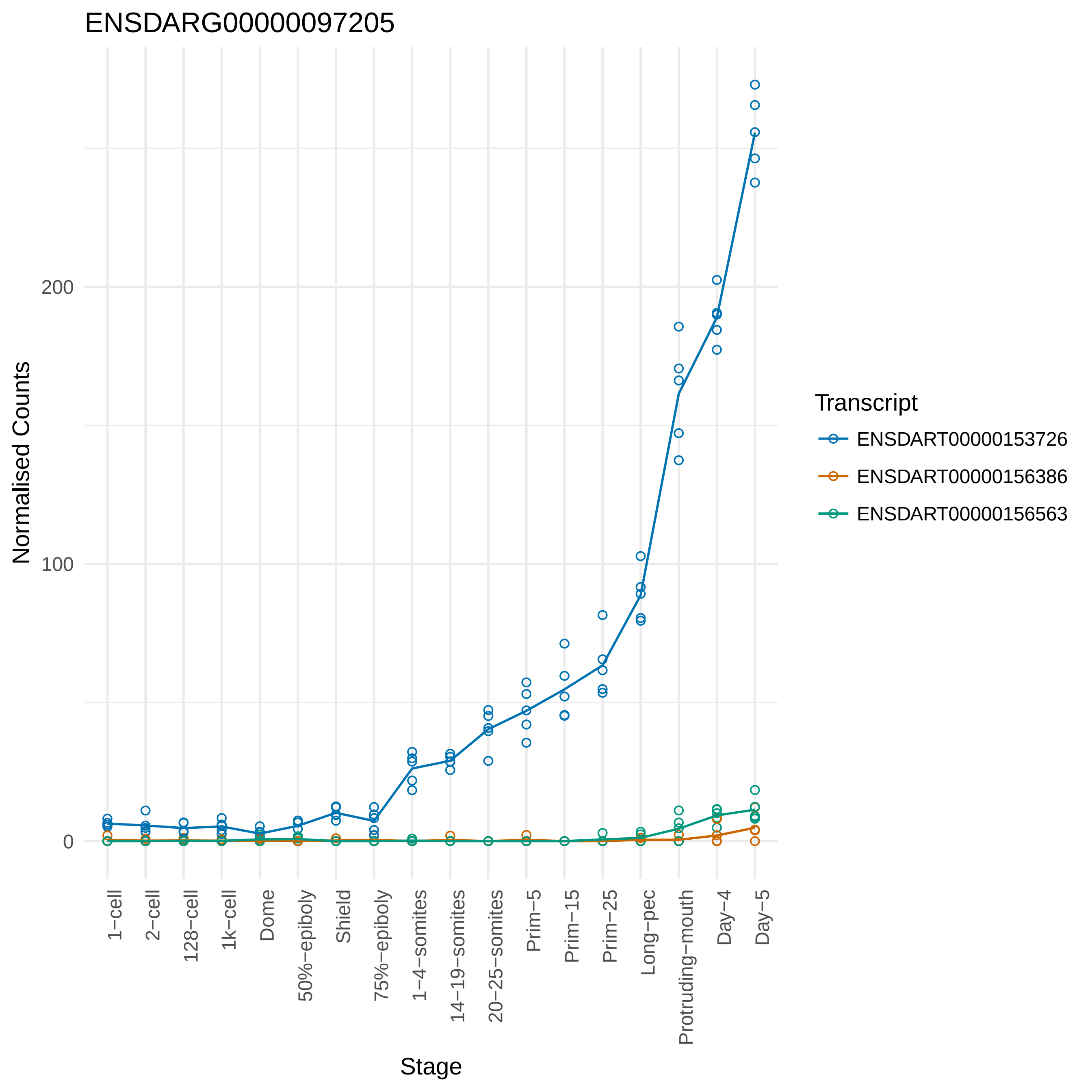
ENSDARG00000097205
Normalised Counts
0
100
200
1−cell
2−cell
128−cell
1k−cell
Dome
50%−epiboly
Shield
75%−epiboly
1−4−somites
14−19−somites
20−25−somites
Prim−5
Prim−15
Prim−25
Long−pec
Protruding−mouth
Day−4
Day−5
Stage
Transcript
ENSDART00000153726
ENSDART00000156386
ENSDART00000156563

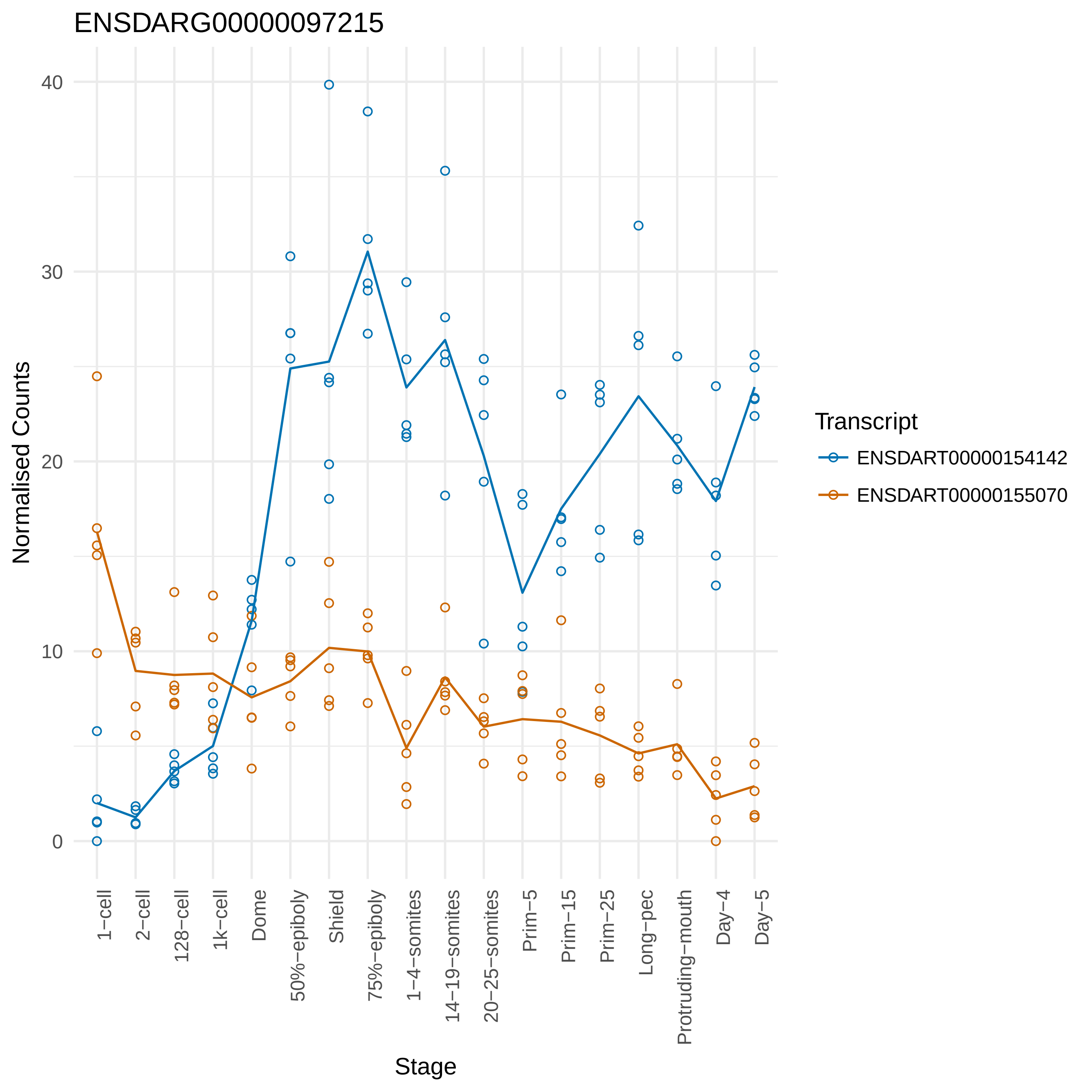

ENSDARG00000097215
Normalised Counts
40
30
20
10
0
1−cell
2−cell
128−cell
1k−cell
Dome
50%−epiboly
Shield
75%−epiboly
1−4−somites
14−19−somites
20−25−somites
Prim−5
Prim−15
Prim−25
Long−pec
Protruding−mouth
Day−4
Day−5
Stage
Transcript
ENSDART00000154142
ENSDART00000155070

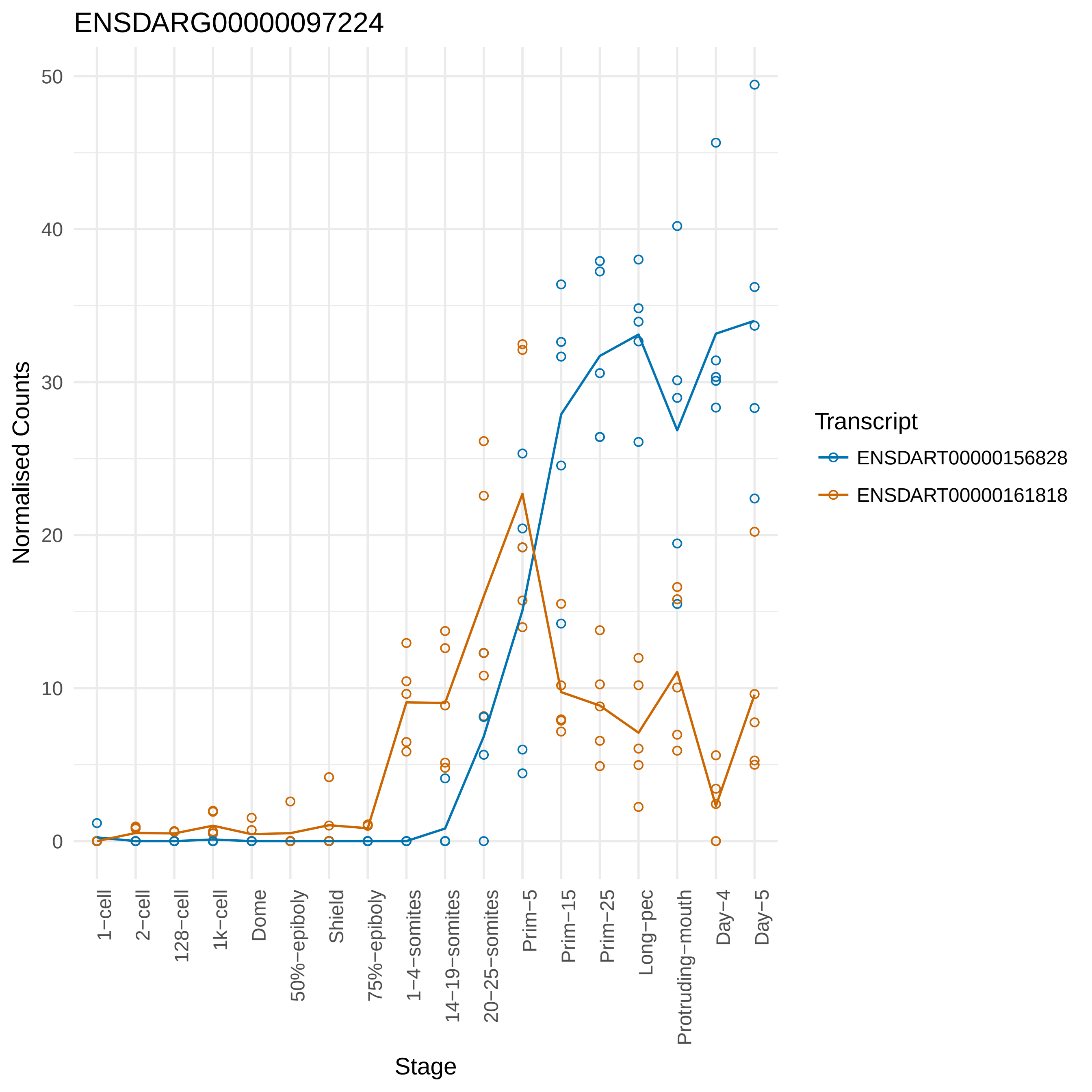

ENSDARG00000097224
Normalised Counts
0
10
20
30
40
50
1−cell
2−cell
128−cell
1k−cell
Dome
50%−epiboly
Shield
75%−epiboly
1−4−somites
14−19−somites
20−25−somites
Prim−5
Prim−15
Prim−25
Long−pec
Protruding−mouth
Day−4
Day−5
Stage
Transcript
ENSDART00000156828
ENSDART00000161818

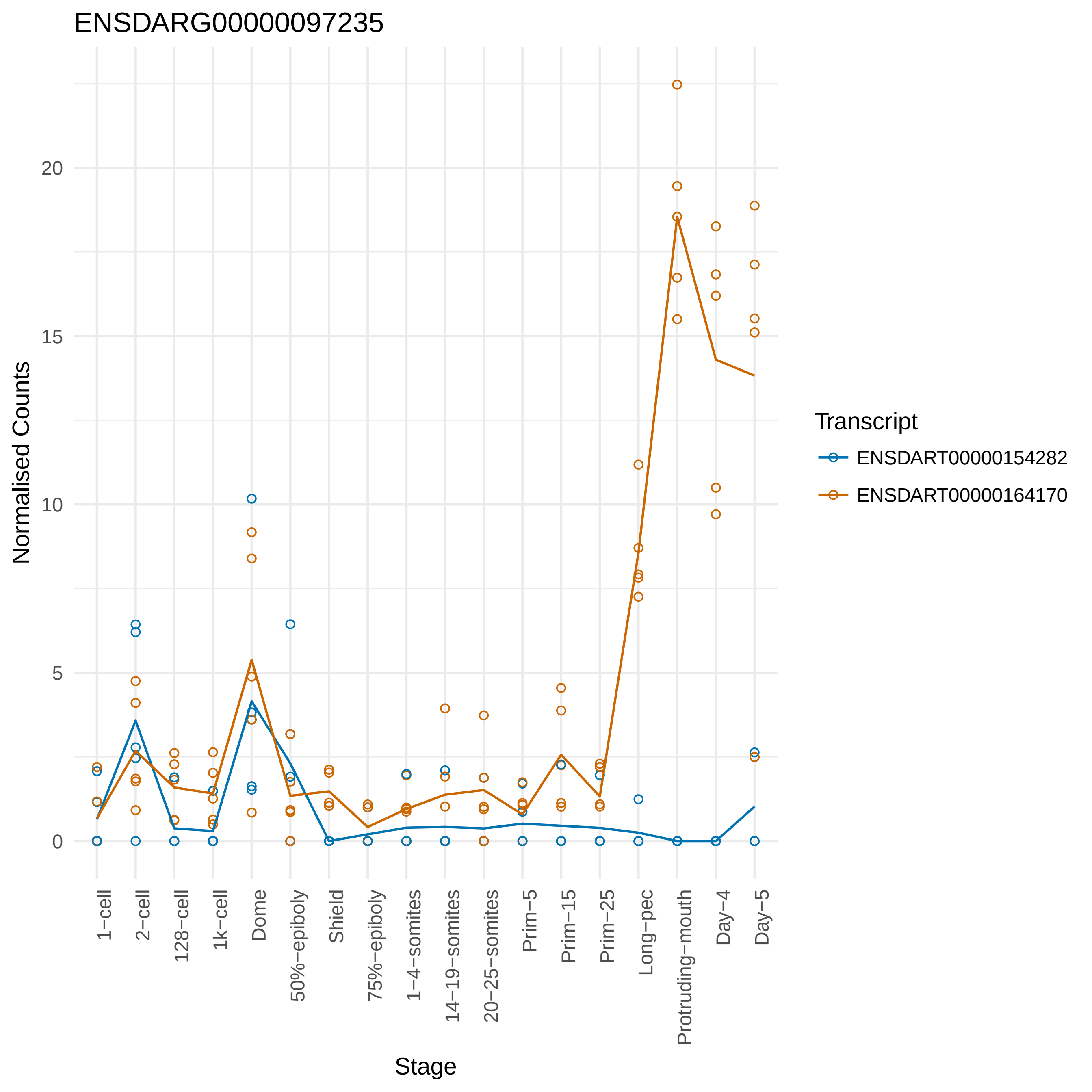

ENSDARG00000097235
Normalised Counts
Stage
Transcript
ENSDART00000154282
ENSDART00000164170
0
5
10
15
20
1−cell
2−cell
128−cell
1k−cell
Dome
50%−epiboly
Shield
75%−epiboly
1−4−somites
14−19−somites
20−25−somites
Prim−5
Prim−15
Prim−25
Long−pec
Protruding−mouth
Day−4
Day−5

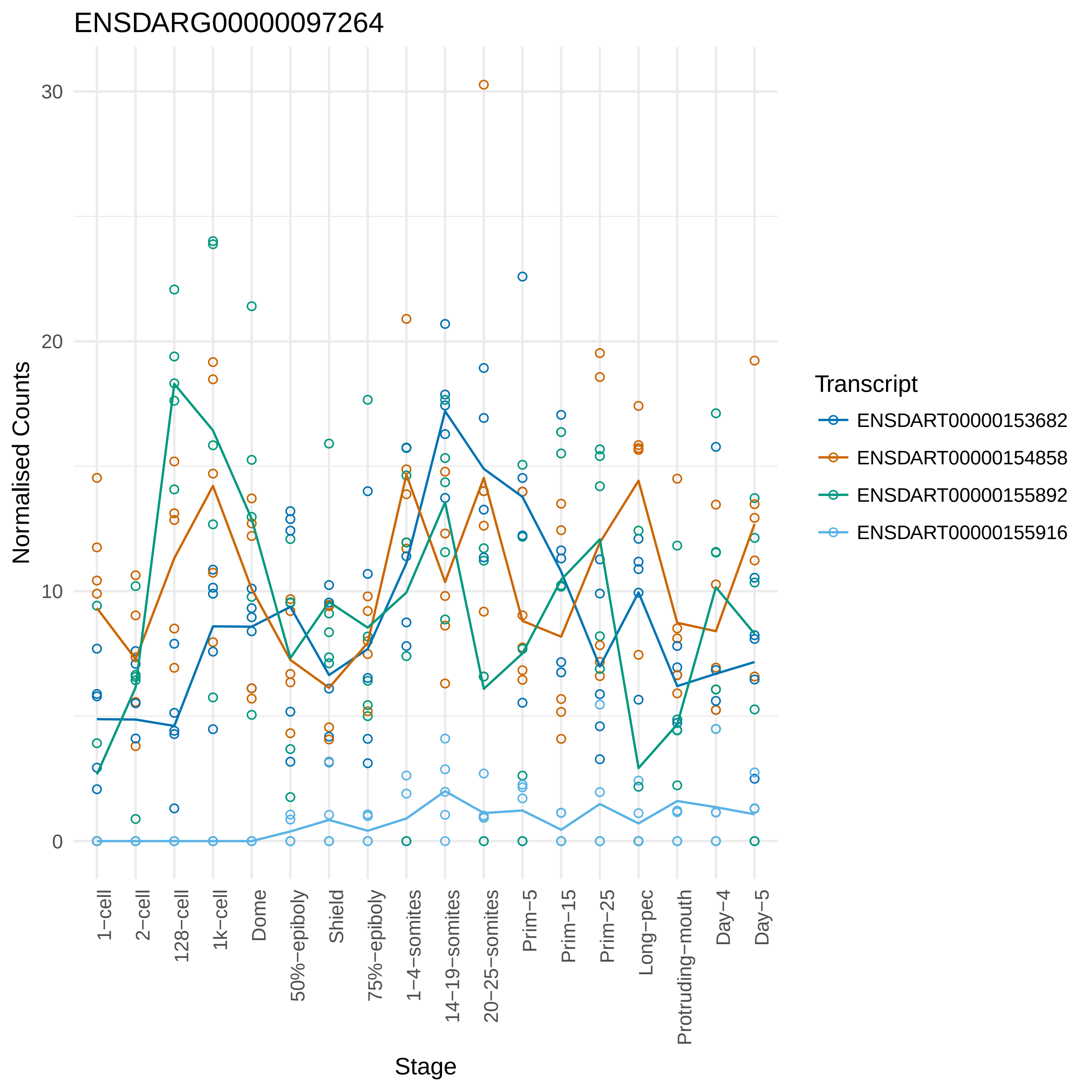

ENSDARG00000097264
Normalised Counts
0
10
20
30
1−cell
2−cell
128−cell
1k−cell
Dome
50%−epiboly
Shield
75%−epiboly
1−4−somites
14−19−somites
20−25−somites
Prim−5
Prim−15
Prim−25
Long−pec
Protruding−mouth
Day−4
Day−5
Stage
Transcript
ENSDART00000153682
ENSDART00000154858
ENSDART00000155892
ENSDART00000155916

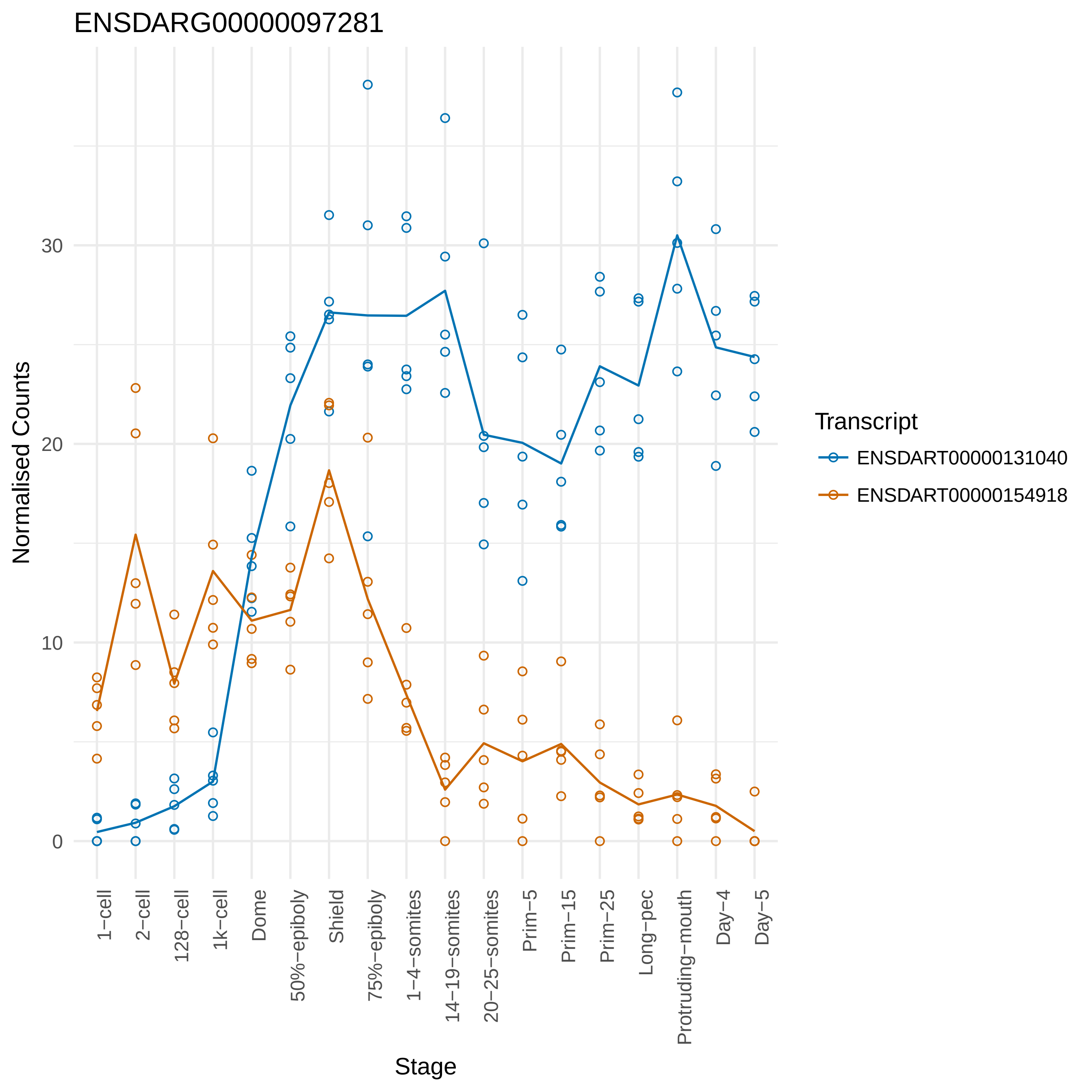

ENSDARG00000097281
Normalised Counts
0
10
20
30
1−cell
2−cell
128−cell
1k−cell
Dome
50%−epiboly
Shield
75%−epiboly
1−4−somites
14−19−somites
20−25−somites
Prim−5
Prim−15
Prim−25
Long−pec
Protruding−mouth
Day−4
Day−5
Stage
Transcript
ENSDART00000131040
ENSDART00000154918

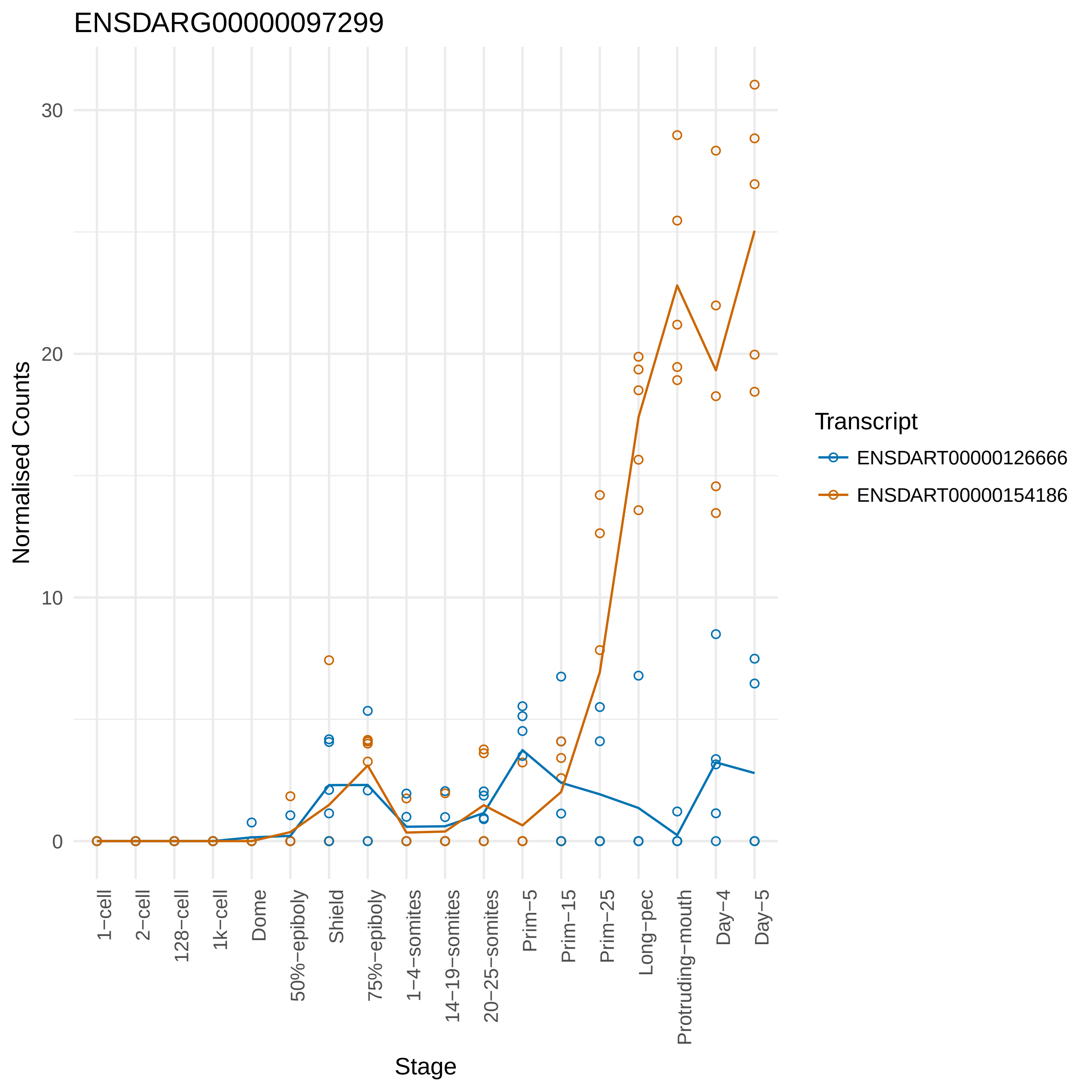
ENSDARG00000097299
Normalised Counts
30
20
10
0
1−cell
2−cell
128−cell
1k−cell
Dome
50%−epiboly
Shield
75%−epiboly
1−4−somites
14−19−somites
20−25−somites
Prim−5
Prim−15
Prim−25
Long−pec
Protruding−mouth
Day−4
Day−5
Stage
Transcript
ENSDART00000126666
ENSDART00000154186

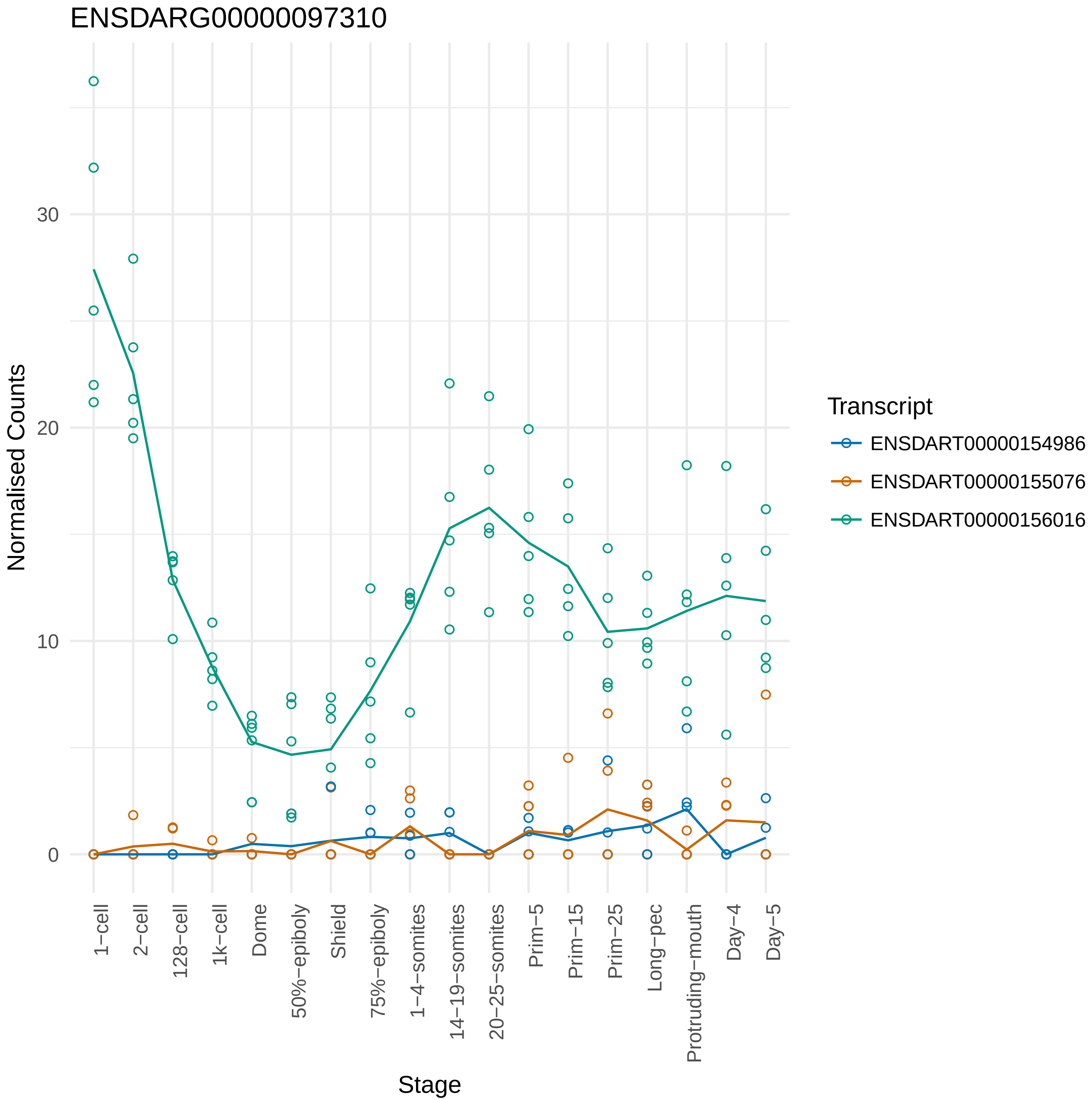

ENSDARG00000097310
Normalised Counts
0
10
20
30
1−cell
2−cell
128−cell
1k−cell
Dome
50%−epiboly
Shield
75%−epiboly
1−4−somites
14−19−somites
20−25−somites
Prim−5
Prim−15
Prim−25
Long−pec
Protruding−mouth
Day−4
Day−5
Stage
Transcript
ENSDART00000154986
ENSDART00000155076
ENSDART00000156016

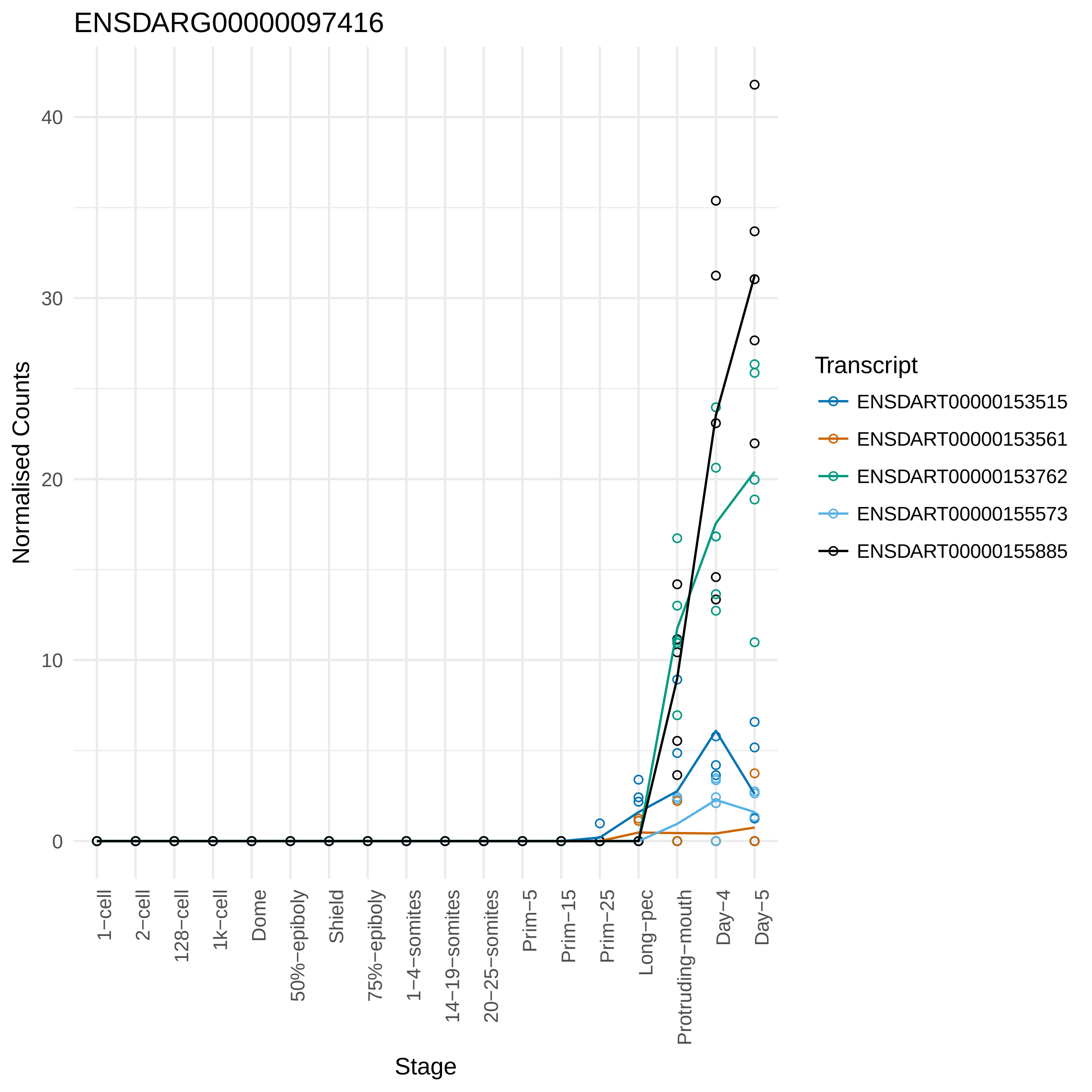

ENSDARG00000097416
Normalised Counts
0
10
20
30
40
1−cell
2−cell
128−cell
1k−cell
Dome
50%−epiboly
Shield
75%−epiboly
1−4−somites
14−19−somites
20−25−somites
Prim−5
Prim−15
Prim−25
Long−pec
Protruding−mouth
Day−4
Day−5
Stage
Transcript
ENSDART00000153515
ENSDART00000153561
ENSDART00000153762
ENSDART00000155573
ENSDART00000155885

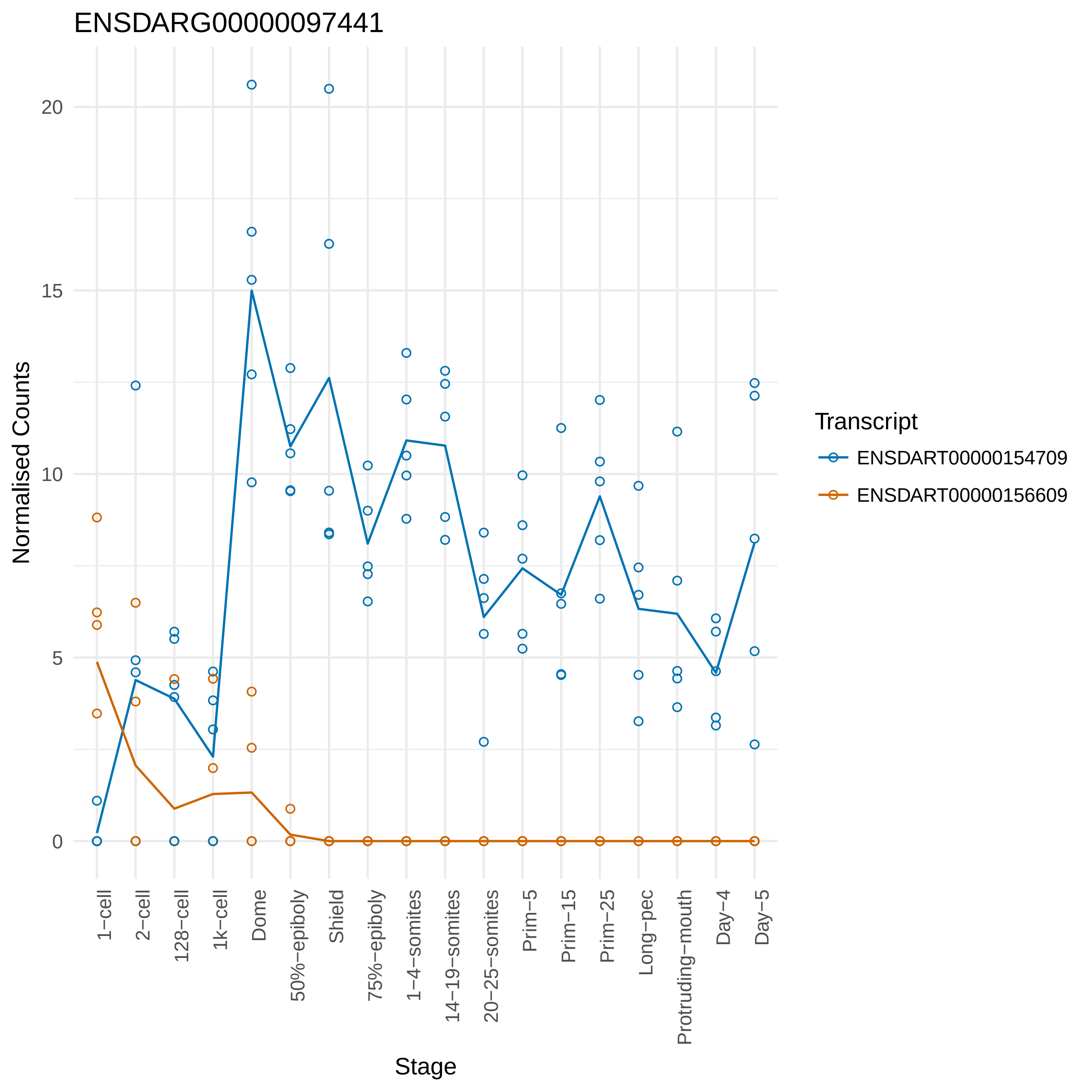

ENSDARG00000097441
Normalised Counts
0
5
10
15
20
1−cell
2−cell
128−cell
1k−cell
Dome
50%−epiboly
Shield
75%−epiboly
1−4−somites
14−19−somites
20−25−somites
Prim−5
Prim−15
Prim−25
Long−pec
Protruding−mouth
Day−4
Day−5
Stage
Transcript
ENSDART00000154709
ENSDART00000156609

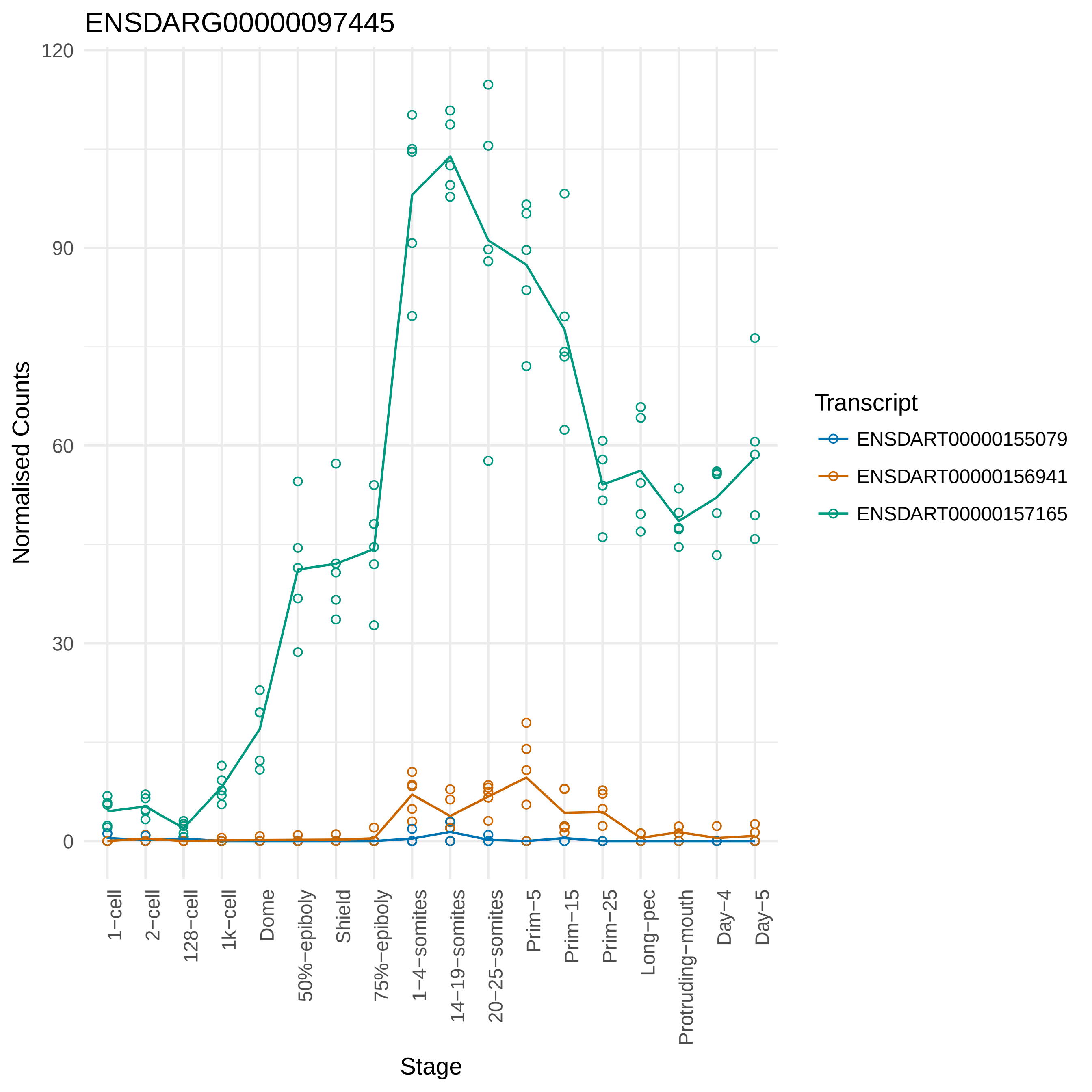

ENSDARG00000097445
Normalised Counts
120
90
60
30
0
1−cell
2−cell
128−cell
1k−cell
Dome
50%−epiboly
Shield
75%−epiboly
1−4−somites
14−19−somites
20−25−somites
Prim−5
Prim−15
Prim−25
Long−pec
Protruding−mouth
Day−4
Day−5
Stage
Transcript
ENSDART00000155079
ENSDART00000156941
ENSDART00000157165

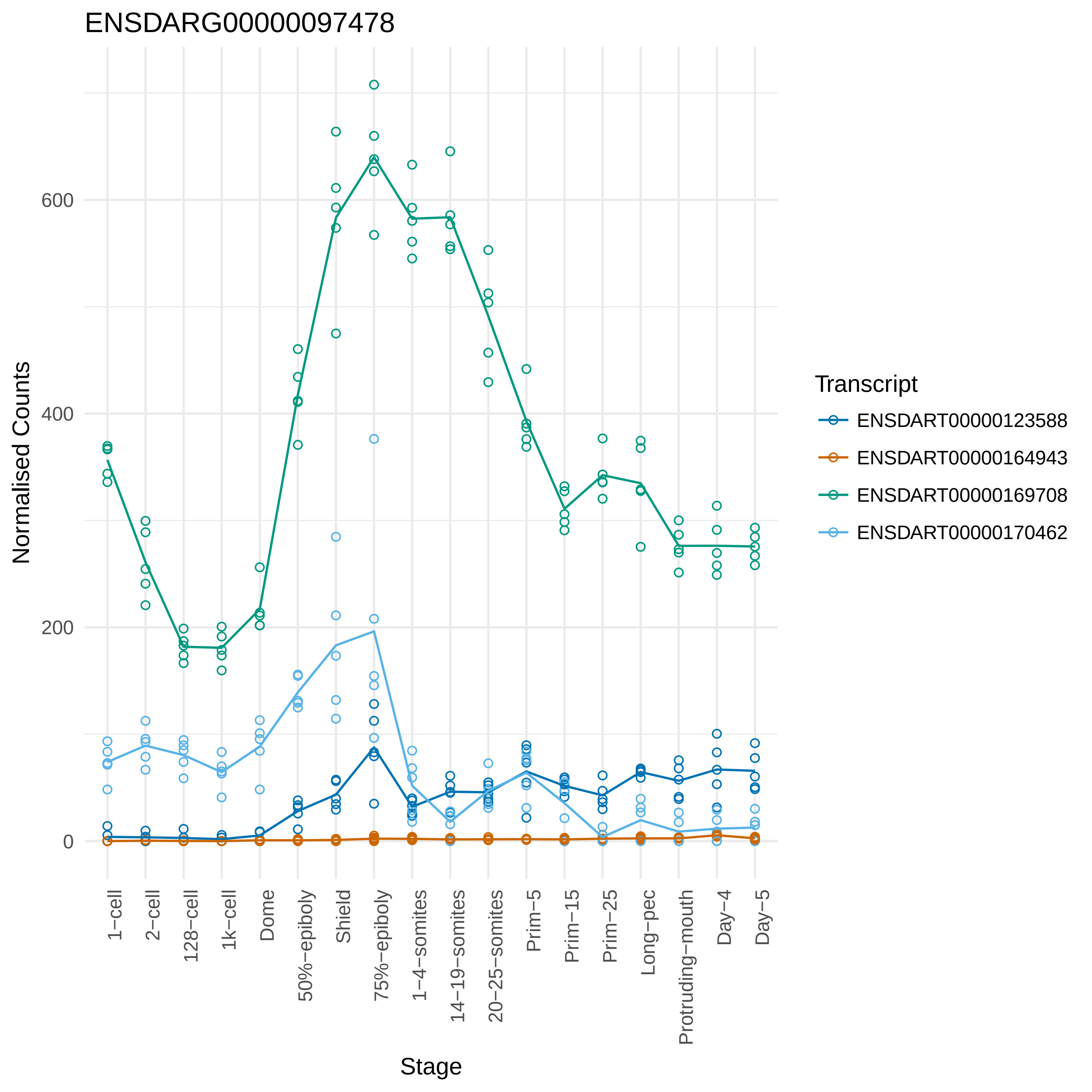

ENSDARG00000097478
Normalised Counts
0
200
400
600
Stage
1−cell
2−cell
128−cell
1k−cell
Dome
50%−epiboly
Shield
75%−epiboly
1−4−somites
14−19−somites
20−25−somites
Prim−5
Prim−15
Prim−25
Long−pec
Protruding−mouth
Day−4
Day−5
Transcript
ENSDART00000123588
ENSDART00000164943
ENSDART00000169708
ENSDART00000170462

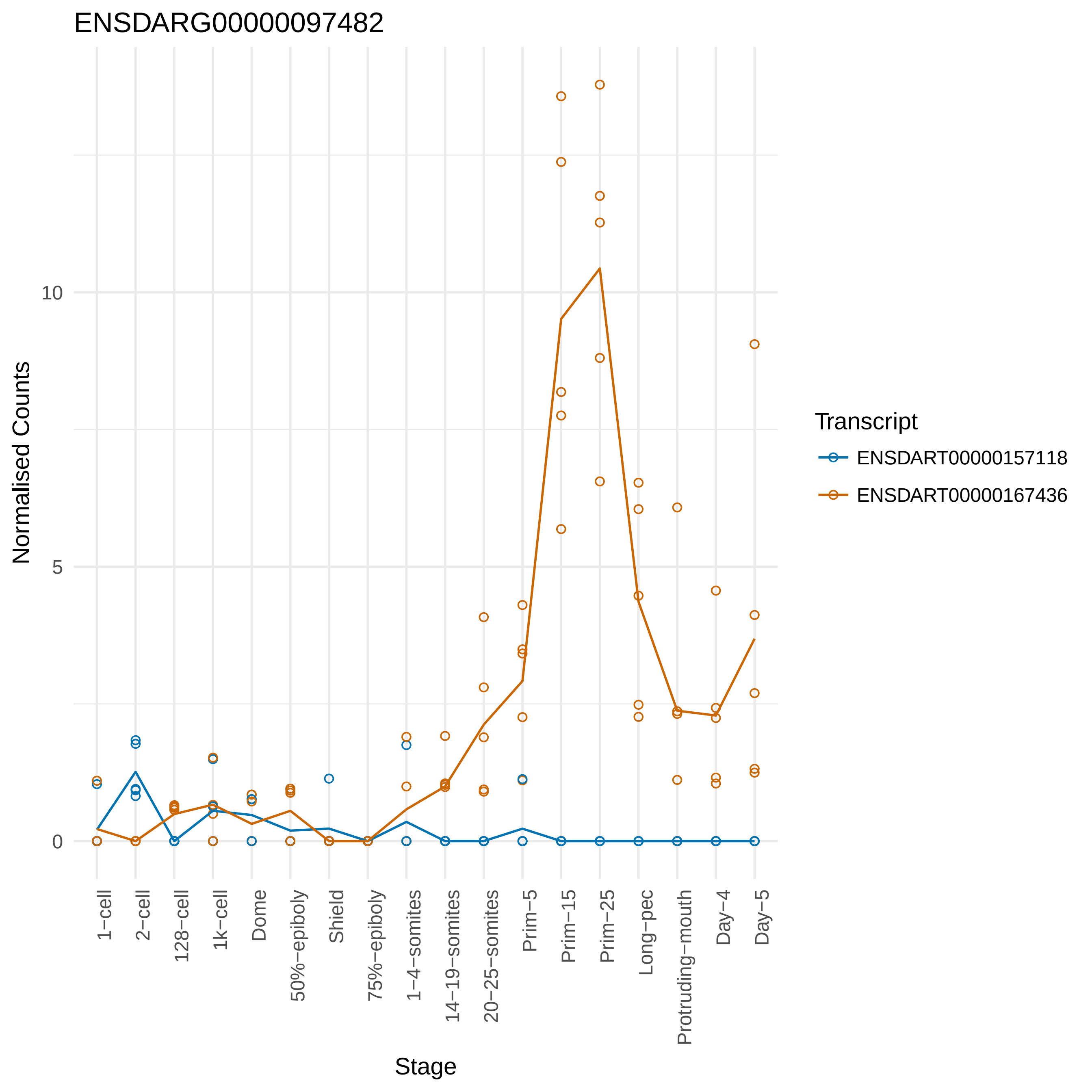

ENSDARG00000097482
Normalised Counts
0
5
10
1−cell
2−cell
128−cell
1k−cell
Dome
50%−epiboly
Shield
75%−epiboly
1−4−somites
14−19−somites
20−25−somites
Prim−5
Prim−15
Prim−25
Long−pec
Protruding−mouth
Day−4
Day−5
Stage
Transcript
ENSDART00000157118
ENSDART00000167436

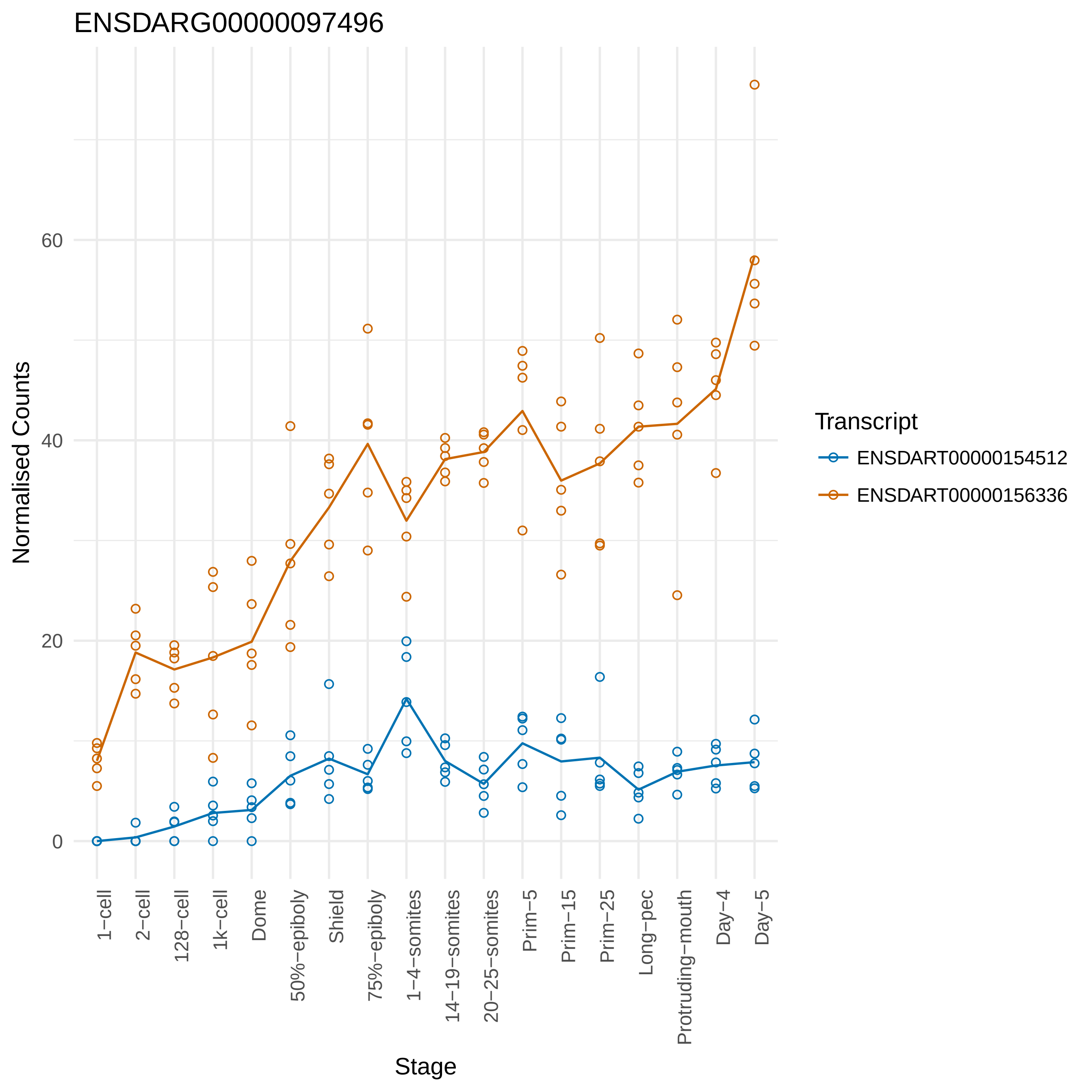
ENSDARG00000097496
Normalised Counts
0
20
40
60
Stage
1−cell
2−cell
128−cell
1k−cell
Dome
50%−epiboly
Shield
75%−epiboly
1−4−somites
14−19−somites
20−25−somites
Prim−5
Prim−15
Prim−25
Long−pec
Protruding−mouth
Day−4
Day−5
Transcript
ENSDART00000154512
ENSDART00000156336

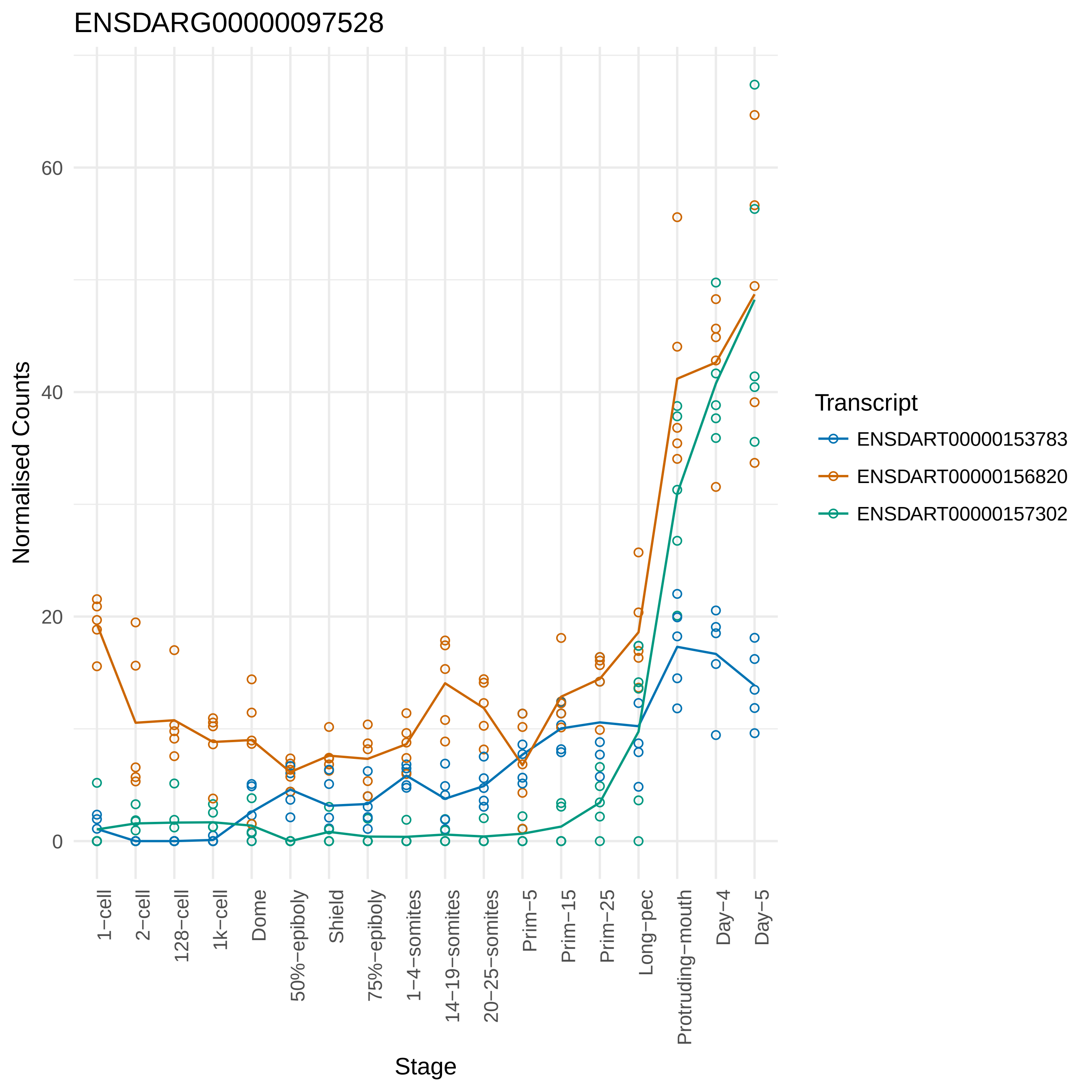

ENSDARG00000097528
Normalised Counts
0
20
40
60
1−cell
2−cell
128−cell
1k−cell
Dome
50%−epiboly
Shield
75%−epiboly
1−4−somites
14−19−somites
20−25−somites
Prim−5
Prim−15
Prim−25
Long−pec
Protruding−mouth
Day−4
Day−5
Stage
Transcript
ENSDART00000153783
ENSDART00000156820
ENSDART00000157302

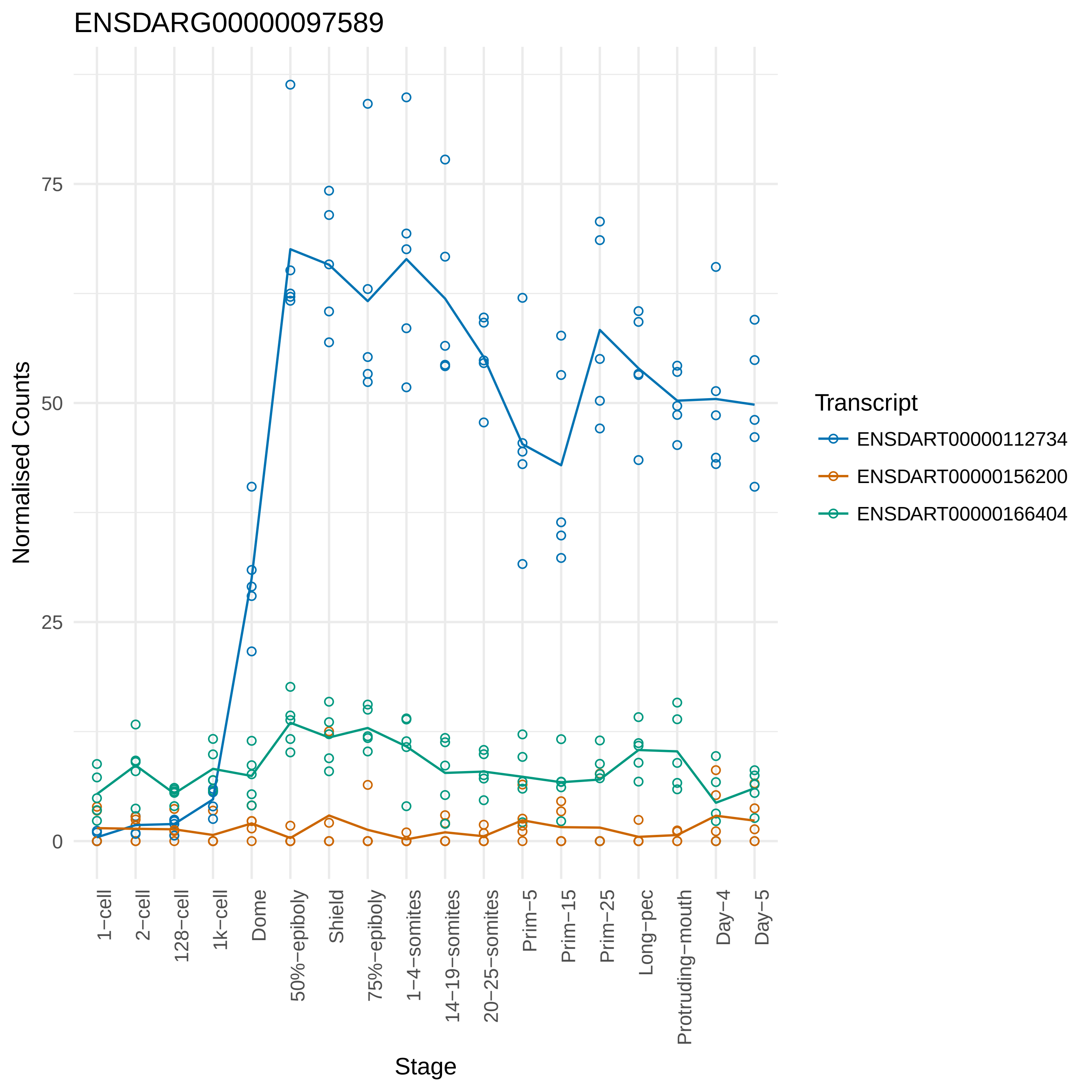

ENSDARG00000097589
Normalised Counts
75
50
25
0
1−cell
2−cell
128−cell
1k−cell
Dome
50%−epiboly
Shield
75%−epiboly
1−4−somites
14−19−somites
20−25−somites
Prim−5
Prim−15
Prim−25
Long−pec
Protruding−mouth
Day−4
Day−5
Stage
Transcript
ENSDART00000112734
ENSDART00000156200
ENSDART00000166404

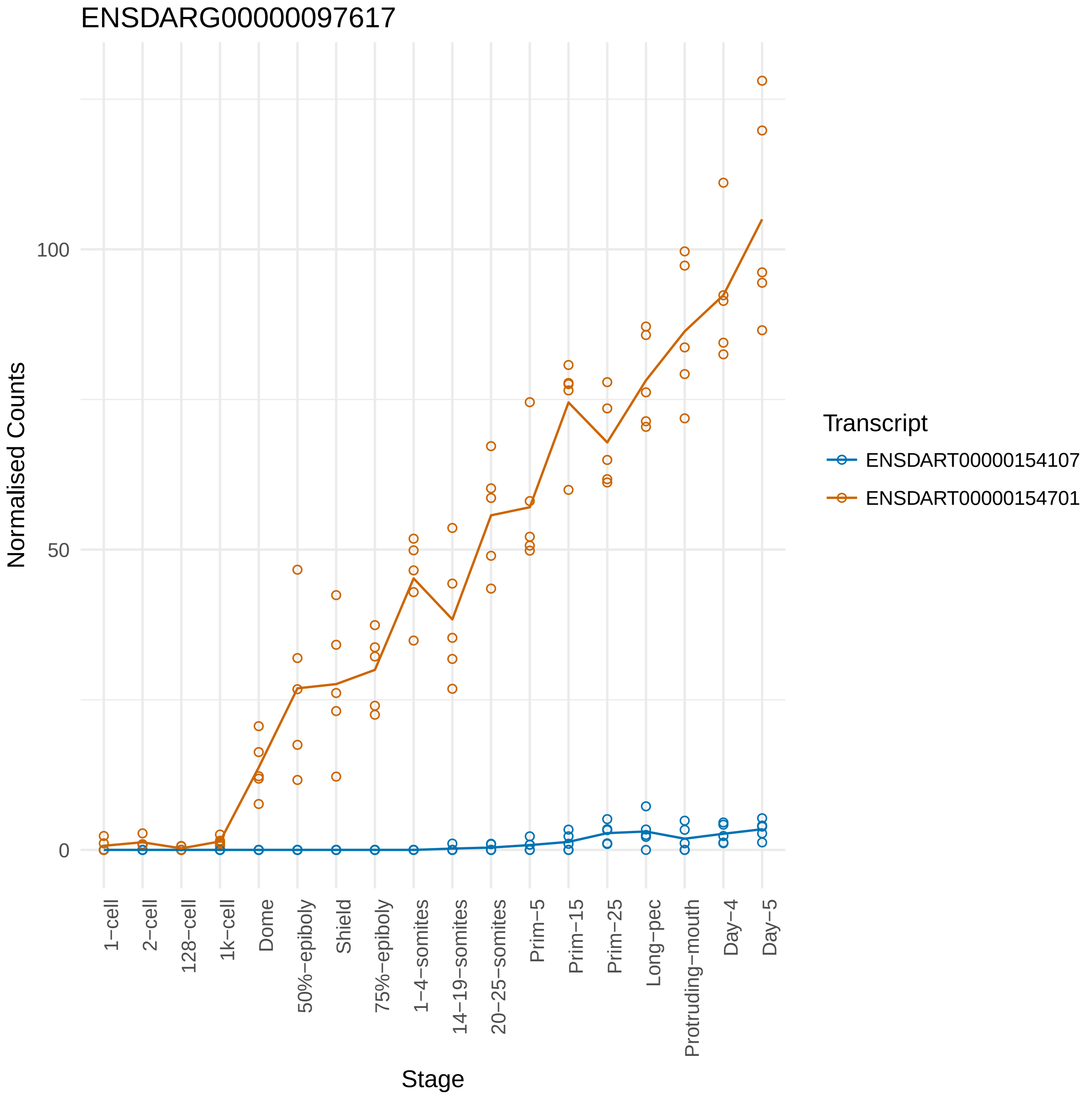

ENSDARG00000097617
Normalised Counts
100
50
0
1−cell
2−cell
128−cell
1k−cell
Dome
50%−epiboly
Shield
75%−epiboly
1−4−somites
14−19−somites
20−25−somites
Prim−5
Prim−15
Prim−25
Long−pec
Protruding−mouth
Day−4
Day−5
Stage
Transcript
ENSDART00000154107
ENSDART00000154701

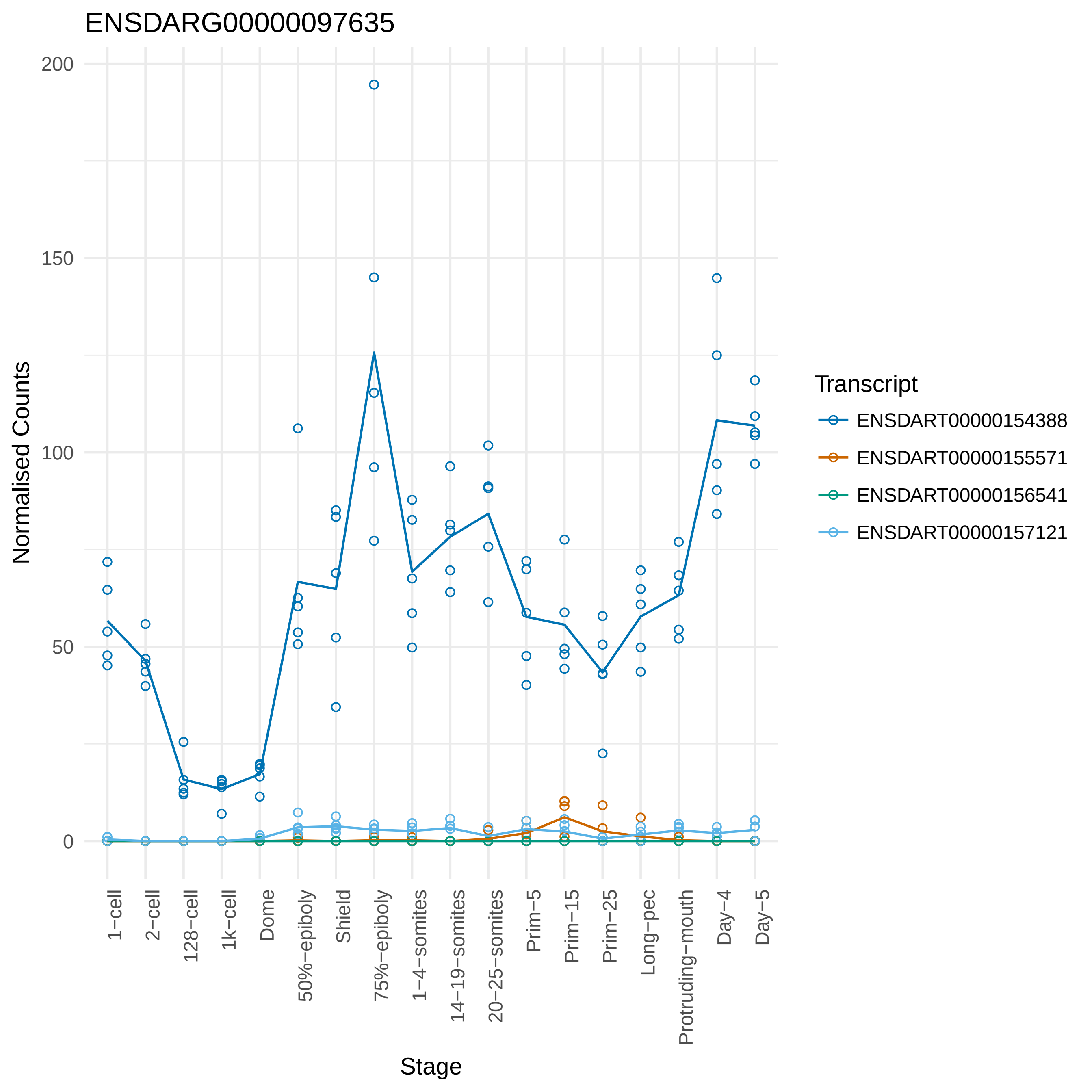

ENSDARG00000097635
Normalised Counts
200
150
100
50
0
1−cell
2−cell
128−cell
1k−cell
Dome
50%−epiboly
Shield
75%−epiboly
1−4−somites
14−19−somites
20−25−somites
Prim−5
Prim−15
Prim−25
Long−pec
Protruding−mouth
Day−4
Day−5
Stage
Transcript
ENSDART00000154388
ENSDART00000155571
ENSDART00000156541
ENSDART00000157121

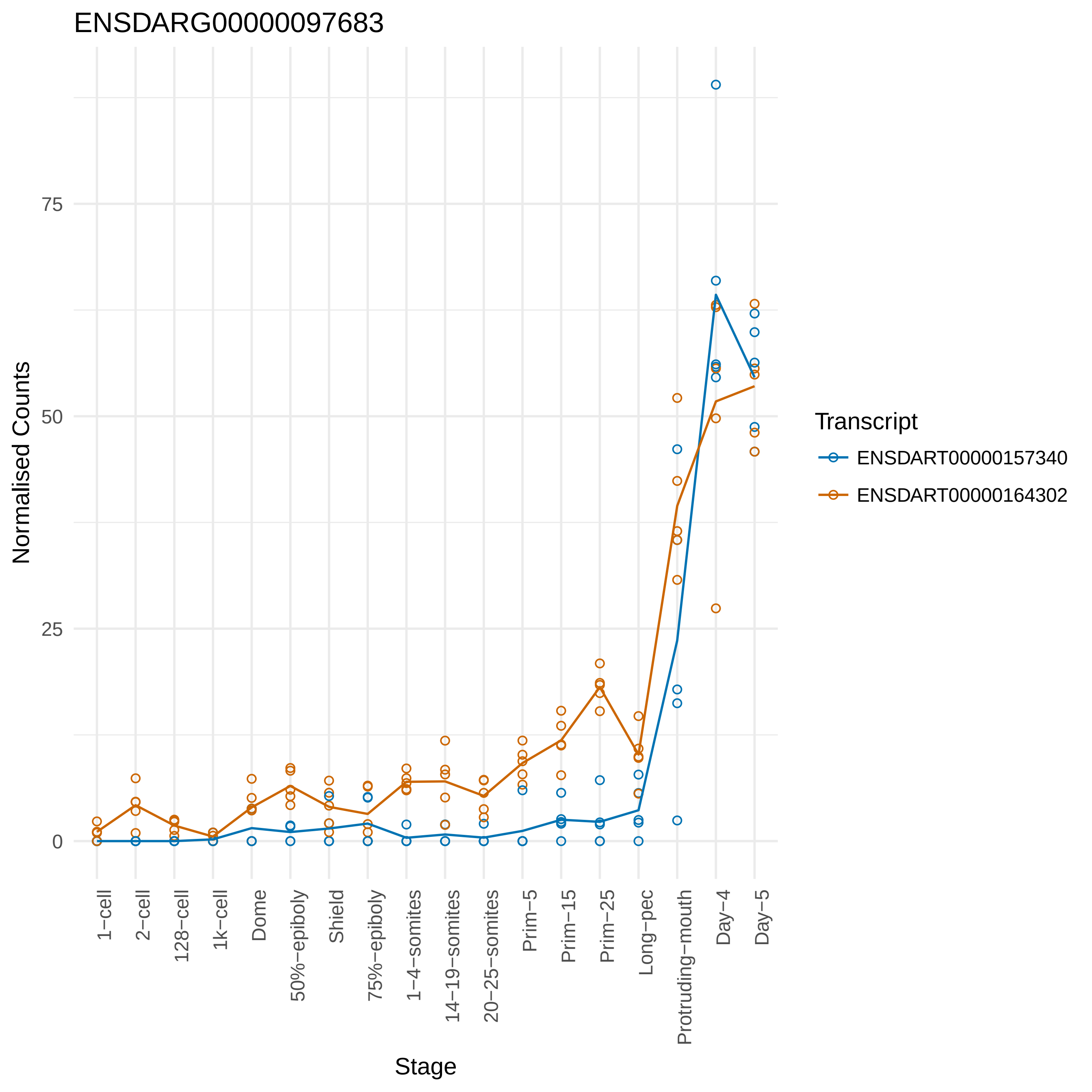

ENSDARG00000097683
Normalised Counts
0
25
50
75
Stage
1−cell
2−cell
128−cell
1k−cell
Dome
50%−epiboly
Shield
75%−epiboly
1−4−somites
14−19−somites
20−25−somites
Prim−5
Prim−15
Prim−25
Long−pec
Protruding−mouth
Day−4
Day−5
Transcript
ENSDART00000157340
ENSDART00000164302

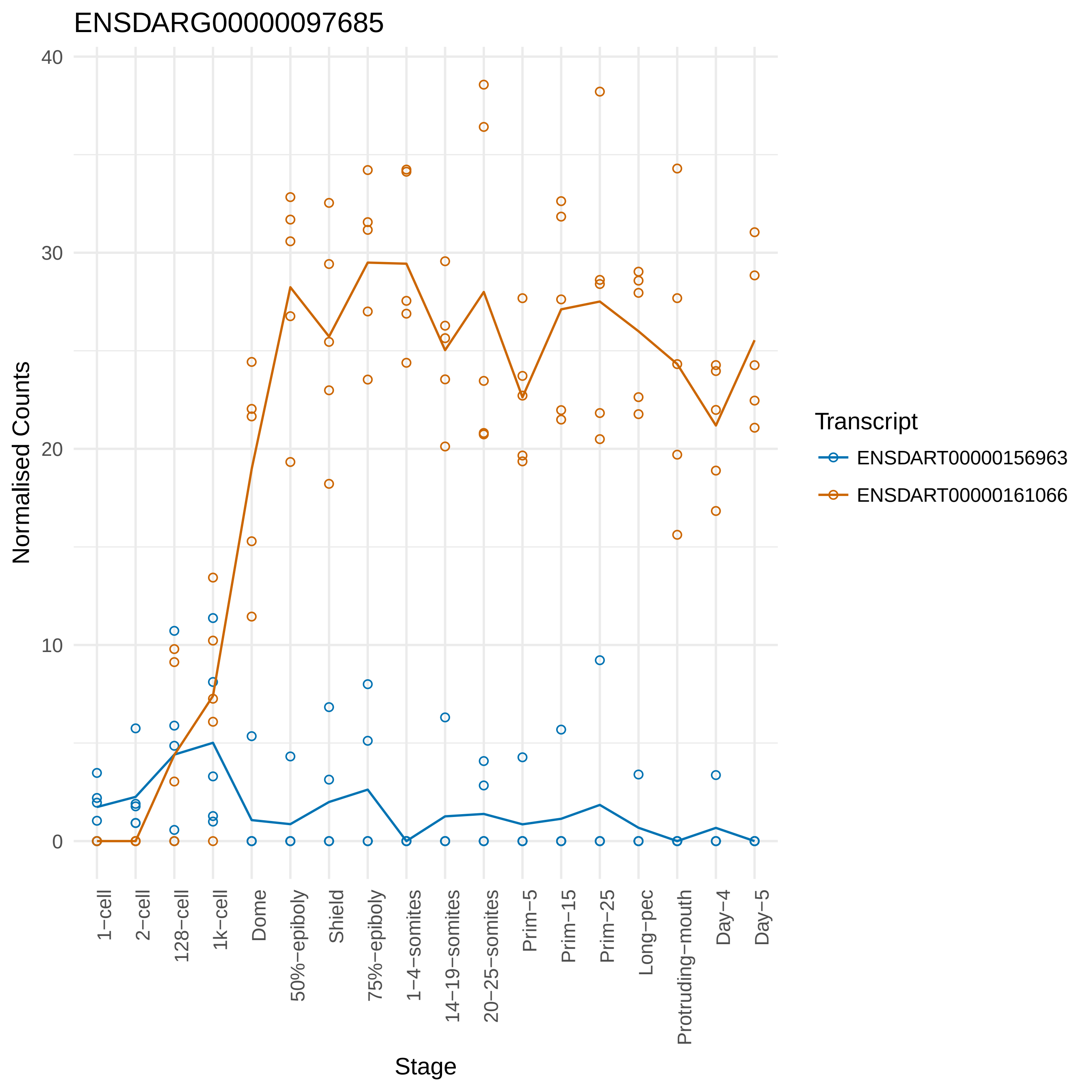

ENSDARG00000097685
Normalised Counts
40
30
20
10
0
1−cell
2−cell
128−cell
1k−cell
Dome
50%−epiboly
Shield
75%−epiboly
1−4−somites
14−19−somites
20−25−somites
Prim−5
Prim−15
Prim−25
Long−pec
Protruding−mouth
Day−4
Day−5
Stage
Transcript
ENSDART00000156963
ENSDART00000161066

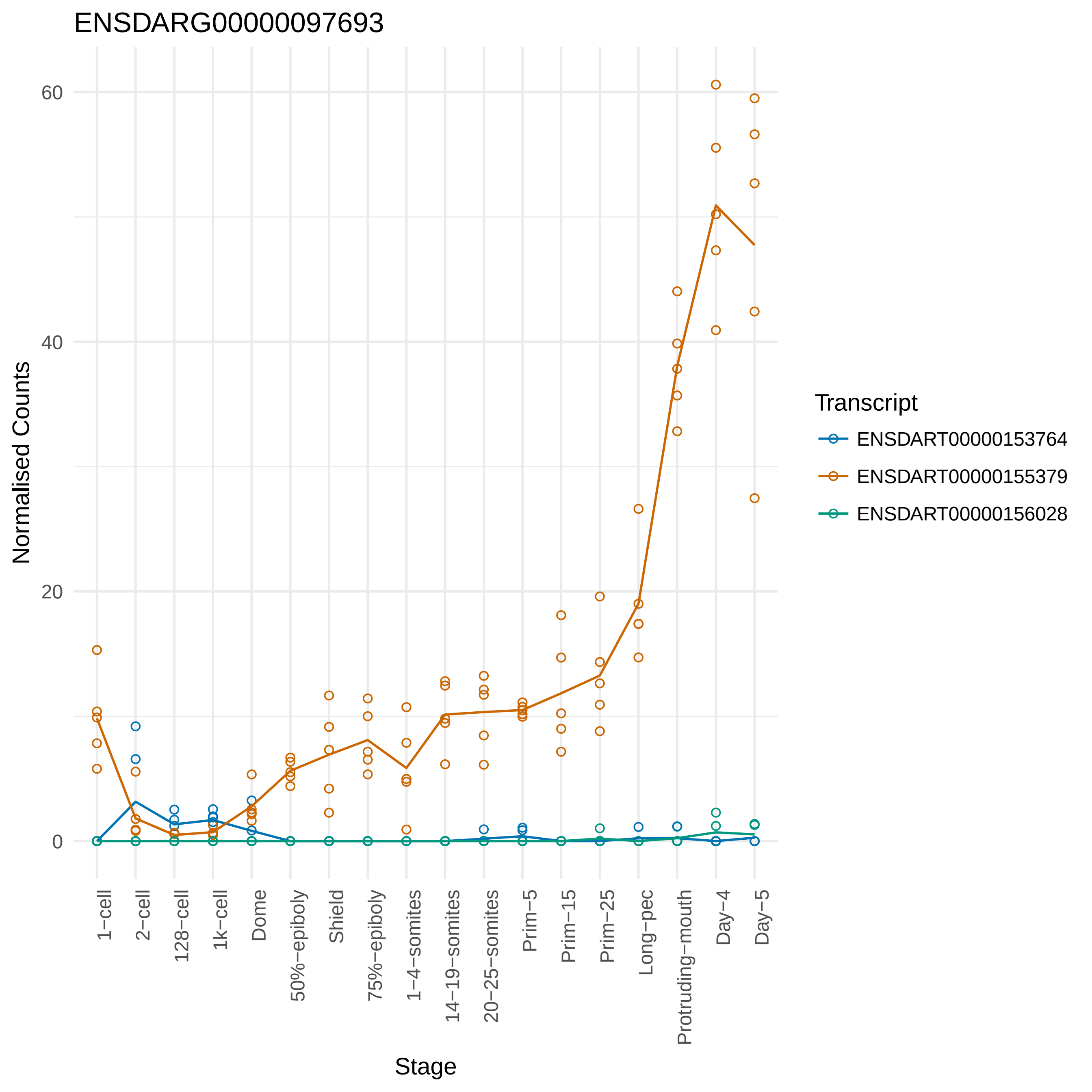
ENSDARG00000097693
Normalised Counts
60
40
20
0
1−cell
2−cell
128−cell
1k−cell
Dome
50%−epiboly
Shield
75%−epiboly
1−4−somites
14−19−somites
20−25−somites
Prim−5
Prim−15
Prim−25
Long−pec
Protruding−mouth
Day−4
Day−5
Stage
Transcript
ENSDART00000153764
ENSDART00000155379
ENSDART00000156028

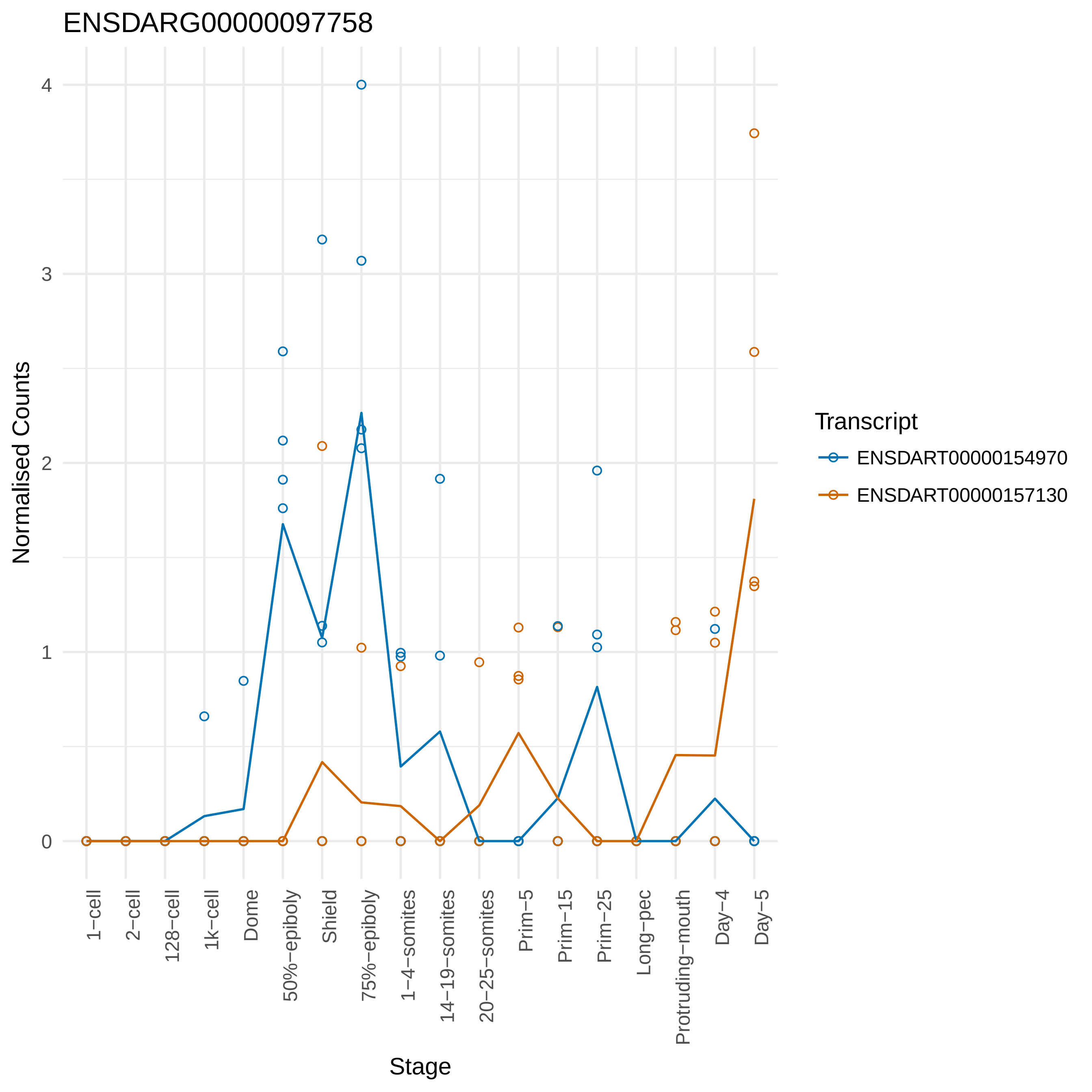
ENSDARG00000097758
Normalised Counts
0
1
2
3
4
Stage
1−cell
2−cell
128−cell
1k−cell
Dome
50%−epiboly
Shield
75%−epiboly
1−4−somites
14−19−somites
20−25−somites
Prim−5
Prim−15
Prim−25
Long−pec
Protruding−mouth
Day−4
Day−5
Transcript
ENSDART00000154970
ENSDART00000157130

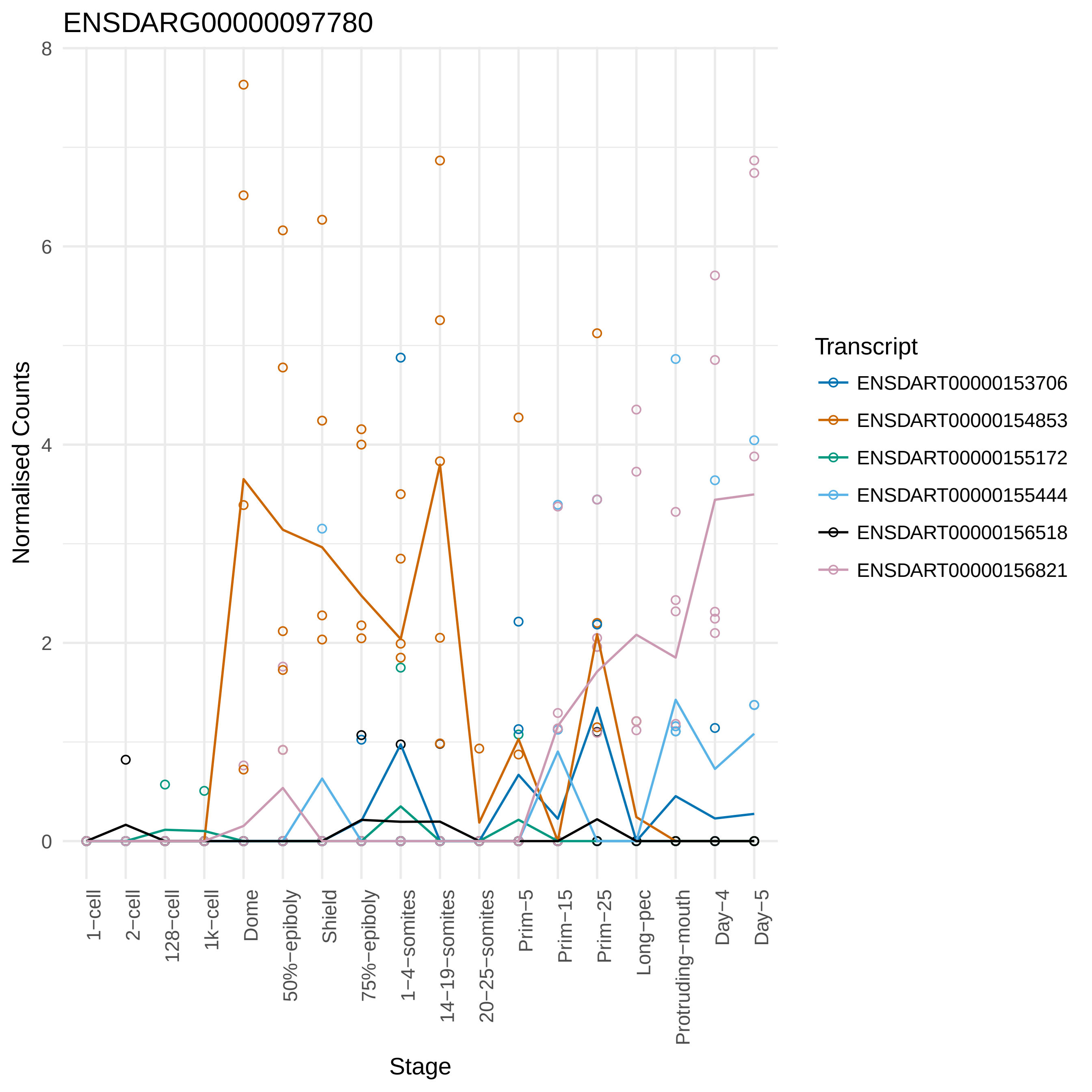
ENSDARG00000097780
Normalised Counts
8
6
4
2
0
1−cell
2−cell
128−cell
1k−cell
Dome
50%−epiboly
Shield
75%−epiboly
1−4−somites
14−19−somites
20−25−somites
Prim−5
Prim−15
Prim−25
Long−pec
Protruding−mouth
Day−4
Day−5
Stage
Transcript
ENSDART00000153706
ENSDART00000154853
ENSDART00000155172
ENSDART00000155444
ENSDART00000156518
ENSDART00000156821

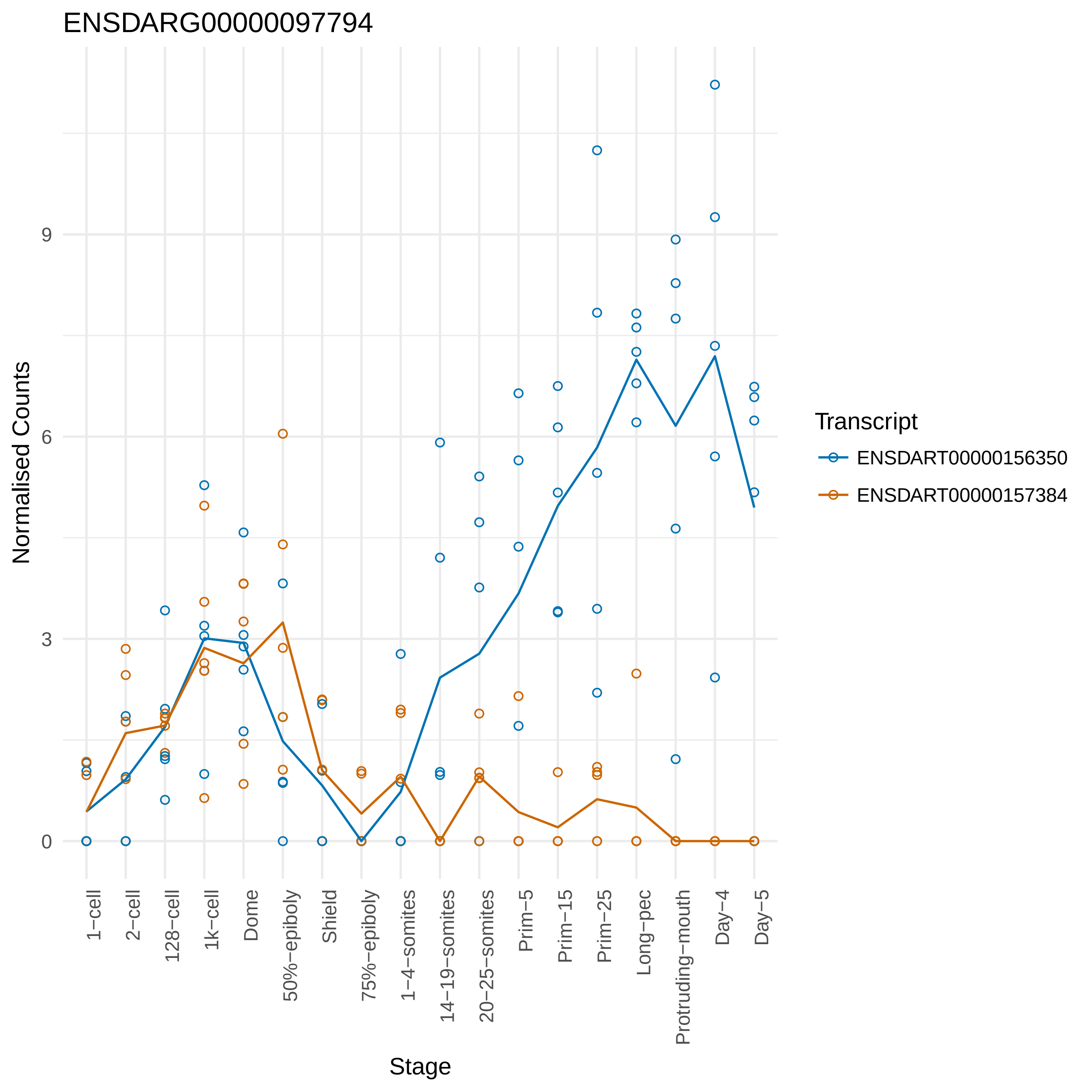

ENSDARG00000097794
Normalised Counts
0
3
6
9
Stage
1−cell
2−cell
128−cell
1k−cell
Dome
50%−epiboly
Shield
75%−epiboly
1−4−somites
14−19−somites
20−25−somites
Prim−5
Prim−15
Prim−25
Long−pec
Protruding−mouth
Day−4
Day−5
Transcript
ENSDART00000156350
ENSDART00000157384

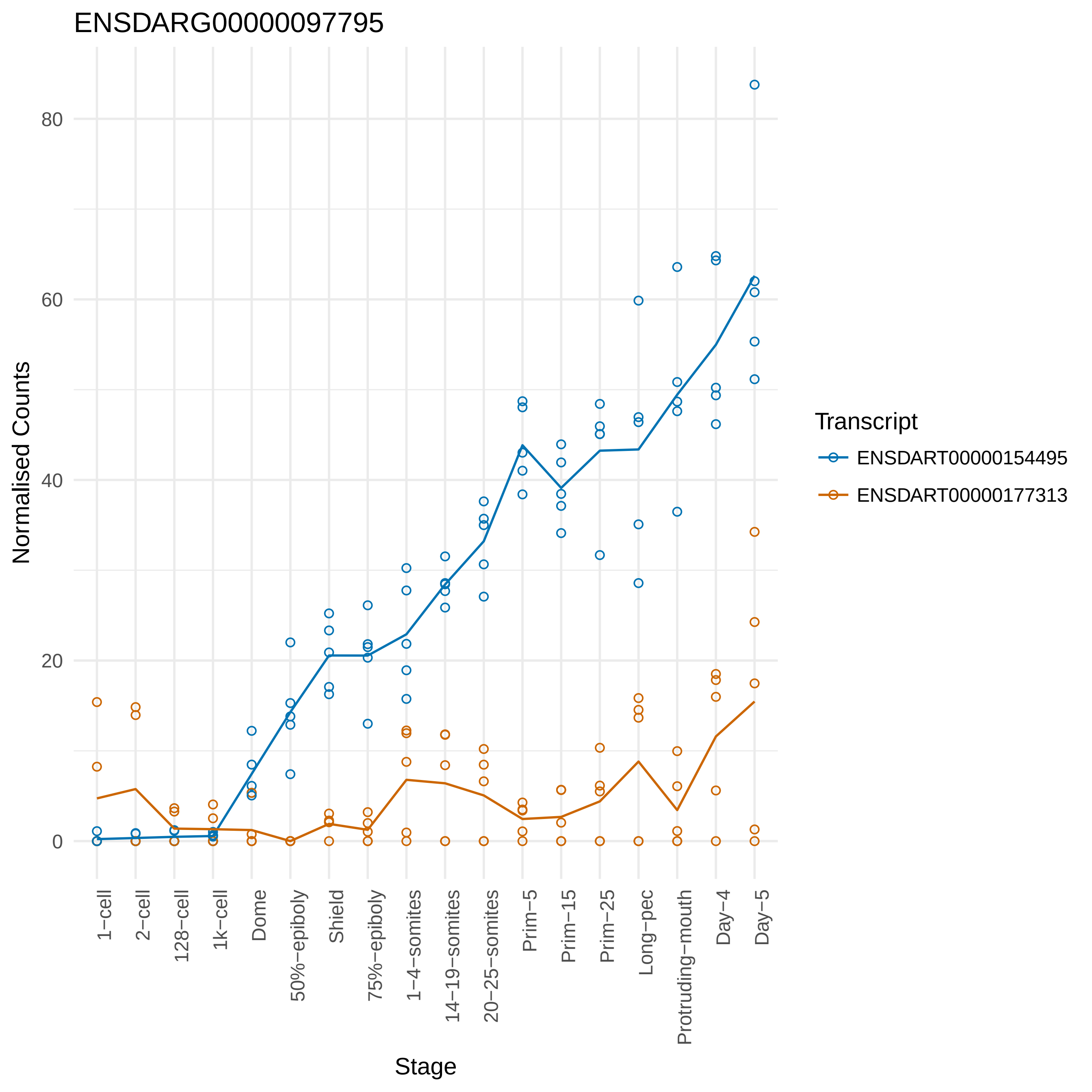

ENSDARG00000097795
Normalised Counts
0
20
40
60
80
1−cell
2−cell
128−cell
1k−cell
Dome
50%−epiboly
Shield
75%−epiboly
1−4−somites
14−19−somites
20−25−somites
Prim−5
Prim−15
Prim−25
Long−pec
Protruding−mouth
Day−4
Day−5
Stage
Transcript
ENSDART00000154495
ENSDART00000177313

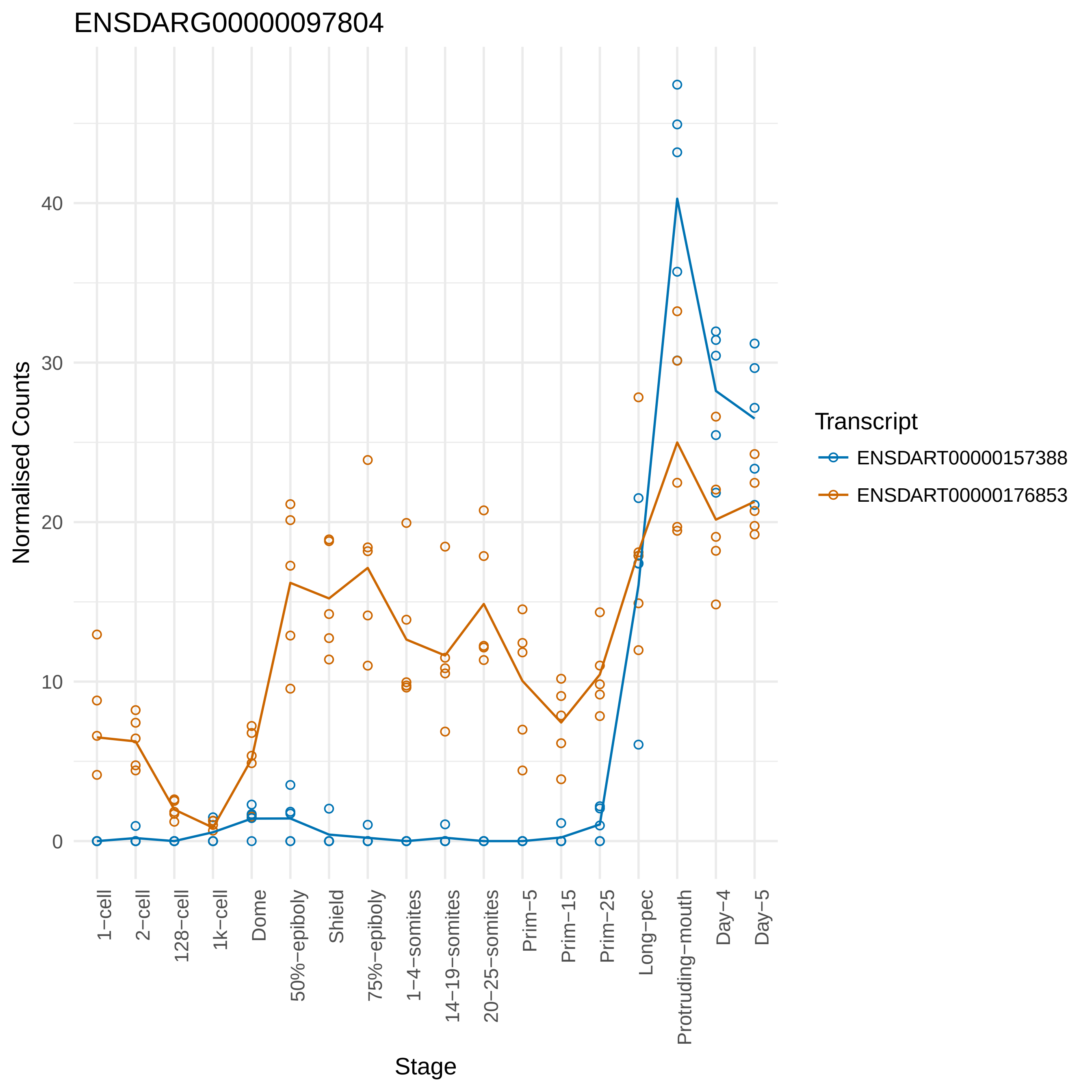

ENSDARG00000097804
Normalised Counts
0
10
20
30
40
1−cell
2−cell
128−cell
1k−cell
Dome
50%−epiboly
Shield
75%−epiboly
1−4−somites
14−19−somites
20−25−somites
Prim−5
Prim−15
Prim−25
Long−pec
Protruding−mouth
Day−4
Day−5
Stage
Transcript
ENSDART00000157388
ENSDART00000176853

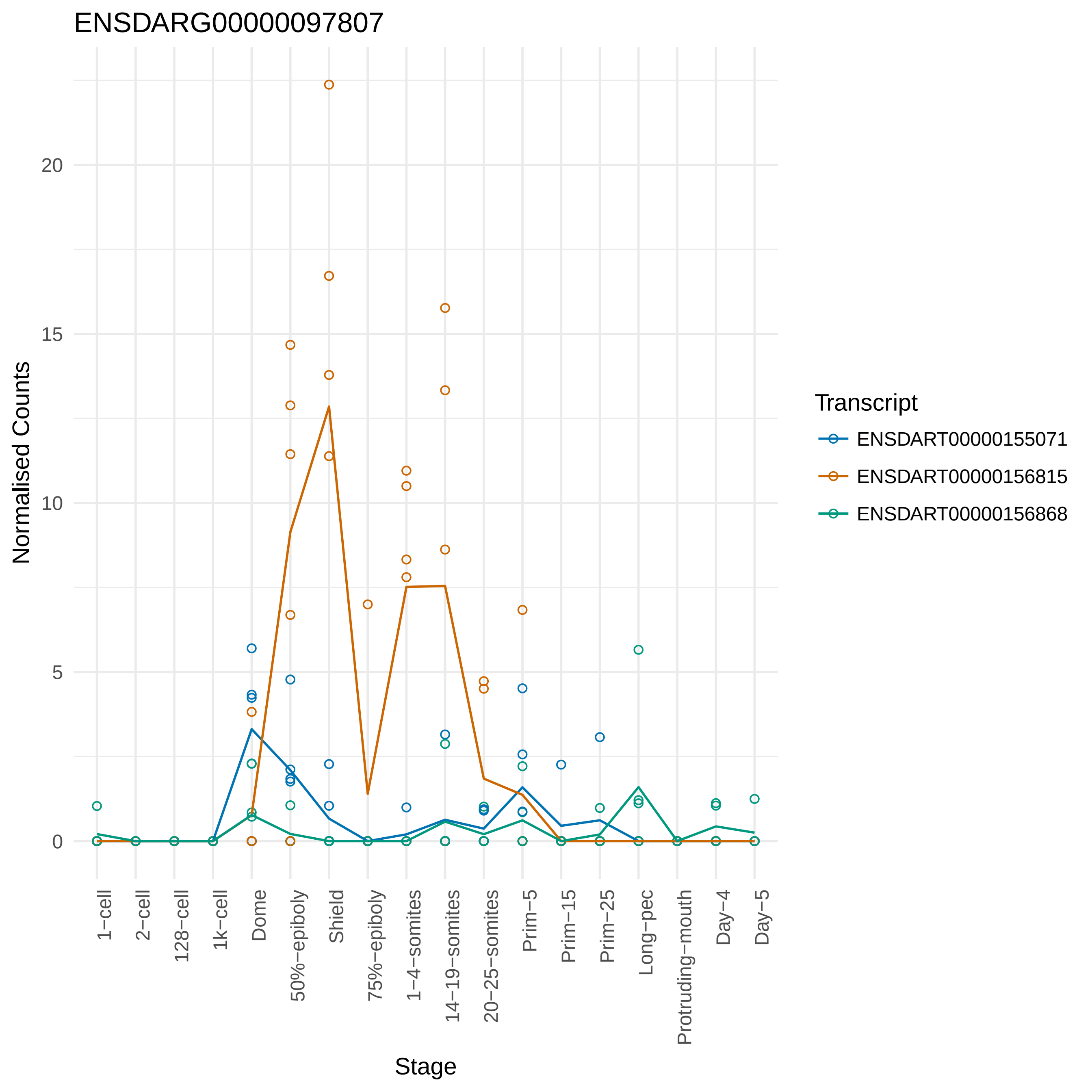
ENSDARG00000097807
Normalised Counts
0
5
10
15
20
1−cell
2−cell
128−cell
1k−cell
Dome
50%−epiboly
Shield
75%−epiboly
1−4−somites
14−19−somites
20−25−somites
Prim−5
Prim−15
Prim−25
Long−pec
Protruding−mouth
Day−4
Day−5
Stage
Transcript
ENSDART00000155071
ENSDART00000156815
ENSDART00000156868

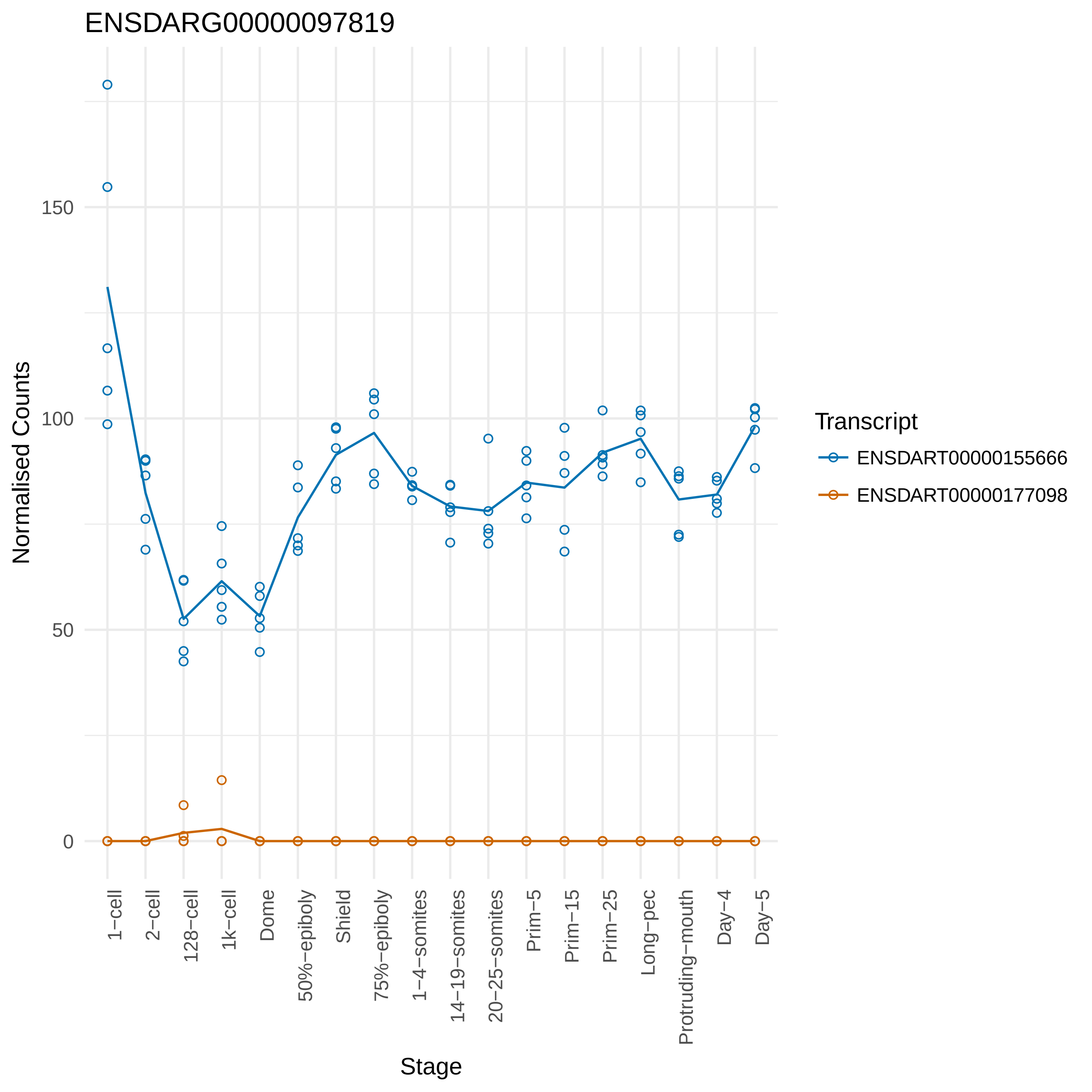

ENSDARG00000097819
Normalised Counts
150
100
50
0
1−cell
2−cell
128−cell
1k−cell
Dome
50%−epiboly
Shield
75%−epiboly
1−4−somites
14−19−somites
20−25−somites
Prim−5
Prim−15
Prim−25
Long−pec
Protruding−mouth
Day−4
Day−5
Stage
Transcript
ENSDART00000155666
ENSDART00000177098

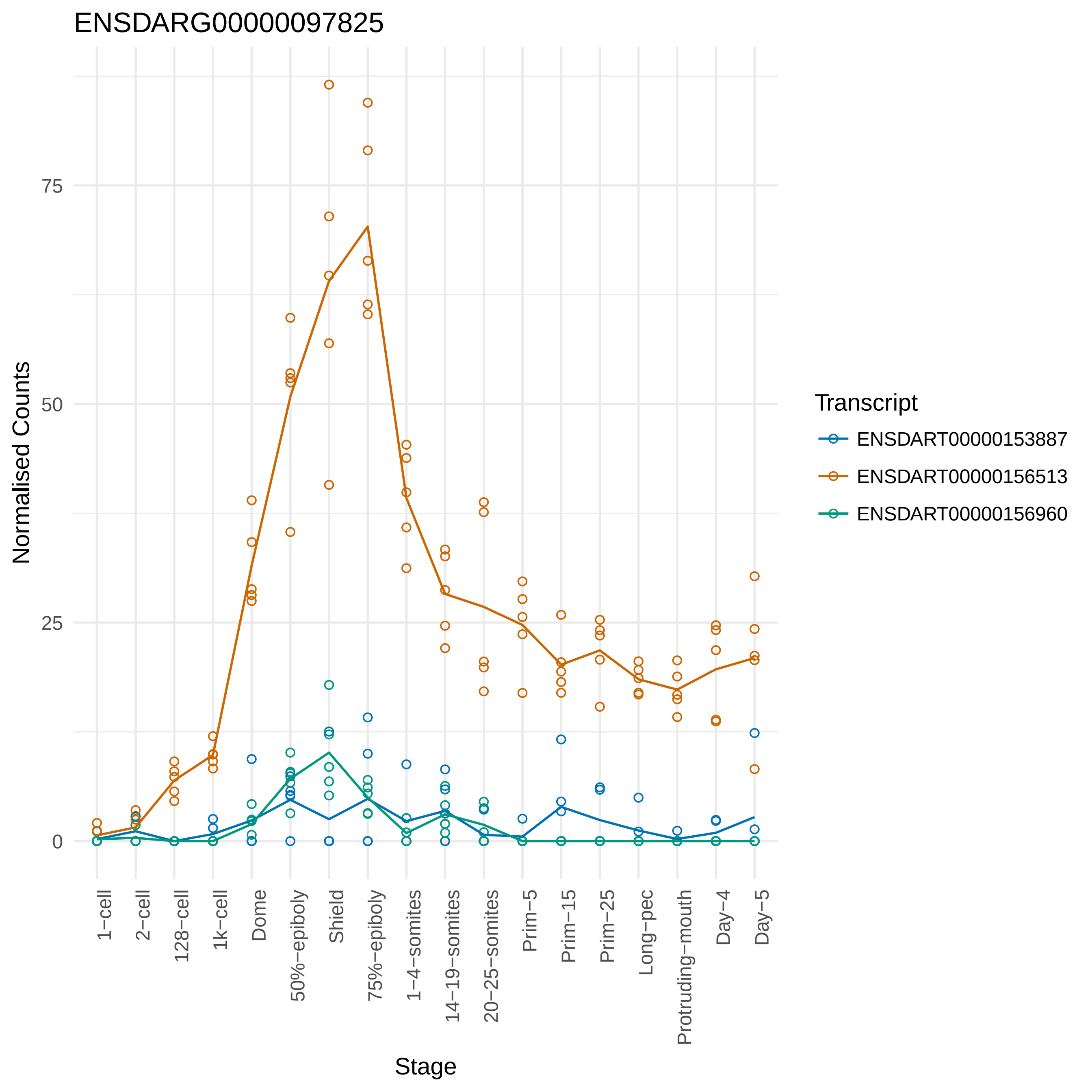
ENSDARG00000097825
Normalised Counts
0
25
50
75
Stage
1−cell
2−cell
128−cell
1k−cell
Dome
50%−epiboly
Shield
75%−epiboly
1−4−somites
14−19−somites
20−25−somites
Prim−5
Prim−15
Prim−25
Long−pec
Protruding−mouth
Day−4
Day−5
Transcript
ENSDART00000153887
ENSDART00000156513
ENSDART00000156960

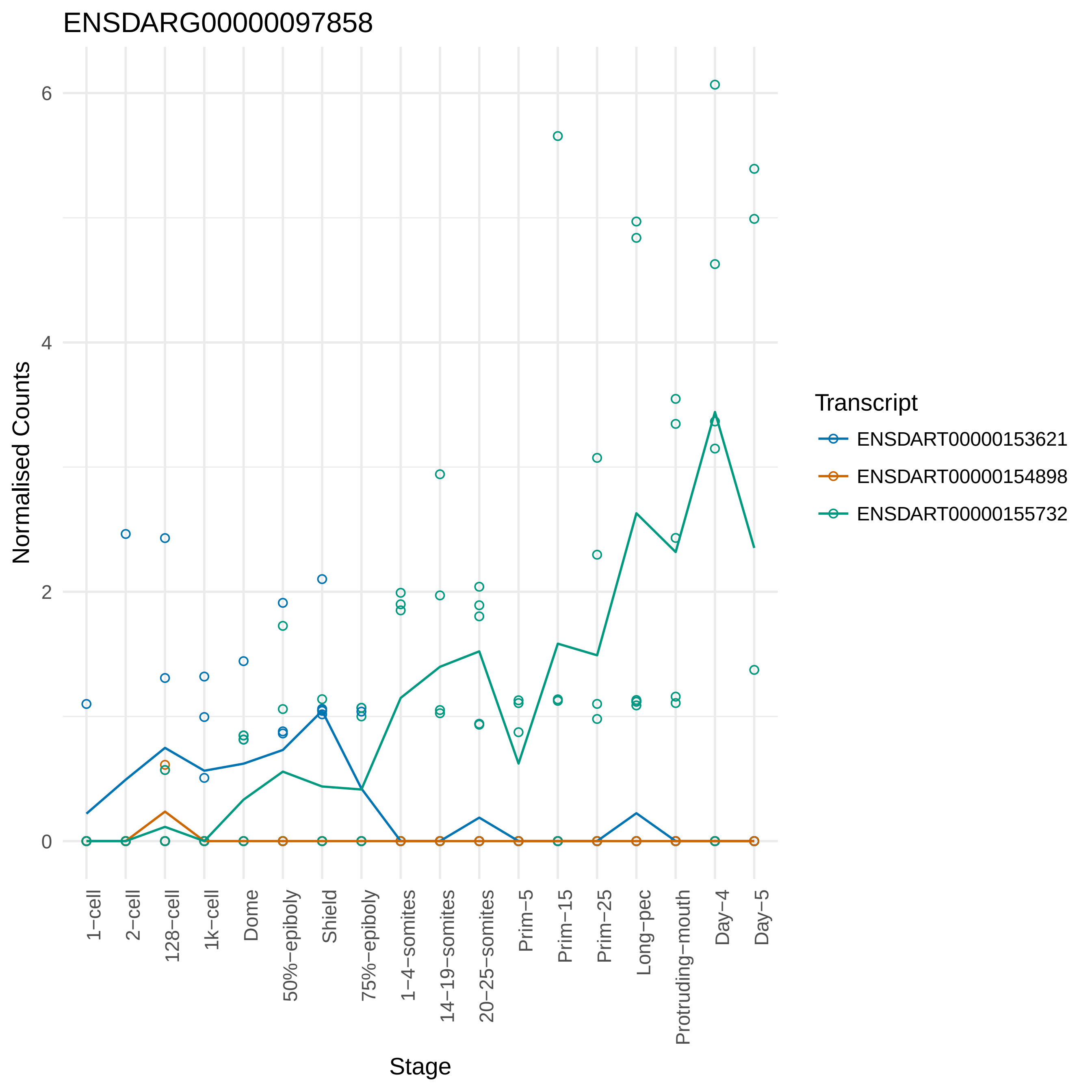

ENSDARG00000097858
Normalised Counts
6
4
2
0
1−cell
2−cell
128−cell
1k−cell
Dome
50%−epiboly
Shield
75%−epiboly
1−4−somites
14−19−somites
20−25−somites
Prim−5
Prim−15
Prim−25
Long−pec
Protruding−mouth
Day−4
Day−5
Stage
Transcript
ENSDART00000153621
ENSDART00000154898
ENSDART00000155732

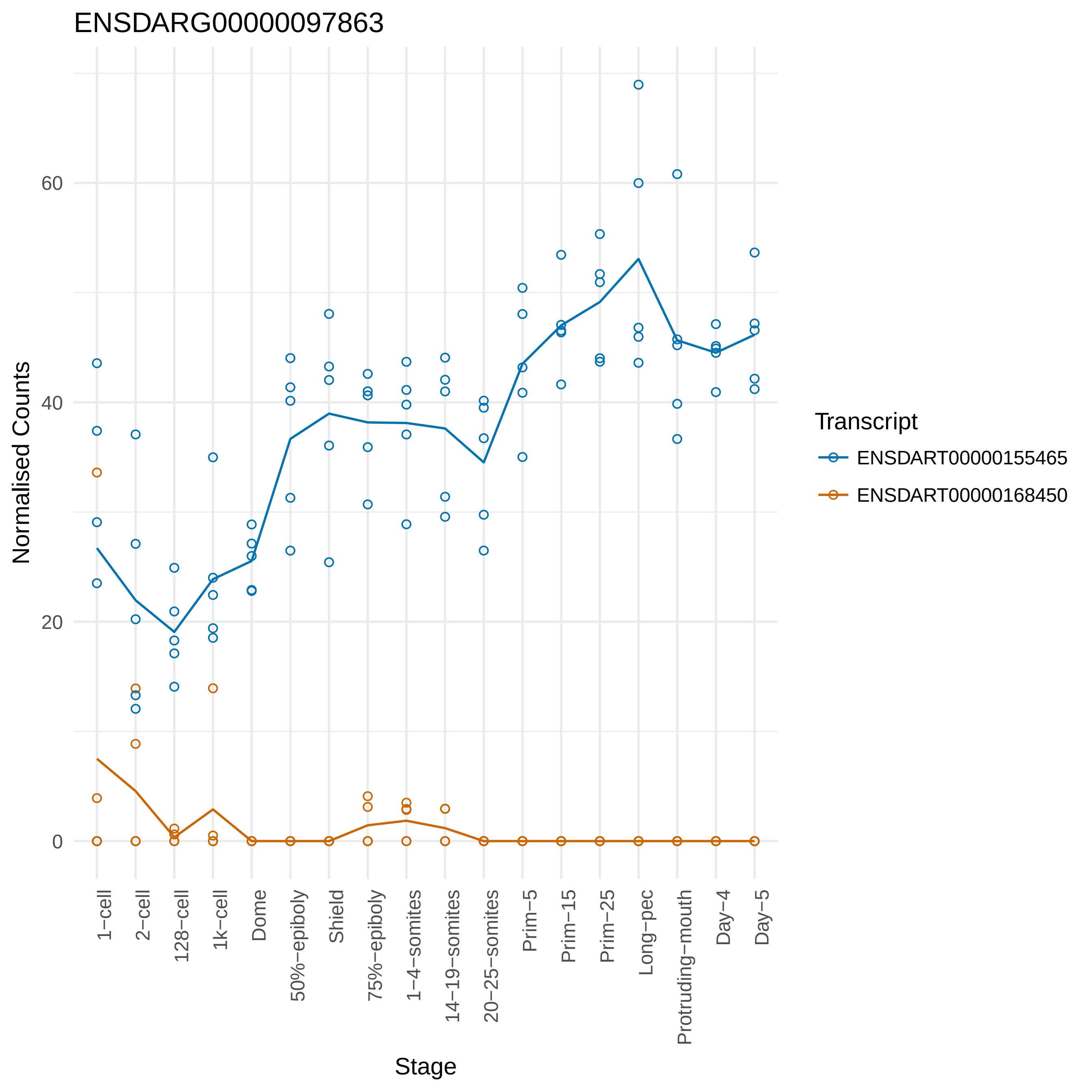
ENSDARG00000097863
Normalised Counts
0
20
40
60
Stage
1−cell
2−cell
128−cell
1k−cell
Dome
50%−epiboly
Shield
75%−epiboly
1−4−somites
14−19−somites
20−25−somites
Prim−5
Prim−15
Prim−25
Long−pec
Protruding−mouth
Day−4
Day−5
Transcript
ENSDART00000155465
ENSDART00000168450

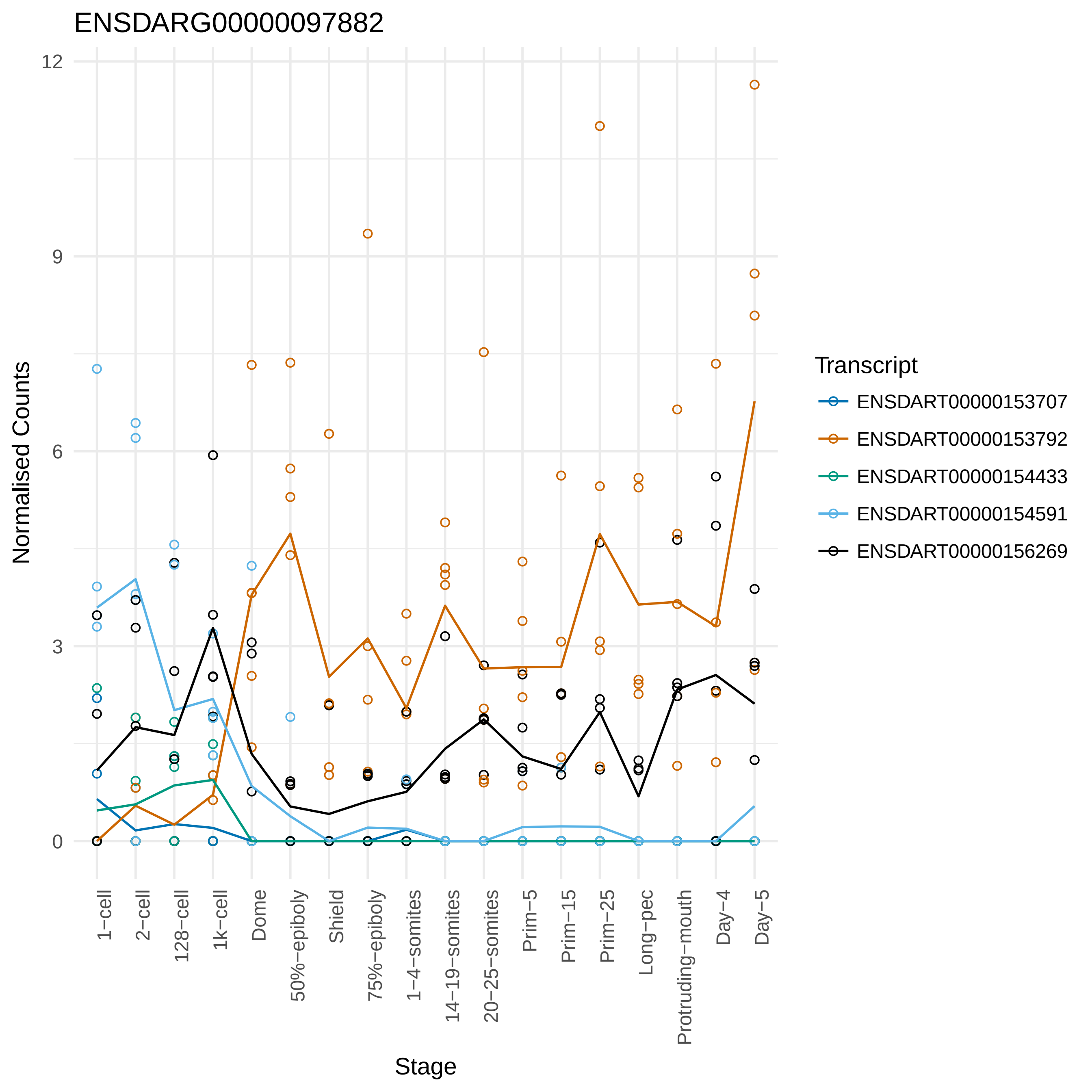

ENSDARG00000097882
Normalised Counts
0
3
6
9
12
1−cell
2−cell
128−cell
1k−cell
Dome
50%−epiboly
Shield
75%−epiboly
1−4−somites
14−19−somites
20−25−somites
Prim−5
Prim−15
Prim−25
Long−pec
Protruding−mouth
Day−4
Day−5
Stage
Transcript
ENSDART00000153707
ENSDART00000153792
ENSDART00000154433
ENSDART00000154591
ENSDART00000156269

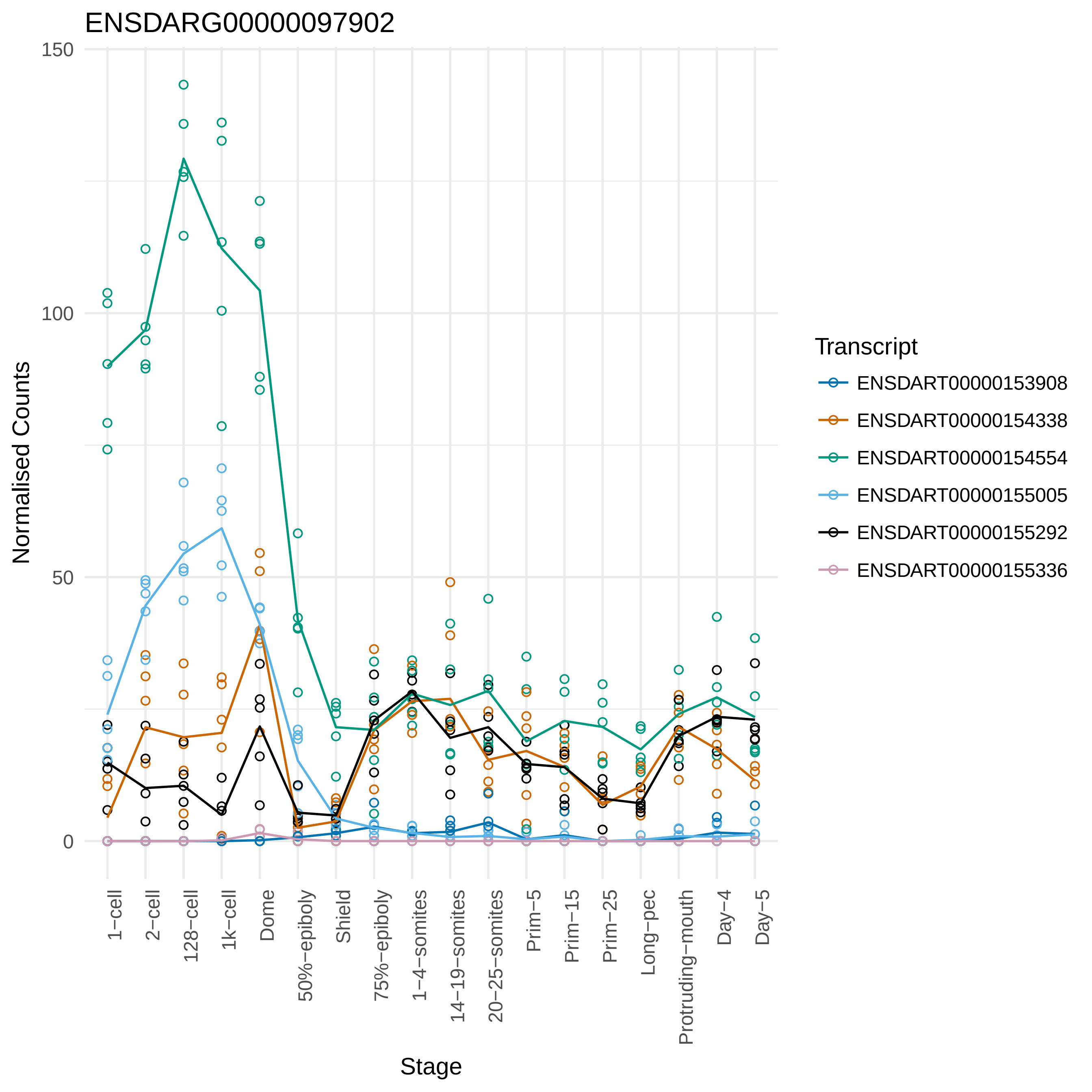

ENSDARG00000097902
Normalised Counts
150
100
50
0
1−cell
2−cell
128−cell
1k−cell
Dome
50%−epiboly
Shield
75%−epiboly
1−4−somites
14−19−somites
20−25−somites
Prim−5
Prim−15
Prim−25
Long−pec
Protruding−mouth
Day−4
Day−5
Stage
Transcript
ENSDART00000153908
ENSDART00000154338
ENSDART00000154554
ENSDART00000155005
ENSDART00000155292
ENSDART00000155336

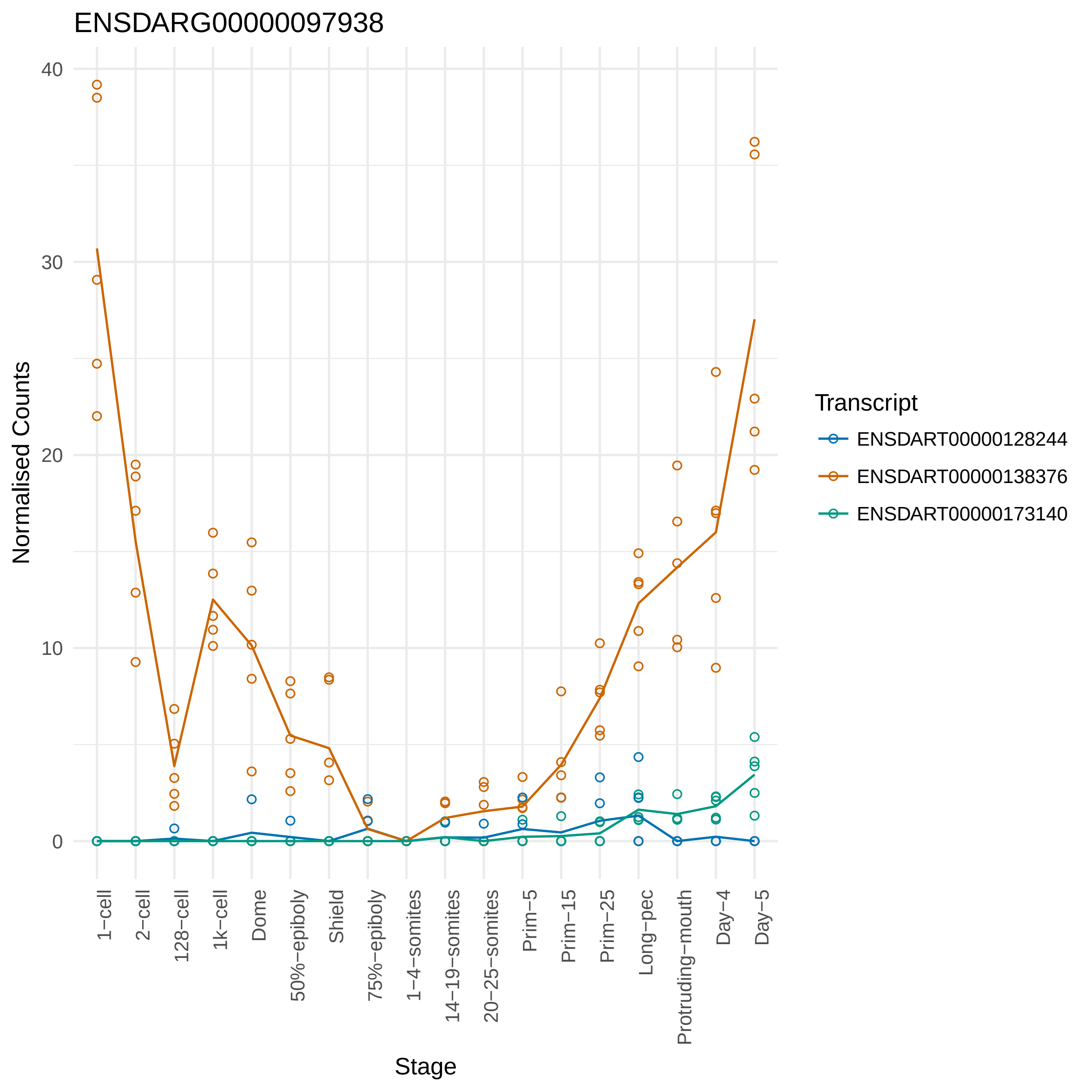

ENSDARG00000097938
Normalised Counts
40
30
20
10
0
1−cell
2−cell
128−cell
1k−cell
Dome
50%−epiboly
Shield
75%−epiboly
1−4−somites
14−19−somites
20−25−somites
Prim−5
Prim−15
Prim−25
Long−pec
Protruding−mouth
Day−4
Day−5
Stage
Transcript
ENSDART00000128244
ENSDART00000138376
ENSDART00000173140

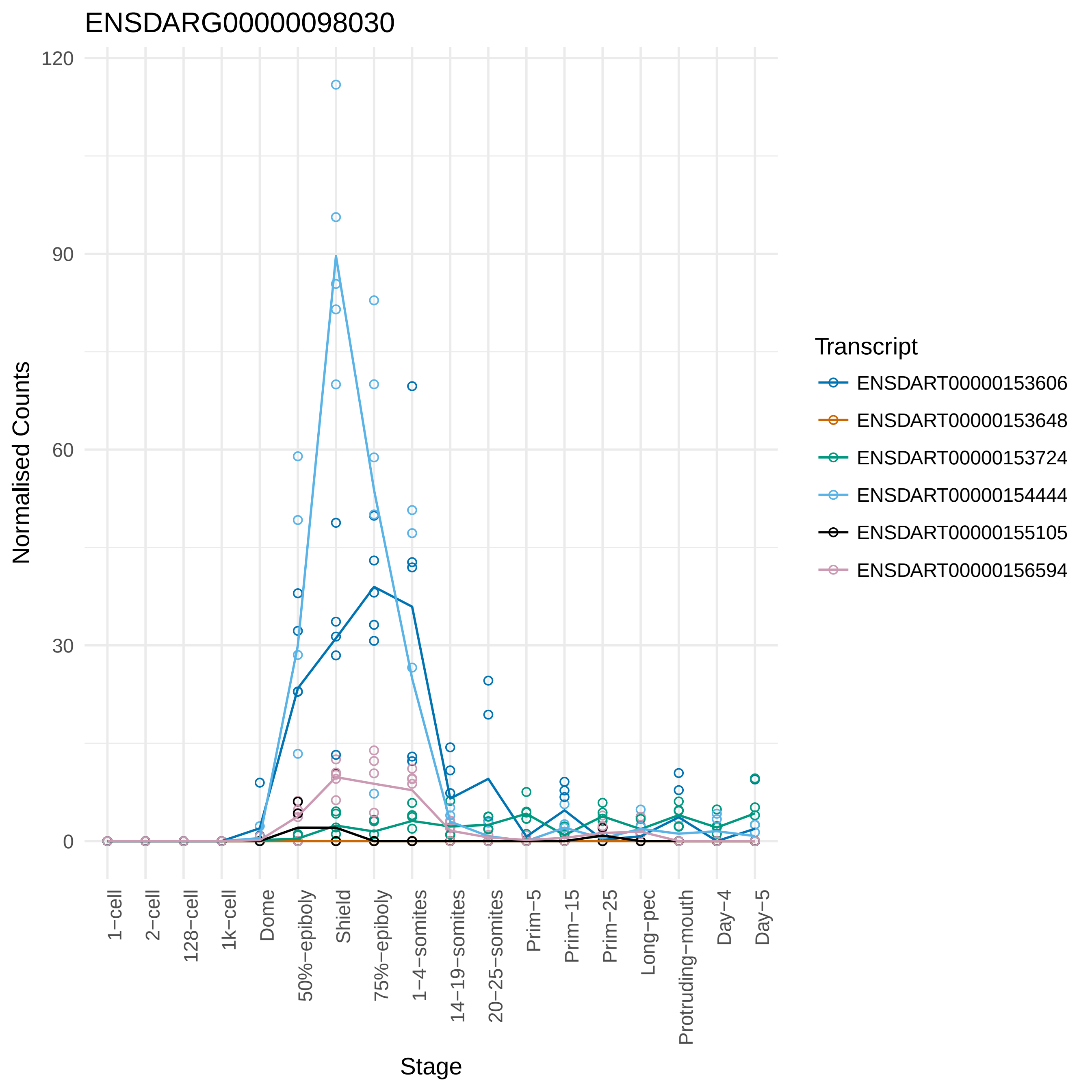

ENSDARG00000098030
Normalised Counts
120
90
60
30
0
1−cell
2−cell
128−cell
1k−cell
Dome
50%−epiboly
Shield
75%−epiboly
1−4−somites
14−19−somites
20−25−somites
Prim−5
Prim−15
Prim−25
Long−pec
Protruding−mouth
Day−4
Day−5
Stage
Transcript
ENSDART00000153606
ENSDART00000153648
ENSDART00000153724
ENSDART00000154444
ENSDART00000155105
ENSDART00000156594

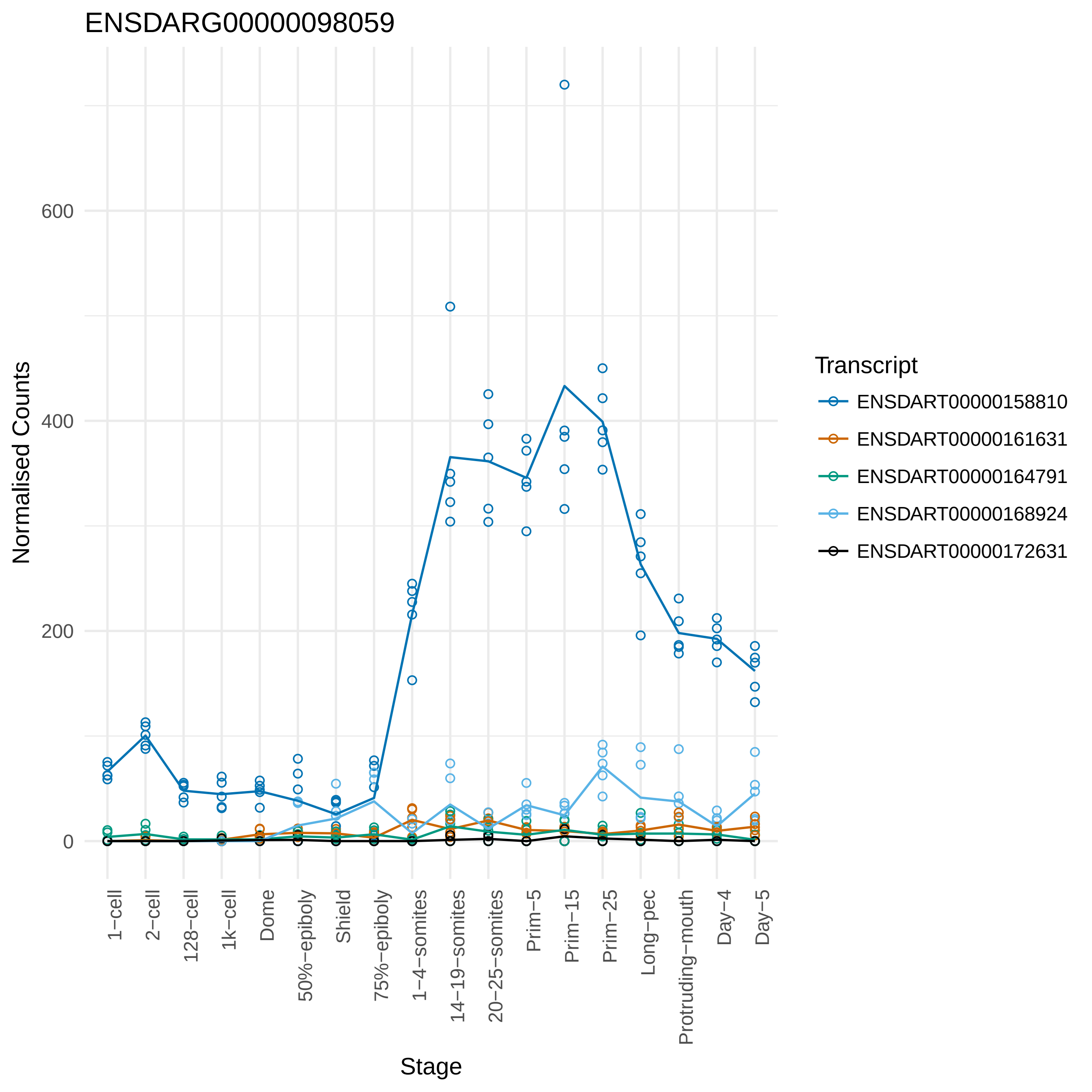
ENSDARG00000098059
Normalised Counts
600
400
200
0
1−cell
2−cell
128−cell
1k−cell
Dome
50%−epiboly
Shield
75%−epiboly
1−4−somites
14−19−somites
20−25−somites
Prim−5
Prim−15
Prim−25
Long−pec
Protruding−mouth
Day−4
Day−5
Stage
Transcript
ENSDART00000158810
ENSDART00000161631
ENSDART00000164791
ENSDART00000168924
ENSDART00000172631

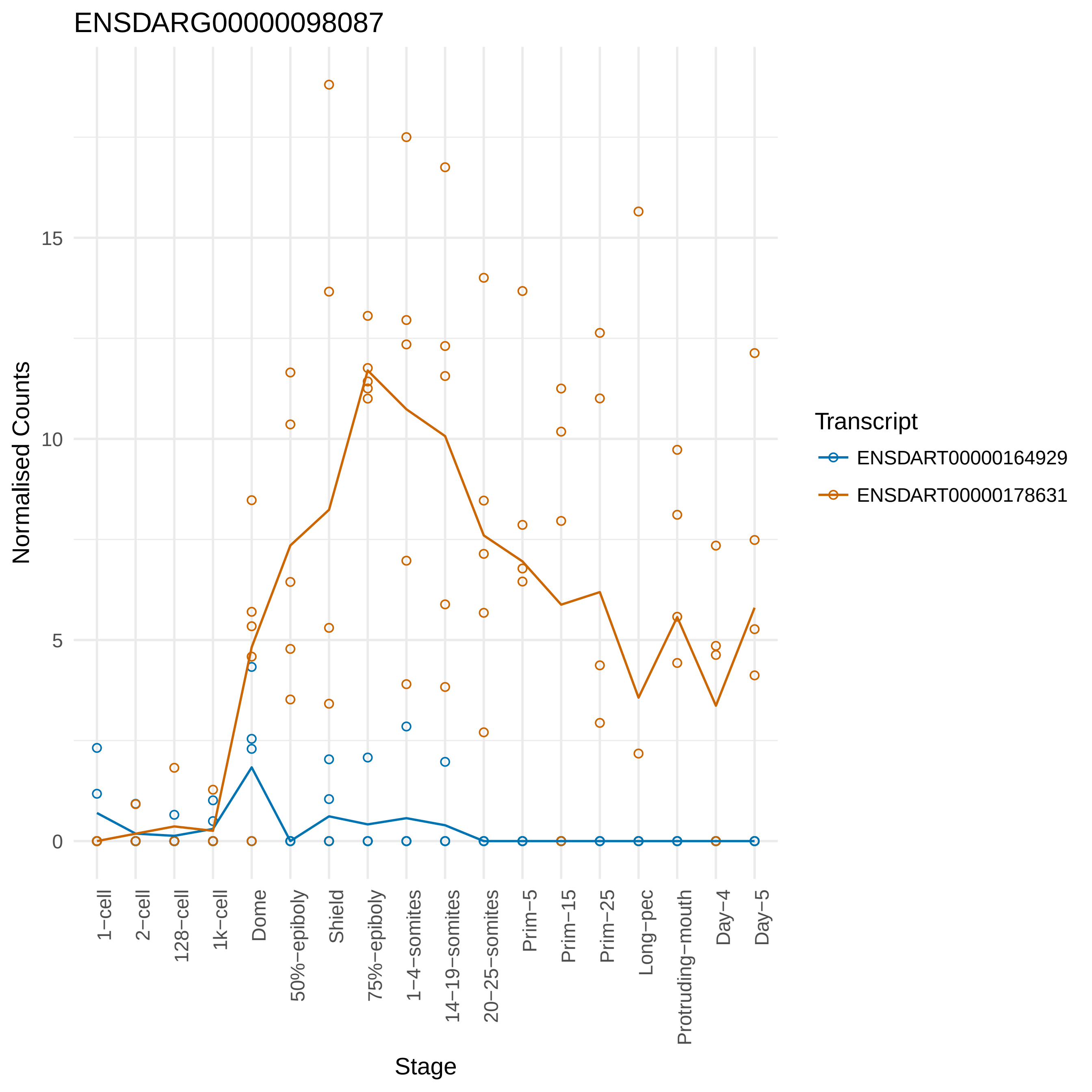

ENSDARG00000098087
Normalised Counts
0
5
10
15
1−cell
2−cell
128−cell
1k−cell
Dome
50%−epiboly
Shield
75%−epiboly
1−4−somites
14−19−somites
20−25−somites
Prim−5
Prim−15
Prim−25
Long−pec
Protruding−mouth
Day−4
Day−5
Stage
Transcript
ENSDART00000164929
ENSDART00000178631

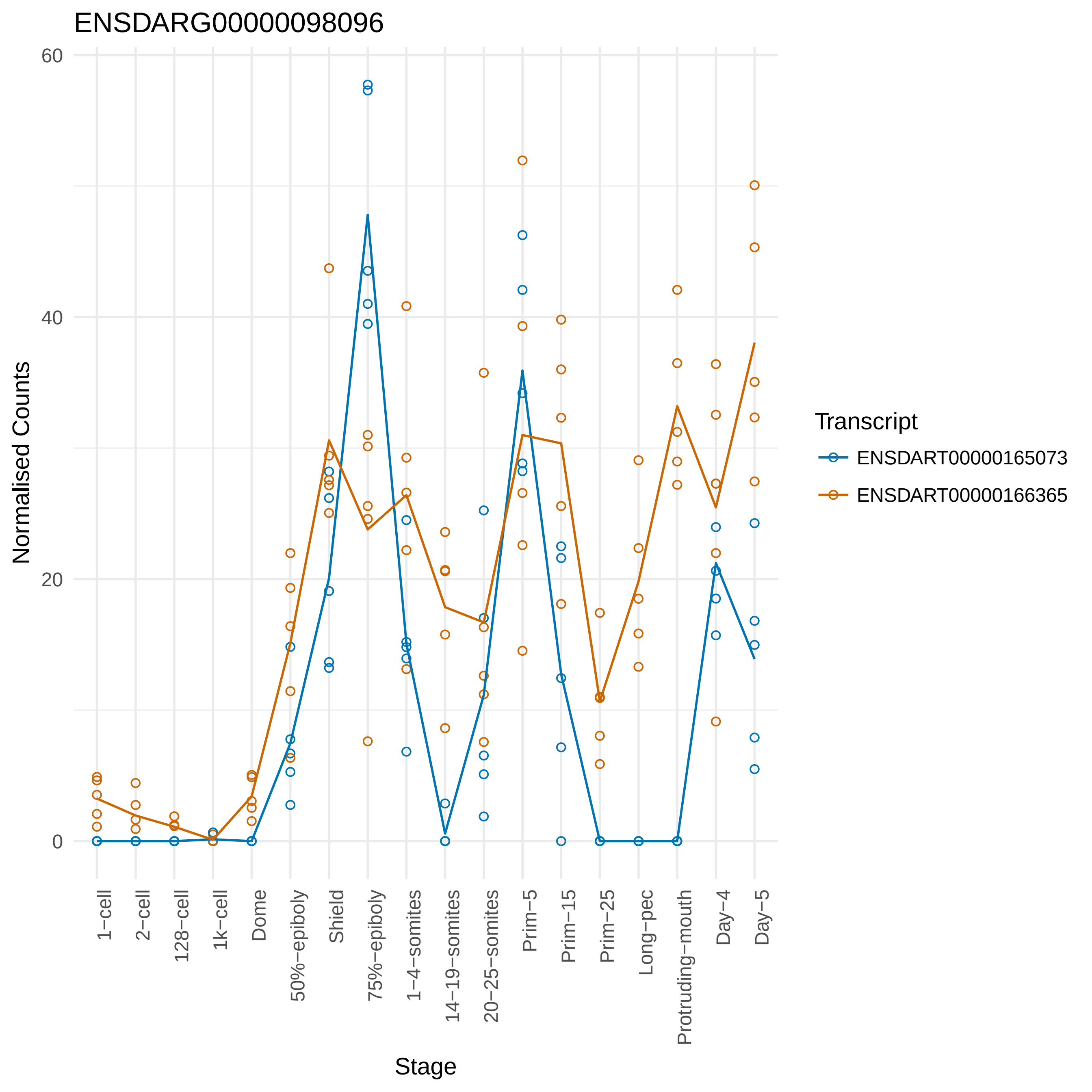

ENSDARG00000098096
Normalised Counts
0
20
40
60
Stage
1−cell
2−cell
128−cell
1k−cell
Dome
50%−epiboly
Shield
75%−epiboly
1−4−somites
14−19−somites
20−25−somites
Prim−5
Prim−15
Prim−25
Long−pec
Protruding−mouth
Day−4
Day−5
Transcript
ENSDART00000165073
ENSDART00000166365

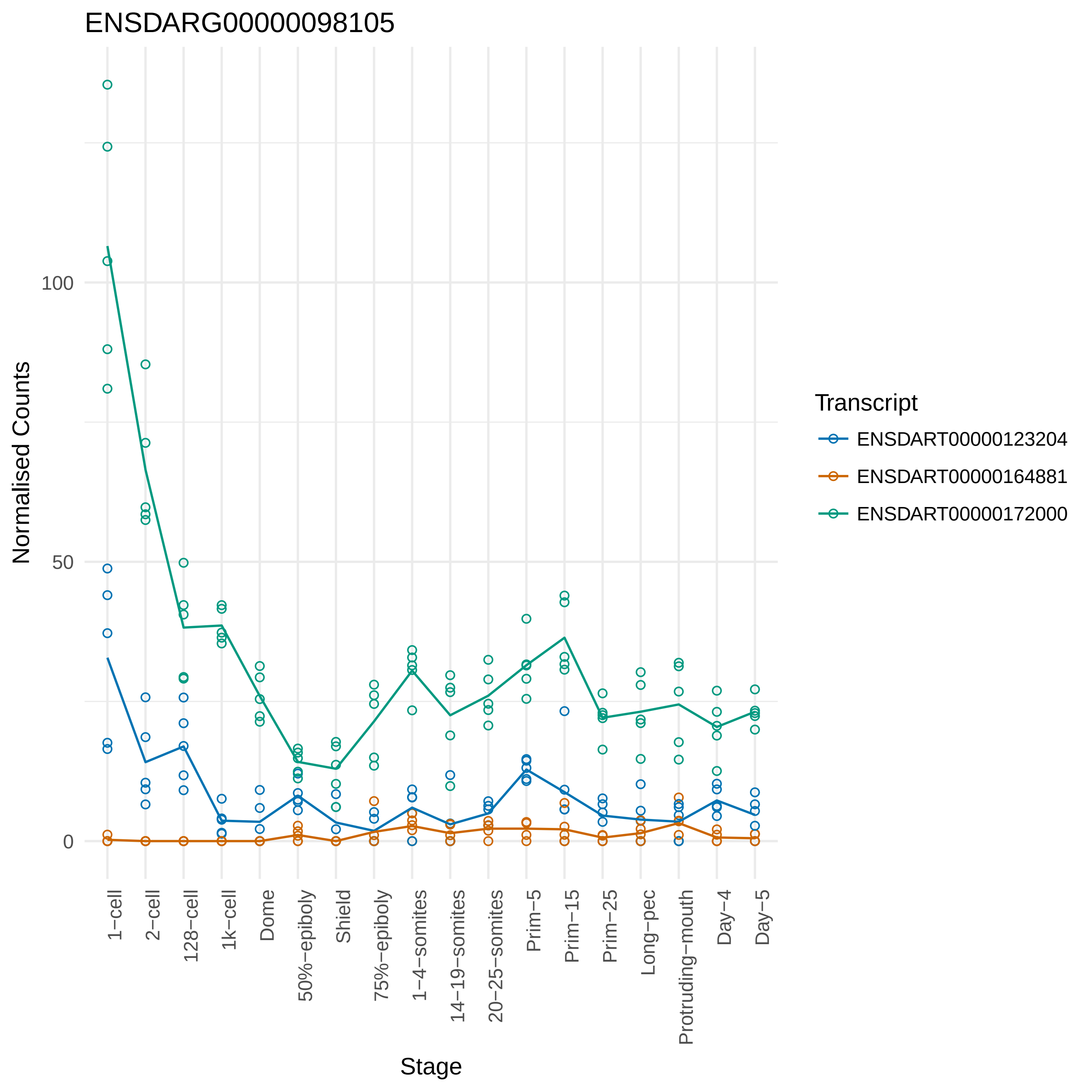
ENSDARG00000098105
Normalised Counts
100
50
0
1−cell
2−cell
128−cell
1k−cell
Dome
50%−epiboly
Shield
75%−epiboly
1−4−somites
14−19−somites
20−25−somites
Prim−5
Prim−15
Prim−25
Long−pec
Protruding−mouth
Day−4
Day−5
Stage
Transcript
ENSDART00000123204
ENSDART00000164881
ENSDART00000172000

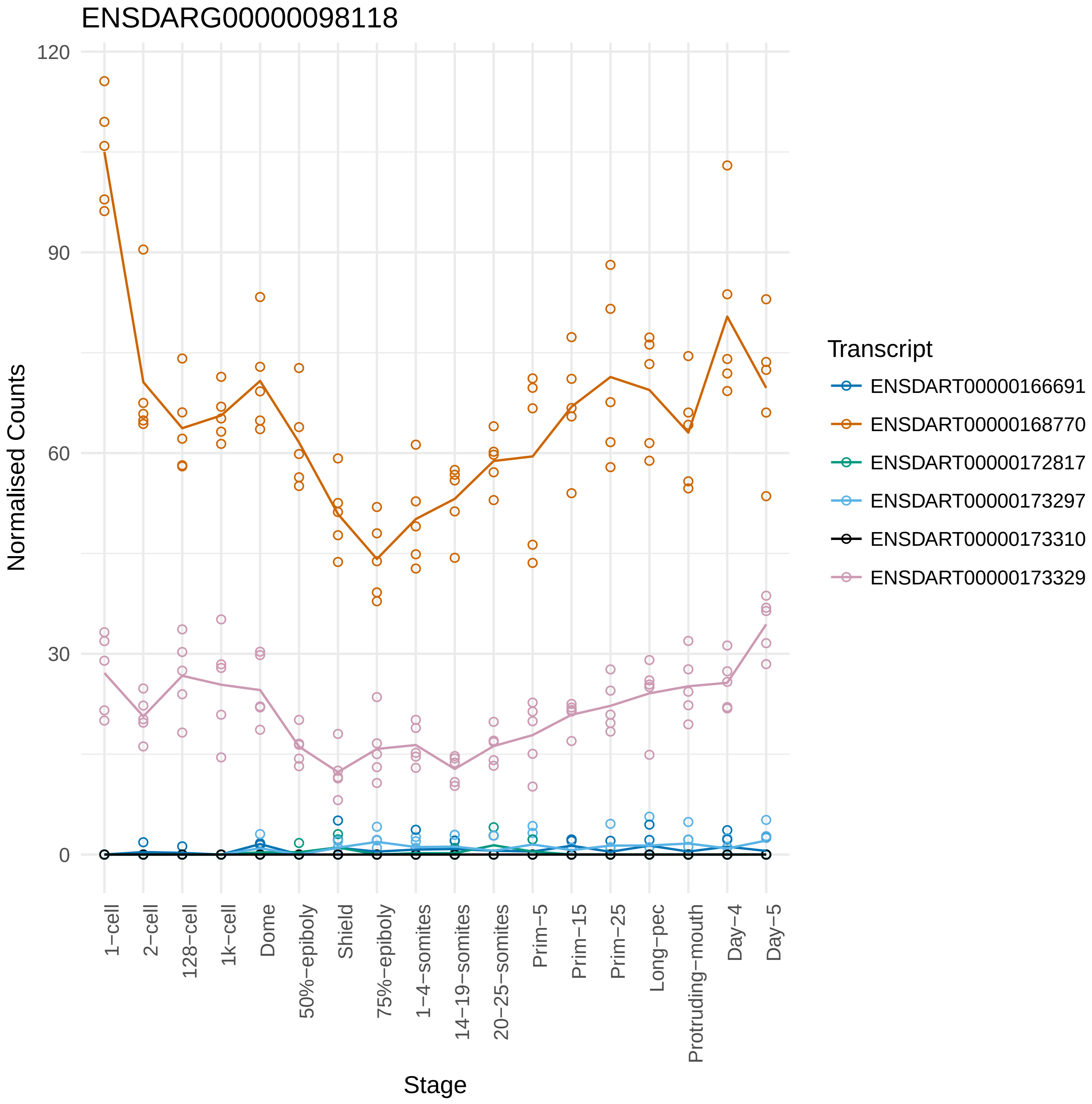

ENSDARG00000098118
Normalised Counts
120
90
60
30
0
1−cell
2−cell
128−cell
1k−cell
Dome
50%−epiboly
Shield
75%−epiboly
1−4−somites
14−19−somites
20−25−somites
Prim−5
Prim−15
Prim−25
Long−pec
Protruding−mouth
Day−4
Day−5
Stage
Transcript
ENSDART00000166691
ENSDART00000168770
ENSDART00000172817
ENSDART00000173297
ENSDART00000173310
ENSDART00000173329

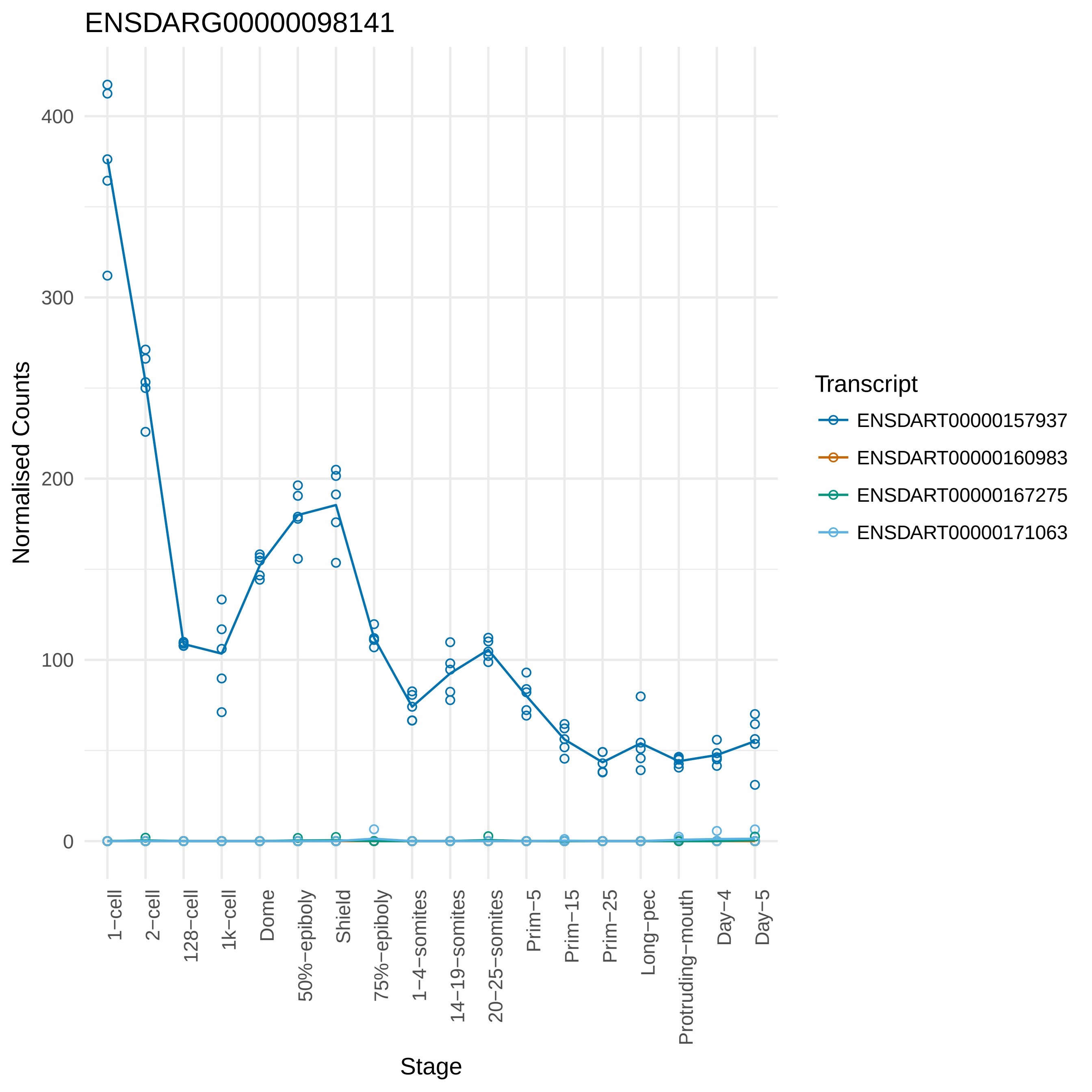
ENSDARG00000098141
Normalised Counts
400
300
200
100
0
1−cell
2−cell
128−cell
1k−cell
Dome
50%−epiboly
Shield
75%−epiboly
1−4−somites
14−19−somites
20−25−somites
Prim−5
Prim−15
Prim−25
Long−pec
Protruding−mouth
Day−4
Day−5
Stage
Transcript
ENSDART00000157937
ENSDART00000160983
ENSDART00000167275
ENSDART00000171063

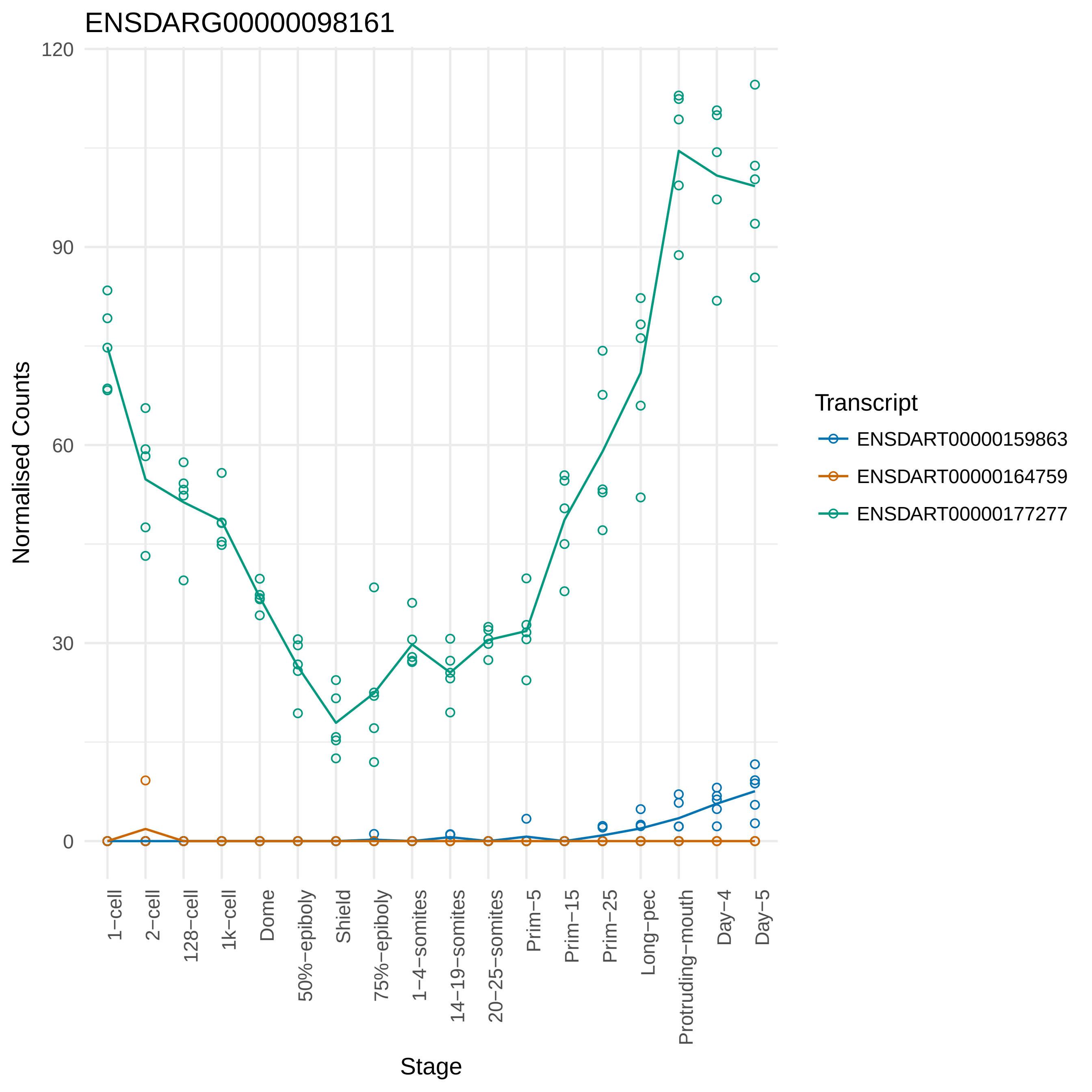
ENSDARG00000098161
Normalised Counts
120
90
60
30
0
1−cell
2−cell
128−cell
1k−cell
Dome
50%−epiboly
Shield
75%−epiboly
1−4−somites
14−19−somites
20−25−somites
Prim−5
Prim−15
Prim−25
Long−pec
Protruding−mouth
Day−4
Day−5
Stage
Transcript
ENSDART00000159863
ENSDART00000164759
ENSDART00000177277

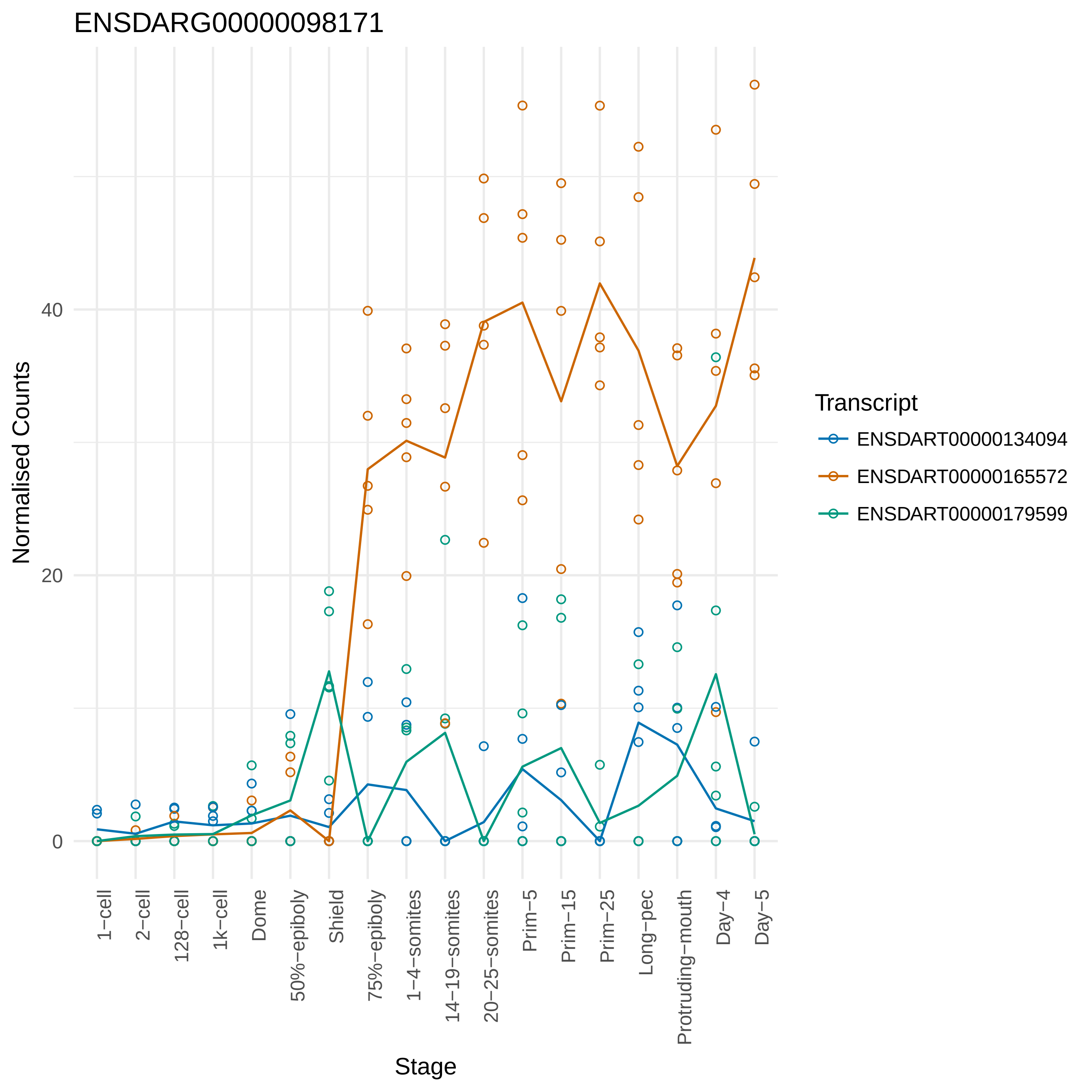

ENSDARG00000098171
Normalised Counts
0
20
40
Stage
1−cell
2−cell
128−cell
1k−cell
Dome
50%−epiboly
Shield
75%−epiboly
1−4−somites
14−19−somites
20−25−somites
Prim−5
Prim−15
Prim−25
Long−pec
Protruding−mouth
Day−4
Day−5
Transcript
ENSDART00000134094
ENSDART00000165572
ENSDART00000179599

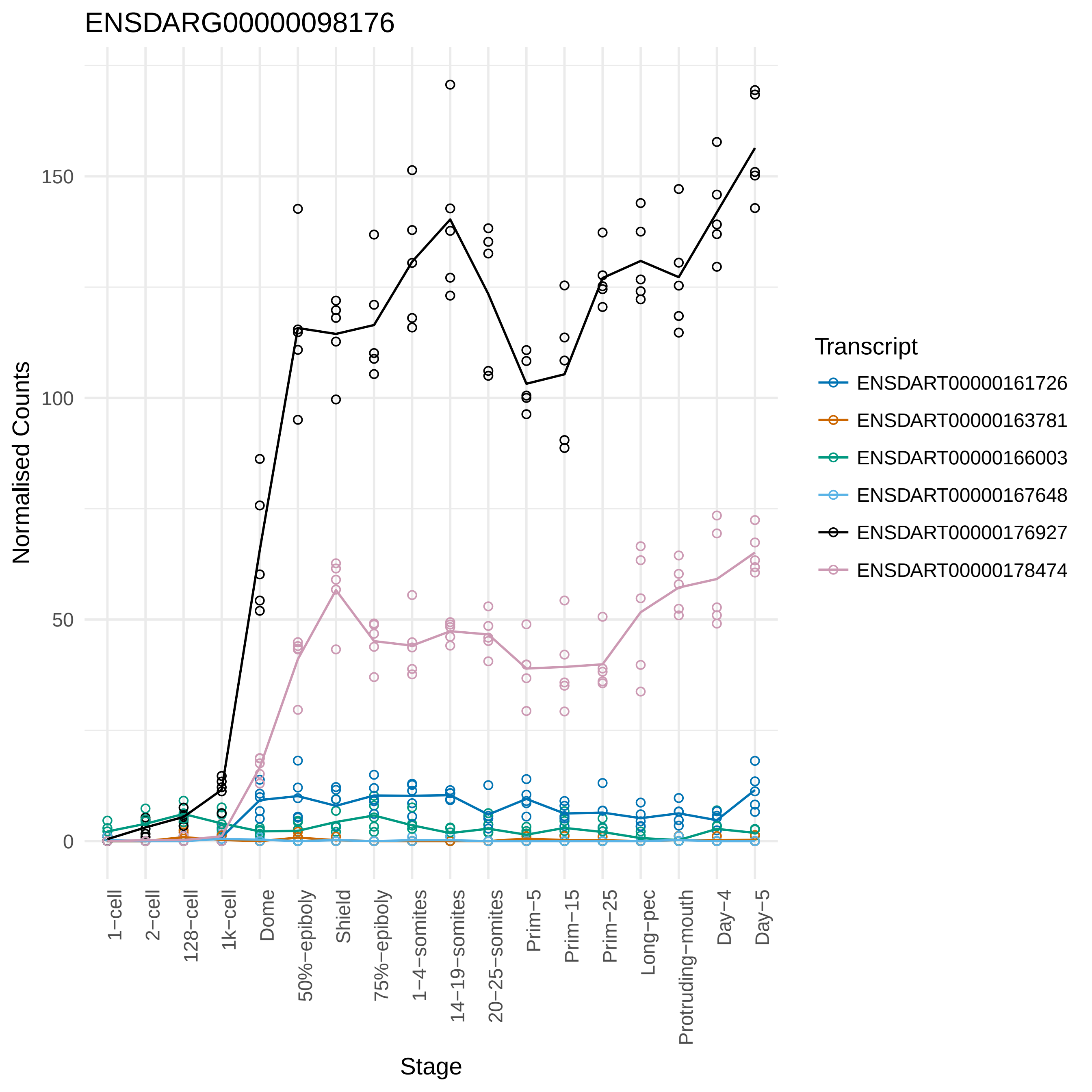
ENSDARG00000098176
Normalised Counts
150
100
50
0
1−cell
2−cell
128−cell
1k−cell
Dome
50%−epiboly
Shield
75%−epiboly
1−4−somites
14−19−somites
20−25−somites
Prim−5
Prim−15
Prim−25
Long−pec
Protruding−mouth
Day−4
Day−5
Stage
Transcript
ENSDART00000161726
ENSDART00000163781
ENSDART00000166003
ENSDART00000167648
ENSDART00000176927
ENSDART00000178474

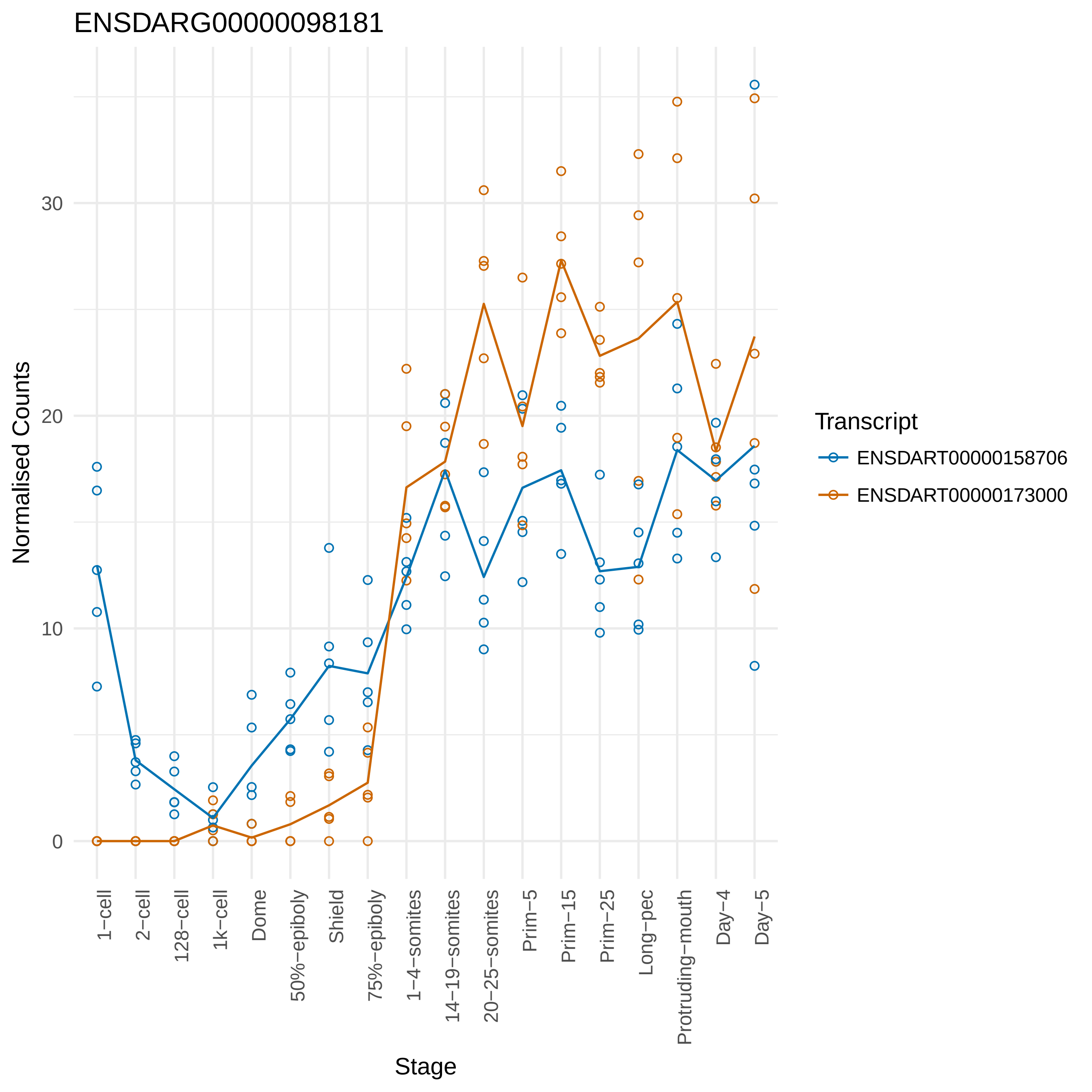
ENSDARG00000098181
Normalised Counts
0
10
20
30
1−cell
2−cell
128−cell
1k−cell
Dome
50%−epiboly
Shield
75%−epiboly
1−4−somites
14−19−somites
20−25−somites
Prim−5
Prim−15
Prim−25
Long−pec
Protruding−mouth
Day−4
Day−5
Stage
Transcript
ENSDART00000158706
ENSDART00000173000

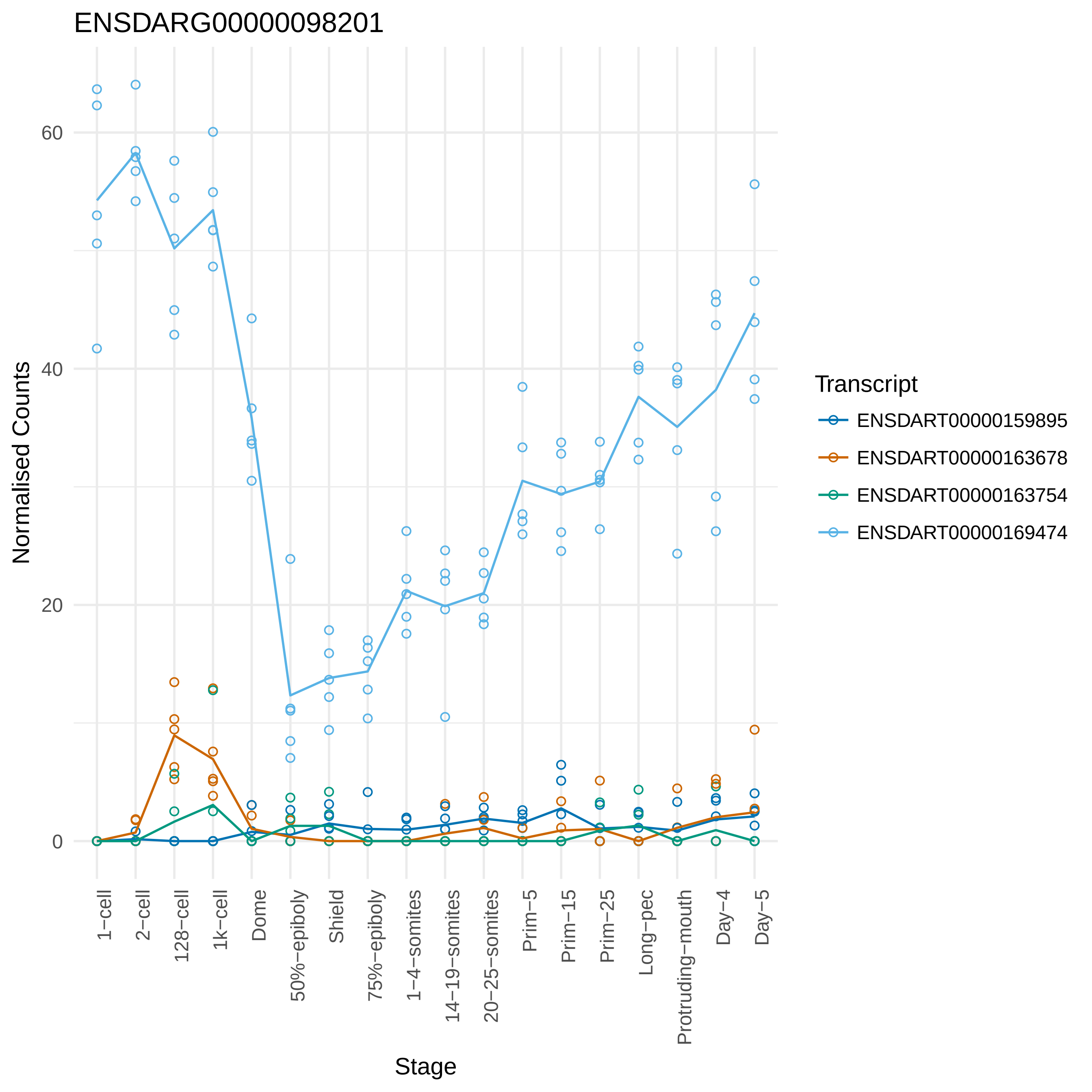

ENSDARG00000098201
Normalised Counts
0
20
40
60
Stage
1−cell
2−cell
128−cell
1k−cell
Dome
50%−epiboly
Shield
75%−epiboly
1−4−somites
14−19−somites
20−25−somites
Prim−5
Prim−15
Prim−25
Long−pec
Protruding−mouth
Day−4
Day−5
Transcript
ENSDART00000159895
ENSDART00000163678
ENSDART00000163754
ENSDART00000169474

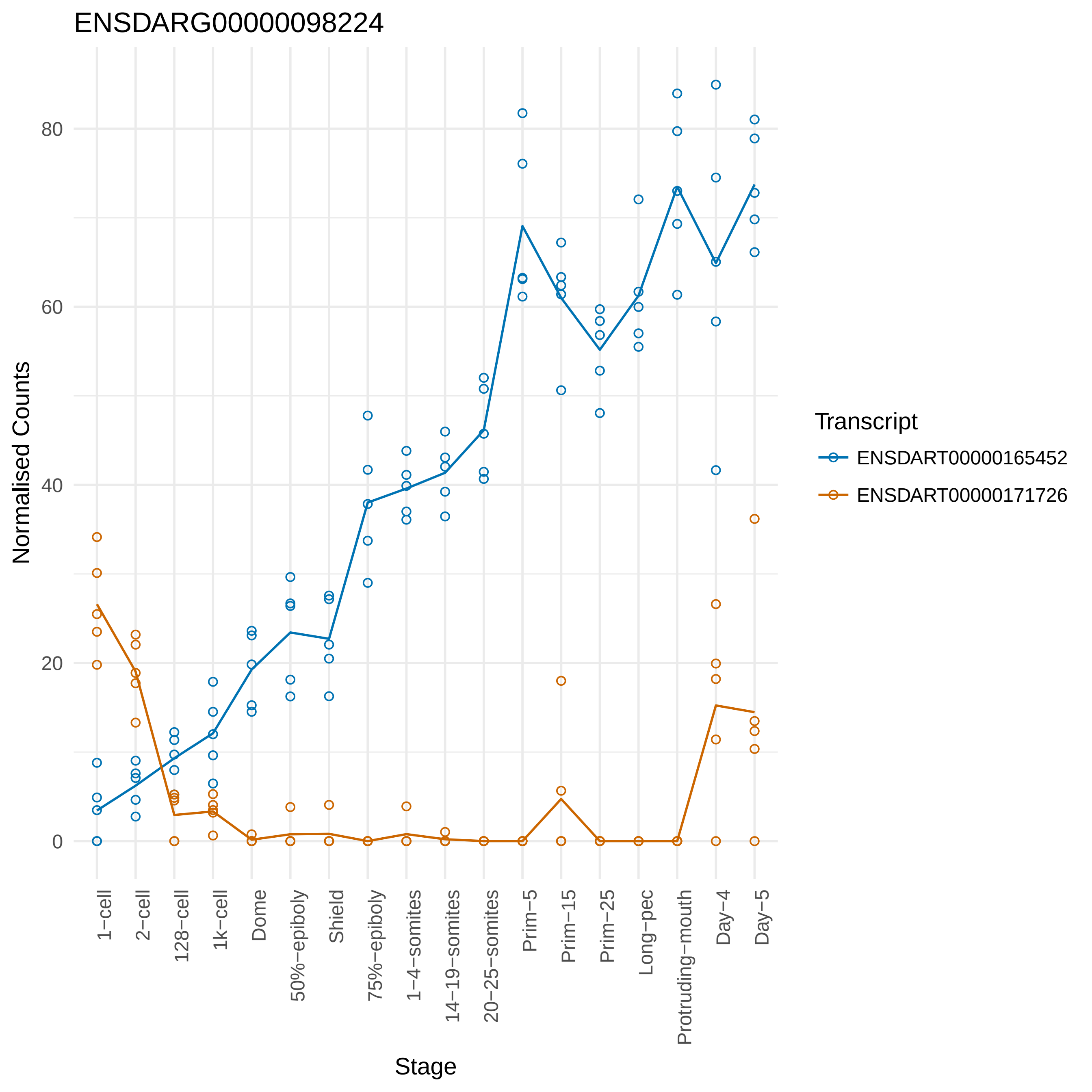

ENSDARG00000098224
Normalised Counts
0
20
40
60
80
Stage
1−cell
2−cell
128−cell
1k−cell
Dome
50%−epiboly
Shield
75%−epiboly
1−4−somites
14−19−somites
20−25−somites
Prim−5
Prim−15
Prim−25
Long−pec
Protruding−mouth
Day−4
Day−5
Transcript
ENSDART00000165452
ENSDART00000171726

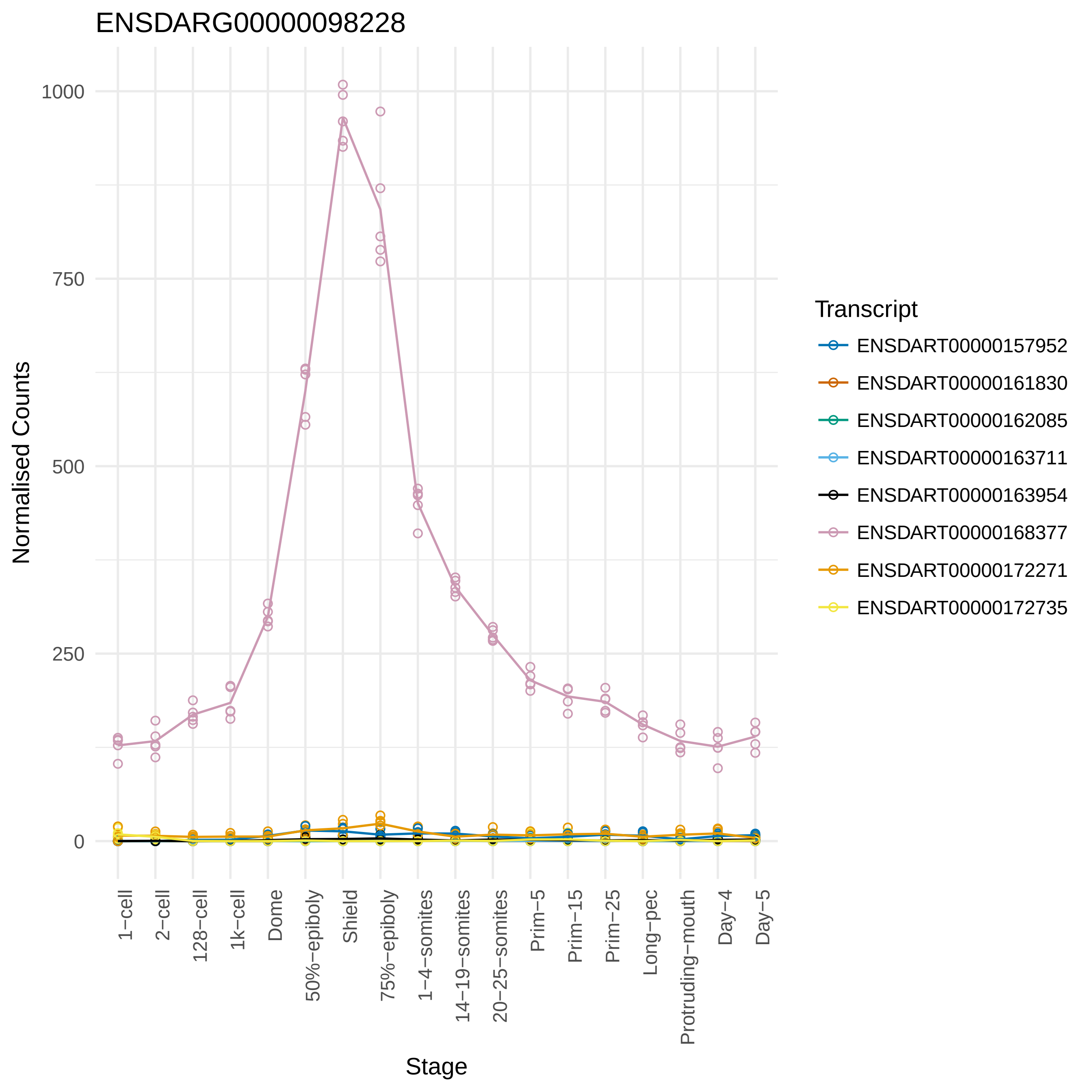

ENSDARG00000098228
Normalised Counts
1000
750
500
250
0
1−cell
2−cell
128−cell
1k−cell
Dome
50%−epiboly
Shield
75%−epiboly
1−4−somites
14−19−somites
20−25−somites
Prim−5
Prim−15
Prim−25
Long−pec
Protruding−mouth
Day−4
Day−5
Stage
Transcript
ENSDART00000157952
ENSDART00000161830
ENSDART00000162085
ENSDART00000163711
ENSDART00000163954
ENSDART00000168377
ENSDART00000172271
ENSDART00000172735

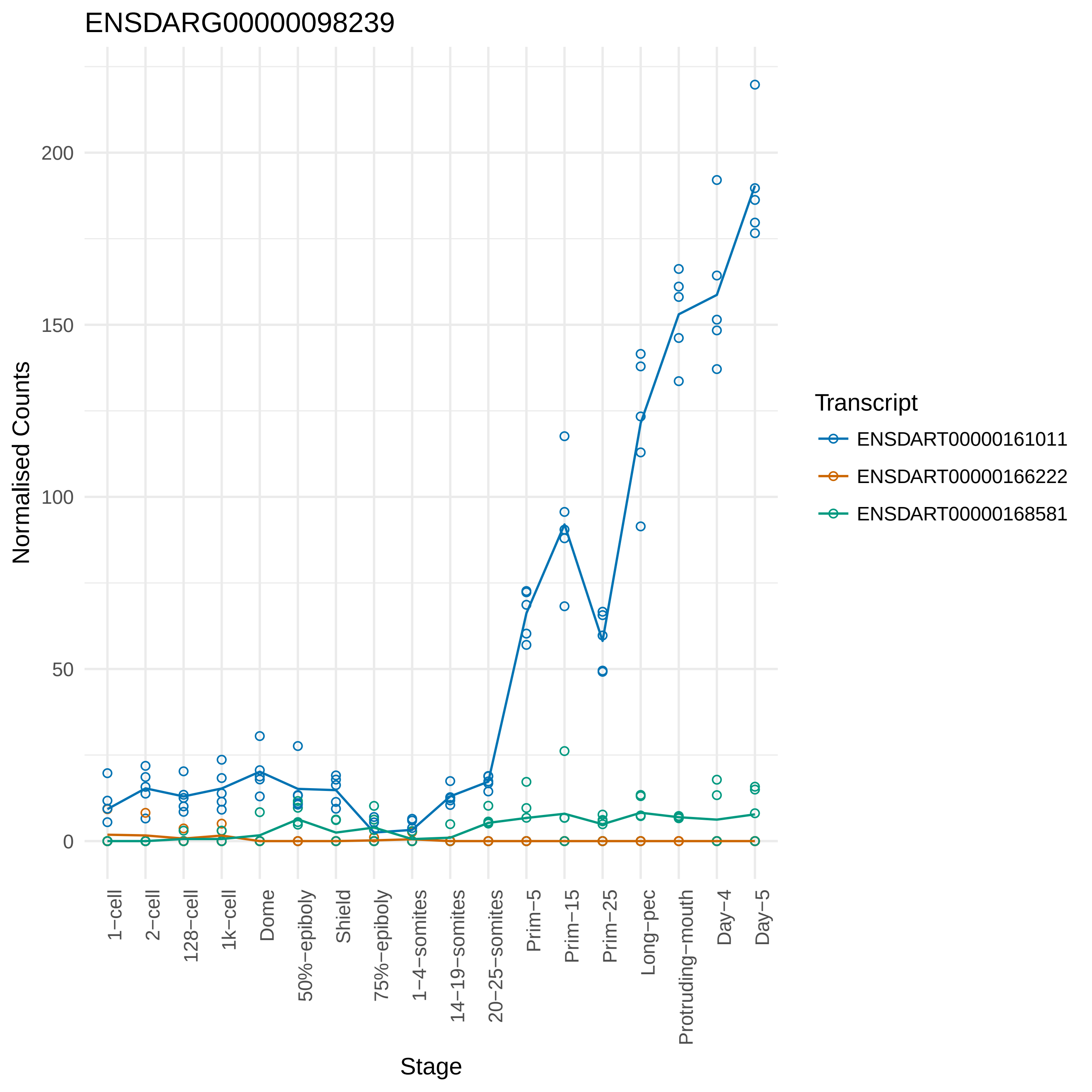

ENSDARG00000098239
Normalised Counts
0
50
100
150
200
1−cell
2−cell
128−cell
1k−cell
Dome
50%−epiboly
Shield
75%−epiboly
1−4−somites
14−19−somites
20−25−somites
Prim−5
Prim−15
Prim−25
Long−pec
Protruding−mouth
Day−4
Day−5
Stage
Transcript
ENSDART00000161011
ENSDART00000166222
ENSDART00000168581

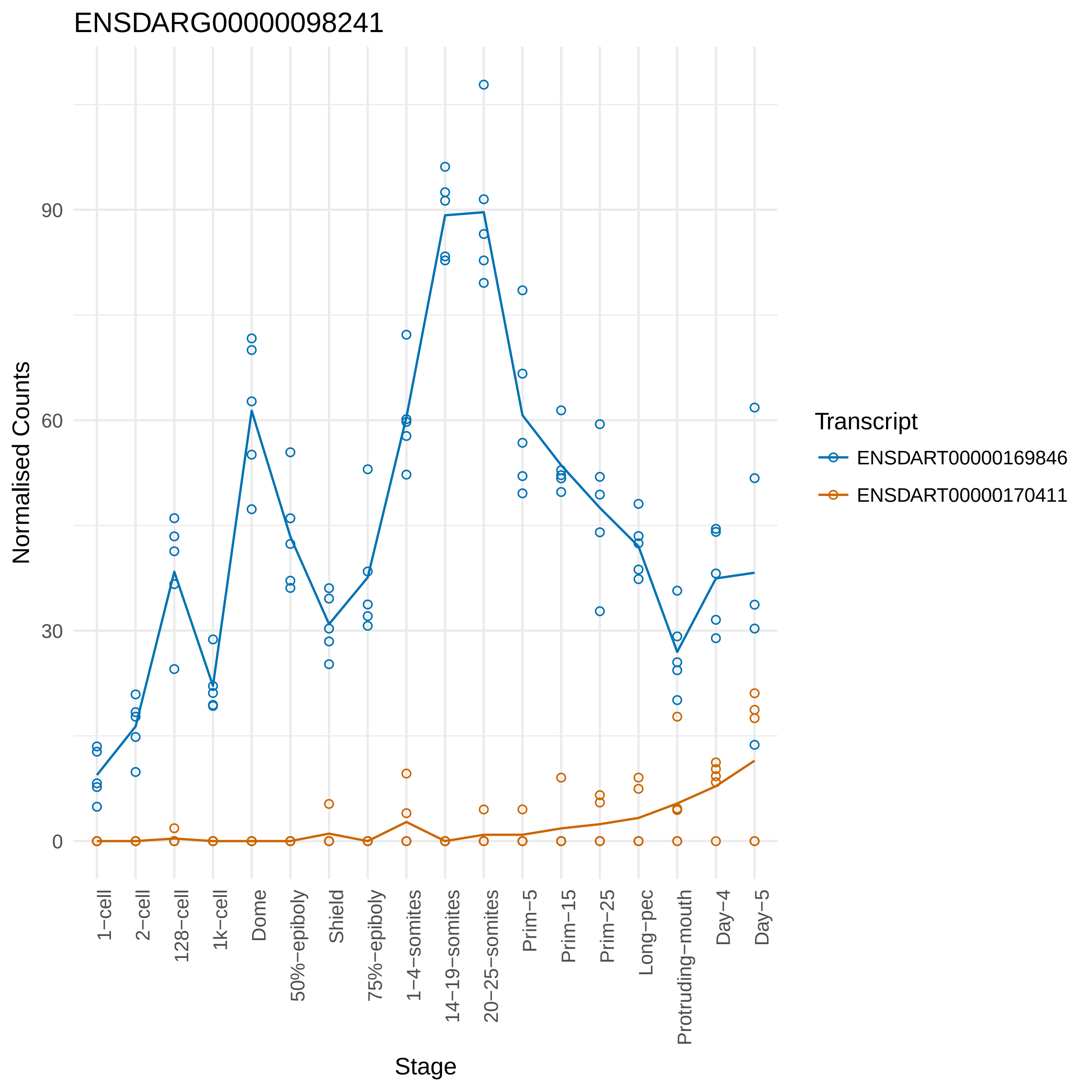

ENSDARG00000098241
Normalised Counts
0
30
60
90
1−cell
2−cell
128−cell
1k−cell
Dome
50%−epiboly
Shield
75%−epiboly
1−4−somites
14−19−somites
20−25−somites
Prim−5
Prim−15
Prim−25
Long−pec
Protruding−mouth
Day−4
Day−5
Stage
Transcript
ENSDART00000169846
ENSDART00000170411

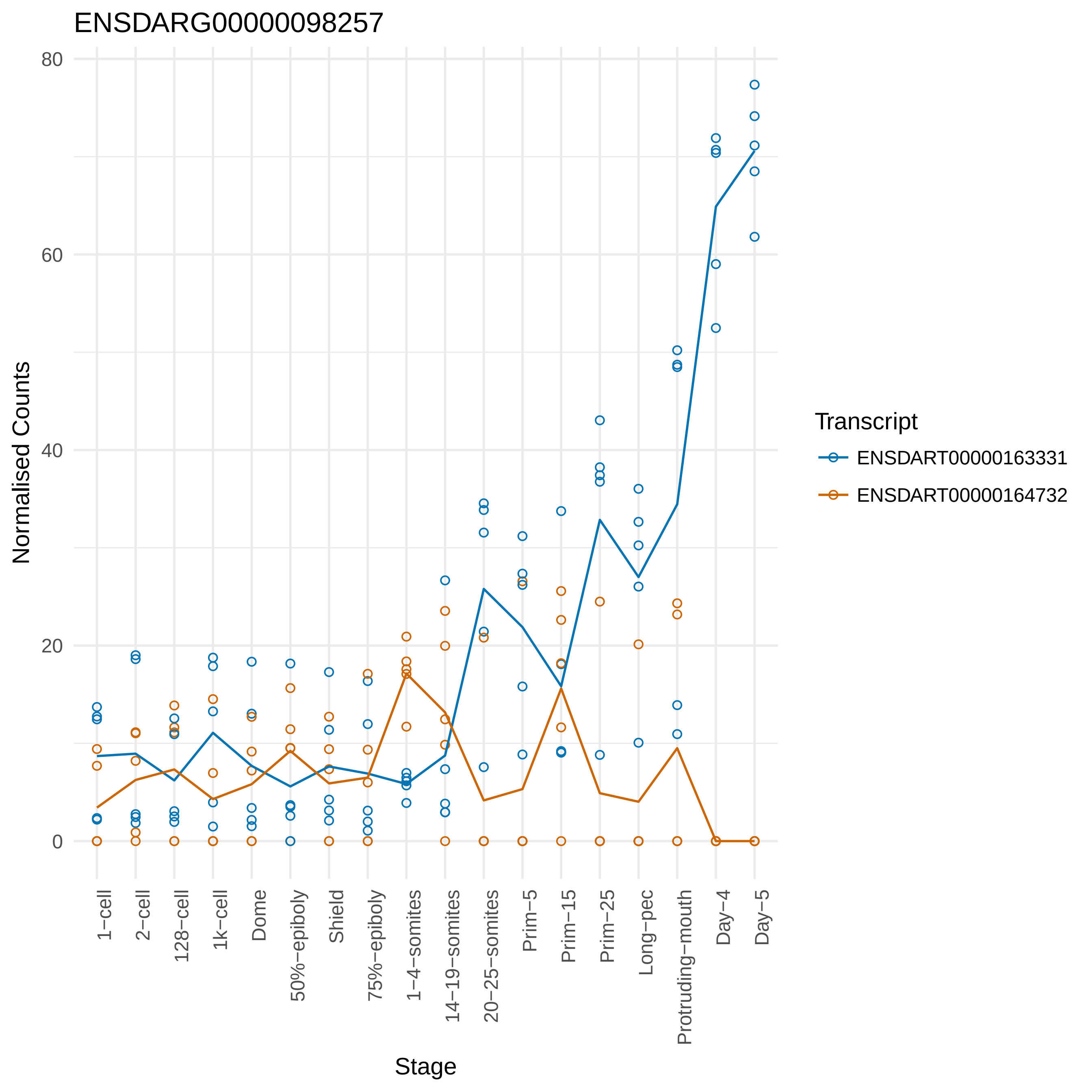

ENSDARG00000098257
Normalised Counts
0
20
40
60
80
Stage
1−cell
2−cell
128−cell
1k−cell
Dome
50%−epiboly
Shield
75%−epiboly
1−4−somites
14−19−somites
20−25−somites
Prim−5
Prim−15
Prim−25
Long−pec
Protruding−mouth
Day−4
Day−5
Transcript
ENSDART00000163331
ENSDART00000164732

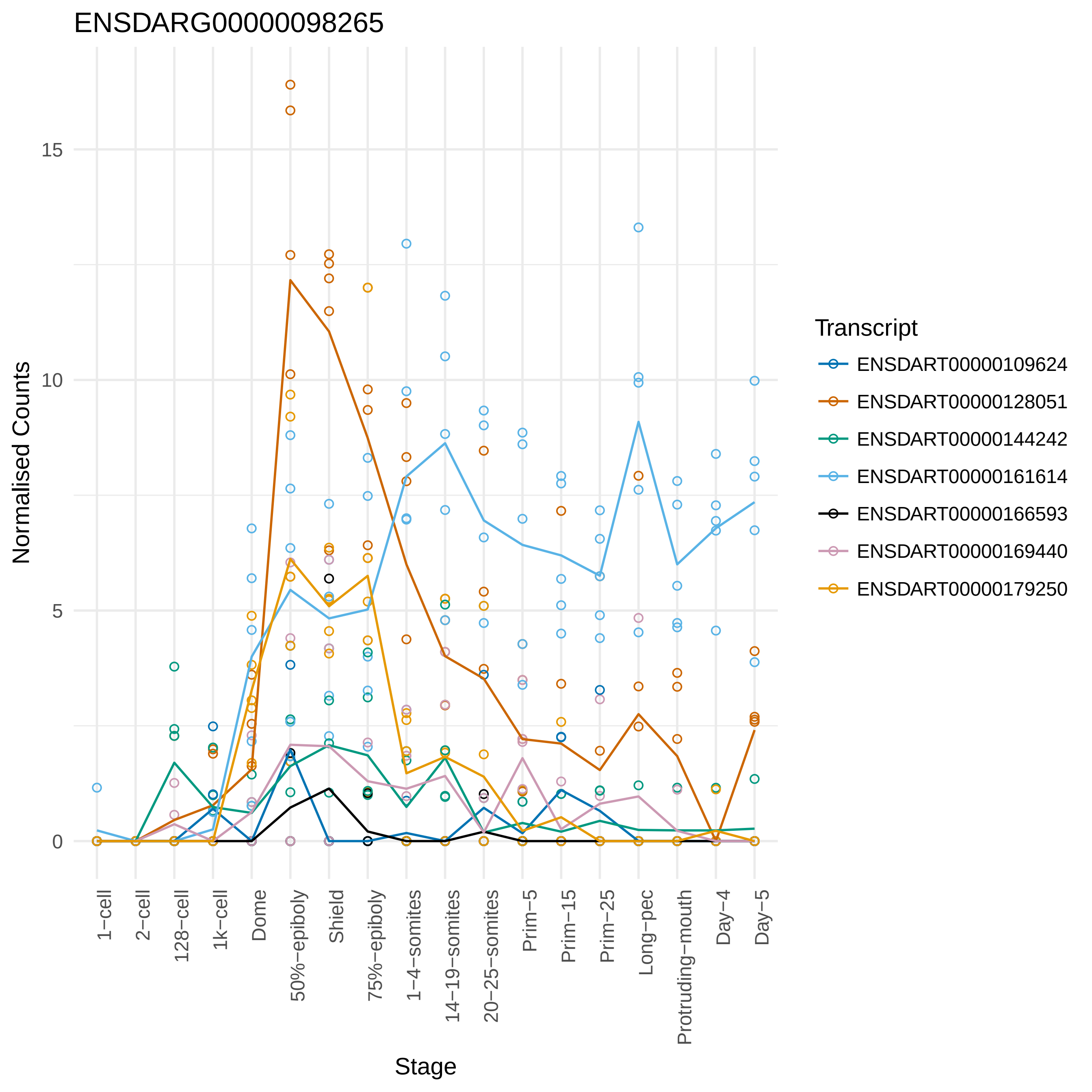

ENSDARG00000098265
Normalised Counts
15
10
5
0
1−cell
2−cell
128−cell
1k−cell
Dome
50%−epiboly
Shield
75%−epiboly
1−4−somites
14−19−somites
20−25−somites
Prim−5
Prim−15
Prim−25
Long−pec
Protruding−mouth
Day−4
Day−5
Stage
Transcript
ENSDART00000109624
ENSDART00000128051
ENSDART00000144242
ENSDART00000161614
ENSDART00000166593
ENSDART00000169440
ENSDART00000179250

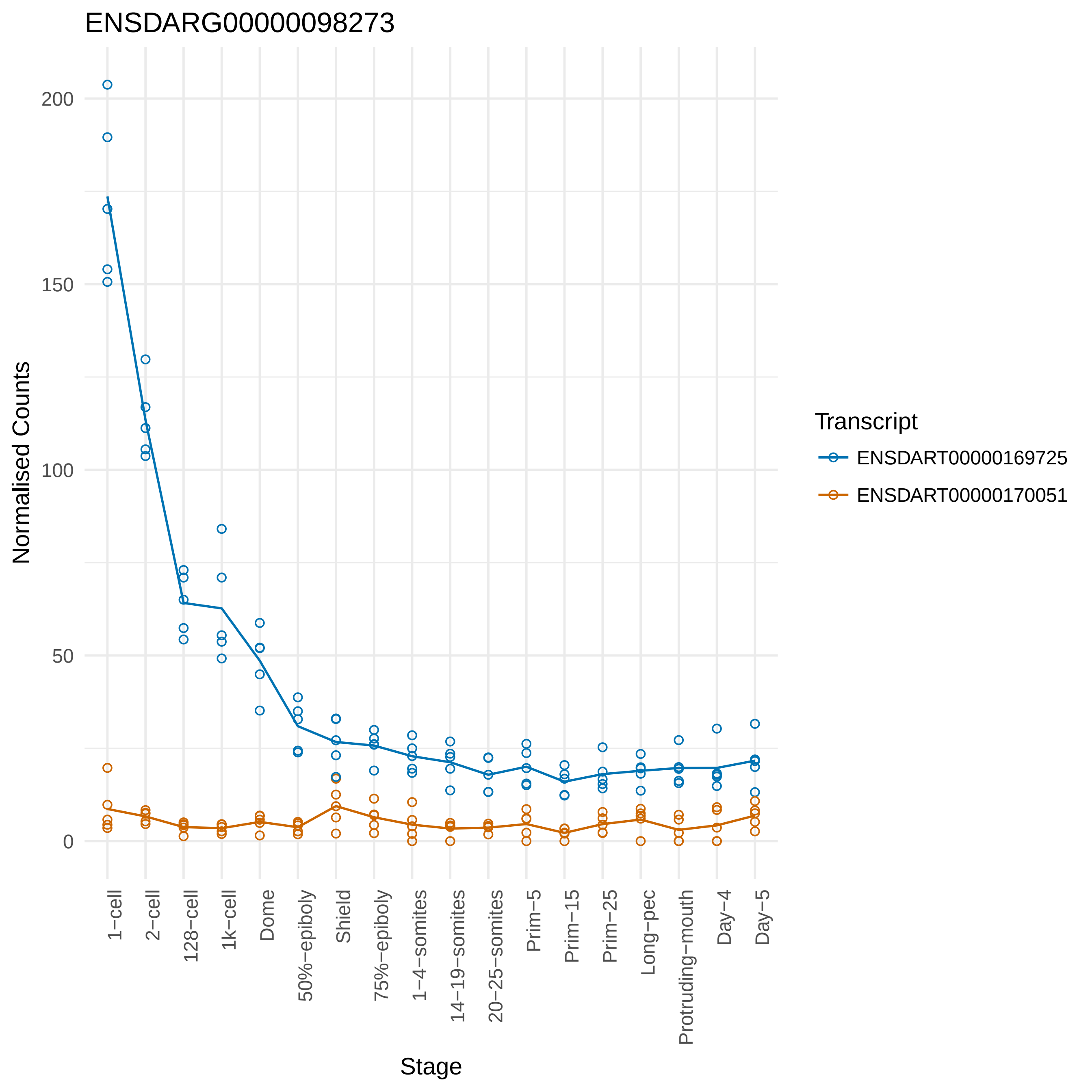

ENSDARG00000098273
Normalised Counts
200
150
100
50
0
1−cell
2−cell
128−cell
1k−cell
Dome
50%−epiboly
Shield
75%−epiboly
1−4−somites
14−19−somites
20−25−somites
Prim−5
Prim−15
Prim−25
Long−pec
Protruding−mouth
Day−4
Day−5
Stage
Transcript
ENSDART00000169725
ENSDART00000170051

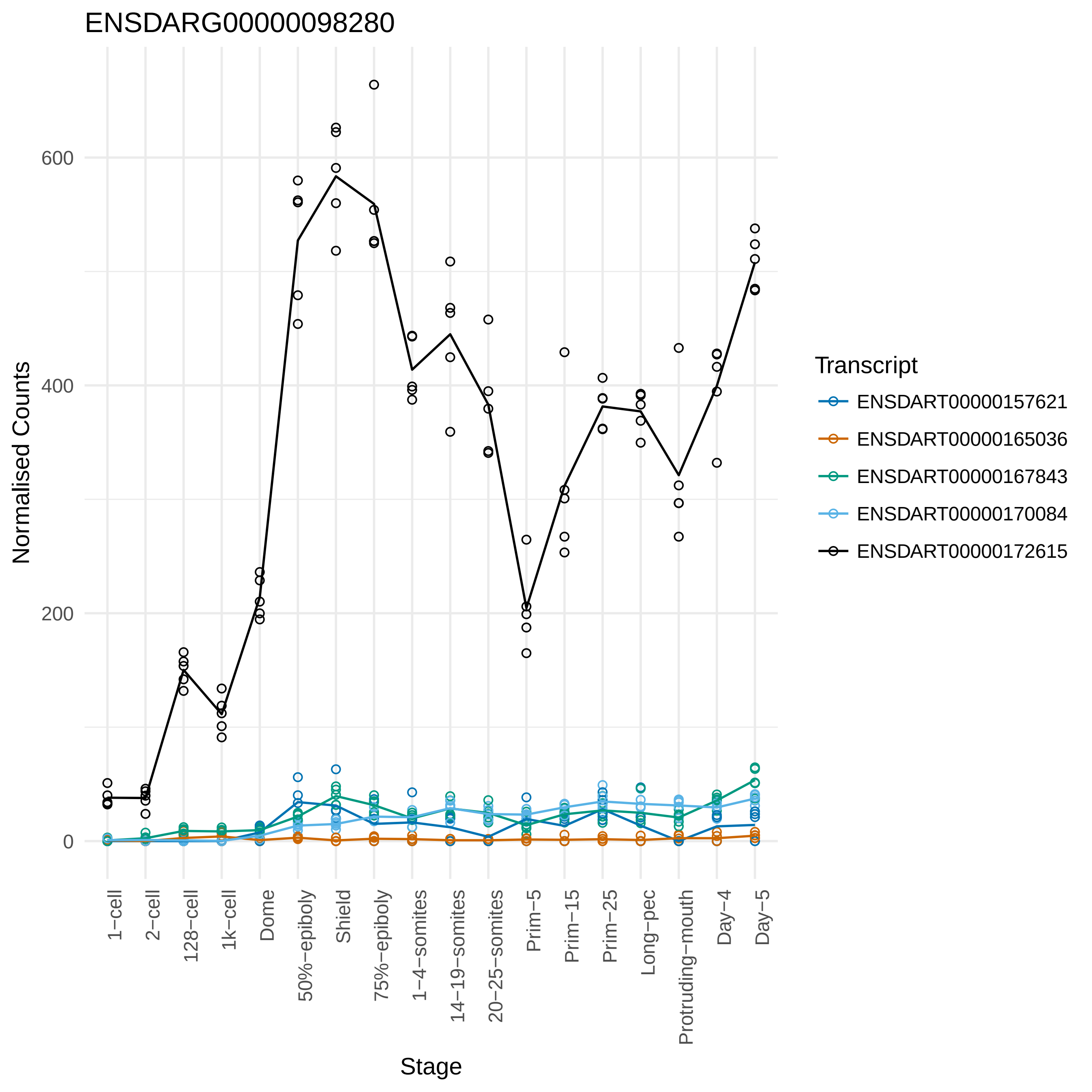
ENSDARG00000098280
Normalised Counts
600
400
200
0
Stage
1−cell
2−cell
128−cell
1k−cell
Dome
50%−epiboly
Shield
75%−epiboly
1−4−somites
14−19−somites
20−25−somites
Prim−5
Prim−15
Prim−25
Long−pec
Protruding−mouth
Day−4
Day−5
Transcript
ENSDART00000157621
ENSDART00000165036
ENSDART00000167843
ENSDART00000170084
ENSDART00000172615

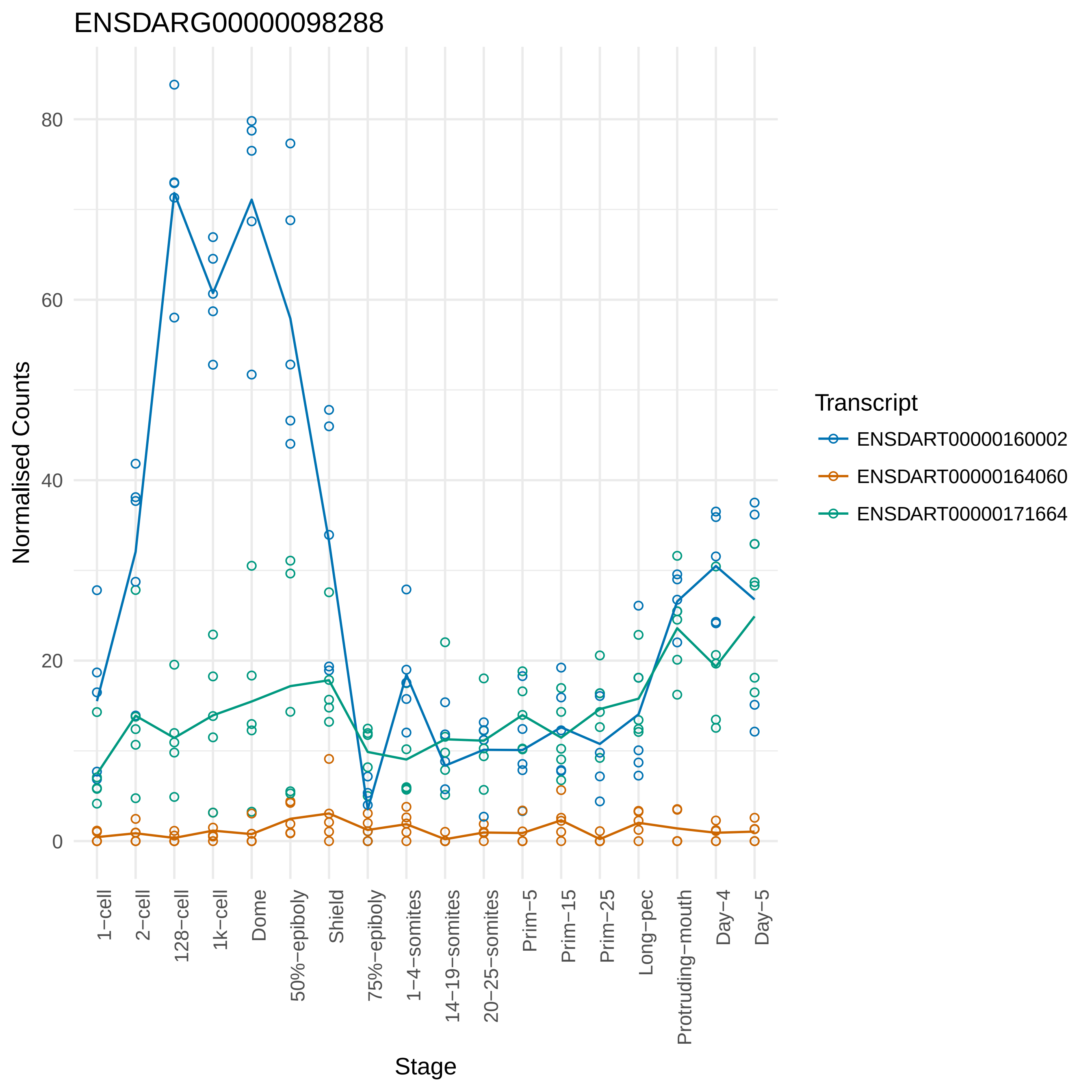

ENSDARG00000098288
Normalised Counts
0
20
40
60
80
1−cell
2−cell
128−cell
1k−cell
Dome
50%−epiboly
Shield
75%−epiboly
1−4−somites
14−19−somites
20−25−somites
Prim−5
Prim−15
Prim−25
Long−pec
Protruding−mouth
Day−4
Day−5
Stage
Transcript
ENSDART00000160002
ENSDART00000164060
ENSDART00000171664

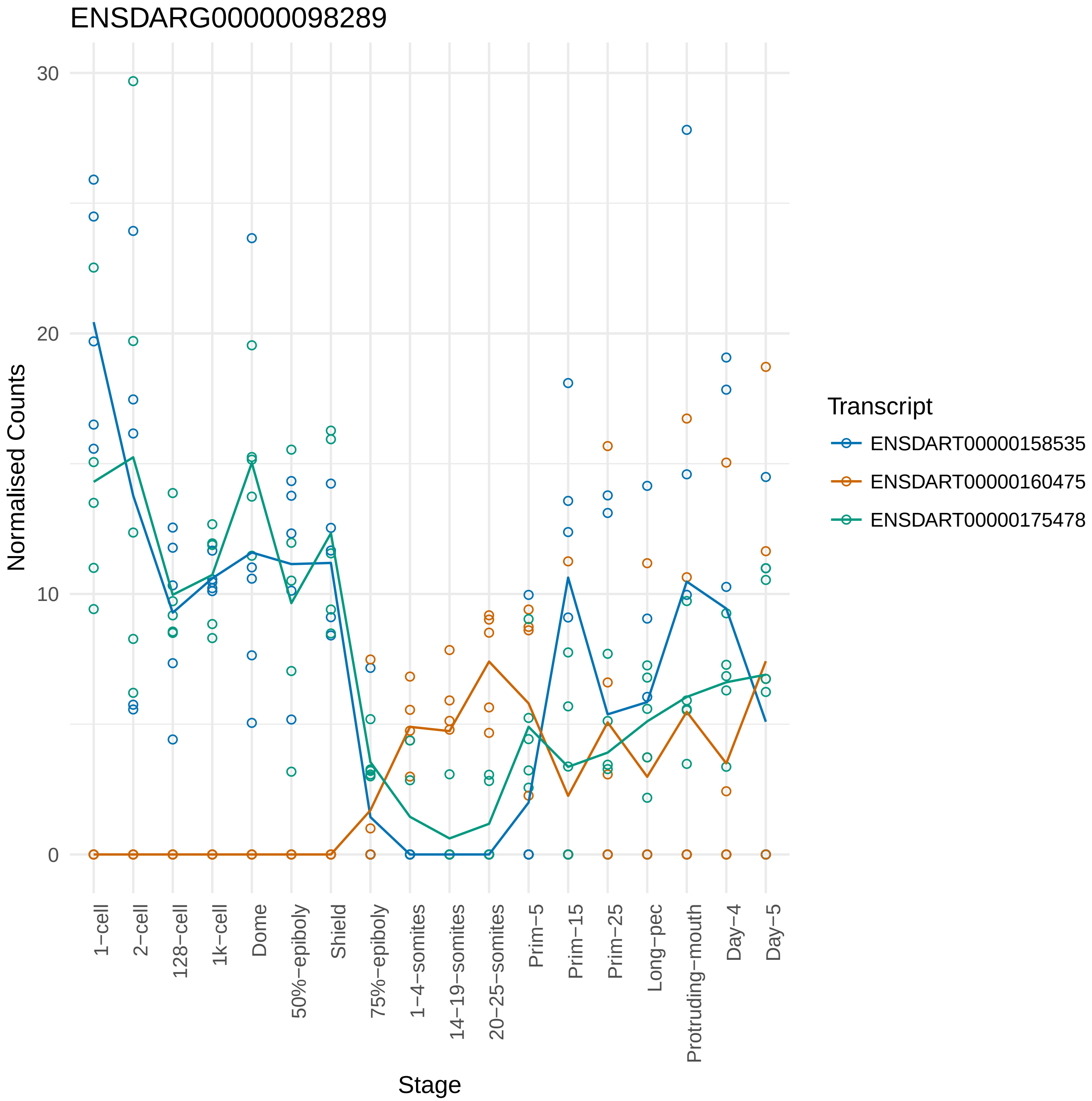
ENSDARG00000098289
Normalised Counts
0
10
20
30
1−cell
2−cell
128−cell
1k−cell
Dome
50%−epiboly
Shield
75%−epiboly
1−4−somites
14−19−somites
20−25−somites
Prim−5
Prim−15
Prim−25
Long−pec
Protruding−mouth
Day−4
Day−5
Stage
Transcript
ENSDART00000158535
ENSDART00000160475
ENSDART00000175478

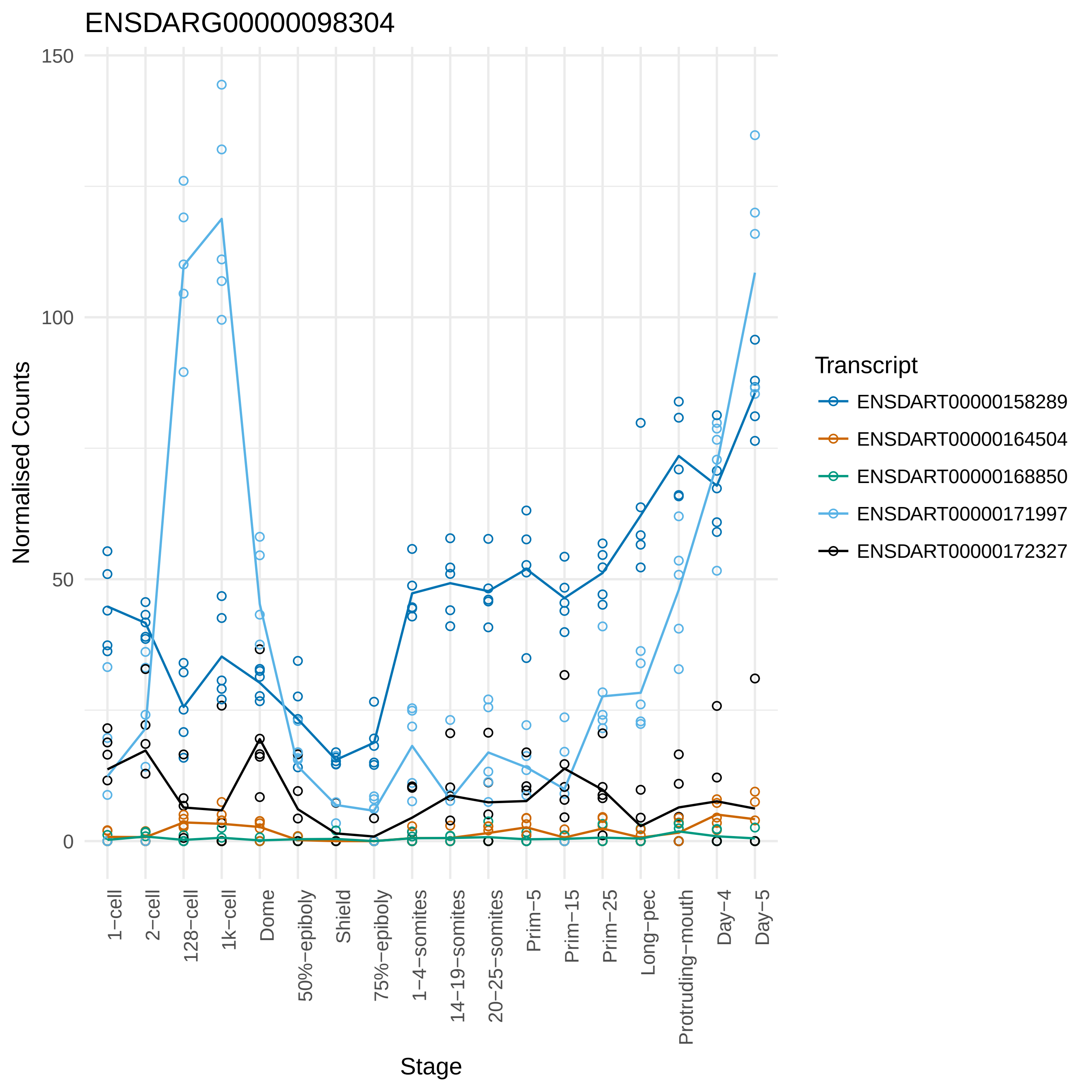
ENSDARG00000098304
150
100
50
0
Normalised Counts
1−cell
2−cell
128−cell
1k−cell
Dome
50%−epiboly
Shield
75%−epiboly
1−4−somites
14−19−somites
20−25−somites
Prim−5
Prim−15
Prim−25
Long−pec
Protruding−mouth
Day−4
Day−5
Stage
Transcript
ENSDART00000158289
ENSDART00000164504
ENSDART00000168850
ENSDART00000171997
ENSDART00000172327

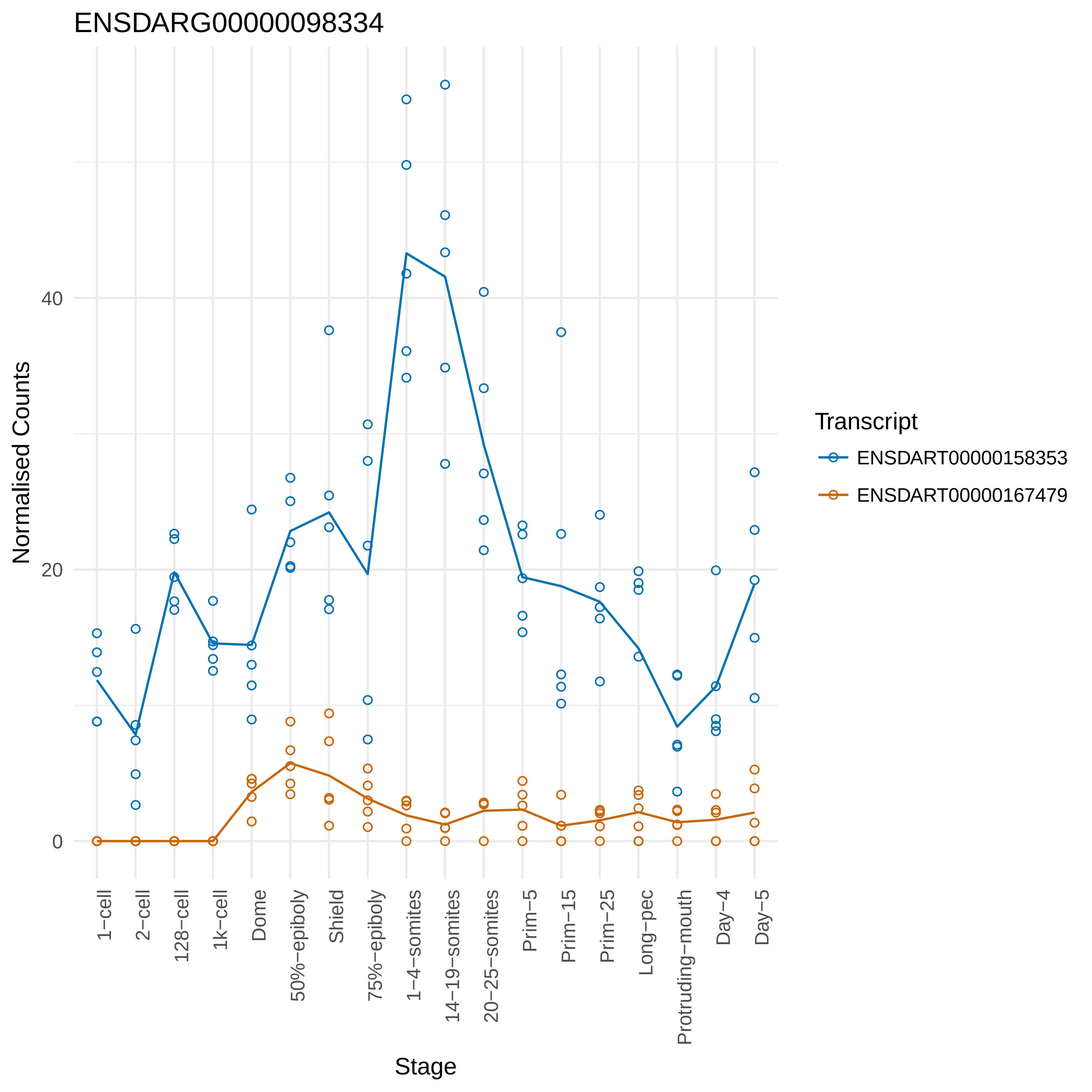
ENSDARG00000098334
Normalised Counts
0
20
40
Stage
1−cell
2−cell
128−cell
1k−cell
Dome
50%−epiboly
Shield
75%−epiboly
1−4−somites
14−19−somites
20−25−somites
Prim−5
Prim−15
Prim−25
Long−pec
Protruding−mouth
Day−4
Day−5
Transcript
ENSDART00000158353
ENSDART00000167479

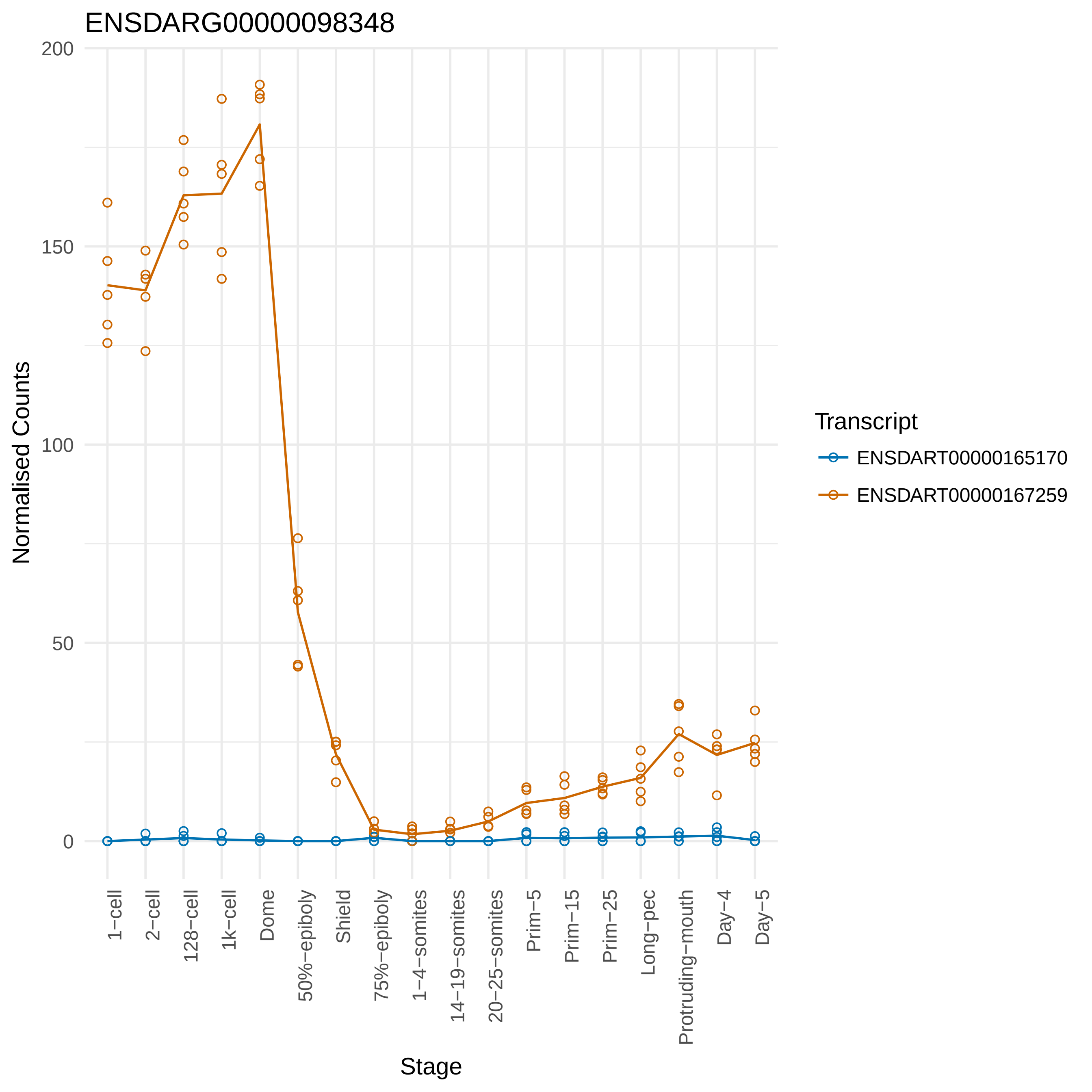

ENSDARG00000098348
Normalised Counts
200
150
100
50
0
1−cell
2−cell
128−cell
1k−cell
Dome
50%−epiboly
Shield
75%−epiboly
1−4−somites
14−19−somites
20−25−somites
Prim−5
Prim−15
Prim−25
Long−pec
Protruding−mouth
Day−4
Day−5
Stage
Transcript
ENSDART00000165170
ENSDART00000167259

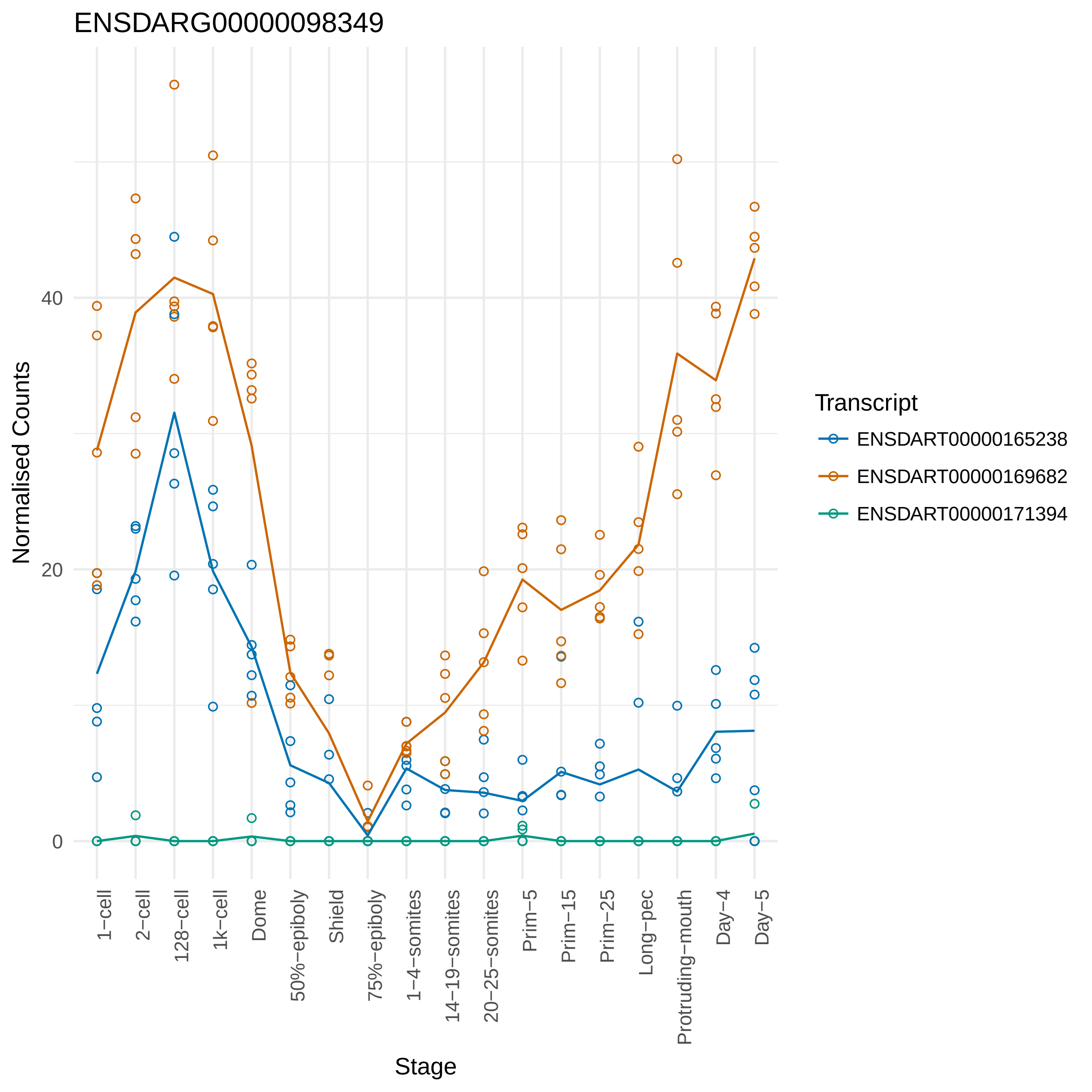
ENSDARG00000098349
Normalised Counts
0
20
40
Stage
1−cell
2−cell
128−cell
1k−cell
Dome
50%−epiboly
Shield
75%−epiboly
1−4−somites
14−19−somites
20−25−somites
Prim−5
Prim−15
Prim−25
Long−pec
Protruding−mouth
Day−4
Day−5
Transcript
ENSDART00000165238
ENSDART00000169682
ENSDART00000171394

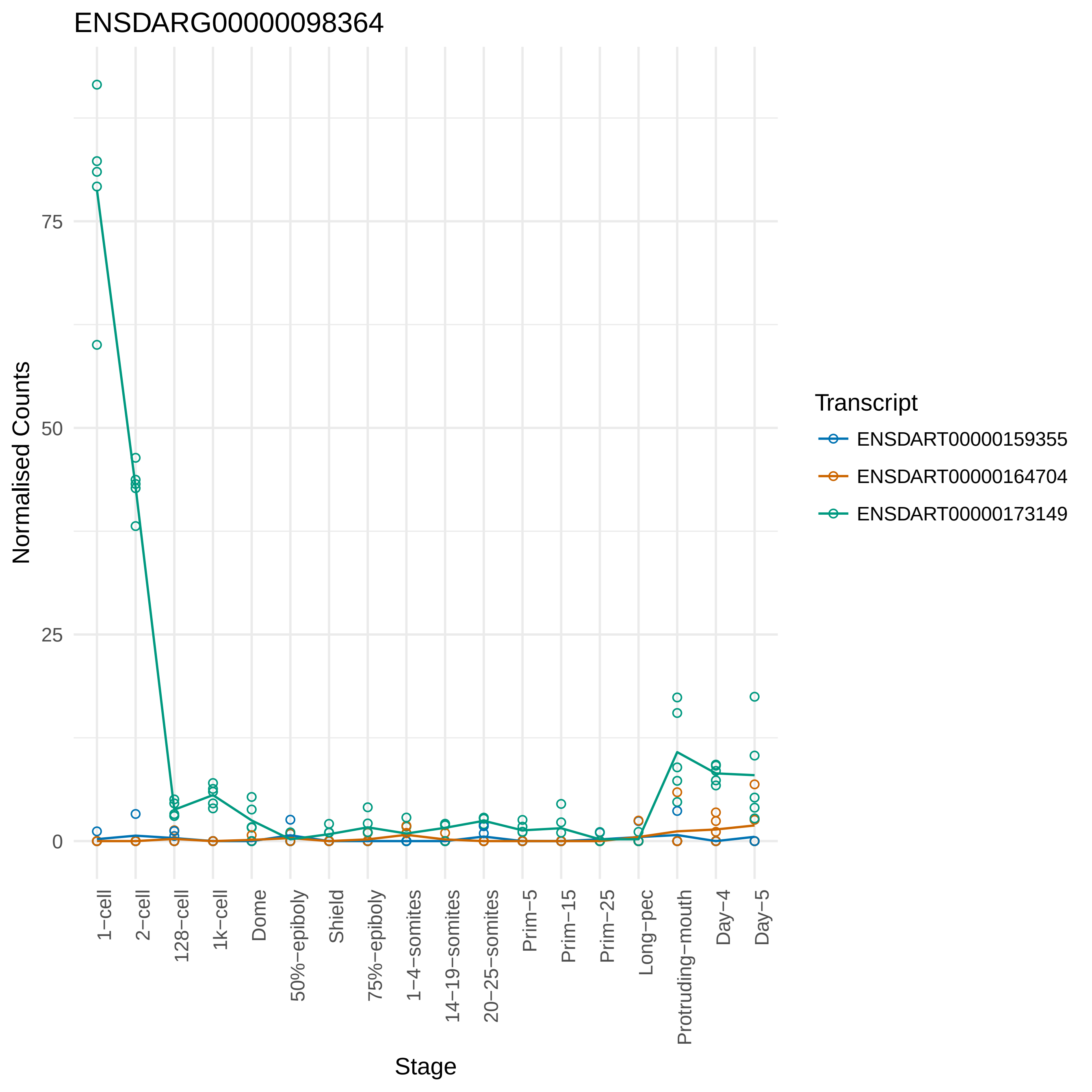
ENSDARG00000098364
Normalised Counts
0
25
50
75
Stage
1−cell
2−cell
128−cell
1k−cell
Dome
50%−epiboly
Shield
75%−epiboly
1−4−somites
14−19−somites
20−25−somites
Prim−5
Prim−15
Prim−25
Long−pec
Protruding−mouth
Day−4
Day−5
Transcript
ENSDART00000159355
ENSDART00000164704
ENSDART00000173149

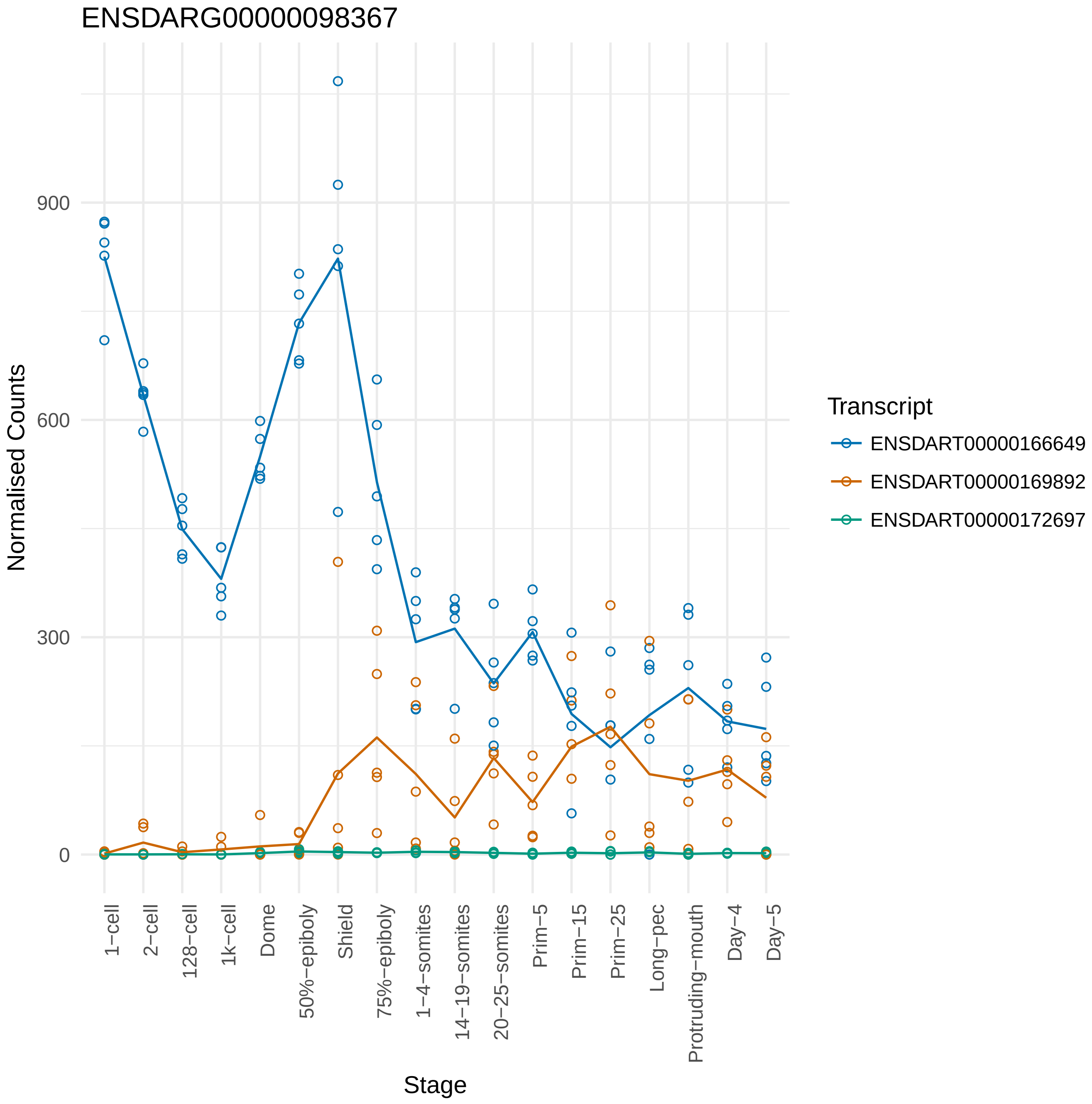
ENSDARG00000098367
Normalised Counts
0
300
600
900
Stage
1−cell
2−cell
128−cell
1k−cell
Dome
50%−epiboly
Shield
75%−epiboly
1−4−somites
14−19−somites
20−25−somites
Prim−5
Prim−15
Prim−25
Long−pec
Protruding−mouth
Day−4
Day−5
Transcript
ENSDART00000166649
ENSDART00000169892
ENSDART00000172697

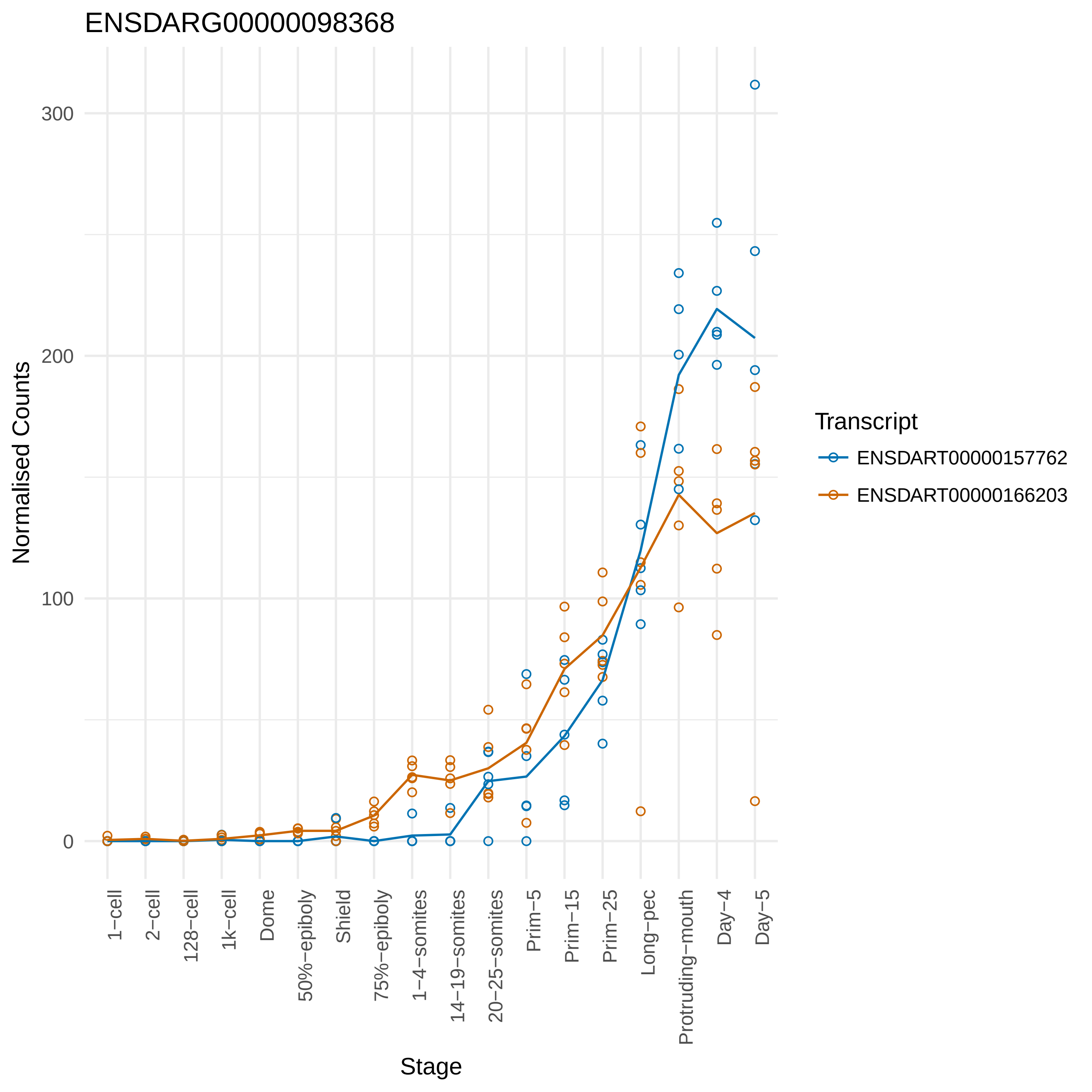

ENSDARG00000098368
Normalised Counts
300
200
100
0
1−cell
2−cell
128−cell
1k−cell
Dome
50%−epiboly
Shield
75%−epiboly
1−4−somites
14−19−somites
20−25−somites
Prim−5
Prim−15
Prim−25
Long−pec
Protruding−mouth
Day−4
Day−5
Stage
Transcript
ENSDART00000157762
ENSDART00000166203

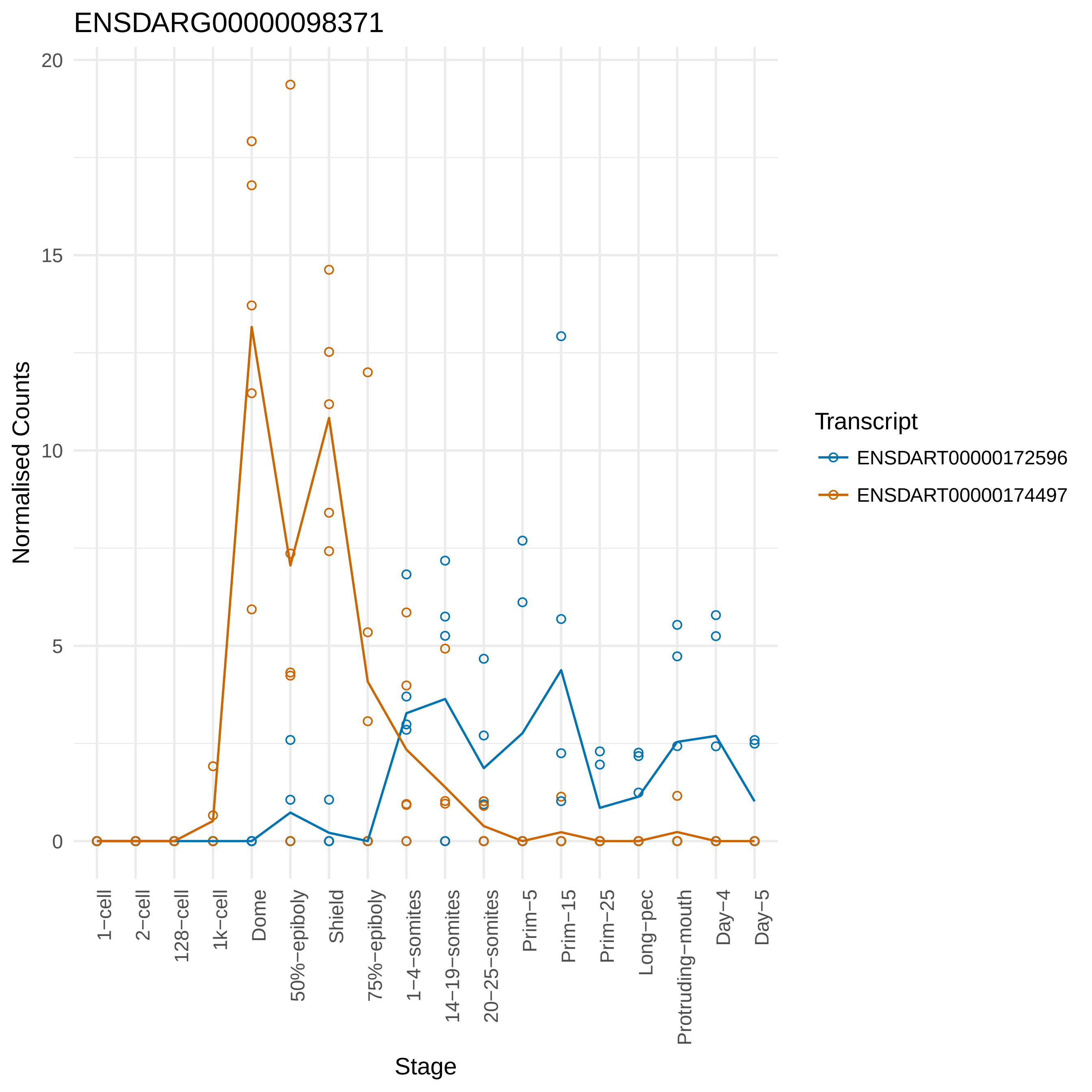
ENSDARG00000098371
Normalised Counts
20
15
10
5
0
1−cell
2−cell
128−cell
1k−cell
Dome
50%−epiboly
Shield
75%−epiboly
1−4−somites
14−19−somites
20−25−somites
Prim−5
Prim−15
Prim−25
Long−pec
Protruding−mouth
Day−4
Day−5
Stage
Transcript
ENSDART00000172596
ENSDART00000174497

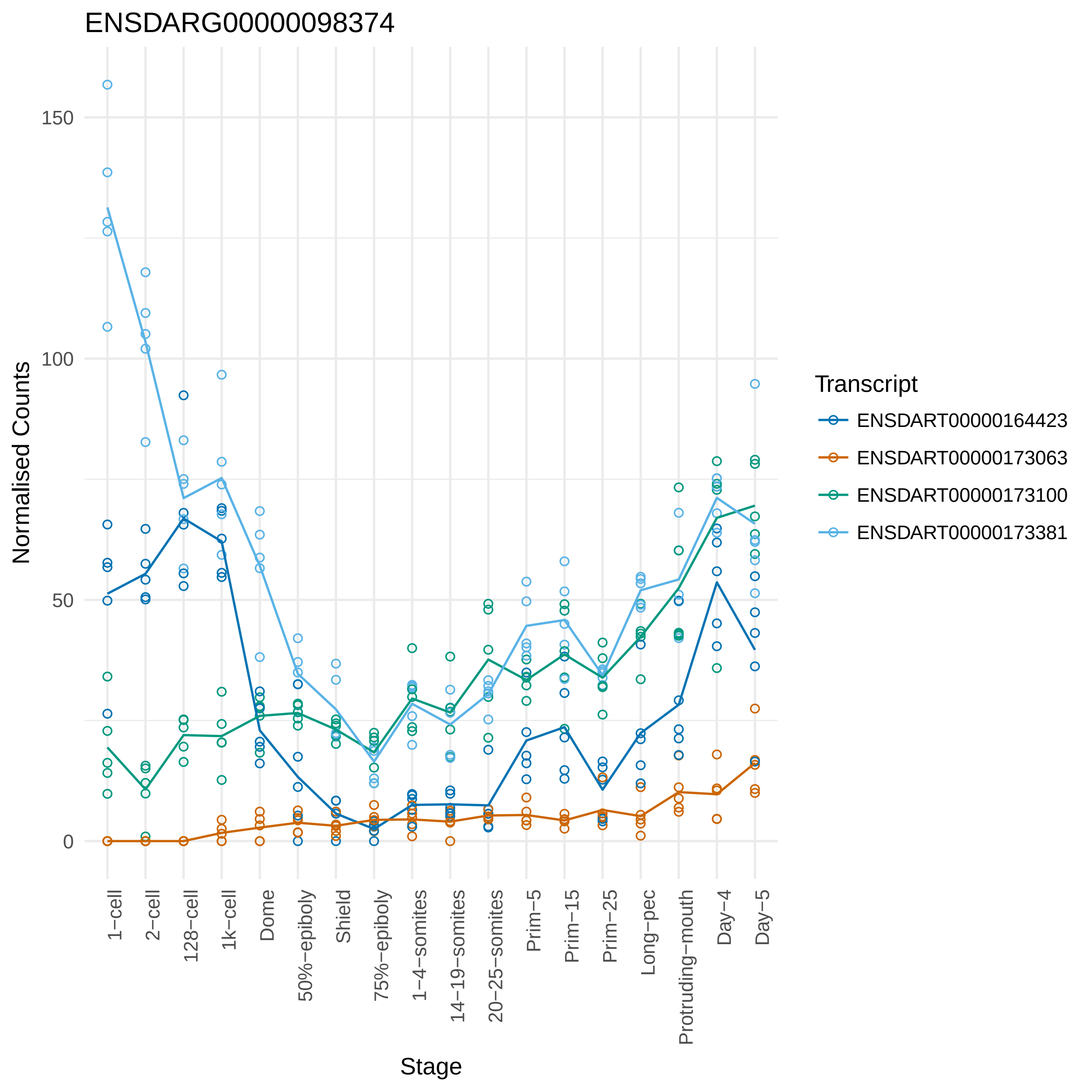

ENSDARG00000098374
Normalised Counts
150
100
50
0
1−cell
2−cell
128−cell
1k−cell
Dome
50%−epiboly
Shield
75%−epiboly
1−4−somites
14−19−somites
20−25−somites
Prim−5
Prim−15
Prim−25
Long−pec
Protruding−mouth
Day−4
Day−5
Stage
Transcript
ENSDART00000164423
ENSDART00000173063
ENSDART00000173100
ENSDART00000173381

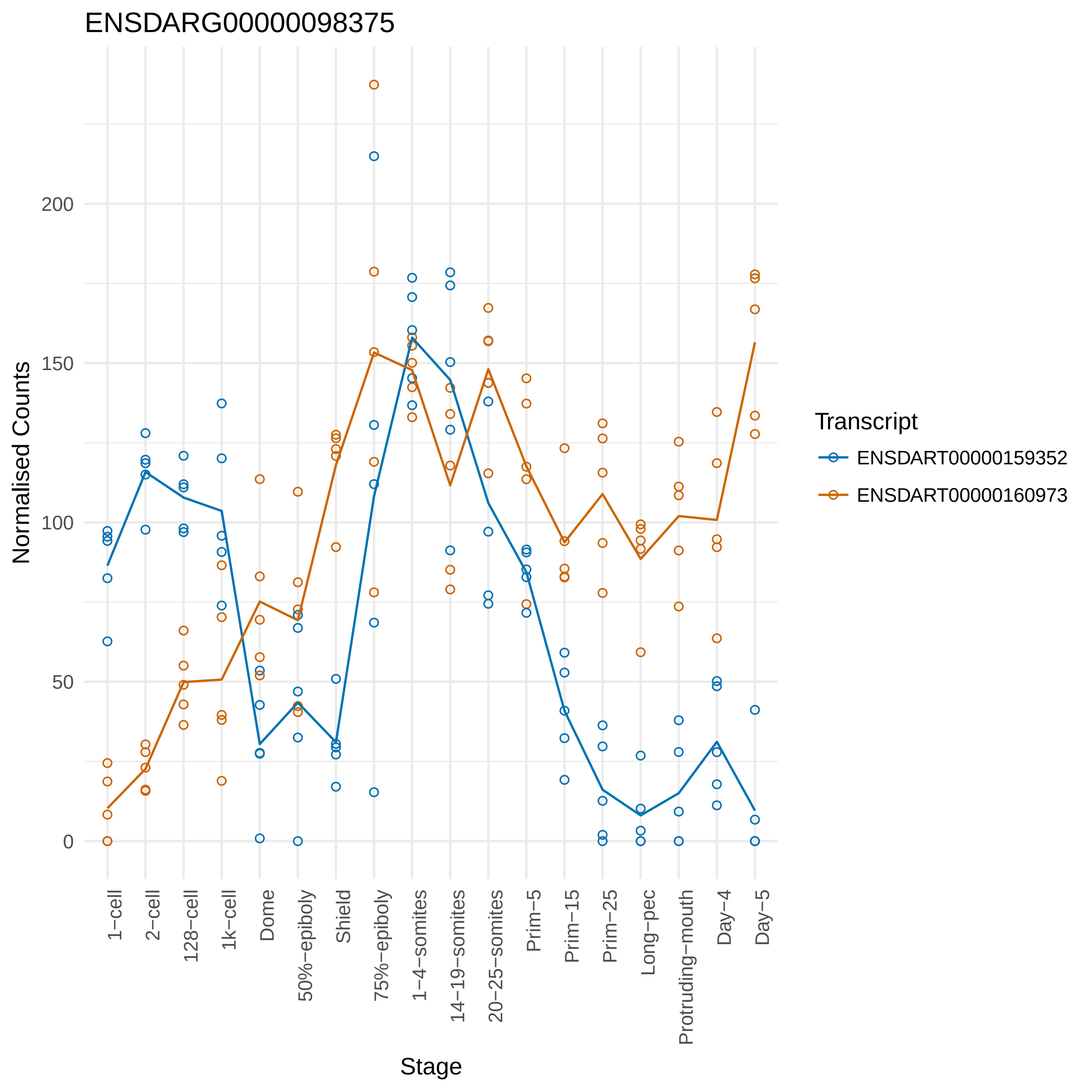
ENSDARG00000098375
Normalised Counts
0
50
100
150
200
Stage
1−cell
2−cell
128−cell
1k−cell
Dome
50%−epiboly
Shield
75%−epiboly
1−4−somites
14−19−somites
20−25−somites
Prim−5
Prim−15
Prim−25
Long−pec
Protruding−mouth
Day−4
Day−5
Transcript
ENSDART00000159352
ENSDART00000160973

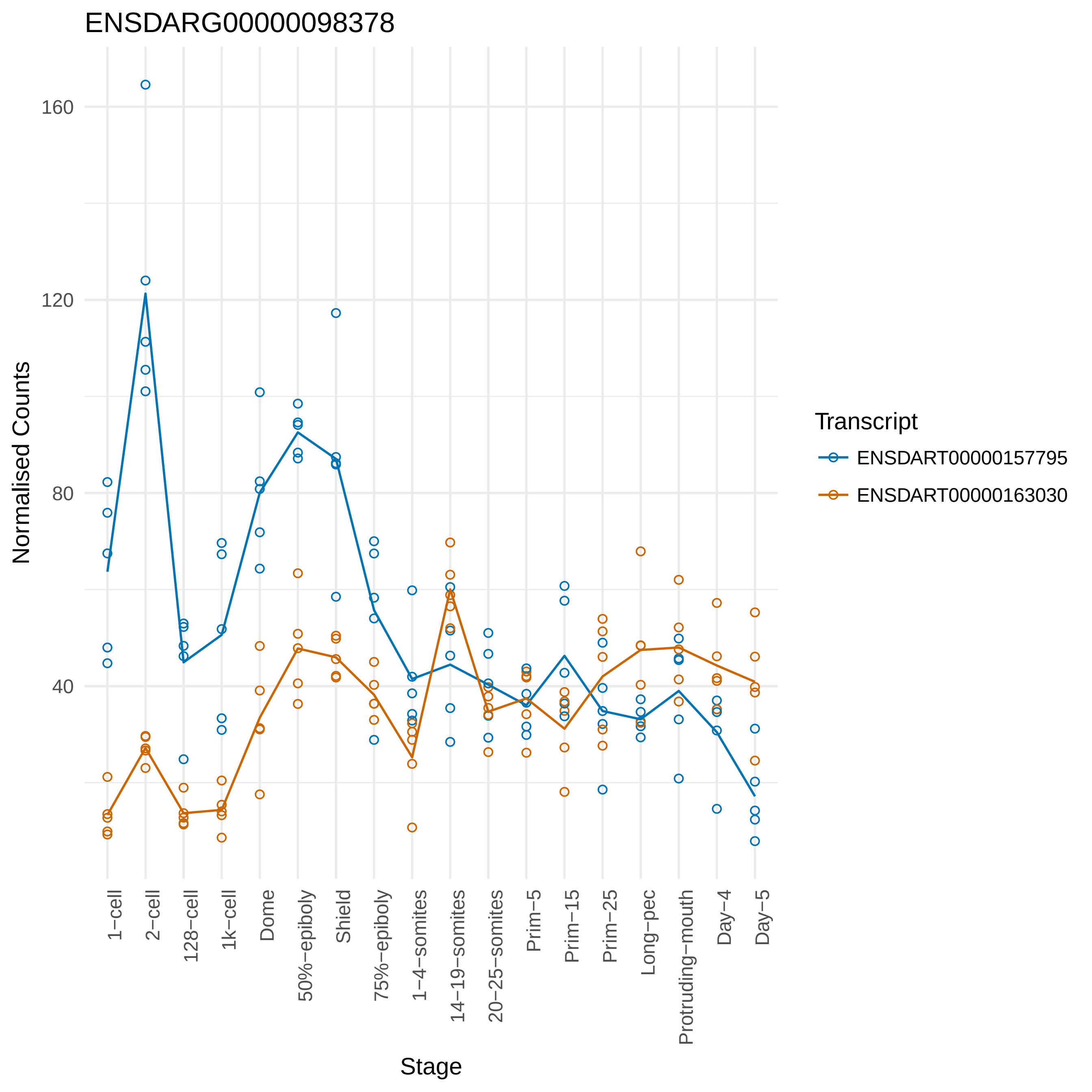

ENSDARG00000098378
Normalised Counts
160
120
80
40
Stage
1−cell
2−cell
128−cell
1k−cell
Dome
50%−epiboly
Shield
75%−epiboly
1−4−somites
14−19−somites
20−25−somites
Prim−5
Prim−15
Prim−25
Long−pec
Protruding−mouth
Day−4
Day−5
Transcript
ENSDART00000157795
ENSDART00000163030

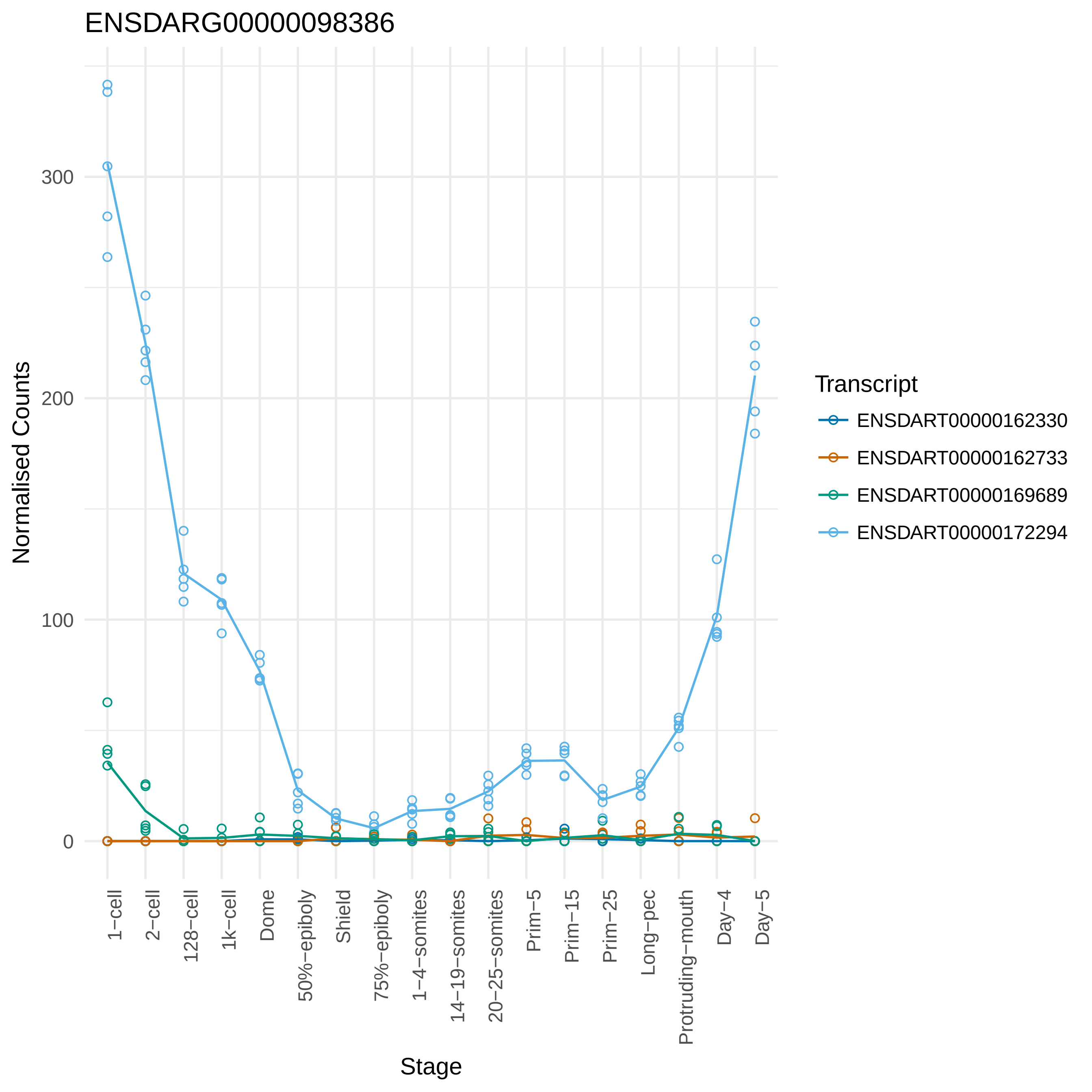

ENSDARG00000098386
Normalised Counts
300
200
100
0
1−cell
2−cell
128−cell
1k−cell
Dome
50%−epiboly
Shield
75%−epiboly
1−4−somites
14−19−somites
20−25−somites
Prim−5
Prim−15
Prim−25
Long−pec
Protruding−mouth
Day−4
Day−5
Stage
Transcript
ENSDART00000162330
ENSDART00000162733
ENSDART00000169689
ENSDART00000172294

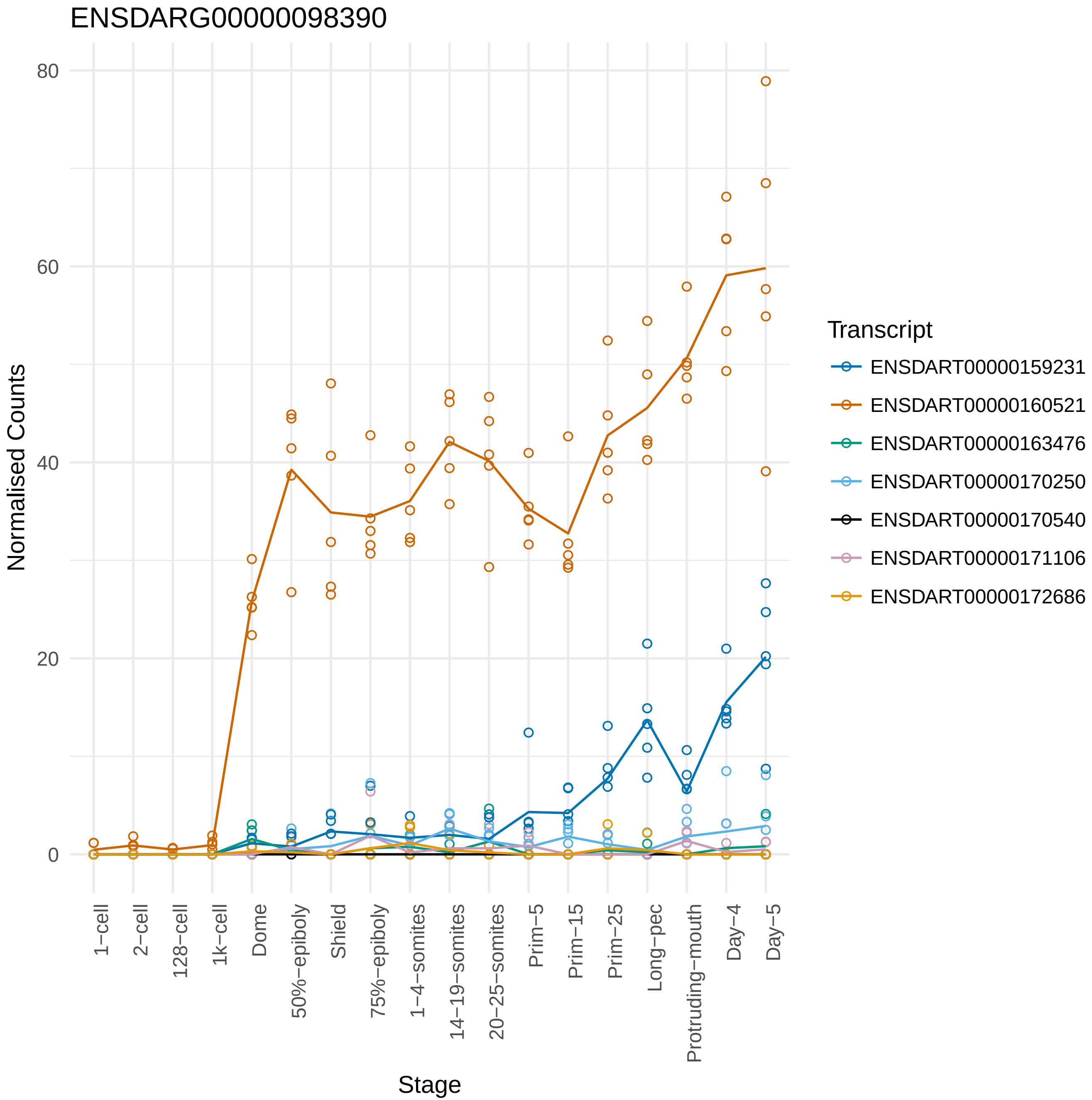
ENSDARG00000098390
Normalised Counts
0
20
40
60
80
Stage
1−cell
2−cell
128−cell
1k−cell
Dome
50%−epiboly
Shield
75%−epiboly
1−4−somites
14−19−somites
20−25−somites
Prim−5
Prim−15
Prim−25
Long−pec
Protruding−mouth
Day−4
Day−5
Transcript
ENSDART00000159231
ENSDART00000160521
ENSDART00000163476
ENSDART00000170250
ENSDART00000170540
ENSDART00000171106
ENSDART00000172686

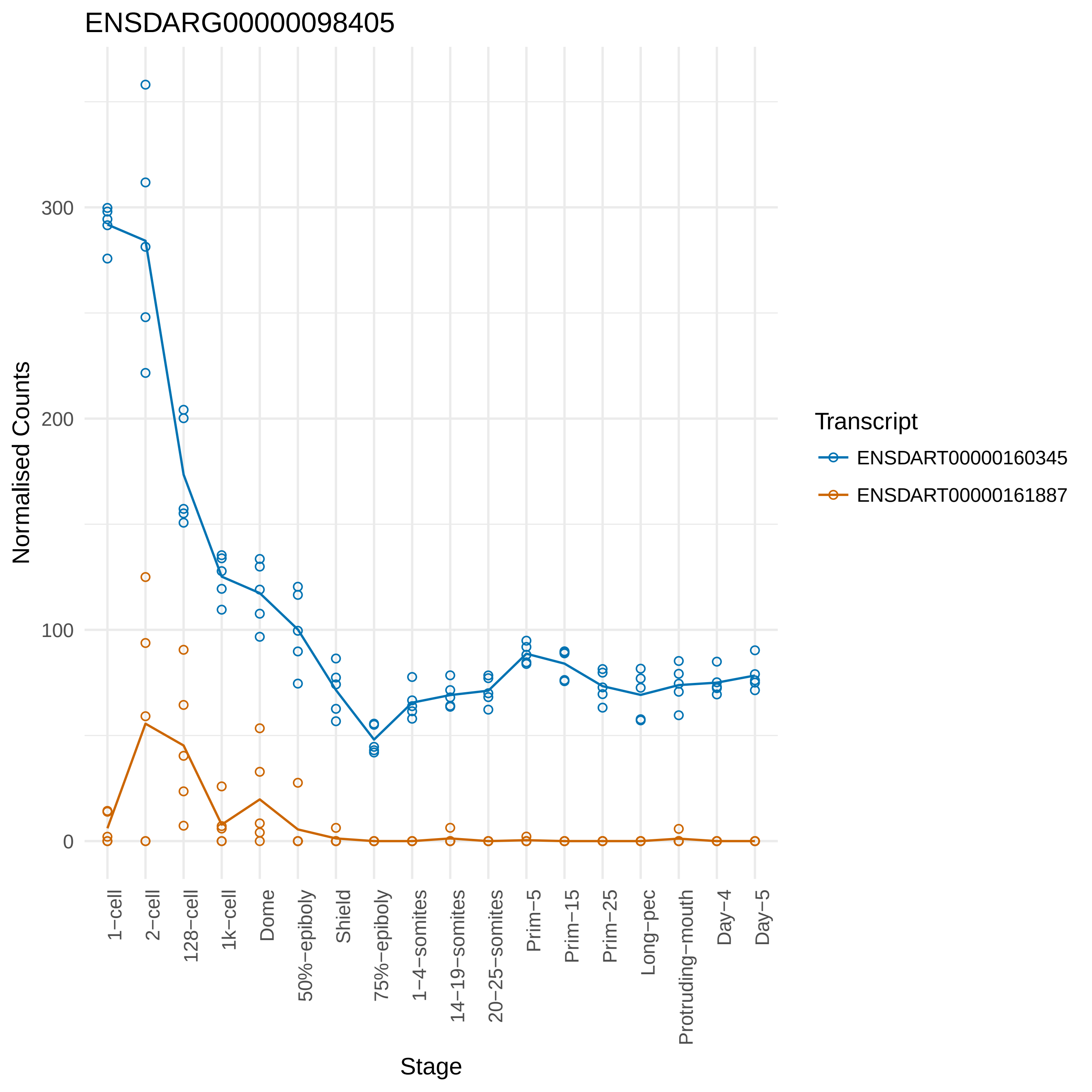

ENSDARG00000098405
Normalised Counts
300
200
100
0
1−cell
2−cell
128−cell
1k−cell
Dome
50%−epiboly
Shield
75%−epiboly
1−4−somites
14−19−somites
20−25−somites
Prim−5
Prim−15
Prim−25
Long−pec
Protruding−mouth
Day−4
Day−5
Stage
Transcript
ENSDART00000160345
ENSDART00000161887

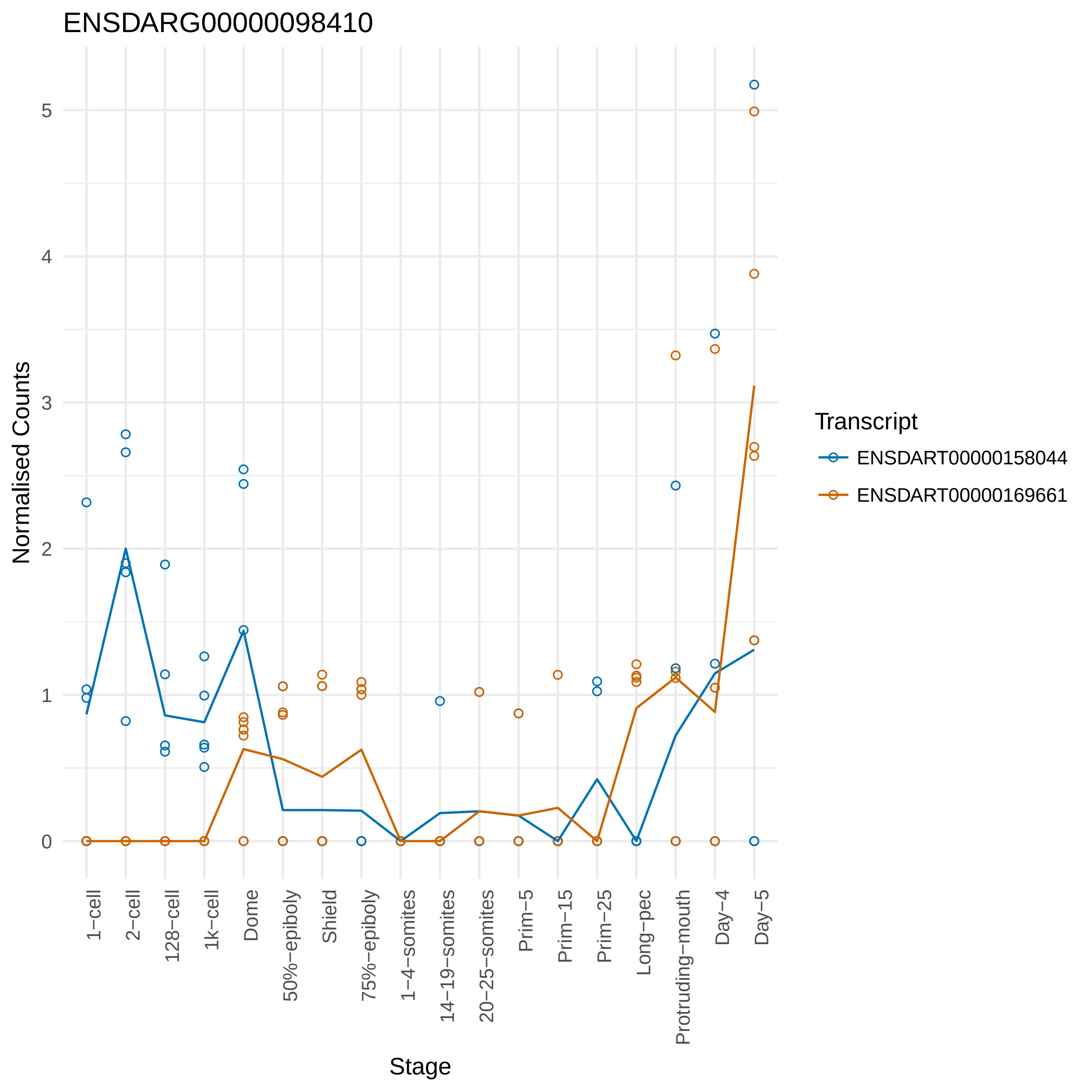

ENSDARG00000098410
Normalised Counts
0
1
2
3
4
5
1−cell
2−cell
128−cell
1k−cell
Dome
50%−epiboly
Shield
75%−epiboly
1−4−somites
14−19−somites
20−25−somites
Prim−5
Prim−15
Prim−25
Long−pec
Protruding−mouth
Day−4
Day−5
Stage
Transcript
ENSDART00000158044
ENSDART00000169661

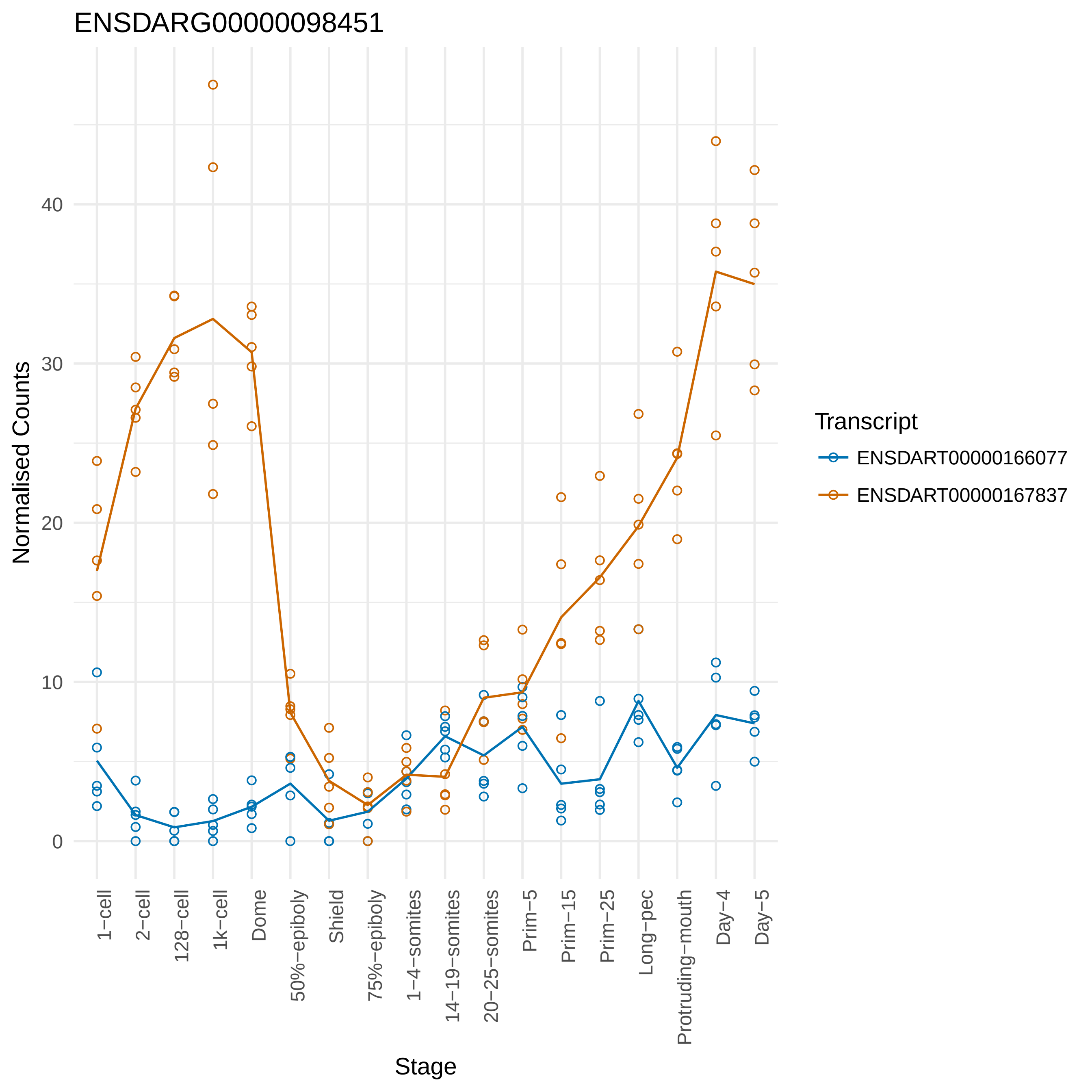

ENSDARG00000098451
Normalised Counts
0
10
20
30
40
1−cell
2−cell
128−cell
1k−cell
Dome
50%−epiboly
Shield
75%−epiboly
1−4−somites
14−19−somites
20−25−somites
Prim−5
Prim−15
Prim−25
Long−pec
Protruding−mouth
Day−4
Day−5
Stage
Transcript
ENSDART00000166077
ENSDART00000167837

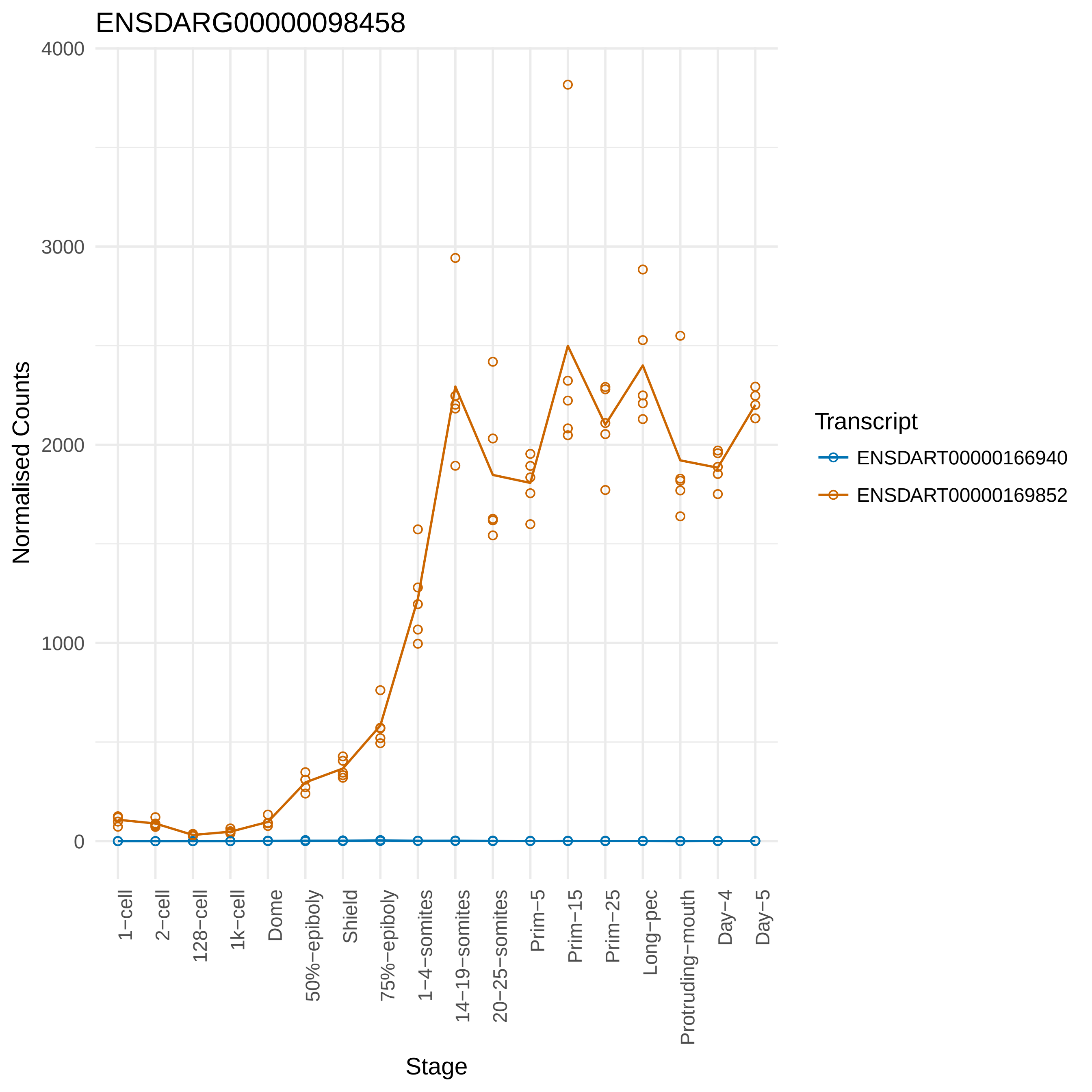
ENSDARG00000098458
Normalised Counts
4000
3000
2000
1000
0
Stage
1−cell
2−cell
128−cell
1k−cell
Dome
50%−epiboly
Shield
75%−epiboly
1−4−somites
14−19−somites
20−25−somites
Prim−5
Prim−15
Prim−25
Long−pec
Protruding−mouth
Day−4
Day−5
Transcript
ENSDART00000166940
ENSDART00000169852

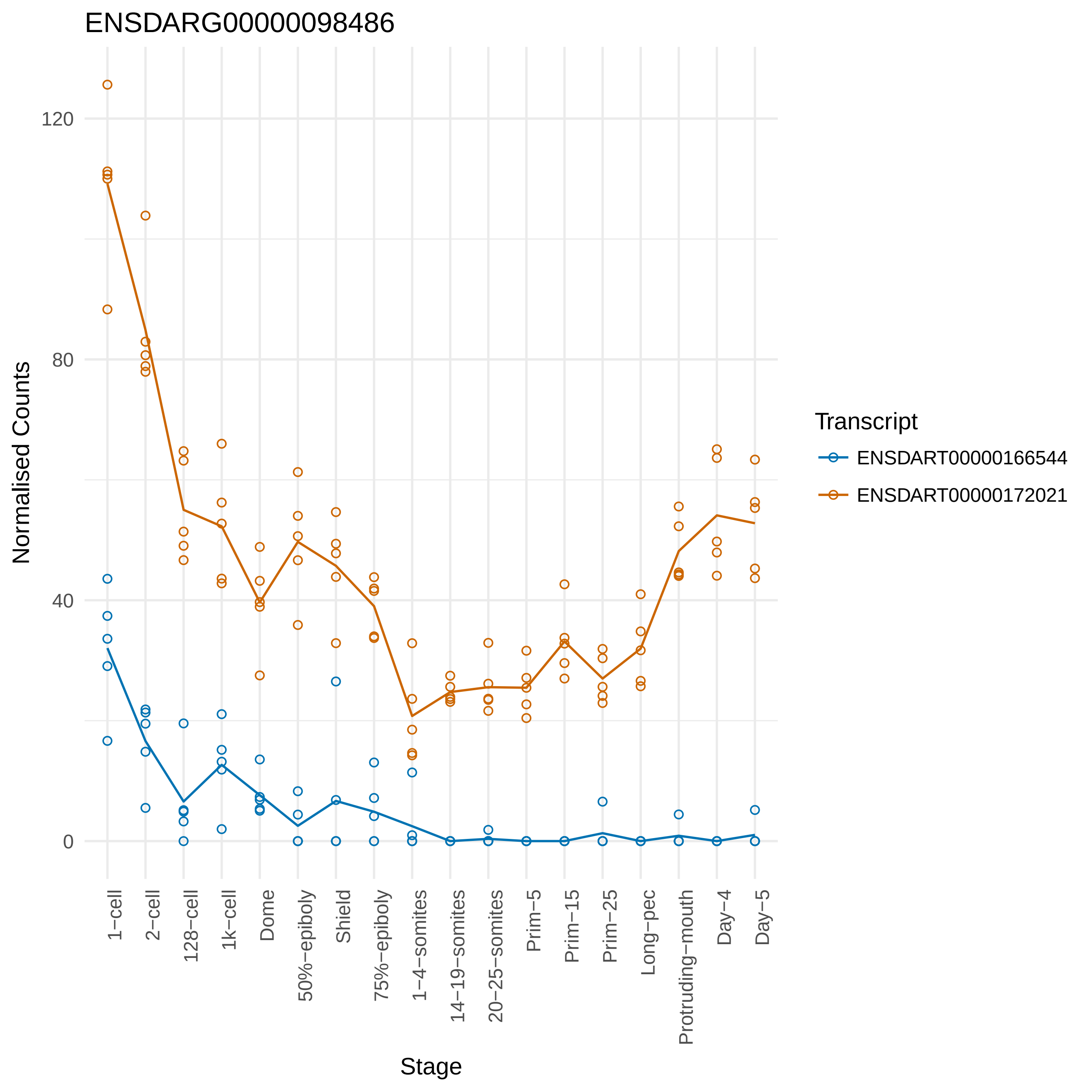
ENSDARG00000098486
Normalised Counts
120
80
40
0
1−cell
2−cell
128−cell
1k−cell
Dome
50%−epiboly
Shield
75%−epiboly
1−4−somites
14−19−somites
20−25−somites
Prim−5
Prim−15
Prim−25
Long−pec
Protruding−mouth
Day−4
Day−5
Stage
Transcript
ENSDART00000166544
ENSDART00000172021

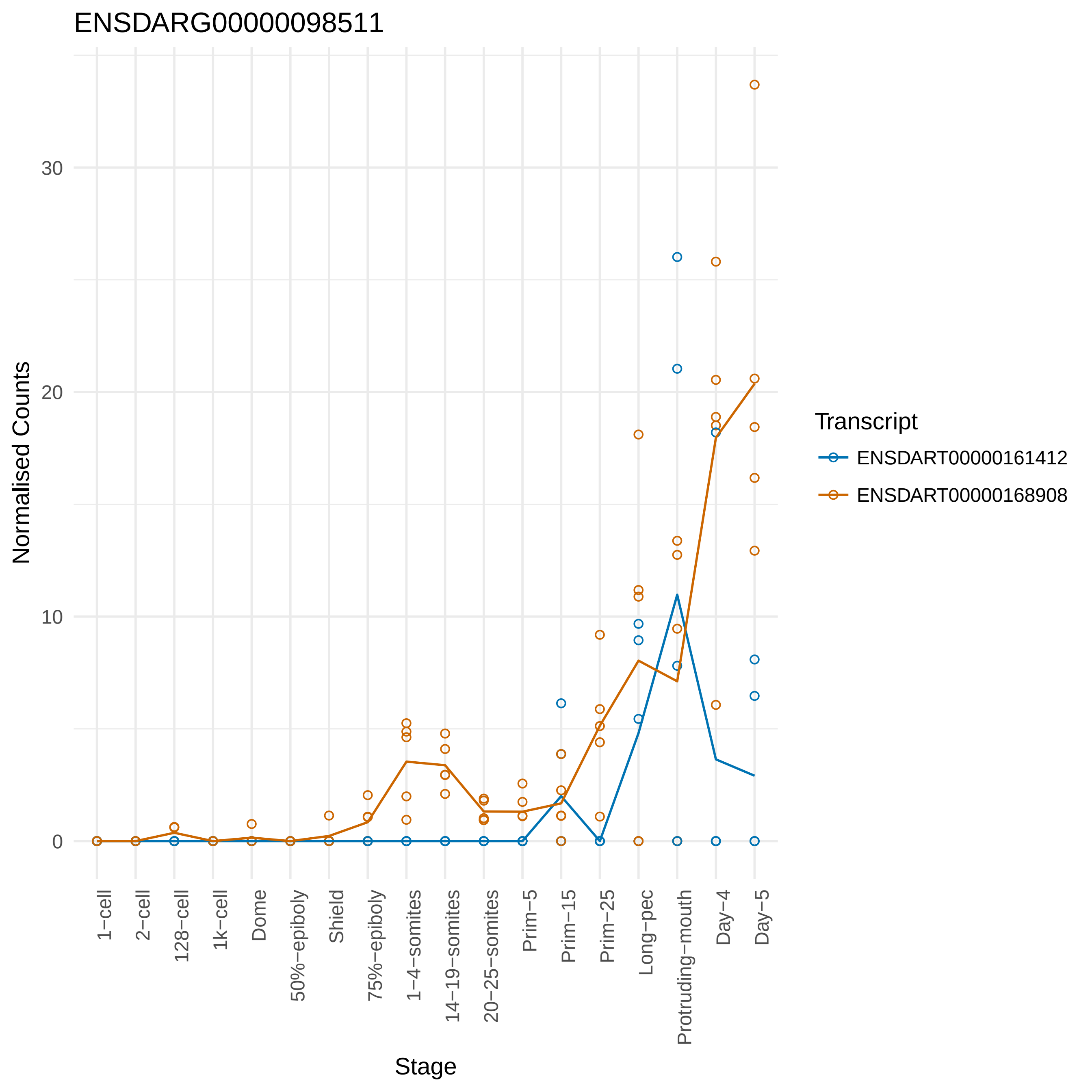

ENSDARG00000098511
Normalised Counts
30
20
10
0
1−cell
2−cell
128−cell
1k−cell
Dome
50%−epiboly
Shield
75%−epiboly
1−4−somites
14−19−somites
20−25−somites
Prim−5
Prim−15
Prim−25
Long−pec
Protruding−mouth
Day−4
Day−5
Stage
Transcript
ENSDART00000161412
ENSDART00000168908

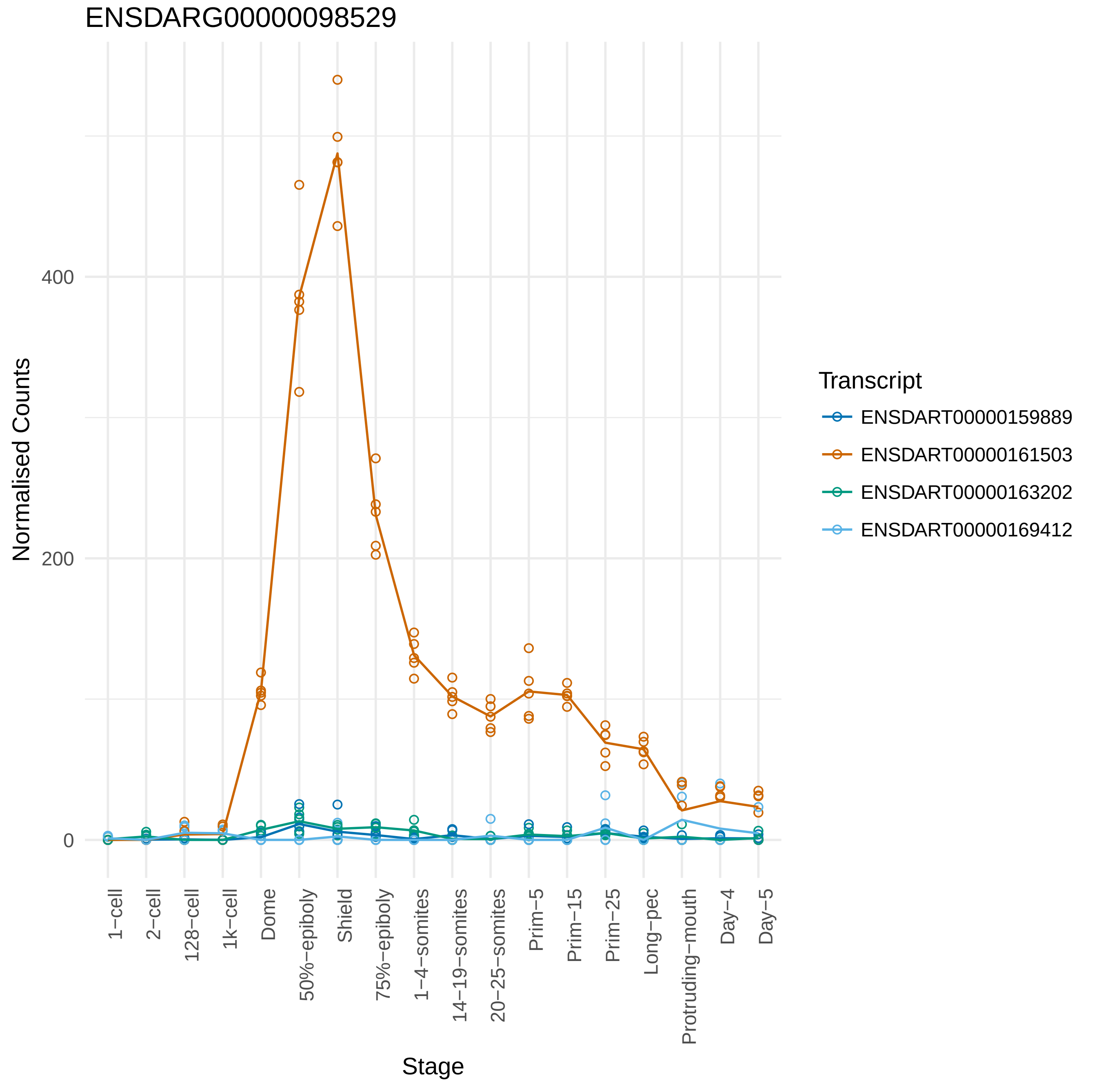

ENSDARG00000098529
Normalised Counts
400
200
0
1−cell
2−cell
128−cell
1k−cell
Dome
50%−epiboly
Shield
75%−epiboly
1−4−somites
14−19−somites
20−25−somites
Prim−5
Prim−15
Prim−25
Long−pec
Protruding−mouth
Day−4
Day−5
Stage
Transcript
ENSDART00000159889
ENSDART00000161503
ENSDART00000163202
ENSDART00000169412

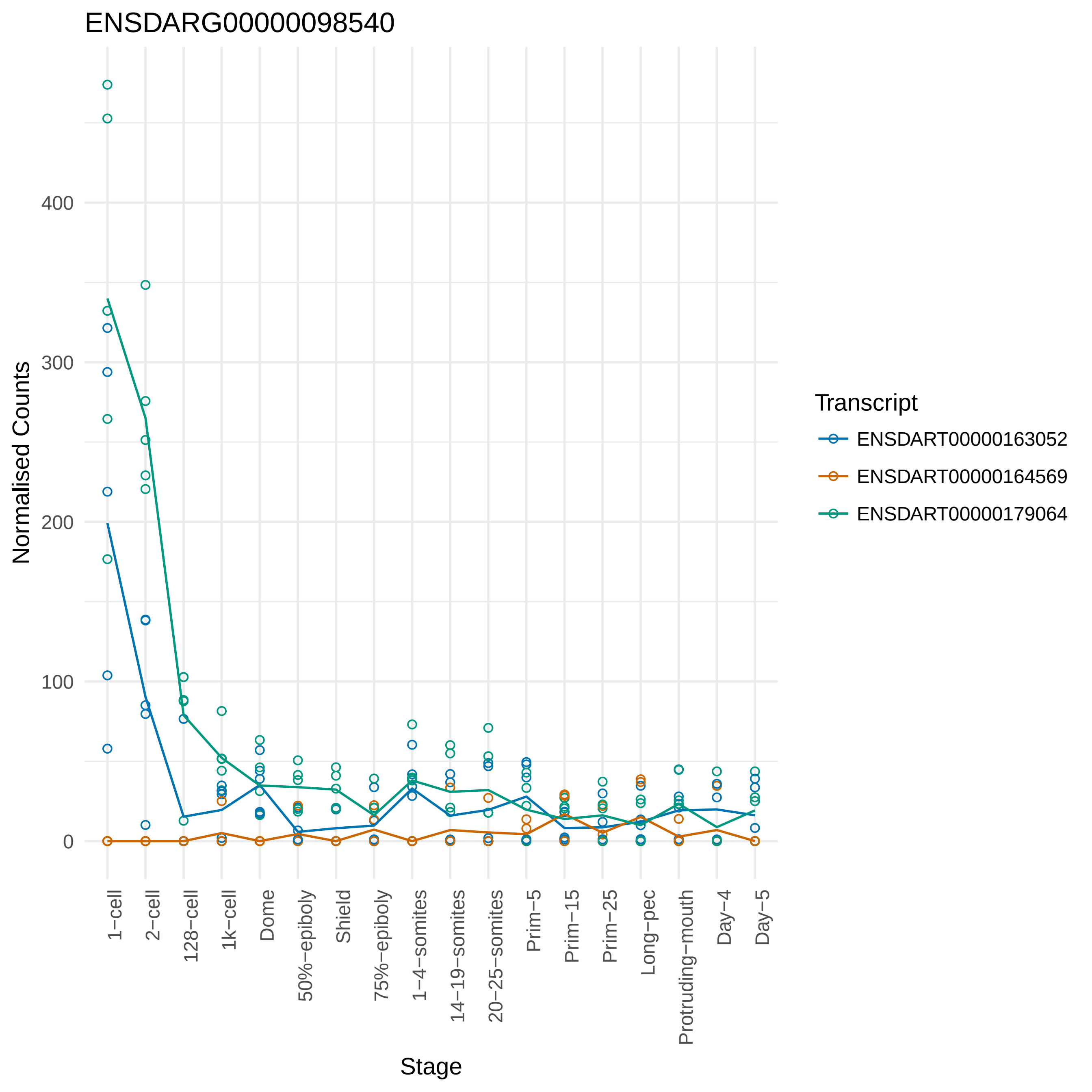

ENSDARG00000098540
Normalised Counts
400
300
200
100
0
1−cell
2−cell
128−cell
1k−cell
Dome
50%−epiboly
Shield
75%−epiboly
1−4−somites
14−19−somites
20−25−somites
Prim−5
Prim−15
Prim−25
Long−pec
Protruding−mouth
Day−4
Day−5
Stage
Transcript
ENSDART00000163052
ENSDART00000164569
ENSDART00000179064

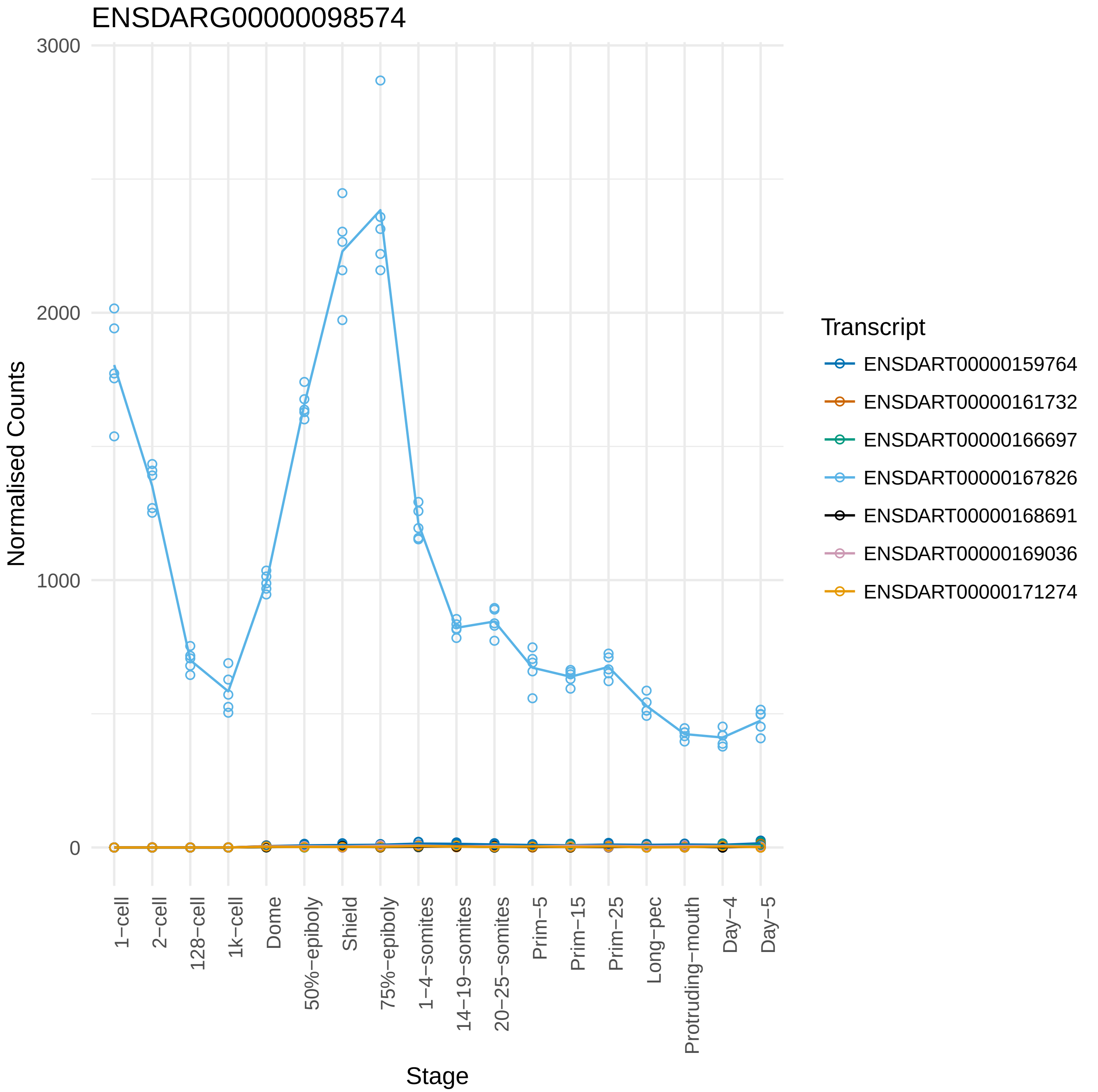
ENSDARG00000098574
Normalised Counts
3000
2000
1000
0
1−cell
2−cell
128−cell
1k−cell
Dome
50%−epiboly
Shield
75%−epiboly
1−4−somites
14−19−somites
20−25−somites
Prim−5
Prim−15
Prim−25
Long−pec
Protruding−mouth
Day−4
Day−5
Stage
Transcript
ENSDART00000159764
ENSDART00000161732
ENSDART00000166697
ENSDART00000167826
ENSDART00000168691
ENSDART00000169036
ENSDART00000171274

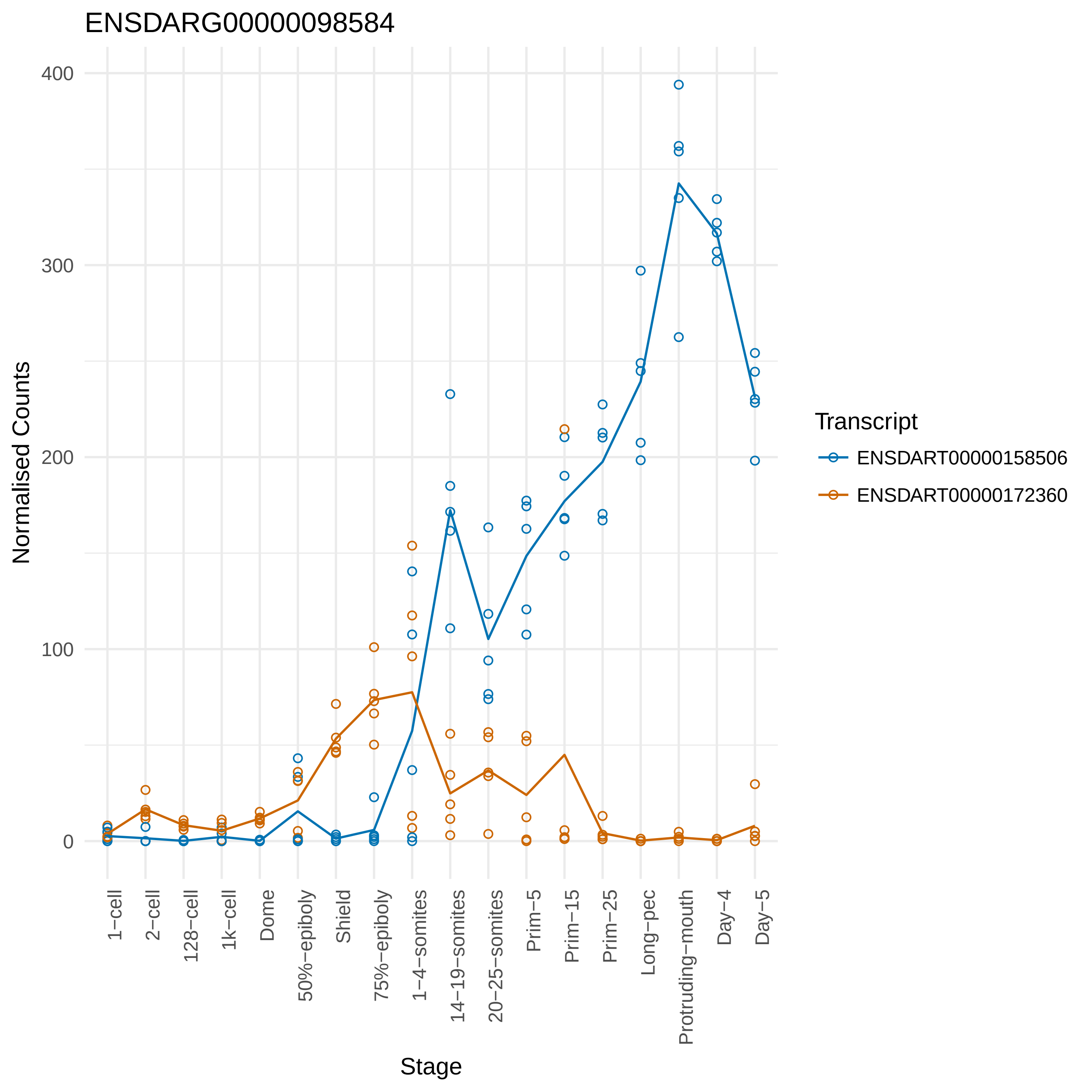
ENSDARG00000098584
Normalised Counts
0
100
200
300
400
1−cell
2−cell
128−cell
1k−cell
Dome
50%−epiboly
Shield
75%−epiboly
1−4−somites
14−19−somites
20−25−somites
Prim−5
Prim−15
Prim−25
Long−pec
Protruding−mouth
Day−4
Day−5
Stage
Transcript
ENSDART00000158506
ENSDART00000172360

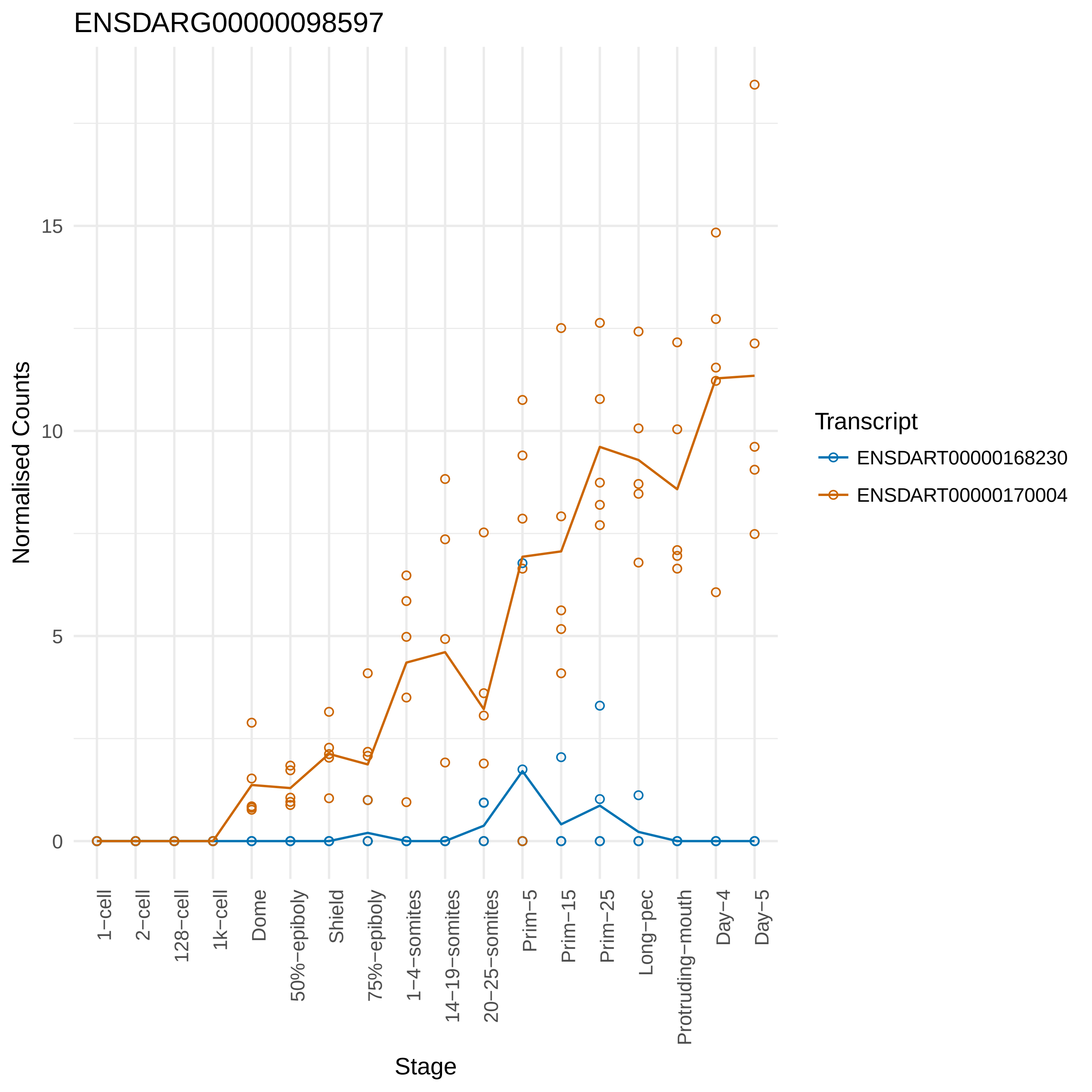
ENSDARG00000098597
Normalised Counts
0
5
10
15
1−cell
2−cell
128−cell
1k−cell
Dome
50%−epiboly
Shield
75%−epiboly
1−4−somites
14−19−somites
20−25−somites
Prim−5
Prim−15
Prim−25
Long−pec
Protruding−mouth
Day−4
Day−5
Stage
Transcript
ENSDART00000168230
ENSDART00000170004

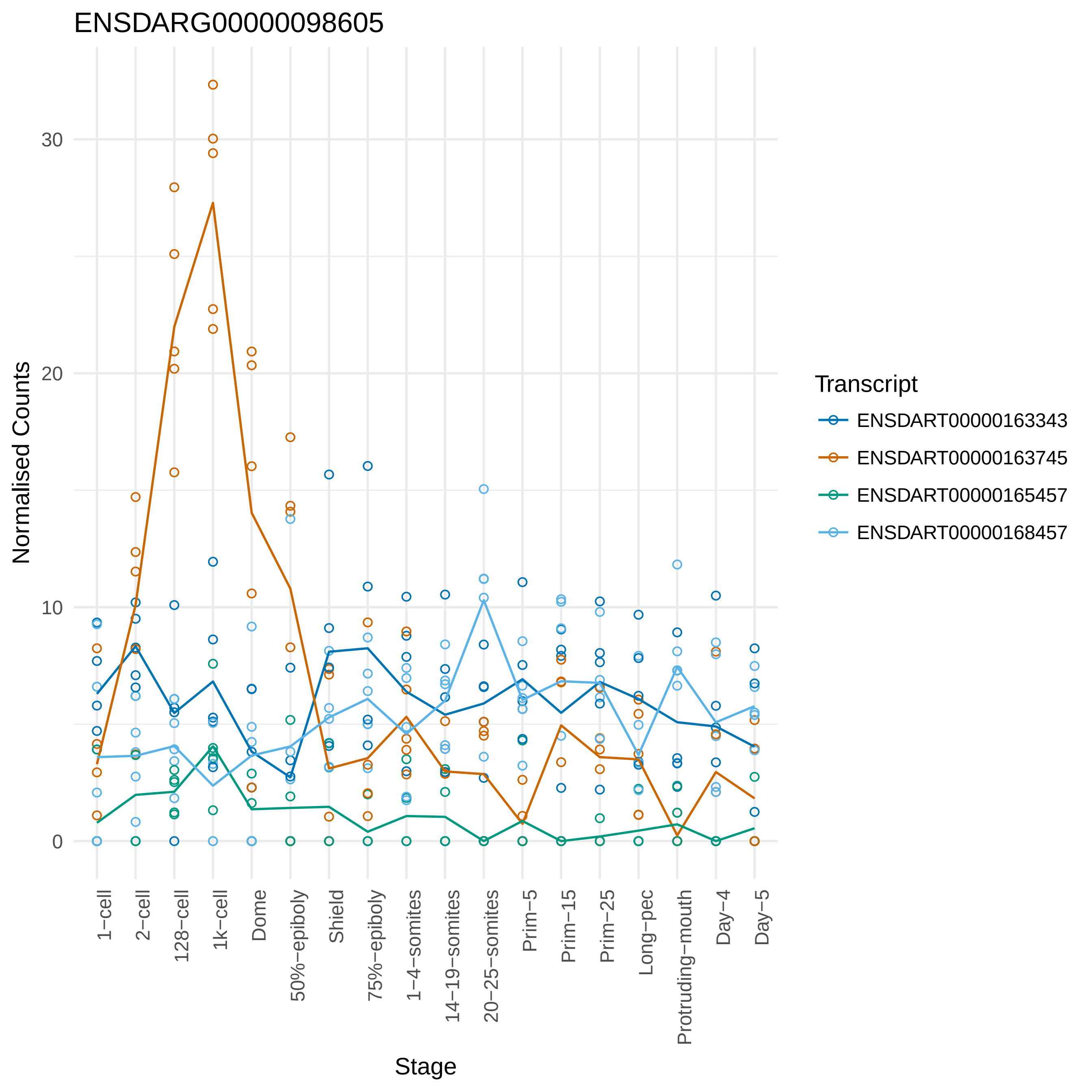

ENSDARG00000098605
Normalised Counts
0
10
20
30
Stage
1−cell
2−cell
128−cell
1k−cell
Dome
50%−epiboly
Shield
75%−epiboly
1−4−somites
14−19−somites
20−25−somites
Prim−5
Prim−15
Prim−25
Long−pec
Protruding−mouth
Day−4
Day−5
Transcript
ENSDART00000163343
ENSDART00000163745
ENSDART00000165457
ENSDART00000168457

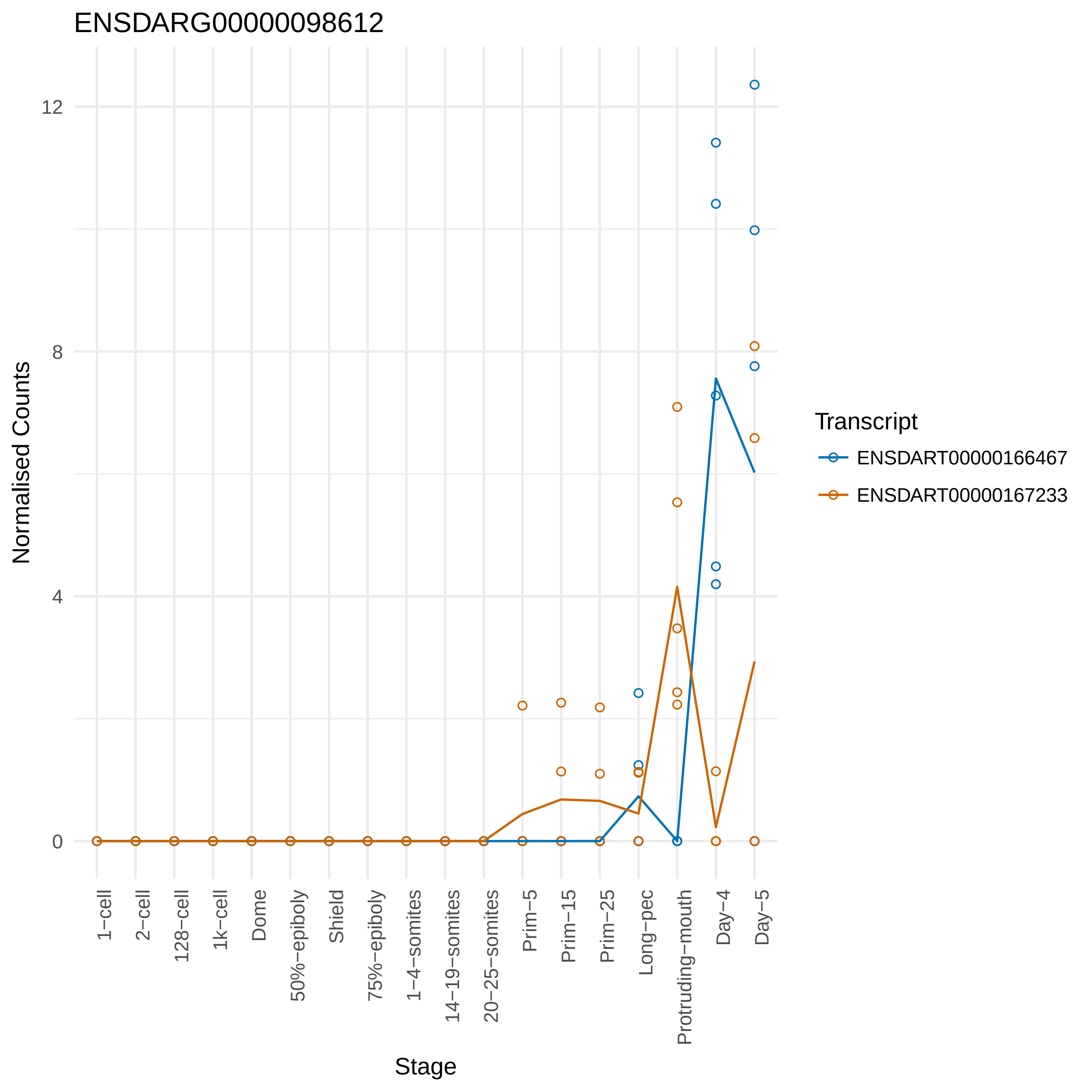

ENSDARG00000098612
Normalised Counts
12
8
4
0
1−cell
2−cell
128−cell
1k−cell
Dome
50%−epiboly
Shield
75%−epiboly
1−4−somites
14−19−somites
20−25−somites
Prim−5
Prim−15
Prim−25
Long−pec
Protruding−mouth
Day−4
Day−5
Stage
Transcript
ENSDART00000166467
ENSDART00000167233

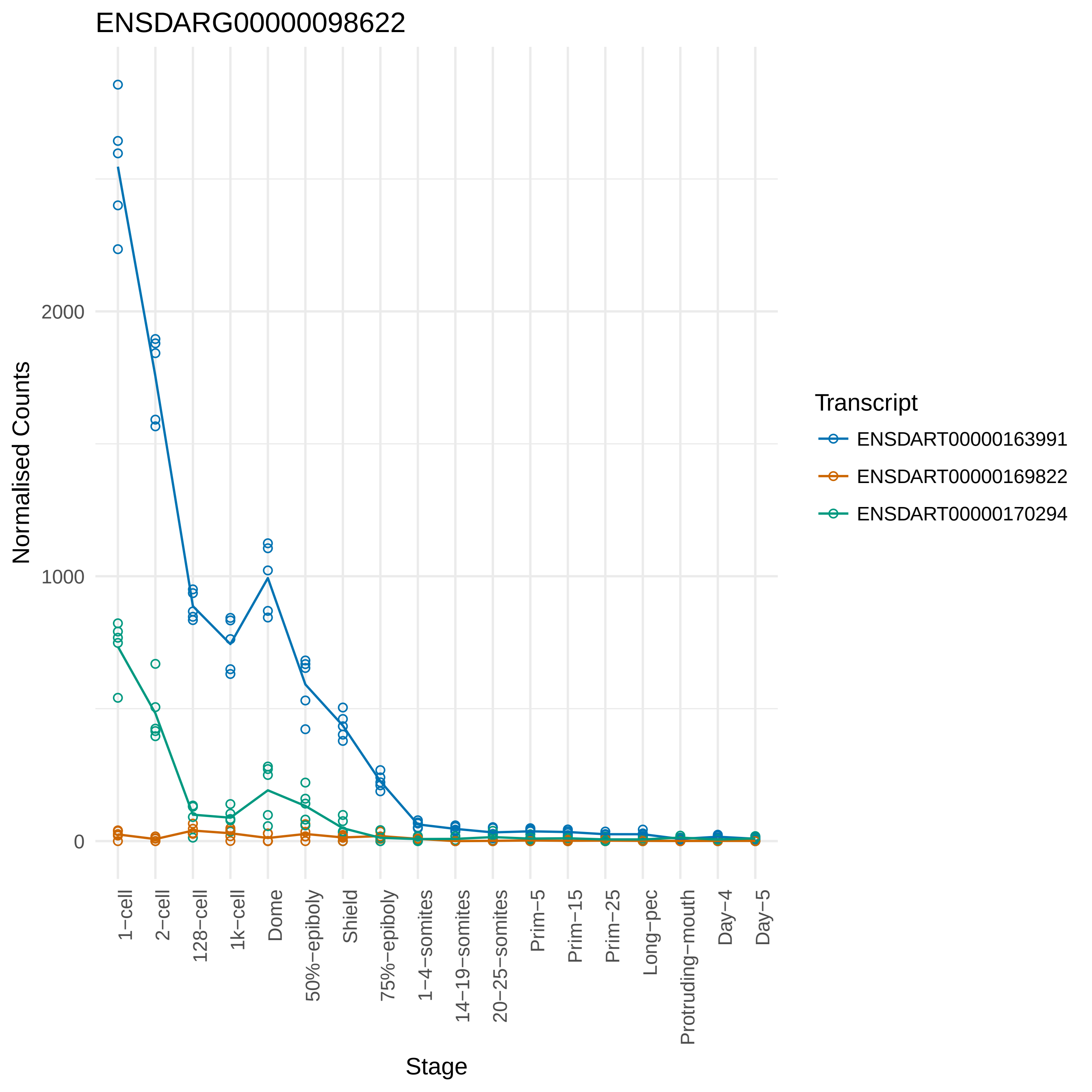
ENSDARG00000098622
Normalised Counts
2000
1000
0
Stage
1−cell
2−cell
128−cell
1k−cell
Dome
50%−epiboly
Shield
75%−epiboly
1−4−somites
14−19−somites
20−25−somites
Prim−5
Prim−15
Prim−25
Long−pec
Protruding−mouth
Day−4
Day−5
Transcript
ENSDART00000163991
ENSDART00000169822
ENSDART00000170294

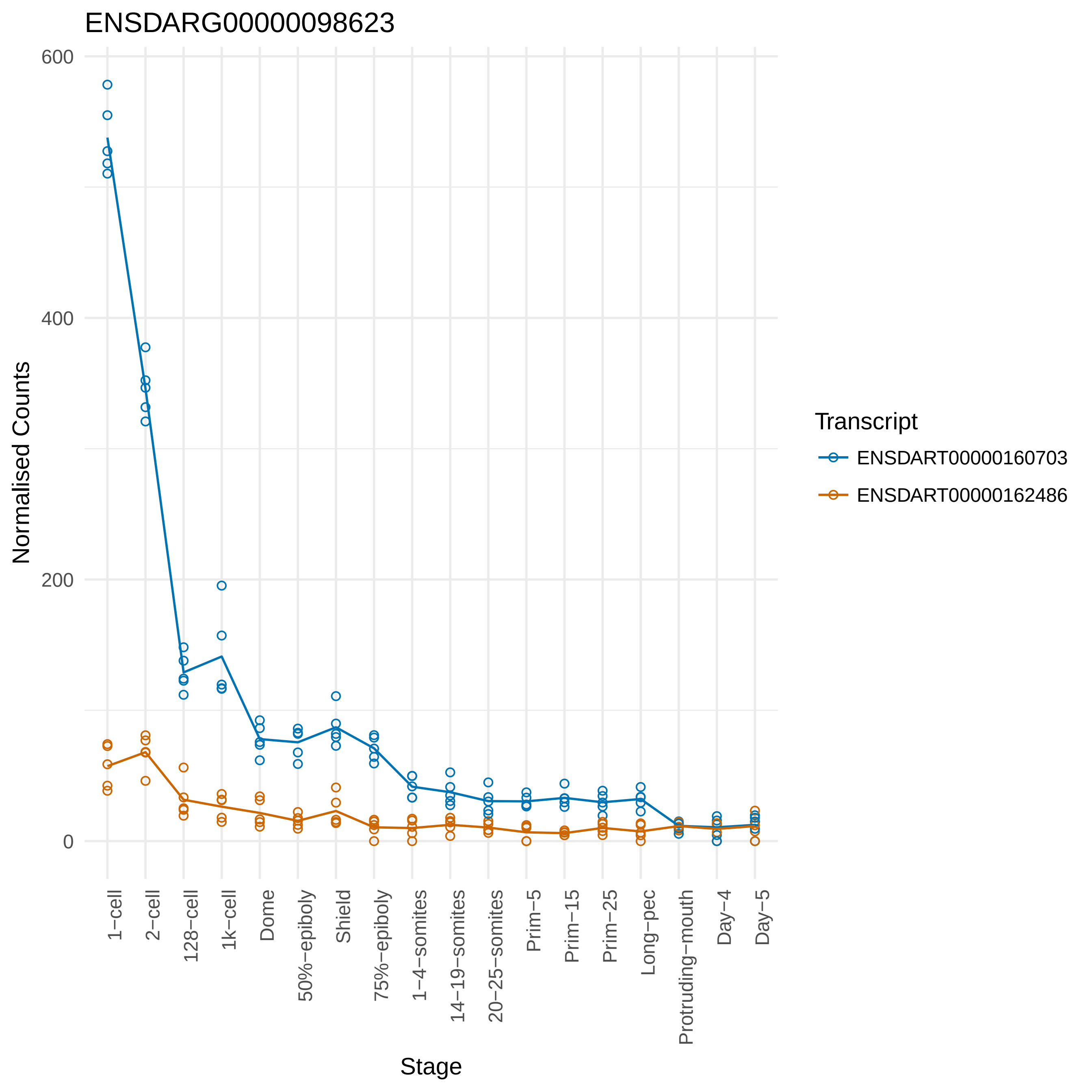

ENSDARG00000098623
Normalised Counts
600
400
200
0
1−cell
2−cell
128−cell
1k−cell
Dome
50%−epiboly
Shield
75%−epiboly
1−4−somites
14−19−somites
20−25−somites
Prim−5
Prim−15
Prim−25
Long−pec
Protruding−mouth
Day−4
Day−5
Stage
Transcript
ENSDART00000160703
ENSDART00000162486

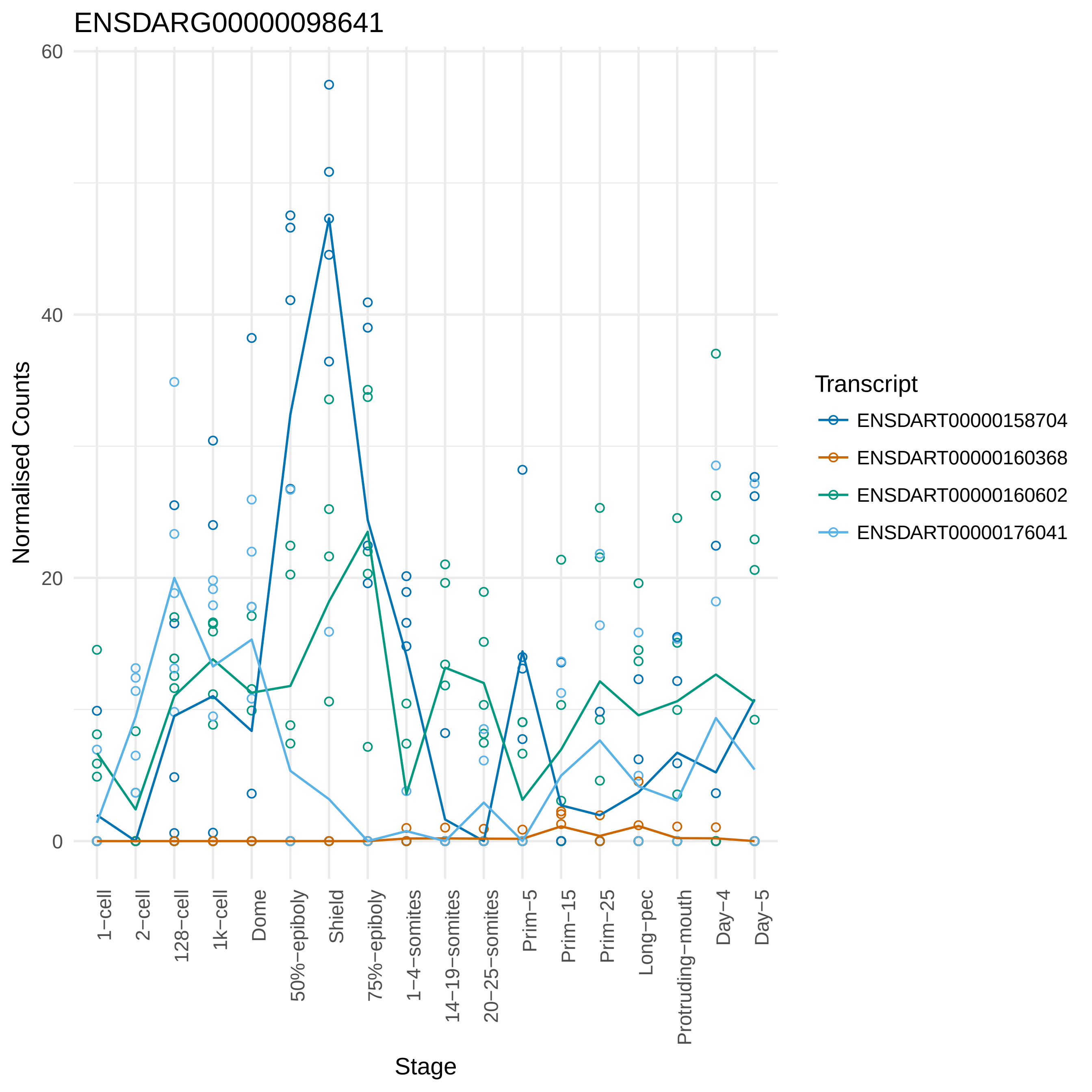

ENSDARG00000098641
Normalised Counts
0
20
40
60
Stage
1−cell
2−cell
128−cell
1k−cell
Dome
50%−epiboly
Shield
75%−epiboly
1−4−somites
14−19−somites
20−25−somites
Prim−5
Prim−15
Prim−25
Long−pec
Protruding−mouth
Day−4
Day−5
Transcript
ENSDART00000158704
ENSDART00000160368
ENSDART00000160602
ENSDART00000176041

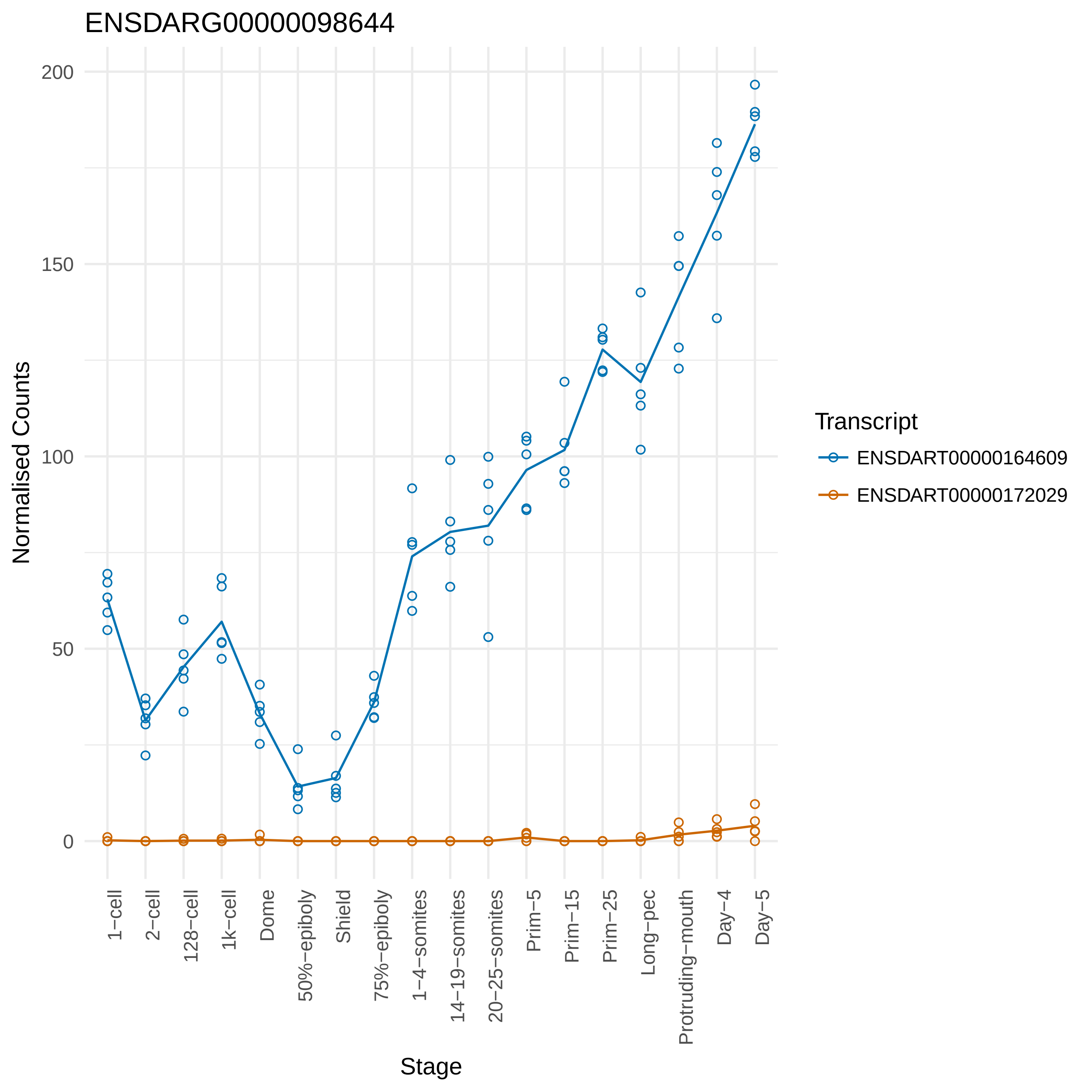

ENSDARG00000098644
Normalised Counts
0
50
100
150
200
Stage
1−cell
2−cell
128−cell
1k−cell
Dome
50%−epiboly
Shield
75%−epiboly
1−4−somites
14−19−somites
20−25−somites
Prim−5
Prim−15
Prim−25
Long−pec
Protruding−mouth
Day−4
Day−5
Transcript
ENSDART00000164609
ENSDART00000172029

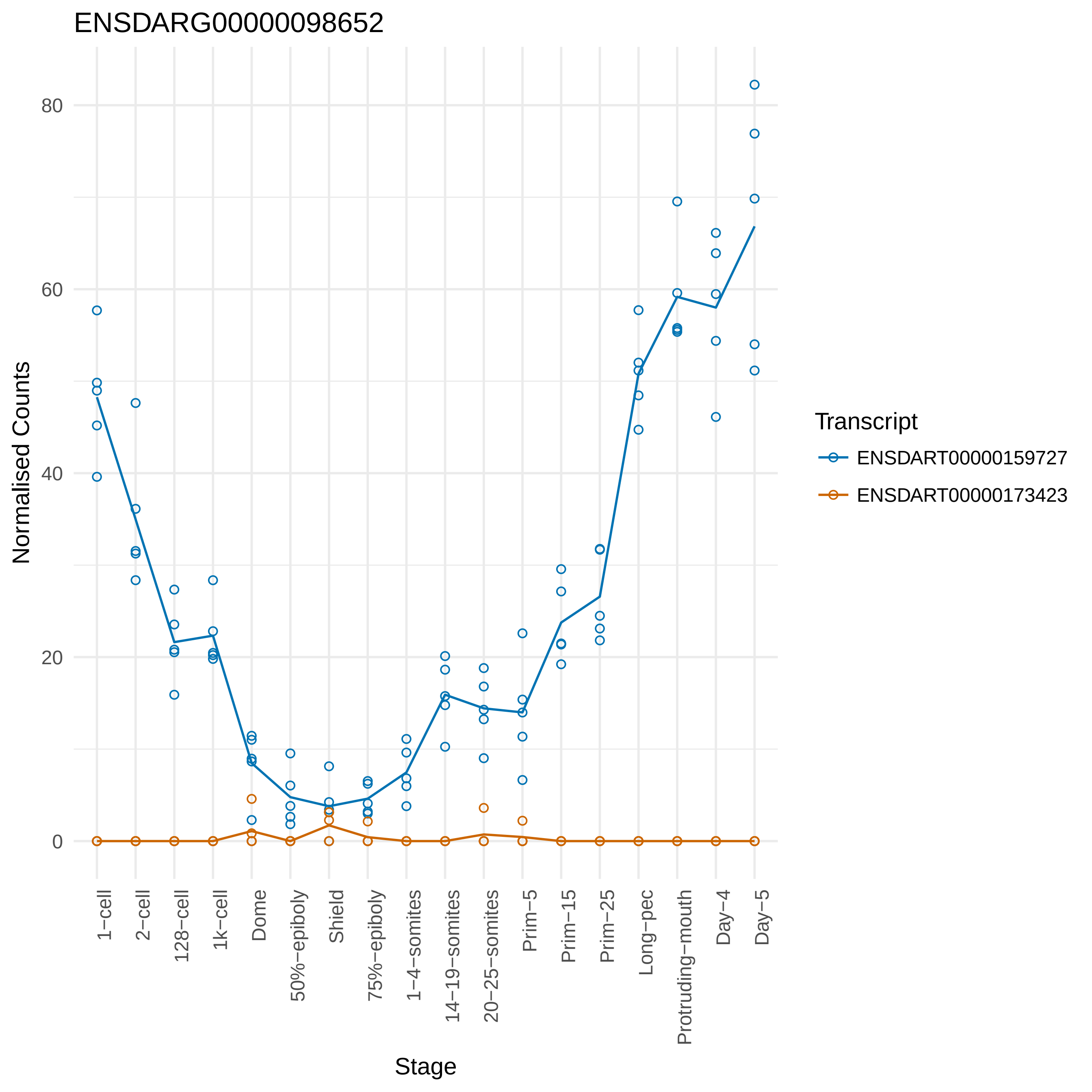
ENSDARG00000098652
Normalised Counts
80
60
40
20
0
Stage
1−cell
2−cell
128−cell
1k−cell
Dome
50%−epiboly
Shield
75%−epiboly
1−4−somites
14−19−somites
20−25−somites
Prim−5
Prim−15
Prim−25
Long−pec
Protruding−mouth
Day−4
Day−5
Transcript
ENSDART00000159727
ENSDART00000173423

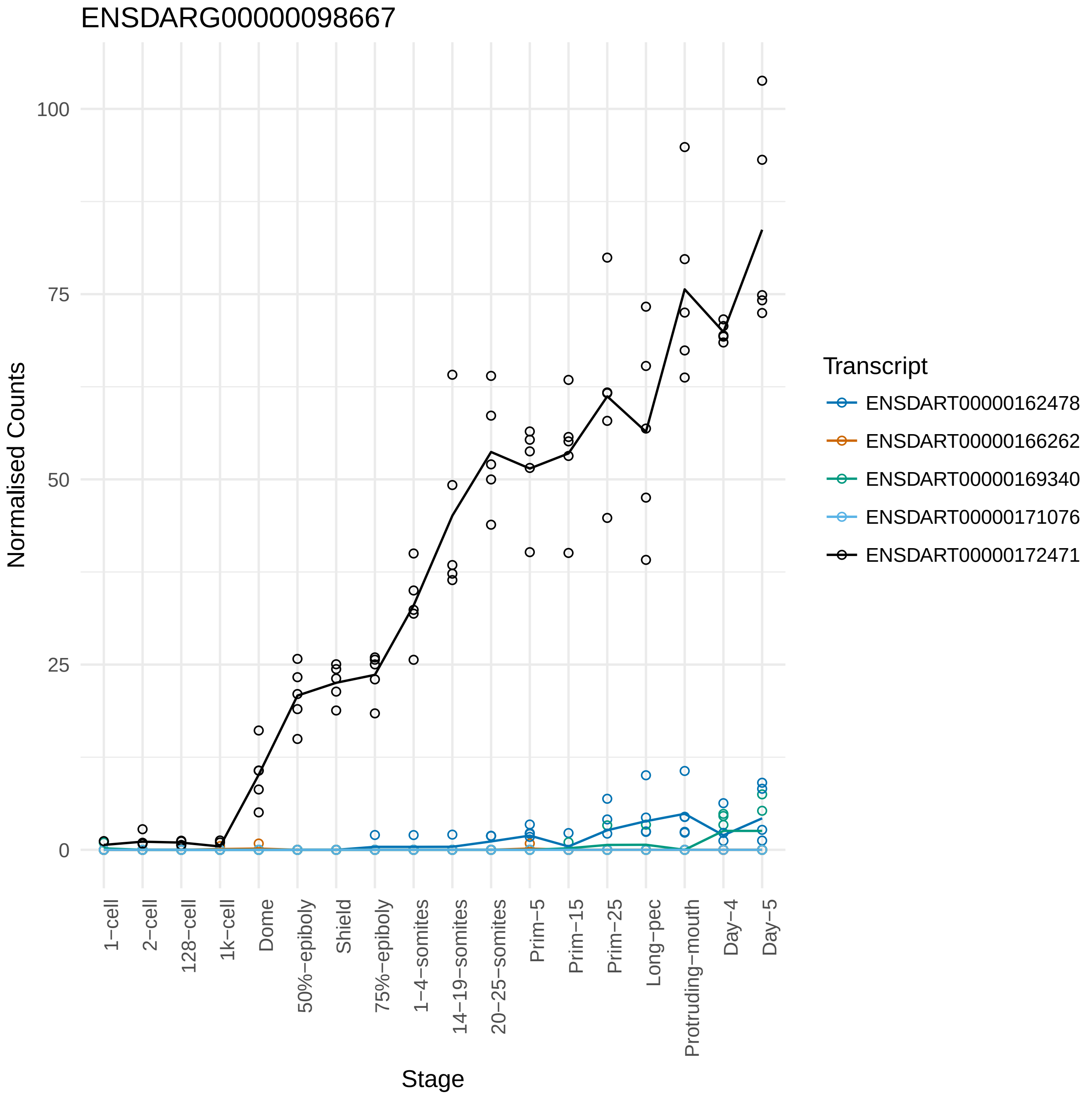
ENSDARG00000098667
Normalised Counts
0
25
50
75
100
Stage
1−cell
2−cell
128−cell
1k−cell
Dome
50%−epiboly
Shield
75%−epiboly
1−4−somites
14−19−somites
20−25−somites
Prim−5
Prim−15
Prim−25
Long−pec
Protruding−mouth
Day−4
Day−5
Transcript
ENSDART00000162478
ENSDART00000166262
ENSDART00000169340
ENSDART00000171076
ENSDART00000172471

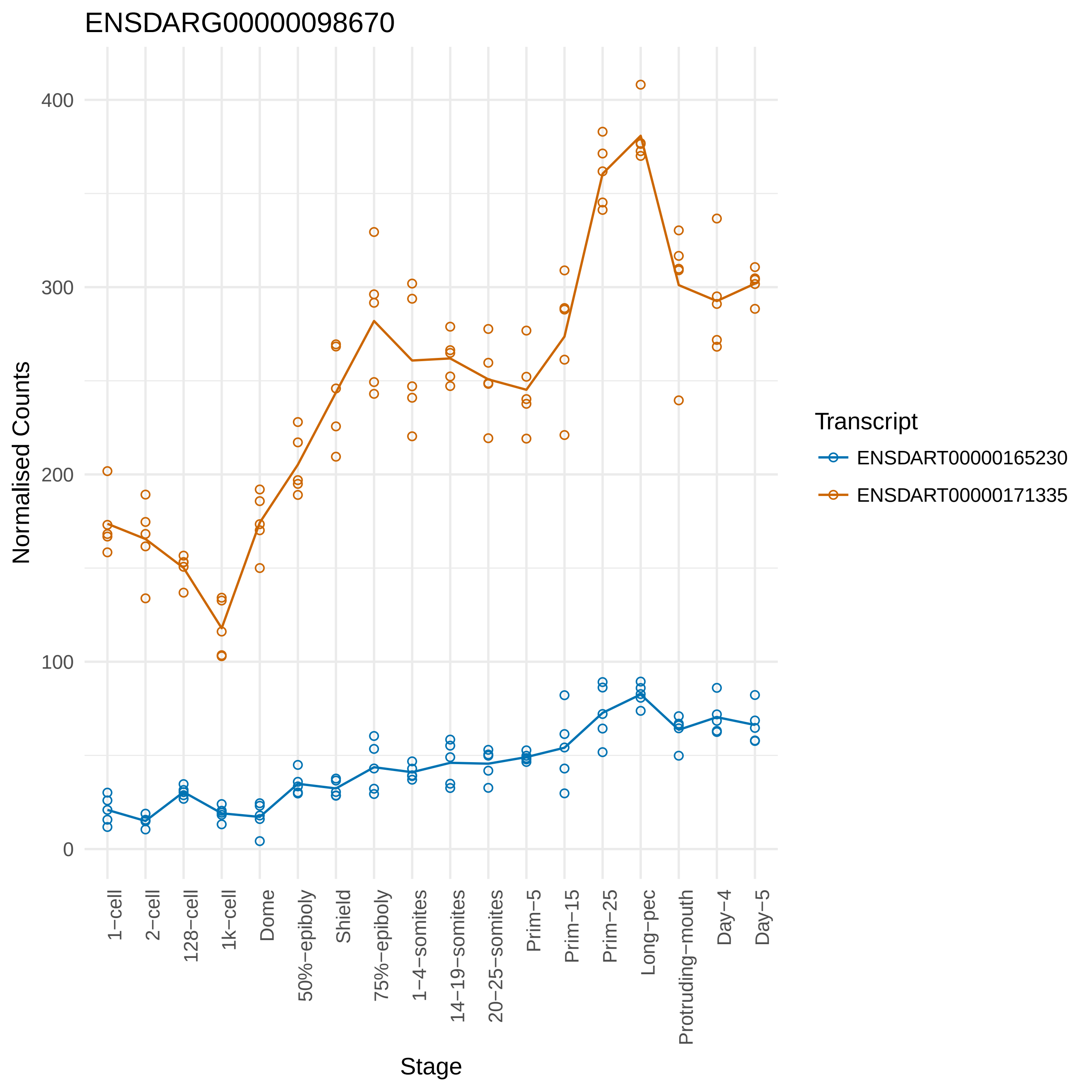

ENSDARG00000098670
Normalised Counts
400
300
200
100
0
1−cell
2−cell
128−cell
1k−cell
Dome
50%−epiboly
Shield
75%−epiboly
1−4−somites
14−19−somites
20−25−somites
Prim−5
Prim−15
Prim−25
Long−pec
Protruding−mouth
Day−4
Day−5
Stage
Transcript
ENSDART00000165230
ENSDART00000171335

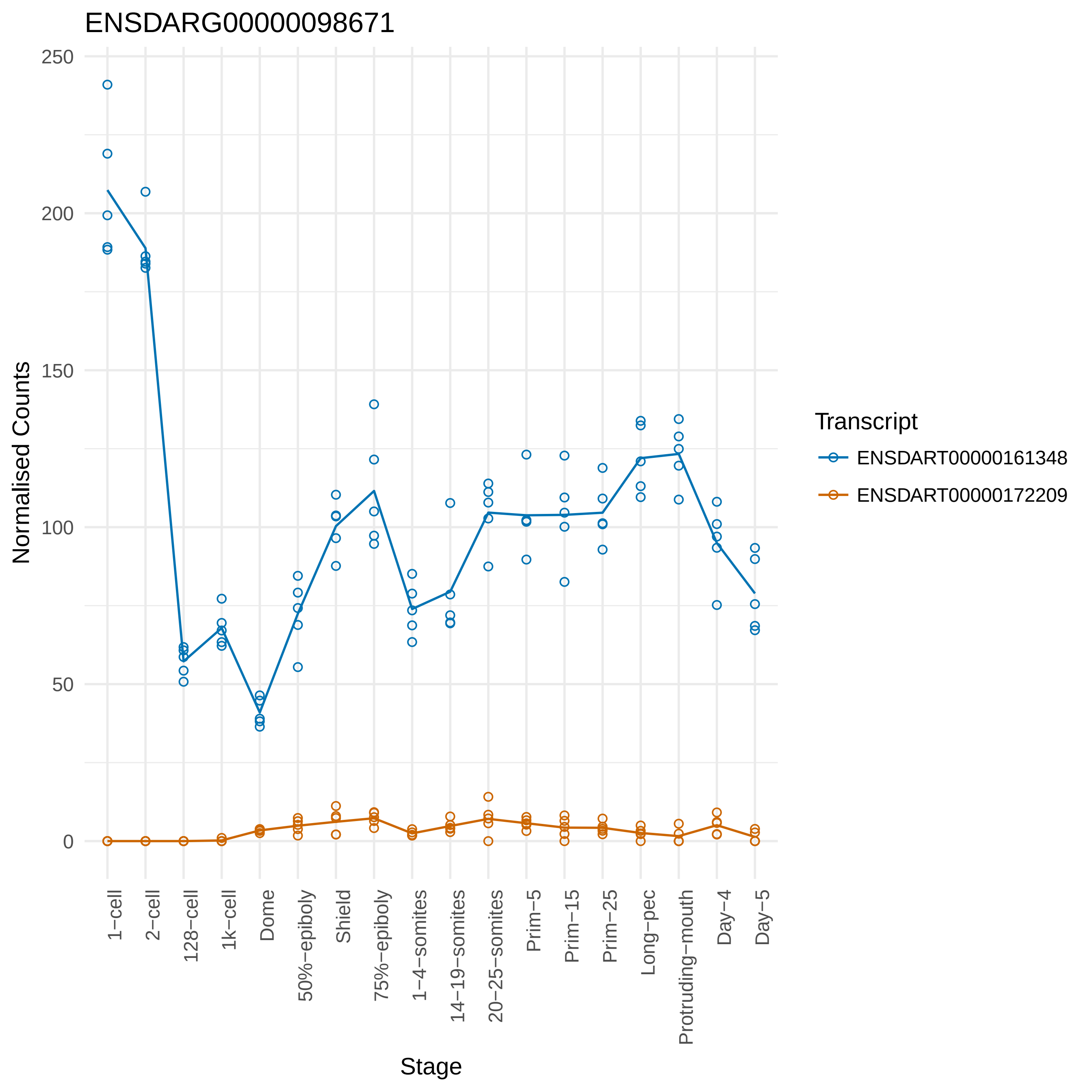

ENSDARG00000098671
Normalised Counts
250
200
150
100
50
0
1−cell
2−cell
128−cell
1k−cell
Dome
50%−epiboly
Shield
75%−epiboly
1−4−somites
14−19−somites
20−25−somites
Prim−5
Prim−15
Prim−25
Long−pec
Protruding−mouth
Day−4
Day−5
Stage
Transcript
ENSDART00000161348
ENSDART00000172209

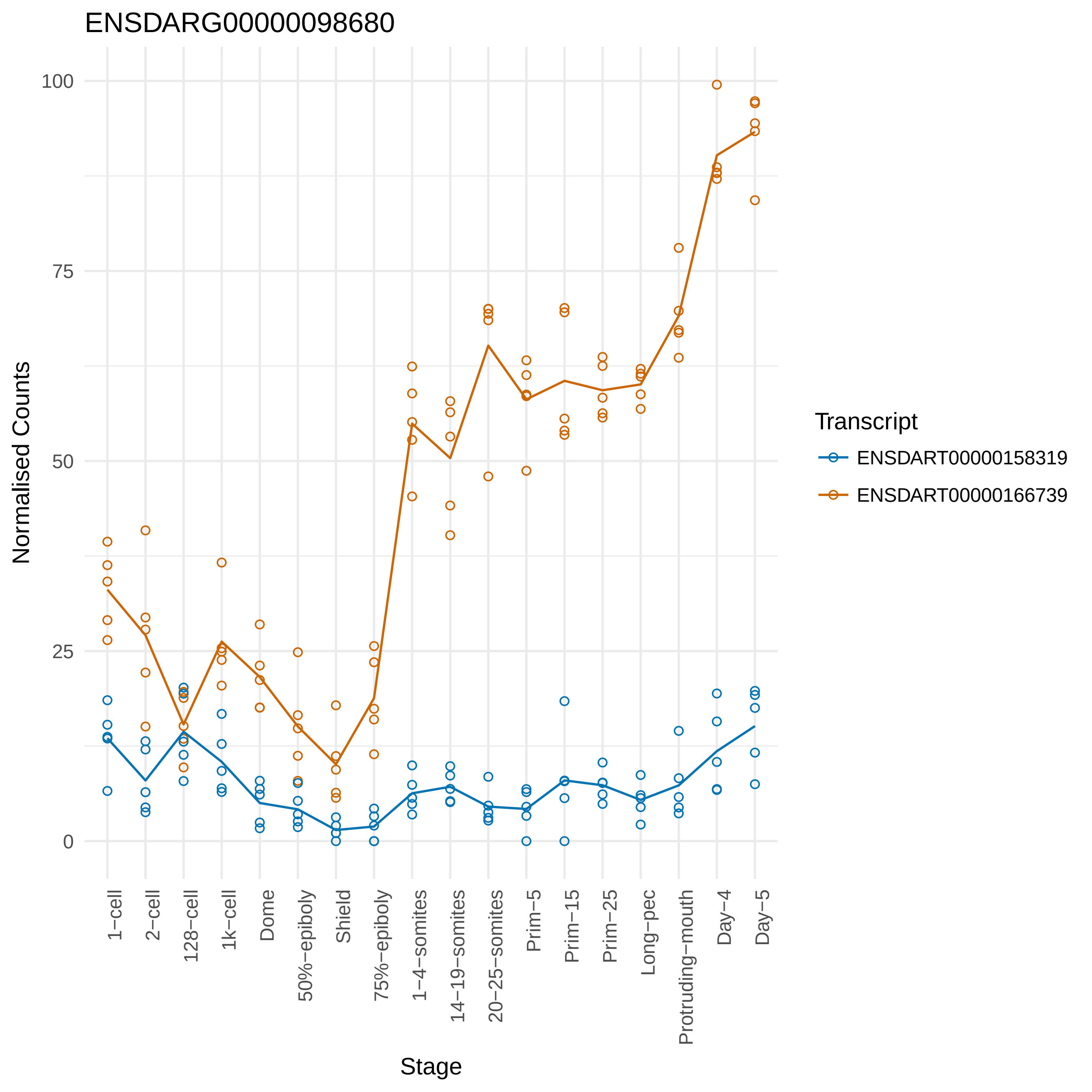

ENSDARG00000098680
Normalised Counts
100
75
50
25
0
1−cell
2−cell
128−cell
1k−cell
Dome
50%−epiboly
Shield
75%−epiboly
1−4−somites
14−19−somites
20−25−somites
Prim−5
Prim−15
Prim−25
Long−pec
Protruding−mouth
Day−4
Day−5
Stage
Transcript
ENSDART00000158319
ENSDART00000166739

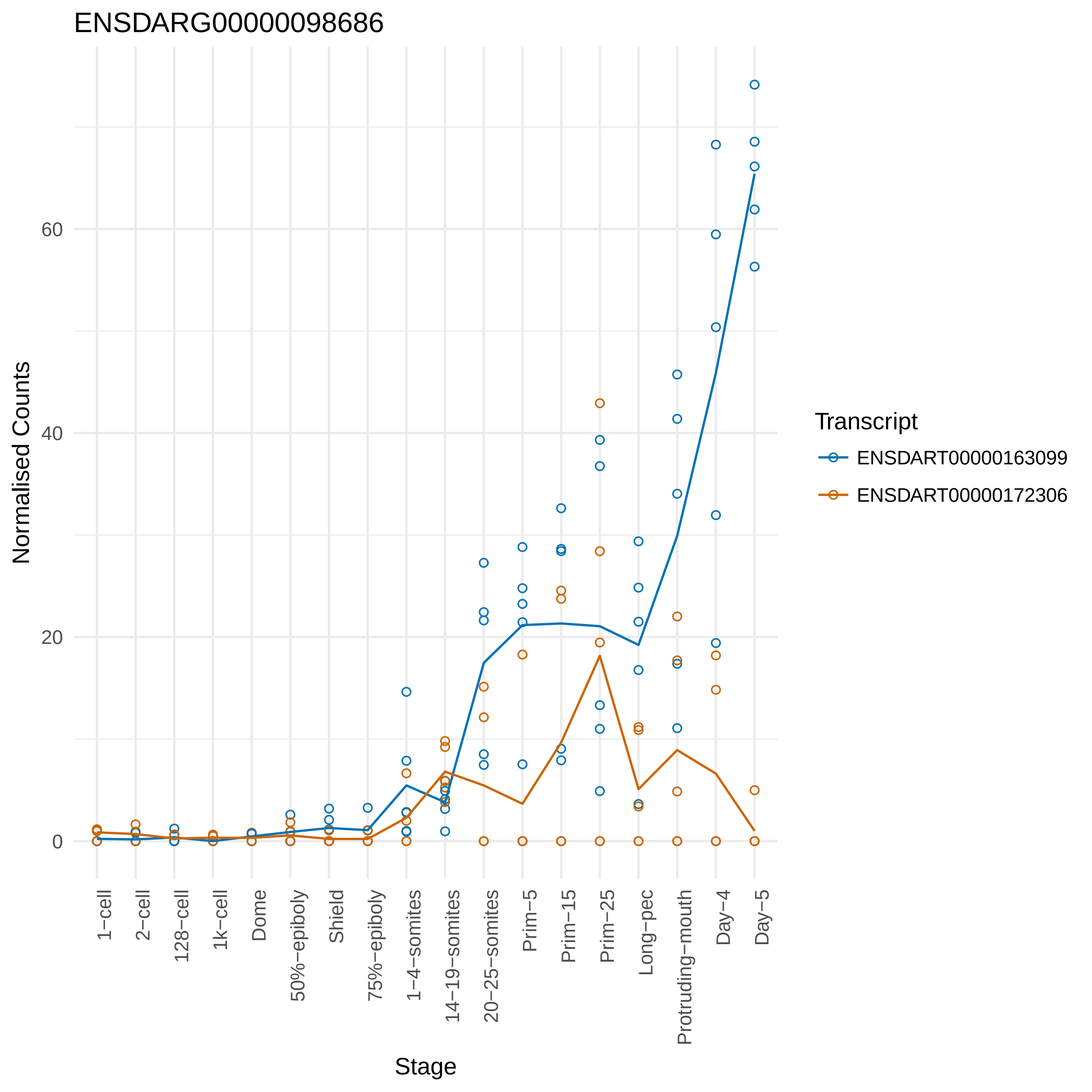

ENSDARG00000098686
Normalised Counts
0
20
40
60
Stage
1−cell
2−cell
128−cell
1k−cell
Dome
50%−epiboly
Shield
75%−epiboly
1−4−somites
14−19−somites
20−25−somites
Prim−5
Prim−15
Prim−25
Long−pec
Protruding−mouth
Day−4
Day−5
Transcript
ENSDART00000163099
ENSDART00000172306

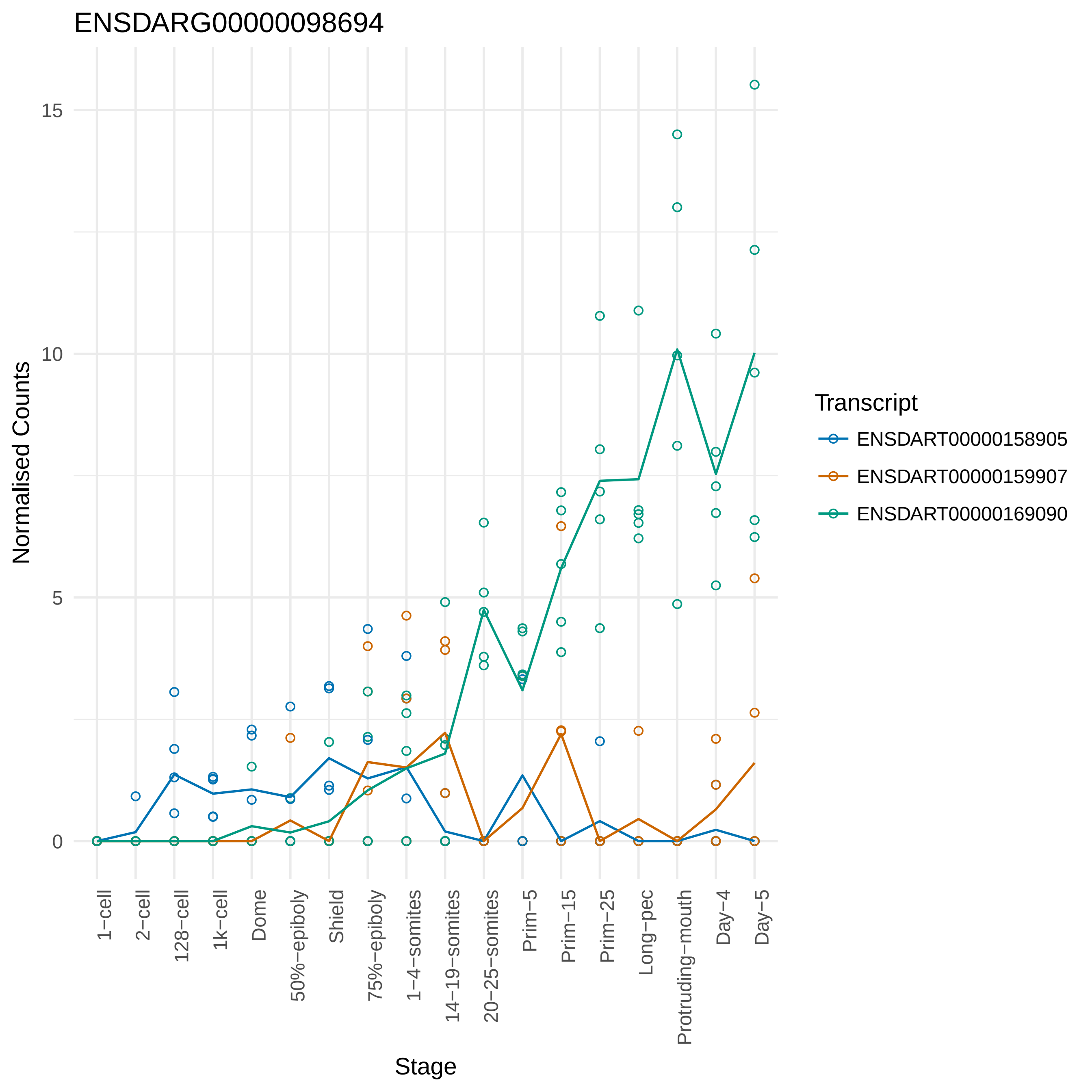

ENSDARG00000098694
Normalised Counts
15
10
5
0
1−cell
2−cell
128−cell
1k−cell
Dome
50%−epiboly
Shield
75%−epiboly
1−4−somites
14−19−somites
20−25−somites
Prim−5
Prim−15
Prim−25
Long−pec
Protruding−mouth
Day−4
Day−5
Stage
Transcript
ENSDART00000158905
ENSDART00000159907
ENSDART00000169090

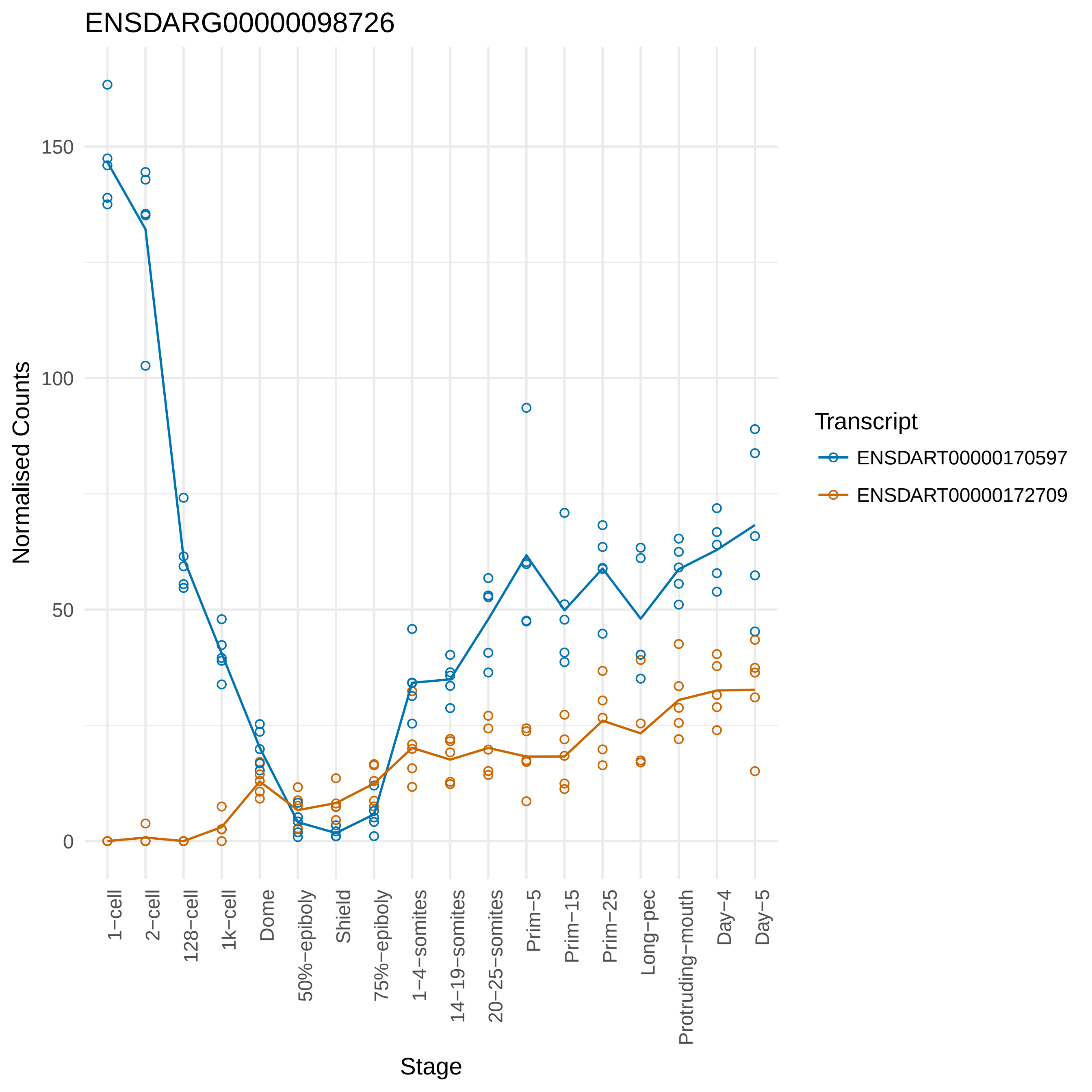

ENSDARG00000098726
Normalised Counts
150
100
50
0
1−cell
2−cell
128−cell
1k−cell
Dome
50%−epiboly
Shield
75%−epiboly
1−4−somites
14−19−somites
20−25−somites
Prim−5
Prim−15
Prim−25
Long−pec
Protruding−mouth
Day−4
Day−5
Stage
Transcript
ENSDART00000170597
ENSDART00000172709

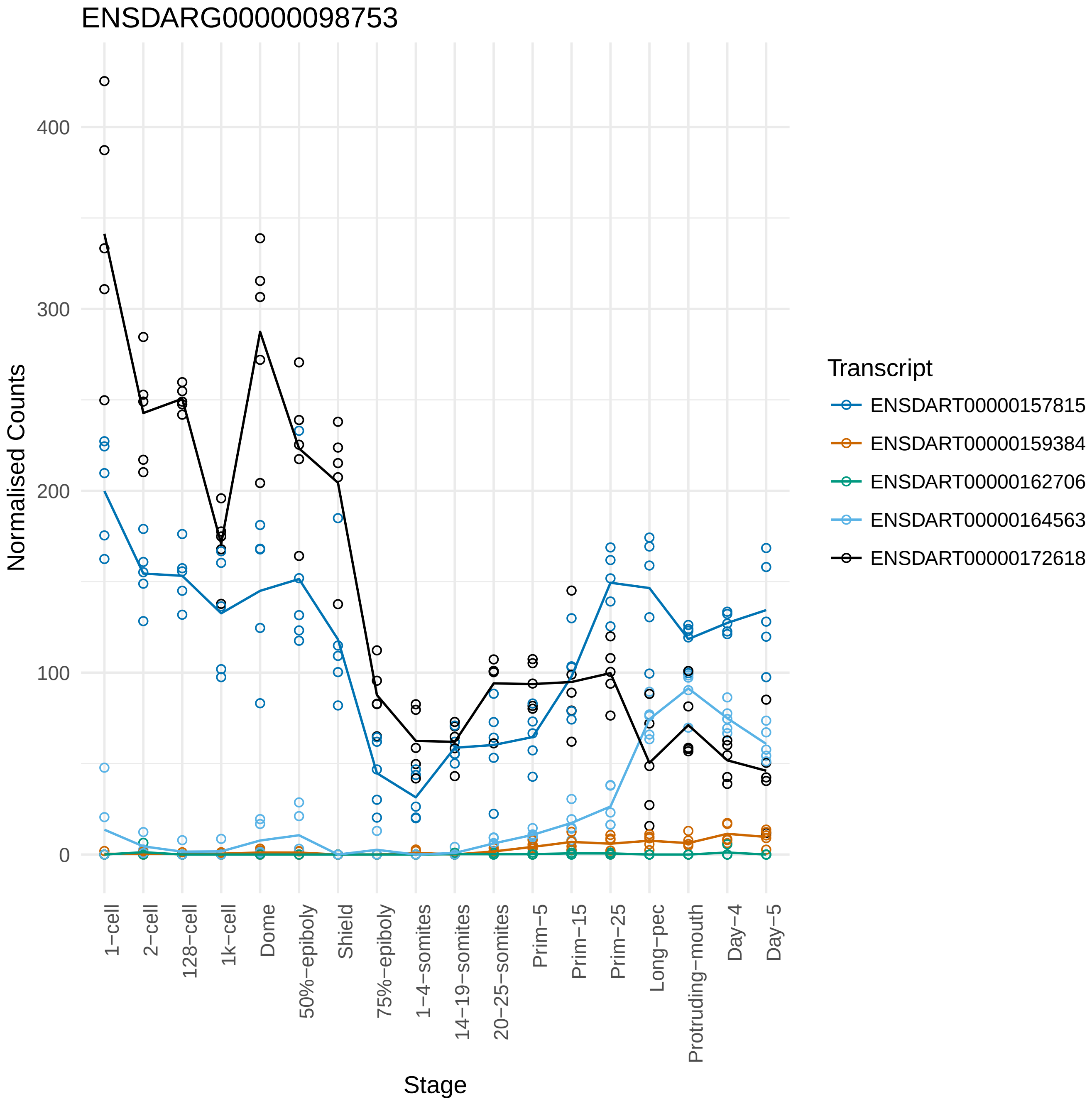

ENSDARG00000098753
Normalised Counts
0
100
200
300
400
Stage
1−cell
2−cell
128−cell
1k−cell
Dome
50%−epiboly
Shield
75%−epiboly
1−4−somites
14−19−somites
20−25−somites
Prim−5
Prim−15
Prim−25
Long−pec
Protruding−mouth
Day−4
Day−5
Transcript
ENSDART00000157815
ENSDART00000159384
ENSDART00000162706
ENSDART00000164563
ENSDART00000172618

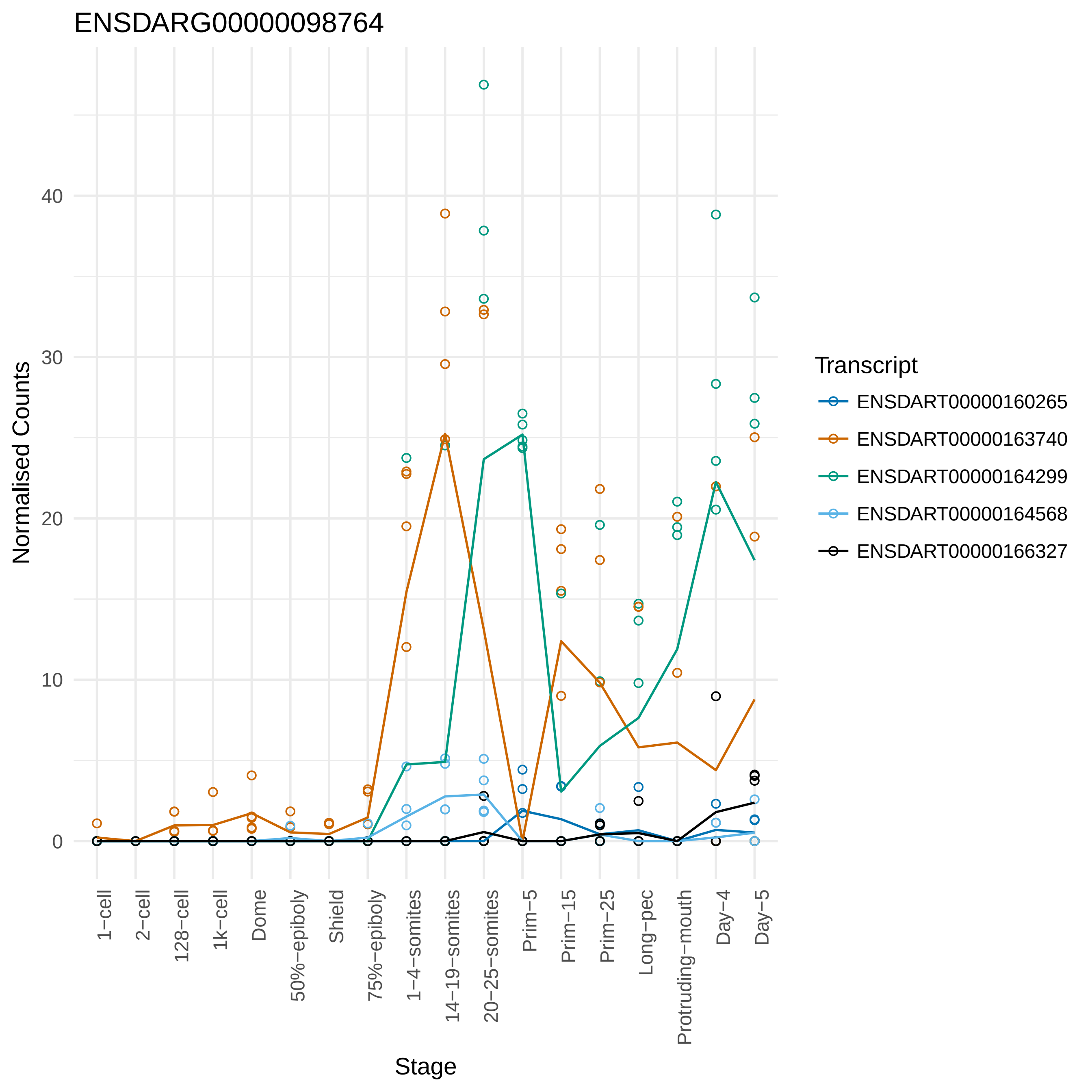

ENSDARG00000098764
Normalised Counts
0
10
20
30
40
1−cell
2−cell
128−cell
1k−cell
Dome
50%−epiboly
Shield
75%−epiboly
1−4−somites
14−19−somites
20−25−somites
Prim−5
Prim−15
Prim−25
Long−pec
Protruding−mouth
Day−4
Day−5
Stage
Transcript
ENSDART00000160265
ENSDART00000163740
ENSDART00000164299
ENSDART00000164568
ENSDART00000166327

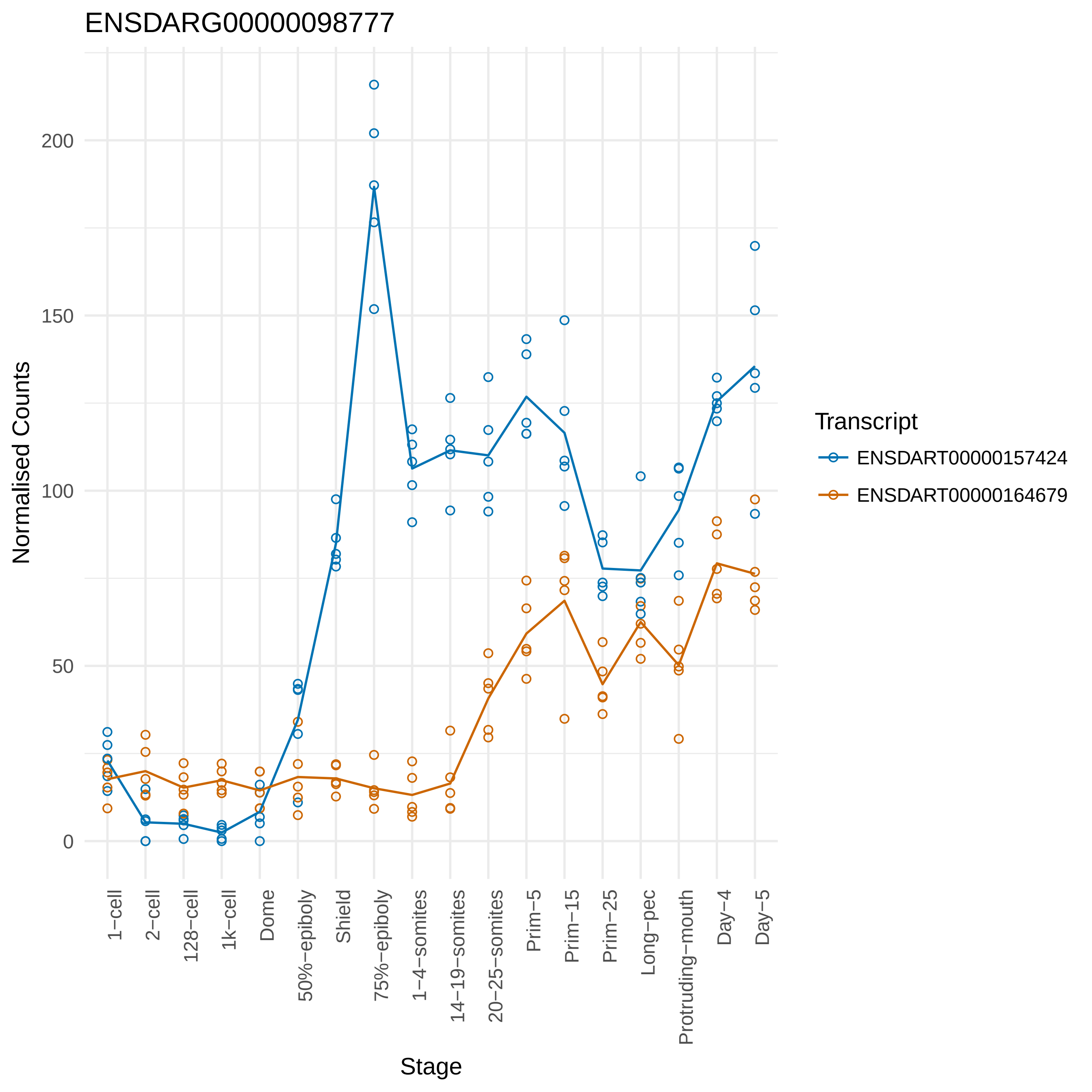

ENSDARG00000098777
Normalised Counts
200
150
100
50
0
1−cell
2−cell
128−cell
1k−cell
Dome
50%−epiboly
Shield
75%−epiboly
1−4−somites
14−19−somites
20−25−somites
Prim−5
Prim−15
Prim−25
Long−pec
Protruding−mouth
Day−4
Day−5
Stage
Transcript
ENSDART00000157424
ENSDART00000164679

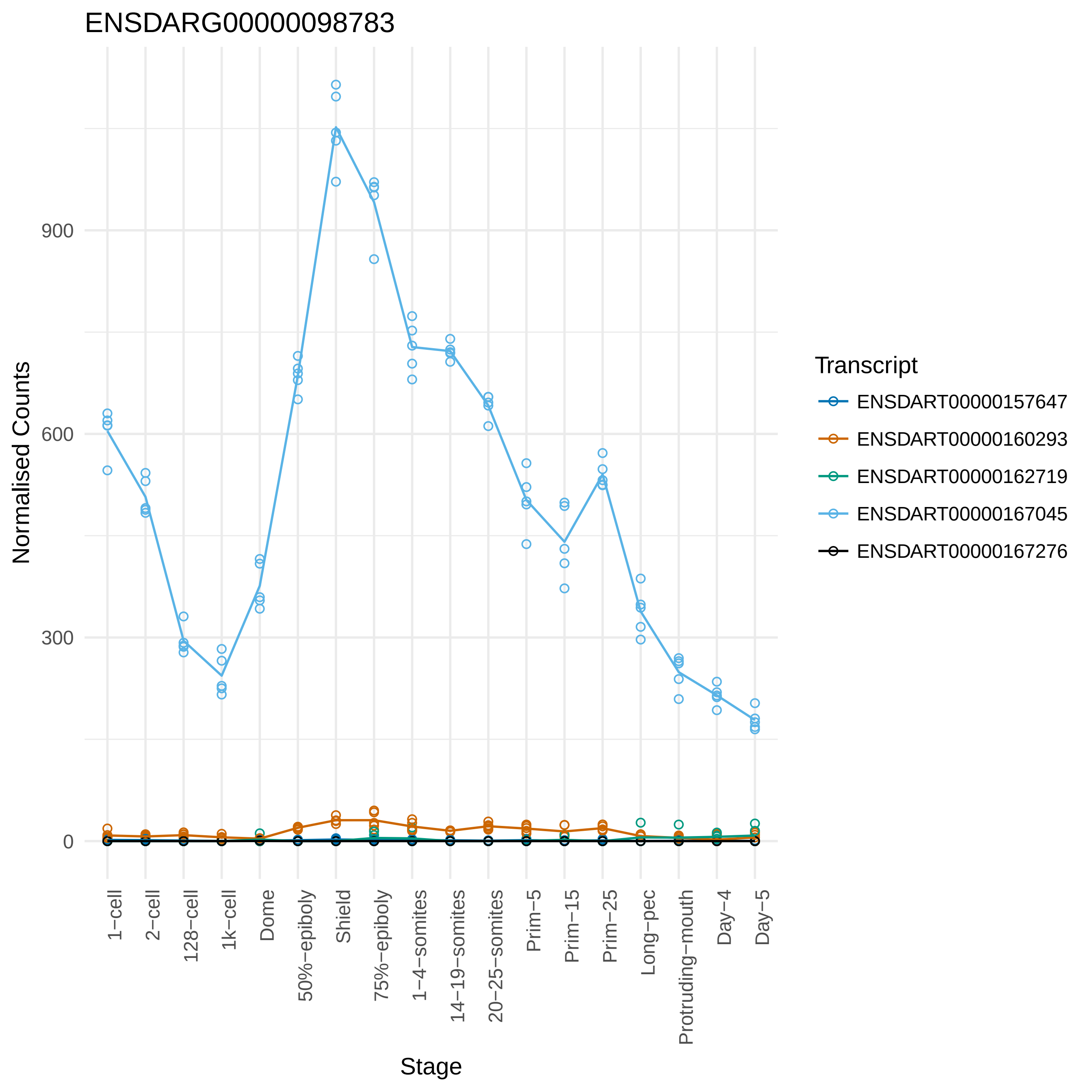

ENSDARG00000098783
Normalised Counts
900
600
300
0
1−cell
2−cell
128−cell
1k−cell
Dome
50%−epiboly
Shield
75%−epiboly
1−4−somites
14−19−somites
20−25−somites
Prim−5
Prim−15
Prim−25
Long−pec
Protruding−mouth
Day−4
Day−5
Stage
Transcript
ENSDART00000157647
ENSDART00000160293
ENSDART00000162719
ENSDART00000167045
ENSDART00000167276

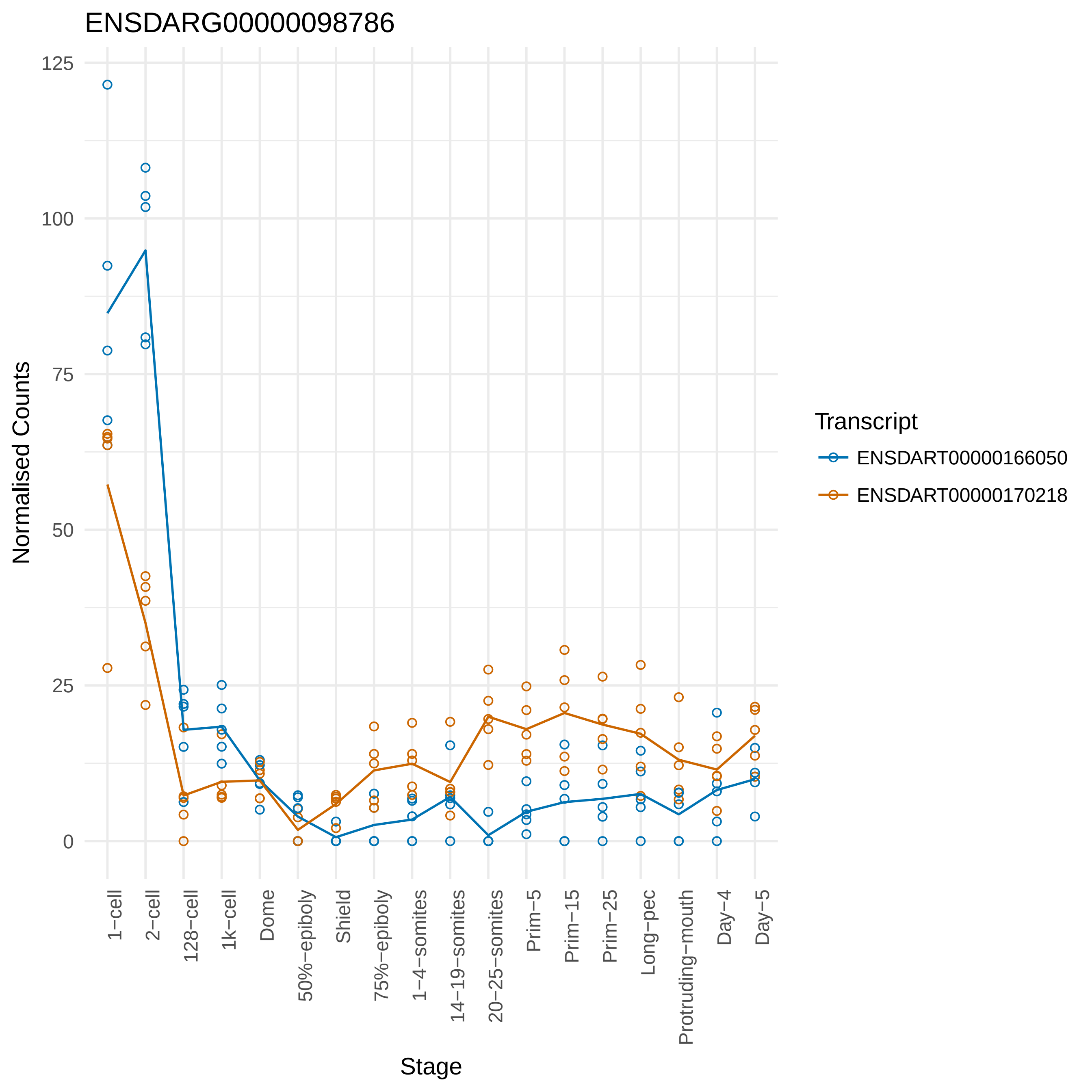
ENSDARG00000098786
Normalised Counts
125
100
75
50
25
0
1−cell
2−cell
128−cell
1k−cell
Dome
50%−epiboly
Shield
75%−epiboly
1−4−somites
14−19−somites
20−25−somites
Prim−5
Prim−15
Prim−25
Long−pec
Protruding−mouth
Day−4
Day−5
Stage
Transcript
ENSDART00000166050
ENSDART00000170218

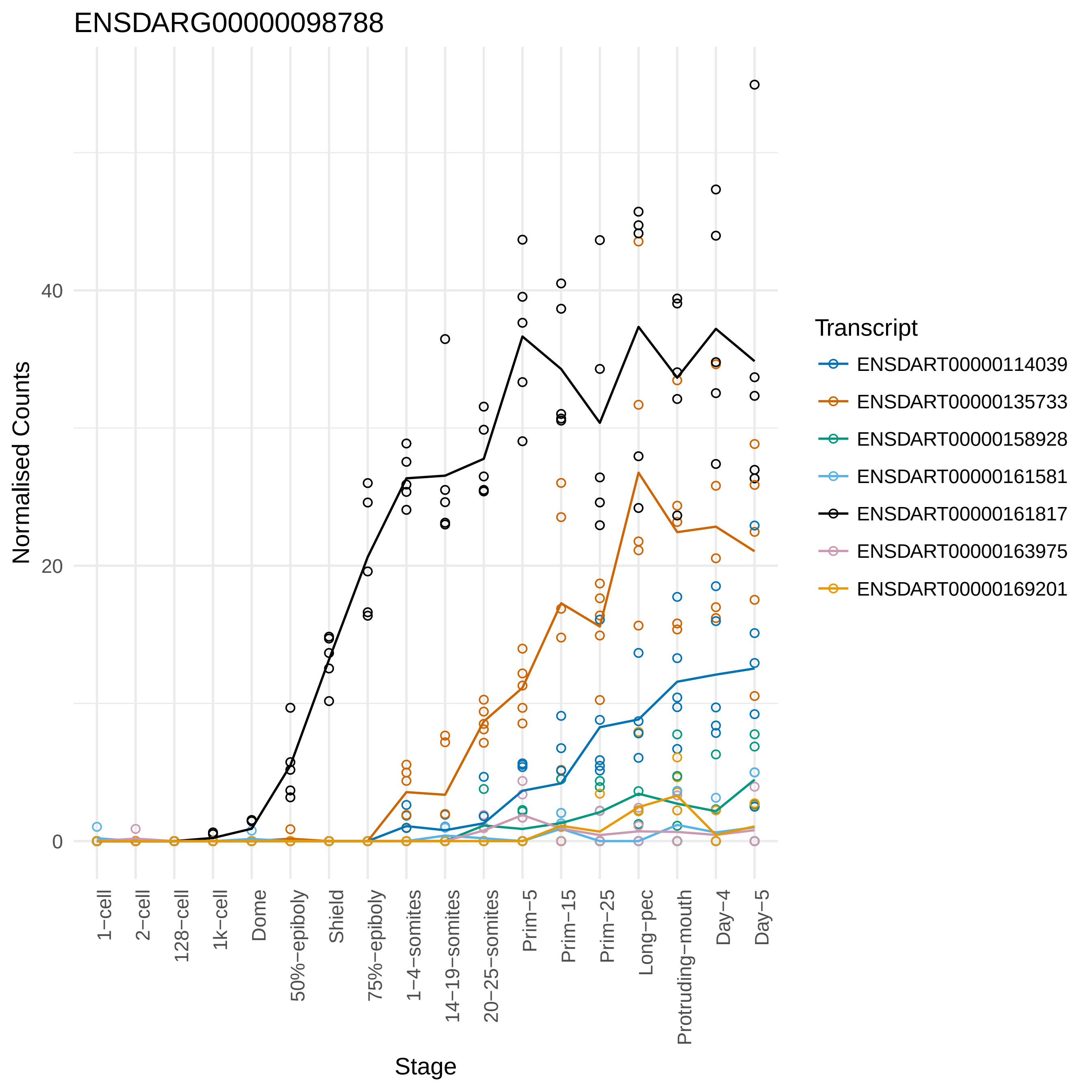

ENSDARG00000098788
Normalised Counts
0
20
40
1−cell
2−cell
128−cell
1k−cell
Dome
50%−epiboly
Shield
75%−epiboly
1−4−somites
14−19−somites
20−25−somites
Prim−5
Prim−15
Prim−25
Long−pec
Protruding−mouth
Day−4
Day−5
Stage
Transcript
ENSDART00000114039
ENSDART00000135733
ENSDART00000158928
ENSDART00000161581
ENSDART00000161817
ENSDART00000163975
ENSDART00000169201

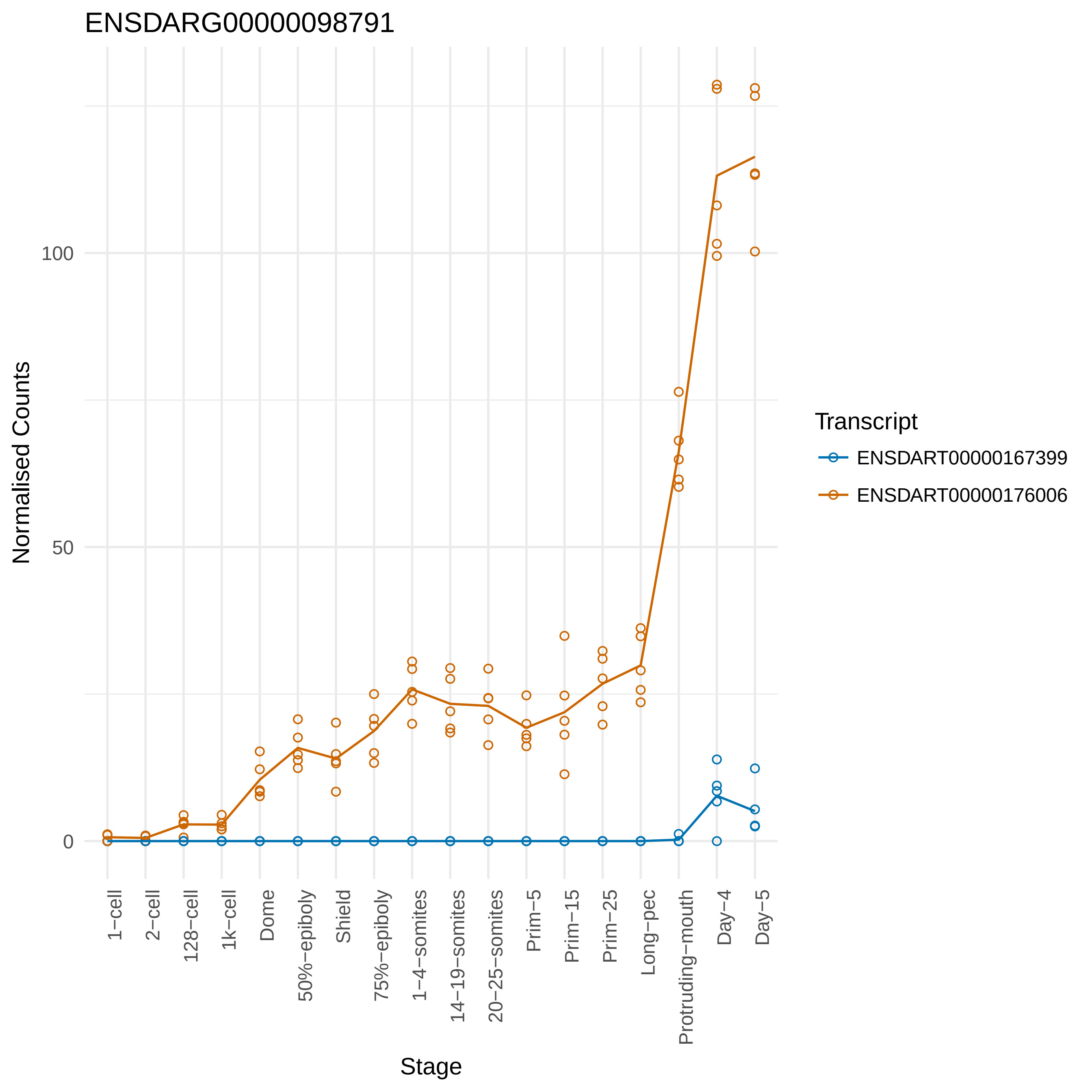
ENSDARG00000098791
Normalised Counts
100
50
0
Stage
1−cell
2−cell
128−cell
1k−cell
Dome
50%−epiboly
Shield
75%−epiboly
1−4−somites
14−19−somites
20−25−somites
Prim−5
Prim−15
Prim−25
Long−pec
Protruding−mouth
Day−4
Day−5
Transcript
ENSDART00000167399
ENSDART00000176006

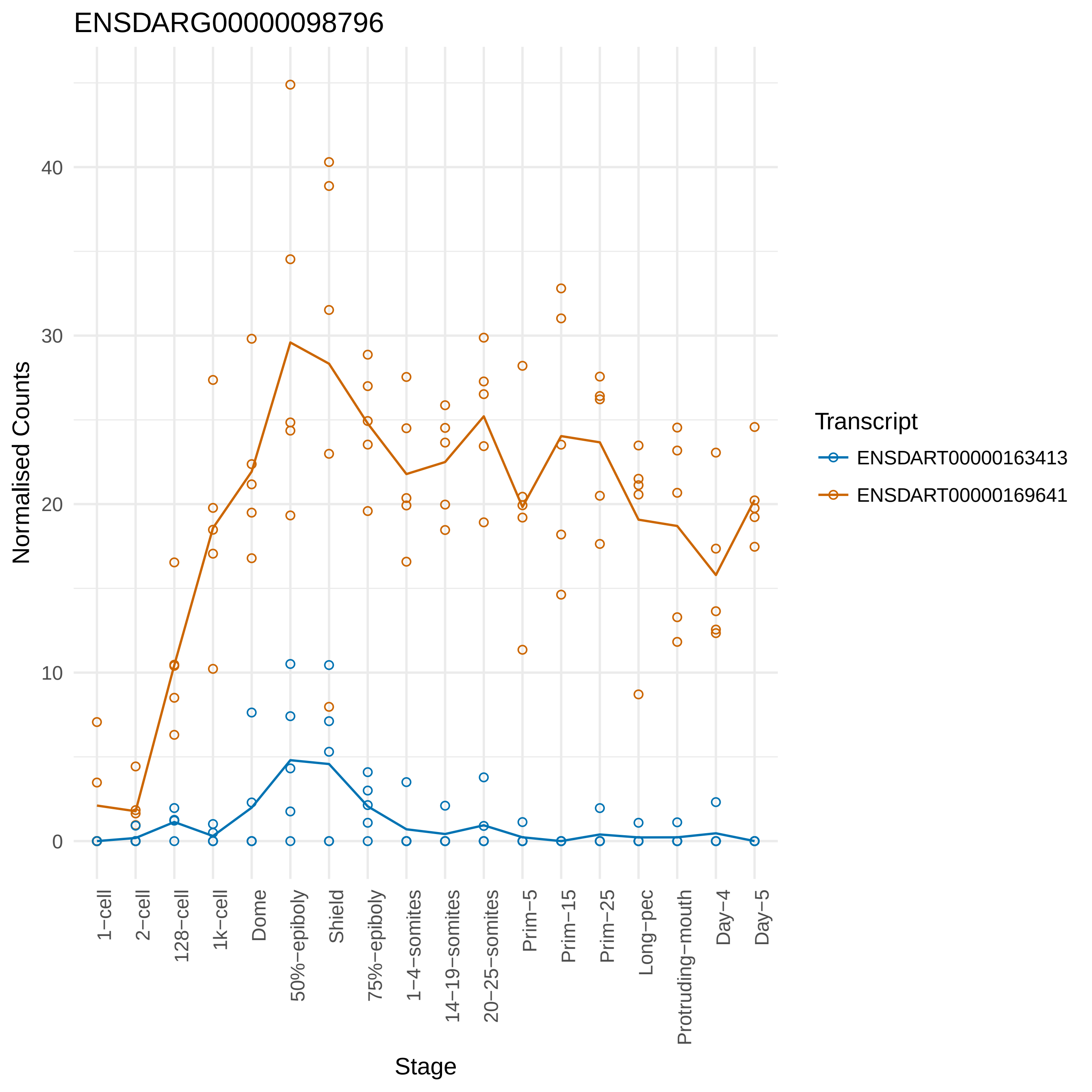

ENSDARG00000098796
Normalised Counts
40
30
20
10
0
1−cell
2−cell
128−cell
1k−cell
Dome
50%−epiboly
Shield
75%−epiboly
1−4−somites
14−19−somites
20−25−somites
Prim−5
Prim−15
Prim−25
Long−pec
Protruding−mouth
Day−4
Day−5
Stage
Transcript
ENSDART00000163413
ENSDART00000169641

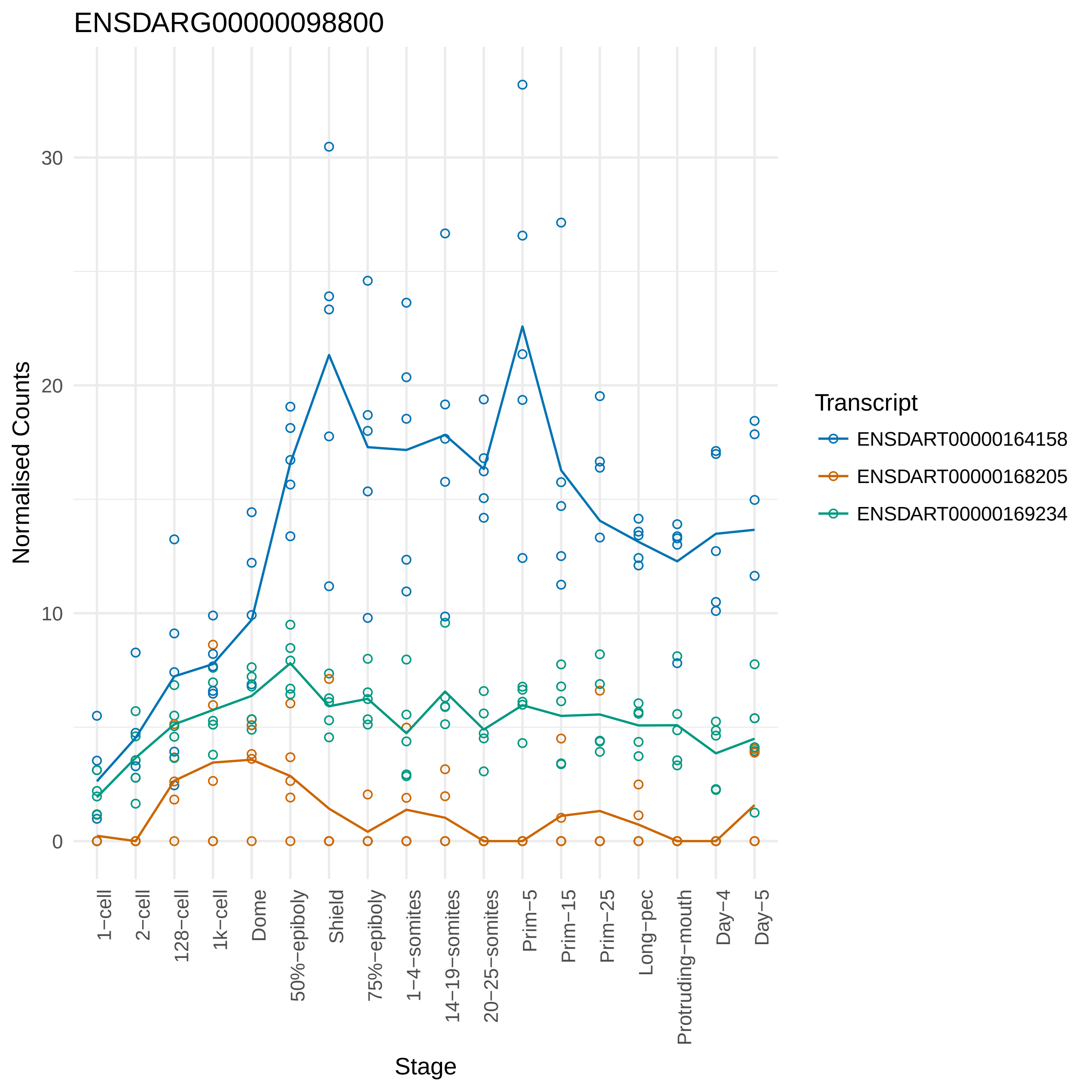

ENSDARG00000098800
Normalised Counts
30
20
10
0
1−cell
2−cell
128−cell
1k−cell
Dome
50%−epiboly
Shield
75%−epiboly
1−4−somites
14−19−somites
20−25−somites
Prim−5
Prim−15
Prim−25
Long−pec
Protruding−mouth
Day−4
Day−5
Stage
Transcript
ENSDART00000164158
ENSDART00000168205
ENSDART00000169234

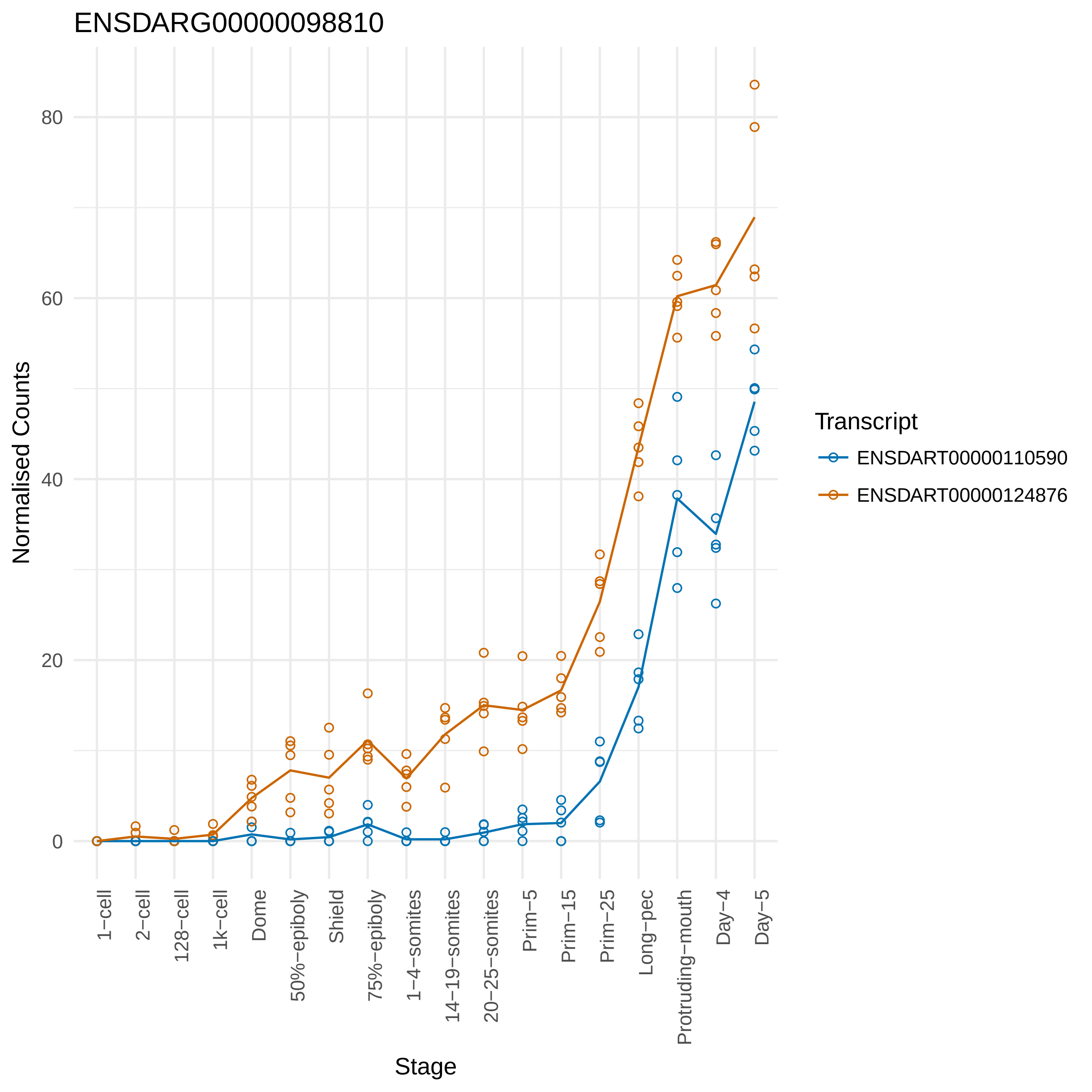
ENSDARG00000098810
Normalised Counts
0
20
40
60
80
Stage
1−cell
2−cell
128−cell
1k−cell
Dome
50%−epiboly
Shield
75%−epiboly
1−4−somites
14−19−somites
20−25−somites
Prim−5
Prim−15
Prim−25
Long−pec
Protruding−mouth
Day−4
Day−5
Transcript
ENSDART00000110590
ENSDART00000124876

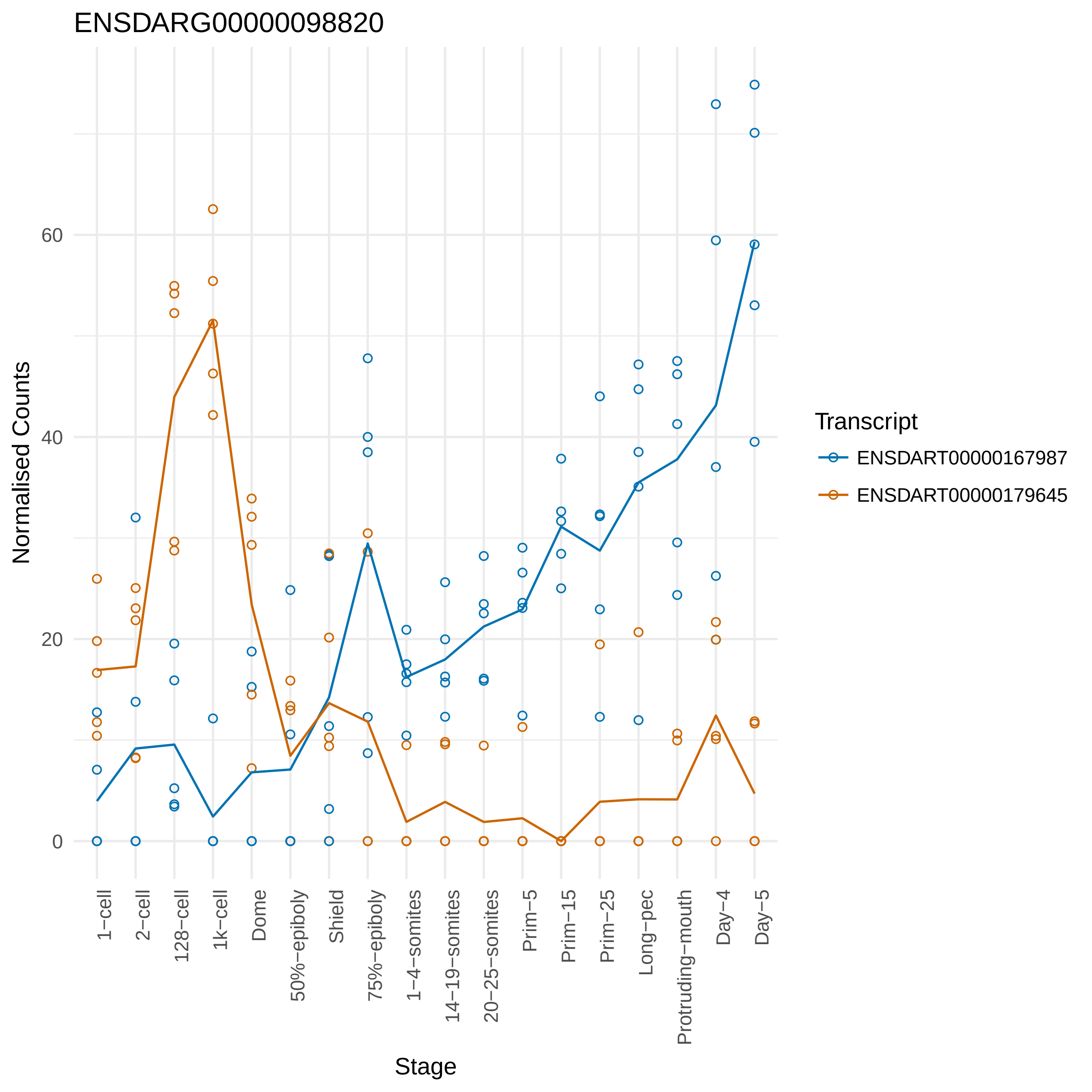

ENSDARG00000098820
Normalised Counts
0
20
40
60
1−cell
2−cell
128−cell
1k−cell
Dome
50%−epiboly
Shield
75%−epiboly
1−4−somites
14−19−somites
20−25−somites
Prim−5
Prim−15
Prim−25
Long−pec
Protruding−mouth
Day−4
Day−5
Stage
Transcript
ENSDART00000167987
ENSDART00000179645

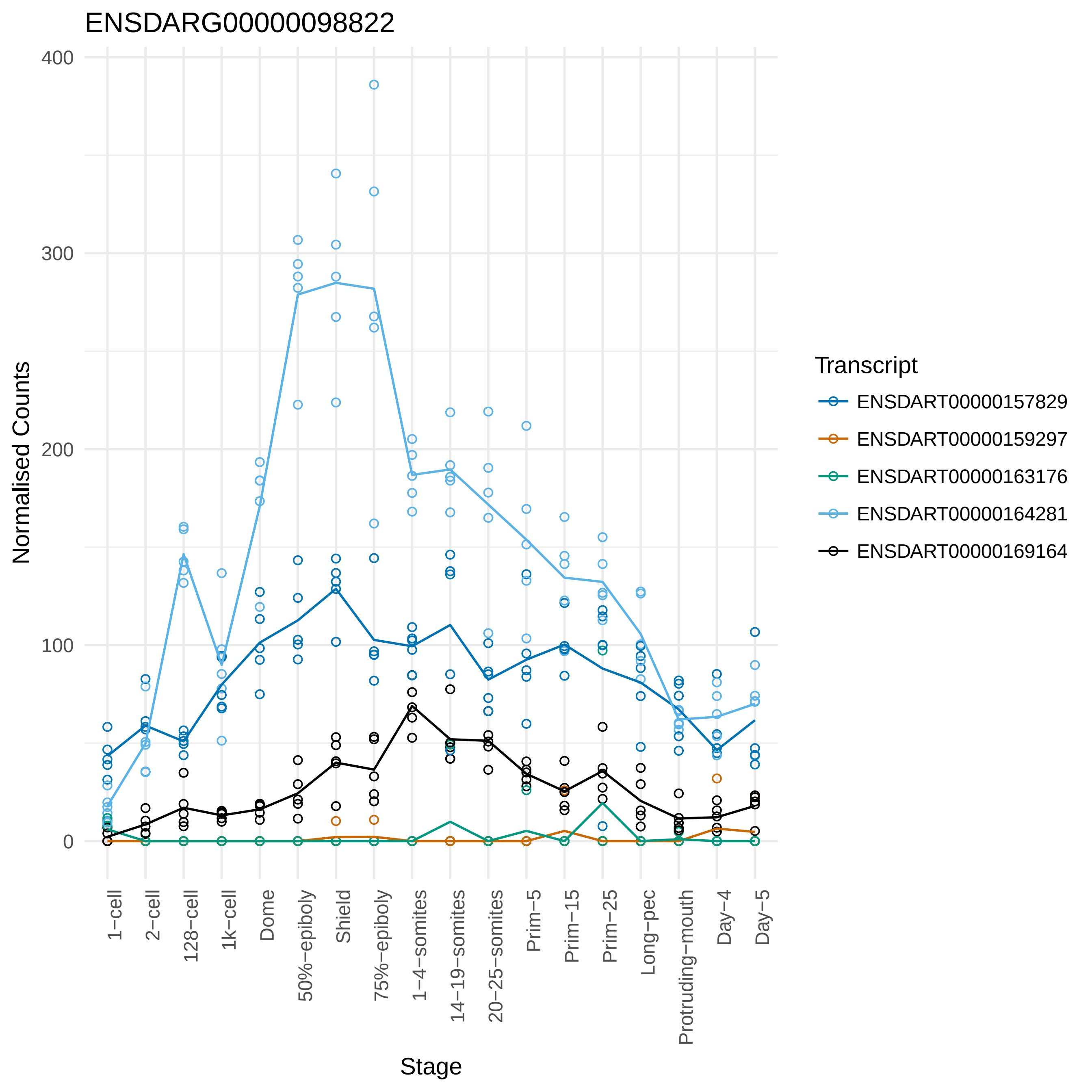

ENSDARG00000098822
Normalised Counts
0
100
200
300
400
1−cell
2−cell
128−cell
1k−cell
Dome
50%−epiboly
Shield
75%−epiboly
1−4−somites
14−19−somites
20−25−somites
Prim−5
Prim−15
Prim−25
Long−pec
Protruding−mouth
Day−4
Day−5
Stage
Transcript
ENSDART00000157829
ENSDART00000159297
ENSDART00000163176
ENSDART00000164281
ENSDART00000169164

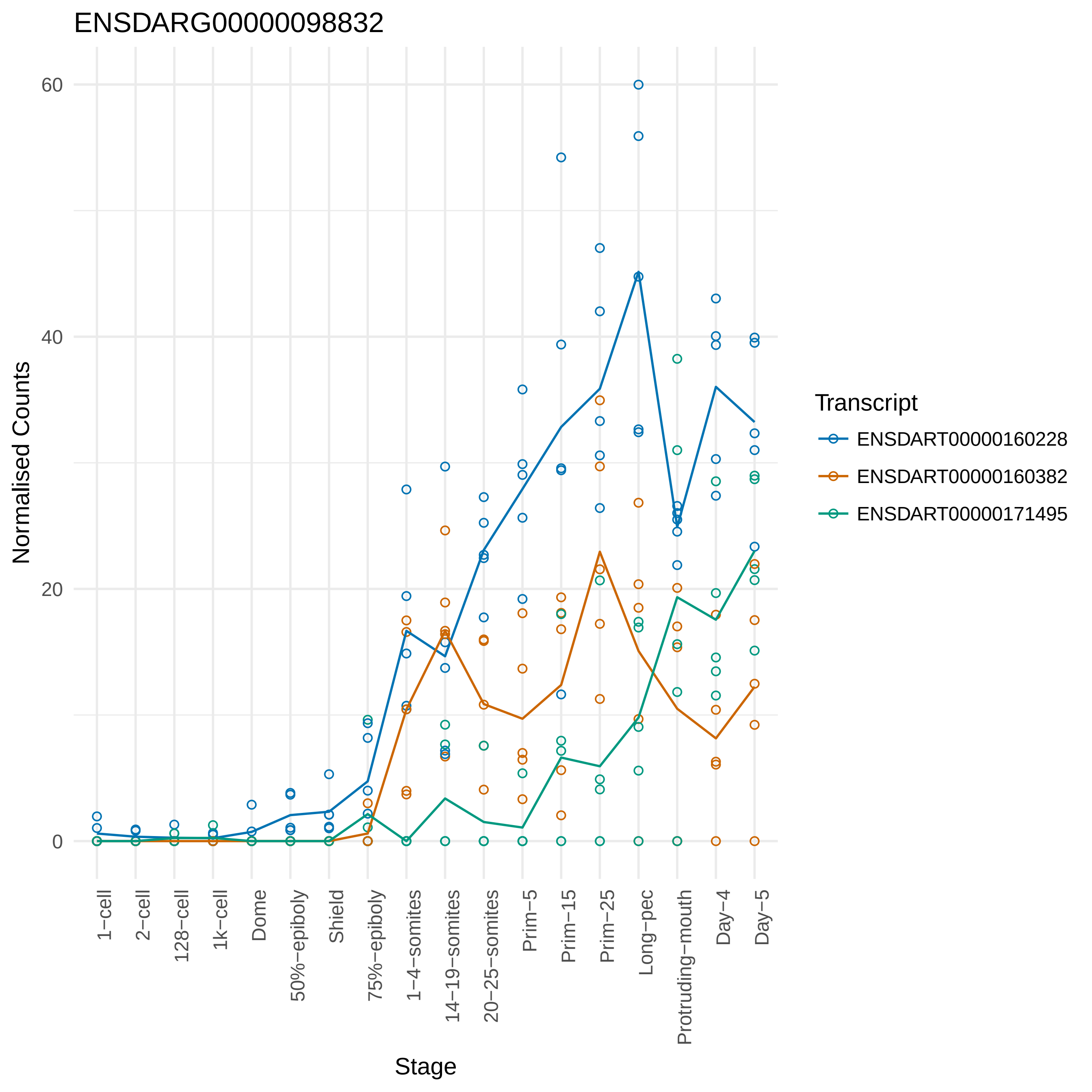
ENSDARG00000098832
Normalised Counts
0
20
40
60
Stage
1−cell
2−cell
128−cell
1k−cell
Dome
50%−epiboly
Shield
75%−epiboly
1−4−somites
14−19−somites
20−25−somites
Prim−5
Prim−15
Prim−25
Long−pec
Protruding−mouth
Day−4
Day−5
Transcript
ENSDART00000160228
ENSDART00000160382
ENSDART00000171495

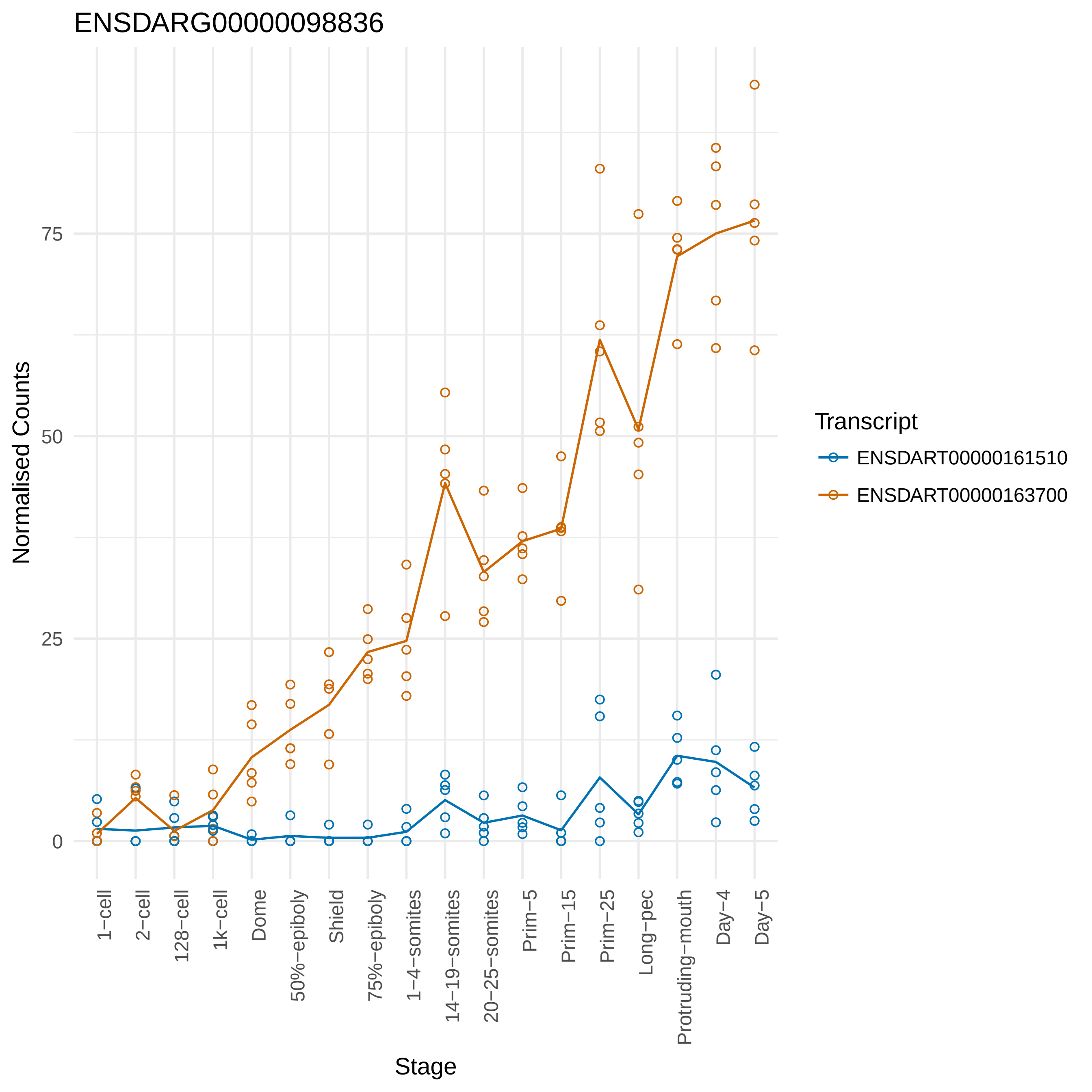
ENSDARG00000098836
Normalised Counts
0
25
50
75
1−cell
2−cell
128−cell
1k−cell
Dome
50%−epiboly
Shield
75%−epiboly
1−4−somites
14−19−somites
20−25−somites
Prim−5
Prim−15
Prim−25
Long−pec
Protruding−mouth
Day−4
Day−5
Stage
Transcript
ENSDART00000161510
ENSDART00000163700

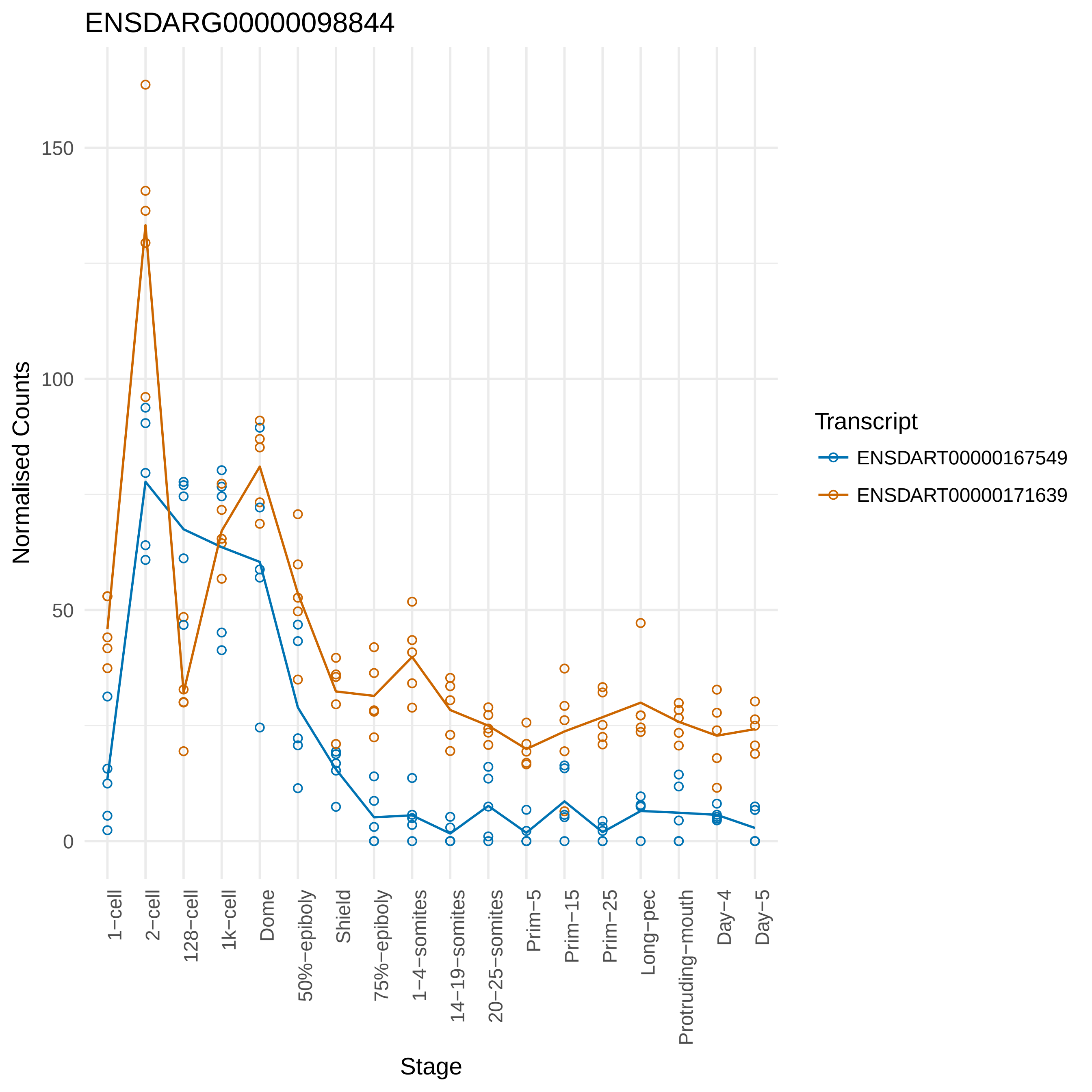

ENSDARG00000098844
Normalised Counts
150
100
50
0
1−cell
2−cell
128−cell
1k−cell
Dome
50%−epiboly
Shield
75%−epiboly
1−4−somites
14−19−somites
20−25−somites
Prim−5
Prim−15
Prim−25
Long−pec
Protruding−mouth
Day−4
Day−5
Stage
Transcript
ENSDART00000167549
ENSDART00000171639

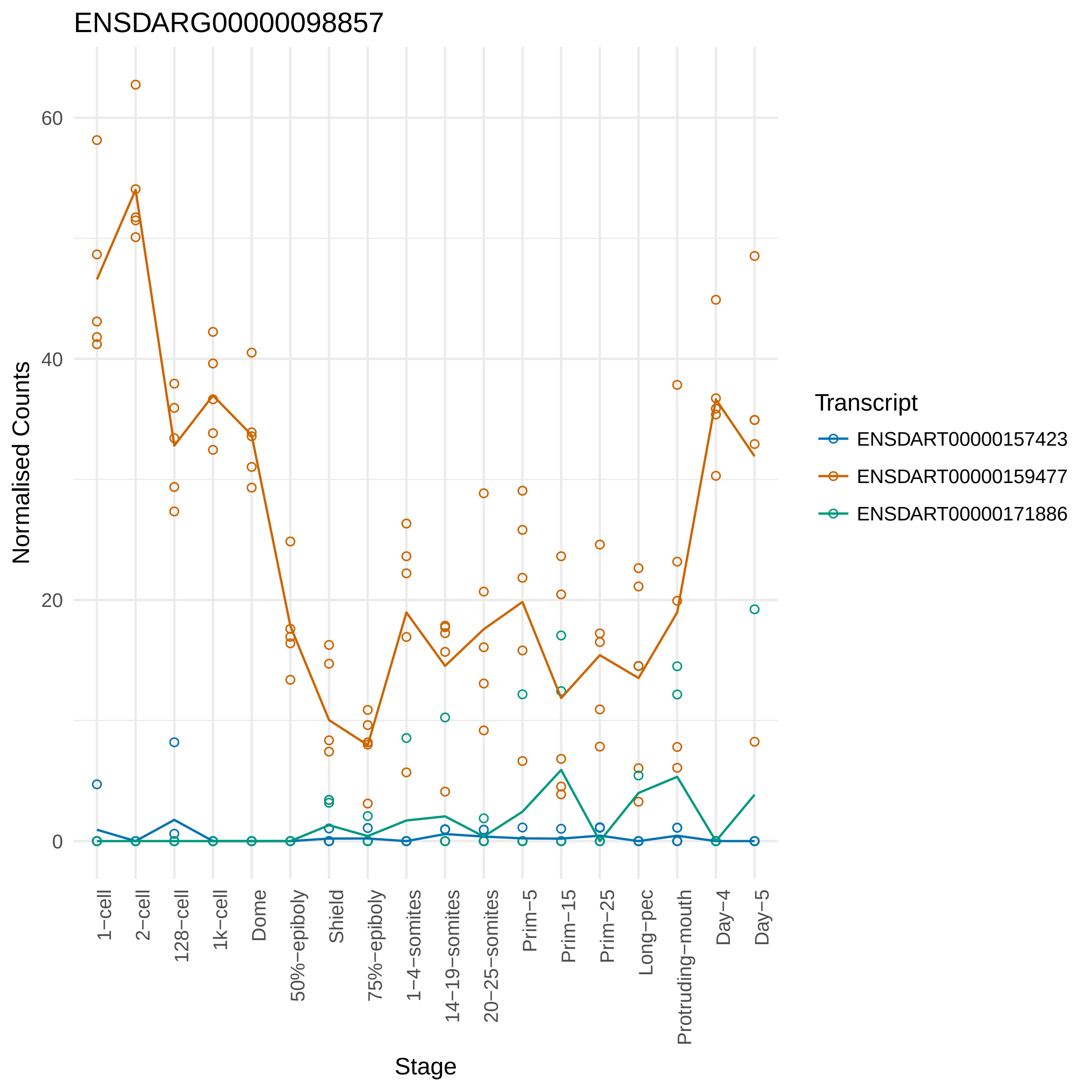
ENSDARG00000098857
Normalised Counts
60
40
20
0
1−cell
2−cell
128−cell
1k−cell
Dome
50%−epiboly
Shield
75%−epiboly
1−4−somites
14−19−somites
20−25−somites
Prim−5
Prim−15
Prim−25
Long−pec
Protruding−mouth
Day−4
Day−5
Stage
Transcript
ENSDART00000157423
ENSDART00000159477
ENSDART00000171886

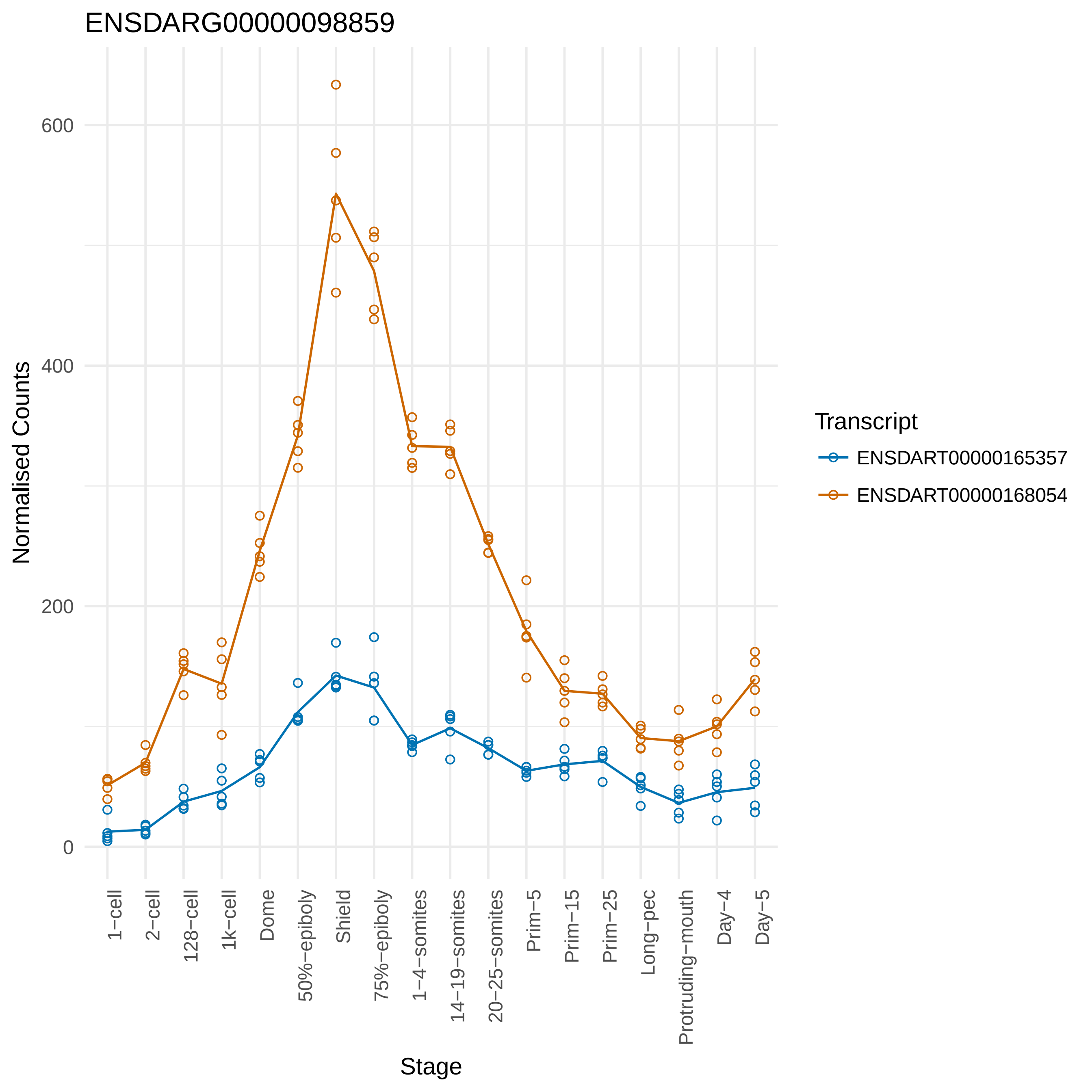

ENSDARG00000098859
Normalised Counts
600
400
200
0
1−cell
2−cell
128−cell
1k−cell
Dome
50%−epiboly
Shield
75%−epiboly
1−4−somites
14−19−somites
20−25−somites
Prim−5
Prim−15
Prim−25
Long−pec
Protruding−mouth
Day−4
Day−5
Stage
Transcript
ENSDART00000165357
ENSDART00000168054

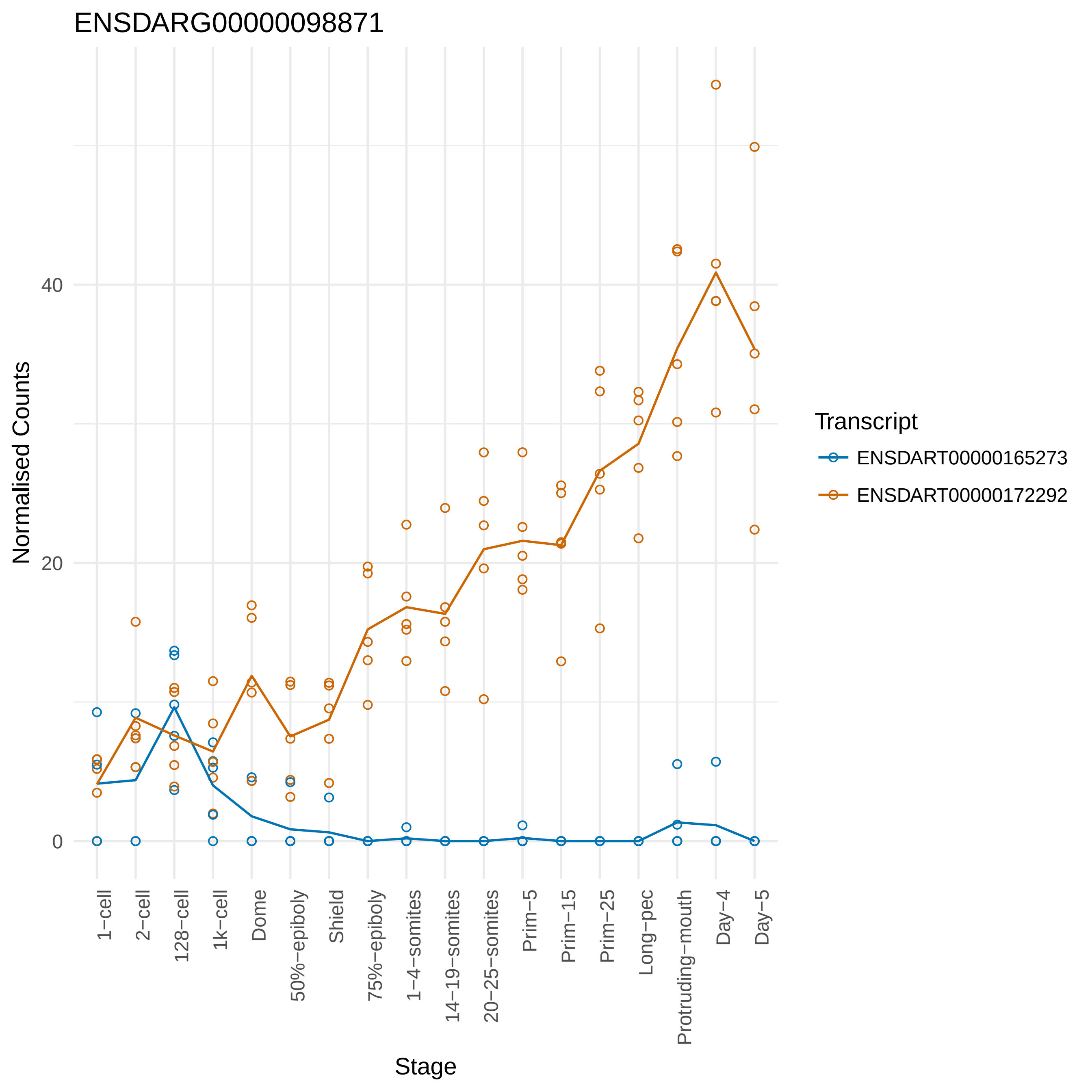

ENSDARG00000098871
Normalised Counts
0
20
40
Stage
1−cell
2−cell
128−cell
1k−cell
Dome
50%−epiboly
Shield
75%−epiboly
1−4−somites
14−19−somites
20−25−somites
Prim−5
Prim−15
Prim−25
Long−pec
Protruding−mouth
Day−4
Day−5
Transcript
ENSDART00000165273
ENSDART00000172292

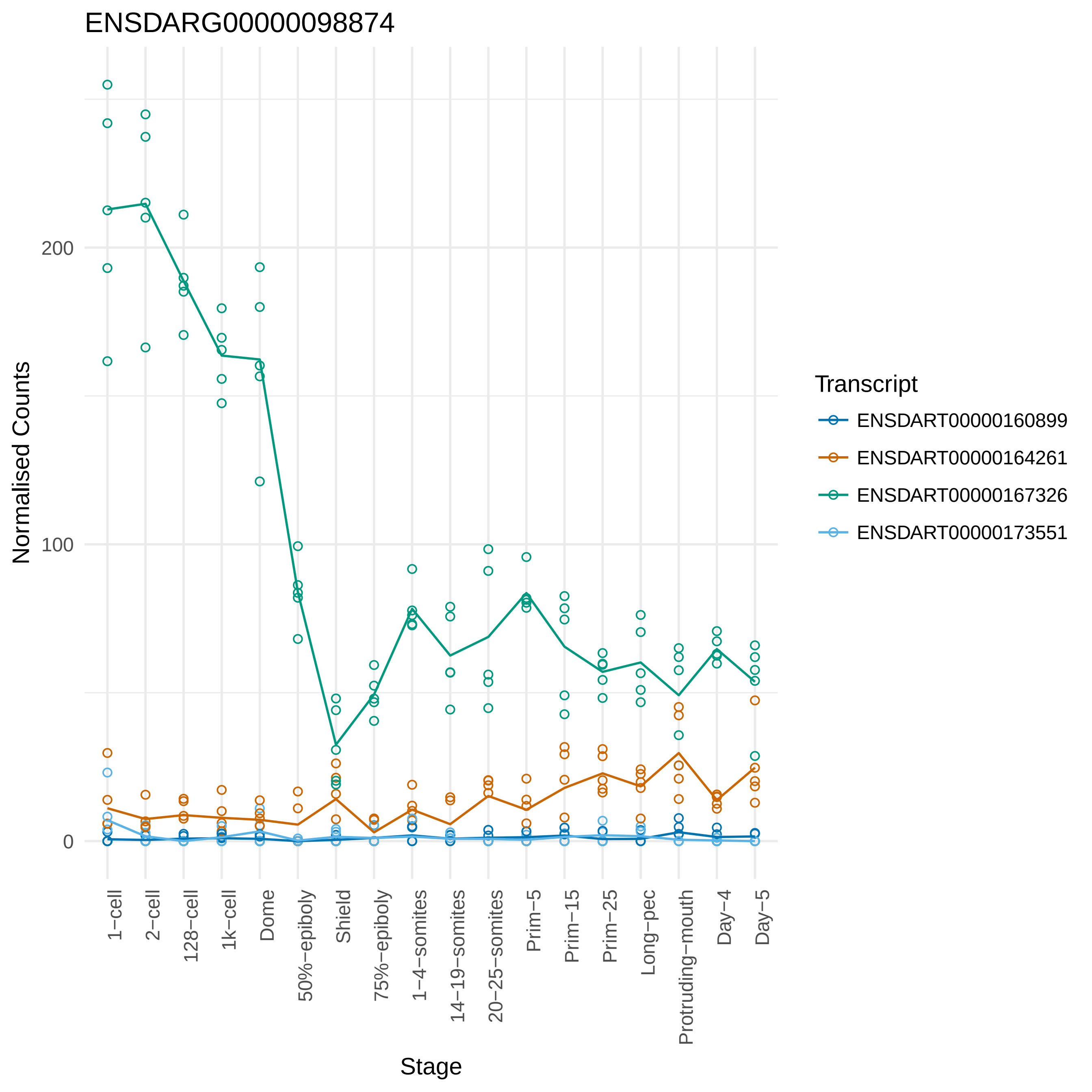

ENSDARG00000098874
Normalised Counts
200
100
0
1−cell
2−cell
128−cell
1k−cell
Dome
50%−epiboly
Shield
75%−epiboly
1−4−somites
14−19−somites
20−25−somites
Prim−5
Prim−15
Prim−25
Long−pec
Protruding−mouth
Day−4
Day−5
Stage
Transcript
ENSDART00000160899
ENSDART00000164261
ENSDART00000167326
ENSDART00000173551

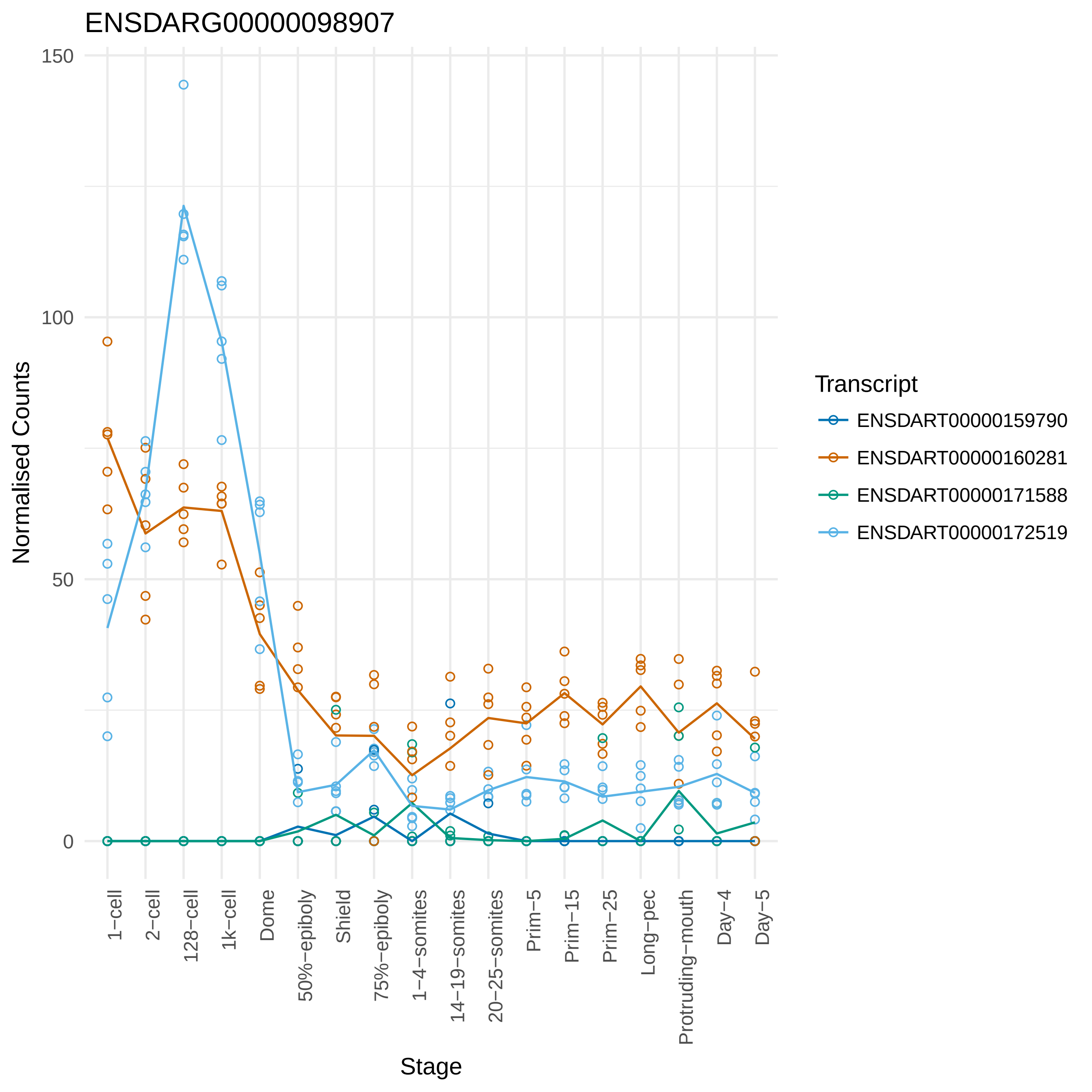

ENSDARG00000098907
Normalised Counts
150
100
50
0
1−cell
2−cell
128−cell
1k−cell
Dome
50%−epiboly
Shield
75%−epiboly
1−4−somites
14−19−somites
20−25−somites
Prim−5
Prim−15
Prim−25
Long−pec
Protruding−mouth
Day−4
Day−5
Stage
Transcript
ENSDART00000159790
ENSDART00000160281
ENSDART00000171588
ENSDART00000172519

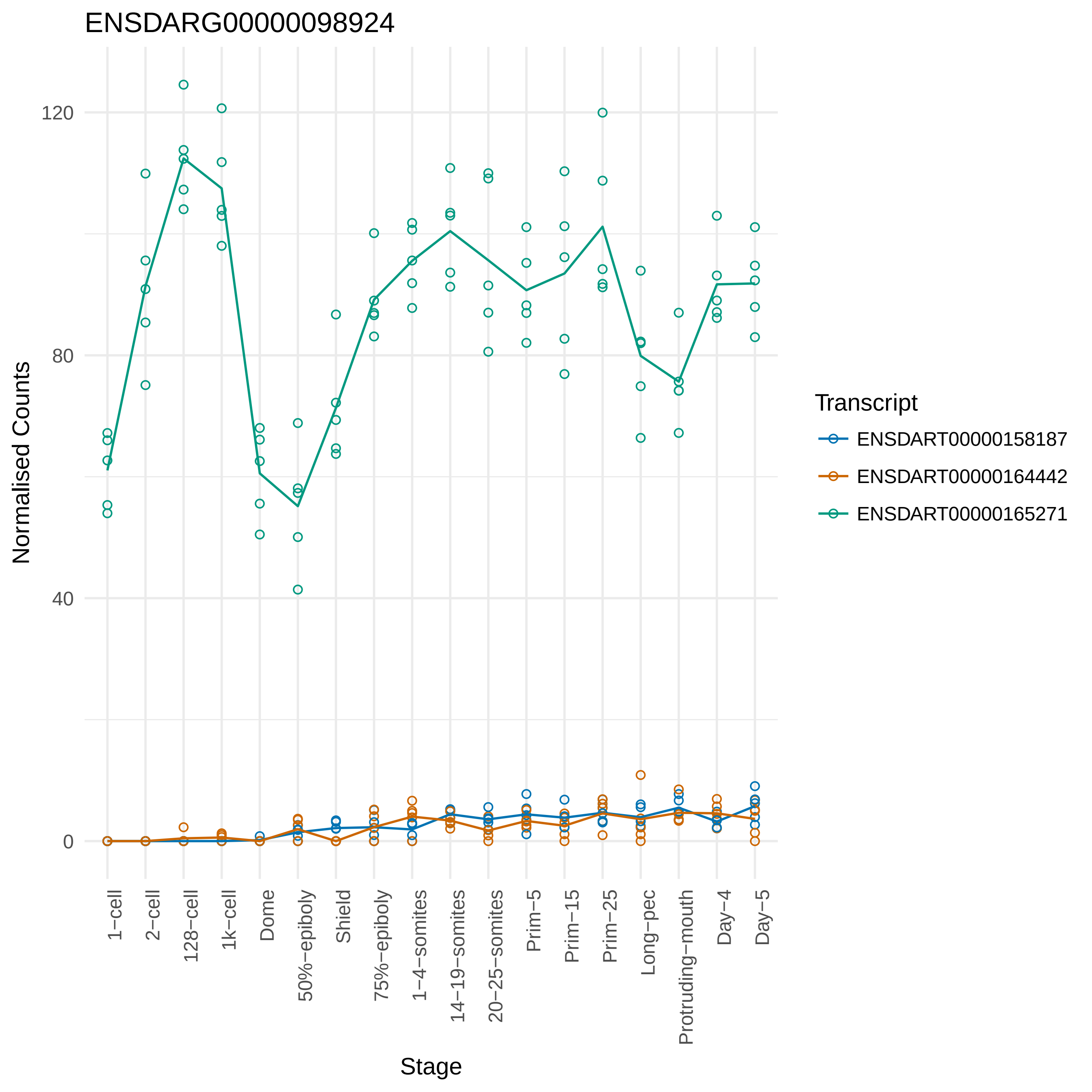

ENSDARG00000098924
Normalised Counts
0
40
80
120
Stage
1−cell
2−cell
128−cell
1k−cell
Dome
50%−epiboly
Shield
75%−epiboly
1−4−somites
14−19−somites
20−25−somites
Prim−5
Prim−15
Prim−25
Long−pec
Protruding−mouth
Day−4
Day−5
Transcript
ENSDART00000158187
ENSDART00000164442
ENSDART00000165271

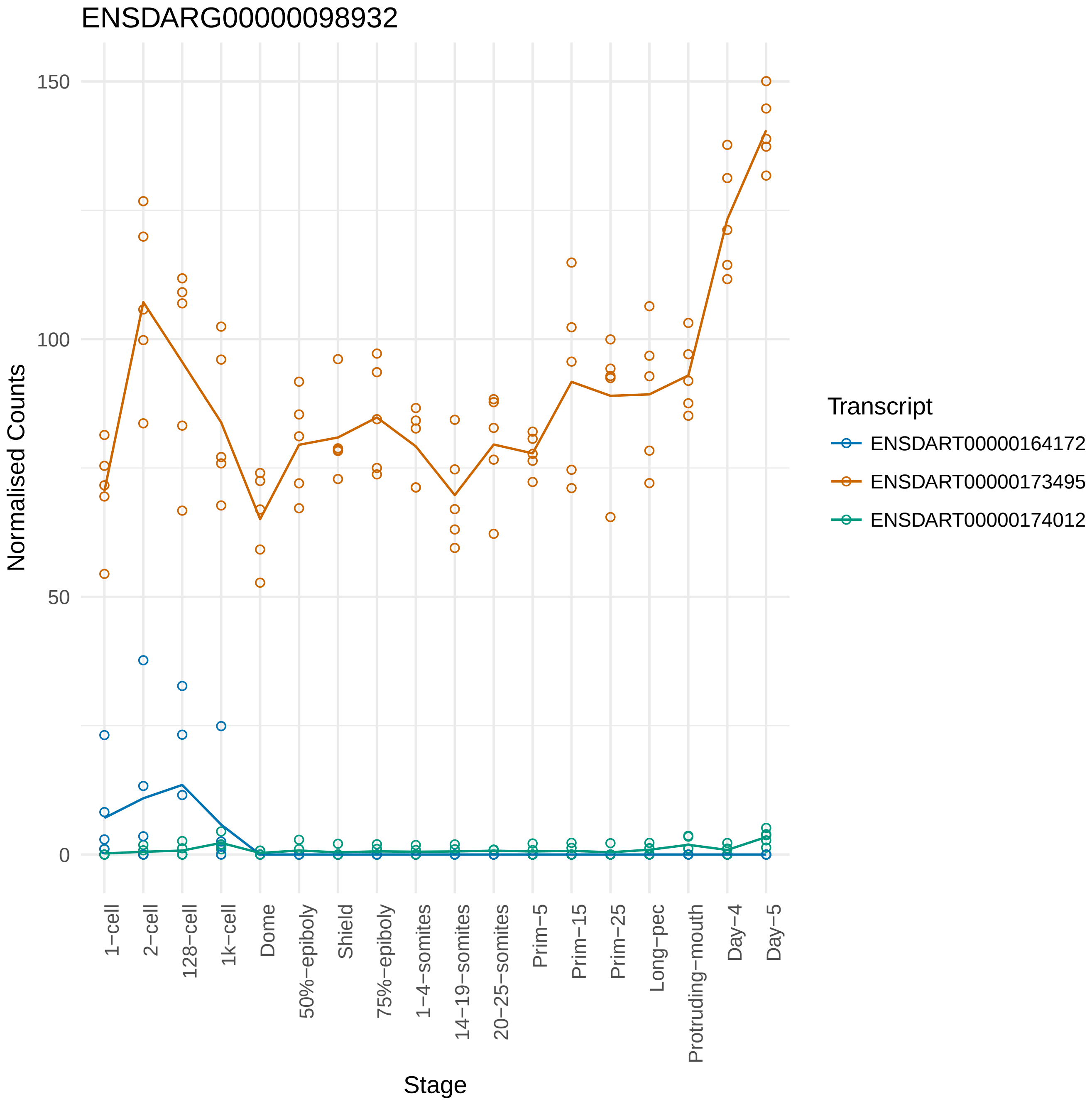
ENSDARG00000098932
Normalised Counts
150
100
50
0
1−cell
2−cell
128−cell
1k−cell
Dome
50%−epiboly
Shield
75%−epiboly
1−4−somites
14−19−somites
20−25−somites
Prim−5
Prim−15
Prim−25
Long−pec
Protruding−mouth
Day−4
Day−5
Stage
Transcript
ENSDART00000164172
ENSDART00000173495
ENSDART00000174012

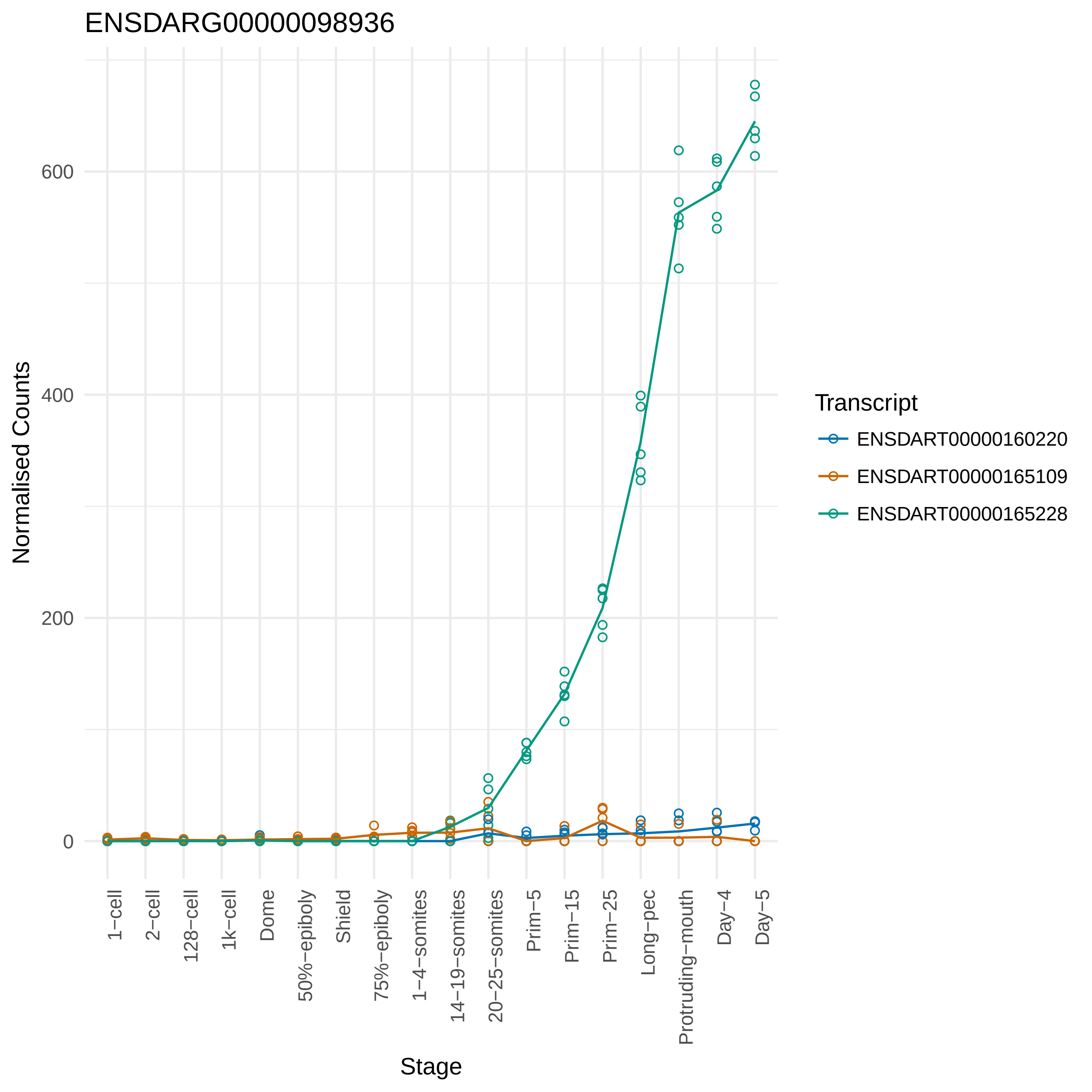
ENSDARG00000098936
Normalised Counts
0
200
400
600
Stage
1−cell
2−cell
128−cell
1k−cell
Dome
50%−epiboly
Shield
75%−epiboly
1−4−somites
14−19−somites
20−25−somites
Prim−5
Prim−15
Prim−25
Long−pec
Protruding−mouth
Day−4
Day−5
Transcript
ENSDART00000160220
ENSDART00000165109
ENSDART00000165228

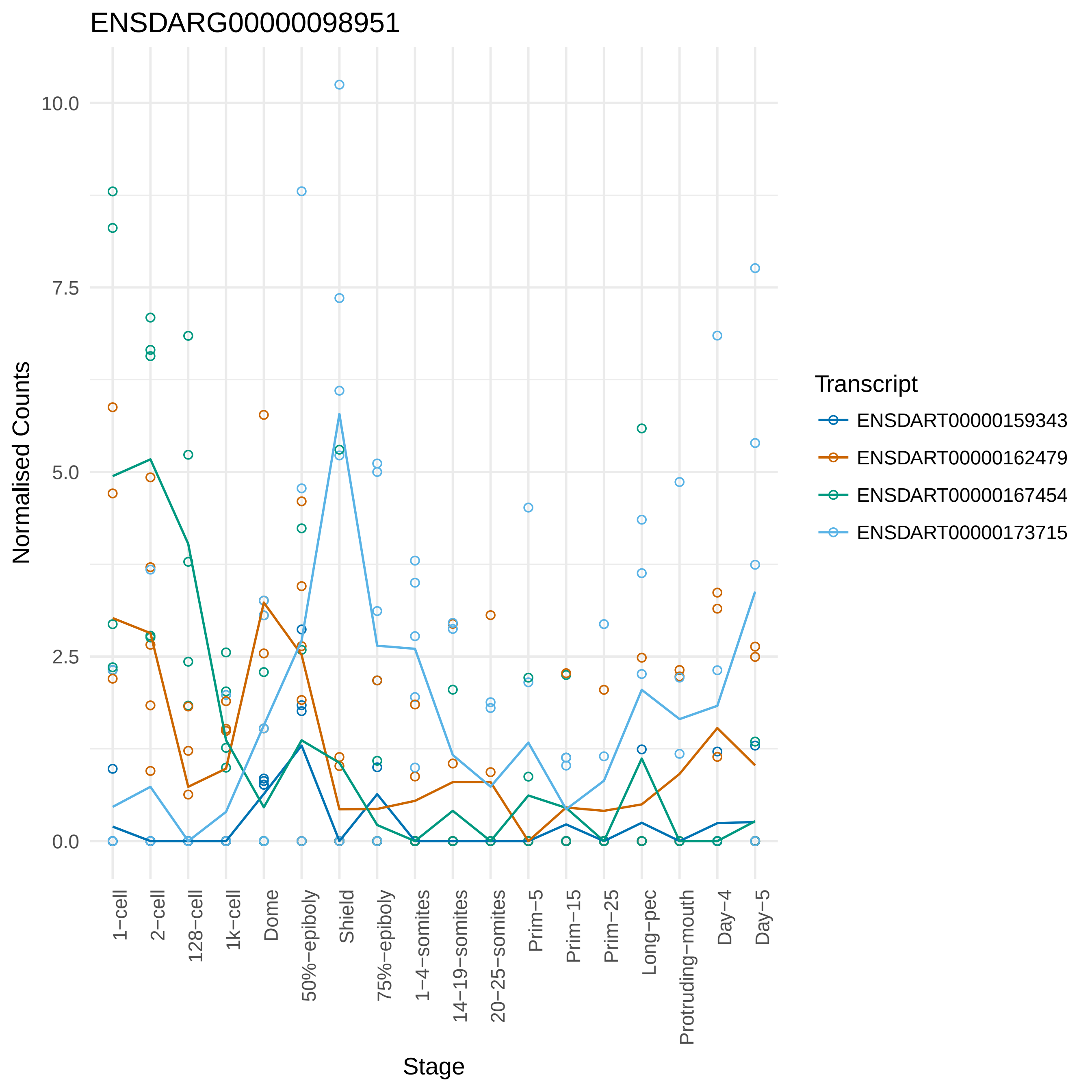

ENSDARG00000098951
Normalised Counts
10.0
7.5
5.0
2.5
0.0
1−cell
2−cell
128−cell
1k−cell
Dome
50%−epiboly
Shield
75%−epiboly
1−4−somites
14−19−somites
20−25−somites
Prim−5
Prim−15
Prim−25
Long−pec
Protruding−mouth
Day−4
Day−5
Stage
Transcript
ENSDART00000159343
ENSDART00000162479
ENSDART00000167454
ENSDART00000173715

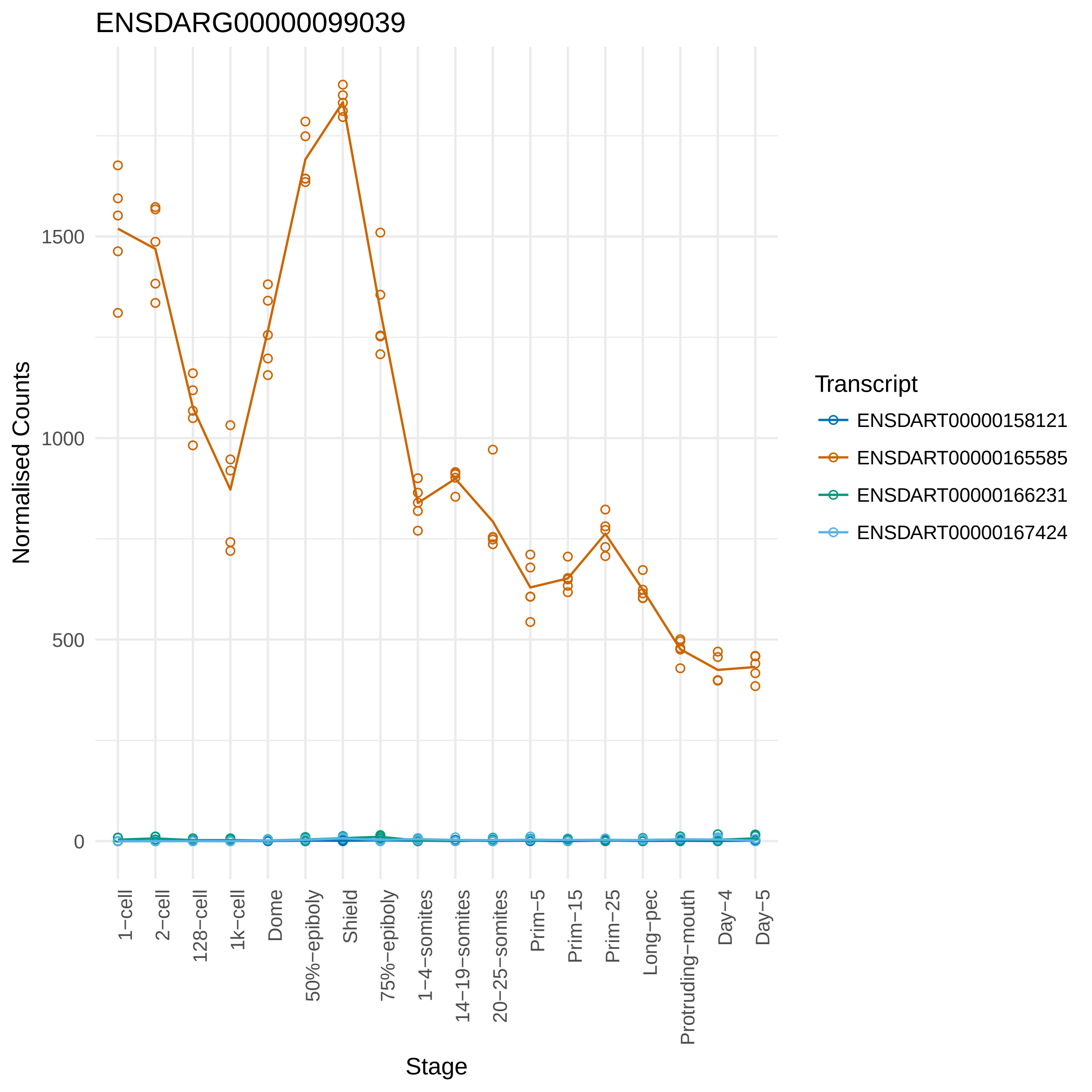

ENSDARG00000099039
Normalised Counts
1500
1000
500
0
1−cell
2−cell
128−cell
1k−cell
Dome
50%−epiboly
Shield
75%−epiboly
1−4−somites
14−19−somites
20−25−somites
Prim−5
Prim−15
Prim−25
Long−pec
Protruding−mouth
Day−4
Day−5
Stage
Transcript
ENSDART00000158121
ENSDART00000165585
ENSDART00000166231
ENSDART00000167424

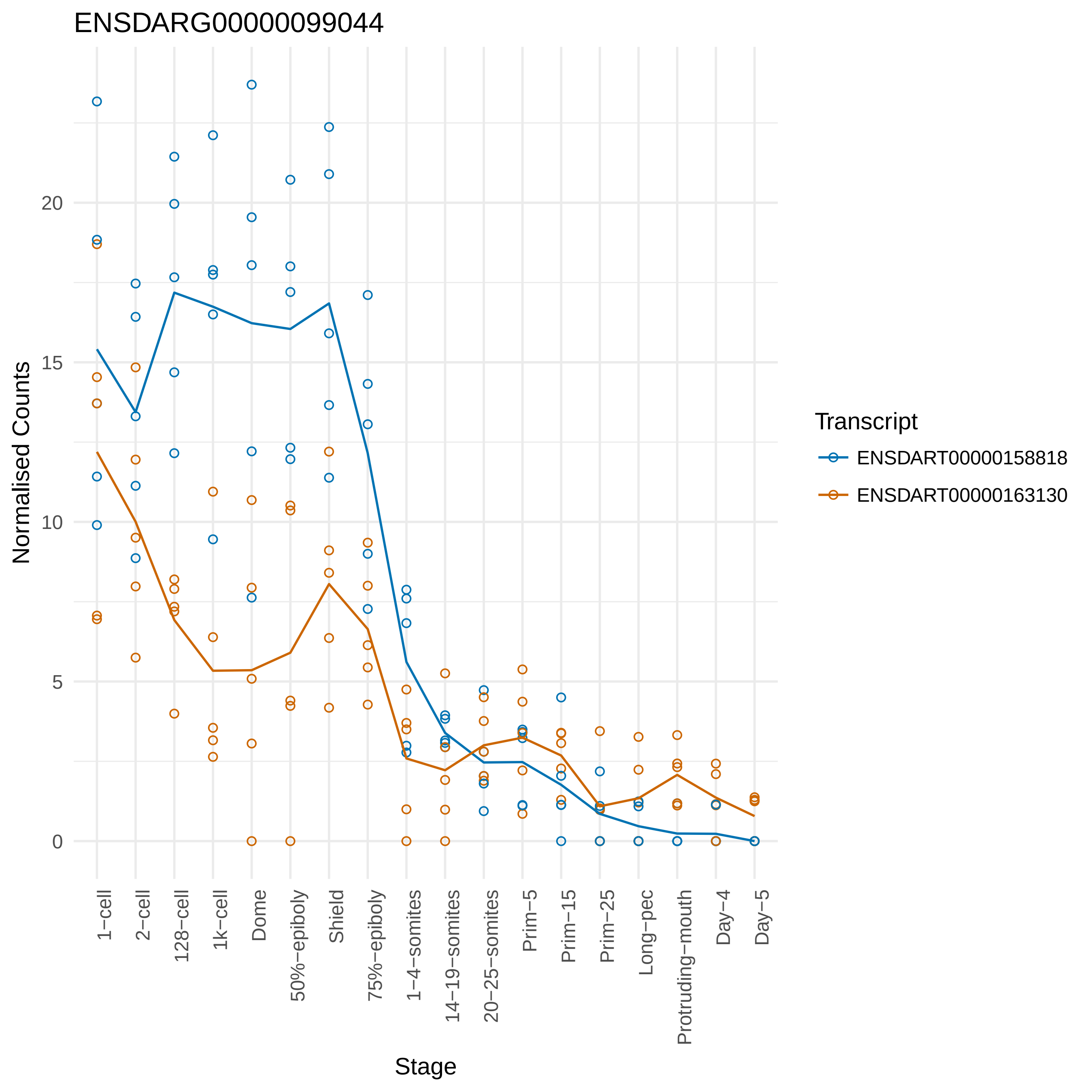

ENSDARG00000099044
Normalised Counts
0
5
10
15
20
1−cell
2−cell
128−cell
1k−cell
Dome
50%−epiboly
Shield
75%−epiboly
1−4−somites
14−19−somites
20−25−somites
Prim−5
Prim−15
Prim−25
Long−pec
Protruding−mouth
Day−4
Day−5
Stage
Transcript
ENSDART00000158818
ENSDART00000163130

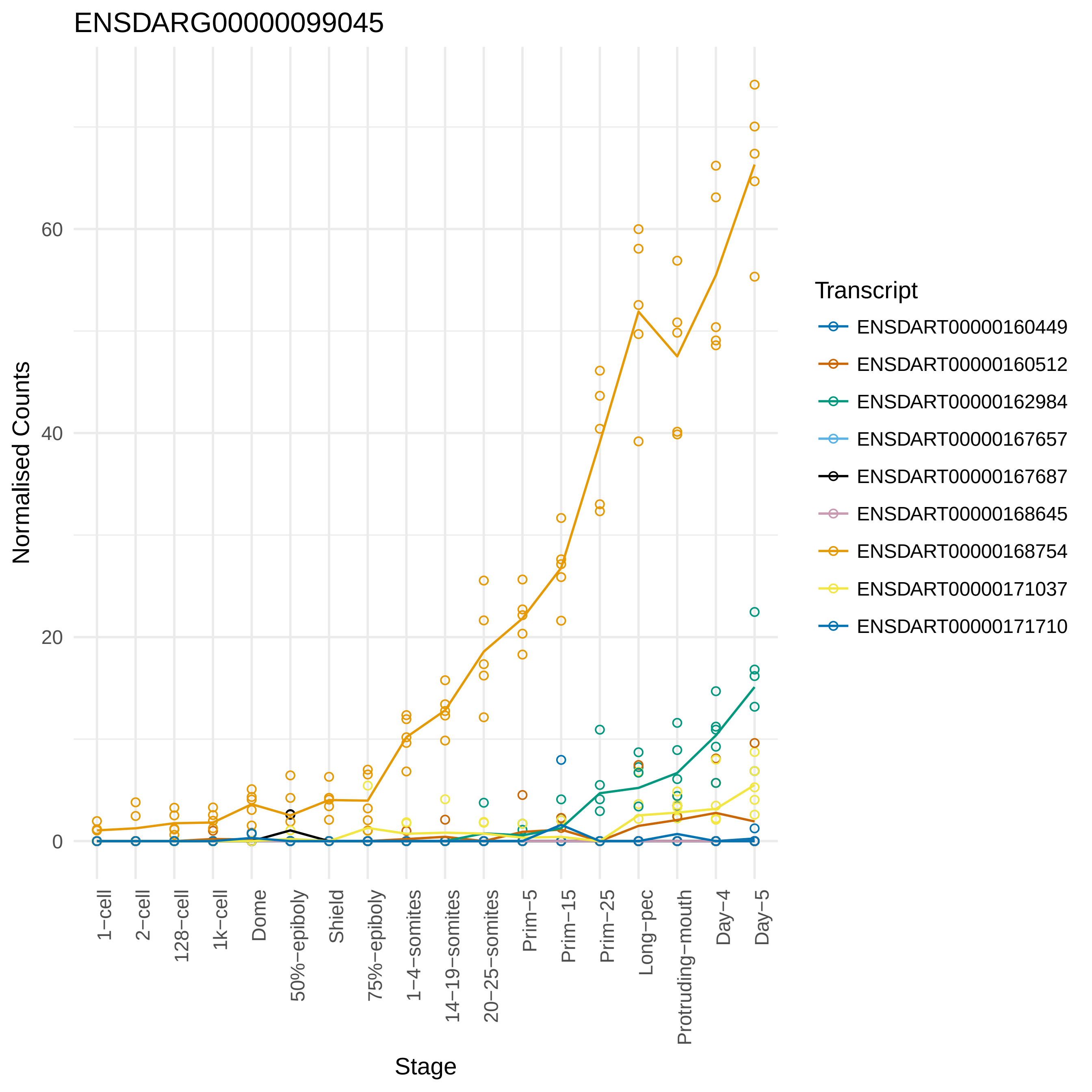
ENSDARG00000099045
Normalised Counts
60
40
20
0
1−cell
2−cell
128−cell
1k−cell
Dome
50%−epiboly
Shield
75%−epiboly
1−4−somites
14−19−somites
20−25−somites
Prim−5
Prim−15
Prim−25
Long−pec
Protruding−mouth
Day−4
Day−5
Stage
Transcript
ENSDART00000160449
ENSDART00000160512
ENSDART00000162984
ENSDART00000167657
ENSDART00000167687
ENSDART00000168645
ENSDART00000168754
ENSDART00000171037
ENSDART00000171710

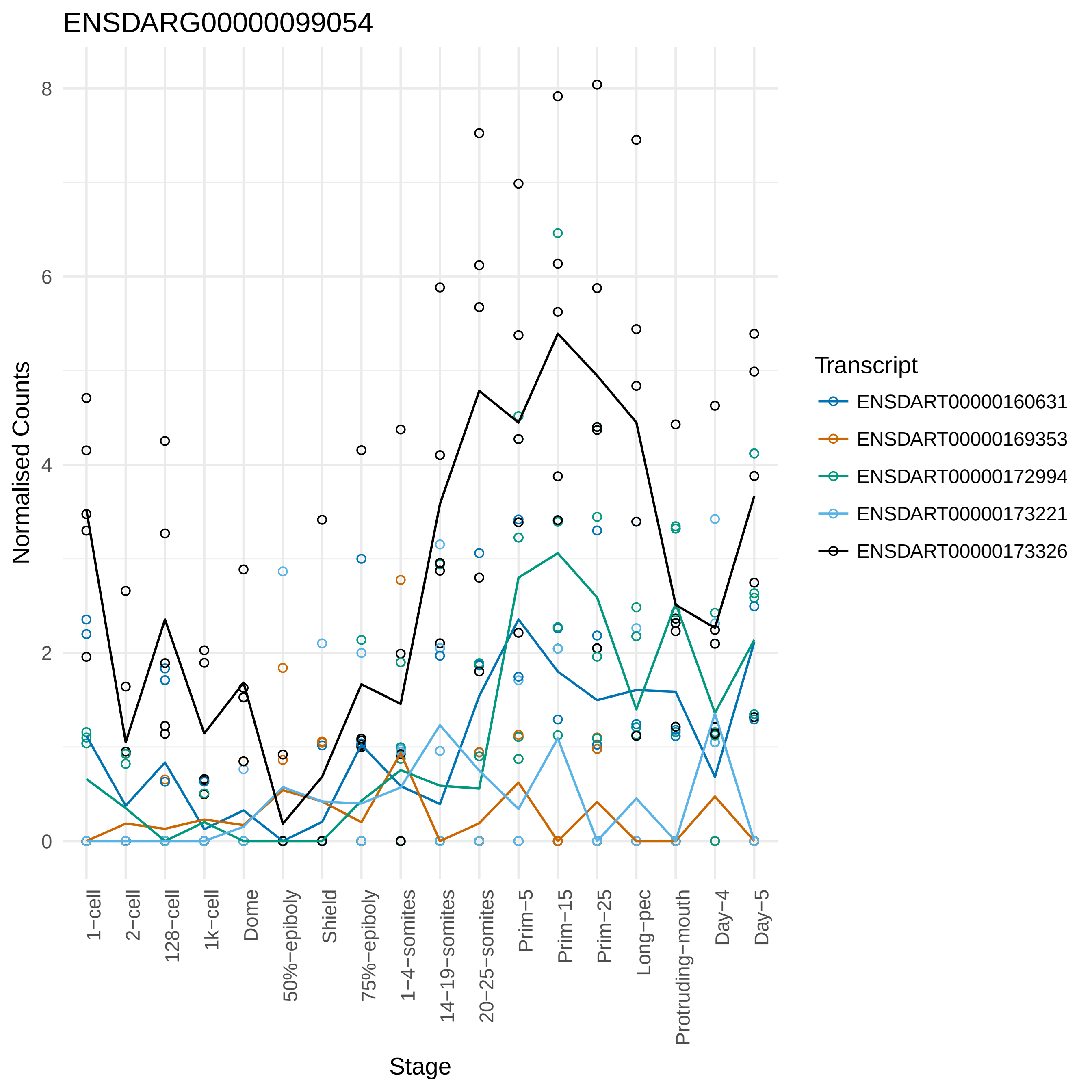

ENSDARG00000099054
Normalised Counts
0
2
4
6
8
1−cell
2−cell
128−cell
1k−cell
Dome
50%−epiboly
Shield
75%−epiboly
1−4−somites
14−19−somites
20−25−somites
Prim−5
Prim−15
Prim−25
Long−pec
Protruding−mouth
Day−4
Day−5
Stage
Transcript
ENSDART00000160631
ENSDART00000169353
ENSDART00000172994
ENSDART00000173221
ENSDART00000173326

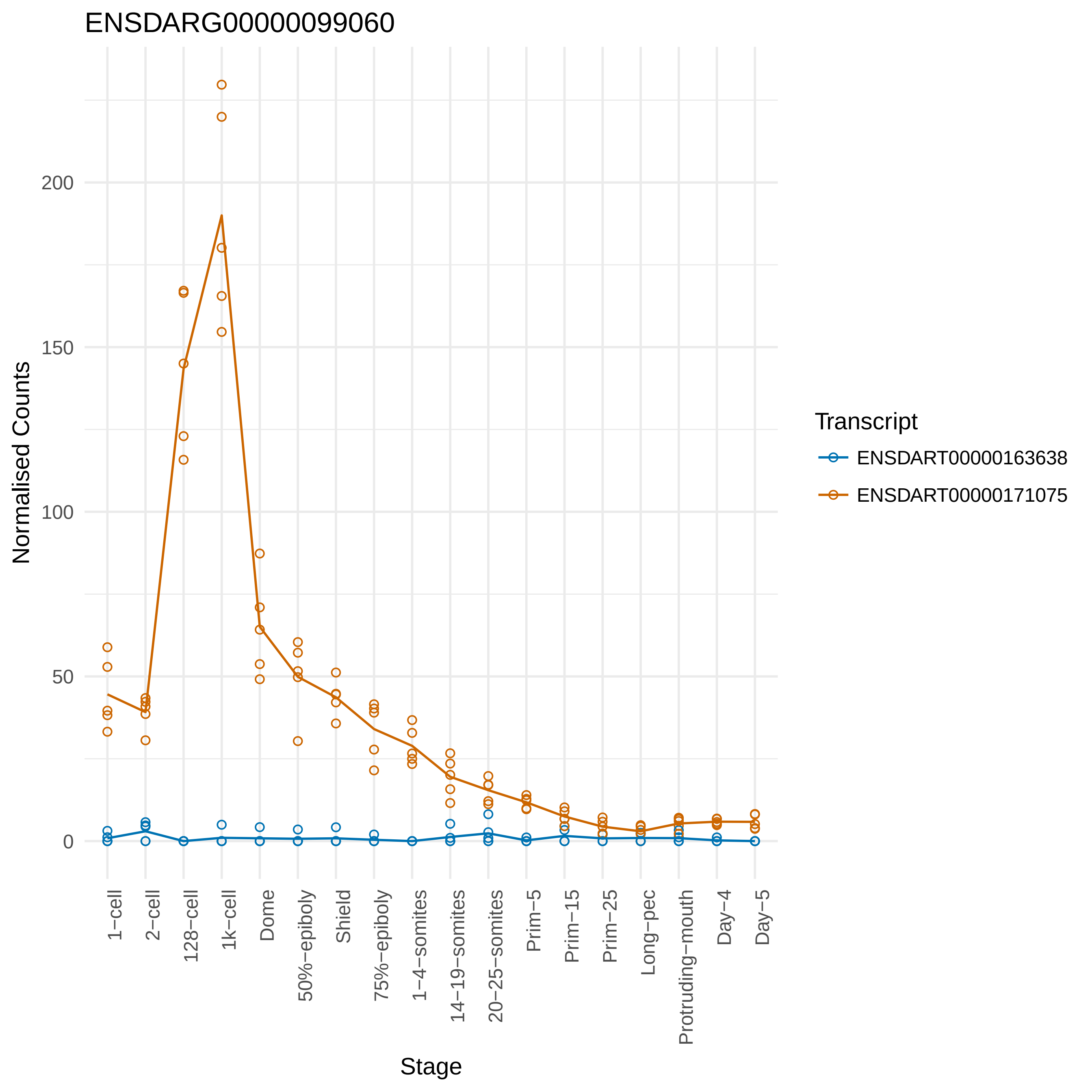

ENSDARG00000099060
Normalised Counts
200
150
100
50
0
1−cell
2−cell
128−cell
1k−cell
Dome
50%−epiboly
Shield
75%−epiboly
1−4−somites
14−19−somites
20−25−somites
Prim−5
Prim−15
Prim−25
Long−pec
Protruding−mouth
Day−4
Day−5
Stage
Transcript
ENSDART00000163638
ENSDART00000171075

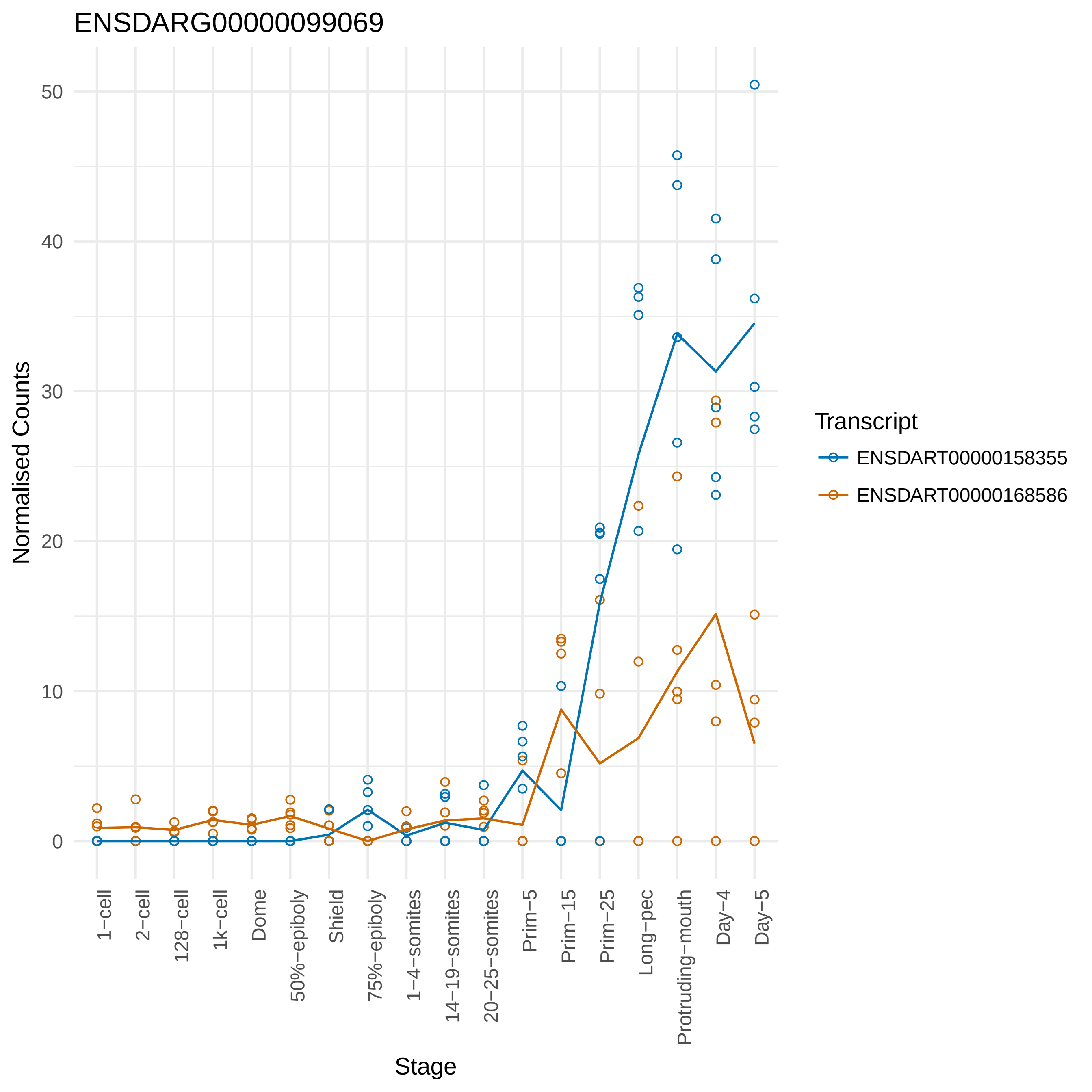

ENSDARG00000099069
Normalised Counts
0
10
20
30
40
50
1−cell
2−cell
128−cell
1k−cell
Dome
50%−epiboly
Shield
75%−epiboly
1−4−somites
14−19−somites
20−25−somites
Prim−5
Prim−15
Prim−25
Long−pec
Protruding−mouth
Day−4
Day−5
Stage
Transcript
ENSDART00000158355
ENSDART00000168586

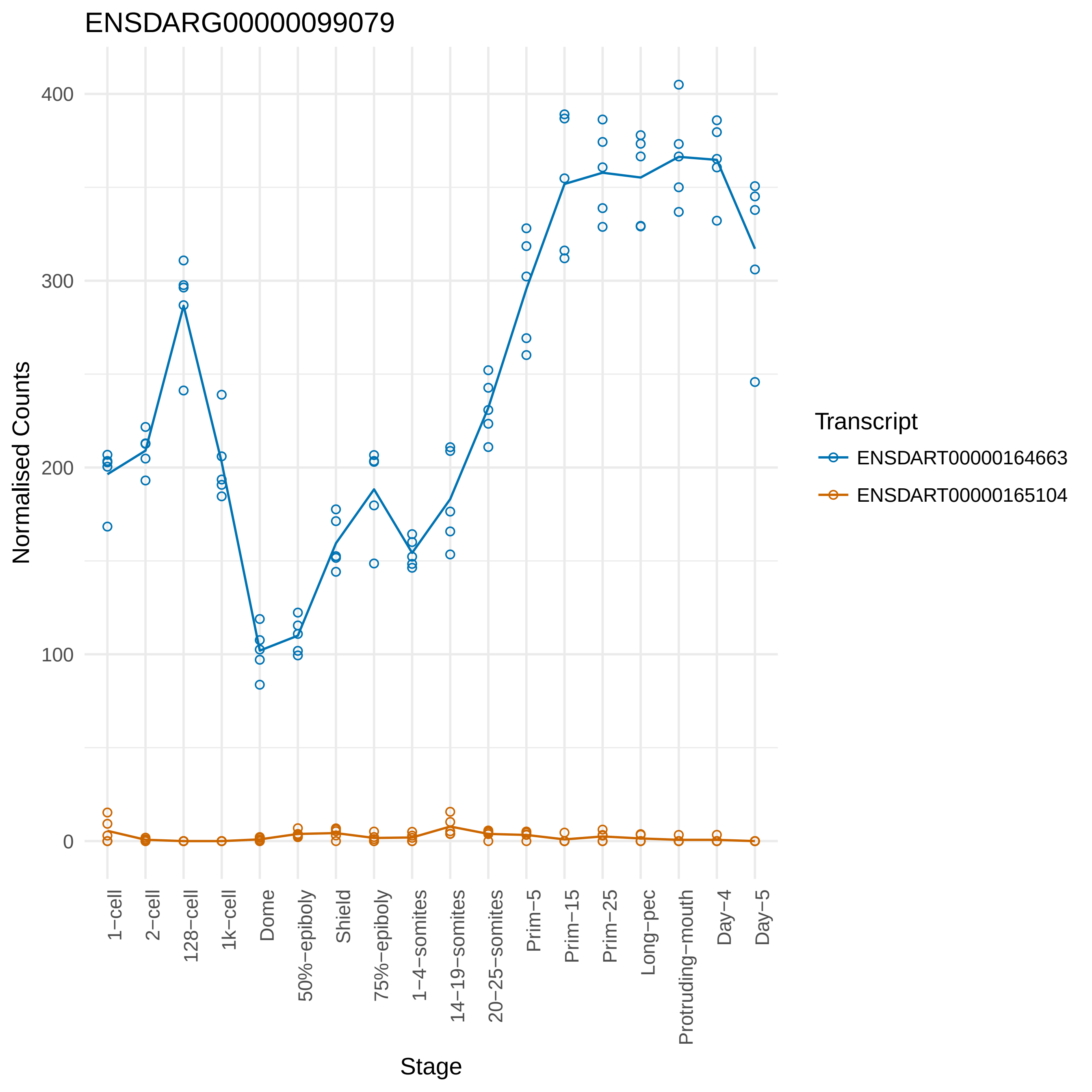

ENSDARG00000099079
Normalised Counts
400
300
200
100
0
1−cell
2−cell
128−cell
1k−cell
Dome
50%−epiboly
Shield
75%−epiboly
1−4−somites
14−19−somites
20−25−somites
Prim−5
Prim−15
Prim−25
Long−pec
Protruding−mouth
Day−4
Day−5
Stage
Transcript
ENSDART00000164663
ENSDART00000165104

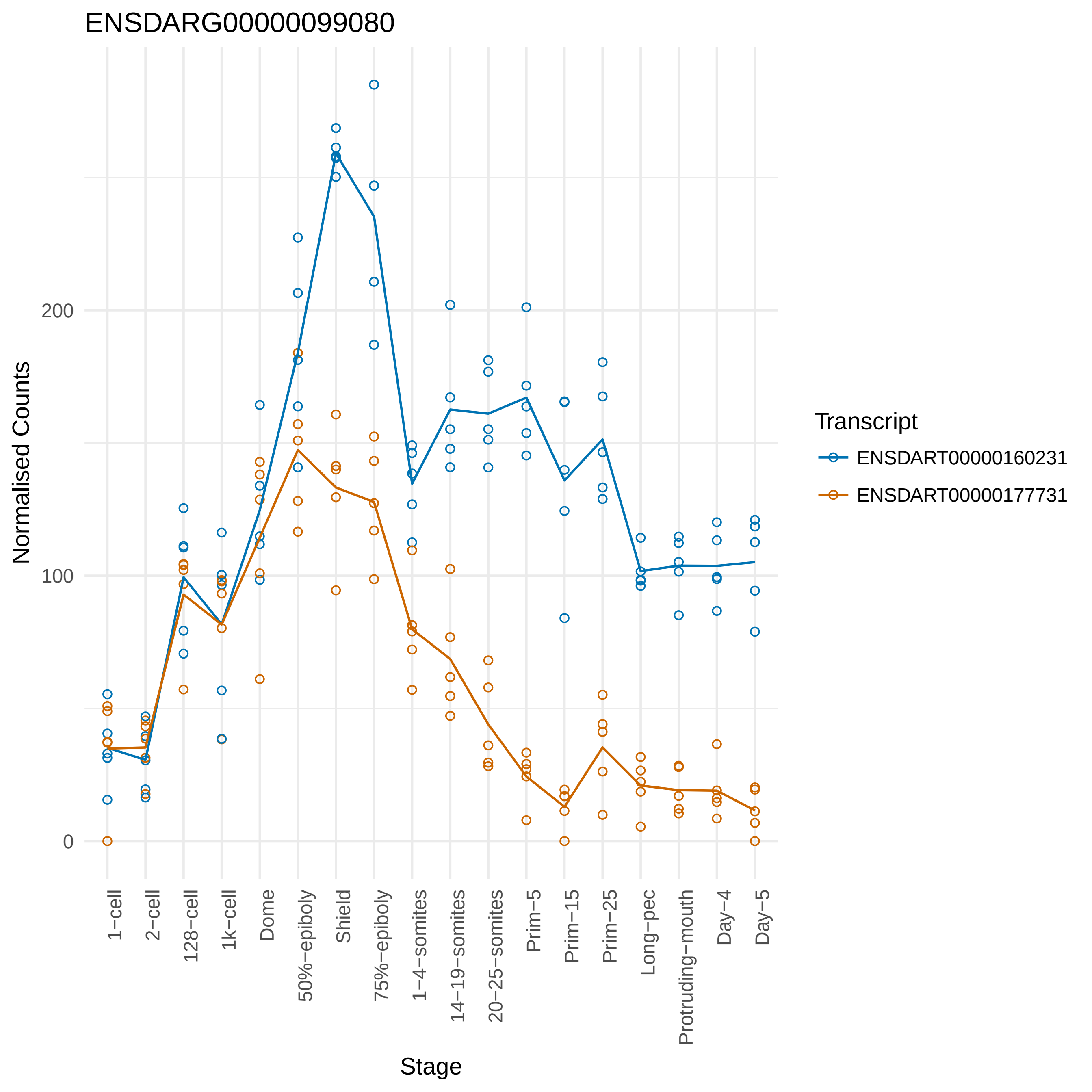
ENSDARG00000099080
Normalised Counts
200
100
0
1−cell
2−cell
128−cell
1k−cell
Dome
50%−epiboly
Shield
75%−epiboly
1−4−somites
14−19−somites
20−25−somites
Prim−5
Prim−15
Prim−25
Long−pec
Protruding−mouth
Day−4
Day−5
Stage
Transcript
ENSDART00000160231
ENSDART00000177731

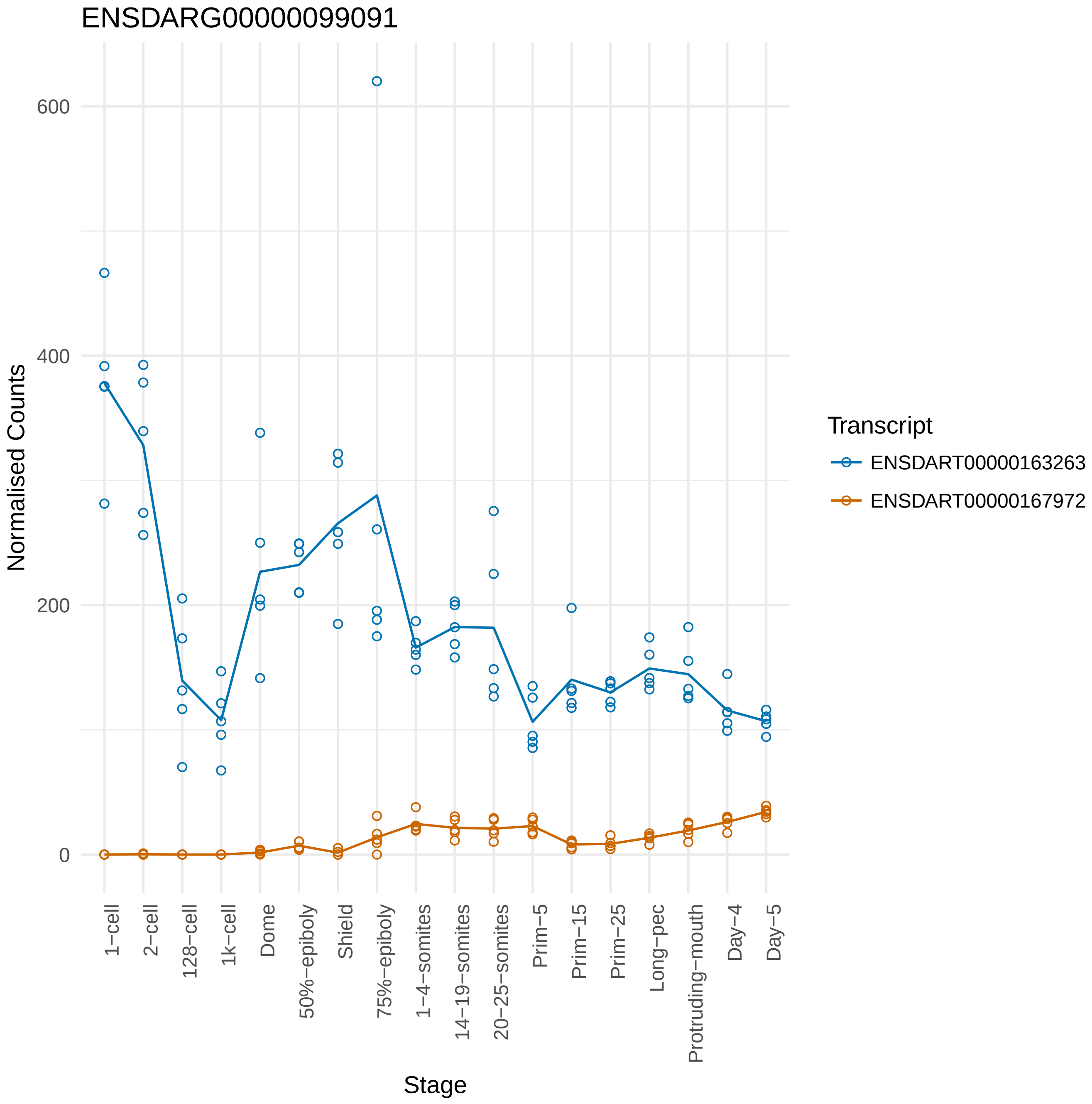
ENSDARG00000099091
Normalised Counts
600
400
200
0
1−cell
2−cell
128−cell
1k−cell
Dome
50%−epiboly
Shield
75%−epiboly
1−4−somites
14−19−somites
20−25−somites
Prim−5
Prim−15
Prim−25
Long−pec
Protruding−mouth
Day−4
Day−5
Stage
Transcript
ENSDART00000163263
ENSDART00000167972

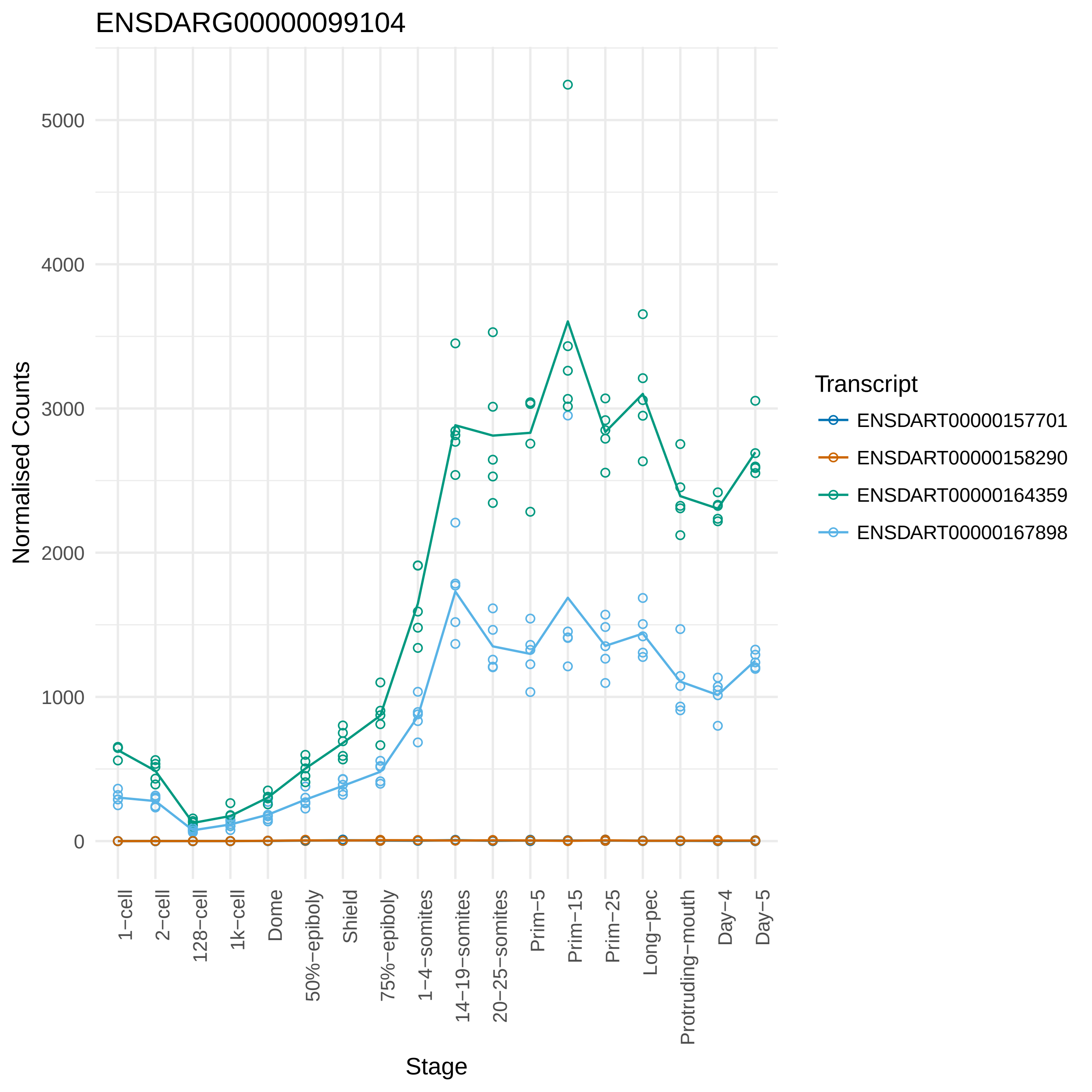

ENSDARG00000099104
Normalised Counts
0
1000
2000
3000
4000
5000
1−cell
2−cell
128−cell
1k−cell
Dome
50%−epiboly
Shield
75%−epiboly
1−4−somites
14−19−somites
20−25−somites
Prim−5
Prim−15
Prim−25
Long−pec
Protruding−mouth
Day−4
Day−5
Stage
Transcript
ENSDART00000157701
ENSDART00000158290
ENSDART00000164359
ENSDART00000167898

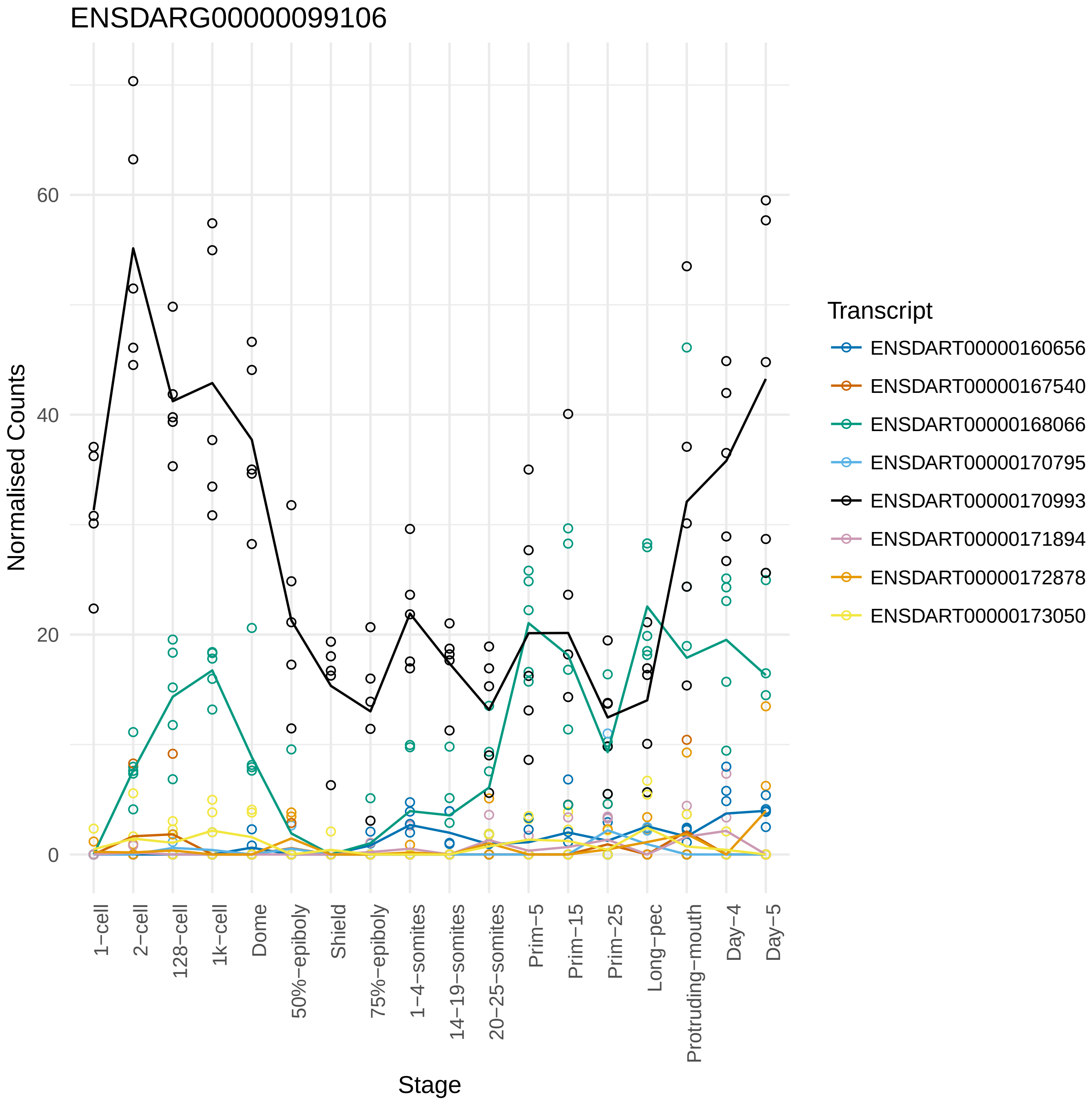

ENSDARG00000099106
Normalised Counts
0
20
40
60
Stage
1−cell
2−cell
128−cell
1k−cell
Dome
50%−epiboly
Shield
75%−epiboly
1−4−somites
14−19−somites
20−25−somites
Prim−5
Prim−15
Prim−25
Long−pec
Protruding−mouth
Day−4
Day−5
Transcript
ENSDART00000160656
ENSDART00000167540
ENSDART00000168066
ENSDART00000170795
ENSDART00000170993
ENSDART00000171894
ENSDART00000172878
ENSDART00000173050

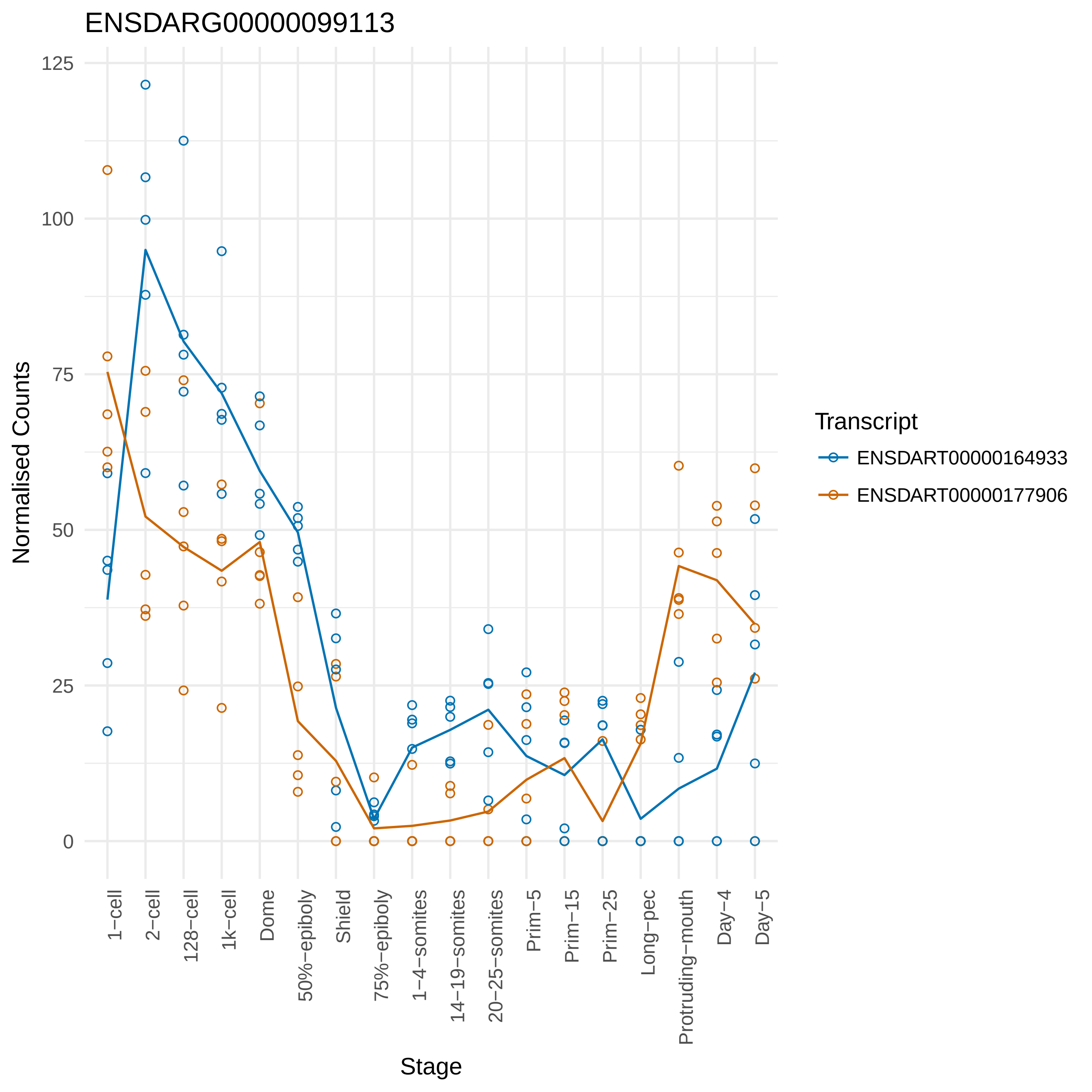

ENSDARG00000099113
Normalised Counts
125
100
75
50
25
0
1−cell
2−cell
128−cell
1k−cell
Dome
50%−epiboly
Shield
75%−epiboly
1−4−somites
14−19−somites
20−25−somites
Prim−5
Prim−15
Prim−25
Long−pec
Protruding−mouth
Day−4
Day−5
Stage
Transcript
ENSDART00000164933
ENSDART00000177906

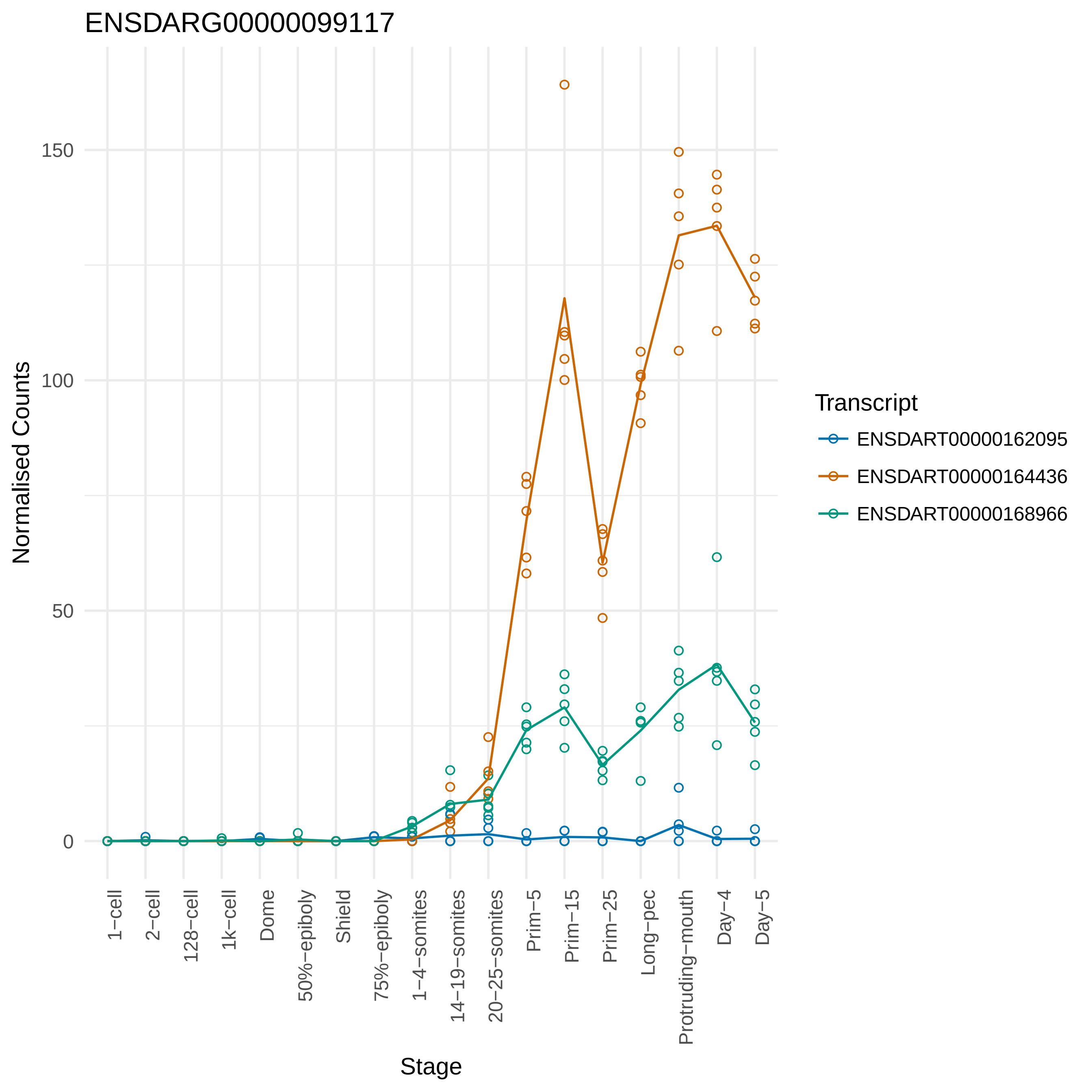

ENSDARG00000099117
Normalised Counts
150
100
50
0
1−cell
2−cell
128−cell
1k−cell
Dome
50%−epiboly
Shield
75%−epiboly
1−4−somites
14−19−somites
20−25−somites
Prim−5
Prim−15
Prim−25
Long−pec
Protruding−mouth
Day−4
Day−5
Stage
Transcript
ENSDART00000162095
ENSDART00000164436
ENSDART00000168966

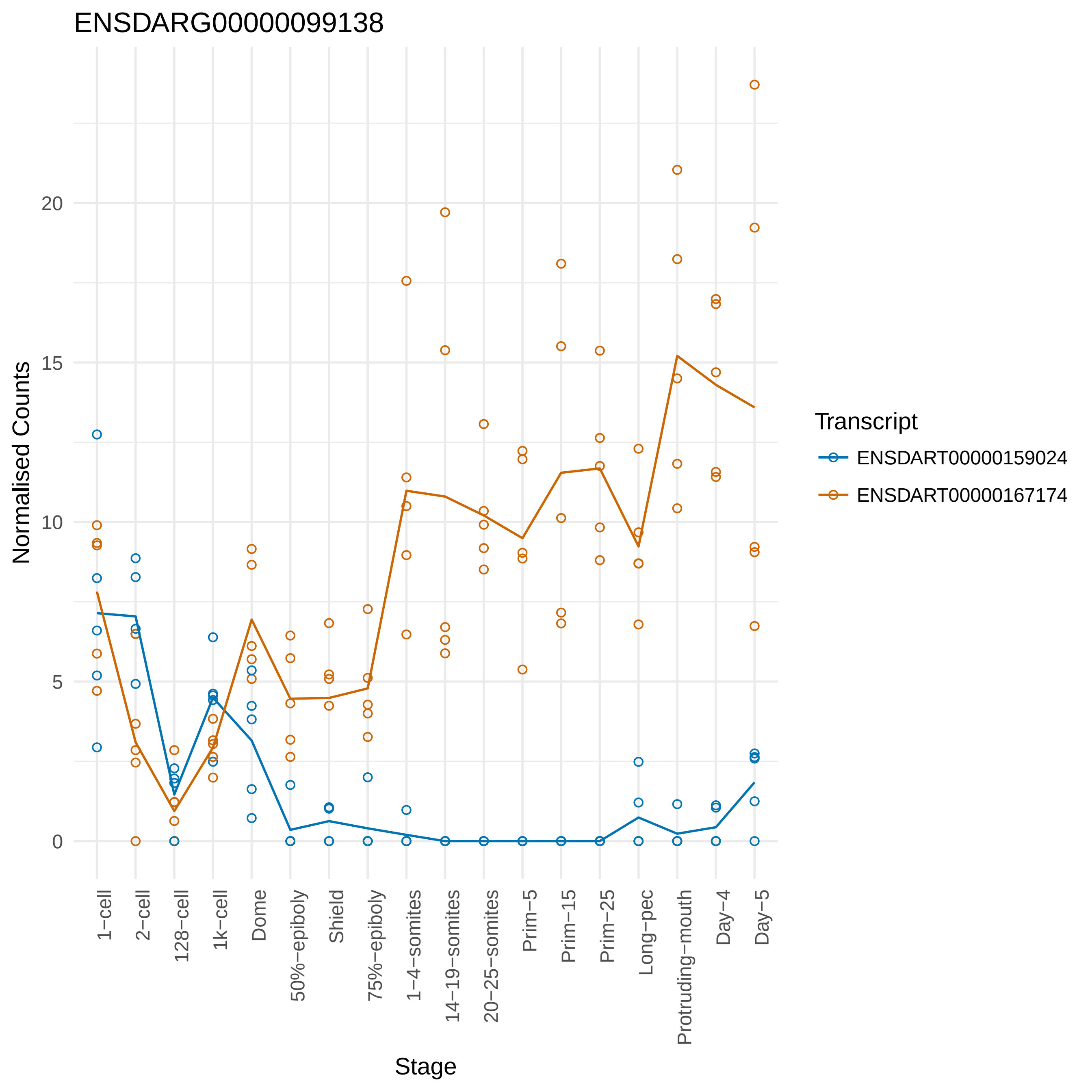
ENSDARG00000099138
Normalised Counts
0
5
10
15
20
1−cell
2−cell
128−cell
1k−cell
Dome
50%−epiboly
Shield
75%−epiboly
1−4−somites
14−19−somites
20−25−somites
Prim−5
Prim−15
Prim−25
Long−pec
Protruding−mouth
Day−4
Day−5
Stage
Transcript
ENSDART00000159024
ENSDART00000167174

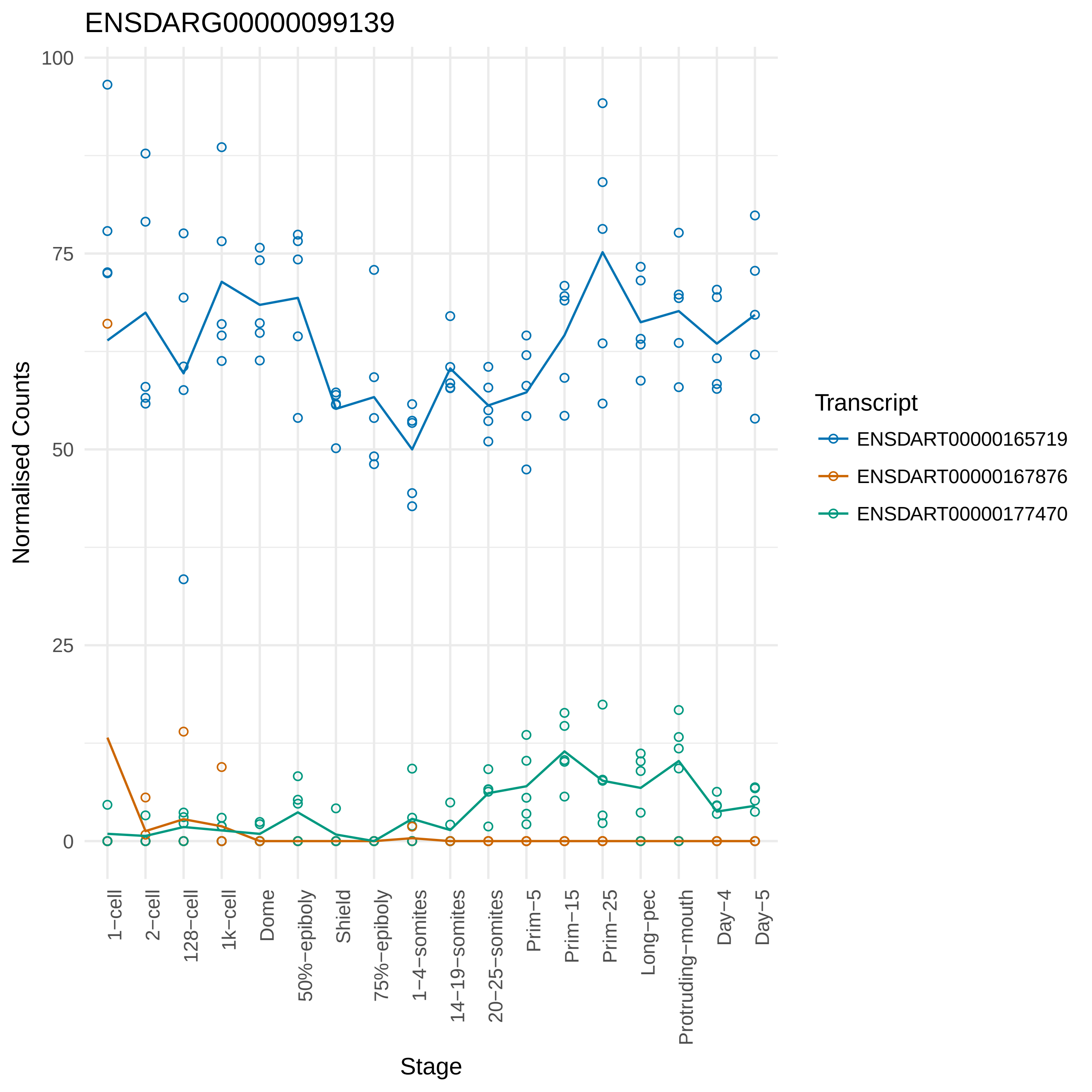

ENSDARG00000099139
Normalised Counts
100
75
50
25
0
1−cell
2−cell
128−cell
1k−cell
Dome
50%−epiboly
Shield
75%−epiboly
1−4−somites
14−19−somites
20−25−somites
Prim−5
Prim−15
Prim−25
Long−pec
Protruding−mouth
Day−4
Day−5
Stage
Transcript
ENSDART00000165719
ENSDART00000167876
ENSDART00000177470

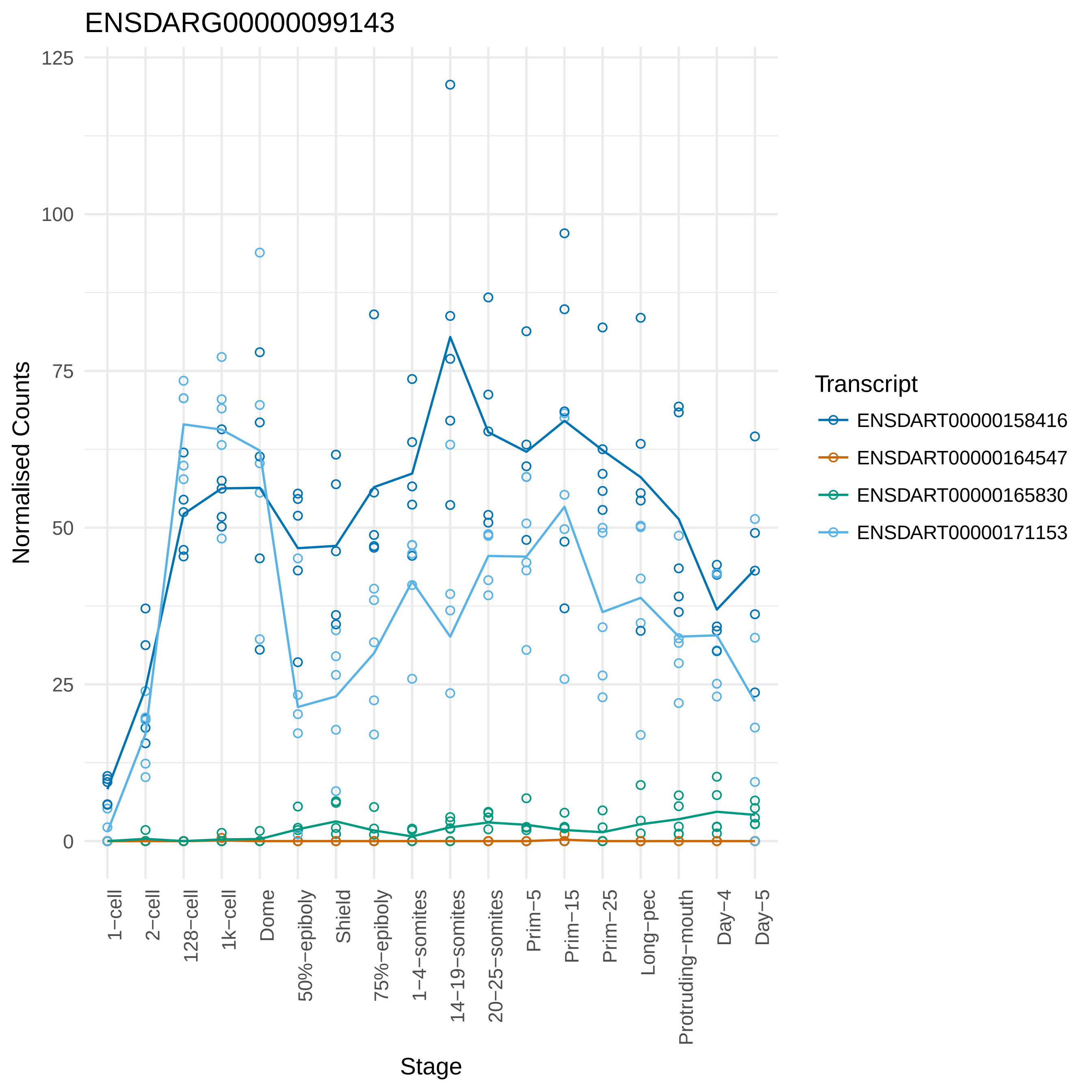
ENSDARG00000099143
Normalised Counts
125
100
75
50
25
0
1−cell
2−cell
128−cell
1k−cell
Dome
50%−epiboly
Shield
75%−epiboly
1−4−somites
14−19−somites
20−25−somites
Prim−5
Prim−15
Prim−25
Long−pec
Protruding−mouth
Day−4
Day−5
Stage
Transcript
ENSDART00000158416
ENSDART00000164547
ENSDART00000165830
ENSDART00000171153

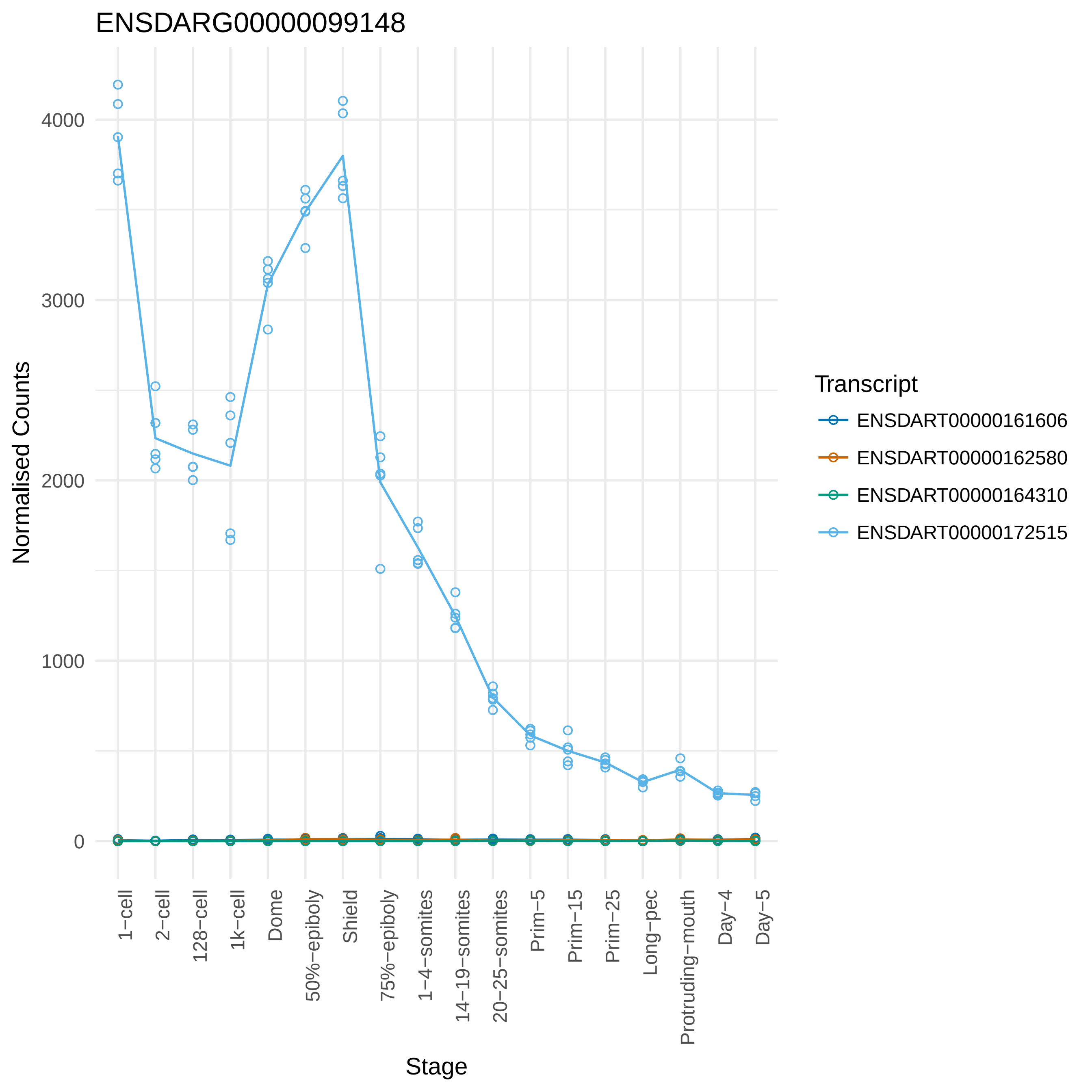

ENSDARG00000099148
Normalised Counts
4000
3000
2000
1000
0
1−cell
2−cell
128−cell
1k−cell
Dome
50%−epiboly
Shield
75%−epiboly
1−4−somites
14−19−somites
20−25−somites
Prim−5
Prim−15
Prim−25
Long−pec
Protruding−mouth
Day−4
Day−5
Stage
Transcript
ENSDART00000161606
ENSDART00000162580
ENSDART00000164310
ENSDART00000172515

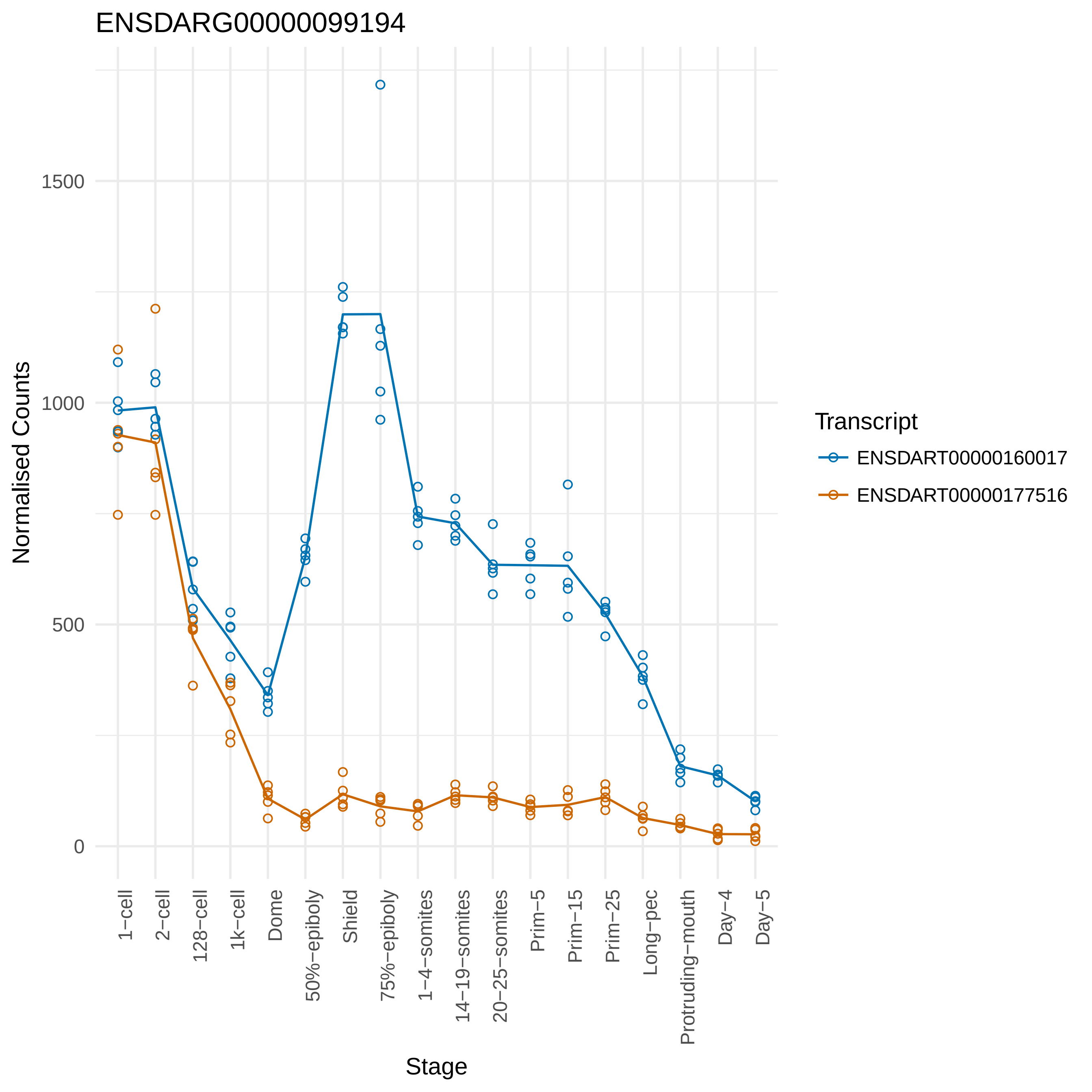
ENSDARG00000099194
Normalised Counts
1500
1000
500
0
1−cell
2−cell
128−cell
1k−cell
Dome
50%−epiboly
Shield
75%−epiboly
1−4−somites
14−19−somites
20−25−somites
Prim−5
Prim−15
Prim−25
Long−pec
Protruding−mouth
Day−4
Day−5
Stage
Transcript
ENSDART00000160017
ENSDART00000177516

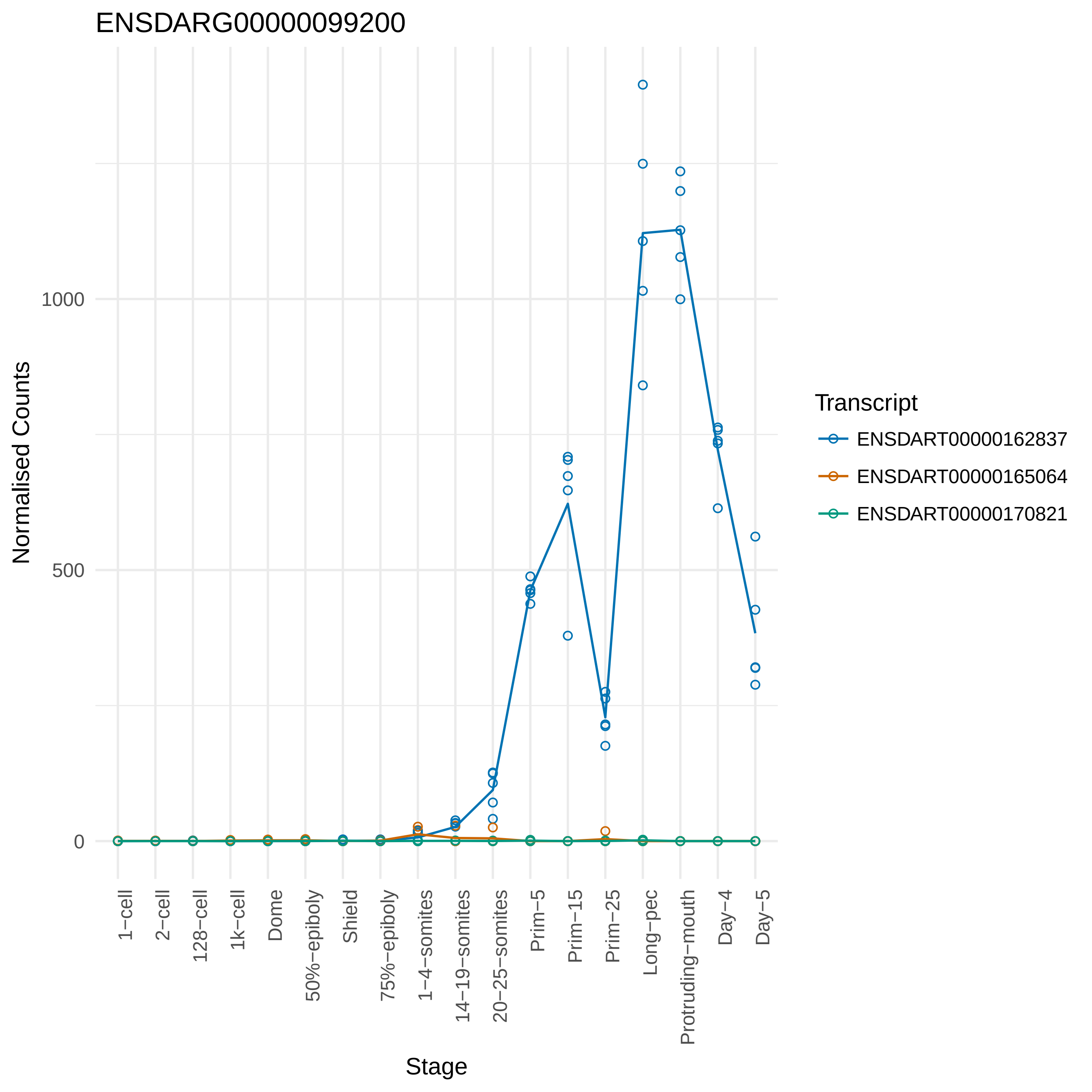

ENSDARG00000099200
Normalised Counts
0
500
1000
Stage
1−cell
2−cell
128−cell
1k−cell
Dome
50%−epiboly
Shield
75%−epiboly
1−4−somites
14−19−somites
20−25−somites
Prim−5
Prim−15
Prim−25
Long−pec
Protruding−mouth
Day−4
Day−5
Transcript
ENSDART00000162837
ENSDART00000165064
ENSDART00000170821

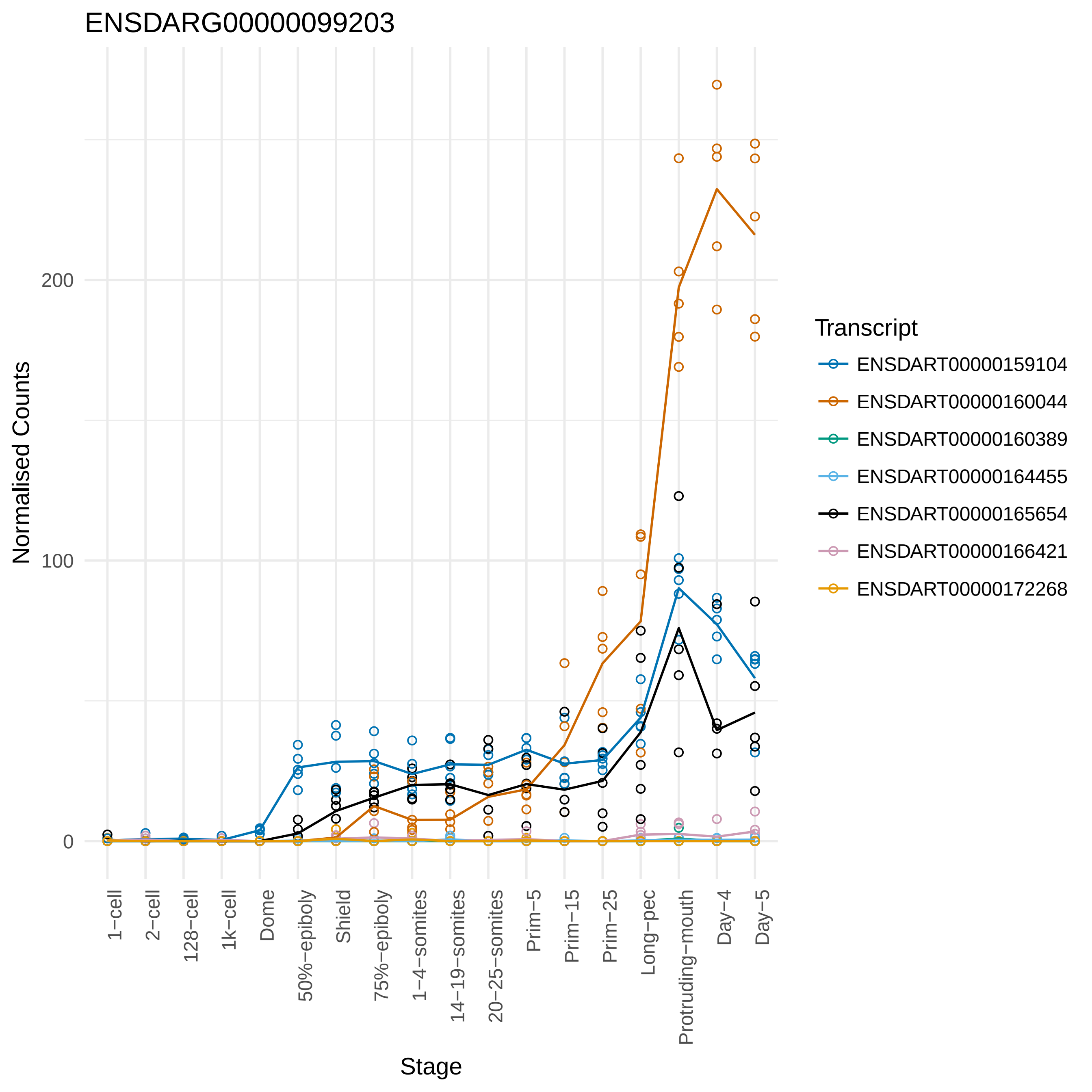
ENSDARG00000099203
Normalised Counts
0
100
200
Stage
1−cell
2−cell
128−cell
1k−cell
Dome
50%−epiboly
Shield
75%−epiboly
1−4−somites
14−19−somites
20−25−somites
Prim−5
Prim−15
Prim−25
Long−pec
Protruding−mouth
Day−4
Day−5
Transcript
ENSDART00000159104
ENSDART00000160044
ENSDART00000160389
ENSDART00000164455
ENSDART00000165654
ENSDART00000166421
ENSDART00000172268

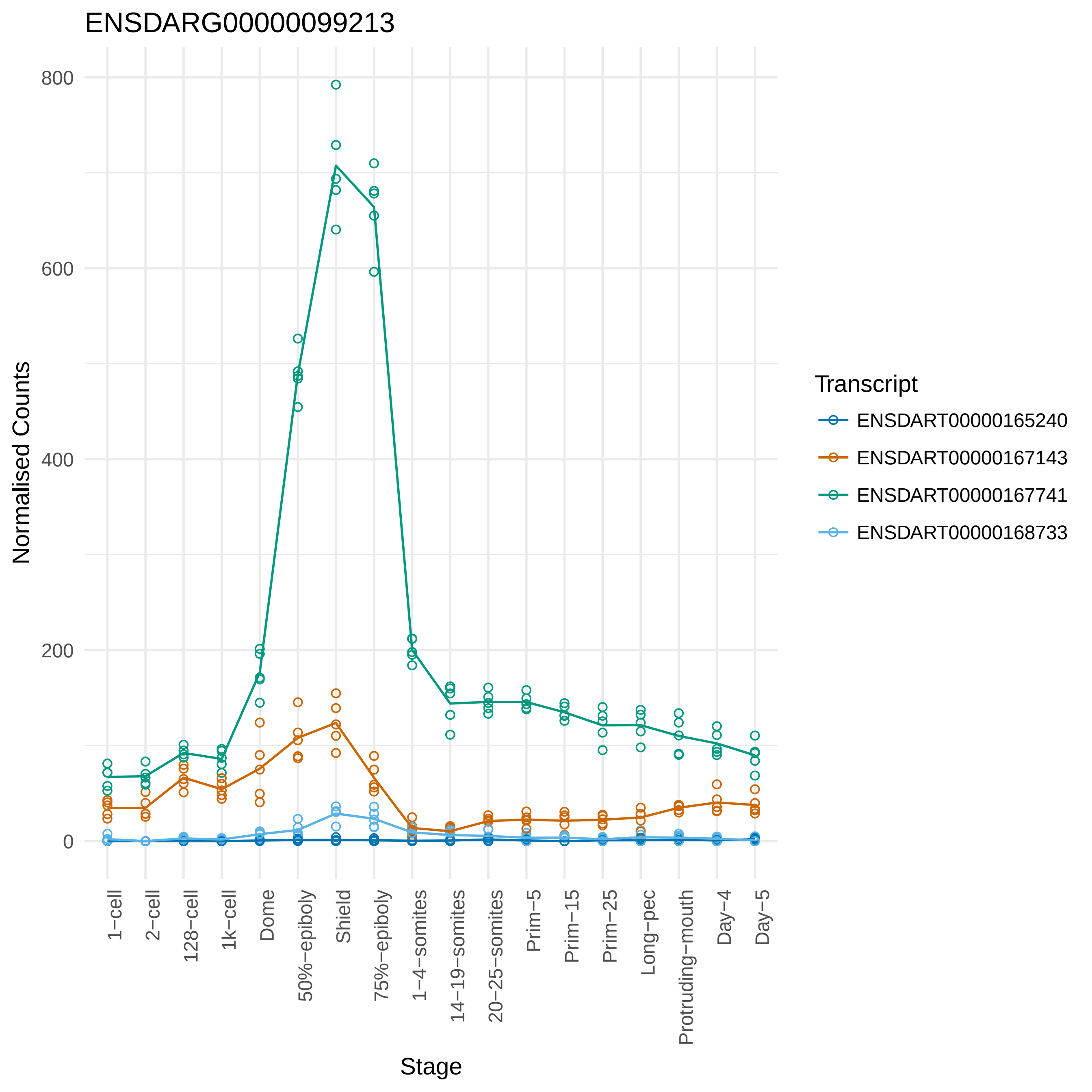

ENSDARG00000099213
Normalised Counts
800
600
400
200
0
1−cell
2−cell
128−cell
1k−cell
Dome
50%−epiboly
Shield
75%−epiboly
1−4−somites
14−19−somites
20−25−somites
Prim−5
Prim−15
Prim−25
Long−pec
Protruding−mouth
Day−4
Day−5
Stage
Transcript
ENSDART00000165240
ENSDART00000167143
ENSDART00000167741
ENSDART00000168733

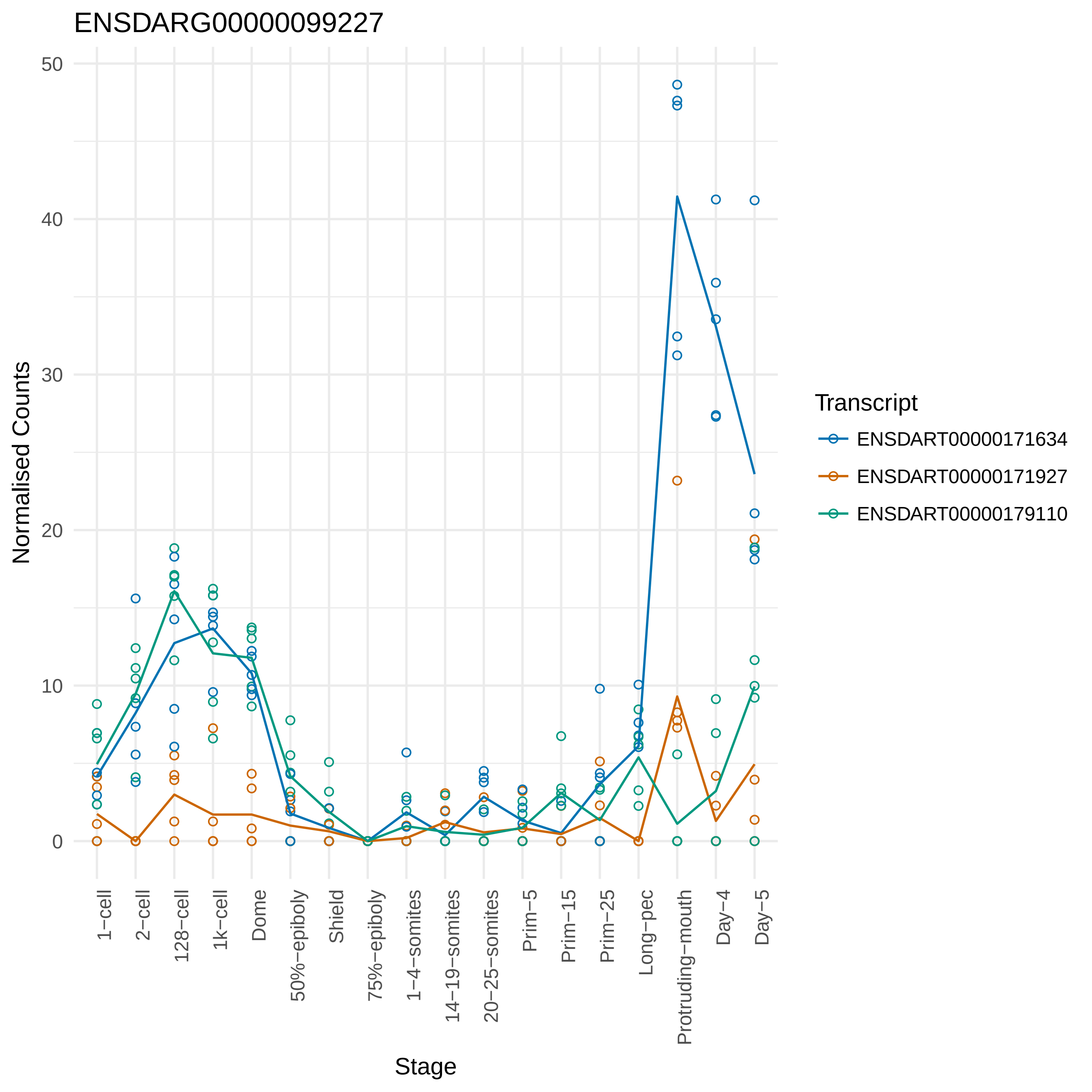

ENSDARG00000099227
Normalised Counts
0
10
20
30
40
50
1−cell
2−cell
128−cell
1k−cell
Dome
50%−epiboly
Shield
75%−epiboly
1−4−somites
14−19−somites
20−25−somites
Prim−5
Prim−15
Prim−25
Long−pec
Protruding−mouth
Day−4
Day−5
Stage
Transcript
ENSDART00000171634
ENSDART00000171927
ENSDART00000179110

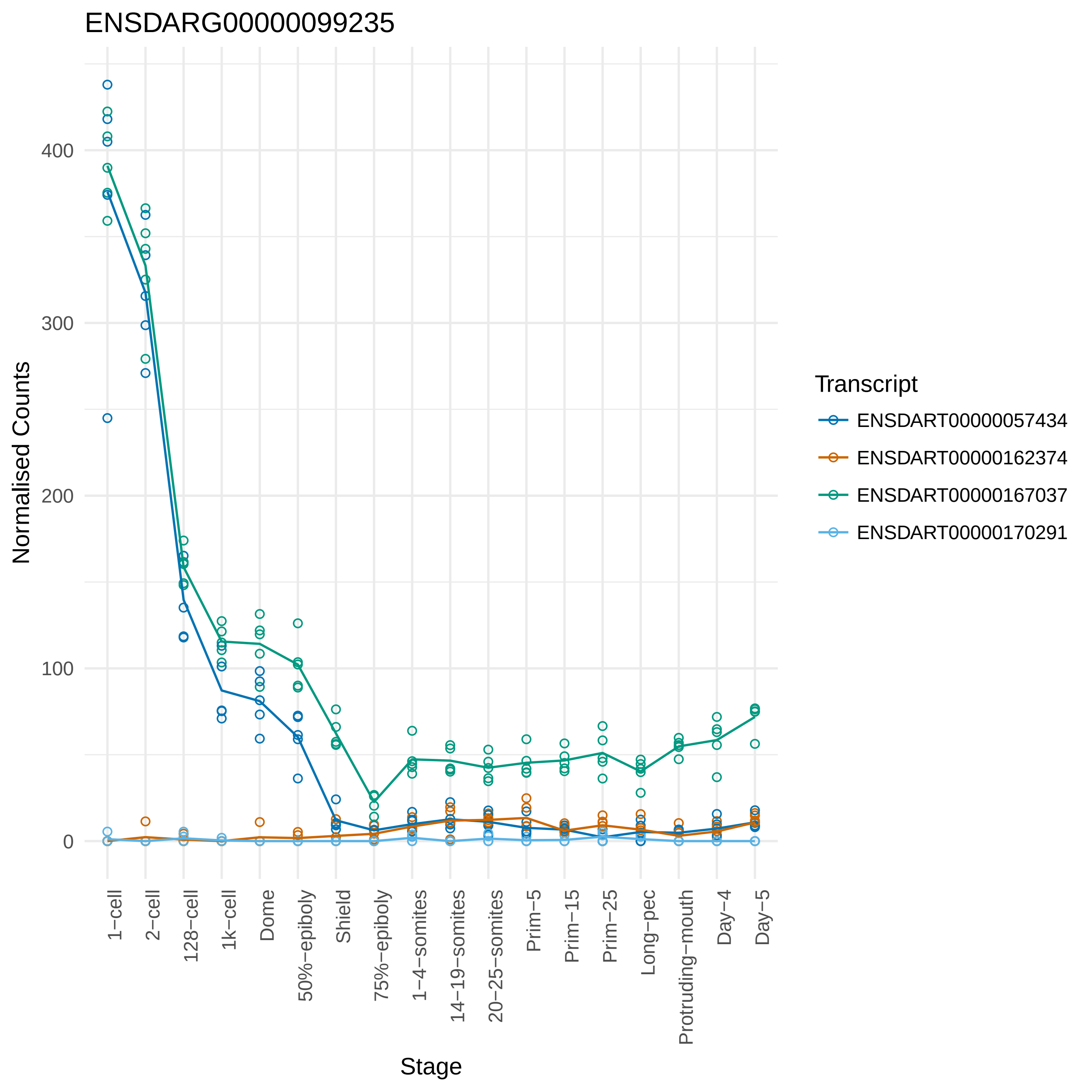
ENSDARG00000099235
Normalised Counts
0
100
200
300
400
Stage
1−cell
2−cell
128−cell
1k−cell
Dome
50%−epiboly
Shield
75%−epiboly
1−4−somites
14−19−somites
20−25−somites
Prim−5
Prim−15
Prim−25
Long−pec
Protruding−mouth
Day−4
Day−5
Transcript
ENSDART00000057434
ENSDART00000162374
ENSDART00000167037
ENSDART00000170291

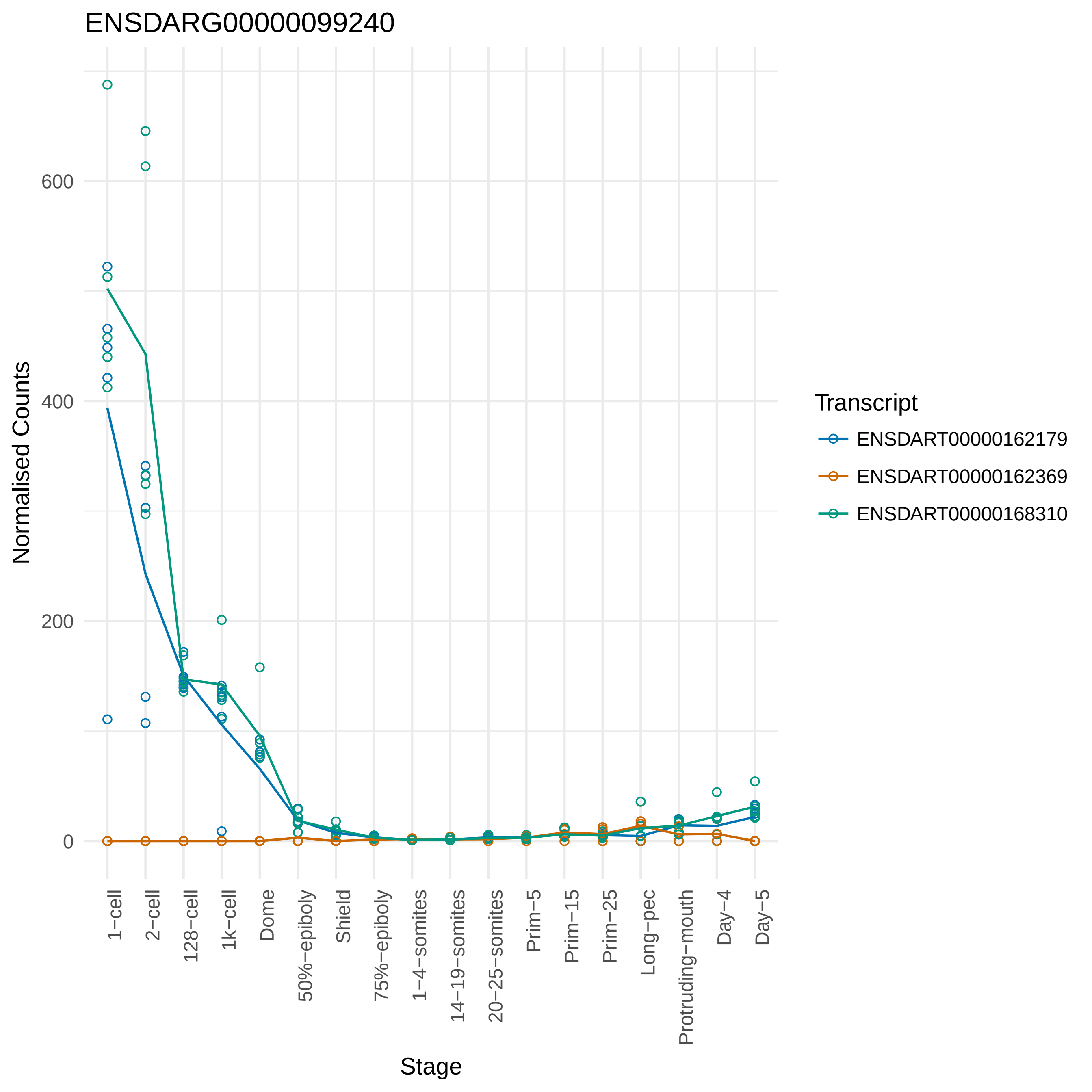
ENSDARG00000099240
Normalised Counts
600
400
200
0
1−cell
2−cell
128−cell
1k−cell
Dome
50%−epiboly
Shield
75%−epiboly
1−4−somites
14−19−somites
20−25−somites
Prim−5
Prim−15
Prim−25
Long−pec
Protruding−mouth
Day−4
Day−5
Stage
Transcript
ENSDART00000162179
ENSDART00000162369
ENSDART00000168310

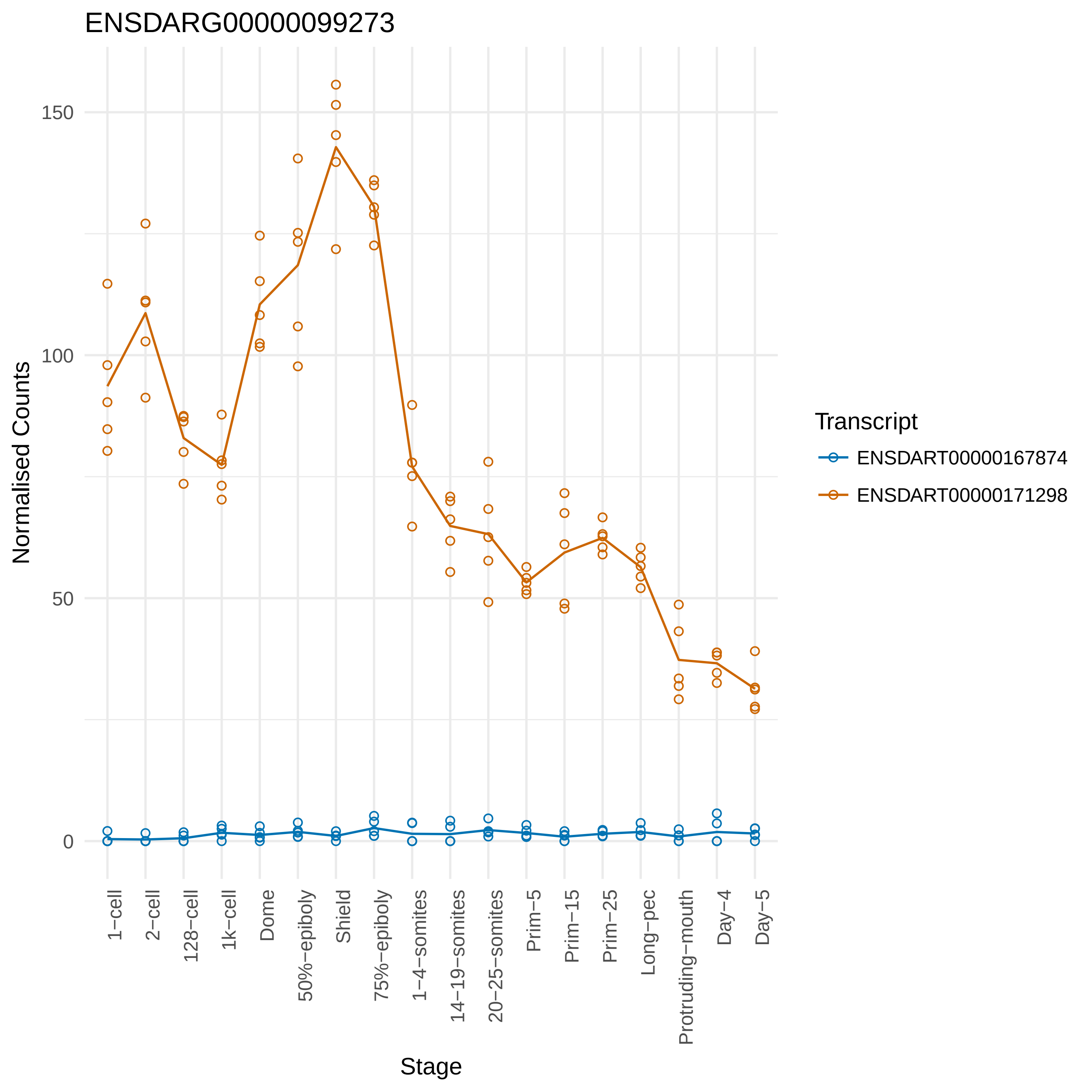

ENSDARG00000099273
Normalised Counts
150
100
50
0
1−cell
2−cell
128−cell
1k−cell
Dome
50%−epiboly
Shield
75%−epiboly
1−4−somites
14−19−somites
20−25−somites
Prim−5
Prim−15
Prim−25
Long−pec
Protruding−mouth
Day−4
Day−5
Stage
Transcript
ENSDART00000167874
ENSDART00000171298

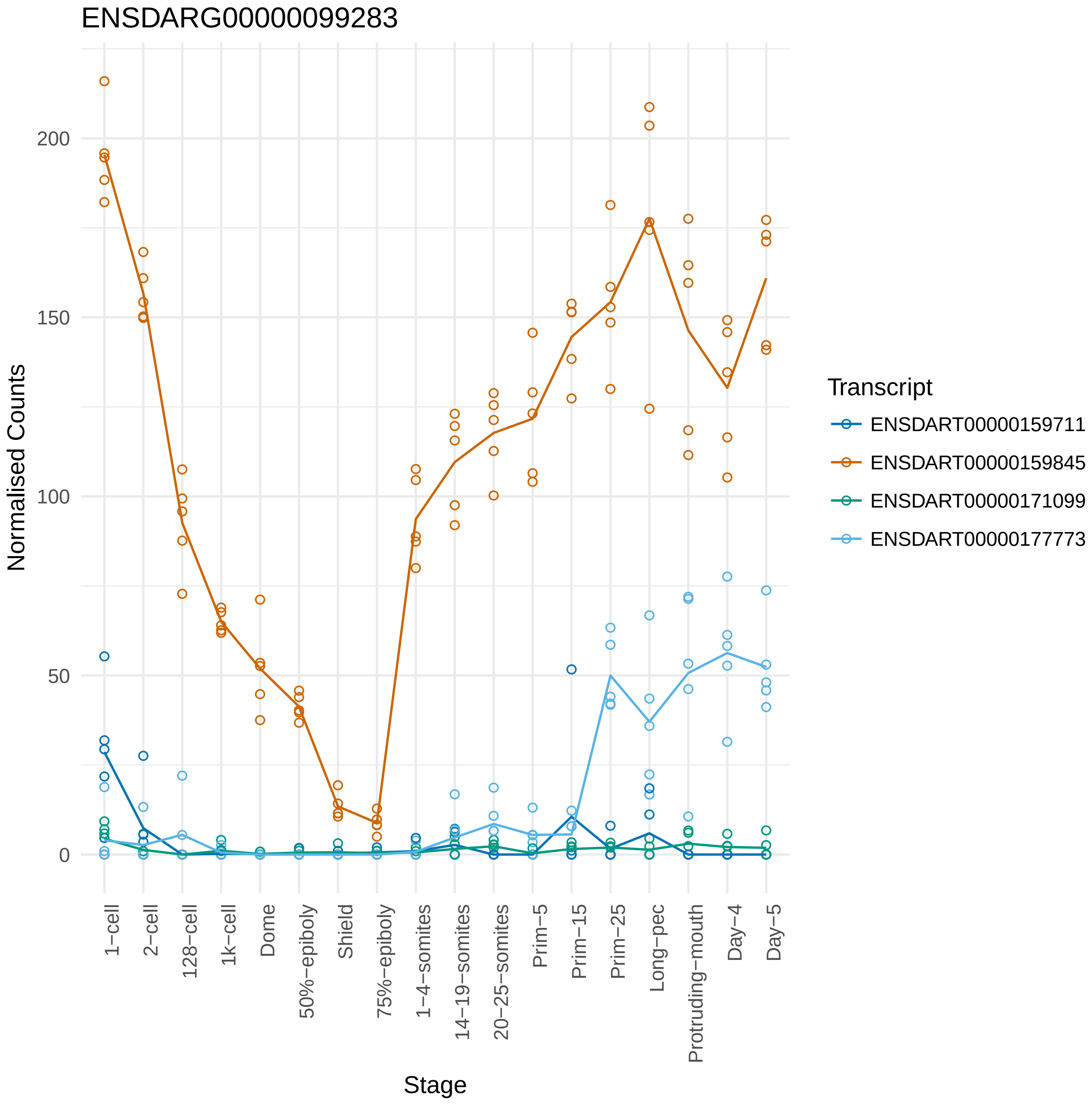

ENSDARG00000099283
Normalised Counts
0
50
100
150
200
Stage
1−cell
2−cell
128−cell
1k−cell
Dome
50%−epiboly
Shield
75%−epiboly
1−4−somites
14−19−somites
20−25−somites
Prim−5
Prim−15
Prim−25
Long−pec
Protruding−mouth
Day−4
Day−5
Transcript
ENSDART00000159711
ENSDART00000159845
ENSDART00000171099
ENSDART00000177773

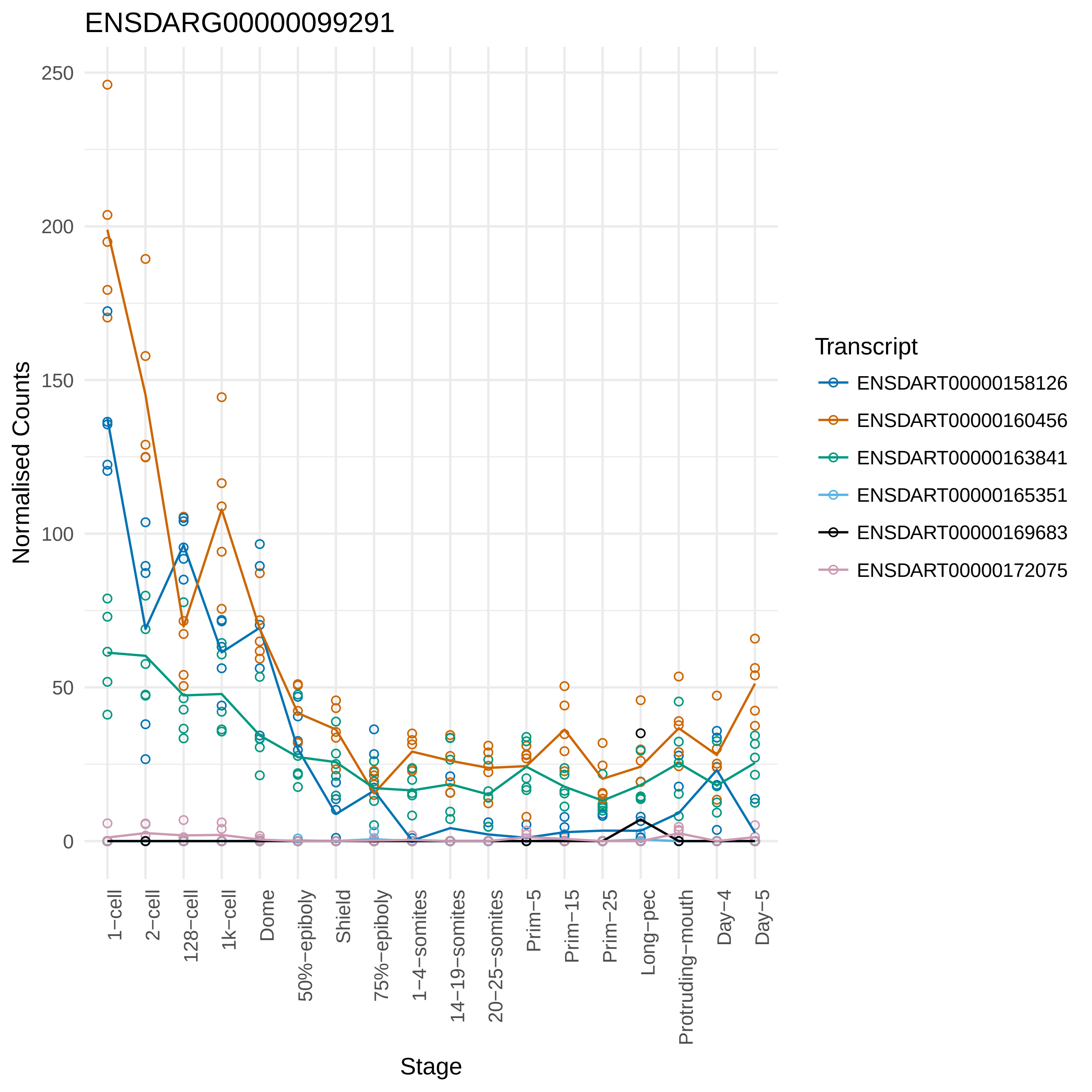

ENSDARG00000099291
Normalised Counts
0
50
100
150
200
250
Stage
1−cell
2−cell
128−cell
1k−cell
Dome
50%−epiboly
Shield
75%−epiboly
1−4−somites
14−19−somites
20−25−somites
Prim−5
Prim−15
Prim−25
Long−pec
Protruding−mouth
Day−4
Day−5
Transcript
ENSDART00000158126
ENSDART00000160456
ENSDART00000163841
ENSDART00000165351
ENSDART00000169683
ENSDART00000172075

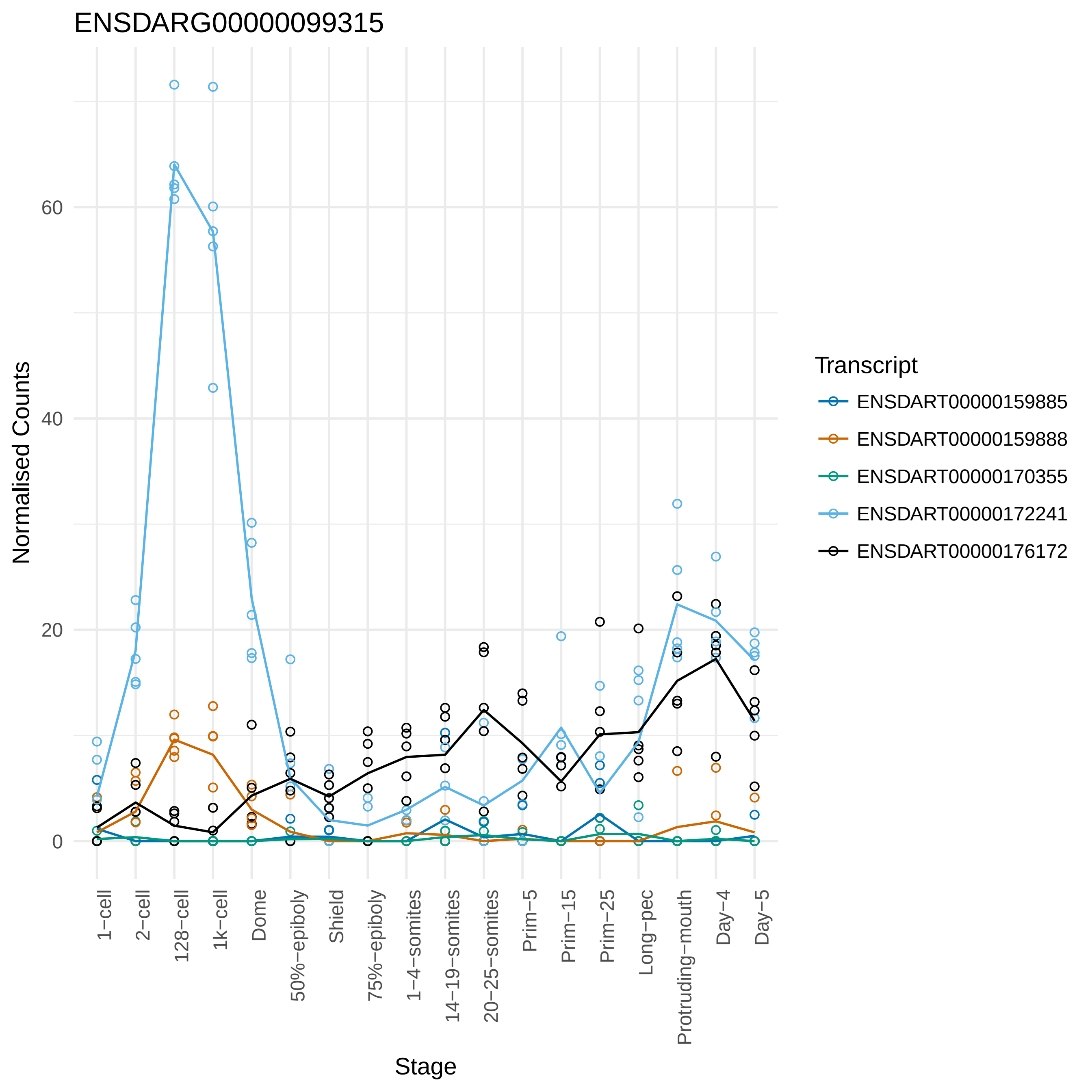
ENSDARG00000099315
Normalised Counts
0
20
40
60
Stage
1−cell
2−cell
128−cell
1k−cell
Dome
50%−epiboly
Shield
75%−epiboly
1−4−somites
14−19−somites
20−25−somites
Prim−5
Prim−15
Prim−25
Long−pec
Protruding−mouth
Day−4
Day−5
Transcript
ENSDART00000159885
ENSDART00000159888
ENSDART00000170355
ENSDART00000172241
ENSDART00000176172

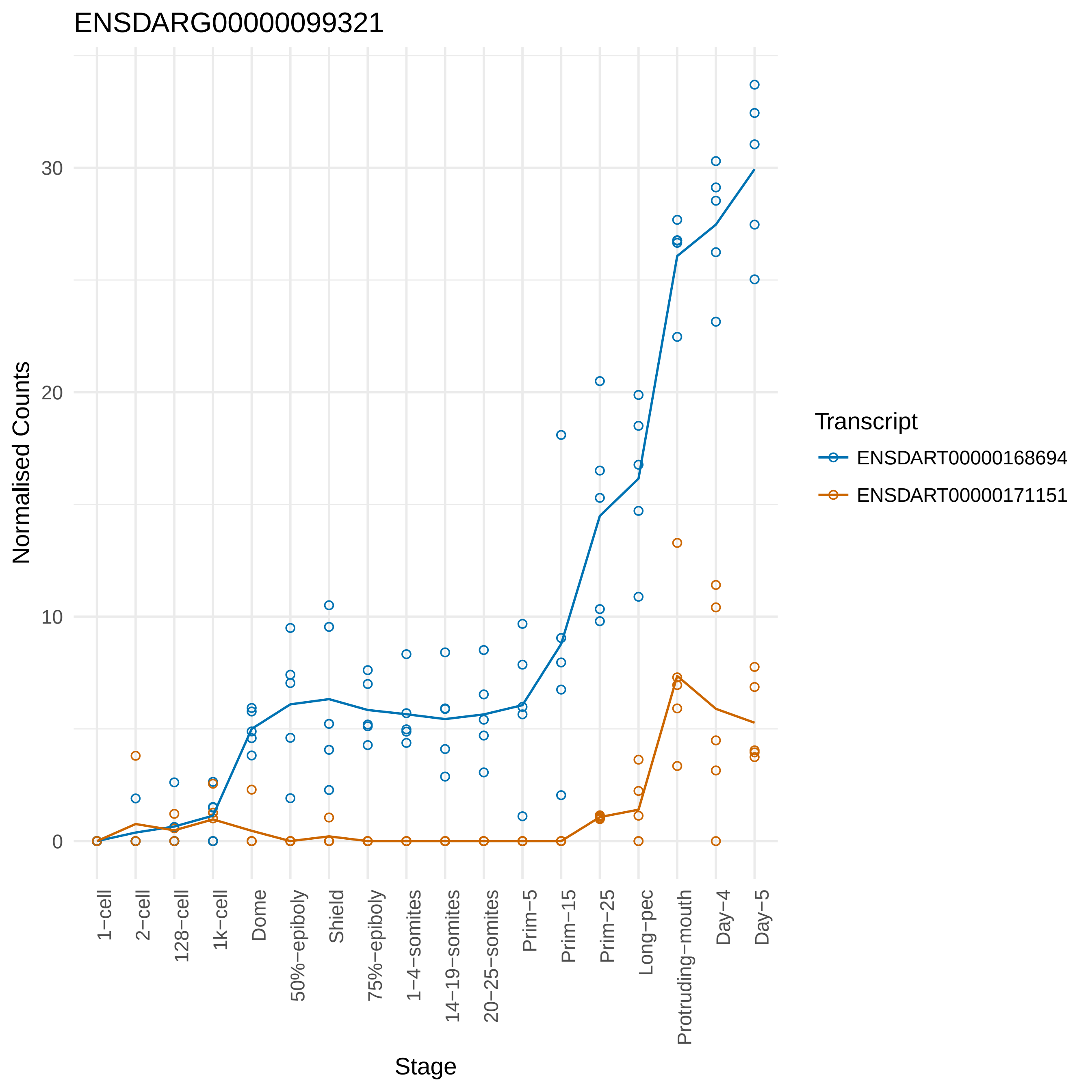

ENSDARG00000099321
Normalised Counts
0
10
20
30
1−cell
2−cell
128−cell
1k−cell
Dome
50%−epiboly
Shield
75%−epiboly
1−4−somites
14−19−somites
20−25−somites
Prim−5
Prim−15
Prim−25
Long−pec
Protruding−mouth
Day−4
Day−5
Stage
Transcript
ENSDART00000168694
ENSDART00000171151

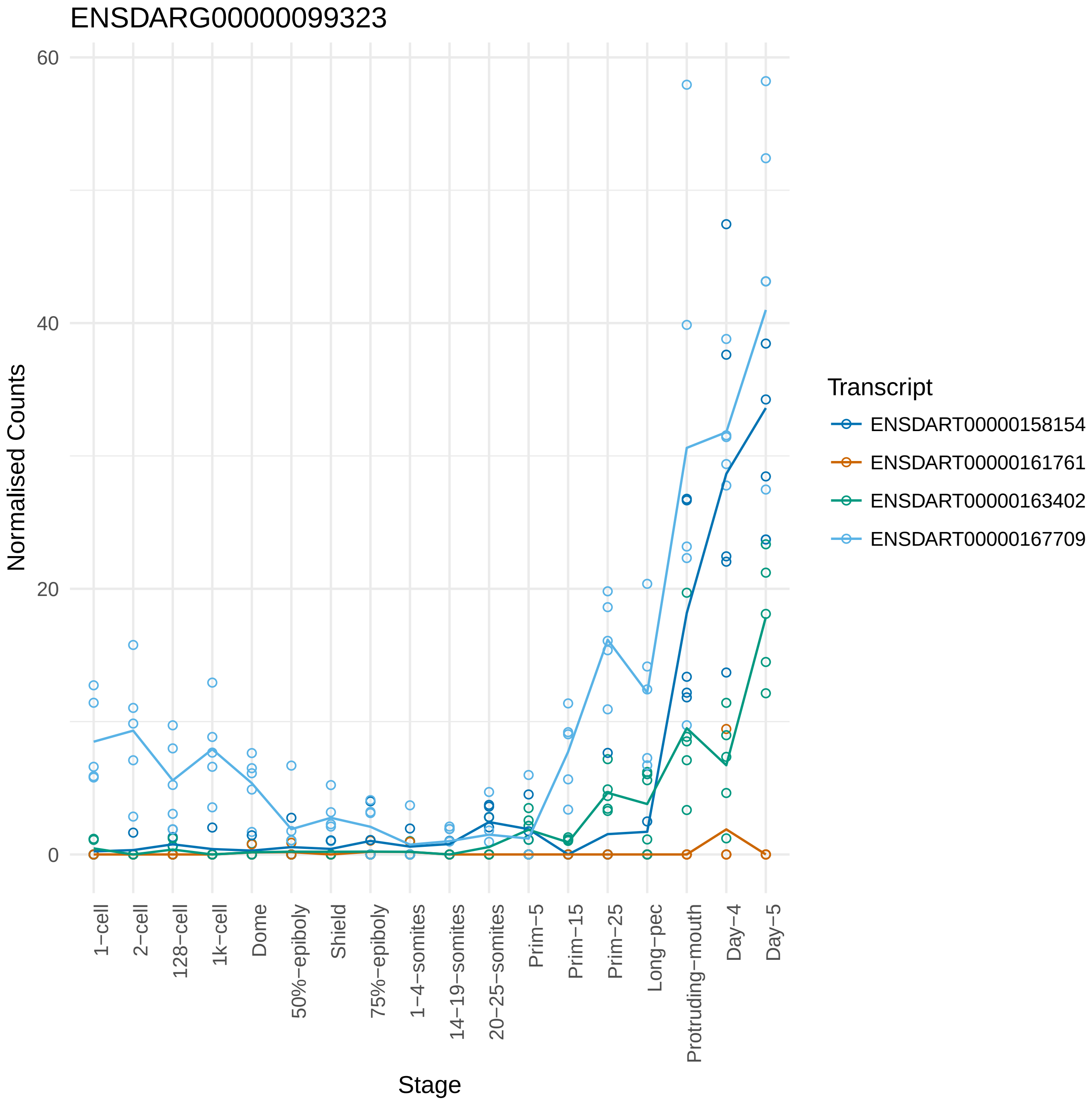

ENSDARG00000099323
Normalised Counts
0
20
40
60
1−cell
2−cell
128−cell
1k−cell
Dome
50%−epiboly
Shield
75%−epiboly
1−4−somites
14−19−somites
20−25−somites
Prim−5
Prim−15
Prim−25
Long−pec
Protruding−mouth
Day−4
Day−5
Stage
Transcript
ENSDART00000158154
ENSDART00000161761
ENSDART00000163402
ENSDART00000167709

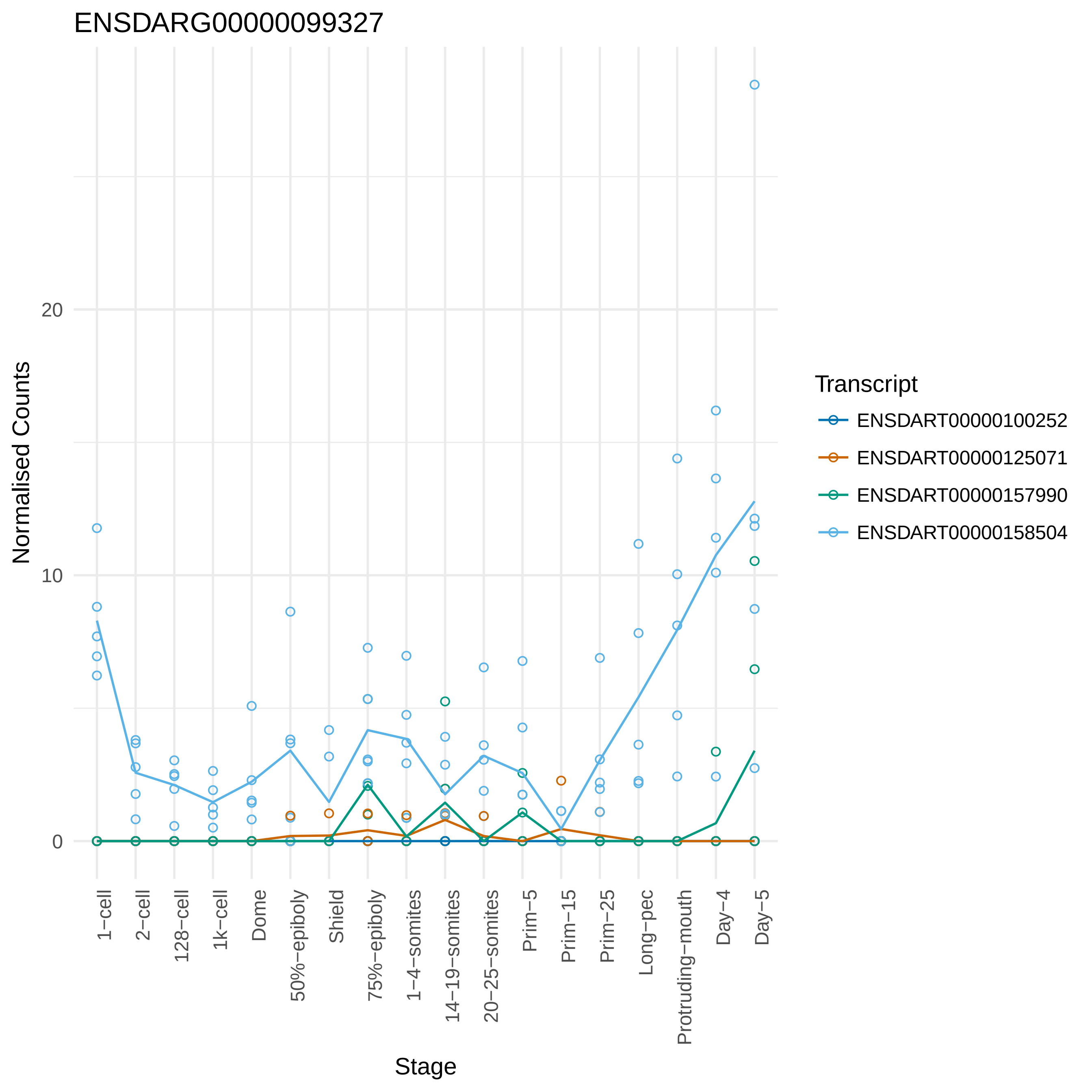

ENSDARG00000099327
Normalised Counts
0
10
20
Stage
1−cell
2−cell
128−cell
1k−cell
Dome
50%−epiboly
Shield
75%−epiboly
1−4−somites
14−19−somites
20−25−somites
Prim−5
Prim−15
Prim−25
Long−pec
Protruding−mouth
Day−4
Day−5
Transcript
ENSDART00000100252
ENSDART00000125071
ENSDART00000157990
ENSDART00000158504

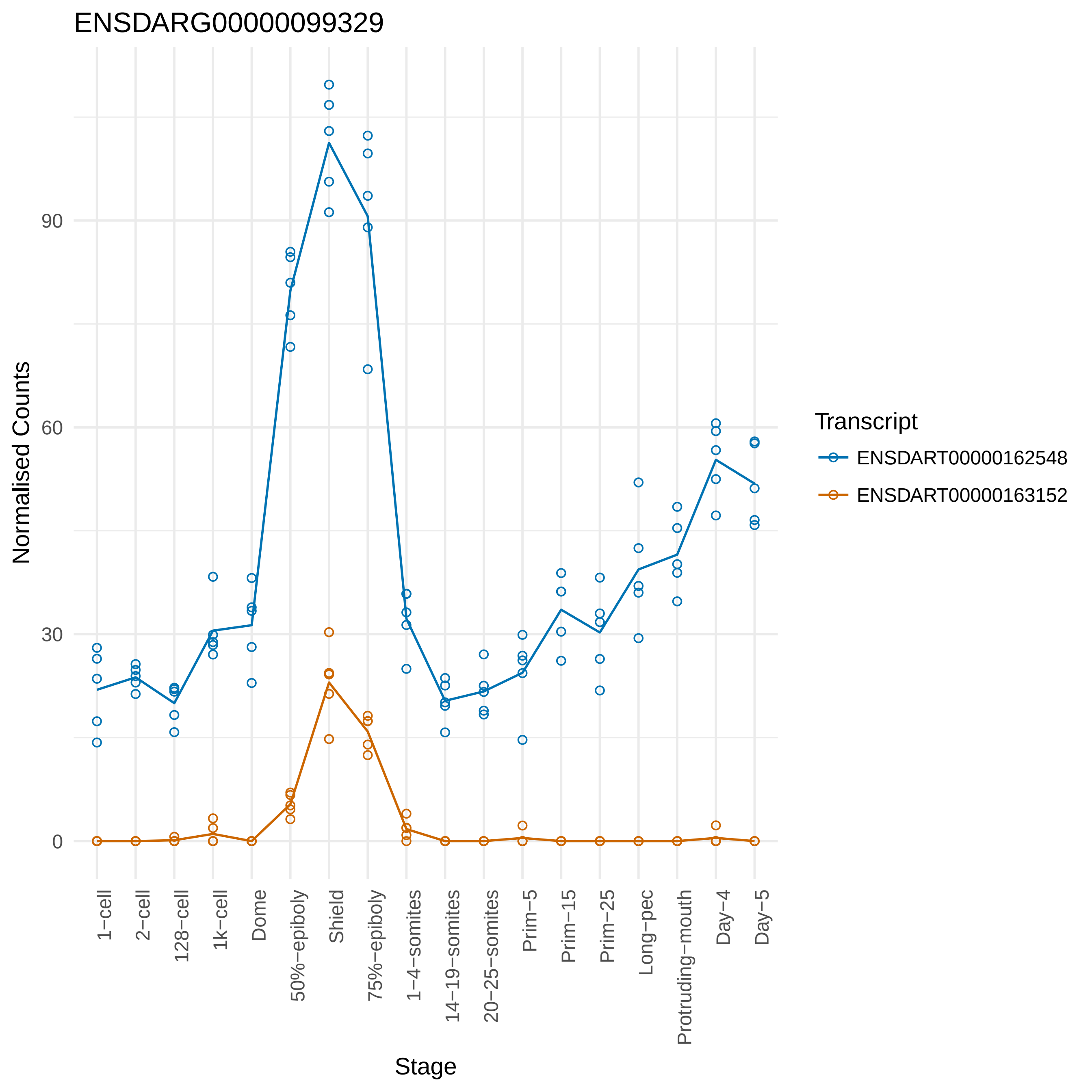
ENSDARG00000099329
Normalised Counts
0
30
60
90
Stage
1−cell
2−cell
128−cell
1k−cell
Dome
50%−epiboly
Shield
75%−epiboly
1−4−somites
14−19−somites
20−25−somites
Prim−5
Prim−15
Prim−25
Long−pec
Protruding−mouth
Day−4
Day−5
Transcript
ENSDART00000162548
ENSDART00000163152

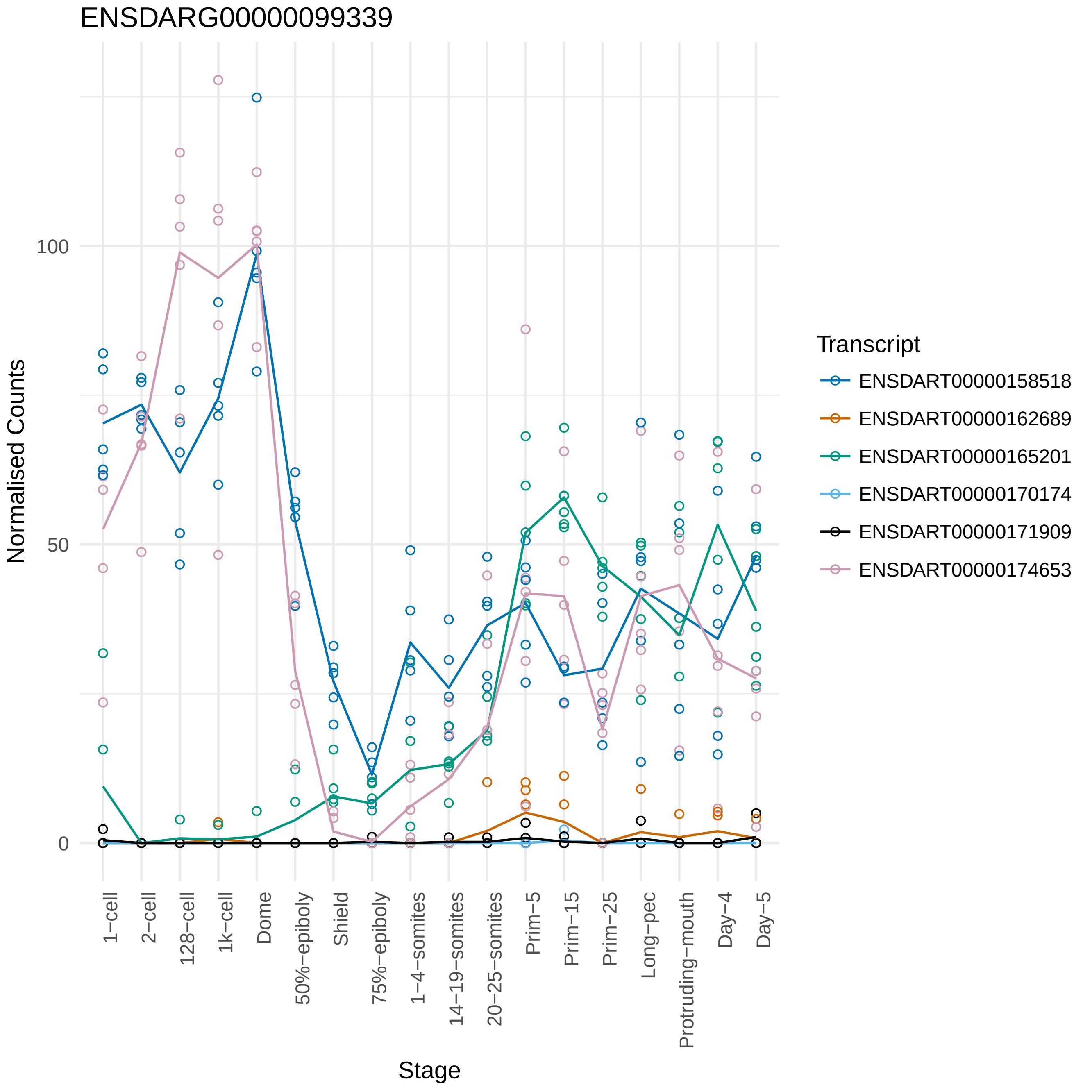
ENSDARG00000099339
Normalised Counts
100
50
0
1−cell
2−cell
128−cell
1k−cell
Dome
50%−epiboly
Shield
75%−epiboly
1−4−somites
14−19−somites
20−25−somites
Prim−5
Prim−15
Prim−25
Long−pec
Protruding−mouth
Day−4
Day−5
Stage
Transcript
ENSDART00000158518
ENSDART00000162689
ENSDART00000165201
ENSDART00000170174
ENSDART00000171909
ENSDART00000174653

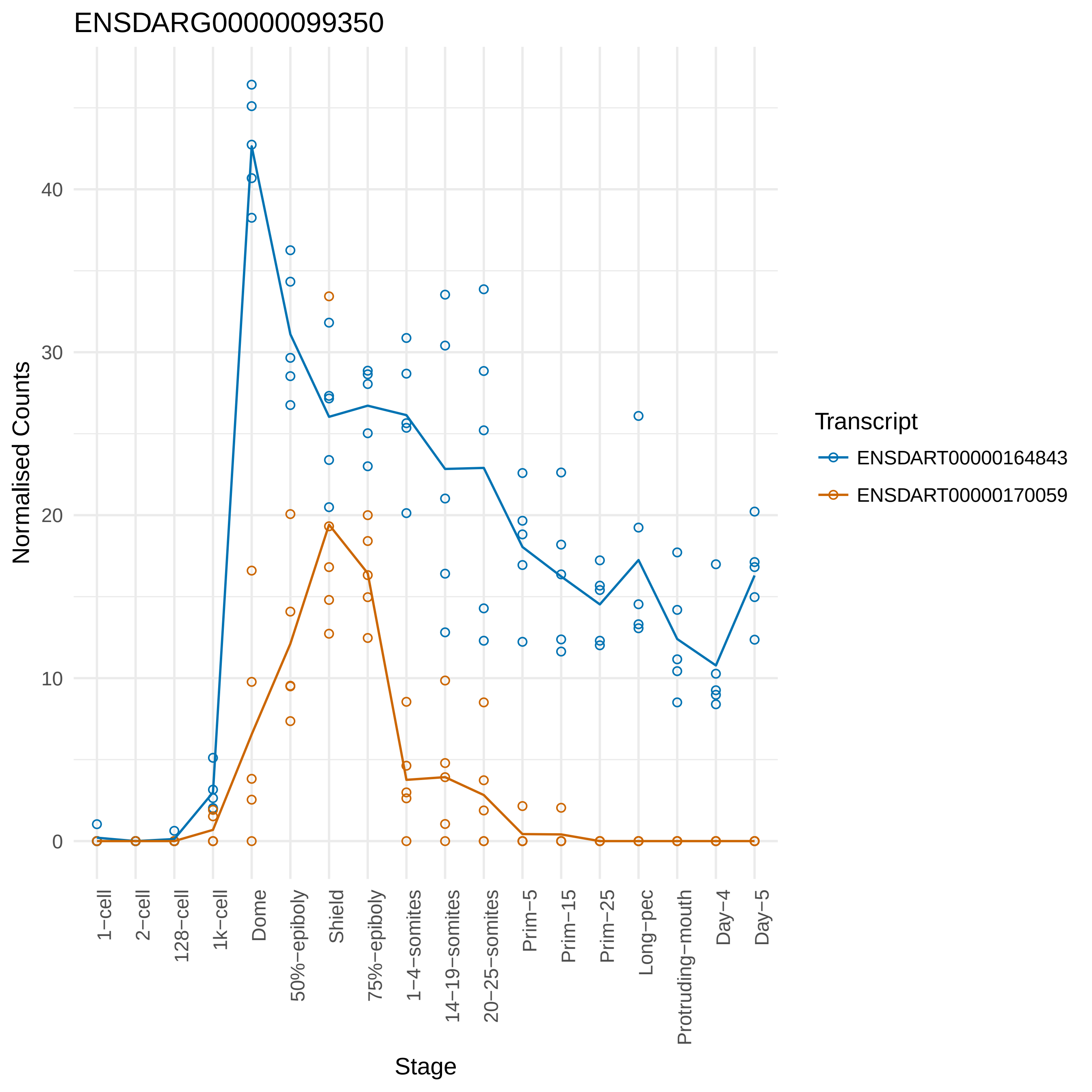
ENSDARG00000099350
Normalised Counts
0
10
20
30
40
Stage
1−cell
2−cell
128−cell
1k−cell
Dome
50%−epiboly
Shield
75%−epiboly
1−4−somites
14−19−somites
20−25−somites
Prim−5
Prim−15
Prim−25
Long−pec
Protruding−mouth
Day−4
Day−5
Transcript
ENSDART00000164843
ENSDART00000170059

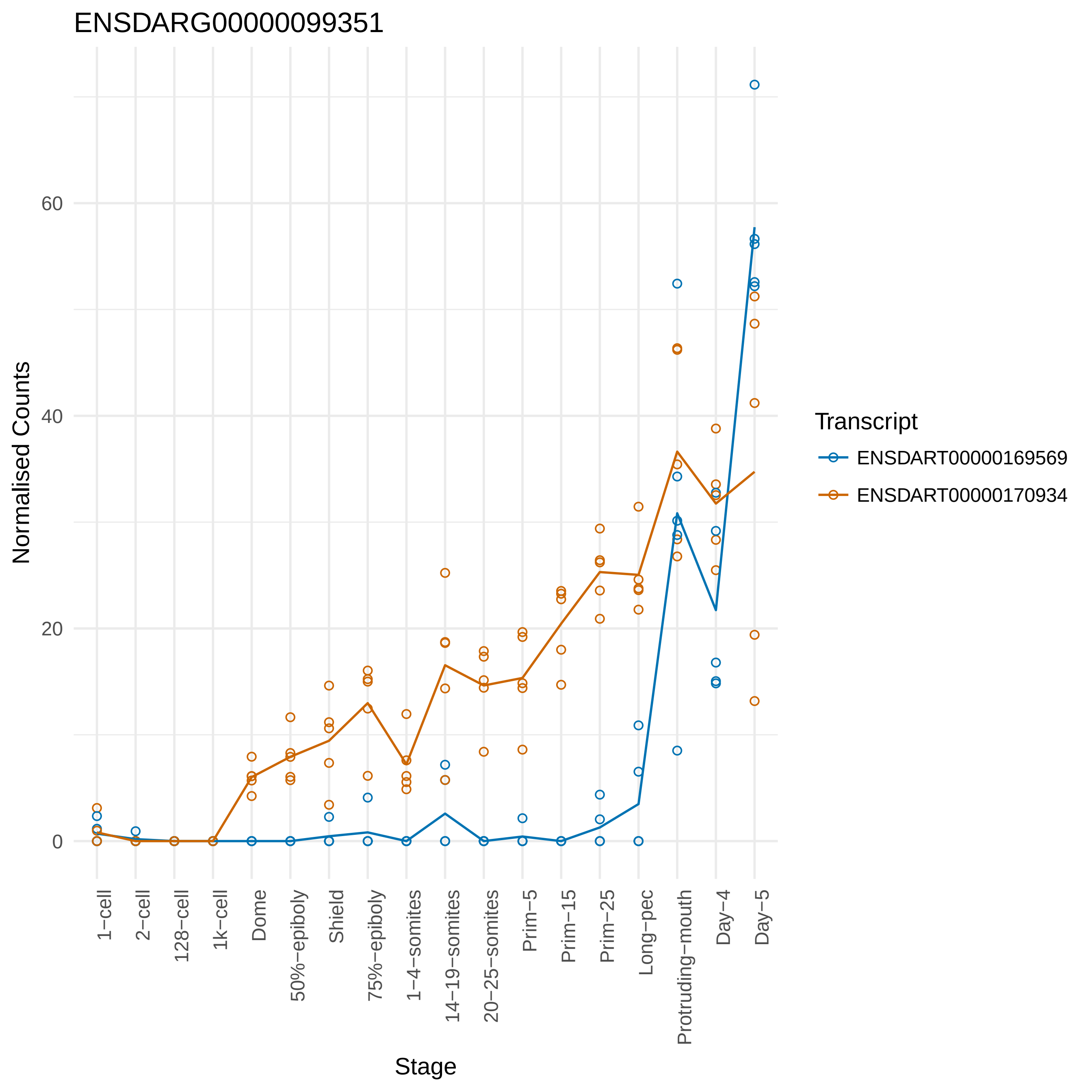

ENSDARG00000099351
Normalised Counts
60
40
20
0
1−cell
2−cell
128−cell
1k−cell
Dome
50%−epiboly
Shield
75%−epiboly
1−4−somites
14−19−somites
20−25−somites
Prim−5
Prim−15
Prim−25
Long−pec
Protruding−mouth
Day−4
Day−5
Stage
Transcript
ENSDART00000169569
ENSDART00000170934

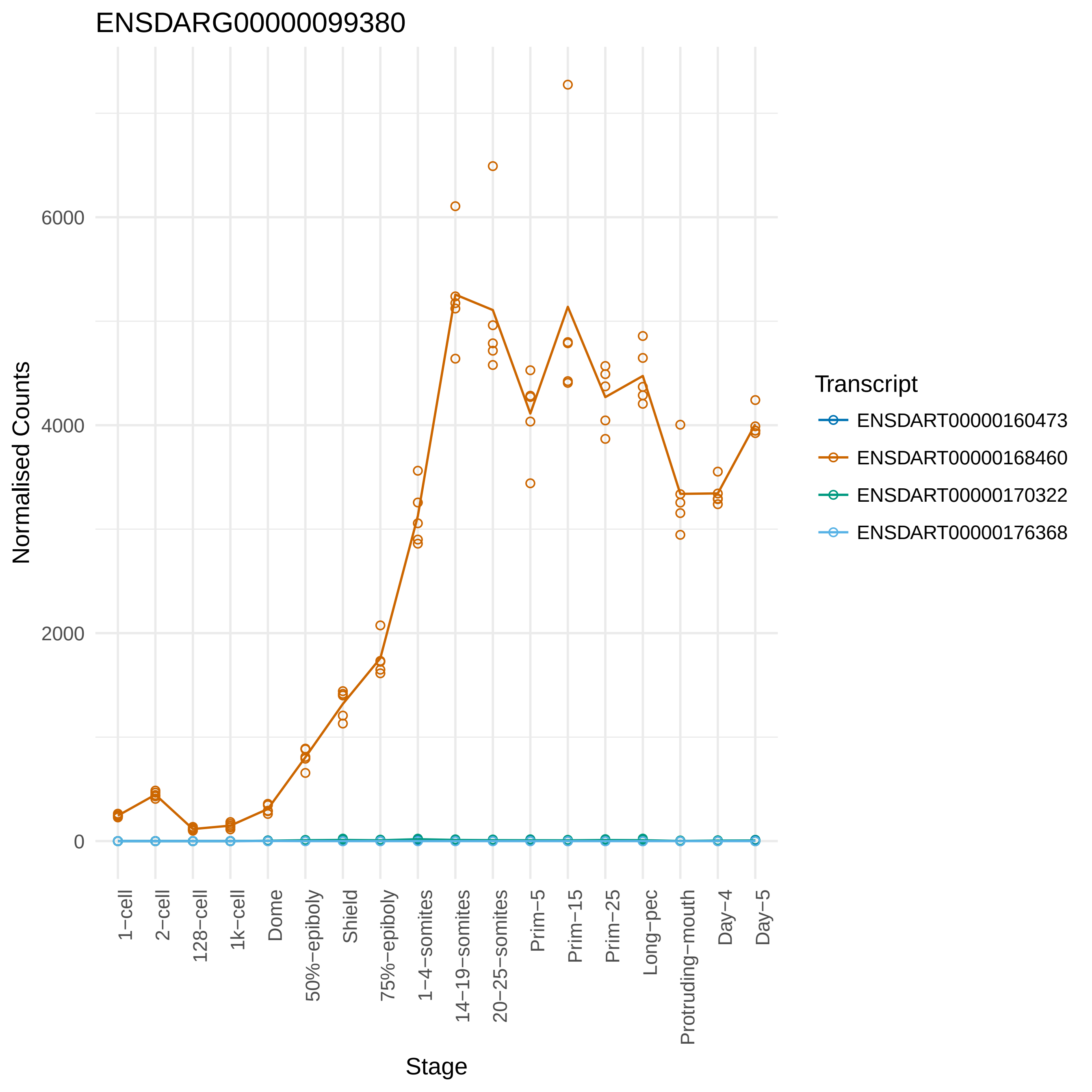
ENSDARG00000099380
Normalised Counts
6000
4000
2000
0
1−cell
2−cell
128−cell
1k−cell
Dome
50%−epiboly
Shield
75%−epiboly
1−4−somites
14−19−somites
20−25−somites
Prim−5
Prim−15
Prim−25
Long−pec
Protruding−mouth
Day−4
Day−5
Stage
Transcript
ENSDART00000160473
ENSDART00000168460
ENSDART00000170322
ENSDART00000176368

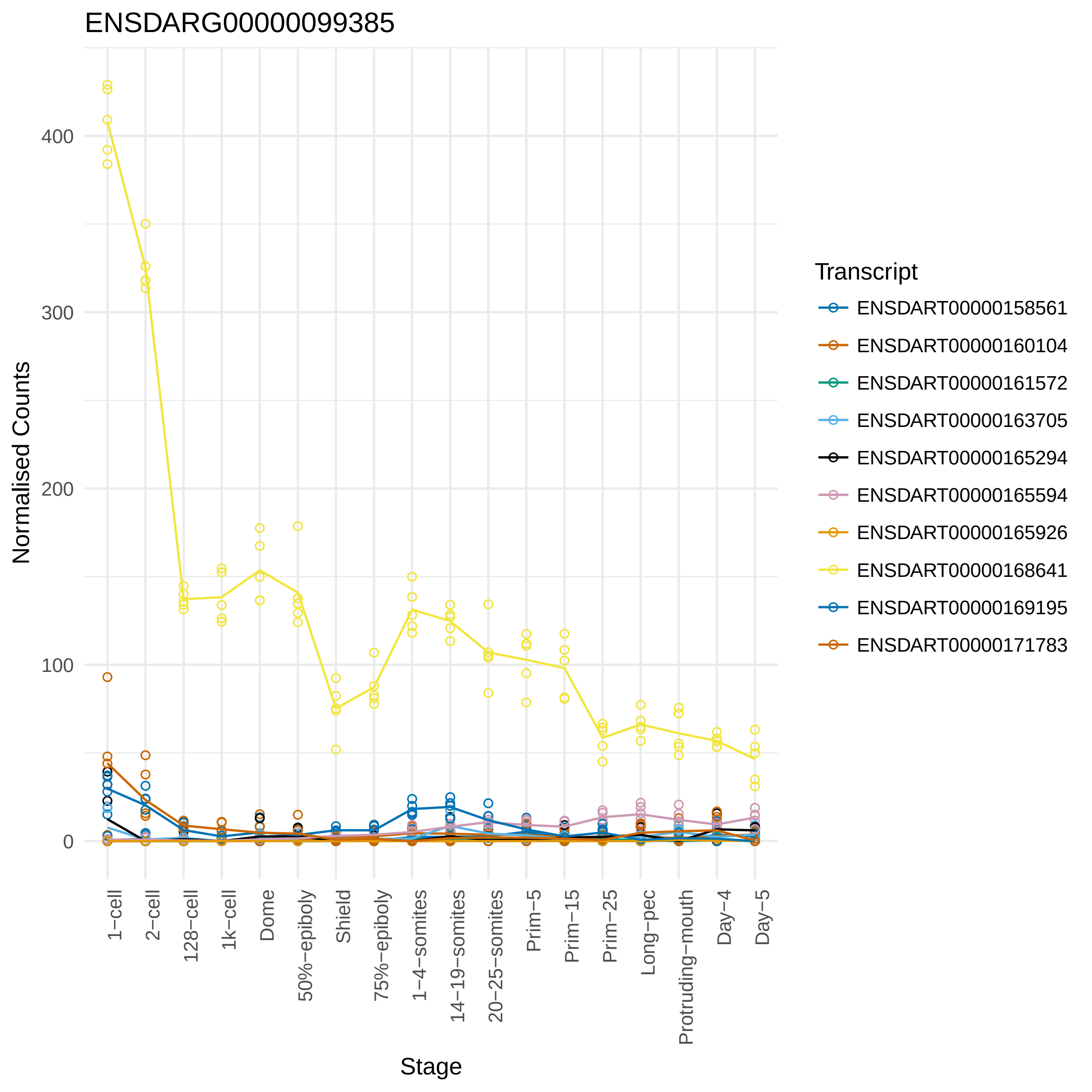

ENSDARG00000099385
Normalised Counts
0
100
200
300
400
Stage
1−cell
2−cell
128−cell
1k−cell
Dome
50%−epiboly
Shield
75%−epiboly
1−4−somites
14−19−somites
20−25−somites
Prim−5
Prim−15
Prim−25
Long−pec
Protruding−mouth
Day−4
Day−5
Transcript
ENSDART00000158561
ENSDART00000160104
ENSDART00000161572
ENSDART00000163705
ENSDART00000165294
ENSDART00000165594
ENSDART00000165926
ENSDART00000168641
ENSDART00000169195
ENSDART00000171783

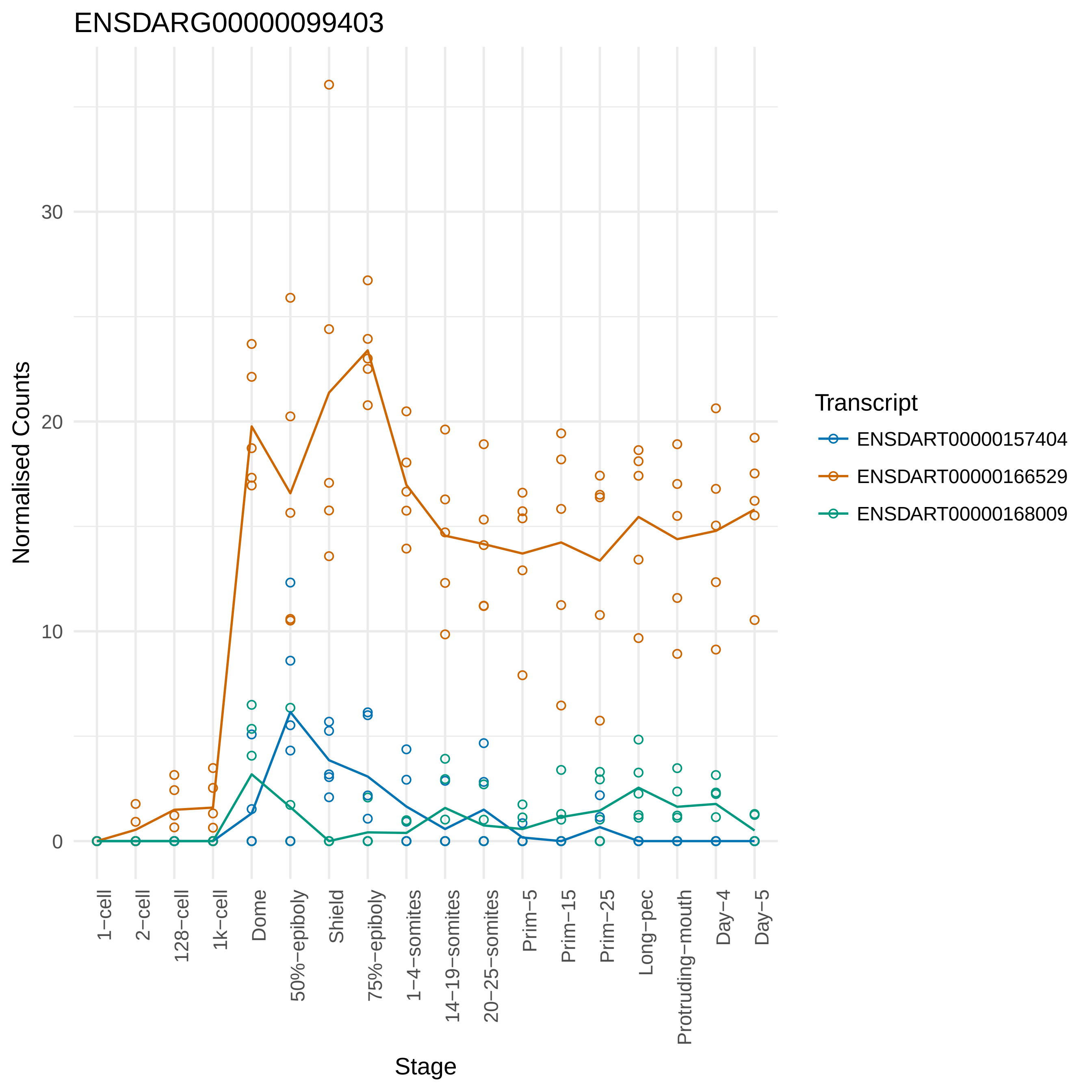
ENSDARG00000099403
Normalised Counts
30
20
10
0
1−cell
2−cell
128−cell
1k−cell
Dome
50%−epiboly
Shield
75%−epiboly
1−4−somites
14−19−somites
20−25−somites
Prim−5
Prim−15
Prim−25
Long−pec
Protruding−mouth
Day−4
Day−5
Stage
Transcript
ENSDART00000157404
ENSDART00000166529
ENSDART00000168009

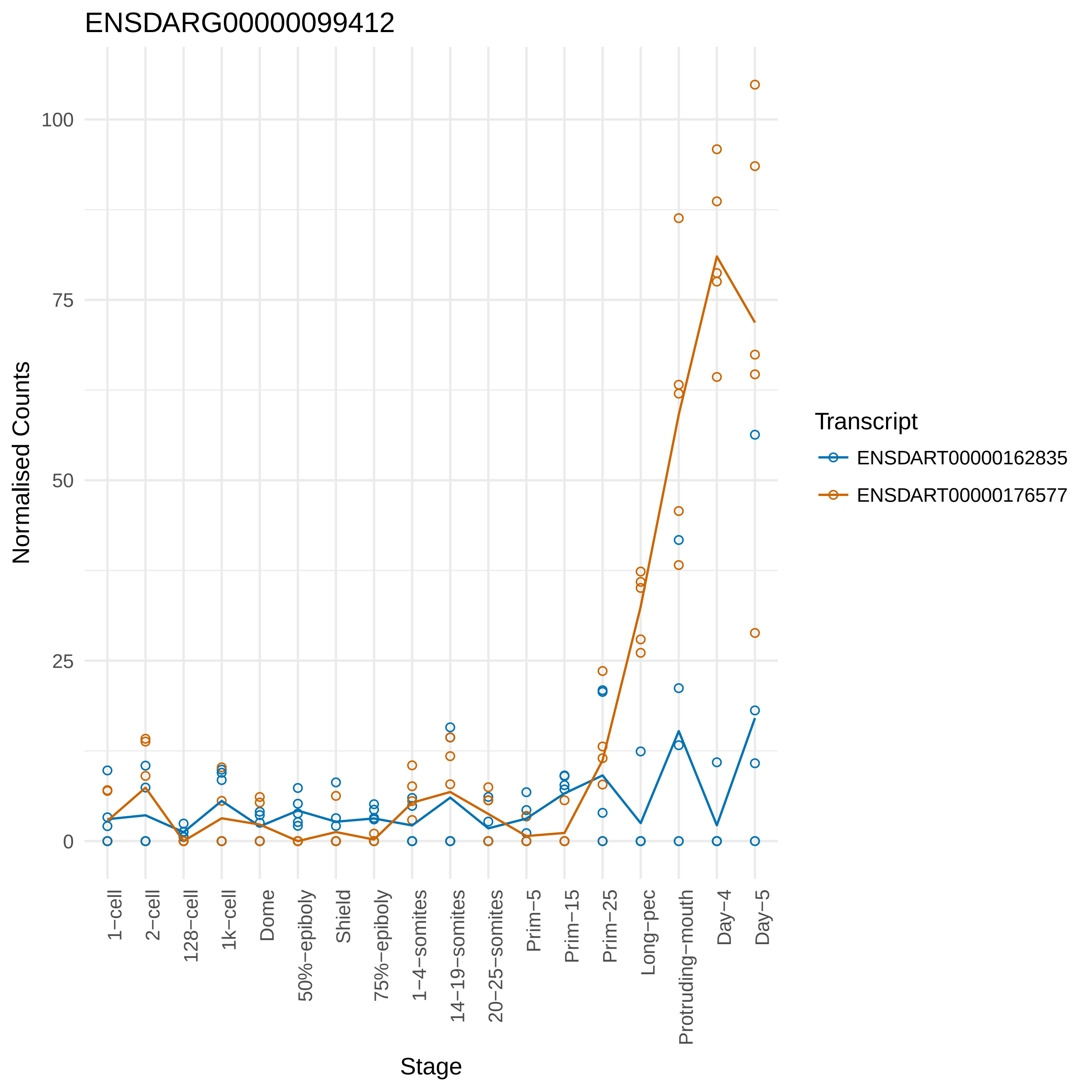
ENSDARG00000099412
Normalised Counts
100
75
50
25
0
1−cell
2−cell
128−cell
1k−cell
Dome
50%−epiboly
Shield
75%−epiboly
1−4−somites
14−19−somites
20−25−somites
Prim−5
Prim−15
Prim−25
Long−pec
Protruding−mouth
Day−4
Day−5
Stage
Transcript
ENSDART00000162835
ENSDART00000176577

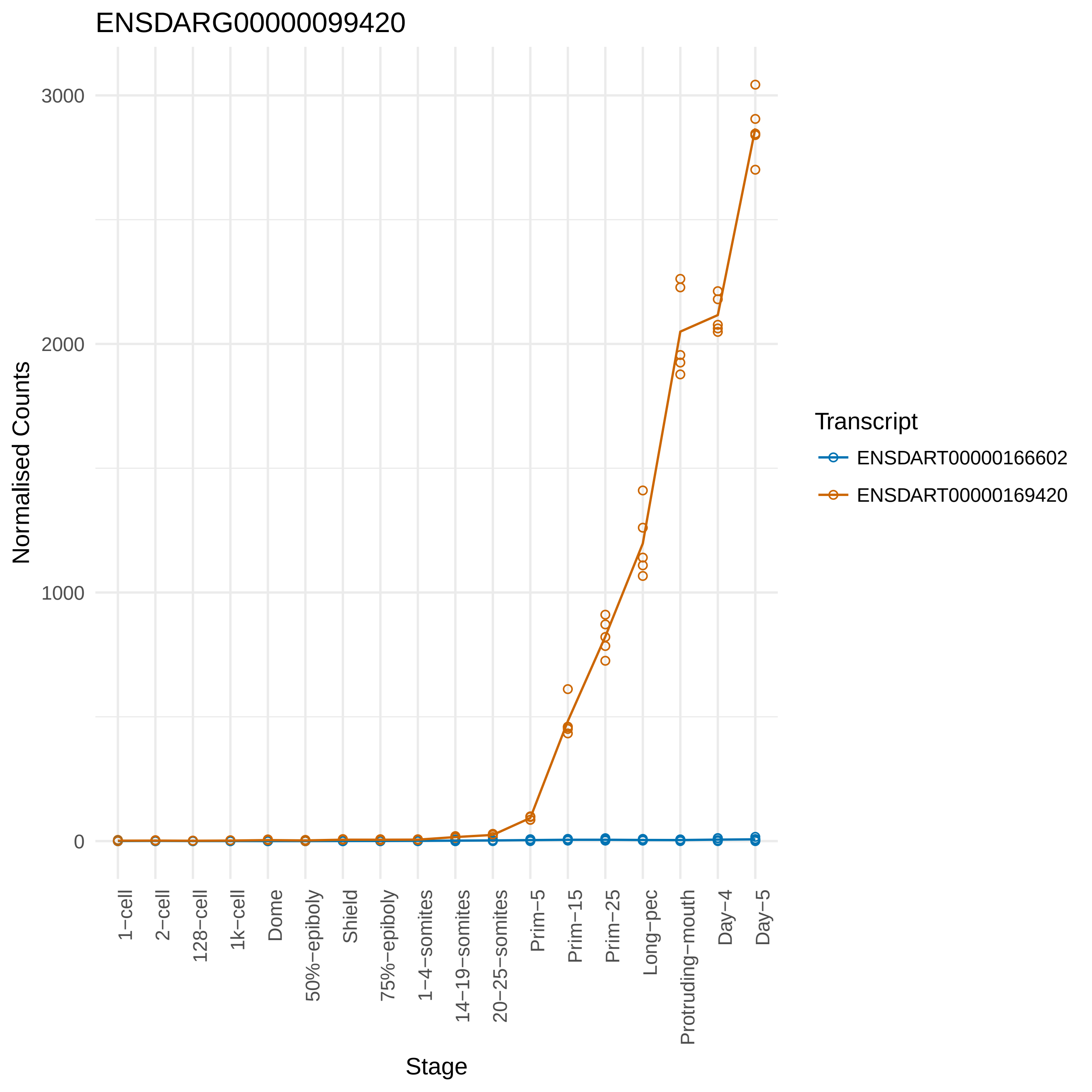
ENSDARG00000099420
Normalised Counts
3000
2000
1000
0
1−cell
2−cell
128−cell
1k−cell
Dome
50%−epiboly
Shield
75%−epiboly
1−4−somites
14−19−somites
20−25−somites
Prim−5
Prim−15
Prim−25
Long−pec
Protruding−mouth
Day−4
Day−5
Stage
Transcript
ENSDART00000166602
ENSDART00000169420

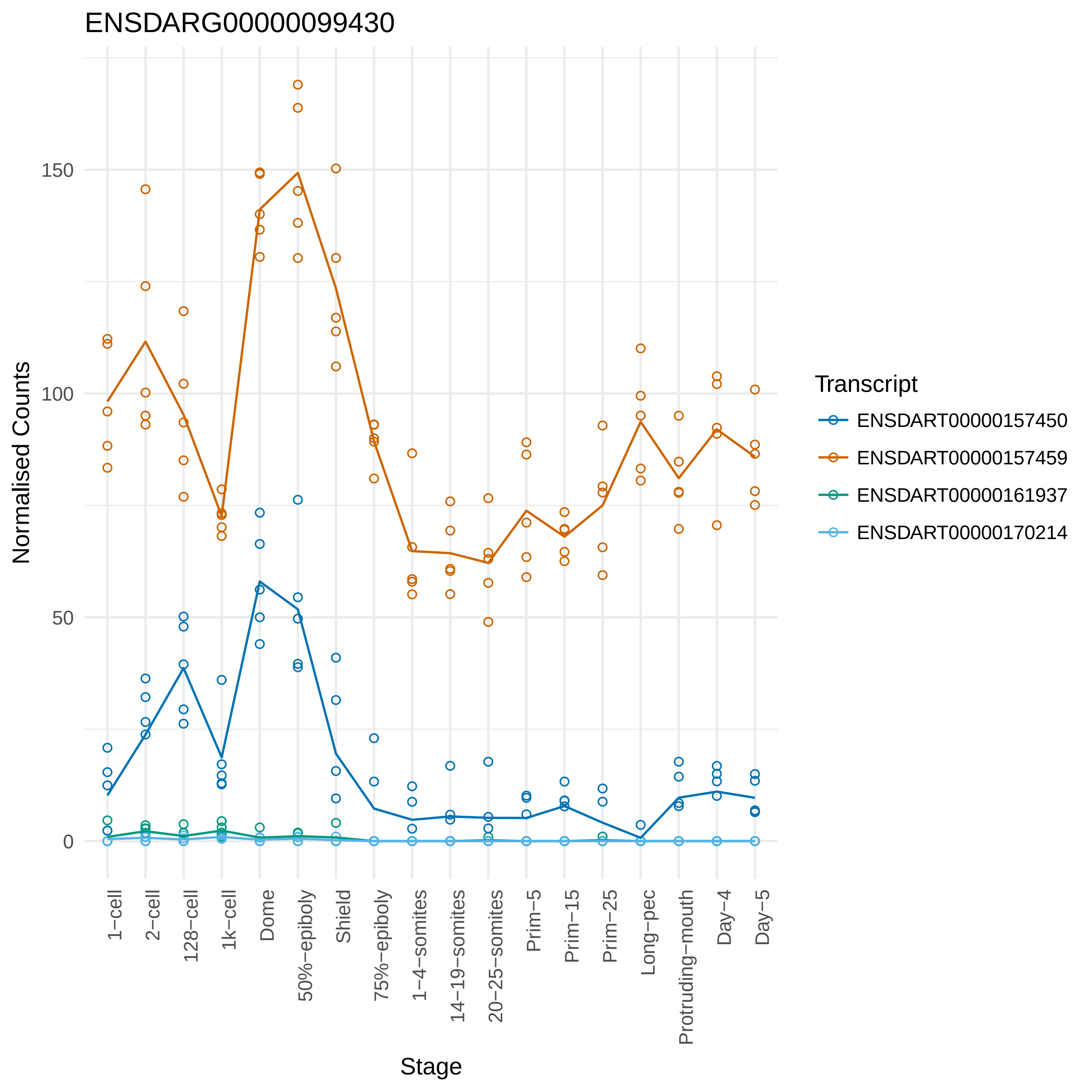

ENSDARG00000099430
Normalised Counts
150
100
50
0
1−cell
2−cell
128−cell
1k−cell
Dome
50%−epiboly
Shield
75%−epiboly
1−4−somites
14−19−somites
20−25−somites
Prim−5
Prim−15
Prim−25
Long−pec
Protruding−mouth
Day−4
Day−5
Stage
Transcript
ENSDART00000157450
ENSDART00000157459
ENSDART00000161937
ENSDART00000170214

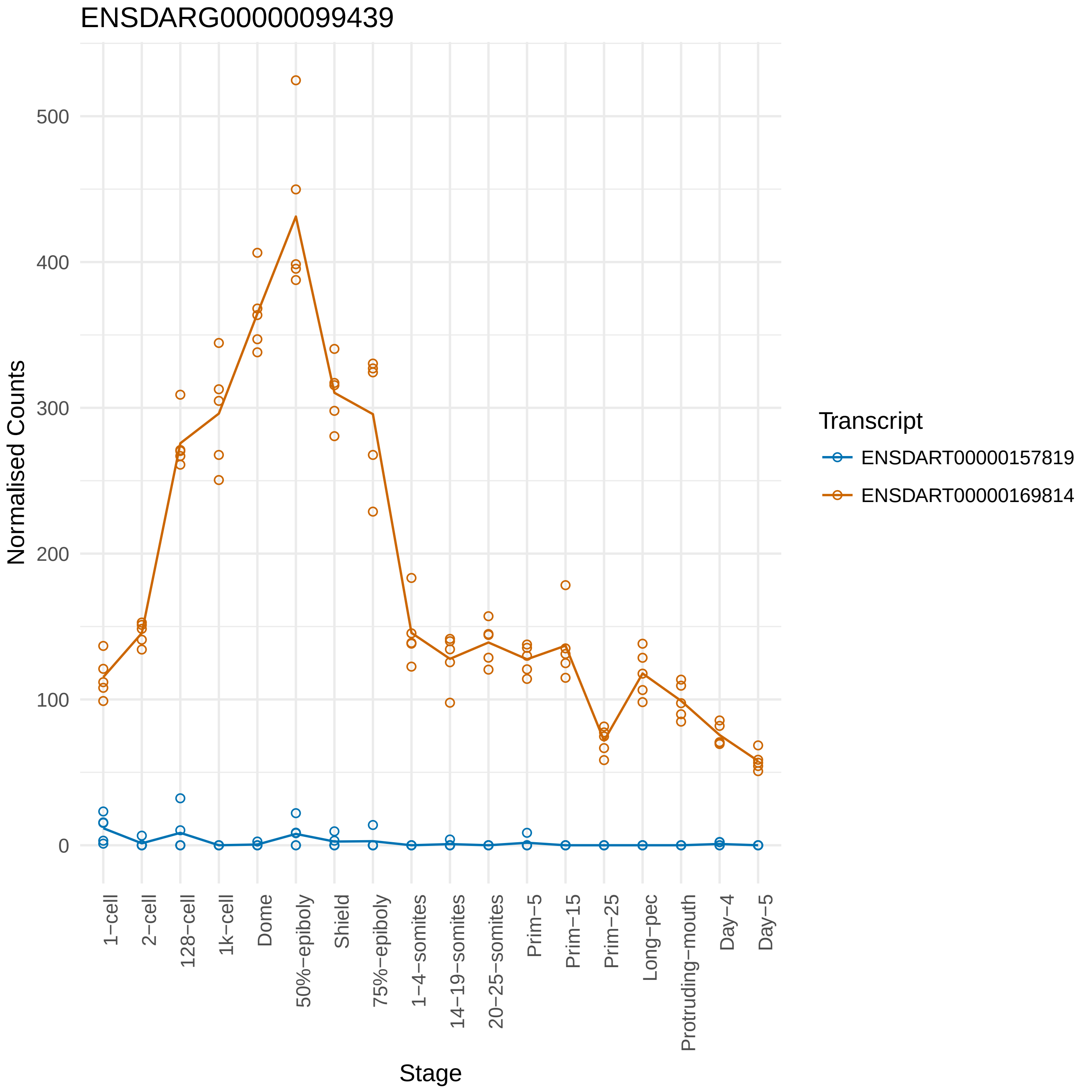
ENSDARG00000099439
Normalised Counts
0
100
200
300
400
500
1−cell
2−cell
128−cell
1k−cell
Dome
50%−epiboly
Shield
75%−epiboly
1−4−somites
14−19−somites
20−25−somites
Prim−5
Prim−15
Prim−25
Long−pec
Protruding−mouth
Day−4
Day−5
Stage
Transcript
ENSDART00000157819
ENSDART00000169814

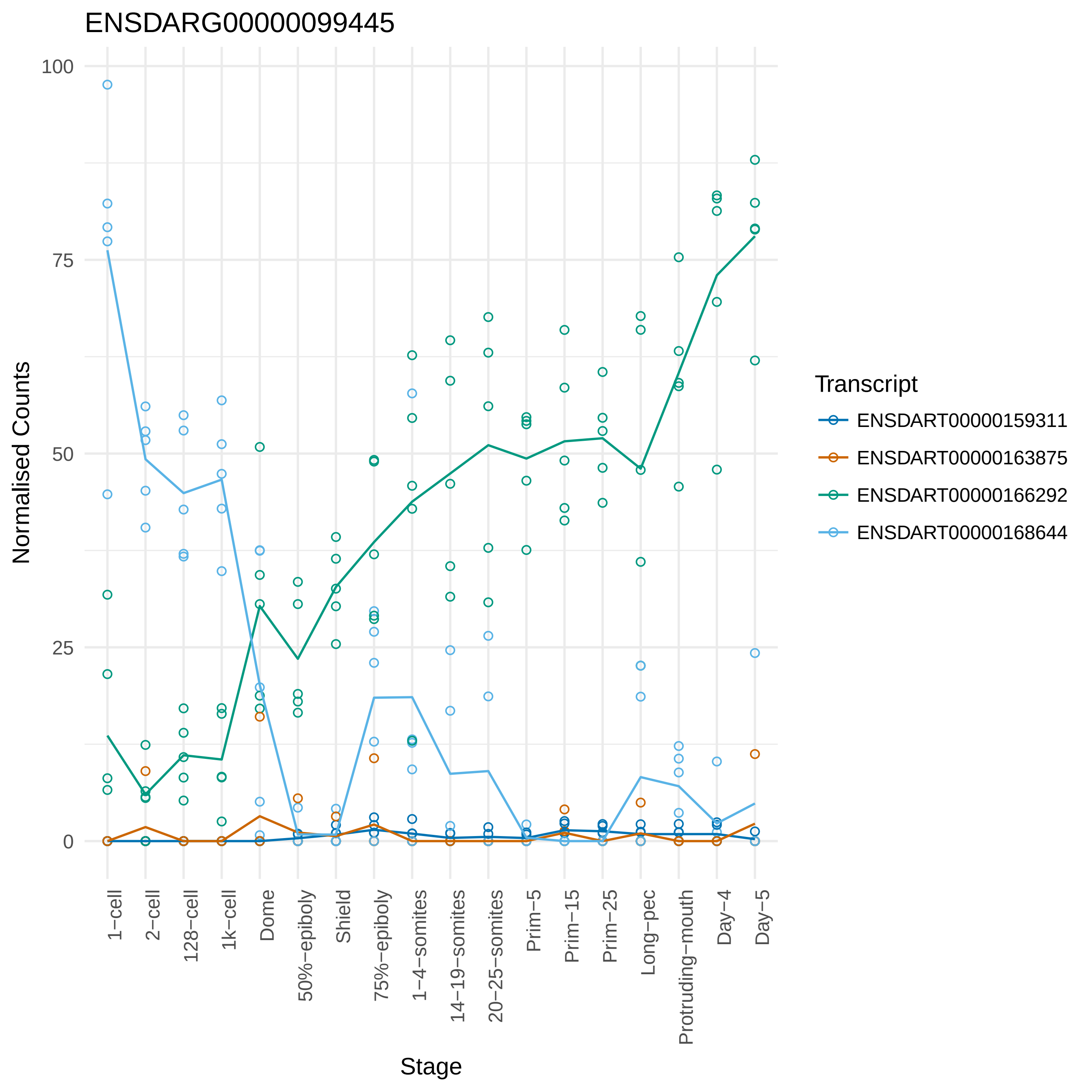
ENSDARG00000099445
Normalised Counts
100
75
50
25
0
1−cell
2−cell
128−cell
1k−cell
Dome
50%−epiboly
Shield
75%−epiboly
1−4−somites
14−19−somites
20−25−somites
Prim−5
Prim−15
Prim−25
Long−pec
Protruding−mouth
Day−4
Day−5
Stage
Transcript
ENSDART00000159311
ENSDART00000163875
ENSDART00000166292
ENSDART00000168644

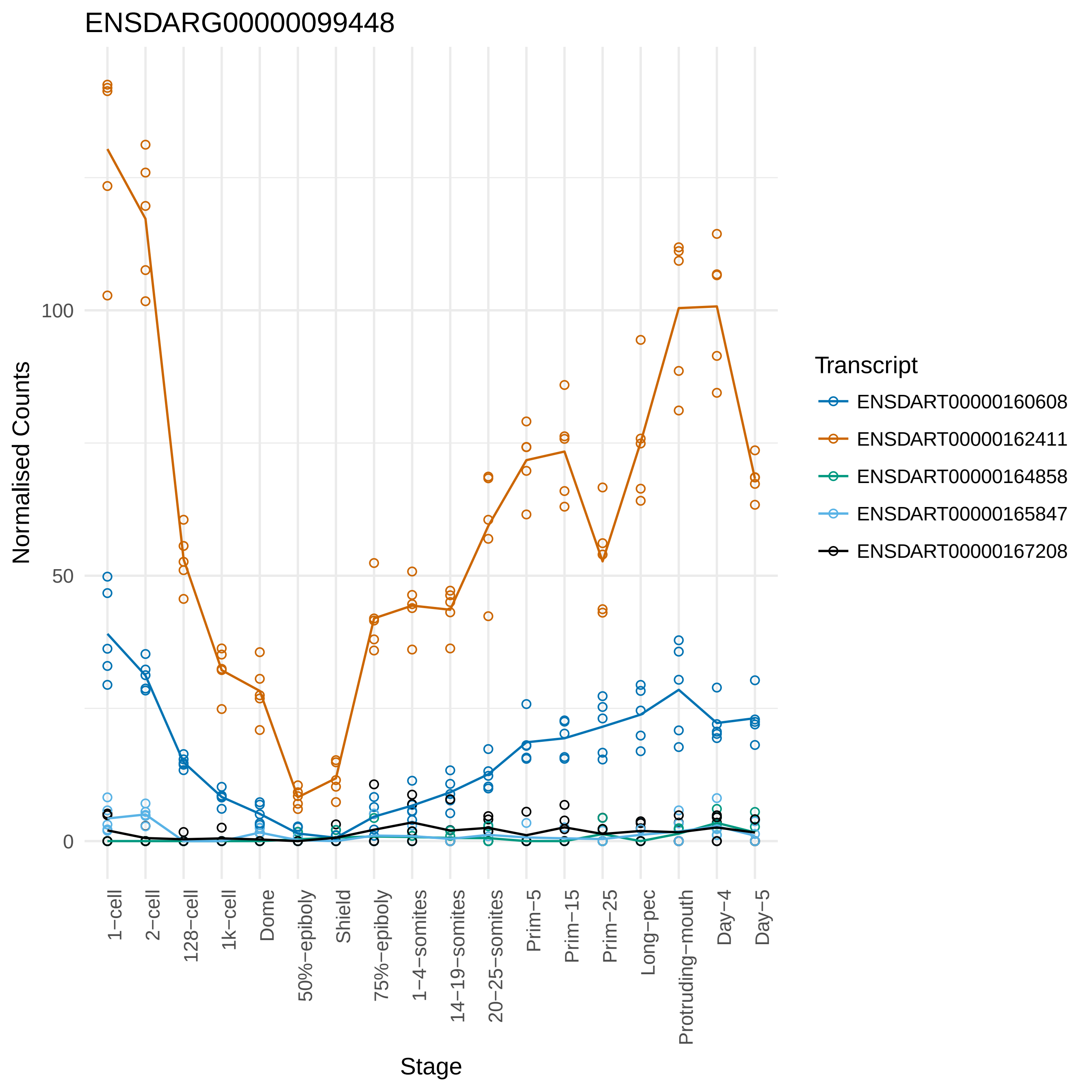
ENSDARG00000099448
Normalised Counts
0
50
100
Stage
1−cell
2−cell
128−cell
1k−cell
Dome
50%−epiboly
Shield
75%−epiboly
1−4−somites
14−19−somites
20−25−somites
Prim−5
Prim−15
Prim−25
Long−pec
Protruding−mouth
Day−4
Day−5
Transcript
ENSDART00000160608
ENSDART00000162411
ENSDART00000164858
ENSDART00000165847
ENSDART00000167208

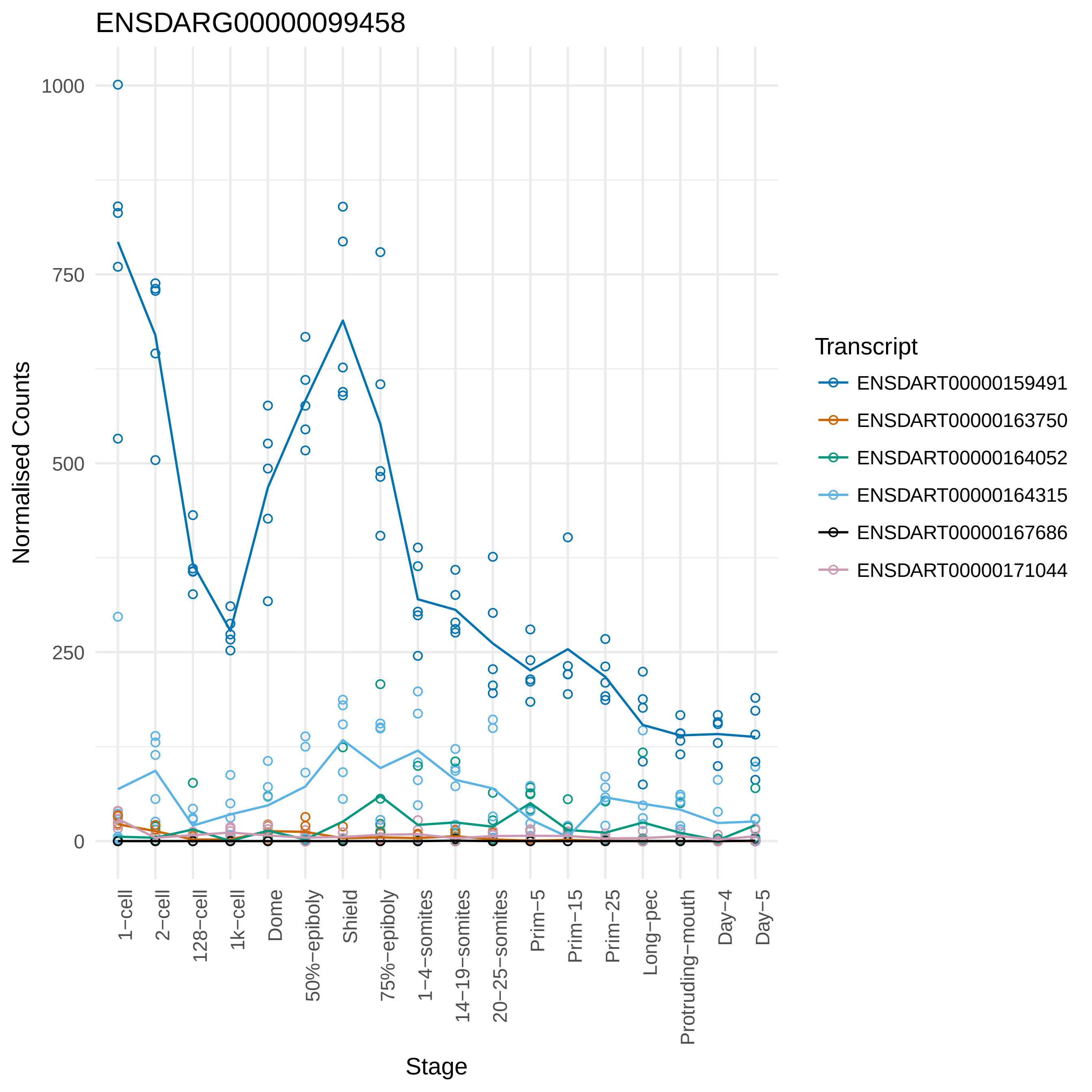

ENSDARG00000099458
Normalised Counts
1000
750
500
250
0
1−cell
2−cell
128−cell
1k−cell
Dome
50%−epiboly
Shield
75%−epiboly
1−4−somites
14−19−somites
20−25−somites
Prim−5
Prim−15
Prim−25
Long−pec
Protruding−mouth
Day−4
Day−5
Stage
Transcript
ENSDART00000159491
ENSDART00000163750
ENSDART00000164052
ENSDART00000164315
ENSDART00000167686
ENSDART00000171044

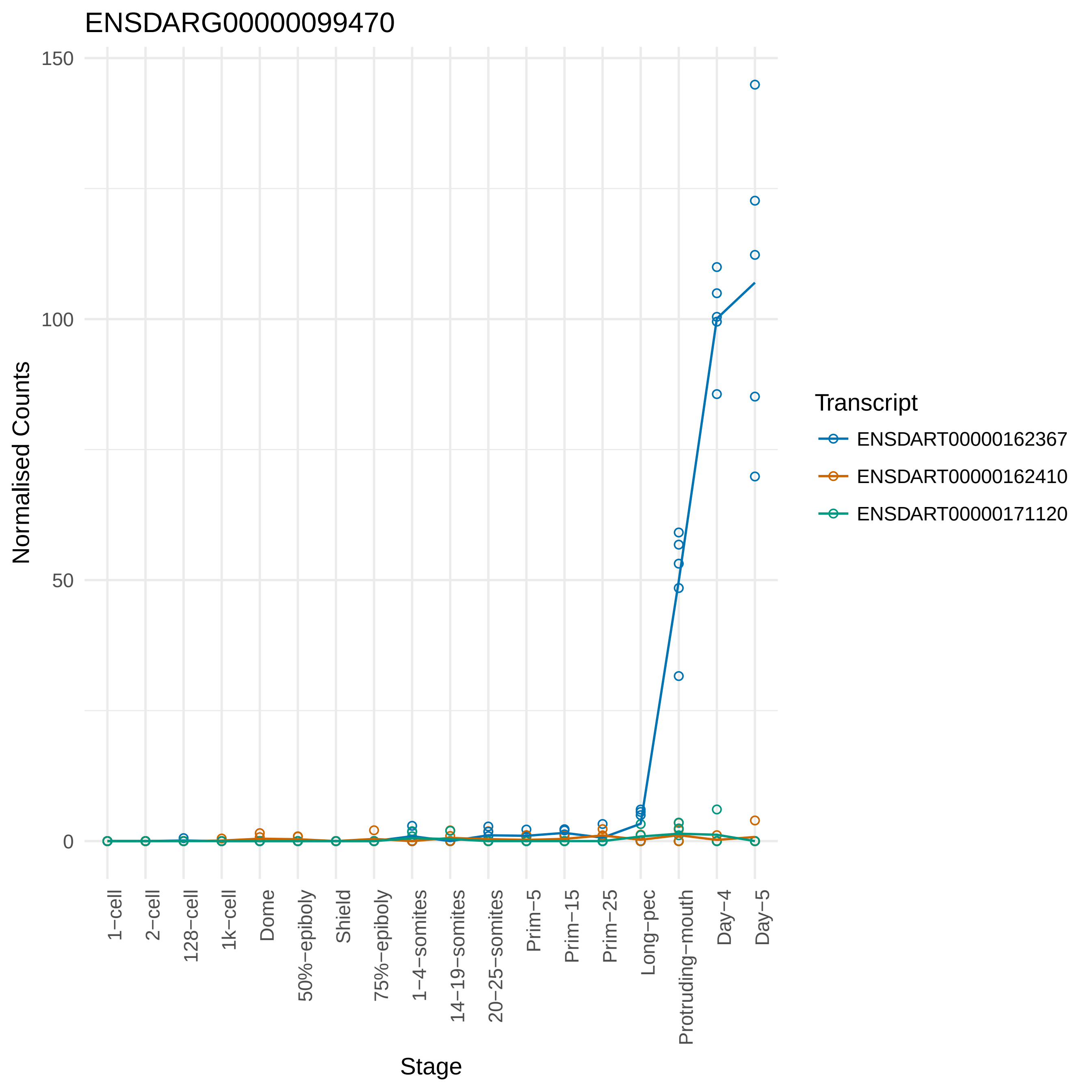

ENSDARG00000099470
Normalised Counts
150
100
50
0
1−cell
2−cell
128−cell
1k−cell
Dome
50%−epiboly
Shield
75%−epiboly
1−4−somites
14−19−somites
20−25−somites
Prim−5
Prim−15
Prim−25
Long−pec
Protruding−mouth
Day−4
Day−5
Stage
Transcript
ENSDART00000162367
ENSDART00000162410
ENSDART00000171120

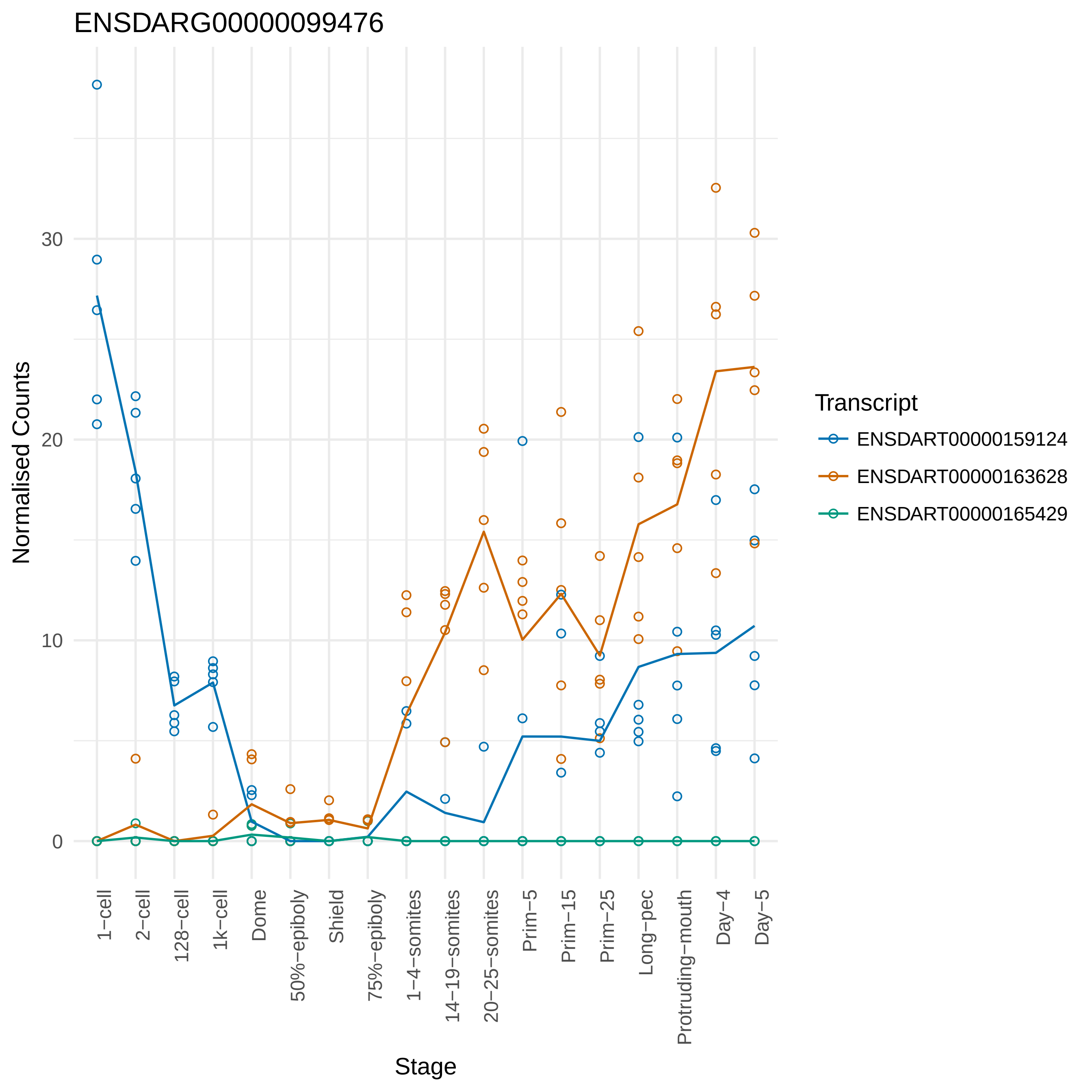

ENSDARG00000099476
Normalised Counts
0
10
20
30
Stage
1−cell
2−cell
128−cell
1k−cell
Dome
50%−epiboly
Shield
75%−epiboly
1−4−somites
14−19−somites
20−25−somites
Prim−5
Prim−15
Prim−25
Long−pec
Protruding−mouth
Day−4
Day−5
Transcript
ENSDART00000159124
ENSDART00000163628
ENSDART00000165429

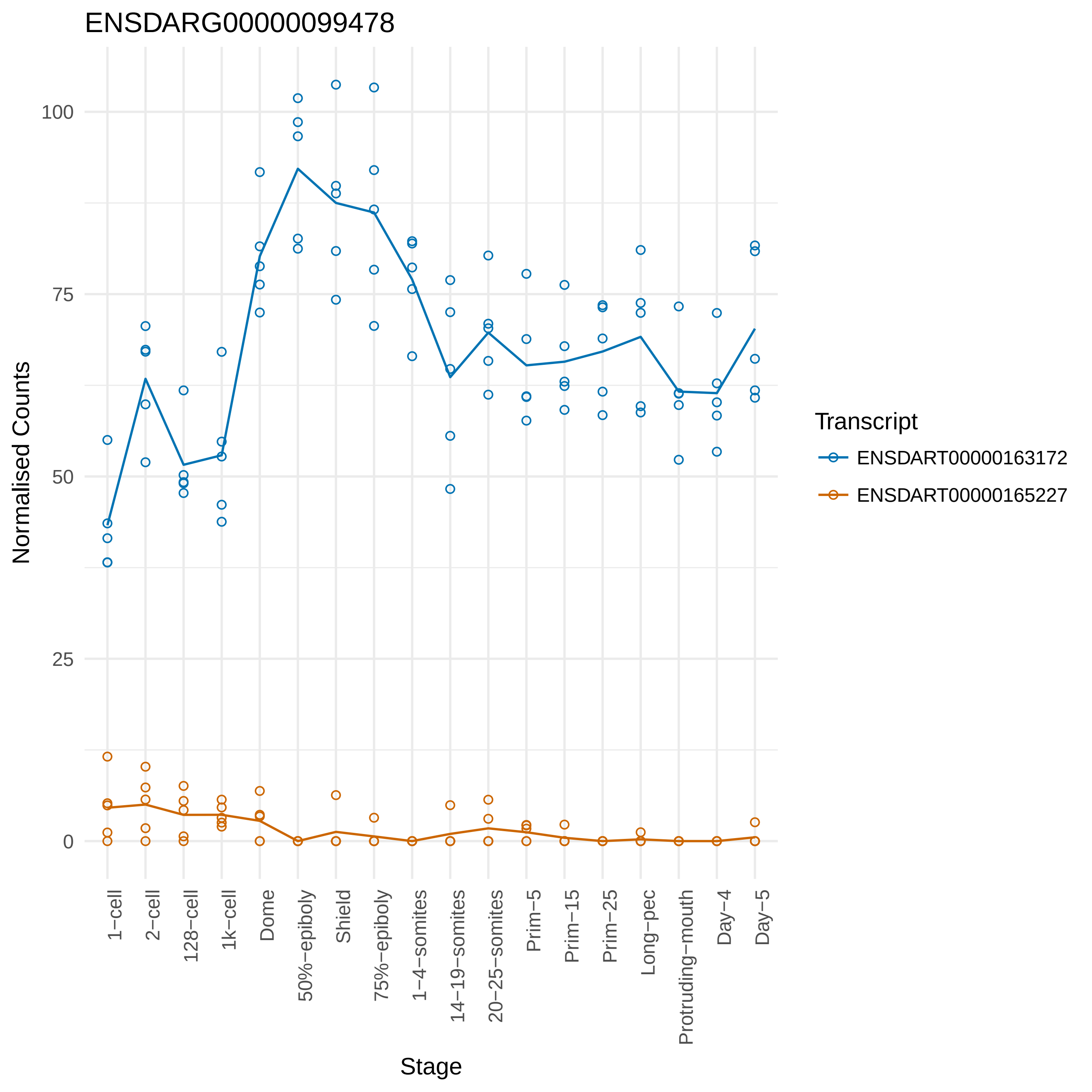
ENSDARG00000099478
Normalised Counts
100
75
50
25
0
1−cell
2−cell
128−cell
1k−cell
Dome
50%−epiboly
Shield
75%−epiboly
1−4−somites
14−19−somites
20−25−somites
Prim−5
Prim−15
Prim−25
Long−pec
Protruding−mouth
Day−4
Day−5
Stage
Transcript
ENSDART00000163172
ENSDART00000165227

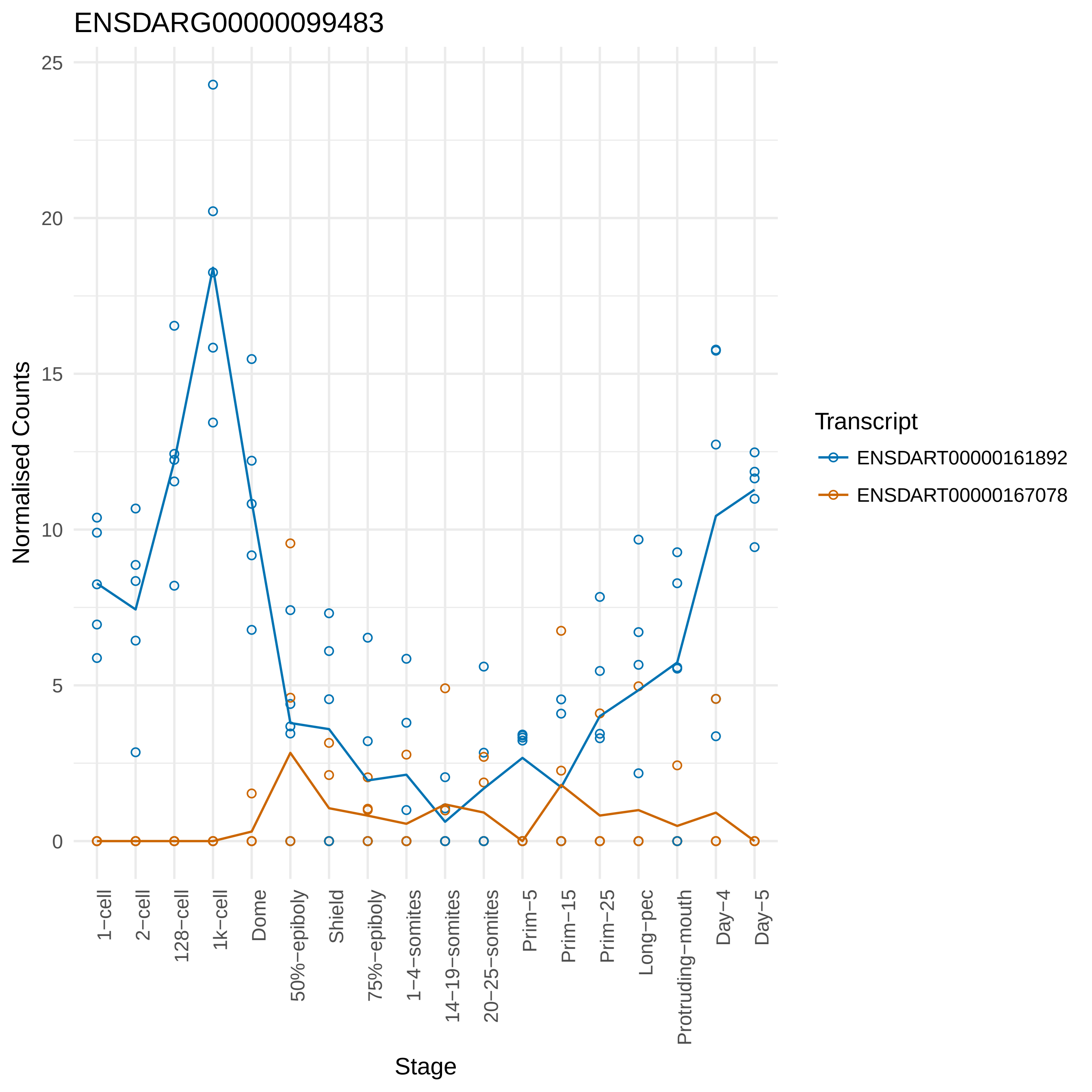

ENSDARG00000099483
Normalised Counts
0
5
10
15
20
25
Stage
1−cell
2−cell
128−cell
1k−cell
Dome
50%−epiboly
Shield
75%−epiboly
1−4−somites
14−19−somites
20−25−somites
Prim−5
Prim−15
Prim−25
Long−pec
Protruding−mouth
Day−4
Day−5
Transcript
ENSDART00000161892
ENSDART00000167078

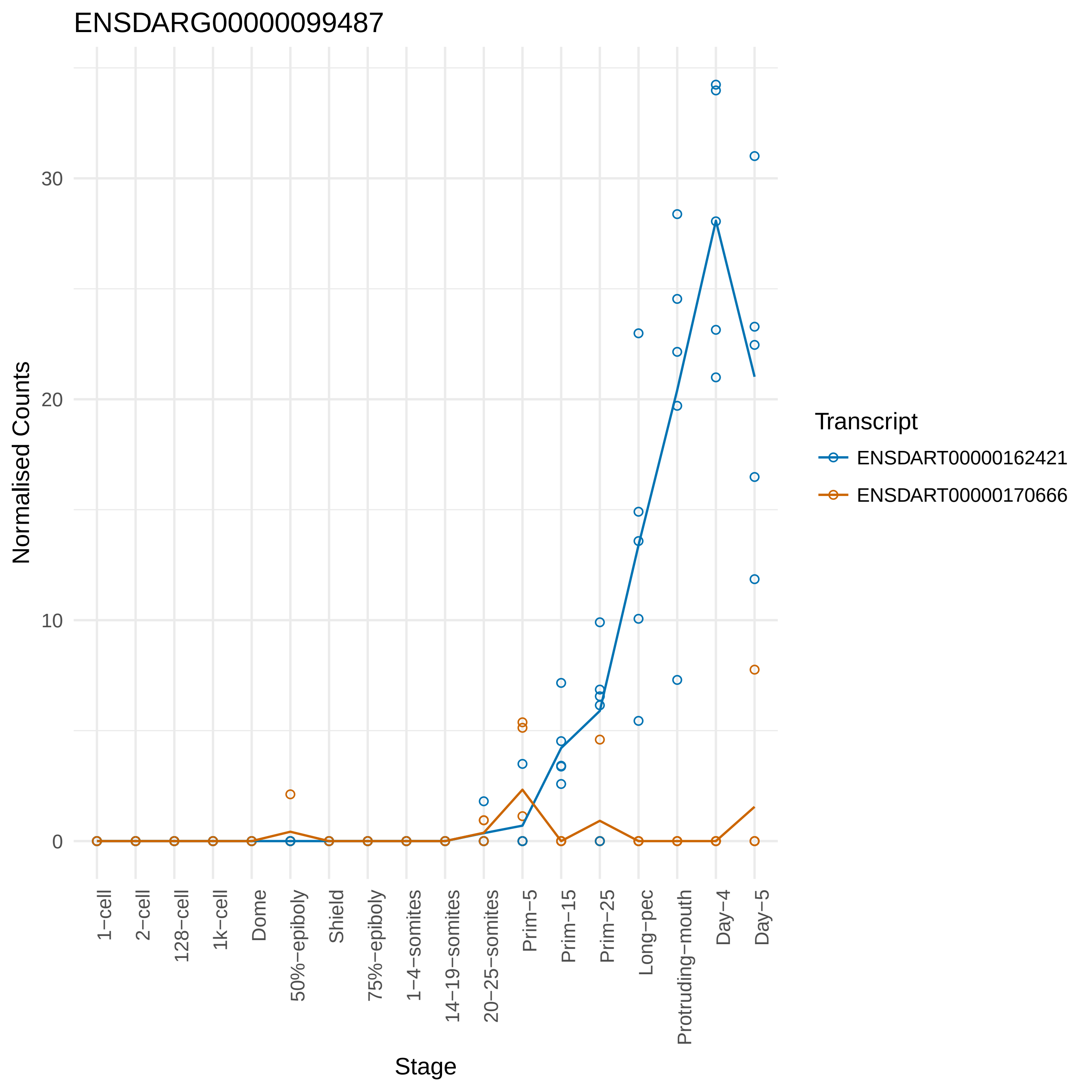

ENSDARG00000099487
Normalised Counts
0
10
20
30
1−cell
2−cell
128−cell
1k−cell
Dome
50%−epiboly
Shield
75%−epiboly
1−4−somites
14−19−somites
20−25−somites
Prim−5
Prim−15
Prim−25
Long−pec
Protruding−mouth
Day−4
Day−5
Stage
Transcript
ENSDART00000162421
ENSDART00000170666

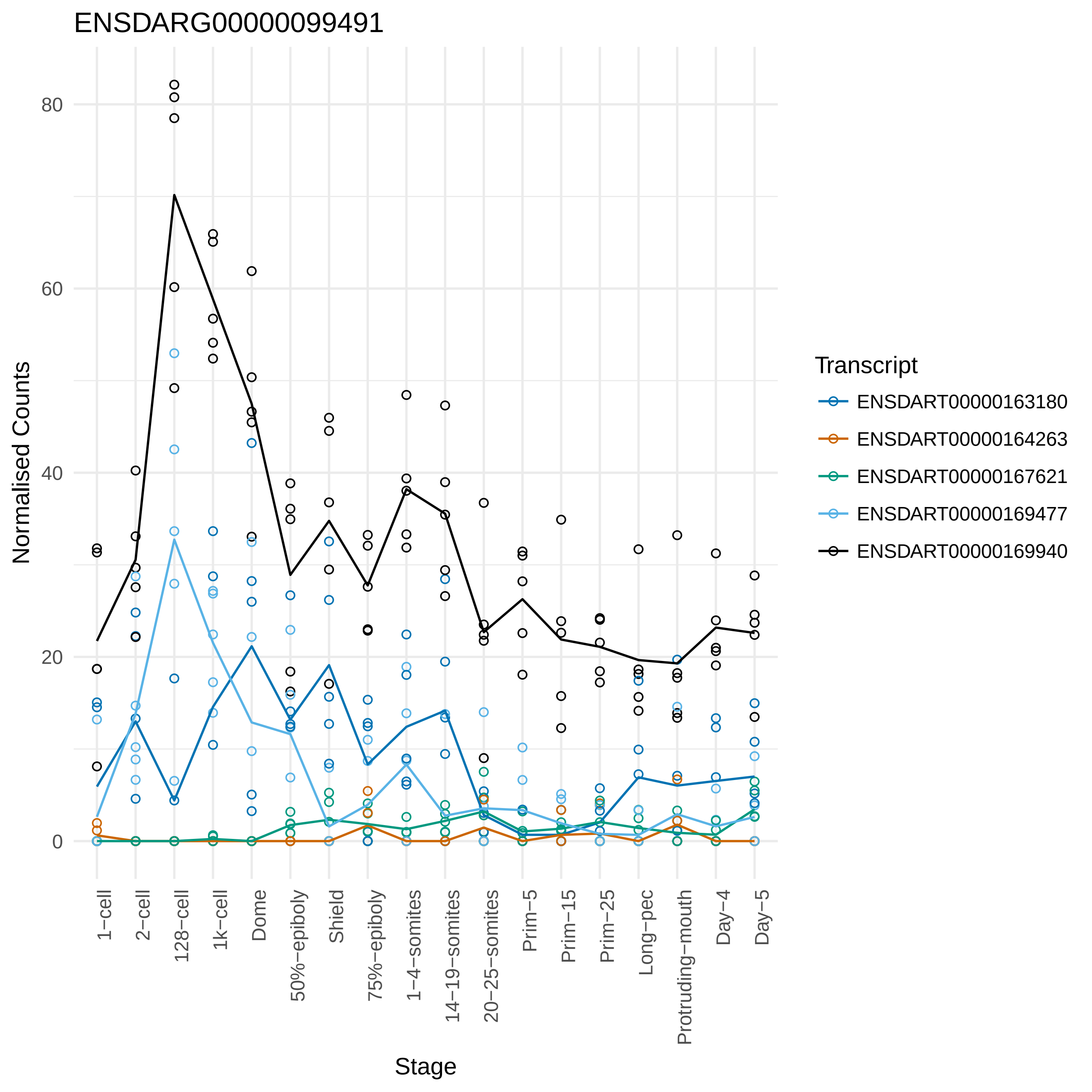

ENSDARG00000099491
Normalised Counts
80
60
40
20
0
1−cell
2−cell
128−cell
1k−cell
Dome
50%−epiboly
Shield
75%−epiboly
1−4−somites
14−19−somites
20−25−somites
Prim−5
Prim−15
Prim−25
Long−pec
Protruding−mouth
Day−4
Day−5
Stage
Transcript
ENSDART00000163180
ENSDART00000164263
ENSDART00000167621
ENSDART00000169477
ENSDART00000169940

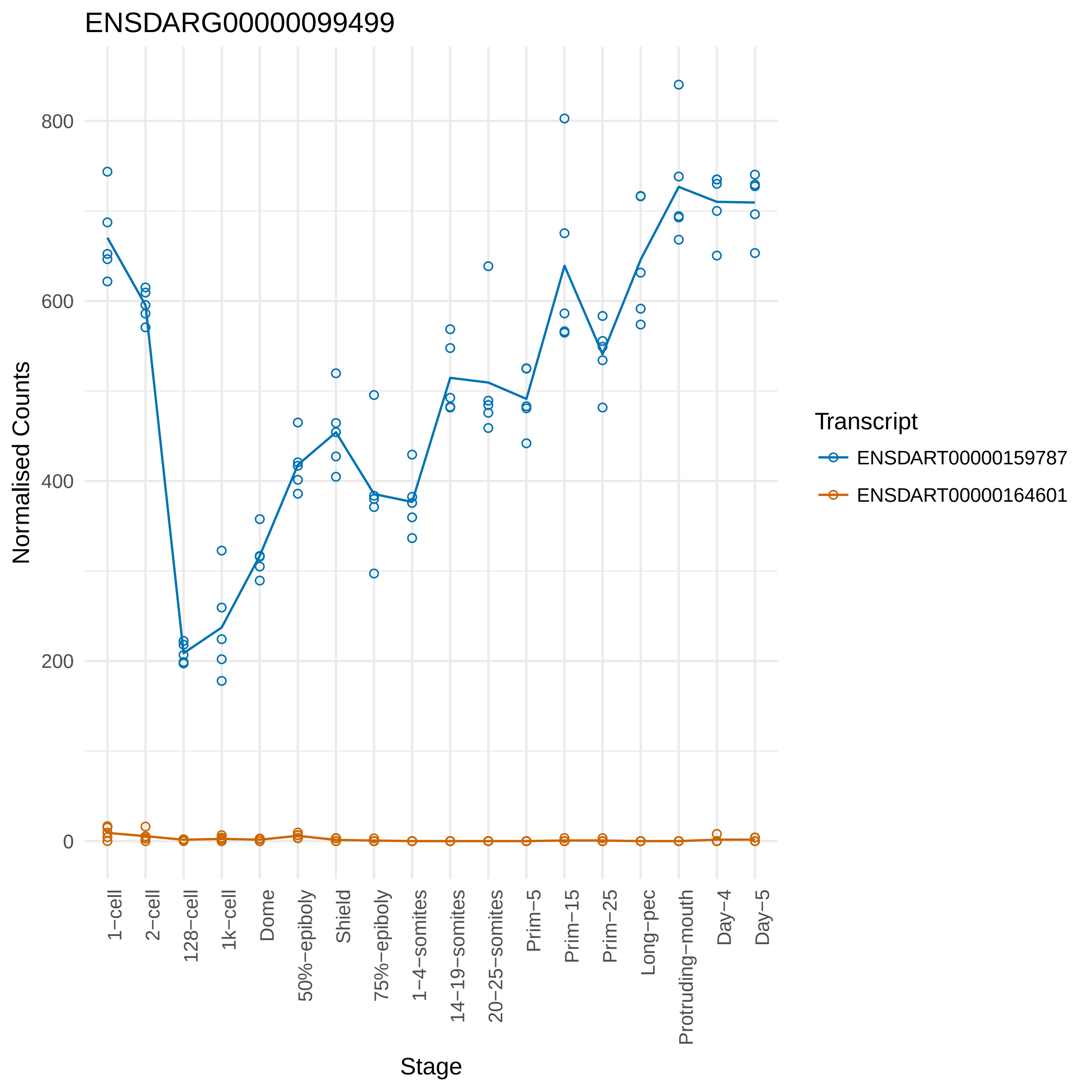

ENSDARG00000099499
Normalised Counts
800
600
400
200
0
1−cell
2−cell
128−cell
1k−cell
Dome
50%−epiboly
Shield
75%−epiboly
1−4−somites
14−19−somites
20−25−somites
Prim−5
Prim−15
Prim−25
Long−pec
Protruding−mouth
Day−4
Day−5
Stage
Transcript
ENSDART00000159787
ENSDART00000164601

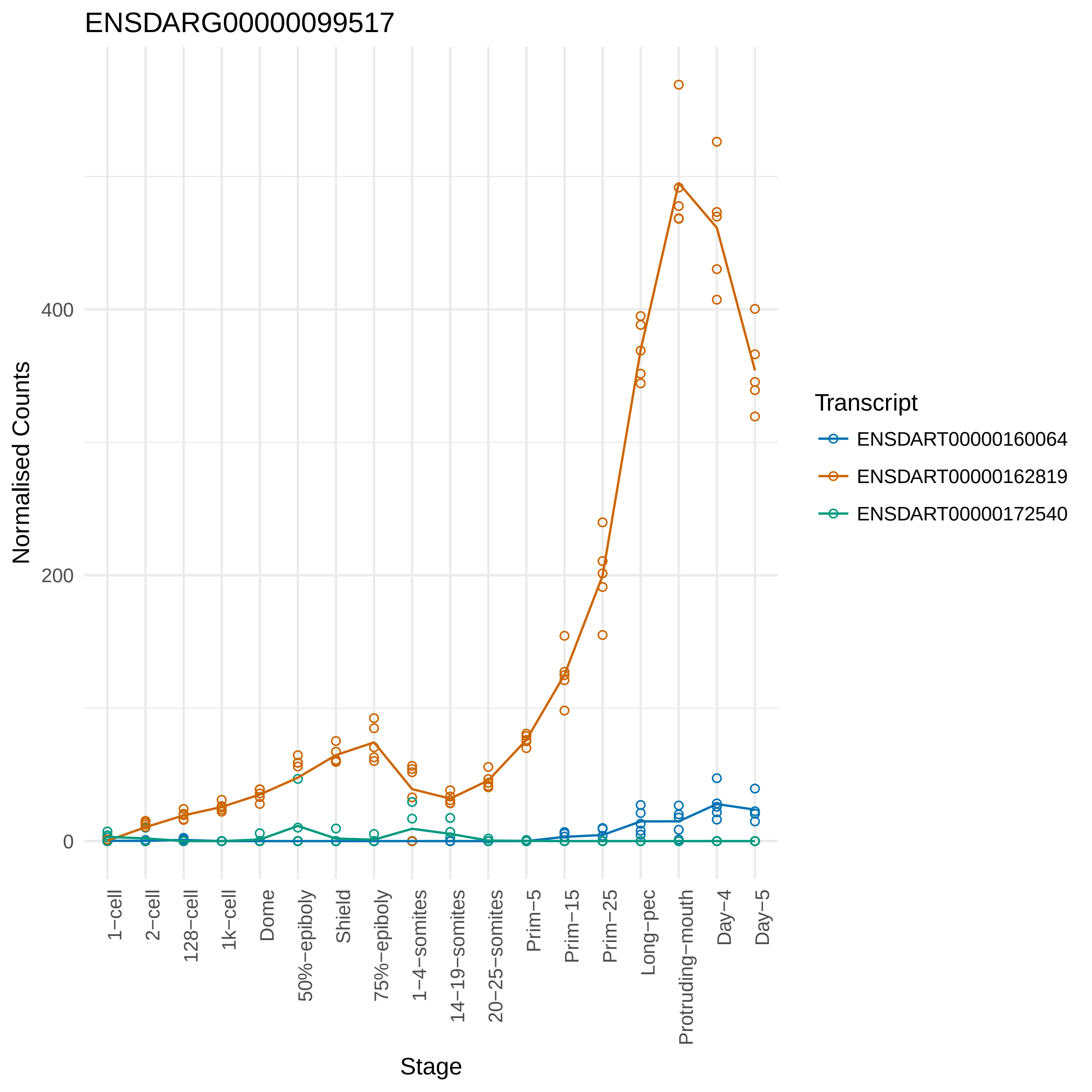
ENSDARG00000099517
Normalised Counts
400
200
0
Stage
1−cell
2−cell
128−cell
1k−cell
Dome
50%−epiboly
Shield
75%−epiboly
1−4−somites
14−19−somites
20−25−somites
Prim−5
Prim−15
Prim−25
Long−pec
Protruding−mouth
Day−4
Day−5
Transcript
ENSDART00000160064
ENSDART00000162819
ENSDART00000172540

ENSDARG00000099524

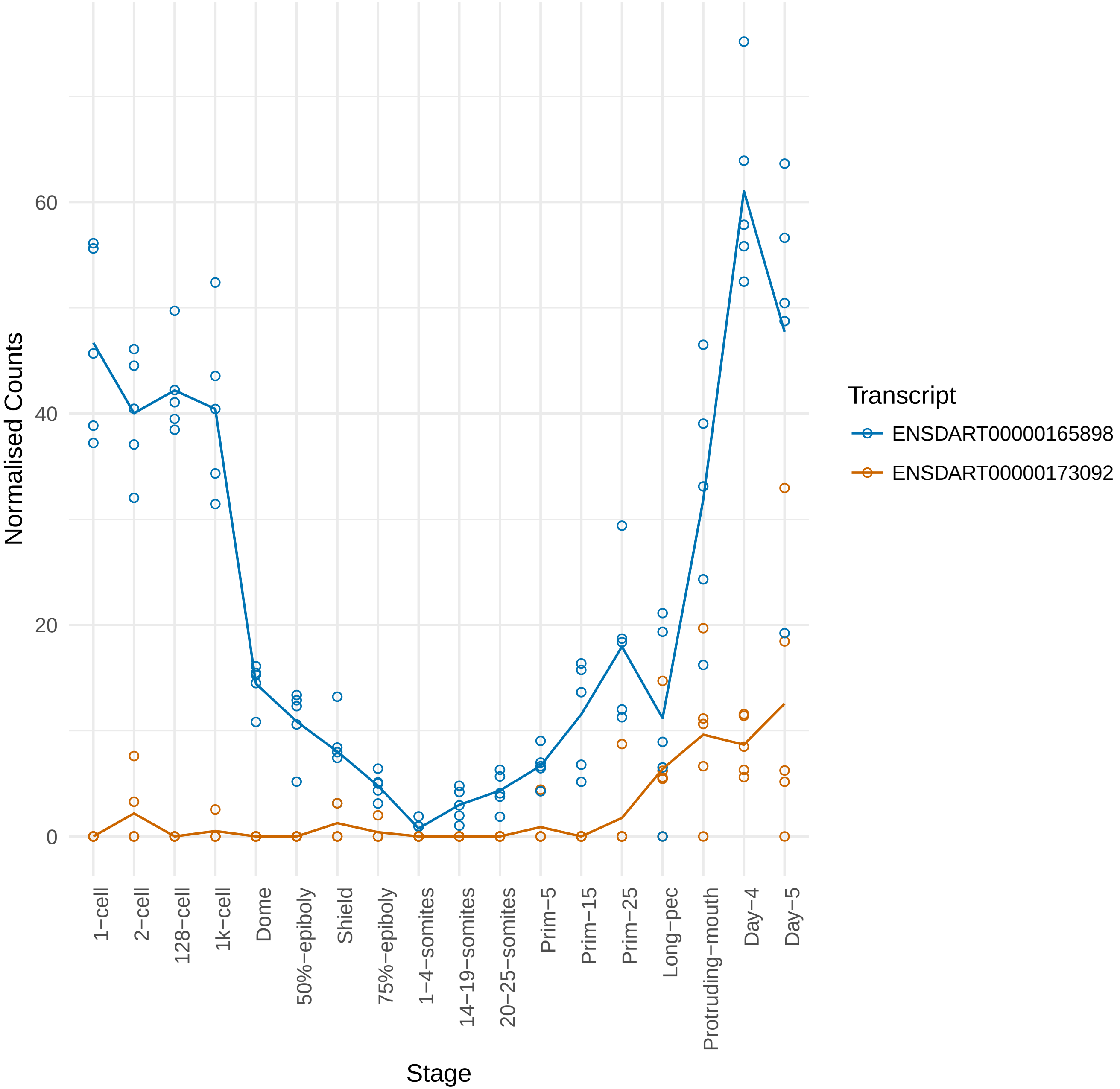

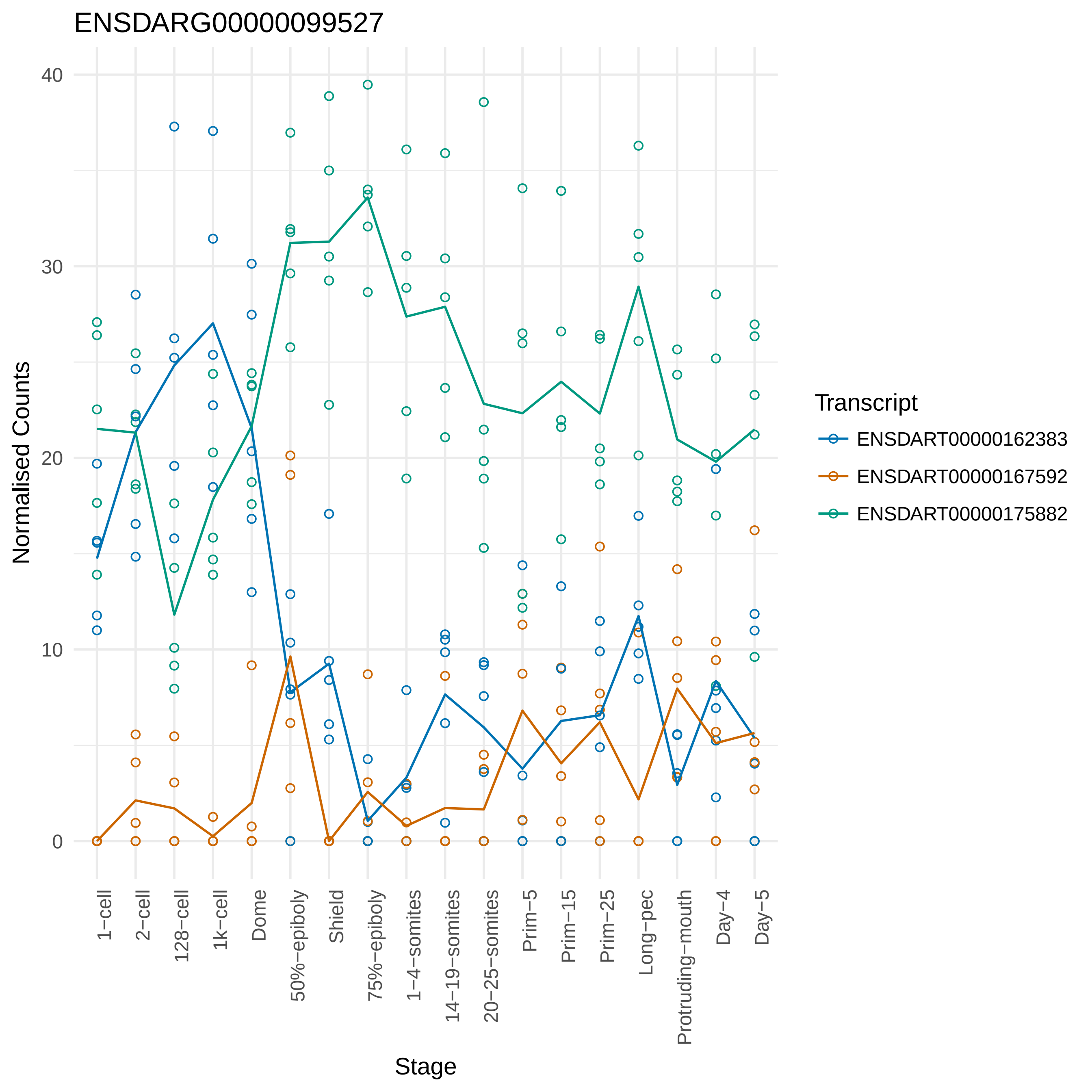

ENSDARG00000099527
Normalised Counts
0
10
20
30
40
1−cell
2−cell
128−cell
1k−cell
Dome
50%−epiboly
Shield
75%−epiboly
1−4−somites
14−19−somites
20−25−somites
Prim−5
Prim−15
Prim−25
Long−pec
Protruding−mouth
Day−4
Day−5
Stage
Transcript
ENSDART00000162383
ENSDART00000167592
ENSDART00000175882

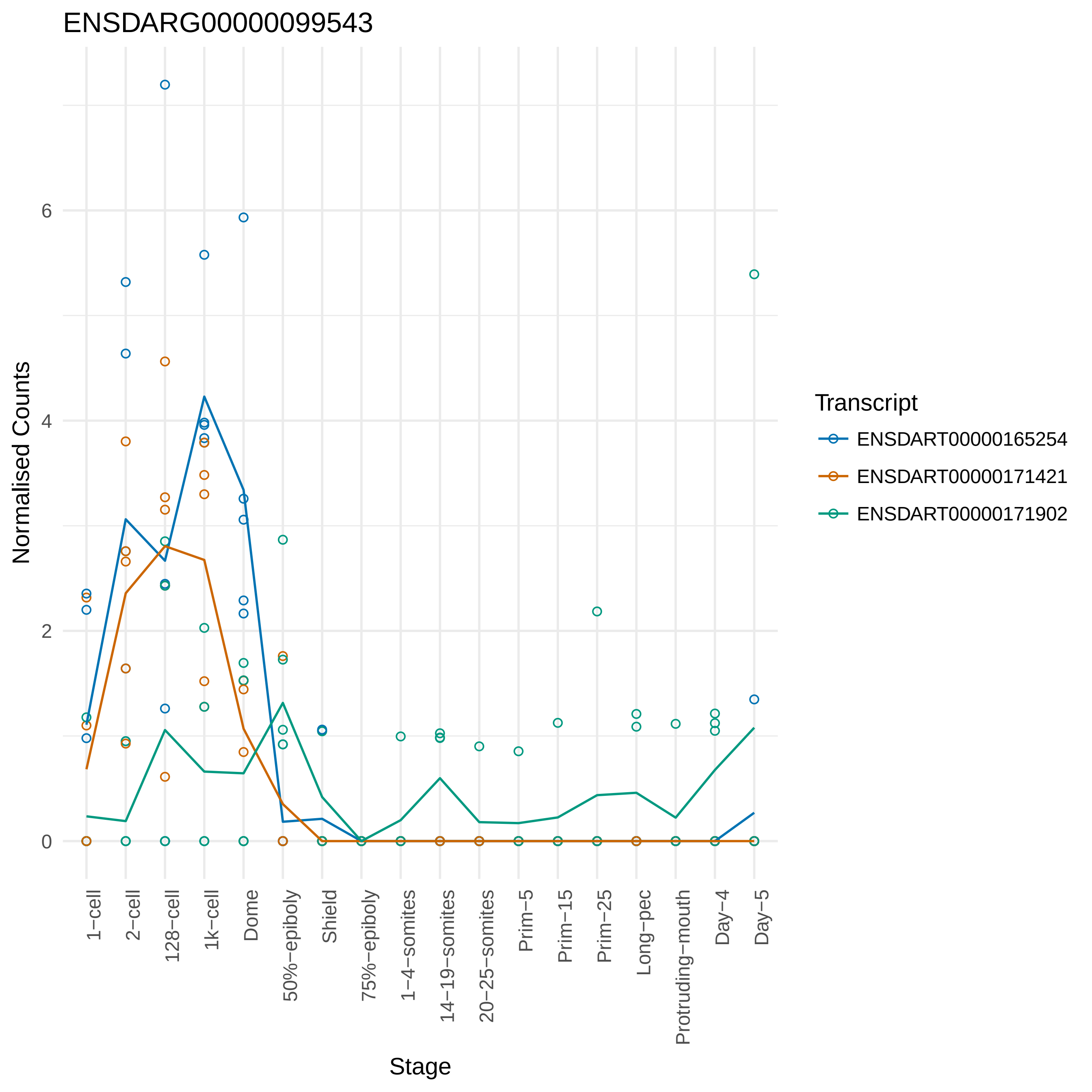

ENSDARG00000099543
Normalised Counts
0
2
4
6
1−cell
2−cell
128−cell
1k−cell
Dome
50%−epiboly
Shield
75%−epiboly
1−4−somites
14−19−somites
20−25−somites
Prim−5
Prim−15
Prim−25
Long−pec
Protruding−mouth
Day−4
Day−5
Stage
Transcript
ENSDART00000165254
ENSDART00000171421
ENSDART00000171902

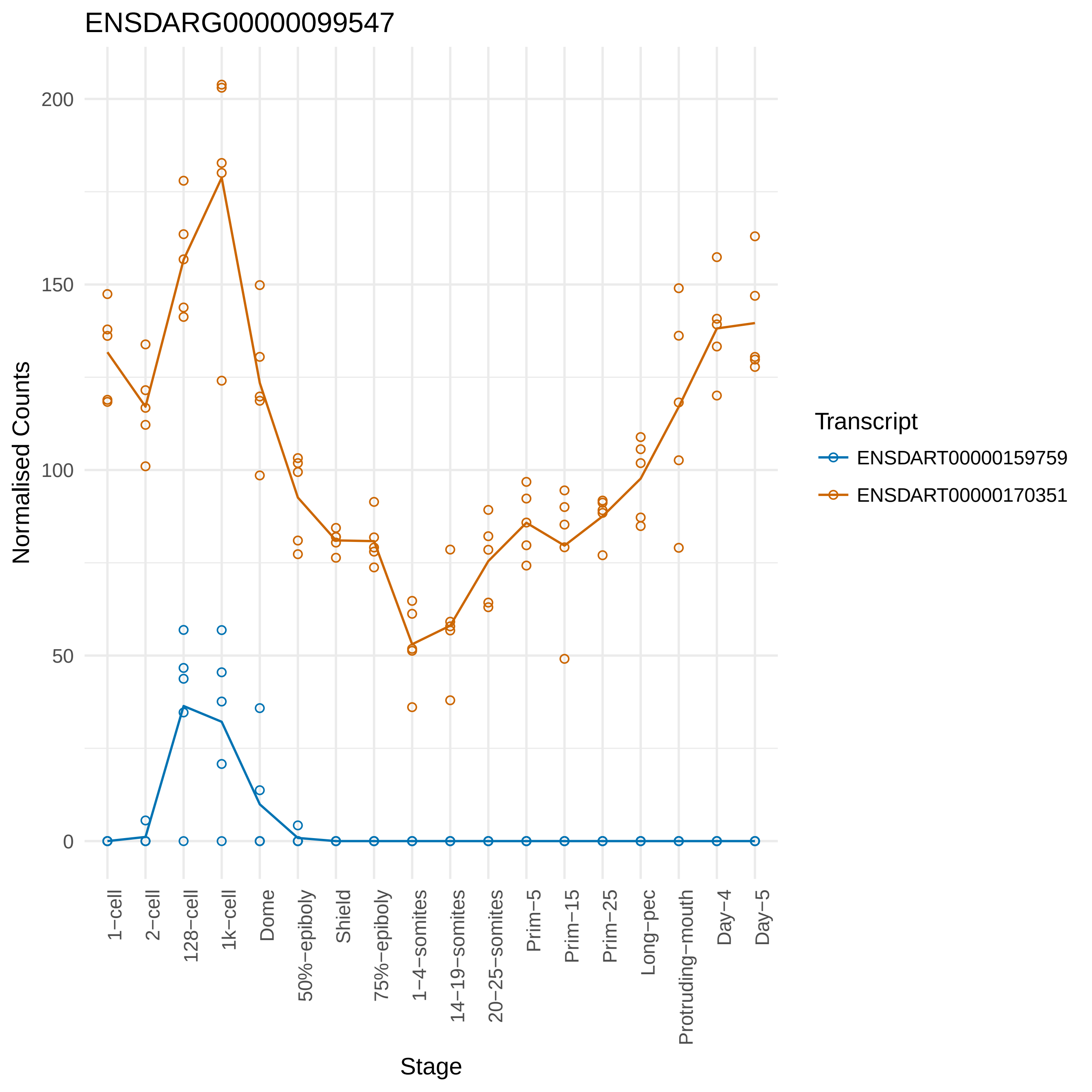

ENSDARG00000099547
Normalised Counts
200
150
100
50
0
1−cell
2−cell
128−cell
1k−cell
Dome
50%−epiboly
Shield
75%−epiboly
1−4−somites
14−19−somites
20−25−somites
Prim−5
Prim−15
Prim−25
Long−pec
Protruding−mouth
Day−4
Day−5
Stage
Transcript
ENSDART00000159759
ENSDART00000170351

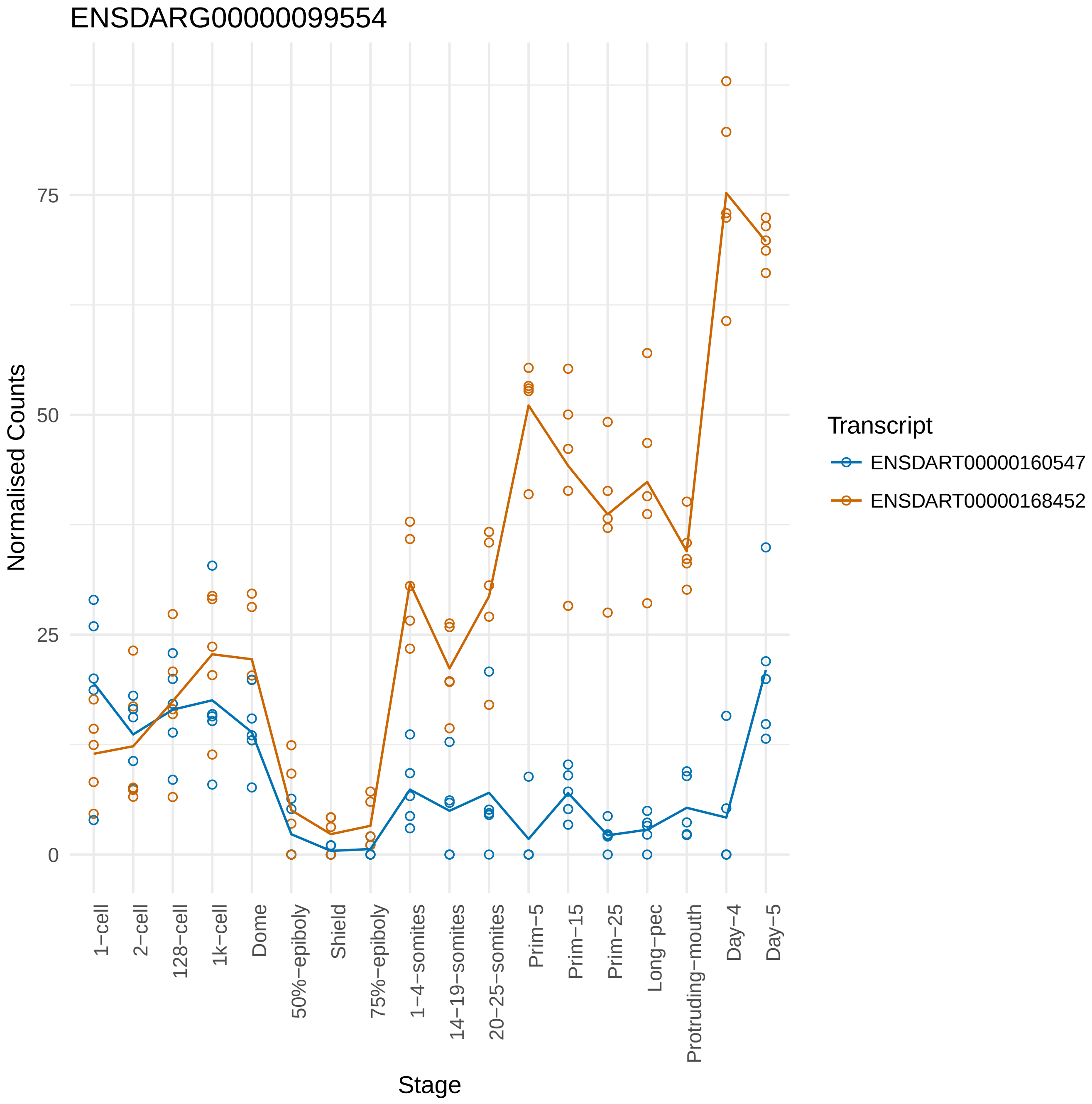

ENSDARG00000099554
Normalised Counts
75
50
25
0
1−cell
2−cell
128−cell
1k−cell
Dome
50%−epiboly
Shield
75%−epiboly
1−4−somites
14−19−somites
20−25−somites
Prim−5
Prim−15
Prim−25
Long−pec
Protruding−mouth
Day−4
Day−5
Stage
Transcript
ENSDART00000160547
ENSDART00000168452

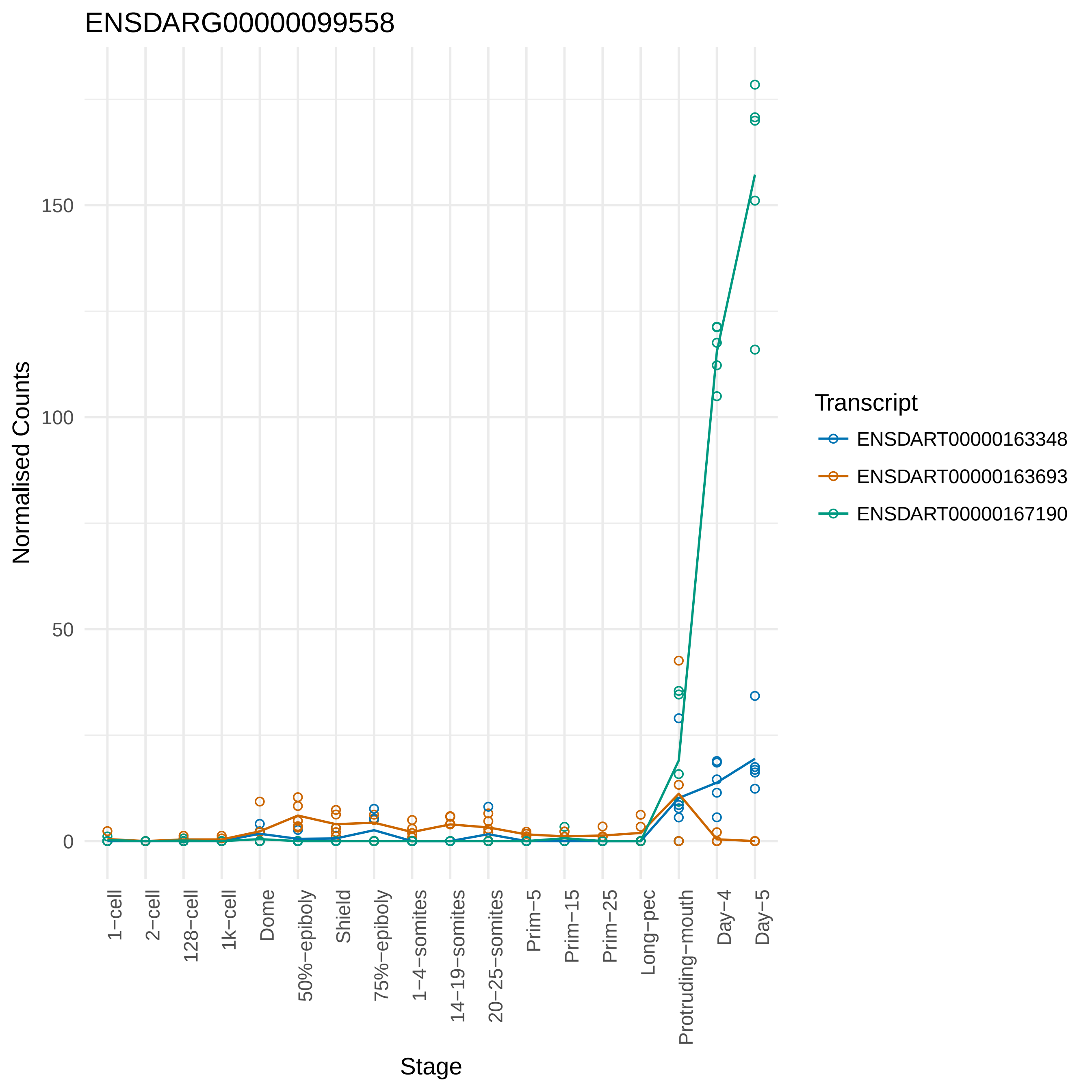

ENSDARG00000099558
Normalised Counts
0
50
100
150
Stage
1−cell
2−cell
128−cell
1k−cell
Dome
50%−epiboly
Shield
75%−epiboly
1−4−somites
14−19−somites
20−25−somites
Prim−5
Prim−15
Prim−25
Long−pec
Protruding−mouth
Day−4
Day−5
Transcript
ENSDART00000163348
ENSDART00000163693
ENSDART00000167190

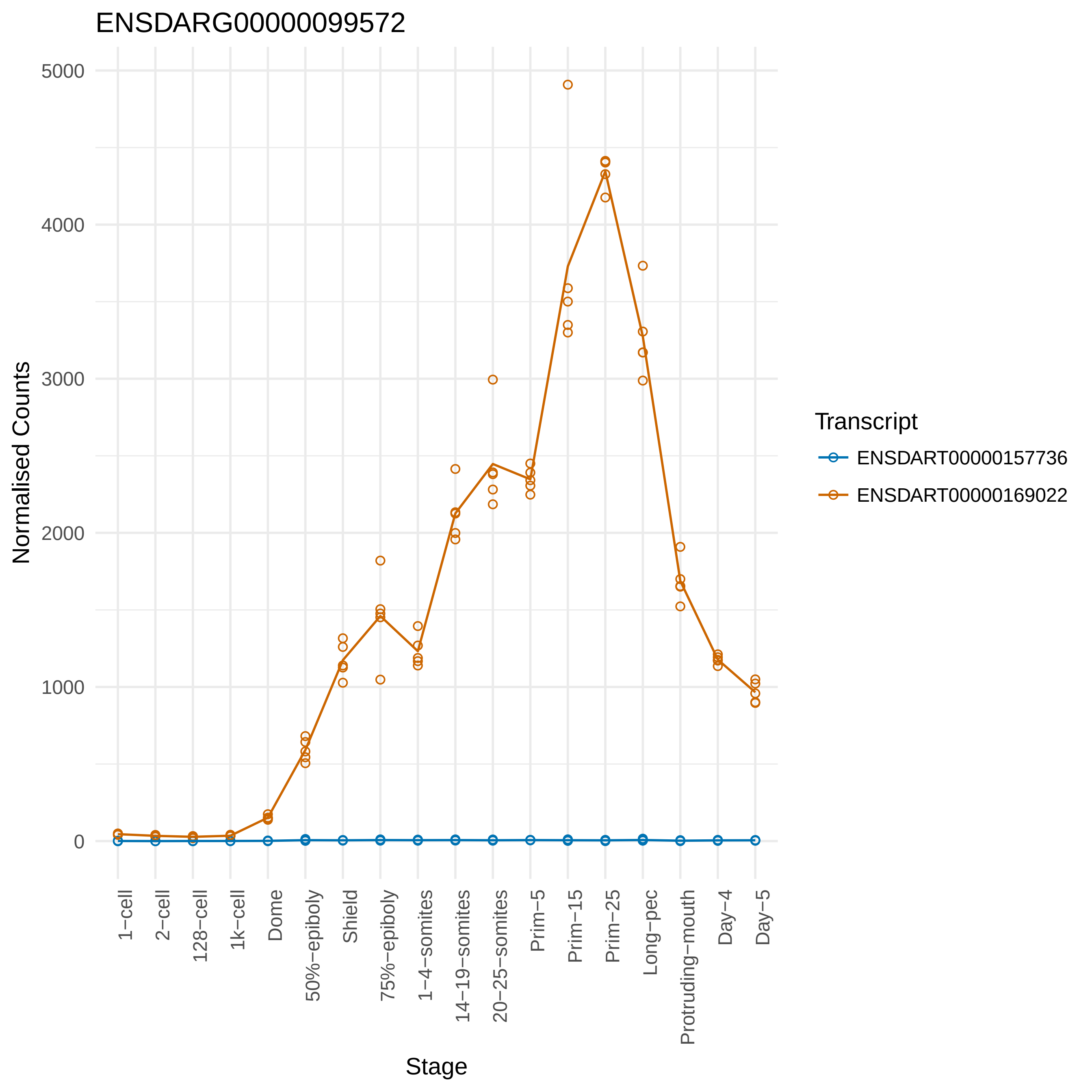
ENSDARG00000099572
Normalised Counts
5000
4000
3000
2000
1000
0
1−cell
2−cell
128−cell
1k−cell
Dome
50%−epiboly
Shield
75%−epiboly
1−4−somites
14−19−somites
20−25−somites
Prim−5
Prim−15
Prim−25
Long−pec
Protruding−mouth
Day−4
Day−5
Stage
Transcript
ENSDART00000157736
ENSDART00000169022

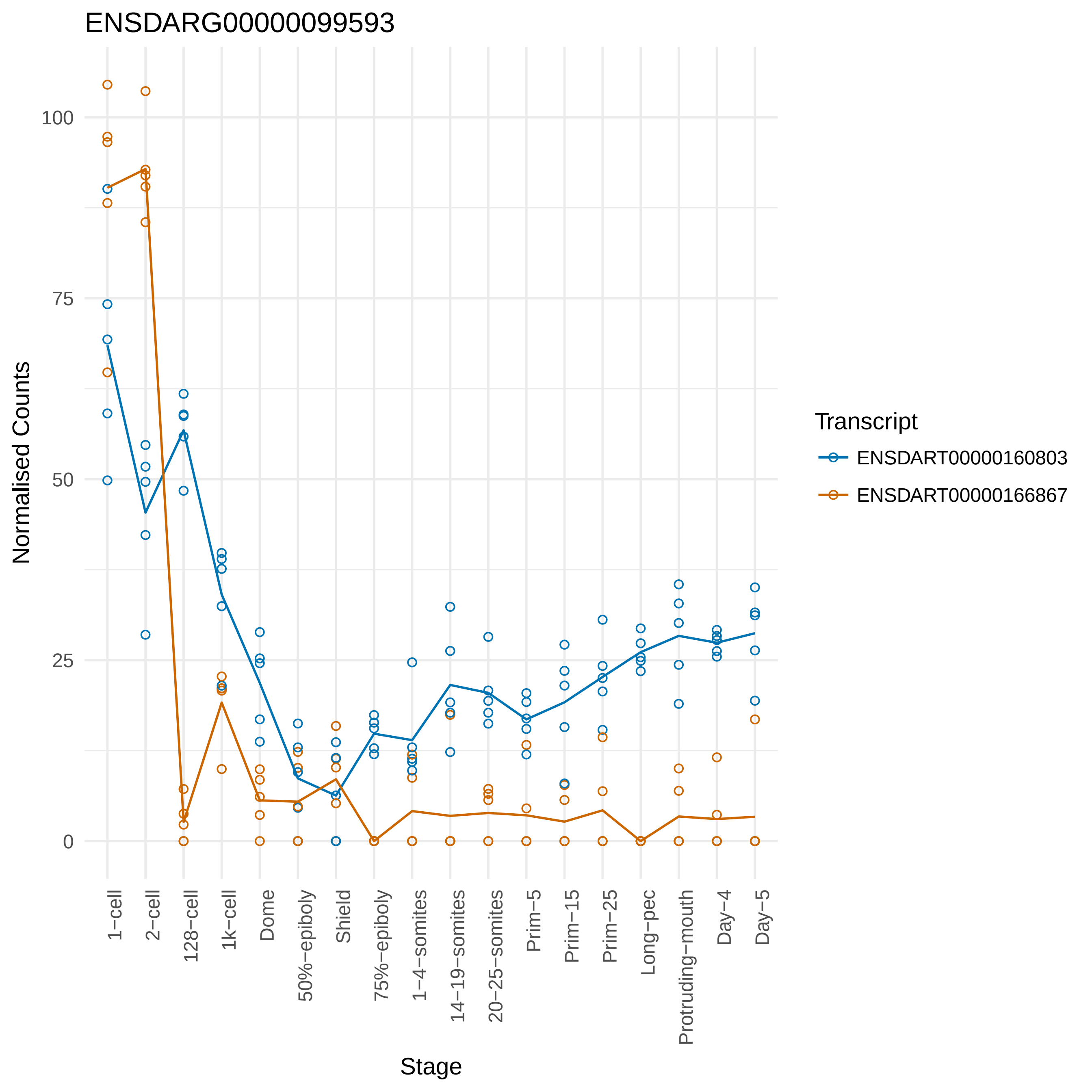

ENSDARG00000099593
Normalised Counts
100
75
50
25
0
1−cell
2−cell
128−cell
1k−cell
Dome
50%−epiboly
Shield
75%−epiboly
1−4−somites
14−19−somites
20−25−somites
Prim−5
Prim−15
Prim−25
Long−pec
Protruding−mouth
Day−4
Day−5
Stage
Transcript
ENSDART00000160803
ENSDART00000166867

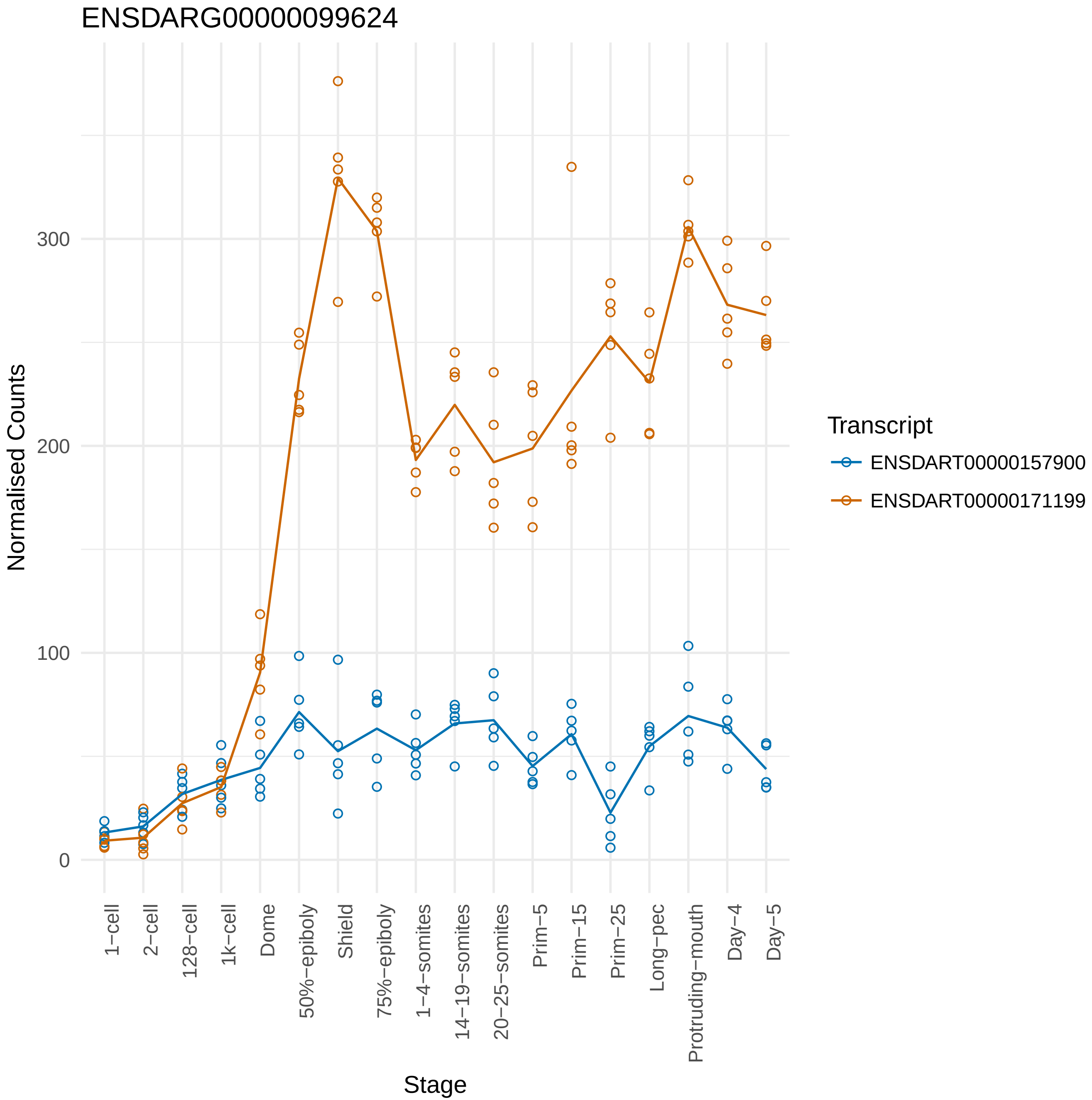

ENSDARG00000099624
Normalised Counts
0
100
200
300
1−cell
2−cell
128−cell
1k−cell
Dome
50%−epiboly
Shield
75%−epiboly
1−4−somites
14−19−somites
20−25−somites
Prim−5
Prim−15
Prim−25
Long−pec
Protruding−mouth
Day−4
Day−5
Stage
Transcript
ENSDART00000157900
ENSDART00000171199

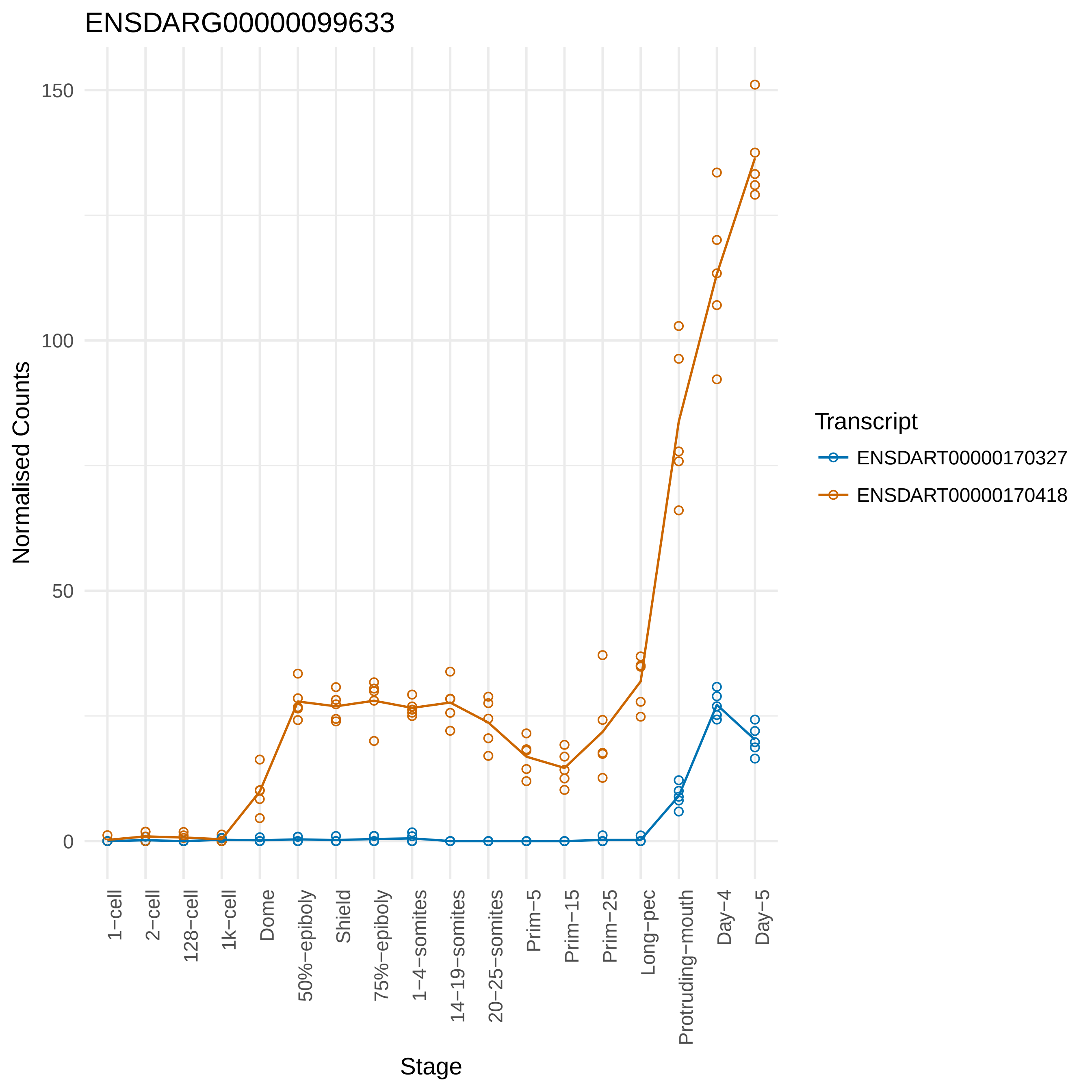

ENSDARG00000099633
Normalised Counts
150
100
50
0
1−cell
2−cell
128−cell
1k−cell
Dome
50%−epiboly
Shield
75%−epiboly
1−4−somites
14−19−somites
20−25−somites
Prim−5
Prim−15
Prim−25
Long−pec
Protruding−mouth
Day−4
Day−5
Stage
Transcript
ENSDART00000170327
ENSDART00000170418

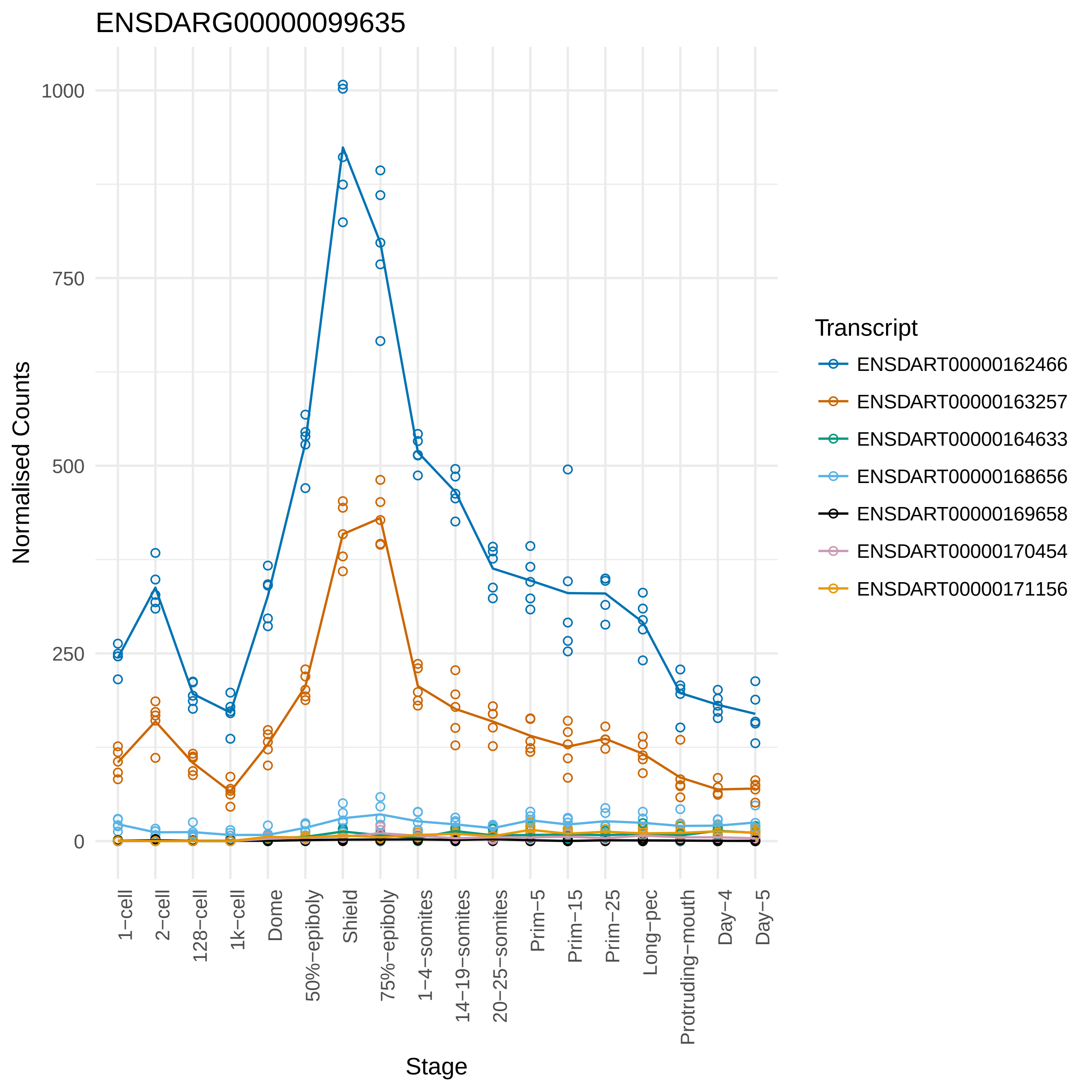

ENSDARG00000099635
Normalised Counts
1000
750
500
250
0
1−cell
2−cell
128−cell
1k−cell
Dome
50%−epiboly
Shield
75%−epiboly
1−4−somites
14−19−somites
20−25−somites
Prim−5
Prim−15
Prim−25
Long−pec
Protruding−mouth
Day−4
Day−5
Stage
Transcript
ENSDART00000162466
ENSDART00000163257
ENSDART00000164633
ENSDART00000168656
ENSDART00000169658
ENSDART00000170454
ENSDART00000171156

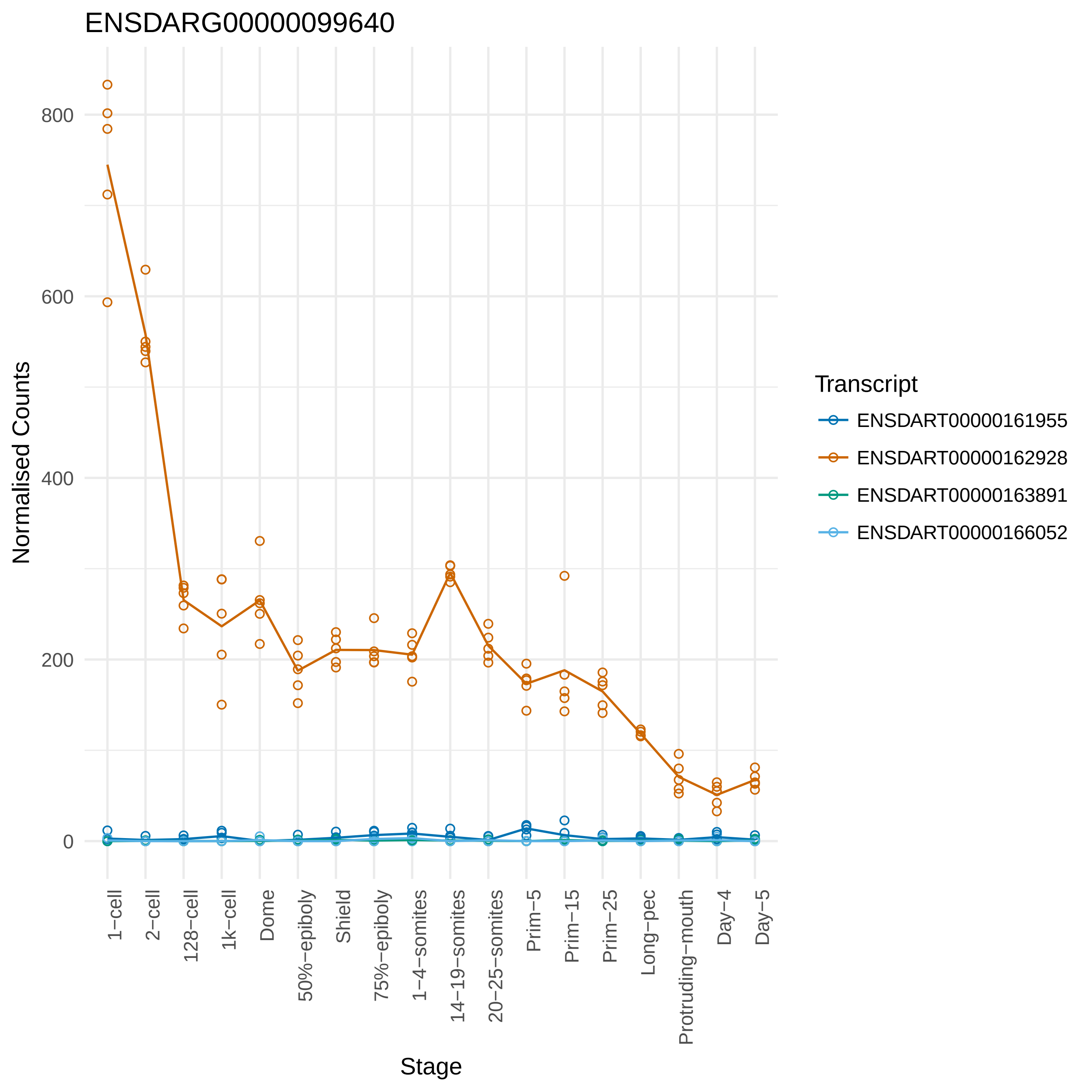

ENSDARG00000099640
Normalised Counts
0
200
400
600
800
1−cell
2−cell
128−cell
1k−cell
Dome
50%−epiboly
Shield
75%−epiboly
1−4−somites
14−19−somites
20−25−somites
Prim−5
Prim−15
Prim−25
Long−pec
Protruding−mouth
Day−4
Day−5
Stage
Transcript
ENSDART00000161955
ENSDART00000162928
ENSDART00000163891
ENSDART00000166052

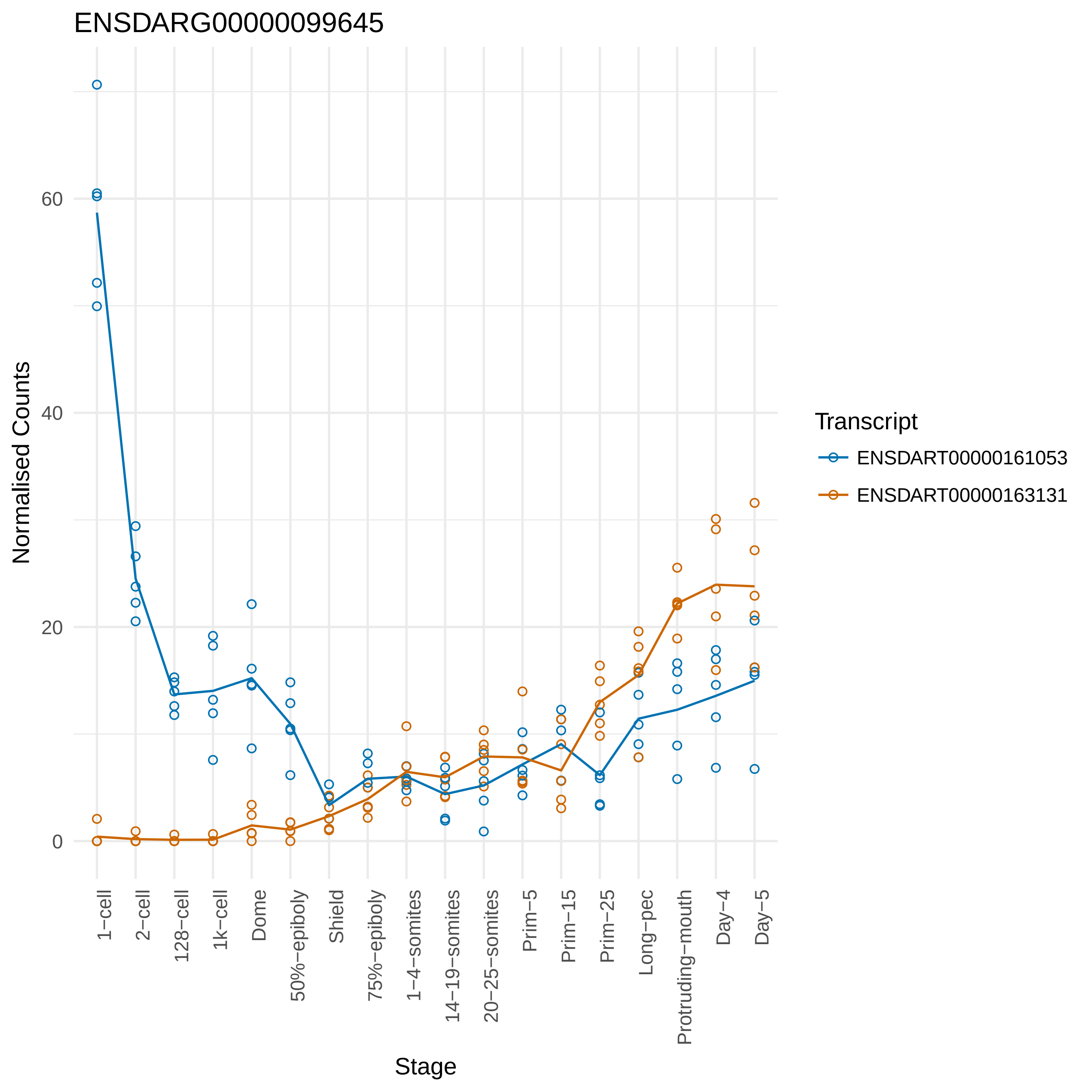

ENSDARG00000099645
Normalised Counts
0
20
40
60
Stage
1−cell
2−cell
128−cell
1k−cell
Dome
50%−epiboly
Shield
75%−epiboly
1−4−somites
14−19−somites
20−25−somites
Prim−5
Prim−15
Prim−25
Long−pec
Protruding−mouth
Day−4
Day−5
Transcript
ENSDART00000161053
ENSDART00000163131

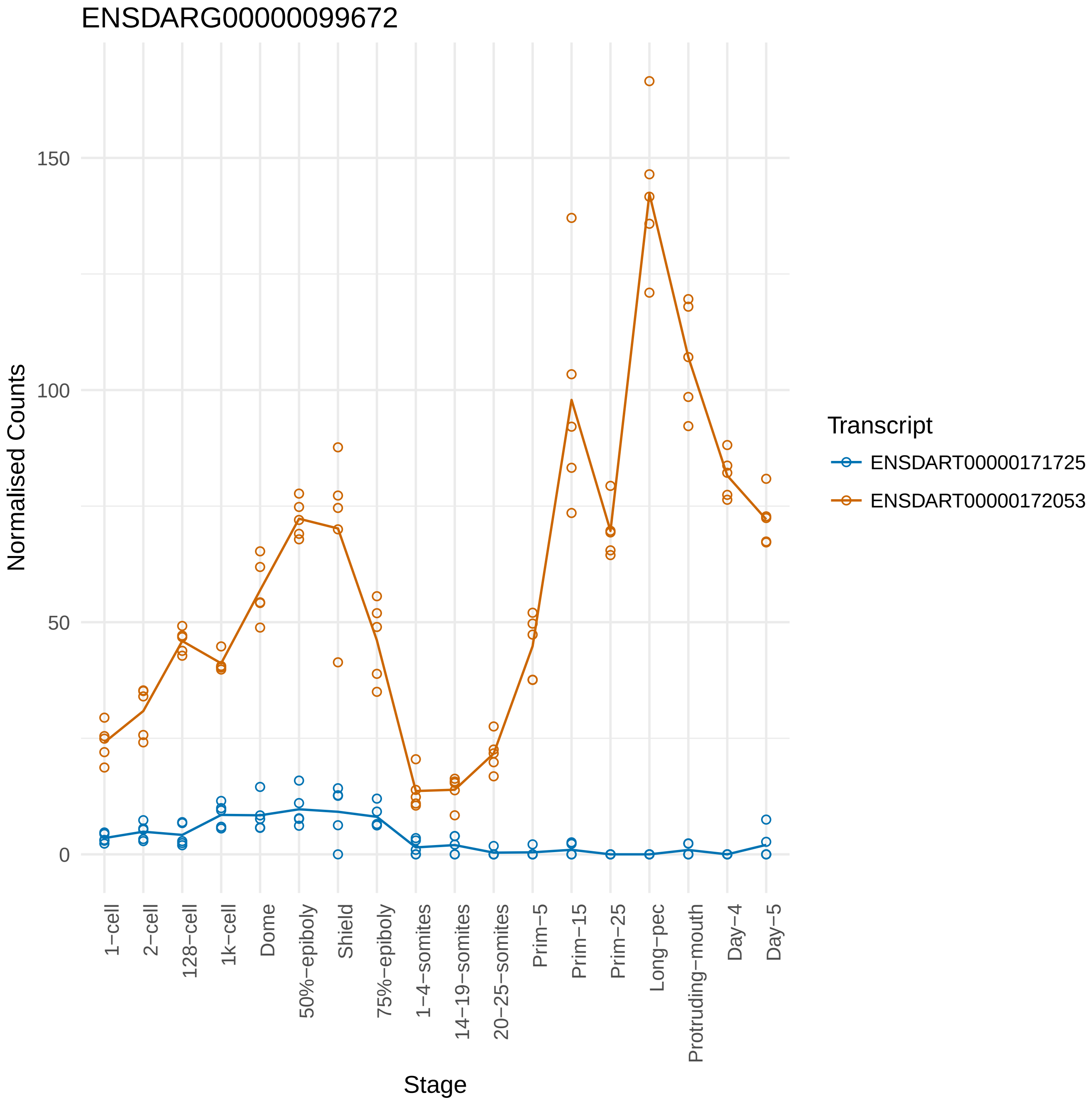

ENSDARG00000099672
Normalised Counts
150
100
50
0
1−cell
2−cell
128−cell
1k−cell
Dome
50%−epiboly
Shield
75%−epiboly
1−4−somites
14−19−somites
20−25−somites
Prim−5
Prim−15
Prim−25
Long−pec
Protruding−mouth
Day−4
Day−5
Stage
Transcript
ENSDART00000171725
ENSDART00000172053

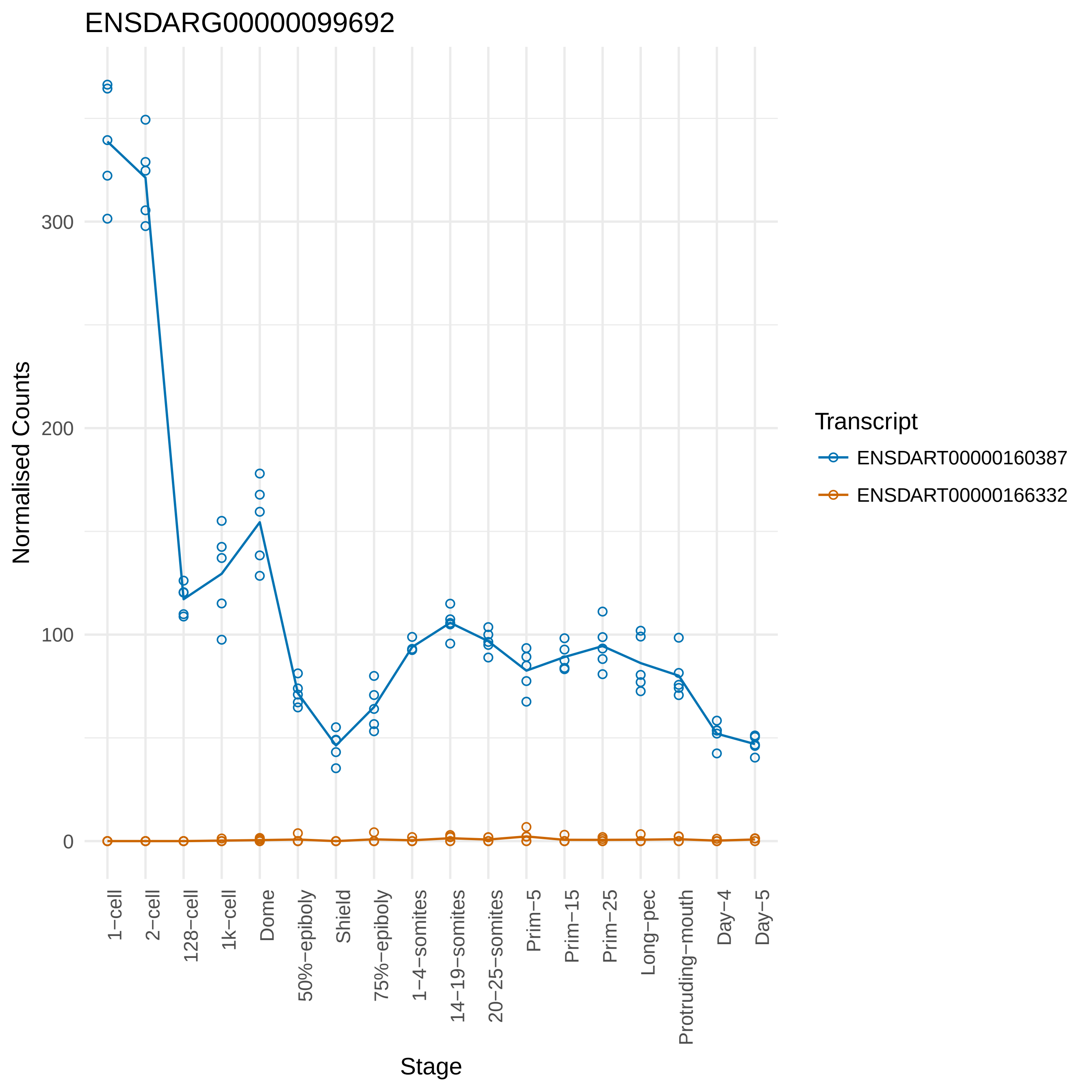
ENSDARG00000099692
Normalised Counts
300
200
100
0
1−cell
2−cell
128−cell
1k−cell
Dome
50%−epiboly
Shield
75%−epiboly
1−4−somites
14−19−somites
20−25−somites
Prim−5
Prim−15
Prim−25
Long−pec
Protruding−mouth
Day−4
Day−5
Stage
Transcript
ENSDART00000160387
ENSDART00000166332

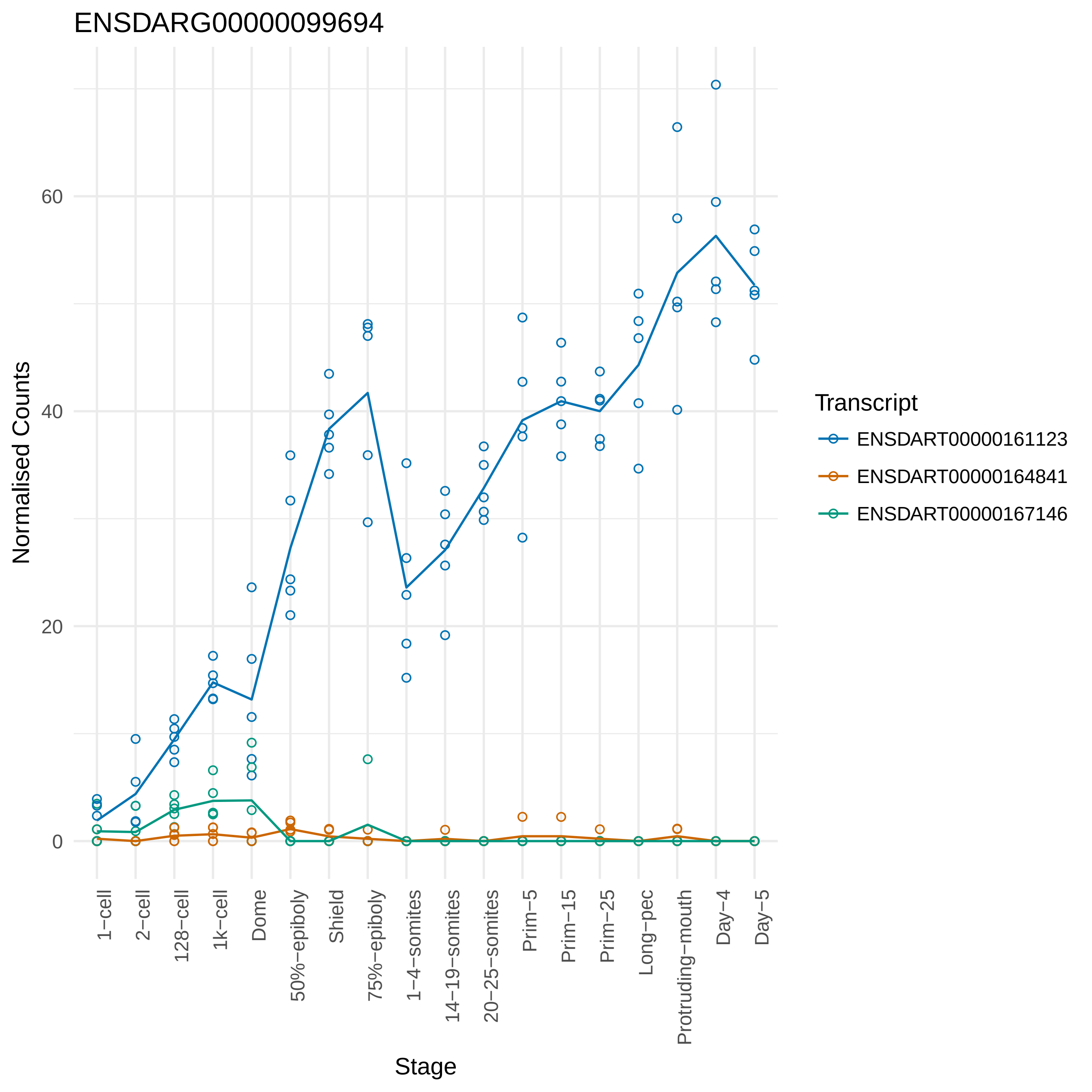
ENSDARG00000099694
Normalised Counts
0
20
40
60
1−cell
2−cell
128−cell
1k−cell
Dome
50%−epiboly
Shield
75%−epiboly
1−4−somites
14−19−somites
20−25−somites
Prim−5
Prim−15
Prim−25
Long−pec
Protruding−mouth
Day−4
Day−5
Stage
Transcript
ENSDART00000161123
ENSDART00000164841
ENSDART00000167146

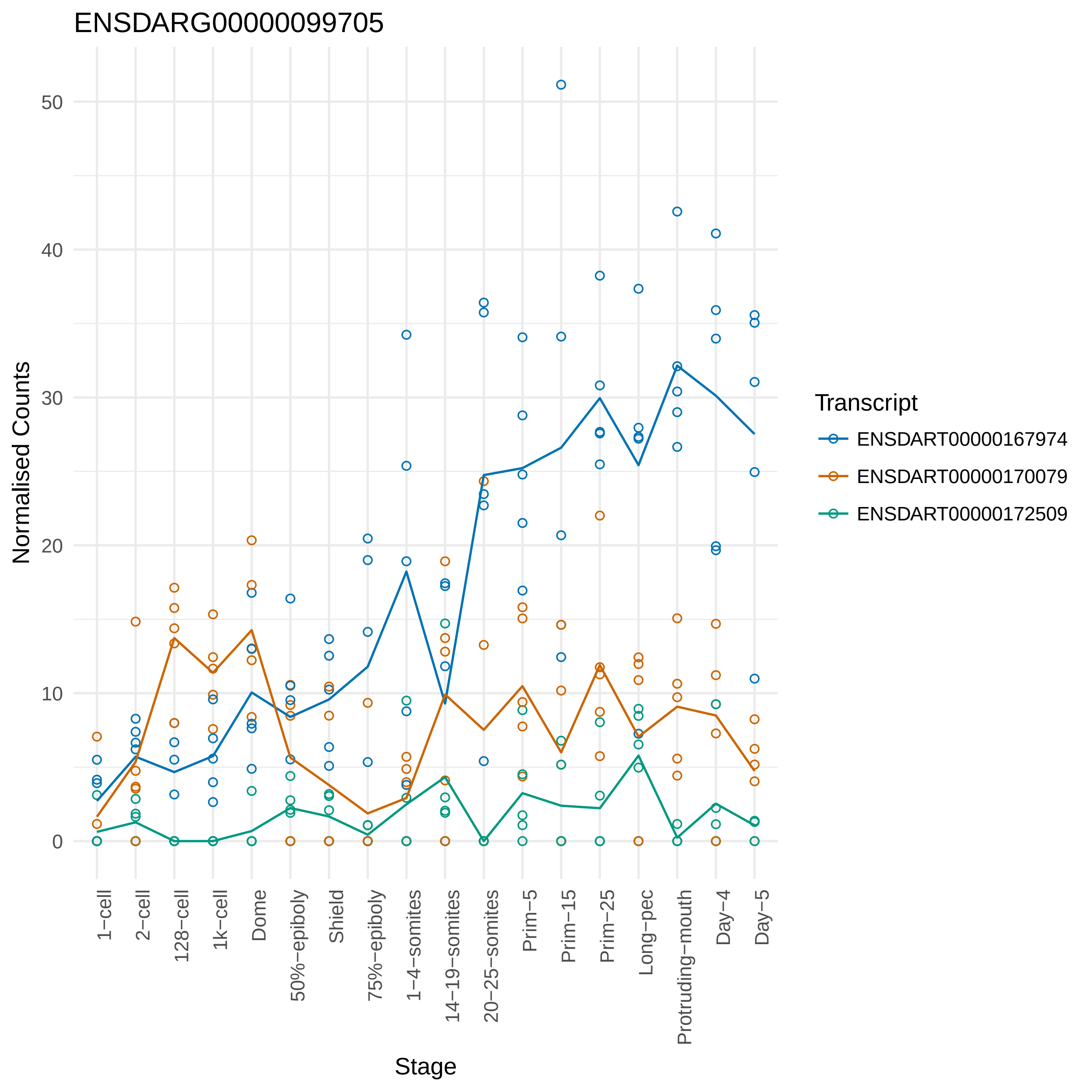
ENSDARG00000099705
Normalised Counts
0
10
20
30
40
50
1−cell
2−cell
128−cell
1k−cell
Dome
50%−epiboly
Shield
75%−epiboly
1−4−somites
14−19−somites
20−25−somites
Prim−5
Prim−15
Prim−25
Long−pec
Protruding−mouth
Day−4
Day−5
Stage
Transcript
ENSDART00000167974
ENSDART00000170079
ENSDART00000172509

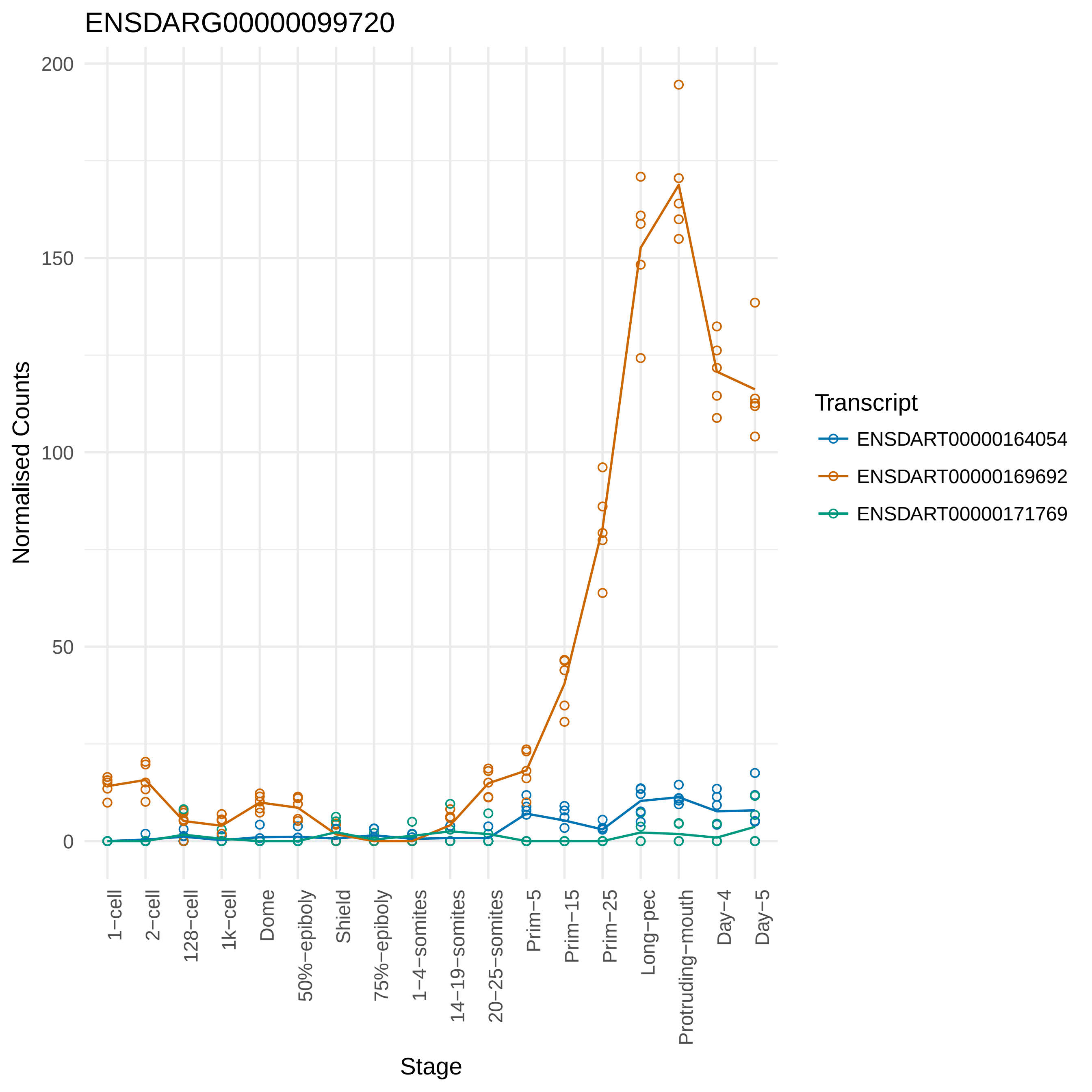

ENSDARG00000099720
Normalised Counts
200
150
100
50
0
1−cell
2−cell
128−cell
1k−cell
Dome
50%−epiboly
Shield
75%−epiboly
1−4−somites
14−19−somites
20−25−somites
Prim−5
Prim−15
Prim−25
Long−pec
Protruding−mouth
Day−4
Day−5
Stage
Transcript
ENSDART00000164054
ENSDART00000169692
ENSDART00000171769

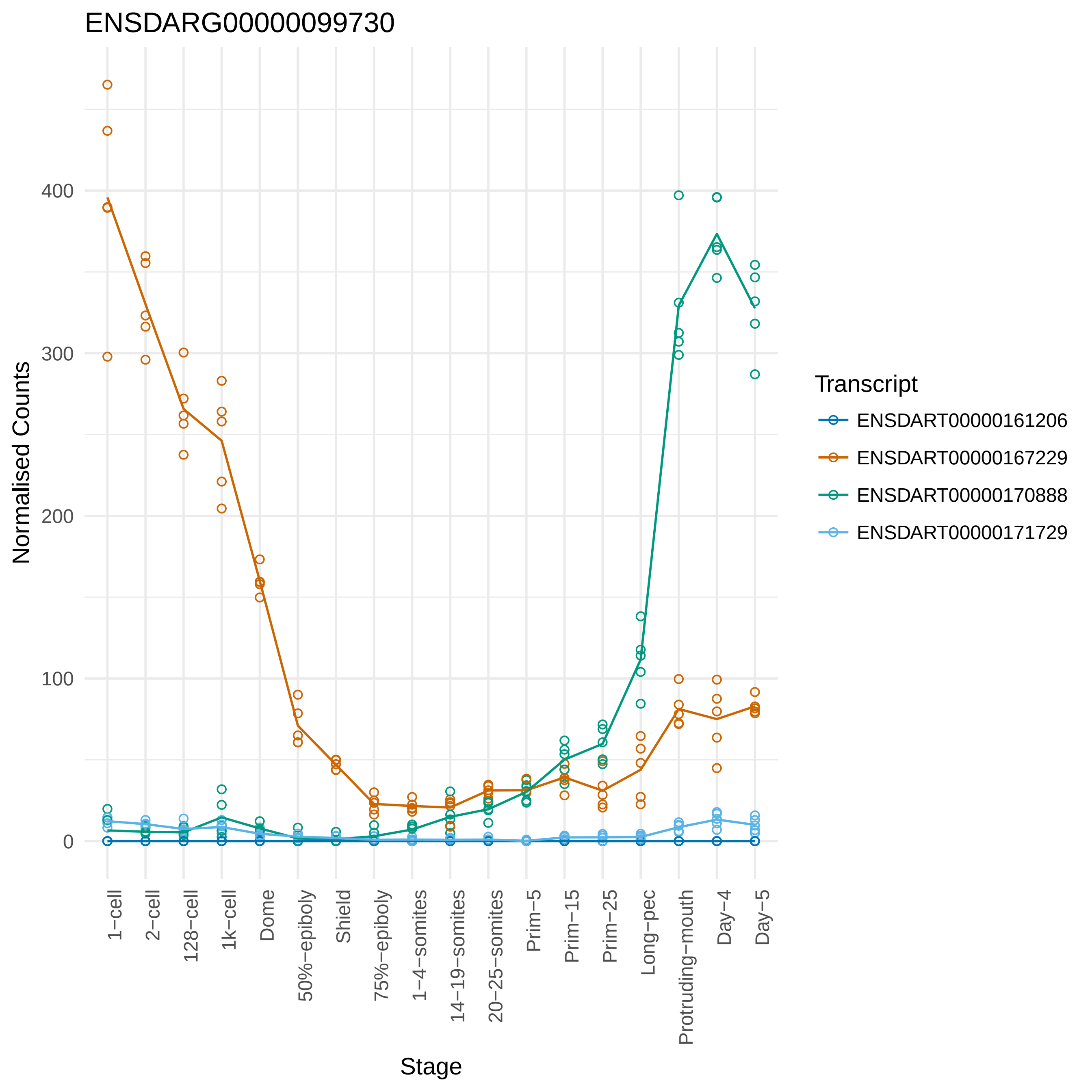

ENSDARG00000099730
Normalised Counts
0
100
200
300
400
Stage
1−cell
2−cell
128−cell
1k−cell
Dome
50%−epiboly
Shield
75%−epiboly
1−4−somites
14−19−somites
20−25−somites
Prim−5
Prim−15
Prim−25
Long−pec
Protruding−mouth
Day−4
Day−5
Transcript
ENSDART00000161206
ENSDART00000167229
ENSDART00000170888
ENSDART00000171729

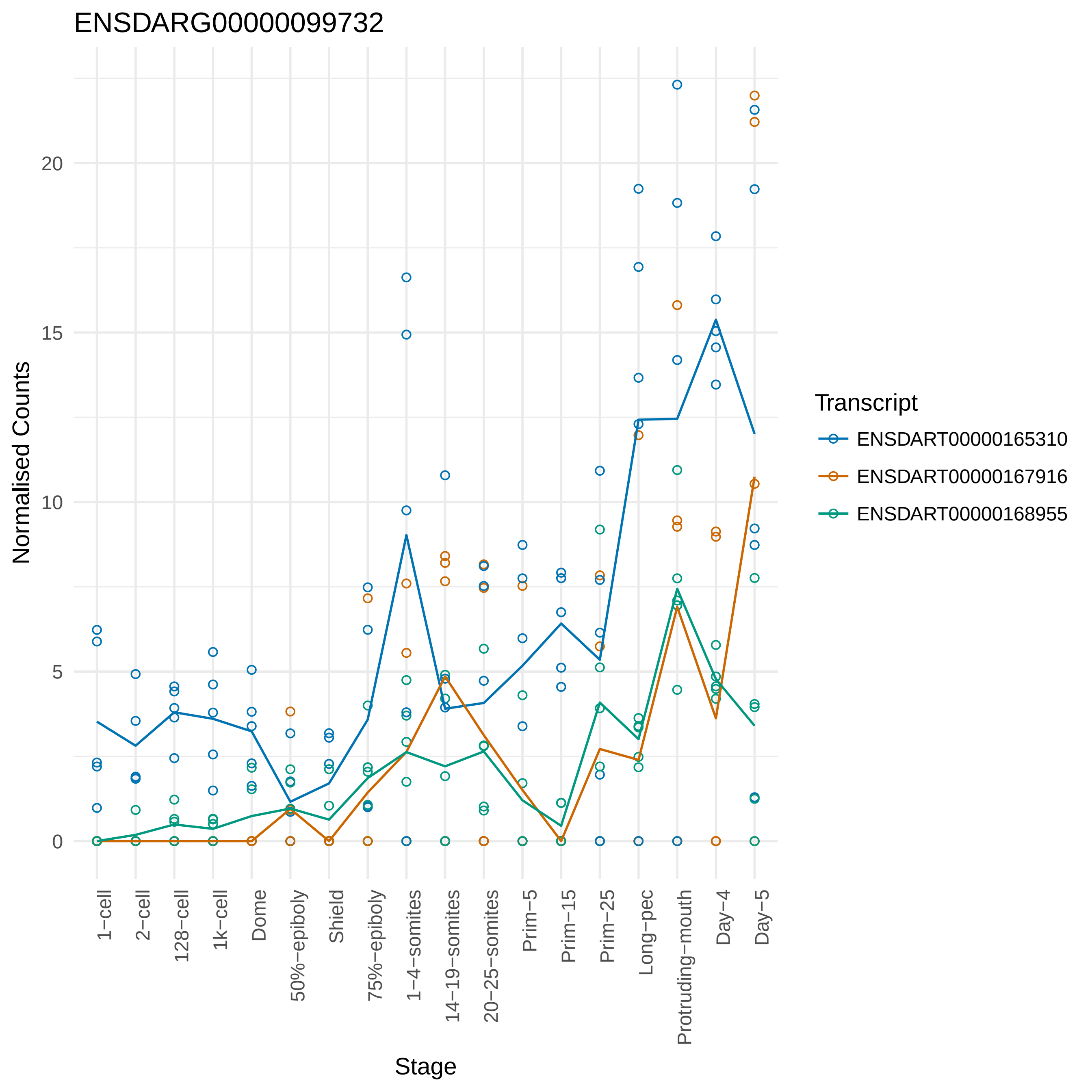

ENSDARG00000099732
Normalised Counts
0
5
10
15
20
Stage
1−cell
2−cell
128−cell
1k−cell
Dome
50%−epiboly
Shield
75%−epiboly
1−4−somites
14−19−somites
20−25−somites
Prim−5
Prim−15
Prim−25
Long−pec
Protruding−mouth
Day−4
Day−5
Transcript
ENSDART00000165310
ENSDART00000167916
ENSDART00000168955

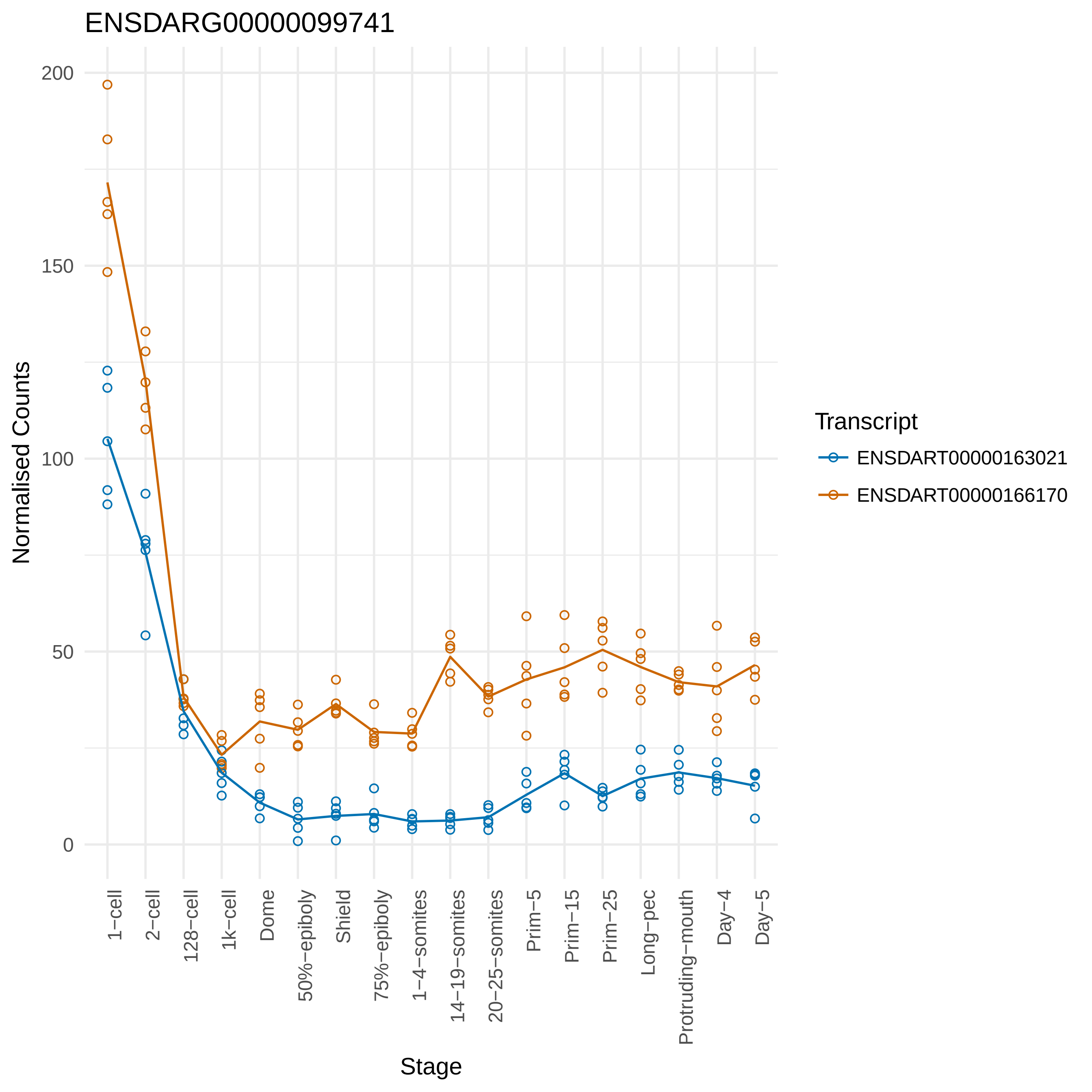
ENSDARG00000099741
Normalised Counts
200
150
100
50
0
1−cell
2−cell
128−cell
1k−cell
Dome
50%−epiboly
Shield
75%−epiboly
1−4−somites
14−19−somites
20−25−somites
Prim−5
Prim−15
Prim−25
Long−pec
Protruding−mouth
Day−4
Day−5
Stage
Transcript
ENSDART00000163021
ENSDART00000166170

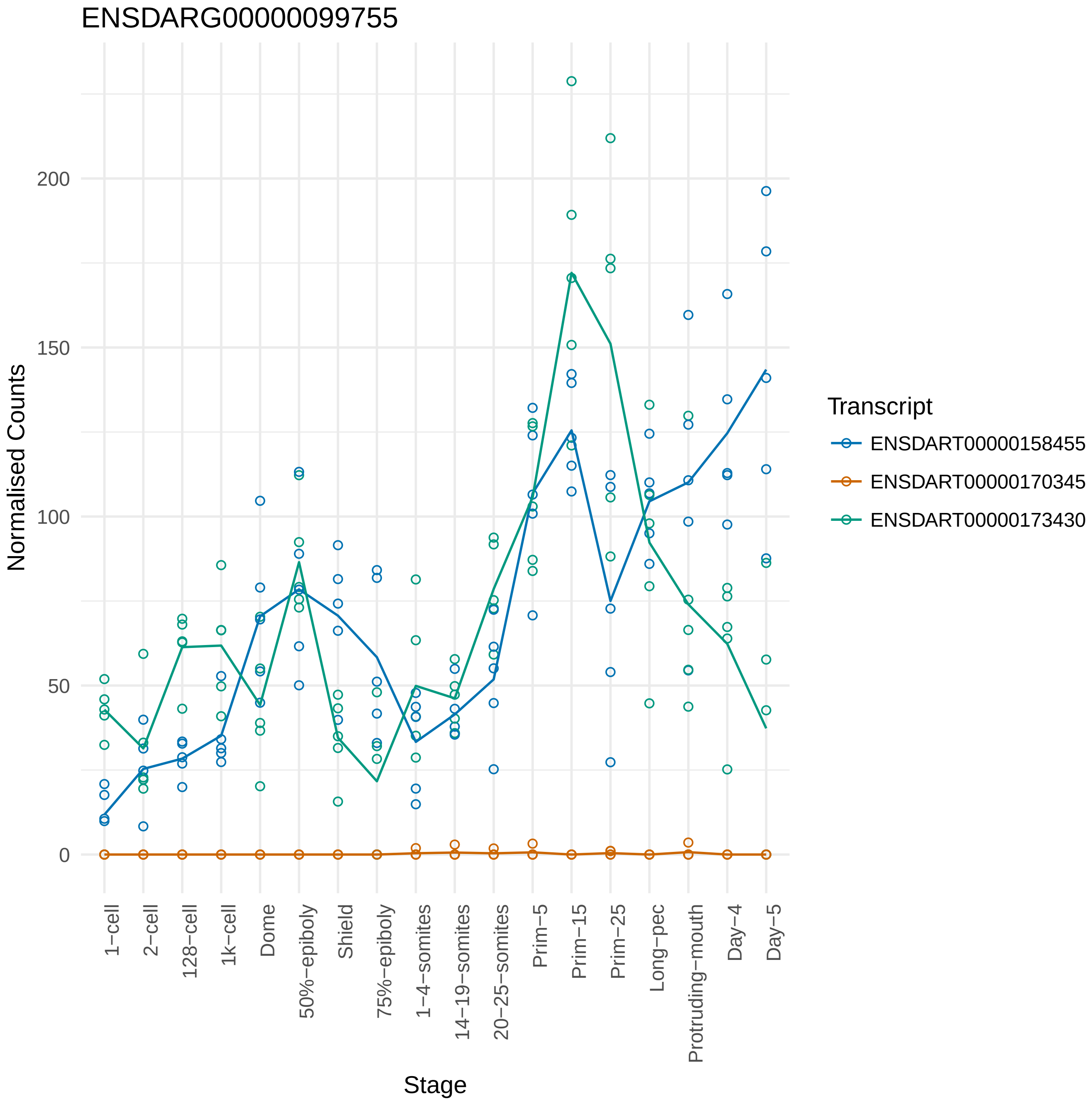

ENSDARG00000099755
Normalised Counts
0
50
100
150
200
Stage
1−cell
2−cell
128−cell
1k−cell
Dome
50%−epiboly
Shield
75%−epiboly
1−4−somites
14−19−somites
20−25−somites
Prim−5
Prim−15
Prim−25
Long−pec
Protruding−mouth
Day−4
Day−5
Transcript
ENSDART00000158455
ENSDART00000170345
ENSDART00000173430

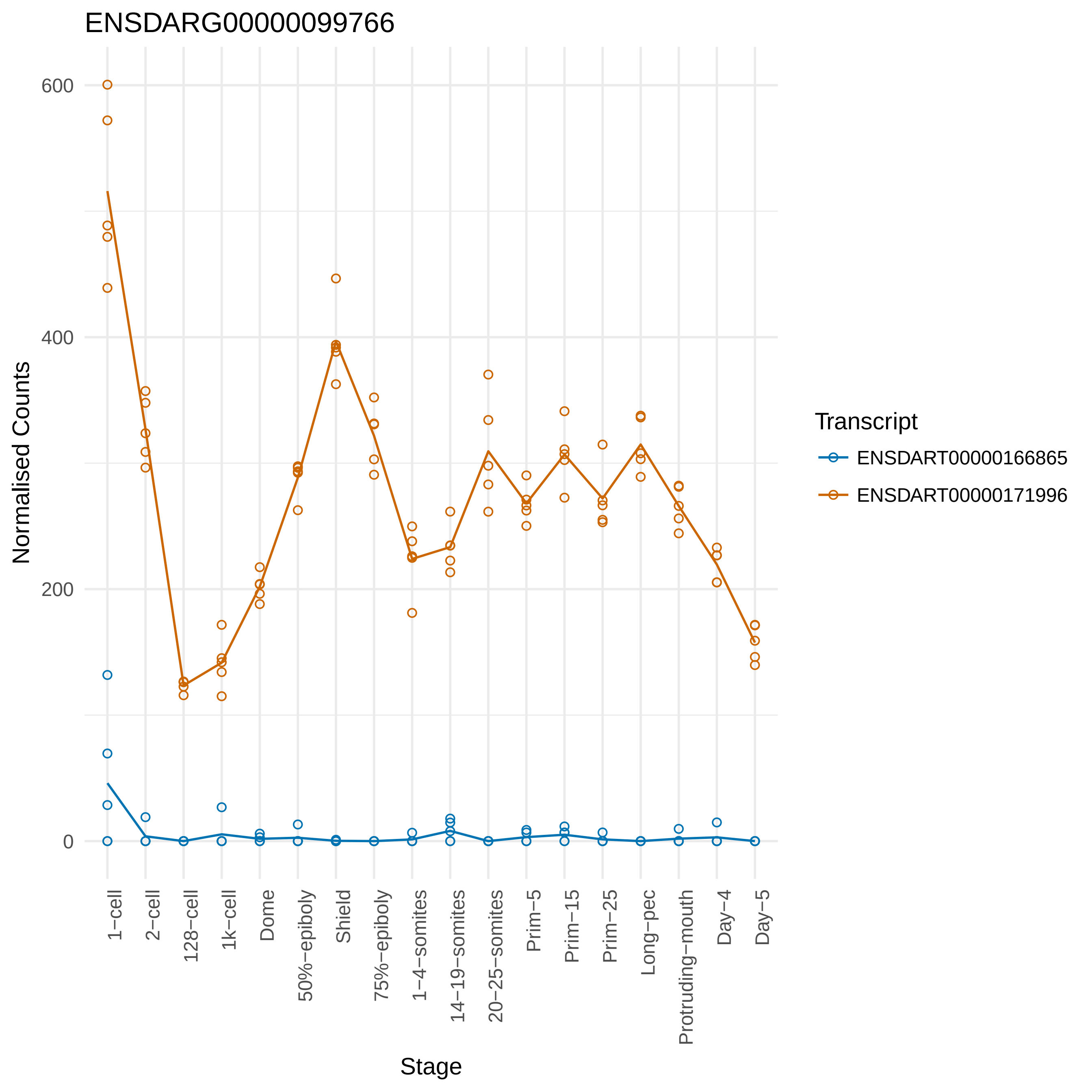
ENSDARG00000099766
Normalised Counts
600
400
200
0
1−cell
2−cell
128−cell
1k−cell
Dome
50%−epiboly
Shield
75%−epiboly
1−4−somites
14−19−somites
20−25−somites
Prim−5
Prim−15
Prim−25
Long−pec
Protruding−mouth
Day−4
Day−5
Stage
Transcript
ENSDART00000166865
ENSDART00000171996

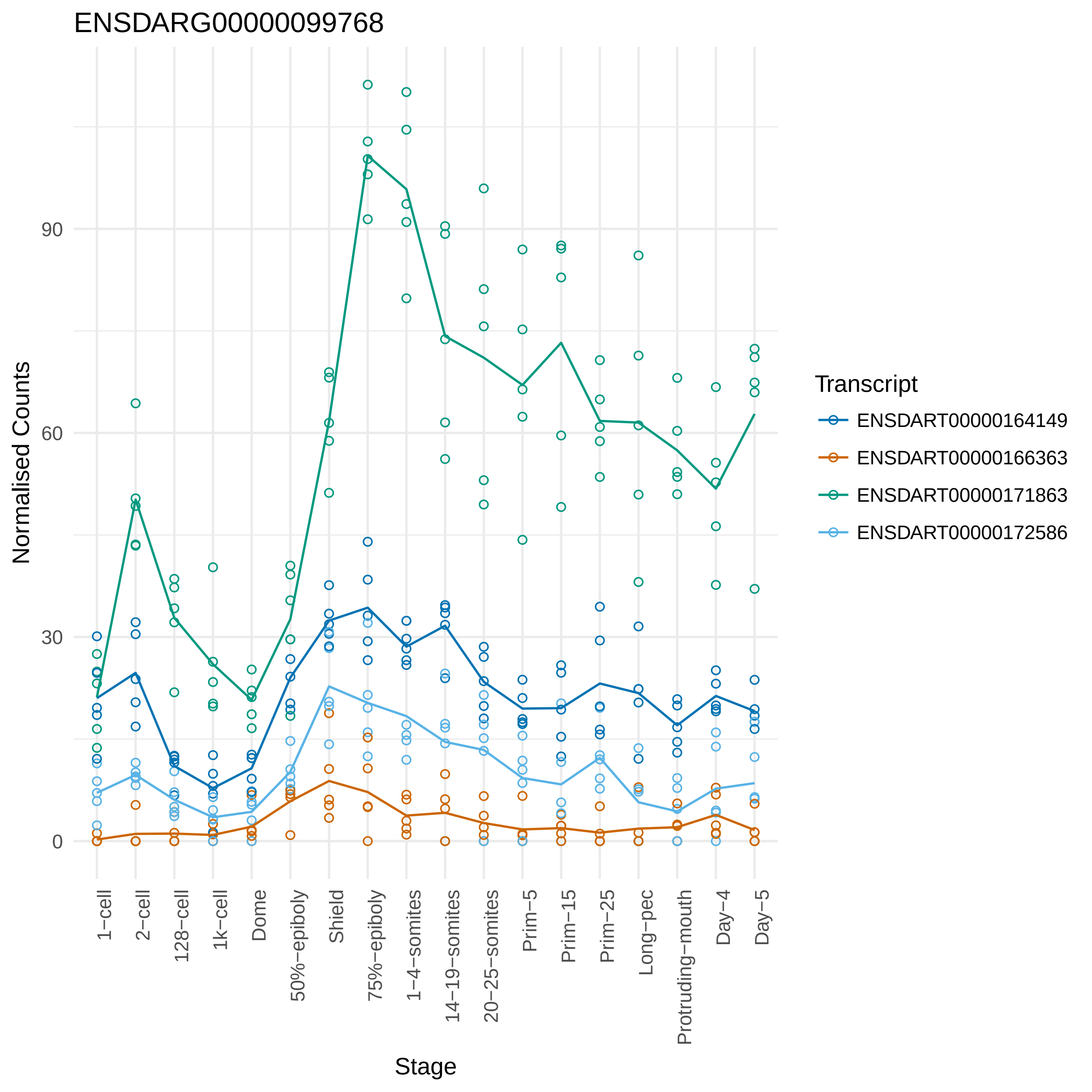
ENSDARG00000099768
Normalised Counts
0
30
60
90
Stage
1−cell
2−cell
128−cell
1k−cell
Dome
50%−epiboly
Shield
75%−epiboly
1−4−somites
14−19−somites
20−25−somites
Prim−5
Prim−15
Prim−25
Long−pec
Protruding−mouth
Day−4
Day−5
Transcript
ENSDART00000164149
ENSDART00000166363
ENSDART00000171863
ENSDART00000172586

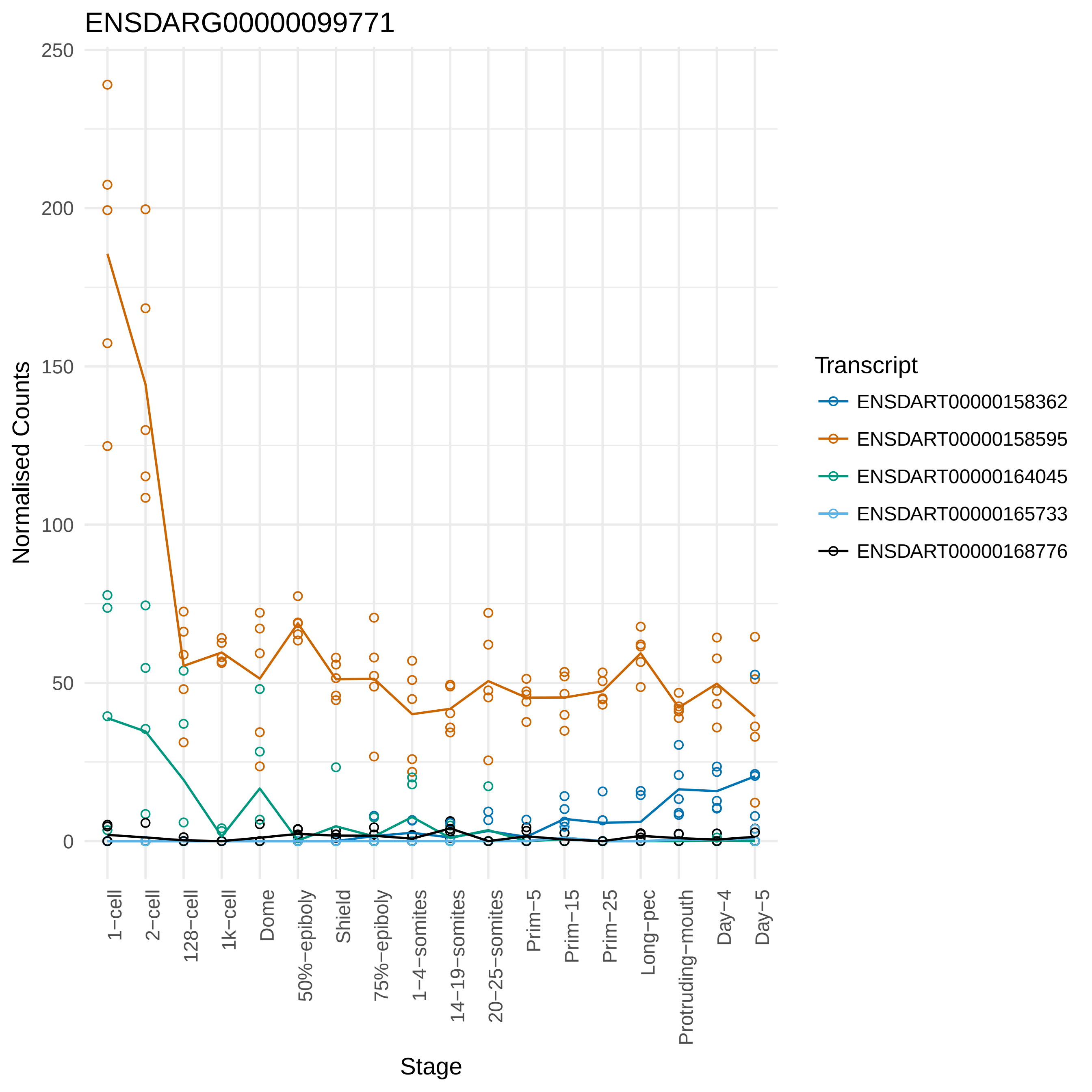
ENSDARG00000099771
Normalised Counts
0
50
100
150
200
250
1−cell
2−cell
128−cell
1k−cell
Dome
50%−epiboly
Shield
75%−epiboly
1−4−somites
14−19−somites
20−25−somites
Prim−5
Prim−15
Prim−25
Long−pec
Protruding−mouth
Day−4
Day−5
Stage
Transcript
ENSDART00000158362
ENSDART00000158595
ENSDART00000164045
ENSDART00000165733
ENSDART00000168776

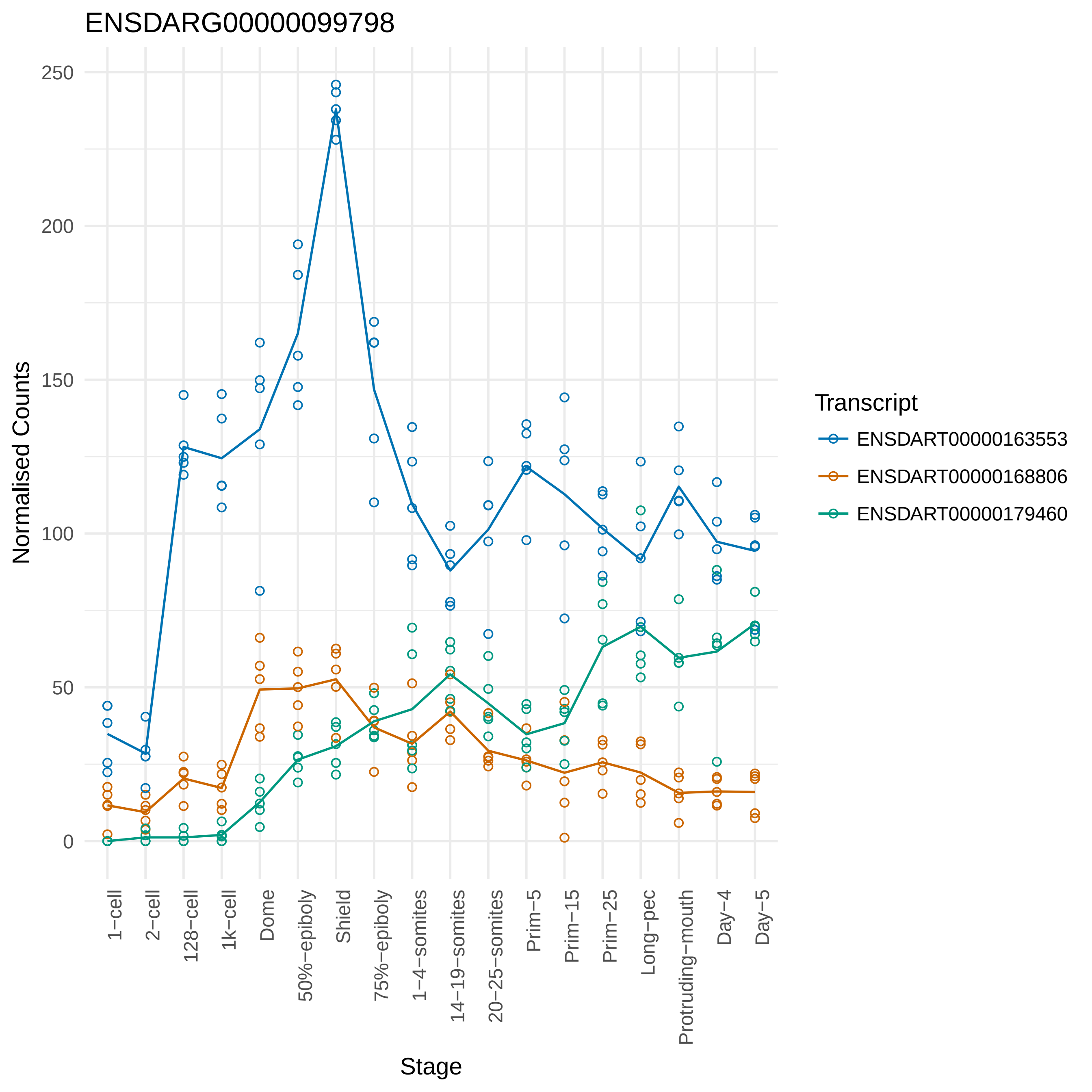

ENSDARG00000099798
Normalised Counts
250
200
150
100
50
0
1−cell
2−cell
128−cell
1k−cell
Dome
50%−epiboly
Shield
75%−epiboly
1−4−somites
14−19−somites
20−25−somites
Prim−5
Prim−15
Prim−25
Long−pec
Protruding−mouth
Day−4
Day−5
Stage
Transcript
ENSDART00000163553
ENSDART00000168806
ENSDART00000179460

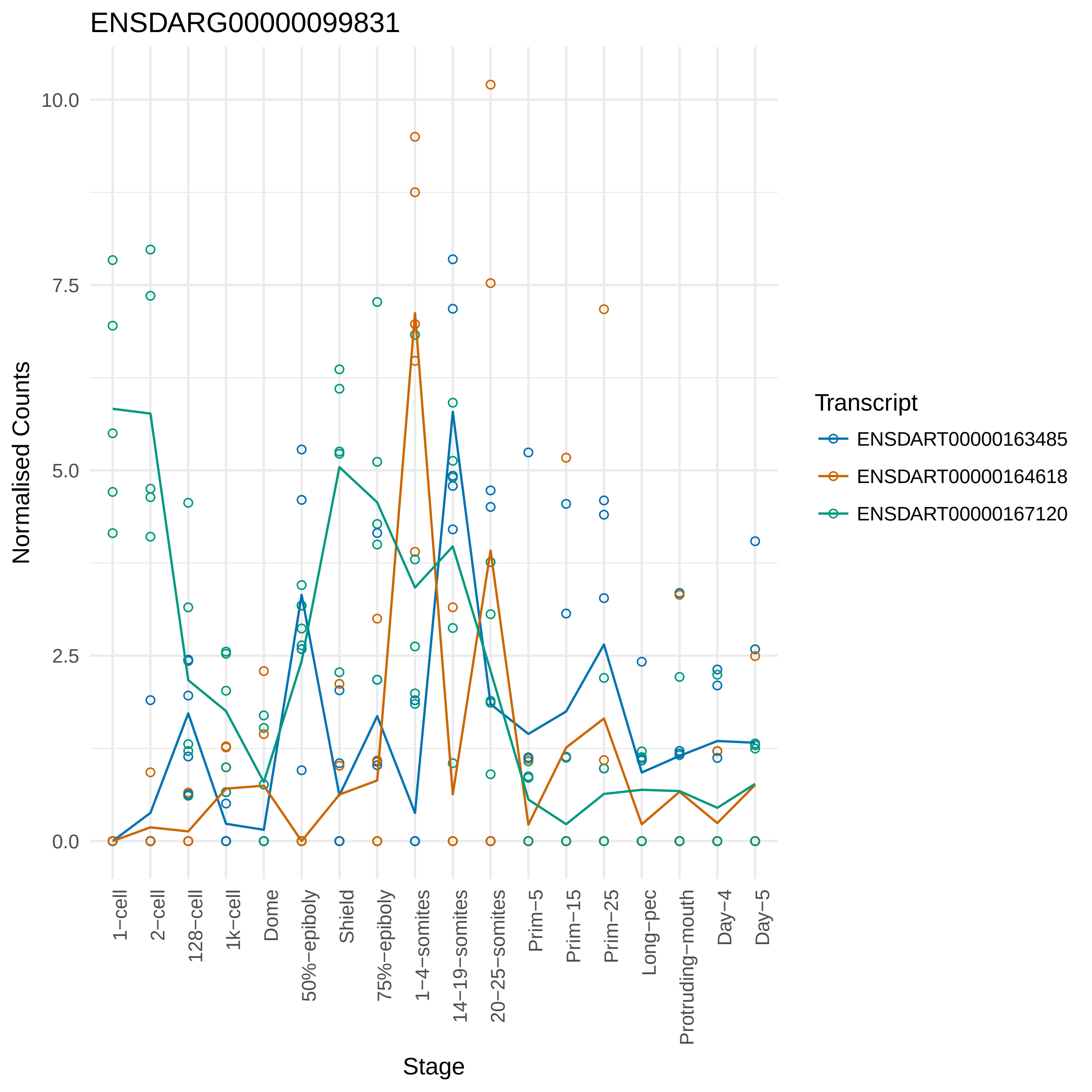
ENSDARG00000099831
Normalised Counts
10.0
7.5
5.0
2.5
0.0
1−cell
2−cell
128−cell
1k−cell
Dome
50%−epiboly
Shield
75%−epiboly
1−4−somites
14−19−somites
20−25−somites
Prim−5
Prim−15
Prim−25
Long−pec
Protruding−mouth
Day−4
Day−5
Stage
Transcript
ENSDART00000163485
ENSDART00000164618
ENSDART00000167120

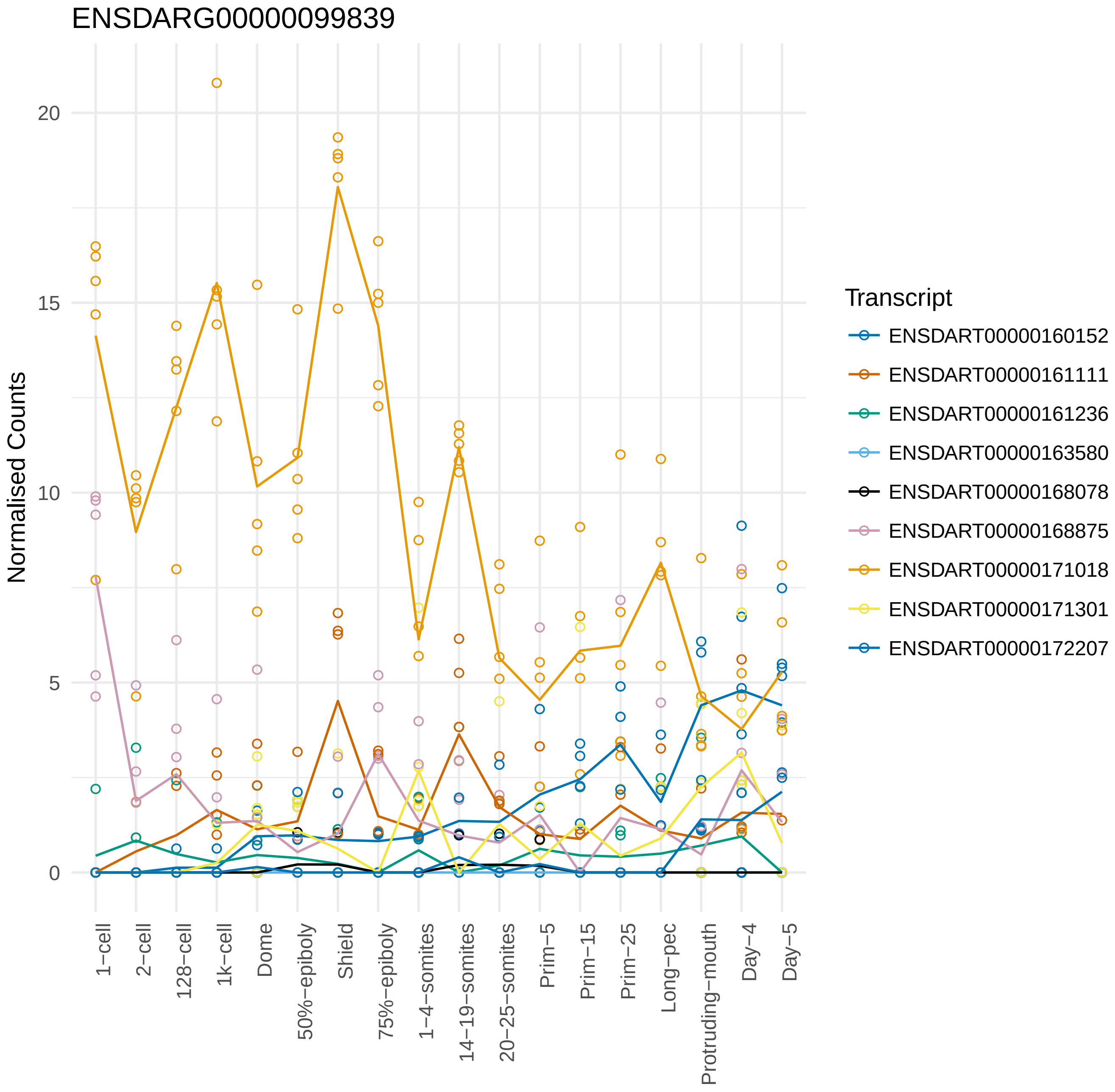
ENSDARG00000099839
Normalised Counts
0
5
10
15
20
Stage
1−cell
2−cell
128−cell
1k−cell
Dome
50%−epiboly
Shield
75%−epiboly
1−4−somites
14−19−somites
20−25−somites
Prim−5
Prim−15
Prim−25
Long−pec
Protruding−mouth
Day−4
Day−5
Transcript
ENSDART00000160152
ENSDART00000161111
ENSDART00000161236
ENSDART00000163580
ENSDART00000168078
ENSDART00000168875
ENSDART00000171018
ENSDART00000171301
ENSDART00000172207

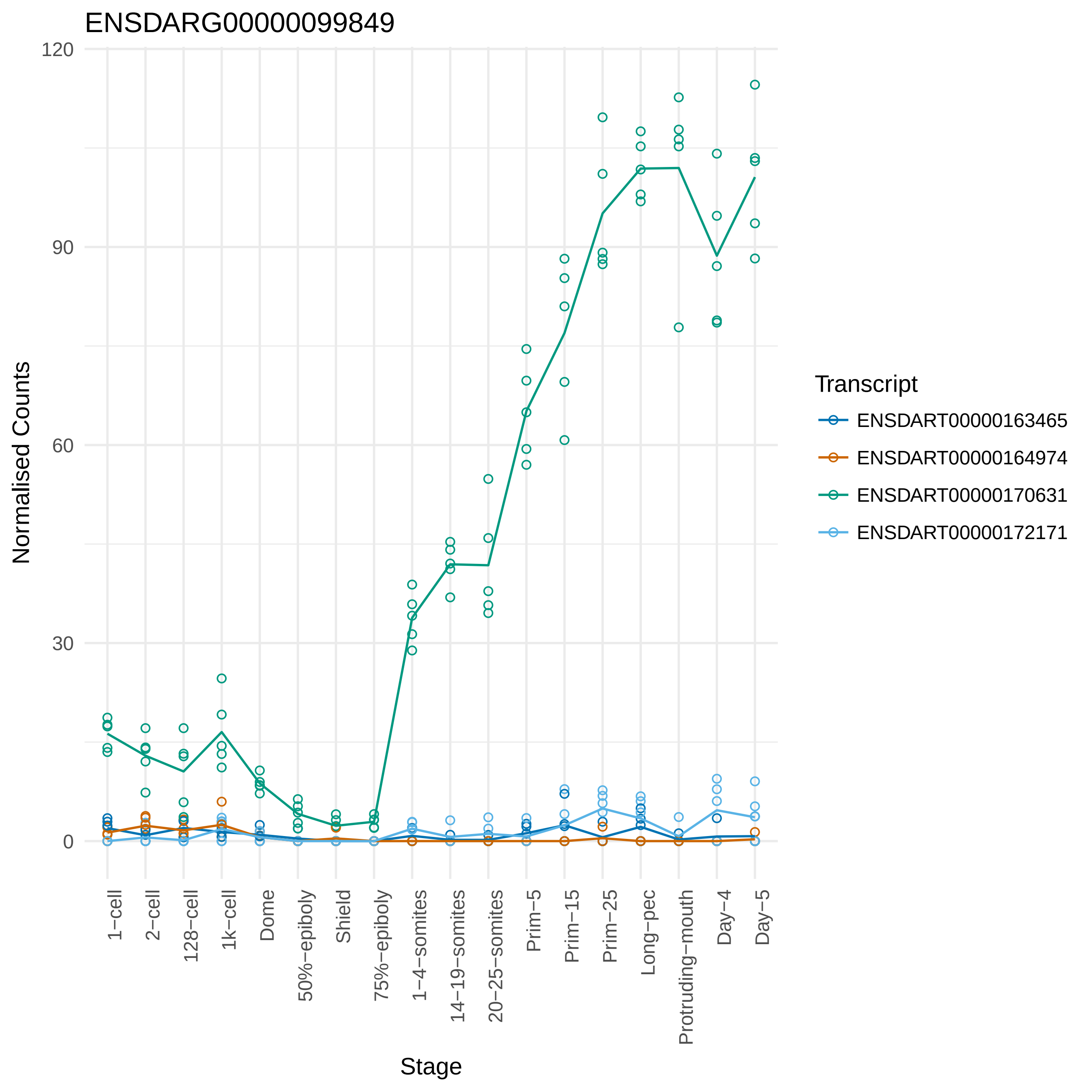

ENSDARG00000099849
Normalised Counts
120
90
60
30
0
1−cell
2−cell
128−cell
1k−cell
Dome
50%−epiboly
Shield
75%−epiboly
1−4−somites
14−19−somites
20−25−somites
Prim−5
Prim−15
Prim−25
Long−pec
Protruding−mouth
Day−4
Day−5
Stage
Transcript
ENSDART00000163465
ENSDART00000164974
ENSDART00000170631
ENSDART00000172171

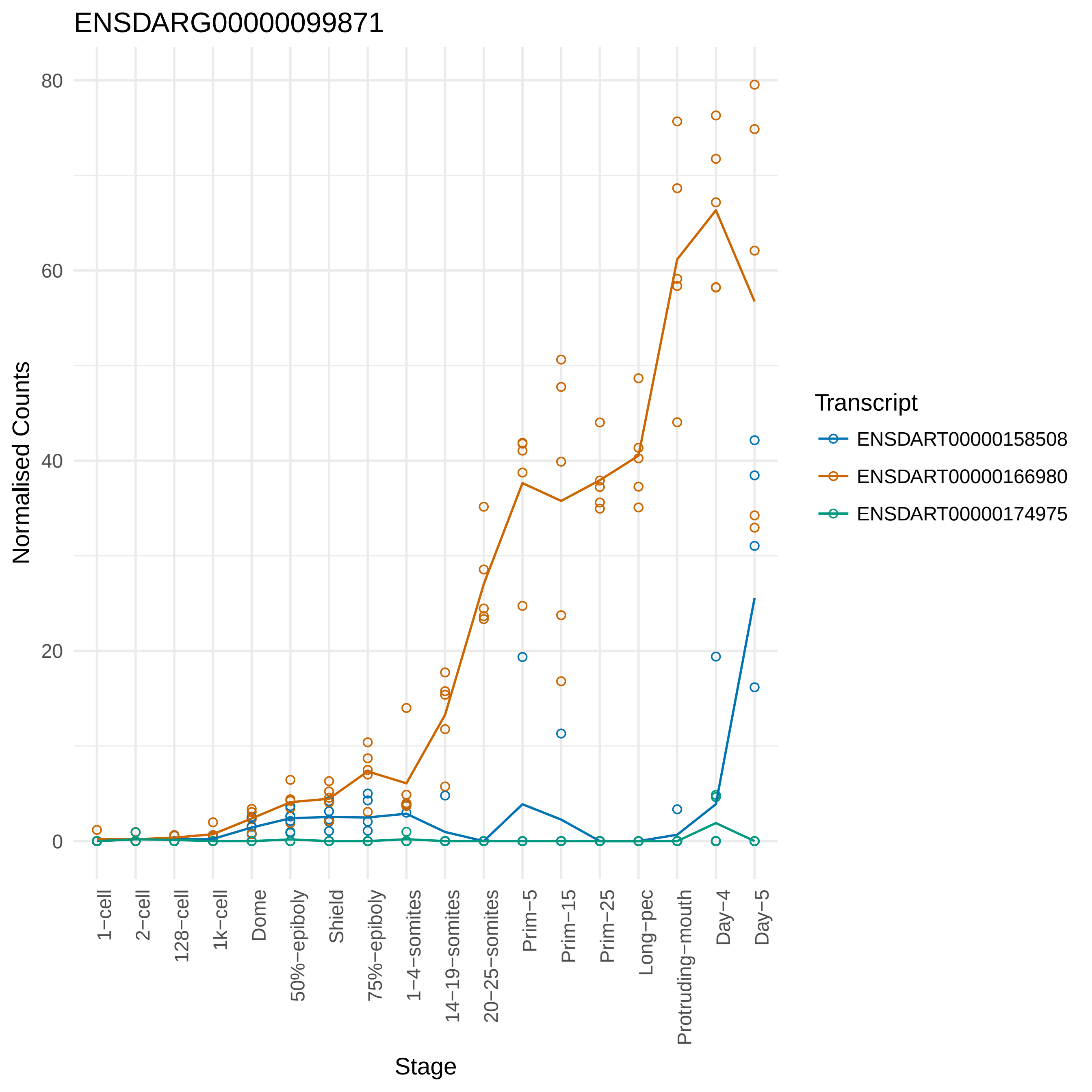
ENSDARG00000099871
Normalised Counts
80
60
40
20
0
1−cell
2−cell
128−cell
1k−cell
Dome
50%−epiboly
Shield
75%−epiboly
1−4−somites
14−19−somites
20−25−somites
Prim−5
Prim−15
Prim−25
Long−pec
Protruding−mouth
Day−4
Day−5
Stage
Transcript
ENSDART00000158508
ENSDART00000166980
ENSDART00000174975

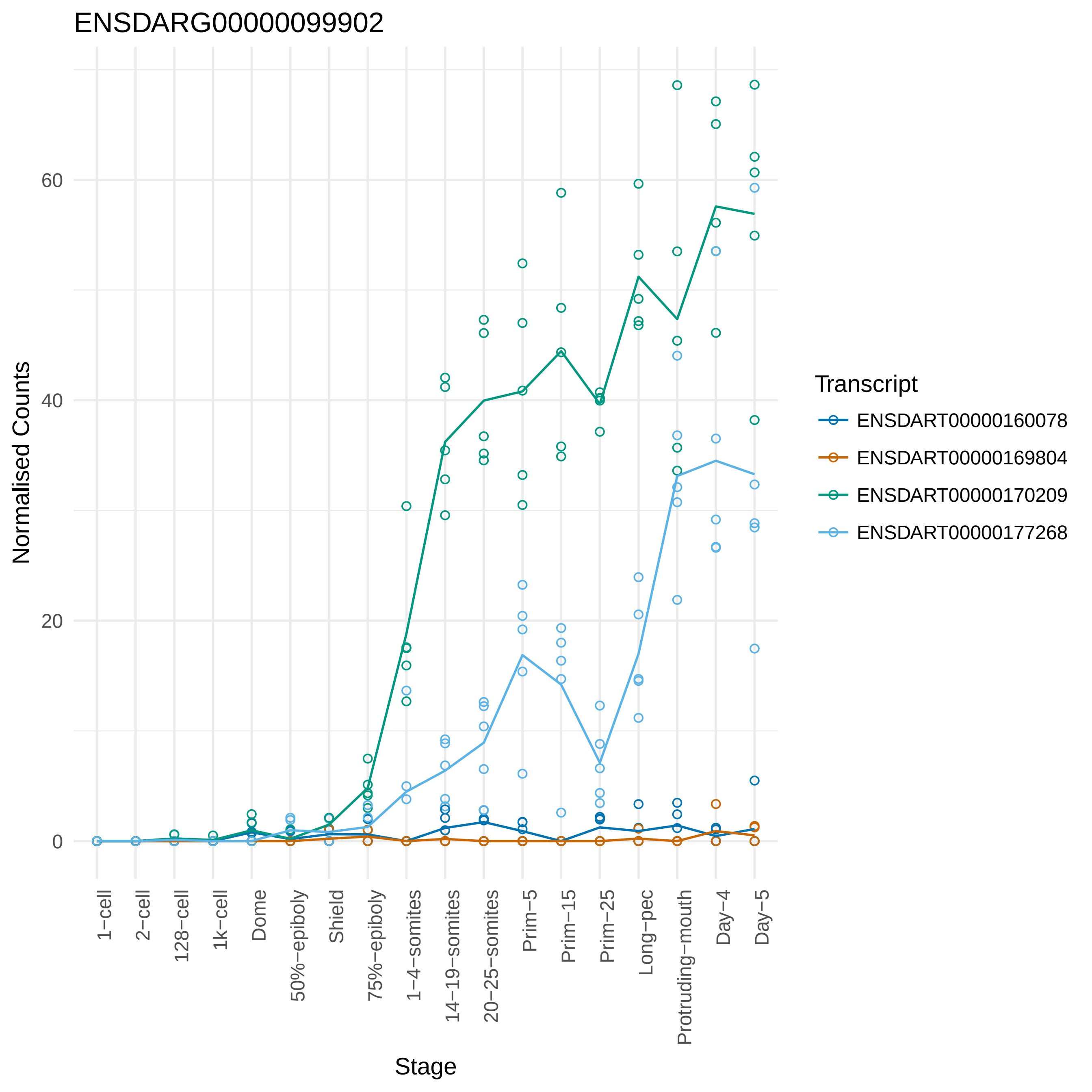

ENSDARG00000099902
Normalised Counts
0
20
40
60
Stage
1−cell
2−cell
128−cell
1k−cell
Dome
50%−epiboly
Shield
75%−epiboly
1−4−somites
14−19−somites
20−25−somites
Prim−5
Prim−15
Prim−25
Long−pec
Protruding−mouth
Day−4
Day−5
Transcript
ENSDART00000160078
ENSDART00000169804
ENSDART00000170209
ENSDART00000177268

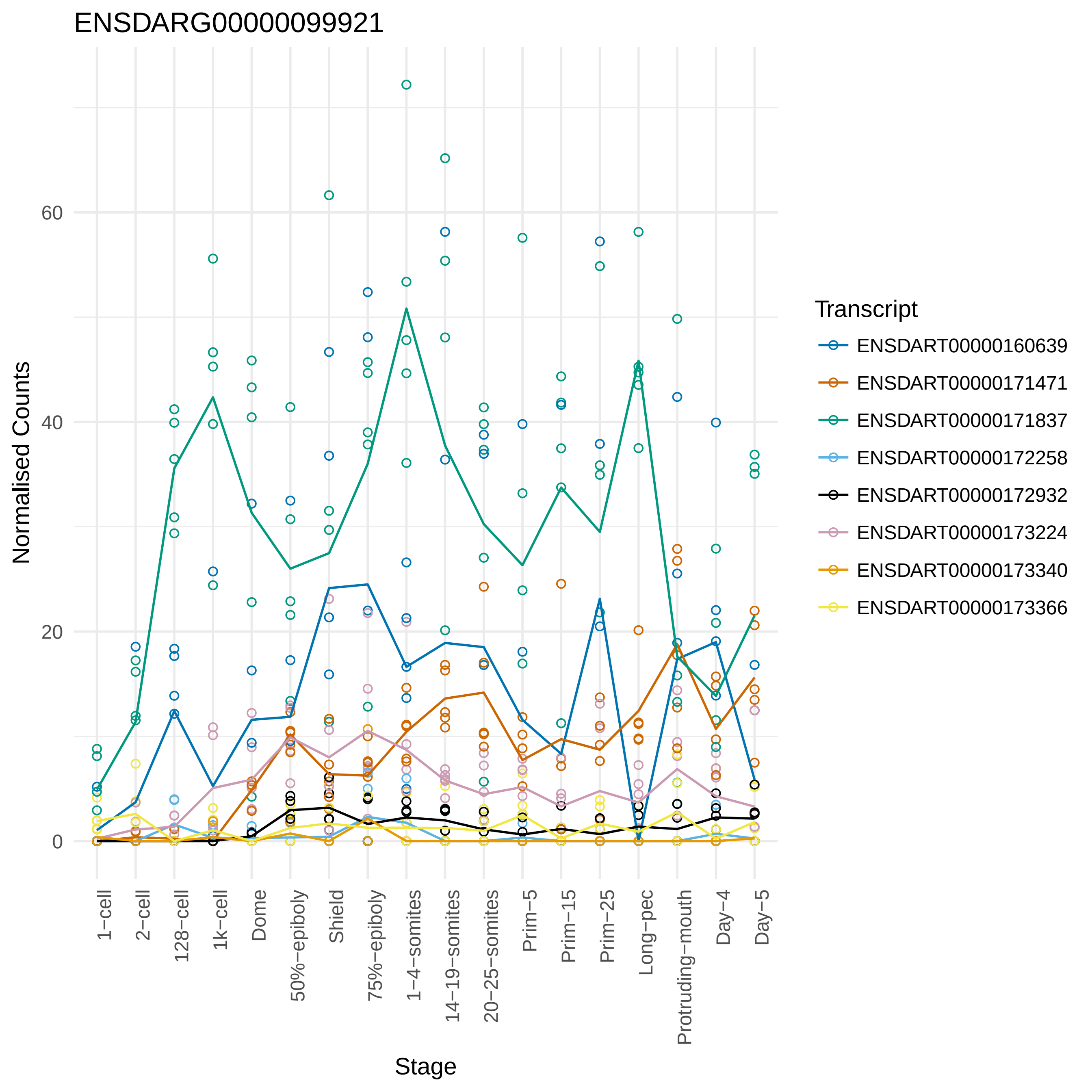

ENSDARG00000099921
Normalised Counts
0
20
40
60
Stage
1−cell
2−cell
128−cell
1k−cell
Dome
50%−epiboly
Shield
75%−epiboly
1−4−somites
14−19−somites
20−25−somites
Prim−5
Prim−15
Prim−25
Long−pec
Protruding−mouth
Day−4
Day−5
Transcript
ENSDART00000160639
ENSDART00000171471
ENSDART00000171837
ENSDART00000172258
ENSDART00000172932
ENSDART00000173224
ENSDART00000173340
ENSDART00000173366

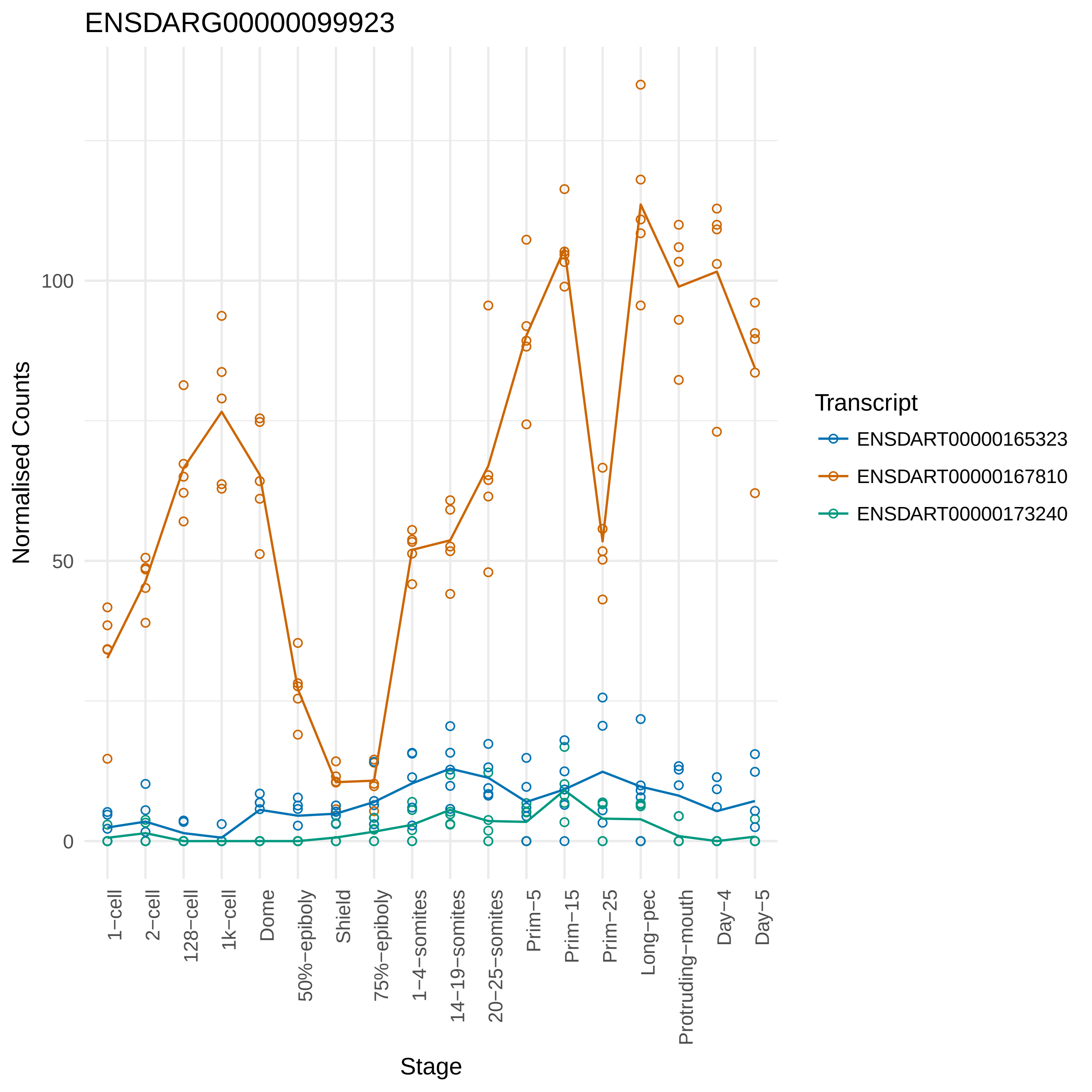

ENSDARG00000099923
Normalised Counts
0
50
100
1−cell
2−cell
128−cell
1k−cell
Dome
50%−epiboly
Shield
75%−epiboly
1−4−somites
14−19−somites
20−25−somites
Prim−5
Prim−15
Prim−25
Long−pec
Protruding−mouth
Day−4
Day−5
Stage
Transcript
ENSDART00000165323
ENSDART00000167810
ENSDART00000173240

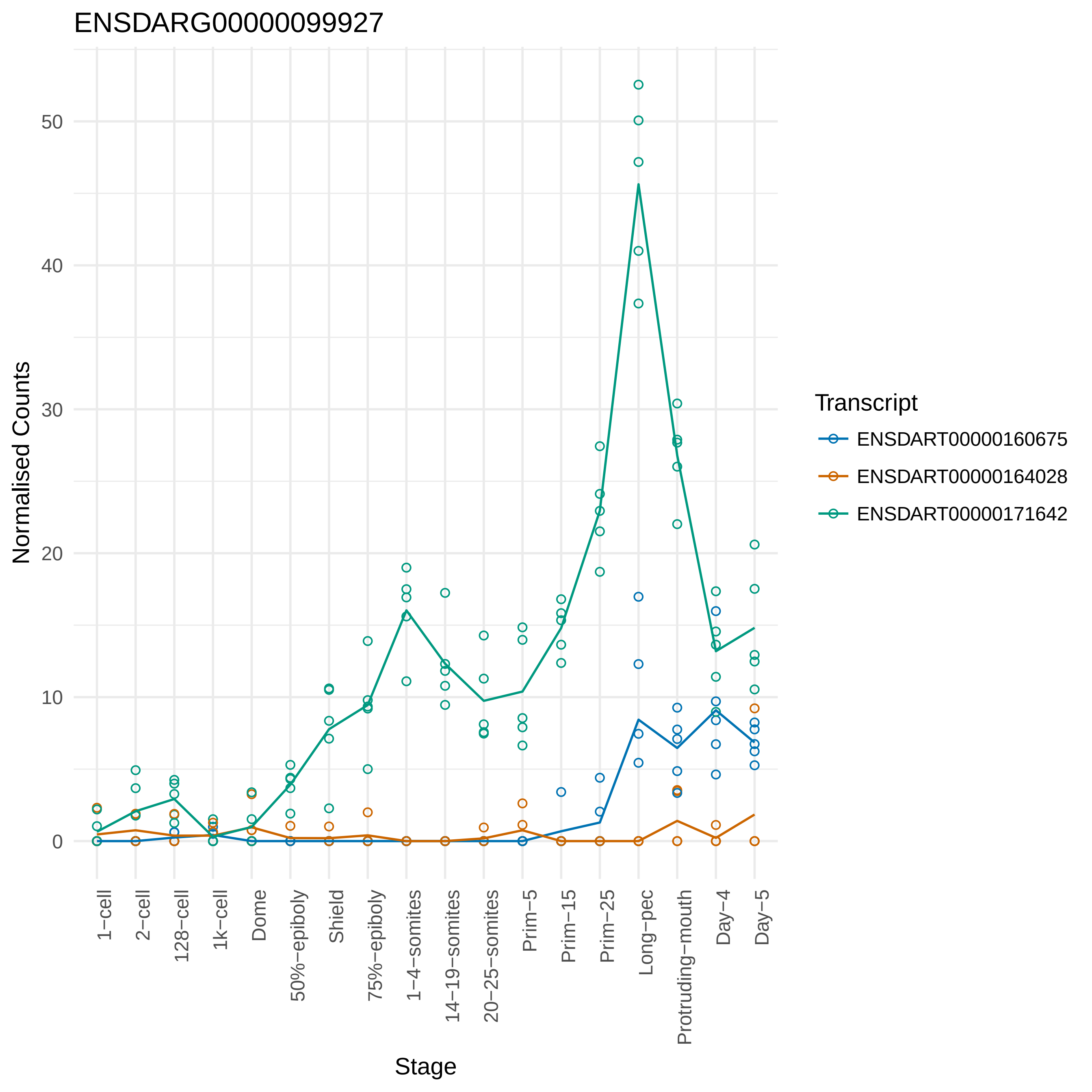

ENSDARG00000099927
Normalised Counts
0
10
20
30
40
50
1−cell
2−cell
128−cell
1k−cell
Dome
50%−epiboly
Shield
75%−epiboly
1−4−somites
14−19−somites
20−25−somites
Prim−5
Prim−15
Prim−25
Long−pec
Protruding−mouth
Day−4
Day−5
Stage
Transcript
ENSDART00000160675
ENSDART00000164028
ENSDART00000171642

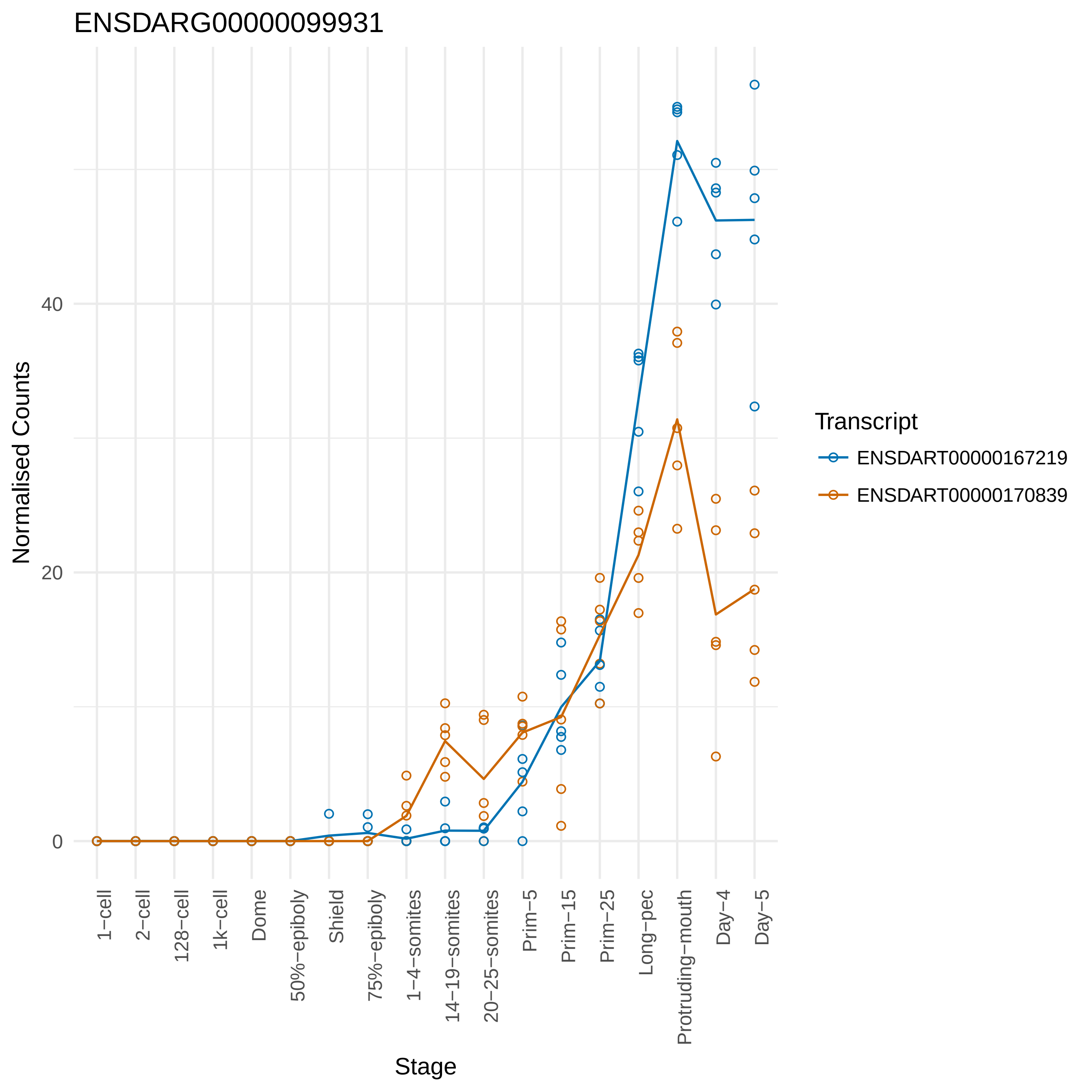

ENSDARG00000099931
Normalised Counts
0
20
40
1−cell
2−cell
128−cell
1k−cell
Dome
50%−epiboly
Shield
75%−epiboly
1−4−somites
14−19−somites
20−25−somites
Prim−5
Prim−15
Prim−25
Long−pec
Protruding−mouth
Day−4
Day−5
Stage
Transcript
ENSDART00000167219
ENSDART00000170839

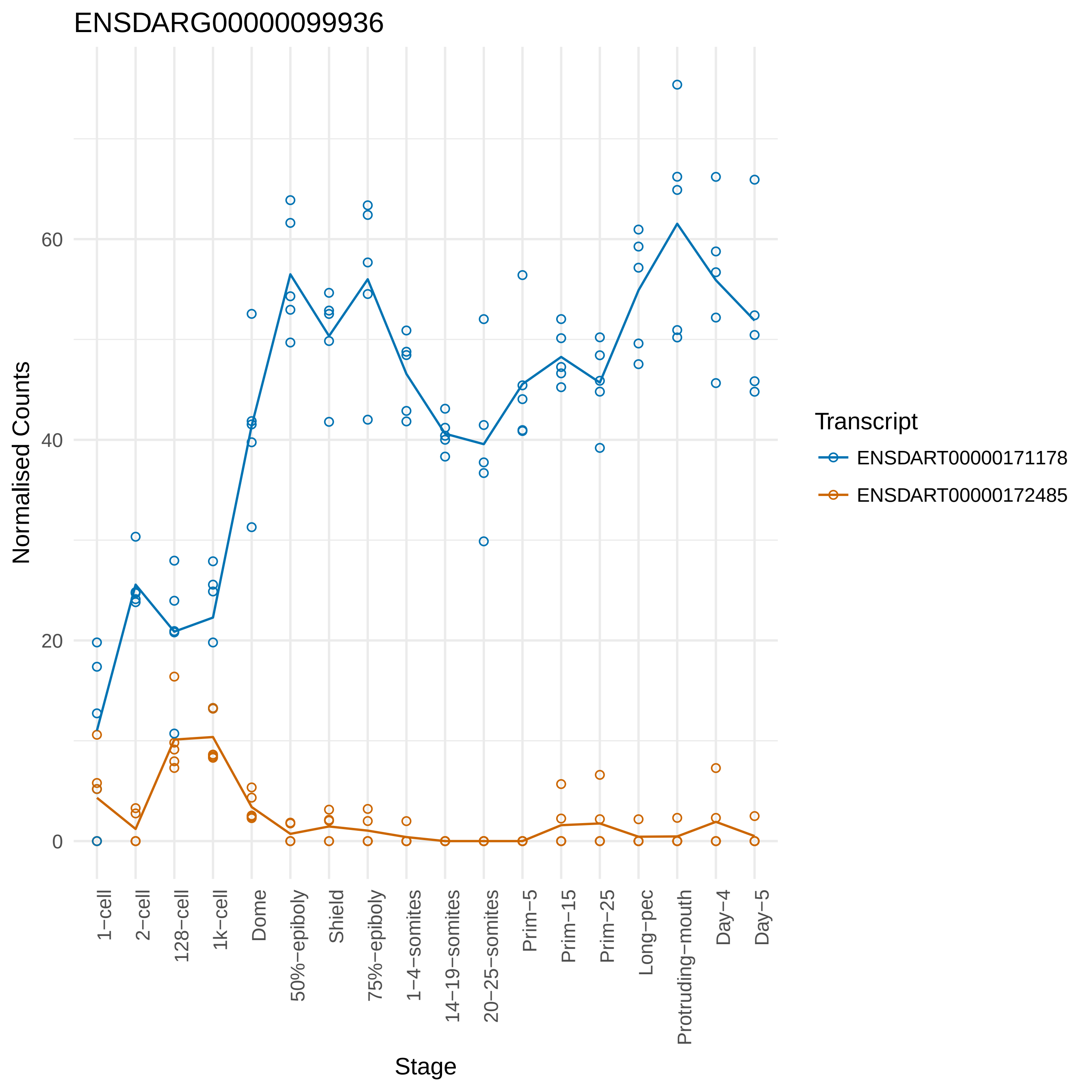

ENSDARG00000099936
Normalised Counts
0
20
40
60
1−cell
2−cell
128−cell
1k−cell
Dome
50%−epiboly
Shield
75%−epiboly
1−4−somites
14−19−somites
20−25−somites
Prim−5
Prim−15
Prim−25
Long−pec
Protruding−mouth
Day−4
Day−5
Stage
Transcript
ENSDART00000171178
ENSDART00000172485

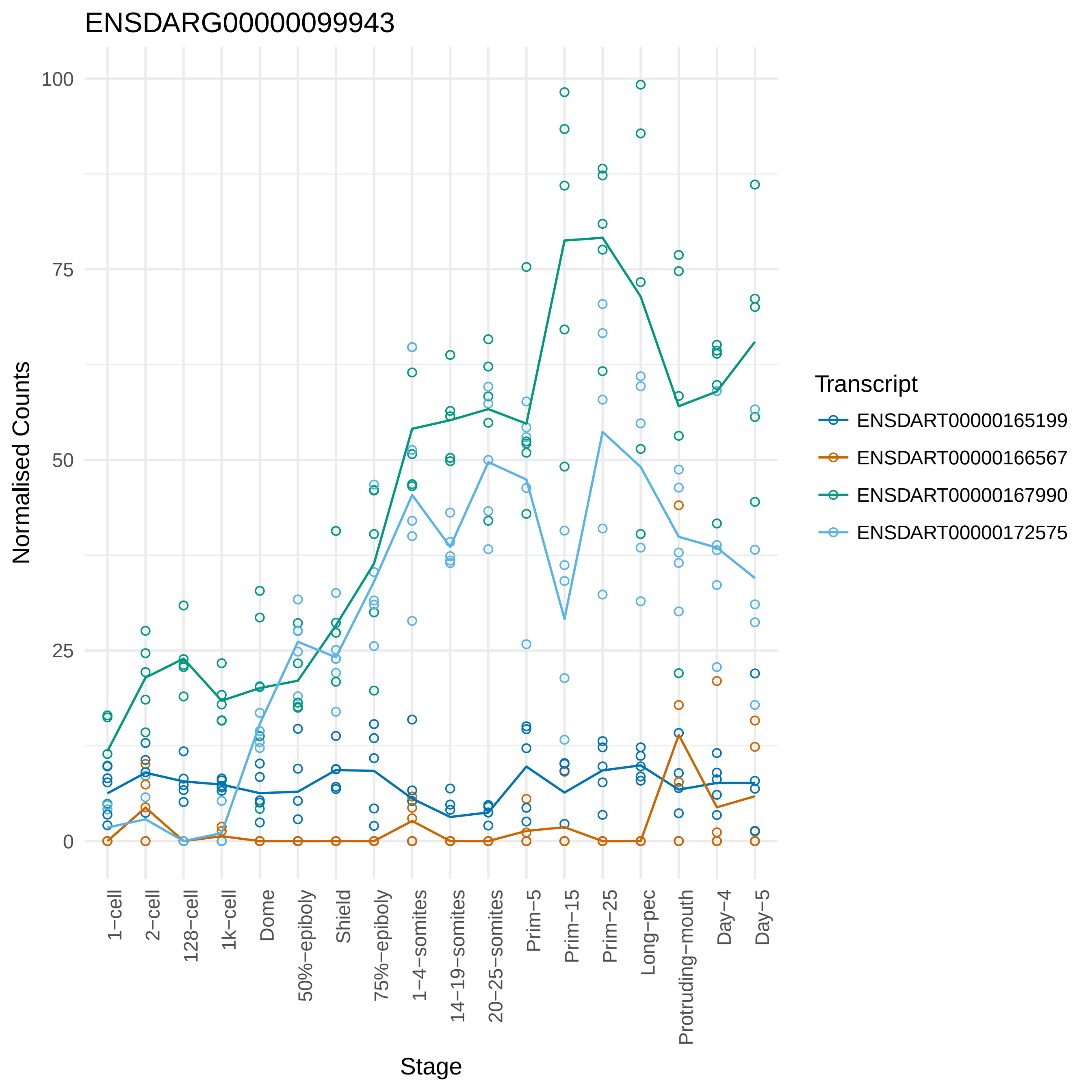
ENSDARG00000099943
Normalised Counts
100
75
50
25
0
1−cell
2−cell
128−cell
1k−cell
Dome
50%−epiboly
Shield
75%−epiboly
1−4−somites
14−19−somites
20−25−somites
Prim−5
Prim−15
Prim−25
Long−pec
Protruding−mouth
Day−4
Day−5
Stage
Transcript
ENSDART00000165199
ENSDART00000166567
ENSDART00000167990
ENSDART00000172575

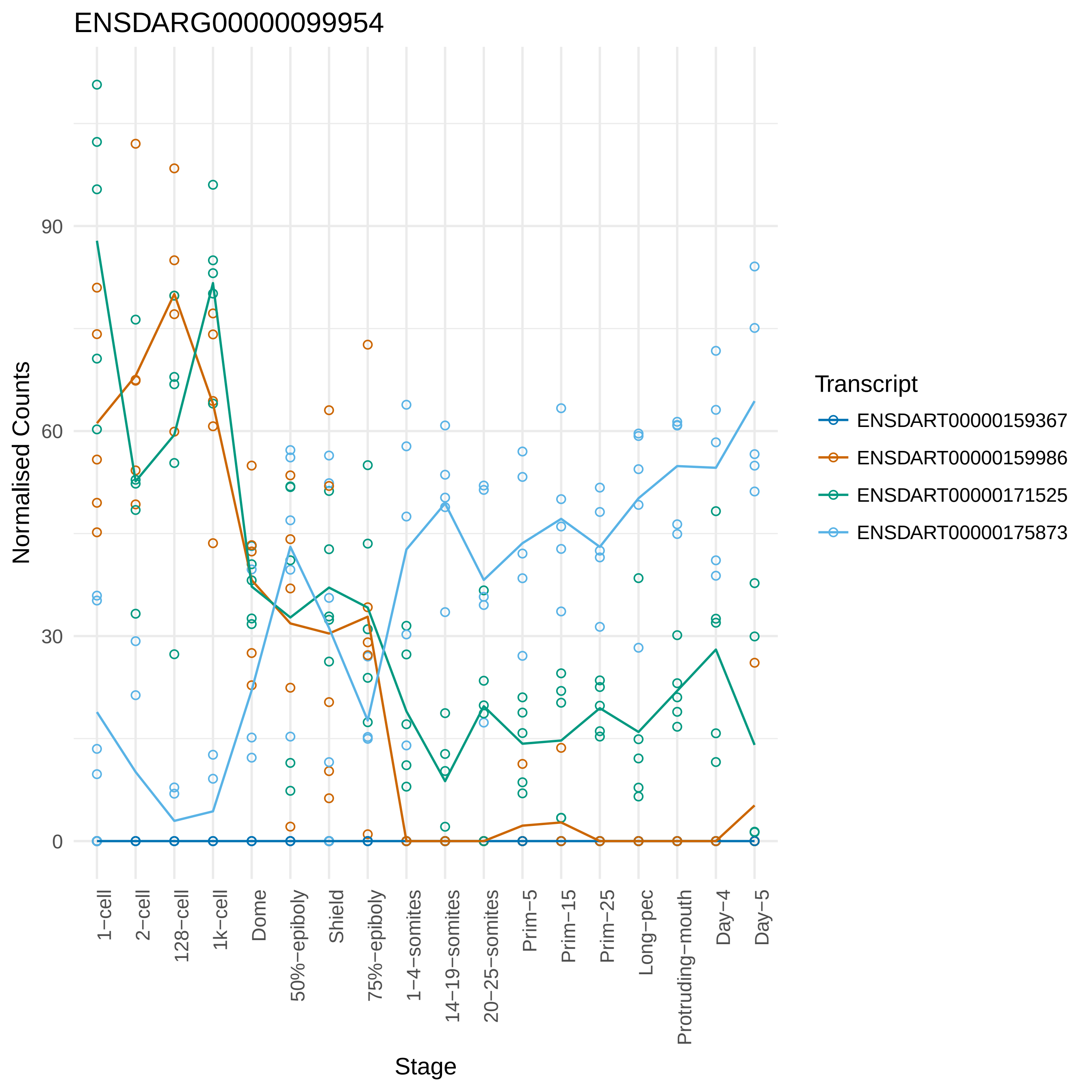

ENSDARG00000099954
Normalised Counts
90
60
30
0
1−cell
2−cell
128−cell
1k−cell
Dome
50%−epiboly
Shield
75%−epiboly
1−4−somites
14−19−somites
20−25−somites
Prim−5
Prim−15
Prim−25
Long−pec
Protruding−mouth
Day−4
Day−5
Stage
Transcript
ENSDART00000159367
ENSDART00000159986
ENSDART00000171525
ENSDART00000175873

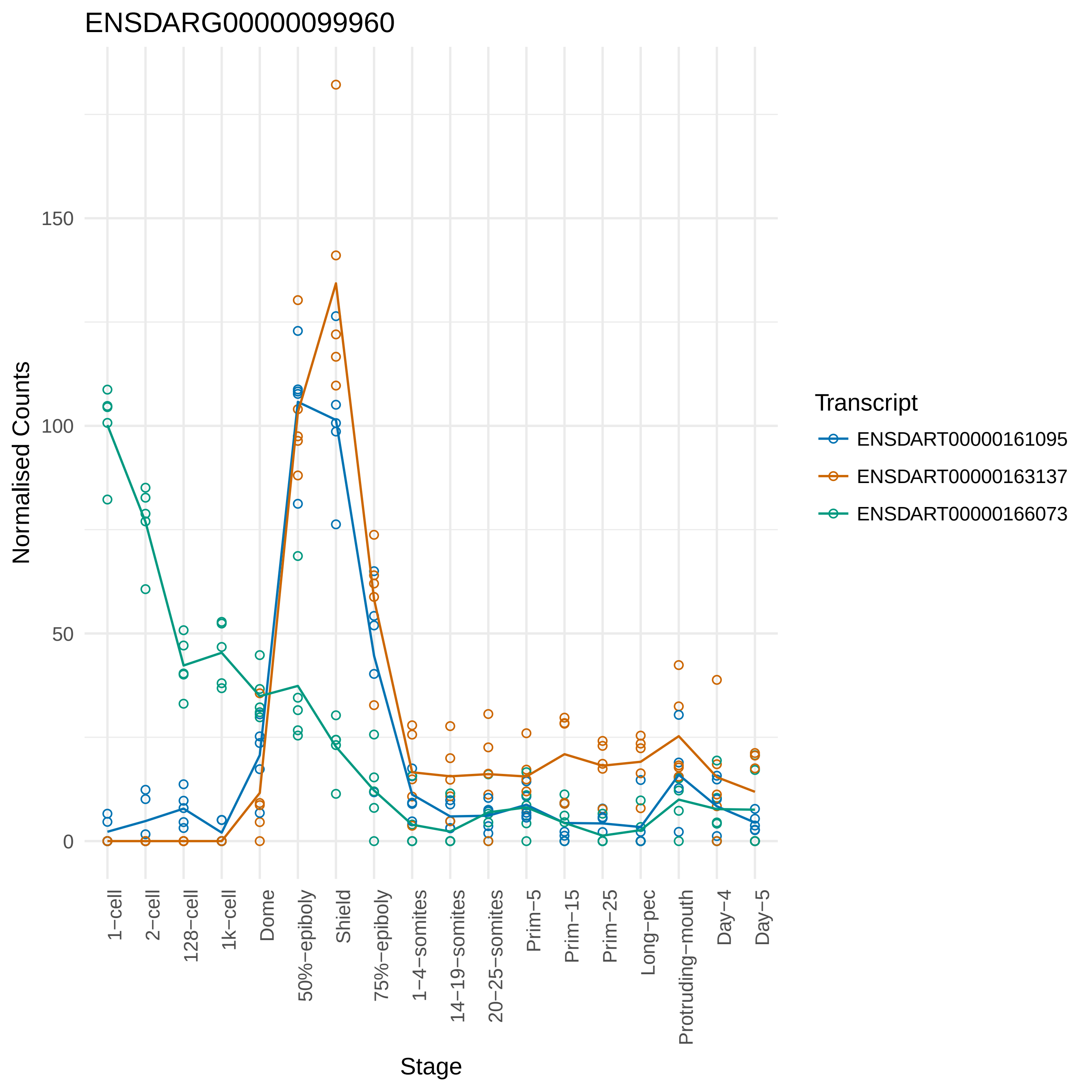

ENSDARG00000099960
Normalised Counts
150
100
50
0
1−cell
2−cell
128−cell
1k−cell
Dome
50%−epiboly
Shield
75%−epiboly
1−4−somites
14−19−somites
20−25−somites
Prim−5
Prim−15
Prim−25
Long−pec
Protruding−mouth
Day−4
Day−5
Stage
Transcript
ENSDART00000161095
ENSDART00000163137
ENSDART00000166073

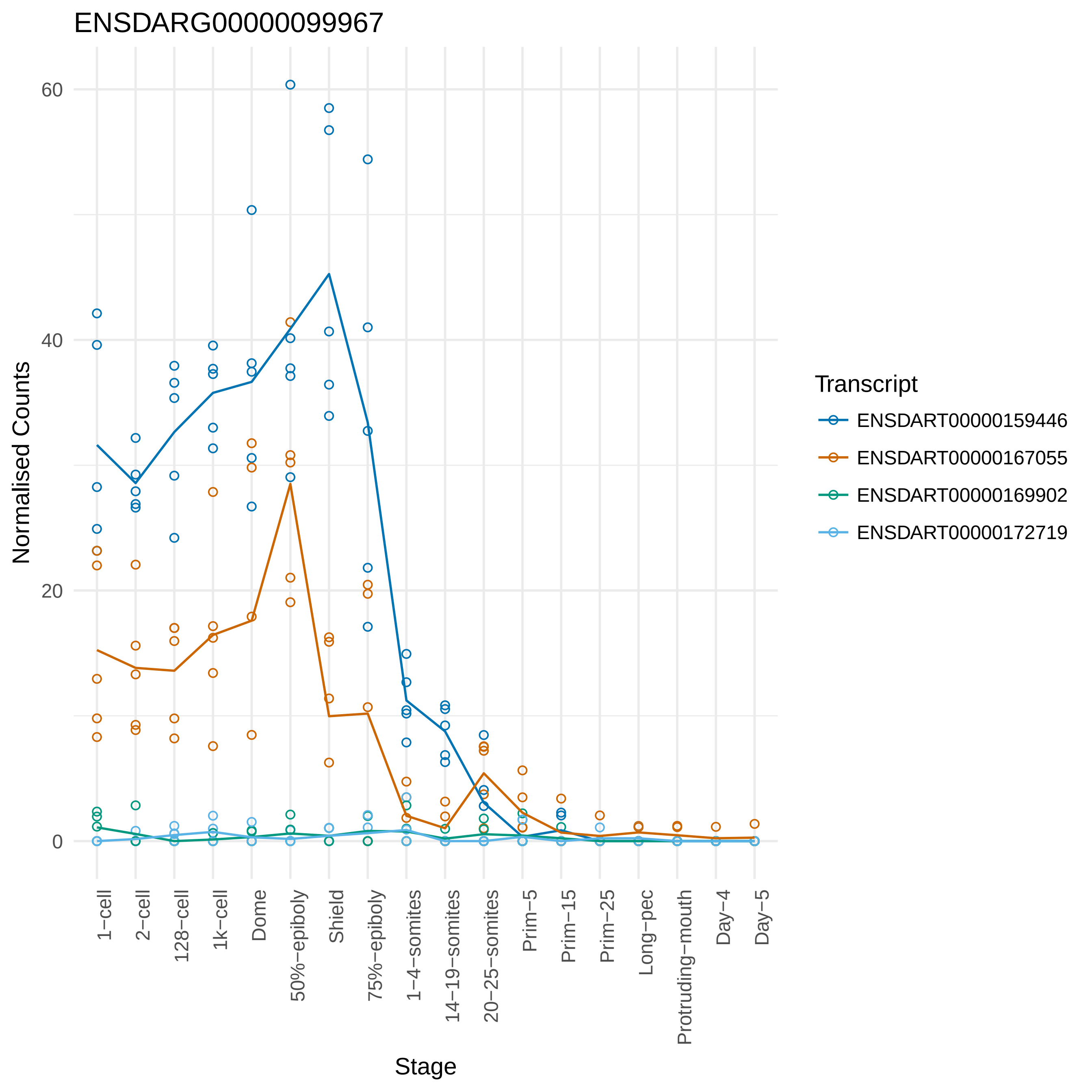

ENSDARG00000099967
Normalised Counts
60
40
20
0
1−cell
2−cell
128−cell
1k−cell
Dome
50%−epiboly
Shield
75%−epiboly
1−4−somites
14−19−somites
20−25−somites
Prim−5
Prim−15
Prim−25
Long−pec
Protruding−mouth
Day−4
Day−5
Stage
Transcript
ENSDART00000159446
ENSDART00000167055
ENSDART00000169902
ENSDART00000172719

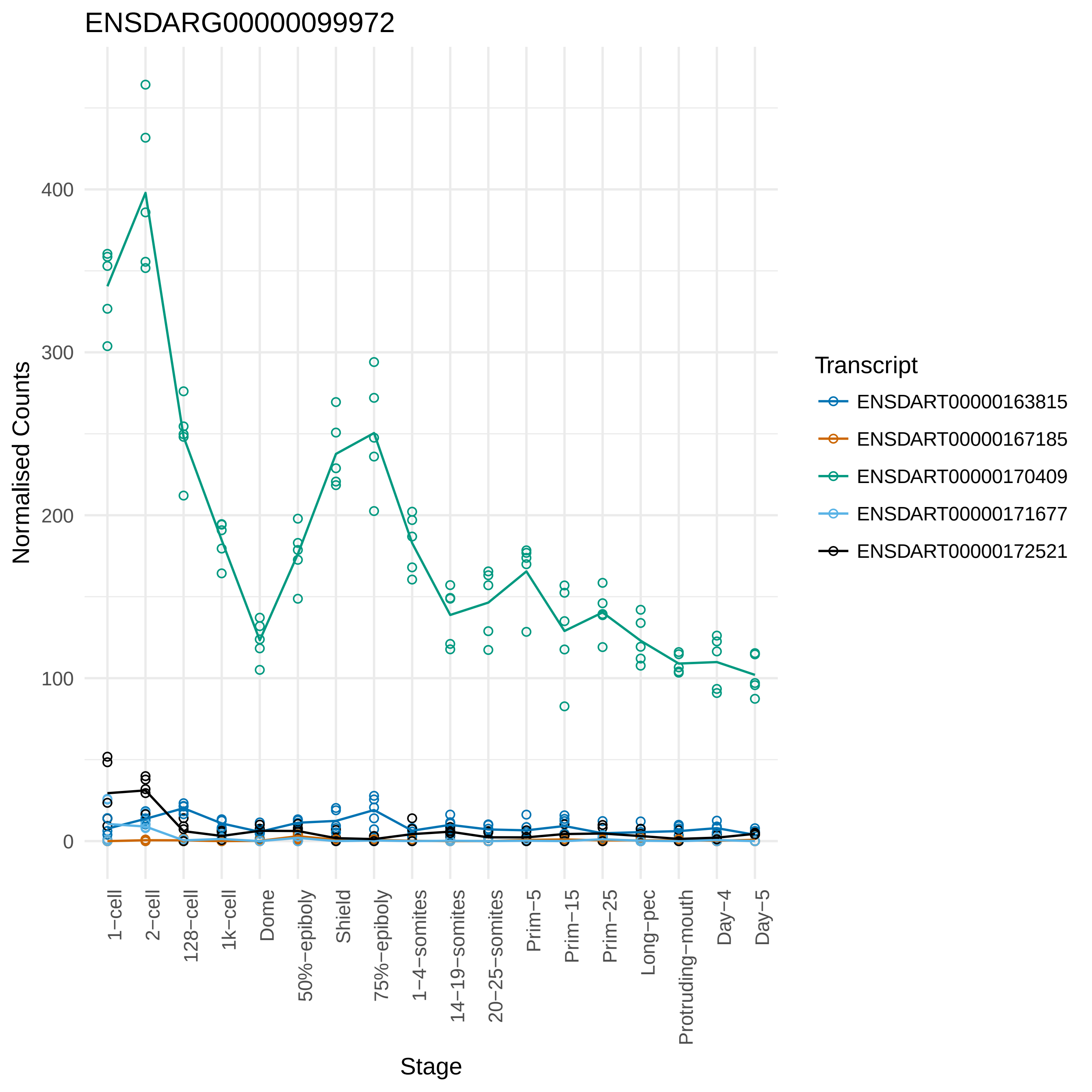

ENSDARG00000099972
Normalised Counts
400
300
200
100
0
1−cell
2−cell
128−cell
1k−cell
Dome
50%−epiboly
Shield
75%−epiboly
1−4−somites
14−19−somites
20−25−somites
Prim−5
Prim−15
Prim−25
Long−pec
Protruding−mouth
Day−4
Day−5
Stage
Transcript
ENSDART00000163815
ENSDART00000167185
ENSDART00000170409
ENSDART00000171677
ENSDART00000172521

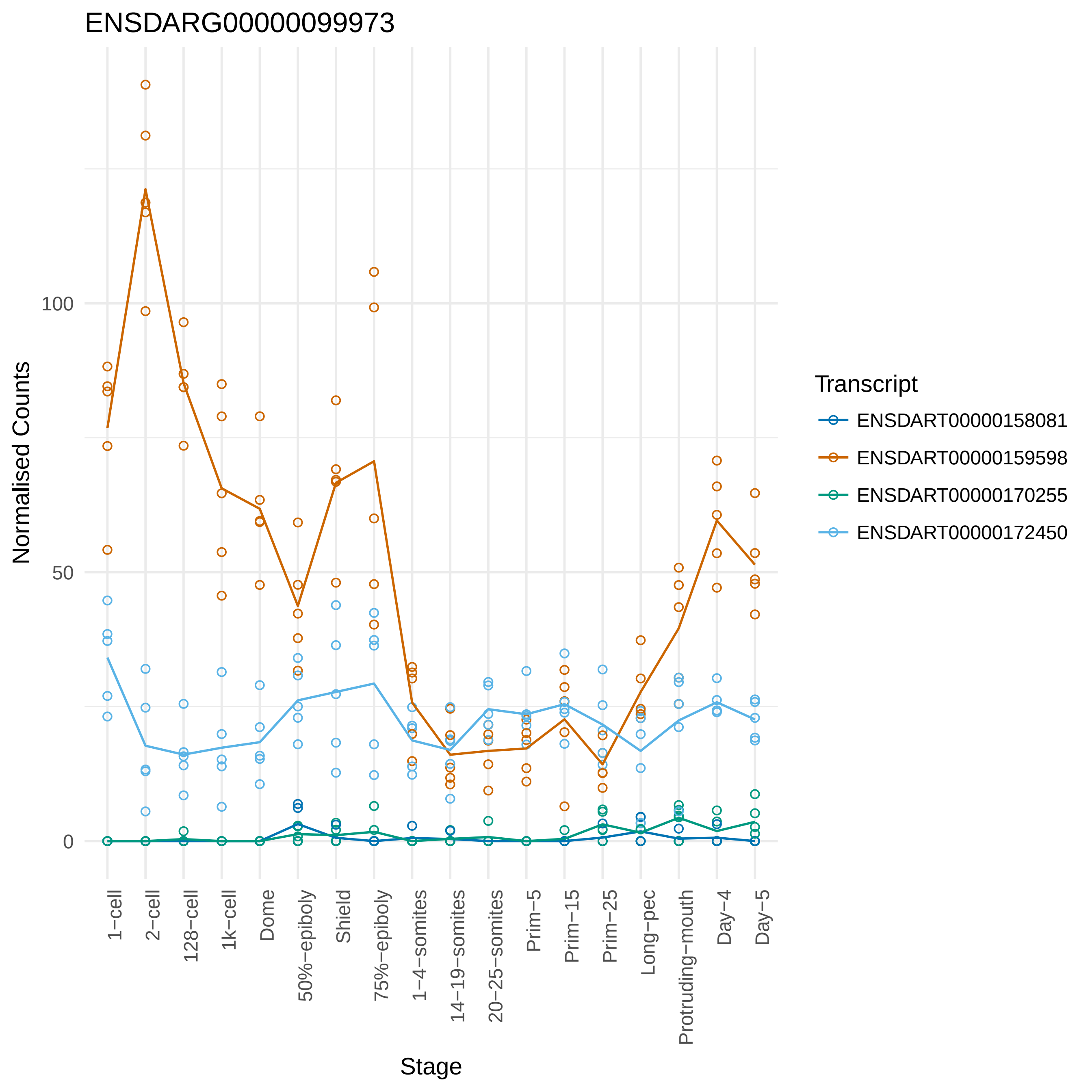
ENSDARG00000099973
Normalised Counts
0
50
100
Stage
1−cell
2−cell
128−cell
1k−cell
Dome
50%−epiboly
Shield
75%−epiboly
1−4−somites
14−19−somites
20−25−somites
Prim−5
Prim−15
Prim−25
Long−pec
Protruding−mouth
Day−4
Day−5
Transcript
ENSDART00000158081
ENSDART00000159598
ENSDART00000170255
ENSDART00000172450

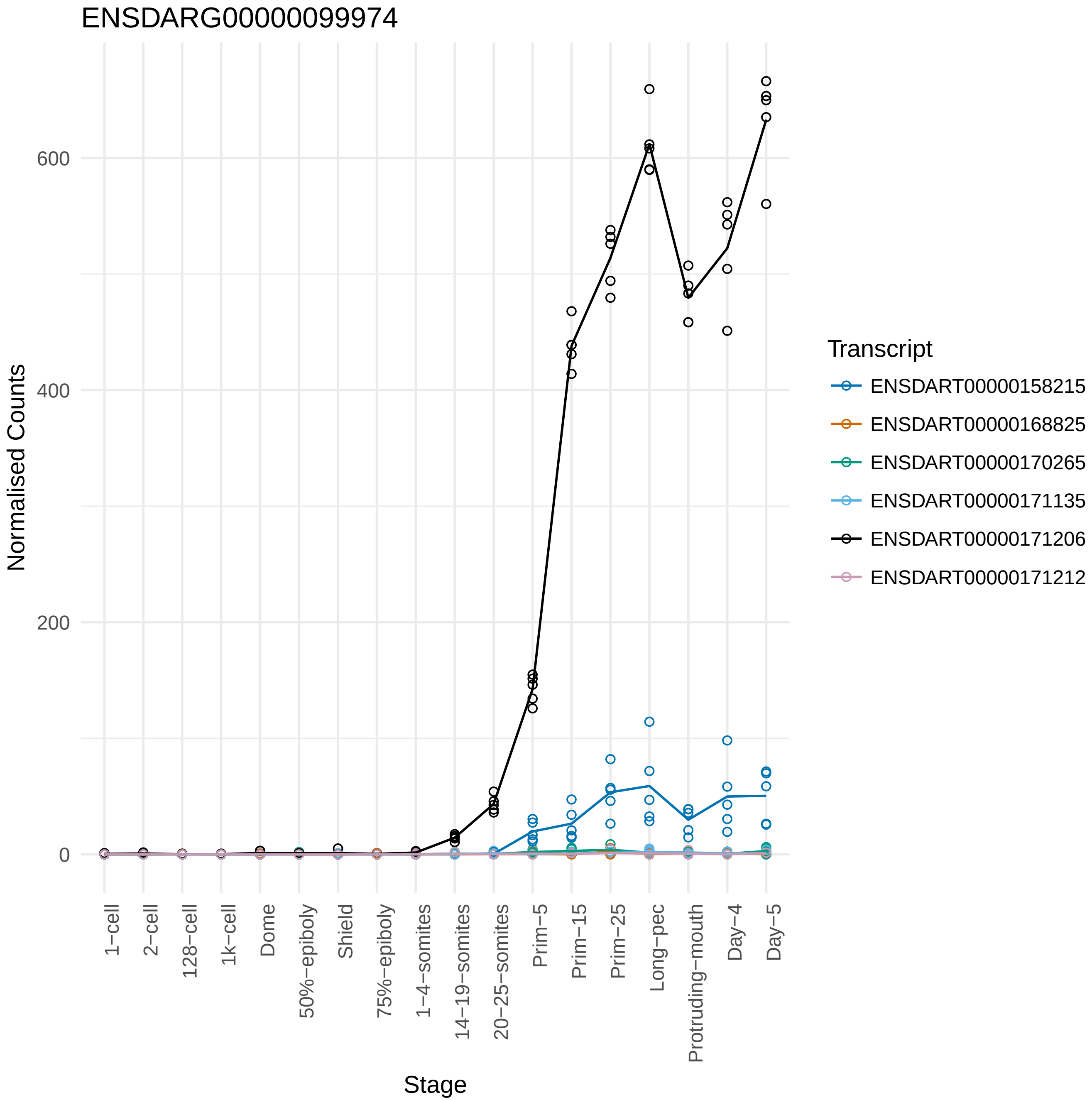
ENSDARG00000099974
Normalised Counts
0
200
400
600
Stage
1−cell
2−cell
128−cell
1k−cell
Dome
50%−epiboly
Shield
75%−epiboly
1−4−somites
14−19−somites
20−25−somites
Prim−5
Prim−15
Prim−25
Long−pec
Protruding−mouth
Day−4
Day−5
Transcript
ENSDART00000158215
ENSDART00000168825
ENSDART00000170265
ENSDART00000171135
ENSDART00000171206
ENSDART00000171212

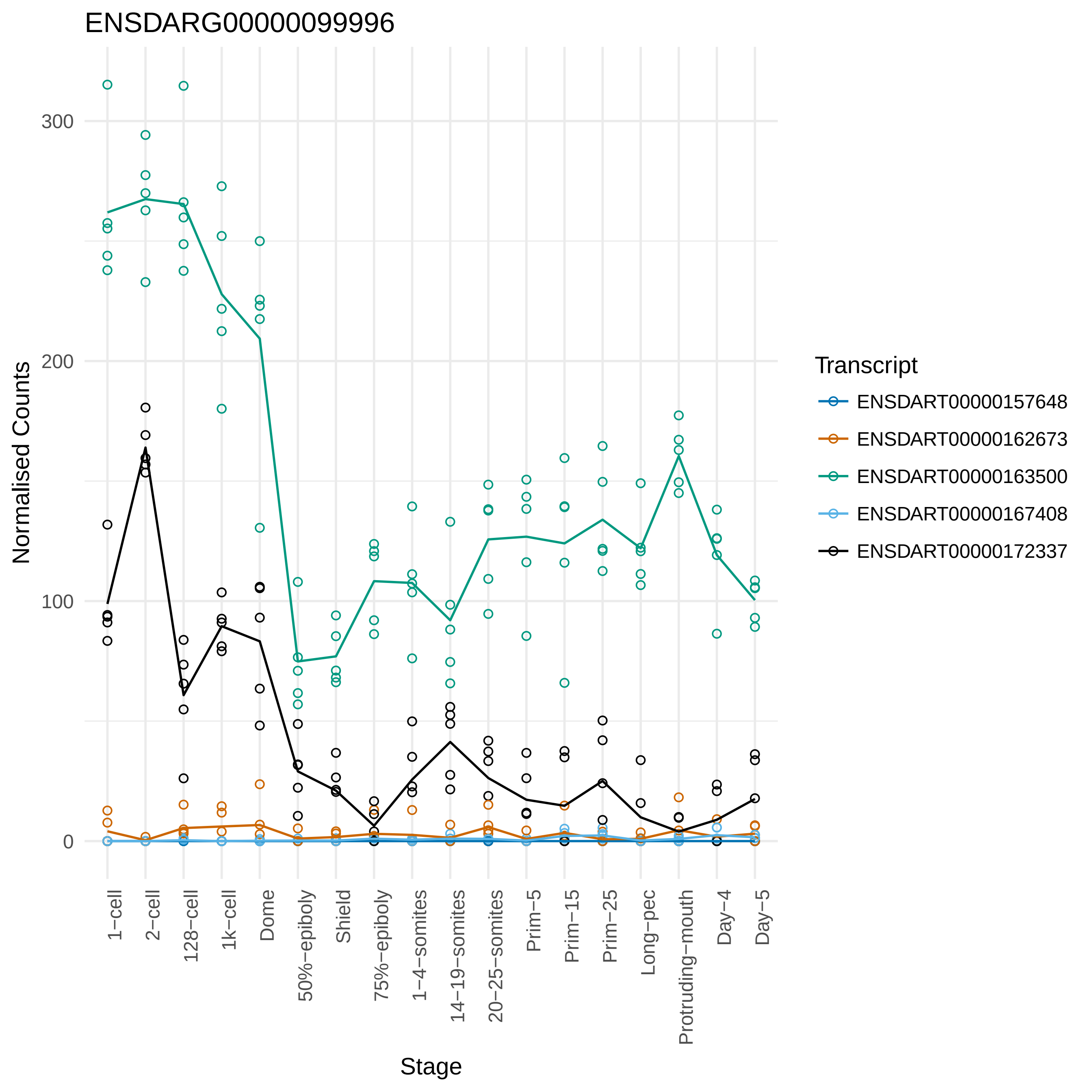
ENSDARG00000099996
Normalised Counts
300
200
100
0
1−cell
2−cell
128−cell
1k−cell
Dome
50%−epiboly
Shield
75%−epiboly
1−4−somites
14−19−somites
20−25−somites
Prim−5
Prim−15
Prim−25
Long−pec
Protruding−mouth
Day−4
Day−5
Stage
Transcript
ENSDART00000157648
ENSDART00000162673
ENSDART00000163500
ENSDART00000167408
ENSDART00000172337

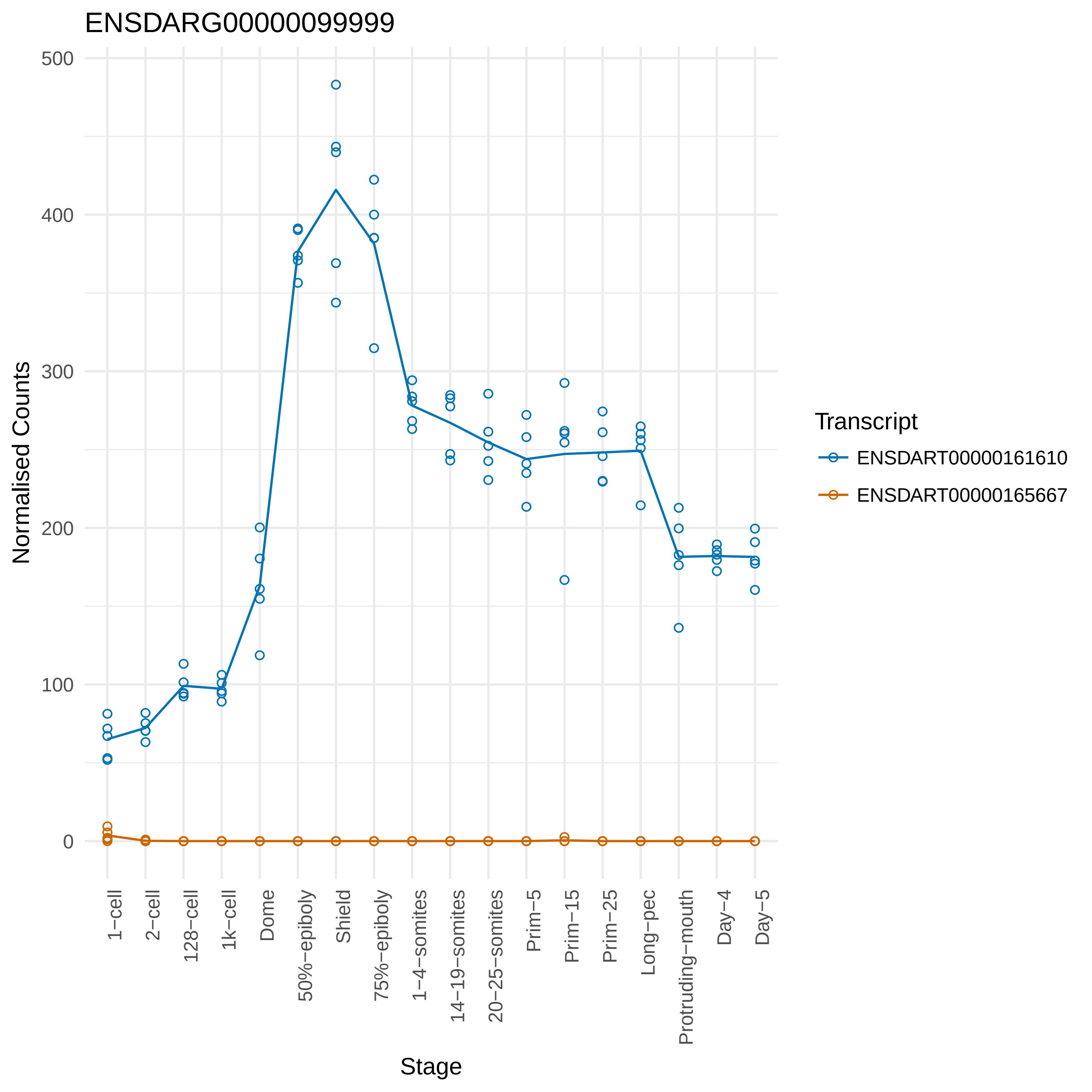

ENSDARG00000099999
Normalised Counts
500
400
300
200
100
0
1−cell
2−cell
128−cell
1k−cell
Dome
50%−epiboly
Shield
75%−epiboly
1−4−somites
14−19−somites
20−25−somites
Prim−5
Prim−15
Prim−25
Long−pec
Protruding−mouth
Day−4
Day−5
Stage
Transcript
ENSDART00000161610
ENSDART00000165667

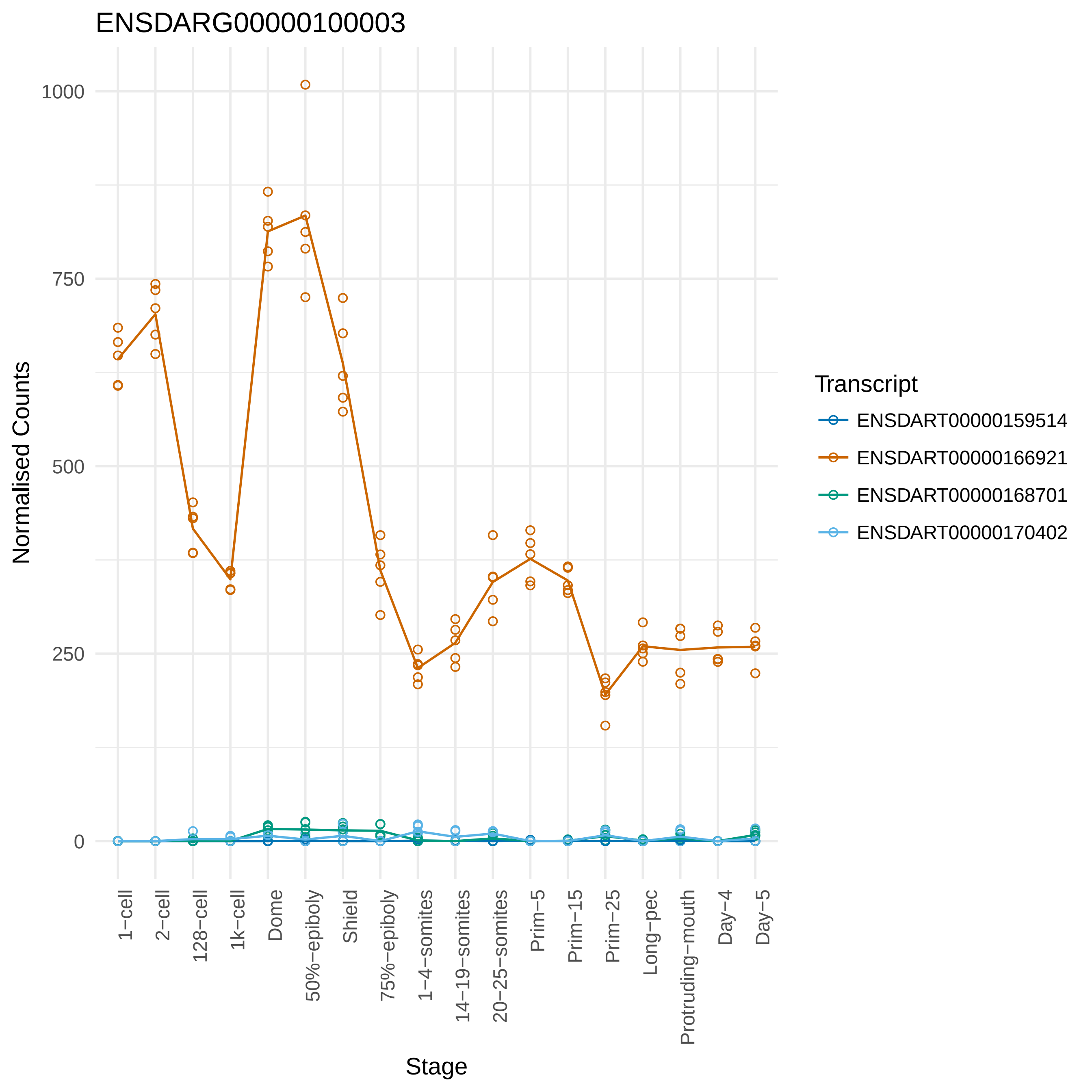

ENSDARG00000100003
Normalised Counts
1000
750
500
250
0
1−cell
2−cell
128−cell
1k−cell
Dome
50%−epiboly
Shield
75%−epiboly
1−4−somites
14−19−somites
20−25−somites
Prim−5
Prim−15
Prim−25
Long−pec
Protruding−mouth
Day−4
Day−5
Stage
Transcript
ENSDART00000159514
ENSDART00000166921
ENSDART00000168701
ENSDART00000170402

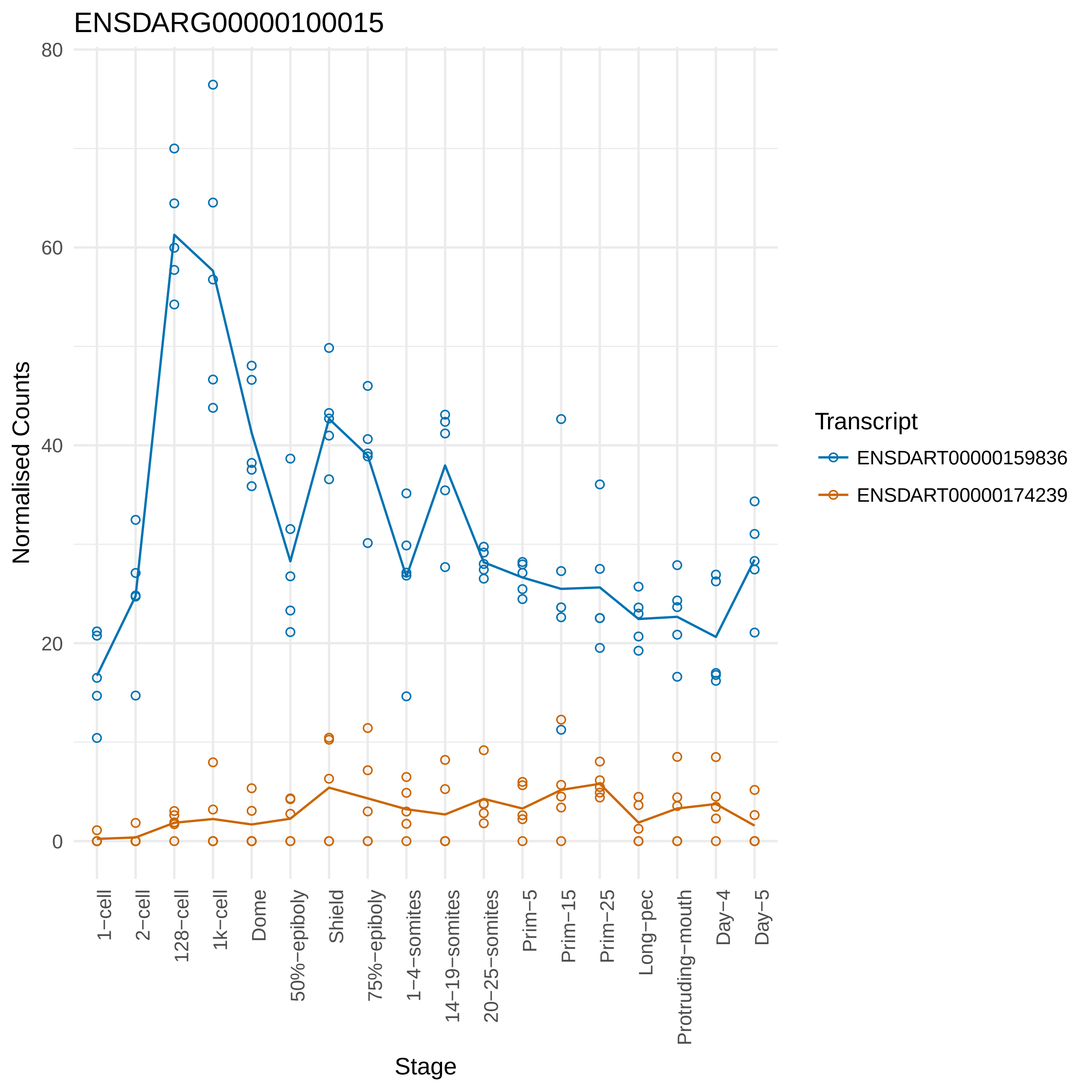
ENSDARG00000100015
Normalised Counts
80
60
40
20
0
1−cell
2−cell
128−cell
1k−cell
Dome
50%−epiboly
Shield
75%−epiboly
1−4−somites
14−19−somites
20−25−somites
Prim−5
Prim−15
Prim−25
Long−pec
Protruding−mouth
Day−4
Day−5
Stage
Transcript
ENSDART00000159836
ENSDART00000174239

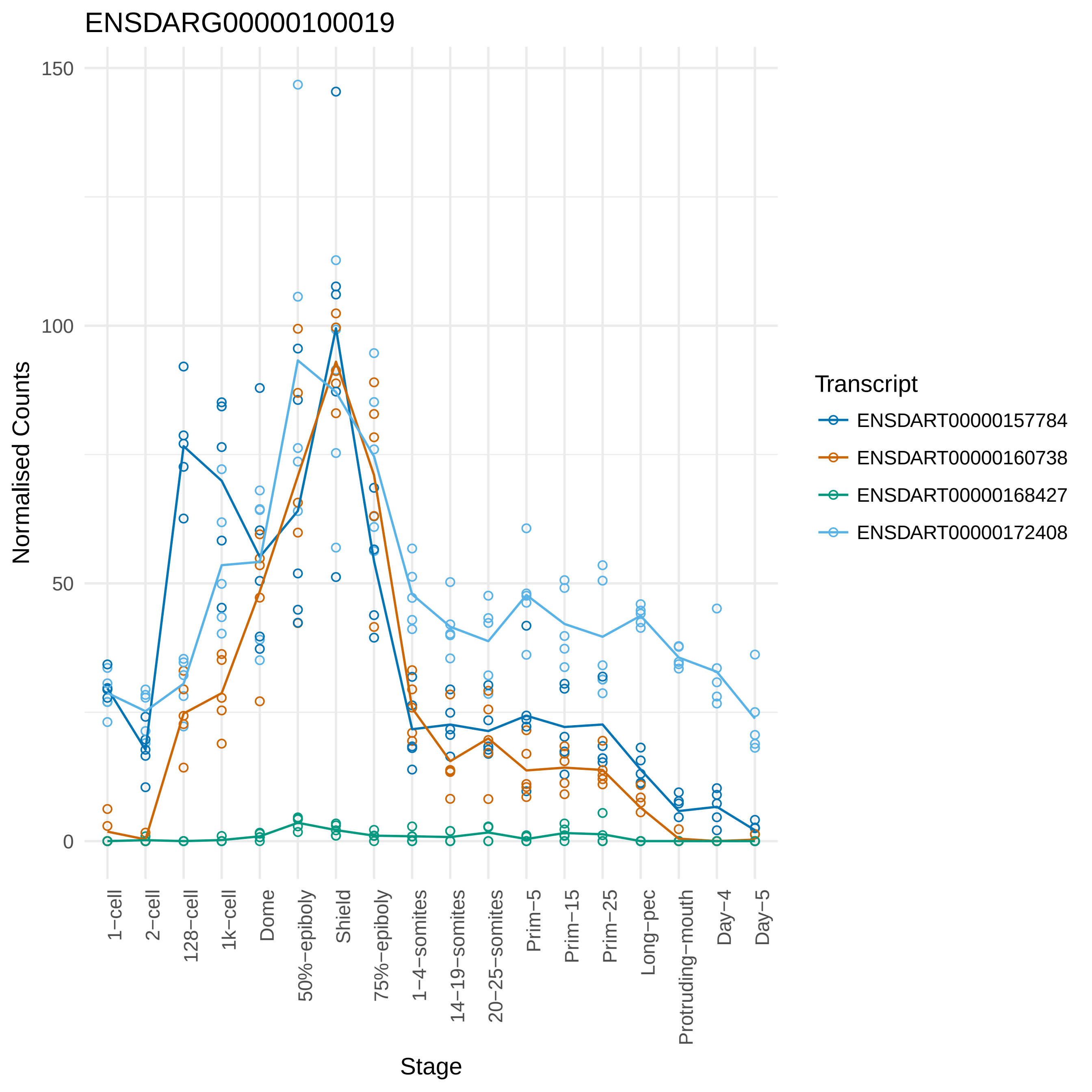

ENSDARG00000100019
Normalised Counts
150
100
50
0
1−cell
2−cell
128−cell
1k−cell
Dome
50%−epiboly
Shield
75%−epiboly
1−4−somites
14−19−somites
20−25−somites
Prim−5
Prim−15
Prim−25
Long−pec
Protruding−mouth
Day−4
Day−5
Stage
Transcript
ENSDART00000157784
ENSDART00000160738
ENSDART00000168427
ENSDART00000172408

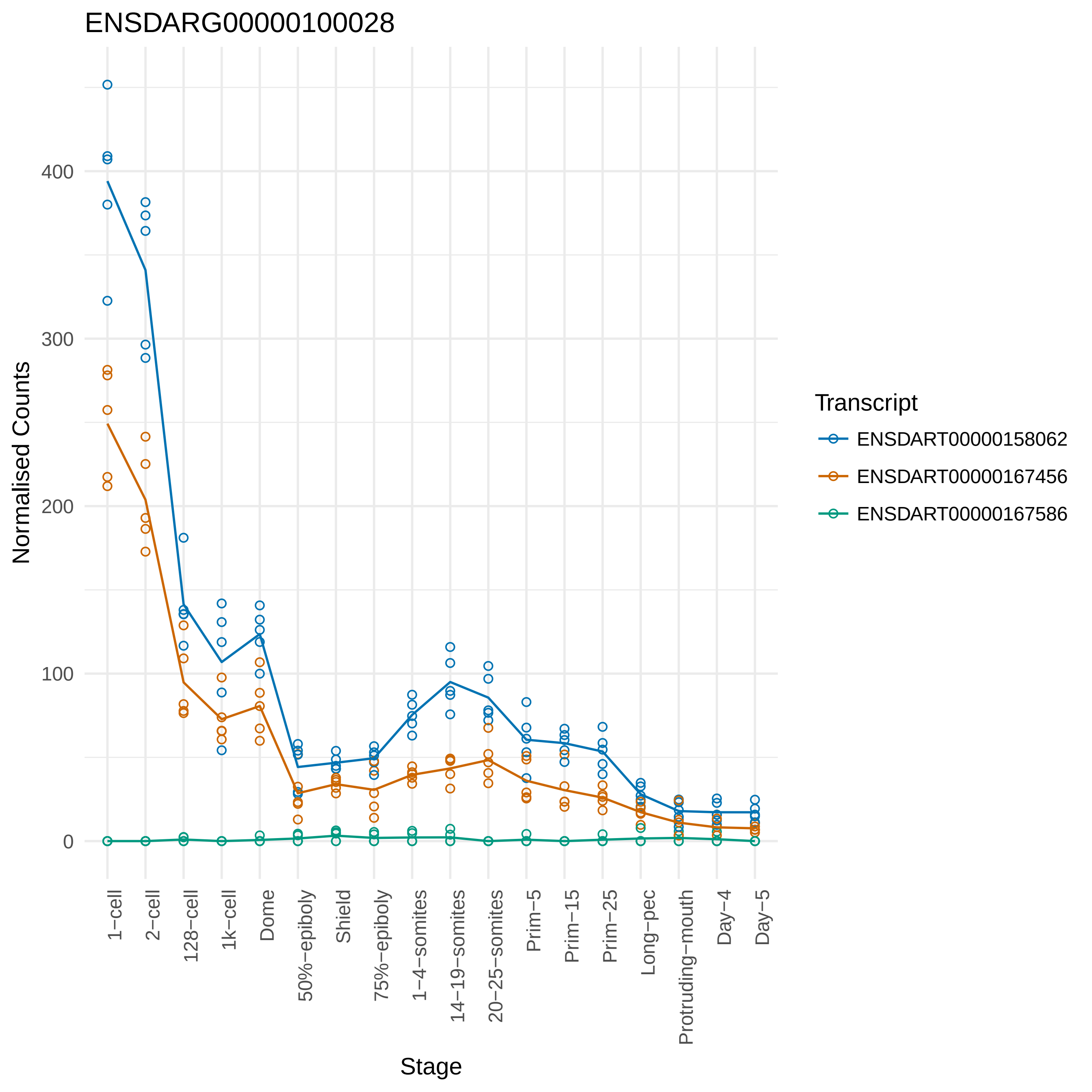

ENSDARG00000100028
Normalised Counts
0
100
200
300
400
Stage
1−cell
2−cell
128−cell
1k−cell
Dome
50%−epiboly
Shield
75%−epiboly
1−4−somites
14−19−somites
20−25−somites
Prim−5
Prim−15
Prim−25
Long−pec
Protruding−mouth
Day−4
Day−5
Transcript
ENSDART00000158062
ENSDART00000167456
ENSDART00000167586

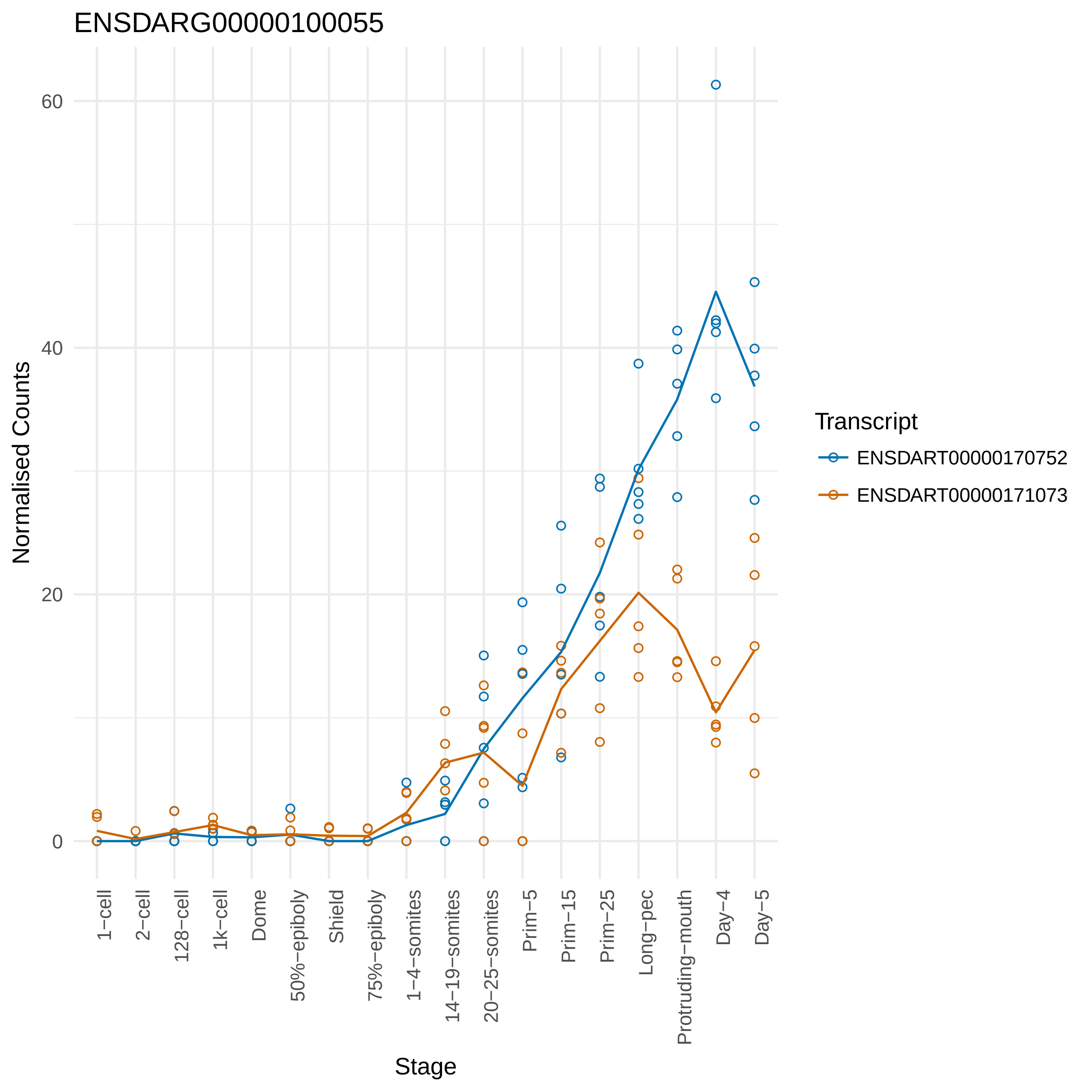

ENSDARG00000100055
Normalised Counts
0
20
40
60
Stage
1−cell
2−cell
128−cell
1k−cell
Dome
50%−epiboly
Shield
75%−epiboly
1−4−somites
14−19−somites
20−25−somites
Prim−5
Prim−15
Prim−25
Long−pec
Protruding−mouth
Day−4
Day−5
Transcript
ENSDART00000170752
ENSDART00000171073

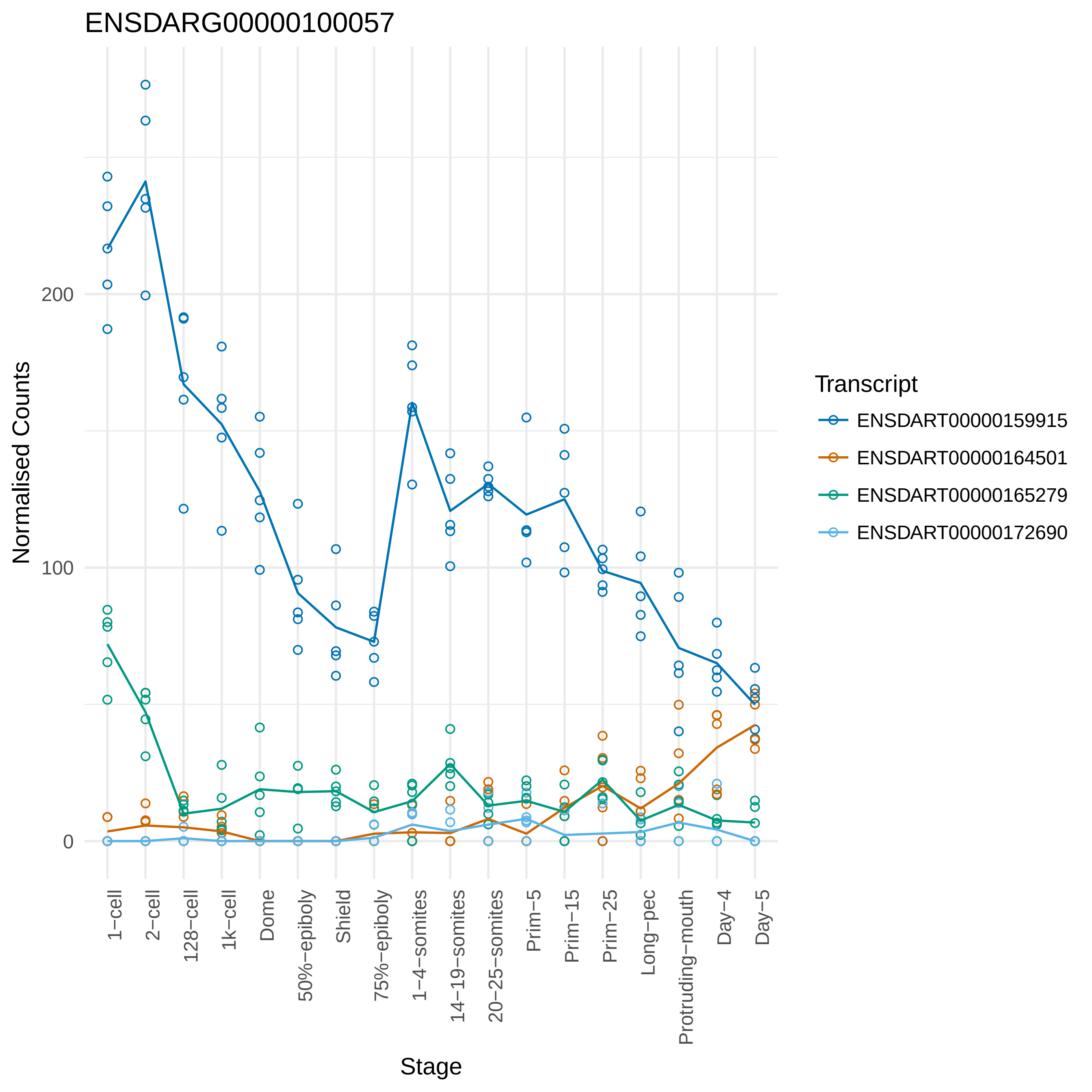
ENSDARG00000100057
Normalised Counts
200
100
0
1−cell
2−cell
128−cell
1k−cell
Dome
50%−epiboly
Shield
75%−epiboly
1−4−somites
14−19−somites
20−25−somites
Prim−5
Prim−15
Prim−25
Long−pec
Protruding−mouth
Day−4
Day−5
Stage
Transcript
ENSDART00000159915
ENSDART00000164501
ENSDART00000165279
ENSDART00000172690

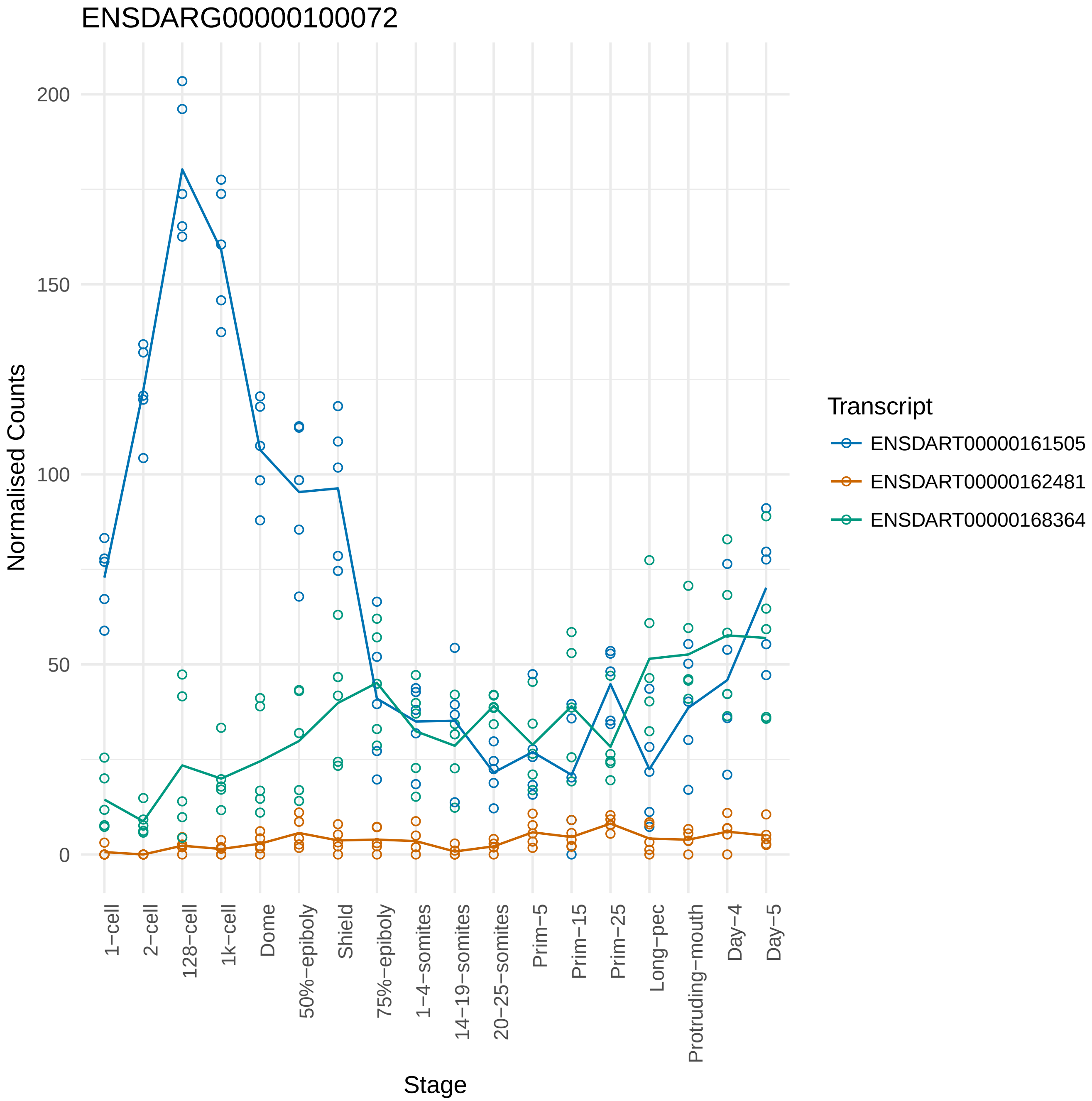

ENSDARG00000100072
Normalised Counts
0
50
100
150
200
1−cell
2−cell
128−cell
1k−cell
Dome
50%−epiboly
Shield
75%−epiboly
1−4−somites
14−19−somites
20−25−somites
Prim−5
Prim−15
Prim−25
Long−pec
Protruding−mouth
Day−4
Day−5
Stage
Transcript
ENSDART00000161505
ENSDART00000162481
ENSDART00000168364

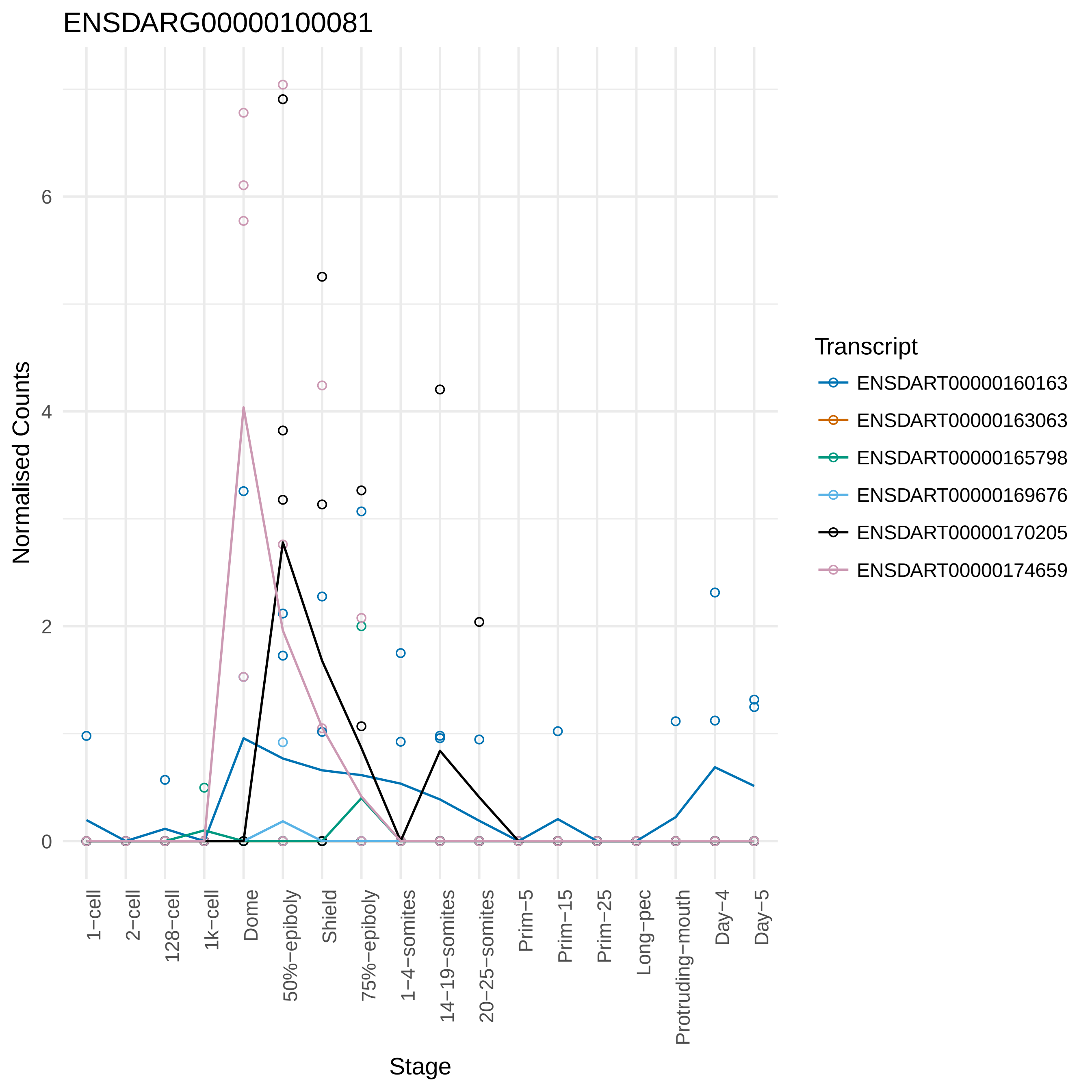
ENSDARG00000100081
Normalised Counts
0
2
4
6
Stage
1−cell
2−cell
128−cell
1k−cell
Dome
50%−epiboly
Shield
75%−epiboly
1−4−somites
14−19−somites
20−25−somites
Prim−5
Prim−15
Prim−25
Long−pec
Protruding−mouth
Day−4
Day−5
Transcript
ENSDART00000160163
ENSDART00000163063
ENSDART00000165798
ENSDART00000169676
ENSDART00000170205
ENSDART00000174659

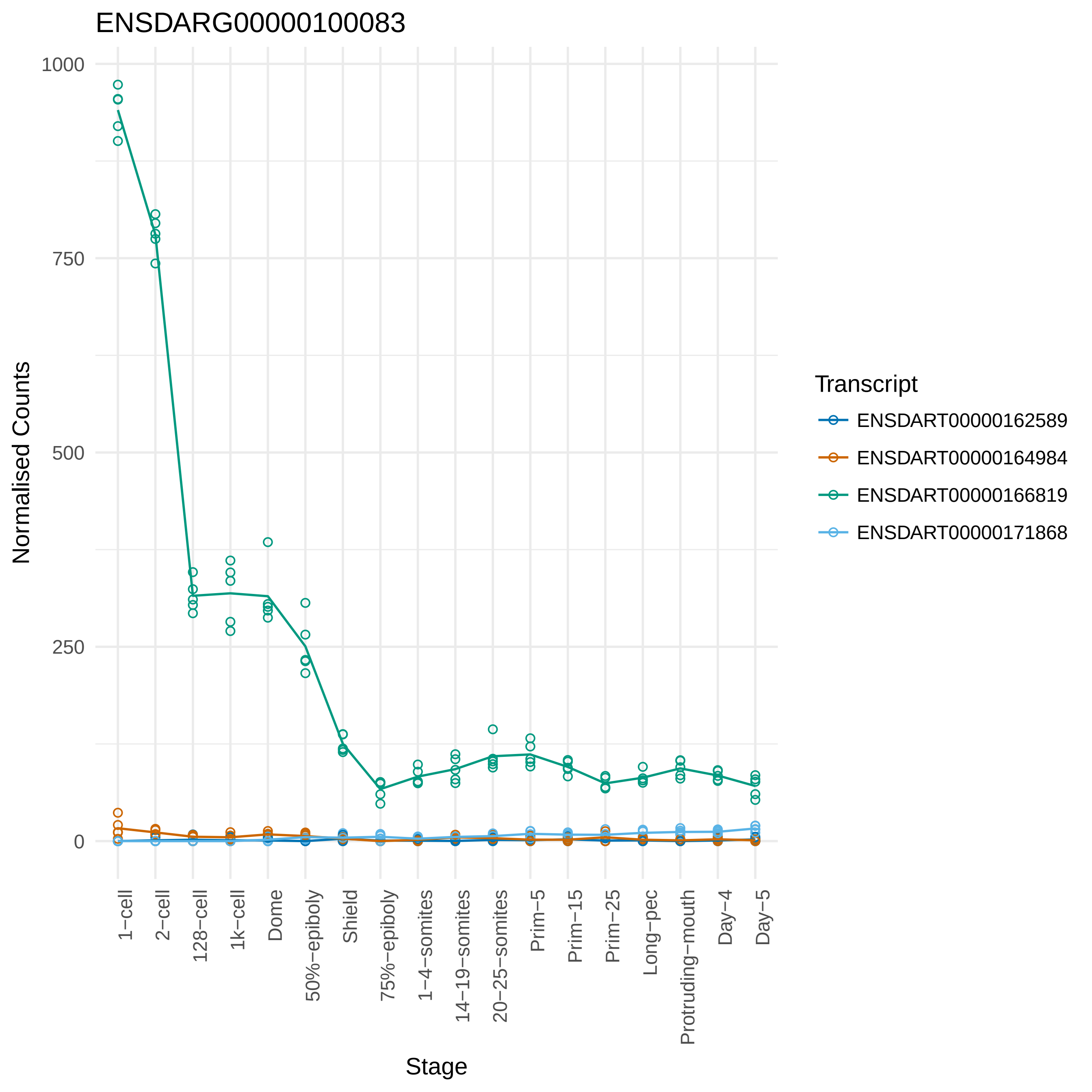
ENSDARG00000100083
Normalised Counts
1000
750
500
250
0
1−cell
2−cell
128−cell
1k−cell
Dome
50%−epiboly
Shield
75%−epiboly
1−4−somites
14−19−somites
20−25−somites
Prim−5
Prim−15
Prim−25
Long−pec
Protruding−mouth
Day−4
Day−5
Stage
Transcript
ENSDART00000162589
ENSDART00000164984
ENSDART00000166819
ENSDART00000171868

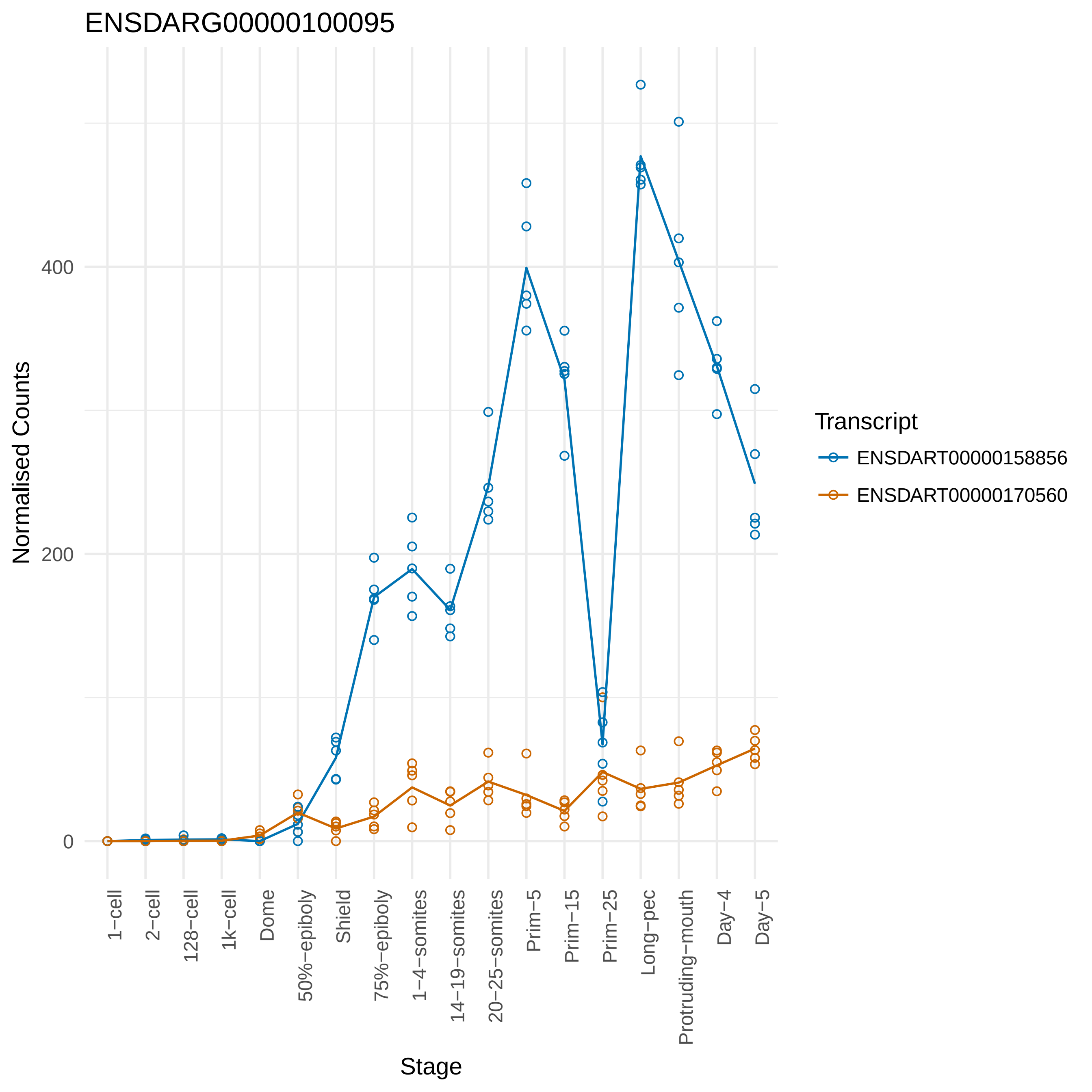

ENSDARG00000100095
Normalised Counts
400
200
0
Stage
1−cell
2−cell
128−cell
1k−cell
Dome
50%−epiboly
Shield
75%−epiboly
1−4−somites
14−19−somites
20−25−somites
Prim−5
Prim−15
Prim−25
Long−pec
Protruding−mouth
Day−4
Day−5
Transcript
ENSDART00000158856
ENSDART00000170560

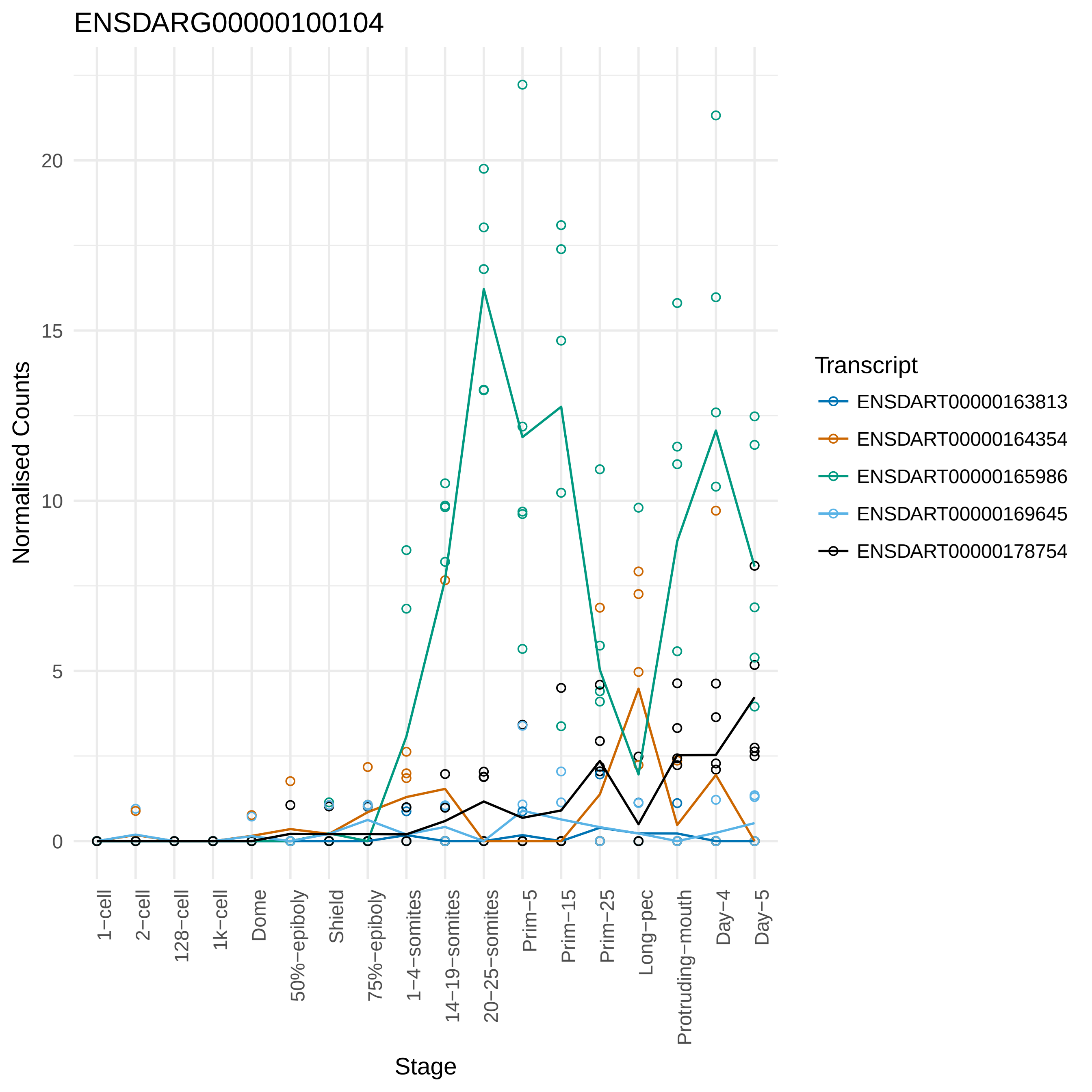

ENSDARG00000100104
Normalised Counts
0
5
10
15
20
Stage
1−cell
2−cell
128−cell
1k−cell
Dome
50%−epiboly
Shield
75%−epiboly
1−4−somites
14−19−somites
20−25−somites
Prim−5
Prim−15
Prim−25
Long−pec
Protruding−mouth
Day−4
Day−5
Transcript
ENSDART00000163813
ENSDART00000164354
ENSDART00000165986
ENSDART00000169645
ENSDART00000178754

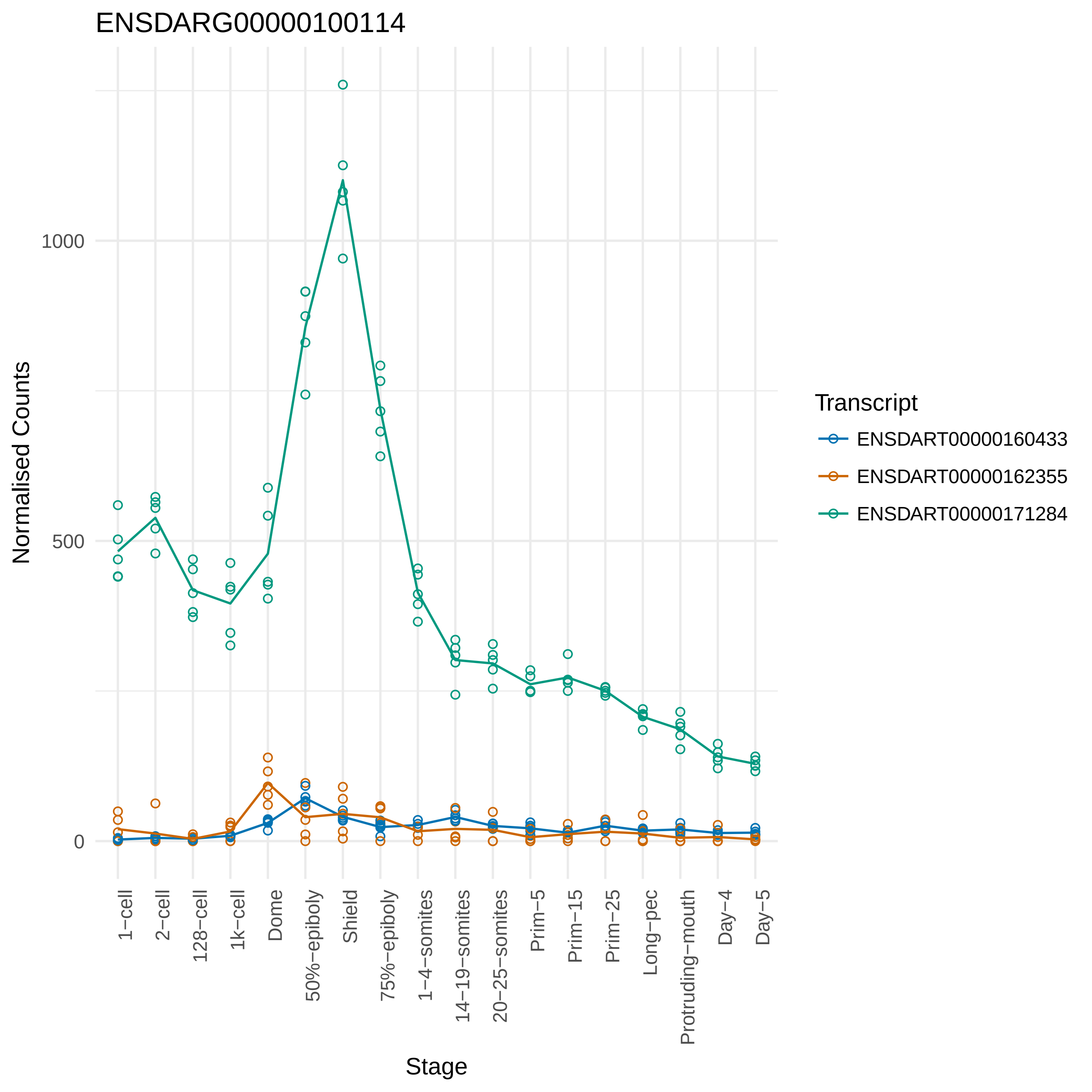

ENSDARG00000100114
Normalised Counts
0
500
1000
Stage
1−cell
2−cell
128−cell
1k−cell
Dome
50%−epiboly
Shield
75%−epiboly
1−4−somites
14−19−somites
20−25−somites
Prim−5
Prim−15
Prim−25
Long−pec
Protruding−mouth
Day−4
Day−5
Transcript
ENSDART00000160433
ENSDART00000162355
ENSDART00000171284

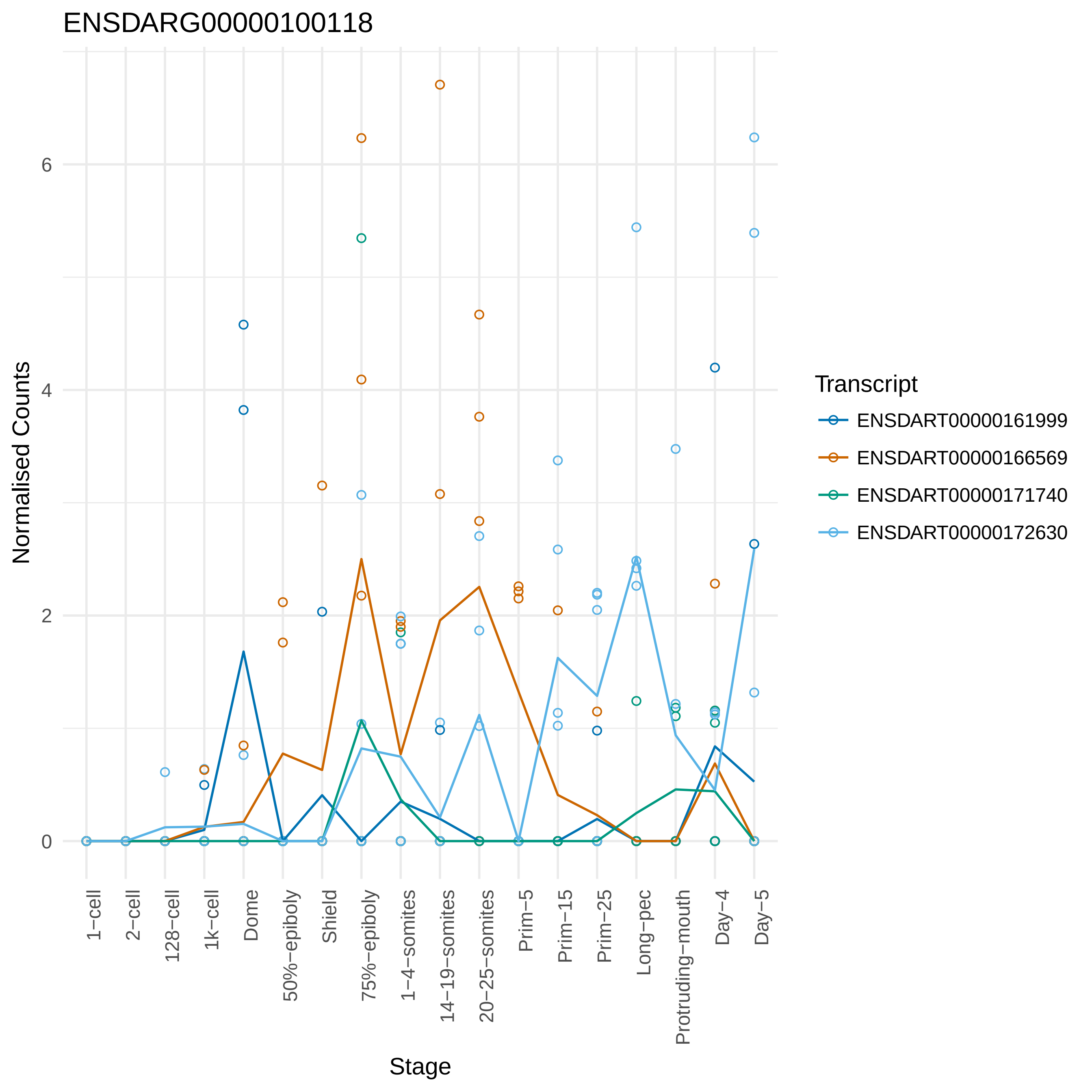
ENSDARG00000100118
Normalised Counts
0
2
4
6
Stage
1−cell
2−cell
128−cell
1k−cell
Dome
50%−epiboly
Shield
75%−epiboly
1−4−somites
14−19−somites
20−25−somites
Prim−5
Prim−15
Prim−25
Long−pec
Protruding−mouth
Day−4
Day−5
Transcript
ENSDART00000161999
ENSDART00000166569
ENSDART00000171740
ENSDART00000172630

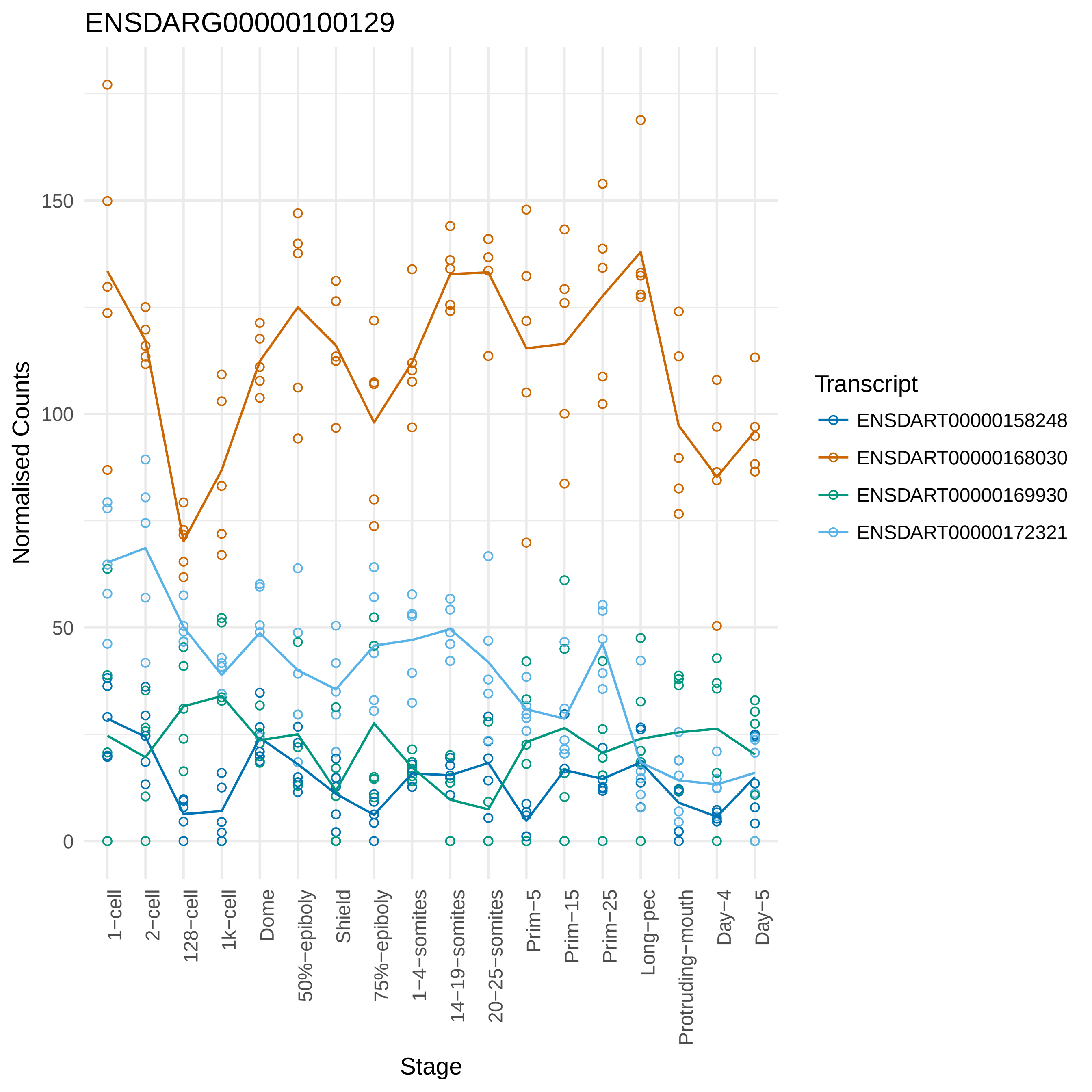

ENSDARG00000100129
Normalised Counts
150
100
50
0
1−cell
2−cell
128−cell
1k−cell
Dome
50%−epiboly
Shield
75%−epiboly
1−4−somites
14−19−somites
20−25−somites
Prim−5
Prim−15
Prim−25
Long−pec
Protruding−mouth
Day−4
Day−5
Stage
Transcript
ENSDART00000158248
ENSDART00000168030
ENSDART00000169930
ENSDART00000172321

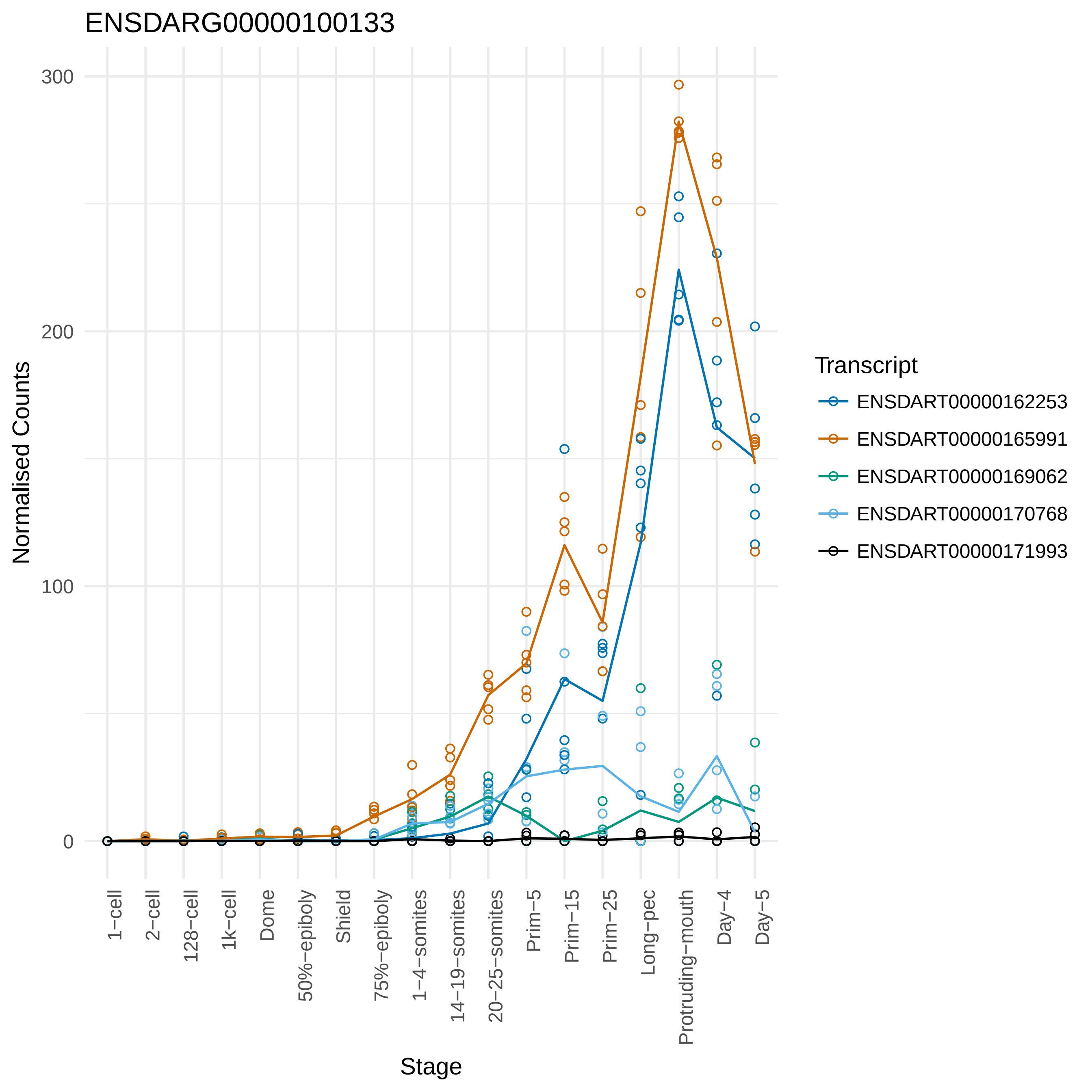
ENSDARG00000100133
Normalised Counts
300
200
100
0
1−cell
2−cell
128−cell
1k−cell
Dome
50%−epiboly
Shield
75%−epiboly
1−4−somites
14−19−somites
20−25−somites
Prim−5
Prim−15
Prim−25
Long−pec
Protruding−mouth
Day−4
Day−5
Stage
Transcript
ENSDART00000162253
ENSDART00000165991
ENSDART00000169062
ENSDART00000170768
ENSDART00000171993

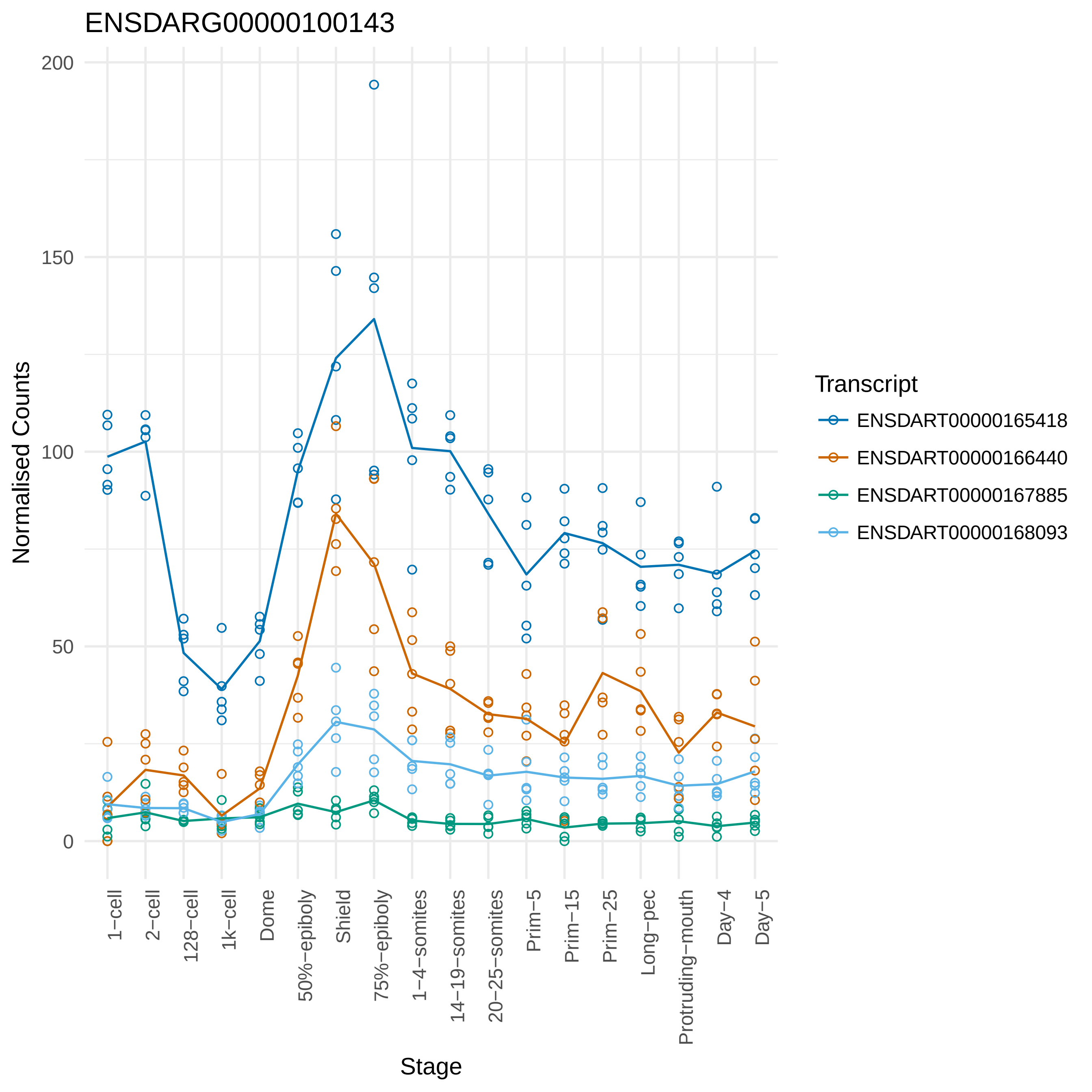
ENSDARG00000100143
Normalised Counts
0
50
100
150
200
1−cell
2−cell
128−cell
1k−cell
Dome
50%−epiboly
Shield
75%−epiboly
1−4−somites
14−19−somites
20−25−somites
Prim−5
Prim−15
Prim−25
Long−pec
Protruding−mouth
Day−4
Day−5
Stage
Transcript
ENSDART00000165418
ENSDART00000166440
ENSDART00000167885
ENSDART00000168093

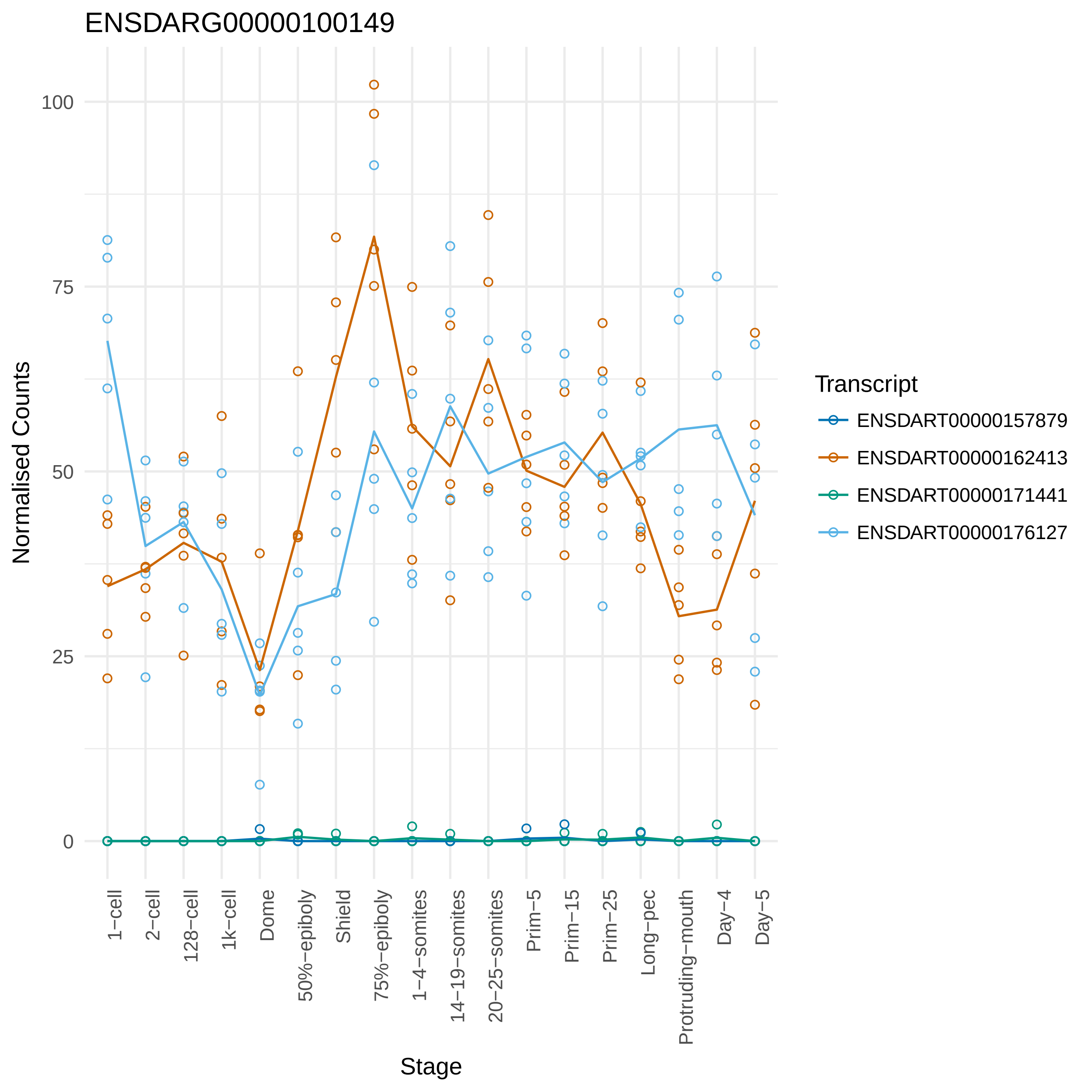

ENSDARG00000100149
Normalised Counts
100
75
50
25
0
1−cell
2−cell
128−cell
1k−cell
Dome
50%−epiboly
Shield
75%−epiboly
1−4−somites
14−19−somites
20−25−somites
Prim−5
Prim−15
Prim−25
Long−pec
Protruding−mouth
Day−4
Day−5
Stage
Transcript
ENSDART00000157879
ENSDART00000162413
ENSDART00000171441
ENSDART00000176127

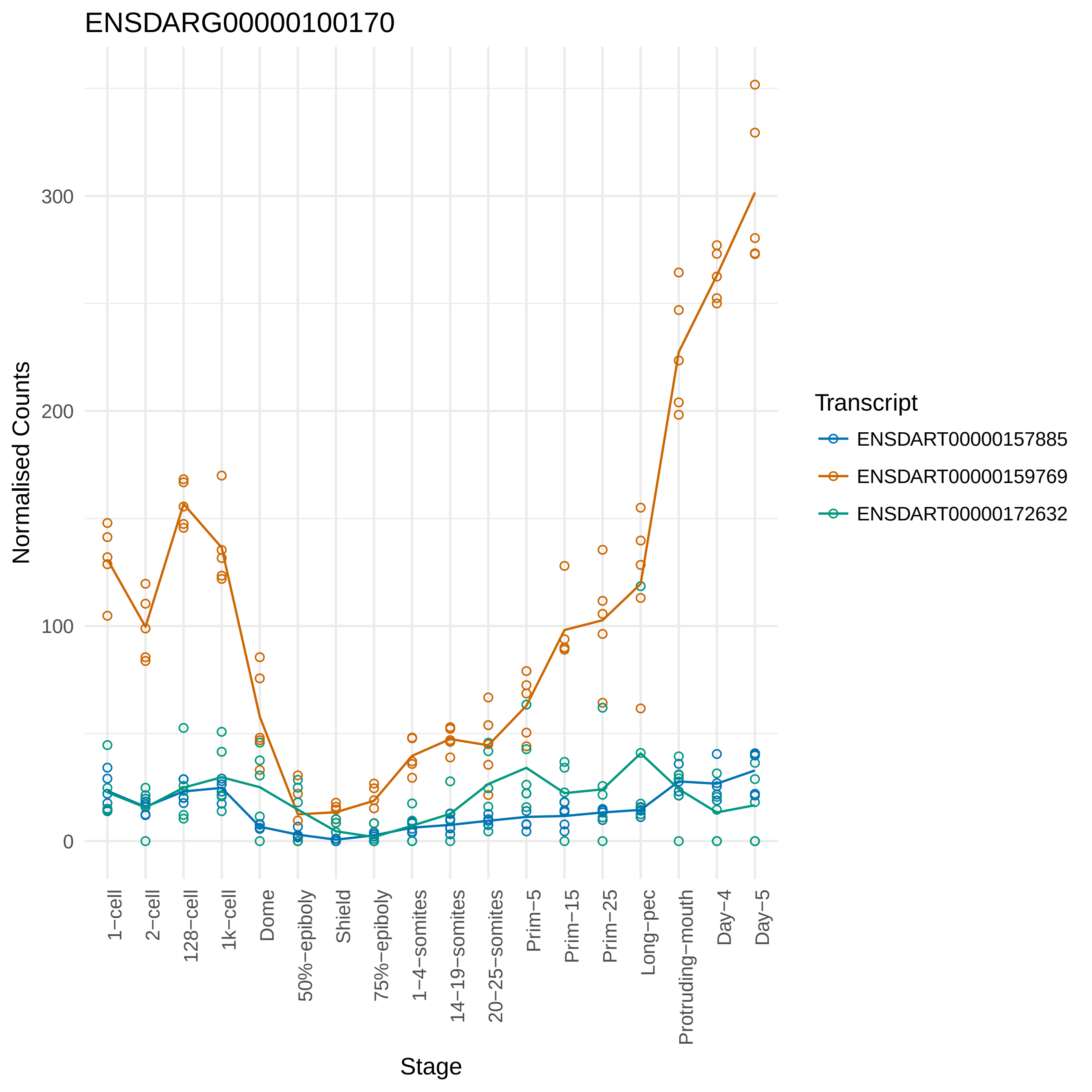

ENSDARG00000100170
Normalised Counts
300
200
100
0
1−cell
2−cell
128−cell
1k−cell
Dome
50%−epiboly
Shield
75%−epiboly
1−4−somites
14−19−somites
20−25−somites
Prim−5
Prim−15
Prim−25
Long−pec
Protruding−mouth
Day−4
Day−5
Stage
Transcript
ENSDART00000157885
ENSDART00000159769
ENSDART00000172632

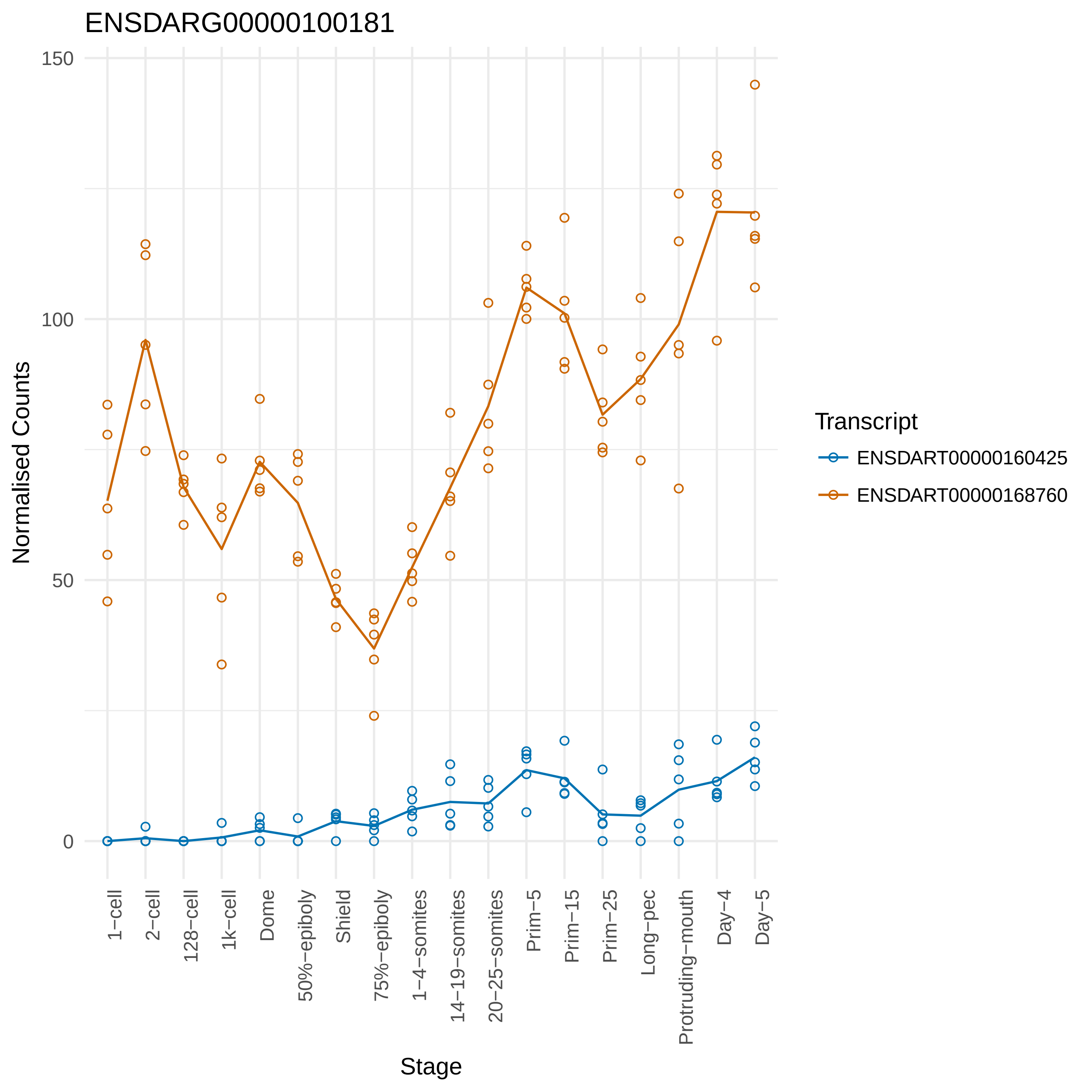
ENSDARG00000100181
Normalised Counts
150
100
50
0
1−cell
2−cell
128−cell
1k−cell
Dome
50%−epiboly
Shield
75%−epiboly
1−4−somites
14−19−somites
20−25−somites
Prim−5
Prim−15
Prim−25
Long−pec
Protruding−mouth
Day−4
Day−5
Stage
Transcript
ENSDART00000160425
ENSDART00000168760

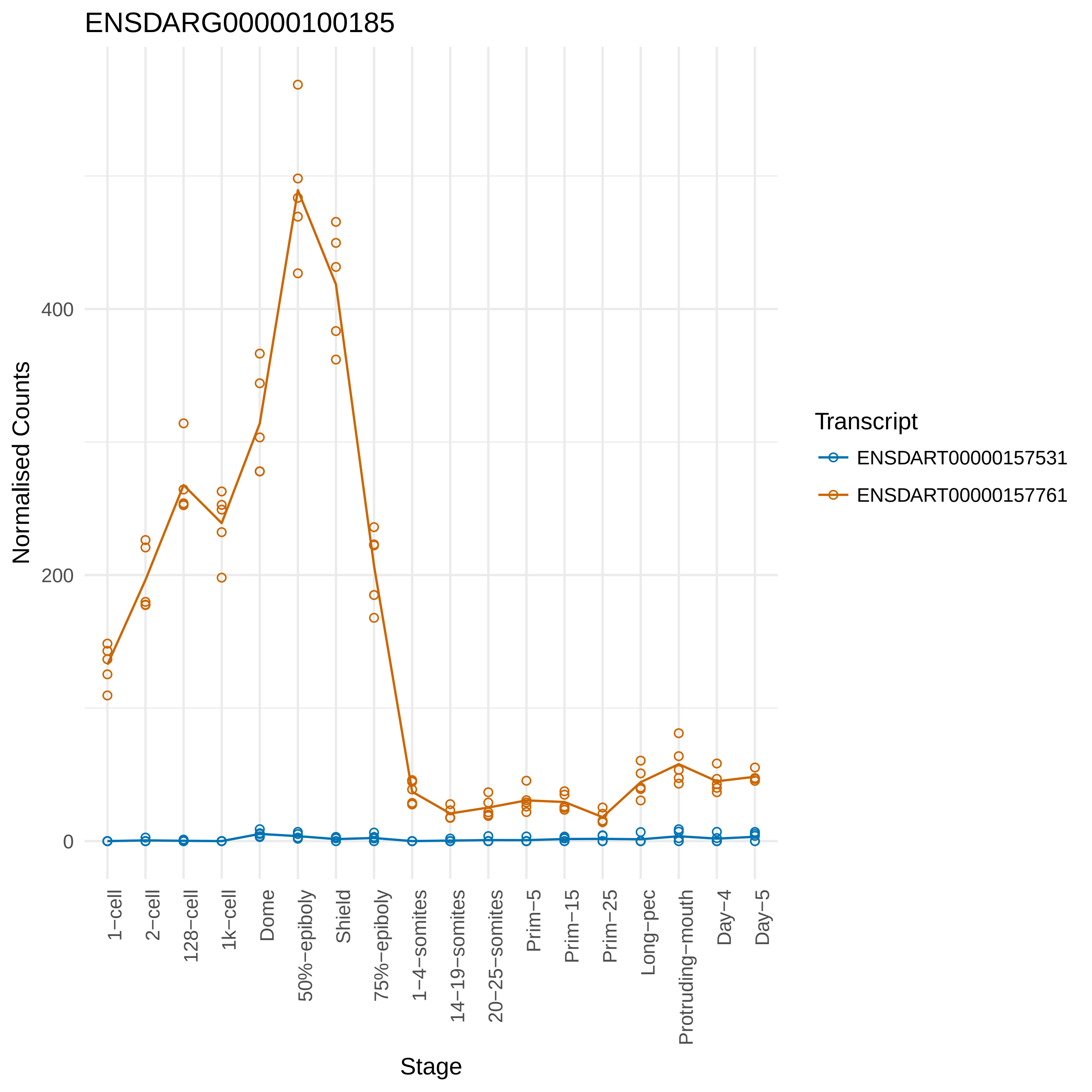
ENSDARG00000100185
Normalised Counts
400
200
0
1−cell
2−cell
128−cell
1k−cell
Dome
50%−epiboly
Shield
75%−epiboly
1−4−somites
14−19−somites
20−25−somites
Prim−5
Prim−15
Prim−25
Long−pec
Protruding−mouth
Day−4
Day−5
Stage
Transcript
ENSDART00000157531
ENSDART00000157761

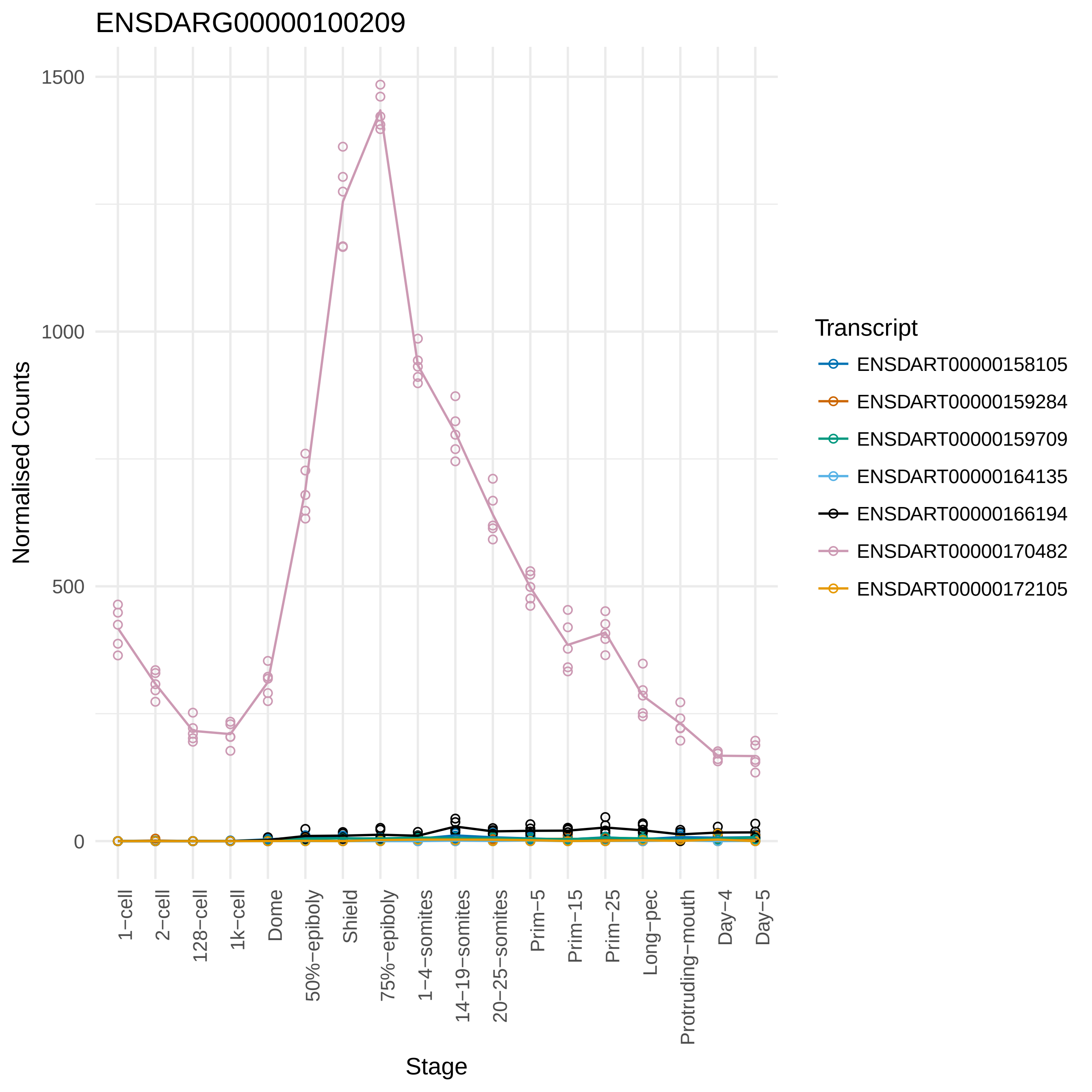
ENSDARG00000100209
Normalised Counts
1500
1000
500
0
1−cell
2−cell
128−cell
1k−cell
Dome
50%−epiboly
Shield
75%−epiboly
1−4−somites
14−19−somites
20−25−somites
Prim−5
Prim−15
Prim−25
Long−pec
Protruding−mouth
Day−4
Day−5
Stage
Transcript
ENSDART00000158105
ENSDART00000159284
ENSDART00000159709
ENSDART00000164135
ENSDART00000166194
ENSDART00000170482
ENSDART00000172105

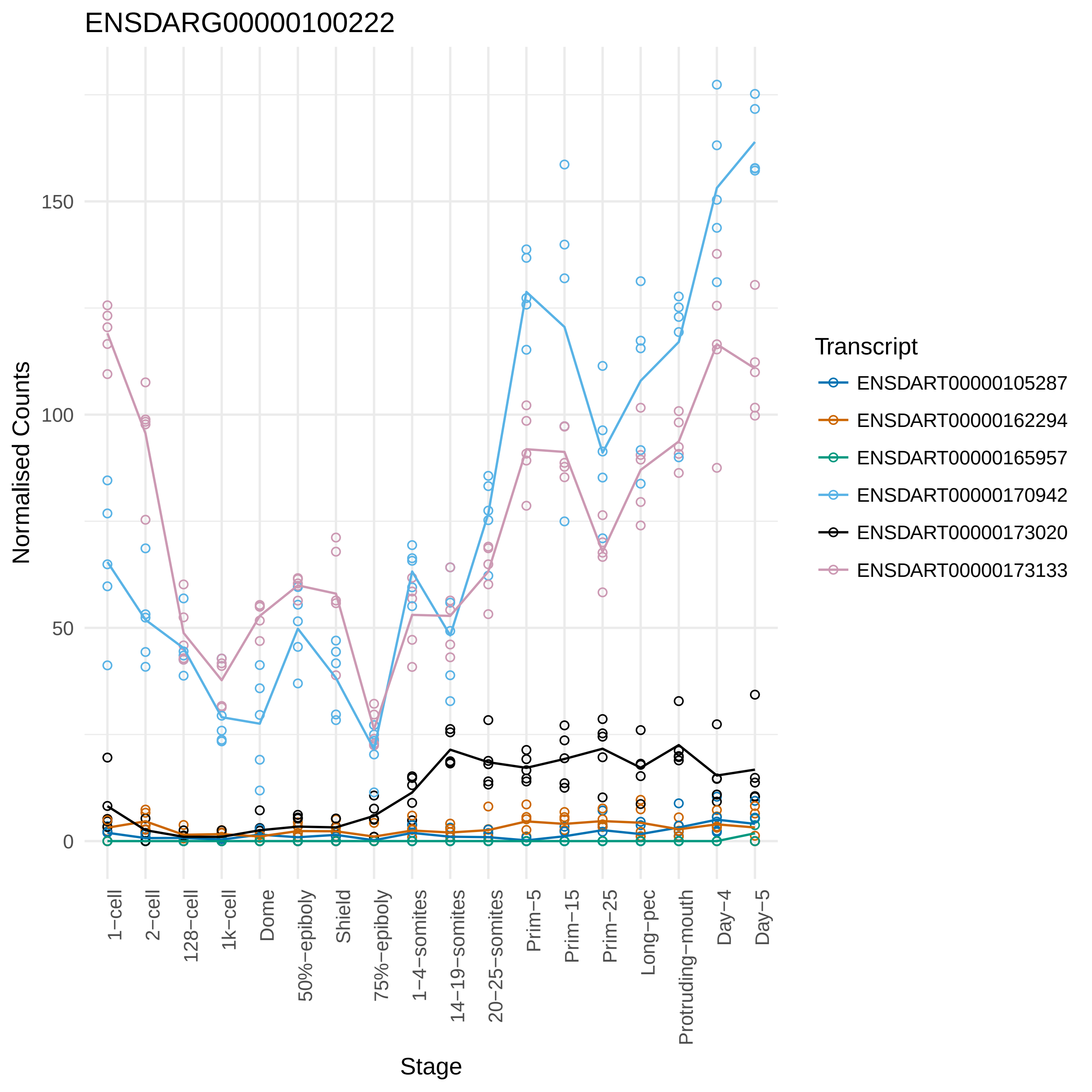

ENSDARG00000100222
Normalised Counts
0
50
100
150
Stage
1−cell
2−cell
128−cell
1k−cell
Dome
50%−epiboly
Shield
75%−epiboly
1−4−somites
14−19−somites
20−25−somites
Prim−5
Prim−15
Prim−25
Long−pec
Protruding−mouth
Day−4
Day−5
Transcript
ENSDART00000105287
ENSDART00000162294
ENSDART00000165957
ENSDART00000170942
ENSDART00000173020
ENSDART00000173133

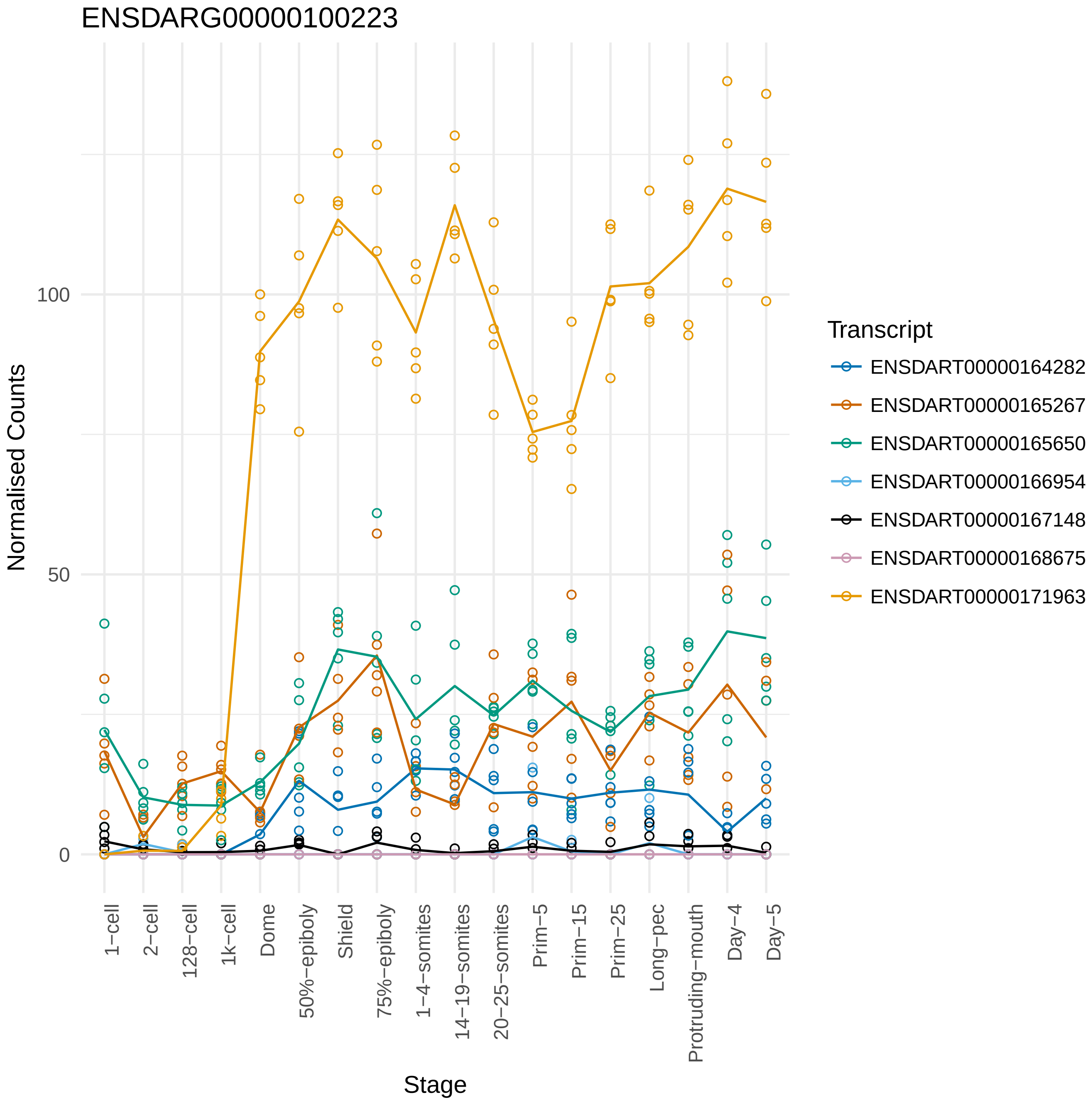

ENSDARG00000100223
Normalised Counts
100
50
0
1−cell
2−cell
128−cell
1k−cell
Dome
50%−epiboly
Shield
75%−epiboly
1−4−somites
14−19−somites
20−25−somites
Prim−5
Prim−15
Prim−25
Long−pec
Protruding−mouth
Day−4
Day−5
Stage
Transcript
ENSDART00000164282
ENSDART00000165267
ENSDART00000165650
ENSDART00000166954
ENSDART00000167148
ENSDART00000168675
ENSDART00000171963

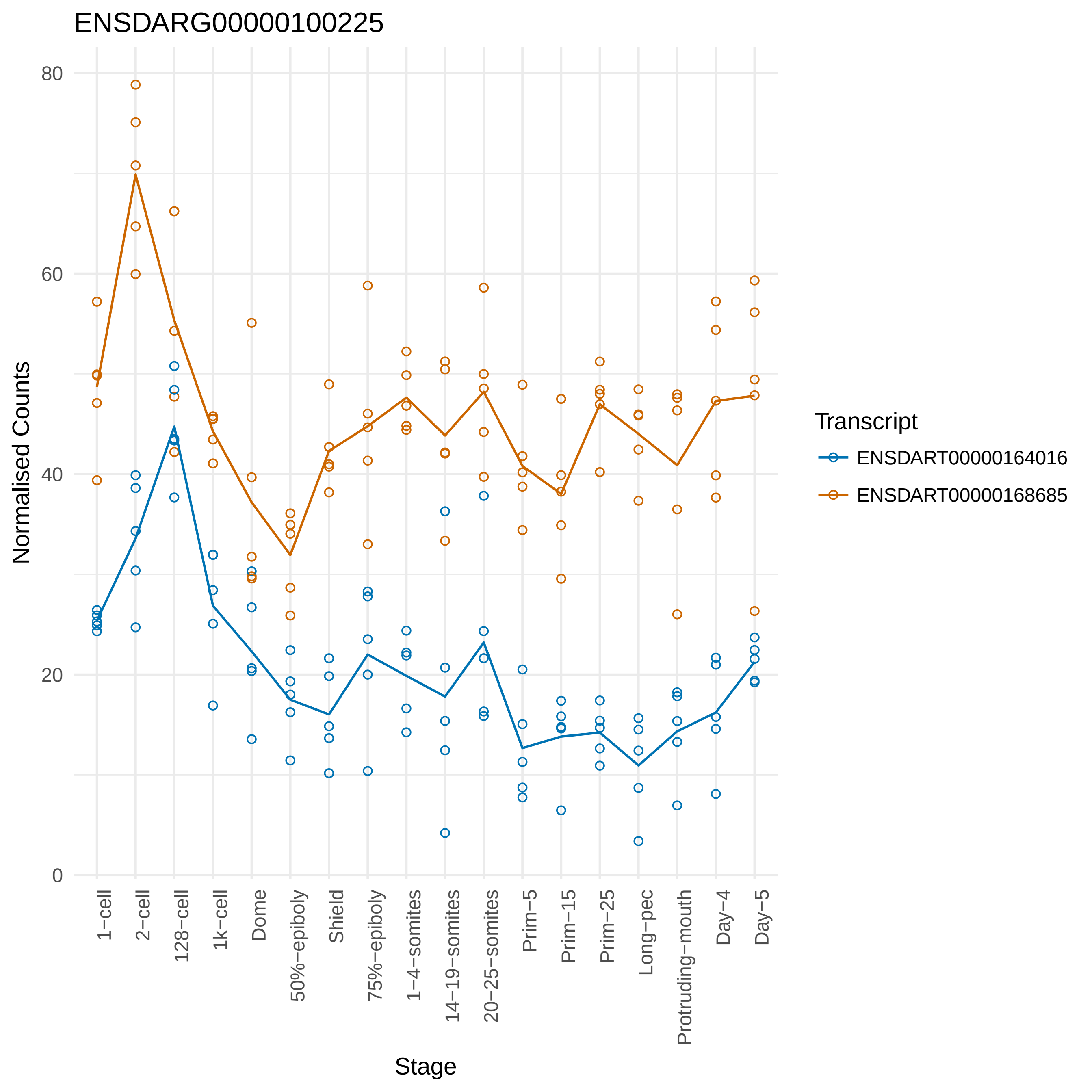

ENSDARG00000100225
Normalised Counts
80
60
40
20
0
1−cell
2−cell
128−cell
1k−cell
Dome
50%−epiboly
Shield
75%−epiboly
1−4−somites
14−19−somites
20−25−somites
Prim−5
Prim−15
Prim−25
Long−pec
Protruding−mouth
Day−4
Day−5
Stage
Transcript
ENSDART00000164016
ENSDART00000168685

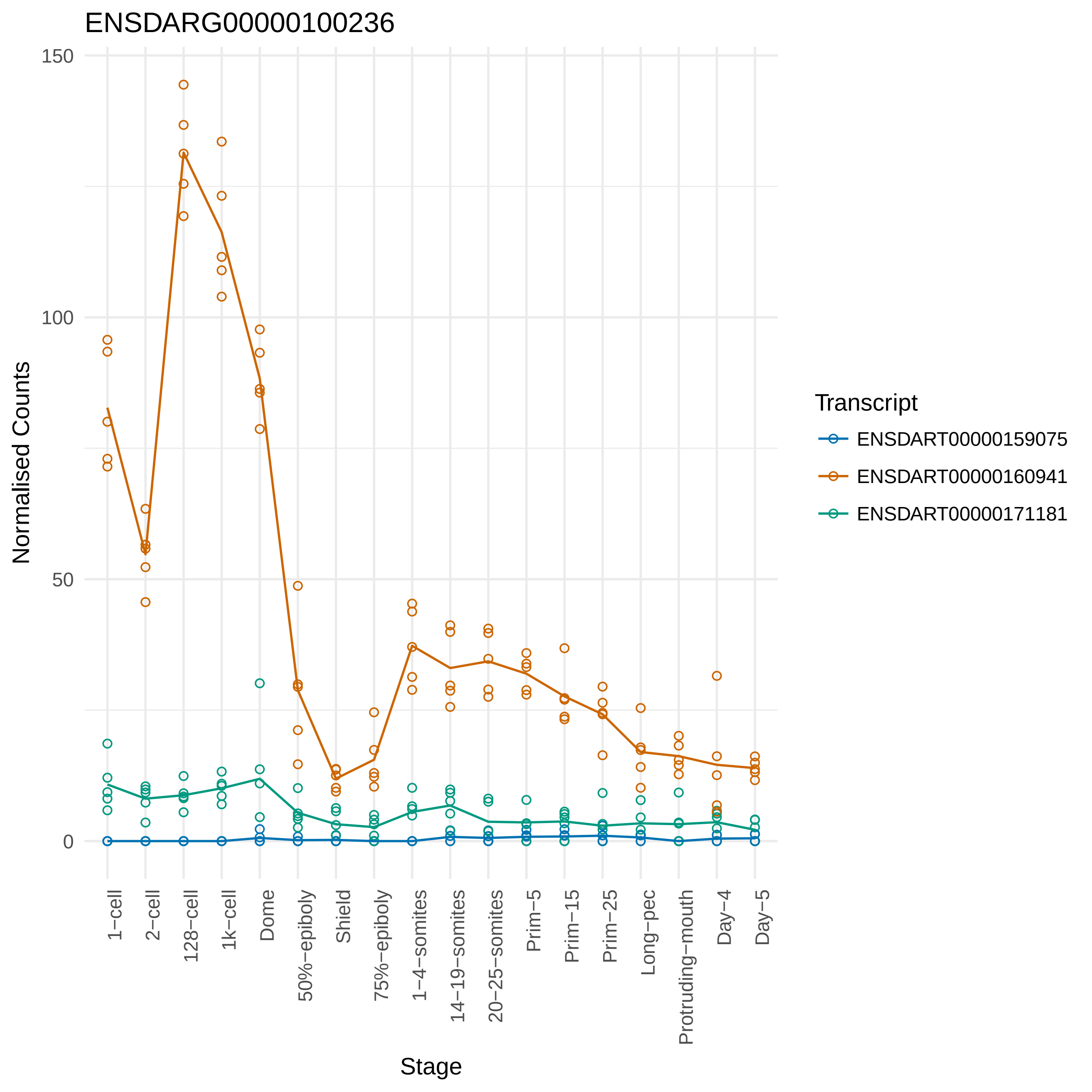

ENSDARG00000100236
Normalised Counts
150
100
50
0
1−cell
2−cell
128−cell
1k−cell
Dome
50%−epiboly
Shield
75%−epiboly
1−4−somites
14−19−somites
20−25−somites
Prim−5
Prim−15
Prim−25
Long−pec
Protruding−mouth
Day−4
Day−5
Stage
Transcript
ENSDART00000159075
ENSDART00000160941
ENSDART00000171181

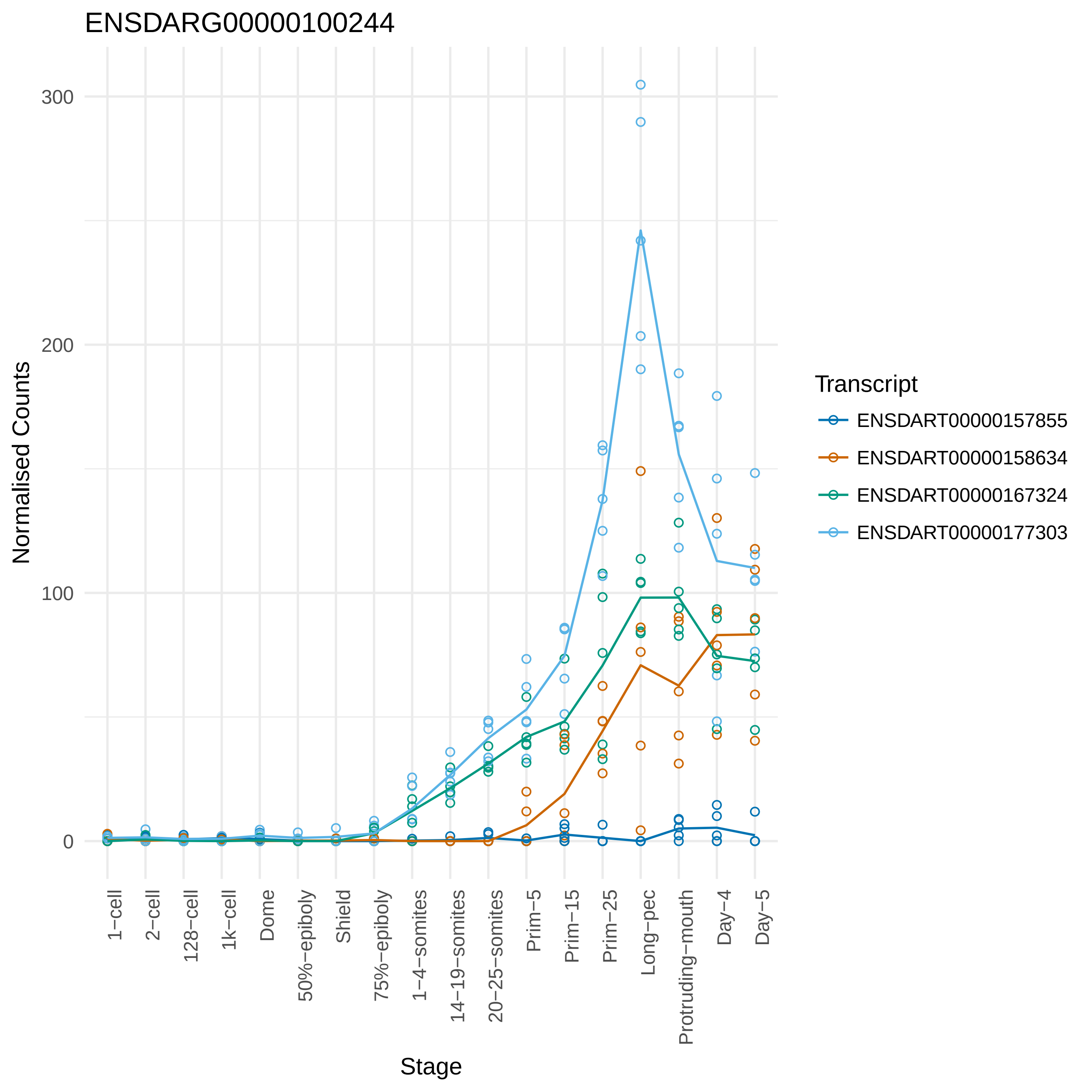

ENSDARG00000100244
Normalised Counts
0
100
200
300
1−cell
2−cell
128−cell
1k−cell
Dome
50%−epiboly
Shield
75%−epiboly
1−4−somites
14−19−somites
20−25−somites
Prim−5
Prim−15
Prim−25
Long−pec
Protruding−mouth
Day−4
Day−5
Stage
Transcript
ENSDART00000157855
ENSDART00000158634
ENSDART00000167324
ENSDART00000177303

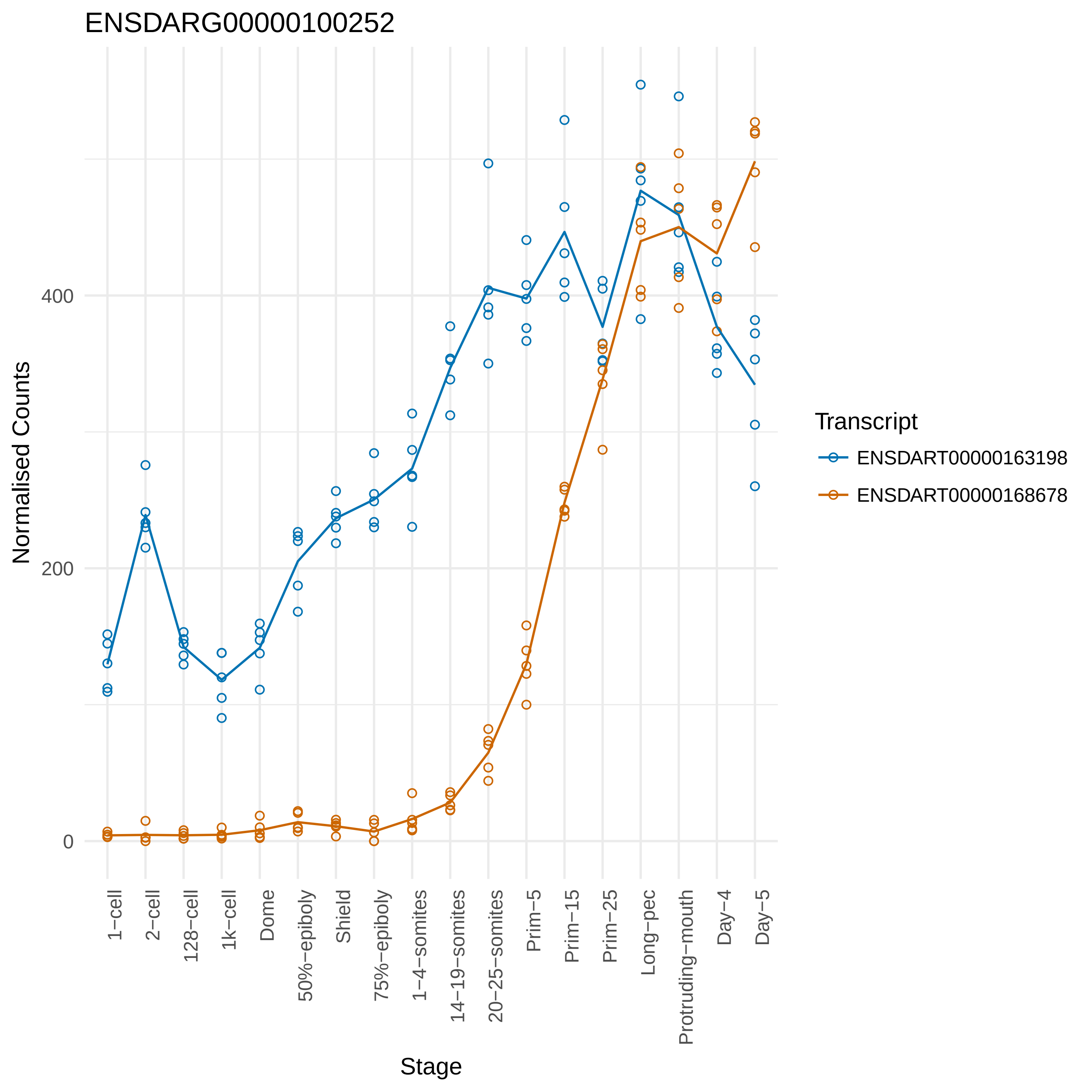
ENSDARG00000100252
Normalised Counts
0
200
400
1−cell
2−cell
128−cell
1k−cell
Dome
50%−epiboly
Shield
75%−epiboly
1−4−somites
14−19−somites
20−25−somites
Prim−5
Prim−15
Prim−25
Long−pec
Protruding−mouth
Day−4
Day−5
Stage
Transcript
ENSDART00000163198
ENSDART00000168678

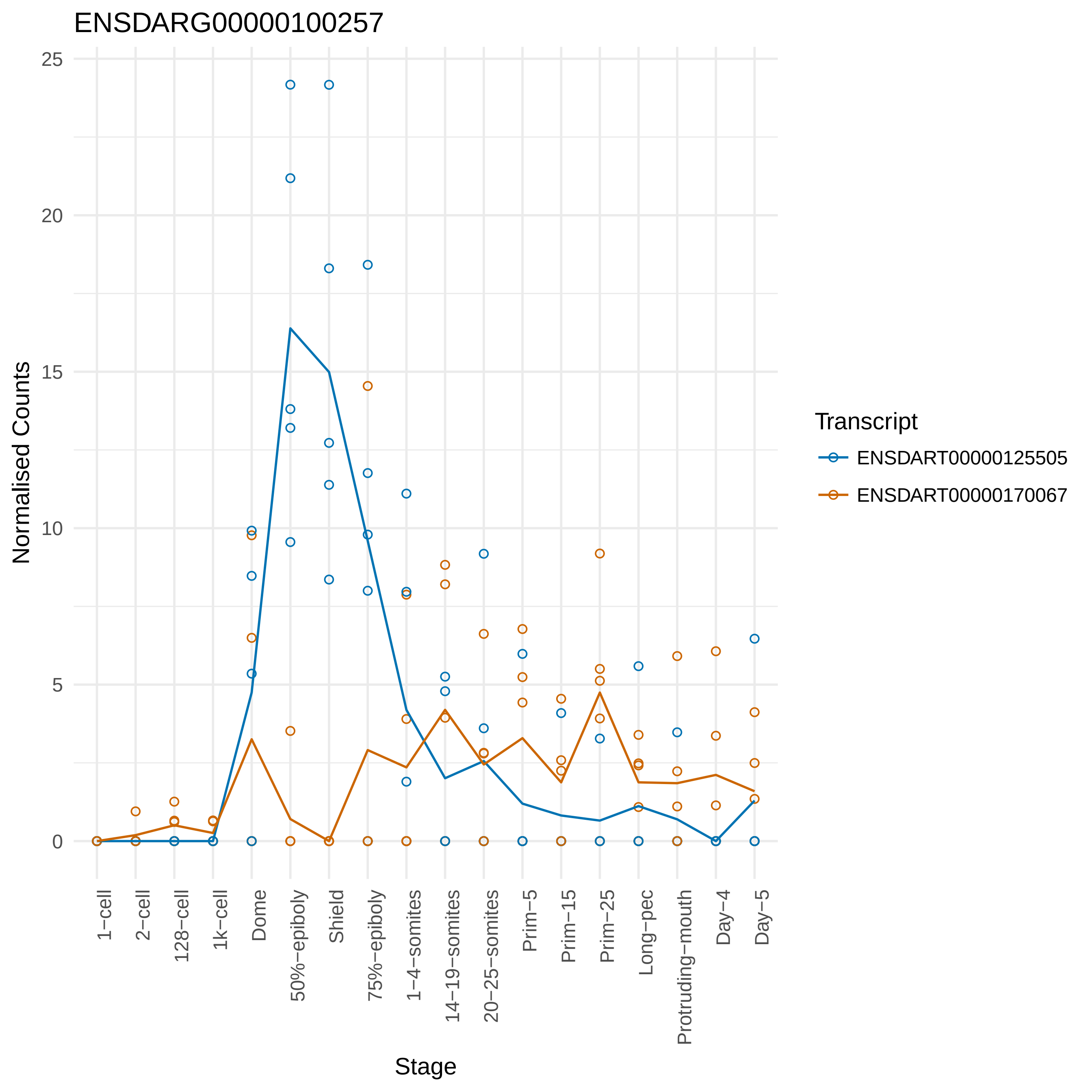

ENSDARG00000100257
Normalised Counts
25
20
15
10
5
0
1−cell
2−cell
128−cell
1k−cell
Dome
50%−epiboly
Shield
75%−epiboly
1−4−somites
14−19−somites
20−25−somites
Prim−5
Prim−15
Prim−25
Long−pec
Protruding−mouth
Day−4
Day−5
Stage
Transcript
ENSDART00000125505
ENSDART00000170067

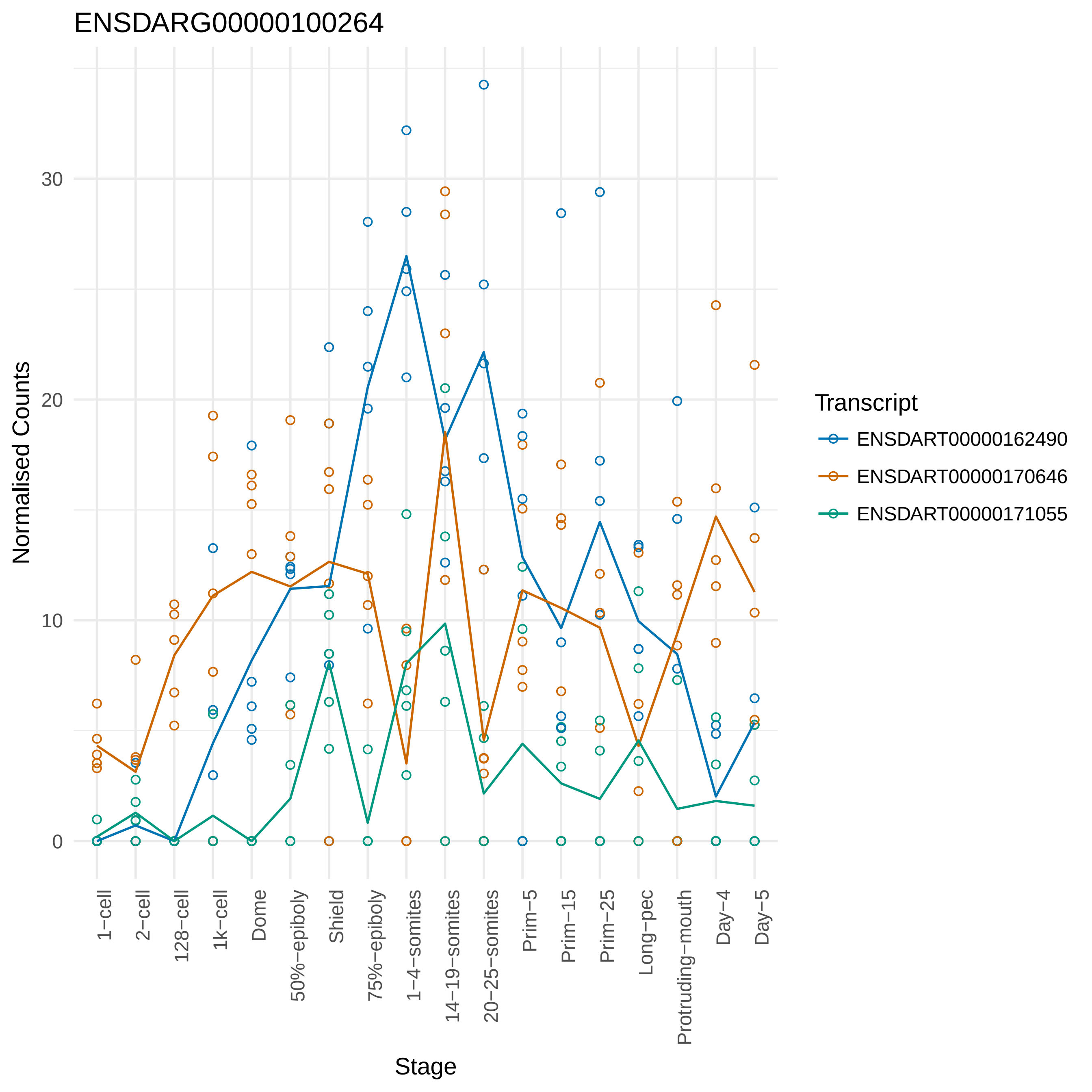

ENSDARG00000100264
Normalised Counts
30
20
10
0
1−cell
2−cell
128−cell
1k−cell
Dome
50%−epiboly
Shield
75%−epiboly
1−4−somites
14−19−somites
20−25−somites
Prim−5
Prim−15
Prim−25
Long−pec
Protruding−mouth
Day−4
Day−5
Stage
Transcript
ENSDART00000162490
ENSDART00000170646
ENSDART00000171055

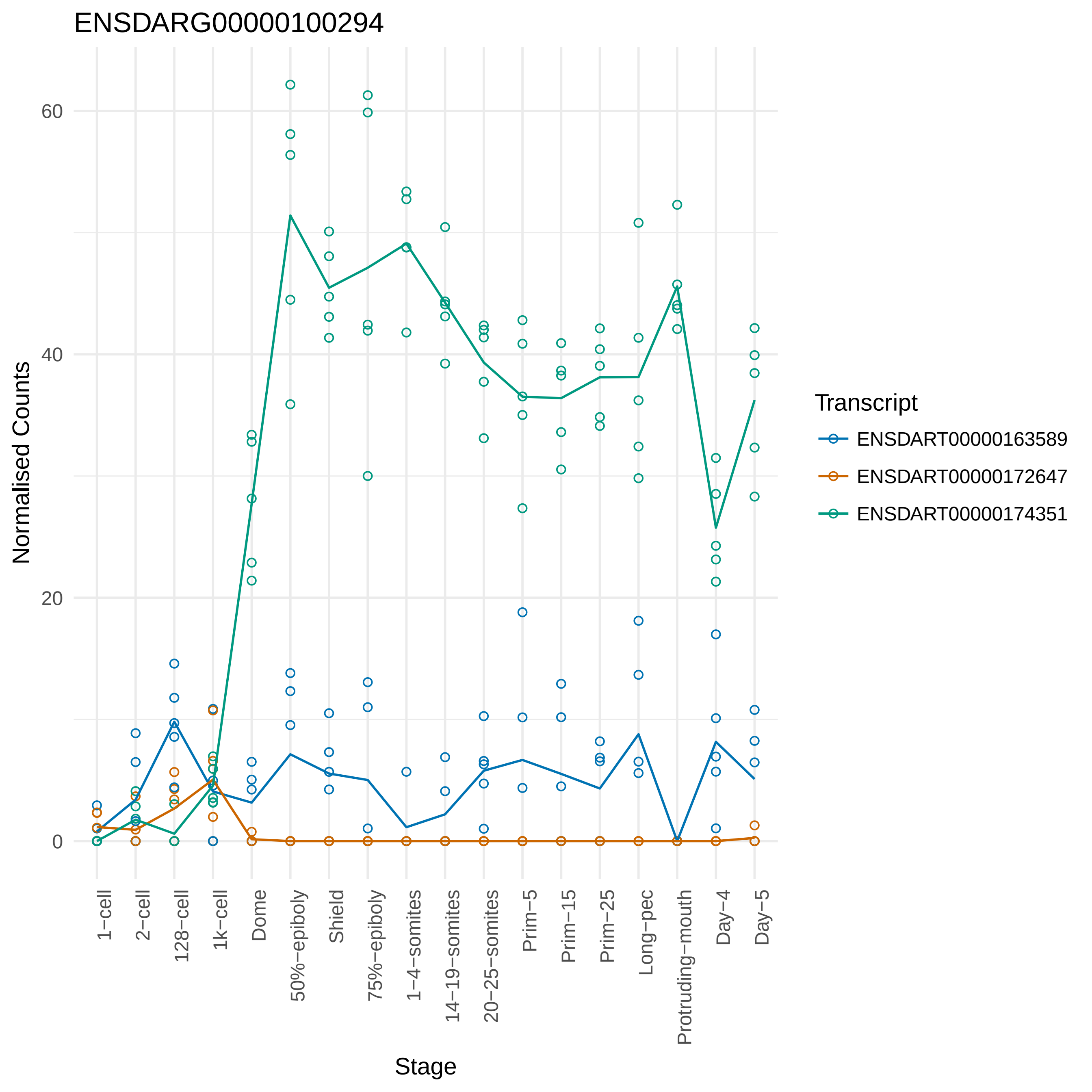

ENSDARG00000100294
Normalised Counts
60
40
20
0
1−cell
2−cell
128−cell
1k−cell
Dome
50%−epiboly
Shield
75%−epiboly
1−4−somites
14−19−somites
20−25−somites
Prim−5
Prim−15
Prim−25
Long−pec
Protruding−mouth
Day−4
Day−5
Stage
Transcript
ENSDART00000163589
ENSDART00000172647
ENSDART00000174351

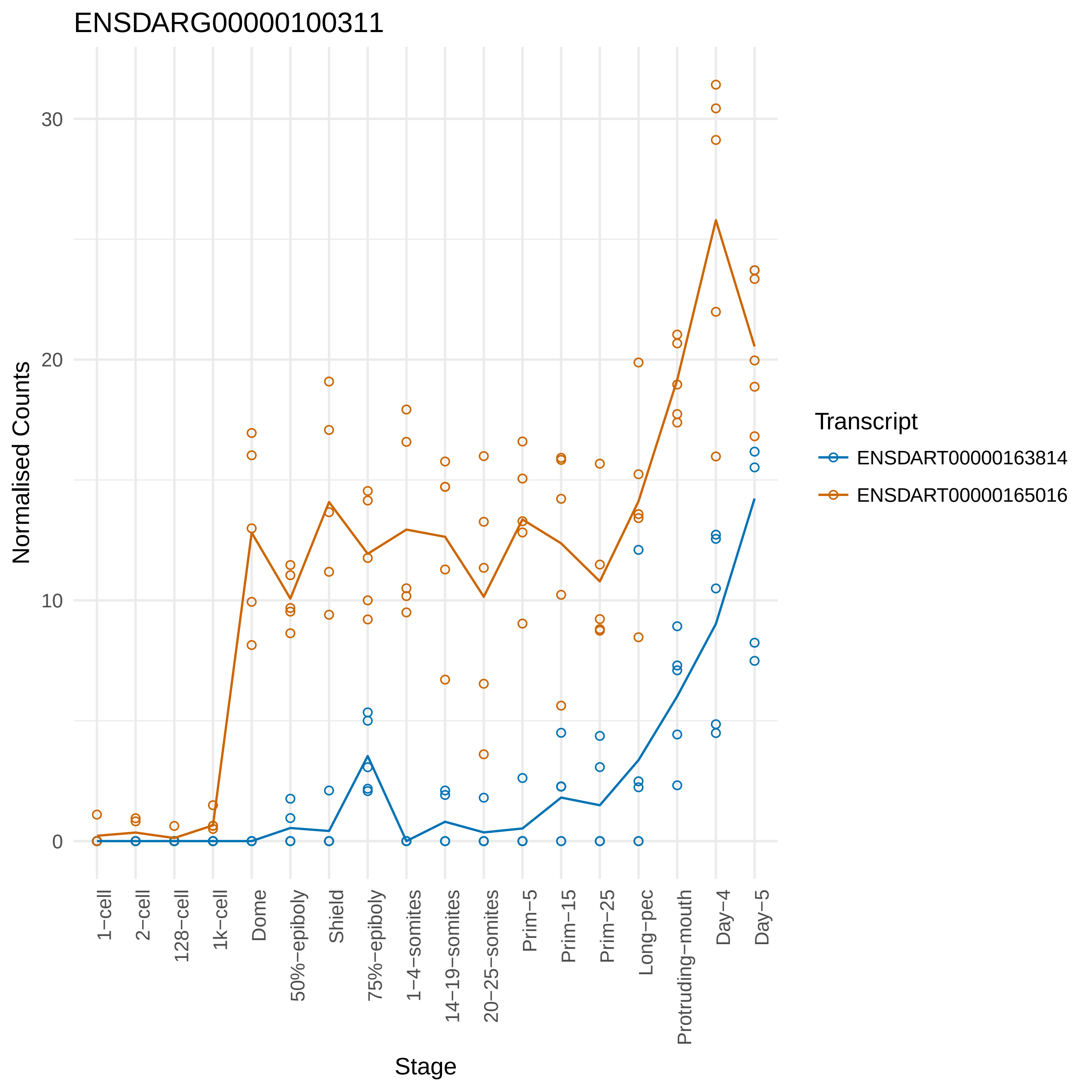

ENSDARG00000100311
Normalised Counts
0
10
20
30
Stage
1−cell
2−cell
128−cell
1k−cell
Dome
50%−epiboly
Shield
75%−epiboly
1−4−somites
14−19−somites
20−25−somites
Prim−5
Prim−15
Prim−25
Long−pec
Protruding−mouth
Day−4
Day−5
Transcript
ENSDART00000163814
ENSDART00000165016

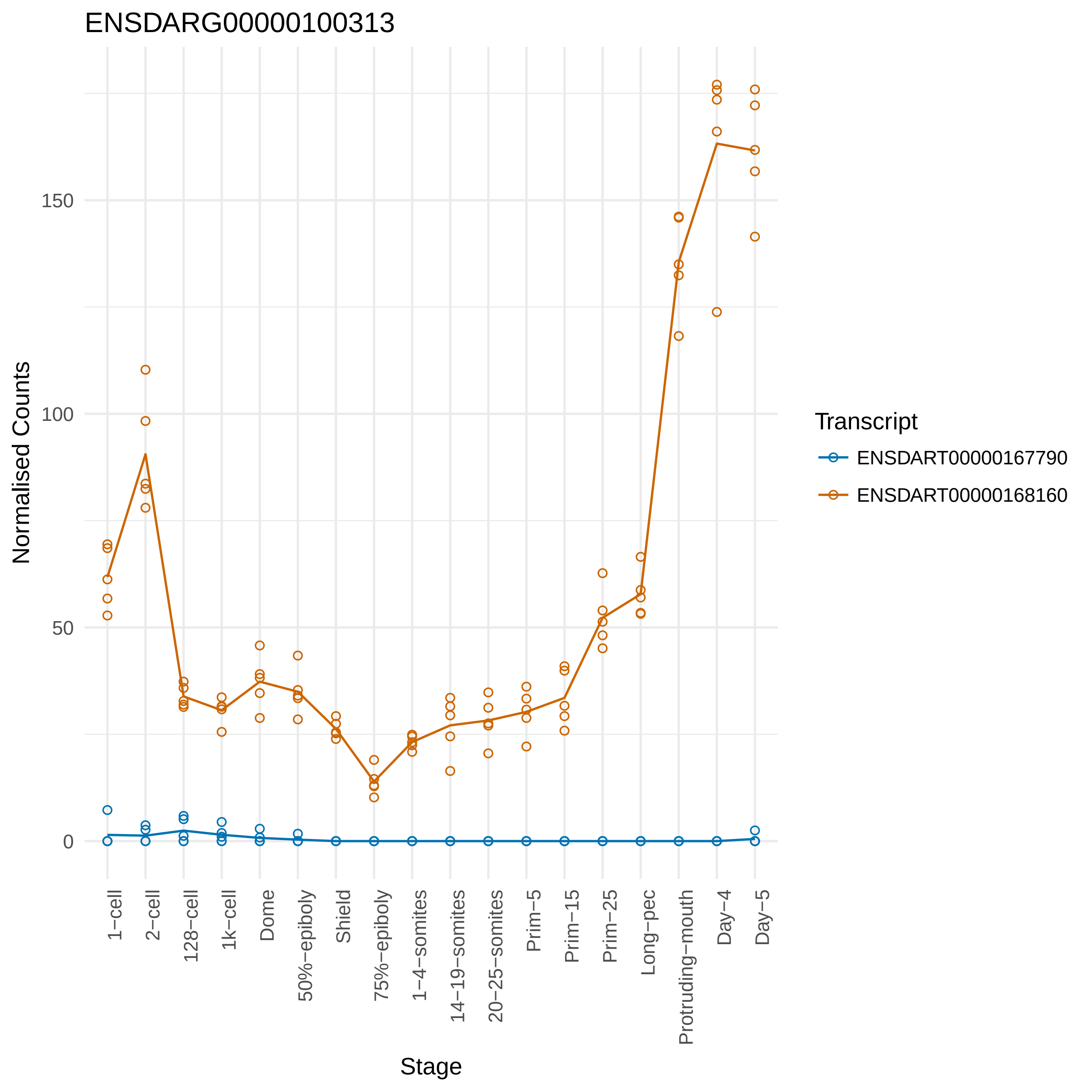

ENSDARG00000100313
Normalised Counts
150
100
50
0
1−cell
2−cell
128−cell
1k−cell
Dome
50%−epiboly
Shield
75%−epiboly
1−4−somites
14−19−somites
20−25−somites
Prim−5
Prim−15
Prim−25
Long−pec
Protruding−mouth
Day−4
Day−5
Stage
Transcript
ENSDART00000167790
ENSDART00000168160

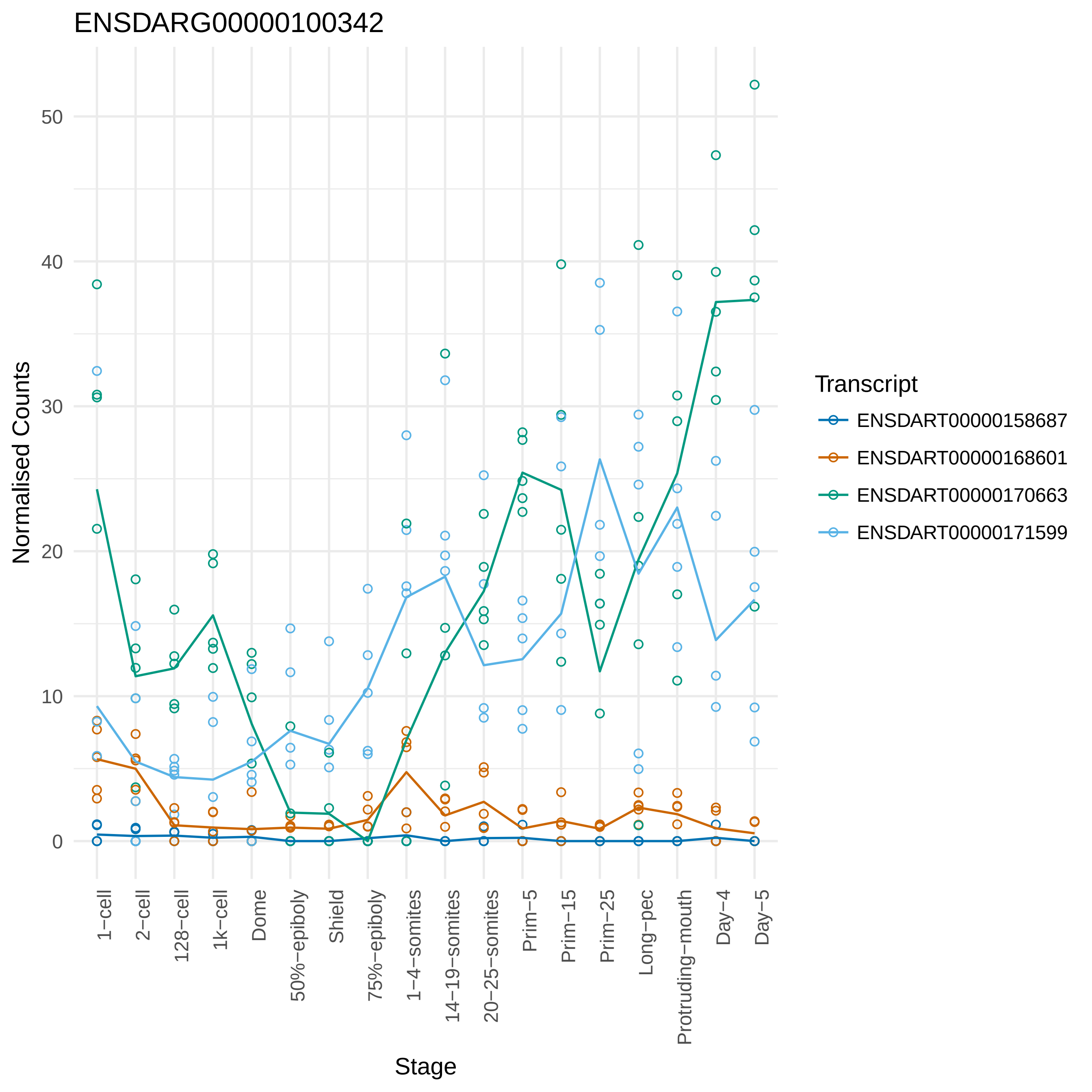

ENSDARG00000100342
Normalised Counts
0
10
20
30
40
50
1−cell
2−cell
128−cell
1k−cell
Dome
50%−epiboly
Shield
75%−epiboly
1−4−somites
14−19−somites
20−25−somites
Prim−5
Prim−15
Prim−25
Long−pec
Protruding−mouth
Day−4
Day−5
Stage
Transcript
ENSDART00000158687
ENSDART00000168601
ENSDART00000170663
ENSDART00000171599

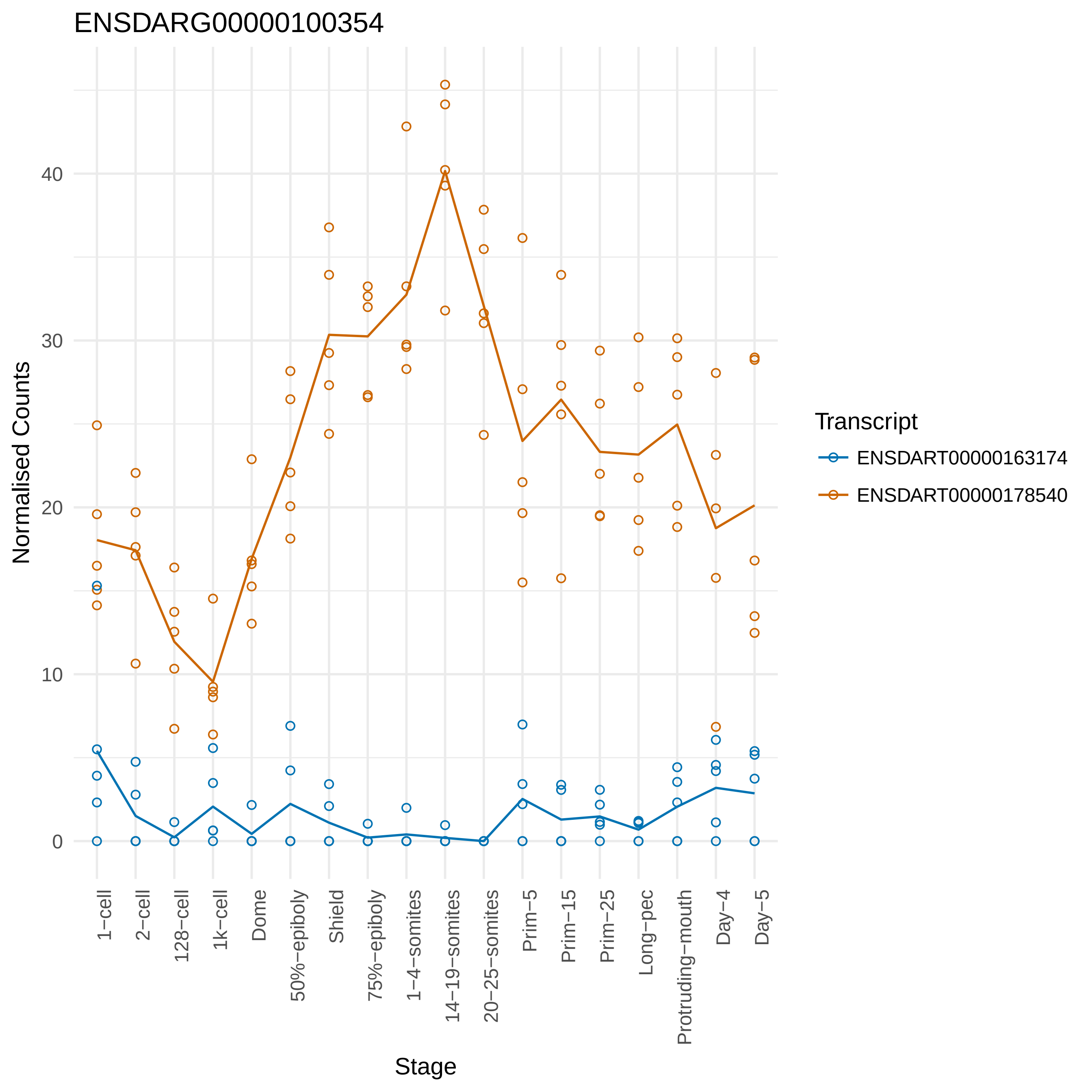
ENSDARG00000100354
Normalised Counts
0
10
20
30
40
1−cell
2−cell
128−cell
1k−cell
Dome
50%−epiboly
Shield
75%−epiboly
1−4−somites
14−19−somites
20−25−somites
Prim−5
Prim−15
Prim−25
Long−pec
Protruding−mouth
Day−4
Day−5
Stage
Transcript
ENSDART00000163174
ENSDART00000178540

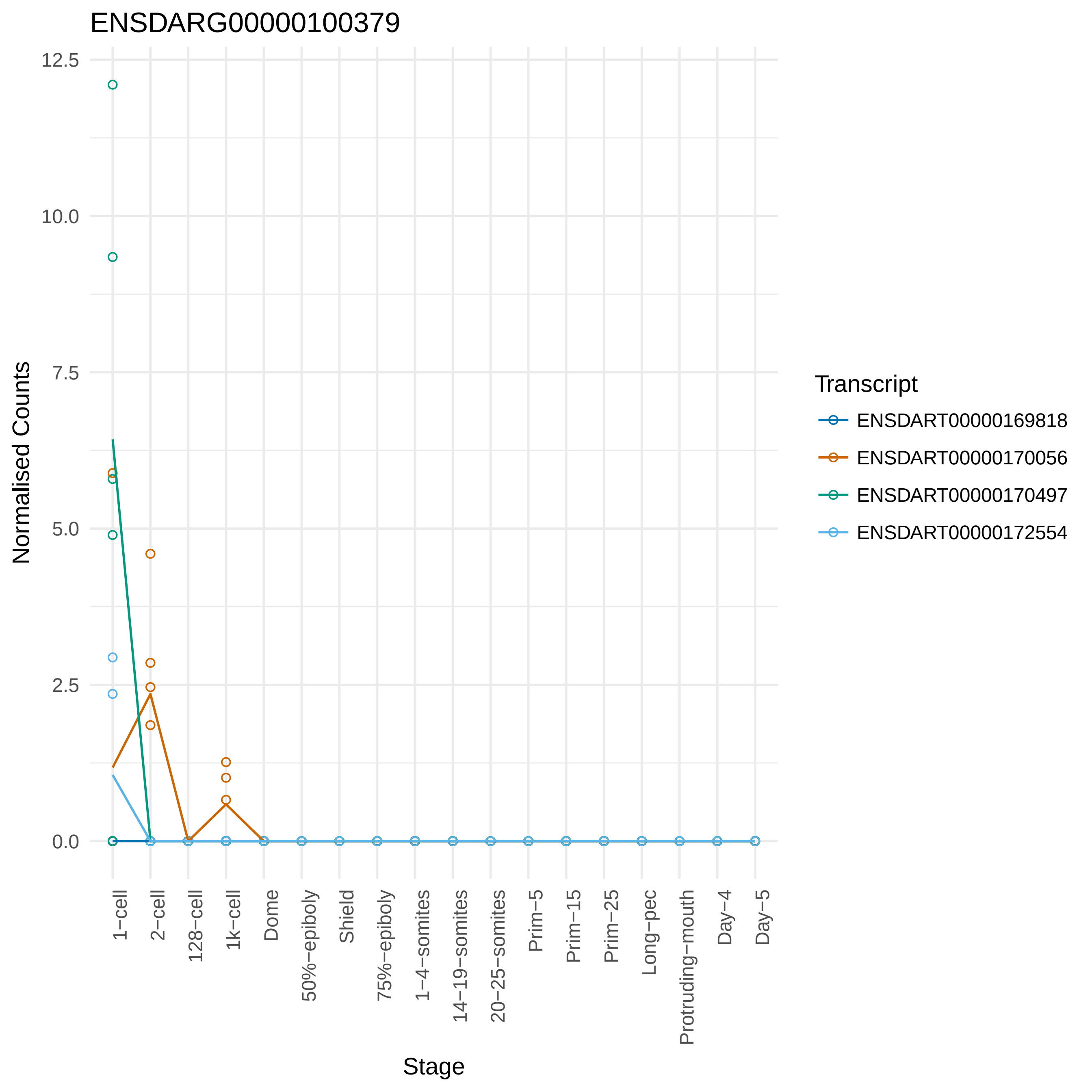

ENSDARG00000100379
Normalised Counts
12.5
10.0
7.5
5.0
2.5
0.0
1−cell
2−cell
128−cell
1k−cell
Dome
50%−epiboly
Shield
75%−epiboly
1−4−somites
14−19−somites
20−25−somites
Prim−5
Prim−15
Prim−25
Long−pec
Protruding−mouth
Day−4
Day−5
Stage
Transcript
ENSDART00000169818
ENSDART00000170056
ENSDART00000170497
ENSDART00000172554

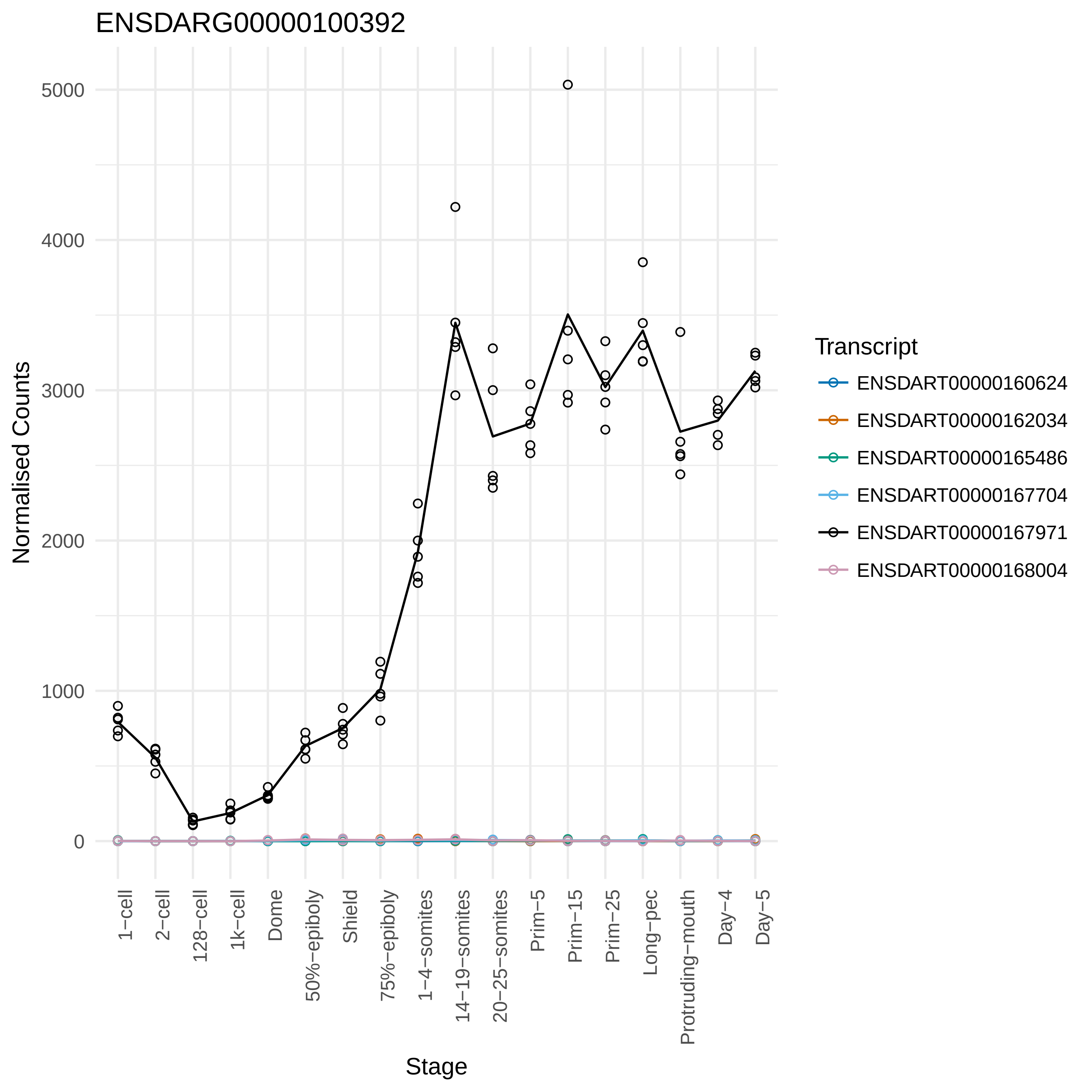

ENSDARG00000100392
Normalised Counts
5000
4000
3000
2000
1000
0
1−cell
2−cell
128−cell
1k−cell
Dome
50%−epiboly
Shield
75%−epiboly
1−4−somites
14−19−somites
20−25−somites
Prim−5
Prim−15
Prim−25
Long−pec
Protruding−mouth
Day−4
Day−5
Stage
Transcript
ENSDART00000160624
ENSDART00000162034
ENSDART00000165486
ENSDART00000167704
ENSDART00000167971
ENSDART00000168004

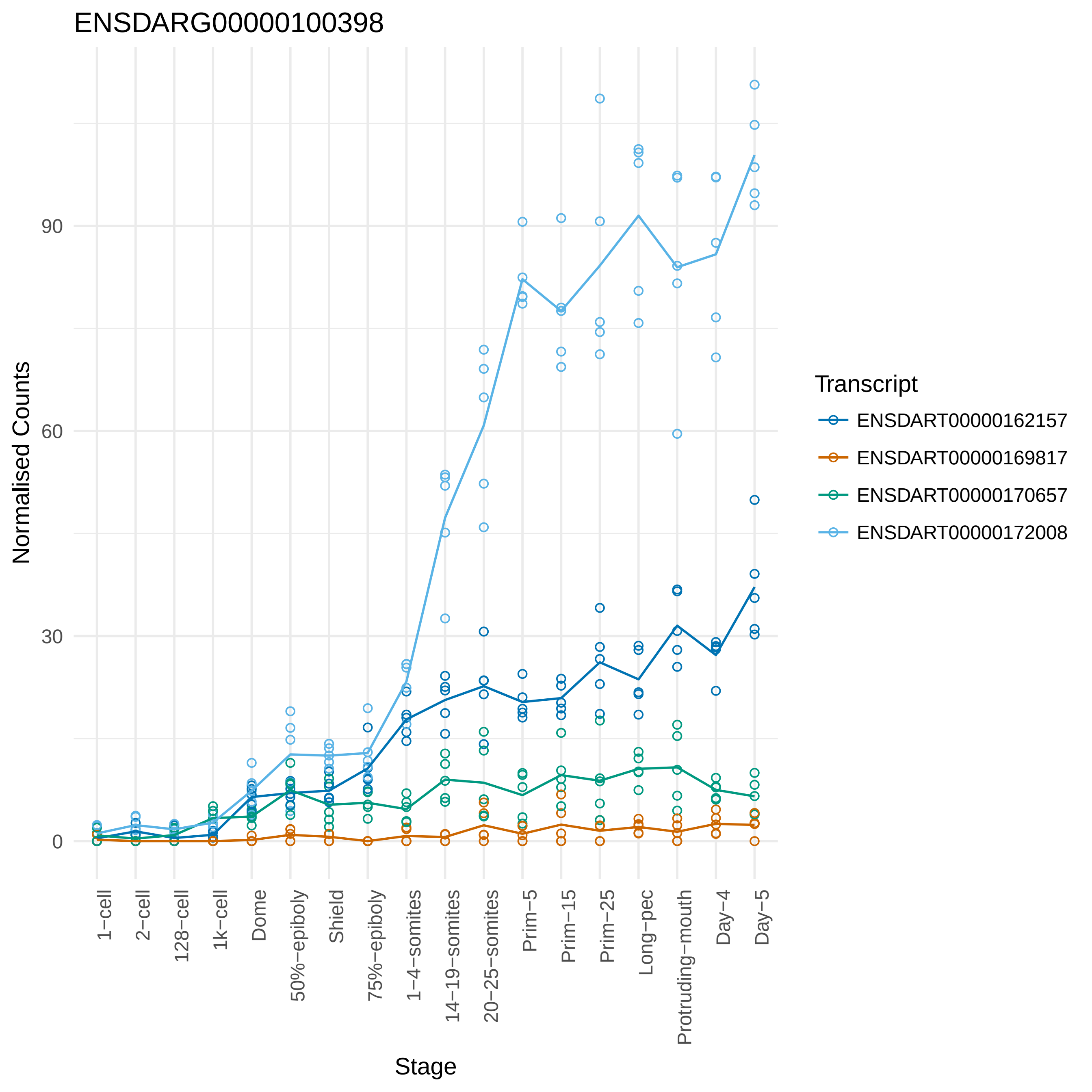

ENSDARG00000100398
Normalised Counts
0
30
60
90
1−cell
2−cell
128−cell
1k−cell
Dome
50%−epiboly
Shield
75%−epiboly
1−4−somites
14−19−somites
20−25−somites
Prim−5
Prim−15
Prim−25
Long−pec
Protruding−mouth
Day−4
Day−5
Stage
Transcript
ENSDART00000162157
ENSDART00000169817
ENSDART00000170657
ENSDART00000172008

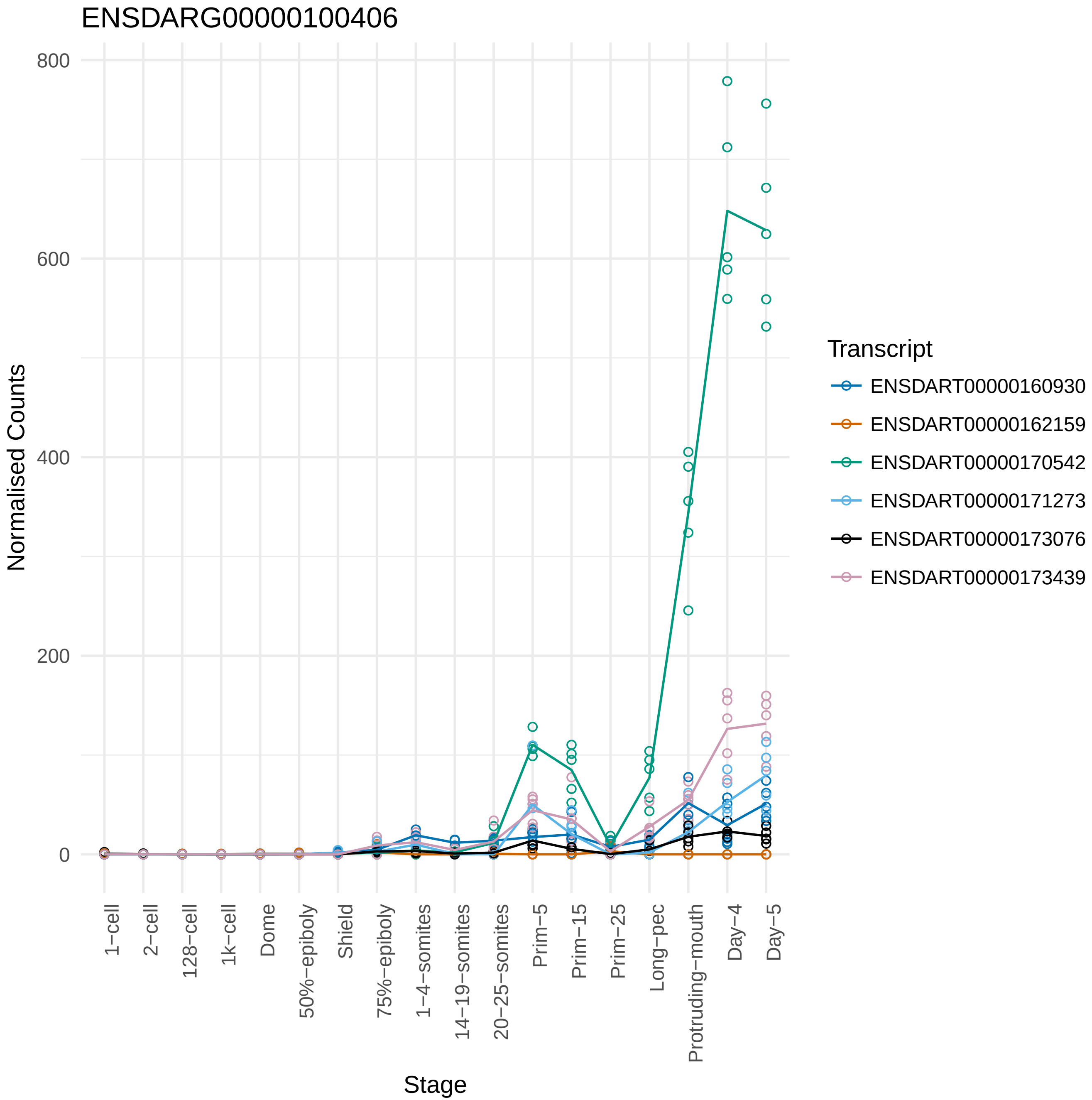
ENSDARG00000100406
Normalised Counts
800
600
400
200
0
1−cell
2−cell
128−cell
1k−cell
Dome
50%−epiboly
Shield
75%−epiboly
1−4−somites
14−19−somites
20−25−somites
Prim−5
Prim−15
Prim−25
Long−pec
Protruding−mouth
Day−4
Day−5
Stage
Transcript
ENSDART00000160930
ENSDART00000162159
ENSDART00000170542
ENSDART00000171273
ENSDART00000173076
ENSDART00000173439

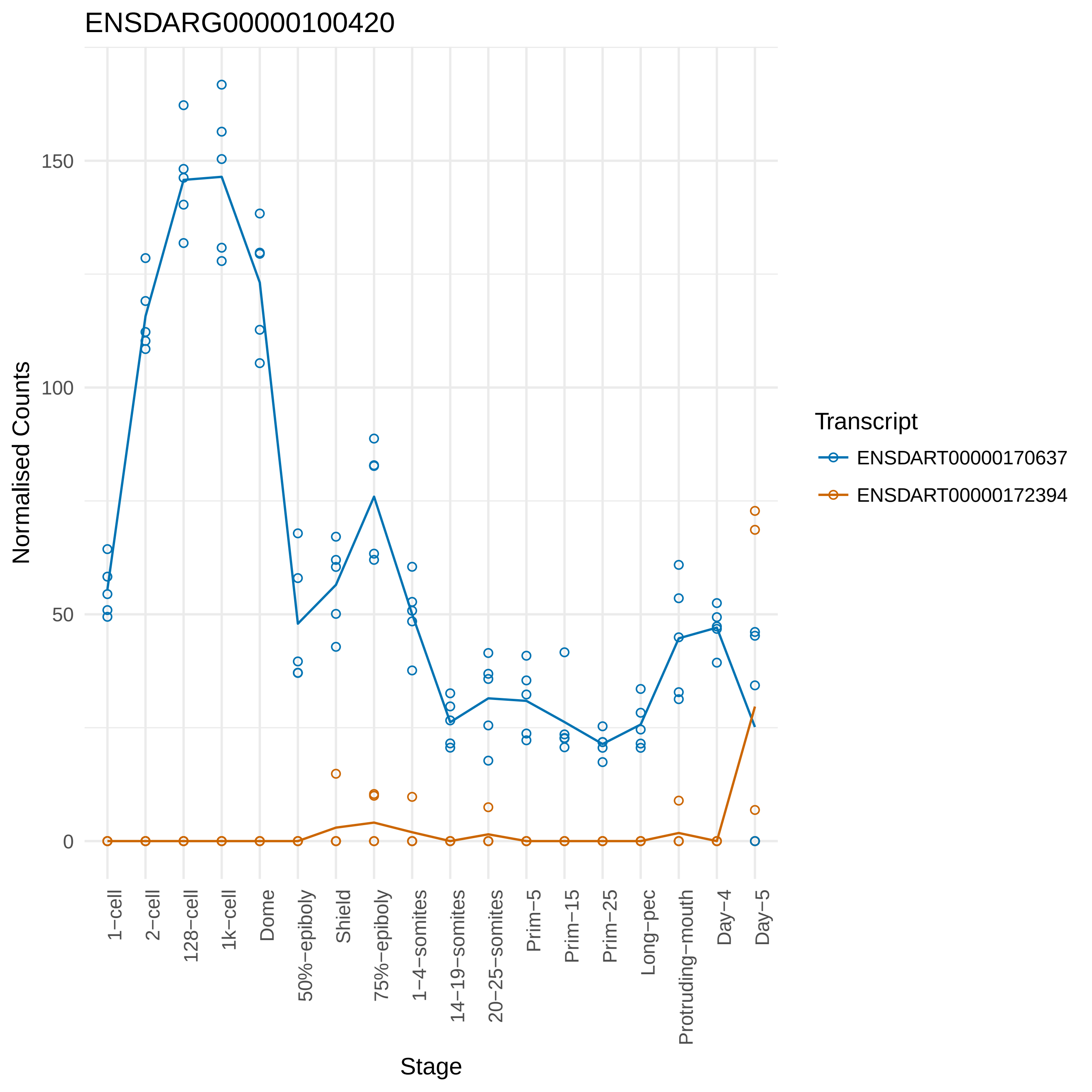

ENSDARG00000100420
Normalised Counts
0
50
100
150
Stage
1−cell
2−cell
128−cell
1k−cell
Dome
50%−epiboly
Shield
75%−epiboly
1−4−somites
14−19−somites
20−25−somites
Prim−5
Prim−15
Prim−25
Long−pec
Protruding−mouth
Day−4
Day−5
Transcript
ENSDART00000170637
ENSDART00000172394

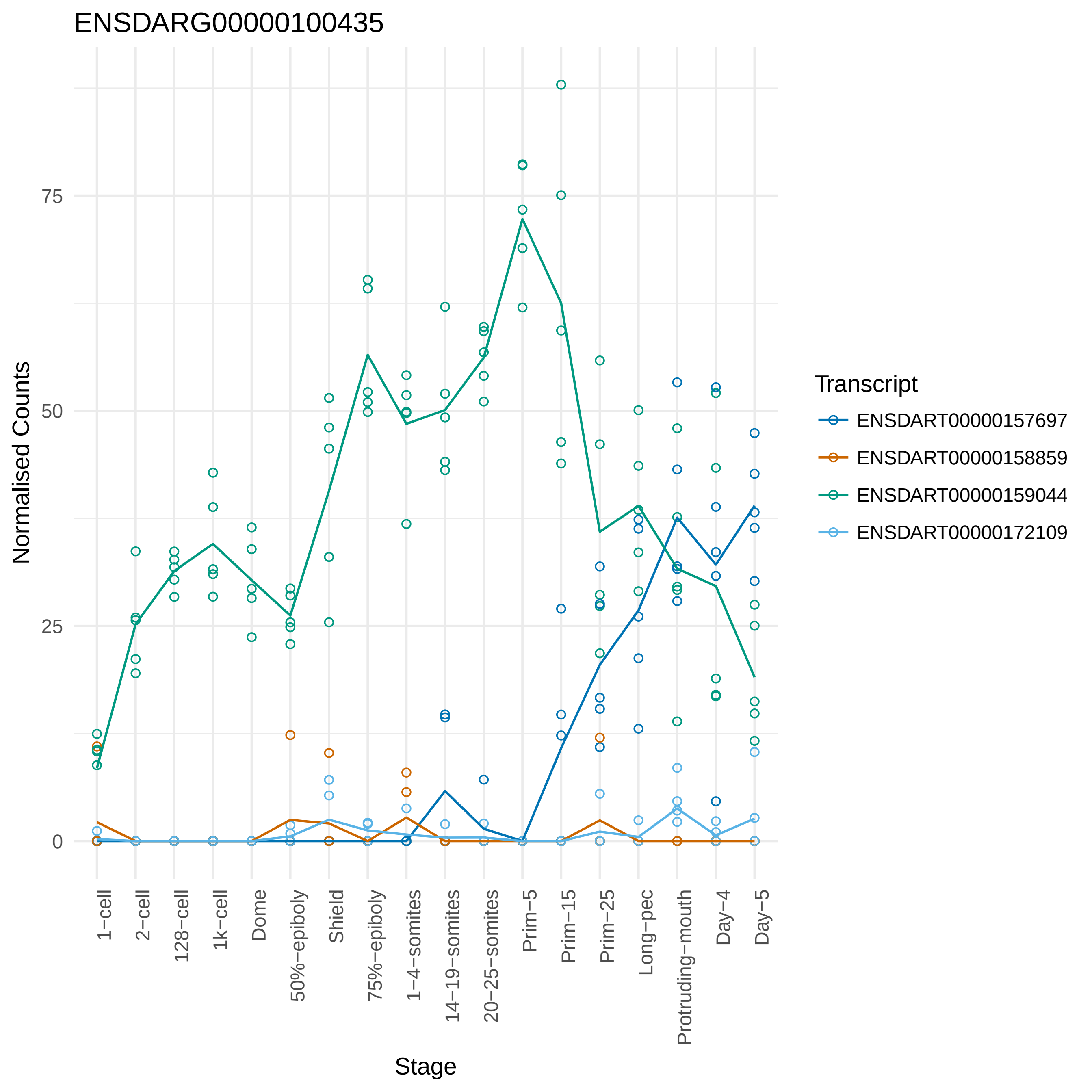

ENSDARG00000100435
Normalised Counts
0
25
50
75
Stage
1−cell
2−cell
128−cell
1k−cell
Dome
50%−epiboly
Shield
75%−epiboly
1−4−somites
14−19−somites
20−25−somites
Prim−5
Prim−15
Prim−25
Long−pec
Protruding−mouth
Day−4
Day−5
Transcript
ENSDART00000157697
ENSDART00000158859
ENSDART00000159044
ENSDART00000172109

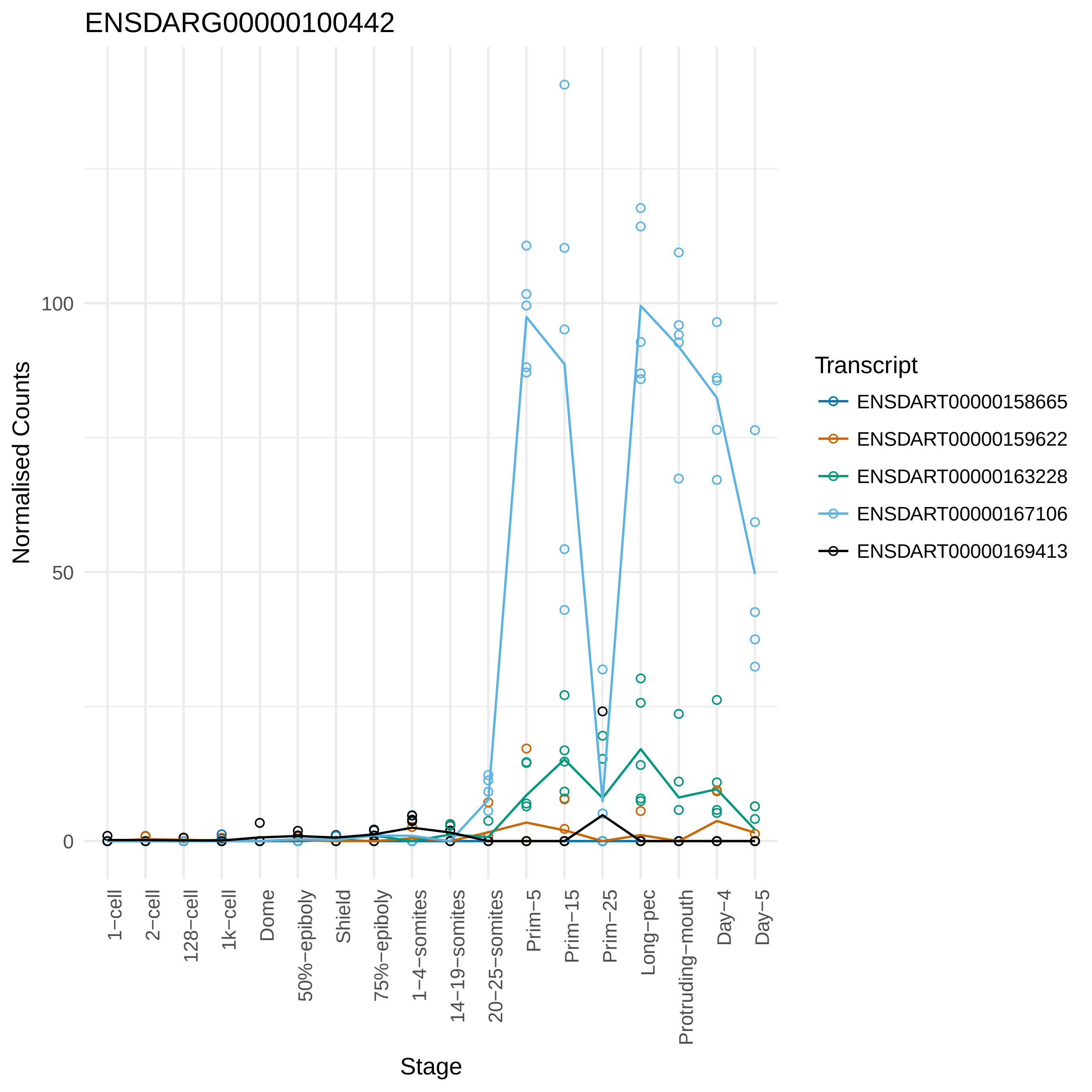

ENSDARG00000100442
Normalised Counts
0
50
100
Stage
1−cell
2−cell
128−cell
1k−cell
Dome
50%−epiboly
Shield
75%−epiboly
1−4−somites
14−19−somites
20−25−somites
Prim−5
Prim−15
Prim−25
Long−pec
Protruding−mouth
Day−4
Day−5
Transcript
ENSDART00000158665
ENSDART00000159622
ENSDART00000163228
ENSDART00000167106
ENSDART00000169413

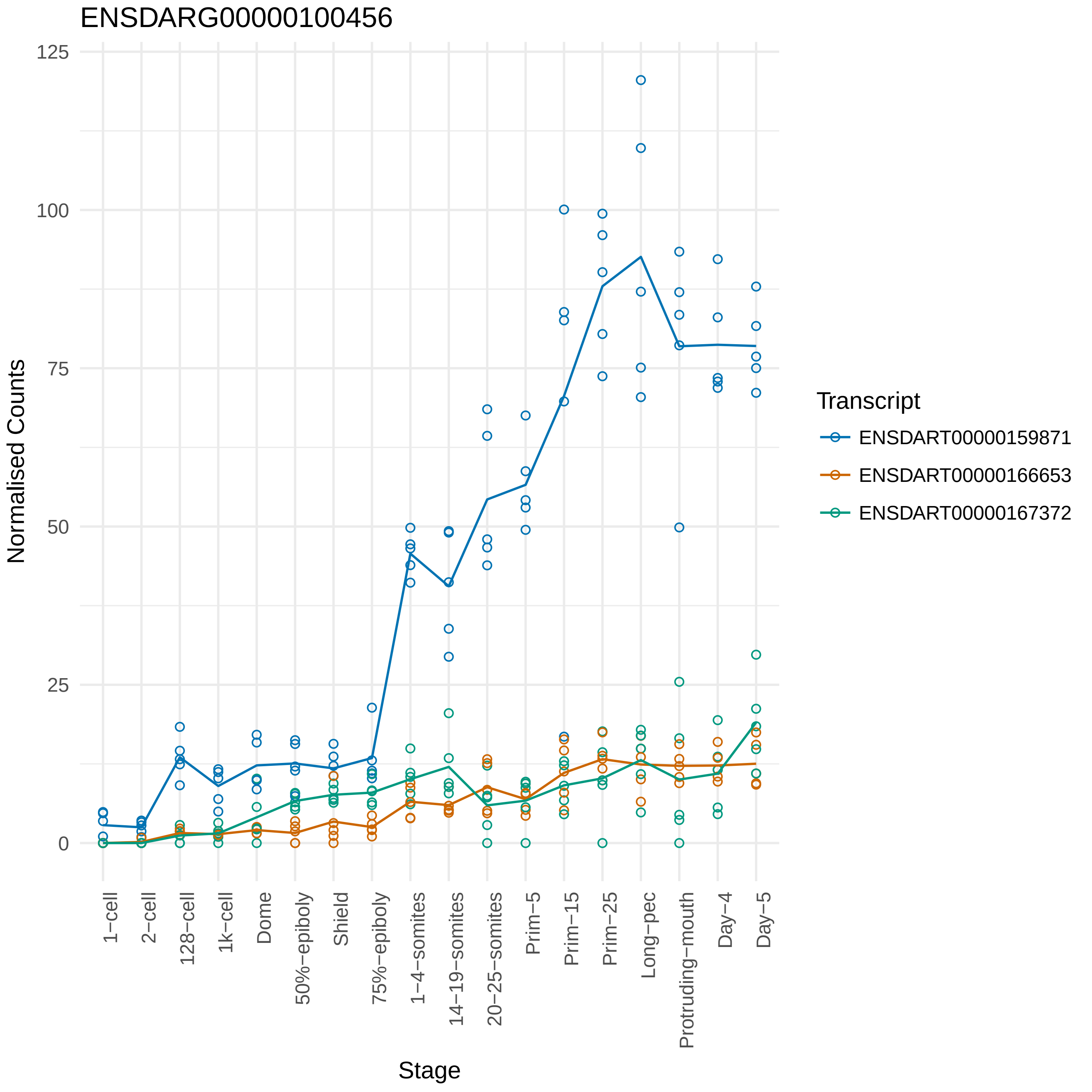
ENSDARG00000100456
Normalised Counts
Stage
125
100
75
50
25
0
1−cell
2−cell
128−cell
1k−cell
Dome
50%−epiboly
Shield
75%−epiboly
1−4−somites
14−19−somites
20−25−somites
Prim−5
Prim−15
Prim−25
Long−pec
Protruding−mouth
Day−4
Day−5
Transcript
ENSDART00000159871
ENSDART00000166653
ENSDART00000167372

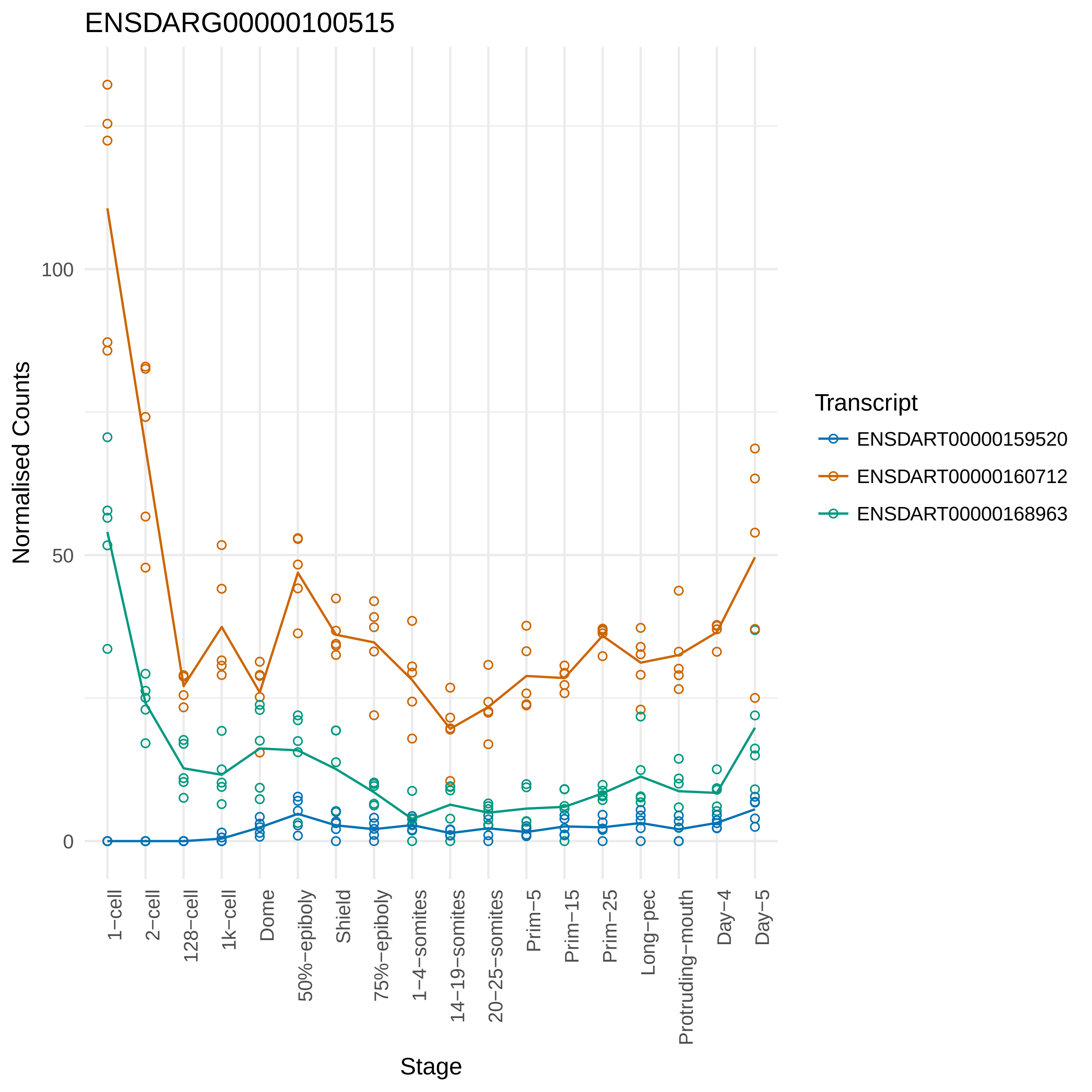

ENSDARG00000100515
Normalised Counts
100
50
0
1−cell
2−cell
128−cell
1k−cell
Dome
50%−epiboly
Shield
75%−epiboly
1−4−somites
14−19−somites
20−25−somites
Prim−5
Prim−15
Prim−25
Long−pec
Protruding−mouth
Day−4
Day−5
Stage
Transcript
ENSDART00000159520
ENSDART00000160712
ENSDART00000168963

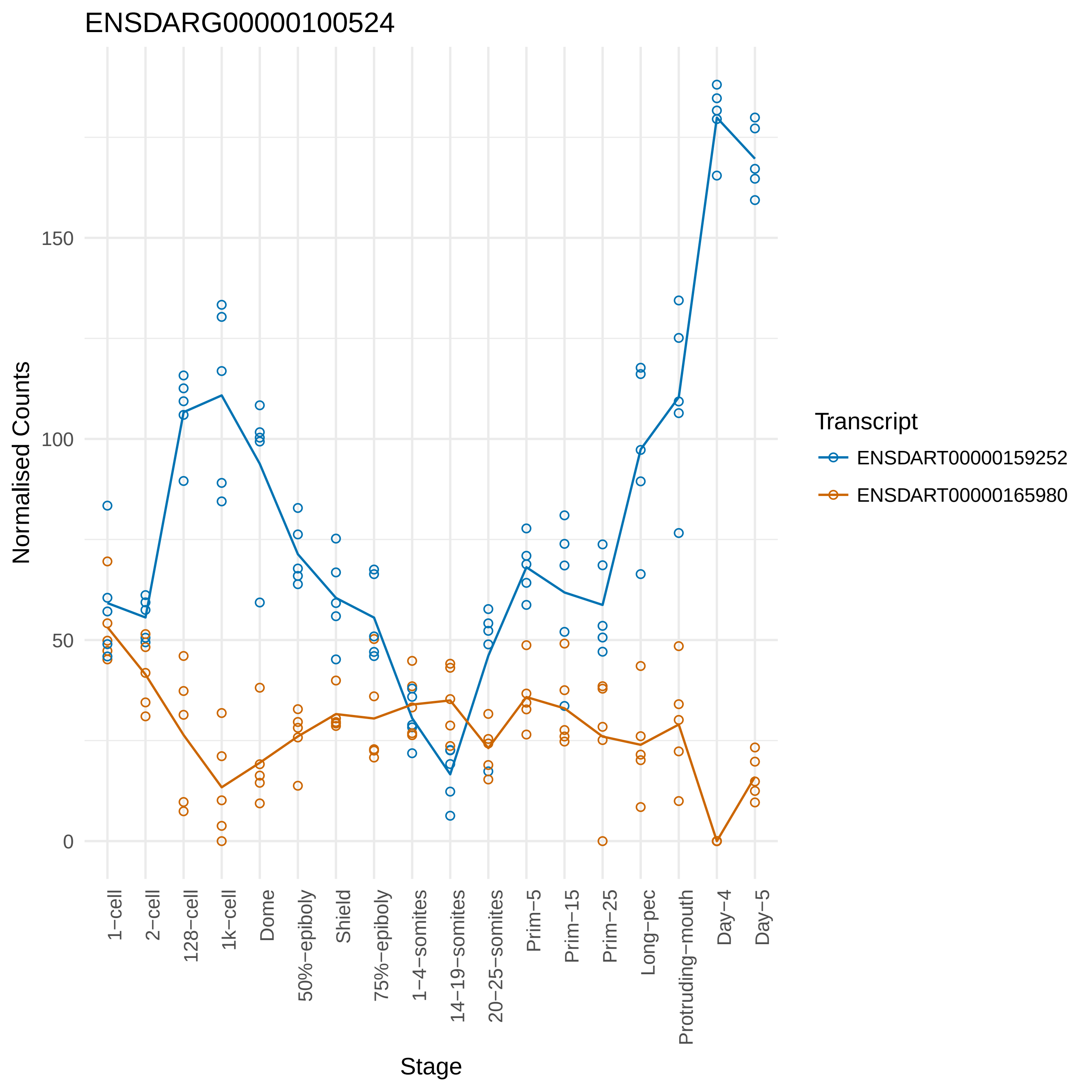

ENSDARG00000100524
Normalised Counts
150
100
50
0
1−cell
2−cell
128−cell
1k−cell
Dome
50%−epiboly
Shield
75%−epiboly
1−4−somites
14−19−somites
20−25−somites
Prim−5
Prim−15
Prim−25
Long−pec
Protruding−mouth
Day−4
Day−5
Stage
Transcript
ENSDART00000159252
ENSDART00000165980

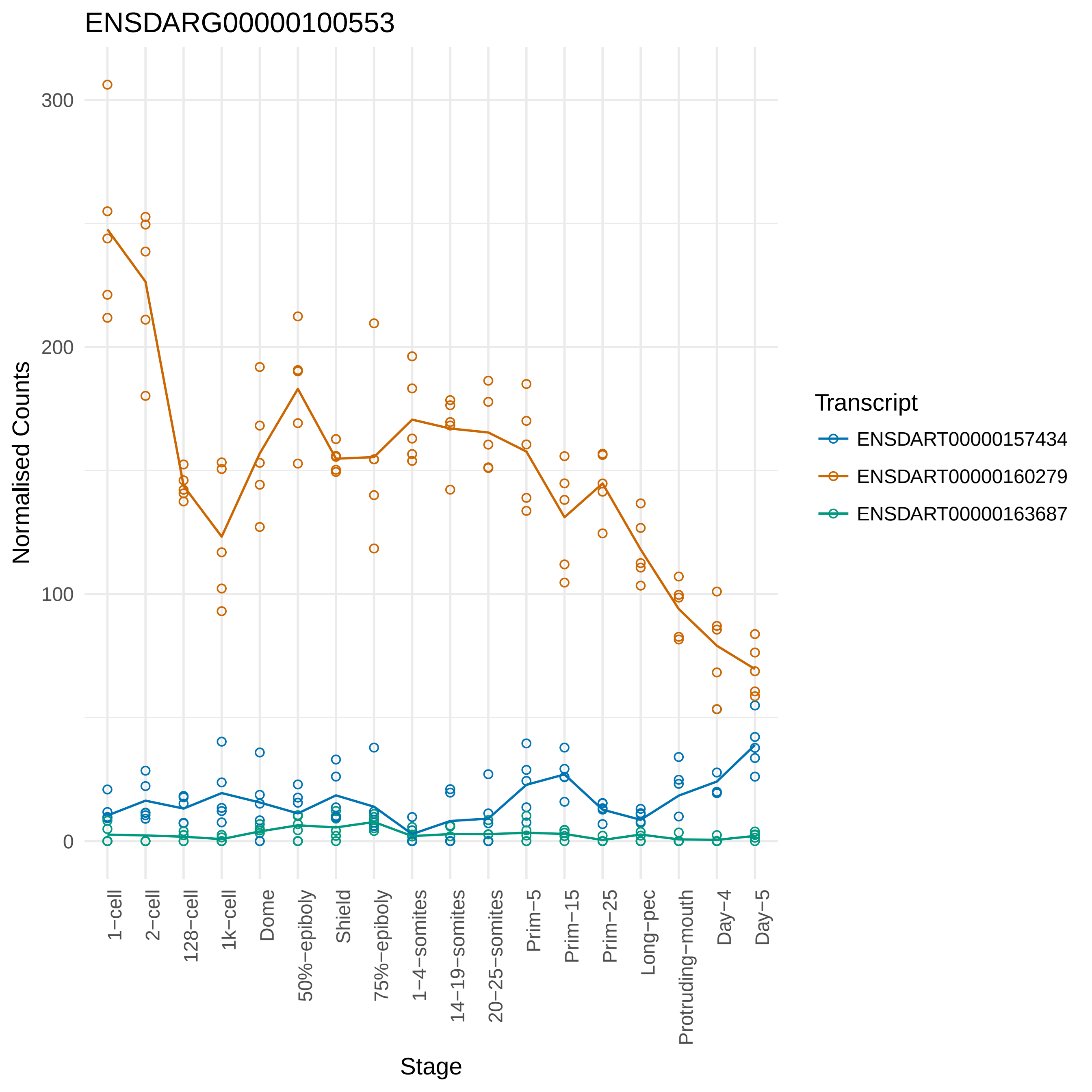

ENSDARG00000100553
Normalised Counts
300
200
100
0
1−cell
2−cell
128−cell
1k−cell
Dome
50%−epiboly
Shield
75%−epiboly
1−4−somites
14−19−somites
20−25−somites
Prim−5
Prim−15
Prim−25
Long−pec
Protruding−mouth
Day−4
Day−5
Stage
Transcript
ENSDART00000157434
ENSDART00000160279
ENSDART00000163687

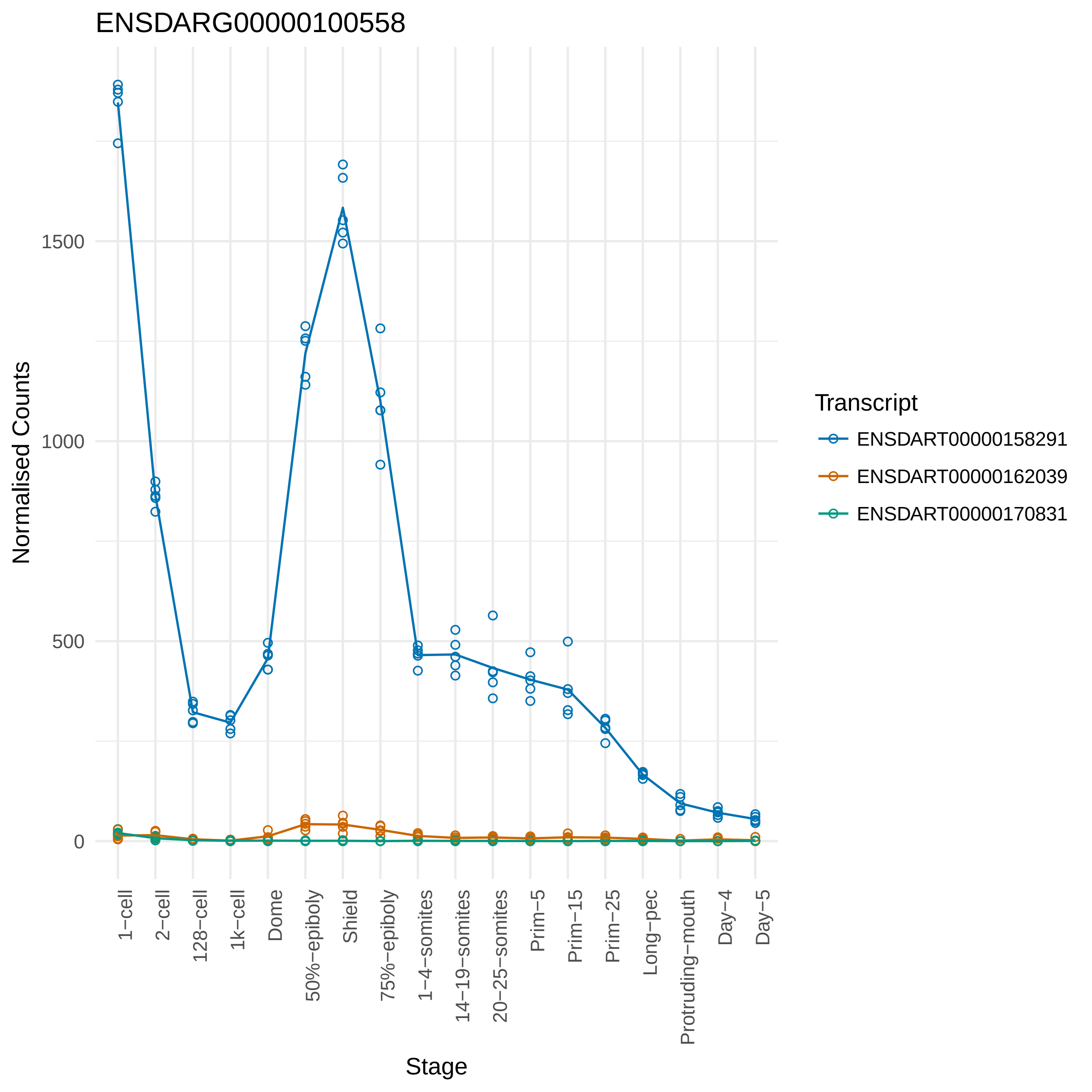

ENSDARG00000100558
Normalised Counts
1500
1000
500
0
1−cell
2−cell
128−cell
1k−cell
Dome
50%−epiboly
Shield
75%−epiboly
1−4−somites
14−19−somites
20−25−somites
Prim−5
Prim−15
Prim−25
Long−pec
Protruding−mouth
Day−4
Day−5
Stage
Transcript
ENSDART00000158291
ENSDART00000162039
ENSDART00000170831

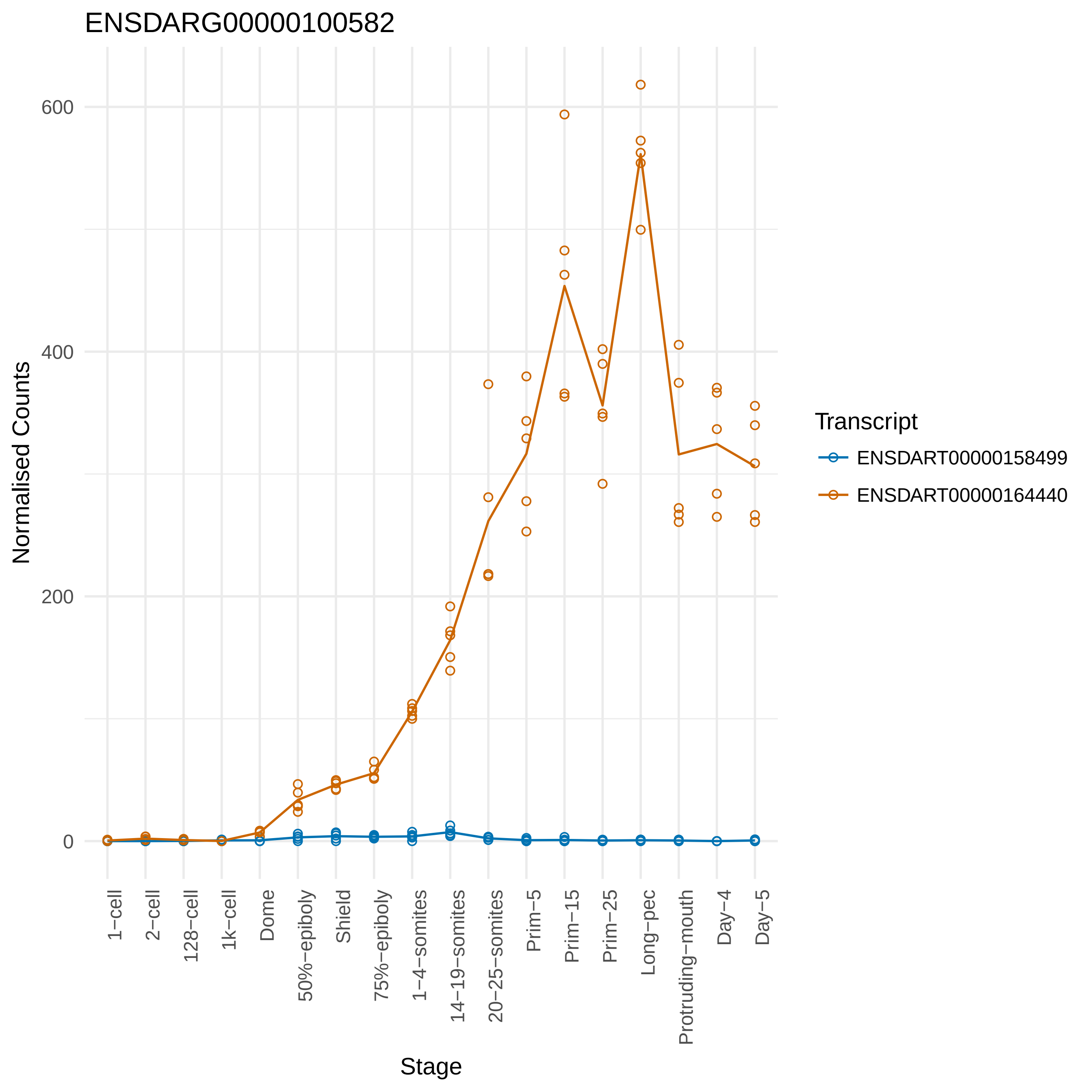
ENSDARG00000100582
Normalised Counts
600
400
200
0
Stage
1−cell
2−cell
128−cell
1k−cell
Dome
50%−epiboly
Shield
75%−epiboly
1−4−somites
14−19−somites
20−25−somites
Prim−5
Prim−15
Prim−25
Long−pec
Protruding−mouth
Day−4
Day−5
Transcript
ENSDART00000158499
ENSDART00000164440

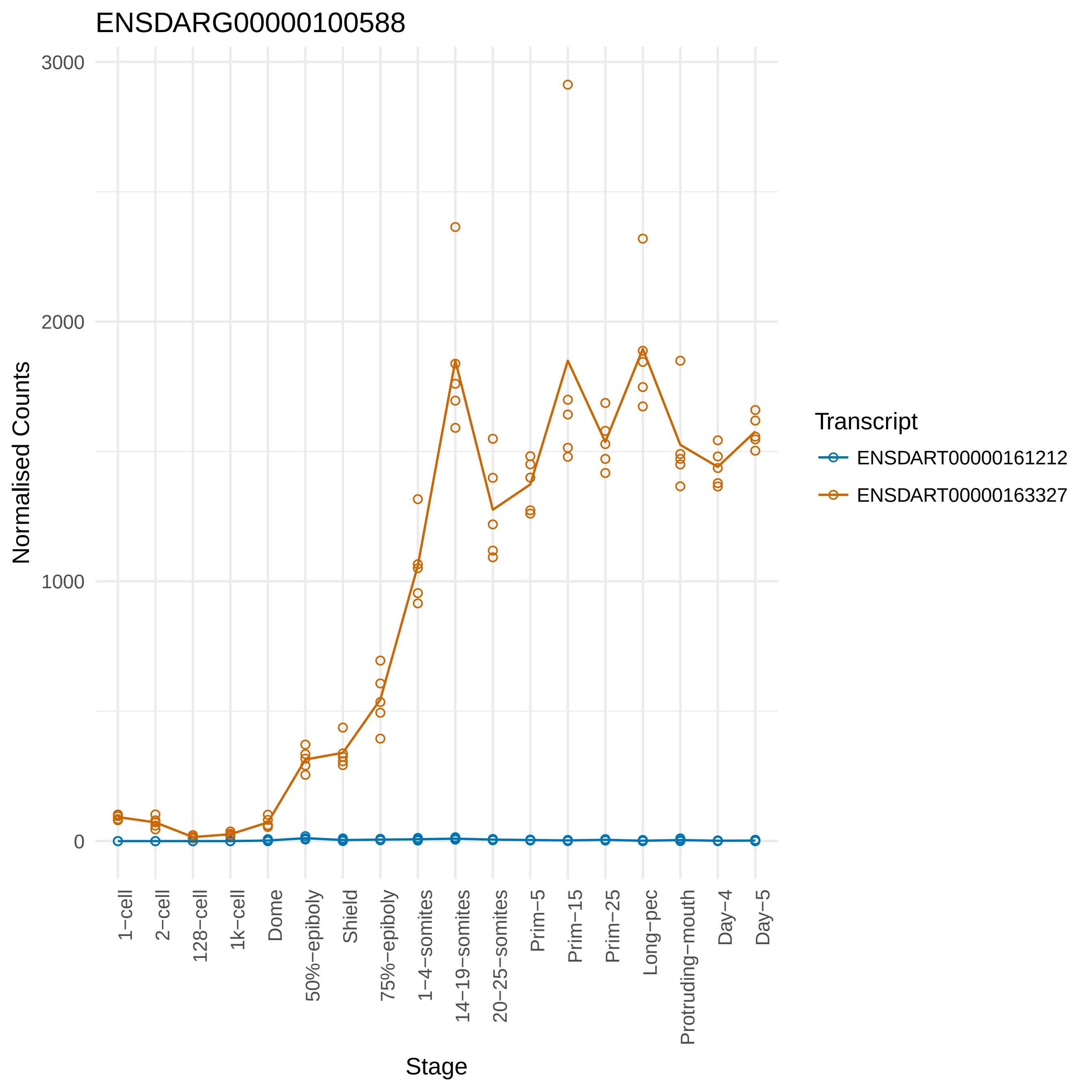

ENSDARG00000100588
Normalised Counts
3000
2000
1000
0
Stage
1−cell
2−cell
128−cell
1k−cell
Dome
50%−epiboly
Shield
75%−epiboly
1−4−somites
14−19−somites
20−25−somites
Prim−5
Prim−15
Prim−25
Long−pec
Protruding−mouth
Day−4
Day−5
Transcript
ENSDART00000161212
ENSDART00000163327

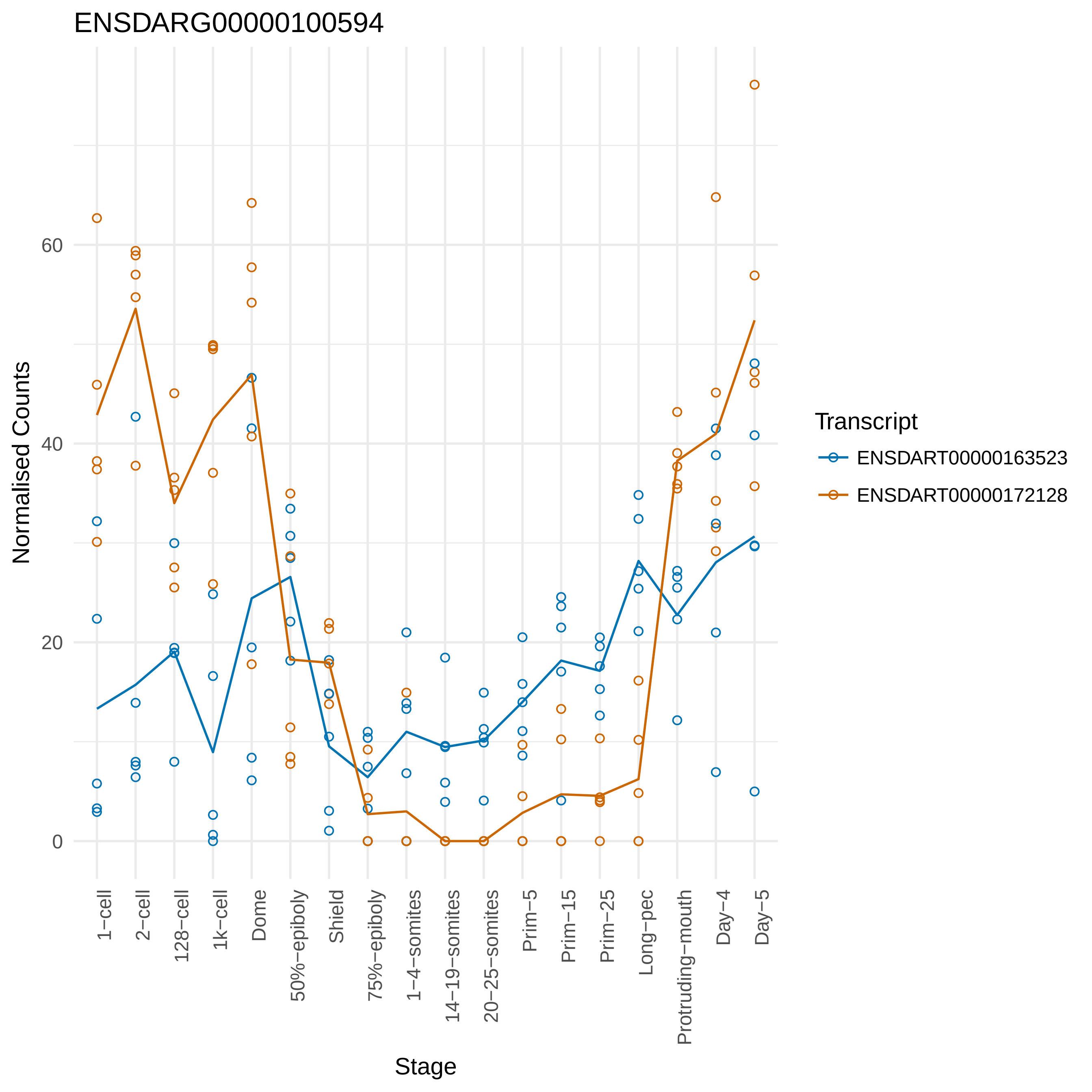
ENSDARG00000100594
Normalised Counts
0
20
40
60
Stage
1−cell
2−cell
128−cell
1k−cell
Dome
50%−epiboly
Shield
75%−epiboly
1−4−somites
14−19−somites
20−25−somites
Prim−5
Prim−15
Prim−25
Long−pec
Protruding−mouth
Day−4
Day−5
Transcript
ENSDART00000163523
ENSDART00000172128

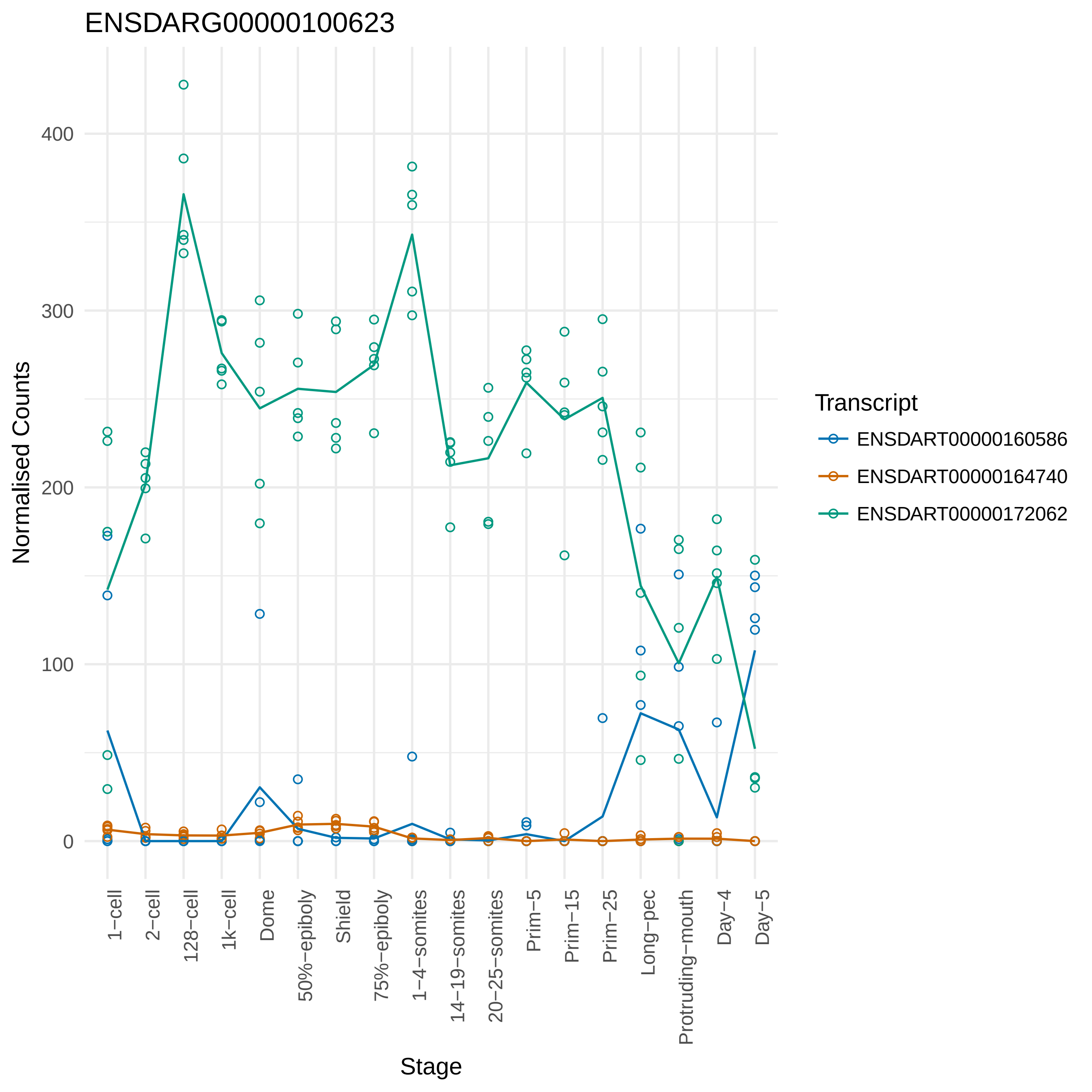

ENSDARG00000100623
Normalised Counts
400
300
200
100
0
1−cell
2−cell
128−cell
1k−cell
Dome
50%−epiboly
Shield
75%−epiboly
1−4−somites
14−19−somites
20−25−somites
Prim−5
Prim−15
Prim−25
Long−pec
Protruding−mouth
Day−4
Day−5
Stage
Transcript
ENSDART00000160586
ENSDART00000164740
ENSDART00000172062

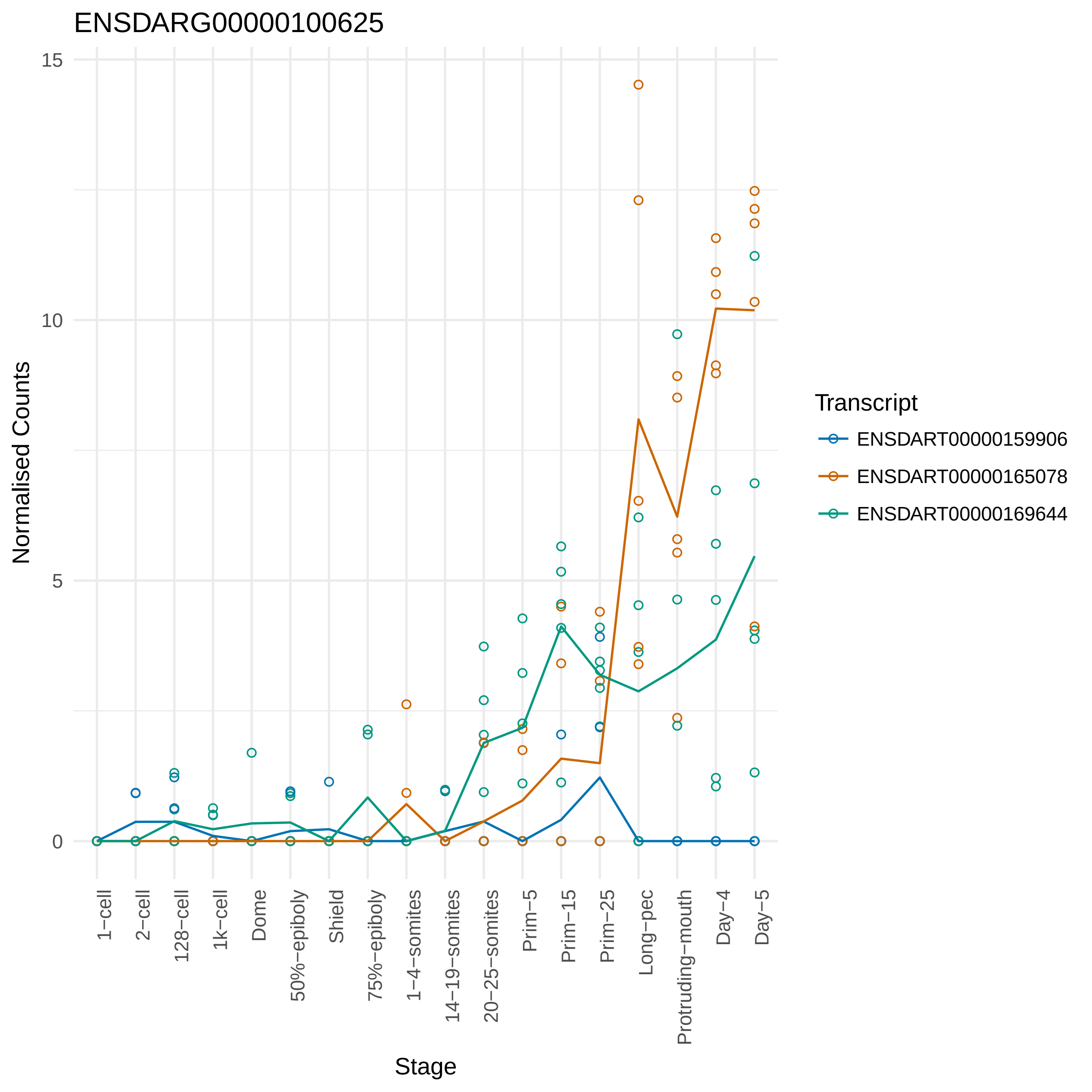

ENSDARG00000100625
Normalised Counts
15
10
5
0
1−cell
2−cell
128−cell
1k−cell
Dome
50%−epiboly
Shield
75%−epiboly
1−4−somites
14−19−somites
20−25−somites
Prim−5
Prim−15
Prim−25
Long−pec
Protruding−mouth
Day−4
Day−5
Stage
Transcript
ENSDART00000159906
ENSDART00000165078
ENSDART00000169644

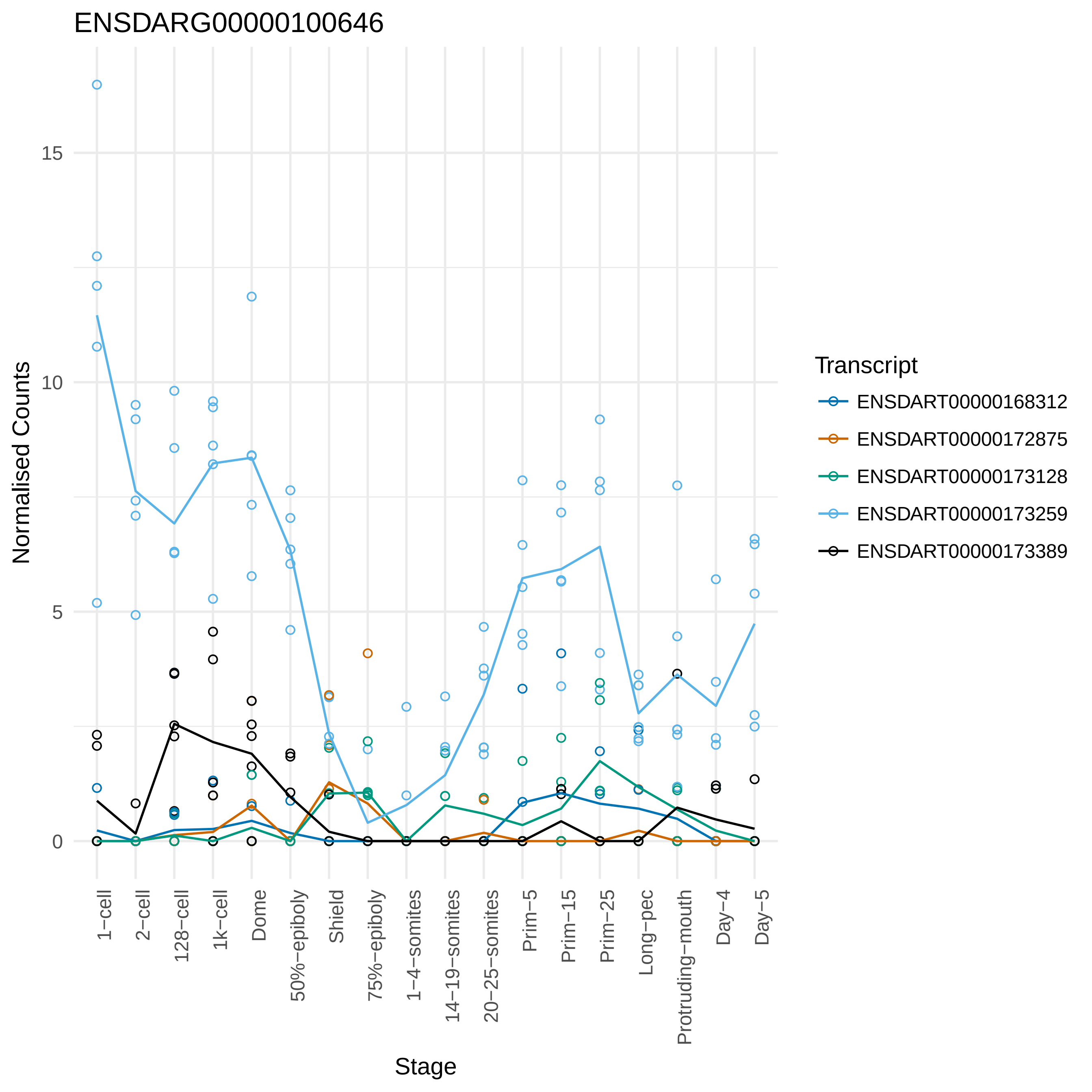
ENSDARG00000100646
Normalised Counts
15
10
5
0
1−cell
2−cell
128−cell
1k−cell
Dome
50%−epiboly
Shield
75%−epiboly
1−4−somites
14−19−somites
20−25−somites
Prim−5
Prim−15
Prim−25
Long−pec
Protruding−mouth
Day−4
Day−5
Stage
Transcript
ENSDART00000168312
ENSDART00000172875
ENSDART00000173128
ENSDART00000173259
ENSDART00000173389

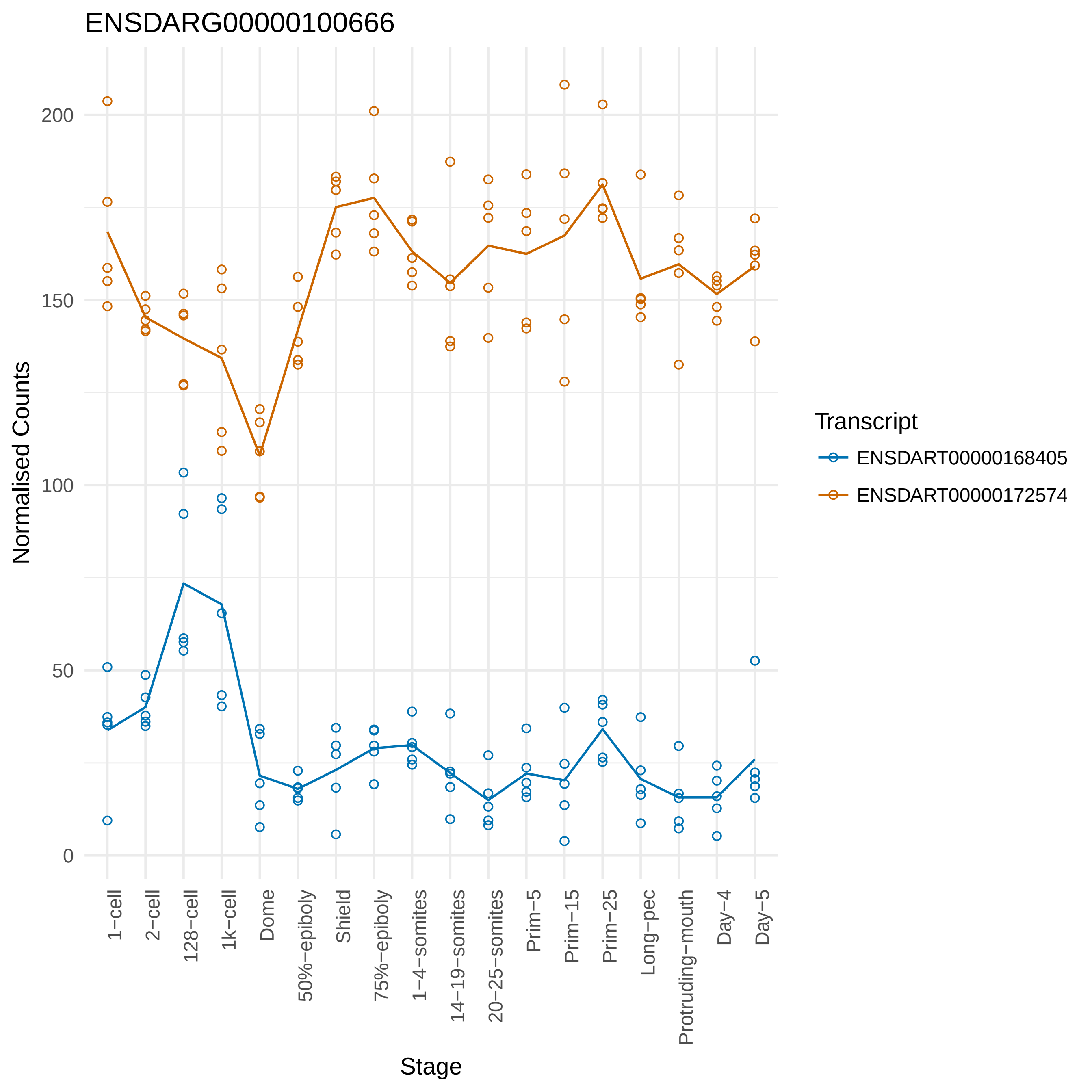
ENSDARG00000100666
Normalised Counts
0
50
100
150
200
Stage
1−cell
2−cell
128−cell
1k−cell
Dome
50%−epiboly
Shield
75%−epiboly
1−4−somites
14−19−somites
20−25−somites
Prim−5
Prim−15
Prim−25
Long−pec
Protruding−mouth
Day−4
Day−5
Transcript
ENSDART00000168405
ENSDART00000172574

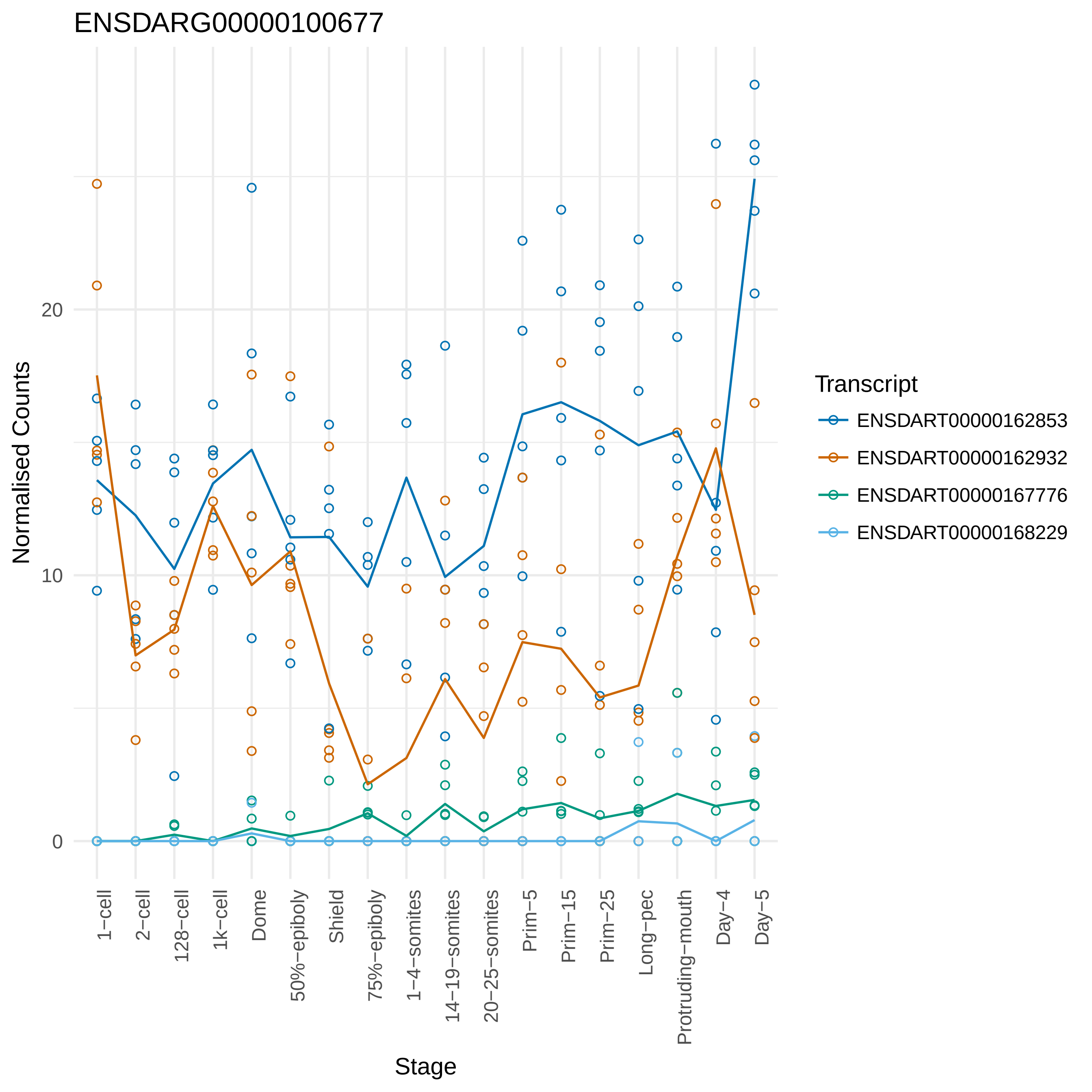

ENSDARG00000100677
Normalised Counts
0
10
20
Stage
1−cell
2−cell
128−cell
1k−cell
Dome
50%−epiboly
Shield
75%−epiboly
1−4−somites
14−19−somites
20−25−somites
Prim−5
Prim−15
Prim−25
Long−pec
Protruding−mouth
Day−4
Day−5
Transcript
ENSDART00000162853
ENSDART00000162932
ENSDART00000167776
ENSDART00000168229

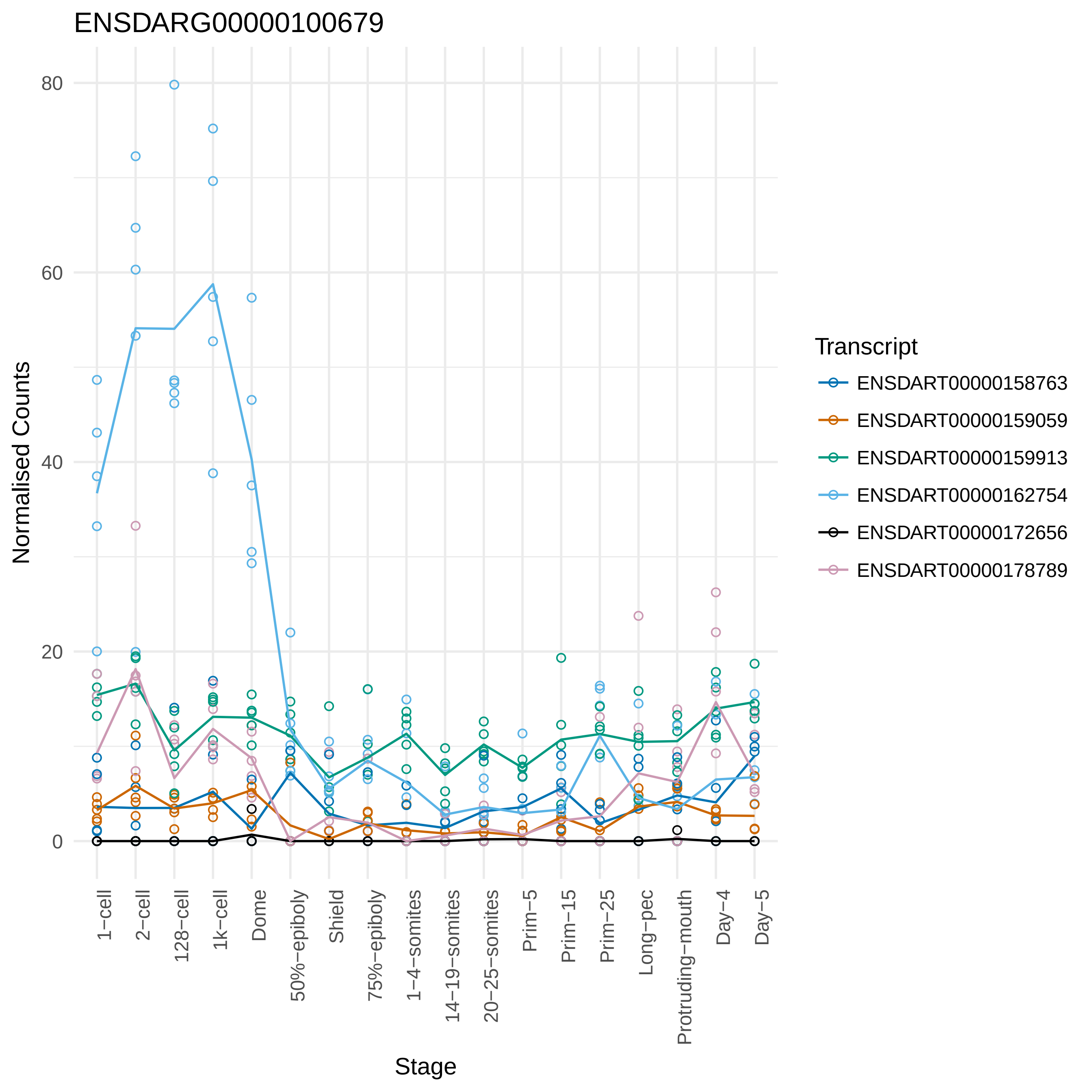
ENSDARG00000100679
Normalised Counts
0
20
40
60
80
1−cell
2−cell
128−cell
1k−cell
Dome
50%−epiboly
Shield
75%−epiboly
1−4−somites
14−19−somites
20−25−somites
Prim−5
Prim−15
Prim−25
Long−pec
Protruding−mouth
Day−4
Day−5
Stage
Transcript
ENSDART00000158763
ENSDART00000159059
ENSDART00000159913
ENSDART00000162754
ENSDART00000172656
ENSDART00000178789

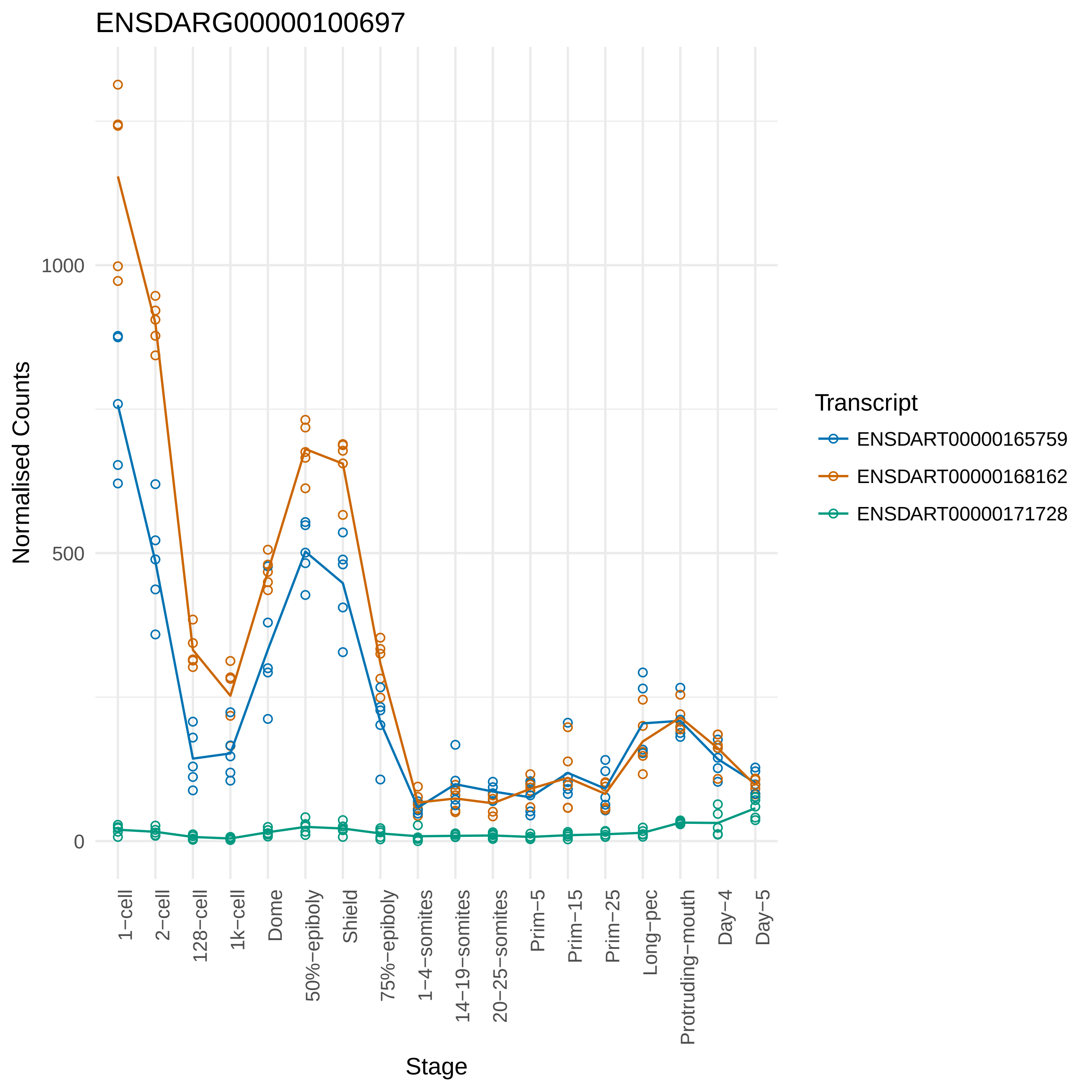
ENSDARG00000100697
Normalised Counts
1000
500
0
Stage
1−cell
2−cell
128−cell
1k−cell
Dome
50%−epiboly
Shield
75%−epiboly
1−4−somites
14−19−somites
20−25−somites
Prim−5
Prim−15
Prim−25
Long−pec
Protruding−mouth
Day−4
Day−5
Transcript
ENSDART00000165759
ENSDART00000168162
ENSDART00000171728

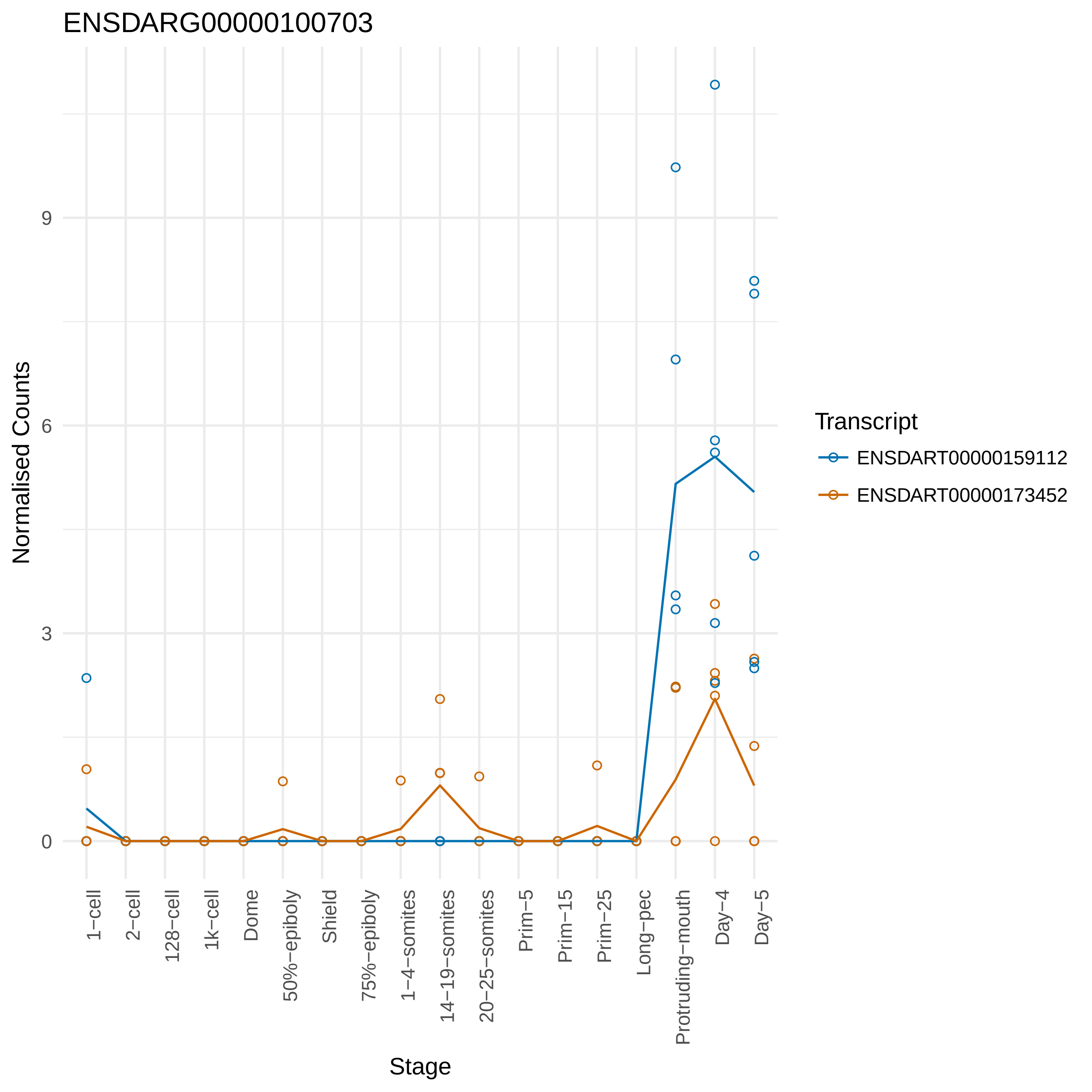

ENSDARG00000100703
Normalised Counts
0
3
6
9
1−cell
2−cell
128−cell
1k−cell
Dome
50%−epiboly
Shield
75%−epiboly
1−4−somites
14−19−somites
20−25−somites
Prim−5
Prim−15
Prim−25
Long−pec
Protruding−mouth
Day−4
Day−5
Stage
Transcript
ENSDART00000159112
ENSDART00000173452

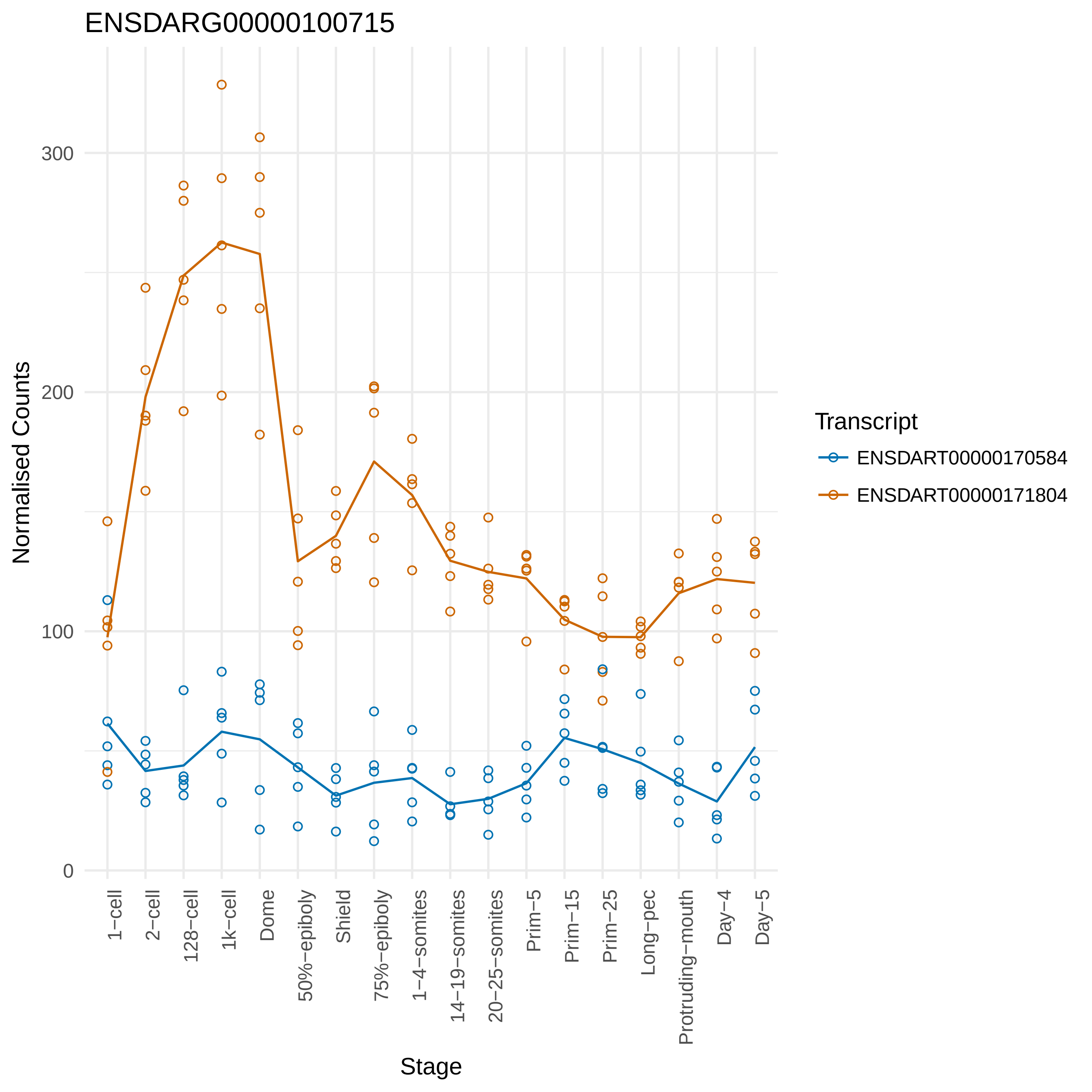
ENSDARG00000100715
Normalised Counts
300
200
100
0
1−cell
2−cell
128−cell
1k−cell
Dome
50%−epiboly
Shield
75%−epiboly
1−4−somites
14−19−somites
20−25−somites
Prim−5
Prim−15
Prim−25
Long−pec
Protruding−mouth
Day−4
Day−5
Stage
Transcript
ENSDART00000170584
ENSDART00000171804

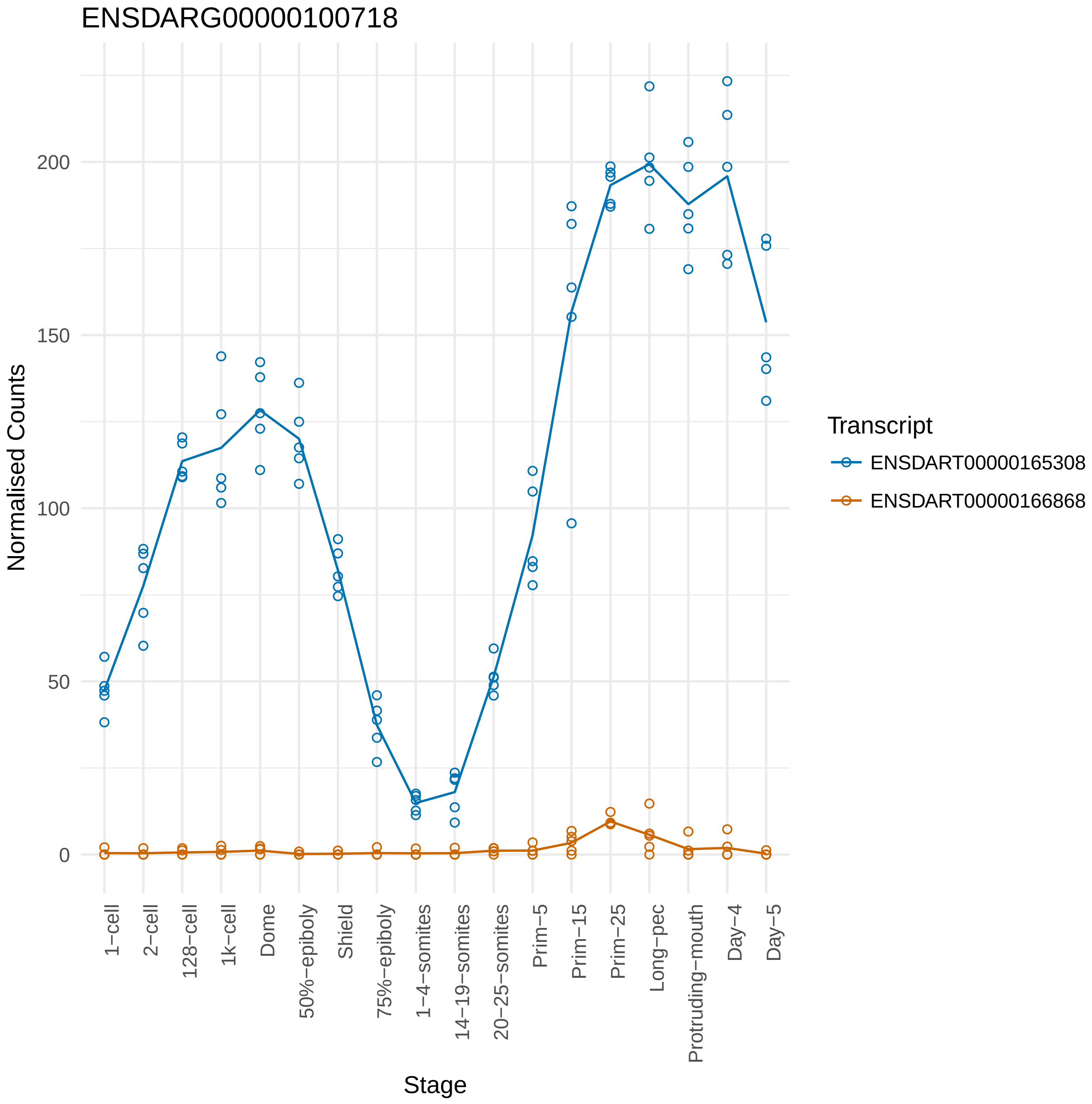

ENSDARG00000100718
Normalised Counts
0
50
100
150
200
Stage
1−cell
2−cell
128−cell
1k−cell
Dome
50%−epiboly
Shield
75%−epiboly
1−4−somites
14−19−somites
20−25−somites
Prim−5
Prim−15
Prim−25
Long−pec
Protruding−mouth
Day−4
Day−5
Transcript
ENSDART00000165308
ENSDART00000166868

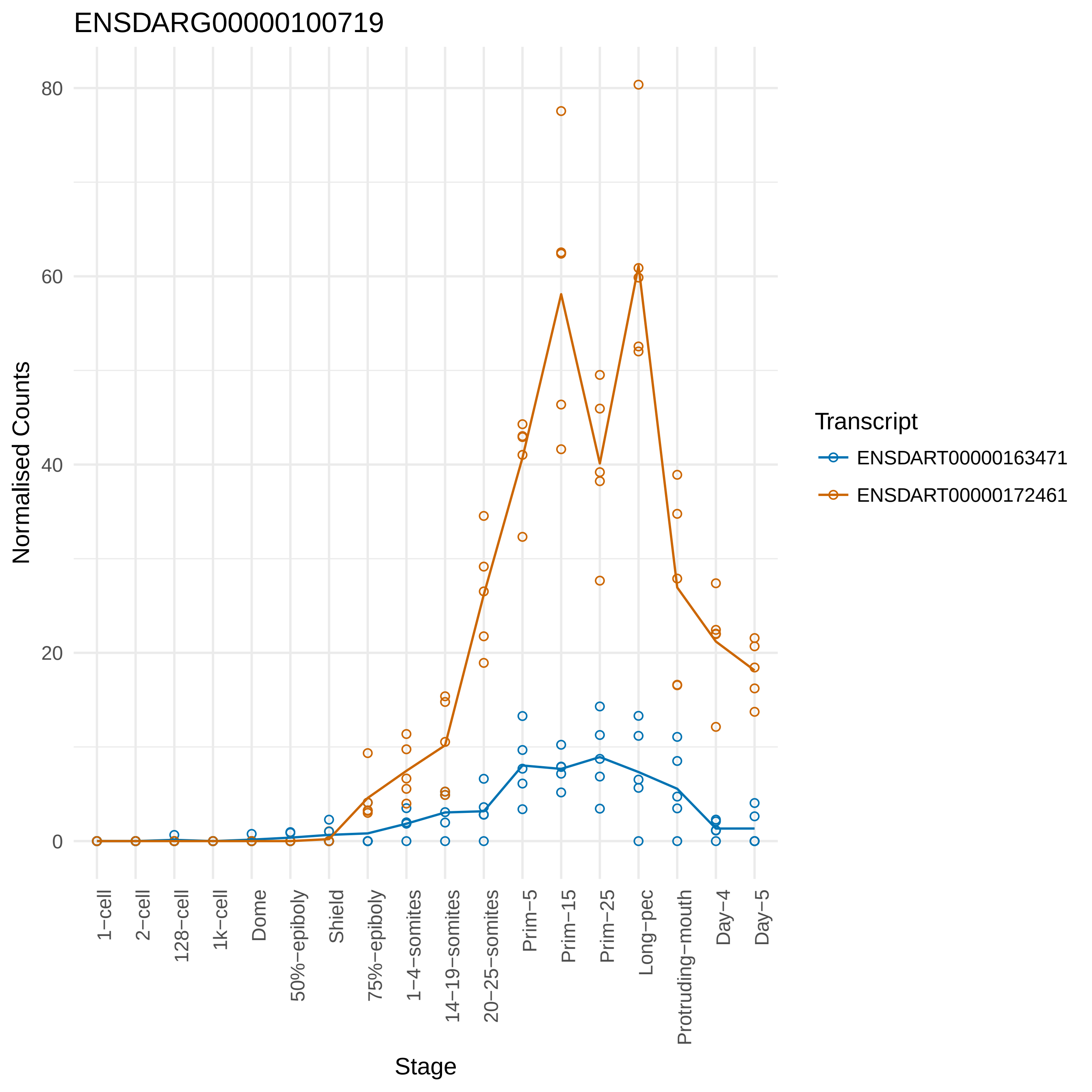

ENSDARG00000100719
Normalised Counts
80
60
40
20
0
1−cell
2−cell
128−cell
1k−cell
Dome
50%−epiboly
Shield
75%−epiboly
1−4−somites
14−19−somites
20−25−somites
Prim−5
Prim−15
Prim−25
Long−pec
Protruding−mouth
Day−4
Day−5
Stage
Transcript
ENSDART00000163471
ENSDART00000172461

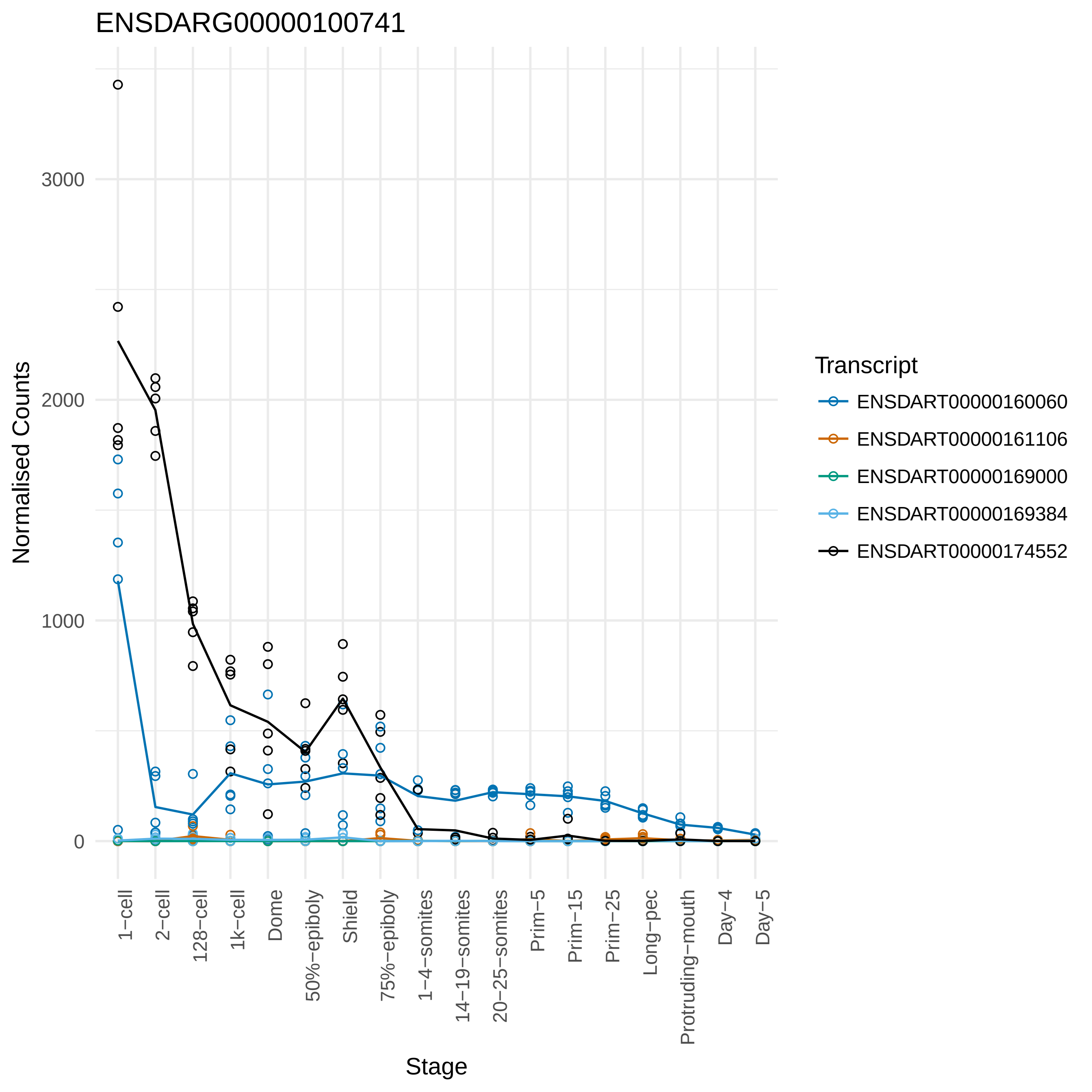

ENSDARG00000100741
Normalised Counts
3000
2000
1000
0
1−cell
2−cell
128−cell
1k−cell
Dome
50%−epiboly
Shield
75%−epiboly
1−4−somites
14−19−somites
20−25−somites
Prim−5
Prim−15
Prim−25
Long−pec
Protruding−mouth
Day−4
Day−5
Stage
Transcript
ENSDART00000160060
ENSDART00000161106
ENSDART00000169000
ENSDART00000169384
ENSDART00000174552

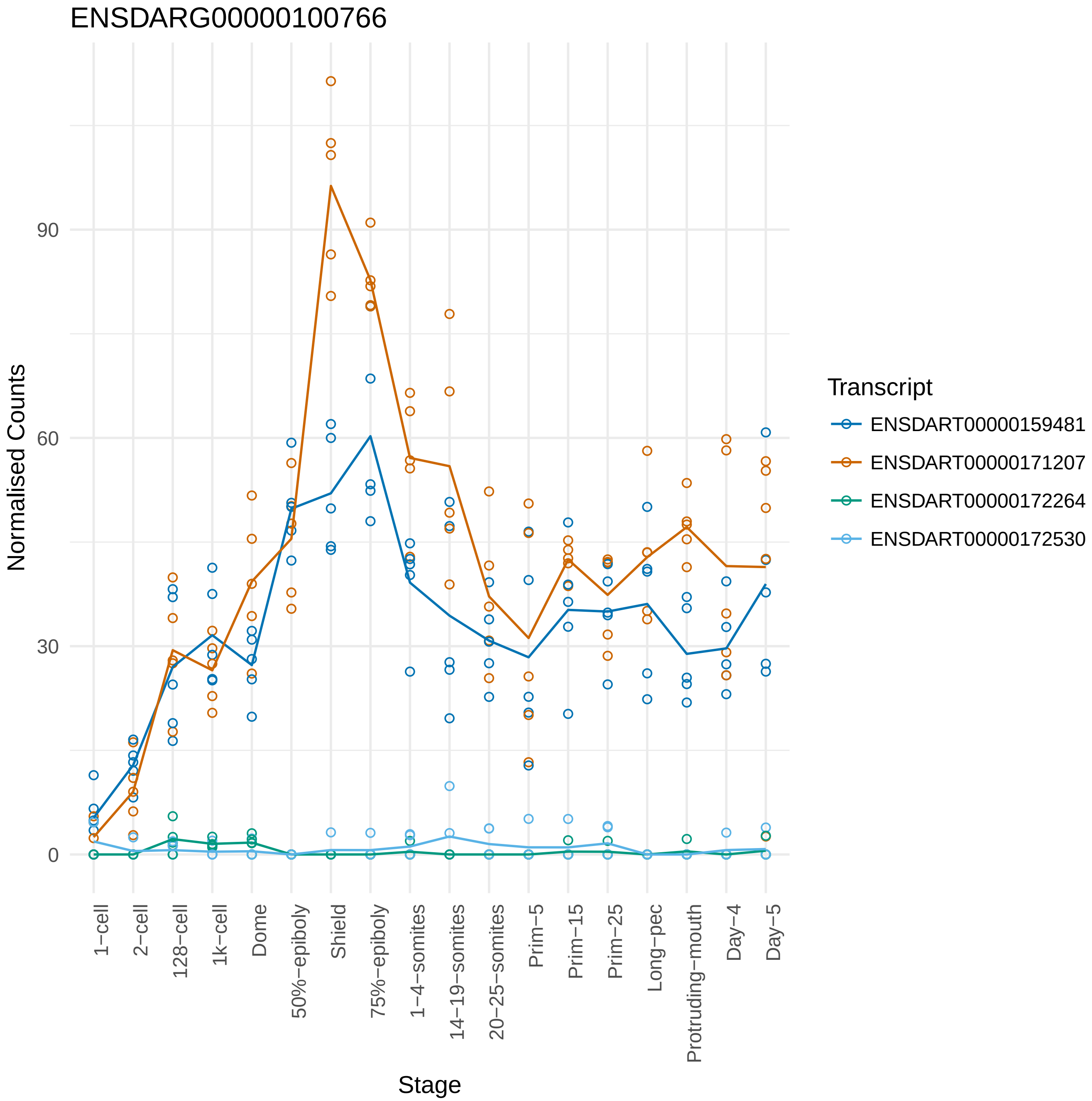

ENSDARG00000100766
Normalised Counts
0
30
60
90
1−cell
2−cell
128−cell
1k−cell
Dome
50%−epiboly
Shield
75%−epiboly
1−4−somites
14−19−somites
20−25−somites
Prim−5
Prim−15
Prim−25
Long−pec
Protruding−mouth
Day−4
Day−5
Stage
Transcript
ENSDART00000159481
ENSDART00000171207
ENSDART00000172264
ENSDART00000172530

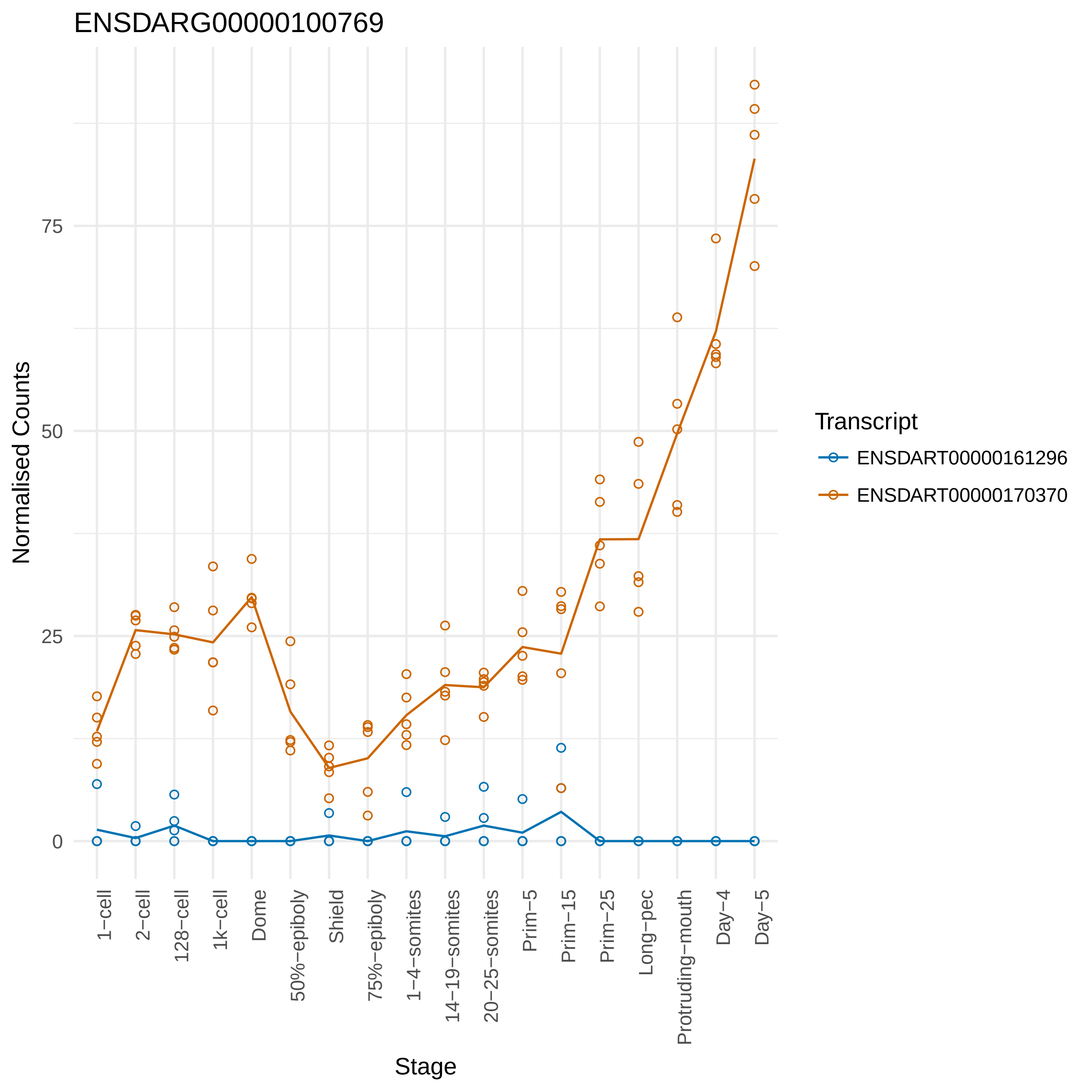
ENSDARG00000100769
Normalised Counts
0
25
50
75
Stage
1−cell
2−cell
128−cell
1k−cell
Dome
50%−epiboly
Shield
75%−epiboly
1−4−somites
14−19−somites
20−25−somites
Prim−5
Prim−15
Prim−25
Long−pec
Protruding−mouth
Day−4
Day−5
Transcript
ENSDART00000161296
ENSDART00000170370

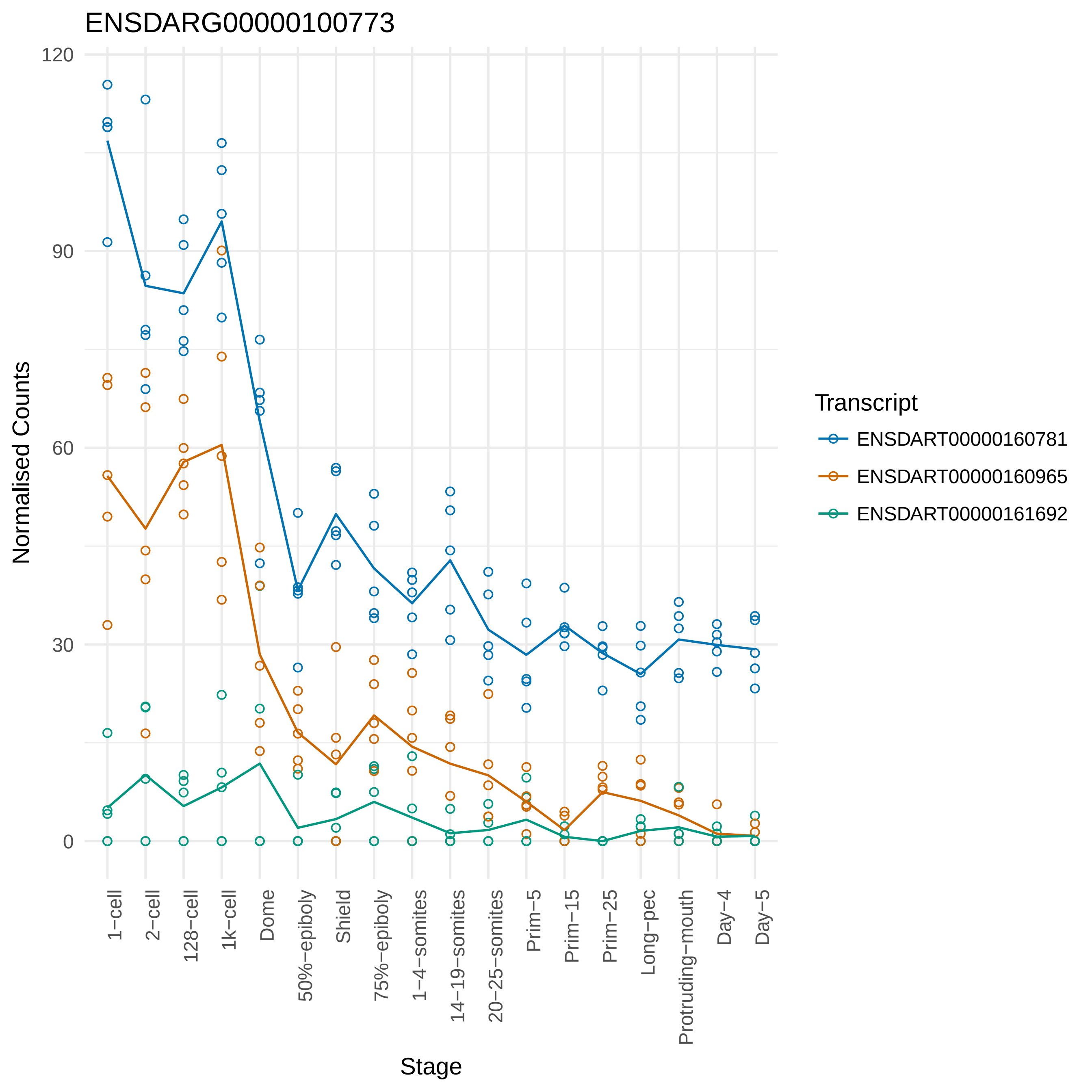
ENSDARG00000100773
Normalised Counts
120
90
60
30
0
1−cell
2−cell
128−cell
1k−cell
Dome
50%−epiboly
Shield
75%−epiboly
1−4−somites
14−19−somites
20−25−somites
Prim−5
Prim−15
Prim−25
Long−pec
Protruding−mouth
Day−4
Day−5
Stage
Transcript
ENSDART00000160781
ENSDART00000160965
ENSDART00000161692

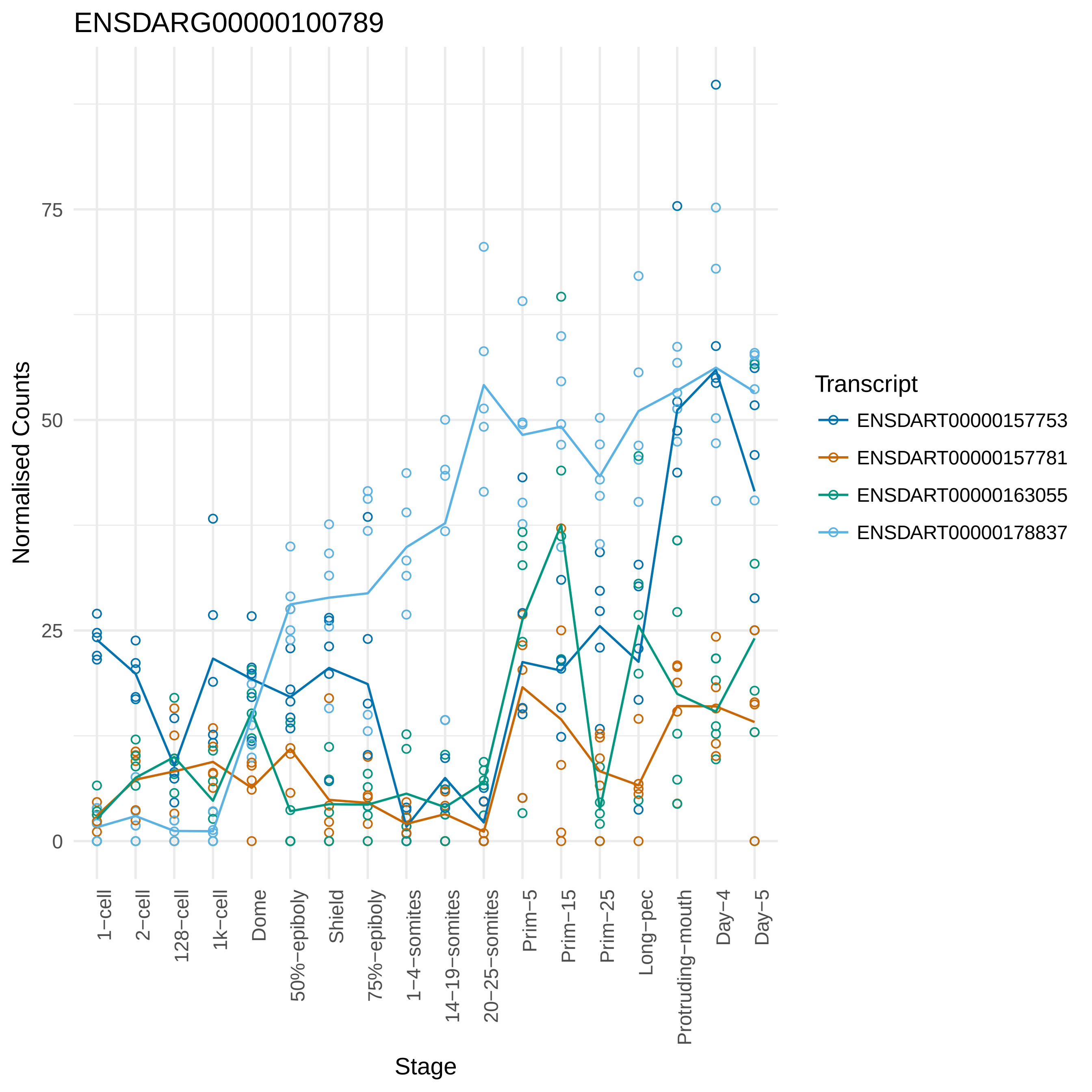
ENSDARG00000100789
Normalised Counts
0
25
50
75
Stage
1−cell
2−cell
128−cell
1k−cell
Dome
50%−epiboly
Shield
75%−epiboly
1−4−somites
14−19−somites
20−25−somites
Prim−5
Prim−15
Prim−25
Long−pec
Protruding−mouth
Day−4
Day−5
Transcript
ENSDART00000157753
ENSDART00000157781
ENSDART00000163055
ENSDART00000178837

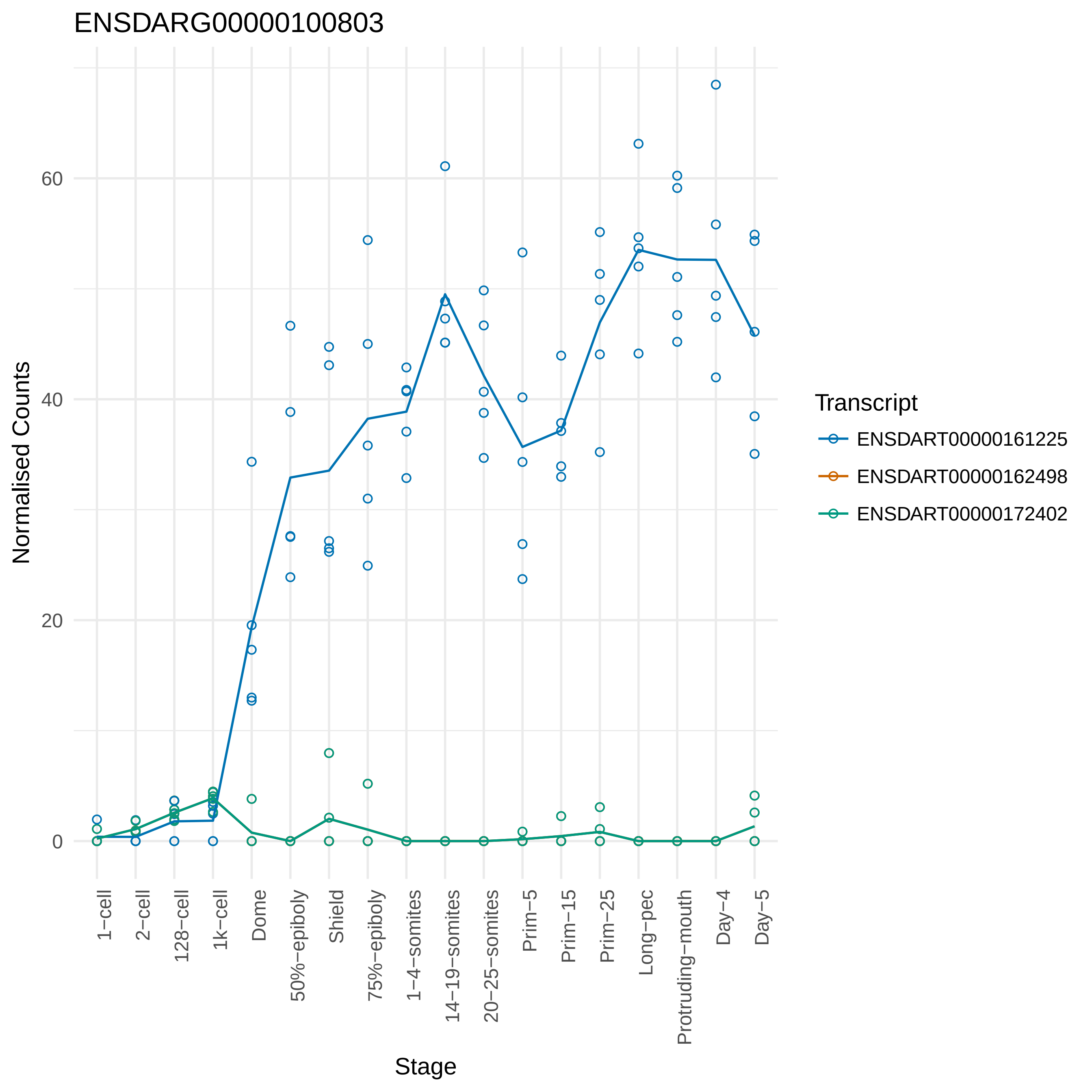
ENSDARG00000100803
Normalised Counts
0
20
40
60
1−cell
2−cell
128−cell
1k−cell
Dome
50%−epiboly
Shield
75%−epiboly
1−4−somites
14−19−somites
20−25−somites
Prim−5
Prim−15
Prim−25
Long−pec
Protruding−mouth
Day−4
Day−5
Stage
Transcript
ENSDART00000161225
ENSDART00000162498
ENSDART00000172402

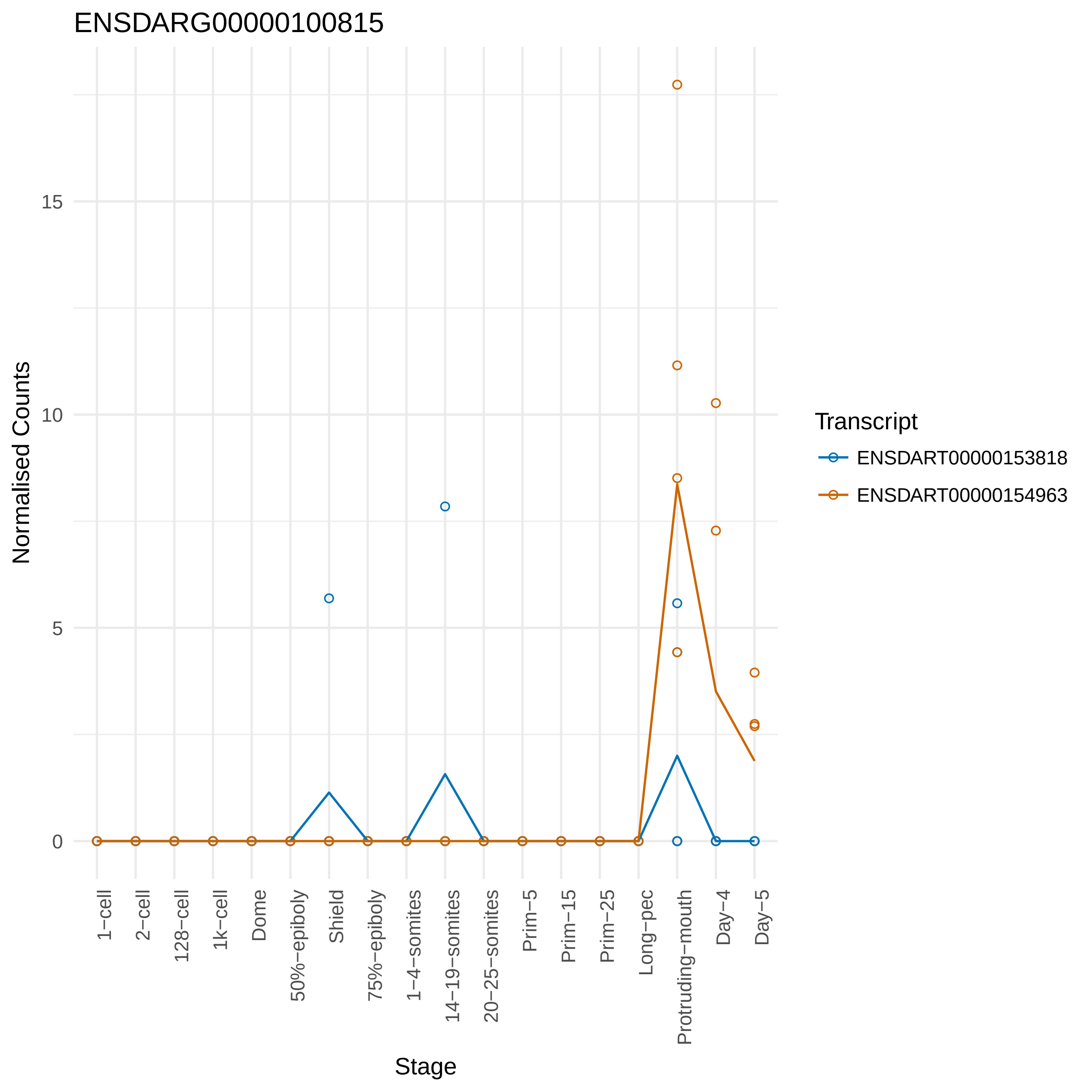

ENSDARG00000100815
Normalised Counts
0
5
10
15
Stage
1−cell
2−cell
128−cell
1k−cell
Dome
50%−epiboly
Shield
75%−epiboly
1−4−somites
14−19−somites
20−25−somites
Prim−5
Prim−15
Prim−25
Long−pec
Protruding−mouth
Day−4
Day−5
Transcript
ENSDART00000153818
ENSDART00000154963

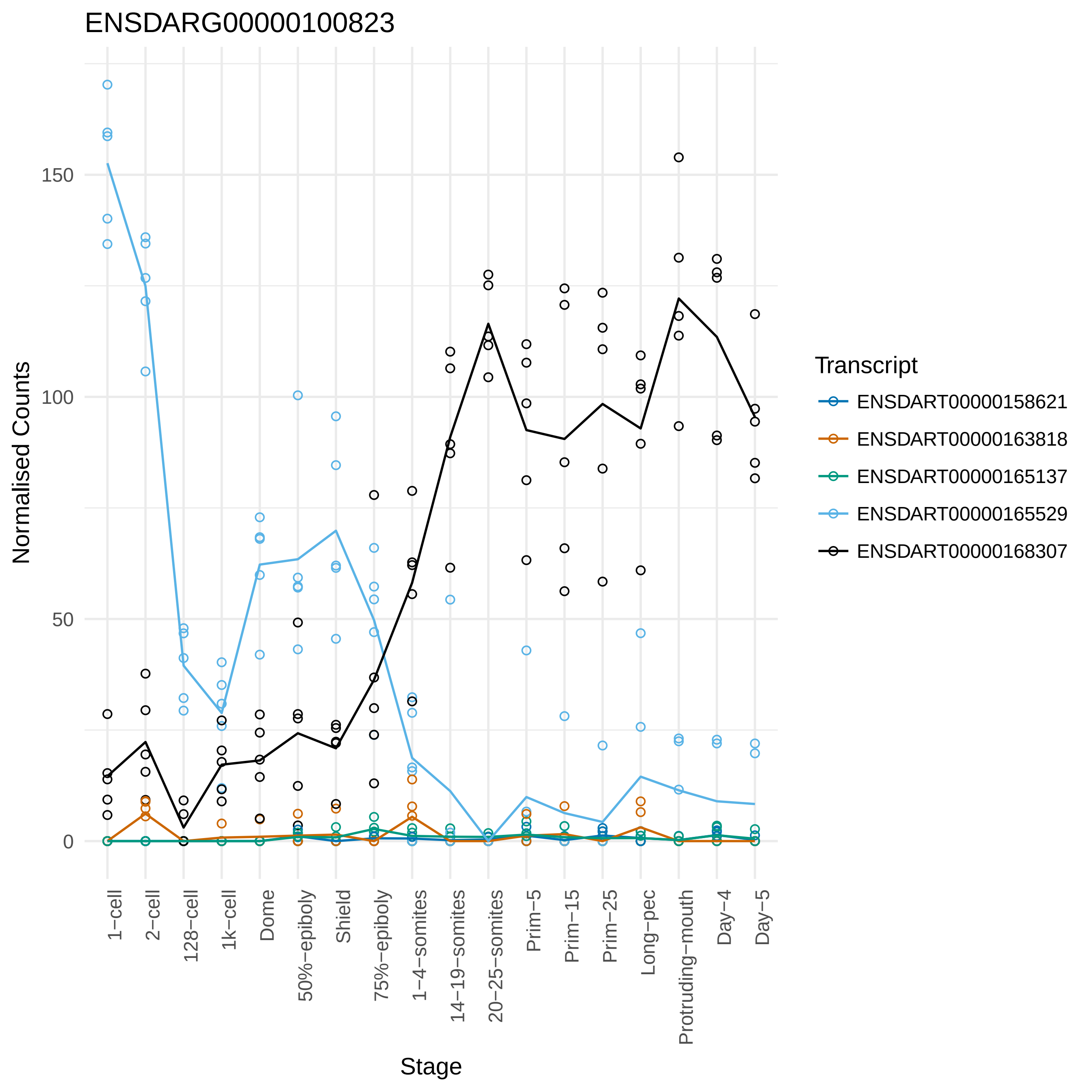
ENSDARG00000100823
Normalised Counts
150
100
50
0
1−cell
2−cell
128−cell
1k−cell
Dome
50%−epiboly
Shield
75%−epiboly
1−4−somites
14−19−somites
20−25−somites
Prim−5
Prim−15
Prim−25
Long−pec
Protruding−mouth
Day−4
Day−5
Stage
Transcript
ENSDART00000158621
ENSDART00000163818
ENSDART00000165137
ENSDART00000165529
ENSDART00000168307

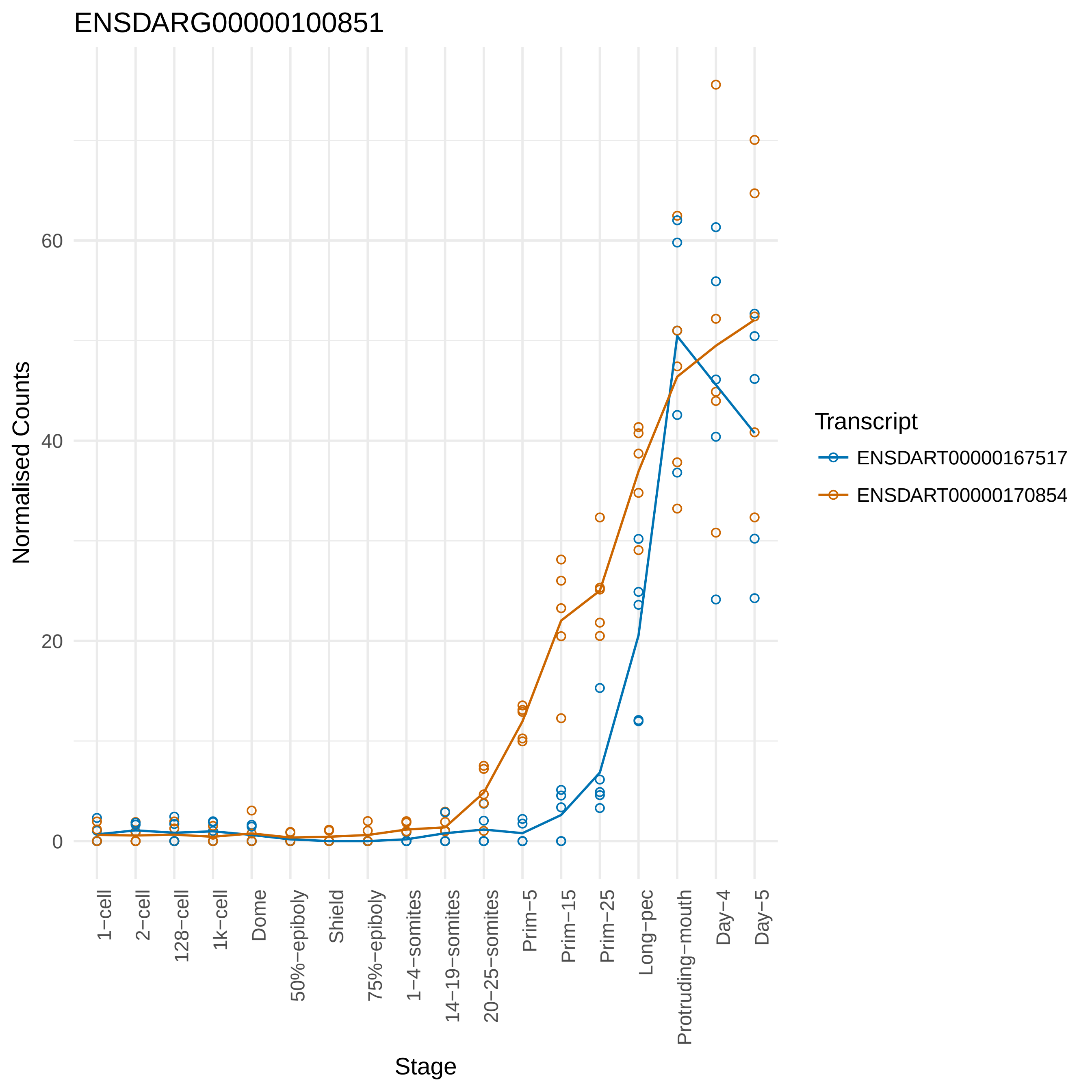
ENSDARG00000100851
Normalised Counts
0
20
40
60
Stage
1−cell
2−cell
128−cell
1k−cell
Dome
50%−epiboly
Shield
75%−epiboly
1−4−somites
14−19−somites
20−25−somites
Prim−5
Prim−15
Prim−25
Long−pec
Protruding−mouth
Day−4
Day−5
Transcript
ENSDART00000167517
ENSDART00000170854

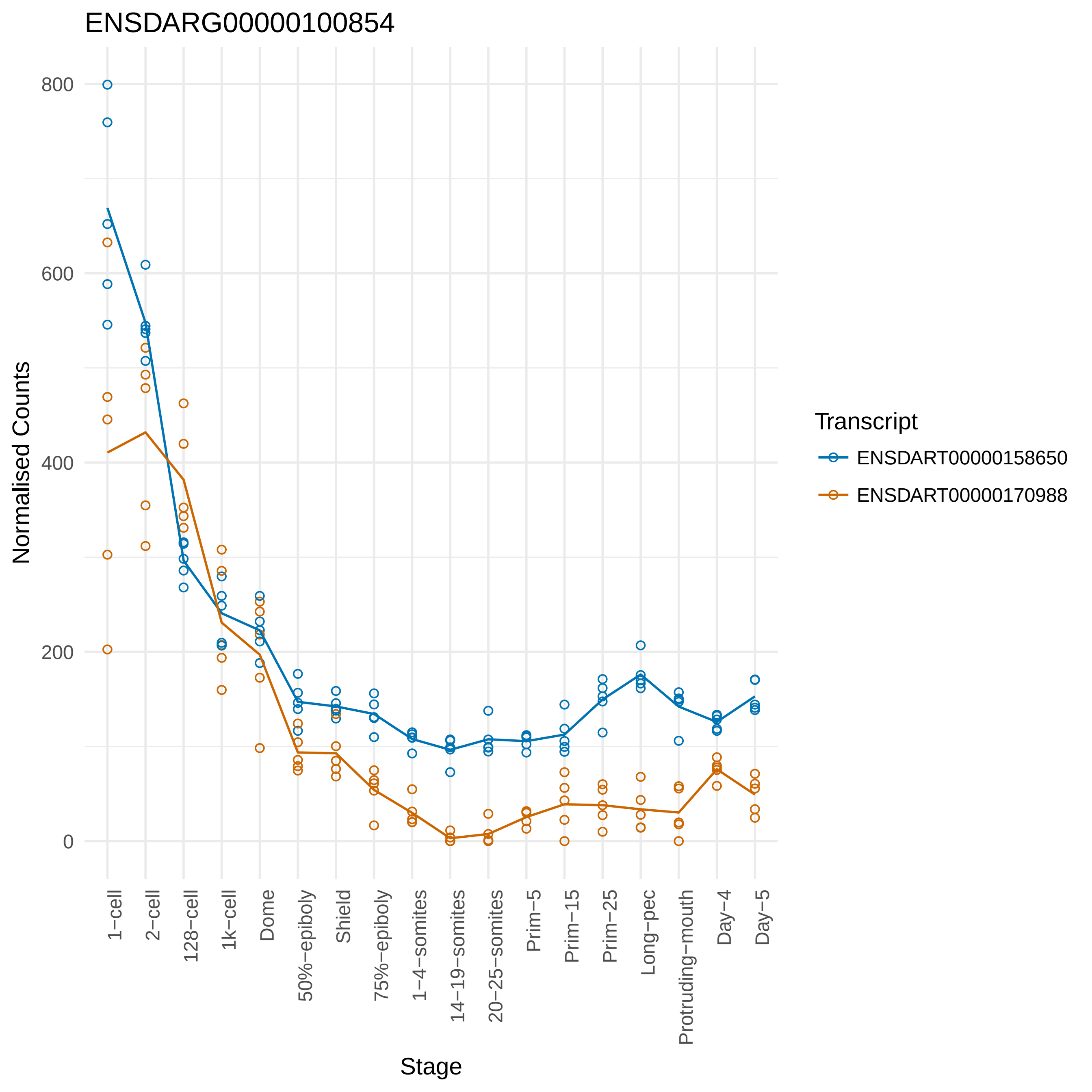

ENSDARG00000100854
Normalised Counts
800
600
400
200
0
1−cell
2−cell
128−cell
1k−cell
Dome
50%−epiboly
Shield
75%−epiboly
1−4−somites
14−19−somites
20−25−somites
Prim−5
Prim−15
Prim−25
Long−pec
Protruding−mouth
Day−4
Day−5
Stage
Transcript
ENSDART00000158650
ENSDART00000170988

ENSDARG00000100882

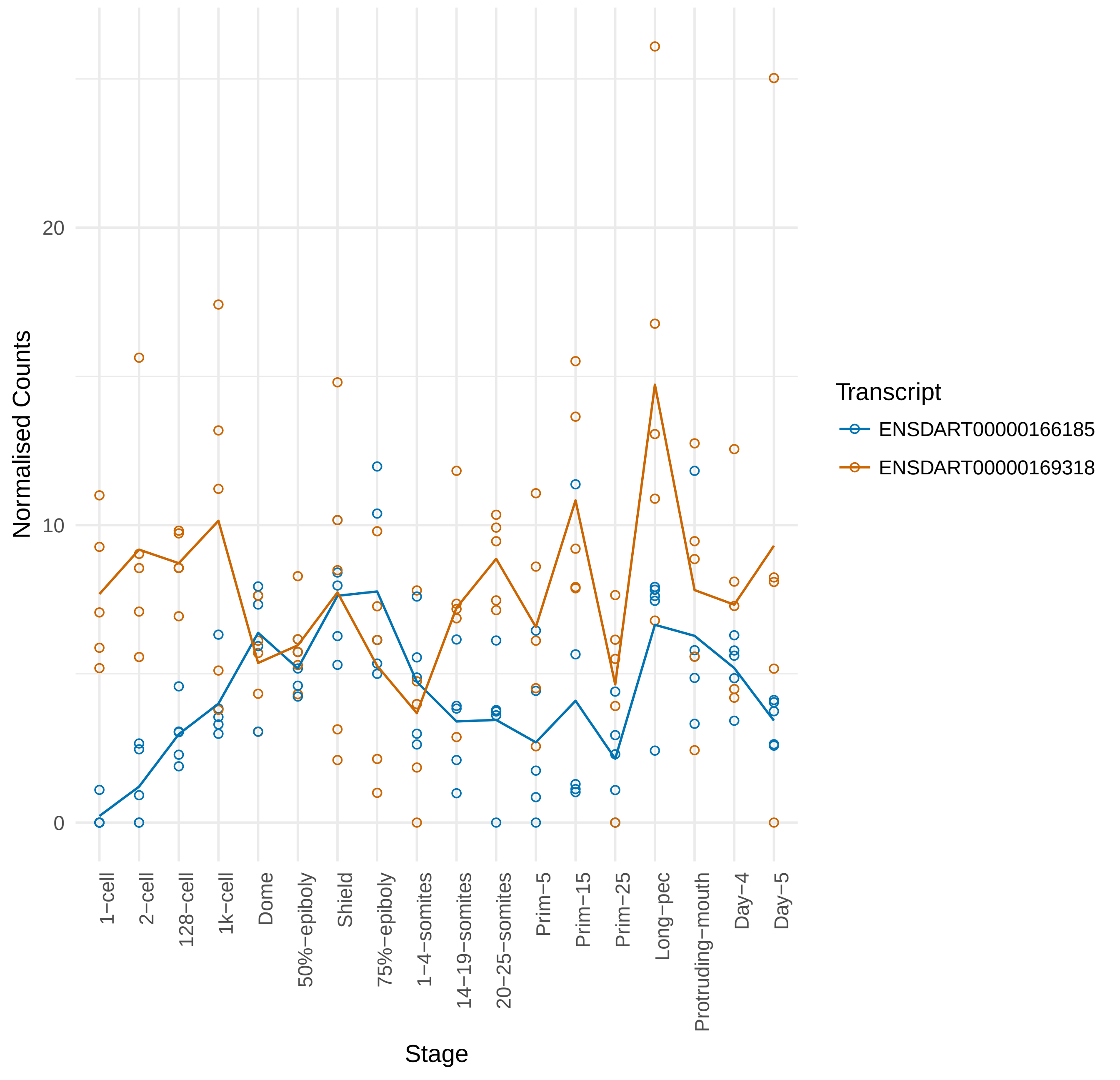

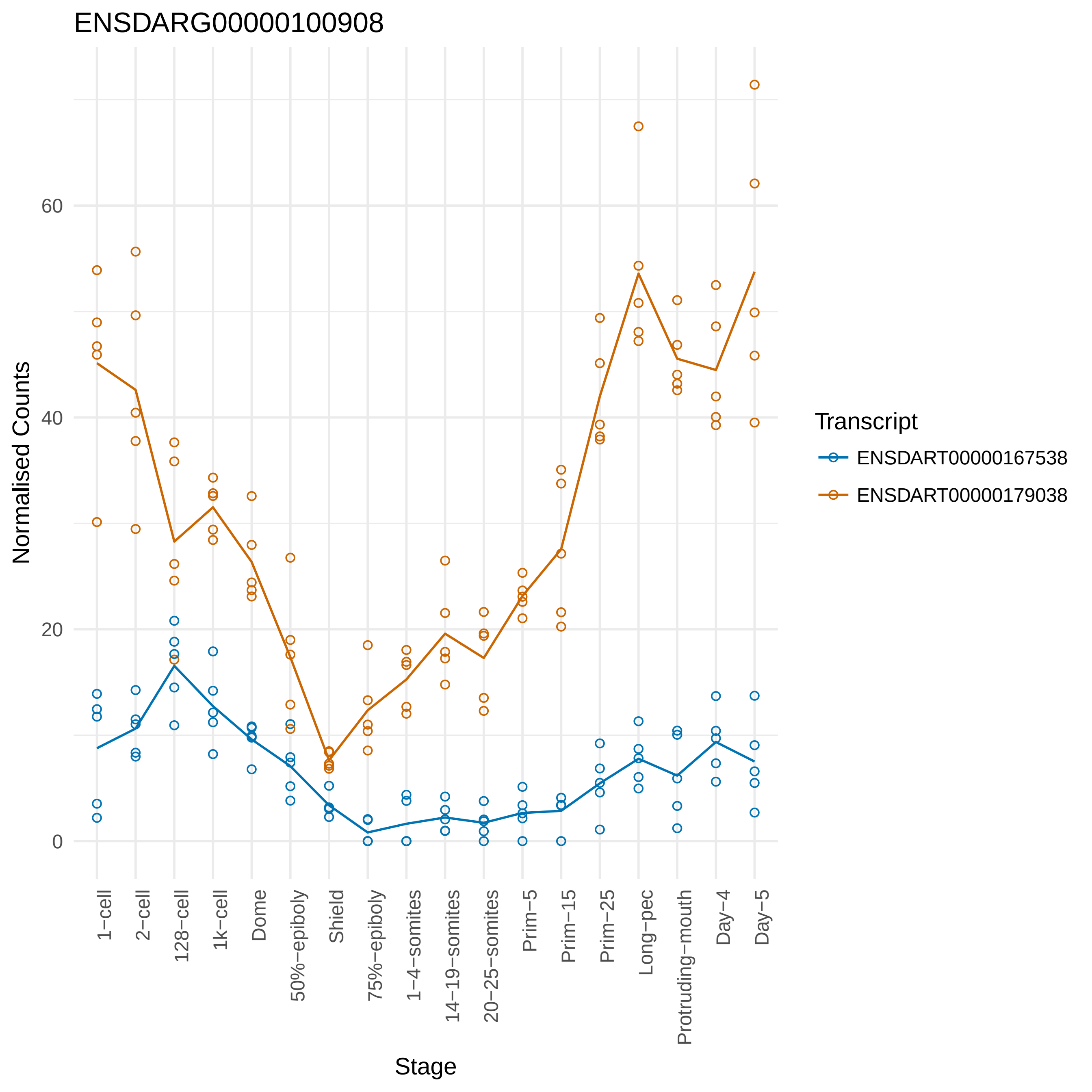

ENSDARG00000100908
Normalised Counts
0
20
40
60
1−cell
2−cell
128−cell
1k−cell
Dome
50%−epiboly
Shield
75%−epiboly
1−4−somites
14−19−somites
20−25−somites
Prim−5
Prim−15
Prim−25
Long−pec
Protruding−mouth
Day−4
Day−5
Stage
Transcript
ENSDART00000167538
ENSDART00000179038

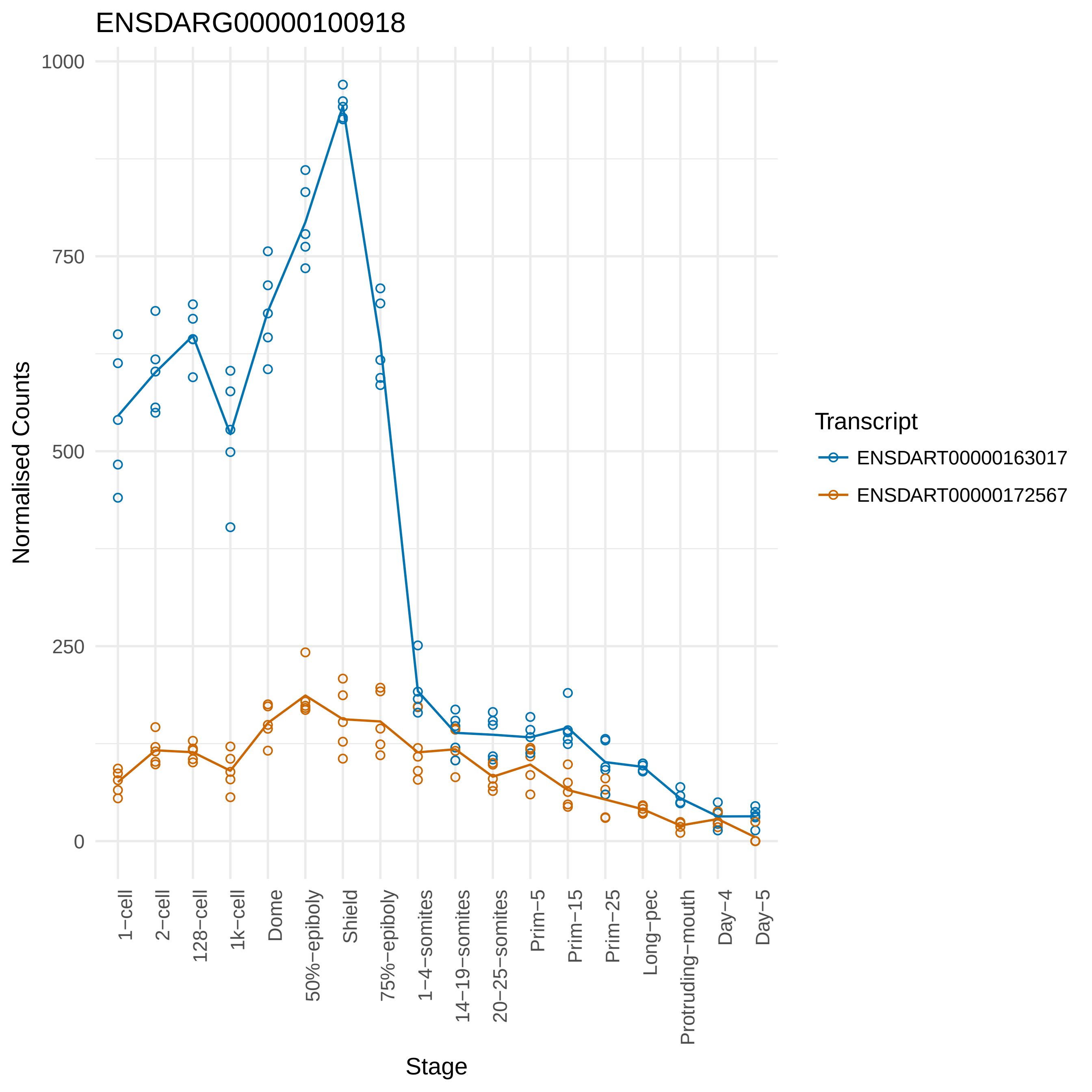
ENSDARG00000100918
Normalised Counts
1000
750
500
250
0
1−cell
2−cell
128−cell
1k−cell
Dome
50%−epiboly
Shield
75%−epiboly
1−4−somites
14−19−somites
20−25−somites
Prim−5
Prim−15
Prim−25
Long−pec
Protruding−mouth
Day−4
Day−5
Stage
Transcript
ENSDART00000163017
ENSDART00000172567

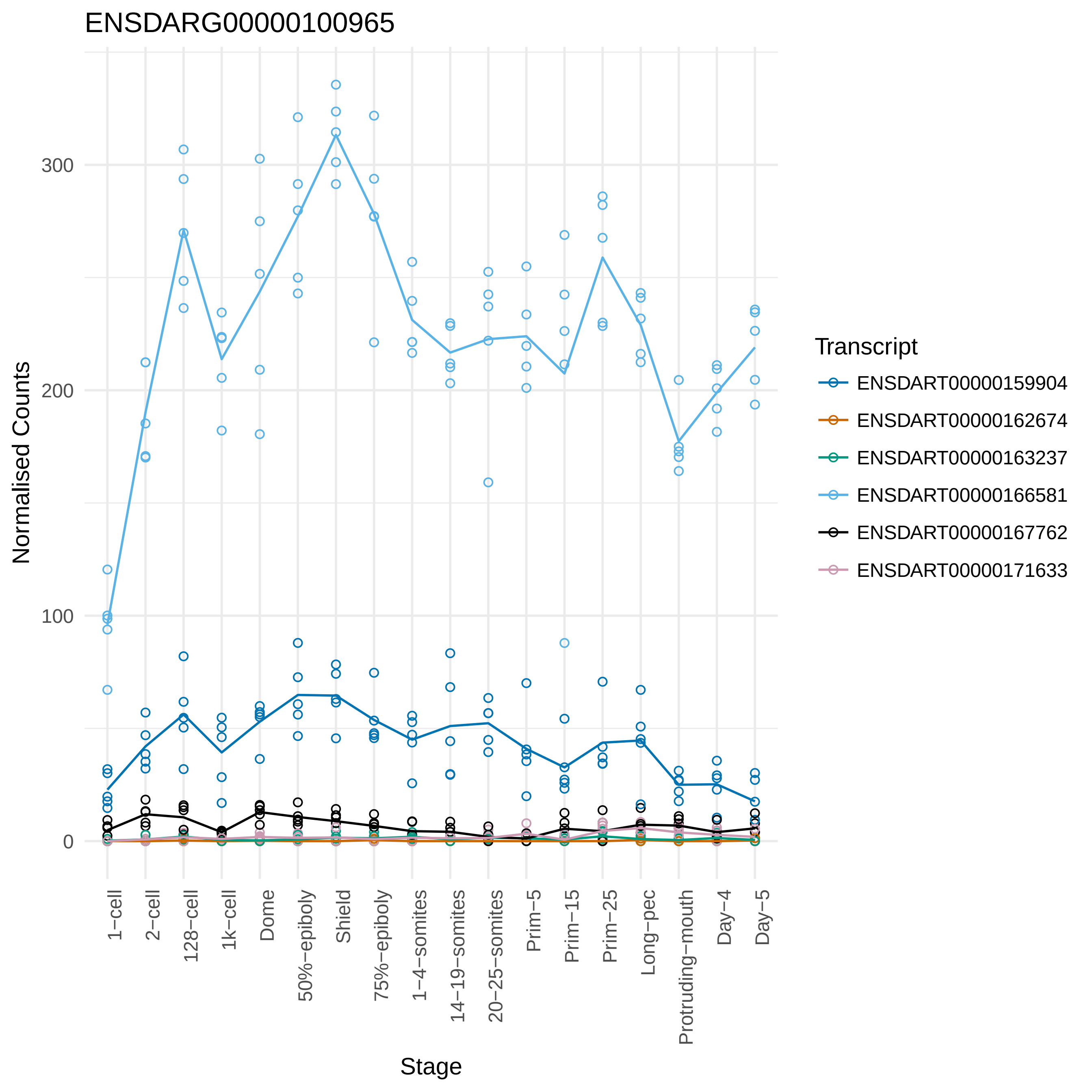

ENSDARG00000100965
Normalised Counts
300
200
100
0
1−cell
2−cell
128−cell
1k−cell
Dome
50%−epiboly
Shield
75%−epiboly
1−4−somites
14−19−somites
20−25−somites
Prim−5
Prim−15
Prim−25
Long−pec
Protruding−mouth
Day−4
Day−5
Stage
Transcript
ENSDART00000159904
ENSDART00000162674
ENSDART00000163237
ENSDART00000166581
ENSDART00000167762
ENSDART00000171633

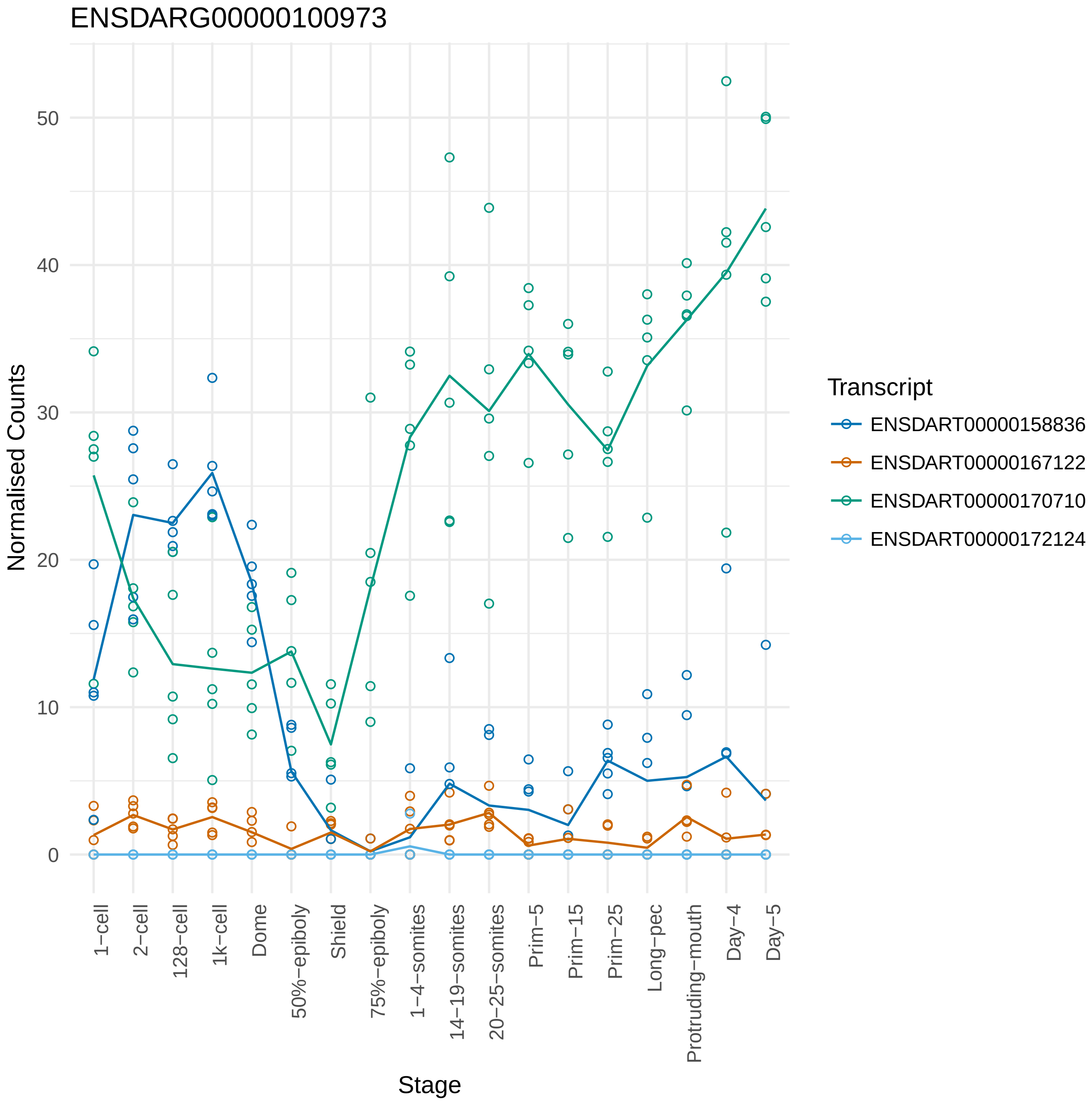
ENSDARG00000100973
Normalised Counts
Stage
Transcript
ENSDART00000158836
ENSDART00000167122
ENSDART00000170710
ENSDART00000172124
0
10
20
30
40
50
1−cell
2−cell
128−cell
1k−cell
Dome
50%−epiboly
Shield
75%−epiboly
1−4−somites
14−19−somites
20−25−somites
Prim−5
Prim−15
Prim−25
Long−pec
Protruding−mouth
Day−4
Day−5

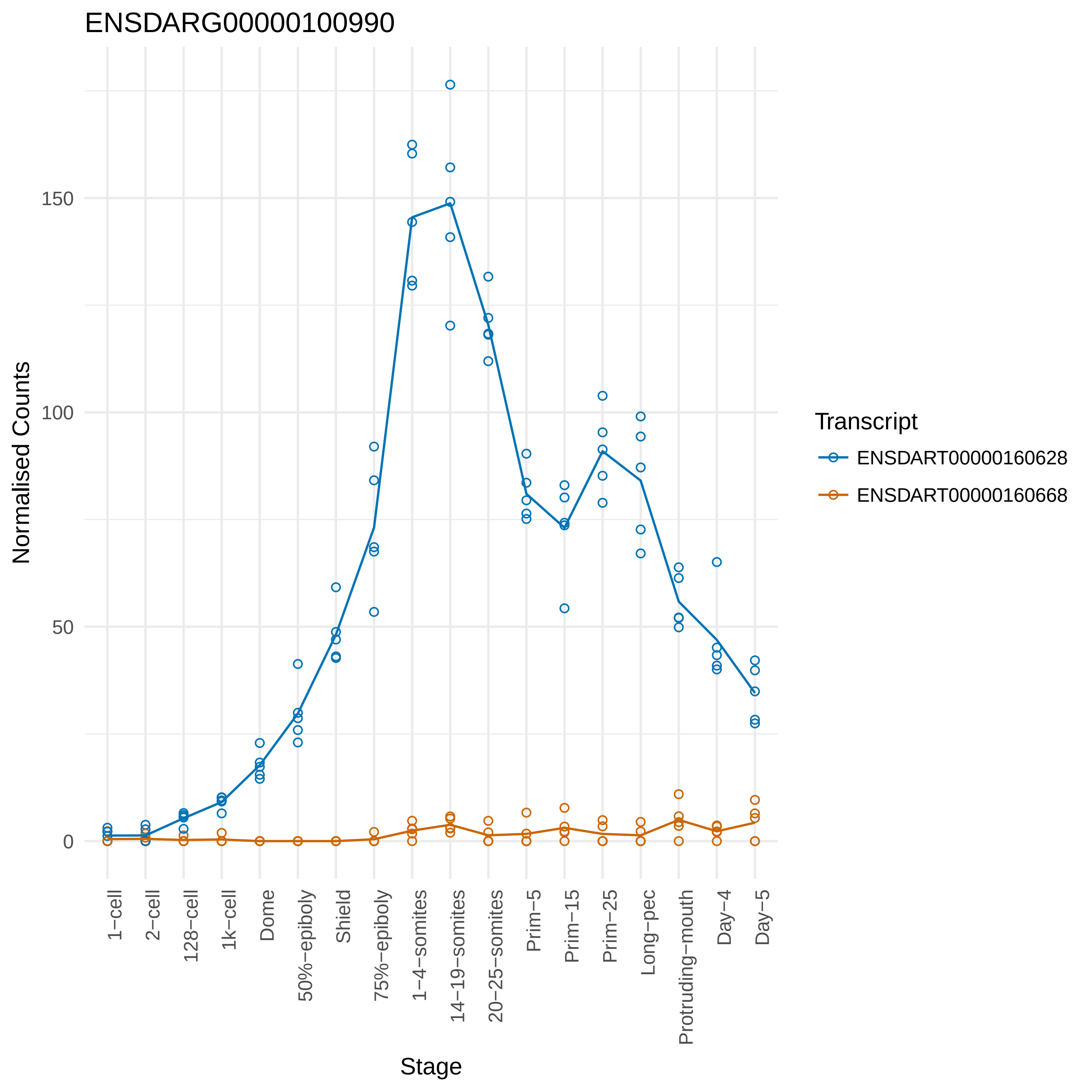
ENSDARG00000100990
Normalised Counts
0
50
100
150
Stage
1−cell
2−cell
128−cell
1k−cell
Dome
50%−epiboly
Shield
75%−epiboly
1−4−somites
14−19−somites
20−25−somites
Prim−5
Prim−15
Prim−25
Long−pec
Protruding−mouth
Day−4
Day−5
Transcript
ENSDART00000160628
ENSDART00000160668

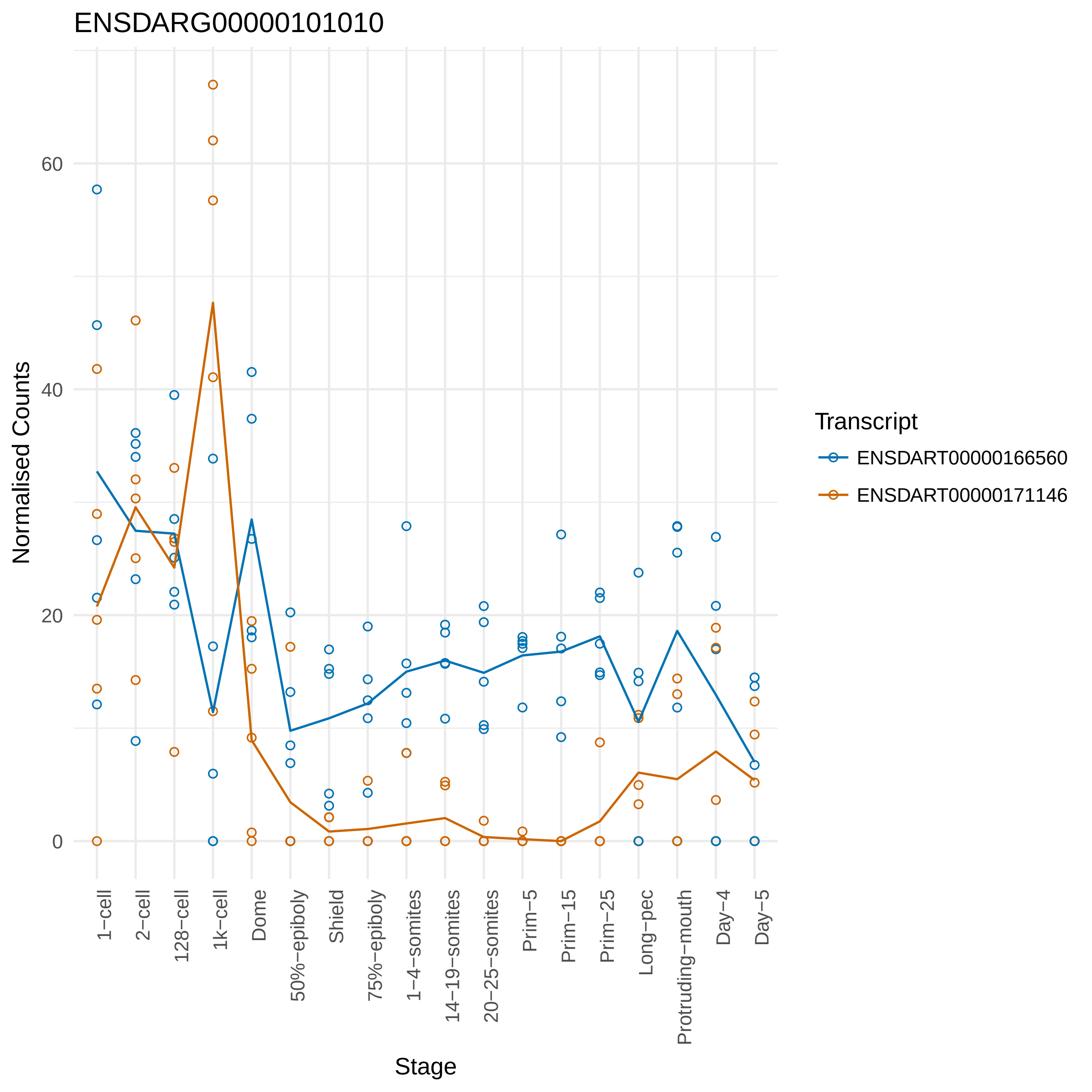

ENSDARG00000101010
Normalised Counts
0
20
40
60
1−cell
2−cell
128−cell
1k−cell
Dome
50%−epiboly
Shield
75%−epiboly
1−4−somites
14−19−somites
20−25−somites
Prim−5
Prim−15
Prim−25
Long−pec
Protruding−mouth
Day−4
Day−5
Stage
Transcript
ENSDART00000166560
ENSDART00000171146

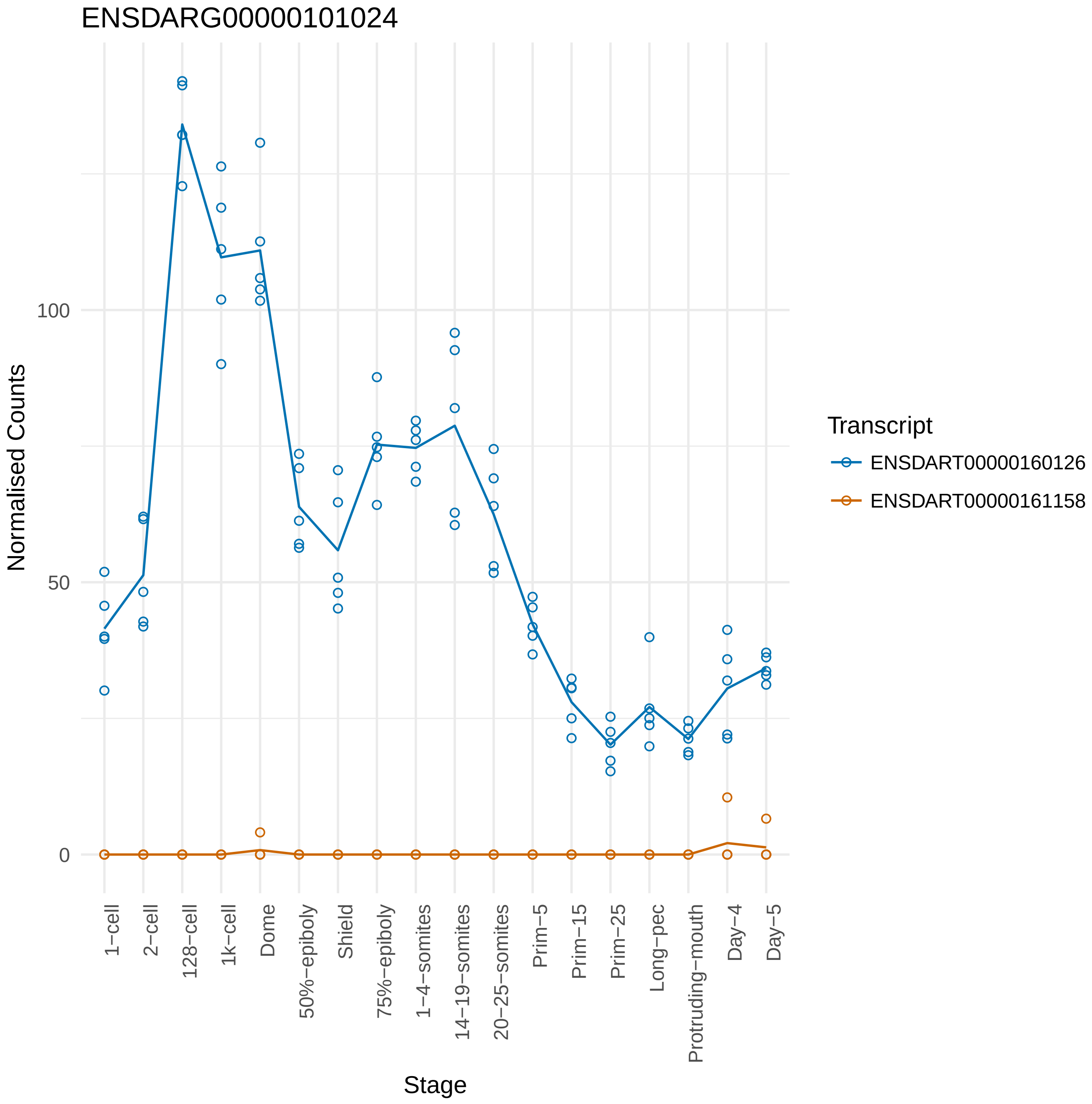

ENSDARG00000101024
Normalised Counts
100
50
0
1−cell
2−cell
128−cell
1k−cell
Dome
50%−epiboly
Shield
75%−epiboly
1−4−somites
14−19−somites
20−25−somites
Prim−5
Prim−15
Prim−25
Long−pec
Protruding−mouth
Day−4
Day−5
Stage
Transcript
ENSDART00000160126
ENSDART00000161158

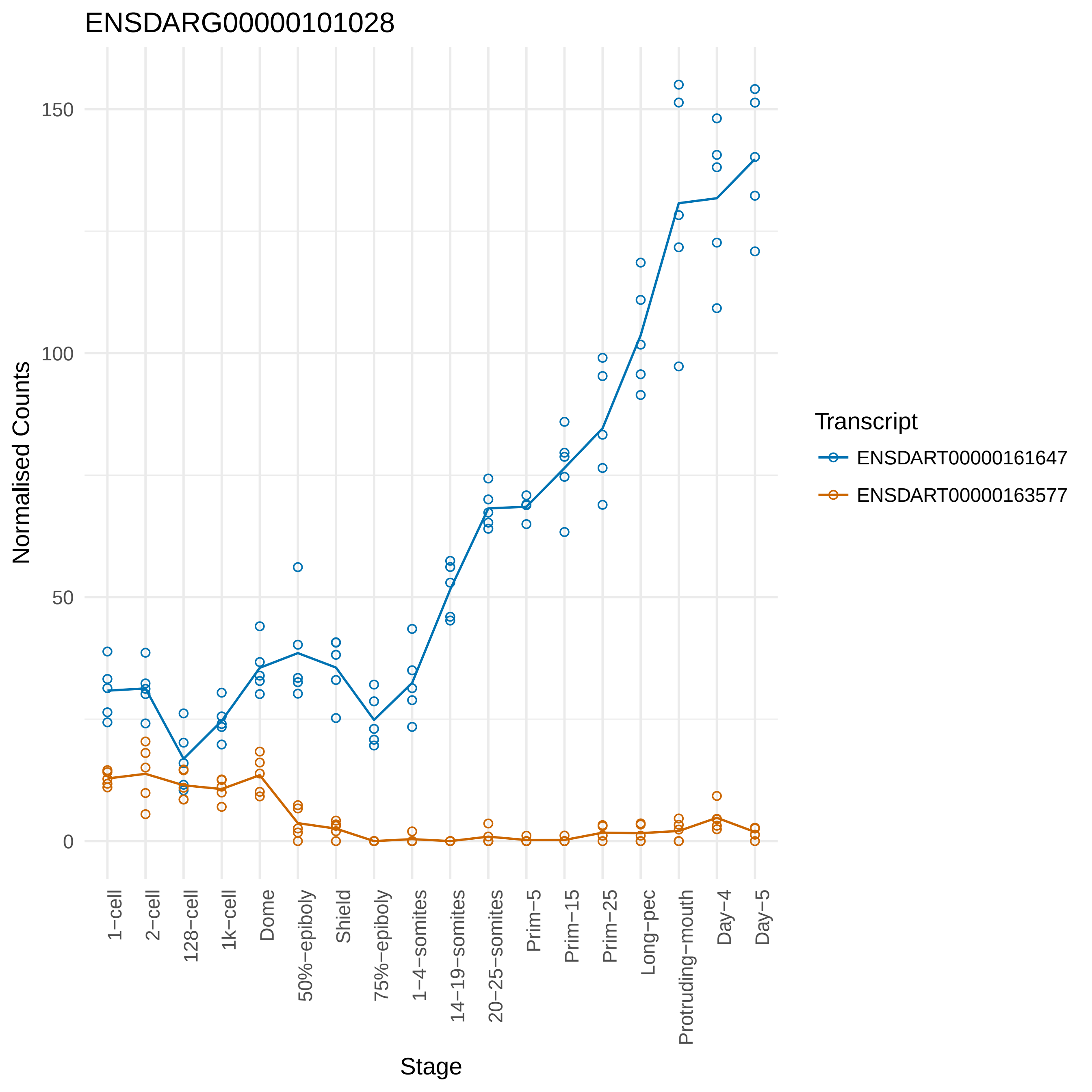
ENSDARG00000101028
Normalised Counts
150
100
50
0
1−cell
2−cell
128−cell
1k−cell
Dome
50%−epiboly
Shield
75%−epiboly
1−4−somites
14−19−somites
20−25−somites
Prim−5
Prim−15
Prim−25
Long−pec
Protruding−mouth
Day−4
Day−5
Stage
Transcript
ENSDART00000161647
ENSDART00000163577

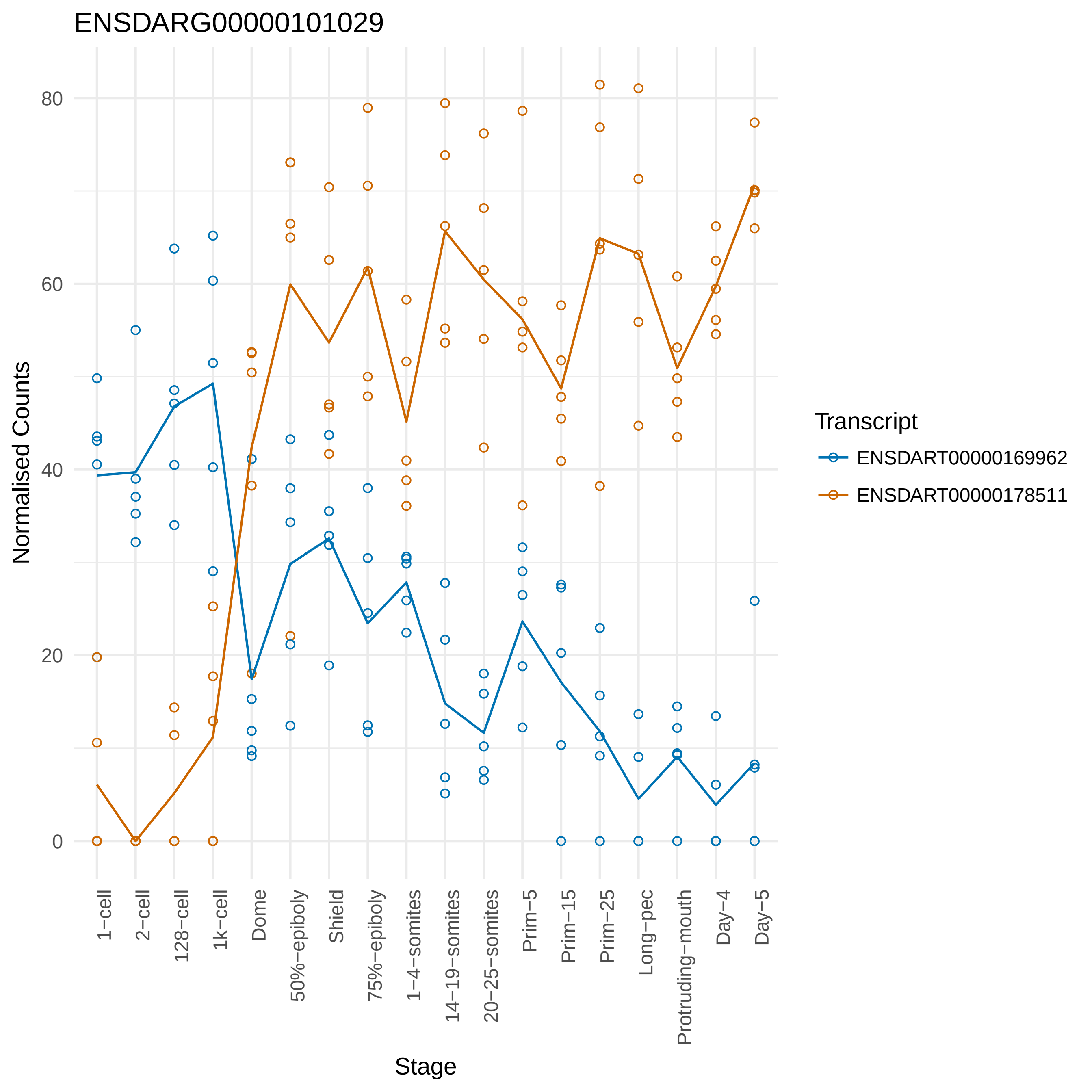

ENSDARG00000101029
Normalised Counts
0
20
40
60
80
1−cell
2−cell
128−cell
1k−cell
Dome
50%−epiboly
Shield
75%−epiboly
1−4−somites
14−19−somites
20−25−somites
Prim−5
Prim−15
Prim−25
Long−pec
Protruding−mouth
Day−4
Day−5
Stage
Transcript
ENSDART00000169962
ENSDART00000178511

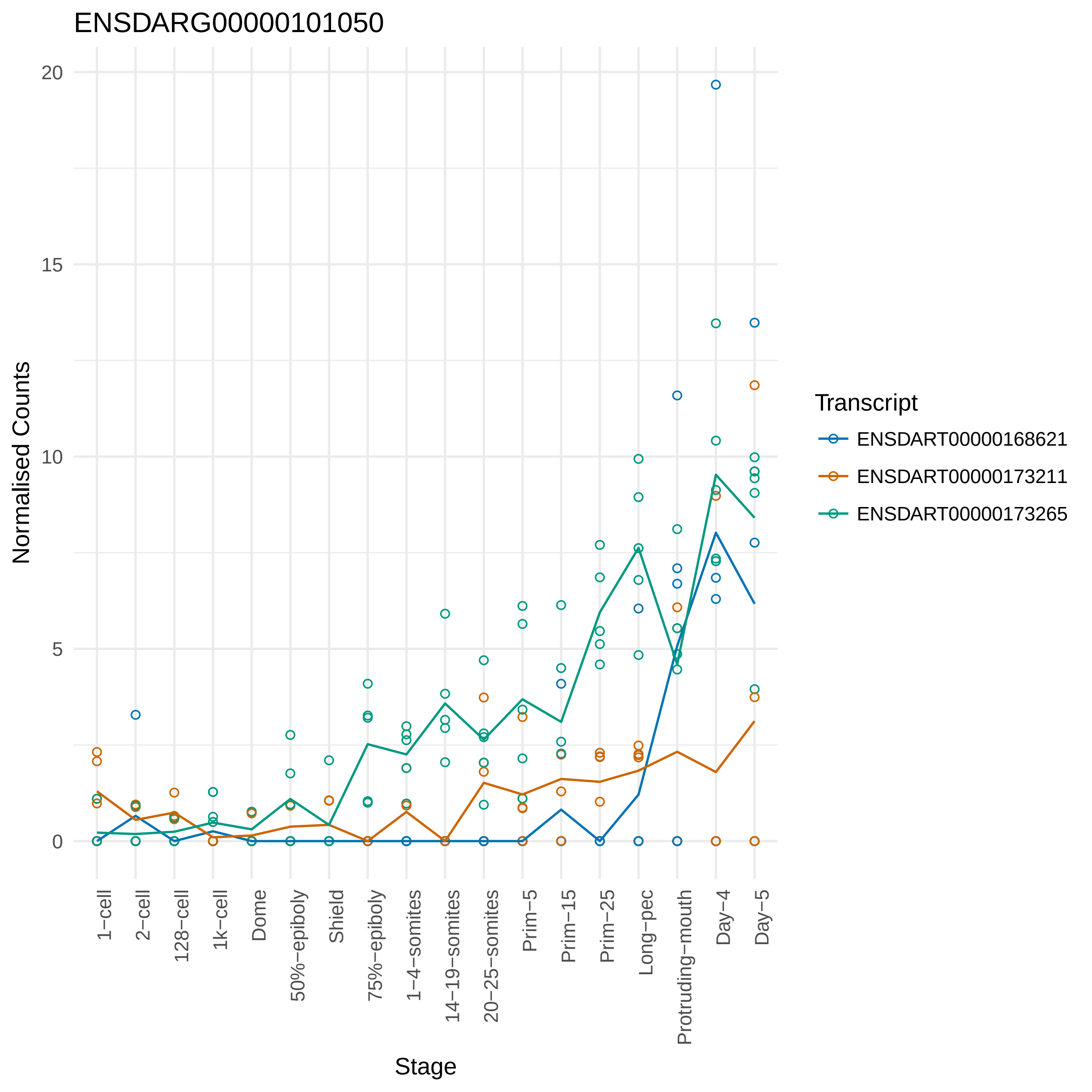
ENSDARG00000101050
Normalised Counts
20
15
10
5
0
1−cell
2−cell
128−cell
1k−cell
Dome
50%−epiboly
Shield
75%−epiboly
1−4−somites
14−19−somites
20−25−somites
Prim−5
Prim−15
Prim−25
Long−pec
Protruding−mouth
Day−4
Day−5
Stage
Transcript
ENSDART00000168621
ENSDART00000173211
ENSDART00000173265

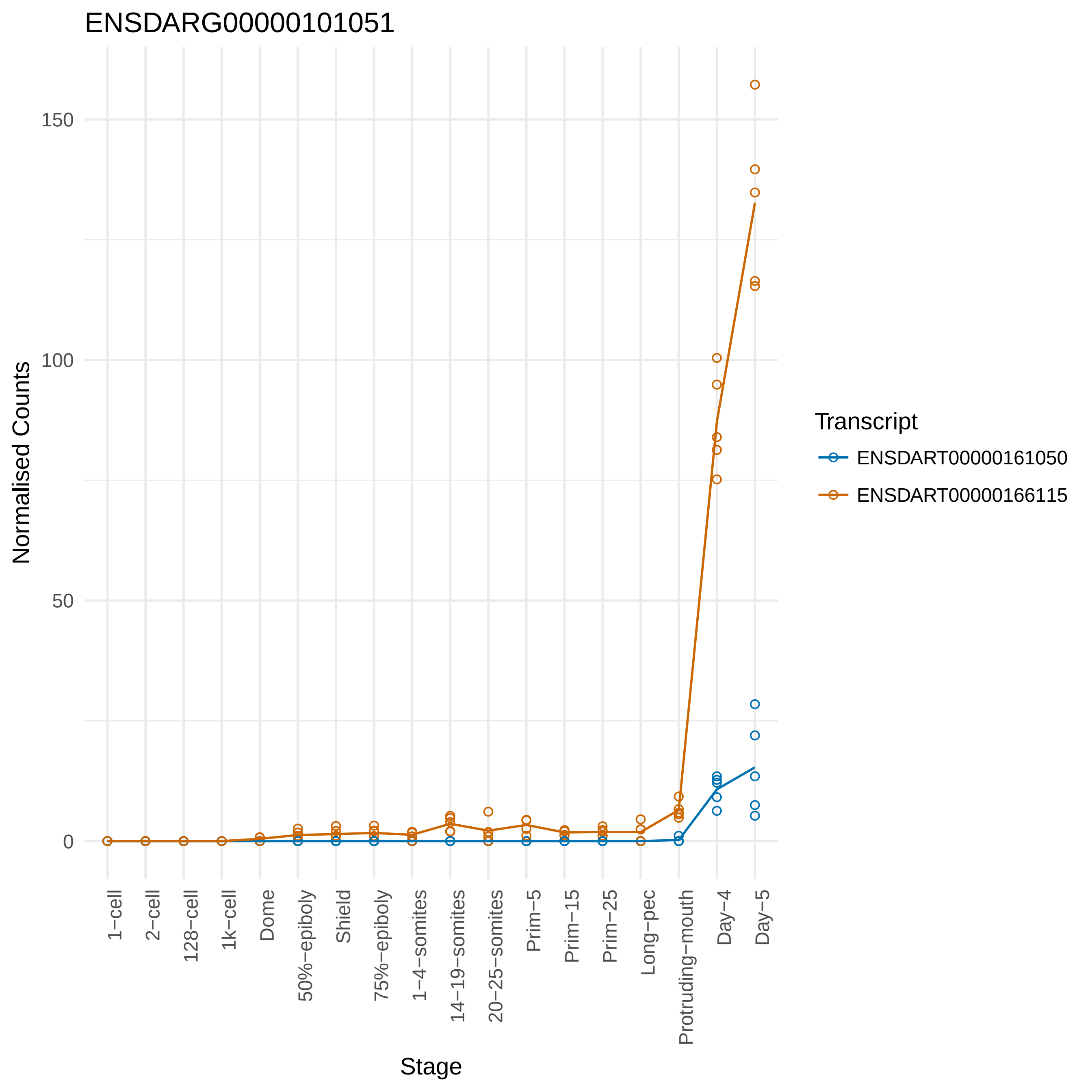

ENSDARG00000101051
Normalised Counts
150
100
50
0
1−cell
2−cell
128−cell
1k−cell
Dome
50%−epiboly
Shield
75%−epiboly
1−4−somites
14−19−somites
20−25−somites
Prim−5
Prim−15
Prim−25
Long−pec
Protruding−mouth
Day−4
Day−5
Stage
Transcript
ENSDART00000161050
ENSDART00000166115

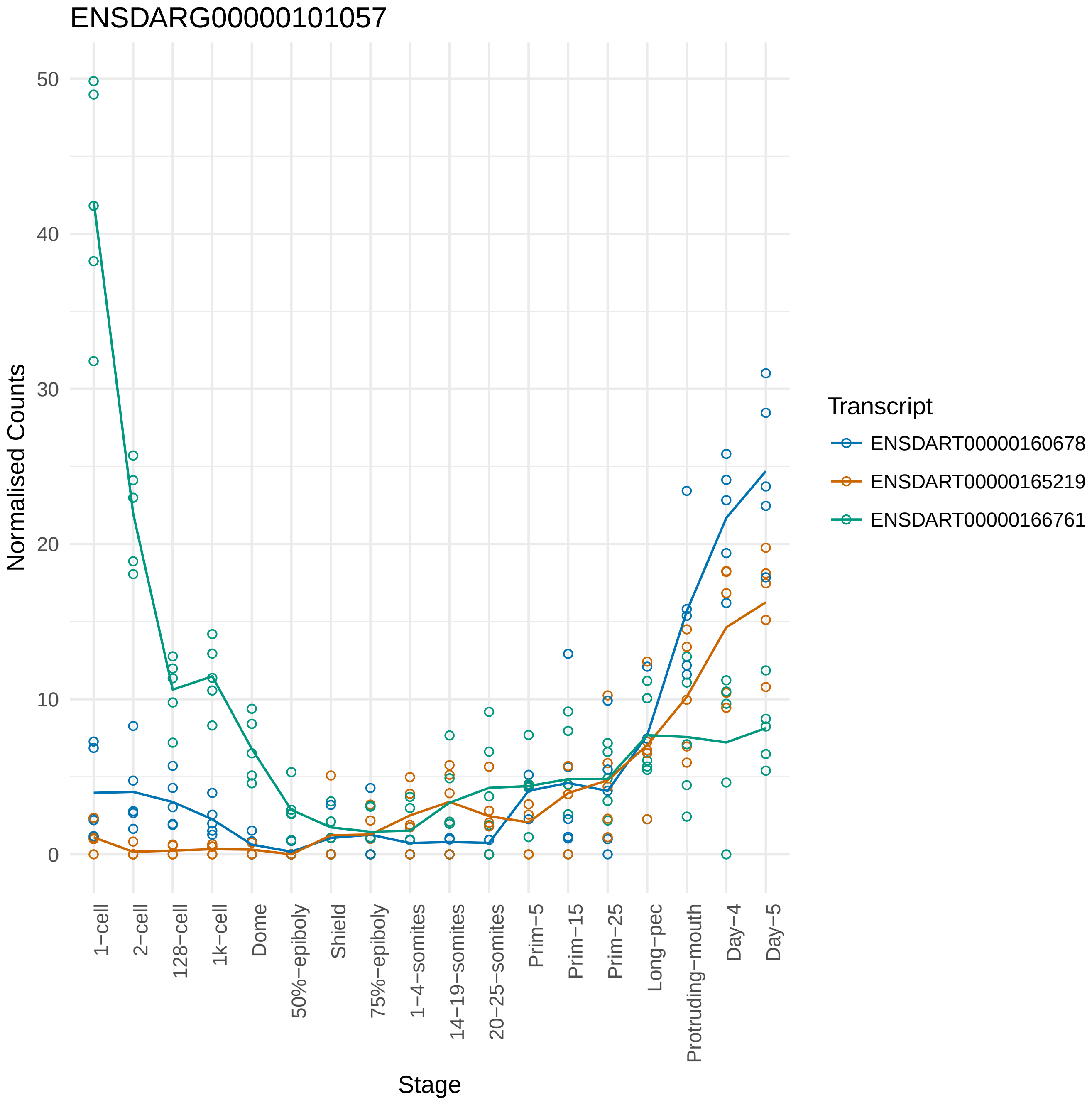

ENSDARG00000101057
Normalised Counts
50
40
30
20
10
0
1−cell
2−cell
128−cell
1k−cell
Dome
50%−epiboly
Shield
75%−epiboly
1−4−somites
14−19−somites
20−25−somites
Prim−5
Prim−15
Prim−25
Long−pec
Protruding−mouth
Day−4
Day−5
Stage
Transcript
ENSDART00000160678
ENSDART00000165219
ENSDART00000166761

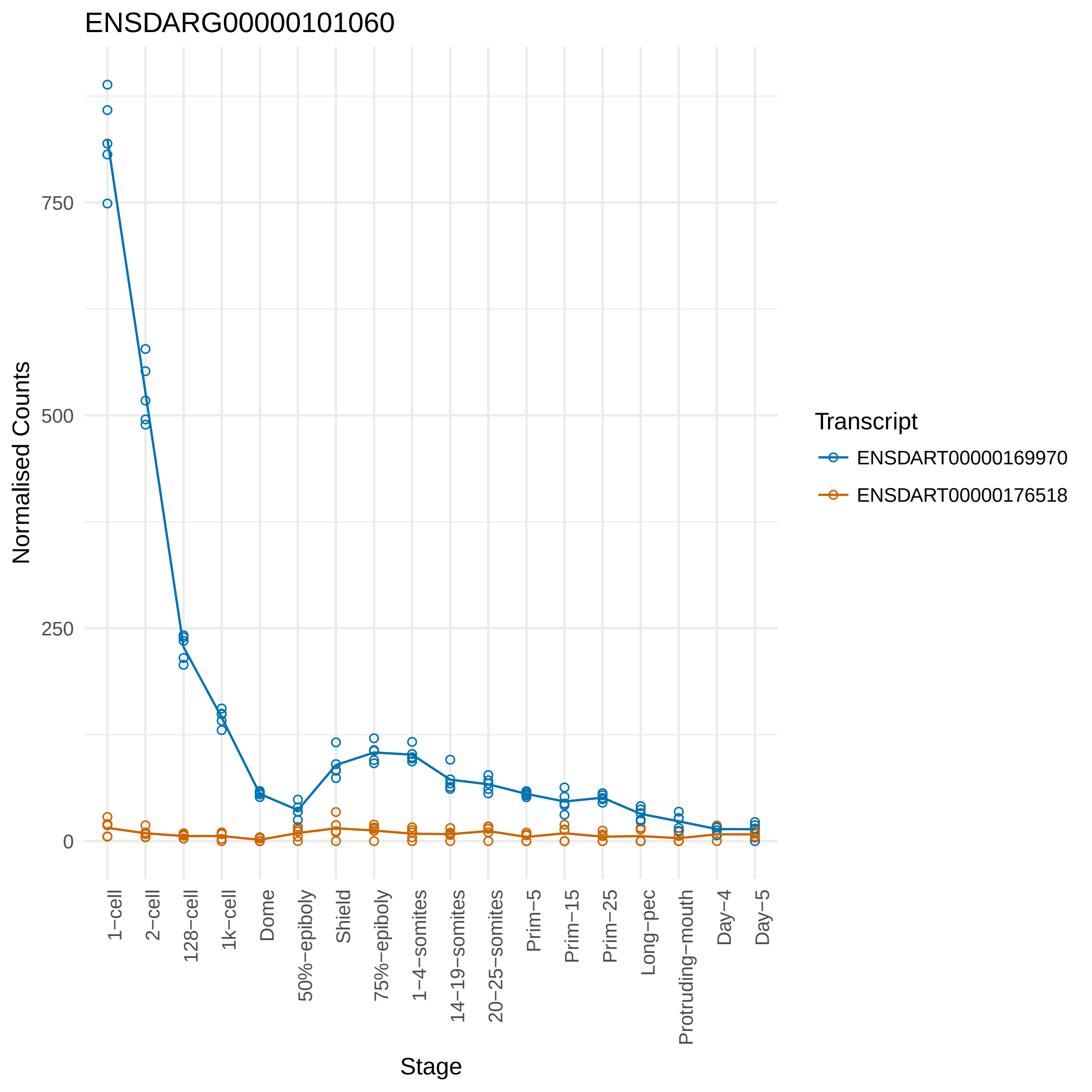

ENSDARG00000101060
Normalised Counts
750
500
250
0
1−cell
2−cell
128−cell
1k−cell
Dome
50%−epiboly
Shield
75%−epiboly
1−4−somites
14−19−somites
20−25−somites
Prim−5
Prim−15
Prim−25
Long−pec
Protruding−mouth
Day−4
Day−5
Stage
Transcript
ENSDART00000169970
ENSDART00000176518

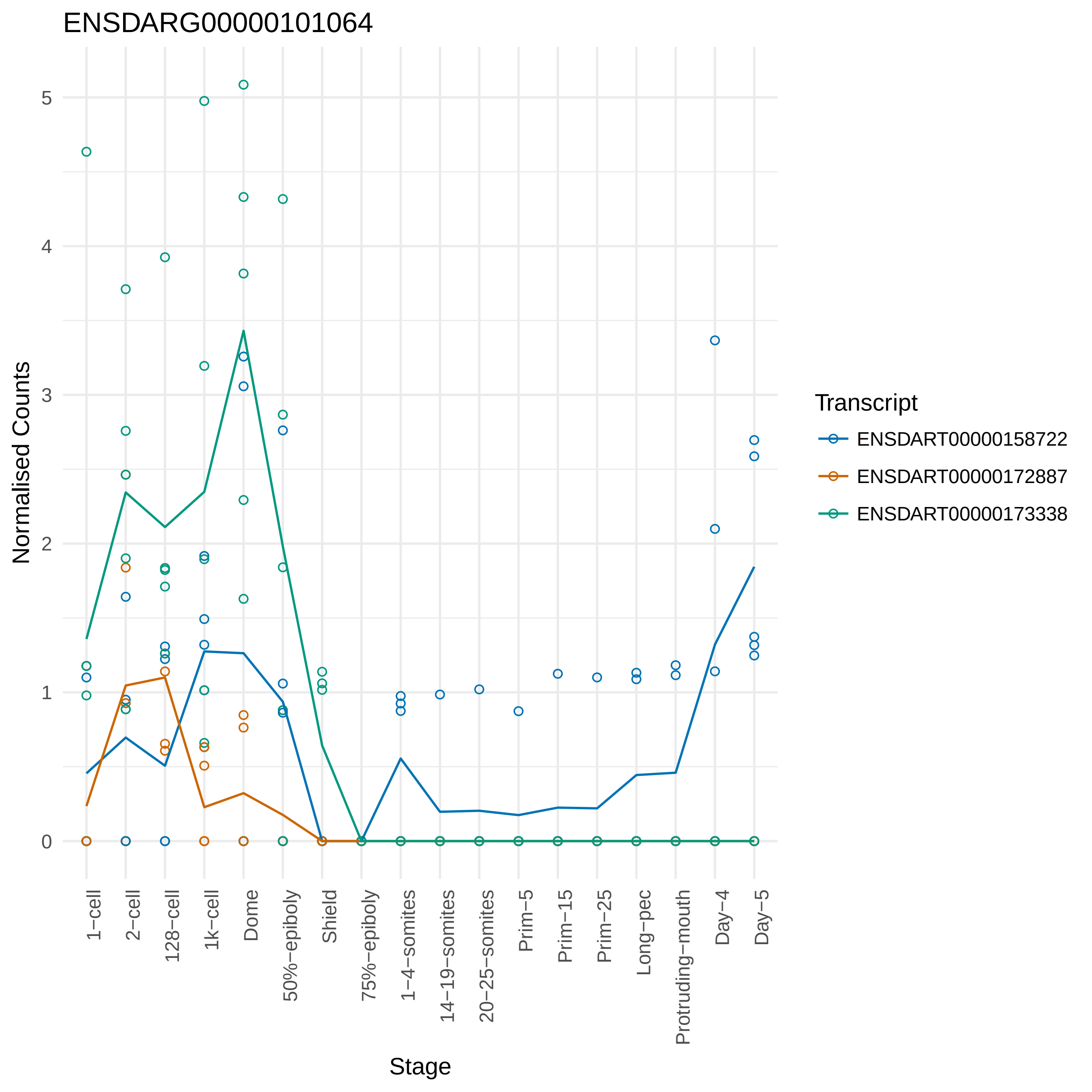

ENSDARG00000101064
Normalised Counts
0
1
2
3
4
5
Stage
1−cell
2−cell
128−cell
1k−cell
Dome
50%−epiboly
Shield
75%−epiboly
1−4−somites
14−19−somites
20−25−somites
Prim−5
Prim−15
Prim−25
Long−pec
Protruding−mouth
Day−4
Day−5
Transcript
ENSDART00000158722
ENSDART00000172887
ENSDART00000173338

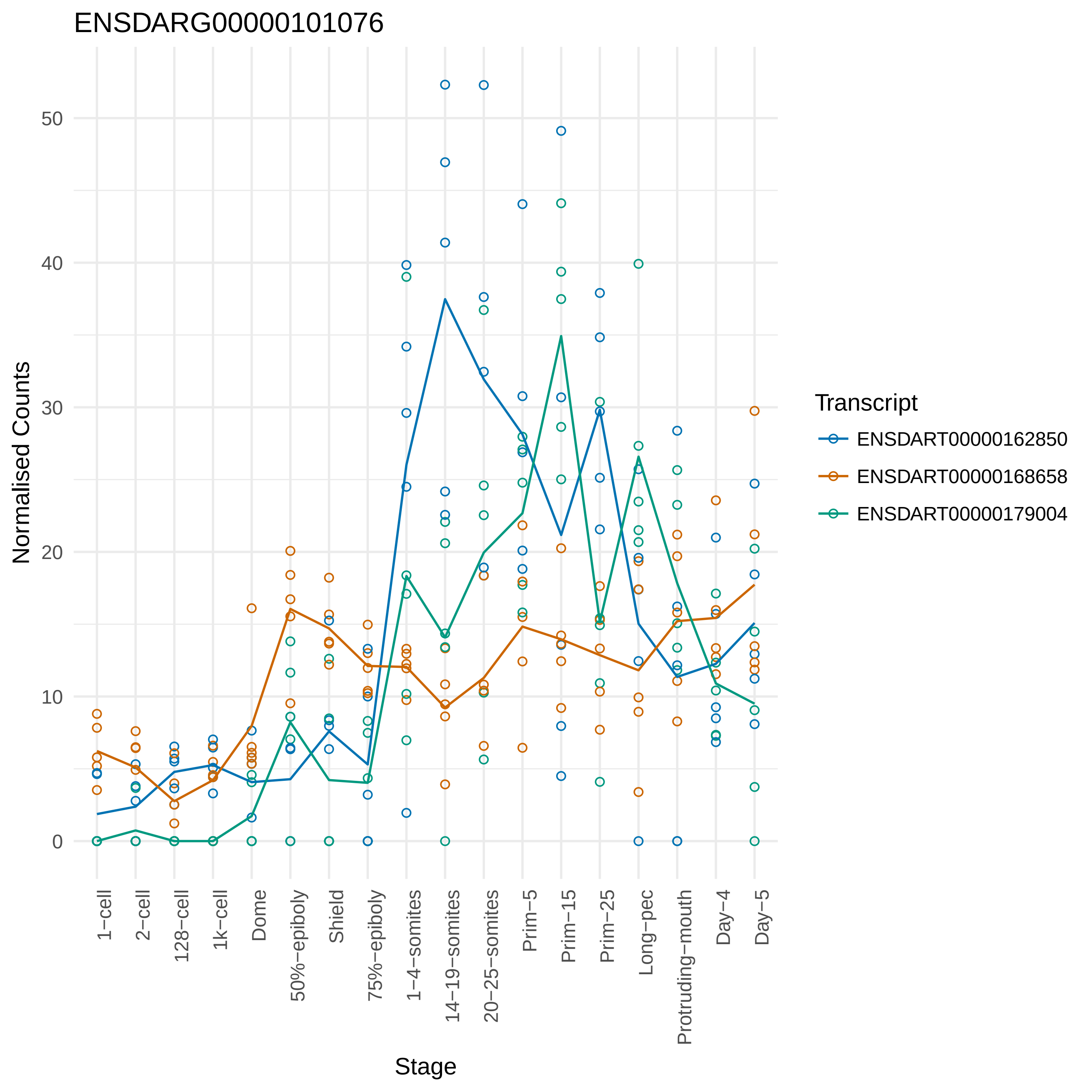
ENSDARG00000101076
Normalised Counts
0
10
20
30
40
50
Stage
1−cell
2−cell
128−cell
1k−cell
Dome
50%−epiboly
Shield
75%−epiboly
1−4−somites
14−19−somites
20−25−somites
Prim−5
Prim−15
Prim−25
Long−pec
Protruding−mouth
Day−4
Day−5
Transcript
ENSDART00000162850
ENSDART00000168658
ENSDART00000179004

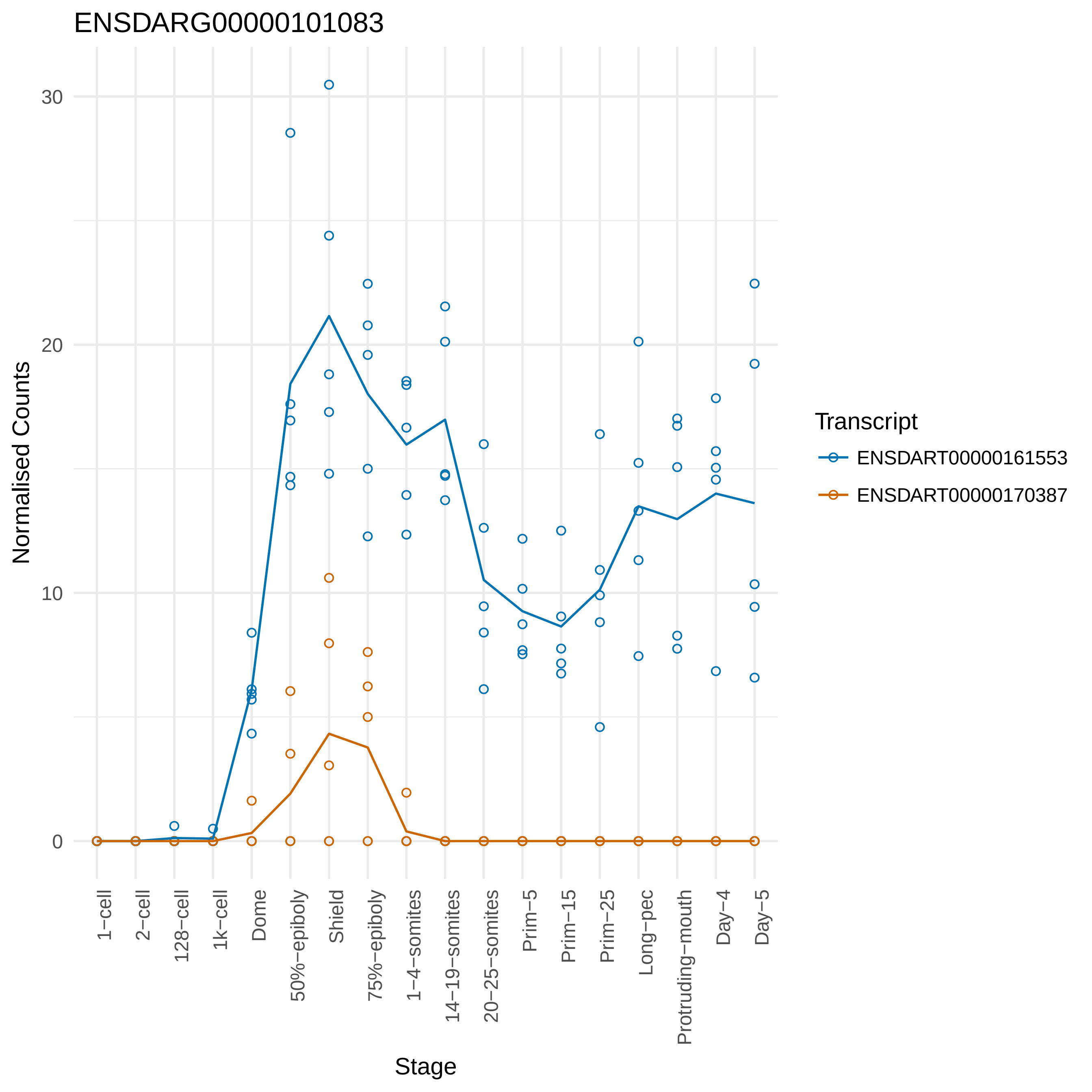

ENSDARG00000101083
Normalised Counts
30
20
10
0
1−cell
2−cell
128−cell
1k−cell
Dome
50%−epiboly
Shield
75%−epiboly
1−4−somites
14−19−somites
20−25−somites
Prim−5
Prim−15
Prim−25
Long−pec
Protruding−mouth
Day−4
Day−5
Stage
Transcript
ENSDART00000161553
ENSDART00000170387

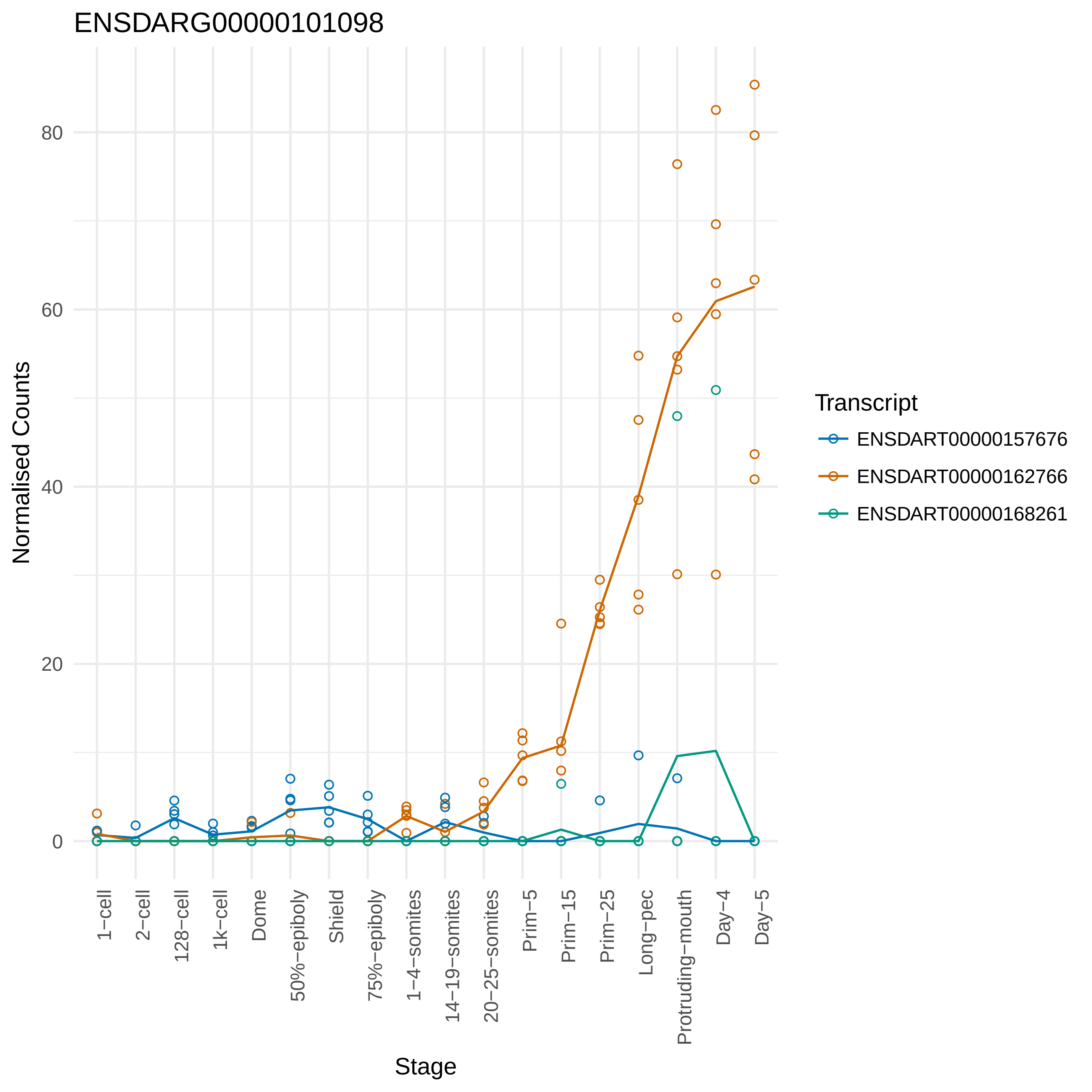

ENSDARG00000101098
Normalised Counts
0
20
40
60
80
Stage
1−cell
2−cell
128−cell
1k−cell
Dome
50%−epiboly
Shield
75%−epiboly
1−4−somites
14−19−somites
20−25−somites
Prim−5
Prim−15
Prim−25
Long−pec
Protruding−mouth
Day−4
Day−5
Transcript
ENSDART00000157676
ENSDART00000162766
ENSDART00000168261

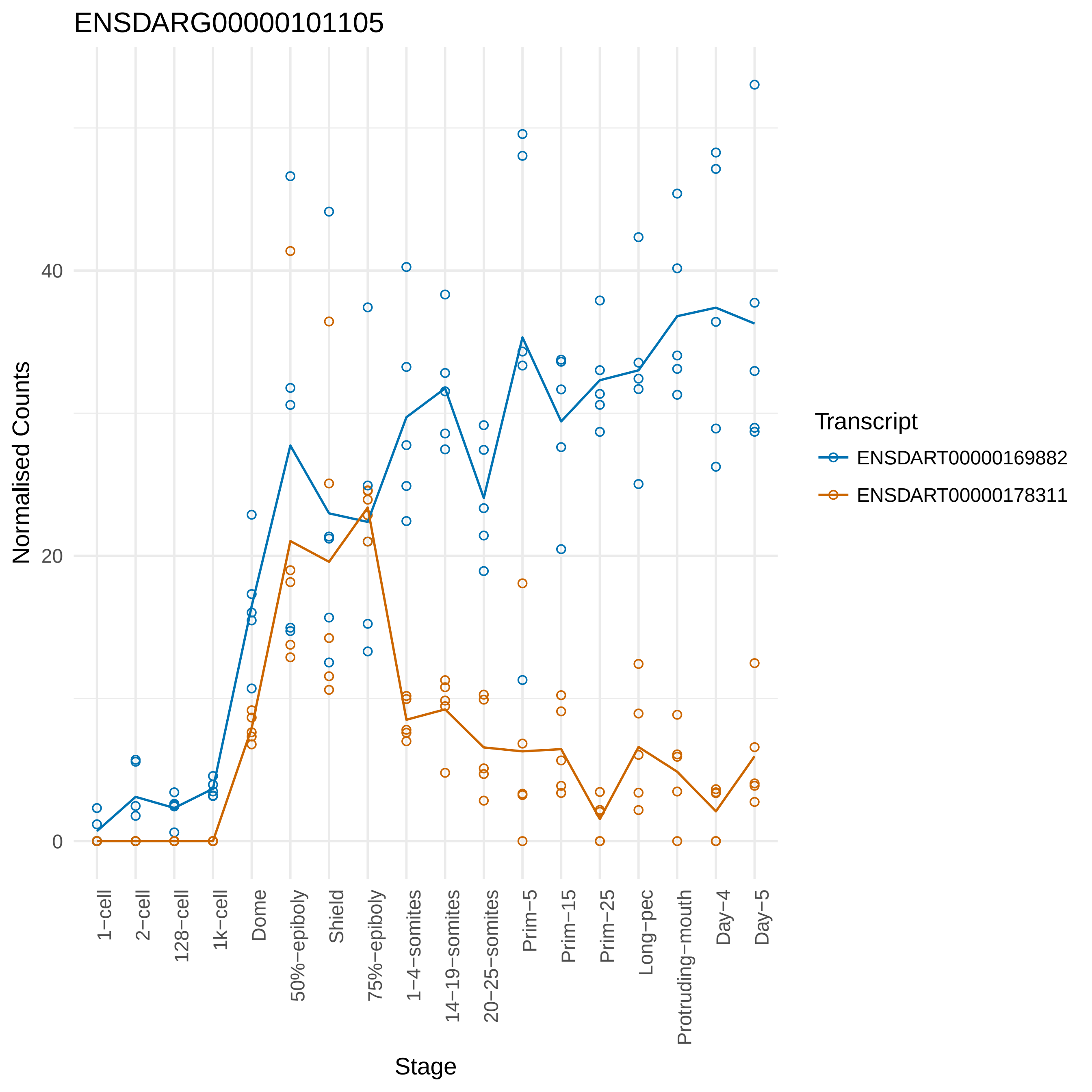

ENSDARG00000101105
Normalised Counts
0
20
40
1−cell
2−cell
128−cell
1k−cell
Dome
50%−epiboly
Shield
75%−epiboly
1−4−somites
14−19−somites
20−25−somites
Prim−5
Prim−15
Prim−25
Long−pec
Protruding−mouth
Day−4
Day−5
Stage
Transcript
ENSDART00000169882
ENSDART00000178311

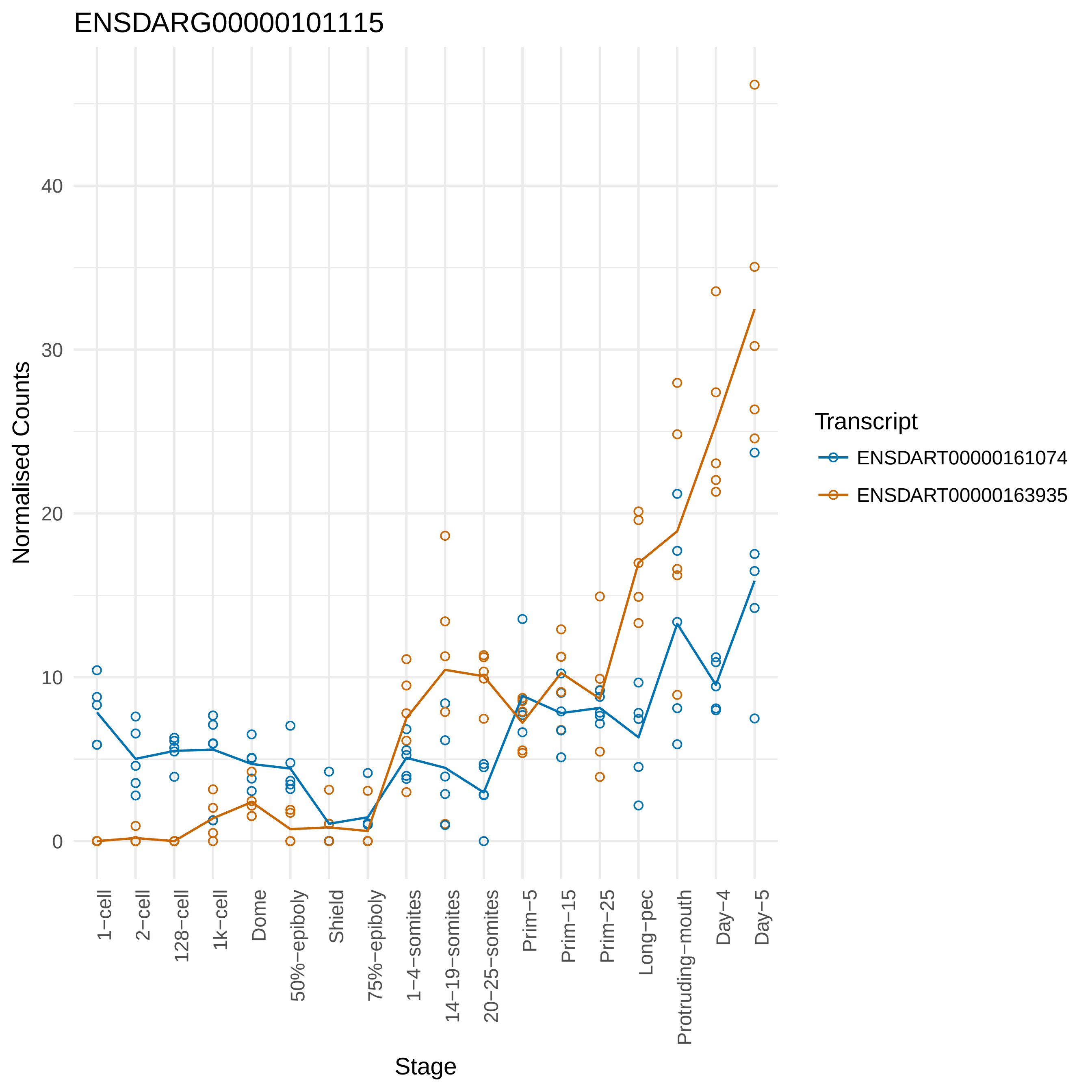

ENSDARG00000101115
Normalised Counts
0
10
20
30
40
1−cell
2−cell
128−cell
1k−cell
Dome
50%−epiboly
Shield
75%−epiboly
1−4−somites
14−19−somites
20−25−somites
Prim−5
Prim−15
Prim−25
Long−pec
Protruding−mouth
Day−4
Day−5
Stage
Transcript
ENSDART00000161074
ENSDART00000163935

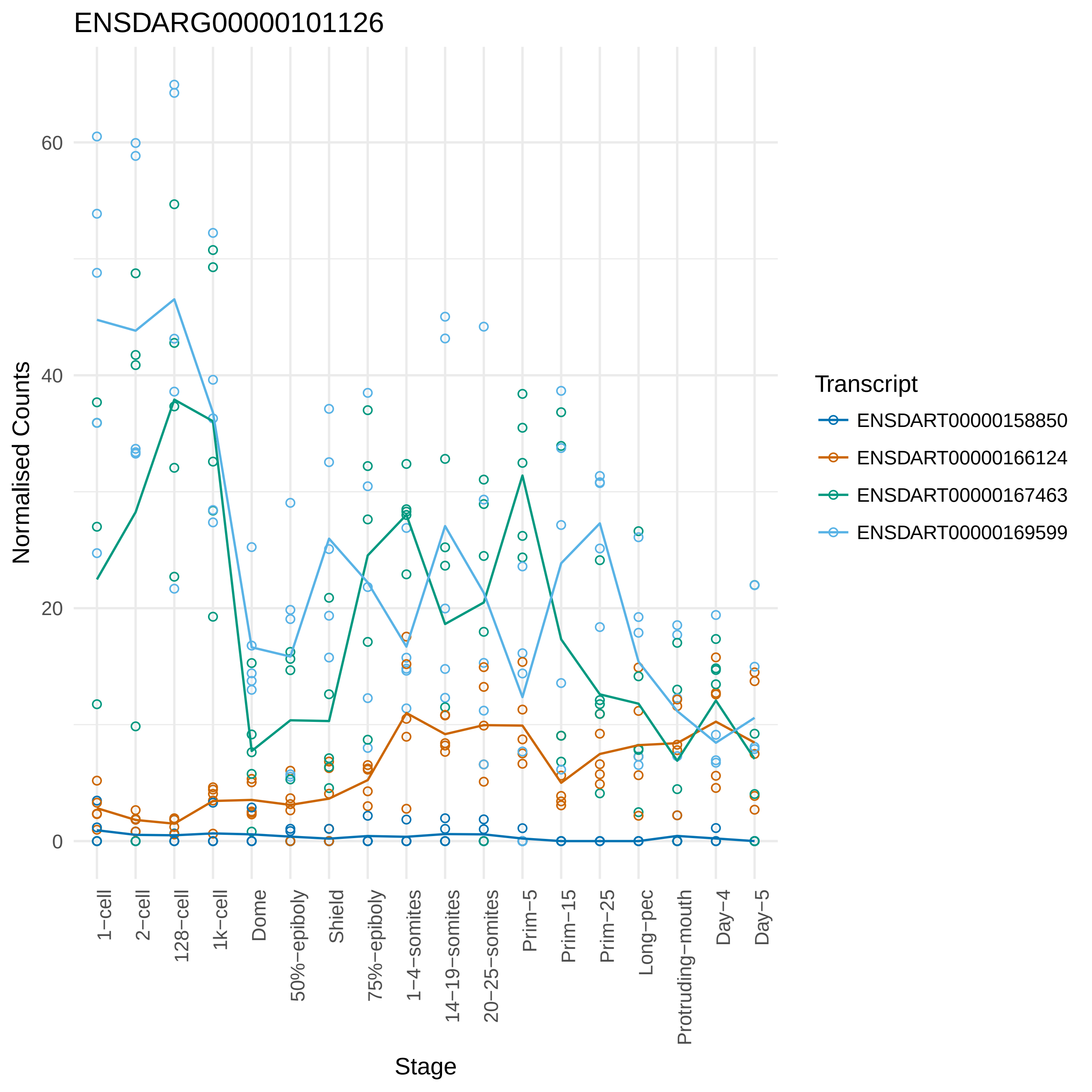

ENSDARG00000101126
Normalised Counts
60
40
20
0
1−cell
2−cell
128−cell
1k−cell
Dome
50%−epiboly
Shield
75%−epiboly
1−4−somites
14−19−somites
20−25−somites
Prim−5
Prim−15
Prim−25
Long−pec
Protruding−mouth
Day−4
Day−5
Stage
Transcript
ENSDART00000158850
ENSDART00000166124
ENSDART00000167463
ENSDART00000169599

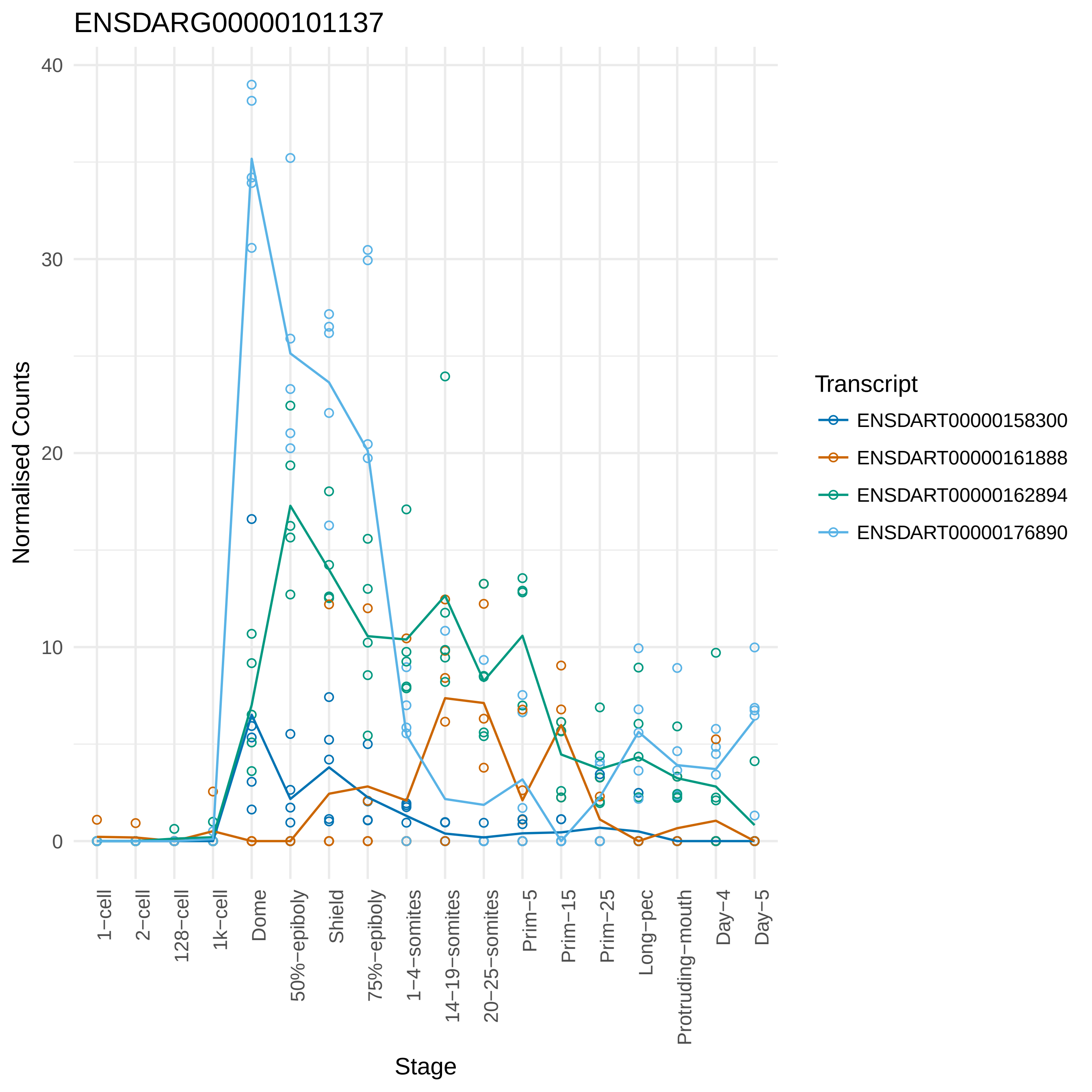
ENSDARG00000101137
Normalised Counts
0
10
20
30
40
1−cell
2−cell
128−cell
1k−cell
Dome
50%−epiboly
Shield
75%−epiboly
1−4−somites
14−19−somites
20−25−somites
Prim−5
Prim−15
Prim−25
Long−pec
Protruding−mouth
Day−4
Day−5
Stage
Transcript
ENSDART00000158300
ENSDART00000161888
ENSDART00000162894
ENSDART00000176890

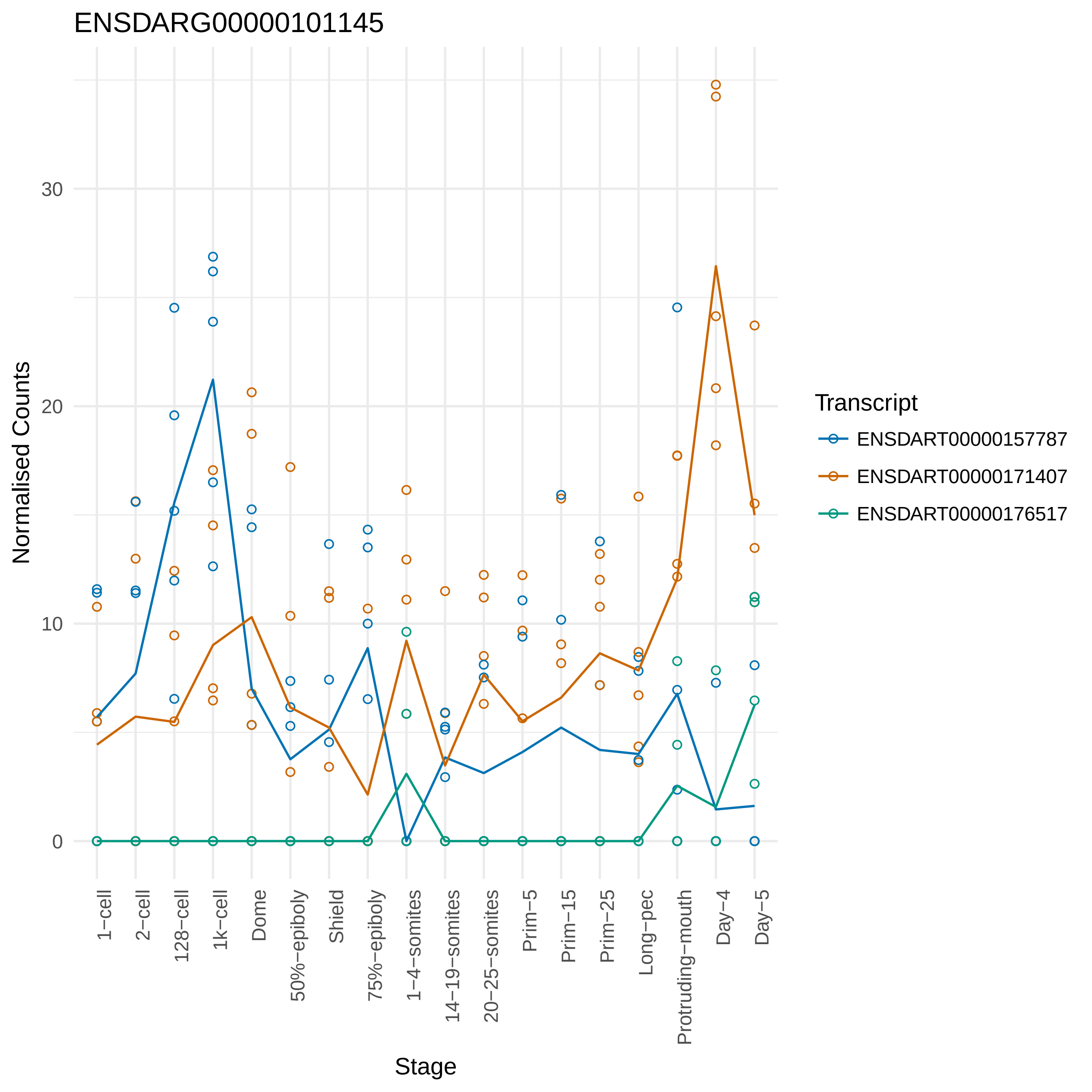

ENSDARG00000101145
Normalised Counts
0
10
20
30
Stage
1−cell
2−cell
128−cell
1k−cell
Dome
50%−epiboly
Shield
75%−epiboly
1−4−somites
14−19−somites
20−25−somites
Prim−5
Prim−15
Prim−25
Long−pec
Protruding−mouth
Day−4
Day−5
Transcript
ENSDART00000157787
ENSDART00000171407
ENSDART00000176517

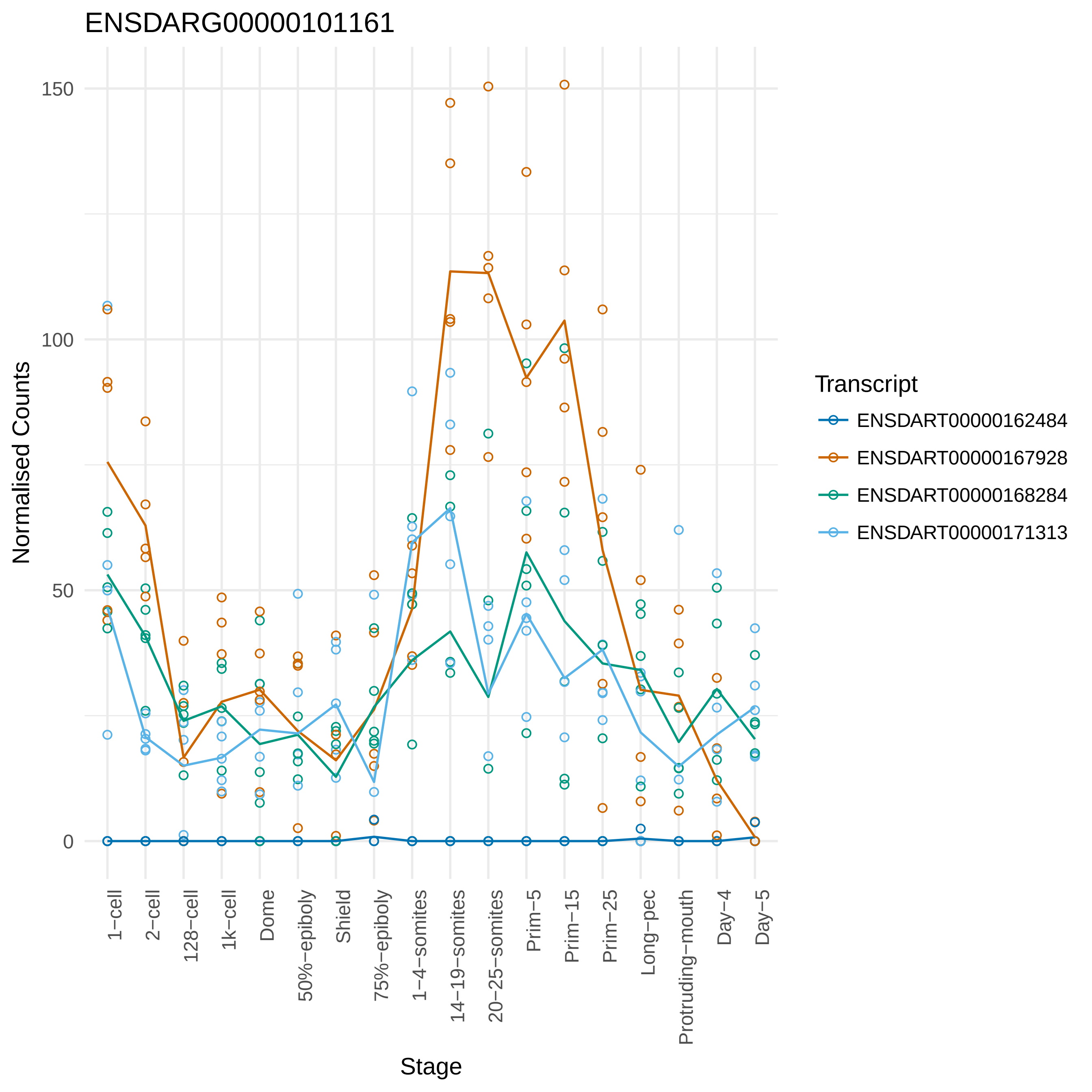

ENSDARG00000101161
Normalised Counts
150
100
50
0
1−cell
2−cell
128−cell
1k−cell
Dome
50%−epiboly
Shield
75%−epiboly
1−4−somites
14−19−somites
20−25−somites
Prim−5
Prim−15
Prim−25
Long−pec
Protruding−mouth
Day−4
Day−5
Stage
Transcript
ENSDART00000162484
ENSDART00000167928
ENSDART00000168284
ENSDART00000171313

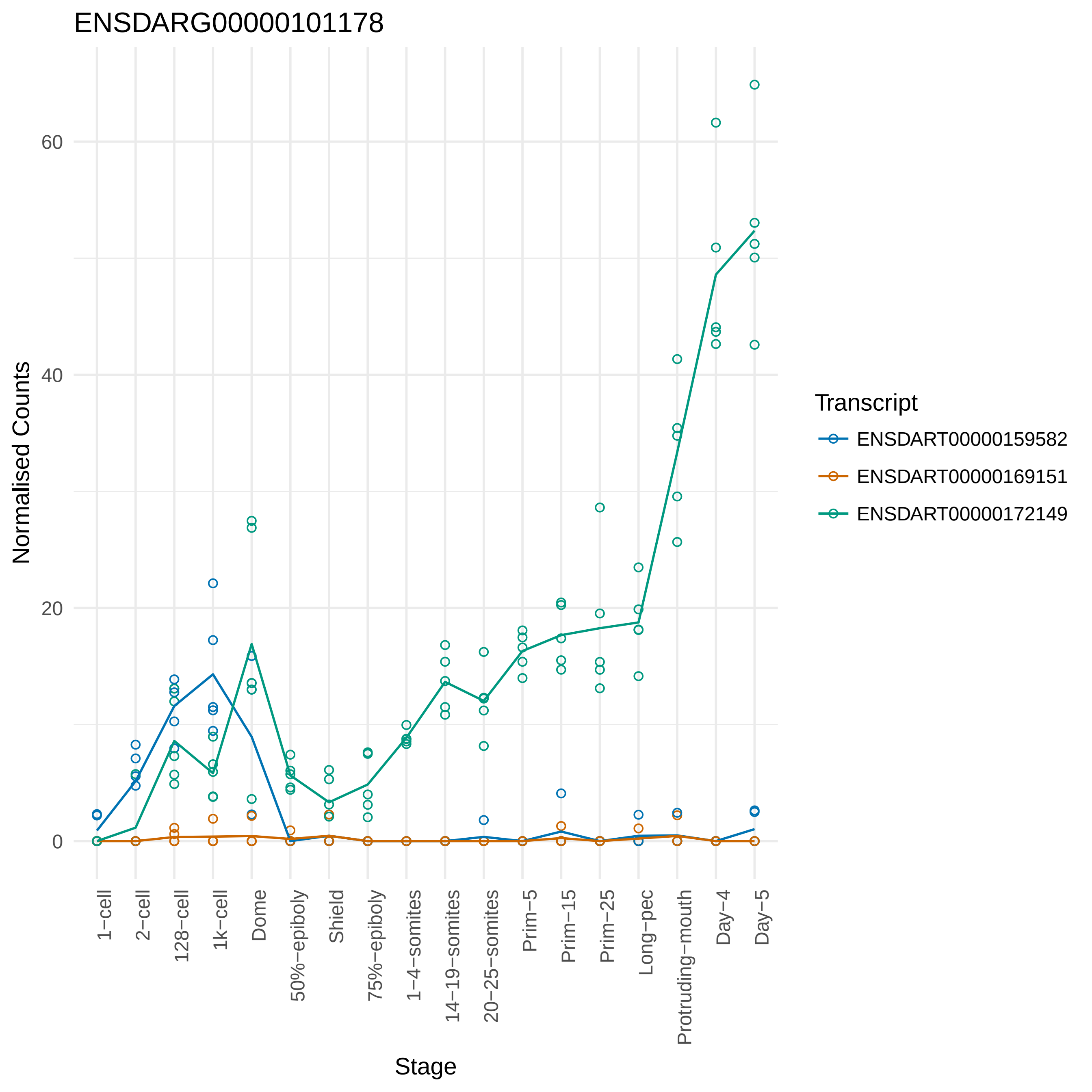
ENSDARG00000101178
Normalised Counts
0
20
40
60
Stage
1−cell
2−cell
128−cell
1k−cell
Dome
50%−epiboly
Shield
75%−epiboly
1−4−somites
14−19−somites
20−25−somites
Prim−5
Prim−15
Prim−25
Long−pec
Protruding−mouth
Day−4
Day−5
Transcript
ENSDART00000159582
ENSDART00000169151
ENSDART00000172149

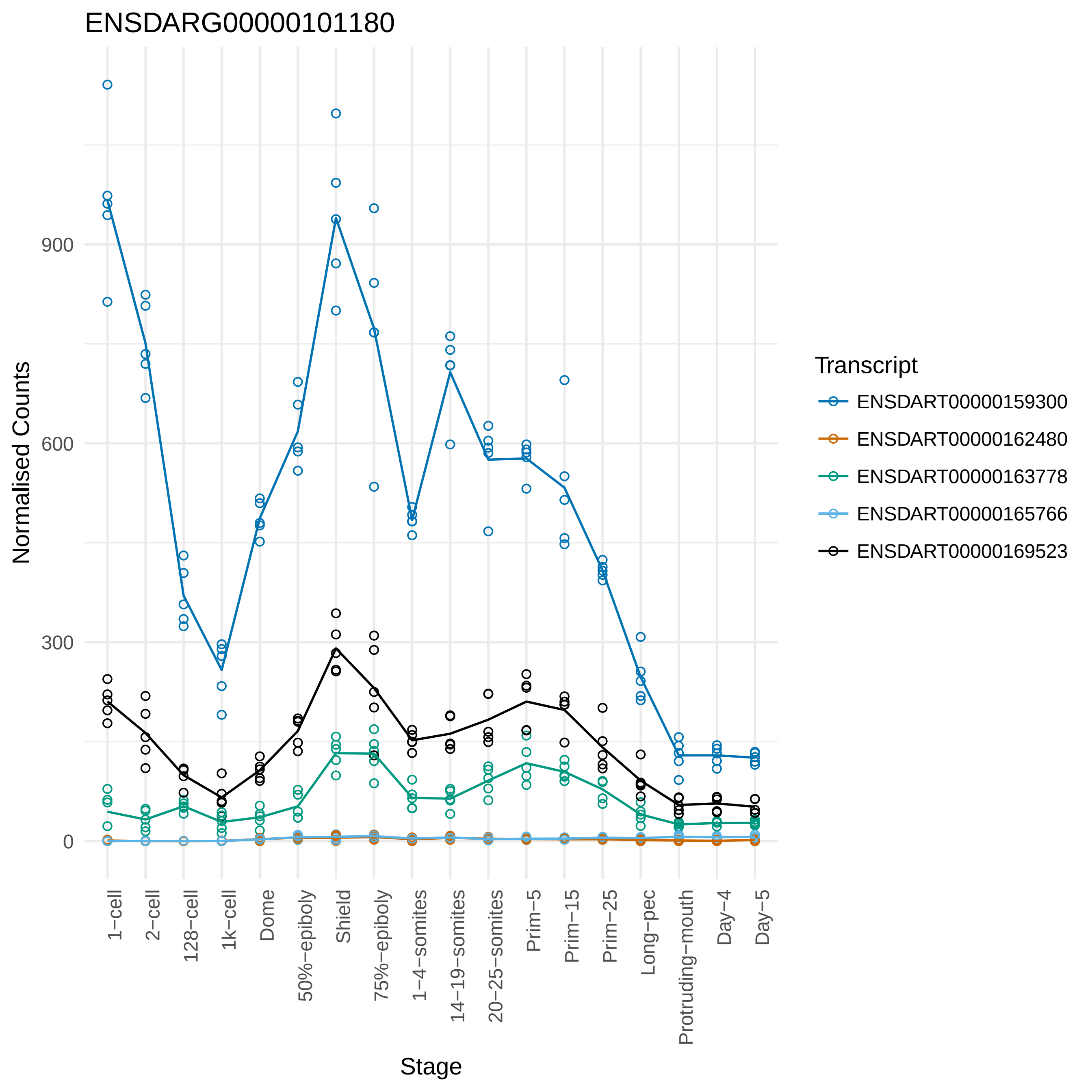

ENSDARG00000101180
Normalised Counts
900
600
300
0
1−cell
2−cell
128−cell
1k−cell
Dome
50%−epiboly
Shield
75%−epiboly
1−4−somites
14−19−somites
20−25−somites
Prim−5
Prim−15
Prim−25
Long−pec
Protruding−mouth
Day−4
Day−5
Stage
Transcript
ENSDART00000159300
ENSDART00000162480
ENSDART00000163778
ENSDART00000165766
ENSDART00000169523

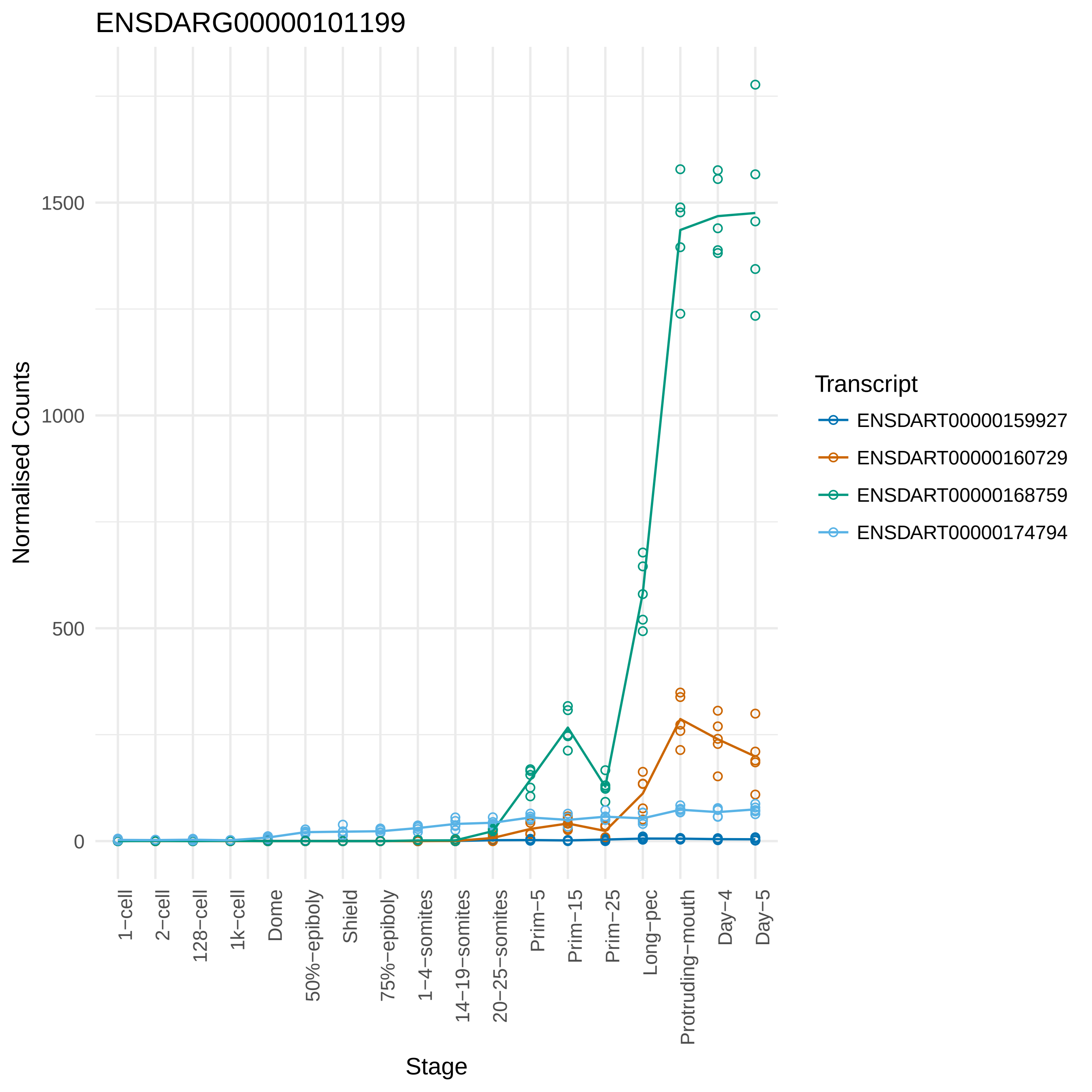
ENSDARG00000101199
Normalised Counts
0
500
1000
1500
Stage
1−cell
2−cell
128−cell
1k−cell
Dome
50%−epiboly
Shield
75%−epiboly
1−4−somites
14−19−somites
20−25−somites
Prim−5
Prim−15
Prim−25
Long−pec
Protruding−mouth
Day−4
Day−5
Transcript
ENSDART00000159927
ENSDART00000160729
ENSDART00000168759
ENSDART00000174794

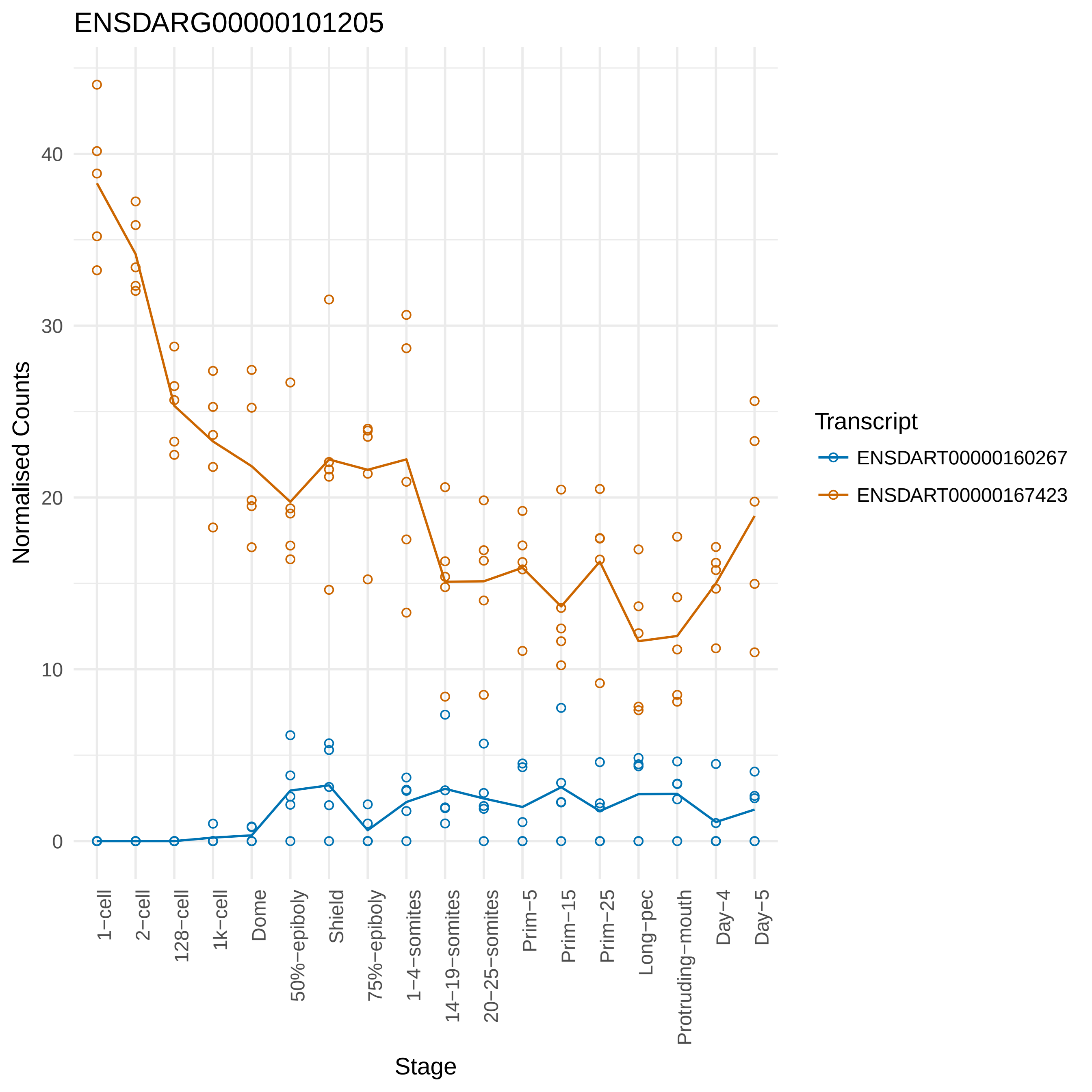

ENSDARG00000101205
Normalised Counts
0
10
20
30
40
1−cell
2−cell
128−cell
1k−cell
Dome
50%−epiboly
Shield
75%−epiboly
1−4−somites
14−19−somites
20−25−somites
Prim−5
Prim−15
Prim−25
Long−pec
Protruding−mouth
Day−4
Day−5
Stage
Transcript
ENSDART00000160267
ENSDART00000167423

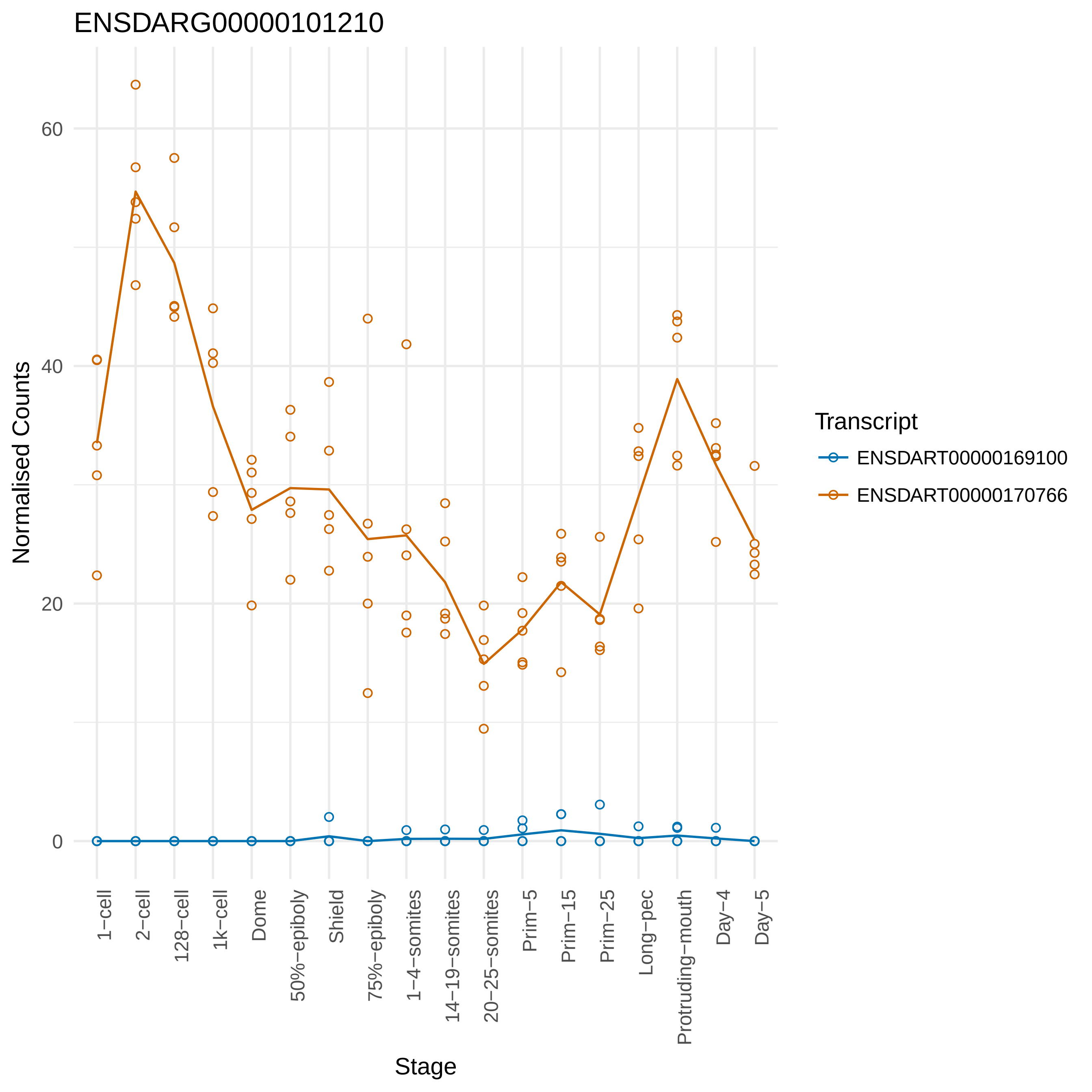
ENSDARG00000101210
Normalised Counts
60
40
20
0
1−cell
2−cell
128−cell
1k−cell
Dome
50%−epiboly
Shield
75%−epiboly
1−4−somites
14−19−somites
20−25−somites
Prim−5
Prim−15
Prim−25
Long−pec
Protruding−mouth
Day−4
Day−5
Stage
Transcript
ENSDART00000169100
ENSDART00000170766

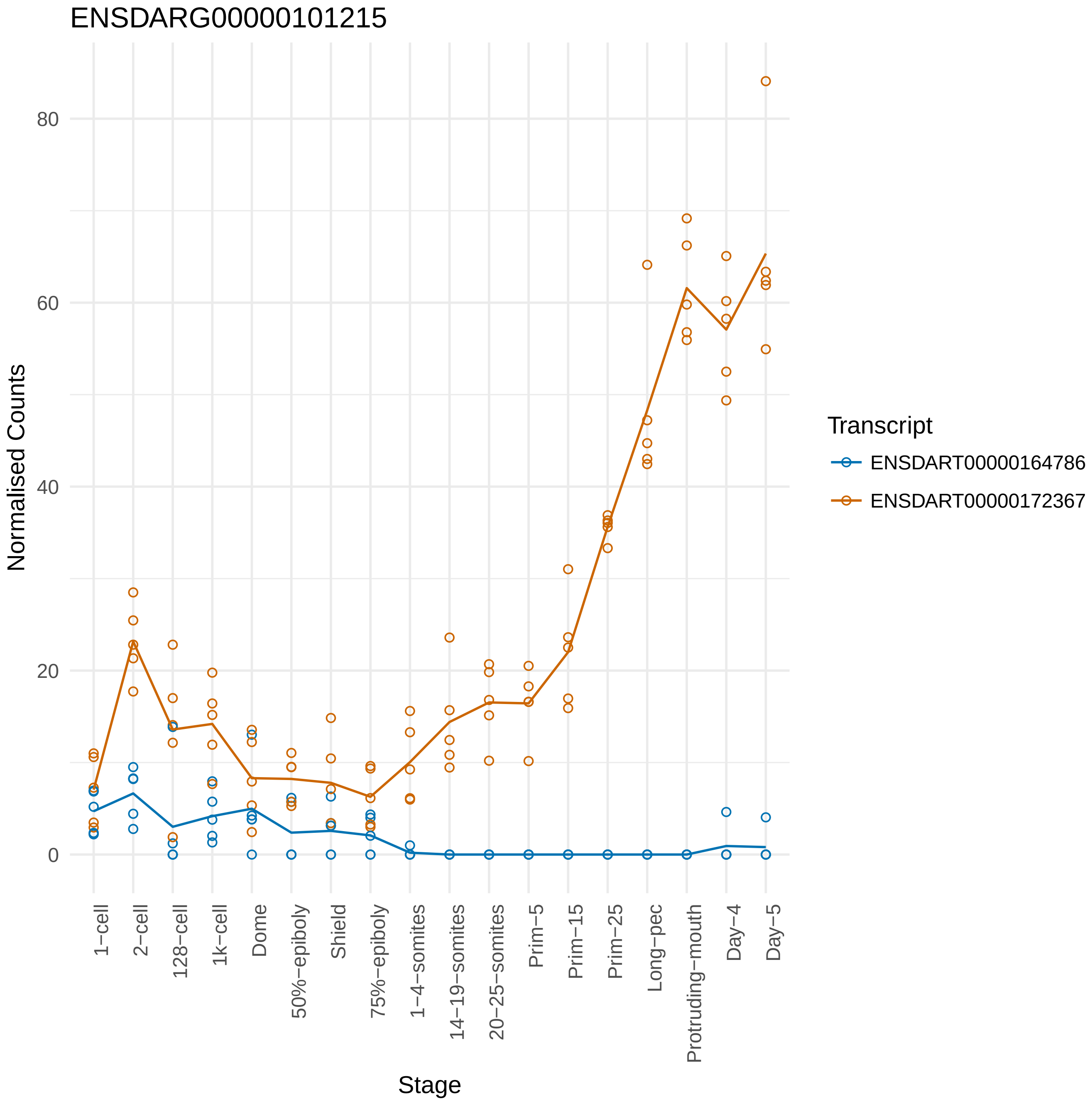
ENSDARG00000101215
Normalised Counts
0
20
40
60
80
1−cell
2−cell
128−cell
1k−cell
Dome
50%−epiboly
Shield
75%−epiboly
1−4−somites
14−19−somites
20−25−somites
Prim−5
Prim−15
Prim−25
Long−pec
Protruding−mouth
Day−4
Day−5
Stage
Transcript
ENSDART00000164786
ENSDART00000172367

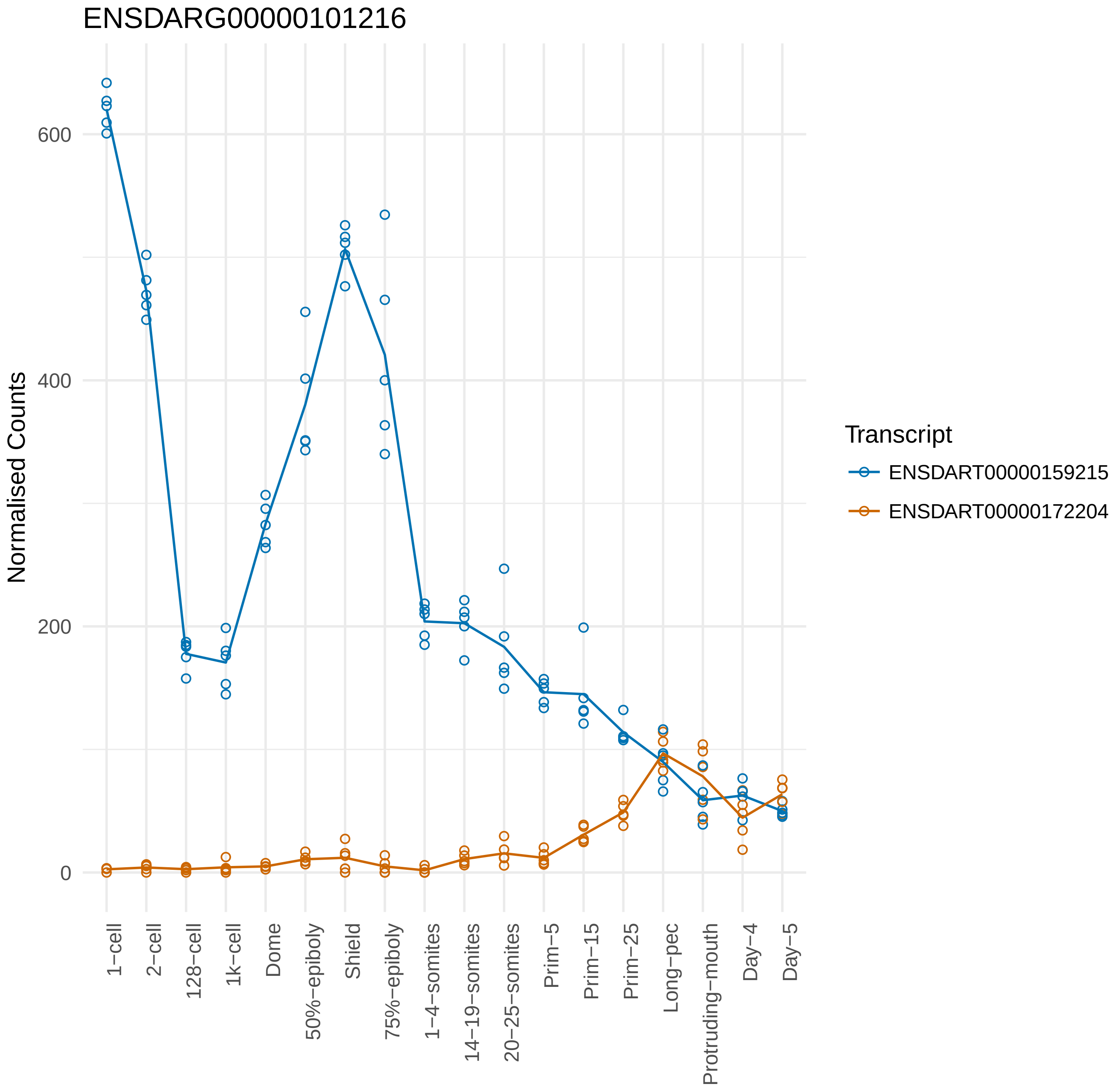
ENSDARG00000101216
Normalised Counts
600
400
200
0
1−cell
2−cell
128−cell
1k−cell
Dome
50%−epiboly
Shield
75%−epiboly
1−4−somites
14−19−somites
20−25−somites
Prim−5
Prim−15
Prim−25
Long−pec
Protruding−mouth
Day−4
Day−5
Stage
Transcript
ENSDART00000159215
ENSDART00000172204

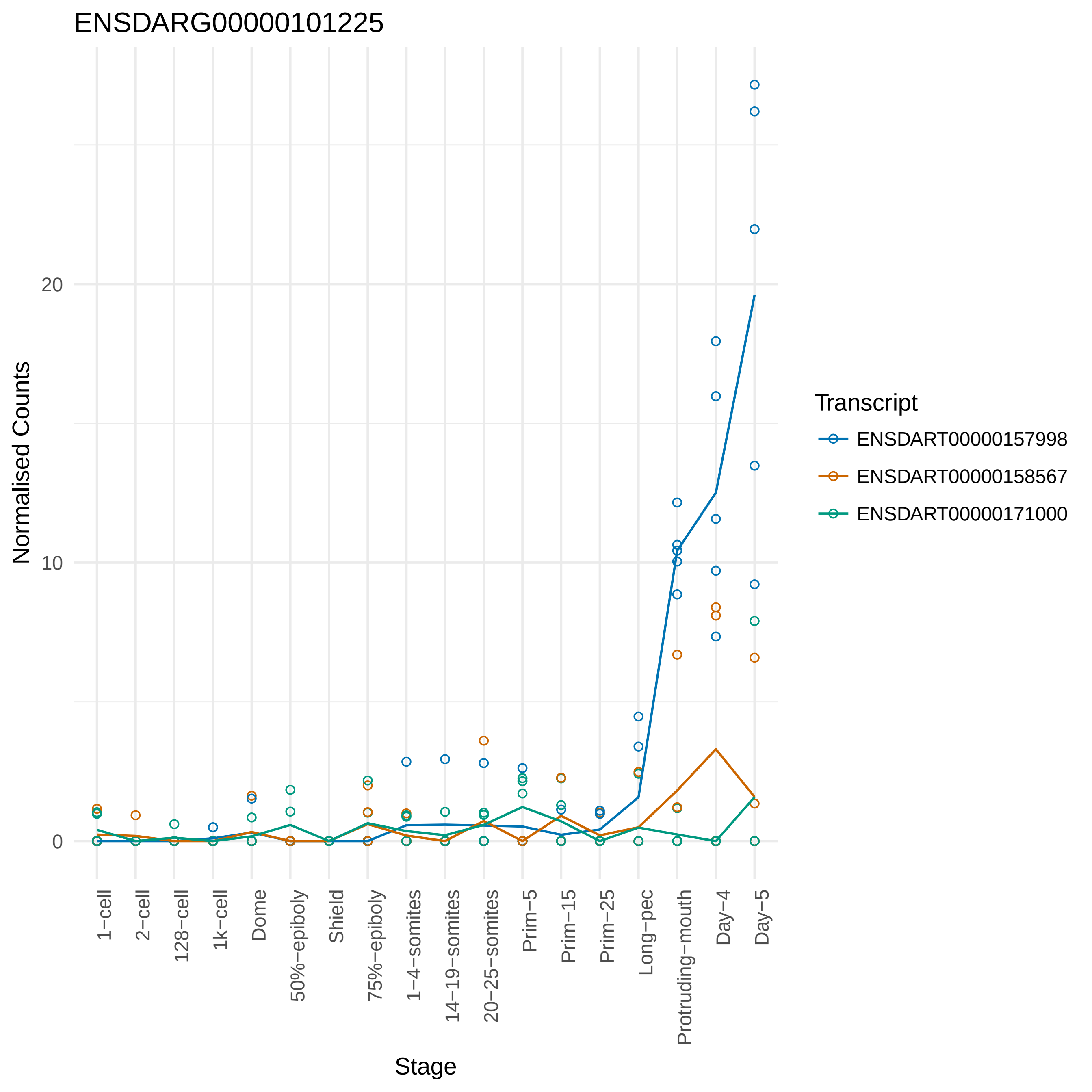

ENSDARG00000101225
Normalised Counts
0
10
20
Stage
1−cell
2−cell
128−cell
1k−cell
Dome
50%−epiboly
Shield
75%−epiboly
1−4−somites
14−19−somites
20−25−somites
Prim−5
Prim−15
Prim−25
Long−pec
Protruding−mouth
Day−4
Day−5
Transcript
ENSDART00000157998
ENSDART00000158567
ENSDART00000171000

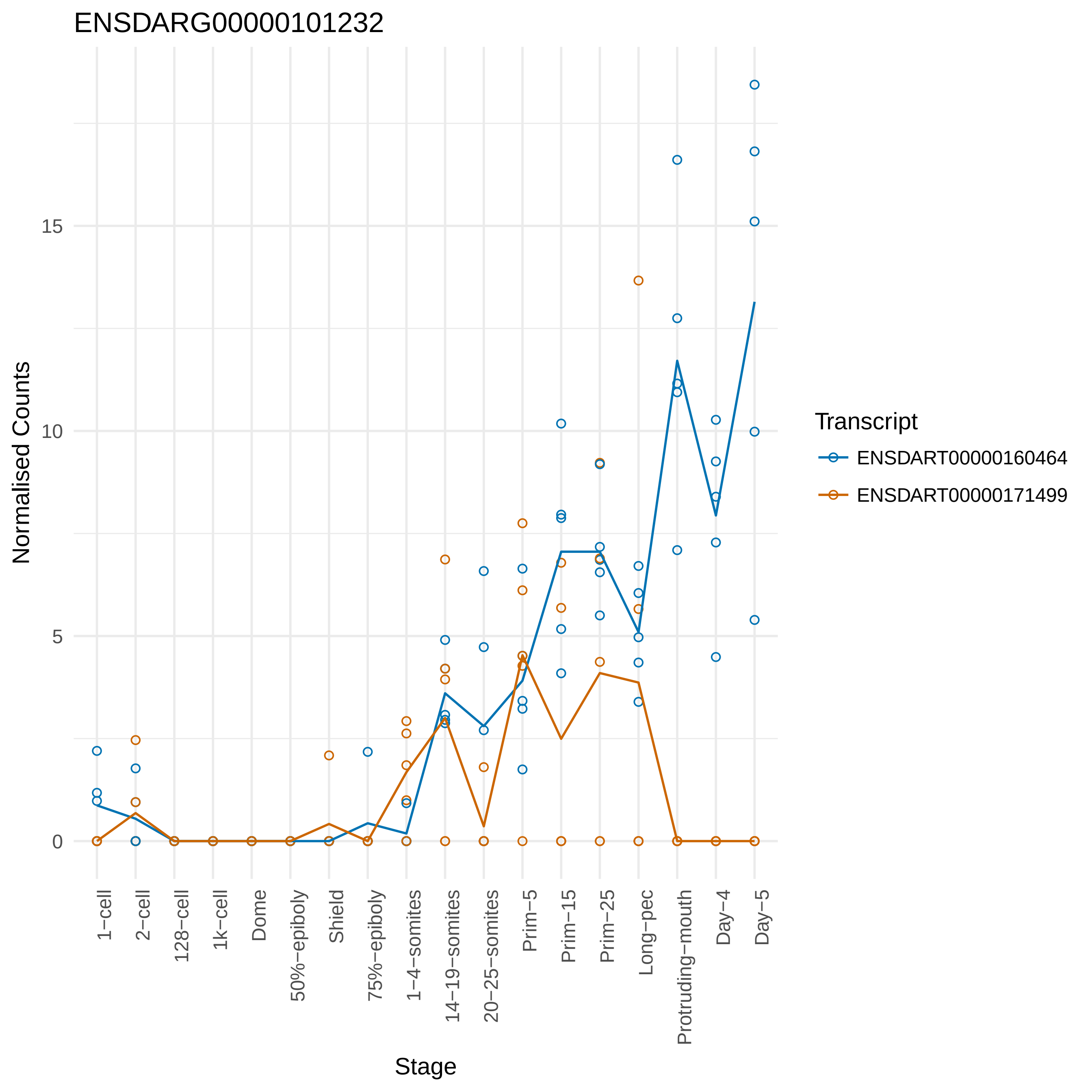

ENSDARG00000101232
Normalised Counts
15
10
5
0
1−cell
2−cell
128−cell
1k−cell
Dome
50%−epiboly
Shield
75%−epiboly
1−4−somites
14−19−somites
20−25−somites
Prim−5
Prim−15
Prim−25
Long−pec
Protruding−mouth
Day−4
Day−5
Stage
Transcript
ENSDART00000160464
ENSDART00000171499

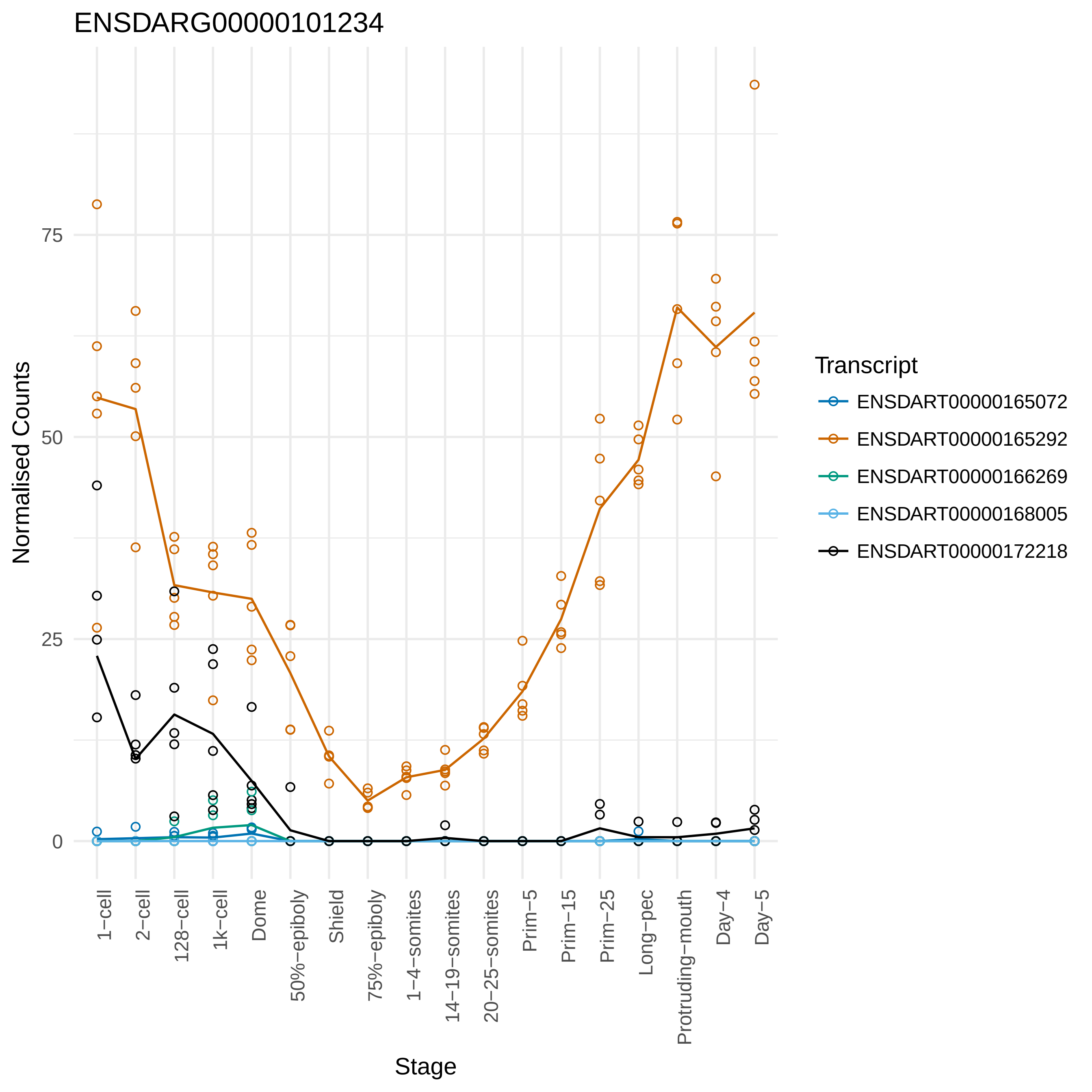

ENSDARG00000101234
Normalised Counts
0
25
50
75
Stage
1−cell
2−cell
128−cell
1k−cell
Dome
50%−epiboly
Shield
75%−epiboly
1−4−somites
14−19−somites
20−25−somites
Prim−5
Prim−15
Prim−25
Long−pec
Protruding−mouth
Day−4
Day−5
Transcript
ENSDART00000165072
ENSDART00000165292
ENSDART00000166269
ENSDART00000168005
ENSDART00000172218

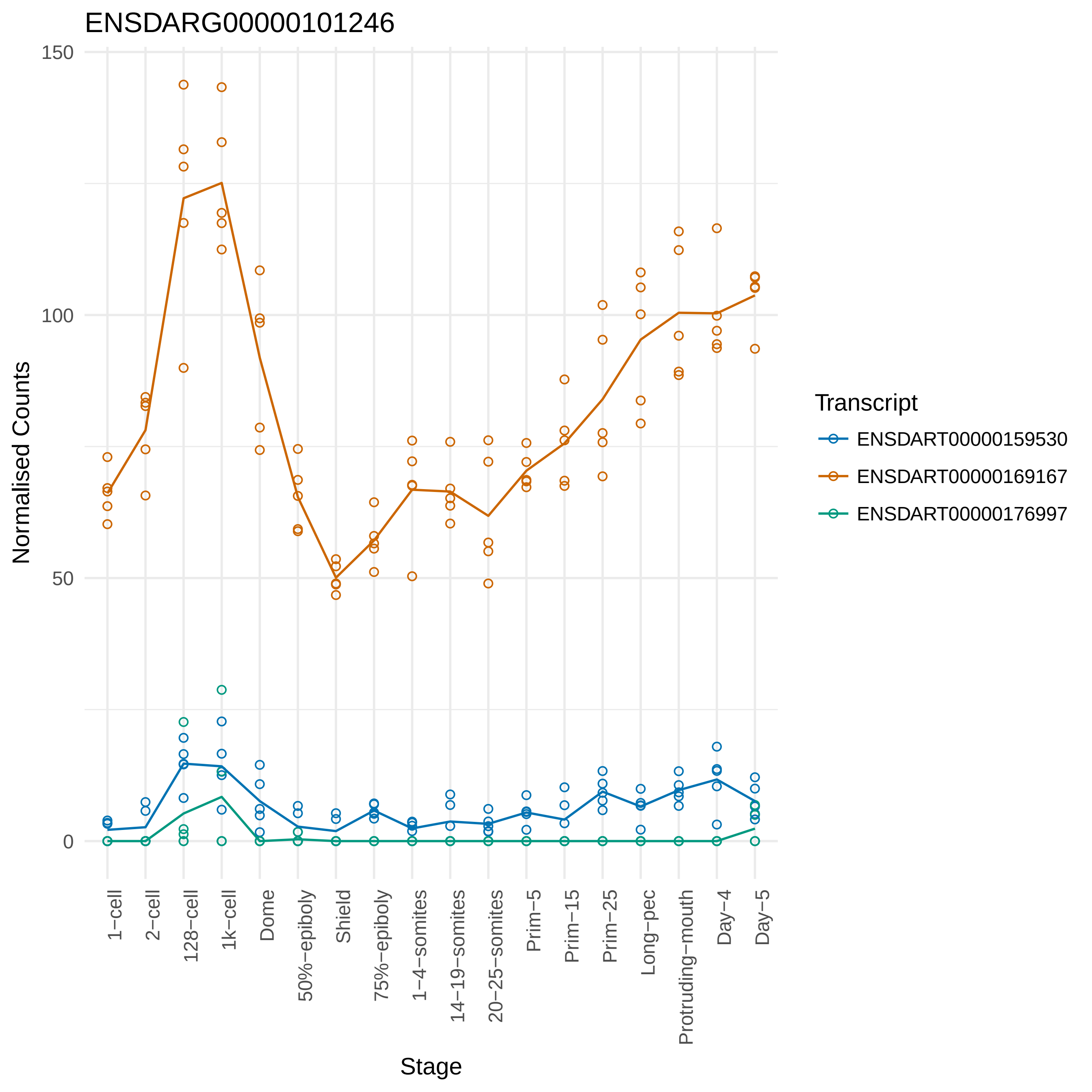
ENSDARG00000101246
Normalised Counts
150
100
50
0
Stage
1−cell
2−cell
128−cell
1k−cell
Dome
50%−epiboly
Shield
75%−epiboly
1−4−somites
14−19−somites
20−25−somites
Prim−5
Prim−15
Prim−25
Long−pec
Protruding−mouth
Day−4
Day−5
Transcript
ENSDART00000159530
ENSDART00000169167
ENSDART00000176997

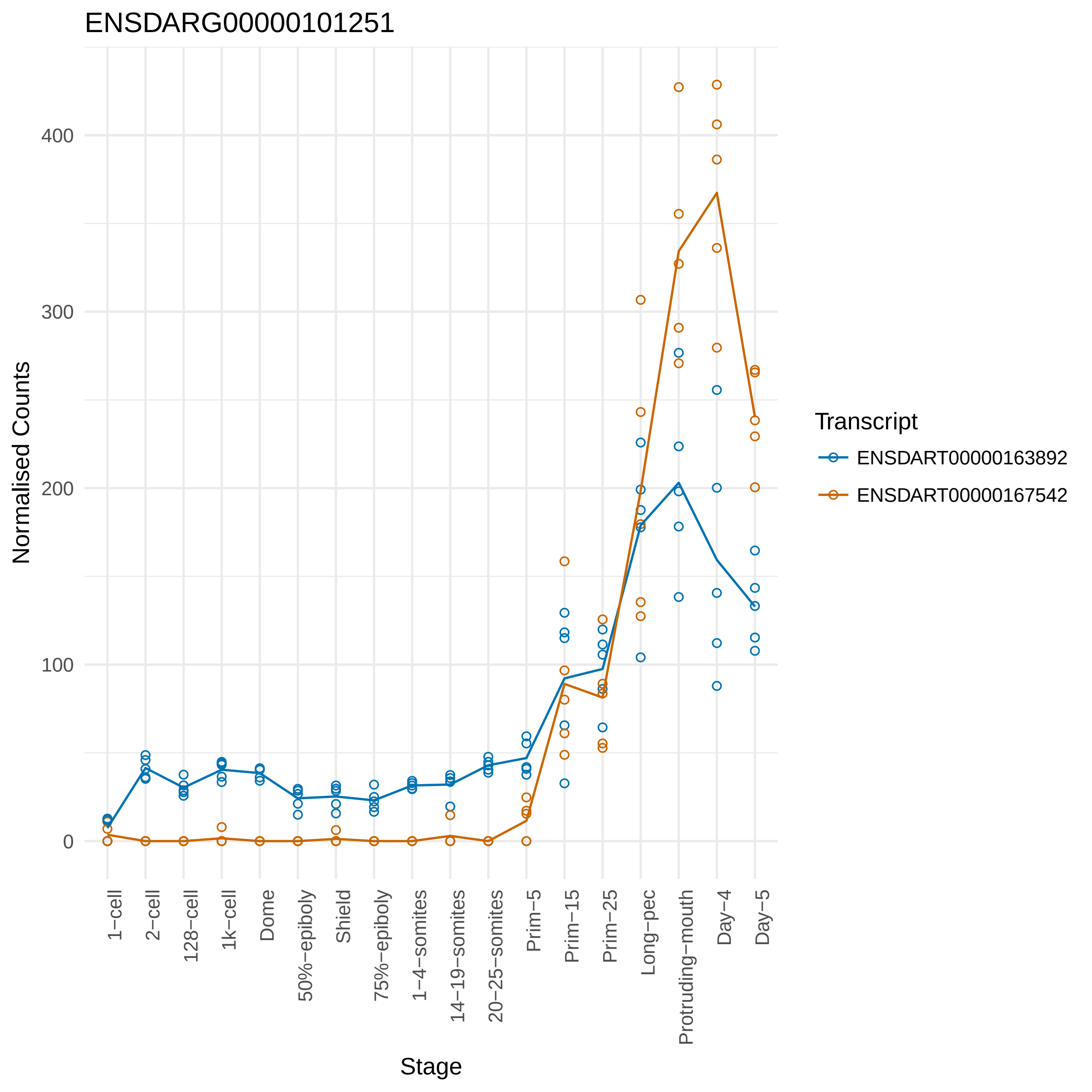

ENSDARG00000101251
Normalised Counts
400
300
200
100
0
1−cell
2−cell
128−cell
1k−cell
Dome
50%−epiboly
Shield
75%−epiboly
1−4−somites
14−19−somites
20−25−somites
Prim−5
Prim−15
Prim−25
Long−pec
Protruding−mouth
Day−4
Day−5
Stage
Transcript
ENSDART00000163892
ENSDART00000167542

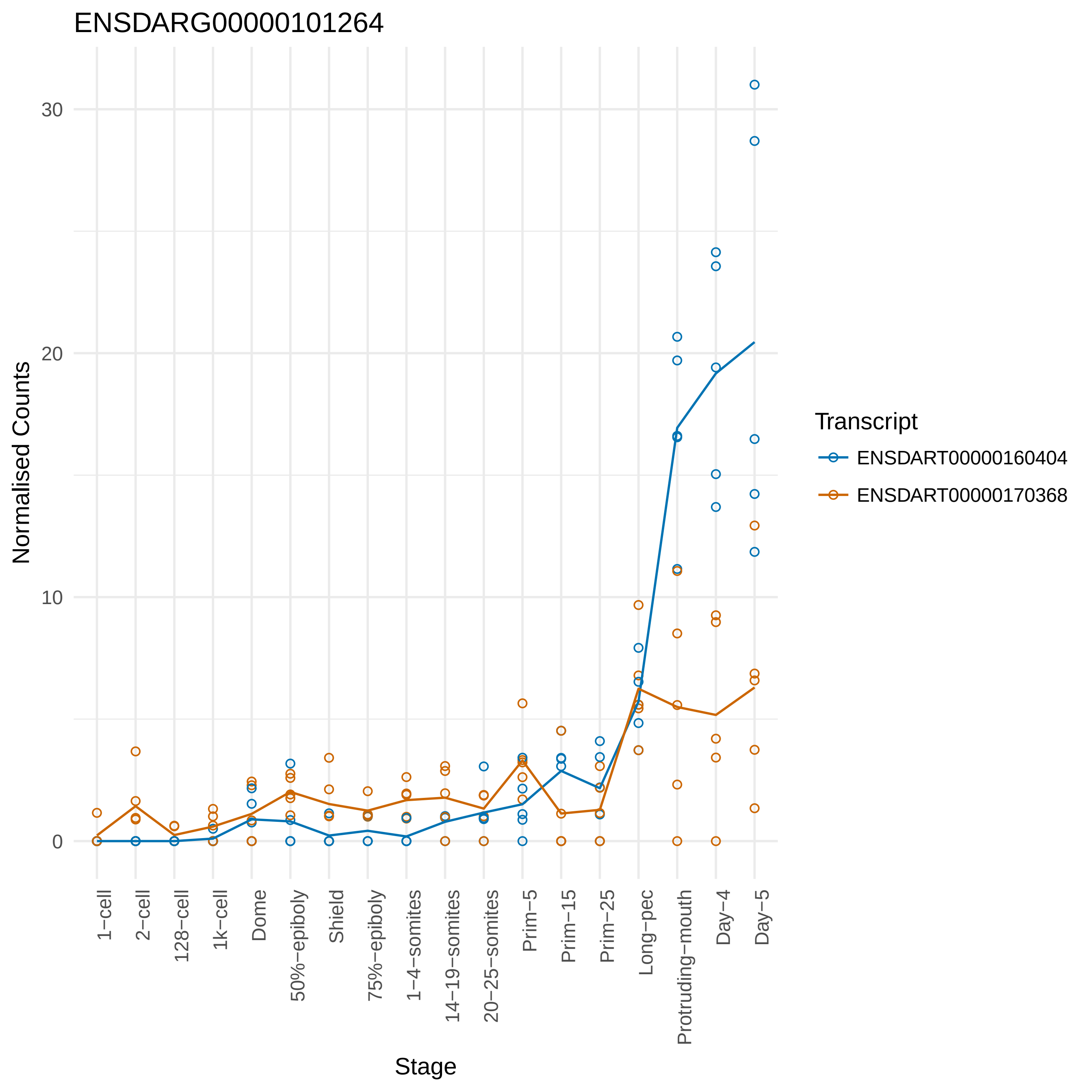
ENSDARG00000101264
Normalised Counts
0
10
20
30
1−cell
2−cell
128−cell
1k−cell
Dome
50%−epiboly
Shield
75%−epiboly
1−4−somites
14−19−somites
20−25−somites
Prim−5
Prim−15
Prim−25
Long−pec
Protruding−mouth
Day−4
Day−5
Stage
Transcript
ENSDART00000160404
ENSDART00000170368

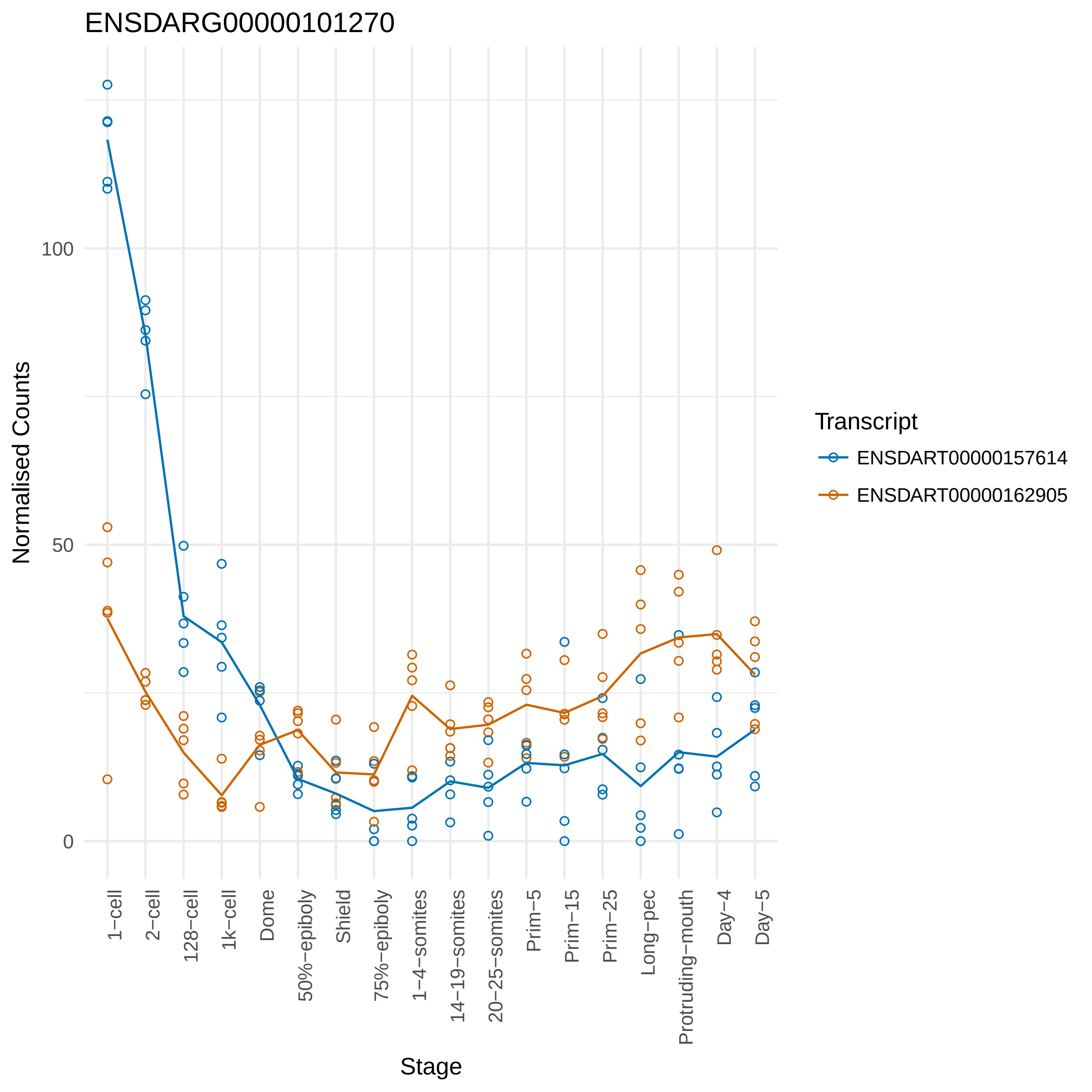
ENSDARG00000101270
Normalised Counts
100
50
0
1−cell
2−cell
128−cell
1k−cell
Dome
50%−epiboly
Shield
75%−epiboly
1−4−somites
14−19−somites
20−25−somites
Prim−5
Prim−15
Prim−25
Long−pec
Protruding−mouth
Day−4
Day−5
Stage
Transcript
ENSDART00000157614
ENSDART00000162905

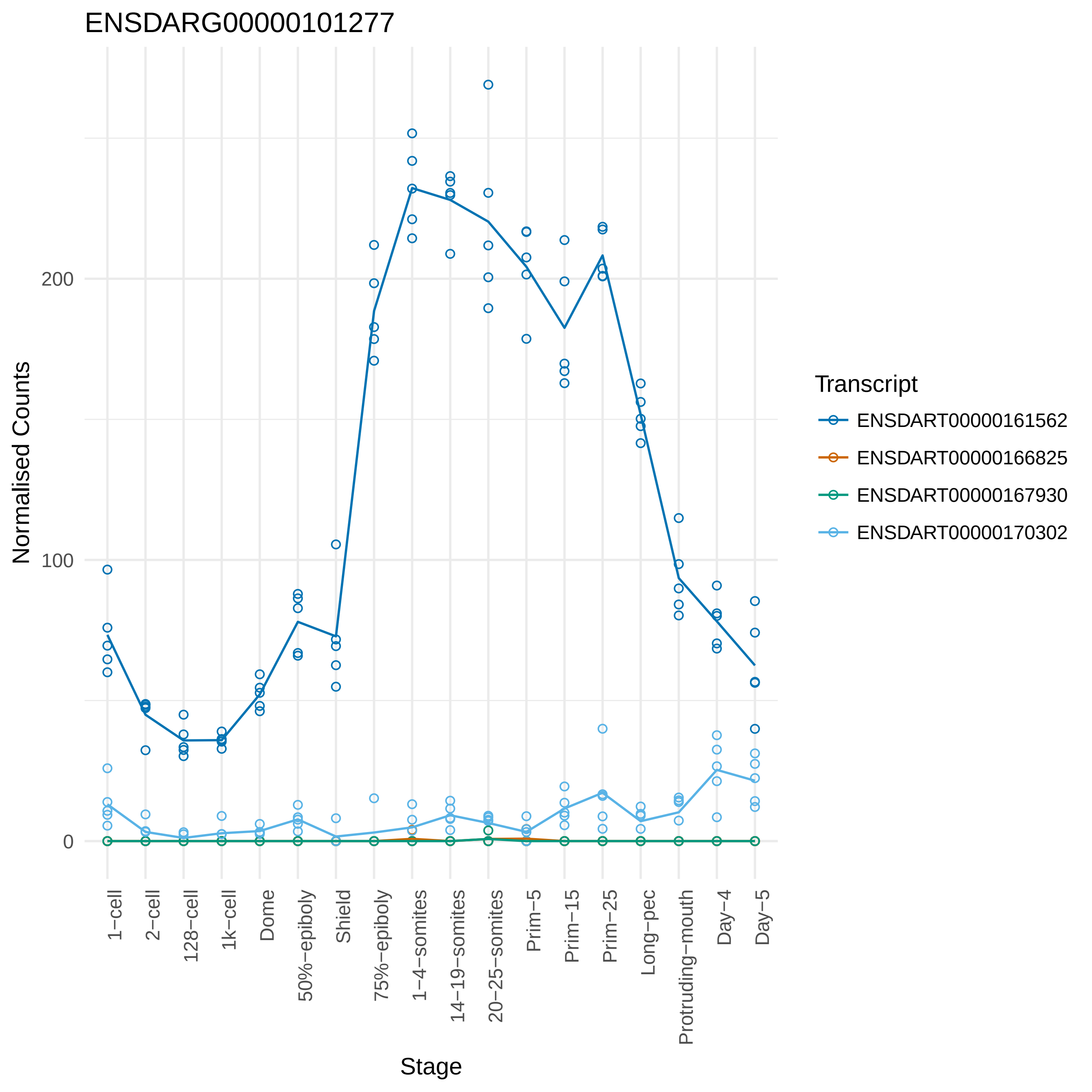
ENSDARG00000101277
Normalised Counts
0
100
200
Stage
1−cell
2−cell
128−cell
1k−cell
Dome
50%−epiboly
Shield
75%−epiboly
1−4−somites
14−19−somites
20−25−somites
Prim−5
Prim−15
Prim−25
Long−pec
Protruding−mouth
Day−4
Day−5
Transcript
ENSDART00000161562
ENSDART00000166825
ENSDART00000167930
ENSDART00000170302

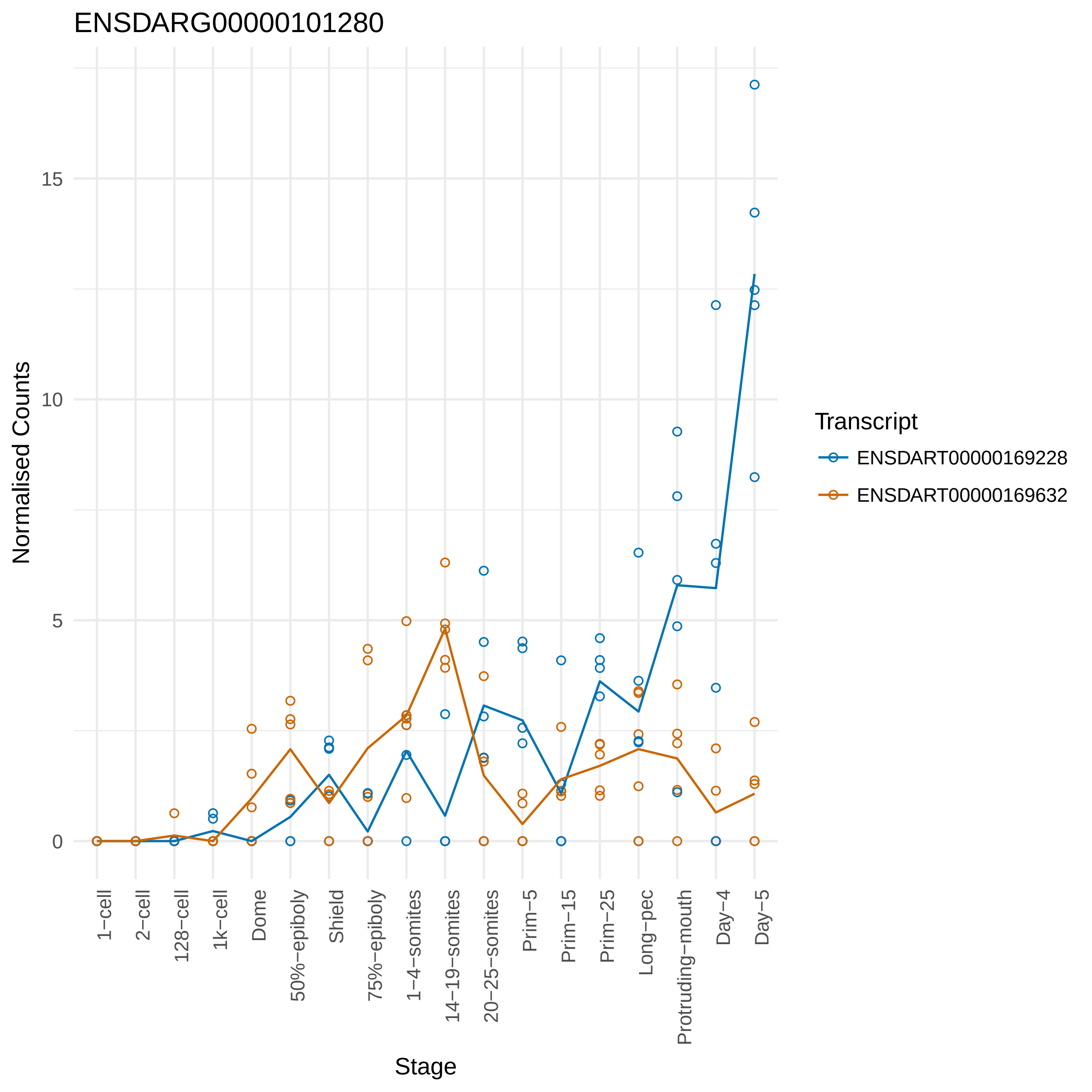
ENSDARG00000101280
Normalised Counts
0
5
10
15
Stage
1−cell
2−cell
128−cell
1k−cell
Dome
50%−epiboly
Shield
75%−epiboly
1−4−somites
14−19−somites
20−25−somites
Prim−5
Prim−15
Prim−25
Long−pec
Protruding−mouth
Day−4
Day−5
Transcript
ENSDART00000169228
ENSDART00000169632

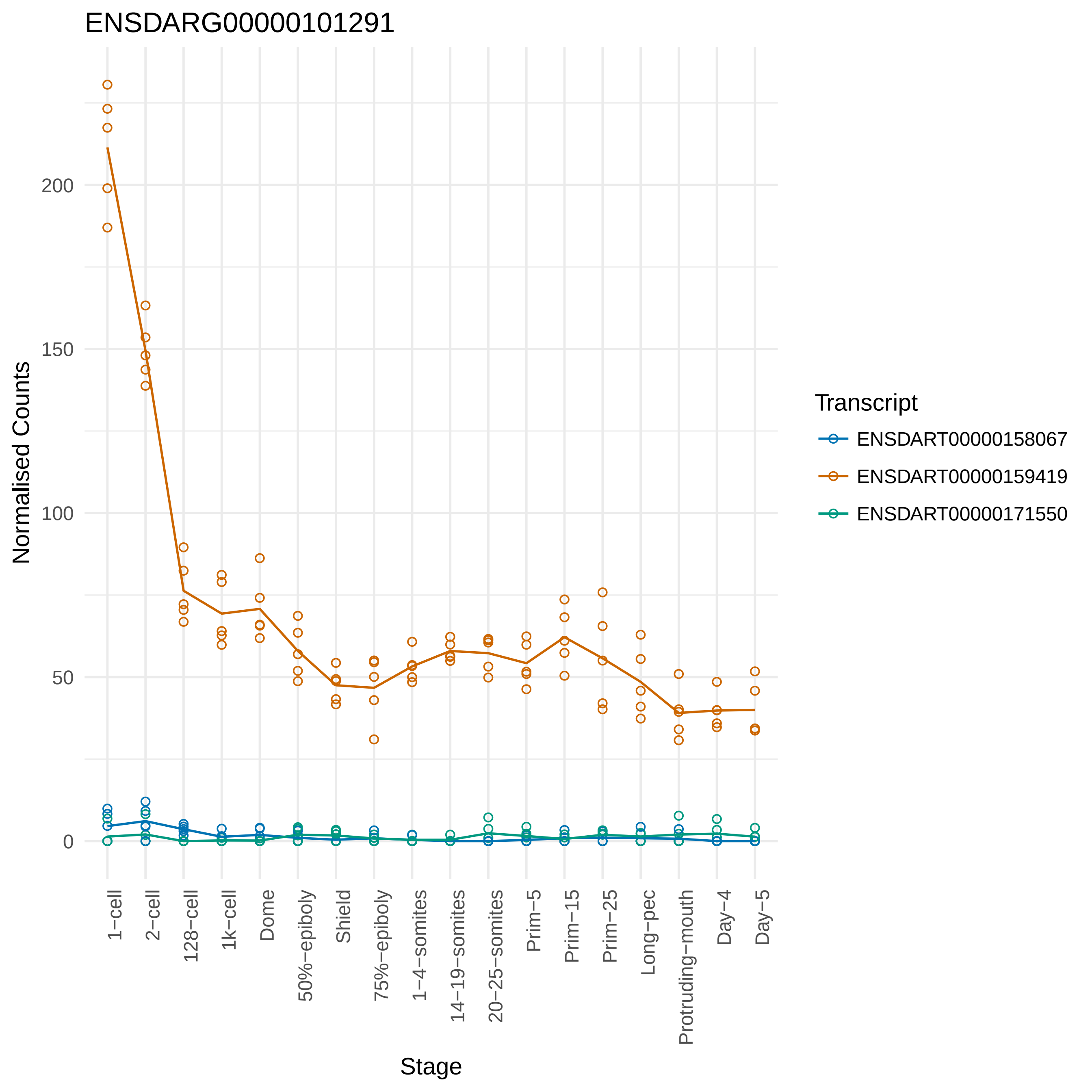
ENSDARG00000101291
Normalised Counts
0
50
100
150
200
1−cell
2−cell
128−cell
1k−cell
Dome
50%−epiboly
Shield
75%−epiboly
1−4−somites
14−19−somites
20−25−somites
Prim−5
Prim−15
Prim−25
Long−pec
Protruding−mouth
Day−4
Day−5
Stage
Transcript
ENSDART00000158067
ENSDART00000159419
ENSDART00000171550

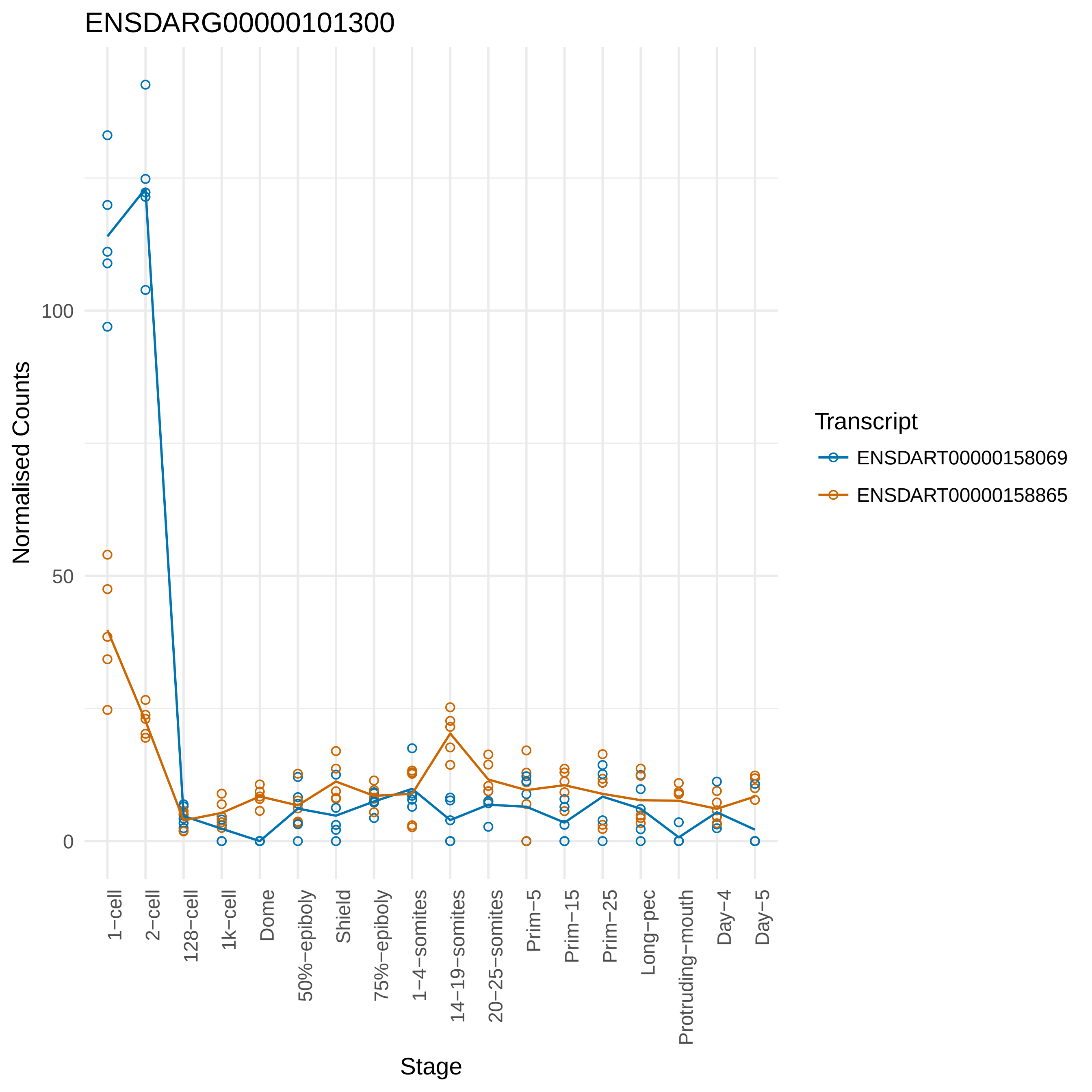

ENSDARG00000101300
Normalised Counts
100
50
0
1−cell
2−cell
128−cell
1k−cell
Dome
50%−epiboly
Shield
75%−epiboly
1−4−somites
14−19−somites
20−25−somites
Prim−5
Prim−15
Prim−25
Long−pec
Protruding−mouth
Day−4
Day−5
Stage
Transcript
ENSDART00000158069
ENSDART00000158865

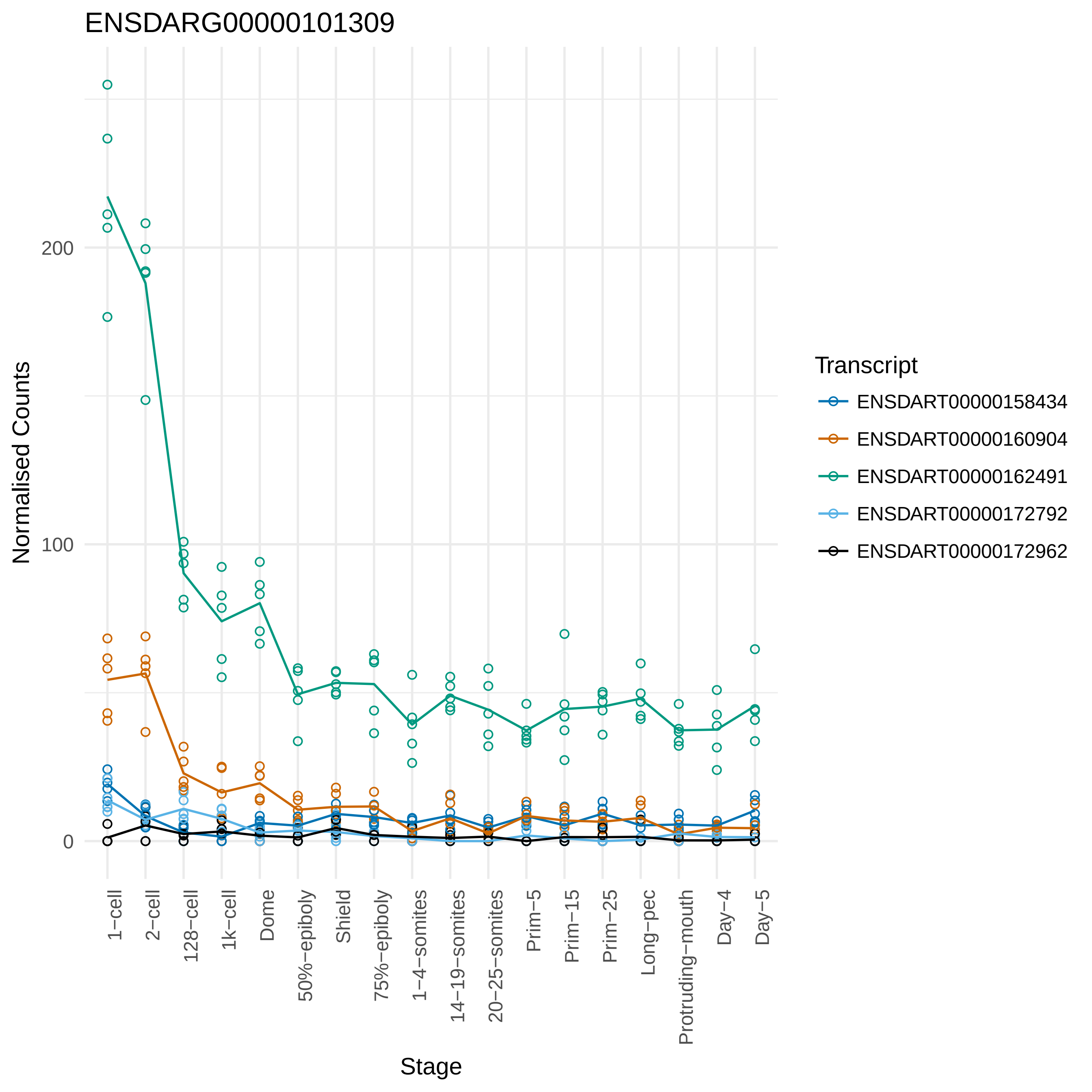

ENSDARG00000101309
Normalised Counts
200
100
0
1−cell
2−cell
128−cell
1k−cell
Dome
50%−epiboly
Shield
75%−epiboly
1−4−somites
14−19−somites
20−25−somites
Prim−5
Prim−15
Prim−25
Long−pec
Protruding−mouth
Day−4
Day−5
Stage
Transcript
ENSDART00000158434
ENSDART00000160904
ENSDART00000162491
ENSDART00000172792
ENSDART00000172962

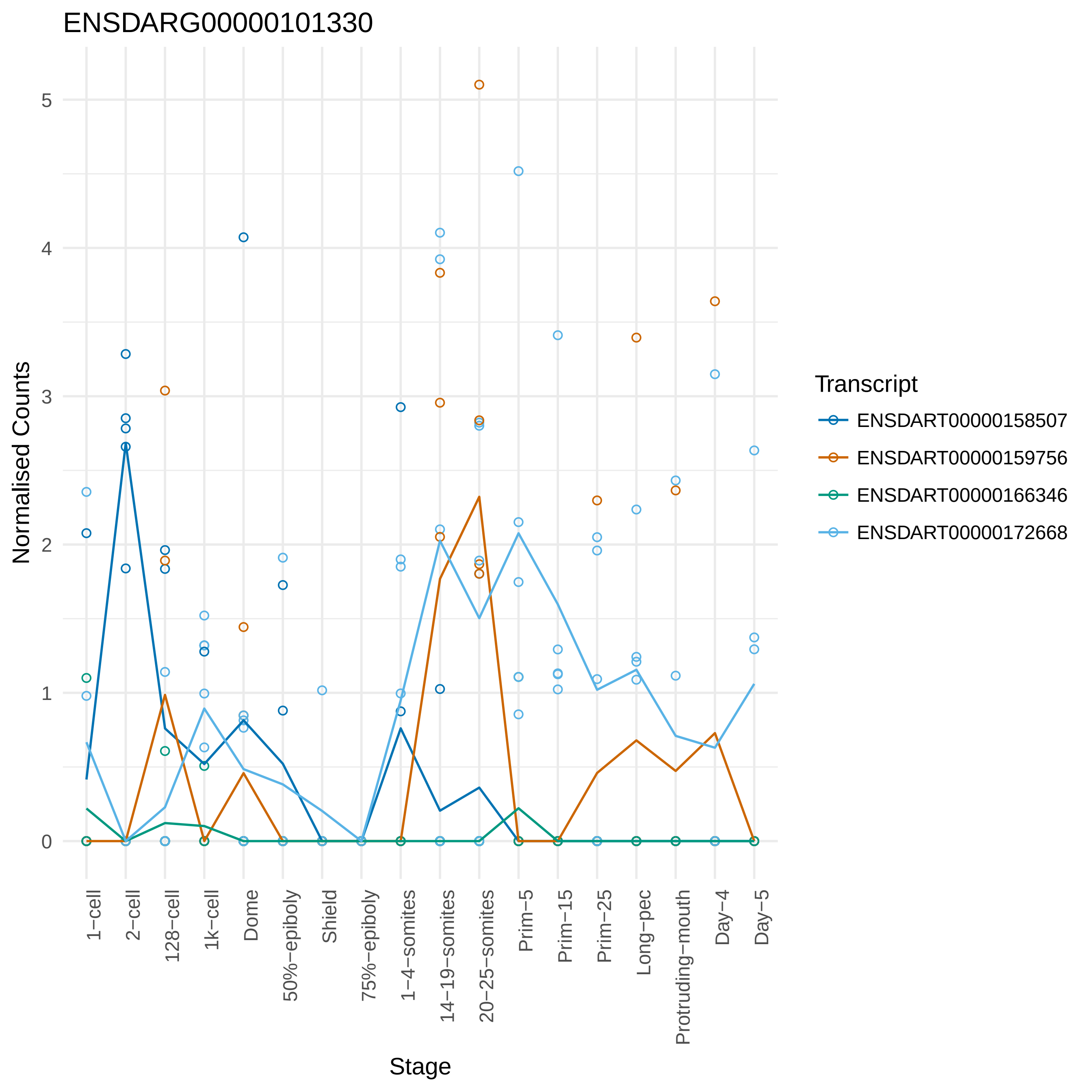
ENSDARG00000101330
Normalised Counts
Stage
0
1
2
3
4
5
1−cell
2−cell
128−cell
1k−cell
Dome
50%−epiboly
Shield
75%−epiboly
1−4−somites
14−19−somites
20−25−somites
Prim−5
Prim−15
Prim−25
Long−pec
Protruding−mouth
Day−4
Day−5
Transcript
ENSDART00000158507
ENSDART00000159756
ENSDART00000166346
ENSDART00000172668

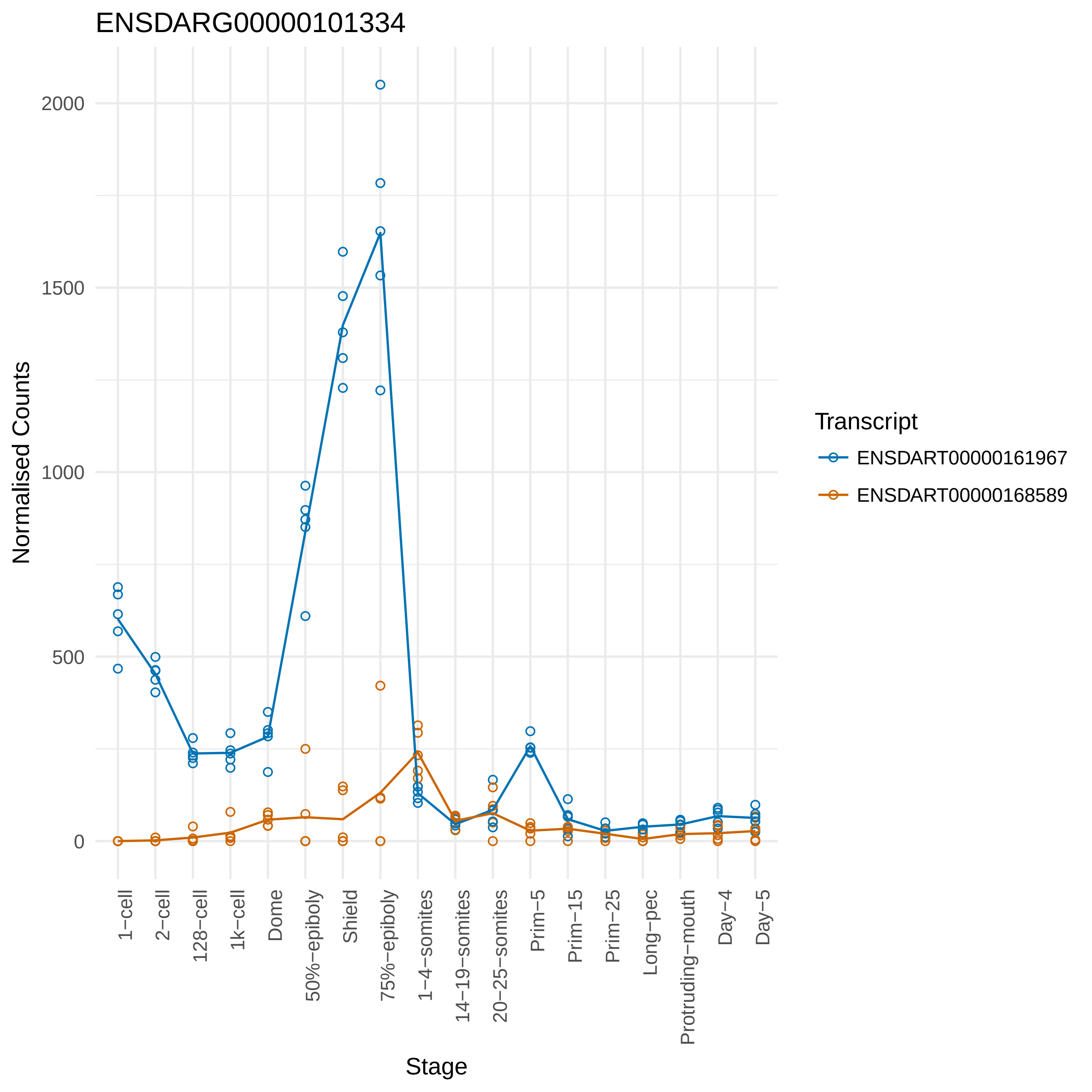
ENSDARG00000101334
Normalised Counts
0
500
1000
1500
2000
1−cell
2−cell
128−cell
1k−cell
Dome
50%−epiboly
Shield
75%−epiboly
1−4−somites
14−19−somites
20−25−somites
Prim−5
Prim−15
Prim−25
Long−pec
Protruding−mouth
Day−4
Day−5
Stage
Transcript
ENSDART00000161967
ENSDART00000168589

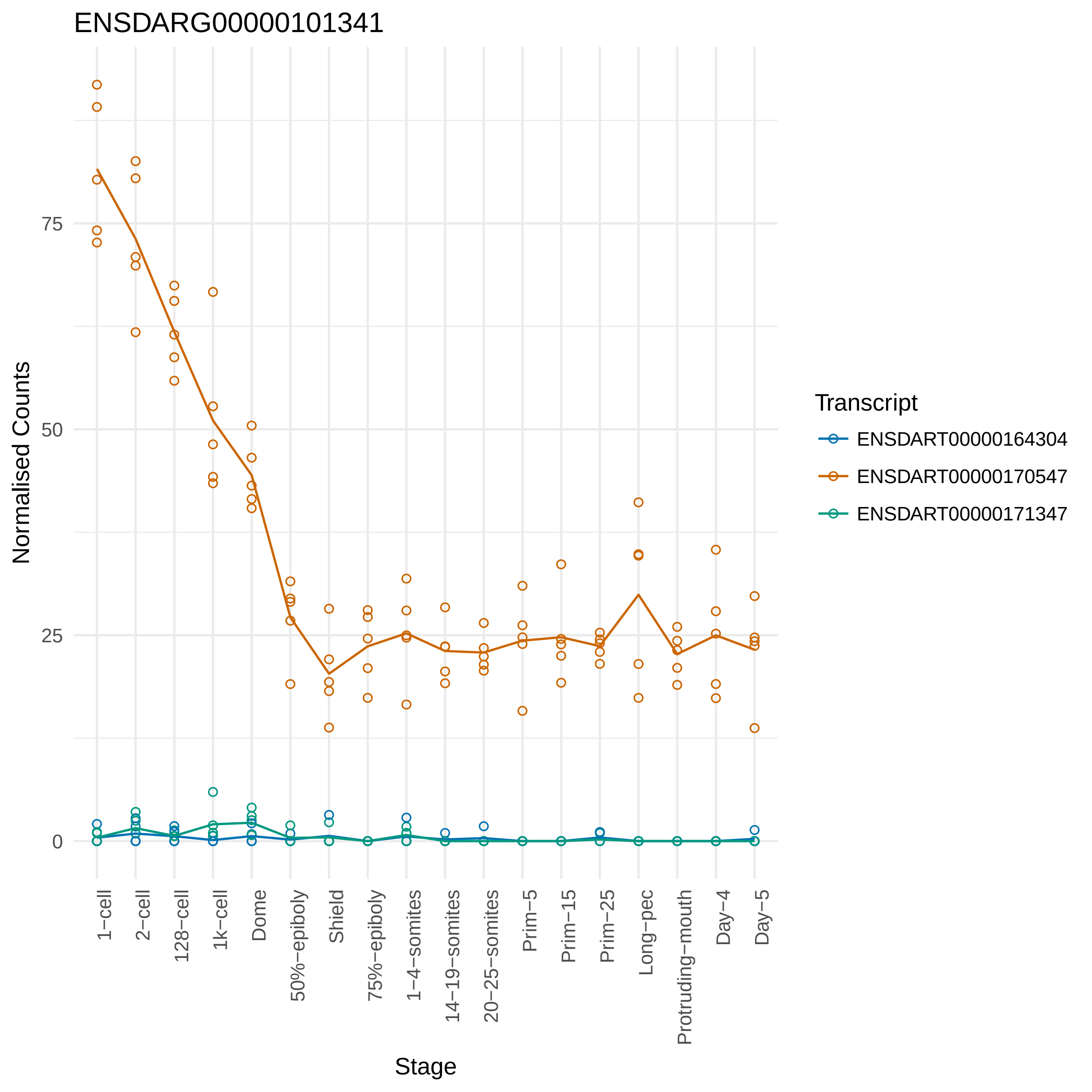

ENSDARG00000101341
Normalised Counts
0
25
50
75
Stage
1−cell
2−cell
128−cell
1k−cell
Dome
50%−epiboly
Shield
75%−epiboly
1−4−somites
14−19−somites
20−25−somites
Prim−5
Prim−15
Prim−25
Long−pec
Protruding−mouth
Day−4
Day−5
Transcript
ENSDART00000164304
ENSDART00000170547
ENSDART00000171347

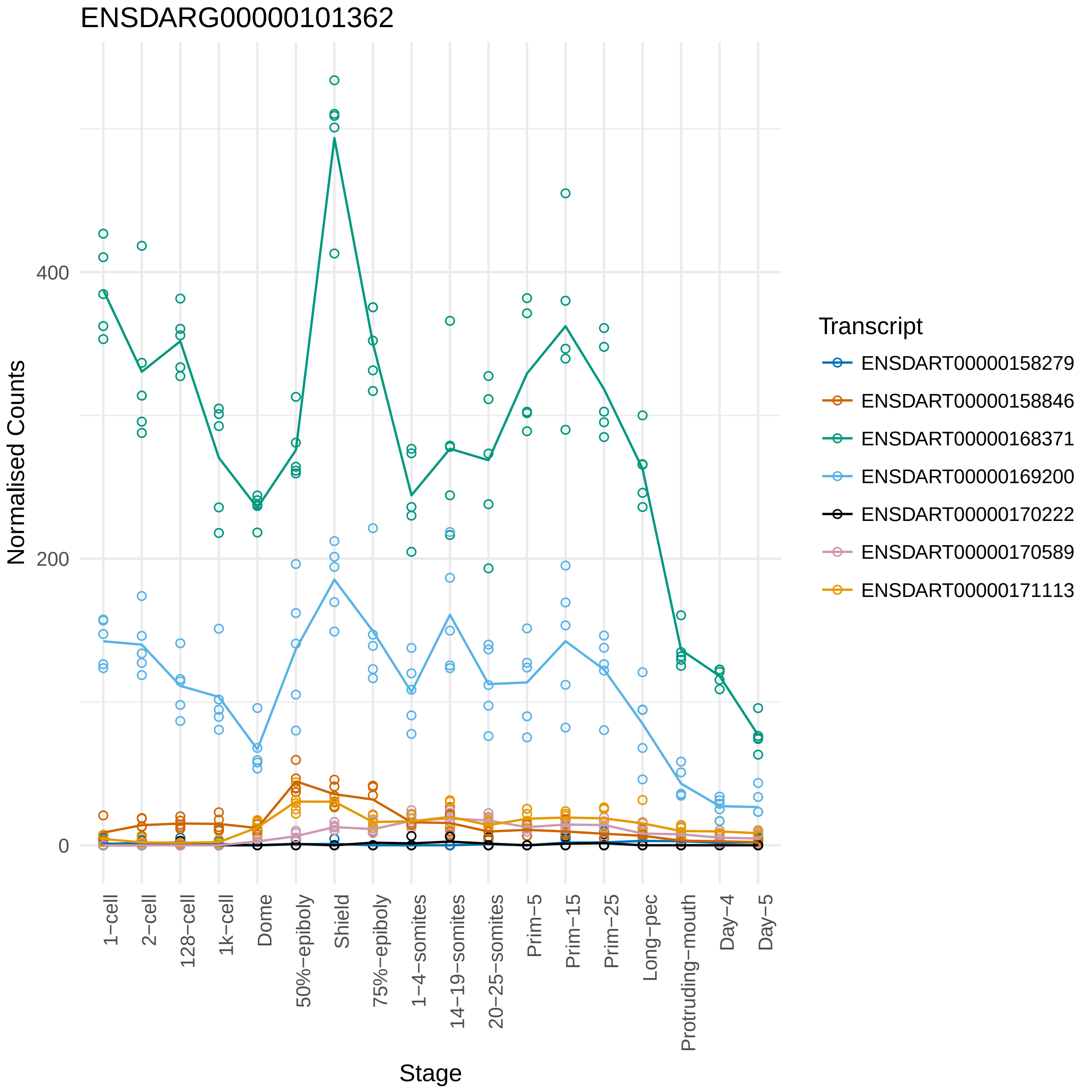
ENSDARG00000101362
Normalised Counts
0
200
400
Stage
1−cell
2−cell
128−cell
1k−cell
Dome
50%−epiboly
Shield
75%−epiboly
1−4−somites
14−19−somites
20−25−somites
Prim−5
Prim−15
Prim−25
Long−pec
Protruding−mouth
Day−4
Day−5
Transcript
ENSDART00000158279
ENSDART00000158846
ENSDART00000168371
ENSDART00000169200
ENSDART00000170222
ENSDART00000170589
ENSDART00000171113

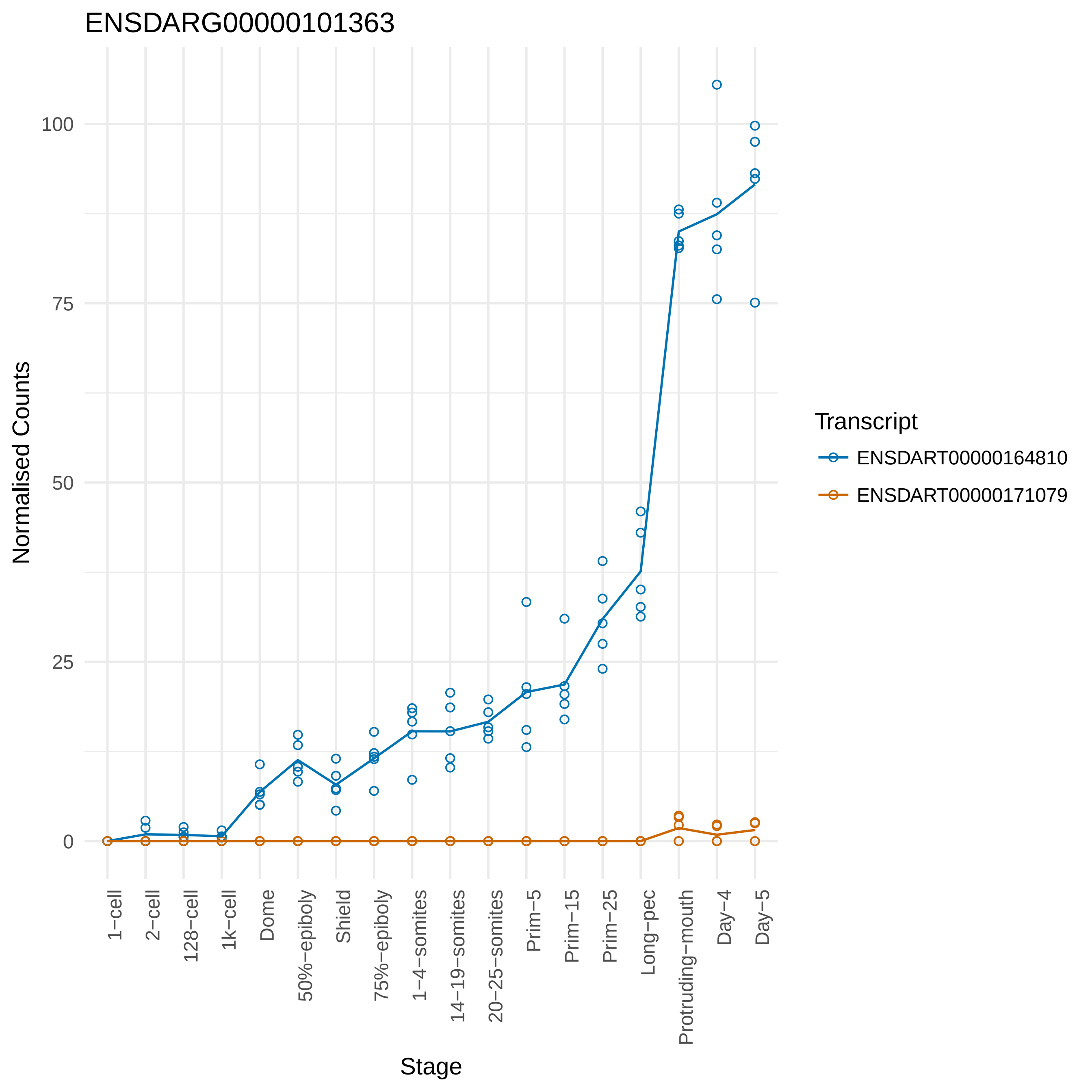
ENSDARG00000101363
Normalised Counts
100
75
50
25
0
1−cell
2−cell
128−cell
1k−cell
Dome
50%−epiboly
Shield
75%−epiboly
1−4−somites
14−19−somites
20−25−somites
Prim−5
Prim−15
Prim−25
Long−pec
Protruding−mouth
Day−4
Day−5
Stage
Transcript
ENSDART00000164810
ENSDART00000171079

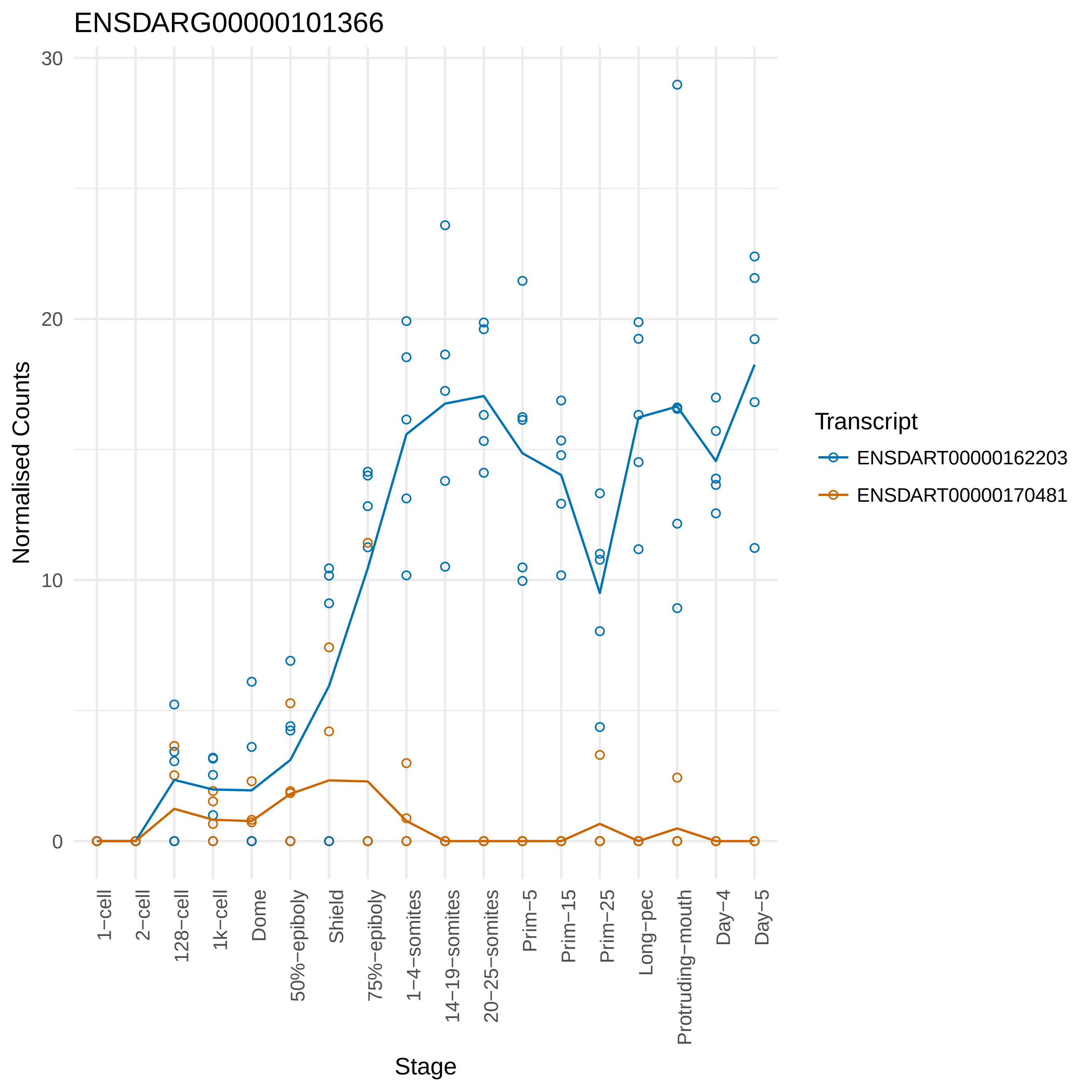

ENSDARG00000101366
Normalised Counts
30
20
10
0
1−cell
2−cell
128−cell
1k−cell
Dome
50%−epiboly
Shield
75%−epiboly
1−4−somites
14−19−somites
20−25−somites
Prim−5
Prim−15
Prim−25
Long−pec
Protruding−mouth
Day−4
Day−5
Stage
Transcript
ENSDART00000162203
ENSDART00000170481

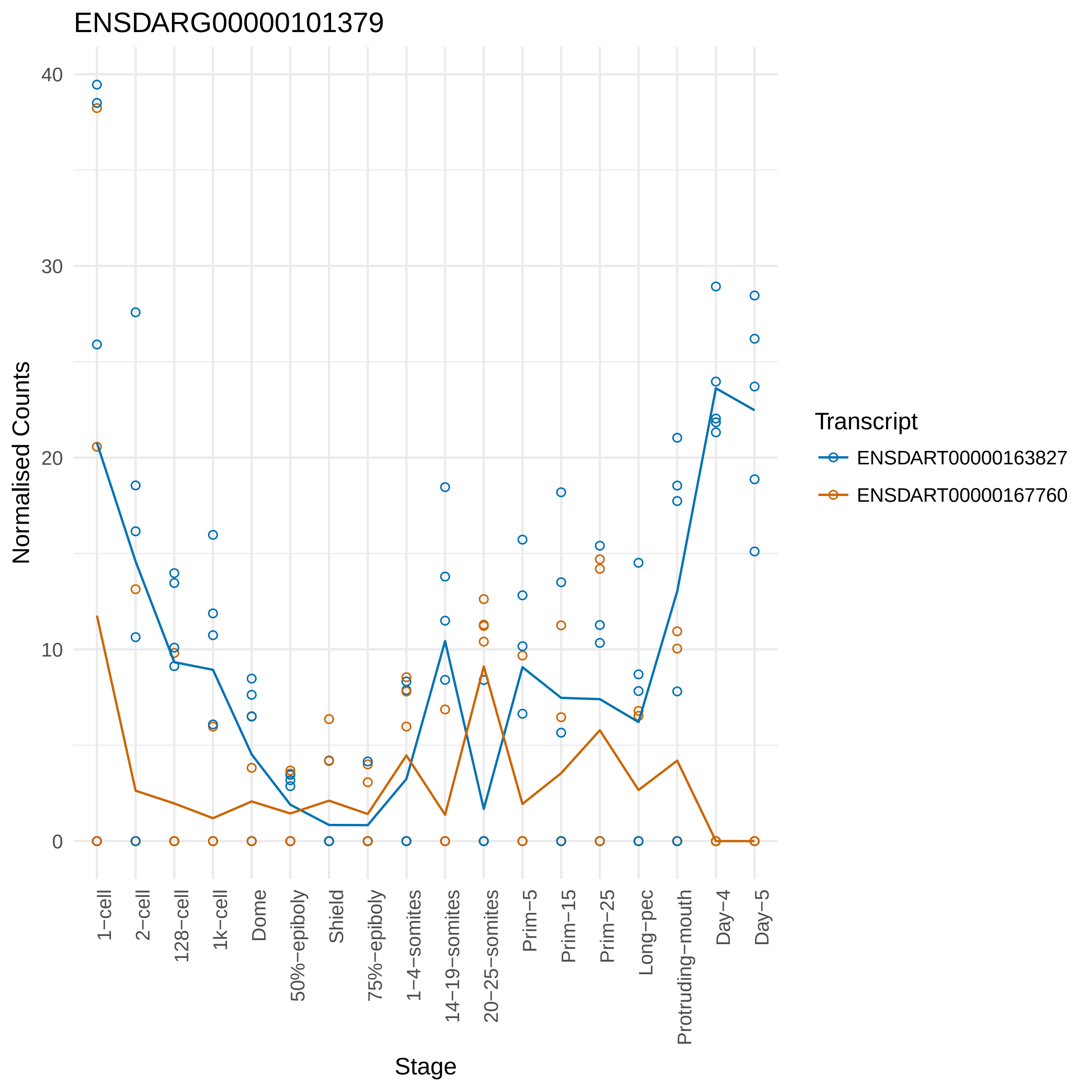
ENSDARG00000101379
Normalised Counts
0
10
20
30
40
1−cell
2−cell
128−cell
1k−cell
Dome
50%−epiboly
Shield
75%−epiboly
1−4−somites
14−19−somites
20−25−somites
Prim−5
Prim−15
Prim−25
Long−pec
Protruding−mouth
Day−4
Day−5
Stage
Transcript
ENSDART00000163827
ENSDART00000167760

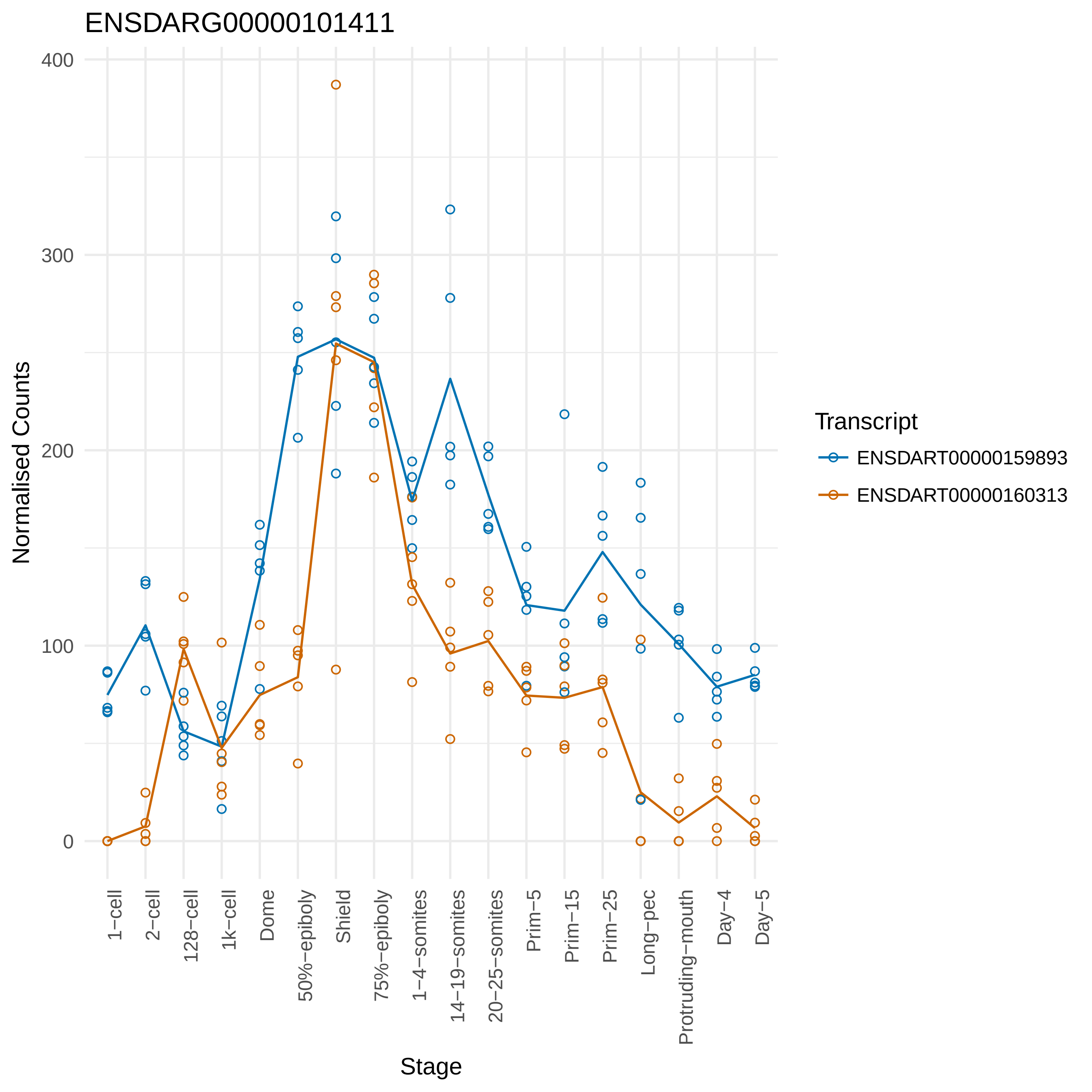
ENSDARG00000101411
Normalised Counts
400
300
200
100
0
1−cell
2−cell
128−cell
1k−cell
Dome
50%−epiboly
Shield
75%−epiboly
1−4−somites
14−19−somites
20−25−somites
Prim−5
Prim−15
Prim−25
Long−pec
Protruding−mouth
Day−4
Day−5
Stage
Transcript
ENSDART00000159893
ENSDART00000160313

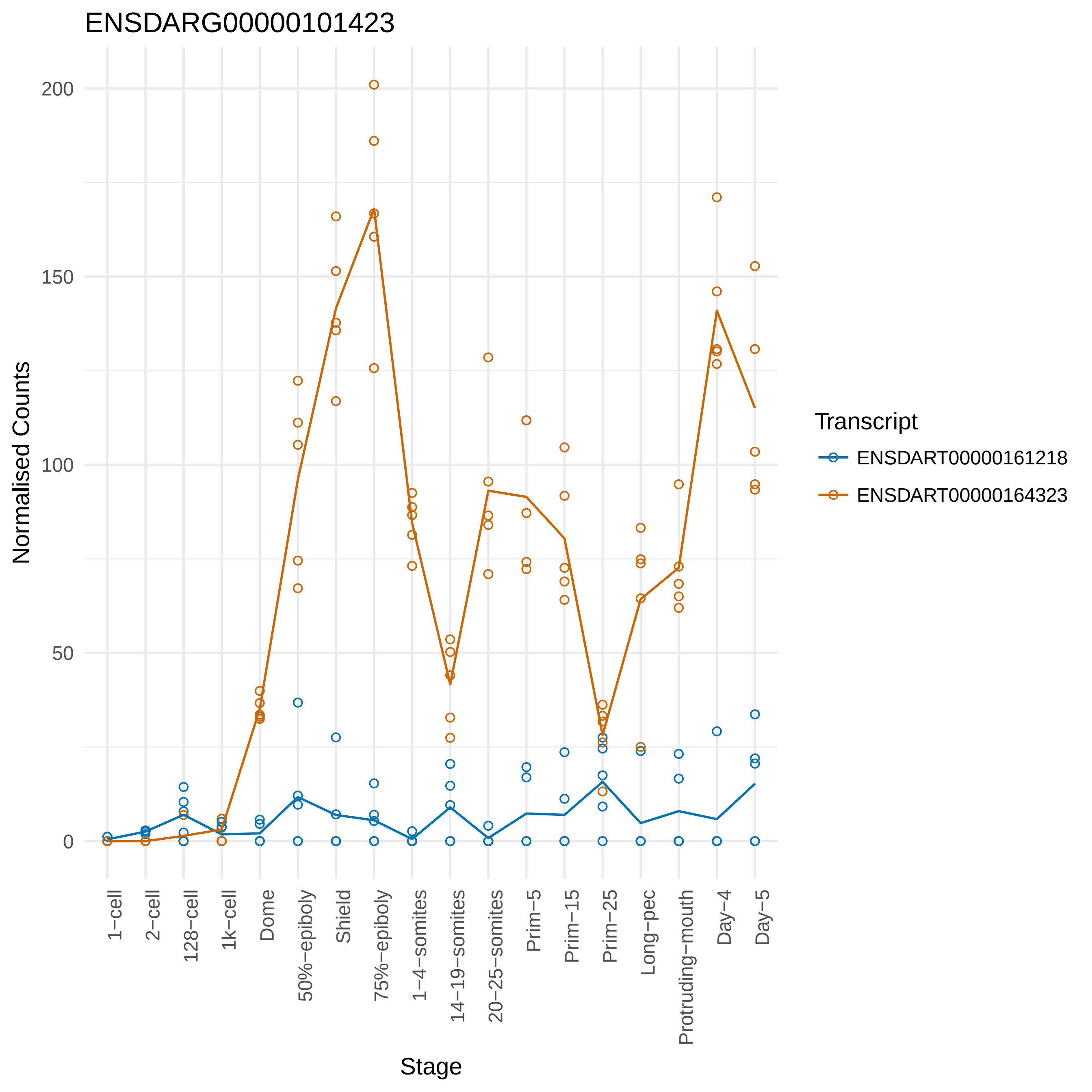

ENSDARG00000101423
Normalised Counts
200
150
100
50
0
Stage
1−cell
2−cell
128−cell
1k−cell
Dome
50%−epiboly
Shield
75%−epiboly
1−4−somites
14−19−somites
20−25−somites
Prim−5
Prim−15
Prim−25
Long−pec
Protruding−mouth
Day−4
Day−5
Transcript
ENSDART00000161218
ENSDART00000164323

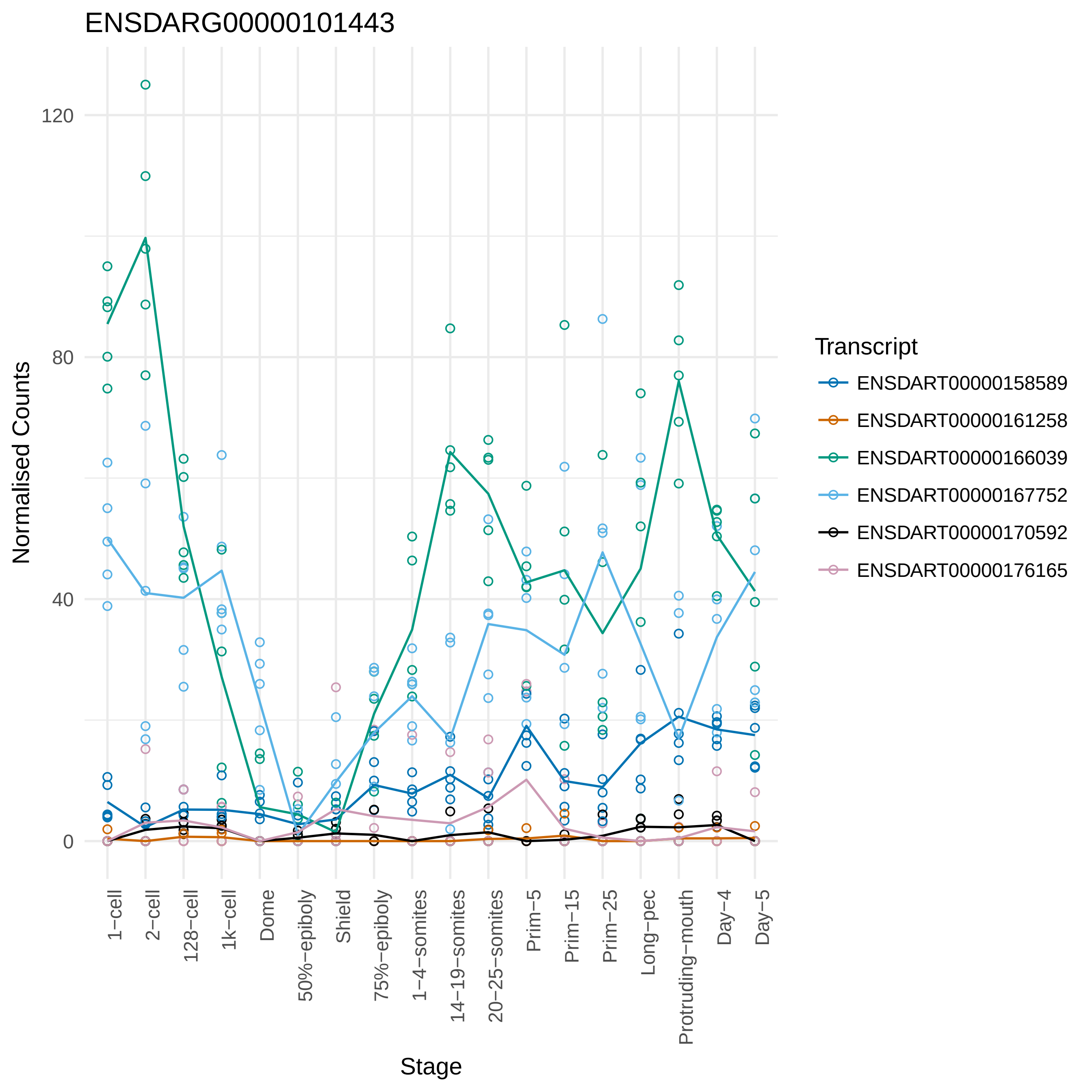

ENSDARG00000101443
Normalised Counts
0
40
80
120
Stage
1−cell
2−cell
128−cell
1k−cell
Dome
50%−epiboly
Shield
75%−epiboly
1−4−somites
14−19−somites
20−25−somites
Prim−5
Prim−15
Prim−25
Long−pec
Protruding−mouth
Day−4
Day−5
Transcript
ENSDART00000158589
ENSDART00000161258
ENSDART00000166039
ENSDART00000167752
ENSDART00000170592
ENSDART00000176165

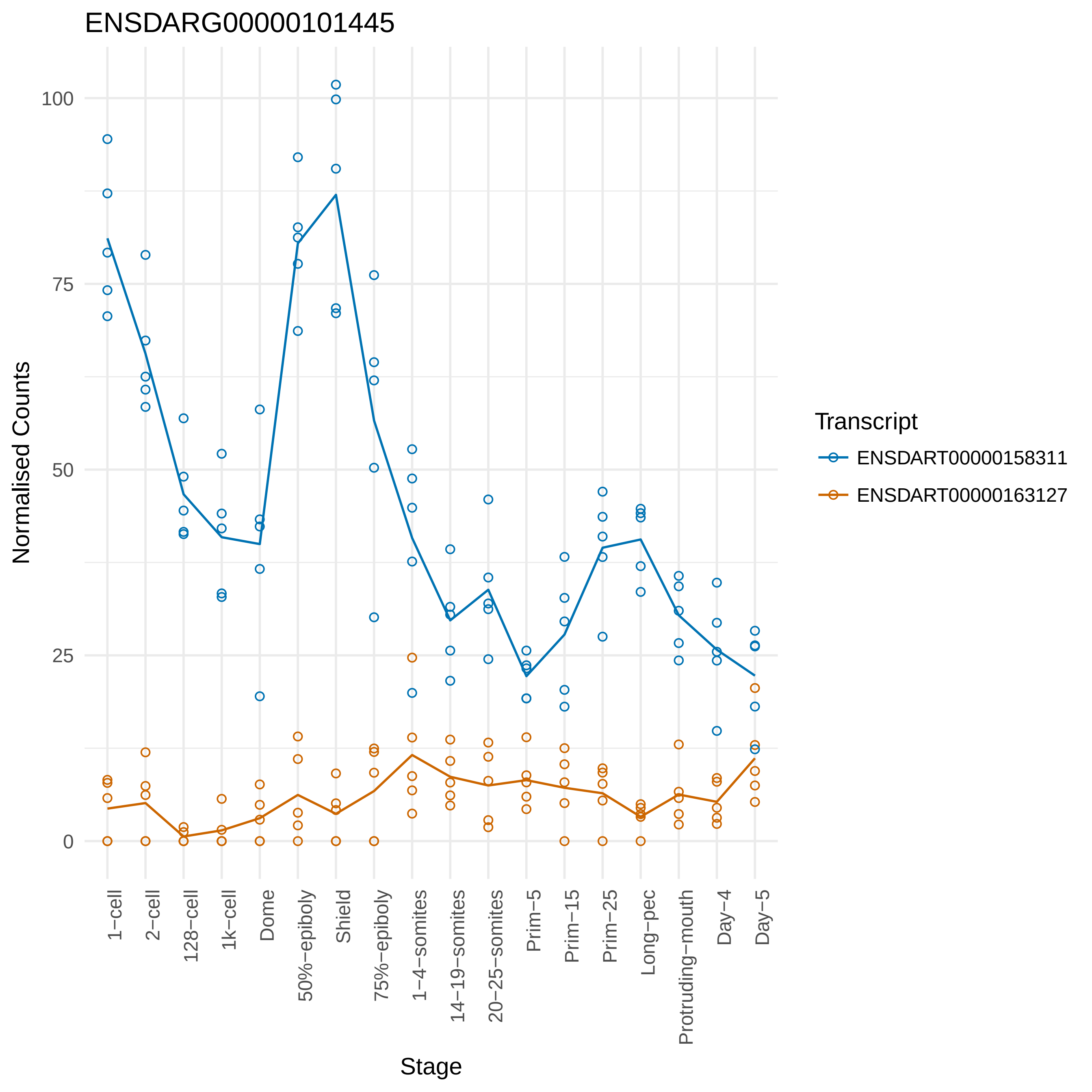
ENSDARG00000101445
Normalised Counts
100
75
50
25
0
1−cell
2−cell
128−cell
1k−cell
Dome
50%−epiboly
Shield
75%−epiboly
1−4−somites
14−19−somites
20−25−somites
Prim−5
Prim−15
Prim−25
Long−pec
Protruding−mouth
Day−4
Day−5
Stage
Transcript
ENSDART00000158311
ENSDART00000163127

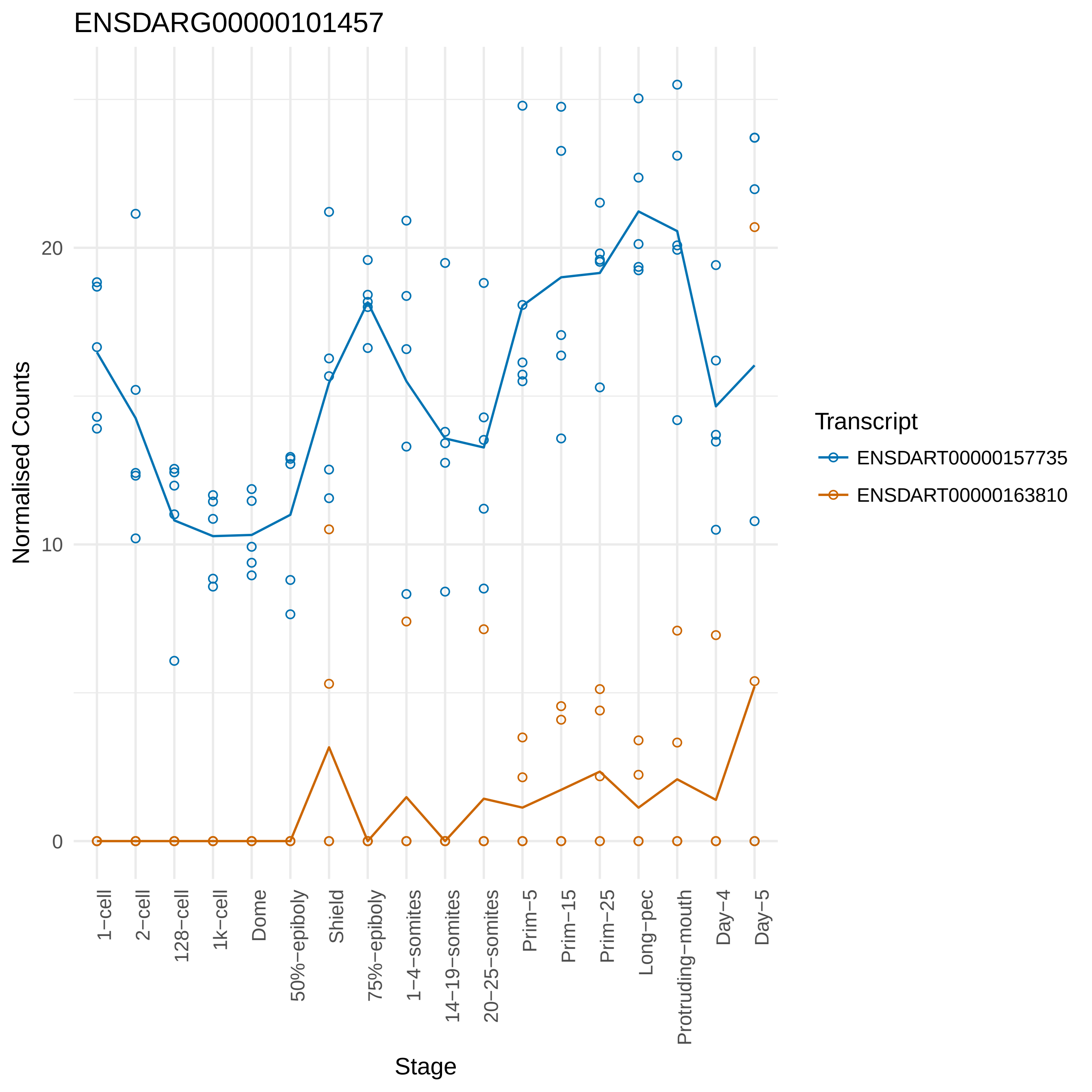

ENSDARG00000101457
Normalised Counts
0
10
20
1−cell
2−cell
128−cell
1k−cell
Dome
50%−epiboly
Shield
75%−epiboly
1−4−somites
14−19−somites
20−25−somites
Prim−5
Prim−15
Prim−25
Long−pec
Protruding−mouth
Day−4
Day−5
Stage
Transcript
ENSDART00000157735
ENSDART00000163810

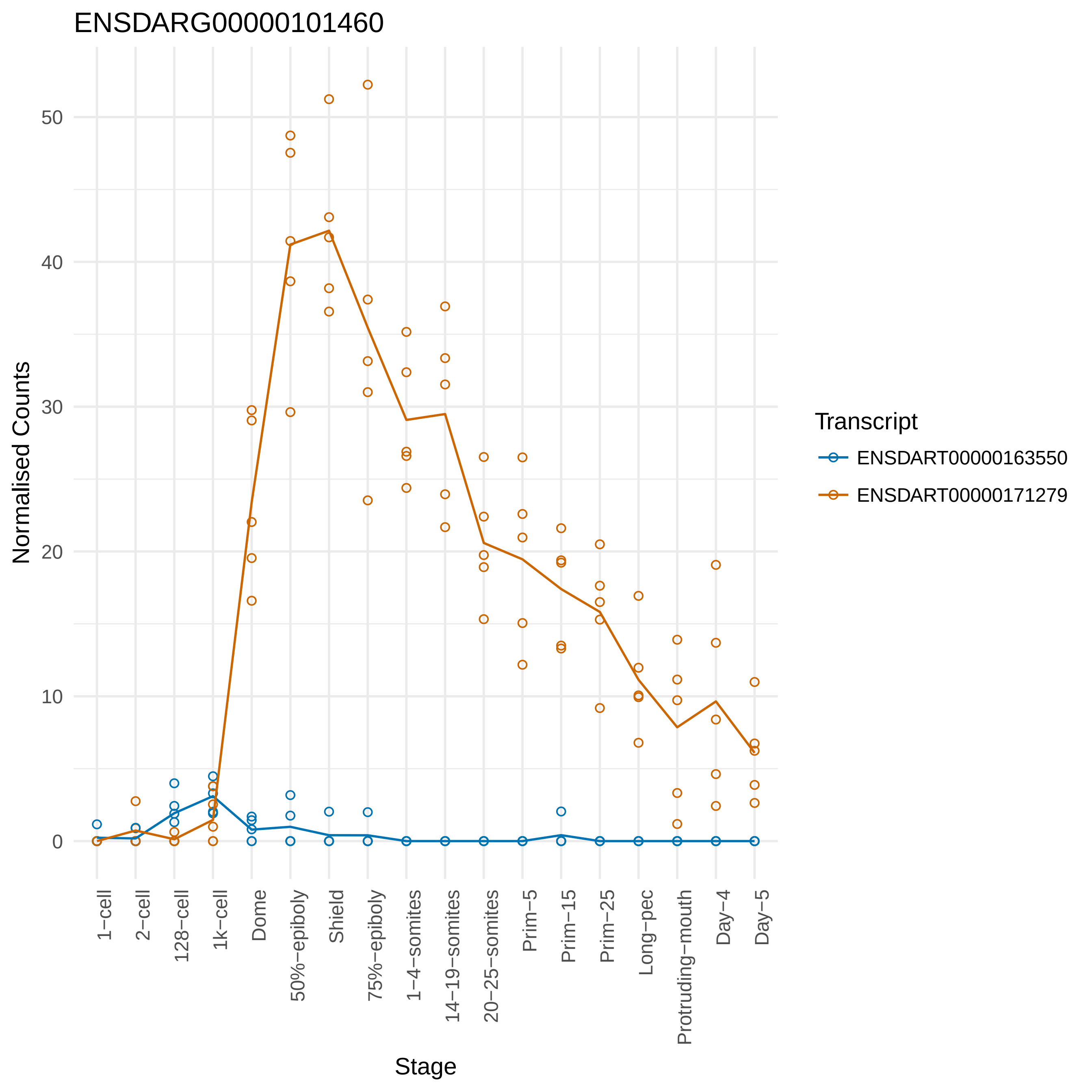

ENSDARG00000101460
Normalised Counts
0
10
20
30
40
50
1−cell
2−cell
128−cell
1k−cell
Dome
50%−epiboly
Shield
75%−epiboly
1−4−somites
14−19−somites
20−25−somites
Prim−5
Prim−15
Prim−25
Long−pec
Protruding−mouth
Day−4
Day−5
Stage
Transcript
ENSDART00000163550
ENSDART00000171279

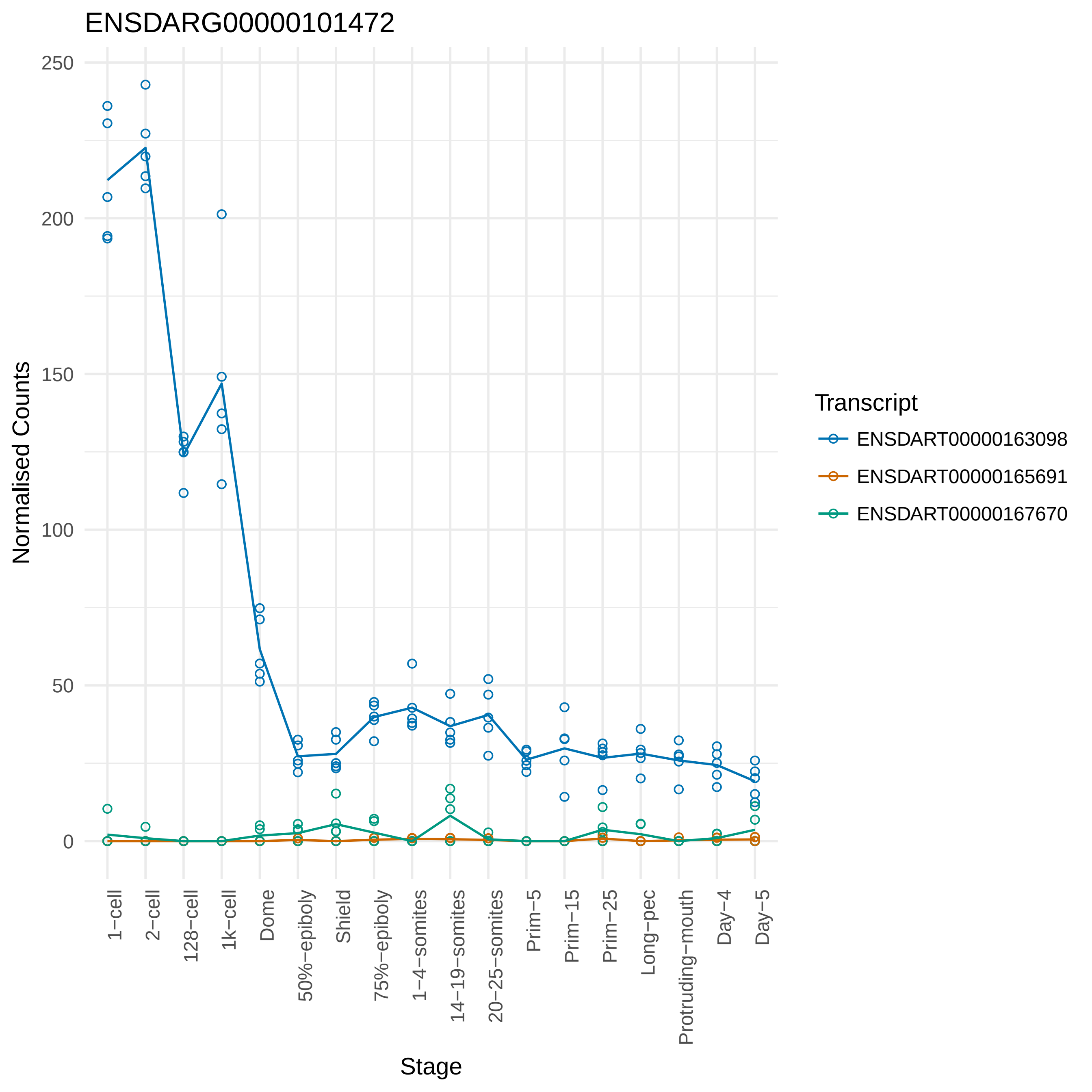

ENSDARG00000101472
Normalised Counts
250
200
150
100
50
0
1−cell
2−cell
128−cell
1k−cell
Dome
50%−epiboly
Shield
75%−epiboly
1−4−somites
14−19−somites
20−25−somites
Prim−5
Prim−15
Prim−25
Long−pec
Protruding−mouth
Day−4
Day−5
Stage
Transcript
ENSDART00000163098
ENSDART00000165691
ENSDART00000167670

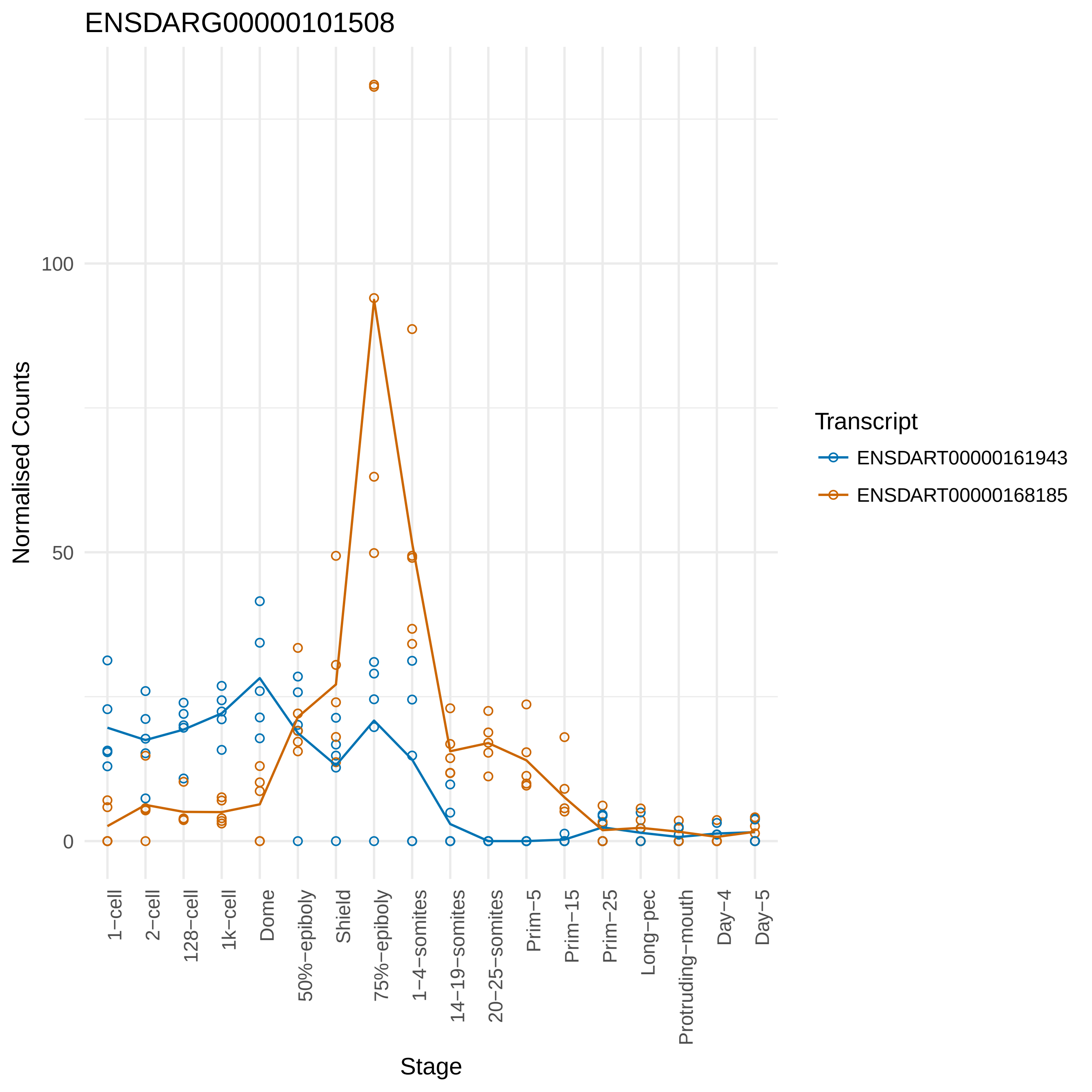

ENSDARG00000101508
Normalised Counts
100
50
0
1−cell
2−cell
128−cell
1k−cell
Dome
50%−epiboly
Shield
75%−epiboly
1−4−somites
14−19−somites
20−25−somites
Prim−5
Prim−15
Prim−25
Long−pec
Protruding−mouth
Day−4
Day−5
Stage
Transcript
ENSDART00000161943
ENSDART00000168185

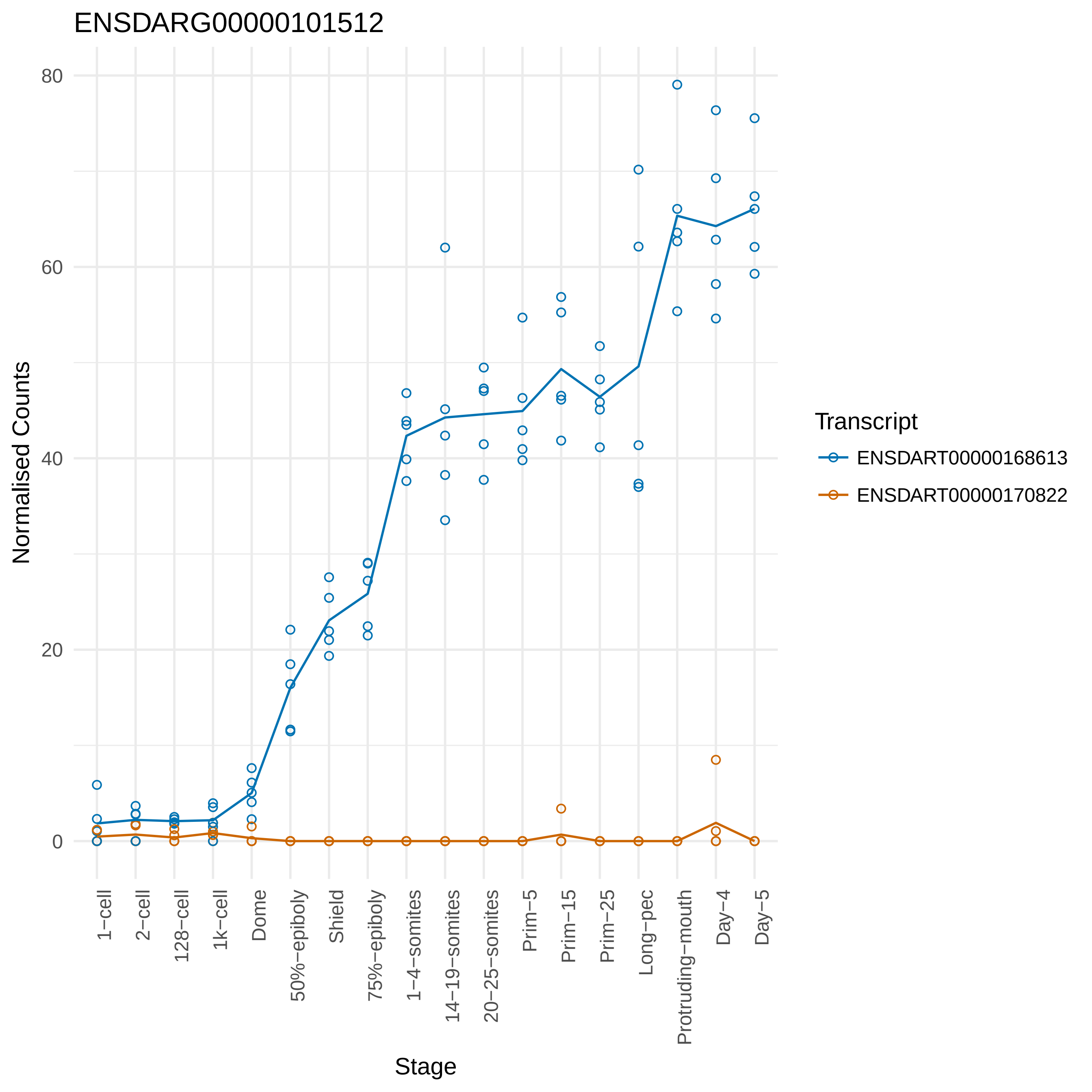

ENSDARG00000101512
Normalised Counts
80
60
40
20
0
1−cell
2−cell
128−cell
1k−cell
Dome
50%−epiboly
Shield
75%−epiboly
1−4−somites
14−19−somites
20−25−somites
Prim−5
Prim−15
Prim−25
Long−pec
Protruding−mouth
Day−4
Day−5
Stage
Transcript
ENSDART00000168613
ENSDART00000170822

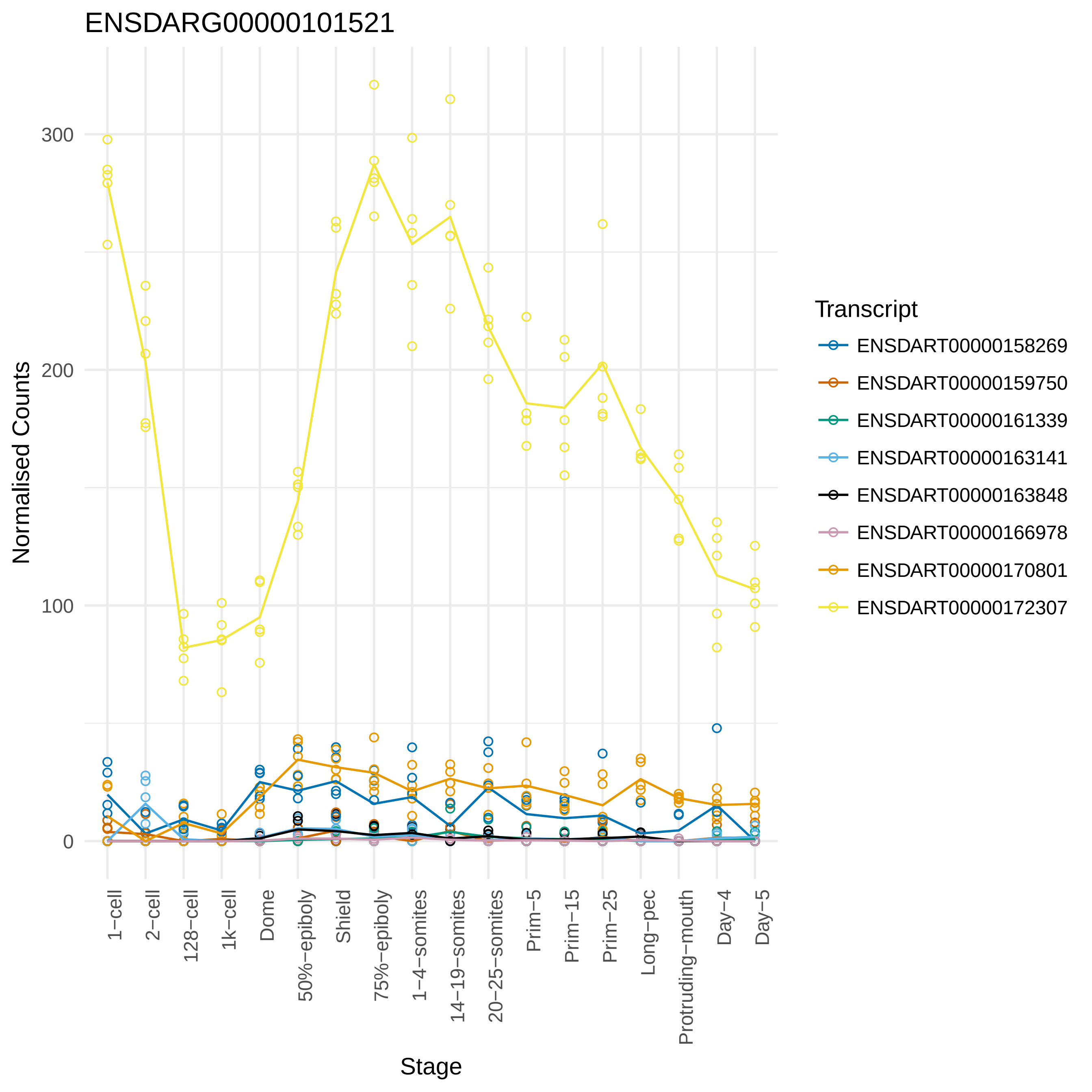

ENSDARG00000101521
Normalised Counts
300
200
100
0
1−cell
2−cell
128−cell
1k−cell
Dome
50%−epiboly
Shield
75%−epiboly
1−4−somites
14−19−somites
20−25−somites
Prim−5
Prim−15
Prim−25
Long−pec
Protruding−mouth
Day−4
Day−5
Stage
Transcript
ENSDART00000158269
ENSDART00000159750
ENSDART00000161339
ENSDART00000163141
ENSDART00000163848
ENSDART00000166978
ENSDART00000170801
ENSDART00000172307

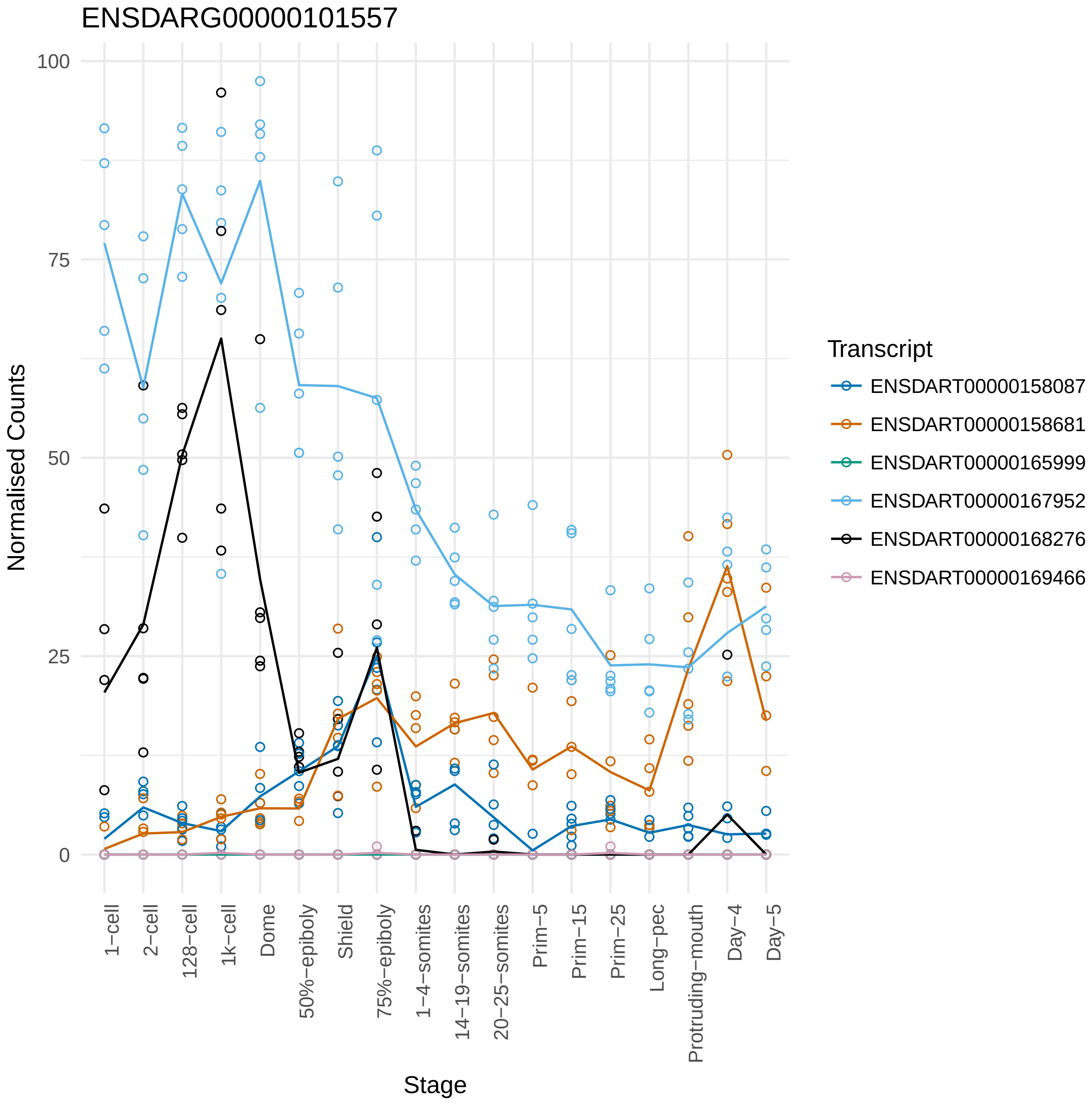

ENSDARG00000101557
Normalised Counts
100
75
50
25
0
1−cell
2−cell
128−cell
1k−cell
Dome
50%−epiboly
Shield
75%−epiboly
1−4−somites
14−19−somites
20−25−somites
Prim−5
Prim−15
Prim−25
Long−pec
Protruding−mouth
Day−4
Day−5
Stage
Transcript
ENSDART00000158087
ENSDART00000158681
ENSDART00000165999
ENSDART00000167952
ENSDART00000168276
ENSDART00000169466

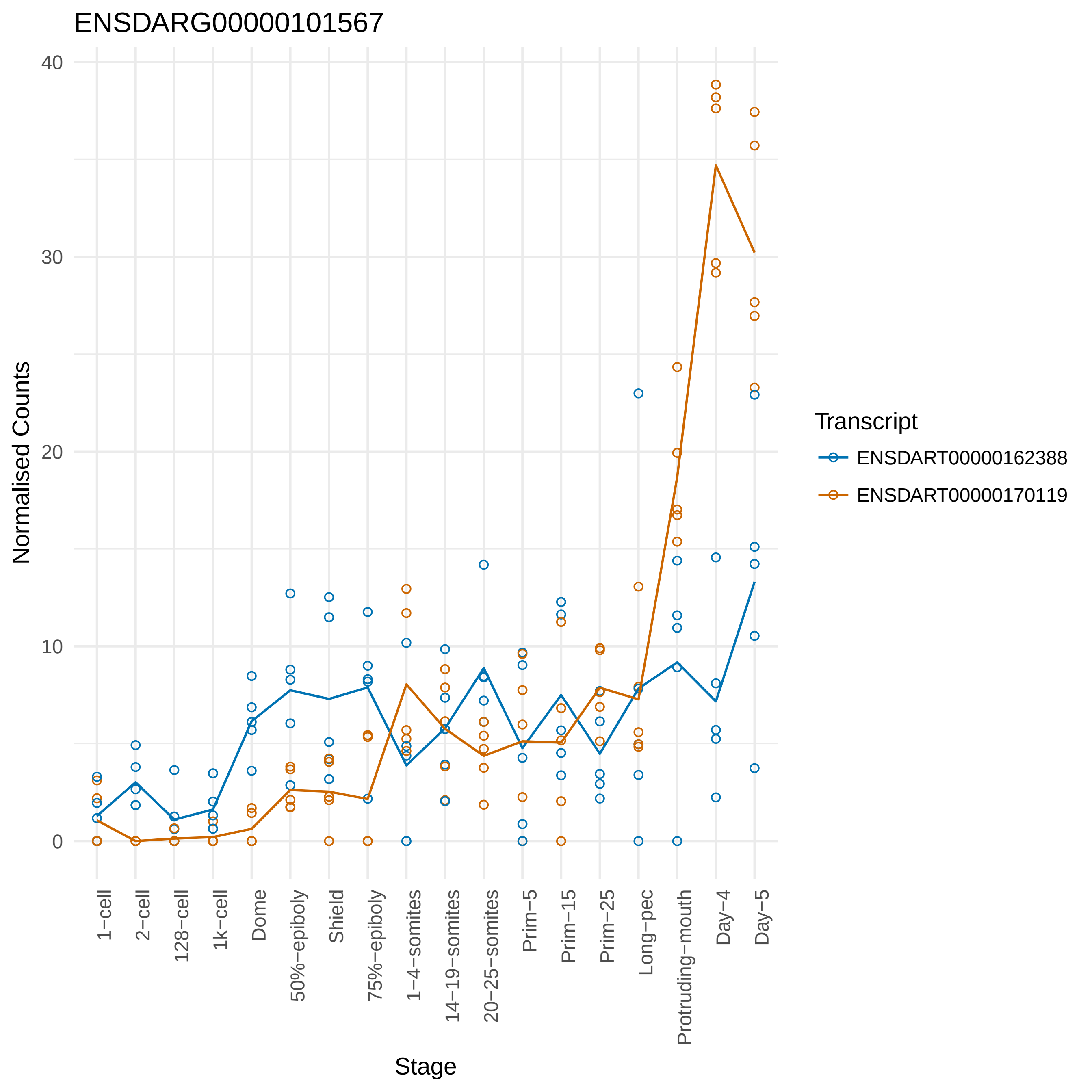
ENSDARG00000101567
Normalised Counts
40
30
20
10
0
1−cell
2−cell
128−cell
1k−cell
Dome
50%−epiboly
Shield
75%−epiboly
1−4−somites
14−19−somites
20−25−somites
Prim−5
Prim−15
Prim−25
Long−pec
Protruding−mouth
Day−4
Day−5
Stage
Transcript
ENSDART00000162388
ENSDART00000170119

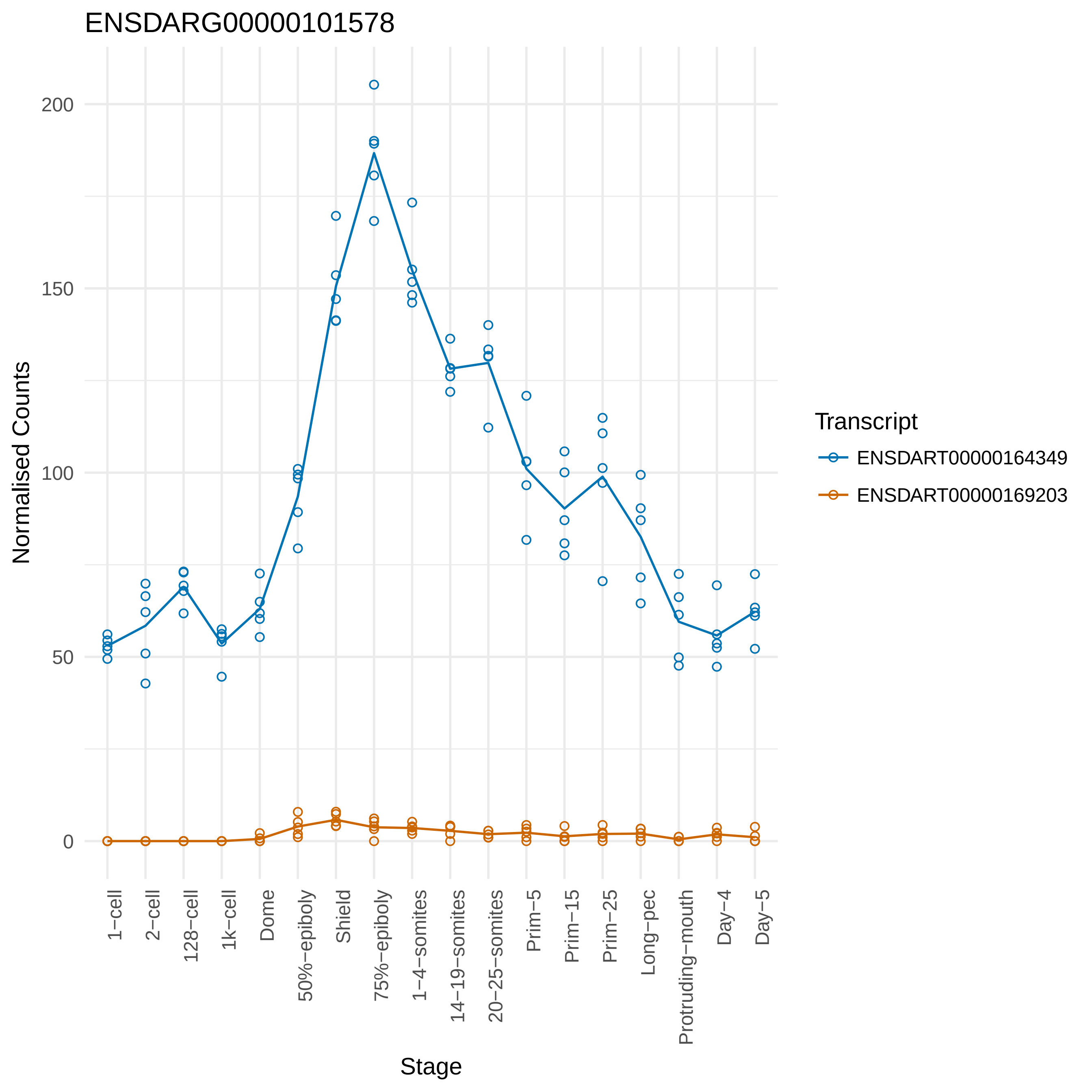

ENSDARG00000101578
Normalised Counts
200
150
100
50
0
1−cell
2−cell
128−cell
1k−cell
Dome
50%−epiboly
Shield
75%−epiboly
1−4−somites
14−19−somites
20−25−somites
Prim−5
Prim−15
Prim−25
Long−pec
Protruding−mouth
Day−4
Day−5
Stage
Transcript
ENSDART00000164349
ENSDART00000169203

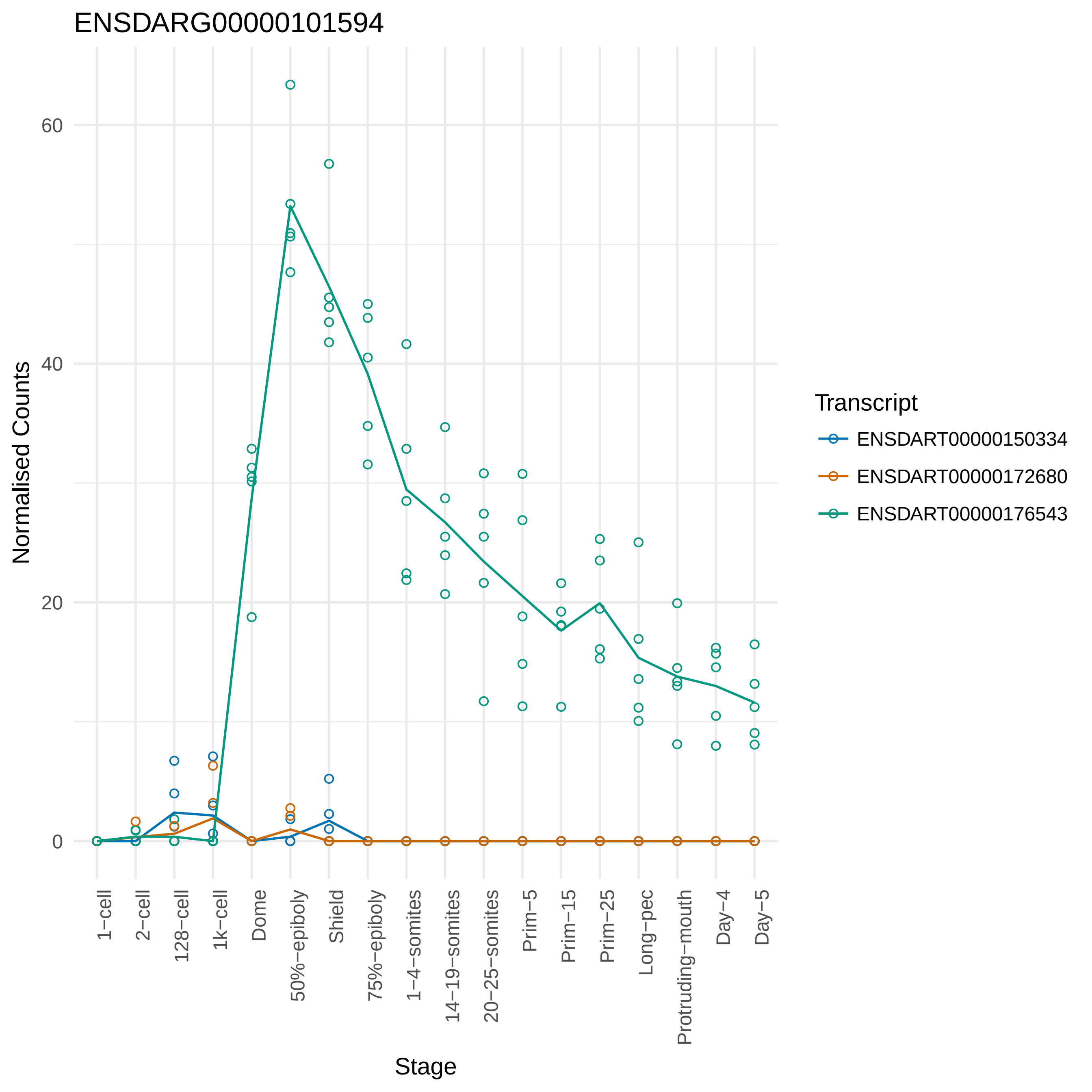

ENSDARG00000101594
Normalised Counts
60
40
20
0
1−cell
2−cell
128−cell
1k−cell
Dome
50%−epiboly
Shield
75%−epiboly
1−4−somites
14−19−somites
20−25−somites
Prim−5
Prim−15
Prim−25
Long−pec
Protruding−mouth
Day−4
Day−5
Stage
Transcript
ENSDART00000150334
ENSDART00000172680
ENSDART00000176543

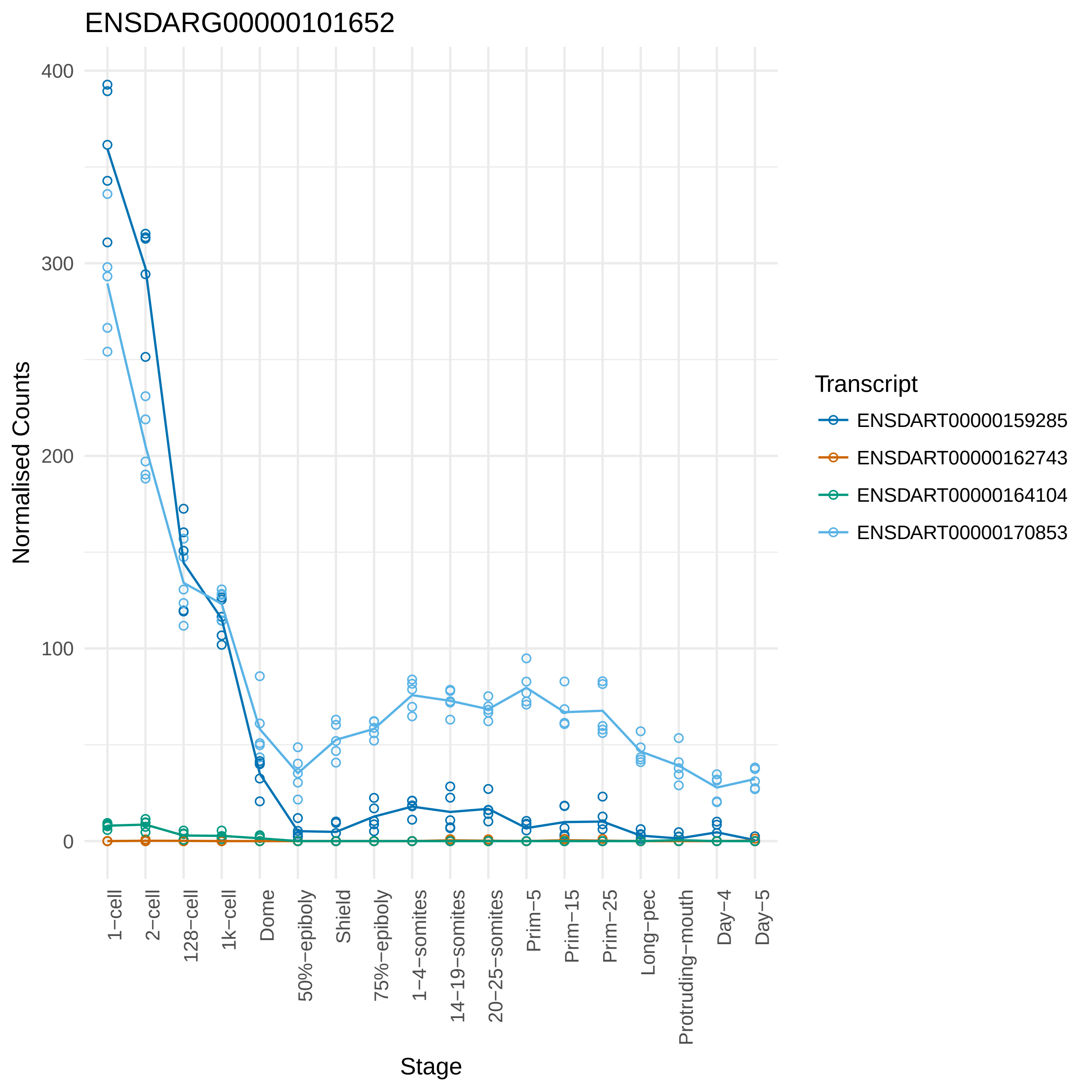
ENSDARG00000101652
Normalised Counts
400
300
200
100
0
1−cell
2−cell
128−cell
1k−cell
Dome
50%−epiboly
Shield
75%−epiboly
1−4−somites
14−19−somites
20−25−somites
Prim−5
Prim−15
Prim−25
Long−pec
Protruding−mouth
Day−4
Day−5
Stage
Transcript
ENSDART00000159285
ENSDART00000162743
ENSDART00000164104
ENSDART00000170853

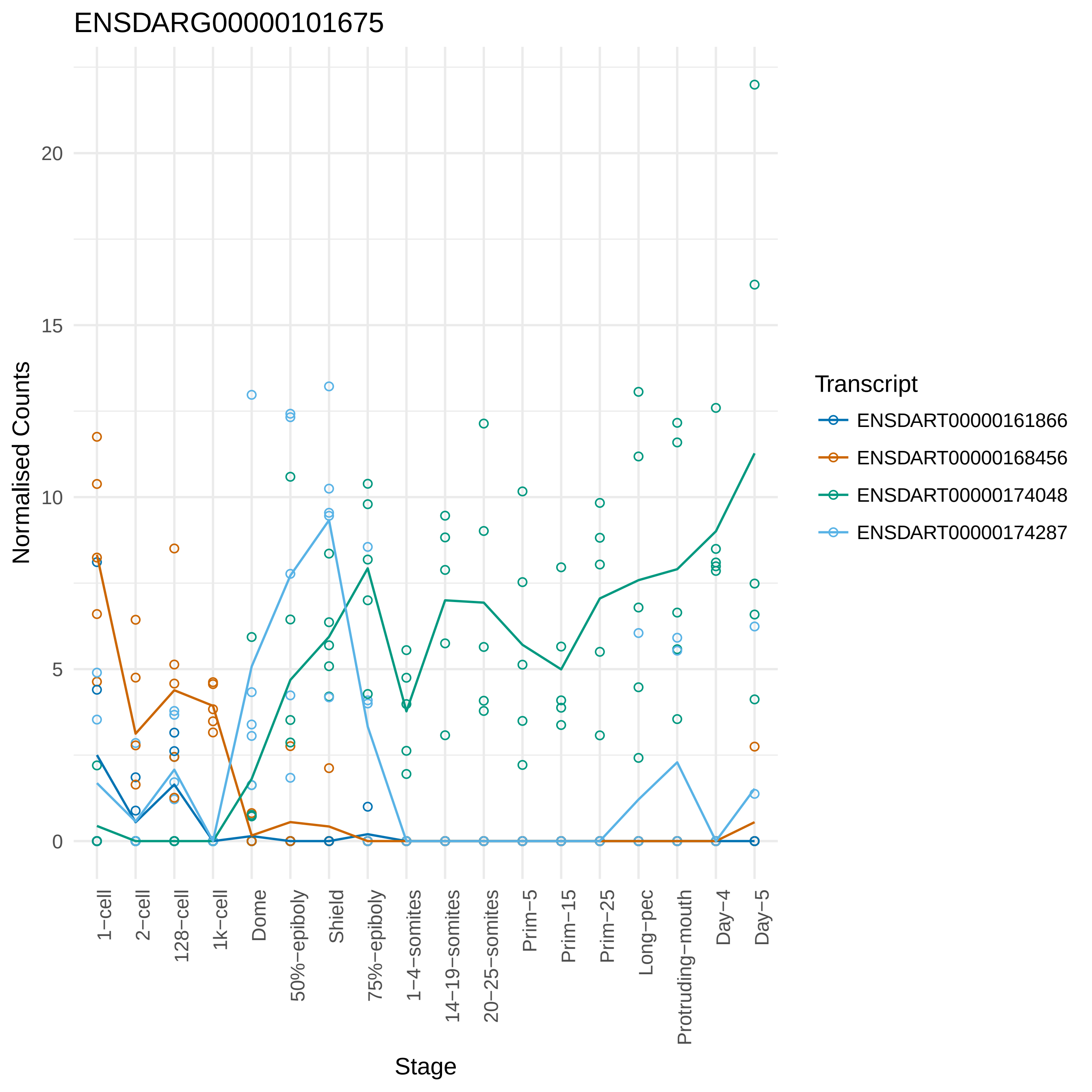
ENSDARG00000101675
Normalised Counts
0
5
10
15
20
Stage
1−cell
2−cell
128−cell
1k−cell
Dome
50%−epiboly
Shield
75%−epiboly
1−4−somites
14−19−somites
20−25−somites
Prim−5
Prim−15
Prim−25
Long−pec
Protruding−mouth
Day−4
Day−5
Transcript
ENSDART00000161866
ENSDART00000168456
ENSDART00000174048
ENSDART00000174287

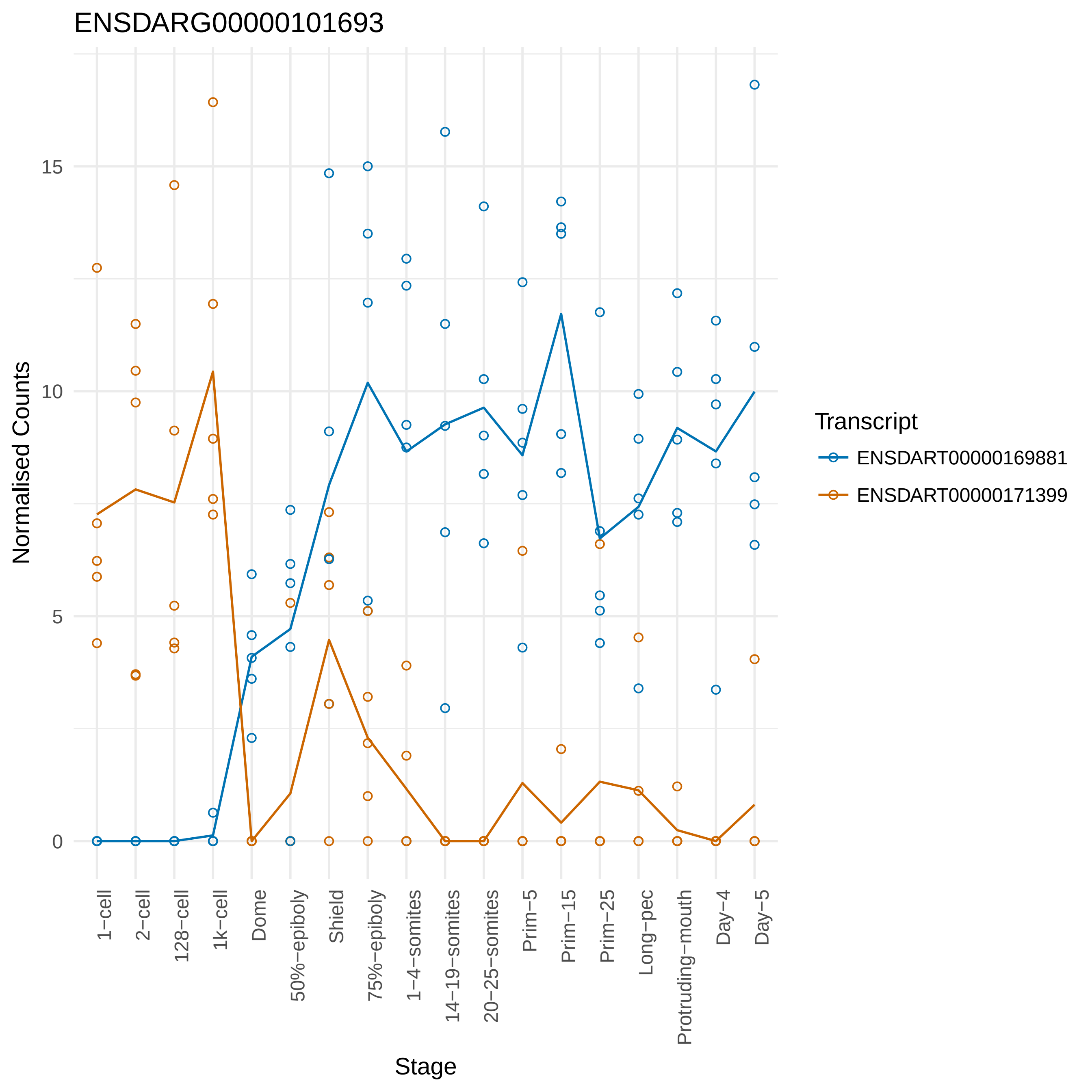

ENSDARG00000101693
Normalised Counts
15
10
5
0
1−cell
2−cell
128−cell
1k−cell
Dome
50%−epiboly
Shield
75%−epiboly
1−4−somites
14−19−somites
20−25−somites
Prim−5
Prim−15
Prim−25
Long−pec
Protruding−mouth
Day−4
Day−5
Stage
Transcript
ENSDART00000169881
ENSDART00000171399

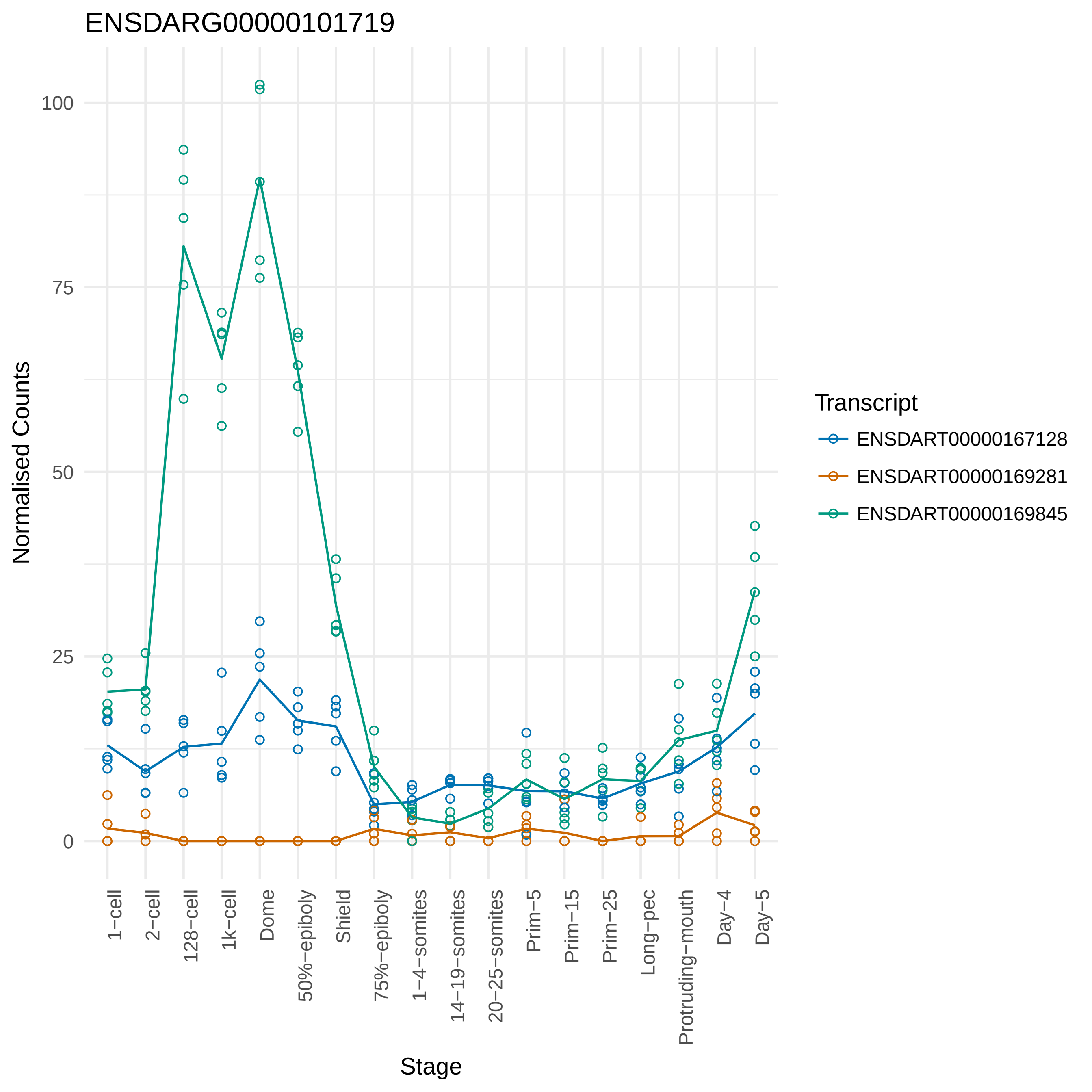

ENSDARG00000101719
Normalised Counts
100
75
50
25
0
1−cell
2−cell
128−cell
1k−cell
Dome
50%−epiboly
Shield
75%−epiboly
1−4−somites
14−19−somites
20−25−somites
Prim−5
Prim−15
Prim−25
Long−pec
Protruding−mouth
Day−4
Day−5
Stage
Transcript
ENSDART00000167128
ENSDART00000169281
ENSDART00000169845

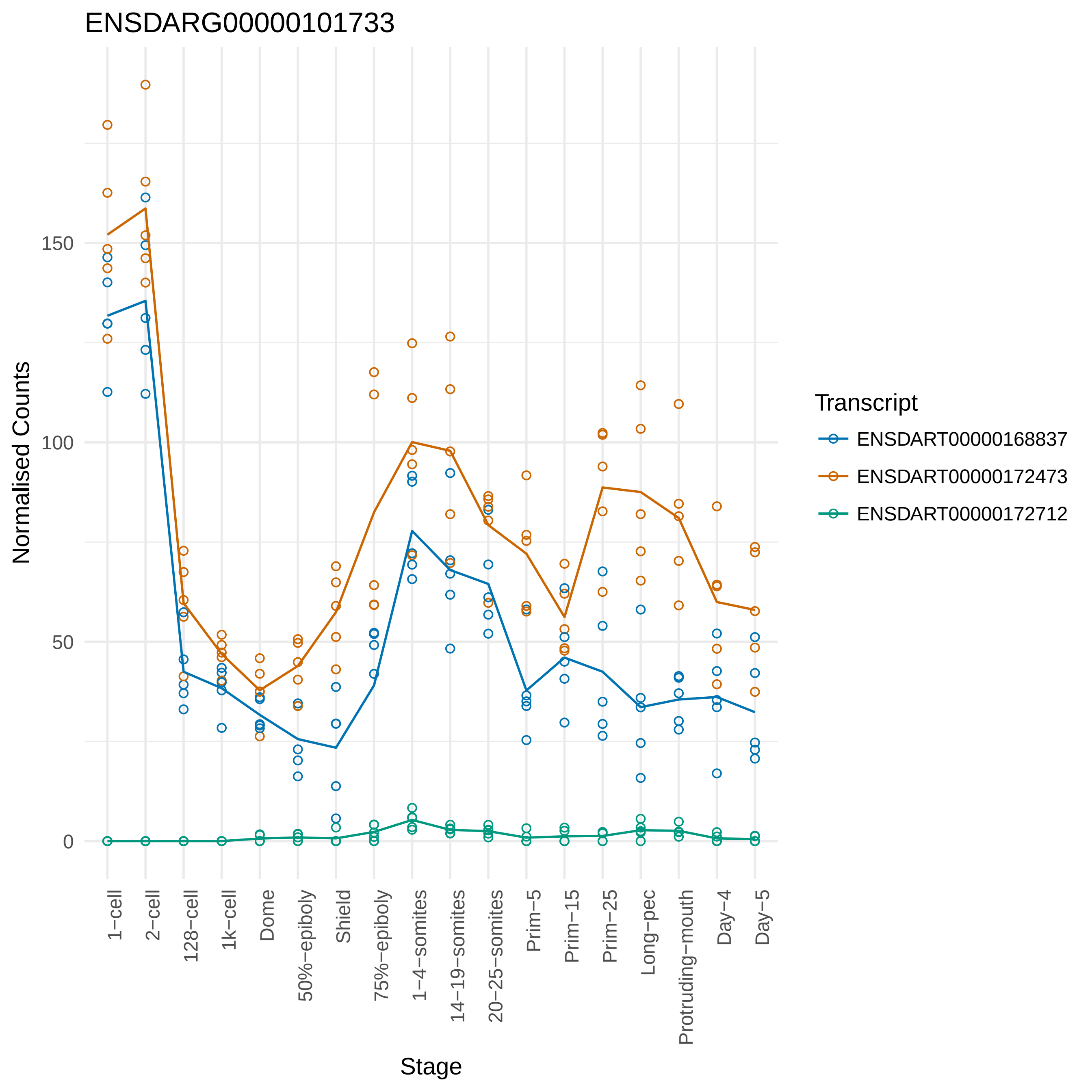

ENSDARG00000101733
Normalised Counts
0
50
100
150
1−cell
2−cell
128−cell
1k−cell
Dome
50%−epiboly
Shield
75%−epiboly
1−4−somites
14−19−somites
20−25−somites
Prim−5
Prim−15
Prim−25
Long−pec
Protruding−mouth
Day−4
Day−5
Stage
Transcript
ENSDART00000168837
ENSDART00000172473
ENSDART00000172712

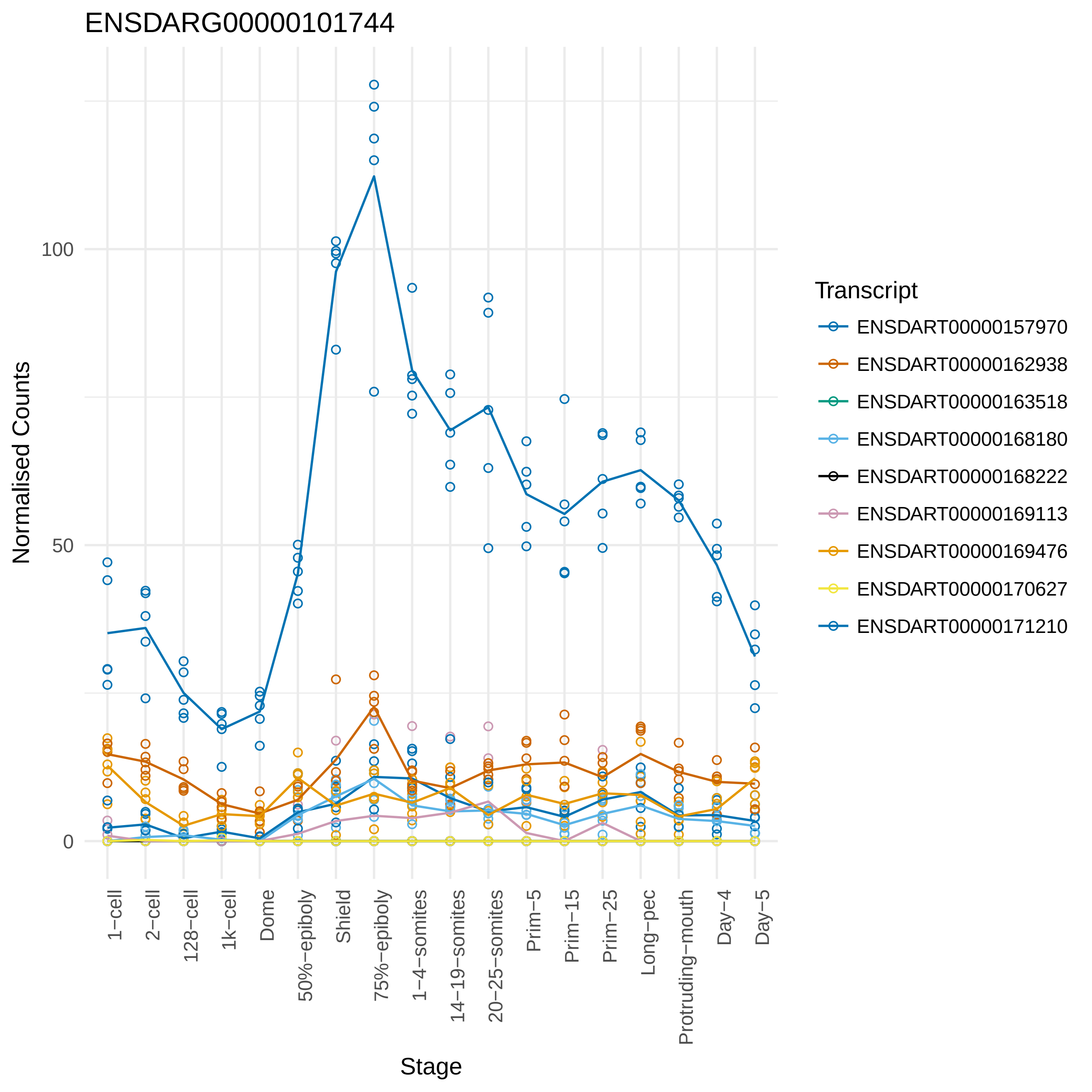

ENSDARG00000101744
Normalised Counts
100
50
0
1−cell
2−cell
128−cell
1k−cell
Dome
50%−epiboly
Shield
75%−epiboly
1−4−somites
14−19−somites
20−25−somites
Prim−5
Prim−15
Prim−25
Long−pec
Protruding−mouth
Day−4
Day−5
Stage
Transcript
ENSDART00000157970
ENSDART00000162938
ENSDART00000163518
ENSDART00000168180
ENSDART00000168222
ENSDART00000169113
ENSDART00000169476
ENSDART00000170627
ENSDART00000171210

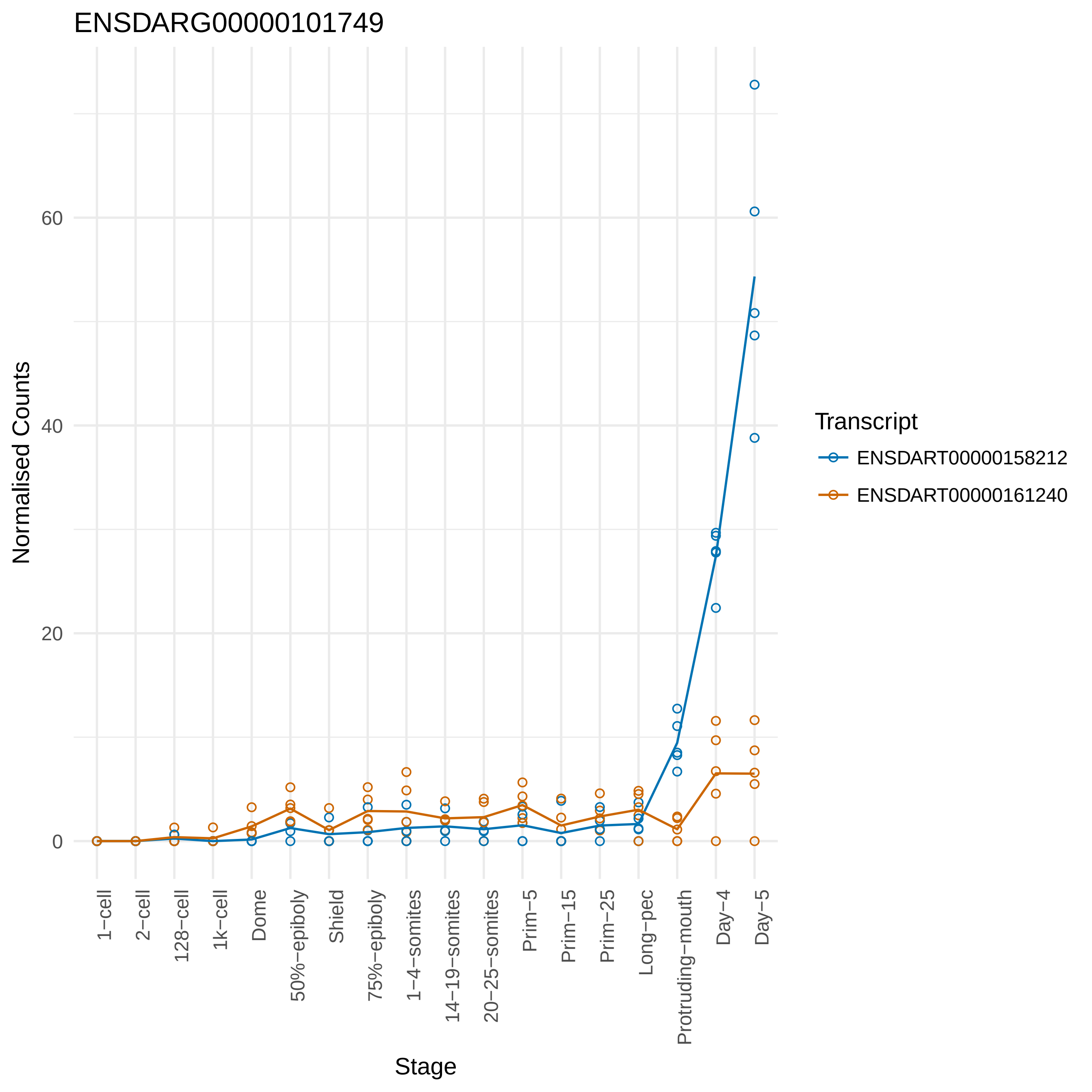

ENSDARG00000101749
Normalised Counts
0
20
40
60
Stage
1−cell
2−cell
128−cell
1k−cell
Dome
50%−epiboly
Shield
75%−epiboly
1−4−somites
14−19−somites
20−25−somites
Prim−5
Prim−15
Prim−25
Long−pec
Protruding−mouth
Day−4
Day−5
Transcript
ENSDART00000158212
ENSDART00000161240

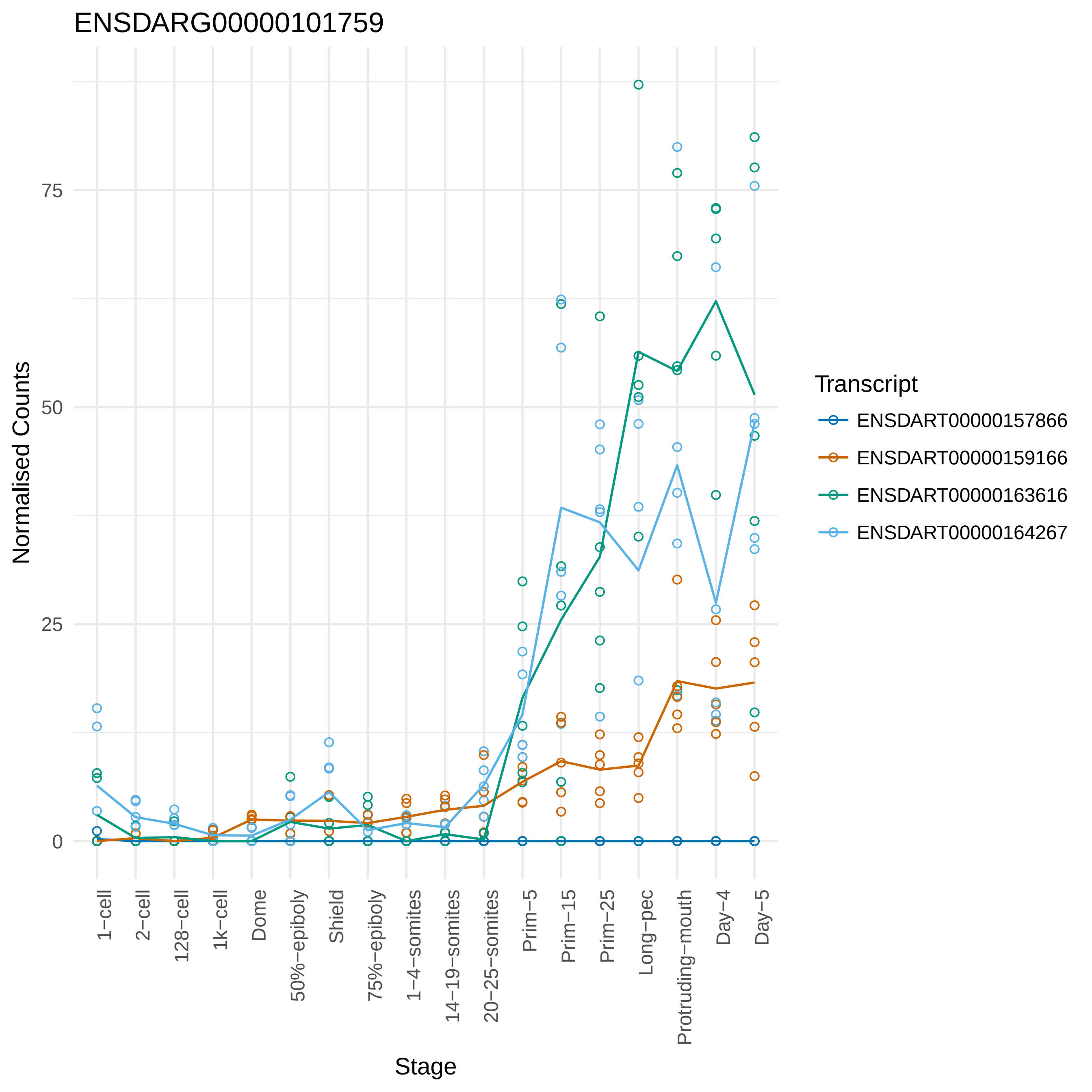

ENSDARG00000101759
Normalised Counts
75
50
25
0
1−cell
2−cell
128−cell
1k−cell
Dome
50%−epiboly
Shield
75%−epiboly
1−4−somites
14−19−somites
20−25−somites
Prim−5
Prim−15
Prim−25
Long−pec
Protruding−mouth
Day−4
Day−5
Stage
Transcript
ENSDART00000157866
ENSDART00000159166
ENSDART00000163616
ENSDART00000164267

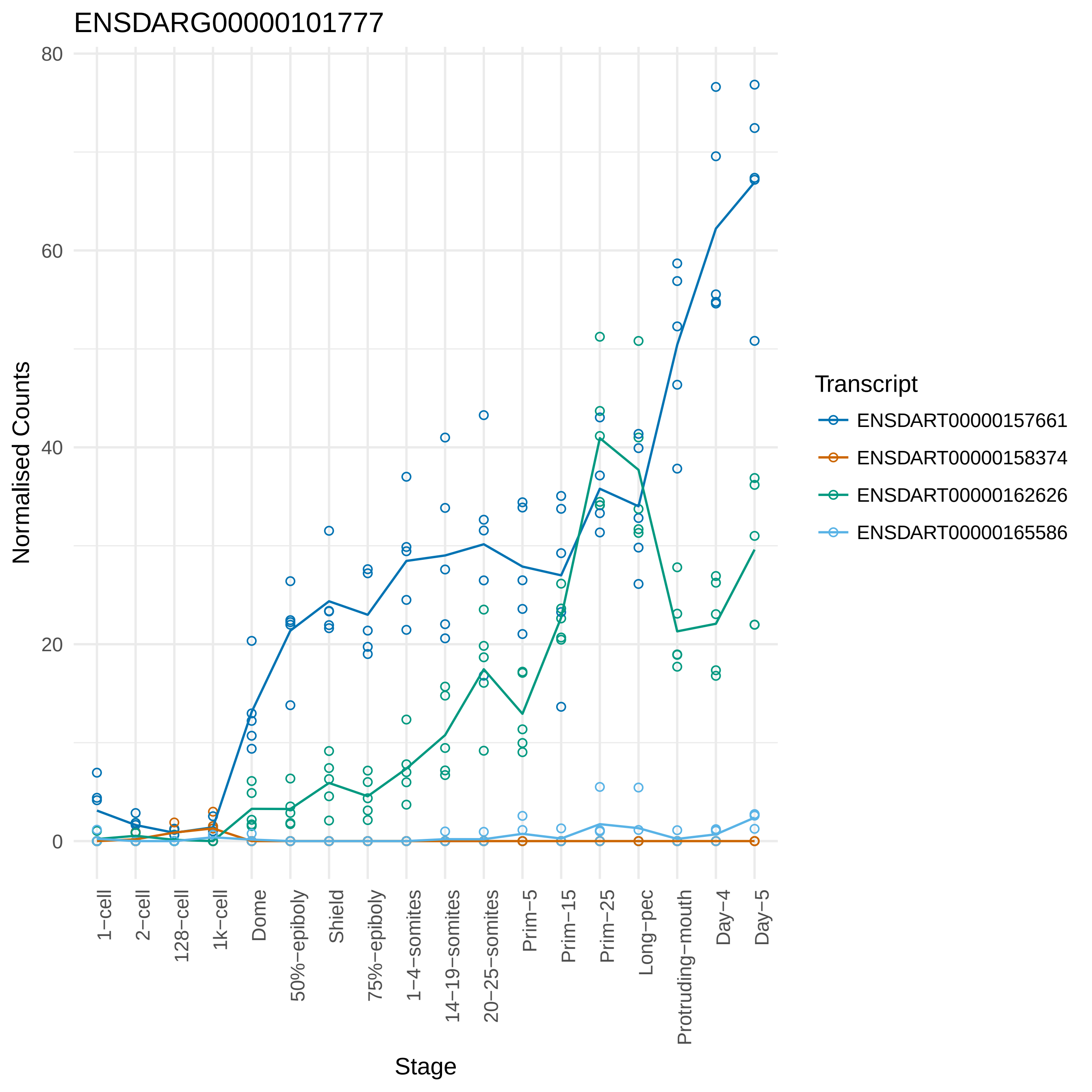
ENSDARG00000101777
Normalised Counts
0
20
40
60
80
1−cell
2−cell
128−cell
1k−cell
Dome
50%−epiboly
Shield
75%−epiboly
1−4−somites
14−19−somites
20−25−somites
Prim−5
Prim−15
Prim−25
Long−pec
Protruding−mouth
Day−4
Day−5
Stage
Transcript
ENSDART00000157661
ENSDART00000158374
ENSDART00000162626
ENSDART00000165586

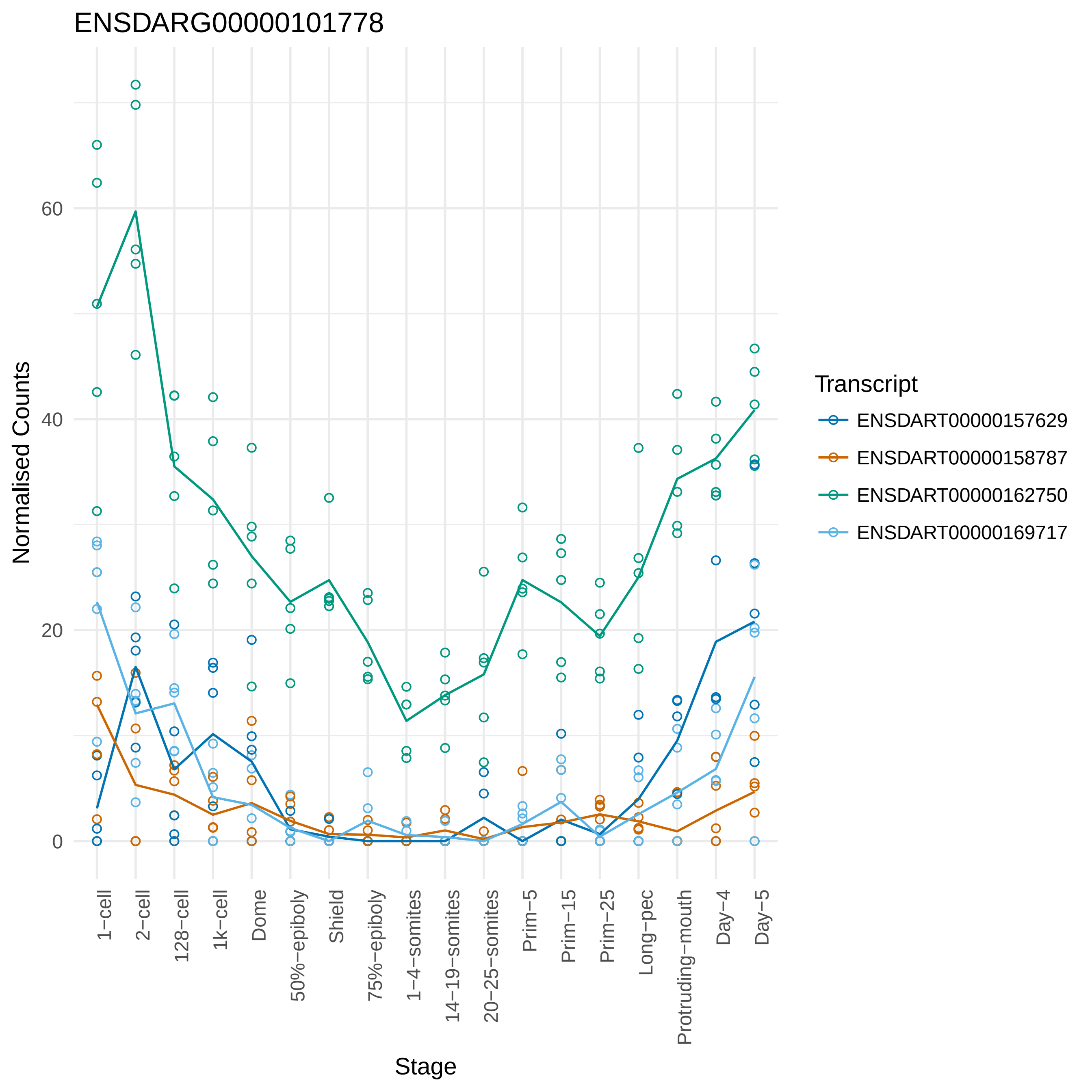

ENSDARG00000101778
Normalised Counts
0
20
40
60
Stage
1−cell
2−cell
128−cell
1k−cell
Dome
50%−epiboly
Shield
75%−epiboly
1−4−somites
14−19−somites
20−25−somites
Prim−5
Prim−15
Prim−25
Long−pec
Protruding−mouth
Day−4
Day−5
Transcript
ENSDART00000157629
ENSDART00000158787
ENSDART00000162750
ENSDART00000169717

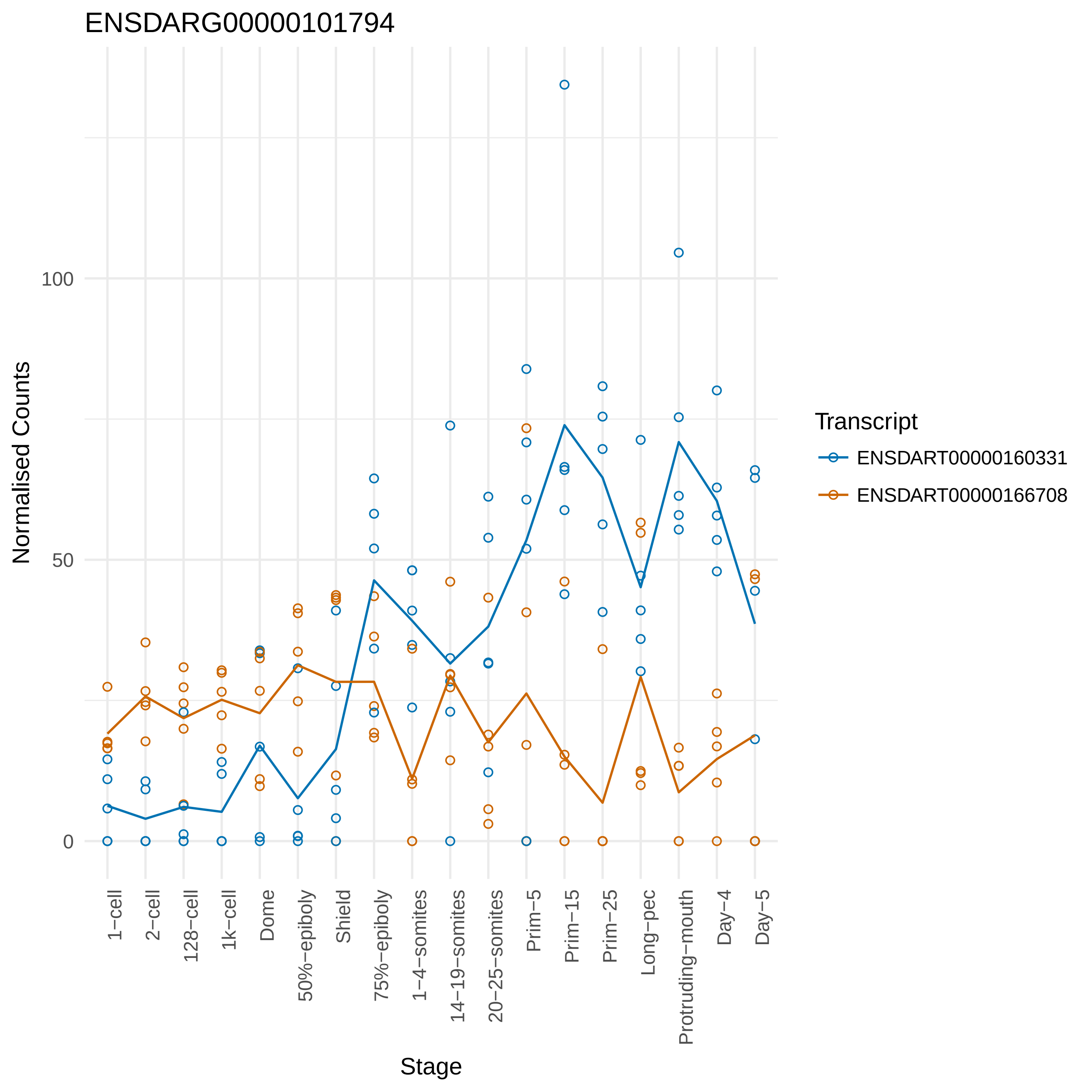
ENSDARG00000101794
Normalised Counts
100
50
0
1−cell
2−cell
128−cell
1k−cell
Dome
50%−epiboly
Shield
75%−epiboly
1−4−somites
14−19−somites
20−25−somites
Prim−5
Prim−15
Prim−25
Long−pec
Protruding−mouth
Day−4
Day−5
Stage
Transcript
ENSDART00000160331
ENSDART00000166708

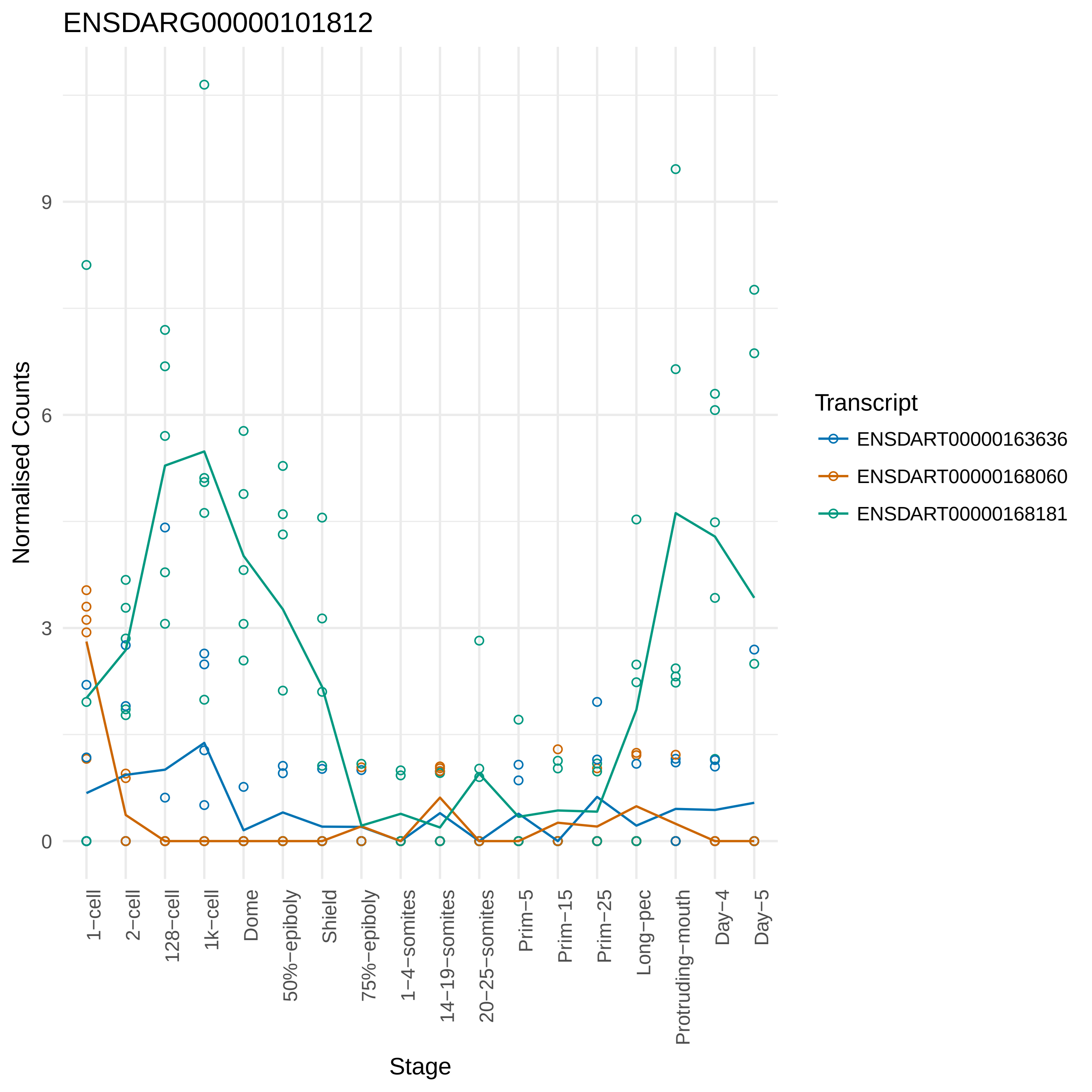

ENSDARG00000101812
Normalised Counts
0
3
6
9
Stage
1−cell
2−cell
128−cell
1k−cell
Dome
50%−epiboly
Shield
75%−epiboly
1−4−somites
14−19−somites
20−25−somites
Prim−5
Prim−15
Prim−25
Long−pec
Protruding−mouth
Day−4
Day−5
Transcript
ENSDART00000163636
ENSDART00000168060
ENSDART00000168181

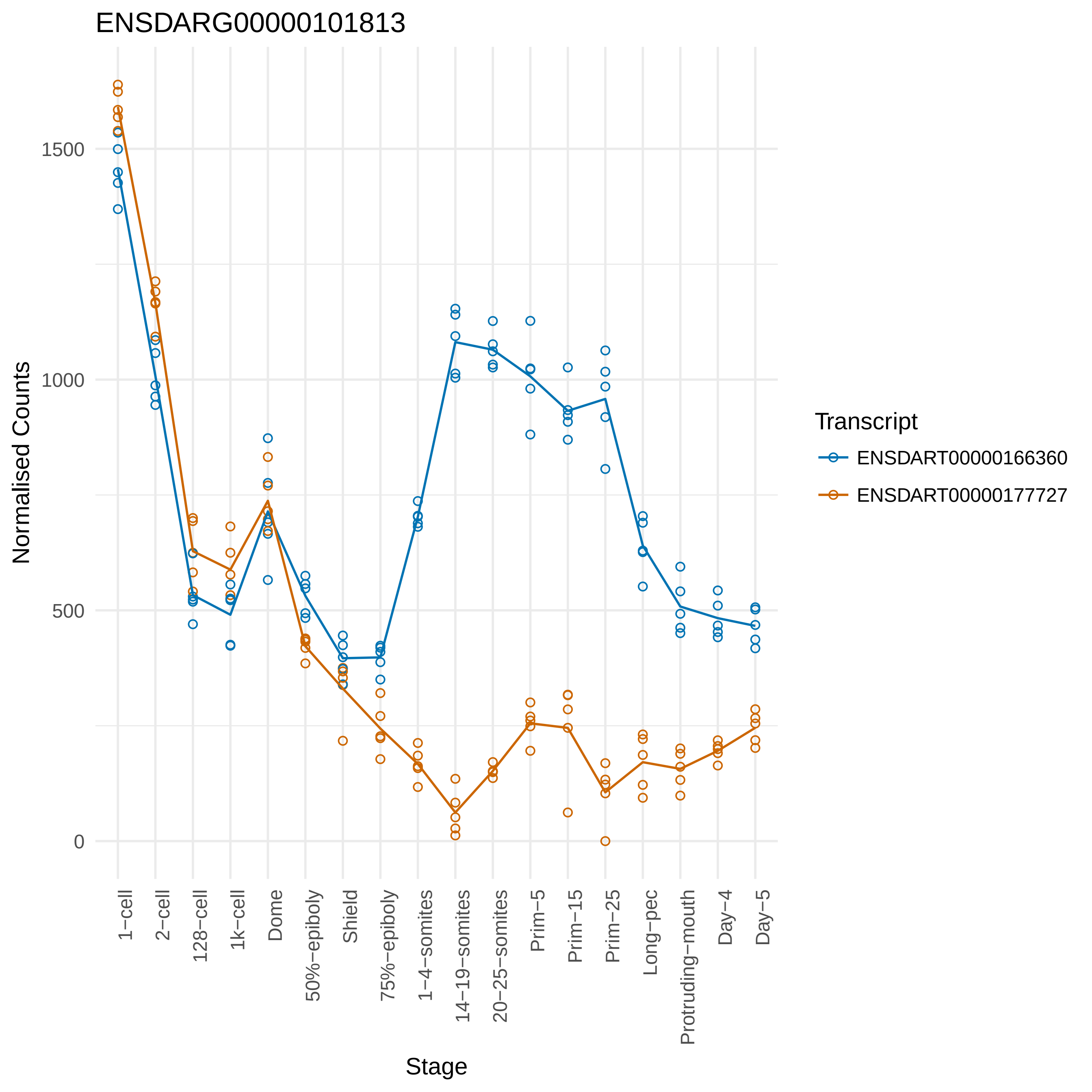
ENSDARG00000101813
Normalised Counts
1500
1000
500
0
1−cell
2−cell
128−cell
1k−cell
Dome
50%−epiboly
Shield
75%−epiboly
1−4−somites
14−19−somites
20−25−somites
Prim−5
Prim−15
Prim−25
Long−pec
Protruding−mouth
Day−4
Day−5
Stage
Transcript
ENSDART00000166360
ENSDART00000177727

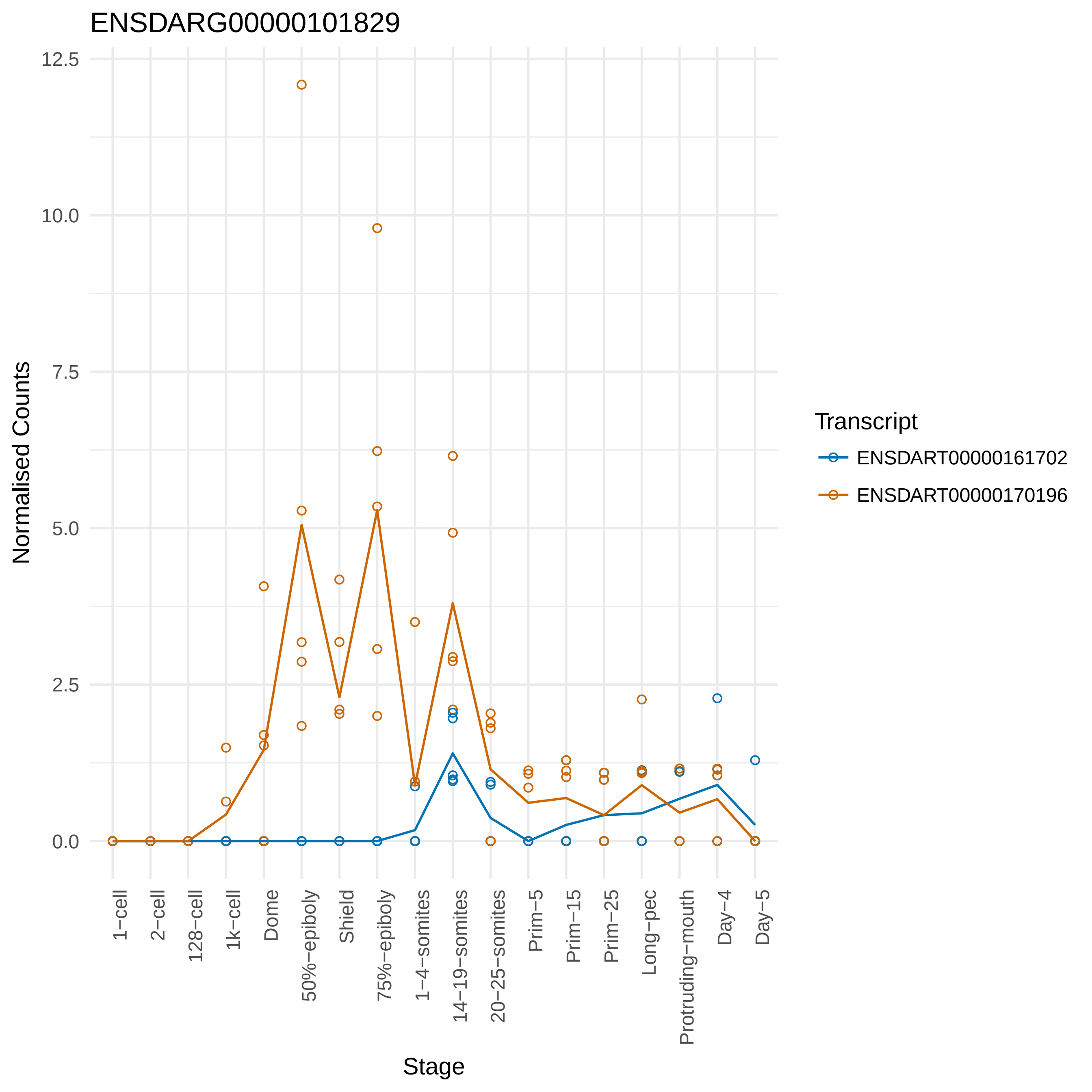

ENSDARG00000101829
12.5
10.0
7.5
5.0
2.5
0.0
Normalised Counts
1−cell
2−cell
128−cell
1k−cell
Dome
50%−epiboly
Shield
75%−epiboly
1−4−somites
14−19−somites
20−25−somites
Prim−5
Prim−15
Prim−25
Long−pec
Protruding−mouth
Day−4
Day−5
Stage
Transcript
ENSDART00000161702
ENSDART00000170196

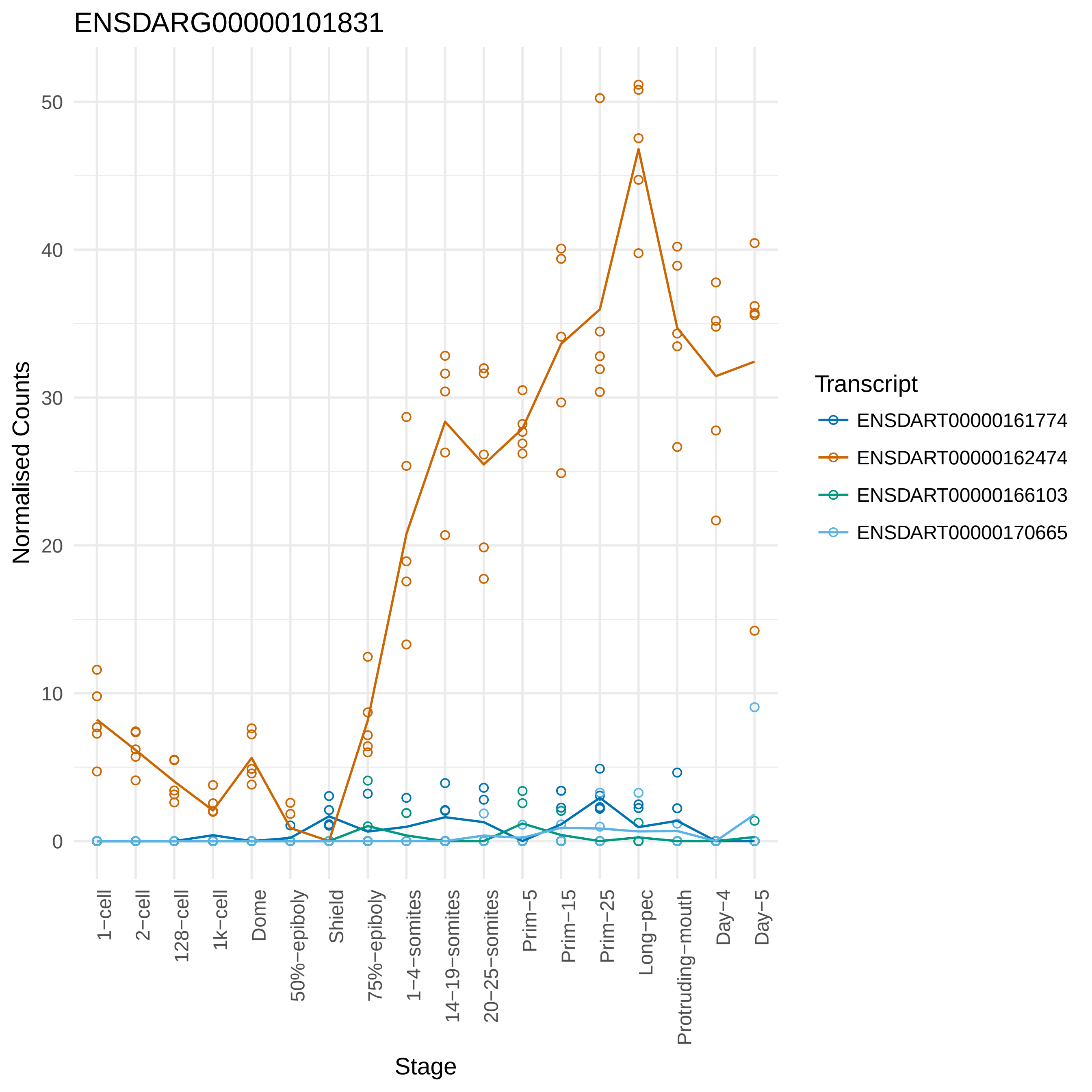

ENSDARG00000101831
Normalised Counts
0
10
20
30
40
50
1−cell
2−cell
128−cell
1k−cell
Dome
50%−epiboly
Shield
75%−epiboly
1−4−somites
14−19−somites
20−25−somites
Prim−5
Prim−15
Prim−25
Long−pec
Protruding−mouth
Day−4
Day−5
Stage
Transcript
ENSDART00000161774
ENSDART00000162474
ENSDART00000166103
ENSDART00000170665

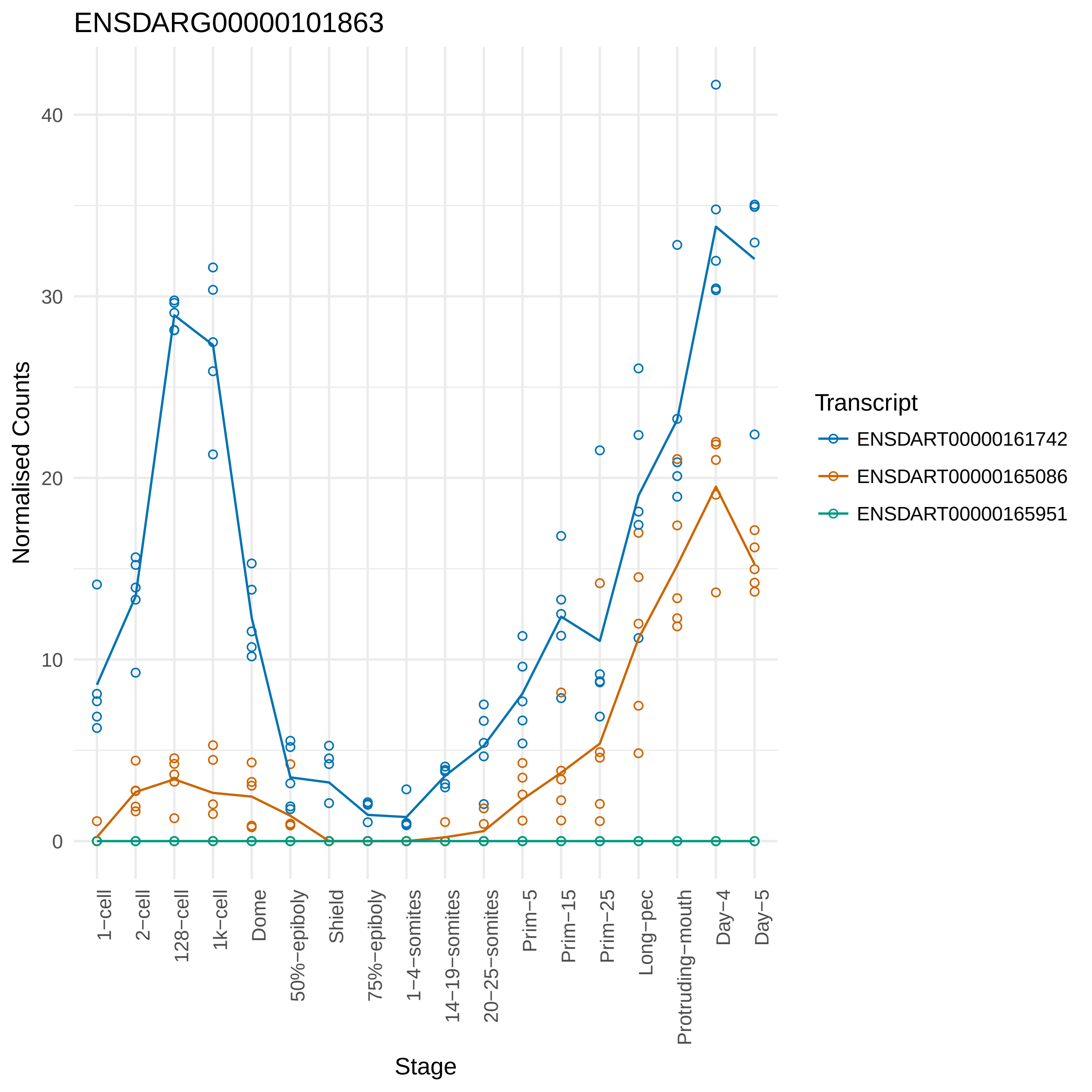

ENSDARG00000101863
Normalised Counts
0
10
20
30
40
1−cell
2−cell
128−cell
1k−cell
Dome
50%−epiboly
Shield
75%−epiboly
1−4−somites
14−19−somites
20−25−somites
Prim−5
Prim−15
Prim−25
Long−pec
Protruding−mouth
Day−4
Day−5
Stage
Transcript
ENSDART00000161742
ENSDART00000165086
ENSDART00000165951

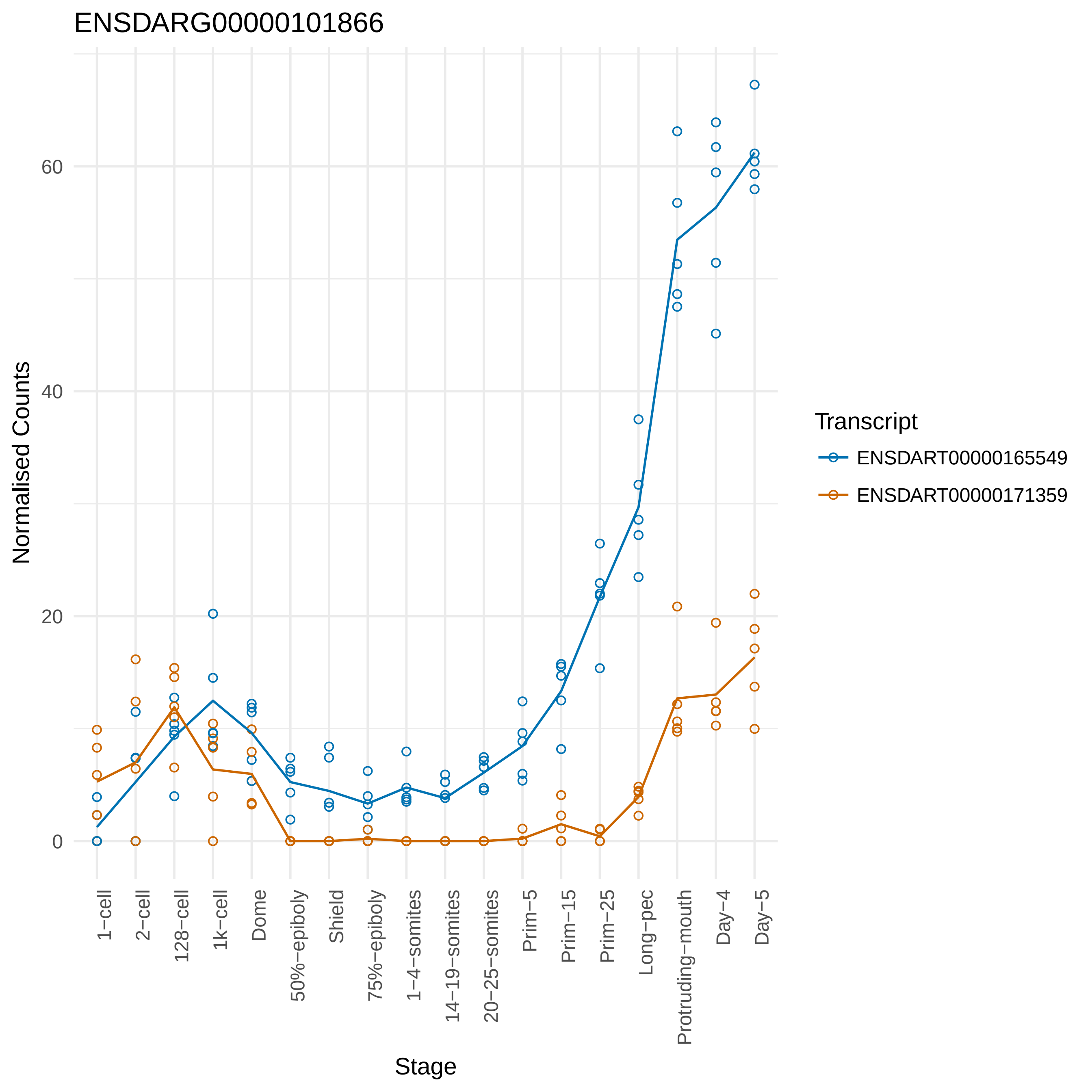

ENSDARG00000101866
Normalised Counts
60
40
20
0
1−cell
2−cell
128−cell
1k−cell
Dome
50%−epiboly
Shield
75%−epiboly
1−4−somites
14−19−somites
20−25−somites
Prim−5
Prim−15
Prim−25
Long−pec
Protruding−mouth
Day−4
Day−5
Stage
Transcript
ENSDART00000165549
ENSDART00000171359

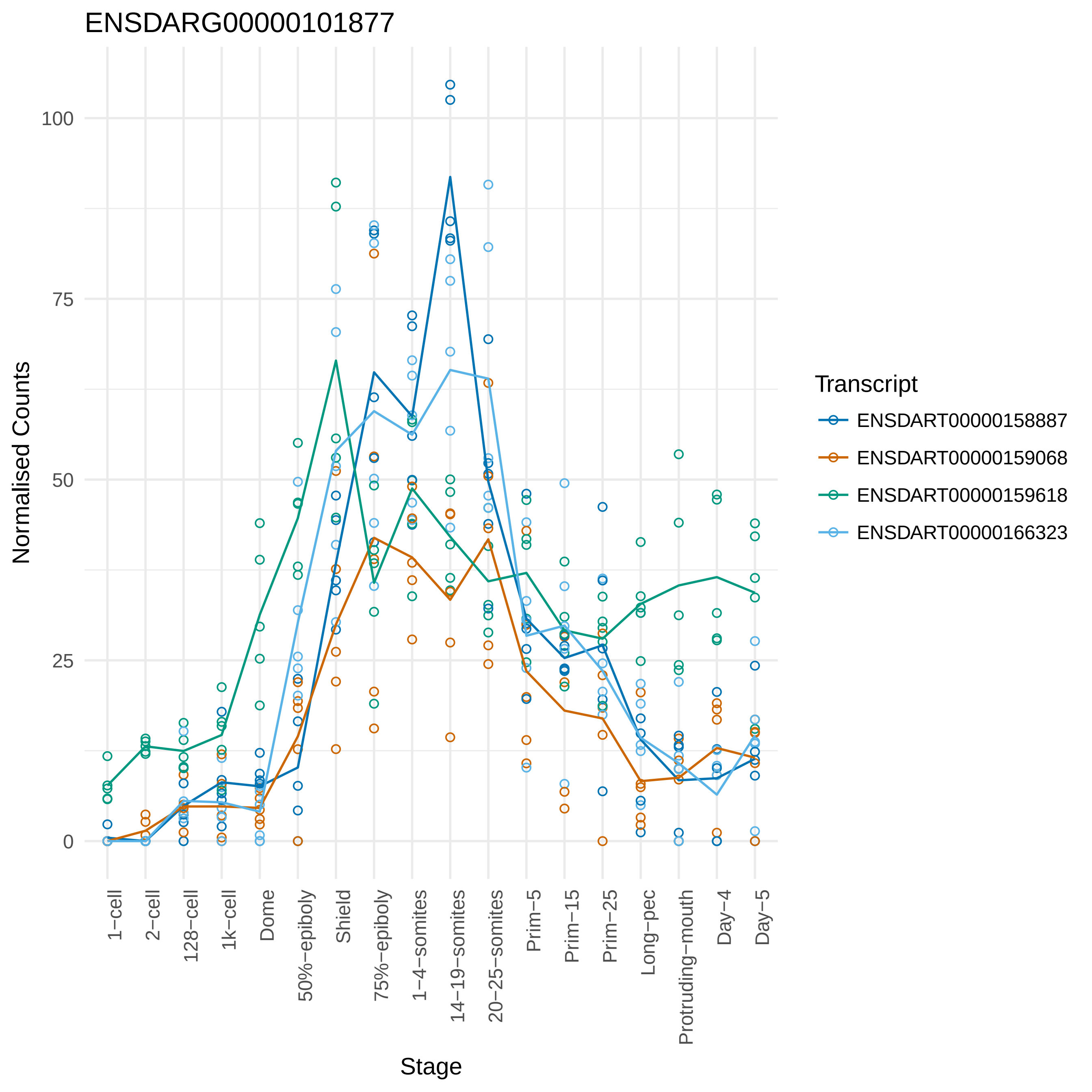

ENSDARG00000101877
Normalised Counts
0
25
50
75
100
1−cell
2−cell
128−cell
1k−cell
Dome
50%−epiboly
Shield
75%−epiboly
1−4−somites
14−19−somites
20−25−somites
Prim−5
Prim−15
Prim−25
Long−pec
Protruding−mouth
Day−4
Day−5
Stage
Transcript
ENSDART00000158887
ENSDART00000159068
ENSDART00000159618
ENSDART00000166323

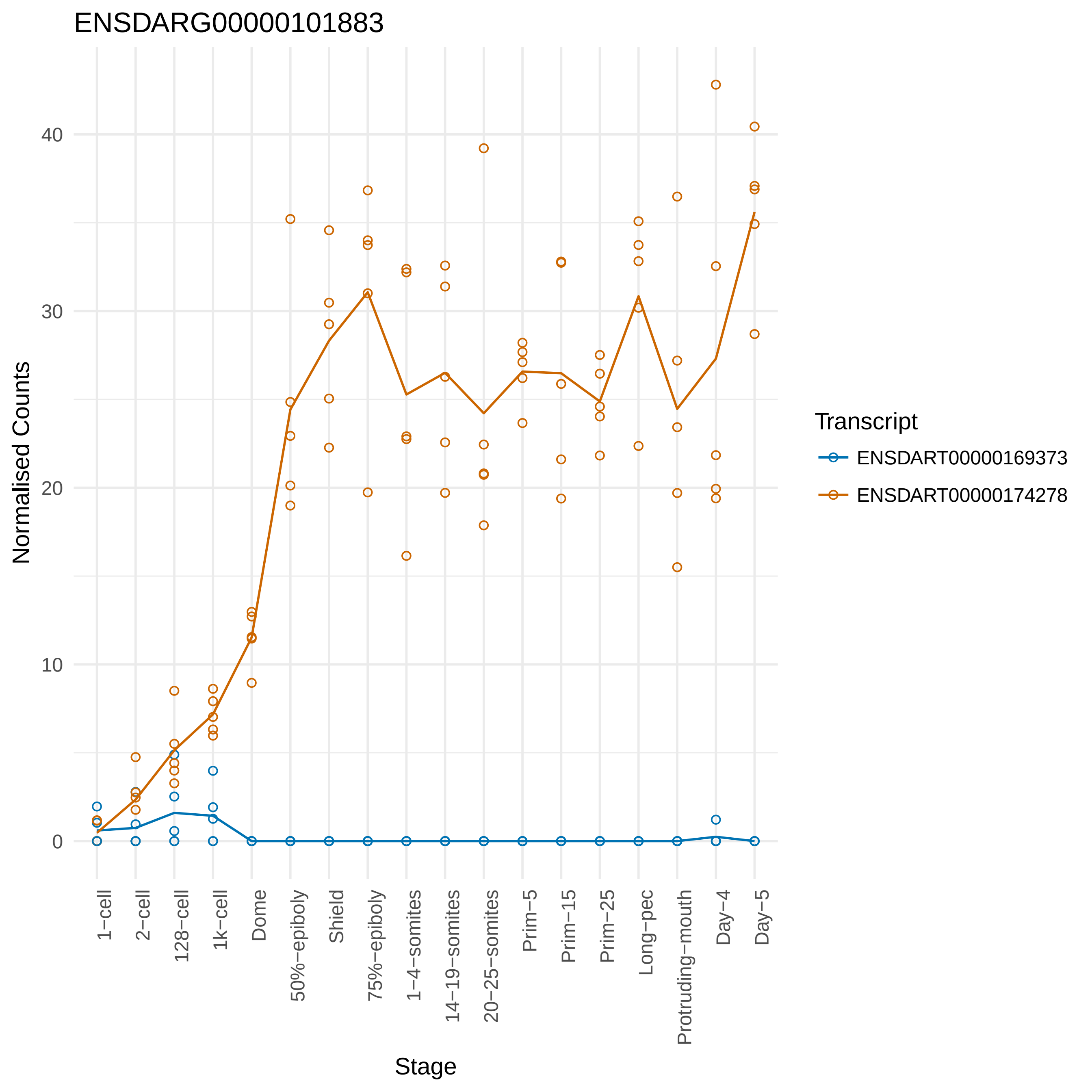

ENSDARG00000101883
Normalised Counts
0
10
20
30
40
Stage
1−cell
2−cell
128−cell
1k−cell
Dome
50%−epiboly
Shield
75%−epiboly
1−4−somites
14−19−somites
20−25−somites
Prim−5
Prim−15
Prim−25
Long−pec
Protruding−mouth
Day−4
Day−5
Transcript
ENSDART00000169373
ENSDART00000174278

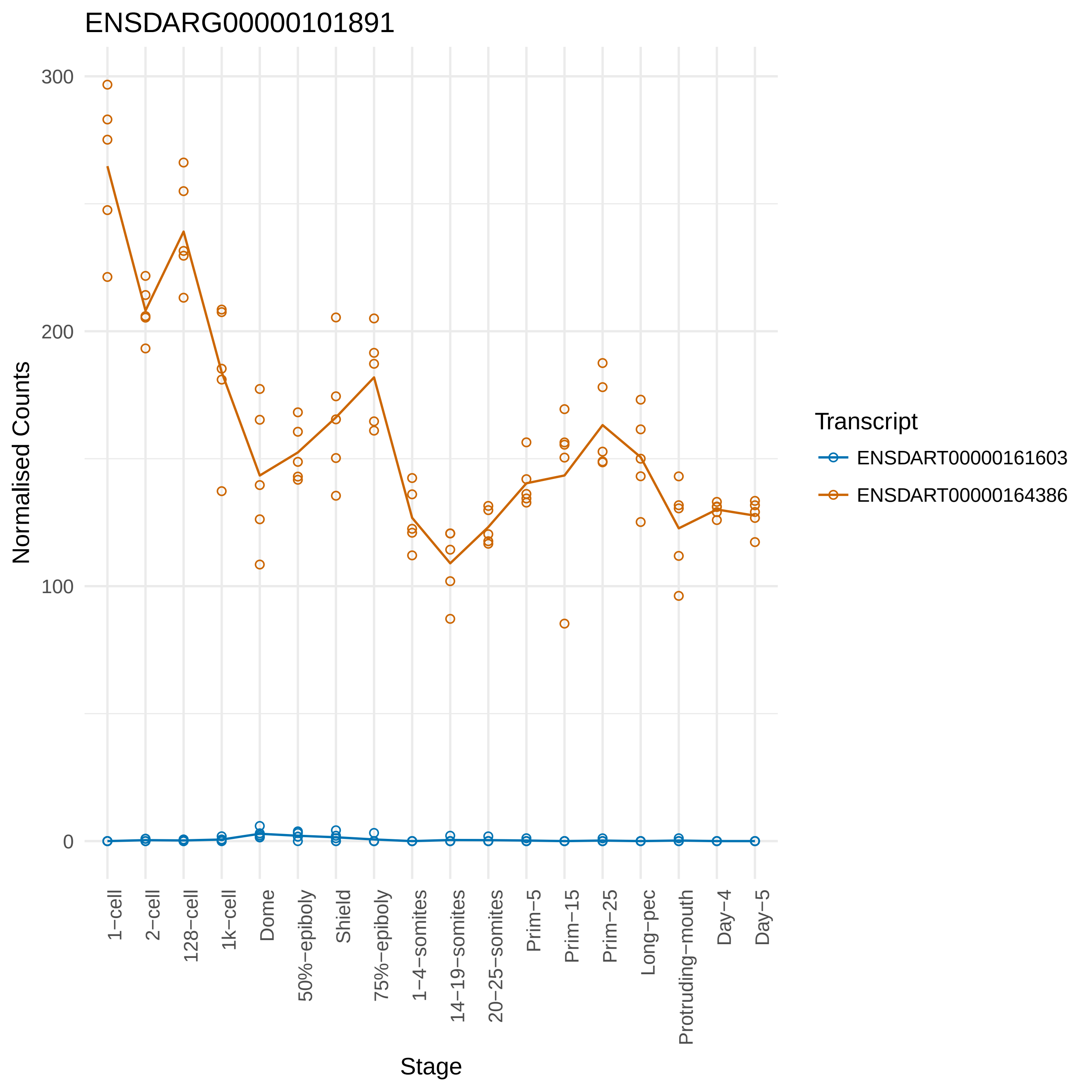
ENSDARG00000101891
Normalised Counts
300
200
100
0
1−cell
2−cell
128−cell
1k−cell
Dome
50%−epiboly
Shield
75%−epiboly
1−4−somites
14−19−somites
20−25−somites
Prim−5
Prim−15
Prim−25
Long−pec
Protruding−mouth
Day−4
Day−5
Stage
Transcript
ENSDART00000161603
ENSDART00000164386

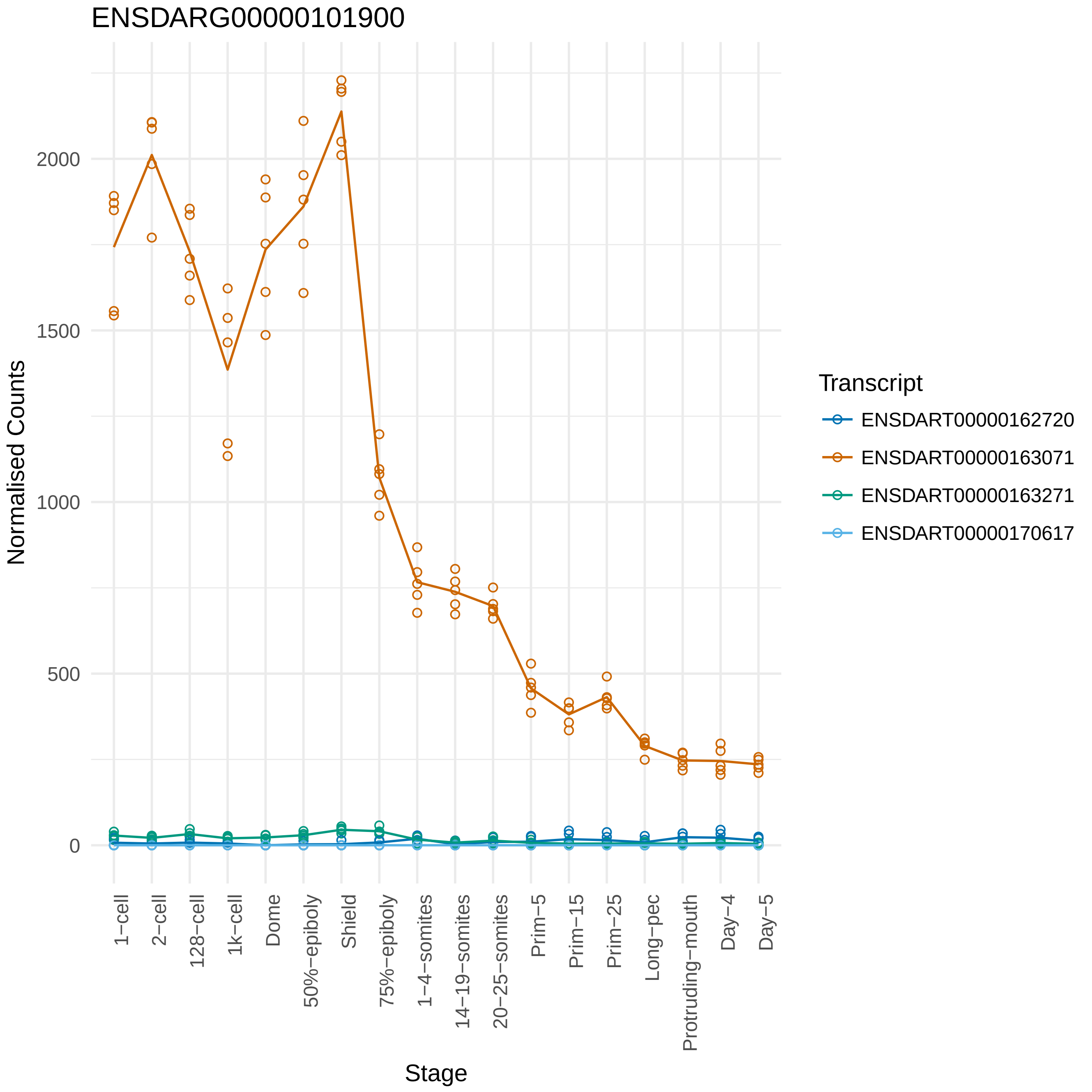

ENSDARG00000101900
Normalised Counts
2000
1500
1000
500
0
1−cell
2−cell
128−cell
1k−cell
Dome
50%−epiboly
Shield
75%−epiboly
1−4−somites
14−19−somites
20−25−somites
Prim−5
Prim−15
Prim−25
Long−pec
Protruding−mouth
Day−4
Day−5
Stage
Transcript
ENSDART00000162720
ENSDART00000163071
ENSDART00000163271
ENSDART00000170617

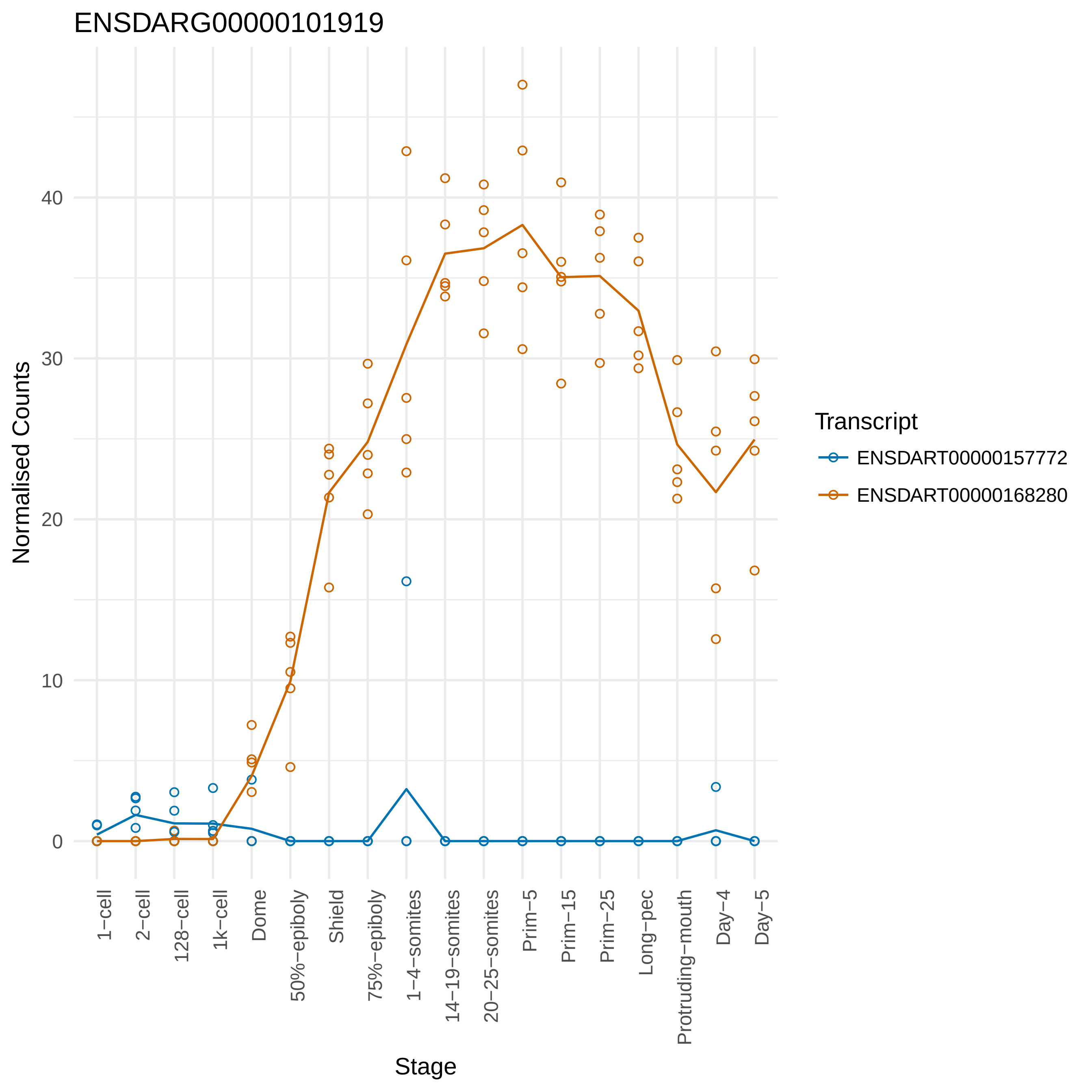
ENSDARG00000101919
Normalised Counts
0
10
20
30
40
Stage
1−cell
2−cell
128−cell
1k−cell
Dome
50%−epiboly
Shield
75%−epiboly
1−4−somites
14−19−somites
20−25−somites
Prim−5
Prim−15
Prim−25
Long−pec
Protruding−mouth
Day−4
Day−5
Transcript
ENSDART00000157772
ENSDART00000168280

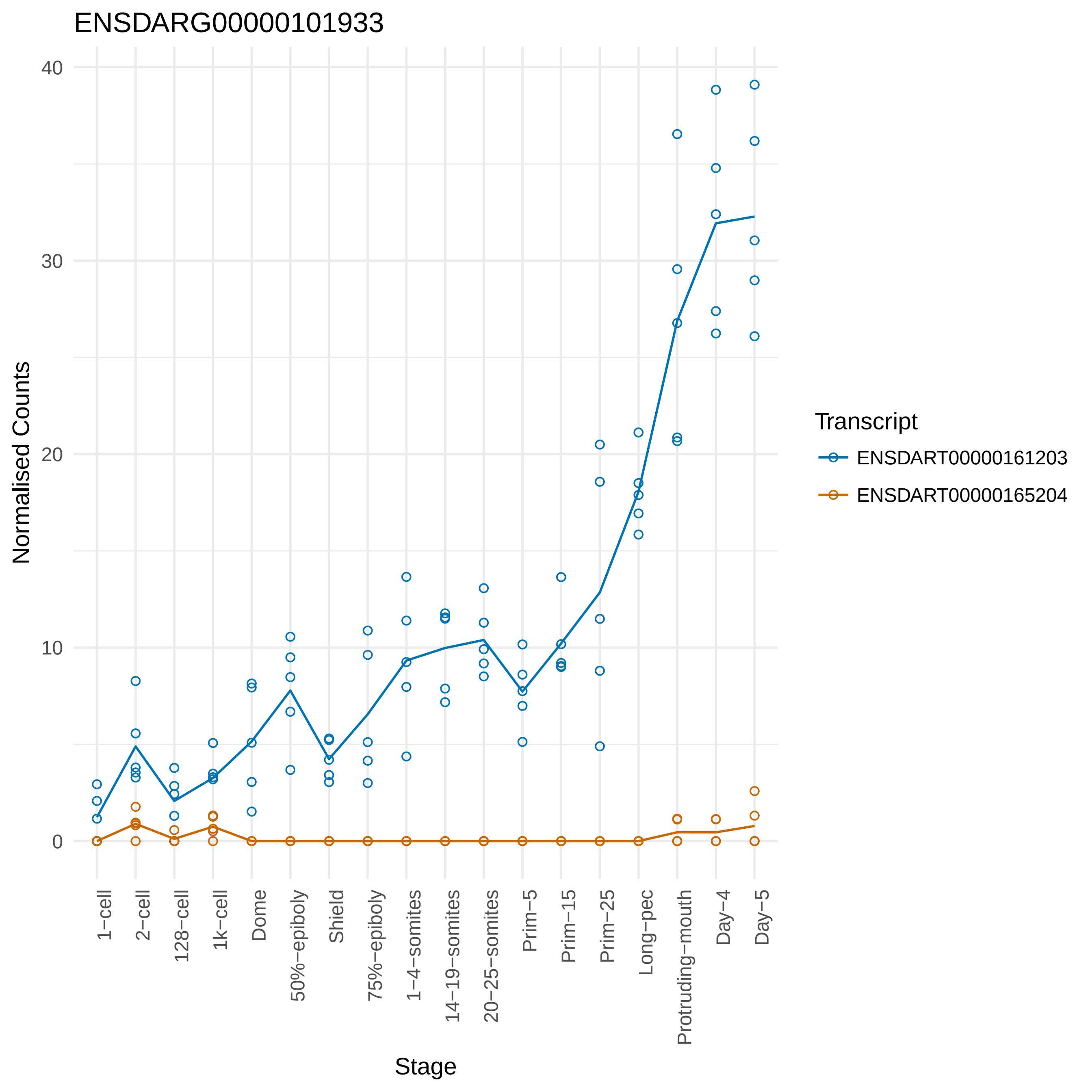
ENSDARG00000101933
Normalised Counts
40
30
20
10
0
1−cell
2−cell
128−cell
1k−cell
Dome
50%−epiboly
Shield
75%−epiboly
1−4−somites
14−19−somites
20−25−somites
Prim−5
Prim−15
Prim−25
Long−pec
Protruding−mouth
Day−4
Day−5
Stage
Transcript
ENSDART00000161203
ENSDART00000165204

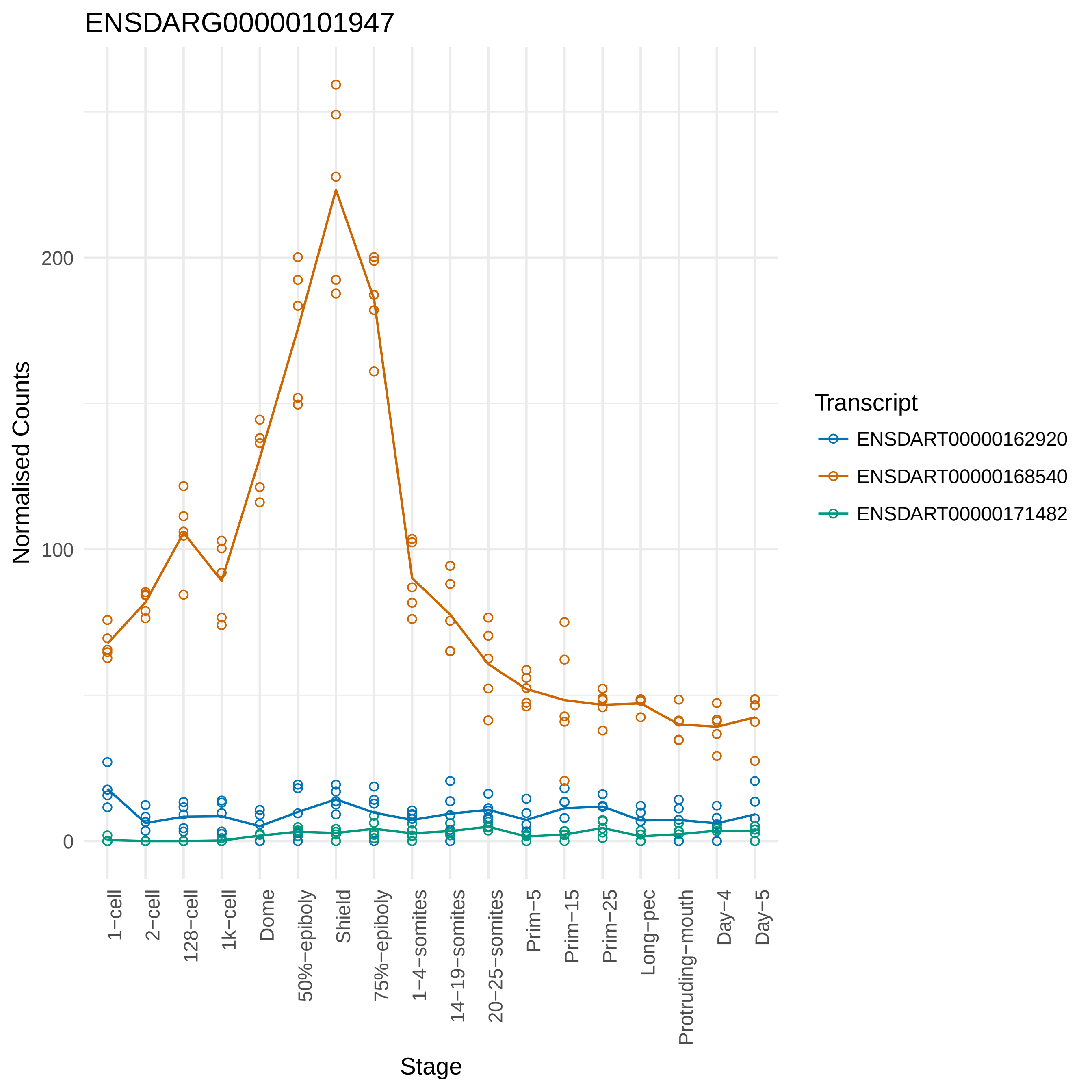

ENSDARG00000101947
Normalised Counts
0
100
200
1−cell
2−cell
128−cell
1k−cell
Dome
50%−epiboly
Shield
75%−epiboly
1−4−somites
14−19−somites
20−25−somites
Prim−5
Prim−15
Prim−25
Long−pec
Protruding−mouth
Day−4
Day−5
Stage
Transcript
ENSDART00000162920
ENSDART00000168540
ENSDART00000171482

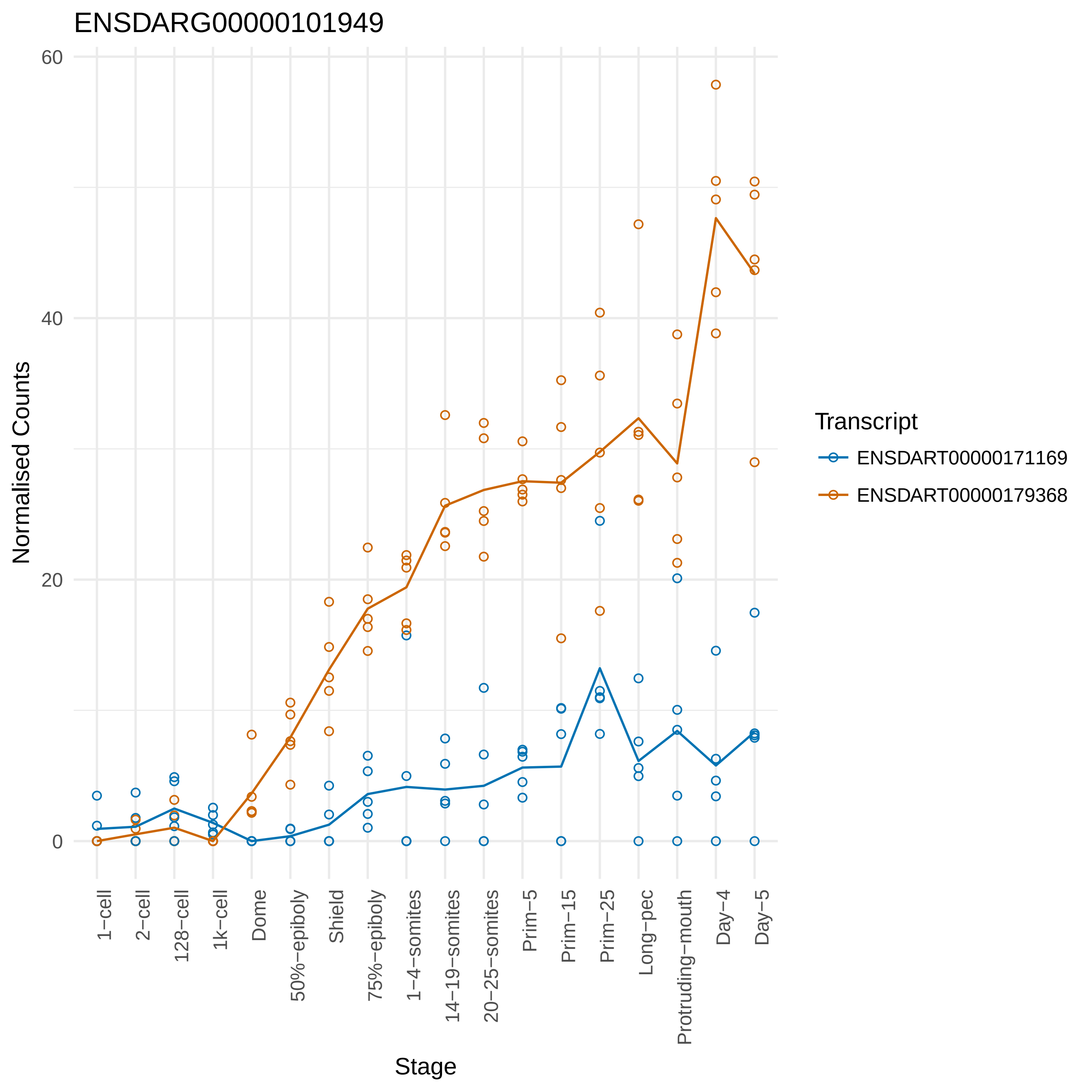

ENSDARG00000101949
Normalised Counts
0
20
40
60
1−cell
2−cell
128−cell
1k−cell
Dome
50%−epiboly
Shield
75%−epiboly
1−4−somites
14−19−somites
20−25−somites
Prim−5
Prim−15
Prim−25
Long−pec
Protruding−mouth
Day−4
Day−5
Stage
Transcript
ENSDART00000171169
ENSDART00000179368

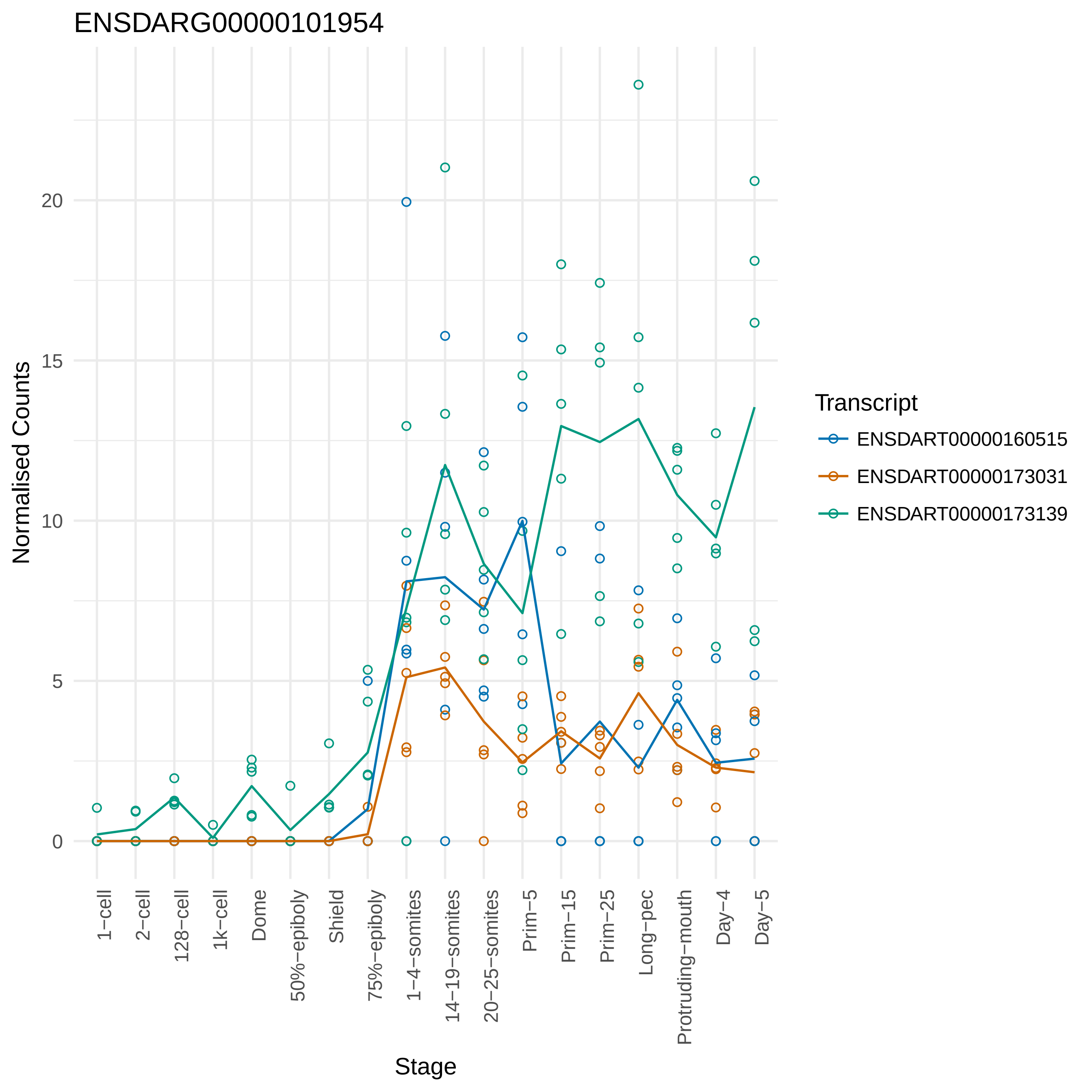

ENSDARG00000101954
Normalised Counts
0
5
10
15
20
Stage
1−cell
2−cell
128−cell
1k−cell
Dome
50%−epiboly
Shield
75%−epiboly
1−4−somites
14−19−somites
20−25−somites
Prim−5
Prim−15
Prim−25
Long−pec
Protruding−mouth
Day−4
Day−5
Transcript
ENSDART00000160515
ENSDART00000173031
ENSDART00000173139

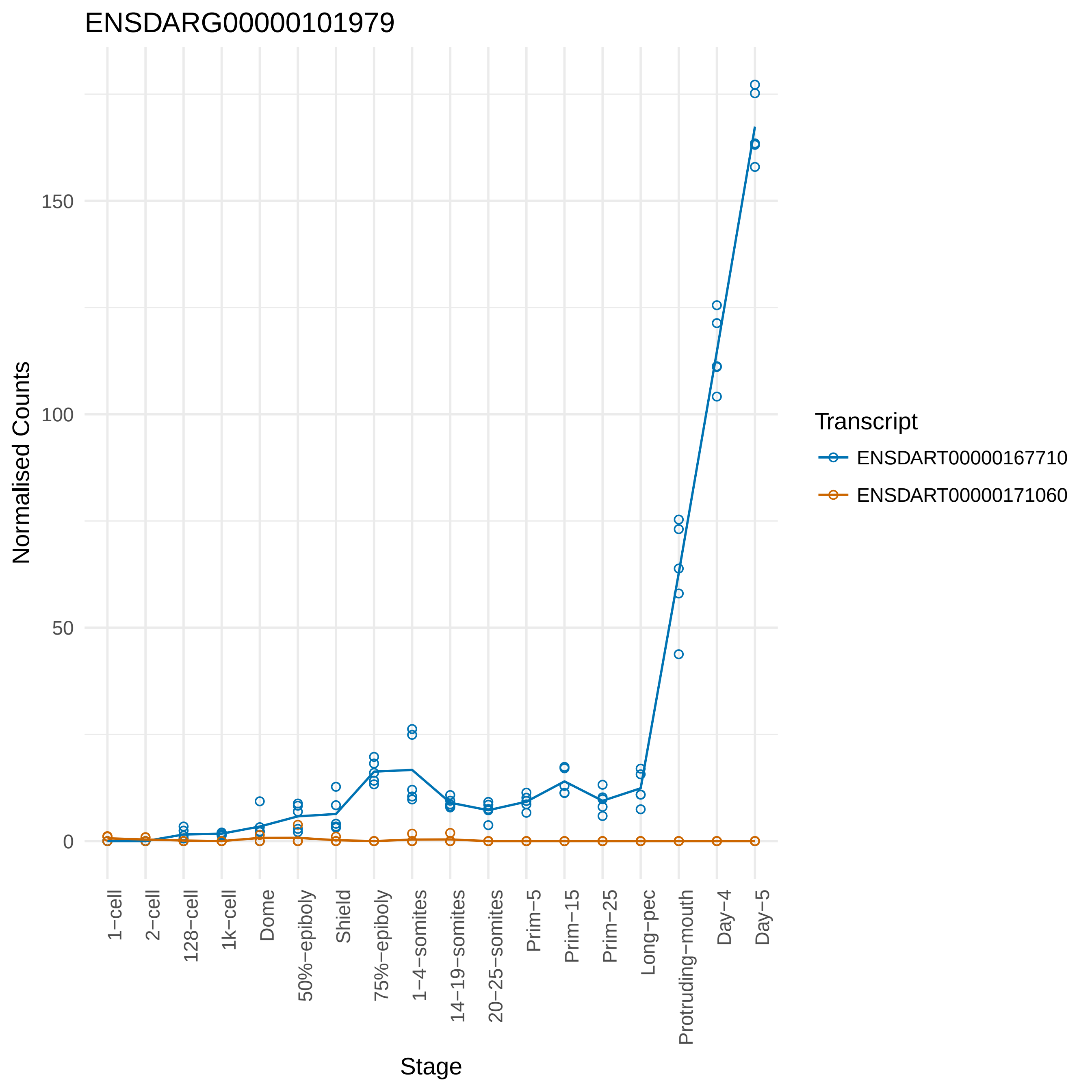
ENSDARG00000101979
Normalised Counts
150
100
50
0
1−cell
2−cell
128−cell
1k−cell
Dome
50%−epiboly
Shield
75%−epiboly
1−4−somites
14−19−somites
20−25−somites
Prim−5
Prim−15
Prim−25
Long−pec
Protruding−mouth
Day−4
Day−5
Stage
Transcript
ENSDART00000167710
ENSDART00000171060

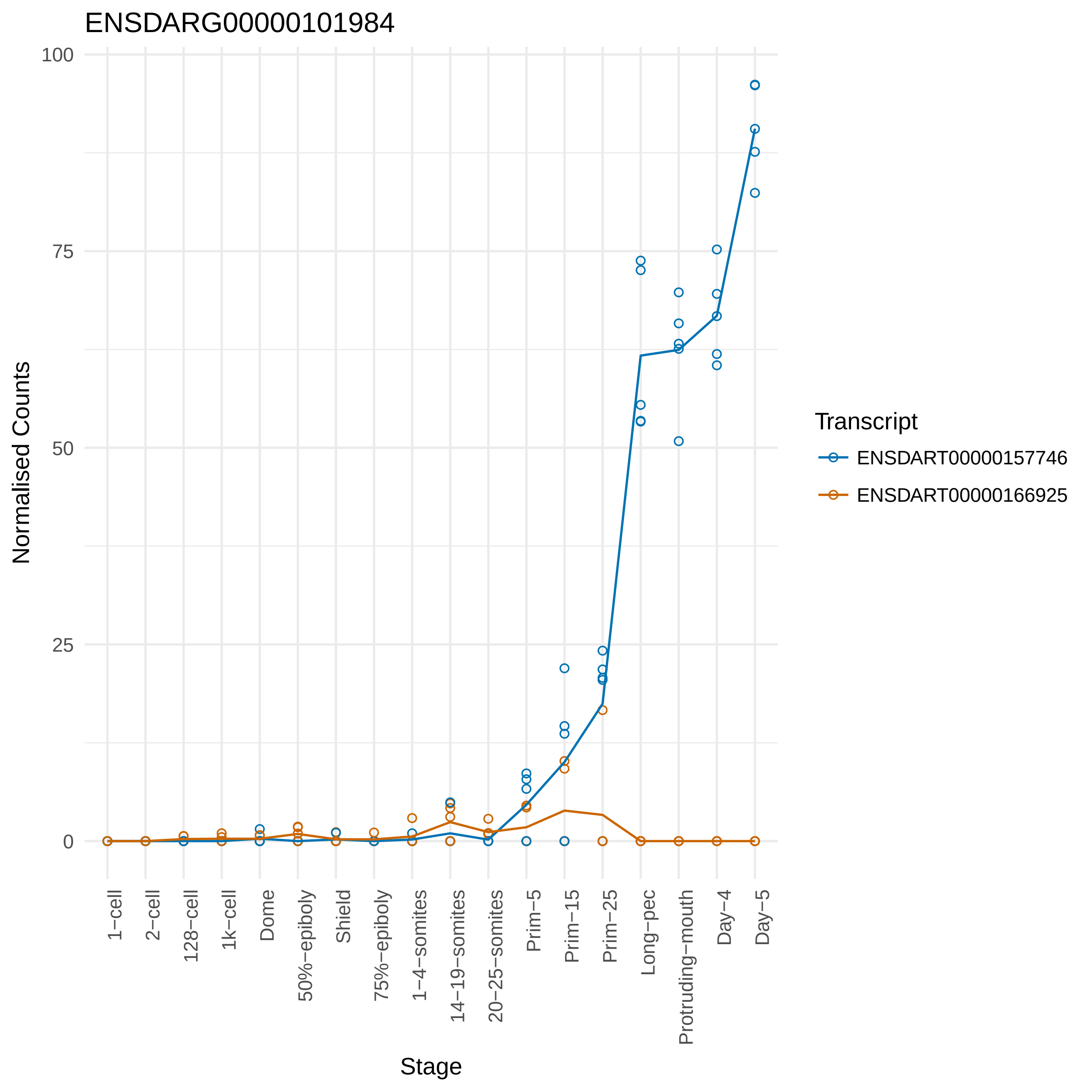

ENSDARG00000101984
Normalised Counts
100
75
50
25
0
1−cell
2−cell
128−cell
1k−cell
Dome
50%−epiboly
Shield
75%−epiboly
1−4−somites
14−19−somites
20−25−somites
Prim−5
Prim−15
Prim−25
Long−pec
Protruding−mouth
Day−4
Day−5
Stage
Transcript
ENSDART00000157746
ENSDART00000166925

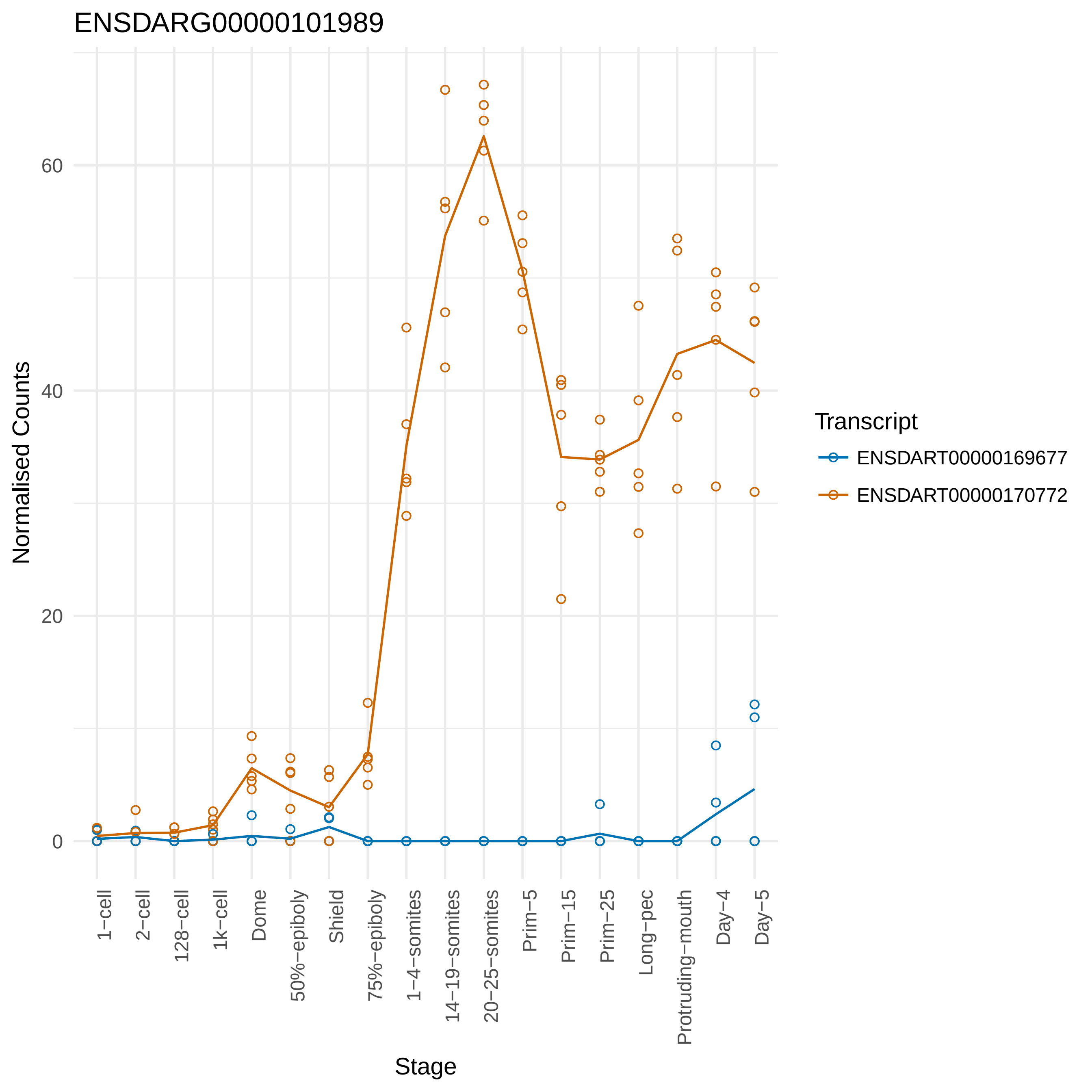
ENSDARG00000101989
Normalised Counts
0
20
40
60
Stage
1−cell
2−cell
128−cell
1k−cell
Dome
50%−epiboly
Shield
75%−epiboly
1−4−somites
14−19−somites
20−25−somites
Prim−5
Prim−15
Prim−25
Long−pec
Protruding−mouth
Day−4
Day−5
Transcript
ENSDART00000169677
ENSDART00000170772

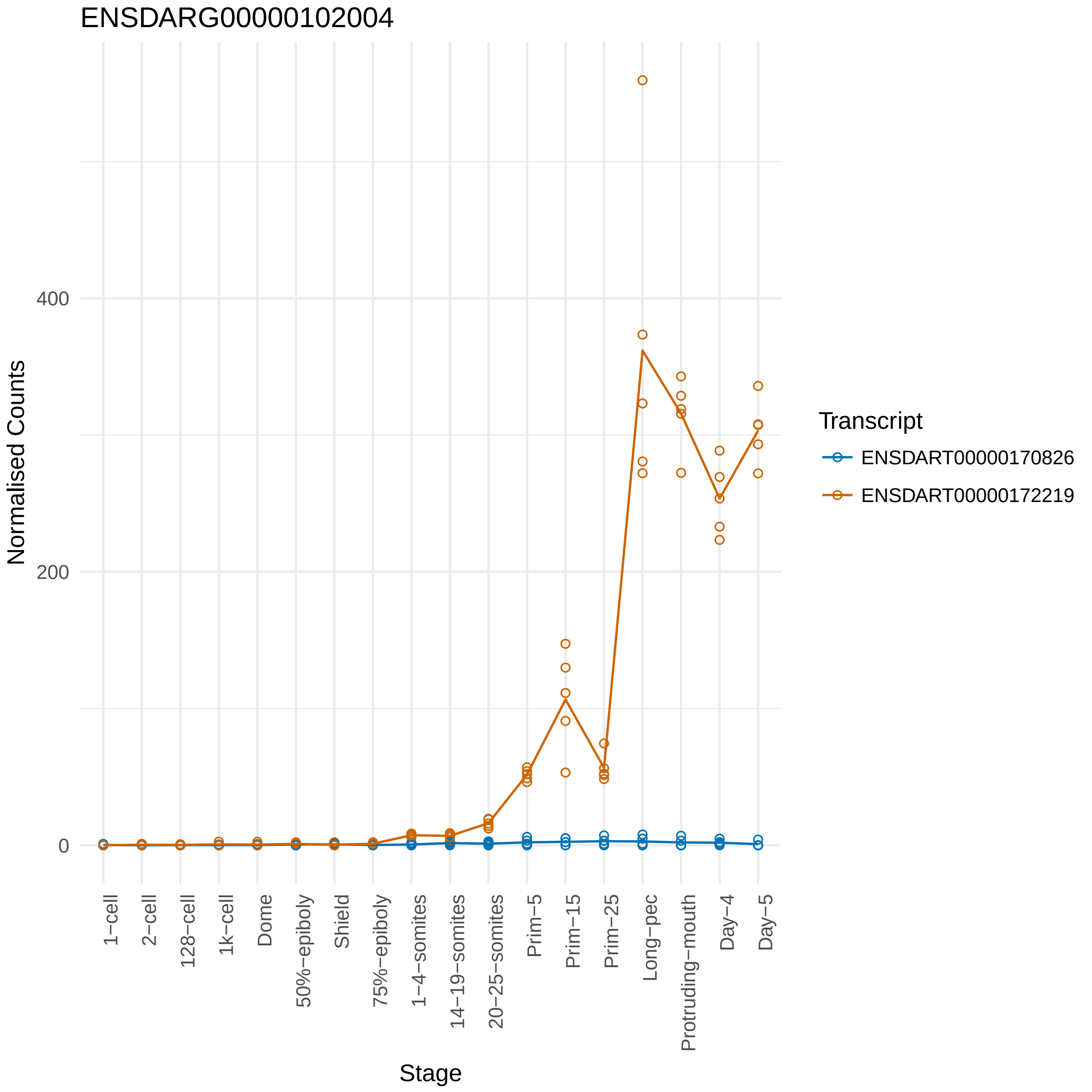
ENSDARG00000102004
Normalised Counts
0
200
400
Stage
1−cell
2−cell
128−cell
1k−cell
Dome
50%−epiboly
Shield
75%−epiboly
1−4−somites
14−19−somites
20−25−somites
Prim−5
Prim−15
Prim−25
Long−pec
Protruding−mouth
Day−4
Day−5
Transcript
ENSDART00000170826
ENSDART00000172219

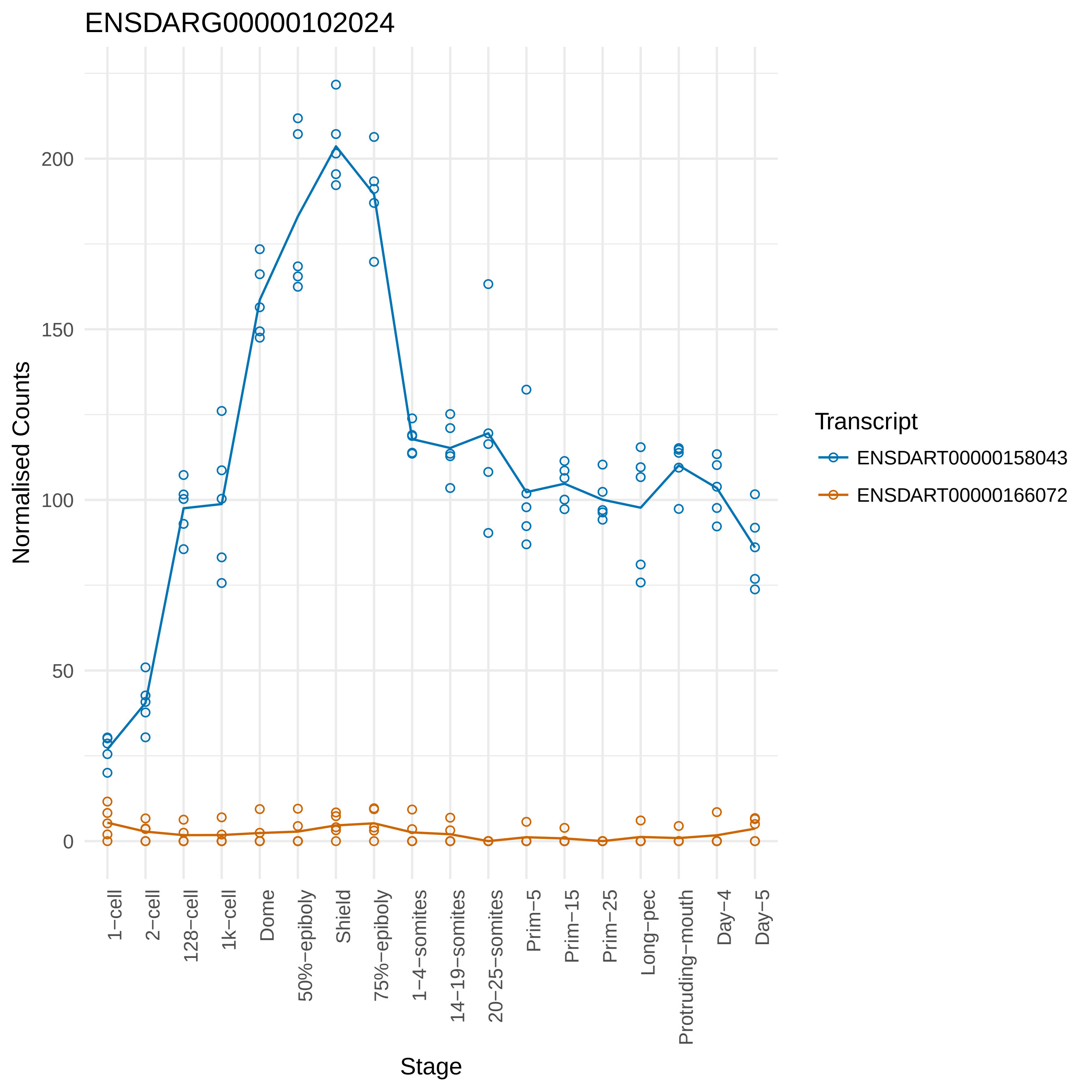

ENSDARG00000102024
Normalised Counts
0
50
100
150
200
Stage
1−cell
2−cell
128−cell
1k−cell
Dome
50%−epiboly
Shield
75%−epiboly
1−4−somites
14−19−somites
20−25−somites
Prim−5
Prim−15
Prim−25
Long−pec
Protruding−mouth
Day−4
Day−5
Transcript
ENSDART00000158043
ENSDART00000166072

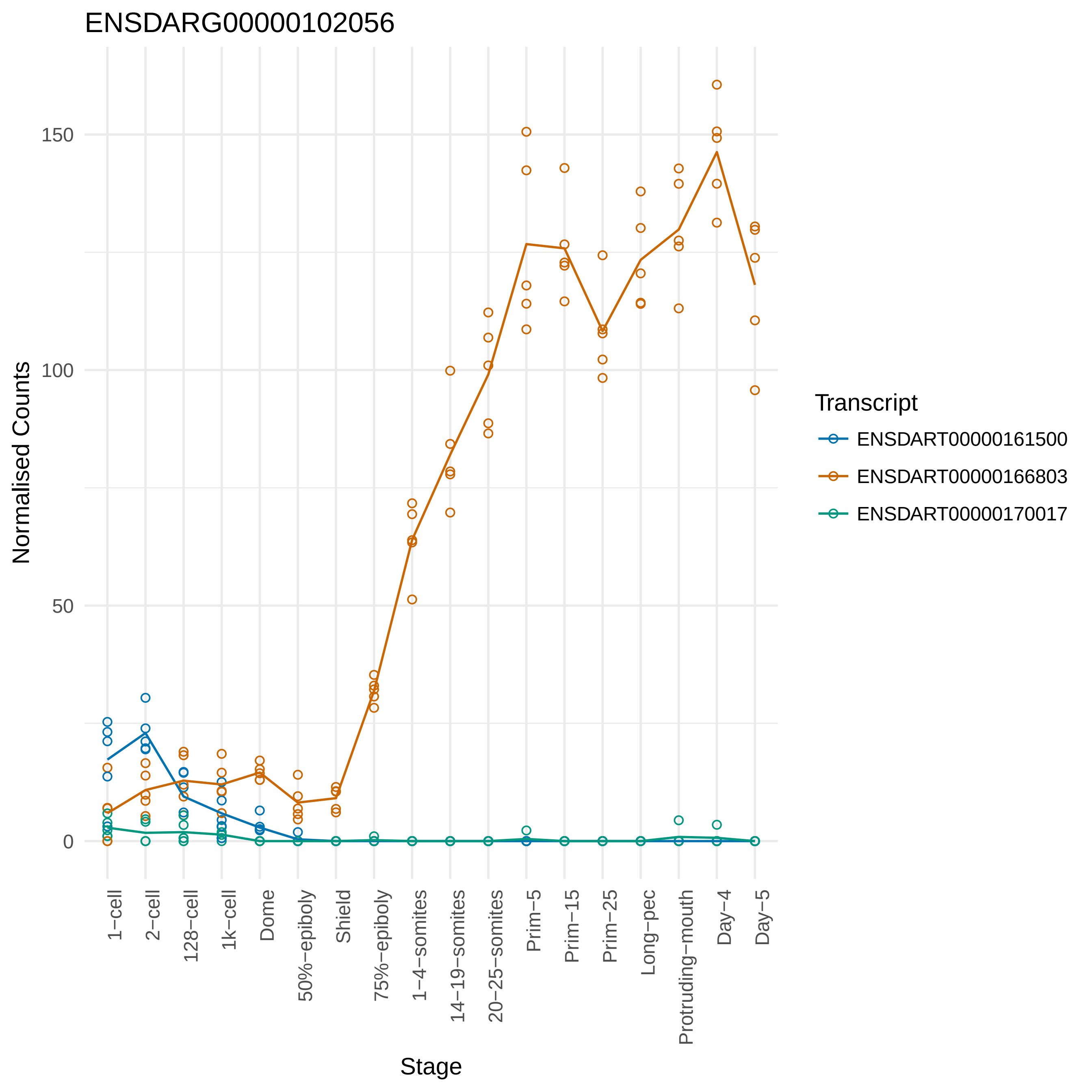

ENSDARG00000102056
Normalised Counts
150
100
50
0
1−cell
2−cell
128−cell
1k−cell
Dome
50%−epiboly
Shield
75%−epiboly
1−4−somites
14−19−somites
20−25−somites
Prim−5
Prim−15
Prim−25
Long−pec
Protruding−mouth
Day−4
Day−5
Stage
Transcript
ENSDART00000161500
ENSDART00000166803
ENSDART00000170017

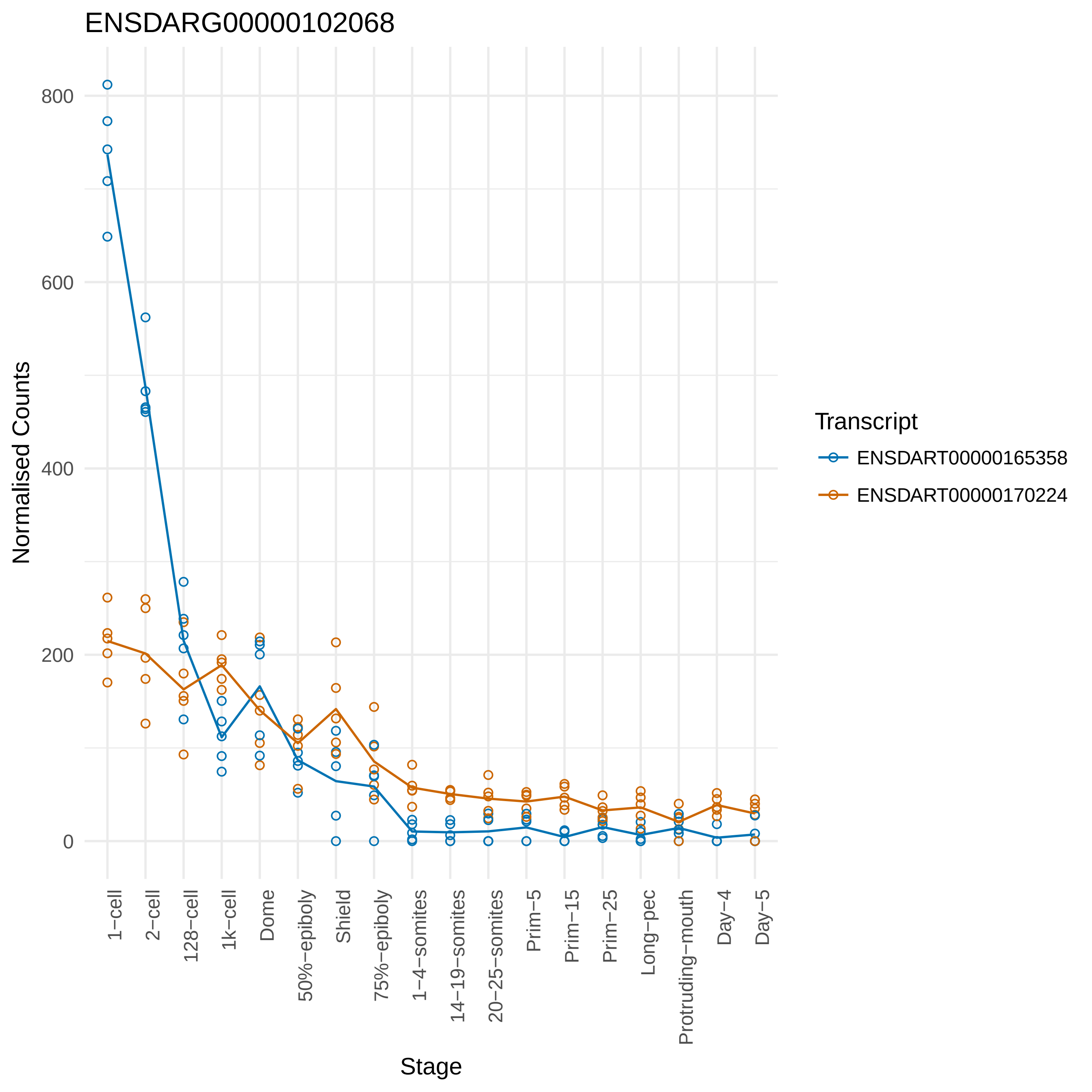

ENSDARG00000102068
Normalised Counts
800
600
400
200
0
1−cell
2−cell
128−cell
1k−cell
Dome
50%−epiboly
Shield
75%−epiboly
1−4−somites
14−19−somites
20−25−somites
Prim−5
Prim−15
Prim−25
Long−pec
Protruding−mouth
Day−4
Day−5
Stage
Transcript
ENSDART00000165358
ENSDART00000170224

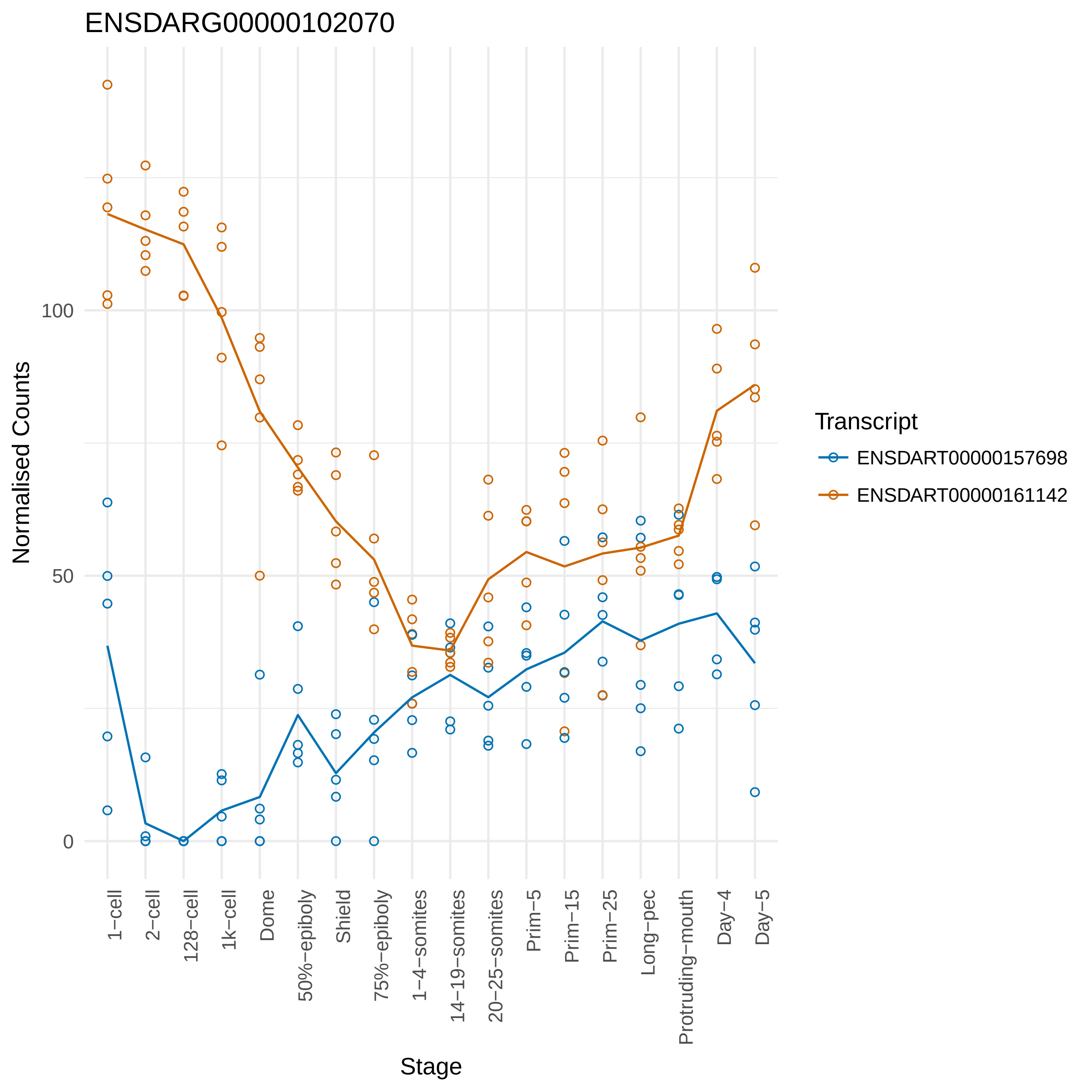
ENSDARG00000102070
Normalised Counts
100
50
0
1−cell
2−cell
128−cell
1k−cell
Dome
50%−epiboly
Shield
75%−epiboly
1−4−somites
14−19−somites
20−25−somites
Prim−5
Prim−15
Prim−25
Long−pec
Protruding−mouth
Day−4
Day−5
Stage
Transcript
ENSDART00000157698
ENSDART00000161142

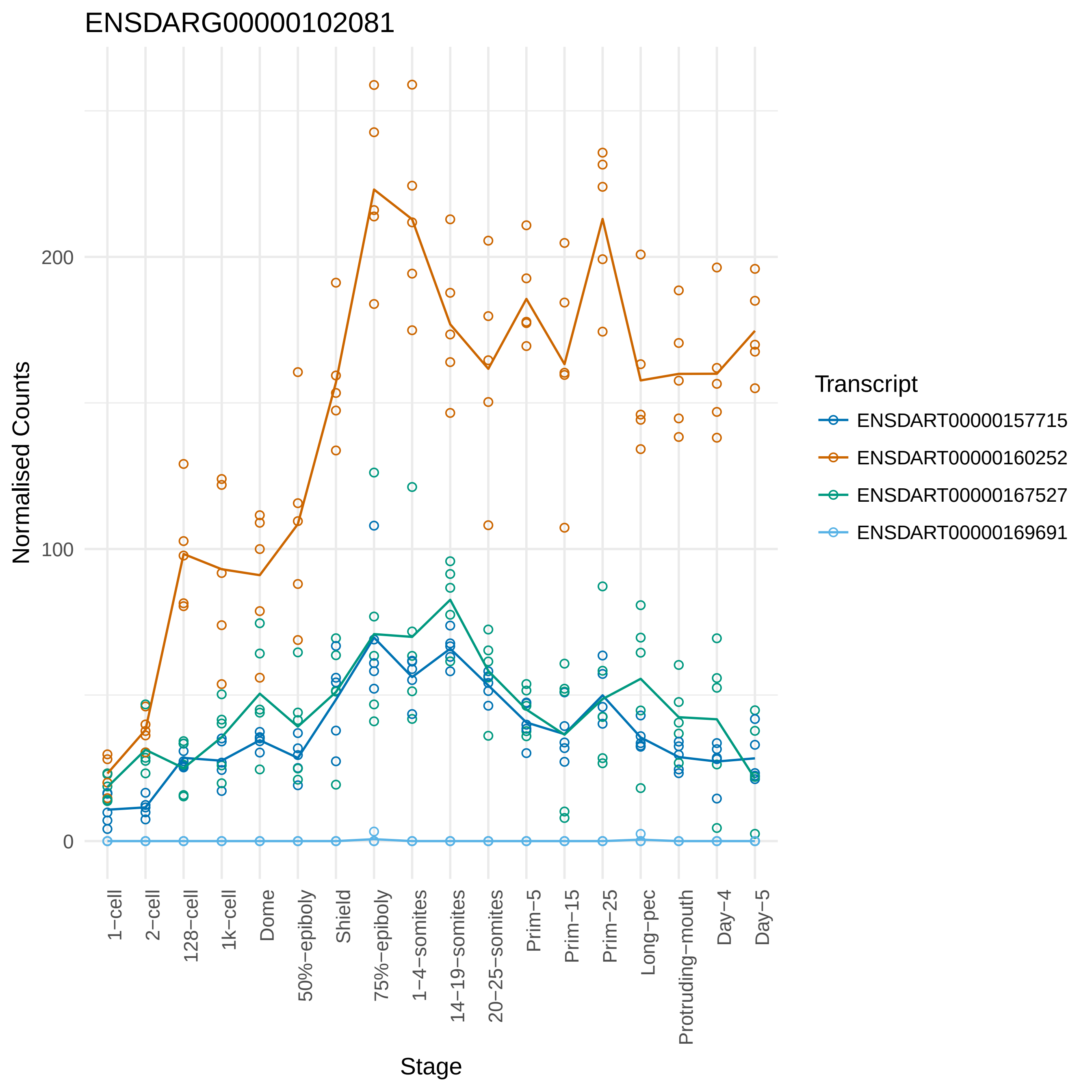

ENSDARG00000102081
Normalised Counts
200
100
0
1−cell
2−cell
128−cell
1k−cell
Dome
50%−epiboly
Shield
75%−epiboly
1−4−somites
14−19−somites
20−25−somites
Prim−5
Prim−15
Prim−25
Long−pec
Protruding−mouth
Day−4
Day−5
Stage
Transcript
ENSDART00000157715
ENSDART00000160252
ENSDART00000167527
ENSDART00000169691

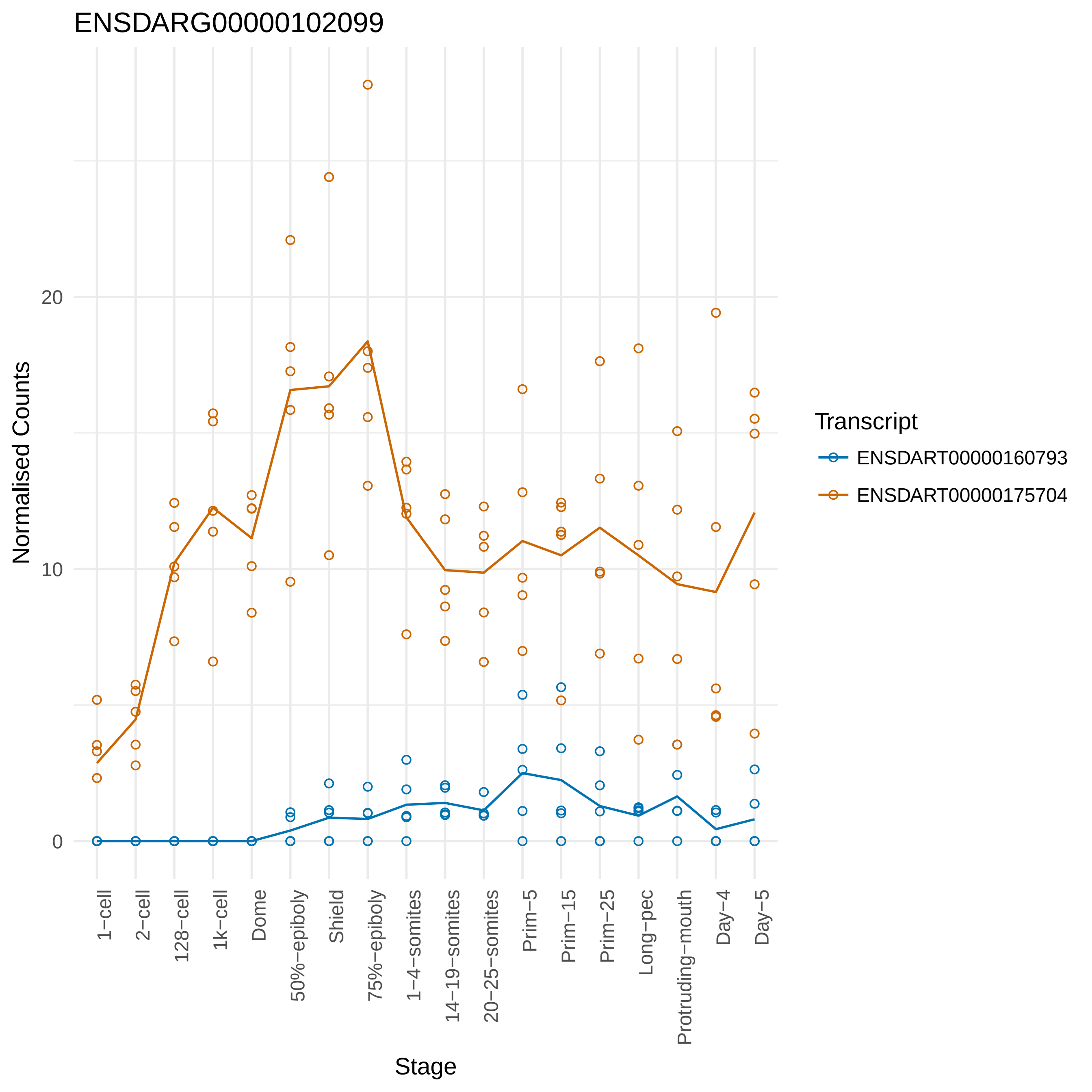

ENSDARG00000102099
Normalised Counts
0
10
20
1−cell
2−cell
128−cell
1k−cell
Dome
50%−epiboly
Shield
75%−epiboly
1−4−somites
14−19−somites
20−25−somites
Prim−5
Prim−15
Prim−25
Long−pec
Protruding−mouth
Day−4
Day−5
Stage
Transcript
ENSDART00000160793
ENSDART00000175704

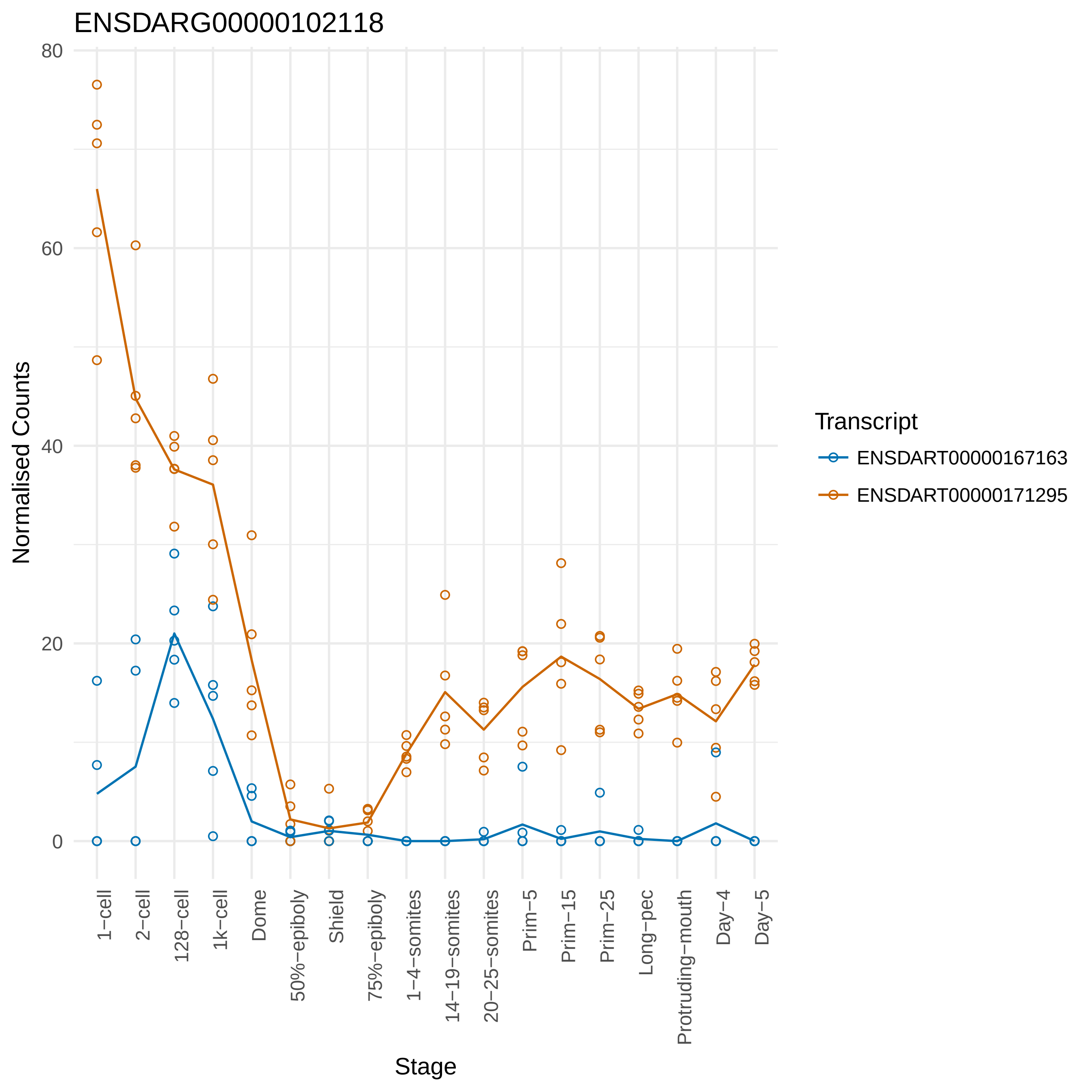

ENSDARG00000102118
Normalised Counts
0
20
40
60
80
Stage
1−cell
2−cell
128−cell
1k−cell
Dome
50%−epiboly
Shield
75%−epiboly
1−4−somites
14−19−somites
20−25−somites
Prim−5
Prim−15
Prim−25
Long−pec
Protruding−mouth
Day−4
Day−5
Transcript
ENSDART00000167163
ENSDART00000171295

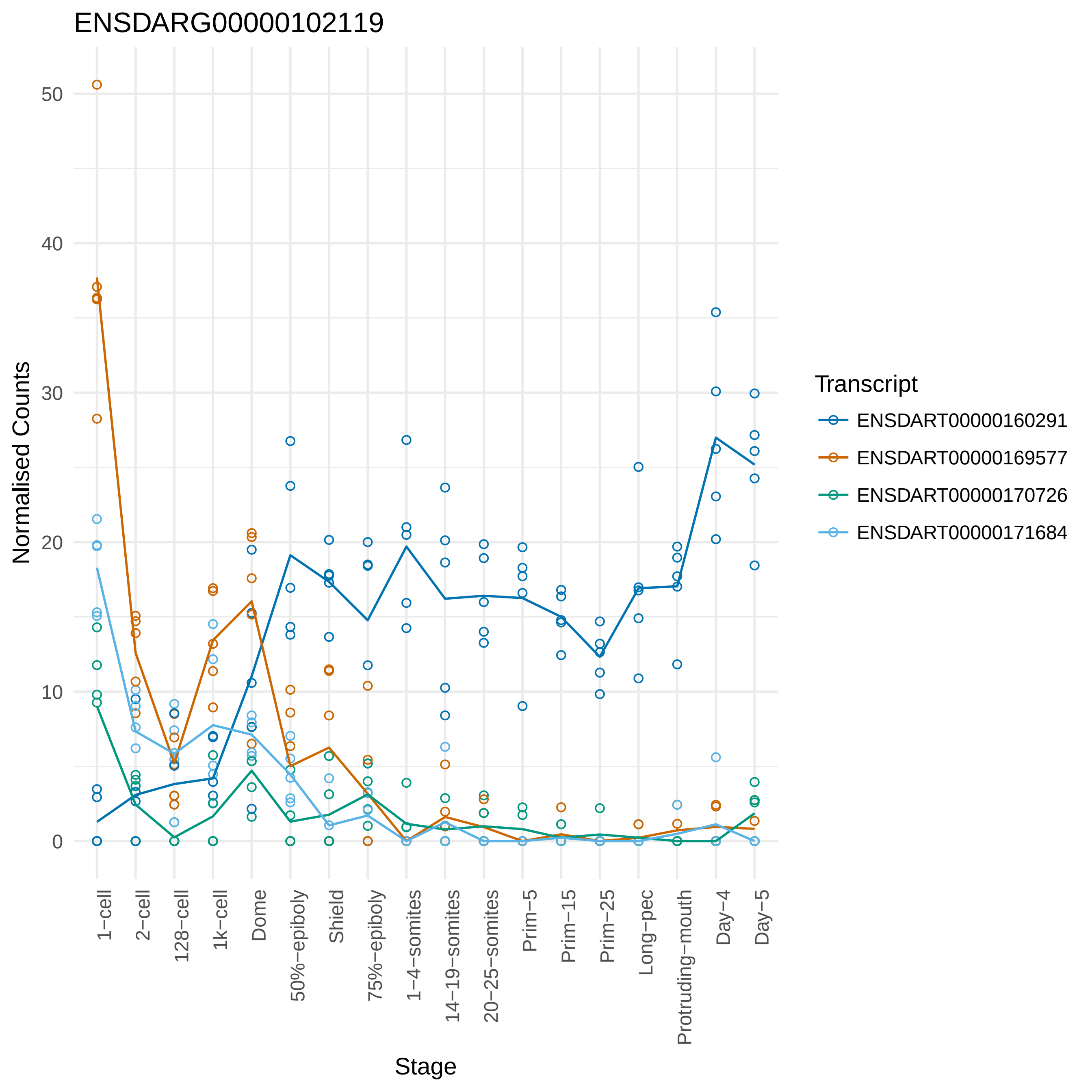
ENSDARG00000102119
Normalised Counts
50
40
30
20
10
0
1−cell
2−cell
128−cell
1k−cell
Dome
50%−epiboly
Shield
75%−epiboly
1−4−somites
14−19−somites
20−25−somites
Prim−5
Prim−15
Prim−25
Long−pec
Protruding−mouth
Day−4
Day−5
Stage
Transcript
ENSDART00000160291
ENSDART00000169577
ENSDART00000170726
ENSDART00000171684

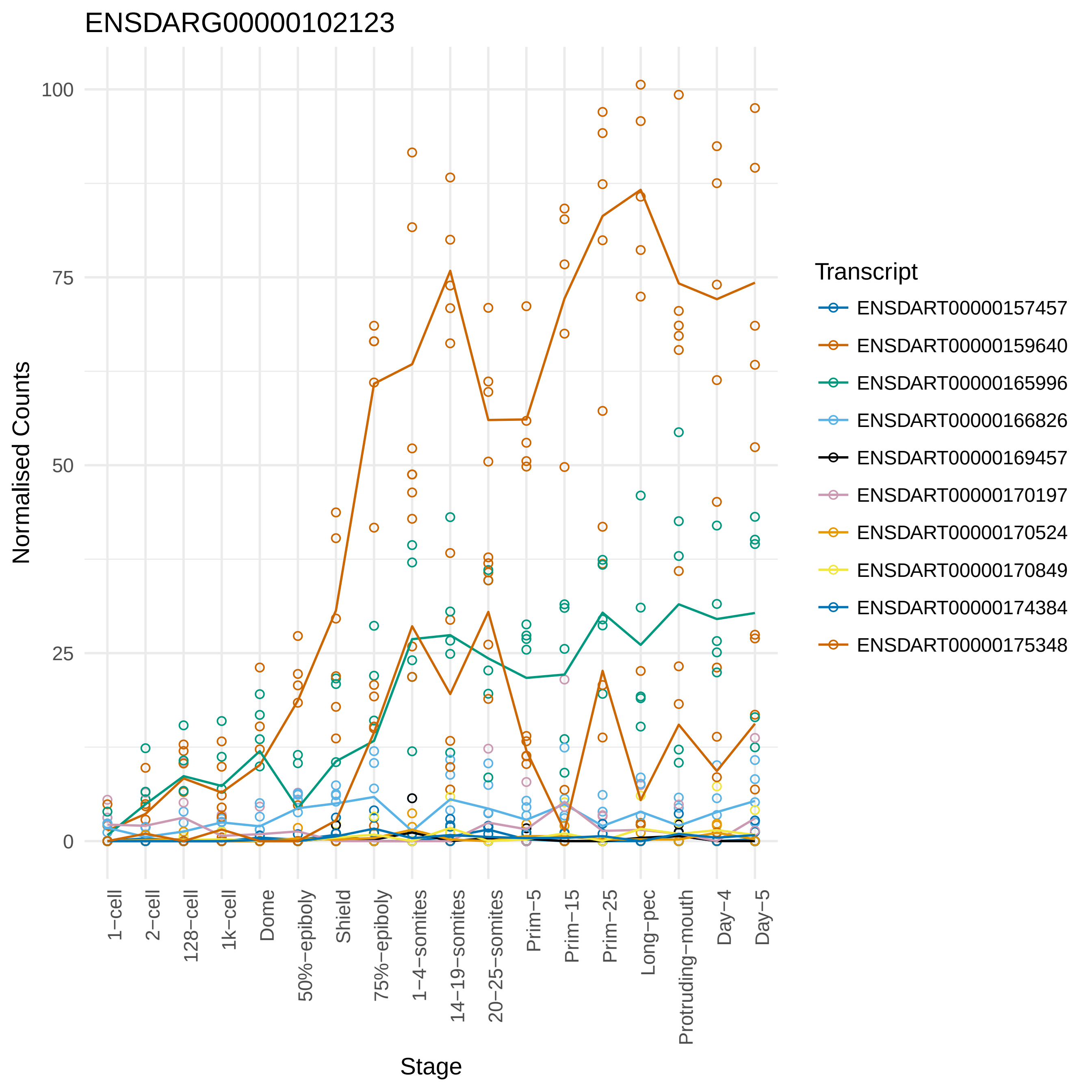

ENSDARG00000102123
Normalised Counts
100
75
50
25
0
1−cell
2−cell
128−cell
1k−cell
Dome
50%−epiboly
Shield
75%−epiboly
1−4−somites
14−19−somites
20−25−somites
Prim−5
Prim−15
Prim−25
Long−pec
Protruding−mouth
Day−4
Day−5
Stage
Transcript
ENSDART00000157457
ENSDART00000159640
ENSDART00000165996
ENSDART00000166826
ENSDART00000169457
ENSDART00000170197
ENSDART00000170524
ENSDART00000170849
ENSDART00000174384
ENSDART00000175348

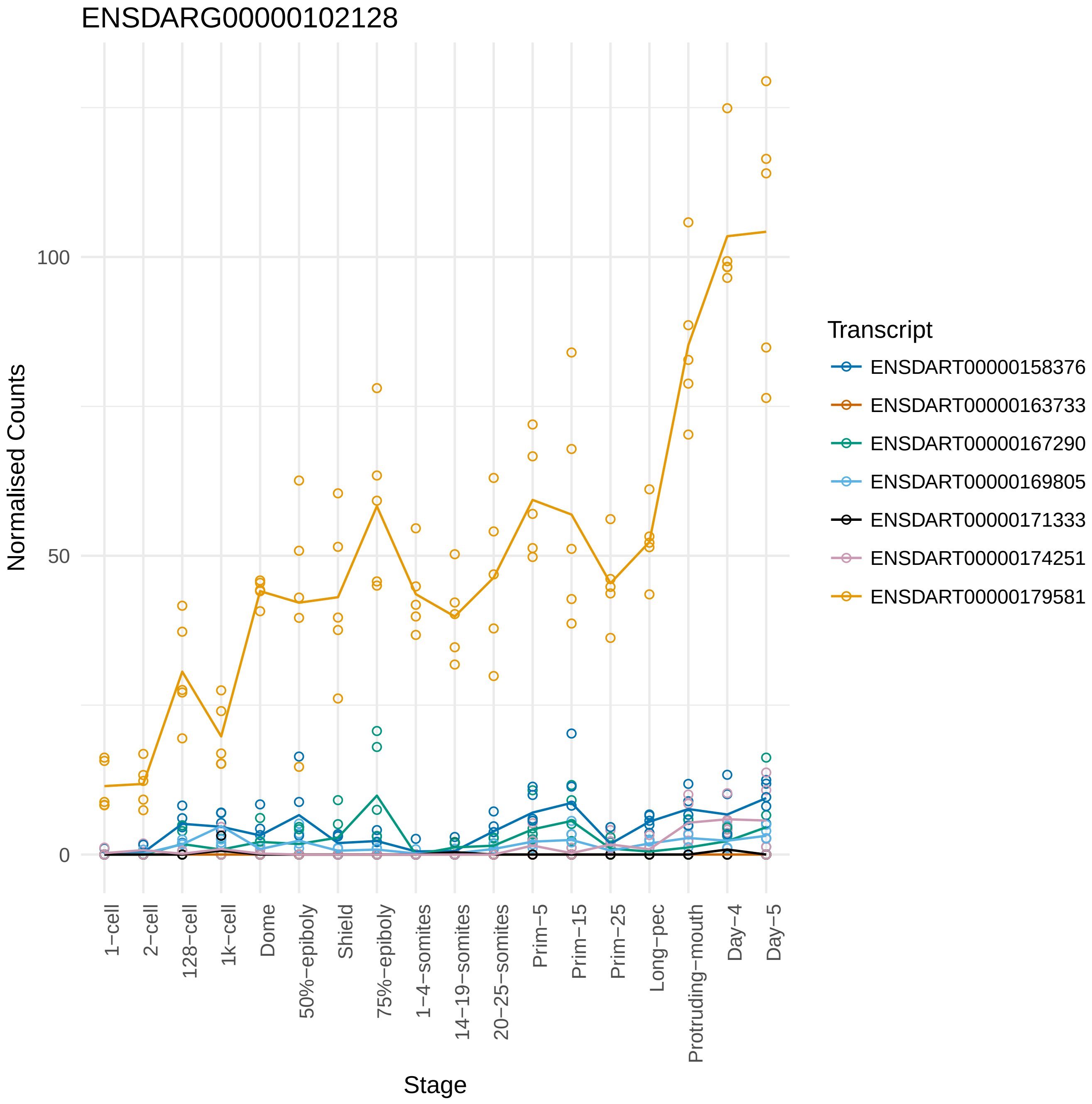
ENSDARG00000102128
Normalised Counts
0
50
100
1−cell
2−cell
128−cell
1k−cell
Dome
50%−epiboly
Shield
75%−epiboly
1−4−somites
14−19−somites
20−25−somites
Prim−5
Prim−15
Prim−25
Long−pec
Protruding−mouth
Day−4
Day−5
Stage
Transcript
ENSDART00000158376
ENSDART00000163733
ENSDART00000167290
ENSDART00000169805
ENSDART00000171333
ENSDART00000174251
ENSDART00000179581

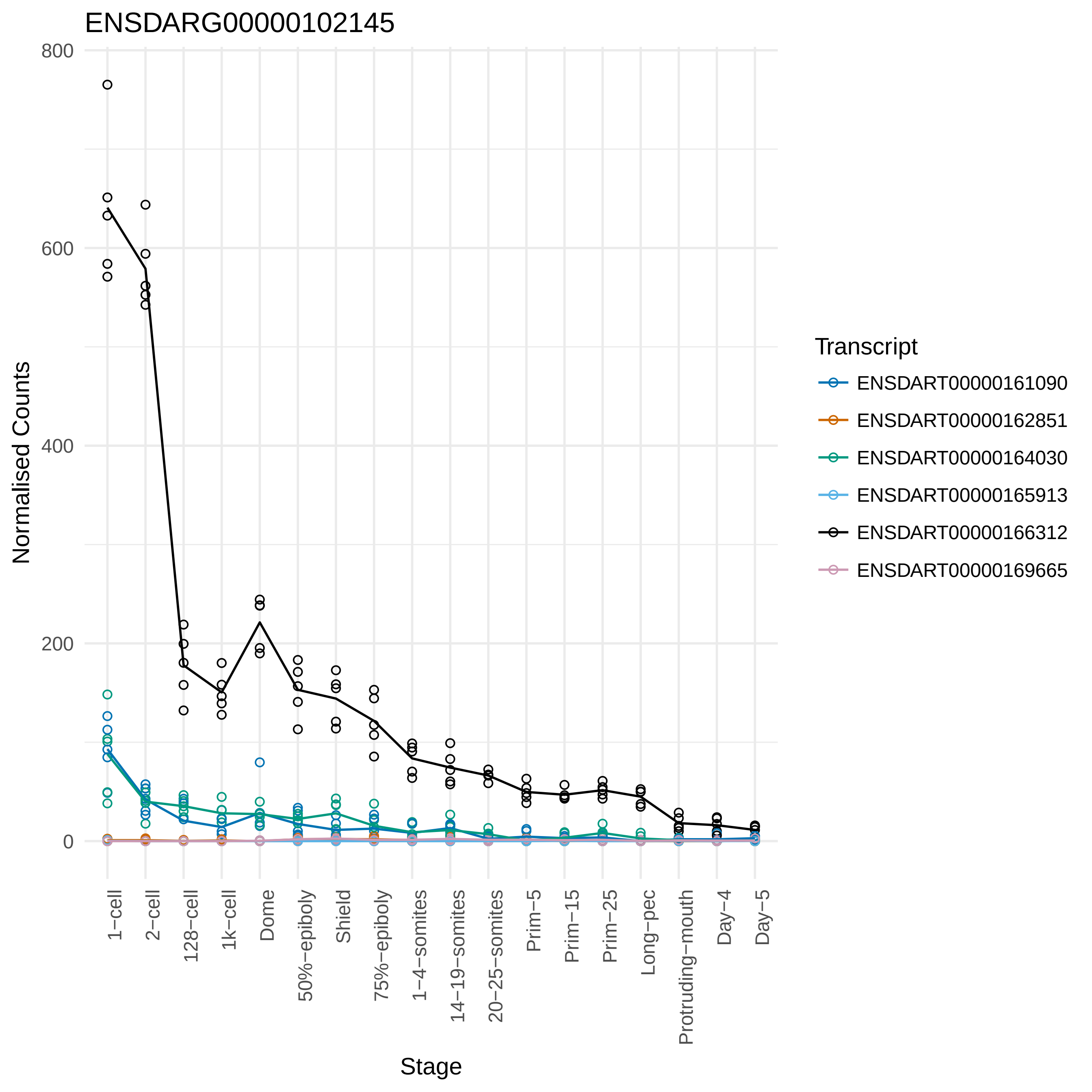
ENSDARG00000102145
Normalised Counts
800
600
400
200
0
1−cell
2−cell
128−cell
1k−cell
Dome
50%−epiboly
Shield
75%−epiboly
1−4−somites
14−19−somites
20−25−somites
Prim−5
Prim−15
Prim−25
Long−pec
Protruding−mouth
Day−4
Day−5
Stage
Transcript
ENSDART00000161090
ENSDART00000162851
ENSDART00000164030
ENSDART00000165913
ENSDART00000166312
ENSDART00000169665

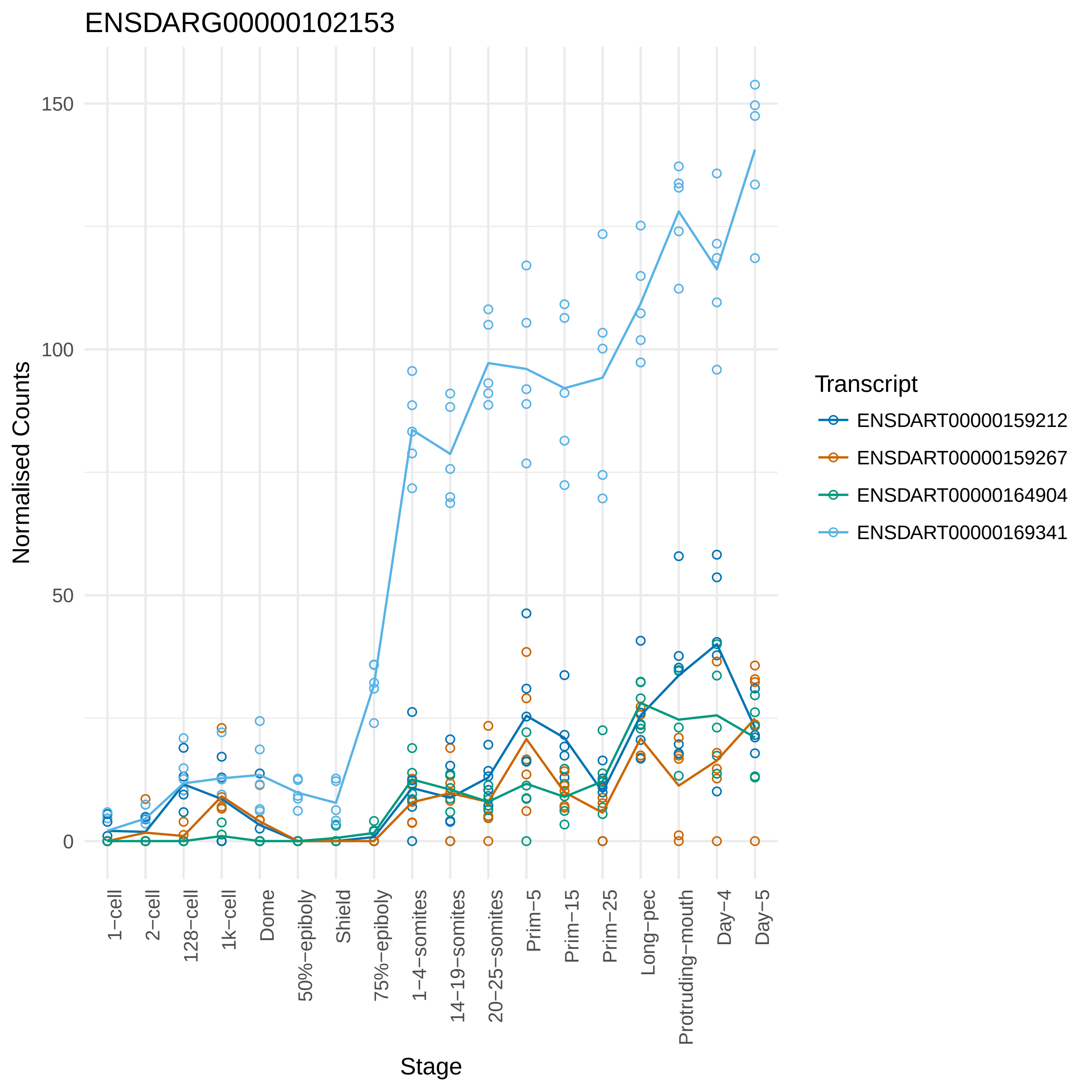

ENSDARG00000102153
Normalised Counts
150
100
50
0
1−cell
2−cell
128−cell
1k−cell
Dome
50%−epiboly
Shield
75%−epiboly
1−4−somites
14−19−somites
20−25−somites
Prim−5
Prim−15
Prim−25
Long−pec
Protruding−mouth
Day−4
Day−5
Stage
Transcript
ENSDART00000159212
ENSDART00000159267
ENSDART00000164904
ENSDART00000169341

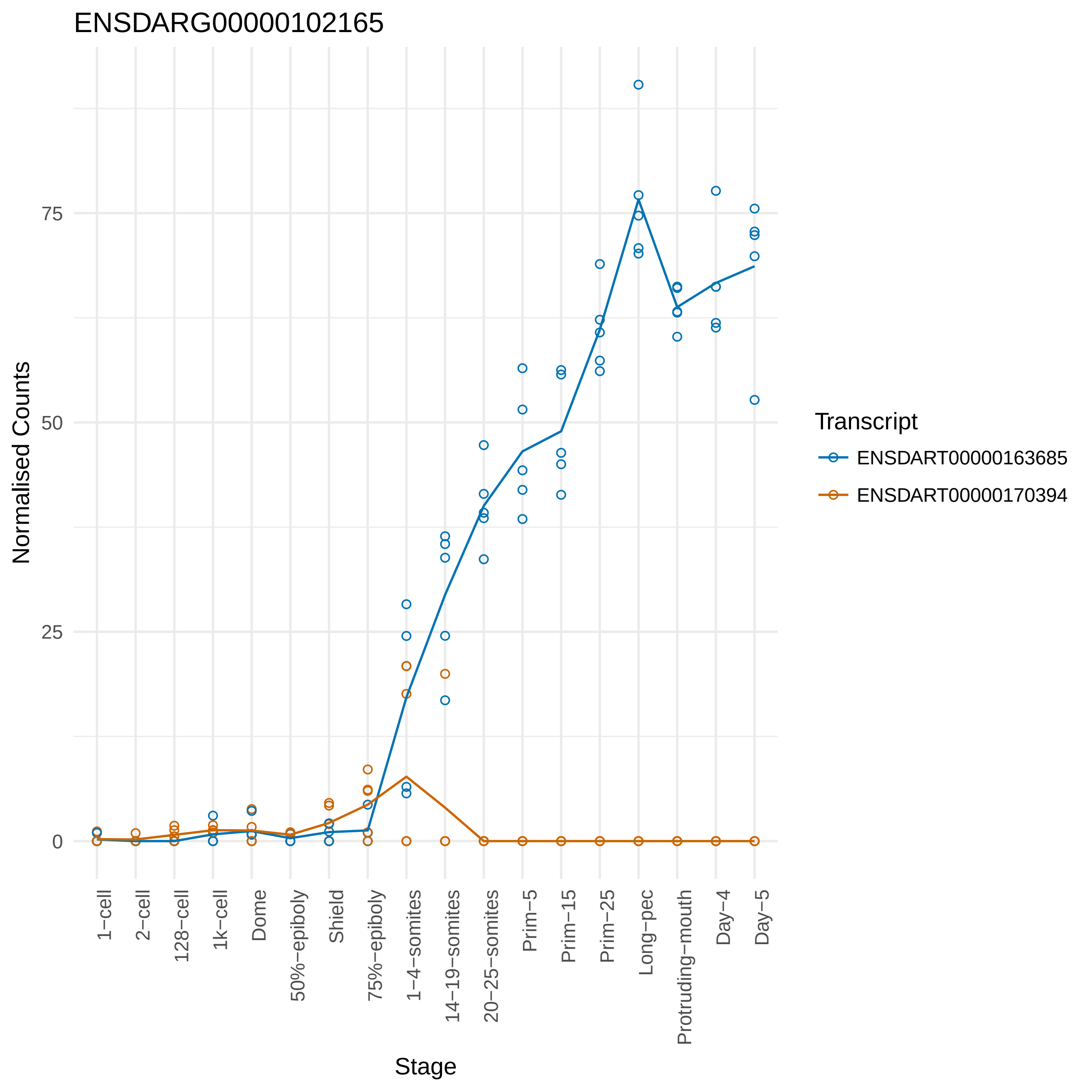
ENSDARG00000102165
Normalised Counts
0
25
50
75
Stage
1−cell
2−cell
128−cell
1k−cell
Dome
50%−epiboly
Shield
75%−epiboly
1−4−somites
14−19−somites
20−25−somites
Prim−5
Prim−15
Prim−25
Long−pec
Protruding−mouth
Day−4
Day−5
Transcript
ENSDART00000163685
ENSDART00000170394

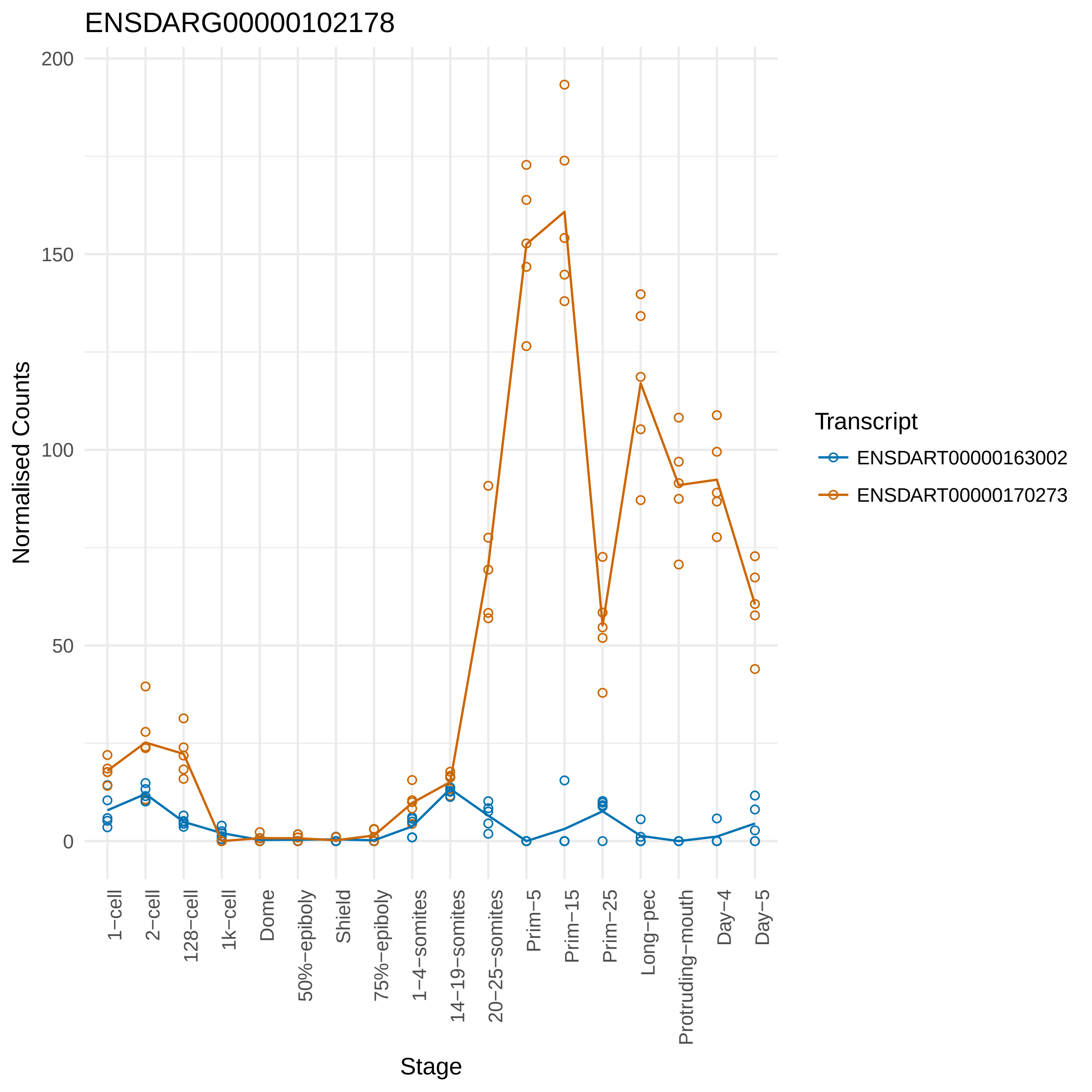
ENSDARG00000102178
Normalised Counts
200
150
100
50
0
1−cell
2−cell
128−cell
1k−cell
Dome
50%−epiboly
Shield
75%−epiboly
1−4−somites
14−19−somites
20−25−somites
Prim−5
Prim−15
Prim−25
Long−pec
Protruding−mouth
Day−4
Day−5
Stage
Transcript
ENSDART00000163002
ENSDART00000170273

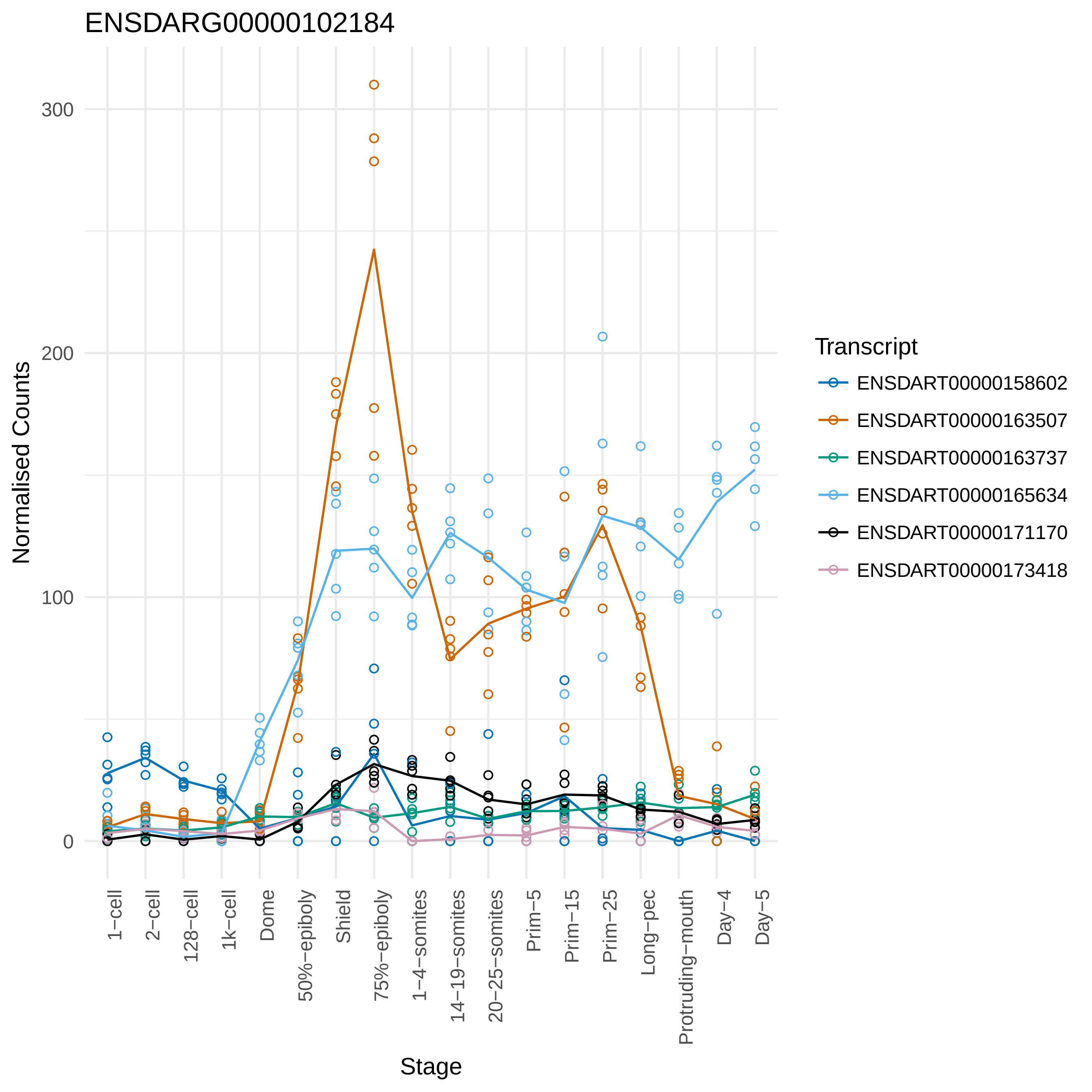
ENSDARG00000102184
Normalised Counts
300
200
100
0
Stage
1−cell
2−cell
128−cell
1k−cell
Dome
50%−epiboly
Shield
75%−epiboly
1−4−somites
14−19−somites
20−25−somites
Prim−5
Prim−15
Prim−25
Long−pec
Protruding−mouth
Day−4
Day−5
Transcript
ENSDART00000158602
ENSDART00000163507
ENSDART00000163737
ENSDART00000165634
ENSDART00000171170
ENSDART00000173418

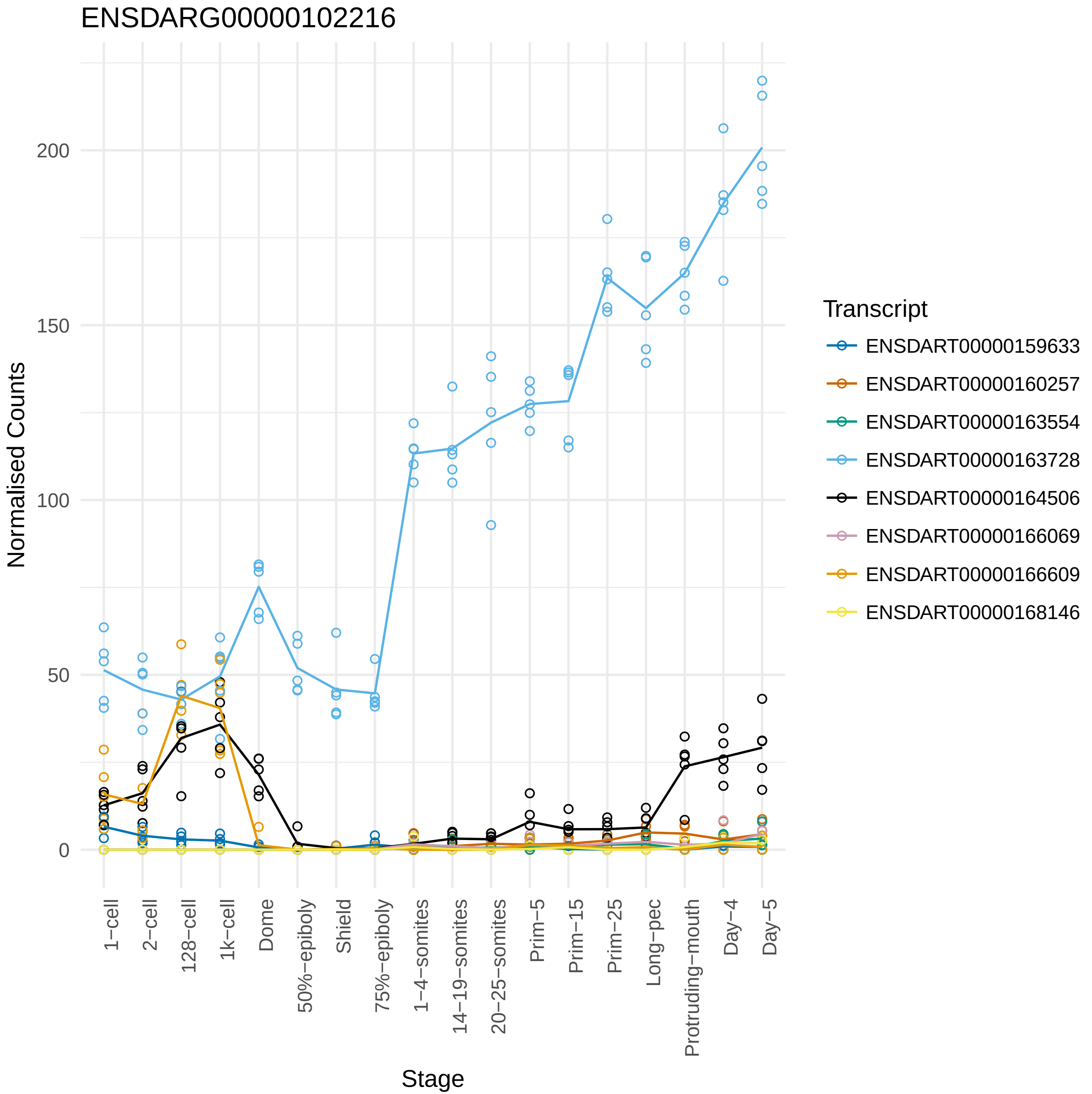

ENSDARG00000102216
Normalised Counts
0
50
100
150
200
Stage
1−cell
2−cell
128−cell
1k−cell
Dome
50%−epiboly
Shield
75%−epiboly
1−4−somites
14−19−somites
20−25−somites
Prim−5
Prim−15
Prim−25
Long−pec
Protruding−mouth
Day−4
Day−5
Transcript
ENSDART00000159633
ENSDART00000160257
ENSDART00000163554
ENSDART00000163728
ENSDART00000164506
ENSDART00000166069
ENSDART00000166609
ENSDART00000168146

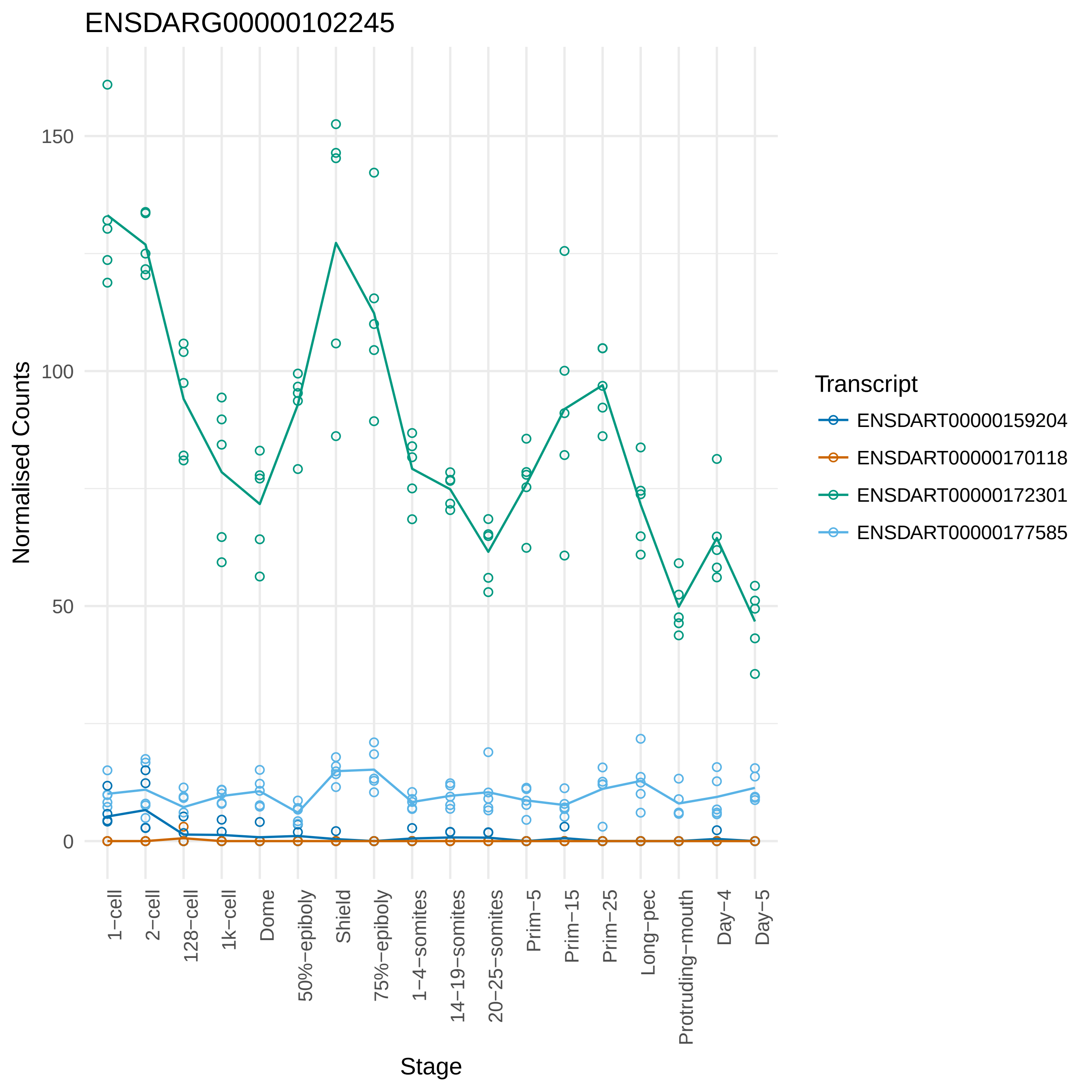

ENSDARG00000102245
Normalised Counts
150
100
50
0
1−cell
2−cell
128−cell
1k−cell
Dome
50%−epiboly
Shield
75%−epiboly
1−4−somites
14−19−somites
20−25−somites
Prim−5
Prim−15
Prim−25
Long−pec
Protruding−mouth
Day−4
Day−5
Stage
Transcript
ENSDART00000159204
ENSDART00000170118
ENSDART00000172301
ENSDART00000177585

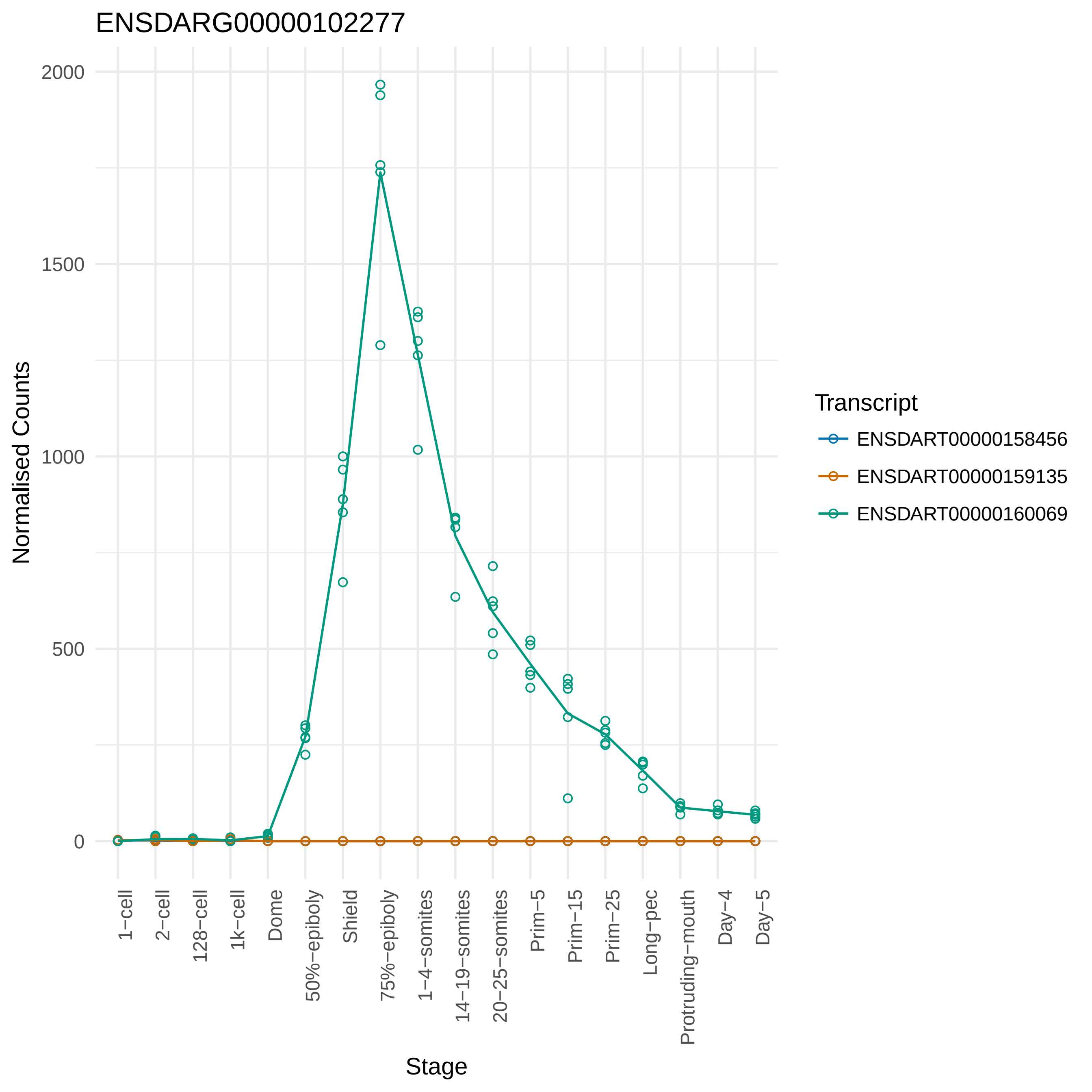

ENSDARG00000102277
Normalised Counts
2000
1500
1000
500
0
1−cell
2−cell
128−cell
1k−cell
Dome
50%−epiboly
Shield
75%−epiboly
1−4−somites
14−19−somites
20−25−somites
Prim−5
Prim−15
Prim−25
Long−pec
Protruding−mouth
Day−4
Day−5
Stage
Transcript
ENSDART00000158456
ENSDART00000159135
ENSDART00000160069

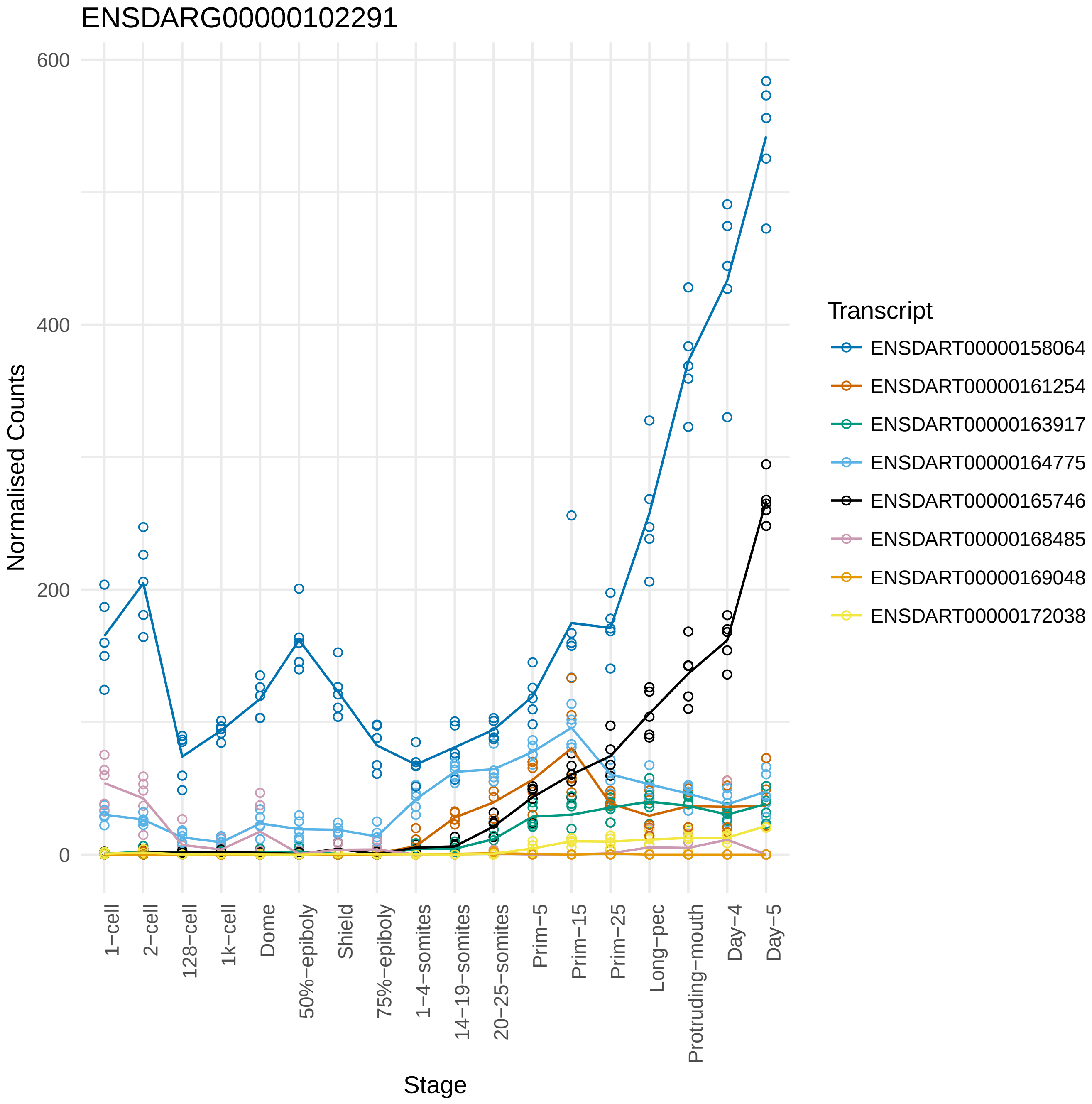
ENSDARG00000102291
Normalised Counts
600
400
200
0
1−cell
2−cell
128−cell
1k−cell
Dome
50%−epiboly
Shield
75%−epiboly
1−4−somites
14−19−somites
20−25−somites
Prim−5
Prim−15
Prim−25
Long−pec
Protruding−mouth
Day−4
Day−5
Stage
Transcript
ENSDART00000158064
ENSDART00000161254
ENSDART00000163917
ENSDART00000164775
ENSDART00000165746
ENSDART00000168485
ENSDART00000169048
ENSDART00000172038

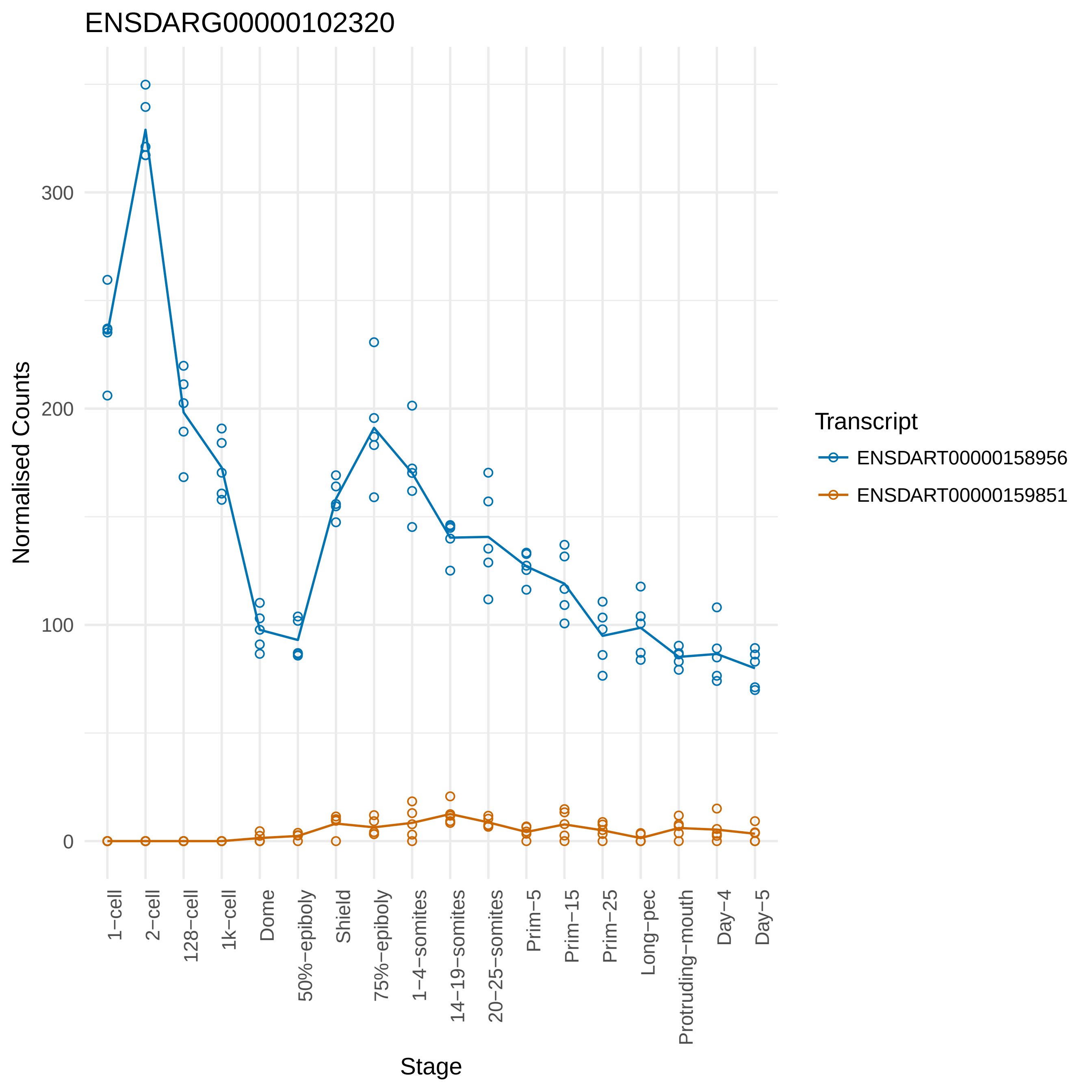

ENSDARG00000102320
Normalised Counts
300
200
100
0
1−cell
2−cell
128−cell
1k−cell
Dome
50%−epiboly
Shield
75%−epiboly
1−4−somites
14−19−somites
20−25−somites
Prim−5
Prim−15
Prim−25
Long−pec
Protruding−mouth
Day−4
Day−5
Stage
Transcript
ENSDART00000158956
ENSDART00000159851

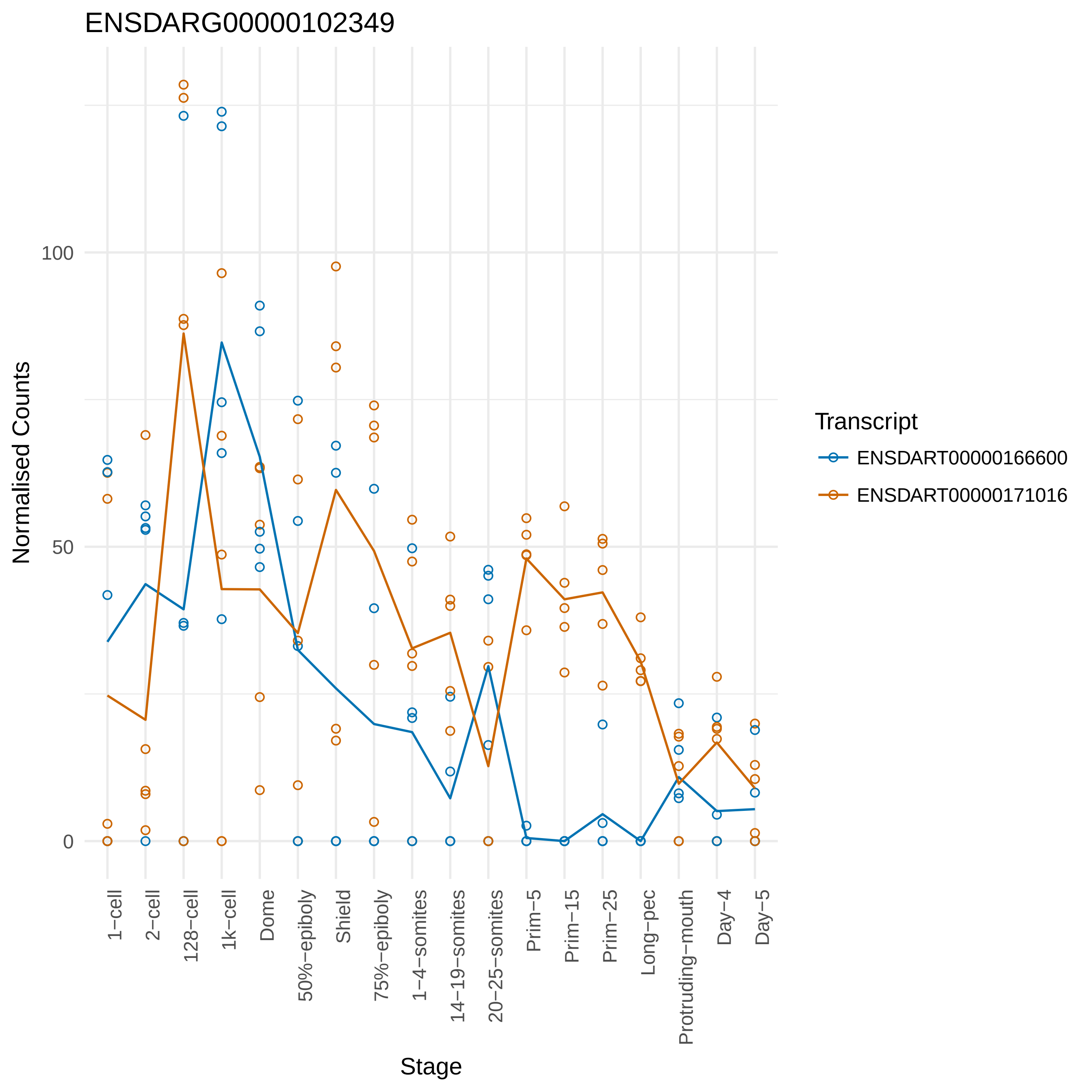

ENSDARG00000102349
Normalised Counts
100
50
0
1−cell
2−cell
128−cell
1k−cell
Dome
50%−epiboly
Shield
75%−epiboly
1−4−somites
14−19−somites
20−25−somites
Prim−5
Prim−15
Prim−25
Long−pec
Protruding−mouth
Day−4
Day−5
Stage
Transcript
ENSDART00000166600
ENSDART00000171016

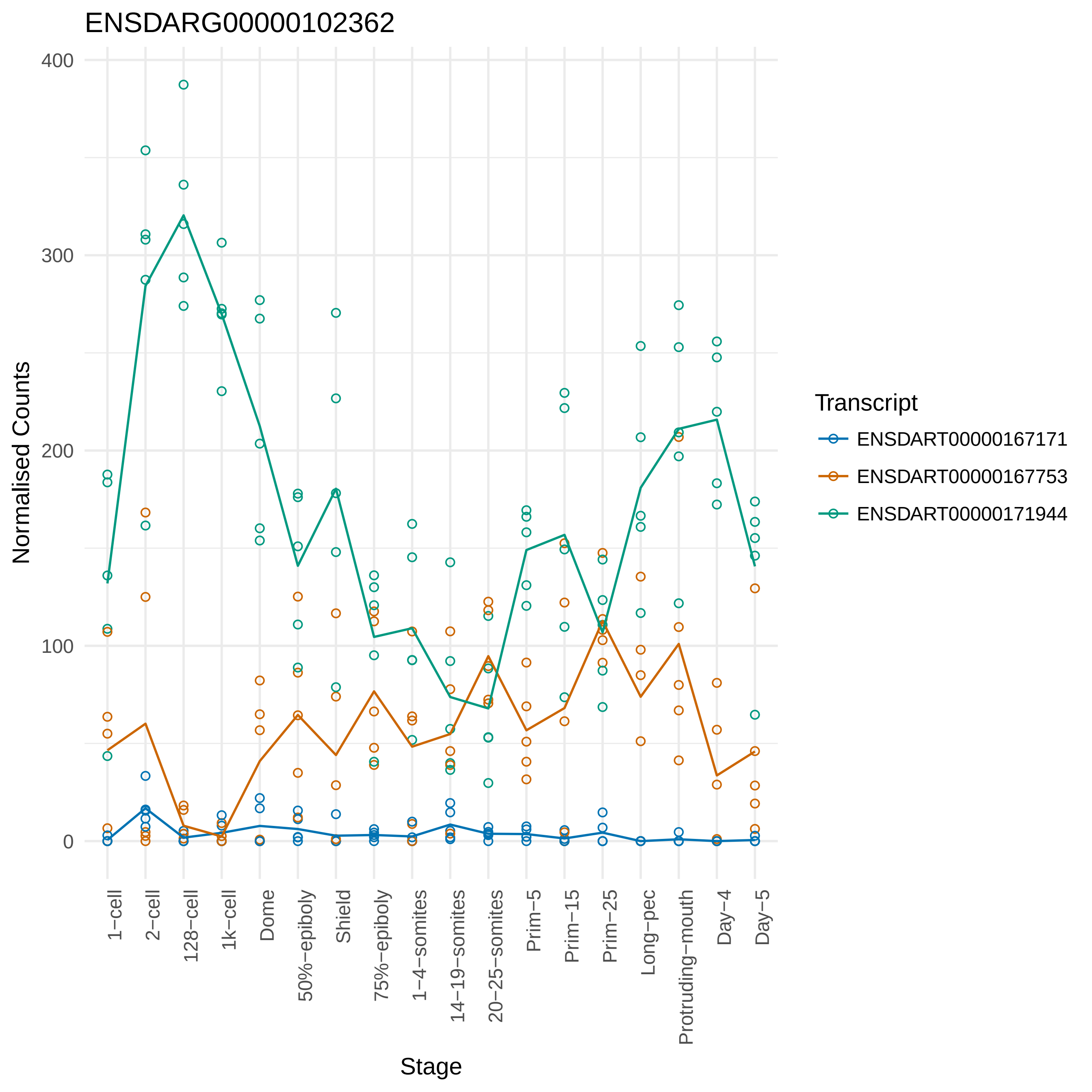
ENSDARG00000102362
Normalised Counts
400
300
200
100
0
1−cell
2−cell
128−cell
1k−cell
Dome
50%−epiboly
Shield
75%−epiboly
1−4−somites
14−19−somites
20−25−somites
Prim−5
Prim−15
Prim−25
Long−pec
Protruding−mouth
Day−4
Day−5
Stage
Transcript
ENSDART00000167171
ENSDART00000167753
ENSDART00000171944

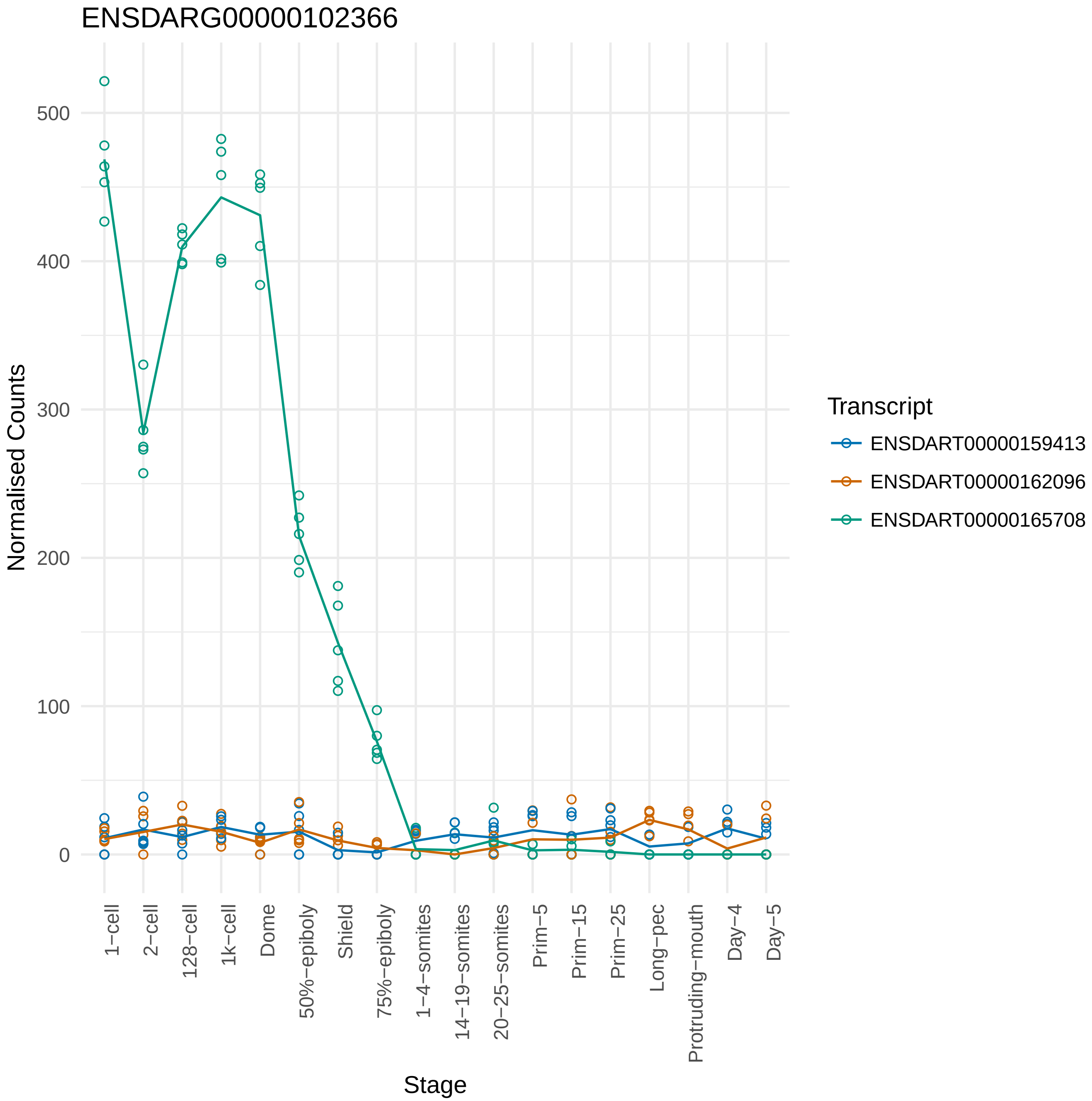
ENSDARG00000102366
Normalised Counts
500
400
300
200
100
0
1−cell
2−cell
128−cell
1k−cell
Dome
50%−epiboly
Shield
75%−epiboly
1−4−somites
14−19−somites
20−25−somites
Prim−5
Prim−15
Prim−25
Long−pec
Protruding−mouth
Day−4
Day−5
Stage
Transcript
ENSDART00000159413
ENSDART00000162096
ENSDART00000165708

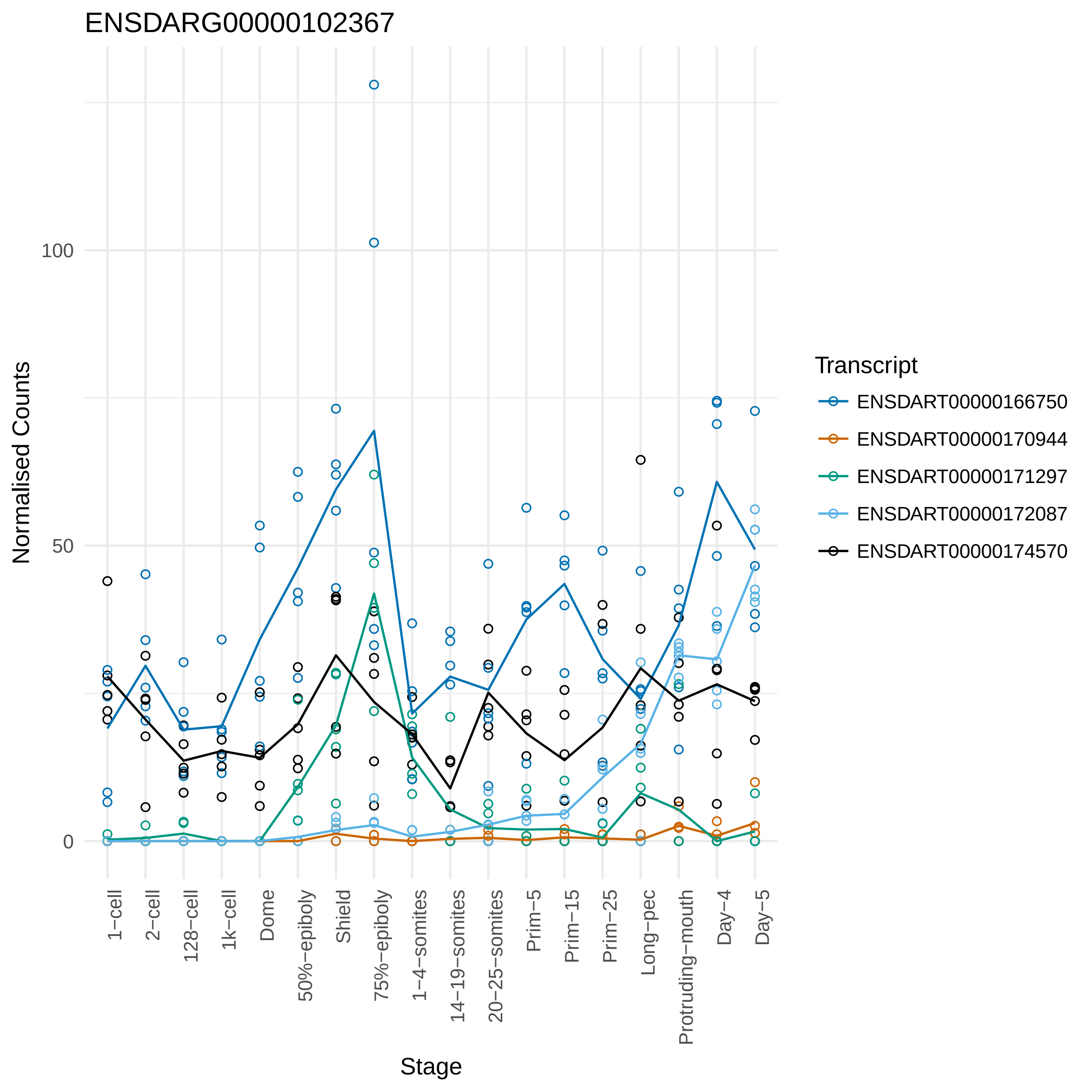

ENSDARG00000102367
Normalised Counts
100
50
0
1−cell
2−cell
128−cell
1k−cell
Dome
50%−epiboly
Shield
75%−epiboly
1−4−somites
14−19−somites
20−25−somites
Prim−5
Prim−15
Prim−25
Long−pec
Protruding−mouth
Day−4
Day−5
Stage
Transcript
ENSDART00000166750
ENSDART00000170944
ENSDART00000171297
ENSDART00000172087
ENSDART00000174570

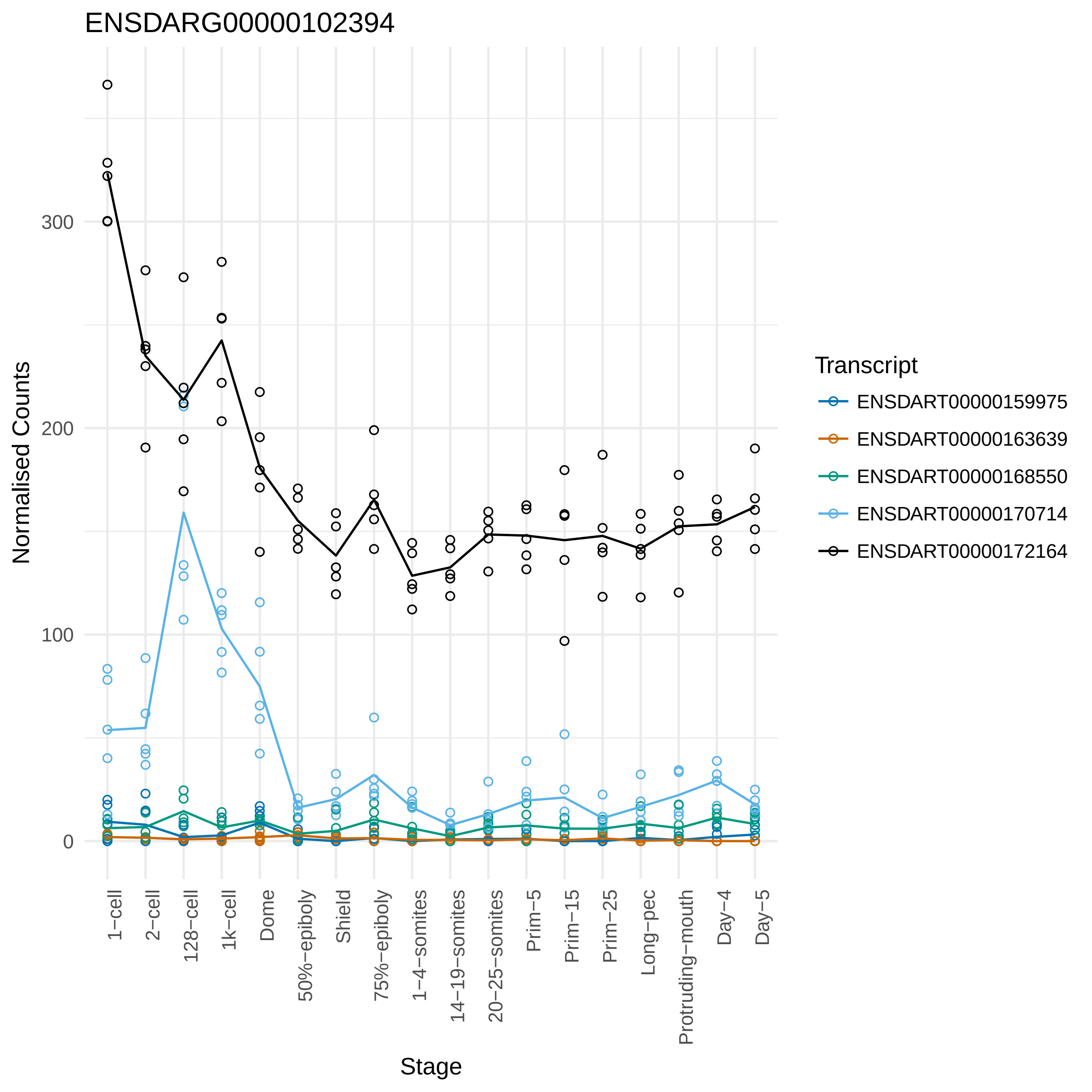

ENSDARG00000102394
Normalised Counts
300
200
100
0
1−cell
2−cell
128−cell
1k−cell
Dome
50%−epiboly
Shield
75%−epiboly
1−4−somites
14−19−somites
20−25−somites
Prim−5
Prim−15
Prim−25
Long−pec
Protruding−mouth
Day−4
Day−5
Stage
Transcript
ENSDART00000159975
ENSDART00000163639
ENSDART00000168550
ENSDART00000170714
ENSDART00000172164

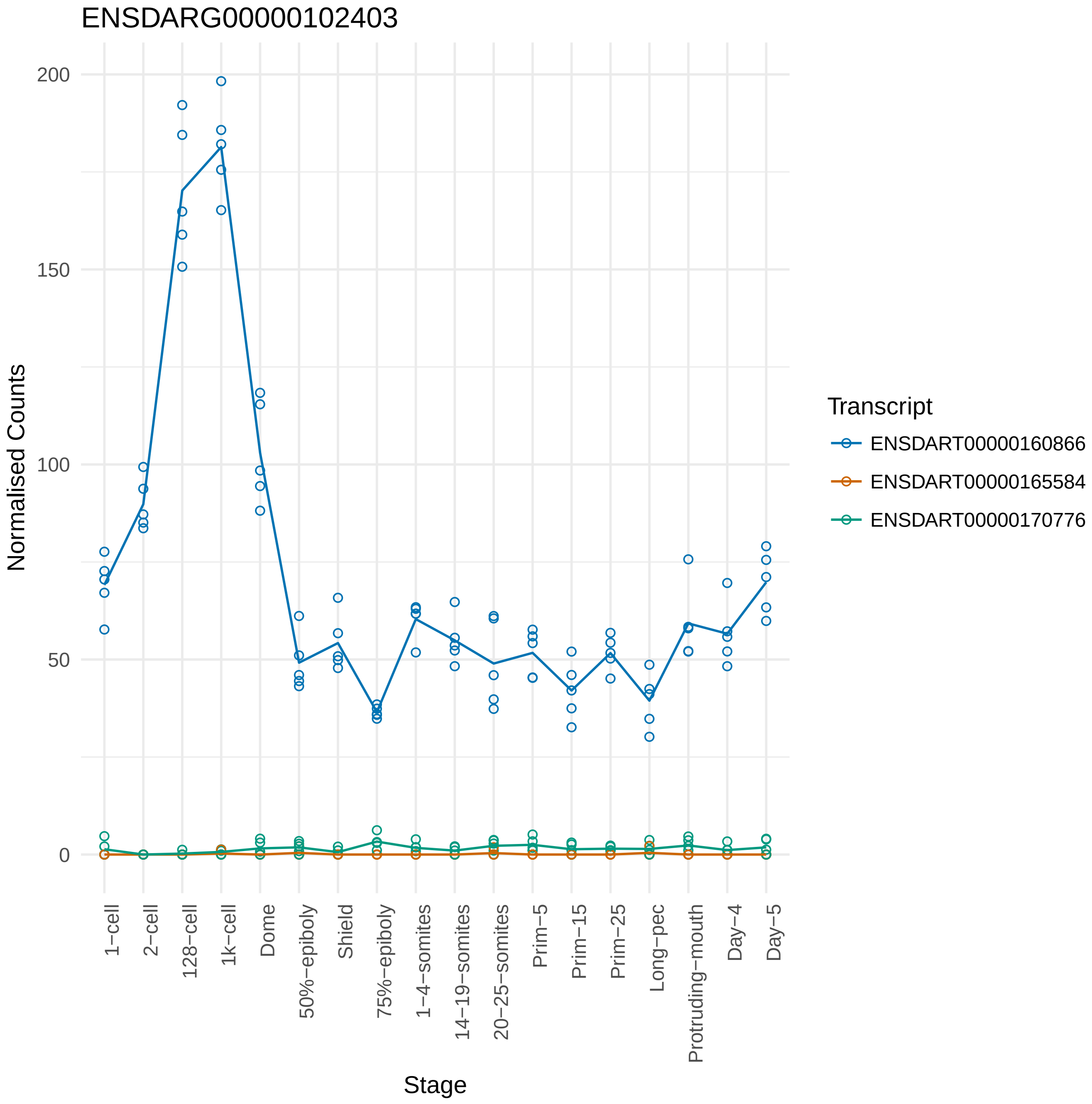

ENSDARG00000102403
Normalised Counts
200
150
100
50
0
1−cell
2−cell
128−cell
1k−cell
Dome
50%−epiboly
Shield
75%−epiboly
1−4−somites
14−19−somites
20−25−somites
Prim−5
Prim−15
Prim−25
Long−pec
Protruding−mouth
Day−4
Day−5
Stage
Transcript
ENSDART00000160866
ENSDART00000165584
ENSDART00000170776

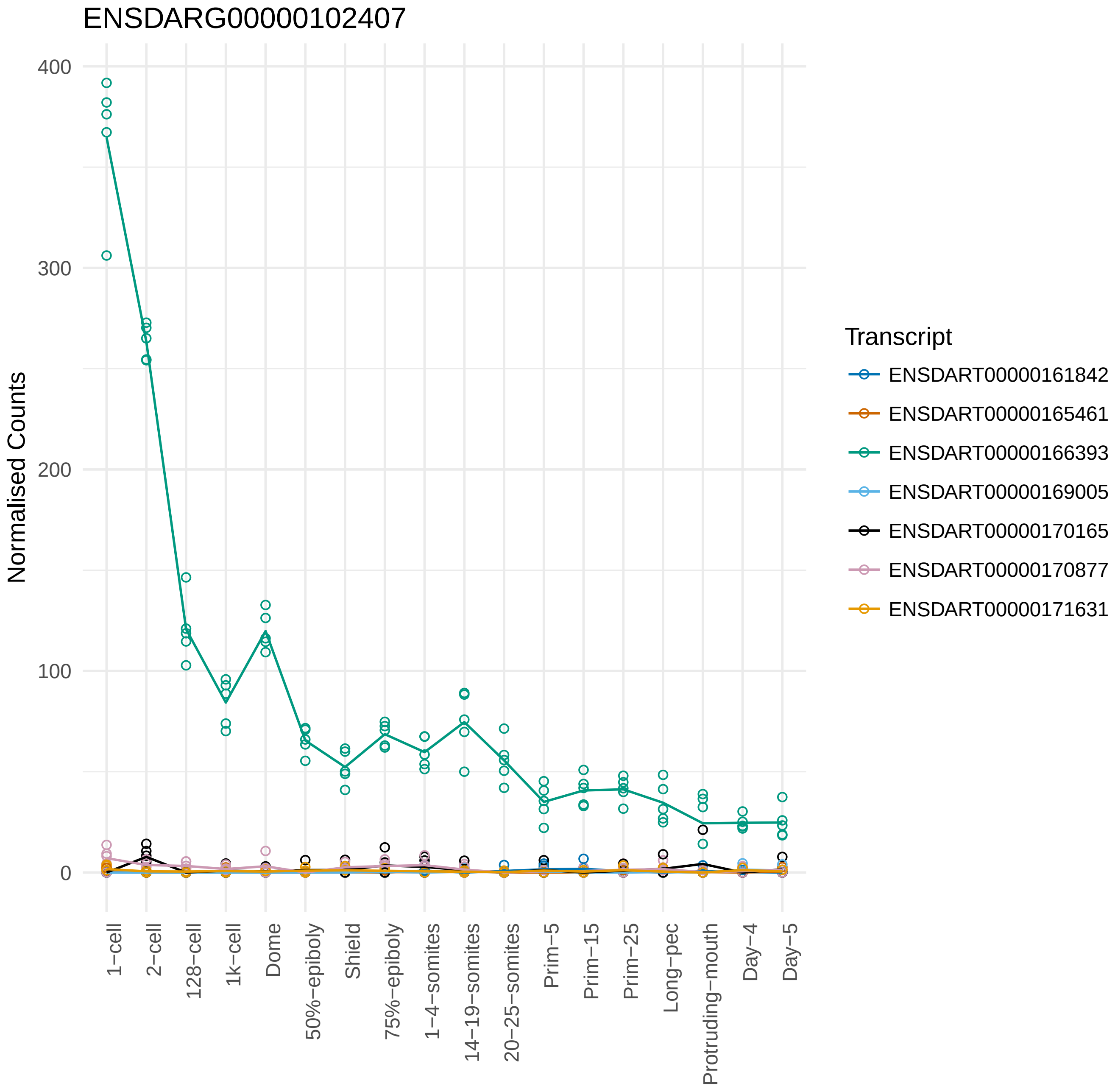

ENSDARG00000102407
Normalised Counts
400
300
200
100
0
1−cell
2−cell
128−cell
1k−cell
Dome
50%−epiboly
Shield
75%−epiboly
1−4−somites
14−19−somites
20−25−somites
Prim−5
Prim−15
Prim−25
Long−pec
Protruding−mouth
Day−4
Day−5
Stage
Transcript
ENSDART00000161842
ENSDART00000165461
ENSDART00000166393
ENSDART00000169005
ENSDART00000170165
ENSDART00000170877
ENSDART00000171631

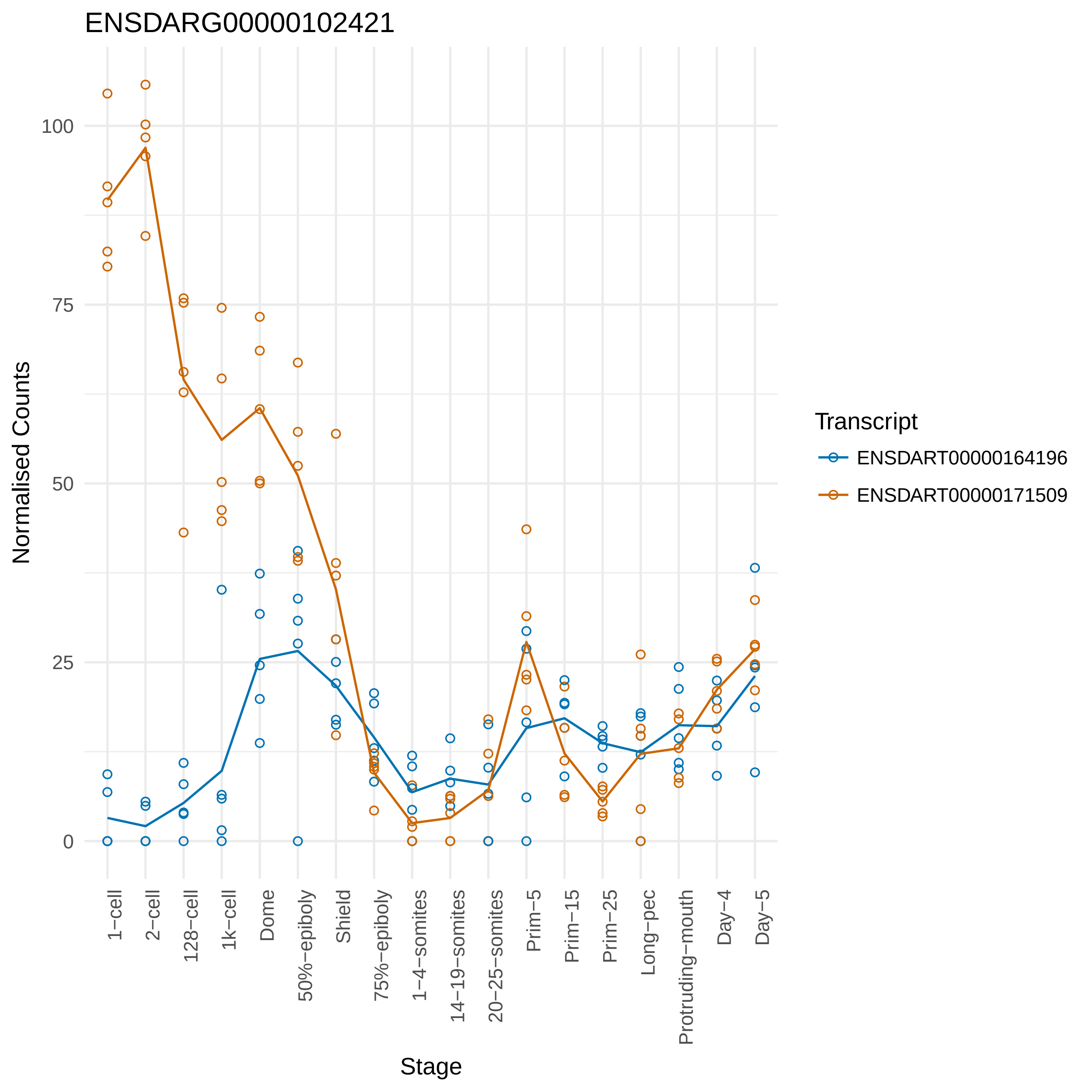
ENSDARG00000102421
Normalised Counts
100
75
50
25
0
1−cell
2−cell
128−cell
1k−cell
Dome
50%−epiboly
Shield
75%−epiboly
1−4−somites
14−19−somites
20−25−somites
Prim−5
Prim−15
Prim−25
Long−pec
Protruding−mouth
Day−4
Day−5
Stage
Transcript
ENSDART00000164196
ENSDART00000171509

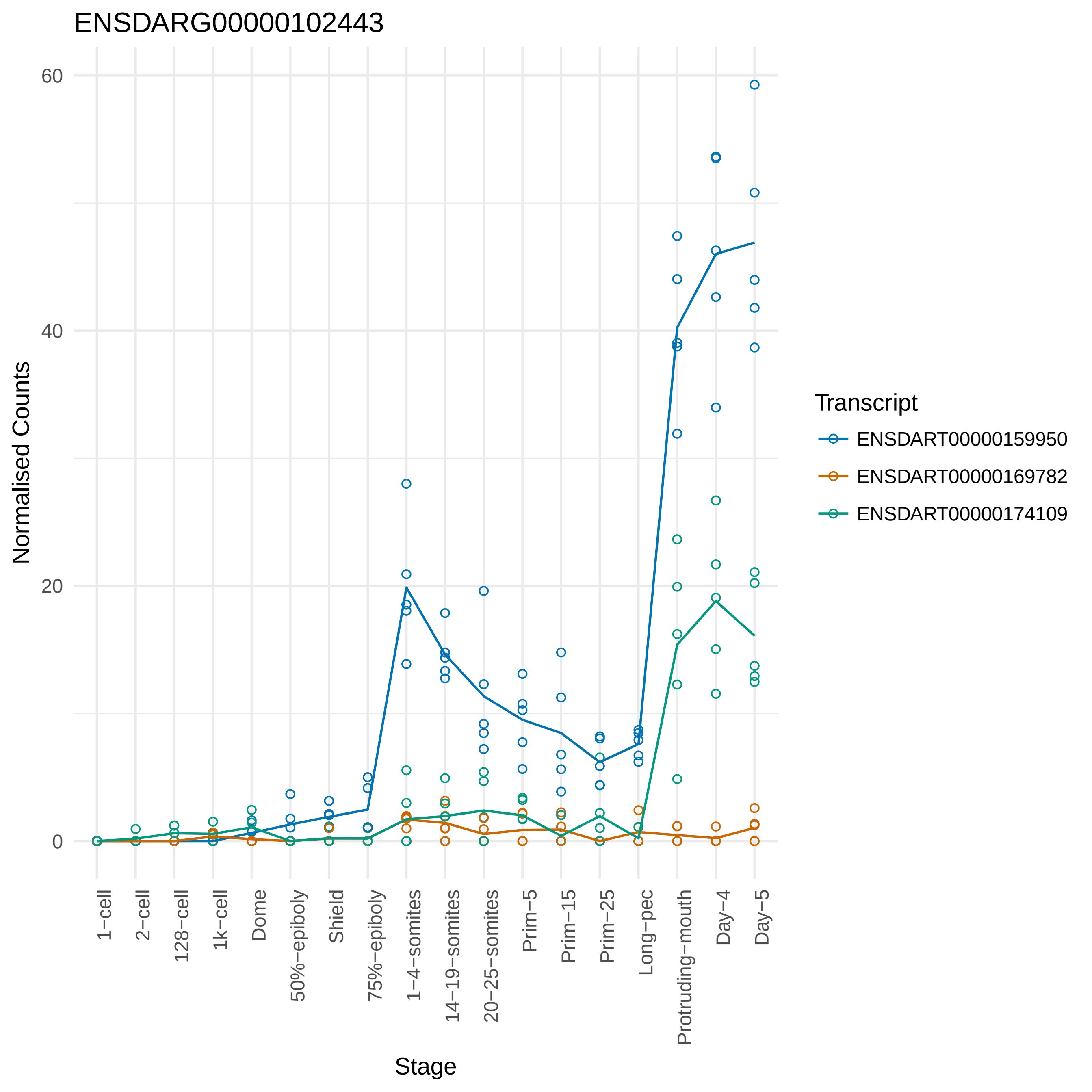
ENSDARG00000102443
Normalised Counts
60
40
20
0
1−cell
2−cell
128−cell
1k−cell
Dome
50%−epiboly
Shield
75%−epiboly
1−4−somites
14−19−somites
20−25−somites
Prim−5
Prim−15
Prim−25
Long−pec
Protruding−mouth
Day−4
Day−5
Stage
Transcript
ENSDART00000159950
ENSDART00000169782
ENSDART00000174109

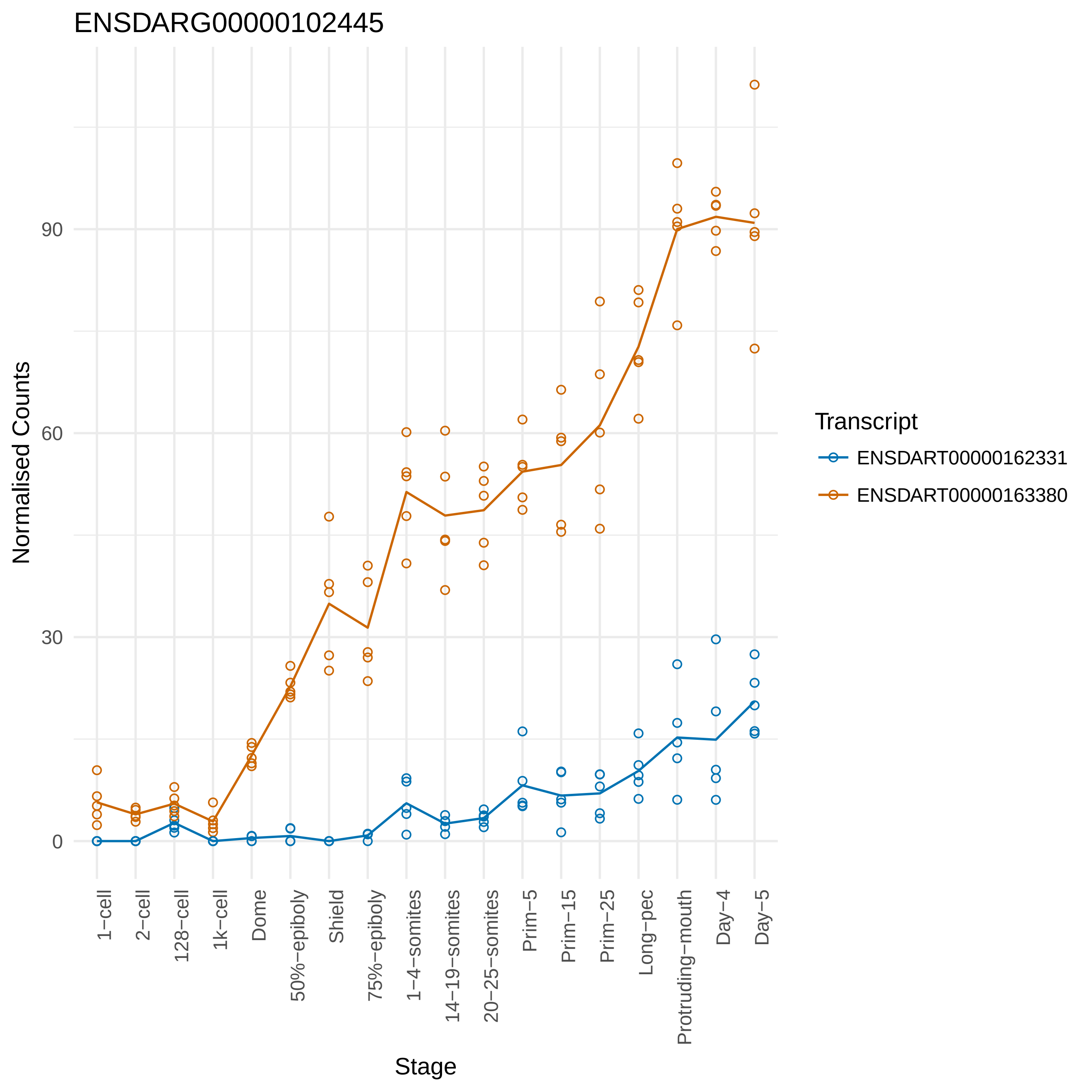

ENSDARG00000102445
Normalised Counts
0
30
60
90
1−cell
2−cell
128−cell
1k−cell
Dome
50%−epiboly
Shield
75%−epiboly
1−4−somites
14−19−somites
20−25−somites
Prim−5
Prim−15
Prim−25
Long−pec
Protruding−mouth
Day−4
Day−5
Stage
Transcript
ENSDART00000162331
ENSDART00000163380

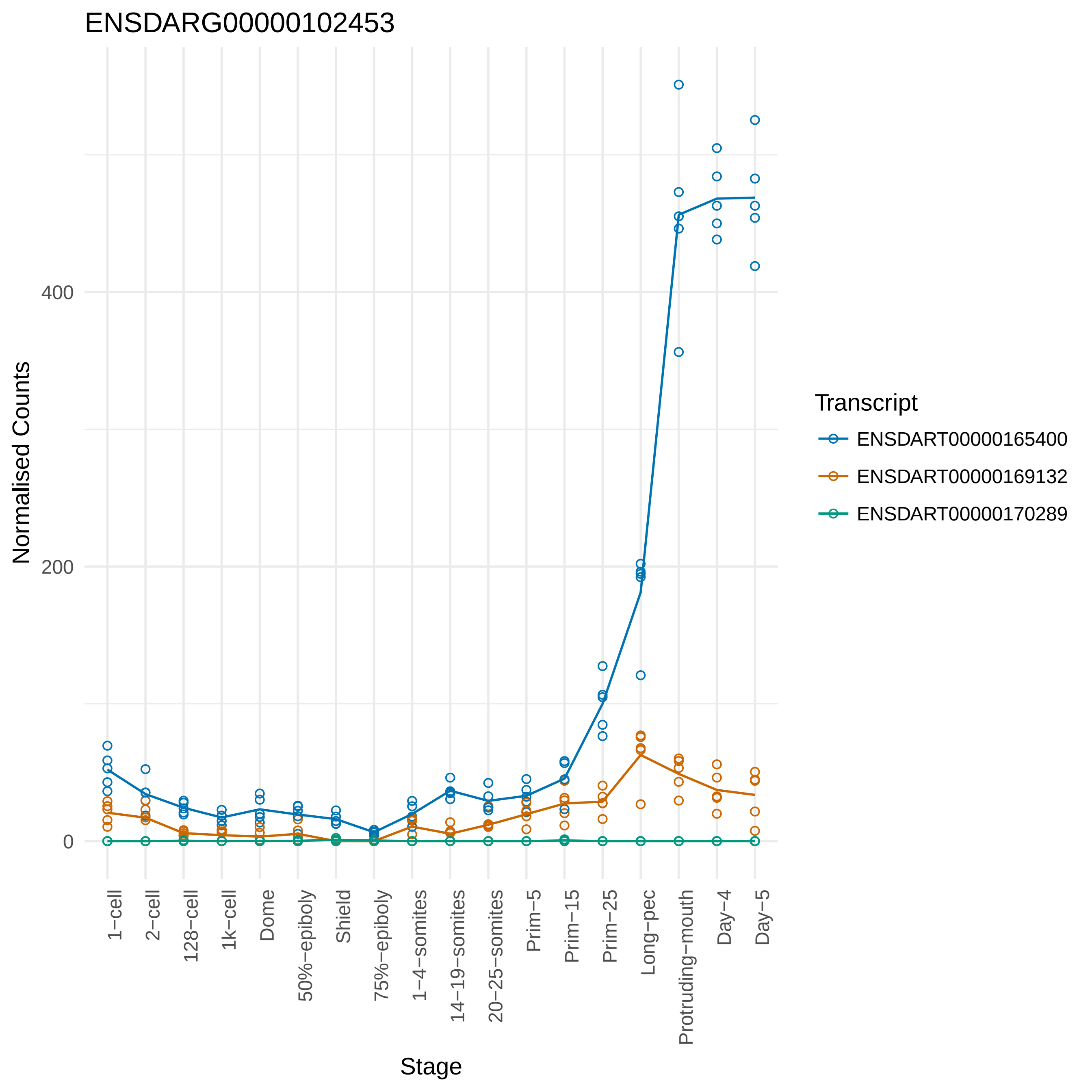
ENSDARG00000102453
Normalised Counts
0
200
400
Stage
1−cell
2−cell
128−cell
1k−cell
Dome
50%−epiboly
Shield
75%−epiboly
1−4−somites
14−19−somites
20−25−somites
Prim−5
Prim−15
Prim−25
Long−pec
Protruding−mouth
Day−4
Day−5
Transcript
ENSDART00000165400
ENSDART00000169132
ENSDART00000170289

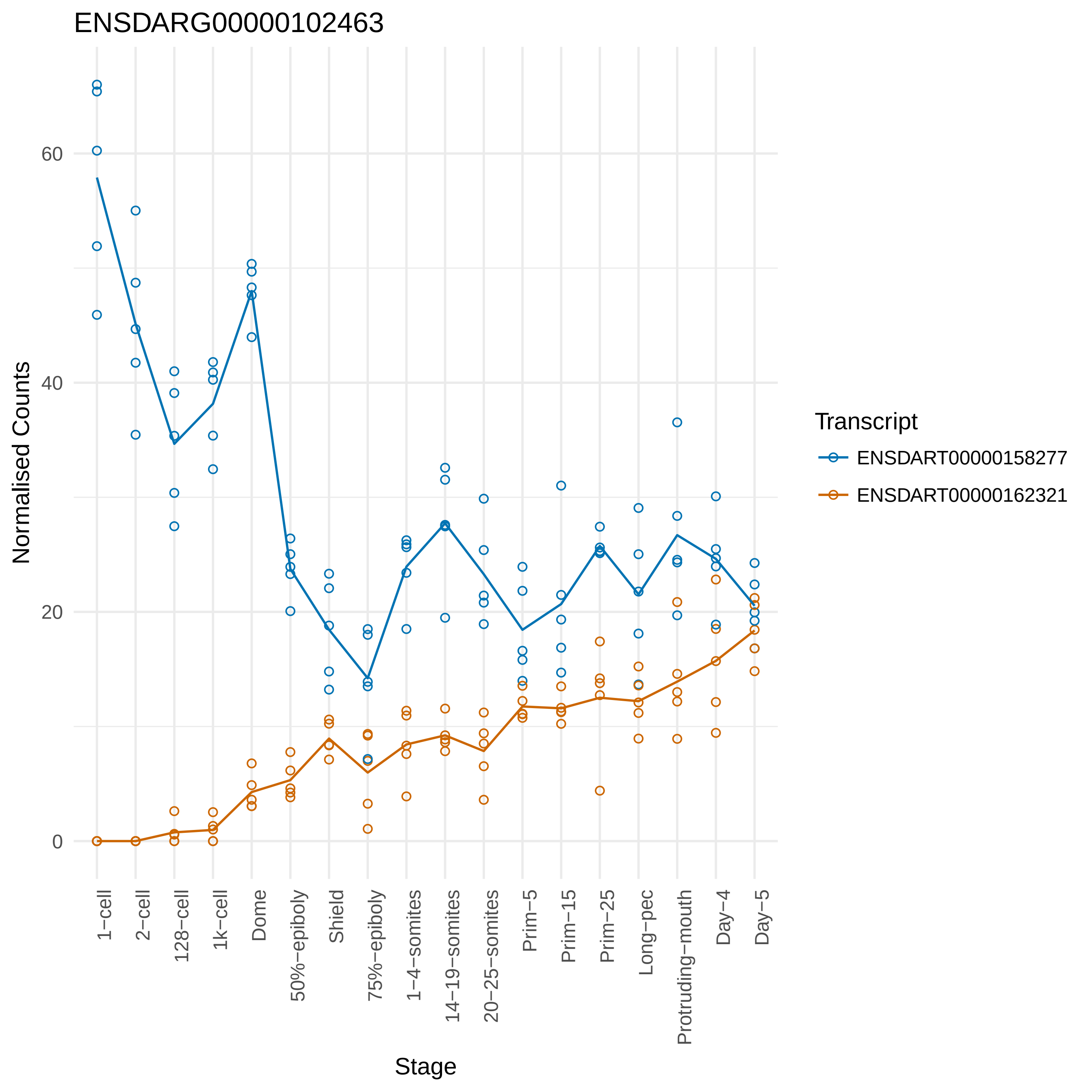

ENSDARG00000102463
Normalised Counts
0
20
40
60
Stage
1−cell
2−cell
128−cell
1k−cell
Dome
50%−epiboly
Shield
75%−epiboly
1−4−somites
14−19−somites
20−25−somites
Prim−5
Prim−15
Prim−25
Long−pec
Protruding−mouth
Day−4
Day−5
Transcript
ENSDART00000158277
ENSDART00000162321

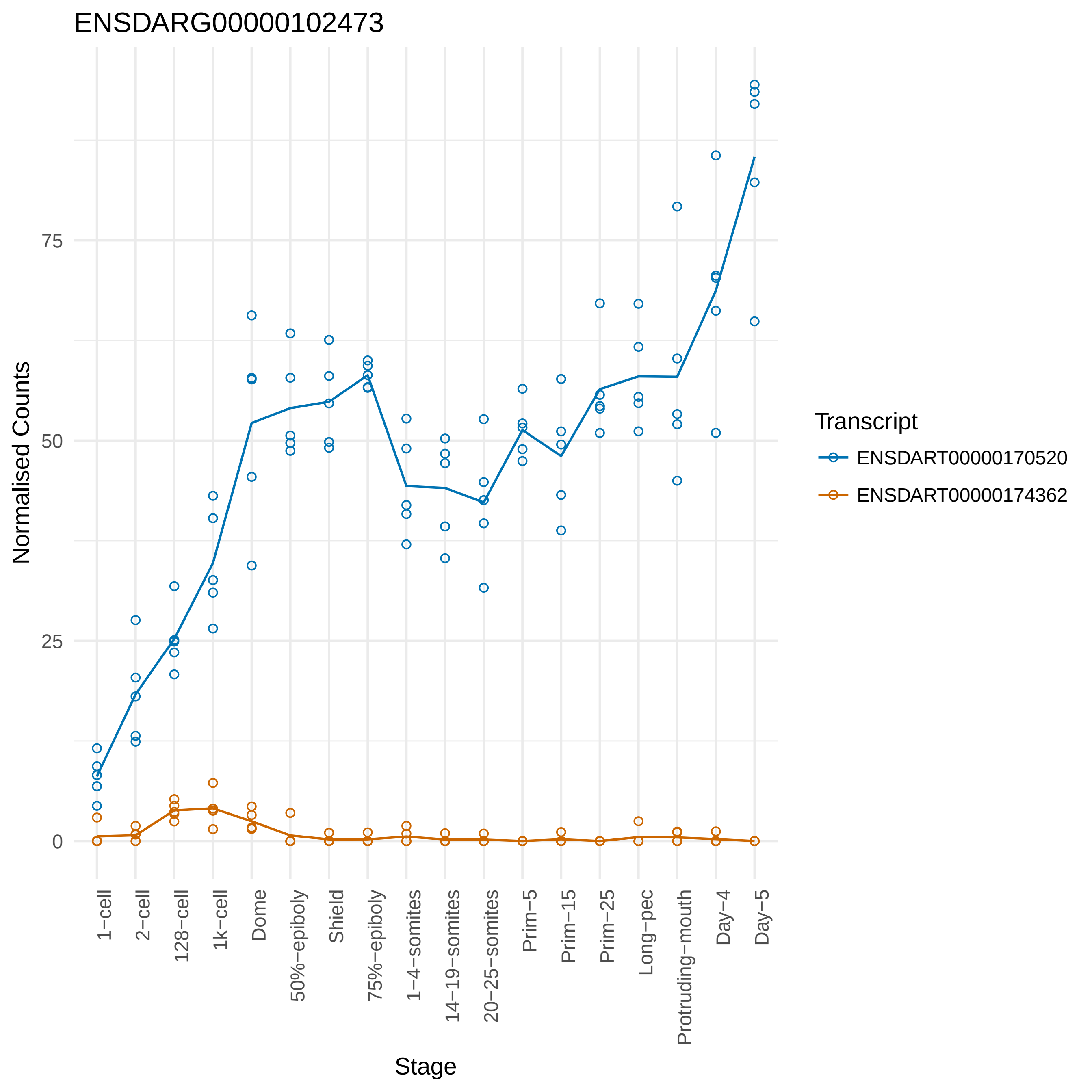

ENSDARG00000102473
Normalised Counts
75
50
25
0
1−cell
2−cell
128−cell
1k−cell
Dome
50%−epiboly
Shield
75%−epiboly
1−4−somites
14−19−somites
20−25−somites
Prim−5
Prim−15
Prim−25
Long−pec
Protruding−mouth
Day−4
Day−5
Stage
Transcript
ENSDART00000170520
ENSDART00000174362

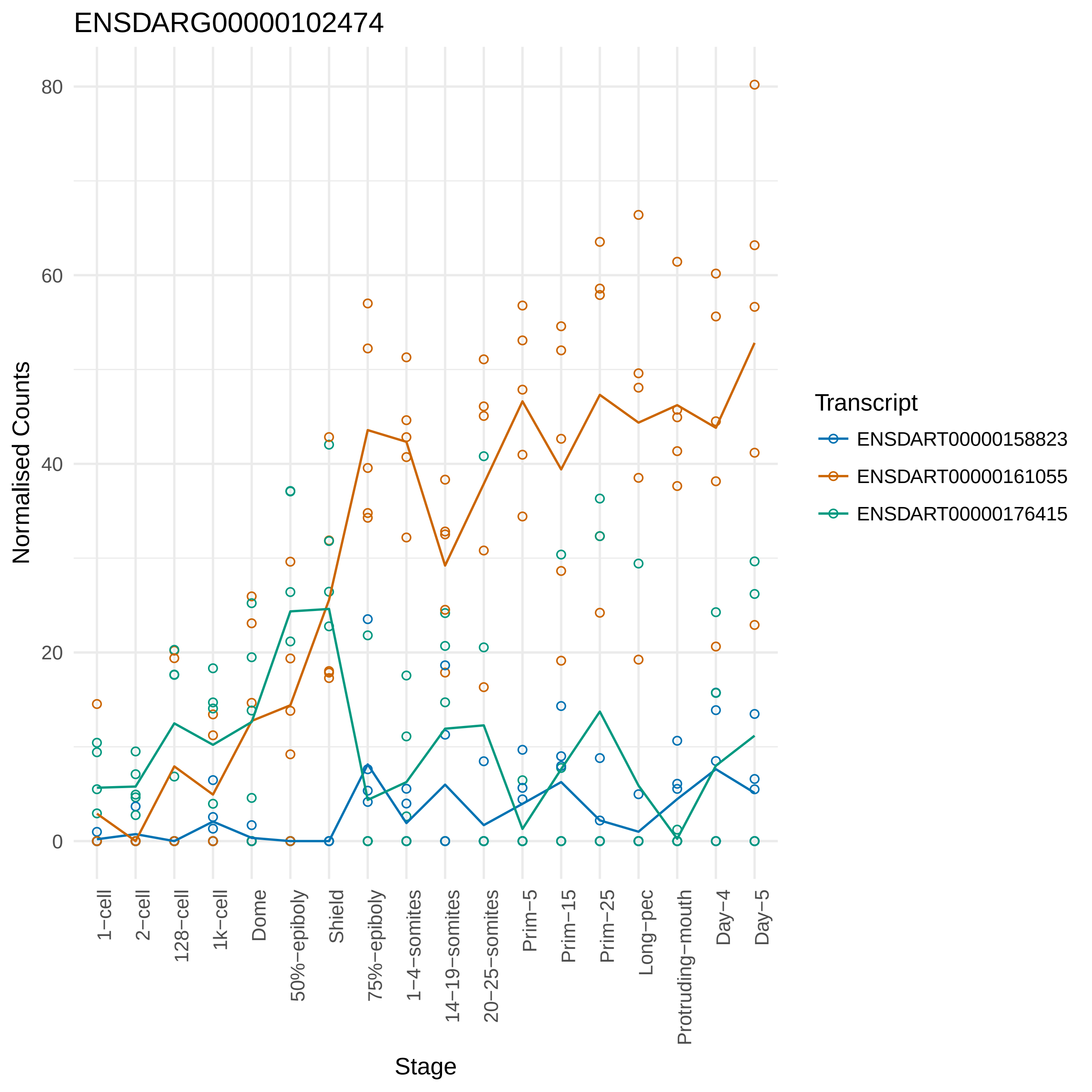
ENSDARG00000102474
Normalised Counts
0
20
40
60
80
Stage
1−cell
2−cell
128−cell
1k−cell
Dome
50%−epiboly
Shield
75%−epiboly
1−4−somites
14−19−somites
20−25−somites
Prim−5
Prim−15
Prim−25
Long−pec
Protruding−mouth
Day−4
Day−5
Transcript
ENSDART00000158823
ENSDART00000161055
ENSDART00000176415

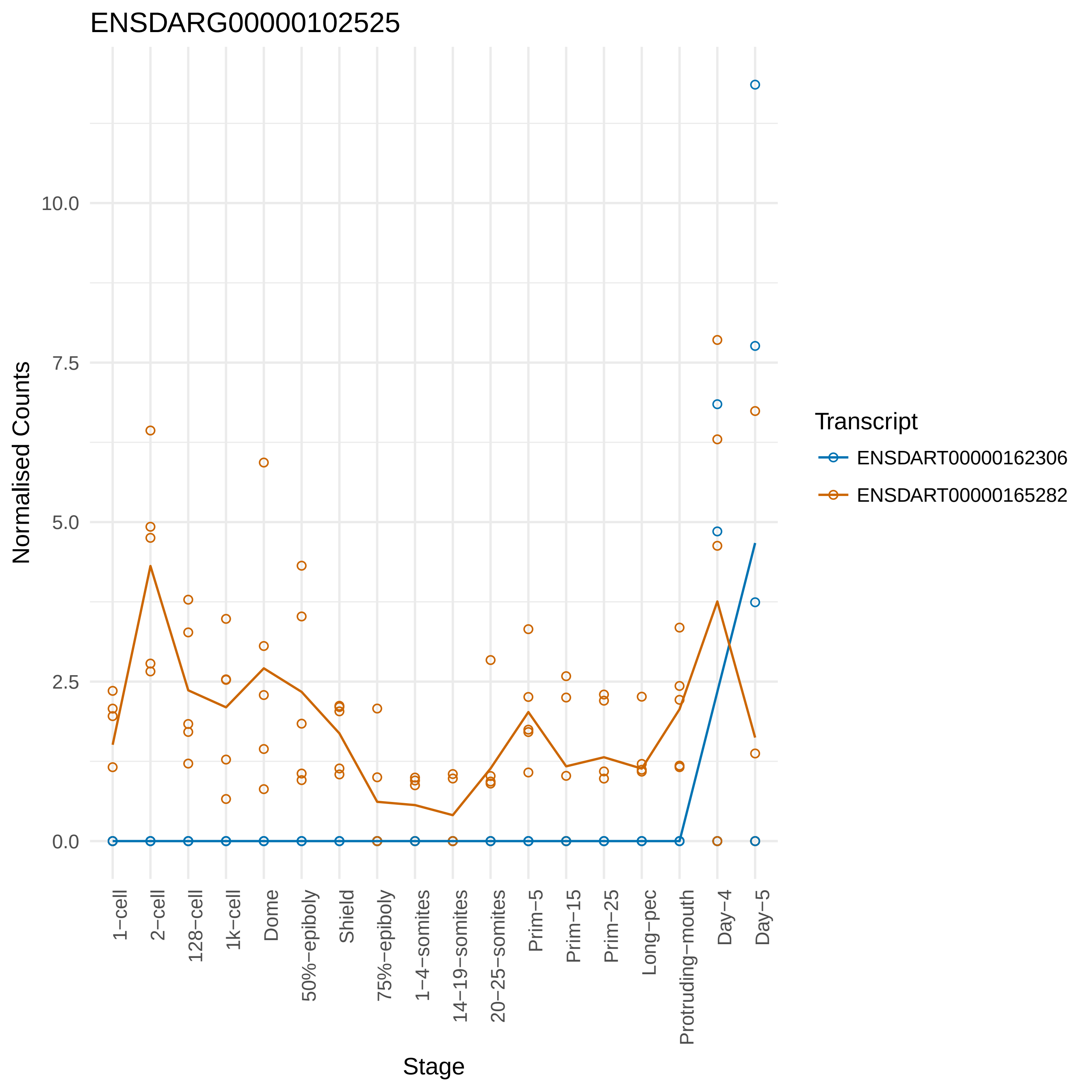
ENSDARG00000102525
Normalised Counts
0.0
2.5
5.0
7.5
10.0
1−cell
2−cell
128−cell
1k−cell
Dome
50%−epiboly
Shield
75%−epiboly
1−4−somites
14−19−somites
20−25−somites
Prim−5
Prim−15
Prim−25
Long−pec
Protruding−mouth
Day−4
Day−5
Stage
Transcript
ENSDART00000162306
ENSDART00000165282

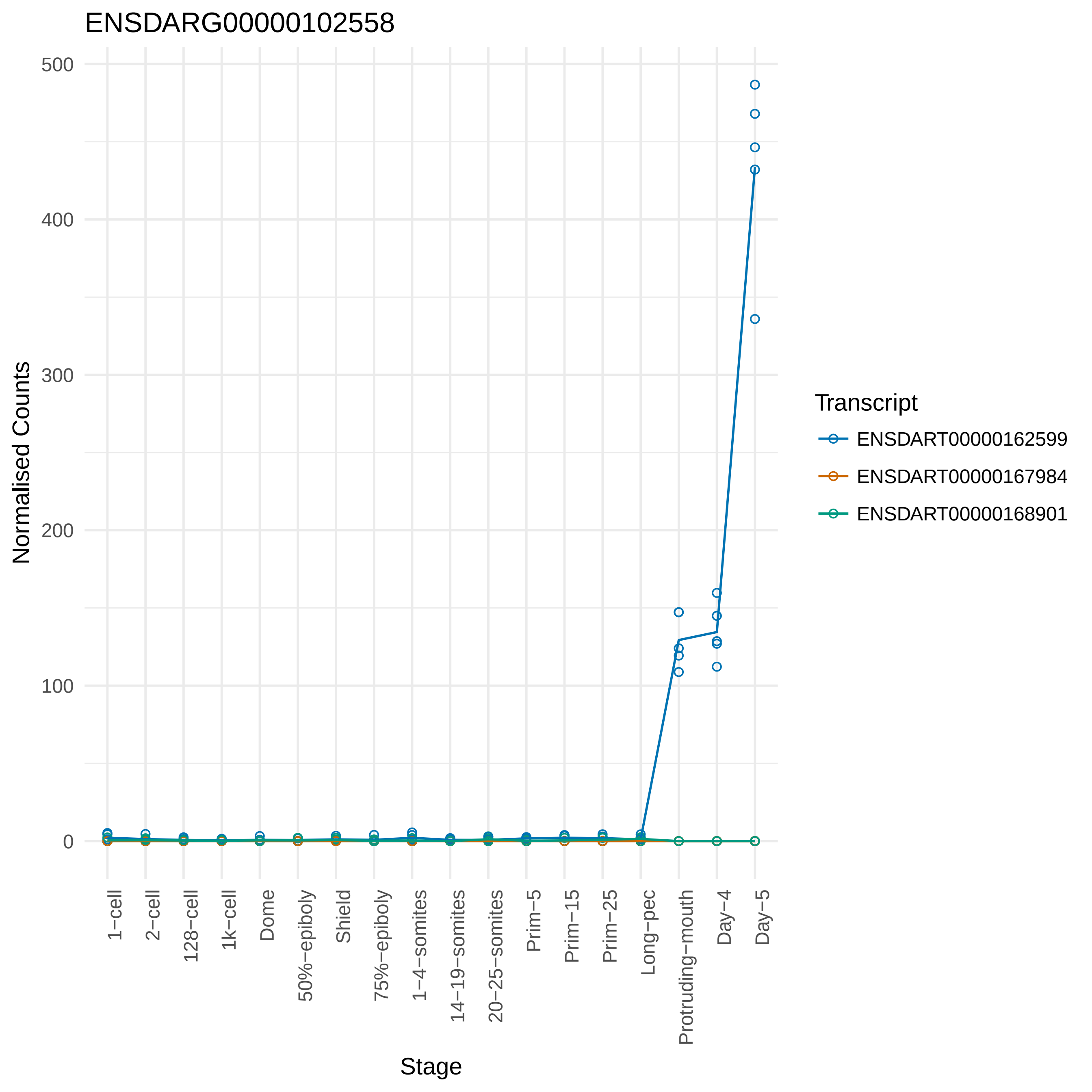

ENSDARG00000102558
Normalised Counts
500
400
300
200
100
0
1−cell
2−cell
128−cell
1k−cell
Dome
50%−epiboly
Shield
75%−epiboly
1−4−somites
14−19−somites
20−25−somites
Prim−5
Prim−15
Prim−25
Long−pec
Protruding−mouth
Day−4
Day−5
Stage
Transcript
ENSDART00000162599
ENSDART00000167984
ENSDART00000168901

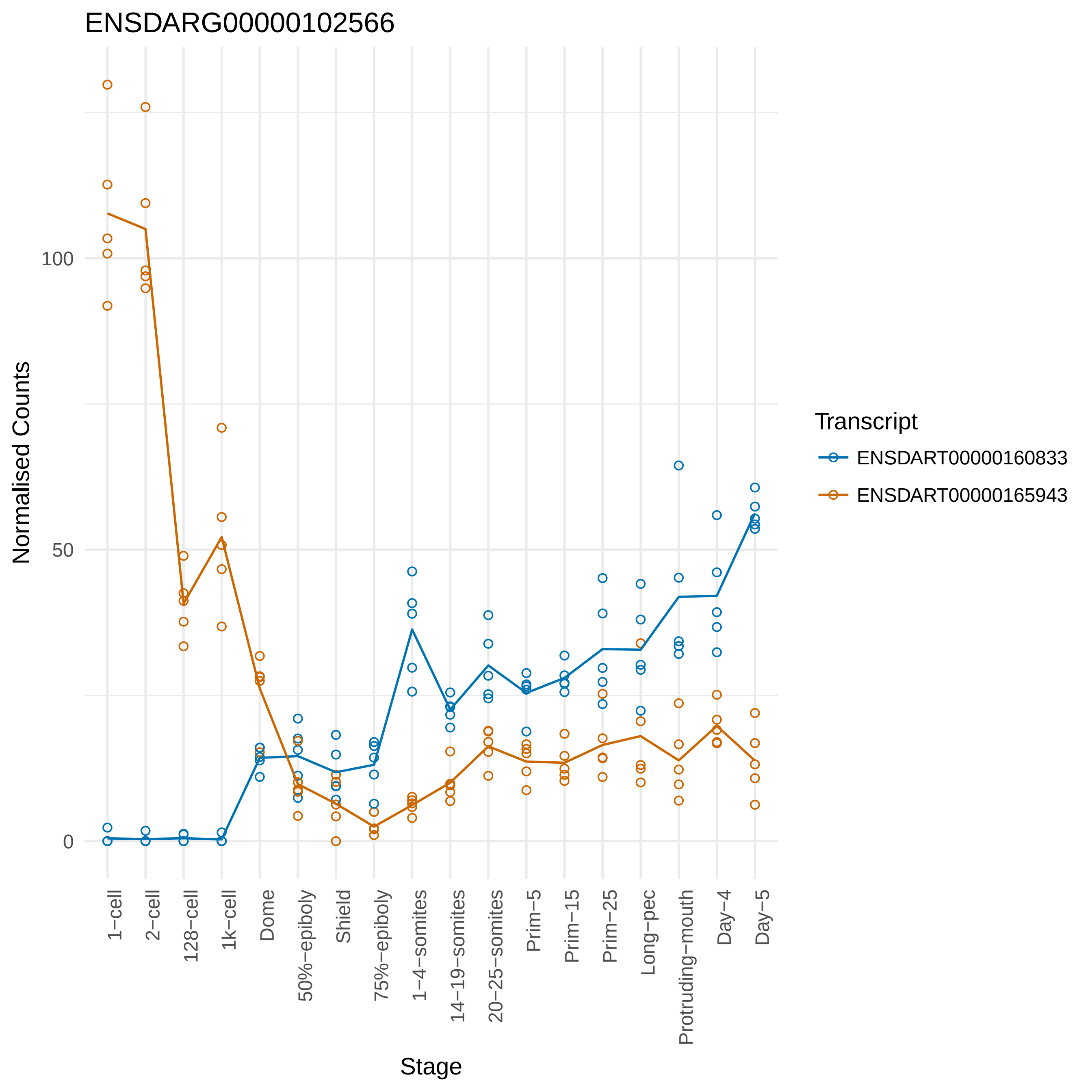

ENSDARG00000102566
Normalised Counts
100
50
0
1−cell
2−cell
128−cell
1k−cell
Dome
50%−epiboly
Shield
75%−epiboly
1−4−somites
14−19−somites
20−25−somites
Prim−5
Prim−15
Prim−25
Long−pec
Protruding−mouth
Day−4
Day−5
Stage
Transcript
ENSDART00000160833
ENSDART00000165943

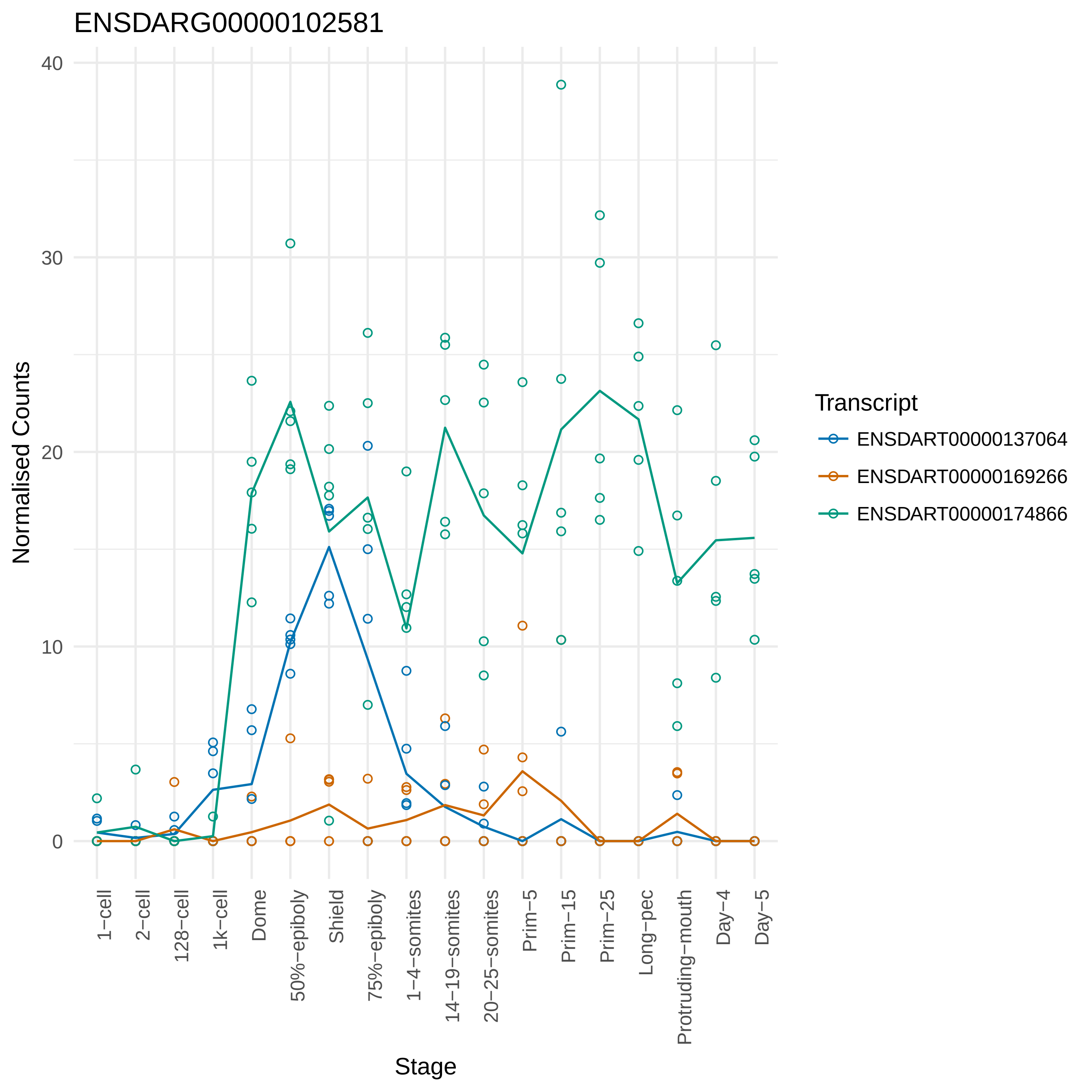

ENSDARG00000102581
Normalised Counts
40
30
20
10
0
1−cell
2−cell
128−cell
1k−cell
Dome
50%−epiboly
Shield
75%−epiboly
1−4−somites
14−19−somites
20−25−somites
Prim−5
Prim−15
Prim−25
Long−pec
Protruding−mouth
Day−4
Day−5
Stage
Transcript
ENSDART00000137064
ENSDART00000169266
ENSDART00000174866

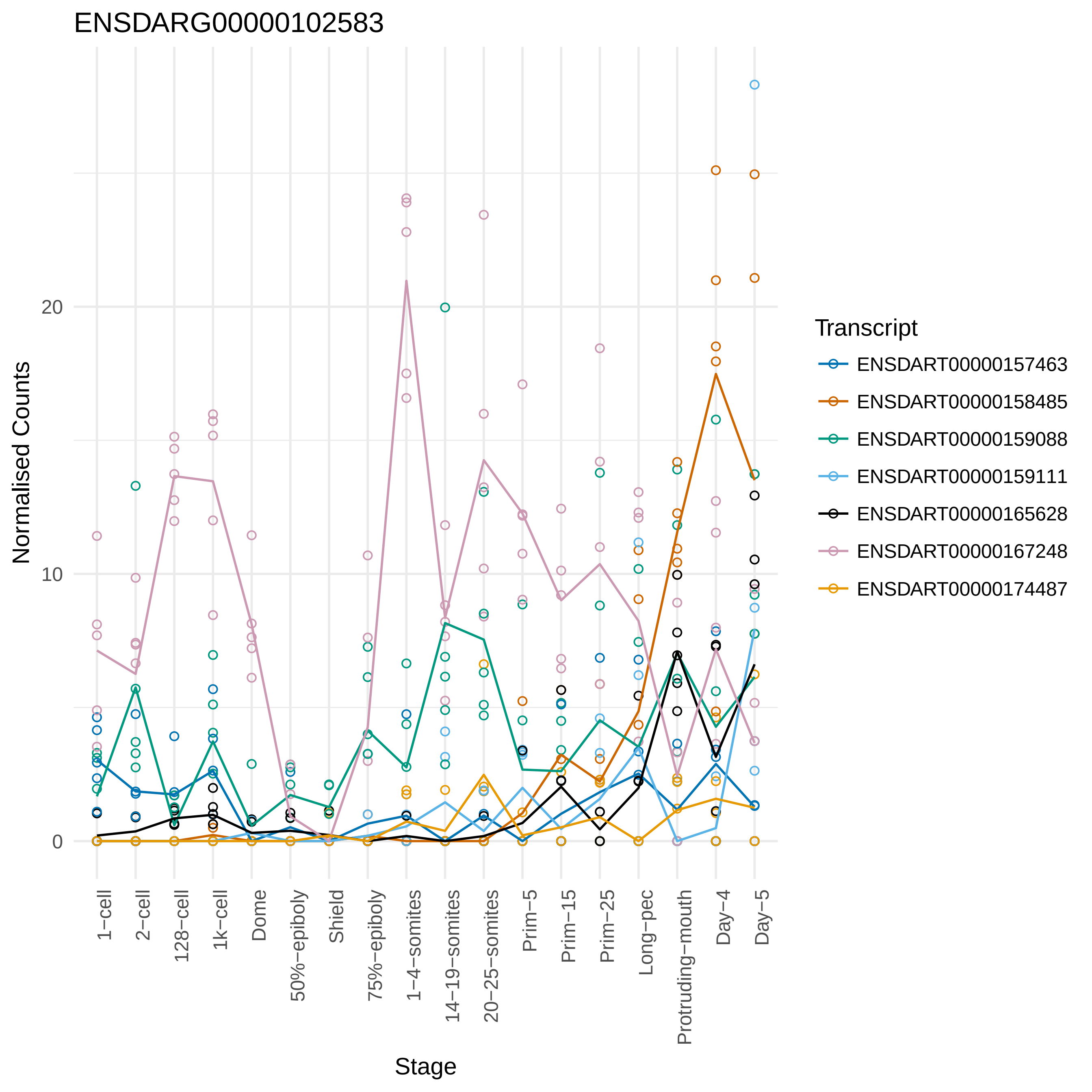

ENSDARG00000102583
Normalised Counts
0
10
20
1−cell
2−cell
128−cell
1k−cell
Dome
50%−epiboly
Shield
75%−epiboly
1−4−somites
14−19−somites
20−25−somites
Prim−5
Prim−15
Prim−25
Long−pec
Protruding−mouth
Day−4
Day−5
Stage
Transcript
ENSDART00000157463
ENSDART00000158485
ENSDART00000159088
ENSDART00000159111
ENSDART00000165628
ENSDART00000167248
ENSDART00000174487

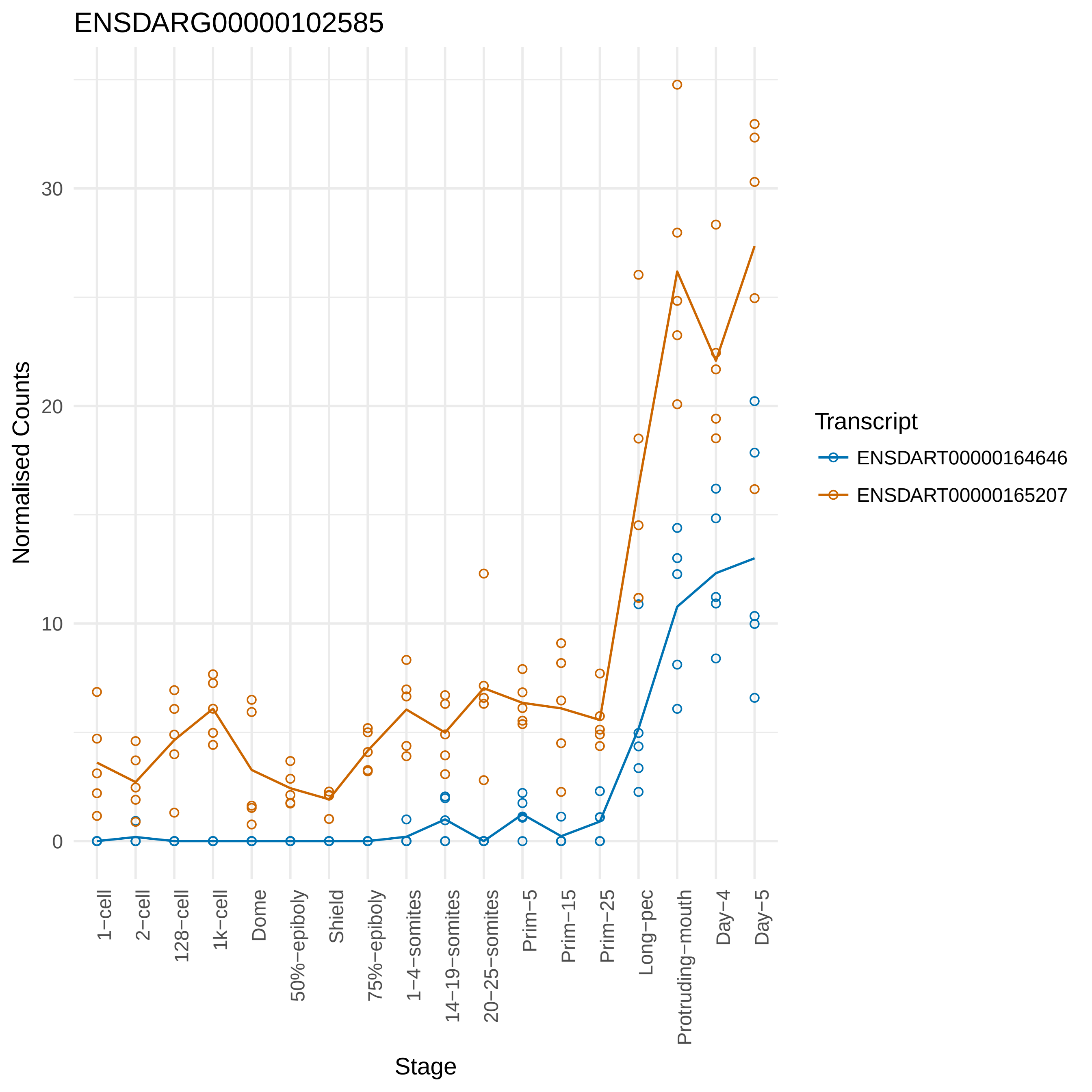

ENSDARG00000102585
Normalised Counts
0
10
20
30
1−cell
2−cell
128−cell
1k−cell
Dome
50%−epiboly
Shield
75%−epiboly
1−4−somites
14−19−somites
20−25−somites
Prim−5
Prim−15
Prim−25
Long−pec
Protruding−mouth
Day−4
Day−5
Stage
Transcript
ENSDART00000164646
ENSDART00000165207

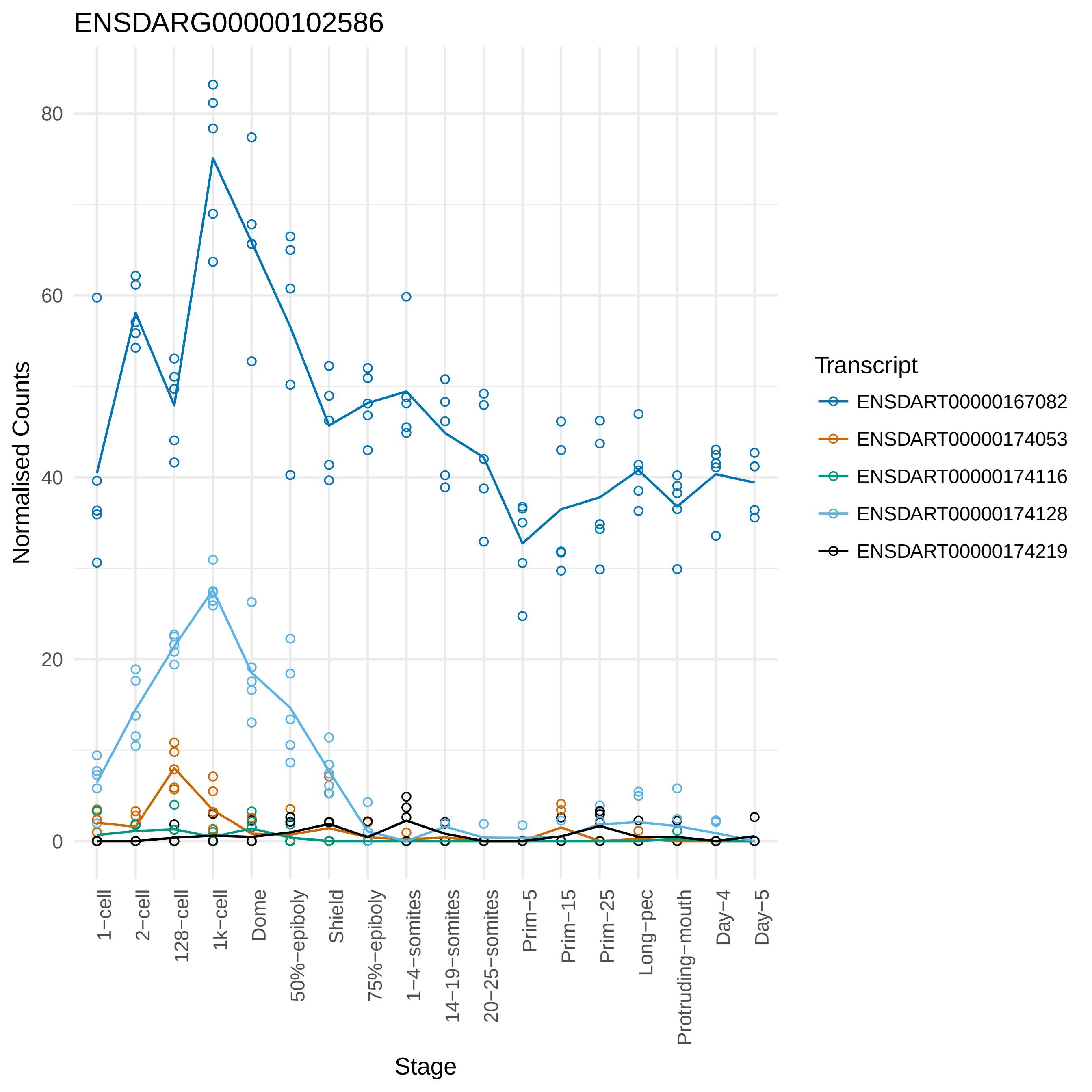

ENSDARG00000102586
Normalised Counts
0
20
40
60
80
1−cell
2−cell
128−cell
1k−cell
Dome
50%−epiboly
Shield
75%−epiboly
1−4−somites
14−19−somites
20−25−somites
Prim−5
Prim−15
Prim−25
Long−pec
Protruding−mouth
Day−4
Day−5
Stage
Transcript
ENSDART00000167082
ENSDART00000174053
ENSDART00000174116
ENSDART00000174128
ENSDART00000174219

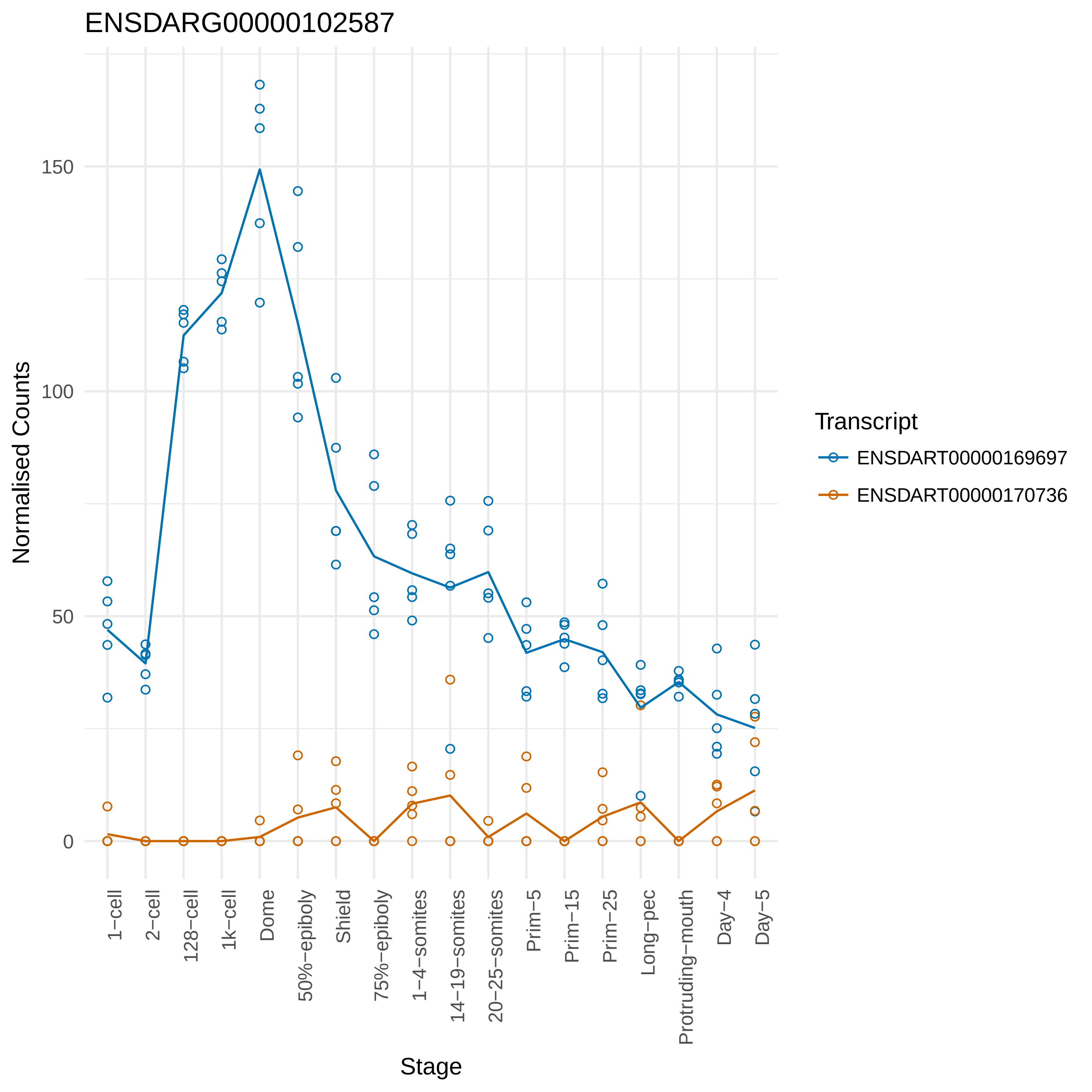

ENSDARG00000102587
Normalised Counts
150
100
50
0
Stage
1−cell
2−cell
128−cell
1k−cell
Dome
50%−epiboly
Shield
75%−epiboly
1−4−somites
14−19−somites
20−25−somites
Prim−5
Prim−15
Prim−25
Long−pec
Protruding−mouth
Day−4
Day−5
Transcript
ENSDART00000169697
ENSDART00000170736

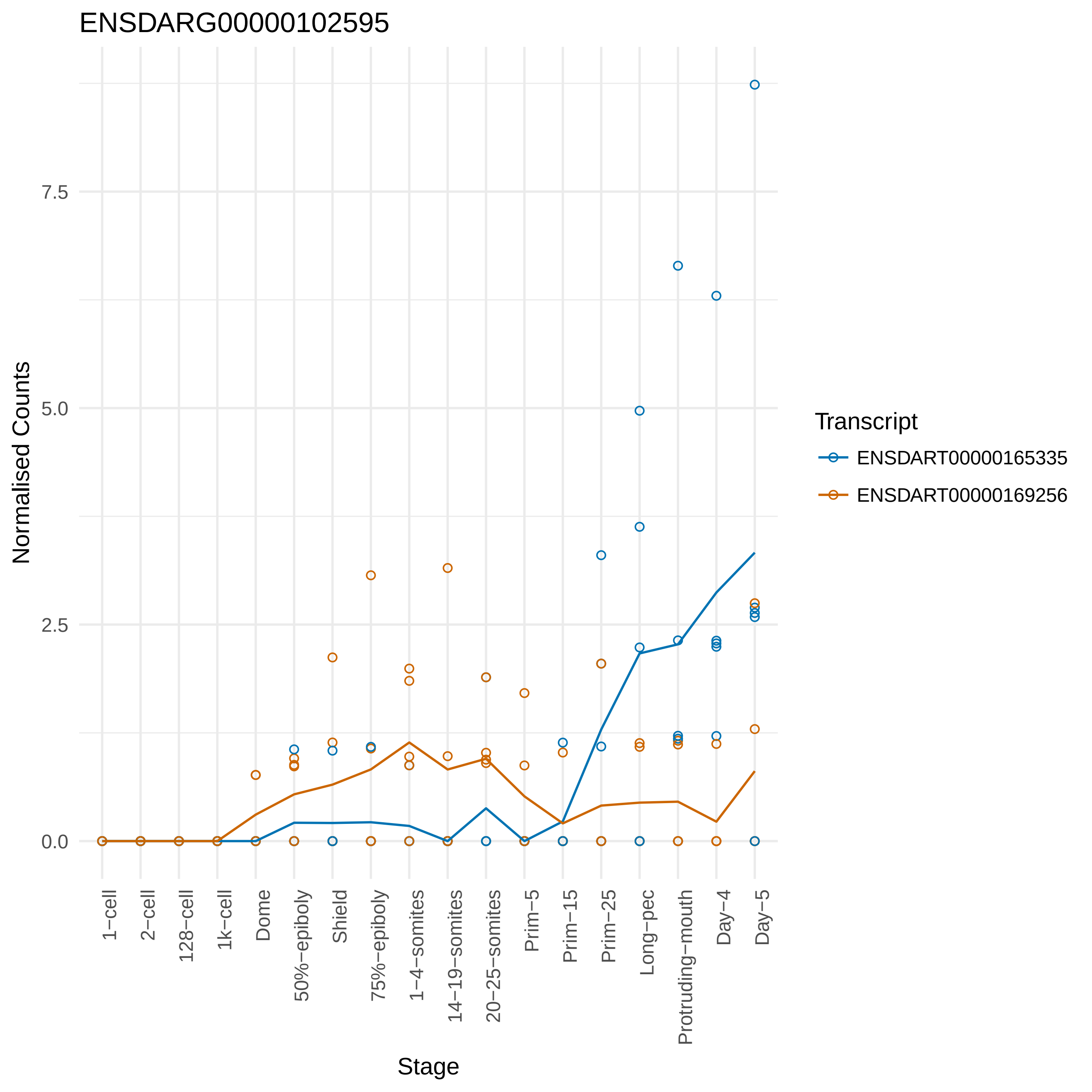

ENSDARG00000102595
Normalised Counts
0.0
2.5
5.0
7.5
Stage
1−cell
2−cell
128−cell
1k−cell
Dome
50%−epiboly
Shield
75%−epiboly
1−4−somites
14−19−somites
20−25−somites
Prim−5
Prim−15
Prim−25
Long−pec
Protruding−mouth
Day−4
Day−5
Transcript
ENSDART00000165335
ENSDART00000169256

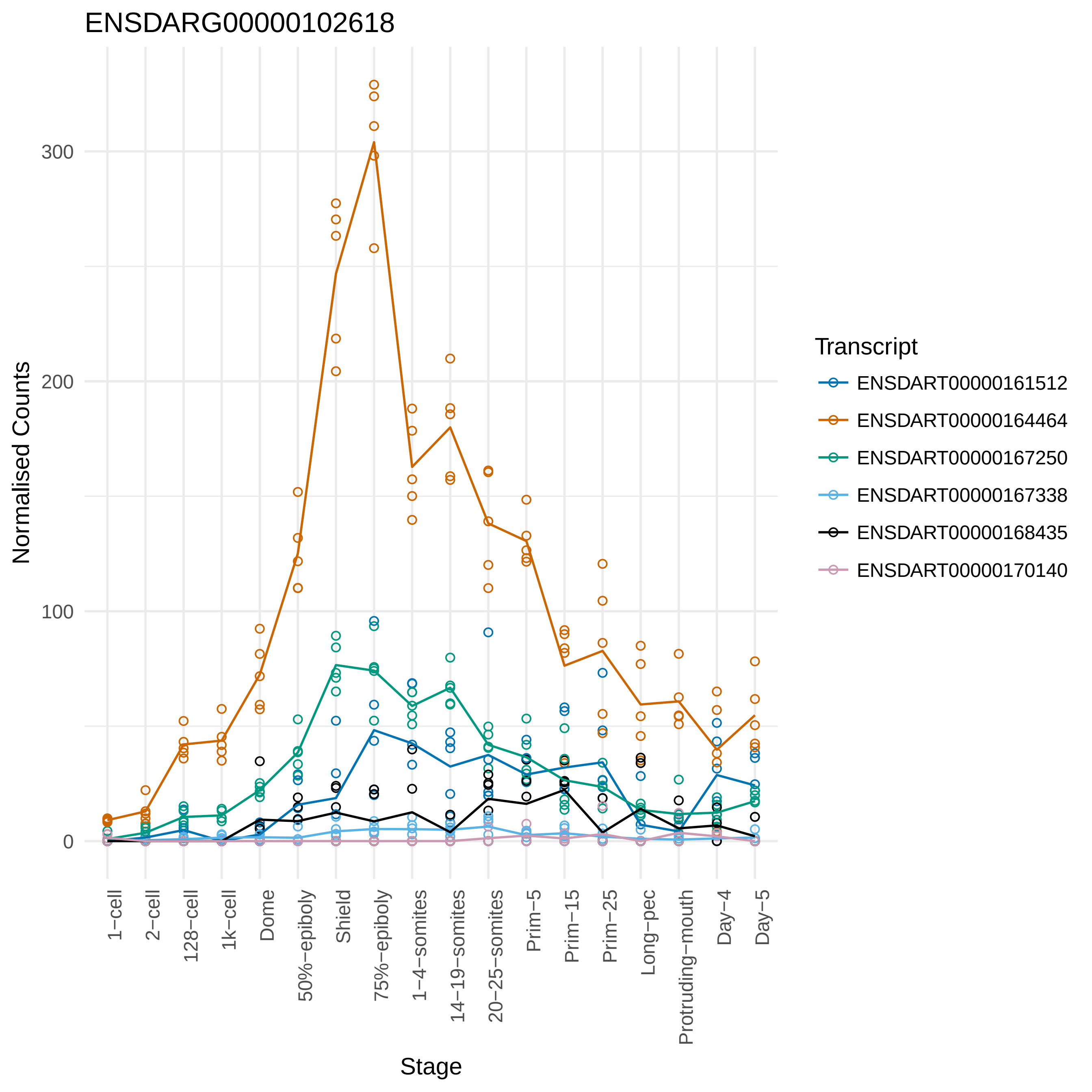

ENSDARG00000102618
Normalised Counts
300
200
100
0
1−cell
2−cell
128−cell
1k−cell
Dome
50%−epiboly
Shield
75%−epiboly
1−4−somites
14−19−somites
20−25−somites
Prim−5
Prim−15
Prim−25
Long−pec
Protruding−mouth
Day−4
Day−5
Stage
Transcript
ENSDART00000161512
ENSDART00000164464
ENSDART00000167250
ENSDART00000167338
ENSDART00000168435
ENSDART00000170140

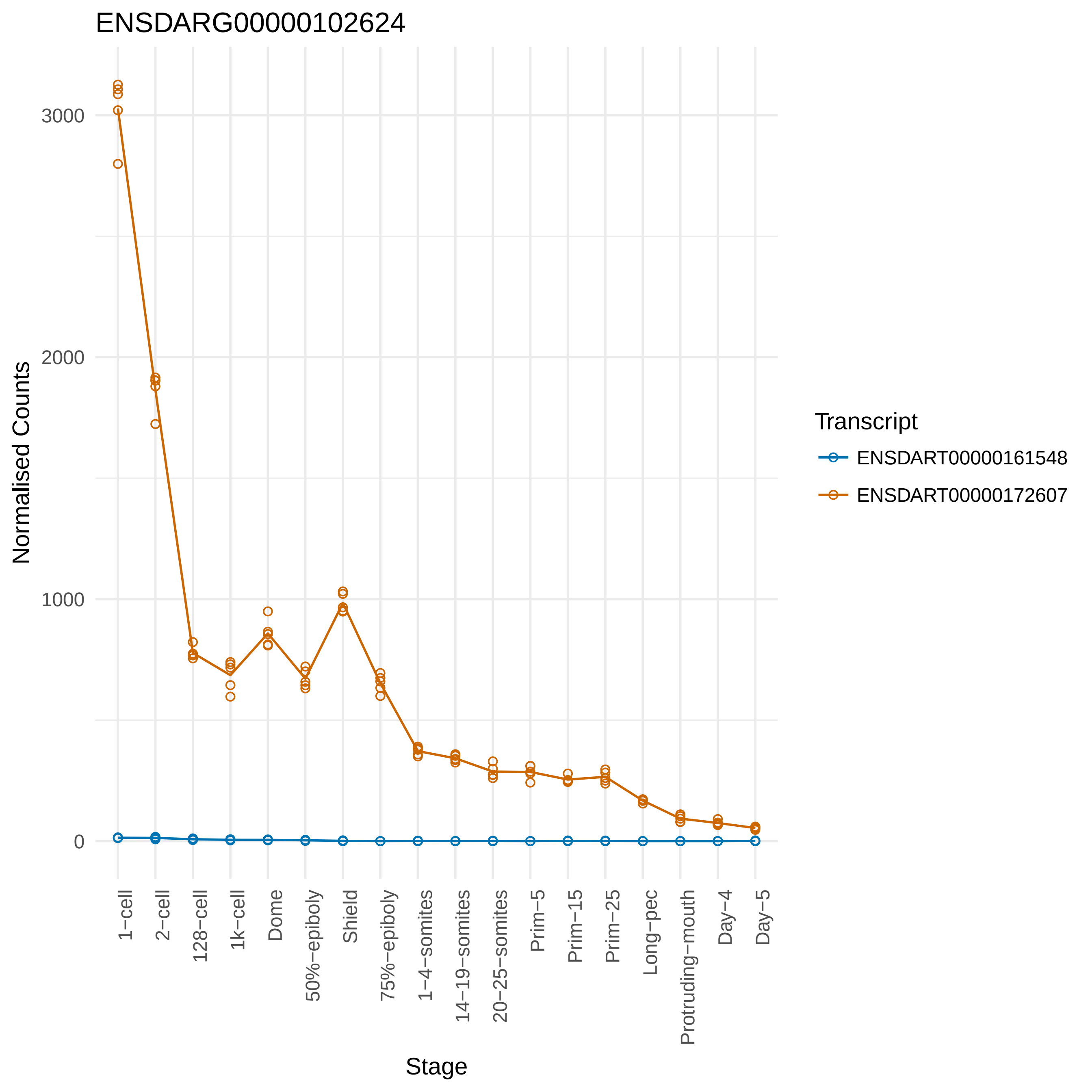

ENSDARG00000102624
Normalised Counts
3000
2000
1000
0
1−cell
2−cell
128−cell
1k−cell
Dome
50%−epiboly
Shield
75%−epiboly
1−4−somites
14−19−somites
20−25−somites
Prim−5
Prim−15
Prim−25
Long−pec
Protruding−mouth
Day−4
Day−5
Stage
Transcript
ENSDART00000161548
ENSDART00000172607

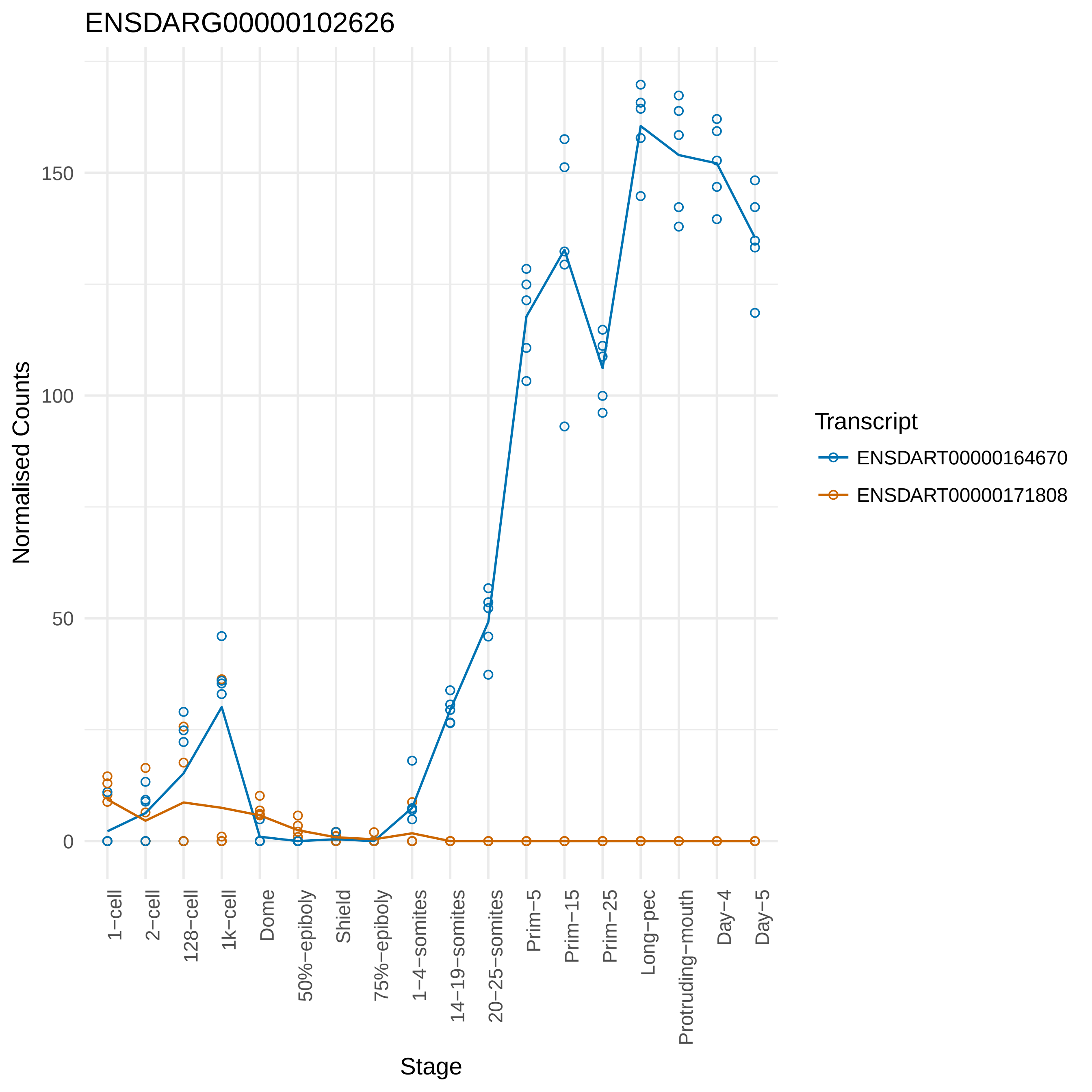

ENSDARG00000102626
Normalised Counts
0
50
100
150
Stage
1−cell
2−cell
128−cell
1k−cell
Dome
50%−epiboly
Shield
75%−epiboly
1−4−somites
14−19−somites
20−25−somites
Prim−5
Prim−15
Prim−25
Long−pec
Protruding−mouth
Day−4
Day−5
Transcript
ENSDART00000164670
ENSDART00000171808

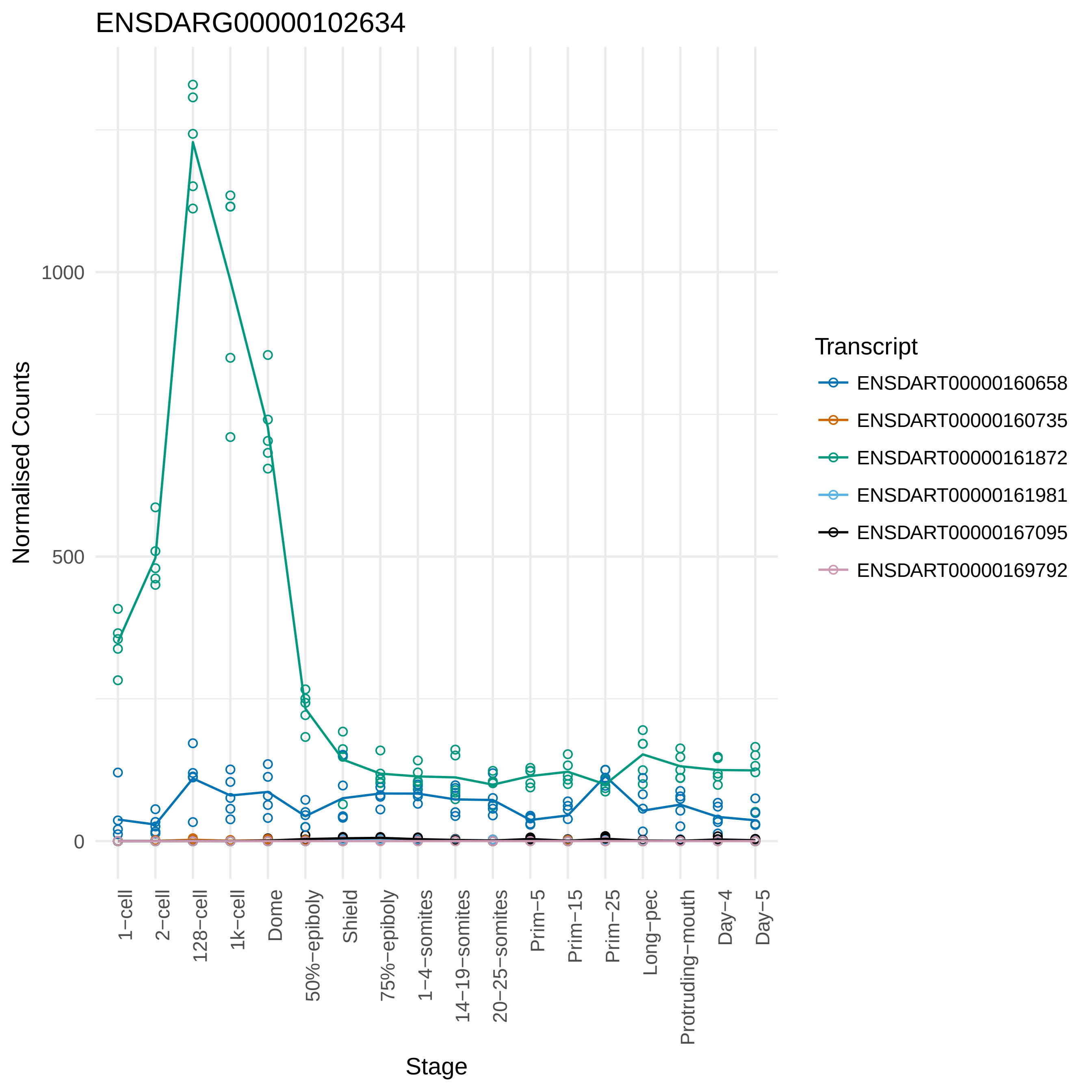

ENSDARG00000102634
Normalised Counts
1000
500
0
1−cell
2−cell
128−cell
1k−cell
Dome
50%−epiboly
Shield
75%−epiboly
1−4−somites
14−19−somites
20−25−somites
Prim−5
Prim−15
Prim−25
Long−pec
Protruding−mouth
Day−4
Day−5
Stage
Transcript
ENSDART00000160658
ENSDART00000160735
ENSDART00000161872
ENSDART00000161981
ENSDART00000167095
ENSDART00000169792

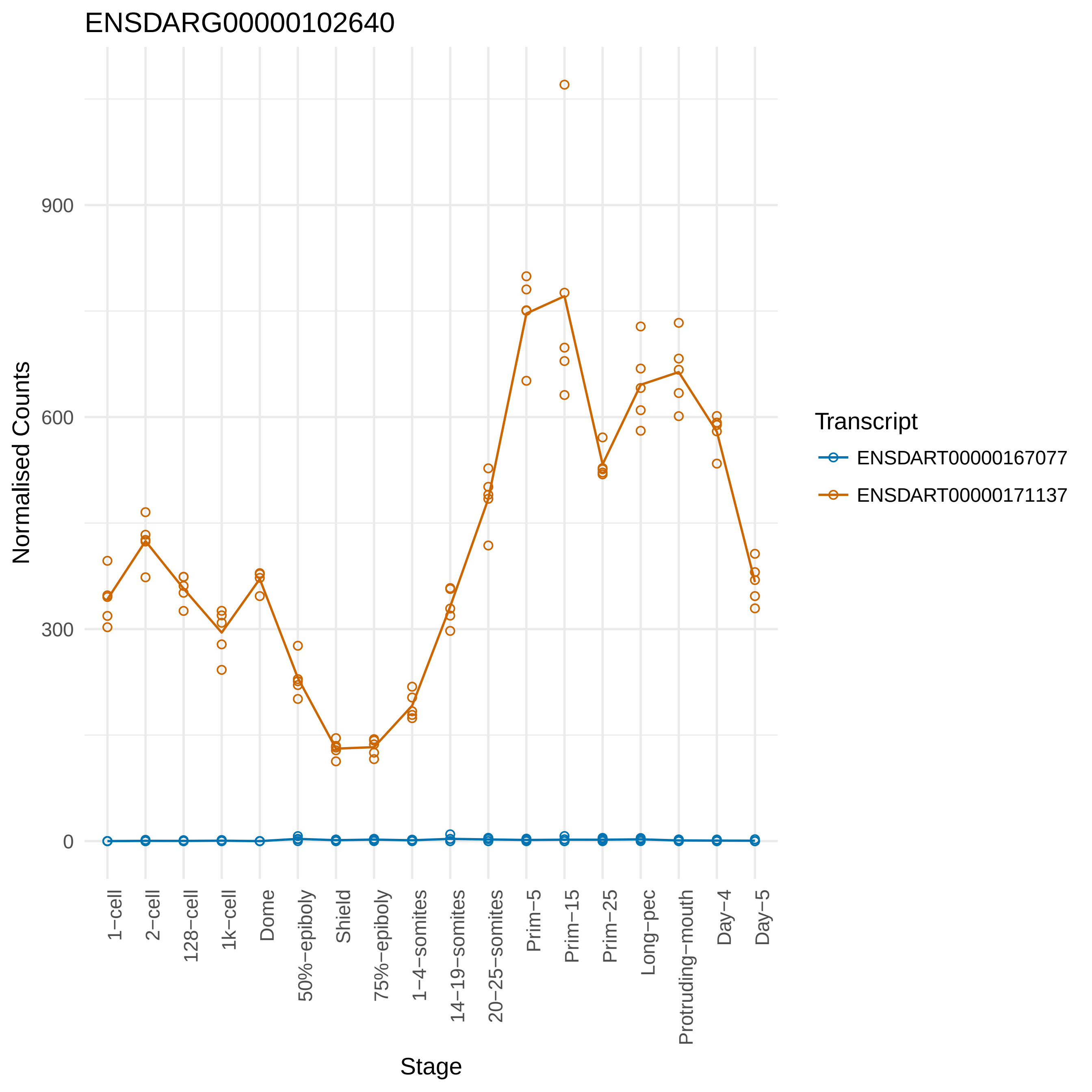

ENSDARG00000102640
Normalised Counts
900
600
300
0
1−cell
2−cell
128−cell
1k−cell
Dome
50%−epiboly
Shield
75%−epiboly
1−4−somites
14−19−somites
20−25−somites
Prim−5
Prim−15
Prim−25
Long−pec
Protruding−mouth
Day−4
Day−5
Stage
Transcript
ENSDART00000167077
ENSDART00000171137

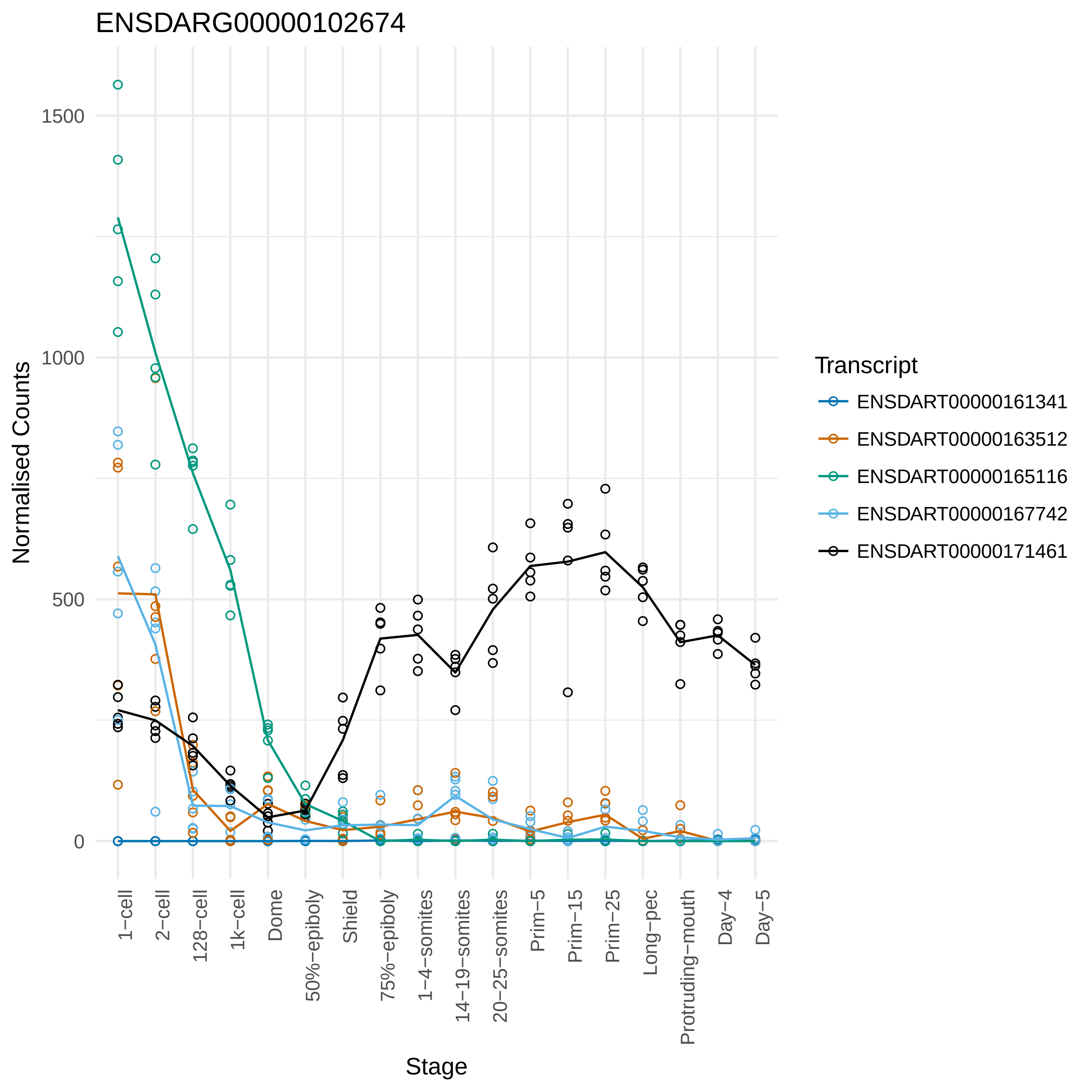

ENSDARG00000102674
Normalised Counts
0
500
1000
1500
1−cell
2−cell
128−cell
1k−cell
Dome
50%−epiboly
Shield
75%−epiboly
1−4−somites
14−19−somites
20−25−somites
Prim−5
Prim−15
Prim−25
Long−pec
Protruding−mouth
Day−4
Day−5
Stage
Transcript
ENSDART00000161341
ENSDART00000163512
ENSDART00000165116
ENSDART00000167742
ENSDART00000171461

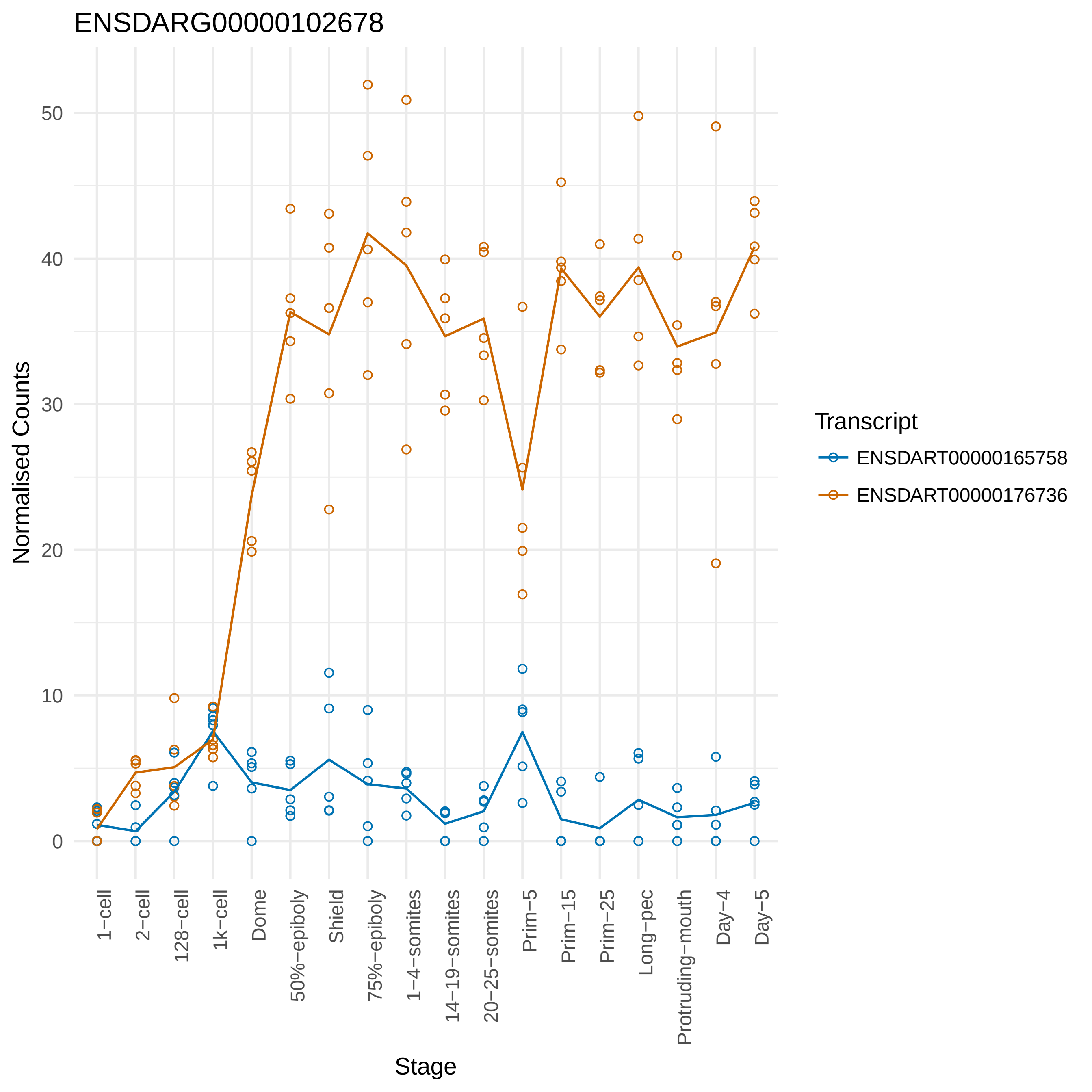
ENSDARG00000102678
Normalised Counts
0
10
20
30
40
50
1−cell
2−cell
128−cell
1k−cell
Dome
50%−epiboly
Shield
75%−epiboly
1−4−somites
14−19−somites
20−25−somites
Prim−5
Prim−15
Prim−25
Long−pec
Protruding−mouth
Day−4
Day−5
Stage
Transcript
ENSDART00000165758
ENSDART00000176736

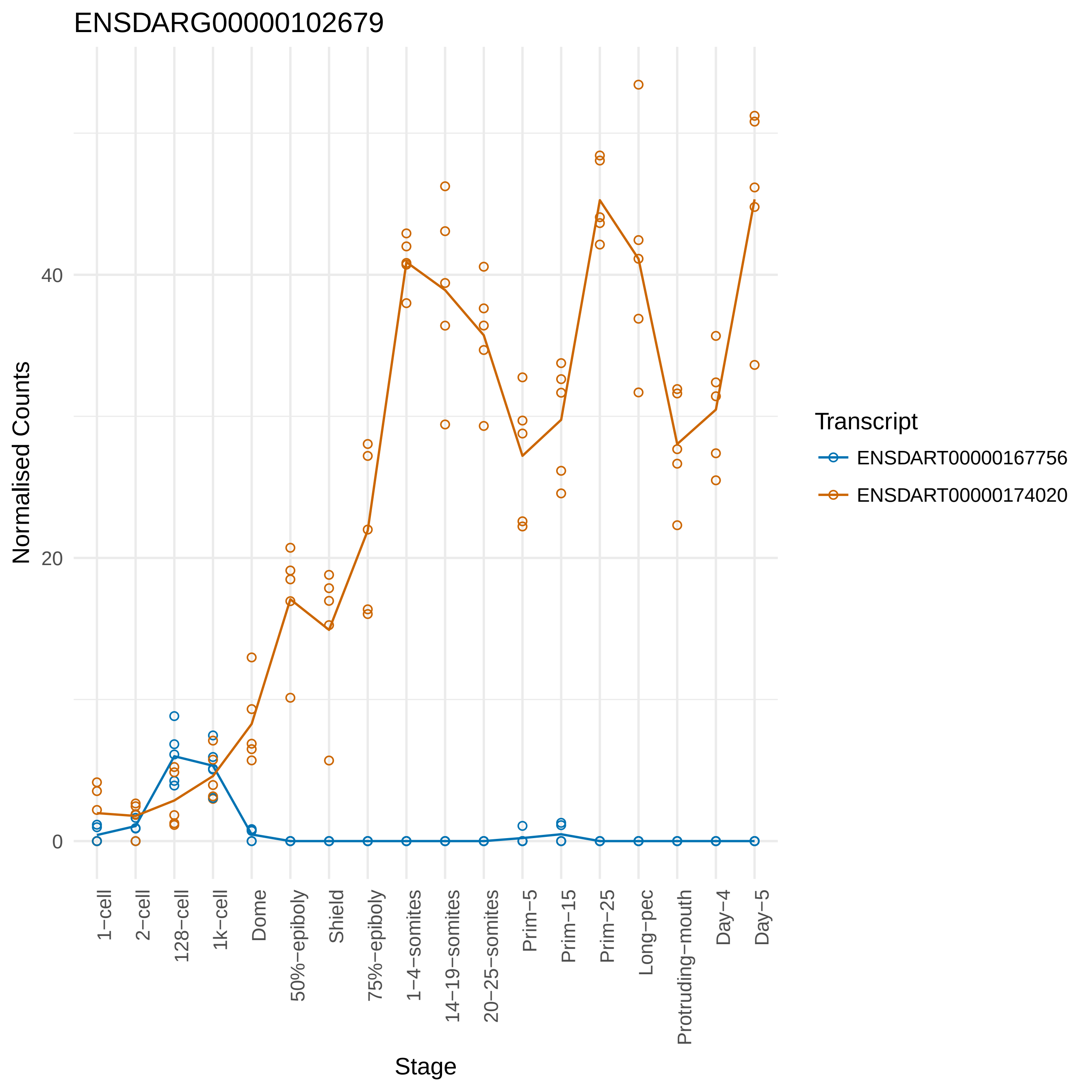

ENSDARG00000102679
Normalised Counts
0
20
40
Stage
1−cell
2−cell
128−cell
1k−cell
Dome
50%−epiboly
Shield
75%−epiboly
1−4−somites
14−19−somites
20−25−somites
Prim−5
Prim−15
Prim−25
Long−pec
Protruding−mouth
Day−4
Day−5
Transcript
ENSDART00000167756
ENSDART00000174020

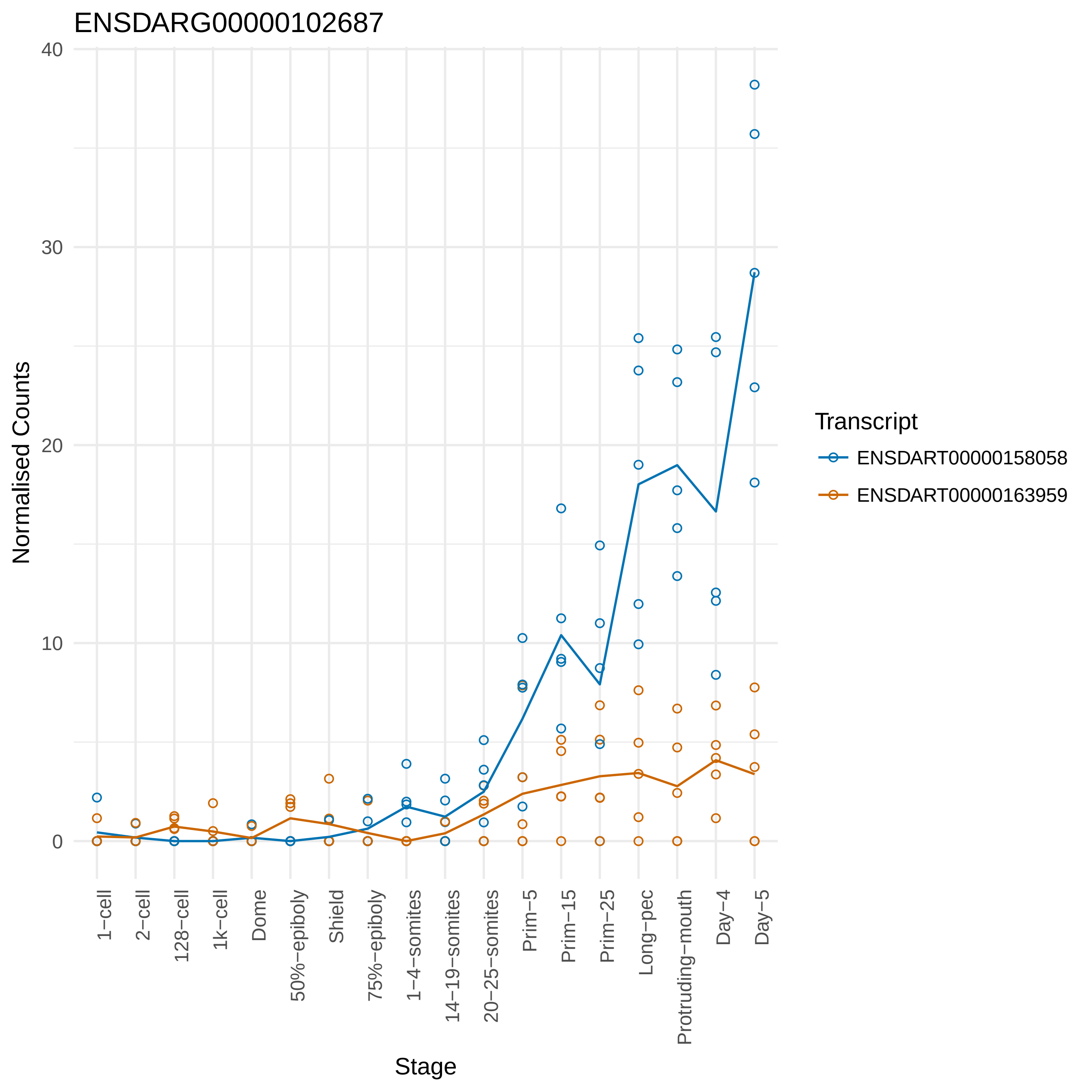

ENSDARG00000102687
Normalised Counts
0
10
20
30
40
1−cell
2−cell
128−cell
1k−cell
Dome
50%−epiboly
Shield
75%−epiboly
1−4−somites
14−19−somites
20−25−somites
Prim−5
Prim−15
Prim−25
Long−pec
Protruding−mouth
Day−4
Day−5
Stage
Transcript
ENSDART00000158058
ENSDART00000163959

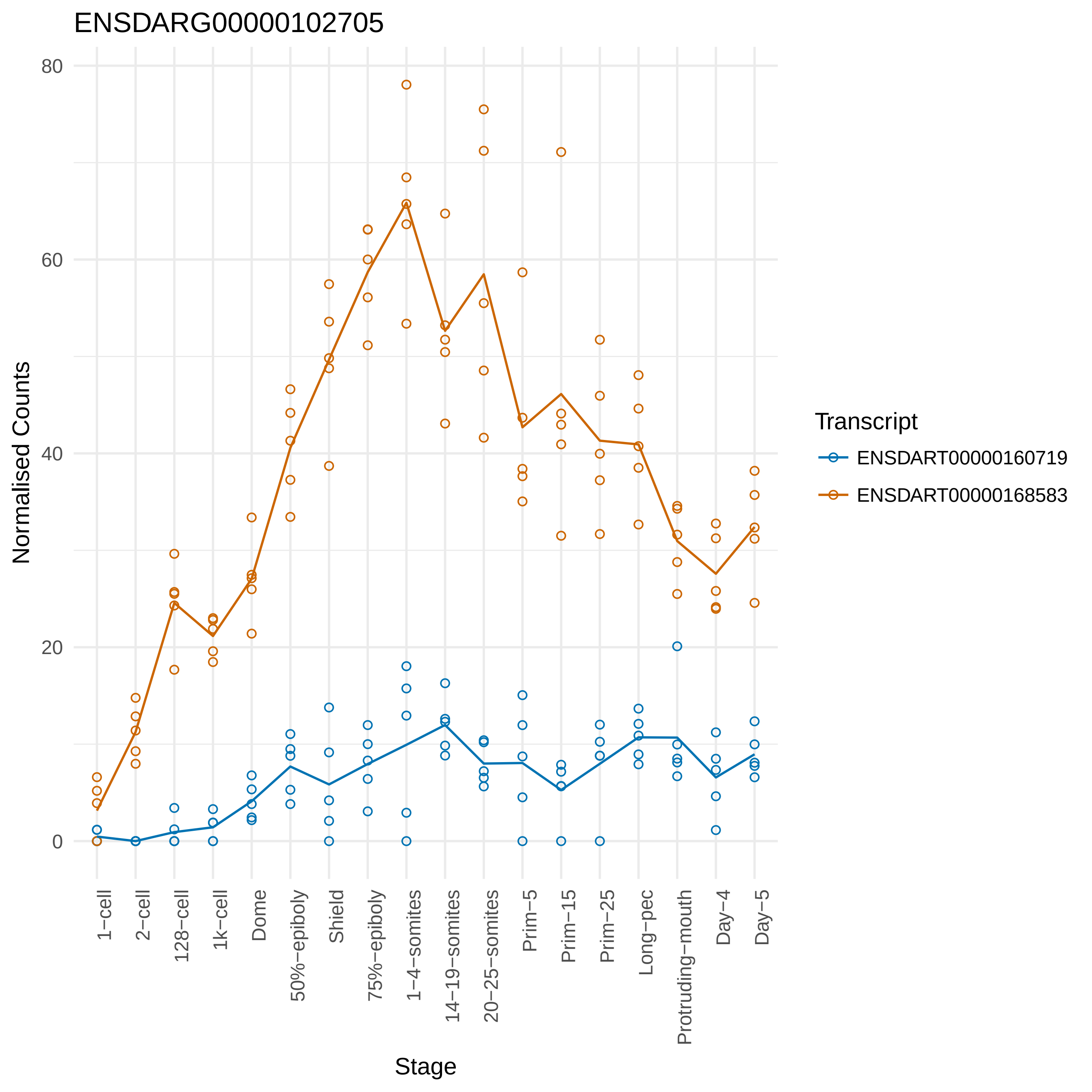

ENSDARG00000102705
Normalised Counts
80
60
40
20
0
1−cell
2−cell
128−cell
1k−cell
Dome
50%−epiboly
Shield
75%−epiboly
1−4−somites
14−19−somites
20−25−somites
Prim−5
Prim−15
Prim−25
Long−pec
Protruding−mouth
Day−4
Day−5
Stage
Transcript
ENSDART00000160719
ENSDART00000168583

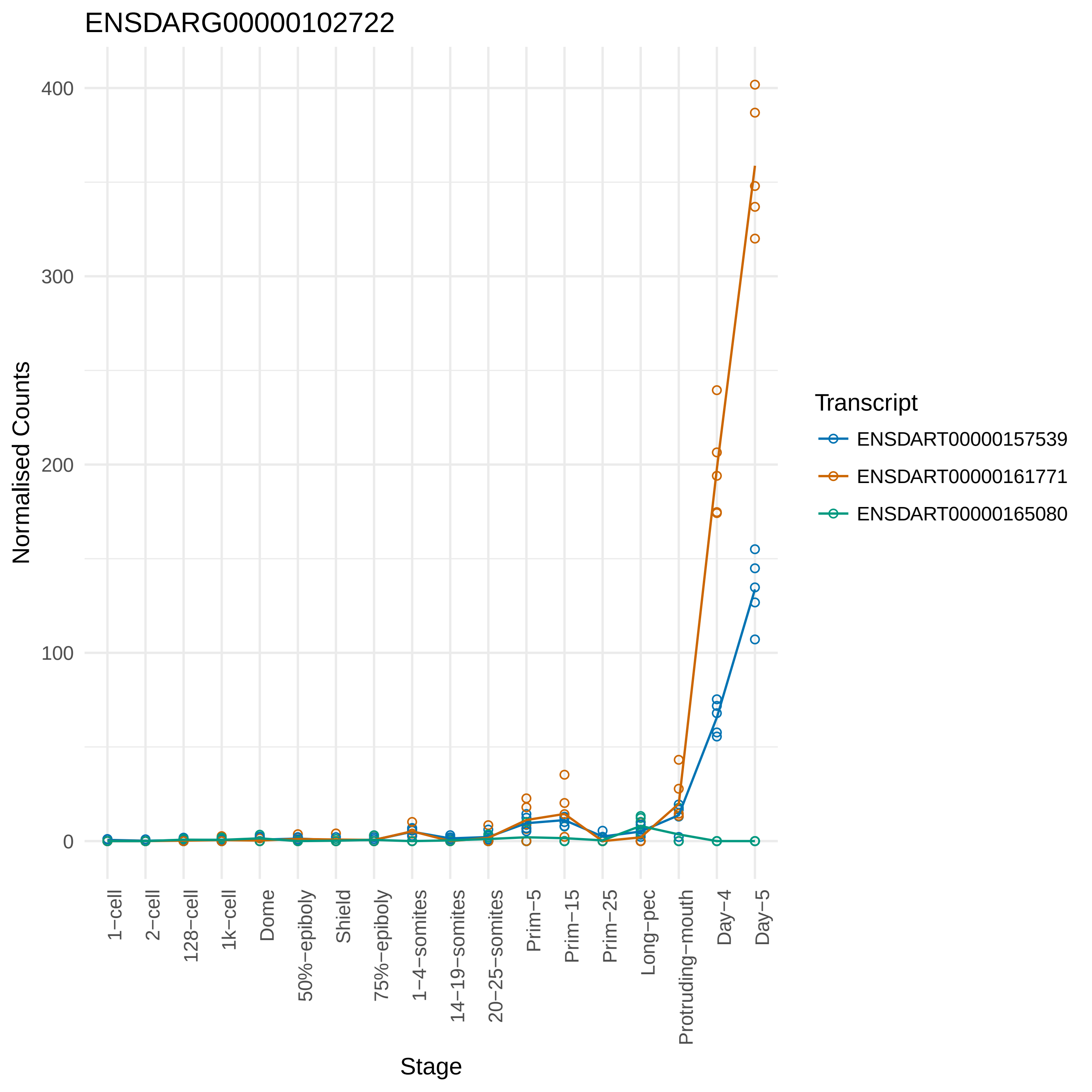

ENSDARG00000102722
Normalised Counts
400
300
200
100
0
1−cell
2−cell
128−cell
1k−cell
Dome
50%−epiboly
Shield
75%−epiboly
1−4−somites
14−19−somites
20−25−somites
Prim−5
Prim−15
Prim−25
Long−pec
Protruding−mouth
Day−4
Day−5
Stage
Transcript
ENSDART00000157539
ENSDART00000161771
ENSDART00000165080

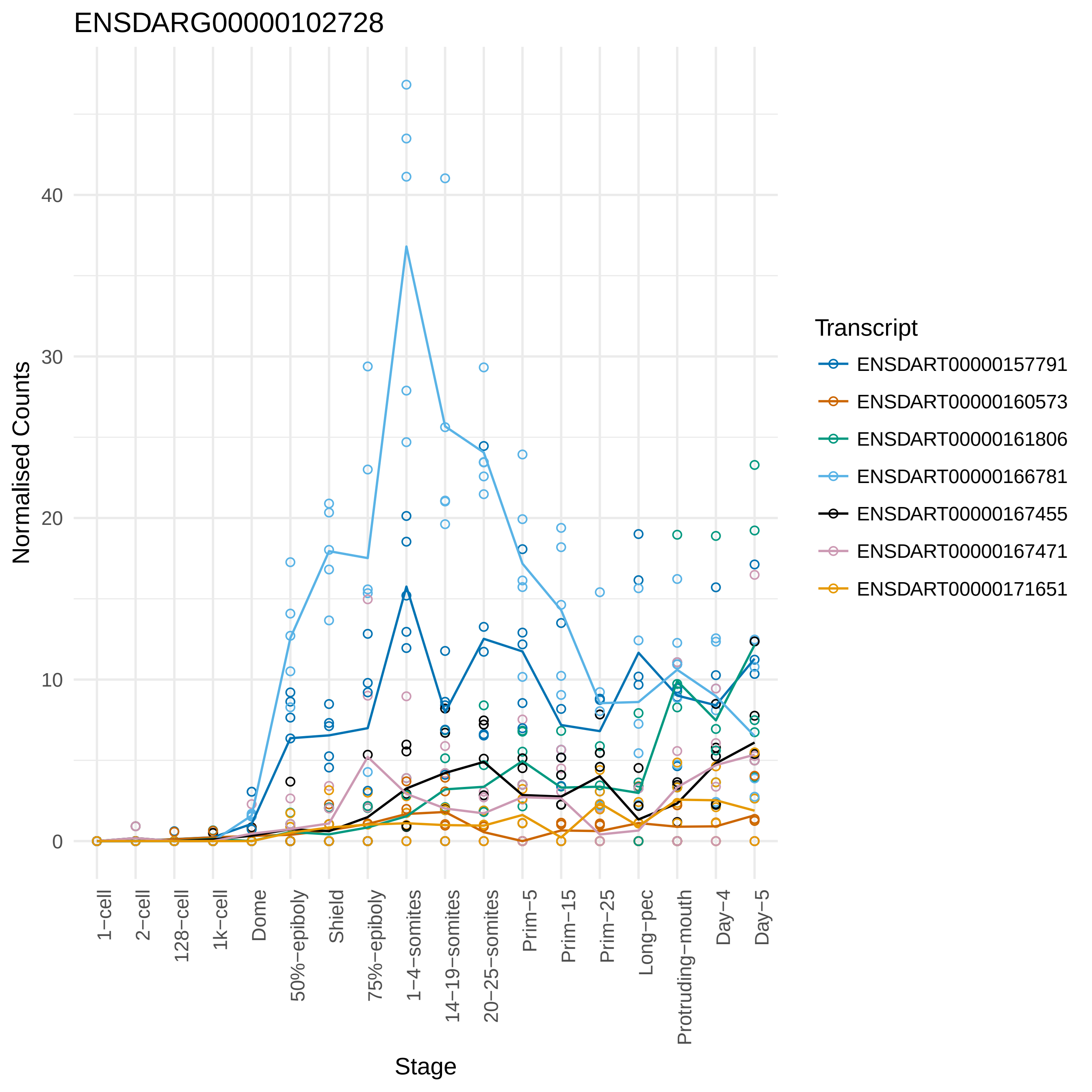

ENSDARG00000102728
Normalised Counts
0
10
20
30
40
1−cell
2−cell
128−cell
1k−cell
Dome
50%−epiboly
Shield
75%−epiboly
1−4−somites
14−19−somites
20−25−somites
Prim−5
Prim−15
Prim−25
Long−pec
Protruding−mouth
Day−4
Day−5
Stage
Transcript
ENSDART00000157791
ENSDART00000160573
ENSDART00000161806
ENSDART00000166781
ENSDART00000167455
ENSDART00000167471
ENSDART00000171651

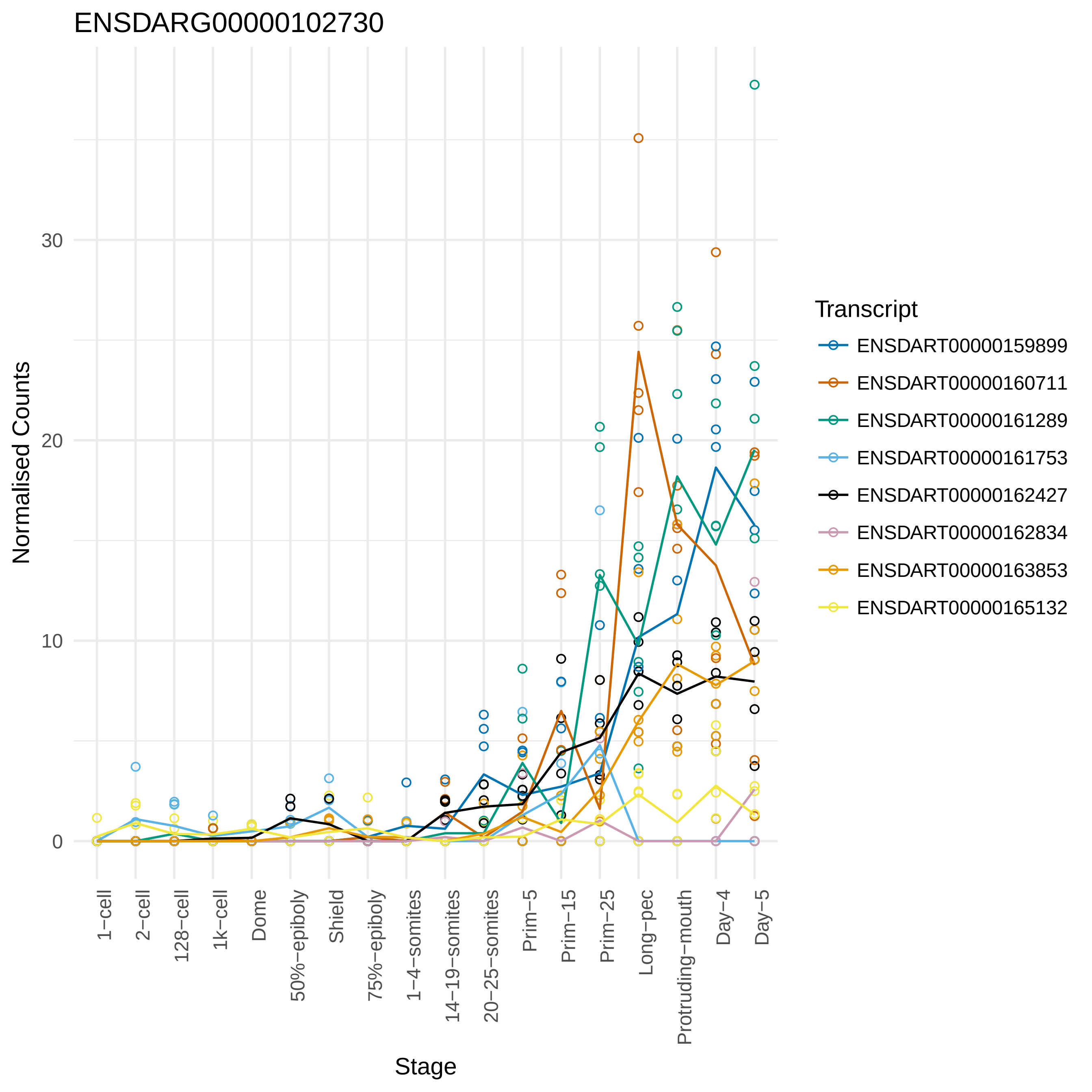

ENSDARG00000102730
Normalised Counts
0
10
20
30
1−cell
2−cell
128−cell
1k−cell
Dome
50%−epiboly
Shield
75%−epiboly
1−4−somites
14−19−somites
20−25−somites
Prim−5
Prim−15
Prim−25
Long−pec
Protruding−mouth
Day−4
Day−5
Stage
Transcript
ENSDART00000159899
ENSDART00000160711
ENSDART00000161289
ENSDART00000161753
ENSDART00000162427
ENSDART00000162834
ENSDART00000163853
ENSDART00000165132

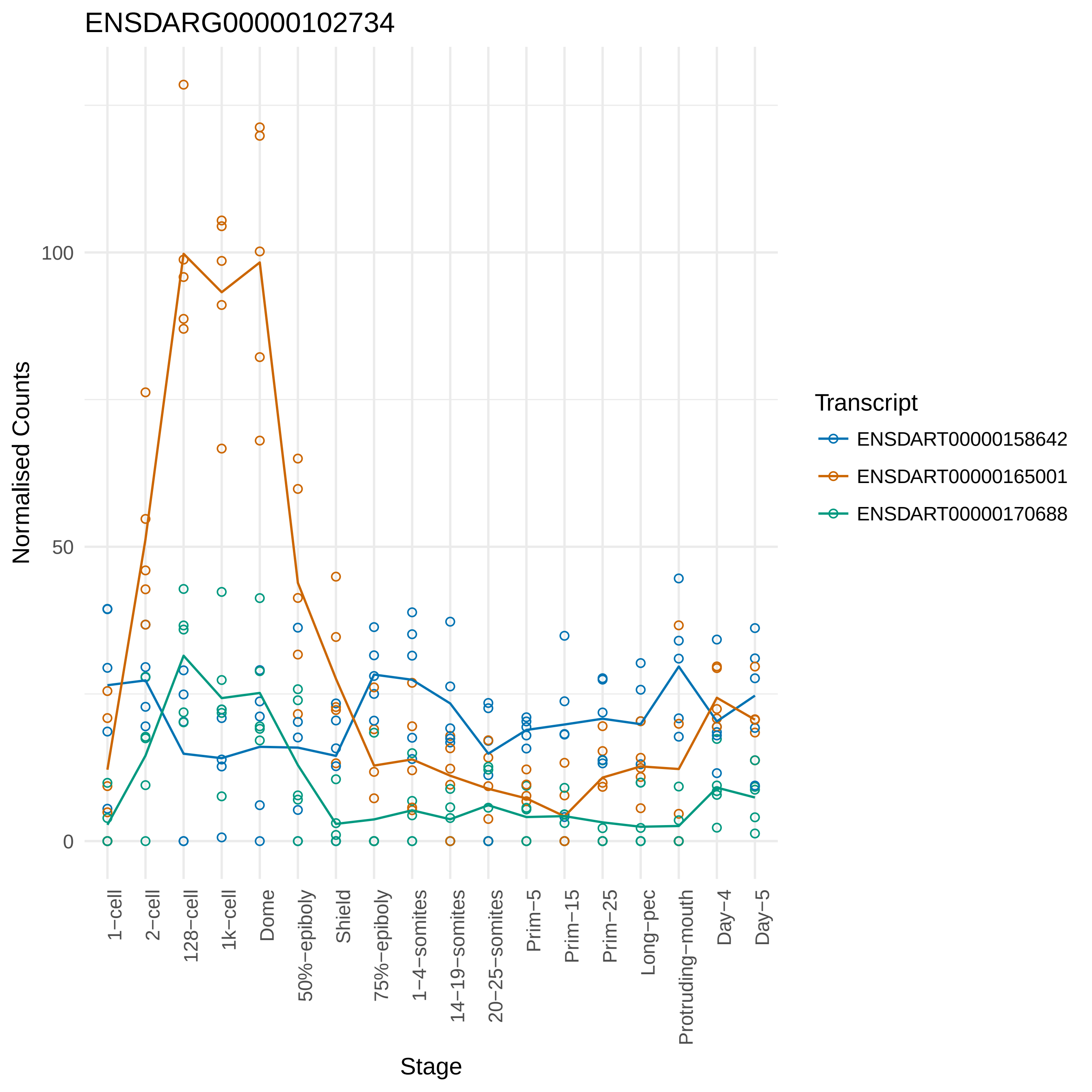

ENSDARG00000102734
Normalised Counts
0
50
100
1−cell
2−cell
128−cell
1k−cell
Dome
50%−epiboly
Shield
75%−epiboly
1−4−somites
14−19−somites
20−25−somites
Prim−5
Prim−15
Prim−25
Long−pec
Protruding−mouth
Day−4
Day−5
Stage
Transcript
ENSDART00000158642
ENSDART00000165001
ENSDART00000170688

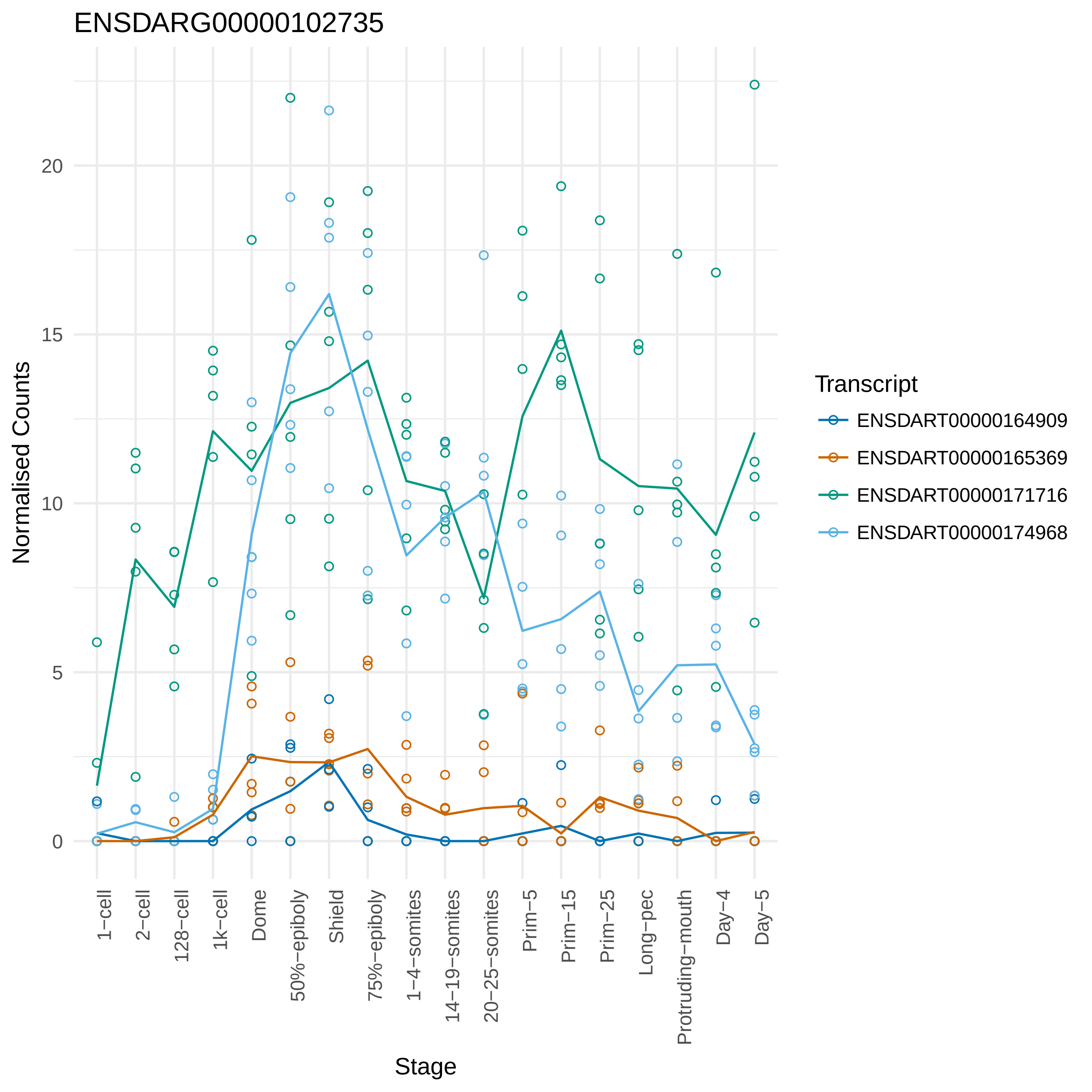
ENSDARG00000102735
Normalised Counts
0
5
10
15
20
Stage
1−cell
2−cell
128−cell
1k−cell
Dome
50%−epiboly
Shield
75%−epiboly
1−4−somites
14−19−somites
20−25−somites
Prim−5
Prim−15
Prim−25
Long−pec
Protruding−mouth
Day−4
Day−5
Transcript
ENSDART00000164909
ENSDART00000165369
ENSDART00000171716
ENSDART00000174968

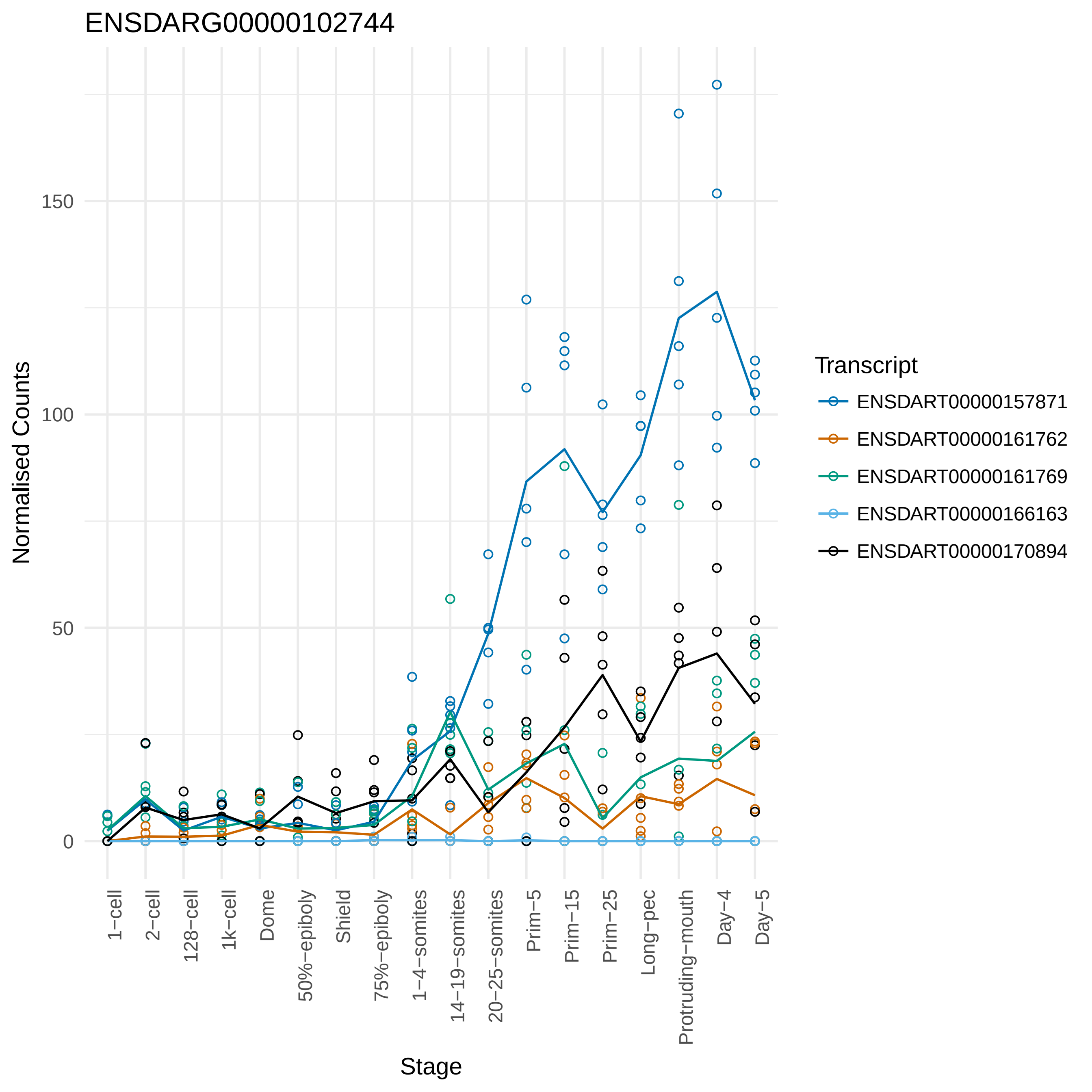
ENSDARG00000102744
Normalised Counts
0
50
100
150
Stage
1−cell
2−cell
128−cell
1k−cell
Dome
50%−epiboly
Shield
75%−epiboly
1−4−somites
14−19−somites
20−25−somites
Prim−5
Prim−15
Prim−25
Long−pec
Protruding−mouth
Day−4
Day−5
Transcript
ENSDART00000157871
ENSDART00000161762
ENSDART00000161769
ENSDART00000166163
ENSDART00000170894

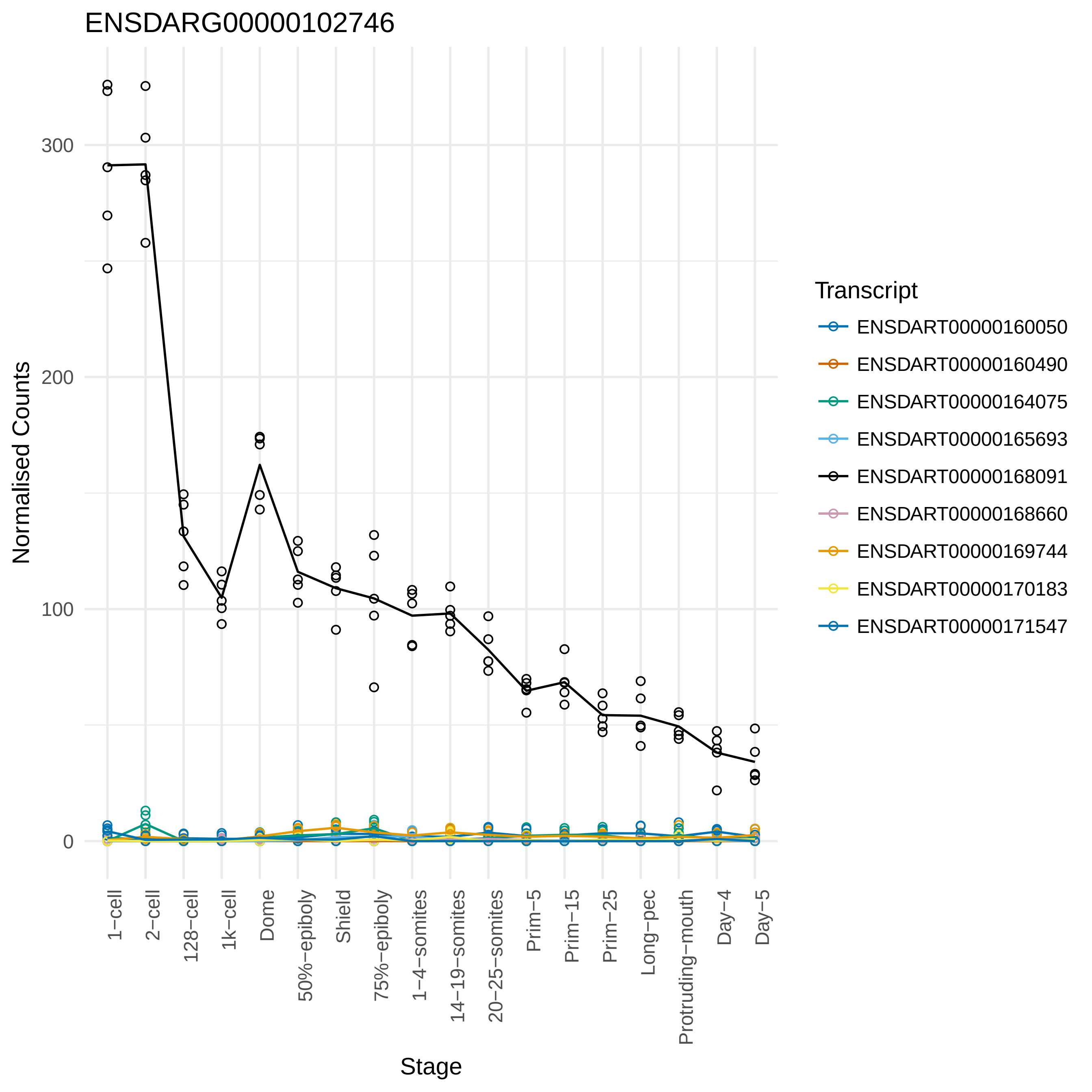

ENSDARG00000102746
Normalised Counts
300
200
100
0
1−cell
2−cell
128−cell
1k−cell
Dome
50%−epiboly
Shield
75%−epiboly
1−4−somites
14−19−somites
20−25−somites
Prim−5
Prim−15
Prim−25
Long−pec
Protruding−mouth
Day−4
Day−5
Stage
Transcript
ENSDART00000160050
ENSDART00000160490
ENSDART00000164075
ENSDART00000165693
ENSDART00000168091
ENSDART00000168660
ENSDART00000169744
ENSDART00000170183
ENSDART00000171547

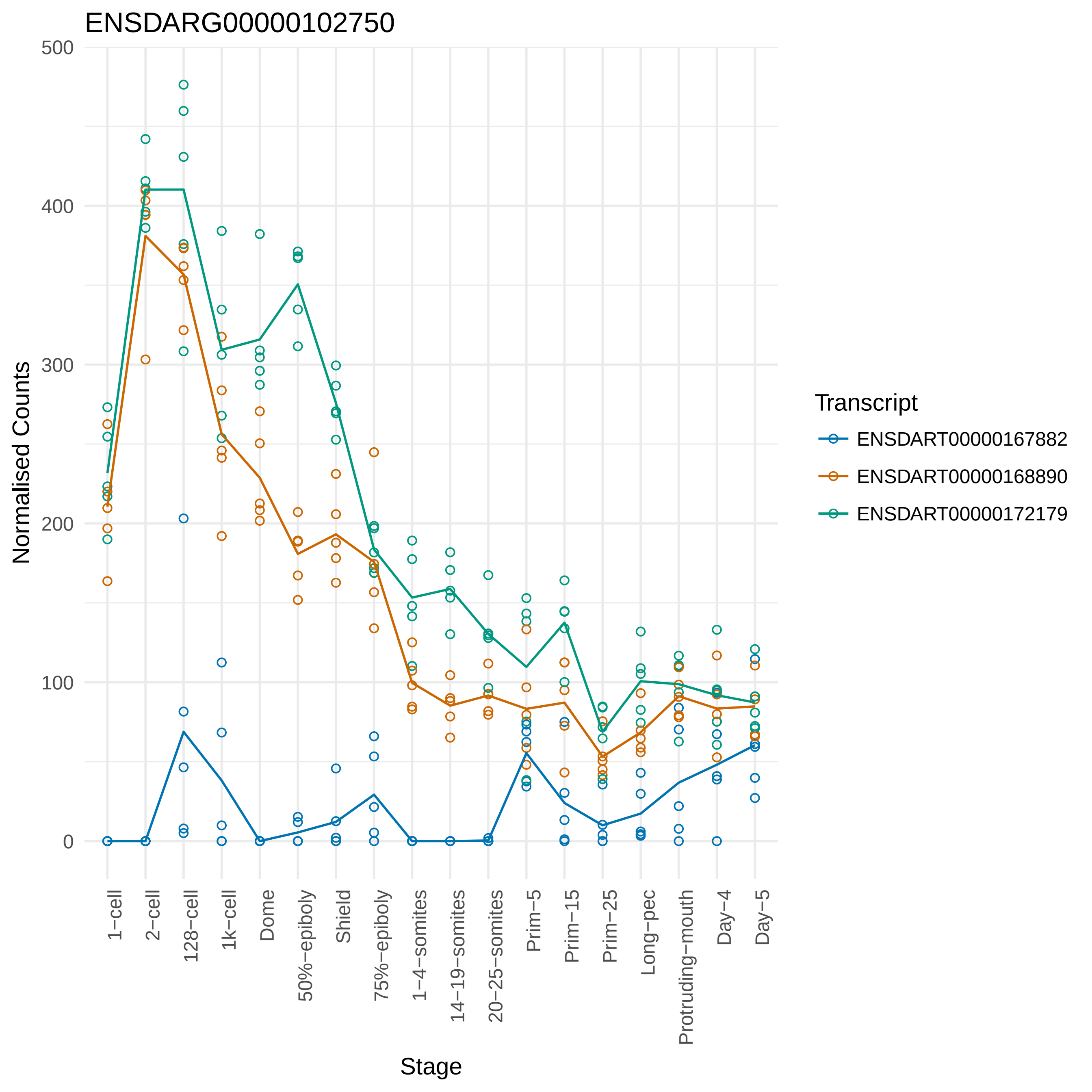
ENSDARG00000102750
Normalised Counts
0
100
200
300
400
500
Stage
1−cell
2−cell
128−cell
1k−cell
Dome
50%−epiboly
Shield
75%−epiboly
1−4−somites
14−19−somites
20−25−somites
Prim−5
Prim−15
Prim−25
Long−pec
Protruding−mouth
Day−4
Day−5
Transcript
ENSDART00000167882
ENSDART00000168890
ENSDART00000172179

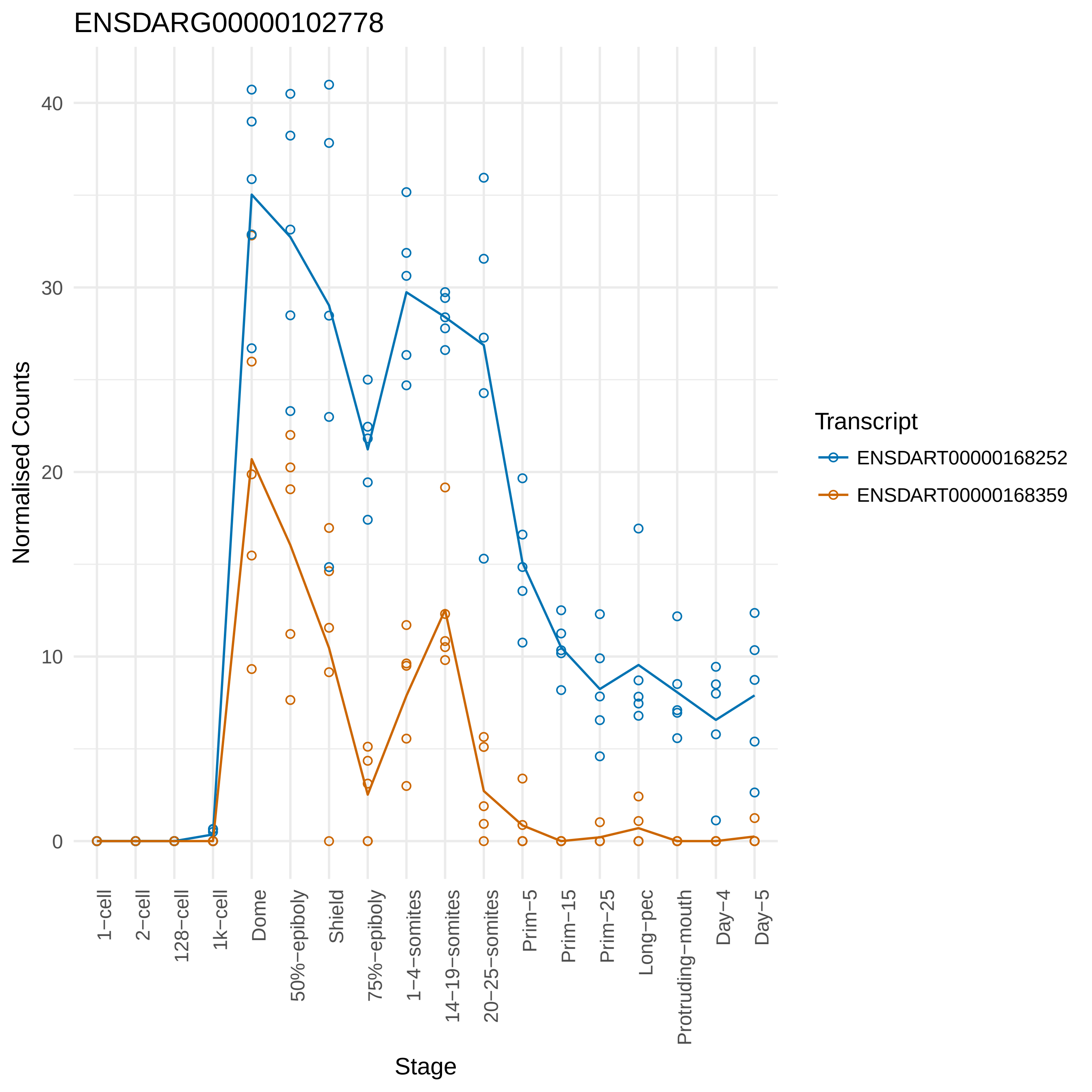

ENSDARG00000102778
Normalised Counts
40
30
20
10
0
1−cell
2−cell
128−cell
1k−cell
Dome
50%−epiboly
Shield
75%−epiboly
1−4−somites
14−19−somites
20−25−somites
Prim−5
Prim−15
Prim−25
Long−pec
Protruding−mouth
Day−4
Day−5
Stage
Transcript
ENSDART00000168252
ENSDART00000168359

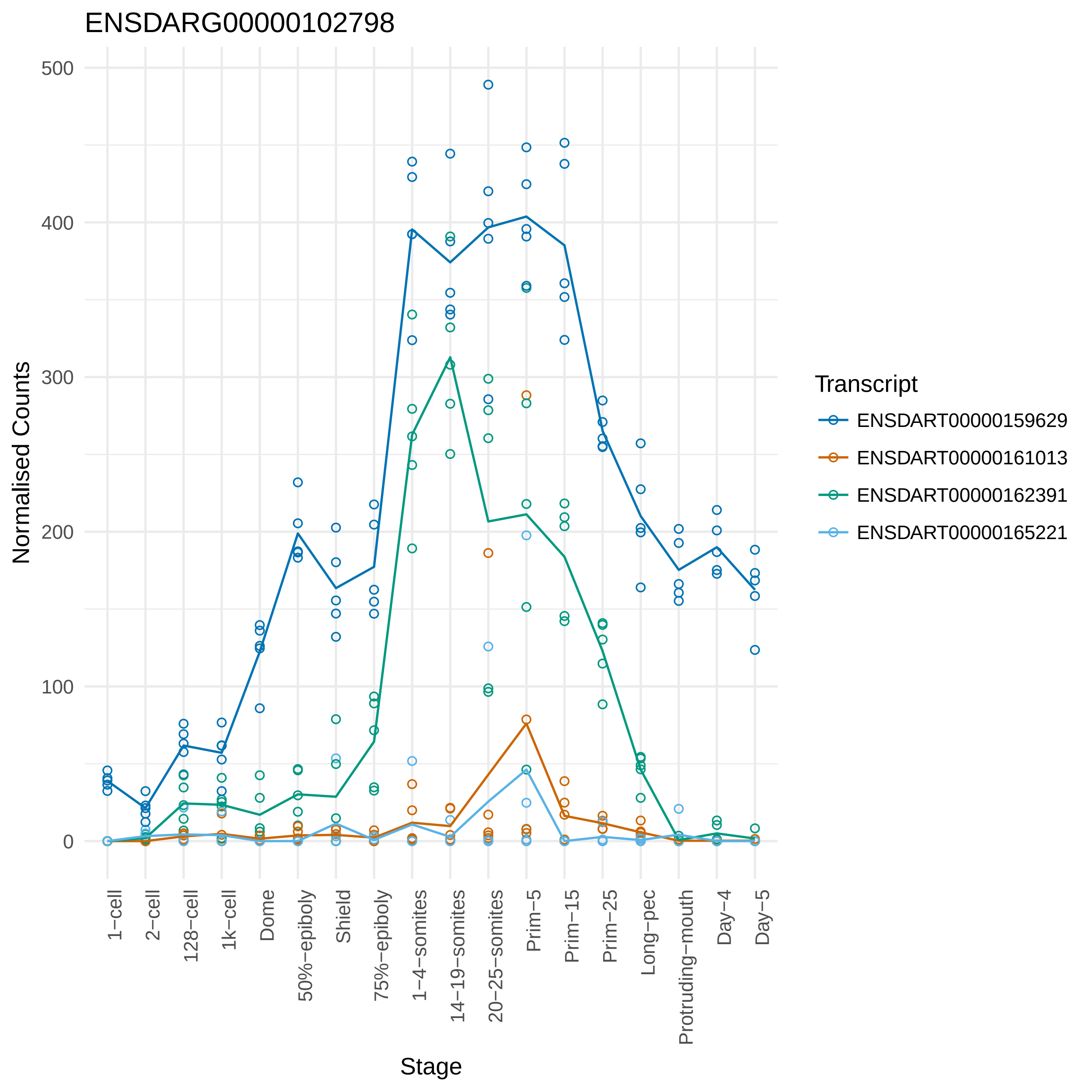

ENSDARG00000102798
Normalised Counts
0
100
200
300
400
500
Stage
1−cell
2−cell
128−cell
1k−cell
Dome
50%−epiboly
Shield
75%−epiboly
1−4−somites
14−19−somites
20−25−somites
Prim−5
Prim−15
Prim−25
Long−pec
Protruding−mouth
Day−4
Day−5
Transcript
ENSDART00000159629
ENSDART00000161013
ENSDART00000162391
ENSDART00000165221

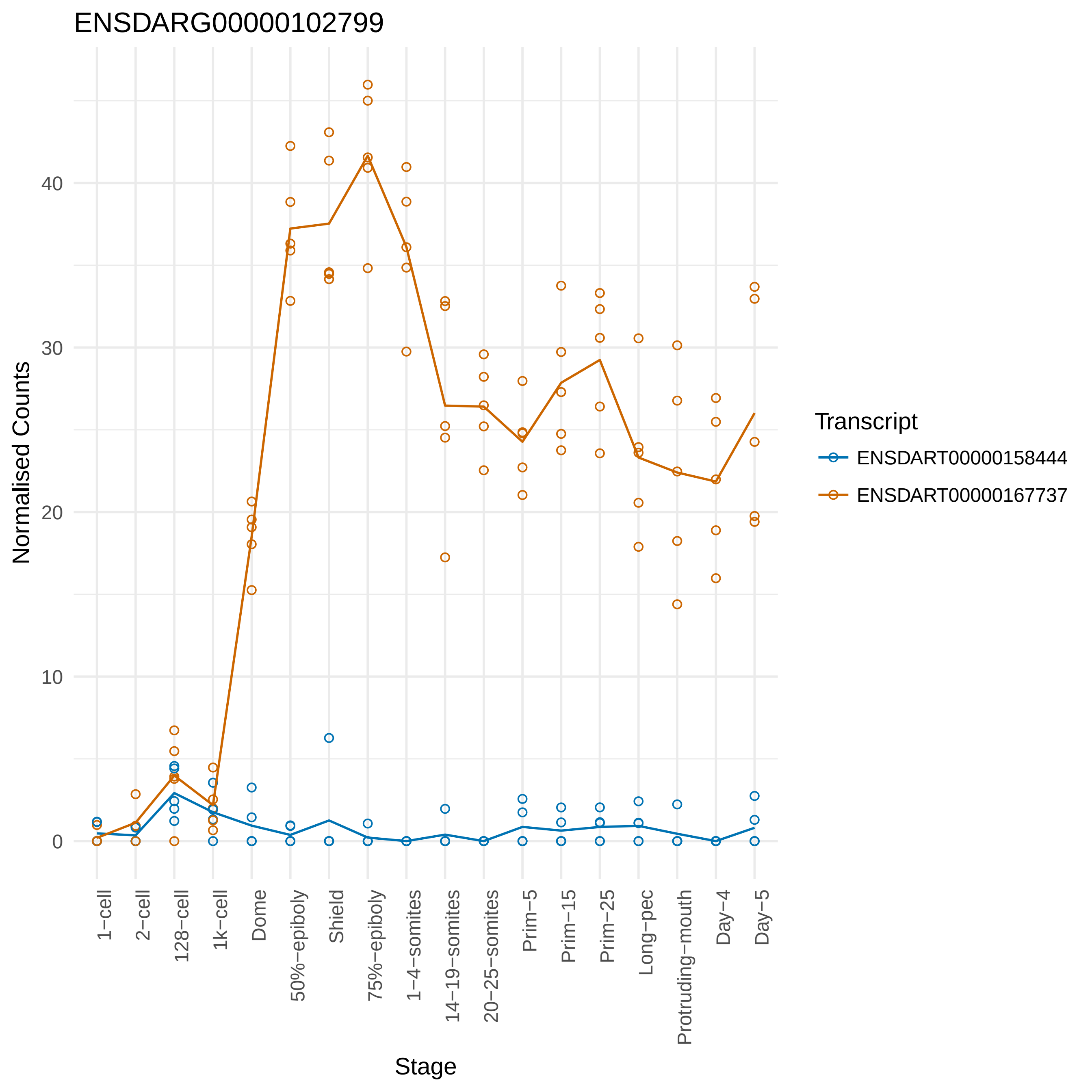

ENSDARG00000102799
Normalised Counts
0
10
20
30
40
1−cell
2−cell
128−cell
1k−cell
Dome
50%−epiboly
Shield
75%−epiboly
1−4−somites
14−19−somites
20−25−somites
Prim−5
Prim−15
Prim−25
Long−pec
Protruding−mouth
Day−4
Day−5
Stage
Transcript
ENSDART00000158444
ENSDART00000167737

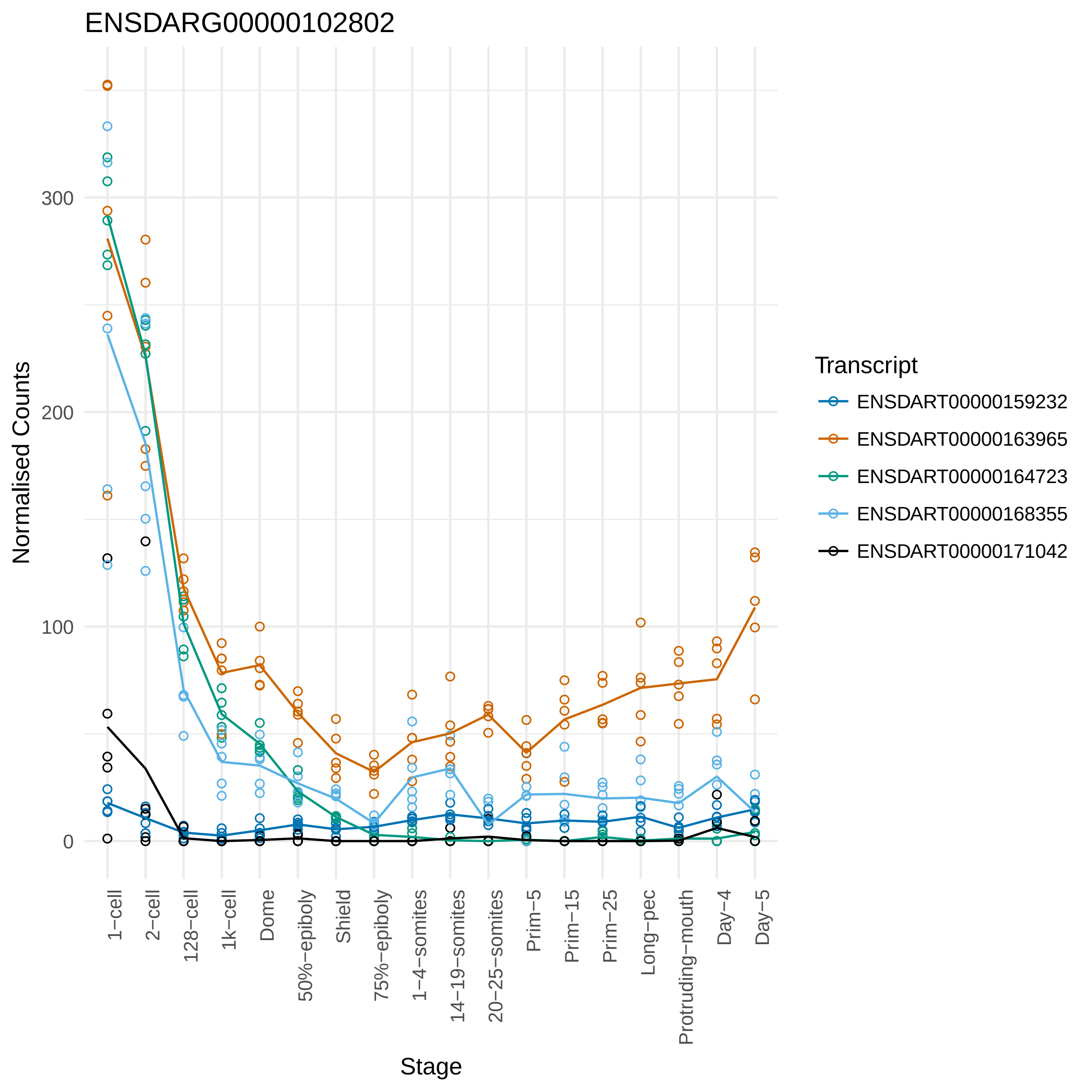

ENSDARG00000102802
Normalised Counts
300
200
100
0
1−cell
2−cell
128−cell
1k−cell
Dome
50%−epiboly
Shield
75%−epiboly
1−4−somites
14−19−somites
20−25−somites
Prim−5
Prim−15
Prim−25
Long−pec
Protruding−mouth
Day−4
Day−5
Stage
Transcript
ENSDART00000159232
ENSDART00000163965
ENSDART00000164723
ENSDART00000168355
ENSDART00000171042

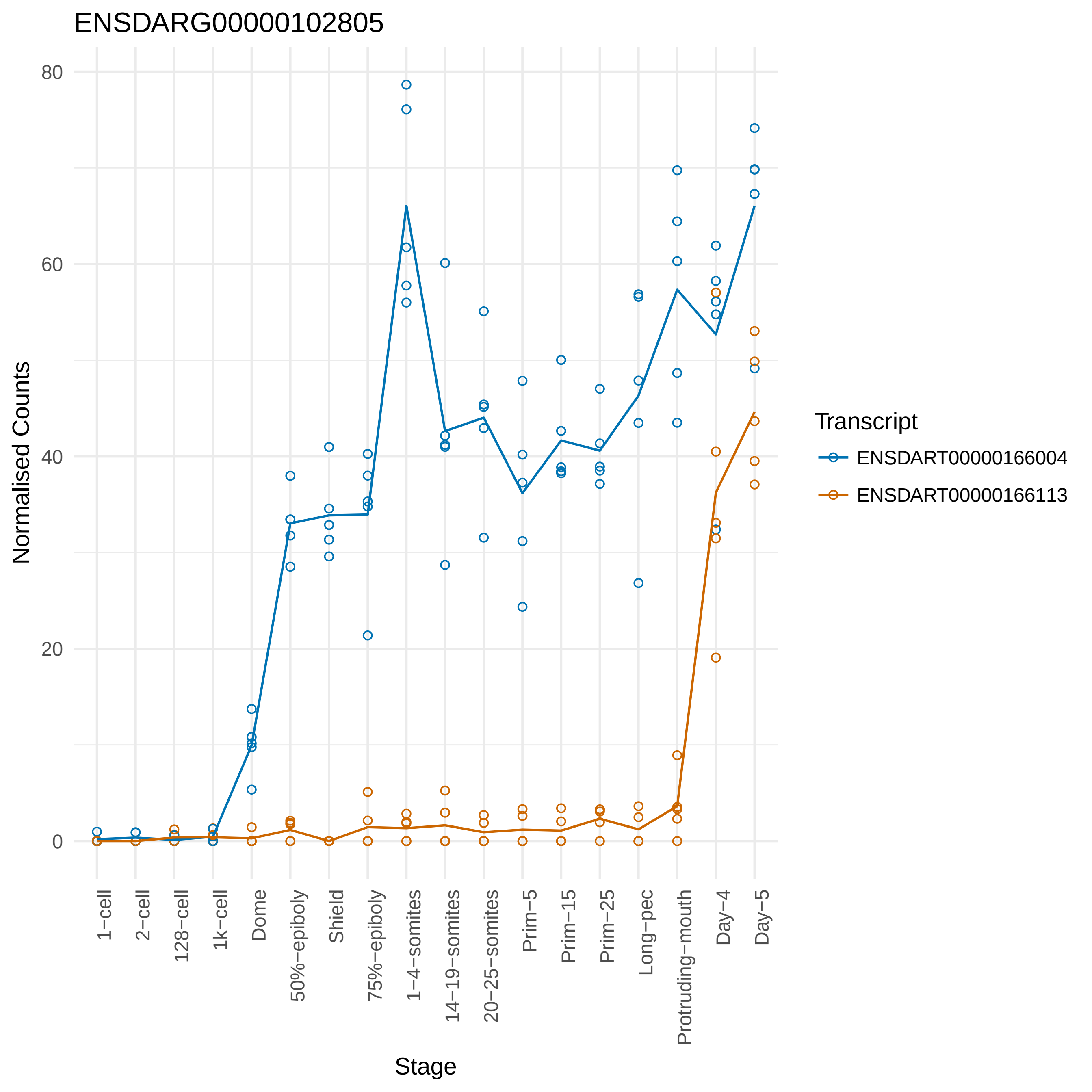
ENSDARG00000102805
Normalised Counts
0
20
40
60
80
1−cell
2−cell
128−cell
1k−cell
Dome
50%−epiboly
Shield
75%−epiboly
1−4−somites
14−19−somites
20−25−somites
Prim−5
Prim−15
Prim−25
Long−pec
Protruding−mouth
Day−4
Day−5
Stage
Transcript
ENSDART00000166004
ENSDART00000166113

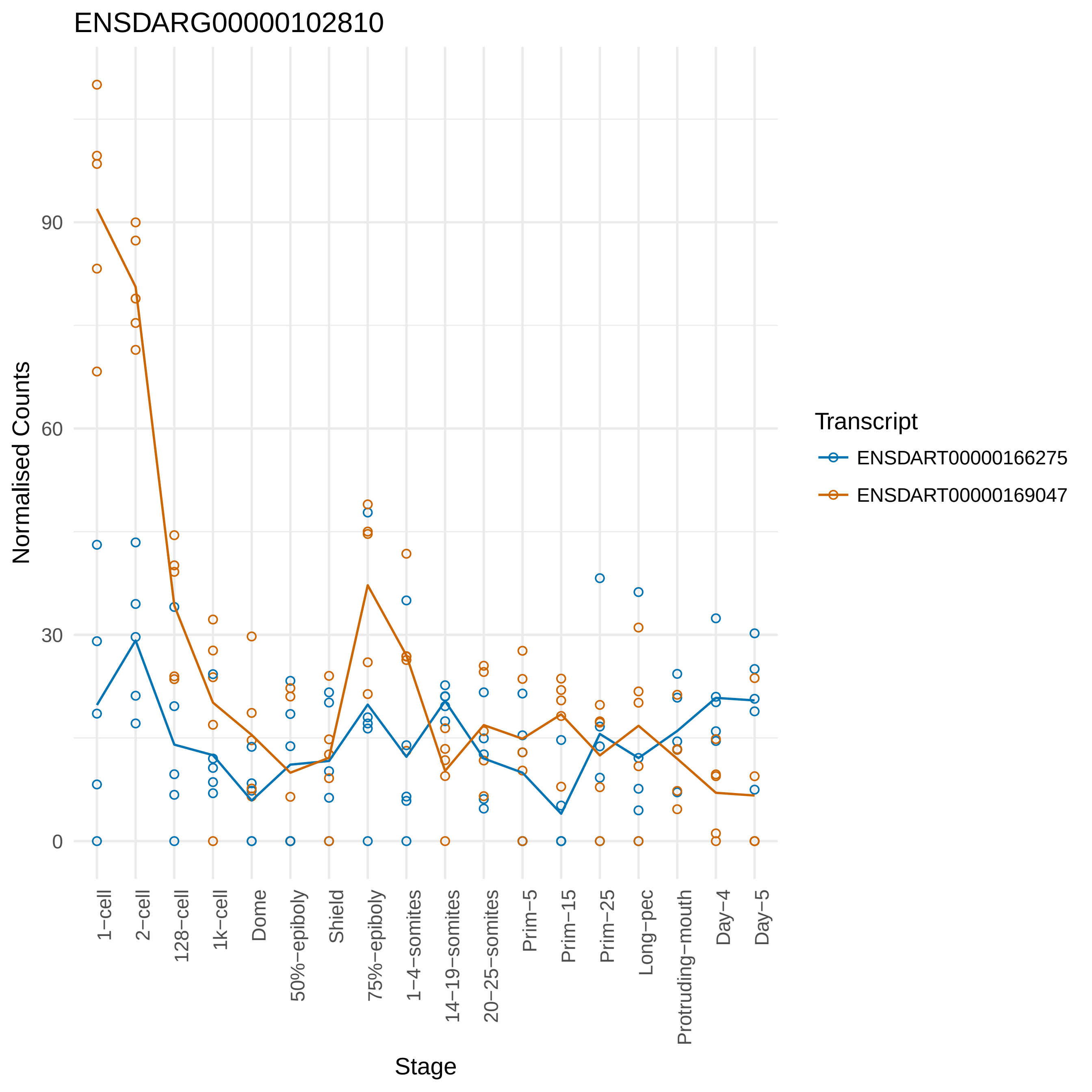
ENSDARG00000102810
Normalised Counts
0
30
60
90
Stage
1−cell
2−cell
128−cell
1k−cell
Dome
50%−epiboly
Shield
75%−epiboly
1−4−somites
14−19−somites
20−25−somites
Prim−5
Prim−15
Prim−25
Long−pec
Protruding−mouth
Day−4
Day−5
Transcript
ENSDART00000166275
ENSDART00000169047

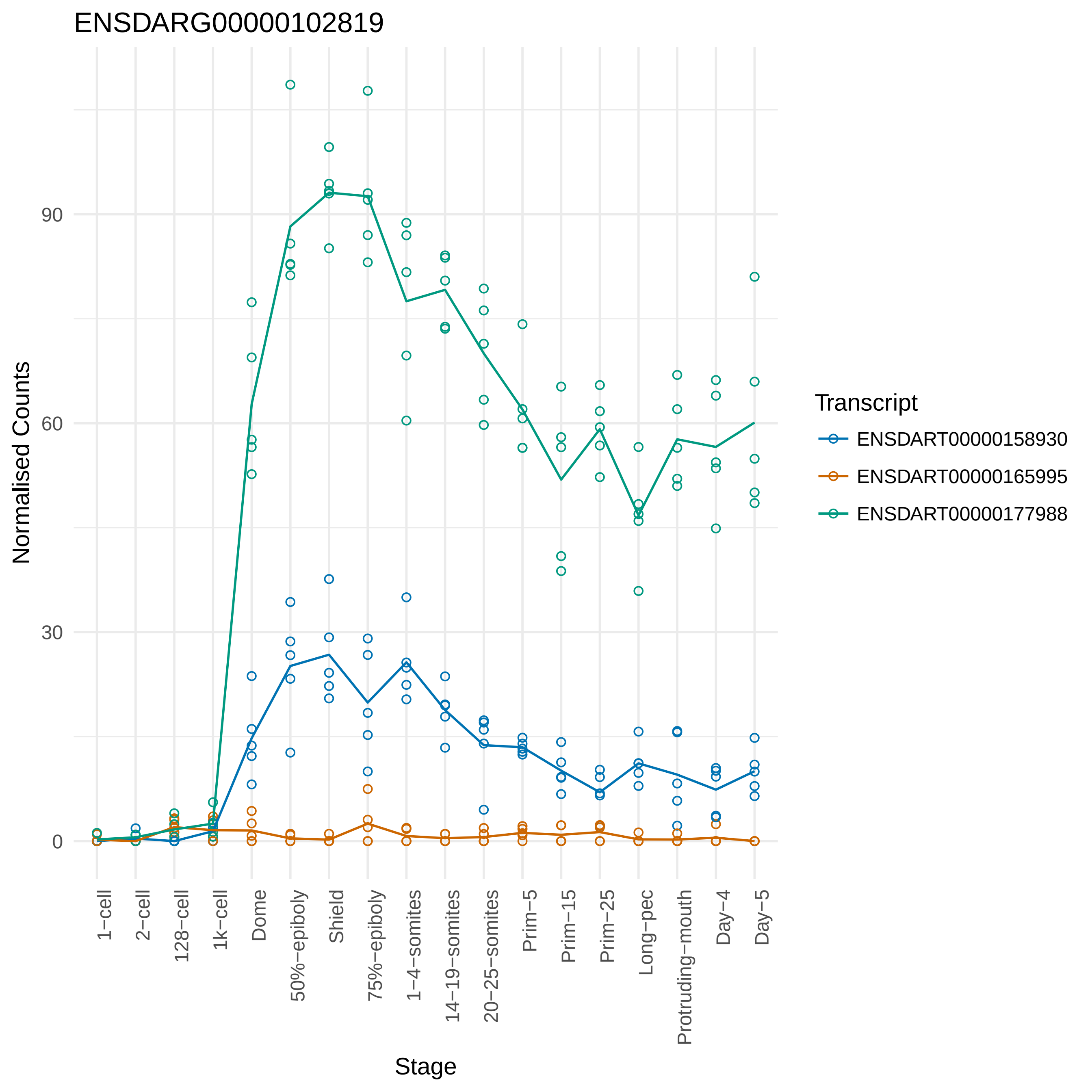

ENSDARG00000102819
Normalised Counts
0
30
60
90
1−cell
2−cell
128−cell
1k−cell
Dome
50%−epiboly
Shield
75%−epiboly
1−4−somites
14−19−somites
20−25−somites
Prim−5
Prim−15
Prim−25
Long−pec
Protruding−mouth
Day−4
Day−5
Stage
Transcript
ENSDART00000158930
ENSDART00000165995
ENSDART00000177988

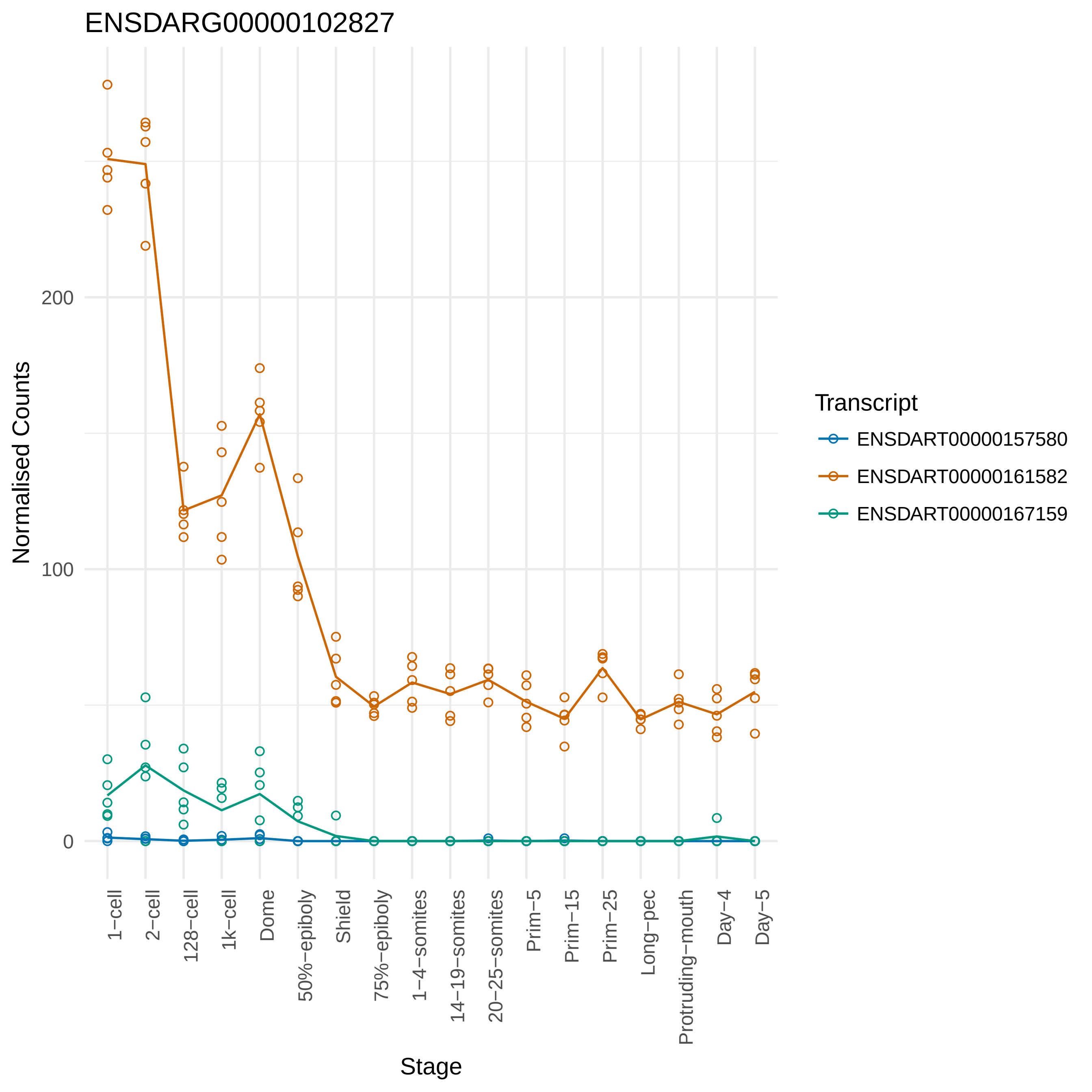

ENSDARG00000102827
Normalised Counts
200
100
0
1−cell
2−cell
128−cell
1k−cell
Dome
50%−epiboly
Shield
75%−epiboly
1−4−somites
14−19−somites
20−25−somites
Prim−5
Prim−15
Prim−25
Long−pec
Protruding−mouth
Day−4
Day−5
Stage
Transcript
ENSDART00000157580
ENSDART00000161582
ENSDART00000167159

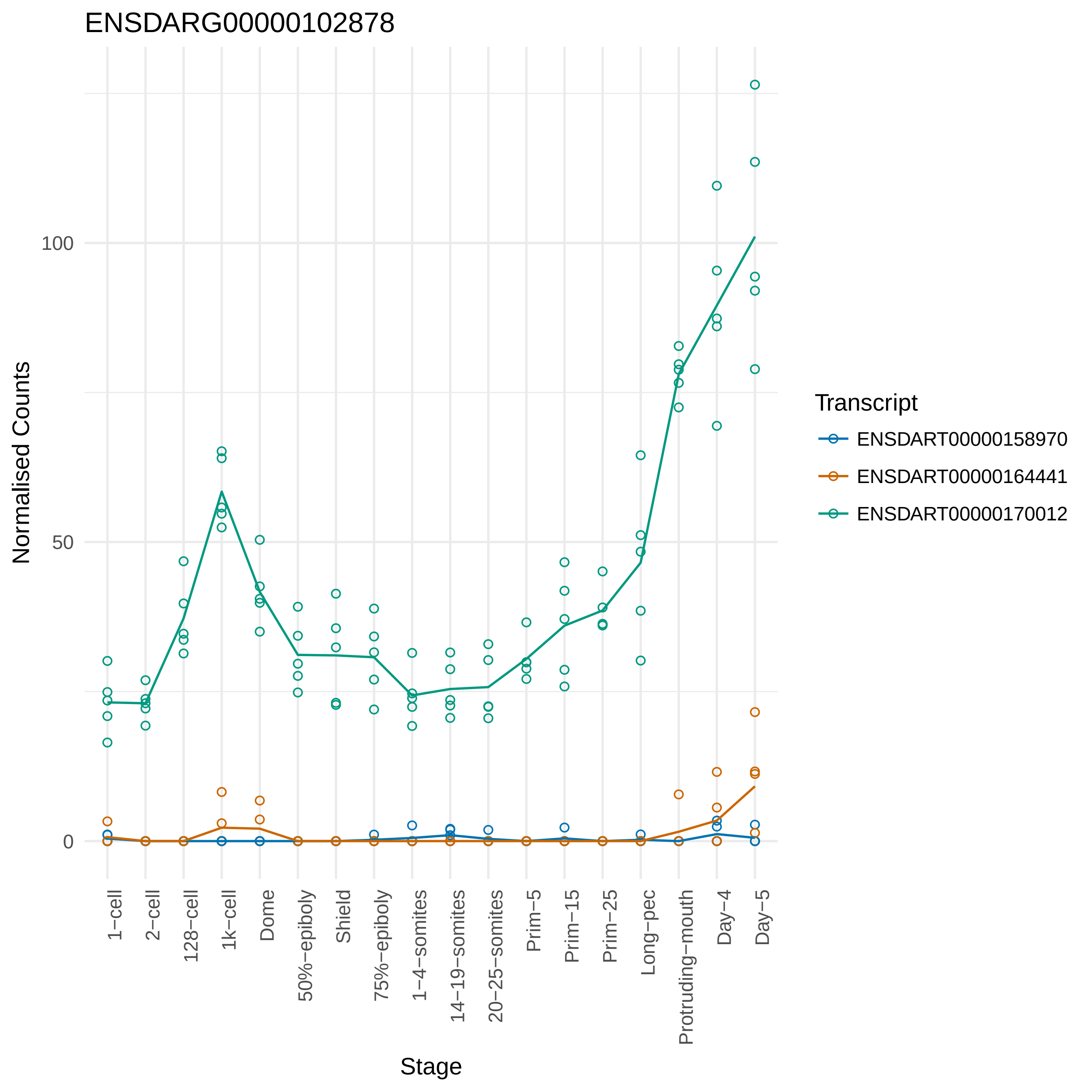

ENSDARG00000102878
Normalised Counts
100
50
0
1−cell
2−cell
128−cell
1k−cell
Dome
50%−epiboly
Shield
75%−epiboly
1−4−somites
14−19−somites
20−25−somites
Prim−5
Prim−15
Prim−25
Long−pec
Protruding−mouth
Day−4
Day−5
Stage
Transcript
ENSDART00000158970
ENSDART00000164441
ENSDART00000170012

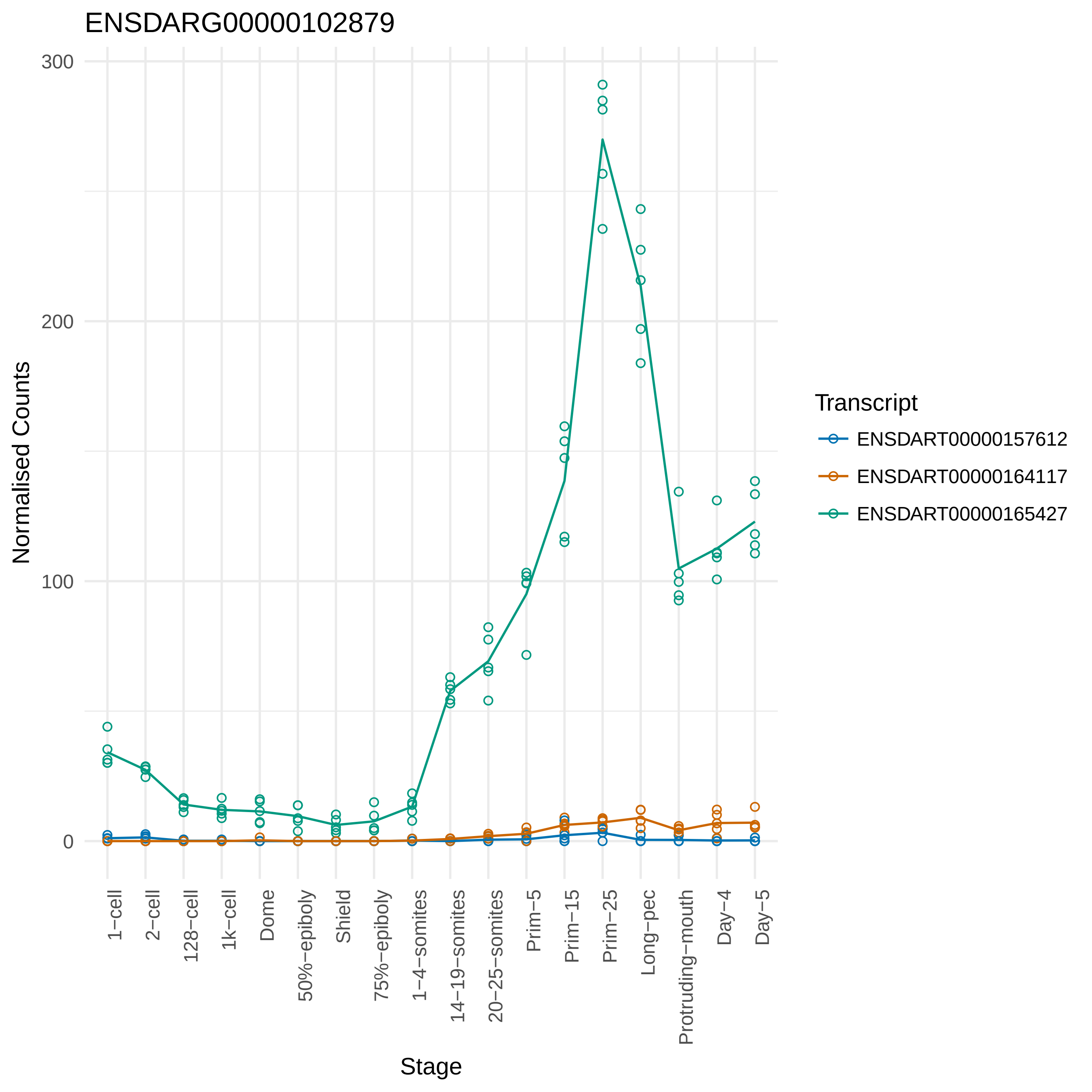
ENSDARG00000102879
Normalised Counts
300
200
100
0
1−cell
2−cell
128−cell
1k−cell
Dome
50%−epiboly
Shield
75%−epiboly
1−4−somites
14−19−somites
20−25−somites
Prim−5
Prim−15
Prim−25
Long−pec
Protruding−mouth
Day−4
Day−5
Stage
Transcript
ENSDART00000157612
ENSDART00000164117
ENSDART00000165427

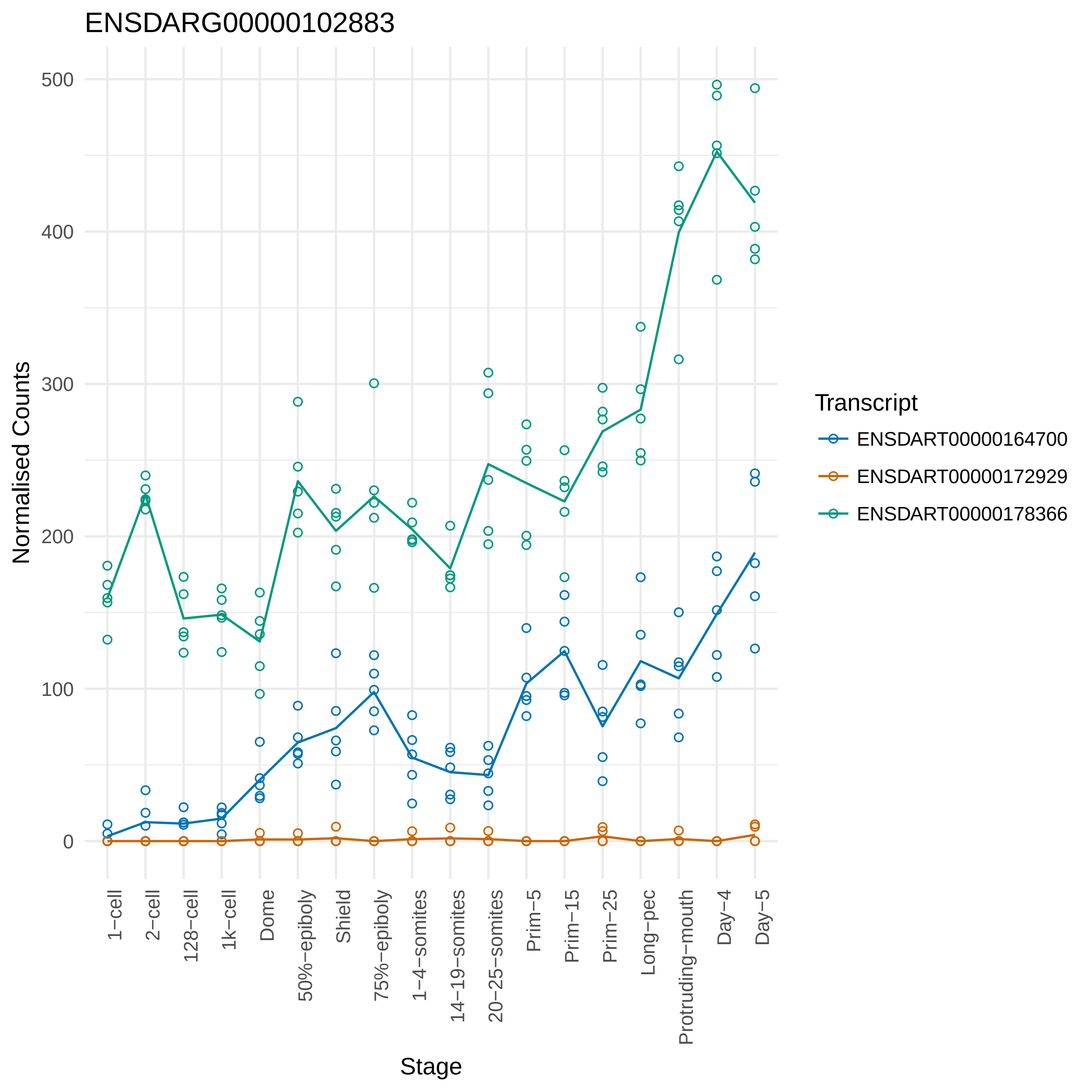

ENSDARG00000102883
Normalised Counts
500
400
300
200
100
0
1−cell
2−cell
128−cell
1k−cell
Dome
50%−epiboly
Shield
75%−epiboly
1−4−somites
14−19−somites
20−25−somites
Prim−5
Prim−15
Prim−25
Long−pec
Protruding−mouth
Day−4
Day−5
Stage
Transcript
ENSDART00000164700
ENSDART00000172929
ENSDART00000178366

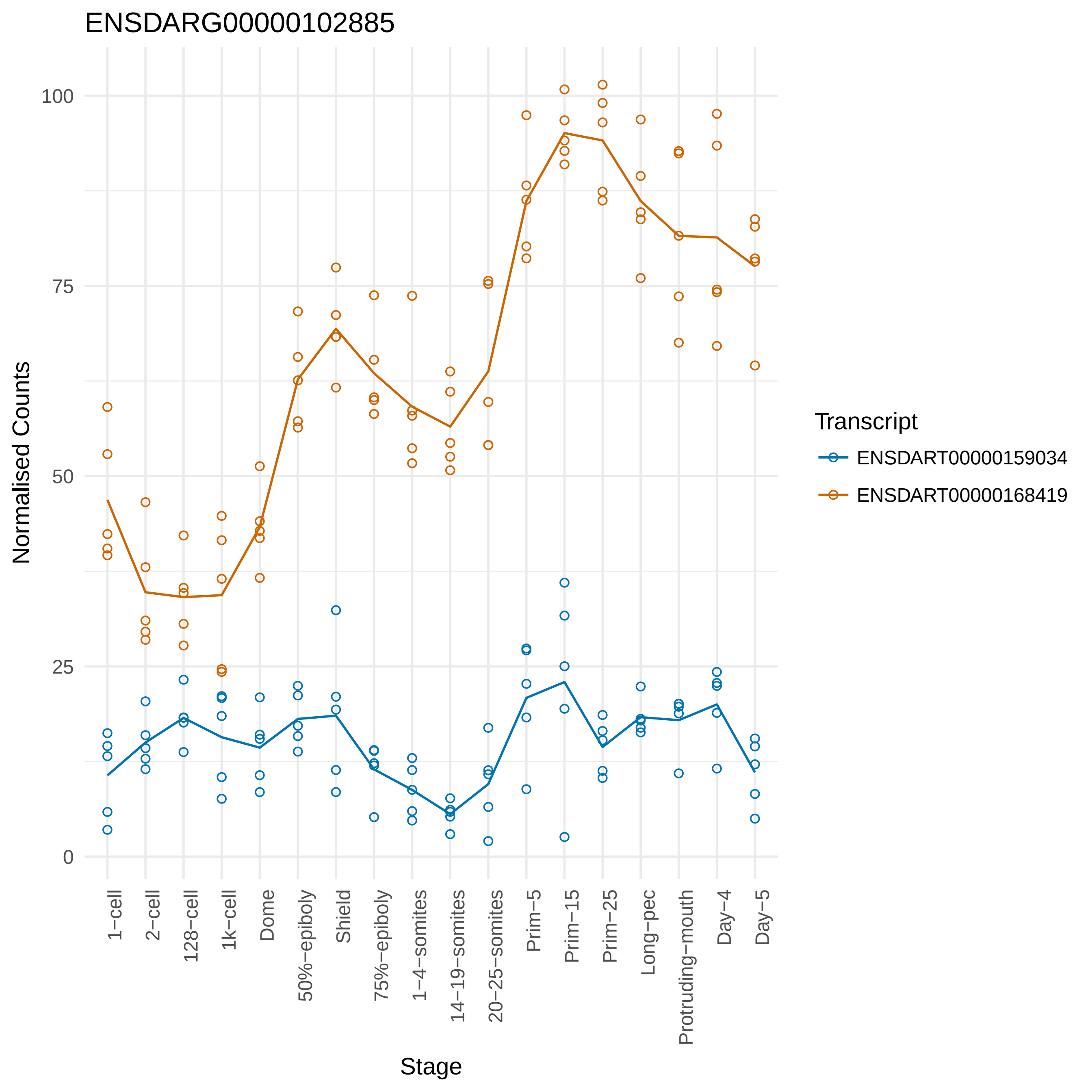

ENSDARG00000102885
Normalised Counts
0
25
50
75
100
1−cell
2−cell
128−cell
1k−cell
Dome
50%−epiboly
Shield
75%−epiboly
1−4−somites
14−19−somites
20−25−somites
Prim−5
Prim−15
Prim−25
Long−pec
Protruding−mouth
Day−4
Day−5
Stage
Transcript
ENSDART00000159034
ENSDART00000168419

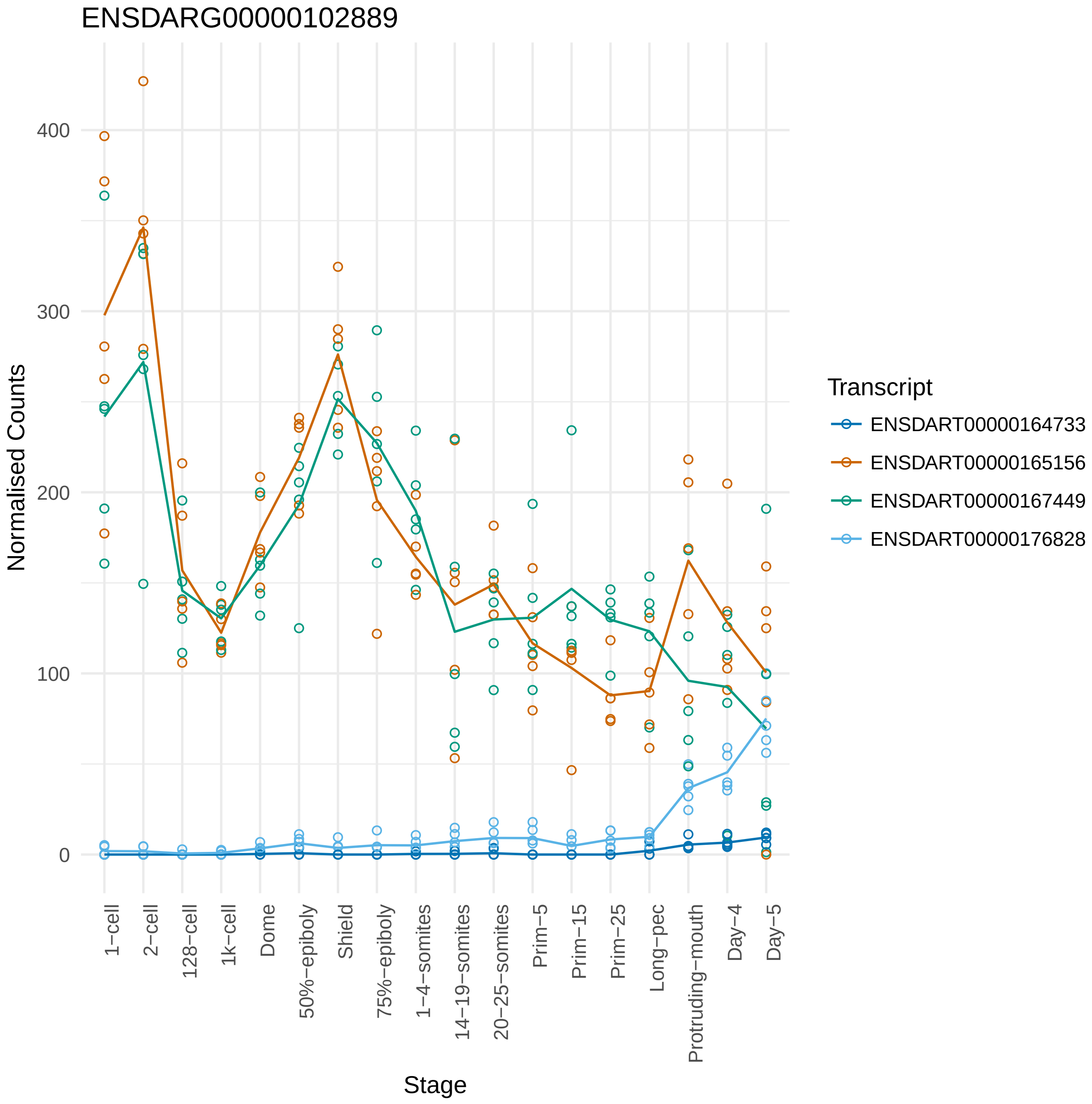
ENSDARG00000102889
Normalised Counts
0
100
200
300
400
1−cell
2−cell
128−cell
1k−cell
Dome
50%−epiboly
Shield
75%−epiboly
1−4−somites
14−19−somites
20−25−somites
Prim−5
Prim−15
Prim−25
Long−pec
Protruding−mouth
Day−4
Day−5
Stage
Transcript
ENSDART00000164733
ENSDART00000165156
ENSDART00000167449
ENSDART00000176828

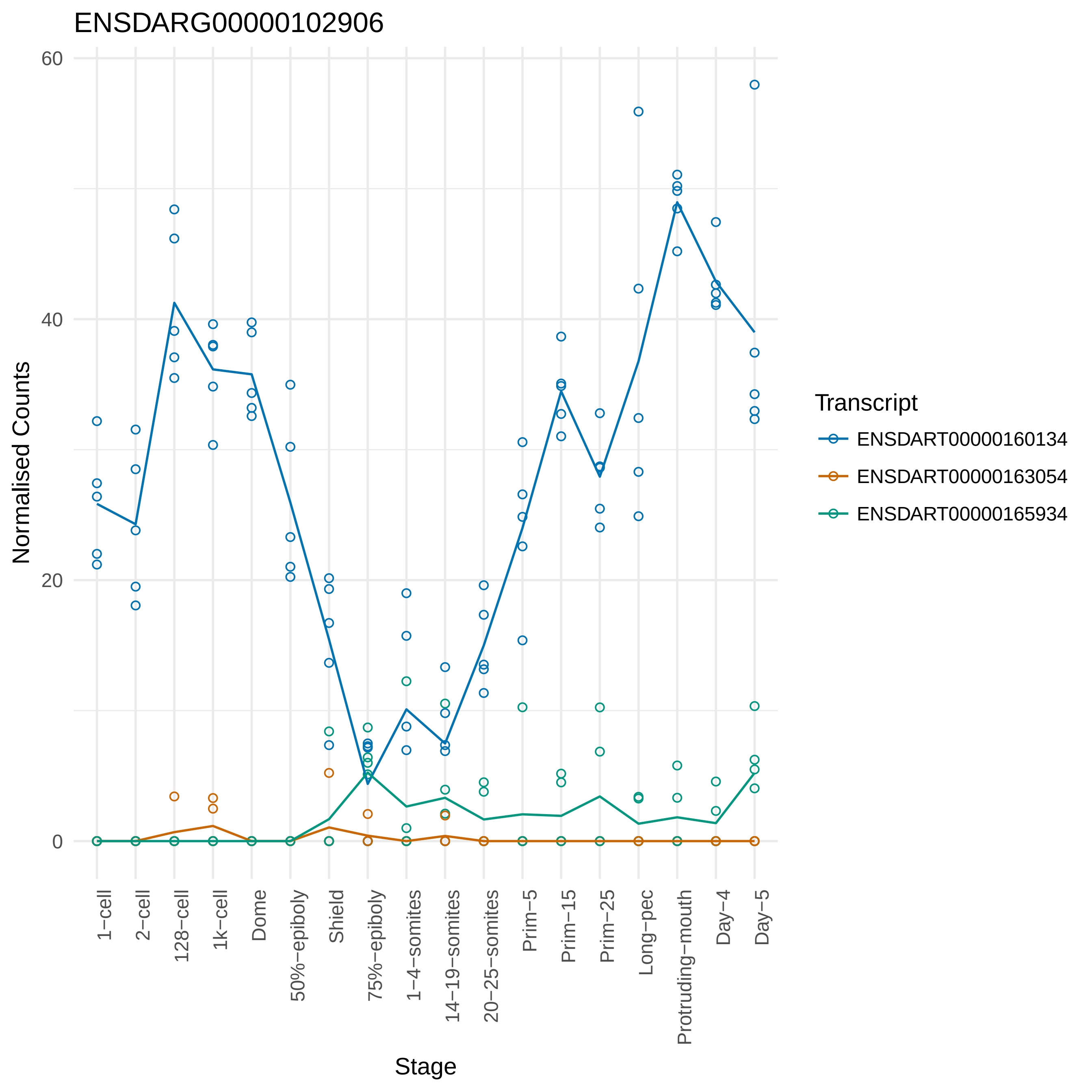

ENSDARG00000102906
Normalised Counts
0
20
40
60
Stage
1−cell
2−cell
128−cell
1k−cell
Dome
50%−epiboly
Shield
75%−epiboly
1−4−somites
14−19−somites
20−25−somites
Prim−5
Prim−15
Prim−25
Long−pec
Protruding−mouth
Day−4
Day−5
Transcript
ENSDART00000160134
ENSDART00000163054
ENSDART00000165934

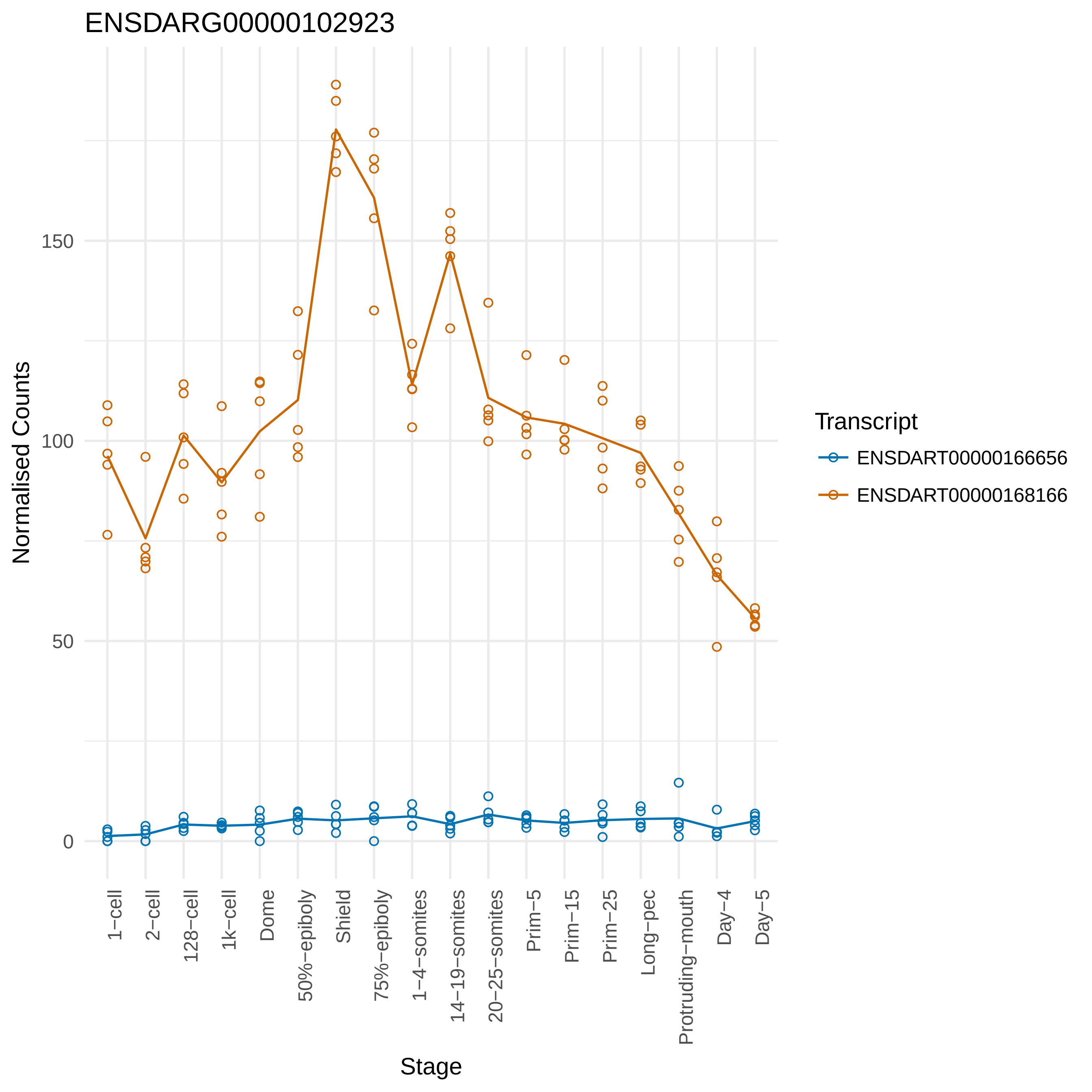

ENSDARG00000102923
Normalised Counts
150
100
50
0
1−cell
2−cell
128−cell
1k−cell
Dome
50%−epiboly
Shield
75%−epiboly
1−4−somites
14−19−somites
20−25−somites
Prim−5
Prim−15
Prim−25
Long−pec
Protruding−mouth
Day−4
Day−5
Stage
Transcript
ENSDART00000166656
ENSDART00000168166

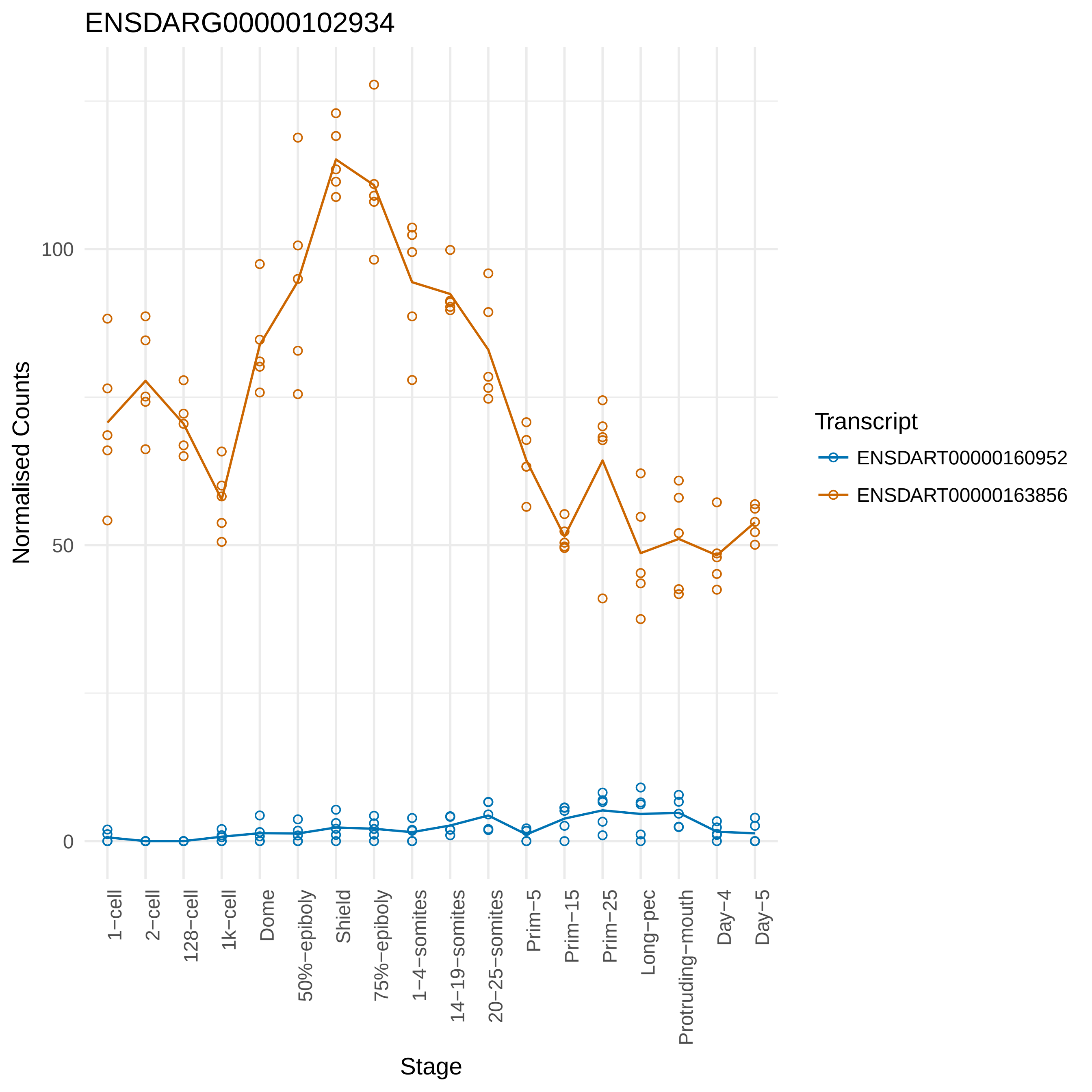

ENSDARG00000102934
Normalised Counts
100
50
0
1−cell
2−cell
128−cell
1k−cell
Dome
50%−epiboly
Shield
75%−epiboly
1−4−somites
14−19−somites
20−25−somites
Prim−5
Prim−15
Prim−25
Long−pec
Protruding−mouth
Day−4
Day−5
Stage
Transcript
ENSDART00000160952
ENSDART00000163856

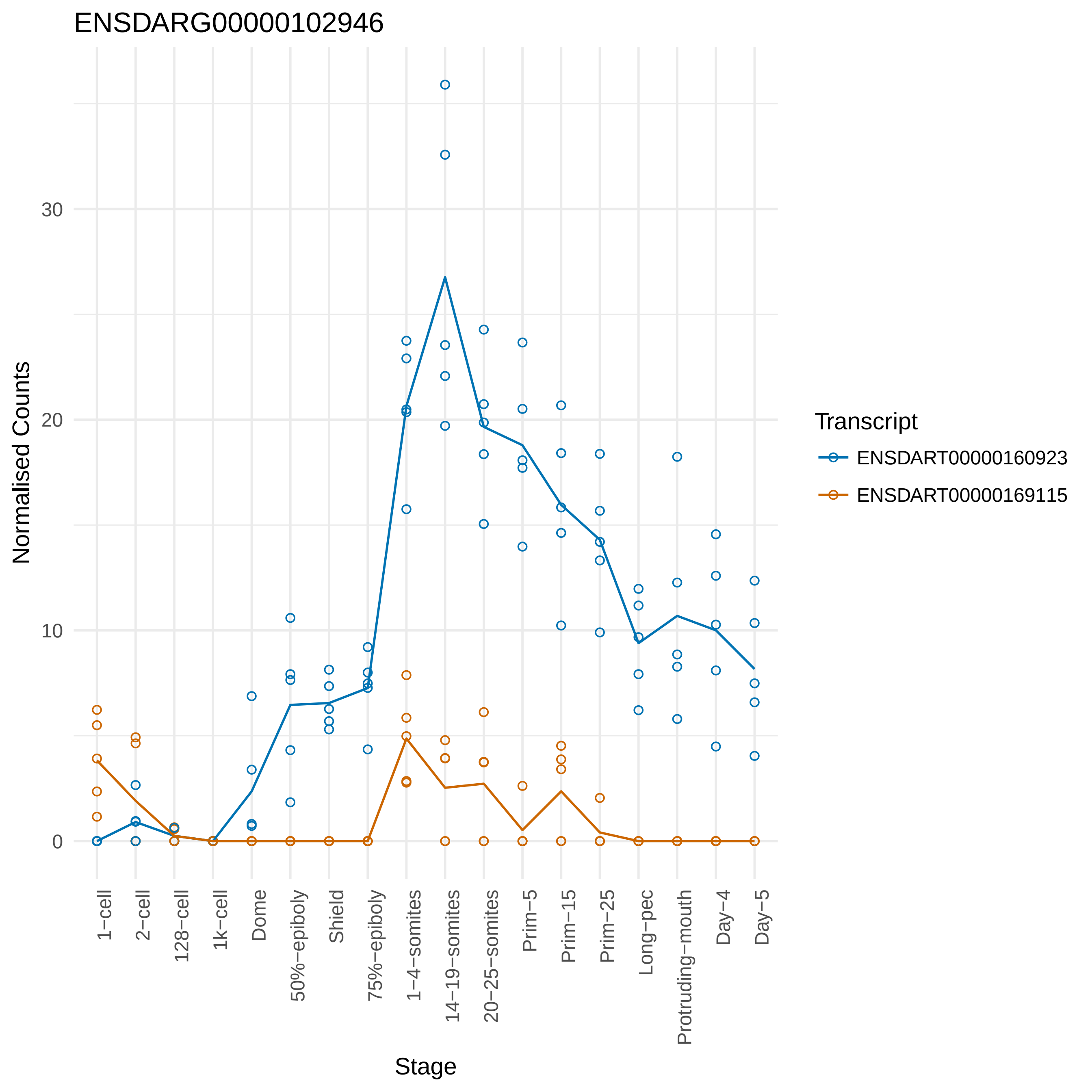

ENSDARG00000102946
Normalised Counts
30
20
10
0
1−cell
2−cell
128−cell
1k−cell
Dome
50%−epiboly
Shield
75%−epiboly
1−4−somites
14−19−somites
20−25−somites
Prim−5
Prim−15
Prim−25
Long−pec
Protruding−mouth
Day−4
Day−5
Stage
Transcript
ENSDART00000160923
ENSDART00000169115

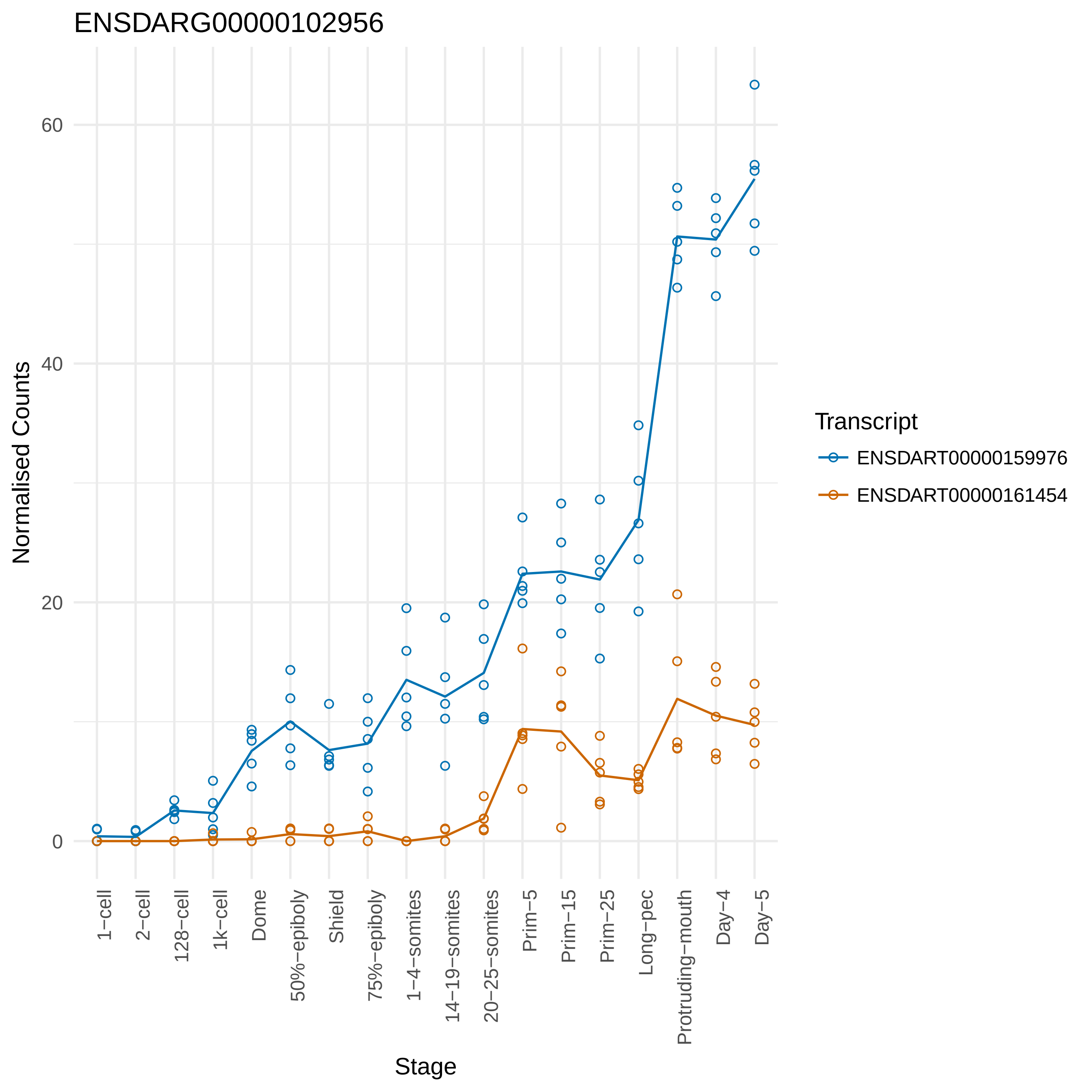
ENSDARG00000102956
Normalised Counts
0
20
40
60
Stage
1−cell
2−cell
128−cell
1k−cell
Dome
50%−epiboly
Shield
75%−epiboly
1−4−somites
14−19−somites
20−25−somites
Prim−5
Prim−15
Prim−25
Long−pec
Protruding−mouth
Day−4
Day−5
Transcript
ENSDART00000159976
ENSDART00000161454

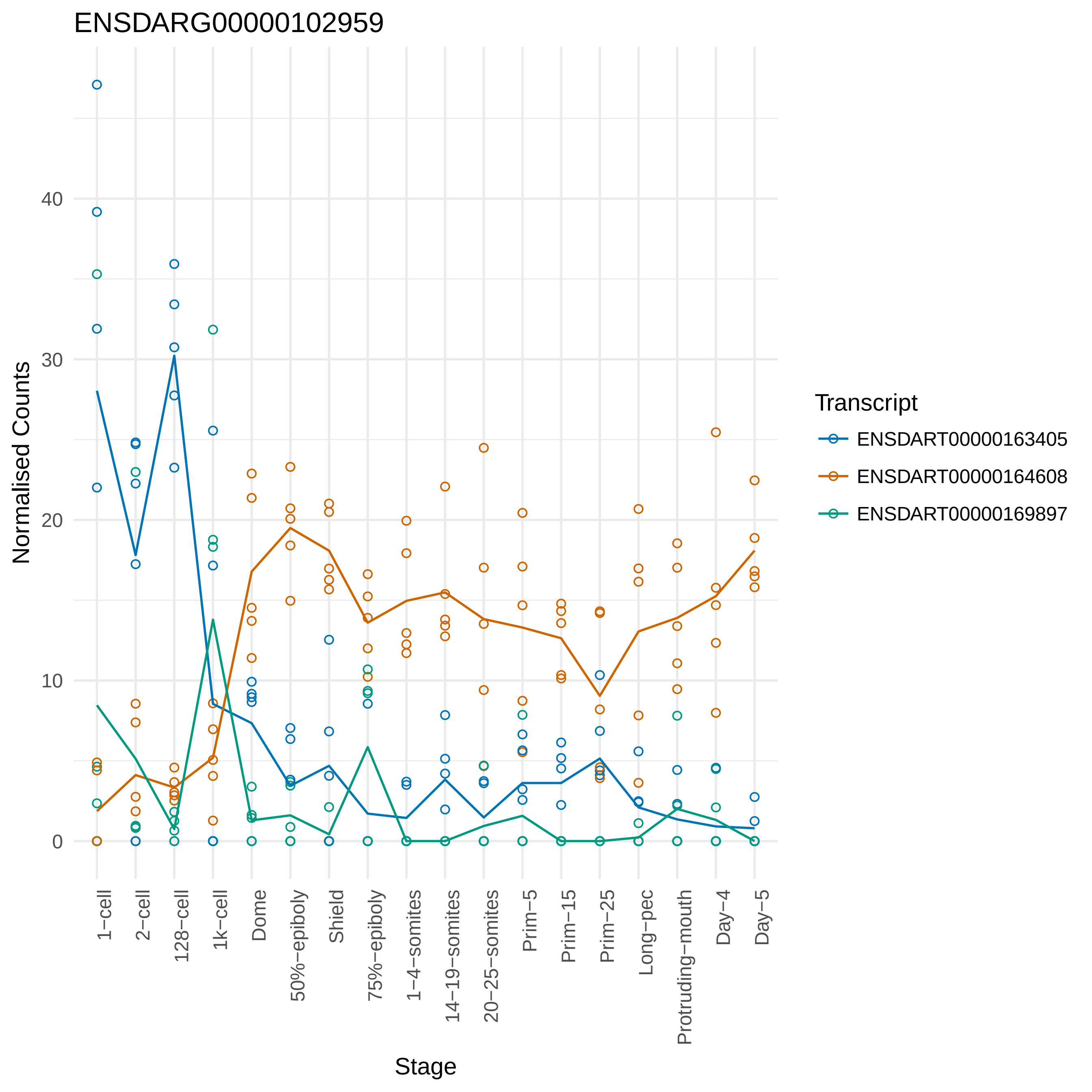
ENSDARG00000102959
Normalised Counts
0
10
20
30
40
Stage
1−cell
2−cell
128−cell
1k−cell
Dome
50%−epiboly
Shield
75%−epiboly
1−4−somites
14−19−somites
20−25−somites
Prim−5
Prim−15
Prim−25
Long−pec
Protruding−mouth
Day−4
Day−5
Transcript
ENSDART00000163405
ENSDART00000164608
ENSDART00000169897

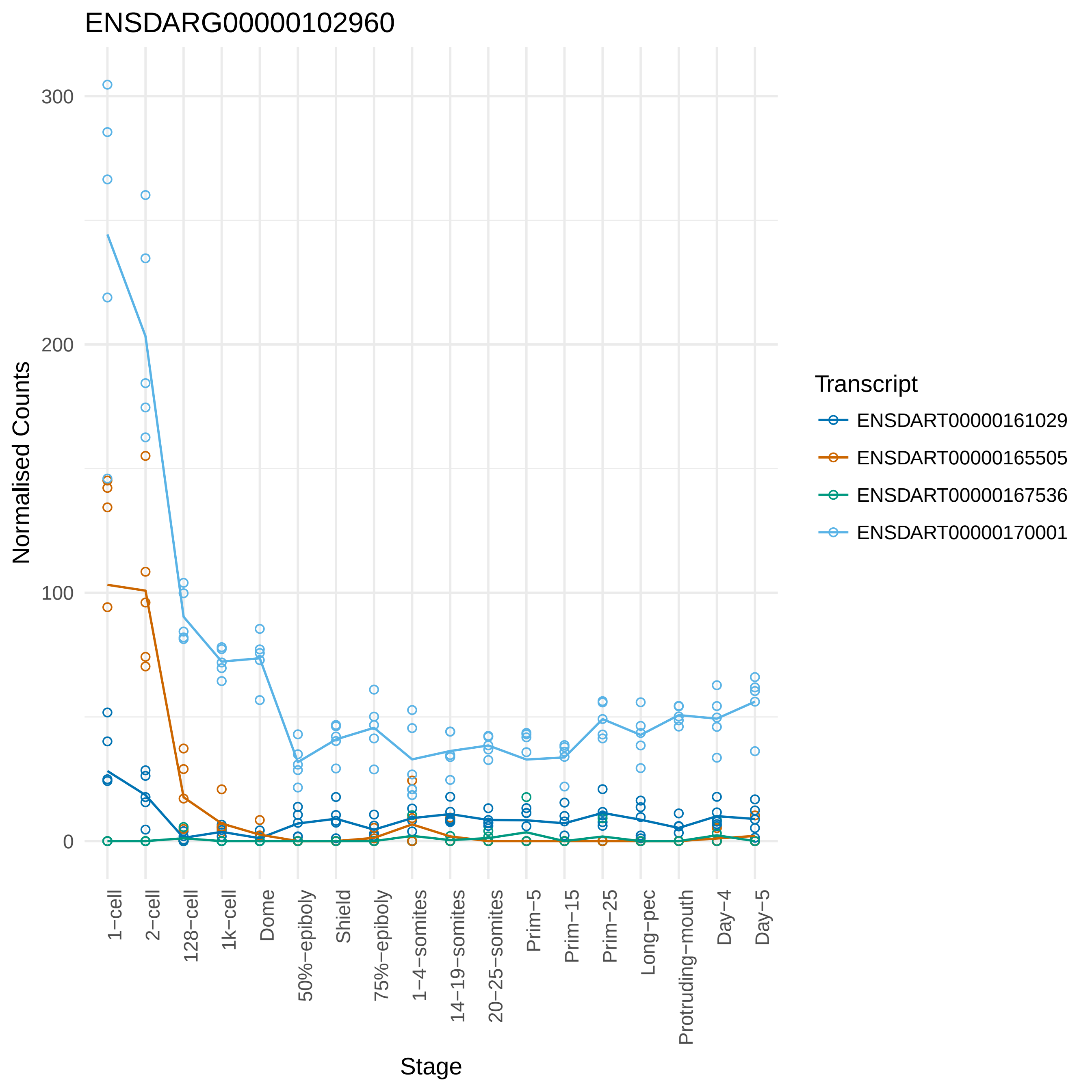

ENSDARG00000102960
Normalised Counts
300
200
100
0
Stage
1−cell
2−cell
128−cell
1k−cell
Dome
50%−epiboly
Shield
75%−epiboly
1−4−somites
14−19−somites
20−25−somites
Prim−5
Prim−15
Prim−25
Long−pec
Protruding−mouth
Day−4
Day−5
Transcript
ENSDART00000161029
ENSDART00000165505
ENSDART00000167536
ENSDART00000170001

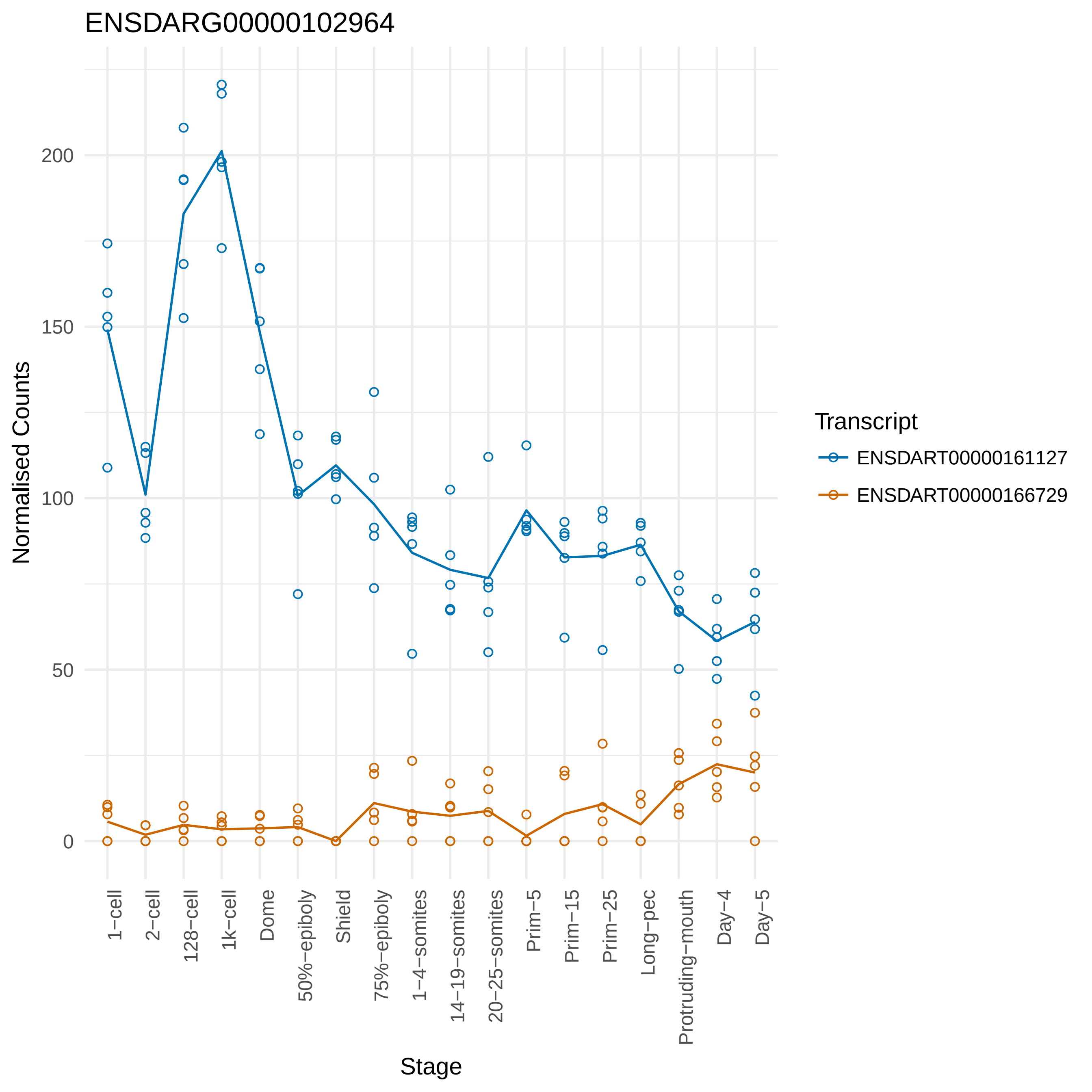
ENSDARG00000102964
Normalised Counts
200
150
100
50
0
1−cell
2−cell
128−cell
1k−cell
Dome
50%−epiboly
Shield
75%−epiboly
1−4−somites
14−19−somites
20−25−somites
Prim−5
Prim−15
Prim−25
Long−pec
Protruding−mouth
Day−4
Day−5
Stage
Transcript
ENSDART00000161127
ENSDART00000166729

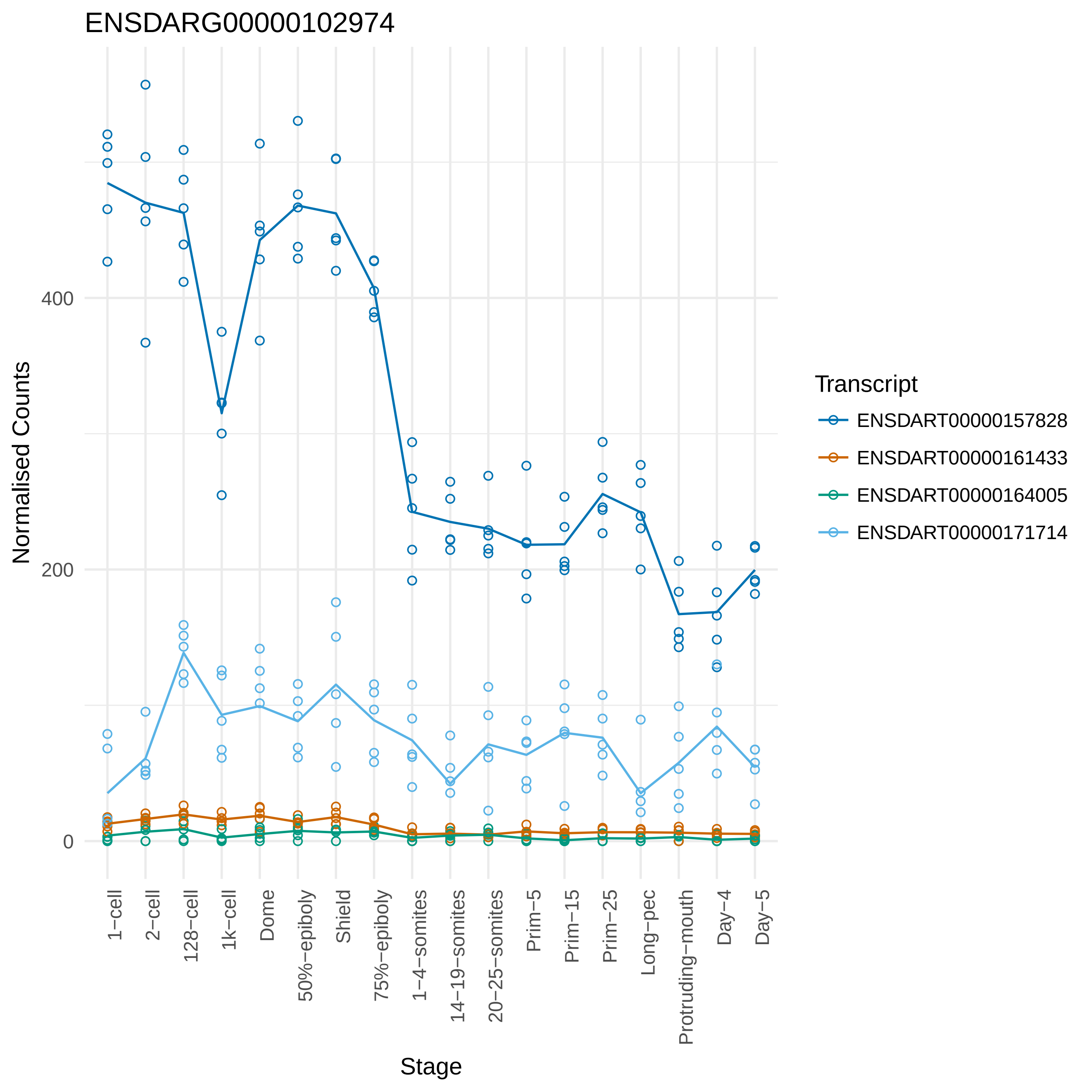

ENSDARG00000102974
Normalised Counts
400
200
0
1−cell
2−cell
128−cell
1k−cell
Dome
50%−epiboly
Shield
75%−epiboly
1−4−somites
14−19−somites
20−25−somites
Prim−5
Prim−15
Prim−25
Long−pec
Protruding−mouth
Day−4
Day−5
Stage
Transcript
ENSDART00000157828
ENSDART00000161433
ENSDART00000164005
ENSDART00000171714

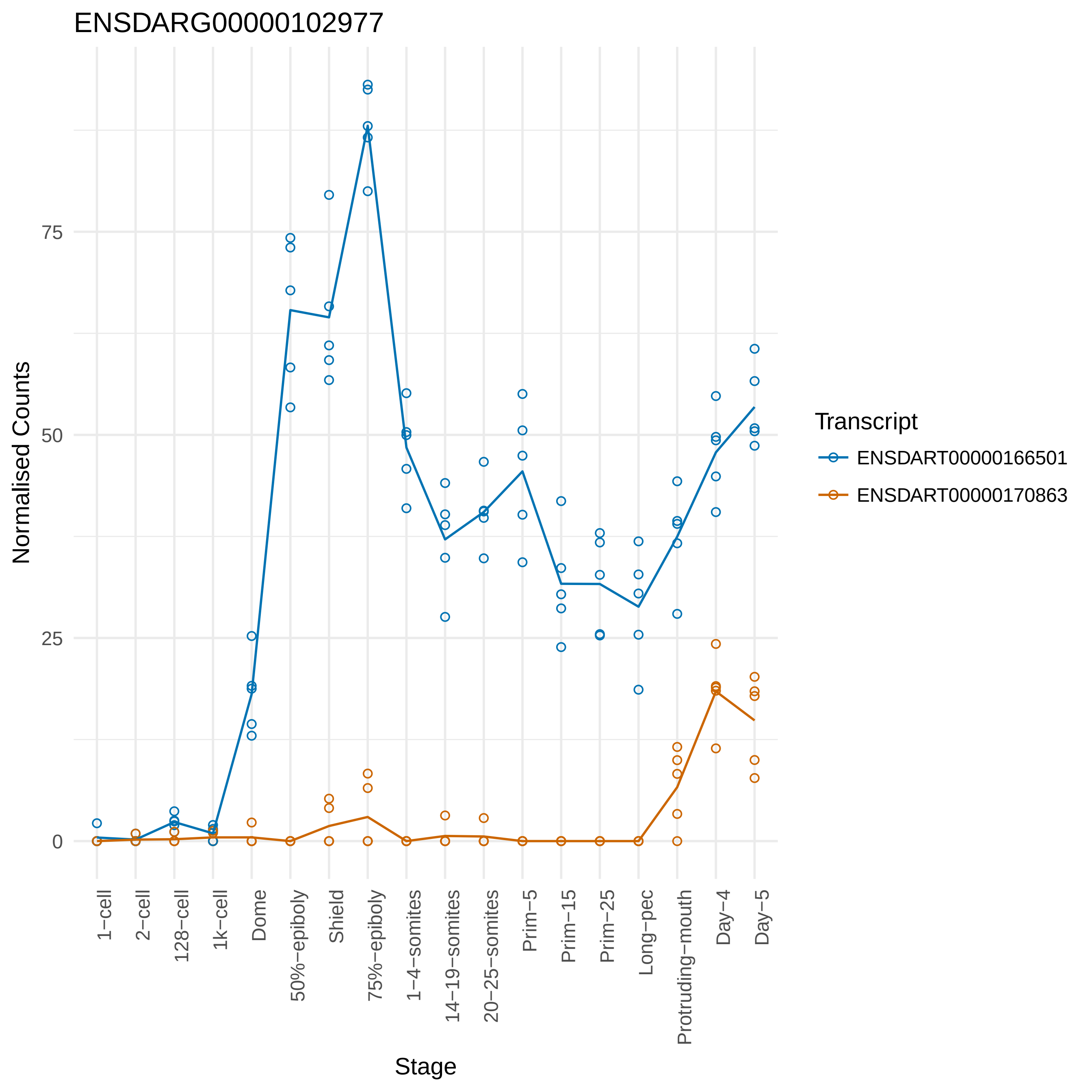

ENSDARG00000102977
Normalised Counts
0
25
50
75
Stage
1−cell
2−cell
128−cell
1k−cell
Dome
50%−epiboly
Shield
75%−epiboly
1−4−somites
14−19−somites
20−25−somites
Prim−5
Prim−15
Prim−25
Long−pec
Protruding−mouth
Day−4
Day−5
Transcript
ENSDART00000166501
ENSDART00000170863

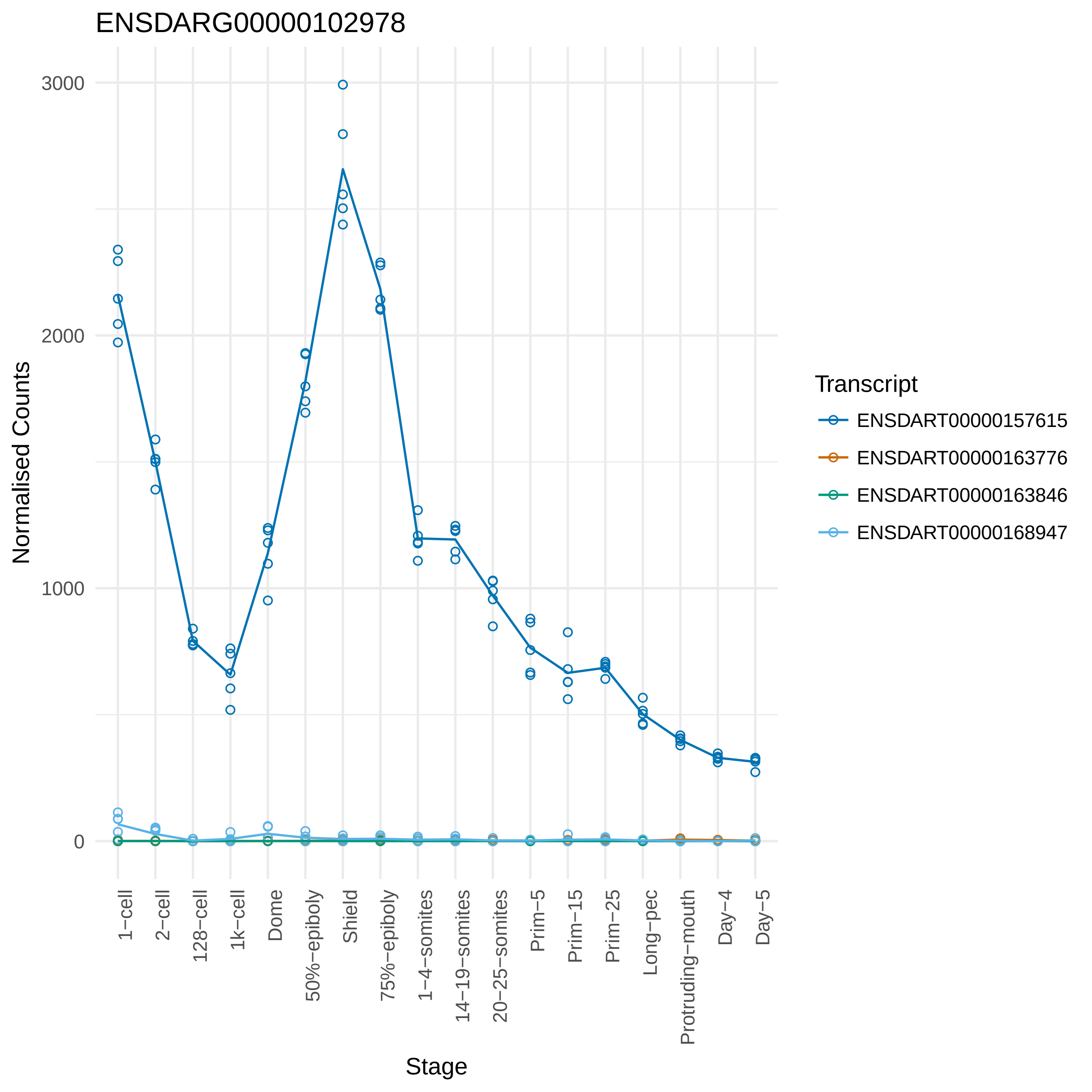
ENSDARG00000102978
Normalised Counts
3000
2000
1000
0
Stage
1−cell
2−cell
128−cell
1k−cell
Dome
50%−epiboly
Shield
75%−epiboly
1−4−somites
14−19−somites
20−25−somites
Prim−5
Prim−15
Prim−25
Long−pec
Protruding−mouth
Day−4
Day−5
Transcript
ENSDART00000157615
ENSDART00000163776
ENSDART00000163846
ENSDART00000168947

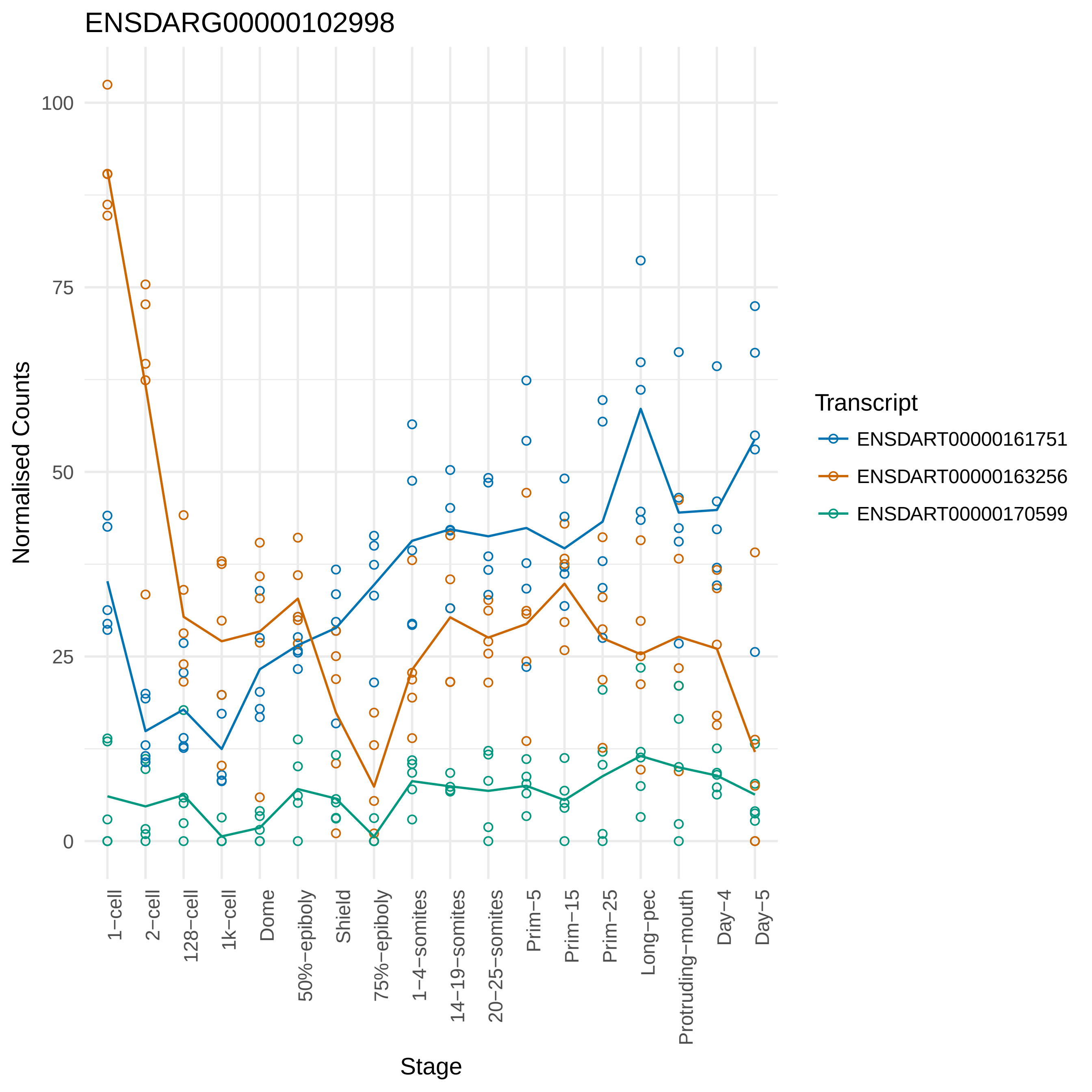

ENSDARG00000102998
Normalised Counts
100
75
50
25
0
1−cell
2−cell
128−cell
1k−cell
Dome
50%−epiboly
Shield
75%−epiboly
1−4−somites
14−19−somites
20−25−somites
Prim−5
Prim−15
Prim−25
Long−pec
Protruding−mouth
Day−4
Day−5
Stage
Transcript
ENSDART00000161751
ENSDART00000163256
ENSDART00000170599

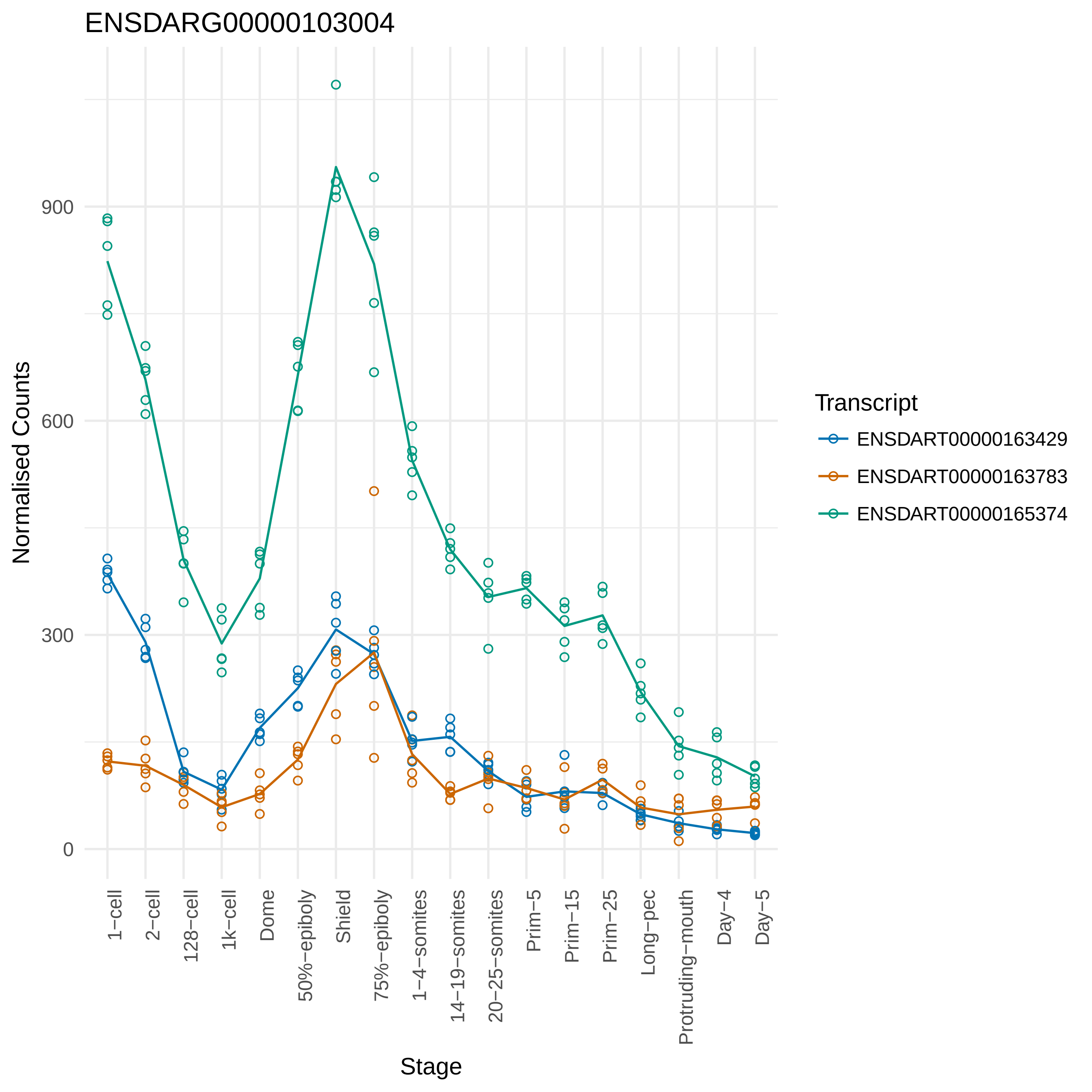
ENSDARG00000103004
Normalised Counts
900
600
300
0
1−cell
2−cell
128−cell
1k−cell
Dome
50%−epiboly
Shield
75%−epiboly
1−4−somites
14−19−somites
20−25−somites
Prim−5
Prim−15
Prim−25
Long−pec
Protruding−mouth
Day−4
Day−5
Stage
Transcript
ENSDART00000163429
ENSDART00000163783
ENSDART00000165374

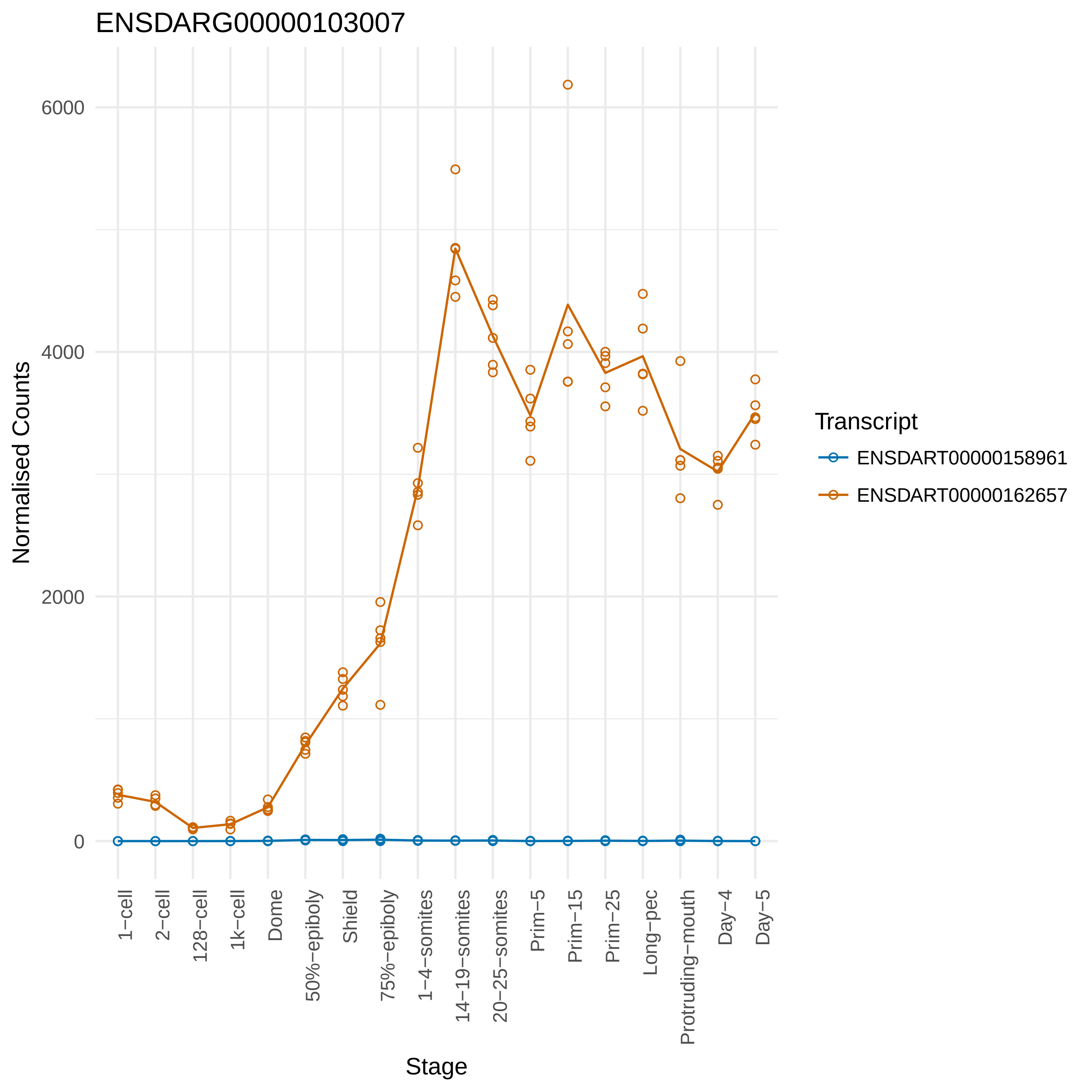

ENSDARG00000103007
Normalised Counts
6000
4000
2000
0
1−cell
2−cell
128−cell
1k−cell
Dome
50%−epiboly
Shield
75%−epiboly
1−4−somites
14−19−somites
20−25−somites
Prim−5
Prim−15
Prim−25
Long−pec
Protruding−mouth
Day−4
Day−5
Stage
Transcript
ENSDART00000158961
ENSDART00000162657

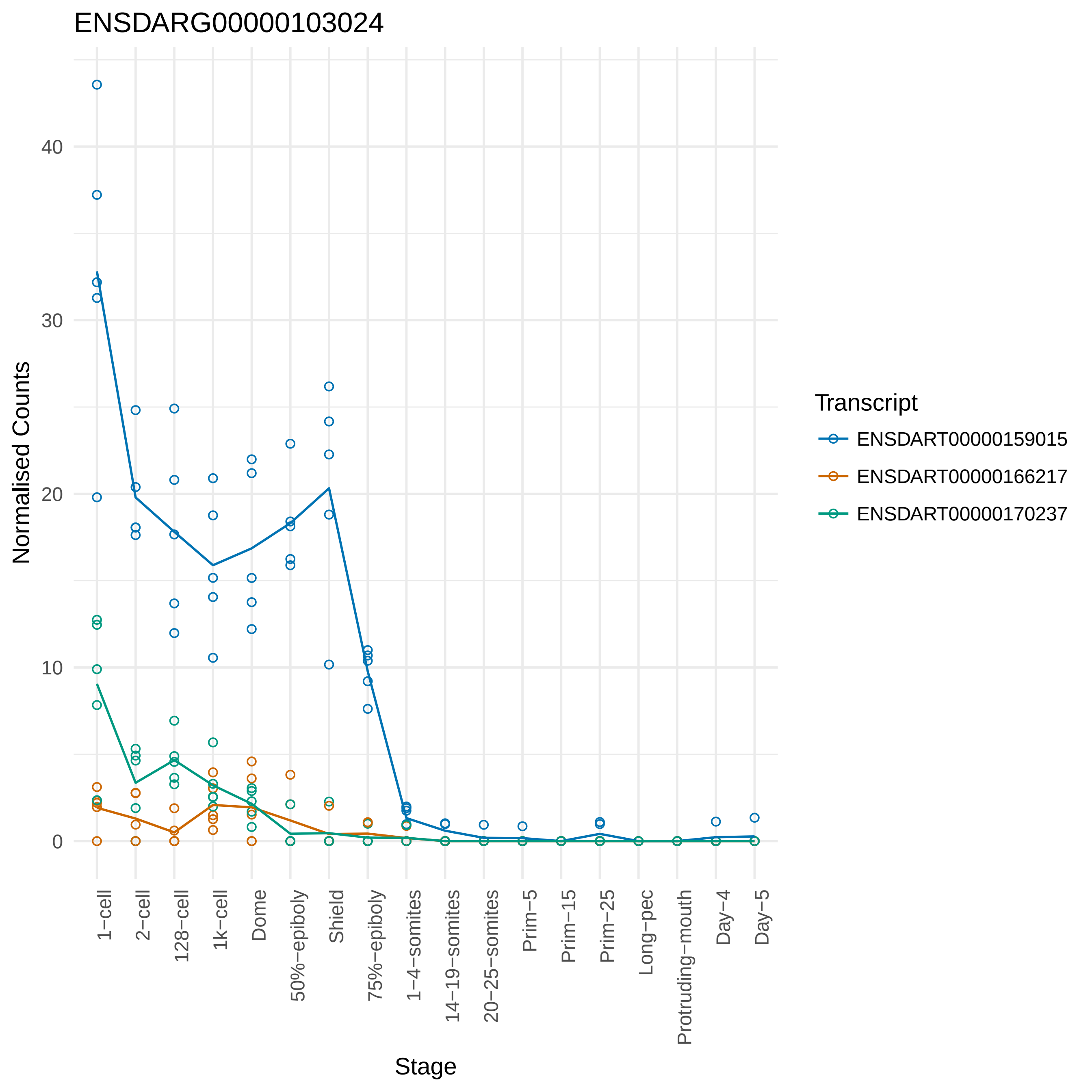
ENSDARG00000103024
Normalised Counts
0
10
20
30
40
Stage
1−cell
2−cell
128−cell
1k−cell
Dome
50%−epiboly
Shield
75%−epiboly
1−4−somites
14−19−somites
20−25−somites
Prim−5
Prim−15
Prim−25
Long−pec
Protruding−mouth
Day−4
Day−5
Transcript
ENSDART00000159015
ENSDART00000166217
ENSDART00000170237

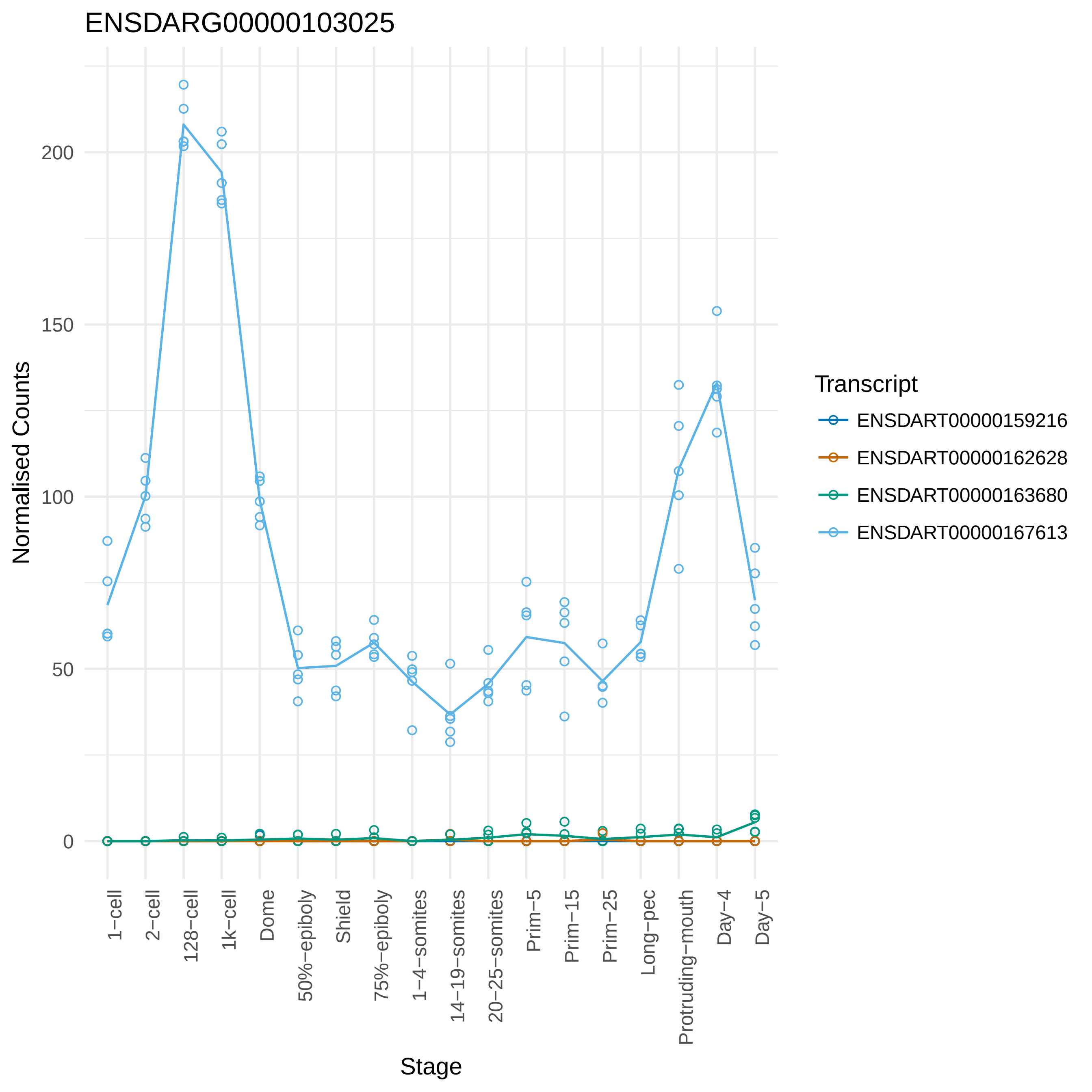
ENSDARG00000103025
Normalised Counts
0
50
100
150
200
Stage
1−cell
2−cell
128−cell
1k−cell
Dome
50%−epiboly
Shield
75%−epiboly
1−4−somites
14−19−somites
20−25−somites
Prim−5
Prim−15
Prim−25
Long−pec
Protruding−mouth
Day−4
Day−5
Transcript
ENSDART00000159216
ENSDART00000162628
ENSDART00000163680
ENSDART00000167613

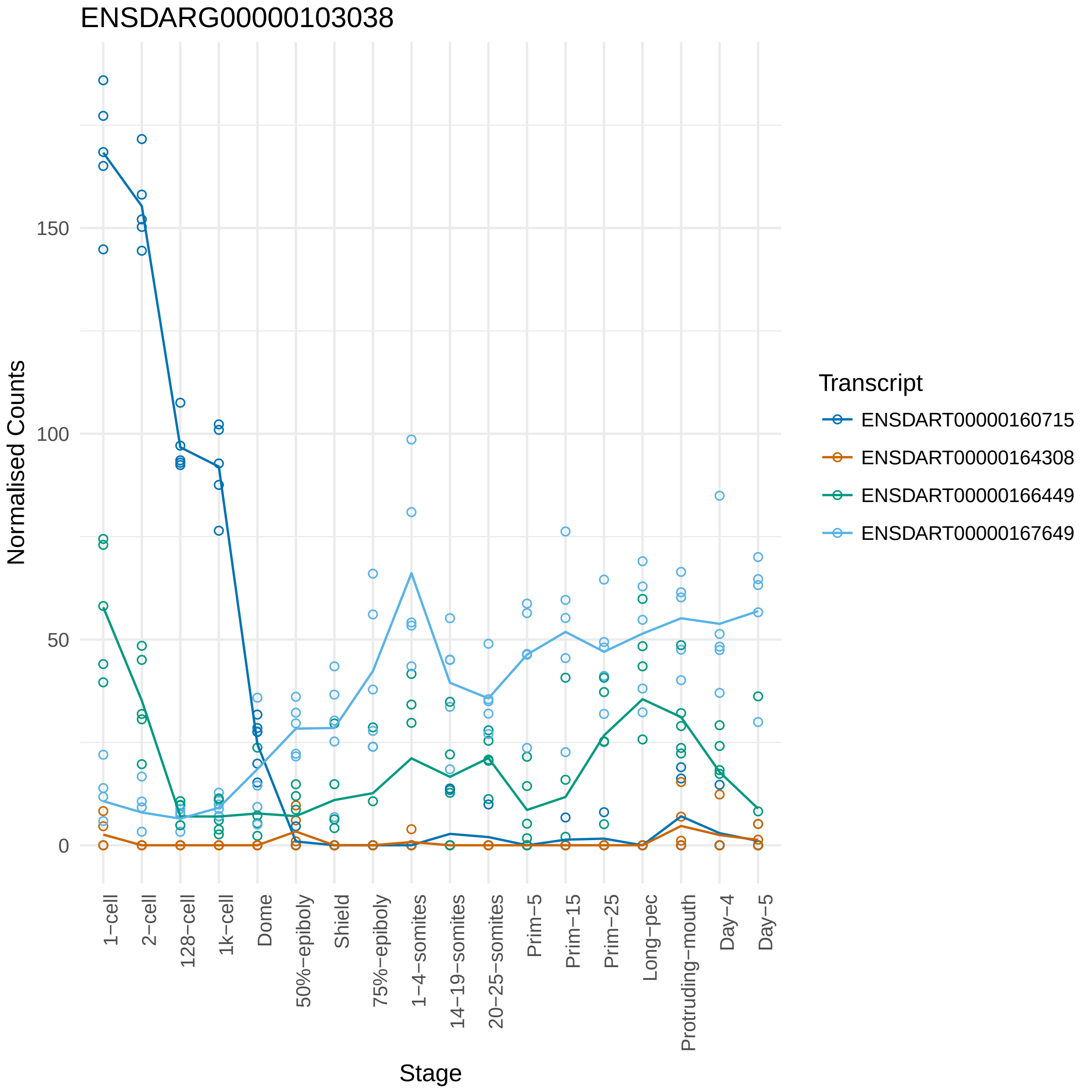

ENSDARG00000103038
Normalised Counts
150
100
50
0
Stage
1−cell
2−cell
128−cell
1k−cell
Dome
50%−epiboly
Shield
75%−epiboly
1−4−somites
14−19−somites
20−25−somites
Prim−5
Prim−15
Prim−25
Long−pec
Protruding−mouth
Day−4
Day−5
Transcript
ENSDART00000160715
ENSDART00000164308
ENSDART00000166449
ENSDART00000167649

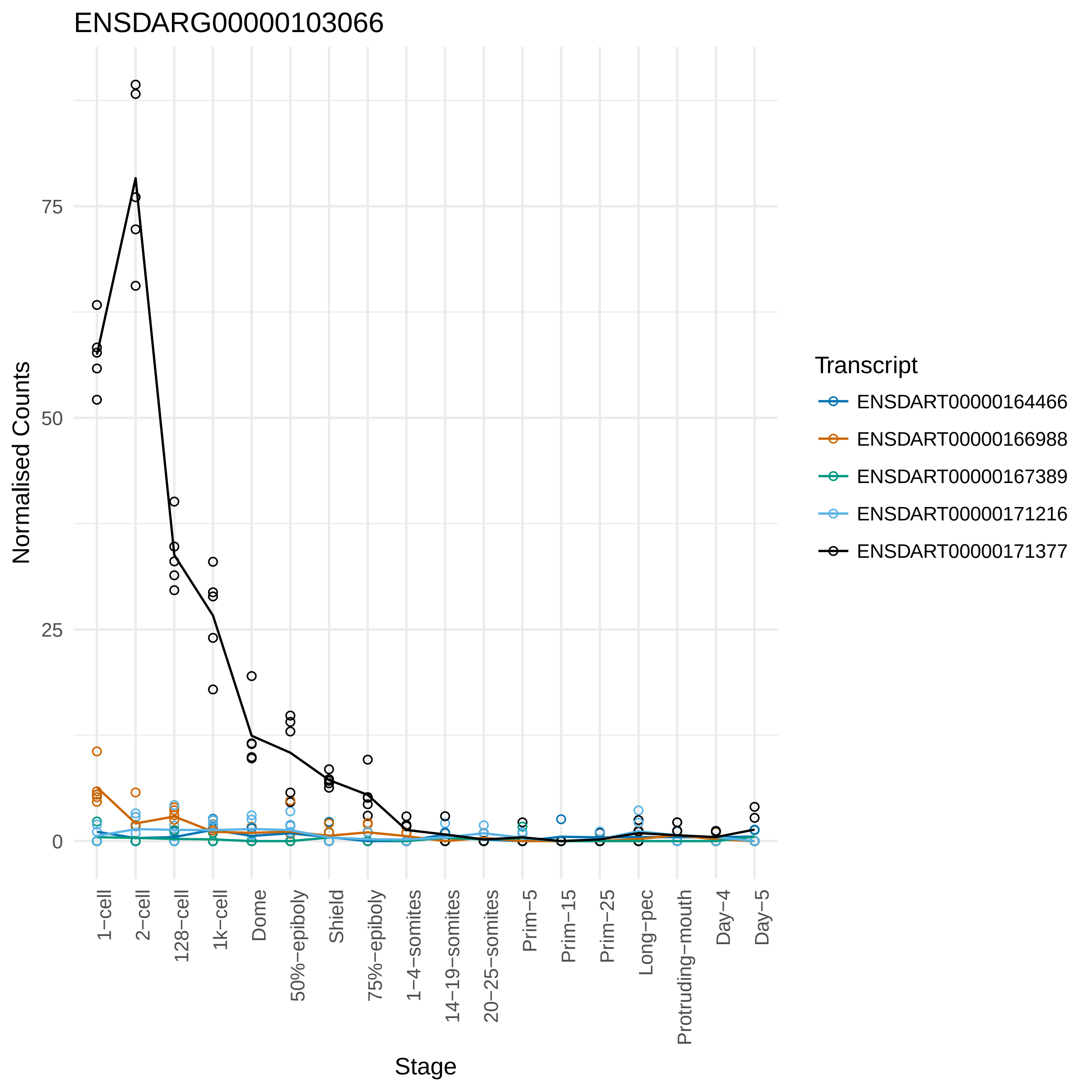

ENSDARG00000103066
Normalised Counts
75
50
25
0
1−cell
2−cell
128−cell
1k−cell
Dome
50%−epiboly
Shield
75%−epiboly
1−4−somites
14−19−somites
20−25−somites
Prim−5
Prim−15
Prim−25
Long−pec
Protruding−mouth
Day−4
Day−5
Stage
Transcript
ENSDART00000164466
ENSDART00000166988
ENSDART00000167389
ENSDART00000171216
ENSDART00000171377

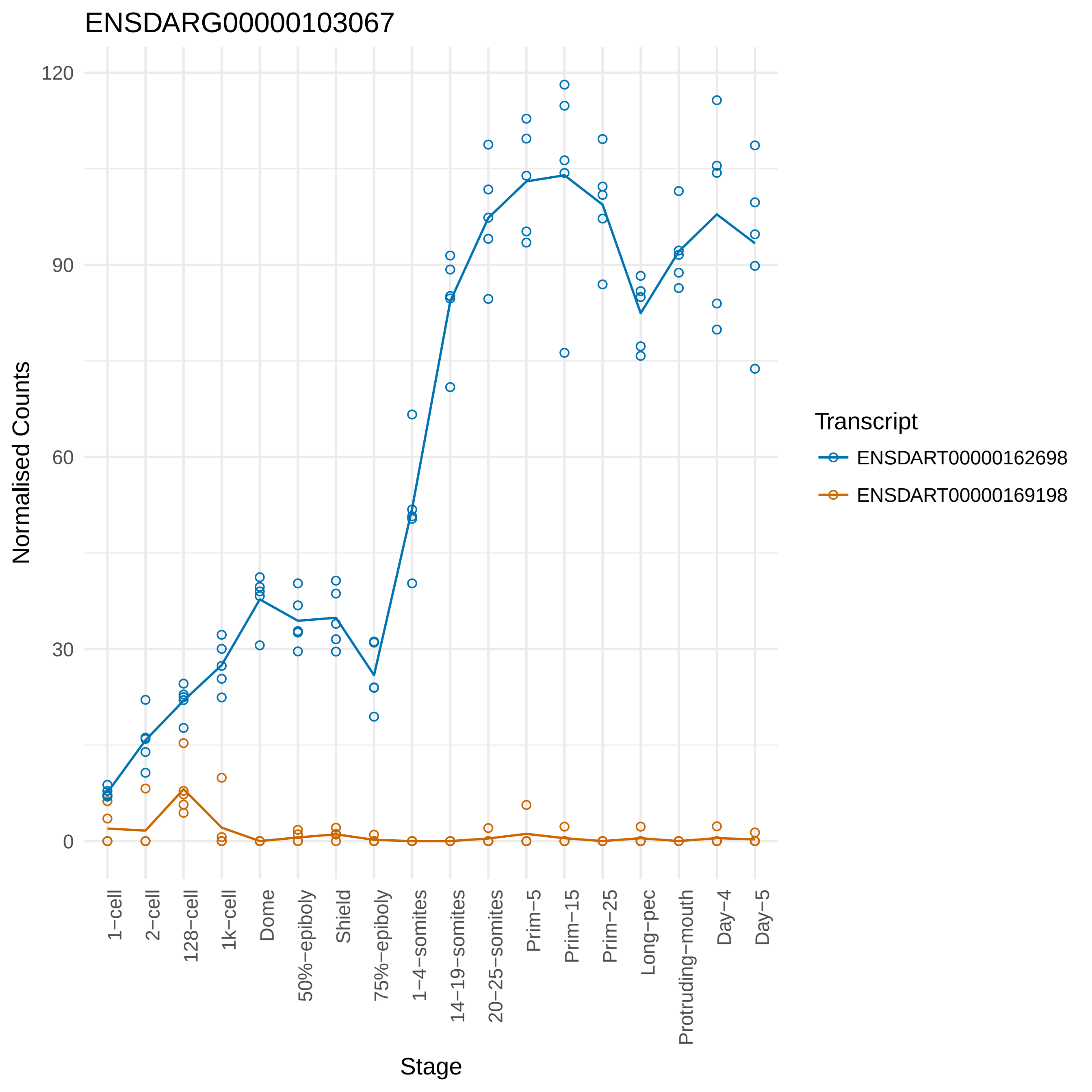
ENSDARG00000103067
Normalised Counts
120
90
60
30
0
1−cell
2−cell
128−cell
1k−cell
Dome
50%−epiboly
Shield
75%−epiboly
1−4−somites
14−19−somites
20−25−somites
Prim−5
Prim−15
Prim−25
Long−pec
Protruding−mouth
Day−4
Day−5
Stage
Transcript
ENSDART00000162698
ENSDART00000169198

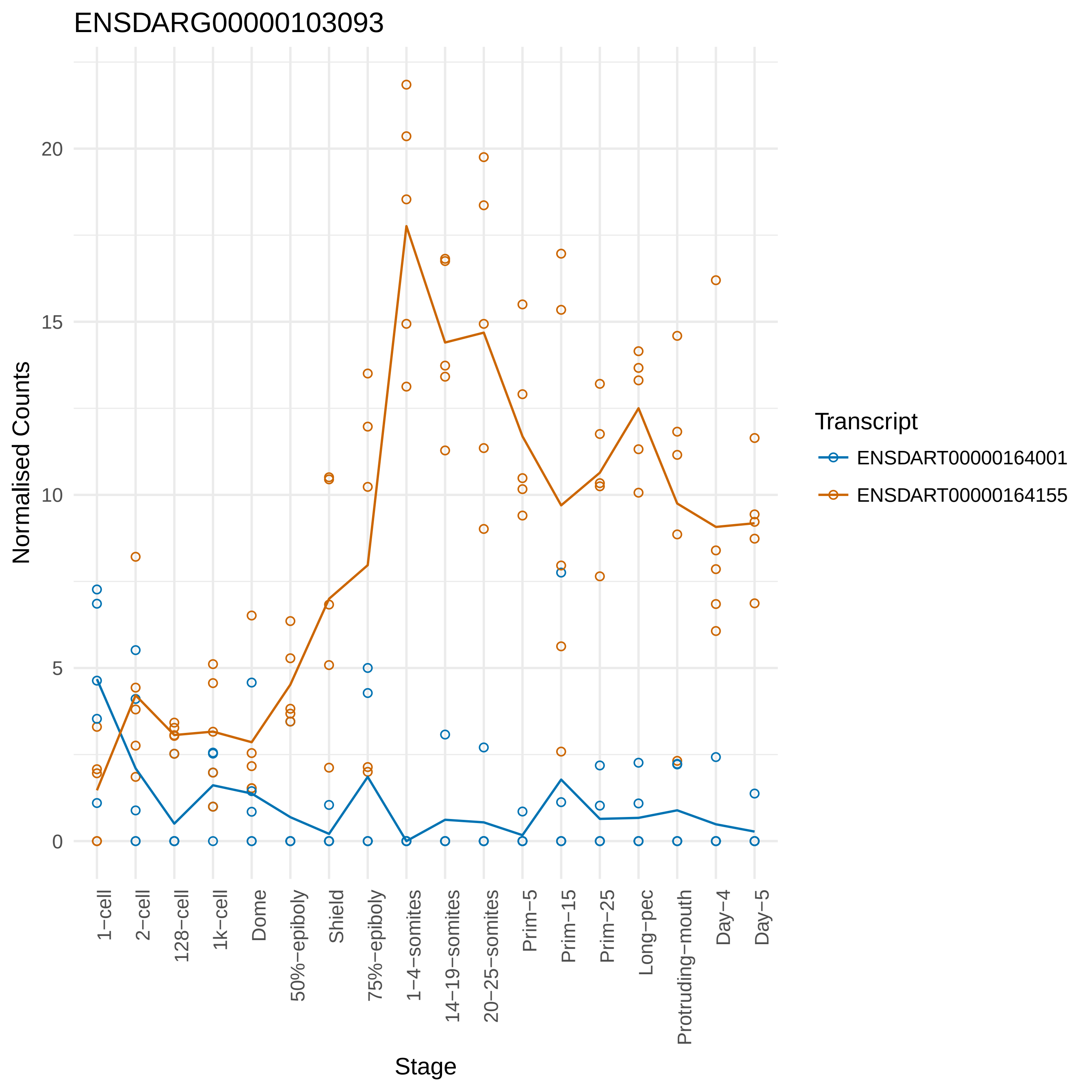
ENSDARG00000103093
Normalised Counts
Stage
20
15
10
5
0
1−cell
2−cell
128−cell
1k−cell
Dome
50%−epiboly
Shield
75%−epiboly
1−4−somites
14−19−somites
20−25−somites
Prim−5
Prim−15
Prim−25
Long−pec
Protruding−mouth
Day−4
Day−5
Transcript
ENSDART00000164001
ENSDART00000164155

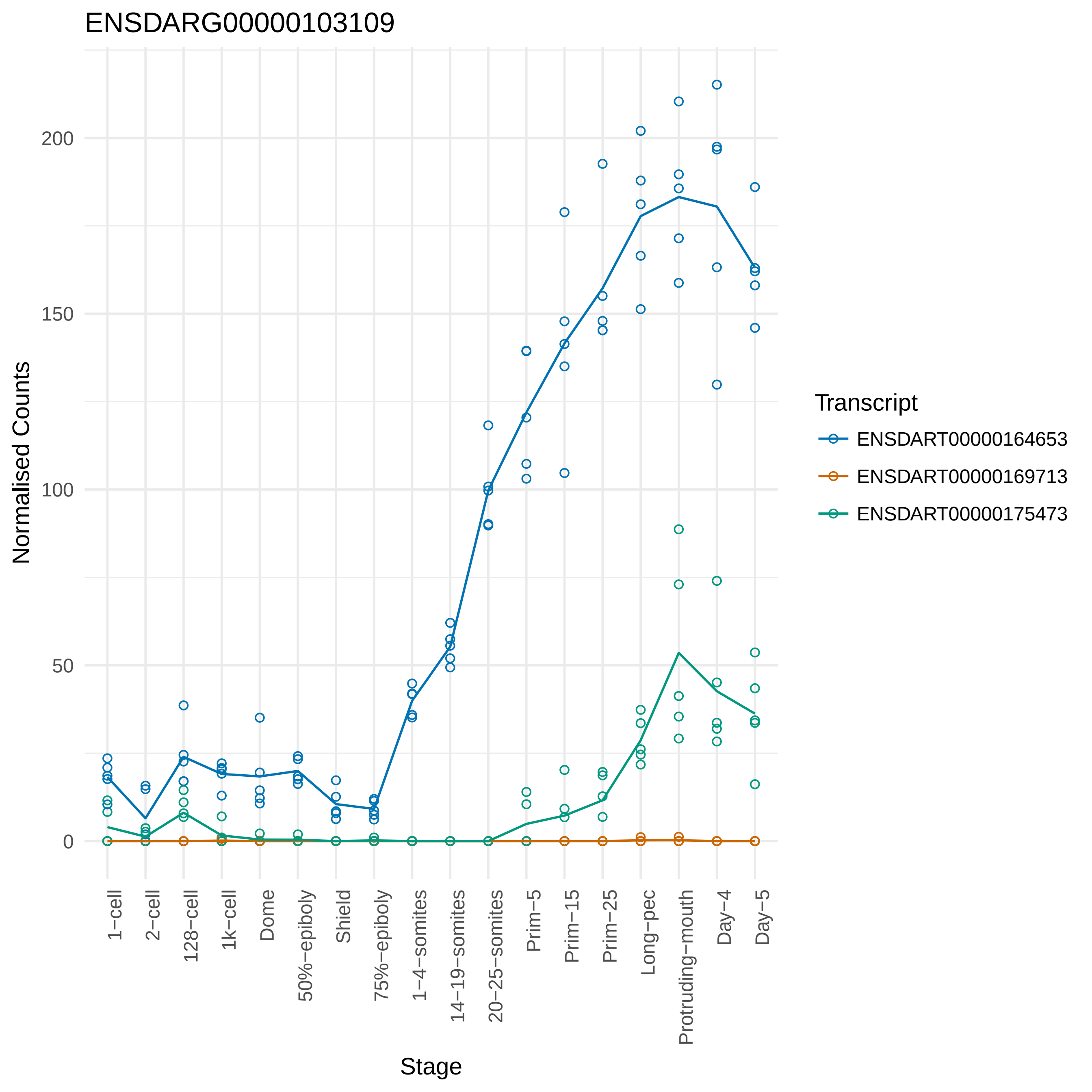

ENSDARG00000103109
Normalised Counts
0
50
100
150
200
1−cell
2−cell
128−cell
1k−cell
Dome
50%−epiboly
Shield
75%−epiboly
1−4−somites
14−19−somites
20−25−somites
Prim−5
Prim−15
Prim−25
Long−pec
Protruding−mouth
Day−4
Day−5
Stage
Transcript
ENSDART00000164653
ENSDART00000169713
ENSDART00000175473

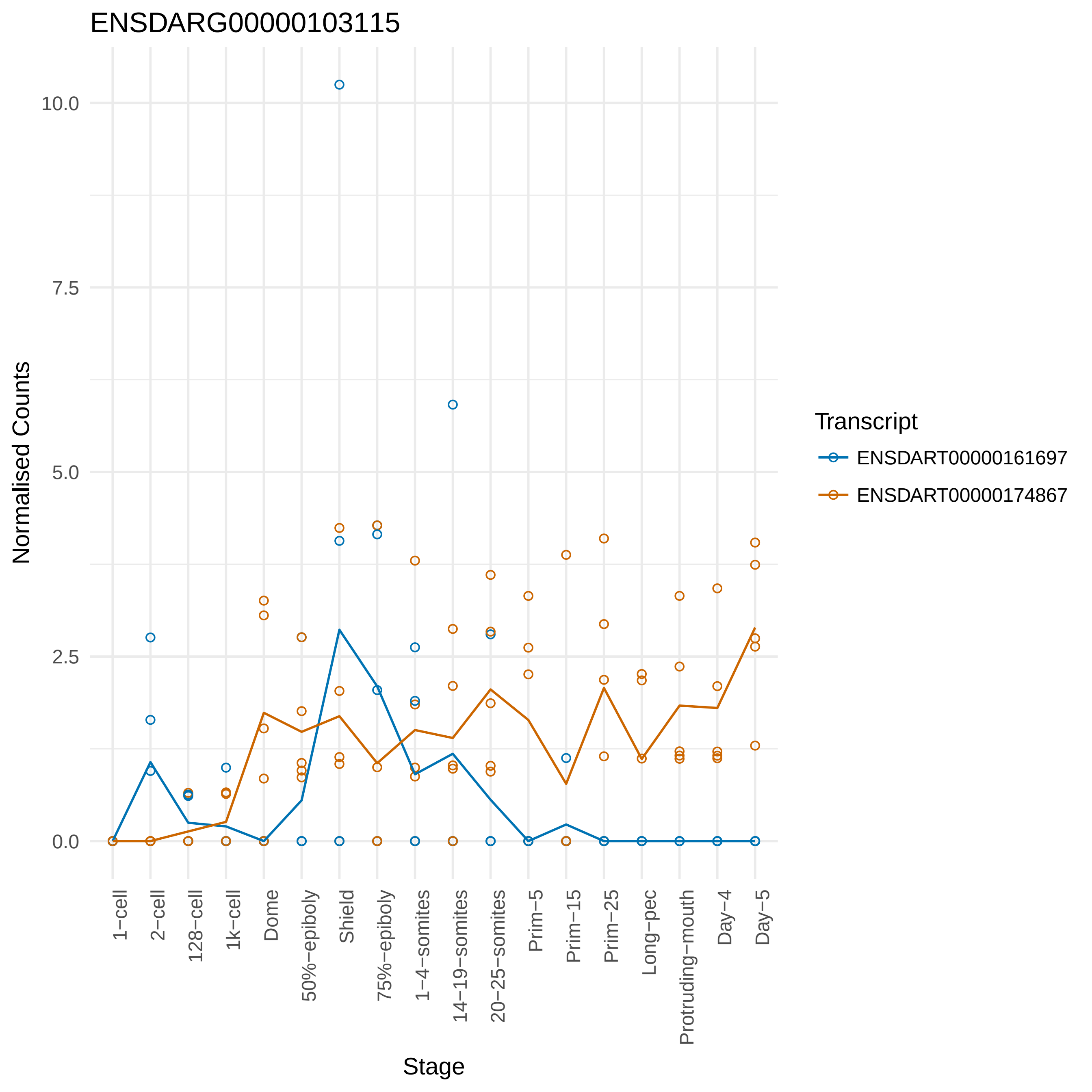

ENSDARG00000103115
Normalised Counts
10.0
7.5
5.0
2.5
0.0
1−cell
2−cell
128−cell
1k−cell
Dome
50%−epiboly
Shield
75%−epiboly
1−4−somites
14−19−somites
20−25−somites
Prim−5
Prim−15
Prim−25
Long−pec
Protruding−mouth
Day−4
Day−5
Stage
Transcript
ENSDART00000161697
ENSDART00000174867

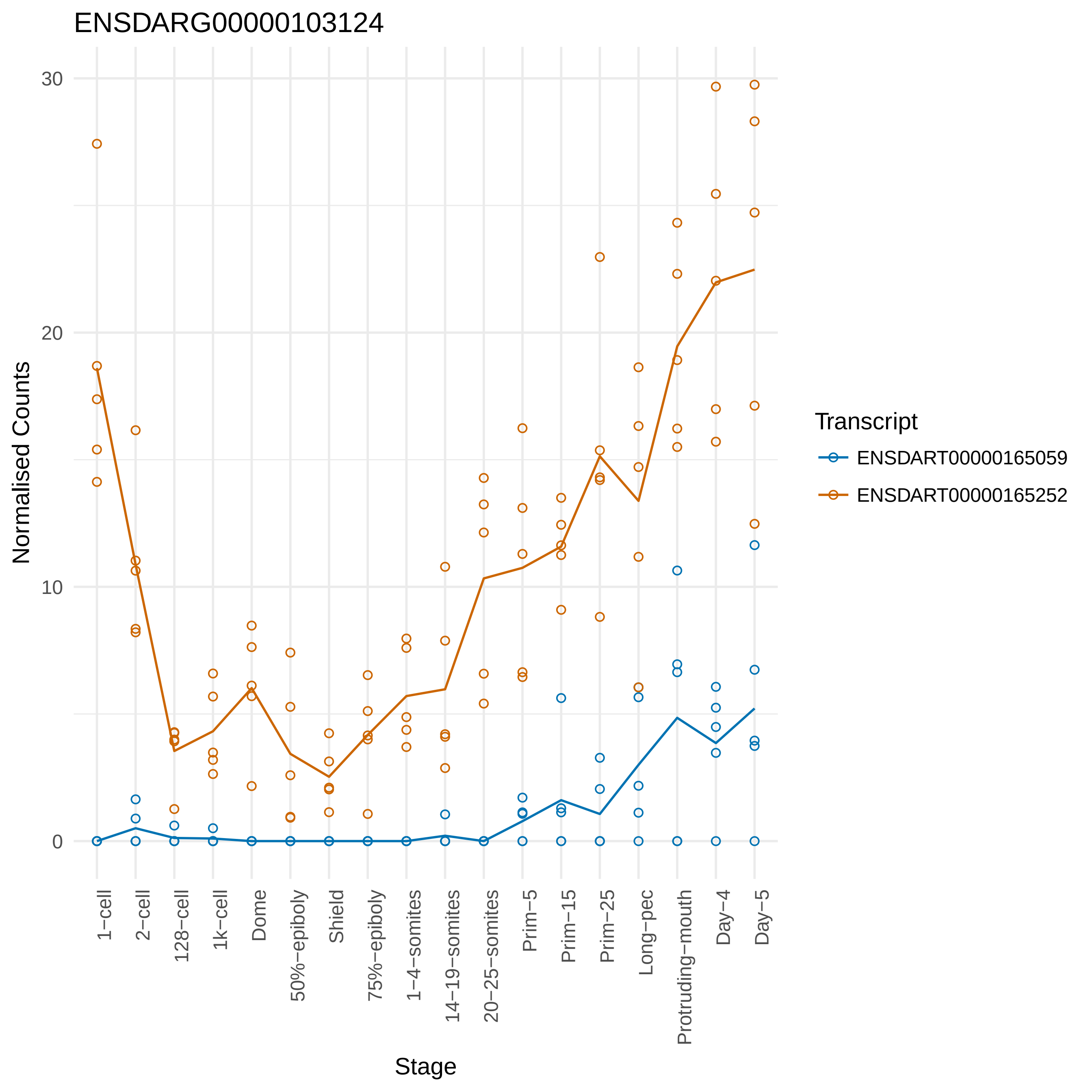
ENSDARG00000103124
Normalised Counts
30
20
10
0
1−cell
2−cell
128−cell
1k−cell
Dome
50%−epiboly
Shield
75%−epiboly
1−4−somites
14−19−somites
20−25−somites
Prim−5
Prim−15
Prim−25
Long−pec
Protruding−mouth
Day−4
Day−5
Stage
Transcript
ENSDART00000165059
ENSDART00000165252

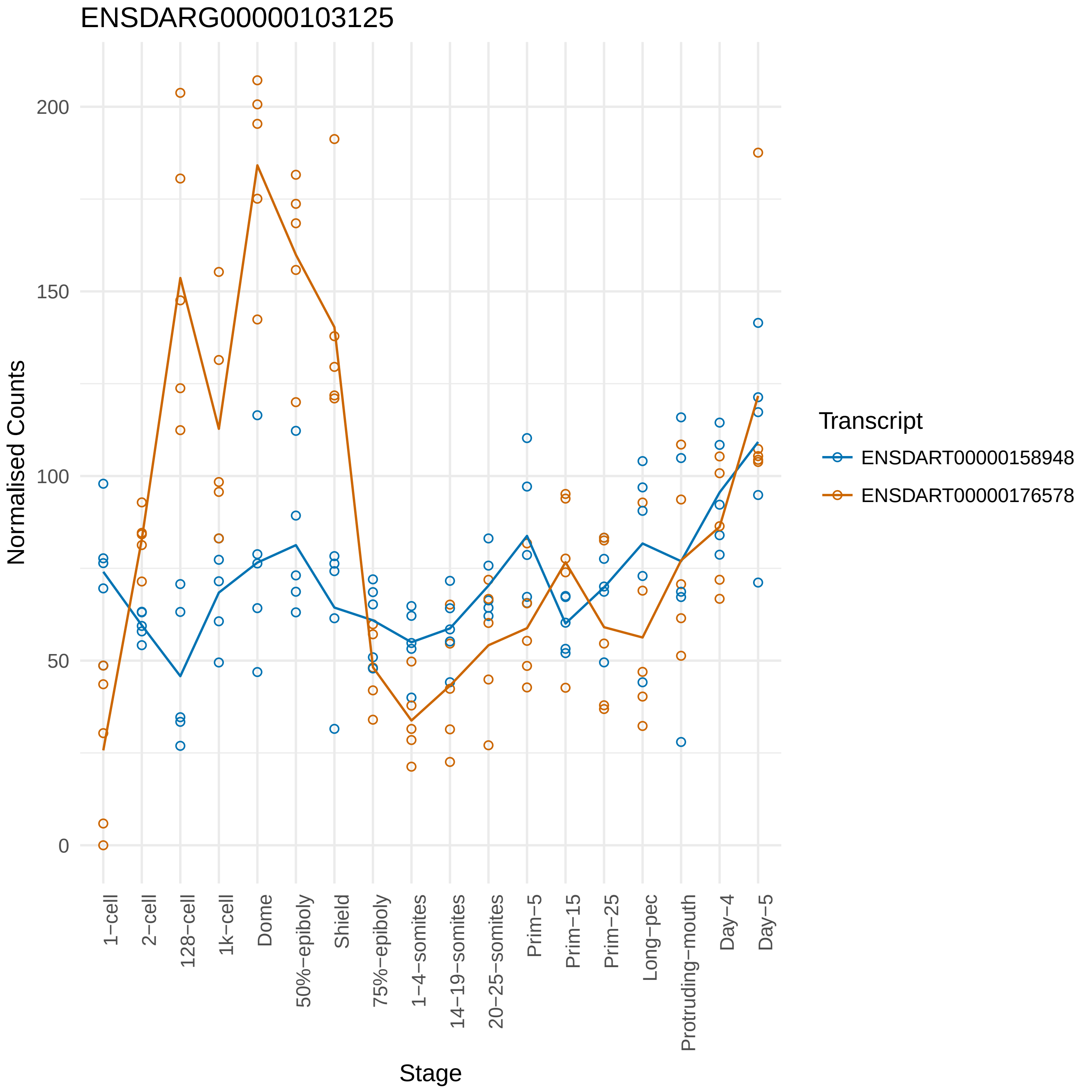

ENSDARG00000103125
Normalised Counts
0
50
100
150
200
Stage
1−cell
2−cell
128−cell
1k−cell
Dome
50%−epiboly
Shield
75%−epiboly
1−4−somites
14−19−somites
20−25−somites
Prim−5
Prim−15
Prim−25
Long−pec
Protruding−mouth
Day−4
Day−5
Transcript
ENSDART00000158948
ENSDART00000176578

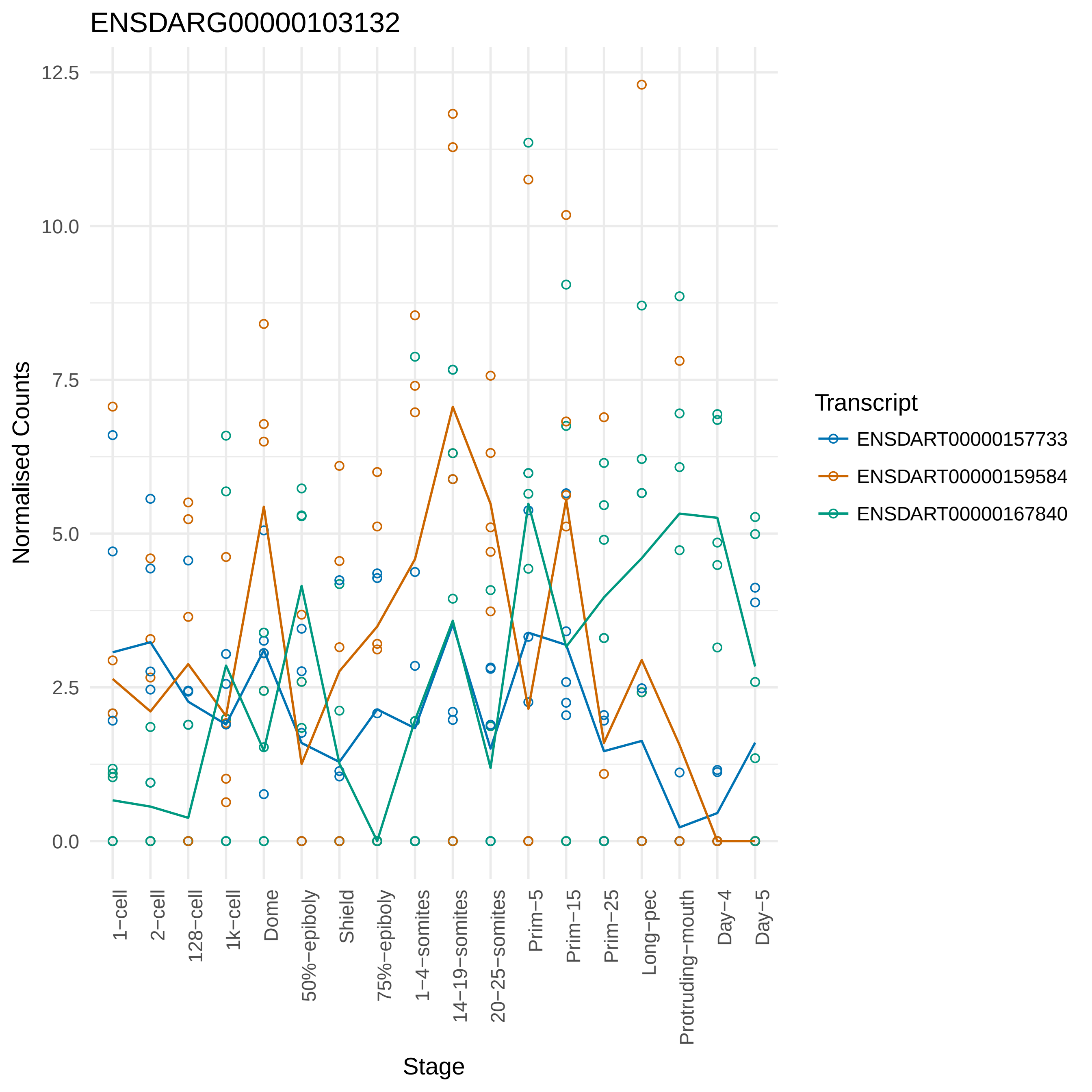
ENSDARG00000103132
Normalised Counts
12.5
10.0
7.5
5.0
2.5
0.0
1−cell
2−cell
128−cell
1k−cell
Dome
50%−epiboly
Shield
75%−epiboly
1−4−somites
14−19−somites
20−25−somites
Prim−5
Prim−15
Prim−25
Long−pec
Protruding−mouth
Day−4
Day−5
Stage
Transcript
ENSDART00000157733
ENSDART00000159584
ENSDART00000167840

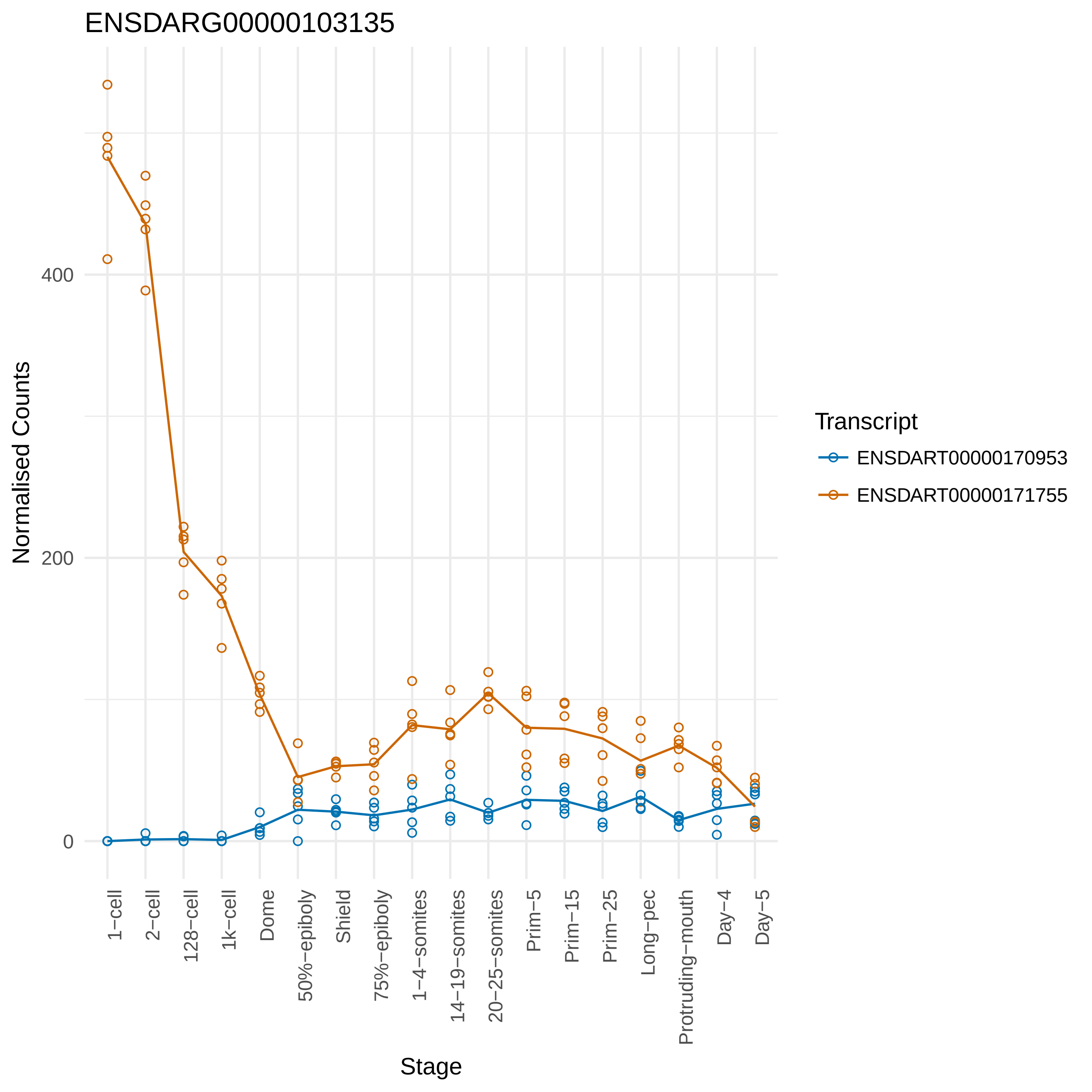
ENSDARG00000103135
Normalised Counts
400
200
0
1−cell
2−cell
128−cell
1k−cell
Dome
50%−epiboly
Shield
75%−epiboly
1−4−somites
14−19−somites
20−25−somites
Prim−5
Prim−15
Prim−25
Long−pec
Protruding−mouth
Day−4
Day−5
Stage
Transcript
ENSDART00000170953
ENSDART00000171755

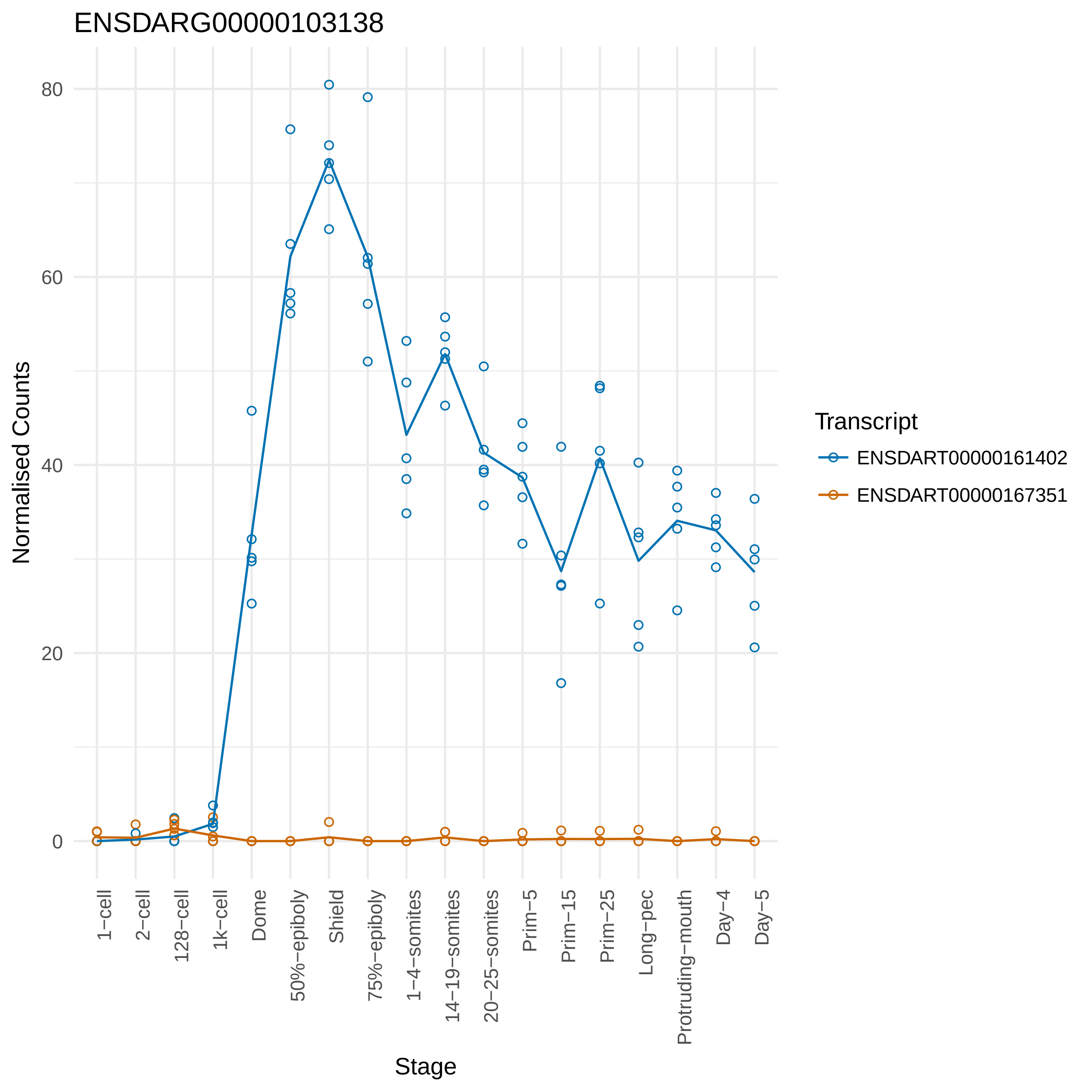

ENSDARG00000103138
Normalised Counts
80
60
40
20
0
1−cell
2−cell
128−cell
1k−cell
Dome
50%−epiboly
Shield
75%−epiboly
1−4−somites
14−19−somites
20−25−somites
Prim−5
Prim−15
Prim−25
Long−pec
Protruding−mouth
Day−4
Day−5
Stage
Transcript
ENSDART00000161402
ENSDART00000167351

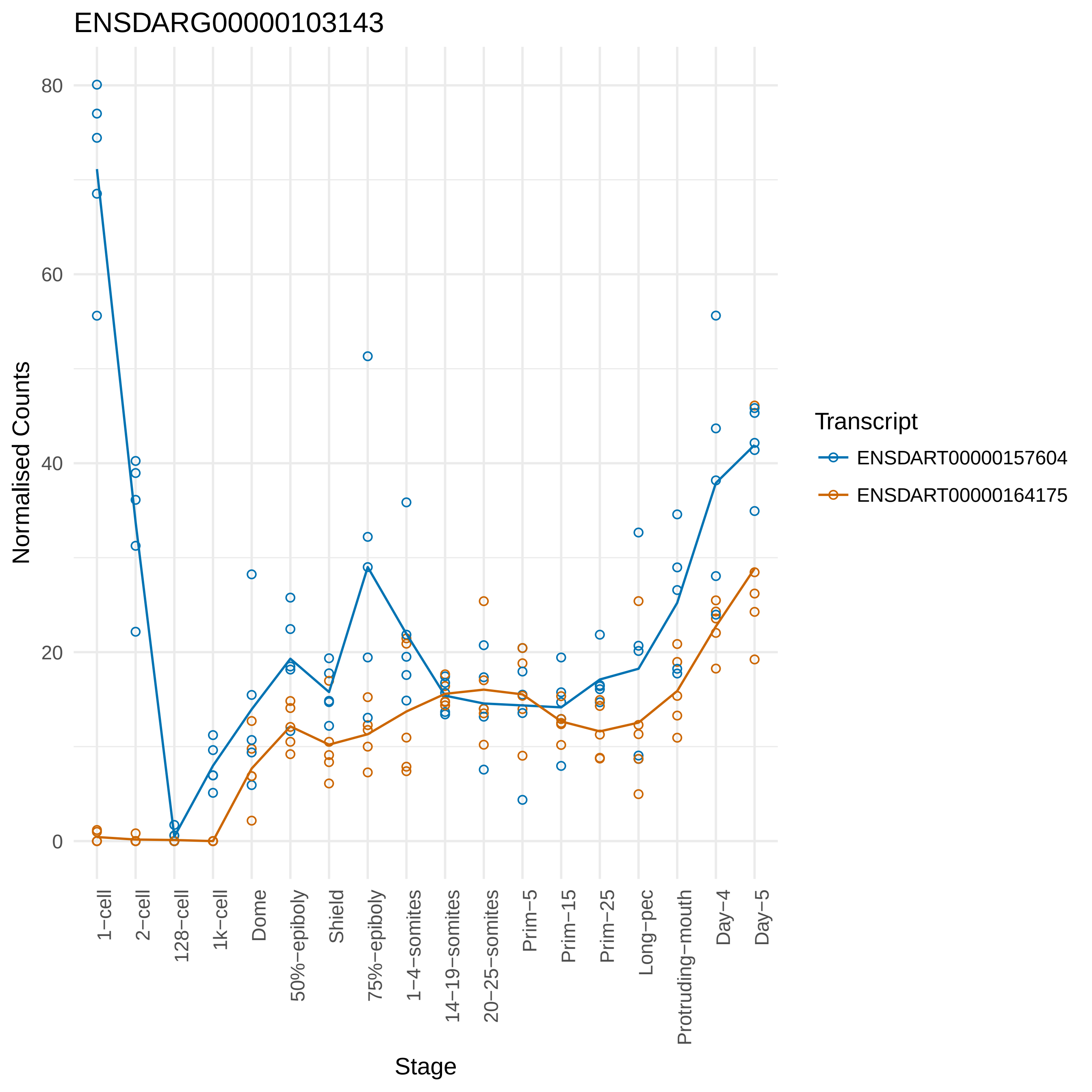
ENSDARG00000103143
Normalised Counts
80
60
40
20
0
1−cell
2−cell
128−cell
1k−cell
Dome
50%−epiboly
Shield
75%−epiboly
1−4−somites
14−19−somites
20−25−somites
Prim−5
Prim−15
Prim−25
Long−pec
Protruding−mouth
Day−4
Day−5
Stage
Transcript
ENSDART00000157604
ENSDART00000164175

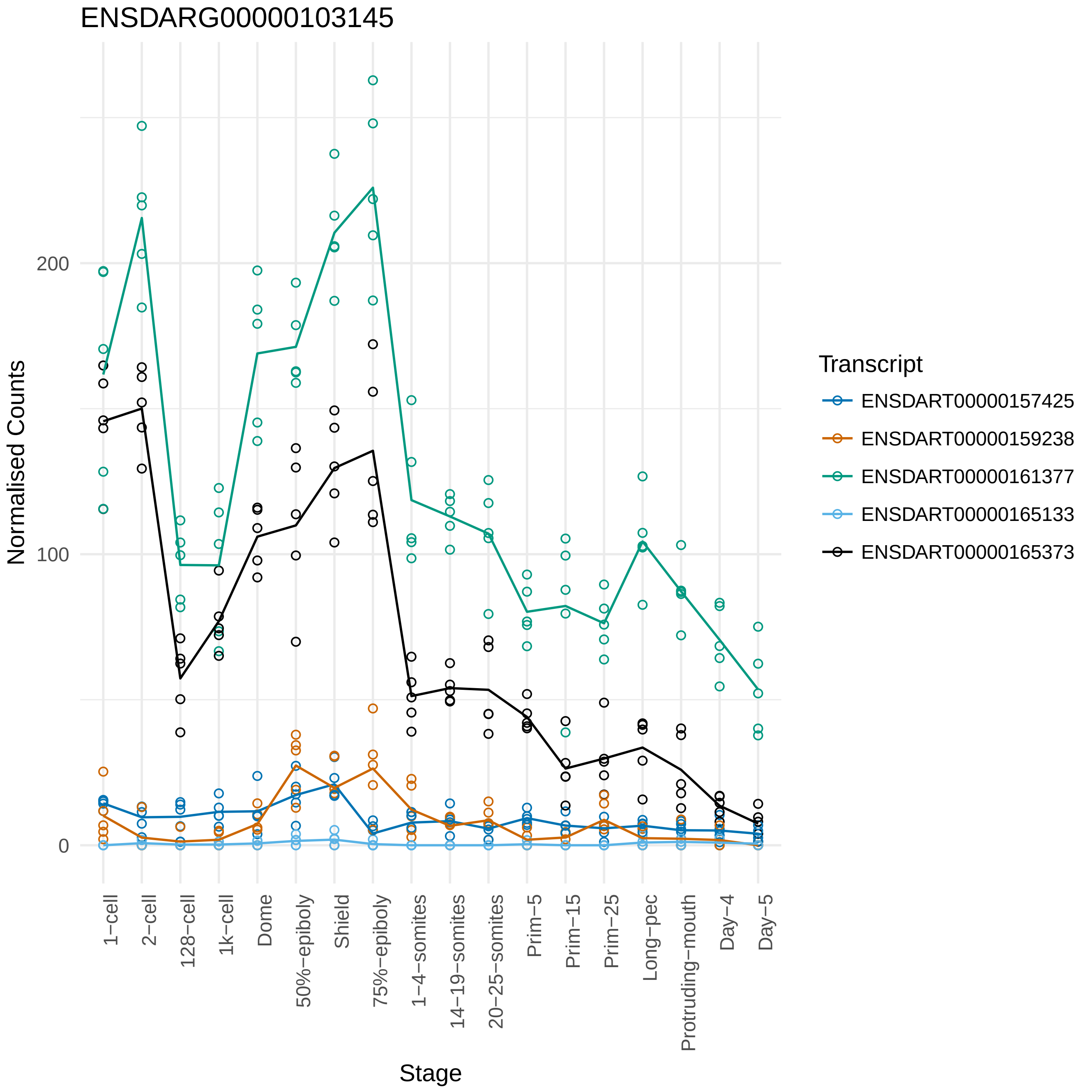
ENSDARG00000103145
Normalised Counts
0
100
200
1−cell
2−cell
128−cell
1k−cell
Dome
50%−epiboly
Shield
75%−epiboly
1−4−somites
14−19−somites
20−25−somites
Prim−5
Prim−15
Prim−25
Long−pec
Protruding−mouth
Day−4
Day−5
Stage
Transcript
ENSDART00000157425
ENSDART00000159238
ENSDART00000161377
ENSDART00000165133
ENSDART00000165373

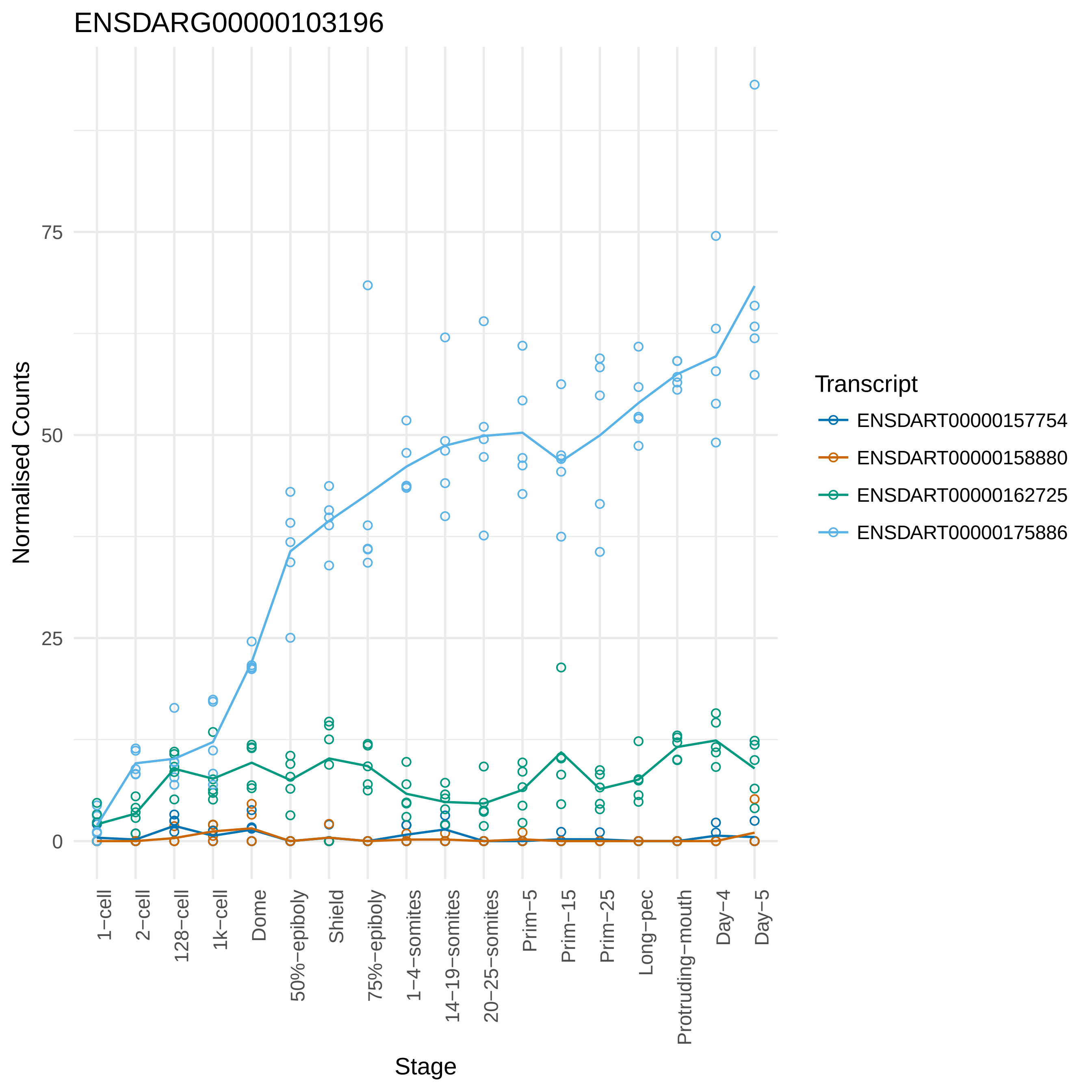
ENSDARG00000103196
Normalised Counts
0
25
50
75
Stage
1−cell
2−cell
128−cell
1k−cell
Dome
50%−epiboly
Shield
75%−epiboly
1−4−somites
14−19−somites
20−25−somites
Prim−5
Prim−15
Prim−25
Long−pec
Protruding−mouth
Day−4
Day−5
Transcript
ENSDART00000157754
ENSDART00000158880
ENSDART00000162725
ENSDART00000175886

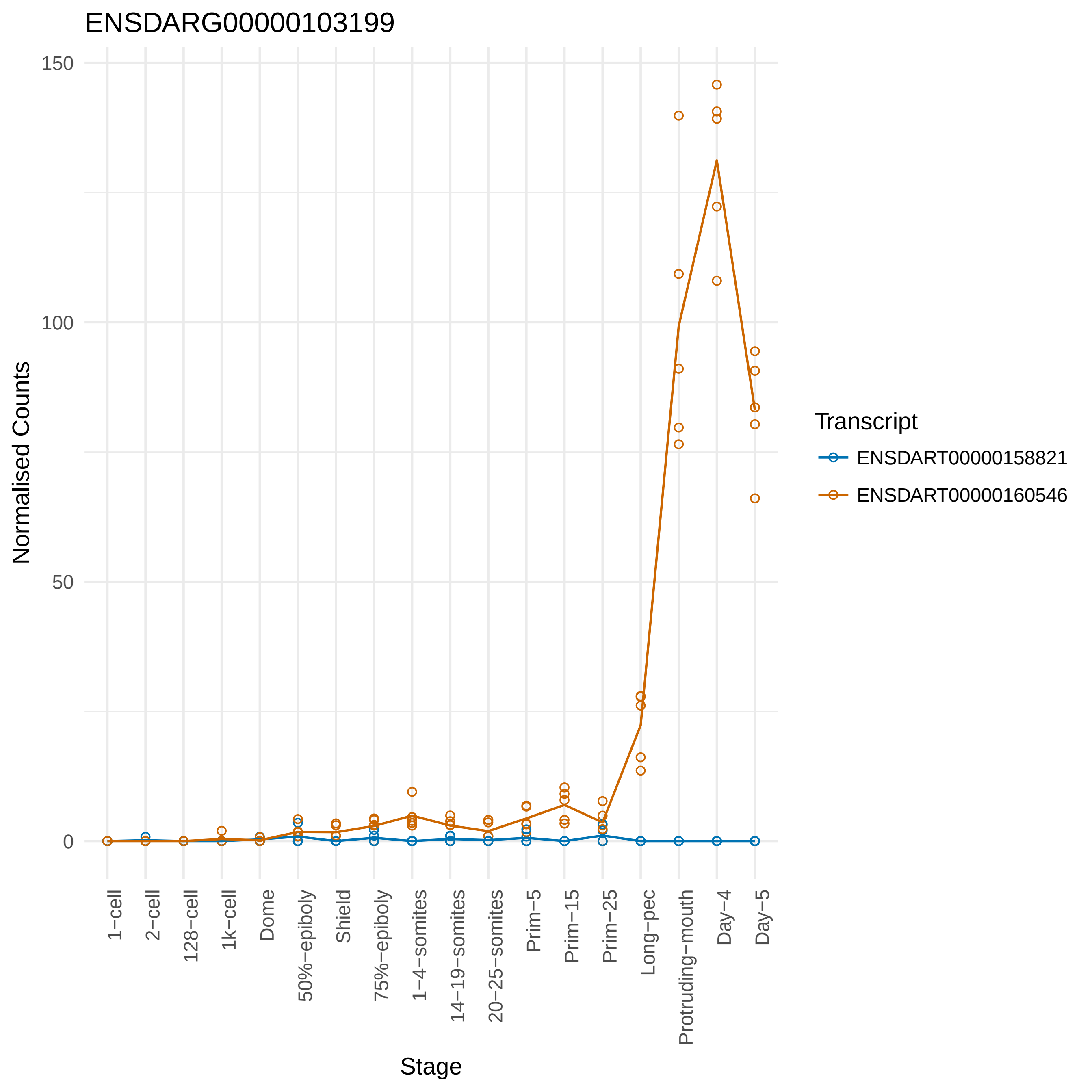
ENSDARG00000103199
Normalised Counts
150
100
50
0
1−cell
2−cell
128−cell
1k−cell
Dome
50%−epiboly
Shield
75%−epiboly
1−4−somites
14−19−somites
20−25−somites
Prim−5
Prim−15
Prim−25
Long−pec
Protruding−mouth
Day−4
Day−5
Stage
Transcript
ENSDART00000158821
ENSDART00000160546

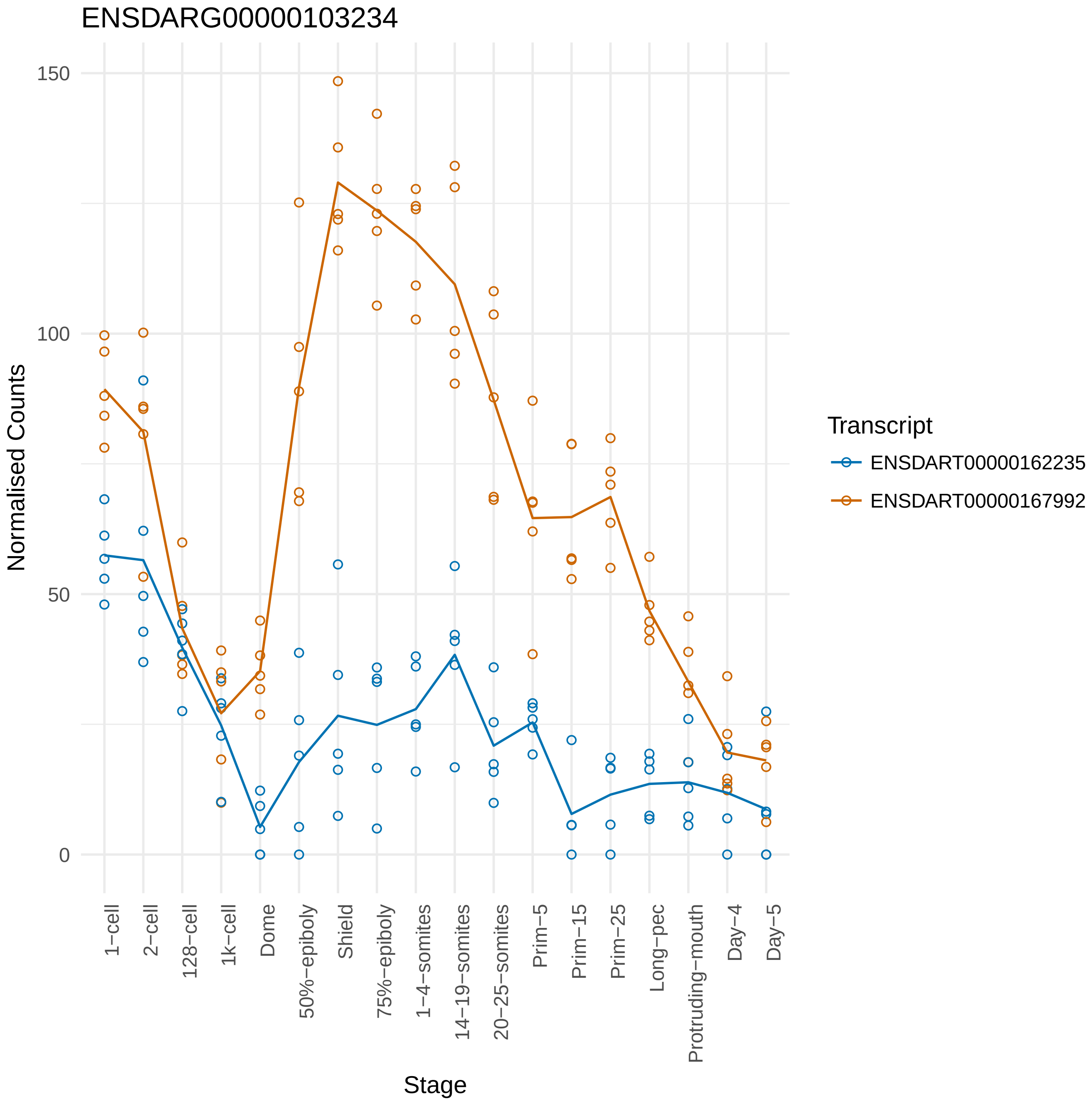

ENSDARG00000103234
Normalised Counts
150
100
50
0
Stage
1−cell
2−cell
128−cell
1k−cell
Dome
50%−epiboly
Shield
75%−epiboly
1−4−somites
14−19−somites
20−25−somites
Prim−5
Prim−15
Prim−25
Long−pec
Protruding−mouth
Day−4
Day−5
Transcript
ENSDART00000162235
ENSDART00000167992

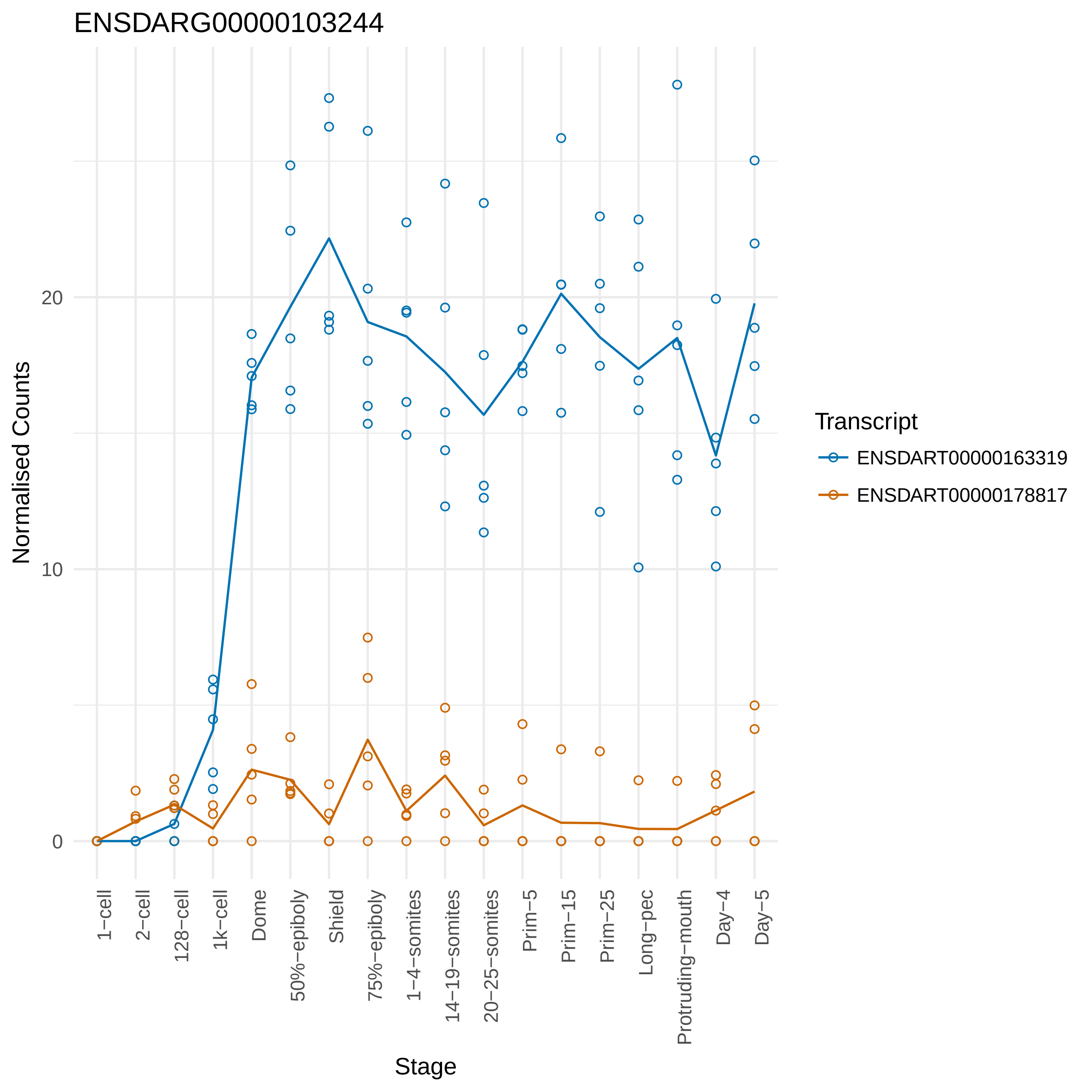

ENSDARG00000103244
Normalised Counts
0
10
20
Stage
1−cell
2−cell
128−cell
1k−cell
Dome
50%−epiboly
Shield
75%−epiboly
1−4−somites
14−19−somites
20−25−somites
Prim−5
Prim−15
Prim−25
Long−pec
Protruding−mouth
Day−4
Day−5
Transcript
ENSDART00000163319
ENSDART00000178817

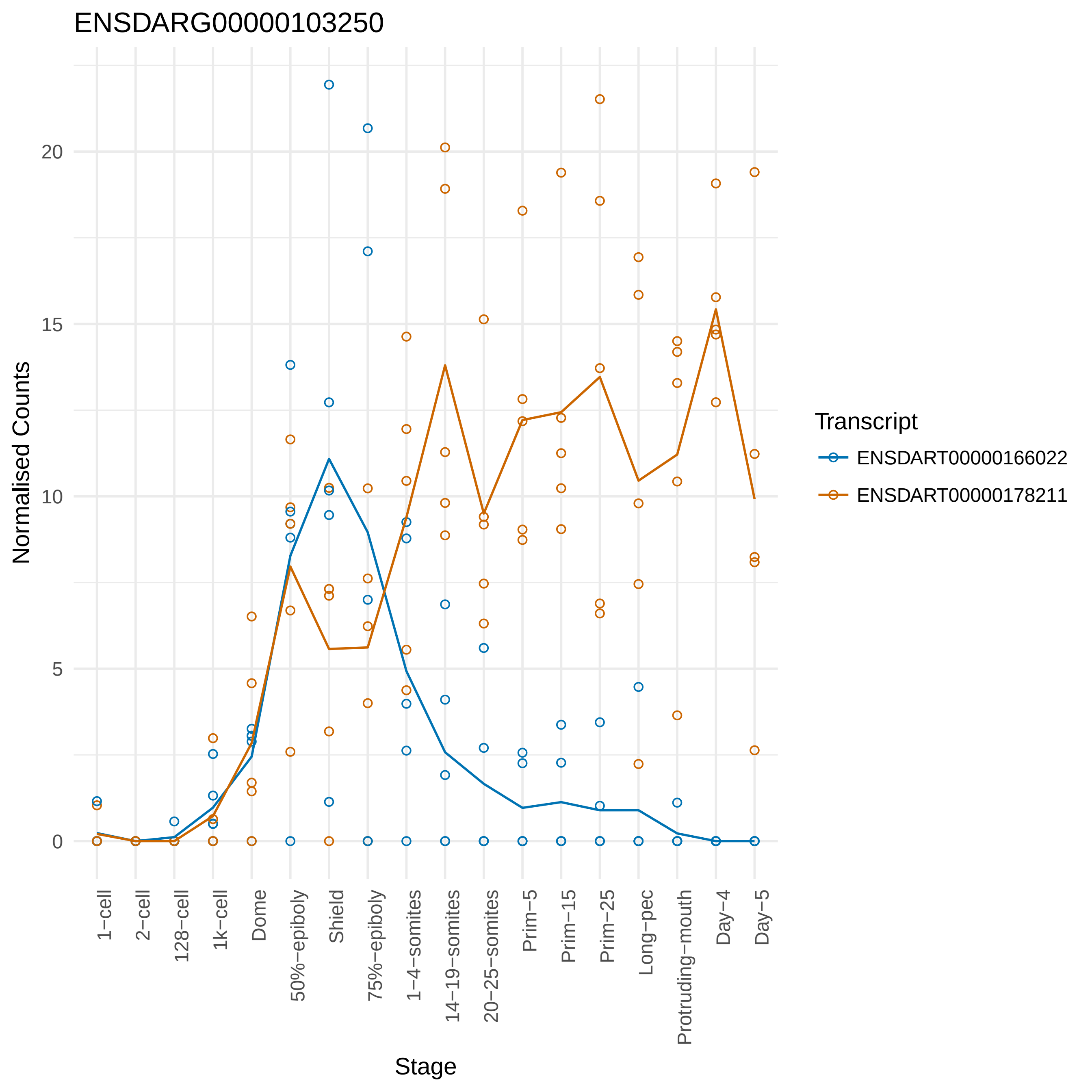
ENSDARG00000103250
Normalised Counts
0
5
10
15
20
Stage
1−cell
2−cell
128−cell
1k−cell
Dome
50%−epiboly
Shield
75%−epiboly
1−4−somites
14−19−somites
20−25−somites
Prim−5
Prim−15
Prim−25
Long−pec
Protruding−mouth
Day−4
Day−5
Transcript
ENSDART00000166022
ENSDART00000178211

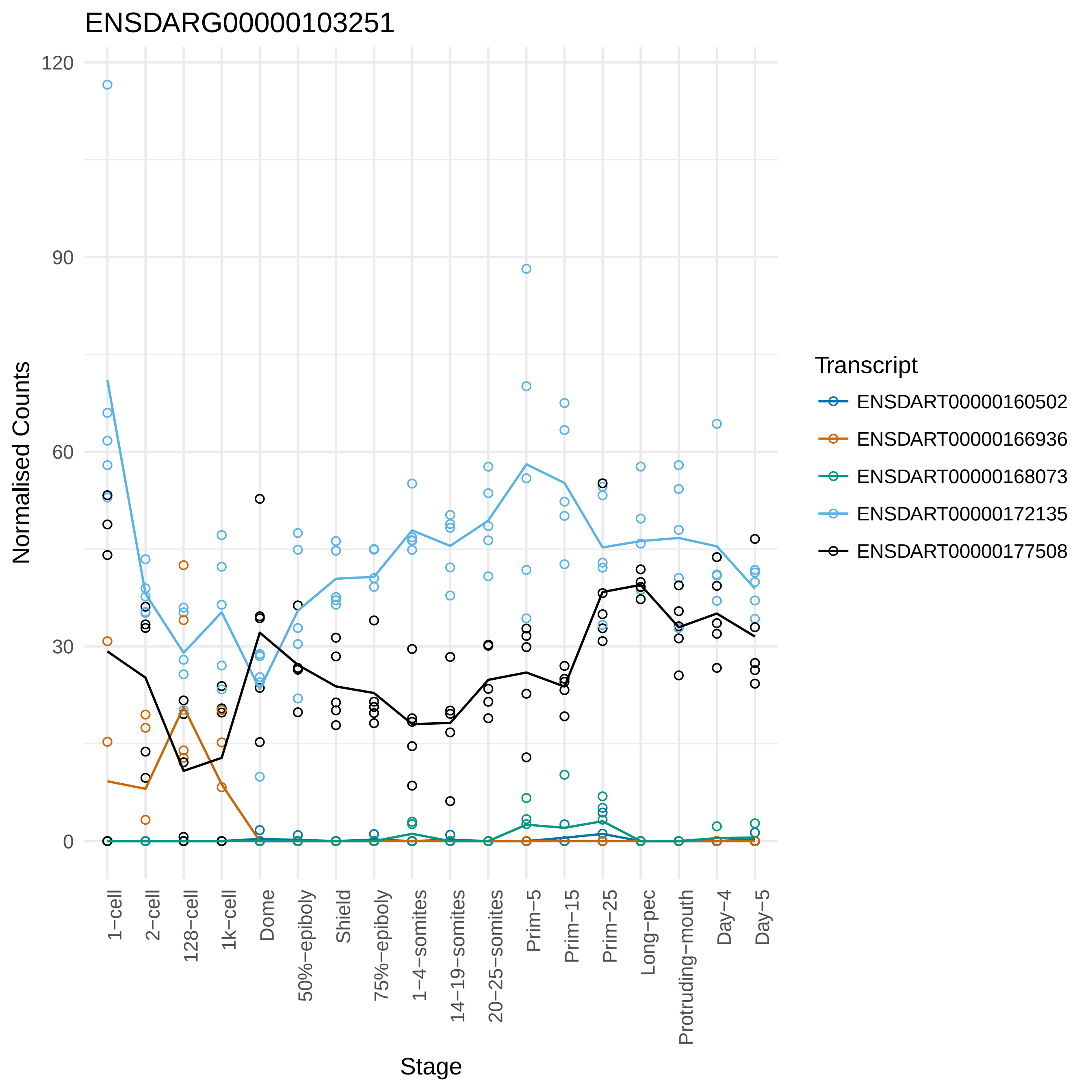
ENSDARG00000103251
Normalised Counts
0
30
60
90
120
1−cell
2−cell
128−cell
1k−cell
Dome
50%−epiboly
Shield
75%−epiboly
1−4−somites
14−19−somites
20−25−somites
Prim−5
Prim−15
Prim−25
Long−pec
Protruding−mouth
Day−4
Day−5
Stage
Transcript
ENSDART00000160502
ENSDART00000166936
ENSDART00000168073
ENSDART00000172135
ENSDART00000177508

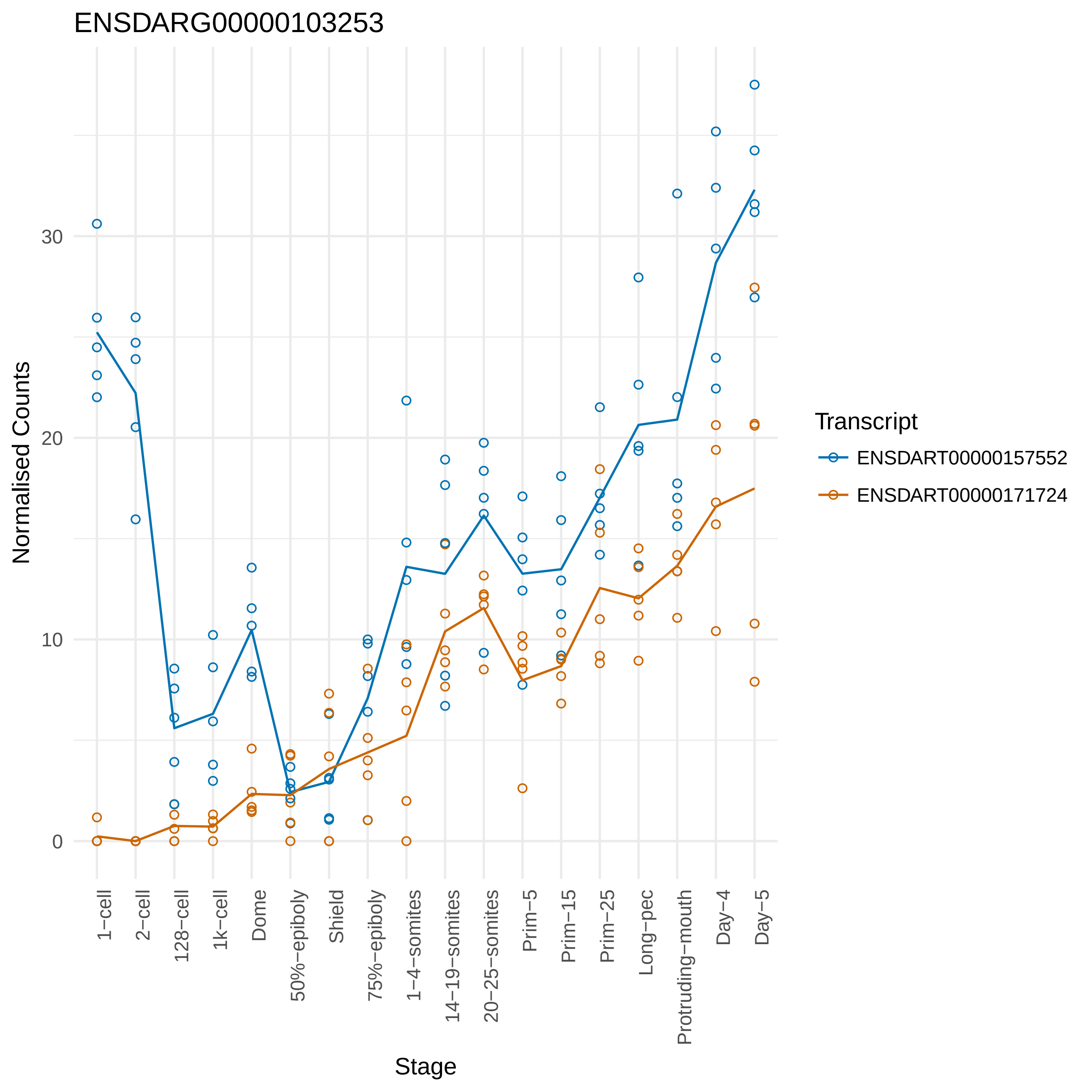
ENSDARG00000103253
Normalised Counts
0
10
20
30
Stage
1−cell
2−cell
128−cell
1k−cell
Dome
50%−epiboly
Shield
75%−epiboly
1−4−somites
14−19−somites
20−25−somites
Prim−5
Prim−15
Prim−25
Long−pec
Protruding−mouth
Day−4
Day−5
Transcript
ENSDART00000157552
ENSDART00000171724

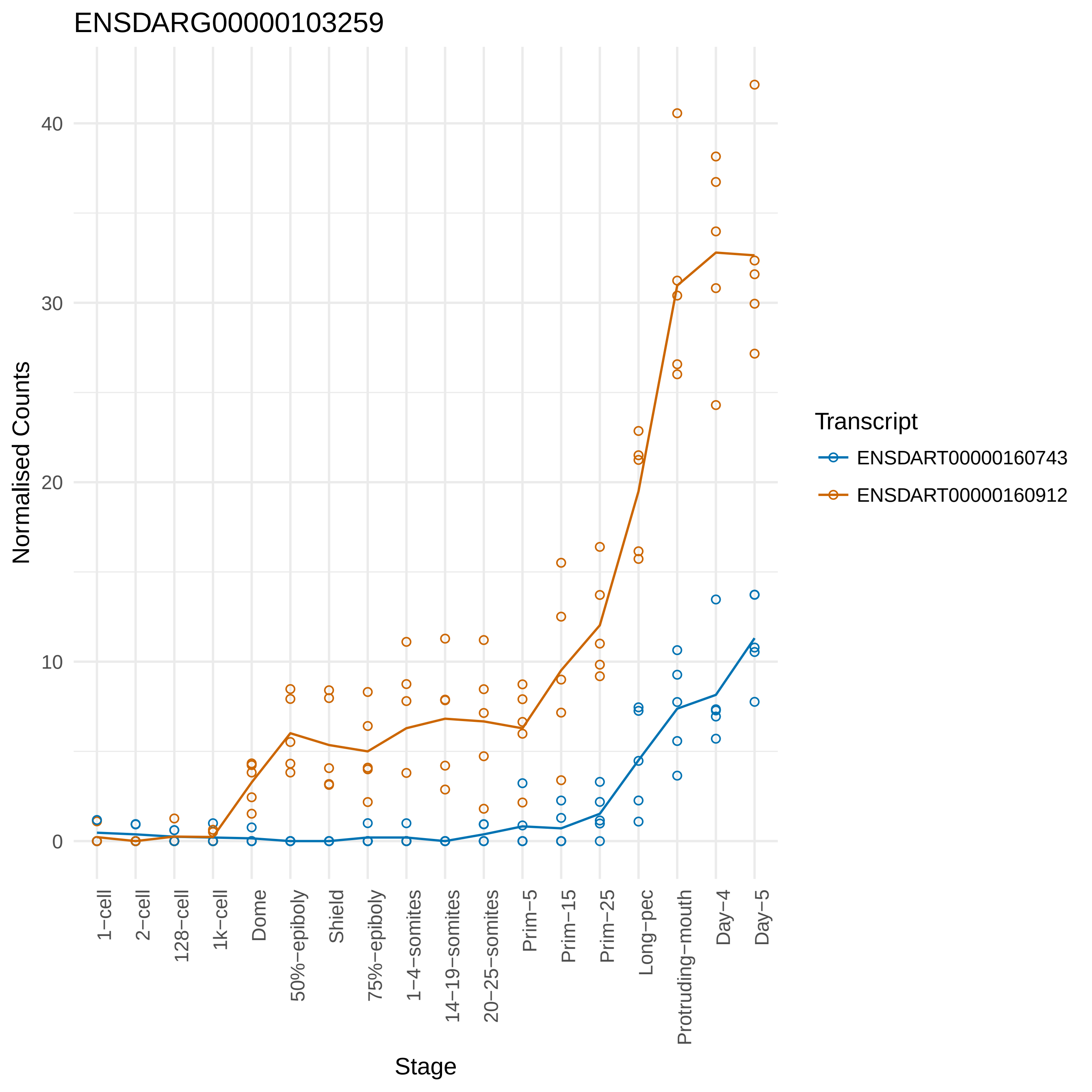

ENSDARG00000103259
Normalised Counts
0
10
20
30
40
Stage
1−cell
2−cell
128−cell
1k−cell
Dome
50%−epiboly
Shield
75%−epiboly
1−4−somites
14−19−somites
20−25−somites
Prim−5
Prim−15
Prim−25
Long−pec
Protruding−mouth
Day−4
Day−5
Transcript
ENSDART00000160743
ENSDART00000160912

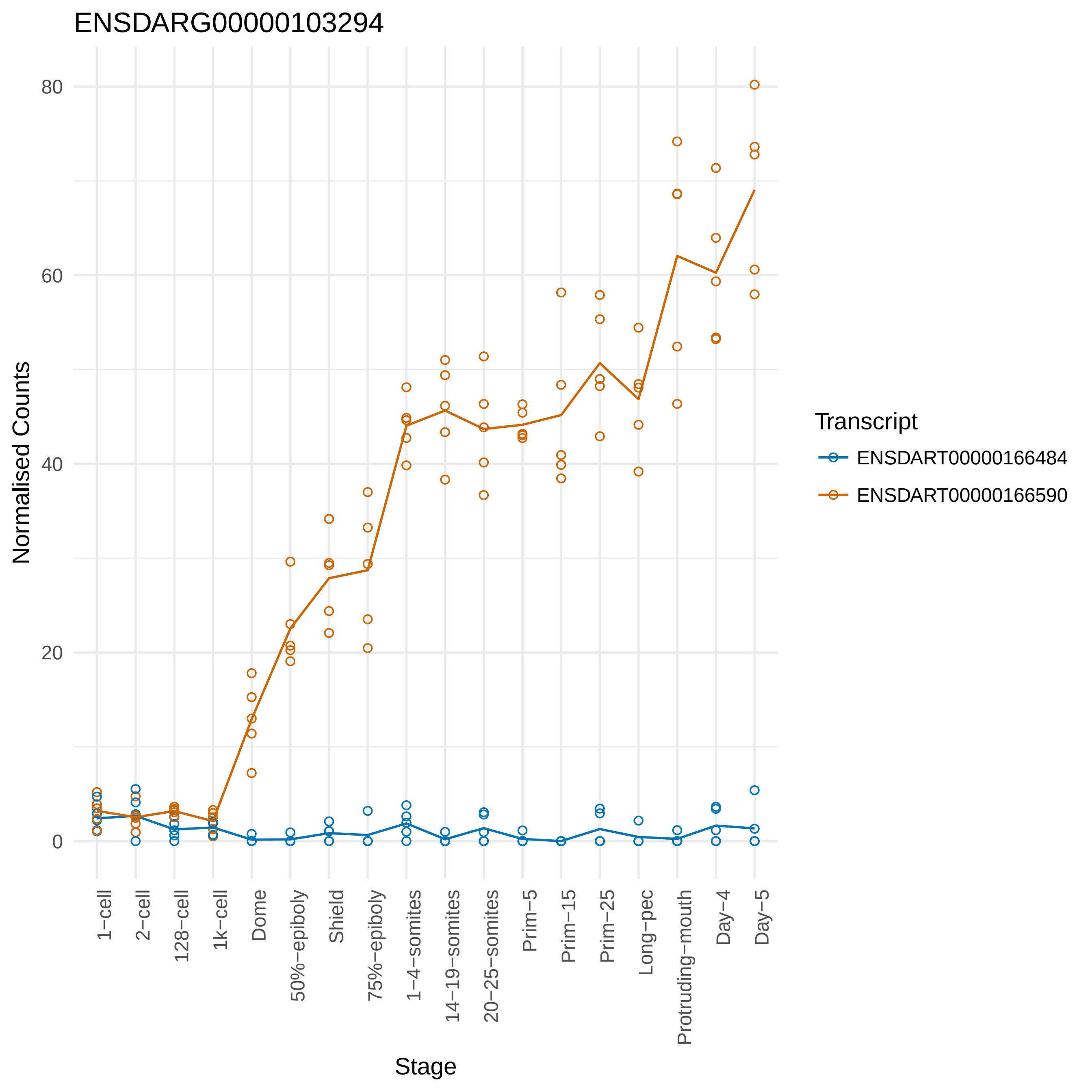

ENSDARG00000103294
Normalised Counts
80
60
40
20
0
1−cell
2−cell
128−cell
1k−cell
Dome
50%−epiboly
Shield
75%−epiboly
1−4−somites
14−19−somites
20−25−somites
Prim−5
Prim−15
Prim−25
Long−pec
Protruding−mouth
Day−4
Day−5
Stage
Transcript
ENSDART00000166484
ENSDART00000166590

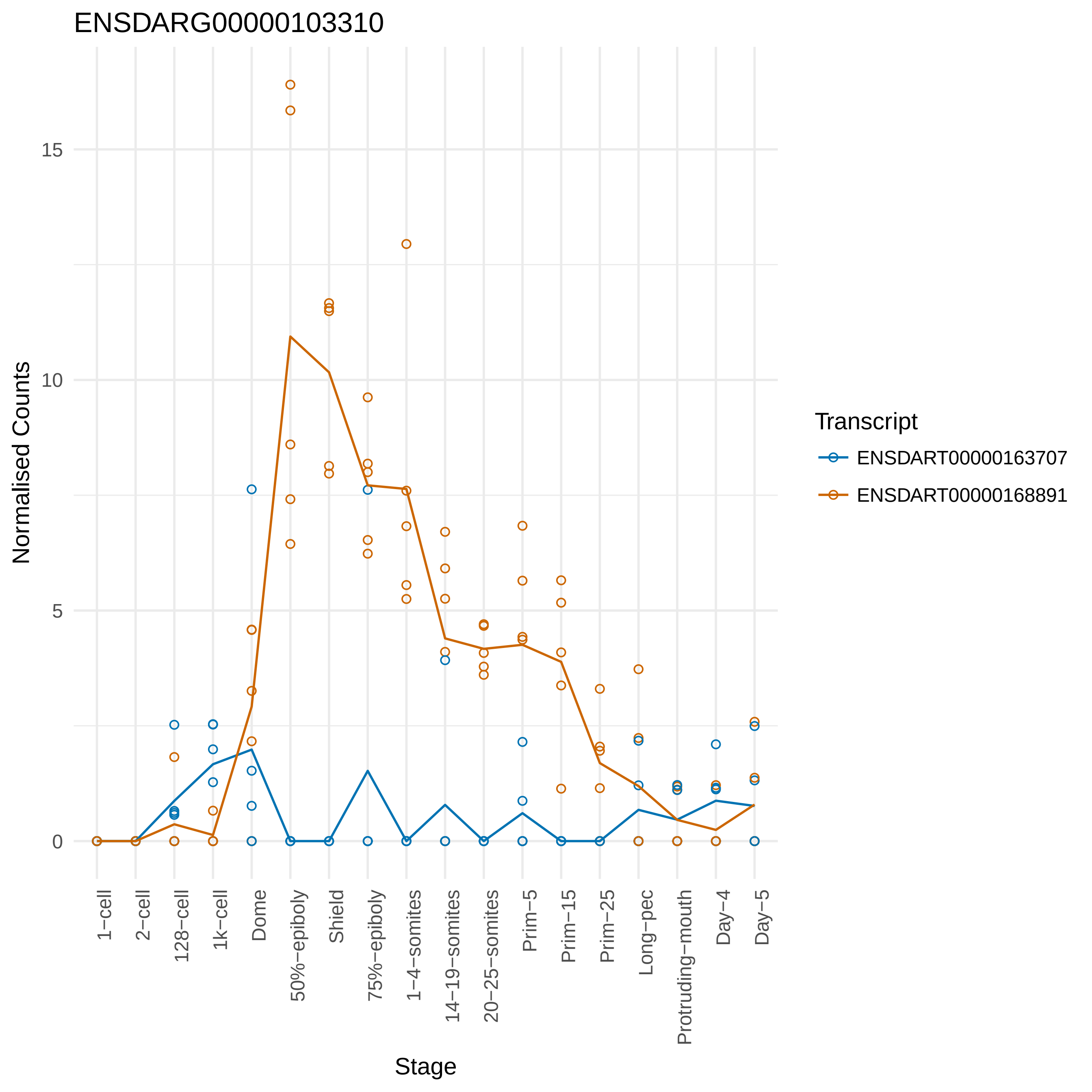

ENSDARG00000103310
Normalised Counts
0
5
10
15
1−cell
2−cell
128−cell
1k−cell
Dome
50%−epiboly
Shield
75%−epiboly
1−4−somites
14−19−somites
20−25−somites
Prim−5
Prim−15
Prim−25
Long−pec
Protruding−mouth
Day−4
Day−5
Stage
Transcript
ENSDART00000163707
ENSDART00000168891

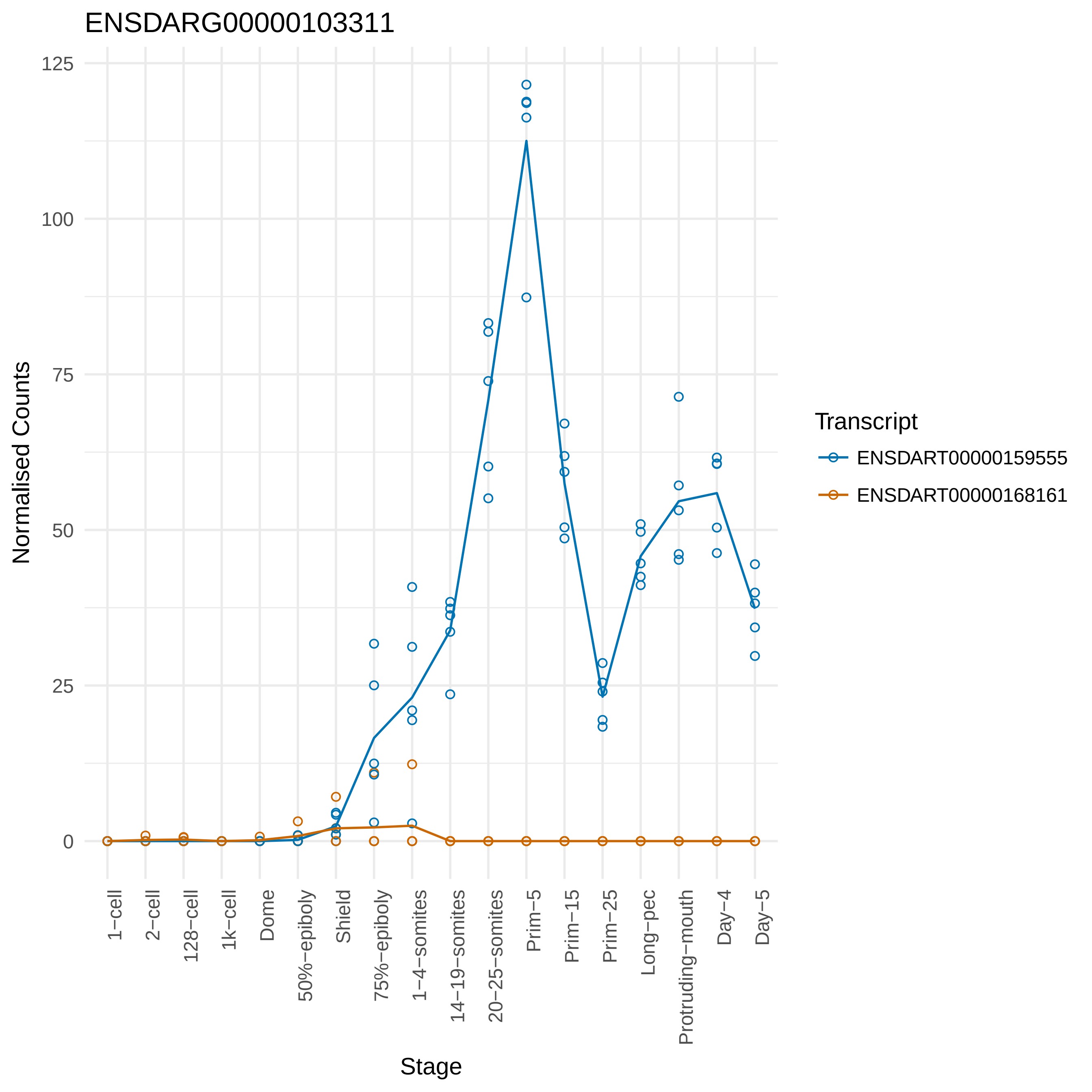

ENSDARG00000103311
Normalised Counts
125
100
75
50
25
0
1−cell
2−cell
128−cell
1k−cell
Dome
50%−epiboly
Shield
75%−epiboly
1−4−somites
14−19−somites
20−25−somites
Prim−5
Prim−15
Prim−25
Long−pec
Protruding−mouth
Day−4
Day−5
Stage
Transcript
ENSDART00000159555
ENSDART00000168161

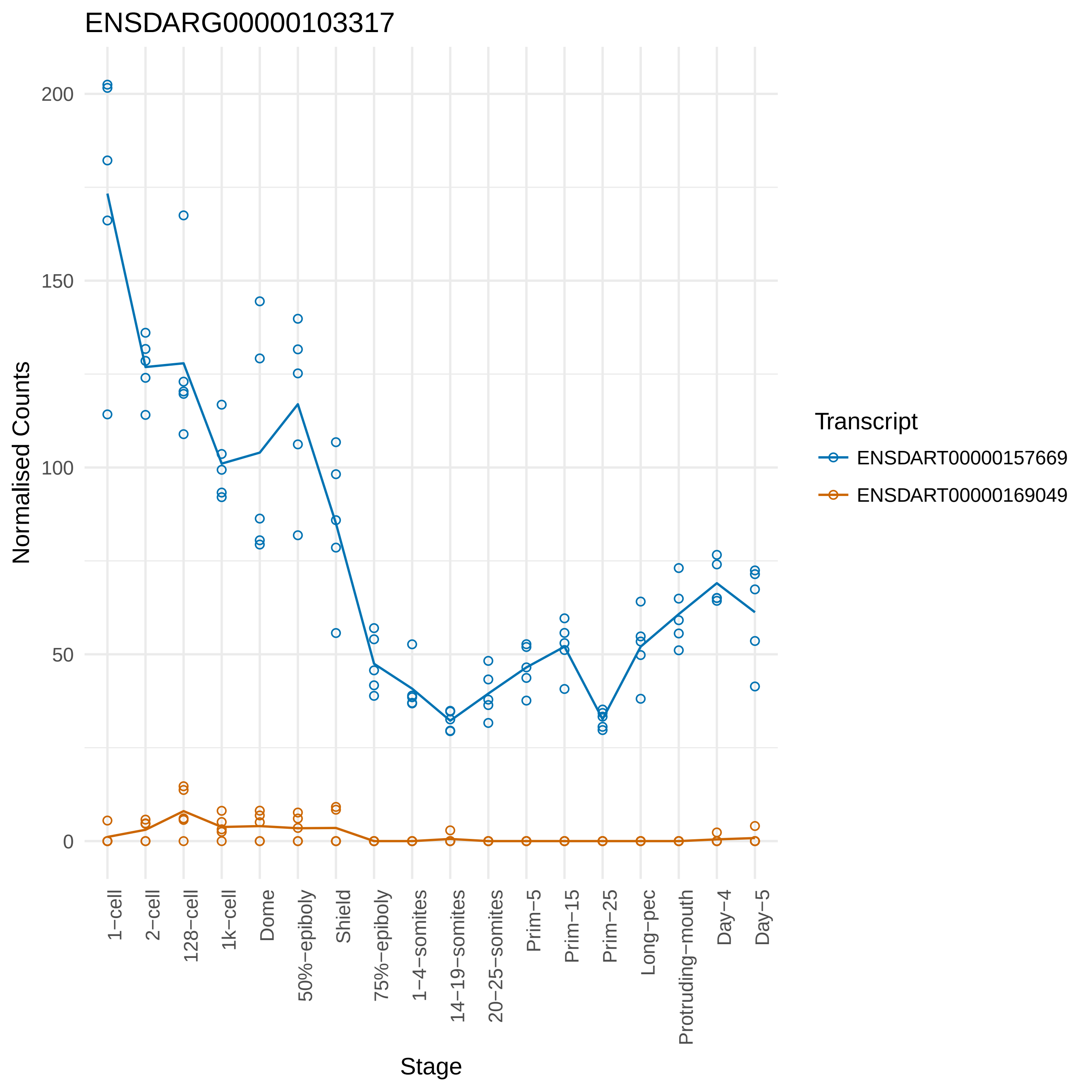

ENSDARG00000103317
Normalised Counts
200
150
100
50
0
1−cell
2−cell
128−cell
1k−cell
Dome
50%−epiboly
Shield
75%−epiboly
1−4−somites
14−19−somites
20−25−somites
Prim−5
Prim−15
Prim−25
Long−pec
Protruding−mouth
Day−4
Day−5
Stage
Transcript
ENSDART00000157669
ENSDART00000169049

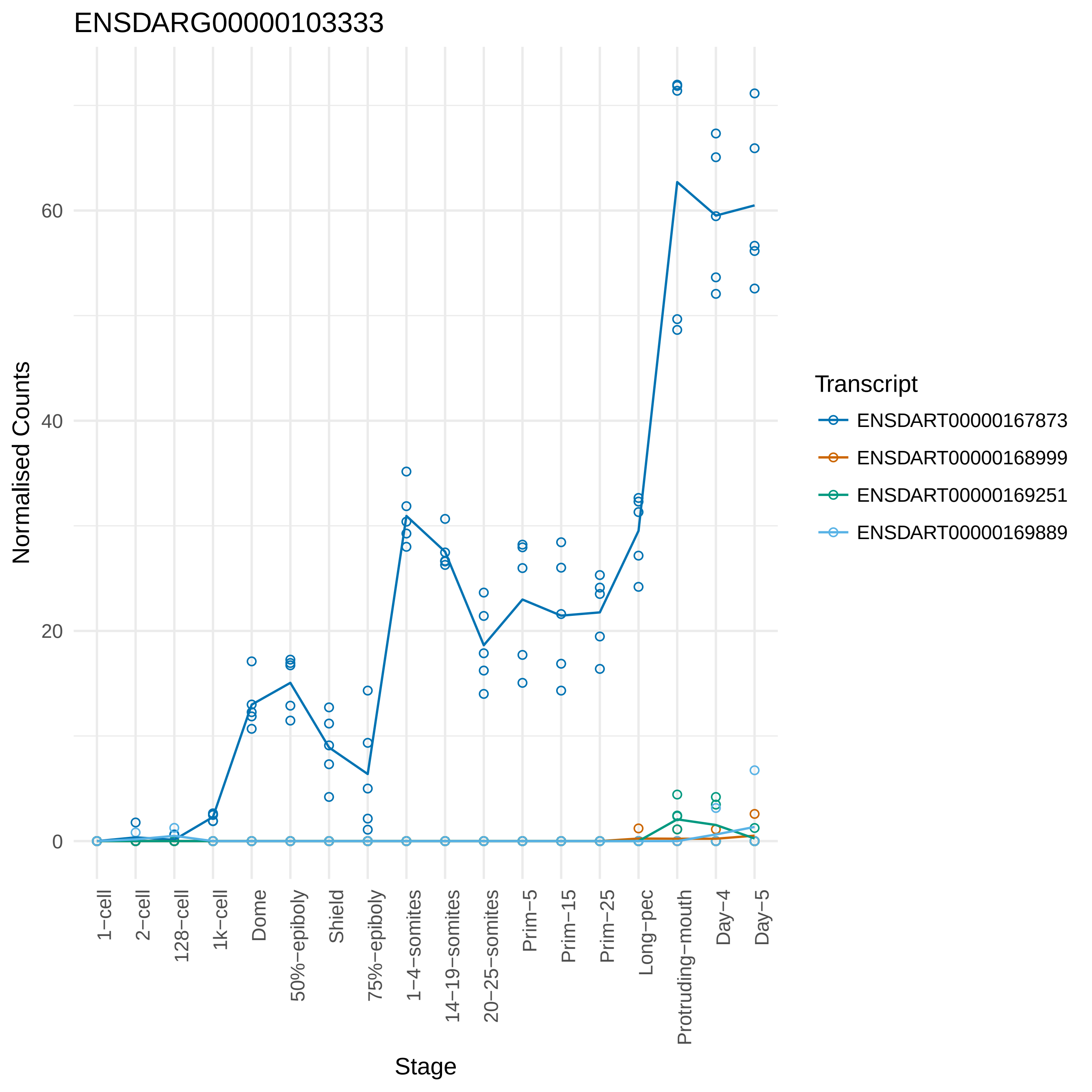

ENSDARG00000103333
Normalised Counts
0
20
40
60
Stage
1−cell
2−cell
128−cell
1k−cell
Dome
50%−epiboly
Shield
75%−epiboly
1−4−somites
14−19−somites
20−25−somites
Prim−5
Prim−15
Prim−25
Long−pec
Protruding−mouth
Day−4
Day−5
Transcript
ENSDART00000167873
ENSDART00000168999
ENSDART00000169251
ENSDART00000169889

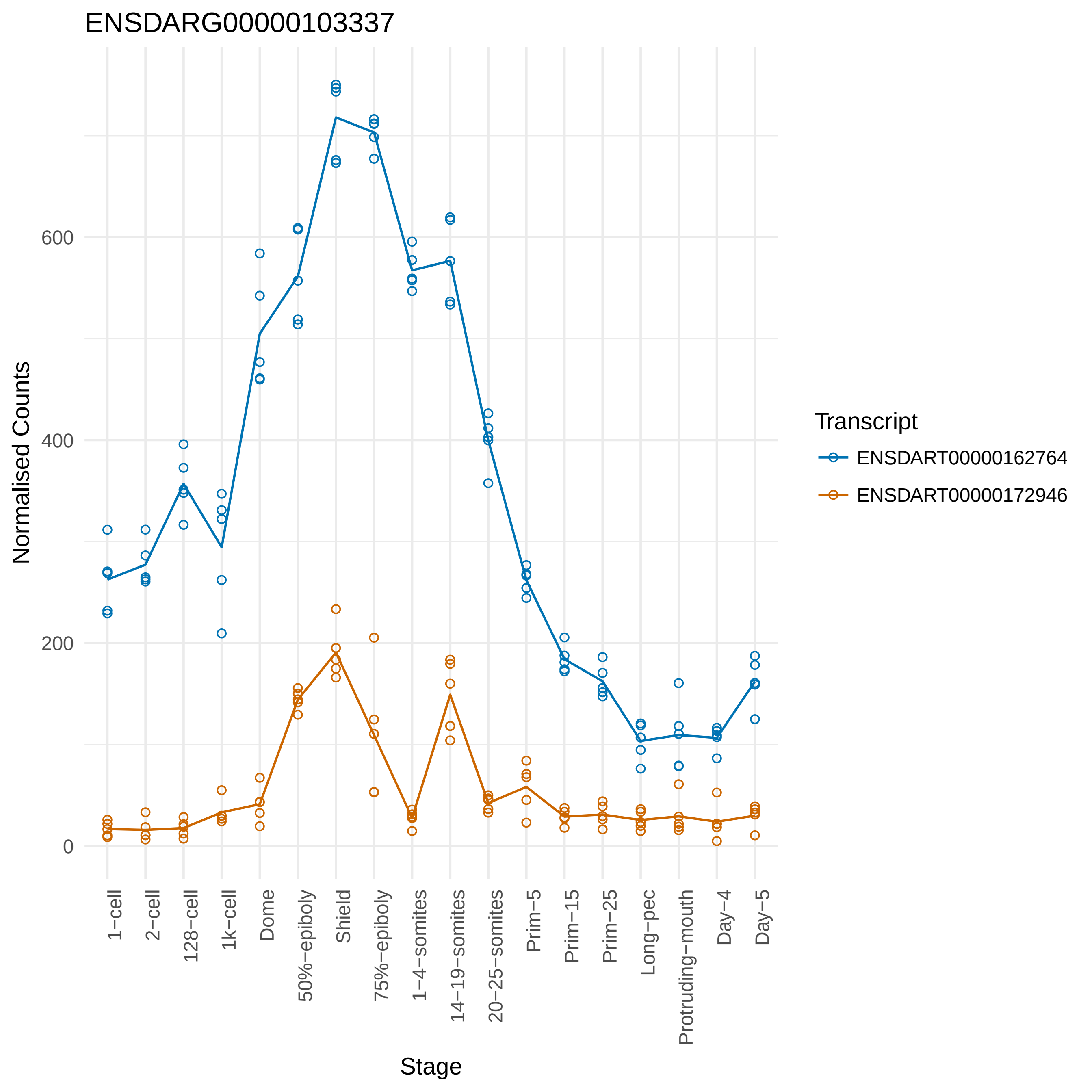
ENSDARG00000103337
Normalised Counts
0
200
400
600
1−cell
2−cell
128−cell
1k−cell
Dome
50%−epiboly
Shield
75%−epiboly
1−4−somites
14−19−somites
20−25−somites
Prim−5
Prim−15
Prim−25
Long−pec
Protruding−mouth
Day−4
Day−5
Stage
Transcript
ENSDART00000162764
ENSDART00000172946

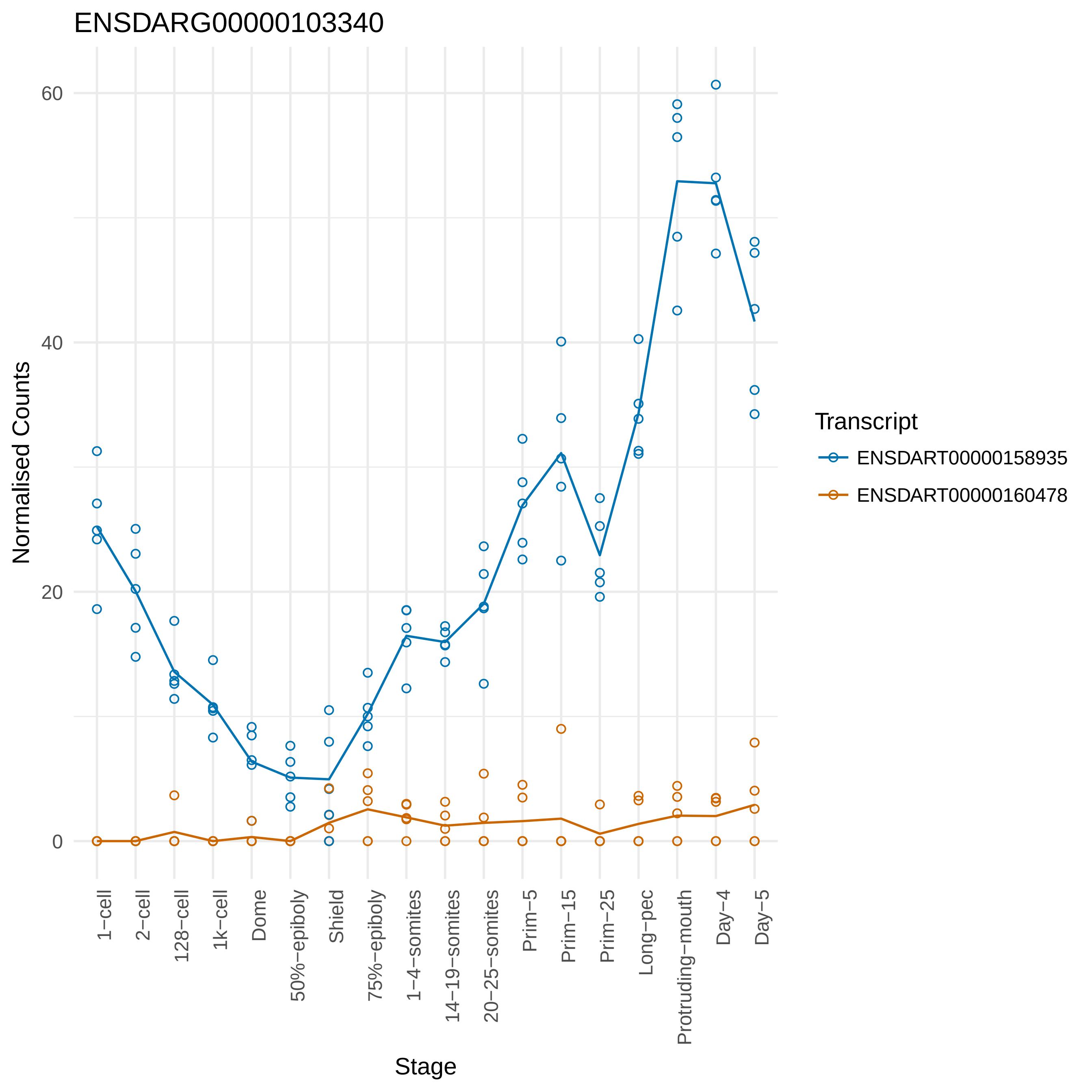

ENSDARG00000103340
Normalised Counts
0
20
40
60
1−cell
2−cell
128−cell
1k−cell
Dome
50%−epiboly
Shield
75%−epiboly
1−4−somites
14−19−somites
20−25−somites
Prim−5
Prim−15
Prim−25
Long−pec
Protruding−mouth
Day−4
Day−5
Stage
Transcript
ENSDART00000158935
ENSDART00000160478

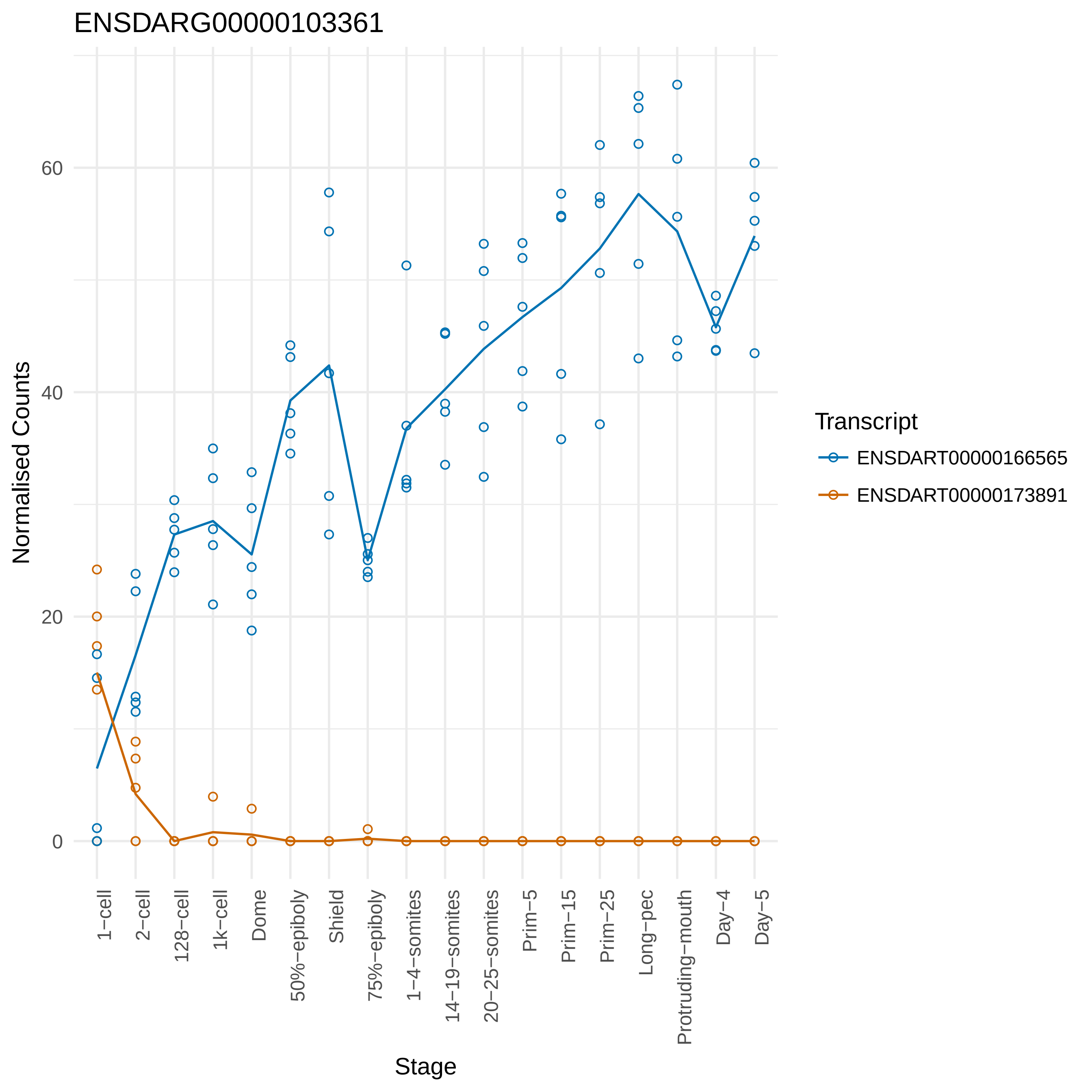

ENSDARG00000103361
Normalised Counts
60
40
20
0
1−cell
2−cell
128−cell
1k−cell
Dome
50%−epiboly
Shield
75%−epiboly
1−4−somites
14−19−somites
20−25−somites
Prim−5
Prim−15
Prim−25
Long−pec
Protruding−mouth
Day−4
Day−5
Stage
Transcript
ENSDART00000166565
ENSDART00000173891

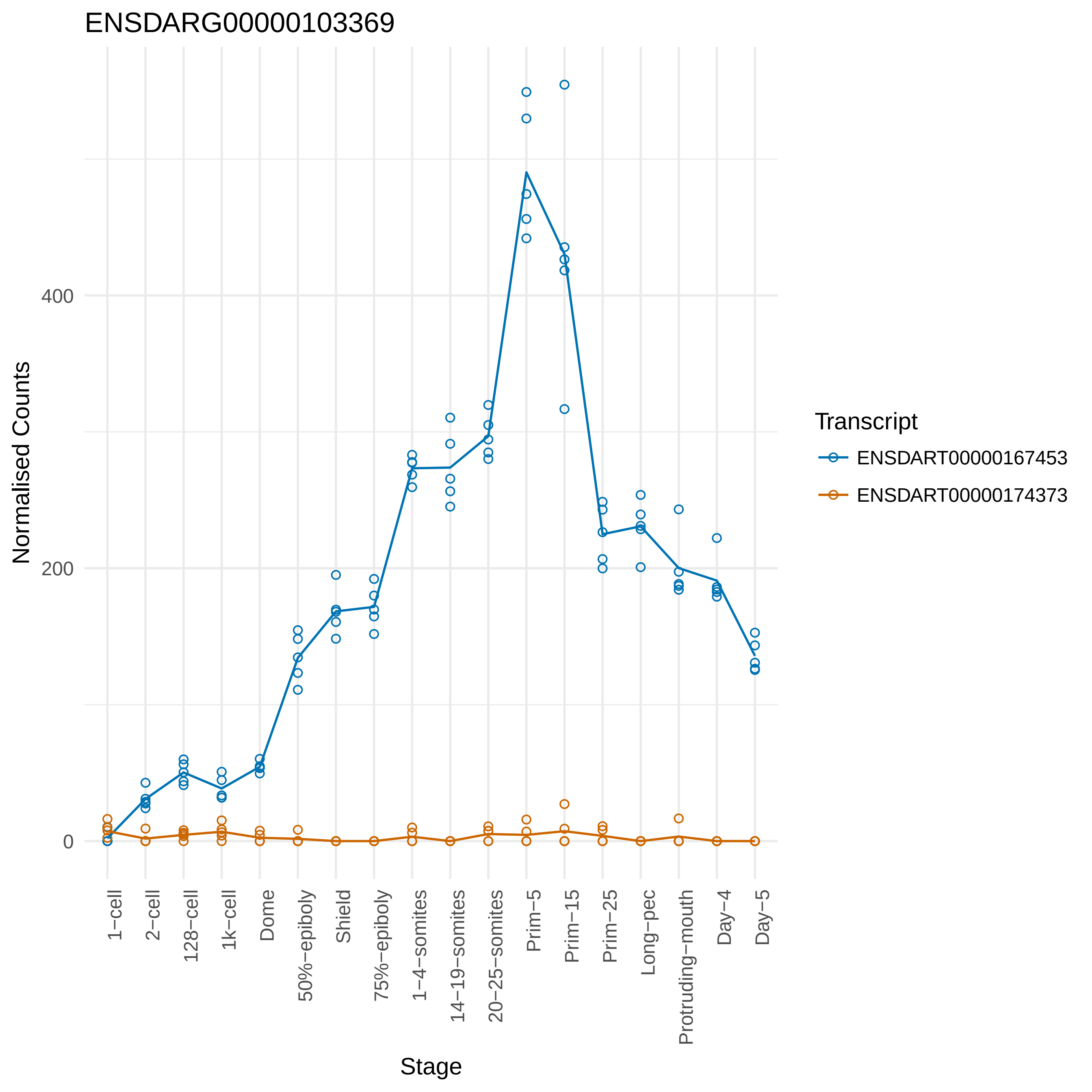

ENSDARG00000103369
Normalised Counts
400
200
0
1−cell
2−cell
128−cell
1k−cell
Dome
50%−epiboly
Shield
75%−epiboly
1−4−somites
14−19−somites
20−25−somites
Prim−5
Prim−15
Prim−25
Long−pec
Protruding−mouth
Day−4
Day−5
Stage
Transcript
ENSDART00000167453
ENSDART00000174373

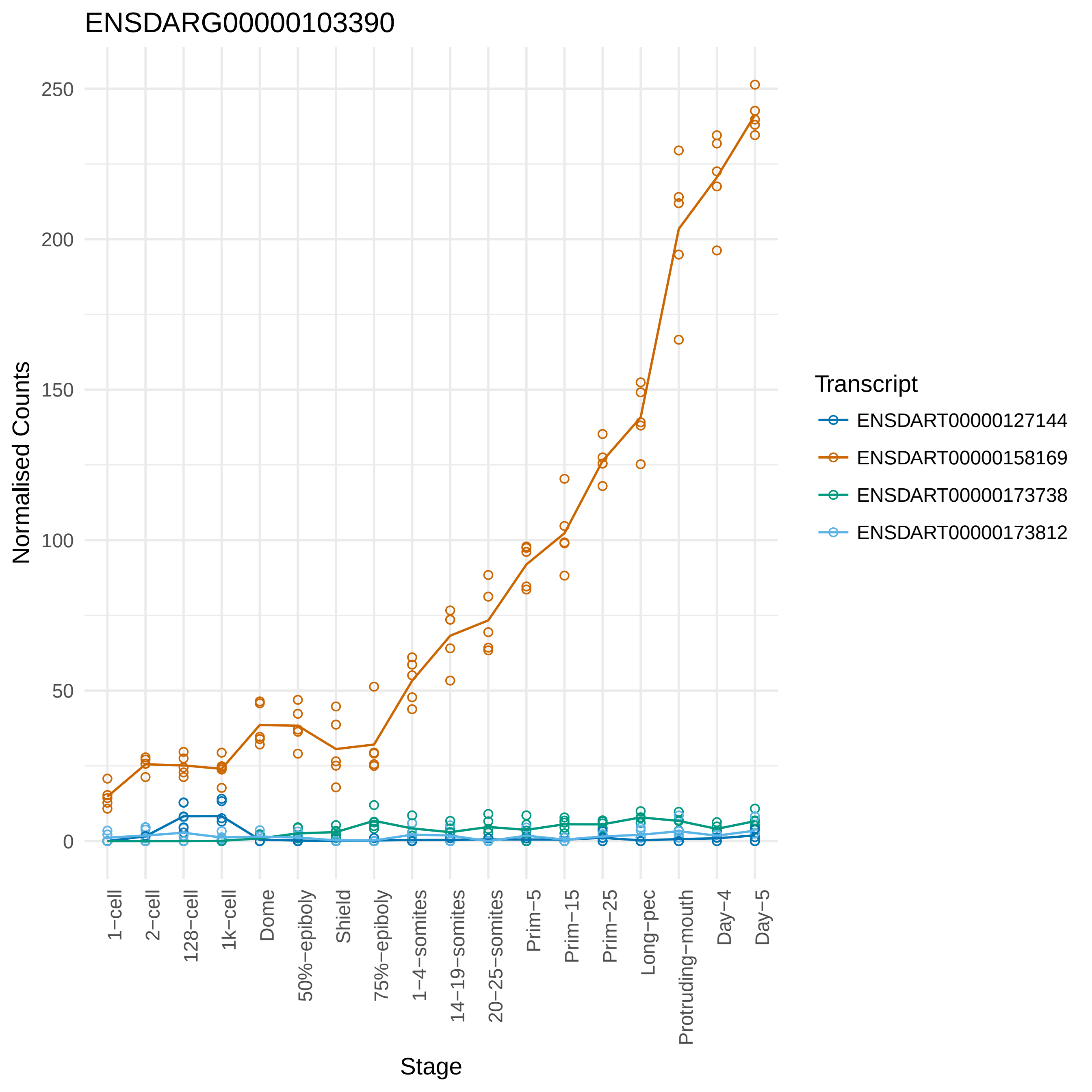
ENSDARG00000103390
Normalised Counts
250
200
150
100
50
0
1−cell
2−cell
128−cell
1k−cell
Dome
50%−epiboly
Shield
75%−epiboly
1−4−somites
14−19−somites
20−25−somites
Prim−5
Prim−15
Prim−25
Long−pec
Protruding−mouth
Day−4
Day−5
Stage
Transcript
ENSDART00000127144
ENSDART00000158169
ENSDART00000173738
ENSDART00000173812

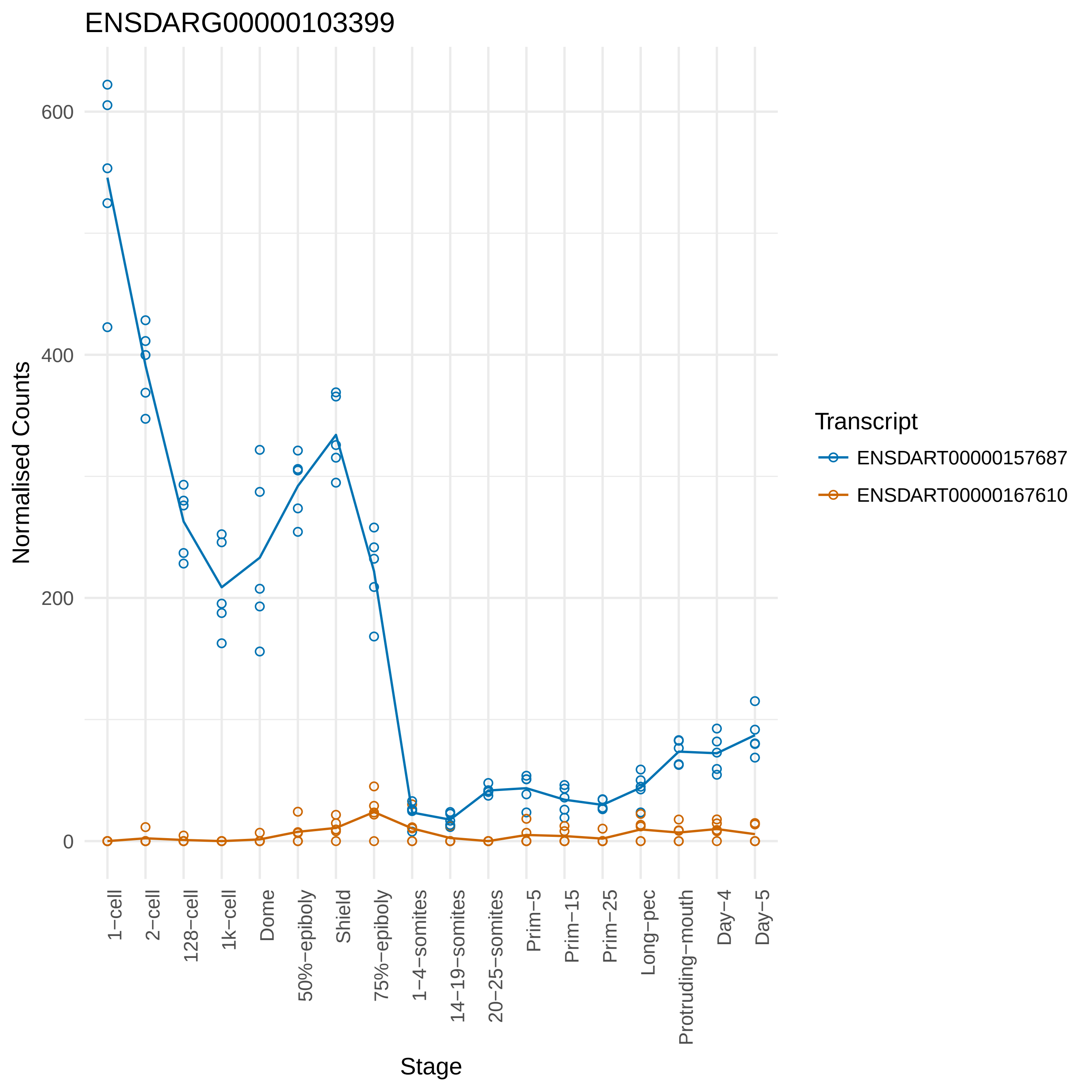

ENSDARG00000103399
Normalised Counts
600
400
200
0
1−cell
2−cell
128−cell
1k−cell
Dome
50%−epiboly
Shield
75%−epiboly
1−4−somites
14−19−somites
20−25−somites
Prim−5
Prim−15
Prim−25
Long−pec
Protruding−mouth
Day−4
Day−5
Stage
Transcript
ENSDART00000157687
ENSDART00000167610

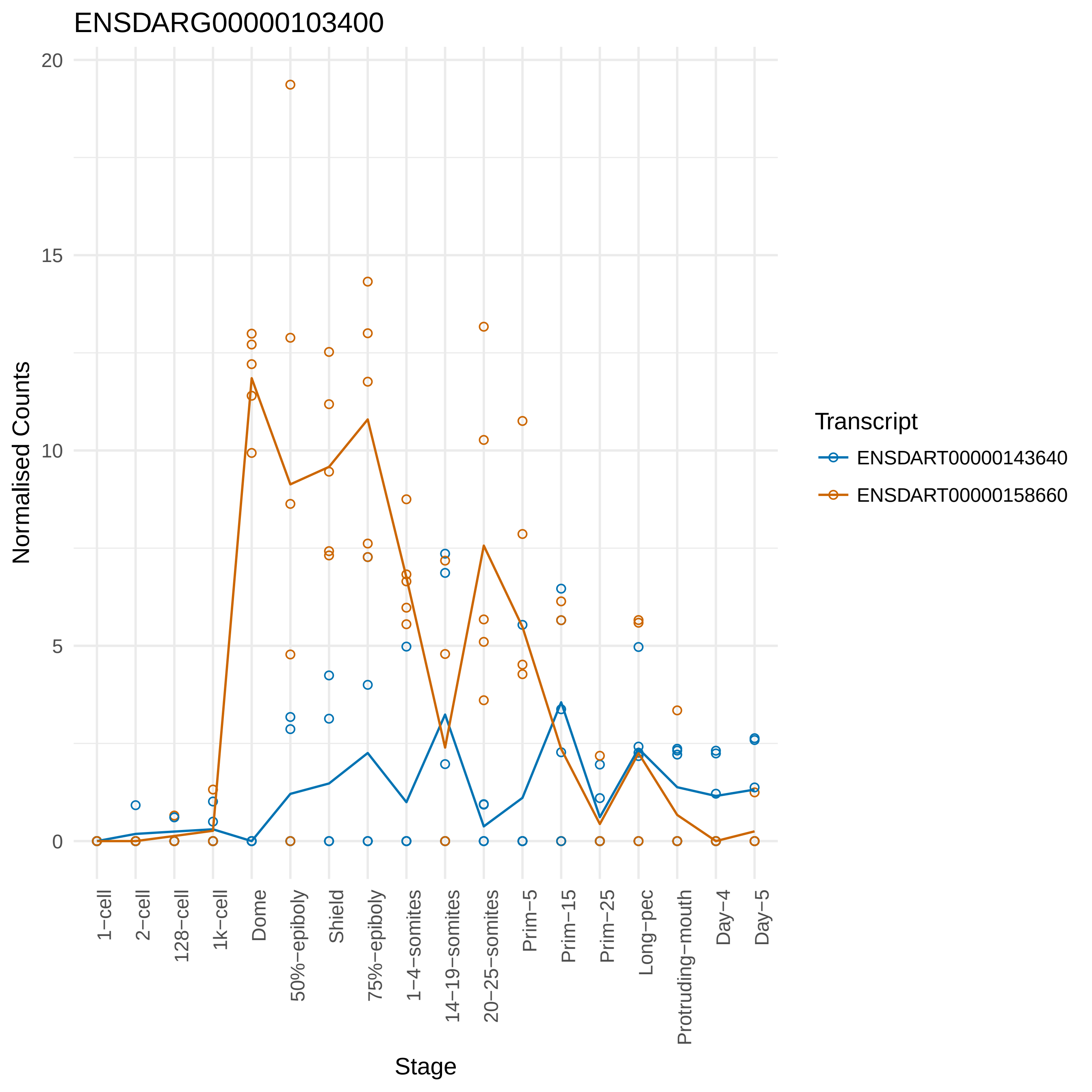
ENSDARG00000103400
Normalised Counts
20
15
10
5
0
1−cell
2−cell
128−cell
1k−cell
Dome
50%−epiboly
Shield
75%−epiboly
1−4−somites
14−19−somites
20−25−somites
Prim−5
Prim−15
Prim−25
Long−pec
Protruding−mouth
Day−4
Day−5
Stage
Transcript
ENSDART00000143640
ENSDART00000158660

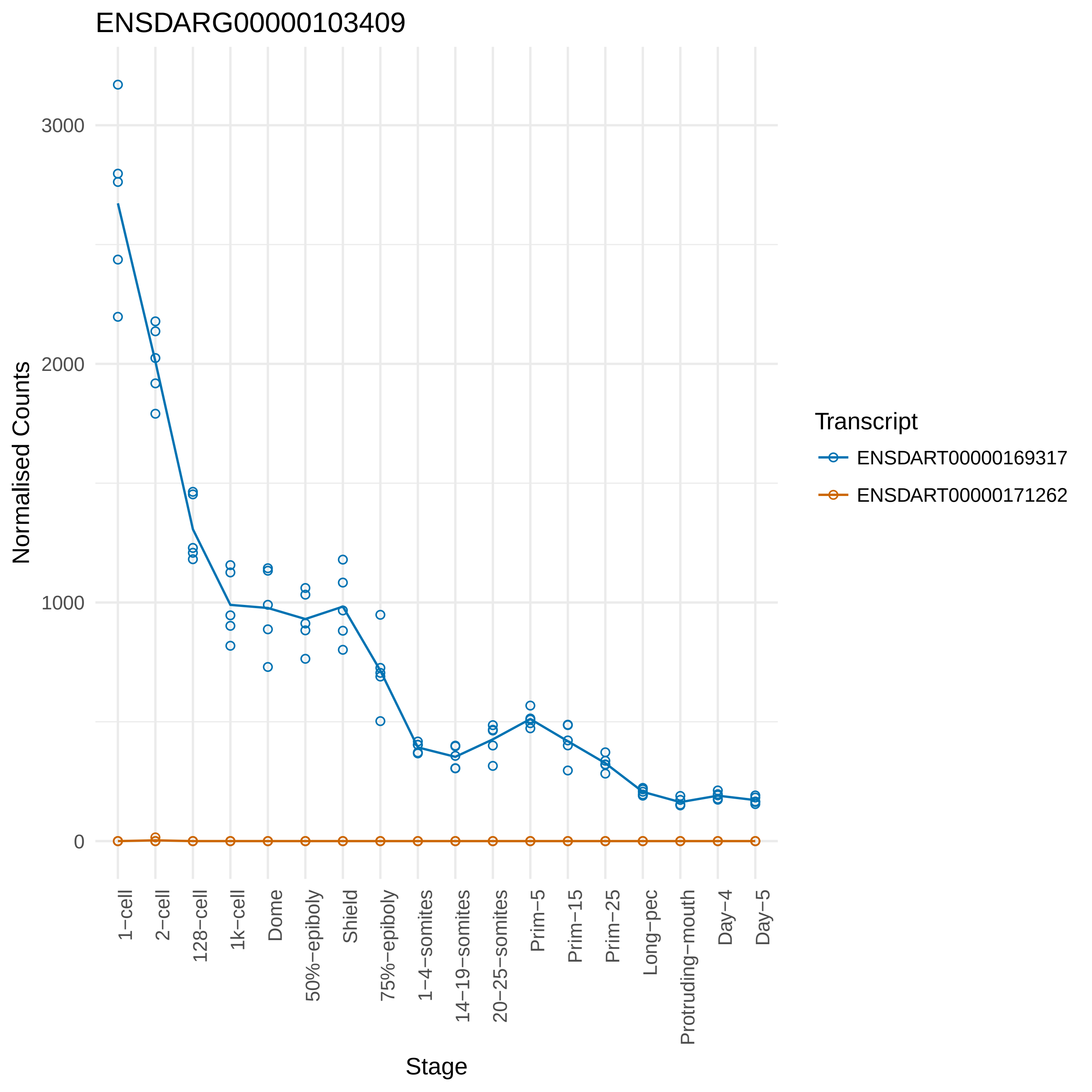

ENSDARG00000103409
Normalised Counts
3000
2000
1000
0
1−cell
2−cell
128−cell
1k−cell
Dome
50%−epiboly
Shield
75%−epiboly
1−4−somites
14−19−somites
20−25−somites
Prim−5
Prim−15
Prim−25
Long−pec
Protruding−mouth
Day−4
Day−5
Stage
Transcript
ENSDART00000169317
ENSDART00000171262

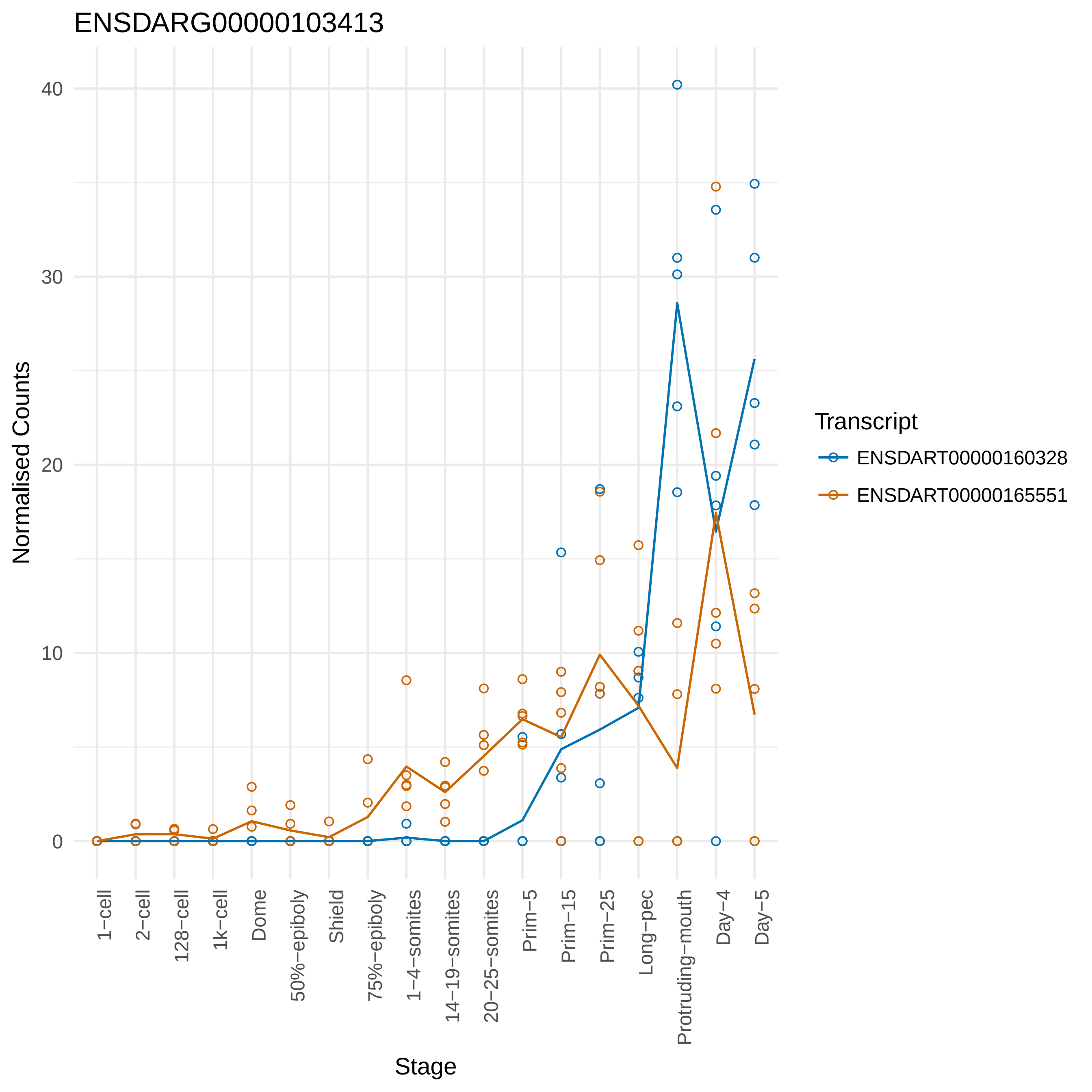

ENSDARG00000103413
Normalised Counts
40
30
20
10
0
1−cell
2−cell
128−cell
1k−cell
Dome
50%−epiboly
Shield
75%−epiboly
1−4−somites
14−19−somites
20−25−somites
Prim−5
Prim−15
Prim−25
Long−pec
Protruding−mouth
Day−4
Day−5
Stage
Transcript
ENSDART00000160328
ENSDART00000165551

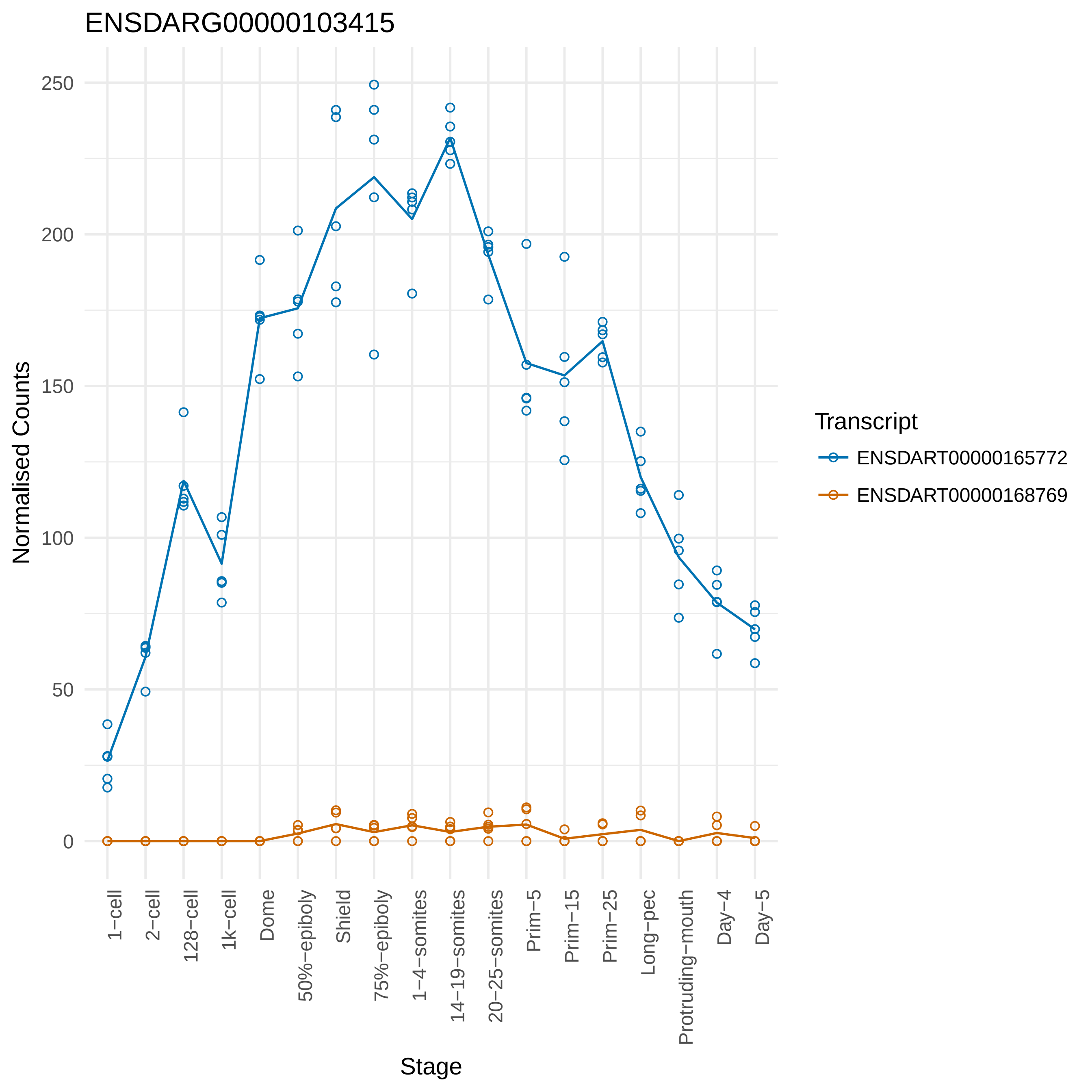

ENSDARG00000103415
Normalised Counts
0
50
100
150
200
250
Stage
1−cell
2−cell
128−cell
1k−cell
Dome
50%−epiboly
Shield
75%−epiboly
1−4−somites
14−19−somites
20−25−somites
Prim−5
Prim−15
Prim−25
Long−pec
Protruding−mouth
Day−4
Day−5
Transcript
ENSDART00000165772
ENSDART00000168769

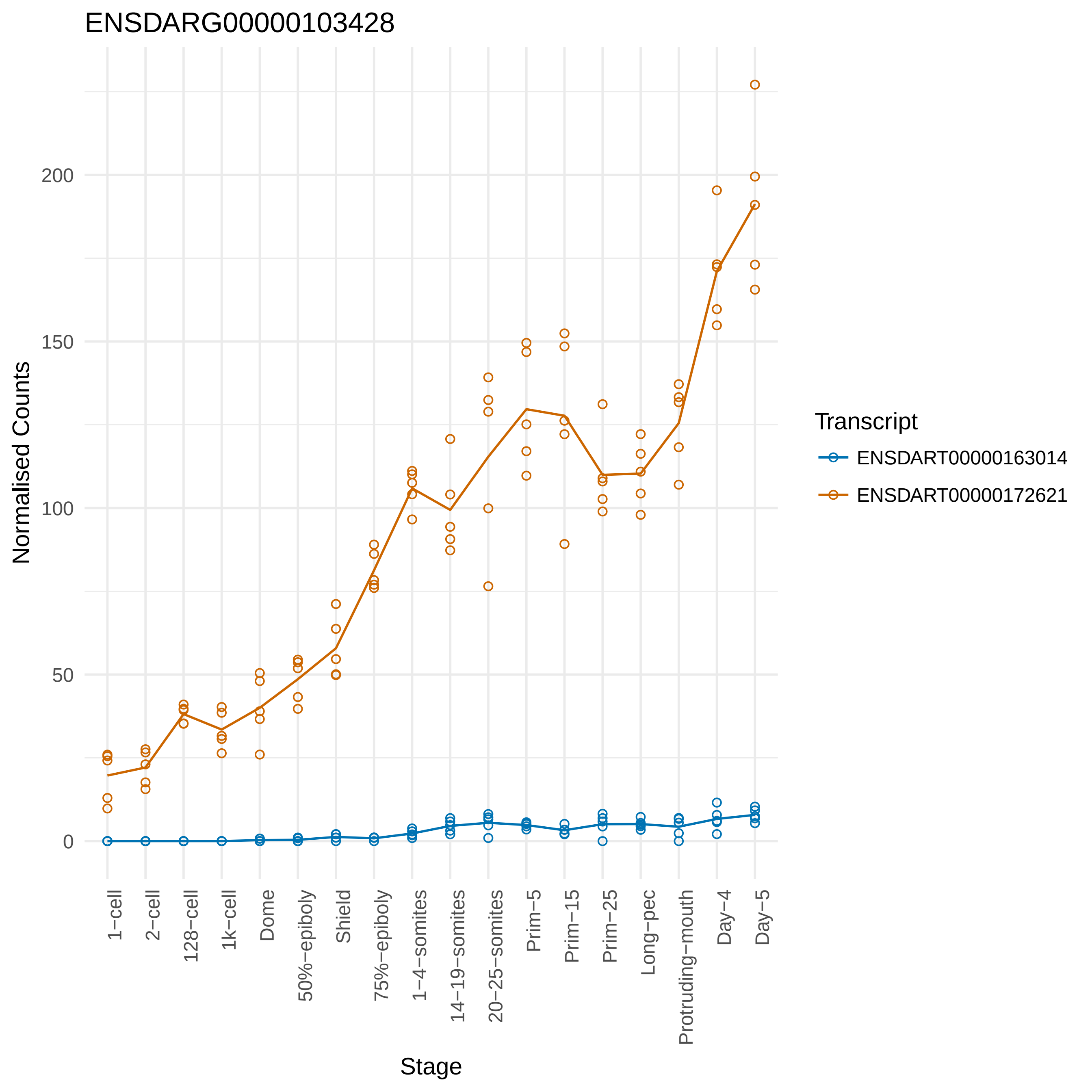

ENSDARG00000103428
Normalised Counts
0
50
100
150
200
1−cell
2−cell
128−cell
1k−cell
Dome
50%−epiboly
Shield
75%−epiboly
1−4−somites
14−19−somites
20−25−somites
Prim−5
Prim−15
Prim−25
Long−pec
Protruding−mouth
Day−4
Day−5
Stage
Transcript
ENSDART00000163014
ENSDART00000172621

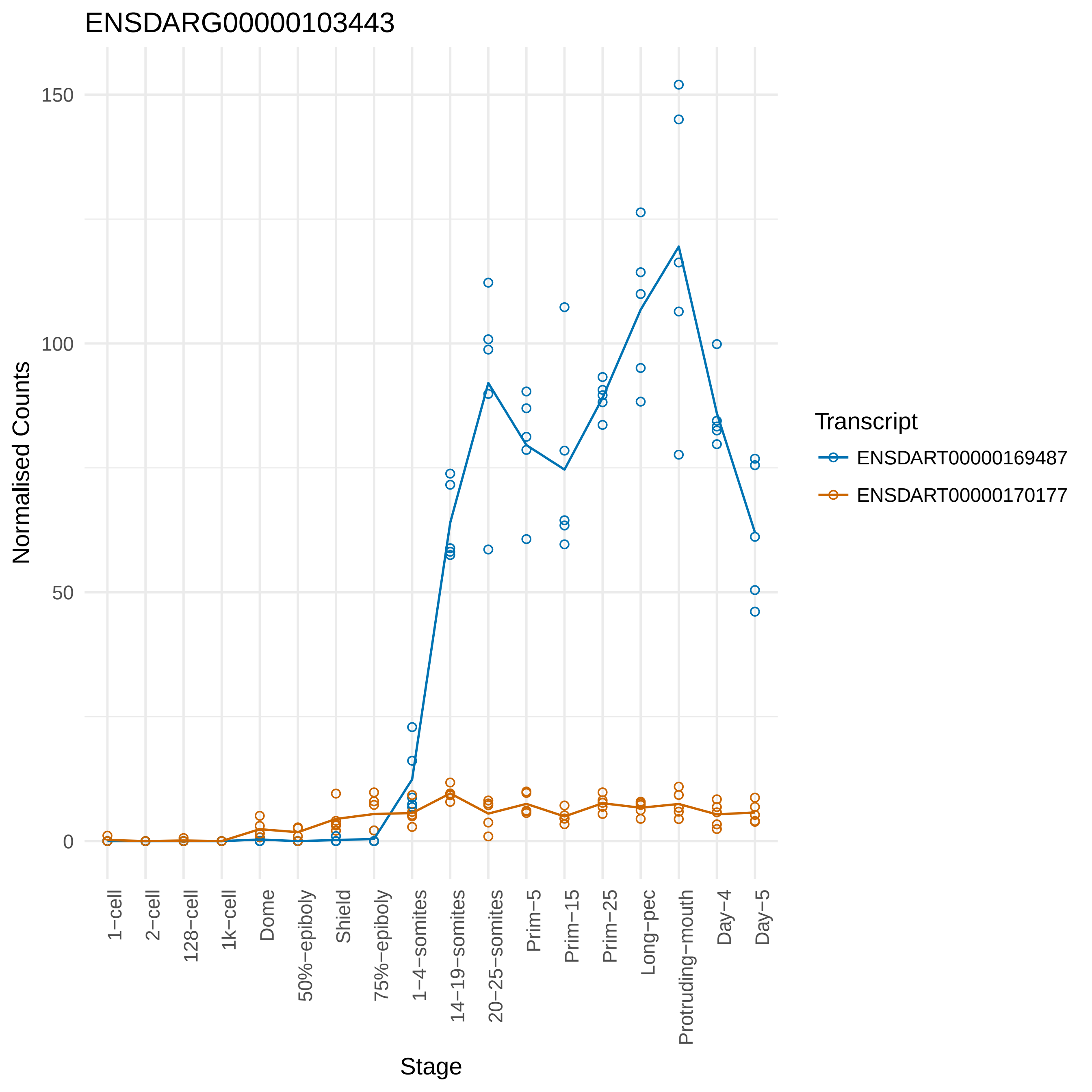

ENSDARG00000103443
Normalised Counts
150
100
50
0
1−cell
2−cell
128−cell
1k−cell
Dome
50%−epiboly
Shield
75%−epiboly
1−4−somites
14−19−somites
20−25−somites
Prim−5
Prim−15
Prim−25
Long−pec
Protruding−mouth
Day−4
Day−5
Stage
Transcript
ENSDART00000169487
ENSDART00000170177

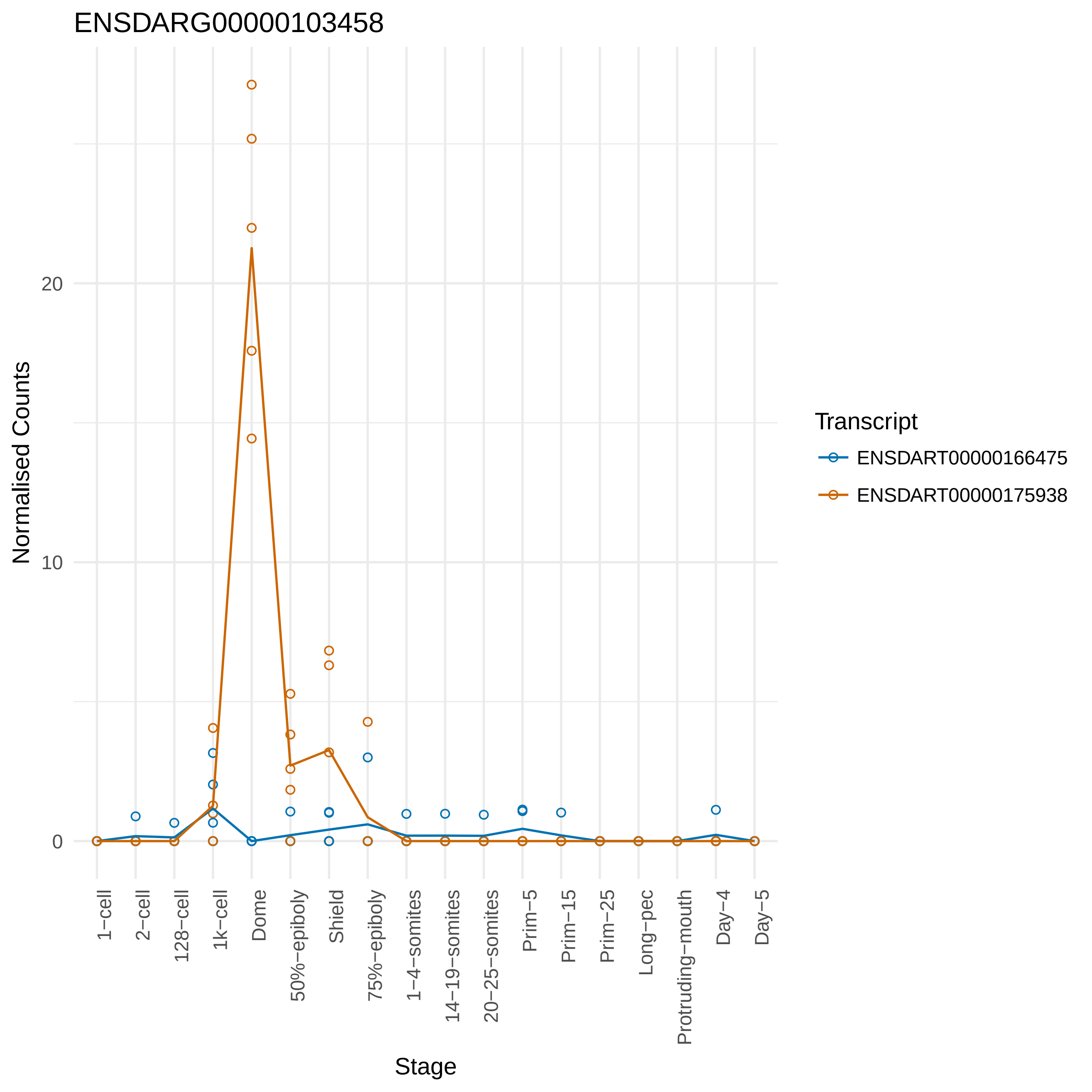

ENSDARG00000103458
Normalised Counts
20
10
0
1−cell
2−cell
128−cell
1k−cell
Dome
50%−epiboly
Shield
75%−epiboly
1−4−somites
14−19−somites
20−25−somites
Prim−5
Prim−15
Prim−25
Long−pec
Protruding−mouth
Day−4
Day−5
Stage
Transcript
ENSDART00000166475
ENSDART00000175938

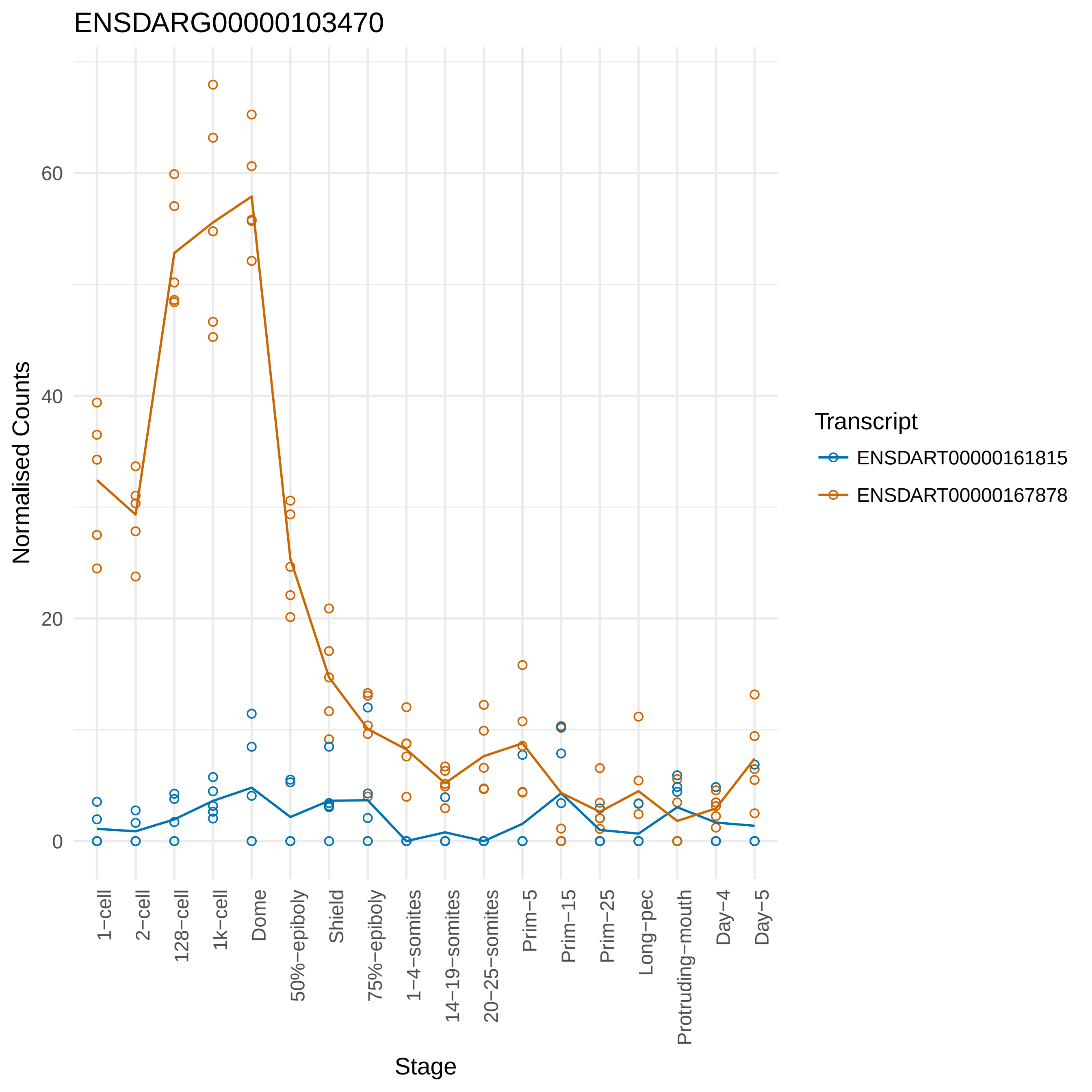

ENSDARG00000103470
Normalised Counts
60
40
20
0
1−cell
2−cell
128−cell
1k−cell
Dome
50%−epiboly
Shield
75%−epiboly
1−4−somites
14−19−somites
20−25−somites
Prim−5
Prim−15
Prim−25
Long−pec
Protruding−mouth
Day−4
Day−5
Stage
Transcript
ENSDART00000161815
ENSDART00000167878

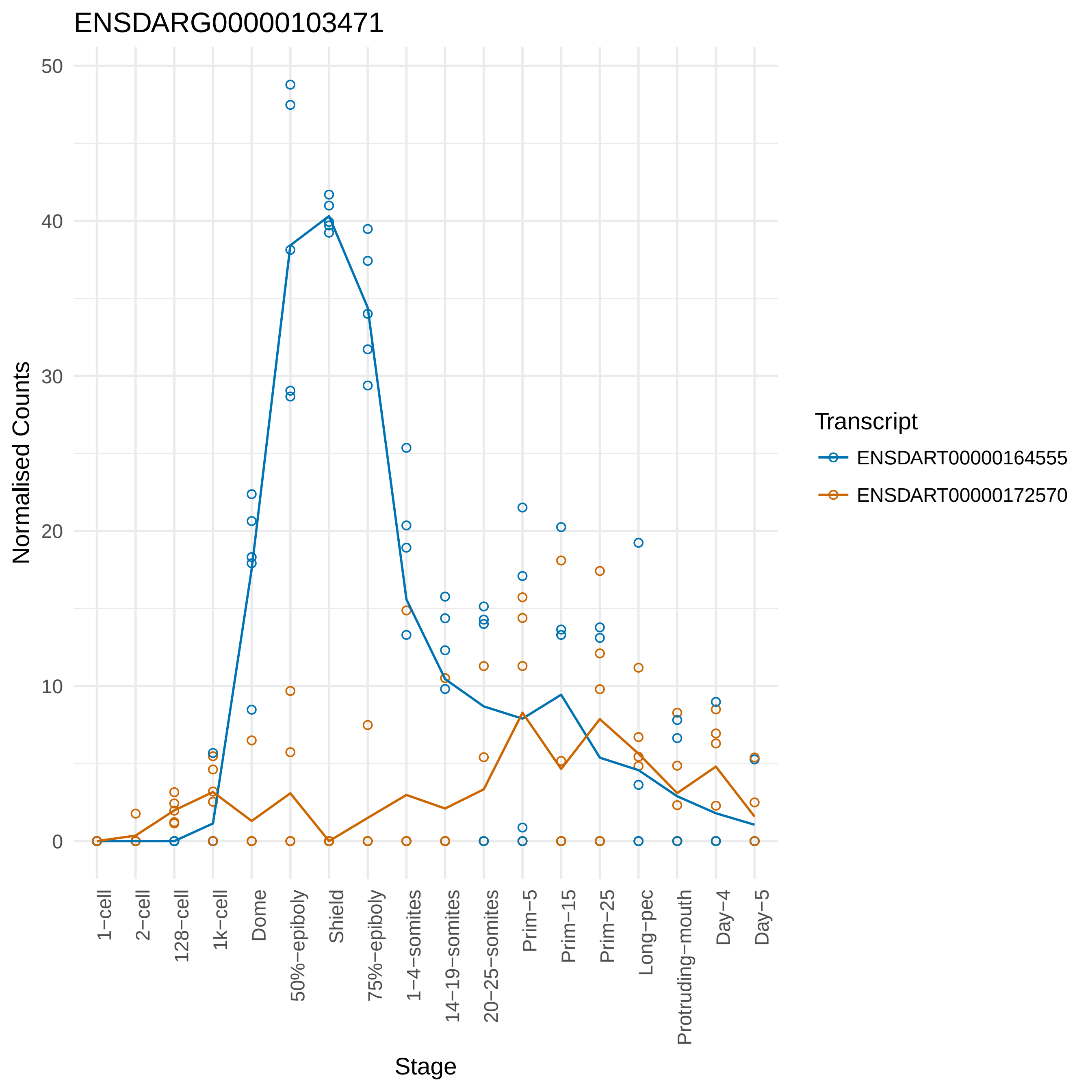
ENSDARG00000103471
Normalised Counts
0
10
20
30
40
50
Stage
1−cell
2−cell
128−cell
1k−cell
Dome
50%−epiboly
Shield
75%−epiboly
1−4−somites
14−19−somites
20−25−somites
Prim−5
Prim−15
Prim−25
Long−pec
Protruding−mouth
Day−4
Day−5
Transcript
ENSDART00000164555
ENSDART00000172570

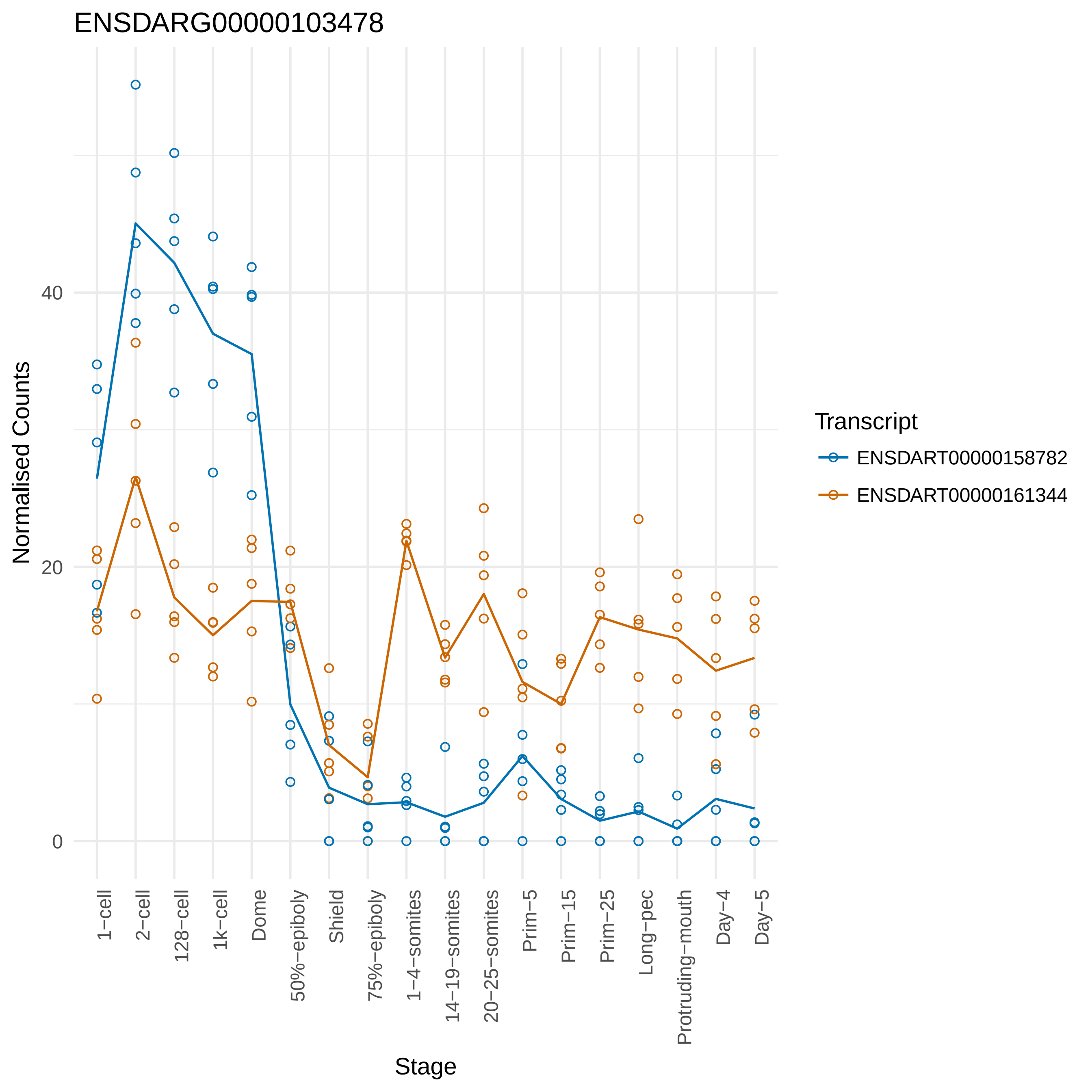

ENSDARG00000103478
Normalised Counts
0
20
40
Stage
1−cell
2−cell
128−cell
1k−cell
Dome
50%−epiboly
Shield
75%−epiboly
1−4−somites
14−19−somites
20−25−somites
Prim−5
Prim−15
Prim−25
Long−pec
Protruding−mouth
Day−4
Day−5
Transcript
ENSDART00000158782
ENSDART00000161344

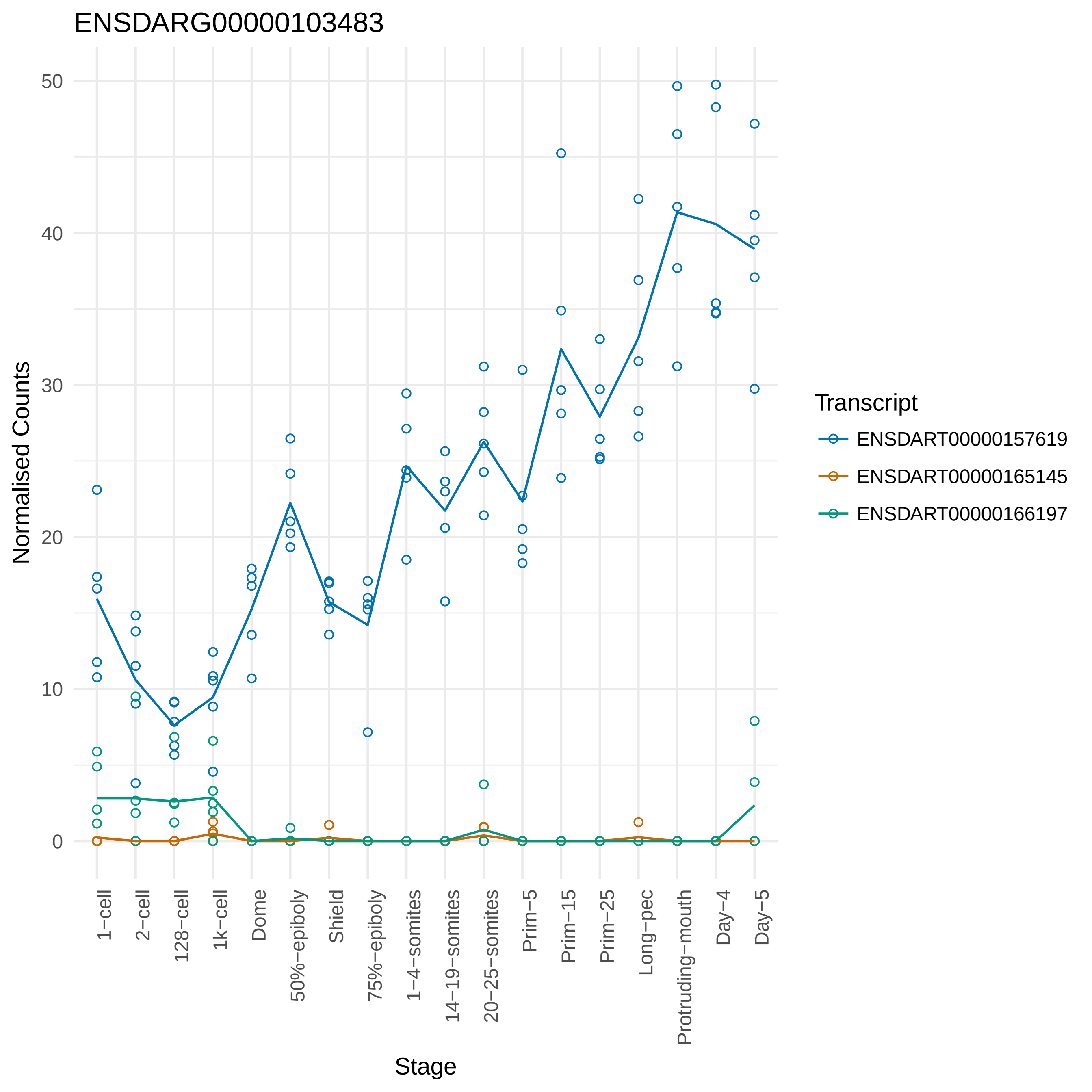

ENSDARG00000103483
Normalised Counts
0
10
20
30
40
50
1−cell
2−cell
128−cell
1k−cell
Dome
50%−epiboly
Shield
75%−epiboly
1−4−somites
14−19−somites
20−25−somites
Prim−5
Prim−15
Prim−25
Long−pec
Protruding−mouth
Day−4
Day−5
Stage
Transcript
ENSDART00000157619
ENSDART00000165145
ENSDART00000166197

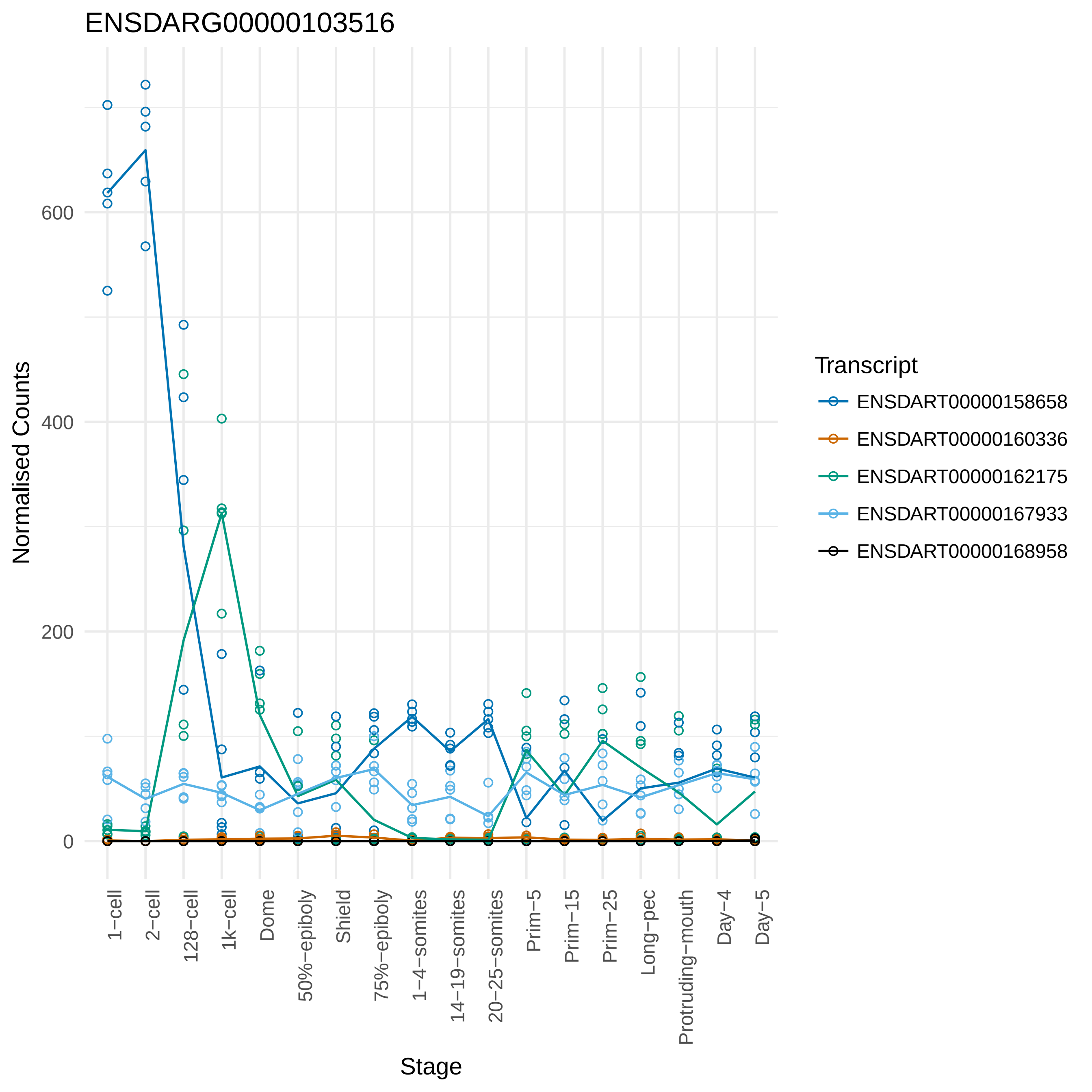
ENSDARG00000103516
Normalised Counts
600
400
200
0
1−cell
2−cell
128−cell
1k−cell
Dome
50%−epiboly
Shield
75%−epiboly
1−4−somites
14−19−somites
20−25−somites
Prim−5
Prim−15
Prim−25
Long−pec
Protruding−mouth
Day−4
Day−5
Stage
Transcript
ENSDART00000158658
ENSDART00000160336
ENSDART00000162175
ENSDART00000167933
ENSDART00000168958

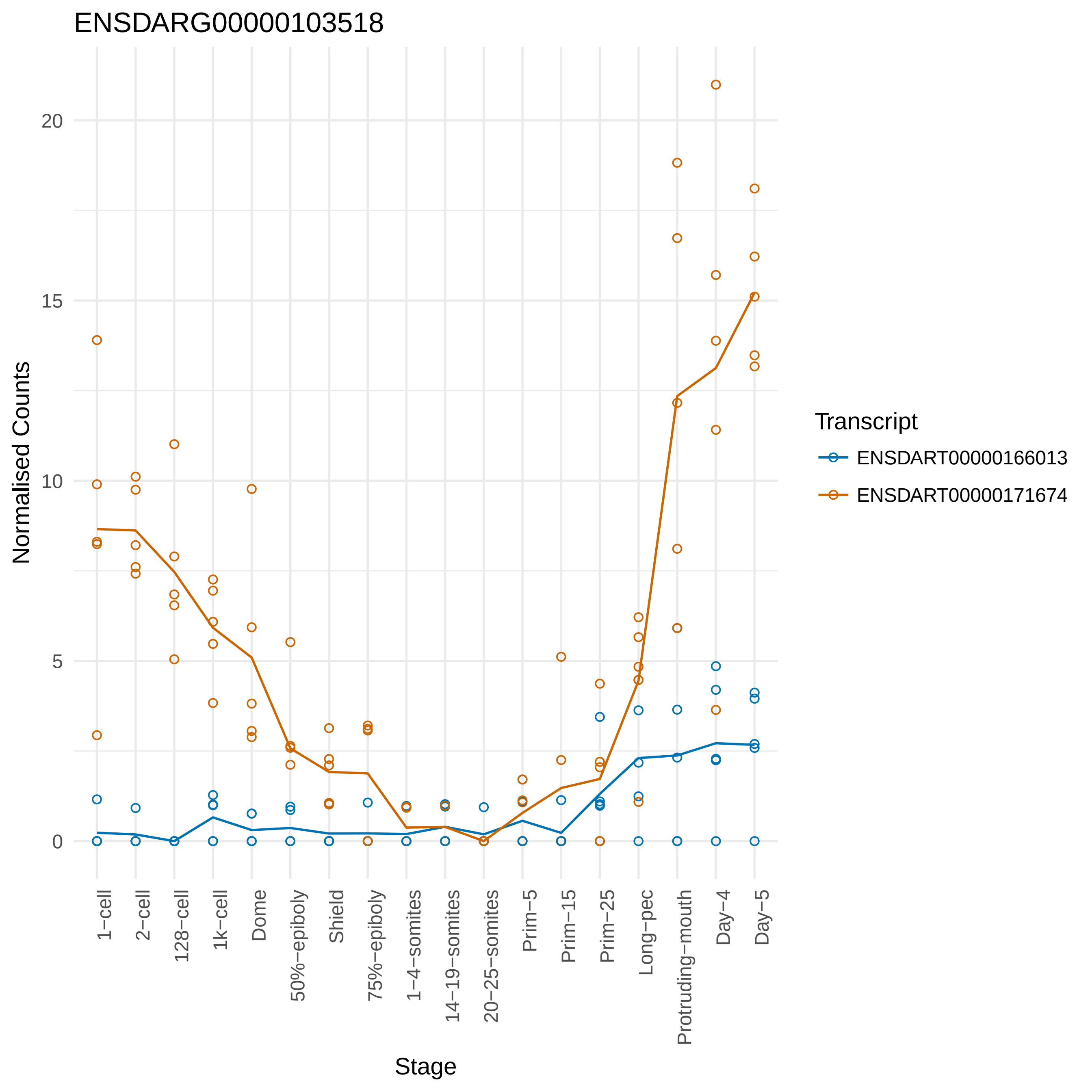

ENSDARG00000103518
Normalised Counts
0
5
10
15
20
1−cell
2−cell
128−cell
1k−cell
Dome
50%−epiboly
Shield
75%−epiboly
1−4−somites
14−19−somites
20−25−somites
Prim−5
Prim−15
Prim−25
Long−pec
Protruding−mouth
Day−4
Day−5
Stage
Transcript
ENSDART00000166013
ENSDART00000171674

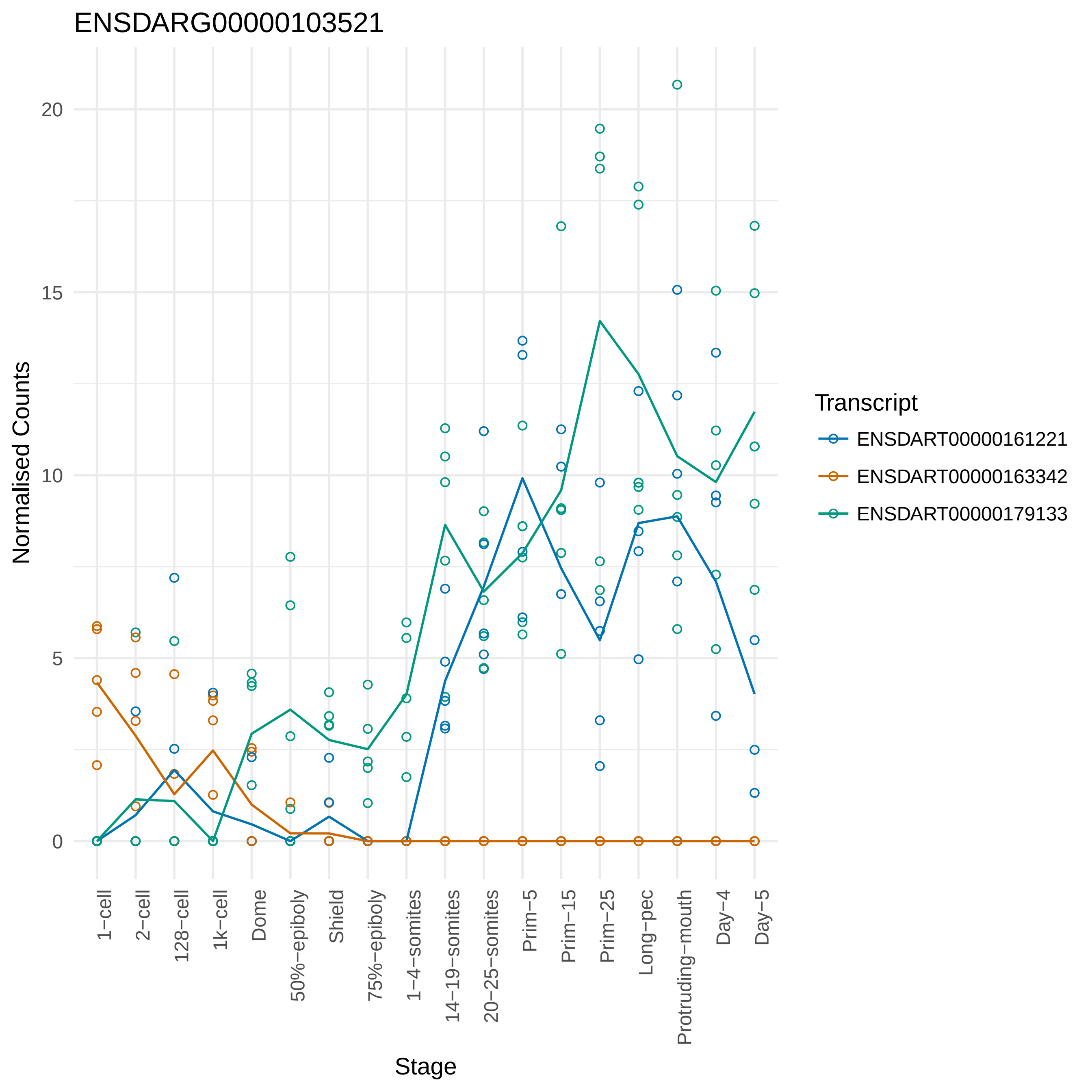

ENSDARG00000103521
Normalised Counts
0
5
10
15
20
1−cell
2−cell
128−cell
1k−cell
Dome
50%−epiboly
Shield
75%−epiboly
1−4−somites
14−19−somites
20−25−somites
Prim−5
Prim−15
Prim−25
Long−pec
Protruding−mouth
Day−4
Day−5
Stage
Transcript
ENSDART00000161221
ENSDART00000163342
ENSDART00000179133

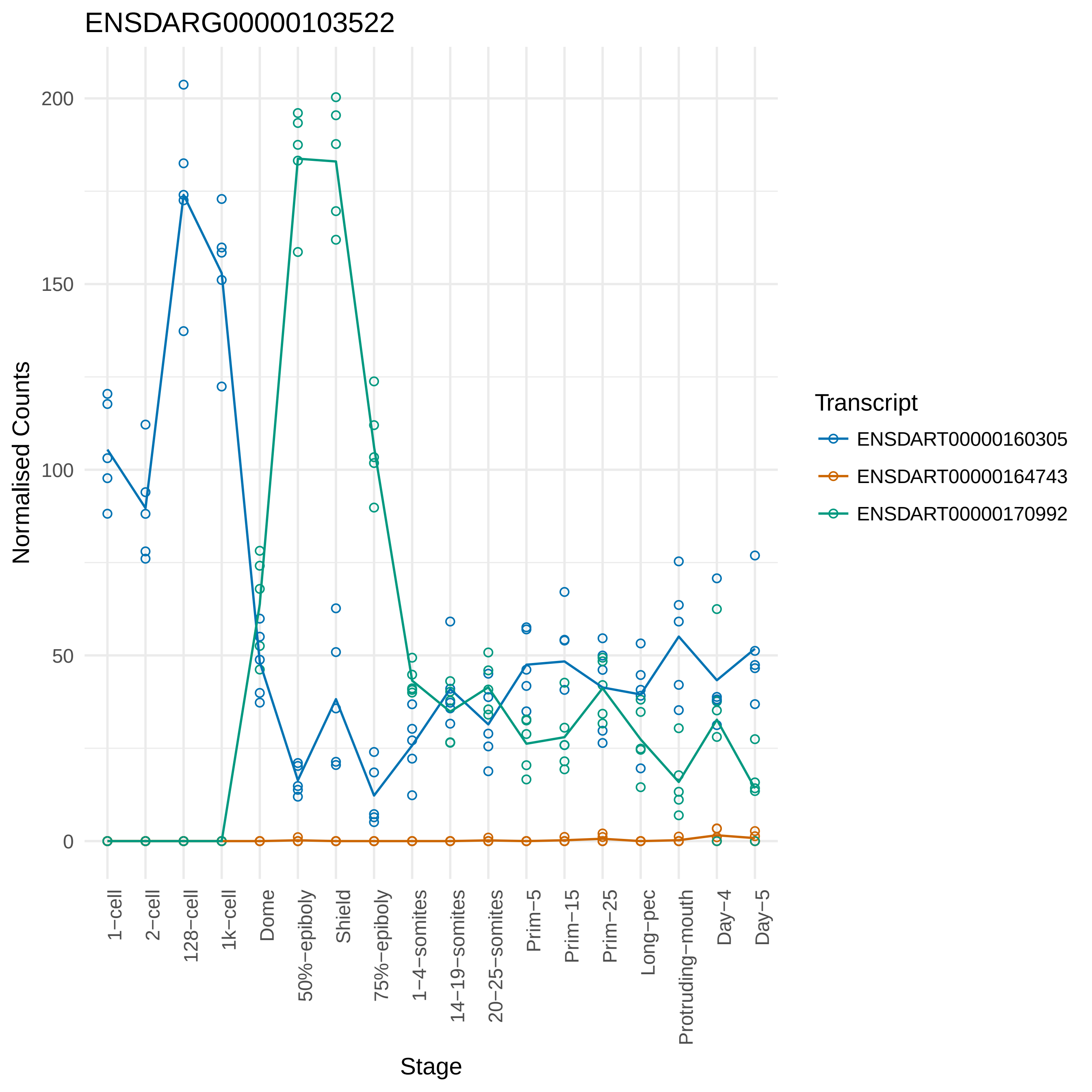

ENSDARG00000103522
Normalised Counts
0
50
100
150
200
1−cell
2−cell
128−cell
1k−cell
Dome
50%−epiboly
Shield
75%−epiboly
1−4−somites
14−19−somites
20−25−somites
Prim−5
Prim−15
Prim−25
Long−pec
Protruding−mouth
Day−4
Day−5
Stage
Transcript
ENSDART00000160305
ENSDART00000164743
ENSDART00000170992

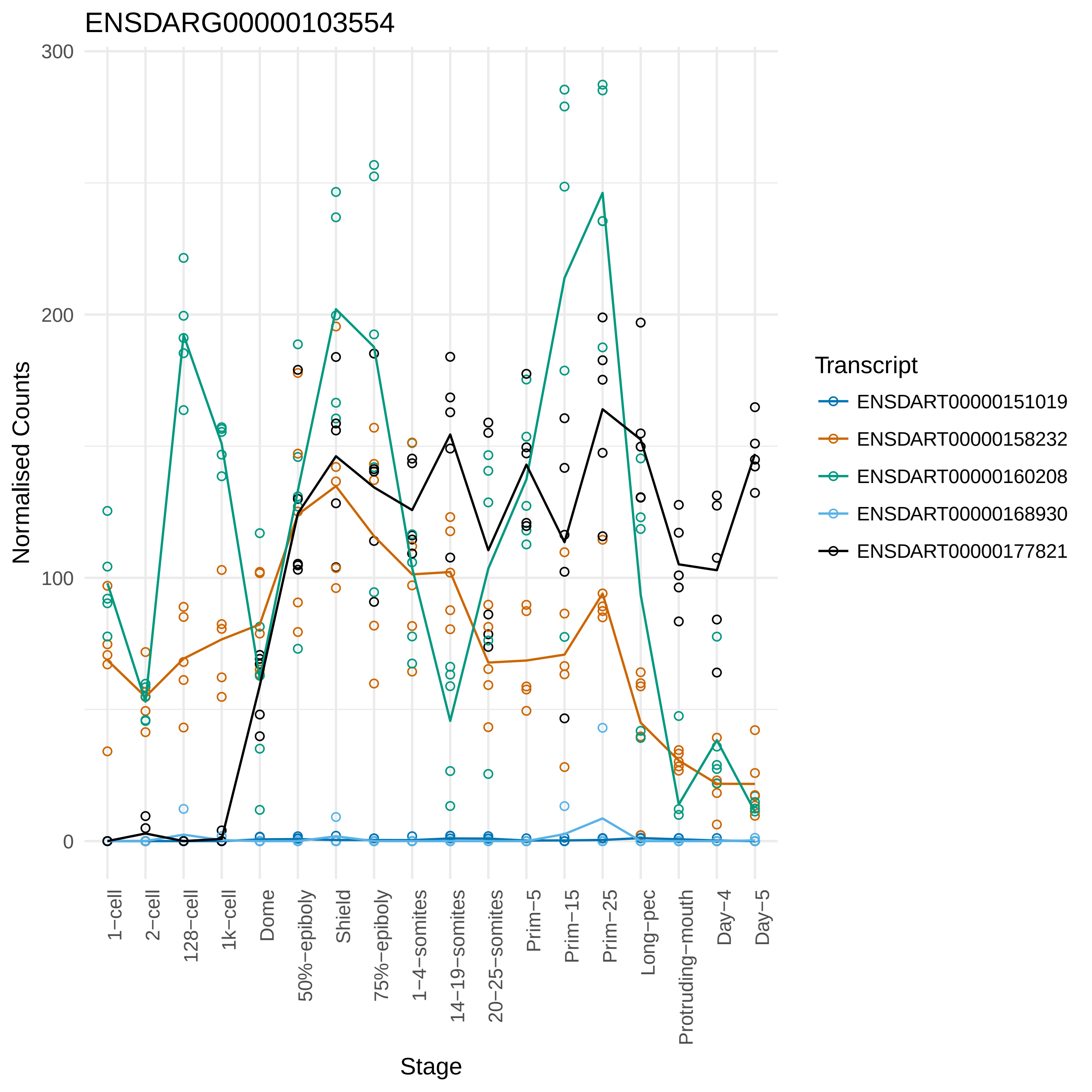

ENSDARG00000103554
300
200
100
0
Normalised Counts
1−cell
2−cell
128−cell
1k−cell
Dome
50%−epiboly
Shield
75%−epiboly
1−4−somites
14−19−somites
20−25−somites
Prim−5
Prim−15
Prim−25
Long−pec
Protruding−mouth
Day−4
Day−5
Stage
Transcript
ENSDART00000151019
ENSDART00000158232
ENSDART00000160208
ENSDART00000168930
ENSDART00000177821

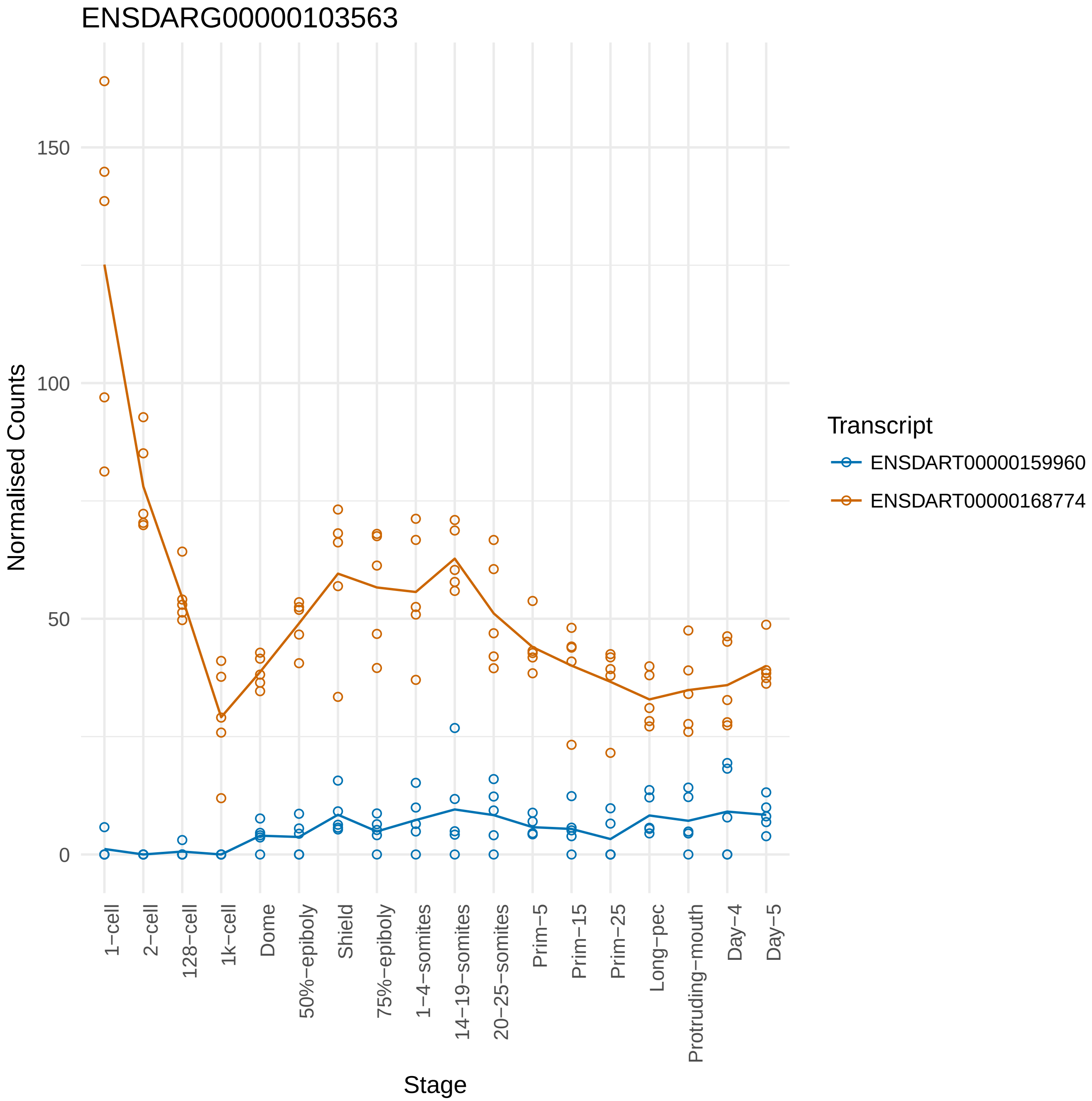

ENSDARG00000103563
Normalised Counts
150
100
50
0
1−cell
2−cell
128−cell
1k−cell
Dome
50%−epiboly
Shield
75%−epiboly
1−4−somites
14−19−somites
20−25−somites
Prim−5
Prim−15
Prim−25
Long−pec
Protruding−mouth
Day−4
Day−5
Stage
Transcript
ENSDART00000159960
ENSDART00000168774

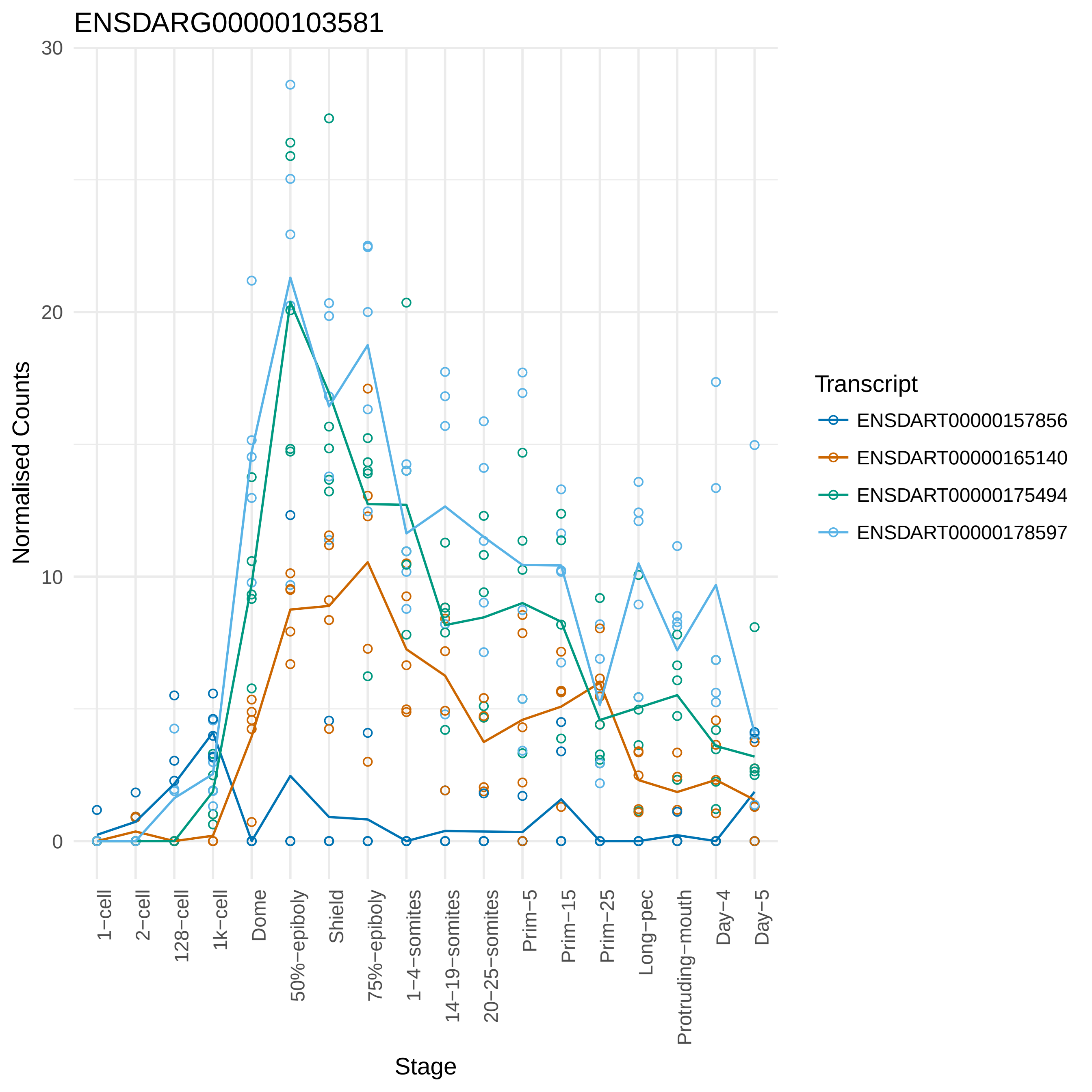
ENSDARG00000103581
30
20
10
0
Normalised Counts
1−cell
2−cell
128−cell
1k−cell
Dome
50%−epiboly
Shield
75%−epiboly
1−4−somites
14−19−somites
20−25−somites
Prim−5
Prim−15
Prim−25
Long−pec
Protruding−mouth
Day−4
Day−5
Stage
Transcript
ENSDART00000157856
ENSDART00000165140
ENSDART00000175494
ENSDART00000178597

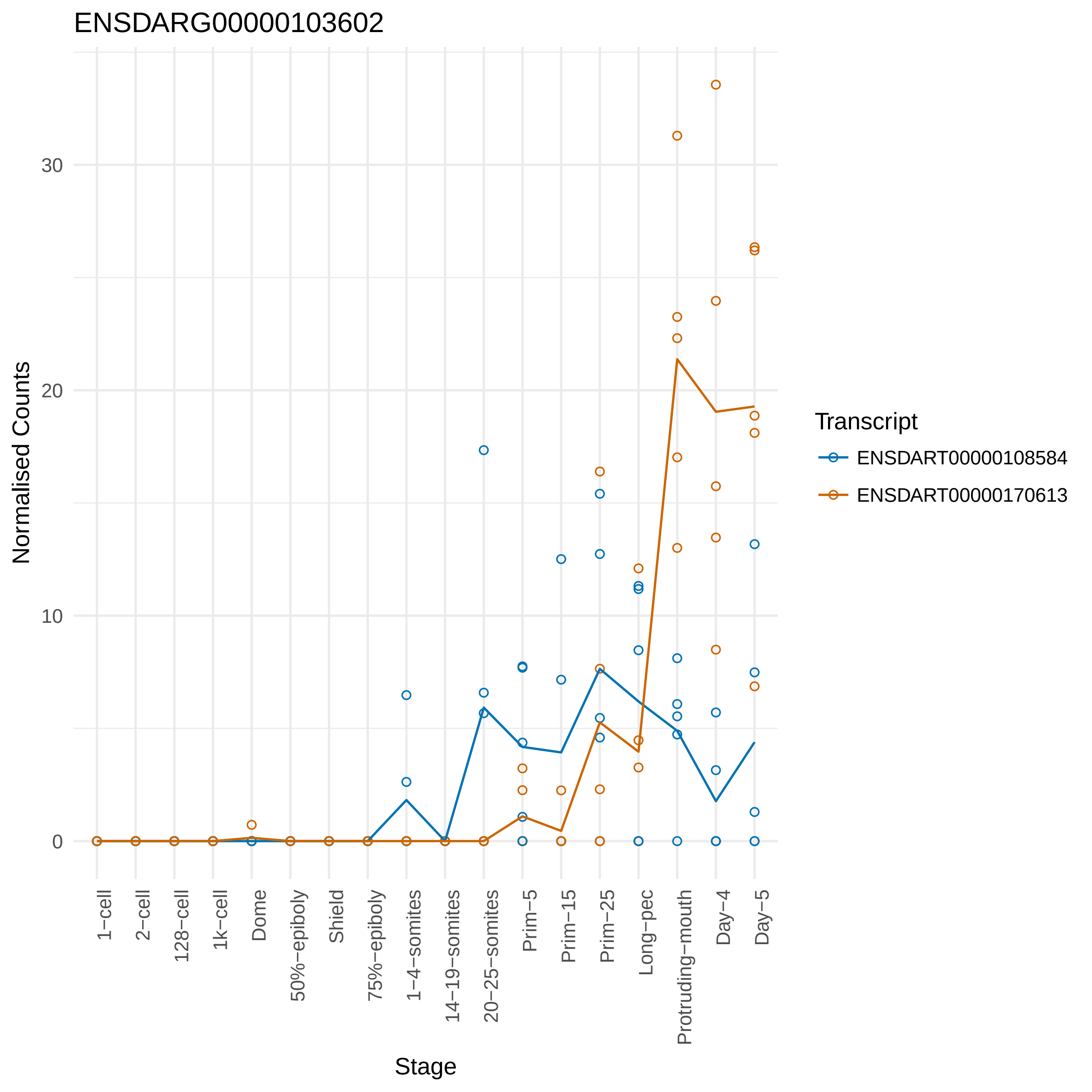

ENSDARG00000103602
Normalised Counts
30
20
10
0
1−cell
2−cell
128−cell
1k−cell
Dome
50%−epiboly
Shield
75%−epiboly
1−4−somites
14−19−somites
20−25−somites
Prim−5
Prim−15
Prim−25
Long−pec
Protruding−mouth
Day−4
Day−5
Stage
Transcript
ENSDART00000108584
ENSDART00000170613

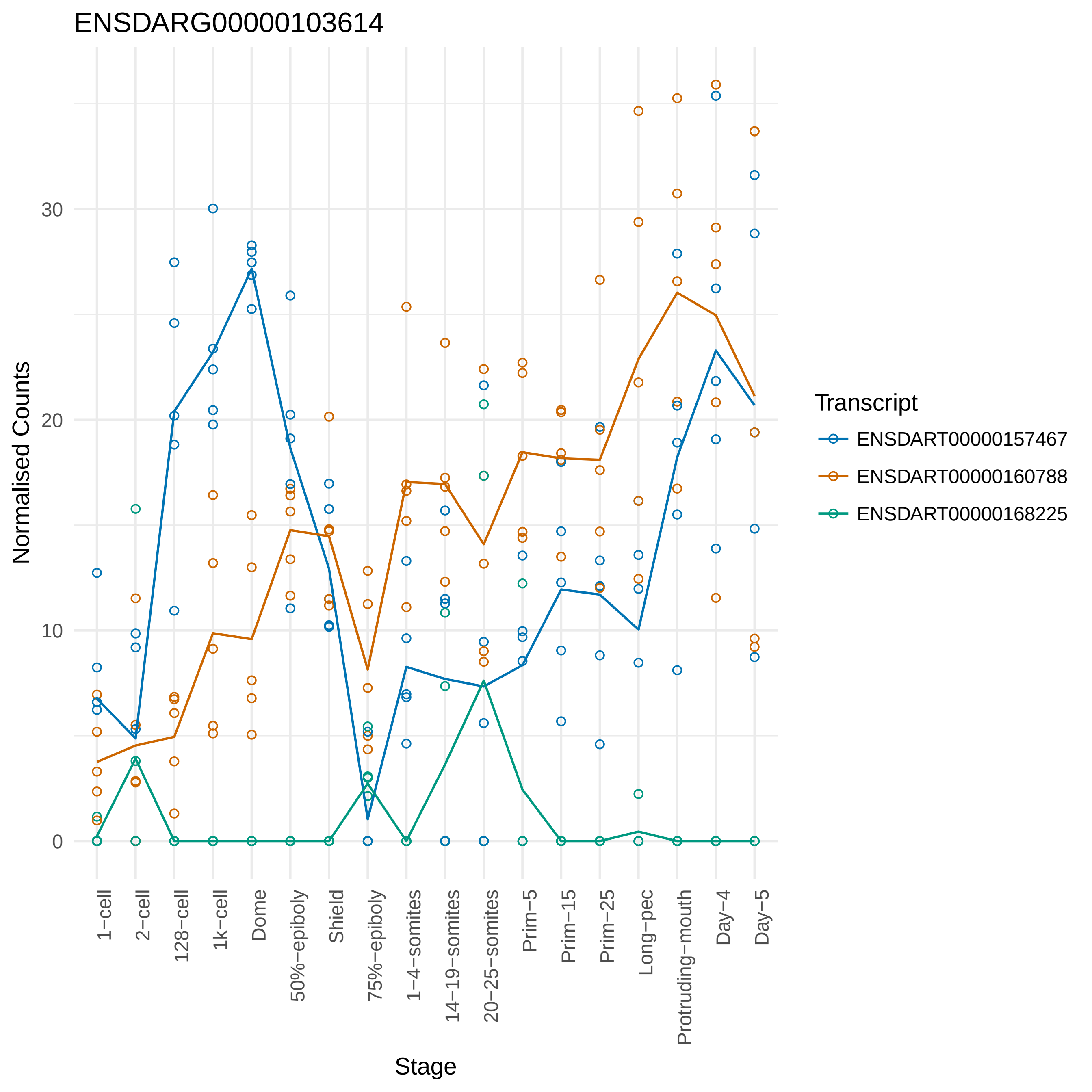
ENSDARG00000103614
Normalised Counts
0
10
20
30
Stage
1−cell
2−cell
128−cell
1k−cell
Dome
50%−epiboly
Shield
75%−epiboly
1−4−somites
14−19−somites
20−25−somites
Prim−5
Prim−15
Prim−25
Long−pec
Protruding−mouth
Day−4
Day−5
Transcript
ENSDART00000157467
ENSDART00000160788
ENSDART00000168225

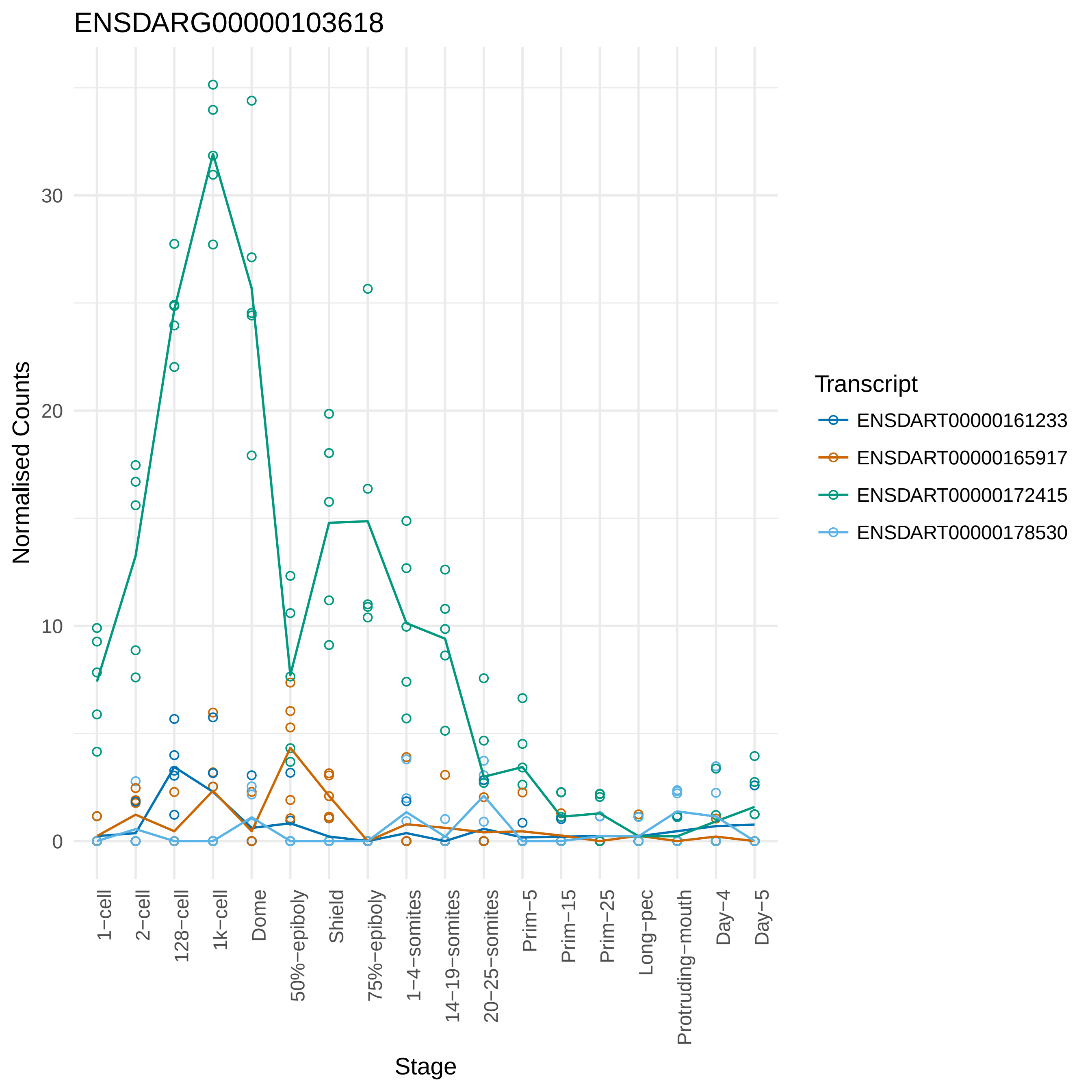

ENSDARG00000103618
Normalised Counts
0
10
20
30
1−cell
2−cell
128−cell
1k−cell
Dome
50%−epiboly
Shield
75%−epiboly
1−4−somites
14−19−somites
20−25−somites
Prim−5
Prim−15
Prim−25
Long−pec
Protruding−mouth
Day−4
Day−5
Stage
Transcript
ENSDART00000161233
ENSDART00000165917
ENSDART00000172415
ENSDART00000178530

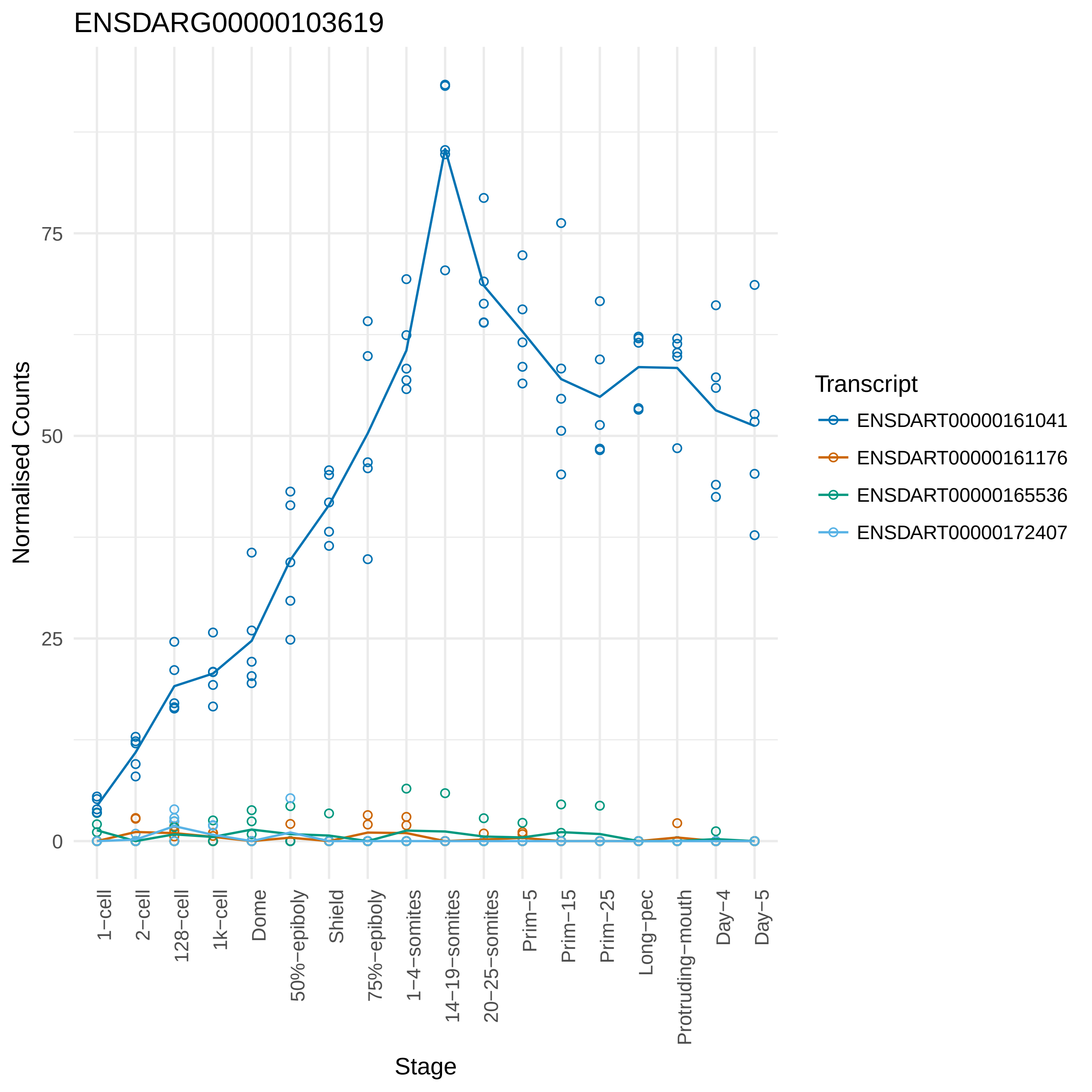

ENSDARG00000103619
Normalised Counts
0
25
50
75
1−cell
2−cell
128−cell
1k−cell
Dome
50%−epiboly
Shield
75%−epiboly
1−4−somites
14−19−somites
20−25−somites
Prim−5
Prim−15
Prim−25
Long−pec
Protruding−mouth
Day−4
Day−5
Stage
Transcript
ENSDART00000161041
ENSDART00000161176
ENSDART00000165536
ENSDART00000172407

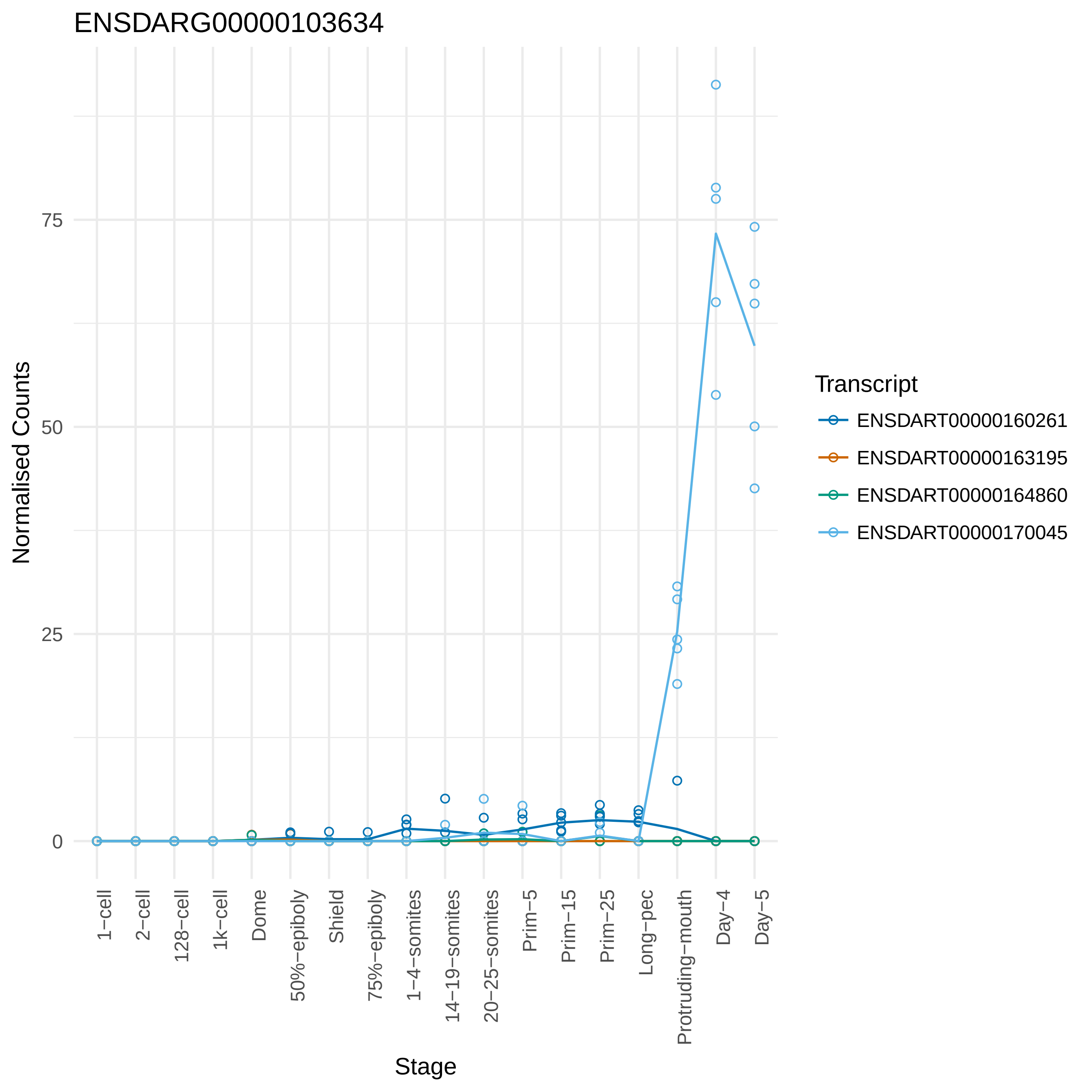
ENSDARG00000103634
Normalised Counts
75
50
25
0
1−cell
2−cell
128−cell
1k−cell
Dome
50%−epiboly
Shield
75%−epiboly
1−4−somites
14−19−somites
20−25−somites
Prim−5
Prim−15
Prim−25
Long−pec
Protruding−mouth
Day−4
Day−5
Stage
Transcript
ENSDART00000160261
ENSDART00000163195
ENSDART00000164860
ENSDART00000170045

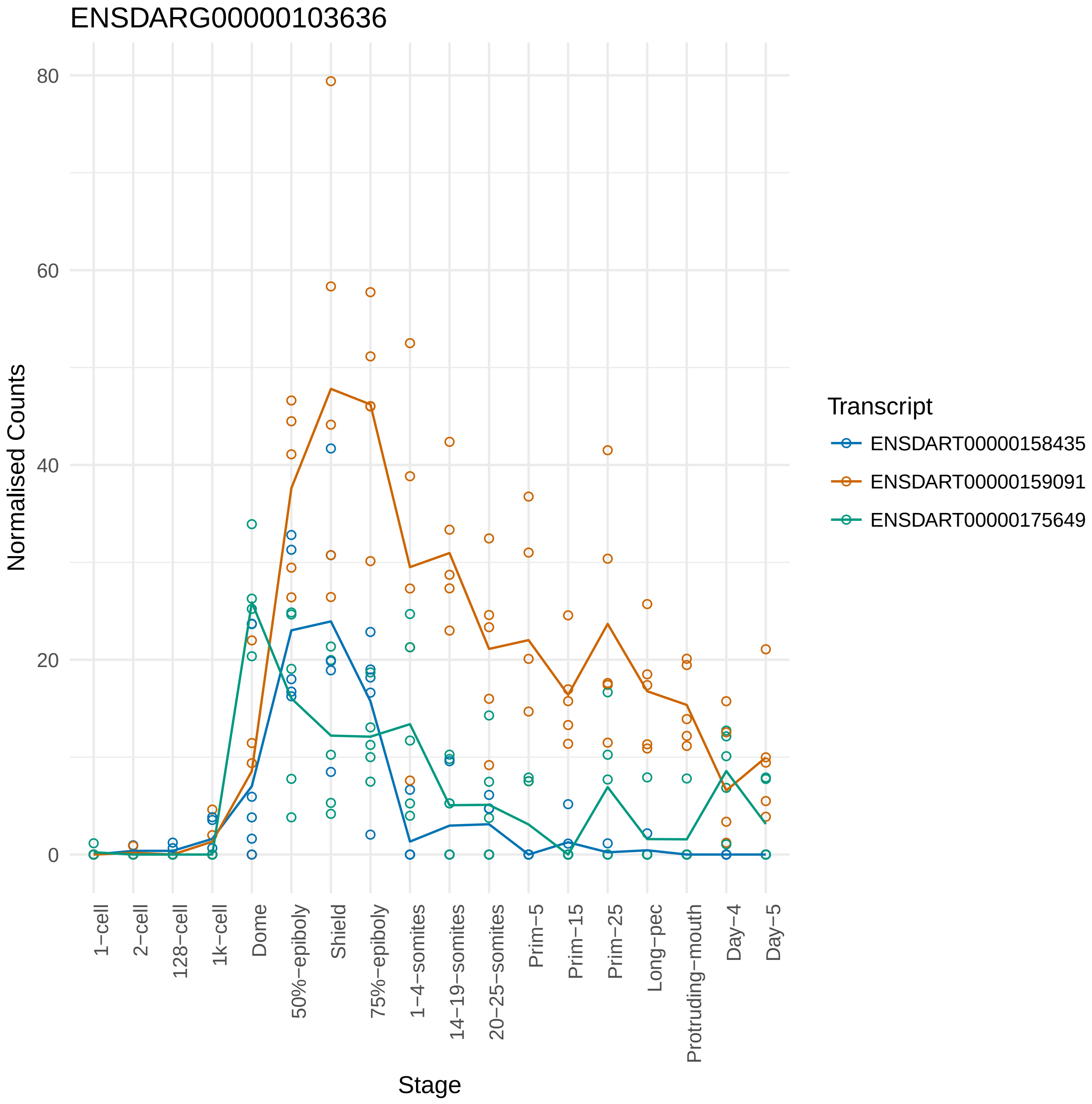

ENSDARG00000103636
Normalised Counts
80
60
40
20
0
1−cell
2−cell
128−cell
1k−cell
Dome
50%−epiboly
Shield
75%−epiboly
1−4−somites
14−19−somites
20−25−somites
Prim−5
Prim−15
Prim−25
Long−pec
Protruding−mouth
Day−4
Day−5
Stage
Transcript
ENSDART00000158435
ENSDART00000159091
ENSDART00000175649

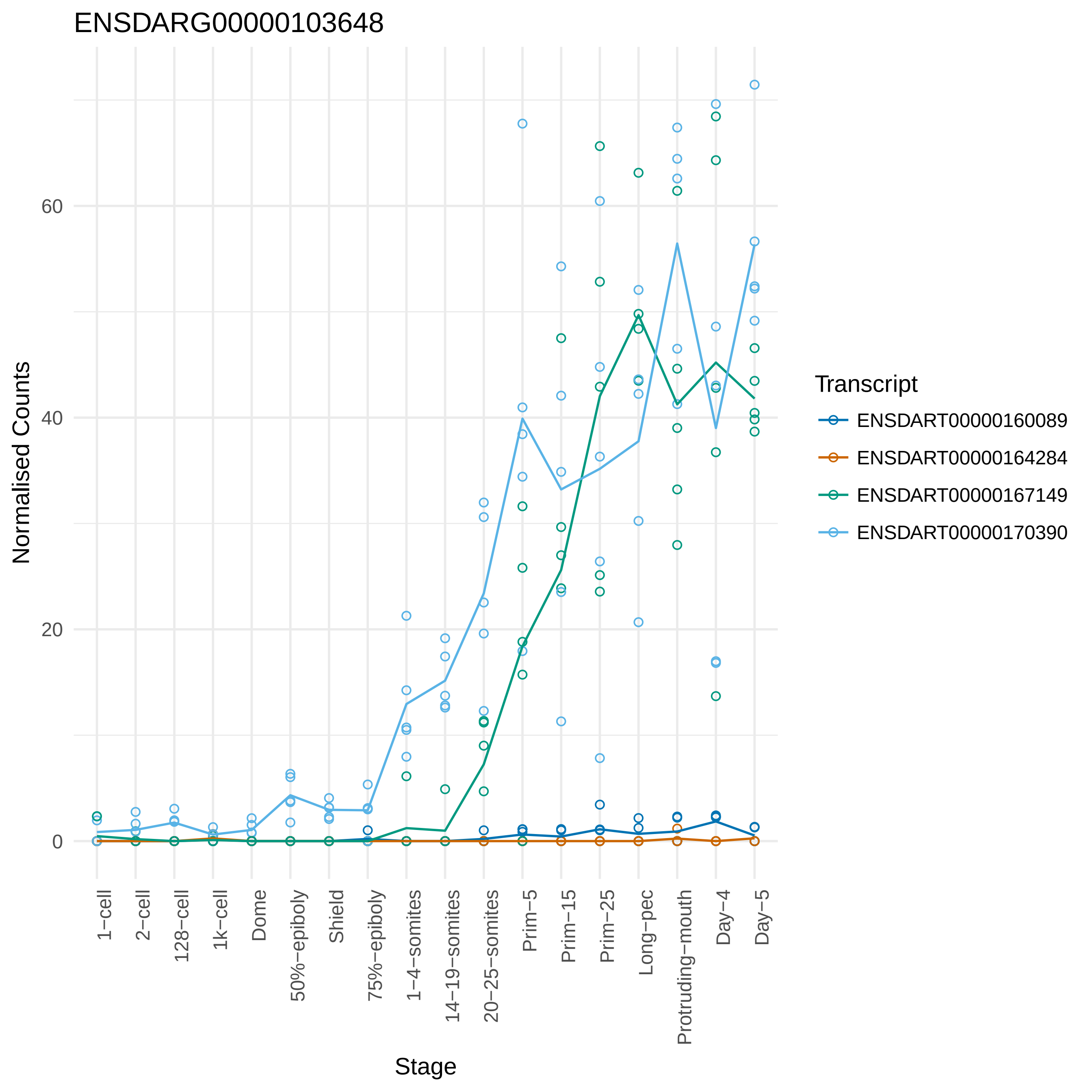

ENSDARG00000103648
Normalised Counts
0
20
40
60
Stage
1−cell
2−cell
128−cell
1k−cell
Dome
50%−epiboly
Shield
75%−epiboly
1−4−somites
14−19−somites
20−25−somites
Prim−5
Prim−15
Prim−25
Long−pec
Protruding−mouth
Day−4
Day−5
Transcript
ENSDART00000160089
ENSDART00000164284
ENSDART00000167149
ENSDART00000170390

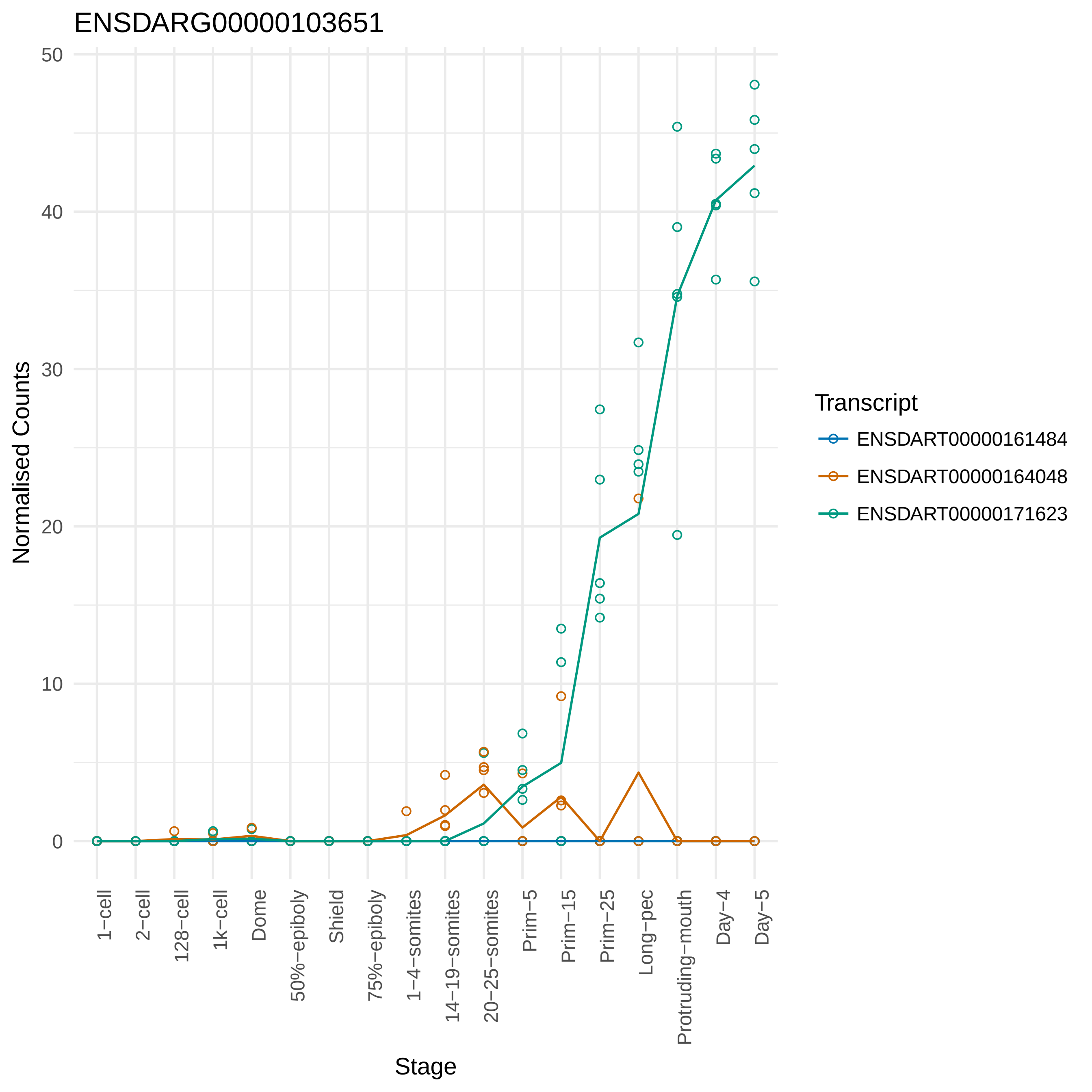
ENSDARG00000103651
Normalised Counts
50
40
30
20
10
0
1−cell
2−cell
128−cell
1k−cell
Dome
50%−epiboly
Shield
75%−epiboly
1−4−somites
14−19−somites
20−25−somites
Prim−5
Prim−15
Prim−25
Long−pec
Protruding−mouth
Day−4
Day−5
Stage
Transcript
ENSDART00000161484
ENSDART00000164048
ENSDART00000171623

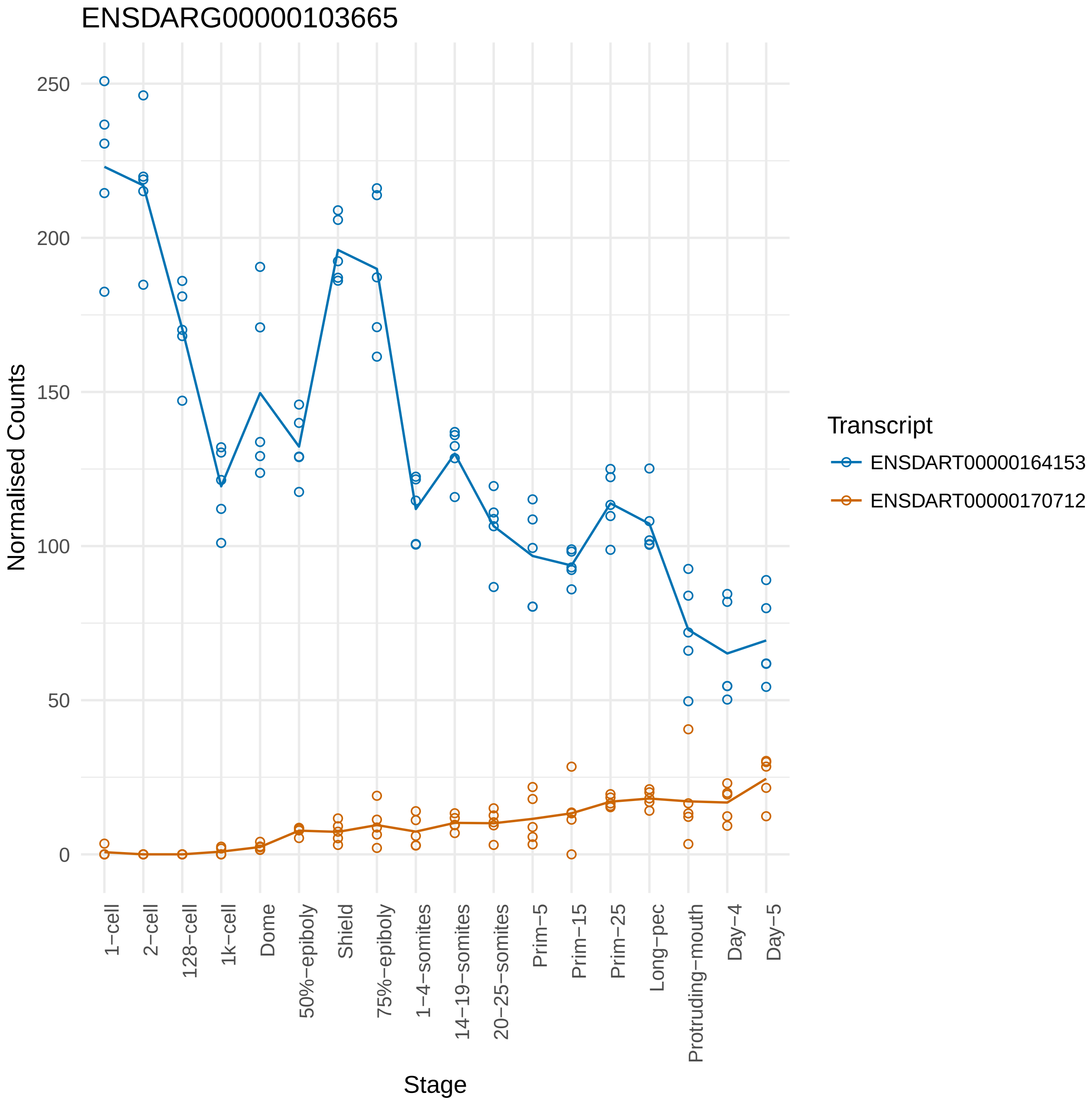

ENSDARG00000103665
Normalised Counts
0
50
100
150
200
250
1−cell
2−cell
128−cell
1k−cell
Dome
50%−epiboly
Shield
75%−epiboly
1−4−somites
14−19−somites
20−25−somites
Prim−5
Prim−15
Prim−25
Long−pec
Protruding−mouth
Day−4
Day−5
Stage
Transcript
ENSDART00000164153
ENSDART00000170712

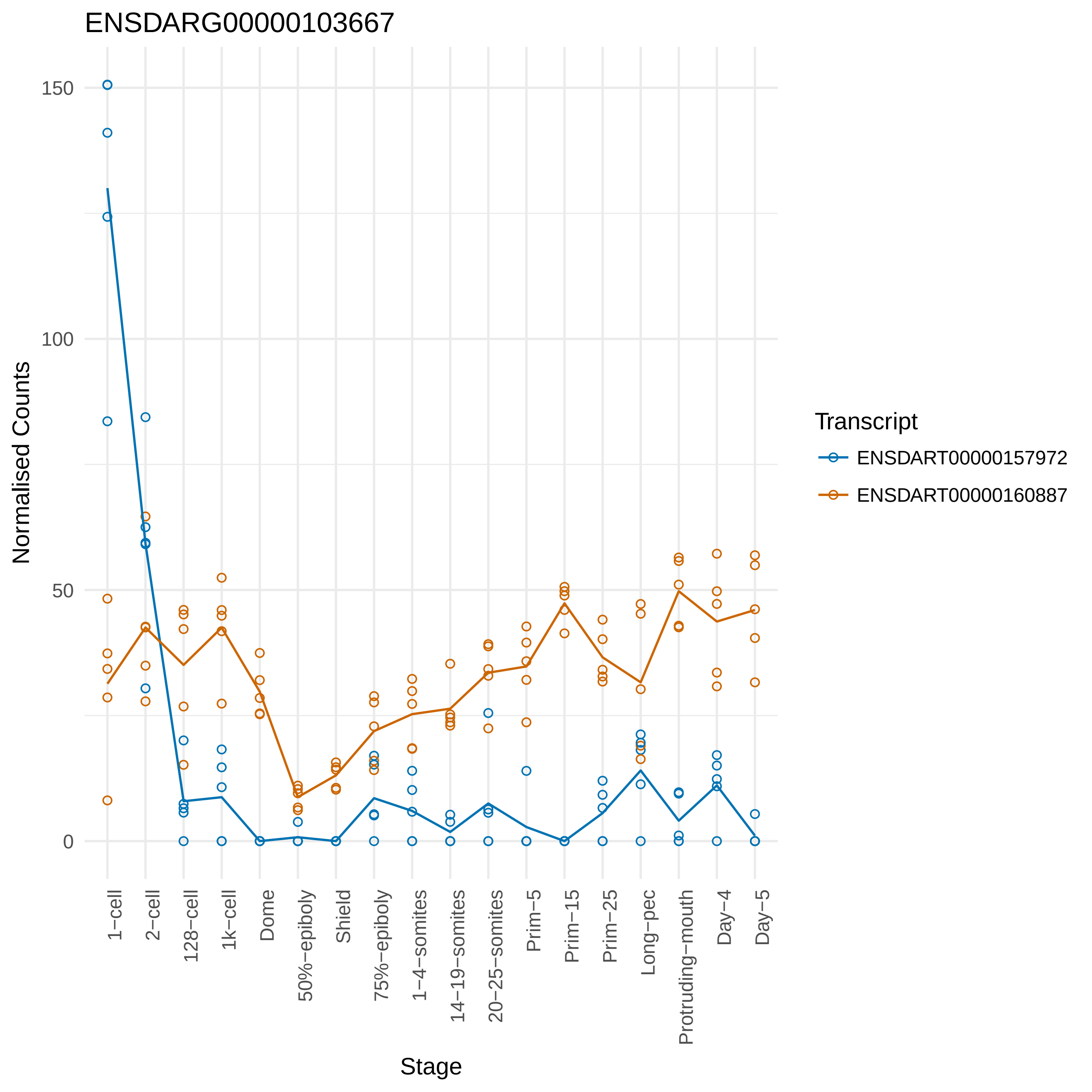

ENSDARG00000103667
Normalised Counts
150
100
50
0
1−cell
2−cell
128−cell
1k−cell
Dome
50%−epiboly
Shield
75%−epiboly
1−4−somites
14−19−somites
20−25−somites
Prim−5
Prim−15
Prim−25
Long−pec
Protruding−mouth
Day−4
Day−5
Stage
Transcript
ENSDART00000157972
ENSDART00000160887

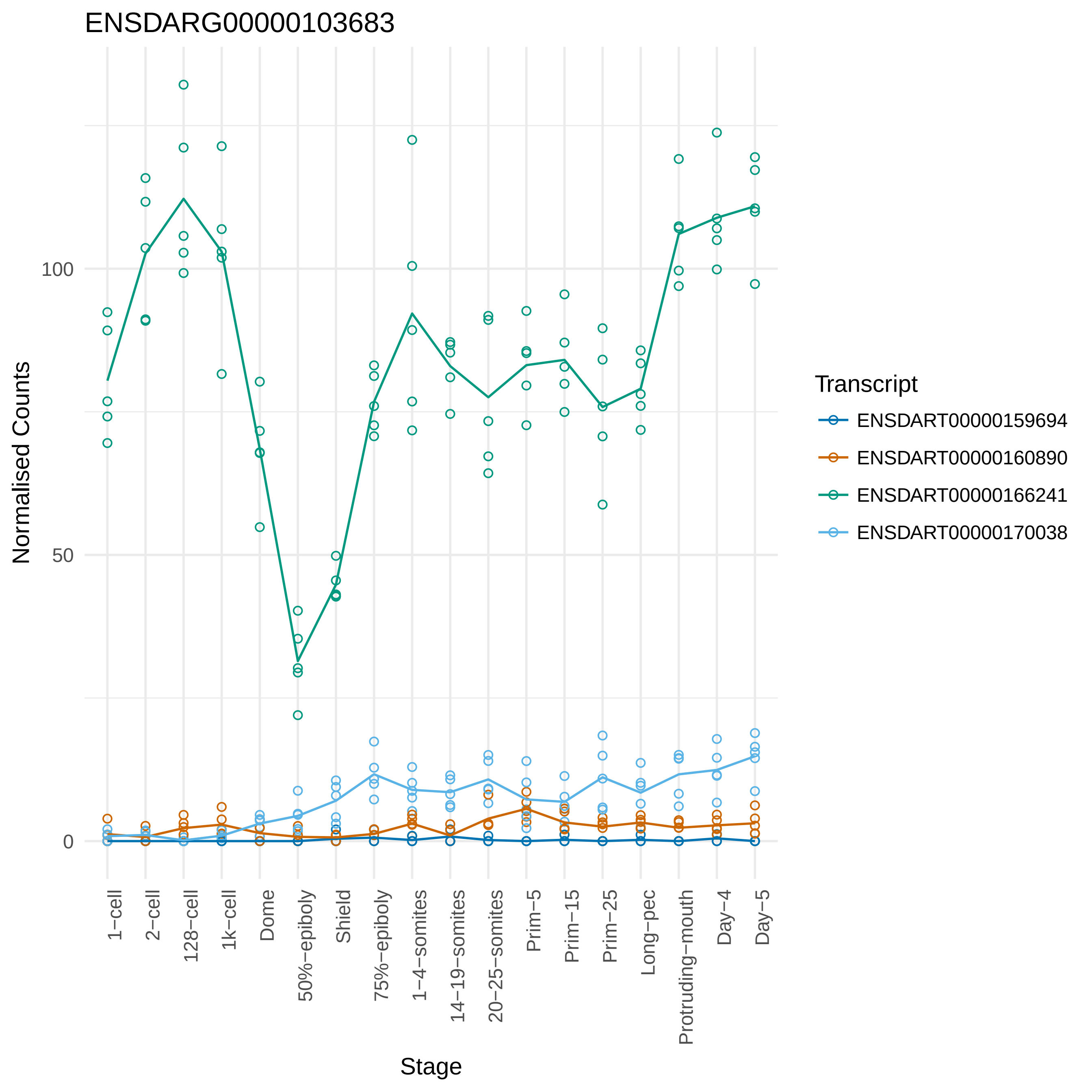

ENSDARG00000103683
Normalised Counts
100
50
0
1−cell
2−cell
128−cell
1k−cell
Dome
50%−epiboly
Shield
75%−epiboly
1−4−somites
14−19−somites
20−25−somites
Prim−5
Prim−15
Prim−25
Long−pec
Protruding−mouth
Day−4
Day−5
Stage
Transcript
ENSDART00000159694
ENSDART00000160890
ENSDART00000166241
ENSDART00000170038

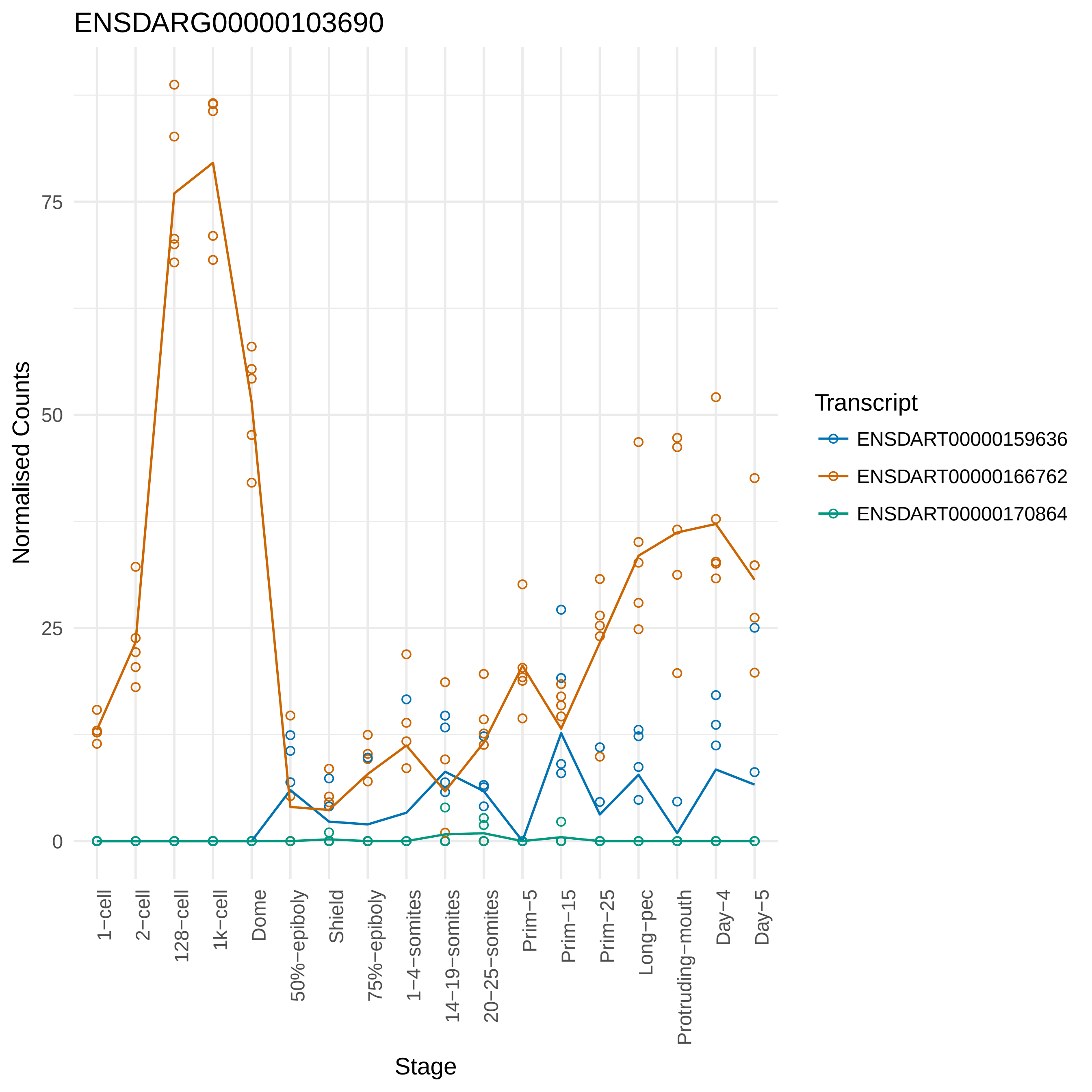

ENSDARG00000103690
Normalised Counts
75
50
25
0
1−cell
2−cell
128−cell
1k−cell
Dome
50%−epiboly
Shield
75%−epiboly
1−4−somites
14−19−somites
20−25−somites
Prim−5
Prim−15
Prim−25
Long−pec
Protruding−mouth
Day−4
Day−5
Stage
Transcript
ENSDART00000159636
ENSDART00000166762
ENSDART00000170864

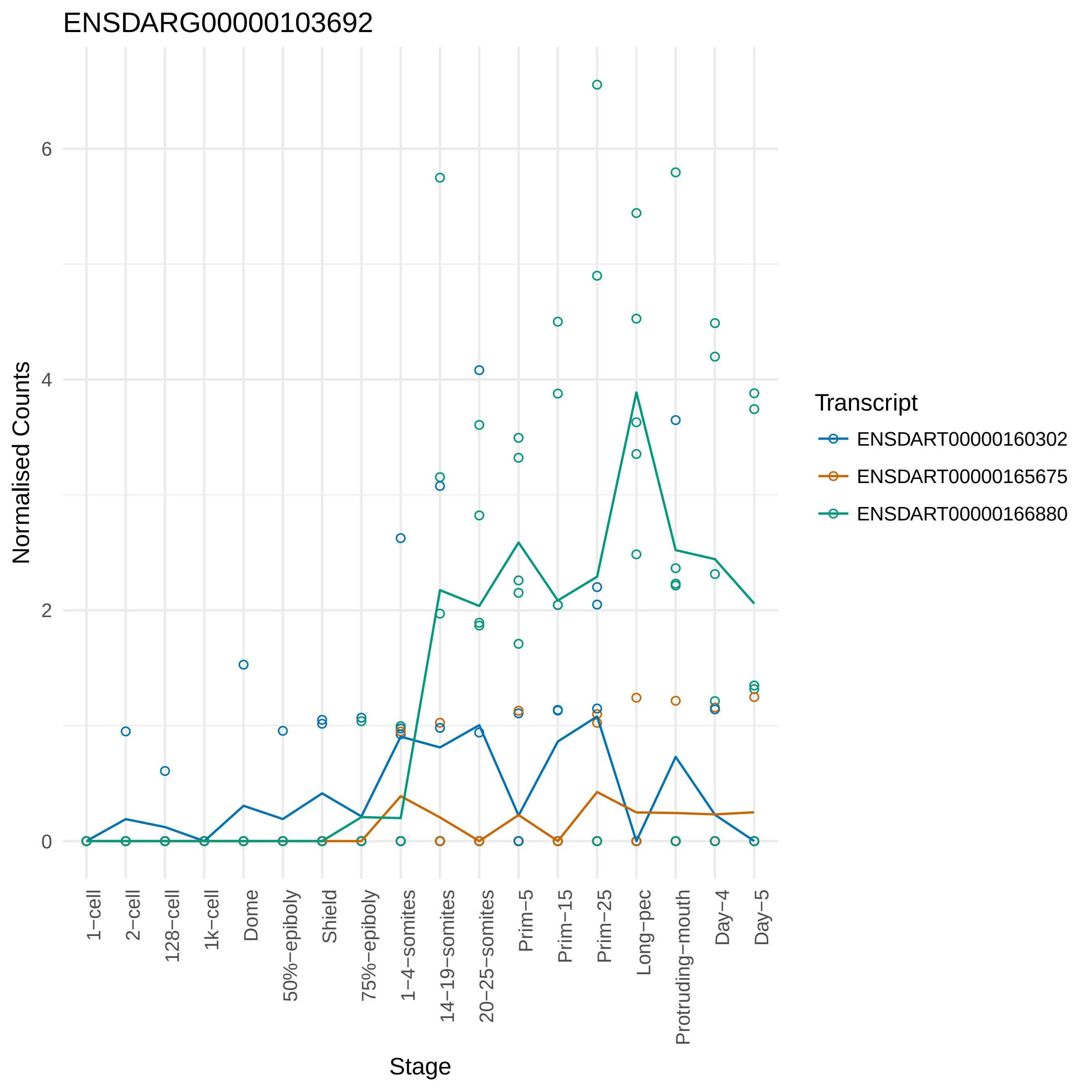

ENSDARG00000103692
Normalised Counts
0
2
4
6
1−cell
2−cell
128−cell
1k−cell
Dome
50%−epiboly
Shield
75%−epiboly
1−4−somites
14−19−somites
20−25−somites
Prim−5
Prim−15
Prim−25
Long−pec
Protruding−mouth
Day−4
Day−5
Stage
Transcript
ENSDART00000160302
ENSDART00000165675
ENSDART00000166880

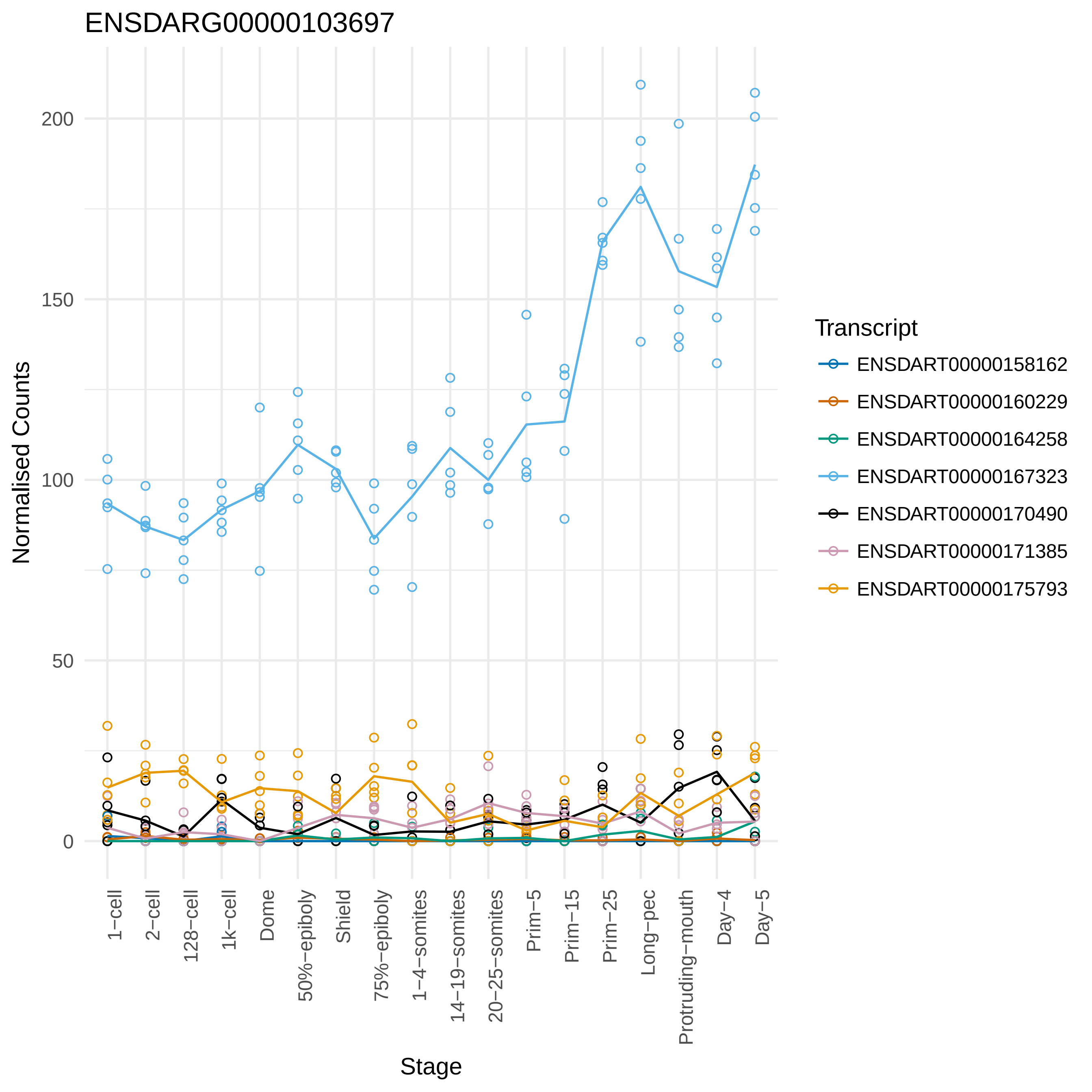

ENSDARG00000103697
Normalised Counts
200
150
100
50
0
1−cell
2−cell
128−cell
1k−cell
Dome
50%−epiboly
Shield
75%−epiboly
1−4−somites
14−19−somites
20−25−somites
Prim−5
Prim−15
Prim−25
Long−pec
Protruding−mouth
Day−4
Day−5
Stage
Transcript
ENSDART00000158162
ENSDART00000160229
ENSDART00000164258
ENSDART00000167323
ENSDART00000170490
ENSDART00000171385
ENSDART00000175793

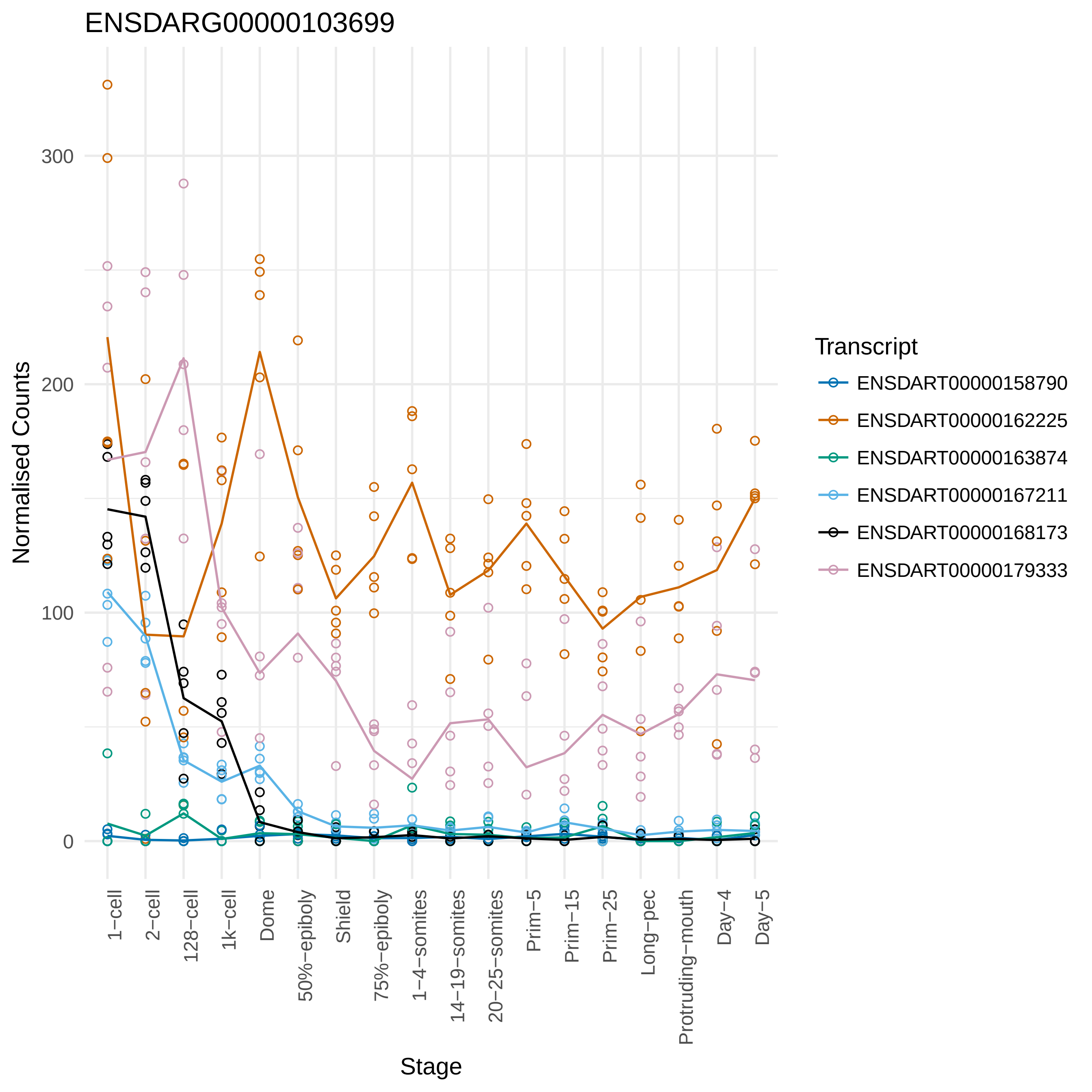

ENSDARG00000103699
Normalised Counts
300
200
100
0
1−cell
2−cell
128−cell
1k−cell
Dome
50%−epiboly
Shield
75%−epiboly
1−4−somites
14−19−somites
20−25−somites
Prim−5
Prim−15
Prim−25
Long−pec
Protruding−mouth
Day−4
Day−5
Stage
Transcript
ENSDART00000158790
ENSDART00000162225
ENSDART00000163874
ENSDART00000167211
ENSDART00000168173
ENSDART00000179333

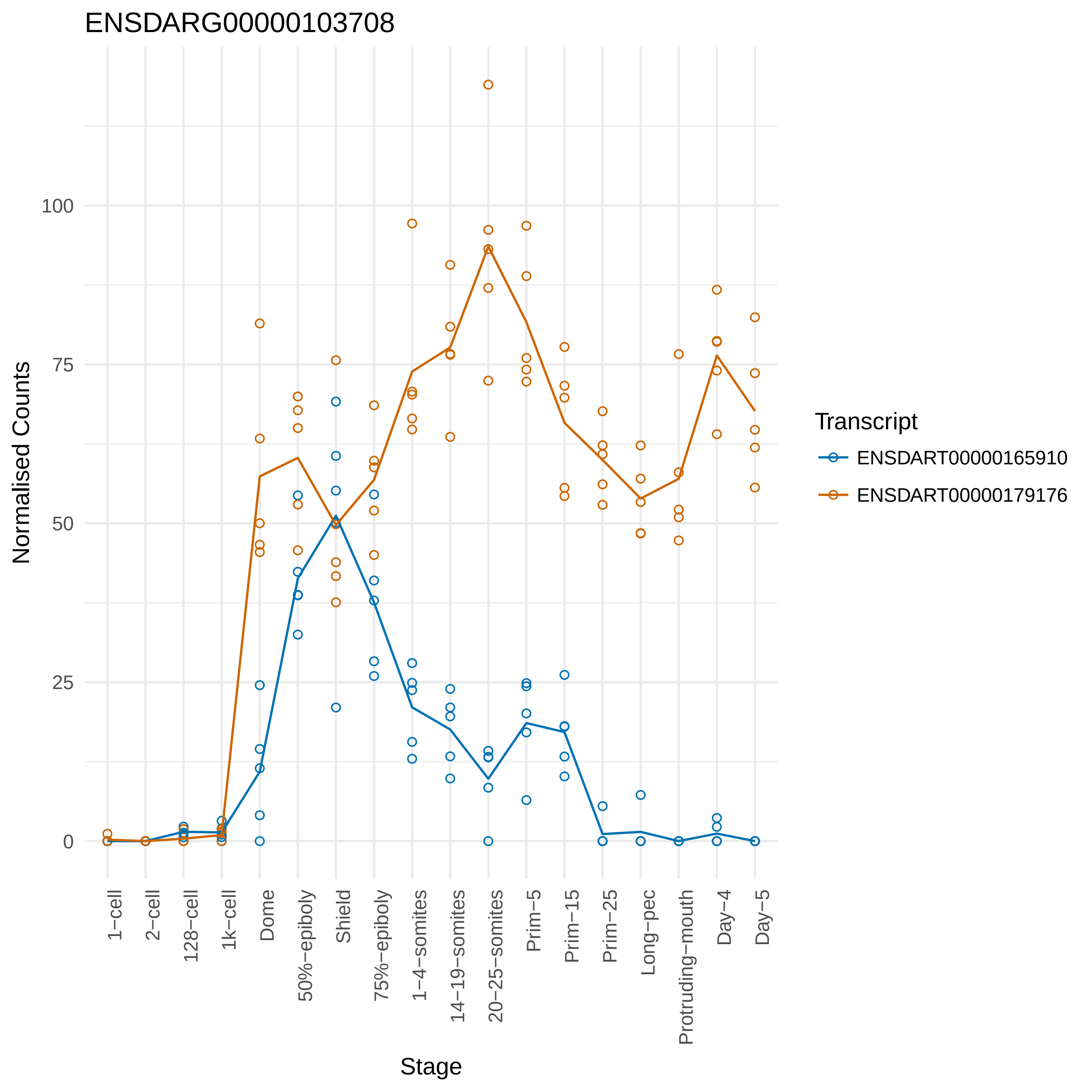
ENSDARG00000103708
Normalised Counts
100
75
50
25
0
1−cell
2−cell
128−cell
1k−cell
Dome
50%−epiboly
Shield
75%−epiboly
1−4−somites
14−19−somites
20−25−somites
Prim−5
Prim−15
Prim−25
Long−pec
Protruding−mouth
Day−4
Day−5
Stage
Transcript
ENSDART00000165910
ENSDART00000179176

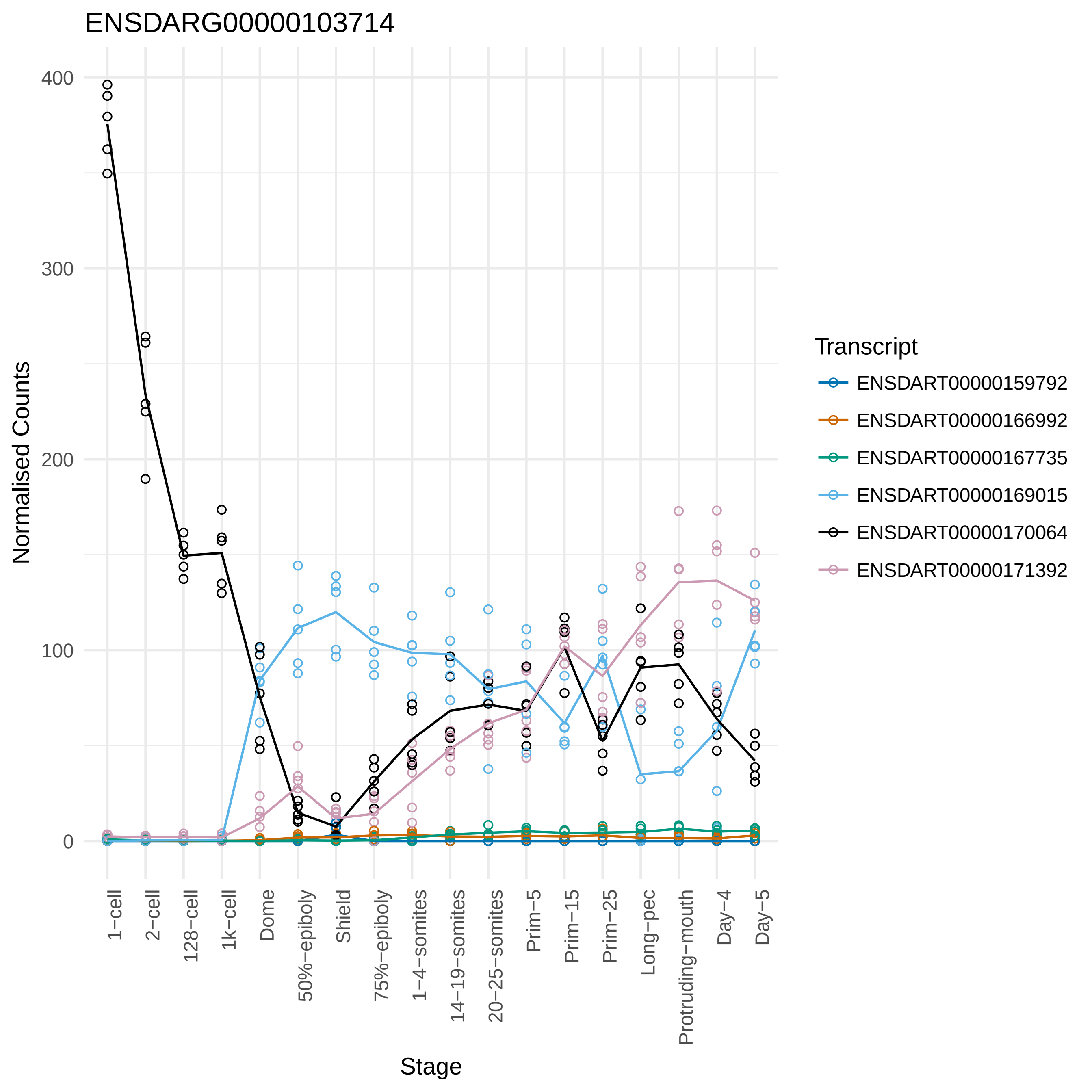
ENSDARG00000103714
Normalised Counts
0
100
200
300
400
1−cell
2−cell
128−cell
1k−cell
Dome
50%−epiboly
Shield
75%−epiboly
1−4−somites
14−19−somites
20−25−somites
Prim−5
Prim−15
Prim−25
Long−pec
Protruding−mouth
Day−4
Day−5
Stage
Transcript
ENSDART00000159792
ENSDART00000166992
ENSDART00000167735
ENSDART00000169015
ENSDART00000170064
ENSDART00000171392

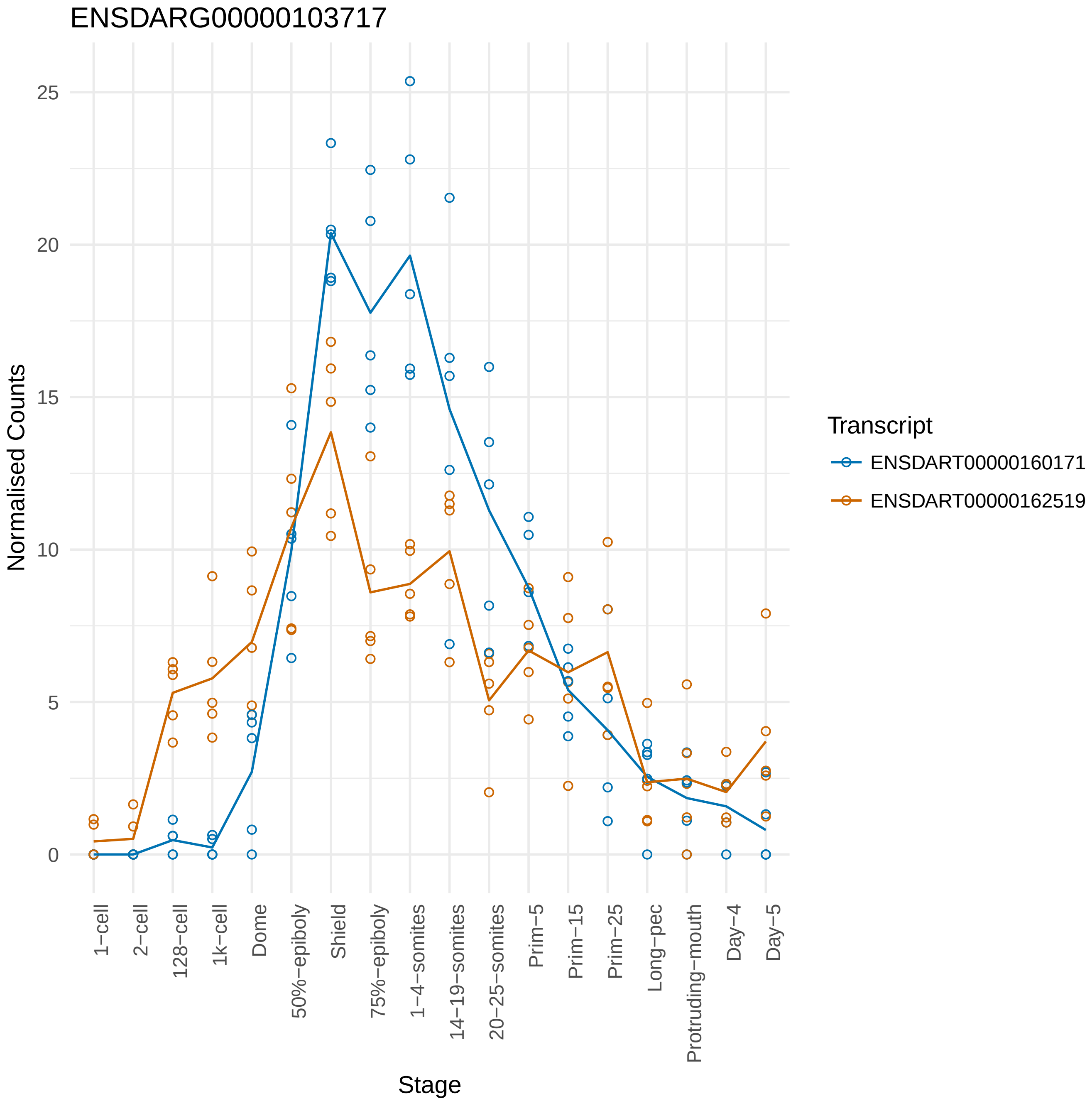

ENSDARG00000103717
Normalised Counts
0
5
10
15
20
25
Stage
1−cell
2−cell
128−cell
1k−cell
Dome
50%−epiboly
Shield
75%−epiboly
1−4−somites
14−19−somites
20−25−somites
Prim−5
Prim−15
Prim−25
Long−pec
Protruding−mouth
Day−4
Day−5
Transcript
ENSDART00000160171
ENSDART00000162519

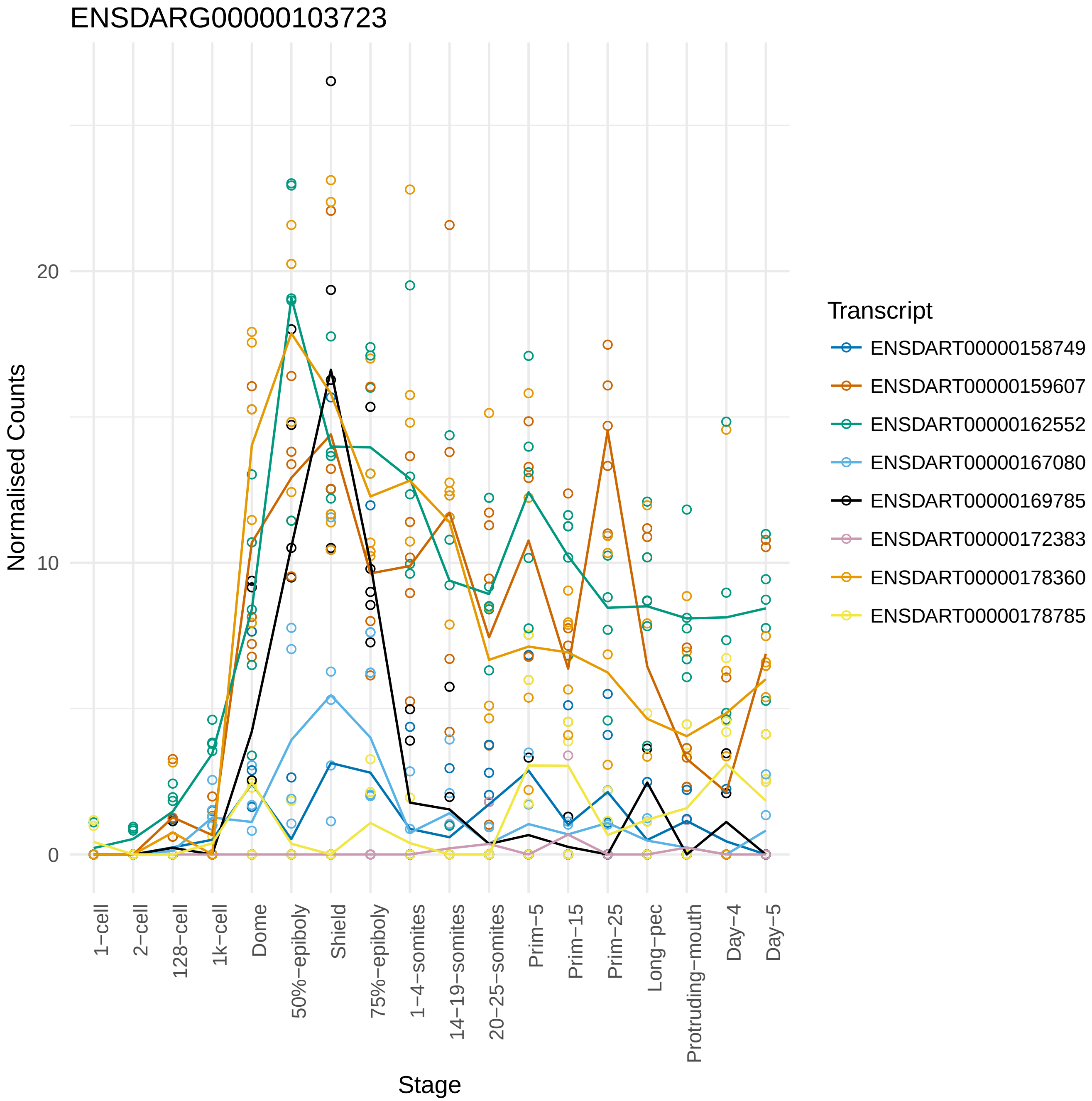

ENSDARG00000103723
Normalised Counts
20
10
0
1−cell
2−cell
128−cell
1k−cell
Dome
50%−epiboly
Shield
75%−epiboly
1−4−somites
14−19−somites
20−25−somites
Prim−5
Prim−15
Prim−25
Long−pec
Protruding−mouth
Day−4
Day−5
Stage
Transcript
ENSDART00000158749
ENSDART00000159607
ENSDART00000162552
ENSDART00000167080
ENSDART00000169785
ENSDART00000172383
ENSDART00000178360
ENSDART00000178785

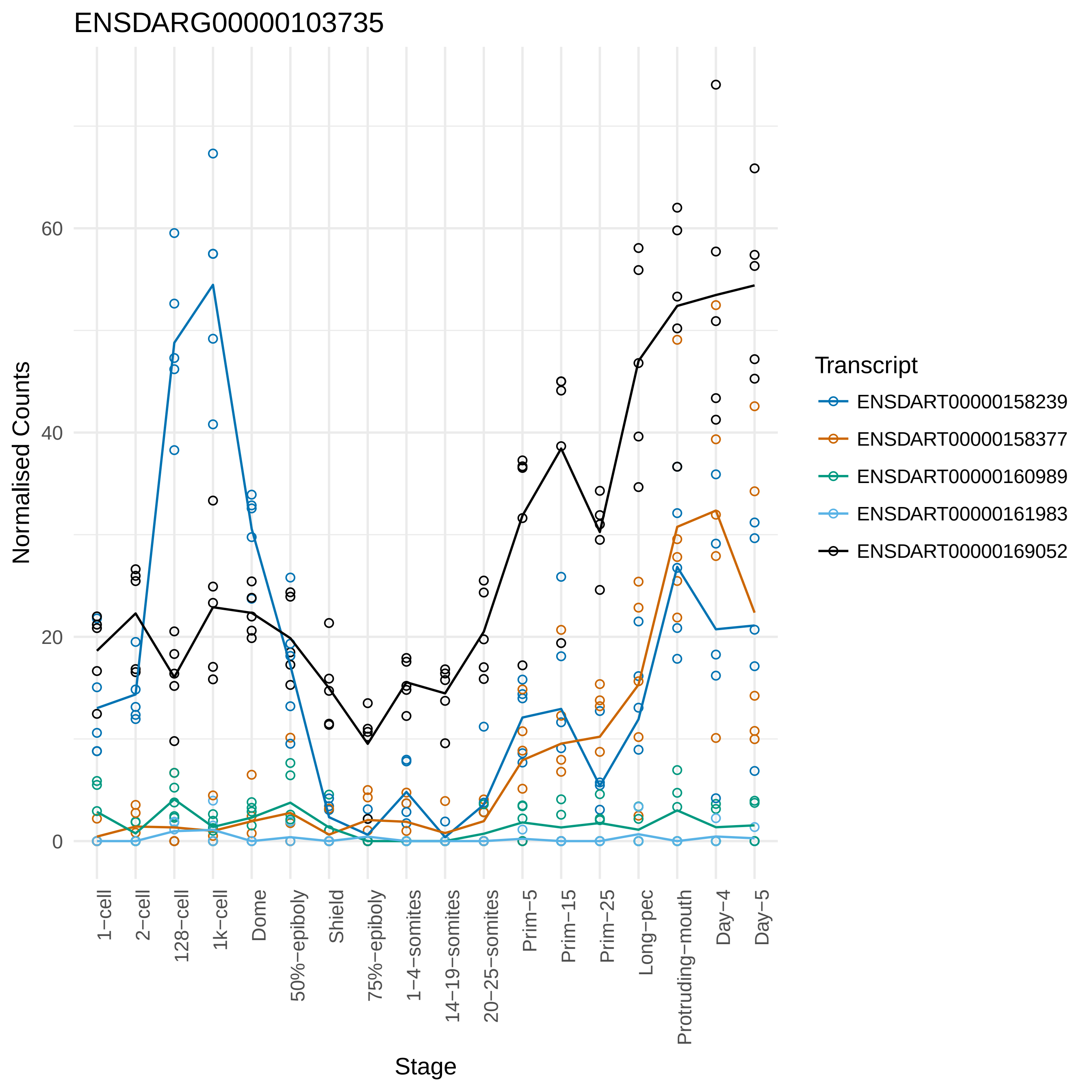

ENSDARG00000103735
Normalised Counts
0
20
40
60
Stage
1−cell
2−cell
128−cell
1k−cell
Dome
50%−epiboly
Shield
75%−epiboly
1−4−somites
14−19−somites
20−25−somites
Prim−5
Prim−15
Prim−25
Long−pec
Protruding−mouth
Day−4
Day−5
Transcript
ENSDART00000158239
ENSDART00000158377
ENSDART00000160989
ENSDART00000161983
ENSDART00000169052

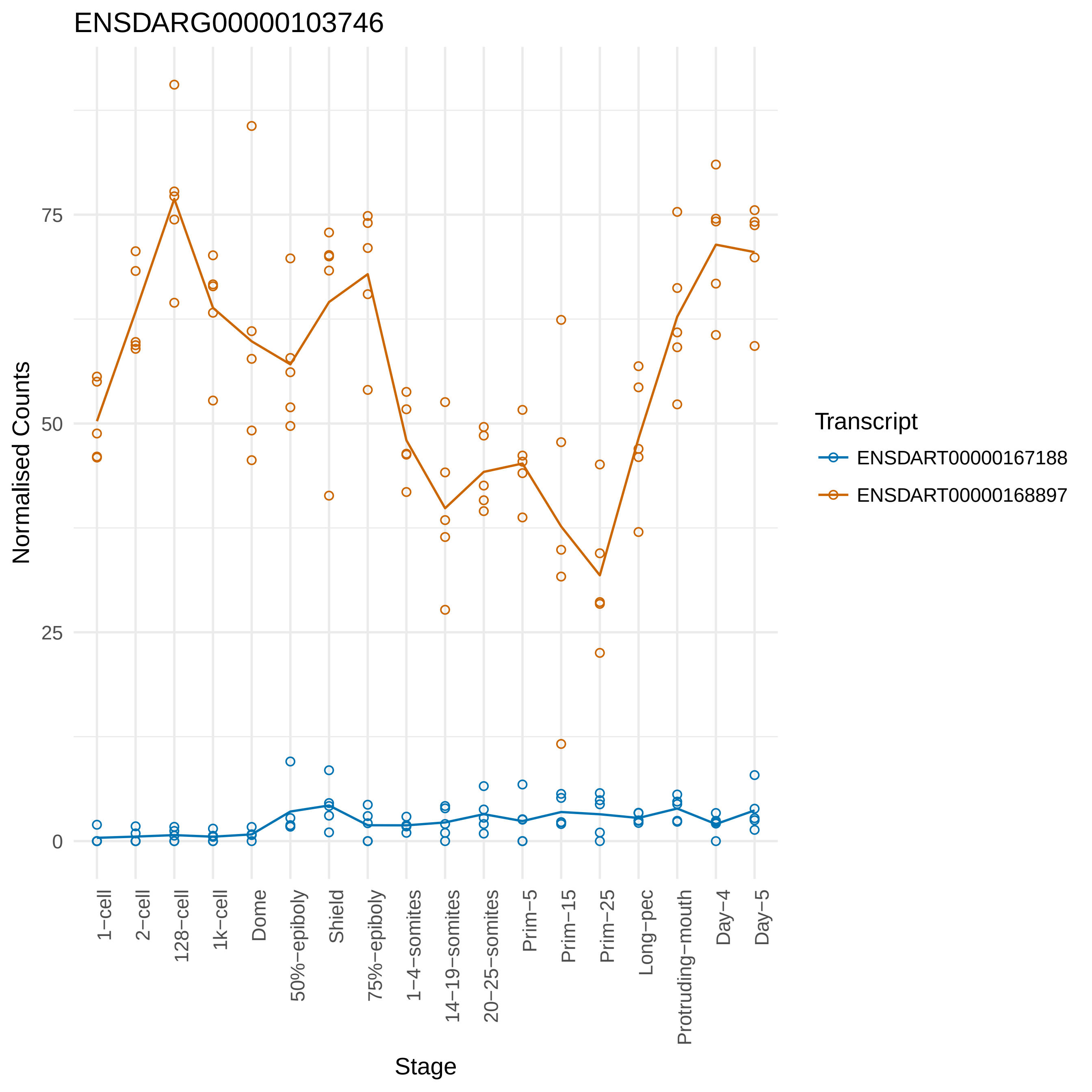

ENSDARG00000103746
Normalised Counts
0
25
50
75
Stage
1−cell
2−cell
128−cell
1k−cell
Dome
50%−epiboly
Shield
75%−epiboly
1−4−somites
14−19−somites
20−25−somites
Prim−5
Prim−15
Prim−25
Long−pec
Protruding−mouth
Day−4
Day−5
Transcript
ENSDART00000167188
ENSDART00000168897

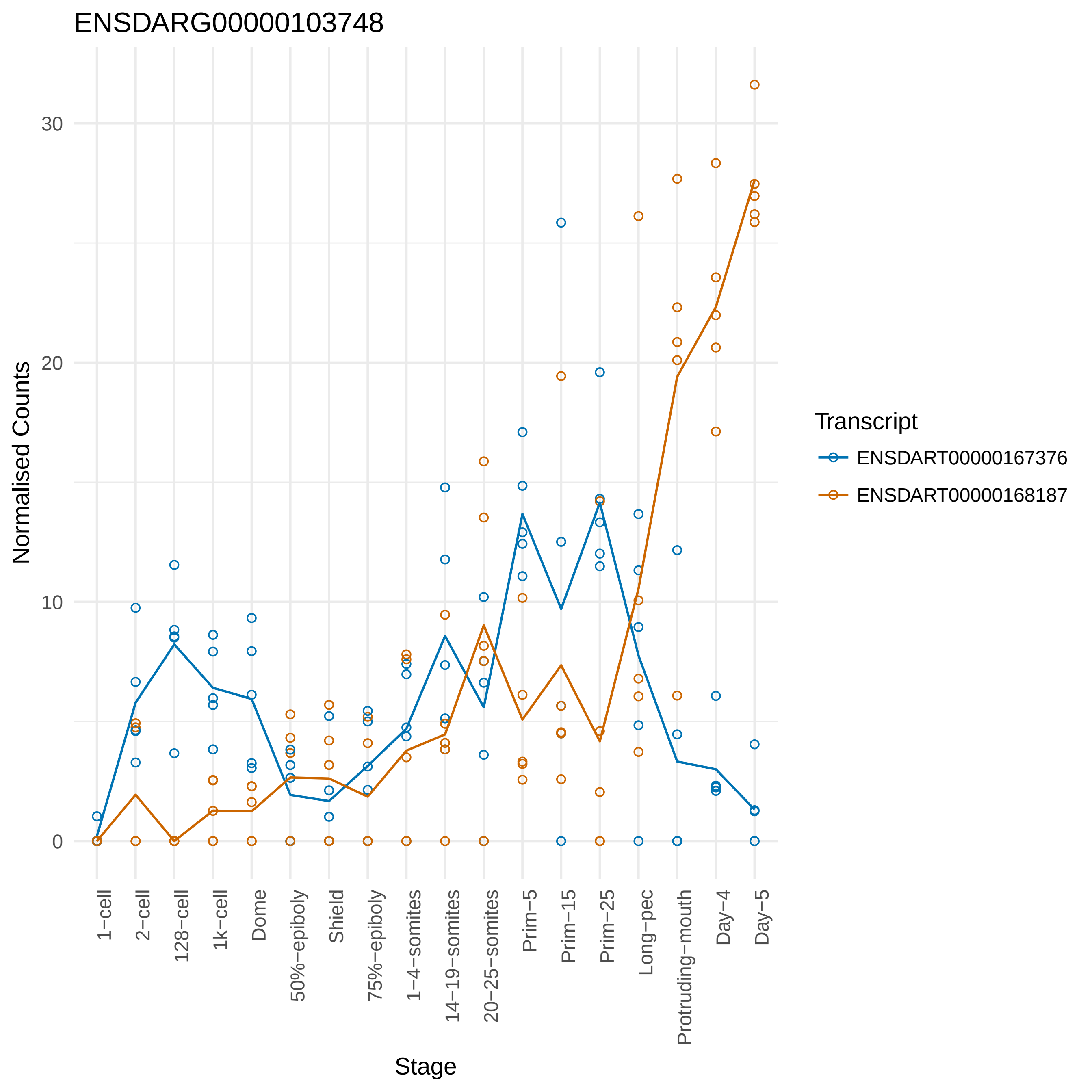

ENSDARG00000103748
Normalised Counts
30
20
10
0
1−cell
2−cell
128−cell
1k−cell
Dome
50%−epiboly
Shield
75%−epiboly
1−4−somites
14−19−somites
20−25−somites
Prim−5
Prim−15
Prim−25
Long−pec
Protruding−mouth
Day−4
Day−5
Stage
Transcript
ENSDART00000167376
ENSDART00000168187

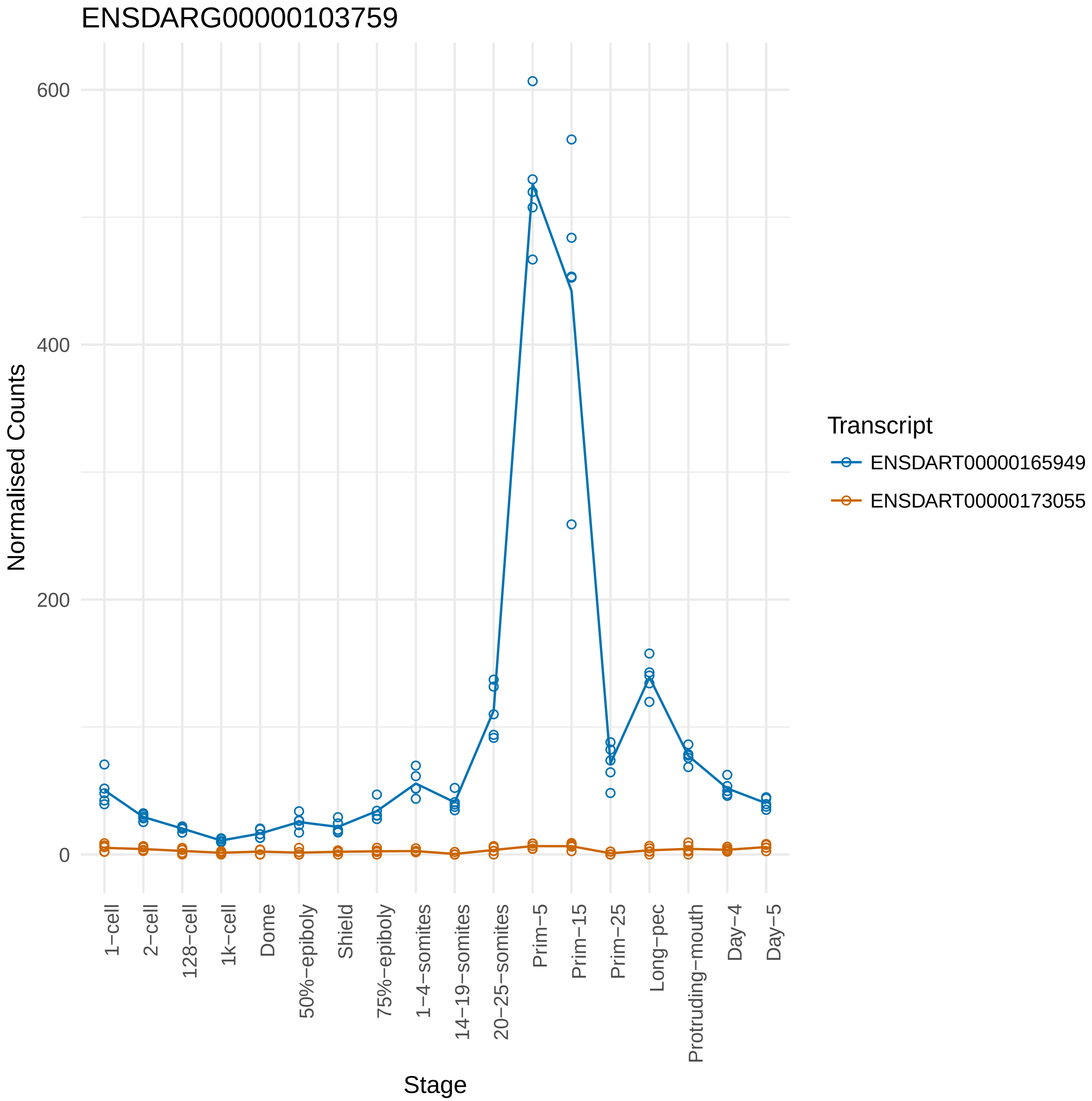

ENSDARG00000103759
Normalised Counts
600
400
200
0
1−cell
2−cell
128−cell
1k−cell
Dome
50%−epiboly
Shield
75%−epiboly
1−4−somites
14−19−somites
20−25−somites
Prim−5
Prim−15
Prim−25
Long−pec
Protruding−mouth
Day−4
Day−5
Stage
Transcript
ENSDART00000165949
ENSDART00000173055

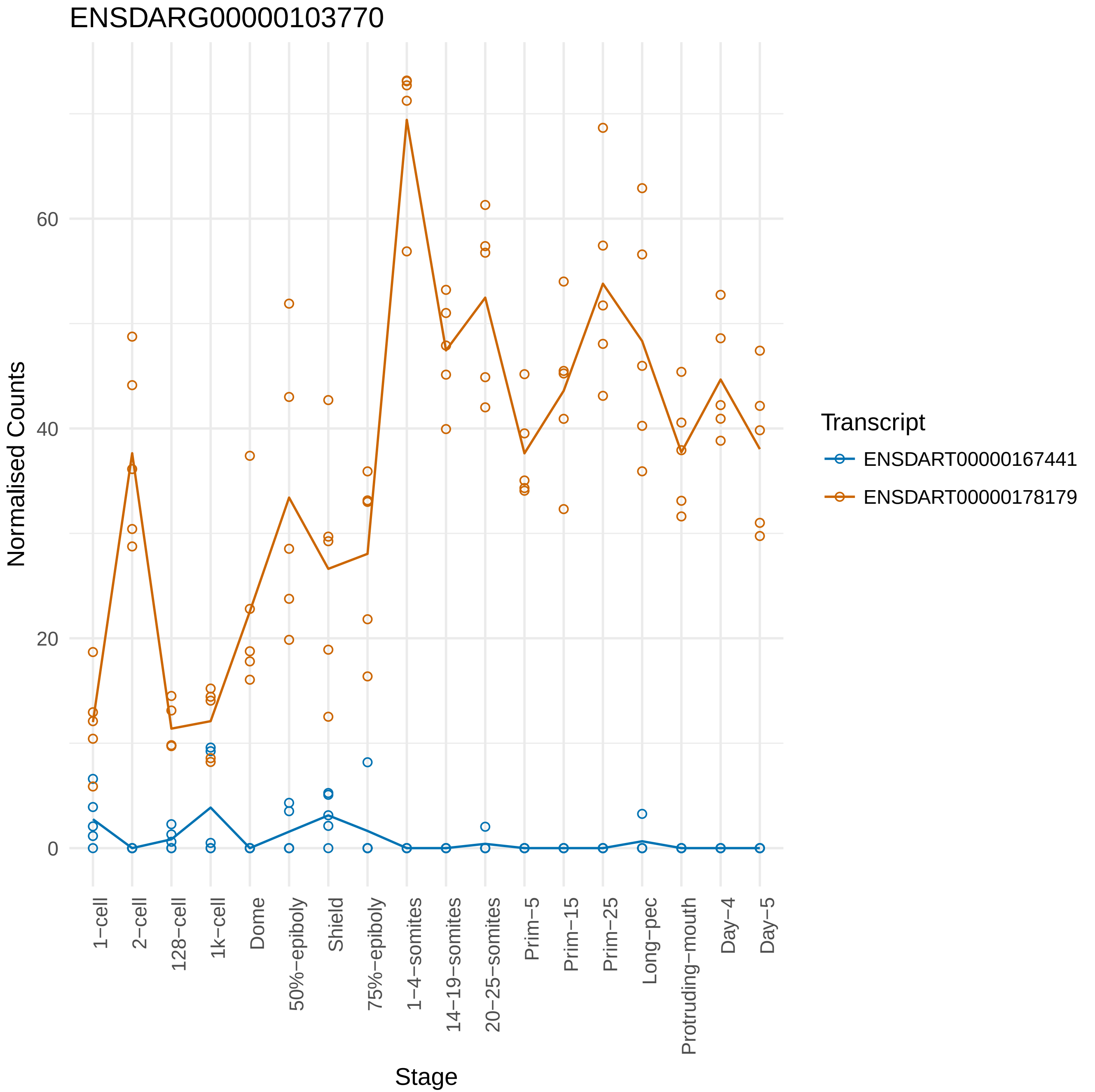
ENSDARG00000103770
Normalised Counts
0
20
40
60
Stage
1−cell
2−cell
128−cell
1k−cell
Dome
50%−epiboly
Shield
75%−epiboly
1−4−somites
14−19−somites
20−25−somites
Prim−5
Prim−15
Prim−25
Long−pec
Protruding−mouth
Day−4
Day−5
Transcript
ENSDART00000167441
ENSDART00000178179

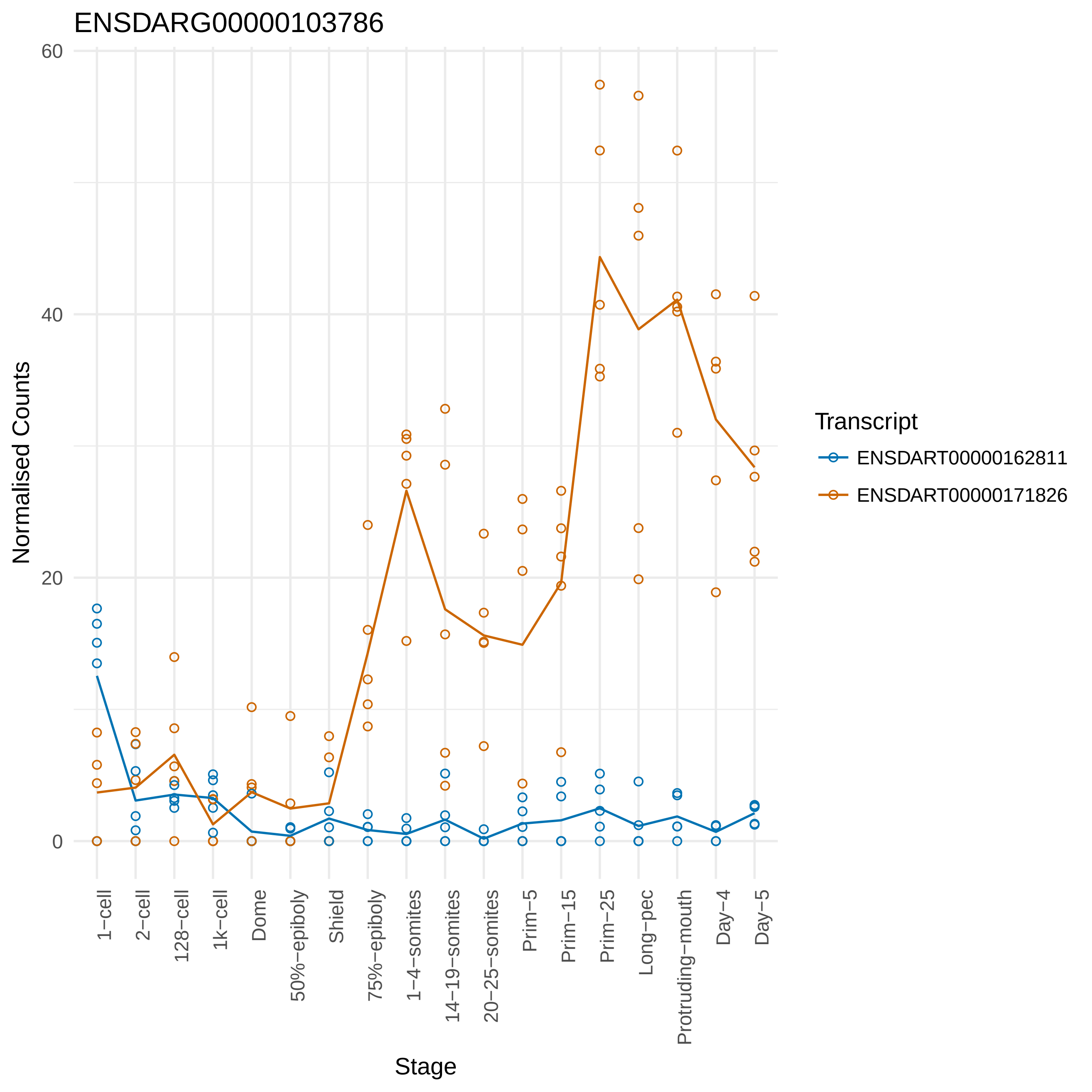

ENSDARG00000103786
Normalised Counts
60
40
20
0
1−cell
2−cell
128−cell
1k−cell
Dome
50%−epiboly
Shield
75%−epiboly
1−4−somites
14−19−somites
20−25−somites
Prim−5
Prim−15
Prim−25
Long−pec
Protruding−mouth
Day−4
Day−5
Stage
Transcript
ENSDART00000162811
ENSDART00000171826

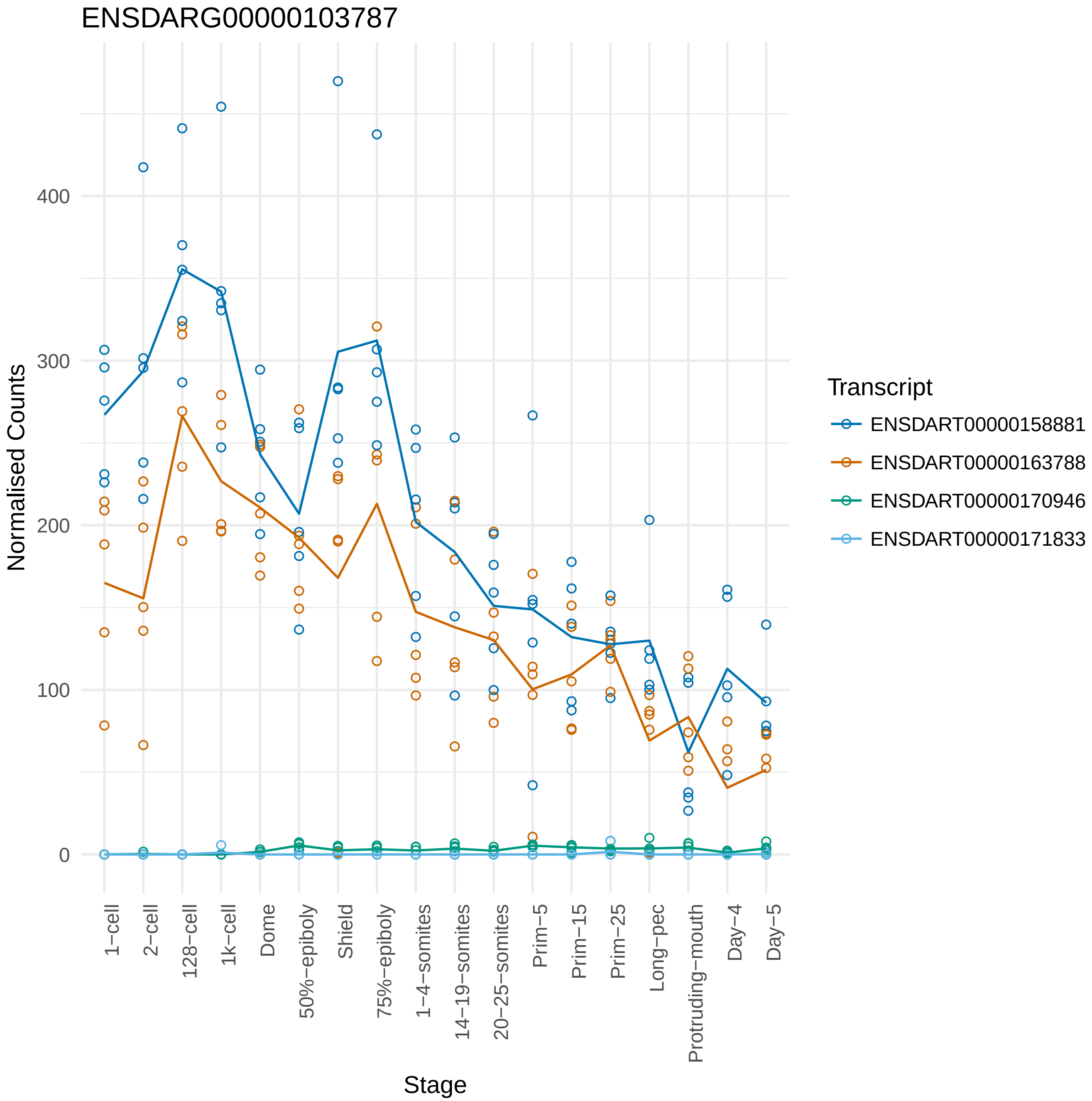
ENSDARG00000103787
Normalised Counts
400
300
200
100
0
1−cell
2−cell
128−cell
1k−cell
Dome
50%−epiboly
Shield
75%−epiboly
1−4−somites
14−19−somites
20−25−somites
Prim−5
Prim−15
Prim−25
Long−pec
Protruding−mouth
Day−4
Day−5
Stage
Transcript
ENSDART00000158881
ENSDART00000163788
ENSDART00000170946
ENSDART00000171833

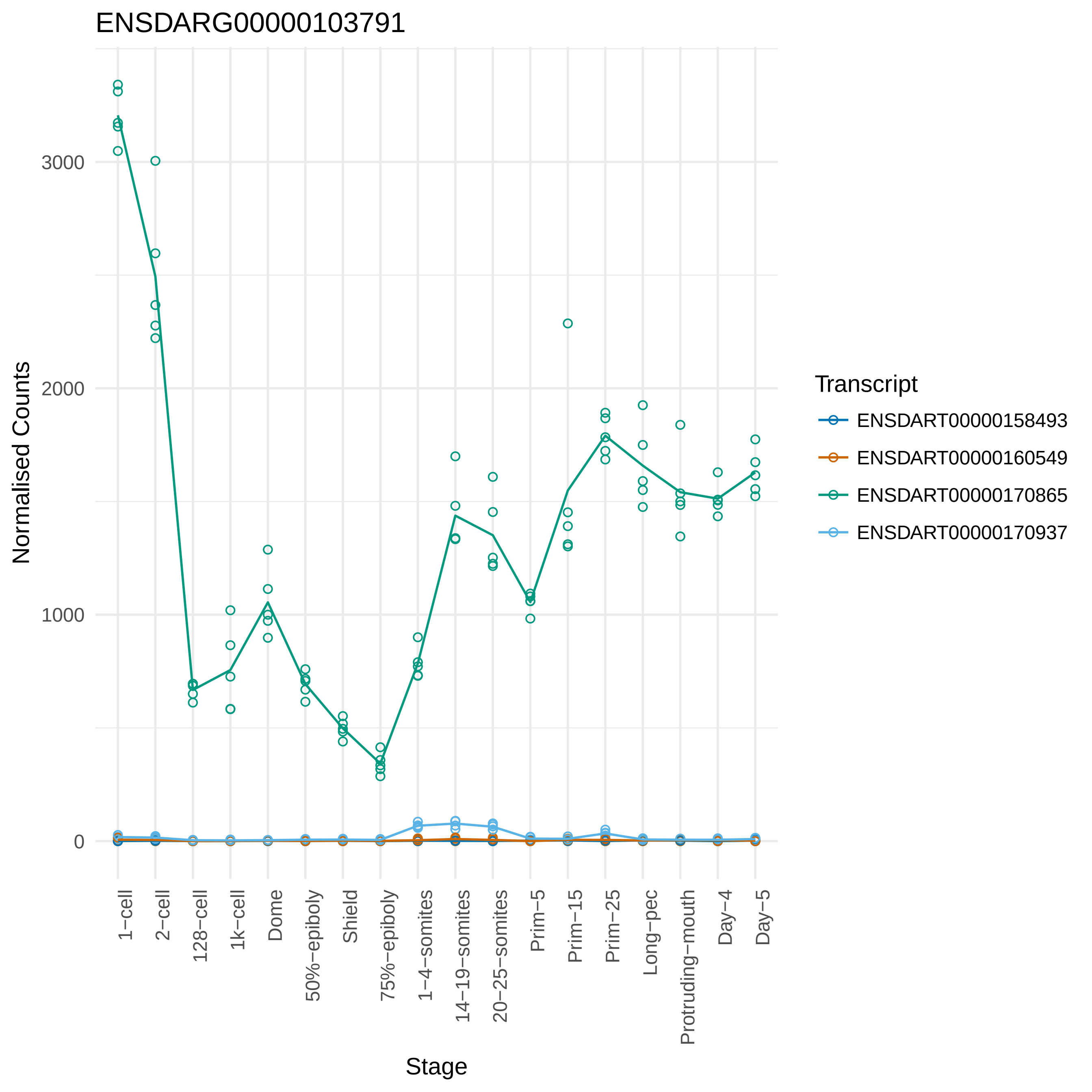

ENSDARG00000103791
Normalised Counts
3000
2000
1000
0
1−cell
2−cell
128−cell
1k−cell
Dome
50%−epiboly
Shield
75%−epiboly
1−4−somites
14−19−somites
20−25−somites
Prim−5
Prim−15
Prim−25
Long−pec
Protruding−mouth
Day−4
Day−5
Stage
Transcript
ENSDART00000158493
ENSDART00000160549
ENSDART00000170865
ENSDART00000170937

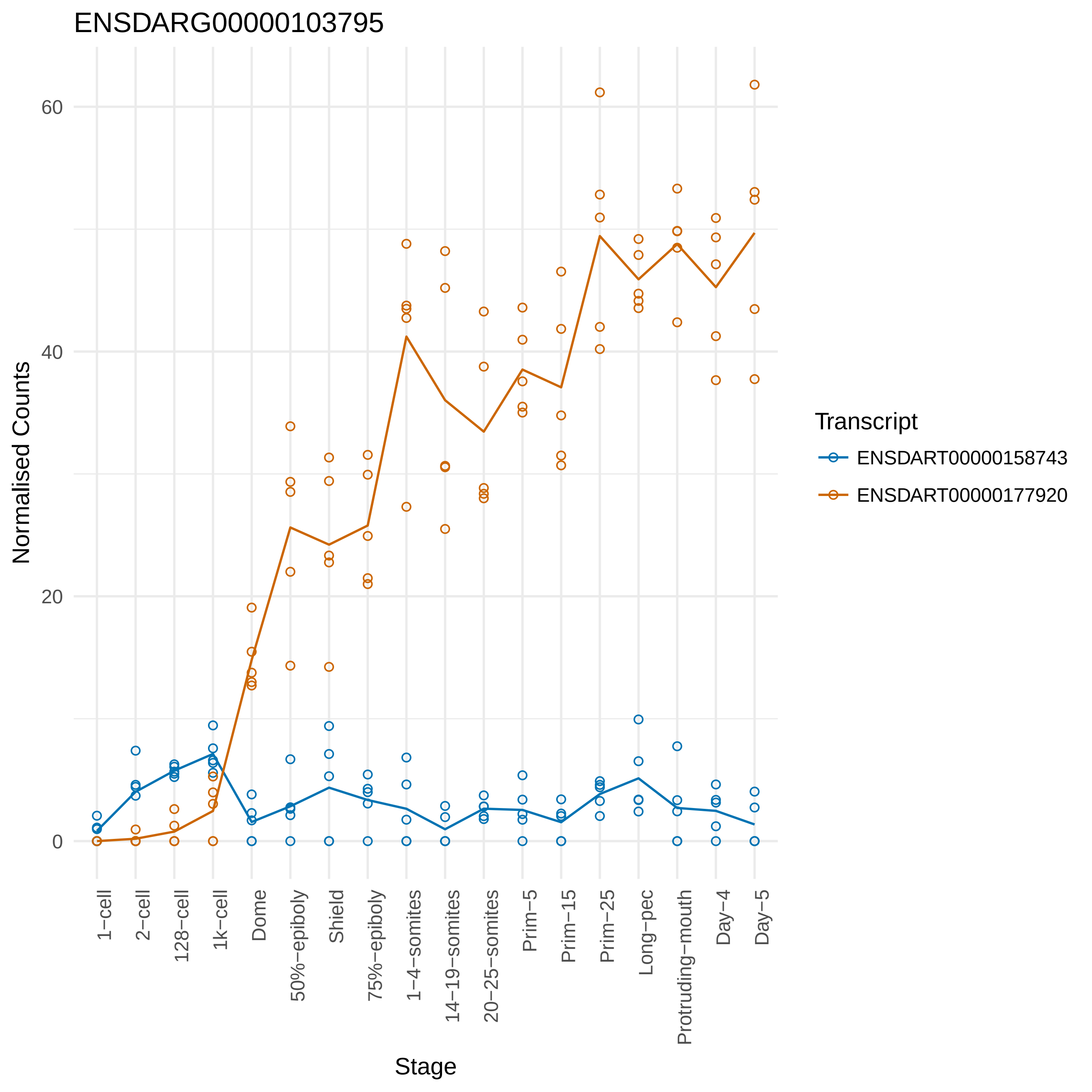

ENSDARG00000103795
Normalised Counts
60
40
20
0
Stage
1−cell
2−cell
128−cell
1k−cell
Dome
50%−epiboly
Shield
75%−epiboly
1−4−somites
14−19−somites
20−25−somites
Prim−5
Prim−15
Prim−25
Long−pec
Protruding−mouth
Day−4
Day−5
Transcript
ENSDART00000158743
ENSDART00000177920

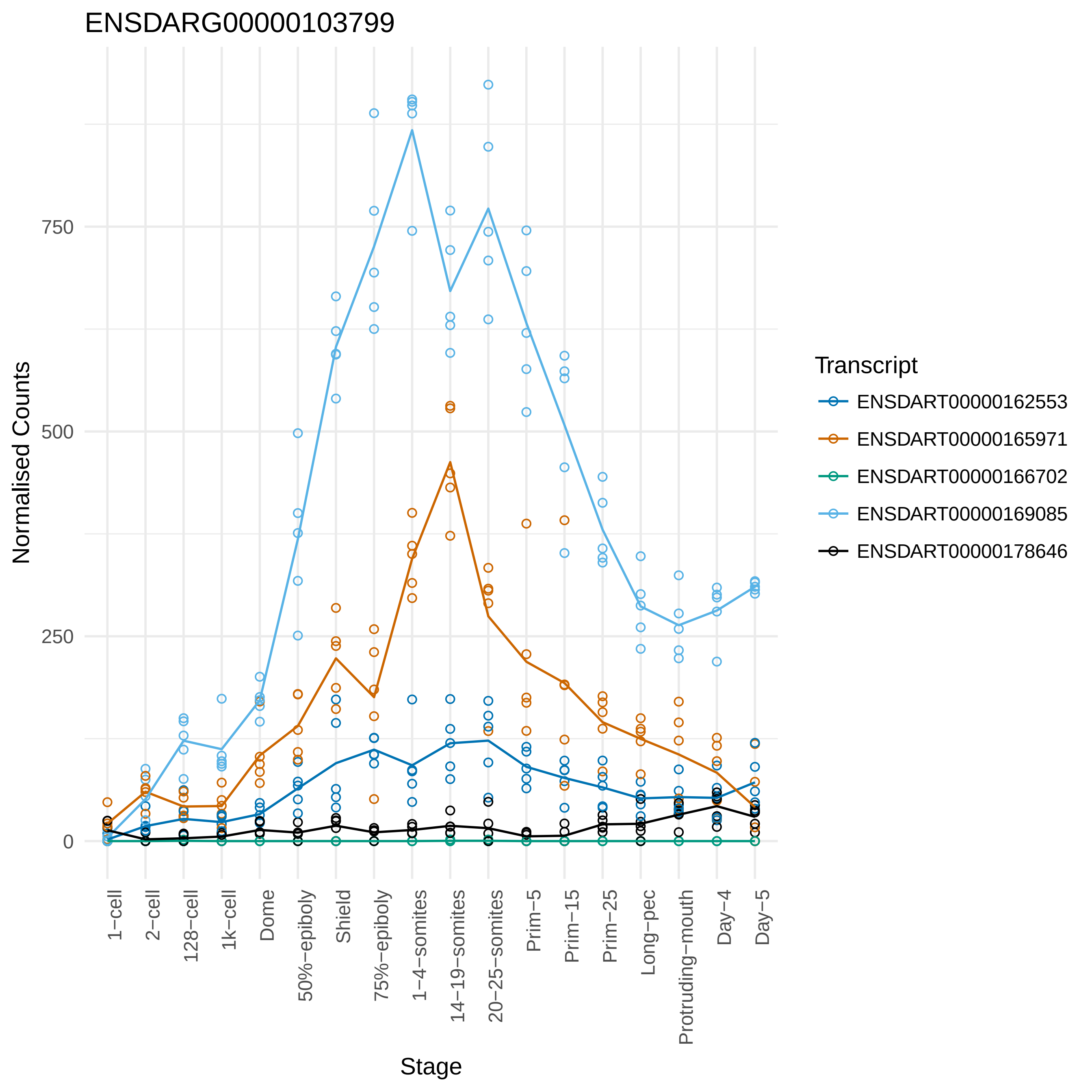

ENSDARG00000103799
Normalised Counts
0
250
500
750
Stage
1−cell
2−cell
128−cell
1k−cell
Dome
50%−epiboly
Shield
75%−epiboly
1−4−somites
14−19−somites
20−25−somites
Prim−5
Prim−15
Prim−25
Long−pec
Protruding−mouth
Day−4
Day−5
Transcript
ENSDART00000162553
ENSDART00000165971
ENSDART00000166702
ENSDART00000169085
ENSDART00000178646

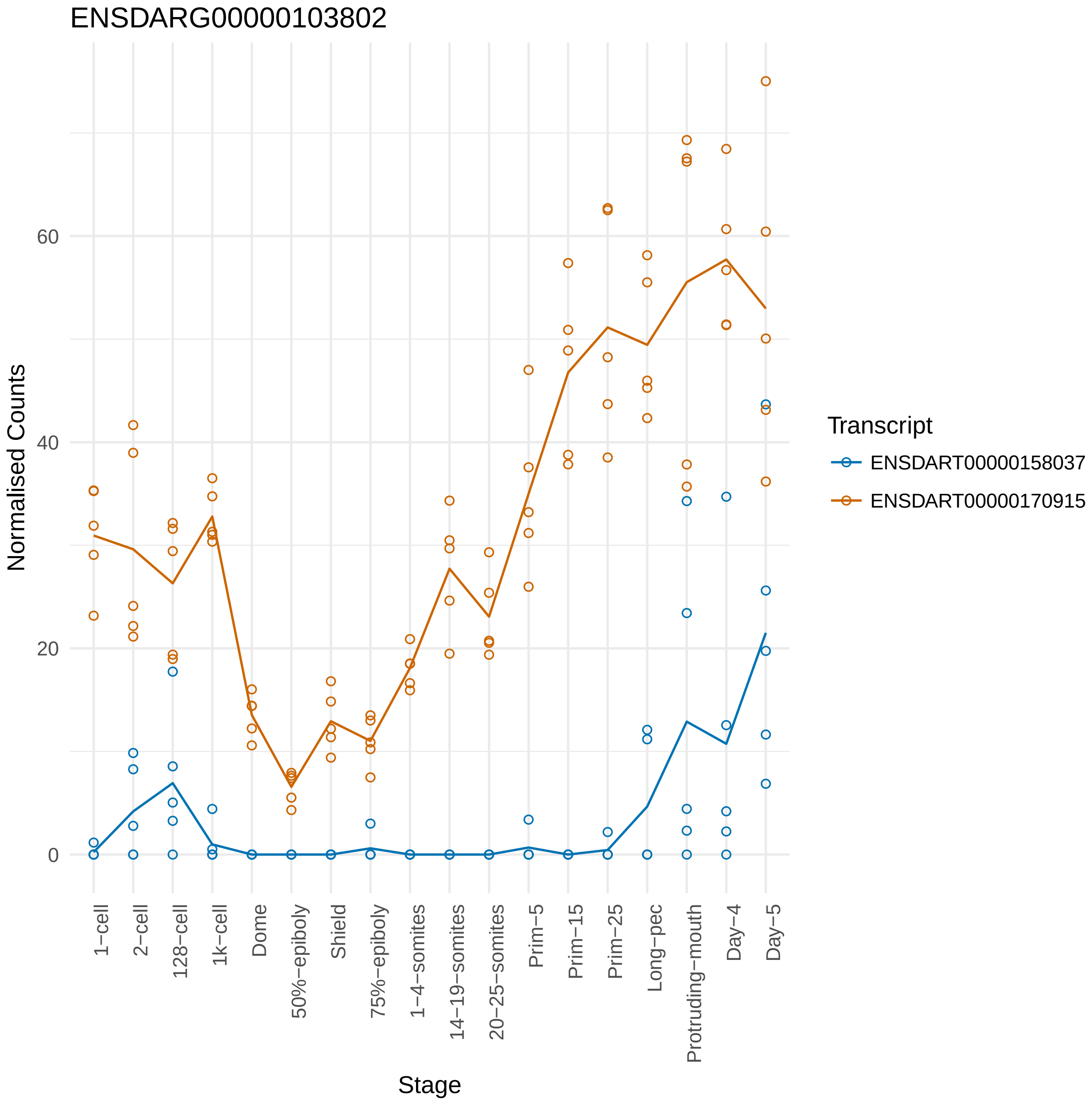

ENSDARG00000103802
Normalised Counts
0
20
40
60
1−cell
2−cell
128−cell
1k−cell
Dome
50%−epiboly
Shield
75%−epiboly
1−4−somites
14−19−somites
20−25−somites
Prim−5
Prim−15
Prim−25
Long−pec
Protruding−mouth
Day−4
Day−5
Stage
Transcript
ENSDART00000158037
ENSDART00000170915

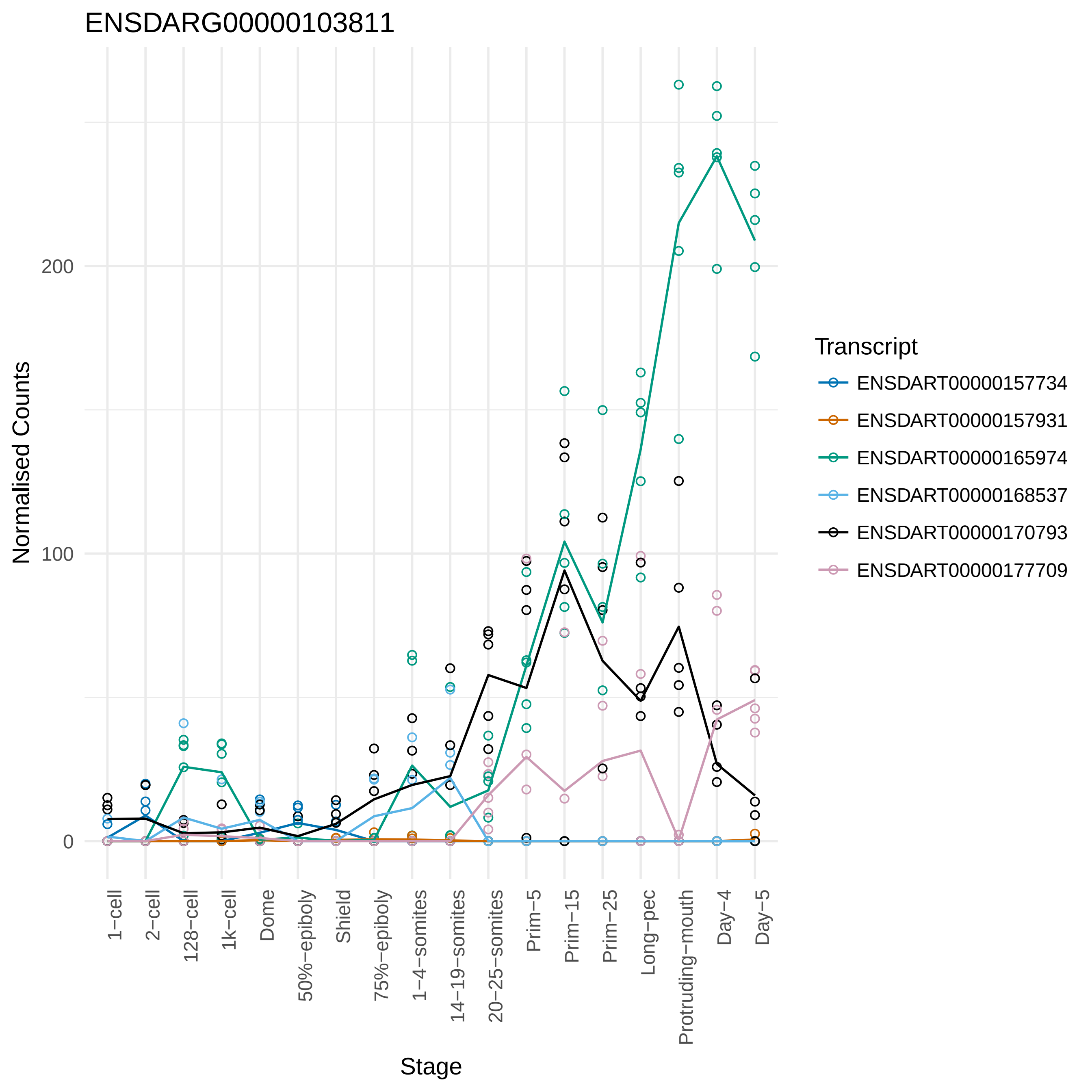

ENSDARG00000103811
Normalised Counts
0
100
200
1−cell
2−cell
128−cell
1k−cell
Dome
50%−epiboly
Shield
75%−epiboly
1−4−somites
14−19−somites
20−25−somites
Prim−5
Prim−15
Prim−25
Long−pec
Protruding−mouth
Day−4
Day−5
Stage
Transcript
ENSDART00000157734
ENSDART00000157931
ENSDART00000165974
ENSDART00000168537
ENSDART00000170793
ENSDART00000177709

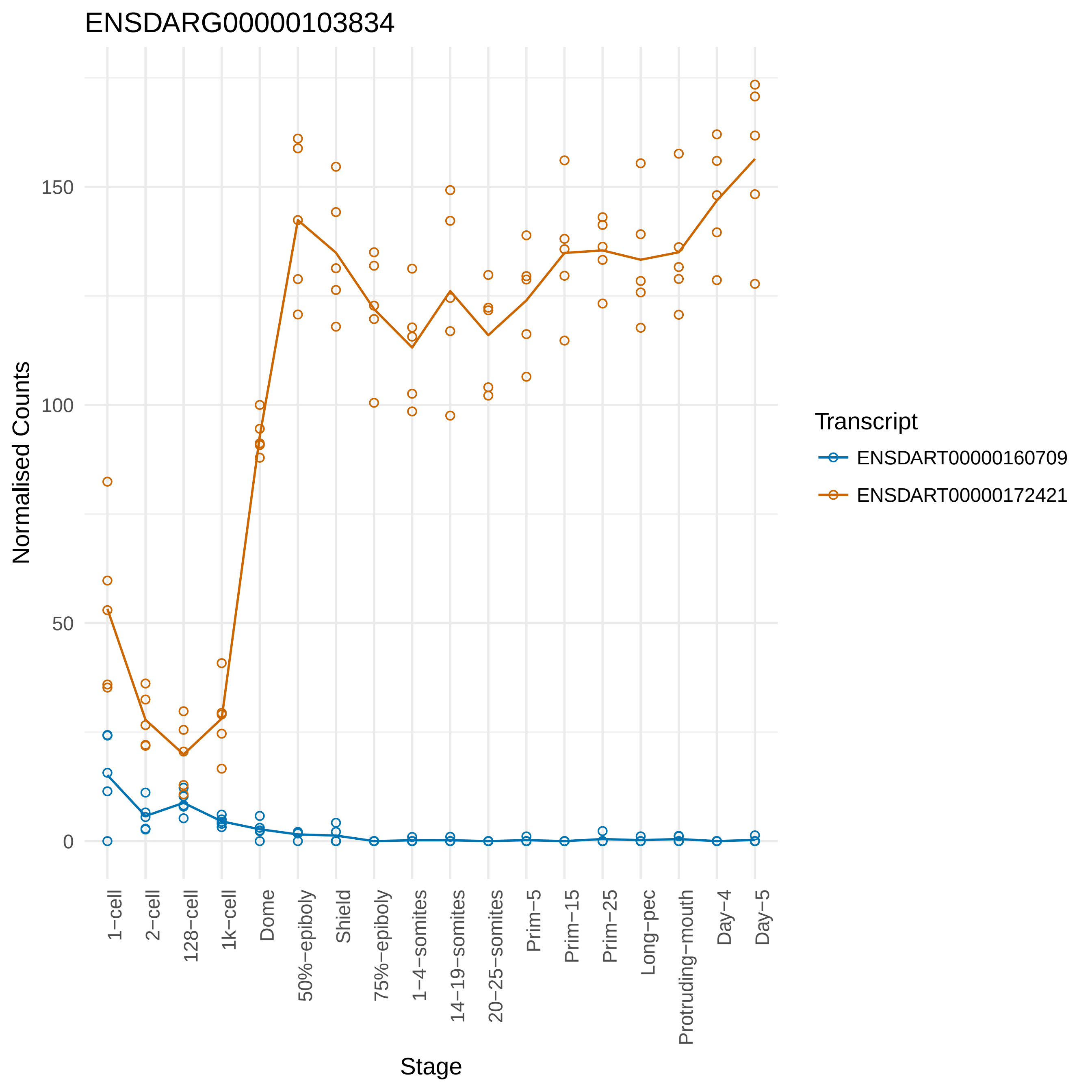

ENSDARG00000103834
Normalised Counts
150
100
50
0
1−cell
2−cell
128−cell
1k−cell
Dome
50%−epiboly
Shield
75%−epiboly
1−4−somites
14−19−somites
20−25−somites
Prim−5
Prim−15
Prim−25
Long−pec
Protruding−mouth
Day−4
Day−5
Stage
Transcript
ENSDART00000160709
ENSDART00000172421

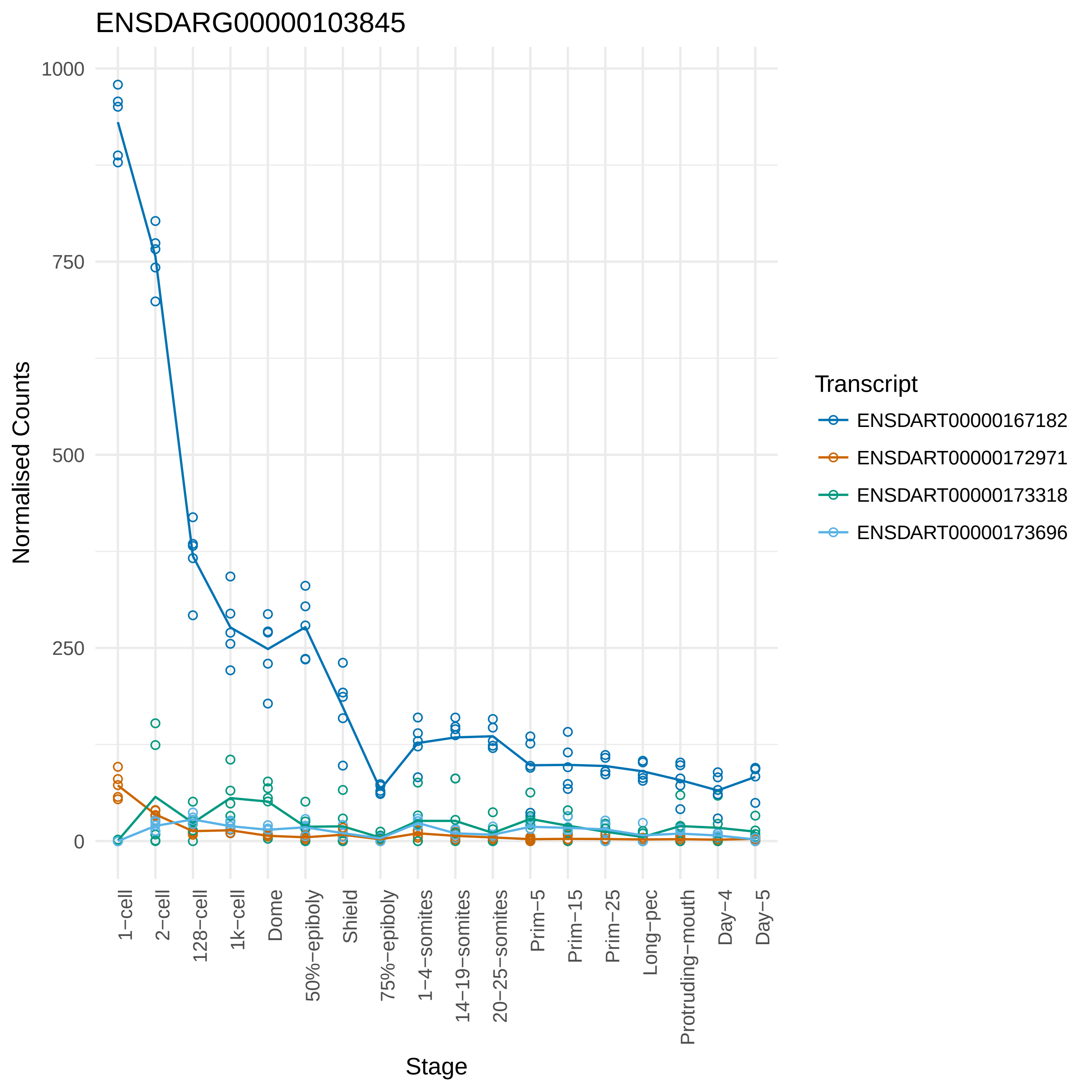

ENSDARG00000103845
1000
750
500
250
0
Normalised Counts
1−cell
2−cell
128−cell
1k−cell
Dome
50%−epiboly
Shield
75%−epiboly
1−4−somites
14−19−somites
20−25−somites
Prim−5
Prim−15
Prim−25
Long−pec
Protruding−mouth
Day−4
Day−5
Stage
Transcript
ENSDART00000167182
ENSDART00000172971
ENSDART00000173318
ENSDART00000173696

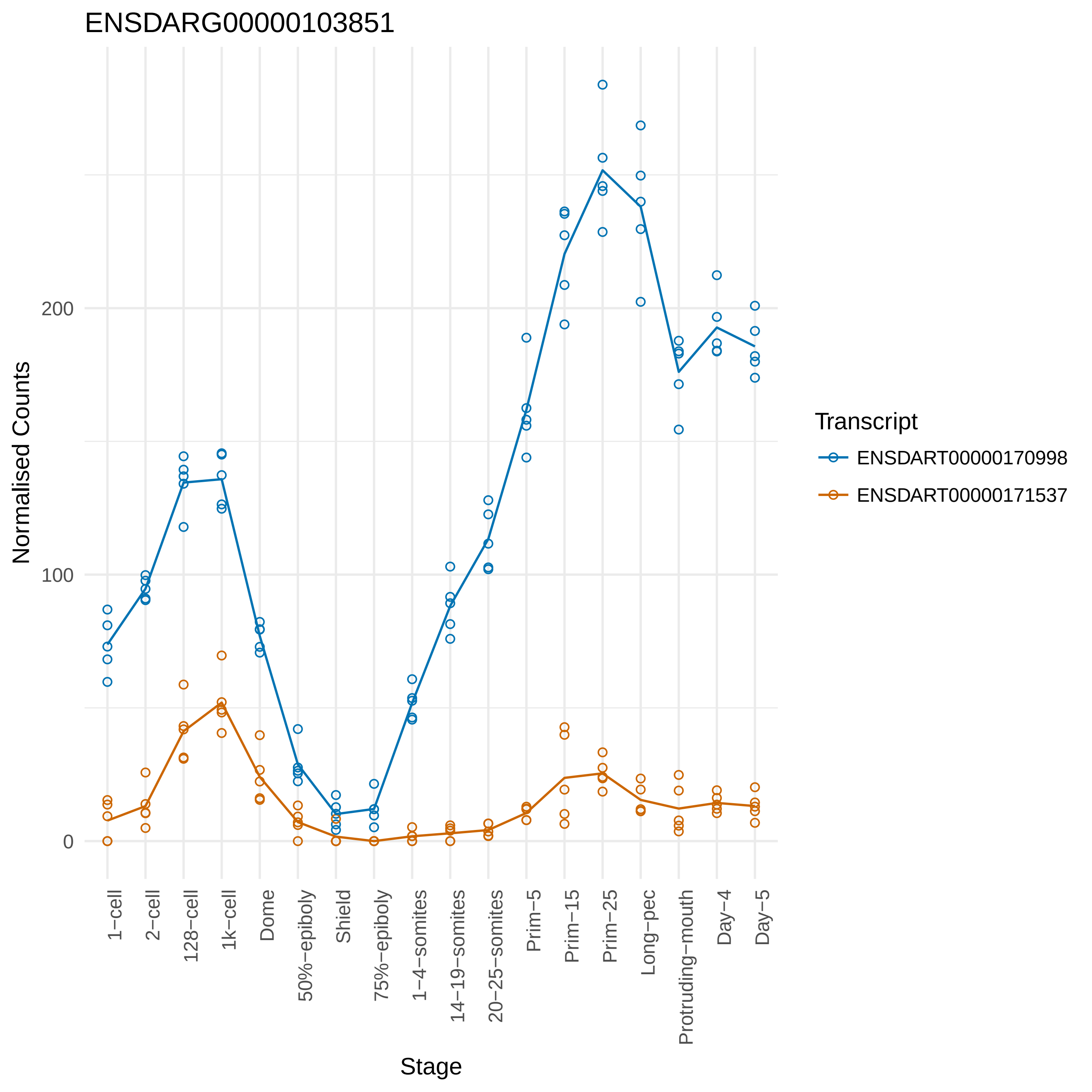
ENSDARG00000103851
Normalised Counts
0
100
200
1−cell
2−cell
128−cell
1k−cell
Dome
50%−epiboly
Shield
75%−epiboly
1−4−somites
14−19−somites
20−25−somites
Prim−5
Prim−15
Prim−25
Long−pec
Protruding−mouth
Day−4
Day−5
Stage
Transcript
ENSDART00000170998
ENSDART00000171537

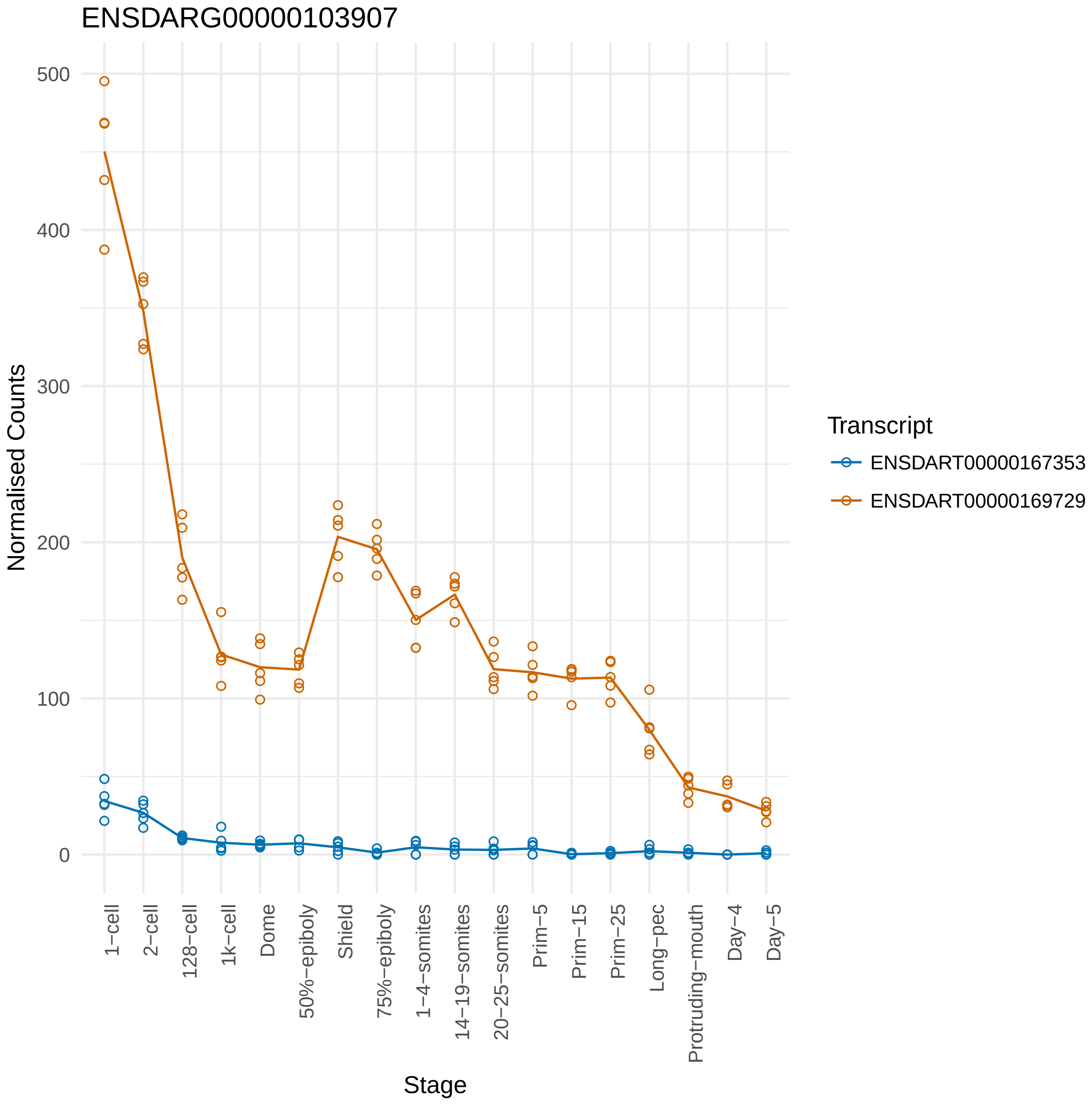
ENSDARG00000103907
Normalised Counts
0
100
200
300
400
500
Stage
1−cell
2−cell
128−cell
1k−cell
Dome
50%−epiboly
Shield
75%−epiboly
1−4−somites
14−19−somites
20−25−somites
Prim−5
Prim−15
Prim−25
Long−pec
Protruding−mouth
Day−4
Day−5
Transcript
ENSDART00000167353
ENSDART00000169729

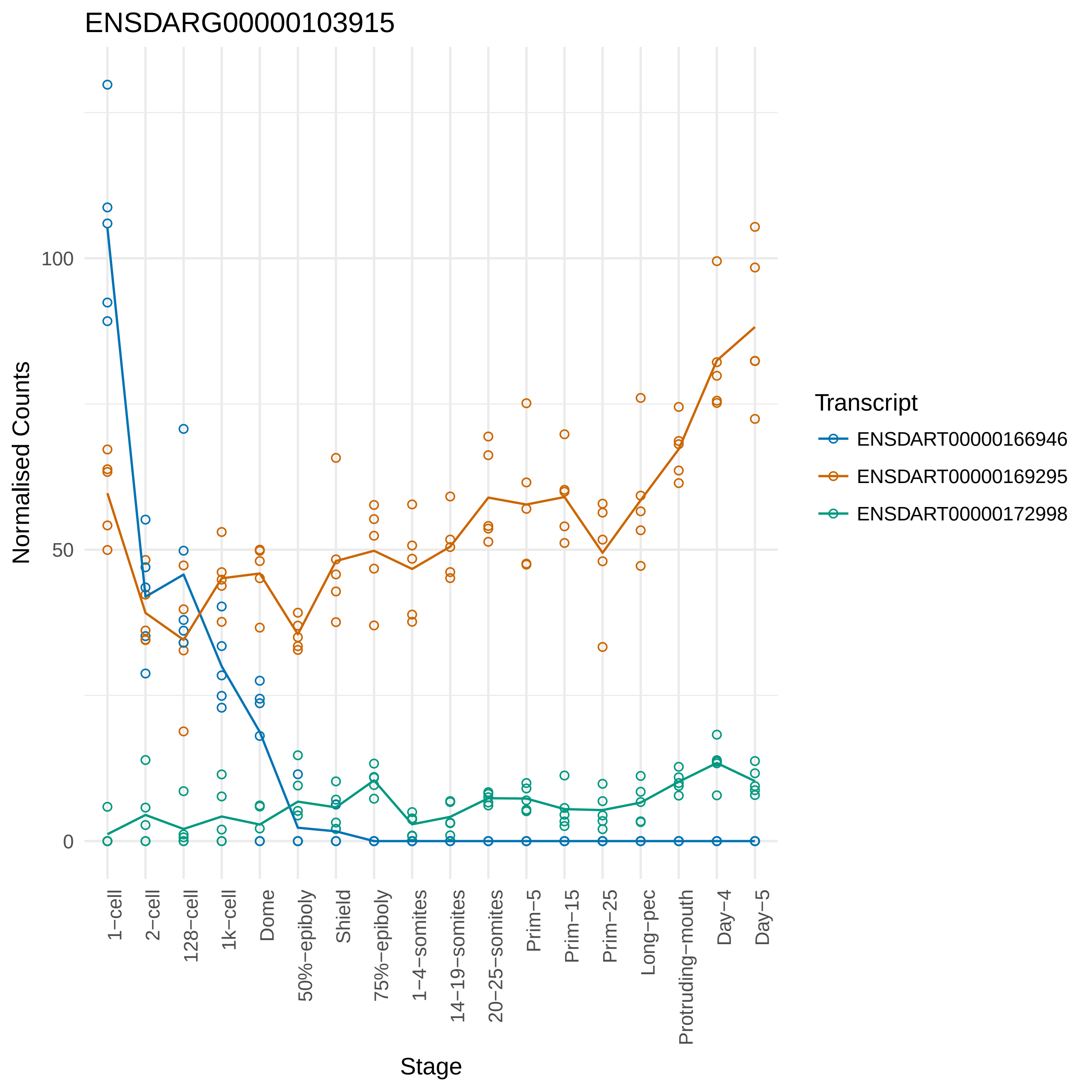
ENSDARG00000103915
Normalised Counts
100
50
0
1−cell
2−cell
128−cell
1k−cell
Dome
50%−epiboly
Shield
75%−epiboly
1−4−somites
14−19−somites
20−25−somites
Prim−5
Prim−15
Prim−25
Long−pec
Protruding−mouth
Day−4
Day−5
Stage
Transcript
ENSDART00000166946
ENSDART00000169295
ENSDART00000172998

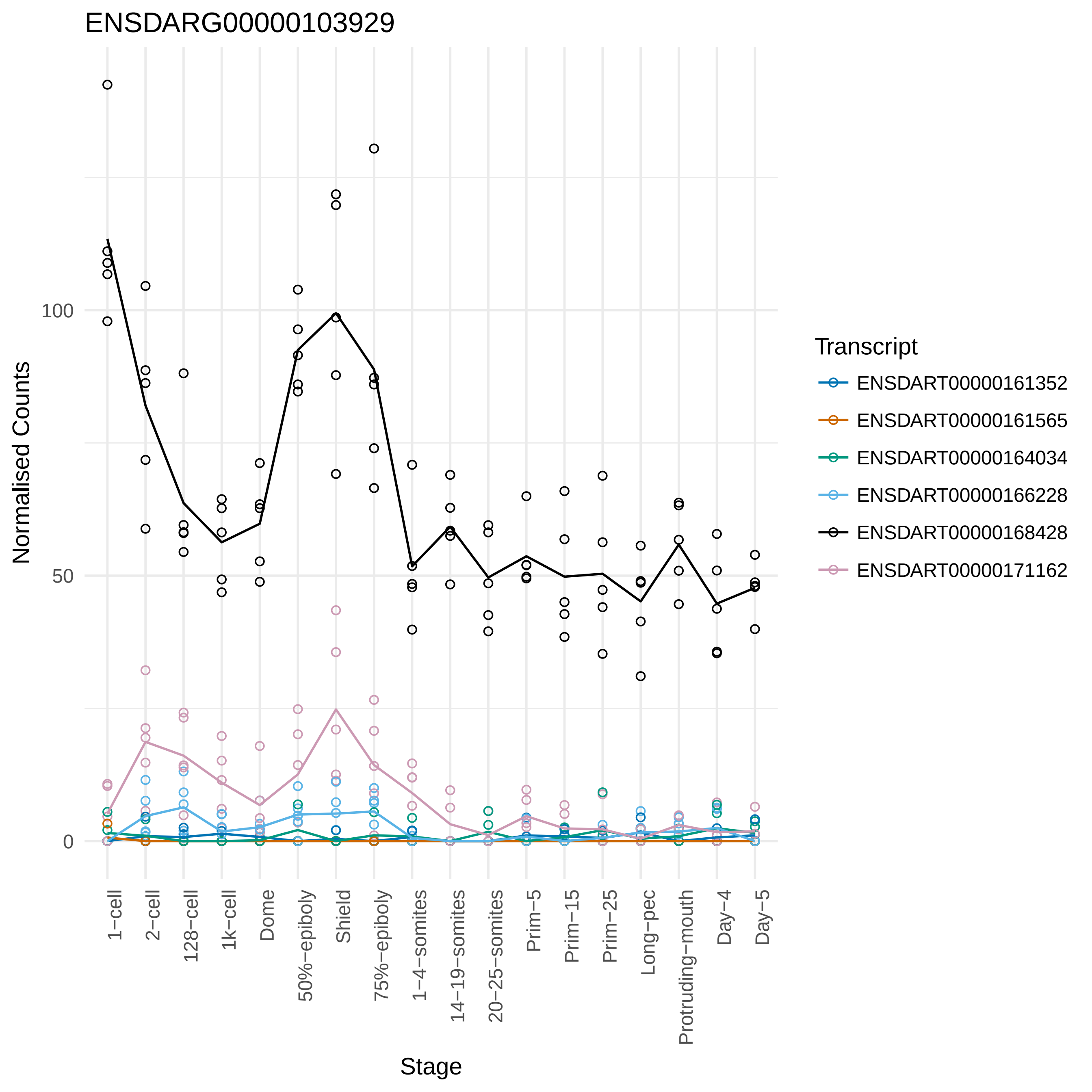

ENSDARG00000103929
Normalised Counts
100
50
0
Stage
1−cell
2−cell
128−cell
1k−cell
Dome
50%−epiboly
Shield
75%−epiboly
1−4−somites
14−19−somites
20−25−somites
Prim−5
Prim−15
Prim−25
Long−pec
Protruding−mouth
Day−4
Day−5
Transcript
ENSDART00000161352
ENSDART00000161565
ENSDART00000164034
ENSDART00000166228
ENSDART00000168428
ENSDART00000171162

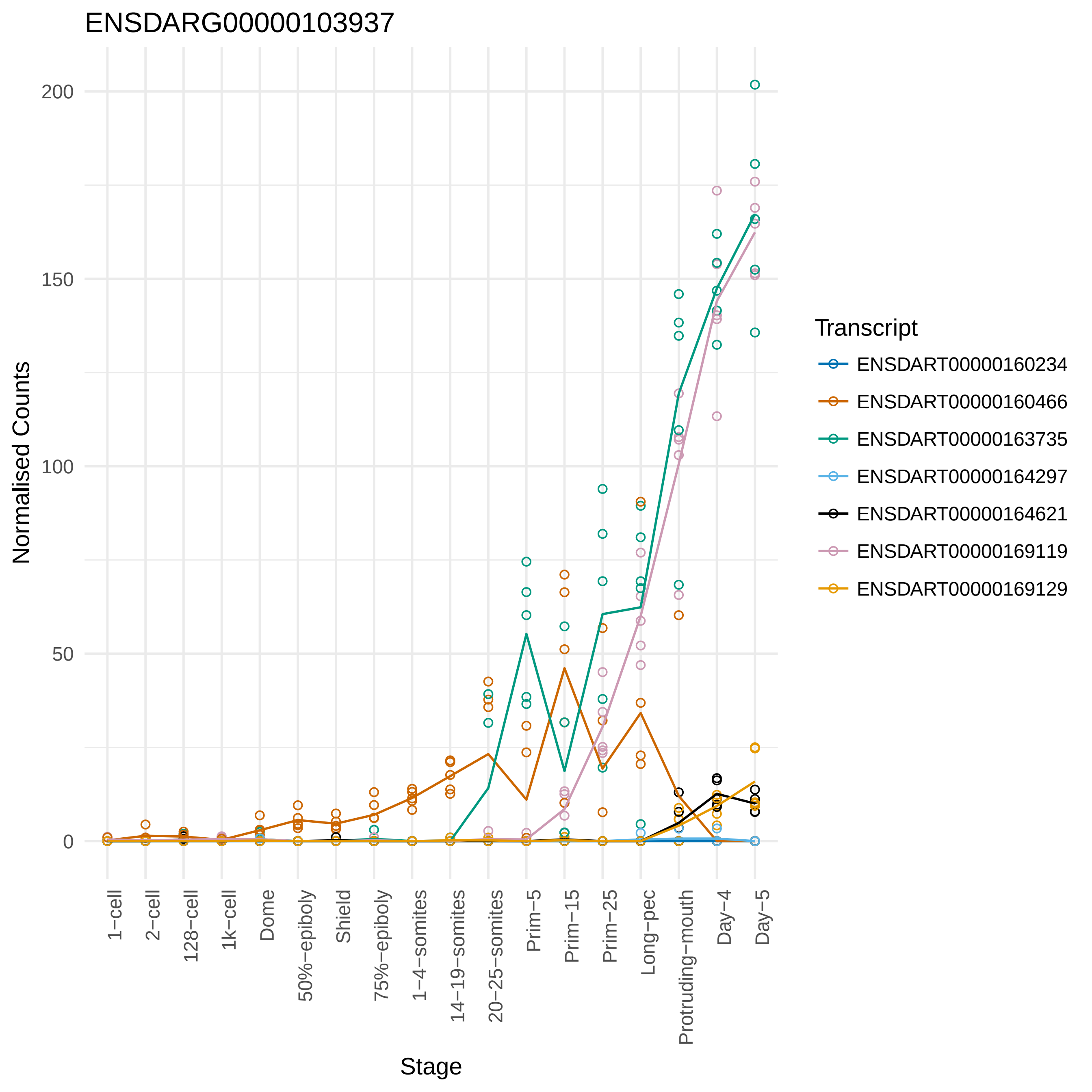
ENSDARG00000103937
Normalised Counts
0
50
100
150
200
Stage
1−cell
2−cell
128−cell
1k−cell
Dome
50%−epiboly
Shield
75%−epiboly
1−4−somites
14−19−somites
20−25−somites
Prim−5
Prim−15
Prim−25
Long−pec
Protruding−mouth
Day−4
Day−5
Transcript
ENSDART00000160234
ENSDART00000160466
ENSDART00000163735
ENSDART00000164297
ENSDART00000164621
ENSDART00000169119
ENSDART00000169129

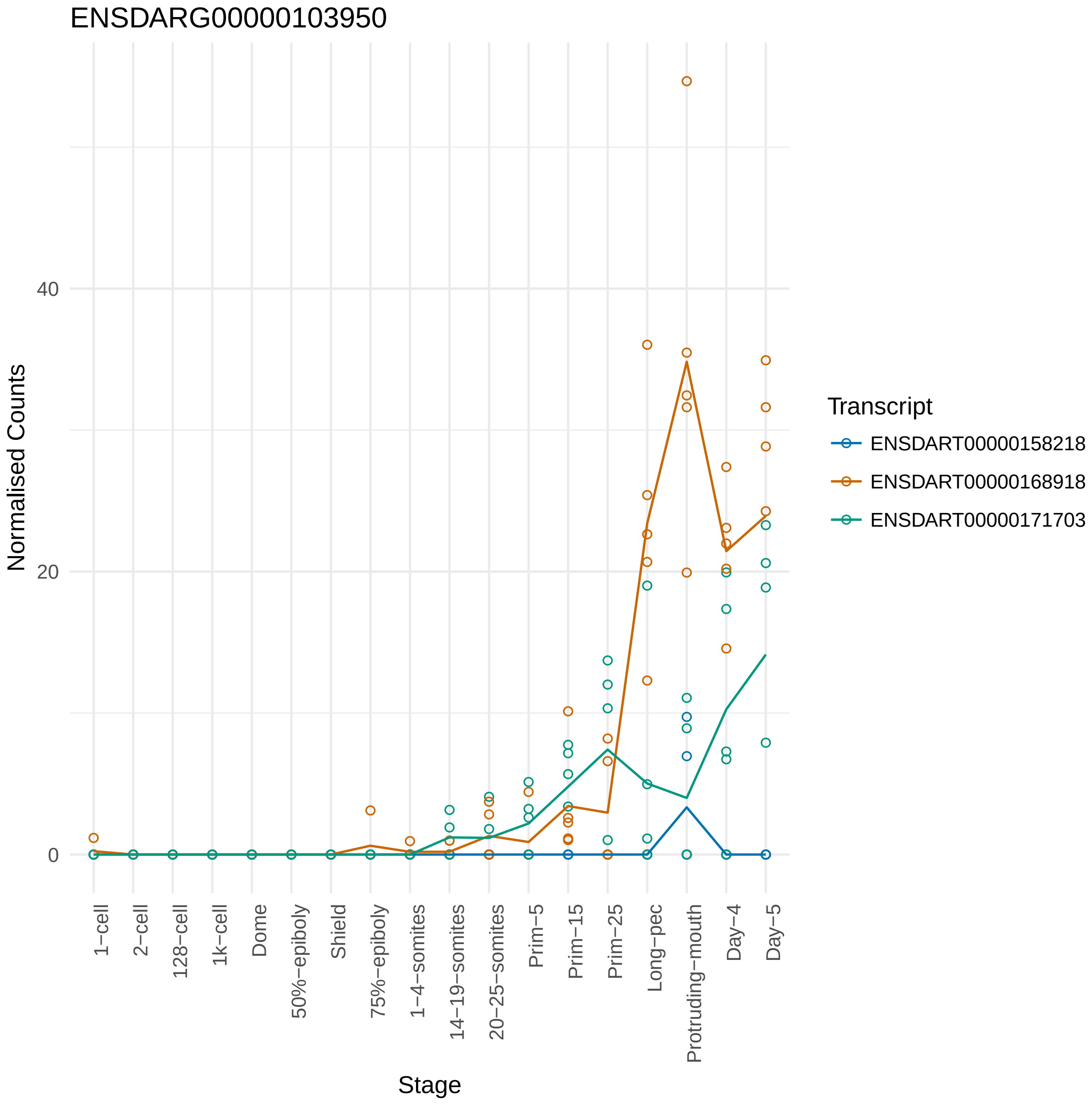

ENSDARG00000103950
Normalised Counts
0
20
40
1−cell
2−cell
128−cell
1k−cell
Dome
50%−epiboly
Shield
75%−epiboly
1−4−somites
14−19−somites
20−25−somites
Prim−5
Prim−15
Prim−25
Long−pec
Protruding−mouth
Day−4
Day−5
Stage
Transcript
ENSDART00000158218
ENSDART00000168918
ENSDART00000171703

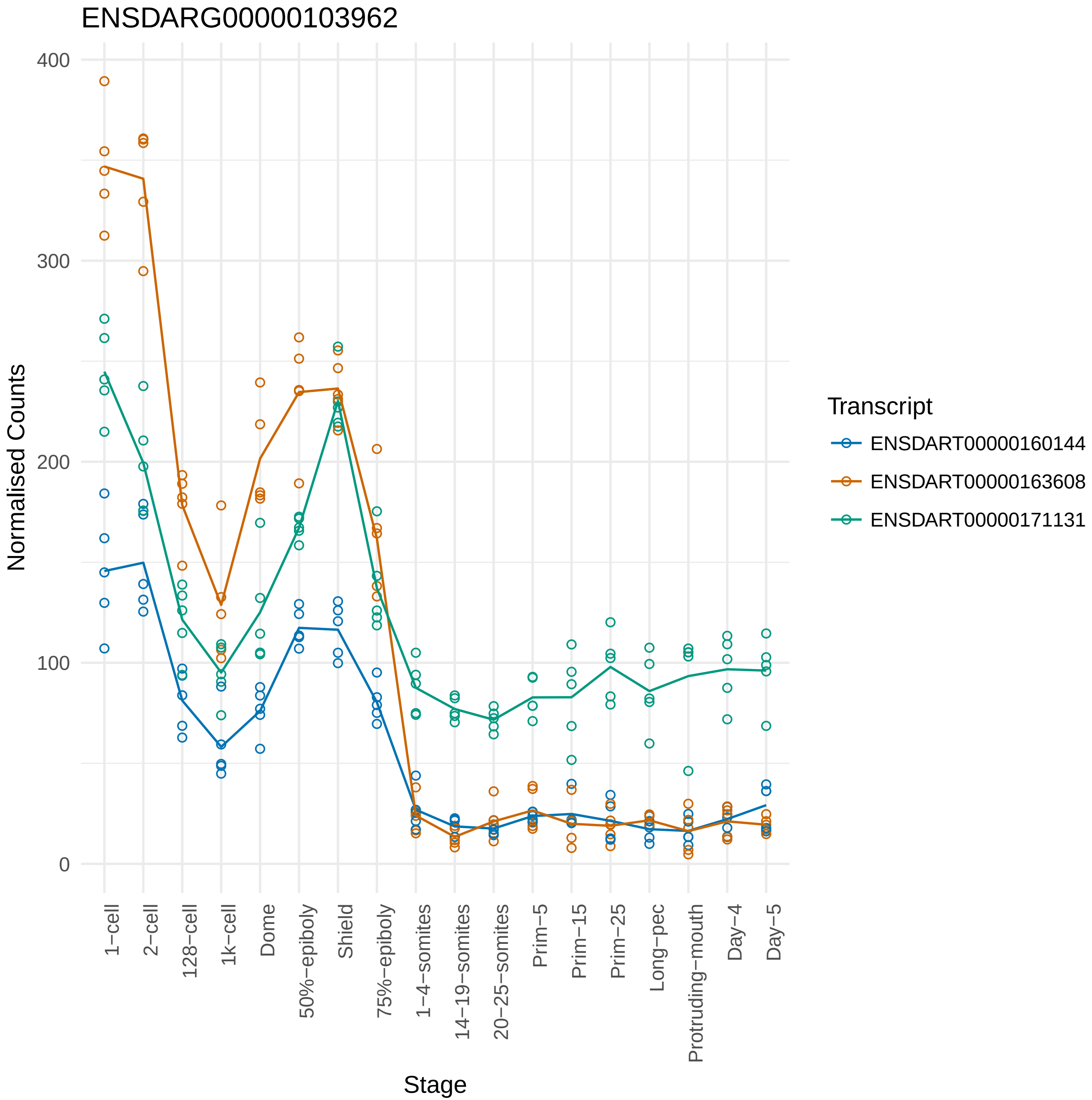
ENSDARG00000103962
Normalised Counts
400
300
200
100
0
1−cell
2−cell
128−cell
1k−cell
Dome
50%−epiboly
Shield
75%−epiboly
1−4−somites
14−19−somites
20−25−somites
Prim−5
Prim−15
Prim−25
Long−pec
Protruding−mouth
Day−4
Day−5
Stage
Transcript
ENSDART00000160144
ENSDART00000163608
ENSDART00000171131

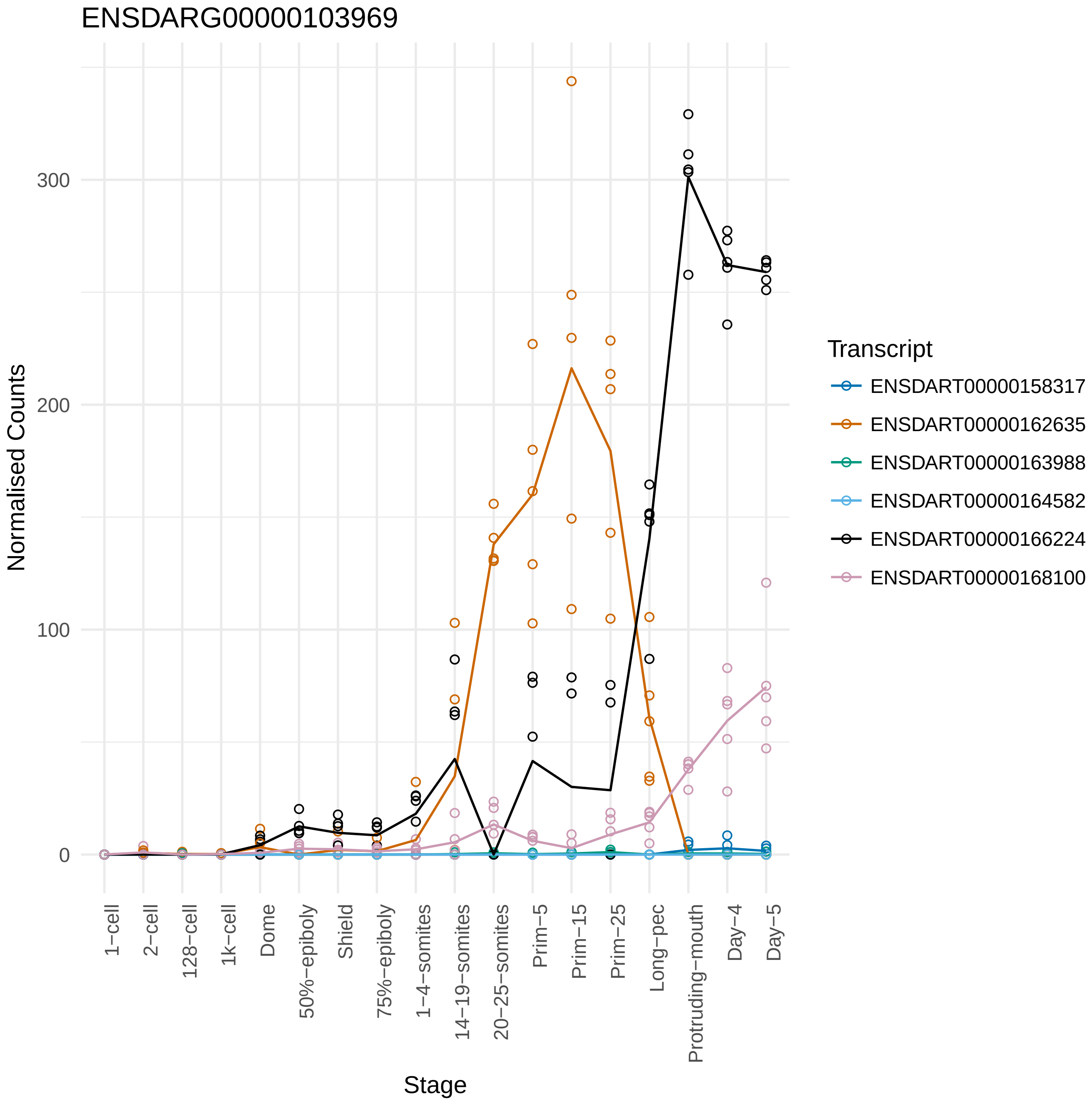
ENSDARG00000103969
Normalised Counts
0
100
200
300
Stage
1−cell
2−cell
128−cell
1k−cell
Dome
50%−epiboly
Shield
75%−epiboly
1−4−somites
14−19−somites
20−25−somites
Prim−5
Prim−15
Prim−25
Long−pec
Protruding−mouth
Day−4
Day−5
Transcript
ENSDART00000158317
ENSDART00000162635
ENSDART00000163988
ENSDART00000164582
ENSDART00000166224
ENSDART00000168100

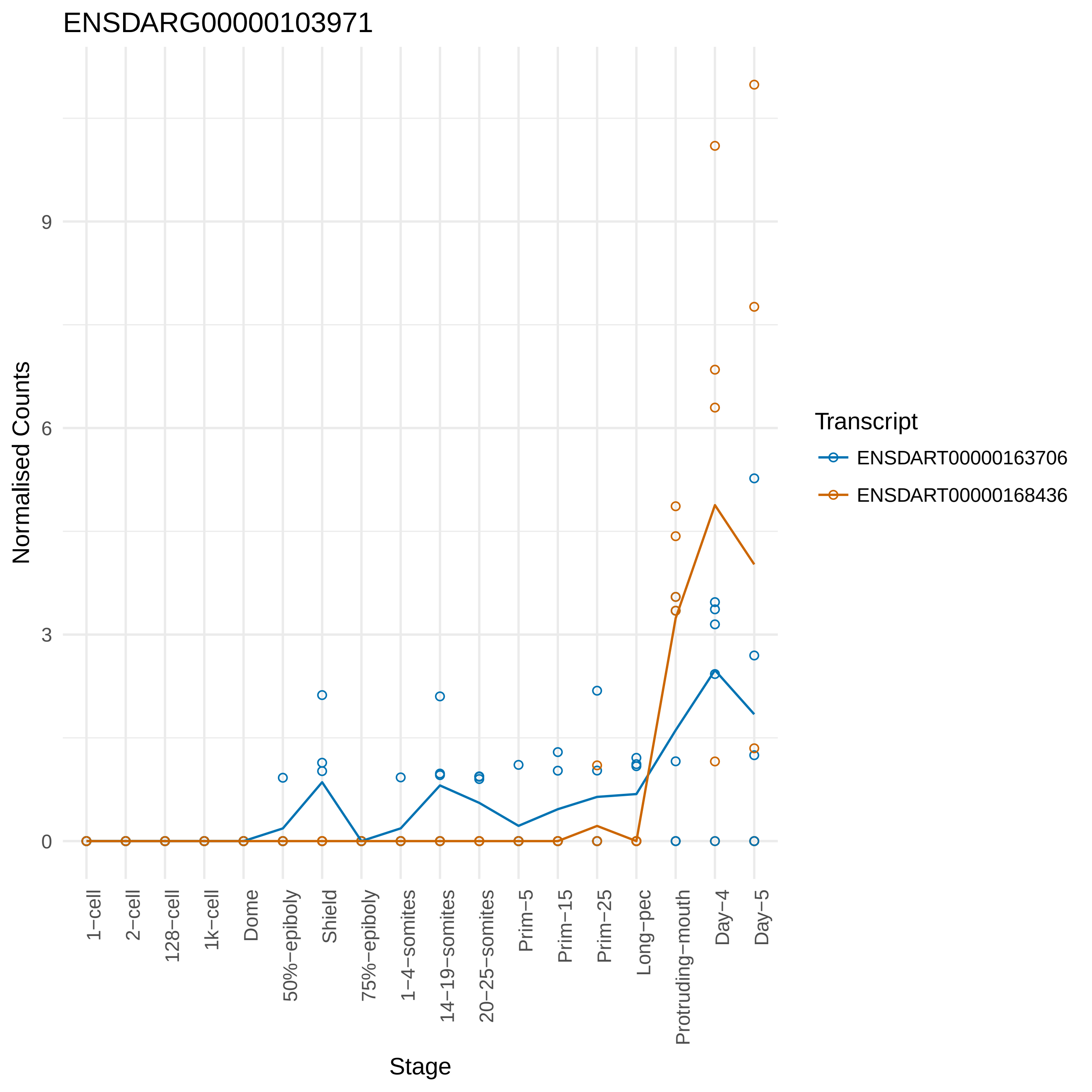

ENSDARG00000103971
Normalised Counts
9
6
3
0
1−cell
2−cell
128−cell
1k−cell
Dome
50%−epiboly
Shield
75%−epiboly
1−4−somites
14−19−somites
20−25−somites
Prim−5
Prim−15
Prim−25
Long−pec
Protruding−mouth
Day−4
Day−5
Stage
Transcript
ENSDART00000163706
ENSDART00000168436

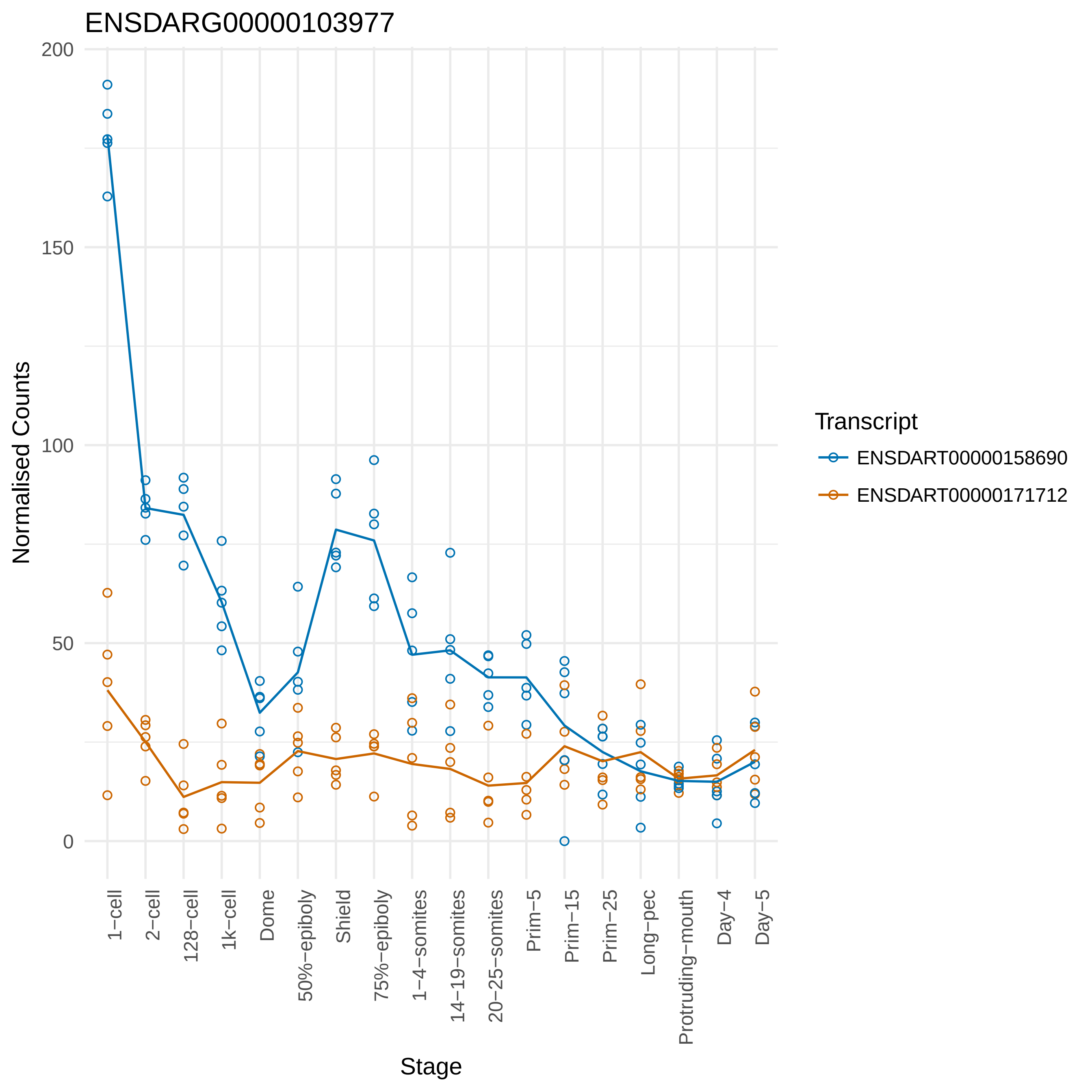
ENSDARG00000103977
Normalised Counts
200
150
100
50
0
1−cell
2−cell
128−cell
1k−cell
Dome
50%−epiboly
Shield
75%−epiboly
1−4−somites
14−19−somites
20−25−somites
Prim−5
Prim−15
Prim−25
Long−pec
Protruding−mouth
Day−4
Day−5
Stage
Transcript
ENSDART00000158690
ENSDART00000171712

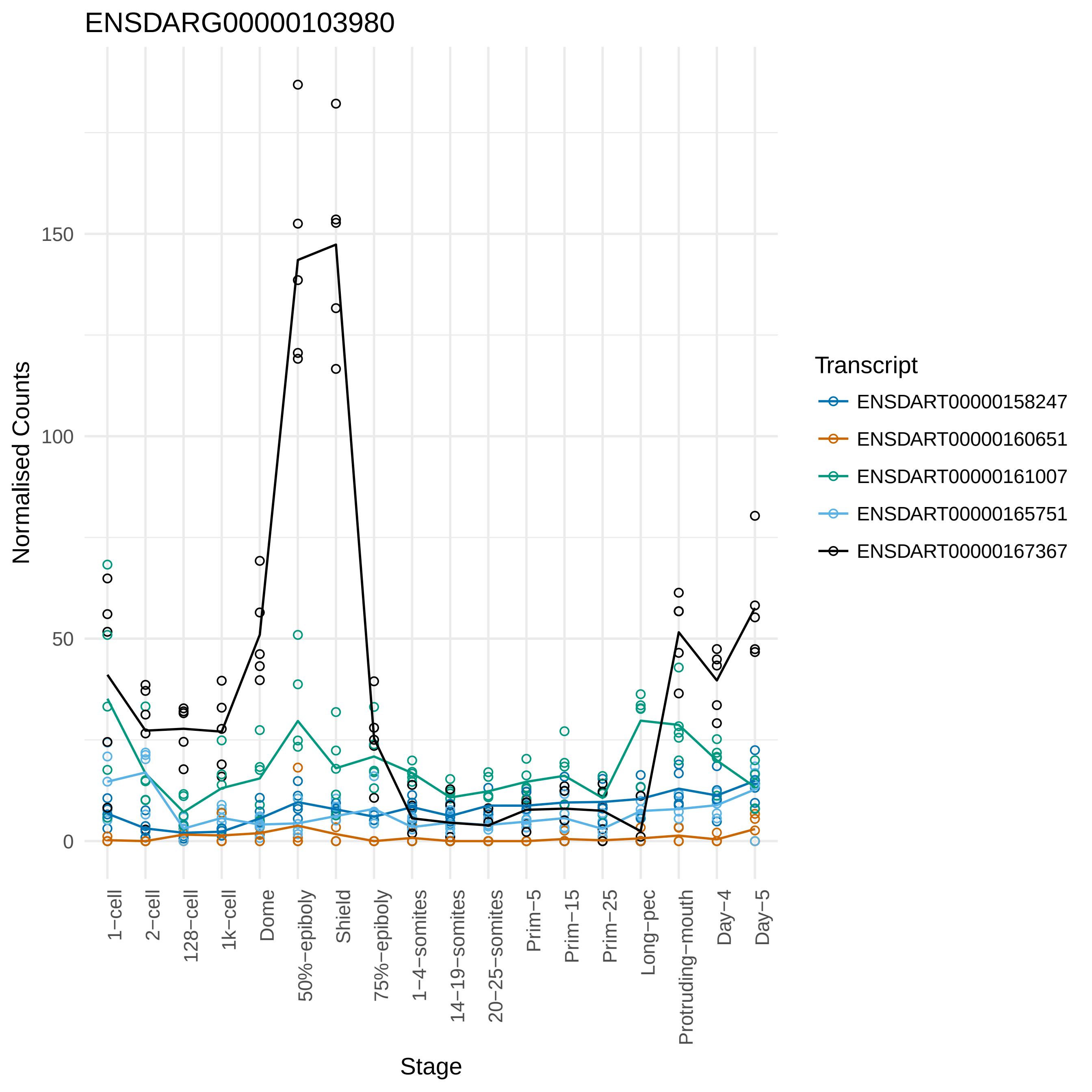

ENSDARG00000103980
Normalised Counts
0
50
100
150
Stage
1−cell
2−cell
128−cell
1k−cell
Dome
50%−epiboly
Shield
75%−epiboly
1−4−somites
14−19−somites
20−25−somites
Prim−5
Prim−15
Prim−25
Long−pec
Protruding−mouth
Day−4
Day−5
Transcript
ENSDART00000158247
ENSDART00000160651
ENSDART00000161007
ENSDART00000165751
ENSDART00000167367

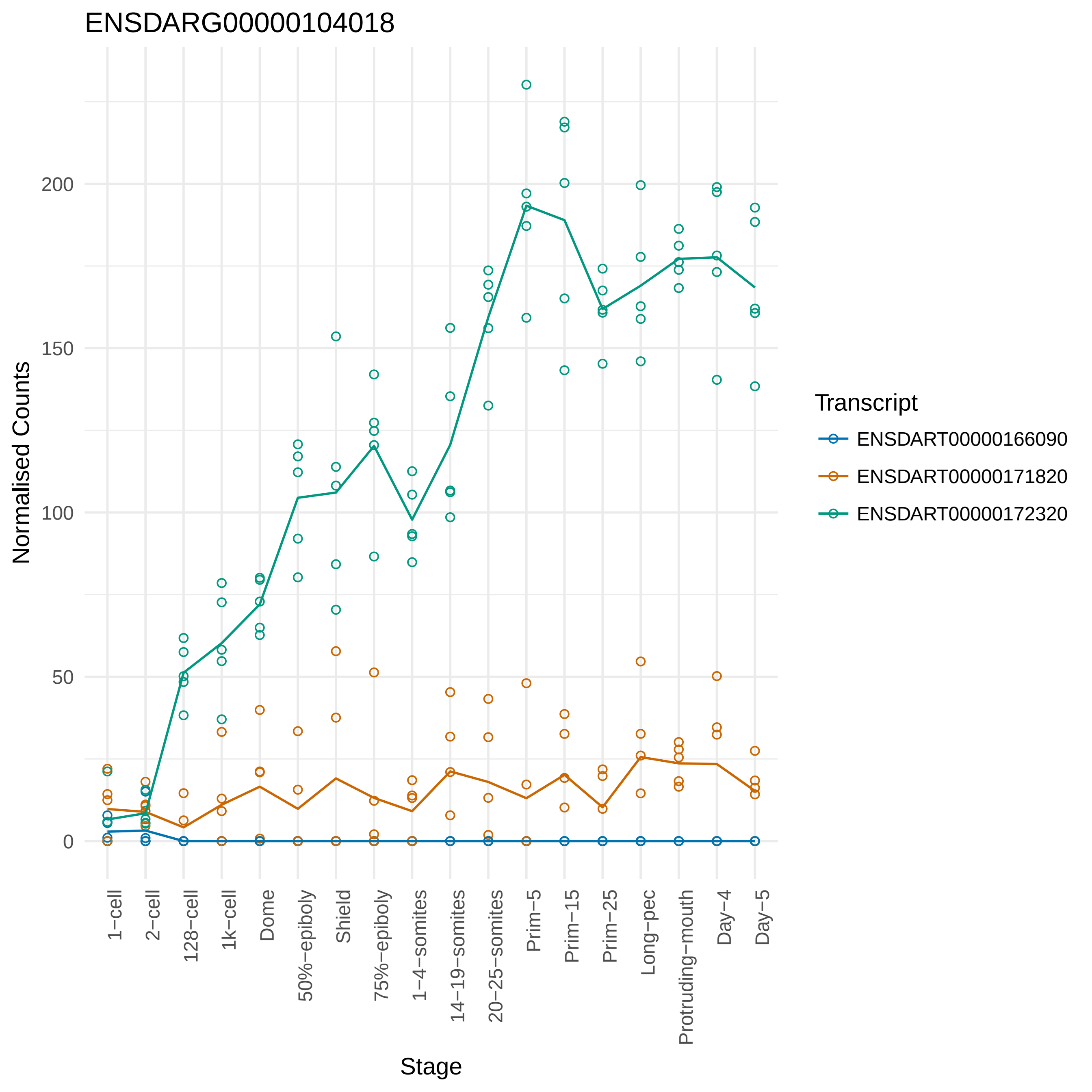

ENSDARG00000104018
Normalised Counts
0
50
100
150
200
Stage
1−cell
2−cell
128−cell
1k−cell
Dome
50%−epiboly
Shield
75%−epiboly
1−4−somites
14−19−somites
20−25−somites
Prim−5
Prim−15
Prim−25
Long−pec
Protruding−mouth
Day−4
Day−5
Transcript
ENSDART00000166090
ENSDART00000171820
ENSDART00000172320

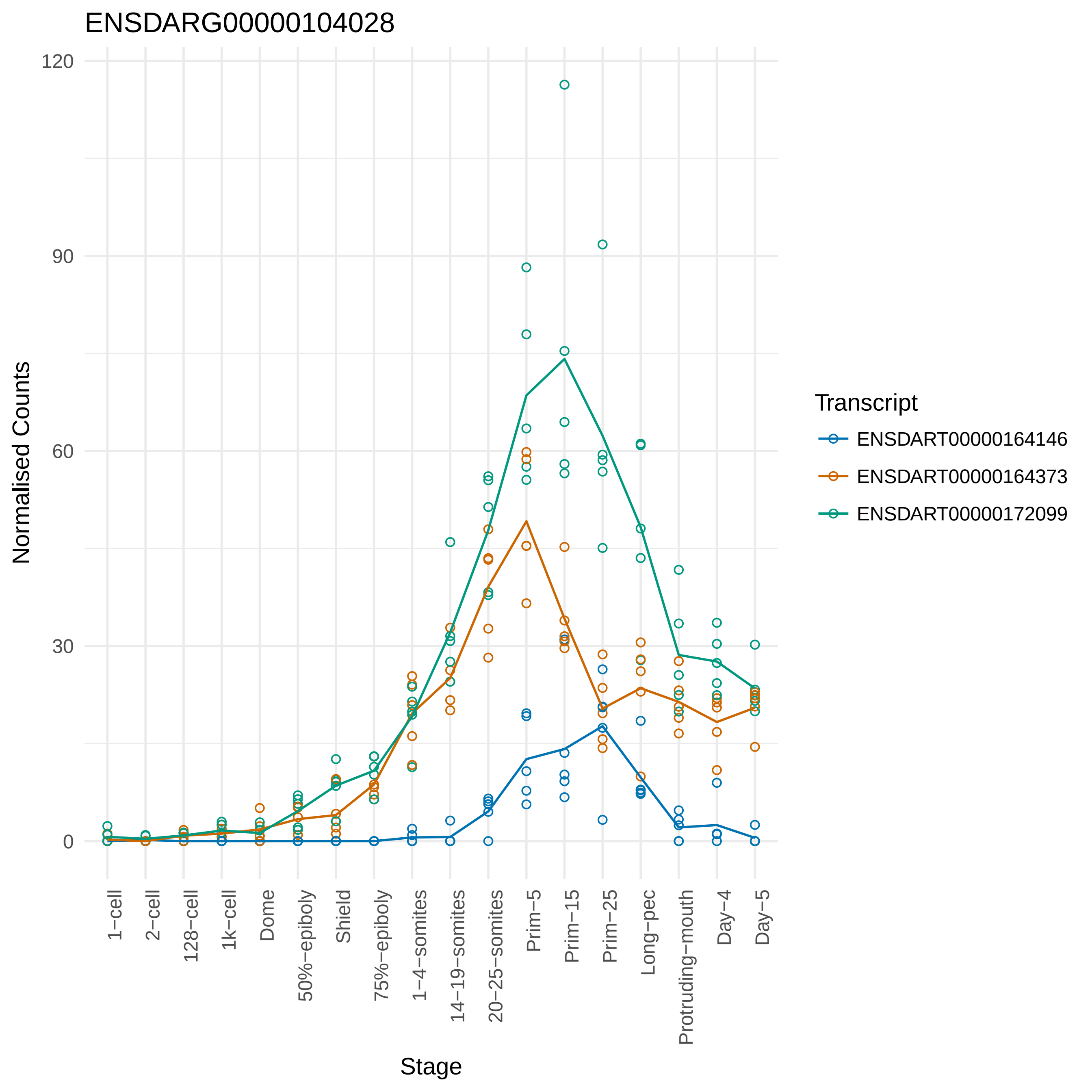

ENSDARG00000104028
Normalised Counts
0
30
60
90
120
1−cell
2−cell
128−cell
1k−cell
Dome
50%−epiboly
Shield
75%−epiboly
1−4−somites
14−19−somites
20−25−somites
Prim−5
Prim−15
Prim−25
Long−pec
Protruding−mouth
Day−4
Day−5
Stage
Transcript
ENSDART00000164146
ENSDART00000164373
ENSDART00000172099

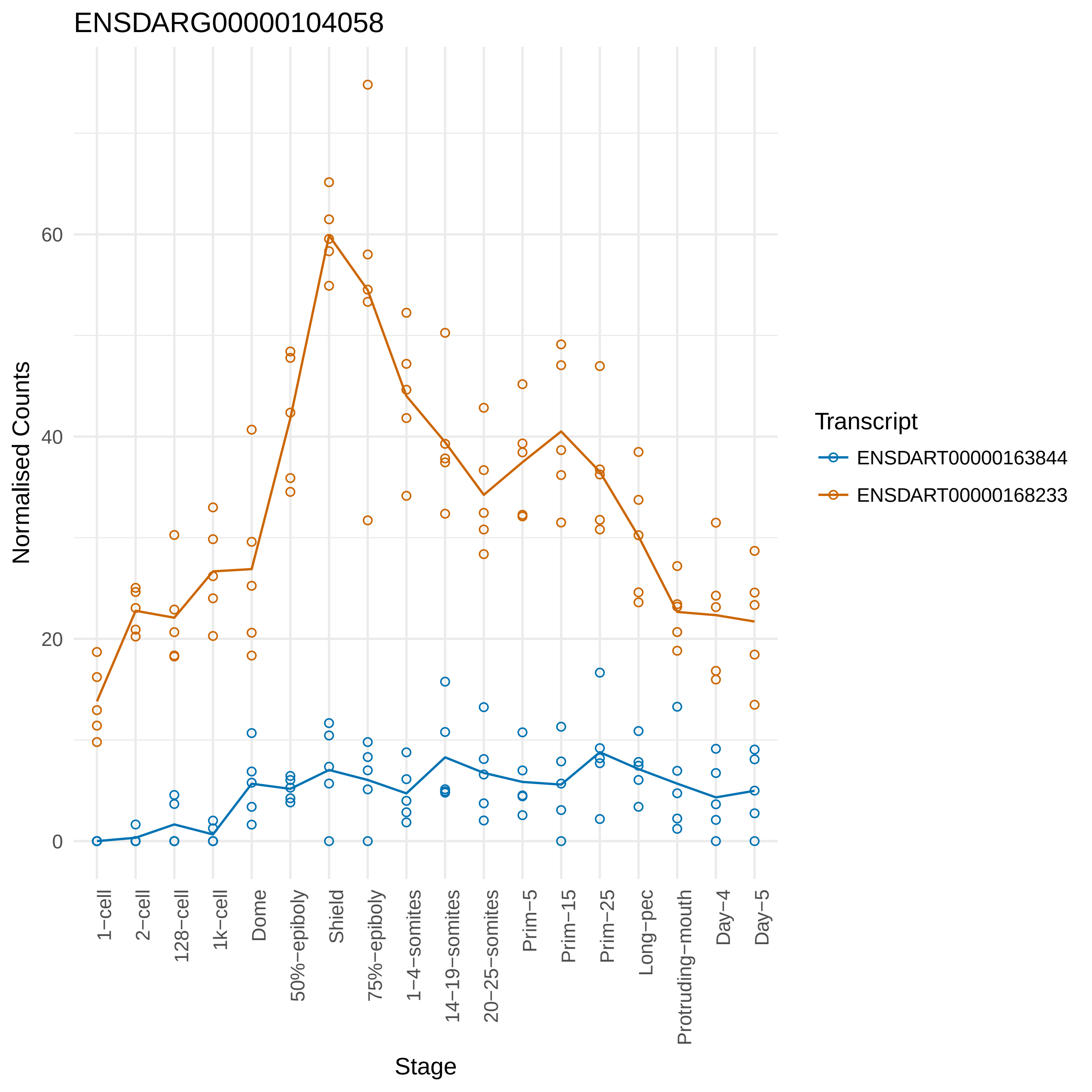
ENSDARG00000104058
Normalised Counts
0
20
40
60
1−cell
2−cell
128−cell
1k−cell
Dome
50%−epiboly
Shield
75%−epiboly
1−4−somites
14−19−somites
20−25−somites
Prim−5
Prim−15
Prim−25
Long−pec
Protruding−mouth
Day−4
Day−5
Stage
Transcript
ENSDART00000163844
ENSDART00000168233

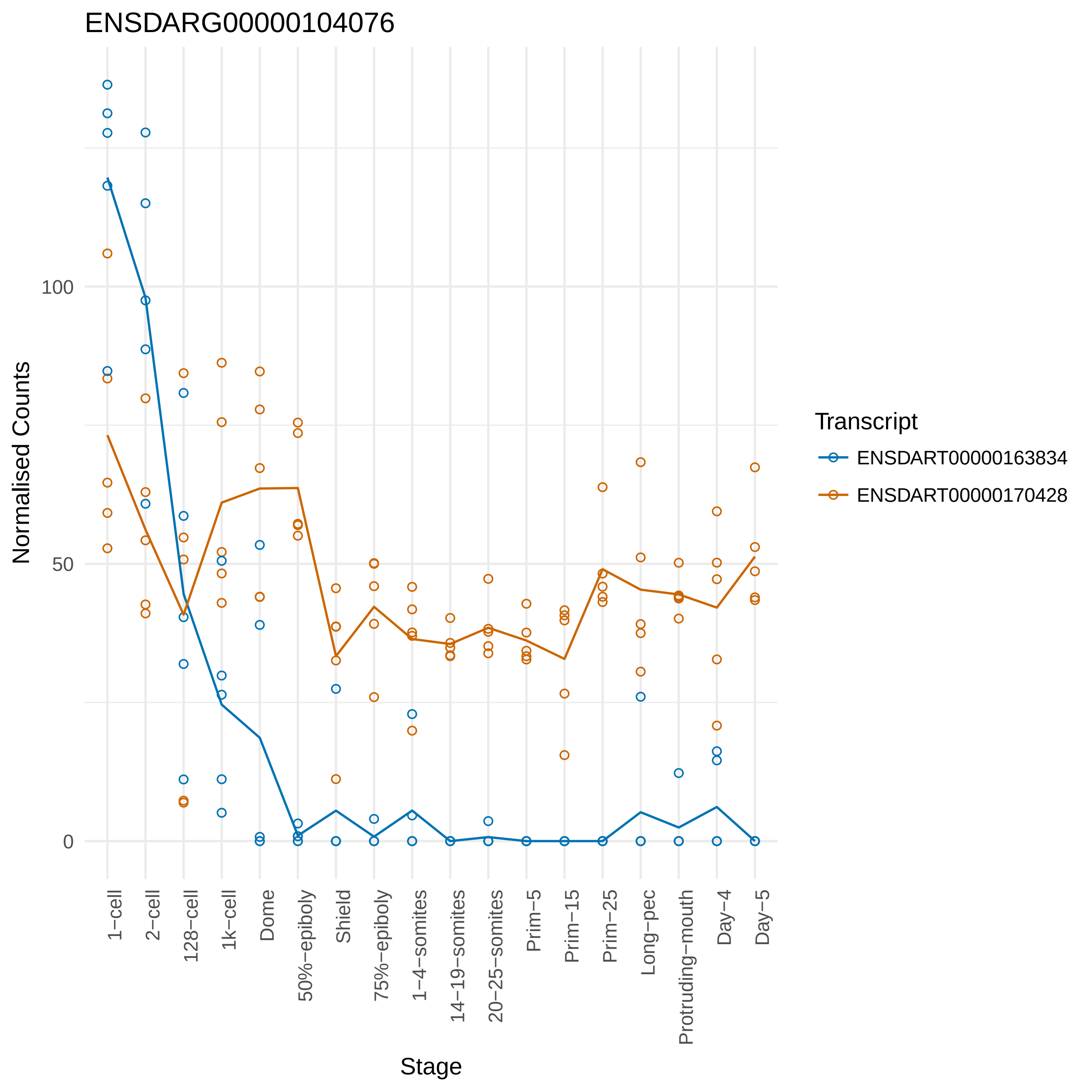
ENSDARG00000104076
Normalised Counts
0
50
100
Stage
1−cell
2−cell
128−cell
1k−cell
Dome
50%−epiboly
Shield
75%−epiboly
1−4−somites
14−19−somites
20−25−somites
Prim−5
Prim−15
Prim−25
Long−pec
Protruding−mouth
Day−4
Day−5
Transcript
ENSDART00000163834
ENSDART00000170428

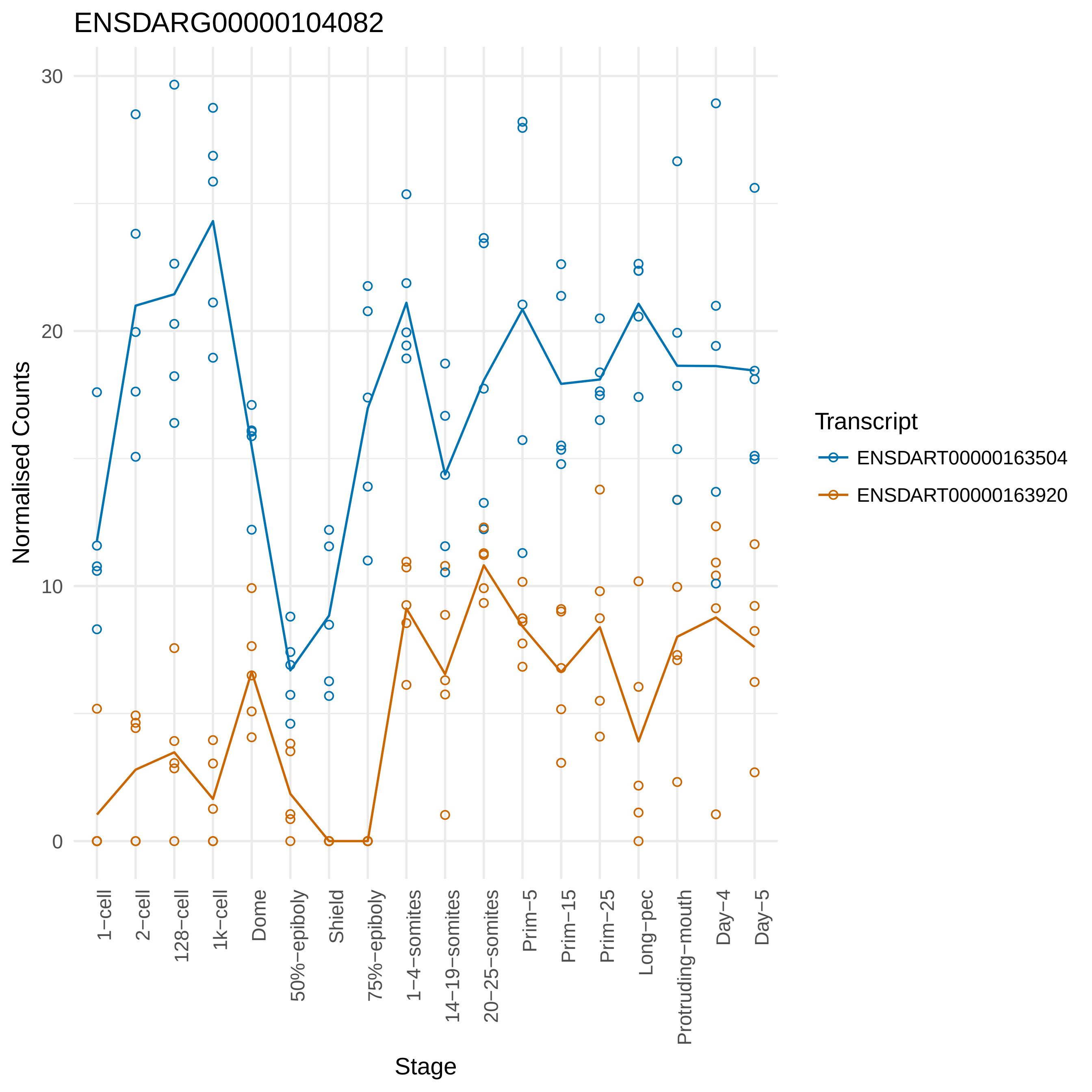
ENSDARG00000104082
Normalised Counts
30
20
10
0
1−cell
2−cell
128−cell
1k−cell
Dome
50%−epiboly
Shield
75%−epiboly
1−4−somites
14−19−somites
20−25−somites
Prim−5
Prim−15
Prim−25
Long−pec
Protruding−mouth
Day−4
Day−5
Stage
Transcript
ENSDART00000163504
ENSDART00000163920

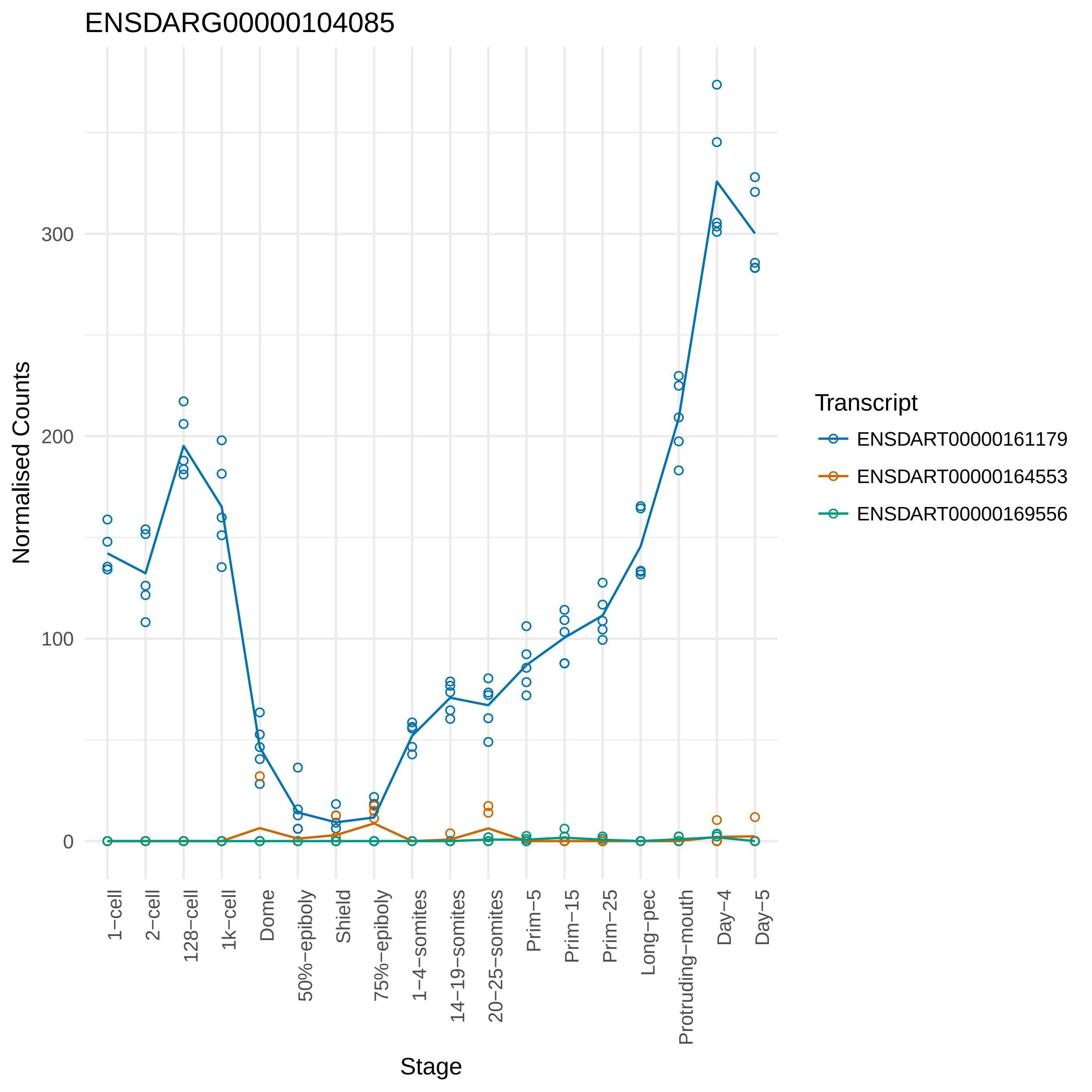
ENSDARG00000104085
Normalised Counts
0
100
200
300
Stage
1−cell
2−cell
128−cell
1k−cell
Dome
50%−epiboly
Shield
75%−epiboly
1−4−somites
14−19−somites
20−25−somites
Prim−5
Prim−15
Prim−25
Long−pec
Protruding−mouth
Day−4
Day−5
Transcript
ENSDART00000161179
ENSDART00000164553
ENSDART00000169556

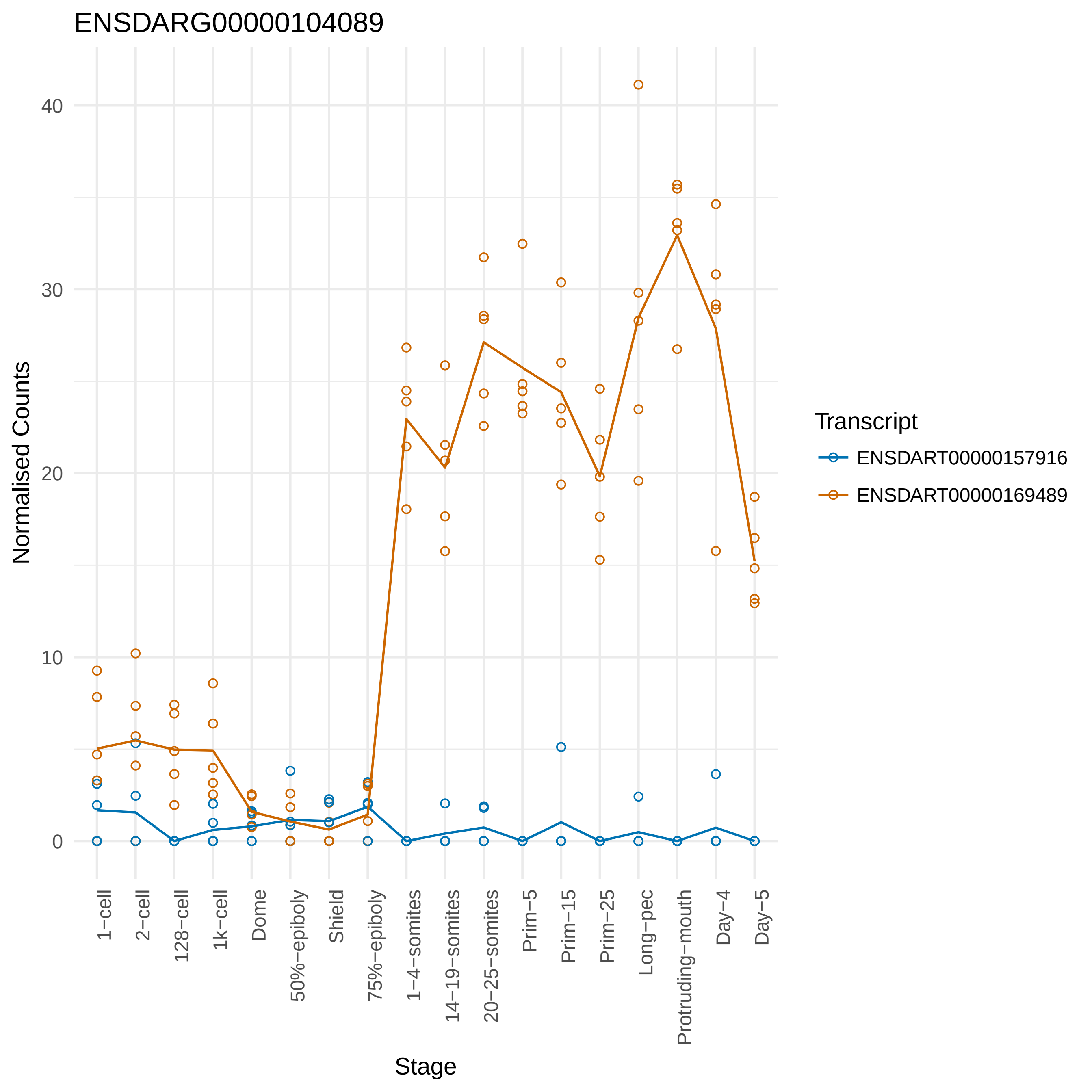

ENSDARG00000104089
Normalised Counts
0
10
20
30
40
1−cell
2−cell
128−cell
1k−cell
Dome
50%−epiboly
Shield
75%−epiboly
1−4−somites
14−19−somites
20−25−somites
Prim−5
Prim−15
Prim−25
Long−pec
Protruding−mouth
Day−4
Day−5
Stage
Transcript
ENSDART00000157916
ENSDART00000169489

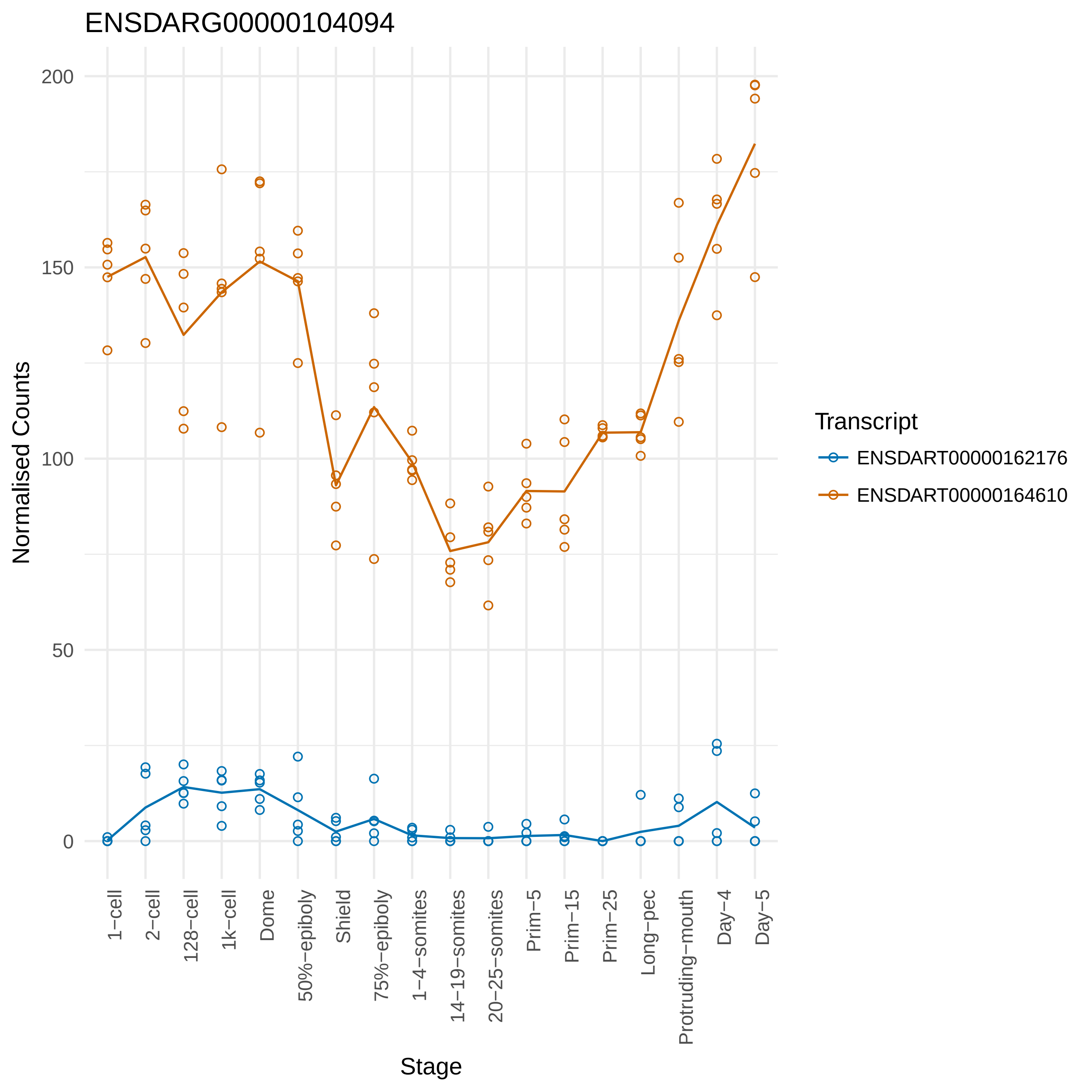
ENSDARG00000104094
Normalised Counts
200
150
100
50
0
1−cell
2−cell
128−cell
1k−cell
Dome
50%−epiboly
Shield
75%−epiboly
1−4−somites
14−19−somites
20−25−somites
Prim−5
Prim−15
Prim−25
Long−pec
Protruding−mouth
Day−4
Day−5
Stage
Transcript
ENSDART00000162176
ENSDART00000164610

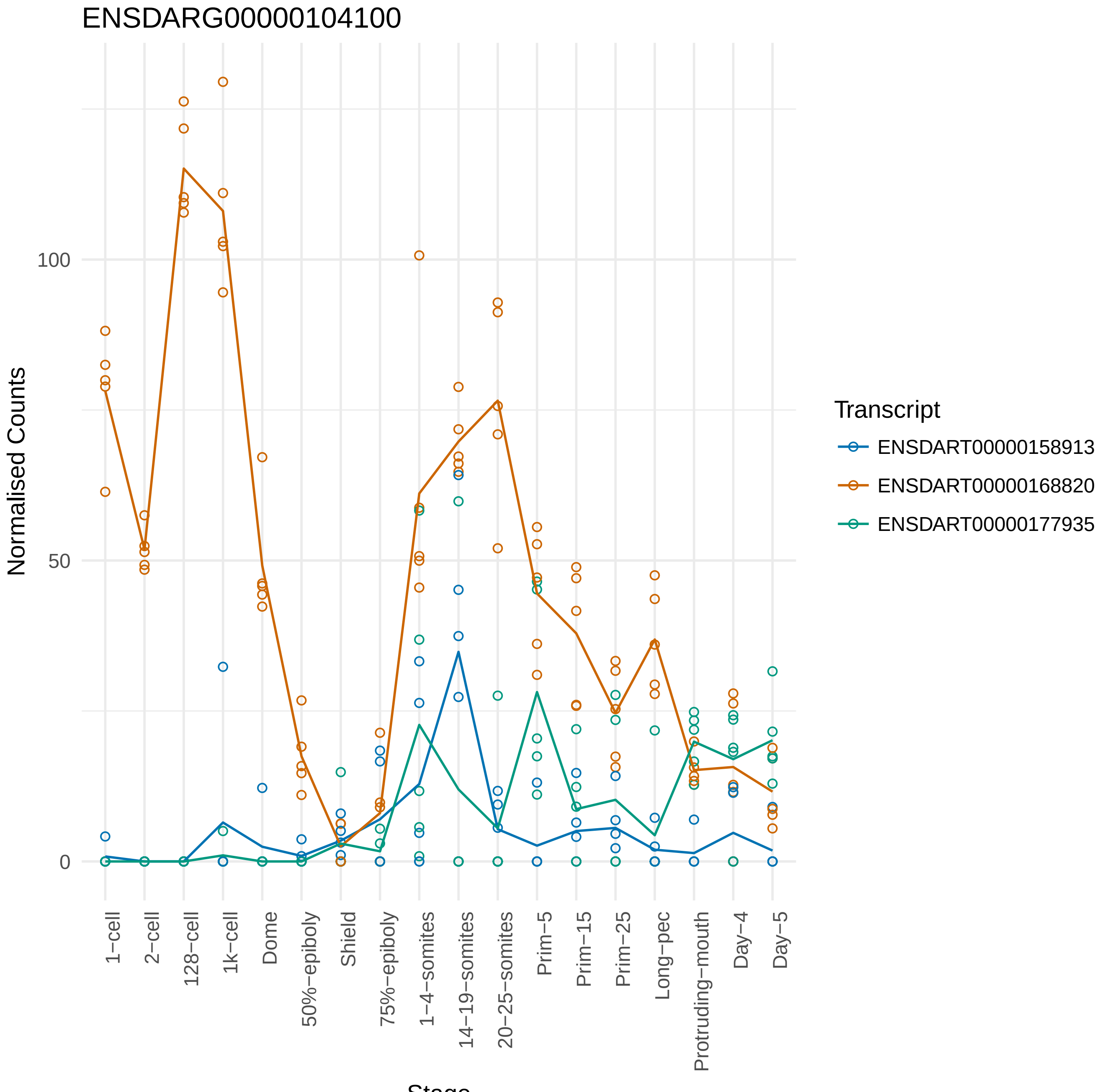

ENSDARG00000104100
Normalised Counts
100
50
0
1−cell
2−cell
128−cell
1k−cell
Dome
50%−epiboly
Shield
75%−epiboly
1−4−somites
14−19−somites
20−25−somites
Prim−5
Prim−15
Prim−25
Long−pec
Protruding−mouth
Day−4
Day−5
Stage
Transcript
ENSDART00000158913
ENSDART00000168820
ENSDART00000177935

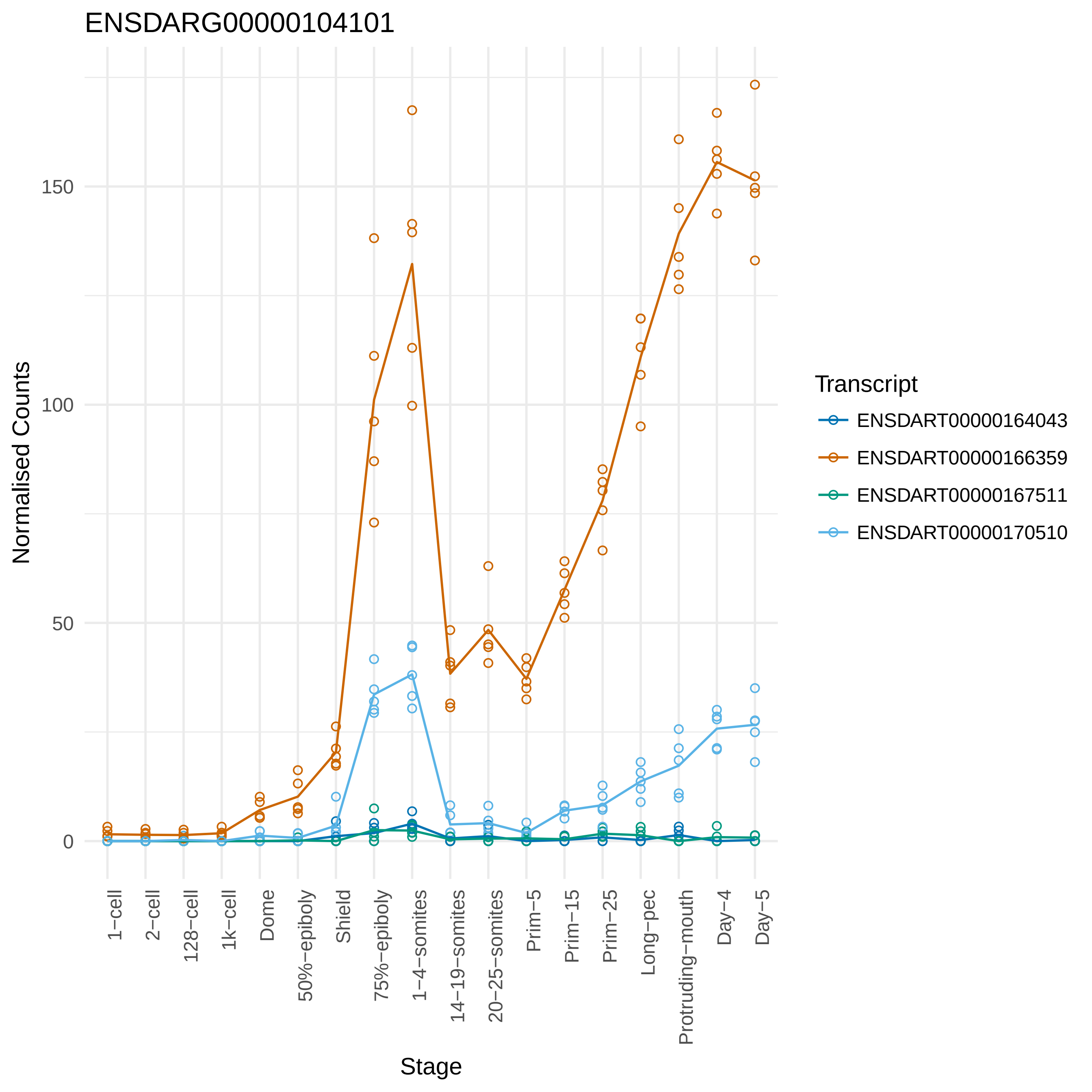

ENSDARG00000104101
Normalised Counts
150
100
50
0
1−cell
2−cell
128−cell
1k−cell
Dome
50%−epiboly
Shield
75%−epiboly
1−4−somites
14−19−somites
20−25−somites
Prim−5
Prim−15
Prim−25
Long−pec
Protruding−mouth
Day−4
Day−5
Stage
Transcript
ENSDART00000164043
ENSDART00000166359
ENSDART00000167511
ENSDART00000170510

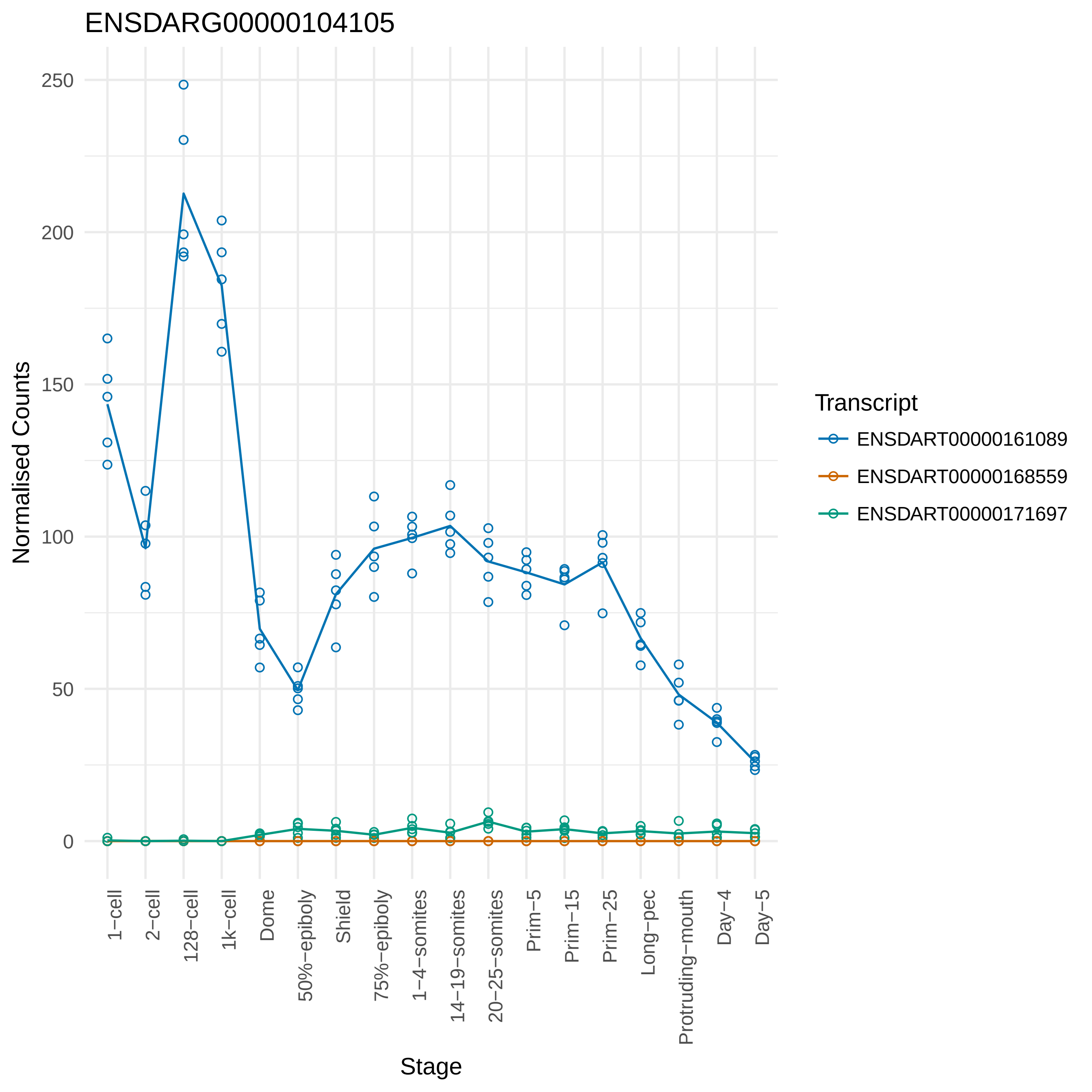
ENSDARG00000104105
Normalised Counts
250
200
150
100
50
0
1−cell
2−cell
128−cell
1k−cell
Dome
50%−epiboly
Shield
75%−epiboly
1−4−somites
14−19−somites
20−25−somites
Prim−5
Prim−15
Prim−25
Long−pec
Protruding−mouth
Day−4
Day−5
Stage
Transcript
ENSDART00000161089
ENSDART00000168559
ENSDART00000171697

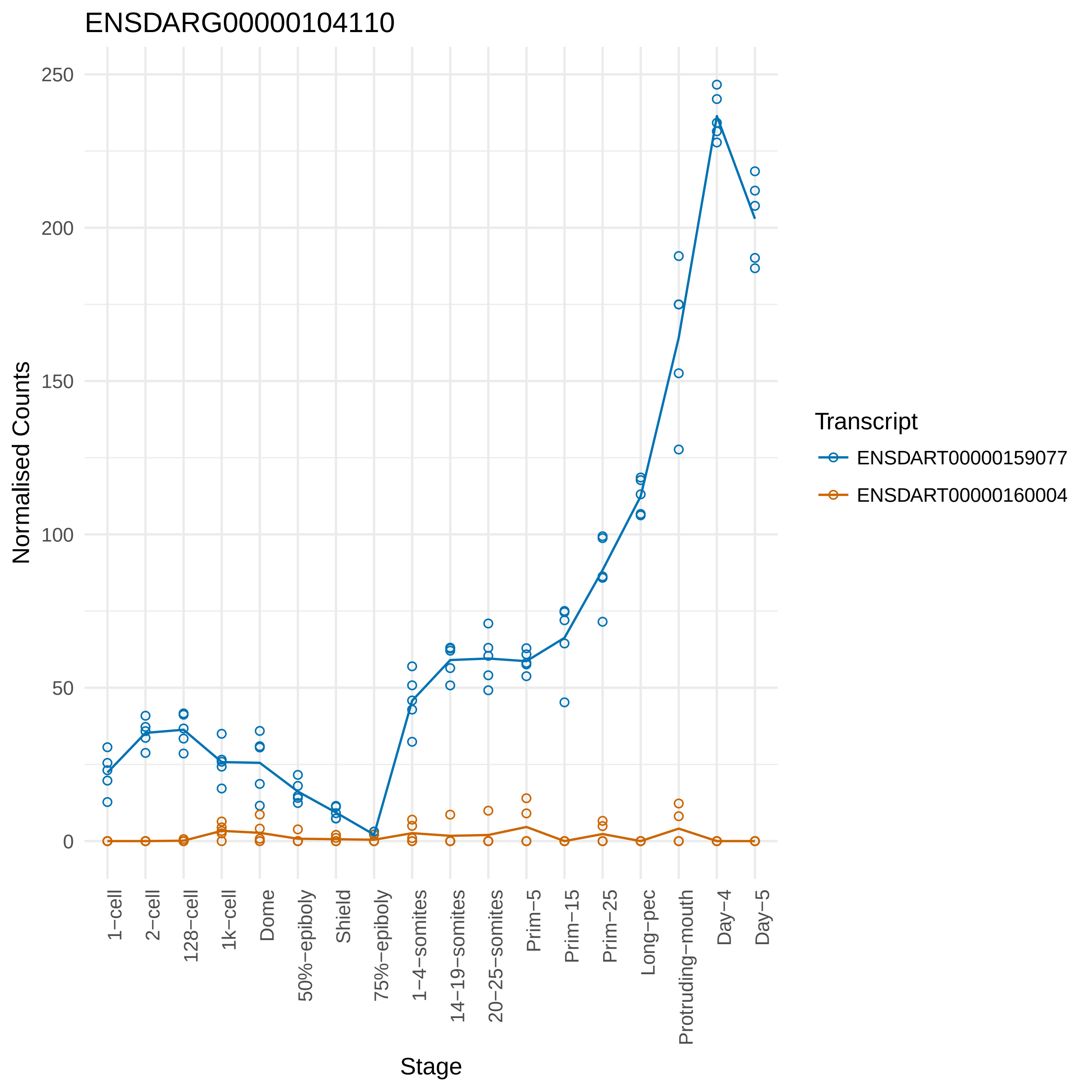

ENSDARG00000104110
Normalised Counts
250
200
150
100
50
0
1−cell
2−cell
128−cell
1k−cell
Dome
50%−epiboly
Shield
75%−epiboly
1−4−somites
14−19−somites
20−25−somites
Prim−5
Prim−15
Prim−25
Long−pec
Protruding−mouth
Day−4
Day−5
Stage
Transcript
ENSDART00000159077
ENSDART00000160004

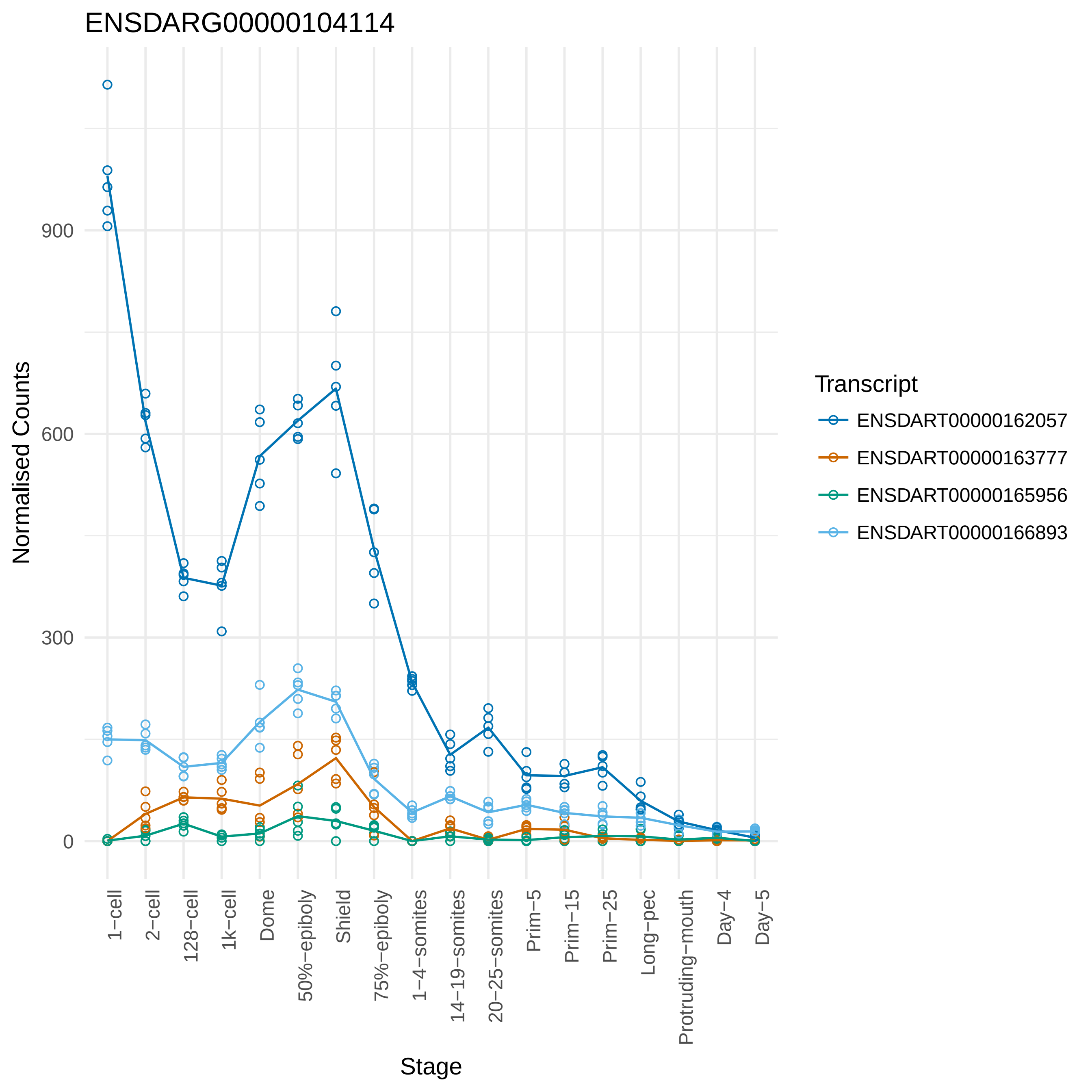
ENSDARG00000104114
Normalised Counts
900
600
300
0
1−cell
2−cell
128−cell
1k−cell
Dome
50%−epiboly
Shield
75%−epiboly
1−4−somites
14−19−somites
20−25−somites
Prim−5
Prim−15
Prim−25
Long−pec
Protruding−mouth
Day−4
Day−5
Stage
Transcript
ENSDART00000162057
ENSDART00000163777
ENSDART00000165956
ENSDART00000166893

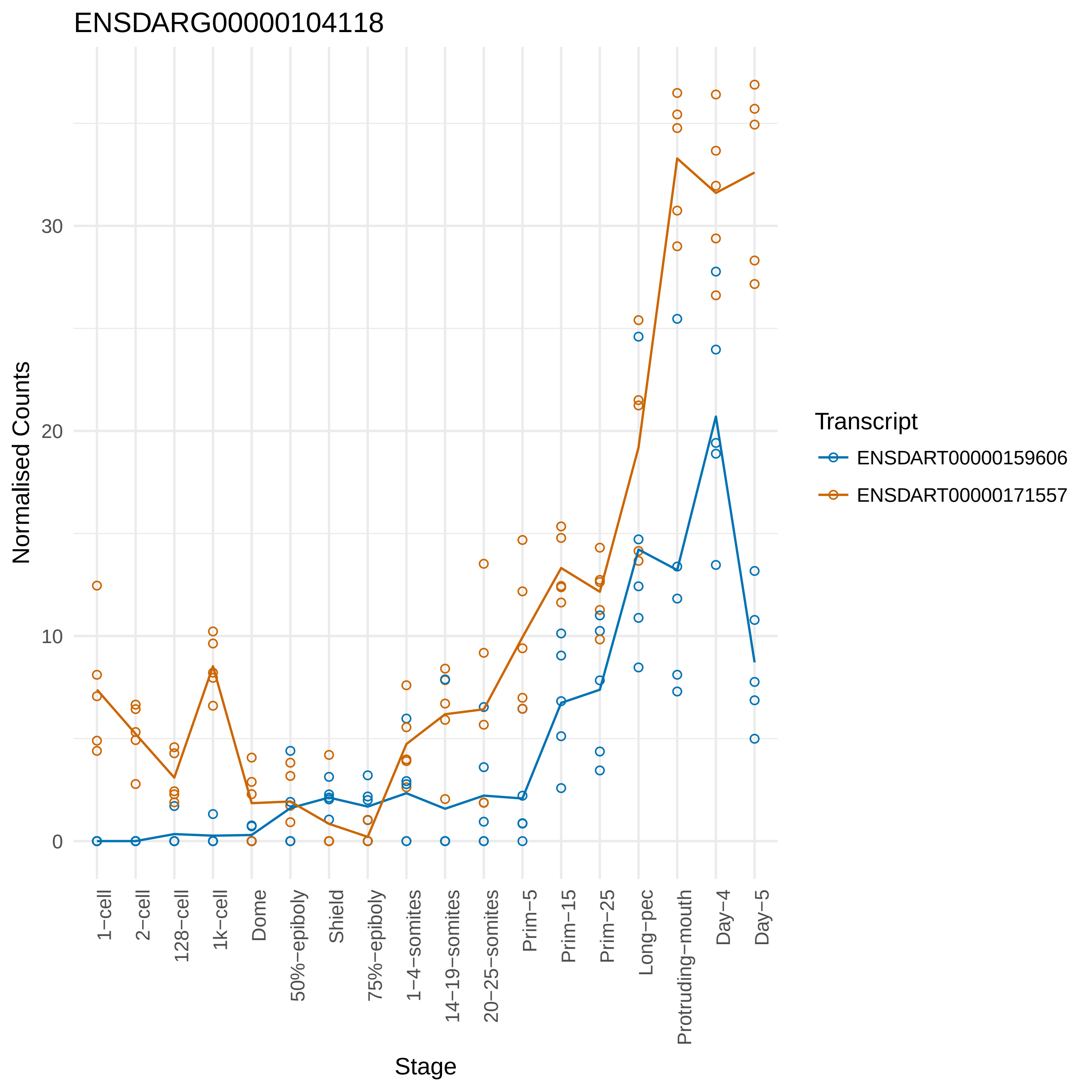
ENSDARG00000104118
Normalised Counts
0
10
20
30
1−cell
2−cell
128−cell
1k−cell
Dome
50%−epiboly
Shield
75%−epiboly
1−4−somites
14−19−somites
20−25−somites
Prim−5
Prim−15
Prim−25
Long−pec
Protruding−mouth
Day−4
Day−5
Stage
Transcript
ENSDART00000159606
ENSDART00000171557

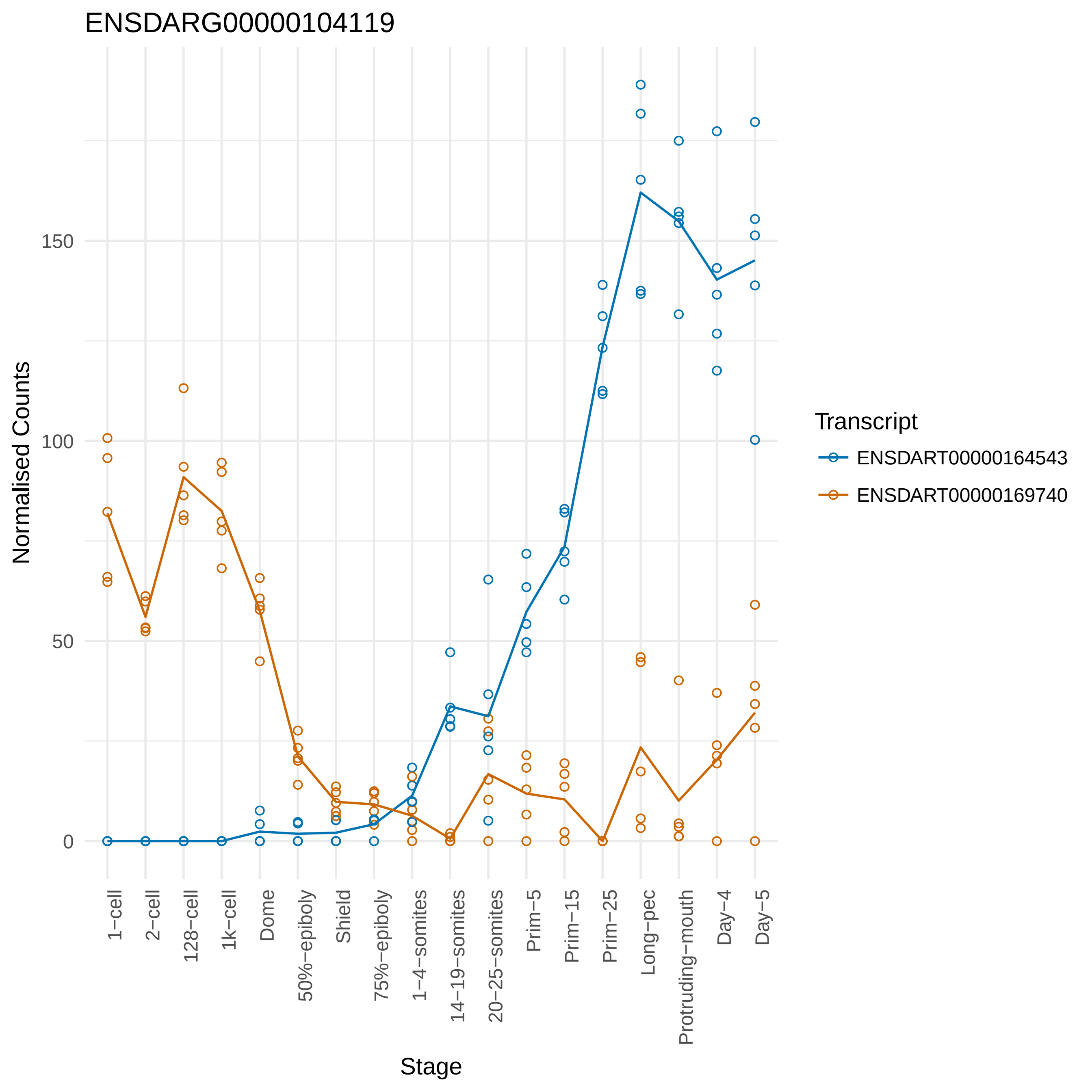
ENSDARG00000104119
Normalised Counts
0
50
100
150
Stage
1−cell
2−cell
128−cell
1k−cell
Dome
50%−epiboly
Shield
75%−epiboly
1−4−somites
14−19−somites
20−25−somites
Prim−5
Prim−15
Prim−25
Long−pec
Protruding−mouth
Day−4
Day−5
Transcript
ENSDART00000164543
ENSDART00000169740

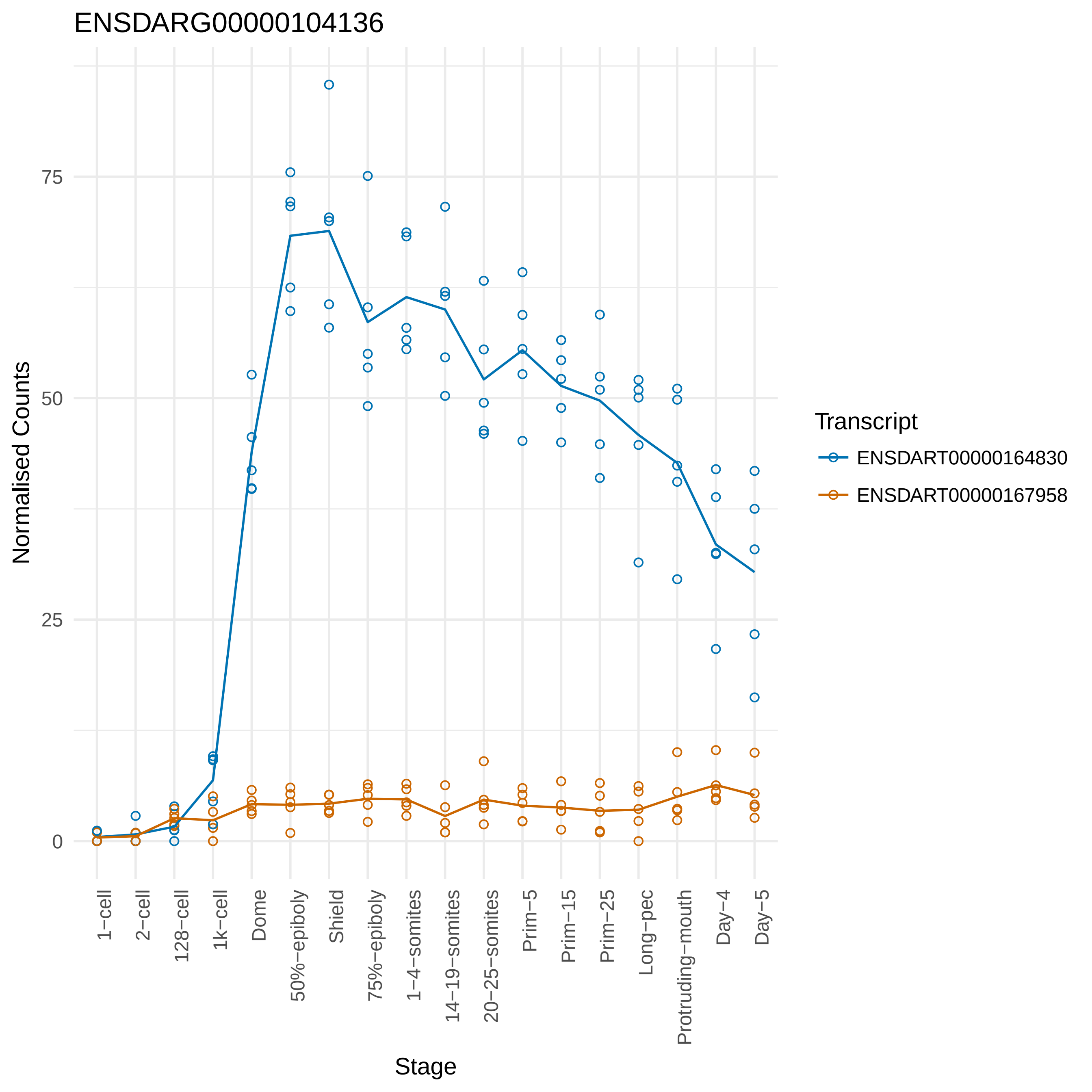

ENSDARG00000104136
Normalised Counts
75
50
25
0
1−cell
2−cell
128−cell
1k−cell
Dome
50%−epiboly
Shield
75%−epiboly
1−4−somites
14−19−somites
20−25−somites
Prim−5
Prim−15
Prim−25
Long−pec
Protruding−mouth
Day−4
Day−5
Stage
Transcript
ENSDART00000164830
ENSDART00000167958

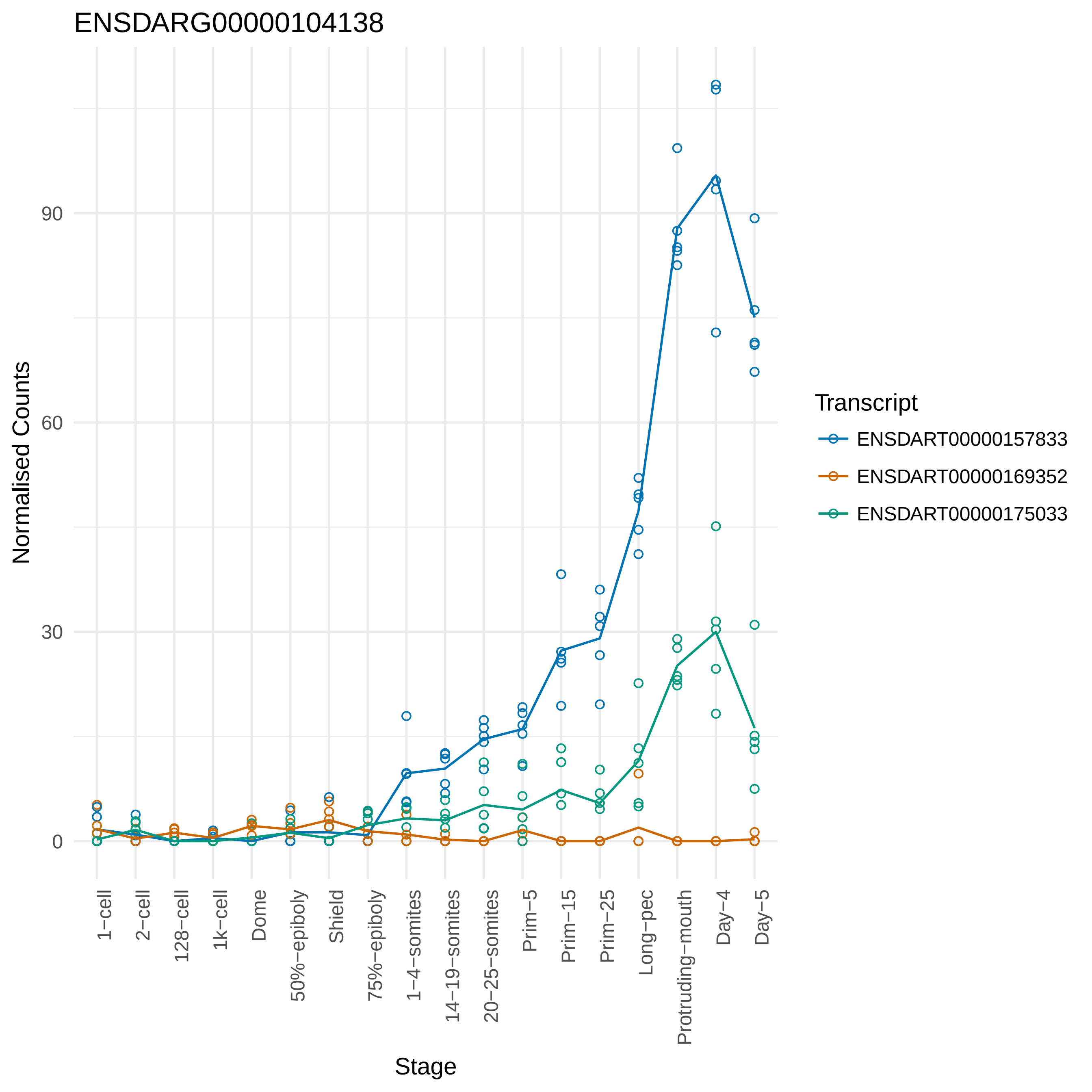

ENSDARG00000104138
Normalised Counts
0
30
60
90
Stage
1−cell
2−cell
128−cell
1k−cell
Dome
50%−epiboly
Shield
75%−epiboly
1−4−somites
14−19−somites
20−25−somites
Prim−5
Prim−15
Prim−25
Long−pec
Protruding−mouth
Day−4
Day−5
Transcript
ENSDART00000157833
ENSDART00000169352
ENSDART00000175033

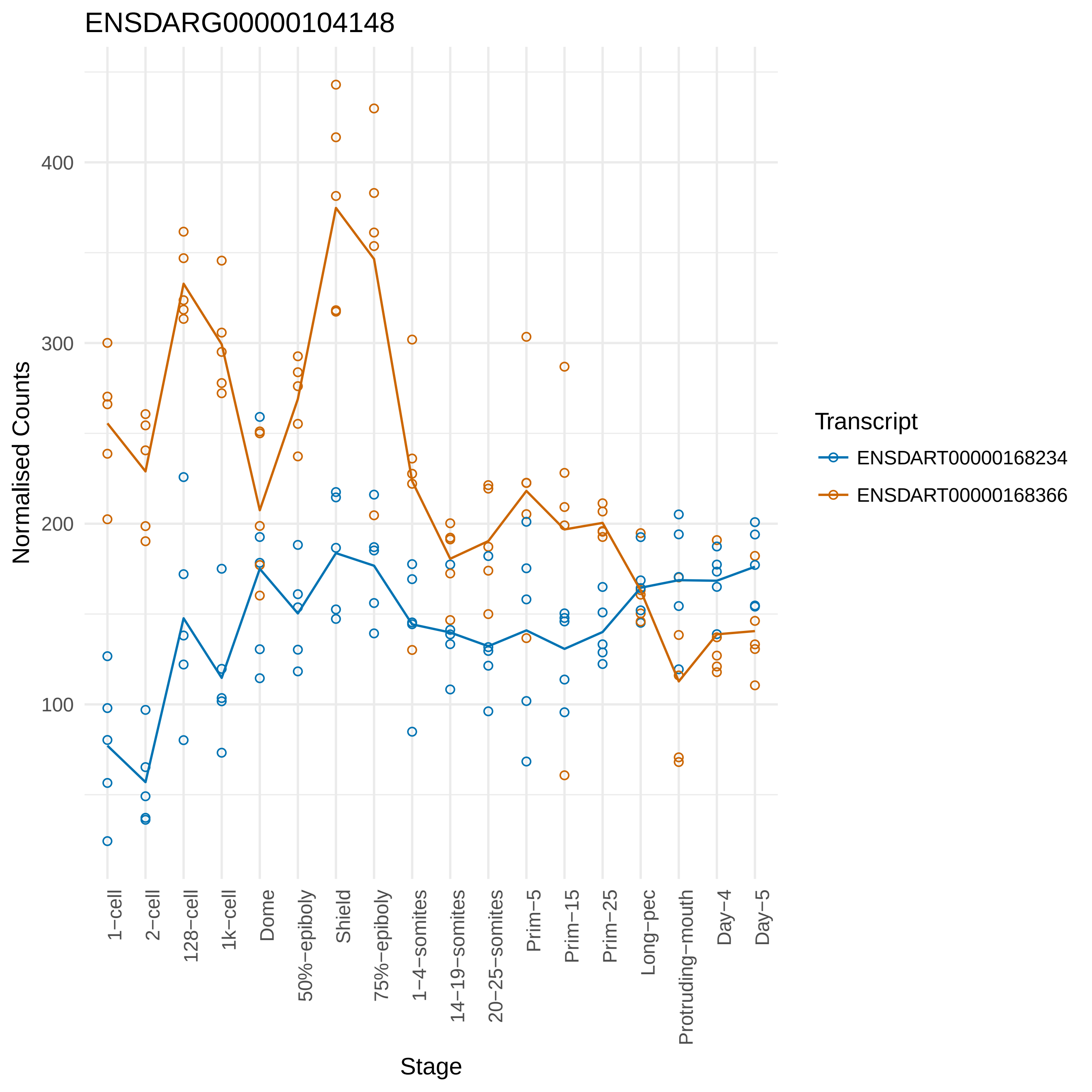
ENSDARG00000104148
Normalised Counts
400
300
200
100
1−cell
2−cell
128−cell
1k−cell
Dome
50%−epiboly
Shield
75%−epiboly
1−4−somites
14−19−somites
20−25−somites
Prim−5
Prim−15
Prim−25
Long−pec
Protruding−mouth
Day−4
Day−5
Stage
Transcript
ENSDART00000168234
ENSDART00000168366

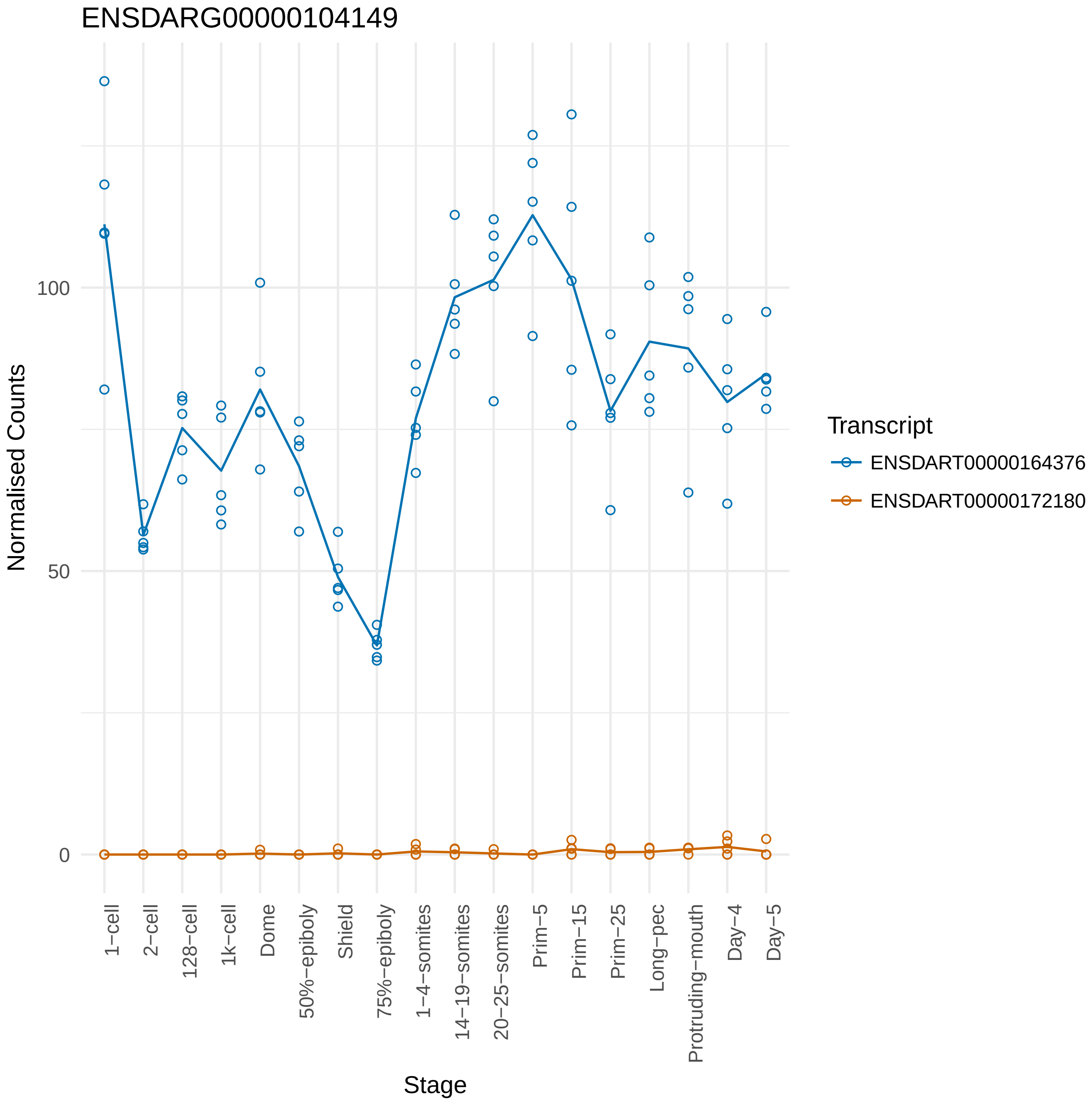

ENSDARG00000104149
Normalised Counts
0
50
100
1−cell
2−cell
128−cell
1k−cell
Dome
50%−epiboly
Shield
75%−epiboly
1−4−somites
14−19−somites
20−25−somites
Prim−5
Prim−15
Prim−25
Long−pec
Protruding−mouth
Day−4
Day−5
Stage
Transcript
ENSDART00000164376
ENSDART00000172180

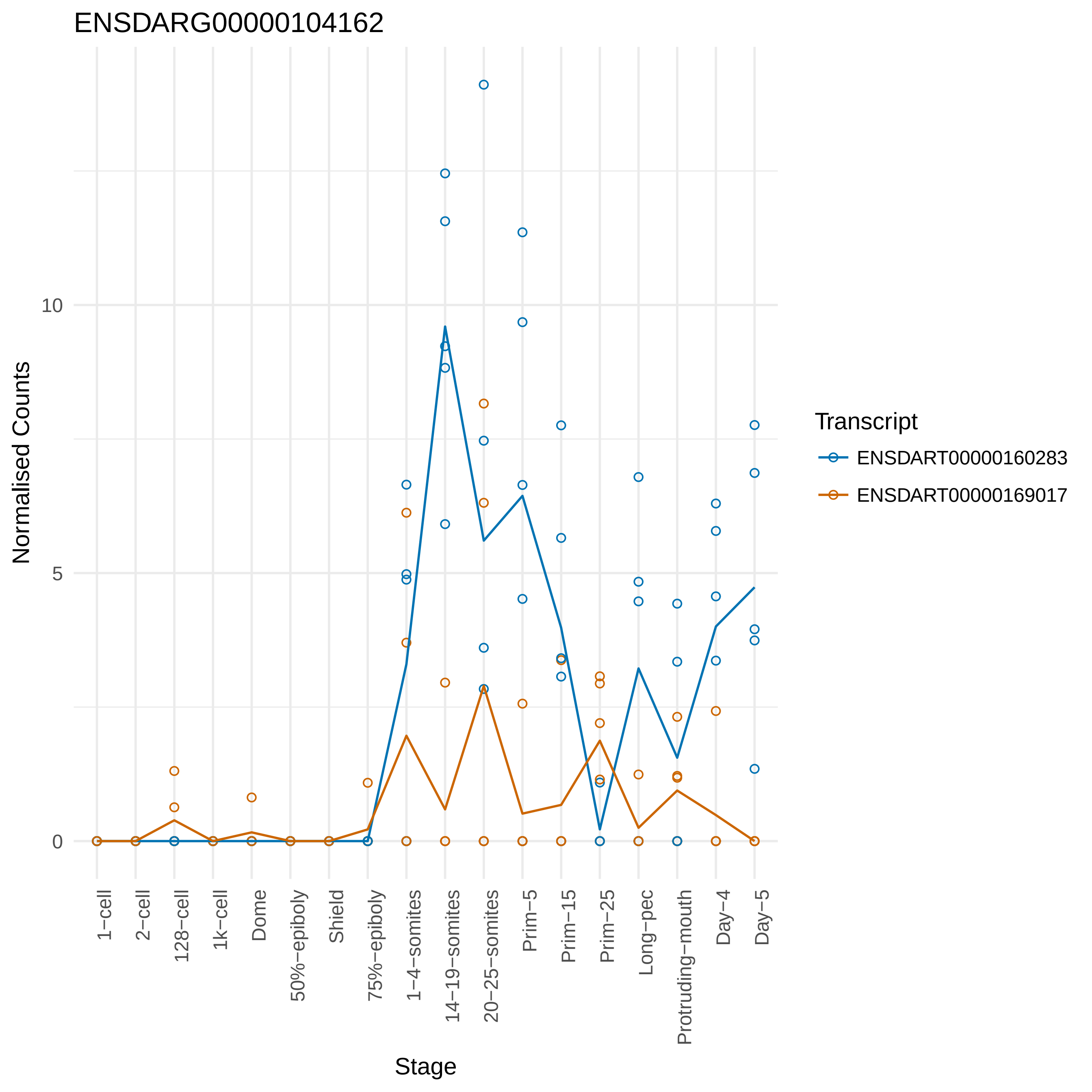

ENSDARG00000104162
Normalised Counts
10
5
0
1−cell
2−cell
128−cell
1k−cell
Dome
50%−epiboly
Shield
75%−epiboly
1−4−somites
14−19−somites
20−25−somites
Prim−5
Prim−15
Prim−25
Long−pec
Protruding−mouth
Day−4
Day−5
Stage
Transcript
ENSDART00000160283
ENSDART00000169017

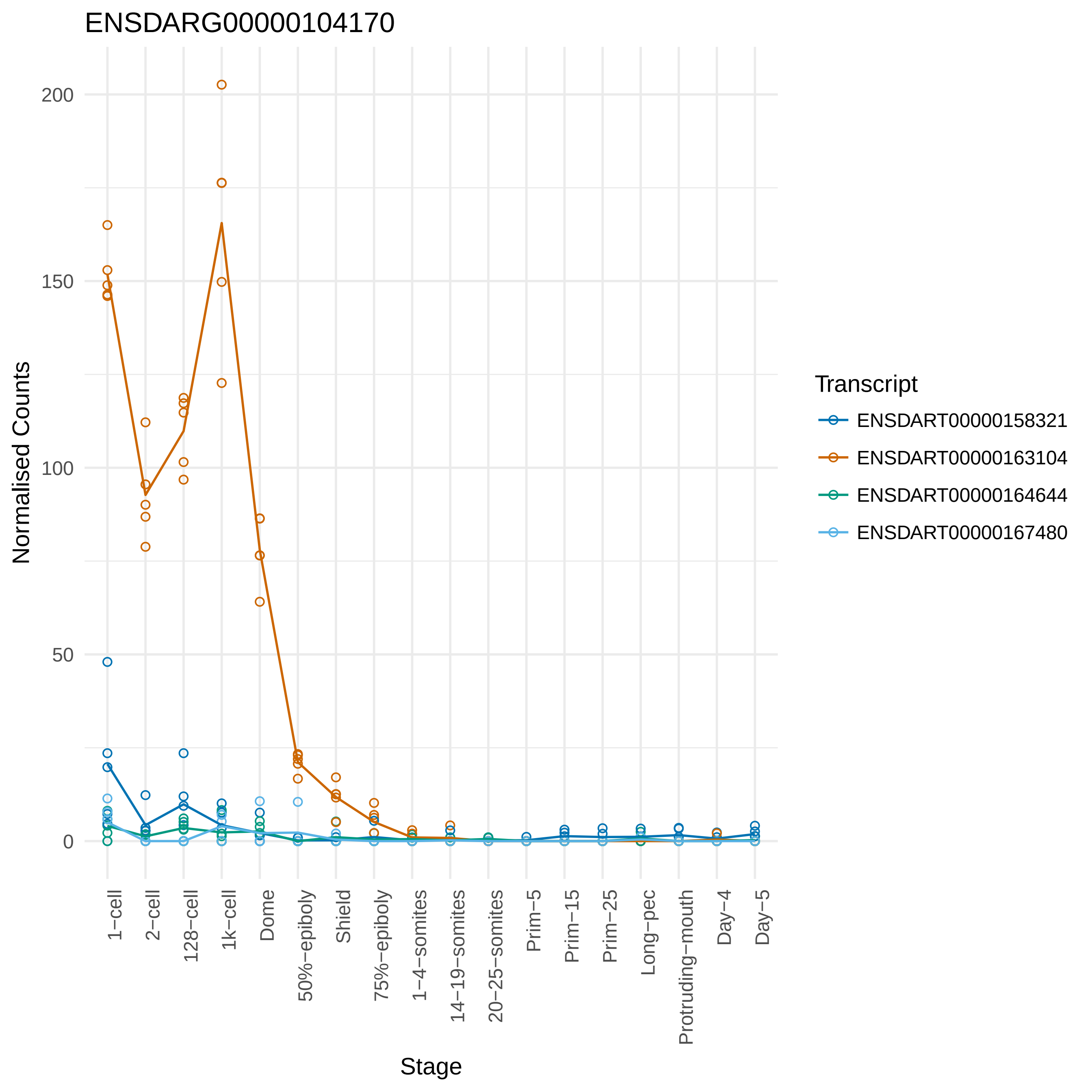

ENSDARG00000104170
Normalised Counts
200
150
100
50
0
1−cell
2−cell
128−cell
1k−cell
Dome
50%−epiboly
Shield
75%−epiboly
1−4−somites
14−19−somites
20−25−somites
Prim−5
Prim−15
Prim−25
Long−pec
Protruding−mouth
Day−4
Day−5
Stage
Transcript
ENSDART00000158321
ENSDART00000163104
ENSDART00000164644
ENSDART00000167480

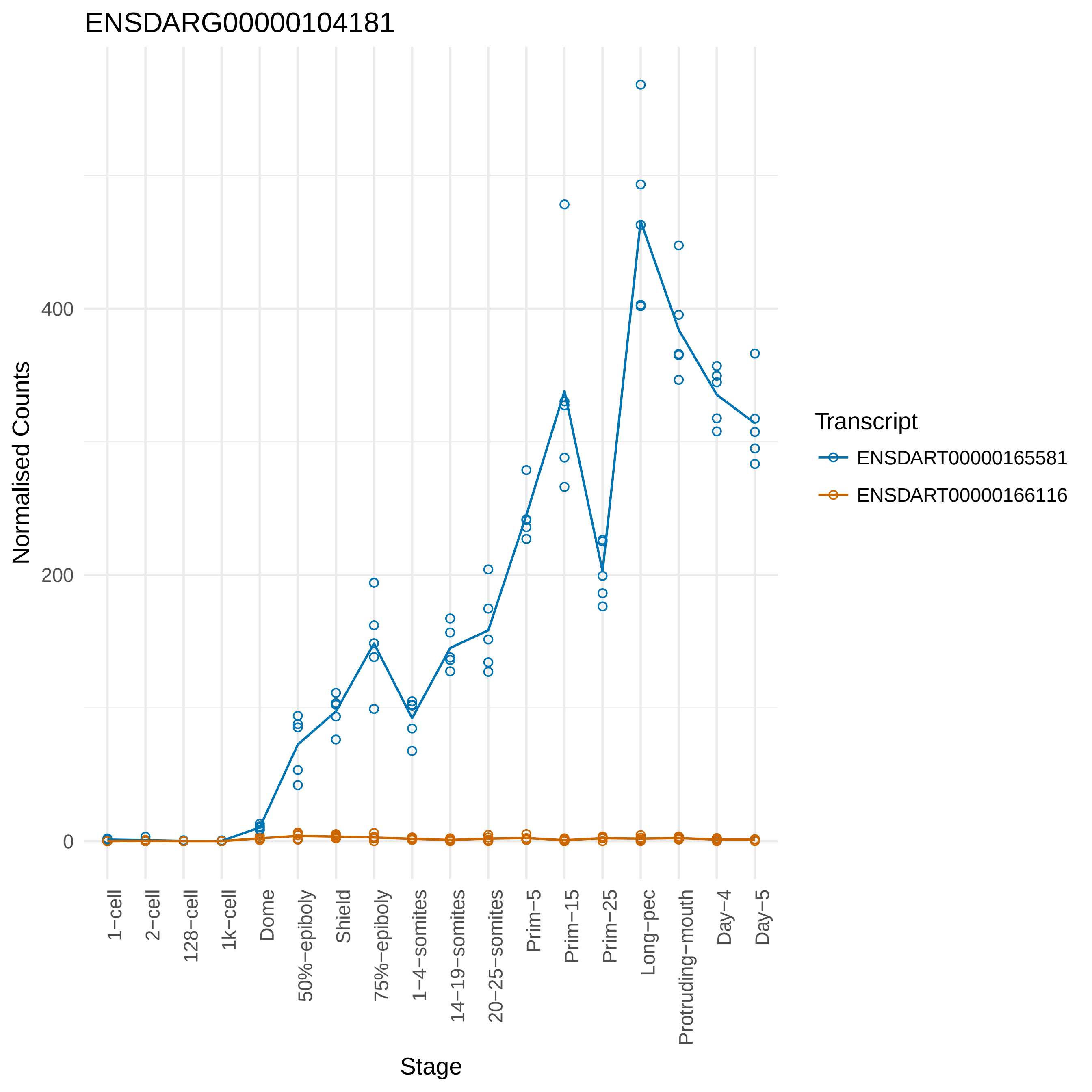
ENSDARG00000104181
Normalised Counts
400
200
0
1−cell
2−cell
128−cell
1k−cell
Dome
50%−epiboly
Shield
75%−epiboly
1−4−somites
14−19−somites
20−25−somites
Prim−5
Prim−15
Prim−25
Long−pec
Protruding−mouth
Day−4
Day−5
Stage
Transcript
ENSDART00000165581
ENSDART00000166116

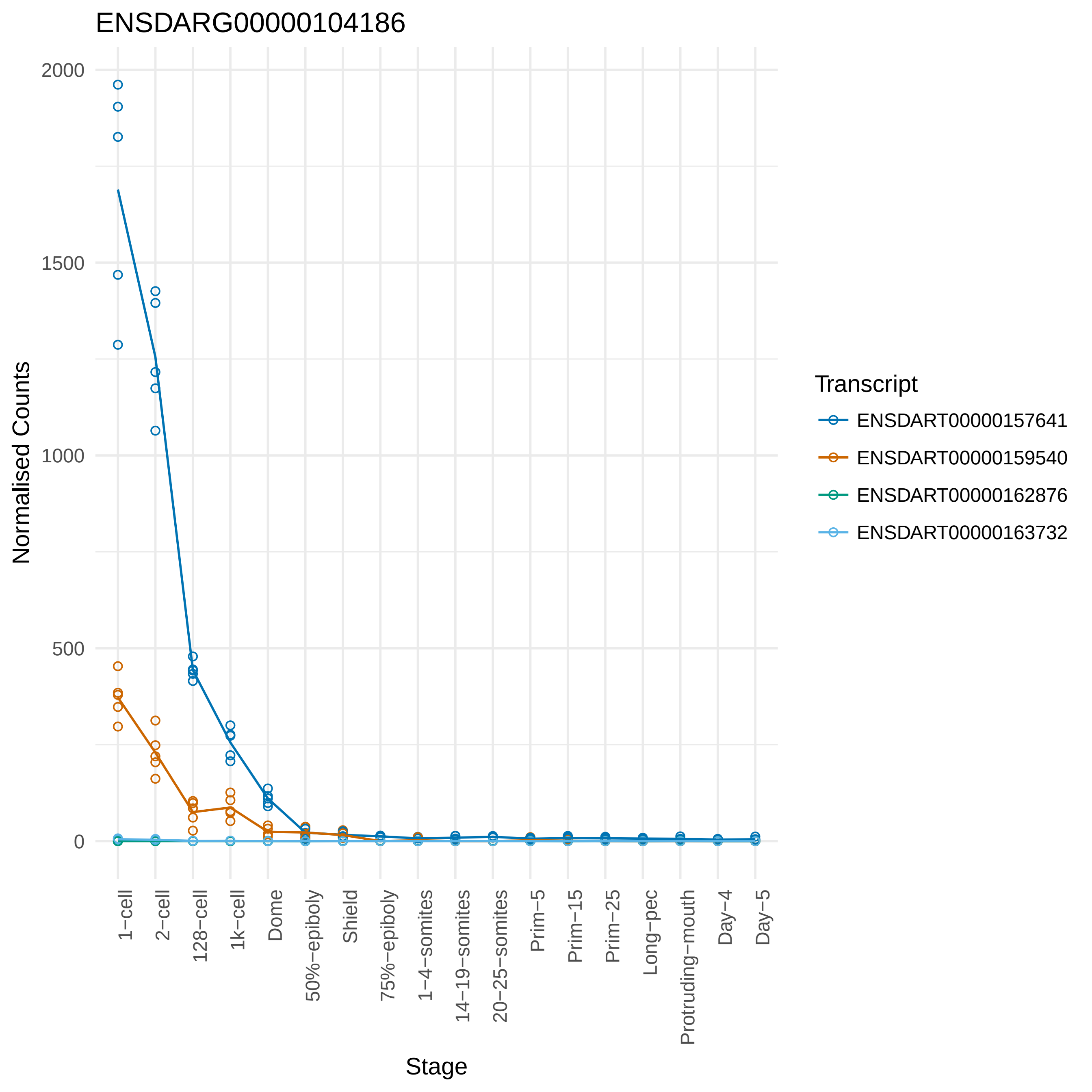

ENSDARG00000104186
Normalised Counts
2000
1500
1000
500
0
1−cell
2−cell
128−cell
1k−cell
Dome
50%−epiboly
Shield
75%−epiboly
1−4−somites
14−19−somites
20−25−somites
Prim−5
Prim−15
Prim−25
Long−pec
Protruding−mouth
Day−4
Day−5
Stage
Transcript
ENSDART00000157641
ENSDART00000159540
ENSDART00000162876
ENSDART00000163732

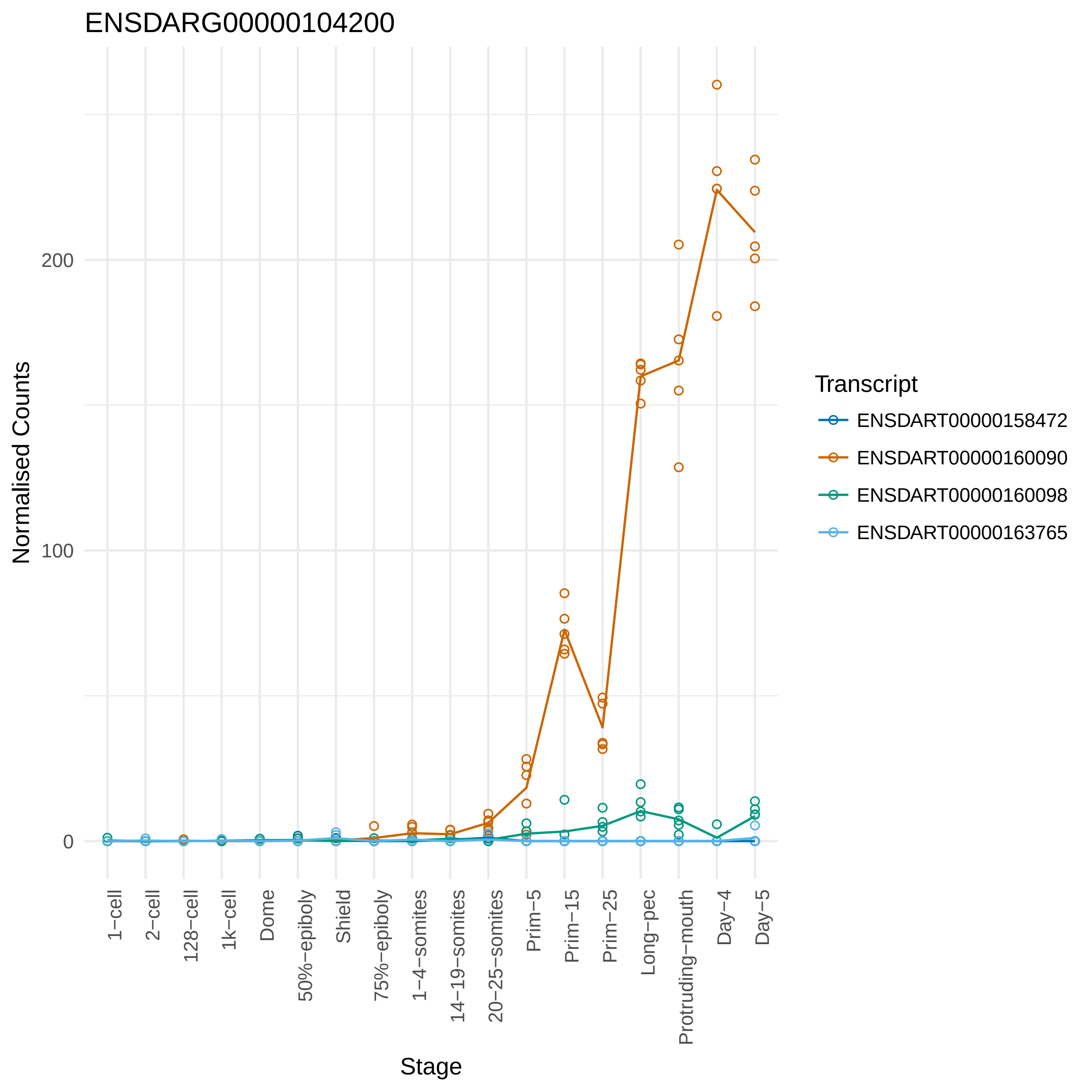

ENSDARG00000104200
Normalised Counts
0
100
200
1−cell
2−cell
128−cell
1k−cell
Dome
50%−epiboly
Shield
75%−epiboly
1−4−somites
14−19−somites
20−25−somites
Prim−5
Prim−15
Prim−25
Long−pec
Protruding−mouth
Day−4
Day−5
Stage
Transcript
ENSDART00000158472
ENSDART00000160090
ENSDART00000160098
ENSDART00000163765

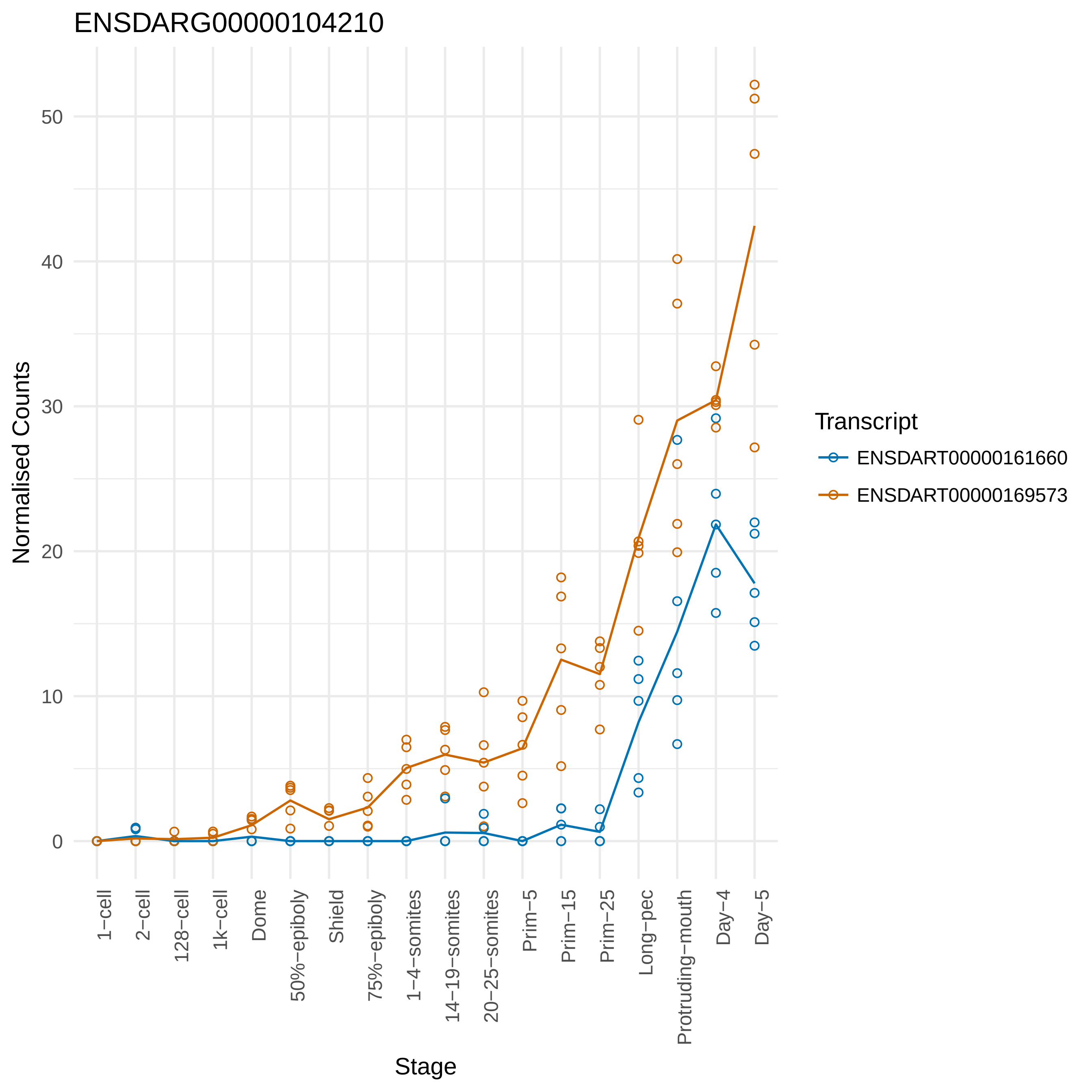

ENSDARG00000104210
Normalised Counts
0
10
20
30
40
50
1−cell
2−cell
128−cell
1k−cell
Dome
50%−epiboly
Shield
75%−epiboly
1−4−somites
14−19−somites
20−25−somites
Prim−5
Prim−15
Prim−25
Long−pec
Protruding−mouth
Day−4
Day−5
Stage
Transcript
ENSDART00000161660
ENSDART00000169573

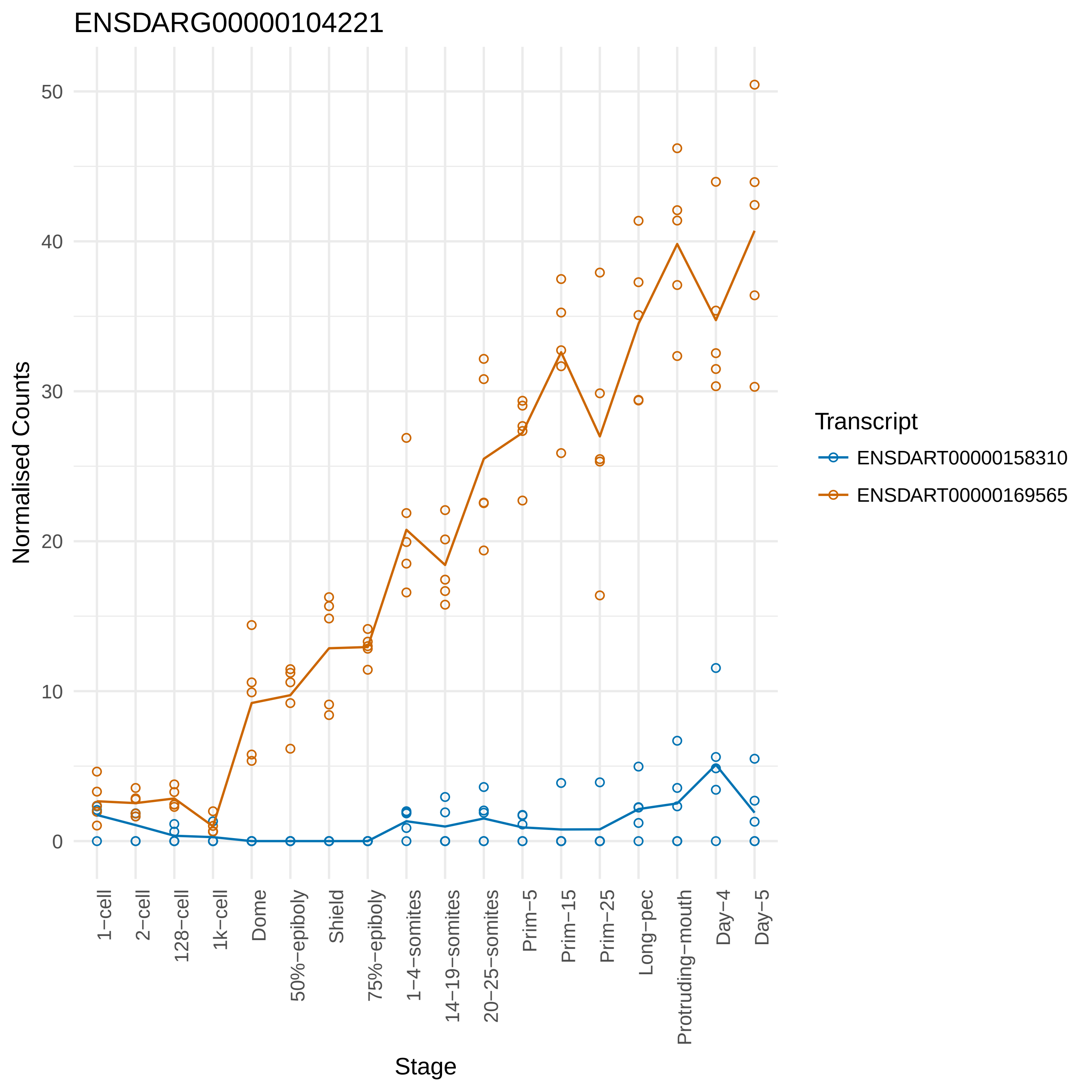

ENSDARG00000104221
Normalised Counts
0
10
20
30
40
50
1−cell
2−cell
128−cell
1k−cell
Dome
50%−epiboly
Shield
75%−epiboly
1−4−somites
14−19−somites
20−25−somites
Prim−5
Prim−15
Prim−25
Long−pec
Protruding−mouth
Day−4
Day−5
Stage
Transcript
ENSDART00000158310
ENSDART00000169565

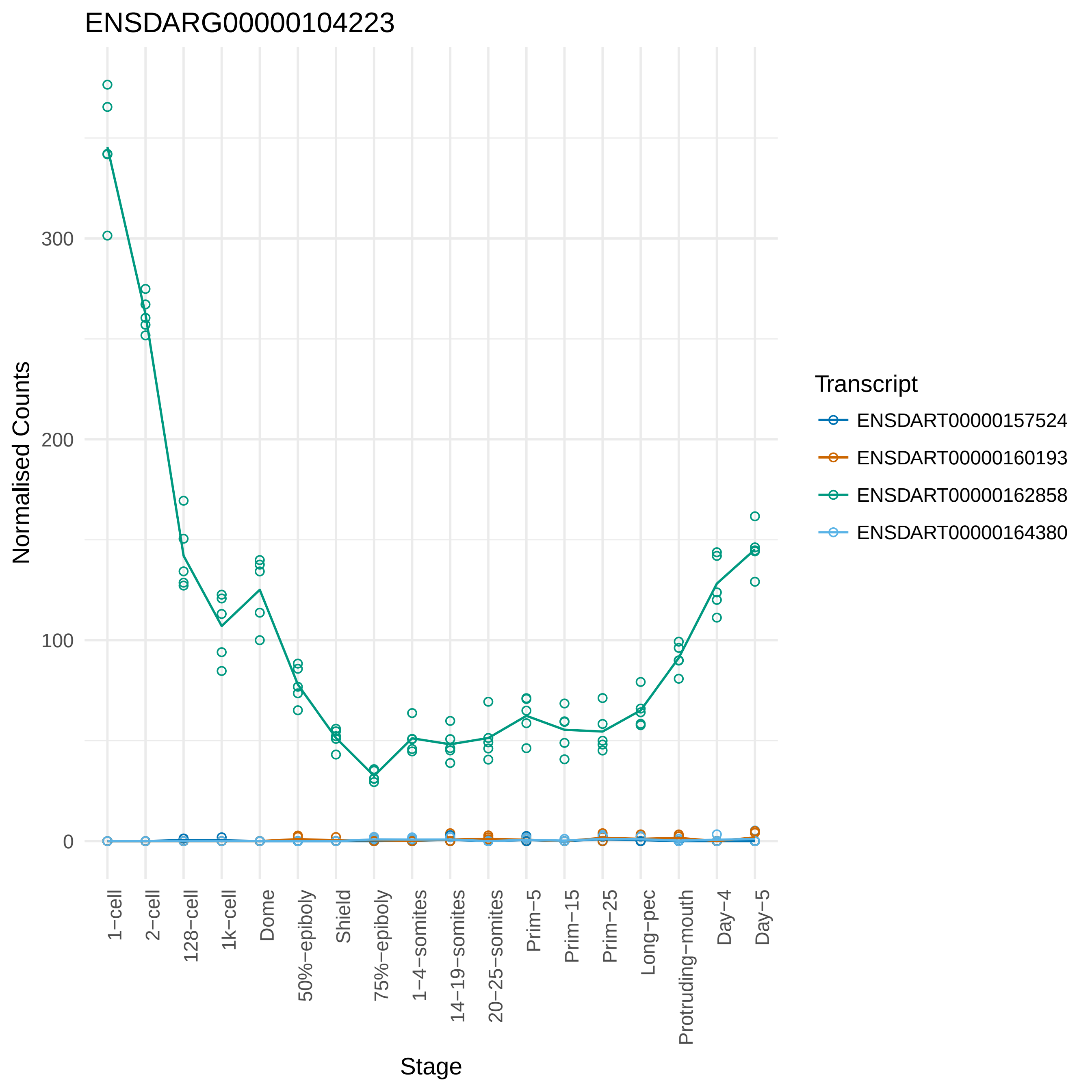

ENSDARG00000104223
Normalised Counts
300
200
100
0
1−cell
2−cell
128−cell
1k−cell
Dome
50%−epiboly
Shield
75%−epiboly
1−4−somites
14−19−somites
20−25−somites
Prim−5
Prim−15
Prim−25
Long−pec
Protruding−mouth
Day−4
Day−5
Stage
Transcript
ENSDART00000157524
ENSDART00000160193
ENSDART00000162858
ENSDART00000164380

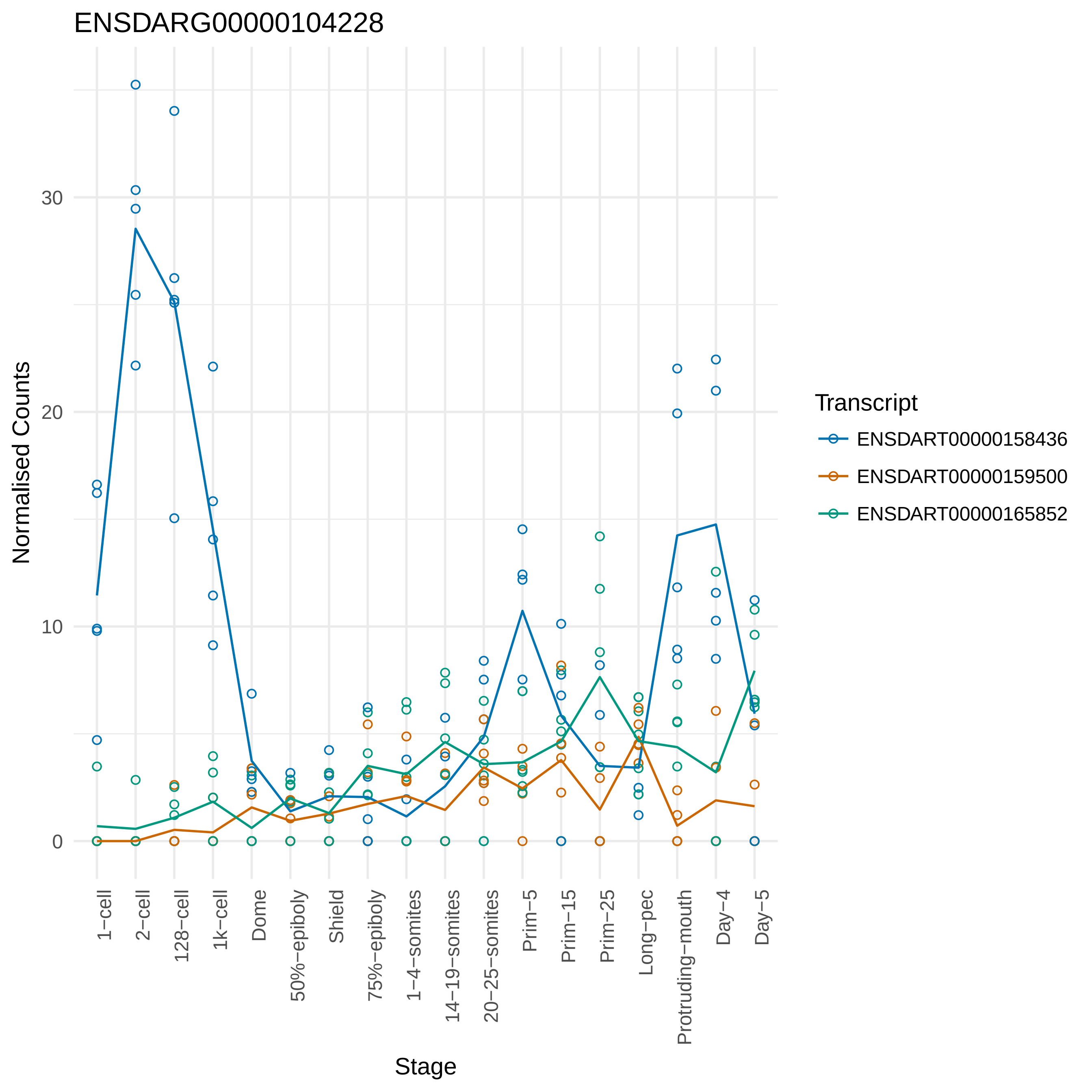
ENSDARG00000104228
Normalised Counts
0
10
20
30
1−cell
2−cell
128−cell
1k−cell
Dome
50%−epiboly
Shield
75%−epiboly
1−4−somites
14−19−somites
20−25−somites
Prim−5
Prim−15
Prim−25
Long−pec
Protruding−mouth
Day−4
Day−5
Stage
Transcript
ENSDART00000158436
ENSDART00000159500
ENSDART00000165852

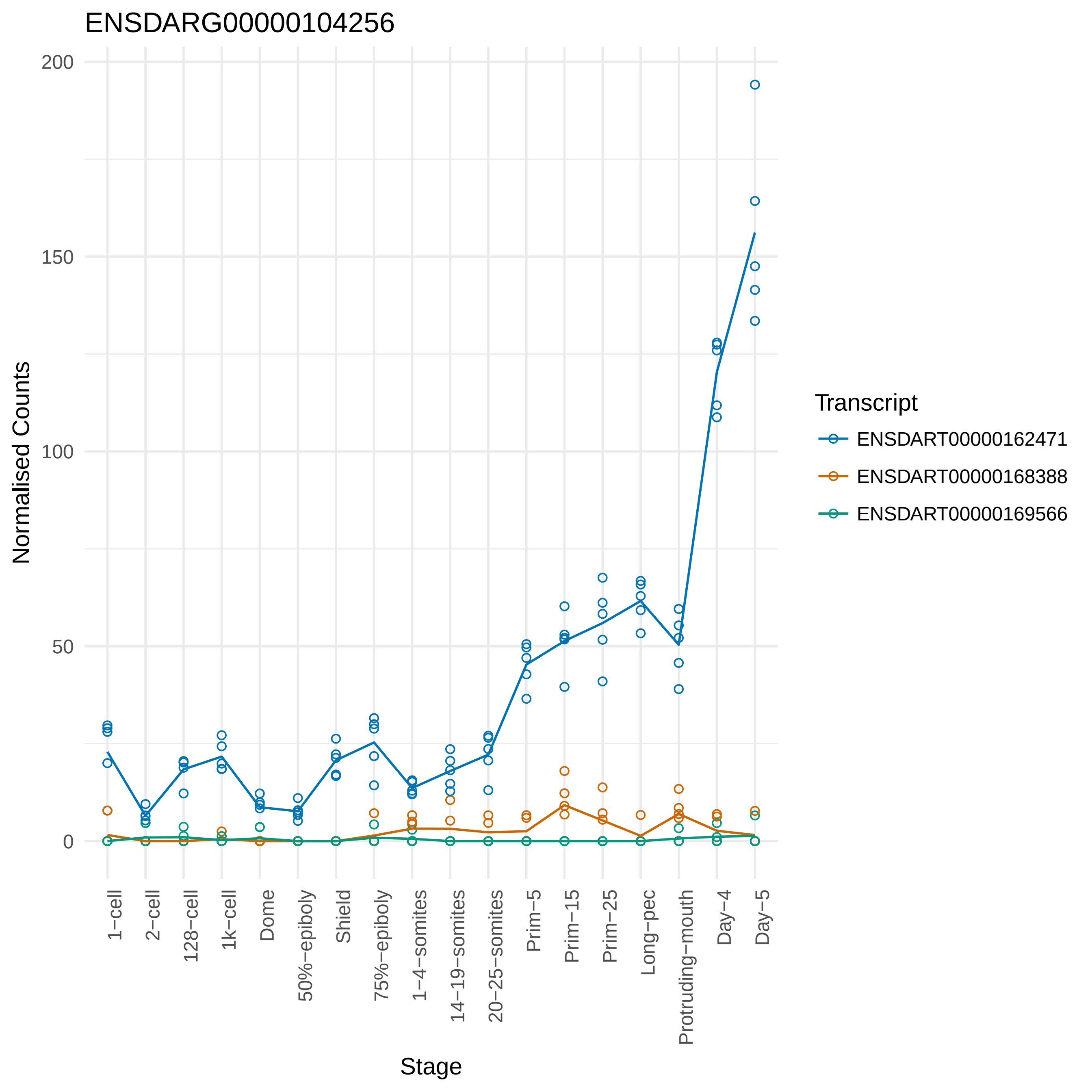

ENSDARG00000104256
Normalised Counts
200
150
100
50
0
1−cell
2−cell
128−cell
1k−cell
Dome
50%−epiboly
Shield
75%−epiboly
1−4−somites
14−19−somites
20−25−somites
Prim−5
Prim−15
Prim−25
Long−pec
Protruding−mouth
Day−4
Day−5
Stage
Transcript
ENSDART00000162471
ENSDART00000168388
ENSDART00000169566

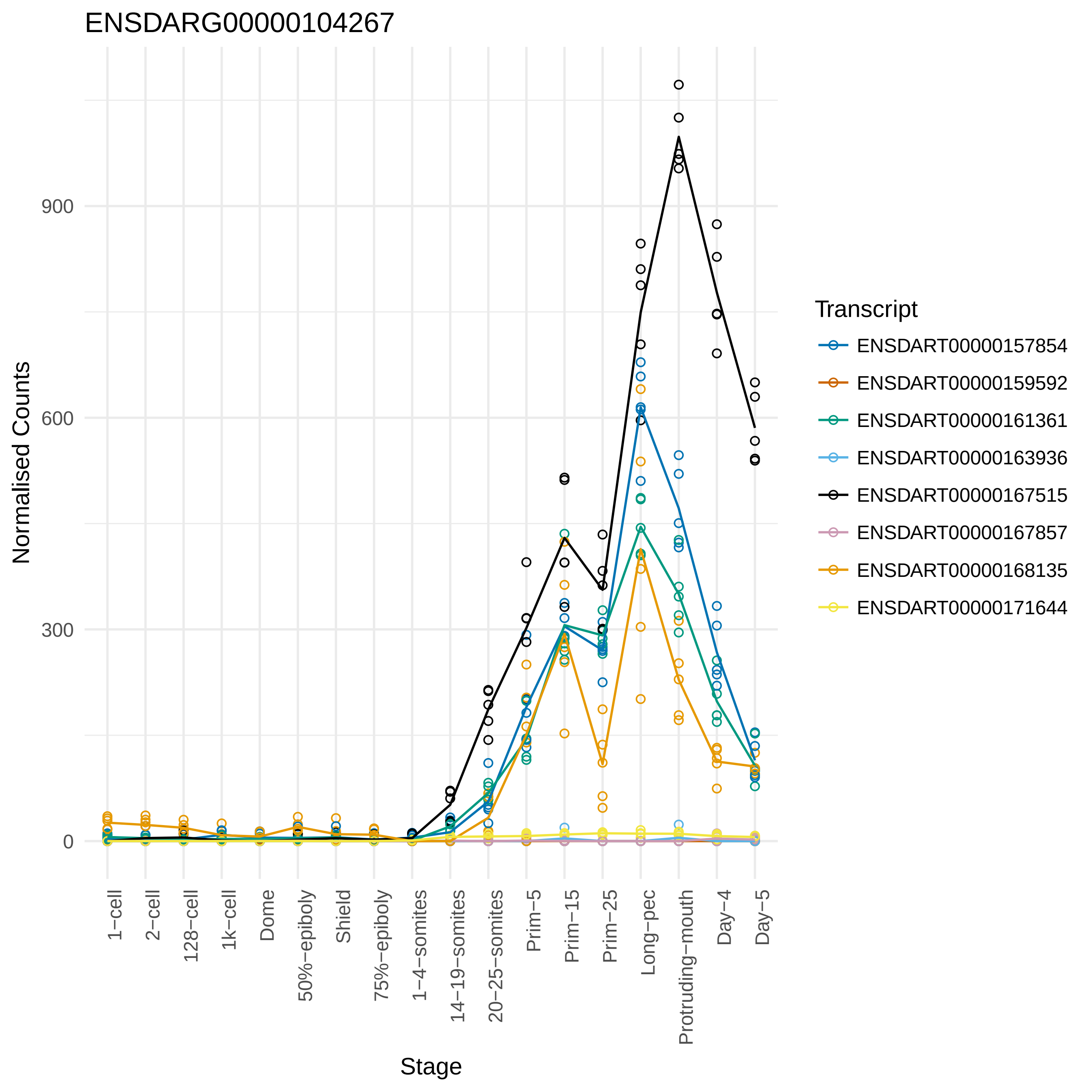

ENSDARG00000104267
Normalised Counts
0
300
600
900
1−cell
2−cell
128−cell
1k−cell
Dome
50%−epiboly
Shield
75%−epiboly
1−4−somites
14−19−somites
20−25−somites
Prim−5
Prim−15
Prim−25
Long−pec
Protruding−mouth
Day−4
Day−5
Stage
Transcript
ENSDART00000157854
ENSDART00000159592
ENSDART00000161361
ENSDART00000163936
ENSDART00000167515
ENSDART00000167857
ENSDART00000168135
ENSDART00000171644

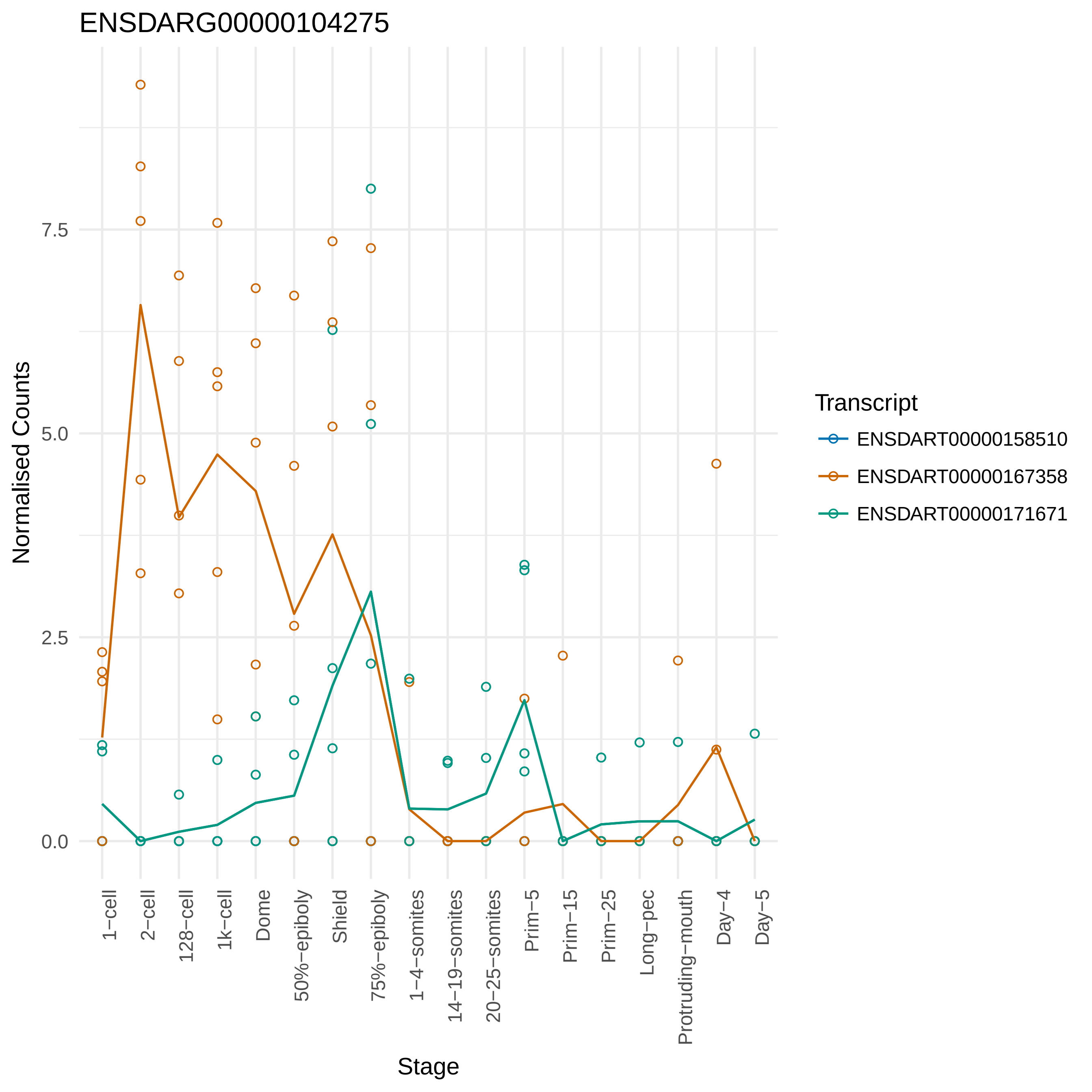
ENSDARG00000104275
Normalised Counts
0.0
2.5
5.0
7.5
Stage
1−cell
2−cell
128−cell
1k−cell
Dome
50%−epiboly
Shield
75%−epiboly
1−4−somites
14−19−somites
20−25−somites
Prim−5
Prim−15
Prim−25
Long−pec
Protruding−mouth
Day−4
Day−5
Transcript
ENSDART00000158510
ENSDART00000167358
ENSDART00000171671

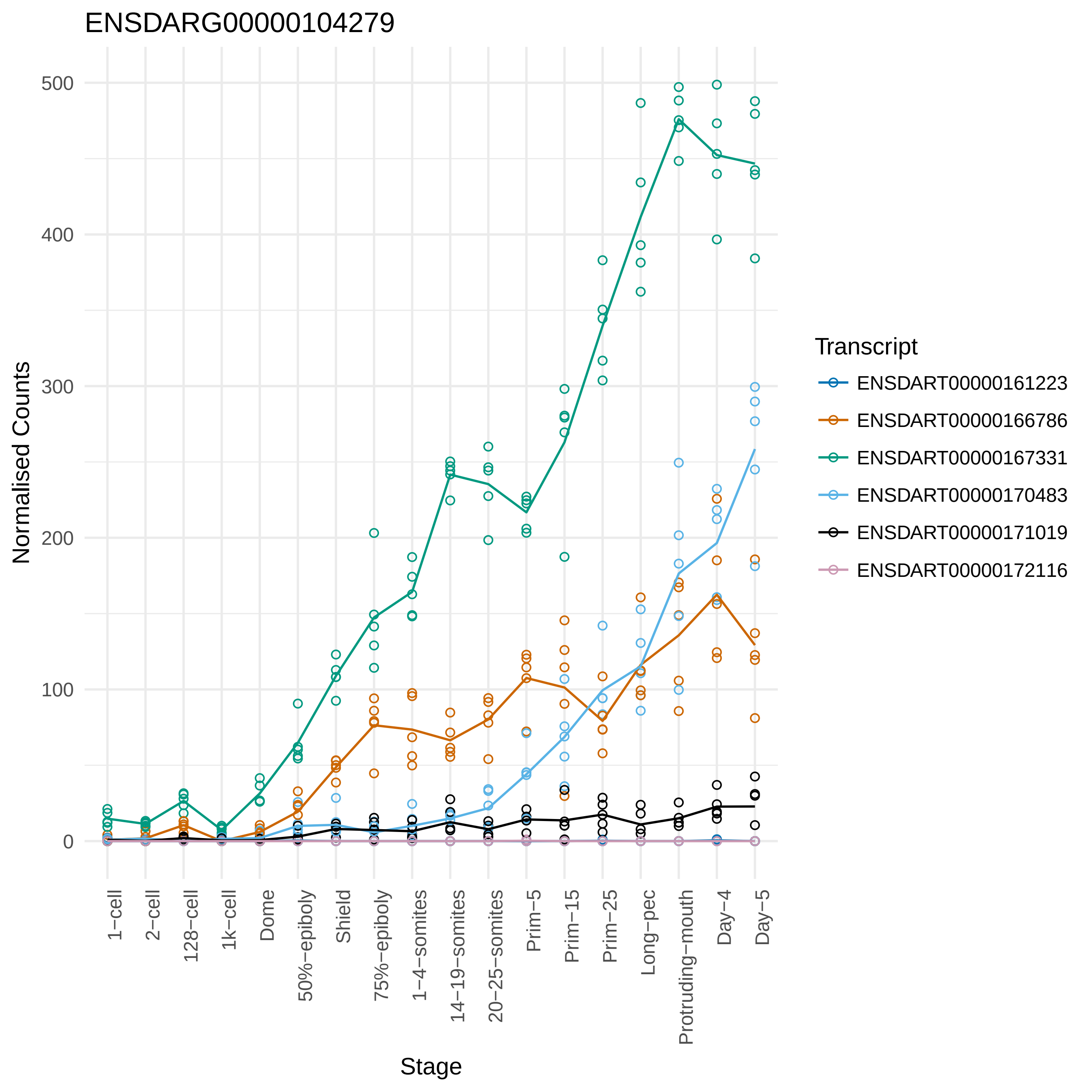
ENSDARG00000104279
Normalised Counts
500
400
300
200
100
0
1−cell
2−cell
128−cell
1k−cell
Dome
50%−epiboly
Shield
75%−epiboly
1−4−somites
14−19−somites
20−25−somites
Prim−5
Prim−15
Prim−25
Long−pec
Protruding−mouth
Day−4
Day−5
Stage
Transcript
ENSDART00000161223
ENSDART00000166786
ENSDART00000167331
ENSDART00000170483
ENSDART00000171019
ENSDART00000172116

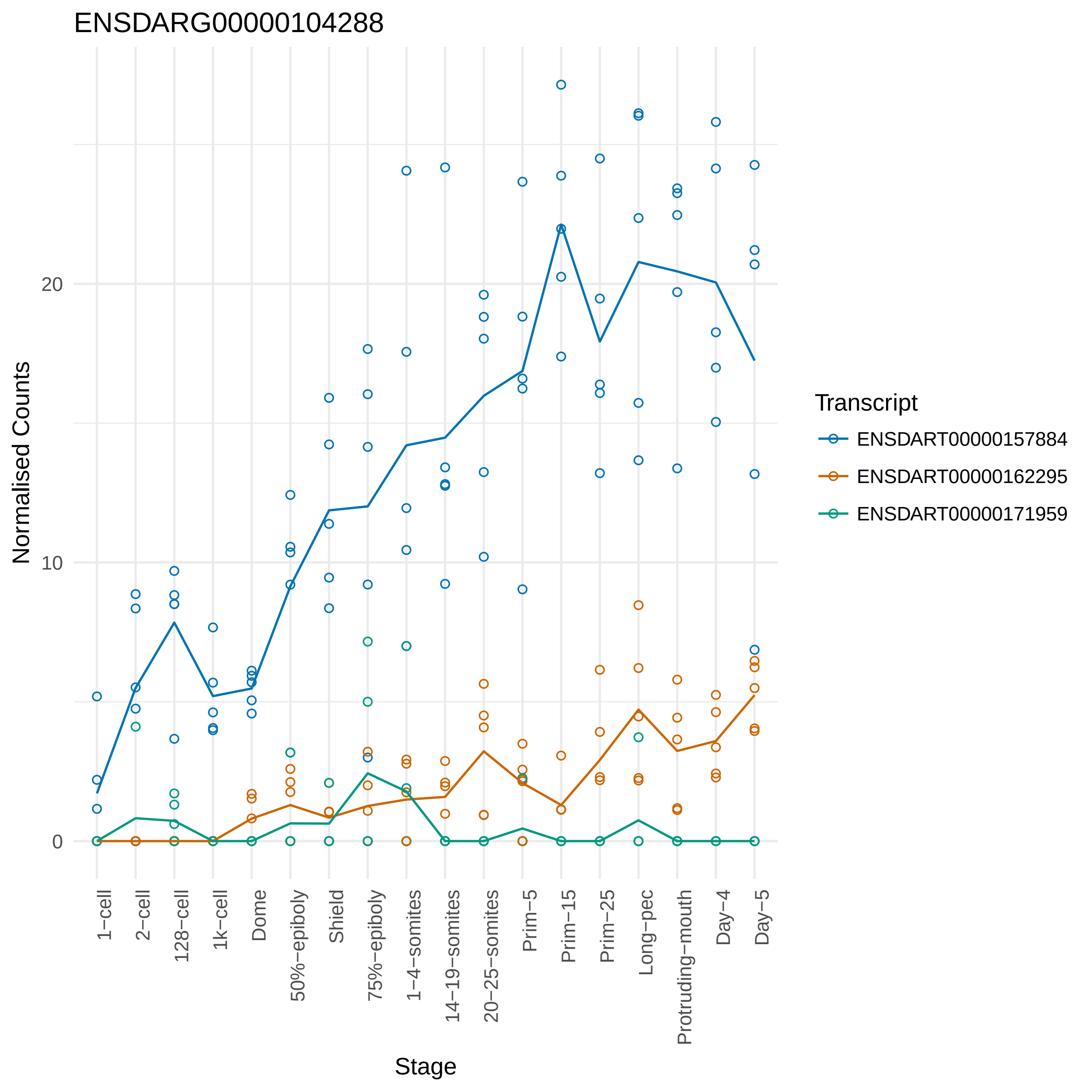

ENSDARG00000104288
Normalised Counts
20
10
0
1−cell
2−cell
128−cell
1k−cell
Dome
50%−epiboly
Shield
75%−epiboly
1−4−somites
14−19−somites
20−25−somites
Prim−5
Prim−15
Prim−25
Long−pec
Protruding−mouth
Day−4
Day−5
Stage
Transcript
ENSDART00000157884
ENSDART00000162295
ENSDART00000171959

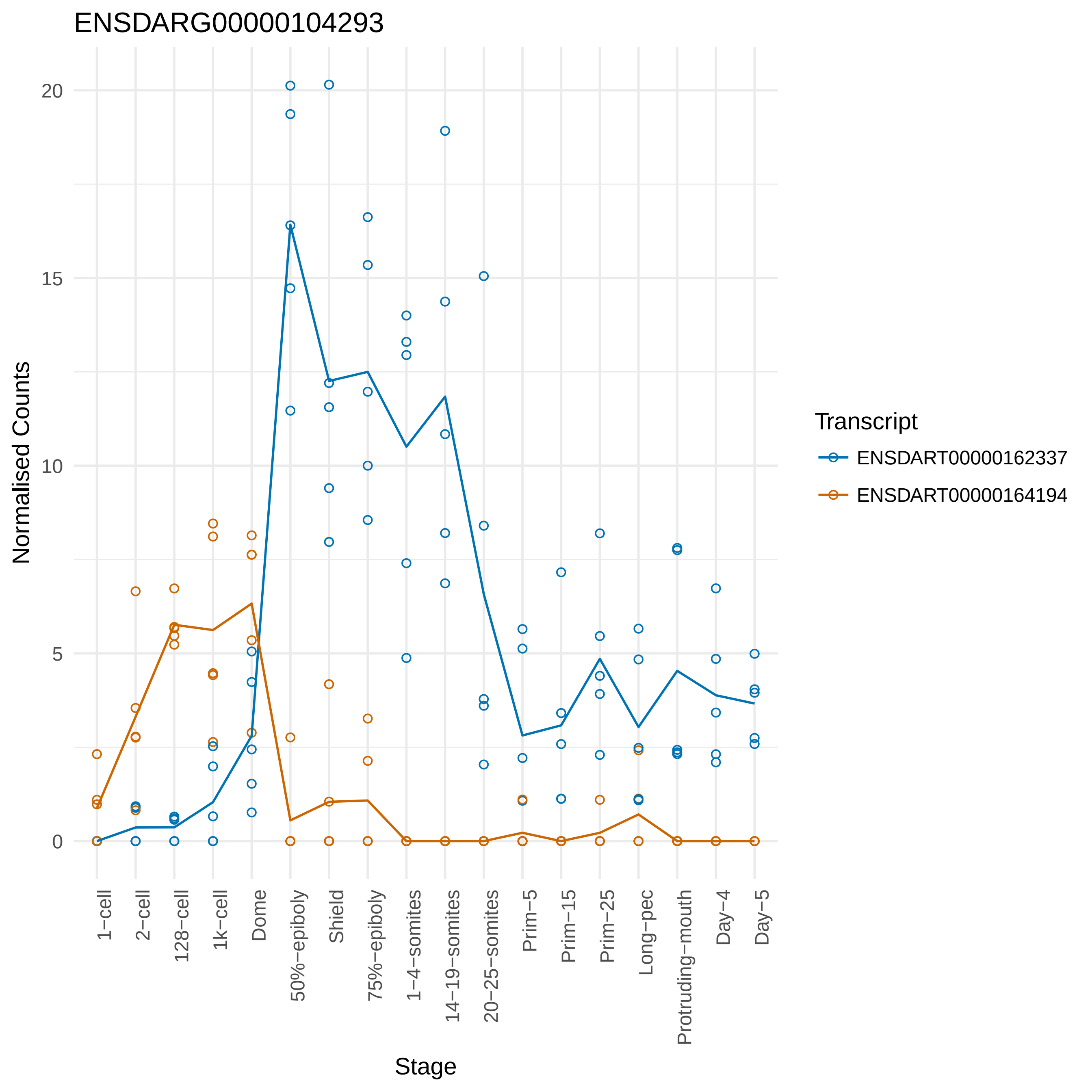

ENSDARG00000104293
Normalised Counts
20
15
10
5
0
1−cell
2−cell
128−cell
1k−cell
Dome
50%−epiboly
Shield
75%−epiboly
1−4−somites
14−19−somites
20−25−somites
Prim−5
Prim−15
Prim−25
Long−pec
Protruding−mouth
Day−4
Day−5
Stage
Transcript
ENSDART00000162337
ENSDART00000164194

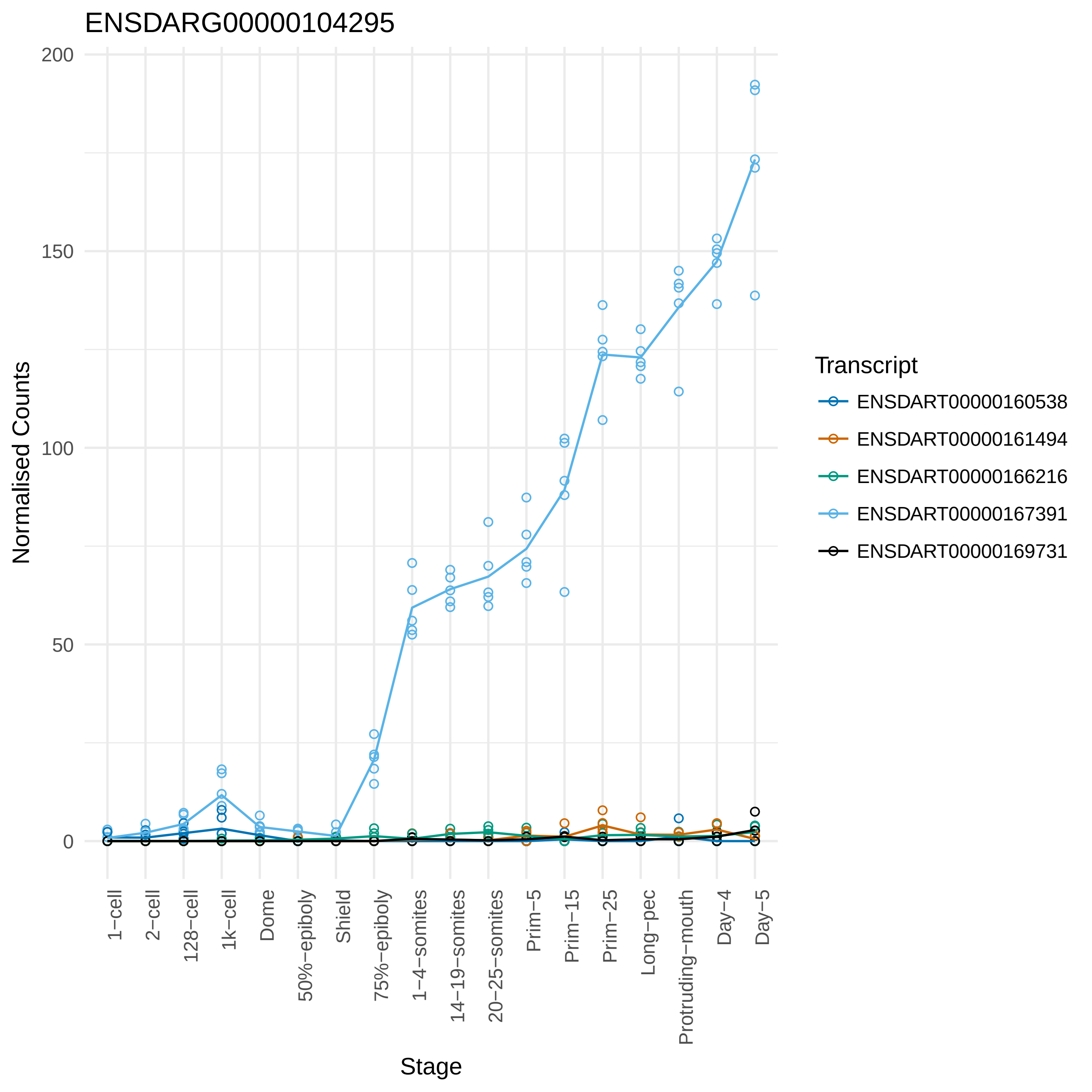

ENSDARG00000104295
Normalised Counts
200
150
100
50
0
1−cell
2−cell
128−cell
1k−cell
Dome
50%−epiboly
Shield
75%−epiboly
1−4−somites
14−19−somites
20−25−somites
Prim−5
Prim−15
Prim−25
Long−pec
Protruding−mouth
Day−4
Day−5
Stage
Transcript
ENSDART00000160538
ENSDART00000161494
ENSDART00000166216
ENSDART00000167391
ENSDART00000169731

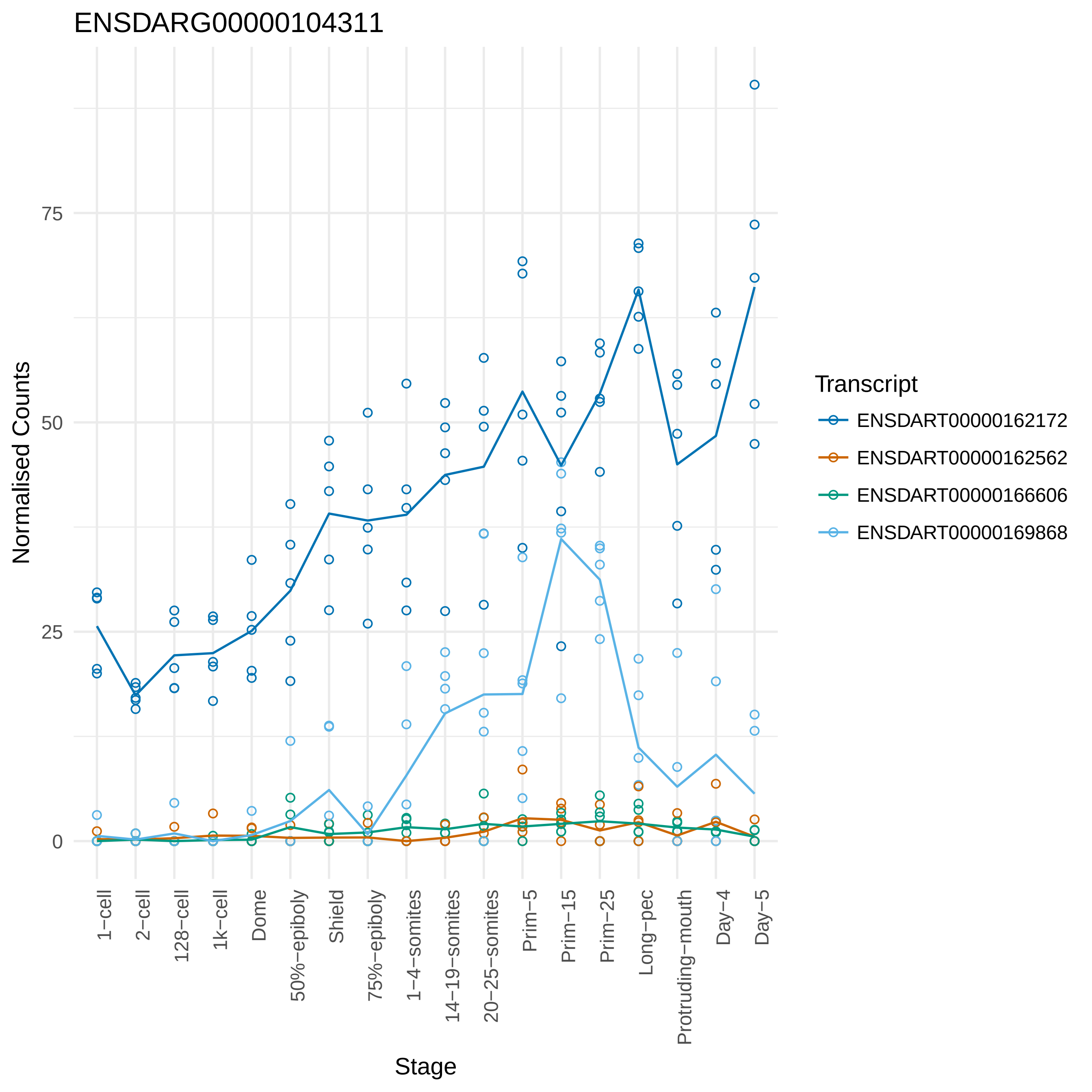

ENSDARG00000104311
Normalised Counts
0
25
50
75
Stage
1−cell
2−cell
128−cell
1k−cell
Dome
50%−epiboly
Shield
75%−epiboly
1−4−somites
14−19−somites
20−25−somites
Prim−5
Prim−15
Prim−25
Long−pec
Protruding−mouth
Day−4
Day−5
Transcript
ENSDART00000162172
ENSDART00000162562
ENSDART00000166606
ENSDART00000169868

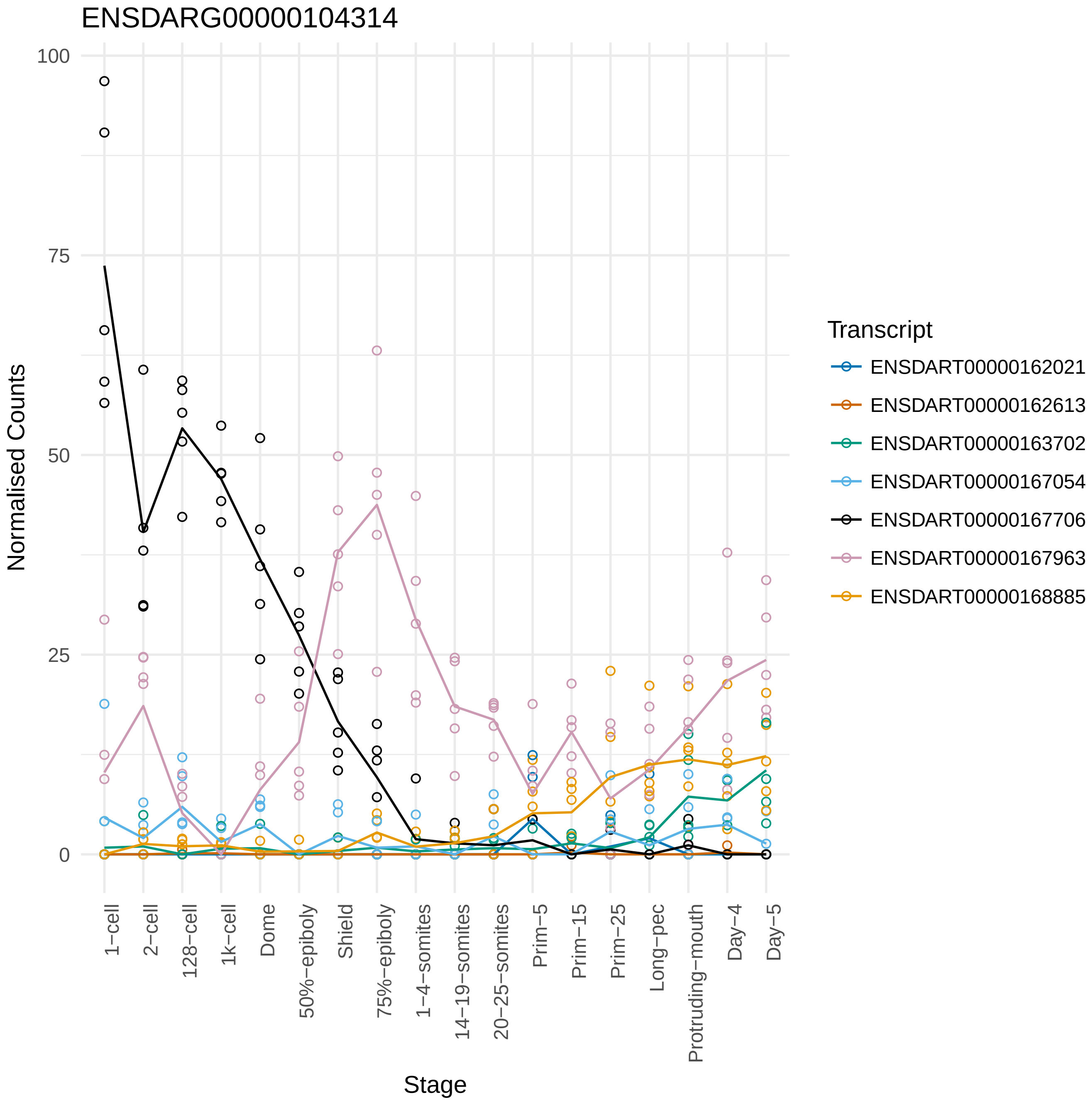
ENSDARG00000104314
100
75
50
25
0
Normalised Counts
1−cell
2−cell
128−cell
1k−cell
Dome
50%−epiboly
Shield
75%−epiboly
1−4−somites
14−19−somites
20−25−somites
Prim−5
Prim−15
Prim−25
Long−pec
Protruding−mouth
Day−4
Day−5
Stage
Transcript
ENSDART00000162021
ENSDART00000162613
ENSDART00000163702
ENSDART00000167054
ENSDART00000167706
ENSDART00000167963
ENSDART00000168885

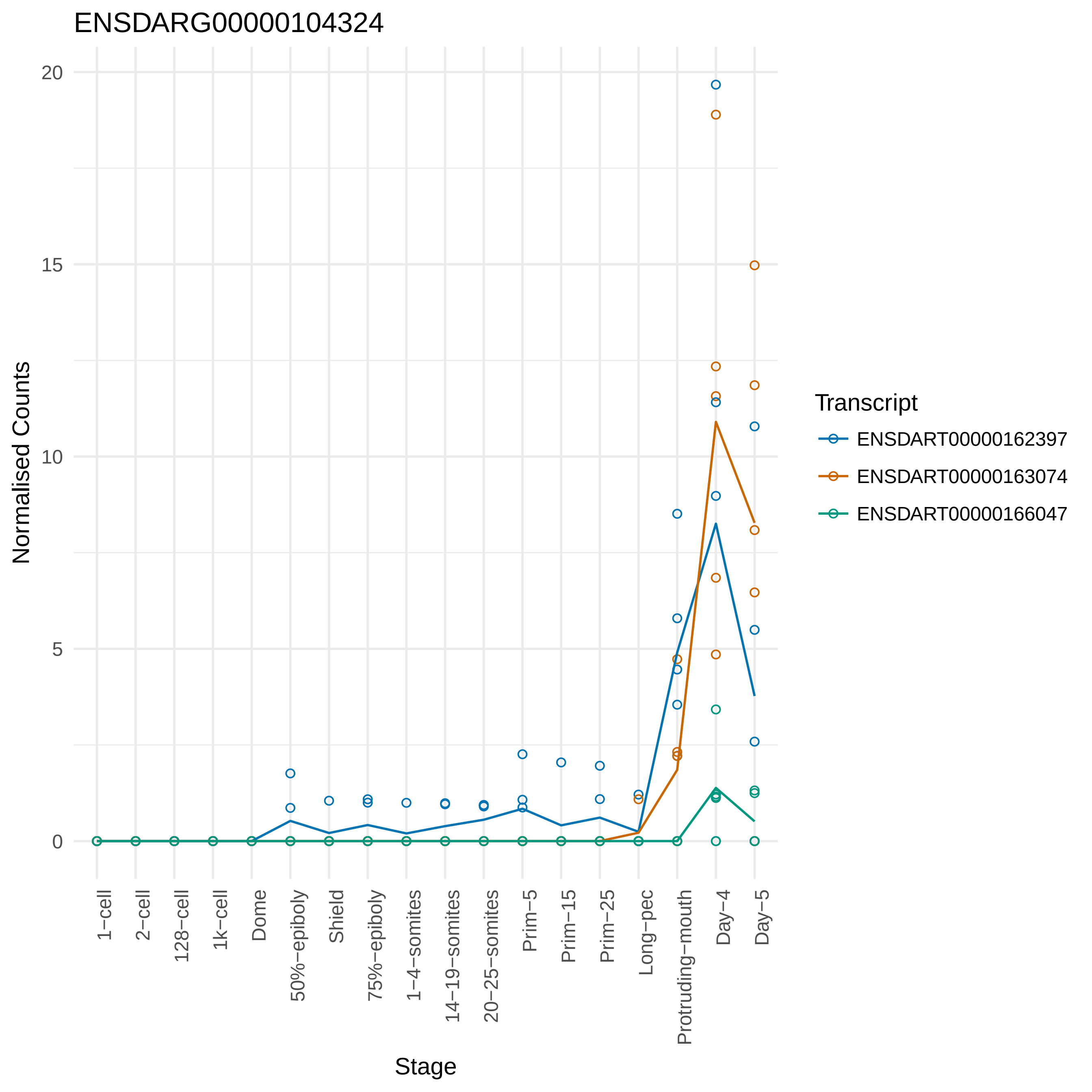
ENSDARG00000104324
Normalised Counts
20
15
10
5
0
1−cell
2−cell
128−cell
1k−cell
Dome
50%−epiboly
Shield
75%−epiboly
1−4−somites
14−19−somites
20−25−somites
Prim−5
Prim−15
Prim−25
Long−pec
Protruding−mouth
Day−4
Day−5
Stage
Transcript
ENSDART00000162397
ENSDART00000163074
ENSDART00000166047

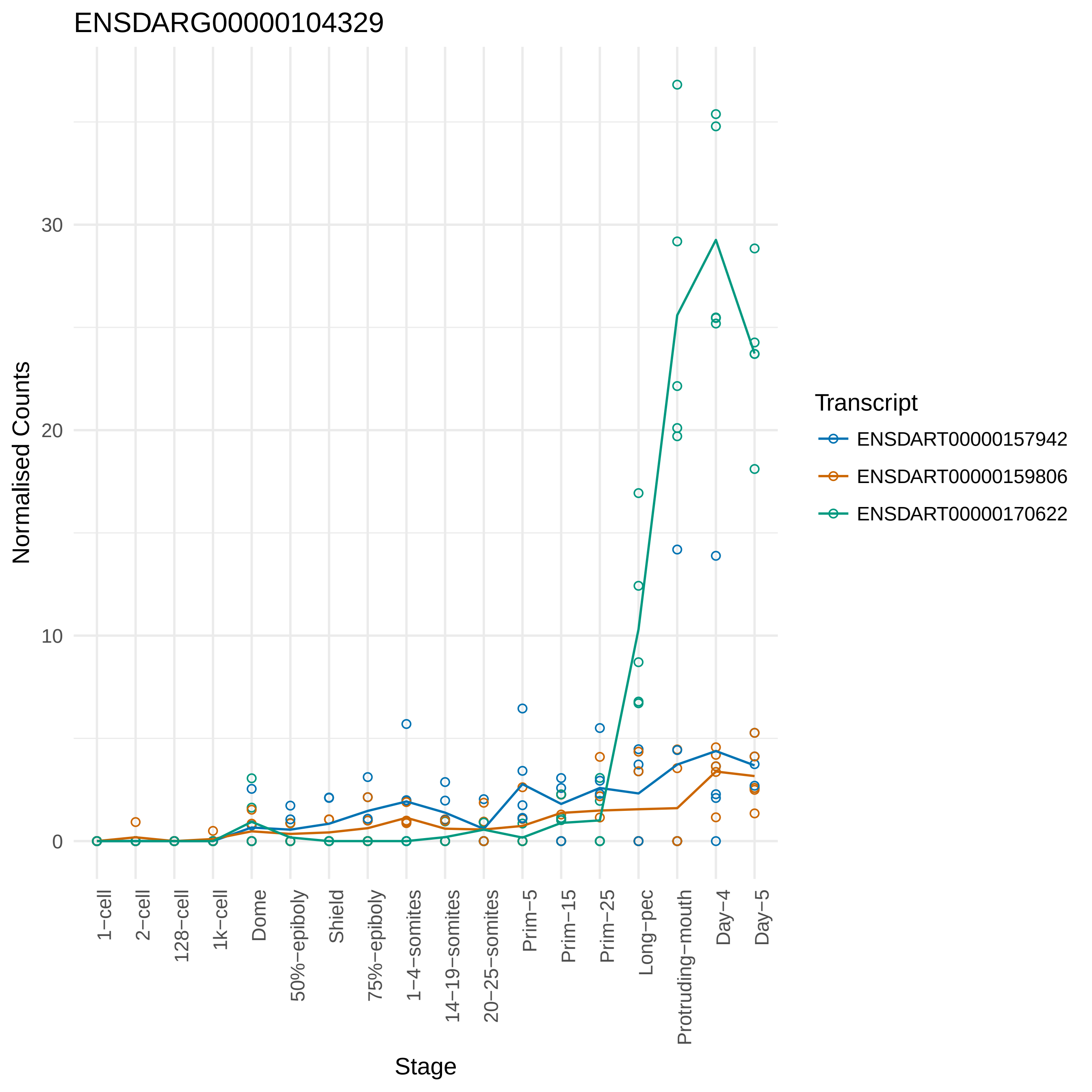

ENSDARG00000104329
Normalised Counts
0
10
20
30
Stage
1−cell
2−cell
128−cell
1k−cell
Dome
50%−epiboly
Shield
75%−epiboly
1−4−somites
14−19−somites
20−25−somites
Prim−5
Prim−15
Prim−25
Long−pec
Protruding−mouth
Day−4
Day−5
Transcript
ENSDART00000157942
ENSDART00000159806
ENSDART00000170622

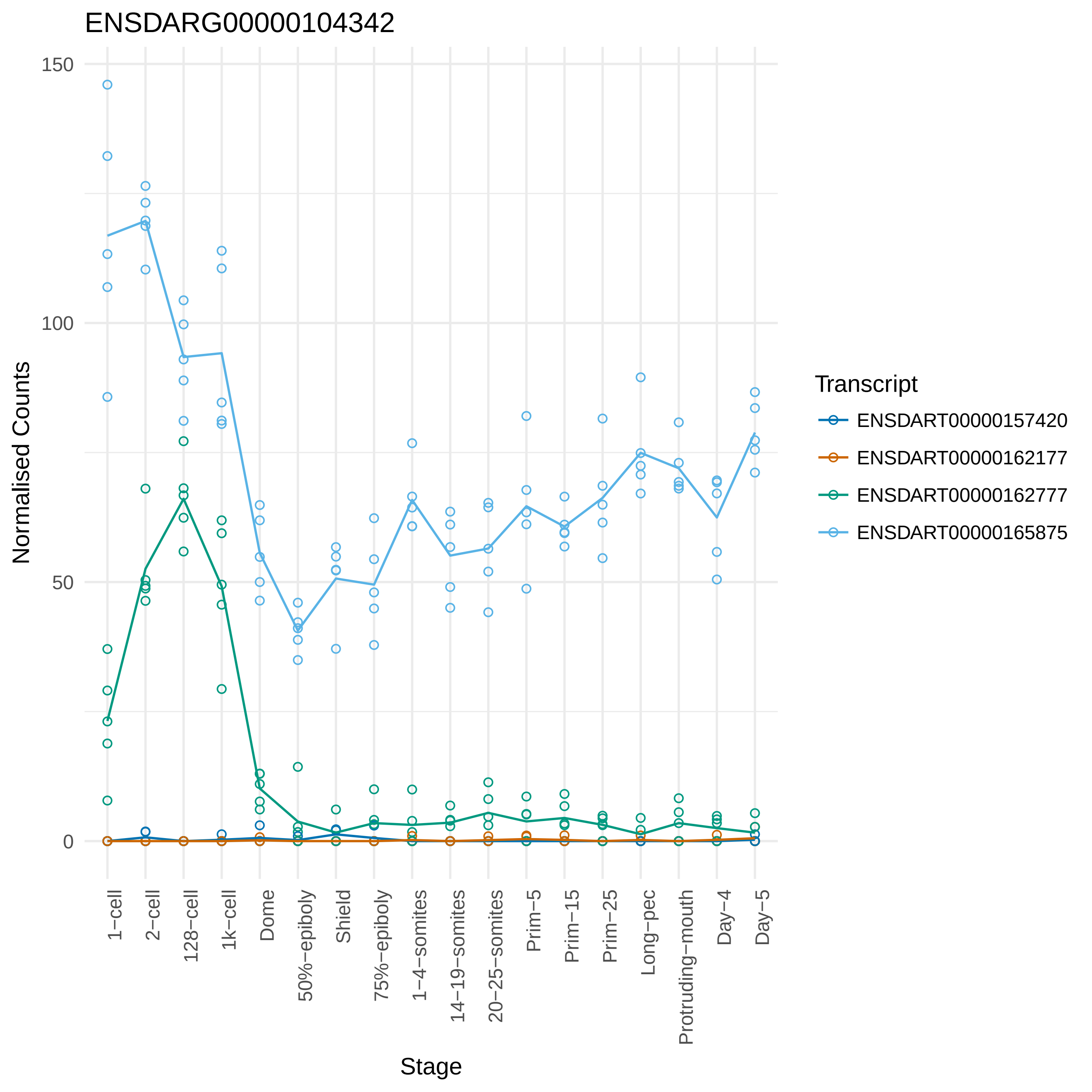
ENSDARG00000104342
Normalised Counts
150
100
50
0
1−cell
2−cell
128−cell
1k−cell
Dome
50%−epiboly
Shield
75%−epiboly
1−4−somites
14−19−somites
20−25−somites
Prim−5
Prim−15
Prim−25
Long−pec
Protruding−mouth
Day−4
Day−5
Stage
Transcript
ENSDART00000157420
ENSDART00000162177
ENSDART00000162777
ENSDART00000165875

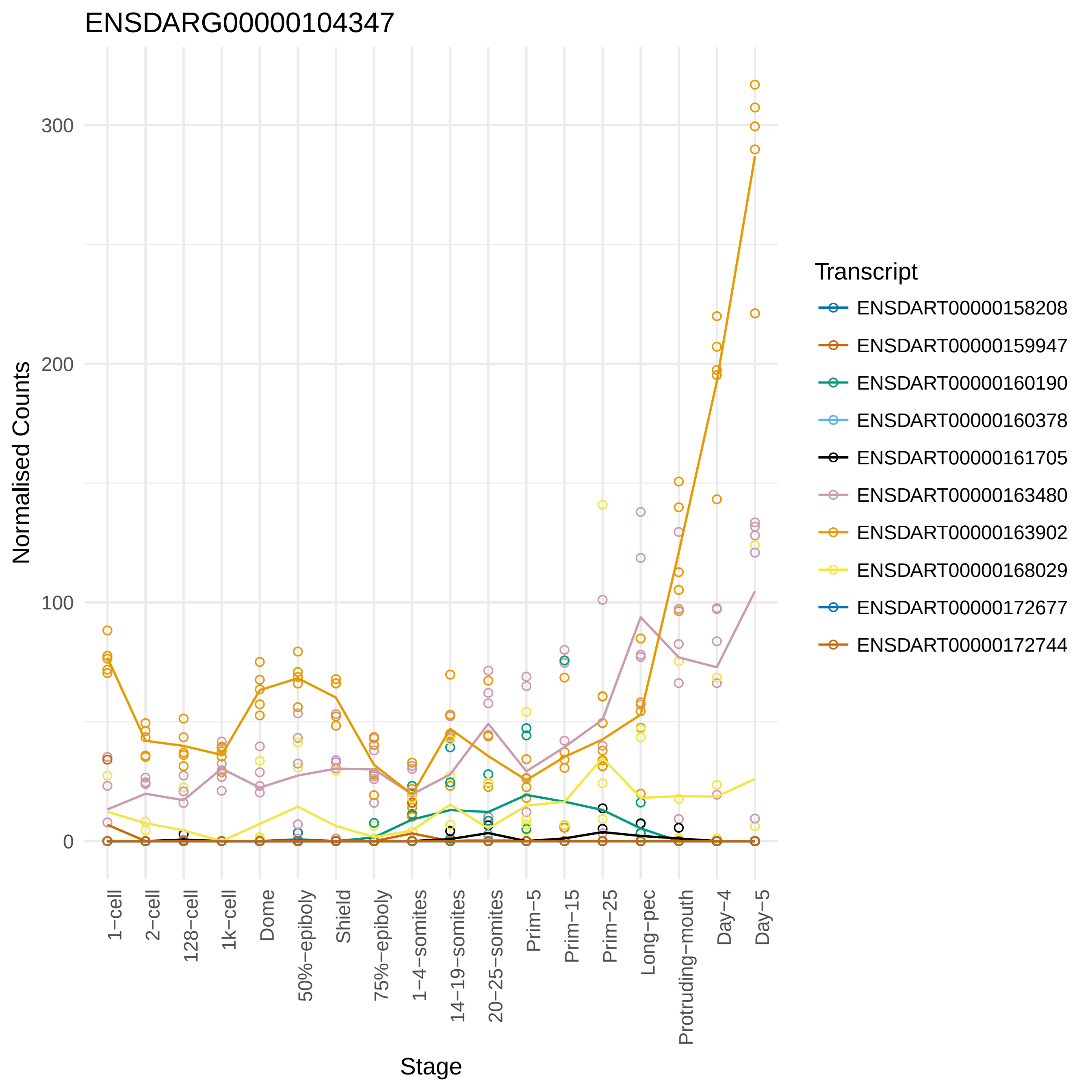

ENSDARG00000104347
Normalised Counts
300
200
100
0
1−cell
2−cell
128−cell
1k−cell
Dome
50%−epiboly
Shield
75%−epiboly
1−4−somites
14−19−somites
20−25−somites
Prim−5
Prim−15
Prim−25
Long−pec
Protruding−mouth
Day−4
Day−5
Stage
Transcript
ENSDART00000158208
ENSDART00000159947
ENSDART00000160190
ENSDART00000160378
ENSDART00000161705
ENSDART00000163480
ENSDART00000163902
ENSDART00000168029
ENSDART00000172677
ENSDART00000172744

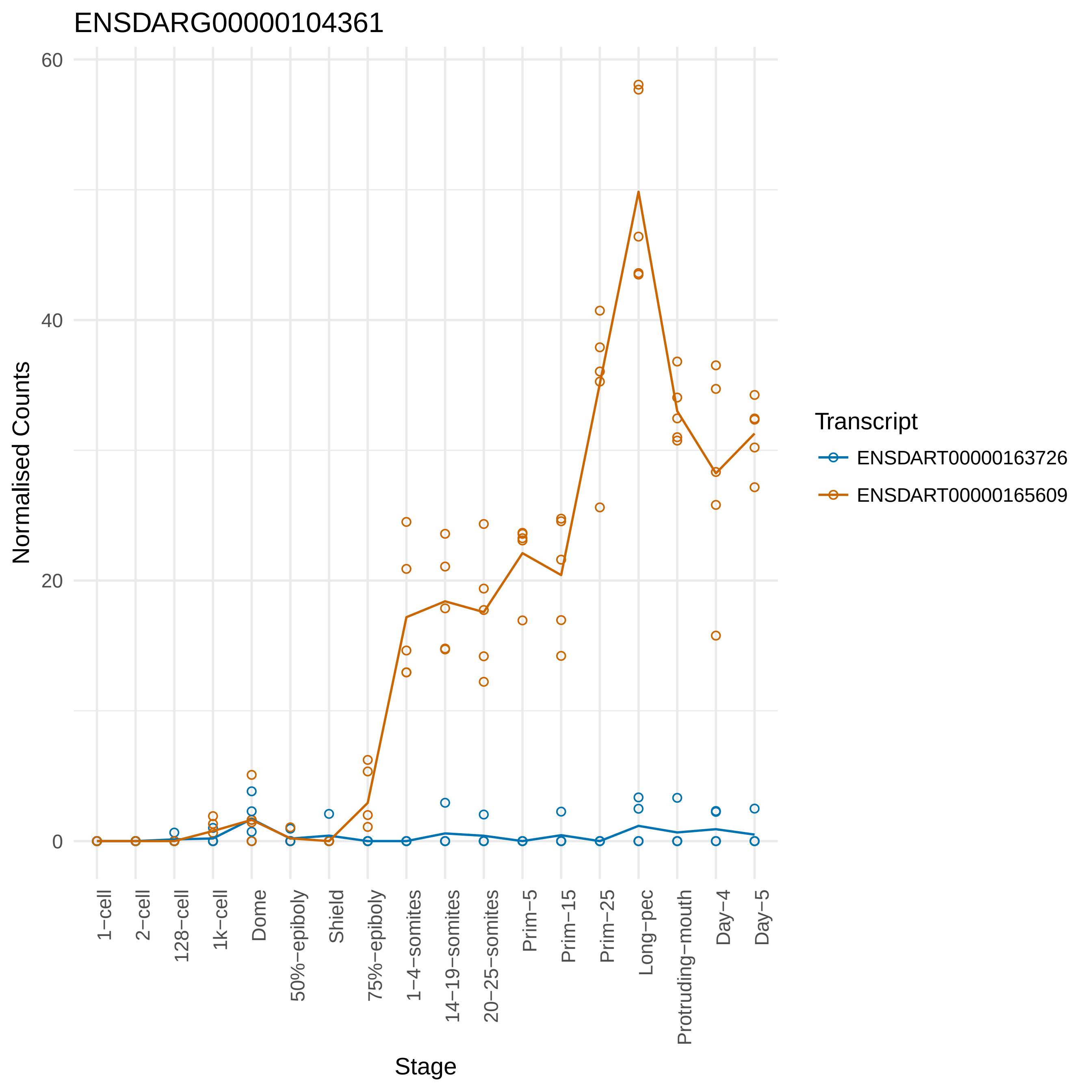

ENSDARG00000104361
Normalised Counts
60
40
20
0
1−cell
2−cell
128−cell
1k−cell
Dome
50%−epiboly
Shield
75%−epiboly
1−4−somites
14−19−somites
20−25−somites
Prim−5
Prim−15
Prim−25
Long−pec
Protruding−mouth
Day−4
Day−5
Stage
Transcript
ENSDART00000163726
ENSDART00000165609

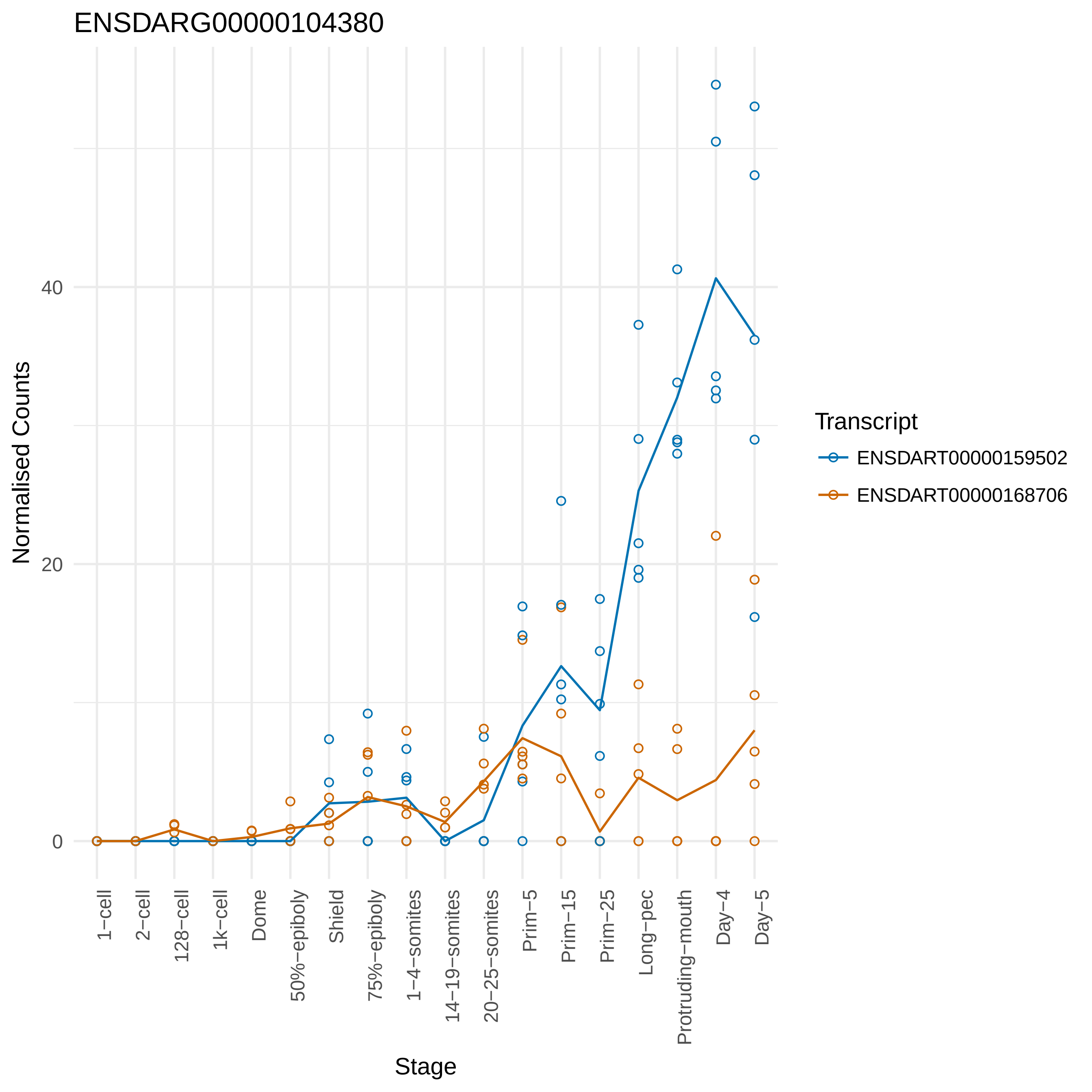
ENSDARG00000104380
Normalised Counts
0
20
40
1−cell
2−cell
128−cell
1k−cell
Dome
50%−epiboly
Shield
75%−epiboly
1−4−somites
14−19−somites
20−25−somites
Prim−5
Prim−15
Prim−25
Long−pec
Protruding−mouth
Day−4
Day−5
Stage
Transcript
ENSDART00000159502
ENSDART00000168706

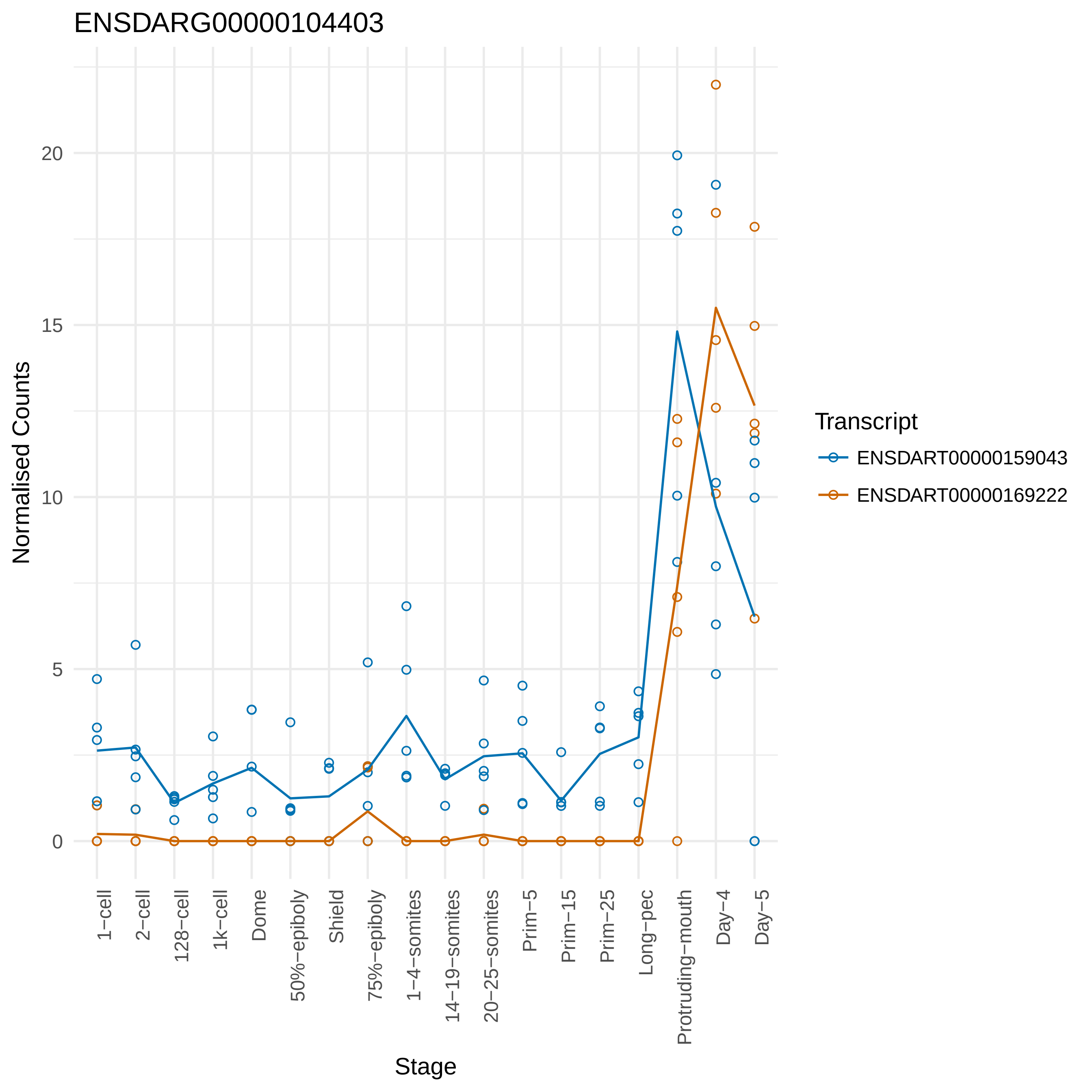
ENSDARG00000104403
Normalised Counts
0
5
10
15
20
1−cell
2−cell
128−cell
1k−cell
Dome
50%−epiboly
Shield
75%−epiboly
1−4−somites
14−19−somites
20−25−somites
Prim−5
Prim−15
Prim−25
Long−pec
Protruding−mouth
Day−4
Day−5
Stage
Transcript
ENSDART00000159043
ENSDART00000169222

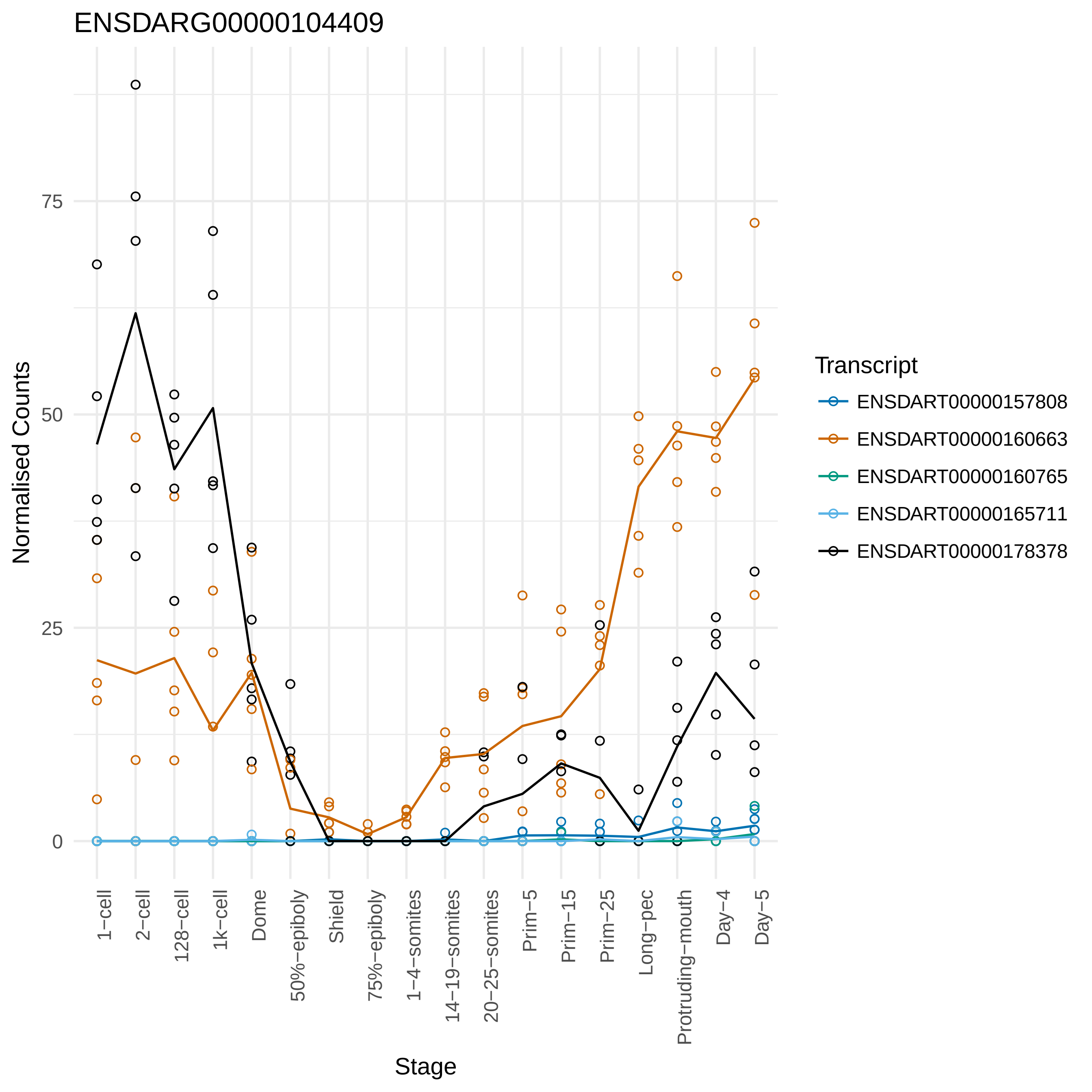

ENSDARG00000104409
Normalised Counts
0
25
50
75
Stage
1−cell
2−cell
128−cell
1k−cell
Dome
50%−epiboly
Shield
75%−epiboly
1−4−somites
14−19−somites
20−25−somites
Prim−5
Prim−15
Prim−25
Long−pec
Protruding−mouth
Day−4
Day−5
Transcript
ENSDART00000157808
ENSDART00000160663
ENSDART00000160765
ENSDART00000165711
ENSDART00000178378

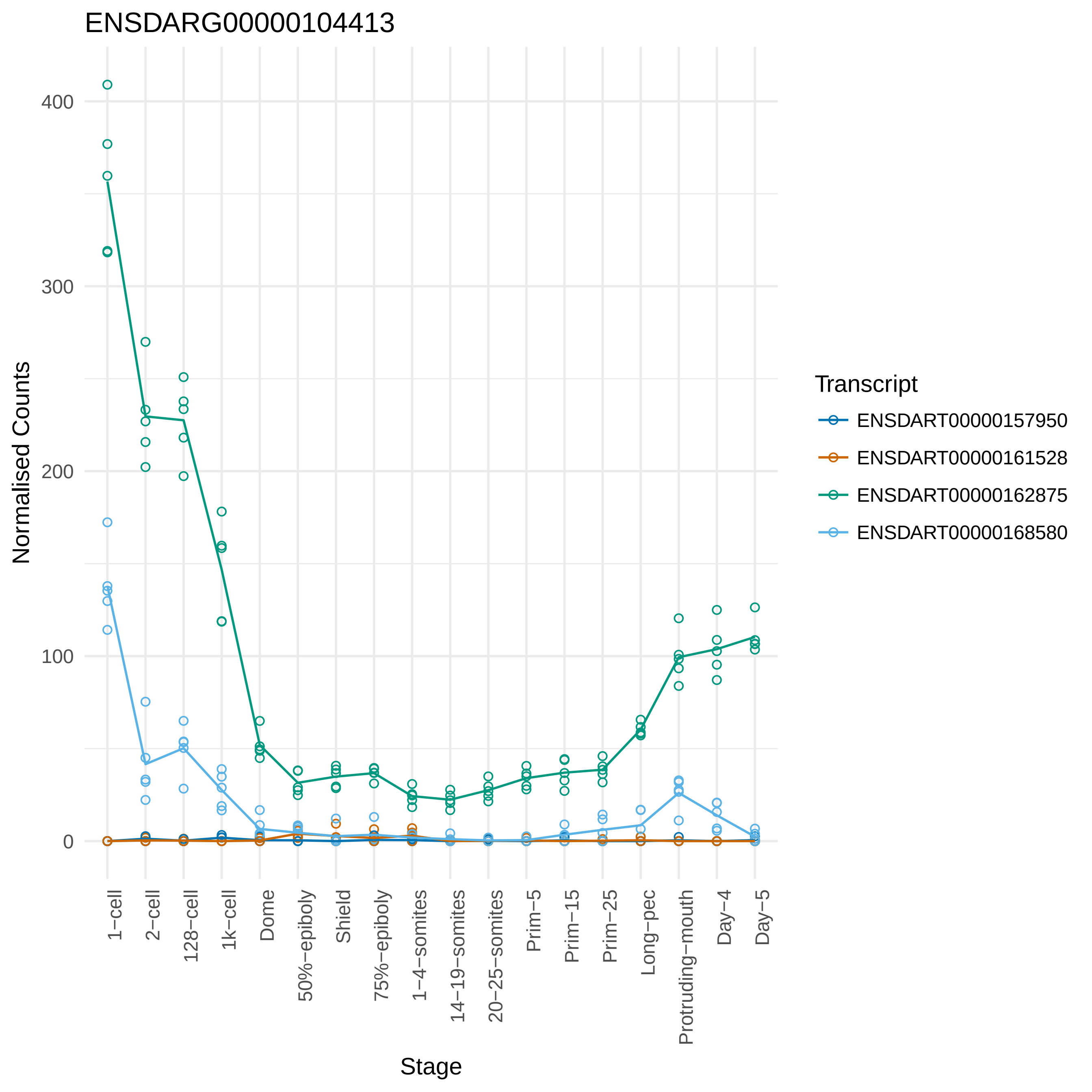

ENSDARG00000104413
Normalised Counts
400
300
200
100
0
1−cell
2−cell
128−cell
1k−cell
Dome
50%−epiboly
Shield
75%−epiboly
1−4−somites
14−19−somites
20−25−somites
Prim−5
Prim−15
Prim−25
Long−pec
Protruding−mouth
Day−4
Day−5
Stage
Transcript
ENSDART00000157950
ENSDART00000161528
ENSDART00000162875
ENSDART00000168580

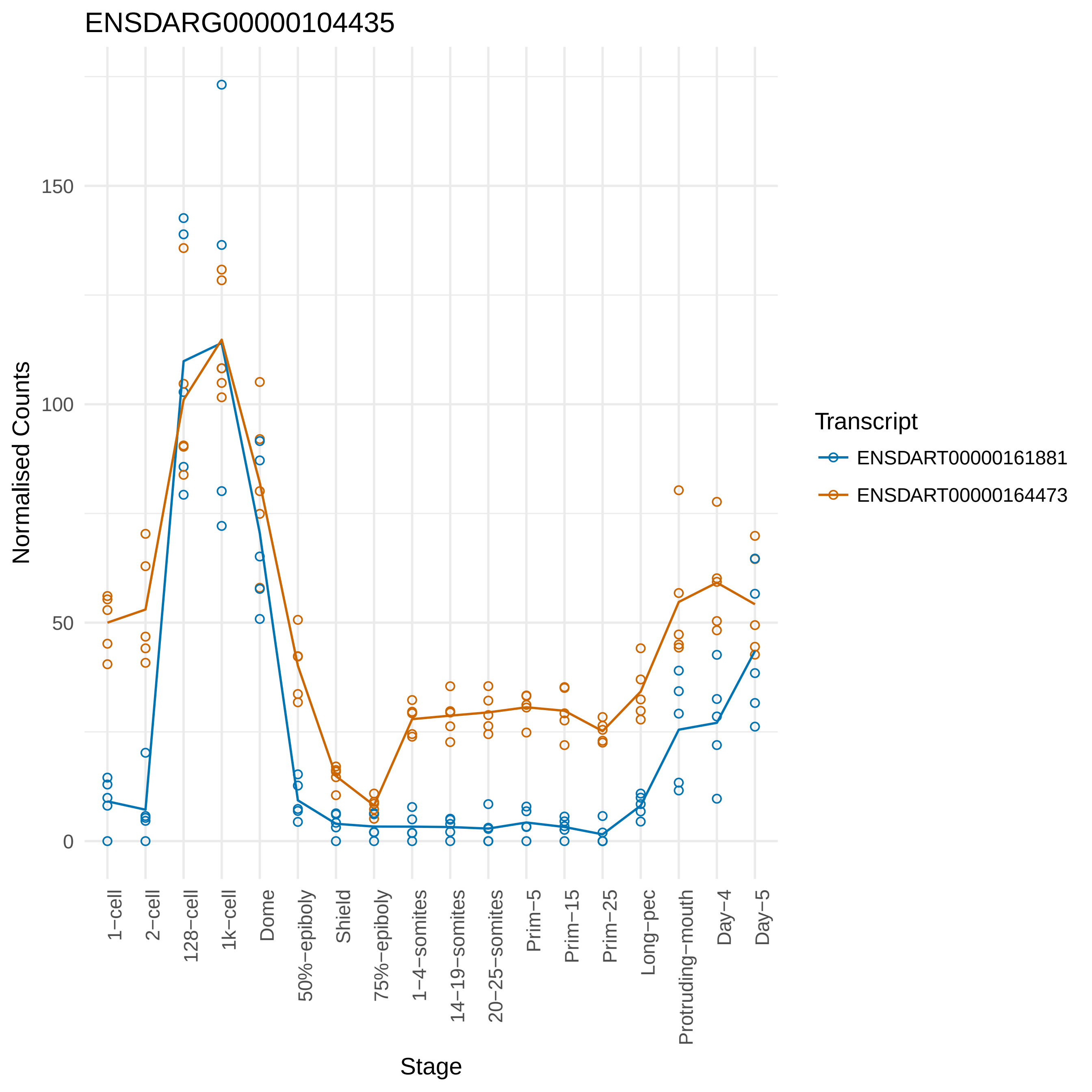
ENSDARG00000104435
Normalised Counts
150
100
50
0
1−cell
2−cell
128−cell
1k−cell
Dome
50%−epiboly
Shield
75%−epiboly
1−4−somites
14−19−somites
20−25−somites
Prim−5
Prim−15
Prim−25
Long−pec
Protruding−mouth
Day−4
Day−5
Stage
Transcript
ENSDART00000161881
ENSDART00000164473

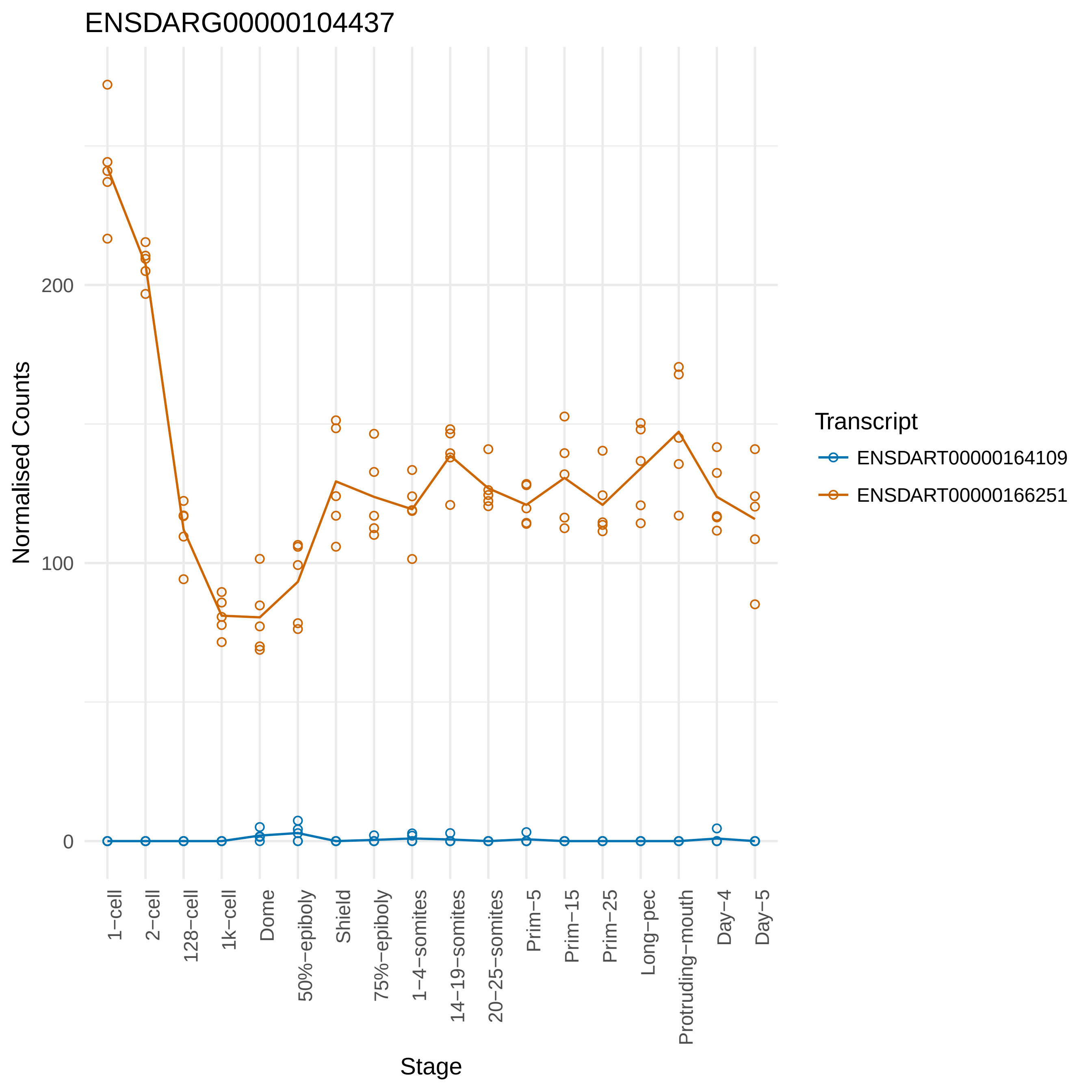
ENSDARG00000104437
Normalised Counts
200
100
0
Stage
1−cell
2−cell
128−cell
1k−cell
Dome
50%−epiboly
Shield
75%−epiboly
1−4−somites
14−19−somites
20−25−somites
Prim−5
Prim−15
Prim−25
Long−pec
Protruding−mouth
Day−4
Day−5
Transcript
ENSDART00000164109
ENSDART00000166251

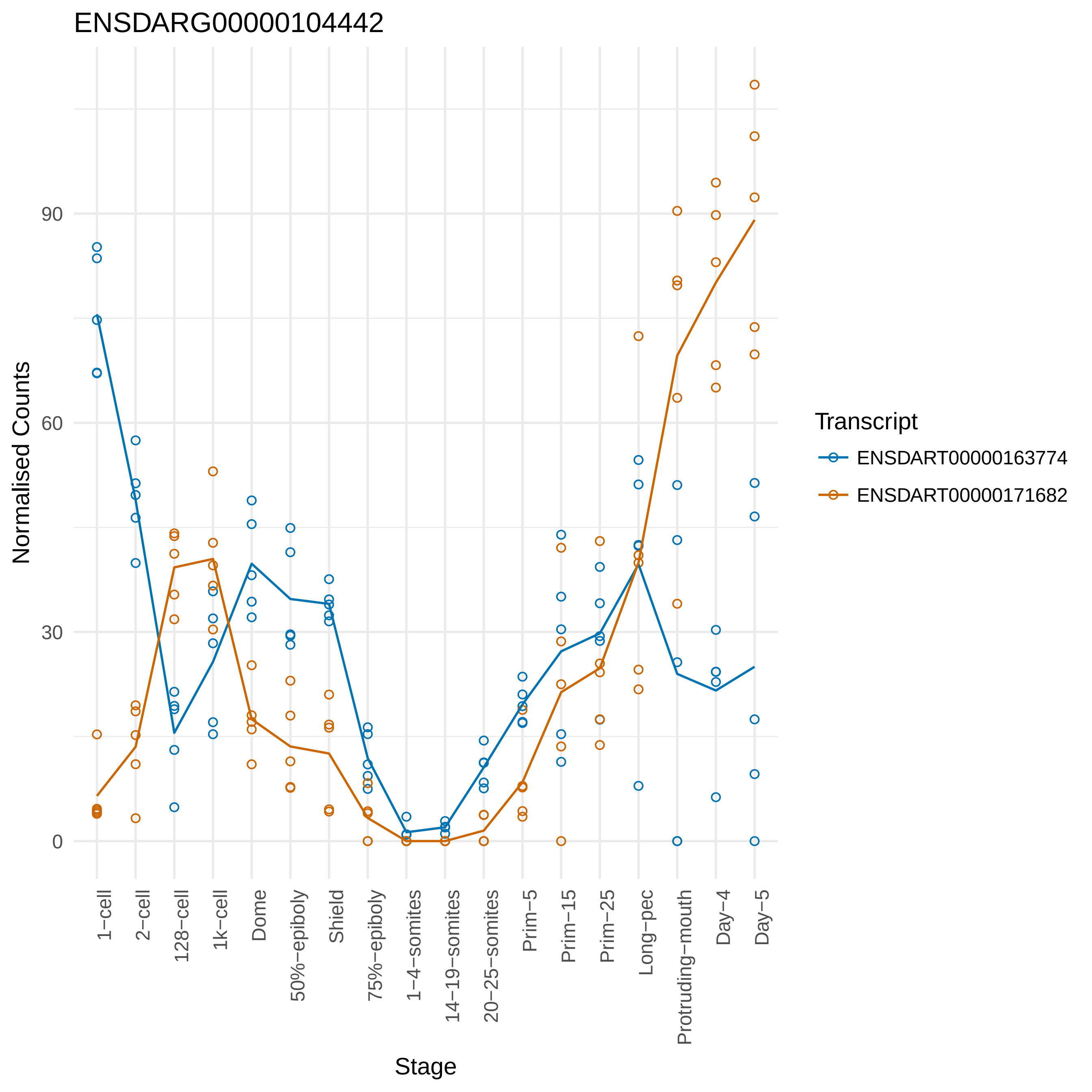

ENSDARG00000104442
Normalised Counts
0
30
60
90
1−cell
2−cell
128−cell
1k−cell
Dome
50%−epiboly
Shield
75%−epiboly
1−4−somites
14−19−somites
20−25−somites
Prim−5
Prim−15
Prim−25
Long−pec
Protruding−mouth
Day−4
Day−5
Stage
Transcript
ENSDART00000163774
ENSDART00000171682

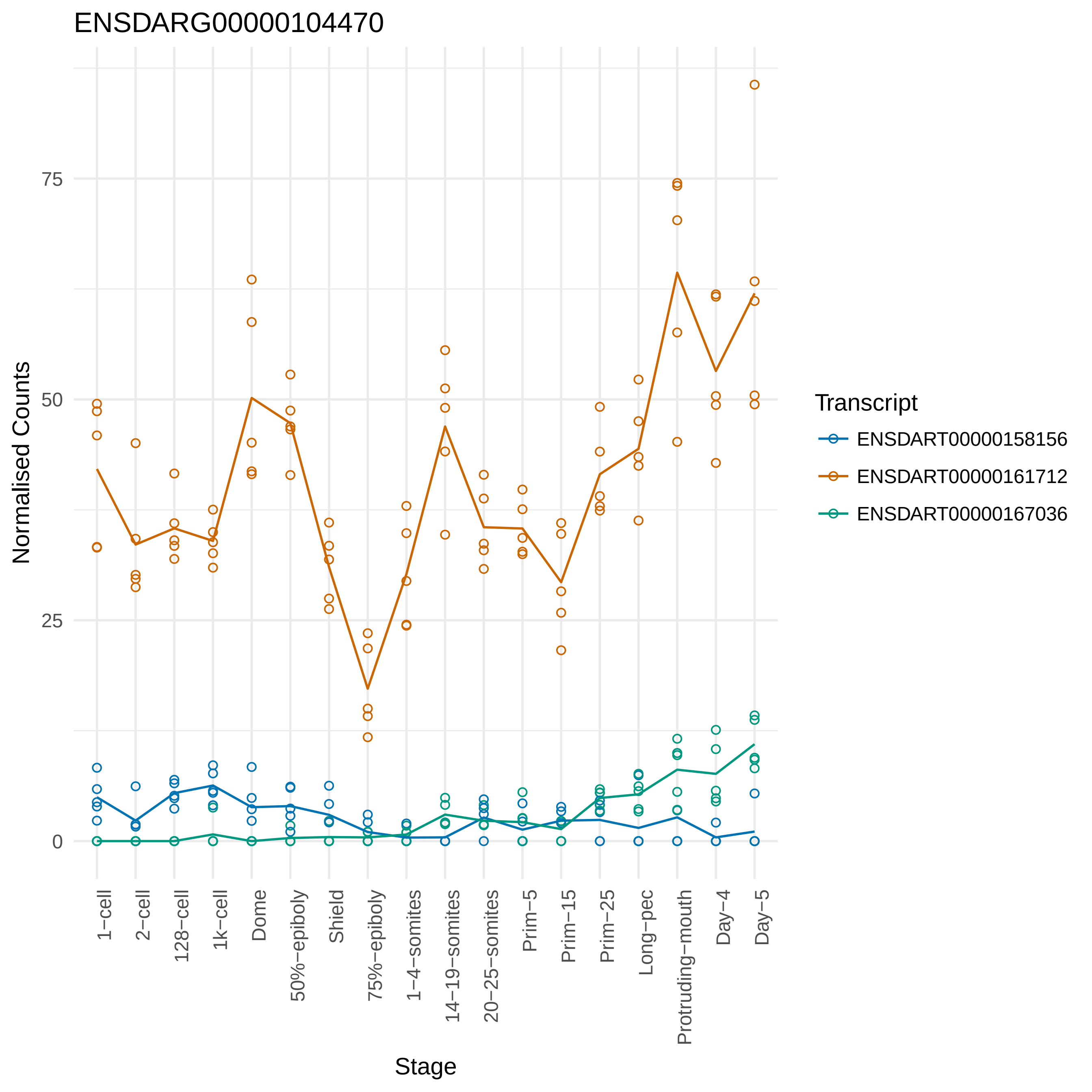

ENSDARG00000104470
Normalised Counts
0
25
50
75
1−cell
2−cell
128−cell
1k−cell
Dome
50%−epiboly
Shield
75%−epiboly
1−4−somites
14−19−somites
20−25−somites
Prim−5
Prim−15
Prim−25
Long−pec
Protruding−mouth
Day−4
Day−5
Stage
Transcript
ENSDART00000158156
ENSDART00000161712
ENSDART00000167036

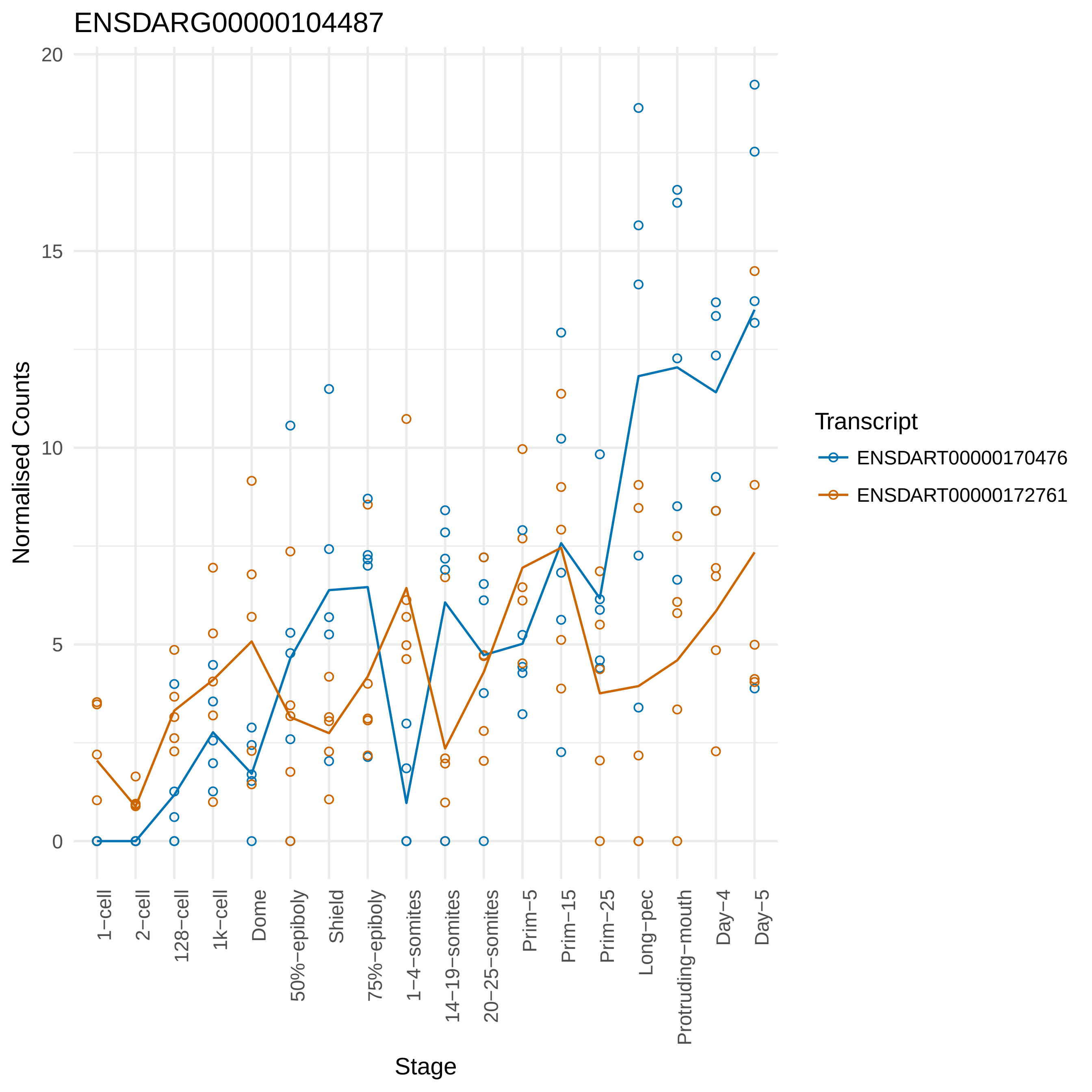

ENSDARG00000104487
Normalised Counts
20
15
10
5
0
1−cell
2−cell
128−cell
1k−cell
Dome
50%−epiboly
Shield
75%−epiboly
1−4−somites
14−19−somites
20−25−somites
Prim−5
Prim−15
Prim−25
Long−pec
Protruding−mouth
Day−4
Day−5
Stage
Transcript
ENSDART00000170476
ENSDART00000172761

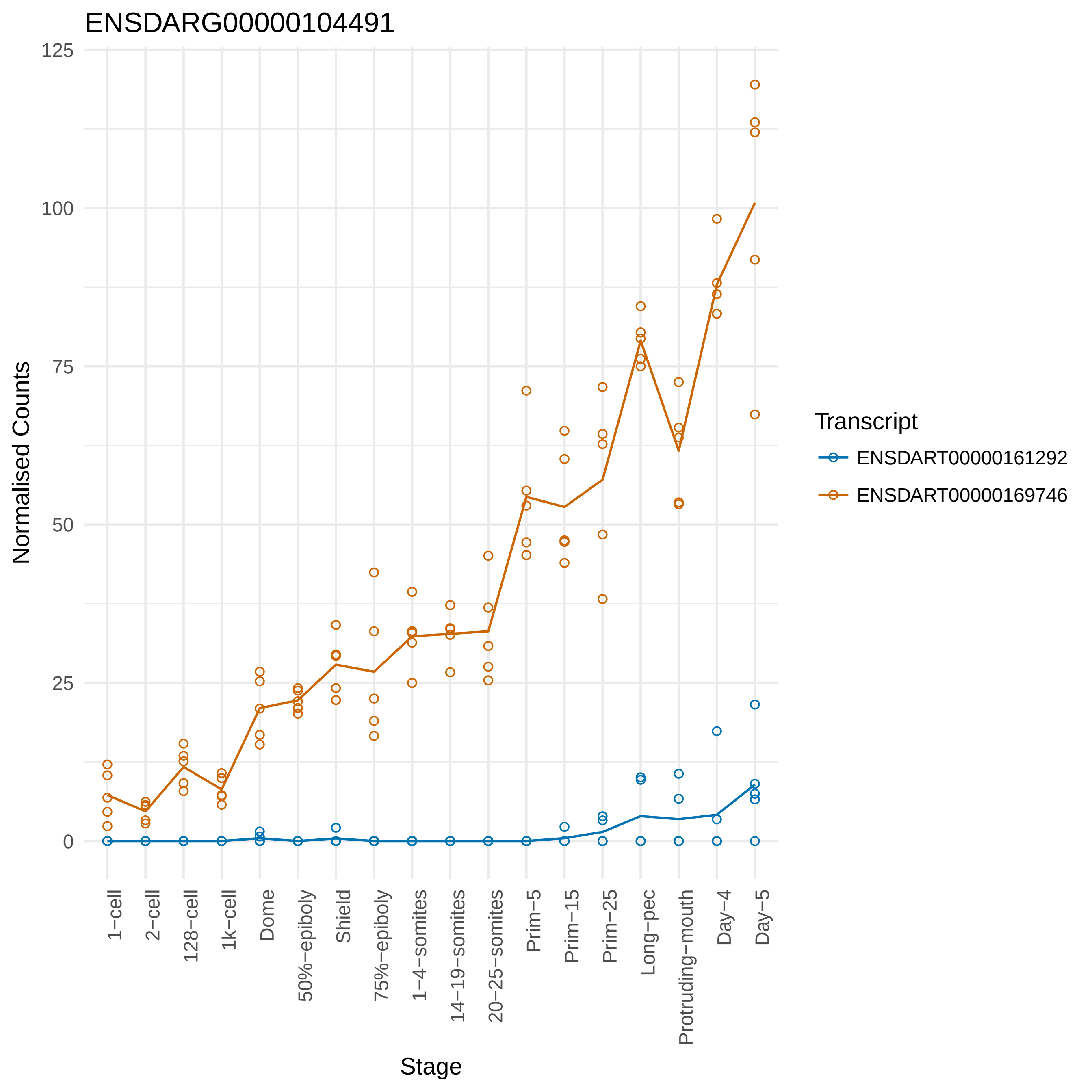

ENSDARG00000104491
Normalised Counts
125
100
75
50
25
0
1−cell
2−cell
128−cell
1k−cell
Dome
50%−epiboly
Shield
75%−epiboly
1−4−somites
14−19−somites
20−25−somites
Prim−5
Prim−15
Prim−25
Long−pec
Protruding−mouth
Day−4
Day−5
Stage
Transcript
ENSDART00000161292
ENSDART00000169746

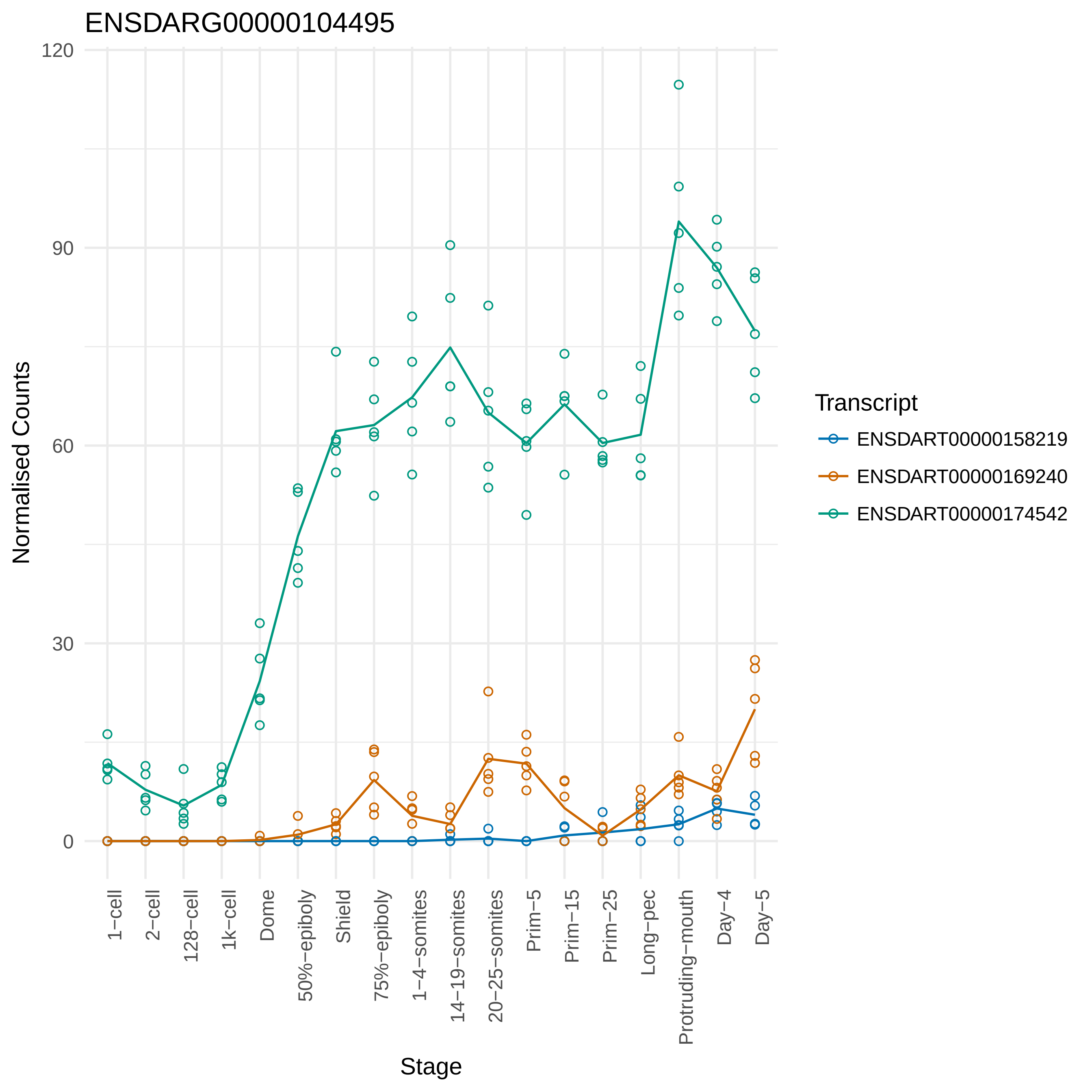

ENSDARG00000104495
Normalised Counts
0
30
60
90
120
1−cell
2−cell
128−cell
1k−cell
Dome
50%−epiboly
Shield
75%−epiboly
1−4−somites
14−19−somites
20−25−somites
Prim−5
Prim−15
Prim−25
Long−pec
Protruding−mouth
Day−4
Day−5
Stage
Transcript
ENSDART00000158219
ENSDART00000169240
ENSDART00000174542

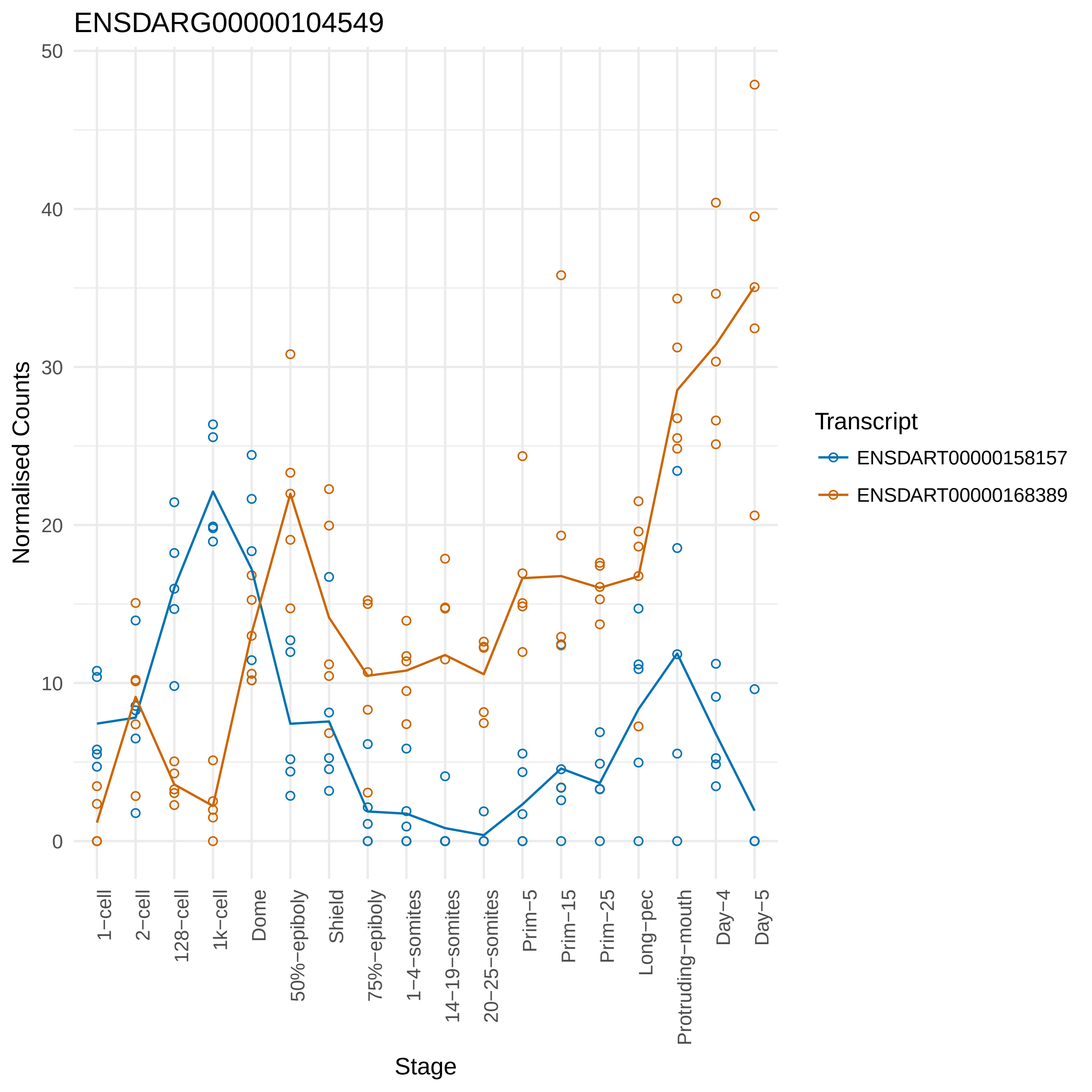

ENSDARG00000104549
Normalised Counts
0
10
20
30
40
50
1−cell
2−cell
128−cell
1k−cell
Dome
50%−epiboly
Shield
75%−epiboly
1−4−somites
14−19−somites
20−25−somites
Prim−5
Prim−15
Prim−25
Long−pec
Protruding−mouth
Day−4
Day−5
Stage
Transcript
ENSDART00000158157
ENSDART00000168389

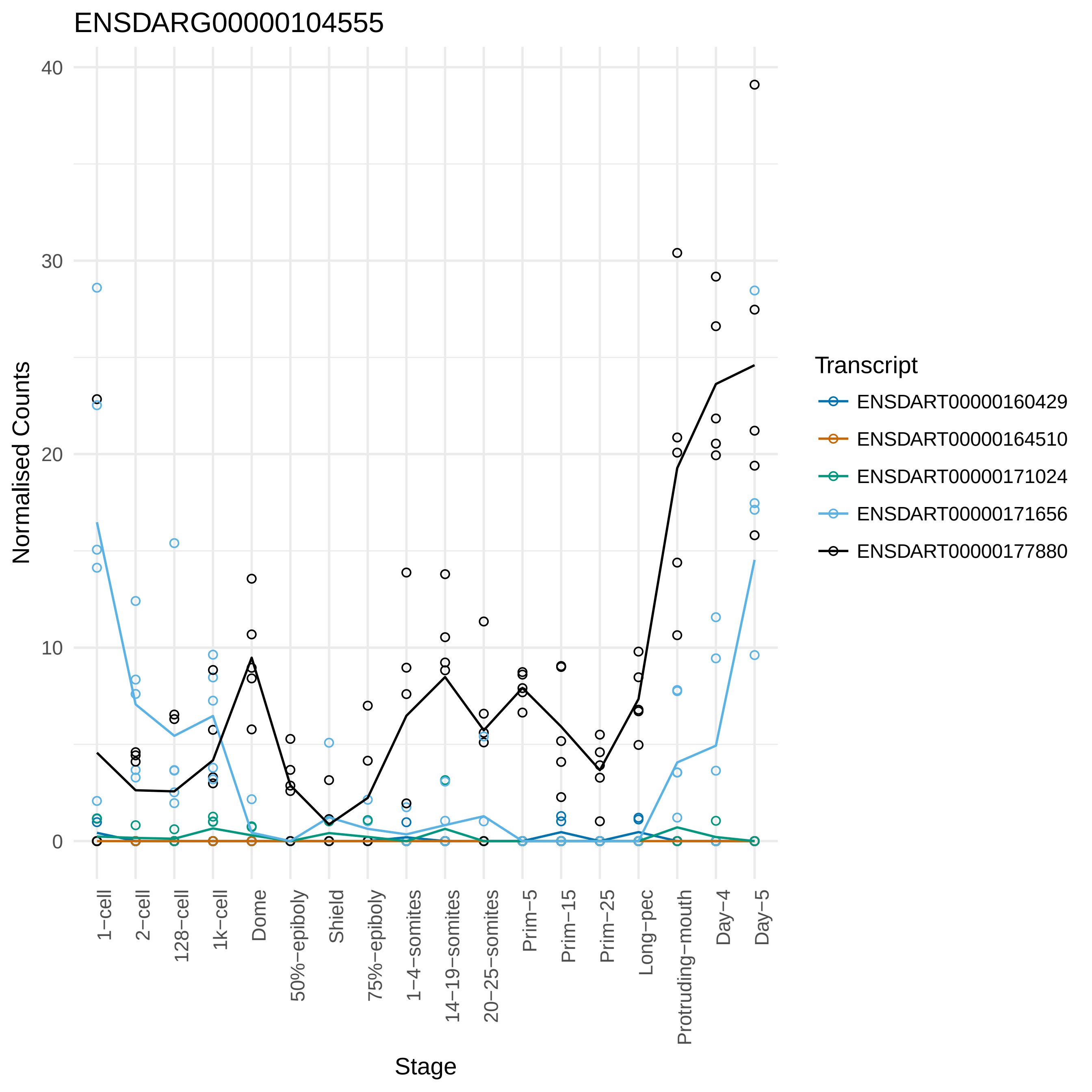
ENSDARG00000104555
Normalised Counts
40
30
20
10
0
Stage
1−cell
2−cell
128−cell
1k−cell
Dome
50%−epiboly
Shield
75%−epiboly
1−4−somites
14−19−somites
20−25−somites
Prim−5
Prim−15
Prim−25
Long−pec
Protruding−mouth
Day−4
Day−5
Transcript
ENSDART00000160429
ENSDART00000164510
ENSDART00000171024
ENSDART00000171656
ENSDART00000177880

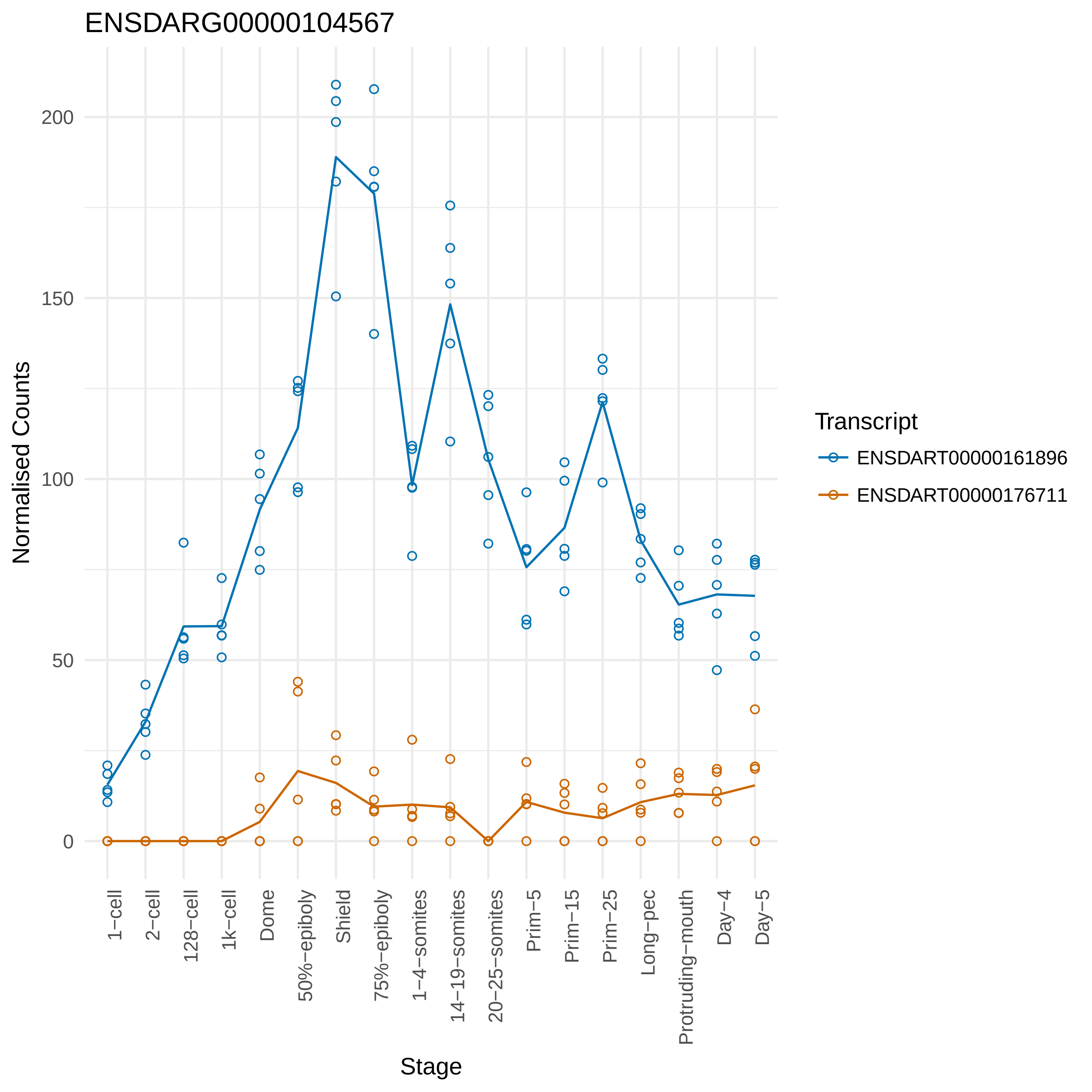
ENSDARG00000104567
Normalised Counts
200
150
100
50
0
1−cell
2−cell
128−cell
1k−cell
Dome
50%−epiboly
Shield
75%−epiboly
1−4−somites
14−19−somites
20−25−somites
Prim−5
Prim−15
Prim−25
Long−pec
Protruding−mouth
Day−4
Day−5
Stage
Transcript
ENSDART00000161896
ENSDART00000176711

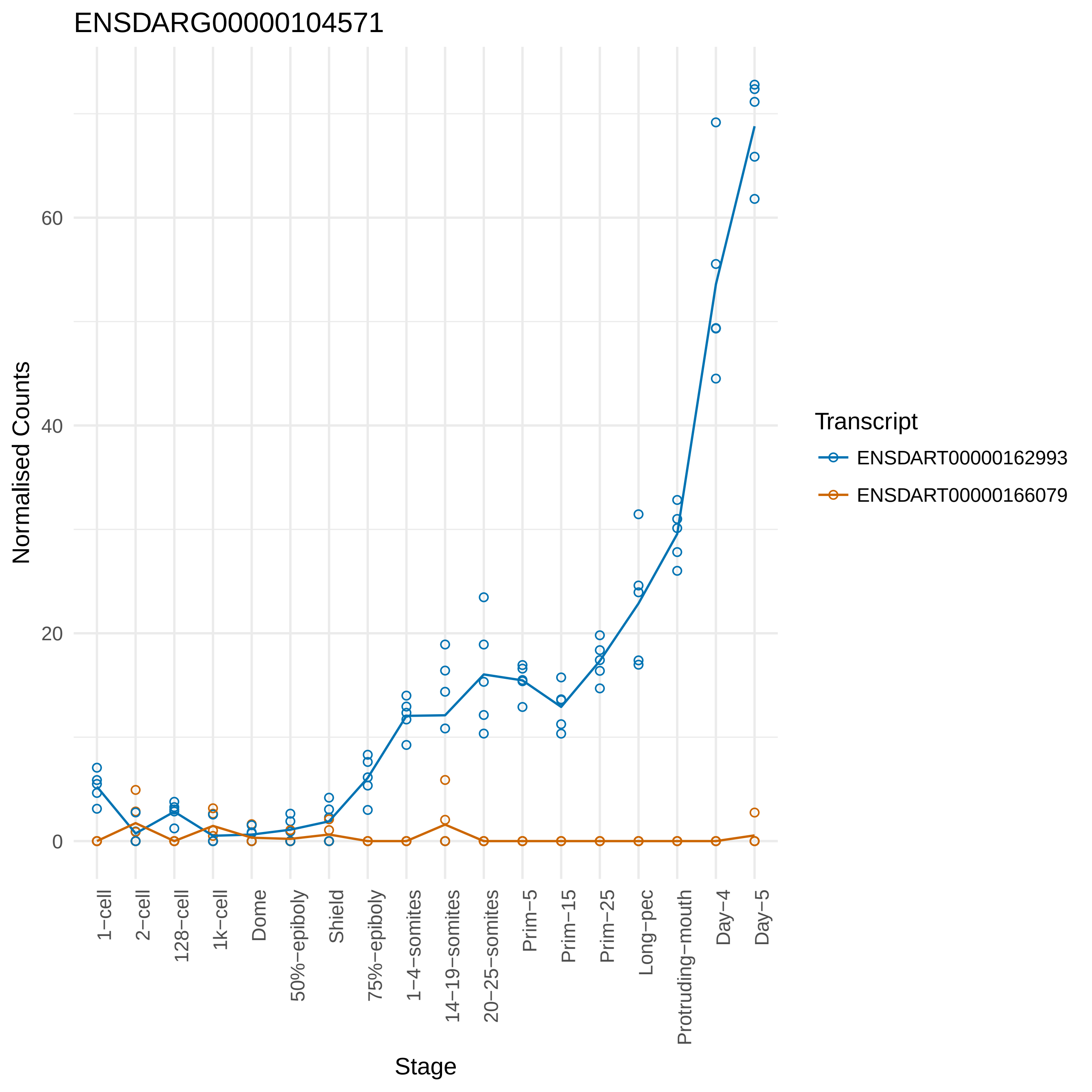
ENSDARG00000104571
Normalised Counts
60
40
20
0
1−cell
2−cell
128−cell
1k−cell
Dome
50%−epiboly
Shield
75%−epiboly
1−4−somites
14−19−somites
20−25−somites
Prim−5
Prim−15
Prim−25
Long−pec
Protruding−mouth
Day−4
Day−5
Stage
Transcript
ENSDART00000162993
ENSDART00000166079

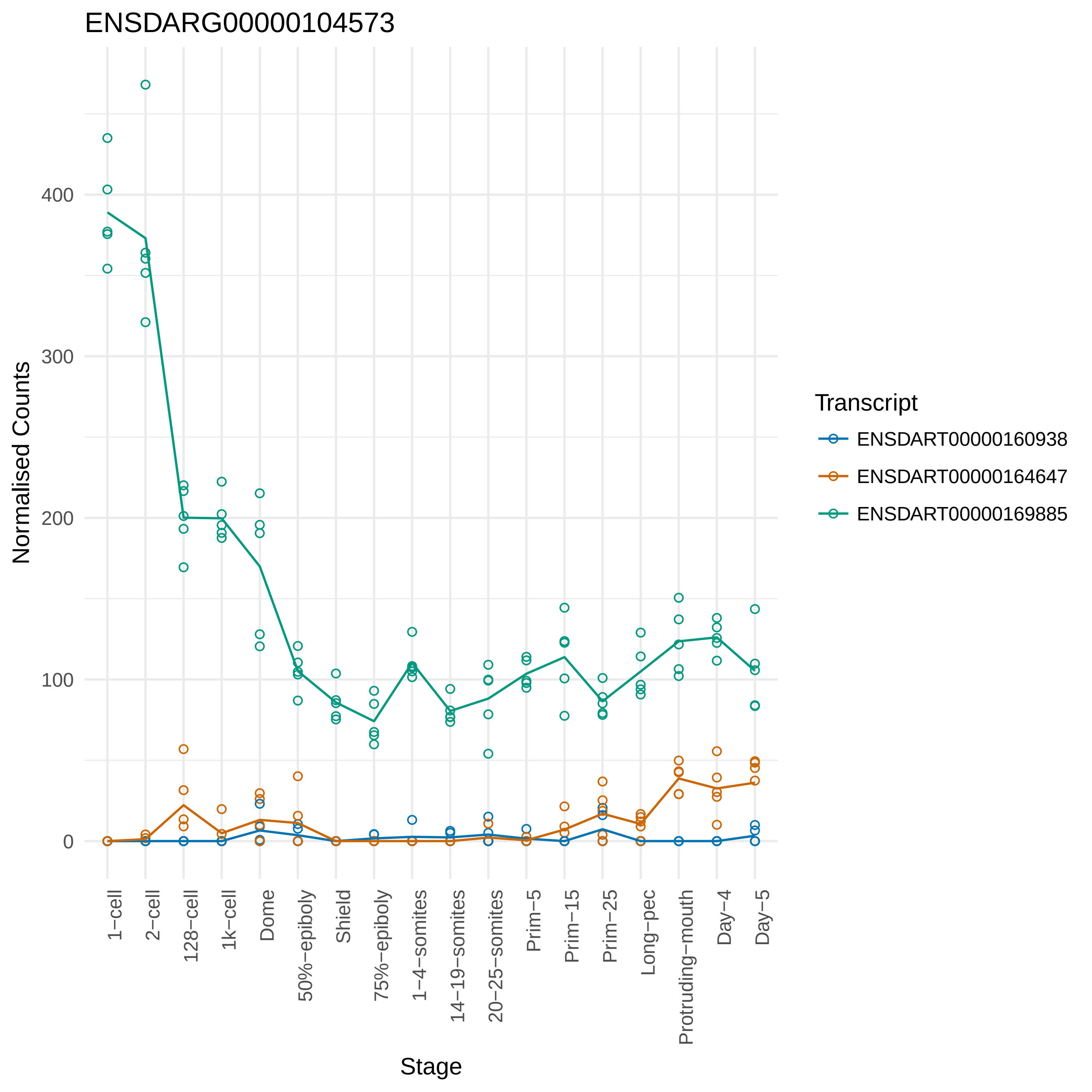

ENSDARG00000104573
Normalised Counts
0
100
200
300
400
Stage
1−cell
2−cell
128−cell
1k−cell
Dome
50%−epiboly
Shield
75%−epiboly
1−4−somites
14−19−somites
20−25−somites
Prim−5
Prim−15
Prim−25
Long−pec
Protruding−mouth
Day−4
Day−5
Transcript
ENSDART00000160938
ENSDART00000164647
ENSDART00000169885

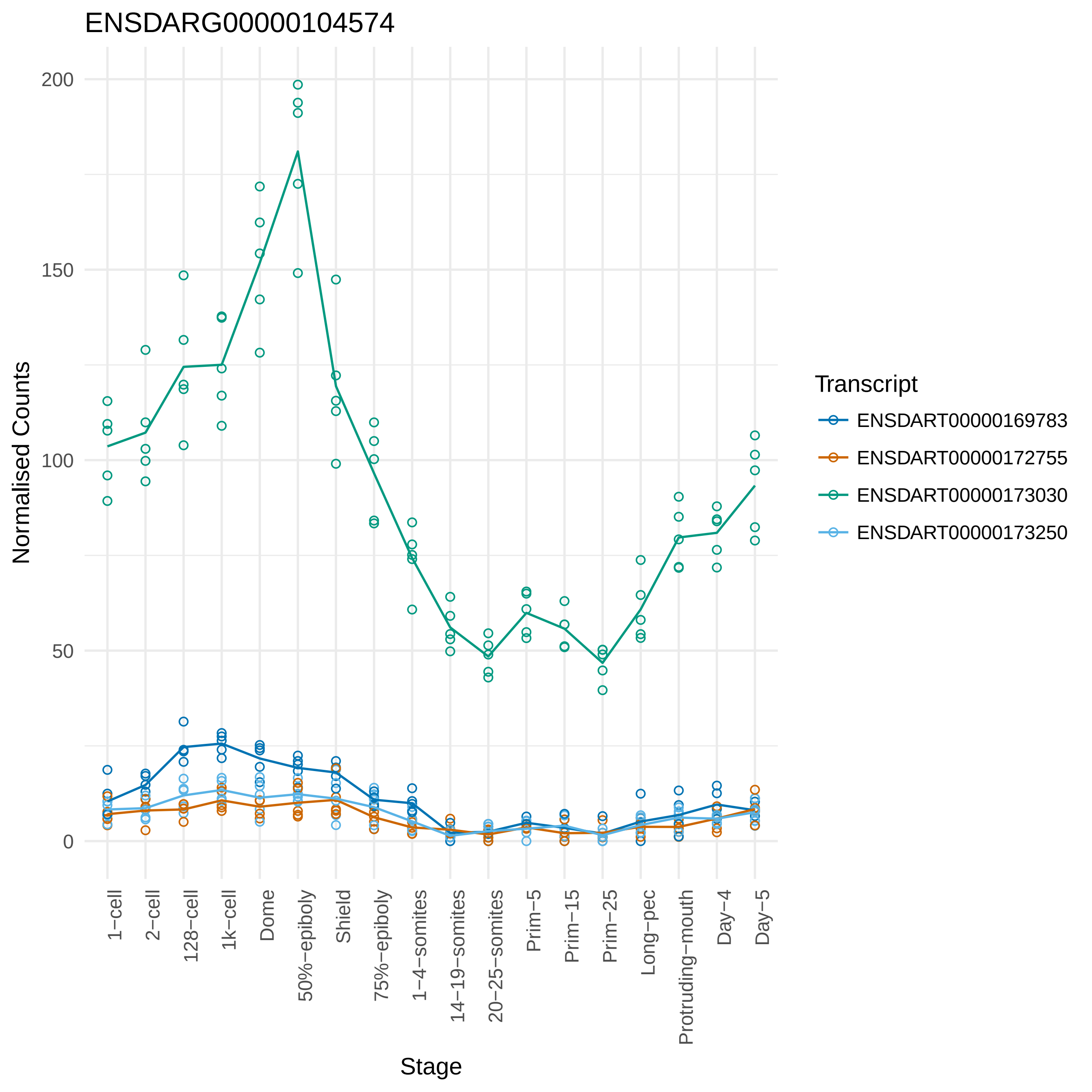

ENSDARG00000104574
Normalised Counts
200
150
100
50
0
Stage
1−cell
2−cell
128−cell
1k−cell
Dome
50%−epiboly
Shield
75%−epiboly
1−4−somites
14−19−somites
20−25−somites
Prim−5
Prim−15
Prim−25
Long−pec
Protruding−mouth
Day−4
Day−5
Transcript
ENSDART00000169783
ENSDART00000172755
ENSDART00000173030
ENSDART00000173250

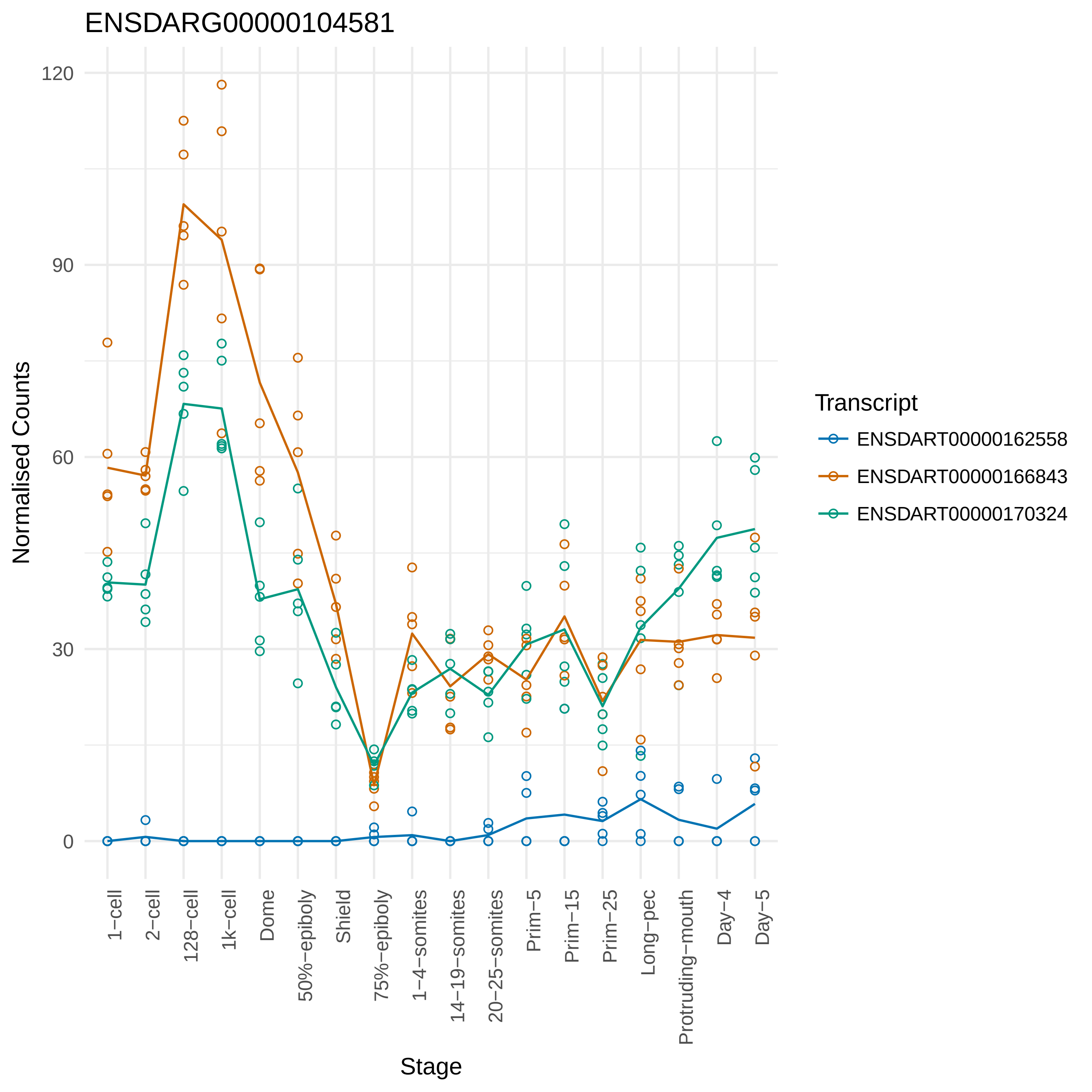
ENSDARG00000104581
Normalised Counts
120
90
60
30
0
1−cell
2−cell
128−cell
1k−cell
Dome
50%−epiboly
Shield
75%−epiboly
1−4−somites
14−19−somites
20−25−somites
Prim−5
Prim−15
Prim−25
Long−pec
Protruding−mouth
Day−4
Day−5
Stage
Transcript
ENSDART00000162558
ENSDART00000166843
ENSDART00000170324

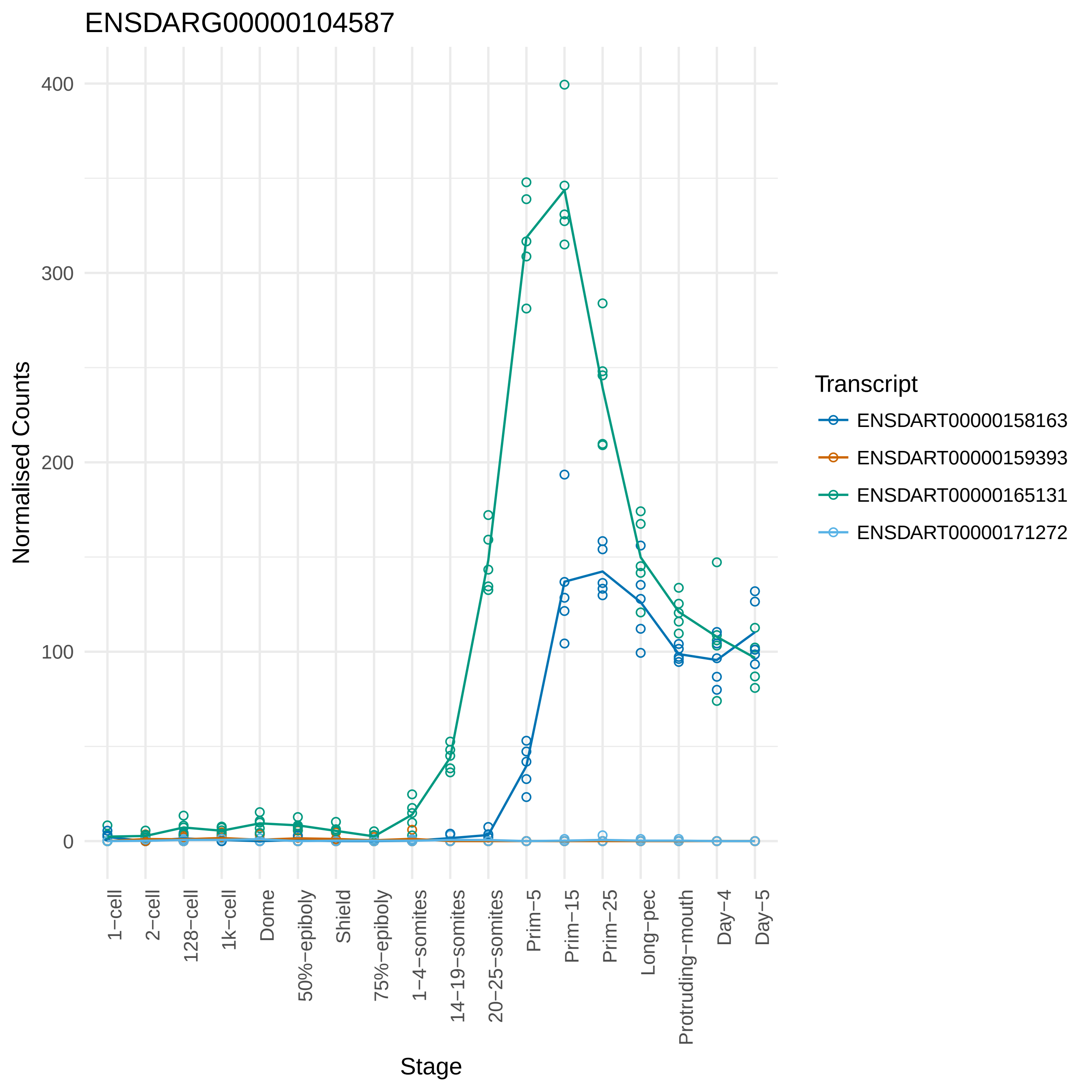
ENSDARG00000104587
Normalised Counts
400
300
200
100
0
1−cell
2−cell
128−cell
1k−cell
Dome
50%−epiboly
Shield
75%−epiboly
1−4−somites
14−19−somites
20−25−somites
Prim−5
Prim−15
Prim−25
Long−pec
Protruding−mouth
Day−4
Day−5
Stage
Transcript
ENSDART00000158163
ENSDART00000159393
ENSDART00000165131
ENSDART00000171272

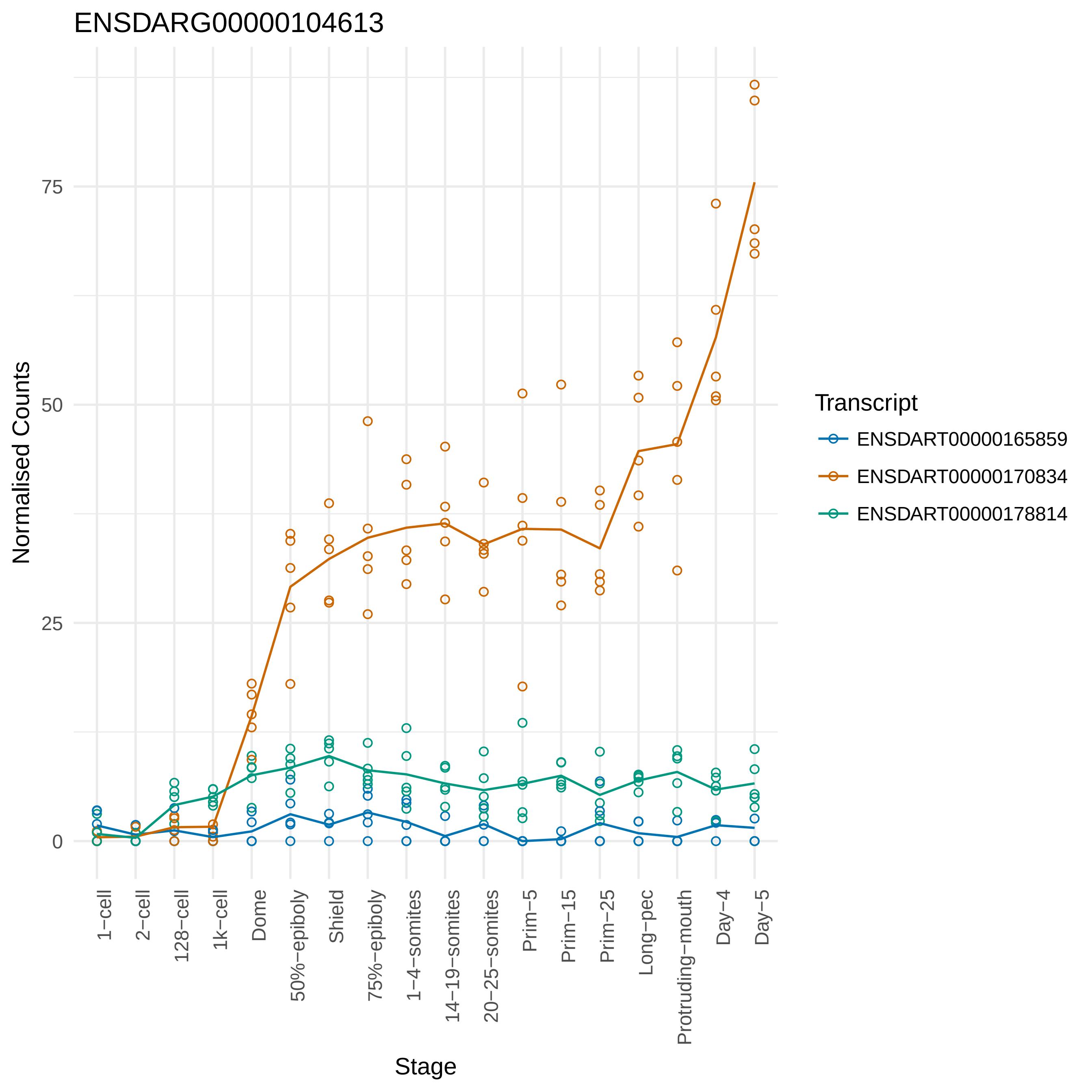

ENSDARG00000104613
Normalised Counts
75
50
25
0
1−cell
2−cell
128−cell
1k−cell
Dome
50%−epiboly
Shield
75%−epiboly
1−4−somites
14−19−somites
20−25−somites
Prim−5
Prim−15
Prim−25
Long−pec
Protruding−mouth
Day−4
Day−5
Stage
Transcript
ENSDART00000165859
ENSDART00000170834
ENSDART00000178814

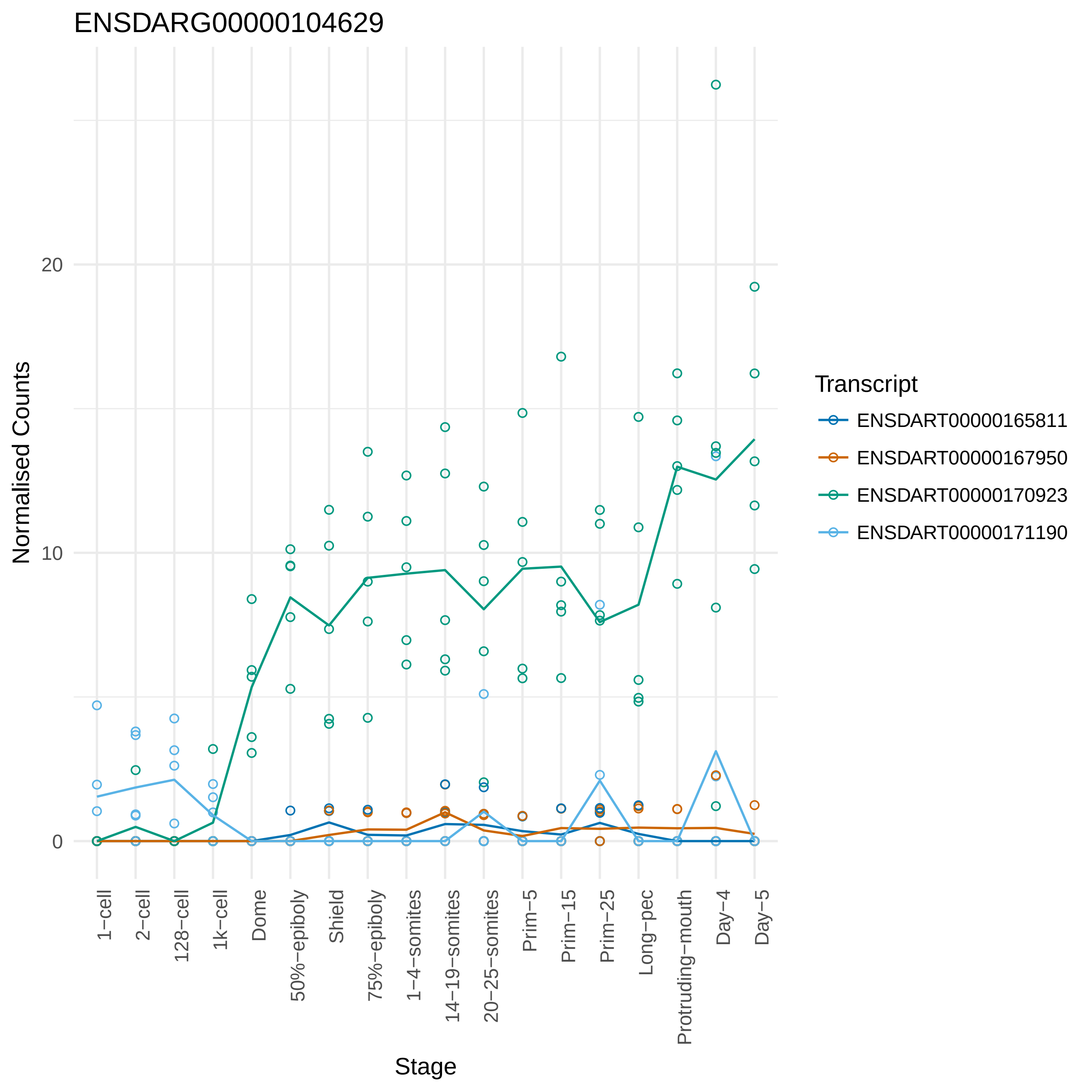

ENSDARG00000104629
Normalised Counts
0
10
20
1−cell
2−cell
128−cell
1k−cell
Dome
50%−epiboly
Shield
75%−epiboly
1−4−somites
14−19−somites
20−25−somites
Prim−5
Prim−15
Prim−25
Long−pec
Protruding−mouth
Day−4
Day−5
Stage
Transcript
ENSDART00000165811
ENSDART00000167950
ENSDART00000170923
ENSDART00000171190

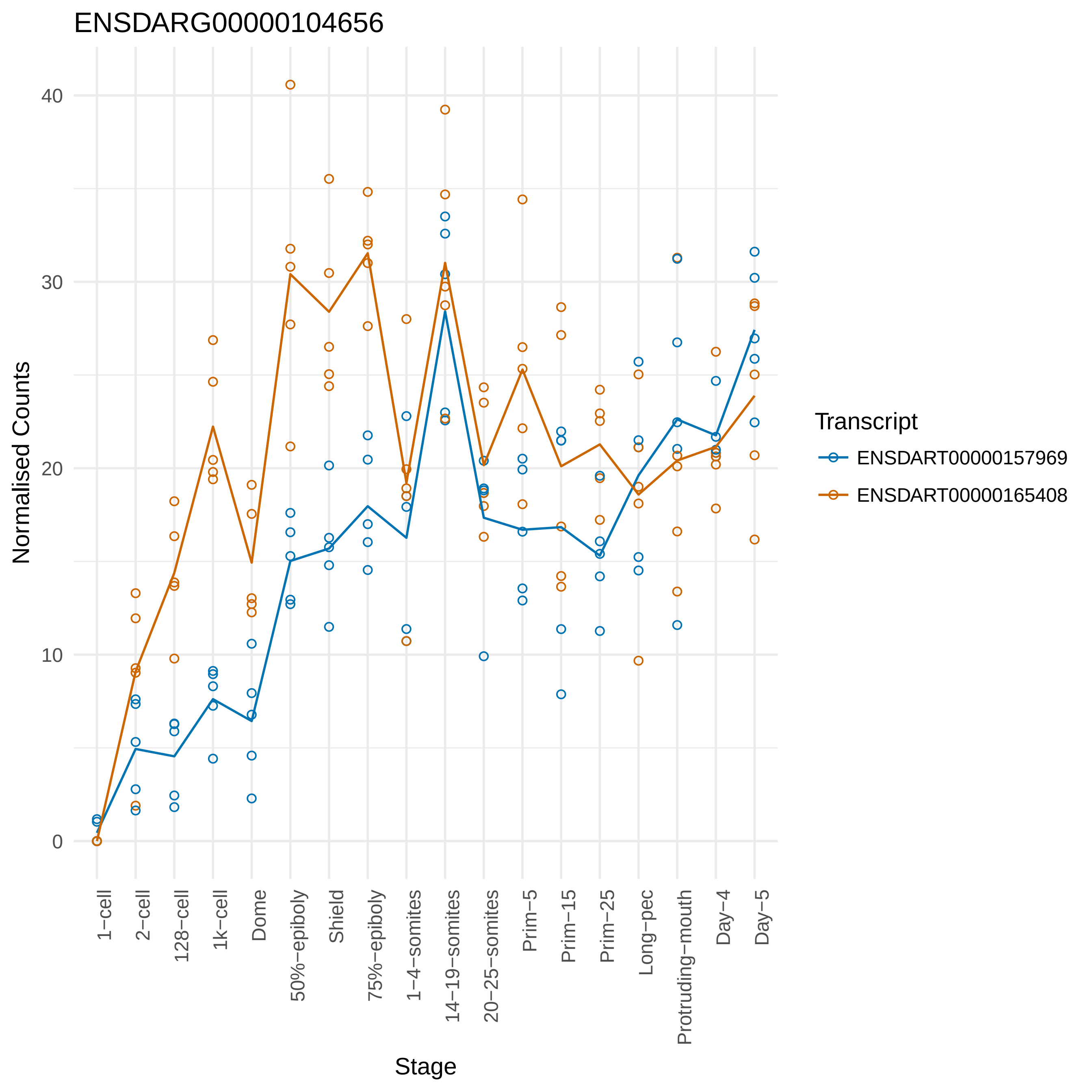
ENSDARG00000104656
Normalised Counts
0
10
20
30
40
Stage
1−cell
2−cell
128−cell
1k−cell
Dome
50%−epiboly
Shield
75%−epiboly
1−4−somites
14−19−somites
20−25−somites
Prim−5
Prim−15
Prim−25
Long−pec
Protruding−mouth
Day−4
Day−5
Transcript
ENSDART00000157969
ENSDART00000165408

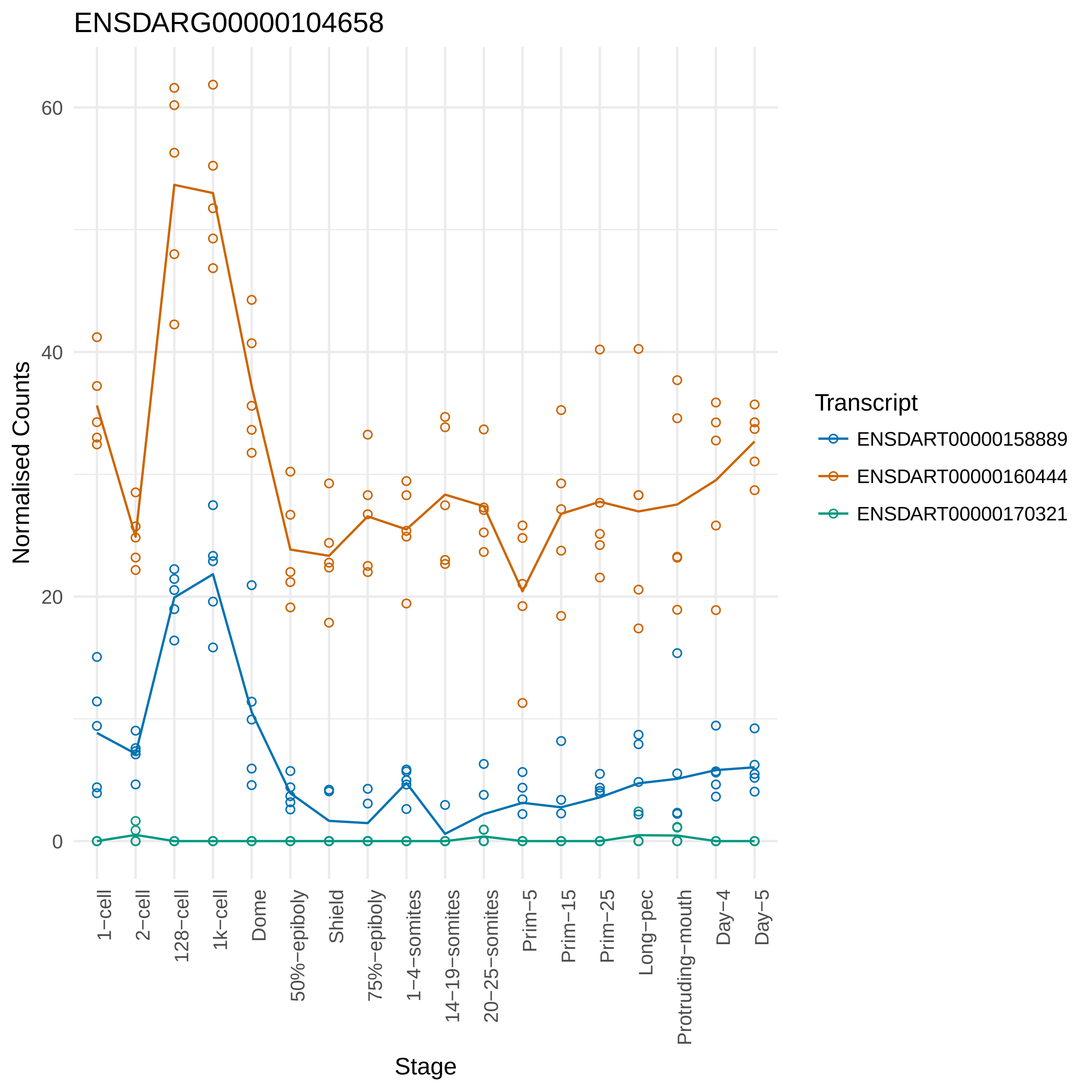

ENSDARG00000104658
Normalised Counts
0
20
40
60
1−cell
2−cell
128−cell
1k−cell
Dome
50%−epiboly
Shield
75%−epiboly
1−4−somites
14−19−somites
20−25−somites
Prim−5
Prim−15
Prim−25
Long−pec
Protruding−mouth
Day−4
Day−5
Stage
Transcript
ENSDART00000158889
ENSDART00000160444
ENSDART00000170321

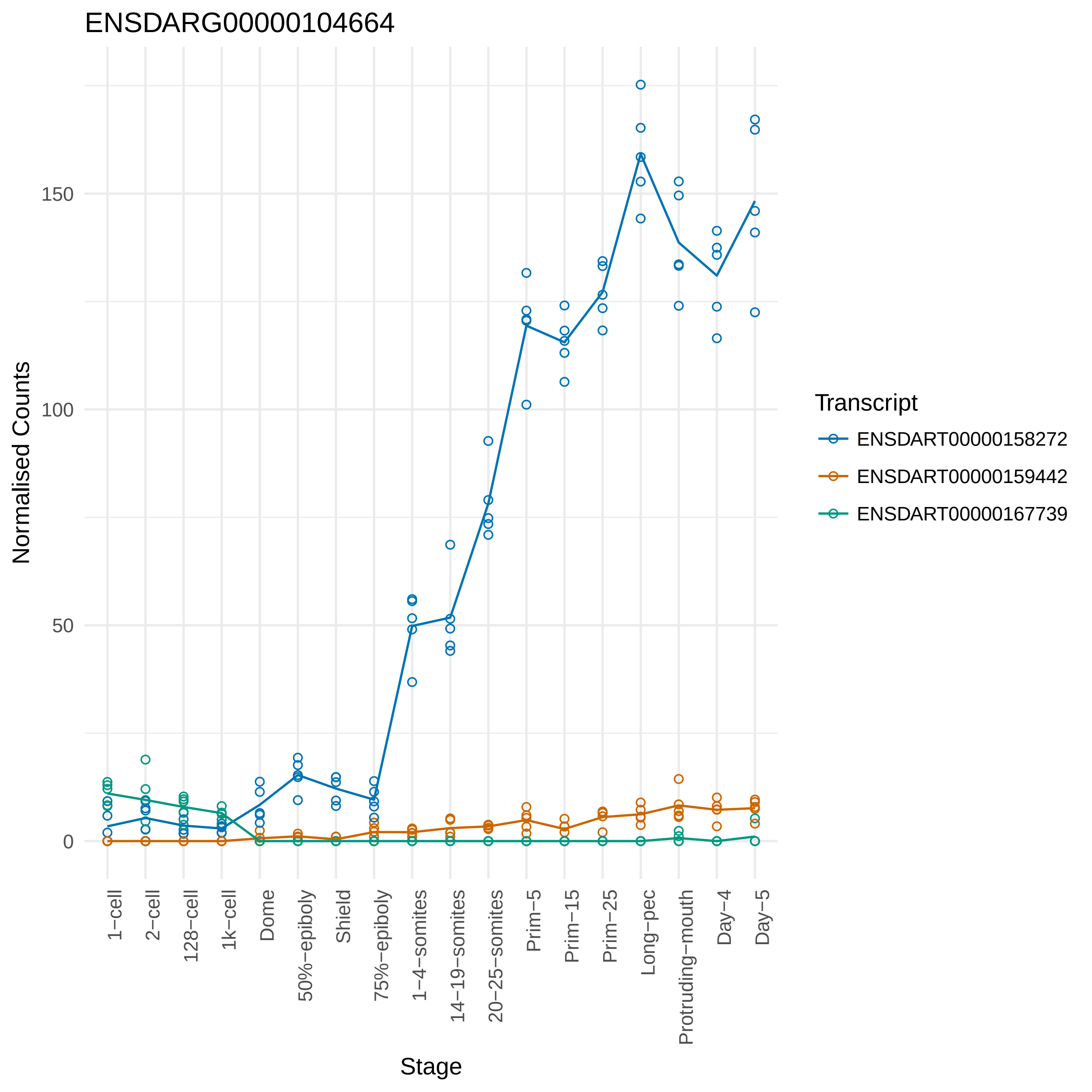
ENSDARG00000104664
Normalised Counts
0
50
100
150
Stage
1−cell
2−cell
128−cell
1k−cell
Dome
50%−epiboly
Shield
75%−epiboly
1−4−somites
14−19−somites
20−25−somites
Prim−5
Prim−15
Prim−25
Long−pec
Protruding−mouth
Day−4
Day−5
Transcript
ENSDART00000158272
ENSDART00000159442
ENSDART00000167739

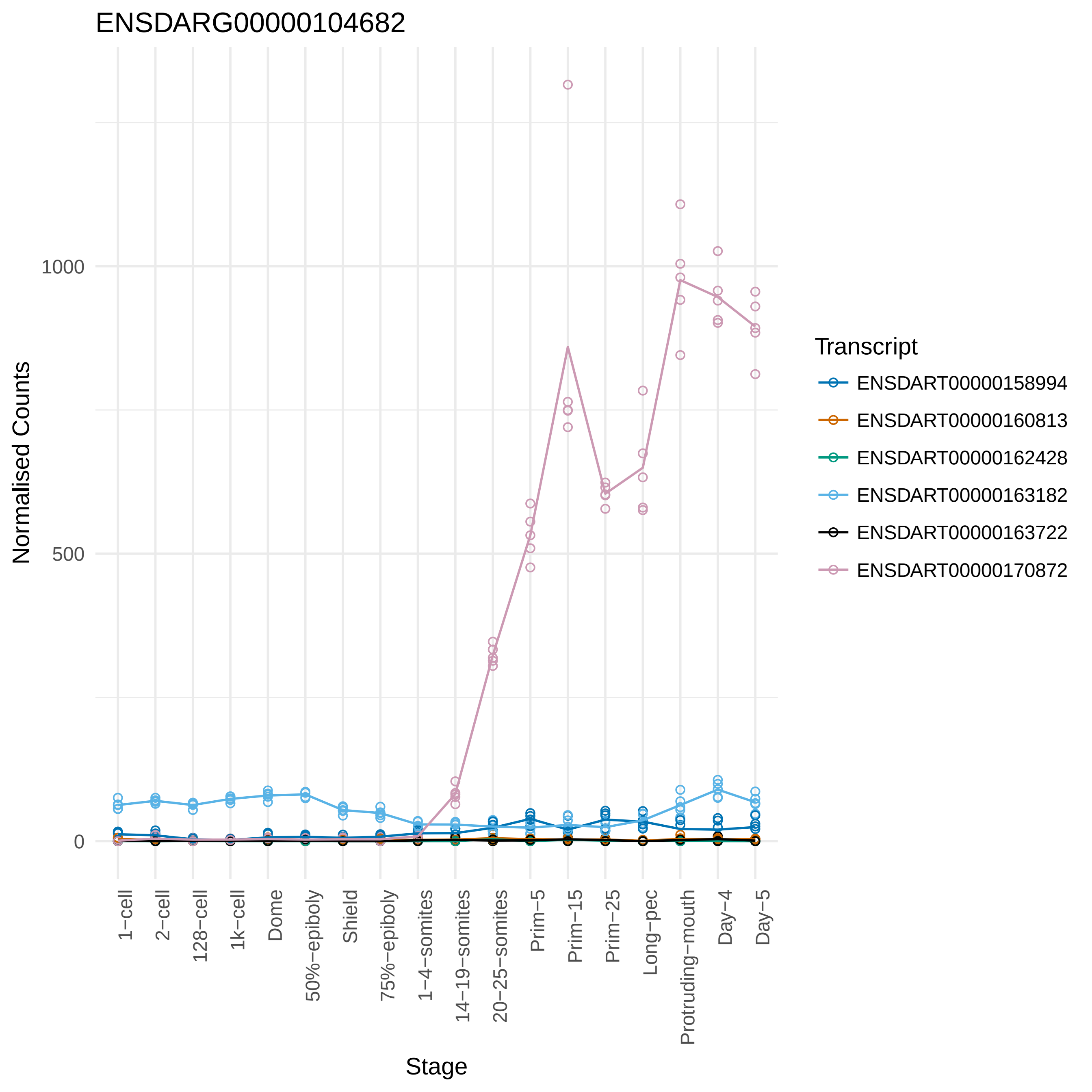
ENSDARG00000104682
Normalised Counts
1000
500
0
1−cell
2−cell
128−cell
1k−cell
Dome
50%−epiboly
Shield
75%−epiboly
1−4−somites
14−19−somites
20−25−somites
Prim−5
Prim−15
Prim−25
Long−pec
Protruding−mouth
Day−4
Day−5
Stage
Transcript
ENSDART00000158994
ENSDART00000160813
ENSDART00000162428
ENSDART00000163182
ENSDART00000163722
ENSDART00000170872

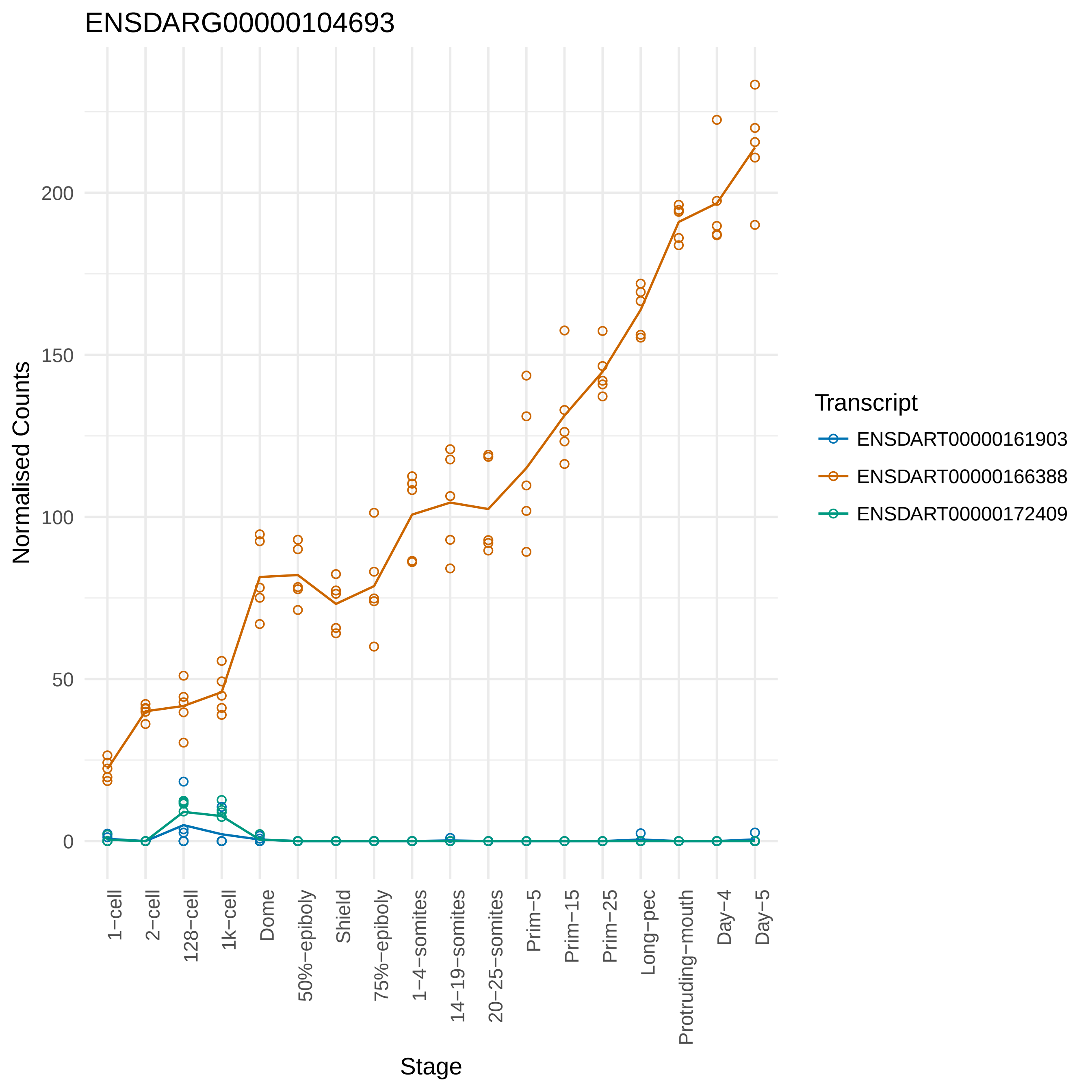

ENSDARG00000104693
Normalised Counts
0
50
100
150
200
Stage
1−cell
2−cell
128−cell
1k−cell
Dome
50%−epiboly
Shield
75%−epiboly
1−4−somites
14−19−somites
20−25−somites
Prim−5
Prim−15
Prim−25
Long−pec
Protruding−mouth
Day−4
Day−5
Transcript
ENSDART00000161903
ENSDART00000166388
ENSDART00000172409

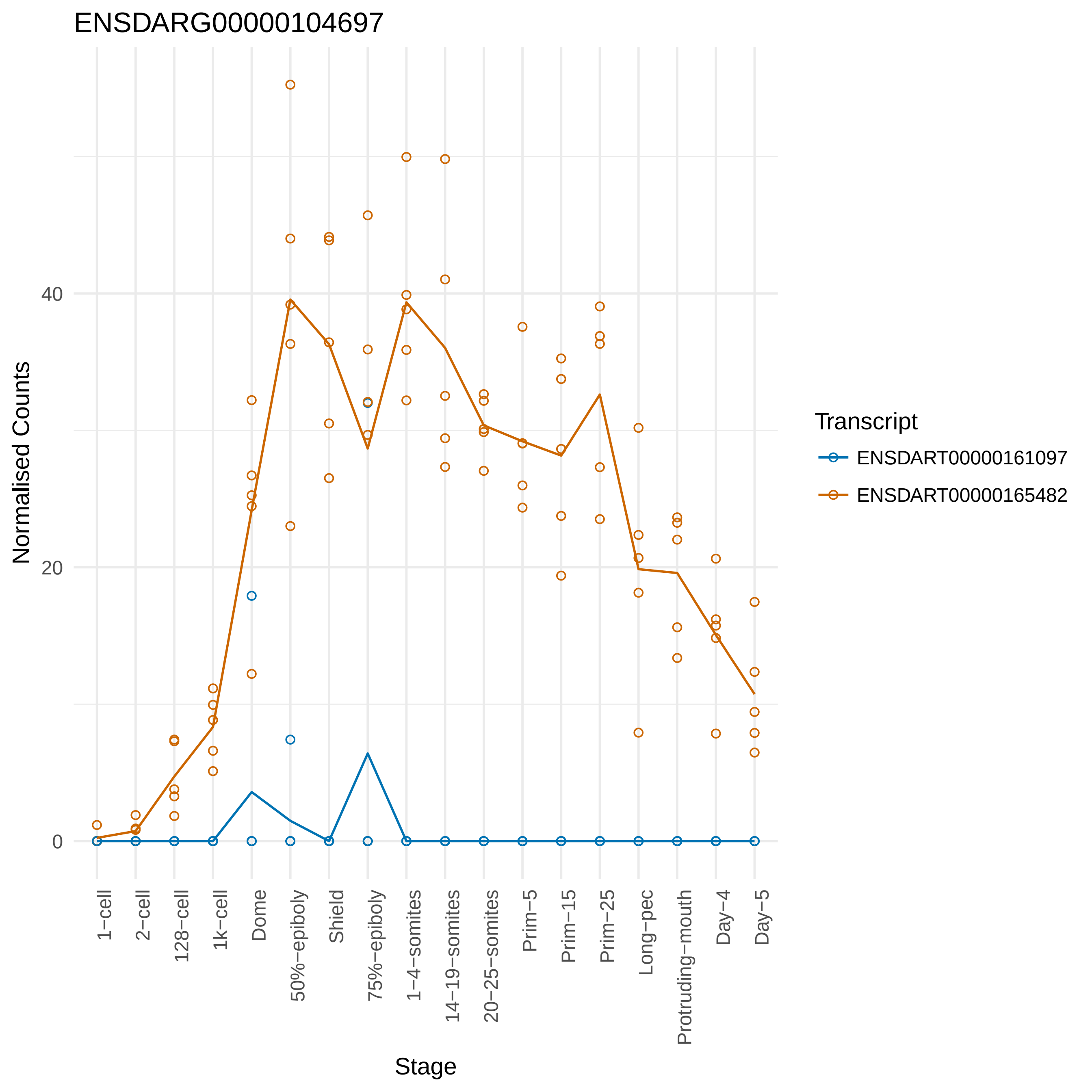

ENSDARG00000104697
Normalised Counts
0
20
40
1−cell
2−cell
128−cell
1k−cell
Dome
50%−epiboly
Shield
75%−epiboly
1−4−somites
14−19−somites
20−25−somites
Prim−5
Prim−15
Prim−25
Long−pec
Protruding−mouth
Day−4
Day−5
Stage
Transcript
ENSDART00000161097
ENSDART00000165482

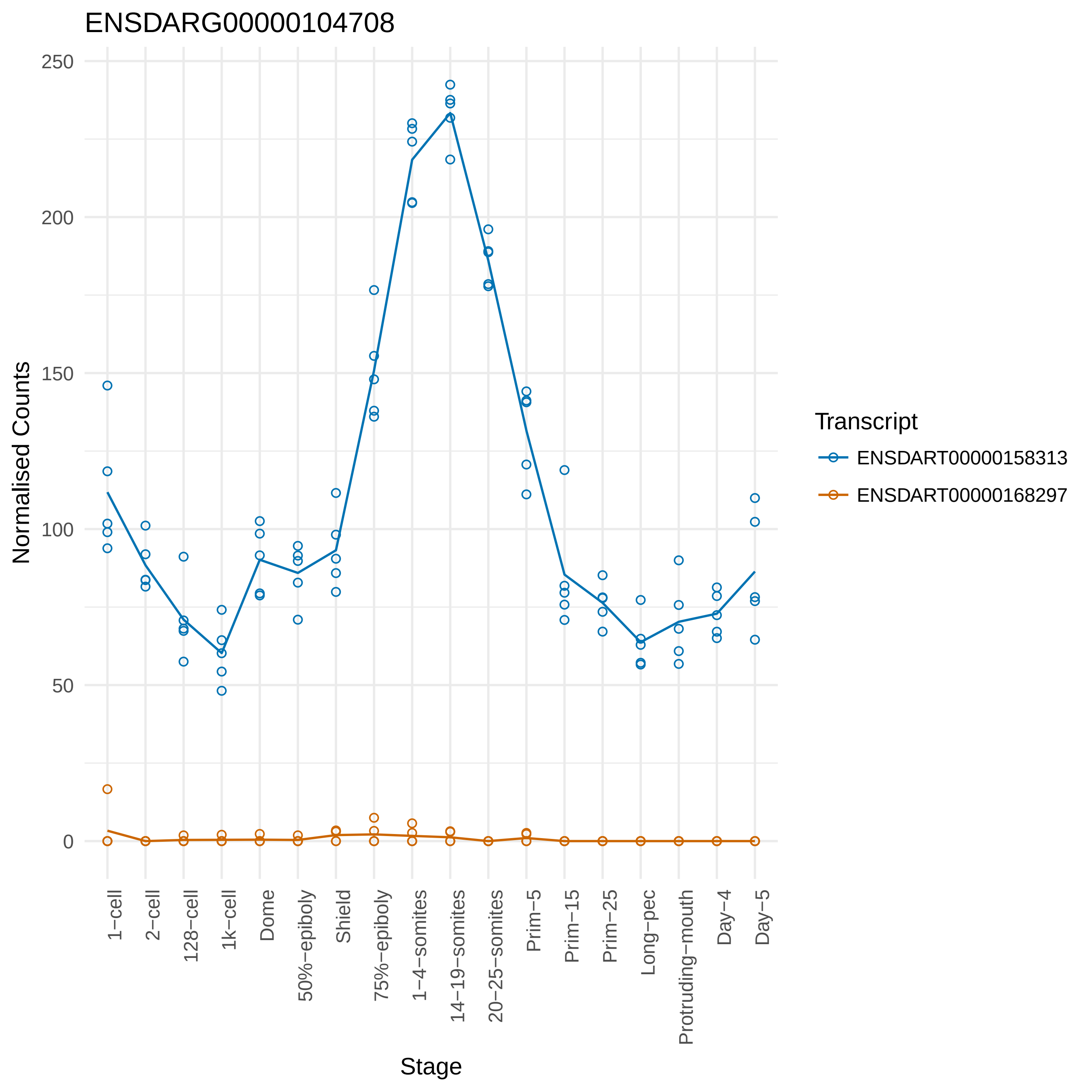

ENSDARG00000104708
Normalised Counts
0
50
100
150
200
250
Stage
1−cell
2−cell
128−cell
1k−cell
Dome
50%−epiboly
Shield
75%−epiboly
1−4−somites
14−19−somites
20−25−somites
Prim−5
Prim−15
Prim−25
Long−pec
Protruding−mouth
Day−4
Day−5
Transcript
ENSDART00000158313
ENSDART00000168297

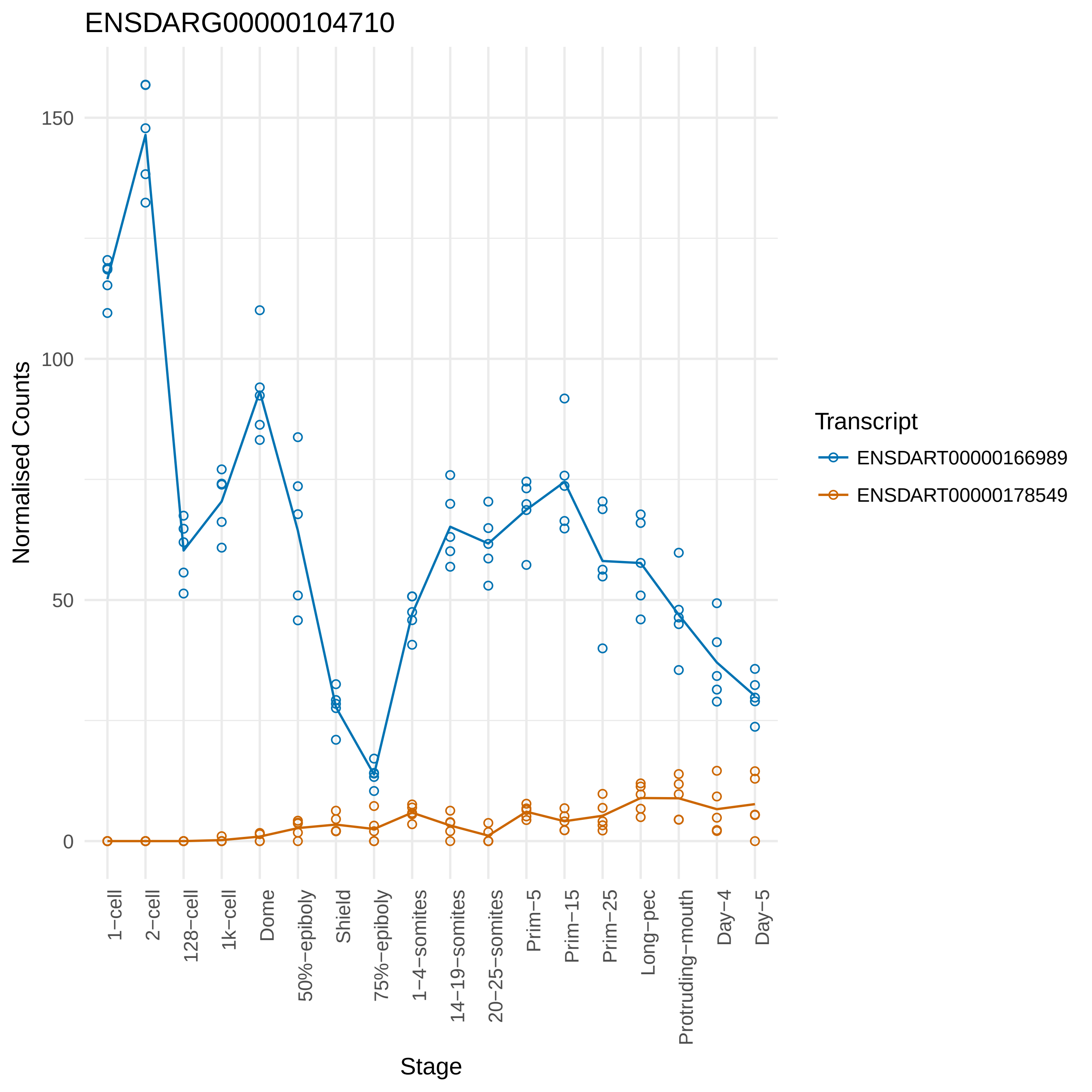

ENSDARG00000104710
Normalised Counts
150
100
50
0
1−cell
2−cell
128−cell
1k−cell
Dome
50%−epiboly
Shield
75%−epiboly
1−4−somites
14−19−somites
20−25−somites
Prim−5
Prim−15
Prim−25
Long−pec
Protruding−mouth
Day−4
Day−5
Stage
Transcript
ENSDART00000166989
ENSDART00000178549

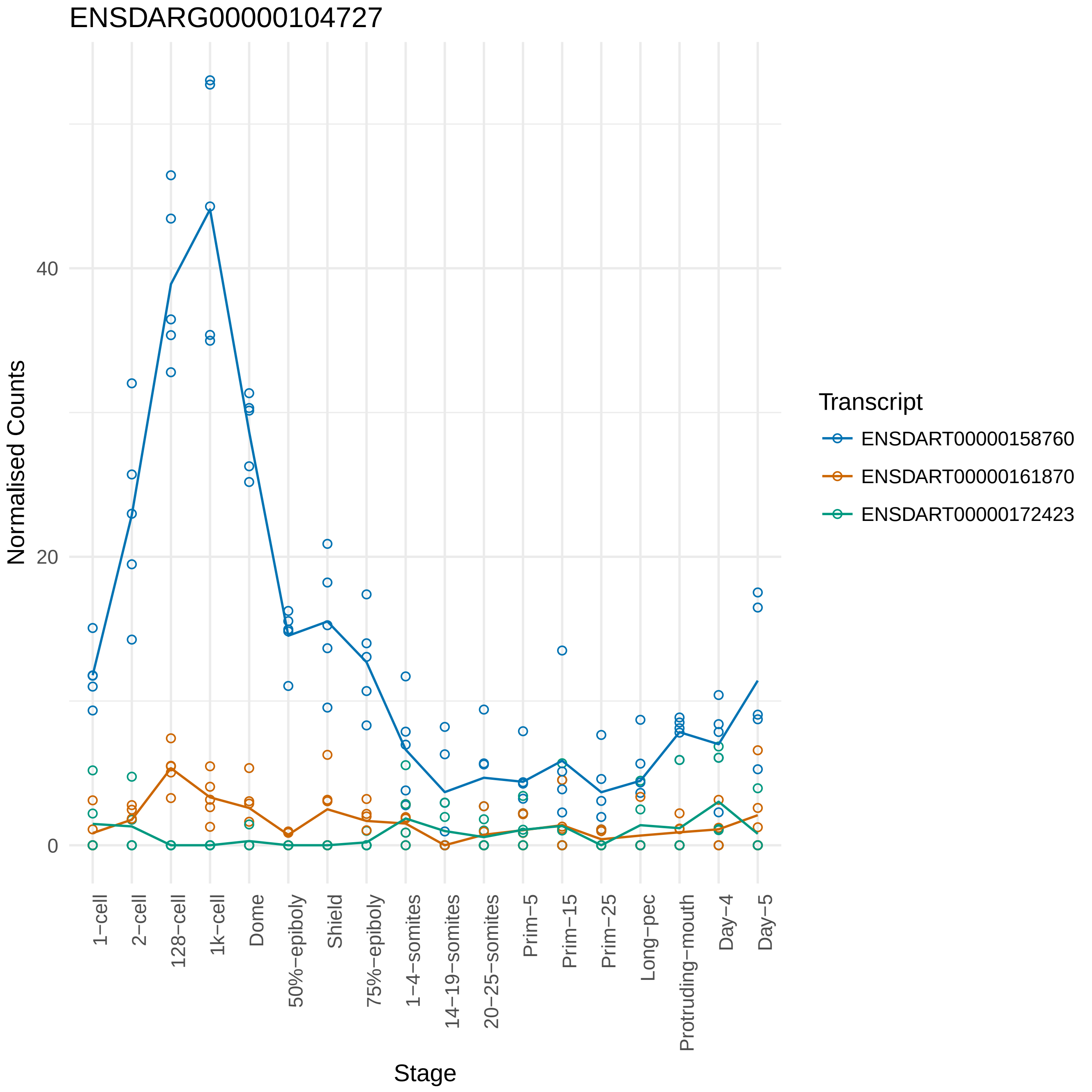
ENSDARG00000104727
Normalised Counts
0
20
40
1−cell
2−cell
128−cell
1k−cell
Dome
50%−epiboly
Shield
75%−epiboly
1−4−somites
14−19−somites
20−25−somites
Prim−5
Prim−15
Prim−25
Long−pec
Protruding−mouth
Day−4
Day−5
Stage
Transcript
ENSDART00000158760
ENSDART00000161870
ENSDART00000172423

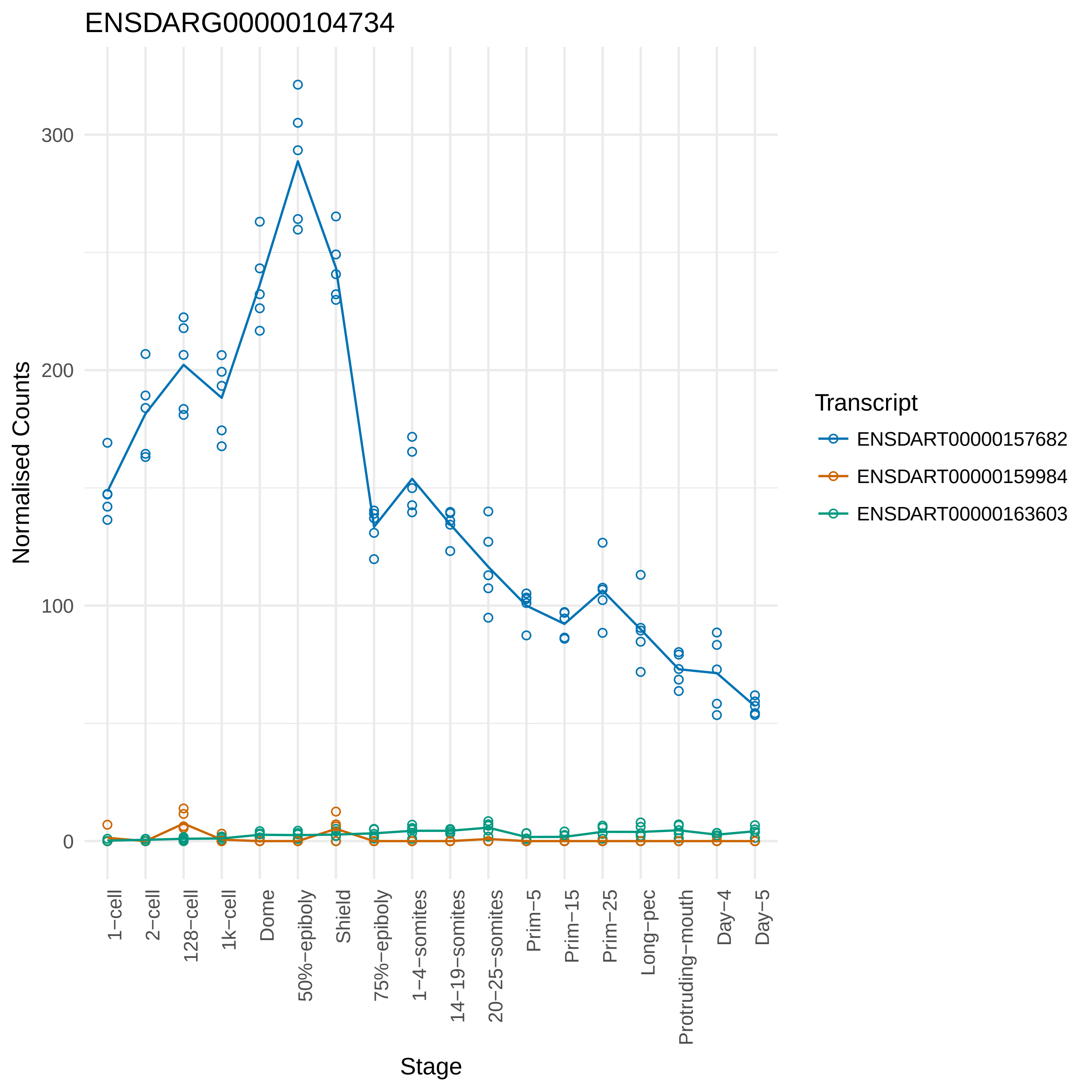

ENSDARG00000104734
Normalised Counts
300
200
100
0
1−cell
2−cell
128−cell
1k−cell
Dome
50%−epiboly
Shield
75%−epiboly
1−4−somites
14−19−somites
20−25−somites
Prim−5
Prim−15
Prim−25
Long−pec
Protruding−mouth
Day−4
Day−5
Stage
Transcript
ENSDART00000157682
ENSDART00000159984
ENSDART00000163603

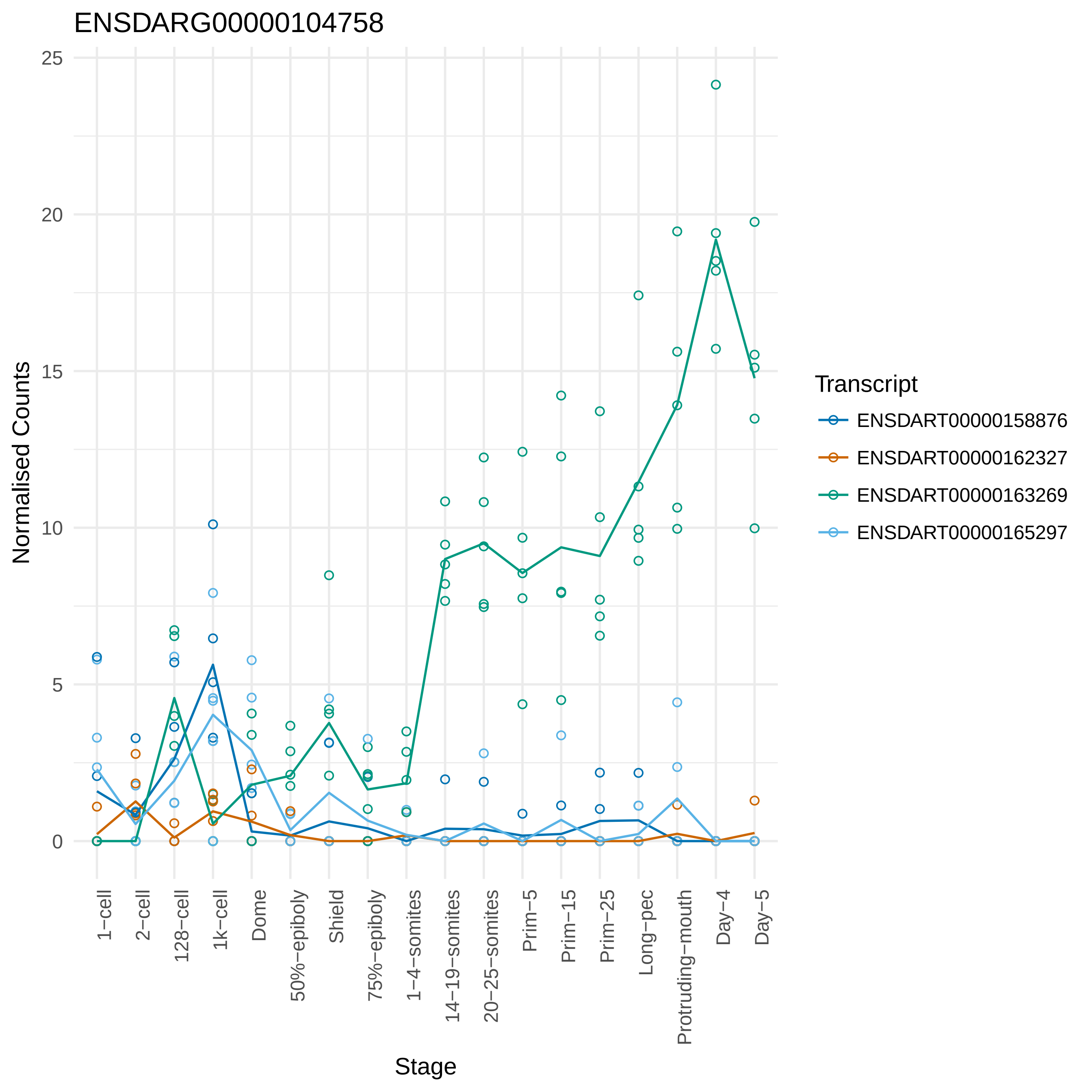

ENSDARG00000104758
Normalised Counts
0
5
10
15
20
25
1−cell
2−cell
128−cell
1k−cell
Dome
50%−epiboly
Shield
75%−epiboly
1−4−somites
14−19−somites
20−25−somites
Prim−5
Prim−15
Prim−25
Long−pec
Protruding−mouth
Day−4
Day−5
Stage
Transcript
ENSDART00000158876
ENSDART00000162327
ENSDART00000163269
ENSDART00000165297

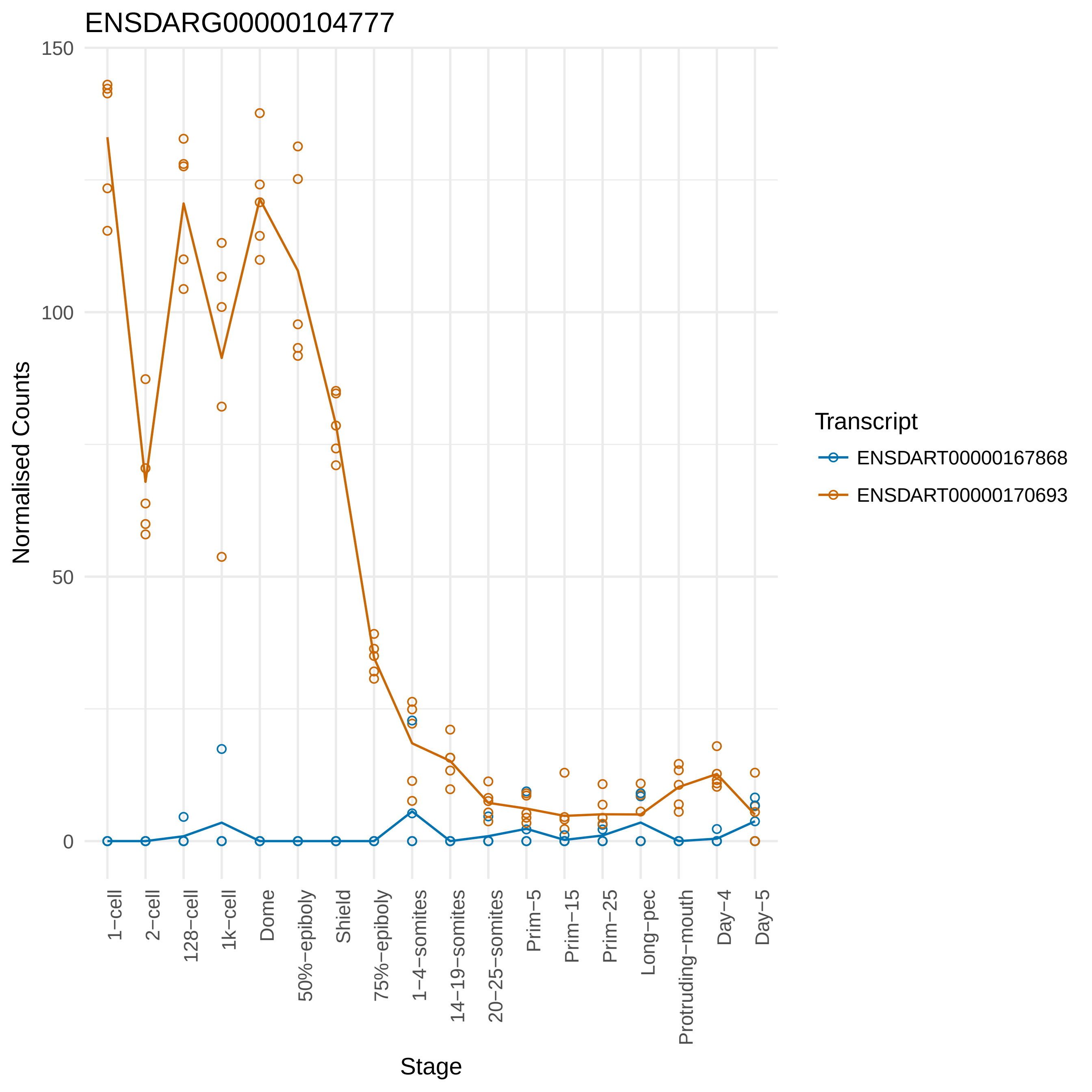

ENSDARG00000104777
150
100
50
0
Normalised Counts
1−cell
2−cell
128−cell
1k−cell
Dome
50%−epiboly
Shield
75%−epiboly
1−4−somites
14−19−somites
20−25−somites
Prim−5
Prim−15
Prim−25
Long−pec
Protruding−mouth
Day−4
Day−5
Stage
Transcript
ENSDART00000167868
ENSDART00000170693

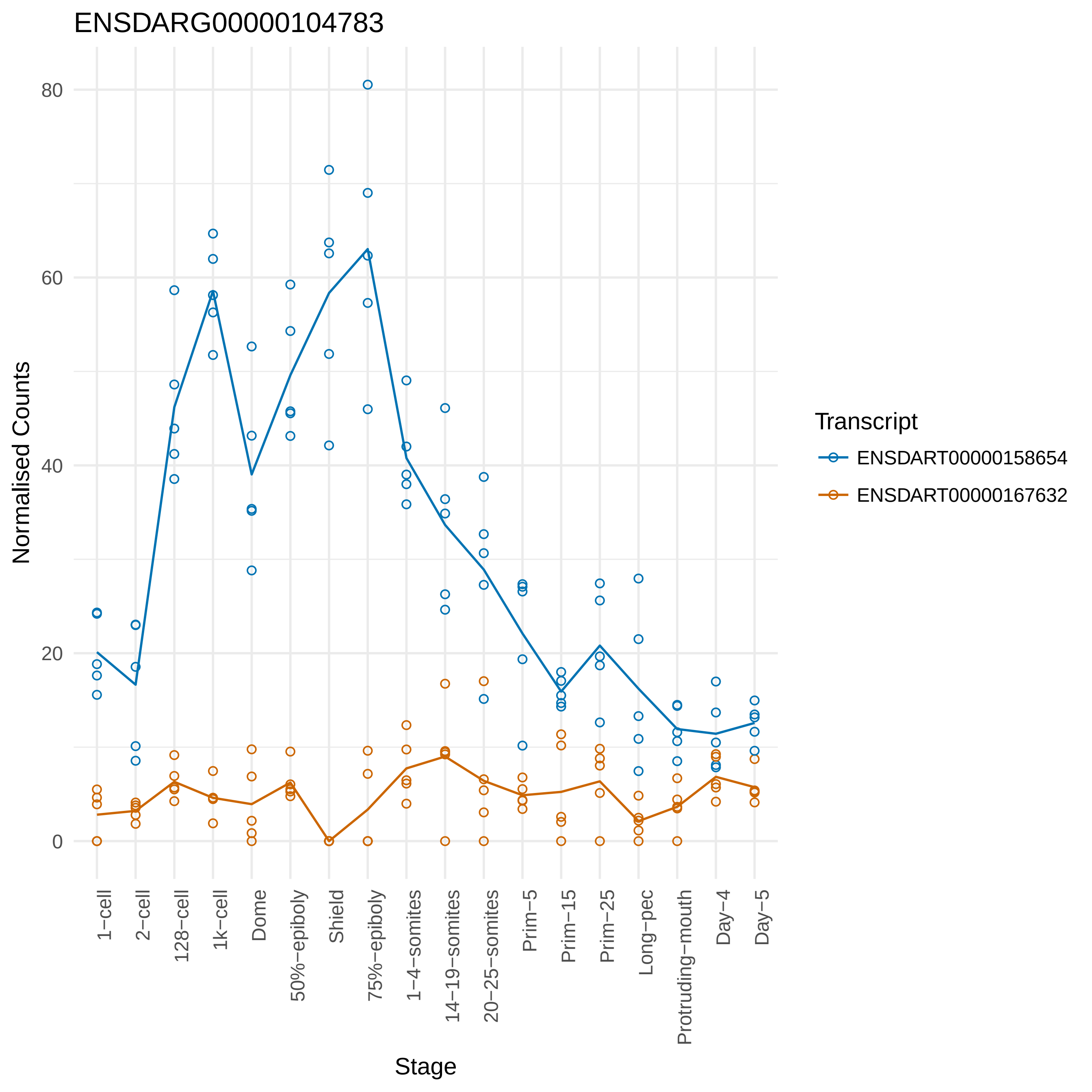

ENSDARG00000104783
Normalised Counts
80
60
40
20
0
1−cell
2−cell
128−cell
1k−cell
Dome
50%−epiboly
Shield
75%−epiboly
1−4−somites
14−19−somites
20−25−somites
Prim−5
Prim−15
Prim−25
Long−pec
Protruding−mouth
Day−4
Day−5
Stage
Transcript
ENSDART00000158654
ENSDART00000167632

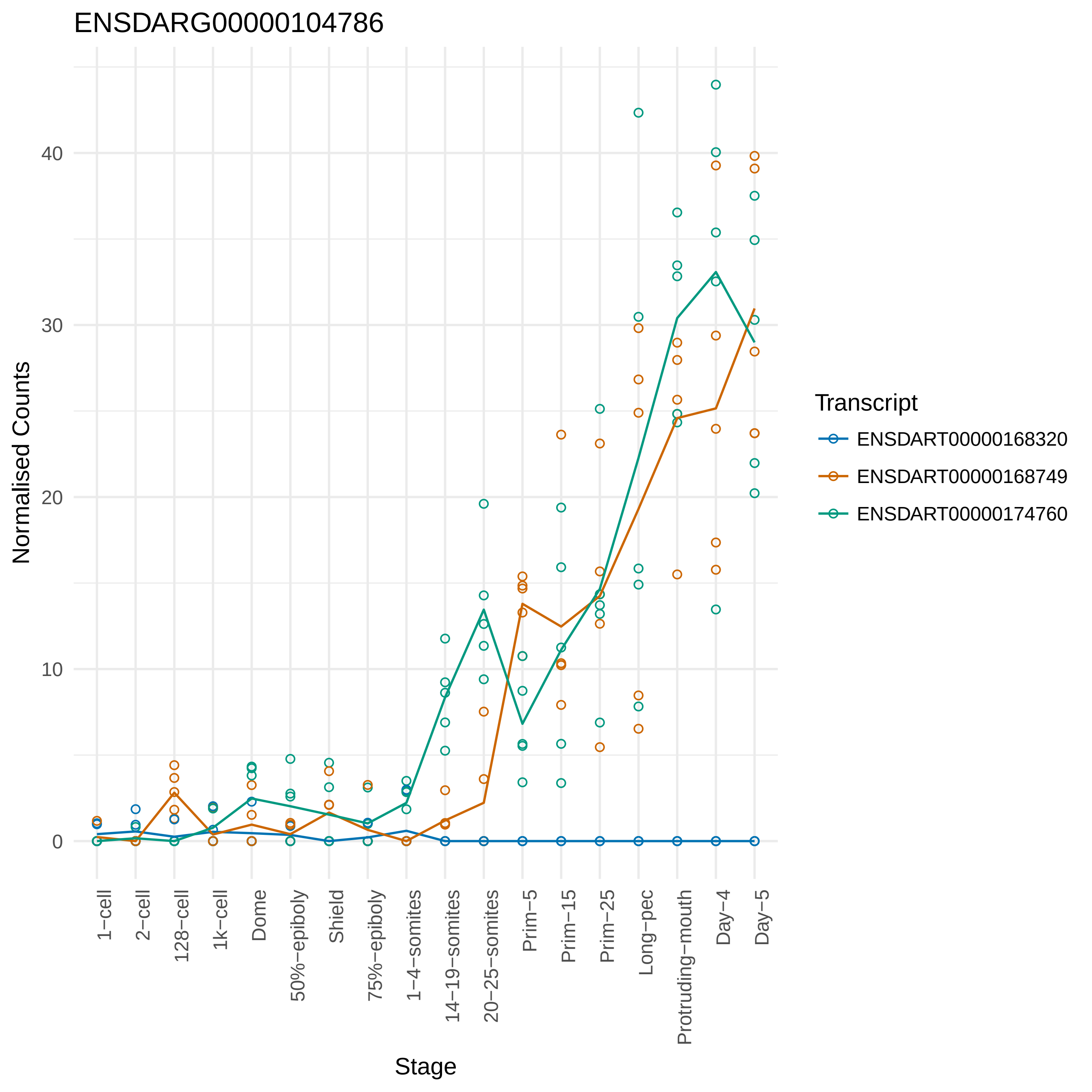
ENSDARG00000104786
Normalised Counts
0
10
20
30
40
Stage
1−cell
2−cell
128−cell
1k−cell
Dome
50%−epiboly
Shield
75%−epiboly
1−4−somites
14−19−somites
20−25−somites
Prim−5
Prim−15
Prim−25
Long−pec
Protruding−mouth
Day−4
Day−5
Transcript
ENSDART00000168320
ENSDART00000168749
ENSDART00000174760

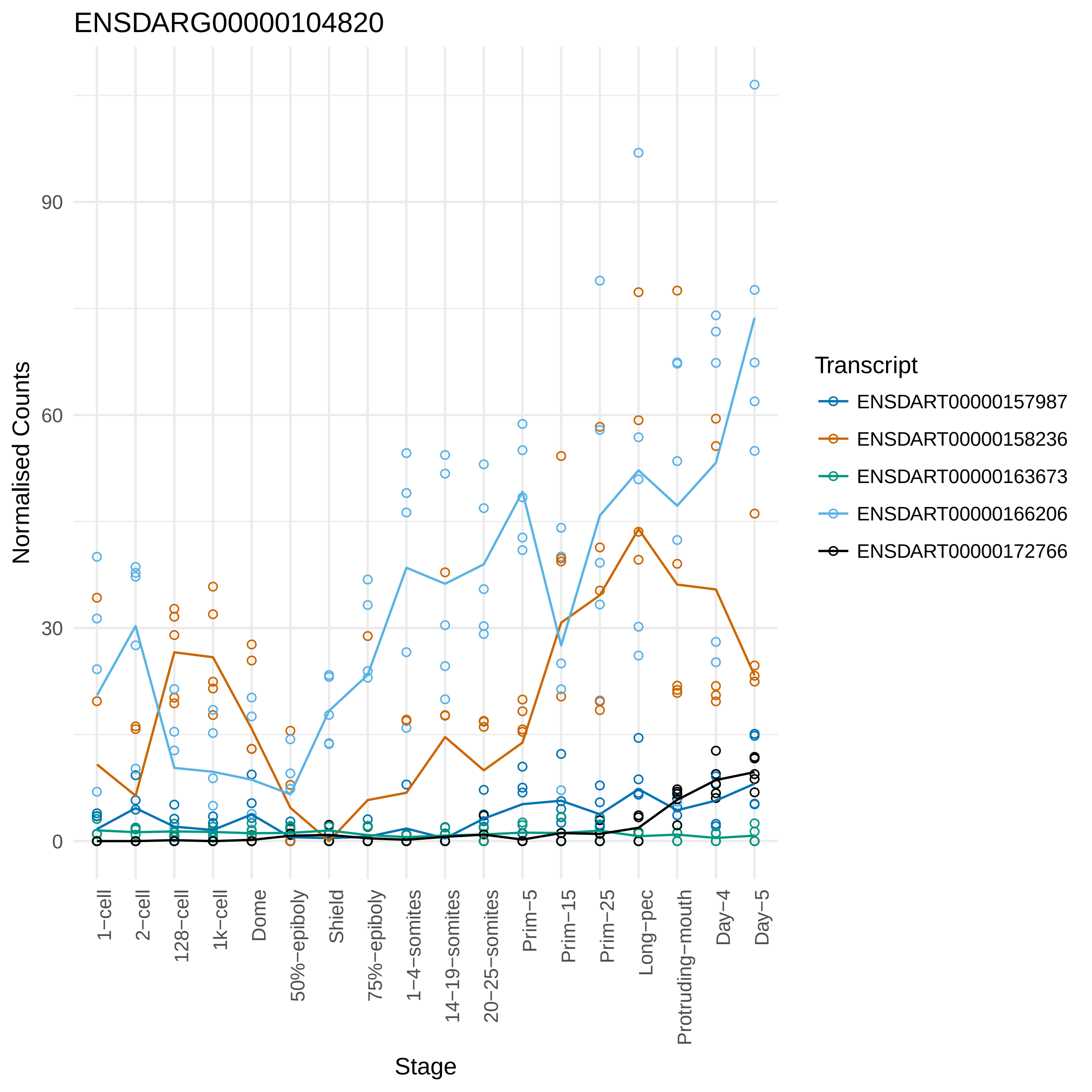

ENSDARG00000104820
Normalised Counts
0
30
60
90
1−cell
2−cell
128−cell
1k−cell
Dome
50%−epiboly
Shield
75%−epiboly
1−4−somites
14−19−somites
20−25−somites
Prim−5
Prim−15
Prim−25
Long−pec
Protruding−mouth
Day−4
Day−5
Stage
Transcript
ENSDART00000157987
ENSDART00000158236
ENSDART00000163673
ENSDART00000166206
ENSDART00000172766

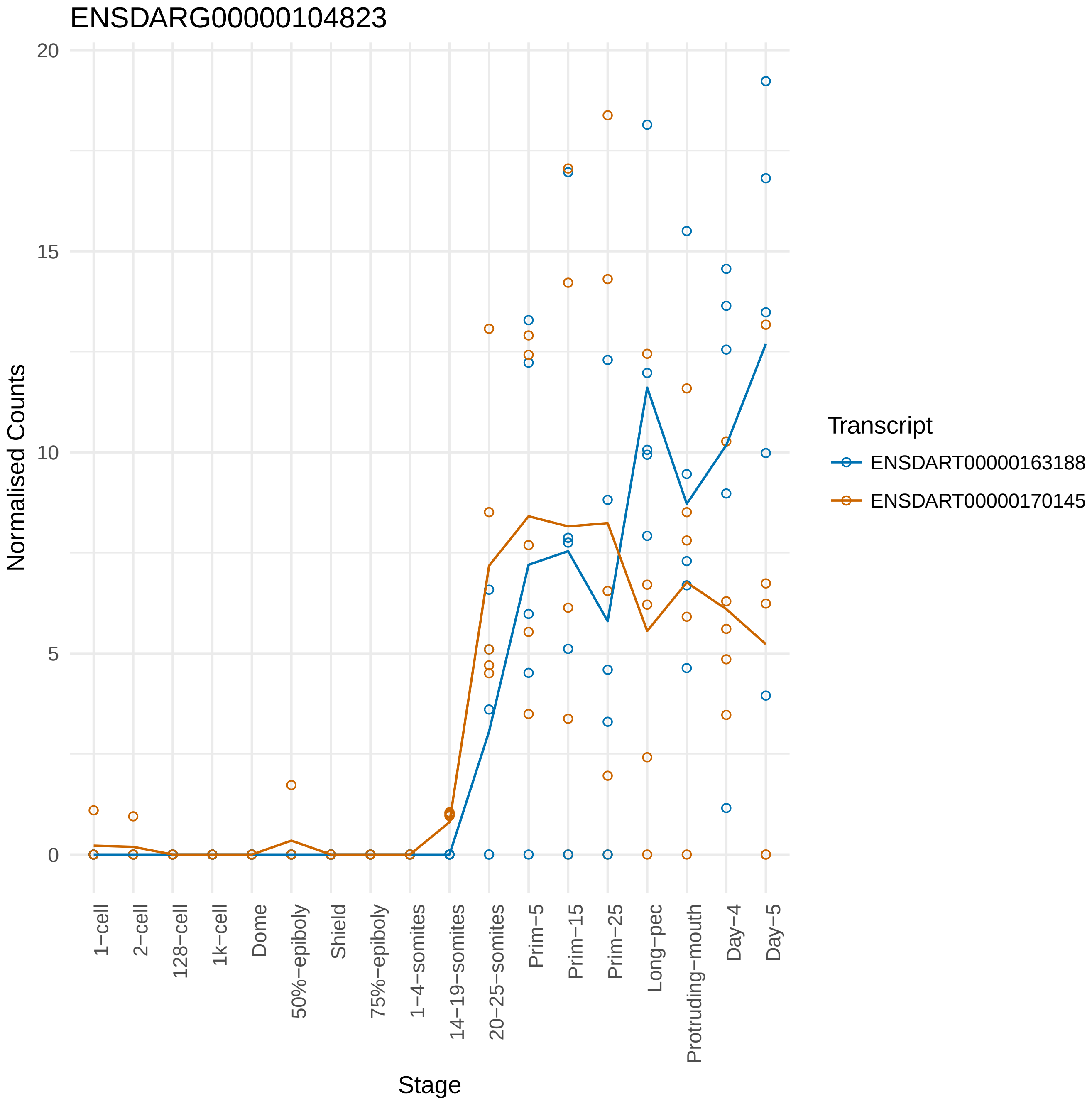
ENSDARG00000104823
20
15
10
5
0
Normalised Counts
1−cell
2−cell
128−cell
1k−cell
Dome
50%−epiboly
Shield
75%−epiboly
1−4−somites
14−19−somites
20−25−somites
Prim−5
Prim−15
Prim−25
Long−pec
Protruding−mouth
Day−4
Day−5
Stage
Transcript
ENSDART00000163188
ENSDART00000170145

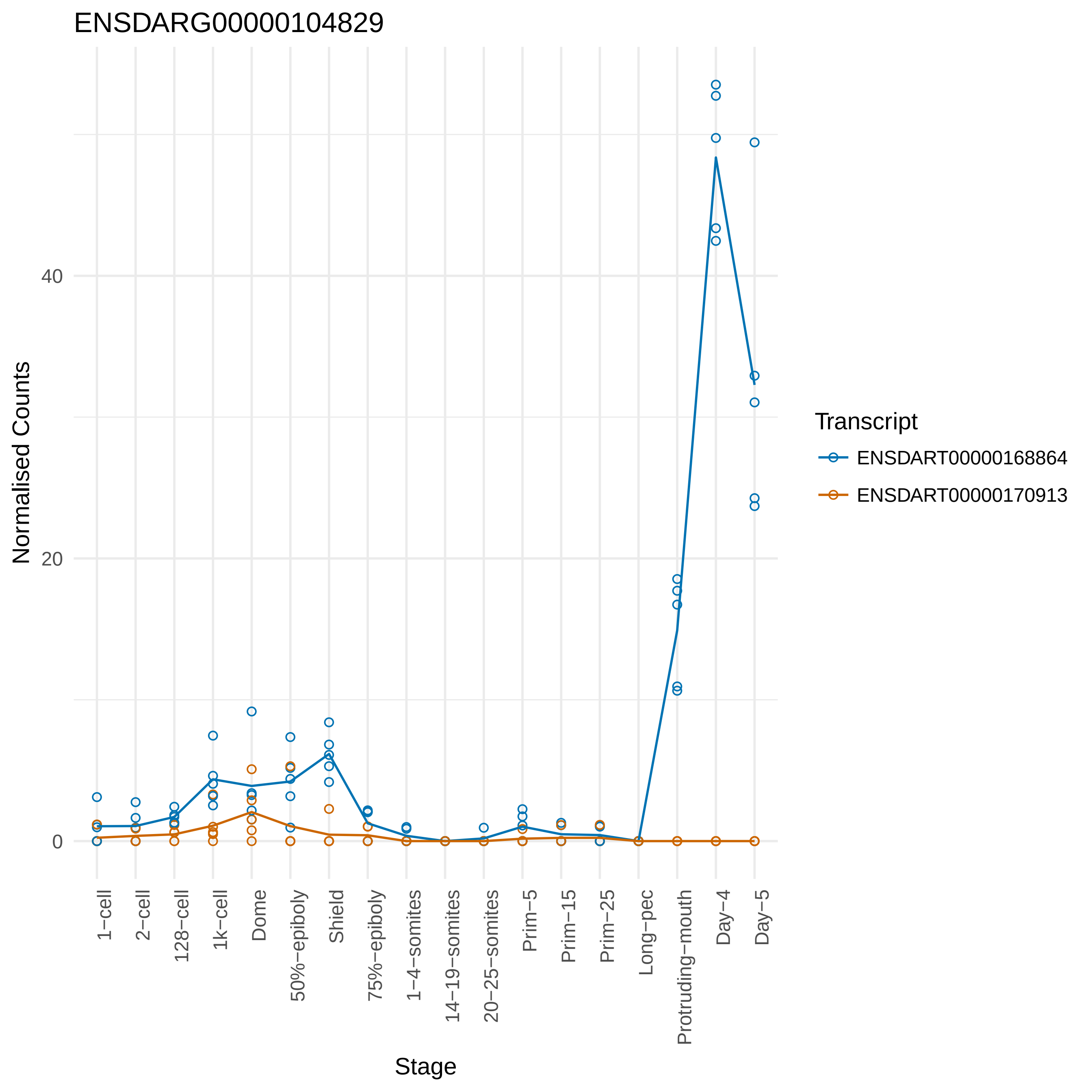
ENSDARG00000104829
Normalised Counts
0
20
40
1−cell
2−cell
128−cell
1k−cell
Dome
50%−epiboly
Shield
75%−epiboly
1−4−somites
14−19−somites
20−25−somites
Prim−5
Prim−15
Prim−25
Long−pec
Protruding−mouth
Day−4
Day−5
Stage
Transcript
ENSDART00000168864
ENSDART00000170913

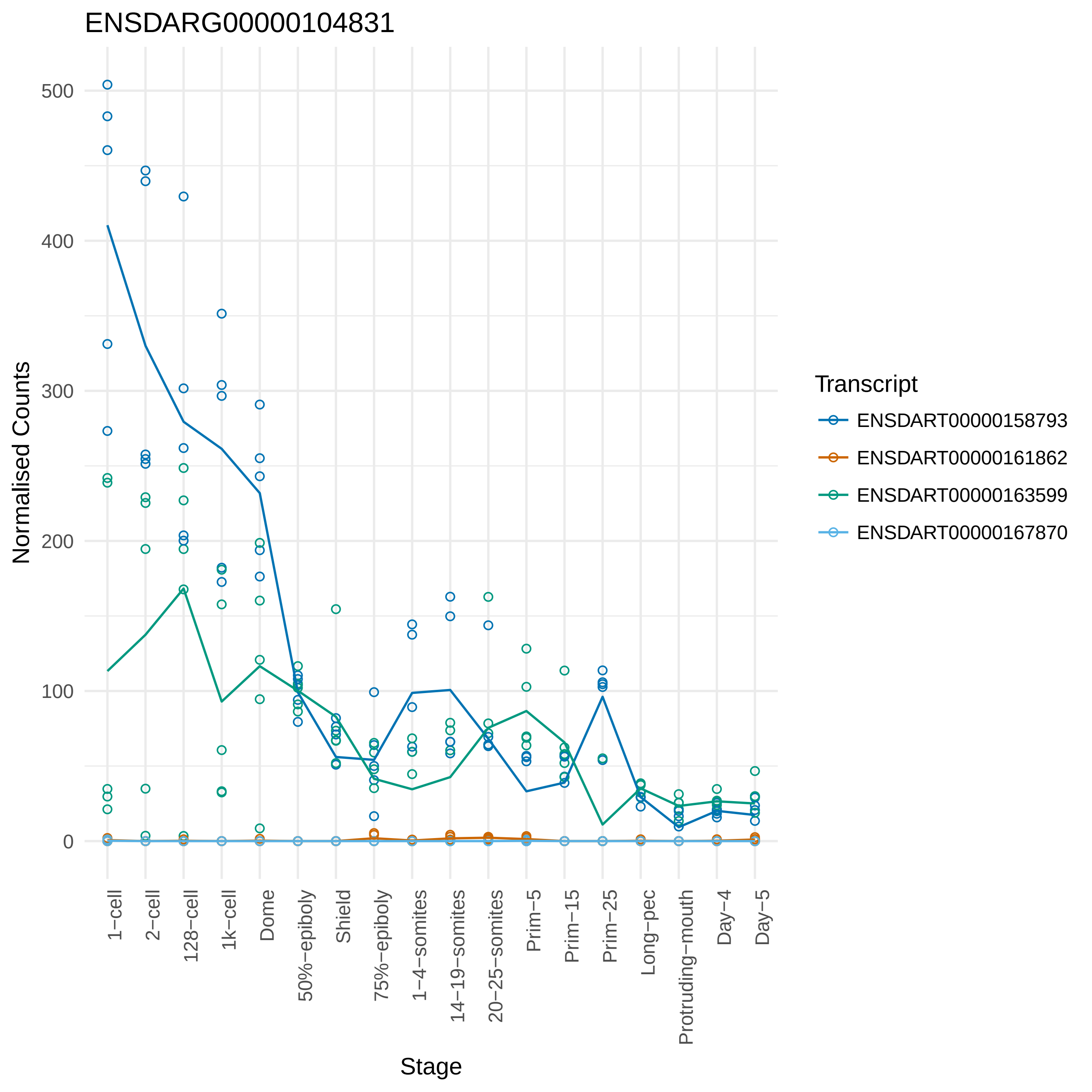
ENSDARG00000104831
Normalised Counts
500
400
300
200
100
0
1−cell
2−cell
128−cell
1k−cell
Dome
50%−epiboly
Shield
75%−epiboly
1−4−somites
14−19−somites
20−25−somites
Prim−5
Prim−15
Prim−25
Long−pec
Protruding−mouth
Day−4
Day−5
Stage
Transcript
ENSDART00000158793
ENSDART00000161862
ENSDART00000163599
ENSDART00000167870

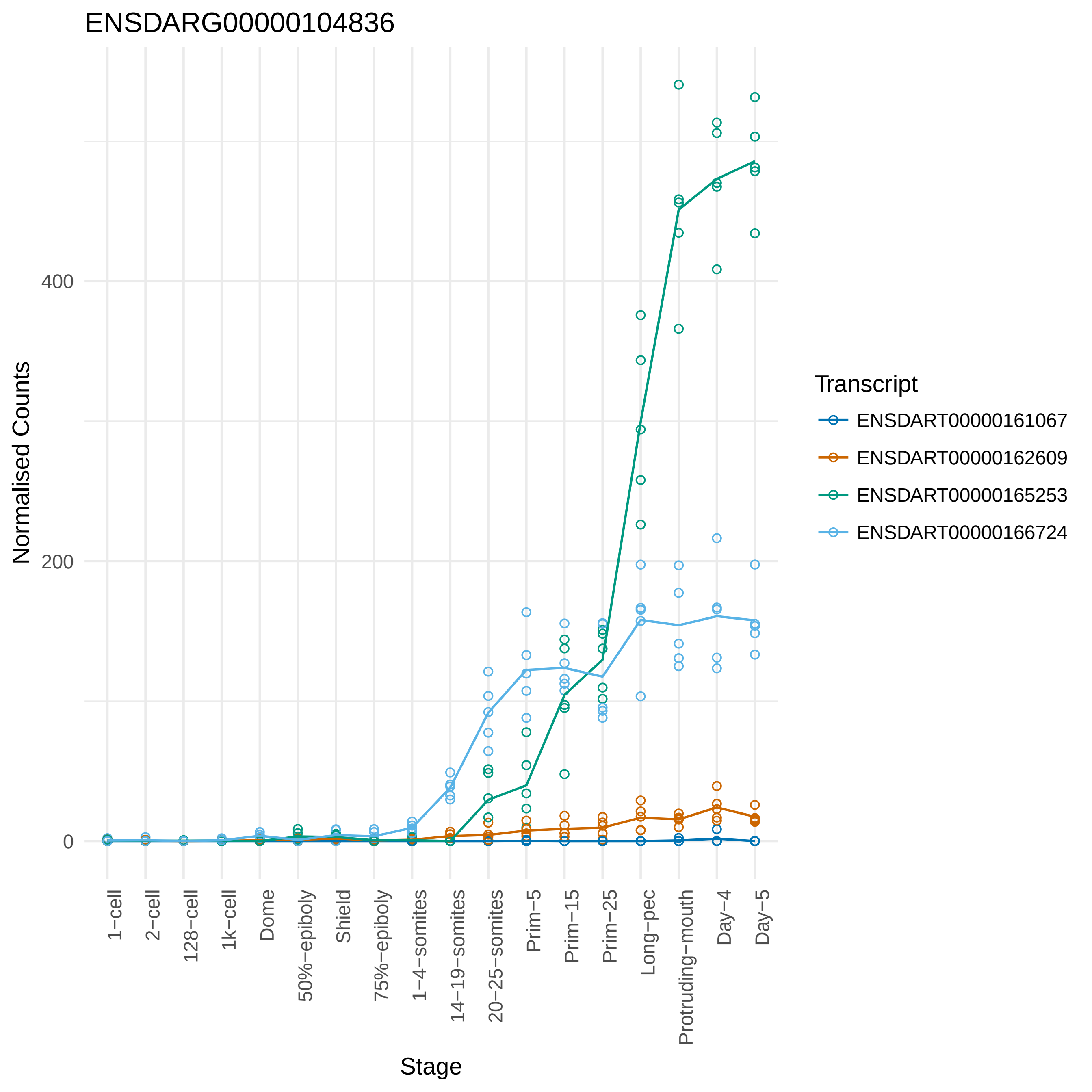

ENSDARG00000104836
Normalised Counts
400
200
0
1−cell
2−cell
128−cell
1k−cell
Dome
50%−epiboly
Shield
75%−epiboly
1−4−somites
14−19−somites
20−25−somites
Prim−5
Prim−15
Prim−25
Long−pec
Protruding−mouth
Day−4
Day−5
Stage
Transcript
ENSDART00000161067
ENSDART00000162609
ENSDART00000165253
ENSDART00000166724

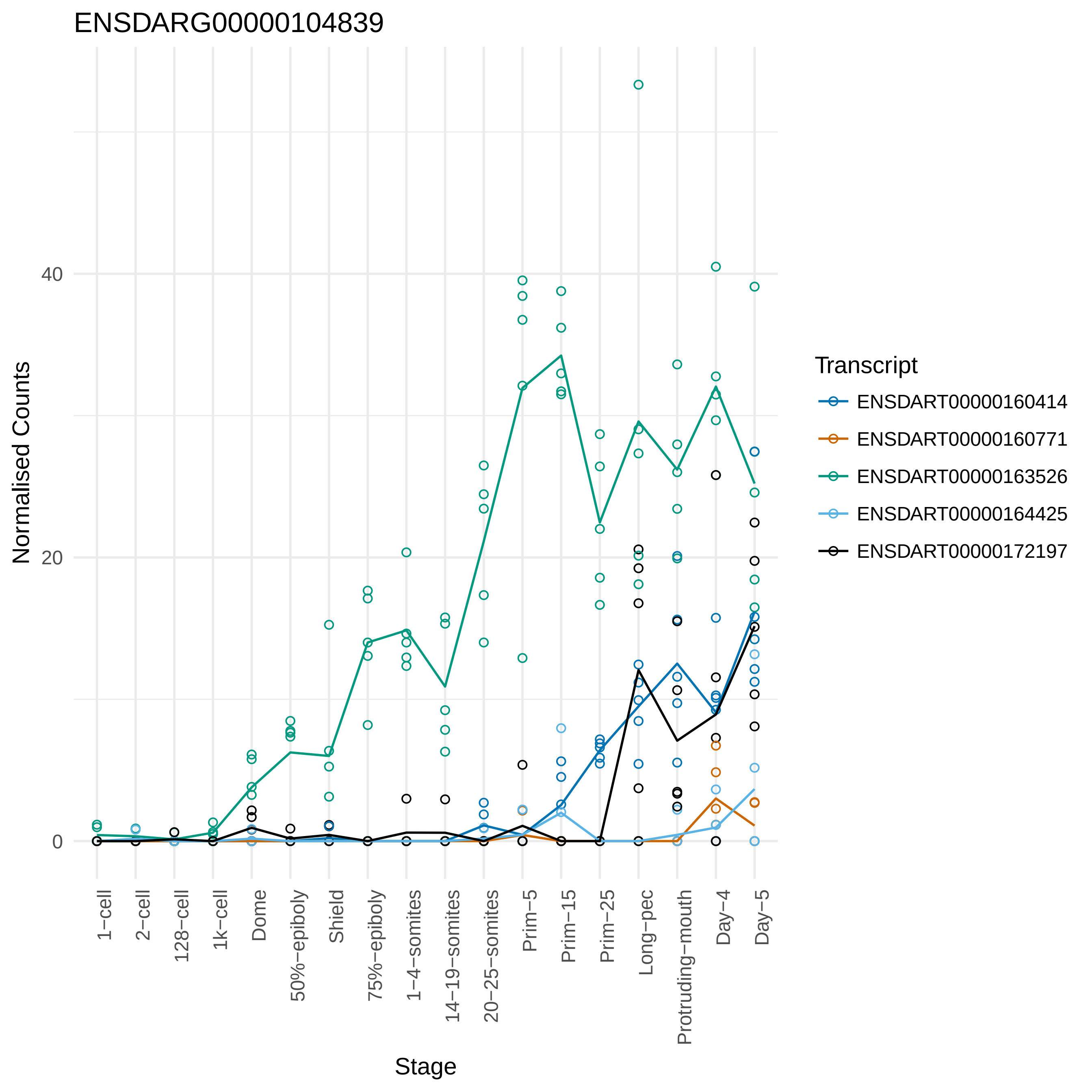

ENSDARG00000104839
Normalised Counts
0
20
40
Stage
1−cell
2−cell
128−cell
1k−cell
Dome
50%−epiboly
Shield
75%−epiboly
1−4−somites
14−19−somites
20−25−somites
Prim−5
Prim−15
Prim−25
Long−pec
Protruding−mouth
Day−4
Day−5
Transcript
ENSDART00000160414
ENSDART00000160771
ENSDART00000163526
ENSDART00000164425
ENSDART00000172197

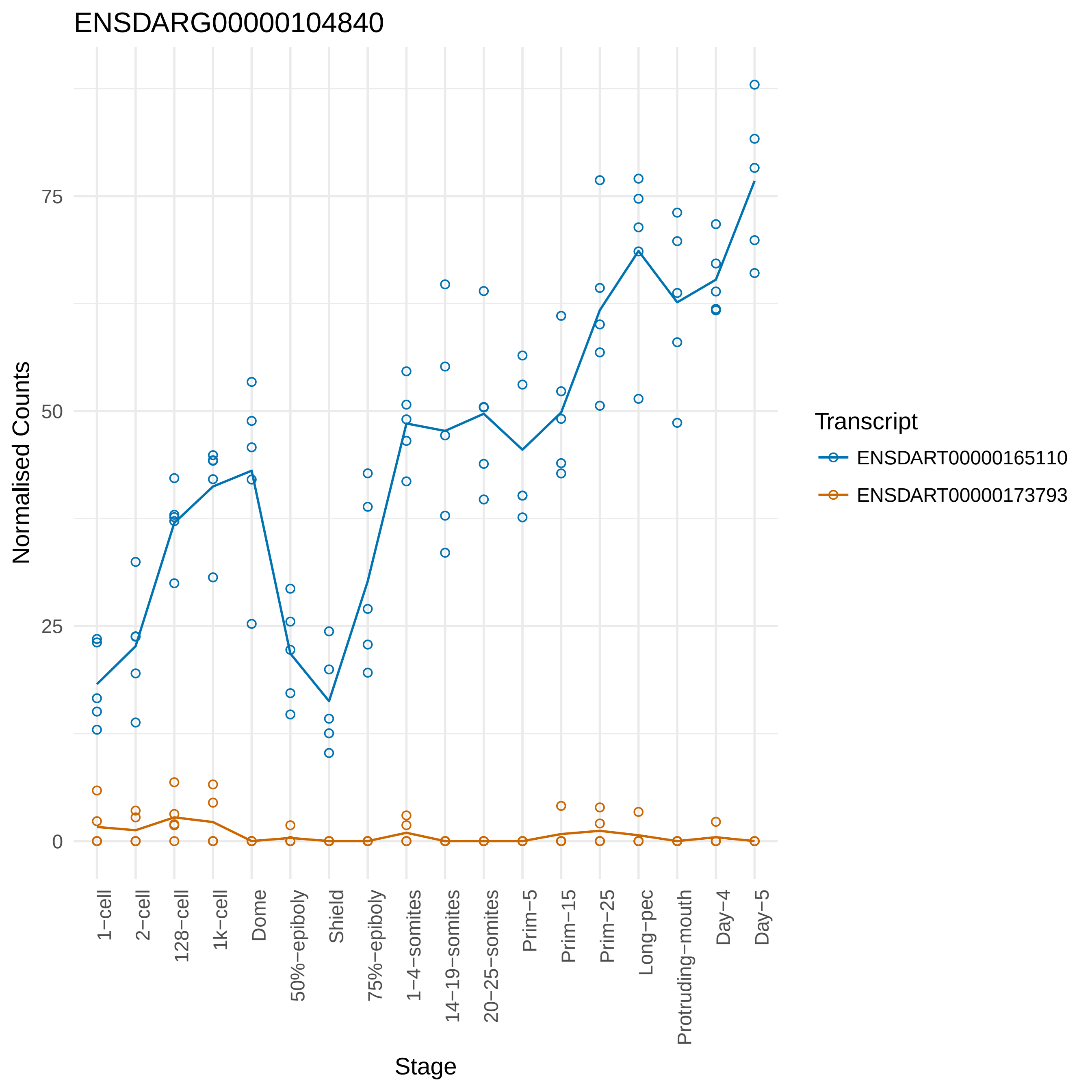

ENSDARG00000104840
Normalised Counts
0
25
50
75
Stage
1−cell
2−cell
128−cell
1k−cell
Dome
50%−epiboly
Shield
75%−epiboly
1−4−somites
14−19−somites
20−25−somites
Prim−5
Prim−15
Prim−25
Long−pec
Protruding−mouth
Day−4
Day−5
Transcript
ENSDART00000165110
ENSDART00000173793

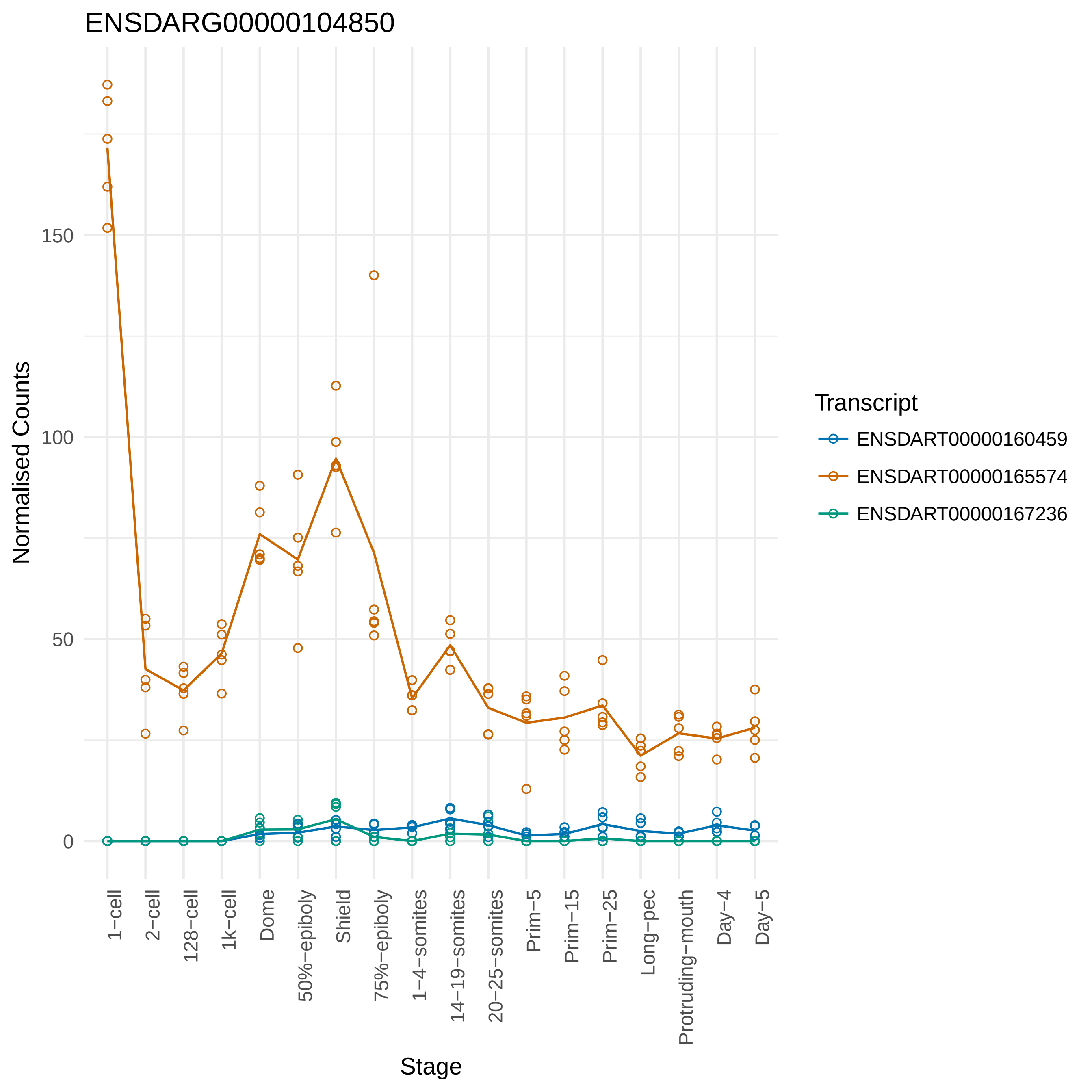

ENSDARG00000104850
Normalised Counts
0
50
100
150
Stage
1−cell
2−cell
128−cell
1k−cell
Dome
50%−epiboly
Shield
75%−epiboly
1−4−somites
14−19−somites
20−25−somites
Prim−5
Prim−15
Prim−25
Long−pec
Protruding−mouth
Day−4
Day−5
Transcript
ENSDART00000160459
ENSDART00000165574
ENSDART00000167236

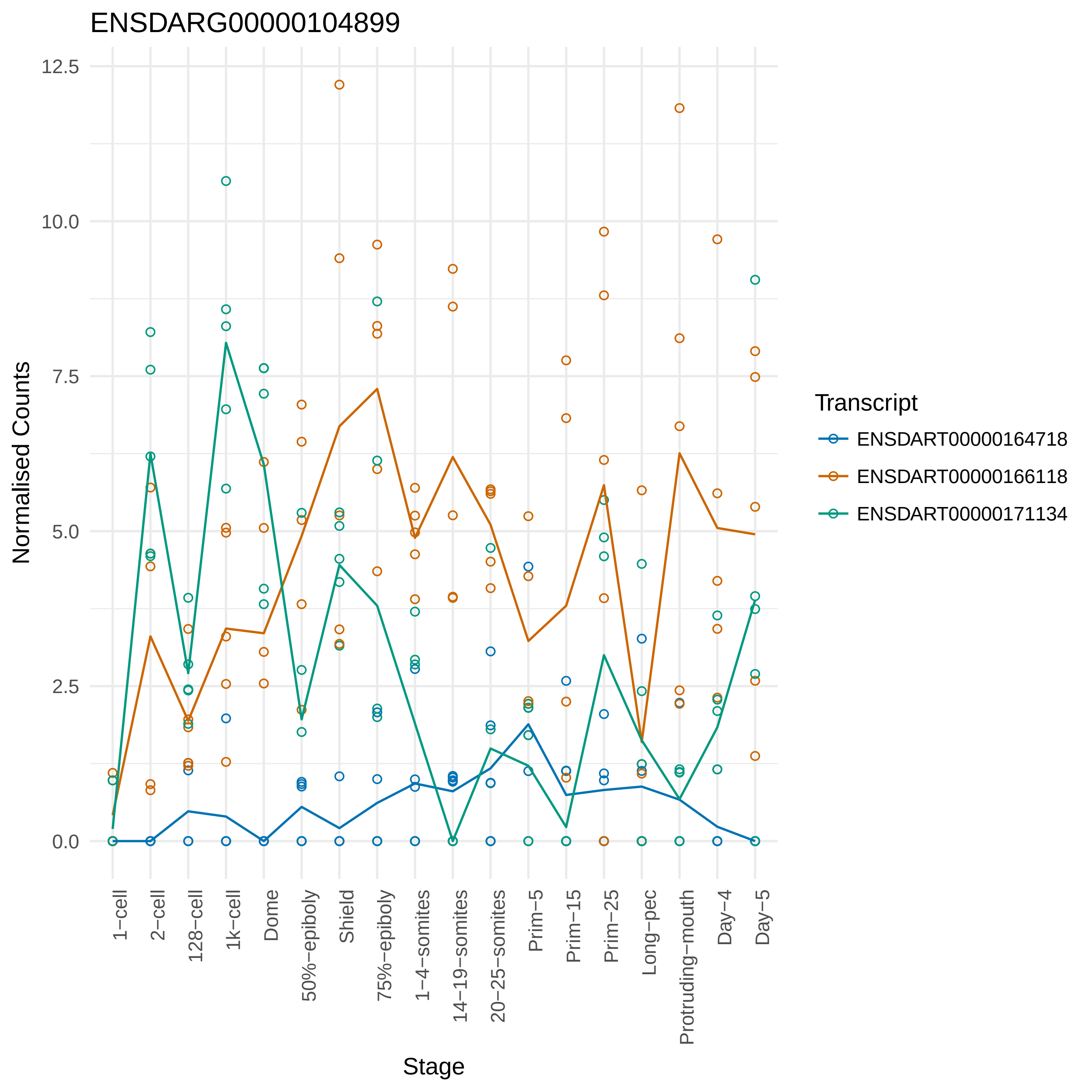

ENSDARG00000104899
Normalised Counts
0.0
2.5
5.0
7.5
10.0
12.5
Stage
1−cell
2−cell
128−cell
1k−cell
Dome
50%−epiboly
Shield
75%−epiboly
1−4−somites
14−19−somites
20−25−somites
Prim−5
Prim−15
Prim−25
Long−pec
Protruding−mouth
Day−4
Day−5
Transcript
ENSDART00000164718
ENSDART00000166118
ENSDART00000171134

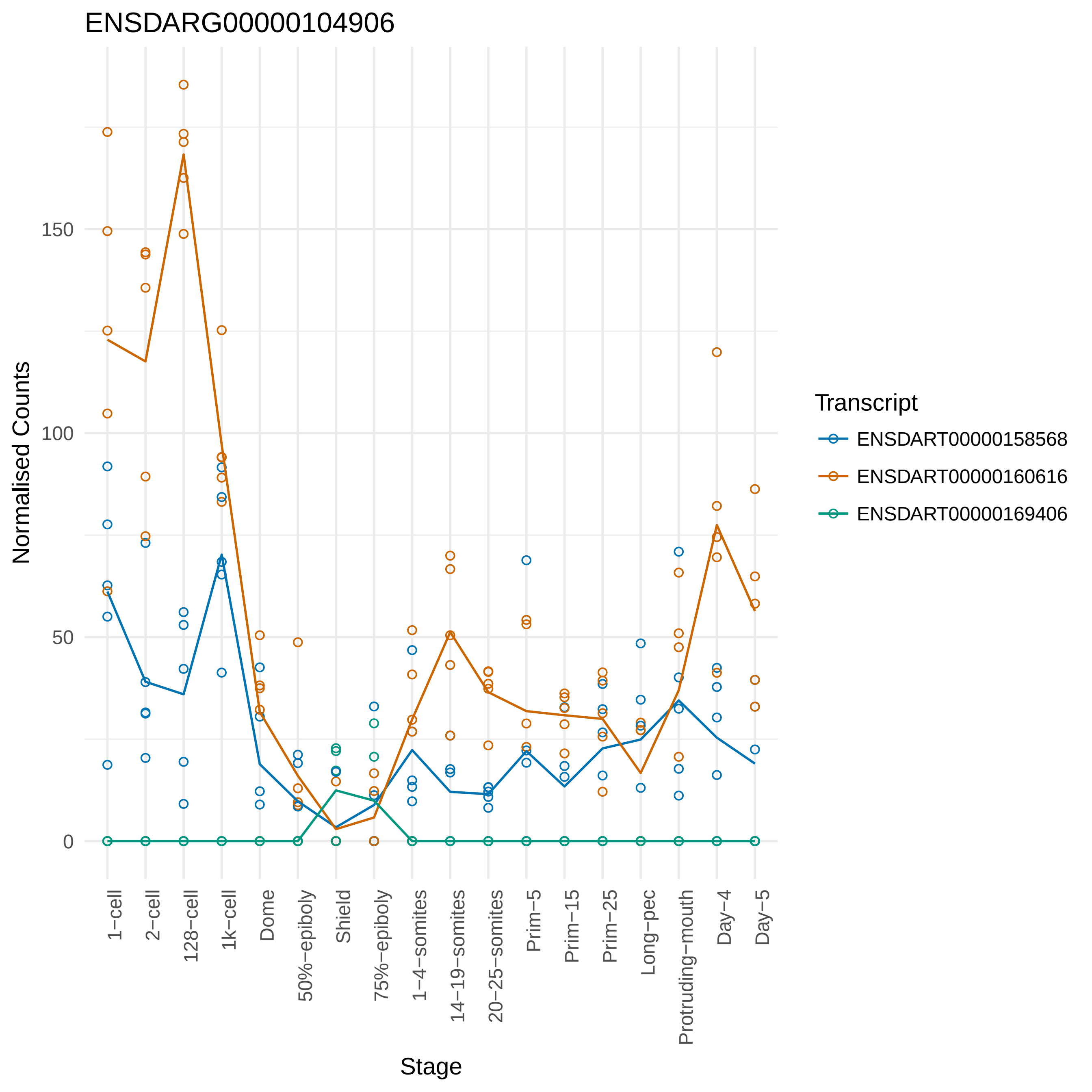

ENSDARG00000104906
Normalised Counts
0
50
100
150
Stage
1−cell
2−cell
128−cell
1k−cell
Dome
50%−epiboly
Shield
75%−epiboly
1−4−somites
14−19−somites
20−25−somites
Prim−5
Prim−15
Prim−25
Long−pec
Protruding−mouth
Day−4
Day−5
Transcript
ENSDART00000158568
ENSDART00000160616
ENSDART00000169406

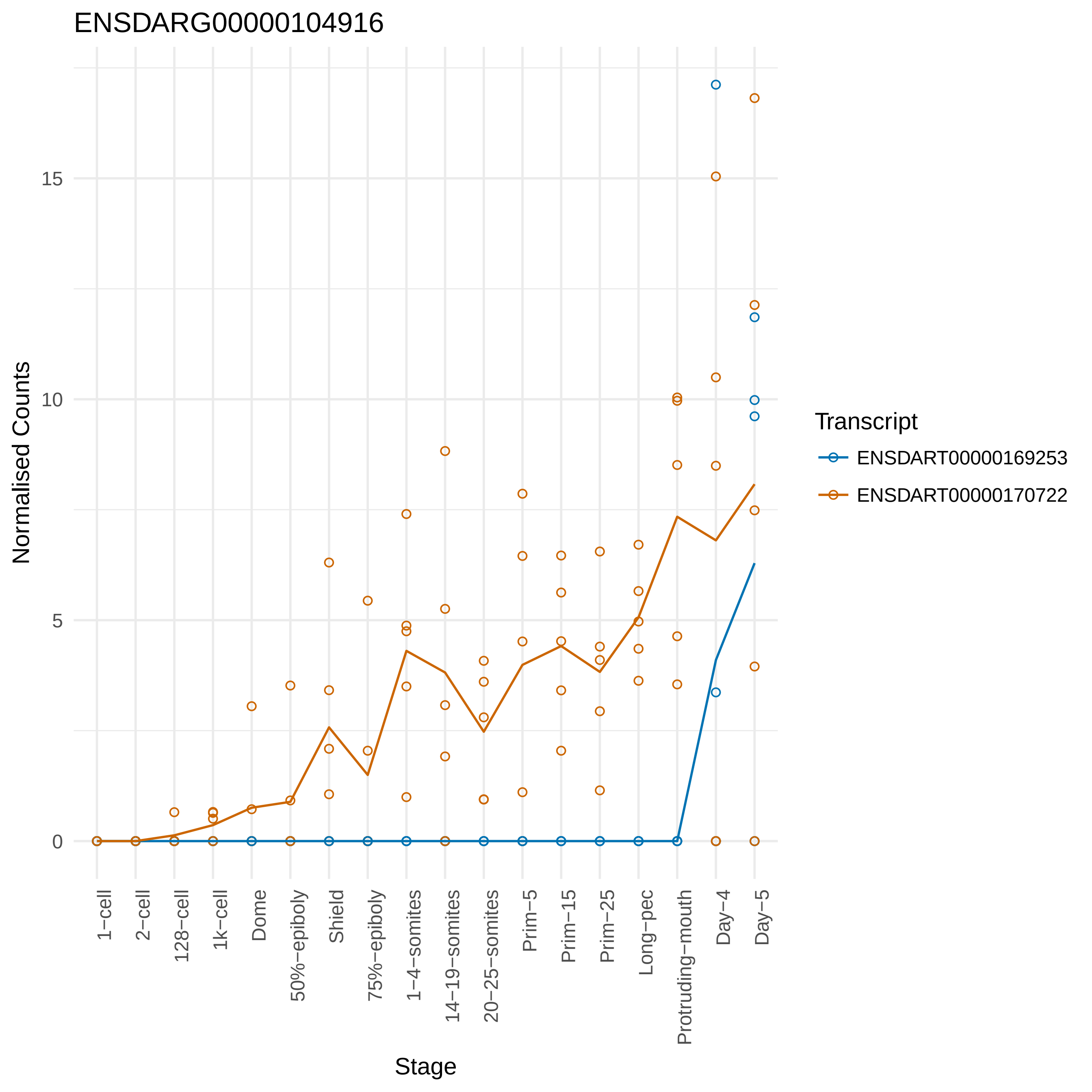
ENSDARG00000104916
Normalised Counts
0
5
10
15
1−cell
2−cell
128−cell
1k−cell
Dome
50%−epiboly
Shield
75%−epiboly
1−4−somites
14−19−somites
20−25−somites
Prim−5
Prim−15
Prim−25
Long−pec
Protruding−mouth
Day−4
Day−5
Stage
Transcript
ENSDART00000169253
ENSDART00000170722

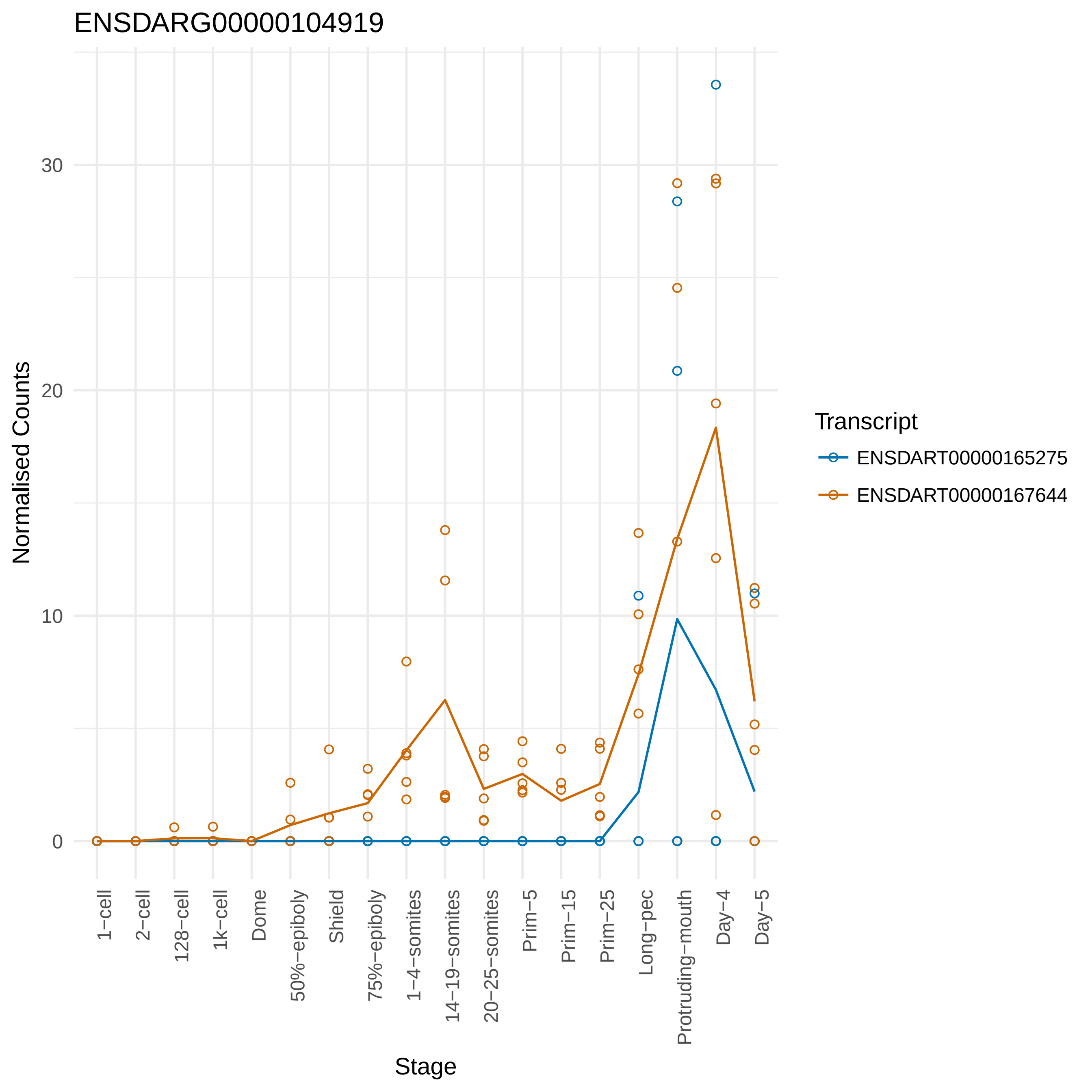

ENSDARG00000104919
Normalised Counts
30
20
10
0
1−cell
2−cell
128−cell
1k−cell
Dome
50%−epiboly
Shield
75%−epiboly
1−4−somites
14−19−somites
20−25−somites
Prim−5
Prim−15
Prim−25
Long−pec
Protruding−mouth
Day−4
Day−5
Stage
Transcript
ENSDART00000165275
ENSDART00000167644

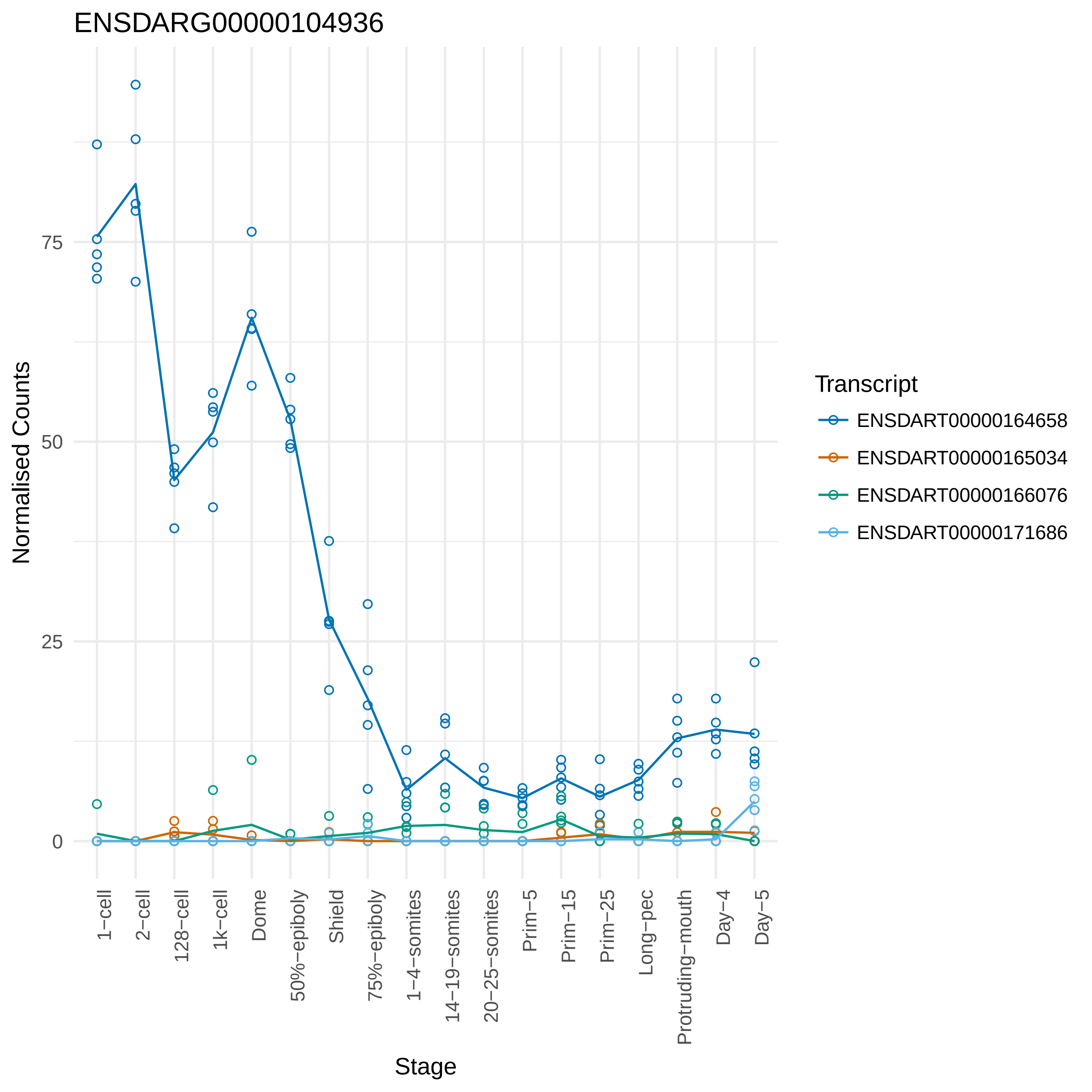
ENSDARG00000104936
Normalised Counts
75
50
25
0
1−cell
2−cell
128−cell
1k−cell
Dome
50%−epiboly
Shield
75%−epiboly
1−4−somites
14−19−somites
20−25−somites
Prim−5
Prim−15
Prim−25
Long−pec
Protruding−mouth
Day−4
Day−5
Stage
Transcript
ENSDART00000164658
ENSDART00000165034
ENSDART00000166076
ENSDART00000171686

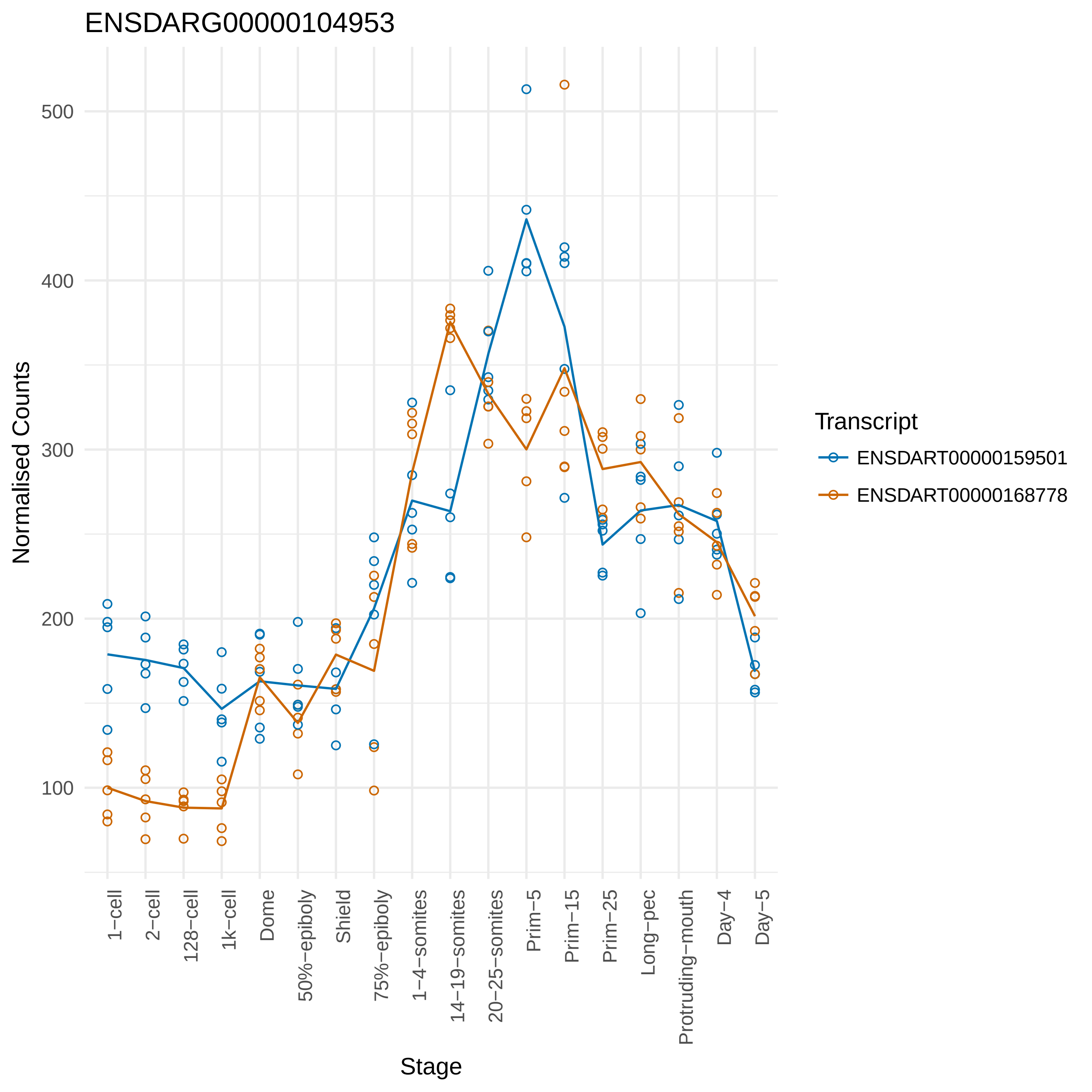

ENSDARG00000104953
Normalised Counts
500
400
300
200
100
Stage
1−cell
2−cell
128−cell
1k−cell
Dome
50%−epiboly
Shield
75%−epiboly
1−4−somites
14−19−somites
20−25−somites
Prim−5
Prim−15
Prim−25
Long−pec
Protruding−mouth
Day−4
Day−5
Transcript
ENSDART00000159501
ENSDART00000168778

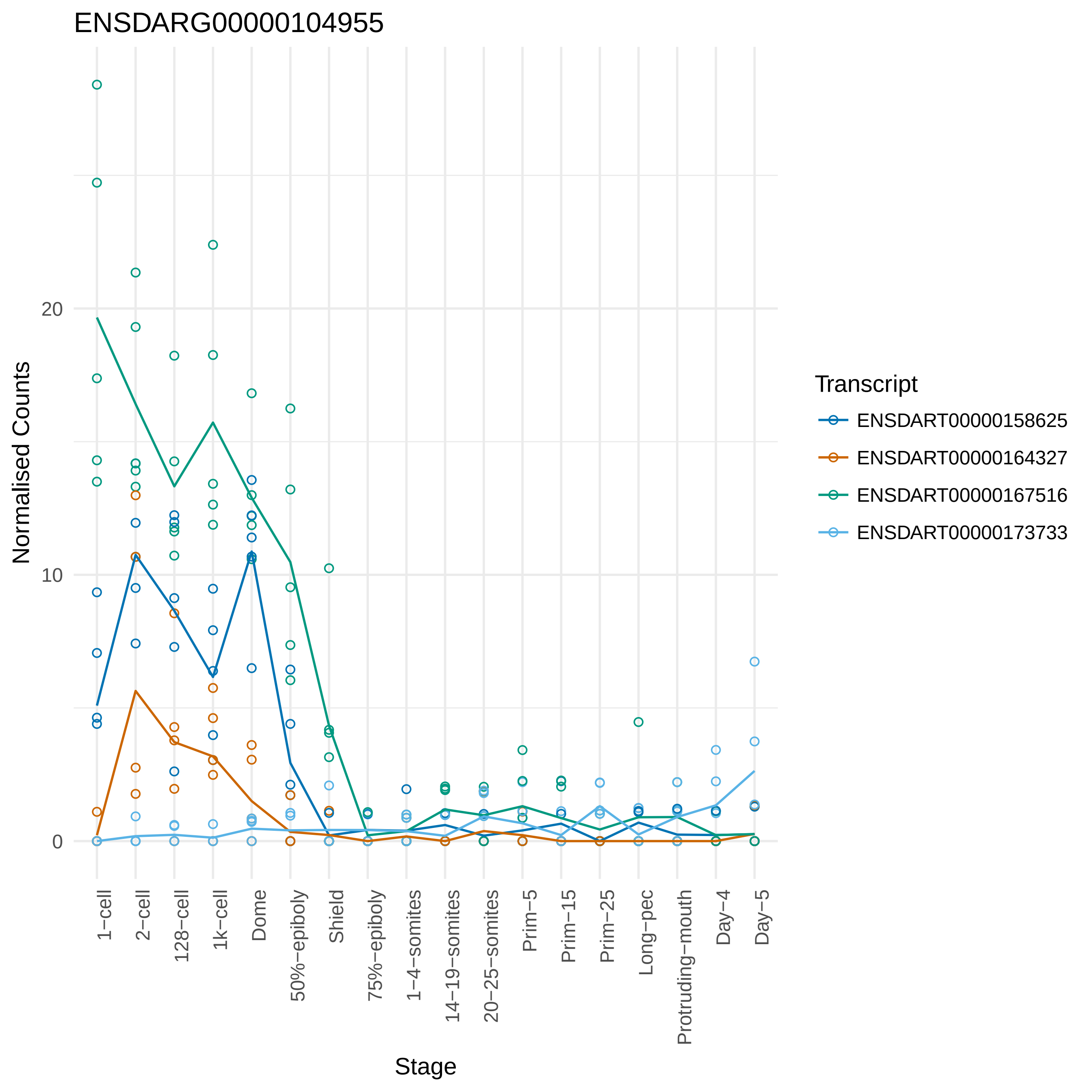

ENSDARG00000104955
Normalised Counts
20
10
0
1−cell
2−cell
128−cell
1k−cell
Dome
50%−epiboly
Shield
75%−epiboly
1−4−somites
14−19−somites
20−25−somites
Prim−5
Prim−15
Prim−25
Long−pec
Protruding−mouth
Day−4
Day−5
Stage
Transcript
ENSDART00000158625
ENSDART00000164327
ENSDART00000167516
ENSDART00000173733

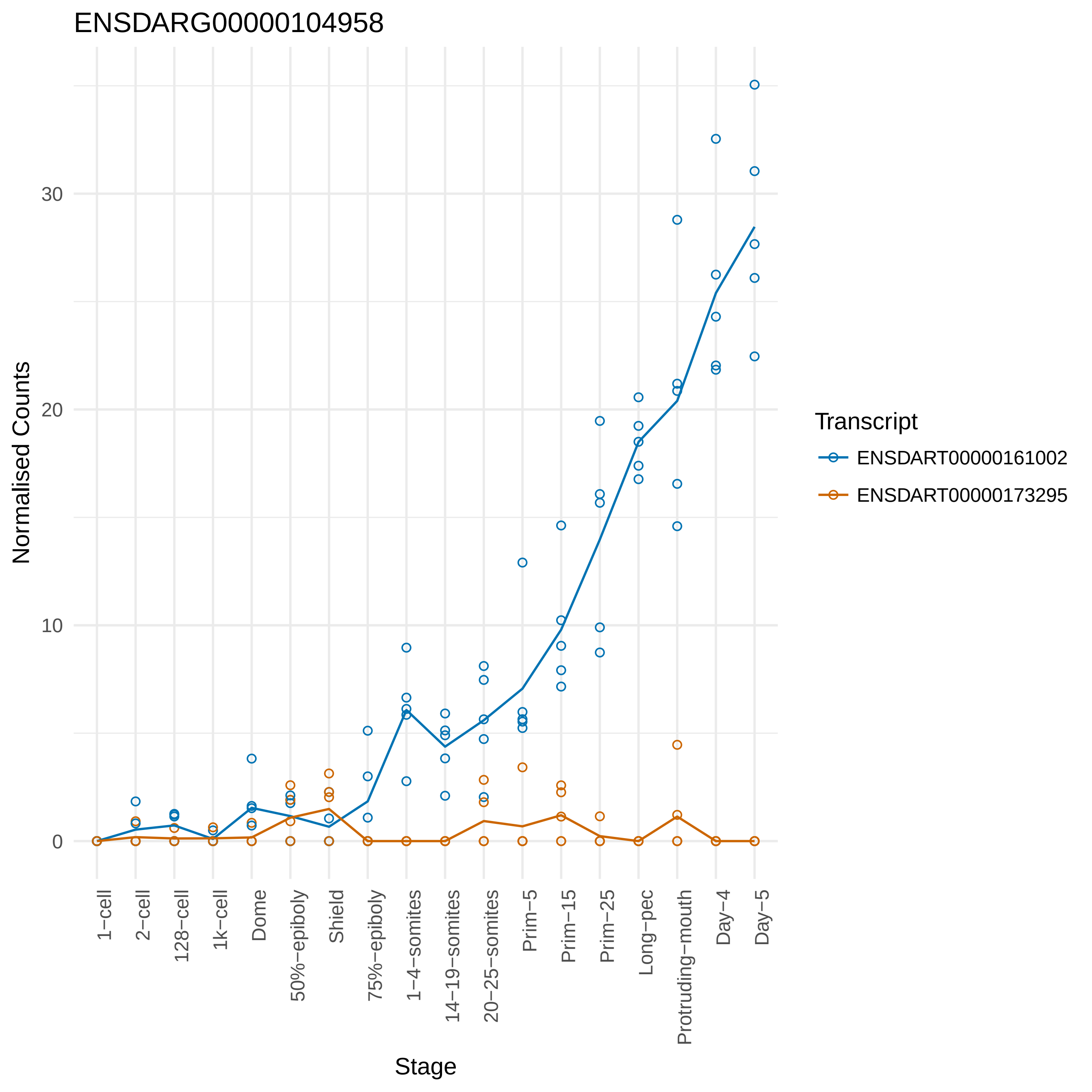

ENSDARG00000104958
Normalised Counts
0
10
20
30
1−cell
2−cell
128−cell
1k−cell
Dome
50%−epiboly
Shield
75%−epiboly
1−4−somites
14−19−somites
20−25−somites
Prim−5
Prim−15
Prim−25
Long−pec
Protruding−mouth
Day−4
Day−5
Stage
Transcript
ENSDART00000161002
ENSDART00000173295

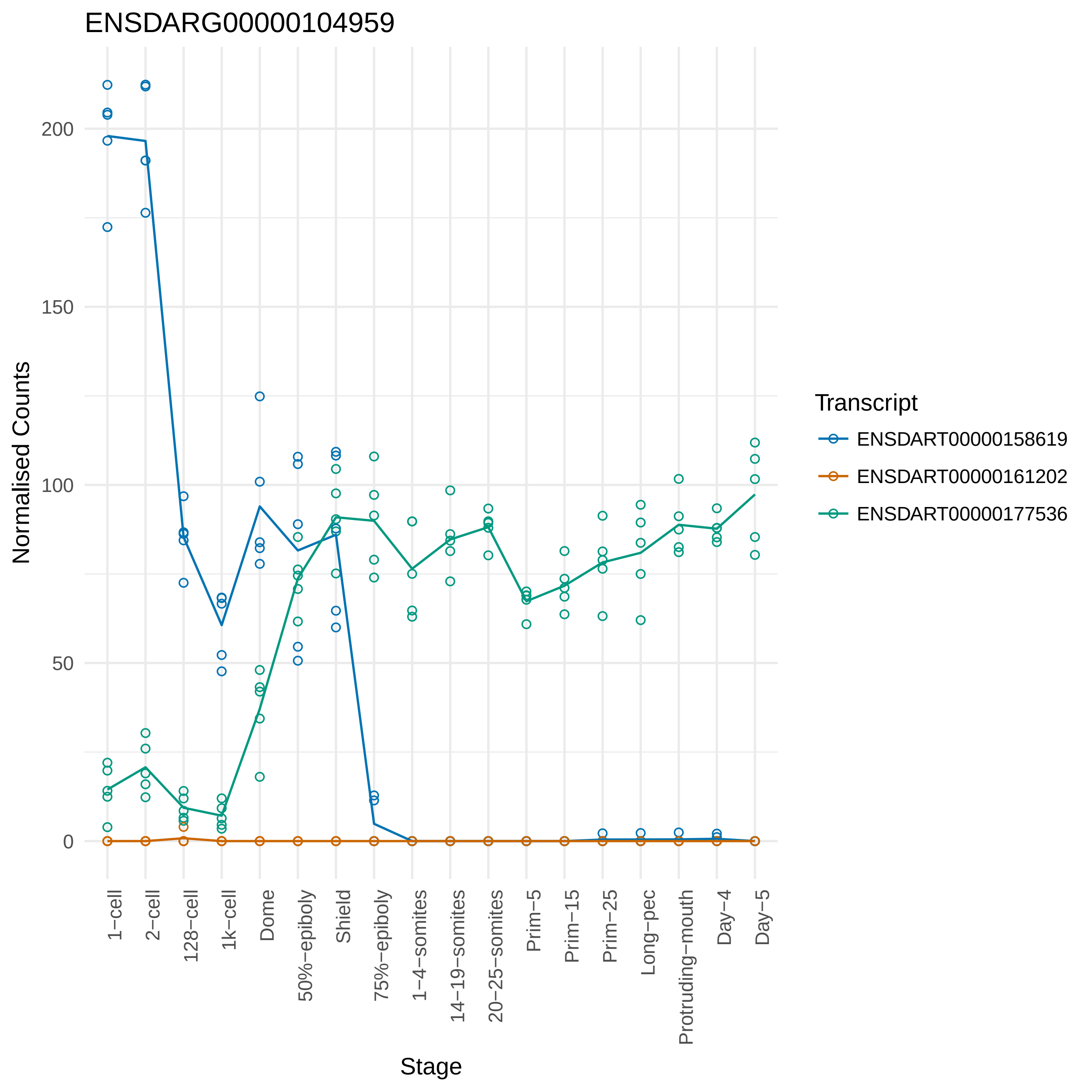
ENSDARG00000104959
Normalised Counts
200
150
100
50
0
1−cell
2−cell
128−cell
1k−cell
Dome
50%−epiboly
Shield
75%−epiboly
1−4−somites
14−19−somites
20−25−somites
Prim−5
Prim−15
Prim−25
Long−pec
Protruding−mouth
Day−4
Day−5
Stage
Transcript
ENSDART00000158619
ENSDART00000161202
ENSDART00000177536

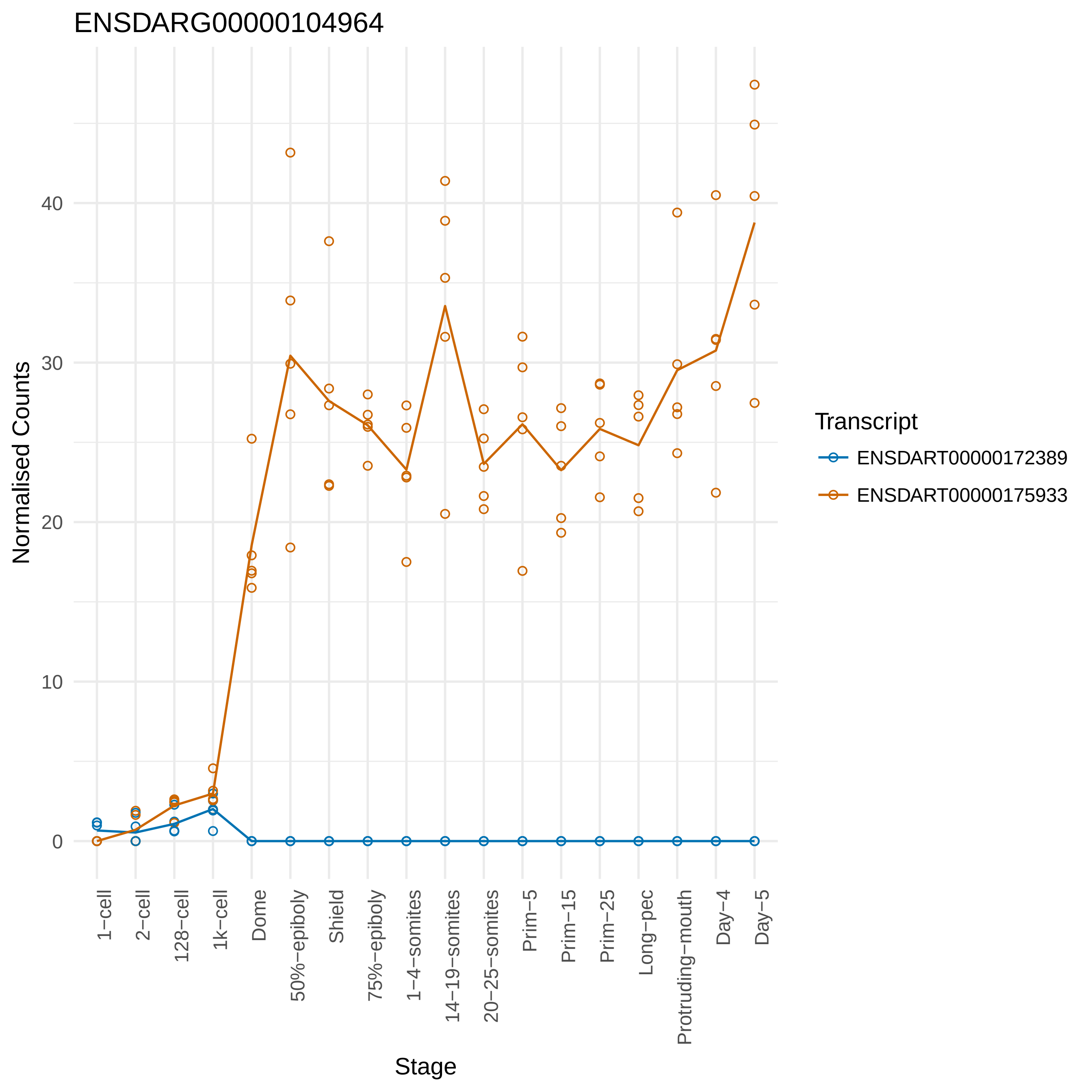
ENSDARG00000104964
Normalised Counts
0
10
20
30
40
Stage
1−cell
2−cell
128−cell
1k−cell
Dome
50%−epiboly
Shield
75%−epiboly
1−4−somites
14−19−somites
20−25−somites
Prim−5
Prim−15
Prim−25
Long−pec
Protruding−mouth
Day−4
Day−5
Transcript
ENSDART00000172389
ENSDART00000175933

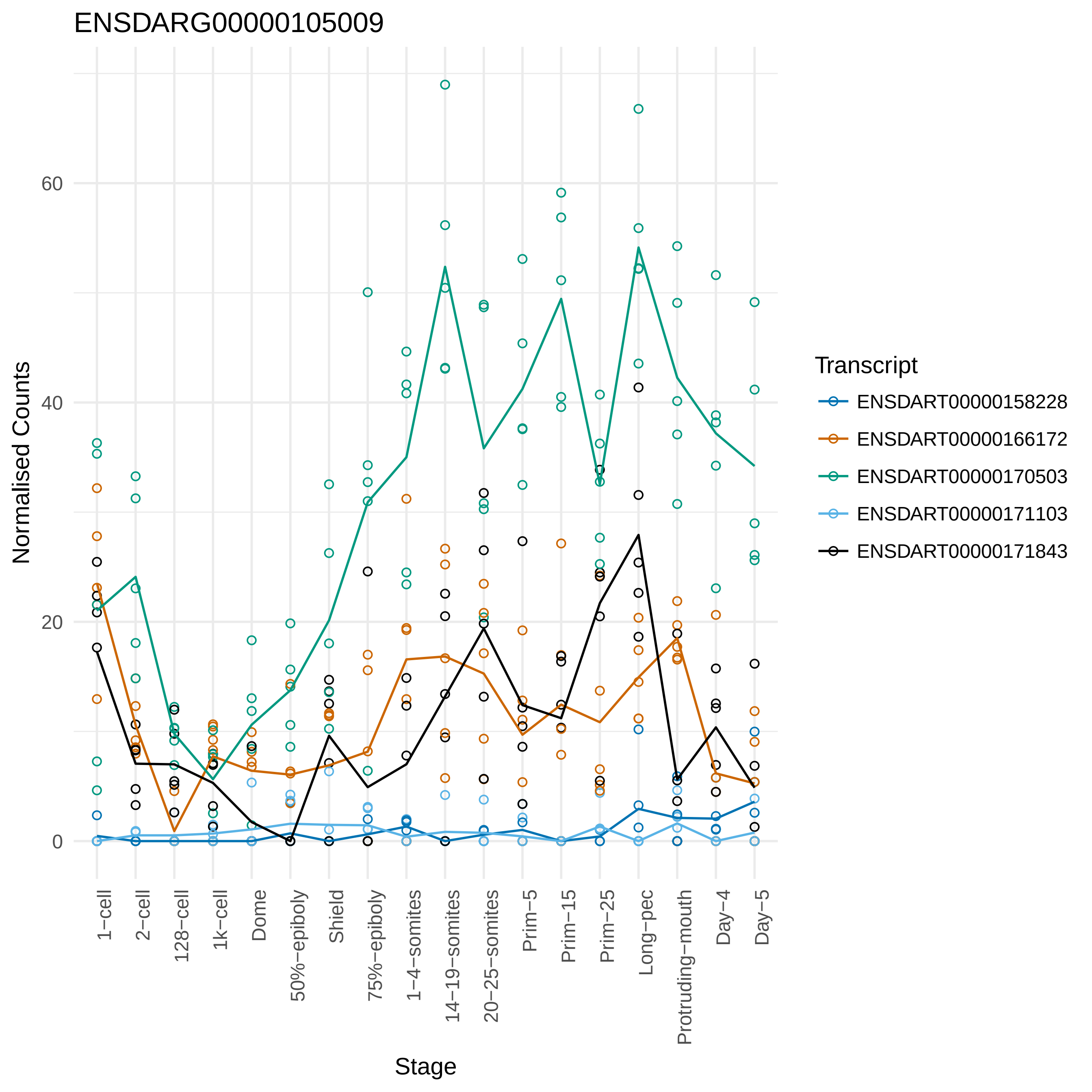

ENSDARG00000105009
Normalised Counts
60
40
20
0
1−cell
2−cell
128−cell
1k−cell
Dome
50%−epiboly
Shield
75%−epiboly
1−4−somites
14−19−somites
20−25−somites
Prim−5
Prim−15
Prim−25
Long−pec
Protruding−mouth
Day−4
Day−5
Stage
Transcript
ENSDART00000158228
ENSDART00000166172
ENSDART00000170503
ENSDART00000171103
ENSDART00000171843

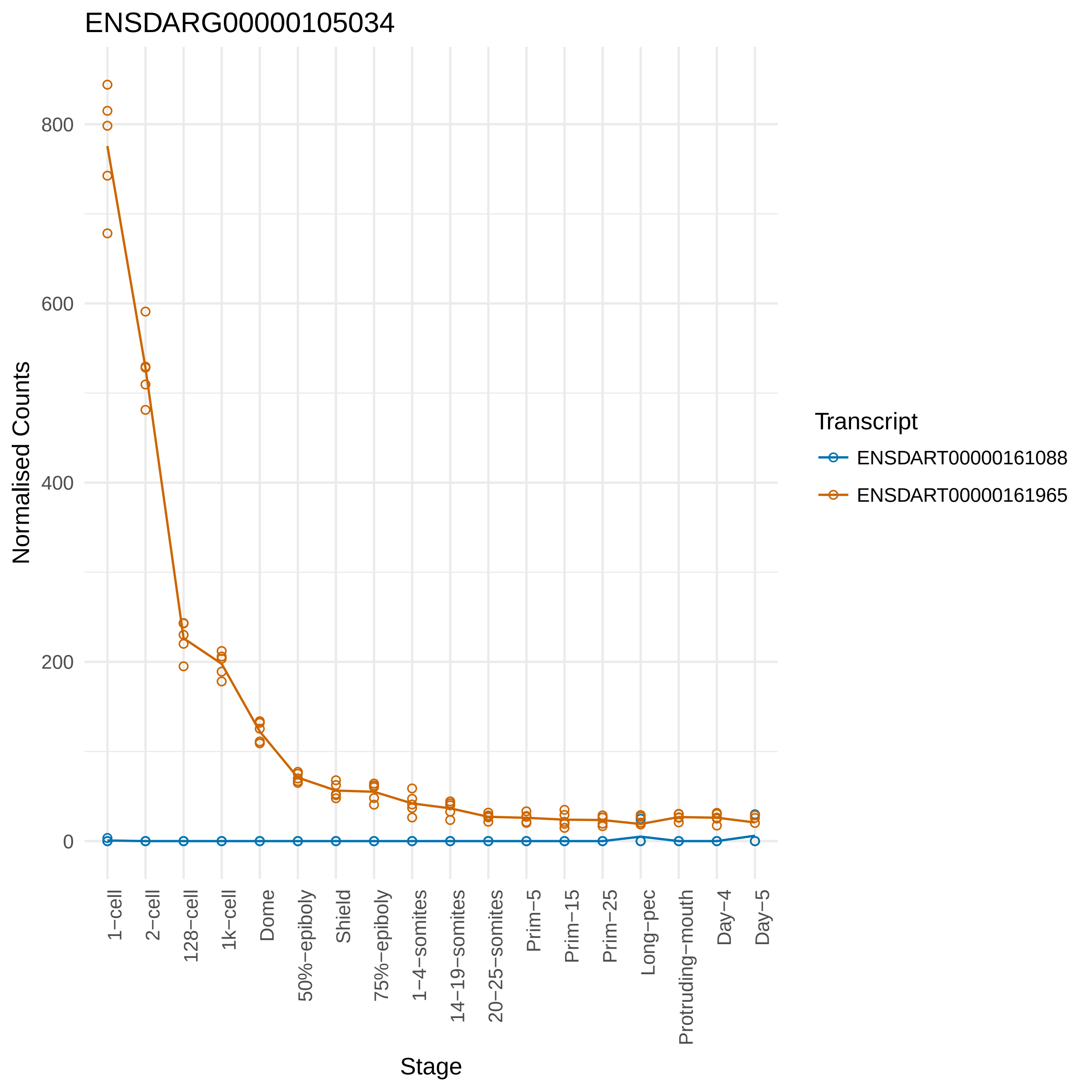

ENSDARG00000105034
Normalised Counts
800
600
400
200
0
1−cell
2−cell
128−cell
1k−cell
Dome
50%−epiboly
Shield
75%−epiboly
1−4−somites
14−19−somites
20−25−somites
Prim−5
Prim−15
Prim−25
Long−pec
Protruding−mouth
Day−4
Day−5
Stage
Transcript
ENSDART00000161088
ENSDART00000161965

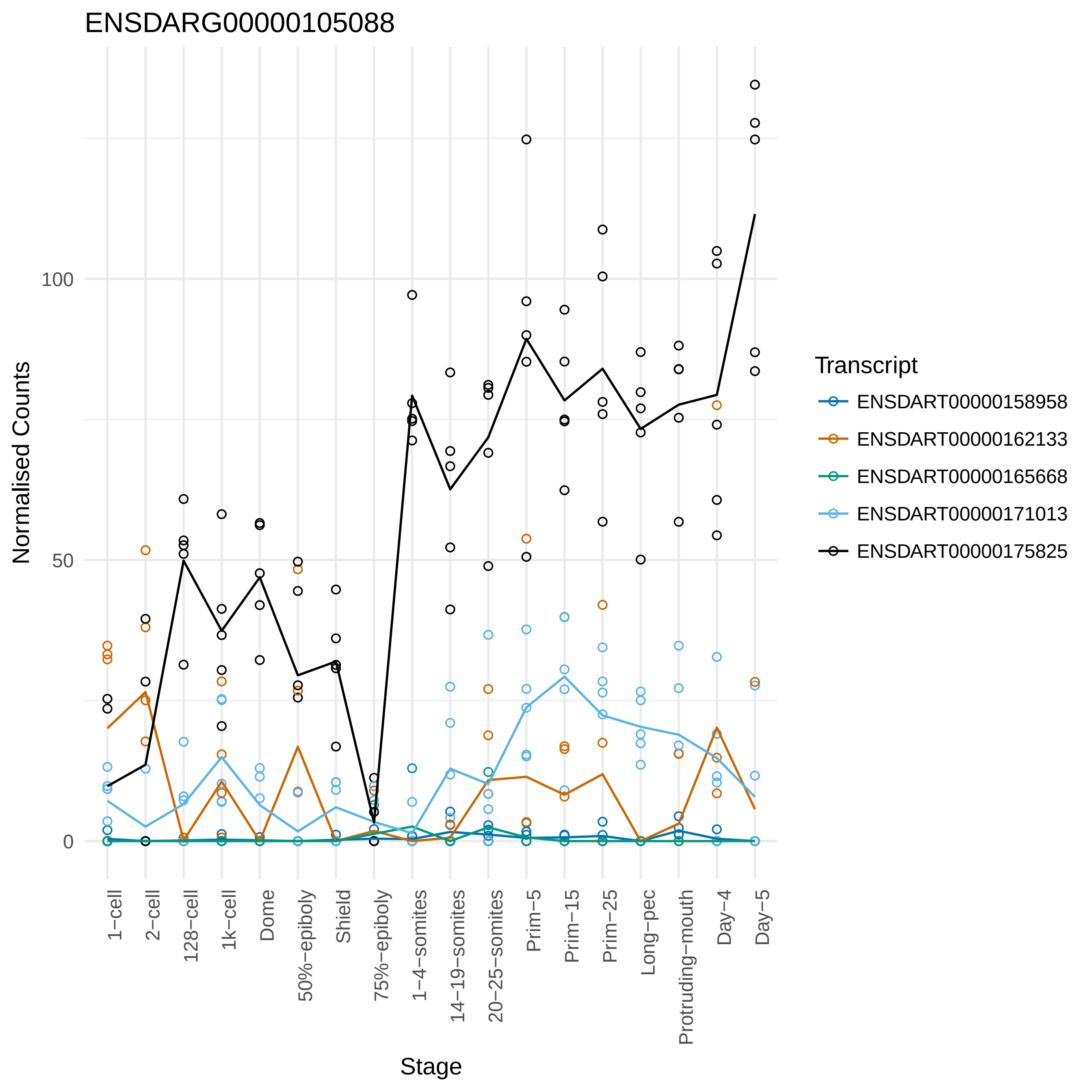
ENSDARG00000105088
Normalised Counts
100
50
0
1−cell
2−cell
128−cell
1k−cell
Dome
50%−epiboly
Shield
75%−epiboly
1−4−somites
14−19−somites
20−25−somites
Prim−5
Prim−15
Prim−25
Long−pec
Protruding−mouth
Day−4
Day−5
Stage
Transcript
ENSDART00000158958
ENSDART00000162133
ENSDART00000165668
ENSDART00000171013
ENSDART00000175825

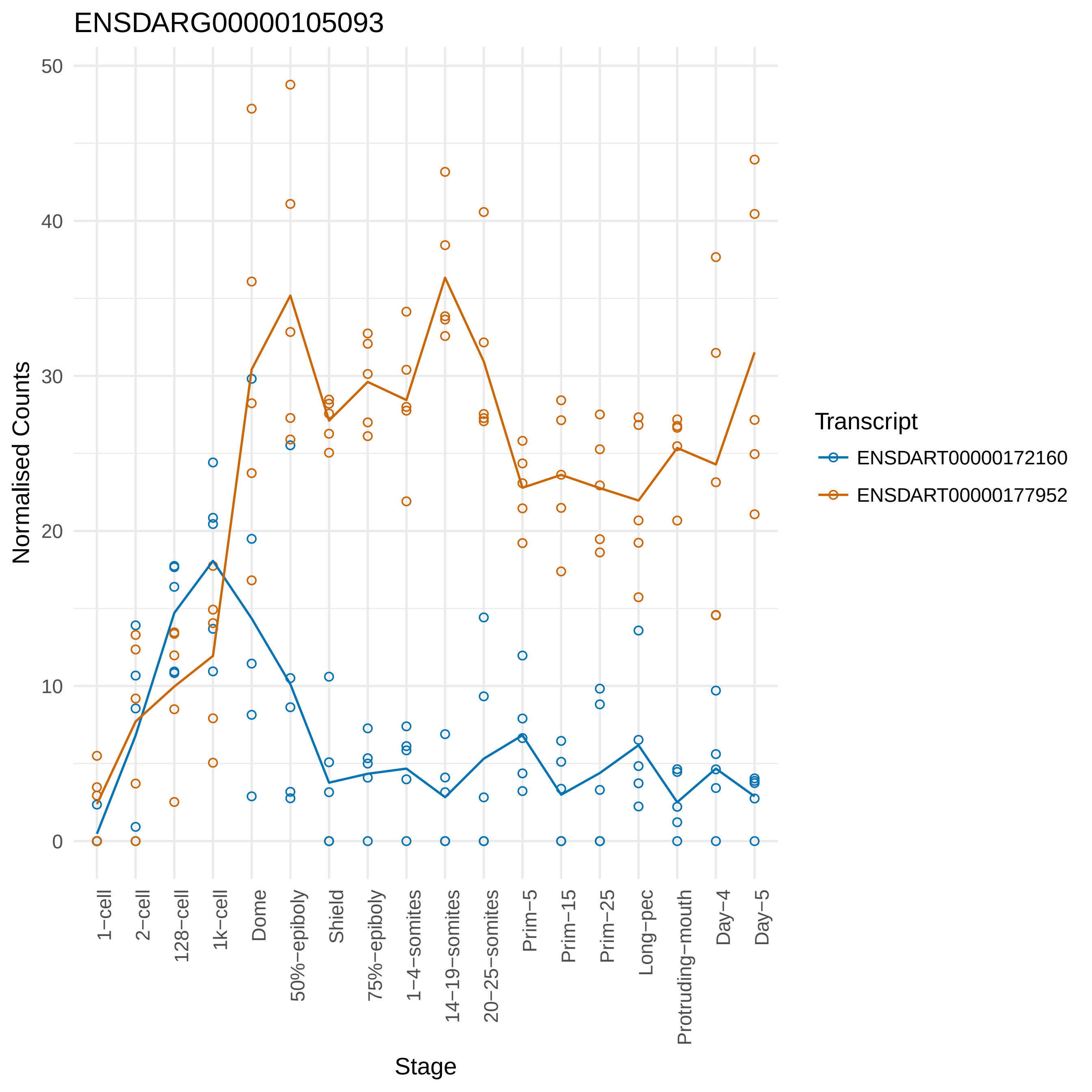
ENSDARG00000105093
Normalised Counts
0
10
20
30
40
50
Stage
1−cell
2−cell
128−cell
1k−cell
Dome
50%−epiboly
Shield
75%−epiboly
1−4−somites
14−19−somites
20−25−somites
Prim−5
Prim−15
Prim−25
Long−pec
Protruding−mouth
Day−4
Day−5
Transcript
ENSDART00000172160
ENSDART00000177952

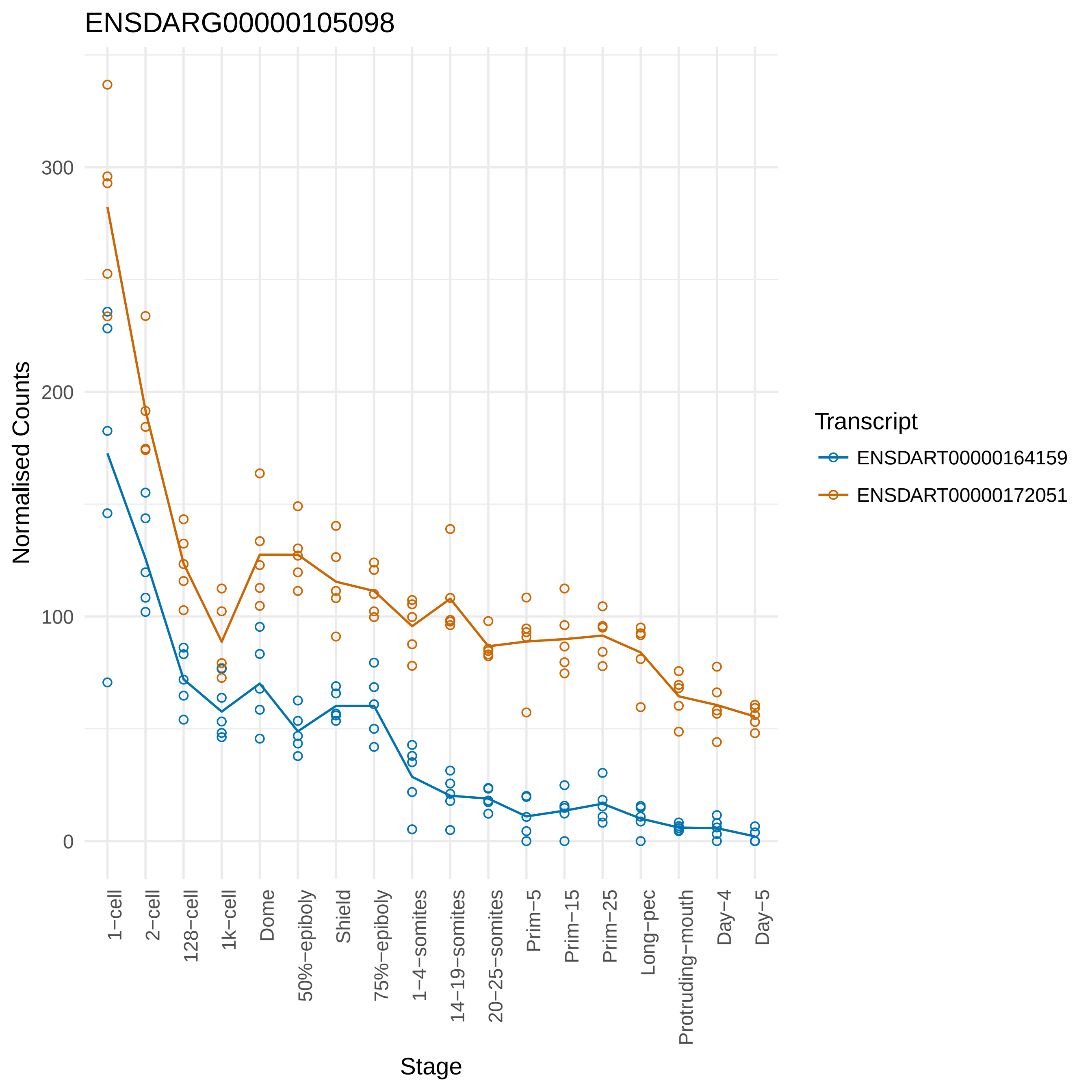
ENSDARG00000105098
Normalised Counts
300
200
100
0
1−cell
2−cell
128−cell
1k−cell
Dome
50%−epiboly
Shield
75%−epiboly
1−4−somites
14−19−somites
20−25−somites
Prim−5
Prim−15
Prim−25
Long−pec
Protruding−mouth
Day−4
Day−5
Stage
Transcript
ENSDART00000164159
ENSDART00000172051

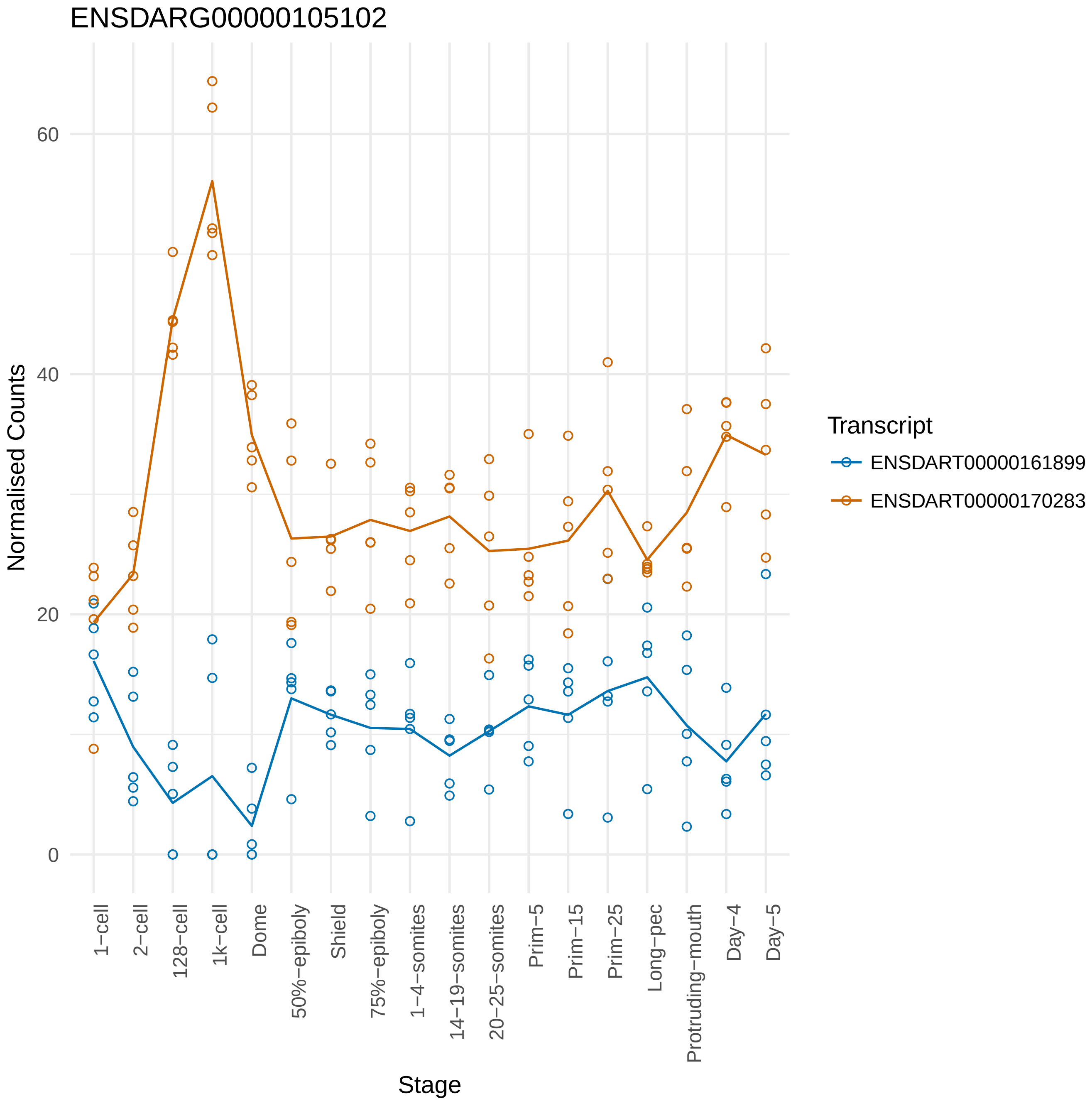

ENSDARG00000105102
Normalised Counts
0
20
40
60
1−cell
2−cell
128−cell
1k−cell
Dome
50%−epiboly
Shield
75%−epiboly
1−4−somites
14−19−somites
20−25−somites
Prim−5
Prim−15
Prim−25
Long−pec
Protruding−mouth
Day−4
Day−5
Stage
Transcript
ENSDART00000161899
ENSDART00000170283

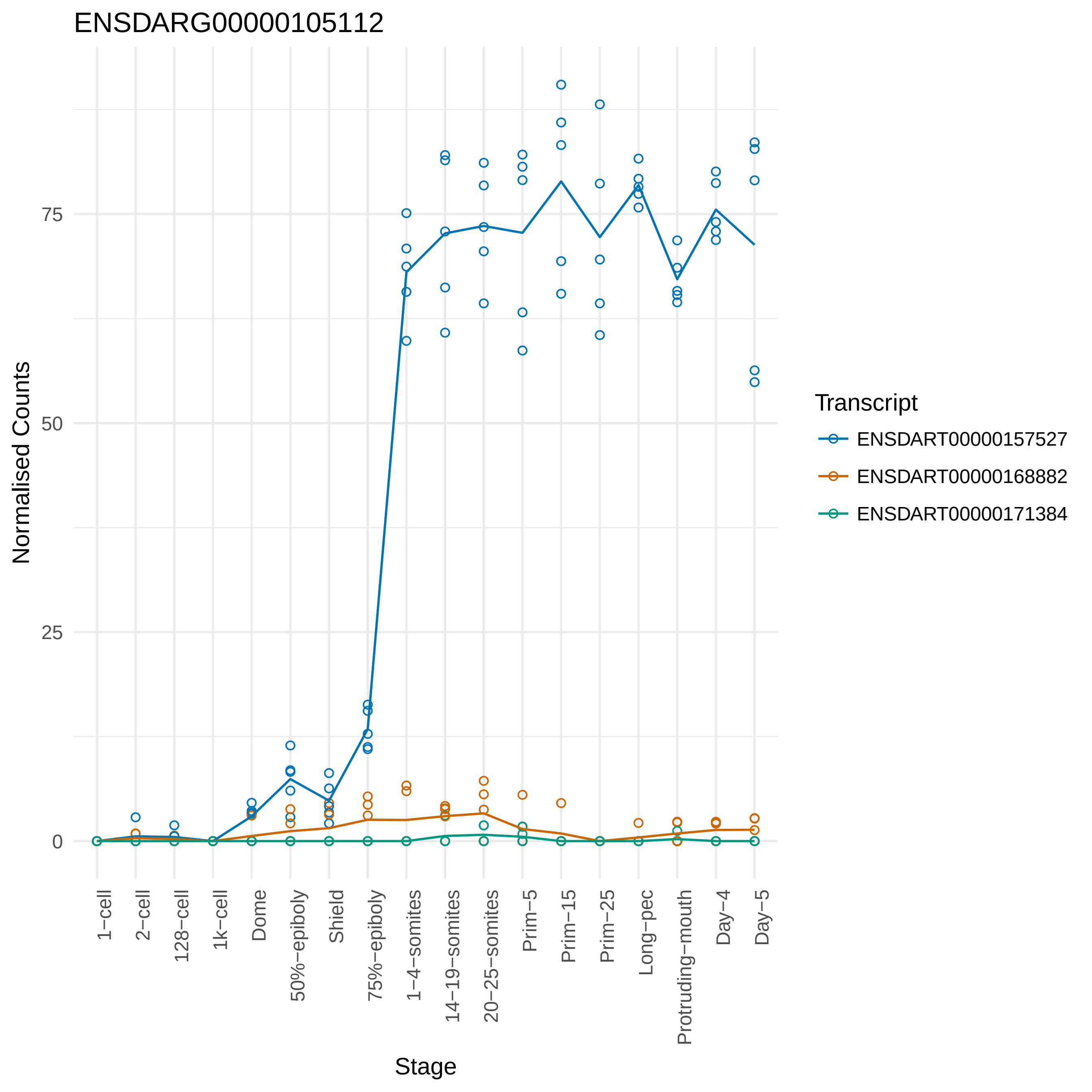

ENSDARG00000105112
Normalised Counts
0
25
50
75
1−cell
2−cell
128−cell
1k−cell
Dome
50%−epiboly
Shield
75%−epiboly
1−4−somites
14−19−somites
20−25−somites
Prim−5
Prim−15
Prim−25
Long−pec
Protruding−mouth
Day−4
Day−5
Stage
Transcript
ENSDART00000157527
ENSDART00000168882
ENSDART00000171384

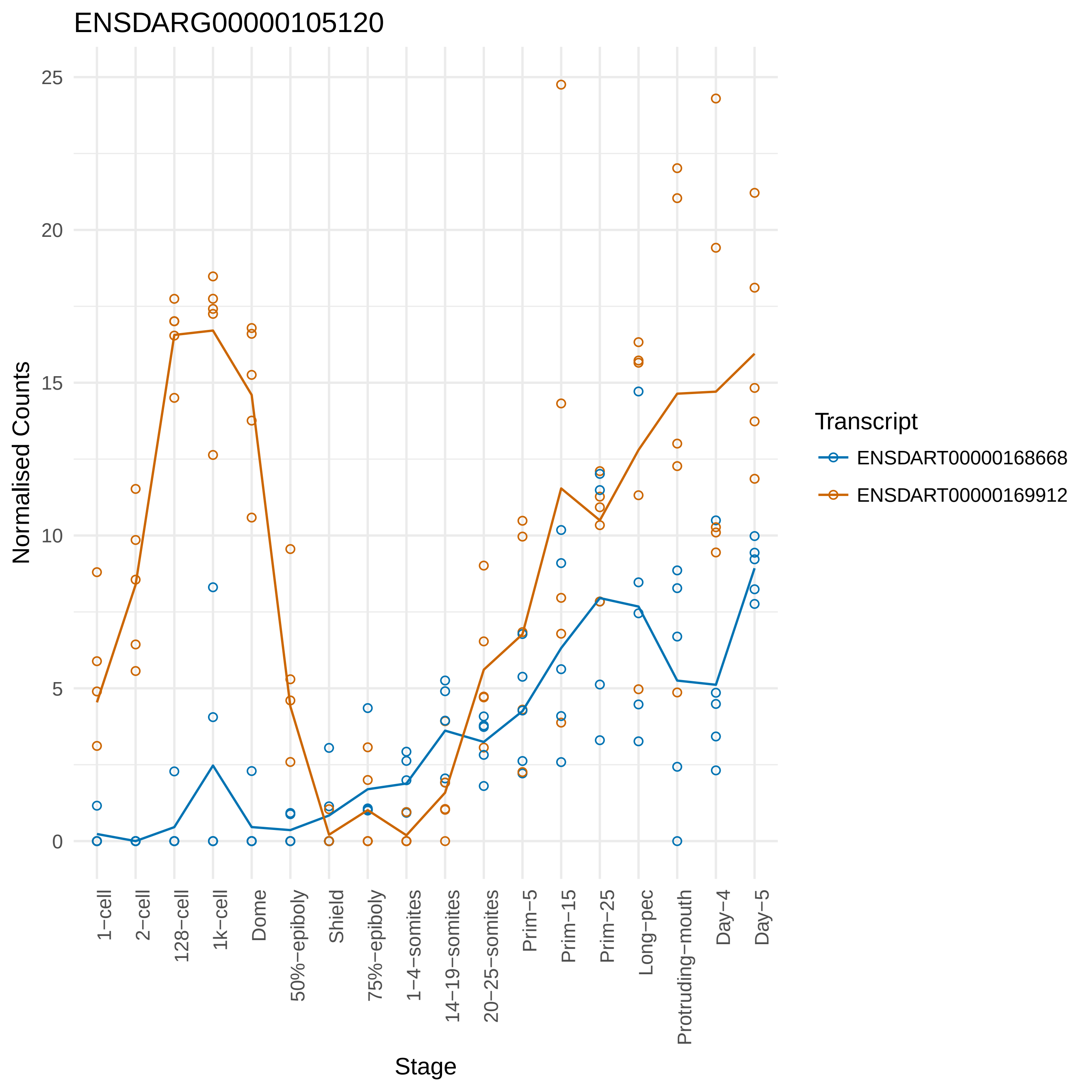

ENSDARG00000105120
Normalised Counts
0
5
10
15
20
25
1−cell
2−cell
128−cell
1k−cell
Dome
50%−epiboly
Shield
75%−epiboly
1−4−somites
14−19−somites
20−25−somites
Prim−5
Prim−15
Prim−25
Long−pec
Protruding−mouth
Day−4
Day−5
Stage
Transcript
ENSDART00000168668
ENSDART00000169912

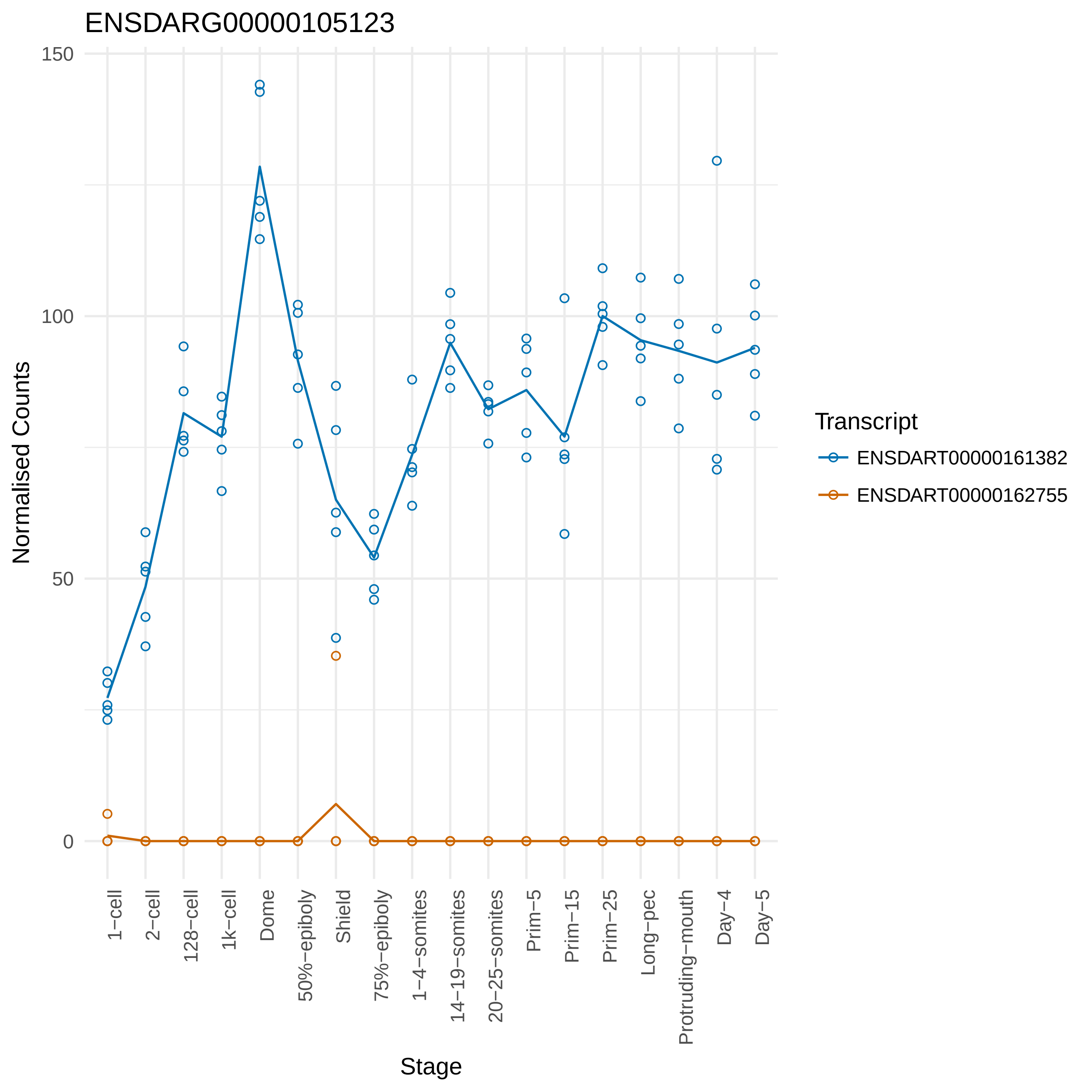
ENSDARG00000105123
Normalised Counts
150
100
50
0
1−cell
2−cell
128−cell
1k−cell
Dome
50%−epiboly
Shield
75%−epiboly
1−4−somites
14−19−somites
20−25−somites
Prim−5
Prim−15
Prim−25
Long−pec
Protruding−mouth
Day−4
Day−5
Stage
Transcript
ENSDART00000161382
ENSDART00000162755

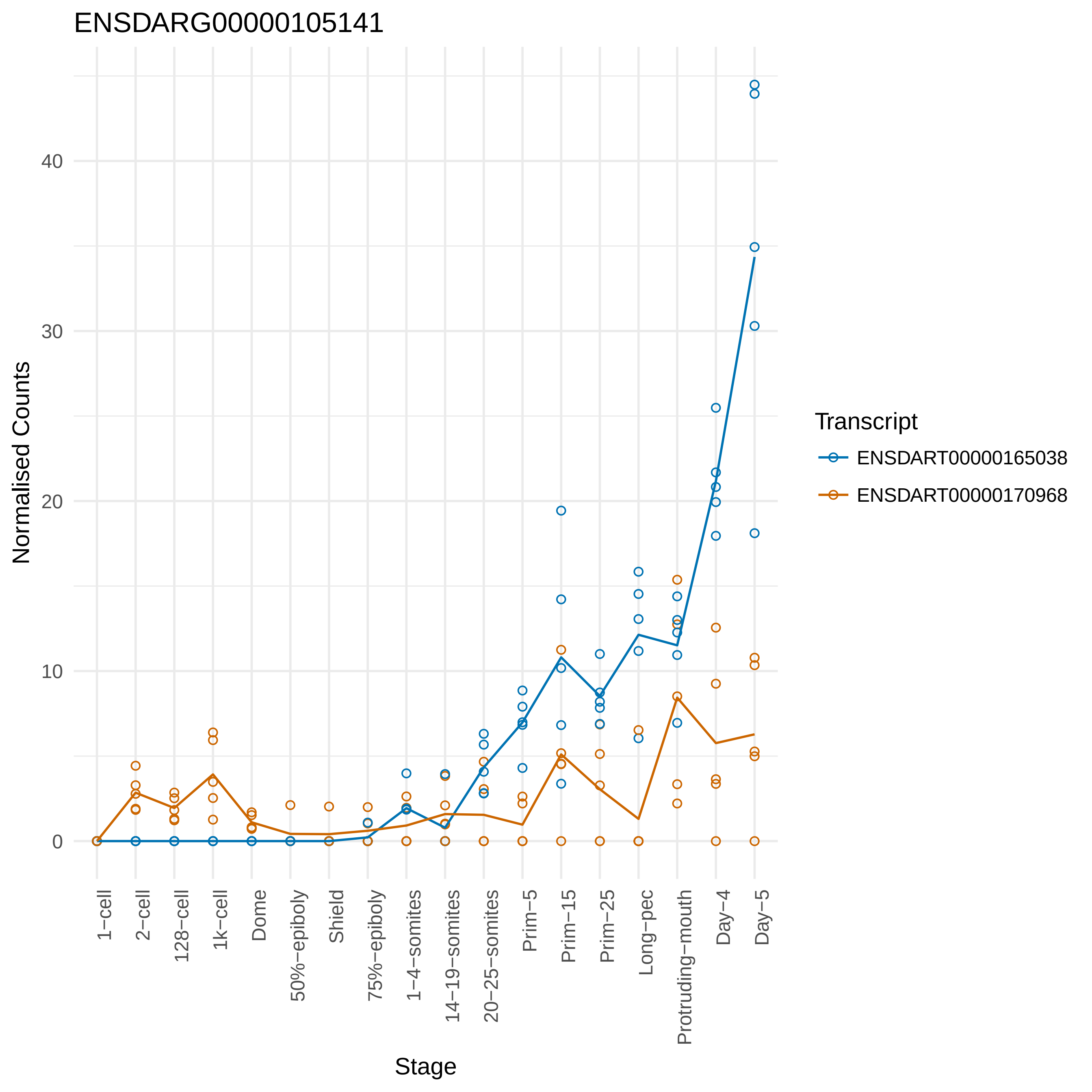
ENSDARG00000105141
Normalised Counts
0
10
20
30
40
Stage
1−cell
2−cell
128−cell
1k−cell
Dome
50%−epiboly
Shield
75%−epiboly
1−4−somites
14−19−somites
20−25−somites
Prim−5
Prim−15
Prim−25
Long−pec
Protruding−mouth
Day−4
Day−5
Transcript
ENSDART00000165038
ENSDART00000170968

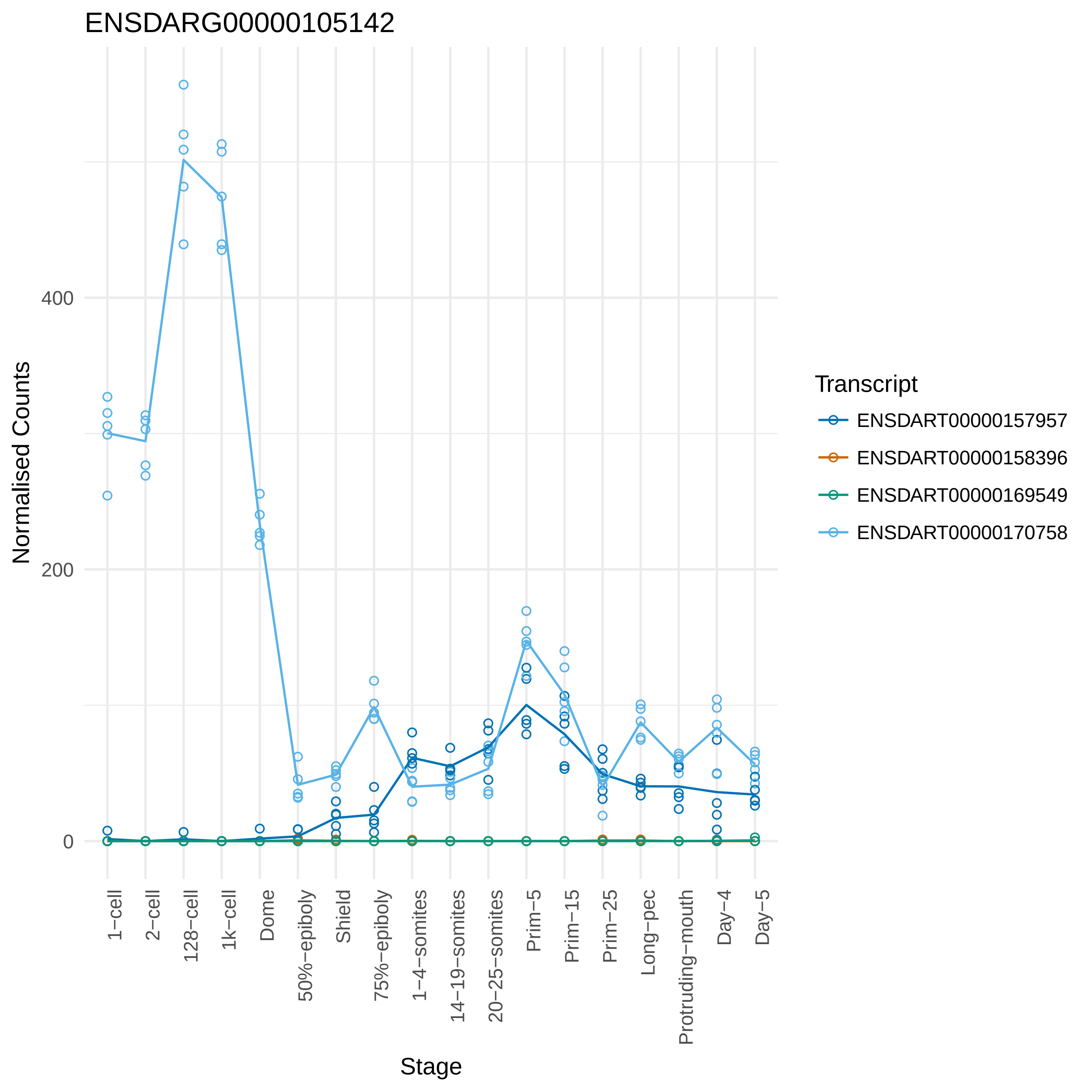

ENSDARG00000105142
Normalised Counts
400
200
0
1−cell
2−cell
128−cell
1k−cell
Dome
50%−epiboly
Shield
75%−epiboly
1−4−somites
14−19−somites
20−25−somites
Prim−5
Prim−15
Prim−25
Long−pec
Protruding−mouth
Day−4
Day−5
Stage
Transcript
ENSDART00000157957
ENSDART00000158396
ENSDART00000169549
ENSDART00000170758

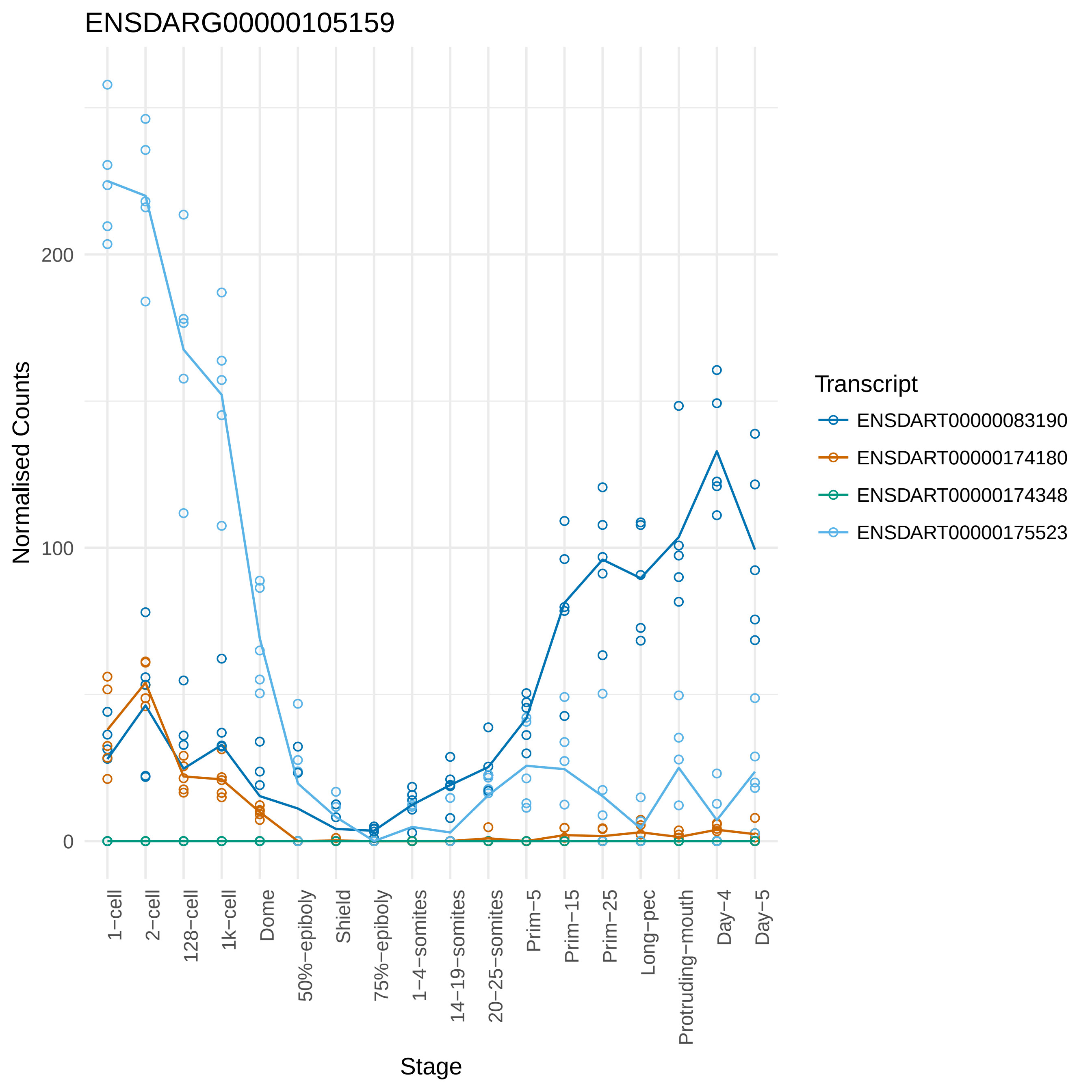

ENSDARG00000105159
Normalised Counts
200
100
0
1−cell
2−cell
128−cell
1k−cell
Dome
50%−epiboly
Shield
75%−epiboly
1−4−somites
14−19−somites
20−25−somites
Prim−5
Prim−15
Prim−25
Long−pec
Protruding−mouth
Day−4
Day−5
Stage
Transcript
ENSDART00000083190
ENSDART00000174180
ENSDART00000174348
ENSDART00000175523

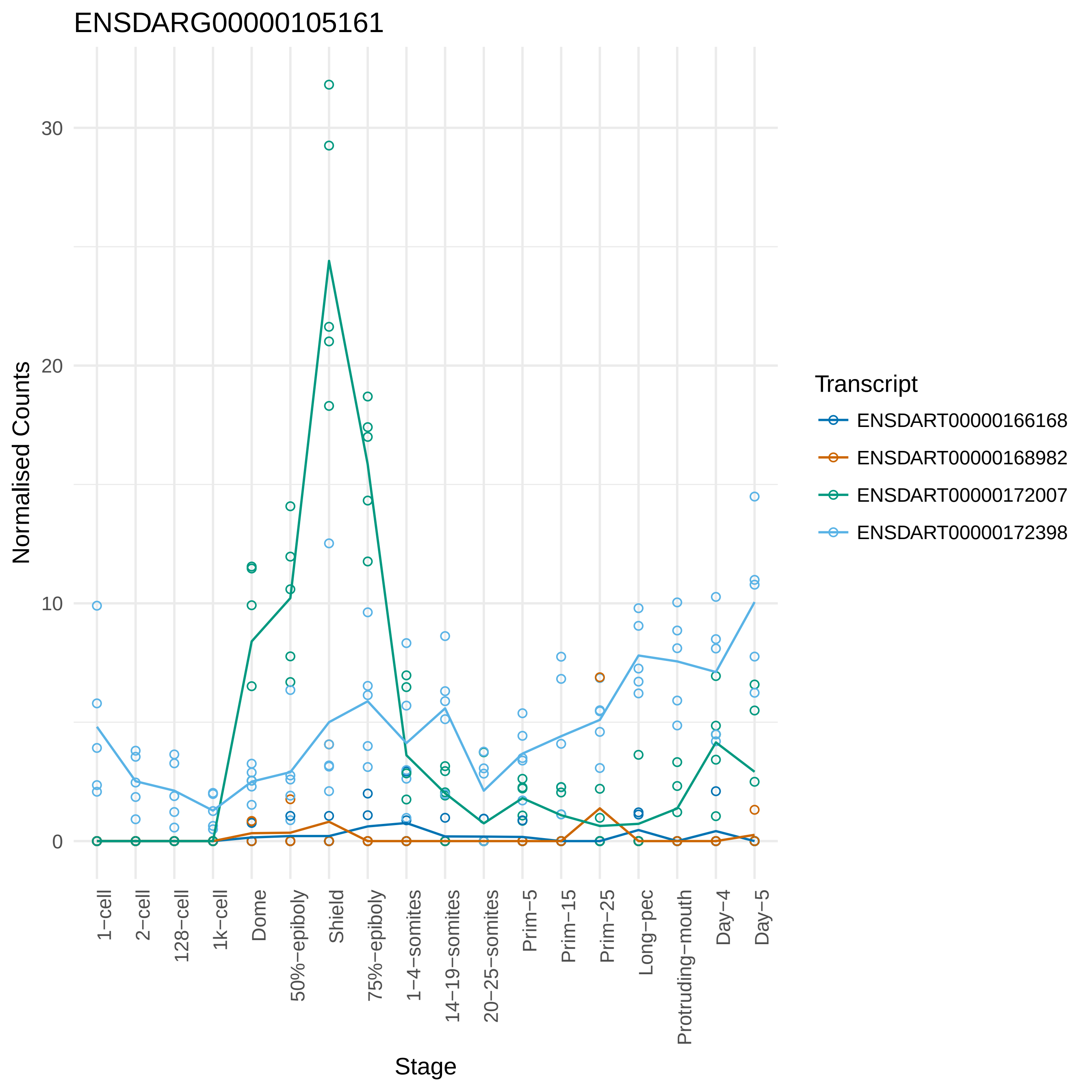
ENSDARG00000105161
Normalised Counts
0
10
20
30
1−cell
2−cell
128−cell
1k−cell
Dome
50%−epiboly
Shield
75%−epiboly
1−4−somites
14−19−somites
20−25−somites
Prim−5
Prim−15
Prim−25
Long−pec
Protruding−mouth
Day−4
Day−5
Stage
Transcript
ENSDART00000166168
ENSDART00000168982
ENSDART00000172007
ENSDART00000172398

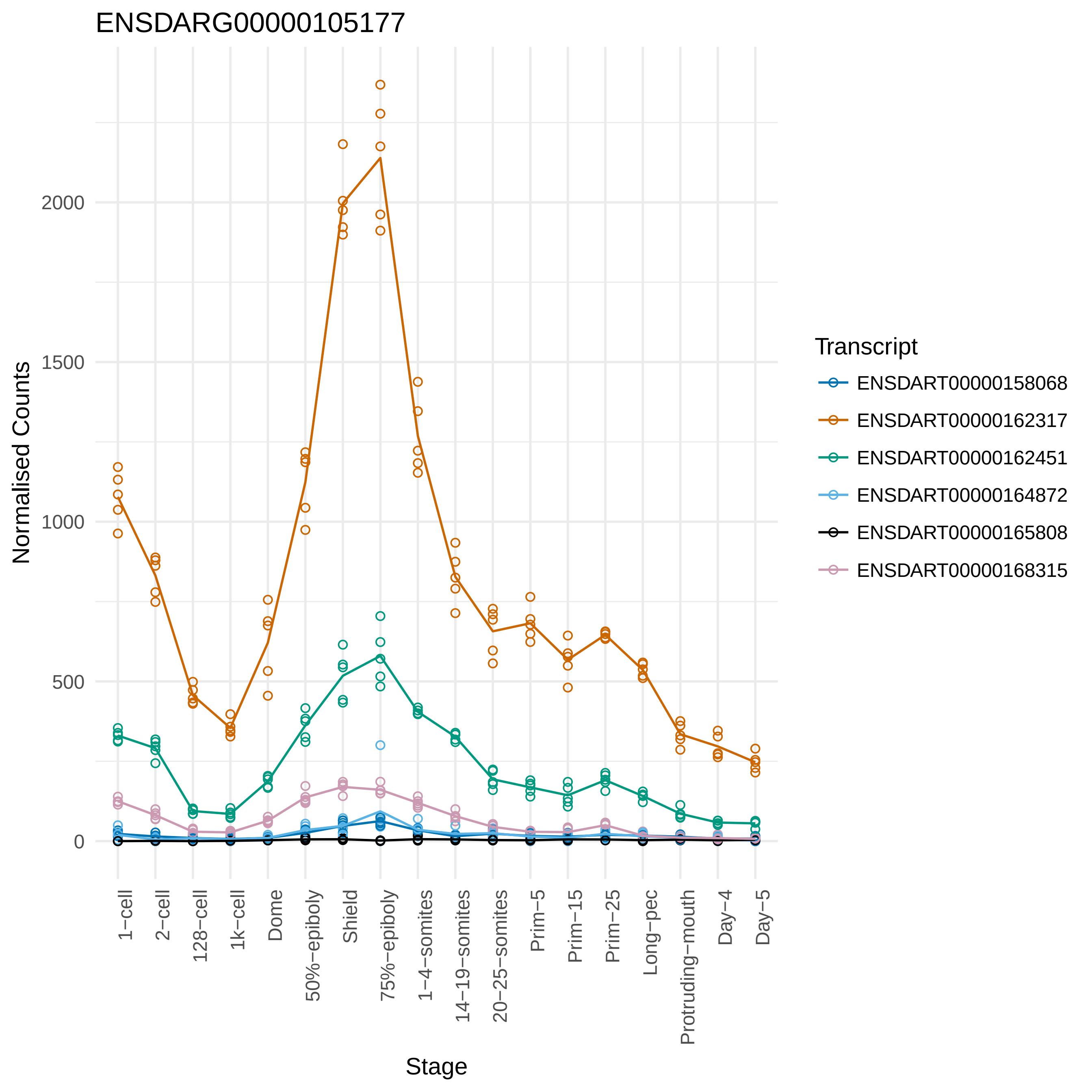

ENSDARG00000105177
Normalised Counts
0
500
1000
1500
2000
Stage
1−cell
2−cell
128−cell
1k−cell
Dome
50%−epiboly
Shield
75%−epiboly
1−4−somites
14−19−somites
20−25−somites
Prim−5
Prim−15
Prim−25
Long−pec
Protruding−mouth
Day−4
Day−5
Transcript
ENSDART00000158068
ENSDART00000162317
ENSDART00000162451
ENSDART00000164872
ENSDART00000165808
ENSDART00000168315

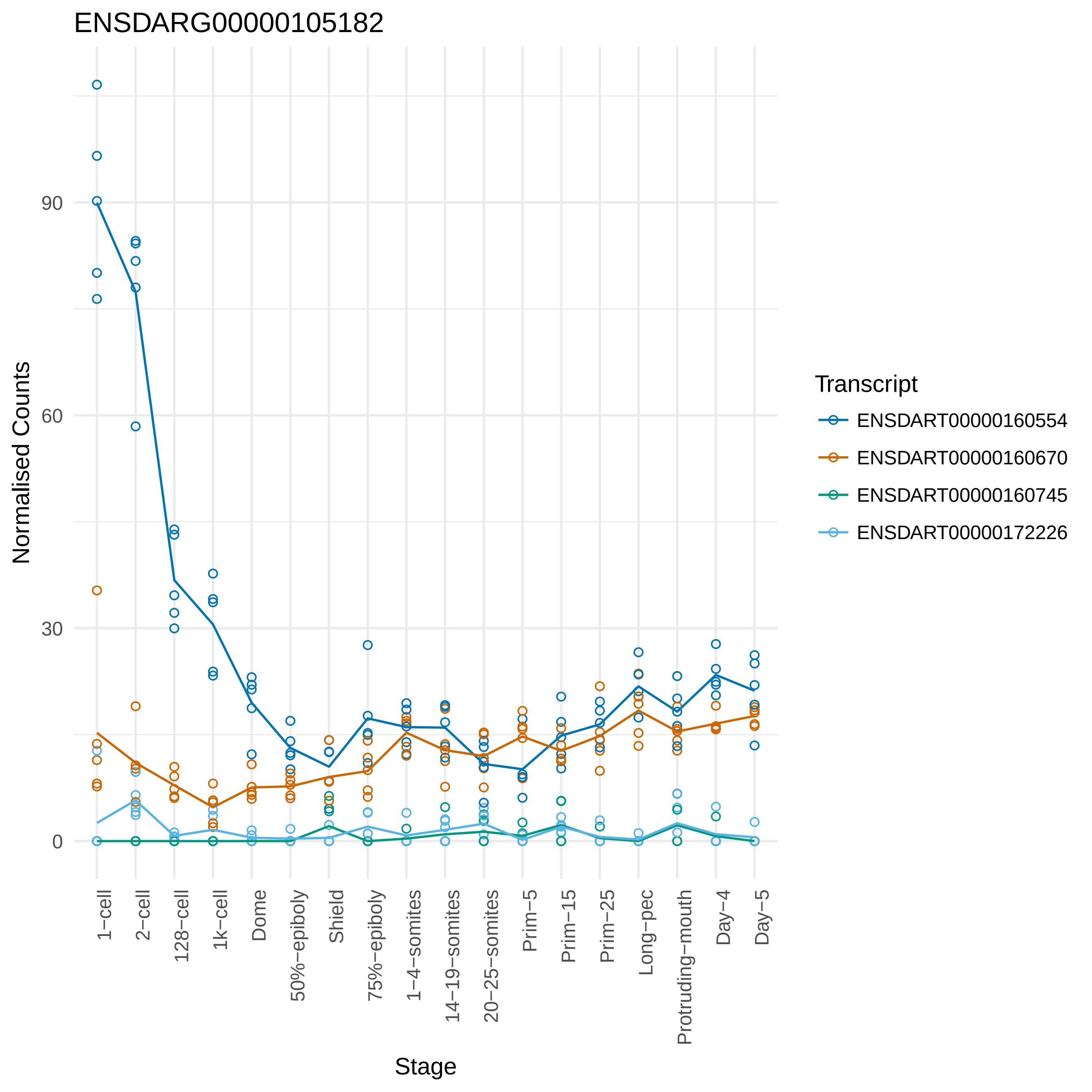
ENSDARG00000105182
Normalised Counts
0
30
60
90
1−cell
2−cell
128−cell
1k−cell
Dome
50%−epiboly
Shield
75%−epiboly
1−4−somites
14−19−somites
20−25−somites
Prim−5
Prim−15
Prim−25
Long−pec
Protruding−mouth
Day−4
Day−5
Stage
Transcript
ENSDART00000160554
ENSDART00000160670
ENSDART00000160745
ENSDART00000172226

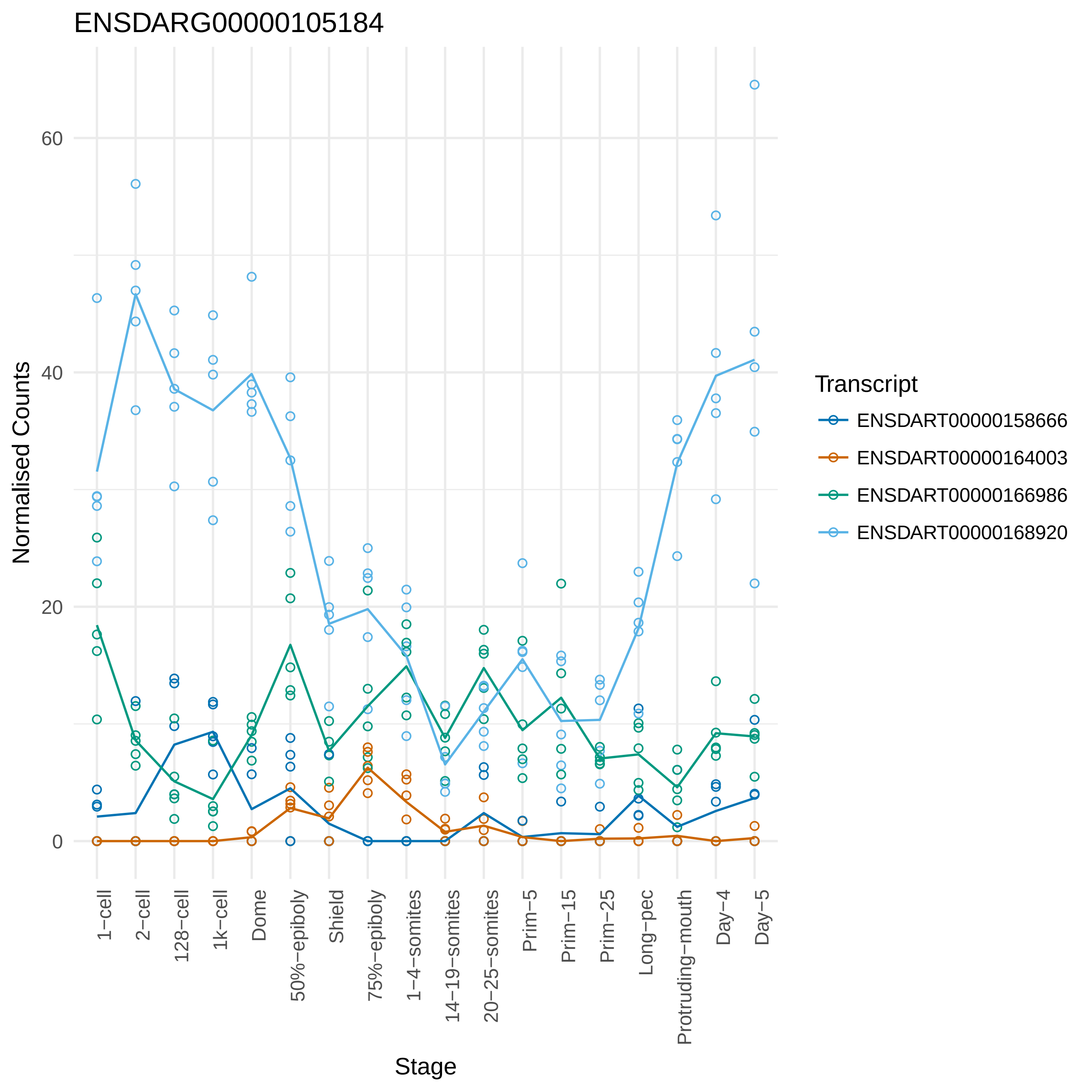
ENSDARG00000105184
Normalised Counts
0
20
40
60
Stage
1−cell
2−cell
128−cell
1k−cell
Dome
50%−epiboly
Shield
75%−epiboly
1−4−somites
14−19−somites
20−25−somites
Prim−5
Prim−15
Prim−25
Long−pec
Protruding−mouth
Day−4
Day−5
Transcript
ENSDART00000158666
ENSDART00000164003
ENSDART00000166986
ENSDART00000168920

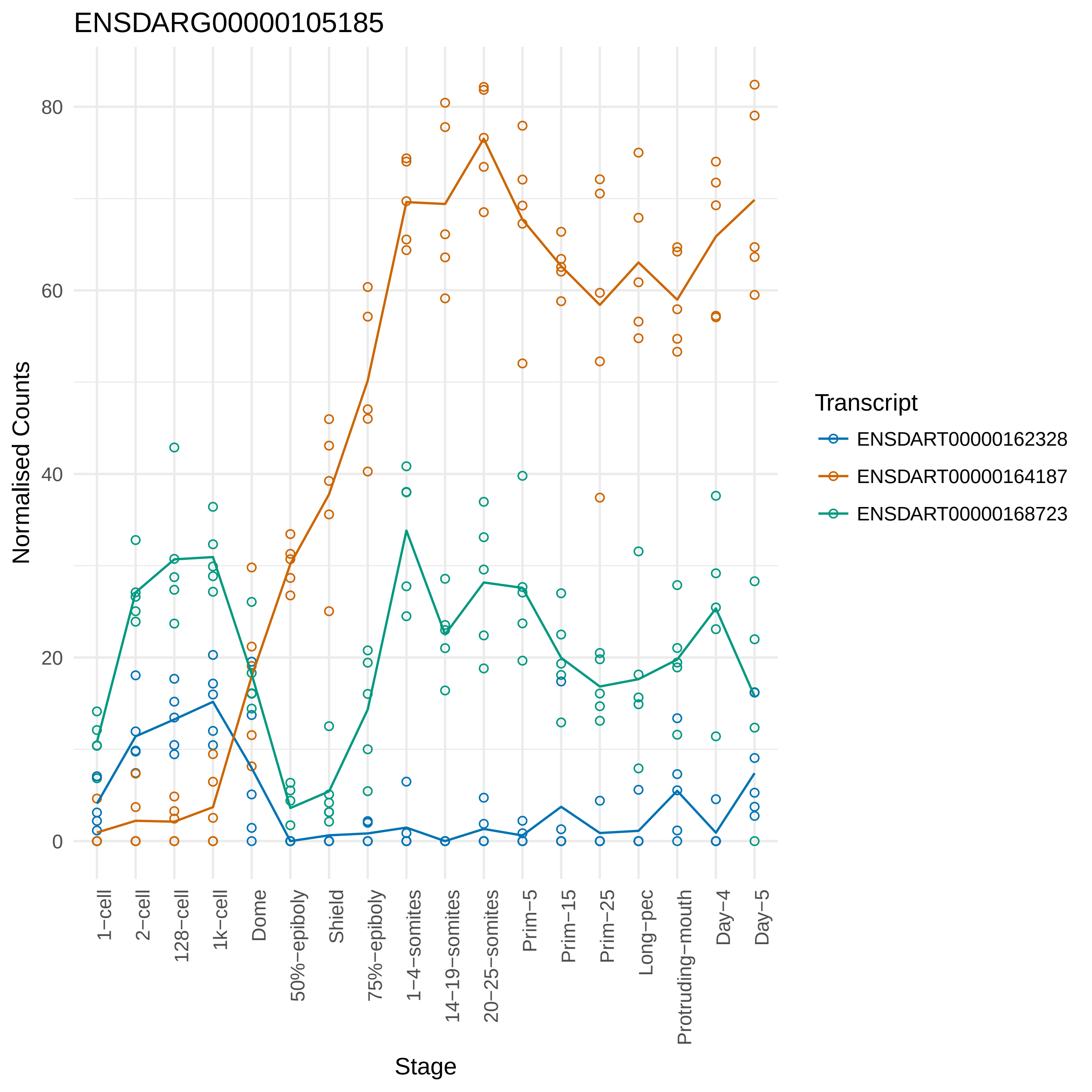

ENSDARG00000105185
Normalised Counts
0
20
40
60
80
Stage
1−cell
2−cell
128−cell
1k−cell
Dome
50%−epiboly
Shield
75%−epiboly
1−4−somites
14−19−somites
20−25−somites
Prim−5
Prim−15
Prim−25
Long−pec
Protruding−mouth
Day−4
Day−5
Transcript
ENSDART00000162328
ENSDART00000164187
ENSDART00000168723

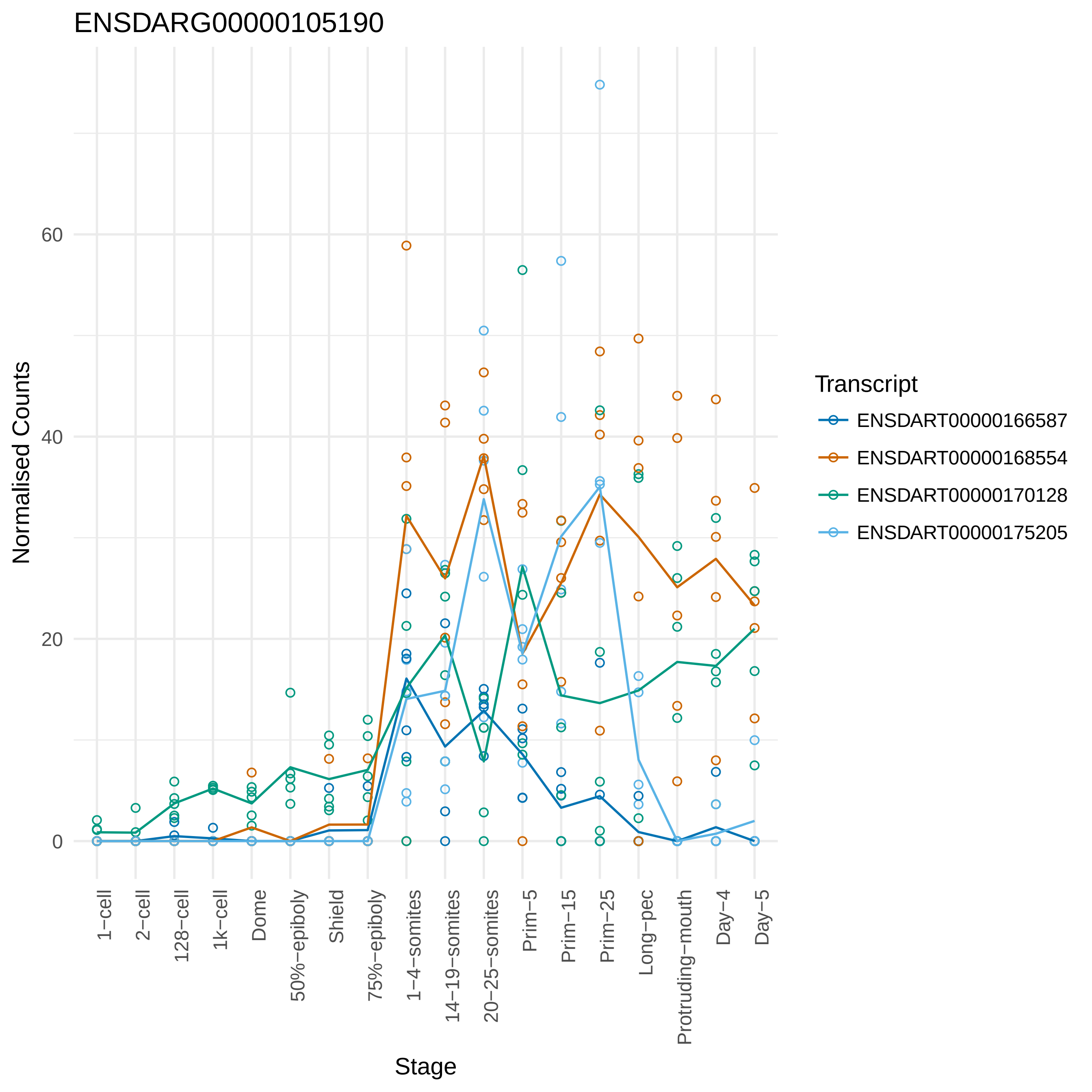

ENSDARG00000105190
Normalised Counts
0
20
40
60
1−cell
2−cell
128−cell
1k−cell
Dome
50%−epiboly
Shield
75%−epiboly
1−4−somites
14−19−somites
20−25−somites
Prim−5
Prim−15
Prim−25
Long−pec
Protruding−mouth
Day−4
Day−5
Stage
Transcript
ENSDART00000166587
ENSDART00000168554
ENSDART00000170128
ENSDART00000175205

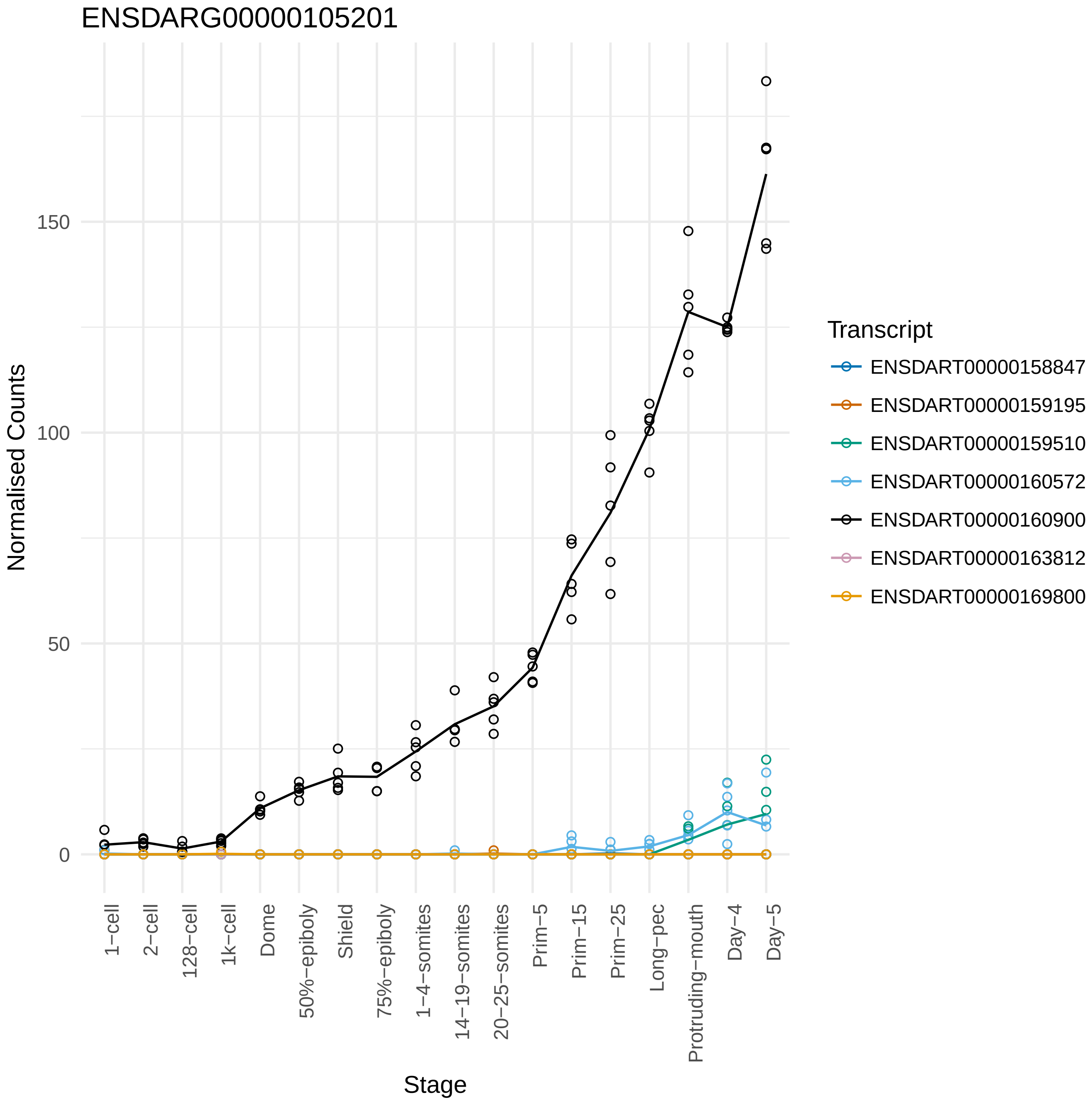

ENSDARG00000105201
Normalised Counts
150
100
50
0
1−cell
2−cell
128−cell
1k−cell
Dome
50%−epiboly
Shield
75%−epiboly
1−4−somites
14−19−somites
20−25−somites
Prim−5
Prim−15
Prim−25
Long−pec
Protruding−mouth
Day−4
Day−5
Stage
Transcript
ENSDART00000158847
ENSDART00000159195
ENSDART00000159510
ENSDART00000160572
ENSDART00000160900
ENSDART00000163812
ENSDART00000169800

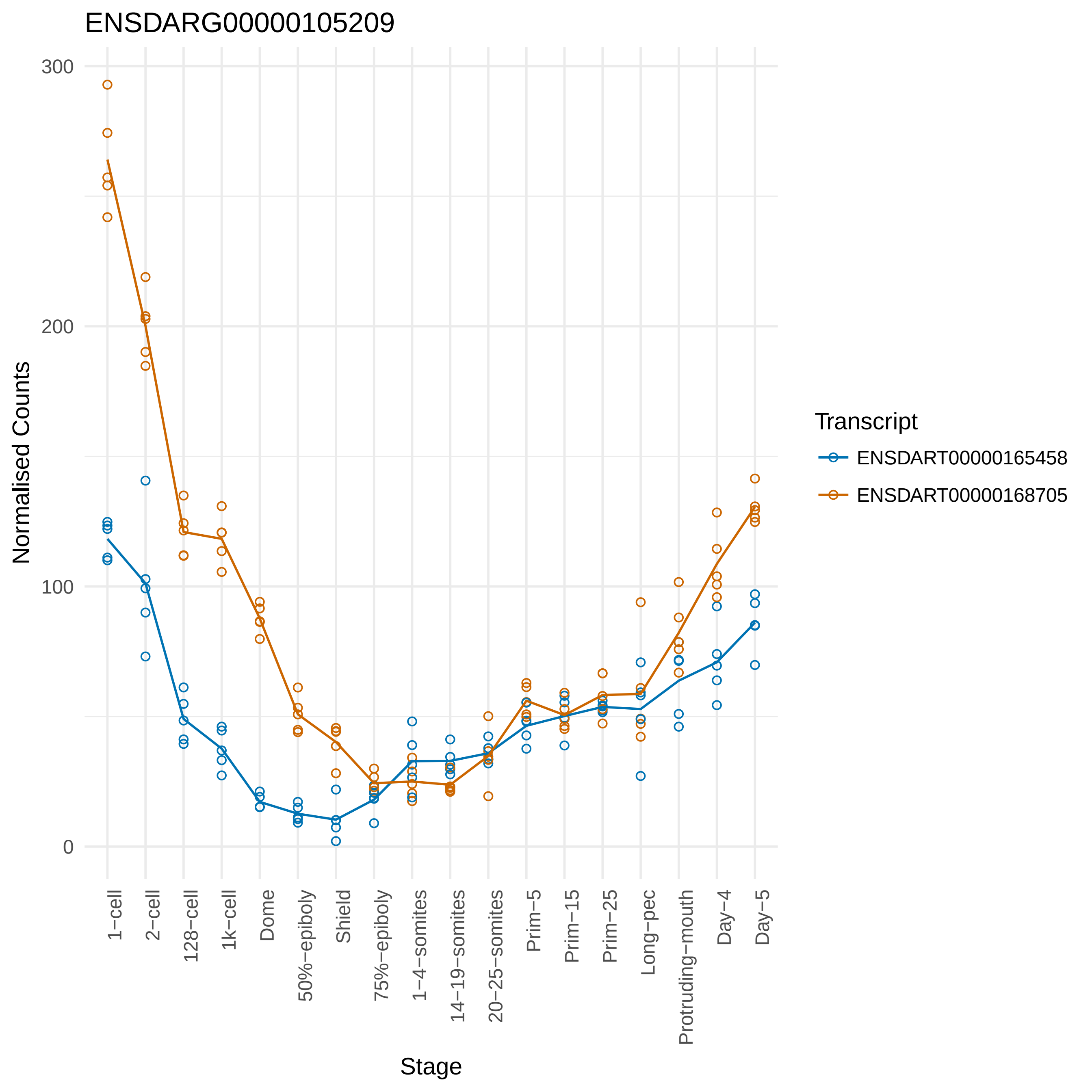
ENSDARG00000105209
Normalised Counts
300
200
100
0
1−cell
2−cell
128−cell
1k−cell
Dome
50%−epiboly
Shield
75%−epiboly
1−4−somites
14−19−somites
20−25−somites
Prim−5
Prim−15
Prim−25
Long−pec
Protruding−mouth
Day−4
Day−5
Stage
Transcript
ENSDART00000165458
ENSDART00000168705

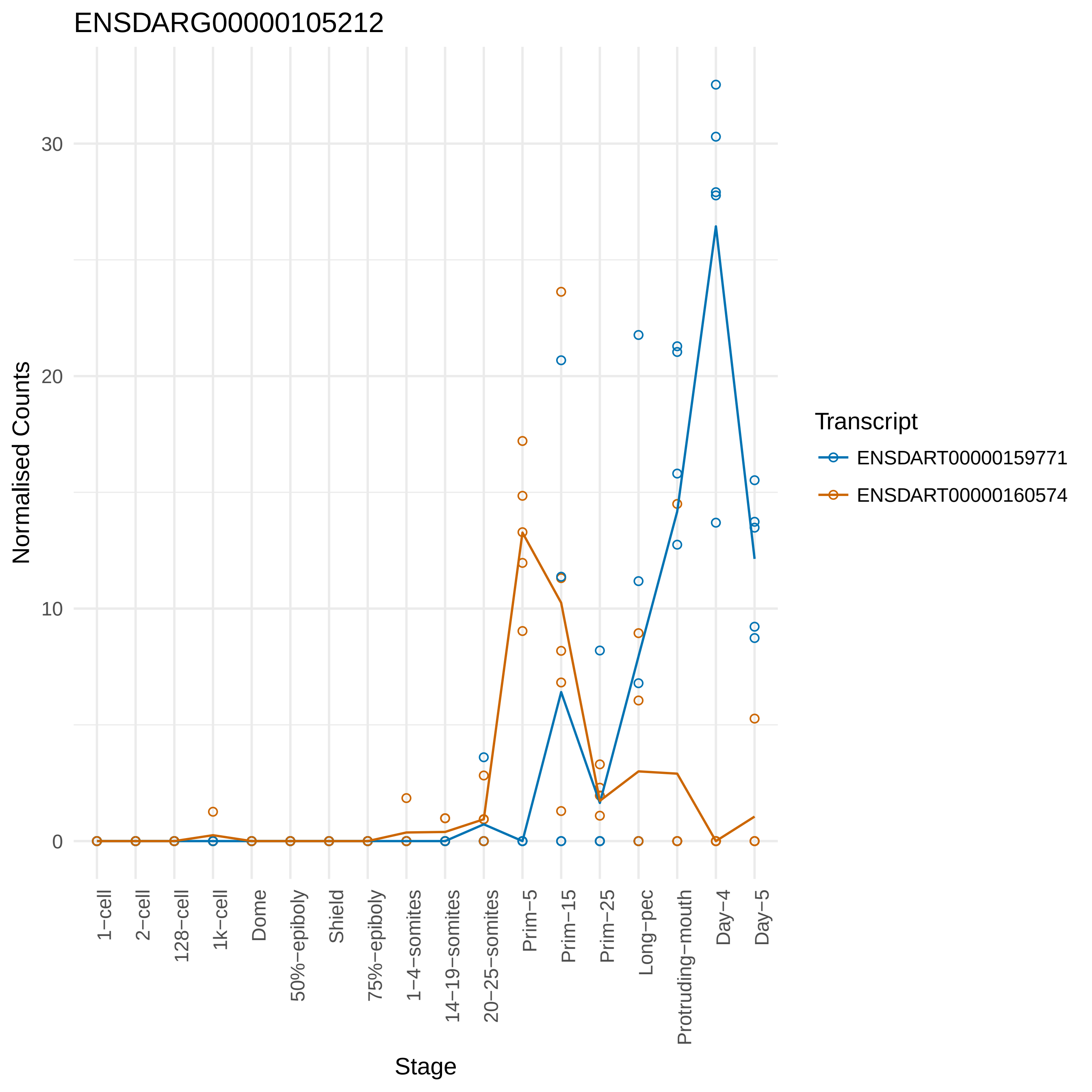
ENSDARG00000105212
Normalised Counts
30
20
10
0
1−cell
2−cell
128−cell
1k−cell
Dome
50%−epiboly
Shield
75%−epiboly
1−4−somites
14−19−somites
20−25−somites
Prim−5
Prim−15
Prim−25
Long−pec
Protruding−mouth
Day−4
Day−5
Stage
Transcript
ENSDART00000159771
ENSDART00000160574

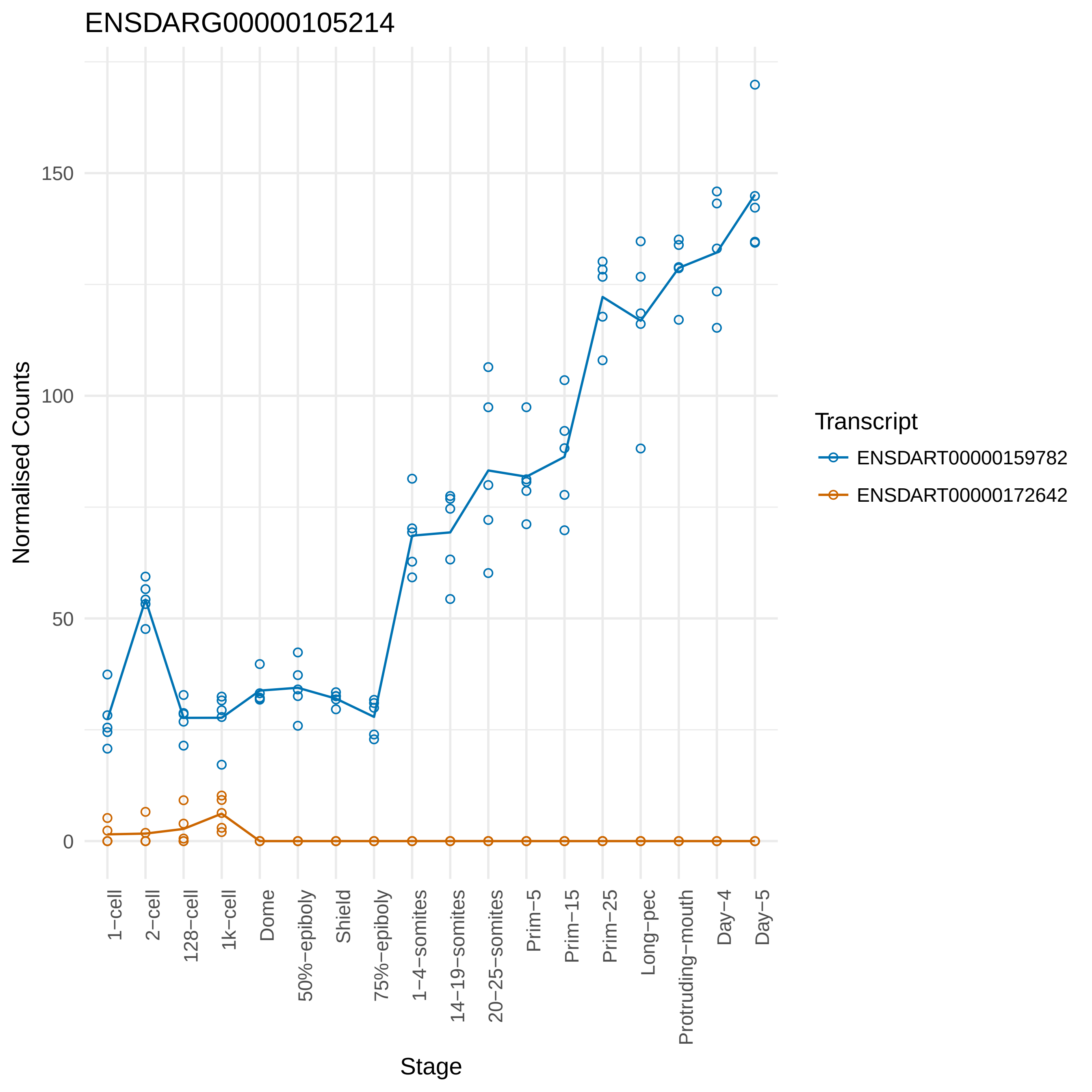

ENSDARG00000105214
Normalised Counts
0
50
100
150
1−cell
2−cell
128−cell
1k−cell
Dome
50%−epiboly
Shield
75%−epiboly
1−4−somites
14−19−somites
20−25−somites
Prim−5
Prim−15
Prim−25
Long−pec
Protruding−mouth
Day−4
Day−5
Stage
Transcript
ENSDART00000159782
ENSDART00000172642

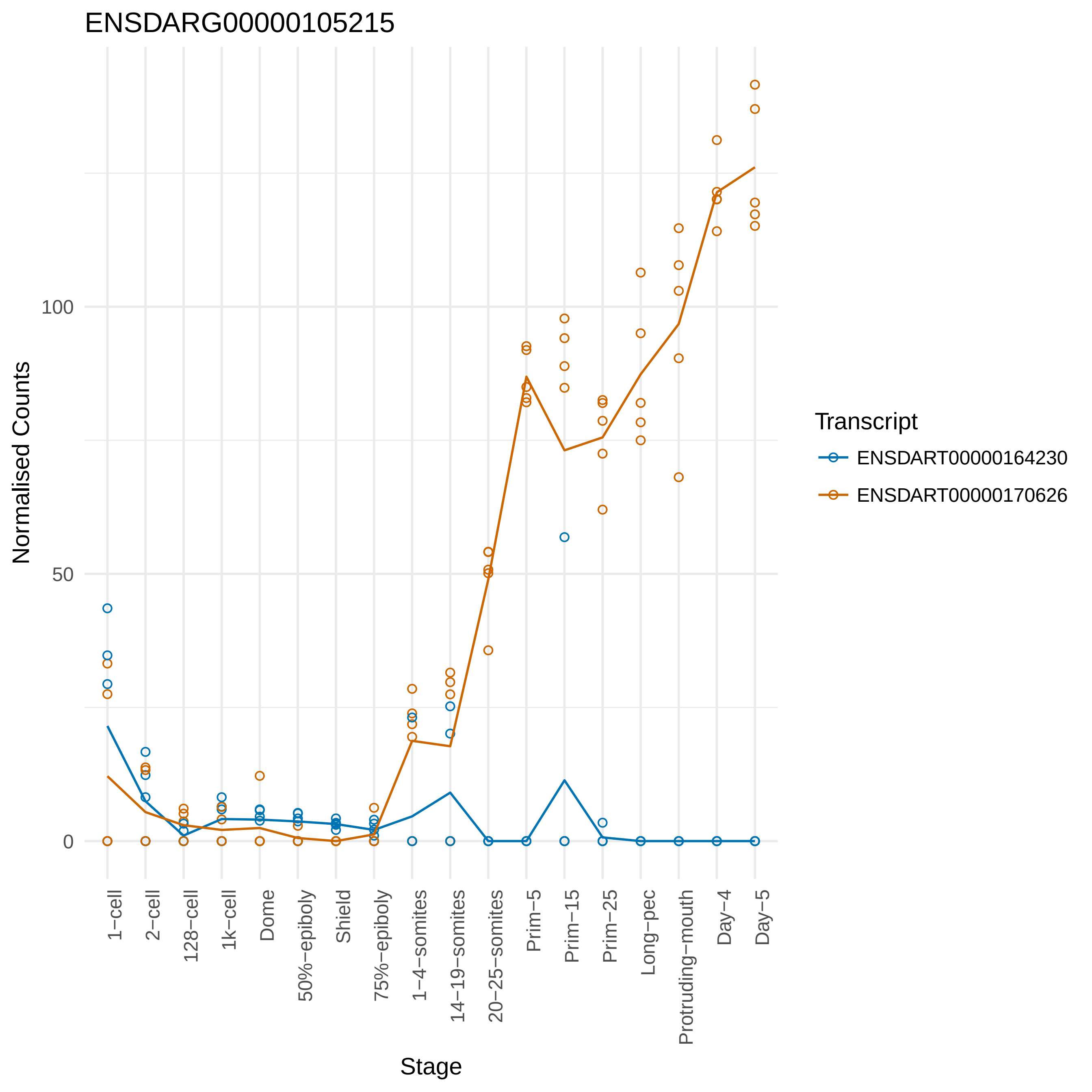

ENSDARG00000105215
Normalised Counts
0
50
100
1−cell
2−cell
128−cell
1k−cell
Dome
50%−epiboly
Shield
75%−epiboly
1−4−somites
14−19−somites
20−25−somites
Prim−5
Prim−15
Prim−25
Long−pec
Protruding−mouth
Day−4
Day−5
Stage
Transcript
ENSDART00000164230
ENSDART00000170626

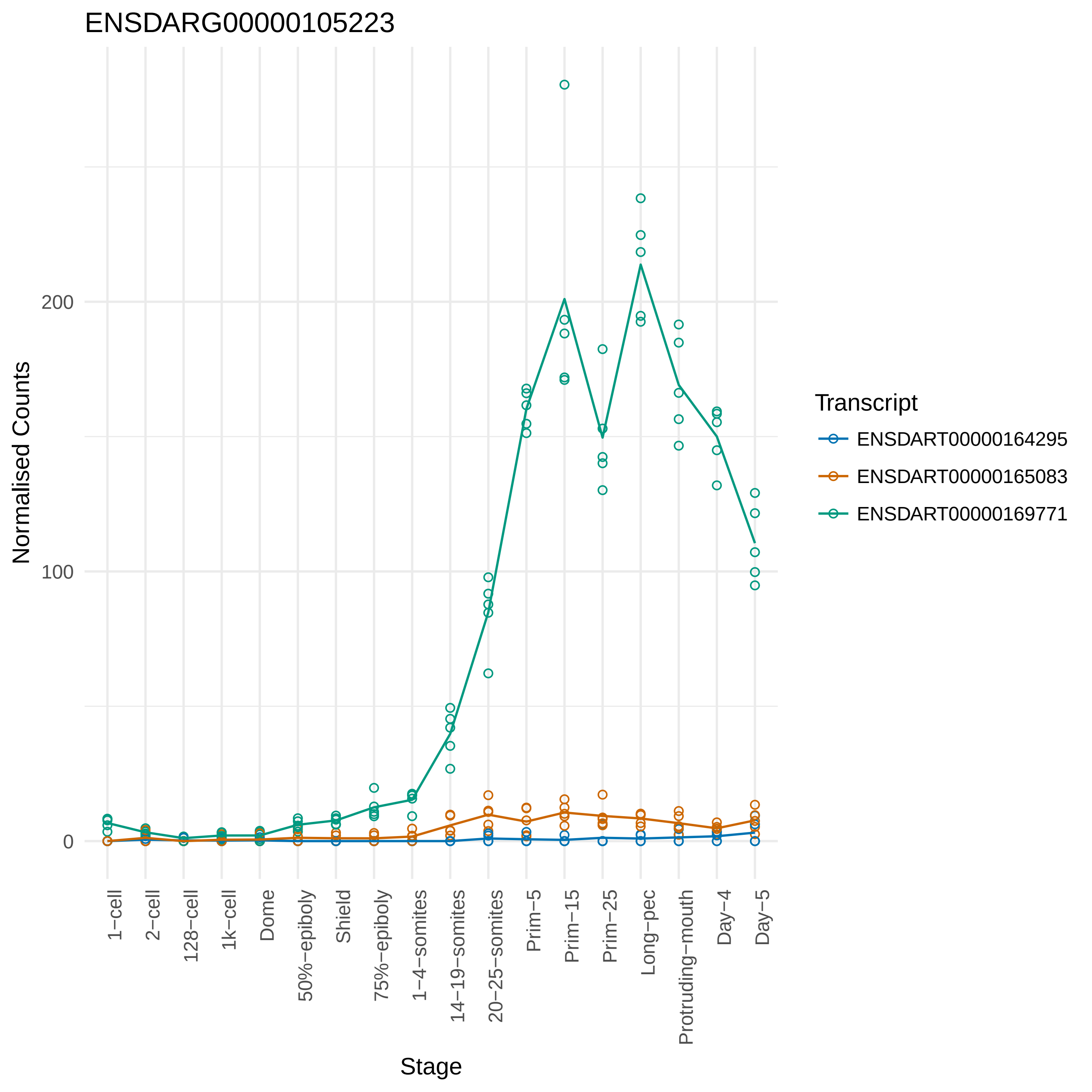

ENSDARG00000105223
Normalised Counts
0
100
200
1−cell
2−cell
128−cell
1k−cell
Dome
50%−epiboly
Shield
75%−epiboly
1−4−somites
14−19−somites
20−25−somites
Prim−5
Prim−15
Prim−25
Long−pec
Protruding−mouth
Day−4
Day−5
Stage
Transcript
ENSDART00000164295
ENSDART00000165083
ENSDART00000169771

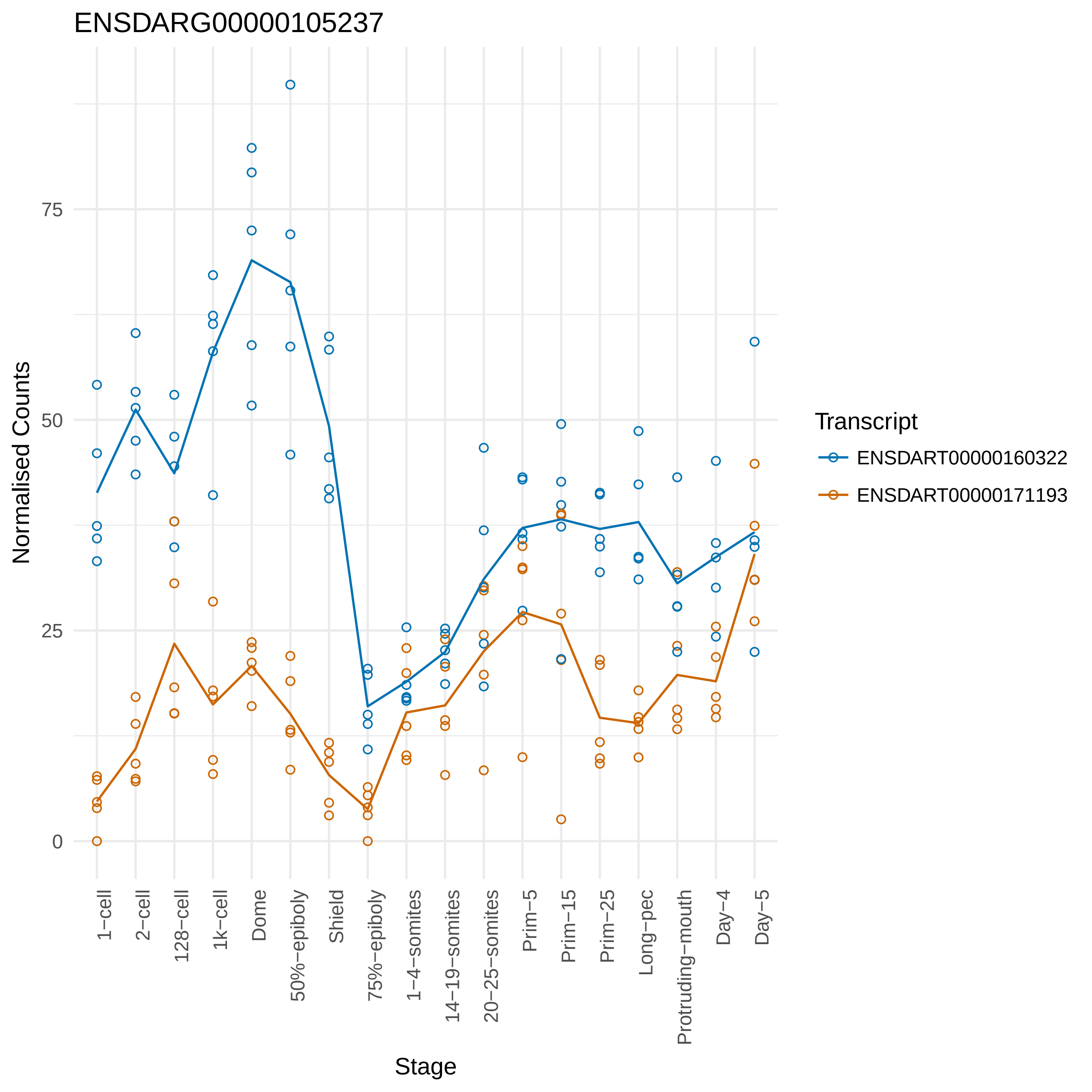
ENSDARG00000105237
Normalised Counts
0
25
50
75
Stage
1−cell
2−cell
128−cell
1k−cell
Dome
50%−epiboly
Shield
75%−epiboly
1−4−somites
14−19−somites
20−25−somites
Prim−5
Prim−15
Prim−25
Long−pec
Protruding−mouth
Day−4
Day−5
Transcript
ENSDART00000160322
ENSDART00000171193

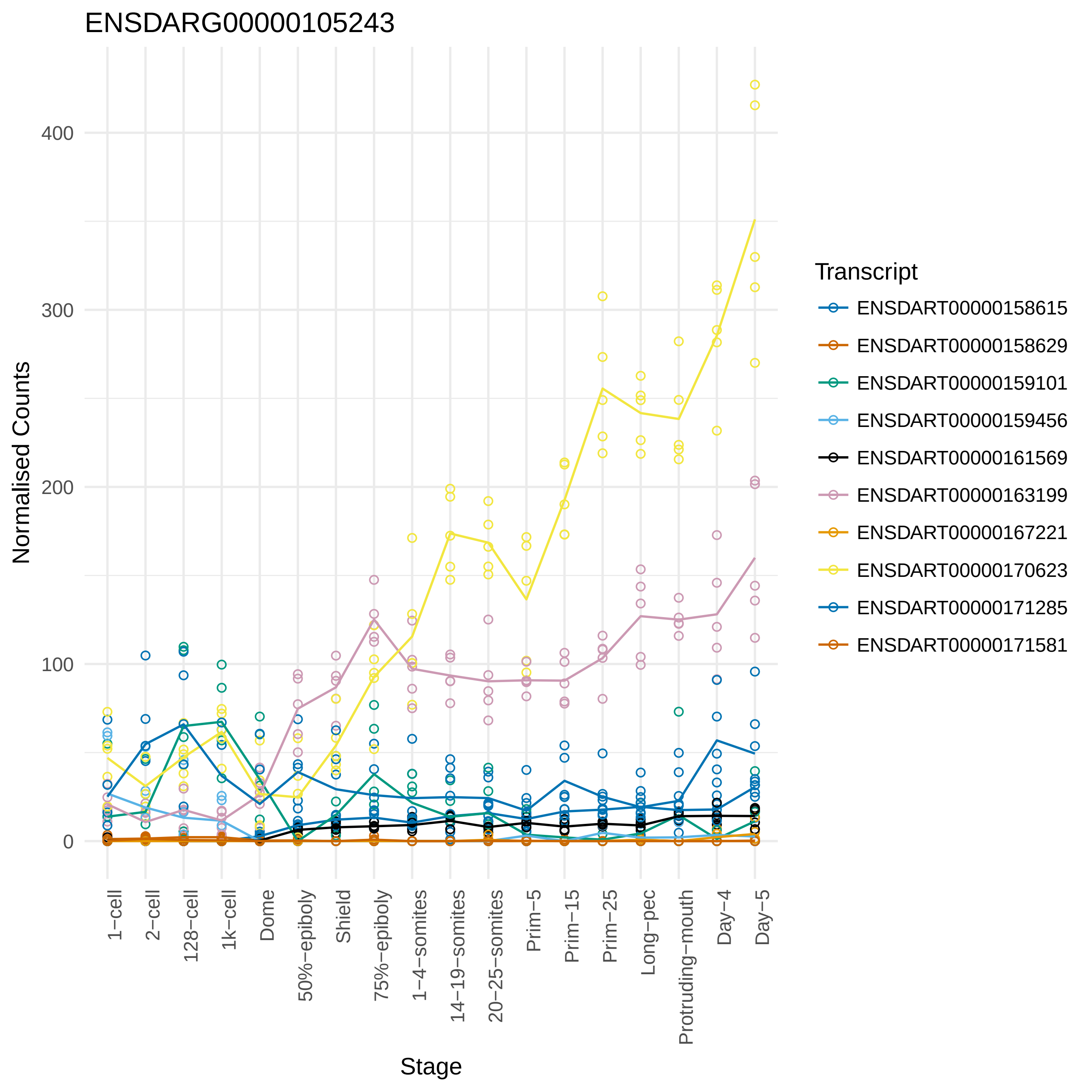

ENSDARG00000105243
Normalised Counts
400
300
200
100
0
1−cell
2−cell
128−cell
1k−cell
Dome
50%−epiboly
Shield
75%−epiboly
1−4−somites
14−19−somites
20−25−somites
Prim−5
Prim−15
Prim−25
Long−pec
Protruding−mouth
Day−4
Day−5
Stage
Transcript
ENSDART00000158615
ENSDART00000158629
ENSDART00000159101
ENSDART00000159456
ENSDART00000161569
ENSDART00000163199
ENSDART00000167221
ENSDART00000170623
ENSDART00000171285
ENSDART00000171581

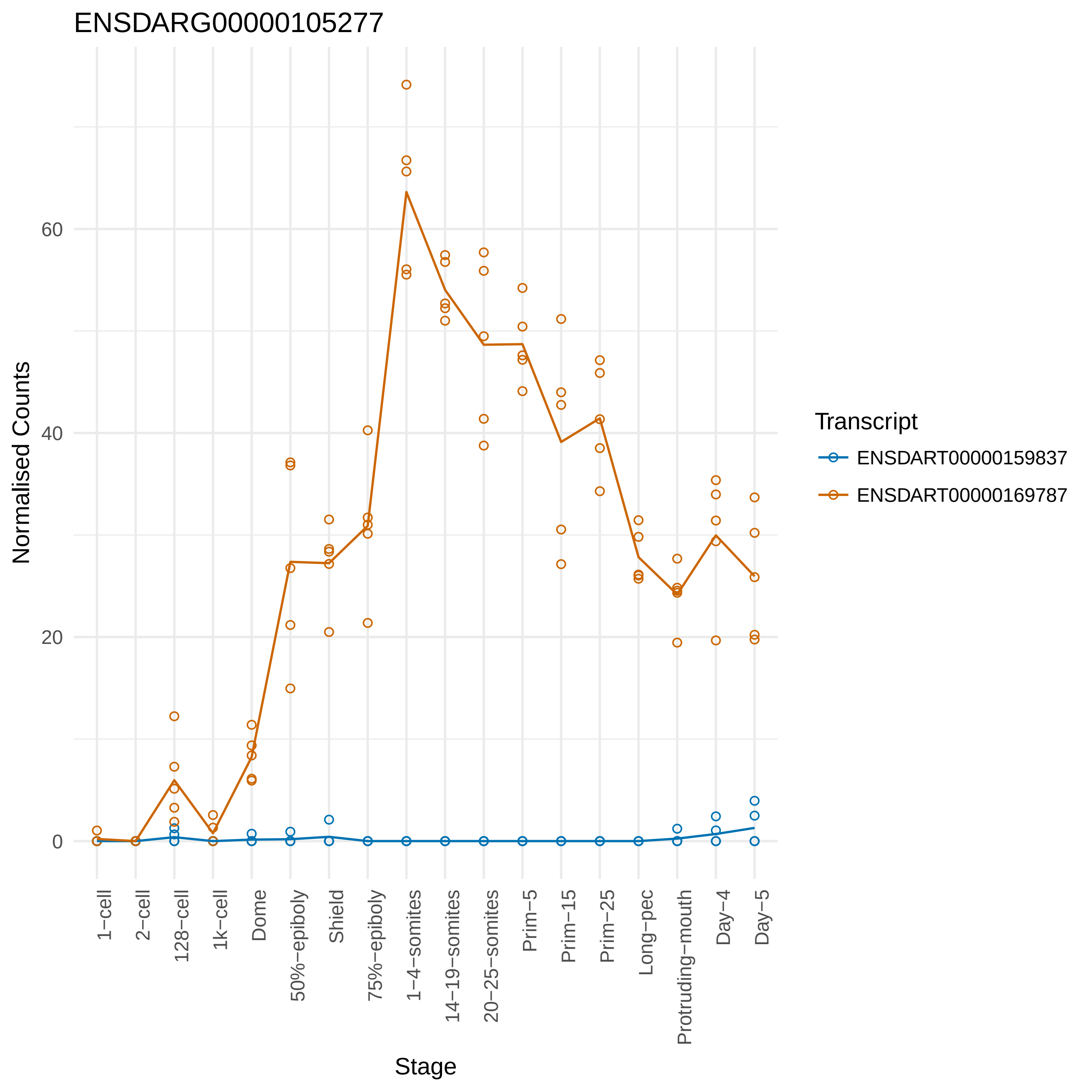

ENSDARG00000105277
Normalised Counts
0
20
40
60
Stage
1−cell
2−cell
128−cell
1k−cell
Dome
50%−epiboly
Shield
75%−epiboly
1−4−somites
14−19−somites
20−25−somites
Prim−5
Prim−15
Prim−25
Long−pec
Protruding−mouth
Day−4
Day−5
Transcript
ENSDART00000159837
ENSDART00000169787

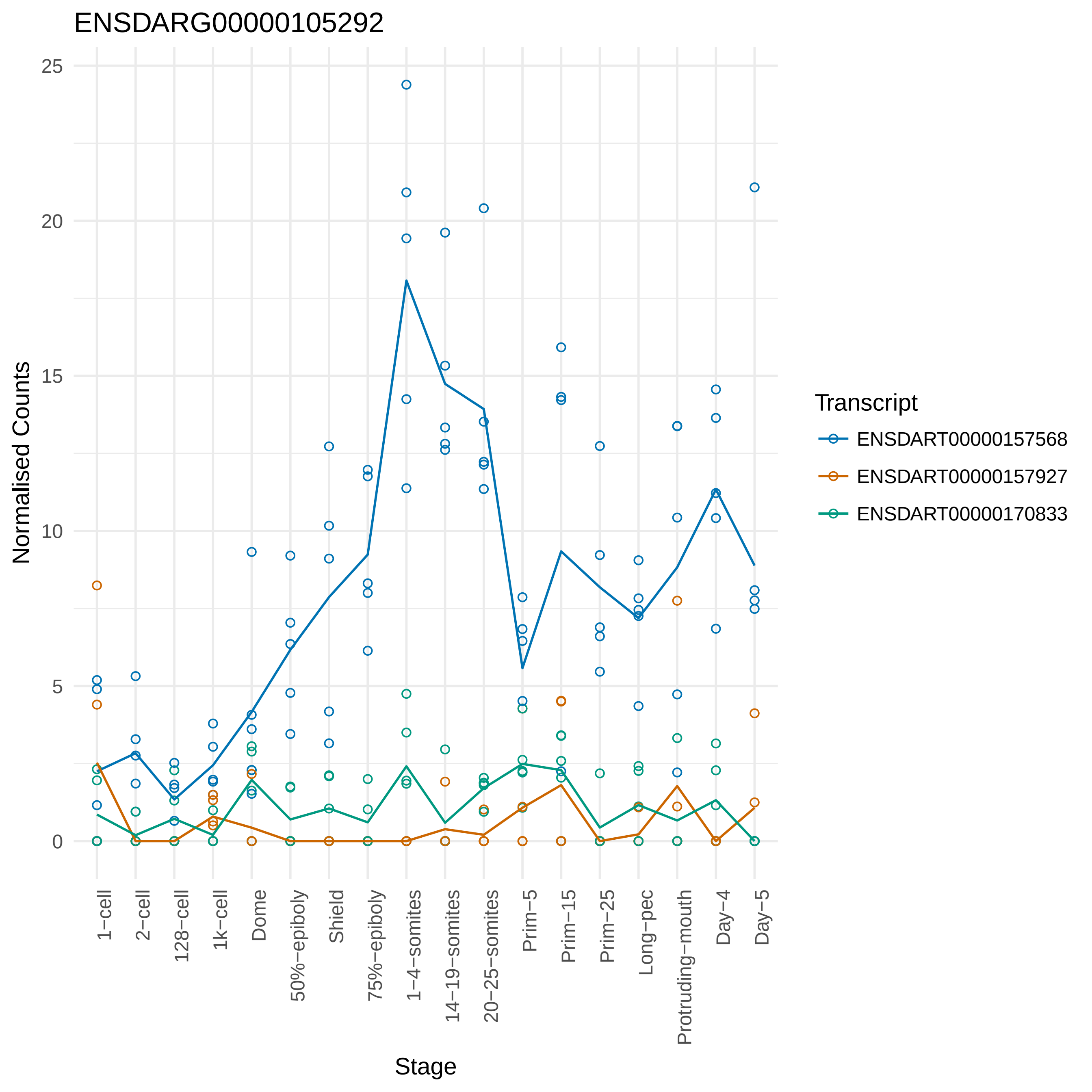

ENSDARG00000105292
Normalised Counts
25
20
15
10
5
0
1−cell
2−cell
128−cell
1k−cell
Dome
50%−epiboly
Shield
75%−epiboly
1−4−somites
14−19−somites
20−25−somites
Prim−5
Prim−15
Prim−25
Long−pec
Protruding−mouth
Day−4
Day−5
Stage
Transcript
ENSDART00000157568
ENSDART00000157927
ENSDART00000170833

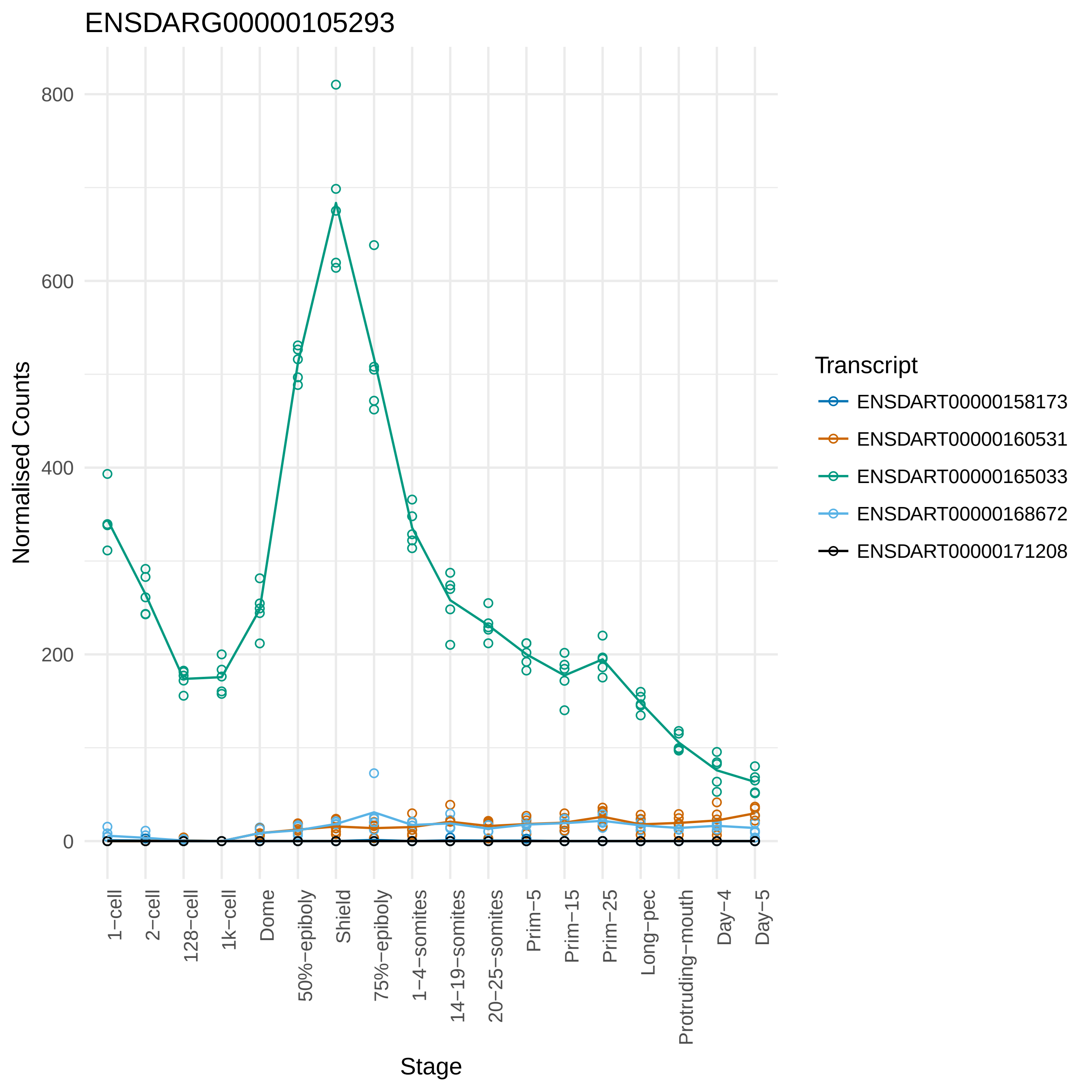

ENSDARG00000105293
Normalised Counts
800
600
400
200
0
1−cell
2−cell
128−cell
1k−cell
Dome
50%−epiboly
Shield
75%−epiboly
1−4−somites
14−19−somites
20−25−somites
Prim−5
Prim−15
Prim−25
Long−pec
Protruding−mouth
Day−4
Day−5
Stage
Transcript
ENSDART00000158173
ENSDART00000160531
ENSDART00000165033
ENSDART00000168672
ENSDART00000171208

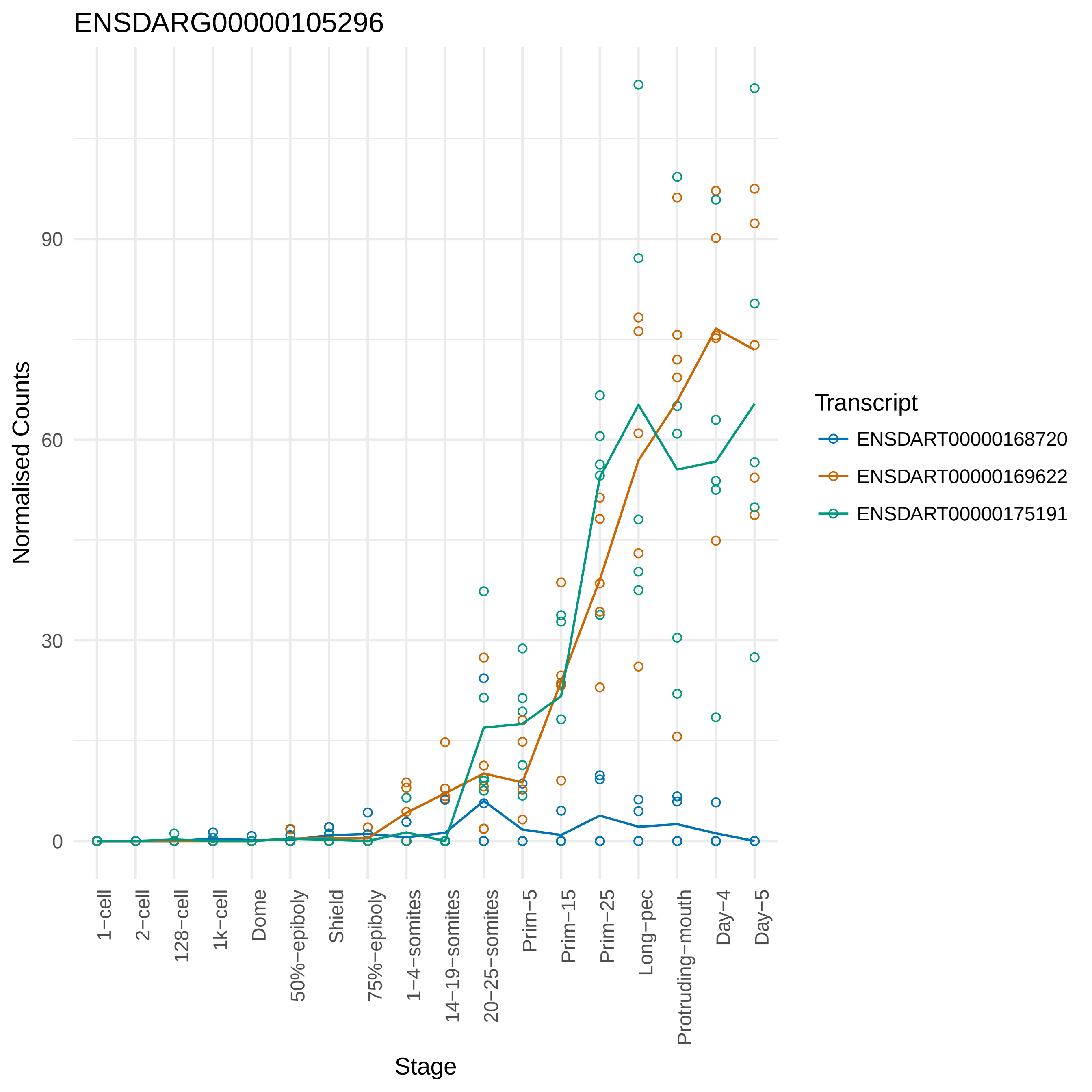

ENSDARG00000105296
Normalised Counts
0
30
60
90
1−cell
2−cell
128−cell
1k−cell
Dome
50%−epiboly
Shield
75%−epiboly
1−4−somites
14−19−somites
20−25−somites
Prim−5
Prim−15
Prim−25
Long−pec
Protruding−mouth
Day−4
Day−5
Stage
Transcript
ENSDART00000168720
ENSDART00000169622
ENSDART00000175191

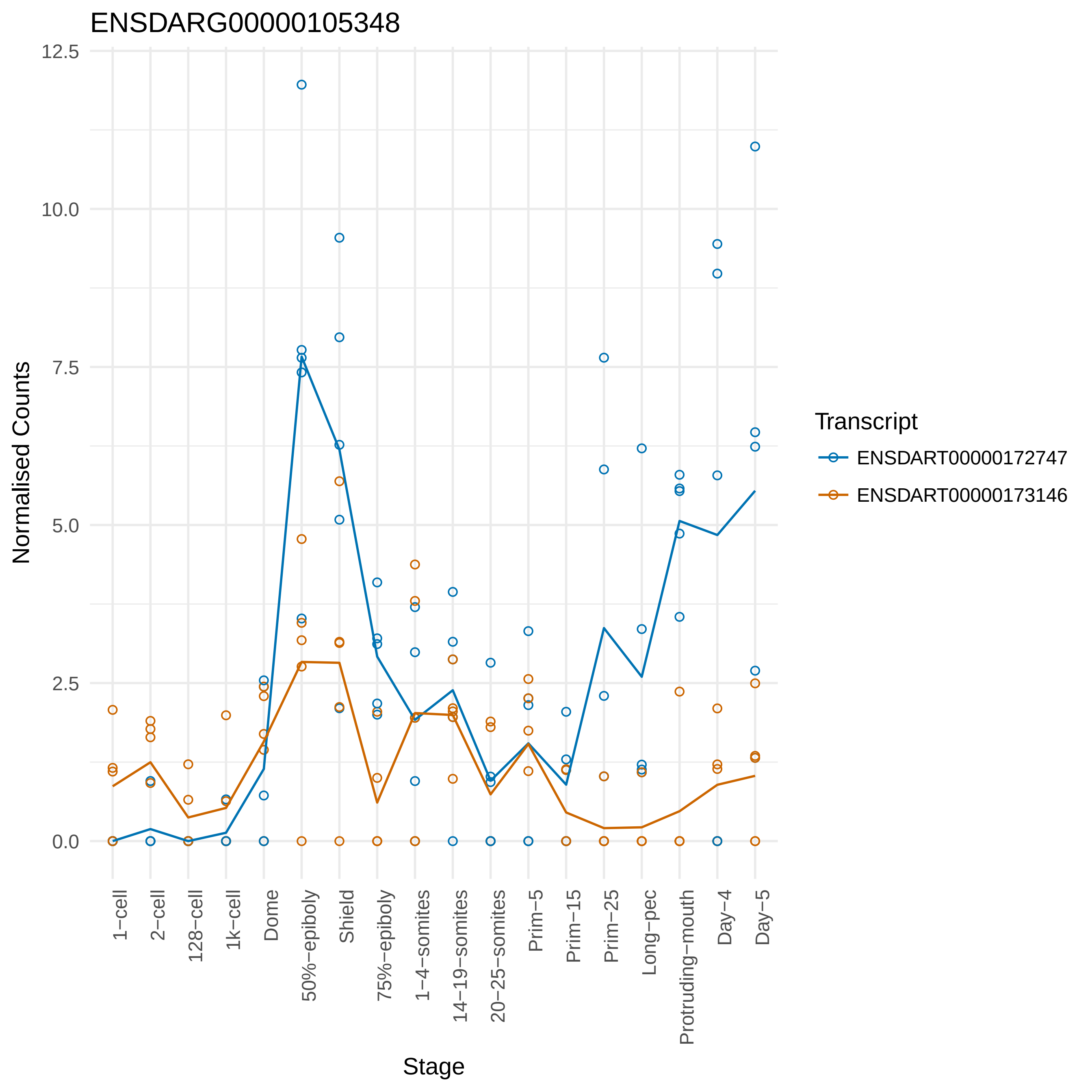

ENSDARG00000105348
Normalised Counts
Stage
Transcript
ENSDART00000172747
ENSDART00000173146
12.5
10.0
7.5
5.0
2.5
0.0
1−cell
2−cell
128−cell
1k−cell
Dome
50%−epiboly
Shield
75%−epiboly
1−4−somites
14−19−somites
20−25−somites
Prim−5
Prim−15
Prim−25
Long−pec
Protruding−mouth
Day−4
Day−5

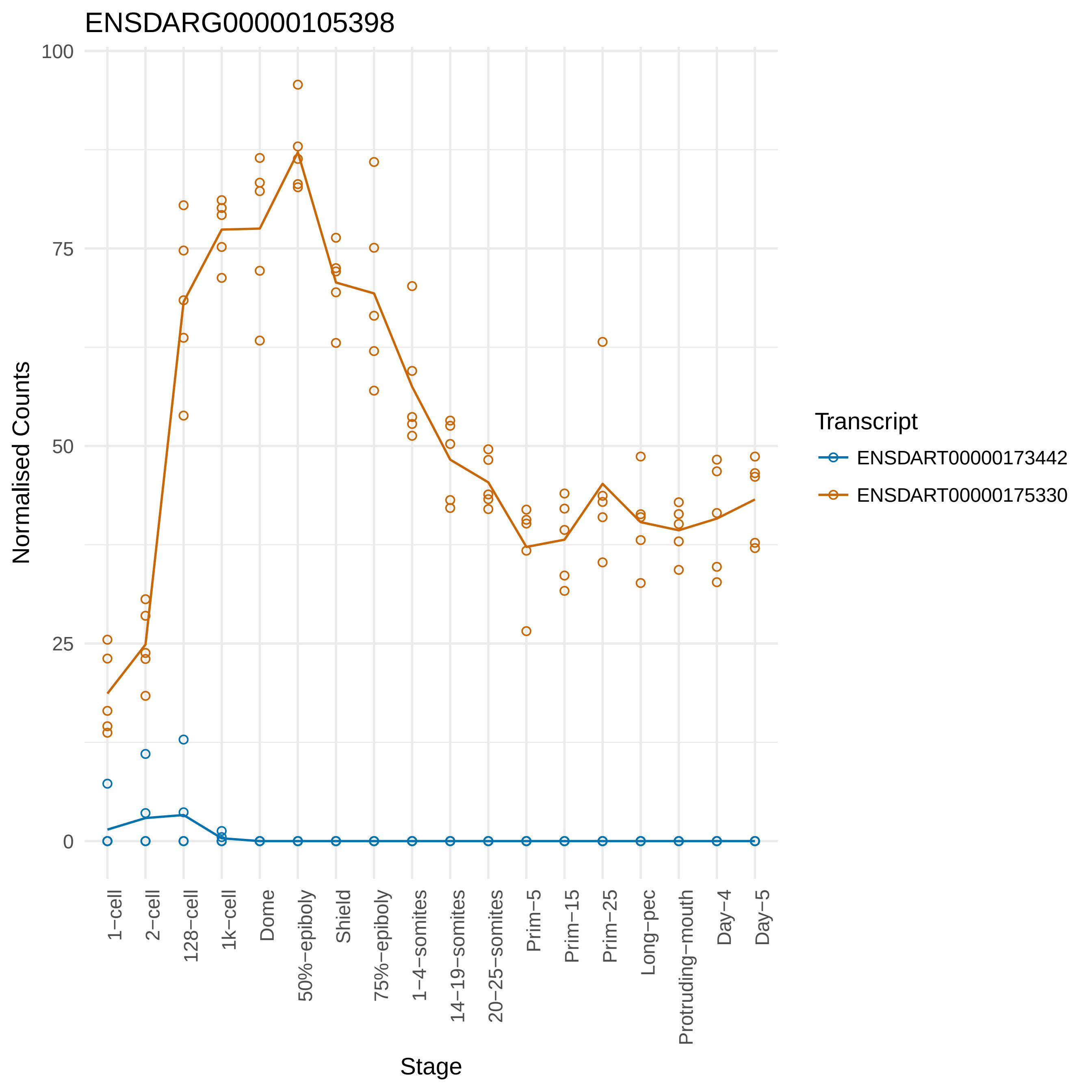

ENSDARG00000105398
Normalised Counts
100
75
50
25
0
Stage
1−cell
2−cell
128−cell
1k−cell
Dome
50%−epiboly
Shield
75%−epiboly
1−4−somites
14−19−somites
20−25−somites
Prim−5
Prim−15
Prim−25
Long−pec
Protruding−mouth
Day−4
Day−5
Transcript
ENSDART00000173442
ENSDART00000175330

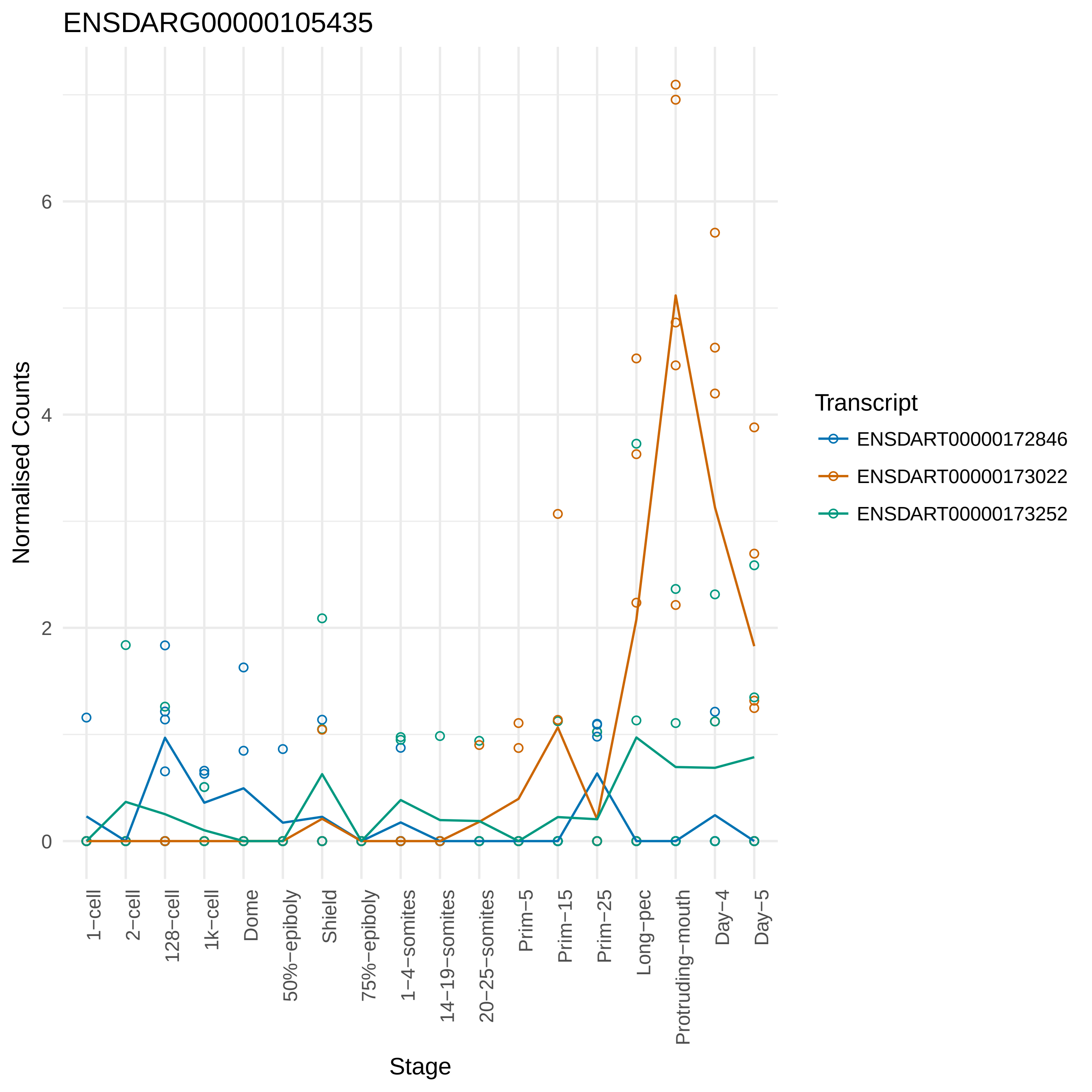

ENSDARG00000105435
Normalised Counts
0
2
4
6
1−cell
2−cell
128−cell
1k−cell
Dome
50%−epiboly
Shield
75%−epiboly
1−4−somites
14−19−somites
20−25−somites
Prim−5
Prim−15
Prim−25
Long−pec
Protruding−mouth
Day−4
Day−5
Stage
Transcript
ENSDART00000172846
ENSDART00000173022
ENSDART00000173252

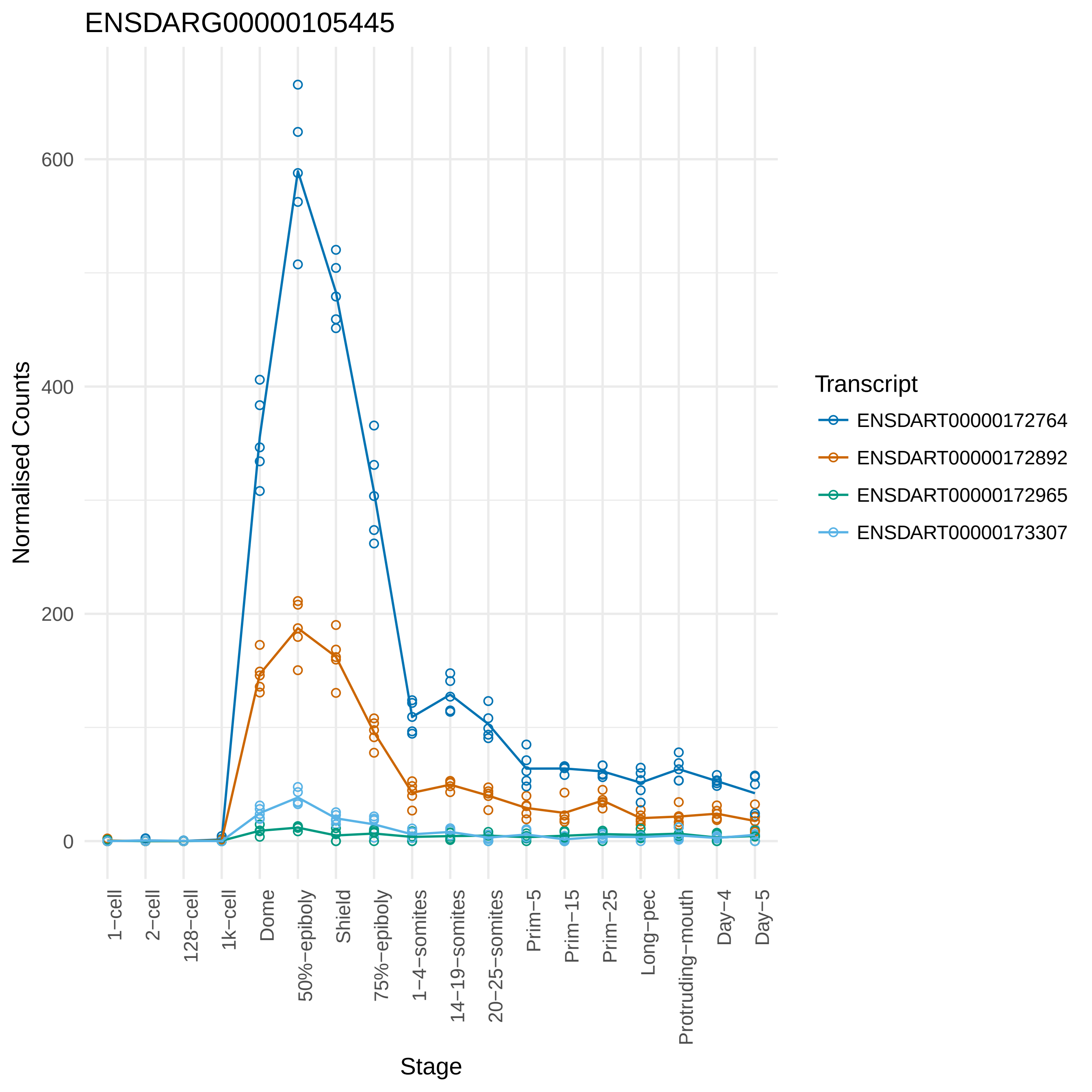
ENSDARG00000105445
Normalised Counts
0
200
400
600
Stage
1−cell
2−cell
128−cell
1k−cell
Dome
50%−epiboly
Shield
75%−epiboly
1−4−somites
14−19−somites
20−25−somites
Prim−5
Prim−15
Prim−25
Long−pec
Protruding−mouth
Day−4
Day−5
Transcript
ENSDART00000172764
ENSDART00000172892
ENSDART00000172965
ENSDART00000173307

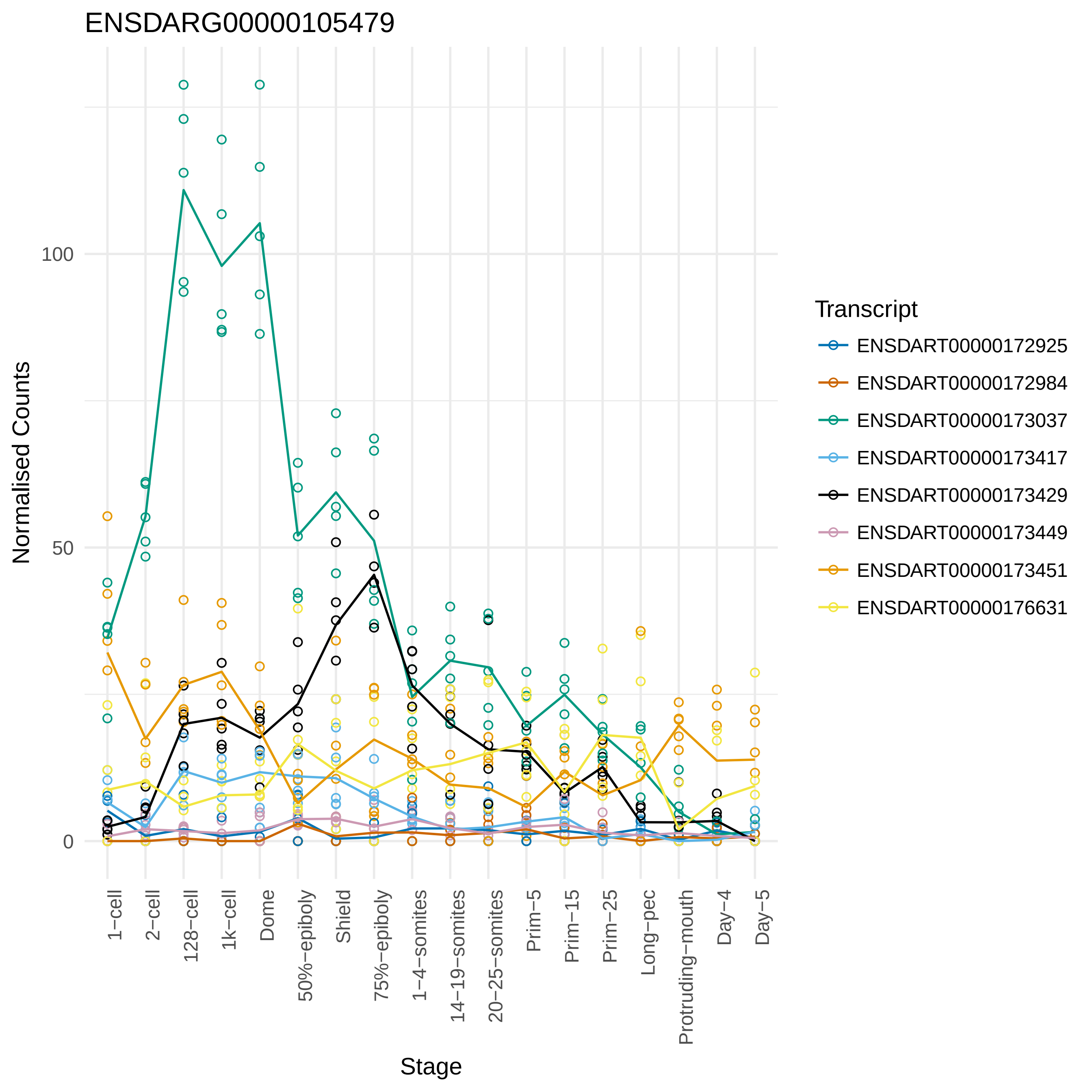

ENSDARG00000105479
Normalised Counts
0
50
100
1−cell
2−cell
128−cell
1k−cell
Dome
50%−epiboly
Shield
75%−epiboly
1−4−somites
14−19−somites
20−25−somites
Prim−5
Prim−15
Prim−25
Long−pec
Protruding−mouth
Day−4
Day−5
Stage
Transcript
ENSDART00000172925
ENSDART00000172984
ENSDART00000173037
ENSDART00000173417
ENSDART00000173429
ENSDART00000173449
ENSDART00000173451
ENSDART00000176631

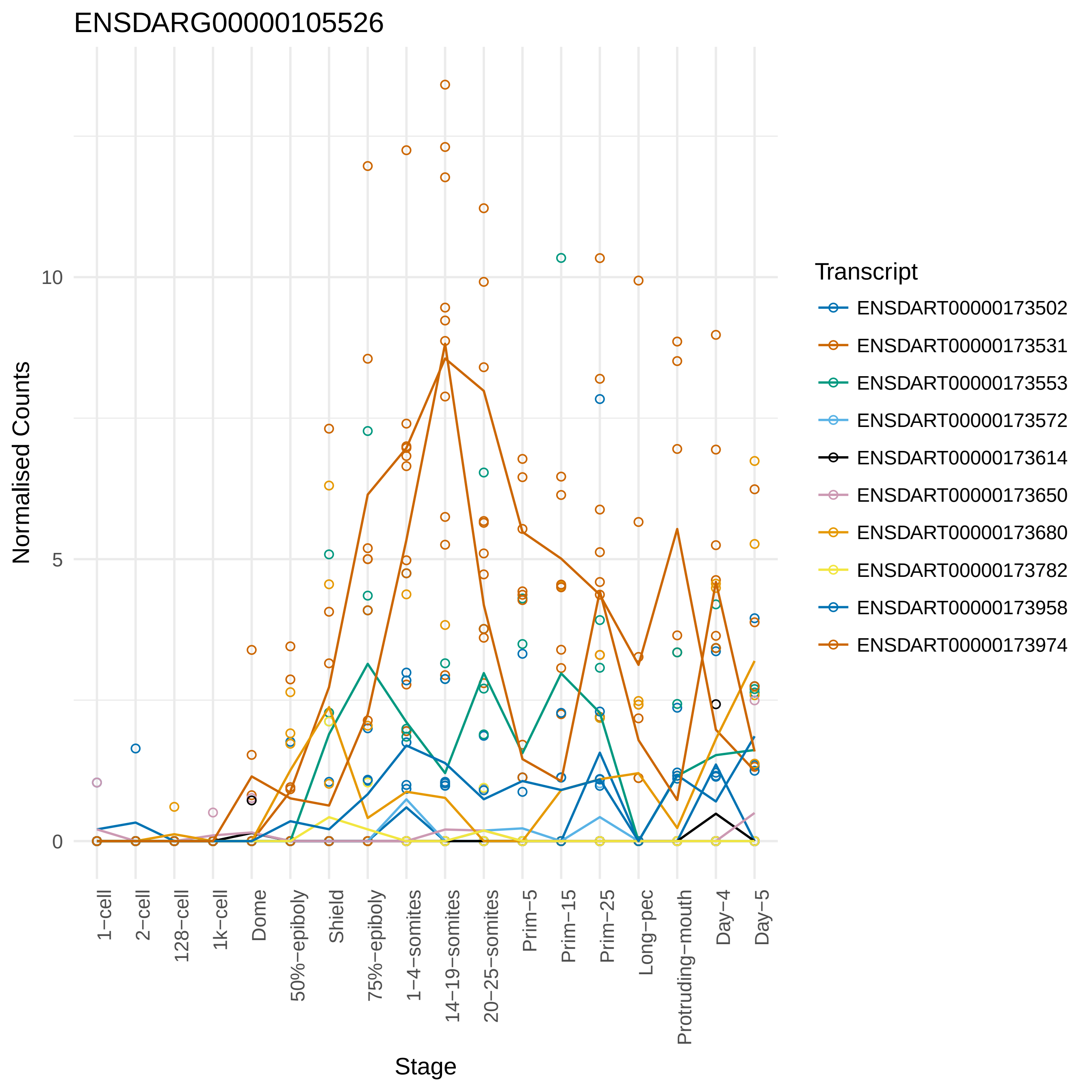

ENSDARG00000105526
Normalised Counts
10
5
0
1−cell
2−cell
128−cell
1k−cell
Dome
50%−epiboly
Shield
75%−epiboly
1−4−somites
14−19−somites
20−25−somites
Prim−5
Prim−15
Prim−25
Long−pec
Protruding−mouth
Day−4
Day−5
Stage
Transcript
ENSDART00000173502
ENSDART00000173531
ENSDART00000173553
ENSDART00000173572
ENSDART00000173614
ENSDART00000173650
ENSDART00000173680
ENSDART00000173782
ENSDART00000173958
ENSDART00000173974

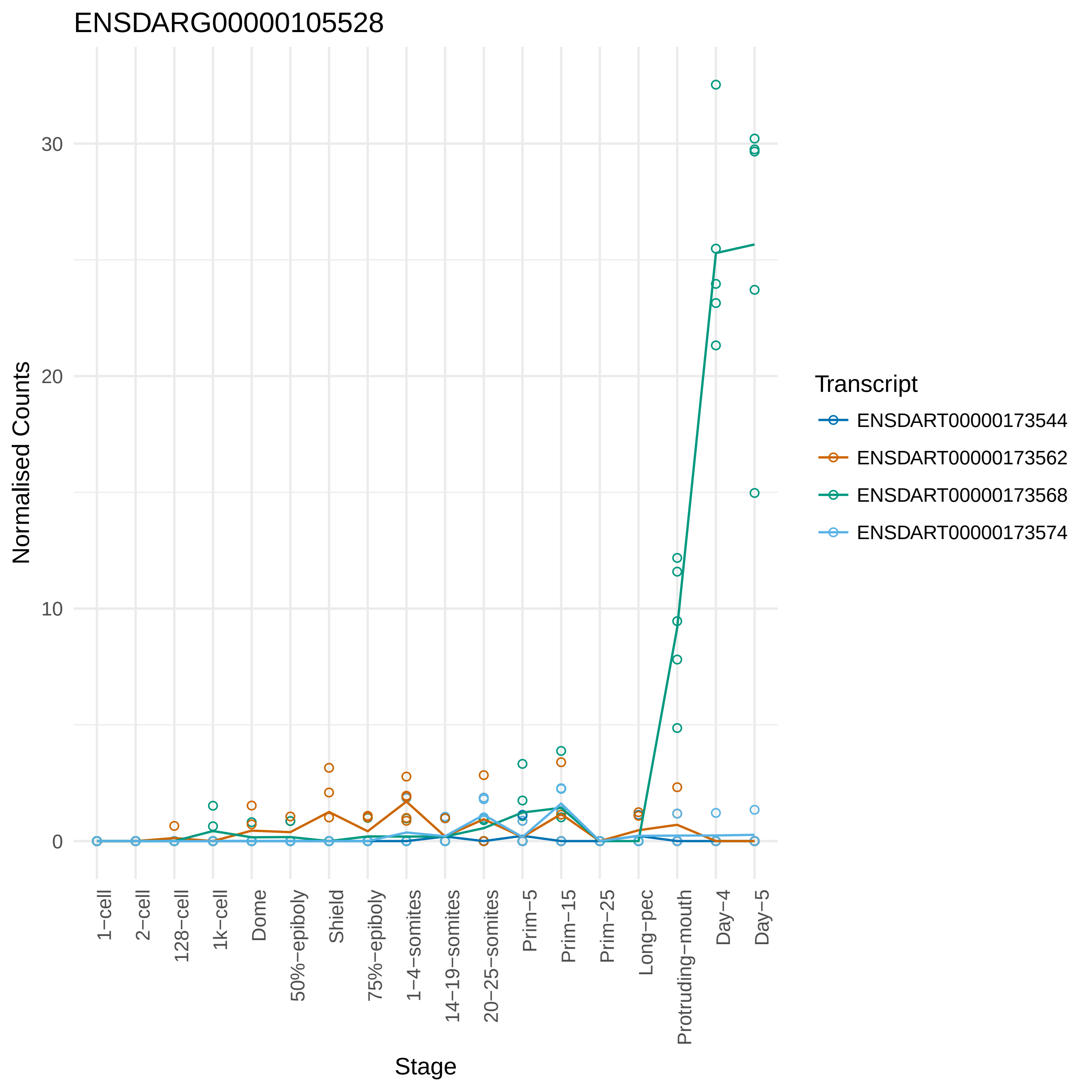

ENSDARG00000105528
Normalised Counts
30
20
10
0
1−cell
2−cell
128−cell
1k−cell
Dome
50%−epiboly
Shield
75%−epiboly
1−4−somites
14−19−somites
20−25−somites
Prim−5
Prim−15
Prim−25
Long−pec
Protruding−mouth
Day−4
Day−5
Stage
Transcript
ENSDART00000173544
ENSDART00000173562
ENSDART00000173568
ENSDART00000173574

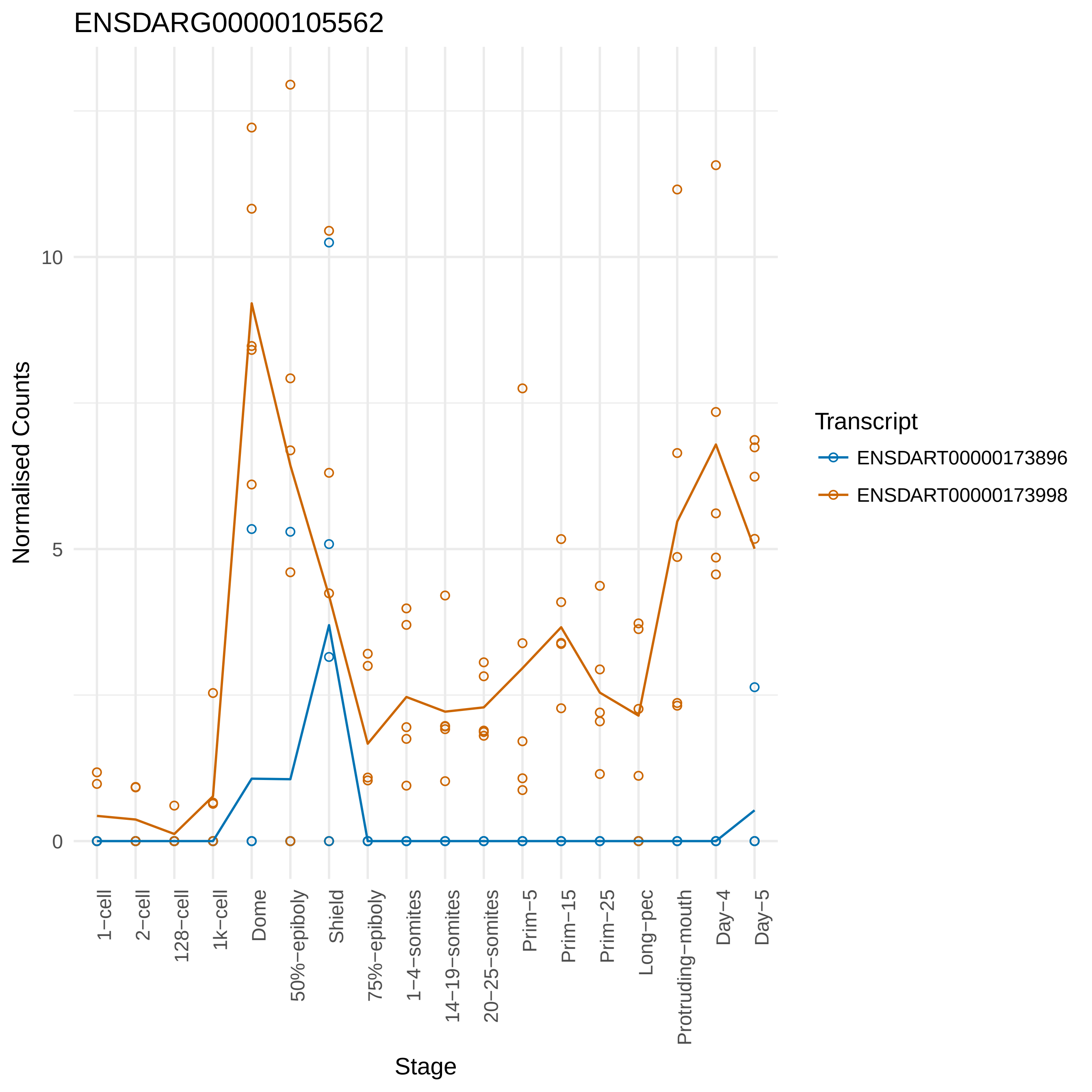
ENSDARG00000105562
Normalised Counts
0
5
10
Stage
1−cell
2−cell
128−cell
1k−cell
Dome
50%−epiboly
Shield
75%−epiboly
1−4−somites
14−19−somites
20−25−somites
Prim−5
Prim−15
Prim−25
Long−pec
Protruding−mouth
Day−4
Day−5
Transcript
ENSDART00000173896
ENSDART00000173998

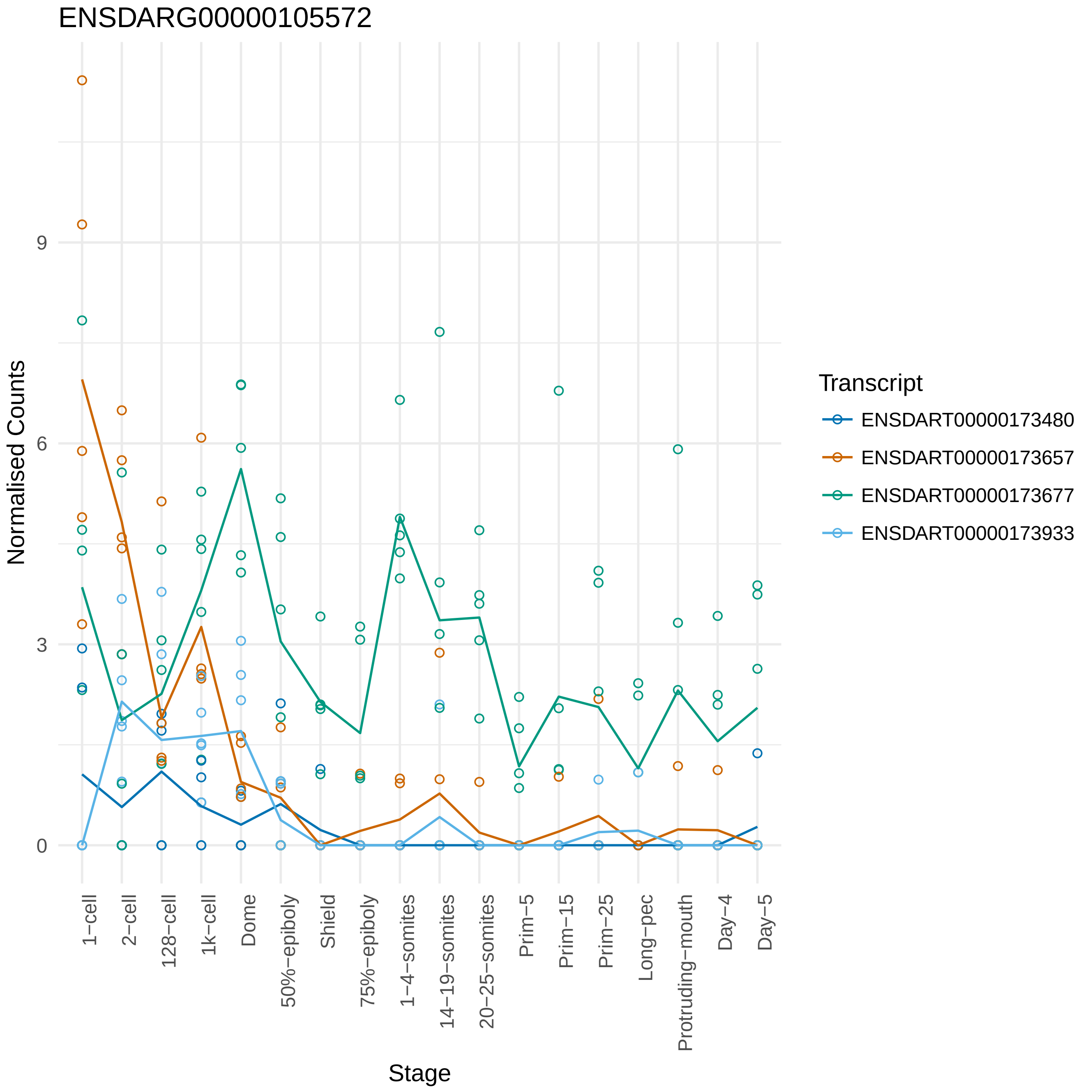

ENSDARG00000105572
Normalised Counts
0
3
6
9
Stage
1−cell
2−cell
128−cell
1k−cell
Dome
50%−epiboly
Shield
75%−epiboly
1−4−somites
14−19−somites
20−25−somites
Prim−5
Prim−15
Prim−25
Long−pec
Protruding−mouth
Day−4
Day−5
Transcript
ENSDART00000173480
ENSDART00000173657
ENSDART00000173677
ENSDART00000173933

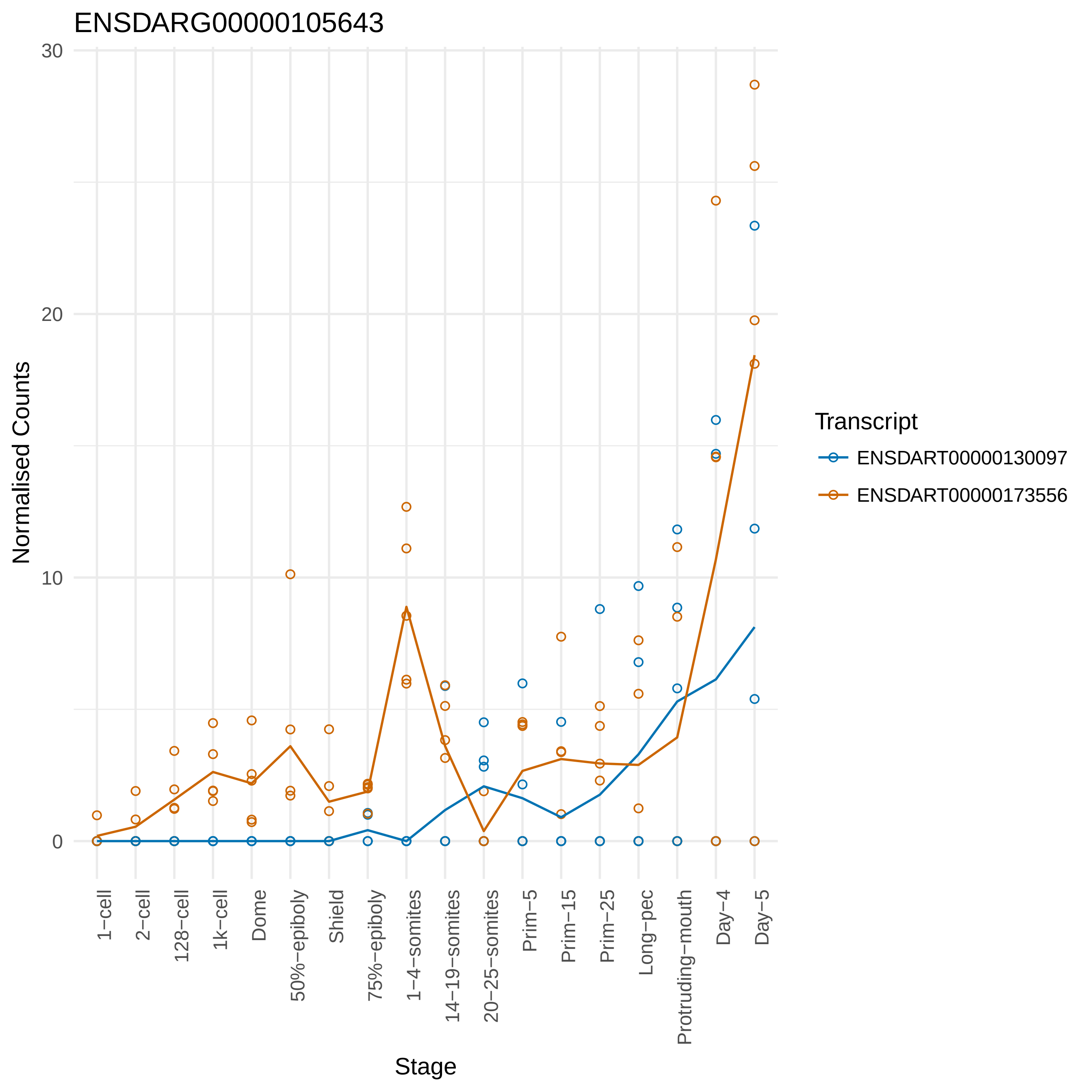

ENSDARG00000105643
Normalised Counts
30
20
10
0
1−cell
2−cell
128−cell
1k−cell
Dome
50%−epiboly
Shield
75%−epiboly
1−4−somites
14−19−somites
20−25−somites
Prim−5
Prim−15
Prim−25
Long−pec
Protruding−mouth
Day−4
Day−5
Stage
Transcript
ENSDART00000130097
ENSDART00000173556

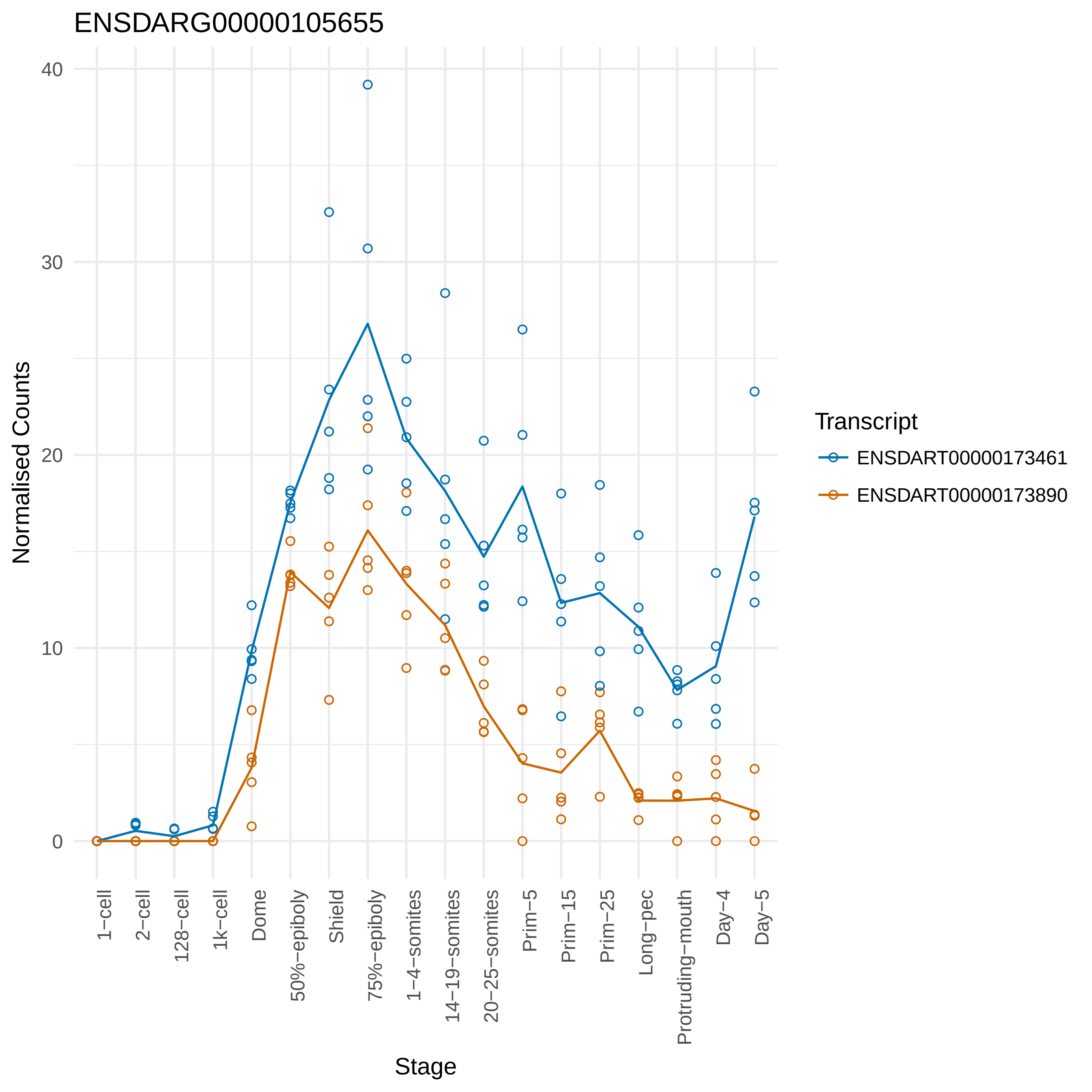

ENSDARG00000105655
Normalised Counts
0
10
20
30
40
1−cell
2−cell
128−cell
1k−cell
Dome
50%−epiboly
Shield
75%−epiboly
1−4−somites
14−19−somites
20−25−somites
Prim−5
Prim−15
Prim−25
Long−pec
Protruding−mouth
Day−4
Day−5
Stage
Transcript
ENSDART00000173461
ENSDART00000173890

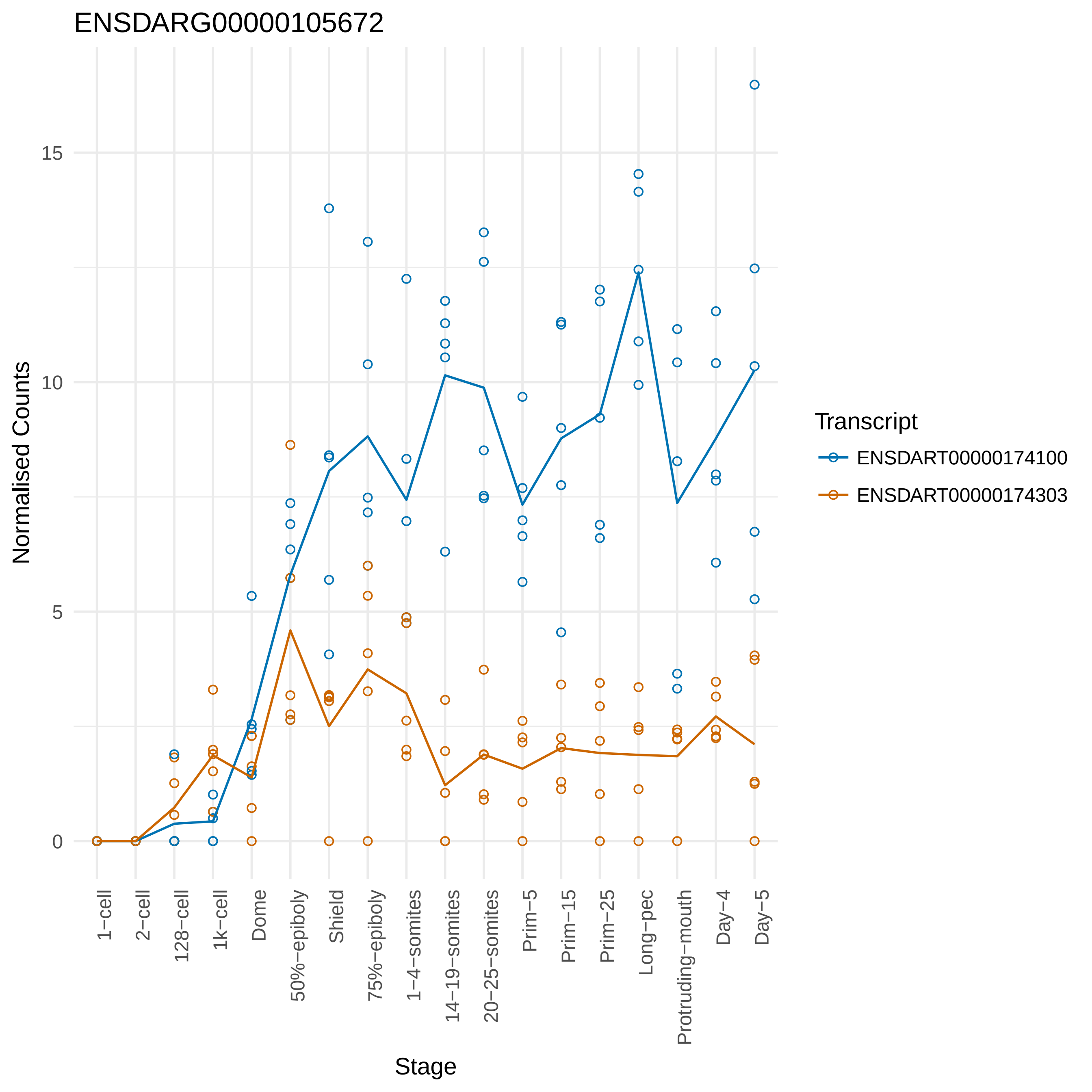
ENSDARG00000105672
Normalised Counts
15
10
5
0
Stage
1−cell
2−cell
128−cell
1k−cell
Dome
50%−epiboly
Shield
75%−epiboly
1−4−somites
14−19−somites
20−25−somites
Prim−5
Prim−15
Prim−25
Long−pec
Protruding−mouth
Day−4
Day−5
Transcript
ENSDART00000174100
ENSDART00000174303

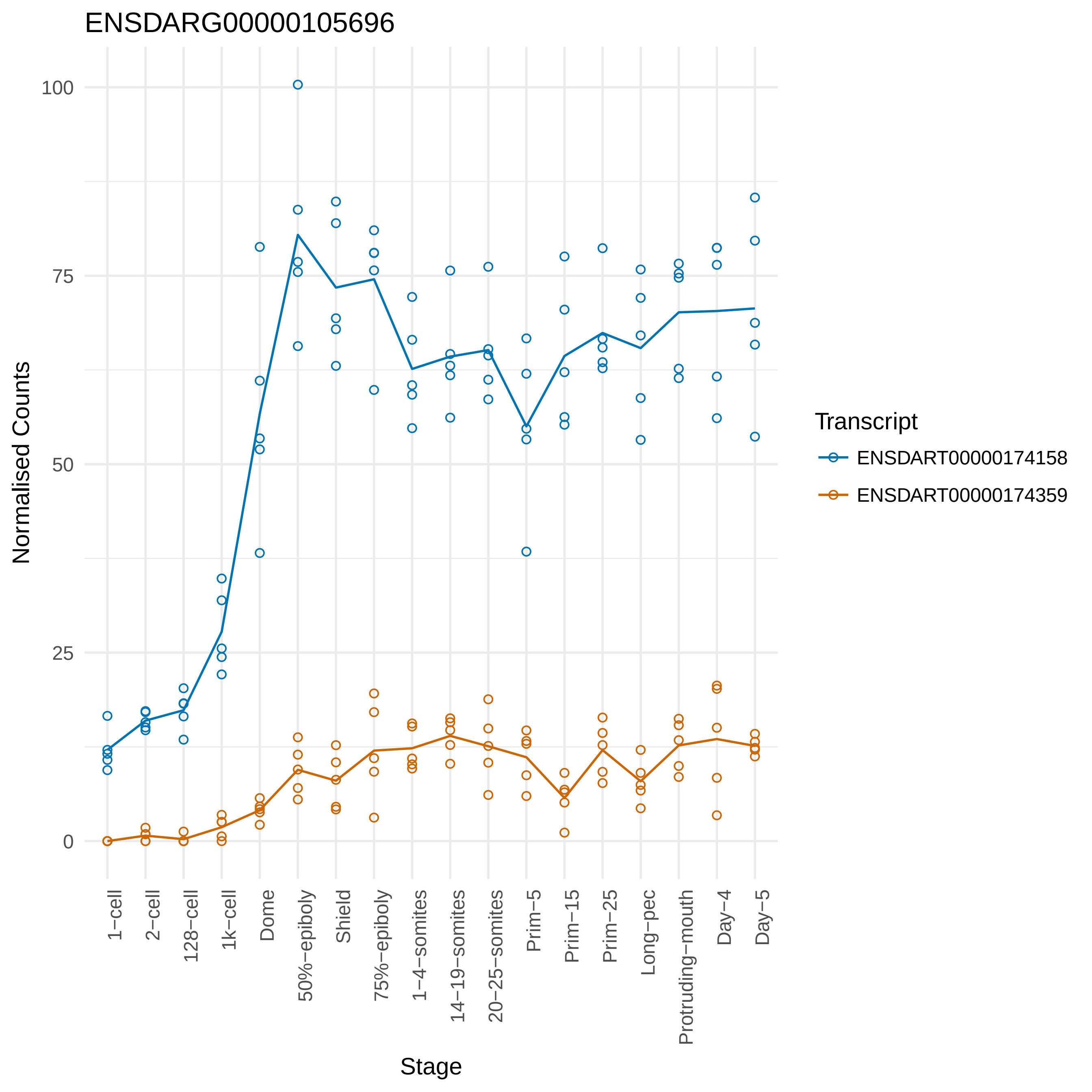
ENSDARG00000105696
Normalised Counts
100
75
50
25
0
Stage
1−cell
2−cell
128−cell
1k−cell
Dome
50%−epiboly
Shield
75%−epiboly
1−4−somites
14−19−somites
20−25−somites
Prim−5
Prim−15
Prim−25
Long−pec
Protruding−mouth
Day−4
Day−5
Transcript
ENSDART00000174158
ENSDART00000174359

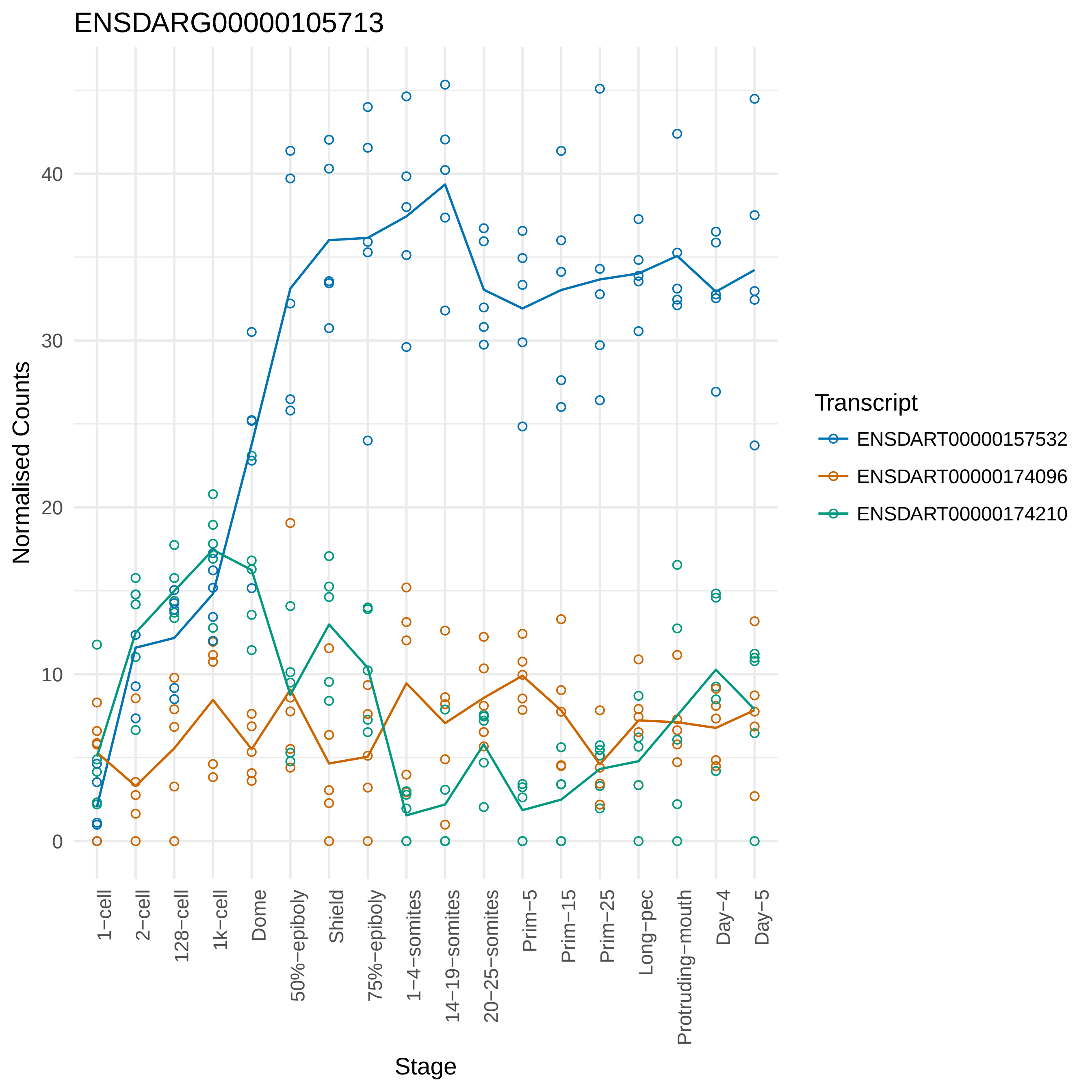

ENSDARG00000105713
Normalised Counts
0
10
20
30
40
Stage
1−cell
2−cell
128−cell
1k−cell
Dome
50%−epiboly
Shield
75%−epiboly
1−4−somites
14−19−somites
20−25−somites
Prim−5
Prim−15
Prim−25
Long−pec
Protruding−mouth
Day−4
Day−5
Transcript
ENSDART00000157532
ENSDART00000174096
ENSDART00000174210

ENSDARG00000105714

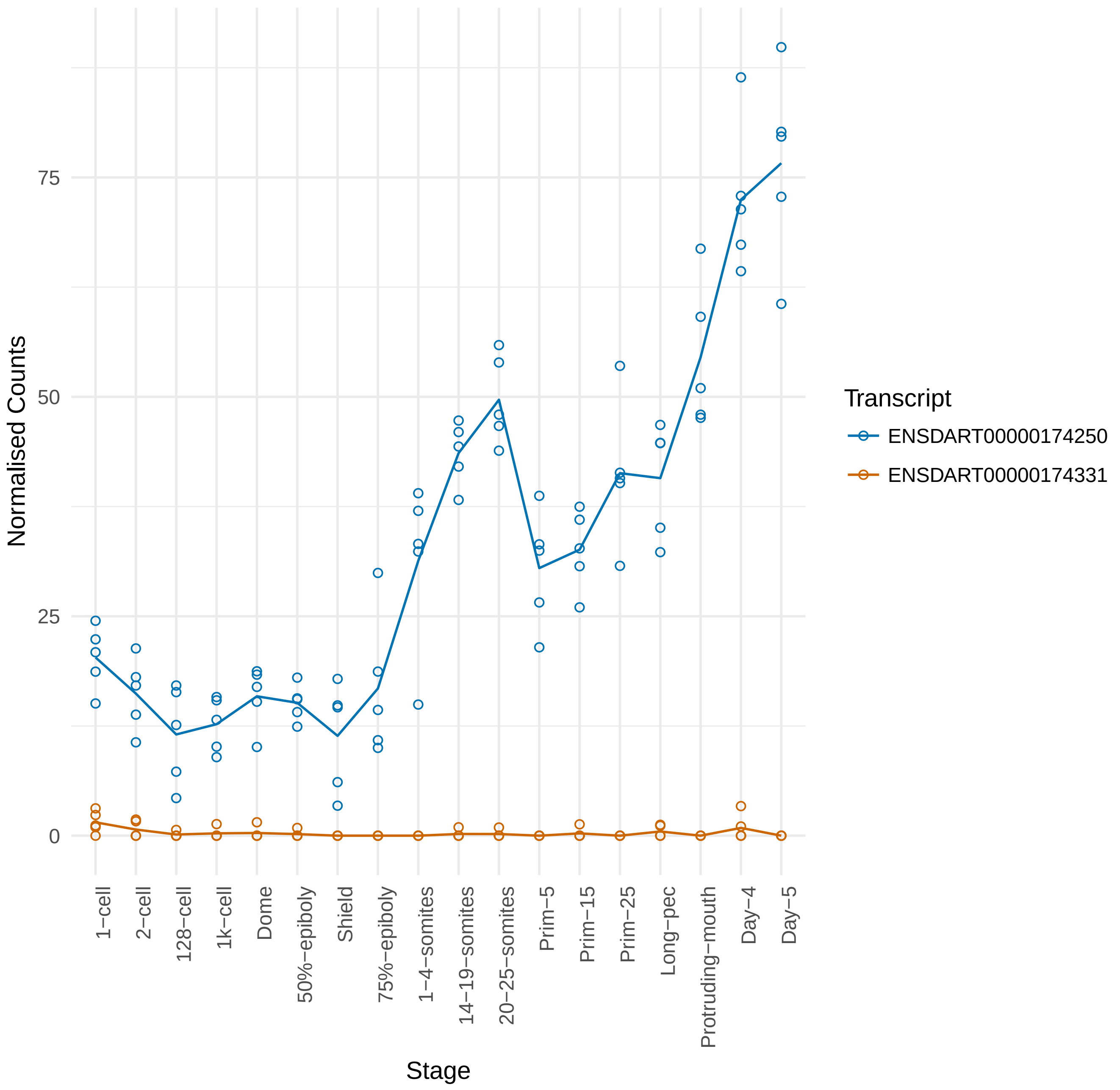

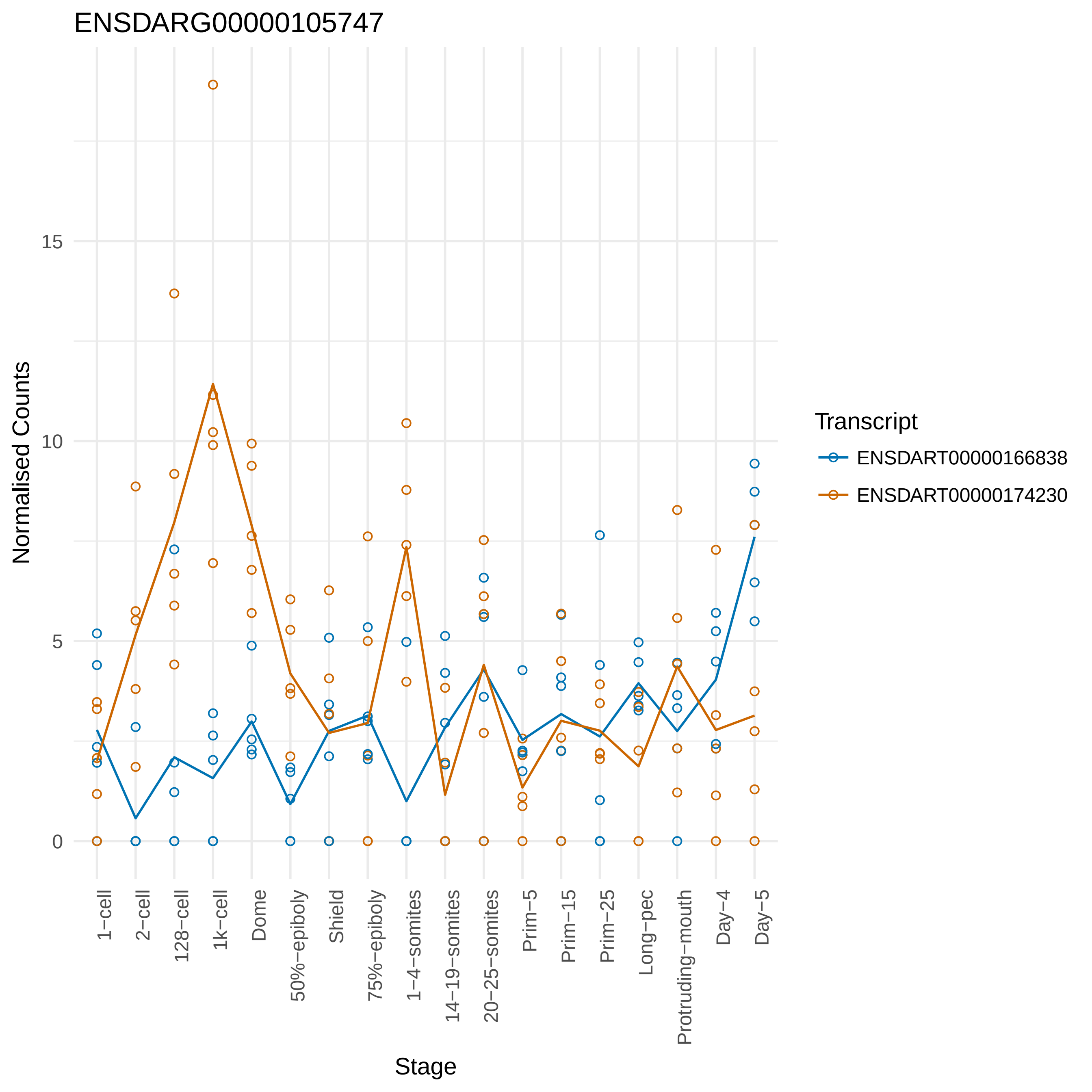

ENSDARG00000105747
Normalised Counts
15
10
5
0
1−cell
2−cell
128−cell
1k−cell
Dome
50%−epiboly
Shield
75%−epiboly
1−4−somites
14−19−somites
20−25−somites
Prim−5
Prim−15
Prim−25
Long−pec
Protruding−mouth
Day−4
Day−5
Stage
Transcript
ENSDART00000166838
ENSDART00000174230

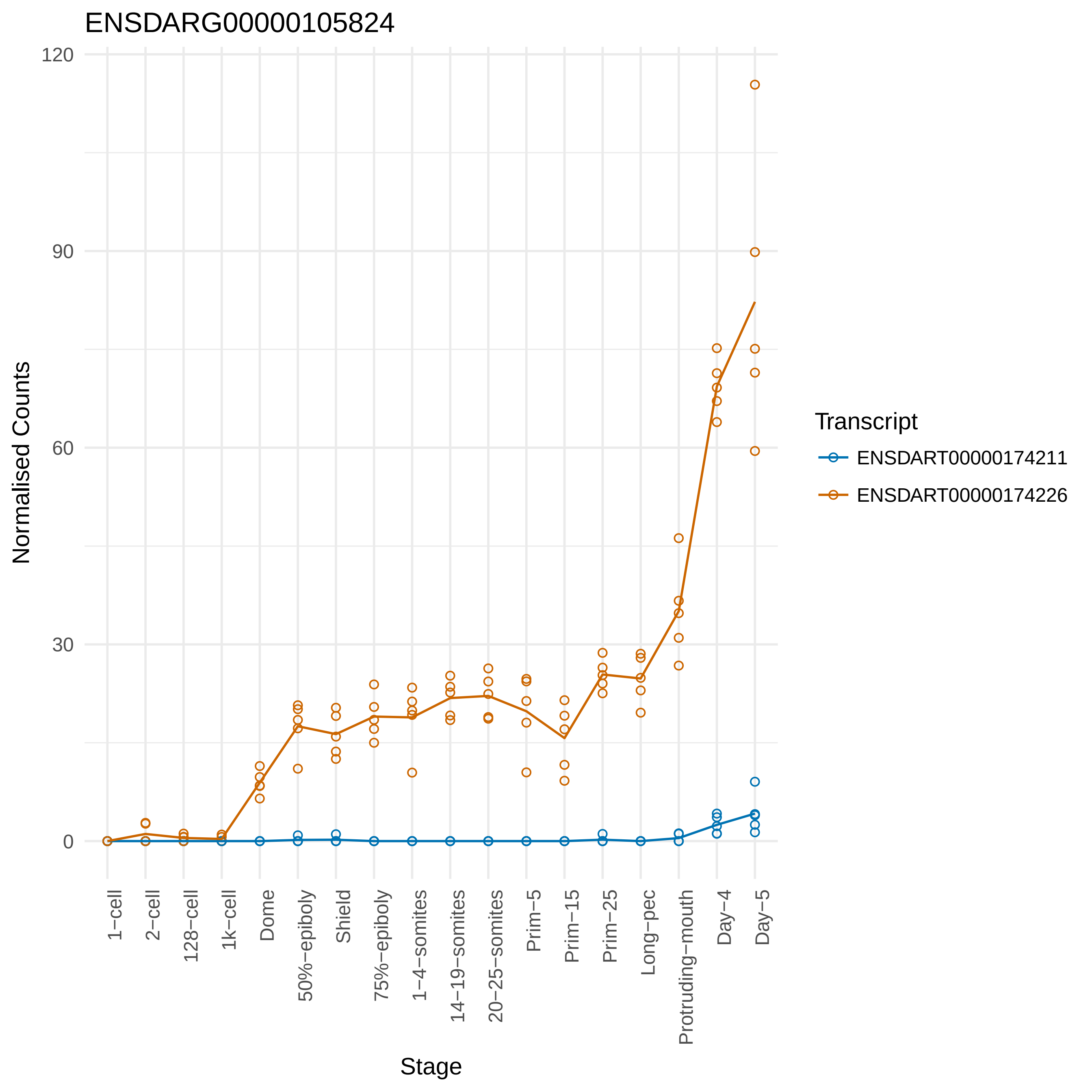

ENSDARG00000105824
120
90
60
30
0
Normalised Counts
1−cell
2−cell
128−cell
1k−cell
Dome
50%−epiboly
Shield
75%−epiboly
1−4−somites
14−19−somites
20−25−somites
Prim−5
Prim−15
Prim−25
Long−pec
Protruding−mouth
Day−4
Day−5
Stage
Transcript
ENSDART00000174211
ENSDART00000174226

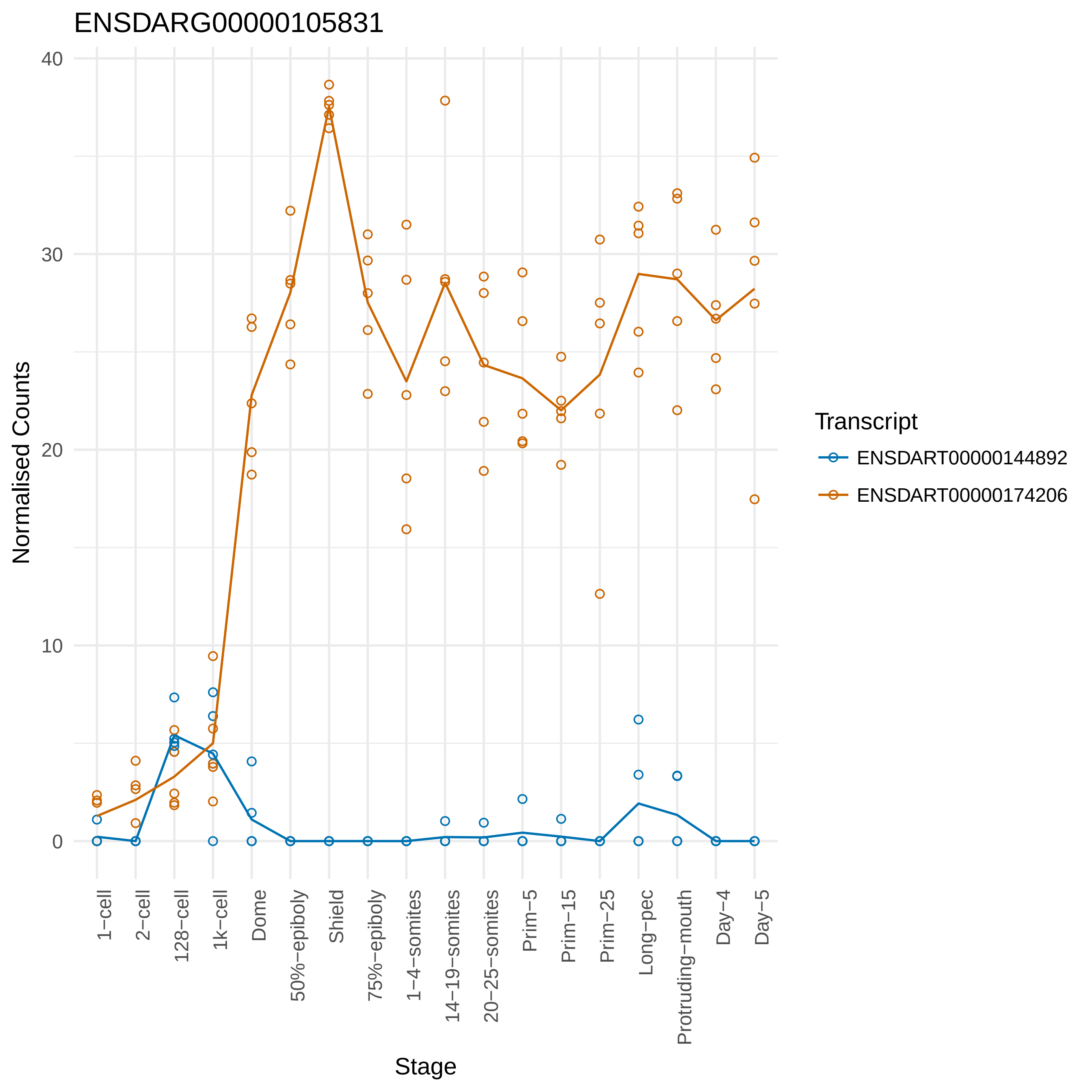
ENSDARG00000105831
Normalised Counts
0
10
20
30
40
1−cell
2−cell
128−cell
1k−cell
Dome
50%−epiboly
Shield
75%−epiboly
1−4−somites
14−19−somites
20−25−somites
Prim−5
Prim−15
Prim−25
Long−pec
Protruding−mouth
Day−4
Day−5
Stage
Transcript
ENSDART00000144892
ENSDART00000174206

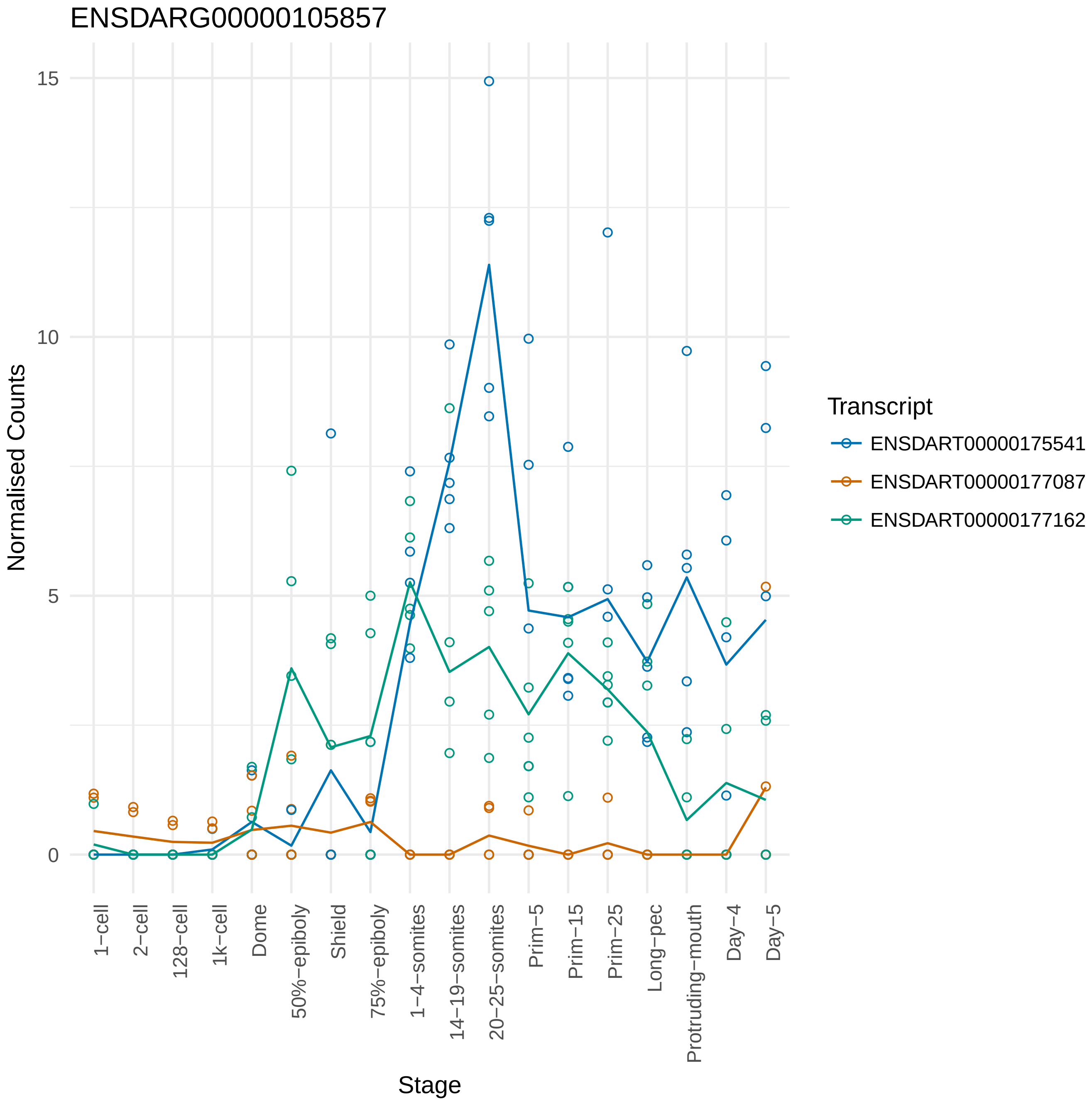
ENSDARG00000105857
Normalised Counts
15
10
5
0
1−cell
2−cell
128−cell
1k−cell
Dome
50%−epiboly
Shield
75%−epiboly
1−4−somites
14−19−somites
20−25−somites
Prim−5
Prim−15
Prim−25
Long−pec
Protruding−mouth
Day−4
Day−5
Stage
Transcript
ENSDART00000175541
ENSDART00000177087
ENSDART00000177162

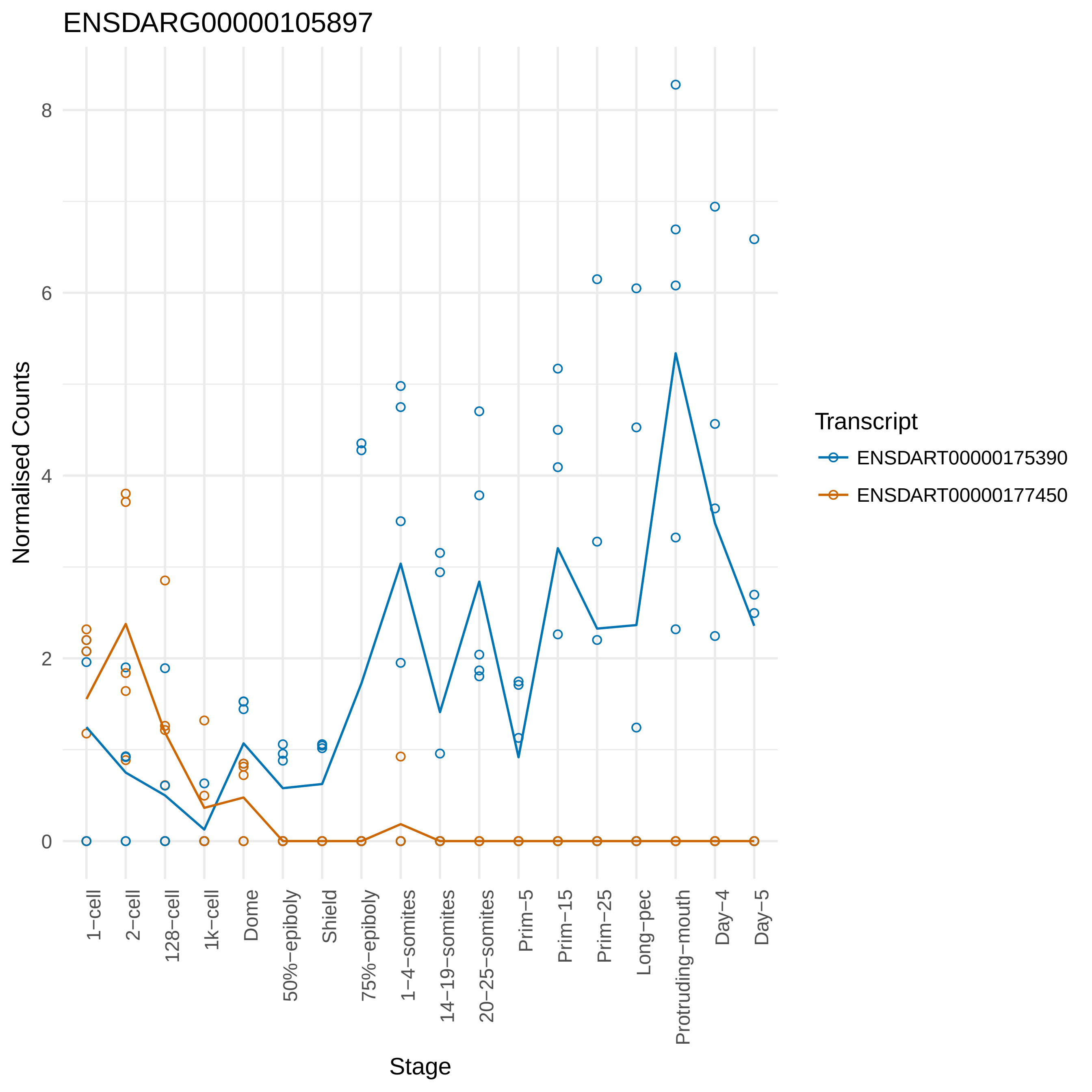
ENSDARG00000105897
Normalised Counts
0
2
4
6
8
Stage
1−cell
2−cell
128−cell
1k−cell
Dome
50%−epiboly
Shield
75%−epiboly
1−4−somites
14−19−somites
20−25−somites
Prim−5
Prim−15
Prim−25
Long−pec
Protruding−mouth
Day−4
Day−5
Transcript
ENSDART00000175390
ENSDART00000177450

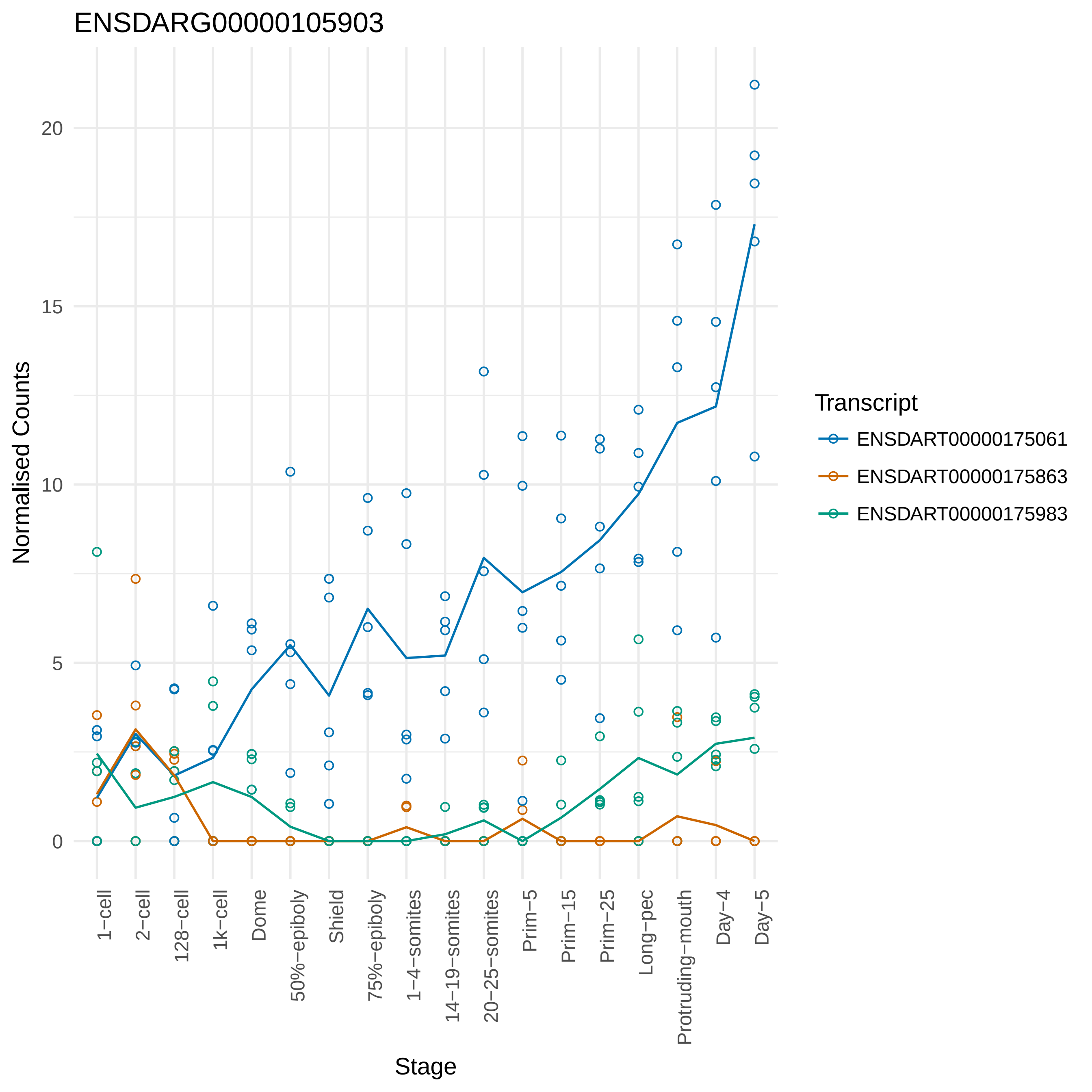

ENSDARG00000105903
Normalised Counts
0
5
10
15
20
Stage
1−cell
2−cell
128−cell
1k−cell
Dome
50%−epiboly
Shield
75%−epiboly
1−4−somites
14−19−somites
20−25−somites
Prim−5
Prim−15
Prim−25
Long−pec
Protruding−mouth
Day−4
Day−5
Transcript
ENSDART00000175061
ENSDART00000175863
ENSDART00000175983

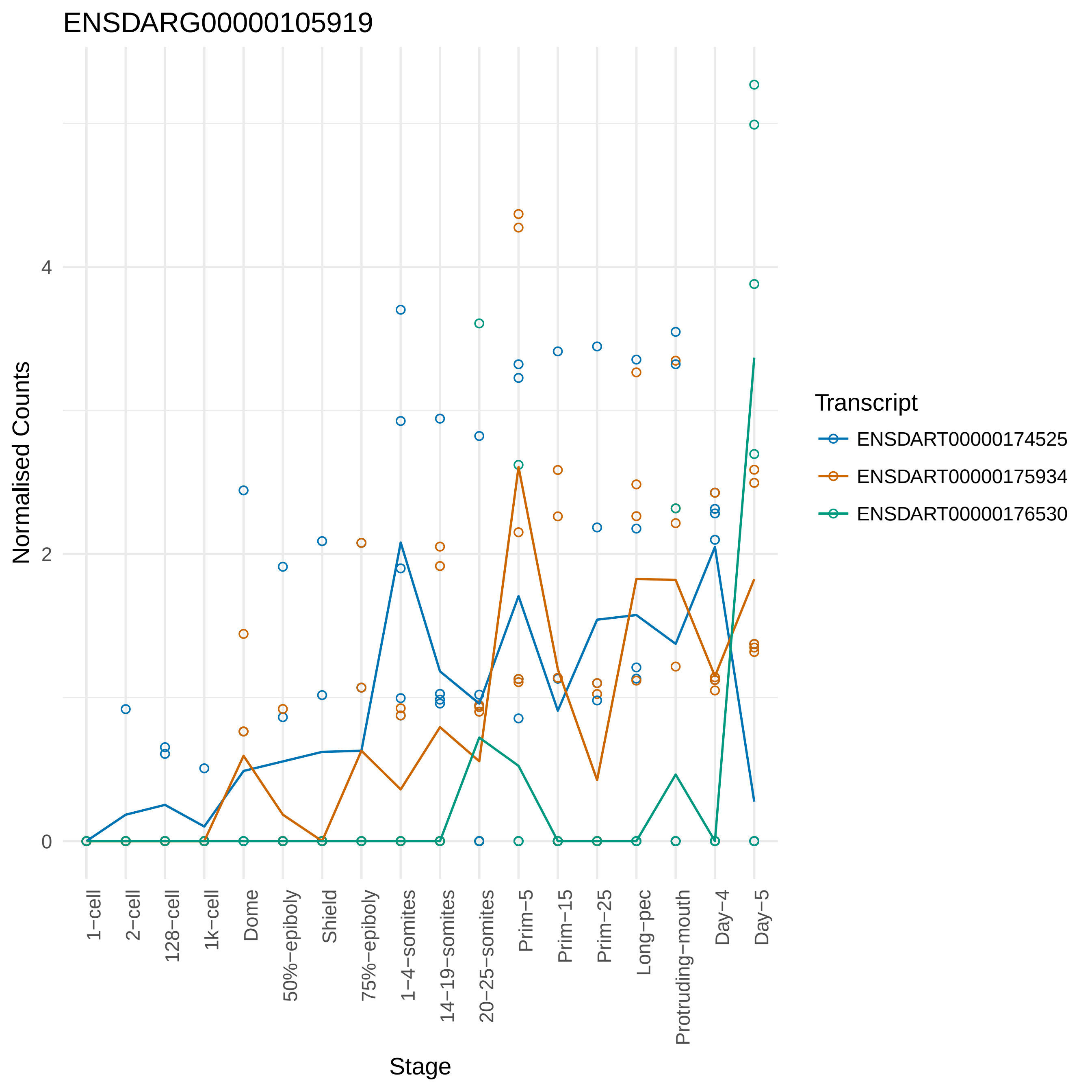
ENSDARG00000105919
Normalised Counts
0
2
4
Stage
1−cell
2−cell
128−cell
1k−cell
Dome
50%−epiboly
Shield
75%−epiboly
1−4−somites
14−19−somites
20−25−somites
Prim−5
Prim−15
Prim−25
Long−pec
Protruding−mouth
Day−4
Day−5
Transcript
ENSDART00000174525
ENSDART00000175934
ENSDART00000176530

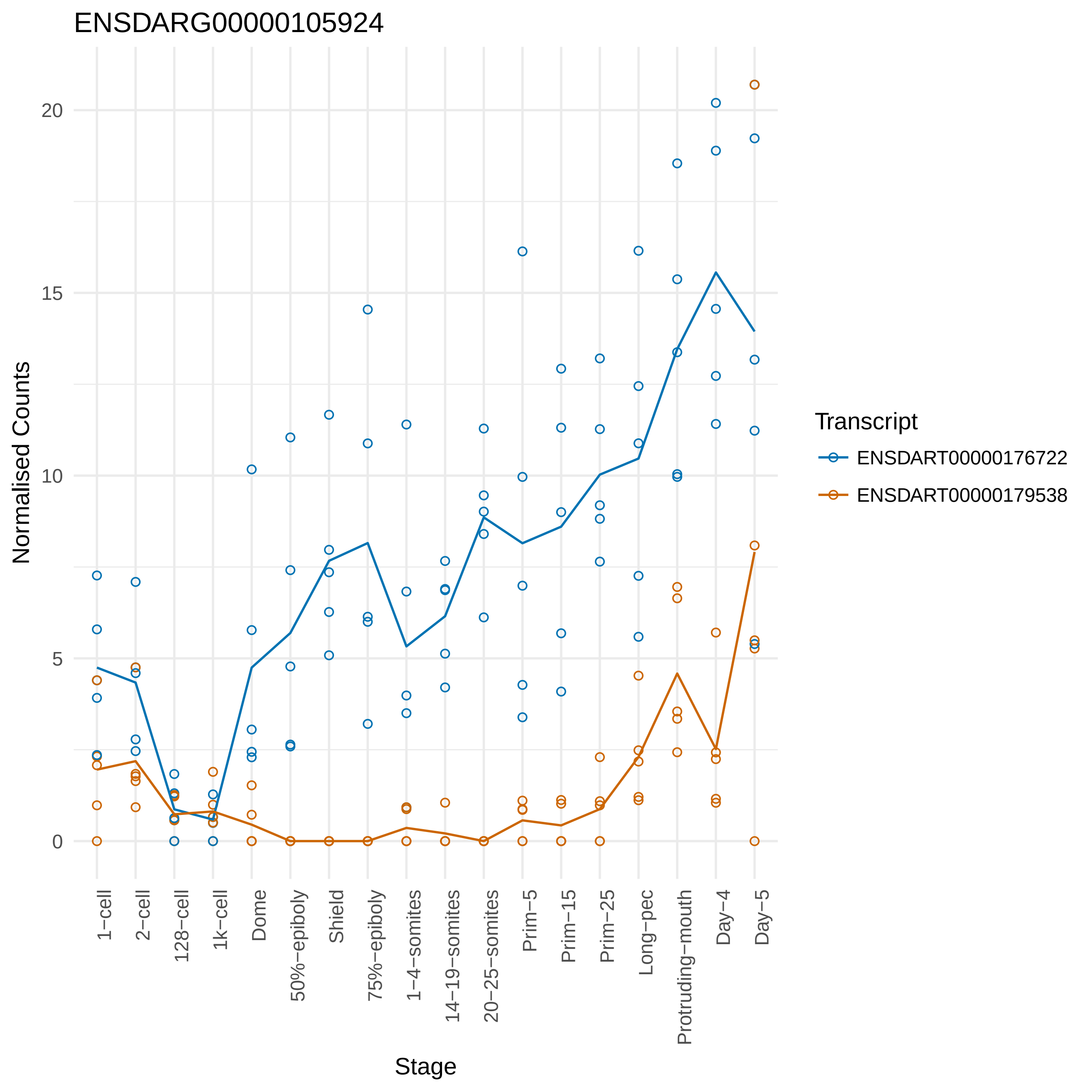

ENSDARG00000105924
Normalised Counts
0
5
10
15
20
1−cell
2−cell
128−cell
1k−cell
Dome
50%−epiboly
Shield
75%−epiboly
1−4−somites
14−19−somites
20−25−somites
Prim−5
Prim−15
Prim−25
Long−pec
Protruding−mouth
Day−4
Day−5
Stage
Transcript
ENSDART00000176722
ENSDART00000179538

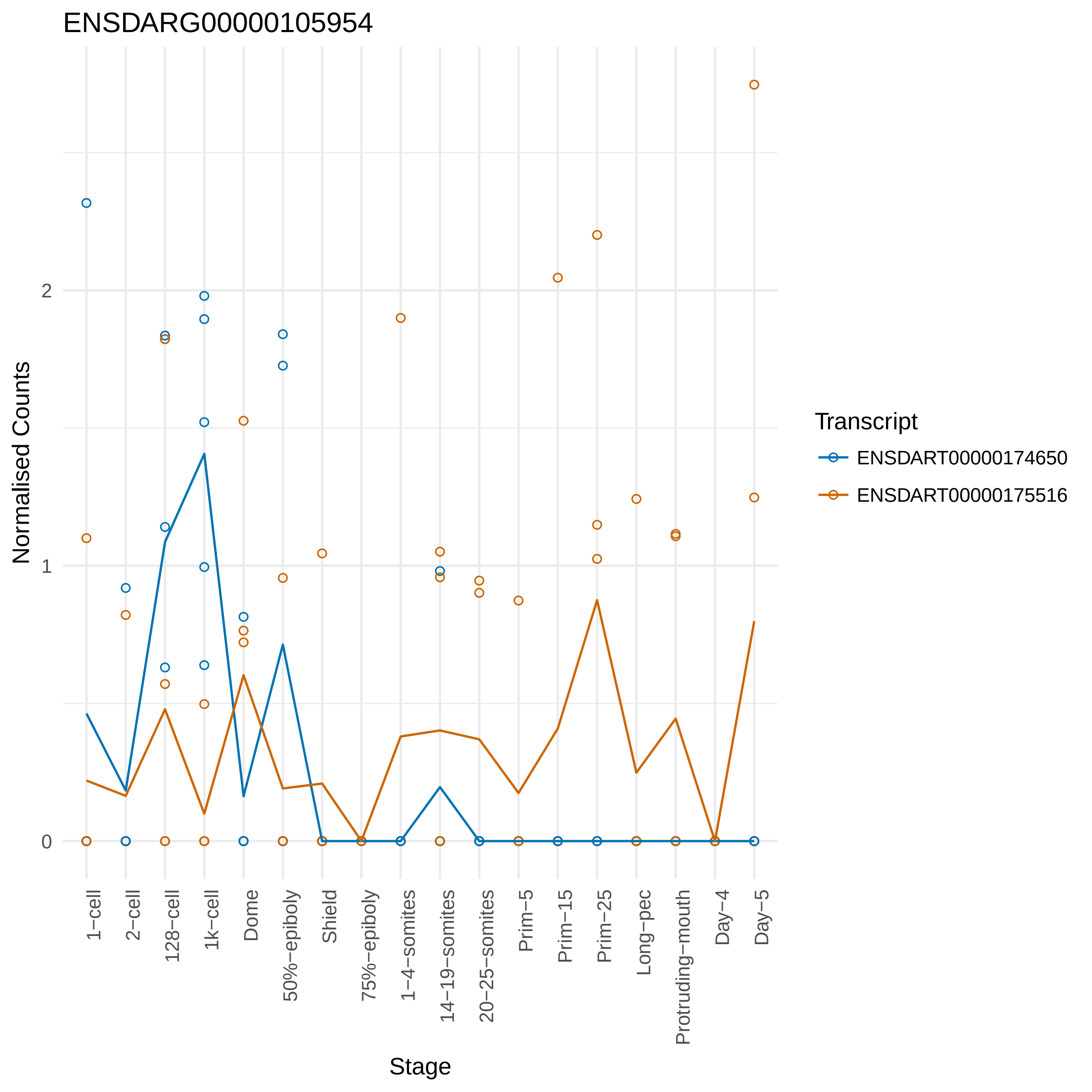

ENSDARG00000105954
Normalised Counts
0
1
2
1−cell
2−cell
128−cell
1k−cell
Dome
50%−epiboly
Shield
75%−epiboly
1−4−somites
14−19−somites
20−25−somites
Prim−5
Prim−15
Prim−25
Long−pec
Protruding−mouth
Day−4
Day−5
Stage
Transcript
ENSDART00000174650
ENSDART00000175516

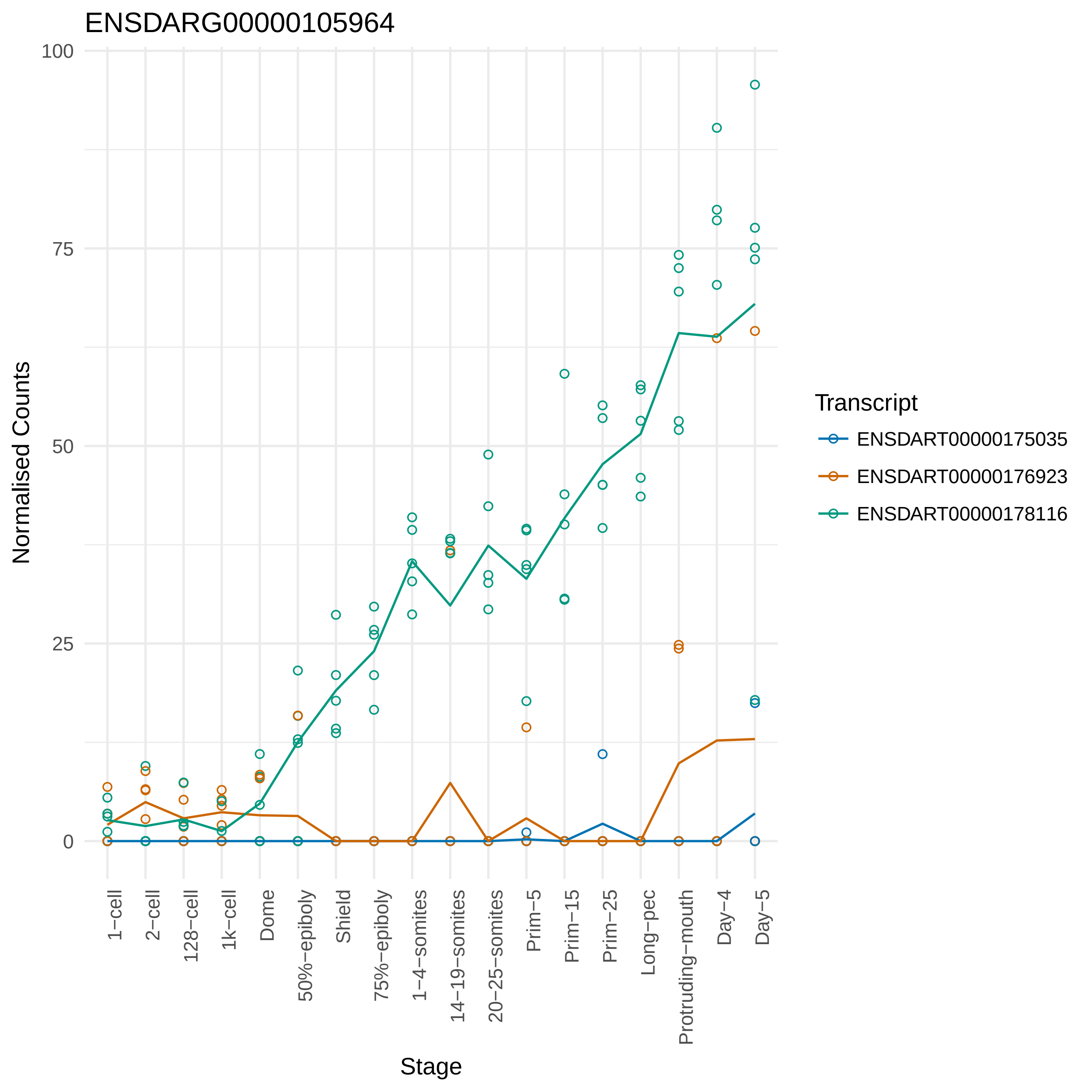
ENSDARG00000105964
Normalised Counts
100
75
50
25
0
1−cell
2−cell
128−cell
1k−cell
Dome
50%−epiboly
Shield
75%−epiboly
1−4−somites
14−19−somites
20−25−somites
Prim−5
Prim−15
Prim−25
Long−pec
Protruding−mouth
Day−4
Day−5
Stage
Transcript
ENSDART00000175035
ENSDART00000176923
ENSDART00000178116

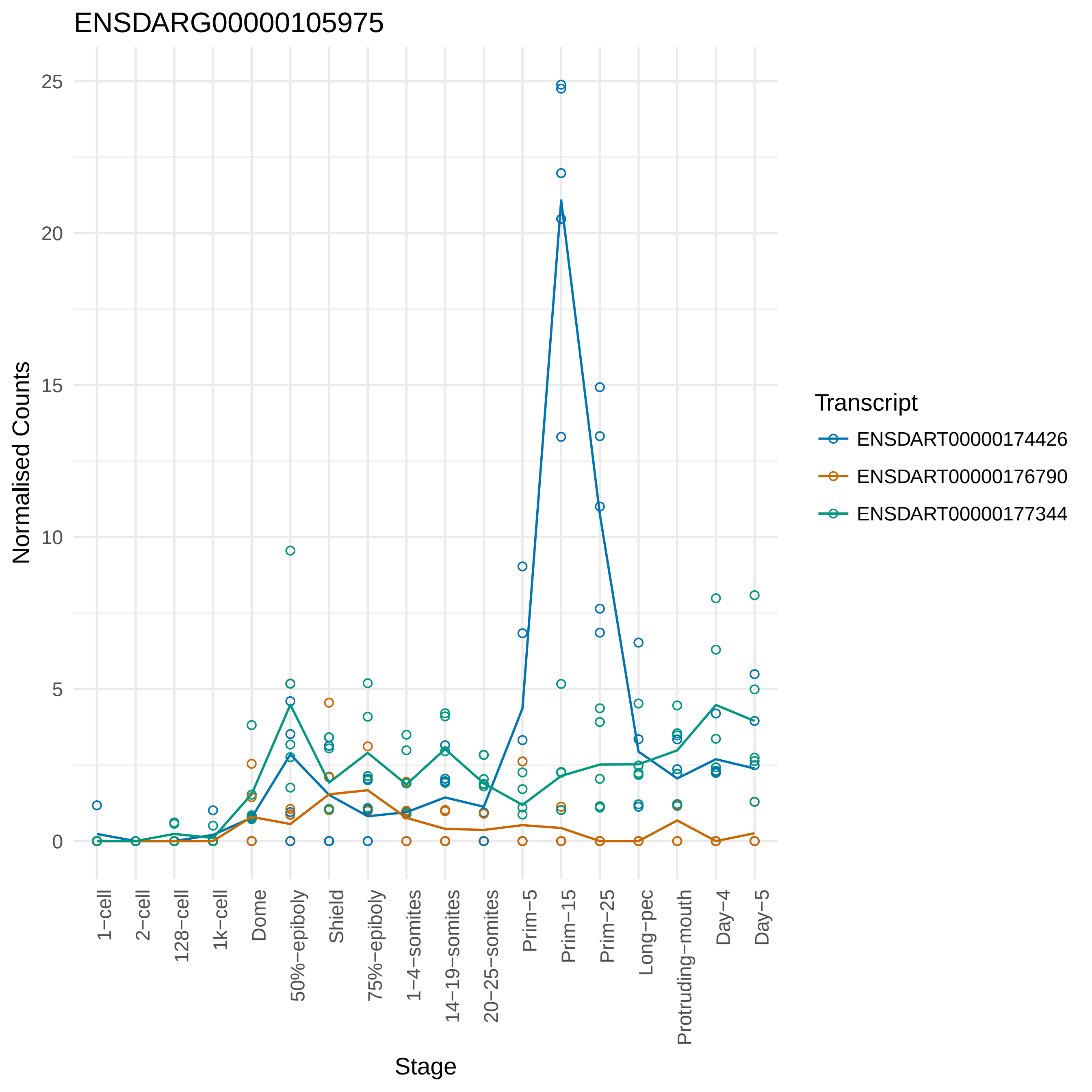
ENSDARG00000105975
Normalised Counts
0
5
10
15
20
25
Stage
1−cell
2−cell
128−cell
1k−cell
Dome
50%−epiboly
Shield
75%−epiboly
1−4−somites
14−19−somites
20−25−somites
Prim−5
Prim−15
Prim−25
Long−pec
Protruding−mouth
Day−4
Day−5
Transcript
ENSDART00000174426
ENSDART00000176790
ENSDART00000177344

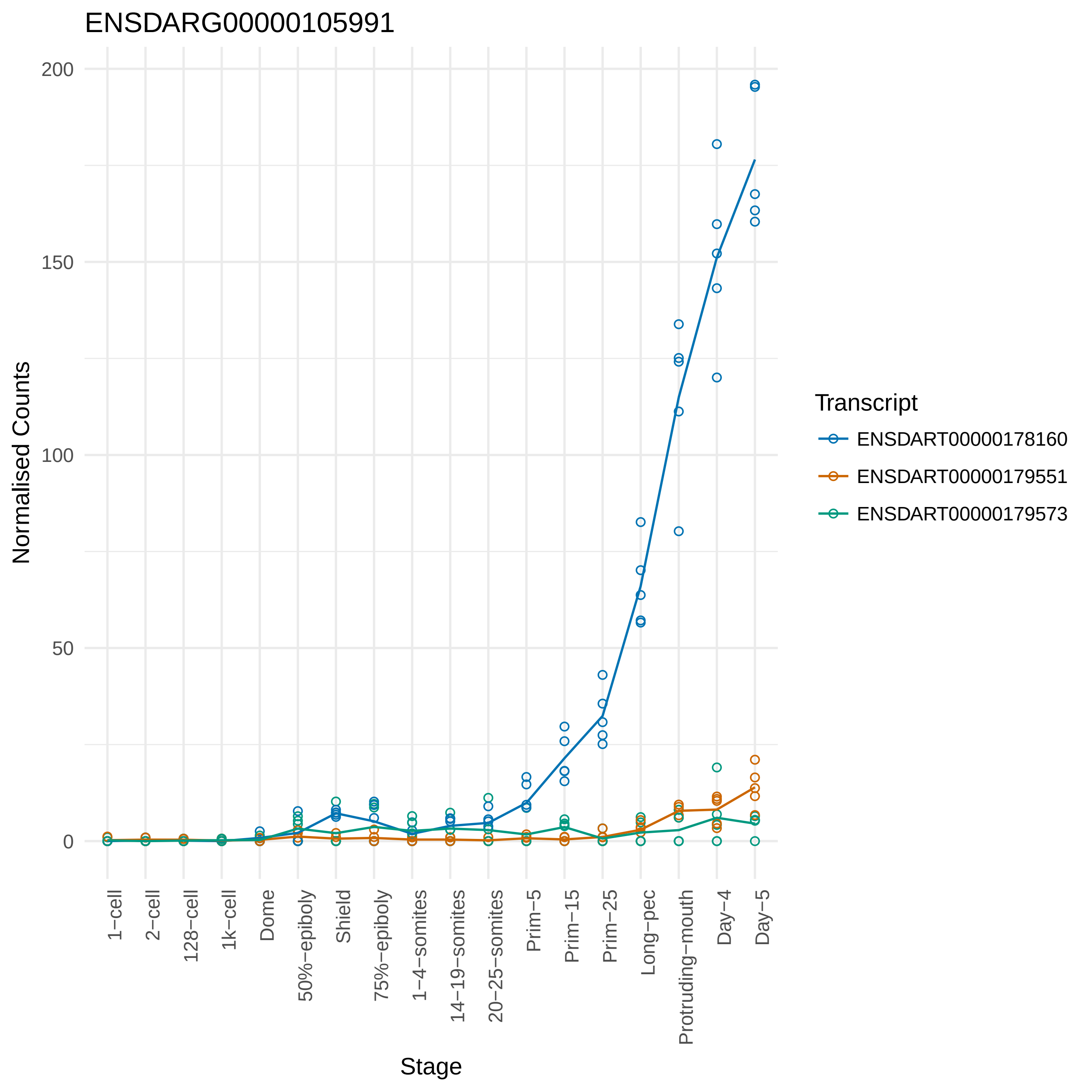

ENSDARG00000105991
Normalised Counts
0
50
100
150
200
1−cell
2−cell
128−cell
1k−cell
Dome
50%−epiboly
Shield
75%−epiboly
1−4−somites
14−19−somites
20−25−somites
Prim−5
Prim−15
Prim−25
Long−pec
Protruding−mouth
Day−4
Day−5
Stage
Transcript
ENSDART00000178160
ENSDART00000179551
ENSDART00000179573

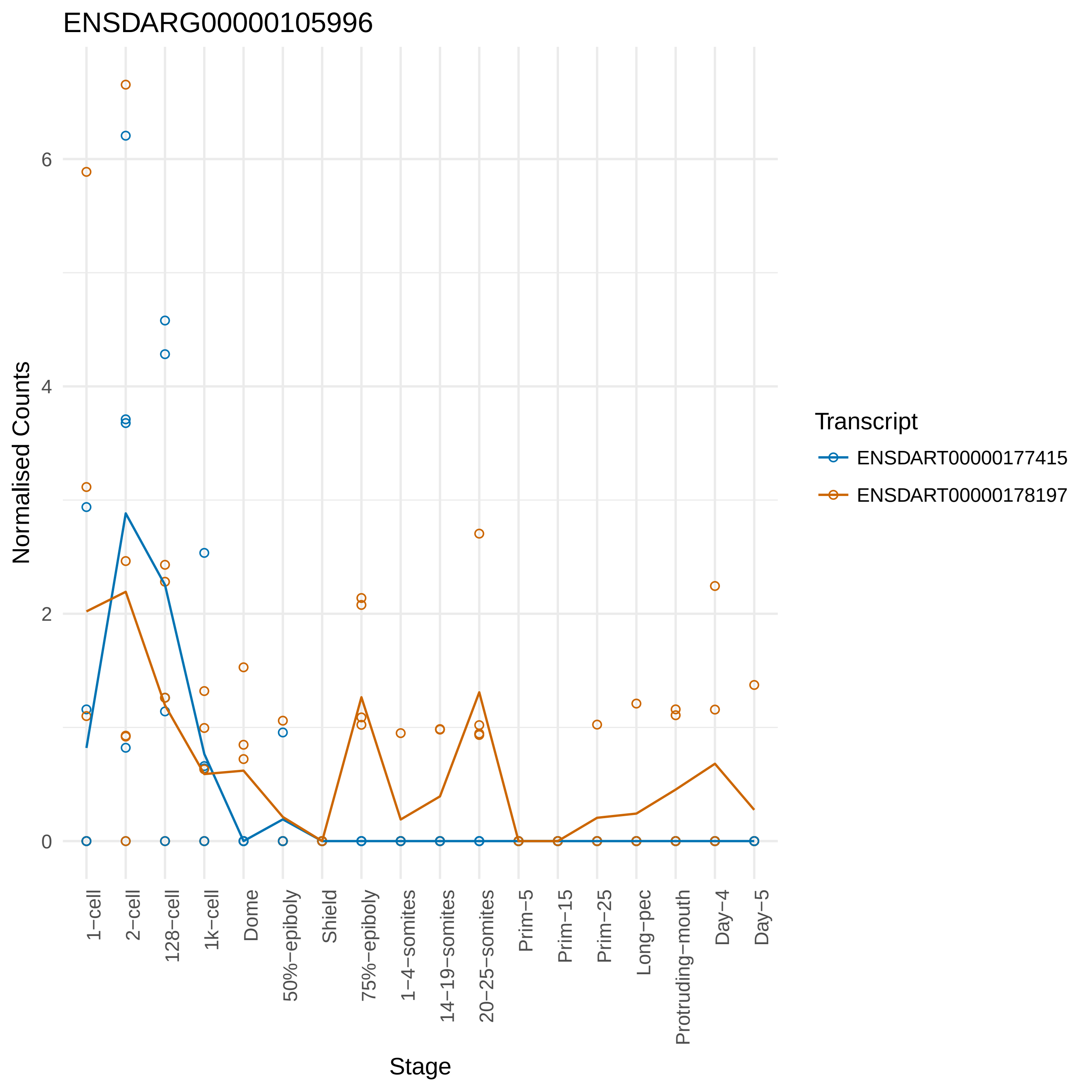
ENSDARG00000105996
Normalised Counts
0
2
4
6
Stage
1−cell
2−cell
128−cell
1k−cell
Dome
50%−epiboly
Shield
75%−epiboly
1−4−somites
14−19−somites
20−25−somites
Prim−5
Prim−15
Prim−25
Long−pec
Protruding−mouth
Day−4
Day−5
Transcript
ENSDART00000177415
ENSDART00000178197

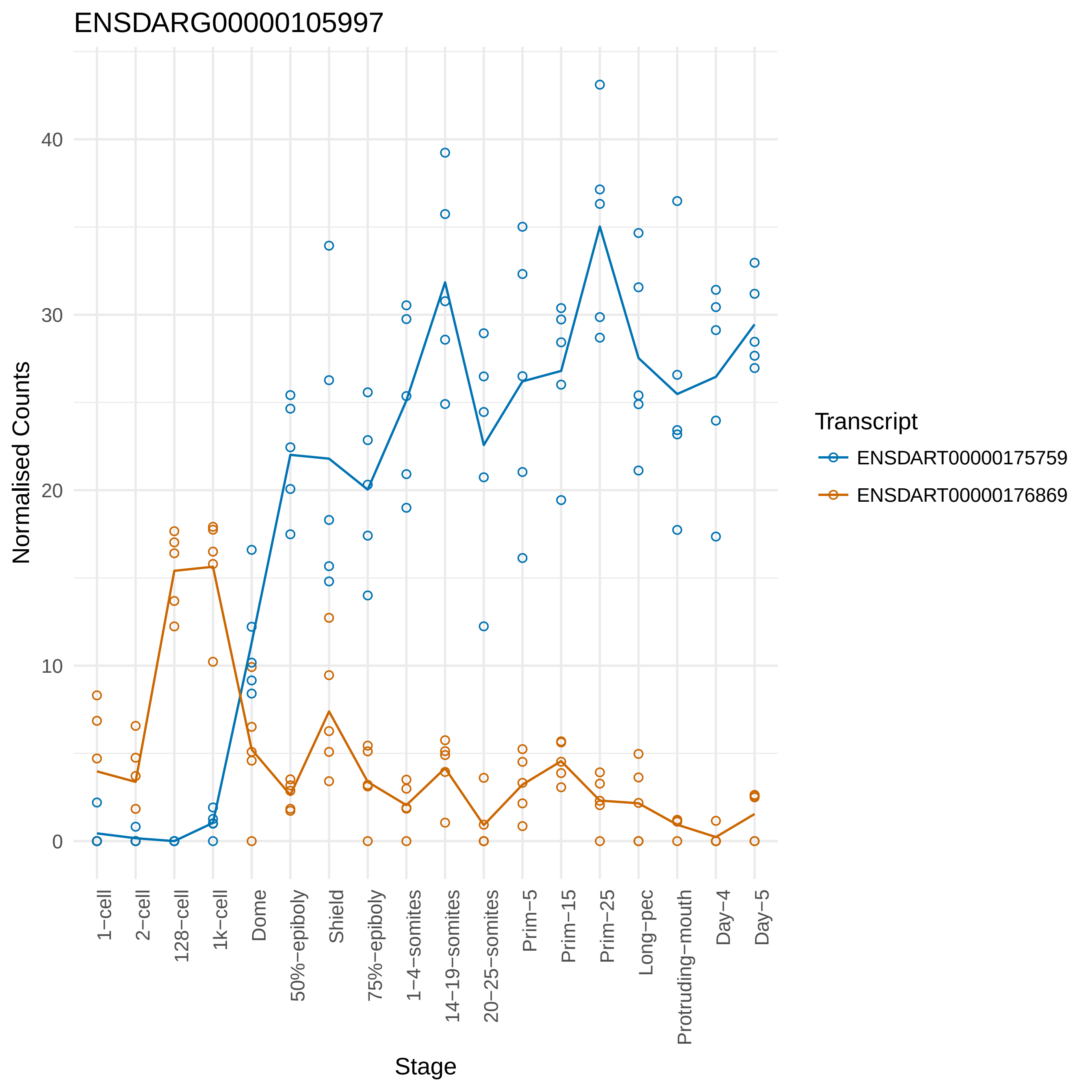
ENSDARG00000105997
Normalised Counts
0
10
20
30
40
1−cell
2−cell
128−cell
1k−cell
Dome
50%−epiboly
Shield
75%−epiboly
1−4−somites
14−19−somites
20−25−somites
Prim−5
Prim−15
Prim−25
Long−pec
Protruding−mouth
Day−4
Day−5
Stage
Transcript
ENSDART00000175759
ENSDART00000176869

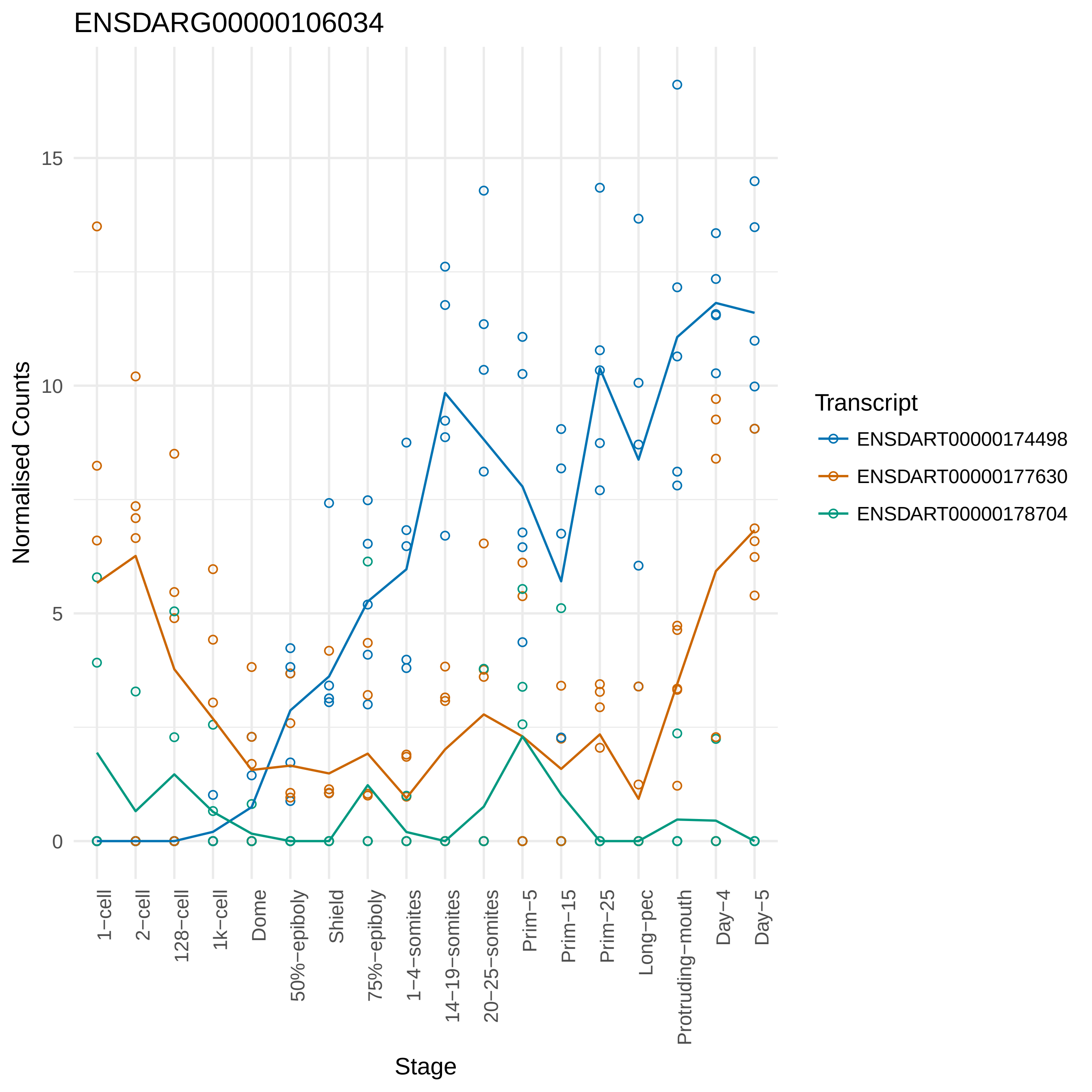

ENSDARG00000106034
Normalised Counts
0
5
10
15
Stage
1−cell
2−cell
128−cell
1k−cell
Dome
50%−epiboly
Shield
75%−epiboly
1−4−somites
14−19−somites
20−25−somites
Prim−5
Prim−15
Prim−25
Long−pec
Protruding−mouth
Day−4
Day−5
Transcript
ENSDART00000174498
ENSDART00000177630
ENSDART00000178704

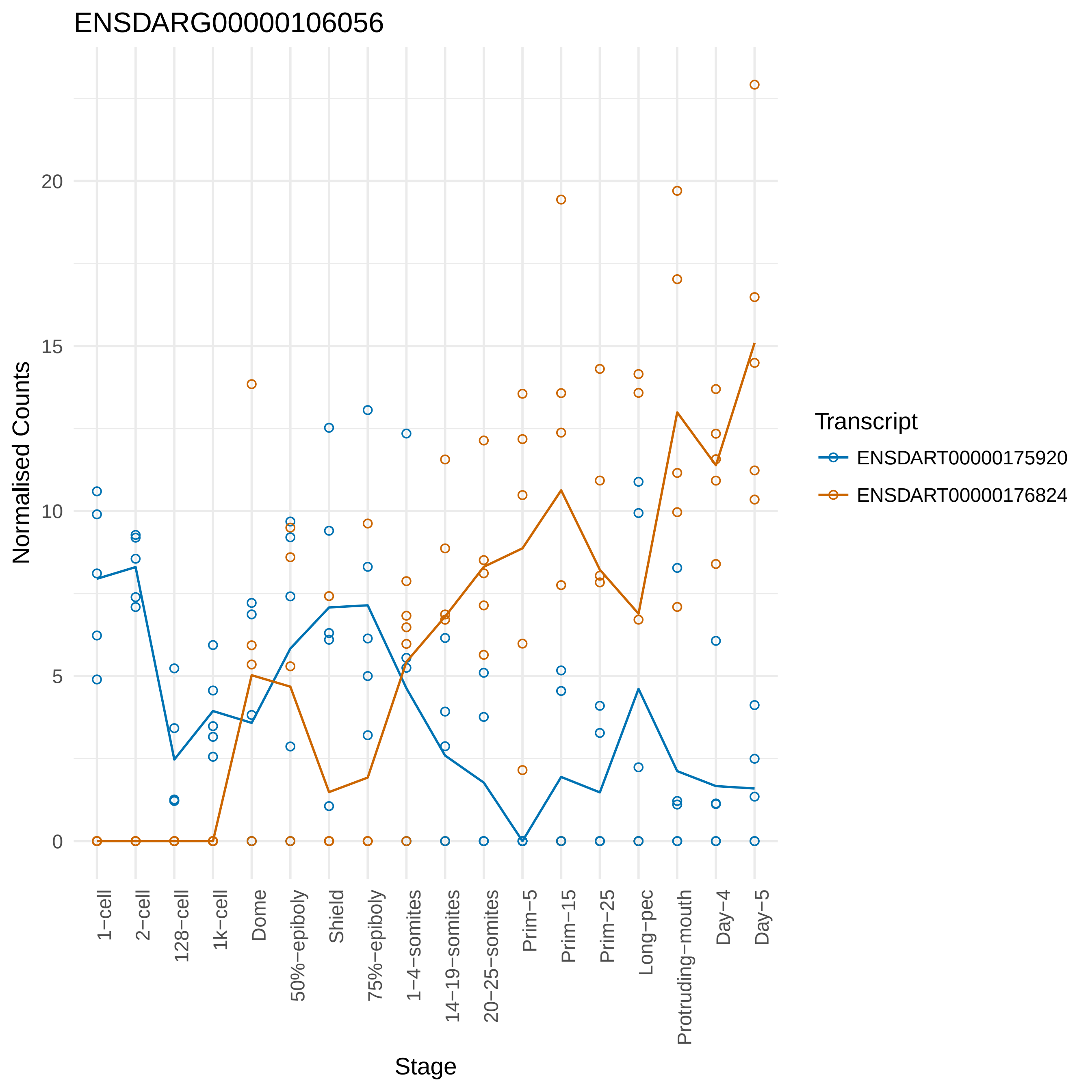

ENSDARG00000106056
Normalised Counts
0
5
10
15
20
Stage
1−cell
2−cell
128−cell
1k−cell
Dome
50%−epiboly
Shield
75%−epiboly
1−4−somites
14−19−somites
20−25−somites
Prim−5
Prim−15
Prim−25
Long−pec
Protruding−mouth
Day−4
Day−5
Transcript
ENSDART00000175920
ENSDART00000176824

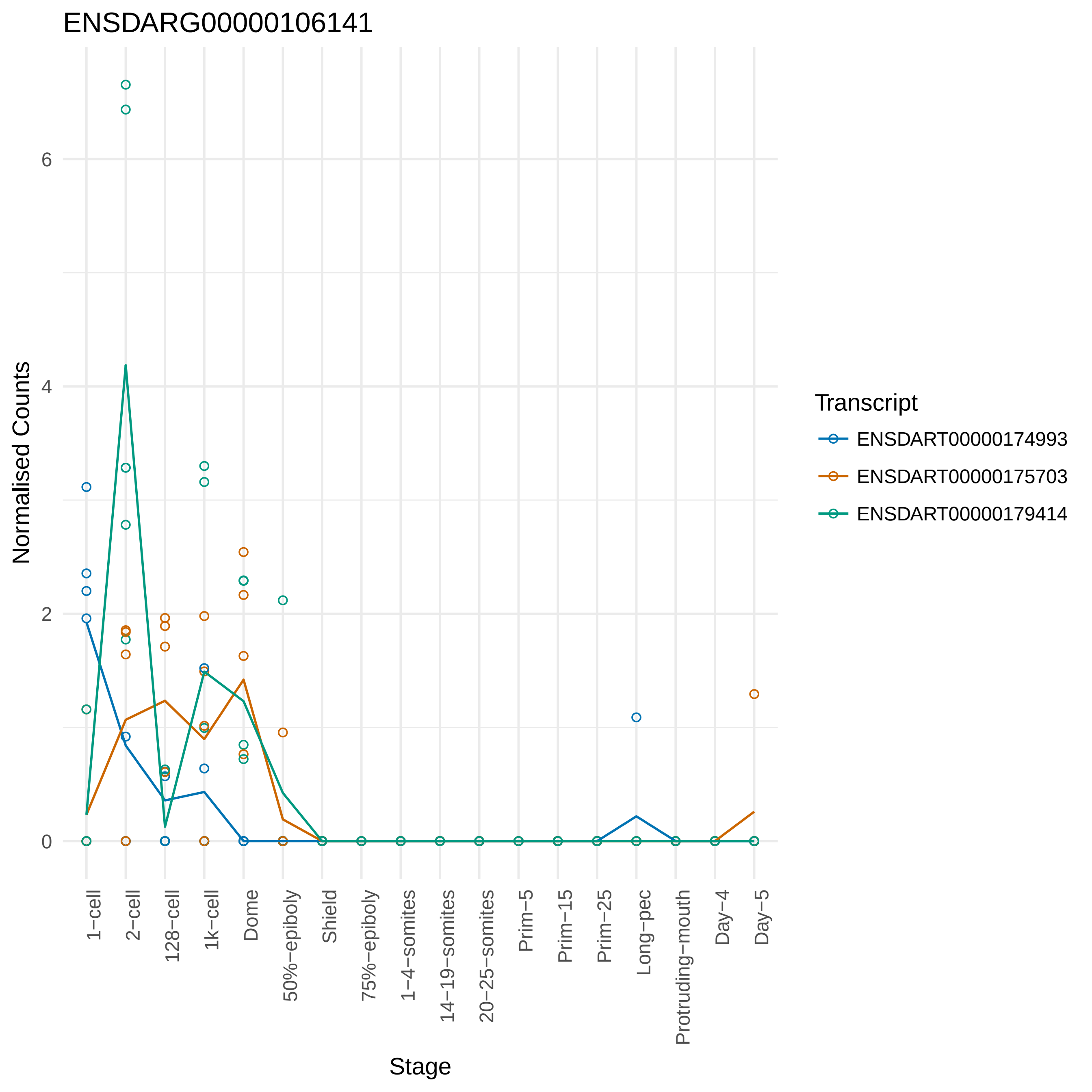
ENSDARG00000106141
Normalised Counts
0
2
4
6
Stage
1−cell
2−cell
128−cell
1k−cell
Dome
50%−epiboly
Shield
75%−epiboly
1−4−somites
14−19−somites
20−25−somites
Prim−5
Prim−15
Prim−25
Long−pec
Protruding−mouth
Day−4
Day−5
Transcript
ENSDART00000174993
ENSDART00000175703
ENSDART00000179414

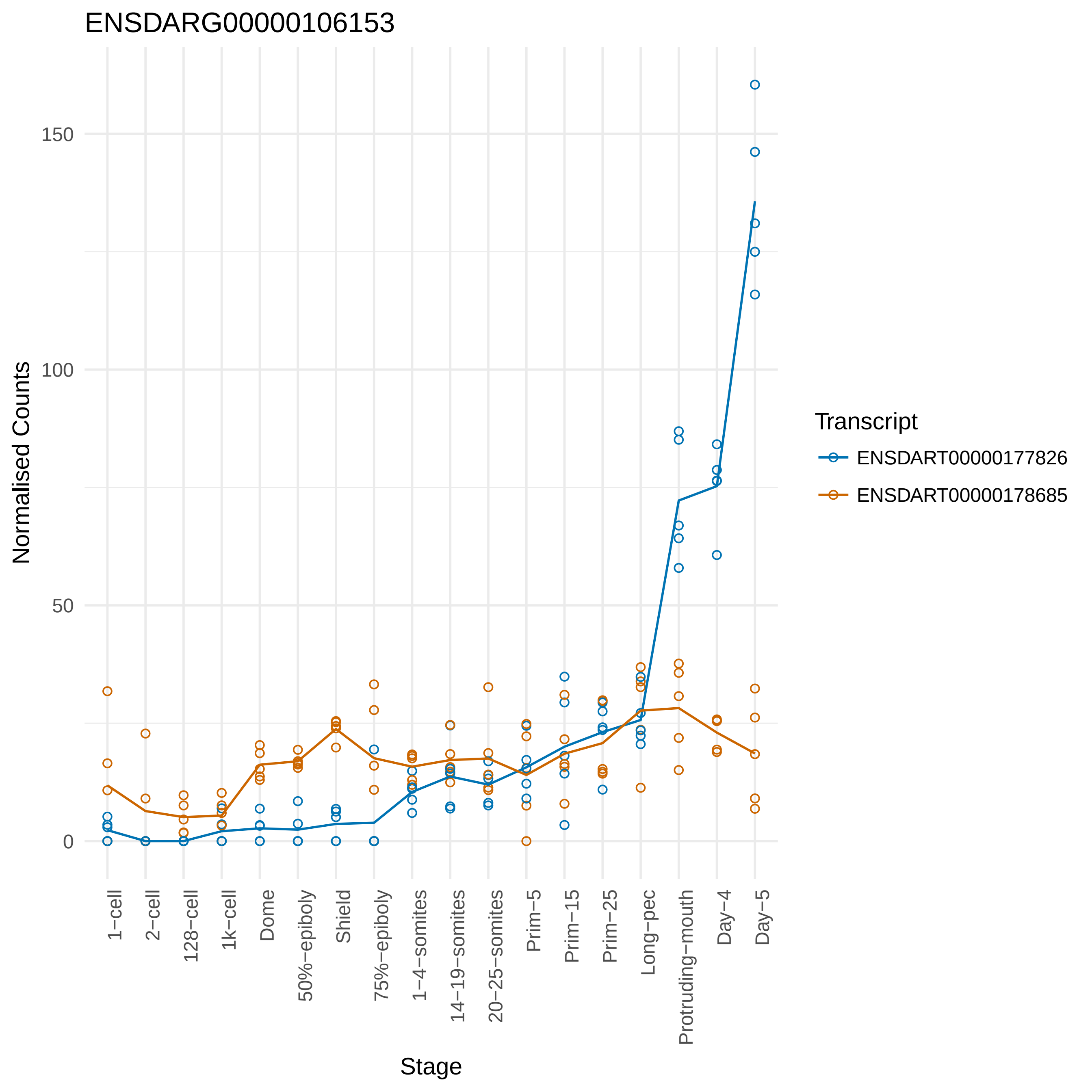
ENSDARG00000106153
Normalised Counts
150
100
50
0
1−cell
2−cell
128−cell
1k−cell
Dome
50%−epiboly
Shield
75%−epiboly
1−4−somites
14−19−somites
20−25−somites
Prim−5
Prim−15
Prim−25
Long−pec
Protruding−mouth
Day−4
Day−5
Stage
Transcript
ENSDART00000177826
ENSDART00000178685

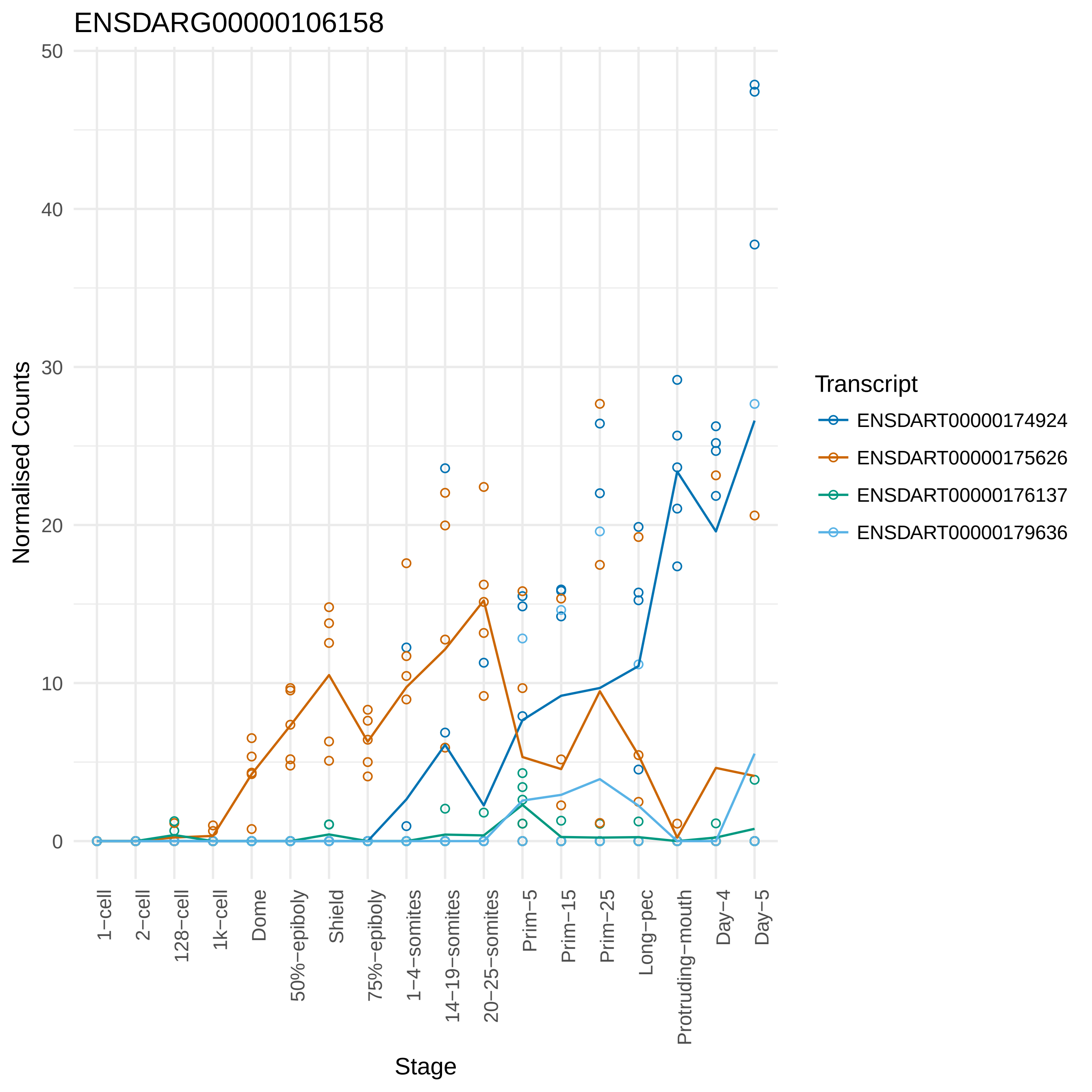

ENSDARG00000106158
Normalised Counts
0
10
20
30
40
50
Stage
1−cell
2−cell
128−cell
1k−cell
Dome
50%−epiboly
Shield
75%−epiboly
1−4−somites
14−19−somites
20−25−somites
Prim−5
Prim−15
Prim−25
Long−pec
Protruding−mouth
Day−4
Day−5
Transcript
ENSDART00000174924
ENSDART00000175626
ENSDART00000176137
ENSDART00000179636

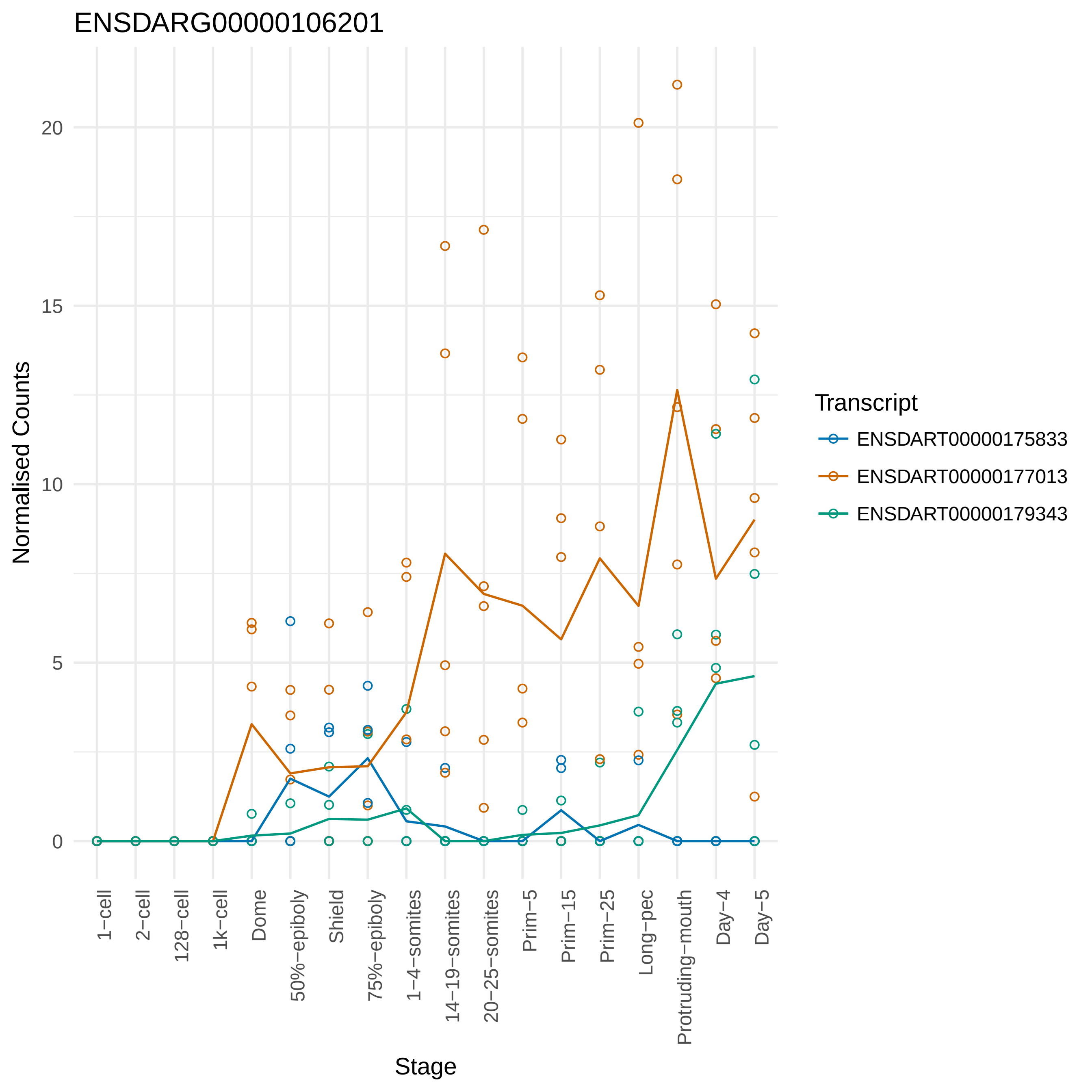

ENSDARG00000106201
Normalised Counts
Stage
Transcript
ENSDART00000175833
ENSDART00000177013
ENSDART00000179343
0
5
10
15
20
1−cell
2−cell
128−cell
1k−cell
Dome
50%−epiboly
Shield
75%−epiboly
1−4−somites
14−19−somites
20−25−somites
Prim−5
Prim−15
Prim−25
Long−pec
Protruding−mouth
Day−4
Day−5

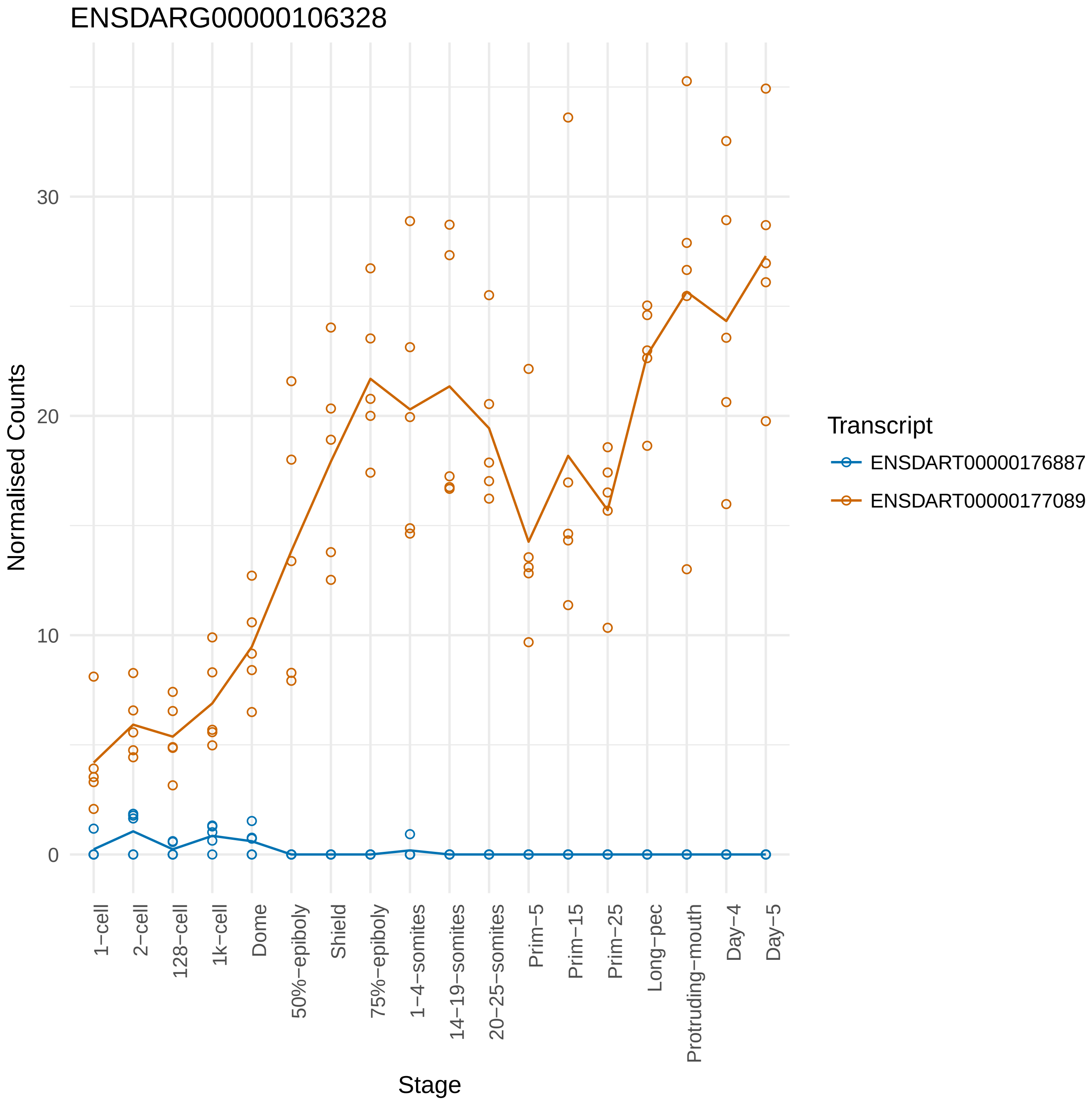
ENSDARG00000106328
Normalised Counts
0
10
20
30
1−cell
2−cell
128−cell
1k−cell
Dome
50%−epiboly
Shield
75%−epiboly
1−4−somites
14−19−somites
20−25−somites
Prim−5
Prim−15
Prim−25
Long−pec
Protruding−mouth
Day−4
Day−5
Stage
Transcript
ENSDART00000176887
ENSDART00000177089

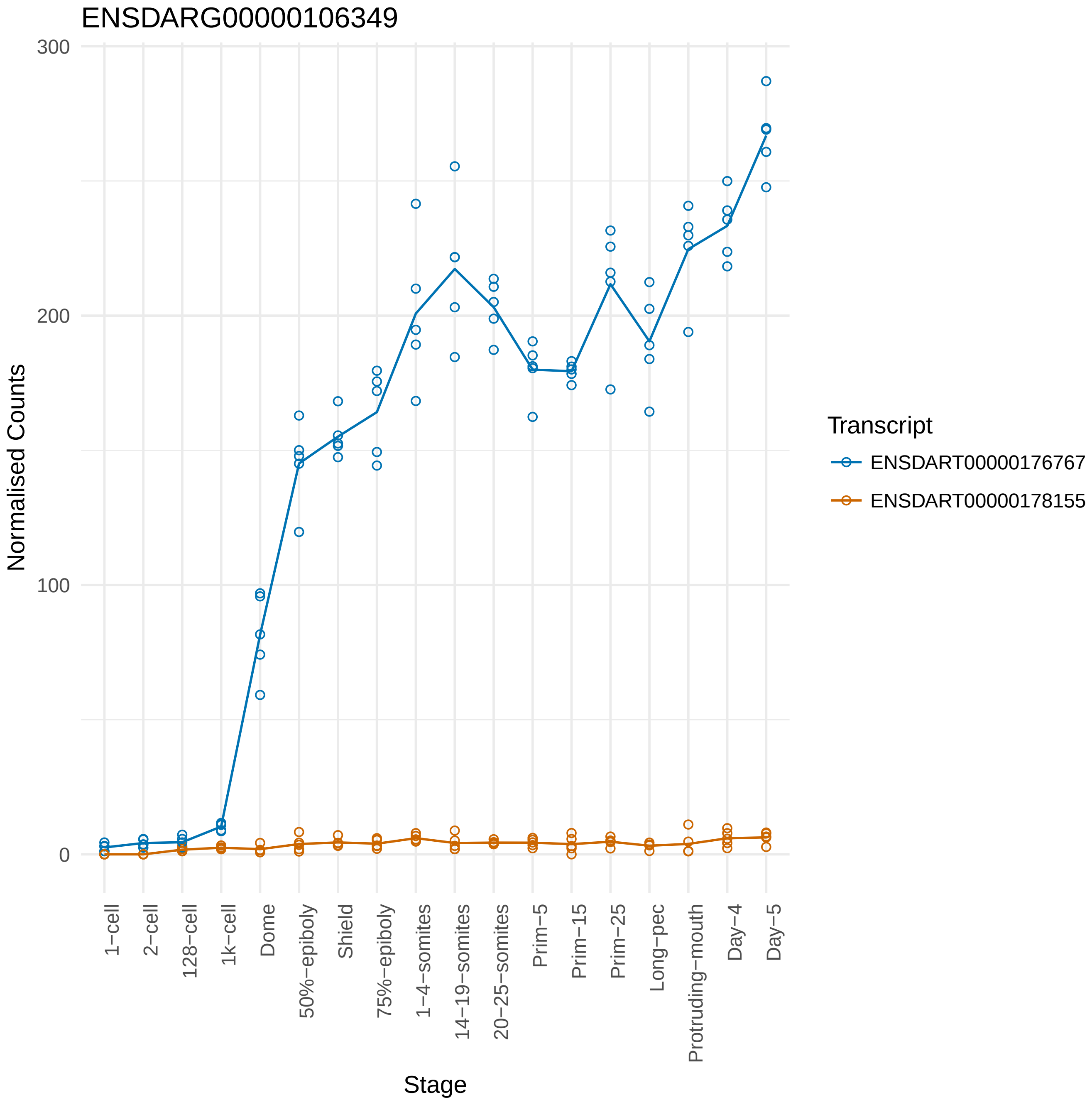
ENSDARG00000106349
Normalised Counts
300
200
100
0
1−cell
2−cell
128−cell
1k−cell
Dome
50%−epiboly
Shield
75%−epiboly
1−4−somites
14−19−somites
20−25−somites
Prim−5
Prim−15
Prim−25
Long−pec
Protruding−mouth
Day−4
Day−5
Stage
Transcript
ENSDART00000176767
ENSDART00000178155

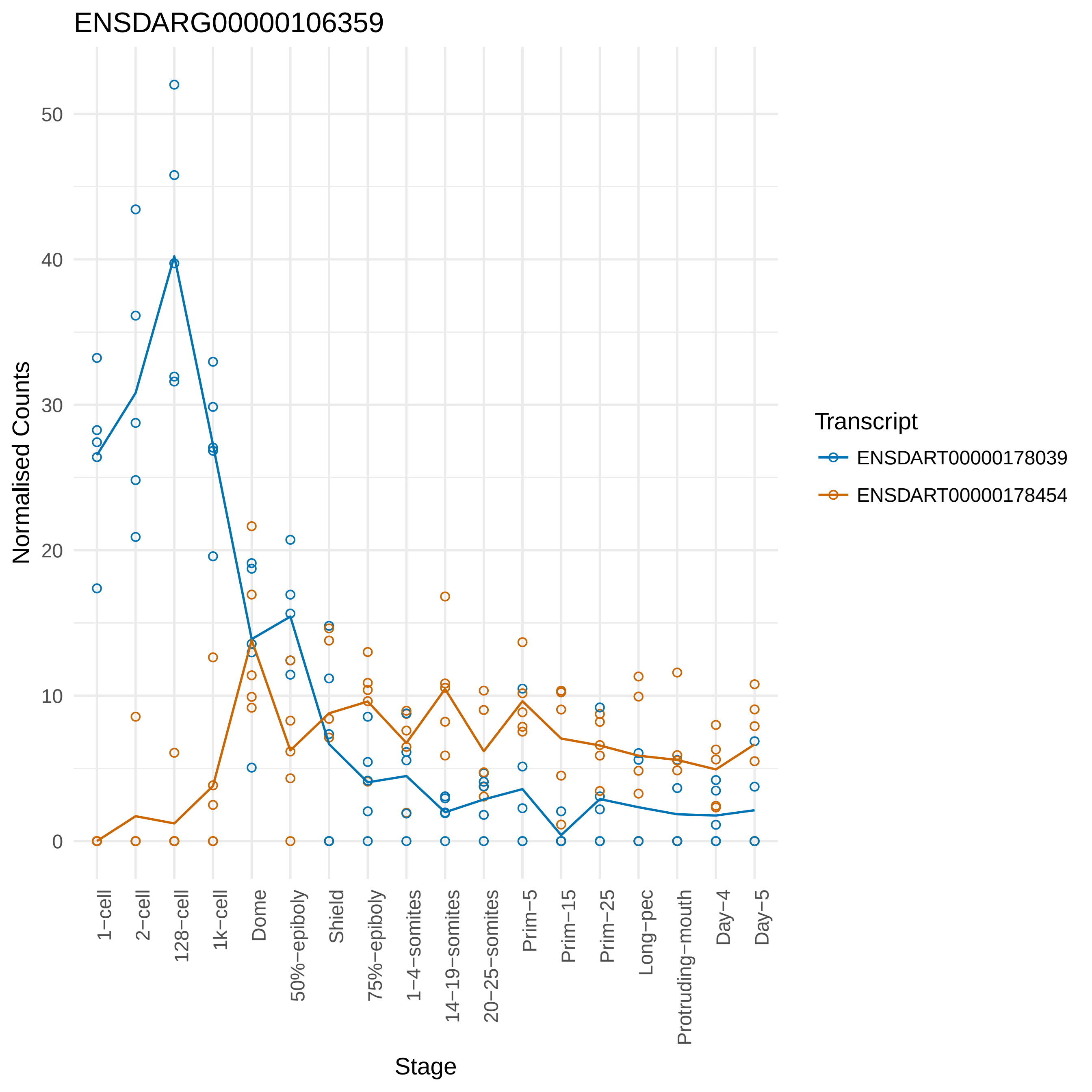

ENSDARG00000106359
Normalised Counts
0
10
20
30
40
50
1−cell
2−cell
128−cell
1k−cell
Dome
50%−epiboly
Shield
75%−epiboly
1−4−somites
14−19−somites
20−25−somites
Prim−5
Prim−15
Prim−25
Long−pec
Protruding−mouth
Day−4
Day−5
Stage
Transcript
ENSDART00000178039
ENSDART00000178454

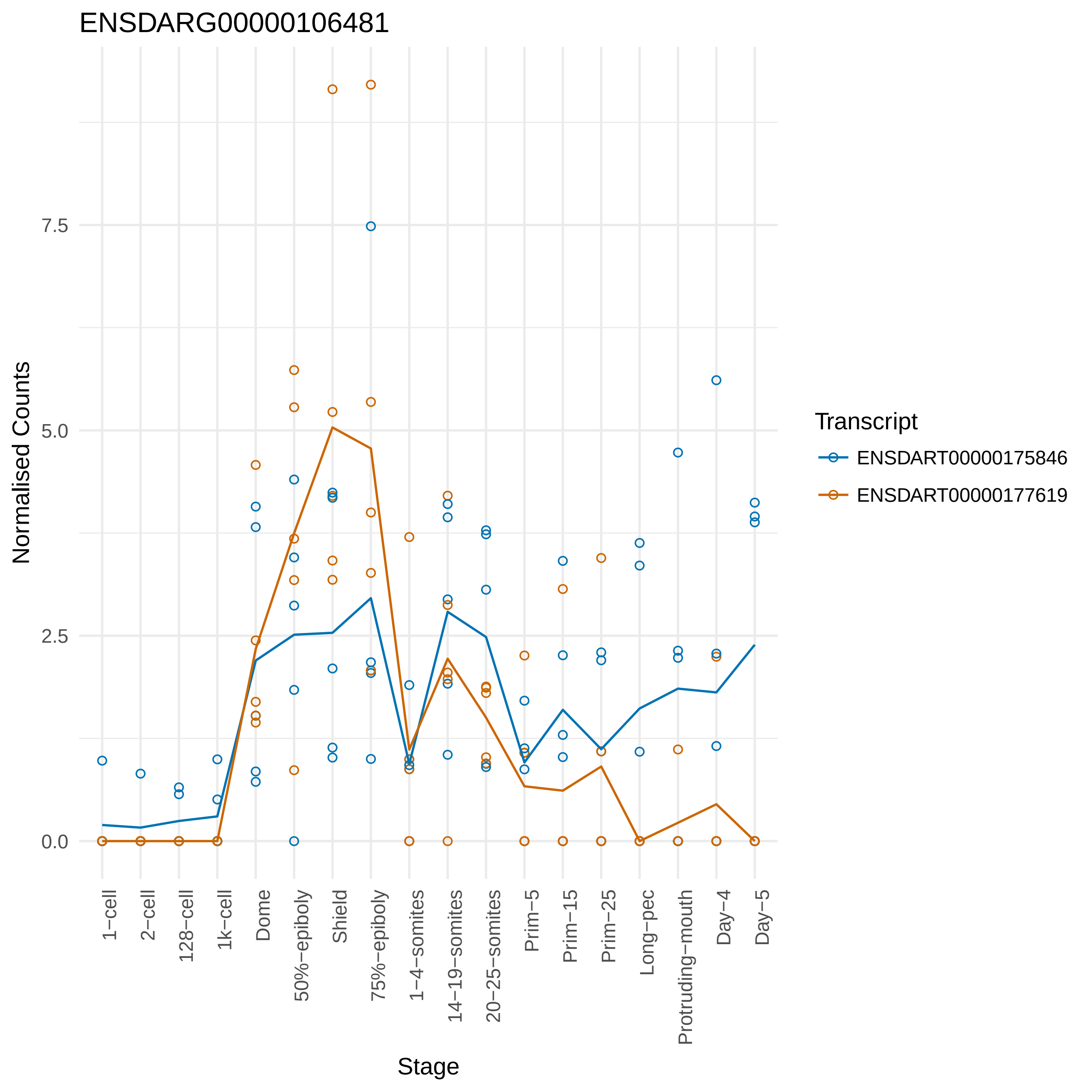

ENSDARG00000106481
Normalised Counts
7.5
5.0
2.5
0.0
1−cell
2−cell
128−cell
1k−cell
Dome
50%−epiboly
Shield
75%−epiboly
1−4−somites
14−19−somites
20−25−somites
Prim−5
Prim−15
Prim−25
Long−pec
Protruding−mouth
Day−4
Day−5
Stage
Transcript
ENSDART00000175846
ENSDART00000177619

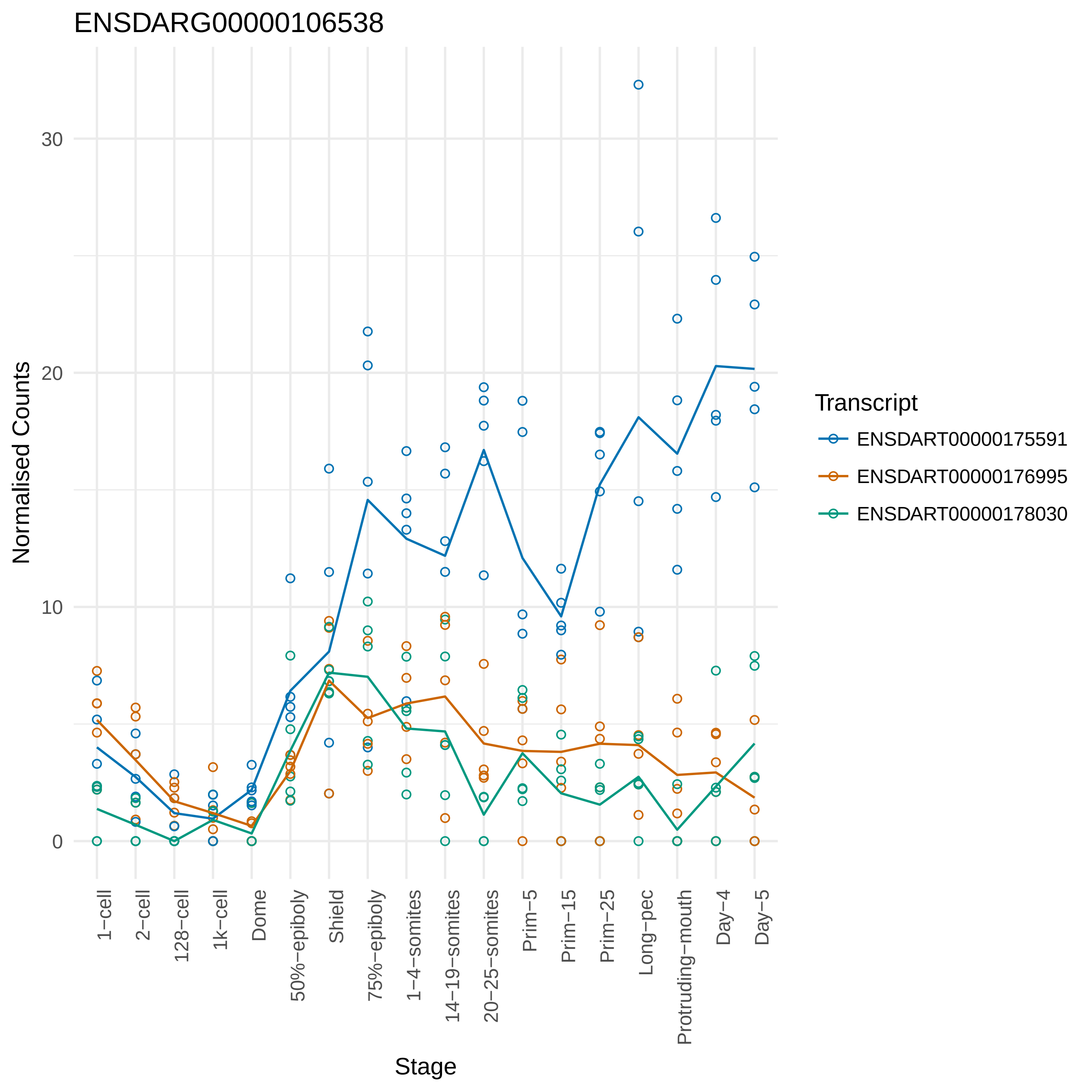
ENSDARG00000106538
Normalised Counts
0
10
20
30
Stage
1−cell
2−cell
128−cell
1k−cell
Dome
50%−epiboly
Shield
75%−epiboly
1−4−somites
14−19−somites
20−25−somites
Prim−5
Prim−15
Prim−25
Long−pec
Protruding−mouth
Day−4
Day−5
Transcript
ENSDART00000175591
ENSDART00000176995
ENSDART00000178030

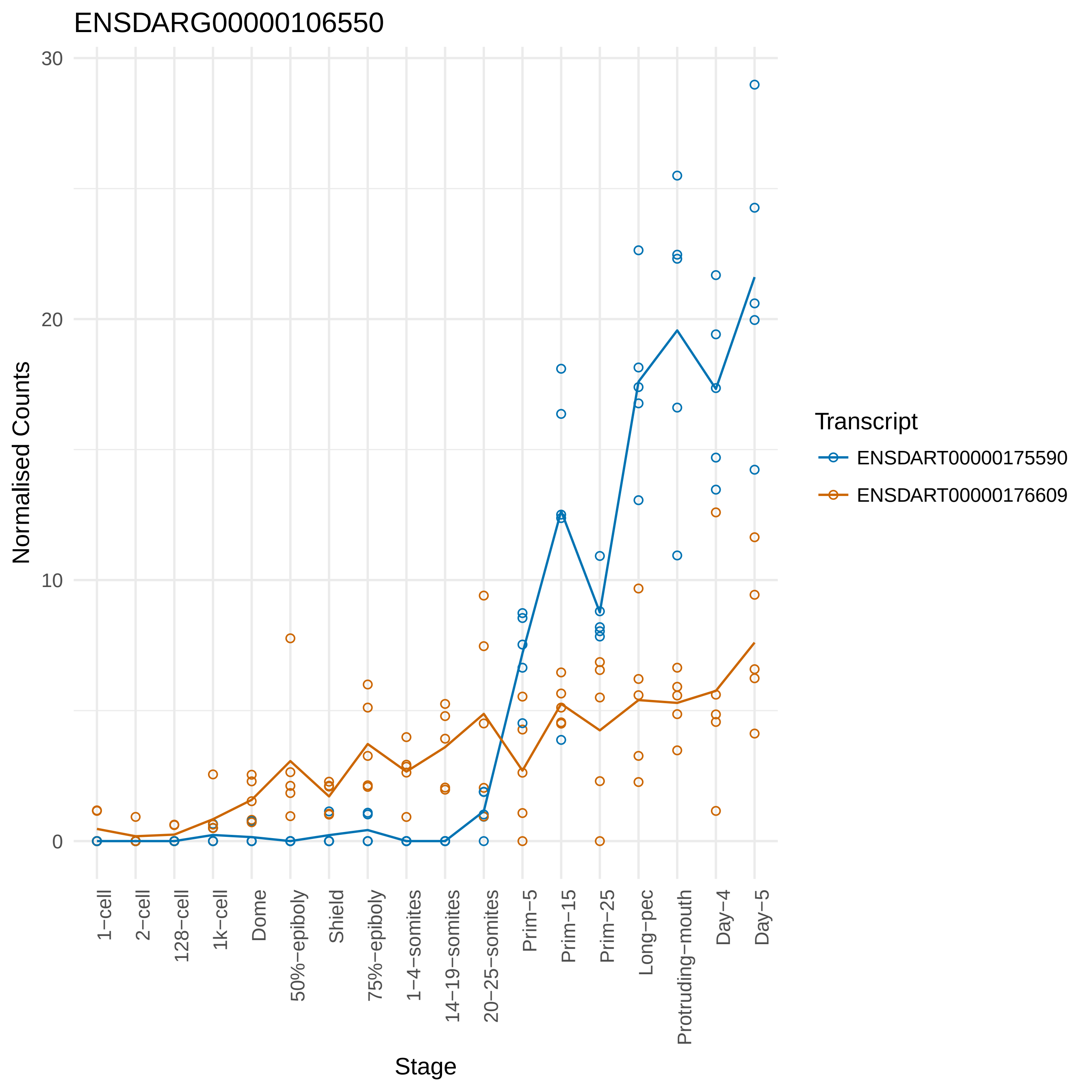

ENSDARG00000106550
Normalised Counts
30
20
10
0
1−cell
2−cell
128−cell
1k−cell
Dome
50%−epiboly
Shield
75%−epiboly
1−4−somites
14−19−somites
20−25−somites
Prim−5
Prim−15
Prim−25
Long−pec
Protruding−mouth
Day−4
Day−5
Stage
Transcript
ENSDART00000175590
ENSDART00000176609

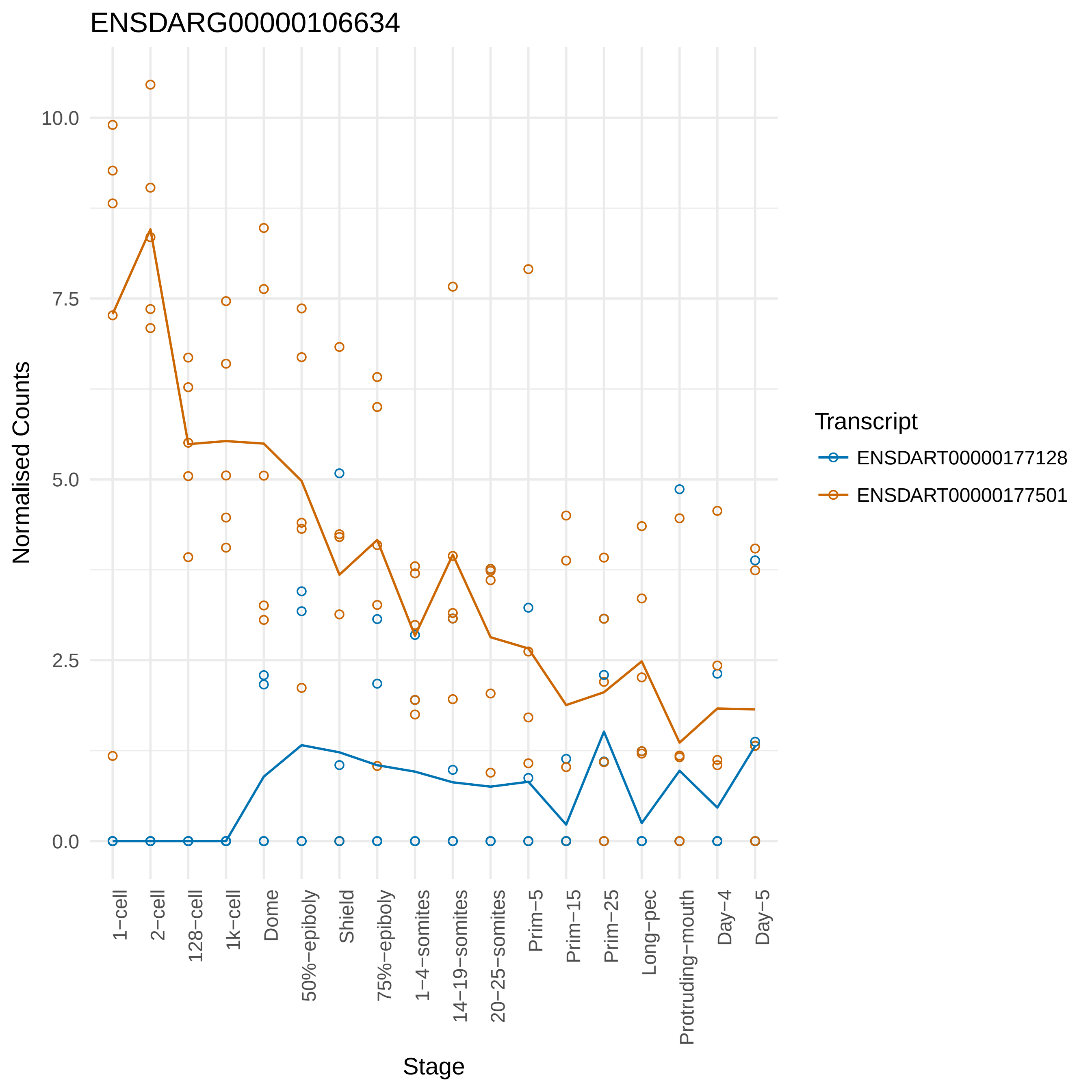
ENSDARG00000106634
Normalised Counts
10.0
7.5
5.0
2.5
0.0
1−cell
2−cell
128−cell
1k−cell
Dome
50%−epiboly
Shield
75%−epiboly
1−4−somites
14−19−somites
20−25−somites
Prim−5
Prim−15
Prim−25
Long−pec
Protruding−mouth
Day−4
Day−5
Stage
Transcript
ENSDART00000177128
ENSDART00000177501

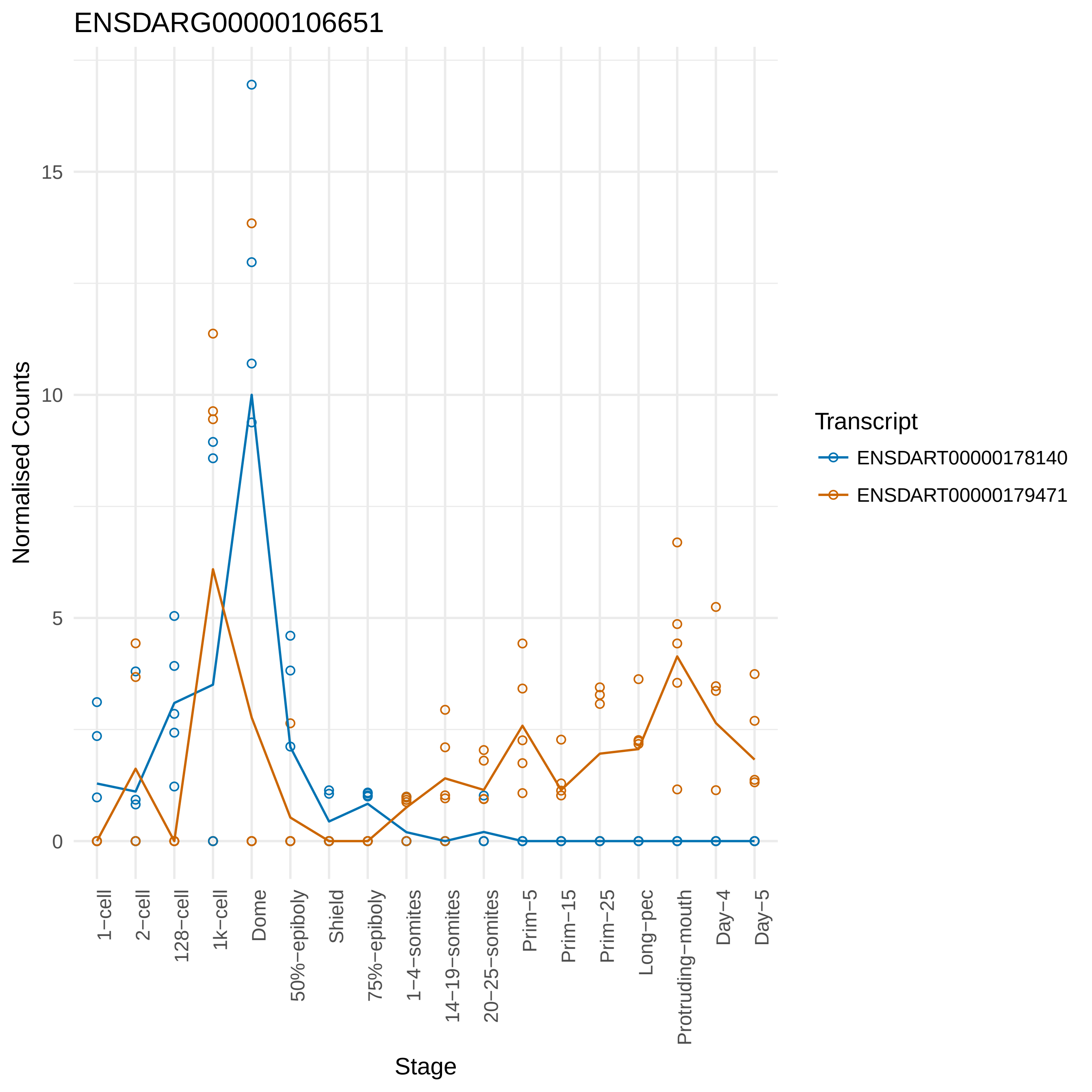

ENSDARG00000106651
Normalised Counts
0
5
10
15
1−cell
2−cell
128−cell
1k−cell
Dome
50%−epiboly
Shield
75%−epiboly
1−4−somites
14−19−somites
20−25−somites
Prim−5
Prim−15
Prim−25
Long−pec
Protruding−mouth
Day−4
Day−5
Stage
Transcript
ENSDART00000178140
ENSDART00000179471

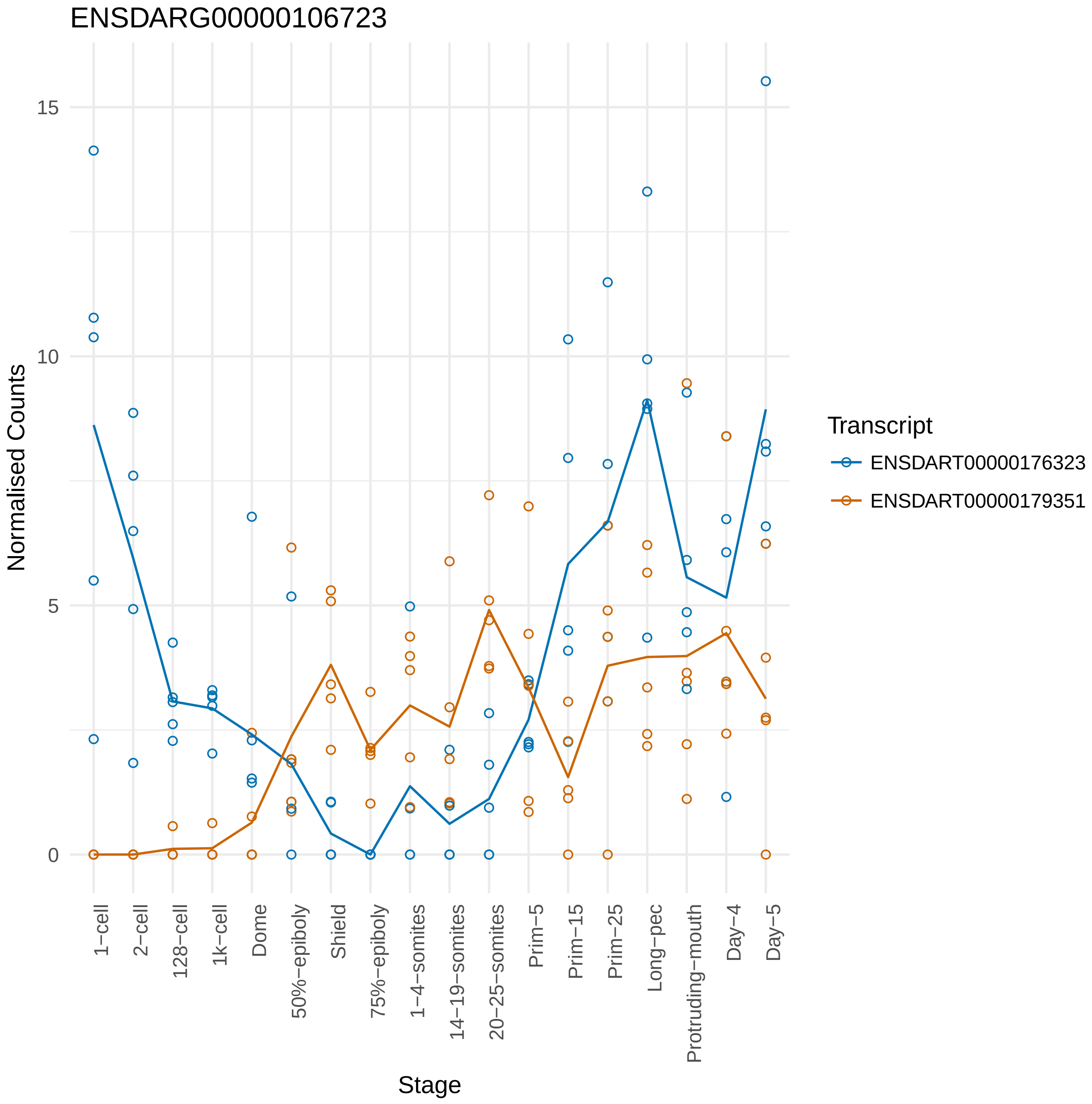

ENSDARG00000106723
Normalised Counts
15
10
5
0
1−cell
2−cell
128−cell
1k−cell
Dome
50%−epiboly
Shield
75%−epiboly
1−4−somites
14−19−somites
20−25−somites
Prim−5
Prim−15
Prim−25
Long−pec
Protruding−mouth
Day−4
Day−5
Stage
Transcript
ENSDART00000176323
ENSDART00000179351

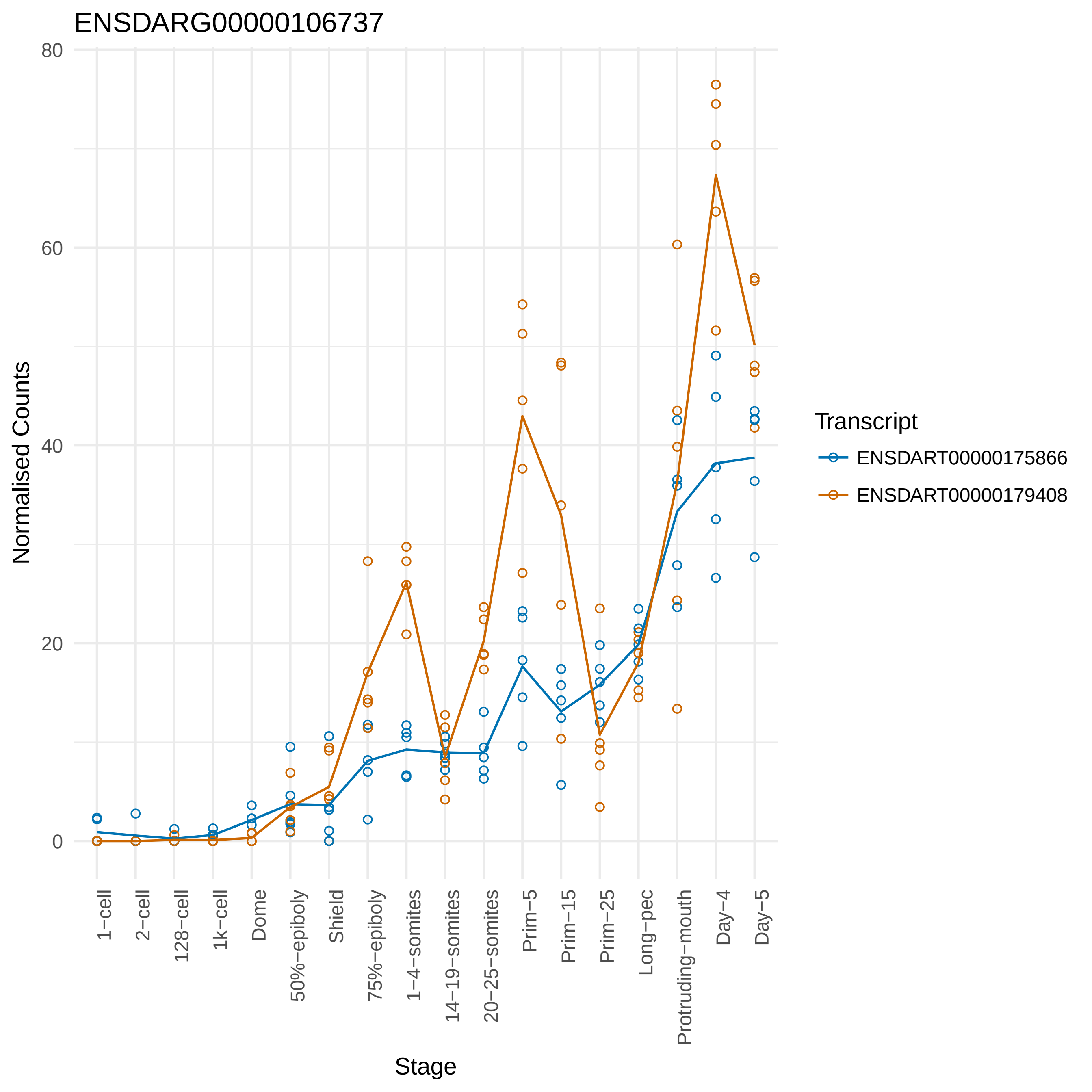
ENSDARG00000106737
Normalised Counts
80
60
40
20
0
1−cell
2−cell
128−cell
1k−cell
Dome
50%−epiboly
Shield
75%−epiboly
1−4−somites
14−19−somites
20−25−somites
Prim−5
Prim−15
Prim−25
Long−pec
Protruding−mouth
Day−4
Day−5
Stage
Transcript
ENSDART00000175866
ENSDART00000179408

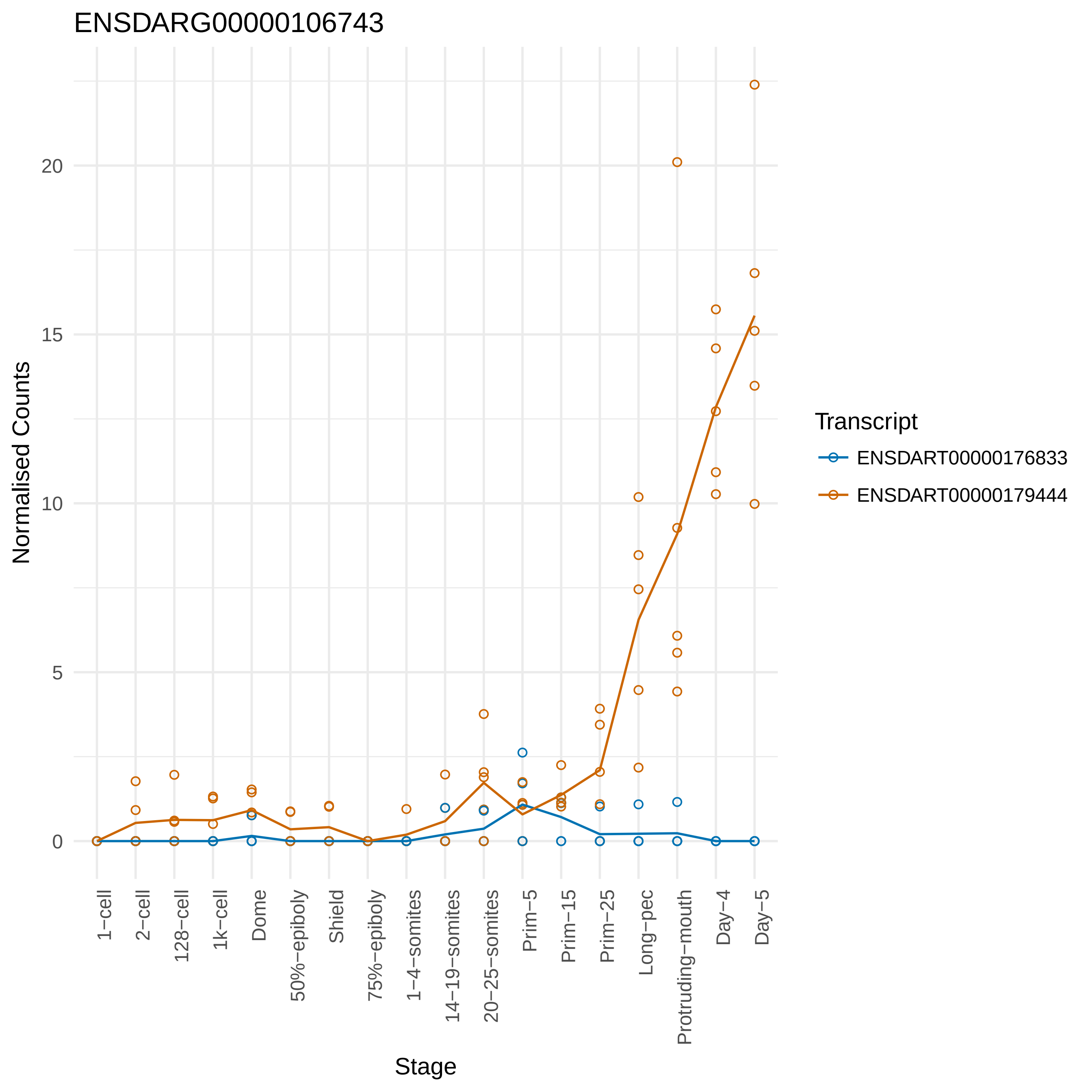

ENSDARG00000106743
Normalised Counts
0
5
10
15
20
Stage
1−cell
2−cell
128−cell
1k−cell
Dome
50%−epiboly
Shield
75%−epiboly
1−4−somites
14−19−somites
20−25−somites
Prim−5
Prim−15
Prim−25
Long−pec
Protruding−mouth
Day−4
Day−5
Transcript
ENSDART00000176833
ENSDART00000179444

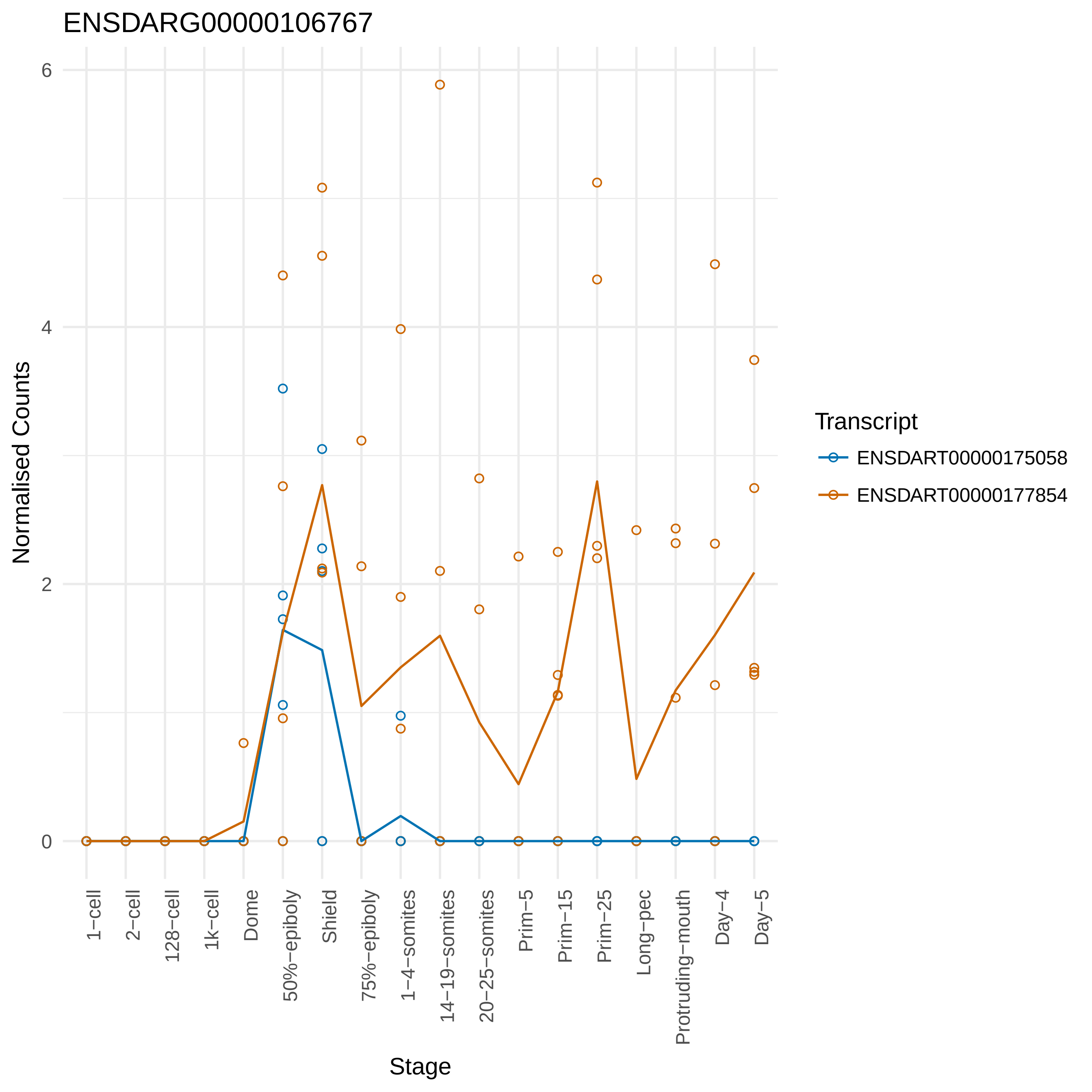
ENSDARG00000106767
Normalised Counts
0
2
4
6
Stage
1−cell
2−cell
128−cell
1k−cell
Dome
50%−epiboly
Shield
75%−epiboly
1−4−somites
14−19−somites
20−25−somites
Prim−5
Prim−15
Prim−25
Long−pec
Protruding−mouth
Day−4
Day−5
Transcript
ENSDART00000175058
ENSDART00000177854

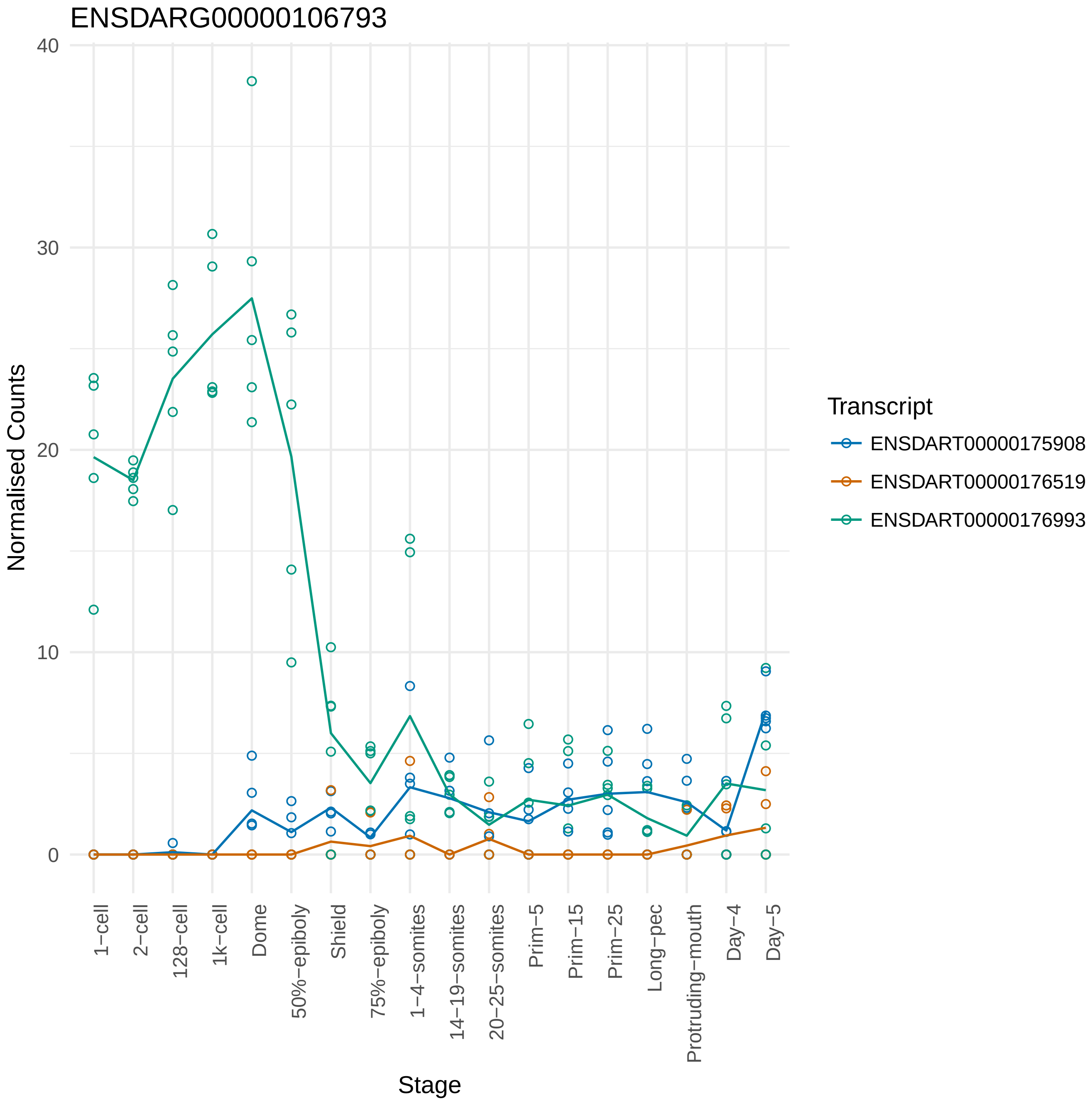

ENSDARG00000106793
Normalised Counts
0
10
20
30
40
Stage
1−cell
2−cell
128−cell
1k−cell
Dome
50%−epiboly
Shield
75%−epiboly
1−4−somites
14−19−somites
20−25−somites
Prim−5
Prim−15
Prim−25
Long−pec
Protruding−mouth
Day−4
Day−5
Transcript
ENSDART00000175908
ENSDART00000176519
ENSDART00000176993

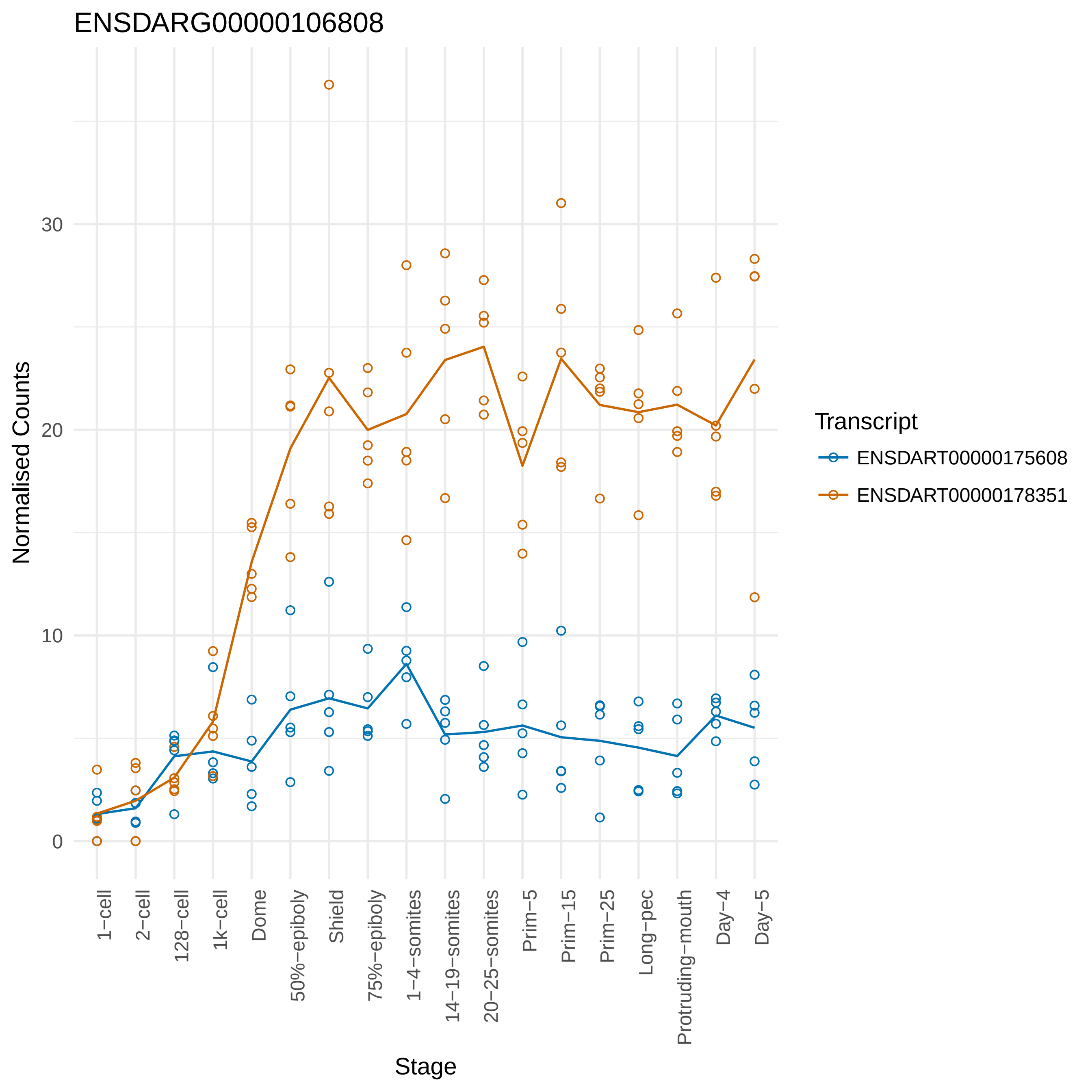
ENSDARG00000106808
Normalised Counts
0
10
20
30
1−cell
2−cell
128−cell
1k−cell
Dome
50%−epiboly
Shield
75%−epiboly
1−4−somites
14−19−somites
20−25−somites
Prim−5
Prim−15
Prim−25
Long−pec
Protruding−mouth
Day−4
Day−5
Stage
Transcript
ENSDART00000175608
ENSDART00000178351

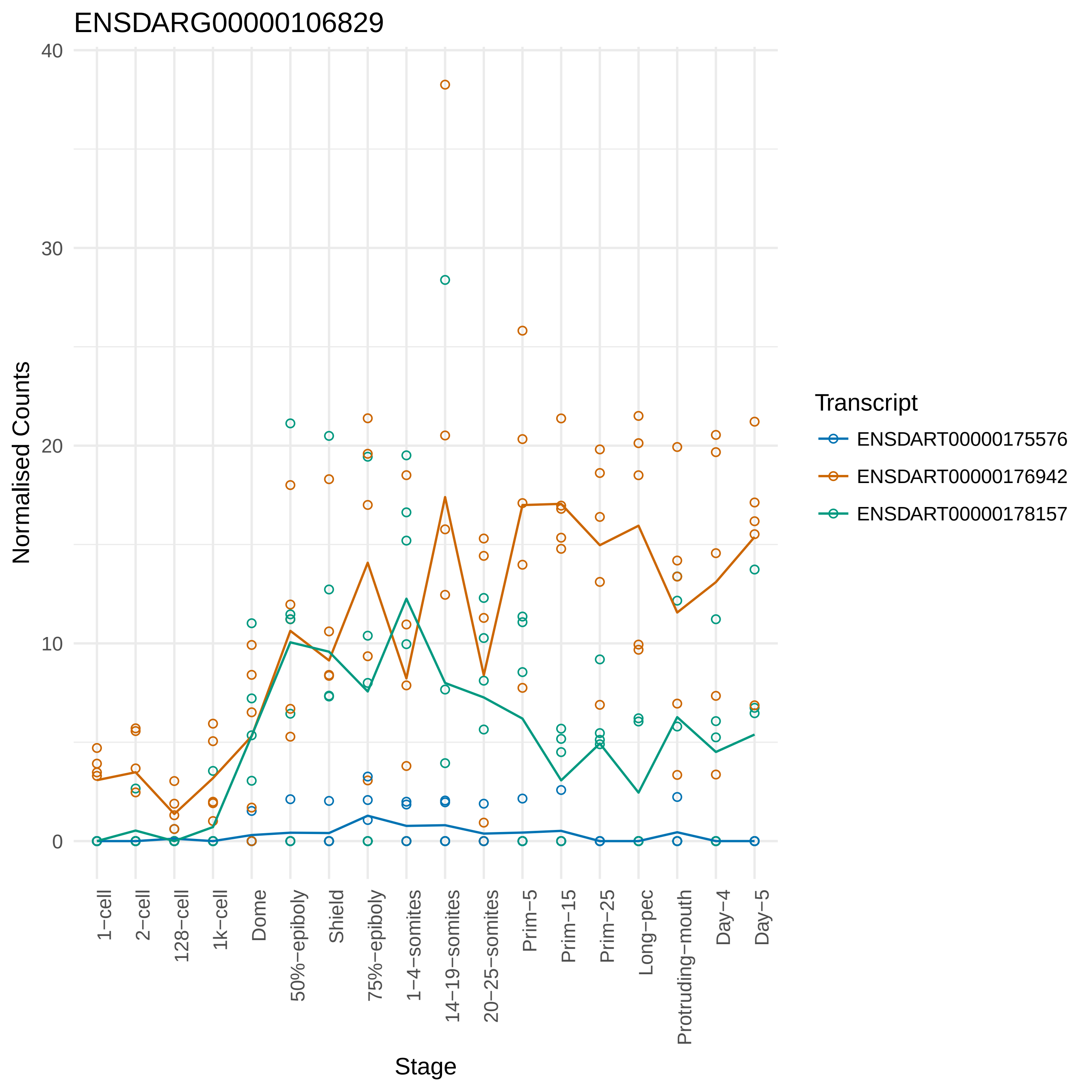

ENSDARG00000106829
Normalised Counts
0
10
20
30
40
Stage
1−cell
2−cell
128−cell
1k−cell
Dome
50%−epiboly
Shield
75%−epiboly
1−4−somites
14−19−somites
20−25−somites
Prim−5
Prim−15
Prim−25
Long−pec
Protruding−mouth
Day−4
Day−5
Transcript
ENSDART00000175576
ENSDART00000176942
ENSDART00000178157

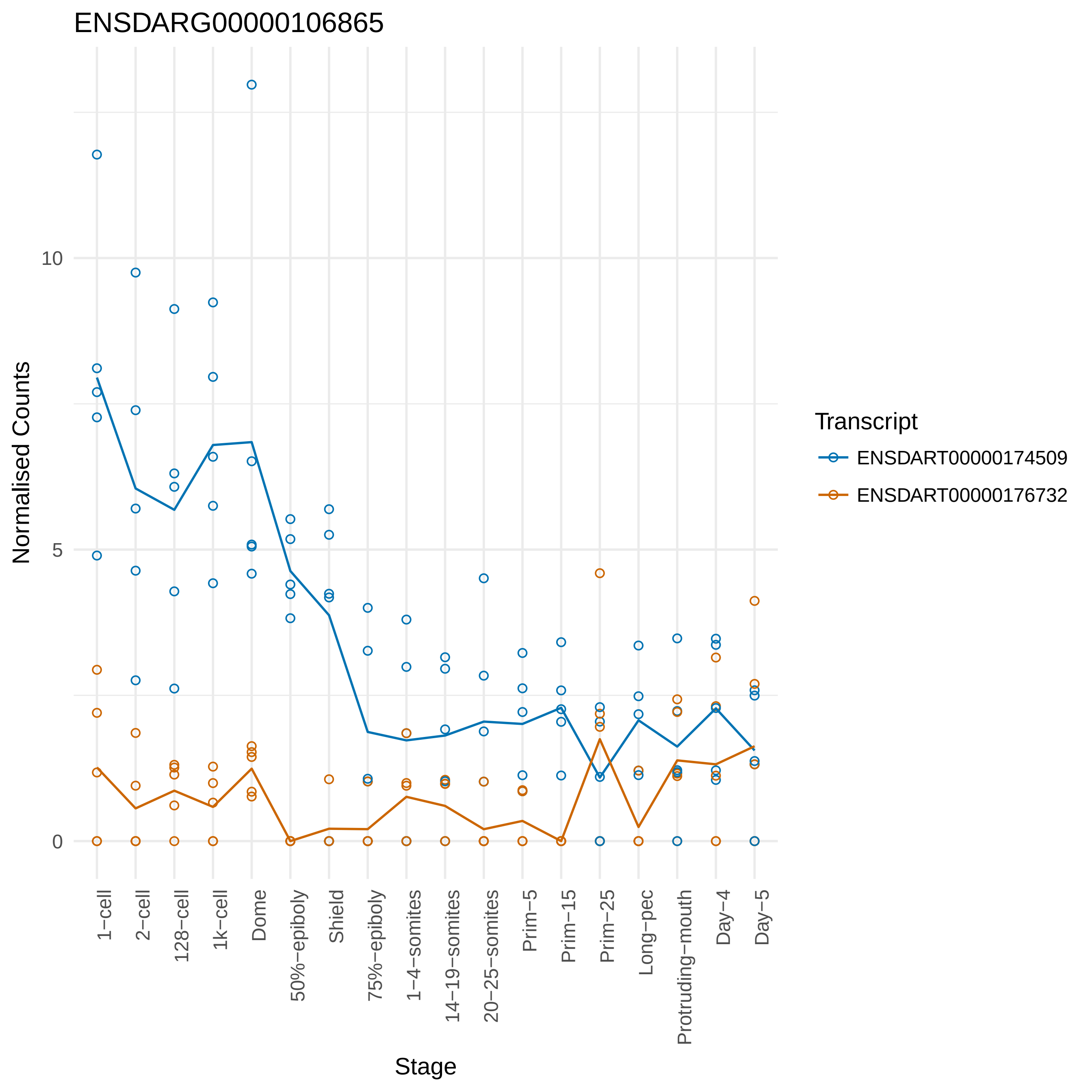
ENSDARG00000106865
Normalised Counts
0
5
10
Stage
1−cell
2−cell
128−cell
1k−cell
Dome
50%−epiboly
Shield
75%−epiboly
1−4−somites
14−19−somites
20−25−somites
Prim−5
Prim−15
Prim−25
Long−pec
Protruding−mouth
Day−4
Day−5
Transcript
ENSDART00000174509
ENSDART00000176732

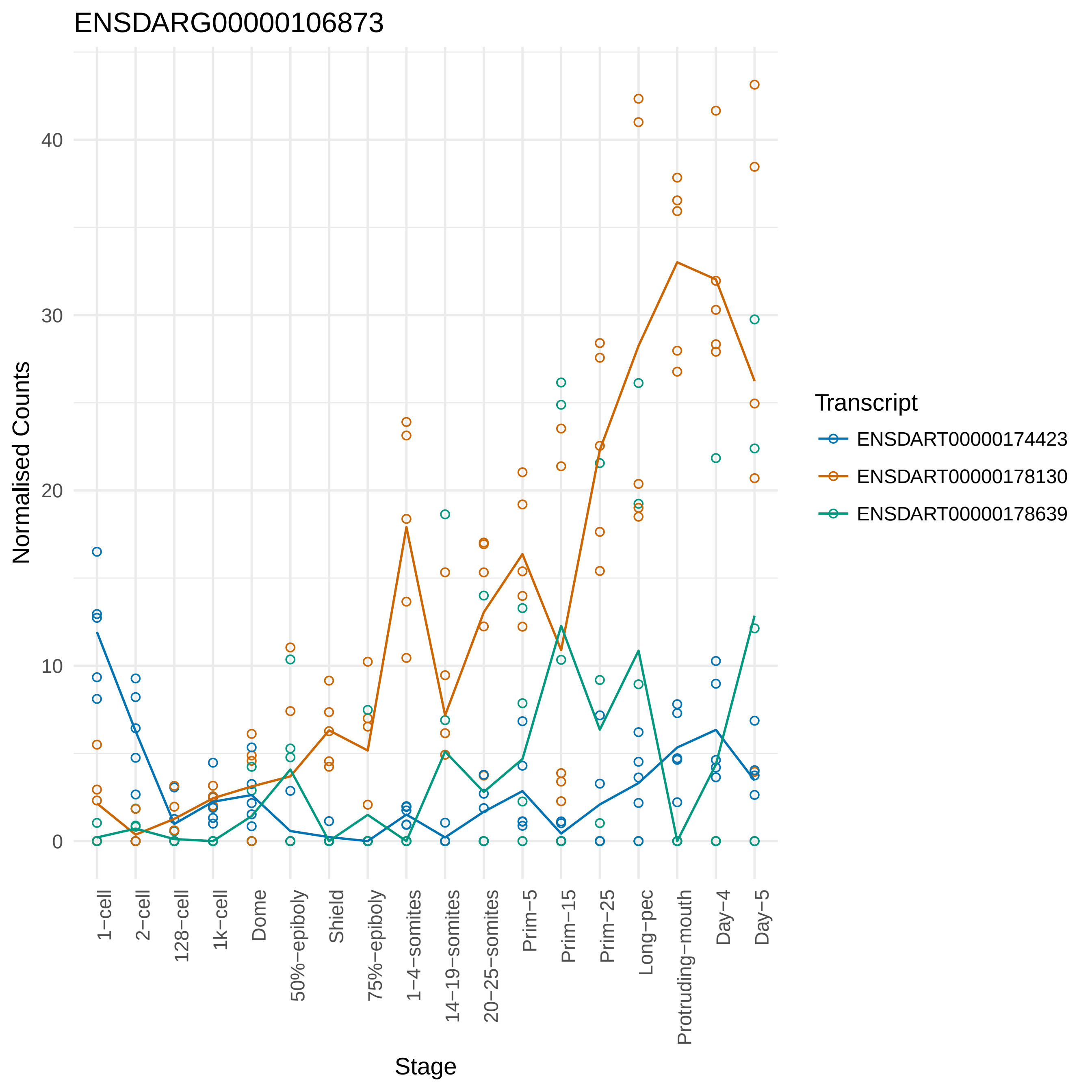

ENSDARG00000106873
Normalised Counts
0
10
20
30
40
Stage
1−cell
2−cell
128−cell
1k−cell
Dome
50%−epiboly
Shield
75%−epiboly
1−4−somites
14−19−somites
20−25−somites
Prim−5
Prim−15
Prim−25
Long−pec
Protruding−mouth
Day−4
Day−5
Transcript
ENSDART00000174423
ENSDART00000178130
ENSDART00000178639

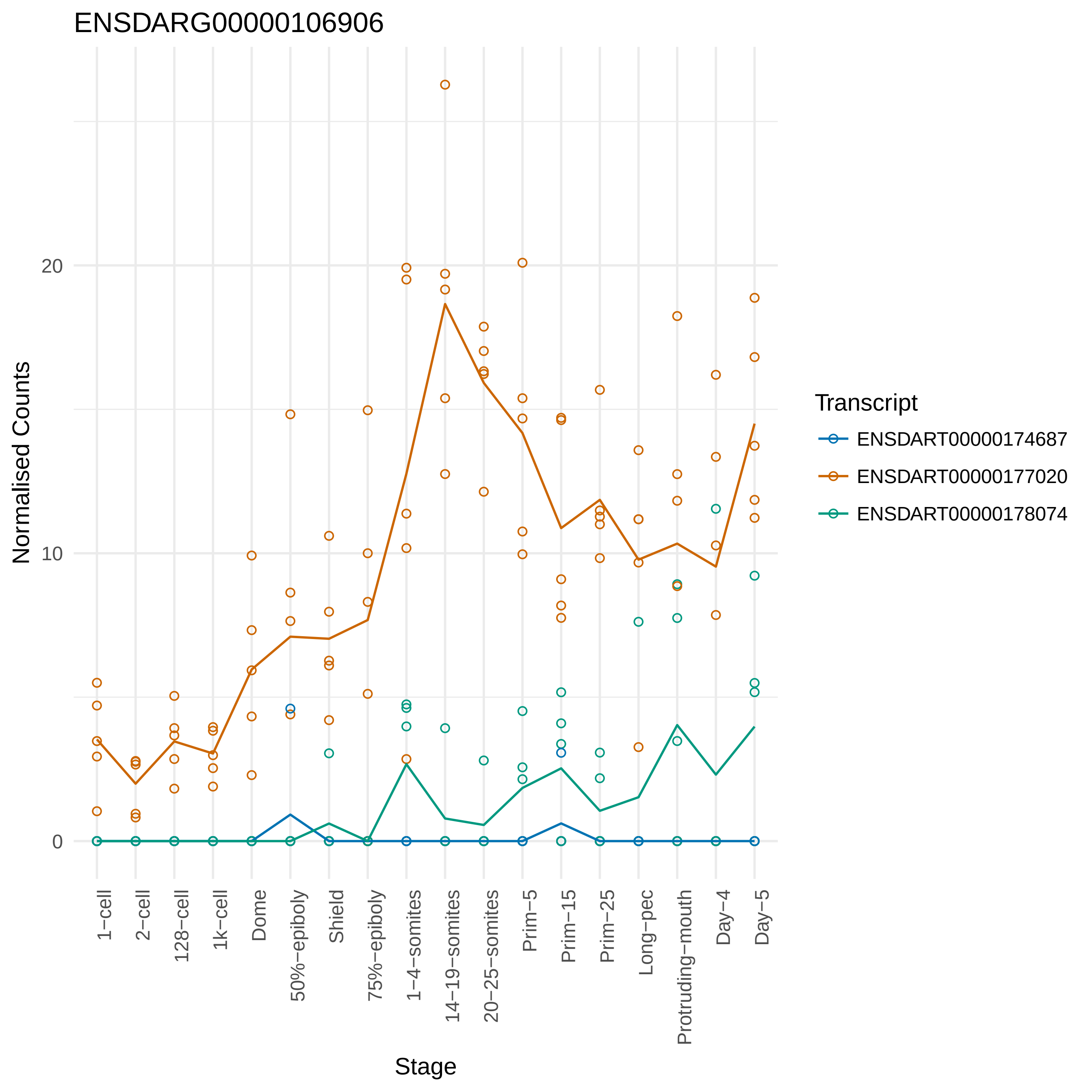
ENSDARG00000106906
Normalised Counts
20
10
0
Stage
1−cell
2−cell
128−cell
1k−cell
Dome
50%−epiboly
Shield
75%−epiboly
1−4−somites
14−19−somites
20−25−somites
Prim−5
Prim−15
Prim−25
Long−pec
Protruding−mouth
Day−4
Day−5
Transcript
ENSDART00000174687
ENSDART00000177020
ENSDART00000178074

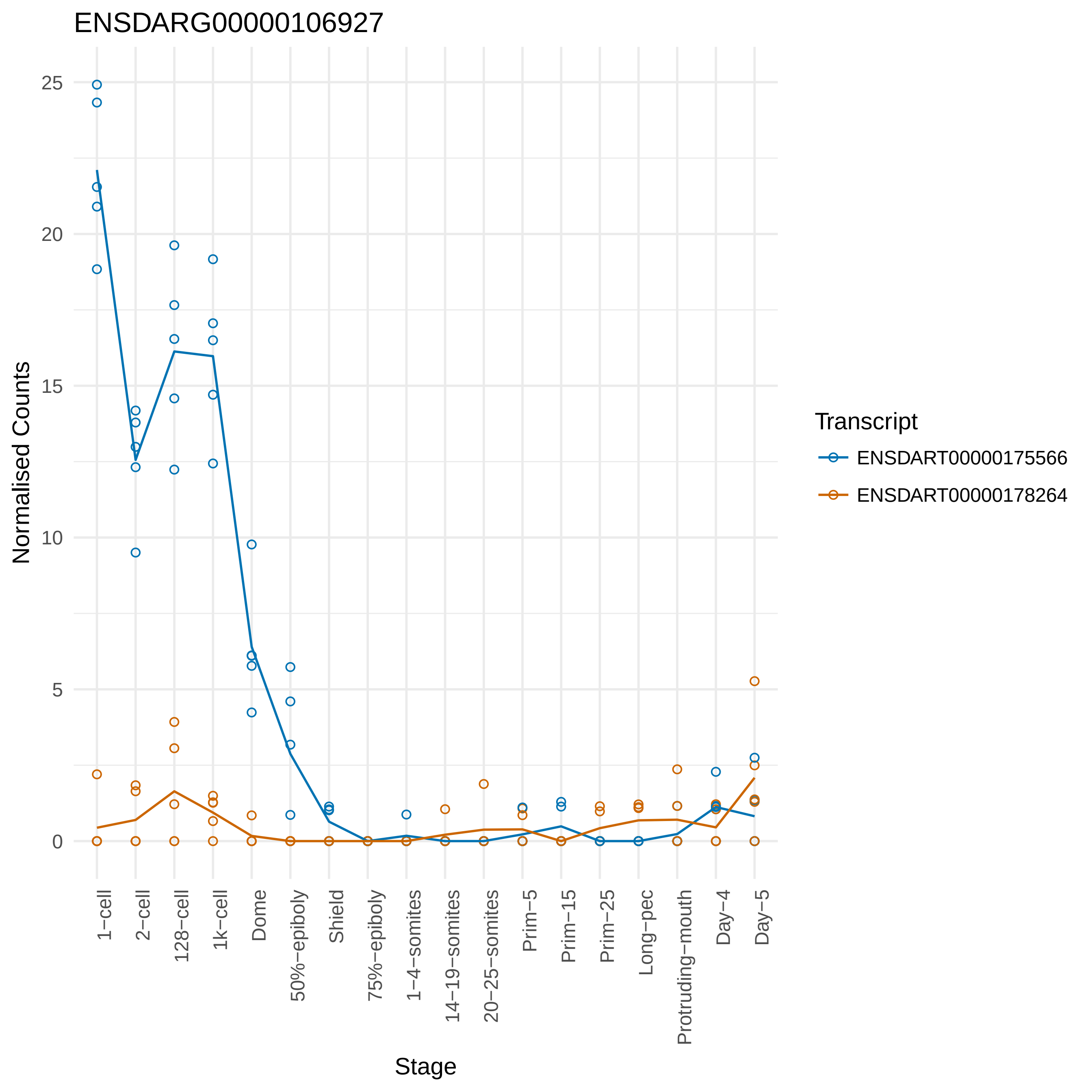

ENSDARG00000106927
Normalised Counts
25
20
15
10
5
0
1−cell
2−cell
128−cell
1k−cell
Dome
50%−epiboly
Shield
75%−epiboly
1−4−somites
14−19−somites
20−25−somites
Prim−5
Prim−15
Prim−25
Long−pec
Protruding−mouth
Day−4
Day−5
Stage
Transcript
ENSDART00000175566
ENSDART00000178264

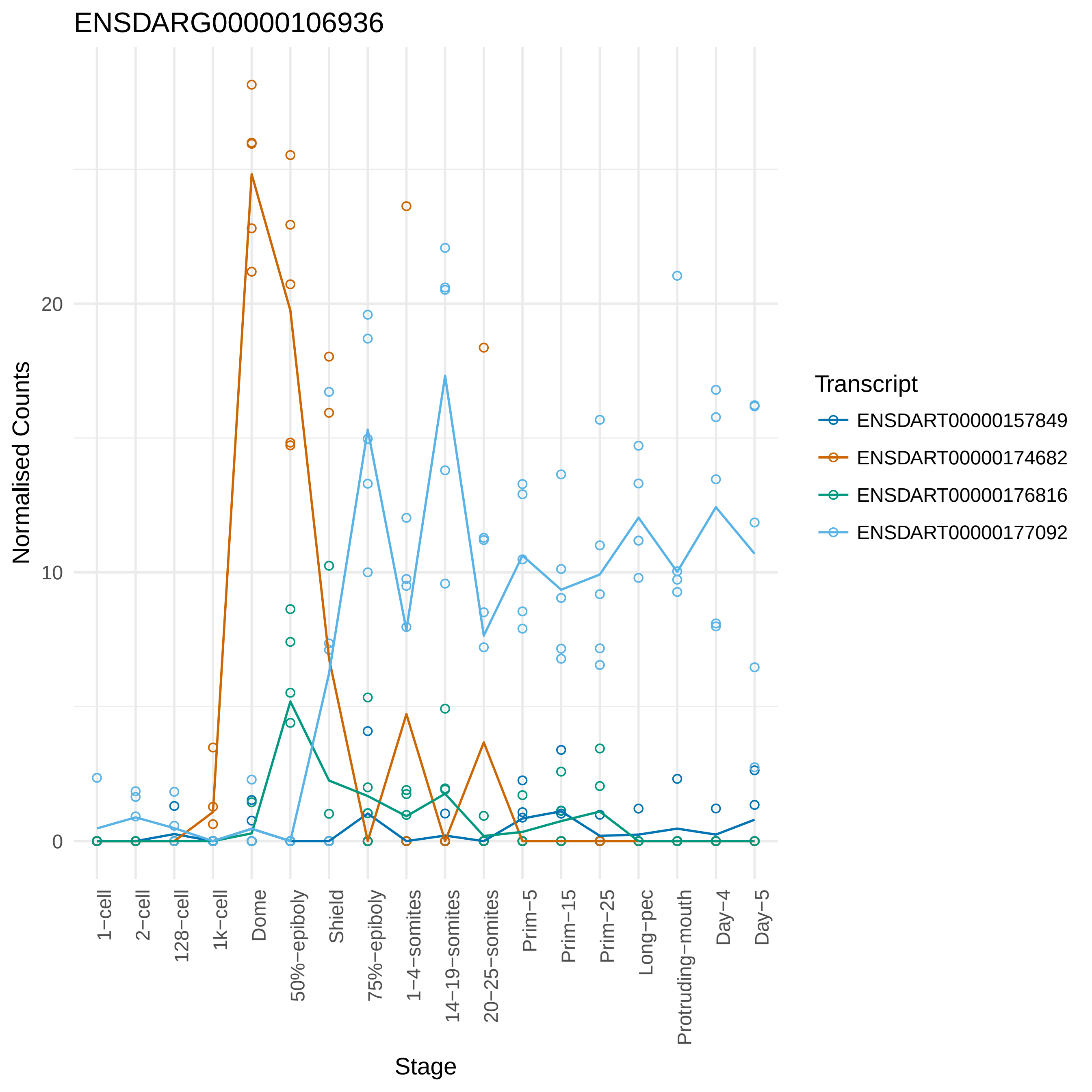

ENSDARG00000106936
Normalised Counts
0
10
20
Stage
1−cell
2−cell
128−cell
1k−cell
Dome
50%−epiboly
Shield
75%−epiboly
1−4−somites
14−19−somites
20−25−somites
Prim−5
Prim−15
Prim−25
Long−pec
Protruding−mouth
Day−4
Day−5
Transcript
ENSDART00000157849
ENSDART00000174682
ENSDART00000176816
ENSDART00000177092

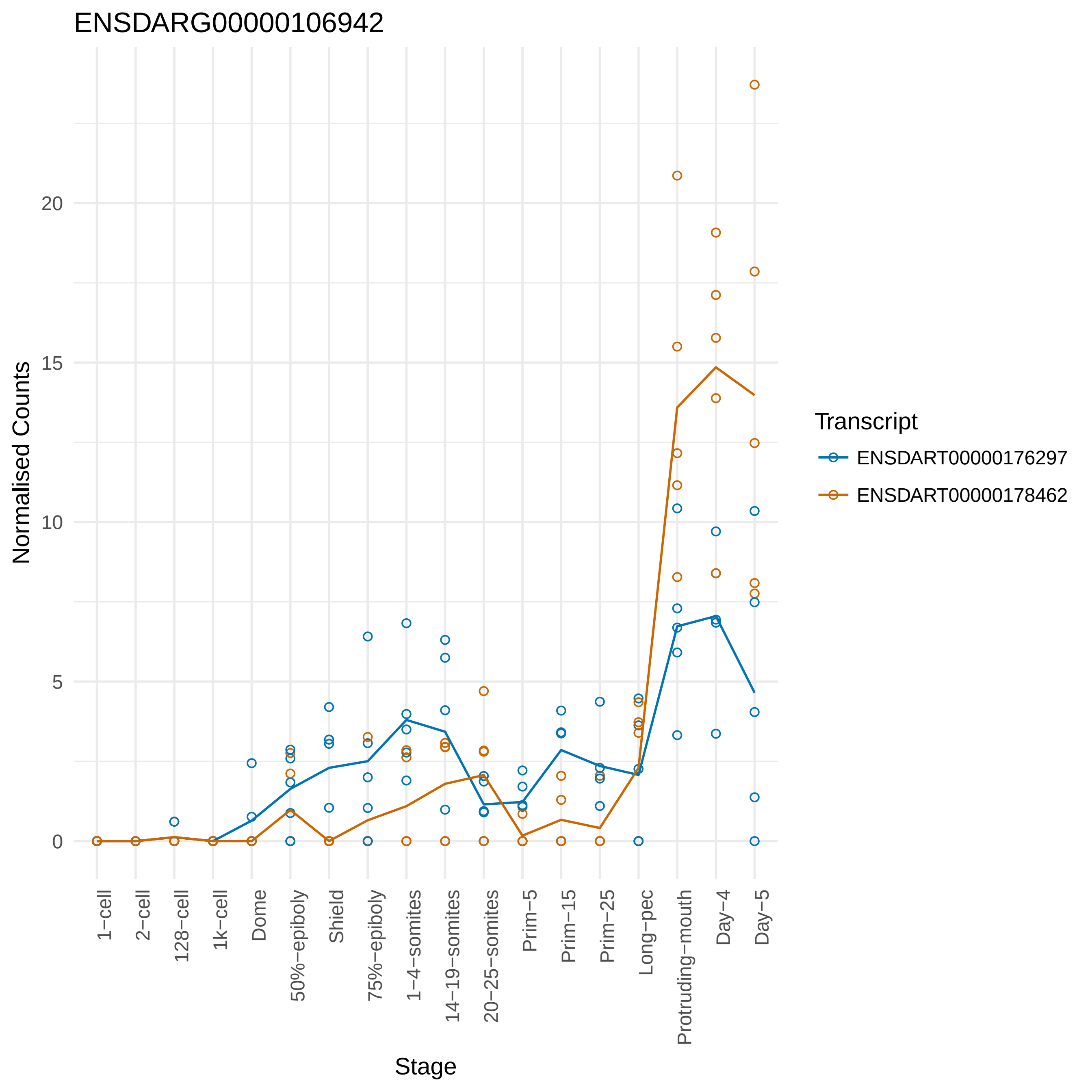

ENSDARG00000106942
Normalised Counts
0
5
10
15
20
Stage
1−cell
2−cell
128−cell
1k−cell
Dome
50%−epiboly
Shield
75%−epiboly
1−4−somites
14−19−somites
20−25−somites
Prim−5
Prim−15
Prim−25
Long−pec
Protruding−mouth
Day−4
Day−5
Transcript
ENSDART00000176297
ENSDART00000178462

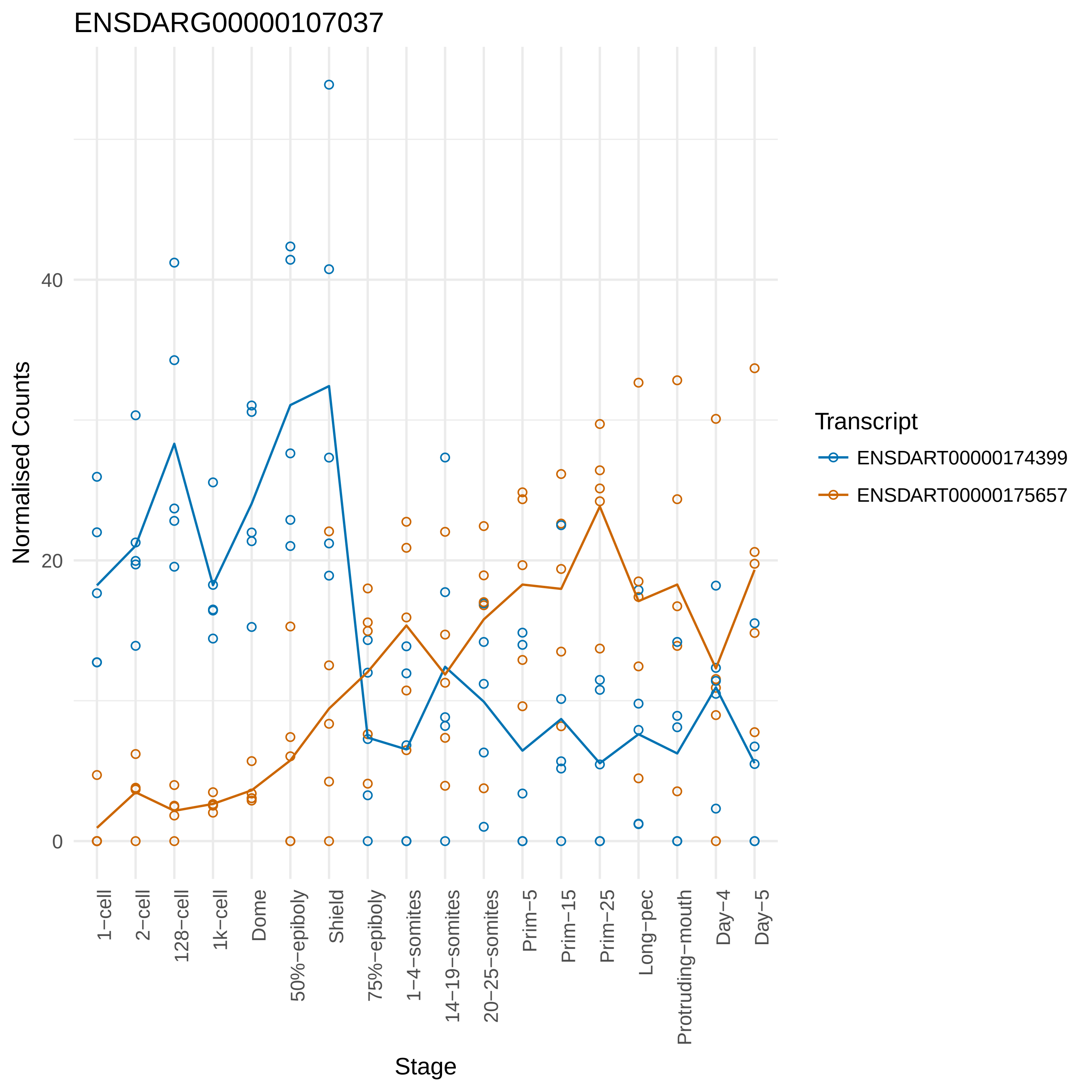
ENSDARG00000107037
Normalised Counts
0
20
40
1−cell
2−cell
128−cell
1k−cell
Dome
50%−epiboly
Shield
75%−epiboly
1−4−somites
14−19−somites
20−25−somites
Prim−5
Prim−15
Prim−25
Long−pec
Protruding−mouth
Day−4
Day−5
Stage
Transcript
ENSDART00000174399
ENSDART00000175657

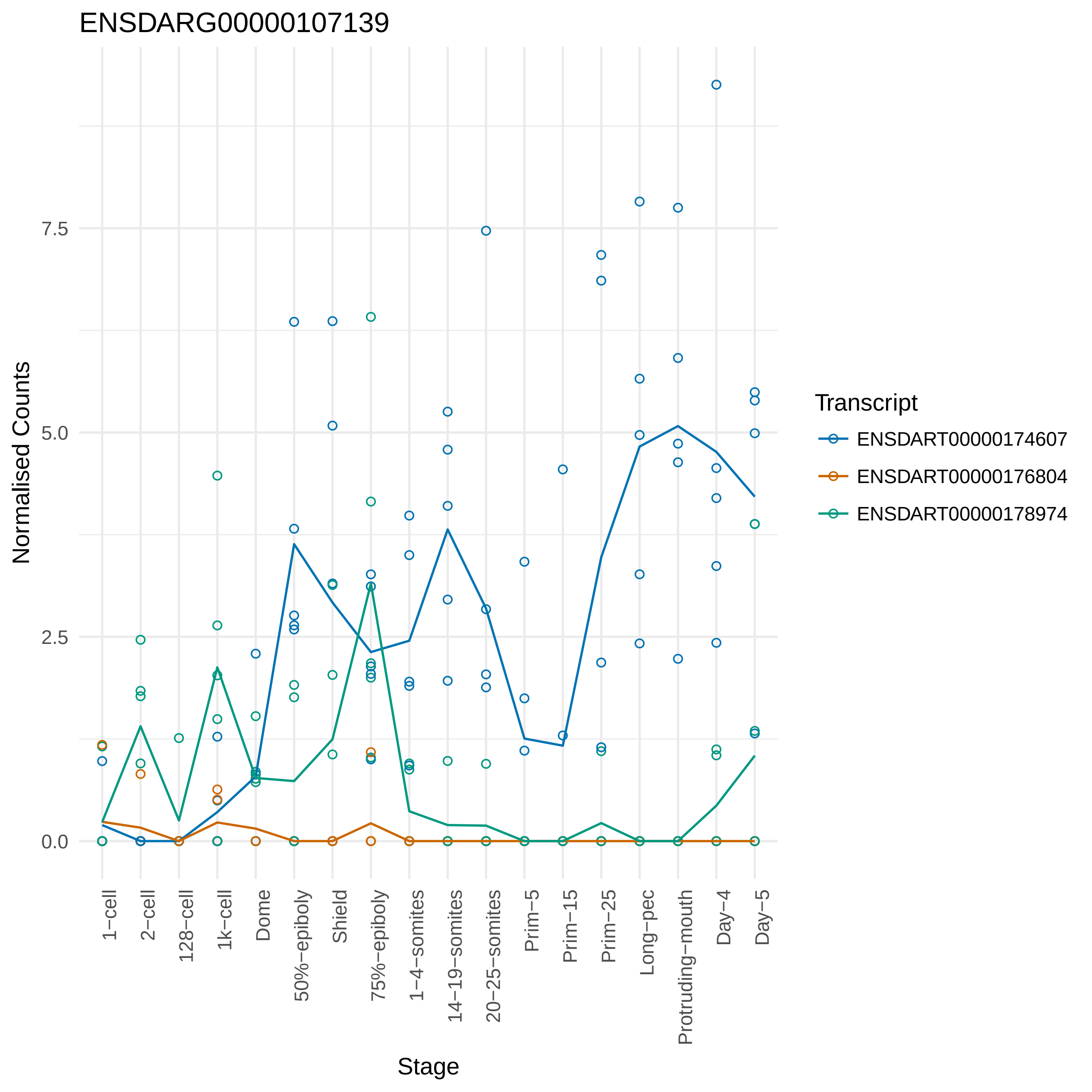

ENSDARG00000107139
Normalised Counts
0.0
2.5
5.0
7.5
Stage
1−cell
2−cell
128−cell
1k−cell
Dome
50%−epiboly
Shield
75%−epiboly
1−4−somites
14−19−somites
20−25−somites
Prim−5
Prim−15
Prim−25
Long−pec
Protruding−mouth
Day−4
Day−5
Transcript
ENSDART00000174607
ENSDART00000176804
ENSDART00000178974

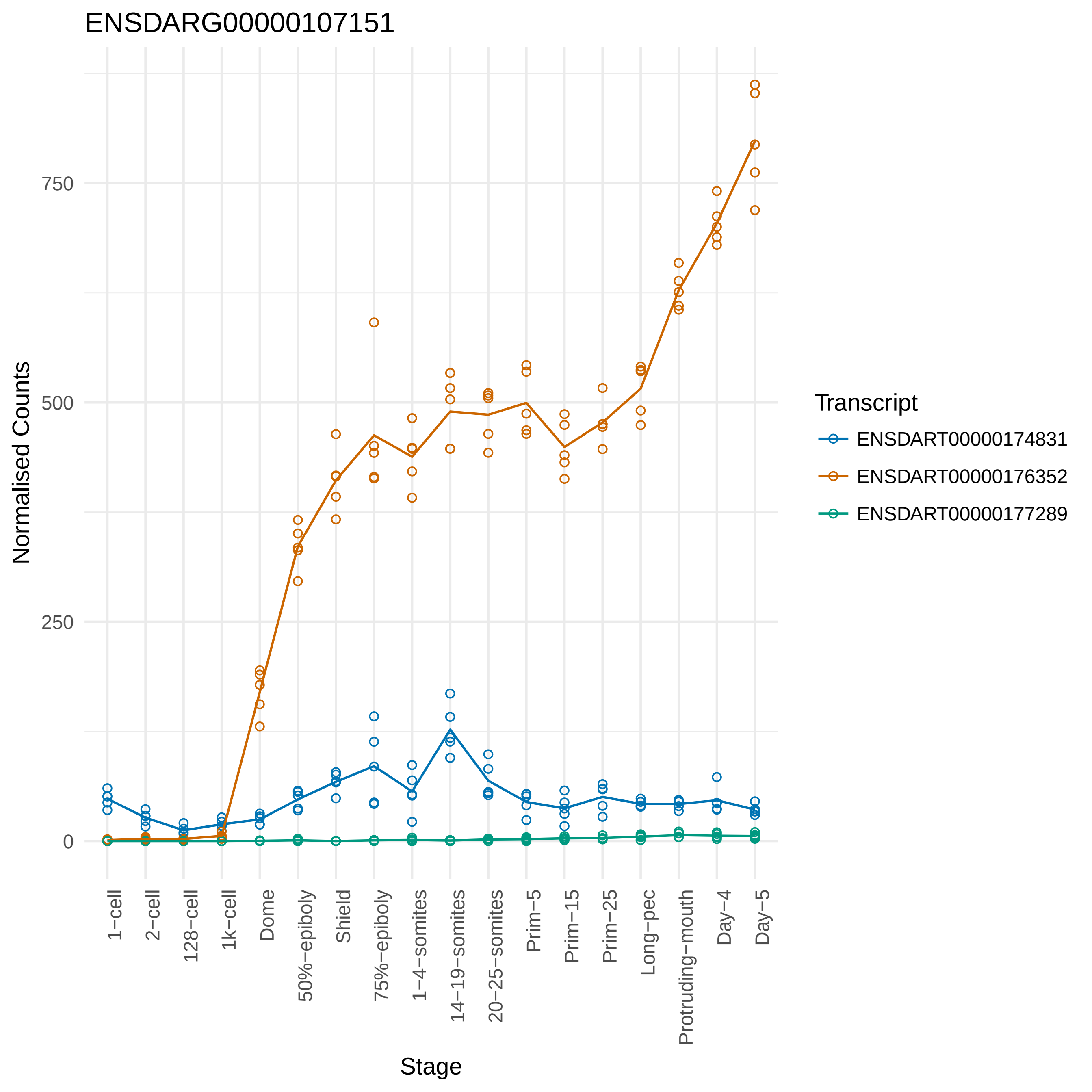
ENSDARG00000107151
Normalised Counts
750
500
250
0
1−cell
2−cell
128−cell
1k−cell
Dome
50%−epiboly
Shield
75%−epiboly
1−4−somites
14−19−somites
20−25−somites
Prim−5
Prim−15
Prim−25
Long−pec
Protruding−mouth
Day−4
Day−5
Stage
Transcript
ENSDART00000174831
ENSDART00000176352
ENSDART00000177289

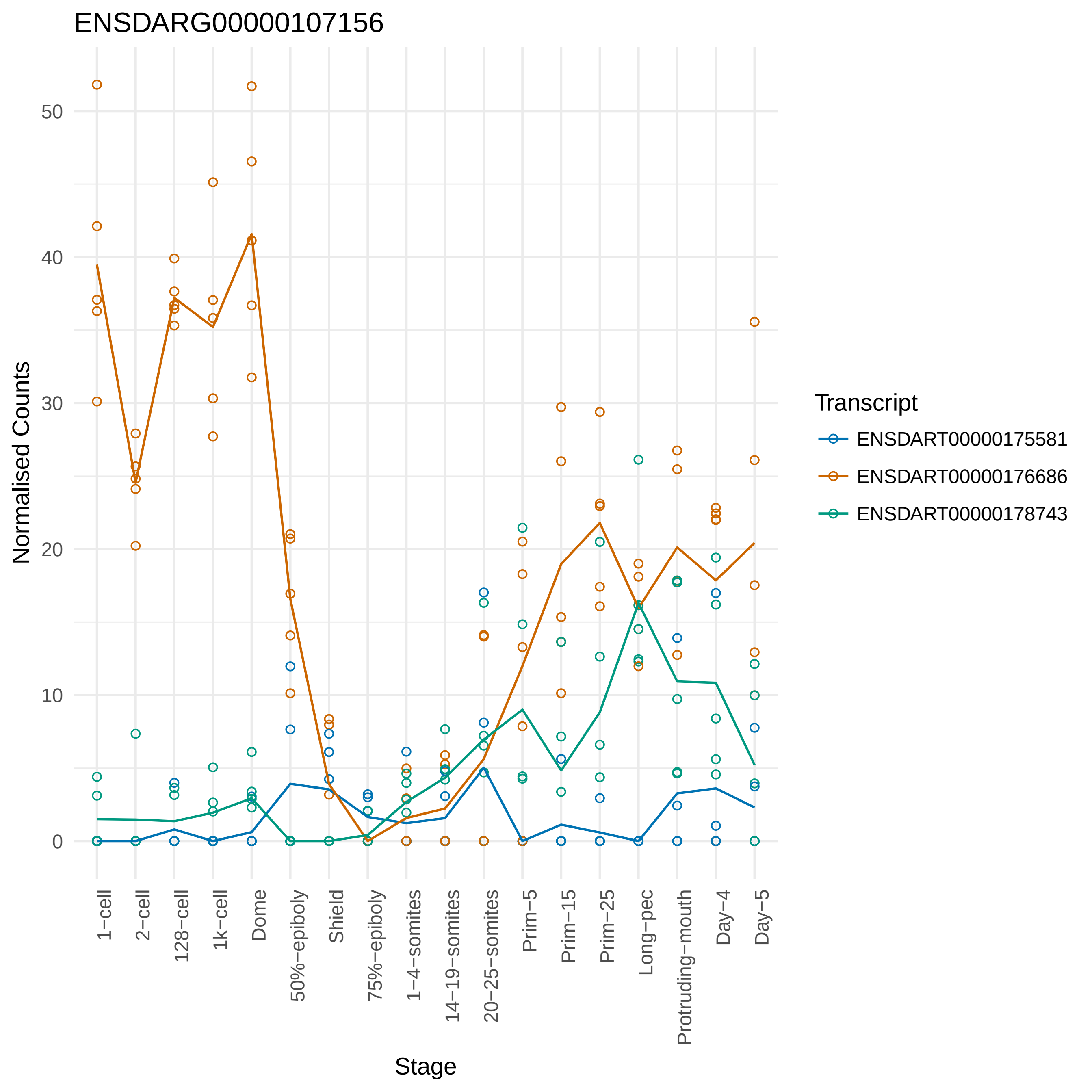

ENSDARG00000107156
Normalised Counts
0
10
20
30
40
50
1−cell
2−cell
128−cell
1k−cell
Dome
50%−epiboly
Shield
75%−epiboly
1−4−somites
14−19−somites
20−25−somites
Prim−5
Prim−15
Prim−25
Long−pec
Protruding−mouth
Day−4
Day−5
Stage
Transcript
ENSDART00000175581
ENSDART00000176686
ENSDART00000178743

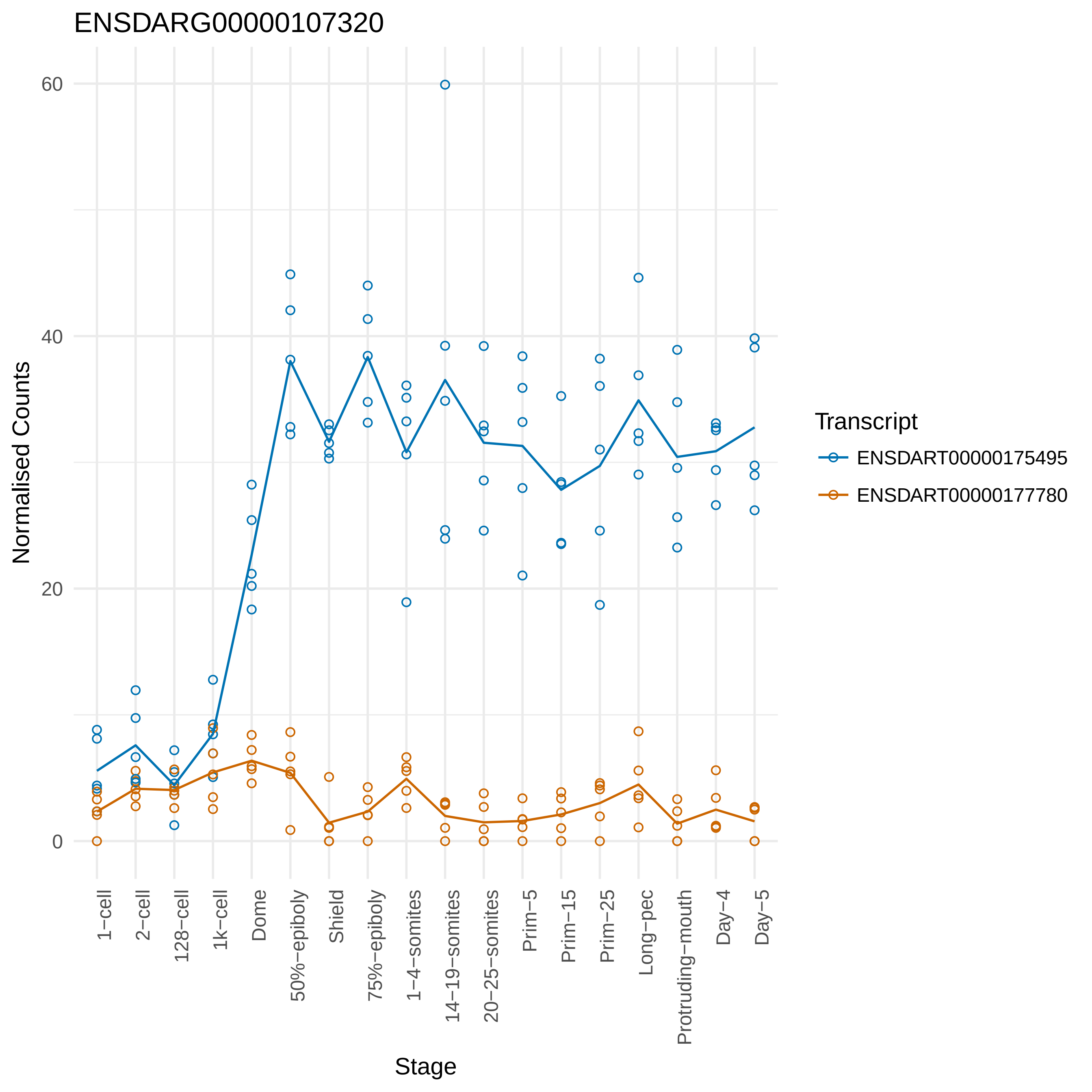

ENSDARG00000107320
Normalised Counts
60
40
20
0
1−cell
2−cell
128−cell
1k−cell
Dome
50%−epiboly
Shield
75%−epiboly
1−4−somites
14−19−somites
20−25−somites
Prim−5
Prim−15
Prim−25
Long−pec
Protruding−mouth
Day−4
Day−5
Stage
Transcript
ENSDART00000175495
ENSDART00000177780

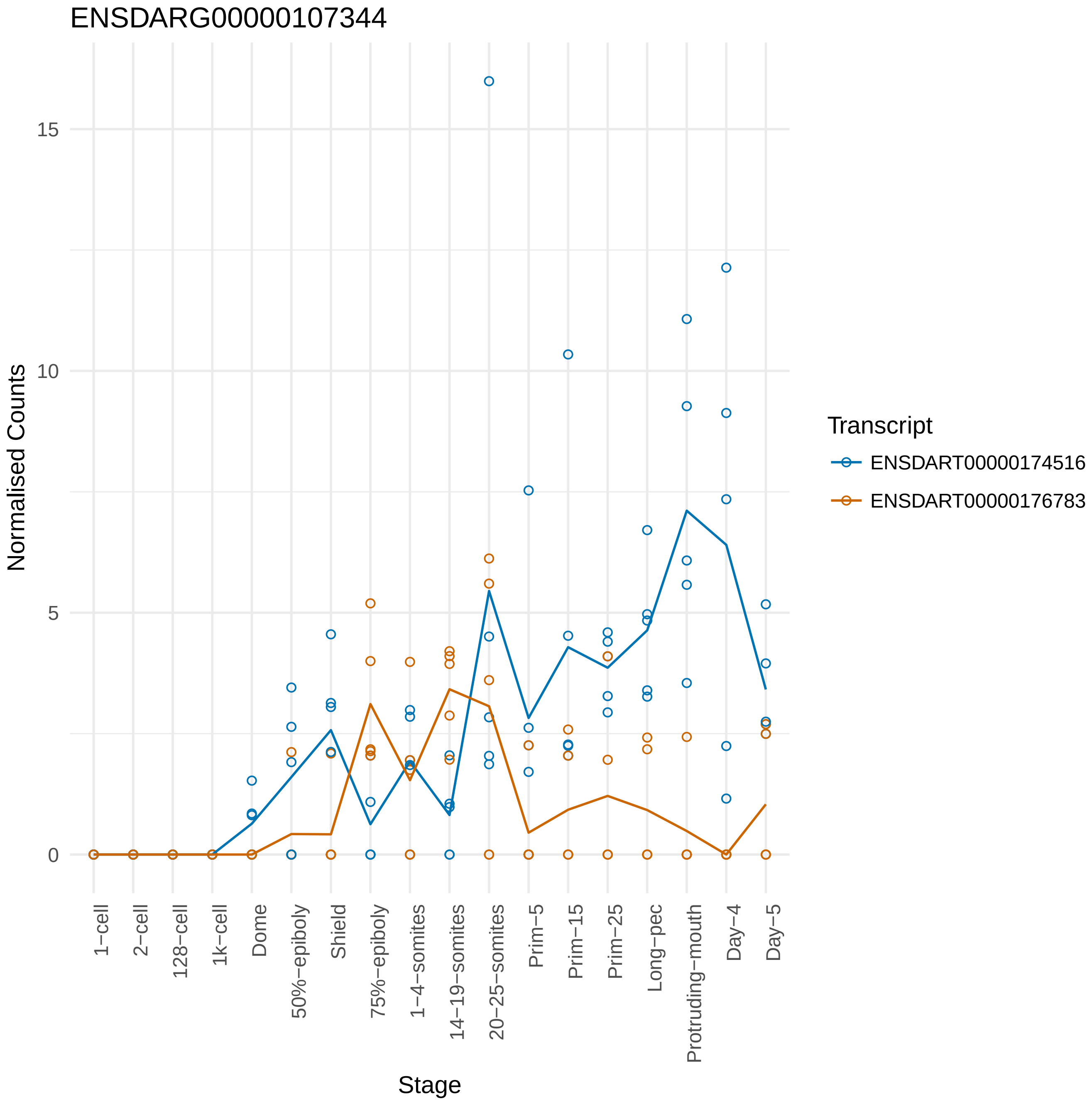

ENSDARG00000107344
Normalised Counts
0
5
10
15
Stage
1−cell
2−cell
128−cell
1k−cell
Dome
50%−epiboly
Shield
75%−epiboly
1−4−somites
14−19−somites
20−25−somites
Prim−5
Prim−15
Prim−25
Long−pec
Protruding−mouth
Day−4
Day−5
Transcript
ENSDART00000174516
ENSDART00000176783

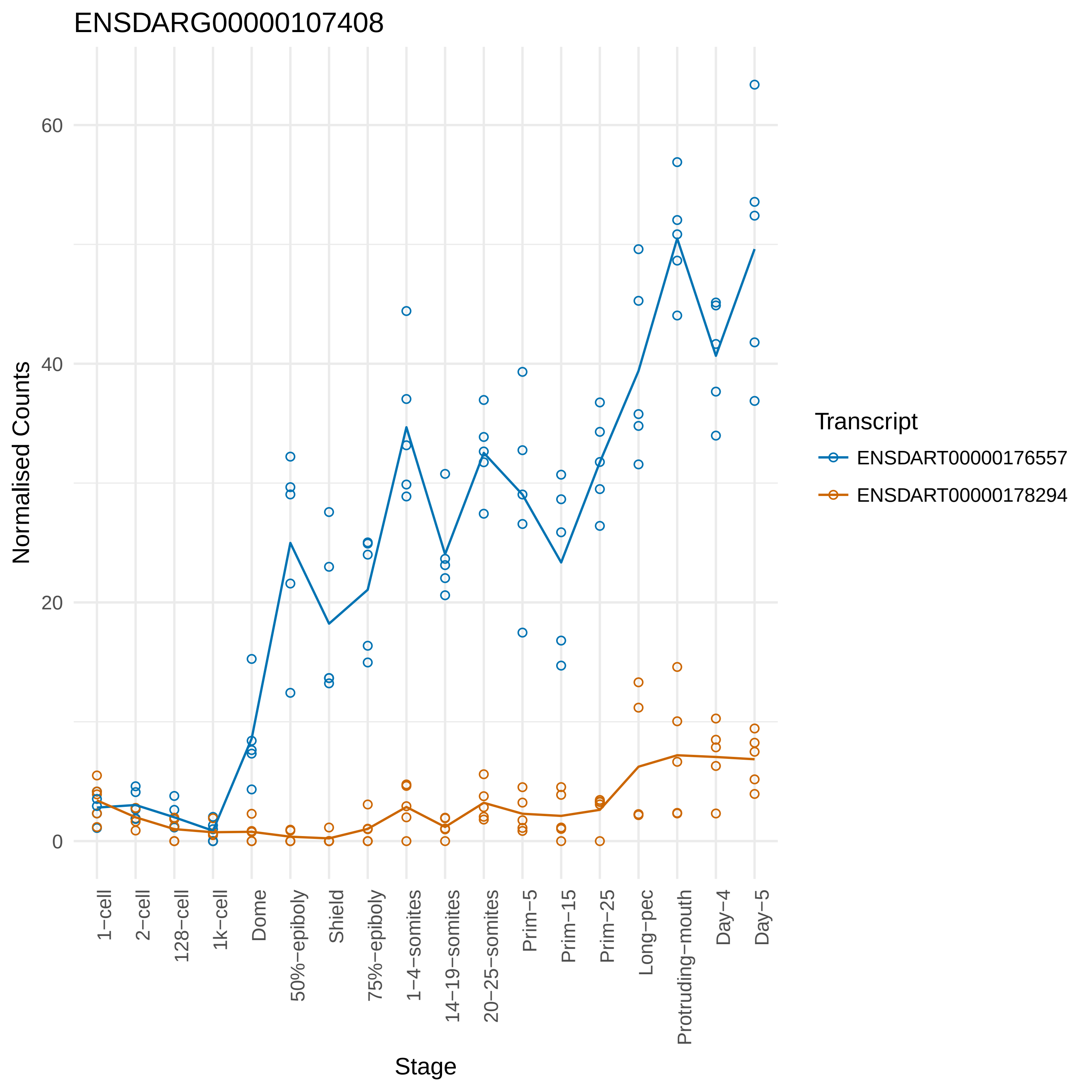

ENSDARG00000107408
Normalised Counts
60
40
20
0
1−cell
2−cell
128−cell
1k−cell
Dome
50%−epiboly
Shield
75%−epiboly
1−4−somites
14−19−somites
20−25−somites
Prim−5
Prim−15
Prim−25
Long−pec
Protruding−mouth
Day−4
Day−5
Stage
Transcript
ENSDART00000176557
ENSDART00000178294

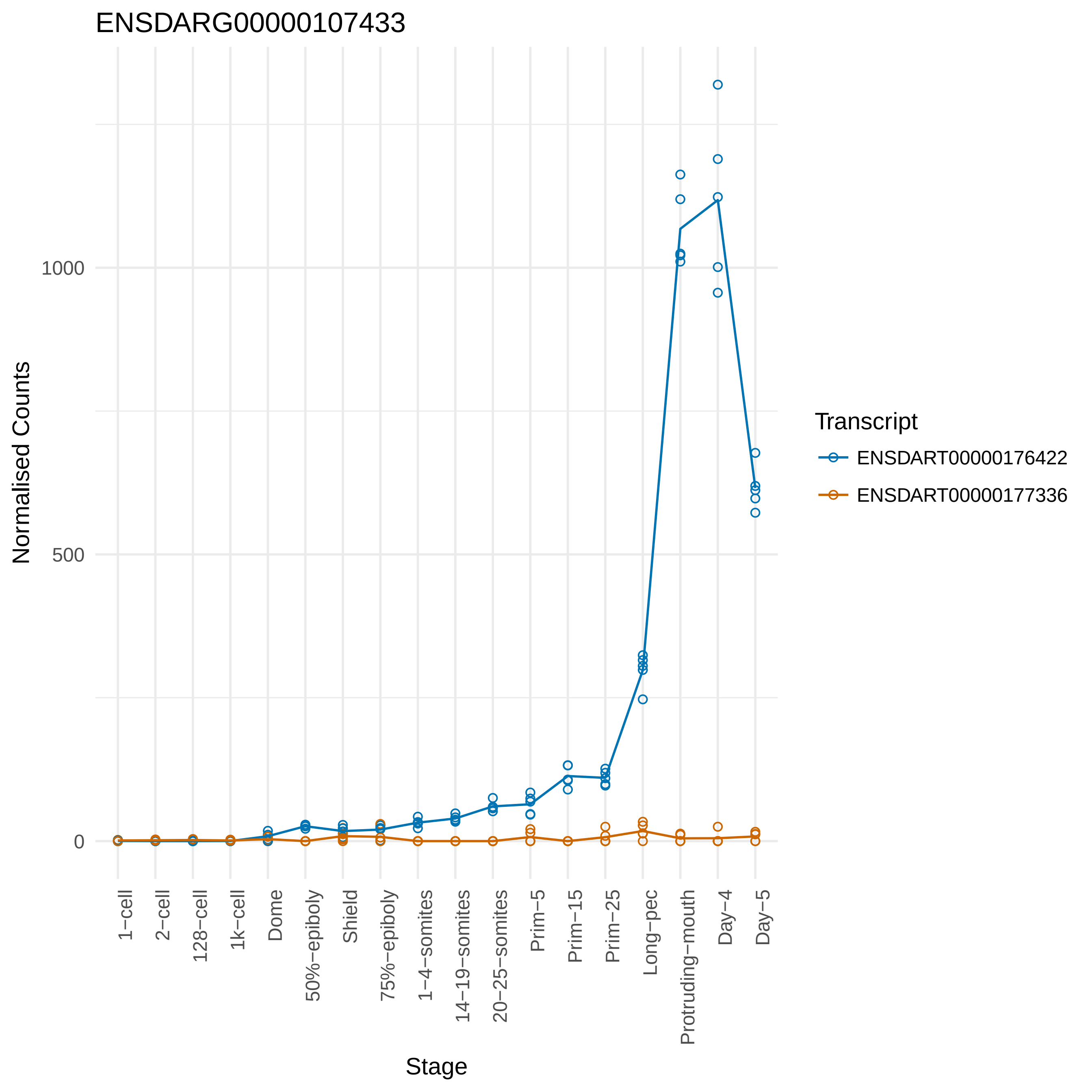
ENSDARG00000107433
Normalised Counts
1000
500
0
Stage
1−cell
2−cell
128−cell
1k−cell
Dome
50%−epiboly
Shield
75%−epiboly
1−4−somites
14−19−somites
20−25−somites
Prim−5
Prim−15
Prim−25
Long−pec
Protruding−mouth
Day−4
Day−5
Transcript
ENSDART00000176422
ENSDART00000177336

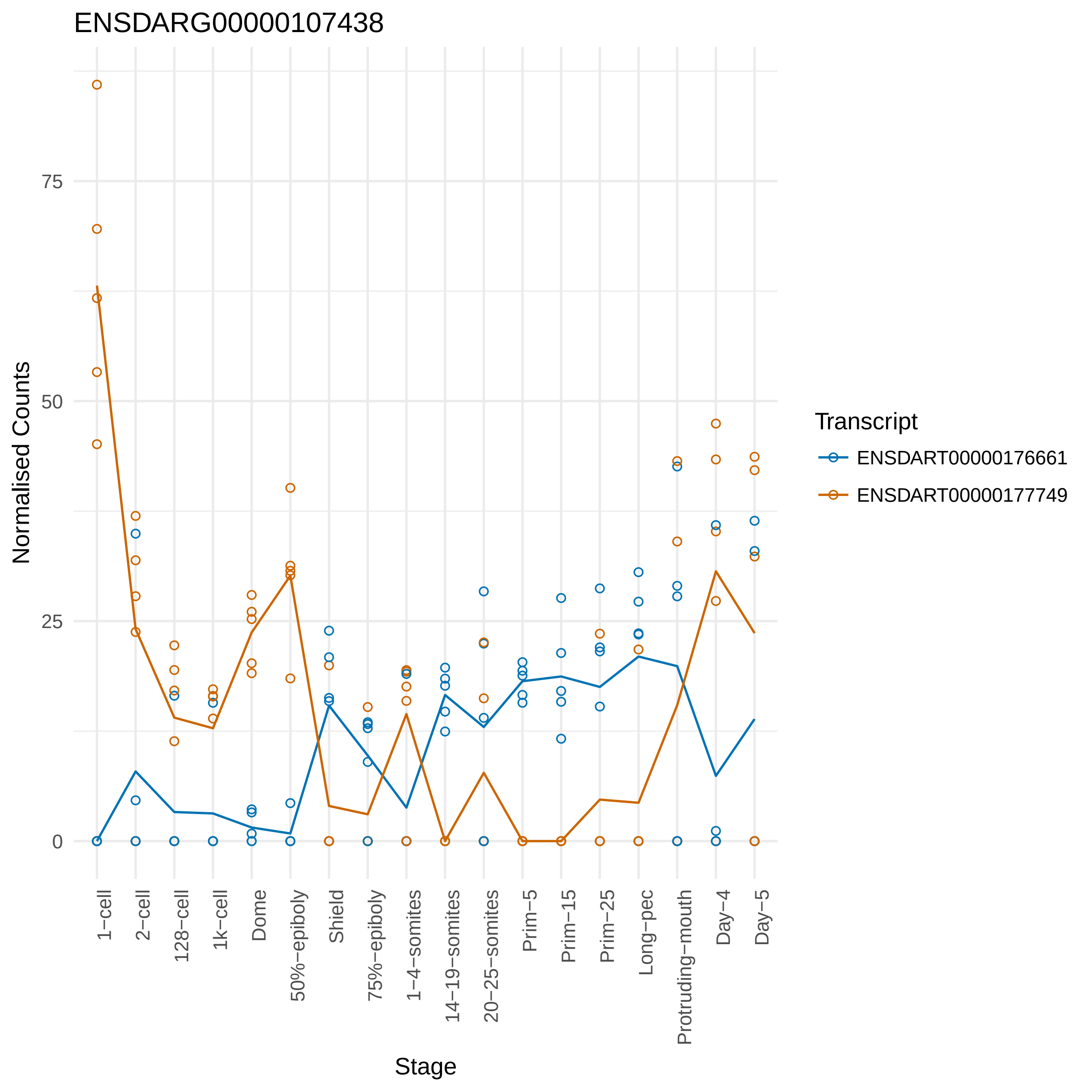
ENSDARG00000107438
Normalised Counts
0
25
50
75
1−cell
2−cell
128−cell
1k−cell
Dome
50%−epiboly
Shield
75%−epiboly
1−4−somites
14−19−somites
20−25−somites
Prim−5
Prim−15
Prim−25
Long−pec
Protruding−mouth
Day−4
Day−5
Stage
Transcript
ENSDART00000176661
ENSDART00000177749

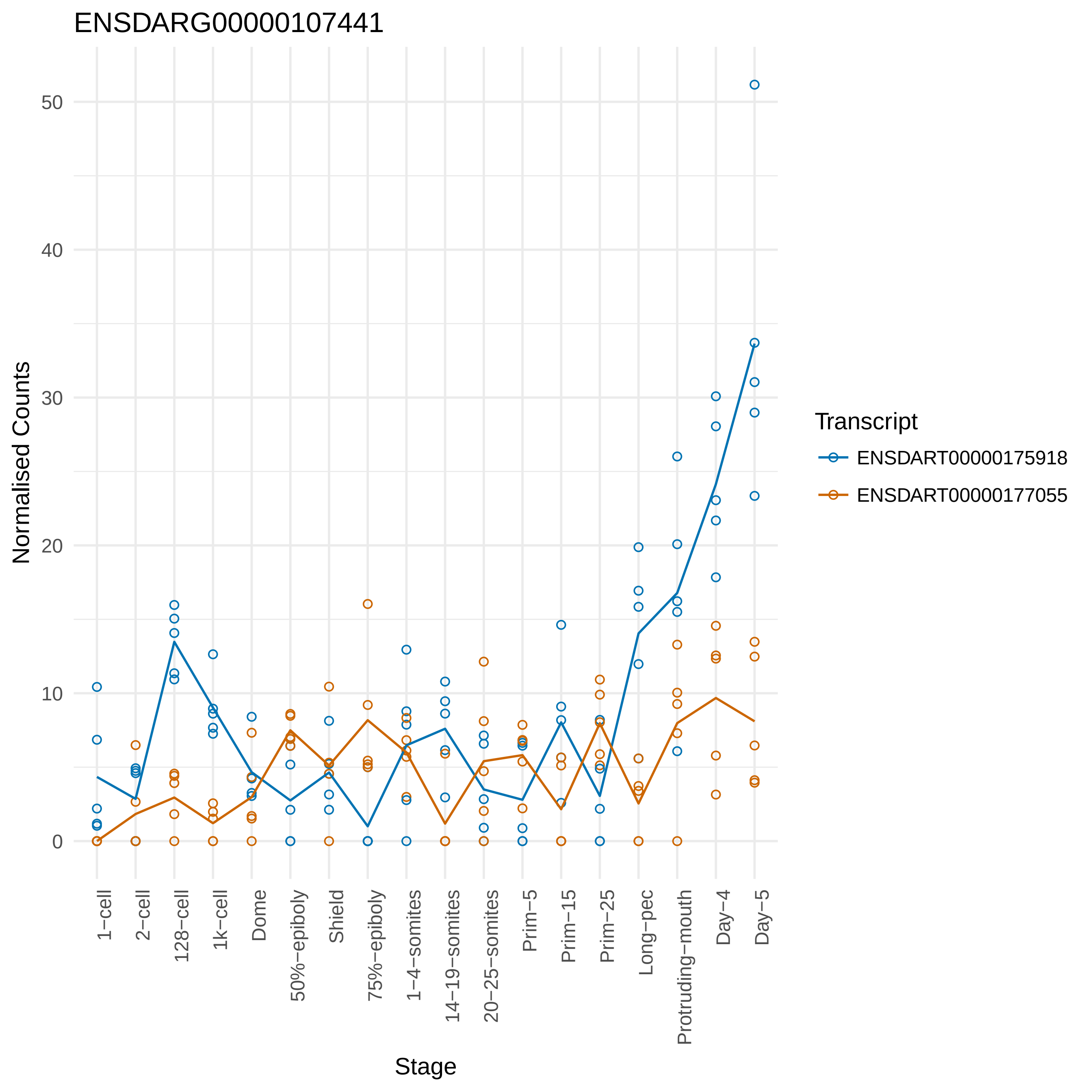

ENSDARG00000107441
Normalised Counts
0
10
20
30
40
50
1−cell
2−cell
128−cell
1k−cell
Dome
50%−epiboly
Shield
75%−epiboly
1−4−somites
14−19−somites
20−25−somites
Prim−5
Prim−15
Prim−25
Long−pec
Protruding−mouth
Day−4
Day−5
Stage
Transcript
ENSDART00000175918
ENSDART00000177055

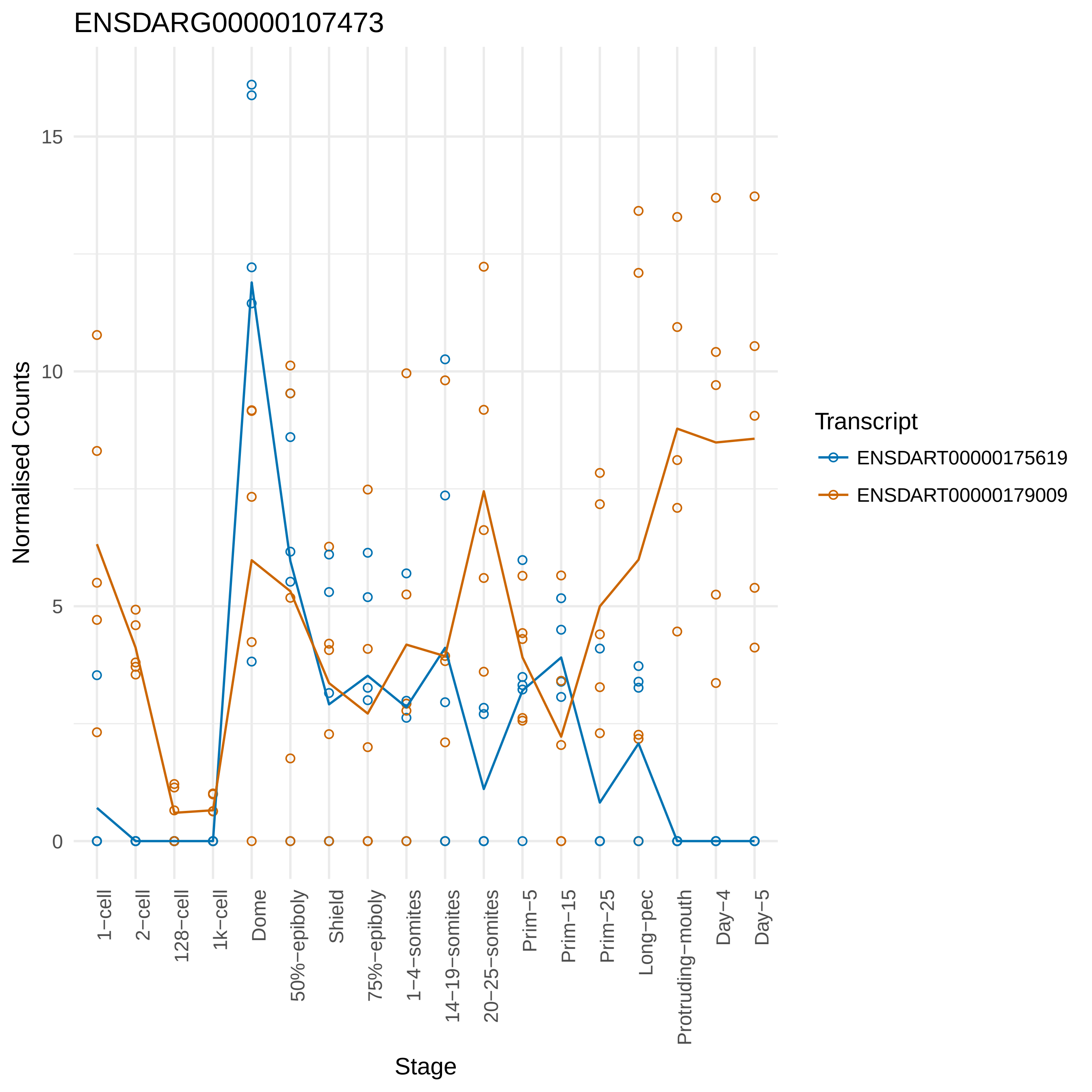

ENSDARG00000107473
Normalised Counts
15
10
5
0
1−cell
2−cell
128−cell
1k−cell
Dome
50%−epiboly
Shield
75%−epiboly
1−4−somites
14−19−somites
20−25−somites
Prim−5
Prim−15
Prim−25
Long−pec
Protruding−mouth
Day−4
Day−5
Stage
Transcript
ENSDART00000175619
ENSDART00000179009

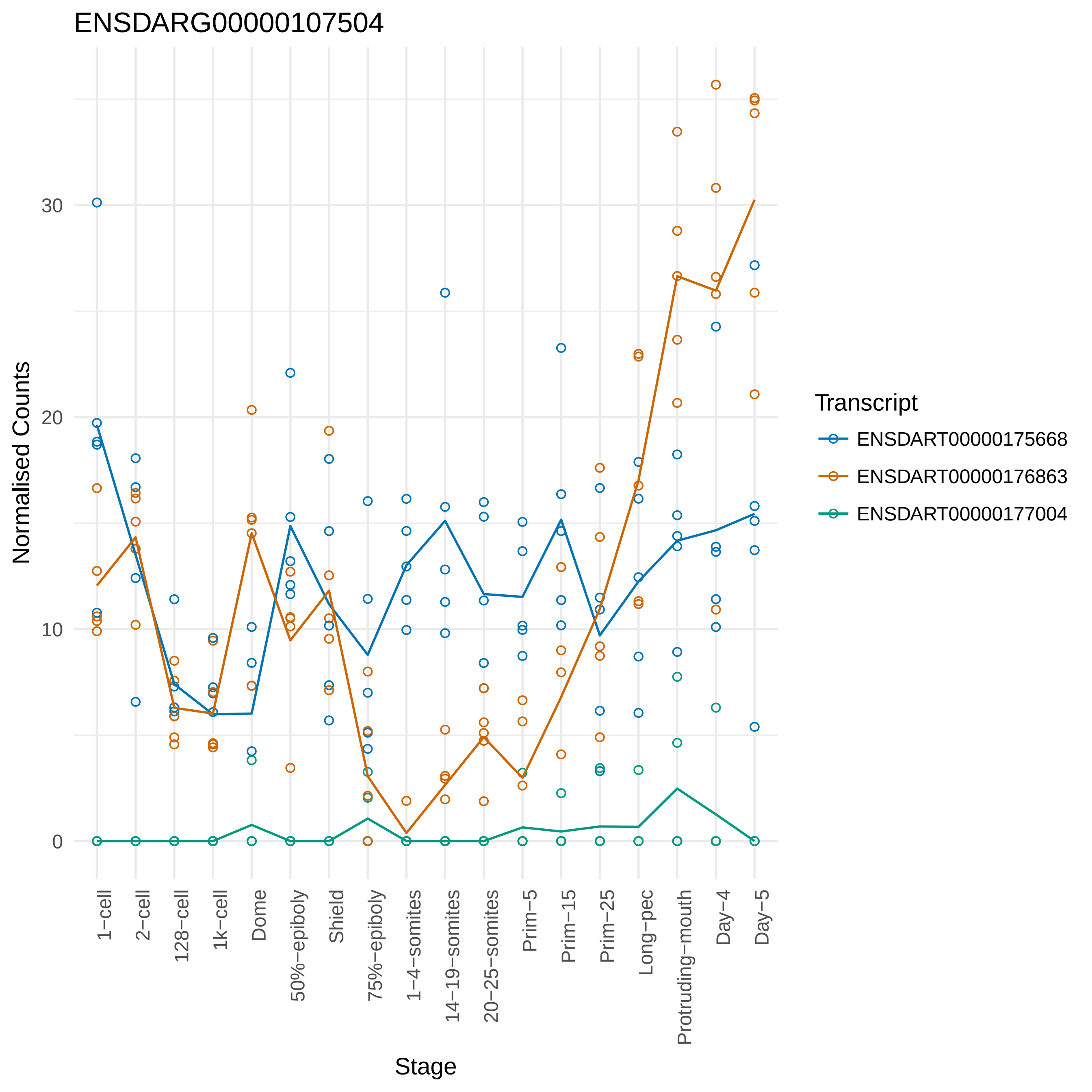
ENSDARG00000107504
Normalised Counts
0
10
20
30
1−cell
2−cell
128−cell
1k−cell
Dome
50%−epiboly
Shield
75%−epiboly
1−4−somites
14−19−somites
20−25−somites
Prim−5
Prim−15
Prim−25
Long−pec
Protruding−mouth
Day−4
Day−5
Stage
Transcript
ENSDART00000175668
ENSDART00000176863
ENSDART00000177004

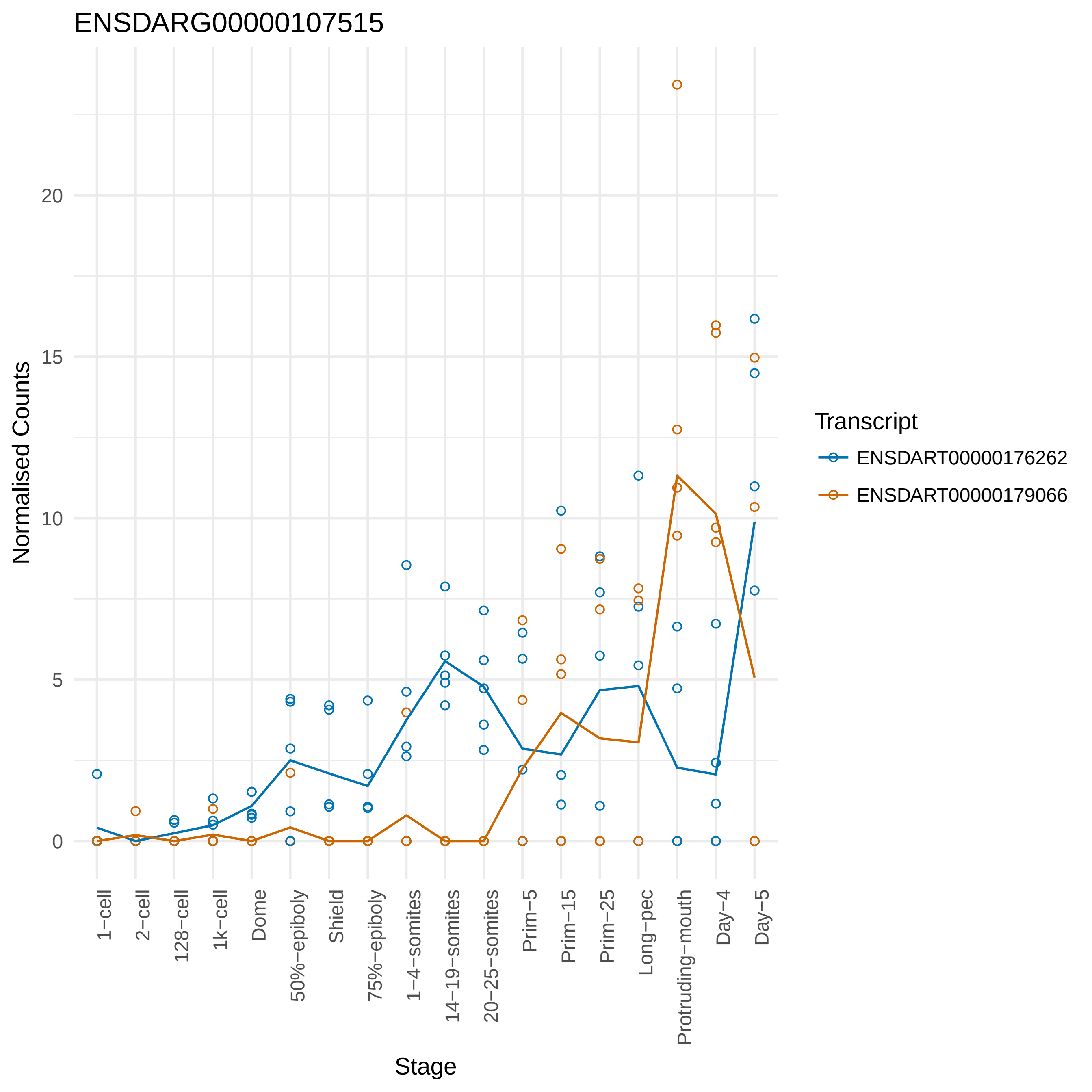
ENSDARG00000107515
Normalised Counts
0
5
10
15
20
Stage
1−cell
2−cell
128−cell
1k−cell
Dome
50%−epiboly
Shield
75%−epiboly
1−4−somites
14−19−somites
20−25−somites
Prim−5
Prim−15
Prim−25
Long−pec
Protruding−mouth
Day−4
Day−5
Transcript
ENSDART00000176262
ENSDART00000179066

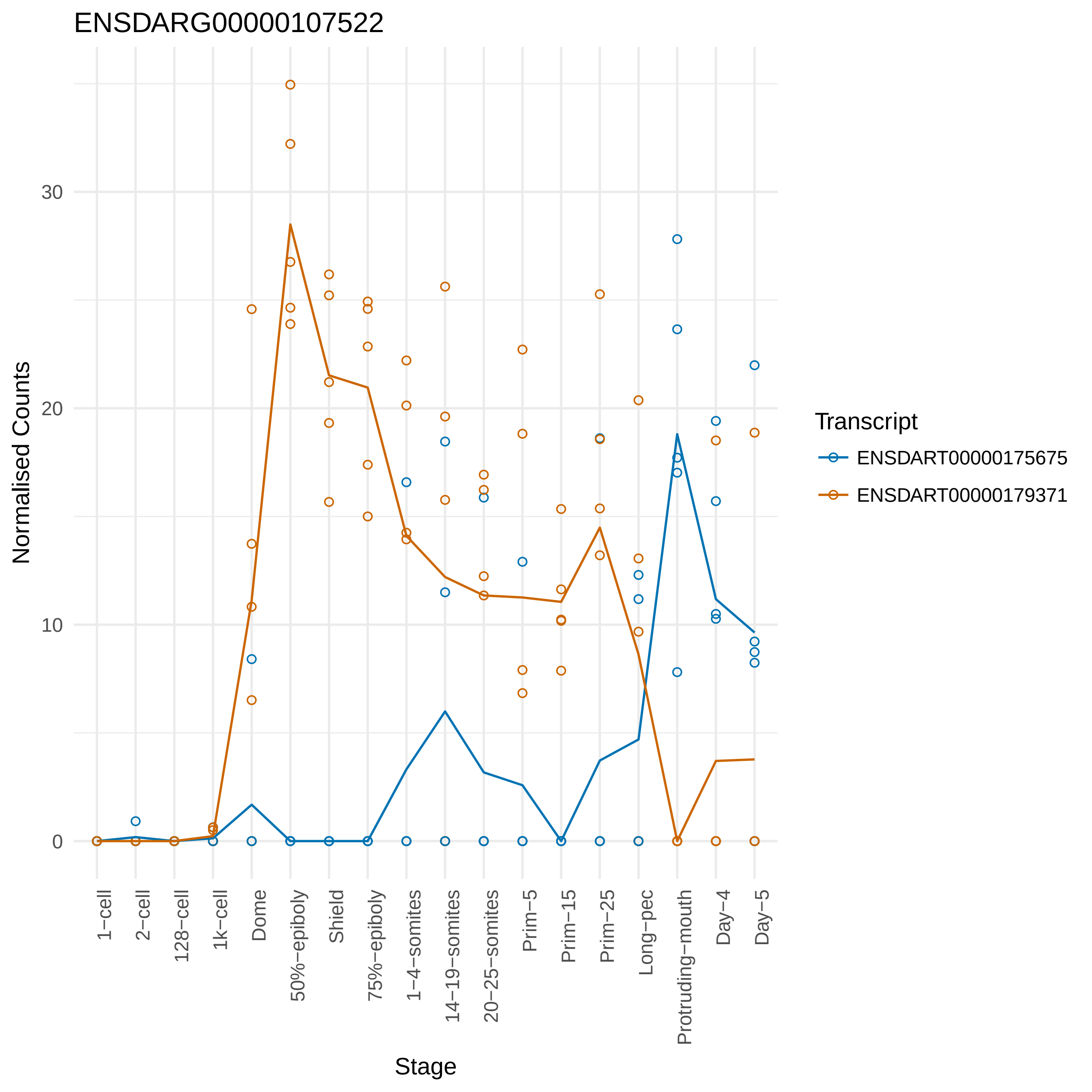

ENSDARG00000107522
Normalised Counts
0
10
20
30
1−cell
2−cell
128−cell
1k−cell
Dome
50%−epiboly
Shield
75%−epiboly
1−4−somites
14−19−somites
20−25−somites
Prim−5
Prim−15
Prim−25
Long−pec
Protruding−mouth
Day−4
Day−5
Stage
Transcript
ENSDART00000175675
ENSDART00000179371

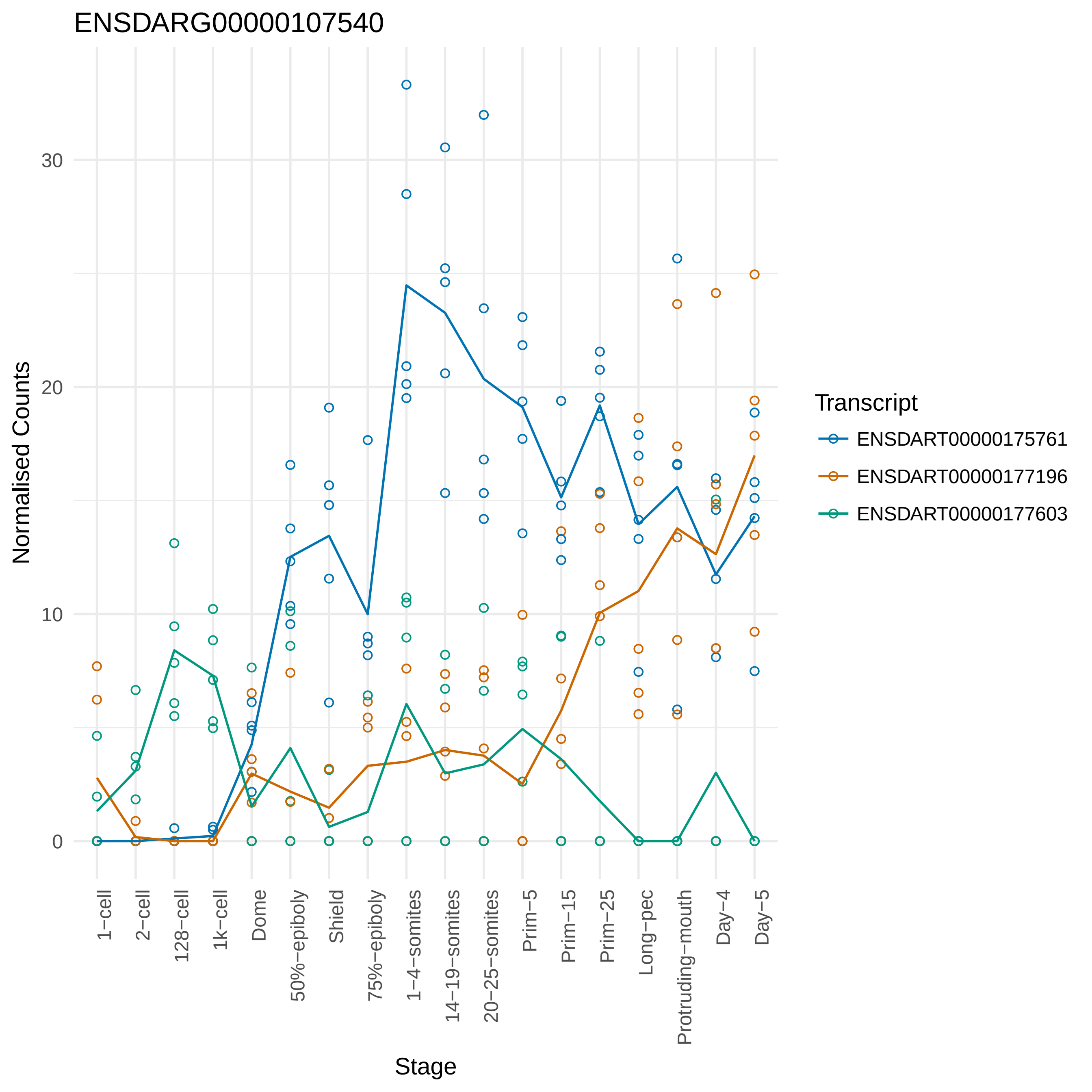

ENSDARG00000107540
Normalised Counts
0
10
20
30
1−cell
2−cell
128−cell
1k−cell
Dome
50%−epiboly
Shield
75%−epiboly
1−4−somites
14−19−somites
20−25−somites
Prim−5
Prim−15
Prim−25
Long−pec
Protruding−mouth
Day−4
Day−5
Stage
Transcript
ENSDART00000175761
ENSDART00000177196
ENSDART00000177603

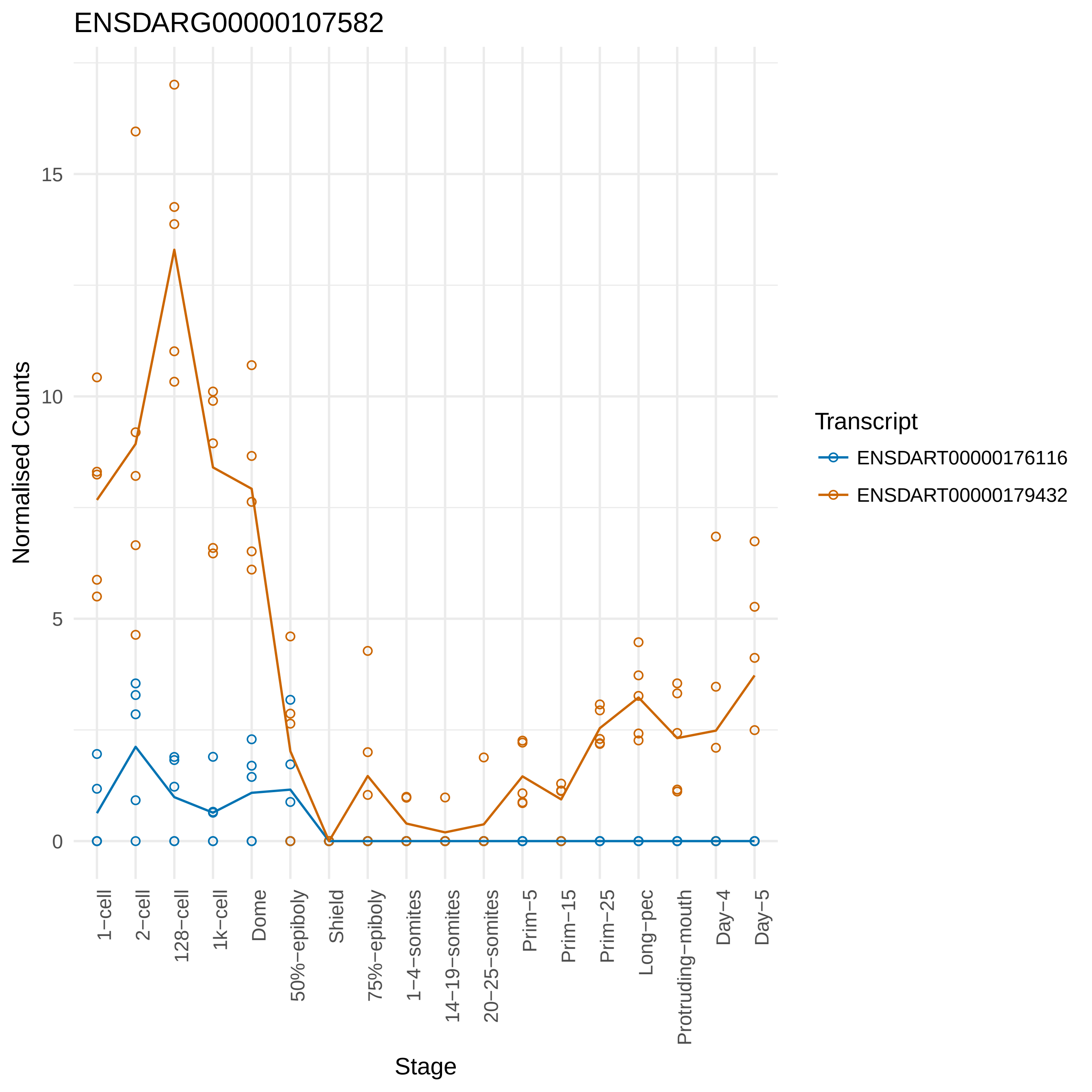

ENSDARG00000107582
Normalised Counts
15
10
5
0
1−cell
2−cell
128−cell
1k−cell
Dome
50%−epiboly
Shield
75%−epiboly
1−4−somites
14−19−somites
20−25−somites
Prim−5
Prim−15
Prim−25
Long−pec
Protruding−mouth
Day−4
Day−5
Stage
Transcript
ENSDART00000176116
ENSDART00000179432

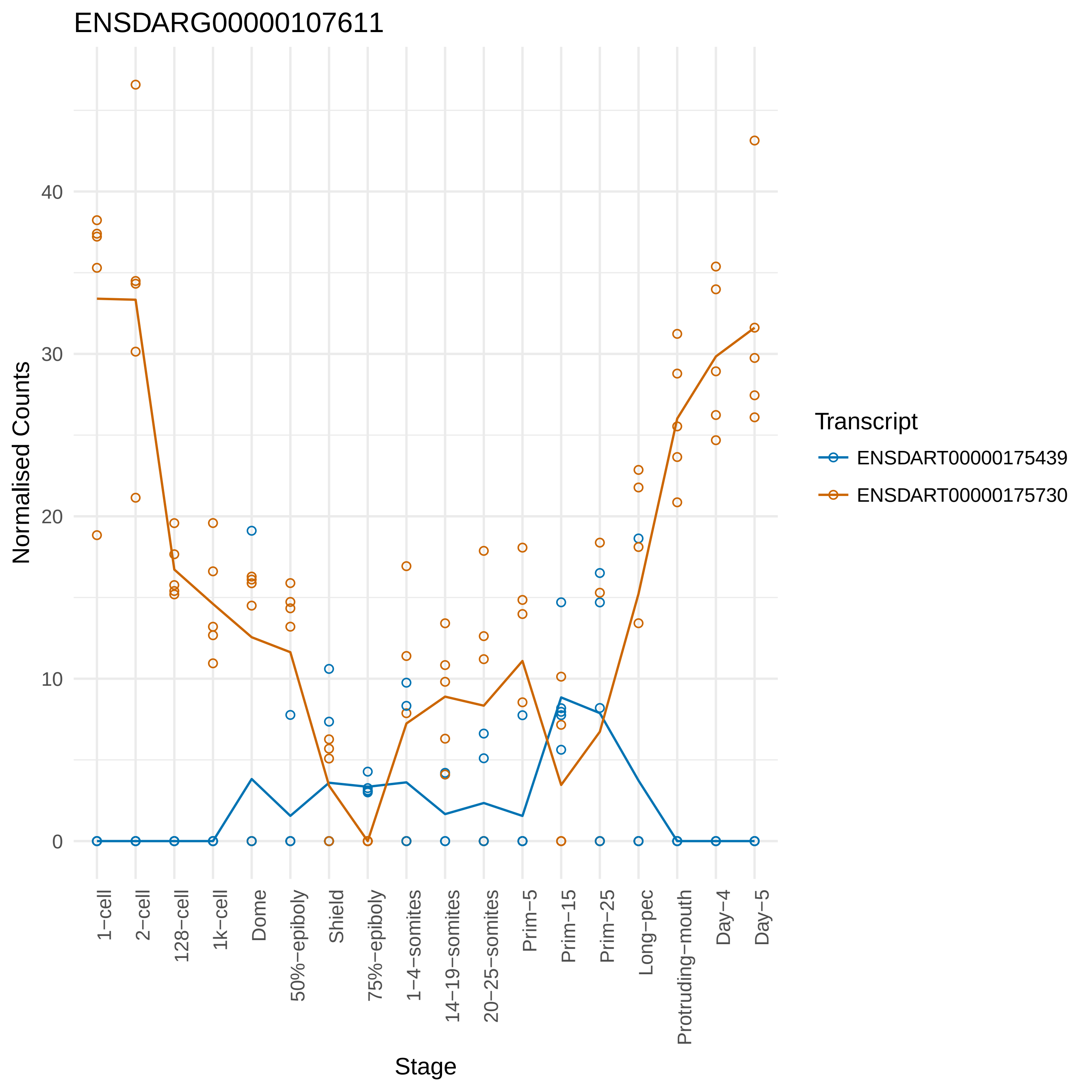

ENSDARG00000107611
Normalised Counts
0
10
20
30
40
1−cell
2−cell
128−cell
1k−cell
Dome
50%−epiboly
Shield
75%−epiboly
1−4−somites
14−19−somites
20−25−somites
Prim−5
Prim−15
Prim−25
Long−pec
Protruding−mouth
Day−4
Day−5
Stage
Transcript
ENSDART00000175439
ENSDART00000175730

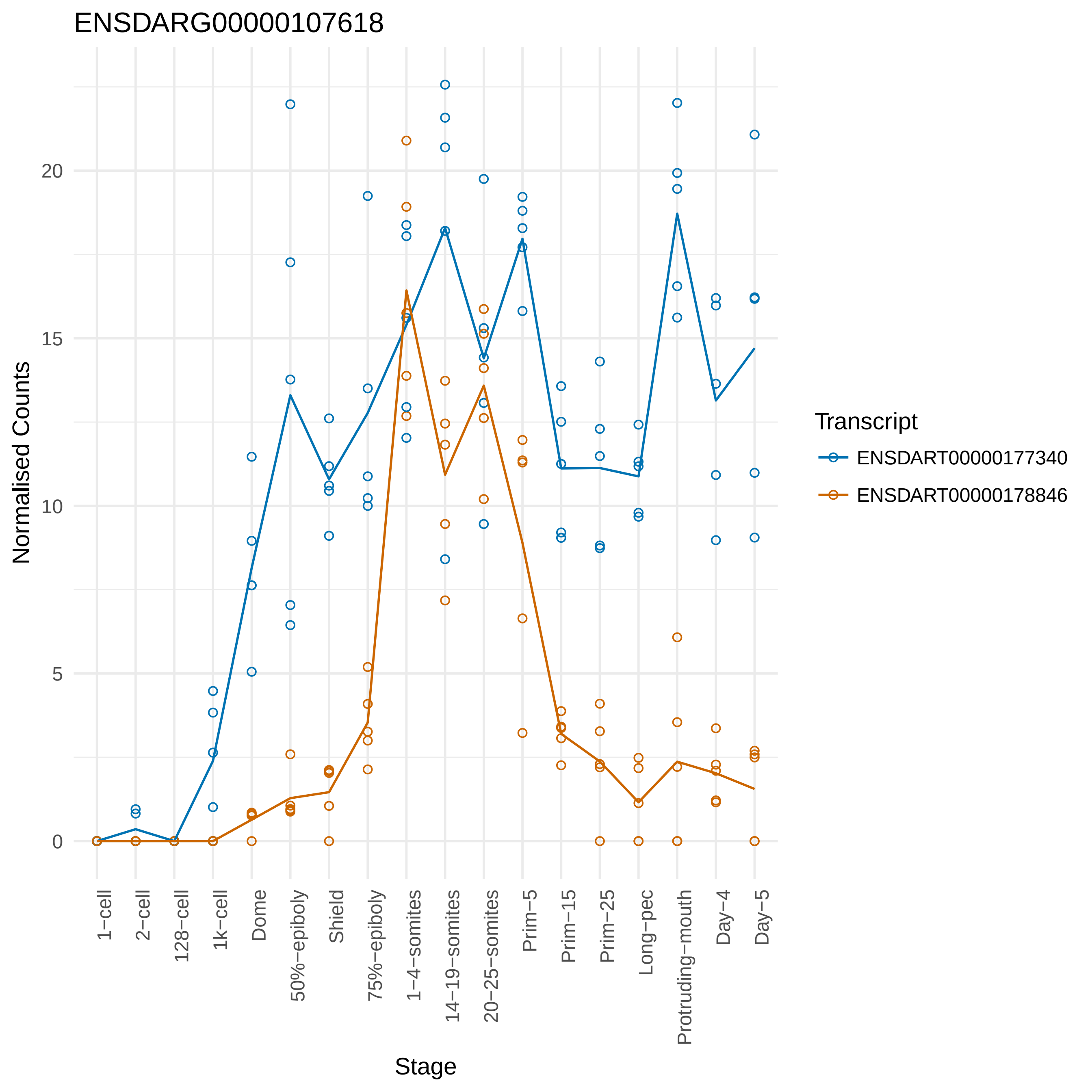
ENSDARG00000107618
Normalised Counts
0
5
10
15
20
Stage
1−cell
2−cell
128−cell
1k−cell
Dome
50%−epiboly
Shield
75%−epiboly
1−4−somites
14−19−somites
20−25−somites
Prim−5
Prim−15
Prim−25
Long−pec
Protruding−mouth
Day−4
Day−5
Transcript
ENSDART00000177340
ENSDART00000178846

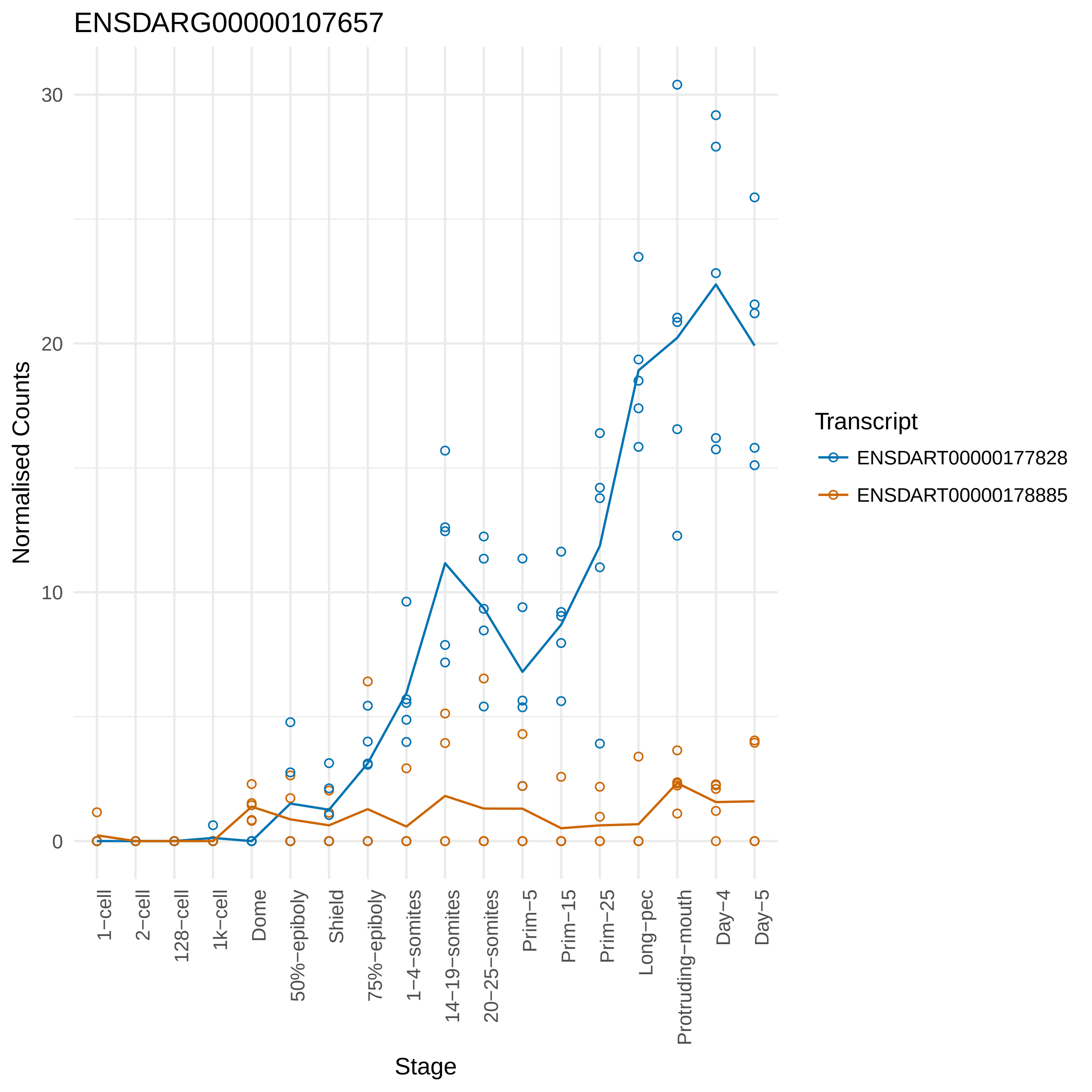

ENSDARG00000107657
Normalised Counts
30
20
10
0
1−cell
2−cell
128−cell
1k−cell
Dome
50%−epiboly
Shield
75%−epiboly
1−4−somites
14−19−somites
20−25−somites
Prim−5
Prim−15
Prim−25
Long−pec
Protruding−mouth
Day−4
Day−5
Stage
Transcript
ENSDART00000177828
ENSDART00000178885

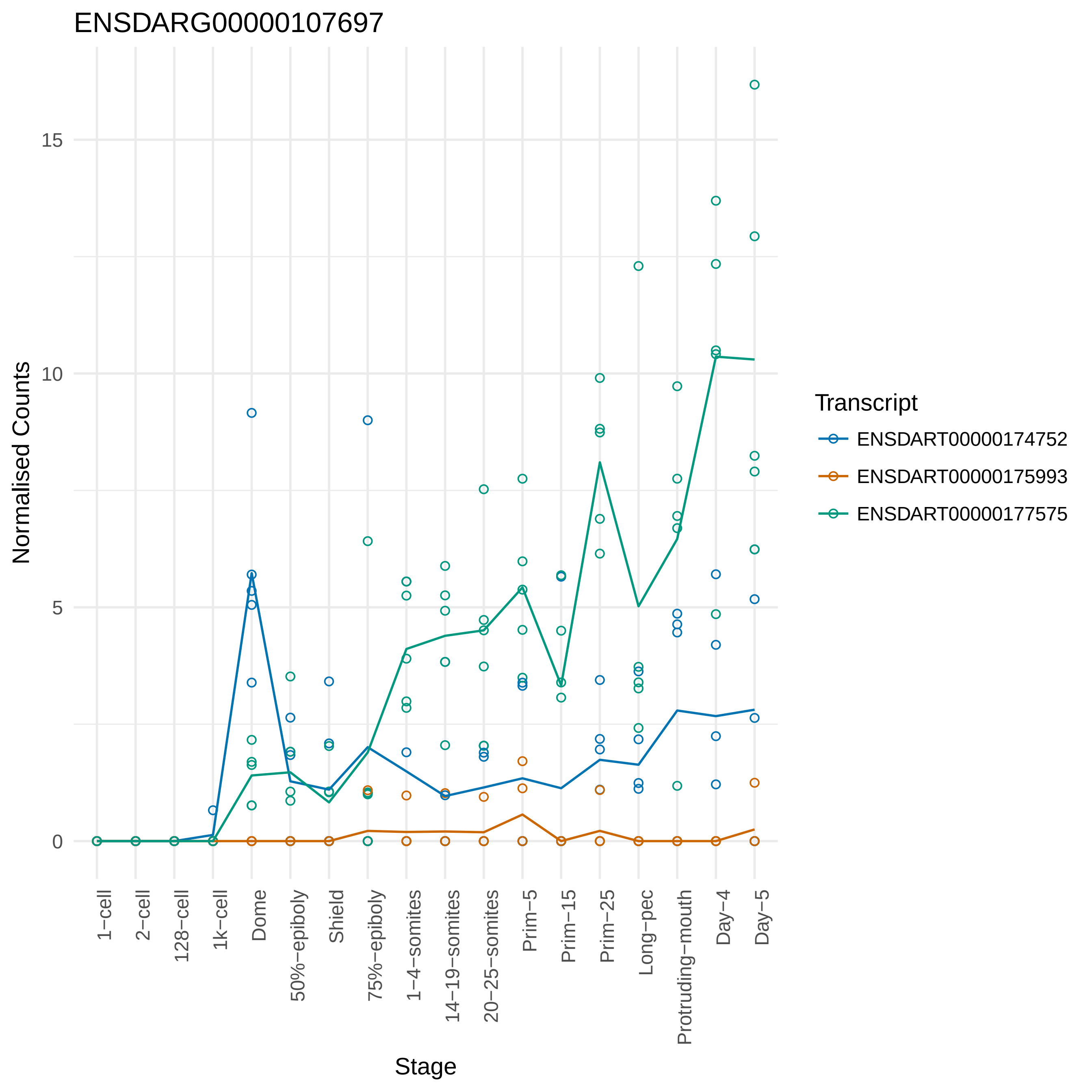
ENSDARG00000107697
Normalised Counts
0
5
10
15
Stage
1−cell
2−cell
128−cell
1k−cell
Dome
50%−epiboly
Shield
75%−epiboly
1−4−somites
14−19−somites
20−25−somites
Prim−5
Prim−15
Prim−25
Long−pec
Protruding−mouth
Day−4
Day−5
Transcript
ENSDART00000174752
ENSDART00000175993
ENSDART00000177575

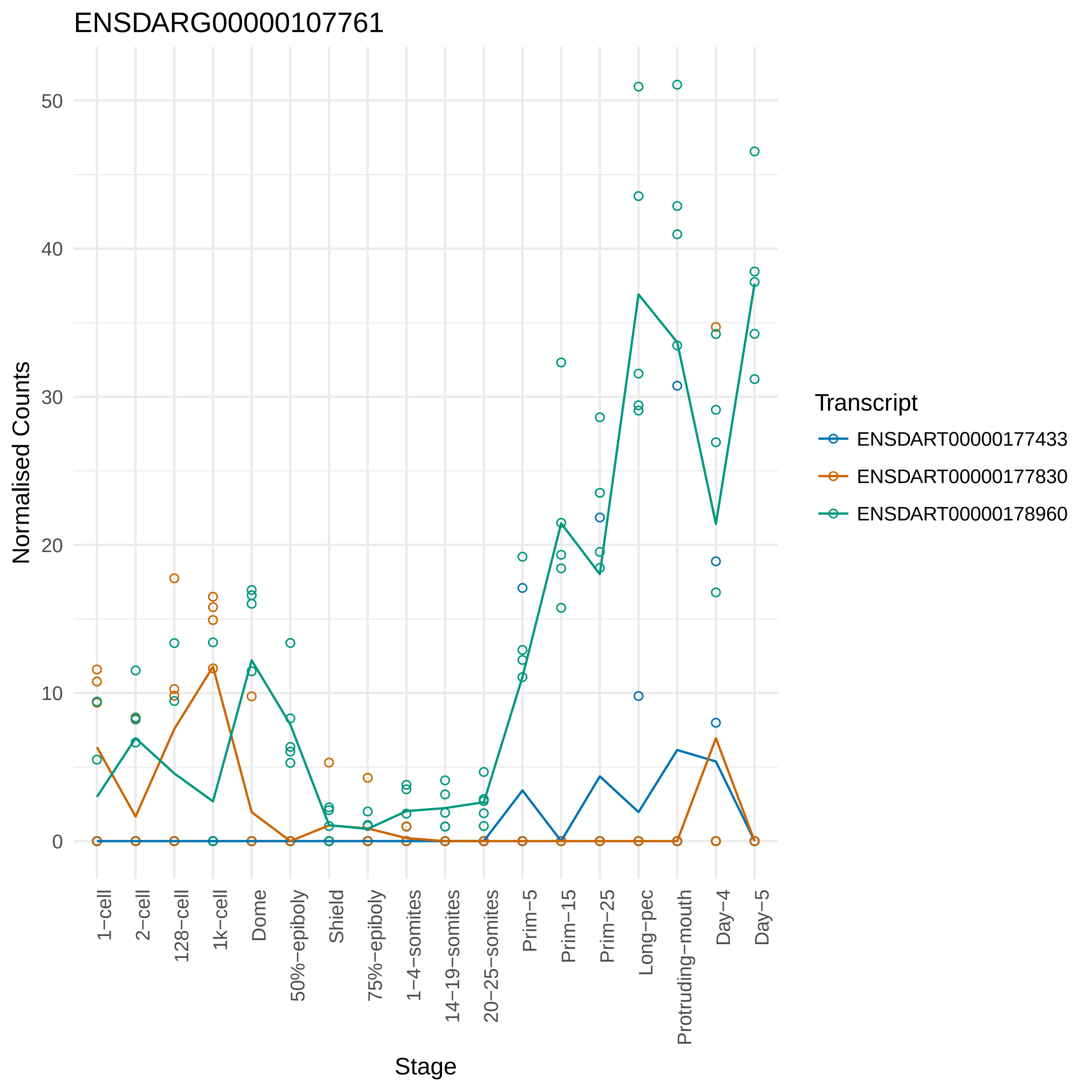

ENSDARG00000107761
Normalised Counts
0
10
20
30
40
50
1−cell
2−cell
128−cell
1k−cell
Dome
50%−epiboly
Shield
75%−epiboly
1−4−somites
14−19−somites
20−25−somites
Prim−5
Prim−15
Prim−25
Long−pec
Protruding−mouth
Day−4
Day−5
Stage
Transcript
ENSDART00000177433
ENSDART00000177830
ENSDART00000178960

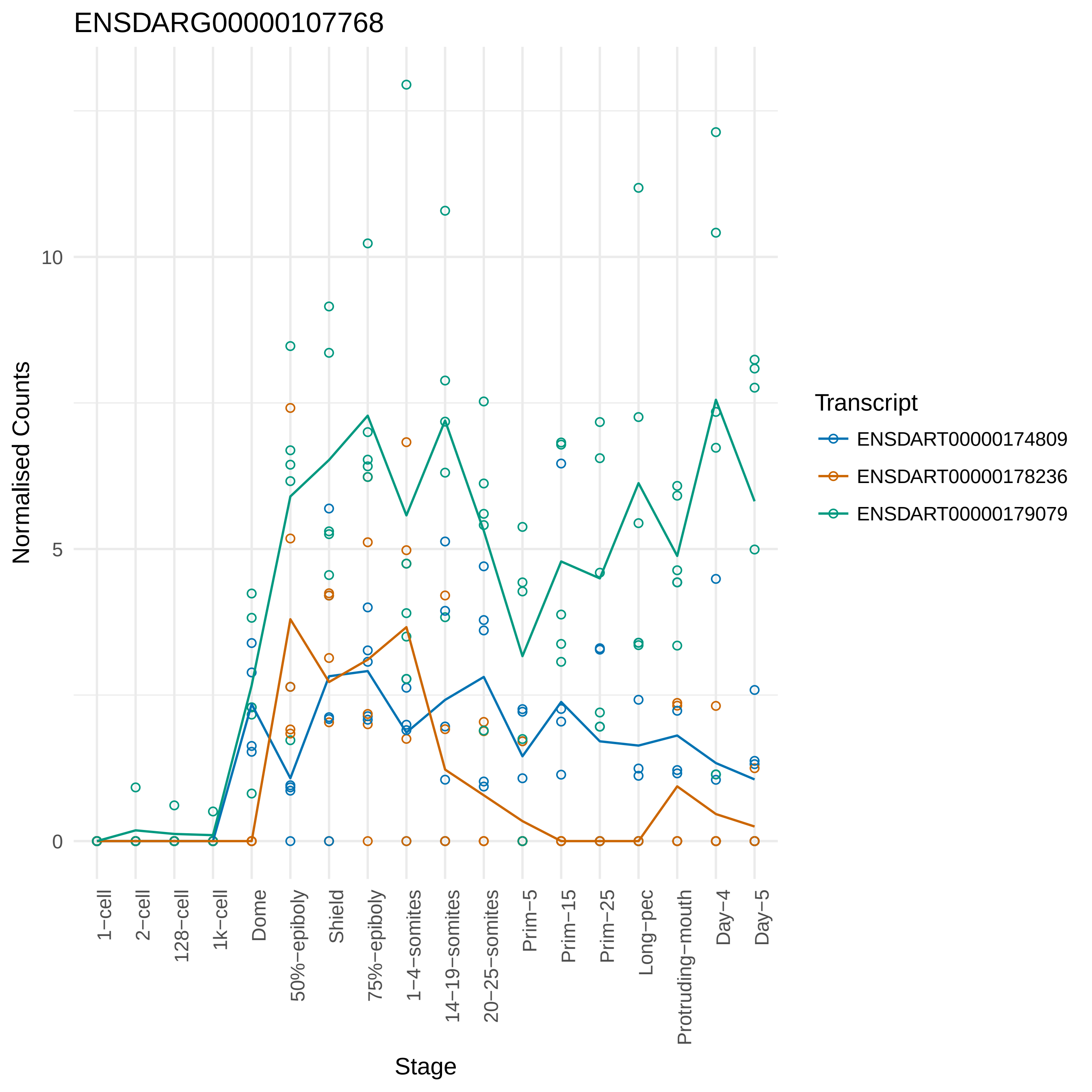
ENSDARG00000107768
Normalised Counts
0
5
10
Stage
1−cell
2−cell
128−cell
1k−cell
Dome
50%−epiboly
Shield
75%−epiboly
1−4−somites
14−19−somites
20−25−somites
Prim−5
Prim−15
Prim−25
Long−pec
Protruding−mouth
Day−4
Day−5
Transcript
ENSDART00000174809
ENSDART00000178236
ENSDART00000179079

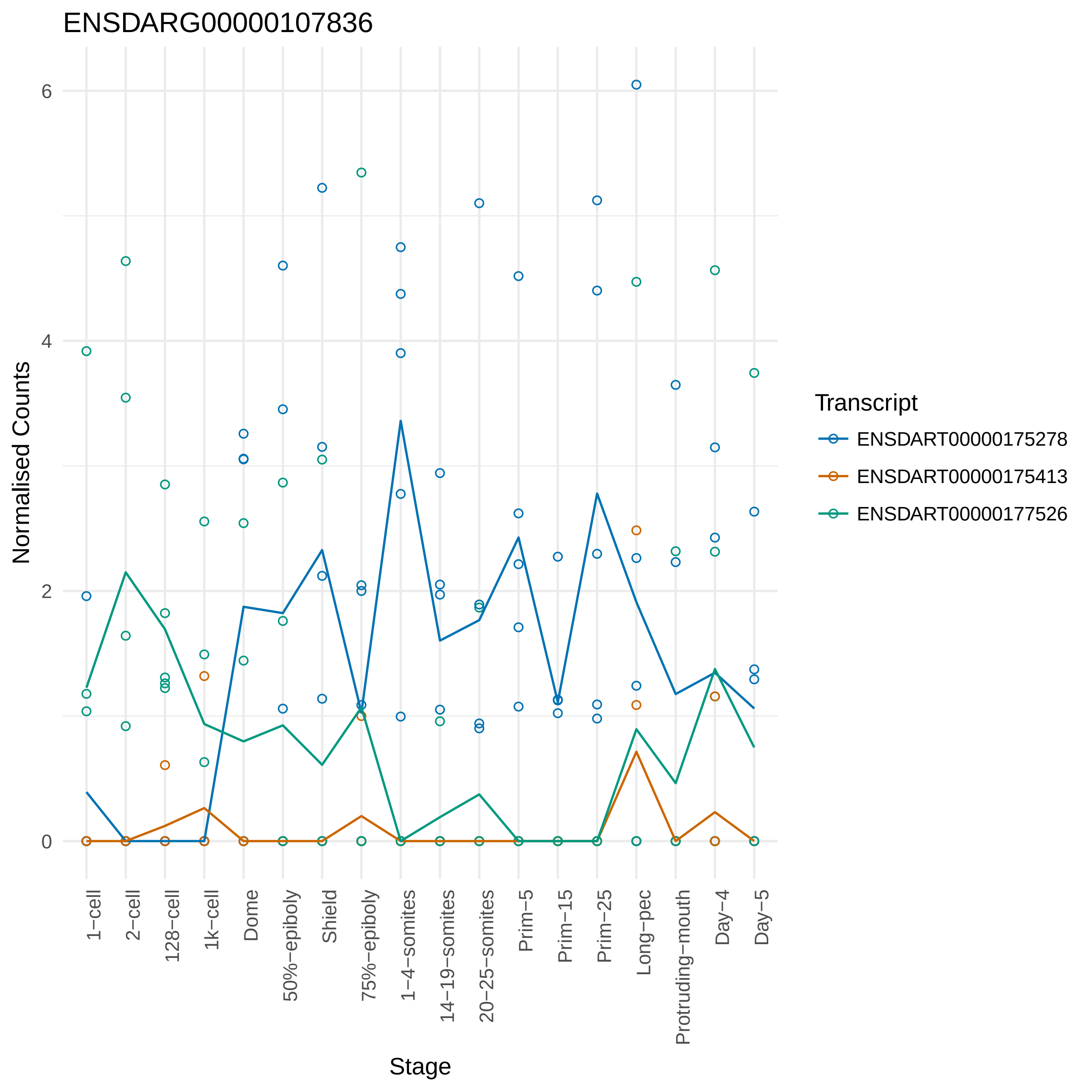

ENSDARG00000107836
Normalised Counts
0
2
4
6
Stage
1−cell
2−cell
128−cell
1k−cell
Dome
50%−epiboly
Shield
75%−epiboly
1−4−somites
14−19−somites
20−25−somites
Prim−5
Prim−15
Prim−25
Long−pec
Protruding−mouth
Day−4
Day−5
Transcript
ENSDART00000175278
ENSDART00000175413
ENSDART00000177526

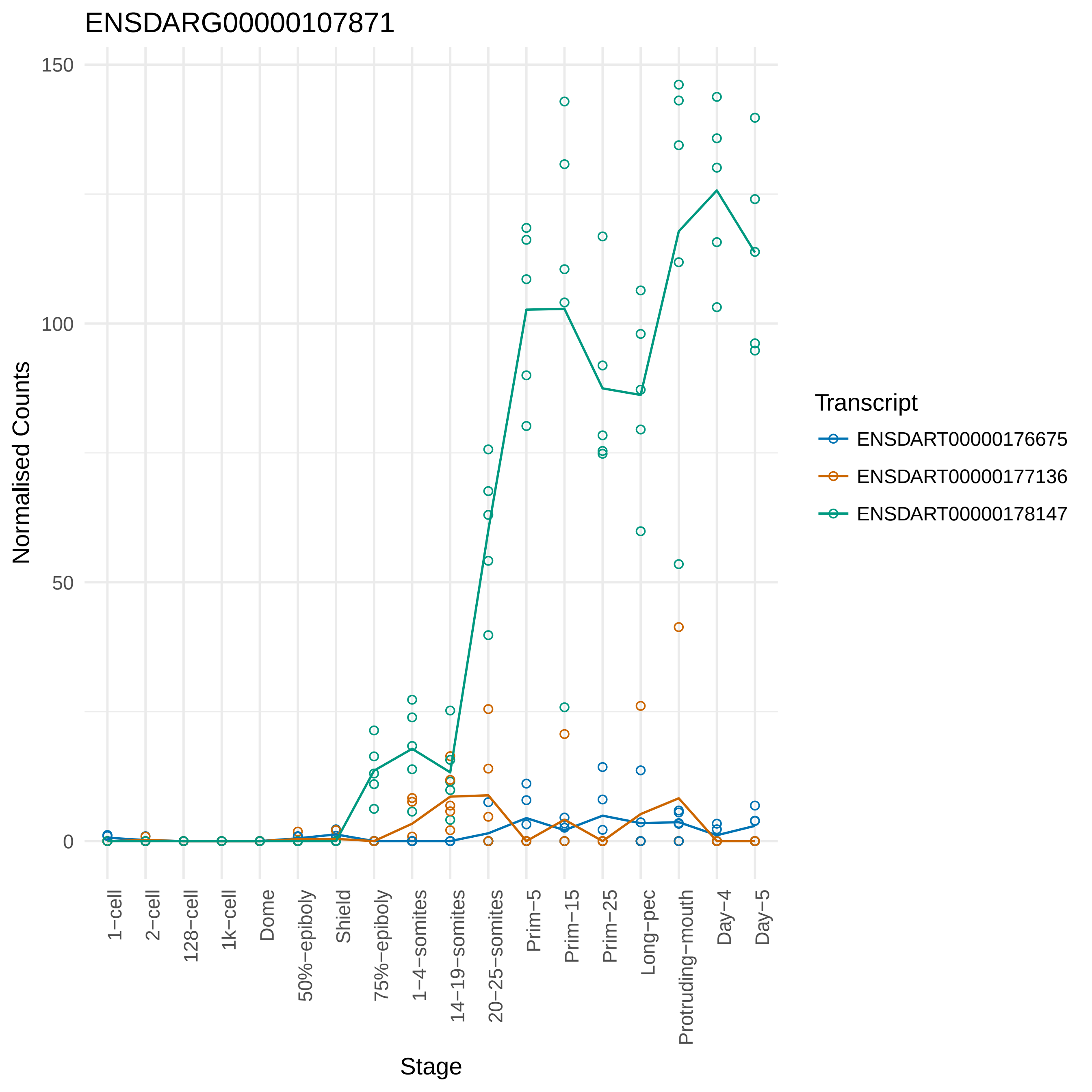

ENSDARG00000107871
Normalised Counts
150
100
50
0
1−cell
2−cell
128−cell
1k−cell
Dome
50%−epiboly
Shield
75%−epiboly
1−4−somites
14−19−somites
20−25−somites
Prim−5
Prim−15
Prim−25
Long−pec
Protruding−mouth
Day−4
Day−5
Stage
Transcript
ENSDART00000176675
ENSDART00000177136
ENSDART00000178147

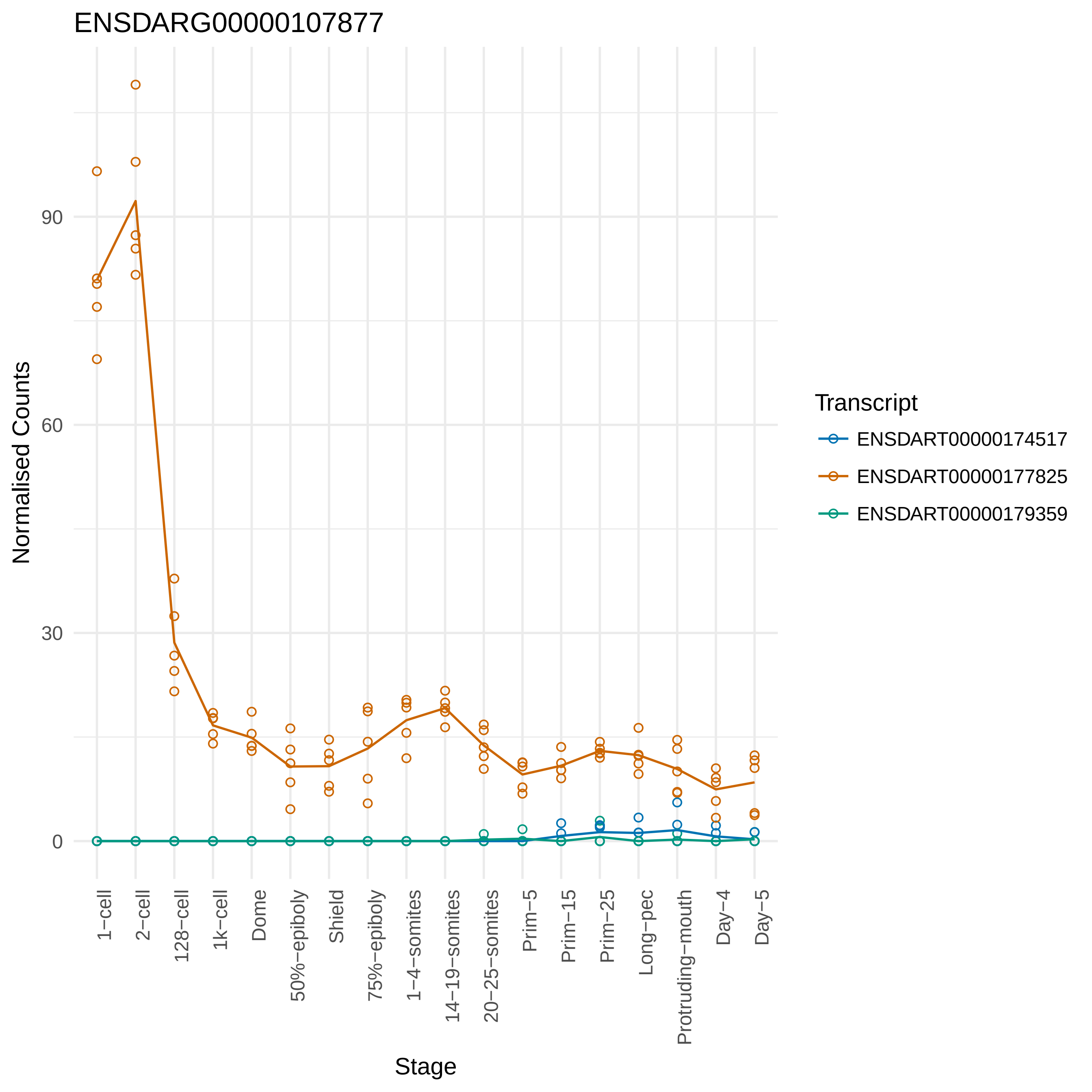

ENSDARG00000107877
Normalised Counts
0
30
60
90
1−cell
2−cell
128−cell
1k−cell
Dome
50%−epiboly
Shield
75%−epiboly
1−4−somites
14−19−somites
20−25−somites
Prim−5
Prim−15
Prim−25
Long−pec
Protruding−mouth
Day−4
Day−5
Stage
Transcript
ENSDART00000174517
ENSDART00000177825
ENSDART00000179359

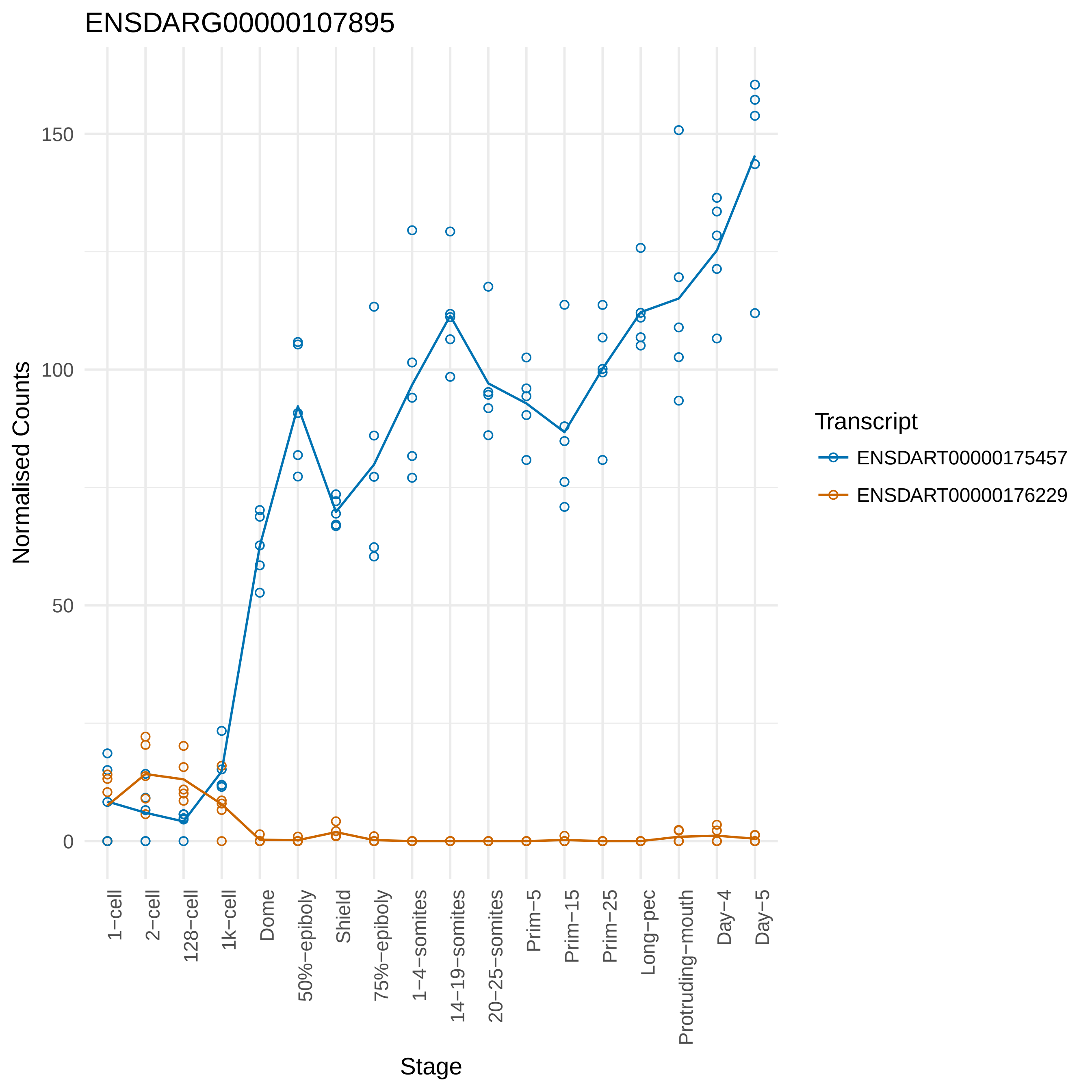

ENSDARG00000107895
Normalised Counts
150
100
50
0
1−cell
2−cell
128−cell
1k−cell
Dome
50%−epiboly
Shield
75%−epiboly
1−4−somites
14−19−somites
20−25−somites
Prim−5
Prim−15
Prim−25
Long−pec
Protruding−mouth
Day−4
Day−5
Stage
Transcript
ENSDART00000175457
ENSDART00000176229

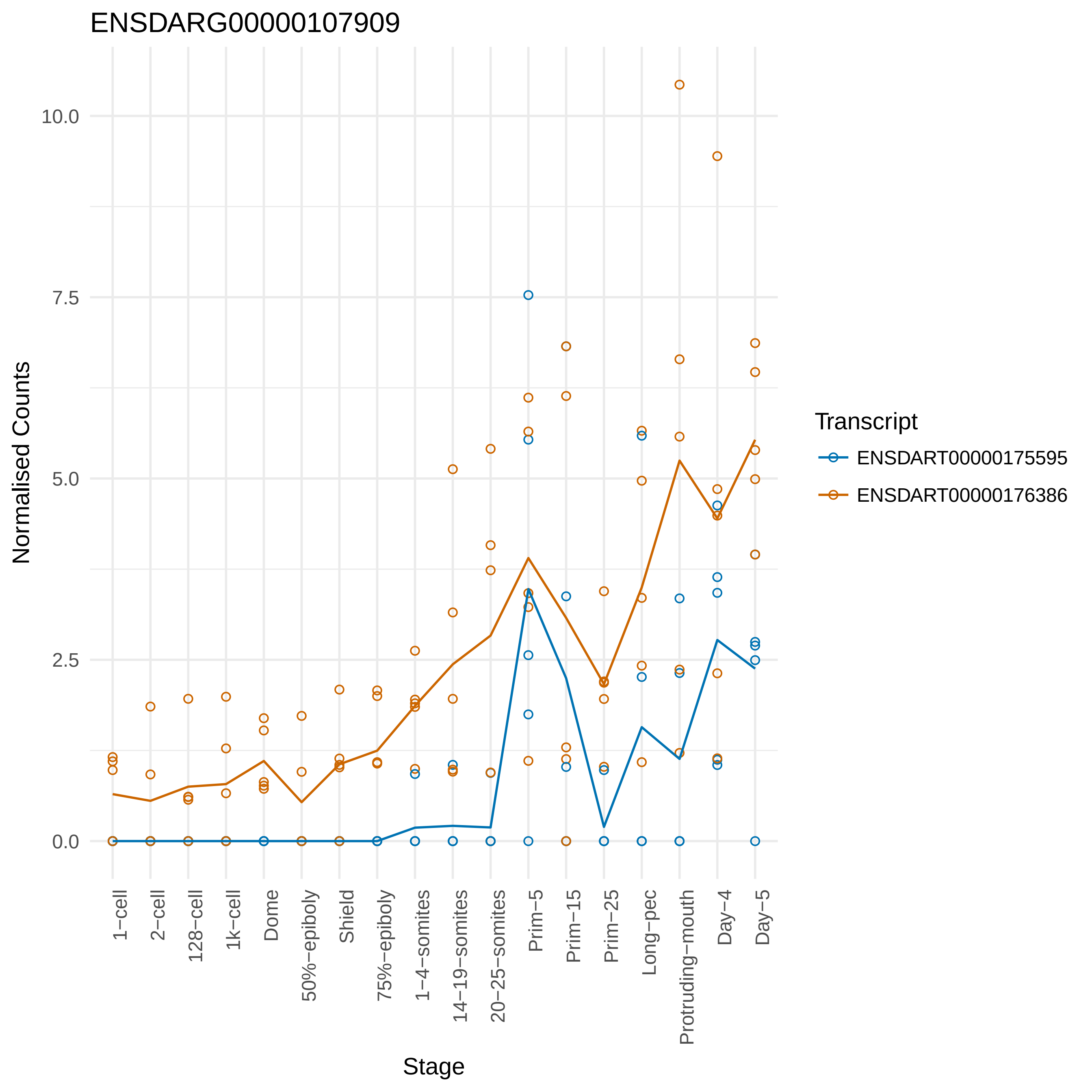
ENSDARG00000107909
Normalised Counts
10.0
7.5
5.0
2.5
0.0
Stage
1−cell
2−cell
128−cell
1k−cell
Dome
50%−epiboly
Shield
75%−epiboly
1−4−somites
14−19−somites
20−25−somites
Prim−5
Prim−15
Prim−25
Long−pec
Protruding−mouth
Day−4
Day−5
Transcript
ENSDART00000175595
ENSDART00000176386

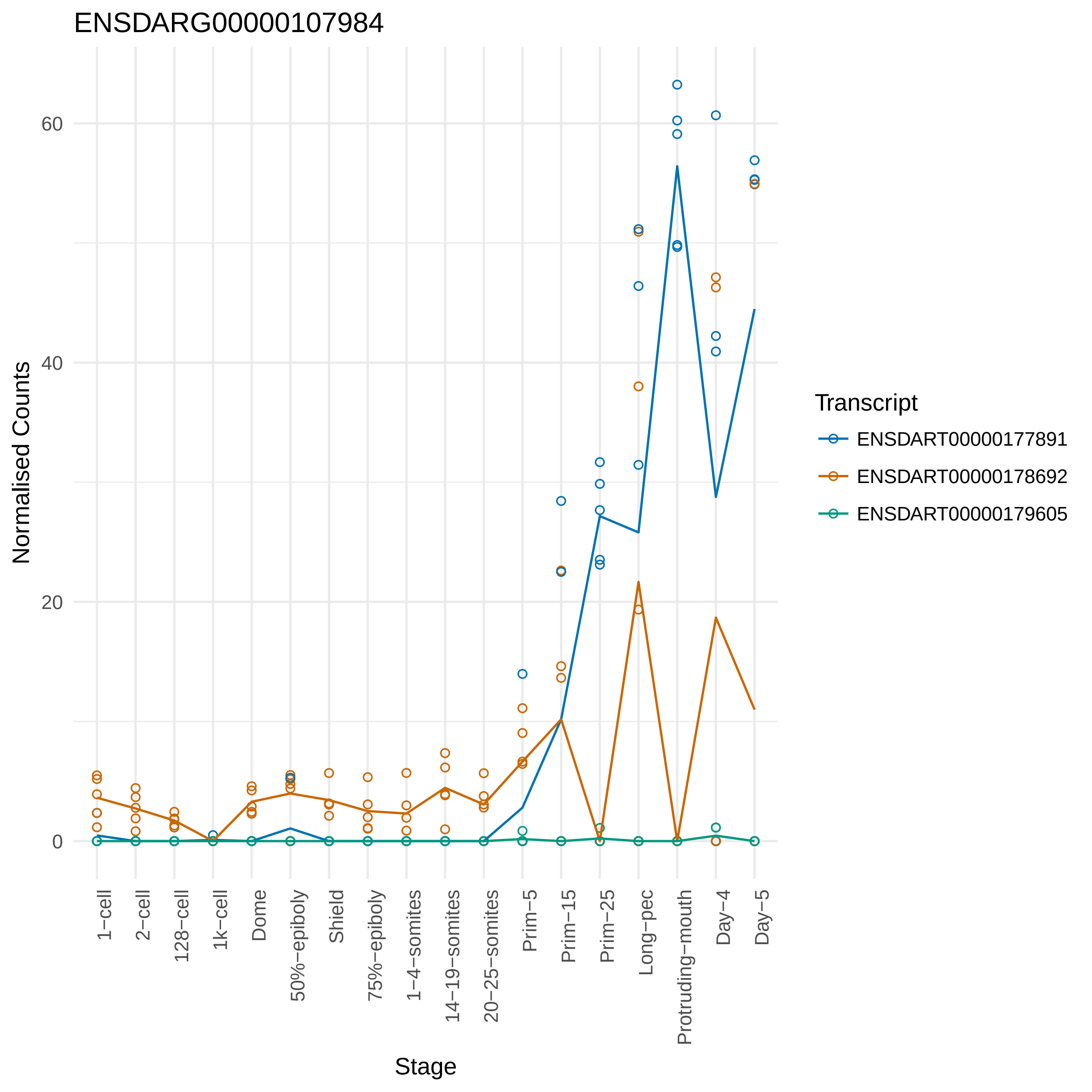
ENSDARG00000107984
Normalised Counts
0
20
40
60
1−cell
2−cell
128−cell
1k−cell
Dome
50%−epiboly
Shield
75%−epiboly
1−4−somites
14−19−somites
20−25−somites
Prim−5
Prim−15
Prim−25
Long−pec
Protruding−mouth
Day−4
Day−5
Stage
Transcript
ENSDART00000177891
ENSDART00000178692
ENSDART00000179605

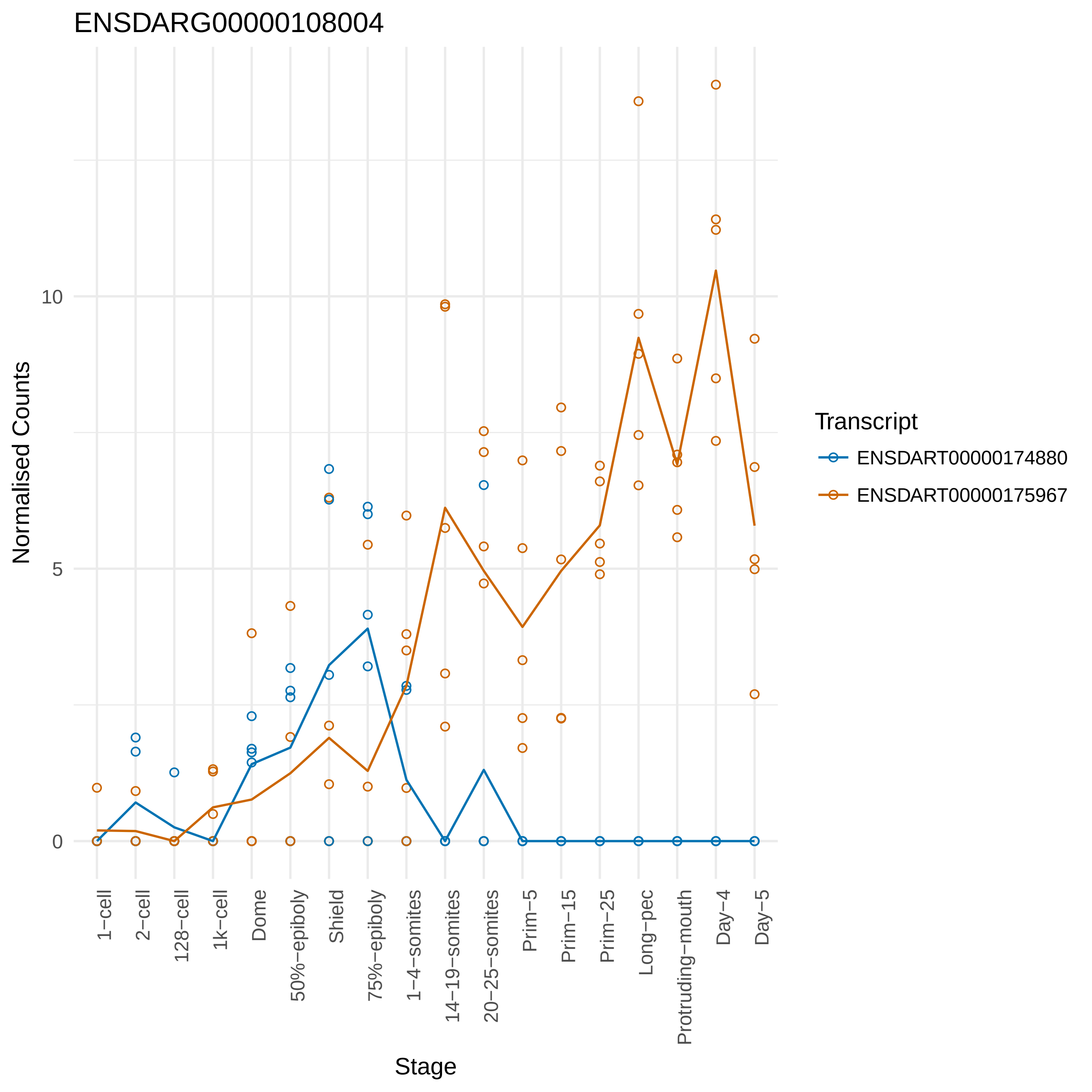

ENSDARG00000108004
Normalised Counts
10
5
0
1−cell
2−cell
128−cell
1k−cell
Dome
50%−epiboly
Shield
75%−epiboly
1−4−somites
14−19−somites
20−25−somites
Prim−5
Prim−15
Prim−25
Long−pec
Protruding−mouth
Day−4
Day−5
Stage
Transcript
ENSDART00000174880
ENSDART00000175967

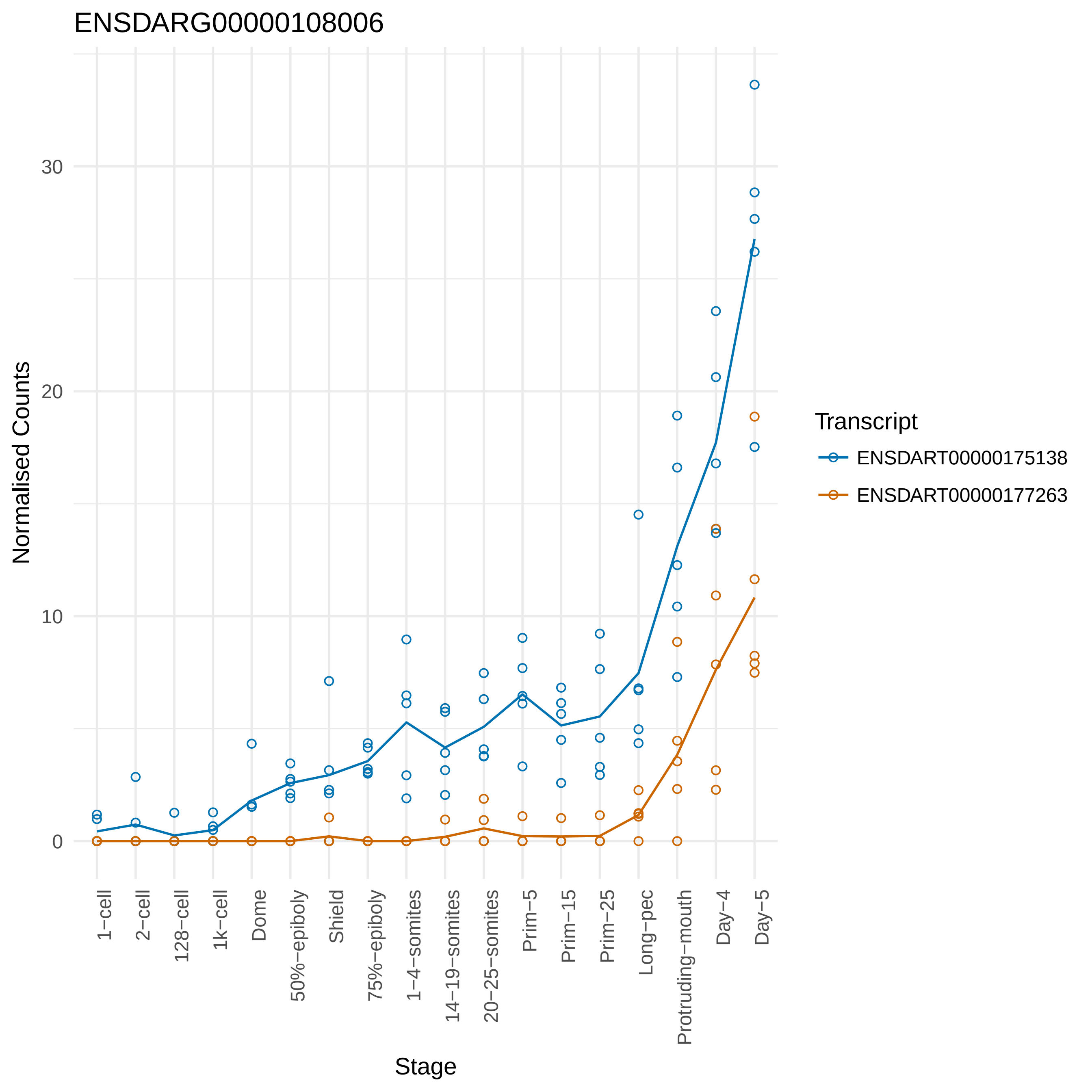

ENSDARG00000108006
Normalised Counts
30
20
10
0
1−cell
2−cell
128−cell
1k−cell
Dome
50%−epiboly
Shield
75%−epiboly
1−4−somites
14−19−somites
20−25−somites
Prim−5
Prim−15
Prim−25
Long−pec
Protruding−mouth
Day−4
Day−5
Stage
Transcript
ENSDART00000175138
ENSDART00000177263

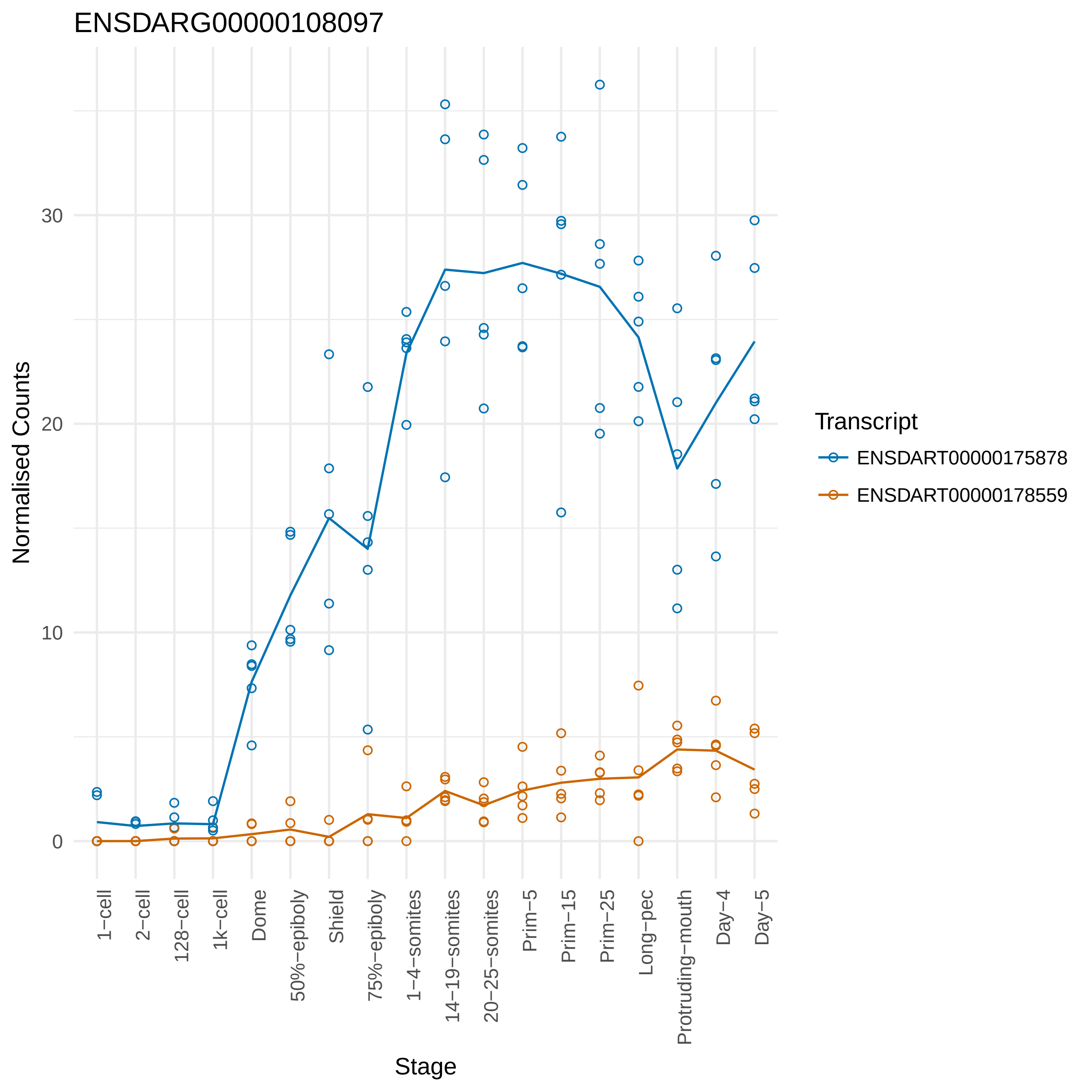

ENSDARG00000108097
Normalised Counts
0
10
20
30
1−cell
2−cell
128−cell
1k−cell
Dome
50%−epiboly
Shield
75%−epiboly
1−4−somites
14−19−somites
20−25−somites
Prim−5
Prim−15
Prim−25
Long−pec
Protruding−mouth
Day−4
Day−5
Stage
Transcript
ENSDART00000175878
ENSDART00000178559

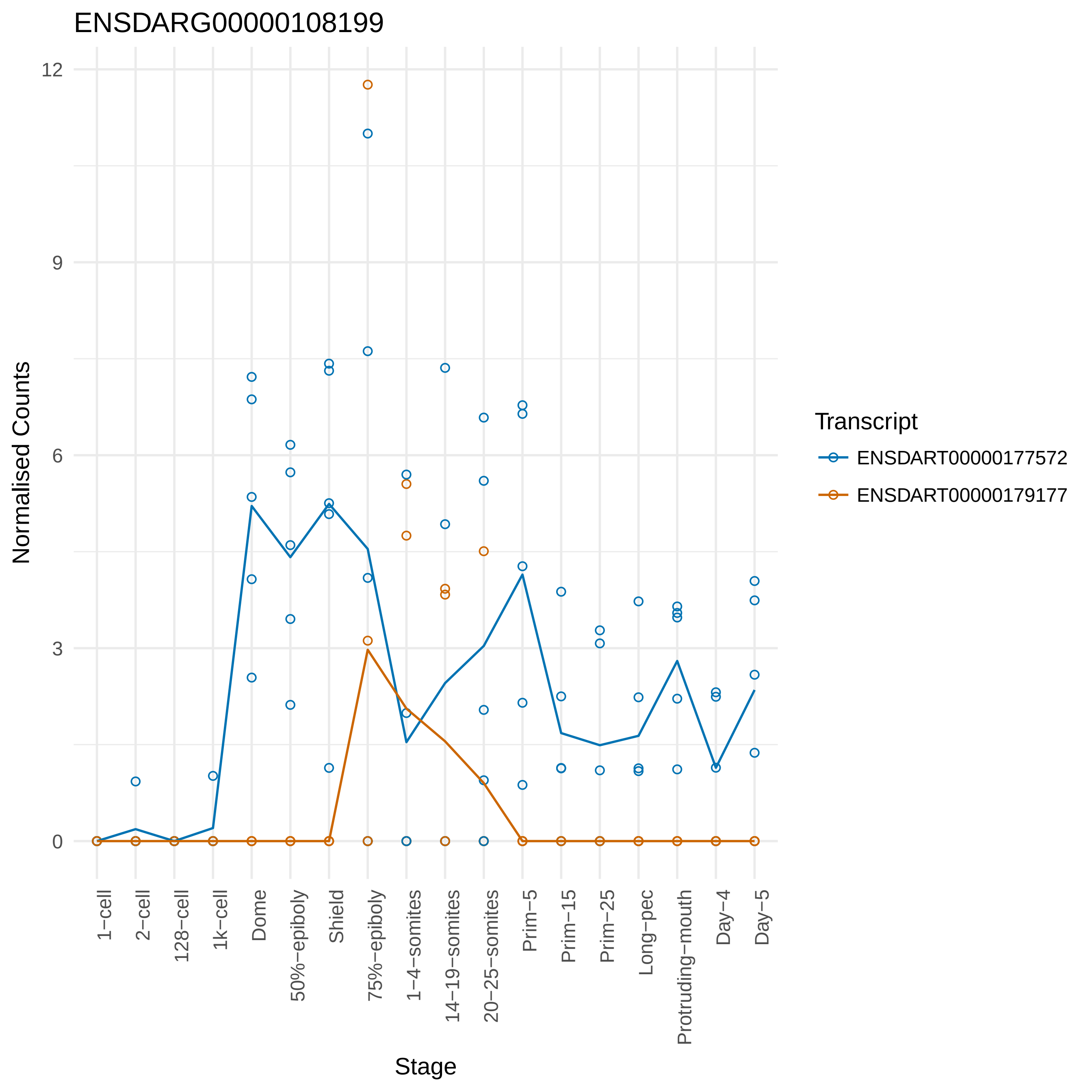
ENSDARG00000108199
Normalised Counts
0
3
6
9
12
Stage
1−cell
2−cell
128−cell
1k−cell
Dome
50%−epiboly
Shield
75%−epiboly
1−4−somites
14−19−somites
20−25−somites
Prim−5
Prim−15
Prim−25
Long−pec
Protruding−mouth
Day−4
Day−5
Transcript
ENSDART00000177572
ENSDART00000179177

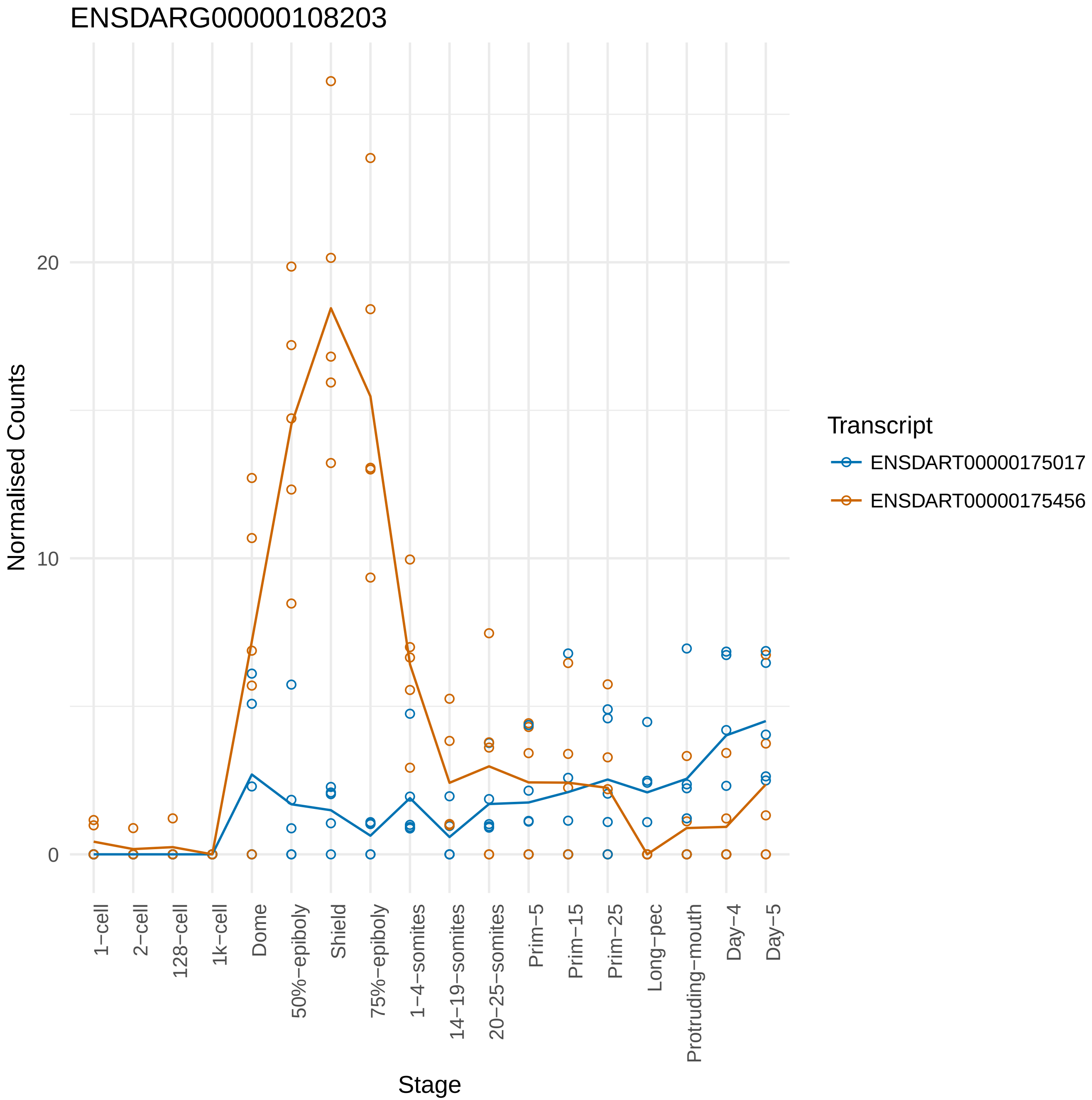

ENSDARG00000108203
Normalised Counts
0
10
20
Stage
1−cell
2−cell
128−cell
1k−cell
Dome
50%−epiboly
Shield
75%−epiboly
1−4−somites
14−19−somites
20−25−somites
Prim−5
Prim−15
Prim−25
Long−pec
Protruding−mouth
Day−4
Day−5
Transcript
ENSDART00000175017
ENSDART00000175456

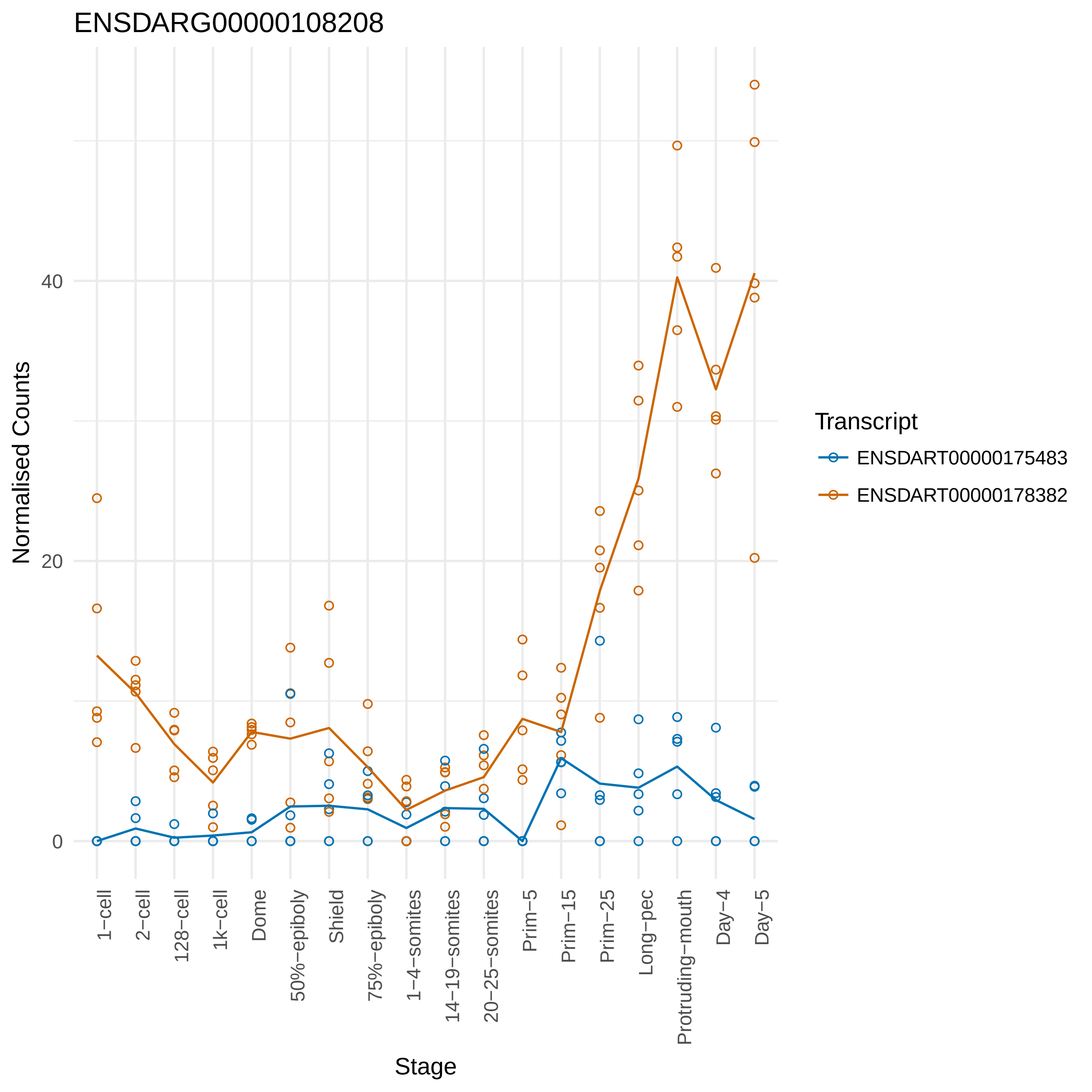
ENSDARG00000108208
Normalised Counts
40
20
0
1−cell
2−cell
128−cell
1k−cell
Dome
50%−epiboly
Shield
75%−epiboly
1−4−somites
14−19−somites
20−25−somites
Prim−5
Prim−15
Prim−25
Long−pec
Protruding−mouth
Day−4
Day−5
Stage
Transcript
ENSDART00000175483
ENSDART00000178382

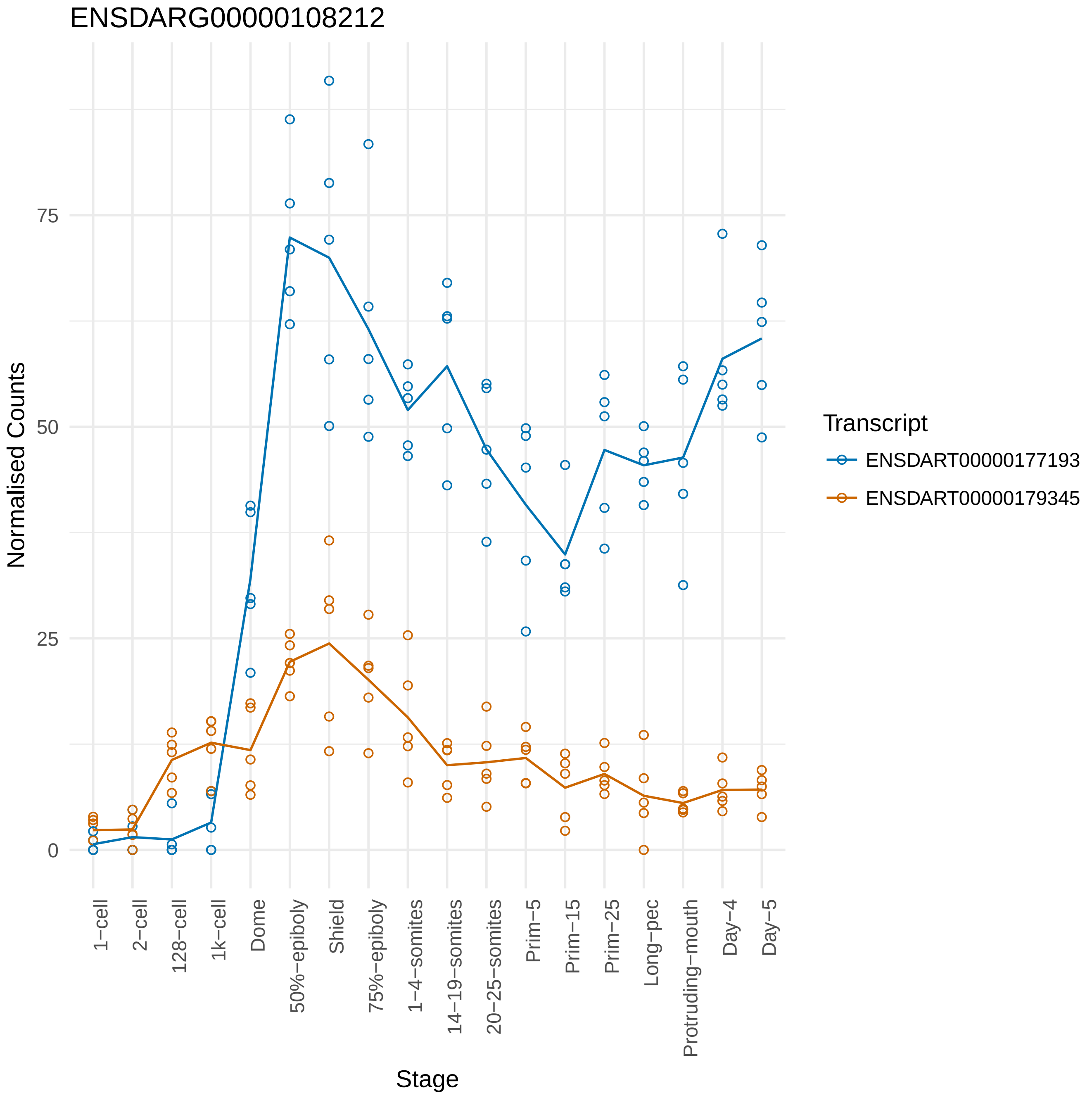

ENSDARG00000108212
Normalised Counts
0
25
50
75
1−cell
2−cell
128−cell
1k−cell
Dome
50%−epiboly
Shield
75%−epiboly
1−4−somites
14−19−somites
20−25−somites
Prim−5
Prim−15
Prim−25
Long−pec
Protruding−mouth
Day−4
Day−5
Stage
Transcript
ENSDART00000177193
ENSDART00000179345

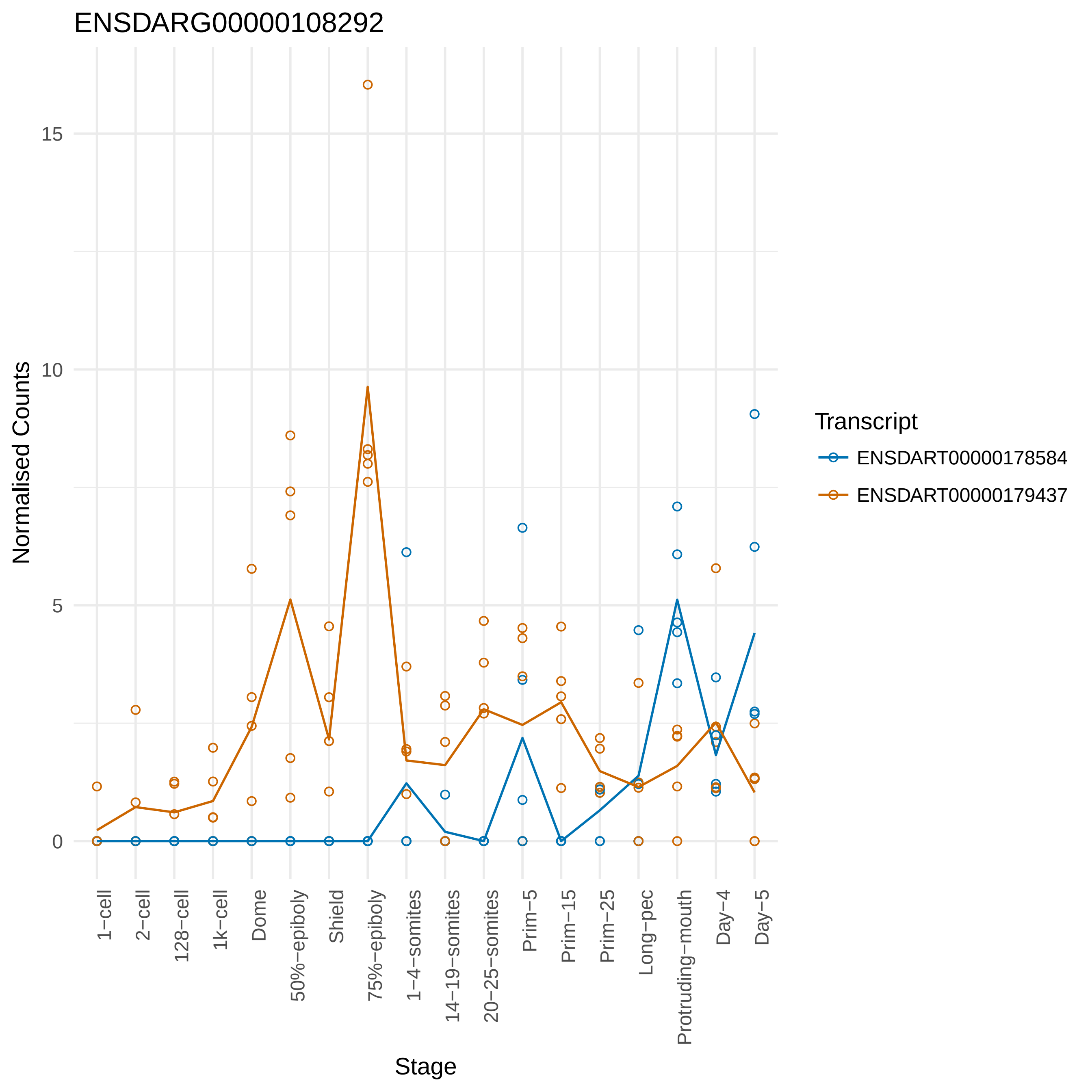

ENSDARG00000108292
Normalised Counts
0
5
10
15
Stage
1−cell
2−cell
128−cell
1k−cell
Dome
50%−epiboly
Shield
75%−epiboly
1−4−somites
14−19−somites
20−25−somites
Prim−5
Prim−15
Prim−25
Long−pec
Protruding−mouth
Day−4
Day−5
Transcript
ENSDART00000178584
ENSDART00000179437

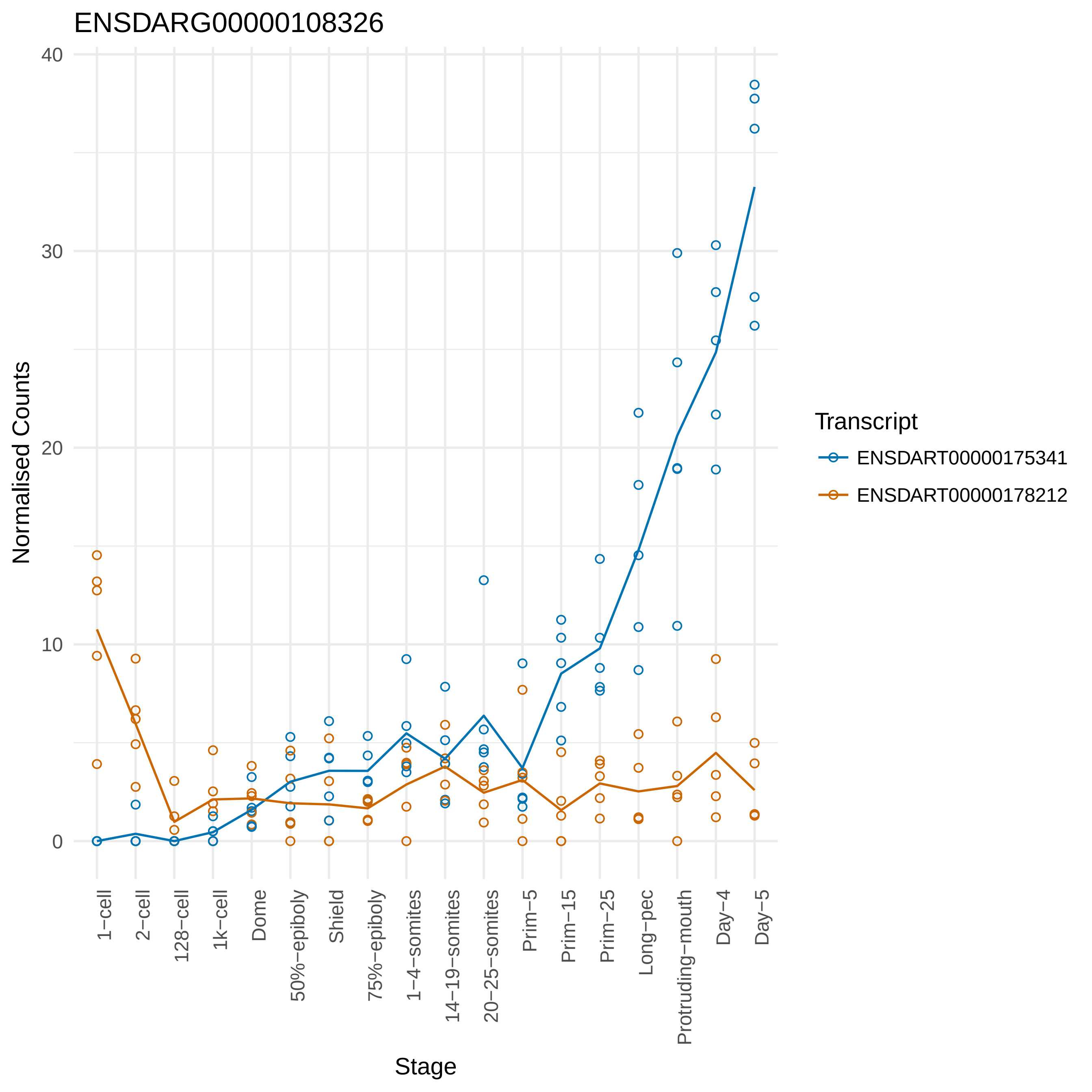

ENSDARG00000108326
Normalised Counts
40
30
20
10
0
1−cell
2−cell
128−cell
1k−cell
Dome
50%−epiboly
Shield
75%−epiboly
1−4−somites
14−19−somites
20−25−somites
Prim−5
Prim−15
Prim−25
Long−pec
Protruding−mouth
Day−4
Day−5
Stage
Transcript
ENSDART00000175341
ENSDART00000178212

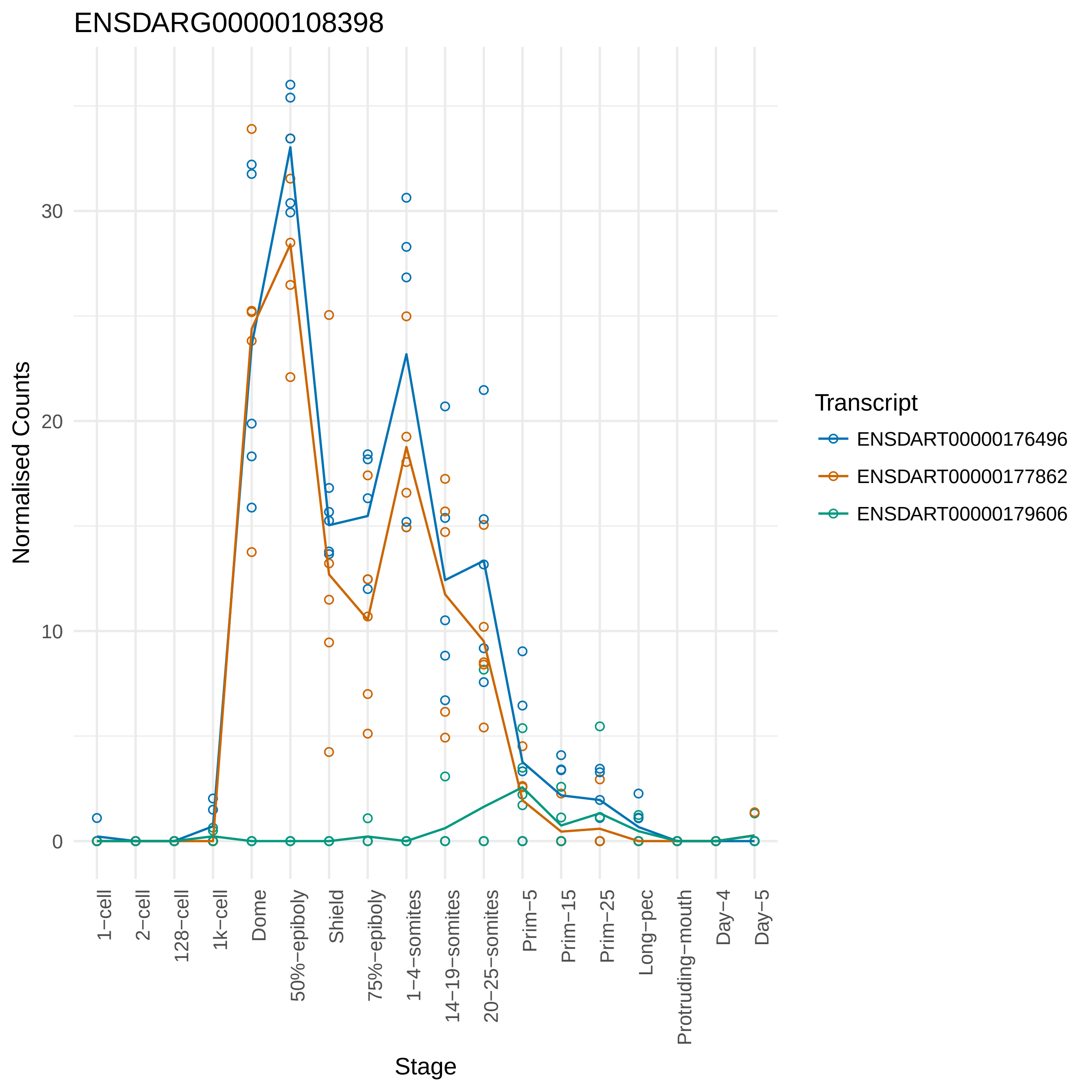

ENSDARG00000108398
Normalised Counts
0
10
20
30
Stage
1−cell
2−cell
128−cell
1k−cell
Dome
50%−epiboly
Shield
75%−epiboly
1−4−somites
14−19−somites
20−25−somites
Prim−5
Prim−15
Prim−25
Long−pec
Protruding−mouth
Day−4
Day−5
Transcript
ENSDART00000176496
ENSDART00000177862
ENSDART00000179606

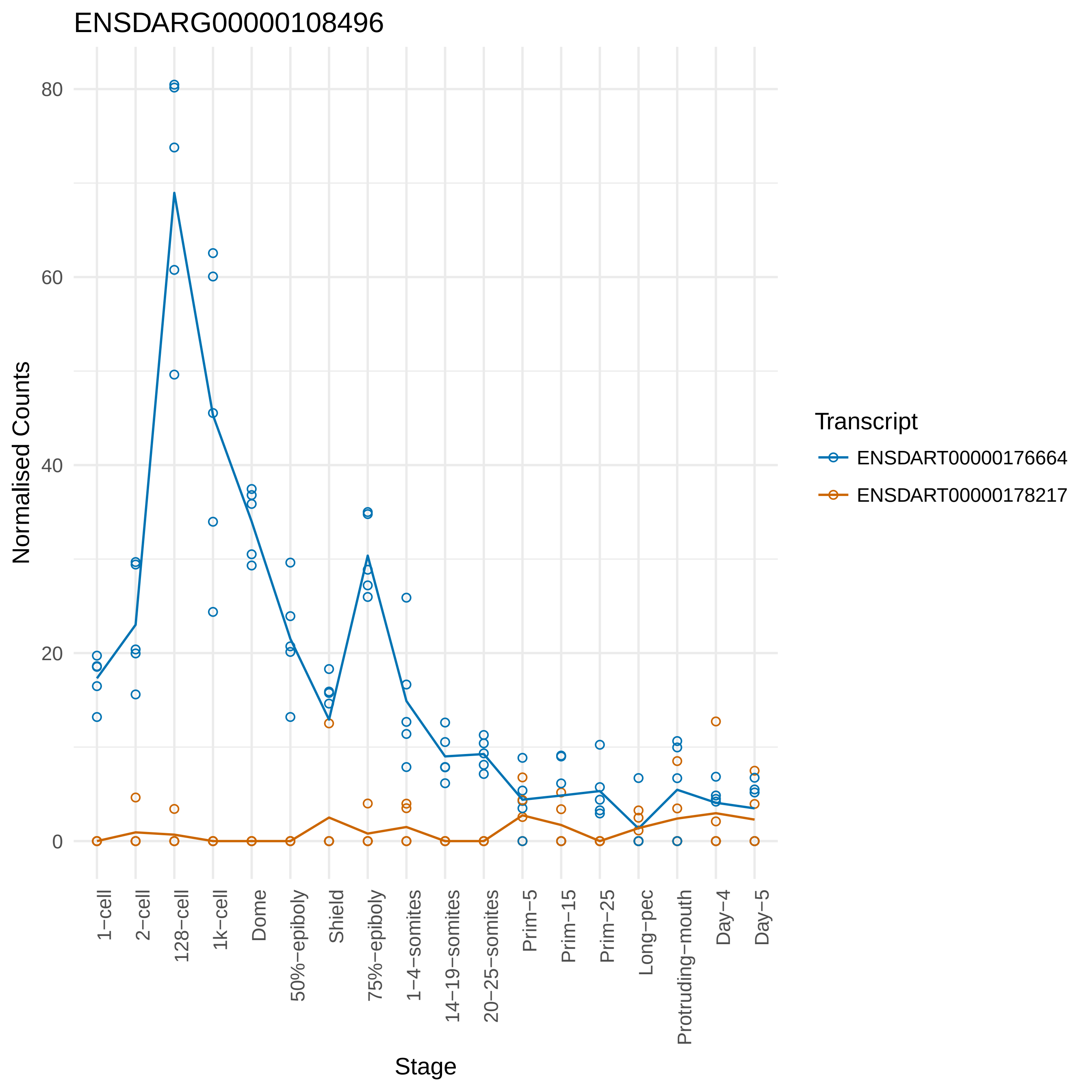

ENSDARG00000108496
Normalised Counts
80
60
40
20
0
1−cell
2−cell
128−cell
1k−cell
Dome
50%−epiboly
Shield
75%−epiboly
1−4−somites
14−19−somites
20−25−somites
Prim−5
Prim−15
Prim−25
Long−pec
Protruding−mouth
Day−4
Day−5
Stage
Transcript
ENSDART00000176664
ENSDART00000178217

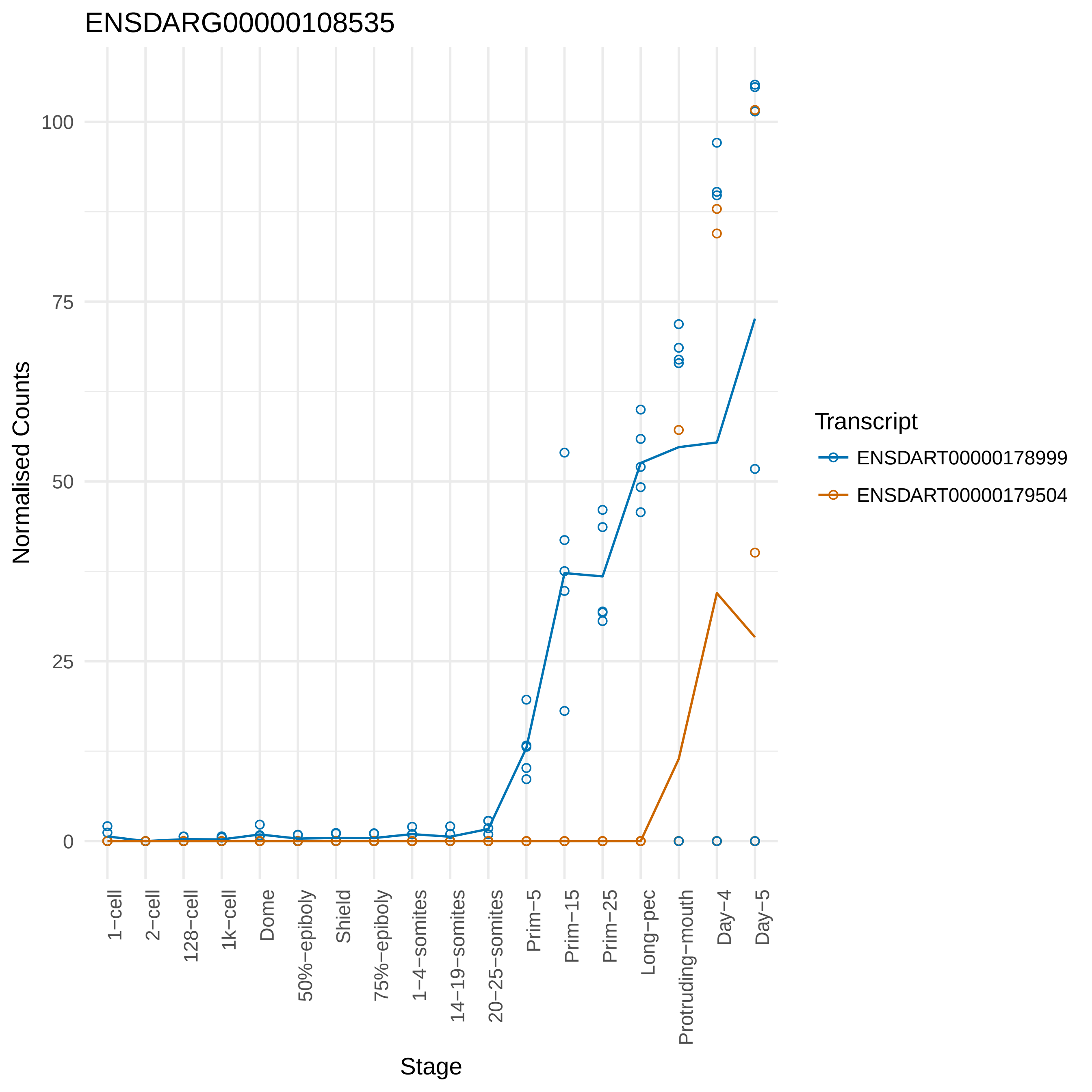

ENSDARG00000108535
Normalised Counts
100
75
50
25
0
Stage
1−cell
2−cell
128−cell
1k−cell
Dome
50%−epiboly
Shield
75%−epiboly
1−4−somites
14−19−somites
20−25−somites
Prim−5
Prim−15
Prim−25
Long−pec
Protruding−mouth
Day−4
Day−5
Transcript
ENSDART00000178999
ENSDART00000179504

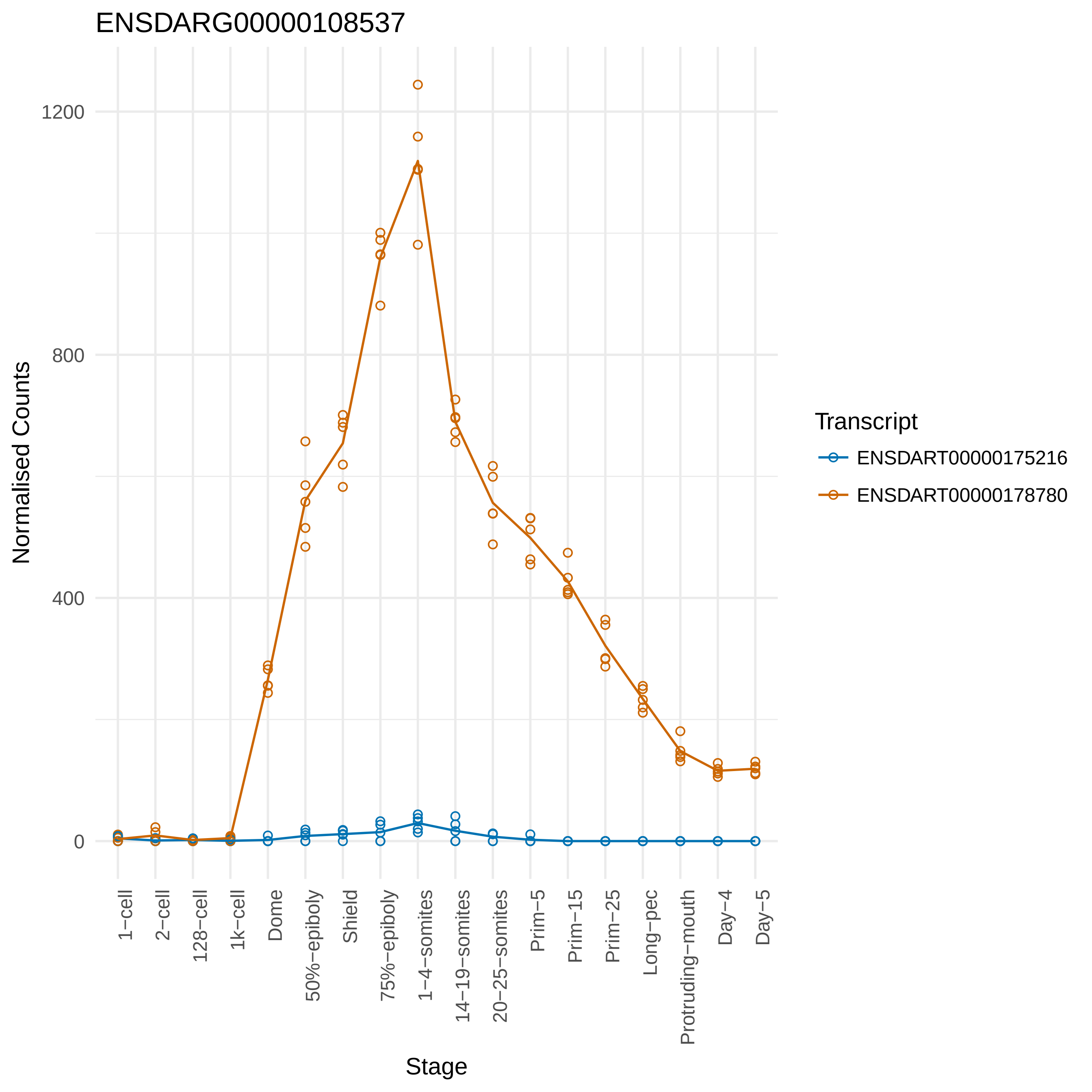
ENSDARG00000108537
Normalised Counts
1200
800
400
0
1−cell
2−cell
128−cell
1k−cell
Dome
50%−epiboly
Shield
75%−epiboly
1−4−somites
14−19−somites
20−25−somites
Prim−5
Prim−15
Prim−25
Long−pec
Protruding−mouth
Day−4
Day−5
Stage
Transcript
ENSDART00000175216
ENSDART00000178780

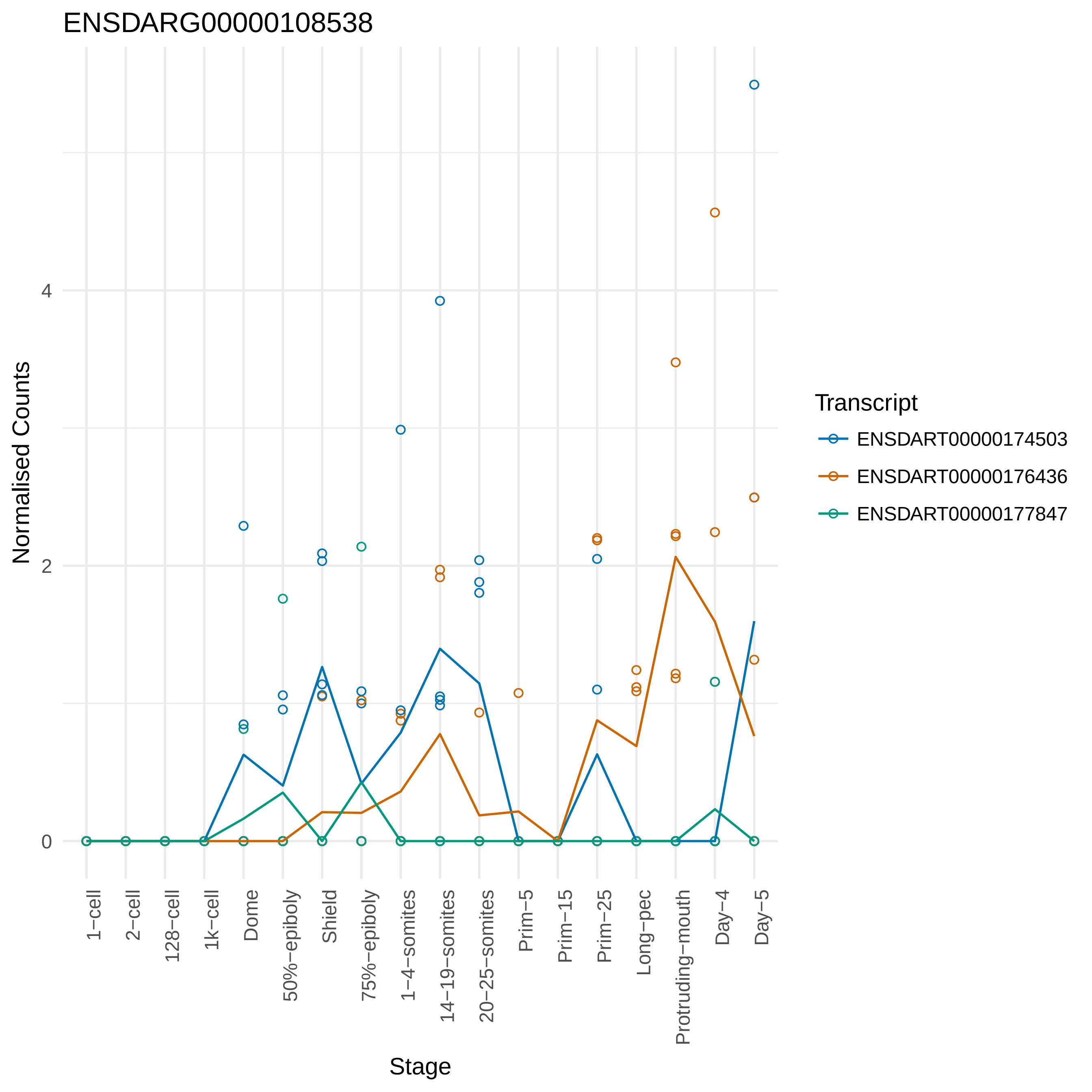

ENSDARG00000108538
Normalised Counts
0
2
4
Stage
1−cell
2−cell
128−cell
1k−cell
Dome
50%−epiboly
Shield
75%−epiboly
1−4−somites
14−19−somites
20−25−somites
Prim−5
Prim−15
Prim−25
Long−pec
Protruding−mouth
Day−4
Day−5
Transcript
ENSDART00000174503
ENSDART00000176436
ENSDART00000177847

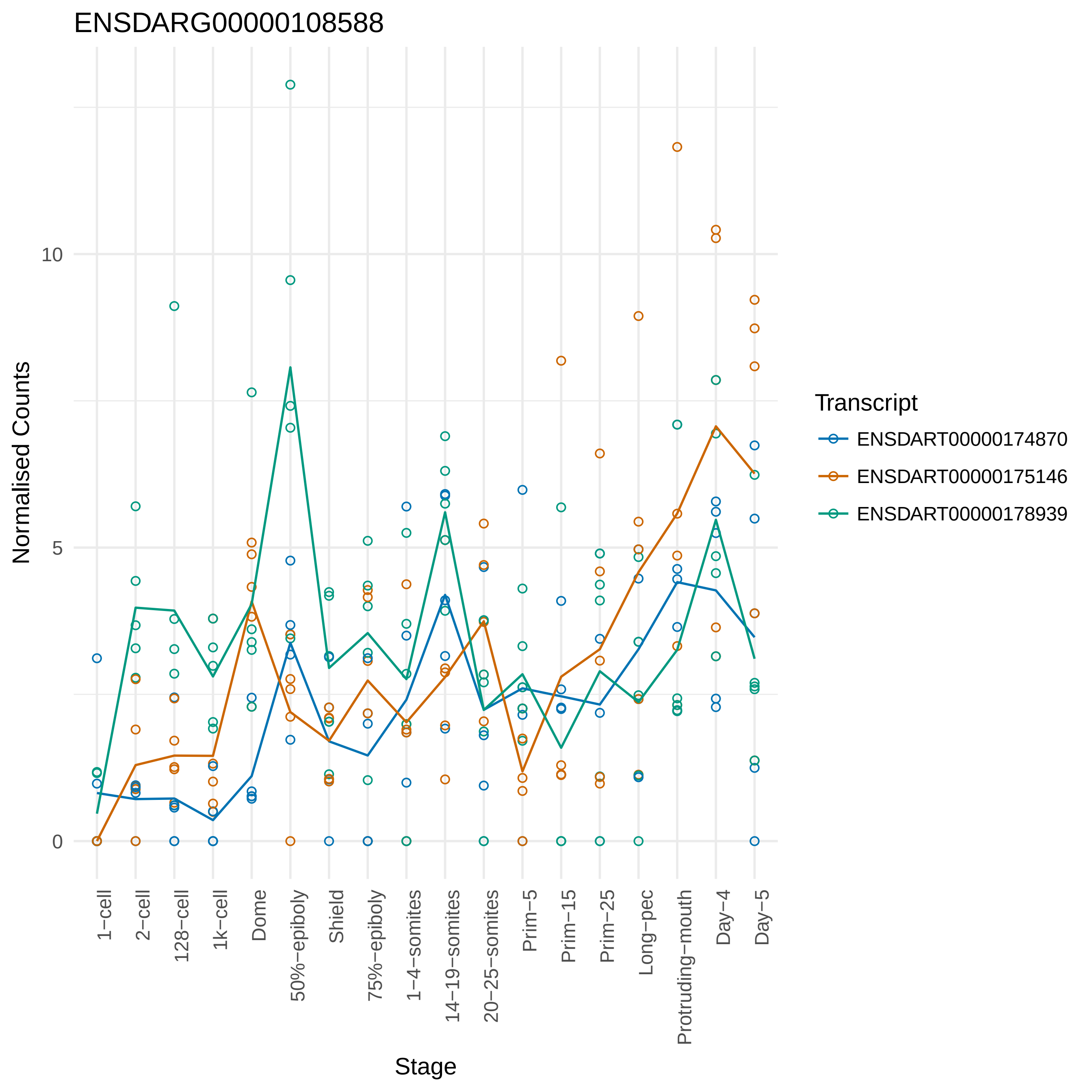

ENSDARG00000108588
Normalised Counts
0
5
10
Stage
1−cell
2−cell
128−cell
1k−cell
Dome
50%−epiboly
Shield
75%−epiboly
1−4−somites
14−19−somites
20−25−somites
Prim−5
Prim−15
Prim−25
Long−pec
Protruding−mouth
Day−4
Day−5
Transcript
ENSDART00000174870
ENSDART00000175146
ENSDART00000178939

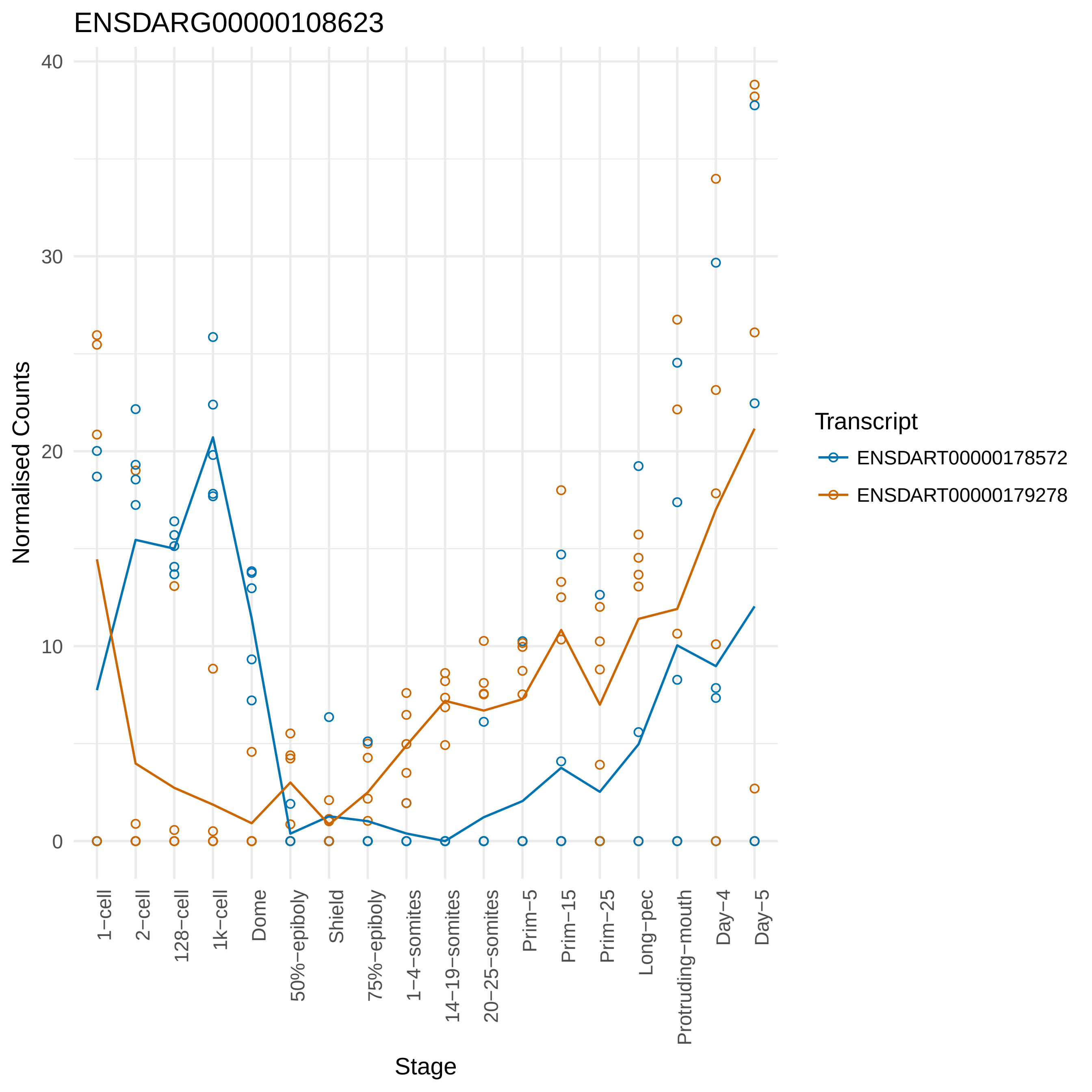
ENSDARG00000108623
Normalised Counts
40
30
20
10
0
Stage
1−cell
2−cell
128−cell
1k−cell
Dome
50%−epiboly
Shield
75%−epiboly
1−4−somites
14−19−somites
20−25−somites
Prim−5
Prim−15
Prim−25
Long−pec
Protruding−mouth
Day−4
Day−5
Transcript
ENSDART00000178572
ENSDART00000179278

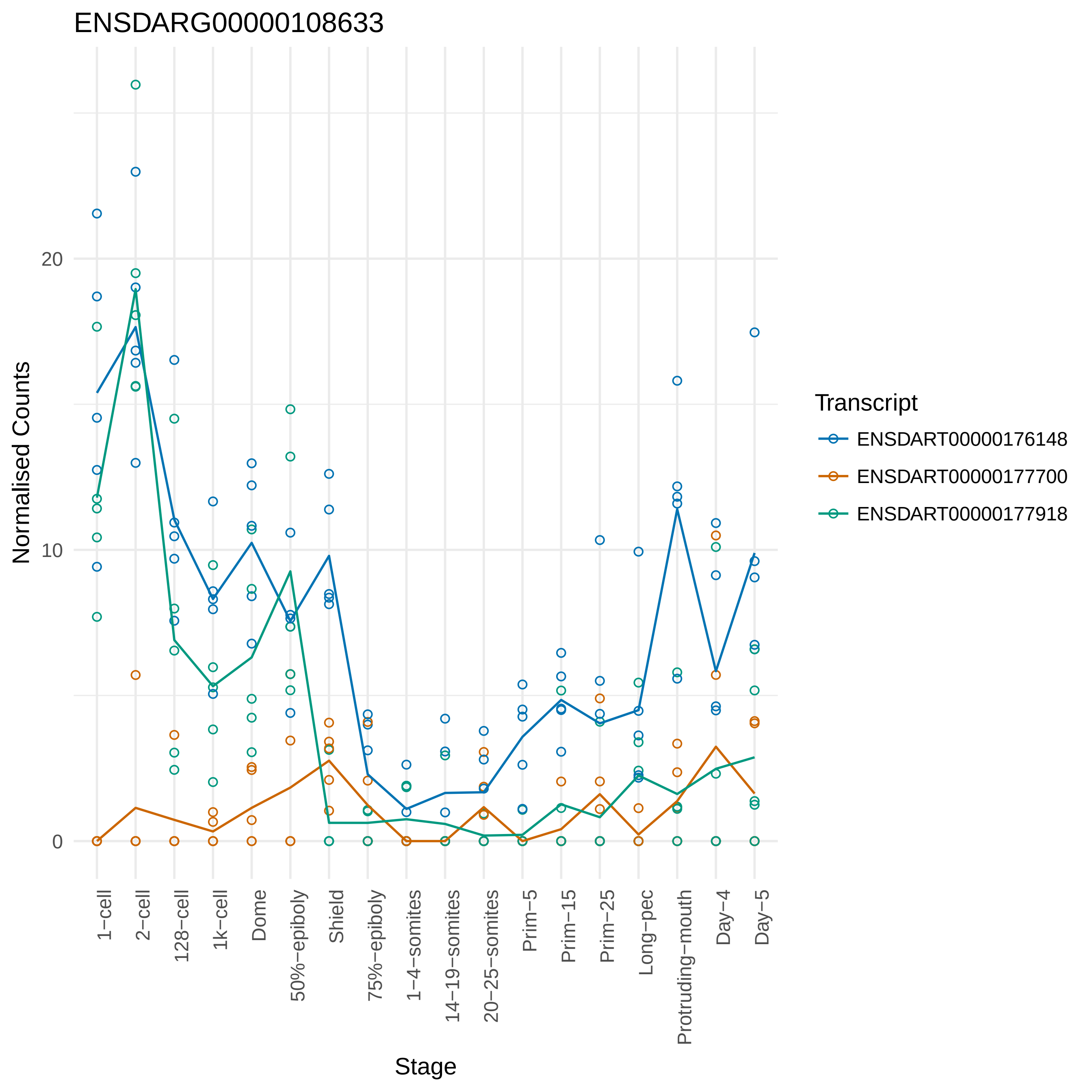

ENSDARG00000108633
Normalised Counts
0
10
20
1−cell
2−cell
128−cell
1k−cell
Dome
50%−epiboly
Shield
75%−epiboly
1−4−somites
14−19−somites
20−25−somites
Prim−5
Prim−15
Prim−25
Long−pec
Protruding−mouth
Day−4
Day−5
Stage
Transcript
ENSDART00000176148
ENSDART00000177700
ENSDART00000177918

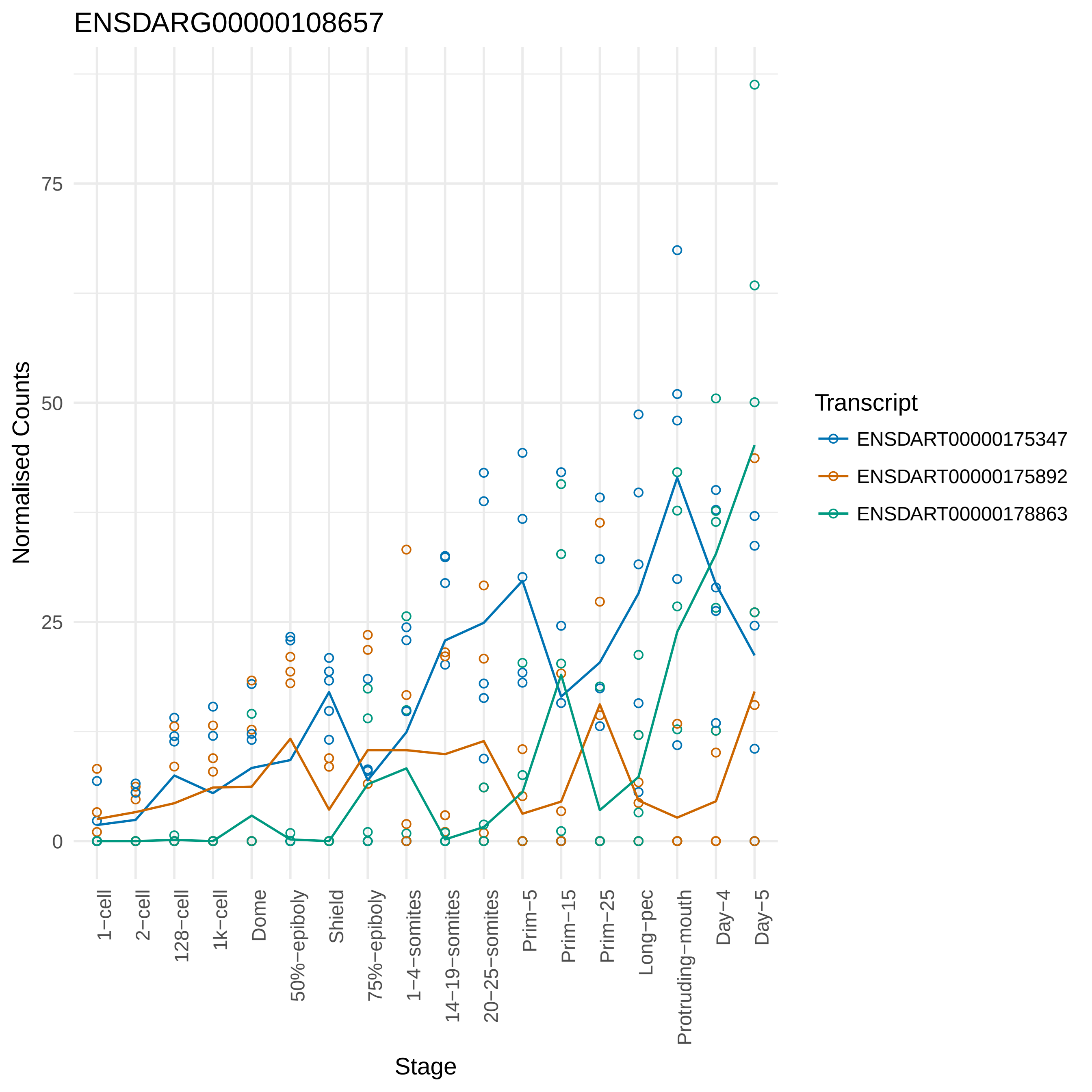
ENSDARG00000108657
Normalised Counts
0
25
50
75
1−cell
2−cell
128−cell
1k−cell
Dome
50%−epiboly
Shield
75%−epiboly
1−4−somites
14−19−somites
20−25−somites
Prim−5
Prim−15
Prim−25
Long−pec
Protruding−mouth
Day−4
Day−5
Stage
Transcript
ENSDART00000175347
ENSDART00000175892
ENSDART00000178863

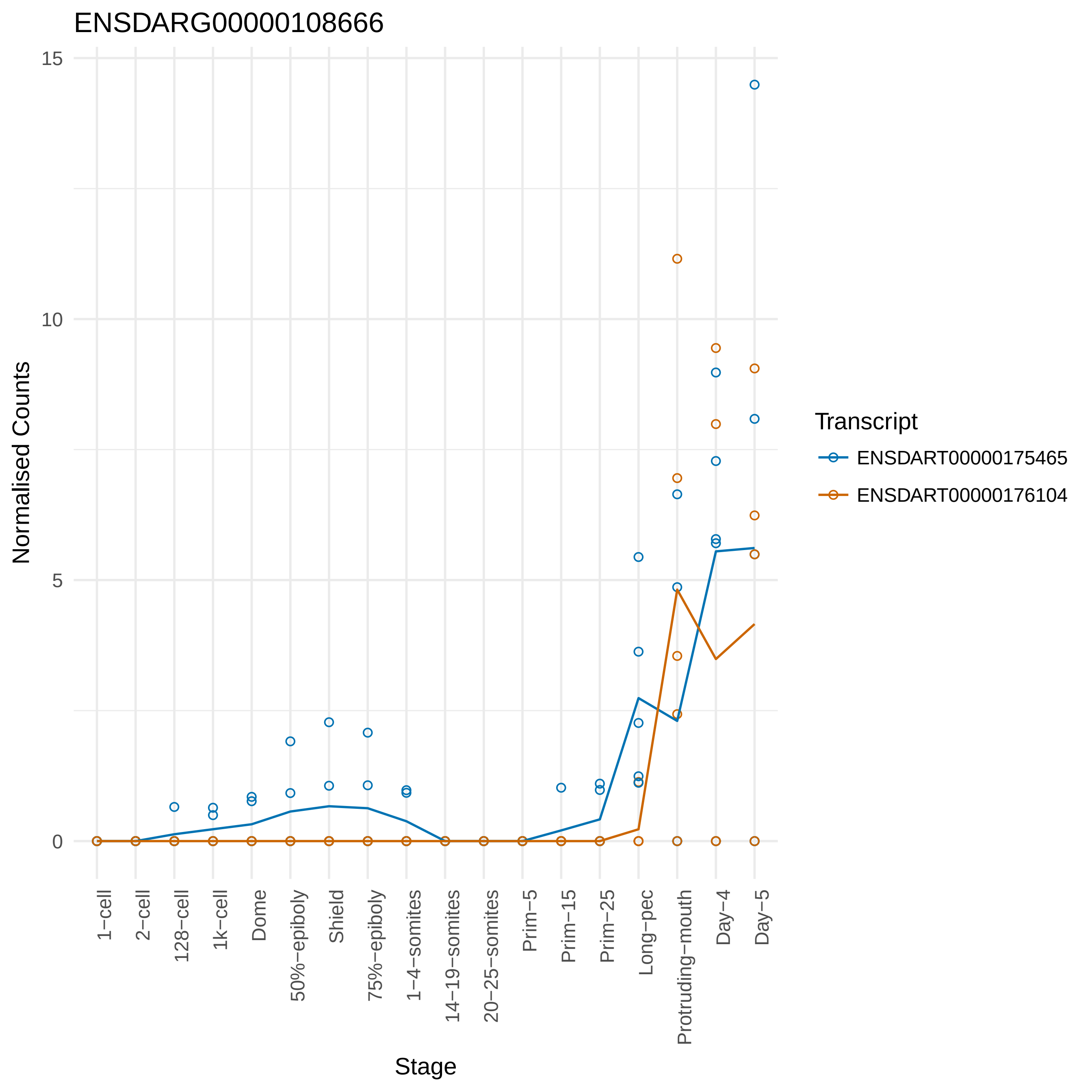
ENSDARG00000108666
Normalised Counts
15
10
5
0
1−cell
2−cell
128−cell
1k−cell
Dome
50%−epiboly
Shield
75%−epiboly
1−4−somites
14−19−somites
20−25−somites
Prim−5
Prim−15
Prim−25
Long−pec
Protruding−mouth
Day−4
Day−5
Stage
Transcript
ENSDART00000175465
ENSDART00000176104

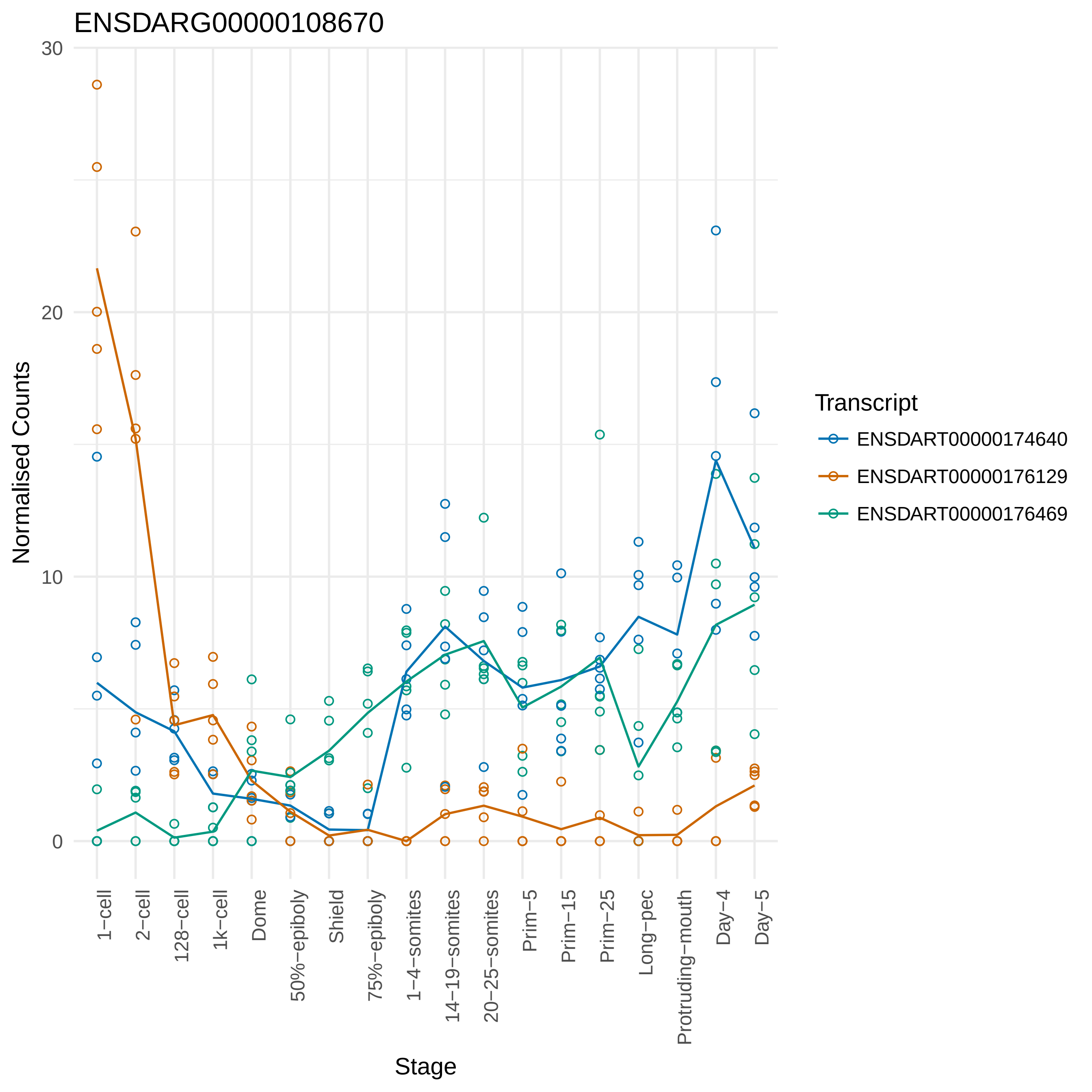

ENSDARG00000108670
30
20
10
0
Normalised Counts
1−cell
2−cell
128−cell
1k−cell
Dome
50%−epiboly
Shield
75%−epiboly
1−4−somites
14−19−somites
20−25−somites
Prim−5
Prim−15
Prim−25
Long−pec
Protruding−mouth
Day−4
Day−5
Stage
Transcript
ENSDART00000174640
ENSDART00000176129
ENSDART00000176469

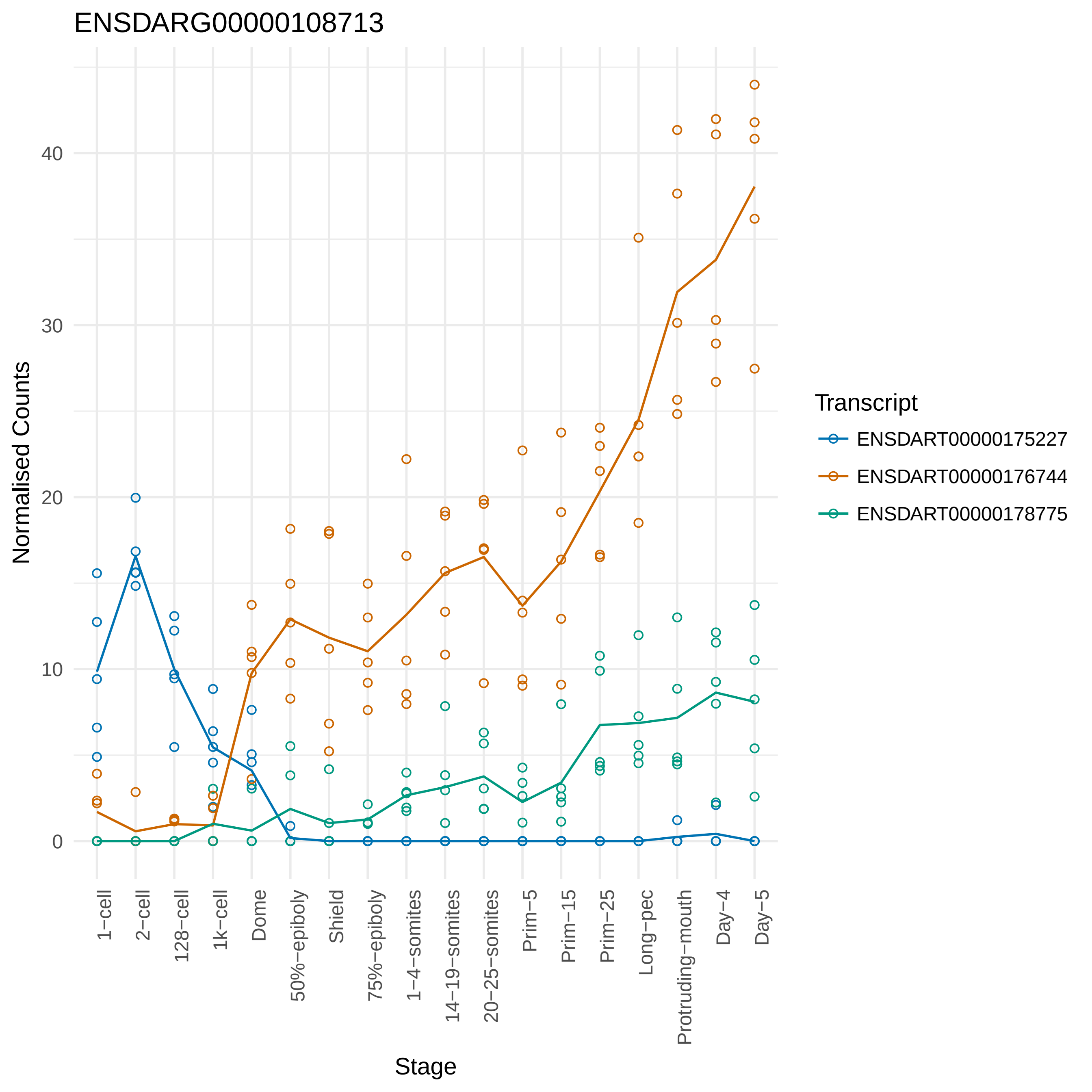

ENSDARG00000108713
Normalised Counts
0
10
20
30
40
1−cell
2−cell
128−cell
1k−cell
Dome
50%−epiboly
Shield
75%−epiboly
1−4−somites
14−19−somites
20−25−somites
Prim−5
Prim−15
Prim−25
Long−pec
Protruding−mouth
Day−4
Day−5
Stage
Transcript
ENSDART00000175227
ENSDART00000176744
ENSDART00000178775

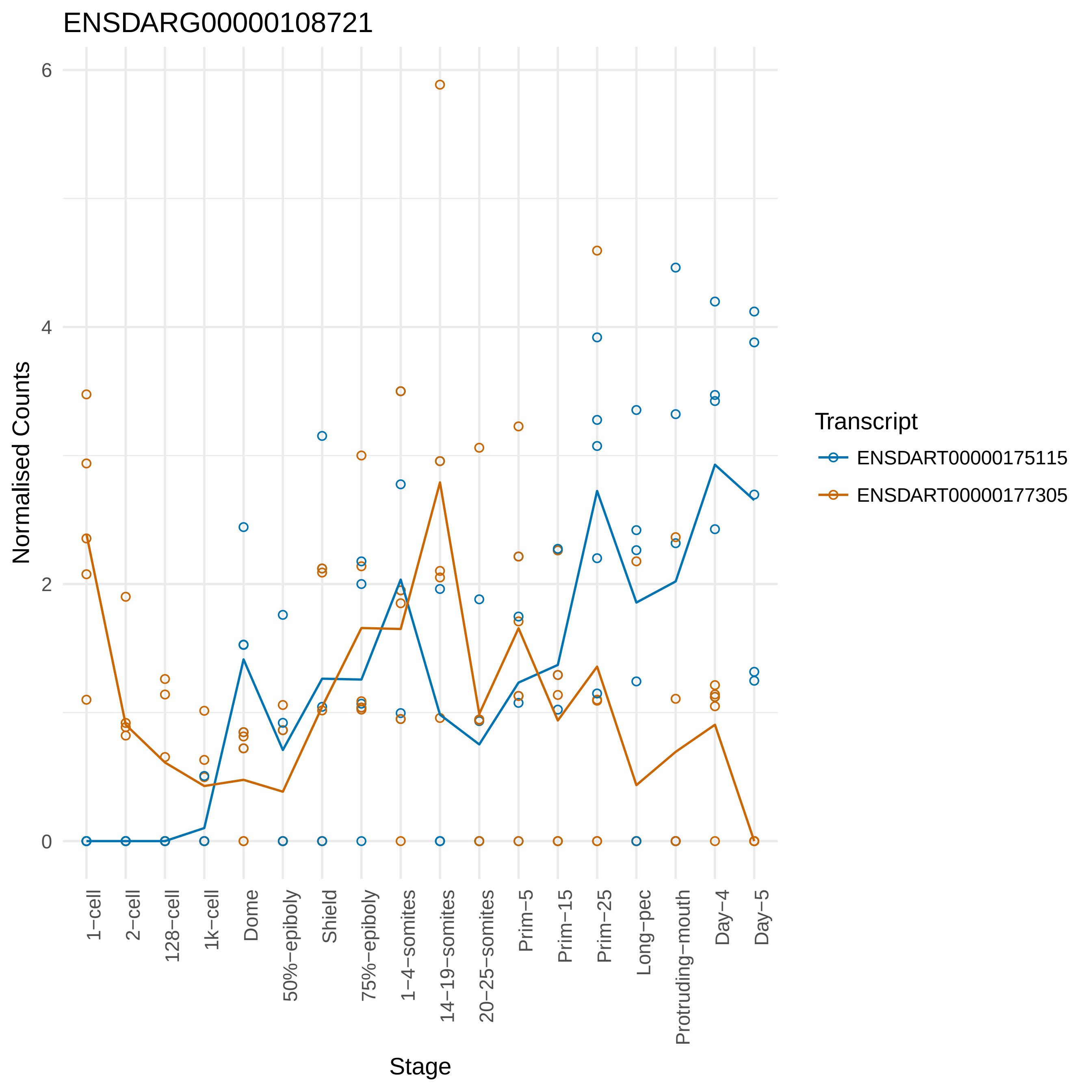

ENSDARG00000108721
Normalised Counts
0
2
4
6
Stage
1−cell
2−cell
128−cell
1k−cell
Dome
50%−epiboly
Shield
75%−epiboly
1−4−somites
14−19−somites
20−25−somites
Prim−5
Prim−15
Prim−25
Long−pec
Protruding−mouth
Day−4
Day−5
Transcript
ENSDART00000175115
ENSDART00000177305

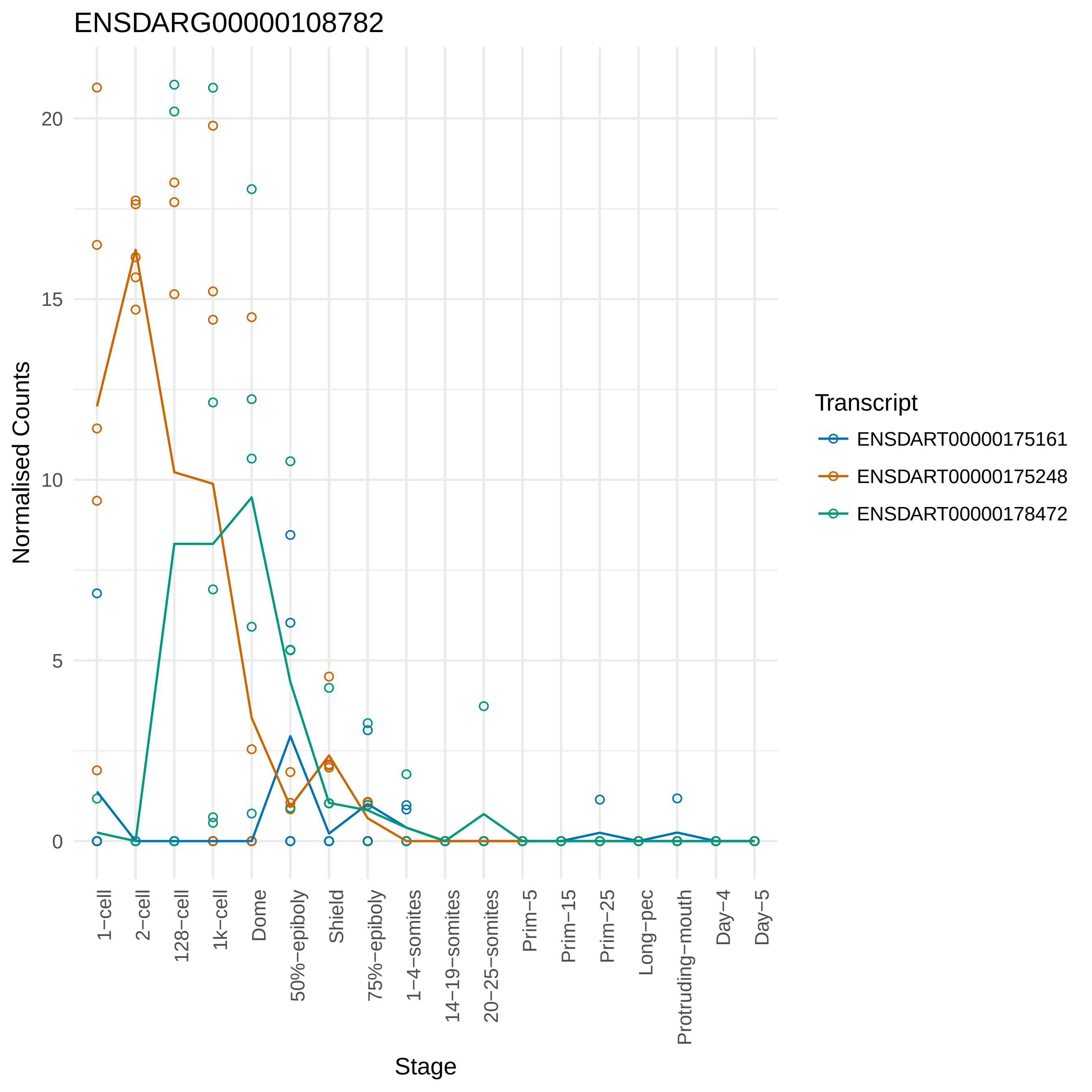

ENSDARG00000108782
Normalised Counts
0
5
10
15
20
1−cell
2−cell
128−cell
1k−cell
Dome
50%−epiboly
Shield
75%−epiboly
1−4−somites
14−19−somites
20−25−somites
Prim−5
Prim−15
Prim−25
Long−pec
Protruding−mouth
Day−4
Day−5
Stage
Transcript
ENSDART00000175161
ENSDART00000175248
ENSDART00000178472

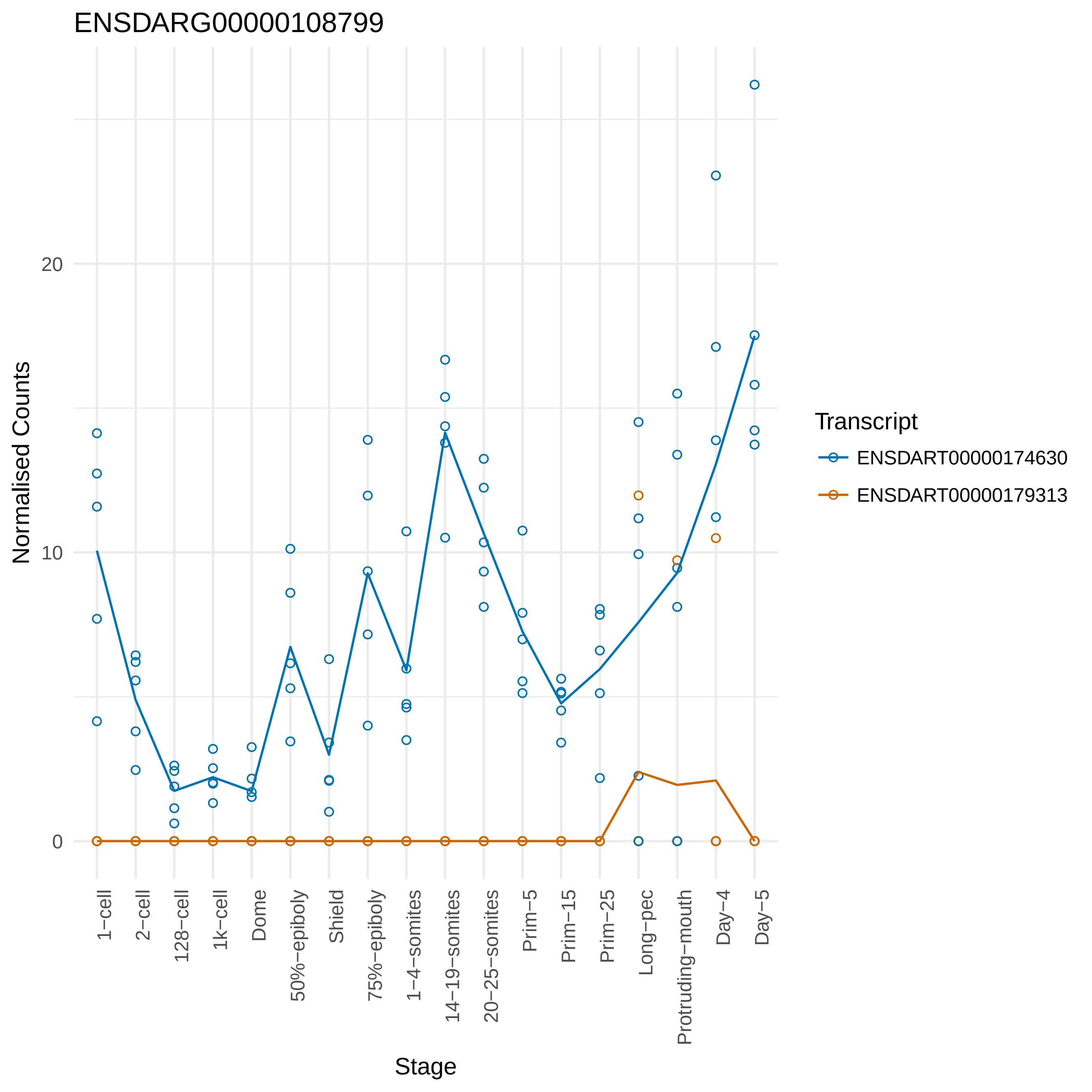

ENSDARG00000108799
Normalised Counts
0
10
20
1−cell
2−cell
128−cell
1k−cell
Dome
50%−epiboly
Shield
75%−epiboly
1−4−somites
14−19−somites
20−25−somites
Prim−5
Prim−15
Prim−25
Long−pec
Protruding−mouth
Day−4
Day−5
Stage
Transcript
ENSDART00000174630
ENSDART00000179313

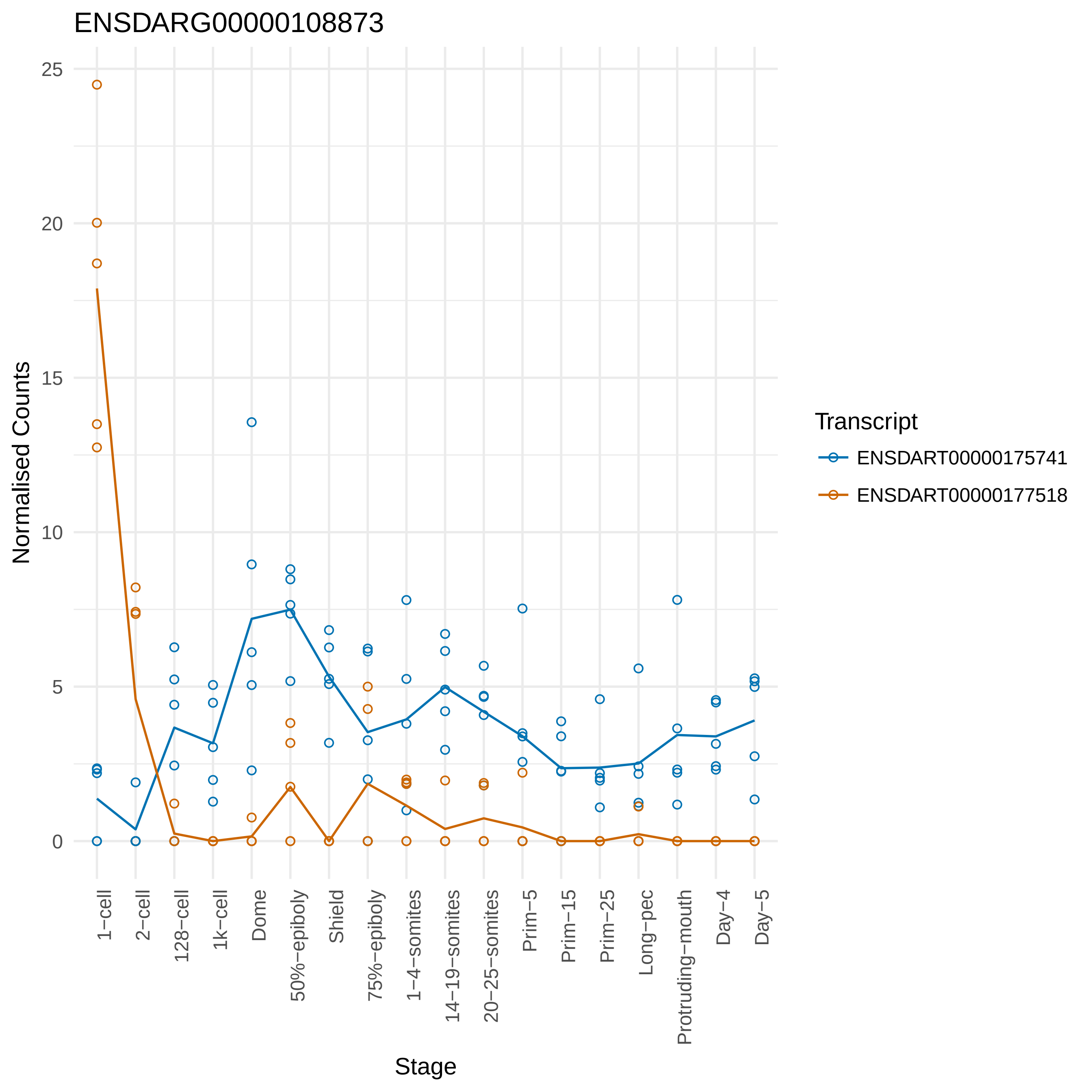
ENSDARG00000108873
Normalised Counts
25
20
15
10
5
0
1−cell
2−cell
128−cell
1k−cell
Dome
50%−epiboly
Shield
75%−epiboly
1−4−somites
14−19−somites
20−25−somites
Prim−5
Prim−15
Prim−25
Long−pec
Protruding−mouth
Day−4
Day−5
Stage
Transcript
ENSDART00000175741
ENSDART00000177518

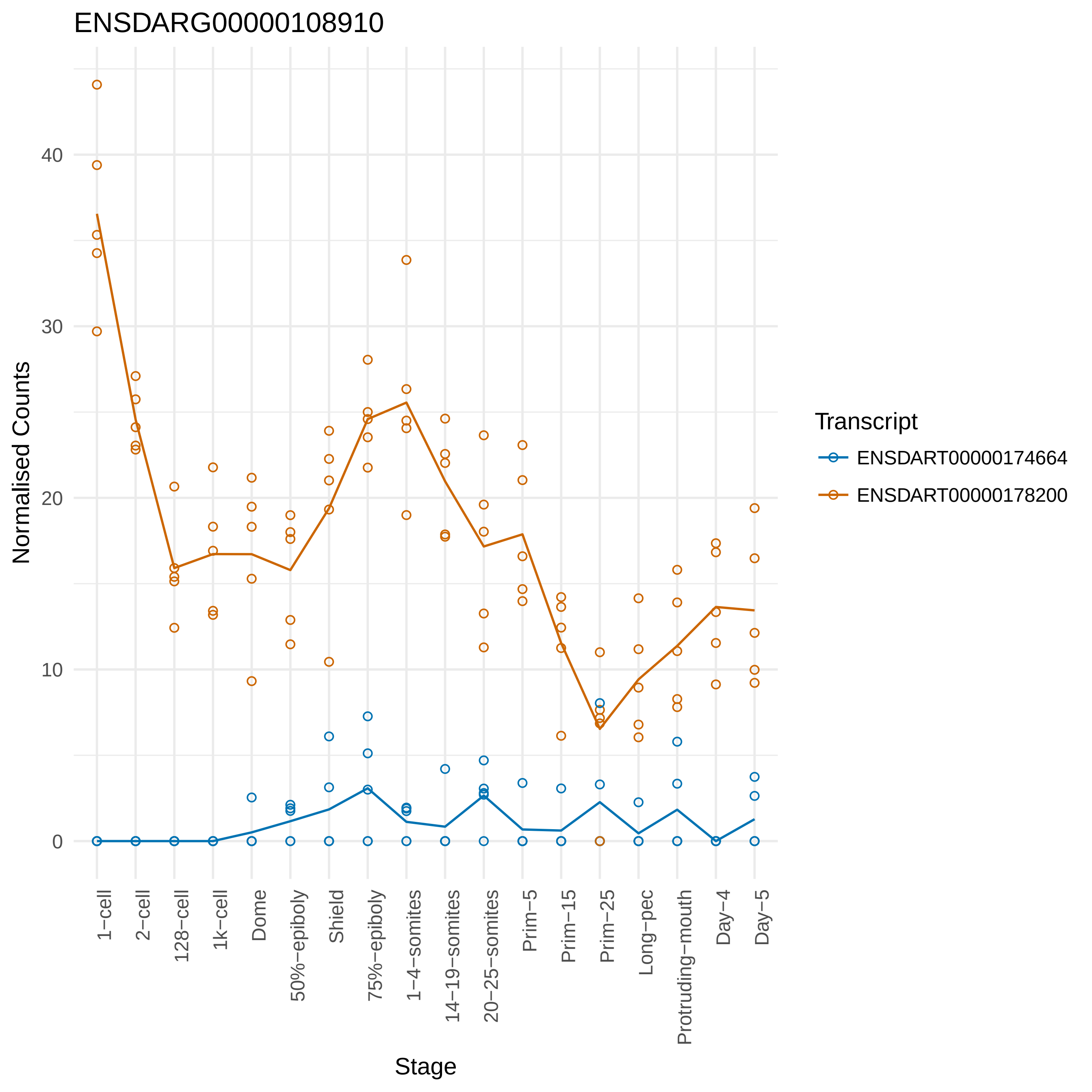

ENSDARG00000108910
Normalised Counts
0
10
20
30
40
1−cell
2−cell
128−cell
1k−cell
Dome
50%−epiboly
Shield
75%−epiboly
1−4−somites
14−19−somites
20−25−somites
Prim−5
Prim−15
Prim−25
Long−pec
Protruding−mouth
Day−4
Day−5
Stage
Transcript
ENSDART00000174664
ENSDART00000178200

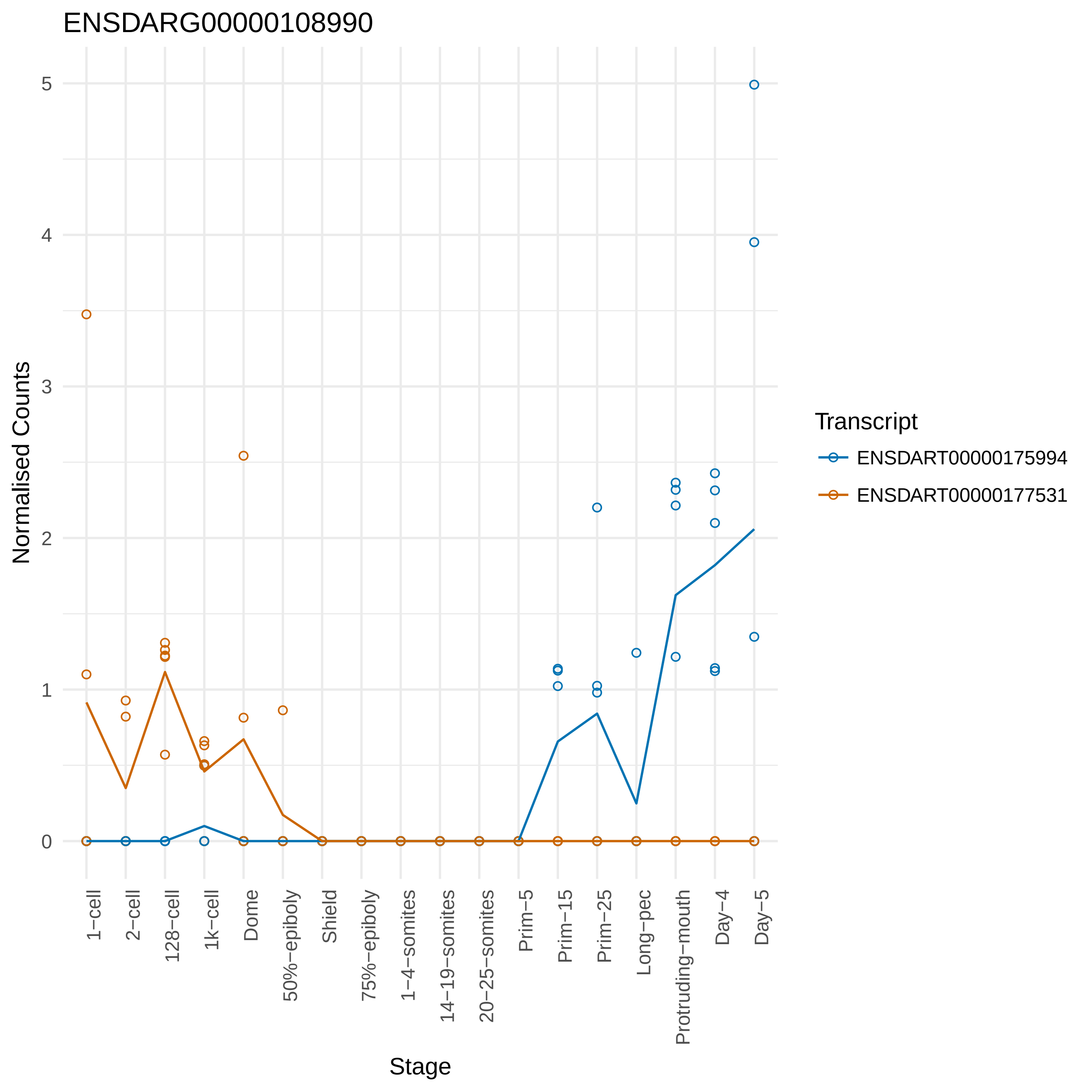

ENSDARG00000108990
Normalised Counts
0
1
2
3
4
5
1−cell
2−cell
128−cell
1k−cell
Dome
50%−epiboly
Shield
75%−epiboly
1−4−somites
14−19−somites
20−25−somites
Prim−5
Prim−15
Prim−25
Long−pec
Protruding−mouth
Day−4
Day−5
Stage
Transcript
ENSDART00000175994
ENSDART00000177531

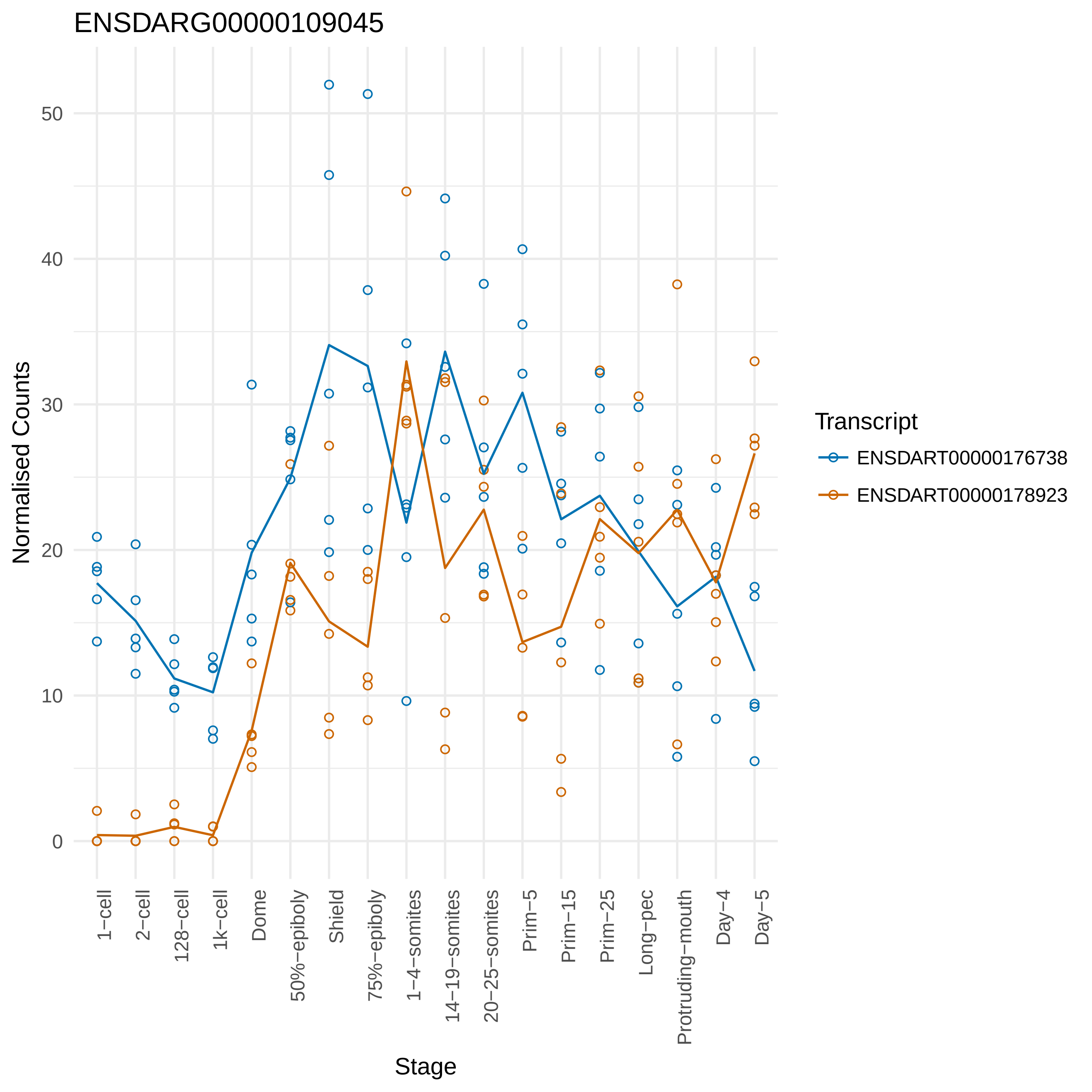
ENSDARG00000109045
Normalised Counts
0
10
20
30
40
50
1−cell
2−cell
128−cell
1k−cell
Dome
50%−epiboly
Shield
75%−epiboly
1−4−somites
14−19−somites
20−25−somites
Prim−5
Prim−15
Prim−25
Long−pec
Protruding−mouth
Day−4
Day−5
Stage
Transcript
ENSDART00000176738
ENSDART00000178923

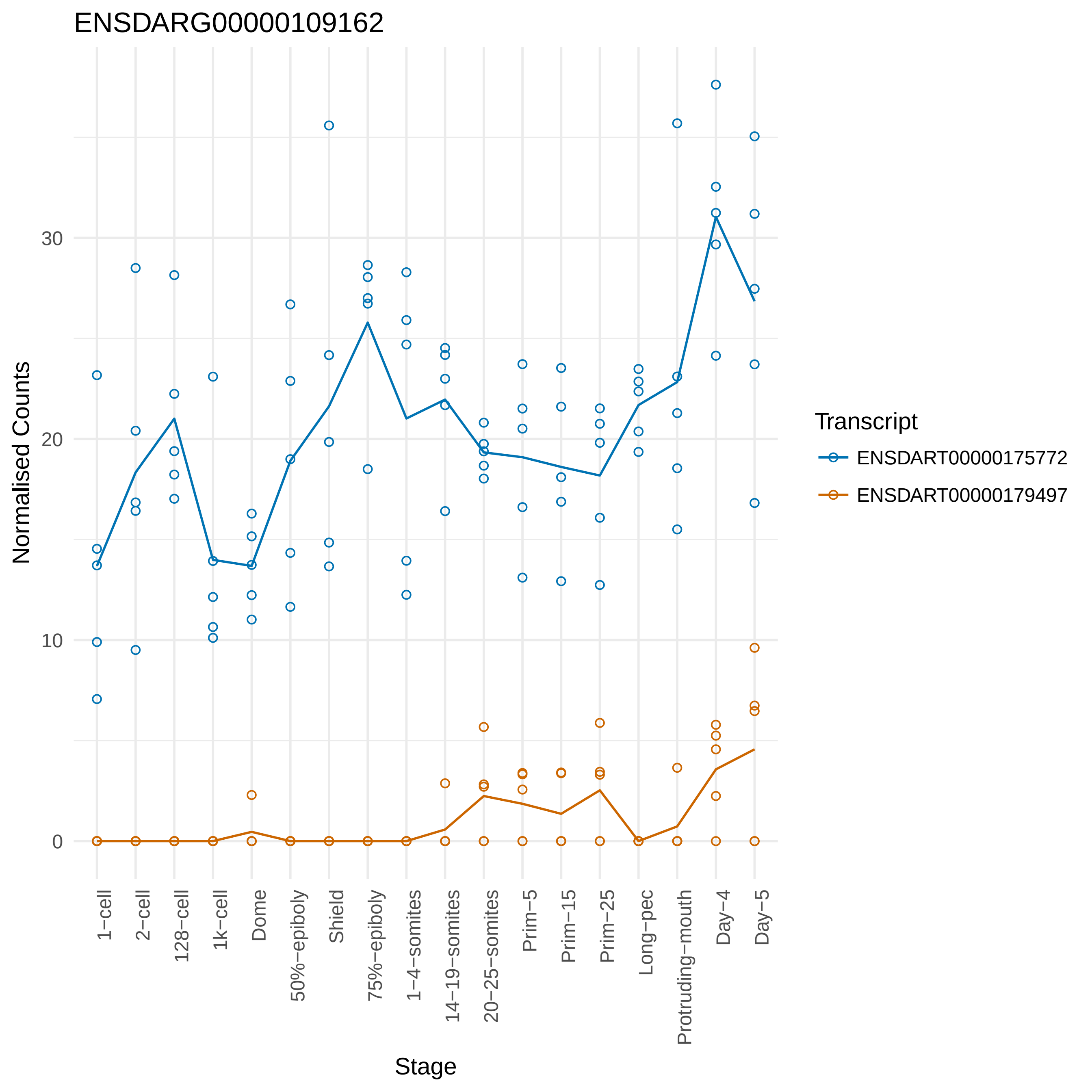

ENSDARG00000109162
Normalised Counts
0
10
20
30
Stage
1−cell
2−cell
128−cell
1k−cell
Dome
50%−epiboly
Shield
75%−epiboly
1−4−somites
14−19−somites
20−25−somites
Prim−5
Prim−15
Prim−25
Long−pec
Protruding−mouth
Day−4
Day−5
Transcript
ENSDART00000175772
ENSDART00000179497

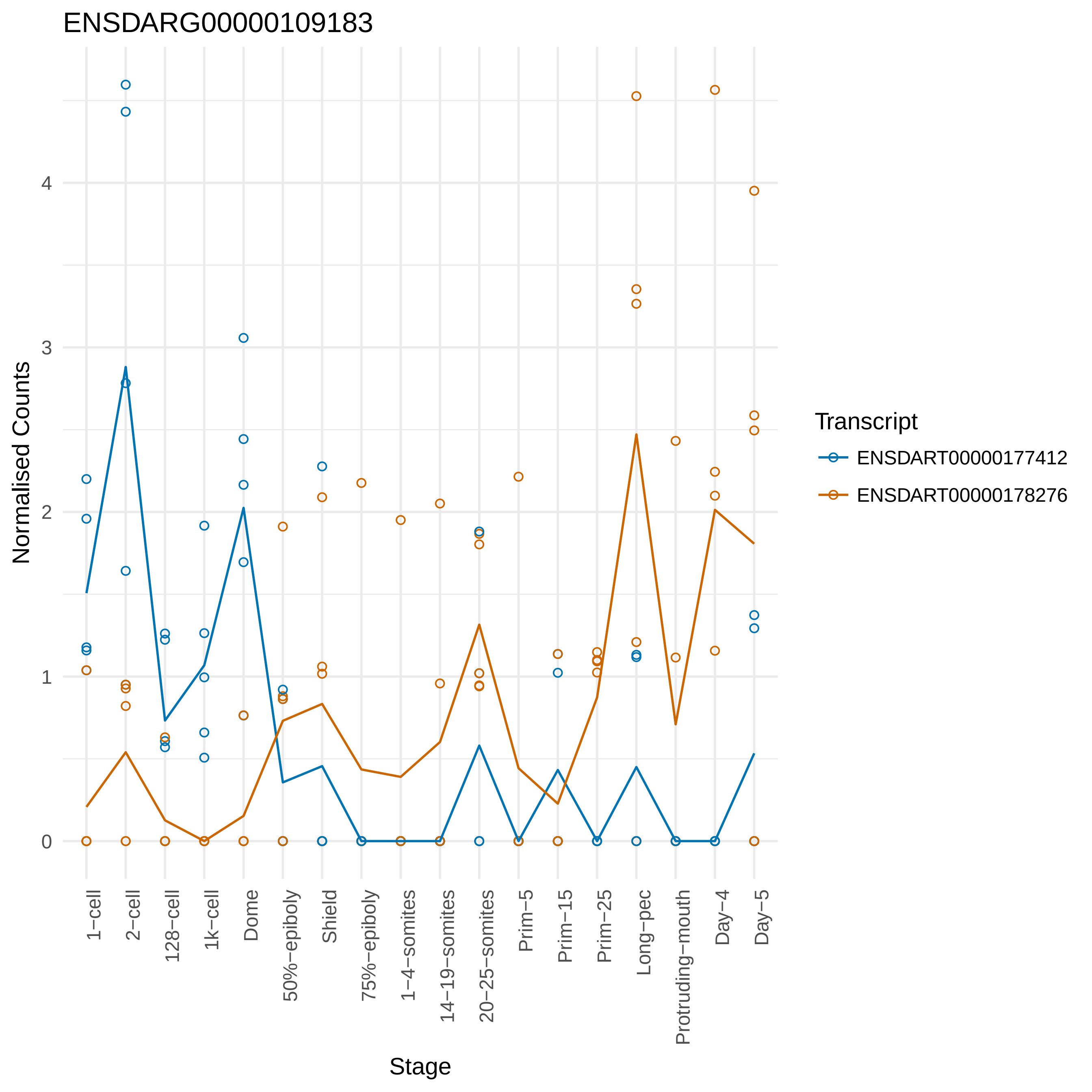
ENSDARG00000109183
Normalised Counts
0
1
2
3
4
Stage
1−cell
2−cell
128−cell
1k−cell
Dome
50%−epiboly
Shield
75%−epiboly
1−4−somites
14−19−somites
20−25−somites
Prim−5
Prim−15
Prim−25
Long−pec
Protruding−mouth
Day−4
Day−5
Transcript
ENSDART00000177412
ENSDART00000178276

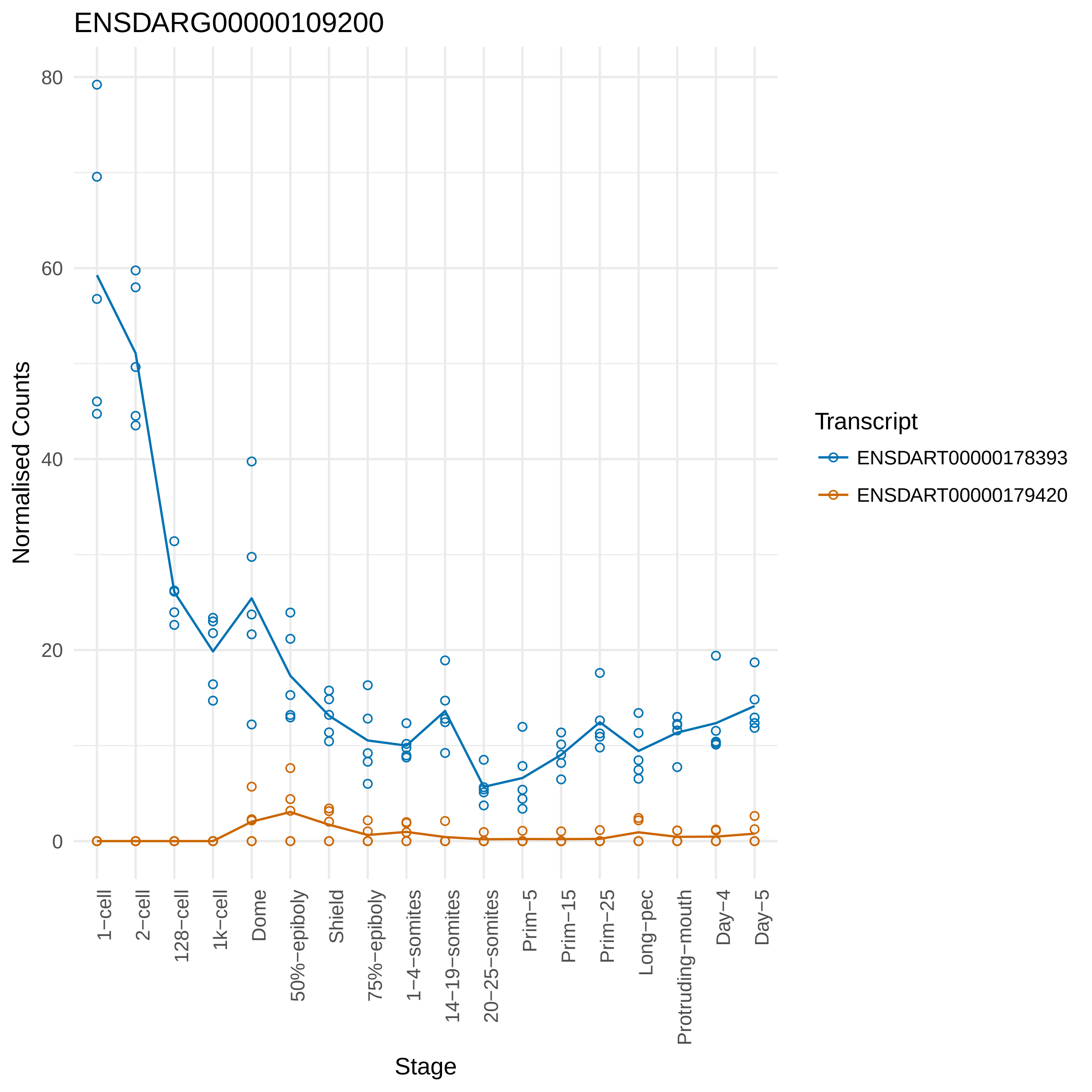
ENSDARG00000109200
Normalised Counts
0
20
40
60
80
1−cell
2−cell
128−cell
1k−cell
Dome
50%−epiboly
Shield
75%−epiboly
1−4−somites
14−19−somites
20−25−somites
Prim−5
Prim−15
Prim−25
Long−pec
Protruding−mouth
Day−4
Day−5
Stage
Transcript
ENSDART00000178393
ENSDART00000179420

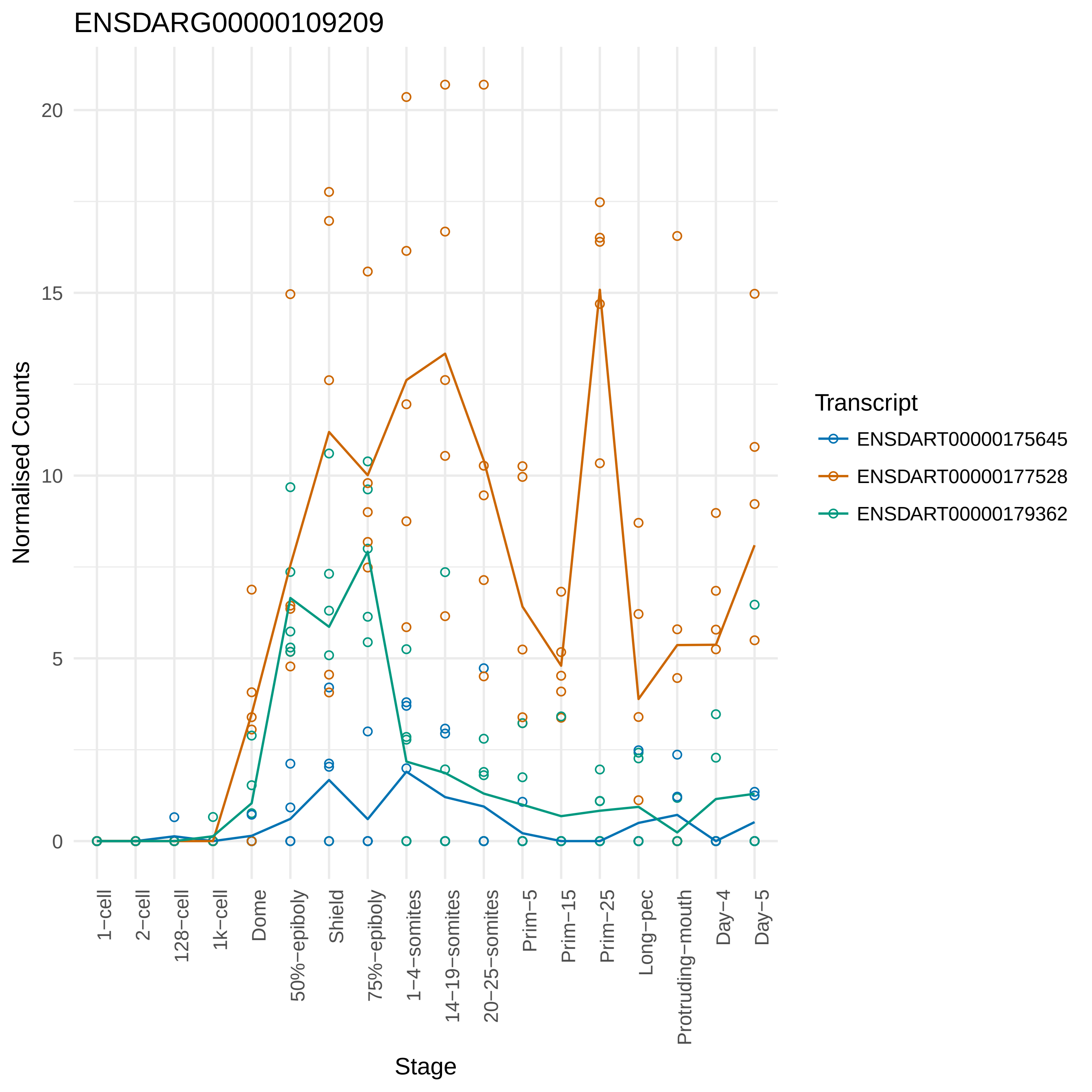

ENSDARG00000109209
Normalised Counts
20
15
10
5
0
1−cell
2−cell
128−cell
1k−cell
Dome
50%−epiboly
Shield
75%−epiboly
1−4−somites
14−19−somites
20−25−somites
Prim−5
Prim−15
Prim−25
Long−pec
Protruding−mouth
Day−4
Day−5
Stage
Transcript
ENSDART00000175645
ENSDART00000177528
ENSDART00000179362
